# Supplementary material for: Genome-Wide Identification and Expression Pattern of the GRAS Gene Family in Pitaya (Selenicereus undatus L.)
Source: Biology (Basel). 2022 Dec 21;12(1):11. doi: 10.3390/biology12010011 (PMC9854919; doi:10.3390/biology12010011)
Supplement: Supplementary file 1 [file biology-12-00011-s001.zip › Supplementary file S1.pdf]

GRAS family transcription factor family protein [Arabidopsis thaliana]

NCBI Reference Sequence: NP_178266.1

GenPept Identical Proteins Graphics

>NP_178266.1 GRAS family transcription factor family protein [Arabidopsis thaliana]

MKRDHHQFQGRLSNHGTSSSSSSISKDKMMMVKKEEDGGGNMDDELLAVLGYKVRSSEMAEVALKLEQLE
TMMSNVQEDGLSHLATDTVHYNPSELYSWLDNMLSELNPPPLPASSNGLDPVLPSPEICGFPASDYDLKV
IPGNAIYQFPAIDSSSSSNNQNKRLKSCSSPDSMVTSTSTGTQIGGVIGTTVTTTTTTTTAAGESTRSVI
LVDSQENGVRLVHALMACAEAIQQNNLTLAEALVKQIGCLAVSQAGAMRKVATYFAEALARRIYRLSPPQ
NQIDHCLSDTLQMHFYETCPYLKFAHFTANQAILEAFEGKKRVHVIDFSMNQGLQWPALMQALALREGGP
PTFRLTGIGPPAPDNSDHLHEVGCKLAQLAEAIHVEFEYRGFVANSLADLDASMLELRPSDTEAVAVNSV
FELHKLLGRPGGIEKVLGVVKQIKPVIFTVVEQESNHNGPVFLDRFTESLHYYSTLFDSLEGVPNSQDKV
MSEVYLGKQICNLVACEGPDRVERHETLSQWGNRFGSSGLAPAHLGSNAFKQASMLLSVFNSGQGYRVEE
SNGCLMLGWHTRPLITTSAWKLSTAAY

221-581 Motif Domain

https://www.ebi.ac.uk/interpro/result/InterProScan/iprscan5-R20221027-080812-0001-93212281-p1m/

Sequence

> Sequence title 46

0	MKRDHHQFQG	RLSNHGTSSS	SSSISKDKMM	MVKKEEDGGG	NMDDELLAVL	GYKVRSSEMA	EVALKLEQLE
70	TMMSNVQEDG	LSHLATDTVH	YNPSELYSWL	DNMLSELNPP	PLPASSNGLD	PVLPSPEICG	FPASDYDLKV
140	IPGNAIYQFP	AIDSSSSSNN	QNKRLKSCSS	PDSMVTSTST	GTQIGGVIGT	TVTTTTTTTT	AAGESTRSVI
210	LVDSQENGVR	LVHALMACAE	AIQQNNLTLA	EALVKQIGCL	AVSQAGAMRK	VATYFAEALA	RRIYRLSPPQ
280	NQIDHCLSDT	LQMHFYETCP	YLKFAHFTAN	QAILEAFEGK	KRVHVIDFSM	NQGLQWPALM	QALALREGGP
350	PTFRLTGIGP	PAPDNSDHLH	EVGCKLAQLA	EAIHVEFEYR	GFVANSLADL	DASMLELRPS	DTEAVAVNSV
420	FELHKLLGRP	GGIEKVLGVV	KQIKPVIFTV	VEQESNHNGP	VFLDRFTESL	HYYSTLFDSL	EGVPNSQDKV
490	MSEVYLGKQI	CNLVACEGPD	RVERHETLSQ	WGNRFGSSGL	APAHLGSNAF	KQASMLLSVF	NSGQGYRVEE
560	SNGCLMLGWH	TRPLITTSAW	KLSTAAY				

GRAS Protein 221-581

LVHALMACAEAIQQNNLTLAEALVKQIGCLAVSQAGAMRKVATYFAEALARRIYRLSPPQNQIDHCLSDTLQMHFYET
CPYLKFAHFTANQAILEAFEGKKRVHVIDFSMNQGLQWPALMQALALREGGPPTFRLTGIGPPAPDNSDHLHEVGCKL
AQLAEAIHVEFEYRGFVANSLADLDASMLELRPSDTEAVAVNSVFELHKLLGRPGGIEKVLGVVKQIKPVIFTVVEQESN
HNGPVFLDRFTESLHYYSTLFDSLEGVPNSQDKVMSEVYLGKQICNLVACEGPDRVERHETLSQWGNRFGSSGLAPAH
LGSNAFKQASMLLSVFNSGQGYRVEESNGCLMLGWHTRPLITTSAWK

Description	Max score	Total score	Query cover	E value	Ident
chr10	564	564	100%	1e-179	75%
chr11	374	906	100%	5e-114	53%
chr03	369	562	100%	6e-112	53%
chr04	224	470	100%	4e-62	35%
chr01	221	969	100%	5e-61	40%
chr08	219	1723	100%	2e-60	53%
chr02	198	1125	100%	3e-53	35%
chr06	192	845	100%	3e-51	36%
chr05	175	822	100%	1e-48	48%
chr07	168	649	100%	6e-43	31%

1. AtPAT1 (119-490)

>AED95624.1 GRAS family transcription factor [Arabidopsis thaliana]
MYKQPRQELEAYYFEPNSVEKLRYLPVNNSRKRFCTLEPFPDSPPYNALSTATYDDTCGSCVTDELNDFK
HKIREIETVMMGPDSLDLLVDCTDSFDSTASQEINGWRSTLEAISRRDLRADLVSCAKAMSENDLMMAHS
MMEKLRQMVSVSGEPIQRLGAYLLEGLVAQLASSGSSIYKALNRCPEPASTELLSYMHILYEVCPYFKFG
YMSANGAIAEAMKEENRVHIIDFQIGQGSQWVTLIQAFAARPGGPPRIRITGIDDMTSAYARGGGLSIVG
NRLAKLAKQFNVPFEFNSVSVSVSEVKPKNLGVRPGEALAVNFAFVLHHMPDESVSTENHRDRLLRMVKS
LSPKVVTLVEQESNTNTAAFFPRFMETMNYYAAMFESIDVTLPRDHKQRINVEQHCLARDVVNIIACEGA
DRVERHELLGKWRSRFGMAGFTPYPLSPLVNSTIKSLLRNYSDKYRLEERDGALYLGWMHRDLVASCAWK

QEINGWRSTLEAISRRDLRADLVSCAKAMSENDLMMAHSMMEKLRQMVSVSGEPIQRLGAYLLEGLVAQLASSGSSI
YKALNRCPEPASTELLSYMHILYEVCPYFKFGYMSANGAIAEAMKEENRVHIIDFQIGQGSQWVTLIQAFAARPGGP
PRIRITGIDDMTSAYARGGGLSIVGNRLAKLAKQFNVPFEFNSVSVSVSEVKPKNLGVRPGEALAVNFAFVLHHMPD
ESVSTENHRDRLLRMVKSLSPKVVTLVEQESNTNTAAFFPRFMETMNYYAAMFESIDVTLPRDHKQRINVEQHCLAR
DVVNIIACEGADRVERHELLGKWRSRFGMAGFTPYPLSPLVNSTIKSLLRNYSDKYRLEERDGALYLGWMHRDLVA

AtGRAS (221-581)

MKRDHHQFQGRLSNHGTSSSSSSISKDKMMMVKKEEDGGGNMDDELLAVLGYKVRSSEMAEVALKLEQLE

TMMSNVQEDGLSHLATDTVHYNPSELYSWLDNMLSELNPPPLPASSNGLDPVLPSPEICGFPASDYDLKV

IPGNAIYQFPAIDSSSSSNNQNKRLKSCSSPDSMVTSTSTGTQIGGVIGTTVTTTTTTTAAGESTRSVI

LVDSQENGVRLVHALMACAEAIQQNNLTLAEALVKQIGCLAVSQAGAMRKVATYFAEALARRIYRLSPPQ

NQIDHCLSDTLQMHFYETCPYLKFAHFTANQAILEAFEGKKRVHVIDFSMNQGLQWPALMQALALREGGP

PTFRLTGIGPPAPDNSDHLHEVGCKLAQLAEAIHVEFEYRGFVANSLADLDASMLELRPSDTEAVAVNSV

FELHKLLGRPGGIEKVLGVVKQIKPVIFTVVEQESNHNGPVFLDRFTESLHYYSTLFDSLEGVPNSQDKV

MSEVYLGKQICNLVACEGPDRVERHETLSQWGNRFGSSGLAPAHLGSNAFKQASMLLSVFNSGQGYRVEE

SNGCLMLGWHTRPLITTSAWKLSTAAY

AtGRAS (221-581)

LVHALMACAEAIQQNNLTLAEALVKQIGCLAVSQAGAMRKVATYFAEALARRIYRLSPPQNQIDHCLSDTLQMHFYE
TCPYLKFAHFTANQAILEAFEGKKRVHVIDFSMNQGLQWPALMQALALREGGPPTFRLTGIGPPAPDNSDHLHEVGC
KLAQLAEAIHVEFEYRGFVANSLADLDASMLELRPSDTEAVAVNSVFELHKLLGRPGGIEKVLGVVKQIKPVIFTVV
EQESNHNGPVFLDRFTESLHYYSTLFDSLEGVPNSQDKVMSEVYLGKQICNLVACEGPDRVERHETLSQWGNRFGSS
GLAPAHLGSNAFKQASMLLSVFNSGQGYRVEESNGCLMLGWHTRPLITTSAWK

AtLAS

MLTSFKSSSSSSEDATATTTENPPPLCIASSSAATSASHHLRRLLFTAANFVSQSNFTAAQNLLSILSLN
SSPHGDSTERLVHLFTKALSVRINRQQQDQTAETVATWTTNEMTMSNSTVFTSSVCKEQFLFRTKNNNSD
FESCYYLWLNQLTPFIRFGHLTANQAILDATETNDNGALHILDLDISQGLQWPPLMQALAERSSNPSSPP
PSLRITGCGRDVTGLNRTGDRLTRFADSLGLQFQFHTLVIVEEDLAGLLLQIRLLALSAVQGETIAVNCV
HFLHKIFNDDGDMIGHFLSAIKSLNSRIVTMAEREANHGDHSFLNRFSEAVDHYMAIFDSLEATLPPNSR
ERLTLEQRWFGKEILDVVAAEETERKQRHRRFEIWEEMMKRFGFVNVPIGSFALSQAKLLLRLHYPSEGY
NLQFLNNSLFLGWQNRPLFSVSSWK

>AtLAS Pfam Domain
SSAATSASHHLRRLLFTAANFVSQSNFTAAQNLLSILSLNSSPHGDSTERLVHLFTKALSVRINRQQQDQTAETVAT
WTTNEMTMSNSTVFTSSVCKEQFLFRTKNNNSDFESCYYLWLNQLTPFIRFGHLTANQAILDATETNDNGALHILDL
DISQGLQWPPLMQALAERSSNPSSPPPSLRITGCGRDVTGLNRTGDRLTRFADSLGLQFQFHTLVIVEEDLAGLLLQ
IRLLALSAVQGETIAVNCVHFLHKIFNDDGDMIGHFLSAIKSLNSRIVTMAEREANHGDHSFLNRFSEAVDHYMAIF
DSLEATLPPNSRERLTLEQRWFGKEILDVVAAEETERKQRHRRFEIWEEMMKRFGFVNVPIGSFALSQAKLLLRLHY
PSEGYNLQFLNNSLFLGWQNRPLFSVSSWK

AtSCL
>CAB10504.1 SCARECROW like protein [Arabidopsis thaliana]
MEATVKIFYYGTGVKSKIRELEVSLLSGDTKVEEFSGFSPAAGKSWNWDELLALTPQLDLKEVLVEAARA
VADGDFATAYGFLDVLEQMVSVSGSPIQRLGTYMAEGLRARLEGSGSNIYKSLKCNEPTGRELMSYMSVL
YEICPYWKFAYTTANVEILEAIAGETRVHIIDFQIAQGSQYMFLIQELAKHPGGPPLLRVTGVDDSQSTY
ARGGGLSLVGERLATLAQSCGVPFEFHDAIMSGCKVQREHLGLEPGFAVVVNFPYVLHHMPDESVSVENH
RDRLLHLIKSLSPKLVTLVEQESNTNTSPFLSRFVETLDYYTAMFESIDAARPRDDKQRISAEQHCVARD
IVNMIACEESERVERHEVLGNGGSE

Pfam >AtSCL

LKEVLVEAARAVADGDFATAYGFLDVLEQMVSVSGSPIQRLGTYMAEGLRARLEGSGSNIYKSLKCNEPTGRELMSY
MSVLYEICPYWKFAYTTANVEILEAIAGETRVHIIDFQIAQGSQYMFLIQELAKHPGGPPLLRVTGVDDSQSTYARG
GGLSLVGERLATLAQSCGVPFEFHDAIMSGCKVQREHLGLEPGFAVVVNFPYVLHHMPDESVSVENHRDRLLHLIKS
LSPKLVTLVEQESNTNTSPFLSRFVETLDYYTAMFESIDAARPRDDKQRISAEQHCVARDIVNMIACEESERVERHE
VL

AtSHR (Short Root Like Protein)

>NP_195480.1 GRAS family transcription factor [Arabidopsis thaliana]
MDTLFRLVSLQQQQQSDSIITNQSSLSRTSTTTTGSPQTAYHYNFPQNDVVEECFNFFMDEEDLSSSSSH
HNHHNHNNPNTYYSPFTTPTQYHPATSSTPSSTAAAAALASPYSSSGHHNDPSAFSIPQTPPSFDFSANA
KWADSVLLEAARAFSDKDTARAQQILWTLNELSSPYGDTEQKLASYFLQALFNRMTGSGERCYRTMVTAA
ATEKTCSFESTRKTVLKFQEVSPWATFGHVAANGAILEAVDGEAKIHIVDISSTFCTQWPTLLEALATRS
DDTPHLRLTTVVVANKFVNDQTASHRMMKEIGNRMEKFARLMGVPFKFNIIHHVGDLSEFDLNELDVKPD
EVLAINCVGAMHGIASRGSPRDAVISSFRRLRPRIVTVVEEEADLVGEEEGGFDDEFLRGFGECLRWFRV
CFESWEESFPRTSNERLMLERAAGRAIVDLVACEPSDSTERRETARKWSRRMRNSGFGAVGYSDEVADDV
RALLRRYKEGVWSMVQCPDAAGIFLCWRDQPVVWASAWRPT

SVLLEAARAFSDKDTARAQQILWTLNELSSPYGDTEQKLASYFLQALFNRMTGSGERCYRTMVTAA
ATEKTCSFESTRKTVLKFQEVSPWATFGHVAANGAILEAVDGEAKIHIVDISSTFCTQWPTLLEALATRS
DDTPHLRLTTVVVANKFVNDQTASHRMMKEIGNRMEKFARLMGVPFKFNIIHHVGDLSEFDLNELDVKPD
EVLAINCVGAMHGIASRGSPRDAVISSFRRLRPRIVTVVEEEADLVGEEEGGFDDEFLRGFGECLRWFRV
CFESWEESFPRTSNERLMLERAAGRAIVDLVACEPSDSTERRETARKWSRRMRNSGFGAVGYSDEVADDV
RALLRRYKEGVWSMVQCPDAAGIFLCWRDQPVVWASAWR

OSRGA3 (RGL3)

>OAO92748.1 RGL3 [Arabidopsis thaliana]
MKRSHQETSVEEEASSMVEKLENGCGGGGDDNMDEFLAVLGYKVRSSDMADVAQKLEQLEIVLSNDIASS
SNAFNDTVHYNPSDLSGWAQSMLSDLNYYPDLDPNRICDLRPITDDDECCSSNSNSNKRIRLGPWCDSVT
SESTRSVVLIEETGVRLVQALVACAEAVQLENLSLADALVKRVGLLAASQAGAMGKVATYFAEALARRIY
RIHPSAAAIDPSFEEILQMNFYDSCPYLKFAHFTANQAILEAVTTSRVVHVIDLGLNQGMQWPALMQALA
LRPGGPPSFRLTGVGNPSNREGIQELGWKLAQLAQAIGVEFKFNGLTTERLSDLEPDMFETRTESETLVV
NSVFELHPVLSQPGSIEKLLATVKAVKPGLVTVVEQEANHNGDVFLDRFNEALHYYSSLFDSLEDGVVIP
SQDRVMSEVYLGRQILNVVATEGSDRIERHETLAQWRKRMGSAGFDPVNLGSDAFKQASLLLALSGGGDG
YRVEENDGSLMLAWQTKPLIAASAWKLAAELRR

LVQALVACAEAVQLENLSLADALVKRVGLLAASQAGAMGKVATYFAEALARRIYRIHPSAAAIDPSFEEILQMNFYD
SCPYLKFAHFTANQAILEAVTTSRVVHVIDLGLNQGMQWPALMQALALRPGGPPSFRLTGVGNPSNREGIQELGWKL
AQLAQAIGVEFKFNGLTTERLSDLEPDMFETRTESETLVVNSVFELHPVLSQPGSIEKLLATVKAVKPGLVTVVEQE
ANHNGDVFLDRFNEALHYYSSLFDSLEDGVVIPSQDRVMSEVYLGRQILNVVATEGSDRIERHETLAQWRKRMGSAG
FDPVNLGSDAFKQASLLLALSGGGDGYRVEENDGSLMLAWQTKPLIAASAWK

SlDELLA
>NP_001234365.1 DELLA protein GAI [Solanum lycopersicum]
MKRDRDRDREREKRAFSNGAVSSGKSKIWEEDEEEKPDAGMDELLAVLGYKVKSSDMADVAQKLEQLEMA
MGTTMEDGITHLSTDTVHKNPSDMAGWVQSMLSSISTNFDMCNQENDVLVSGCGSSSSIIDFSQNHRTST
ISDDDLRAIPGGAVFNSDSNKRHRSTTSSFSTTSSSMVTDSSATRPVVLVDSQETGVRLVHTLMACAEAV
QQENLTLADQLVRHIGILAVSQSGAMRKVATYFAEALARRIYKIYPQDSMESSYTDVLQMHFYETCPYLK
FAHFTANQAILEAFTGCNKVHVIDFSLKQGMQWPALMQALALRPGGPPAFRLTGIGPPQPDNTDALQQVG
WKLAQLAETIGVEFEFRGFVANSLADLDATILDIRPSETEAVAINSVFELHRLLSRPGAIEKVLNSIKQI
NPKIVTLVEQEANHNAGVFIDRFNEALHYYSTMFDSLESSGSSSSASPTGILPQPPVNNQDLVMSEVYLG
RQICNVVACEGSDRVERHETLNQWRVRMNSSGFDPVHLGSNAFKQASMLLALFAGGDGYRVEENDGCLML
GWHTRPLIATSAWKLLPDSGTGAGEVEL

VDSQETGVRLVHTLMACAEAVQQENLTLADQLVRHIGILAVSQSGAMRKVATYFAEALARRIYKIYPQDSMESSYTD
VLQMHFYETCPYLKFAHFTANQAILEAFTGCNKVHVIDFSLKQGMQWPALMQALALRPGGPPAFRLTGIGPPQPDNT
DALQQVGWKLAQLAETIGVEFEFRGFVANSLADLDATILDIRPSETEAVAINSVFELHRLLSRPGAIEKVLNSIKQI
NPKIVTLVEQEANHNAGVFIDRFNEALHYYSTMFDSLESSGSSSSASPTGILPQPPVNNQDLVMSEVYLGRQICNVV
ACEGSDRVERHETLNQWRVRMNSSGFDPVHLGSNAFKQASMLLALFAGGDGYRVEENDGCLMLGWHTRPLIATSAWK

OsSLR1
DELLA protein SLR1, putative, expressed >LOC_Os06g03710.1
OsSLR1
MLAGCSFSSSRHQMSTAQRFDILPCGFSKRGSRGDGAAPRVAGDARSGATTCSFRTHPAP
PVTQSVSWGAKPEPGGNGNGAHRAVKRAHDEDAVEEYGPIVRAKRTRMGGDGDEVWFHQS
IAGTMQATAAGEGEEAEEEKVFLVPSAAAFPHGMAAAGPSLAAAKKEEYSKSPSDSSSSS
GTDGGSSAMMPPPQPPEFDARNGVPAPGQAEREALELVRALTACADSLSAGNHEAANYYL
ARLGEMASPAGPTPMHRVAAYFTEALALRVVRMWPHMFDIGPPRELTDDAFGGGDDDAMA
LRILNAITPIPRFLHFTLNERLLREFEGHERVHVIDFDIKQGLQWPGLLQSLAARAVPPA
HVRITGVGESRQELQETGARLARVAAALGLAFEFHAVVDRLEDVRLWMLHVKRGECVAVN
CVLAMHRLLRDDAALTDFLGLARSTGATILLLGEHEGGGLNSGRWEARFARALRYYAAAF
DAVDAAGLPEASPARAKAEEMFAREIRNAVAFEGPERFERHESFAGWRRRMEDGGGFKNA
GIGEREAMQGRMIARMFGPDKYTVQAHGGGGSGGGEALTLRWLDQPLYTVTAWTPAGDGA
GGSTVSASTTASHSQQS

GQAEREALELVRALTACADSLSAGNHEAANYYLARLGEMASPAGPTPMHRVAAYFTEALALRVVRMWPHMFDIG
PPRELTDDAFGGGDDDAMALRILNAITPIPRFLHFTLNERLLREFEGHERVHVIDFDIKQGLQWPGLLQSLAAR
AVPPAHVRITGVGESRQELQETGARLARVAAALGLAFEFHAVVDRLEDVRLWMLHVKRGECVAVNCVLAMHRLL

RDDAALTDFLGLARSTGATILLLGEHEGGGLNSGRWEARFARALRYYAAAFDAVDAAGLPEASPARAKAEEMFA
REIRNAVAFEGPERFERHESFAGWRRRMEDGGGFKNAGIGEREAMQGRMIARMFGPDKYTVQAHGGGGSGGGEA
LTLRWLDQPLYTVTAWT

Medicago Trunctula

```
> >MtrunA17_Chr1g0159461

MSLIISPELADASYGNAKLYSLKGADVTPGLSSHDFAPDKHRSMYMNDSYSSESYEKYFLDSPTEELIEPSSSGISGNSV

HADGDSSYQLRANSGGSMITDNPFDTYVSSTRQRDAYQSNFESDFLESESPDCFEFDGDMRLKIQELERALLGDNNDECD

DDEENMFRTVQSMEIDPDFAECANPIQNKSHHDSPKESLSSDSNLSSTSSTKEISQNFPRTPKQLLYECASALSEGNEVK

ASAMIDDLRQLVSIQGEPSDRIAAYMVEGLAARLASSGKCIYKALKCKEPPSSDRLAAMQILFEVCPCFKFGFIAANGAI

SEAIKDDKKVHIIDFDINQGSQYITLIQTLASRPGKPPYVRLTGVDDPESVQRSVGGLSIIGQRLEKLAEVLGLPFEFRA

VASRSSIVTPSMLNCRPGEALVVNFAFQLHHMPDESVSTVNERDQLLRMVKSLNPKLVTVVEQDVNTNTSPFLPRFVEAY

NYYSAVFESLDATLPRESQDRVNVERQCLARDIVNVIACEGEDRIERYEVAGKWRARMKMAGFTSSPMSTNVKEAIRELI

RQYCDKYKIIEEMGGLHFGWEDKNLIVASAWK
```

>MtGRAS

LSSDSNLSSTSSTKEISQNFPRTPKQLLYECASALSEGNEVKASAMIDDLRQLVSIQGEPSDRIAAYMVEGLAA
RLASSGKCIYKALKCKEPPSSDRLAAMQILFEVCPCFKFGFIAANGAISEAIKDDKKVHIIDFDINQGSQYITL
IQTLASRPGKPPYVRLTGVDDPESVQRSVGGLSIIGQRLEKLAEVLGLPFEFRAVASRSSIVTPSMLNCRPGEA
LVVNFAFQLHHMPDESVSTVNERDQLLRMVKSLNPKLVTVVEQDVNTNTSPFLPRFVEAYNYYSAVFESLDATL
PRESQDRVNVERQCLARDIVNVIACEGEDRIERYEVAGKWRARMKMAGFTSSPMSTNVKEAIRELIRQYCDKYK
IIEEMGGLHFGWEDKNLIV

>AFT82684.1 GRAS23 GRAS type transcription factor, partial [Zea mays subsp. mays]
MVMDAGLHEPCALLPGSKRDSHMLVYPQIAAAANGFTAQELESLLFLSSDGITLAAGSGGGGGGSYLNV
APTTVVPPAPTNRASPPRDVSAAPPSVGQPDDSEAFSDIVLGYINRMLMAEDIDDKFEHYPEHPAVLAAE
KPFLEILAERPSSSGGSAVESPGGSSVGNSCYSARSCTCATATAASDAFGAVLTPALDFPSAAFLQPPQL
YQDLSPEGSVVDAGGAWPYDSTEFYQLHSNPSSFASSNGSCATISDGFESLLSSPGVMPDVGFTDFAVQS
QQAMQFCRGFEEASKFLPDESKLVIDLEKPASVTSLVANIKGENRFAEVKAEKADVQAEIHRGKKHFYGD
DLDAEEGRRSKHSAPAIDTDHLVREMMDKVLLCNGETCSKGVKELREALQHDVAKNSHGVHGKGSGHGKG
RGKKQPKKEVVDLETLLVHCAQSVATDDRRGATELLKQIRQHASPNGDGDQRLAHCFANGLEARLAGNGS
QIYKSLIMTRFPCTDVLKAYQLYLAACPFKKISHFFANQTIMNAVEKAKKVHIVDYGIYYGFQWPCLIQR
LSTRPGGPPRLRITAIDTPHPGFRPAERIEETGRYLKDYAETFNVPFEFRAIPSRFEAVQIEDLHIAKDE
LLIVNSMFKFKTLMDESVVAESPRNMVLSTIRKMNPHLFIHGIINGSYNAPFFASRFREALYHYSAIFDM
LETNIPRDNEQRLLIESALFGREAINVISCEGLERMERPETYKQWQVRYQRAGFRQLPINQDIMKRAREK
VRCYHKDFLIDEDNRWLLQGWKGRIVLALSTWKPDHRSP

PAIDTDHLVREMMDKVLLCNGETCSKGVKELREALQHDVAKNSHGVHGKGSGHGKGRGKKQPKKEVVDLETLLV
HCAQSVATDDRRGATELLKQIRQHASPNGDGDQRLAHCFANGLEARLAGNGSQIYKSLIMTRFPCTDVLKAYQL
YLAACPFKKISHFFANQTIMNAVEKAKKVHIVDYGIYYGFQWPCLIQRLSTRPGGPPRLRITAIDTPHPGFRPA
ERIEETGRYLKDYAETFNVPFEFRAIPSRFEAVQIEDLHIAKDELLIVNSMFKFKTLMDESVVAESPRNMVLST
IRKMNPHLFIHGIINGSYNAPFFASRFREALYHYSAIFDMLETNIPRDNEQRLLIESALFGREAINVISCEGLE
RMERPETYKQWQVRYQRAGFRQLPINQDIMKRAREKVRCYHKDFLIDEDNRWLLQGWKGRIVL
