# Supplementary material for: Genome-Wide Identification and Expression Pattern of the GRAS Gene Family in Pitaya (Selenicereus undatus L.)
Source: Biology (Basel). 2022 Dec 21;12(1):11. doi: 10.3390/biology12010011 (PMC9854919; doi:10.3390/biology12010011)
Supplement: Supplementary file 1 [file biology-12-00011-s001.zip › Supplementary file S5/HU01G00472.1_plantcare.html]

Content-Type: text/html; charset=ISO-8859-1


PlantCARE


Webmaster Firefox specific output  
To save the result:
click on the frame with the right mouse button and save the source code as a text file with extension .html  
REFERENCE:PlantCARE: a database of plant cis-acting regulatory elements and a portal to tools for in silico analysis of promoter sequences.  
Lescot, M., Déhais, P., Moreau, Y., De Moor, B., Rouzé ,P.,and Rombauts, S.  
Nucleic Acids Res., Database issue(2002), 30(1):325-327.   


---

>HU01G00472.1   
+ -Up\_Stream \_Len000GTCTCT GTAACACAAT TCATTAAATC CATCCAAAGG GGATCATAAA TAACCAAACT   
  
  
+ TTGTTGGAAA CTGCTTTGAT GTGGGCTTTC TCTTTCAGCA TTTATGTTAT ACATATATAT GGTTATAAAG   
  
  
+ TTACAAAAGT ATGACCCGAC ACGACAATTC AATCCGAATC CGACTCGAAG TTACTGAAAG TAATCTGAAT   
  
  
+ ACGATTTGAC AGCTTAGTTT AAAAACCTAT TACTTCTCAA TTTTGAGTAA ATCTAAGCTA TTTTTAATGC   
  
  
+ TGAATAGATC GACCCGATTC GACCCGTTTT CCGACTCTAT ATGGTTATGG TGCCCCTAAA AAAAATTAAA   
  
  
+ AAAGAAAAAG AATAGAAAAA TATAAAATAA AACTGAGATT TCTGAACACT CTCTCCCCTA GTCTTGTGGG   
  
  
+ CATGGAAAGT GGTTGGAGAT GGAGATGTTT GAGTTTCATT TTCTGATATT ATTTTTTAGT CATTAGAAGT   
  
  
+ ACAAAGTCTA AACTCAACAA GAGCATGAGG GGAGAATGGT TGTTGTAGCC AATGGAAAAT AAAAAGAACA   
  
  
+ TTCACTCTTC TTGTGCTTTA TTTATGGTGA TAATTTGTTA CTTGTATTTT TAAAAATGAT TAAATATATT   
  
  
+ GATCCTTTGA TATTATGTTG AAATAATAAA AATATAAAAA TTAGAAAATA TTTTACCTCG TGAAATTTTT   
  
  
+ TTTCTGATTA ATCATTGAAA TTATGGTGAA AAATAAAGGA ACATCTCTTG TAAACACTCT ATCCGTTTAC   
  
  
+ GTTTGACCAC AAACTTGCTA TCCACAAGCA TCTCAACCTT TGCCTTTAAC CCAATTGTGG GTTGATGAAA   
  
  
+ TCTAACCATT ATAGGATTAG ATGGTAGTCG TTAATCCTGT GAAACAAGTT TTTGGTTATG ATGTATCCAA   
  
  
+ TTATGAGGGC CTGATAACTC TTGCCTCGGC ATTTGCGTGA TAATGACGTG GTACTAATAG CAATTATTTT   
  
  
+ TCGCAAATAT TCGAAATTTT TTAAGAATAA ACGCAAAATT ATTGATCTTG ACAAATGATT TTATTTTTTT   
  
  
+ GTTGGGAAAA GGATAAACAT CTAGTGTCAA TTGTTGATGA CCTGATAGCT CTCGATTTCT TATTTATGAG   
  
  
+ TTATGTTGAT ATGACACTAA TTGTAATTAT TTGTTGTAAA CTTAAACATT TTTAAAAATC AAAGCAAAAT   
  
  
+ TTGTTAATTA CAATAAATCA ATCTACTTTT ACTATGATCT TATTTGGTGT AATTATTGAT CTTGAATTTT   
  
  
+ TTTTTTTCTT TTTGAAAAAG ATAAGCATCG AATTTTTTCA AAAATAAAAA GGGTGAAGGA AGAAAAAATG   
  
  
+ GAAACAGTTA CCGAGTAAGG AAGGACAGAG CGCGAGAGGA CAAGACACAA AGAGAGGGGA GAGAGAGAGA   
  
  
+ GAAGGGGGCA ATGGCAGTGG GGAAGGTCGA GGAGCACCTT TCAACTTCAA GGCTCAACAC CCCCCCCCCC   
  
  
+ CCCCTCTTTC CTCATTTTGC GACGTTAGCA TTTGACATTT TGCTTTCAAC CGTCCCTCGG CATCATCAGC   
  
  
+ CCTGCCCTTG CTCAGCTTTT CCCATCTTTT TTTGGCTTTT TGTTTGACTC CCTCTATAAC TCTATTCACG   
  
  
+ TCGAGCCTTG TAAAAATTTT ACATATTTTA TTTTATTTTA CTTTTTTAGG TTTATCCCTC TCTATTCTTG   
  
  
+ ACCTACGGGC ATTTTTTATT TTCTTTGGGT TTGAGACTTG TGAACTTTTT GCACATTTTC TCACAAAAAT   
  
  
+ TTATCTTGTC TTTTTCACTT TCAACGTTCC TTCTCTCTCT CTCTCTCTCT CTCTCTCTCT CTCACTCACA   
  
  
+ TCCTAGACAG ACGTCCTTTT TAAAGAATTT ATTTAACAAG TTAGAAGAAA CCGTTGTTTA AGTAGAACCA   
  
  
+ CCCTGTTTGG ATGCCAAAGG AAATACCAGT CAAGAAAAAT CACGACAATC ACAACACATC CATTTCGTTT   
  
  
+ ACATCTCATC CTCATCCTCA TCTTCCTCAT CTCTACCCAT CTCCCTCTTT CTGTATGGCT GCTTCTTCTA   
  
  
+ CTTTGTTCCC TGACCCTAAC AATACTAGTT TAGTCCTCCC TTATTATTAT TATCCTCCTC CTTCCTCCTC   
  
  
+ TTACTATAAT GGTAACCCTT ACCTTCCTCT TCCTAACACC ACTGCTATTC CTACTCCTTC AACAACCTCT   
  
  
+ CCGGTATTTT CTTCCGCCAC CTACTCGGAG TTGAATACTG ATTGTCAAAC TCAACTGCCC ATGTCTAACC   
  
  
+ TCCCCGGAAA GATCGTTCGG AAGCGGAGTG CTGCTGAAAT GGAGCAGCGC TCCGCTCCGA TTGCGGGTGA   
  
  
+ TTACCCGCCA ACTCATCAGC GGGTAATCAC CCGCCACAAT GAGGCGGCCT CATCAGTGTC TTTCATTGAC   
  
  
+ TCCTCACTCT CGCCACTTCA CCTCTTCAAC GGCTCCAATT CGACGACCCC ATTAGCCCCC GACCGTCCTA   
  
  
+ ATGATCCGGG TCTGAGTCAG GGTCCGGATC TGCCTCTTGT GTGCGGATTC TCCGGACTCC CTTTATTCCC   
  
  
+ ACCGGTGGAG AGAAGACCTT CCATCCCCGC CACGACCACT GCTGCCGCGG CGATCGCGGT GGTTCCTGTG   
  
  
+ GAGGAGGATG ATCCGACGTG GATGGATAGC ATCATAAAAG AGCTGATTCA AAGCTCAAAC TCGATCTCAA   
  
  
+ TCCCACAGTT GATTCAGAAC GTTAGAGATA TCATCTACCC ATGTAATCCT AATCTGGGGT CCGCCATTGA   
  
  
+ GTTCCGCCTC CGCTCGCTCG CCGCCGACCC CCTCATTGCT CCGCCGCCAC TTCCGCCCTT CCACCACCAC   
  
  
+ CAACACCACC TCAATCAACA AATCACTCTT CCTCGAATCA GCAACACTAA CAACAACAAT CATGTGAGCG   
  
  
+ TGTACGCCAA CAAAGGACCC GGATACTTCA ACTTGGGTCC GGGTCAGGGT CAGGGTCCGA TTAACATCGA   
  
  
+ CCAAGCTAAC CTTTCCTTCC CTCCTGATTC CACCGCCTGT TGGGGTGTCA GTGTCACACC GCCACCGTCT   
  
  
+ TCCGCCGCCG GTTCCGGCTC CGGTGGAAGC GGCAGTAGTA GTGGTAAATC AAACCCTAAC CCTAATCCCA   
  
  
+ ACCCGAACCC AAACCCAAAC CCAAACCCGA ACAACAAAGC TCAGGATGTT CAACTCCAAA CTCCACAACA   
  
  
+ ACAGCAACAG CAACAGCAAC AAATCCCCGT GGATCAGGAG CAAGACCCGG CGGCTCCACC GGCGACGGAA   
  
  
+ GCATCGCCAC CATCTCCAAG AGCGGCGGCA CCACCGCCGG CGGCAGTGAA AGCAAGAGAA AGAGAGGAGA   
  
  
+ TGCGGCAAAG GAAGCGCGAC GAAGAGGGTC TCCACCTCCT AACCCTCCTC CTCCAATGCG CAGAAGCAGT   
  
  
+ ATCCGGCGAC AAATACGAAG AAGCGAACAA GATGCTTCTA GAAATCTCGG AGTGGGCCAC CCCATTCGGC   
  
  
+ ACCTCCGCCC AACGCGTCGC CGCGTACTTC TCGGAAGCCA TGTCAGCCCG TCTCGTATCC TCCTGCCTCG   
  
  
+ GCATCTACGC CGCCCTCCCC ACCGTCCCAC ACTACGTCAA GCTCCTCTCC GCCTTCCAAG TCTTCAATGG   
  
  
+ CATCAGCCCA TTCGTCAAAT TCTCTCACTT CACTGCAAAC CAAGCAATCC AAGAGGCCTT CCAGAGGGAA   
  
  
+ GACAGGGTCC ACATCATCGA CCTCGATATC ATGCAGGGGC TCCAGTGGCC CGGGCTGTTC CACATCCTCG   
  
  
+ CGTCCCGGCC AGGTGGGCCT CCCTTCGTAA GGCTCACCGG GCTCGGGACC TCCATGGAGG CGCTCGAGGC   
  
  
+ CACCGGAAAA AGGCTCTCAG ACTTCGCCGA GAAGTTGGGG TTGCCCTTTG AGTTTATACC CGTGGCGGAG   
  
  
+ AAGATTGGAA ATTTGGACTT GGAAAGGTTG CATGTTAGTA AAAGGGAAGC TCTTGCTGTG CATTGGTTAC   
  
  
+ AGCACTCTTT GTATGATGTT ACTGGCTCTG ATACTAATAC ACTTGGCCTT CTTCAAAGGT TGGCGCCAAA   
  
  
+ AGTGGTGACG GTGGTGGAGC AAGACCTAAG CCGAACAGGC TCTTTCCTAG GAAGGTTTGT AGAGGCGATC   
  
  
+ CACTACTATT CAGCCCTATT TGACTCCTTA GGAGCGAGTT ATGGAGAGGA CAGTGAGGAG AGGCATGTGG   
  
  
+ TTGAGCAACA GCTCCTTTCT AGGGAGATTC GAAACATTCT GGCCGTTGGT GGGCCCTCAA GGACCGGGGA   
  
  
+ GCCCAAGTTT GCGAGCTGGA GGGAGAAGCT ACAACAGTCC GGCTTTAGGG GAATCTCATT GGCAGGCAAC   
  
  
+ GCCGCTGCCC AGGCCACCTT GCTCCTCGGC ATGTTCCCTT CTGATGGGTA TACTTTAATC GAGGACAGTG   
  
  
+ GCACACTTAA GCTCGGGTGG AAGGACTTGT GCCTCCTGAC TGCTTCGGCC TGGAGGCCTT CCCATGCTCA   
  
  
+ TACTATGAGC ACCGTCTGTA CTCGGAGCCA ATA  

- -Up\_Stream \_Len000CAGAGA CATTGTGTTA AGTAATTTAG GTAGGTTTCC CCTAGTATTT ATTGGTTTGA   
  
  
- AACAACCTTT GACGAAACTA CACCCGAAAG AGAAAGTCGT AAATACAATA TGTATATATA CCAATATTTC   
  
  
- AATGTTTTCA TACTGGGCTG TGCTGTTAAG TTAGGCTTAG GCTGAGCTTC AATGACTTTC ATTAGACTTA   
  
  
- TGCTAAACTG TCGAATCAAA TTTTTGGATA ATGAAGAGTT AAAACTCATT TAGATTCGAT AAAAATTACG   
  
  
- ACTTATCTAG CTGGGCTAAG CTGGGCAAAA GGCTGAGATA TACCAATACC ACGGGGATTT TTTTTAATTT   
  
  
- TTTCTTTTTC TTATCTTTTT ATATTTTATT TTGACTCTAA AGACTTGTGA GAGAGGGGAT CAGAACACCC   
  
  
- GTACCTTTCA CCAACCTCTA CCTCTACAAA CTCAAAGTAA AAGACTATAA TAAAAAATCA GTAATCTTCA   
  
  
- TGTTTCAGAT TTGAGTTGTT CTCGTACTCC CCTCTTACCA ACAACATCGG TTACCTTTTA TTTTTCTTGT   
  
  
- AAGTGAGAAG AACACGAAAT AAATACCACT ATTAAACAAT GAACATAAAA ATTTTTACTA ATTTATATAA   
  
  
- CTAGGAAACT ATAATACAAC TTTATTATTT TTATATTTTT AATCTTTTAT AAAATGGAGC ACTTTAAAAA   
  
  
- AAAGACTAAT TAGTAACTTT AATACCACTT TTTATTTCCT TGTAGAGAAC ATTTGTGAGA TAGGCAAATG   
  
  
- CAAACTGGTG TTTGAACGAT AGGTGTTCGT AGAGTTGGAA ACGGAAATTG GGTTAACACC CAACTACTTT   
  
  
- AGATTGGTAA TATCCTAATC TACCATCAGC AATTAGGACA CTTTGTTCAA AAACCAATAC TACATAGGTT   
  
  
- AATACTCCCG GACTATTGAG AACGGAGCCG TAAACGCACT ATTACTGCAC CATGATTATC GTTAATAAAA   
  
  
- AGCGTTTATA AGCTTTAAAA AATTCTTATT TGCGTTTTAA TAACTAGAAC TGTTTACTAA AATAAAAAAA   
  
  
- CAACCCTTTT CCTATTTGTA GATCACAGTT AACAACTACT GGACTATCGA GAGCTAAAGA ATAAATACTC   
  
  
- AATACAACTA TACTGTGATT AACATTAATA AACAACATTT GAATTTGTAA AAATTTTTAG TTTCGTTTTA   
  
  
- AACAATTAAT GTTATTTAGT TAGATGAAAA TGATACTAGA ATAAACCACA TTAATAACTA GAACTTAAAA   
  
  
- AAAAAAAGAA AAACTTTTTC TATTCGTAGC TTAAAAAAGT TTTTATTTTT CCCACTTCCT TCTTTTTTAC   
  
  
- CTTTGTCAAT GGCTCATTCC TTCCTGTCTC GCGCTCTCCT GTTCTGTGTT TCTCTCCCCT CTCTCTCTCT   
  
  
- CTTCCCCCGT TACCGTCACC CCTTCCAGCT CCTCGTGGAA AGTTGAAGTT CCGAGTTGTG GGGGGGGGGG   
  
  
- GGGGAGAAAG GAGTAAAACG CTGCAATCGT AAACTGTAAA ACGAAAGTTG GCAGGGAGCC GTAGTAGTCG   
  
  
- GGACGGGAAC GAGTCGAAAA GGGTAGAAAA AAACCGAAAA ACAAACTGAG GGAGATATTG AGATAAGTGC   
  
  
- AGCTCGGAAC ATTTTTAAAA TGTATAAAAT AAAATAAAAT GAAAAAATCC AAATAGGGAG AGATAAGAAC   
  
  
- TGGATGCCCG TAAAAAATAA AAGAAACCCA AACTCTGAAC ACTTGAAAAA CGTGTAAAAG AGTGTTTTTA   
  
  
- AATAGAACAG AAAAAGTGAA AGTTGCAAGG AAGAGAGAGA GAGAGAGAGA GAGAGAGAGA GAGTGAGTGT   
  
  
- AGGATCTGTC TGCAGGAAAA ATTTCTTAAA TAAATTGTTC AATCTTCTTT GGCAACAAAT TCATCTTGGT   
  
  
- GGGACAAACC TACGGTTTCC TTTATGGTCA GTTCTTTTTA GTGCTGTTAG TGTTGTGTAG GTAAAGCAAA   
  
  
- TGTAGAGTAG GAGTAGGAGT AGAAGGAGTA GAGATGGGTA GAGGGAGAAA GACATACCGA CGAAGAAGAT   
  
  
- GAAACAAGGG ACTGGGATTG TTATGATCAA ATCAGGAGGG AATAATAATA ATAGGAGGAG GAAGGAGGAG   
  
  
- AATGATATTA CCATTGGGAA TGGAAGGAGA AGGATTGTGG TGACGATAAG GATGAGGAAG TTGTTGGAGA   
  
  
- GGCCATAAAA GAAGGCGGTG GATGAGCCTC AACTTATGAC TAACAGTTTG AGTTGACGGG TACAGATTGG   
  
  
- AGGGGCCTTT CTAGCAAGCC TTCGCCTCAC GACGACTTTA CCTCGTCGCG AGGCGAGGCT AACGCCCACT   
  
  
- AATGGGCGGT TGAGTAGTCG CCCATTAGTG GGCGGTGTTA CTCCGCCGGA GTAGTCACAG AAAGTAACTG   
  
  
- AGGAGTGAGA GCGGTGAAGT GGAGAAGTTG CCGAGGTTAA GCTGCTGGGG TAATCGGGGG CTGGCAGGAT   
  
  
- TACTAGGCCC AGACTCAGTC CCAGGCCTAG ACGGAGAACA CACGCCTAAG AGGCCTGAGG GAAATAAGGG   
  
  
- TGGCCACCTC TCTTCTGGAA GGTAGGGGCG GTGCTGGTGA CGACGGCGCC GCTAGCGCCA CCAAGGACAC   
  
  
- CTCCTCCTAC TAGGCTGCAC CTACCTATCG TAGTATTTTC TCGACTAAGT TTCGAGTTTG AGCTAGAGTT   
  
  
- AGGGTGTCAA CTAAGTCTTG CAATCTCTAT AGTAGATGGG TACATTAGGA TTAGACCCCA GGCGGTAACT   
  
  
- CAAGGCGGAG GCGAGCGAGC GGCGGCTGGG GGAGTAACGA GGCGGCGGTG AAGGCGGGAA GGTGGTGGTG   
  
  
- GTTGTGGTGG AGTTAGTTGT TTAGTGAGAA GGAGCTTAGT CGTTGTGATT GTTGTTGTTA GTACACTCGC   
  
  
- ACATGCGGTT GTTTCCTGGG CCTATGAAGT TGAACCCAGG CCCAGTCCCA GTCCCAGGCT AATTGTAGCT   
  
  
- GGTTCGATTG GAAAGGAAGG GAGGACTAAG GTGGCGGACA ACCCCACAGT CACAGTGTGG CGGTGGCAGA   
  
  
- AGGCGGCGGC CAAGGCCGAG GCCACCTTCG CCGTCATCAT CACCATTTAG TTTGGGATTG GGATTAGGGT   
  
  
- TGGGCTTGGG TTTGGGTTTG GGTTTGGGCT TGTTGTTTCG AGTCCTACAA GTTGAGGTTT GAGGTGTTGT   
  
  
- TGTCGTTGTC GTTGTCGTTG TTTAGGGGCA CCTAGTCCTC GTTCTGGGCC GCCGAGGTGG CCGCTGCCTT   
  
  
- CGTAGCGGTG GTAGAGGTTC TCGCCGCCGT GGTGGCGGCC GCCGTCACTT TCGTTCTCTT TCTCTCCTCT   
  
  
- ACGCCGTTTC CTTCGCGCTG CTTCTCCCAG AGGTGGAGGA TTGGGAGGAG GAGGTTACGC GTCTTCGTCA   
  
  
- TAGGCCGCTG TTTATGCTTC TTCGCTTGTT CTACGAAGAT CTTTAGAGCC TCACCCGGTG GGGTAAGCCG   
  
  
- TGGAGGCGGG TTGCGCAGCG GCGCATGAAG AGCCTTCGGT ACAGTCGGGC AGAGCATAGG AGGACGGAGC   
  
  
- CGTAGATGCG GCGGGAGGGG TGGCAGGGTG TGATGCAGTT CGAGGAGAGG CGGAAGGTTC AGAAGTTACC   
  
  
- GTAGTCGGGT AAGCAGTTTA AGAGAGTGAA GTGACGTTTG GTTCGTTAGG TTCTCCGGAA GGTCTCCCTT   
  
  
- CTGTCCCAGG TGTAGTAGCT GGAGCTATAG TACGTCCCCG AGGTCACCGG GCCCGACAAG GTGTAGGAGC   
  
  
- GCAGGGCCGG TCCACCCGGA GGGAAGCATT CCGAGTGGCC CGAGCCCTGG AGGTACCTCC GCGAGCTCCG   
  
  
- GTGGCCTTTT TCCGAGAGTC TGAAGCGGCT CTTCAACCCC AACGGGAAAC TCAAATATGG GCACCGCCTC   
  
  
- TTCTAACCTT TAAACCTGAA CCTTTCCAAC GTACAATCAT TTTCCCTTCG AGAACGACAC GTAACCAATG   
  
  
- TCGTGAGAAA CATACTACAA TGACCGAGAC TATGATTATG TGAACCGGAA GAAGTTTCCA ACCGCGGTTT   
  
  
- TCACCACTGC CACCACCTCG TTCTGGATTC GGCTTGTCCG AGAAAGGATC CTTCCAAACA TCTCCGCTAG   
  
  
- GTGATGATAA GTCGGGATAA ACTGAGGAAT CCTCGCTCAA TACCTCTCCT GTCACTCCTC TCCGTACACC   
  
  
- AACTCGTTGT CGAGGAAAGA TCCCTCTAAG CTTTGTAAGA CCGGCAACCA CCCGGGAGTT CCTGGCCCCT   
  
  
- CGGGTTCAAA CGCTCGACCT CCCTCTTCGA TGTTGTCAGG CCGAAATCCC CTTAGAGTAA CCGTCCGTTG   
  
  
- CGGCGACGGG TCCGGTGGAA CGAGGAGCCG TACAAGGGAA GACTACCCAT ATGAAATTAG CTCCTGTCAC   
  
  
- CGTGTGAATT CGAGCCCACC TTCCTGAACA CGGAGGACTG ACGAAGCCGG ACCTCCGGAA GGGTACGAGT   
  
  
- ATGATACTCG TGGCAGACAT GAGCCTCGGT TAT

  
  
Motifs Found  

+   

| Site Name | Organism | Position | Strand | Matrix score. | sequence | function |
| --- | --- | --- | --- | --- | --- | --- |
|  | organism | 405 | + | 4 | motif\_sequence | short\_function |
|  | organism | 439 | - | 4 | motif\_sequence | short\_function |
|  | organism | 445 | - | 4 | motif\_sequence | short\_function |
|  | organism | 560 | - | 4 | motif\_sequence | short\_function |
|  | organism | 569 | + | 4 | motif\_sequence | short\_function |
|  | organism | 743 | - | 4 | motif\_sequence | short\_function |
|  | organism | 1215 | + | 4 | motif\_sequence | short\_function |
|  | organism | 1357 | - | 4 | motif\_sequence | short\_function |
|  | organism | 1392 | - | 4 | motif\_sequence | short\_function |
|  | organism | 1735 | - | 4 | motif\_sequence | short\_function |
|  | organism | 1984 | + | 4 | motif\_sequence | short\_function |
|  | organism | 1995 | + | 4 | motif\_sequence | short\_function |
|  | organism | 2003 | + | 4 | motif\_sequence | short\_function |
|  | organism | 2131 | + | 4 | motif\_sequence | short\_function |
|  | organism | 2171 | + | 4 | motif\_sequence | short\_function |
|  | organism | 2406 | + | 4 | motif\_sequence | short\_function |
|  | organism | 2482 | + | 4 | motif\_sequence | short\_function |
|  | organism | 2531 | - | 4 | motif\_sequence | short\_function |
|  | organism | 2697 | + | 4 | motif\_sequence | short\_function |
|  | organism | 2830 | + | 4 | motif\_sequence | short\_function |
|  | organism | 3130 | + | 4 | motif\_sequence | short\_function |
|  | organism | 3236 | + | 4 | motif\_sequence | short\_function |
|  | organism | 3290 | - | 4 | motif\_sequence | short\_function |
|  | organism | 3315 | - | 4 | motif\_sequence | short\_function |
|  | organism | 3507 | + | 4 | motif\_sequence | short\_function |
|  | organism | 3549 | + | 4 | motif\_sequence | short\_function |
|  | organism | 3699 | + | 4 | motif\_sequence | short\_function |
|  | organism | 3854 | - | 4 | motif\_sequence | short\_function |
|  | organism | 3910 | + | 4 | motif\_sequence | short\_function |
|  | organism | 4027 | - | 4 | motif\_sequence | short\_function |
|  | organism | 4053 | - | 4 | motif\_sequence | short\_function |
|  | organism | 4107 | - | 4 | motif\_sequence | short\_function |
|  | organism | 4112 | - | 4 | motif\_sequence | short\_function |
|  | organism | 4121 | - | 4 | motif\_sequence | short\_function |
|  | organism | 4157 | - | 4 | motif\_sequence | short\_function |
|  | organism | 4305 | + | 4 | motif\_sequence | short\_function |
|  | organism | 4337 | - | 4 | motif\_sequence | short\_function |
|  | organism | 4430 | + | 4 | motif\_sequence | short\_function |

>HU01G00472.1   
+ -Up\_Stream \_Len000GTCTCT GTAACACAAT TCATTAAATC CATCCAAAGG GGATCATAAA TAACCAAACT   
  
  
+ TTGTTGGAAA CTGCTTTGAT GTGGGCTTTC TCTTTCAGCA TTTATGTTAT ACATATATAT GGTTATAAAG   
  
  
+ TTACAAAAGT ATGACCCGAC ACGACAATTC AATCCGAATC CGACTCGAAG TTACTGAAAG TAATCTGAAT   
  
  
+ ACGATTTGAC AGCTTAGTTT AAAAACCTAT TACTTCTCAA TTTTGAGTAA ATCTAAGCTA TTTTTAATGC   
  
  
+ TGAATAGATC GACCCGATTC GACCCGTTTT CCGACTCTAT ATGGTTATGG TGCCCCTAAA AAAAATTAAA   
  
  
+ AAAGAAAAAG AATAGAAAAA TATAAAATAA AACTGAGATT TCTGAACACT CTCTCCCCTA GTCTTGTGGG   
  
  
+ CATGGAAAGT GGTTGGAGAT GGAGATGTTT GAGTTTCATT TTCTGATATT ATTTTTTAGT CATTAGAAGT   
  
  
+ ACAAAGTCTA AACTCAACAA GAGCATGAGG GGAGAATGGT TGTTGTAGCC AATGGAAAAT AAAAAGAACA   
  
  
+ TTCACTCTTC TTGTGCTTTA TTTATGGTGA TAATTTGTTA CTTGTATTTT TAAAAATGAT TAAATATATT   
  
  
+ GATCCTTTGA TATTATGTTG AAATAATAAA AATATAAAAA TTAGAAAATA TTTTACCTCG TGAAATTTTT   
  
  
+ TTTCTGATTA ATCATTGAAA TTATGGTGAA AAATAAAGGA ACATCTCTTG TAAACACTCT ATCCGTTTAC   
  
  
+ GTTTGACCAC AAACTTGCTA TCCACAAGCA TCTCAACCTT TGCCTTTAAC CCAATTGTGG GTTGATGAAA   
  
  
+ TCTAACCATT ATAGGATTAG ATGGTAGTCG TTAATCCTGT GAAACAAGTT TTTGGTTATG ATGTATCCAA   
  
  
+ TTATGAGGGC CTGATAACTC TTGCCTCGGC ATTTGCGTGA TAATGACGTG GTACTAATAG CAATTATTTT   
  
  
+ TCGCAAATAT TCGAAATTTT TTAAGAATAA ACGCAAAATT ATTGATCTTG ACAAATGATT TTATTTTTTT   
  
  
+ GTTGGGAAAA GGATAAACAT CTAGTGTCAA TTGTTGATGA CCTGATAGCT CTCGATTTCT TATTTATGAG   
  
  
+ TTATGTTGAT ATGACACTAA TTGTAATTAT TTGTTGTAAA CTTAAACATT TTTAAAAATC AAAGCAAAAT   
  
  
+ TTGTTAATTA CAATAAATCA ATCTACTTTT ACTATGATCT TATTTGGTGT AATTATTGAT CTTGAATTTT   
  
  
+ TTTTTTTCTT TTTGAAAAAG ATAAGCATCG AATTTTTTCA AAAATAAAAA GGGTGAAGGA AGAAAAAATG   
  
  
+ GAAACAGTTA CCGAGTAAGG AAGGACAGAG CGCGAGAGGA CAAGACACAA AGAGAGGGGA GAGAGAGAGA   
  
  
+ GAAGGGGGCA ATGGCAGTGG GGAAGGTCGA GGAGCACCTT TCAACTTCAA GGCTCAACAC CCCCCCCCCC   
  
  
+ CCCCTCTTTC CTCATTTTGC GACGTTAGCA TTTGACATTT TGCTTTCAAC CGTCCCTCGG CATCATCAGC   
  
  
+ CCTGCCCTTG CTCAGCTTTT CCCATCTTTT TTTGGCTTTT TGTTTGACTC CCTCTATAAC TCTATTCACG   
  
  
+ TCGAGCCTTG TAAAAATTTT ACATATTTTA TTTTATTTTA CTTTTTTAGG TTTATCCCTC TCTATTCTTG   
  
  
+ ACCTACGGGC ATTTTTTATT TTCTTTGGGT TTGAGACTTG TGAACTTTTT GCACATTTTC TCACAAAAAT   
  
  
+ TTATCTTGTC TTTTTCACTT TCAACGTTCC TTCTCTCTCT CTCTCTCTCT CTCTCTCTCT CTCACTCACA   
  
  
+ TCCTAGACAG ACGTCCTTTT TAAAGAATTT ATTTAACAAG TTAGAAGAAA CCGTTGTTTA AGTAGAACCA   
  
  
+ CCCTGTTTGG ATGCCAAAGG AAATACCAGT CAAGAAAAAT CACGACAATC ACAACACATC CATTTCGTTT   
  
  
+ ACATCTCATC CTCATCCTCA TCTTCCTCAT CTCTACCCAT CTCCCTCTTT CTGTATGGCT GCTTCTTCTA   
  
  
+ CTTTGTTCCC TGACCCTAAC AATACTAGTT TAGTCCTCCC TTATTATTAT TATCCTCCTC CTTCCTCCTC   
  
  
+ TTACTATAAT GGTAACCCTT ACCTTCCTCT TCCTAACACC ACTGCTATTC CTACTCCTTC AACAACCTCT   
  
  
+ CCGGTATTTT CTTCCGCCAC CTACTCGGAG TTGAATACTG ATTGTCAAAC TCAACTGCCC ATGTCTAACC   
  
  
+ TCCCCGGAAA GATCGTTCGG AAGCGGAGTG CTGCTGAAAT GGAGCAGCGC TCCGCTCCGA TTGCGGGTGA   
  
  
+ TTACCCGCCA ACTCATCAGC GGGTAATCAC CCGCCACAAT GAGGCGGCCT CATCAGTGTC TTTCATTGAC   
  
  
+ TCCTCACTCT CGCCACTTCA CCTCTTCAAC GGCTCCAATT CGACGACCCC ATTAGCCCCC GACCGTCCTA   
  
  
+ ATGATCCGGG TCTGAGTCAG GGTCCGGATC TGCCTCTTGT GTGCGGATTC TCCGGACTCC CTTTATTCCC   
  
  
+ ACCGGTGGAG AGAAGACCTT CCATCCCCGC CACGACCACT GCTGCCGCGG CGATCGCGGT GGTTCCTGTG   
  
  
+ GAGGAGGATG ATCCGACGTG GATGGATAGC ATCATAAAAG AGCTGATTCA AAGCTCAAAC TCGATCTCAA   
  
  
+ TCCCACAGTT GATTCAGAAC GTTAGAGATA TCATCTACCC ATGTAATCCT AATCTGGGGT CCGCCATTGA   
  
  
+ GTTCCGCCTC CGCTCGCTCG CCGCCGACCC CCTCATTGCT CCGCCGCCAC TTCCGCCCTT CCACCACCAC   
  
  
+ CAACACCACC TCAATCAACA AATCACTCTT CCTCGAATCA GCAACACTAA CAACAACAAT CATGTGAGCG   
  
  
+ TGTACGCCAA CAAAGGACCC GGATACTTCA ACTTGGGTCC GGGTCAGGGT CAGGGTCCGA TTAACATCGA   
  
  
+ CCAAGCTAAC CTTTCCTTCC CTCCTGATTC CACCGCCTGT TGGGGTGTCA GTGTCACACC GCCACCGTCT   
  
  
+ TCCGCCGCCG GTTCCGGCTC CGGTGGAAGC GGCAGTAGTA GTGGTAAATC AAACCCTAAC CCTAATCCCA   
  
  
+ ACCCGAACCC AAACCCAAAC CCAAACCCGA ACAACAAAGC TCAGGATGTT CAACTCCAAA CTCCACAACA   
  
  
+ ACAGCAACAG CAACAGCAAC AAATCCCCGT GGATCAGGAG CAAGACCCGG CGGCTCCACC GGCGACGGAA   
  
  
+ GCATCGCCAC CATCTCCAAG AGCGGCGGCA CCACCGCCGG CGGCAGTGAA AGCAAGAGAA AGAGAGGAGA   
  
  
+ TGCGGCAAAG GAAGCGCGAC GAAGAGGGTC TCCACCTCCT AACCCTCCTC CTCCAATGCG CAGAAGCAGT   
  
  
+ ATCCGGCGAC AAATACGAAG AAGCGAACAA GATGCTTCTA GAAATCTCGG AGTGGGCCAC CCCATTCGGC   
  
  
+ ACCTCCGCCC AACGCGTCGC CGCGTACTTC TCGGAAGCCA TGTCAGCCCG TCTCGTATCC TCCTGCCTCG   
  
  
+ GCATCTACGC CGCCCTCCCC ACCGTCCCAC ACTACGTCAA GCTCCTCTCC GCCTTCCAAG TCTTCAATGG   
  
  
+ CATCAGCCCA TTCGTCAAAT TCTCTCACTT CACTGCAAAC CAAGCAATCC AAGAGGCCTT CCAGAGGGAA   
  
  
+ GACAGGGTCC ACATCATCGA CCTCGATATC ATGCAGGGGC TCCAGTGGCC CGGGCTGTTC CACATCCTCG   
  
  
+ CGTCCCGGCC AGGTGGGCCT CCCTTCGTAA GGCTCACCGG GCTCGGGACC TCCATGGAGG CGCTCGAGGC   
  
  
+ CACCGGAAAA AGGCTCTCAG ACTTCGCCGA GAAGTTGGGG TTGCCCTTTG AGTTTATACC CGTGGCGGAG   
  
  
+ AAGATTGGAA ATTTGGACTT GGAAAGGTTG CATGTTAGTA AAAGGGAAGC TCTTGCTGTG CATTGGTTAC   
  
  
+ AGCACTCTTT GTATGATGTT ACTGGCTCTG ATACTAATAC ACTTGGCCTT CTTCAAAGGT TGGCGCCAAA   
  
  
+ AGTGGTGACG GTGGTGGAGC AAGACCTAAG CCGAACAGGC TCTTTCCTAG GAAGGTTTGT AGAGGCGATC   
  
  
+ CACTACTATT CAGCCCTATT TGACTCCTTA GGAGCGAGTT ATGGAGAGGA CAGTGAGGAG AGGCATGTGG   
  
  
+ TTGAGCAACA GCTCCTTTCT AGGGAGATTC GAAACATTCT GGCCGTTGGT GGGCCCTCAA GGACCGGGGA   
  
  
+ GCCCAAGTTT GCGAGCTGGA GGGAGAAGCT ACAACAGTCC GGCTTTAGGG GAATCTCATT GGCAGGCAAC   
  
  
+ GCCGCTGCCC AGGCCACCTT GCTCCTCGGC ATGTTCCCTT CTGATGGGTA TACTTTAATC GAGGACAGTG   
  
  
+ GCACACTTAA GCTCGGGTGG AAGGACTTGT GCCTCCTGAC TGCTTCGGCC TGGAGGCCTT CCCATGCTCA   
  
  
+ TACTATGAGC ACCGTCTGTA CTCGGAGCCA ATA  

- -Up\_Stream \_Len000CAGAGA CATTGTGTTA AGTAATTTAG GTAGGTTTCC CCTAGTATTT ATTGGTTTGA   
  
  
- AACAACCTTT GACGAAACTA CACCCGAAAG AGAAAGTCGT AAATACAATA TGTATATATA CCAATATTTC   
  
  
- AATGTTTTCA TACTGGGCTG TGCTGTTAAG TTAGGCTTAG GCTGAGCTTC AATGACTTTC ATTAGACTTA   
  
  
- TGCTAAACTG TCGAATCAAA TTTTTGGATA ATGAAGAGTT AAAACTCATT TAGATTCGAT AAAAATTACG   
  
  
- ACTTATCTAG CTGGGCTAAG CTGGGCAAAA GGCTGAGATA TACCAATACC ACGGGGATTT TTTTTAATTT   
  
  
- TTTCTTTTTC TTATCTTTTT ATATTTTATT TTGACTCTAA AGACTTGTGA GAGAGGGGAT CAGAACACCC   
  
  
- GTACCTTTCA CCAACCTCTA CCTCTACAAA CTCAAAGTAA AAGACTATAA TAAAAAATCA GTAATCTTCA   
  
  
- TGTTTCAGAT TTGAGTTGTT CTCGTACTCC CCTCTTACCA ACAACATCGG TTACCTTTTA TTTTTCTTGT   
  
  
- AAGTGAGAAG AACACGAAAT AAATACCACT ATTAAACAAT GAACATAAAA ATTTTTACTA ATTTATATAA   
  
  
- CTAGGAAACT ATAATACAAC TTTATTATTT TTATATTTTT AATCTTTTAT AAAATGGAGC ACTTTAAAAA   
  
  
- AAAGACTAAT TAGTAACTTT AATACCACTT TTTATTTCCT TGTAGAGAAC ATTTGTGAGA TAGGCAAATG   
  
  
- CAAACTGGTG TTTGAACGAT AGGTGTTCGT AGAGTTGGAA ACGGAAATTG GGTTAACACC CAACTACTTT   
  
  
- AGATTGGTAA TATCCTAATC TACCATCAGC AATTAGGACA CTTTGTTCAA AAACCAATAC TACATAGGTT   
  
  
- AATACTCCCG GACTATTGAG AACGGAGCCG TAAACGCACT ATTACTGCAC CATGATTATC GTTAATAAAA   
  
  
- AGCGTTTATA AGCTTTAAAA AATTCTTATT TGCGTTTTAA TAACTAGAAC TGTTTACTAA AATAAAAAAA   
  
  
- CAACCCTTTT CCTATTTGTA GATCACAGTT AACAACTACT GGACTATCGA GAGCTAAAGA ATAAATACTC   
  
  
- AATACAACTA TACTGTGATT AACATTAATA AACAACATTT GAATTTGTAA AAATTTTTAG TTTCGTTTTA   
  
  
- AACAATTAAT GTTATTTAGT TAGATGAAAA TGATACTAGA ATAAACCACA TTAATAACTA GAACTTAAAA   
  
  
- AAAAAAAGAA AAACTTTTTC TATTCGTAGC TTAAAAAAGT TTTTATTTTT CCCACTTCCT TCTTTTTTAC   
  
  
- CTTTGTCAAT GGCTCATTCC TTCCTGTCTC GCGCTCTCCT GTTCTGTGTT TCTCTCCCCT CTCTCTCTCT   
  
  
- CTTCCCCCGT TACCGTCACC CCTTCCAGCT CCTCGTGGAA AGTTGAAGTT CCGAGTTGTG GGGGGGGGGG   
  
  
- GGGGAGAAAG GAGTAAAACG CTGCAATCGT AAACTGTAAA ACGAAAGTTG GCAGGGAGCC GTAGTAGTCG   
  
  
- GGACGGGAAC GAGTCGAAAA GGGTAGAAAA AAACCGAAAA ACAAACTGAG GGAGATATTG AGATAAGTGC   
  
  
- AGCTCGGAAC ATTTTTAAAA TGTATAAAAT AAAATAAAAT GAAAAAATCC AAATAGGGAG AGATAAGAAC   
  
  
- TGGATGCCCG TAAAAAATAA AAGAAACCCA AACTCTGAAC ACTTGAAAAA CGTGTAAAAG AGTGTTTTTA   
  
  
- AATAGAACAG AAAAAGTGAA AGTTGCAAGG AAGAGAGAGA GAGAGAGAGA GAGAGAGAGA GAGTGAGTGT   
  
  
- AGGATCTGTC TGCAGGAAAA ATTTCTTAAA TAAATTGTTC AATCTTCTTT GGCAACAAAT TCATCTTGGT   
  
  
- GGGACAAACC TACGGTTTCC TTTATGGTCA GTTCTTTTTA GTGCTGTTAG TGTTGTGTAG GTAAAGCAAA   
  
  
- TGTAGAGTAG GAGTAGGAGT AGAAGGAGTA GAGATGGGTA GAGGGAGAAA GACATACCGA CGAAGAAGAT   
  
  
- GAAACAAGGG ACTGGGATTG TTATGATCAA ATCAGGAGGG AATAATAATA ATAGGAGGAG GAAGGAGGAG   
  
  
- AATGATATTA CCATTGGGAA TGGAAGGAGA AGGATTGTGG TGACGATAAG GATGAGGAAG TTGTTGGAGA   
  
  
- GGCCATAAAA GAAGGCGGTG GATGAGCCTC AACTTATGAC TAACAGTTTG AGTTGACGGG TACAGATTGG   
  
  
- AGGGGCCTTT CTAGCAAGCC TTCGCCTCAC GACGACTTTA CCTCGTCGCG AGGCGAGGCT AACGCCCACT   
  
  
- AATGGGCGGT TGAGTAGTCG CCCATTAGTG GGCGGTGTTA CTCCGCCGGA GTAGTCACAG AAAGTAACTG   
  
  
- AGGAGTGAGA GCGGTGAAGT GGAGAAGTTG CCGAGGTTAA GCTGCTGGGG TAATCGGGGG CTGGCAGGAT   
  
  
- TACTAGGCCC AGACTCAGTC CCAGGCCTAG ACGGAGAACA CACGCCTAAG AGGCCTGAGG GAAATAAGGG   
  
  
- TGGCCACCTC TCTTCTGGAA GGTAGGGGCG GTGCTGGTGA CGACGGCGCC GCTAGCGCCA CCAAGGACAC   
  
  
- CTCCTCCTAC TAGGCTGCAC CTACCTATCG TAGTATTTTC TCGACTAAGT TTCGAGTTTG AGCTAGAGTT   
  
  
- AGGGTGTCAA CTAAGTCTTG CAATCTCTAT AGTAGATGGG TACATTAGGA TTAGACCCCA GGCGGTAACT   
  
  
- CAAGGCGGAG GCGAGCGAGC GGCGGCTGGG GGAGTAACGA GGCGGCGGTG AAGGCGGGAA GGTGGTGGTG   
  
  
- GTTGTGGTGG AGTTAGTTGT TTAGTGAGAA GGAGCTTAGT CGTTGTGATT GTTGTTGTTA GTACACTCGC   
  
  
- ACATGCGGTT GTTTCCTGGG CCTATGAAGT TGAACCCAGG CCCAGTCCCA GTCCCAGGCT AATTGTAGCT   
  
  
- GGTTCGATTG GAAAGGAAGG GAGGACTAAG GTGGCGGACA ACCCCACAGT CACAGTGTGG CGGTGGCAGA   
  
  
- AGGCGGCGGC CAAGGCCGAG GCCACCTTCG CCGTCATCAT CACCATTTAG TTTGGGATTG GGATTAGGGT   
  
  
- TGGGCTTGGG TTTGGGTTTG GGTTTGGGCT TGTTGTTTCG AGTCCTACAA GTTGAGGTTT GAGGTGTTGT   
  
  
- TGTCGTTGTC GTTGTCGTTG TTTAGGGGCA CCTAGTCCTC GTTCTGGGCC GCCGAGGTGG CCGCTGCCTT   
  
  
- CGTAGCGGTG GTAGAGGTTC TCGCCGCCGT GGTGGCGGCC GCCGTCACTT TCGTTCTCTT TCTCTCCTCT   
  
  
- ACGCCGTTTC CTTCGCGCTG CTTCTCCCAG AGGTGGAGGA TTGGGAGGAG GAGGTTACGC GTCTTCGTCA   
  
  
- TAGGCCGCTG TTTATGCTTC TTCGCTTGTT CTACGAAGAT CTTTAGAGCC TCACCCGGTG GGGTAAGCCG   
  
  
- TGGAGGCGGG TTGCGCAGCG GCGCATGAAG AGCCTTCGGT ACAGTCGGGC AGAGCATAGG AGGACGGAGC   
  
  
- CGTAGATGCG GCGGGAGGGG TGGCAGGGTG TGATGCAGTT CGAGGAGAGG CGGAAGGTTC AGAAGTTACC   
  
  
- GTAGTCGGGT AAGCAGTTTA AGAGAGTGAA GTGACGTTTG GTTCGTTAGG TTCTCCGGAA GGTCTCCCTT   
  
  
- CTGTCCCAGG TGTAGTAGCT GGAGCTATAG TACGTCCCCG AGGTCACCGG GCCCGACAAG GTGTAGGAGC   
  
  
- GCAGGGCCGG TCCACCCGGA GGGAAGCATT CCGAGTGGCC CGAGCCCTGG AGGTACCTCC GCGAGCTCCG   
  
  
- GTGGCCTTTT TCCGAGAGTC TGAAGCGGCT CTTCAACCCC AACGGGAAAC TCAAATATGG GCACCGCCTC   
  
  
- TTCTAACCTT TAAACCTGAA CCTTTCCAAC GTACAATCAT TTTCCCTTCG AGAACGACAC GTAACCAATG   
  
  
- TCGTGAGAAA CATACTACAA TGACCGAGAC TATGATTATG TGAACCGGAA GAAGTTTCCA ACCGCGGTTT   
  
  
- TCACCACTGC CACCACCTCG TTCTGGATTC GGCTTGTCCG AGAAAGGATC CTTCCAAACA TCTCCGCTAG   
  
  
- GTGATGATAA GTCGGGATAA ACTGAGGAAT CCTCGCTCAA TACCTCTCCT GTCACTCCTC TCCGTACACC   
  
  
- AACTCGTTGT CGAGGAAAGA TCCCTCTAAG CTTTGTAAGA CCGGCAACCA CCCGGGAGTT CCTGGCCCCT   
  
  
- CGGGTTCAAA CGCTCGACCT CCCTCTTCGA TGTTGTCAGG CCGAAATCCC CTTAGAGTAA CCGTCCGTTG   
  
  
- CGGCGACGGG TCCGGTGGAA CGAGGAGCCG TACAAGGGAA GACTACCCAT ATGAAATTAG CTCCTGTCAC   
  
  
- CGTGTGAATT CGAGCCCACC TTCCTGAACA CGGAGGACTG ACGAAGCCGG ACCTCCGGAA GGGTACGAGT   
  
  
- ATGATACTCG TGGCAGACAT GAGCCTCGGT TAT

+     A-box

| Site Name | Organism | Position | Strand | Matrix score. | sequence | function |
| --- | --- | --- | --- | --- | --- | --- |
| A-box | Petroselinum crispum | 1524 | + | 6 | CCGTCC | cis-acting regulatory element |
| A-box | Petroselinum crispum | 3526 | + | 6 | CCGTCC | cis-acting regulatory element |
| A-box | Petroselinum crispum | 2447 | + | 6 | CCGTCC | cis-acting regulatory element |

>HU01G00472.1   
+ -Up\_Stream \_Len000GTCTCT GTAACACAAT TCATTAAATC CATCCAAAGG GGATCATAAA TAACCAAACT   
  
  
+ TTGTTGGAAA CTGCTTTGAT GTGGGCTTTC TCTTTCAGCA TTTATGTTAT ACATATATAT GGTTATAAAG   
  
  
+ TTACAAAAGT ATGACCCGAC ACGACAATTC AATCCGAATC CGACTCGAAG TTACTGAAAG TAATCTGAAT   
  
  
+ ACGATTTGAC AGCTTAGTTT AAAAACCTAT TACTTCTCAA TTTTGAGTAA ATCTAAGCTA TTTTTAATGC   
  
  
+ TGAATAGATC GACCCGATTC GACCCGTTTT CCGACTCTAT ATGGTTATGG TGCCCCTAAA AAAAATTAAA   
  
  
+ AAAGAAAAAG AATAGAAAAA TATAAAATAA AACTGAGATT TCTGAACACT CTCTCCCCTA GTCTTGTGGG   
  
  
+ CATGGAAAGT GGTTGGAGAT GGAGATGTTT GAGTTTCATT TTCTGATATT ATTTTTTAGT CATTAGAAGT   
  
  
+ ACAAAGTCTA AACTCAACAA GAGCATGAGG GGAGAATGGT TGTTGTAGCC AATGGAAAAT AAAAAGAACA   
  
  
+ TTCACTCTTC TTGTGCTTTA TTTATGGTGA TAATTTGTTA CTTGTATTTT TAAAAATGAT TAAATATATT   
  
  
+ GATCCTTTGA TATTATGTTG AAATAATAAA AATATAAAAA TTAGAAAATA TTTTACCTCG TGAAATTTTT   
  
  
+ TTTCTGATTA ATCATTGAAA TTATGGTGAA AAATAAAGGA ACATCTCTTG TAAACACTCT ATCCGTTTAC   
  
  
+ GTTTGACCAC AAACTTGCTA TCCACAAGCA TCTCAACCTT TGCCTTTAAC CCAATTGTGG GTTGATGAAA   
  
  
+ TCTAACCATT ATAGGATTAG ATGGTAGTCG TTAATCCTGT GAAACAAGTT TTTGGTTATG ATGTATCCAA   
  
  
+ TTATGAGGGC CTGATAACTC TTGCCTCGGC ATTTGCGTGA TAATGACGTG GTACTAATAG CAATTATTTT   
  
  
+ TCGCAAATAT TCGAAATTTT TTAAGAATAA ACGCAAAATT ATTGATCTTG ACAAATGATT TTATTTTTTT   
  
  
+ GTTGGGAAAA GGATAAACAT CTAGTGTCAA TTGTTGATGA CCTGATAGCT CTCGATTTCT TATTTATGAG   
  
  
+ TTATGTTGAT ATGACACTAA TTGTAATTAT TTGTTGTAAA CTTAAACATT TTTAAAAATC AAAGCAAAAT   
  
  
+ TTGTTAATTA CAATAAATCA ATCTACTTTT ACTATGATCT TATTTGGTGT AATTATTGAT CTTGAATTTT   
  
  
+ TTTTTTTCTT TTTGAAAAAG ATAAGCATCG AATTTTTTCA AAAATAAAAA GGGTGAAGGA AGAAAAAATG   
  
  
+ GAAACAGTTA CCGAGTAAGG AAGGACAGAG CGCGAGAGGA CAAGACACAA AGAGAGGGGA GAGAGAGAGA   
  
  
+ GAAGGGGGCA ATGGCAGTGG GGAAGGTCGA GGAGCACCTT TCAACTTCAA GGCTCAACAC CCCCCCCCCC   
  
  
+ CCCCTCTTTC CTCATTTTGC GACGTTAGCA TTTGACATTT TGCTTTCAAC CGTCCCTCGG CATCATCAGC   
  
  
+ CCTGCCCTTG CTCAGCTTTT CCCATCTTTT TTTGGCTTTT TGTTTGACTC CCTCTATAAC TCTATTCACG   
  
  
+ TCGAGCCTTG TAAAAATTTT ACATATTTTA TTTTATTTTA CTTTTTTAGG TTTATCCCTC TCTATTCTTG   
  
  
+ ACCTACGGGC ATTTTTTATT TTCTTTGGGT TTGAGACTTG TGAACTTTTT GCACATTTTC TCACAAAAAT   
  
  
+ TTATCTTGTC TTTTTCACTT TCAACGTTCC TTCTCTCTCT CTCTCTCTCT CTCTCTCTCT CTCACTCACA   
  
  
+ TCCTAGACAG ACGTCCTTTT TAAAGAATTT ATTTAACAAG TTAGAAGAAA CCGTTGTTTA AGTAGAACCA   
  
  
+ CCCTGTTTGG ATGCCAAAGG AAATACCAGT CAAGAAAAAT CACGACAATC ACAACACATC CATTTCGTTT   
  
  
+ ACATCTCATC CTCATCCTCA TCTTCCTCAT CTCTACCCAT CTCCCTCTTT CTGTATGGCT GCTTCTTCTA   
  
  
+ CTTTGTTCCC TGACCCTAAC AATACTAGTT TAGTCCTCCC TTATTATTAT TATCCTCCTC CTTCCTCCTC   
  
  
+ TTACTATAAT GGTAACCCTT ACCTTCCTCT TCCTAACACC ACTGCTATTC CTACTCCTTC AACAACCTCT   
  
  
+ CCGGTATTTT CTTCCGCCAC CTACTCGGAG TTGAATACTG ATTGTCAAAC TCAACTGCCC ATGTCTAACC   
  
  
+ TCCCCGGAAA GATCGTTCGG AAGCGGAGTG CTGCTGAAAT GGAGCAGCGC TCCGCTCCGA TTGCGGGTGA   
  
  
+ TTACCCGCCA ACTCATCAGC GGGTAATCAC CCGCCACAAT GAGGCGGCCT CATCAGTGTC TTTCATTGAC   
  
  
+ TCCTCACTCT CGCCACTTCA CCTCTTCAAC GGCTCCAATT CGACGACCCC ATTAGCCCCC GACCGTCCTA   
  
  
+ ATGATCCGGG TCTGAGTCAG GGTCCGGATC TGCCTCTTGT GTGCGGATTC TCCGGACTCC CTTTATTCCC   
  
  
+ ACCGGTGGAG AGAAGACCTT CCATCCCCGC CACGACCACT GCTGCCGCGG CGATCGCGGT GGTTCCTGTG   
  
  
+ GAGGAGGATG ATCCGACGTG GATGGATAGC ATCATAAAAG AGCTGATTCA AAGCTCAAAC TCGATCTCAA   
  
  
+ TCCCACAGTT GATTCAGAAC GTTAGAGATA TCATCTACCC ATGTAATCCT AATCTGGGGT CCGCCATTGA   
  
  
+ GTTCCGCCTC CGCTCGCTCG CCGCCGACCC CCTCATTGCT CCGCCGCCAC TTCCGCCCTT CCACCACCAC   
  
  
+ CAACACCACC TCAATCAACA AATCACTCTT CCTCGAATCA GCAACACTAA CAACAACAAT CATGTGAGCG   
  
  
+ TGTACGCCAA CAAAGGACCC GGATACTTCA ACTTGGGTCC GGGTCAGGGT CAGGGTCCGA TTAACATCGA   
  
  
+ CCAAGCTAAC CTTTCCTTCC CTCCTGATTC CACCGCCTGT TGGGGTGTCA GTGTCACACC GCCACCGTCT   
  
  
+ TCCGCCGCCG GTTCCGGCTC CGGTGGAAGC GGCAGTAGTA GTGGTAAATC AAACCCTAAC CCTAATCCCA   
  
  
+ ACCCGAACCC AAACCCAAAC CCAAACCCGA ACAACAAAGC TCAGGATGTT CAACTCCAAA CTCCACAACA   
  
  
+ ACAGCAACAG CAACAGCAAC AAATCCCCGT GGATCAGGAG CAAGACCCGG CGGCTCCACC GGCGACGGAA   
  
  
+ GCATCGCCAC CATCTCCAAG AGCGGCGGCA CCACCGCCGG CGGCAGTGAA AGCAAGAGAA AGAGAGGAGA   
  
  
+ TGCGGCAAAG GAAGCGCGAC GAAGAGGGTC TCCACCTCCT AACCCTCCTC CTCCAATGCG CAGAAGCAGT   
  
  
+ ATCCGGCGAC AAATACGAAG AAGCGAACAA GATGCTTCTA GAAATCTCGG AGTGGGCCAC CCCATTCGGC   
  
  
+ ACCTCCGCCC AACGCGTCGC CGCGTACTTC TCGGAAGCCA TGTCAGCCCG TCTCGTATCC TCCTGCCTCG   
  
  
+ GCATCTACGC CGCCCTCCCC ACCGTCCCAC ACTACGTCAA GCTCCTCTCC GCCTTCCAAG TCTTCAATGG   
  
  
+ CATCAGCCCA TTCGTCAAAT TCTCTCACTT CACTGCAAAC CAAGCAATCC AAGAGGCCTT CCAGAGGGAA   
  
  
+ GACAGGGTCC ACATCATCGA CCTCGATATC ATGCAGGGGC TCCAGTGGCC CGGGCTGTTC CACATCCTCG   
  
  
+ CGTCCCGGCC AGGTGGGCCT CCCTTCGTAA GGCTCACCGG GCTCGGGACC TCCATGGAGG CGCTCGAGGC   
  
  
+ CACCGGAAAA AGGCTCTCAG ACTTCGCCGA GAAGTTGGGG TTGCCCTTTG AGTTTATACC CGTGGCGGAG   
  
  
+ AAGATTGGAA ATTTGGACTT GGAAAGGTTG CATGTTAGTA AAAGGGAAGC TCTTGCTGTG CATTGGTTAC   
  
  
+ AGCACTCTTT GTATGATGTT ACTGGCTCTG ATACTAATAC ACTTGGCCTT CTTCAAAGGT TGGCGCCAAA   
  
  
+ AGTGGTGACG GTGGTGGAGC AAGACCTAAG CCGAACAGGC TCTTTCCTAG GAAGGTTTGT AGAGGCGATC   
  
  
+ CACTACTATT CAGCCCTATT TGACTCCTTA GGAGCGAGTT ATGGAGAGGA CAGTGAGGAG AGGCATGTGG   
  
  
+ TTGAGCAACA GCTCCTTTCT AGGGAGATTC GAAACATTCT GGCCGTTGGT GGGCCCTCAA GGACCGGGGA   
  
  
+ GCCCAAGTTT GCGAGCTGGA GGGAGAAGCT ACAACAGTCC GGCTTTAGGG GAATCTCATT GGCAGGCAAC   
  
  
+ GCCGCTGCCC AGGCCACCTT GCTCCTCGGC ATGTTCCCTT CTGATGGGTA TACTTTAATC GAGGACAGTG   
  
  
+ GCACACTTAA GCTCGGGTGG AAGGACTTGT GCCTCCTGAC TGCTTCGGCC TGGAGGCCTT CCCATGCTCA   
  
  
+ TACTATGAGC ACCGTCTGTA CTCGGAGCCA ATA  

- -Up\_Stream \_Len000CAGAGA CATTGTGTTA AGTAATTTAG GTAGGTTTCC CCTAGTATTT ATTGGTTTGA   
  
  
- AACAACCTTT GACGAAACTA CACCCGAAAG AGAAAGTCGT AAATACAATA TGTATATATA CCAATATTTC   
  
  
- AATGTTTTCA TACTGGGCTG TGCTGTTAAG TTAGGCTTAG GCTGAGCTTC AATGACTTTC ATTAGACTTA   
  
  
- TGCTAAACTG TCGAATCAAA TTTTTGGATA ATGAAGAGTT AAAACTCATT TAGATTCGAT AAAAATTACG   
  
  
- ACTTATCTAG CTGGGCTAAG CTGGGCAAAA GGCTGAGATA TACCAATACC ACGGGGATTT TTTTTAATTT   
  
  
- TTTCTTTTTC TTATCTTTTT ATATTTTATT TTGACTCTAA AGACTTGTGA GAGAGGGGAT CAGAACACCC   
  
  
- GTACCTTTCA CCAACCTCTA CCTCTACAAA CTCAAAGTAA AAGACTATAA TAAAAAATCA GTAATCTTCA   
  
  
- TGTTTCAGAT TTGAGTTGTT CTCGTACTCC CCTCTTACCA ACAACATCGG TTACCTTTTA TTTTTCTTGT   
  
  
- AAGTGAGAAG AACACGAAAT AAATACCACT ATTAAACAAT GAACATAAAA ATTTTTACTA ATTTATATAA   
  
  
- CTAGGAAACT ATAATACAAC TTTATTATTT TTATATTTTT AATCTTTTAT AAAATGGAGC ACTTTAAAAA   
  
  
- AAAGACTAAT TAGTAACTTT AATACCACTT TTTATTTCCT TGTAGAGAAC ATTTGTGAGA TAGGCAAATG   
  
  
- CAAACTGGTG TTTGAACGAT AGGTGTTCGT AGAGTTGGAA ACGGAAATTG GGTTAACACC CAACTACTTT   
  
  
- AGATTGGTAA TATCCTAATC TACCATCAGC AATTAGGACA CTTTGTTCAA AAACCAATAC TACATAGGTT   
  
  
- AATACTCCCG GACTATTGAG AACGGAGCCG TAAACGCACT ATTACTGCAC CATGATTATC GTTAATAAAA   
  
  
- AGCGTTTATA AGCTTTAAAA AATTCTTATT TGCGTTTTAA TAACTAGAAC TGTTTACTAA AATAAAAAAA   
  
  
- CAACCCTTTT CCTATTTGTA GATCACAGTT AACAACTACT GGACTATCGA GAGCTAAAGA ATAAATACTC   
  
  
- AATACAACTA TACTGTGATT AACATTAATA AACAACATTT GAATTTGTAA AAATTTTTAG TTTCGTTTTA   
  
  
- AACAATTAAT GTTATTTAGT TAGATGAAAA TGATACTAGA ATAAACCACA TTAATAACTA GAACTTAAAA   
  
  
- AAAAAAAGAA AAACTTTTTC TATTCGTAGC TTAAAAAAGT TTTTATTTTT CCCACTTCCT TCTTTTTTAC   
  
  
- CTTTGTCAAT GGCTCATTCC TTCCTGTCTC GCGCTCTCCT GTTCTGTGTT TCTCTCCCCT CTCTCTCTCT   
  
  
- CTTCCCCCGT TACCGTCACC CCTTCCAGCT CCTCGTGGAA AGTTGAAGTT CCGAGTTGTG GGGGGGGGGG   
  
  
- GGGGAGAAAG GAGTAAAACG CTGCAATCGT AAACTGTAAA ACGAAAGTTG GCAGGGAGCC GTAGTAGTCG   
  
  
- GGACGGGAAC GAGTCGAAAA GGGTAGAAAA AAACCGAAAA ACAAACTGAG GGAGATATTG AGATAAGTGC   
  
  
- AGCTCGGAAC ATTTTTAAAA TGTATAAAAT AAAATAAAAT GAAAAAATCC AAATAGGGAG AGATAAGAAC   
  
  
- TGGATGCCCG TAAAAAATAA AAGAAACCCA AACTCTGAAC ACTTGAAAAA CGTGTAAAAG AGTGTTTTTA   
  
  
- AATAGAACAG AAAAAGTGAA AGTTGCAAGG AAGAGAGAGA GAGAGAGAGA GAGAGAGAGA GAGTGAGTGT   
  
  
- AGGATCTGTC TGCAGGAAAA ATTTCTTAAA TAAATTGTTC AATCTTCTTT GGCAACAAAT TCATCTTGGT   
  
  
- GGGACAAACC TACGGTTTCC TTTATGGTCA GTTCTTTTTA GTGCTGTTAG TGTTGTGTAG GTAAAGCAAA   
  
  
- TGTAGAGTAG GAGTAGGAGT AGAAGGAGTA GAGATGGGTA GAGGGAGAAA GACATACCGA CGAAGAAGAT   
  
  
- GAAACAAGGG ACTGGGATTG TTATGATCAA ATCAGGAGGG AATAATAATA ATAGGAGGAG GAAGGAGGAG   
  
  
- AATGATATTA CCATTGGGAA TGGAAGGAGA AGGATTGTGG TGACGATAAG GATGAGGAAG TTGTTGGAGA   
  
  
- GGCCATAAAA GAAGGCGGTG GATGAGCCTC AACTTATGAC TAACAGTTTG AGTTGACGGG TACAGATTGG   
  
  
- AGGGGCCTTT CTAGCAAGCC TTCGCCTCAC GACGACTTTA CCTCGTCGCG AGGCGAGGCT AACGCCCACT   
  
  
- AATGGGCGGT TGAGTAGTCG CCCATTAGTG GGCGGTGTTA CTCCGCCGGA GTAGTCACAG AAAGTAACTG   
  
  
- AGGAGTGAGA GCGGTGAAGT GGAGAAGTTG CCGAGGTTAA GCTGCTGGGG TAATCGGGGG CTGGCAGGAT   
  
  
- TACTAGGCCC AGACTCAGTC CCAGGCCTAG ACGGAGAACA CACGCCTAAG AGGCCTGAGG GAAATAAGGG   
  
  
- TGGCCACCTC TCTTCTGGAA GGTAGGGGCG GTGCTGGTGA CGACGGCGCC GCTAGCGCCA CCAAGGACAC   
  
  
- CTCCTCCTAC TAGGCTGCAC CTACCTATCG TAGTATTTTC TCGACTAAGT TTCGAGTTTG AGCTAGAGTT   
  
  
- AGGGTGTCAA CTAAGTCTTG CAATCTCTAT AGTAGATGGG TACATTAGGA TTAGACCCCA GGCGGTAACT   
  
  
- CAAGGCGGAG GCGAGCGAGC GGCGGCTGGG GGAGTAACGA GGCGGCGGTG AAGGCGGGAA GGTGGTGGTG   
  
  
- GTTGTGGTGG AGTTAGTTGT TTAGTGAGAA GGAGCTTAGT CGTTGTGATT GTTGTTGTTA GTACACTCGC   
  
  
- ACATGCGGTT GTTTCCTGGG CCTATGAAGT TGAACCCAGG CCCAGTCCCA GTCCCAGGCT AATTGTAGCT   
  
  
- GGTTCGATTG GAAAGGAAGG GAGGACTAAG GTGGCGGACA ACCCCACAGT CACAGTGTGG CGGTGGCAGA   
  
  
- AGGCGGCGGC CAAGGCCGAG GCCACCTTCG CCGTCATCAT CACCATTTAG TTTGGGATTG GGATTAGGGT   
  
  
- TGGGCTTGGG TTTGGGTTTG GGTTTGGGCT TGTTGTTTCG AGTCCTACAA GTTGAGGTTT GAGGTGTTGT   
  
  
- TGTCGTTGTC GTTGTCGTTG TTTAGGGGCA CCTAGTCCTC GTTCTGGGCC GCCGAGGTGG CCGCTGCCTT   
  
  
- CGTAGCGGTG GTAGAGGTTC TCGCCGCCGT GGTGGCGGCC GCCGTCACTT TCGTTCTCTT TCTCTCCTCT   
  
  
- ACGCCGTTTC CTTCGCGCTG CTTCTCCCAG AGGTGGAGGA TTGGGAGGAG GAGGTTACGC GTCTTCGTCA   
  
  
- TAGGCCGCTG TTTATGCTTC TTCGCTTGTT CTACGAAGAT CTTTAGAGCC TCACCCGGTG GGGTAAGCCG   
  
  
- TGGAGGCGGG TTGCGCAGCG GCGCATGAAG AGCCTTCGGT ACAGTCGGGC AGAGCATAGG AGGACGGAGC   
  
  
- CGTAGATGCG GCGGGAGGGG TGGCAGGGTG TGATGCAGTT CGAGGAGAGG CGGAAGGTTC AGAAGTTACC   
  
  
- GTAGTCGGGT AAGCAGTTTA AGAGAGTGAA GTGACGTTTG GTTCGTTAGG TTCTCCGGAA GGTCTCCCTT   
  
  
- CTGTCCCAGG TGTAGTAGCT GGAGCTATAG TACGTCCCCG AGGTCACCGG GCCCGACAAG GTGTAGGAGC   
  
  
- GCAGGGCCGG TCCACCCGGA GGGAAGCATT CCGAGTGGCC CGAGCCCTGG AGGTACCTCC GCGAGCTCCG   
  
  
- GTGGCCTTTT TCCGAGAGTC TGAAGCGGCT CTTCAACCCC AACGGGAAAC TCAAATATGG GCACCGCCTC   
  
  
- TTCTAACCTT TAAACCTGAA CCTTTCCAAC GTACAATCAT TTTCCCTTCG AGAACGACAC GTAACCAATG   
  
  
- TCGTGAGAAA CATACTACAA TGACCGAGAC TATGATTATG TGAACCGGAA GAAGTTTCCA ACCGCGGTTT   
  
  
- TCACCACTGC CACCACCTCG TTCTGGATTC GGCTTGTCCG AGAAAGGATC CTTCCAAACA TCTCCGCTAG   
  
  
- GTGATGATAA GTCGGGATAA ACTGAGGAAT CCTCGCTCAA TACCTCTCCT GTCACTCCTC TCCGTACACC   
  
  
- AACTCGTTGT CGAGGAAAGA TCCCTCTAAG CTTTGTAAGA CCGGCAACCA CCCGGGAGTT CCTGGCCCCT   
  
  
- CGGGTTCAAA CGCTCGACCT CCCTCTTCGA TGTTGTCAGG CCGAAATCCC CTTAGAGTAA CCGTCCGTTG   
  
  
- CGGCGACGGG TCCGGTGGAA CGAGGAGCCG TACAAGGGAA GACTACCCAT ATGAAATTAG CTCCTGTCAC   
  
  
- CGTGTGAATT CGAGCCCACC TTCCTGAACA CGGAGGACTG ACGAAGCCGG ACCTCCGGAA GGGTACGAGT   
  
  
- ATGATACTCG TGGCAGACAT GAGCCTCGGT TAT

+     ABRE

| Site Name | Organism | Position | Strand | Matrix score. | sequence | function |
| --- | --- | --- | --- | --- | --- | --- |
| ABRE | Arabidopsis thaliana | 960 | + | 5 | ACGTG | cis-acting element involved in the abscisic acid responsiveness |
| ABRE | Arabidopsis thaliana | 2610 | + | 5 | ACGTG | cis-acting element involved in the abscisic acid responsiveness |
| ABRE | Arabidopsis thaliana | 1611 | - | 5 | ACGTG | cis-acting element involved in the abscisic acid responsiveness |

>HU01G00472.1   
+ -Up\_Stream \_Len000GTCTCT GTAACACAAT TCATTAAATC CATCCAAAGG GGATCATAAA TAACCAAACT   
  
  
+ TTGTTGGAAA CTGCTTTGAT GTGGGCTTTC TCTTTCAGCA TTTATGTTAT ACATATATAT GGTTATAAAG   
  
  
+ TTACAAAAGT ATGACCCGAC ACGACAATTC AATCCGAATC CGACTCGAAG TTACTGAAAG TAATCTGAAT   
  
  
+ ACGATTTGAC AGCTTAGTTT AAAAACCTAT TACTTCTCAA TTTTGAGTAA ATCTAAGCTA TTTTTAATGC   
  
  
+ TGAATAGATC GACCCGATTC GACCCGTTTT CCGACTCTAT ATGGTTATGG TGCCCCTAAA AAAAATTAAA   
  
  
+ AAAGAAAAAG AATAGAAAAA TATAAAATAA AACTGAGATT TCTGAACACT CTCTCCCCTA GTCTTGTGGG   
  
  
+ CATGGAAAGT GGTTGGAGAT GGAGATGTTT GAGTTTCATT TTCTGATATT ATTTTTTAGT CATTAGAAGT   
  
  
+ ACAAAGTCTA AACTCAACAA GAGCATGAGG GGAGAATGGT TGTTGTAGCC AATGGAAAAT AAAAAGAACA   
  
  
+ TTCACTCTTC TTGTGCTTTA TTTATGGTGA TAATTTGTTA CTTGTATTTT TAAAAATGAT TAAATATATT   
  
  
+ GATCCTTTGA TATTATGTTG AAATAATAAA AATATAAAAA TTAGAAAATA TTTTACCTCG TGAAATTTTT   
  
  
+ TTTCTGATTA ATCATTGAAA TTATGGTGAA AAATAAAGGA ACATCTCTTG TAAACACTCT ATCCGTTTAC   
  
  
+ GTTTGACCAC AAACTTGCTA TCCACAAGCA TCTCAACCTT TGCCTTTAAC CCAATTGTGG GTTGATGAAA   
  
  
+ TCTAACCATT ATAGGATTAG ATGGTAGTCG TTAATCCTGT GAAACAAGTT TTTGGTTATG ATGTATCCAA   
  
  
+ TTATGAGGGC CTGATAACTC TTGCCTCGGC ATTTGCGTGA TAATGACGTG GTACTAATAG CAATTATTTT   
  
  
+ TCGCAAATAT TCGAAATTTT TTAAGAATAA ACGCAAAATT ATTGATCTTG ACAAATGATT TTATTTTTTT   
  
  
+ GTTGGGAAAA GGATAAACAT CTAGTGTCAA TTGTTGATGA CCTGATAGCT CTCGATTTCT TATTTATGAG   
  
  
+ TTATGTTGAT ATGACACTAA TTGTAATTAT TTGTTGTAAA CTTAAACATT TTTAAAAATC AAAGCAAAAT   
  
  
+ TTGTTAATTA CAATAAATCA ATCTACTTTT ACTATGATCT TATTTGGTGT AATTATTGAT CTTGAATTTT   
  
  
+ TTTTTTTCTT TTTGAAAAAG ATAAGCATCG AATTTTTTCA AAAATAAAAA GGGTGAAGGA AGAAAAAATG   
  
  
+ GAAACAGTTA CCGAGTAAGG AAGGACAGAG CGCGAGAGGA CAAGACACAA AGAGAGGGGA GAGAGAGAGA   
  
  
+ GAAGGGGGCA ATGGCAGTGG GGAAGGTCGA GGAGCACCTT TCAACTTCAA GGCTCAACAC CCCCCCCCCC   
  
  
+ CCCCTCTTTC CTCATTTTGC GACGTTAGCA TTTGACATTT TGCTTTCAAC CGTCCCTCGG CATCATCAGC   
  
  
+ CCTGCCCTTG CTCAGCTTTT CCCATCTTTT TTTGGCTTTT TGTTTGACTC CCTCTATAAC TCTATTCACG   
  
  
+ TCGAGCCTTG TAAAAATTTT ACATATTTTA TTTTATTTTA CTTTTTTAGG TTTATCCCTC TCTATTCTTG   
  
  
+ ACCTACGGGC ATTTTTTATT TTCTTTGGGT TTGAGACTTG TGAACTTTTT GCACATTTTC TCACAAAAAT   
  
  
+ TTATCTTGTC TTTTTCACTT TCAACGTTCC TTCTCTCTCT CTCTCTCTCT CTCTCTCTCT CTCACTCACA   
  
  
+ TCCTAGACAG ACGTCCTTTT TAAAGAATTT ATTTAACAAG TTAGAAGAAA CCGTTGTTTA AGTAGAACCA   
  
  
+ CCCTGTTTGG ATGCCAAAGG AAATACCAGT CAAGAAAAAT CACGACAATC ACAACACATC CATTTCGTTT   
  
  
+ ACATCTCATC CTCATCCTCA TCTTCCTCAT CTCTACCCAT CTCCCTCTTT CTGTATGGCT GCTTCTTCTA   
  
  
+ CTTTGTTCCC TGACCCTAAC AATACTAGTT TAGTCCTCCC TTATTATTAT TATCCTCCTC CTTCCTCCTC   
  
  
+ TTACTATAAT GGTAACCCTT ACCTTCCTCT TCCTAACACC ACTGCTATTC CTACTCCTTC AACAACCTCT   
  
  
+ CCGGTATTTT CTTCCGCCAC CTACTCGGAG TTGAATACTG ATTGTCAAAC TCAACTGCCC ATGTCTAACC   
  
  
+ TCCCCGGAAA GATCGTTCGG AAGCGGAGTG CTGCTGAAAT GGAGCAGCGC TCCGCTCCGA TTGCGGGTGA   
  
  
+ TTACCCGCCA ACTCATCAGC GGGTAATCAC CCGCCACAAT GAGGCGGCCT CATCAGTGTC TTTCATTGAC   
  
  
+ TCCTCACTCT CGCCACTTCA CCTCTTCAAC GGCTCCAATT CGACGACCCC ATTAGCCCCC GACCGTCCTA   
  
  
+ ATGATCCGGG TCTGAGTCAG GGTCCGGATC TGCCTCTTGT GTGCGGATTC TCCGGACTCC CTTTATTCCC   
  
  
+ ACCGGTGGAG AGAAGACCTT CCATCCCCGC CACGACCACT GCTGCCGCGG CGATCGCGGT GGTTCCTGTG   
  
  
+ GAGGAGGATG ATCCGACGTG GATGGATAGC ATCATAAAAG AGCTGATTCA AAGCTCAAAC TCGATCTCAA   
  
  
+ TCCCACAGTT GATTCAGAAC GTTAGAGATA TCATCTACCC ATGTAATCCT AATCTGGGGT CCGCCATTGA   
  
  
+ GTTCCGCCTC CGCTCGCTCG CCGCCGACCC CCTCATTGCT CCGCCGCCAC TTCCGCCCTT CCACCACCAC   
  
  
+ CAACACCACC TCAATCAACA AATCACTCTT CCTCGAATCA GCAACACTAA CAACAACAAT CATGTGAGCG   
  
  
+ TGTACGCCAA CAAAGGACCC GGATACTTCA ACTTGGGTCC GGGTCAGGGT CAGGGTCCGA TTAACATCGA   
  
  
+ CCAAGCTAAC CTTTCCTTCC CTCCTGATTC CACCGCCTGT TGGGGTGTCA GTGTCACACC GCCACCGTCT   
  
  
+ TCCGCCGCCG GTTCCGGCTC CGGTGGAAGC GGCAGTAGTA GTGGTAAATC AAACCCTAAC CCTAATCCCA   
  
  
+ ACCCGAACCC AAACCCAAAC CCAAACCCGA ACAACAAAGC TCAGGATGTT CAACTCCAAA CTCCACAACA   
  
  
+ ACAGCAACAG CAACAGCAAC AAATCCCCGT GGATCAGGAG CAAGACCCGG CGGCTCCACC GGCGACGGAA   
  
  
+ GCATCGCCAC CATCTCCAAG AGCGGCGGCA CCACCGCCGG CGGCAGTGAA AGCAAGAGAA AGAGAGGAGA   
  
  
+ TGCGGCAAAG GAAGCGCGAC GAAGAGGGTC TCCACCTCCT AACCCTCCTC CTCCAATGCG CAGAAGCAGT   
  
  
+ ATCCGGCGAC AAATACGAAG AAGCGAACAA GATGCTTCTA GAAATCTCGG AGTGGGCCAC CCCATTCGGC   
  
  
+ ACCTCCGCCC AACGCGTCGC CGCGTACTTC TCGGAAGCCA TGTCAGCCCG TCTCGTATCC TCCTGCCTCG   
  
  
+ GCATCTACGC CGCCCTCCCC ACCGTCCCAC ACTACGTCAA GCTCCTCTCC GCCTTCCAAG TCTTCAATGG   
  
  
+ CATCAGCCCA TTCGTCAAAT TCTCTCACTT CACTGCAAAC CAAGCAATCC AAGAGGCCTT CCAGAGGGAA   
  
  
+ GACAGGGTCC ACATCATCGA CCTCGATATC ATGCAGGGGC TCCAGTGGCC CGGGCTGTTC CACATCCTCG   
  
  
+ CGTCCCGGCC AGGTGGGCCT CCCTTCGTAA GGCTCACCGG GCTCGGGACC TCCATGGAGG CGCTCGAGGC   
  
  
+ CACCGGAAAA AGGCTCTCAG ACTTCGCCGA GAAGTTGGGG TTGCCCTTTG AGTTTATACC CGTGGCGGAG   
  
  
+ AAGATTGGAA ATTTGGACTT GGAAAGGTTG CATGTTAGTA AAAGGGAAGC TCTTGCTGTG CATTGGTTAC   
  
  
+ AGCACTCTTT GTATGATGTT ACTGGCTCTG ATACTAATAC ACTTGGCCTT CTTCAAAGGT TGGCGCCAAA   
  
  
+ AGTGGTGACG GTGGTGGAGC AAGACCTAAG CCGAACAGGC TCTTTCCTAG GAAGGTTTGT AGAGGCGATC   
  
  
+ CACTACTATT CAGCCCTATT TGACTCCTTA GGAGCGAGTT ATGGAGAGGA CAGTGAGGAG AGGCATGTGG   
  
  
+ TTGAGCAACA GCTCCTTTCT AGGGAGATTC GAAACATTCT GGCCGTTGGT GGGCCCTCAA GGACCGGGGA   
  
  
+ GCCCAAGTTT GCGAGCTGGA GGGAGAAGCT ACAACAGTCC GGCTTTAGGG GAATCTCATT GGCAGGCAAC   
  
  
+ GCCGCTGCCC AGGCCACCTT GCTCCTCGGC ATGTTCCCTT CTGATGGGTA TACTTTAATC GAGGACAGTG   
  
  
+ GCACACTTAA GCTCGGGTGG AAGGACTTGT GCCTCCTGAC TGCTTCGGCC TGGAGGCCTT CCCATGCTCA   
  
  
+ TACTATGAGC ACCGTCTGTA CTCGGAGCCA ATA  

- -Up\_Stream \_Len000CAGAGA CATTGTGTTA AGTAATTTAG GTAGGTTTCC CCTAGTATTT ATTGGTTTGA   
  
  
- AACAACCTTT GACGAAACTA CACCCGAAAG AGAAAGTCGT AAATACAATA TGTATATATA CCAATATTTC   
  
  
- AATGTTTTCA TACTGGGCTG TGCTGTTAAG TTAGGCTTAG GCTGAGCTTC AATGACTTTC ATTAGACTTA   
  
  
- TGCTAAACTG TCGAATCAAA TTTTTGGATA ATGAAGAGTT AAAACTCATT TAGATTCGAT AAAAATTACG   
  
  
- ACTTATCTAG CTGGGCTAAG CTGGGCAAAA GGCTGAGATA TACCAATACC ACGGGGATTT TTTTTAATTT   
  
  
- TTTCTTTTTC TTATCTTTTT ATATTTTATT TTGACTCTAA AGACTTGTGA GAGAGGGGAT CAGAACACCC   
  
  
- GTACCTTTCA CCAACCTCTA CCTCTACAAA CTCAAAGTAA AAGACTATAA TAAAAAATCA GTAATCTTCA   
  
  
- TGTTTCAGAT TTGAGTTGTT CTCGTACTCC CCTCTTACCA ACAACATCGG TTACCTTTTA TTTTTCTTGT   
  
  
- AAGTGAGAAG AACACGAAAT AAATACCACT ATTAAACAAT GAACATAAAA ATTTTTACTA ATTTATATAA   
  
  
- CTAGGAAACT ATAATACAAC TTTATTATTT TTATATTTTT AATCTTTTAT AAAATGGAGC ACTTTAAAAA   
  
  
- AAAGACTAAT TAGTAACTTT AATACCACTT TTTATTTCCT TGTAGAGAAC ATTTGTGAGA TAGGCAAATG   
  
  
- CAAACTGGTG TTTGAACGAT AGGTGTTCGT AGAGTTGGAA ACGGAAATTG GGTTAACACC CAACTACTTT   
  
  
- AGATTGGTAA TATCCTAATC TACCATCAGC AATTAGGACA CTTTGTTCAA AAACCAATAC TACATAGGTT   
  
  
- AATACTCCCG GACTATTGAG AACGGAGCCG TAAACGCACT ATTACTGCAC CATGATTATC GTTAATAAAA   
  
  
- AGCGTTTATA AGCTTTAAAA AATTCTTATT TGCGTTTTAA TAACTAGAAC TGTTTACTAA AATAAAAAAA   
  
  
- CAACCCTTTT CCTATTTGTA GATCACAGTT AACAACTACT GGACTATCGA GAGCTAAAGA ATAAATACTC   
  
  
- AATACAACTA TACTGTGATT AACATTAATA AACAACATTT GAATTTGTAA AAATTTTTAG TTTCGTTTTA   
  
  
- AACAATTAAT GTTATTTAGT TAGATGAAAA TGATACTAGA ATAAACCACA TTAATAACTA GAACTTAAAA   
  
  
- AAAAAAAGAA AAACTTTTTC TATTCGTAGC TTAAAAAAGT TTTTATTTTT CCCACTTCCT TCTTTTTTAC   
  
  
- CTTTGTCAAT GGCTCATTCC TTCCTGTCTC GCGCTCTCCT GTTCTGTGTT TCTCTCCCCT CTCTCTCTCT   
  
  
- CTTCCCCCGT TACCGTCACC CCTTCCAGCT CCTCGTGGAA AGTTGAAGTT CCGAGTTGTG GGGGGGGGGG   
  
  
- GGGGAGAAAG GAGTAAAACG CTGCAATCGT AAACTGTAAA ACGAAAGTTG GCAGGGAGCC GTAGTAGTCG   
  
  
- GGACGGGAAC GAGTCGAAAA GGGTAGAAAA AAACCGAAAA ACAAACTGAG GGAGATATTG AGATAAGTGC   
  
  
- AGCTCGGAAC ATTTTTAAAA TGTATAAAAT AAAATAAAAT GAAAAAATCC AAATAGGGAG AGATAAGAAC   
  
  
- TGGATGCCCG TAAAAAATAA AAGAAACCCA AACTCTGAAC ACTTGAAAAA CGTGTAAAAG AGTGTTTTTA   
  
  
- AATAGAACAG AAAAAGTGAA AGTTGCAAGG AAGAGAGAGA GAGAGAGAGA GAGAGAGAGA GAGTGAGTGT   
  
  
- AGGATCTGTC TGCAGGAAAA ATTTCTTAAA TAAATTGTTC AATCTTCTTT GGCAACAAAT TCATCTTGGT   
  
  
- GGGACAAACC TACGGTTTCC TTTATGGTCA GTTCTTTTTA GTGCTGTTAG TGTTGTGTAG GTAAAGCAAA   
  
  
- TGTAGAGTAG GAGTAGGAGT AGAAGGAGTA GAGATGGGTA GAGGGAGAAA GACATACCGA CGAAGAAGAT   
  
  
- GAAACAAGGG ACTGGGATTG TTATGATCAA ATCAGGAGGG AATAATAATA ATAGGAGGAG GAAGGAGGAG   
  
  
- AATGATATTA CCATTGGGAA TGGAAGGAGA AGGATTGTGG TGACGATAAG GATGAGGAAG TTGTTGGAGA   
  
  
- GGCCATAAAA GAAGGCGGTG GATGAGCCTC AACTTATGAC TAACAGTTTG AGTTGACGGG TACAGATTGG   
  
  
- AGGGGCCTTT CTAGCAAGCC TTCGCCTCAC GACGACTTTA CCTCGTCGCG AGGCGAGGCT AACGCCCACT   
  
  
- AATGGGCGGT TGAGTAGTCG CCCATTAGTG GGCGGTGTTA CTCCGCCGGA GTAGTCACAG AAAGTAACTG   
  
  
- AGGAGTGAGA GCGGTGAAGT GGAGAAGTTG CCGAGGTTAA GCTGCTGGGG TAATCGGGGG CTGGCAGGAT   
  
  
- TACTAGGCCC AGACTCAGTC CCAGGCCTAG ACGGAGAACA CACGCCTAAG AGGCCTGAGG GAAATAAGGG   
  
  
- TGGCCACCTC TCTTCTGGAA GGTAGGGGCG GTGCTGGTGA CGACGGCGCC GCTAGCGCCA CCAAGGACAC   
  
  
- CTCCTCCTAC TAGGCTGCAC CTACCTATCG TAGTATTTTC TCGACTAAGT TTCGAGTTTG AGCTAGAGTT   
  
  
- AGGGTGTCAA CTAAGTCTTG CAATCTCTAT AGTAGATGGG TACATTAGGA TTAGACCCCA GGCGGTAACT   
  
  
- CAAGGCGGAG GCGAGCGAGC GGCGGCTGGG GGAGTAACGA GGCGGCGGTG AAGGCGGGAA GGTGGTGGTG   
  
  
- GTTGTGGTGG AGTTAGTTGT TTAGTGAGAA GGAGCTTAGT CGTTGTGATT GTTGTTGTTA GTACACTCGC   
  
  
- ACATGCGGTT GTTTCCTGGG CCTATGAAGT TGAACCCAGG CCCAGTCCCA GTCCCAGGCT AATTGTAGCT   
  
  
- GGTTCGATTG GAAAGGAAGG GAGGACTAAG GTGGCGGACA ACCCCACAGT CACAGTGTGG CGGTGGCAGA   
  
  
- AGGCGGCGGC CAAGGCCGAG GCCACCTTCG CCGTCATCAT CACCATTTAG TTTGGGATTG GGATTAGGGT   
  
  
- TGGGCTTGGG TTTGGGTTTG GGTTTGGGCT TGTTGTTTCG AGTCCTACAA GTTGAGGTTT GAGGTGTTGT   
  
  
- TGTCGTTGTC GTTGTCGTTG TTTAGGGGCA CCTAGTCCTC GTTCTGGGCC GCCGAGGTGG CCGCTGCCTT   
  
  
- CGTAGCGGTG GTAGAGGTTC TCGCCGCCGT GGTGGCGGCC GCCGTCACTT TCGTTCTCTT TCTCTCCTCT   
  
  
- ACGCCGTTTC CTTCGCGCTG CTTCTCCCAG AGGTGGAGGA TTGGGAGGAG GAGGTTACGC GTCTTCGTCA   
  
  
- TAGGCCGCTG TTTATGCTTC TTCGCTTGTT CTACGAAGAT CTTTAGAGCC TCACCCGGTG GGGTAAGCCG   
  
  
- TGGAGGCGGG TTGCGCAGCG GCGCATGAAG AGCCTTCGGT ACAGTCGGGC AGAGCATAGG AGGACGGAGC   
  
  
- CGTAGATGCG GCGGGAGGGG TGGCAGGGTG TGATGCAGTT CGAGGAGAGG CGGAAGGTTC AGAAGTTACC   
  
  
- GTAGTCGGGT AAGCAGTTTA AGAGAGTGAA GTGACGTTTG GTTCGTTAGG TTCTCCGGAA GGTCTCCCTT   
  
  
- CTGTCCCAGG TGTAGTAGCT GGAGCTATAG TACGTCCCCG AGGTCACCGG GCCCGACAAG GTGTAGGAGC   
  
  
- GCAGGGCCGG TCCACCCGGA GGGAAGCATT CCGAGTGGCC CGAGCCCTGG AGGTACCTCC GCGAGCTCCG   
  
  
- GTGGCCTTTT TCCGAGAGTC TGAAGCGGCT CTTCAACCCC AACGGGAAAC TCAAATATGG GCACCGCCTC   
  
  
- TTCTAACCTT TAAACCTGAA CCTTTCCAAC GTACAATCAT TTTCCCTTCG AGAACGACAC GTAACCAATG   
  
  
- TCGTGAGAAA CATACTACAA TGACCGAGAC TATGATTATG TGAACCGGAA GAAGTTTCCA ACCGCGGTTT   
  
  
- TCACCACTGC CACCACCTCG TTCTGGATTC GGCTTGTCCG AGAAAGGATC CTTCCAAACA TCTCCGCTAG   
  
  
- GTGATGATAA GTCGGGATAA ACTGAGGAAT CCTCGCTCAA TACCTCTCCT GTCACTCCTC TCCGTACACC   
  
  
- AACTCGTTGT CGAGGAAAGA TCCCTCTAAG CTTTGTAAGA CCGGCAACCA CCCGGGAGTT CCTGGCCCCT   
  
  
- CGGGTTCAAA CGCTCGACCT CCCTCTTCGA TGTTGTCAGG CCGAAATCCC CTTAGAGTAA CCGTCCGTTG   
  
  
- CGGCGACGGG TCCGGTGGAA CGAGGAGCCG TACAAGGGAA GACTACCCAT ATGAAATTAG CTCCTGTCAC   
  
  
- CGTGTGAATT CGAGCCCACC TTCCTGAACA CGGAGGACTG ACGAAGCCGG ACCTCCGGAA GGGTACGAGT   
  
  
- ATGATACTCG TGGCAGACAT GAGCCTCGGT TAT

+     ARE

| Site Name | Organism | Position | Strand | Matrix score. | sequence | function |
| --- | --- | --- | --- | --- | --- | --- |
| ARE | Zea mays | 3611 | + | 6 | AAACCA | cis-acting regulatory element essential for the anaerobic induction |

>HU01G00472.1   
+ -Up\_Stream \_Len000GTCTCT GTAACACAAT TCATTAAATC CATCCAAAGG GGATCATAAA TAACCAAACT   
  
  
+ TTGTTGGAAA CTGCTTTGAT GTGGGCTTTC TCTTTCAGCA TTTATGTTAT ACATATATAT GGTTATAAAG   
  
  
+ TTACAAAAGT ATGACCCGAC ACGACAATTC AATCCGAATC CGACTCGAAG TTACTGAAAG TAATCTGAAT   
  
  
+ ACGATTTGAC AGCTTAGTTT AAAAACCTAT TACTTCTCAA TTTTGAGTAA ATCTAAGCTA TTTTTAATGC   
  
  
+ TGAATAGATC GACCCGATTC GACCCGTTTT CCGACTCTAT ATGGTTATGG TGCCCCTAAA AAAAATTAAA   
  
  
+ AAAGAAAAAG AATAGAAAAA TATAAAATAA AACTGAGATT TCTGAACACT CTCTCCCCTA GTCTTGTGGG   
  
  
+ CATGGAAAGT GGTTGGAGAT GGAGATGTTT GAGTTTCATT TTCTGATATT ATTTTTTAGT CATTAGAAGT   
  
  
+ ACAAAGTCTA AACTCAACAA GAGCATGAGG GGAGAATGGT TGTTGTAGCC AATGGAAAAT AAAAAGAACA   
  
  
+ TTCACTCTTC TTGTGCTTTA TTTATGGTGA TAATTTGTTA CTTGTATTTT TAAAAATGAT TAAATATATT   
  
  
+ GATCCTTTGA TATTATGTTG AAATAATAAA AATATAAAAA TTAGAAAATA TTTTACCTCG TGAAATTTTT   
  
  
+ TTTCTGATTA ATCATTGAAA TTATGGTGAA AAATAAAGGA ACATCTCTTG TAAACACTCT ATCCGTTTAC   
  
  
+ GTTTGACCAC AAACTTGCTA TCCACAAGCA TCTCAACCTT TGCCTTTAAC CCAATTGTGG GTTGATGAAA   
  
  
+ TCTAACCATT ATAGGATTAG ATGGTAGTCG TTAATCCTGT GAAACAAGTT TTTGGTTATG ATGTATCCAA   
  
  
+ TTATGAGGGC CTGATAACTC TTGCCTCGGC ATTTGCGTGA TAATGACGTG GTACTAATAG CAATTATTTT   
  
  
+ TCGCAAATAT TCGAAATTTT TTAAGAATAA ACGCAAAATT ATTGATCTTG ACAAATGATT TTATTTTTTT   
  
  
+ GTTGGGAAAA GGATAAACAT CTAGTGTCAA TTGTTGATGA CCTGATAGCT CTCGATTTCT TATTTATGAG   
  
  
+ TTATGTTGAT ATGACACTAA TTGTAATTAT TTGTTGTAAA CTTAAACATT TTTAAAAATC AAAGCAAAAT   
  
  
+ TTGTTAATTA CAATAAATCA ATCTACTTTT ACTATGATCT TATTTGGTGT AATTATTGAT CTTGAATTTT   
  
  
+ TTTTTTTCTT TTTGAAAAAG ATAAGCATCG AATTTTTTCA AAAATAAAAA GGGTGAAGGA AGAAAAAATG   
  
  
+ GAAACAGTTA CCGAGTAAGG AAGGACAGAG CGCGAGAGGA CAAGACACAA AGAGAGGGGA GAGAGAGAGA   
  
  
+ GAAGGGGGCA ATGGCAGTGG GGAAGGTCGA GGAGCACCTT TCAACTTCAA GGCTCAACAC CCCCCCCCCC   
  
  
+ CCCCTCTTTC CTCATTTTGC GACGTTAGCA TTTGACATTT TGCTTTCAAC CGTCCCTCGG CATCATCAGC   
  
  
+ CCTGCCCTTG CTCAGCTTTT CCCATCTTTT TTTGGCTTTT TGTTTGACTC CCTCTATAAC TCTATTCACG   
  
  
+ TCGAGCCTTG TAAAAATTTT ACATATTTTA TTTTATTTTA CTTTTTTAGG TTTATCCCTC TCTATTCTTG   
  
  
+ ACCTACGGGC ATTTTTTATT TTCTTTGGGT TTGAGACTTG TGAACTTTTT GCACATTTTC TCACAAAAAT   
  
  
+ TTATCTTGTC TTTTTCACTT TCAACGTTCC TTCTCTCTCT CTCTCTCTCT CTCTCTCTCT CTCACTCACA   
  
  
+ TCCTAGACAG ACGTCCTTTT TAAAGAATTT ATTTAACAAG TTAGAAGAAA CCGTTGTTTA AGTAGAACCA   
  
  
+ CCCTGTTTGG ATGCCAAAGG AAATACCAGT CAAGAAAAAT CACGACAATC ACAACACATC CATTTCGTTT   
  
  
+ ACATCTCATC CTCATCCTCA TCTTCCTCAT CTCTACCCAT CTCCCTCTTT CTGTATGGCT GCTTCTTCTA   
  
  
+ CTTTGTTCCC TGACCCTAAC AATACTAGTT TAGTCCTCCC TTATTATTAT TATCCTCCTC CTTCCTCCTC   
  
  
+ TTACTATAAT GGTAACCCTT ACCTTCCTCT TCCTAACACC ACTGCTATTC CTACTCCTTC AACAACCTCT   
  
  
+ CCGGTATTTT CTTCCGCCAC CTACTCGGAG TTGAATACTG ATTGTCAAAC TCAACTGCCC ATGTCTAACC   
  
  
+ TCCCCGGAAA GATCGTTCGG AAGCGGAGTG CTGCTGAAAT GGAGCAGCGC TCCGCTCCGA TTGCGGGTGA   
  
  
+ TTACCCGCCA ACTCATCAGC GGGTAATCAC CCGCCACAAT GAGGCGGCCT CATCAGTGTC TTTCATTGAC   
  
  
+ TCCTCACTCT CGCCACTTCA CCTCTTCAAC GGCTCCAATT CGACGACCCC ATTAGCCCCC GACCGTCCTA   
  
  
+ ATGATCCGGG TCTGAGTCAG GGTCCGGATC TGCCTCTTGT GTGCGGATTC TCCGGACTCC CTTTATTCCC   
  
  
+ ACCGGTGGAG AGAAGACCTT CCATCCCCGC CACGACCACT GCTGCCGCGG CGATCGCGGT GGTTCCTGTG   
  
  
+ GAGGAGGATG ATCCGACGTG GATGGATAGC ATCATAAAAG AGCTGATTCA AAGCTCAAAC TCGATCTCAA   
  
  
+ TCCCACAGTT GATTCAGAAC GTTAGAGATA TCATCTACCC ATGTAATCCT AATCTGGGGT CCGCCATTGA   
  
  
+ GTTCCGCCTC CGCTCGCTCG CCGCCGACCC CCTCATTGCT CCGCCGCCAC TTCCGCCCTT CCACCACCAC   
  
  
+ CAACACCACC TCAATCAACA AATCACTCTT CCTCGAATCA GCAACACTAA CAACAACAAT CATGTGAGCG   
  
  
+ TGTACGCCAA CAAAGGACCC GGATACTTCA ACTTGGGTCC GGGTCAGGGT CAGGGTCCGA TTAACATCGA   
  
  
+ CCAAGCTAAC CTTTCCTTCC CTCCTGATTC CACCGCCTGT TGGGGTGTCA GTGTCACACC GCCACCGTCT   
  
  
+ TCCGCCGCCG GTTCCGGCTC CGGTGGAAGC GGCAGTAGTA GTGGTAAATC AAACCCTAAC CCTAATCCCA   
  
  
+ ACCCGAACCC AAACCCAAAC CCAAACCCGA ACAACAAAGC TCAGGATGTT CAACTCCAAA CTCCACAACA   
  
  
+ ACAGCAACAG CAACAGCAAC AAATCCCCGT GGATCAGGAG CAAGACCCGG CGGCTCCACC GGCGACGGAA   
  
  
+ GCATCGCCAC CATCTCCAAG AGCGGCGGCA CCACCGCCGG CGGCAGTGAA AGCAAGAGAA AGAGAGGAGA   
  
  
+ TGCGGCAAAG GAAGCGCGAC GAAGAGGGTC TCCACCTCCT AACCCTCCTC CTCCAATGCG CAGAAGCAGT   
  
  
+ ATCCGGCGAC AAATACGAAG AAGCGAACAA GATGCTTCTA GAAATCTCGG AGTGGGCCAC CCCATTCGGC   
  
  
+ ACCTCCGCCC AACGCGTCGC CGCGTACTTC TCGGAAGCCA TGTCAGCCCG TCTCGTATCC TCCTGCCTCG   
  
  
+ GCATCTACGC CGCCCTCCCC ACCGTCCCAC ACTACGTCAA GCTCCTCTCC GCCTTCCAAG TCTTCAATGG   
  
  
+ CATCAGCCCA TTCGTCAAAT TCTCTCACTT CACTGCAAAC CAAGCAATCC AAGAGGCCTT CCAGAGGGAA   
  
  
+ GACAGGGTCC ACATCATCGA CCTCGATATC ATGCAGGGGC TCCAGTGGCC CGGGCTGTTC CACATCCTCG   
  
  
+ CGTCCCGGCC AGGTGGGCCT CCCTTCGTAA GGCTCACCGG GCTCGGGACC TCCATGGAGG CGCTCGAGGC   
  
  
+ CACCGGAAAA AGGCTCTCAG ACTTCGCCGA GAAGTTGGGG TTGCCCTTTG AGTTTATACC CGTGGCGGAG   
  
  
+ AAGATTGGAA ATTTGGACTT GGAAAGGTTG CATGTTAGTA AAAGGGAAGC TCTTGCTGTG CATTGGTTAC   
  
  
+ AGCACTCTTT GTATGATGTT ACTGGCTCTG ATACTAATAC ACTTGGCCTT CTTCAAAGGT TGGCGCCAAA   
  
  
+ AGTGGTGACG GTGGTGGAGC AAGACCTAAG CCGAACAGGC TCTTTCCTAG GAAGGTTTGT AGAGGCGATC   
  
  
+ CACTACTATT CAGCCCTATT TGACTCCTTA GGAGCGAGTT ATGGAGAGGA CAGTGAGGAG AGGCATGTGG   
  
  
+ TTGAGCAACA GCTCCTTTCT AGGGAGATTC GAAACATTCT GGCCGTTGGT GGGCCCTCAA GGACCGGGGA   
  
  
+ GCCCAAGTTT GCGAGCTGGA GGGAGAAGCT ACAACAGTCC GGCTTTAGGG GAATCTCATT GGCAGGCAAC   
  
  
+ GCCGCTGCCC AGGCCACCTT GCTCCTCGGC ATGTTCCCTT CTGATGGGTA TACTTTAATC GAGGACAGTG   
  
  
+ GCACACTTAA GCTCGGGTGG AAGGACTTGT GCCTCCTGAC TGCTTCGGCC TGGAGGCCTT CCCATGCTCA   
  
  
+ TACTATGAGC ACCGTCTGTA CTCGGAGCCA ATA  

- -Up\_Stream \_Len000CAGAGA CATTGTGTTA AGTAATTTAG GTAGGTTTCC CCTAGTATTT ATTGGTTTGA   
  
  
- AACAACCTTT GACGAAACTA CACCCGAAAG AGAAAGTCGT AAATACAATA TGTATATATA CCAATATTTC   
  
  
- AATGTTTTCA TACTGGGCTG TGCTGTTAAG TTAGGCTTAG GCTGAGCTTC AATGACTTTC ATTAGACTTA   
  
  
- TGCTAAACTG TCGAATCAAA TTTTTGGATA ATGAAGAGTT AAAACTCATT TAGATTCGAT AAAAATTACG   
  
  
- ACTTATCTAG CTGGGCTAAG CTGGGCAAAA GGCTGAGATA TACCAATACC ACGGGGATTT TTTTTAATTT   
  
  
- TTTCTTTTTC TTATCTTTTT ATATTTTATT TTGACTCTAA AGACTTGTGA GAGAGGGGAT CAGAACACCC   
  
  
- GTACCTTTCA CCAACCTCTA CCTCTACAAA CTCAAAGTAA AAGACTATAA TAAAAAATCA GTAATCTTCA   
  
  
- TGTTTCAGAT TTGAGTTGTT CTCGTACTCC CCTCTTACCA ACAACATCGG TTACCTTTTA TTTTTCTTGT   
  
  
- AAGTGAGAAG AACACGAAAT AAATACCACT ATTAAACAAT GAACATAAAA ATTTTTACTA ATTTATATAA   
  
  
- CTAGGAAACT ATAATACAAC TTTATTATTT TTATATTTTT AATCTTTTAT AAAATGGAGC ACTTTAAAAA   
  
  
- AAAGACTAAT TAGTAACTTT AATACCACTT TTTATTTCCT TGTAGAGAAC ATTTGTGAGA TAGGCAAATG   
  
  
- CAAACTGGTG TTTGAACGAT AGGTGTTCGT AGAGTTGGAA ACGGAAATTG GGTTAACACC CAACTACTTT   
  
  
- AGATTGGTAA TATCCTAATC TACCATCAGC AATTAGGACA CTTTGTTCAA AAACCAATAC TACATAGGTT   
  
  
- AATACTCCCG GACTATTGAG AACGGAGCCG TAAACGCACT ATTACTGCAC CATGATTATC GTTAATAAAA   
  
  
- AGCGTTTATA AGCTTTAAAA AATTCTTATT TGCGTTTTAA TAACTAGAAC TGTTTACTAA AATAAAAAAA   
  
  
- CAACCCTTTT CCTATTTGTA GATCACAGTT AACAACTACT GGACTATCGA GAGCTAAAGA ATAAATACTC   
  
  
- AATACAACTA TACTGTGATT AACATTAATA AACAACATTT GAATTTGTAA AAATTTTTAG TTTCGTTTTA   
  
  
- AACAATTAAT GTTATTTAGT TAGATGAAAA TGATACTAGA ATAAACCACA TTAATAACTA GAACTTAAAA   
  
  
- AAAAAAAGAA AAACTTTTTC TATTCGTAGC TTAAAAAAGT TTTTATTTTT CCCACTTCCT TCTTTTTTAC   
  
  
- CTTTGTCAAT GGCTCATTCC TTCCTGTCTC GCGCTCTCCT GTTCTGTGTT TCTCTCCCCT CTCTCTCTCT   
  
  
- CTTCCCCCGT TACCGTCACC CCTTCCAGCT CCTCGTGGAA AGTTGAAGTT CCGAGTTGTG GGGGGGGGGG   
  
  
- GGGGAGAAAG GAGTAAAACG CTGCAATCGT AAACTGTAAA ACGAAAGTTG GCAGGGAGCC GTAGTAGTCG   
  
  
- GGACGGGAAC GAGTCGAAAA GGGTAGAAAA AAACCGAAAA ACAAACTGAG GGAGATATTG AGATAAGTGC   
  
  
- AGCTCGGAAC ATTTTTAAAA TGTATAAAAT AAAATAAAAT GAAAAAATCC AAATAGGGAG AGATAAGAAC   
  
  
- TGGATGCCCG TAAAAAATAA AAGAAACCCA AACTCTGAAC ACTTGAAAAA CGTGTAAAAG AGTGTTTTTA   
  
  
- AATAGAACAG AAAAAGTGAA AGTTGCAAGG AAGAGAGAGA GAGAGAGAGA GAGAGAGAGA GAGTGAGTGT   
  
  
- AGGATCTGTC TGCAGGAAAA ATTTCTTAAA TAAATTGTTC AATCTTCTTT GGCAACAAAT TCATCTTGGT   
  
  
- GGGACAAACC TACGGTTTCC TTTATGGTCA GTTCTTTTTA GTGCTGTTAG TGTTGTGTAG GTAAAGCAAA   
  
  
- TGTAGAGTAG GAGTAGGAGT AGAAGGAGTA GAGATGGGTA GAGGGAGAAA GACATACCGA CGAAGAAGAT   
  
  
- GAAACAAGGG ACTGGGATTG TTATGATCAA ATCAGGAGGG AATAATAATA ATAGGAGGAG GAAGGAGGAG   
  
  
- AATGATATTA CCATTGGGAA TGGAAGGAGA AGGATTGTGG TGACGATAAG GATGAGGAAG TTGTTGGAGA   
  
  
- GGCCATAAAA GAAGGCGGTG GATGAGCCTC AACTTATGAC TAACAGTTTG AGTTGACGGG TACAGATTGG   
  
  
- AGGGGCCTTT CTAGCAAGCC TTCGCCTCAC GACGACTTTA CCTCGTCGCG AGGCGAGGCT AACGCCCACT   
  
  
- AATGGGCGGT TGAGTAGTCG CCCATTAGTG GGCGGTGTTA CTCCGCCGGA GTAGTCACAG AAAGTAACTG   
  
  
- AGGAGTGAGA GCGGTGAAGT GGAGAAGTTG CCGAGGTTAA GCTGCTGGGG TAATCGGGGG CTGGCAGGAT   
  
  
- TACTAGGCCC AGACTCAGTC CCAGGCCTAG ACGGAGAACA CACGCCTAAG AGGCCTGAGG GAAATAAGGG   
  
  
- TGGCCACCTC TCTTCTGGAA GGTAGGGGCG GTGCTGGTGA CGACGGCGCC GCTAGCGCCA CCAAGGACAC   
  
  
- CTCCTCCTAC TAGGCTGCAC CTACCTATCG TAGTATTTTC TCGACTAAGT TTCGAGTTTG AGCTAGAGTT   
  
  
- AGGGTGTCAA CTAAGTCTTG CAATCTCTAT AGTAGATGGG TACATTAGGA TTAGACCCCA GGCGGTAACT   
  
  
- CAAGGCGGAG GCGAGCGAGC GGCGGCTGGG GGAGTAACGA GGCGGCGGTG AAGGCGGGAA GGTGGTGGTG   
  
  
- GTTGTGGTGG AGTTAGTTGT TTAGTGAGAA GGAGCTTAGT CGTTGTGATT GTTGTTGTTA GTACACTCGC   
  
  
- ACATGCGGTT GTTTCCTGGG CCTATGAAGT TGAACCCAGG CCCAGTCCCA GTCCCAGGCT AATTGTAGCT   
  
  
- GGTTCGATTG GAAAGGAAGG GAGGACTAAG GTGGCGGACA ACCCCACAGT CACAGTGTGG CGGTGGCAGA   
  
  
- AGGCGGCGGC CAAGGCCGAG GCCACCTTCG CCGTCATCAT CACCATTTAG TTTGGGATTG GGATTAGGGT   
  
  
- TGGGCTTGGG TTTGGGTTTG GGTTTGGGCT TGTTGTTTCG AGTCCTACAA GTTGAGGTTT GAGGTGTTGT   
  
  
- TGTCGTTGTC GTTGTCGTTG TTTAGGGGCA CCTAGTCCTC GTTCTGGGCC GCCGAGGTGG CCGCTGCCTT   
  
  
- CGTAGCGGTG GTAGAGGTTC TCGCCGCCGT GGTGGCGGCC GCCGTCACTT TCGTTCTCTT TCTCTCCTCT   
  
  
- ACGCCGTTTC CTTCGCGCTG CTTCTCCCAG AGGTGGAGGA TTGGGAGGAG GAGGTTACGC GTCTTCGTCA   
  
  
- TAGGCCGCTG TTTATGCTTC TTCGCTTGTT CTACGAAGAT CTTTAGAGCC TCACCCGGTG GGGTAAGCCG   
  
  
- TGGAGGCGGG TTGCGCAGCG GCGCATGAAG AGCCTTCGGT ACAGTCGGGC AGAGCATAGG AGGACGGAGC   
  
  
- CGTAGATGCG GCGGGAGGGG TGGCAGGGTG TGATGCAGTT CGAGGAGAGG CGGAAGGTTC AGAAGTTACC   
  
  
- GTAGTCGGGT AAGCAGTTTA AGAGAGTGAA GTGACGTTTG GTTCGTTAGG TTCTCCGGAA GGTCTCCCTT   
  
  
- CTGTCCCAGG TGTAGTAGCT GGAGCTATAG TACGTCCCCG AGGTCACCGG GCCCGACAAG GTGTAGGAGC   
  
  
- GCAGGGCCGG TCCACCCGGA GGGAAGCATT CCGAGTGGCC CGAGCCCTGG AGGTACCTCC GCGAGCTCCG   
  
  
- GTGGCCTTTT TCCGAGAGTC TGAAGCGGCT CTTCAACCCC AACGGGAAAC TCAAATATGG GCACCGCCTC   
  
  
- TTCTAACCTT TAAACCTGAA CCTTTCCAAC GTACAATCAT TTTCCCTTCG AGAACGACAC GTAACCAATG   
  
  
- TCGTGAGAAA CATACTACAA TGACCGAGAC TATGATTATG TGAACCGGAA GAAGTTTCCA ACCGCGGTTT   
  
  
- TCACCACTGC CACCACCTCG TTCTGGATTC GGCTTGTCCG AGAAAGGATC CTTCCAAACA TCTCCGCTAG   
  
  
- GTGATGATAA GTCGGGATAA ACTGAGGAAT CCTCGCTCAA TACCTCTCCT GTCACTCCTC TCCGTACACC   
  
  
- AACTCGTTGT CGAGGAAAGA TCCCTCTAAG CTTTGTAAGA CCGGCAACCA CCCGGGAGTT CCTGGCCCCT   
  
  
- CGGGTTCAAA CGCTCGACCT CCCTCTTCGA TGTTGTCAGG CCGAAATCCC CTTAGAGTAA CCGTCCGTTG   
  
  
- CGGCGACGGG TCCGGTGGAA CGAGGAGCCG TACAAGGGAA GACTACCCAT ATGAAATTAG CTCCTGTCAC   
  
  
- CGTGTGAATT CGAGCCCACC TTCCTGAACA CGGAGGACTG ACGAAGCCGG ACCTCCGGAA GGGTACGAGT   
  
  
- ATGATACTCG TGGCAGACAT GAGCCTCGGT TAT

+     ATC-motif

| Site Name | Organism | Position | Strand | Matrix score. | sequence | function |
| --- | --- | --- | --- | --- | --- | --- |
| ATC-motif | Spinacia oleracea | 203 | + | 8 | AGTAATCT | part of a conserved DNA module involved in light responsiveness |
| ATC-motif | Pisum sativum | 2617 | - | 9 | TGCTATCCA | part of a conserved DNA module involved in light responsiveness |
| ATC-motif | Pisum sativum | 790 | + | 9 | TGCTATCCA | part of a conserved DNA module involved in light responsiveness |

>HU01G00472.1   
+ -Up\_Stream \_Len000GTCTCT GTAACACAAT TCATTAAATC CATCCAAAGG GGATCATAAA TAACCAAACT   
  
  
+ TTGTTGGAAA CTGCTTTGAT GTGGGCTTTC TCTTTCAGCA TTTATGTTAT ACATATATAT GGTTATAAAG   
  
  
+ TTACAAAAGT ATGACCCGAC ACGACAATTC AATCCGAATC CGACTCGAAG TTACTGAAAG TAATCTGAAT   
  
  
+ ACGATTTGAC AGCTTAGTTT AAAAACCTAT TACTTCTCAA TTTTGAGTAA ATCTAAGCTA TTTTTAATGC   
  
  
+ TGAATAGATC GACCCGATTC GACCCGTTTT CCGACTCTAT ATGGTTATGG TGCCCCTAAA AAAAATTAAA   
  
  
+ AAAGAAAAAG AATAGAAAAA TATAAAATAA AACTGAGATT TCTGAACACT CTCTCCCCTA GTCTTGTGGG   
  
  
+ CATGGAAAGT GGTTGGAGAT GGAGATGTTT GAGTTTCATT TTCTGATATT ATTTTTTAGT CATTAGAAGT   
  
  
+ ACAAAGTCTA AACTCAACAA GAGCATGAGG GGAGAATGGT TGTTGTAGCC AATGGAAAAT AAAAAGAACA   
  
  
+ TTCACTCTTC TTGTGCTTTA TTTATGGTGA TAATTTGTTA CTTGTATTTT TAAAAATGAT TAAATATATT   
  
  
+ GATCCTTTGA TATTATGTTG AAATAATAAA AATATAAAAA TTAGAAAATA TTTTACCTCG TGAAATTTTT   
  
  
+ TTTCTGATTA ATCATTGAAA TTATGGTGAA AAATAAAGGA ACATCTCTTG TAAACACTCT ATCCGTTTAC   
  
  
+ GTTTGACCAC AAACTTGCTA TCCACAAGCA TCTCAACCTT TGCCTTTAAC CCAATTGTGG GTTGATGAAA   
  
  
+ TCTAACCATT ATAGGATTAG ATGGTAGTCG TTAATCCTGT GAAACAAGTT TTTGGTTATG ATGTATCCAA   
  
  
+ TTATGAGGGC CTGATAACTC TTGCCTCGGC ATTTGCGTGA TAATGACGTG GTACTAATAG CAATTATTTT   
  
  
+ TCGCAAATAT TCGAAATTTT TTAAGAATAA ACGCAAAATT ATTGATCTTG ACAAATGATT TTATTTTTTT   
  
  
+ GTTGGGAAAA GGATAAACAT CTAGTGTCAA TTGTTGATGA CCTGATAGCT CTCGATTTCT TATTTATGAG   
  
  
+ TTATGTTGAT ATGACACTAA TTGTAATTAT TTGTTGTAAA CTTAAACATT TTTAAAAATC AAAGCAAAAT   
  
  
+ TTGTTAATTA CAATAAATCA ATCTACTTTT ACTATGATCT TATTTGGTGT AATTATTGAT CTTGAATTTT   
  
  
+ TTTTTTTCTT TTTGAAAAAG ATAAGCATCG AATTTTTTCA AAAATAAAAA GGGTGAAGGA AGAAAAAATG   
  
  
+ GAAACAGTTA CCGAGTAAGG AAGGACAGAG CGCGAGAGGA CAAGACACAA AGAGAGGGGA GAGAGAGAGA   
  
  
+ GAAGGGGGCA ATGGCAGTGG GGAAGGTCGA GGAGCACCTT TCAACTTCAA GGCTCAACAC CCCCCCCCCC   
  
  
+ CCCCTCTTTC CTCATTTTGC GACGTTAGCA TTTGACATTT TGCTTTCAAC CGTCCCTCGG CATCATCAGC   
  
  
+ CCTGCCCTTG CTCAGCTTTT CCCATCTTTT TTTGGCTTTT TGTTTGACTC CCTCTATAAC TCTATTCACG   
  
  
+ TCGAGCCTTG TAAAAATTTT ACATATTTTA TTTTATTTTA CTTTTTTAGG TTTATCCCTC TCTATTCTTG   
  
  
+ ACCTACGGGC ATTTTTTATT TTCTTTGGGT TTGAGACTTG TGAACTTTTT GCACATTTTC TCACAAAAAT   
  
  
+ TTATCTTGTC TTTTTCACTT TCAACGTTCC TTCTCTCTCT CTCTCTCTCT CTCTCTCTCT CTCACTCACA   
  
  
+ TCCTAGACAG ACGTCCTTTT TAAAGAATTT ATTTAACAAG TTAGAAGAAA CCGTTGTTTA AGTAGAACCA   
  
  
+ CCCTGTTTGG ATGCCAAAGG AAATACCAGT CAAGAAAAAT CACGACAATC ACAACACATC CATTTCGTTT   
  
  
+ ACATCTCATC CTCATCCTCA TCTTCCTCAT CTCTACCCAT CTCCCTCTTT CTGTATGGCT GCTTCTTCTA   
  
  
+ CTTTGTTCCC TGACCCTAAC AATACTAGTT TAGTCCTCCC TTATTATTAT TATCCTCCTC CTTCCTCCTC   
  
  
+ TTACTATAAT GGTAACCCTT ACCTTCCTCT TCCTAACACC ACTGCTATTC CTACTCCTTC AACAACCTCT   
  
  
+ CCGGTATTTT CTTCCGCCAC CTACTCGGAG TTGAATACTG ATTGTCAAAC TCAACTGCCC ATGTCTAACC   
  
  
+ TCCCCGGAAA GATCGTTCGG AAGCGGAGTG CTGCTGAAAT GGAGCAGCGC TCCGCTCCGA TTGCGGGTGA   
  
  
+ TTACCCGCCA ACTCATCAGC GGGTAATCAC CCGCCACAAT GAGGCGGCCT CATCAGTGTC TTTCATTGAC   
  
  
+ TCCTCACTCT CGCCACTTCA CCTCTTCAAC GGCTCCAATT CGACGACCCC ATTAGCCCCC GACCGTCCTA   
  
  
+ ATGATCCGGG TCTGAGTCAG GGTCCGGATC TGCCTCTTGT GTGCGGATTC TCCGGACTCC CTTTATTCCC   
  
  
+ ACCGGTGGAG AGAAGACCTT CCATCCCCGC CACGACCACT GCTGCCGCGG CGATCGCGGT GGTTCCTGTG   
  
  
+ GAGGAGGATG ATCCGACGTG GATGGATAGC ATCATAAAAG AGCTGATTCA AAGCTCAAAC TCGATCTCAA   
  
  
+ TCCCACAGTT GATTCAGAAC GTTAGAGATA TCATCTACCC ATGTAATCCT AATCTGGGGT CCGCCATTGA   
  
  
+ GTTCCGCCTC CGCTCGCTCG CCGCCGACCC CCTCATTGCT CCGCCGCCAC TTCCGCCCTT CCACCACCAC   
  
  
+ CAACACCACC TCAATCAACA AATCACTCTT CCTCGAATCA GCAACACTAA CAACAACAAT CATGTGAGCG   
  
  
+ TGTACGCCAA CAAAGGACCC GGATACTTCA ACTTGGGTCC GGGTCAGGGT CAGGGTCCGA TTAACATCGA   
  
  
+ CCAAGCTAAC CTTTCCTTCC CTCCTGATTC CACCGCCTGT TGGGGTGTCA GTGTCACACC GCCACCGTCT   
  
  
+ TCCGCCGCCG GTTCCGGCTC CGGTGGAAGC GGCAGTAGTA GTGGTAAATC AAACCCTAAC CCTAATCCCA   
  
  
+ ACCCGAACCC AAACCCAAAC CCAAACCCGA ACAACAAAGC TCAGGATGTT CAACTCCAAA CTCCACAACA   
  
  
+ ACAGCAACAG CAACAGCAAC AAATCCCCGT GGATCAGGAG CAAGACCCGG CGGCTCCACC GGCGACGGAA   
  
  
+ GCATCGCCAC CATCTCCAAG AGCGGCGGCA CCACCGCCGG CGGCAGTGAA AGCAAGAGAA AGAGAGGAGA   
  
  
+ TGCGGCAAAG GAAGCGCGAC GAAGAGGGTC TCCACCTCCT AACCCTCCTC CTCCAATGCG CAGAAGCAGT   
  
  
+ ATCCGGCGAC AAATACGAAG AAGCGAACAA GATGCTTCTA GAAATCTCGG AGTGGGCCAC CCCATTCGGC   
  
  
+ ACCTCCGCCC AACGCGTCGC CGCGTACTTC TCGGAAGCCA TGTCAGCCCG TCTCGTATCC TCCTGCCTCG   
  
  
+ GCATCTACGC CGCCCTCCCC ACCGTCCCAC ACTACGTCAA GCTCCTCTCC GCCTTCCAAG TCTTCAATGG   
  
  
+ CATCAGCCCA TTCGTCAAAT TCTCTCACTT CACTGCAAAC CAAGCAATCC AAGAGGCCTT CCAGAGGGAA   
  
  
+ GACAGGGTCC ACATCATCGA CCTCGATATC ATGCAGGGGC TCCAGTGGCC CGGGCTGTTC CACATCCTCG   
  
  
+ CGTCCCGGCC AGGTGGGCCT CCCTTCGTAA GGCTCACCGG GCTCGGGACC TCCATGGAGG CGCTCGAGGC   
  
  
+ CACCGGAAAA AGGCTCTCAG ACTTCGCCGA GAAGTTGGGG TTGCCCTTTG AGTTTATACC CGTGGCGGAG   
  
  
+ AAGATTGGAA ATTTGGACTT GGAAAGGTTG CATGTTAGTA AAAGGGAAGC TCTTGCTGTG CATTGGTTAC   
  
  
+ AGCACTCTTT GTATGATGTT ACTGGCTCTG ATACTAATAC ACTTGGCCTT CTTCAAAGGT TGGCGCCAAA   
  
  
+ AGTGGTGACG GTGGTGGAGC AAGACCTAAG CCGAACAGGC TCTTTCCTAG GAAGGTTTGT AGAGGCGATC   
  
  
+ CACTACTATT CAGCCCTATT TGACTCCTTA GGAGCGAGTT ATGGAGAGGA CAGTGAGGAG AGGCATGTGG   
  
  
+ TTGAGCAACA GCTCCTTTCT AGGGAGATTC GAAACATTCT GGCCGTTGGT GGGCCCTCAA GGACCGGGGA   
  
  
+ GCCCAAGTTT GCGAGCTGGA GGGAGAAGCT ACAACAGTCC GGCTTTAGGG GAATCTCATT GGCAGGCAAC   
  
  
+ GCCGCTGCCC AGGCCACCTT GCTCCTCGGC ATGTTCCCTT CTGATGGGTA TACTTTAATC GAGGACAGTG   
  
  
+ GCACACTTAA GCTCGGGTGG AAGGACTTGT GCCTCCTGAC TGCTTCGGCC TGGAGGCCTT CCCATGCTCA   
  
  
+ TACTATGAGC ACCGTCTGTA CTCGGAGCCA ATA  

- -Up\_Stream \_Len000CAGAGA CATTGTGTTA AGTAATTTAG GTAGGTTTCC CCTAGTATTT ATTGGTTTGA   
  
  
- AACAACCTTT GACGAAACTA CACCCGAAAG AGAAAGTCGT AAATACAATA TGTATATATA CCAATATTTC   
  
  
- AATGTTTTCA TACTGGGCTG TGCTGTTAAG TTAGGCTTAG GCTGAGCTTC AATGACTTTC ATTAGACTTA   
  
  
- TGCTAAACTG TCGAATCAAA TTTTTGGATA ATGAAGAGTT AAAACTCATT TAGATTCGAT AAAAATTACG   
  
  
- ACTTATCTAG CTGGGCTAAG CTGGGCAAAA GGCTGAGATA TACCAATACC ACGGGGATTT TTTTTAATTT   
  
  
- TTTCTTTTTC TTATCTTTTT ATATTTTATT TTGACTCTAA AGACTTGTGA GAGAGGGGAT CAGAACACCC   
  
  
- GTACCTTTCA CCAACCTCTA CCTCTACAAA CTCAAAGTAA AAGACTATAA TAAAAAATCA GTAATCTTCA   
  
  
- TGTTTCAGAT TTGAGTTGTT CTCGTACTCC CCTCTTACCA ACAACATCGG TTACCTTTTA TTTTTCTTGT   
  
  
- AAGTGAGAAG AACACGAAAT AAATACCACT ATTAAACAAT GAACATAAAA ATTTTTACTA ATTTATATAA   
  
  
- CTAGGAAACT ATAATACAAC TTTATTATTT TTATATTTTT AATCTTTTAT AAAATGGAGC ACTTTAAAAA   
  
  
- AAAGACTAAT TAGTAACTTT AATACCACTT TTTATTTCCT TGTAGAGAAC ATTTGTGAGA TAGGCAAATG   
  
  
- CAAACTGGTG TTTGAACGAT AGGTGTTCGT AGAGTTGGAA ACGGAAATTG GGTTAACACC CAACTACTTT   
  
  
- AGATTGGTAA TATCCTAATC TACCATCAGC AATTAGGACA CTTTGTTCAA AAACCAATAC TACATAGGTT   
  
  
- AATACTCCCG GACTATTGAG AACGGAGCCG TAAACGCACT ATTACTGCAC CATGATTATC GTTAATAAAA   
  
  
- AGCGTTTATA AGCTTTAAAA AATTCTTATT TGCGTTTTAA TAACTAGAAC TGTTTACTAA AATAAAAAAA   
  
  
- CAACCCTTTT CCTATTTGTA GATCACAGTT AACAACTACT GGACTATCGA GAGCTAAAGA ATAAATACTC   
  
  
- AATACAACTA TACTGTGATT AACATTAATA AACAACATTT GAATTTGTAA AAATTTTTAG TTTCGTTTTA   
  
  
- AACAATTAAT GTTATTTAGT TAGATGAAAA TGATACTAGA ATAAACCACA TTAATAACTA GAACTTAAAA   
  
  
- AAAAAAAGAA AAACTTTTTC TATTCGTAGC TTAAAAAAGT TTTTATTTTT CCCACTTCCT TCTTTTTTAC   
  
  
- CTTTGTCAAT GGCTCATTCC TTCCTGTCTC GCGCTCTCCT GTTCTGTGTT TCTCTCCCCT CTCTCTCTCT   
  
  
- CTTCCCCCGT TACCGTCACC CCTTCCAGCT CCTCGTGGAA AGTTGAAGTT CCGAGTTGTG GGGGGGGGGG   
  
  
- GGGGAGAAAG GAGTAAAACG CTGCAATCGT AAACTGTAAA ACGAAAGTTG GCAGGGAGCC GTAGTAGTCG   
  
  
- GGACGGGAAC GAGTCGAAAA GGGTAGAAAA AAACCGAAAA ACAAACTGAG GGAGATATTG AGATAAGTGC   
  
  
- AGCTCGGAAC ATTTTTAAAA TGTATAAAAT AAAATAAAAT GAAAAAATCC AAATAGGGAG AGATAAGAAC   
  
  
- TGGATGCCCG TAAAAAATAA AAGAAACCCA AACTCTGAAC ACTTGAAAAA CGTGTAAAAG AGTGTTTTTA   
  
  
- AATAGAACAG AAAAAGTGAA AGTTGCAAGG AAGAGAGAGA GAGAGAGAGA GAGAGAGAGA GAGTGAGTGT   
  
  
- AGGATCTGTC TGCAGGAAAA ATTTCTTAAA TAAATTGTTC AATCTTCTTT GGCAACAAAT TCATCTTGGT   
  
  
- GGGACAAACC TACGGTTTCC TTTATGGTCA GTTCTTTTTA GTGCTGTTAG TGTTGTGTAG GTAAAGCAAA   
  
  
- TGTAGAGTAG GAGTAGGAGT AGAAGGAGTA GAGATGGGTA GAGGGAGAAA GACATACCGA CGAAGAAGAT   
  
  
- GAAACAAGGG ACTGGGATTG TTATGATCAA ATCAGGAGGG AATAATAATA ATAGGAGGAG GAAGGAGGAG   
  
  
- AATGATATTA CCATTGGGAA TGGAAGGAGA AGGATTGTGG TGACGATAAG GATGAGGAAG TTGTTGGAGA   
  
  
- GGCCATAAAA GAAGGCGGTG GATGAGCCTC AACTTATGAC TAACAGTTTG AGTTGACGGG TACAGATTGG   
  
  
- AGGGGCCTTT CTAGCAAGCC TTCGCCTCAC GACGACTTTA CCTCGTCGCG AGGCGAGGCT AACGCCCACT   
  
  
- AATGGGCGGT TGAGTAGTCG CCCATTAGTG GGCGGTGTTA CTCCGCCGGA GTAGTCACAG AAAGTAACTG   
  
  
- AGGAGTGAGA GCGGTGAAGT GGAGAAGTTG CCGAGGTTAA GCTGCTGGGG TAATCGGGGG CTGGCAGGAT   
  
  
- TACTAGGCCC AGACTCAGTC CCAGGCCTAG ACGGAGAACA CACGCCTAAG AGGCCTGAGG GAAATAAGGG   
  
  
- TGGCCACCTC TCTTCTGGAA GGTAGGGGCG GTGCTGGTGA CGACGGCGCC GCTAGCGCCA CCAAGGACAC   
  
  
- CTCCTCCTAC TAGGCTGCAC CTACCTATCG TAGTATTTTC TCGACTAAGT TTCGAGTTTG AGCTAGAGTT   
  
  
- AGGGTGTCAA CTAAGTCTTG CAATCTCTAT AGTAGATGGG TACATTAGGA TTAGACCCCA GGCGGTAACT   
  
  
- CAAGGCGGAG GCGAGCGAGC GGCGGCTGGG GGAGTAACGA GGCGGCGGTG AAGGCGGGAA GGTGGTGGTG   
  
  
- GTTGTGGTGG AGTTAGTTGT TTAGTGAGAA GGAGCTTAGT CGTTGTGATT GTTGTTGTTA GTACACTCGC   
  
  
- ACATGCGGTT GTTTCCTGGG CCTATGAAGT TGAACCCAGG CCCAGTCCCA GTCCCAGGCT AATTGTAGCT   
  
  
- GGTTCGATTG GAAAGGAAGG GAGGACTAAG GTGGCGGACA ACCCCACAGT CACAGTGTGG CGGTGGCAGA   
  
  
- AGGCGGCGGC CAAGGCCGAG GCCACCTTCG CCGTCATCAT CACCATTTAG TTTGGGATTG GGATTAGGGT   
  
  
- TGGGCTTGGG TTTGGGTTTG GGTTTGGGCT TGTTGTTTCG AGTCCTACAA GTTGAGGTTT GAGGTGTTGT   
  
  
- TGTCGTTGTC GTTGTCGTTG TTTAGGGGCA CCTAGTCCTC GTTCTGGGCC GCCGAGGTGG CCGCTGCCTT   
  
  
- CGTAGCGGTG GTAGAGGTTC TCGCCGCCGT GGTGGCGGCC GCCGTCACTT TCGTTCTCTT TCTCTCCTCT   
  
  
- ACGCCGTTTC CTTCGCGCTG CTTCTCCCAG AGGTGGAGGA TTGGGAGGAG GAGGTTACGC GTCTTCGTCA   
  
  
- TAGGCCGCTG TTTATGCTTC TTCGCTTGTT CTACGAAGAT CTTTAGAGCC TCACCCGGTG GGGTAAGCCG   
  
  
- TGGAGGCGGG TTGCGCAGCG GCGCATGAAG AGCCTTCGGT ACAGTCGGGC AGAGCATAGG AGGACGGAGC   
  
  
- CGTAGATGCG GCGGGAGGGG TGGCAGGGTG TGATGCAGTT CGAGGAGAGG CGGAAGGTTC AGAAGTTACC   
  
  
- GTAGTCGGGT AAGCAGTTTA AGAGAGTGAA GTGACGTTTG GTTCGTTAGG TTCTCCGGAA GGTCTCCCTT   
  
  
- CTGTCCCAGG TGTAGTAGCT GGAGCTATAG TACGTCCCCG AGGTCACCGG GCCCGACAAG GTGTAGGAGC   
  
  
- GCAGGGCCGG TCCACCCGGA GGGAAGCATT CCGAGTGGCC CGAGCCCTGG AGGTACCTCC GCGAGCTCCG   
  
  
- GTGGCCTTTT TCCGAGAGTC TGAAGCGGCT CTTCAACCCC AACGGGAAAC TCAAATATGG GCACCGCCTC   
  
  
- TTCTAACCTT TAAACCTGAA CCTTTCCAAC GTACAATCAT TTTCCCTTCG AGAACGACAC GTAACCAATG   
  
  
- TCGTGAGAAA CATACTACAA TGACCGAGAC TATGATTATG TGAACCGGAA GAAGTTTCCA ACCGCGGTTT   
  
  
- TCACCACTGC CACCACCTCG TTCTGGATTC GGCTTGTCCG AGAAAGGATC CTTCCAAACA TCTCCGCTAG   
  
  
- GTGATGATAA GTCGGGATAA ACTGAGGAAT CCTCGCTCAA TACCTCTCCT GTCACTCCTC TCCGTACACC   
  
  
- AACTCGTTGT CGAGGAAAGA TCCCTCTAAG CTTTGTAAGA CCGGCAACCA CCCGGGAGTT CCTGGCCCCT   
  
  
- CGGGTTCAAA CGCTCGACCT CCCTCTTCGA TGTTGTCAGG CCGAAATCCC CTTAGAGTAA CCGTCCGTTG   
  
  
- CGGCGACGGG TCCGGTGGAA CGAGGAGCCG TACAAGGGAA GACTACCCAT ATGAAATTAG CTCCTGTCAC   
  
  
- CGTGTGAATT CGAGCCCACC TTCCTGAACA CGGAGGACTG ACGAAGCCGG ACCTCCGGAA GGGTACGAGT   
  
  
- ATGATACTCG TGGCAGACAT GAGCCTCGGT TAT

+     ATCT-motif

| Site Name | Organism | Position | Strand | Matrix score. | sequence | function |
| --- | --- | --- | --- | --- | --- | --- |
| ATCT-motif | Pisum sativum | 858 | - | 9 | AATCTAATCC | part of a conserved DNA module involved in light responsiveness |

>HU01G00472.1   
+ -Up\_Stream \_Len000GTCTCT GTAACACAAT TCATTAAATC CATCCAAAGG GGATCATAAA TAACCAAACT   
  
  
+ TTGTTGGAAA CTGCTTTGAT GTGGGCTTTC TCTTTCAGCA TTTATGTTAT ACATATATAT GGTTATAAAG   
  
  
+ TTACAAAAGT ATGACCCGAC ACGACAATTC AATCCGAATC CGACTCGAAG TTACTGAAAG TAATCTGAAT   
  
  
+ ACGATTTGAC AGCTTAGTTT AAAAACCTAT TACTTCTCAA TTTTGAGTAA ATCTAAGCTA TTTTTAATGC   
  
  
+ TGAATAGATC GACCCGATTC GACCCGTTTT CCGACTCTAT ATGGTTATGG TGCCCCTAAA AAAAATTAAA   
  
  
+ AAAGAAAAAG AATAGAAAAA TATAAAATAA AACTGAGATT TCTGAACACT CTCTCCCCTA GTCTTGTGGG   
  
  
+ CATGGAAAGT GGTTGGAGAT GGAGATGTTT GAGTTTCATT TTCTGATATT ATTTTTTAGT CATTAGAAGT   
  
  
+ ACAAAGTCTA AACTCAACAA GAGCATGAGG GGAGAATGGT TGTTGTAGCC AATGGAAAAT AAAAAGAACA   
  
  
+ TTCACTCTTC TTGTGCTTTA TTTATGGTGA TAATTTGTTA CTTGTATTTT TAAAAATGAT TAAATATATT   
  
  
+ GATCCTTTGA TATTATGTTG AAATAATAAA AATATAAAAA TTAGAAAATA TTTTACCTCG TGAAATTTTT   
  
  
+ TTTCTGATTA ATCATTGAAA TTATGGTGAA AAATAAAGGA ACATCTCTTG TAAACACTCT ATCCGTTTAC   
  
  
+ GTTTGACCAC AAACTTGCTA TCCACAAGCA TCTCAACCTT TGCCTTTAAC CCAATTGTGG GTTGATGAAA   
  
  
+ TCTAACCATT ATAGGATTAG ATGGTAGTCG TTAATCCTGT GAAACAAGTT TTTGGTTATG ATGTATCCAA   
  
  
+ TTATGAGGGC CTGATAACTC TTGCCTCGGC ATTTGCGTGA TAATGACGTG GTACTAATAG CAATTATTTT   
  
  
+ TCGCAAATAT TCGAAATTTT TTAAGAATAA ACGCAAAATT ATTGATCTTG ACAAATGATT TTATTTTTTT   
  
  
+ GTTGGGAAAA GGATAAACAT CTAGTGTCAA TTGTTGATGA CCTGATAGCT CTCGATTTCT TATTTATGAG   
  
  
+ TTATGTTGAT ATGACACTAA TTGTAATTAT TTGTTGTAAA CTTAAACATT TTTAAAAATC AAAGCAAAAT   
  
  
+ TTGTTAATTA CAATAAATCA ATCTACTTTT ACTATGATCT TATTTGGTGT AATTATTGAT CTTGAATTTT   
  
  
+ TTTTTTTCTT TTTGAAAAAG ATAAGCATCG AATTTTTTCA AAAATAAAAA GGGTGAAGGA AGAAAAAATG   
  
  
+ GAAACAGTTA CCGAGTAAGG AAGGACAGAG CGCGAGAGGA CAAGACACAA AGAGAGGGGA GAGAGAGAGA   
  
  
+ GAAGGGGGCA ATGGCAGTGG GGAAGGTCGA GGAGCACCTT TCAACTTCAA GGCTCAACAC CCCCCCCCCC   
  
  
+ CCCCTCTTTC CTCATTTTGC GACGTTAGCA TTTGACATTT TGCTTTCAAC CGTCCCTCGG CATCATCAGC   
  
  
+ CCTGCCCTTG CTCAGCTTTT CCCATCTTTT TTTGGCTTTT TGTTTGACTC CCTCTATAAC TCTATTCACG   
  
  
+ TCGAGCCTTG TAAAAATTTT ACATATTTTA TTTTATTTTA CTTTTTTAGG TTTATCCCTC TCTATTCTTG   
  
  
+ ACCTACGGGC ATTTTTTATT TTCTTTGGGT TTGAGACTTG TGAACTTTTT GCACATTTTC TCACAAAAAT   
  
  
+ TTATCTTGTC TTTTTCACTT TCAACGTTCC TTCTCTCTCT CTCTCTCTCT CTCTCTCTCT CTCACTCACA   
  
  
+ TCCTAGACAG ACGTCCTTTT TAAAGAATTT ATTTAACAAG TTAGAAGAAA CCGTTGTTTA AGTAGAACCA   
  
  
+ CCCTGTTTGG ATGCCAAAGG AAATACCAGT CAAGAAAAAT CACGACAATC ACAACACATC CATTTCGTTT   
  
  
+ ACATCTCATC CTCATCCTCA TCTTCCTCAT CTCTACCCAT CTCCCTCTTT CTGTATGGCT GCTTCTTCTA   
  
  
+ CTTTGTTCCC TGACCCTAAC AATACTAGTT TAGTCCTCCC TTATTATTAT TATCCTCCTC CTTCCTCCTC   
  
  
+ TTACTATAAT GGTAACCCTT ACCTTCCTCT TCCTAACACC ACTGCTATTC CTACTCCTTC AACAACCTCT   
  
  
+ CCGGTATTTT CTTCCGCCAC CTACTCGGAG TTGAATACTG ATTGTCAAAC TCAACTGCCC ATGTCTAACC   
  
  
+ TCCCCGGAAA GATCGTTCGG AAGCGGAGTG CTGCTGAAAT GGAGCAGCGC TCCGCTCCGA TTGCGGGTGA   
  
  
+ TTACCCGCCA ACTCATCAGC GGGTAATCAC CCGCCACAAT GAGGCGGCCT CATCAGTGTC TTTCATTGAC   
  
  
+ TCCTCACTCT CGCCACTTCA CCTCTTCAAC GGCTCCAATT CGACGACCCC ATTAGCCCCC GACCGTCCTA   
  
  
+ ATGATCCGGG TCTGAGTCAG GGTCCGGATC TGCCTCTTGT GTGCGGATTC TCCGGACTCC CTTTATTCCC   
  
  
+ ACCGGTGGAG AGAAGACCTT CCATCCCCGC CACGACCACT GCTGCCGCGG CGATCGCGGT GGTTCCTGTG   
  
  
+ GAGGAGGATG ATCCGACGTG GATGGATAGC ATCATAAAAG AGCTGATTCA AAGCTCAAAC TCGATCTCAA   
  
  
+ TCCCACAGTT GATTCAGAAC GTTAGAGATA TCATCTACCC ATGTAATCCT AATCTGGGGT CCGCCATTGA   
  
  
+ GTTCCGCCTC CGCTCGCTCG CCGCCGACCC CCTCATTGCT CCGCCGCCAC TTCCGCCCTT CCACCACCAC   
  
  
+ CAACACCACC TCAATCAACA AATCACTCTT CCTCGAATCA GCAACACTAA CAACAACAAT CATGTGAGCG   
  
  
+ TGTACGCCAA CAAAGGACCC GGATACTTCA ACTTGGGTCC GGGTCAGGGT CAGGGTCCGA TTAACATCGA   
  
  
+ CCAAGCTAAC CTTTCCTTCC CTCCTGATTC CACCGCCTGT TGGGGTGTCA GTGTCACACC GCCACCGTCT   
  
  
+ TCCGCCGCCG GTTCCGGCTC CGGTGGAAGC GGCAGTAGTA GTGGTAAATC AAACCCTAAC CCTAATCCCA   
  
  
+ ACCCGAACCC AAACCCAAAC CCAAACCCGA ACAACAAAGC TCAGGATGTT CAACTCCAAA CTCCACAACA   
  
  
+ ACAGCAACAG CAACAGCAAC AAATCCCCGT GGATCAGGAG CAAGACCCGG CGGCTCCACC GGCGACGGAA   
  
  
+ GCATCGCCAC CATCTCCAAG AGCGGCGGCA CCACCGCCGG CGGCAGTGAA AGCAAGAGAA AGAGAGGAGA   
  
  
+ TGCGGCAAAG GAAGCGCGAC GAAGAGGGTC TCCACCTCCT AACCCTCCTC CTCCAATGCG CAGAAGCAGT   
  
  
+ ATCCGGCGAC AAATACGAAG AAGCGAACAA GATGCTTCTA GAAATCTCGG AGTGGGCCAC CCCATTCGGC   
  
  
+ ACCTCCGCCC AACGCGTCGC CGCGTACTTC TCGGAAGCCA TGTCAGCCCG TCTCGTATCC TCCTGCCTCG   
  
  
+ GCATCTACGC CGCCCTCCCC ACCGTCCCAC ACTACGTCAA GCTCCTCTCC GCCTTCCAAG TCTTCAATGG   
  
  
+ CATCAGCCCA TTCGTCAAAT TCTCTCACTT CACTGCAAAC CAAGCAATCC AAGAGGCCTT CCAGAGGGAA   
  
  
+ GACAGGGTCC ACATCATCGA CCTCGATATC ATGCAGGGGC TCCAGTGGCC CGGGCTGTTC CACATCCTCG   
  
  
+ CGTCCCGGCC AGGTGGGCCT CCCTTCGTAA GGCTCACCGG GCTCGGGACC TCCATGGAGG CGCTCGAGGC   
  
  
+ CACCGGAAAA AGGCTCTCAG ACTTCGCCGA GAAGTTGGGG TTGCCCTTTG AGTTTATACC CGTGGCGGAG   
  
  
+ AAGATTGGAA ATTTGGACTT GGAAAGGTTG CATGTTAGTA AAAGGGAAGC TCTTGCTGTG CATTGGTTAC   
  
  
+ AGCACTCTTT GTATGATGTT ACTGGCTCTG ATACTAATAC ACTTGGCCTT CTTCAAAGGT TGGCGCCAAA   
  
  
+ AGTGGTGACG GTGGTGGAGC AAGACCTAAG CCGAACAGGC TCTTTCCTAG GAAGGTTTGT AGAGGCGATC   
  
  
+ CACTACTATT CAGCCCTATT TGACTCCTTA GGAGCGAGTT ATGGAGAGGA CAGTGAGGAG AGGCATGTGG   
  
  
+ TTGAGCAACA GCTCCTTTCT AGGGAGATTC GAAACATTCT GGCCGTTGGT GGGCCCTCAA GGACCGGGGA   
  
  
+ GCCCAAGTTT GCGAGCTGGA GGGAGAAGCT ACAACAGTCC GGCTTTAGGG GAATCTCATT GGCAGGCAAC   
  
  
+ GCCGCTGCCC AGGCCACCTT GCTCCTCGGC ATGTTCCCTT CTGATGGGTA TACTTTAATC GAGGACAGTG   
  
  
+ GCACACTTAA GCTCGGGTGG AAGGACTTGT GCCTCCTGAC TGCTTCGGCC TGGAGGCCTT CCCATGCTCA   
  
  
+ TACTATGAGC ACCGTCTGTA CTCGGAGCCA ATA  

- -Up\_Stream \_Len000CAGAGA CATTGTGTTA AGTAATTTAG GTAGGTTTCC CCTAGTATTT ATTGGTTTGA   
  
  
- AACAACCTTT GACGAAACTA CACCCGAAAG AGAAAGTCGT AAATACAATA TGTATATATA CCAATATTTC   
  
  
- AATGTTTTCA TACTGGGCTG TGCTGTTAAG TTAGGCTTAG GCTGAGCTTC AATGACTTTC ATTAGACTTA   
  
  
- TGCTAAACTG TCGAATCAAA TTTTTGGATA ATGAAGAGTT AAAACTCATT TAGATTCGAT AAAAATTACG   
  
  
- ACTTATCTAG CTGGGCTAAG CTGGGCAAAA GGCTGAGATA TACCAATACC ACGGGGATTT TTTTTAATTT   
  
  
- TTTCTTTTTC TTATCTTTTT ATATTTTATT TTGACTCTAA AGACTTGTGA GAGAGGGGAT CAGAACACCC   
  
  
- GTACCTTTCA CCAACCTCTA CCTCTACAAA CTCAAAGTAA AAGACTATAA TAAAAAATCA GTAATCTTCA   
  
  
- TGTTTCAGAT TTGAGTTGTT CTCGTACTCC CCTCTTACCA ACAACATCGG TTACCTTTTA TTTTTCTTGT   
  
  
- AAGTGAGAAG AACACGAAAT AAATACCACT ATTAAACAAT GAACATAAAA ATTTTTACTA ATTTATATAA   
  
  
- CTAGGAAACT ATAATACAAC TTTATTATTT TTATATTTTT AATCTTTTAT AAAATGGAGC ACTTTAAAAA   
  
  
- AAAGACTAAT TAGTAACTTT AATACCACTT TTTATTTCCT TGTAGAGAAC ATTTGTGAGA TAGGCAAATG   
  
  
- CAAACTGGTG TTTGAACGAT AGGTGTTCGT AGAGTTGGAA ACGGAAATTG GGTTAACACC CAACTACTTT   
  
  
- AGATTGGTAA TATCCTAATC TACCATCAGC AATTAGGACA CTTTGTTCAA AAACCAATAC TACATAGGTT   
  
  
- AATACTCCCG GACTATTGAG AACGGAGCCG TAAACGCACT ATTACTGCAC CATGATTATC GTTAATAAAA   
  
  
- AGCGTTTATA AGCTTTAAAA AATTCTTATT TGCGTTTTAA TAACTAGAAC TGTTTACTAA AATAAAAAAA   
  
  
- CAACCCTTTT CCTATTTGTA GATCACAGTT AACAACTACT GGACTATCGA GAGCTAAAGA ATAAATACTC   
  
  
- AATACAACTA TACTGTGATT AACATTAATA AACAACATTT GAATTTGTAA AAATTTTTAG TTTCGTTTTA   
  
  
- AACAATTAAT GTTATTTAGT TAGATGAAAA TGATACTAGA ATAAACCACA TTAATAACTA GAACTTAAAA   
  
  
- AAAAAAAGAA AAACTTTTTC TATTCGTAGC TTAAAAAAGT TTTTATTTTT CCCACTTCCT TCTTTTTTAC   
  
  
- CTTTGTCAAT GGCTCATTCC TTCCTGTCTC GCGCTCTCCT GTTCTGTGTT TCTCTCCCCT CTCTCTCTCT   
  
  
- CTTCCCCCGT TACCGTCACC CCTTCCAGCT CCTCGTGGAA AGTTGAAGTT CCGAGTTGTG GGGGGGGGGG   
  
  
- GGGGAGAAAG GAGTAAAACG CTGCAATCGT AAACTGTAAA ACGAAAGTTG GCAGGGAGCC GTAGTAGTCG   
  
  
- GGACGGGAAC GAGTCGAAAA GGGTAGAAAA AAACCGAAAA ACAAACTGAG GGAGATATTG AGATAAGTGC   
  
  
- AGCTCGGAAC ATTTTTAAAA TGTATAAAAT AAAATAAAAT GAAAAAATCC AAATAGGGAG AGATAAGAAC   
  
  
- TGGATGCCCG TAAAAAATAA AAGAAACCCA AACTCTGAAC ACTTGAAAAA CGTGTAAAAG AGTGTTTTTA   
  
  
- AATAGAACAG AAAAAGTGAA AGTTGCAAGG AAGAGAGAGA GAGAGAGAGA GAGAGAGAGA GAGTGAGTGT   
  
  
- AGGATCTGTC TGCAGGAAAA ATTTCTTAAA TAAATTGTTC AATCTTCTTT GGCAACAAAT TCATCTTGGT   
  
  
- GGGACAAACC TACGGTTTCC TTTATGGTCA GTTCTTTTTA GTGCTGTTAG TGTTGTGTAG GTAAAGCAAA   
  
  
- TGTAGAGTAG GAGTAGGAGT AGAAGGAGTA GAGATGGGTA GAGGGAGAAA GACATACCGA CGAAGAAGAT   
  
  
- GAAACAAGGG ACTGGGATTG TTATGATCAA ATCAGGAGGG AATAATAATA ATAGGAGGAG GAAGGAGGAG   
  
  
- AATGATATTA CCATTGGGAA TGGAAGGAGA AGGATTGTGG TGACGATAAG GATGAGGAAG TTGTTGGAGA   
  
  
- GGCCATAAAA GAAGGCGGTG GATGAGCCTC AACTTATGAC TAACAGTTTG AGTTGACGGG TACAGATTGG   
  
  
- AGGGGCCTTT CTAGCAAGCC TTCGCCTCAC GACGACTTTA CCTCGTCGCG AGGCGAGGCT AACGCCCACT   
  
  
- AATGGGCGGT TGAGTAGTCG CCCATTAGTG GGCGGTGTTA CTCCGCCGGA GTAGTCACAG AAAGTAACTG   
  
  
- AGGAGTGAGA GCGGTGAAGT GGAGAAGTTG CCGAGGTTAA GCTGCTGGGG TAATCGGGGG CTGGCAGGAT   
  
  
- TACTAGGCCC AGACTCAGTC CCAGGCCTAG ACGGAGAACA CACGCCTAAG AGGCCTGAGG GAAATAAGGG   
  
  
- TGGCCACCTC TCTTCTGGAA GGTAGGGGCG GTGCTGGTGA CGACGGCGCC GCTAGCGCCA CCAAGGACAC   
  
  
- CTCCTCCTAC TAGGCTGCAC CTACCTATCG TAGTATTTTC TCGACTAAGT TTCGAGTTTG AGCTAGAGTT   
  
  
- AGGGTGTCAA CTAAGTCTTG CAATCTCTAT AGTAGATGGG TACATTAGGA TTAGACCCCA GGCGGTAACT   
  
  
- CAAGGCGGAG GCGAGCGAGC GGCGGCTGGG GGAGTAACGA GGCGGCGGTG AAGGCGGGAA GGTGGTGGTG   
  
  
- GTTGTGGTGG AGTTAGTTGT TTAGTGAGAA GGAGCTTAGT CGTTGTGATT GTTGTTGTTA GTACACTCGC   
  
  
- ACATGCGGTT GTTTCCTGGG CCTATGAAGT TGAACCCAGG CCCAGTCCCA GTCCCAGGCT AATTGTAGCT   
  
  
- GGTTCGATTG GAAAGGAAGG GAGGACTAAG GTGGCGGACA ACCCCACAGT CACAGTGTGG CGGTGGCAGA   
  
  
- AGGCGGCGGC CAAGGCCGAG GCCACCTTCG CCGTCATCAT CACCATTTAG TTTGGGATTG GGATTAGGGT   
  
  
- TGGGCTTGGG TTTGGGTTTG GGTTTGGGCT TGTTGTTTCG AGTCCTACAA GTTGAGGTTT GAGGTGTTGT   
  
  
- TGTCGTTGTC GTTGTCGTTG TTTAGGGGCA CCTAGTCCTC GTTCTGGGCC GCCGAGGTGG CCGCTGCCTT   
  
  
- CGTAGCGGTG GTAGAGGTTC TCGCCGCCGT GGTGGCGGCC GCCGTCACTT TCGTTCTCTT TCTCTCCTCT   
  
  
- ACGCCGTTTC CTTCGCGCTG CTTCTCCCAG AGGTGGAGGA TTGGGAGGAG GAGGTTACGC GTCTTCGTCA   
  
  
- TAGGCCGCTG TTTATGCTTC TTCGCTTGTT CTACGAAGAT CTTTAGAGCC TCACCCGGTG GGGTAAGCCG   
  
  
- TGGAGGCGGG TTGCGCAGCG GCGCATGAAG AGCCTTCGGT ACAGTCGGGC AGAGCATAGG AGGACGGAGC   
  
  
- CGTAGATGCG GCGGGAGGGG TGGCAGGGTG TGATGCAGTT CGAGGAGAGG CGGAAGGTTC AGAAGTTACC   
  
  
- GTAGTCGGGT AAGCAGTTTA AGAGAGTGAA GTGACGTTTG GTTCGTTAGG TTCTCCGGAA GGTCTCCCTT   
  
  
- CTGTCCCAGG TGTAGTAGCT GGAGCTATAG TACGTCCCCG AGGTCACCGG GCCCGACAAG GTGTAGGAGC   
  
  
- GCAGGGCCGG TCCACCCGGA GGGAAGCATT CCGAGTGGCC CGAGCCCTGG AGGTACCTCC GCGAGCTCCG   
  
  
- GTGGCCTTTT TCCGAGAGTC TGAAGCGGCT CTTCAACCCC AACGGGAAAC TCAAATATGG GCACCGCCTC   
  
  
- TTCTAACCTT TAAACCTGAA CCTTTCCAAC GTACAATCAT TTTCCCTTCG AGAACGACAC GTAACCAATG   
  
  
- TCGTGAGAAA CATACTACAA TGACCGAGAC TATGATTATG TGAACCGGAA GAAGTTTCCA ACCGCGGTTT   
  
  
- TCACCACTGC CACCACCTCG TTCTGGATTC GGCTTGTCCG AGAAAGGATC CTTCCAAACA TCTCCGCTAG   
  
  
- GTGATGATAA GTCGGGATAA ACTGAGGAAT CCTCGCTCAA TACCTCTCCT GTCACTCCTC TCCGTACACC   
  
  
- AACTCGTTGT CGAGGAAAGA TCCCTCTAAG CTTTGTAAGA CCGGCAACCA CCCGGGAGTT CCTGGCCCCT   
  
  
- CGGGTTCAAA CGCTCGACCT CCCTCTTCGA TGTTGTCAGG CCGAAATCCC CTTAGAGTAA CCGTCCGTTG   
  
  
- CGGCGACGGG TCCGGTGGAA CGAGGAGCCG TACAAGGGAA GACTACCCAT ATGAAATTAG CTCCTGTCAC   
  
  
- CGTGTGAATT CGAGCCCACC TTCCTGAACA CGGAGGACTG ACGAAGCCGG ACCTCCGGAA GGGTACGAGT   
  
  
- ATGATACTCG TGGCAGACAT GAGCCTCGGT TAT

+     AT~TATA-box

| Site Name | Organism | Position | Strand | Matrix score. | sequence | function |
| --- | --- | --- | --- | --- | --- | --- |
| AT~TATA-box | Arabidopsis thaliana | 128 | + | 6 | TATATA |  |

>HU01G00472.1   
+ -Up\_Stream \_Len000GTCTCT GTAACACAAT TCATTAAATC CATCCAAAGG GGATCATAAA TAACCAAACT   
  
  
+ TTGTTGGAAA CTGCTTTGAT GTGGGCTTTC TCTTTCAGCA TTTATGTTAT ACATATATAT GGTTATAAAG   
  
  
+ TTACAAAAGT ATGACCCGAC ACGACAATTC AATCCGAATC CGACTCGAAG TTACTGAAAG TAATCTGAAT   
  
  
+ ACGATTTGAC AGCTTAGTTT AAAAACCTAT TACTTCTCAA TTTTGAGTAA ATCTAAGCTA TTTTTAATGC   
  
  
+ TGAATAGATC GACCCGATTC GACCCGTTTT CCGACTCTAT ATGGTTATGG TGCCCCTAAA AAAAATTAAA   
  
  
+ AAAGAAAAAG AATAGAAAAA TATAAAATAA AACTGAGATT TCTGAACACT CTCTCCCCTA GTCTTGTGGG   
  
  
+ CATGGAAAGT GGTTGGAGAT GGAGATGTTT GAGTTTCATT TTCTGATATT ATTTTTTAGT CATTAGAAGT   
  
  
+ ACAAAGTCTA AACTCAACAA GAGCATGAGG GGAGAATGGT TGTTGTAGCC AATGGAAAAT AAAAAGAACA   
  
  
+ TTCACTCTTC TTGTGCTTTA TTTATGGTGA TAATTTGTTA CTTGTATTTT TAAAAATGAT TAAATATATT   
  
  
+ GATCCTTTGA TATTATGTTG AAATAATAAA AATATAAAAA TTAGAAAATA TTTTACCTCG TGAAATTTTT   
  
  
+ TTTCTGATTA ATCATTGAAA TTATGGTGAA AAATAAAGGA ACATCTCTTG TAAACACTCT ATCCGTTTAC   
  
  
+ GTTTGACCAC AAACTTGCTA TCCACAAGCA TCTCAACCTT TGCCTTTAAC CCAATTGTGG GTTGATGAAA   
  
  
+ TCTAACCATT ATAGGATTAG ATGGTAGTCG TTAATCCTGT GAAACAAGTT TTTGGTTATG ATGTATCCAA   
  
  
+ TTATGAGGGC CTGATAACTC TTGCCTCGGC ATTTGCGTGA TAATGACGTG GTACTAATAG CAATTATTTT   
  
  
+ TCGCAAATAT TCGAAATTTT TTAAGAATAA ACGCAAAATT ATTGATCTTG ACAAATGATT TTATTTTTTT   
  
  
+ GTTGGGAAAA GGATAAACAT CTAGTGTCAA TTGTTGATGA CCTGATAGCT CTCGATTTCT TATTTATGAG   
  
  
+ TTATGTTGAT ATGACACTAA TTGTAATTAT TTGTTGTAAA CTTAAACATT TTTAAAAATC AAAGCAAAAT   
  
  
+ TTGTTAATTA CAATAAATCA ATCTACTTTT ACTATGATCT TATTTGGTGT AATTATTGAT CTTGAATTTT   
  
  
+ TTTTTTTCTT TTTGAAAAAG ATAAGCATCG AATTTTTTCA AAAATAAAAA GGGTGAAGGA AGAAAAAATG   
  
  
+ GAAACAGTTA CCGAGTAAGG AAGGACAGAG CGCGAGAGGA CAAGACACAA AGAGAGGGGA GAGAGAGAGA   
  
  
+ GAAGGGGGCA ATGGCAGTGG GGAAGGTCGA GGAGCACCTT TCAACTTCAA GGCTCAACAC CCCCCCCCCC   
  
  
+ CCCCTCTTTC CTCATTTTGC GACGTTAGCA TTTGACATTT TGCTTTCAAC CGTCCCTCGG CATCATCAGC   
  
  
+ CCTGCCCTTG CTCAGCTTTT CCCATCTTTT TTTGGCTTTT TGTTTGACTC CCTCTATAAC TCTATTCACG   
  
  
+ TCGAGCCTTG TAAAAATTTT ACATATTTTA TTTTATTTTA CTTTTTTAGG TTTATCCCTC TCTATTCTTG   
  
  
+ ACCTACGGGC ATTTTTTATT TTCTTTGGGT TTGAGACTTG TGAACTTTTT GCACATTTTC TCACAAAAAT   
  
  
+ TTATCTTGTC TTTTTCACTT TCAACGTTCC TTCTCTCTCT CTCTCTCTCT CTCTCTCTCT CTCACTCACA   
  
  
+ TCCTAGACAG ACGTCCTTTT TAAAGAATTT ATTTAACAAG TTAGAAGAAA CCGTTGTTTA AGTAGAACCA   
  
  
+ CCCTGTTTGG ATGCCAAAGG AAATACCAGT CAAGAAAAAT CACGACAATC ACAACACATC CATTTCGTTT   
  
  
+ ACATCTCATC CTCATCCTCA TCTTCCTCAT CTCTACCCAT CTCCCTCTTT CTGTATGGCT GCTTCTTCTA   
  
  
+ CTTTGTTCCC TGACCCTAAC AATACTAGTT TAGTCCTCCC TTATTATTAT TATCCTCCTC CTTCCTCCTC   
  
  
+ TTACTATAAT GGTAACCCTT ACCTTCCTCT TCCTAACACC ACTGCTATTC CTACTCCTTC AACAACCTCT   
  
  
+ CCGGTATTTT CTTCCGCCAC CTACTCGGAG TTGAATACTG ATTGTCAAAC TCAACTGCCC ATGTCTAACC   
  
  
+ TCCCCGGAAA GATCGTTCGG AAGCGGAGTG CTGCTGAAAT GGAGCAGCGC TCCGCTCCGA TTGCGGGTGA   
  
  
+ TTACCCGCCA ACTCATCAGC GGGTAATCAC CCGCCACAAT GAGGCGGCCT CATCAGTGTC TTTCATTGAC   
  
  
+ TCCTCACTCT CGCCACTTCA CCTCTTCAAC GGCTCCAATT CGACGACCCC ATTAGCCCCC GACCGTCCTA   
  
  
+ ATGATCCGGG TCTGAGTCAG GGTCCGGATC TGCCTCTTGT GTGCGGATTC TCCGGACTCC CTTTATTCCC   
  
  
+ ACCGGTGGAG AGAAGACCTT CCATCCCCGC CACGACCACT GCTGCCGCGG CGATCGCGGT GGTTCCTGTG   
  
  
+ GAGGAGGATG ATCCGACGTG GATGGATAGC ATCATAAAAG AGCTGATTCA AAGCTCAAAC TCGATCTCAA   
  
  
+ TCCCACAGTT GATTCAGAAC GTTAGAGATA TCATCTACCC ATGTAATCCT AATCTGGGGT CCGCCATTGA   
  
  
+ GTTCCGCCTC CGCTCGCTCG CCGCCGACCC CCTCATTGCT CCGCCGCCAC TTCCGCCCTT CCACCACCAC   
  
  
+ CAACACCACC TCAATCAACA AATCACTCTT CCTCGAATCA GCAACACTAA CAACAACAAT CATGTGAGCG   
  
  
+ TGTACGCCAA CAAAGGACCC GGATACTTCA ACTTGGGTCC GGGTCAGGGT CAGGGTCCGA TTAACATCGA   
  
  
+ CCAAGCTAAC CTTTCCTTCC CTCCTGATTC CACCGCCTGT TGGGGTGTCA GTGTCACACC GCCACCGTCT   
  
  
+ TCCGCCGCCG GTTCCGGCTC CGGTGGAAGC GGCAGTAGTA GTGGTAAATC AAACCCTAAC CCTAATCCCA   
  
  
+ ACCCGAACCC AAACCCAAAC CCAAACCCGA ACAACAAAGC TCAGGATGTT CAACTCCAAA CTCCACAACA   
  
  
+ ACAGCAACAG CAACAGCAAC AAATCCCCGT GGATCAGGAG CAAGACCCGG CGGCTCCACC GGCGACGGAA   
  
  
+ GCATCGCCAC CATCTCCAAG AGCGGCGGCA CCACCGCCGG CGGCAGTGAA AGCAAGAGAA AGAGAGGAGA   
  
  
+ TGCGGCAAAG GAAGCGCGAC GAAGAGGGTC TCCACCTCCT AACCCTCCTC CTCCAATGCG CAGAAGCAGT   
  
  
+ ATCCGGCGAC AAATACGAAG AAGCGAACAA GATGCTTCTA GAAATCTCGG AGTGGGCCAC CCCATTCGGC   
  
  
+ ACCTCCGCCC AACGCGTCGC CGCGTACTTC TCGGAAGCCA TGTCAGCCCG TCTCGTATCC TCCTGCCTCG   
  
  
+ GCATCTACGC CGCCCTCCCC ACCGTCCCAC ACTACGTCAA GCTCCTCTCC GCCTTCCAAG TCTTCAATGG   
  
  
+ CATCAGCCCA TTCGTCAAAT TCTCTCACTT CACTGCAAAC CAAGCAATCC AAGAGGCCTT CCAGAGGGAA   
  
  
+ GACAGGGTCC ACATCATCGA CCTCGATATC ATGCAGGGGC TCCAGTGGCC CGGGCTGTTC CACATCCTCG   
  
  
+ CGTCCCGGCC AGGTGGGCCT CCCTTCGTAA GGCTCACCGG GCTCGGGACC TCCATGGAGG CGCTCGAGGC   
  
  
+ CACCGGAAAA AGGCTCTCAG ACTTCGCCGA GAAGTTGGGG TTGCCCTTTG AGTTTATACC CGTGGCGGAG   
  
  
+ AAGATTGGAA ATTTGGACTT GGAAAGGTTG CATGTTAGTA AAAGGGAAGC TCTTGCTGTG CATTGGTTAC   
  
  
+ AGCACTCTTT GTATGATGTT ACTGGCTCTG ATACTAATAC ACTTGGCCTT CTTCAAAGGT TGGCGCCAAA   
  
  
+ AGTGGTGACG GTGGTGGAGC AAGACCTAAG CCGAACAGGC TCTTTCCTAG GAAGGTTTGT AGAGGCGATC   
  
  
+ CACTACTATT CAGCCCTATT TGACTCCTTA GGAGCGAGTT ATGGAGAGGA CAGTGAGGAG AGGCATGTGG   
  
  
+ TTGAGCAACA GCTCCTTTCT AGGGAGATTC GAAACATTCT GGCCGTTGGT GGGCCCTCAA GGACCGGGGA   
  
  
+ GCCCAAGTTT GCGAGCTGGA GGGAGAAGCT ACAACAGTCC GGCTTTAGGG GAATCTCATT GGCAGGCAAC   
  
  
+ GCCGCTGCCC AGGCCACCTT GCTCCTCGGC ATGTTCCCTT CTGATGGGTA TACTTTAATC GAGGACAGTG   
  
  
+ GCACACTTAA GCTCGGGTGG AAGGACTTGT GCCTCCTGAC TGCTTCGGCC TGGAGGCCTT CCCATGCTCA   
  
  
+ TACTATGAGC ACCGTCTGTA CTCGGAGCCA ATA  

- -Up\_Stream \_Len000CAGAGA CATTGTGTTA AGTAATTTAG GTAGGTTTCC CCTAGTATTT ATTGGTTTGA   
  
  
- AACAACCTTT GACGAAACTA CACCCGAAAG AGAAAGTCGT AAATACAATA TGTATATATA CCAATATTTC   
  
  
- AATGTTTTCA TACTGGGCTG TGCTGTTAAG TTAGGCTTAG GCTGAGCTTC AATGACTTTC ATTAGACTTA   
  
  
- TGCTAAACTG TCGAATCAAA TTTTTGGATA ATGAAGAGTT AAAACTCATT TAGATTCGAT AAAAATTACG   
  
  
- ACTTATCTAG CTGGGCTAAG CTGGGCAAAA GGCTGAGATA TACCAATACC ACGGGGATTT TTTTTAATTT   
  
  
- TTTCTTTTTC TTATCTTTTT ATATTTTATT TTGACTCTAA AGACTTGTGA GAGAGGGGAT CAGAACACCC   
  
  
- GTACCTTTCA CCAACCTCTA CCTCTACAAA CTCAAAGTAA AAGACTATAA TAAAAAATCA GTAATCTTCA   
  
  
- TGTTTCAGAT TTGAGTTGTT CTCGTACTCC CCTCTTACCA ACAACATCGG TTACCTTTTA TTTTTCTTGT   
  
  
- AAGTGAGAAG AACACGAAAT AAATACCACT ATTAAACAAT GAACATAAAA ATTTTTACTA ATTTATATAA   
  
  
- CTAGGAAACT ATAATACAAC TTTATTATTT TTATATTTTT AATCTTTTAT AAAATGGAGC ACTTTAAAAA   
  
  
- AAAGACTAAT TAGTAACTTT AATACCACTT TTTATTTCCT TGTAGAGAAC ATTTGTGAGA TAGGCAAATG   
  
  
- CAAACTGGTG TTTGAACGAT AGGTGTTCGT AGAGTTGGAA ACGGAAATTG GGTTAACACC CAACTACTTT   
  
  
- AGATTGGTAA TATCCTAATC TACCATCAGC AATTAGGACA CTTTGTTCAA AAACCAATAC TACATAGGTT   
  
  
- AATACTCCCG GACTATTGAG AACGGAGCCG TAAACGCACT ATTACTGCAC CATGATTATC GTTAATAAAA   
  
  
- AGCGTTTATA AGCTTTAAAA AATTCTTATT TGCGTTTTAA TAACTAGAAC TGTTTACTAA AATAAAAAAA   
  
  
- CAACCCTTTT CCTATTTGTA GATCACAGTT AACAACTACT GGACTATCGA GAGCTAAAGA ATAAATACTC   
  
  
- AATACAACTA TACTGTGATT AACATTAATA AACAACATTT GAATTTGTAA AAATTTTTAG TTTCGTTTTA   
  
  
- AACAATTAAT GTTATTTAGT TAGATGAAAA TGATACTAGA ATAAACCACA TTAATAACTA GAACTTAAAA   
  
  
- AAAAAAAGAA AAACTTTTTC TATTCGTAGC TTAAAAAAGT TTTTATTTTT CCCACTTCCT TCTTTTTTAC   
  
  
- CTTTGTCAAT GGCTCATTCC TTCCTGTCTC GCGCTCTCCT GTTCTGTGTT TCTCTCCCCT CTCTCTCTCT   
  
  
- CTTCCCCCGT TACCGTCACC CCTTCCAGCT CCTCGTGGAA AGTTGAAGTT CCGAGTTGTG GGGGGGGGGG   
  
  
- GGGGAGAAAG GAGTAAAACG CTGCAATCGT AAACTGTAAA ACGAAAGTTG GCAGGGAGCC GTAGTAGTCG   
  
  
- GGACGGGAAC GAGTCGAAAA GGGTAGAAAA AAACCGAAAA ACAAACTGAG GGAGATATTG AGATAAGTGC   
  
  
- AGCTCGGAAC ATTTTTAAAA TGTATAAAAT AAAATAAAAT GAAAAAATCC AAATAGGGAG AGATAAGAAC   
  
  
- TGGATGCCCG TAAAAAATAA AAGAAACCCA AACTCTGAAC ACTTGAAAAA CGTGTAAAAG AGTGTTTTTA   
  
  
- AATAGAACAG AAAAAGTGAA AGTTGCAAGG AAGAGAGAGA GAGAGAGAGA GAGAGAGAGA GAGTGAGTGT   
  
  
- AGGATCTGTC TGCAGGAAAA ATTTCTTAAA TAAATTGTTC AATCTTCTTT GGCAACAAAT TCATCTTGGT   
  
  
- GGGACAAACC TACGGTTTCC TTTATGGTCA GTTCTTTTTA GTGCTGTTAG TGTTGTGTAG GTAAAGCAAA   
  
  
- TGTAGAGTAG GAGTAGGAGT AGAAGGAGTA GAGATGGGTA GAGGGAGAAA GACATACCGA CGAAGAAGAT   
  
  
- GAAACAAGGG ACTGGGATTG TTATGATCAA ATCAGGAGGG AATAATAATA ATAGGAGGAG GAAGGAGGAG   
  
  
- AATGATATTA CCATTGGGAA TGGAAGGAGA AGGATTGTGG TGACGATAAG GATGAGGAAG TTGTTGGAGA   
  
  
- GGCCATAAAA GAAGGCGGTG GATGAGCCTC AACTTATGAC TAACAGTTTG AGTTGACGGG TACAGATTGG   
  
  
- AGGGGCCTTT CTAGCAAGCC TTCGCCTCAC GACGACTTTA CCTCGTCGCG AGGCGAGGCT AACGCCCACT   
  
  
- AATGGGCGGT TGAGTAGTCG CCCATTAGTG GGCGGTGTTA CTCCGCCGGA GTAGTCACAG AAAGTAACTG   
  
  
- AGGAGTGAGA GCGGTGAAGT GGAGAAGTTG CCGAGGTTAA GCTGCTGGGG TAATCGGGGG CTGGCAGGAT   
  
  
- TACTAGGCCC AGACTCAGTC CCAGGCCTAG ACGGAGAACA CACGCCTAAG AGGCCTGAGG GAAATAAGGG   
  
  
- TGGCCACCTC TCTTCTGGAA GGTAGGGGCG GTGCTGGTGA CGACGGCGCC GCTAGCGCCA CCAAGGACAC   
  
  
- CTCCTCCTAC TAGGCTGCAC CTACCTATCG TAGTATTTTC TCGACTAAGT TTCGAGTTTG AGCTAGAGTT   
  
  
- AGGGTGTCAA CTAAGTCTTG CAATCTCTAT AGTAGATGGG TACATTAGGA TTAGACCCCA GGCGGTAACT   
  
  
- CAAGGCGGAG GCGAGCGAGC GGCGGCTGGG GGAGTAACGA GGCGGCGGTG AAGGCGGGAA GGTGGTGGTG   
  
  
- GTTGTGGTGG AGTTAGTTGT TTAGTGAGAA GGAGCTTAGT CGTTGTGATT GTTGTTGTTA GTACACTCGC   
  
  
- ACATGCGGTT GTTTCCTGGG CCTATGAAGT TGAACCCAGG CCCAGTCCCA GTCCCAGGCT AATTGTAGCT   
  
  
- GGTTCGATTG GAAAGGAAGG GAGGACTAAG GTGGCGGACA ACCCCACAGT CACAGTGTGG CGGTGGCAGA   
  
  
- AGGCGGCGGC CAAGGCCGAG GCCACCTTCG CCGTCATCAT CACCATTTAG TTTGGGATTG GGATTAGGGT   
  
  
- TGGGCTTGGG TTTGGGTTTG GGTTTGGGCT TGTTGTTTCG AGTCCTACAA GTTGAGGTTT GAGGTGTTGT   
  
  
- TGTCGTTGTC GTTGTCGTTG TTTAGGGGCA CCTAGTCCTC GTTCTGGGCC GCCGAGGTGG CCGCTGCCTT   
  
  
- CGTAGCGGTG GTAGAGGTTC TCGCCGCCGT GGTGGCGGCC GCCGTCACTT TCGTTCTCTT TCTCTCCTCT   
  
  
- ACGCCGTTTC CTTCGCGCTG CTTCTCCCAG AGGTGGAGGA TTGGGAGGAG GAGGTTACGC GTCTTCGTCA   
  
  
- TAGGCCGCTG TTTATGCTTC TTCGCTTGTT CTACGAAGAT CTTTAGAGCC TCACCCGGTG GGGTAAGCCG   
  
  
- TGGAGGCGGG TTGCGCAGCG GCGCATGAAG AGCCTTCGGT ACAGTCGGGC AGAGCATAGG AGGACGGAGC   
  
  
- CGTAGATGCG GCGGGAGGGG TGGCAGGGTG TGATGCAGTT CGAGGAGAGG CGGAAGGTTC AGAAGTTACC   
  
  
- GTAGTCGGGT AAGCAGTTTA AGAGAGTGAA GTGACGTTTG GTTCGTTAGG TTCTCCGGAA GGTCTCCCTT   
  
  
- CTGTCCCAGG TGTAGTAGCT GGAGCTATAG TACGTCCCCG AGGTCACCGG GCCCGACAAG GTGTAGGAGC   
  
  
- GCAGGGCCGG TCCACCCGGA GGGAAGCATT CCGAGTGGCC CGAGCCCTGG AGGTACCTCC GCGAGCTCCG   
  
  
- GTGGCCTTTT TCCGAGAGTC TGAAGCGGCT CTTCAACCCC AACGGGAAAC TCAAATATGG GCACCGCCTC   
  
  
- TTCTAACCTT TAAACCTGAA CCTTTCCAAC GTACAATCAT TTTCCCTTCG AGAACGACAC GTAACCAATG   
  
  
- TCGTGAGAAA CATACTACAA TGACCGAGAC TATGATTATG TGAACCGGAA GAAGTTTCCA ACCGCGGTTT   
  
  
- TCACCACTGC CACCACCTCG TTCTGGATTC GGCTTGTCCG AGAAAGGATC CTTCCAAACA TCTCCGCTAG   
  
  
- GTGATGATAA GTCGGGATAA ACTGAGGAAT CCTCGCTCAA TACCTCTCCT GTCACTCCTC TCCGTACACC   
  
  
- AACTCGTTGT CGAGGAAAGA TCCCTCTAAG CTTTGTAAGA CCGGCAACCA CCCGGGAGTT CCTGGCCCCT   
  
  
- CGGGTTCAAA CGCTCGACCT CCCTCTTCGA TGTTGTCAGG CCGAAATCCC CTTAGAGTAA CCGTCCGTTG   
  
  
- CGGCGACGGG TCCGGTGGAA CGAGGAGCCG TACAAGGGAA GACTACCCAT ATGAAATTAG CTCCTGTCAC   
  
  
- CGTGTGAATT CGAGCCCACC TTCCTGAACA CGGAGGACTG ACGAAGCCGG ACCTCCGGAA GGGTACGAGT   
  
  
- ATGATACTCG TGGCAGACAT GAGCCTCGGT TAT

+     Box 4

| Site Name | Organism | Position | Strand | Matrix score. | sequence | function |
| --- | --- | --- | --- | --- | --- | --- |
| Box 4 | Petroselinum crispum | 711 | + | 6 | ATTAAT | part of a conserved DNA module involved in light responsiveness |

>HU01G00472.1   
+ -Up\_Stream \_Len000GTCTCT GTAACACAAT TCATTAAATC CATCCAAAGG GGATCATAAA TAACCAAACT   
  
  
+ TTGTTGGAAA CTGCTTTGAT GTGGGCTTTC TCTTTCAGCA TTTATGTTAT ACATATATAT GGTTATAAAG   
  
  
+ TTACAAAAGT ATGACCCGAC ACGACAATTC AATCCGAATC CGACTCGAAG TTACTGAAAG TAATCTGAAT   
  
  
+ ACGATTTGAC AGCTTAGTTT AAAAACCTAT TACTTCTCAA TTTTGAGTAA ATCTAAGCTA TTTTTAATGC   
  
  
+ TGAATAGATC GACCCGATTC GACCCGTTTT CCGACTCTAT ATGGTTATGG TGCCCCTAAA AAAAATTAAA   
  
  
+ AAAGAAAAAG AATAGAAAAA TATAAAATAA AACTGAGATT TCTGAACACT CTCTCCCCTA GTCTTGTGGG   
  
  
+ CATGGAAAGT GGTTGGAGAT GGAGATGTTT GAGTTTCATT TTCTGATATT ATTTTTTAGT CATTAGAAGT   
  
  
+ ACAAAGTCTA AACTCAACAA GAGCATGAGG GGAGAATGGT TGTTGTAGCC AATGGAAAAT AAAAAGAACA   
  
  
+ TTCACTCTTC TTGTGCTTTA TTTATGGTGA TAATTTGTTA CTTGTATTTT TAAAAATGAT TAAATATATT   
  
  
+ GATCCTTTGA TATTATGTTG AAATAATAAA AATATAAAAA TTAGAAAATA TTTTACCTCG TGAAATTTTT   
  
  
+ TTTCTGATTA ATCATTGAAA TTATGGTGAA AAATAAAGGA ACATCTCTTG TAAACACTCT ATCCGTTTAC   
  
  
+ GTTTGACCAC AAACTTGCTA TCCACAAGCA TCTCAACCTT TGCCTTTAAC CCAATTGTGG GTTGATGAAA   
  
  
+ TCTAACCATT ATAGGATTAG ATGGTAGTCG TTAATCCTGT GAAACAAGTT TTTGGTTATG ATGTATCCAA   
  
  
+ TTATGAGGGC CTGATAACTC TTGCCTCGGC ATTTGCGTGA TAATGACGTG GTACTAATAG CAATTATTTT   
  
  
+ TCGCAAATAT TCGAAATTTT TTAAGAATAA ACGCAAAATT ATTGATCTTG ACAAATGATT TTATTTTTTT   
  
  
+ GTTGGGAAAA GGATAAACAT CTAGTGTCAA TTGTTGATGA CCTGATAGCT CTCGATTTCT TATTTATGAG   
  
  
+ TTATGTTGAT ATGACACTAA TTGTAATTAT TTGTTGTAAA CTTAAACATT TTTAAAAATC AAAGCAAAAT   
  
  
+ TTGTTAATTA CAATAAATCA ATCTACTTTT ACTATGATCT TATTTGGTGT AATTATTGAT CTTGAATTTT   
  
  
+ TTTTTTTCTT TTTGAAAAAG ATAAGCATCG AATTTTTTCA AAAATAAAAA GGGTGAAGGA AGAAAAAATG   
  
  
+ GAAACAGTTA CCGAGTAAGG AAGGACAGAG CGCGAGAGGA CAAGACACAA AGAGAGGGGA GAGAGAGAGA   
  
  
+ GAAGGGGGCA ATGGCAGTGG GGAAGGTCGA GGAGCACCTT TCAACTTCAA GGCTCAACAC CCCCCCCCCC   
  
  
+ CCCCTCTTTC CTCATTTTGC GACGTTAGCA TTTGACATTT TGCTTTCAAC CGTCCCTCGG CATCATCAGC   
  
  
+ CCTGCCCTTG CTCAGCTTTT CCCATCTTTT TTTGGCTTTT TGTTTGACTC CCTCTATAAC TCTATTCACG   
  
  
+ TCGAGCCTTG TAAAAATTTT ACATATTTTA TTTTATTTTA CTTTTTTAGG TTTATCCCTC TCTATTCTTG   
  
  
+ ACCTACGGGC ATTTTTTATT TTCTTTGGGT TTGAGACTTG TGAACTTTTT GCACATTTTC TCACAAAAAT   
  
  
+ TTATCTTGTC TTTTTCACTT TCAACGTTCC TTCTCTCTCT CTCTCTCTCT CTCTCTCTCT CTCACTCACA   
  
  
+ TCCTAGACAG ACGTCCTTTT TAAAGAATTT ATTTAACAAG TTAGAAGAAA CCGTTGTTTA AGTAGAACCA   
  
  
+ CCCTGTTTGG ATGCCAAAGG AAATACCAGT CAAGAAAAAT CACGACAATC ACAACACATC CATTTCGTTT   
  
  
+ ACATCTCATC CTCATCCTCA TCTTCCTCAT CTCTACCCAT CTCCCTCTTT CTGTATGGCT GCTTCTTCTA   
  
  
+ CTTTGTTCCC TGACCCTAAC AATACTAGTT TAGTCCTCCC TTATTATTAT TATCCTCCTC CTTCCTCCTC   
  
  
+ TTACTATAAT GGTAACCCTT ACCTTCCTCT TCCTAACACC ACTGCTATTC CTACTCCTTC AACAACCTCT   
  
  
+ CCGGTATTTT CTTCCGCCAC CTACTCGGAG TTGAATACTG ATTGTCAAAC TCAACTGCCC ATGTCTAACC   
  
  
+ TCCCCGGAAA GATCGTTCGG AAGCGGAGTG CTGCTGAAAT GGAGCAGCGC TCCGCTCCGA TTGCGGGTGA   
  
  
+ TTACCCGCCA ACTCATCAGC GGGTAATCAC CCGCCACAAT GAGGCGGCCT CATCAGTGTC TTTCATTGAC   
  
  
+ TCCTCACTCT CGCCACTTCA CCTCTTCAAC GGCTCCAATT CGACGACCCC ATTAGCCCCC GACCGTCCTA   
  
  
+ ATGATCCGGG TCTGAGTCAG GGTCCGGATC TGCCTCTTGT GTGCGGATTC TCCGGACTCC CTTTATTCCC   
  
  
+ ACCGGTGGAG AGAAGACCTT CCATCCCCGC CACGACCACT GCTGCCGCGG CGATCGCGGT GGTTCCTGTG   
  
  
+ GAGGAGGATG ATCCGACGTG GATGGATAGC ATCATAAAAG AGCTGATTCA AAGCTCAAAC TCGATCTCAA   
  
  
+ TCCCACAGTT GATTCAGAAC GTTAGAGATA TCATCTACCC ATGTAATCCT AATCTGGGGT CCGCCATTGA   
  
  
+ GTTCCGCCTC CGCTCGCTCG CCGCCGACCC CCTCATTGCT CCGCCGCCAC TTCCGCCCTT CCACCACCAC   
  
  
+ CAACACCACC TCAATCAACA AATCACTCTT CCTCGAATCA GCAACACTAA CAACAACAAT CATGTGAGCG   
  
  
+ TGTACGCCAA CAAAGGACCC GGATACTTCA ACTTGGGTCC GGGTCAGGGT CAGGGTCCGA TTAACATCGA   
  
  
+ CCAAGCTAAC CTTTCCTTCC CTCCTGATTC CACCGCCTGT TGGGGTGTCA GTGTCACACC GCCACCGTCT   
  
  
+ TCCGCCGCCG GTTCCGGCTC CGGTGGAAGC GGCAGTAGTA GTGGTAAATC AAACCCTAAC CCTAATCCCA   
  
  
+ ACCCGAACCC AAACCCAAAC CCAAACCCGA ACAACAAAGC TCAGGATGTT CAACTCCAAA CTCCACAACA   
  
  
+ ACAGCAACAG CAACAGCAAC AAATCCCCGT GGATCAGGAG CAAGACCCGG CGGCTCCACC GGCGACGGAA   
  
  
+ GCATCGCCAC CATCTCCAAG AGCGGCGGCA CCACCGCCGG CGGCAGTGAA AGCAAGAGAA AGAGAGGAGA   
  
  
+ TGCGGCAAAG GAAGCGCGAC GAAGAGGGTC TCCACCTCCT AACCCTCCTC CTCCAATGCG CAGAAGCAGT   
  
  
+ ATCCGGCGAC AAATACGAAG AAGCGAACAA GATGCTTCTA GAAATCTCGG AGTGGGCCAC CCCATTCGGC   
  
  
+ ACCTCCGCCC AACGCGTCGC CGCGTACTTC TCGGAAGCCA TGTCAGCCCG TCTCGTATCC TCCTGCCTCG   
  
  
+ GCATCTACGC CGCCCTCCCC ACCGTCCCAC ACTACGTCAA GCTCCTCTCC GCCTTCCAAG TCTTCAATGG   
  
  
+ CATCAGCCCA TTCGTCAAAT TCTCTCACTT CACTGCAAAC CAAGCAATCC AAGAGGCCTT CCAGAGGGAA   
  
  
+ GACAGGGTCC ACATCATCGA CCTCGATATC ATGCAGGGGC TCCAGTGGCC CGGGCTGTTC CACATCCTCG   
  
  
+ CGTCCCGGCC AGGTGGGCCT CCCTTCGTAA GGCTCACCGG GCTCGGGACC TCCATGGAGG CGCTCGAGGC   
  
  
+ CACCGGAAAA AGGCTCTCAG ACTTCGCCGA GAAGTTGGGG TTGCCCTTTG AGTTTATACC CGTGGCGGAG   
  
  
+ AAGATTGGAA ATTTGGACTT GGAAAGGTTG CATGTTAGTA AAAGGGAAGC TCTTGCTGTG CATTGGTTAC   
  
  
+ AGCACTCTTT GTATGATGTT ACTGGCTCTG ATACTAATAC ACTTGGCCTT CTTCAAAGGT TGGCGCCAAA   
  
  
+ AGTGGTGACG GTGGTGGAGC AAGACCTAAG CCGAACAGGC TCTTTCCTAG GAAGGTTTGT AGAGGCGATC   
  
  
+ CACTACTATT CAGCCCTATT TGACTCCTTA GGAGCGAGTT ATGGAGAGGA CAGTGAGGAG AGGCATGTGG   
  
  
+ TTGAGCAACA GCTCCTTTCT AGGGAGATTC GAAACATTCT GGCCGTTGGT GGGCCCTCAA GGACCGGGGA   
  
  
+ GCCCAAGTTT GCGAGCTGGA GGGAGAAGCT ACAACAGTCC GGCTTTAGGG GAATCTCATT GGCAGGCAAC   
  
  
+ GCCGCTGCCC AGGCCACCTT GCTCCTCGGC ATGTTCCCTT CTGATGGGTA TACTTTAATC GAGGACAGTG   
  
  
+ GCACACTTAA GCTCGGGTGG AAGGACTTGT GCCTCCTGAC TGCTTCGGCC TGGAGGCCTT CCCATGCTCA   
  
  
+ TACTATGAGC ACCGTCTGTA CTCGGAGCCA ATA  

- -Up\_Stream \_Len000CAGAGA CATTGTGTTA AGTAATTTAG GTAGGTTTCC CCTAGTATTT ATTGGTTTGA   
  
  
- AACAACCTTT GACGAAACTA CACCCGAAAG AGAAAGTCGT AAATACAATA TGTATATATA CCAATATTTC   
  
  
- AATGTTTTCA TACTGGGCTG TGCTGTTAAG TTAGGCTTAG GCTGAGCTTC AATGACTTTC ATTAGACTTA   
  
  
- TGCTAAACTG TCGAATCAAA TTTTTGGATA ATGAAGAGTT AAAACTCATT TAGATTCGAT AAAAATTACG   
  
  
- ACTTATCTAG CTGGGCTAAG CTGGGCAAAA GGCTGAGATA TACCAATACC ACGGGGATTT TTTTTAATTT   
  
  
- TTTCTTTTTC TTATCTTTTT ATATTTTATT TTGACTCTAA AGACTTGTGA GAGAGGGGAT CAGAACACCC   
  
  
- GTACCTTTCA CCAACCTCTA CCTCTACAAA CTCAAAGTAA AAGACTATAA TAAAAAATCA GTAATCTTCA   
  
  
- TGTTTCAGAT TTGAGTTGTT CTCGTACTCC CCTCTTACCA ACAACATCGG TTACCTTTTA TTTTTCTTGT   
  
  
- AAGTGAGAAG AACACGAAAT AAATACCACT ATTAAACAAT GAACATAAAA ATTTTTACTA ATTTATATAA   
  
  
- CTAGGAAACT ATAATACAAC TTTATTATTT TTATATTTTT AATCTTTTAT AAAATGGAGC ACTTTAAAAA   
  
  
- AAAGACTAAT TAGTAACTTT AATACCACTT TTTATTTCCT TGTAGAGAAC ATTTGTGAGA TAGGCAAATG   
  
  
- CAAACTGGTG TTTGAACGAT AGGTGTTCGT AGAGTTGGAA ACGGAAATTG GGTTAACACC CAACTACTTT   
  
  
- AGATTGGTAA TATCCTAATC TACCATCAGC AATTAGGACA CTTTGTTCAA AAACCAATAC TACATAGGTT   
  
  
- AATACTCCCG GACTATTGAG AACGGAGCCG TAAACGCACT ATTACTGCAC CATGATTATC GTTAATAAAA   
  
  
- AGCGTTTATA AGCTTTAAAA AATTCTTATT TGCGTTTTAA TAACTAGAAC TGTTTACTAA AATAAAAAAA   
  
  
- CAACCCTTTT CCTATTTGTA GATCACAGTT AACAACTACT GGACTATCGA GAGCTAAAGA ATAAATACTC   
  
  
- AATACAACTA TACTGTGATT AACATTAATA AACAACATTT GAATTTGTAA AAATTTTTAG TTTCGTTTTA   
  
  
- AACAATTAAT GTTATTTAGT TAGATGAAAA TGATACTAGA ATAAACCACA TTAATAACTA GAACTTAAAA   
  
  
- AAAAAAAGAA AAACTTTTTC TATTCGTAGC TTAAAAAAGT TTTTATTTTT CCCACTTCCT TCTTTTTTAC   
  
  
- CTTTGTCAAT GGCTCATTCC TTCCTGTCTC GCGCTCTCCT GTTCTGTGTT TCTCTCCCCT CTCTCTCTCT   
  
  
- CTTCCCCCGT TACCGTCACC CCTTCCAGCT CCTCGTGGAA AGTTGAAGTT CCGAGTTGTG GGGGGGGGGG   
  
  
- GGGGAGAAAG GAGTAAAACG CTGCAATCGT AAACTGTAAA ACGAAAGTTG GCAGGGAGCC GTAGTAGTCG   
  
  
- GGACGGGAAC GAGTCGAAAA GGGTAGAAAA AAACCGAAAA ACAAACTGAG GGAGATATTG AGATAAGTGC   
  
  
- AGCTCGGAAC ATTTTTAAAA TGTATAAAAT AAAATAAAAT GAAAAAATCC AAATAGGGAG AGATAAGAAC   
  
  
- TGGATGCCCG TAAAAAATAA AAGAAACCCA AACTCTGAAC ACTTGAAAAA CGTGTAAAAG AGTGTTTTTA   
  
  
- AATAGAACAG AAAAAGTGAA AGTTGCAAGG AAGAGAGAGA GAGAGAGAGA GAGAGAGAGA GAGTGAGTGT   
  
  
- AGGATCTGTC TGCAGGAAAA ATTTCTTAAA TAAATTGTTC AATCTTCTTT GGCAACAAAT TCATCTTGGT   
  
  
- GGGACAAACC TACGGTTTCC TTTATGGTCA GTTCTTTTTA GTGCTGTTAG TGTTGTGTAG GTAAAGCAAA   
  
  
- TGTAGAGTAG GAGTAGGAGT AGAAGGAGTA GAGATGGGTA GAGGGAGAAA GACATACCGA CGAAGAAGAT   
  
  
- GAAACAAGGG ACTGGGATTG TTATGATCAA ATCAGGAGGG AATAATAATA ATAGGAGGAG GAAGGAGGAG   
  
  
- AATGATATTA CCATTGGGAA TGGAAGGAGA AGGATTGTGG TGACGATAAG GATGAGGAAG TTGTTGGAGA   
  
  
- GGCCATAAAA GAAGGCGGTG GATGAGCCTC AACTTATGAC TAACAGTTTG AGTTGACGGG TACAGATTGG   
  
  
- AGGGGCCTTT CTAGCAAGCC TTCGCCTCAC GACGACTTTA CCTCGTCGCG AGGCGAGGCT AACGCCCACT   
  
  
- AATGGGCGGT TGAGTAGTCG CCCATTAGTG GGCGGTGTTA CTCCGCCGGA GTAGTCACAG AAAGTAACTG   
  
  
- AGGAGTGAGA GCGGTGAAGT GGAGAAGTTG CCGAGGTTAA GCTGCTGGGG TAATCGGGGG CTGGCAGGAT   
  
  
- TACTAGGCCC AGACTCAGTC CCAGGCCTAG ACGGAGAACA CACGCCTAAG AGGCCTGAGG GAAATAAGGG   
  
  
- TGGCCACCTC TCTTCTGGAA GGTAGGGGCG GTGCTGGTGA CGACGGCGCC GCTAGCGCCA CCAAGGACAC   
  
  
- CTCCTCCTAC TAGGCTGCAC CTACCTATCG TAGTATTTTC TCGACTAAGT TTCGAGTTTG AGCTAGAGTT   
  
  
- AGGGTGTCAA CTAAGTCTTG CAATCTCTAT AGTAGATGGG TACATTAGGA TTAGACCCCA GGCGGTAACT   
  
  
- CAAGGCGGAG GCGAGCGAGC GGCGGCTGGG GGAGTAACGA GGCGGCGGTG AAGGCGGGAA GGTGGTGGTG   
  
  
- GTTGTGGTGG AGTTAGTTGT TTAGTGAGAA GGAGCTTAGT CGTTGTGATT GTTGTTGTTA GTACACTCGC   
  
  
- ACATGCGGTT GTTTCCTGGG CCTATGAAGT TGAACCCAGG CCCAGTCCCA GTCCCAGGCT AATTGTAGCT   
  
  
- GGTTCGATTG GAAAGGAAGG GAGGACTAAG GTGGCGGACA ACCCCACAGT CACAGTGTGG CGGTGGCAGA   
  
  
- AGGCGGCGGC CAAGGCCGAG GCCACCTTCG CCGTCATCAT CACCATTTAG TTTGGGATTG GGATTAGGGT   
  
  
- TGGGCTTGGG TTTGGGTTTG GGTTTGGGCT TGTTGTTTCG AGTCCTACAA GTTGAGGTTT GAGGTGTTGT   
  
  
- TGTCGTTGTC GTTGTCGTTG TTTAGGGGCA CCTAGTCCTC GTTCTGGGCC GCCGAGGTGG CCGCTGCCTT   
  
  
- CGTAGCGGTG GTAGAGGTTC TCGCCGCCGT GGTGGCGGCC GCCGTCACTT TCGTTCTCTT TCTCTCCTCT   
  
  
- ACGCCGTTTC CTTCGCGCTG CTTCTCCCAG AGGTGGAGGA TTGGGAGGAG GAGGTTACGC GTCTTCGTCA   
  
  
- TAGGCCGCTG TTTATGCTTC TTCGCTTGTT CTACGAAGAT CTTTAGAGCC TCACCCGGTG GGGTAAGCCG   
  
  
- TGGAGGCGGG TTGCGCAGCG GCGCATGAAG AGCCTTCGGT ACAGTCGGGC AGAGCATAGG AGGACGGAGC   
  
  
- CGTAGATGCG GCGGGAGGGG TGGCAGGGTG TGATGCAGTT CGAGGAGAGG CGGAAGGTTC AGAAGTTACC   
  
  
- GTAGTCGGGT AAGCAGTTTA AGAGAGTGAA GTGACGTTTG GTTCGTTAGG TTCTCCGGAA GGTCTCCCTT   
  
  
- CTGTCCCAGG TGTAGTAGCT GGAGCTATAG TACGTCCCCG AGGTCACCGG GCCCGACAAG GTGTAGGAGC   
  
  
- GCAGGGCCGG TCCACCCGGA GGGAAGCATT CCGAGTGGCC CGAGCCCTGG AGGTACCTCC GCGAGCTCCG   
  
  
- GTGGCCTTTT TCCGAGAGTC TGAAGCGGCT CTTCAACCCC AACGGGAAAC TCAAATATGG GCACCGCCTC   
  
  
- TTCTAACCTT TAAACCTGAA CCTTTCCAAC GTACAATCAT TTTCCCTTCG AGAACGACAC GTAACCAATG   
  
  
- TCGTGAGAAA CATACTACAA TGACCGAGAC TATGATTATG TGAACCGGAA GAAGTTTCCA ACCGCGGTTT   
  
  
- TCACCACTGC CACCACCTCG TTCTGGATTC GGCTTGTCCG AGAAAGGATC CTTCCAAACA TCTCCGCTAG   
  
  
- GTGATGATAA GTCGGGATAA ACTGAGGAAT CCTCGCTCAA TACCTCTCCT GTCACTCCTC TCCGTACACC   
  
  
- AACTCGTTGT CGAGGAAAGA TCCCTCTAAG CTTTGTAAGA CCGGCAACCA CCCGGGAGTT CCTGGCCCCT   
  
  
- CGGGTTCAAA CGCTCGACCT CCCTCTTCGA TGTTGTCAGG CCGAAATCCC CTTAGAGTAA CCGTCCGTTG   
  
  
- CGGCGACGGG TCCGGTGGAA CGAGGAGCCG TACAAGGGAA GACTACCCAT ATGAAATTAG CTCCTGTCAC   
  
  
- CGTGTGAATT CGAGCCCACC TTCCTGAACA CGGAGGACTG ACGAAGCCGG ACCTCCGGAA GGGTACGAGT   
  
  
- ATGATACTCG TGGCAGACAT GAGCCTCGGT TAT

+     CAAT-box

| Site Name | Organism | Position | Strand | Matrix score. | sequence | function |
| --- | --- | --- | --- | --- | --- | --- |
| CAAT-box | Nicotiana glutinosa | 544 | + | 4 | CAAT |  |
| CAAT-box | Arabidopsis thaliana | 4262 | - | 5 | CCAAT | common cis-acting element in promoter and enhancer regions |
| CAAT-box | Pisum sativum | 945 | - | 5 | CAAAT | common cis-acting element in promoter and enhancer regions |
| CAAT-box | Arabidopsis thaliana | 4442 | + | 5 | CCAAT | common cis-acting element in promoter and enhancer regions |
| CAAT-box | Nicotiana glutinosa | 912 | + | 4 | CAAT |  |
| CAAT-box | Arabidopsis thaliana | 3916 | - | 5 | CCAAT | common cis-acting element in promoter and enhancer regions |
| CAAT-box | Arabidopsis thaliana | 911 | + | 5 | CCAAT | common cis-acting element in promoter and enhancer regions |
| CAAT-box | Arabidopsis thaliana | 3858 | - | 5 | CCAAT | common cis-acting element in promoter and enhancer regions |
| CAAT-box | Pisum sativum | 597 | - | 5 | CAAAT | common cis-acting element in promoter and enhancer regions |
| CAAT-box | Pisum sativum | 3590 | + | 5 | CAAAT | common cis-acting element in promoter and enhancer regions |
| CAAT-box | Nicotiana glutinosa | 828 | - | 4 | CAAT |  |
| CAAT-box | Nicotiana glutinosa | 4443 | + | 4 | CAAT |  |
| CAAT-box | Arabidopsis thaliana | 825 | + | 5 | CCAAT | common cis-acting element in promoter and enhancer regions |
| CAAT-box | Nicotiana glutinosa | 975 | + | 4 | CAAT |  |
| CAAT-box | Nicotiana glutinosa | 826 | + | 4 | CAAT |  |
| CAAT-box | Pisum sativum | 4082 | - | 5 | CAAAT | common cis-acting element in promoter and enhancer regions |
| CAAT-box | Nicotiana glutinosa | 252 | + | 4 | CAAT |  |
| CAAT-box | Pisum sativum | 3865 | - | 5 | CAAAT | common cis-acting element in promoter and enhancer regions |
| CAAT-box | Arabidopsis thaliana | 543 | + | 5 | CCAAT | common cis-acting element in promoter and enhancer regions |
| CAAT-box | Pisum sativum | 3374 | + | 5 | CAAAT | common cis-acting element in promoter and enhancer regions |
| CAAT-box | Nicotiana glutinosa | 632 | - | 4 | CAAT |  |
| CAAT-box | Nicotiana glutinosa | 3619 | + | 4 | CAAT |  |
| CAAT-box | Nicotiana glutinosa | 174 | + | 4 | CAAT |  |
| CAAT-box | Pisum sativum | 988 | + | 5 | CAAAT | common cis-acting element in promoter and enhancer regions |
| CAAT-box | Nicotiana glutinosa | 718 | - | 4 | CAAT |  |
| CAAT-box | Nicotiana glutinosa | 3569 | + | 4 | CAAT |  |
| CAAT-box | Pisum sativum | 218 | - | 5 | CAAAT | common cis-acting element in promoter and enhancer regions |
| CAAT-box | Nicotiana glutinosa | 1025 | - | 4 | CAAT |  |
| CAAT-box | Nicotiana glutinosa | 31 | + | 4 | CAAT |  |
| CAAT-box | Pisum sativum | 1036 | + | 5 | CAAAT | common cis-acting element in promoter and enhancer regions |
| CAAT-box | Nicotiana glutinosa | 169 | + | 4 | CAAT |  |
| CAAT-box | Nicotiana glutinosa | 1082 | + | 4 | CAAT |  |
| CAAT-box | Nicotiana glutinosa | 1084 | - | 4 | CAAT |  |
| CAAT-box | Nicotiana glutinosa | 1144 | - | 4 | CAAT |  |
| CAAT-box | Pisum sativum | 1153 | - | 5 | CAAAT | common cis-acting element in promoter and enhancer regions |
| CAAT-box | Pisum sativum | 1193 | - | 5 | CAAAT | common cis-acting element in promoter and enhancer regions |
| CAAT-box | Nicotiana glutinosa | 1205 | + | 4 | CAAT |  |
| CAAT-box | Nicotiana glutinosa | 1213 | + | 4 | CAAT |  |
| CAAT-box | Pisum sativum | 1236 | - | 5 | CAAAT | common cis-acting element in promoter and enhancer regions |
| CAAT-box | Nicotiana glutinosa | 1249 | - | 4 | CAAT |  |
| CAAT-box | Nicotiana glutinosa | 1413 | + | 4 | CAAT |  |
| CAAT-box | Pisum sativum | 1504 | - | 5 | CAAAT | common cis-acting element in promoter and enhancer regions |
| CAAT-box | Nicotiana glutinosa | 1940 | + | 4 | CAAT |  |
| CAAT-box | Nicotiana glutinosa | 2054 | + | 4 | CAAT |  |
| CAAT-box | Nicotiana glutinosa | 2215 | - | 4 | CAAT |  |
| CAAT-box | Nicotiana glutinosa | 2304 | - | 4 | CAAT |  |
| CAAT-box | Nicotiana glutinosa | 2351 | + | 4 | CAAT |  |
| CAAT-box | Nicotiana glutinosa | 2379 | - | 4 | CAAT |  |
| CAAT-box | Arabidopsis thaliana | 2419 | + | 5 | CCAAT | common cis-acting element in promoter and enhancer regions |
| CAAT-box | Nicotiana glutinosa | 2420 | + | 4 | CAAT |  |
| CAAT-box | Nicotiana glutinosa | 2662 | + | 4 | CAAT |  |
| CAAT-box | Nicotiana glutinosa | 2730 | - | 4 | CAAT |  |
| CAAT-box | Nicotiana glutinosa | 2769 | - | 4 | CAAT |  |
| CAAT-box | Nicotiana glutinosa | 2816 | + | 4 | CAAT |  |
| CAAT-box | Pisum sativum | 2823 | + | 5 | CAAAT | common cis-acting element in promoter and enhancer regions |
| CAAT-box | Nicotiana glutinosa | 2861 | + | 4 | CAAT |  |
| CAAT-box | Pisum sativum | 3174 | + | 5 | CAAAT | common cis-acting element in promoter and enhancer regions |
| CAAT-box | Arabidopsis thaliana | 3347 | + | 5 | CCAAT | common cis-acting element in promoter and enhancer regions |
| CAAT-box | Nicotiana glutinosa | 3348 | + | 4 | CAAT |  |

>HU01G00472.1   
+ -Up\_Stream \_Len000GTCTCT GTAACACAAT TCATTAAATC CATCCAAAGG GGATCATAAA TAACCAAACT   
  
  
+ TTGTTGGAAA CTGCTTTGAT GTGGGCTTTC TCTTTCAGCA TTTATGTTAT ACATATATAT GGTTATAAAG   
  
  
+ TTACAAAAGT ATGACCCGAC ACGACAATTC AATCCGAATC CGACTCGAAG TTACTGAAAG TAATCTGAAT   
  
  
+ ACGATTTGAC AGCTTAGTTT AAAAACCTAT TACTTCTCAA TTTTGAGTAA ATCTAAGCTA TTTTTAATGC   
  
  
+ TGAATAGATC GACCCGATTC GACCCGTTTT CCGACTCTAT ATGGTTATGG TGCCCCTAAA AAAAATTAAA   
  
  
+ AAAGAAAAAG AATAGAAAAA TATAAAATAA AACTGAGATT TCTGAACACT CTCTCCCCTA GTCTTGTGGG   
  
  
+ CATGGAAAGT GGTTGGAGAT GGAGATGTTT GAGTTTCATT TTCTGATATT ATTTTTTAGT CATTAGAAGT   
  
  
+ ACAAAGTCTA AACTCAACAA GAGCATGAGG GGAGAATGGT TGTTGTAGCC AATGGAAAAT AAAAAGAACA   
  
  
+ TTCACTCTTC TTGTGCTTTA TTTATGGTGA TAATTTGTTA CTTGTATTTT TAAAAATGAT TAAATATATT   
  
  
+ GATCCTTTGA TATTATGTTG AAATAATAAA AATATAAAAA TTAGAAAATA TTTTACCTCG TGAAATTTTT   
  
  
+ TTTCTGATTA ATCATTGAAA TTATGGTGAA AAATAAAGGA ACATCTCTTG TAAACACTCT ATCCGTTTAC   
  
  
+ GTTTGACCAC AAACTTGCTA TCCACAAGCA TCTCAACCTT TGCCTTTAAC CCAATTGTGG GTTGATGAAA   
  
  
+ TCTAACCATT ATAGGATTAG ATGGTAGTCG TTAATCCTGT GAAACAAGTT TTTGGTTATG ATGTATCCAA   
  
  
+ TTATGAGGGC CTGATAACTC TTGCCTCGGC ATTTGCGTGA TAATGACGTG GTACTAATAG CAATTATTTT   
  
  
+ TCGCAAATAT TCGAAATTTT TTAAGAATAA ACGCAAAATT ATTGATCTTG ACAAATGATT TTATTTTTTT   
  
  
+ GTTGGGAAAA GGATAAACAT CTAGTGTCAA TTGTTGATGA CCTGATAGCT CTCGATTTCT TATTTATGAG   
  
  
+ TTATGTTGAT ATGACACTAA TTGTAATTAT TTGTTGTAAA CTTAAACATT TTTAAAAATC AAAGCAAAAT   
  
  
+ TTGTTAATTA CAATAAATCA ATCTACTTTT ACTATGATCT TATTTGGTGT AATTATTGAT CTTGAATTTT   
  
  
+ TTTTTTTCTT TTTGAAAAAG ATAAGCATCG AATTTTTTCA AAAATAAAAA GGGTGAAGGA AGAAAAAATG   
  
  
+ GAAACAGTTA CCGAGTAAGG AAGGACAGAG CGCGAGAGGA CAAGACACAA AGAGAGGGGA GAGAGAGAGA   
  
  
+ GAAGGGGGCA ATGGCAGTGG GGAAGGTCGA GGAGCACCTT TCAACTTCAA GGCTCAACAC CCCCCCCCCC   
  
  
+ CCCCTCTTTC CTCATTTTGC GACGTTAGCA TTTGACATTT TGCTTTCAAC CGTCCCTCGG CATCATCAGC   
  
  
+ CCTGCCCTTG CTCAGCTTTT CCCATCTTTT TTTGGCTTTT TGTTTGACTC CCTCTATAAC TCTATTCACG   
  
  
+ TCGAGCCTTG TAAAAATTTT ACATATTTTA TTTTATTTTA CTTTTTTAGG TTTATCCCTC TCTATTCTTG   
  
  
+ ACCTACGGGC ATTTTTTATT TTCTTTGGGT TTGAGACTTG TGAACTTTTT GCACATTTTC TCACAAAAAT   
  
  
+ TTATCTTGTC TTTTTCACTT TCAACGTTCC TTCTCTCTCT CTCTCTCTCT CTCTCTCTCT CTCACTCACA   
  
  
+ TCCTAGACAG ACGTCCTTTT TAAAGAATTT ATTTAACAAG TTAGAAGAAA CCGTTGTTTA AGTAGAACCA   
  
  
+ CCCTGTTTGG ATGCCAAAGG AAATACCAGT CAAGAAAAAT CACGACAATC ACAACACATC CATTTCGTTT   
  
  
+ ACATCTCATC CTCATCCTCA TCTTCCTCAT CTCTACCCAT CTCCCTCTTT CTGTATGGCT GCTTCTTCTA   
  
  
+ CTTTGTTCCC TGACCCTAAC AATACTAGTT TAGTCCTCCC TTATTATTAT TATCCTCCTC CTTCCTCCTC   
  
  
+ TTACTATAAT GGTAACCCTT ACCTTCCTCT TCCTAACACC ACTGCTATTC CTACTCCTTC AACAACCTCT   
  
  
+ CCGGTATTTT CTTCCGCCAC CTACTCGGAG TTGAATACTG ATTGTCAAAC TCAACTGCCC ATGTCTAACC   
  
  
+ TCCCCGGAAA GATCGTTCGG AAGCGGAGTG CTGCTGAAAT GGAGCAGCGC TCCGCTCCGA TTGCGGGTGA   
  
  
+ TTACCCGCCA ACTCATCAGC GGGTAATCAC CCGCCACAAT GAGGCGGCCT CATCAGTGTC TTTCATTGAC   
  
  
+ TCCTCACTCT CGCCACTTCA CCTCTTCAAC GGCTCCAATT CGACGACCCC ATTAGCCCCC GACCGTCCTA   
  
  
+ ATGATCCGGG TCTGAGTCAG GGTCCGGATC TGCCTCTTGT GTGCGGATTC TCCGGACTCC CTTTATTCCC   
  
  
+ ACCGGTGGAG AGAAGACCTT CCATCCCCGC CACGACCACT GCTGCCGCGG CGATCGCGGT GGTTCCTGTG   
  
  
+ GAGGAGGATG ATCCGACGTG GATGGATAGC ATCATAAAAG AGCTGATTCA AAGCTCAAAC TCGATCTCAA   
  
  
+ TCCCACAGTT GATTCAGAAC GTTAGAGATA TCATCTACCC ATGTAATCCT AATCTGGGGT CCGCCATTGA   
  
  
+ GTTCCGCCTC CGCTCGCTCG CCGCCGACCC CCTCATTGCT CCGCCGCCAC TTCCGCCCTT CCACCACCAC   
  
  
+ CAACACCACC TCAATCAACA AATCACTCTT CCTCGAATCA GCAACACTAA CAACAACAAT CATGTGAGCG   
  
  
+ TGTACGCCAA CAAAGGACCC GGATACTTCA ACTTGGGTCC GGGTCAGGGT CAGGGTCCGA TTAACATCGA   
  
  
+ CCAAGCTAAC CTTTCCTTCC CTCCTGATTC CACCGCCTGT TGGGGTGTCA GTGTCACACC GCCACCGTCT   
  
  
+ TCCGCCGCCG GTTCCGGCTC CGGTGGAAGC GGCAGTAGTA GTGGTAAATC AAACCCTAAC CCTAATCCCA   
  
  
+ ACCCGAACCC AAACCCAAAC CCAAACCCGA ACAACAAAGC TCAGGATGTT CAACTCCAAA CTCCACAACA   
  
  
+ ACAGCAACAG CAACAGCAAC AAATCCCCGT GGATCAGGAG CAAGACCCGG CGGCTCCACC GGCGACGGAA   
  
  
+ GCATCGCCAC CATCTCCAAG AGCGGCGGCA CCACCGCCGG CGGCAGTGAA AGCAAGAGAA AGAGAGGAGA   
  
  
+ TGCGGCAAAG GAAGCGCGAC GAAGAGGGTC TCCACCTCCT AACCCTCCTC CTCCAATGCG CAGAAGCAGT   
  
  
+ ATCCGGCGAC AAATACGAAG AAGCGAACAA GATGCTTCTA GAAATCTCGG AGTGGGCCAC CCCATTCGGC   
  
  
+ ACCTCCGCCC AACGCGTCGC CGCGTACTTC TCGGAAGCCA TGTCAGCCCG TCTCGTATCC TCCTGCCTCG   
  
  
+ GCATCTACGC CGCCCTCCCC ACCGTCCCAC ACTACGTCAA GCTCCTCTCC GCCTTCCAAG TCTTCAATGG   
  
  
+ CATCAGCCCA TTCGTCAAAT TCTCTCACTT CACTGCAAAC CAAGCAATCC AAGAGGCCTT CCAGAGGGAA   
  
  
+ GACAGGGTCC ACATCATCGA CCTCGATATC ATGCAGGGGC TCCAGTGGCC CGGGCTGTTC CACATCCTCG   
  
  
+ CGTCCCGGCC AGGTGGGCCT CCCTTCGTAA GGCTCACCGG GCTCGGGACC TCCATGGAGG CGCTCGAGGC   
  
  
+ CACCGGAAAA AGGCTCTCAG ACTTCGCCGA GAAGTTGGGG TTGCCCTTTG AGTTTATACC CGTGGCGGAG   
  
  
+ AAGATTGGAA ATTTGGACTT GGAAAGGTTG CATGTTAGTA AAAGGGAAGC TCTTGCTGTG CATTGGTTAC   
  
  
+ AGCACTCTTT GTATGATGTT ACTGGCTCTG ATACTAATAC ACTTGGCCTT CTTCAAAGGT TGGCGCCAAA   
  
  
+ AGTGGTGACG GTGGTGGAGC AAGACCTAAG CCGAACAGGC TCTTTCCTAG GAAGGTTTGT AGAGGCGATC   
  
  
+ CACTACTATT CAGCCCTATT TGACTCCTTA GGAGCGAGTT ATGGAGAGGA CAGTGAGGAG AGGCATGTGG   
  
  
+ TTGAGCAACA GCTCCTTTCT AGGGAGATTC GAAACATTCT GGCCGTTGGT GGGCCCTCAA GGACCGGGGA   
  
  
+ GCCCAAGTTT GCGAGCTGGA GGGAGAAGCT ACAACAGTCC GGCTTTAGGG GAATCTCATT GGCAGGCAAC   
  
  
+ GCCGCTGCCC AGGCCACCTT GCTCCTCGGC ATGTTCCCTT CTGATGGGTA TACTTTAATC GAGGACAGTG   
  
  
+ GCACACTTAA GCTCGGGTGG AAGGACTTGT GCCTCCTGAC TGCTTCGGCC TGGAGGCCTT CCCATGCTCA   
  
  
+ TACTATGAGC ACCGTCTGTA CTCGGAGCCA ATA  

- -Up\_Stream \_Len000CAGAGA CATTGTGTTA AGTAATTTAG GTAGGTTTCC CCTAGTATTT ATTGGTTTGA   
  
  
- AACAACCTTT GACGAAACTA CACCCGAAAG AGAAAGTCGT AAATACAATA TGTATATATA CCAATATTTC   
  
  
- AATGTTTTCA TACTGGGCTG TGCTGTTAAG TTAGGCTTAG GCTGAGCTTC AATGACTTTC ATTAGACTTA   
  
  
- TGCTAAACTG TCGAATCAAA TTTTTGGATA ATGAAGAGTT AAAACTCATT TAGATTCGAT AAAAATTACG   
  
  
- ACTTATCTAG CTGGGCTAAG CTGGGCAAAA GGCTGAGATA TACCAATACC ACGGGGATTT TTTTTAATTT   
  
  
- TTTCTTTTTC TTATCTTTTT ATATTTTATT TTGACTCTAA AGACTTGTGA GAGAGGGGAT CAGAACACCC   
  
  
- GTACCTTTCA CCAACCTCTA CCTCTACAAA CTCAAAGTAA AAGACTATAA TAAAAAATCA GTAATCTTCA   
  
  
- TGTTTCAGAT TTGAGTTGTT CTCGTACTCC CCTCTTACCA ACAACATCGG TTACCTTTTA TTTTTCTTGT   
  
  
- AAGTGAGAAG AACACGAAAT AAATACCACT ATTAAACAAT GAACATAAAA ATTTTTACTA ATTTATATAA   
  
  
- CTAGGAAACT ATAATACAAC TTTATTATTT TTATATTTTT AATCTTTTAT AAAATGGAGC ACTTTAAAAA   
  
  
- AAAGACTAAT TAGTAACTTT AATACCACTT TTTATTTCCT TGTAGAGAAC ATTTGTGAGA TAGGCAAATG   
  
  
- CAAACTGGTG TTTGAACGAT AGGTGTTCGT AGAGTTGGAA ACGGAAATTG GGTTAACACC CAACTACTTT   
  
  
- AGATTGGTAA TATCCTAATC TACCATCAGC AATTAGGACA CTTTGTTCAA AAACCAATAC TACATAGGTT   
  
  
- AATACTCCCG GACTATTGAG AACGGAGCCG TAAACGCACT ATTACTGCAC CATGATTATC GTTAATAAAA   
  
  
- AGCGTTTATA AGCTTTAAAA AATTCTTATT TGCGTTTTAA TAACTAGAAC TGTTTACTAA AATAAAAAAA   
  
  
- CAACCCTTTT CCTATTTGTA GATCACAGTT AACAACTACT GGACTATCGA GAGCTAAAGA ATAAATACTC   
  
  
- AATACAACTA TACTGTGATT AACATTAATA AACAACATTT GAATTTGTAA AAATTTTTAG TTTCGTTTTA   
  
  
- AACAATTAAT GTTATTTAGT TAGATGAAAA TGATACTAGA ATAAACCACA TTAATAACTA GAACTTAAAA   
  
  
- AAAAAAAGAA AAACTTTTTC TATTCGTAGC TTAAAAAAGT TTTTATTTTT CCCACTTCCT TCTTTTTTAC   
  
  
- CTTTGTCAAT GGCTCATTCC TTCCTGTCTC GCGCTCTCCT GTTCTGTGTT TCTCTCCCCT CTCTCTCTCT   
  
  
- CTTCCCCCGT TACCGTCACC CCTTCCAGCT CCTCGTGGAA AGTTGAAGTT CCGAGTTGTG GGGGGGGGGG   
  
  
- GGGGAGAAAG GAGTAAAACG CTGCAATCGT AAACTGTAAA ACGAAAGTTG GCAGGGAGCC GTAGTAGTCG   
  
  
- GGACGGGAAC GAGTCGAAAA GGGTAGAAAA AAACCGAAAA ACAAACTGAG GGAGATATTG AGATAAGTGC   
  
  
- AGCTCGGAAC ATTTTTAAAA TGTATAAAAT AAAATAAAAT GAAAAAATCC AAATAGGGAG AGATAAGAAC   
  
  
- TGGATGCCCG TAAAAAATAA AAGAAACCCA AACTCTGAAC ACTTGAAAAA CGTGTAAAAG AGTGTTTTTA   
  
  
- AATAGAACAG AAAAAGTGAA AGTTGCAAGG AAGAGAGAGA GAGAGAGAGA GAGAGAGAGA GAGTGAGTGT   
  
  
- AGGATCTGTC TGCAGGAAAA ATTTCTTAAA TAAATTGTTC AATCTTCTTT GGCAACAAAT TCATCTTGGT   
  
  
- GGGACAAACC TACGGTTTCC TTTATGGTCA GTTCTTTTTA GTGCTGTTAG TGTTGTGTAG GTAAAGCAAA   
  
  
- TGTAGAGTAG GAGTAGGAGT AGAAGGAGTA GAGATGGGTA GAGGGAGAAA GACATACCGA CGAAGAAGAT   
  
  
- GAAACAAGGG ACTGGGATTG TTATGATCAA ATCAGGAGGG AATAATAATA ATAGGAGGAG GAAGGAGGAG   
  
  
- AATGATATTA CCATTGGGAA TGGAAGGAGA AGGATTGTGG TGACGATAAG GATGAGGAAG TTGTTGGAGA   
  
  
- GGCCATAAAA GAAGGCGGTG GATGAGCCTC AACTTATGAC TAACAGTTTG AGTTGACGGG TACAGATTGG   
  
  
- AGGGGCCTTT CTAGCAAGCC TTCGCCTCAC GACGACTTTA CCTCGTCGCG AGGCGAGGCT AACGCCCACT   
  
  
- AATGGGCGGT TGAGTAGTCG CCCATTAGTG GGCGGTGTTA CTCCGCCGGA GTAGTCACAG AAAGTAACTG   
  
  
- AGGAGTGAGA GCGGTGAAGT GGAGAAGTTG CCGAGGTTAA GCTGCTGGGG TAATCGGGGG CTGGCAGGAT   
  
  
- TACTAGGCCC AGACTCAGTC CCAGGCCTAG ACGGAGAACA CACGCCTAAG AGGCCTGAGG GAAATAAGGG   
  
  
- TGGCCACCTC TCTTCTGGAA GGTAGGGGCG GTGCTGGTGA CGACGGCGCC GCTAGCGCCA CCAAGGACAC   
  
  
- CTCCTCCTAC TAGGCTGCAC CTACCTATCG TAGTATTTTC TCGACTAAGT TTCGAGTTTG AGCTAGAGTT   
  
  
- AGGGTGTCAA CTAAGTCTTG CAATCTCTAT AGTAGATGGG TACATTAGGA TTAGACCCCA GGCGGTAACT   
  
  
- CAAGGCGGAG GCGAGCGAGC GGCGGCTGGG GGAGTAACGA GGCGGCGGTG AAGGCGGGAA GGTGGTGGTG   
  
  
- GTTGTGGTGG AGTTAGTTGT TTAGTGAGAA GGAGCTTAGT CGTTGTGATT GTTGTTGTTA GTACACTCGC   
  
  
- ACATGCGGTT GTTTCCTGGG CCTATGAAGT TGAACCCAGG CCCAGTCCCA GTCCCAGGCT AATTGTAGCT   
  
  
- GGTTCGATTG GAAAGGAAGG GAGGACTAAG GTGGCGGACA ACCCCACAGT CACAGTGTGG CGGTGGCAGA   
  
  
- AGGCGGCGGC CAAGGCCGAG GCCACCTTCG CCGTCATCAT CACCATTTAG TTTGGGATTG GGATTAGGGT   
  
  
- TGGGCTTGGG TTTGGGTTTG GGTTTGGGCT TGTTGTTTCG AGTCCTACAA GTTGAGGTTT GAGGTGTTGT   
  
  
- TGTCGTTGTC GTTGTCGTTG TTTAGGGGCA CCTAGTCCTC GTTCTGGGCC GCCGAGGTGG CCGCTGCCTT   
  
  
- CGTAGCGGTG GTAGAGGTTC TCGCCGCCGT GGTGGCGGCC GCCGTCACTT TCGTTCTCTT TCTCTCCTCT   
  
  
- ACGCCGTTTC CTTCGCGCTG CTTCTCCCAG AGGTGGAGGA TTGGGAGGAG GAGGTTACGC GTCTTCGTCA   
  
  
- TAGGCCGCTG TTTATGCTTC TTCGCTTGTT CTACGAAGAT CTTTAGAGCC TCACCCGGTG GGGTAAGCCG   
  
  
- TGGAGGCGGG TTGCGCAGCG GCGCATGAAG AGCCTTCGGT ACAGTCGGGC AGAGCATAGG AGGACGGAGC   
  
  
- CGTAGATGCG GCGGGAGGGG TGGCAGGGTG TGATGCAGTT CGAGGAGAGG CGGAAGGTTC AGAAGTTACC   
  
  
- GTAGTCGGGT AAGCAGTTTA AGAGAGTGAA GTGACGTTTG GTTCGTTAGG TTCTCCGGAA GGTCTCCCTT   
  
  
- CTGTCCCAGG TGTAGTAGCT GGAGCTATAG TACGTCCCCG AGGTCACCGG GCCCGACAAG GTGTAGGAGC   
  
  
- GCAGGGCCGG TCCACCCGGA GGGAAGCATT CCGAGTGGCC CGAGCCCTGG AGGTACCTCC GCGAGCTCCG   
  
  
- GTGGCCTTTT TCCGAGAGTC TGAAGCGGCT CTTCAACCCC AACGGGAAAC TCAAATATGG GCACCGCCTC   
  
  
- TTCTAACCTT TAAACCTGAA CCTTTCCAAC GTACAATCAT TTTCCCTTCG AGAACGACAC GTAACCAATG   
  
  
- TCGTGAGAAA CATACTACAA TGACCGAGAC TATGATTATG TGAACCGGAA GAAGTTTCCA ACCGCGGTTT   
  
  
- TCACCACTGC CACCACCTCG TTCTGGATTC GGCTTGTCCG AGAAAGGATC CTTCCAAACA TCTCCGCTAG   
  
  
- GTGATGATAA GTCGGGATAA ACTGAGGAAT CCTCGCTCAA TACCTCTCCT GTCACTCCTC TCCGTACACC   
  
  
- AACTCGTTGT CGAGGAAAGA TCCCTCTAAG CTTTGTAAGA CCGGCAACCA CCCGGGAGTT CCTGGCCCCT   
  
  
- CGGGTTCAAA CGCTCGACCT CCCTCTTCGA TGTTGTCAGG CCGAAATCCC CTTAGAGTAA CCGTCCGTTG   
  
  
- CGGCGACGGG TCCGGTGGAA CGAGGAGCCG TACAAGGGAA GACTACCCAT ATGAAATTAG CTCCTGTCAC   
  
  
- CGTGTGAATT CGAGCCCACC TTCCTGAACA CGGAGGACTG ACGAAGCCGG ACCTCCGGAA GGGTACGAGT   
  
  
- ATGATACTCG TGGCAGACAT GAGCCTCGGT TAT

+     CAT-box

| Site Name | Organism | Position | Strand | Matrix score. | sequence | function |
| --- | --- | --- | --- | --- | --- | --- |
| CAT-box | Arabidopsis thaliana | 2396 | + | 6 | GCCACT | cis-acting regulatory element related to meristem expression |
| CAT-box | Arabidopsis thaliana | 3688 | - | 6 | GCCACT | cis-acting regulatory element related to meristem expression |
| CAT-box | Arabidopsis thaliana | 2780 | + | 6 | GCCACT | cis-acting regulatory element related to meristem expression |
| CAT-box | Arabidopsis thaliana | 4341 | - | 6 | GCCACT | cis-acting regulatory element related to meristem expression |

>HU01G00472.1   
+ -Up\_Stream \_Len000GTCTCT GTAACACAAT TCATTAAATC CATCCAAAGG GGATCATAAA TAACCAAACT   
  
  
+ TTGTTGGAAA CTGCTTTGAT GTGGGCTTTC TCTTTCAGCA TTTATGTTAT ACATATATAT GGTTATAAAG   
  
  
+ TTACAAAAGT ATGACCCGAC ACGACAATTC AATCCGAATC CGACTCGAAG TTACTGAAAG TAATCTGAAT   
  
  
+ ACGATTTGAC AGCTTAGTTT AAAAACCTAT TACTTCTCAA TTTTGAGTAA ATCTAAGCTA TTTTTAATGC   
  
  
+ TGAATAGATC GACCCGATTC GACCCGTTTT CCGACTCTAT ATGGTTATGG TGCCCCTAAA AAAAATTAAA   
  
  
+ AAAGAAAAAG AATAGAAAAA TATAAAATAA AACTGAGATT TCTGAACACT CTCTCCCCTA GTCTTGTGGG   
  
  
+ CATGGAAAGT GGTTGGAGAT GGAGATGTTT GAGTTTCATT TTCTGATATT ATTTTTTAGT CATTAGAAGT   
  
  
+ ACAAAGTCTA AACTCAACAA GAGCATGAGG GGAGAATGGT TGTTGTAGCC AATGGAAAAT AAAAAGAACA   
  
  
+ TTCACTCTTC TTGTGCTTTA TTTATGGTGA TAATTTGTTA CTTGTATTTT TAAAAATGAT TAAATATATT   
  
  
+ GATCCTTTGA TATTATGTTG AAATAATAAA AATATAAAAA TTAGAAAATA TTTTACCTCG TGAAATTTTT   
  
  
+ TTTCTGATTA ATCATTGAAA TTATGGTGAA AAATAAAGGA ACATCTCTTG TAAACACTCT ATCCGTTTAC   
  
  
+ GTTTGACCAC AAACTTGCTA TCCACAAGCA TCTCAACCTT TGCCTTTAAC CCAATTGTGG GTTGATGAAA   
  
  
+ TCTAACCATT ATAGGATTAG ATGGTAGTCG TTAATCCTGT GAAACAAGTT TTTGGTTATG ATGTATCCAA   
  
  
+ TTATGAGGGC CTGATAACTC TTGCCTCGGC ATTTGCGTGA TAATGACGTG GTACTAATAG CAATTATTTT   
  
  
+ TCGCAAATAT TCGAAATTTT TTAAGAATAA ACGCAAAATT ATTGATCTTG ACAAATGATT TTATTTTTTT   
  
  
+ GTTGGGAAAA GGATAAACAT CTAGTGTCAA TTGTTGATGA CCTGATAGCT CTCGATTTCT TATTTATGAG   
  
  
+ TTATGTTGAT ATGACACTAA TTGTAATTAT TTGTTGTAAA CTTAAACATT TTTAAAAATC AAAGCAAAAT   
  
  
+ TTGTTAATTA CAATAAATCA ATCTACTTTT ACTATGATCT TATTTGGTGT AATTATTGAT CTTGAATTTT   
  
  
+ TTTTTTTCTT TTTGAAAAAG ATAAGCATCG AATTTTTTCA AAAATAAAAA GGGTGAAGGA AGAAAAAATG   
  
  
+ GAAACAGTTA CCGAGTAAGG AAGGACAGAG CGCGAGAGGA CAAGACACAA AGAGAGGGGA GAGAGAGAGA   
  
  
+ GAAGGGGGCA ATGGCAGTGG GGAAGGTCGA GGAGCACCTT TCAACTTCAA GGCTCAACAC CCCCCCCCCC   
  
  
+ CCCCTCTTTC CTCATTTTGC GACGTTAGCA TTTGACATTT TGCTTTCAAC CGTCCCTCGG CATCATCAGC   
  
  
+ CCTGCCCTTG CTCAGCTTTT CCCATCTTTT TTTGGCTTTT TGTTTGACTC CCTCTATAAC TCTATTCACG   
  
  
+ TCGAGCCTTG TAAAAATTTT ACATATTTTA TTTTATTTTA CTTTTTTAGG TTTATCCCTC TCTATTCTTG   
  
  
+ ACCTACGGGC ATTTTTTATT TTCTTTGGGT TTGAGACTTG TGAACTTTTT GCACATTTTC TCACAAAAAT   
  
  
+ TTATCTTGTC TTTTTCACTT TCAACGTTCC TTCTCTCTCT CTCTCTCTCT CTCTCTCTCT CTCACTCACA   
  
  
+ TCCTAGACAG ACGTCCTTTT TAAAGAATTT ATTTAACAAG TTAGAAGAAA CCGTTGTTTA AGTAGAACCA   
  
  
+ CCCTGTTTGG ATGCCAAAGG AAATACCAGT CAAGAAAAAT CACGACAATC ACAACACATC CATTTCGTTT   
  
  
+ ACATCTCATC CTCATCCTCA TCTTCCTCAT CTCTACCCAT CTCCCTCTTT CTGTATGGCT GCTTCTTCTA   
  
  
+ CTTTGTTCCC TGACCCTAAC AATACTAGTT TAGTCCTCCC TTATTATTAT TATCCTCCTC CTTCCTCCTC   
  
  
+ TTACTATAAT GGTAACCCTT ACCTTCCTCT TCCTAACACC ACTGCTATTC CTACTCCTTC AACAACCTCT   
  
  
+ CCGGTATTTT CTTCCGCCAC CTACTCGGAG TTGAATACTG ATTGTCAAAC TCAACTGCCC ATGTCTAACC   
  
  
+ TCCCCGGAAA GATCGTTCGG AAGCGGAGTG CTGCTGAAAT GGAGCAGCGC TCCGCTCCGA TTGCGGGTGA   
  
  
+ TTACCCGCCA ACTCATCAGC GGGTAATCAC CCGCCACAAT GAGGCGGCCT CATCAGTGTC TTTCATTGAC   
  
  
+ TCCTCACTCT CGCCACTTCA CCTCTTCAAC GGCTCCAATT CGACGACCCC ATTAGCCCCC GACCGTCCTA   
  
  
+ ATGATCCGGG TCTGAGTCAG GGTCCGGATC TGCCTCTTGT GTGCGGATTC TCCGGACTCC CTTTATTCCC   
  
  
+ ACCGGTGGAG AGAAGACCTT CCATCCCCGC CACGACCACT GCTGCCGCGG CGATCGCGGT GGTTCCTGTG   
  
  
+ GAGGAGGATG ATCCGACGTG GATGGATAGC ATCATAAAAG AGCTGATTCA AAGCTCAAAC TCGATCTCAA   
  
  
+ TCCCACAGTT GATTCAGAAC GTTAGAGATA TCATCTACCC ATGTAATCCT AATCTGGGGT CCGCCATTGA   
  
  
+ GTTCCGCCTC CGCTCGCTCG CCGCCGACCC CCTCATTGCT CCGCCGCCAC TTCCGCCCTT CCACCACCAC   
  
  
+ CAACACCACC TCAATCAACA AATCACTCTT CCTCGAATCA GCAACACTAA CAACAACAAT CATGTGAGCG   
  
  
+ TGTACGCCAA CAAAGGACCC GGATACTTCA ACTTGGGTCC GGGTCAGGGT CAGGGTCCGA TTAACATCGA   
  
  
+ CCAAGCTAAC CTTTCCTTCC CTCCTGATTC CACCGCCTGT TGGGGTGTCA GTGTCACACC GCCACCGTCT   
  
  
+ TCCGCCGCCG GTTCCGGCTC CGGTGGAAGC GGCAGTAGTA GTGGTAAATC AAACCCTAAC CCTAATCCCA   
  
  
+ ACCCGAACCC AAACCCAAAC CCAAACCCGA ACAACAAAGC TCAGGATGTT CAACTCCAAA CTCCACAACA   
  
  
+ ACAGCAACAG CAACAGCAAC AAATCCCCGT GGATCAGGAG CAAGACCCGG CGGCTCCACC GGCGACGGAA   
  
  
+ GCATCGCCAC CATCTCCAAG AGCGGCGGCA CCACCGCCGG CGGCAGTGAA AGCAAGAGAA AGAGAGGAGA   
  
  
+ TGCGGCAAAG GAAGCGCGAC GAAGAGGGTC TCCACCTCCT AACCCTCCTC CTCCAATGCG CAGAAGCAGT   
  
  
+ ATCCGGCGAC AAATACGAAG AAGCGAACAA GATGCTTCTA GAAATCTCGG AGTGGGCCAC CCCATTCGGC   
  
  
+ ACCTCCGCCC AACGCGTCGC CGCGTACTTC TCGGAAGCCA TGTCAGCCCG TCTCGTATCC TCCTGCCTCG   
  
  
+ GCATCTACGC CGCCCTCCCC ACCGTCCCAC ACTACGTCAA GCTCCTCTCC GCCTTCCAAG TCTTCAATGG   
  
  
+ CATCAGCCCA TTCGTCAAAT TCTCTCACTT CACTGCAAAC CAAGCAATCC AAGAGGCCTT CCAGAGGGAA   
  
  
+ GACAGGGTCC ACATCATCGA CCTCGATATC ATGCAGGGGC TCCAGTGGCC CGGGCTGTTC CACATCCTCG   
  
  
+ CGTCCCGGCC AGGTGGGCCT CCCTTCGTAA GGCTCACCGG GCTCGGGACC TCCATGGAGG CGCTCGAGGC   
  
  
+ CACCGGAAAA AGGCTCTCAG ACTTCGCCGA GAAGTTGGGG TTGCCCTTTG AGTTTATACC CGTGGCGGAG   
  
  
+ AAGATTGGAA ATTTGGACTT GGAAAGGTTG CATGTTAGTA AAAGGGAAGC TCTTGCTGTG CATTGGTTAC   
  
  
+ AGCACTCTTT GTATGATGTT ACTGGCTCTG ATACTAATAC ACTTGGCCTT CTTCAAAGGT TGGCGCCAAA   
  
  
+ AGTGGTGACG GTGGTGGAGC AAGACCTAAG CCGAACAGGC TCTTTCCTAG GAAGGTTTGT AGAGGCGATC   
  
  
+ CACTACTATT CAGCCCTATT TGACTCCTTA GGAGCGAGTT ATGGAGAGGA CAGTGAGGAG AGGCATGTGG   
  
  
+ TTGAGCAACA GCTCCTTTCT AGGGAGATTC GAAACATTCT GGCCGTTGGT GGGCCCTCAA GGACCGGGGA   
  
  
+ GCCCAAGTTT GCGAGCTGGA GGGAGAAGCT ACAACAGTCC GGCTTTAGGG GAATCTCATT GGCAGGCAAC   
  
  
+ GCCGCTGCCC AGGCCACCTT GCTCCTCGGC ATGTTCCCTT CTGATGGGTA TACTTTAATC GAGGACAGTG   
  
  
+ GCACACTTAA GCTCGGGTGG AAGGACTTGT GCCTCCTGAC TGCTTCGGCC TGGAGGCCTT CCCATGCTCA   
  
  
+ TACTATGAGC ACCGTCTGTA CTCGGAGCCA ATA  

- -Up\_Stream \_Len000CAGAGA CATTGTGTTA AGTAATTTAG GTAGGTTTCC CCTAGTATTT ATTGGTTTGA   
  
  
- AACAACCTTT GACGAAACTA CACCCGAAAG AGAAAGTCGT AAATACAATA TGTATATATA CCAATATTTC   
  
  
- AATGTTTTCA TACTGGGCTG TGCTGTTAAG TTAGGCTTAG GCTGAGCTTC AATGACTTTC ATTAGACTTA   
  
  
- TGCTAAACTG TCGAATCAAA TTTTTGGATA ATGAAGAGTT AAAACTCATT TAGATTCGAT AAAAATTACG   
  
  
- ACTTATCTAG CTGGGCTAAG CTGGGCAAAA GGCTGAGATA TACCAATACC ACGGGGATTT TTTTTAATTT   
  
  
- TTTCTTTTTC TTATCTTTTT ATATTTTATT TTGACTCTAA AGACTTGTGA GAGAGGGGAT CAGAACACCC   
  
  
- GTACCTTTCA CCAACCTCTA CCTCTACAAA CTCAAAGTAA AAGACTATAA TAAAAAATCA GTAATCTTCA   
  
  
- TGTTTCAGAT TTGAGTTGTT CTCGTACTCC CCTCTTACCA ACAACATCGG TTACCTTTTA TTTTTCTTGT   
  
  
- AAGTGAGAAG AACACGAAAT AAATACCACT ATTAAACAAT GAACATAAAA ATTTTTACTA ATTTATATAA   
  
  
- CTAGGAAACT ATAATACAAC TTTATTATTT TTATATTTTT AATCTTTTAT AAAATGGAGC ACTTTAAAAA   
  
  
- AAAGACTAAT TAGTAACTTT AATACCACTT TTTATTTCCT TGTAGAGAAC ATTTGTGAGA TAGGCAAATG   
  
  
- CAAACTGGTG TTTGAACGAT AGGTGTTCGT AGAGTTGGAA ACGGAAATTG GGTTAACACC CAACTACTTT   
  
  
- AGATTGGTAA TATCCTAATC TACCATCAGC AATTAGGACA CTTTGTTCAA AAACCAATAC TACATAGGTT   
  
  
- AATACTCCCG GACTATTGAG AACGGAGCCG TAAACGCACT ATTACTGCAC CATGATTATC GTTAATAAAA   
  
  
- AGCGTTTATA AGCTTTAAAA AATTCTTATT TGCGTTTTAA TAACTAGAAC TGTTTACTAA AATAAAAAAA   
  
  
- CAACCCTTTT CCTATTTGTA GATCACAGTT AACAACTACT GGACTATCGA GAGCTAAAGA ATAAATACTC   
  
  
- AATACAACTA TACTGTGATT AACATTAATA AACAACATTT GAATTTGTAA AAATTTTTAG TTTCGTTTTA   
  
  
- AACAATTAAT GTTATTTAGT TAGATGAAAA TGATACTAGA ATAAACCACA TTAATAACTA GAACTTAAAA   
  
  
- AAAAAAAGAA AAACTTTTTC TATTCGTAGC TTAAAAAAGT TTTTATTTTT CCCACTTCCT TCTTTTTTAC   
  
  
- CTTTGTCAAT GGCTCATTCC TTCCTGTCTC GCGCTCTCCT GTTCTGTGTT TCTCTCCCCT CTCTCTCTCT   
  
  
- CTTCCCCCGT TACCGTCACC CCTTCCAGCT CCTCGTGGAA AGTTGAAGTT CCGAGTTGTG GGGGGGGGGG   
  
  
- GGGGAGAAAG GAGTAAAACG CTGCAATCGT AAACTGTAAA ACGAAAGTTG GCAGGGAGCC GTAGTAGTCG   
  
  
- GGACGGGAAC GAGTCGAAAA GGGTAGAAAA AAACCGAAAA ACAAACTGAG GGAGATATTG AGATAAGTGC   
  
  
- AGCTCGGAAC ATTTTTAAAA TGTATAAAAT AAAATAAAAT GAAAAAATCC AAATAGGGAG AGATAAGAAC   
  
  
- TGGATGCCCG TAAAAAATAA AAGAAACCCA AACTCTGAAC ACTTGAAAAA CGTGTAAAAG AGTGTTTTTA   
  
  
- AATAGAACAG AAAAAGTGAA AGTTGCAAGG AAGAGAGAGA GAGAGAGAGA GAGAGAGAGA GAGTGAGTGT   
  
  
- AGGATCTGTC TGCAGGAAAA ATTTCTTAAA TAAATTGTTC AATCTTCTTT GGCAACAAAT TCATCTTGGT   
  
  
- GGGACAAACC TACGGTTTCC TTTATGGTCA GTTCTTTTTA GTGCTGTTAG TGTTGTGTAG GTAAAGCAAA   
  
  
- TGTAGAGTAG GAGTAGGAGT AGAAGGAGTA GAGATGGGTA GAGGGAGAAA GACATACCGA CGAAGAAGAT   
  
  
- GAAACAAGGG ACTGGGATTG TTATGATCAA ATCAGGAGGG AATAATAATA ATAGGAGGAG GAAGGAGGAG   
  
  
- AATGATATTA CCATTGGGAA TGGAAGGAGA AGGATTGTGG TGACGATAAG GATGAGGAAG TTGTTGGAGA   
  
  
- GGCCATAAAA GAAGGCGGTG GATGAGCCTC AACTTATGAC TAACAGTTTG AGTTGACGGG TACAGATTGG   
  
  
- AGGGGCCTTT CTAGCAAGCC TTCGCCTCAC GACGACTTTA CCTCGTCGCG AGGCGAGGCT AACGCCCACT   
  
  
- AATGGGCGGT TGAGTAGTCG CCCATTAGTG GGCGGTGTTA CTCCGCCGGA GTAGTCACAG AAAGTAACTG   
  
  
- AGGAGTGAGA GCGGTGAAGT GGAGAAGTTG CCGAGGTTAA GCTGCTGGGG TAATCGGGGG CTGGCAGGAT   
  
  
- TACTAGGCCC AGACTCAGTC CCAGGCCTAG ACGGAGAACA CACGCCTAAG AGGCCTGAGG GAAATAAGGG   
  
  
- TGGCCACCTC TCTTCTGGAA GGTAGGGGCG GTGCTGGTGA CGACGGCGCC GCTAGCGCCA CCAAGGACAC   
  
  
- CTCCTCCTAC TAGGCTGCAC CTACCTATCG TAGTATTTTC TCGACTAAGT TTCGAGTTTG AGCTAGAGTT   
  
  
- AGGGTGTCAA CTAAGTCTTG CAATCTCTAT AGTAGATGGG TACATTAGGA TTAGACCCCA GGCGGTAACT   
  
  
- CAAGGCGGAG GCGAGCGAGC GGCGGCTGGG GGAGTAACGA GGCGGCGGTG AAGGCGGGAA GGTGGTGGTG   
  
  
- GTTGTGGTGG AGTTAGTTGT TTAGTGAGAA GGAGCTTAGT CGTTGTGATT GTTGTTGTTA GTACACTCGC   
  
  
- ACATGCGGTT GTTTCCTGGG CCTATGAAGT TGAACCCAGG CCCAGTCCCA GTCCCAGGCT AATTGTAGCT   
  
  
- GGTTCGATTG GAAAGGAAGG GAGGACTAAG GTGGCGGACA ACCCCACAGT CACAGTGTGG CGGTGGCAGA   
  
  
- AGGCGGCGGC CAAGGCCGAG GCCACCTTCG CCGTCATCAT CACCATTTAG TTTGGGATTG GGATTAGGGT   
  
  
- TGGGCTTGGG TTTGGGTTTG GGTTTGGGCT TGTTGTTTCG AGTCCTACAA GTTGAGGTTT GAGGTGTTGT   
  
  
- TGTCGTTGTC GTTGTCGTTG TTTAGGGGCA CCTAGTCCTC GTTCTGGGCC GCCGAGGTGG CCGCTGCCTT   
  
  
- CGTAGCGGTG GTAGAGGTTC TCGCCGCCGT GGTGGCGGCC GCCGTCACTT TCGTTCTCTT TCTCTCCTCT   
  
  
- ACGCCGTTTC CTTCGCGCTG CTTCTCCCAG AGGTGGAGGA TTGGGAGGAG GAGGTTACGC GTCTTCGTCA   
  
  
- TAGGCCGCTG TTTATGCTTC TTCGCTTGTT CTACGAAGAT CTTTAGAGCC TCACCCGGTG GGGTAAGCCG   
  
  
- TGGAGGCGGG TTGCGCAGCG GCGCATGAAG AGCCTTCGGT ACAGTCGGGC AGAGCATAGG AGGACGGAGC   
  
  
- CGTAGATGCG GCGGGAGGGG TGGCAGGGTG TGATGCAGTT CGAGGAGAGG CGGAAGGTTC AGAAGTTACC   
  
  
- GTAGTCGGGT AAGCAGTTTA AGAGAGTGAA GTGACGTTTG GTTCGTTAGG TTCTCCGGAA GGTCTCCCTT   
  
  
- CTGTCCCAGG TGTAGTAGCT GGAGCTATAG TACGTCCCCG AGGTCACCGG GCCCGACAAG GTGTAGGAGC   
  
  
- GCAGGGCCGG TCCACCCGGA GGGAAGCATT CCGAGTGGCC CGAGCCCTGG AGGTACCTCC GCGAGCTCCG   
  
  
- GTGGCCTTTT TCCGAGAGTC TGAAGCGGCT CTTCAACCCC AACGGGAAAC TCAAATATGG GCACCGCCTC   
  
  
- TTCTAACCTT TAAACCTGAA CCTTTCCAAC GTACAATCAT TTTCCCTTCG AGAACGACAC GTAACCAATG   
  
  
- TCGTGAGAAA CATACTACAA TGACCGAGAC TATGATTATG TGAACCGGAA GAAGTTTCCA ACCGCGGTTT   
  
  
- TCACCACTGC CACCACCTCG TTCTGGATTC GGCTTGTCCG AGAAAGGATC CTTCCAAACA TCTCCGCTAG   
  
  
- GTGATGATAA GTCGGGATAA ACTGAGGAAT CCTCGCTCAA TACCTCTCCT GTCACTCCTC TCCGTACACC   
  
  
- AACTCGTTGT CGAGGAAAGA TCCCTCTAAG CTTTGTAAGA CCGGCAACCA CCCGGGAGTT CCTGGCCCCT   
  
  
- CGGGTTCAAA CGCTCGACCT CCCTCTTCGA TGTTGTCAGG CCGAAATCCC CTTAGAGTAA CCGTCCGTTG   
  
  
- CGGCGACGGG TCCGGTGGAA CGAGGAGCCG TACAAGGGAA GACTACCCAT ATGAAATTAG CTCCTGTCAC   
  
  
- CGTGTGAATT CGAGCCCACC TTCCTGAACA CGGAGGACTG ACGAAGCCGG ACCTCCGGAA GGGTACGAGT   
  
  
- ATGATACTCG TGGCAGACAT GAGCCTCGGT TAT

+     CCAAT-box

| Site Name | Organism | Position | Strand | Matrix score. | sequence | function |
| --- | --- | --- | --- | --- | --- | --- |
| CCAAT-box | Hordeum vulgare | 1875 | - | 6 | CAACGG | MYBHv1 binding site |
| CCAAT-box | Hordeum vulgare | 4177 | - | 6 | CAACGG | MYBHv1 binding site |
| CCAAT-box | Hordeum vulgare | 2411 | + | 6 | CAACGG | MYBHv1 binding site |

>HU01G00472.1   
+ -Up\_Stream \_Len000GTCTCT GTAACACAAT TCATTAAATC CATCCAAAGG GGATCATAAA TAACCAAACT   
  
  
+ TTGTTGGAAA CTGCTTTGAT GTGGGCTTTC TCTTTCAGCA TTTATGTTAT ACATATATAT GGTTATAAAG   
  
  
+ TTACAAAAGT ATGACCCGAC ACGACAATTC AATCCGAATC CGACTCGAAG TTACTGAAAG TAATCTGAAT   
  
  
+ ACGATTTGAC AGCTTAGTTT AAAAACCTAT TACTTCTCAA TTTTGAGTAA ATCTAAGCTA TTTTTAATGC   
  
  
+ TGAATAGATC GACCCGATTC GACCCGTTTT CCGACTCTAT ATGGTTATGG TGCCCCTAAA AAAAATTAAA   
  
  
+ AAAGAAAAAG AATAGAAAAA TATAAAATAA AACTGAGATT TCTGAACACT CTCTCCCCTA GTCTTGTGGG   
  
  
+ CATGGAAAGT GGTTGGAGAT GGAGATGTTT GAGTTTCATT TTCTGATATT ATTTTTTAGT CATTAGAAGT   
  
  
+ ACAAAGTCTA AACTCAACAA GAGCATGAGG GGAGAATGGT TGTTGTAGCC AATGGAAAAT AAAAAGAACA   
  
  
+ TTCACTCTTC TTGTGCTTTA TTTATGGTGA TAATTTGTTA CTTGTATTTT TAAAAATGAT TAAATATATT   
  
  
+ GATCCTTTGA TATTATGTTG AAATAATAAA AATATAAAAA TTAGAAAATA TTTTACCTCG TGAAATTTTT   
  
  
+ TTTCTGATTA ATCATTGAAA TTATGGTGAA AAATAAAGGA ACATCTCTTG TAAACACTCT ATCCGTTTAC   
  
  
+ GTTTGACCAC AAACTTGCTA TCCACAAGCA TCTCAACCTT TGCCTTTAAC CCAATTGTGG GTTGATGAAA   
  
  
+ TCTAACCATT ATAGGATTAG ATGGTAGTCG TTAATCCTGT GAAACAAGTT TTTGGTTATG ATGTATCCAA   
  
  
+ TTATGAGGGC CTGATAACTC TTGCCTCGGC ATTTGCGTGA TAATGACGTG GTACTAATAG CAATTATTTT   
  
  
+ TCGCAAATAT TCGAAATTTT TTAAGAATAA ACGCAAAATT ATTGATCTTG ACAAATGATT TTATTTTTTT   
  
  
+ GTTGGGAAAA GGATAAACAT CTAGTGTCAA TTGTTGATGA CCTGATAGCT CTCGATTTCT TATTTATGAG   
  
  
+ TTATGTTGAT ATGACACTAA TTGTAATTAT TTGTTGTAAA CTTAAACATT TTTAAAAATC AAAGCAAAAT   
  
  
+ TTGTTAATTA CAATAAATCA ATCTACTTTT ACTATGATCT TATTTGGTGT AATTATTGAT CTTGAATTTT   
  
  
+ TTTTTTTCTT TTTGAAAAAG ATAAGCATCG AATTTTTTCA AAAATAAAAA GGGTGAAGGA AGAAAAAATG   
  
  
+ GAAACAGTTA CCGAGTAAGG AAGGACAGAG CGCGAGAGGA CAAGACACAA AGAGAGGGGA GAGAGAGAGA   
  
  
+ GAAGGGGGCA ATGGCAGTGG GGAAGGTCGA GGAGCACCTT TCAACTTCAA GGCTCAACAC CCCCCCCCCC   
  
  
+ CCCCTCTTTC CTCATTTTGC GACGTTAGCA TTTGACATTT TGCTTTCAAC CGTCCCTCGG CATCATCAGC   
  
  
+ CCTGCCCTTG CTCAGCTTTT CCCATCTTTT TTTGGCTTTT TGTTTGACTC CCTCTATAAC TCTATTCACG   
  
  
+ TCGAGCCTTG TAAAAATTTT ACATATTTTA TTTTATTTTA CTTTTTTAGG TTTATCCCTC TCTATTCTTG   
  
  
+ ACCTACGGGC ATTTTTTATT TTCTTTGGGT TTGAGACTTG TGAACTTTTT GCACATTTTC TCACAAAAAT   
  
  
+ TTATCTTGTC TTTTTCACTT TCAACGTTCC TTCTCTCTCT CTCTCTCTCT CTCTCTCTCT CTCACTCACA   
  
  
+ TCCTAGACAG ACGTCCTTTT TAAAGAATTT ATTTAACAAG TTAGAAGAAA CCGTTGTTTA AGTAGAACCA   
  
  
+ CCCTGTTTGG ATGCCAAAGG AAATACCAGT CAAGAAAAAT CACGACAATC ACAACACATC CATTTCGTTT   
  
  
+ ACATCTCATC CTCATCCTCA TCTTCCTCAT CTCTACCCAT CTCCCTCTTT CTGTATGGCT GCTTCTTCTA   
  
  
+ CTTTGTTCCC TGACCCTAAC AATACTAGTT TAGTCCTCCC TTATTATTAT TATCCTCCTC CTTCCTCCTC   
  
  
+ TTACTATAAT GGTAACCCTT ACCTTCCTCT TCCTAACACC ACTGCTATTC CTACTCCTTC AACAACCTCT   
  
  
+ CCGGTATTTT CTTCCGCCAC CTACTCGGAG TTGAATACTG ATTGTCAAAC TCAACTGCCC ATGTCTAACC   
  
  
+ TCCCCGGAAA GATCGTTCGG AAGCGGAGTG CTGCTGAAAT GGAGCAGCGC TCCGCTCCGA TTGCGGGTGA   
  
  
+ TTACCCGCCA ACTCATCAGC GGGTAATCAC CCGCCACAAT GAGGCGGCCT CATCAGTGTC TTTCATTGAC   
  
  
+ TCCTCACTCT CGCCACTTCA CCTCTTCAAC GGCTCCAATT CGACGACCCC ATTAGCCCCC GACCGTCCTA   
  
  
+ ATGATCCGGG TCTGAGTCAG GGTCCGGATC TGCCTCTTGT GTGCGGATTC TCCGGACTCC CTTTATTCCC   
  
  
+ ACCGGTGGAG AGAAGACCTT CCATCCCCGC CACGACCACT GCTGCCGCGG CGATCGCGGT GGTTCCTGTG   
  
  
+ GAGGAGGATG ATCCGACGTG GATGGATAGC ATCATAAAAG AGCTGATTCA AAGCTCAAAC TCGATCTCAA   
  
  
+ TCCCACAGTT GATTCAGAAC GTTAGAGATA TCATCTACCC ATGTAATCCT AATCTGGGGT CCGCCATTGA   
  
  
+ GTTCCGCCTC CGCTCGCTCG CCGCCGACCC CCTCATTGCT CCGCCGCCAC TTCCGCCCTT CCACCACCAC   
  
  
+ CAACACCACC TCAATCAACA AATCACTCTT CCTCGAATCA GCAACACTAA CAACAACAAT CATGTGAGCG   
  
  
+ TGTACGCCAA CAAAGGACCC GGATACTTCA ACTTGGGTCC GGGTCAGGGT CAGGGTCCGA TTAACATCGA   
  
  
+ CCAAGCTAAC CTTTCCTTCC CTCCTGATTC CACCGCCTGT TGGGGTGTCA GTGTCACACC GCCACCGTCT   
  
  
+ TCCGCCGCCG GTTCCGGCTC CGGTGGAAGC GGCAGTAGTA GTGGTAAATC AAACCCTAAC CCTAATCCCA   
  
  
+ ACCCGAACCC AAACCCAAAC CCAAACCCGA ACAACAAAGC TCAGGATGTT CAACTCCAAA CTCCACAACA   
  
  
+ ACAGCAACAG CAACAGCAAC AAATCCCCGT GGATCAGGAG CAAGACCCGG CGGCTCCACC GGCGACGGAA   
  
  
+ GCATCGCCAC CATCTCCAAG AGCGGCGGCA CCACCGCCGG CGGCAGTGAA AGCAAGAGAA AGAGAGGAGA   
  
  
+ TGCGGCAAAG GAAGCGCGAC GAAGAGGGTC TCCACCTCCT AACCCTCCTC CTCCAATGCG CAGAAGCAGT   
  
  
+ ATCCGGCGAC AAATACGAAG AAGCGAACAA GATGCTTCTA GAAATCTCGG AGTGGGCCAC CCCATTCGGC   
  
  
+ ACCTCCGCCC AACGCGTCGC CGCGTACTTC TCGGAAGCCA TGTCAGCCCG TCTCGTATCC TCCTGCCTCG   
  
  
+ GCATCTACGC CGCCCTCCCC ACCGTCCCAC ACTACGTCAA GCTCCTCTCC GCCTTCCAAG TCTTCAATGG   
  
  
+ CATCAGCCCA TTCGTCAAAT TCTCTCACTT CACTGCAAAC CAAGCAATCC AAGAGGCCTT CCAGAGGGAA   
  
  
+ GACAGGGTCC ACATCATCGA CCTCGATATC ATGCAGGGGC TCCAGTGGCC CGGGCTGTTC CACATCCTCG   
  
  
+ CGTCCCGGCC AGGTGGGCCT CCCTTCGTAA GGCTCACCGG GCTCGGGACC TCCATGGAGG CGCTCGAGGC   
  
  
+ CACCGGAAAA AGGCTCTCAG ACTTCGCCGA GAAGTTGGGG TTGCCCTTTG AGTTTATACC CGTGGCGGAG   
  
  
+ AAGATTGGAA ATTTGGACTT GGAAAGGTTG CATGTTAGTA AAAGGGAAGC TCTTGCTGTG CATTGGTTAC   
  
  
+ AGCACTCTTT GTATGATGTT ACTGGCTCTG ATACTAATAC ACTTGGCCTT CTTCAAAGGT TGGCGCCAAA   
  
  
+ AGTGGTGACG GTGGTGGAGC AAGACCTAAG CCGAACAGGC TCTTTCCTAG GAAGGTTTGT AGAGGCGATC   
  
  
+ CACTACTATT CAGCCCTATT TGACTCCTTA GGAGCGAGTT ATGGAGAGGA CAGTGAGGAG AGGCATGTGG   
  
  
+ TTGAGCAACA GCTCCTTTCT AGGGAGATTC GAAACATTCT GGCCGTTGGT GGGCCCTCAA GGACCGGGGA   
  
  
+ GCCCAAGTTT GCGAGCTGGA GGGAGAAGCT ACAACAGTCC GGCTTTAGGG GAATCTCATT GGCAGGCAAC   
  
  
+ GCCGCTGCCC AGGCCACCTT GCTCCTCGGC ATGTTCCCTT CTGATGGGTA TACTTTAATC GAGGACAGTG   
  
  
+ GCACACTTAA GCTCGGGTGG AAGGACTTGT GCCTCCTGAC TGCTTCGGCC TGGAGGCCTT CCCATGCTCA   
  
  
+ TACTATGAGC ACCGTCTGTA CTCGGAGCCA ATA  

- -Up\_Stream \_Len000CAGAGA CATTGTGTTA AGTAATTTAG GTAGGTTTCC CCTAGTATTT ATTGGTTTGA   
  
  
- AACAACCTTT GACGAAACTA CACCCGAAAG AGAAAGTCGT AAATACAATA TGTATATATA CCAATATTTC   
  
  
- AATGTTTTCA TACTGGGCTG TGCTGTTAAG TTAGGCTTAG GCTGAGCTTC AATGACTTTC ATTAGACTTA   
  
  
- TGCTAAACTG TCGAATCAAA TTTTTGGATA ATGAAGAGTT AAAACTCATT TAGATTCGAT AAAAATTACG   
  
  
- ACTTATCTAG CTGGGCTAAG CTGGGCAAAA GGCTGAGATA TACCAATACC ACGGGGATTT TTTTTAATTT   
  
  
- TTTCTTTTTC TTATCTTTTT ATATTTTATT TTGACTCTAA AGACTTGTGA GAGAGGGGAT CAGAACACCC   
  
  
- GTACCTTTCA CCAACCTCTA CCTCTACAAA CTCAAAGTAA AAGACTATAA TAAAAAATCA GTAATCTTCA   
  
  
- TGTTTCAGAT TTGAGTTGTT CTCGTACTCC CCTCTTACCA ACAACATCGG TTACCTTTTA TTTTTCTTGT   
  
  
- AAGTGAGAAG AACACGAAAT AAATACCACT ATTAAACAAT GAACATAAAA ATTTTTACTA ATTTATATAA   
  
  
- CTAGGAAACT ATAATACAAC TTTATTATTT TTATATTTTT AATCTTTTAT AAAATGGAGC ACTTTAAAAA   
  
  
- AAAGACTAAT TAGTAACTTT AATACCACTT TTTATTTCCT TGTAGAGAAC ATTTGTGAGA TAGGCAAATG   
  
  
- CAAACTGGTG TTTGAACGAT AGGTGTTCGT AGAGTTGGAA ACGGAAATTG GGTTAACACC CAACTACTTT   
  
  
- AGATTGGTAA TATCCTAATC TACCATCAGC AATTAGGACA CTTTGTTCAA AAACCAATAC TACATAGGTT   
  
  
- AATACTCCCG GACTATTGAG AACGGAGCCG TAAACGCACT ATTACTGCAC CATGATTATC GTTAATAAAA   
  
  
- AGCGTTTATA AGCTTTAAAA AATTCTTATT TGCGTTTTAA TAACTAGAAC TGTTTACTAA AATAAAAAAA   
  
  
- CAACCCTTTT CCTATTTGTA GATCACAGTT AACAACTACT GGACTATCGA GAGCTAAAGA ATAAATACTC   
  
  
- AATACAACTA TACTGTGATT AACATTAATA AACAACATTT GAATTTGTAA AAATTTTTAG TTTCGTTTTA   
  
  
- AACAATTAAT GTTATTTAGT TAGATGAAAA TGATACTAGA ATAAACCACA TTAATAACTA GAACTTAAAA   
  
  
- AAAAAAAGAA AAACTTTTTC TATTCGTAGC TTAAAAAAGT TTTTATTTTT CCCACTTCCT TCTTTTTTAC   
  
  
- CTTTGTCAAT GGCTCATTCC TTCCTGTCTC GCGCTCTCCT GTTCTGTGTT TCTCTCCCCT CTCTCTCTCT   
  
  
- CTTCCCCCGT TACCGTCACC CCTTCCAGCT CCTCGTGGAA AGTTGAAGTT CCGAGTTGTG GGGGGGGGGG   
  
  
- GGGGAGAAAG GAGTAAAACG CTGCAATCGT AAACTGTAAA ACGAAAGTTG GCAGGGAGCC GTAGTAGTCG   
  
  
- GGACGGGAAC GAGTCGAAAA GGGTAGAAAA AAACCGAAAA ACAAACTGAG GGAGATATTG AGATAAGTGC   
  
  
- AGCTCGGAAC ATTTTTAAAA TGTATAAAAT AAAATAAAAT GAAAAAATCC AAATAGGGAG AGATAAGAAC   
  
  
- TGGATGCCCG TAAAAAATAA AAGAAACCCA AACTCTGAAC ACTTGAAAAA CGTGTAAAAG AGTGTTTTTA   
  
  
- AATAGAACAG AAAAAGTGAA AGTTGCAAGG AAGAGAGAGA GAGAGAGAGA GAGAGAGAGA GAGTGAGTGT   
  
  
- AGGATCTGTC TGCAGGAAAA ATTTCTTAAA TAAATTGTTC AATCTTCTTT GGCAACAAAT TCATCTTGGT   
  
  
- GGGACAAACC TACGGTTTCC TTTATGGTCA GTTCTTTTTA GTGCTGTTAG TGTTGTGTAG GTAAAGCAAA   
  
  
- TGTAGAGTAG GAGTAGGAGT AGAAGGAGTA GAGATGGGTA GAGGGAGAAA GACATACCGA CGAAGAAGAT   
  
  
- GAAACAAGGG ACTGGGATTG TTATGATCAA ATCAGGAGGG AATAATAATA ATAGGAGGAG GAAGGAGGAG   
  
  
- AATGATATTA CCATTGGGAA TGGAAGGAGA AGGATTGTGG TGACGATAAG GATGAGGAAG TTGTTGGAGA   
  
  
- GGCCATAAAA GAAGGCGGTG GATGAGCCTC AACTTATGAC TAACAGTTTG AGTTGACGGG TACAGATTGG   
  
  
- AGGGGCCTTT CTAGCAAGCC TTCGCCTCAC GACGACTTTA CCTCGTCGCG AGGCGAGGCT AACGCCCACT   
  
  
- AATGGGCGGT TGAGTAGTCG CCCATTAGTG GGCGGTGTTA CTCCGCCGGA GTAGTCACAG AAAGTAACTG   
  
  
- AGGAGTGAGA GCGGTGAAGT GGAGAAGTTG CCGAGGTTAA GCTGCTGGGG TAATCGGGGG CTGGCAGGAT   
  
  
- TACTAGGCCC AGACTCAGTC CCAGGCCTAG ACGGAGAACA CACGCCTAAG AGGCCTGAGG GAAATAAGGG   
  
  
- TGGCCACCTC TCTTCTGGAA GGTAGGGGCG GTGCTGGTGA CGACGGCGCC GCTAGCGCCA CCAAGGACAC   
  
  
- CTCCTCCTAC TAGGCTGCAC CTACCTATCG TAGTATTTTC TCGACTAAGT TTCGAGTTTG AGCTAGAGTT   
  
  
- AGGGTGTCAA CTAAGTCTTG CAATCTCTAT AGTAGATGGG TACATTAGGA TTAGACCCCA GGCGGTAACT   
  
  
- CAAGGCGGAG GCGAGCGAGC GGCGGCTGGG GGAGTAACGA GGCGGCGGTG AAGGCGGGAA GGTGGTGGTG   
  
  
- GTTGTGGTGG AGTTAGTTGT TTAGTGAGAA GGAGCTTAGT CGTTGTGATT GTTGTTGTTA GTACACTCGC   
  
  
- ACATGCGGTT GTTTCCTGGG CCTATGAAGT TGAACCCAGG CCCAGTCCCA GTCCCAGGCT AATTGTAGCT   
  
  
- GGTTCGATTG GAAAGGAAGG GAGGACTAAG GTGGCGGACA ACCCCACAGT CACAGTGTGG CGGTGGCAGA   
  
  
- AGGCGGCGGC CAAGGCCGAG GCCACCTTCG CCGTCATCAT CACCATTTAG TTTGGGATTG GGATTAGGGT   
  
  
- TGGGCTTGGG TTTGGGTTTG GGTTTGGGCT TGTTGTTTCG AGTCCTACAA GTTGAGGTTT GAGGTGTTGT   
  
  
- TGTCGTTGTC GTTGTCGTTG TTTAGGGGCA CCTAGTCCTC GTTCTGGGCC GCCGAGGTGG CCGCTGCCTT   
  
  
- CGTAGCGGTG GTAGAGGTTC TCGCCGCCGT GGTGGCGGCC GCCGTCACTT TCGTTCTCTT TCTCTCCTCT   
  
  
- ACGCCGTTTC CTTCGCGCTG CTTCTCCCAG AGGTGGAGGA TTGGGAGGAG GAGGTTACGC GTCTTCGTCA   
  
  
- TAGGCCGCTG TTTATGCTTC TTCGCTTGTT CTACGAAGAT CTTTAGAGCC TCACCCGGTG GGGTAAGCCG   
  
  
- TGGAGGCGGG TTGCGCAGCG GCGCATGAAG AGCCTTCGGT ACAGTCGGGC AGAGCATAGG AGGACGGAGC   
  
  
- CGTAGATGCG GCGGGAGGGG TGGCAGGGTG TGATGCAGTT CGAGGAGAGG CGGAAGGTTC AGAAGTTACC   
  
  
- GTAGTCGGGT AAGCAGTTTA AGAGAGTGAA GTGACGTTTG GTTCGTTAGG TTCTCCGGAA GGTCTCCCTT   
  
  
- CTGTCCCAGG TGTAGTAGCT GGAGCTATAG TACGTCCCCG AGGTCACCGG GCCCGACAAG GTGTAGGAGC   
  
  
- GCAGGGCCGG TCCACCCGGA GGGAAGCATT CCGAGTGGCC CGAGCCCTGG AGGTACCTCC GCGAGCTCCG   
  
  
- GTGGCCTTTT TCCGAGAGTC TGAAGCGGCT CTTCAACCCC AACGGGAAAC TCAAATATGG GCACCGCCTC   
  
  
- TTCTAACCTT TAAACCTGAA CCTTTCCAAC GTACAATCAT TTTCCCTTCG AGAACGACAC GTAACCAATG   
  
  
- TCGTGAGAAA CATACTACAA TGACCGAGAC TATGATTATG TGAACCGGAA GAAGTTTCCA ACCGCGGTTT   
  
  
- TCACCACTGC CACCACCTCG TTCTGGATTC GGCTTGTCCG AGAAAGGATC CTTCCAAACA TCTCCGCTAG   
  
  
- GTGATGATAA GTCGGGATAA ACTGAGGAAT CCTCGCTCAA TACCTCTCCT GTCACTCCTC TCCGTACACC   
  
  
- AACTCGTTGT CGAGGAAAGA TCCCTCTAAG CTTTGTAAGA CCGGCAACCA CCCGGGAGTT CCTGGCCCCT   
  
  
- CGGGTTCAAA CGCTCGACCT CCCTCTTCGA TGTTGTCAGG CCGAAATCCC CTTAGAGTAA CCGTCCGTTG   
  
  
- CGGCGACGGG TCCGGTGGAA CGAGGAGCCG TACAAGGGAA GACTACCCAT ATGAAATTAG CTCCTGTCAC   
  
  
- CGTGTGAATT CGAGCCCACC TTCCTGAACA CGGAGGACTG ACGAAGCCGG ACCTCCGGAA GGGTACGAGT   
  
  
- ATGATACTCG TGGCAGACAT GAGCCTCGGT TAT

+     CCGTCC motif

| Site Name | Organism | Position | Strand | Matrix score. | sequence | function |
| --- | --- | --- | --- | --- | --- | --- |
| CCGTCC motif | Nicotiana tabacum | 1524 | + | 6 | CCGTCC |  |
| CCGTCC motif | Nicotiana tabacum | 3526 | + | 6 | CCGTCC |  |
| CCGTCC motif | Nicotiana tabacum | 2447 | + | 6 | CCGTCC |  |

>HU01G00472.1   
+ -Up\_Stream \_Len000GTCTCT GTAACACAAT TCATTAAATC CATCCAAAGG GGATCATAAA TAACCAAACT   
  
  
+ TTGTTGGAAA CTGCTTTGAT GTGGGCTTTC TCTTTCAGCA TTTATGTTAT ACATATATAT GGTTATAAAG   
  
  
+ TTACAAAAGT ATGACCCGAC ACGACAATTC AATCCGAATC CGACTCGAAG TTACTGAAAG TAATCTGAAT   
  
  
+ ACGATTTGAC AGCTTAGTTT AAAAACCTAT TACTTCTCAA TTTTGAGTAA ATCTAAGCTA TTTTTAATGC   
  
  
+ TGAATAGATC GACCCGATTC GACCCGTTTT CCGACTCTAT ATGGTTATGG TGCCCCTAAA AAAAATTAAA   
  
  
+ AAAGAAAAAG AATAGAAAAA TATAAAATAA AACTGAGATT TCTGAACACT CTCTCCCCTA GTCTTGTGGG   
  
  
+ CATGGAAAGT GGTTGGAGAT GGAGATGTTT GAGTTTCATT TTCTGATATT ATTTTTTAGT CATTAGAAGT   
  
  
+ ACAAAGTCTA AACTCAACAA GAGCATGAGG GGAGAATGGT TGTTGTAGCC AATGGAAAAT AAAAAGAACA   
  
  
+ TTCACTCTTC TTGTGCTTTA TTTATGGTGA TAATTTGTTA CTTGTATTTT TAAAAATGAT TAAATATATT   
  
  
+ GATCCTTTGA TATTATGTTG AAATAATAAA AATATAAAAA TTAGAAAATA TTTTACCTCG TGAAATTTTT   
  
  
+ TTTCTGATTA ATCATTGAAA TTATGGTGAA AAATAAAGGA ACATCTCTTG TAAACACTCT ATCCGTTTAC   
  
  
+ GTTTGACCAC AAACTTGCTA TCCACAAGCA TCTCAACCTT TGCCTTTAAC CCAATTGTGG GTTGATGAAA   
  
  
+ TCTAACCATT ATAGGATTAG ATGGTAGTCG TTAATCCTGT GAAACAAGTT TTTGGTTATG ATGTATCCAA   
  
  
+ TTATGAGGGC CTGATAACTC TTGCCTCGGC ATTTGCGTGA TAATGACGTG GTACTAATAG CAATTATTTT   
  
  
+ TCGCAAATAT TCGAAATTTT TTAAGAATAA ACGCAAAATT ATTGATCTTG ACAAATGATT TTATTTTTTT   
  
  
+ GTTGGGAAAA GGATAAACAT CTAGTGTCAA TTGTTGATGA CCTGATAGCT CTCGATTTCT TATTTATGAG   
  
  
+ TTATGTTGAT ATGACACTAA TTGTAATTAT TTGTTGTAAA CTTAAACATT TTTAAAAATC AAAGCAAAAT   
  
  
+ TTGTTAATTA CAATAAATCA ATCTACTTTT ACTATGATCT TATTTGGTGT AATTATTGAT CTTGAATTTT   
  
  
+ TTTTTTTCTT TTTGAAAAAG ATAAGCATCG AATTTTTTCA AAAATAAAAA GGGTGAAGGA AGAAAAAATG   
  
  
+ GAAACAGTTA CCGAGTAAGG AAGGACAGAG CGCGAGAGGA CAAGACACAA AGAGAGGGGA GAGAGAGAGA   
  
  
+ GAAGGGGGCA ATGGCAGTGG GGAAGGTCGA GGAGCACCTT TCAACTTCAA GGCTCAACAC CCCCCCCCCC   
  
  
+ CCCCTCTTTC CTCATTTTGC GACGTTAGCA TTTGACATTT TGCTTTCAAC CGTCCCTCGG CATCATCAGC   
  
  
+ CCTGCCCTTG CTCAGCTTTT CCCATCTTTT TTTGGCTTTT TGTTTGACTC CCTCTATAAC TCTATTCACG   
  
  
+ TCGAGCCTTG TAAAAATTTT ACATATTTTA TTTTATTTTA CTTTTTTAGG TTTATCCCTC TCTATTCTTG   
  
  
+ ACCTACGGGC ATTTTTTATT TTCTTTGGGT TTGAGACTTG TGAACTTTTT GCACATTTTC TCACAAAAAT   
  
  
+ TTATCTTGTC TTTTTCACTT TCAACGTTCC TTCTCTCTCT CTCTCTCTCT CTCTCTCTCT CTCACTCACA   
  
  
+ TCCTAGACAG ACGTCCTTTT TAAAGAATTT ATTTAACAAG TTAGAAGAAA CCGTTGTTTA AGTAGAACCA   
  
  
+ CCCTGTTTGG ATGCCAAAGG AAATACCAGT CAAGAAAAAT CACGACAATC ACAACACATC CATTTCGTTT   
  
  
+ ACATCTCATC CTCATCCTCA TCTTCCTCAT CTCTACCCAT CTCCCTCTTT CTGTATGGCT GCTTCTTCTA   
  
  
+ CTTTGTTCCC TGACCCTAAC AATACTAGTT TAGTCCTCCC TTATTATTAT TATCCTCCTC CTTCCTCCTC   
  
  
+ TTACTATAAT GGTAACCCTT ACCTTCCTCT TCCTAACACC ACTGCTATTC CTACTCCTTC AACAACCTCT   
  
  
+ CCGGTATTTT CTTCCGCCAC CTACTCGGAG TTGAATACTG ATTGTCAAAC TCAACTGCCC ATGTCTAACC   
  
  
+ TCCCCGGAAA GATCGTTCGG AAGCGGAGTG CTGCTGAAAT GGAGCAGCGC TCCGCTCCGA TTGCGGGTGA   
  
  
+ TTACCCGCCA ACTCATCAGC GGGTAATCAC CCGCCACAAT GAGGCGGCCT CATCAGTGTC TTTCATTGAC   
  
  
+ TCCTCACTCT CGCCACTTCA CCTCTTCAAC GGCTCCAATT CGACGACCCC ATTAGCCCCC GACCGTCCTA   
  
  
+ ATGATCCGGG TCTGAGTCAG GGTCCGGATC TGCCTCTTGT GTGCGGATTC TCCGGACTCC CTTTATTCCC   
  
  
+ ACCGGTGGAG AGAAGACCTT CCATCCCCGC CACGACCACT GCTGCCGCGG CGATCGCGGT GGTTCCTGTG   
  
  
+ GAGGAGGATG ATCCGACGTG GATGGATAGC ATCATAAAAG AGCTGATTCA AAGCTCAAAC TCGATCTCAA   
  
  
+ TCCCACAGTT GATTCAGAAC GTTAGAGATA TCATCTACCC ATGTAATCCT AATCTGGGGT CCGCCATTGA   
  
  
+ GTTCCGCCTC CGCTCGCTCG CCGCCGACCC CCTCATTGCT CCGCCGCCAC TTCCGCCCTT CCACCACCAC   
  
  
+ CAACACCACC TCAATCAACA AATCACTCTT CCTCGAATCA GCAACACTAA CAACAACAAT CATGTGAGCG   
  
  
+ TGTACGCCAA CAAAGGACCC GGATACTTCA ACTTGGGTCC GGGTCAGGGT CAGGGTCCGA TTAACATCGA   
  
  
+ CCAAGCTAAC CTTTCCTTCC CTCCTGATTC CACCGCCTGT TGGGGTGTCA GTGTCACACC GCCACCGTCT   
  
  
+ TCCGCCGCCG GTTCCGGCTC CGGTGGAAGC GGCAGTAGTA GTGGTAAATC AAACCCTAAC CCTAATCCCA   
  
  
+ ACCCGAACCC AAACCCAAAC CCAAACCCGA ACAACAAAGC TCAGGATGTT CAACTCCAAA CTCCACAACA   
  
  
+ ACAGCAACAG CAACAGCAAC AAATCCCCGT GGATCAGGAG CAAGACCCGG CGGCTCCACC GGCGACGGAA   
  
  
+ GCATCGCCAC CATCTCCAAG AGCGGCGGCA CCACCGCCGG CGGCAGTGAA AGCAAGAGAA AGAGAGGAGA   
  
  
+ TGCGGCAAAG GAAGCGCGAC GAAGAGGGTC TCCACCTCCT AACCCTCCTC CTCCAATGCG CAGAAGCAGT   
  
  
+ ATCCGGCGAC AAATACGAAG AAGCGAACAA GATGCTTCTA GAAATCTCGG AGTGGGCCAC CCCATTCGGC   
  
  
+ ACCTCCGCCC AACGCGTCGC CGCGTACTTC TCGGAAGCCA TGTCAGCCCG TCTCGTATCC TCCTGCCTCG   
  
  
+ GCATCTACGC CGCCCTCCCC ACCGTCCCAC ACTACGTCAA GCTCCTCTCC GCCTTCCAAG TCTTCAATGG   
  
  
+ CATCAGCCCA TTCGTCAAAT TCTCTCACTT CACTGCAAAC CAAGCAATCC AAGAGGCCTT CCAGAGGGAA   
  
  
+ GACAGGGTCC ACATCATCGA CCTCGATATC ATGCAGGGGC TCCAGTGGCC CGGGCTGTTC CACATCCTCG   
  
  
+ CGTCCCGGCC AGGTGGGCCT CCCTTCGTAA GGCTCACCGG GCTCGGGACC TCCATGGAGG CGCTCGAGGC   
  
  
+ CACCGGAAAA AGGCTCTCAG ACTTCGCCGA GAAGTTGGGG TTGCCCTTTG AGTTTATACC CGTGGCGGAG   
  
  
+ AAGATTGGAA ATTTGGACTT GGAAAGGTTG CATGTTAGTA AAAGGGAAGC TCTTGCTGTG CATTGGTTAC   
  
  
+ AGCACTCTTT GTATGATGTT ACTGGCTCTG ATACTAATAC ACTTGGCCTT CTTCAAAGGT TGGCGCCAAA   
  
  
+ AGTGGTGACG GTGGTGGAGC AAGACCTAAG CCGAACAGGC TCTTTCCTAG GAAGGTTTGT AGAGGCGATC   
  
  
+ CACTACTATT CAGCCCTATT TGACTCCTTA GGAGCGAGTT ATGGAGAGGA CAGTGAGGAG AGGCATGTGG   
  
  
+ TTGAGCAACA GCTCCTTTCT AGGGAGATTC GAAACATTCT GGCCGTTGGT GGGCCCTCAA GGACCGGGGA   
  
  
+ GCCCAAGTTT GCGAGCTGGA GGGAGAAGCT ACAACAGTCC GGCTTTAGGG GAATCTCATT GGCAGGCAAC   
  
  
+ GCCGCTGCCC AGGCCACCTT GCTCCTCGGC ATGTTCCCTT CTGATGGGTA TACTTTAATC GAGGACAGTG   
  
  
+ GCACACTTAA GCTCGGGTGG AAGGACTTGT GCCTCCTGAC TGCTTCGGCC TGGAGGCCTT CCCATGCTCA   
  
  
+ TACTATGAGC ACCGTCTGTA CTCGGAGCCA ATA  

- -Up\_Stream \_Len000CAGAGA CATTGTGTTA AGTAATTTAG GTAGGTTTCC CCTAGTATTT ATTGGTTTGA   
  
  
- AACAACCTTT GACGAAACTA CACCCGAAAG AGAAAGTCGT AAATACAATA TGTATATATA CCAATATTTC   
  
  
- AATGTTTTCA TACTGGGCTG TGCTGTTAAG TTAGGCTTAG GCTGAGCTTC AATGACTTTC ATTAGACTTA   
  
  
- TGCTAAACTG TCGAATCAAA TTTTTGGATA ATGAAGAGTT AAAACTCATT TAGATTCGAT AAAAATTACG   
  
  
- ACTTATCTAG CTGGGCTAAG CTGGGCAAAA GGCTGAGATA TACCAATACC ACGGGGATTT TTTTTAATTT   
  
  
- TTTCTTTTTC TTATCTTTTT ATATTTTATT TTGACTCTAA AGACTTGTGA GAGAGGGGAT CAGAACACCC   
  
  
- GTACCTTTCA CCAACCTCTA CCTCTACAAA CTCAAAGTAA AAGACTATAA TAAAAAATCA GTAATCTTCA   
  
  
- TGTTTCAGAT TTGAGTTGTT CTCGTACTCC CCTCTTACCA ACAACATCGG TTACCTTTTA TTTTTCTTGT   
  
  
- AAGTGAGAAG AACACGAAAT AAATACCACT ATTAAACAAT GAACATAAAA ATTTTTACTA ATTTATATAA   
  
  
- CTAGGAAACT ATAATACAAC TTTATTATTT TTATATTTTT AATCTTTTAT AAAATGGAGC ACTTTAAAAA   
  
  
- AAAGACTAAT TAGTAACTTT AATACCACTT TTTATTTCCT TGTAGAGAAC ATTTGTGAGA TAGGCAAATG   
  
  
- CAAACTGGTG TTTGAACGAT AGGTGTTCGT AGAGTTGGAA ACGGAAATTG GGTTAACACC CAACTACTTT   
  
  
- AGATTGGTAA TATCCTAATC TACCATCAGC AATTAGGACA CTTTGTTCAA AAACCAATAC TACATAGGTT   
  
  
- AATACTCCCG GACTATTGAG AACGGAGCCG TAAACGCACT ATTACTGCAC CATGATTATC GTTAATAAAA   
  
  
- AGCGTTTATA AGCTTTAAAA AATTCTTATT TGCGTTTTAA TAACTAGAAC TGTTTACTAA AATAAAAAAA   
  
  
- CAACCCTTTT CCTATTTGTA GATCACAGTT AACAACTACT GGACTATCGA GAGCTAAAGA ATAAATACTC   
  
  
- AATACAACTA TACTGTGATT AACATTAATA AACAACATTT GAATTTGTAA AAATTTTTAG TTTCGTTTTA   
  
  
- AACAATTAAT GTTATTTAGT TAGATGAAAA TGATACTAGA ATAAACCACA TTAATAACTA GAACTTAAAA   
  
  
- AAAAAAAGAA AAACTTTTTC TATTCGTAGC TTAAAAAAGT TTTTATTTTT CCCACTTCCT TCTTTTTTAC   
  
  
- CTTTGTCAAT GGCTCATTCC TTCCTGTCTC GCGCTCTCCT GTTCTGTGTT TCTCTCCCCT CTCTCTCTCT   
  
  
- CTTCCCCCGT TACCGTCACC CCTTCCAGCT CCTCGTGGAA AGTTGAAGTT CCGAGTTGTG GGGGGGGGGG   
  
  
- GGGGAGAAAG GAGTAAAACG CTGCAATCGT AAACTGTAAA ACGAAAGTTG GCAGGGAGCC GTAGTAGTCG   
  
  
- GGACGGGAAC GAGTCGAAAA GGGTAGAAAA AAACCGAAAA ACAAACTGAG GGAGATATTG AGATAAGTGC   
  
  
- AGCTCGGAAC ATTTTTAAAA TGTATAAAAT AAAATAAAAT GAAAAAATCC AAATAGGGAG AGATAAGAAC   
  
  
- TGGATGCCCG TAAAAAATAA AAGAAACCCA AACTCTGAAC ACTTGAAAAA CGTGTAAAAG AGTGTTTTTA   
  
  
- AATAGAACAG AAAAAGTGAA AGTTGCAAGG AAGAGAGAGA GAGAGAGAGA GAGAGAGAGA GAGTGAGTGT   
  
  
- AGGATCTGTC TGCAGGAAAA ATTTCTTAAA TAAATTGTTC AATCTTCTTT GGCAACAAAT TCATCTTGGT   
  
  
- GGGACAAACC TACGGTTTCC TTTATGGTCA GTTCTTTTTA GTGCTGTTAG TGTTGTGTAG GTAAAGCAAA   
  
  
- TGTAGAGTAG GAGTAGGAGT AGAAGGAGTA GAGATGGGTA GAGGGAGAAA GACATACCGA CGAAGAAGAT   
  
  
- GAAACAAGGG ACTGGGATTG TTATGATCAA ATCAGGAGGG AATAATAATA ATAGGAGGAG GAAGGAGGAG   
  
  
- AATGATATTA CCATTGGGAA TGGAAGGAGA AGGATTGTGG TGACGATAAG GATGAGGAAG TTGTTGGAGA   
  
  
- GGCCATAAAA GAAGGCGGTG GATGAGCCTC AACTTATGAC TAACAGTTTG AGTTGACGGG TACAGATTGG   
  
  
- AGGGGCCTTT CTAGCAAGCC TTCGCCTCAC GACGACTTTA CCTCGTCGCG AGGCGAGGCT AACGCCCACT   
  
  
- AATGGGCGGT TGAGTAGTCG CCCATTAGTG GGCGGTGTTA CTCCGCCGGA GTAGTCACAG AAAGTAACTG   
  
  
- AGGAGTGAGA GCGGTGAAGT GGAGAAGTTG CCGAGGTTAA GCTGCTGGGG TAATCGGGGG CTGGCAGGAT   
  
  
- TACTAGGCCC AGACTCAGTC CCAGGCCTAG ACGGAGAACA CACGCCTAAG AGGCCTGAGG GAAATAAGGG   
  
  
- TGGCCACCTC TCTTCTGGAA GGTAGGGGCG GTGCTGGTGA CGACGGCGCC GCTAGCGCCA CCAAGGACAC   
  
  
- CTCCTCCTAC TAGGCTGCAC CTACCTATCG TAGTATTTTC TCGACTAAGT TTCGAGTTTG AGCTAGAGTT   
  
  
- AGGGTGTCAA CTAAGTCTTG CAATCTCTAT AGTAGATGGG TACATTAGGA TTAGACCCCA GGCGGTAACT   
  
  
- CAAGGCGGAG GCGAGCGAGC GGCGGCTGGG GGAGTAACGA GGCGGCGGTG AAGGCGGGAA GGTGGTGGTG   
  
  
- GTTGTGGTGG AGTTAGTTGT TTAGTGAGAA GGAGCTTAGT CGTTGTGATT GTTGTTGTTA GTACACTCGC   
  
  
- ACATGCGGTT GTTTCCTGGG CCTATGAAGT TGAACCCAGG CCCAGTCCCA GTCCCAGGCT AATTGTAGCT   
  
  
- GGTTCGATTG GAAAGGAAGG GAGGACTAAG GTGGCGGACA ACCCCACAGT CACAGTGTGG CGGTGGCAGA   
  
  
- AGGCGGCGGC CAAGGCCGAG GCCACCTTCG CCGTCATCAT CACCATTTAG TTTGGGATTG GGATTAGGGT   
  
  
- TGGGCTTGGG TTTGGGTTTG GGTTTGGGCT TGTTGTTTCG AGTCCTACAA GTTGAGGTTT GAGGTGTTGT   
  
  
- TGTCGTTGTC GTTGTCGTTG TTTAGGGGCA CCTAGTCCTC GTTCTGGGCC GCCGAGGTGG CCGCTGCCTT   
  
  
- CGTAGCGGTG GTAGAGGTTC TCGCCGCCGT GGTGGCGGCC GCCGTCACTT TCGTTCTCTT TCTCTCCTCT   
  
  
- ACGCCGTTTC CTTCGCGCTG CTTCTCCCAG AGGTGGAGGA TTGGGAGGAG GAGGTTACGC GTCTTCGTCA   
  
  
- TAGGCCGCTG TTTATGCTTC TTCGCTTGTT CTACGAAGAT CTTTAGAGCC TCACCCGGTG GGGTAAGCCG   
  
  
- TGGAGGCGGG TTGCGCAGCG GCGCATGAAG AGCCTTCGGT ACAGTCGGGC AGAGCATAGG AGGACGGAGC   
  
  
- CGTAGATGCG GCGGGAGGGG TGGCAGGGTG TGATGCAGTT CGAGGAGAGG CGGAAGGTTC AGAAGTTACC   
  
  
- GTAGTCGGGT AAGCAGTTTA AGAGAGTGAA GTGACGTTTG GTTCGTTAGG TTCTCCGGAA GGTCTCCCTT   
  
  
- CTGTCCCAGG TGTAGTAGCT GGAGCTATAG TACGTCCCCG AGGTCACCGG GCCCGACAAG GTGTAGGAGC   
  
  
- GCAGGGCCGG TCCACCCGGA GGGAAGCATT CCGAGTGGCC CGAGCCCTGG AGGTACCTCC GCGAGCTCCG   
  
  
- GTGGCCTTTT TCCGAGAGTC TGAAGCGGCT CTTCAACCCC AACGGGAAAC TCAAATATGG GCACCGCCTC   
  
  
- TTCTAACCTT TAAACCTGAA CCTTTCCAAC GTACAATCAT TTTCCCTTCG AGAACGACAC GTAACCAATG   
  
  
- TCGTGAGAAA CATACTACAA TGACCGAGAC TATGATTATG TGAACCGGAA GAAGTTTCCA ACCGCGGTTT   
  
  
- TCACCACTGC CACCACCTCG TTCTGGATTC GGCTTGTCCG AGAAAGGATC CTTCCAAACA TCTCCGCTAG   
  
  
- GTGATGATAA GTCGGGATAA ACTGAGGAAT CCTCGCTCAA TACCTCTCCT GTCACTCCTC TCCGTACACC   
  
  
- AACTCGTTGT CGAGGAAAGA TCCCTCTAAG CTTTGTAAGA CCGGCAACCA CCCGGGAGTT CCTGGCCCCT   
  
  
- CGGGTTCAAA CGCTCGACCT CCCTCTTCGA TGTTGTCAGG CCGAAATCCC CTTAGAGTAA CCGTCCGTTG   
  
  
- CGGCGACGGG TCCGGTGGAA CGAGGAGCCG TACAAGGGAA GACTACCCAT ATGAAATTAG CTCCTGTCAC   
  
  
- CGTGTGAATT CGAGCCCACC TTCCTGAACA CGGAGGACTG ACGAAGCCGG ACCTCCGGAA GGGTACGAGT   
  
  
- ATGATACTCG TGGCAGACAT GAGCCTCGGT TAT

+     CCGTCC-box

| Site Name | Organism | Position | Strand | Matrix score. | sequence | function |
| --- | --- | --- | --- | --- | --- | --- |
| CCGTCC-box | Petroselinum hortense | 1524 | + | 6 | CCGTCC |  |
| CCGTCC-box | Petroselinum hortense | 3526 | + | 6 | CCGTCC |  |
| CCGTCC-box | Petroselinum hortense | 2447 | + | 6 | CCGTCC |  |

>HU01G00472.1   
+ -Up\_Stream \_Len000GTCTCT GTAACACAAT TCATTAAATC CATCCAAAGG GGATCATAAA TAACCAAACT   
  
  
+ TTGTTGGAAA CTGCTTTGAT GTGGGCTTTC TCTTTCAGCA TTTATGTTAT ACATATATAT GGTTATAAAG   
  
  
+ TTACAAAAGT ATGACCCGAC ACGACAATTC AATCCGAATC CGACTCGAAG TTACTGAAAG TAATCTGAAT   
  
  
+ ACGATTTGAC AGCTTAGTTT AAAAACCTAT TACTTCTCAA TTTTGAGTAA ATCTAAGCTA TTTTTAATGC   
  
  
+ TGAATAGATC GACCCGATTC GACCCGTTTT CCGACTCTAT ATGGTTATGG TGCCCCTAAA AAAAATTAAA   
  
  
+ AAAGAAAAAG AATAGAAAAA TATAAAATAA AACTGAGATT TCTGAACACT CTCTCCCCTA GTCTTGTGGG   
  
  
+ CATGGAAAGT GGTTGGAGAT GGAGATGTTT GAGTTTCATT TTCTGATATT ATTTTTTAGT CATTAGAAGT   
  
  
+ ACAAAGTCTA AACTCAACAA GAGCATGAGG GGAGAATGGT TGTTGTAGCC AATGGAAAAT AAAAAGAACA   
  
  
+ TTCACTCTTC TTGTGCTTTA TTTATGGTGA TAATTTGTTA CTTGTATTTT TAAAAATGAT TAAATATATT   
  
  
+ GATCCTTTGA TATTATGTTG AAATAATAAA AATATAAAAA TTAGAAAATA TTTTACCTCG TGAAATTTTT   
  
  
+ TTTCTGATTA ATCATTGAAA TTATGGTGAA AAATAAAGGA ACATCTCTTG TAAACACTCT ATCCGTTTAC   
  
  
+ GTTTGACCAC AAACTTGCTA TCCACAAGCA TCTCAACCTT TGCCTTTAAC CCAATTGTGG GTTGATGAAA   
  
  
+ TCTAACCATT ATAGGATTAG ATGGTAGTCG TTAATCCTGT GAAACAAGTT TTTGGTTATG ATGTATCCAA   
  
  
+ TTATGAGGGC CTGATAACTC TTGCCTCGGC ATTTGCGTGA TAATGACGTG GTACTAATAG CAATTATTTT   
  
  
+ TCGCAAATAT TCGAAATTTT TTAAGAATAA ACGCAAAATT ATTGATCTTG ACAAATGATT TTATTTTTTT   
  
  
+ GTTGGGAAAA GGATAAACAT CTAGTGTCAA TTGTTGATGA CCTGATAGCT CTCGATTTCT TATTTATGAG   
  
  
+ TTATGTTGAT ATGACACTAA TTGTAATTAT TTGTTGTAAA CTTAAACATT TTTAAAAATC AAAGCAAAAT   
  
  
+ TTGTTAATTA CAATAAATCA ATCTACTTTT ACTATGATCT TATTTGGTGT AATTATTGAT CTTGAATTTT   
  
  
+ TTTTTTTCTT TTTGAAAAAG ATAAGCATCG AATTTTTTCA AAAATAAAAA GGGTGAAGGA AGAAAAAATG   
  
  
+ GAAACAGTTA CCGAGTAAGG AAGGACAGAG CGCGAGAGGA CAAGACACAA AGAGAGGGGA GAGAGAGAGA   
  
  
+ GAAGGGGGCA ATGGCAGTGG GGAAGGTCGA GGAGCACCTT TCAACTTCAA GGCTCAACAC CCCCCCCCCC   
  
  
+ CCCCTCTTTC CTCATTTTGC GACGTTAGCA TTTGACATTT TGCTTTCAAC CGTCCCTCGG CATCATCAGC   
  
  
+ CCTGCCCTTG CTCAGCTTTT CCCATCTTTT TTTGGCTTTT TGTTTGACTC CCTCTATAAC TCTATTCACG   
  
  
+ TCGAGCCTTG TAAAAATTTT ACATATTTTA TTTTATTTTA CTTTTTTAGG TTTATCCCTC TCTATTCTTG   
  
  
+ ACCTACGGGC ATTTTTTATT TTCTTTGGGT TTGAGACTTG TGAACTTTTT GCACATTTTC TCACAAAAAT   
  
  
+ TTATCTTGTC TTTTTCACTT TCAACGTTCC TTCTCTCTCT CTCTCTCTCT CTCTCTCTCT CTCACTCACA   
  
  
+ TCCTAGACAG ACGTCCTTTT TAAAGAATTT ATTTAACAAG TTAGAAGAAA CCGTTGTTTA AGTAGAACCA   
  
  
+ CCCTGTTTGG ATGCCAAAGG AAATACCAGT CAAGAAAAAT CACGACAATC ACAACACATC CATTTCGTTT   
  
  
+ ACATCTCATC CTCATCCTCA TCTTCCTCAT CTCTACCCAT CTCCCTCTTT CTGTATGGCT GCTTCTTCTA   
  
  
+ CTTTGTTCCC TGACCCTAAC AATACTAGTT TAGTCCTCCC TTATTATTAT TATCCTCCTC CTTCCTCCTC   
  
  
+ TTACTATAAT GGTAACCCTT ACCTTCCTCT TCCTAACACC ACTGCTATTC CTACTCCTTC AACAACCTCT   
  
  
+ CCGGTATTTT CTTCCGCCAC CTACTCGGAG TTGAATACTG ATTGTCAAAC TCAACTGCCC ATGTCTAACC   
  
  
+ TCCCCGGAAA GATCGTTCGG AAGCGGAGTG CTGCTGAAAT GGAGCAGCGC TCCGCTCCGA TTGCGGGTGA   
  
  
+ TTACCCGCCA ACTCATCAGC GGGTAATCAC CCGCCACAAT GAGGCGGCCT CATCAGTGTC TTTCATTGAC   
  
  
+ TCCTCACTCT CGCCACTTCA CCTCTTCAAC GGCTCCAATT CGACGACCCC ATTAGCCCCC GACCGTCCTA   
  
  
+ ATGATCCGGG TCTGAGTCAG GGTCCGGATC TGCCTCTTGT GTGCGGATTC TCCGGACTCC CTTTATTCCC   
  
  
+ ACCGGTGGAG AGAAGACCTT CCATCCCCGC CACGACCACT GCTGCCGCGG CGATCGCGGT GGTTCCTGTG   
  
  
+ GAGGAGGATG ATCCGACGTG GATGGATAGC ATCATAAAAG AGCTGATTCA AAGCTCAAAC TCGATCTCAA   
  
  
+ TCCCACAGTT GATTCAGAAC GTTAGAGATA TCATCTACCC ATGTAATCCT AATCTGGGGT CCGCCATTGA   
  
  
+ GTTCCGCCTC CGCTCGCTCG CCGCCGACCC CCTCATTGCT CCGCCGCCAC TTCCGCCCTT CCACCACCAC   
  
  
+ CAACACCACC TCAATCAACA AATCACTCTT CCTCGAATCA GCAACACTAA CAACAACAAT CATGTGAGCG   
  
  
+ TGTACGCCAA CAAAGGACCC GGATACTTCA ACTTGGGTCC GGGTCAGGGT CAGGGTCCGA TTAACATCGA   
  
  
+ CCAAGCTAAC CTTTCCTTCC CTCCTGATTC CACCGCCTGT TGGGGTGTCA GTGTCACACC GCCACCGTCT   
  
  
+ TCCGCCGCCG GTTCCGGCTC CGGTGGAAGC GGCAGTAGTA GTGGTAAATC AAACCCTAAC CCTAATCCCA   
  
  
+ ACCCGAACCC AAACCCAAAC CCAAACCCGA ACAACAAAGC TCAGGATGTT CAACTCCAAA CTCCACAACA   
  
  
+ ACAGCAACAG CAACAGCAAC AAATCCCCGT GGATCAGGAG CAAGACCCGG CGGCTCCACC GGCGACGGAA   
  
  
+ GCATCGCCAC CATCTCCAAG AGCGGCGGCA CCACCGCCGG CGGCAGTGAA AGCAAGAGAA AGAGAGGAGA   
  
  
+ TGCGGCAAAG GAAGCGCGAC GAAGAGGGTC TCCACCTCCT AACCCTCCTC CTCCAATGCG CAGAAGCAGT   
  
  
+ ATCCGGCGAC AAATACGAAG AAGCGAACAA GATGCTTCTA GAAATCTCGG AGTGGGCCAC CCCATTCGGC   
  
  
+ ACCTCCGCCC AACGCGTCGC CGCGTACTTC TCGGAAGCCA TGTCAGCCCG TCTCGTATCC TCCTGCCTCG   
  
  
+ GCATCTACGC CGCCCTCCCC ACCGTCCCAC ACTACGTCAA GCTCCTCTCC GCCTTCCAAG TCTTCAATGG   
  
  
+ CATCAGCCCA TTCGTCAAAT TCTCTCACTT CACTGCAAAC CAAGCAATCC AAGAGGCCTT CCAGAGGGAA   
  
  
+ GACAGGGTCC ACATCATCGA CCTCGATATC ATGCAGGGGC TCCAGTGGCC CGGGCTGTTC CACATCCTCG   
  
  
+ CGTCCCGGCC AGGTGGGCCT CCCTTCGTAA GGCTCACCGG GCTCGGGACC TCCATGGAGG CGCTCGAGGC   
  
  
+ CACCGGAAAA AGGCTCTCAG ACTTCGCCGA GAAGTTGGGG TTGCCCTTTG AGTTTATACC CGTGGCGGAG   
  
  
+ AAGATTGGAA ATTTGGACTT GGAAAGGTTG CATGTTAGTA AAAGGGAAGC TCTTGCTGTG CATTGGTTAC   
  
  
+ AGCACTCTTT GTATGATGTT ACTGGCTCTG ATACTAATAC ACTTGGCCTT CTTCAAAGGT TGGCGCCAAA   
  
  
+ AGTGGTGACG GTGGTGGAGC AAGACCTAAG CCGAACAGGC TCTTTCCTAG GAAGGTTTGT AGAGGCGATC   
  
  
+ CACTACTATT CAGCCCTATT TGACTCCTTA GGAGCGAGTT ATGGAGAGGA CAGTGAGGAG AGGCATGTGG   
  
  
+ TTGAGCAACA GCTCCTTTCT AGGGAGATTC GAAACATTCT GGCCGTTGGT GGGCCCTCAA GGACCGGGGA   
  
  
+ GCCCAAGTTT GCGAGCTGGA GGGAGAAGCT ACAACAGTCC GGCTTTAGGG GAATCTCATT GGCAGGCAAC   
  
  
+ GCCGCTGCCC AGGCCACCTT GCTCCTCGGC ATGTTCCCTT CTGATGGGTA TACTTTAATC GAGGACAGTG   
  
  
+ GCACACTTAA GCTCGGGTGG AAGGACTTGT GCCTCCTGAC TGCTTCGGCC TGGAGGCCTT CCCATGCTCA   
  
  
+ TACTATGAGC ACCGTCTGTA CTCGGAGCCA ATA  

- -Up\_Stream \_Len000CAGAGA CATTGTGTTA AGTAATTTAG GTAGGTTTCC CCTAGTATTT ATTGGTTTGA   
  
  
- AACAACCTTT GACGAAACTA CACCCGAAAG AGAAAGTCGT AAATACAATA TGTATATATA CCAATATTTC   
  
  
- AATGTTTTCA TACTGGGCTG TGCTGTTAAG TTAGGCTTAG GCTGAGCTTC AATGACTTTC ATTAGACTTA   
  
  
- TGCTAAACTG TCGAATCAAA TTTTTGGATA ATGAAGAGTT AAAACTCATT TAGATTCGAT AAAAATTACG   
  
  
- ACTTATCTAG CTGGGCTAAG CTGGGCAAAA GGCTGAGATA TACCAATACC ACGGGGATTT TTTTTAATTT   
  
  
- TTTCTTTTTC TTATCTTTTT ATATTTTATT TTGACTCTAA AGACTTGTGA GAGAGGGGAT CAGAACACCC   
  
  
- GTACCTTTCA CCAACCTCTA CCTCTACAAA CTCAAAGTAA AAGACTATAA TAAAAAATCA GTAATCTTCA   
  
  
- TGTTTCAGAT TTGAGTTGTT CTCGTACTCC CCTCTTACCA ACAACATCGG TTACCTTTTA TTTTTCTTGT   
  
  
- AAGTGAGAAG AACACGAAAT AAATACCACT ATTAAACAAT GAACATAAAA ATTTTTACTA ATTTATATAA   
  
  
- CTAGGAAACT ATAATACAAC TTTATTATTT TTATATTTTT AATCTTTTAT AAAATGGAGC ACTTTAAAAA   
  
  
- AAAGACTAAT TAGTAACTTT AATACCACTT TTTATTTCCT TGTAGAGAAC ATTTGTGAGA TAGGCAAATG   
  
  
- CAAACTGGTG TTTGAACGAT AGGTGTTCGT AGAGTTGGAA ACGGAAATTG GGTTAACACC CAACTACTTT   
  
  
- AGATTGGTAA TATCCTAATC TACCATCAGC AATTAGGACA CTTTGTTCAA AAACCAATAC TACATAGGTT   
  
  
- AATACTCCCG GACTATTGAG AACGGAGCCG TAAACGCACT ATTACTGCAC CATGATTATC GTTAATAAAA   
  
  
- AGCGTTTATA AGCTTTAAAA AATTCTTATT TGCGTTTTAA TAACTAGAAC TGTTTACTAA AATAAAAAAA   
  
  
- CAACCCTTTT CCTATTTGTA GATCACAGTT AACAACTACT GGACTATCGA GAGCTAAAGA ATAAATACTC   
  
  
- AATACAACTA TACTGTGATT AACATTAATA AACAACATTT GAATTTGTAA AAATTTTTAG TTTCGTTTTA   
  
  
- AACAATTAAT GTTATTTAGT TAGATGAAAA TGATACTAGA ATAAACCACA TTAATAACTA GAACTTAAAA   
  
  
- AAAAAAAGAA AAACTTTTTC TATTCGTAGC TTAAAAAAGT TTTTATTTTT CCCACTTCCT TCTTTTTTAC   
  
  
- CTTTGTCAAT GGCTCATTCC TTCCTGTCTC GCGCTCTCCT GTTCTGTGTT TCTCTCCCCT CTCTCTCTCT   
  
  
- CTTCCCCCGT TACCGTCACC CCTTCCAGCT CCTCGTGGAA AGTTGAAGTT CCGAGTTGTG GGGGGGGGGG   
  
  
- GGGGAGAAAG GAGTAAAACG CTGCAATCGT AAACTGTAAA ACGAAAGTTG GCAGGGAGCC GTAGTAGTCG   
  
  
- GGACGGGAAC GAGTCGAAAA GGGTAGAAAA AAACCGAAAA ACAAACTGAG GGAGATATTG AGATAAGTGC   
  
  
- AGCTCGGAAC ATTTTTAAAA TGTATAAAAT AAAATAAAAT GAAAAAATCC AAATAGGGAG AGATAAGAAC   
  
  
- TGGATGCCCG TAAAAAATAA AAGAAACCCA AACTCTGAAC ACTTGAAAAA CGTGTAAAAG AGTGTTTTTA   
  
  
- AATAGAACAG AAAAAGTGAA AGTTGCAAGG AAGAGAGAGA GAGAGAGAGA GAGAGAGAGA GAGTGAGTGT   
  
  
- AGGATCTGTC TGCAGGAAAA ATTTCTTAAA TAAATTGTTC AATCTTCTTT GGCAACAAAT TCATCTTGGT   
  
  
- GGGACAAACC TACGGTTTCC TTTATGGTCA GTTCTTTTTA GTGCTGTTAG TGTTGTGTAG GTAAAGCAAA   
  
  
- TGTAGAGTAG GAGTAGGAGT AGAAGGAGTA GAGATGGGTA GAGGGAGAAA GACATACCGA CGAAGAAGAT   
  
  
- GAAACAAGGG ACTGGGATTG TTATGATCAA ATCAGGAGGG AATAATAATA ATAGGAGGAG GAAGGAGGAG   
  
  
- AATGATATTA CCATTGGGAA TGGAAGGAGA AGGATTGTGG TGACGATAAG GATGAGGAAG TTGTTGGAGA   
  
  
- GGCCATAAAA GAAGGCGGTG GATGAGCCTC AACTTATGAC TAACAGTTTG AGTTGACGGG TACAGATTGG   
  
  
- AGGGGCCTTT CTAGCAAGCC TTCGCCTCAC GACGACTTTA CCTCGTCGCG AGGCGAGGCT AACGCCCACT   
  
  
- AATGGGCGGT TGAGTAGTCG CCCATTAGTG GGCGGTGTTA CTCCGCCGGA GTAGTCACAG AAAGTAACTG   
  
  
- AGGAGTGAGA GCGGTGAAGT GGAGAAGTTG CCGAGGTTAA GCTGCTGGGG TAATCGGGGG CTGGCAGGAT   
  
  
- TACTAGGCCC AGACTCAGTC CCAGGCCTAG ACGGAGAACA CACGCCTAAG AGGCCTGAGG GAAATAAGGG   
  
  
- TGGCCACCTC TCTTCTGGAA GGTAGGGGCG GTGCTGGTGA CGACGGCGCC GCTAGCGCCA CCAAGGACAC   
  
  
- CTCCTCCTAC TAGGCTGCAC CTACCTATCG TAGTATTTTC TCGACTAAGT TTCGAGTTTG AGCTAGAGTT   
  
  
- AGGGTGTCAA CTAAGTCTTG CAATCTCTAT AGTAGATGGG TACATTAGGA TTAGACCCCA GGCGGTAACT   
  
  
- CAAGGCGGAG GCGAGCGAGC GGCGGCTGGG GGAGTAACGA GGCGGCGGTG AAGGCGGGAA GGTGGTGGTG   
  
  
- GTTGTGGTGG AGTTAGTTGT TTAGTGAGAA GGAGCTTAGT CGTTGTGATT GTTGTTGTTA GTACACTCGC   
  
  
- ACATGCGGTT GTTTCCTGGG CCTATGAAGT TGAACCCAGG CCCAGTCCCA GTCCCAGGCT AATTGTAGCT   
  
  
- GGTTCGATTG GAAAGGAAGG GAGGACTAAG GTGGCGGACA ACCCCACAGT CACAGTGTGG CGGTGGCAGA   
  
  
- AGGCGGCGGC CAAGGCCGAG GCCACCTTCG CCGTCATCAT CACCATTTAG TTTGGGATTG GGATTAGGGT   
  
  
- TGGGCTTGGG TTTGGGTTTG GGTTTGGGCT TGTTGTTTCG AGTCCTACAA GTTGAGGTTT GAGGTGTTGT   
  
  
- TGTCGTTGTC GTTGTCGTTG TTTAGGGGCA CCTAGTCCTC GTTCTGGGCC GCCGAGGTGG CCGCTGCCTT   
  
  
- CGTAGCGGTG GTAGAGGTTC TCGCCGCCGT GGTGGCGGCC GCCGTCACTT TCGTTCTCTT TCTCTCCTCT   
  
  
- ACGCCGTTTC CTTCGCGCTG CTTCTCCCAG AGGTGGAGGA TTGGGAGGAG GAGGTTACGC GTCTTCGTCA   
  
  
- TAGGCCGCTG TTTATGCTTC TTCGCTTGTT CTACGAAGAT CTTTAGAGCC TCACCCGGTG GGGTAAGCCG   
  
  
- TGGAGGCGGG TTGCGCAGCG GCGCATGAAG AGCCTTCGGT ACAGTCGGGC AGAGCATAGG AGGACGGAGC   
  
  
- CGTAGATGCG GCGGGAGGGG TGGCAGGGTG TGATGCAGTT CGAGGAGAGG CGGAAGGTTC AGAAGTTACC   
  
  
- GTAGTCGGGT AAGCAGTTTA AGAGAGTGAA GTGACGTTTG GTTCGTTAGG TTCTCCGGAA GGTCTCCCTT   
  
  
- CTGTCCCAGG TGTAGTAGCT GGAGCTATAG TACGTCCCCG AGGTCACCGG GCCCGACAAG GTGTAGGAGC   
  
  
- GCAGGGCCGG TCCACCCGGA GGGAAGCATT CCGAGTGGCC CGAGCCCTGG AGGTACCTCC GCGAGCTCCG   
  
  
- GTGGCCTTTT TCCGAGAGTC TGAAGCGGCT CTTCAACCCC AACGGGAAAC TCAAATATGG GCACCGCCTC   
  
  
- TTCTAACCTT TAAACCTGAA CCTTTCCAAC GTACAATCAT TTTCCCTTCG AGAACGACAC GTAACCAATG   
  
  
- TCGTGAGAAA CATACTACAA TGACCGAGAC TATGATTATG TGAACCGGAA GAAGTTTCCA ACCGCGGTTT   
  
  
- TCACCACTGC CACCACCTCG TTCTGGATTC GGCTTGTCCG AGAAAGGATC CTTCCAAACA TCTCCGCTAG   
  
  
- GTGATGATAA GTCGGGATAA ACTGAGGAAT CCTCGCTCAA TACCTCTCCT GTCACTCCTC TCCGTACACC   
  
  
- AACTCGTTGT CGAGGAAAGA TCCCTCTAAG CTTTGTAAGA CCGGCAACCA CCCGGGAGTT CCTGGCCCCT   
  
  
- CGGGTTCAAA CGCTCGACCT CCCTCTTCGA TGTTGTCAGG CCGAAATCCC CTTAGAGTAA CCGTCCGTTG   
  
  
- CGGCGACGGG TCCGGTGGAA CGAGGAGCCG TACAAGGGAA GACTACCCAT ATGAAATTAG CTCCTGTCAC   
  
  
- CGTGTGAATT CGAGCCCACC TTCCTGAACA CGGAGGACTG ACGAAGCCGG ACCTCCGGAA GGGTACGAGT   
  
  
- ATGATACTCG TGGCAGACAT GAGCCTCGGT TAT

+     CGTCA-motif

| Site Name | Organism | Position | Strand | Matrix score. | sequence | function |
| --- | --- | --- | --- | --- | --- | --- |
| CGTCA-motif | Hordeum vulgare | 958 | - | 5 | CGTCA | cis-acting regulatory element involved in the MeJA-responsiveness |
| CGTCA-motif | Hordeum vulgare | 3587 | + | 5 | CGTCA | cis-acting regulatory element involved in the MeJA-responsiveness |
| CGTCA-motif | Hordeum vulgare | 3539 | + | 5 | CGTCA | cis-acting regulatory element involved in the MeJA-responsiveness |
| CGTCA-motif | Hordeum vulgare | 4000 | - | 5 | CGTCA | cis-acting regulatory element involved in the MeJA-responsiveness |

>HU01G00472.1   
+ -Up\_Stream \_Len000GTCTCT GTAACACAAT TCATTAAATC CATCCAAAGG GGATCATAAA TAACCAAACT   
  
  
+ TTGTTGGAAA CTGCTTTGAT GTGGGCTTTC TCTTTCAGCA TTTATGTTAT ACATATATAT GGTTATAAAG   
  
  
+ TTACAAAAGT ATGACCCGAC ACGACAATTC AATCCGAATC CGACTCGAAG TTACTGAAAG TAATCTGAAT   
  
  
+ ACGATTTGAC AGCTTAGTTT AAAAACCTAT TACTTCTCAA TTTTGAGTAA ATCTAAGCTA TTTTTAATGC   
  
  
+ TGAATAGATC GACCCGATTC GACCCGTTTT CCGACTCTAT ATGGTTATGG TGCCCCTAAA AAAAATTAAA   
  
  
+ AAAGAAAAAG AATAGAAAAA TATAAAATAA AACTGAGATT TCTGAACACT CTCTCCCCTA GTCTTGTGGG   
  
  
+ CATGGAAAGT GGTTGGAGAT GGAGATGTTT GAGTTTCATT TTCTGATATT ATTTTTTAGT CATTAGAAGT   
  
  
+ ACAAAGTCTA AACTCAACAA GAGCATGAGG GGAGAATGGT TGTTGTAGCC AATGGAAAAT AAAAAGAACA   
  
  
+ TTCACTCTTC TTGTGCTTTA TTTATGGTGA TAATTTGTTA CTTGTATTTT TAAAAATGAT TAAATATATT   
  
  
+ GATCCTTTGA TATTATGTTG AAATAATAAA AATATAAAAA TTAGAAAATA TTTTACCTCG TGAAATTTTT   
  
  
+ TTTCTGATTA ATCATTGAAA TTATGGTGAA AAATAAAGGA ACATCTCTTG TAAACACTCT ATCCGTTTAC   
  
  
+ GTTTGACCAC AAACTTGCTA TCCACAAGCA TCTCAACCTT TGCCTTTAAC CCAATTGTGG GTTGATGAAA   
  
  
+ TCTAACCATT ATAGGATTAG ATGGTAGTCG TTAATCCTGT GAAACAAGTT TTTGGTTATG ATGTATCCAA   
  
  
+ TTATGAGGGC CTGATAACTC TTGCCTCGGC ATTTGCGTGA TAATGACGTG GTACTAATAG CAATTATTTT   
  
  
+ TCGCAAATAT TCGAAATTTT TTAAGAATAA ACGCAAAATT ATTGATCTTG ACAAATGATT TTATTTTTTT   
  
  
+ GTTGGGAAAA GGATAAACAT CTAGTGTCAA TTGTTGATGA CCTGATAGCT CTCGATTTCT TATTTATGAG   
  
  
+ TTATGTTGAT ATGACACTAA TTGTAATTAT TTGTTGTAAA CTTAAACATT TTTAAAAATC AAAGCAAAAT   
  
  
+ TTGTTAATTA CAATAAATCA ATCTACTTTT ACTATGATCT TATTTGGTGT AATTATTGAT CTTGAATTTT   
  
  
+ TTTTTTTCTT TTTGAAAAAG ATAAGCATCG AATTTTTTCA AAAATAAAAA GGGTGAAGGA AGAAAAAATG   
  
  
+ GAAACAGTTA CCGAGTAAGG AAGGACAGAG CGCGAGAGGA CAAGACACAA AGAGAGGGGA GAGAGAGAGA   
  
  
+ GAAGGGGGCA ATGGCAGTGG GGAAGGTCGA GGAGCACCTT TCAACTTCAA GGCTCAACAC CCCCCCCCCC   
  
  
+ CCCCTCTTTC CTCATTTTGC GACGTTAGCA TTTGACATTT TGCTTTCAAC CGTCCCTCGG CATCATCAGC   
  
  
+ CCTGCCCTTG CTCAGCTTTT CCCATCTTTT TTTGGCTTTT TGTTTGACTC CCTCTATAAC TCTATTCACG   
  
  
+ TCGAGCCTTG TAAAAATTTT ACATATTTTA TTTTATTTTA CTTTTTTAGG TTTATCCCTC TCTATTCTTG   
  
  
+ ACCTACGGGC ATTTTTTATT TTCTTTGGGT TTGAGACTTG TGAACTTTTT GCACATTTTC TCACAAAAAT   
  
  
+ TTATCTTGTC TTTTTCACTT TCAACGTTCC TTCTCTCTCT CTCTCTCTCT CTCTCTCTCT CTCACTCACA   
  
  
+ TCCTAGACAG ACGTCCTTTT TAAAGAATTT ATTTAACAAG TTAGAAGAAA CCGTTGTTTA AGTAGAACCA   
  
  
+ CCCTGTTTGG ATGCCAAAGG AAATACCAGT CAAGAAAAAT CACGACAATC ACAACACATC CATTTCGTTT   
  
  
+ ACATCTCATC CTCATCCTCA TCTTCCTCAT CTCTACCCAT CTCCCTCTTT CTGTATGGCT GCTTCTTCTA   
  
  
+ CTTTGTTCCC TGACCCTAAC AATACTAGTT TAGTCCTCCC TTATTATTAT TATCCTCCTC CTTCCTCCTC   
  
  
+ TTACTATAAT GGTAACCCTT ACCTTCCTCT TCCTAACACC ACTGCTATTC CTACTCCTTC AACAACCTCT   
  
  
+ CCGGTATTTT CTTCCGCCAC CTACTCGGAG TTGAATACTG ATTGTCAAAC TCAACTGCCC ATGTCTAACC   
  
  
+ TCCCCGGAAA GATCGTTCGG AAGCGGAGTG CTGCTGAAAT GGAGCAGCGC TCCGCTCCGA TTGCGGGTGA   
  
  
+ TTACCCGCCA ACTCATCAGC GGGTAATCAC CCGCCACAAT GAGGCGGCCT CATCAGTGTC TTTCATTGAC   
  
  
+ TCCTCACTCT CGCCACTTCA CCTCTTCAAC GGCTCCAATT CGACGACCCC ATTAGCCCCC GACCGTCCTA   
  
  
+ ATGATCCGGG TCTGAGTCAG GGTCCGGATC TGCCTCTTGT GTGCGGATTC TCCGGACTCC CTTTATTCCC   
  
  
+ ACCGGTGGAG AGAAGACCTT CCATCCCCGC CACGACCACT GCTGCCGCGG CGATCGCGGT GGTTCCTGTG   
  
  
+ GAGGAGGATG ATCCGACGTG GATGGATAGC ATCATAAAAG AGCTGATTCA AAGCTCAAAC TCGATCTCAA   
  
  
+ TCCCACAGTT GATTCAGAAC GTTAGAGATA TCATCTACCC ATGTAATCCT AATCTGGGGT CCGCCATTGA   
  
  
+ GTTCCGCCTC CGCTCGCTCG CCGCCGACCC CCTCATTGCT CCGCCGCCAC TTCCGCCCTT CCACCACCAC   
  
  
+ CAACACCACC TCAATCAACA AATCACTCTT CCTCGAATCA GCAACACTAA CAACAACAAT CATGTGAGCG   
  
  
+ TGTACGCCAA CAAAGGACCC GGATACTTCA ACTTGGGTCC GGGTCAGGGT CAGGGTCCGA TTAACATCGA   
  
  
+ CCAAGCTAAC CTTTCCTTCC CTCCTGATTC CACCGCCTGT TGGGGTGTCA GTGTCACACC GCCACCGTCT   
  
  
+ TCCGCCGCCG GTTCCGGCTC CGGTGGAAGC GGCAGTAGTA GTGGTAAATC AAACCCTAAC CCTAATCCCA   
  
  
+ ACCCGAACCC AAACCCAAAC CCAAACCCGA ACAACAAAGC TCAGGATGTT CAACTCCAAA CTCCACAACA   
  
  
+ ACAGCAACAG CAACAGCAAC AAATCCCCGT GGATCAGGAG CAAGACCCGG CGGCTCCACC GGCGACGGAA   
  
  
+ GCATCGCCAC CATCTCCAAG AGCGGCGGCA CCACCGCCGG CGGCAGTGAA AGCAAGAGAA AGAGAGGAGA   
  
  
+ TGCGGCAAAG GAAGCGCGAC GAAGAGGGTC TCCACCTCCT AACCCTCCTC CTCCAATGCG CAGAAGCAGT   
  
  
+ ATCCGGCGAC AAATACGAAG AAGCGAACAA GATGCTTCTA GAAATCTCGG AGTGGGCCAC CCCATTCGGC   
  
  
+ ACCTCCGCCC AACGCGTCGC CGCGTACTTC TCGGAAGCCA TGTCAGCCCG TCTCGTATCC TCCTGCCTCG   
  
  
+ GCATCTACGC CGCCCTCCCC ACCGTCCCAC ACTACGTCAA GCTCCTCTCC GCCTTCCAAG TCTTCAATGG   
  
  
+ CATCAGCCCA TTCGTCAAAT TCTCTCACTT CACTGCAAAC CAAGCAATCC AAGAGGCCTT CCAGAGGGAA   
  
  
+ GACAGGGTCC ACATCATCGA CCTCGATATC ATGCAGGGGC TCCAGTGGCC CGGGCTGTTC CACATCCTCG   
  
  
+ CGTCCCGGCC AGGTGGGCCT CCCTTCGTAA GGCTCACCGG GCTCGGGACC TCCATGGAGG CGCTCGAGGC   
  
  
+ CACCGGAAAA AGGCTCTCAG ACTTCGCCGA GAAGTTGGGG TTGCCCTTTG AGTTTATACC CGTGGCGGAG   
  
  
+ AAGATTGGAA ATTTGGACTT GGAAAGGTTG CATGTTAGTA AAAGGGAAGC TCTTGCTGTG CATTGGTTAC   
  
  
+ AGCACTCTTT GTATGATGTT ACTGGCTCTG ATACTAATAC ACTTGGCCTT CTTCAAAGGT TGGCGCCAAA   
  
  
+ AGTGGTGACG GTGGTGGAGC AAGACCTAAG CCGAACAGGC TCTTTCCTAG GAAGGTTTGT AGAGGCGATC   
  
  
+ CACTACTATT CAGCCCTATT TGACTCCTTA GGAGCGAGTT ATGGAGAGGA CAGTGAGGAG AGGCATGTGG   
  
  
+ TTGAGCAACA GCTCCTTTCT AGGGAGATTC GAAACATTCT GGCCGTTGGT GGGCCCTCAA GGACCGGGGA   
  
  
+ GCCCAAGTTT GCGAGCTGGA GGGAGAAGCT ACAACAGTCC GGCTTTAGGG GAATCTCATT GGCAGGCAAC   
  
  
+ GCCGCTGCCC AGGCCACCTT GCTCCTCGGC ATGTTCCCTT CTGATGGGTA TACTTTAATC GAGGACAGTG   
  
  
+ GCACACTTAA GCTCGGGTGG AAGGACTTGT GCCTCCTGAC TGCTTCGGCC TGGAGGCCTT CCCATGCTCA   
  
  
+ TACTATGAGC ACCGTCTGTA CTCGGAGCCA ATA  

- -Up\_Stream \_Len000CAGAGA CATTGTGTTA AGTAATTTAG GTAGGTTTCC CCTAGTATTT ATTGGTTTGA   
  
  
- AACAACCTTT GACGAAACTA CACCCGAAAG AGAAAGTCGT AAATACAATA TGTATATATA CCAATATTTC   
  
  
- AATGTTTTCA TACTGGGCTG TGCTGTTAAG TTAGGCTTAG GCTGAGCTTC AATGACTTTC ATTAGACTTA   
  
  
- TGCTAAACTG TCGAATCAAA TTTTTGGATA ATGAAGAGTT AAAACTCATT TAGATTCGAT AAAAATTACG   
  
  
- ACTTATCTAG CTGGGCTAAG CTGGGCAAAA GGCTGAGATA TACCAATACC ACGGGGATTT TTTTTAATTT   
  
  
- TTTCTTTTTC TTATCTTTTT ATATTTTATT TTGACTCTAA AGACTTGTGA GAGAGGGGAT CAGAACACCC   
  
  
- GTACCTTTCA CCAACCTCTA CCTCTACAAA CTCAAAGTAA AAGACTATAA TAAAAAATCA GTAATCTTCA   
  
  
- TGTTTCAGAT TTGAGTTGTT CTCGTACTCC CCTCTTACCA ACAACATCGG TTACCTTTTA TTTTTCTTGT   
  
  
- AAGTGAGAAG AACACGAAAT AAATACCACT ATTAAACAAT GAACATAAAA ATTTTTACTA ATTTATATAA   
  
  
- CTAGGAAACT ATAATACAAC TTTATTATTT TTATATTTTT AATCTTTTAT AAAATGGAGC ACTTTAAAAA   
  
  
- AAAGACTAAT TAGTAACTTT AATACCACTT TTTATTTCCT TGTAGAGAAC ATTTGTGAGA TAGGCAAATG   
  
  
- CAAACTGGTG TTTGAACGAT AGGTGTTCGT AGAGTTGGAA ACGGAAATTG GGTTAACACC CAACTACTTT   
  
  
- AGATTGGTAA TATCCTAATC TACCATCAGC AATTAGGACA CTTTGTTCAA AAACCAATAC TACATAGGTT   
  
  
- AATACTCCCG GACTATTGAG AACGGAGCCG TAAACGCACT ATTACTGCAC CATGATTATC GTTAATAAAA   
  
  
- AGCGTTTATA AGCTTTAAAA AATTCTTATT TGCGTTTTAA TAACTAGAAC TGTTTACTAA AATAAAAAAA   
  
  
- CAACCCTTTT CCTATTTGTA GATCACAGTT AACAACTACT GGACTATCGA GAGCTAAAGA ATAAATACTC   
  
  
- AATACAACTA TACTGTGATT AACATTAATA AACAACATTT GAATTTGTAA AAATTTTTAG TTTCGTTTTA   
  
  
- AACAATTAAT GTTATTTAGT TAGATGAAAA TGATACTAGA ATAAACCACA TTAATAACTA GAACTTAAAA   
  
  
- AAAAAAAGAA AAACTTTTTC TATTCGTAGC TTAAAAAAGT TTTTATTTTT CCCACTTCCT TCTTTTTTAC   
  
  
- CTTTGTCAAT GGCTCATTCC TTCCTGTCTC GCGCTCTCCT GTTCTGTGTT TCTCTCCCCT CTCTCTCTCT   
  
  
- CTTCCCCCGT TACCGTCACC CCTTCCAGCT CCTCGTGGAA AGTTGAAGTT CCGAGTTGTG GGGGGGGGGG   
  
  
- GGGGAGAAAG GAGTAAAACG CTGCAATCGT AAACTGTAAA ACGAAAGTTG GCAGGGAGCC GTAGTAGTCG   
  
  
- GGACGGGAAC GAGTCGAAAA GGGTAGAAAA AAACCGAAAA ACAAACTGAG GGAGATATTG AGATAAGTGC   
  
  
- AGCTCGGAAC ATTTTTAAAA TGTATAAAAT AAAATAAAAT GAAAAAATCC AAATAGGGAG AGATAAGAAC   
  
  
- TGGATGCCCG TAAAAAATAA AAGAAACCCA AACTCTGAAC ACTTGAAAAA CGTGTAAAAG AGTGTTTTTA   
  
  
- AATAGAACAG AAAAAGTGAA AGTTGCAAGG AAGAGAGAGA GAGAGAGAGA GAGAGAGAGA GAGTGAGTGT   
  
  
- AGGATCTGTC TGCAGGAAAA ATTTCTTAAA TAAATTGTTC AATCTTCTTT GGCAACAAAT TCATCTTGGT   
  
  
- GGGACAAACC TACGGTTTCC TTTATGGTCA GTTCTTTTTA GTGCTGTTAG TGTTGTGTAG GTAAAGCAAA   
  
  
- TGTAGAGTAG GAGTAGGAGT AGAAGGAGTA GAGATGGGTA GAGGGAGAAA GACATACCGA CGAAGAAGAT   
  
  
- GAAACAAGGG ACTGGGATTG TTATGATCAA ATCAGGAGGG AATAATAATA ATAGGAGGAG GAAGGAGGAG   
  
  
- AATGATATTA CCATTGGGAA TGGAAGGAGA AGGATTGTGG TGACGATAAG GATGAGGAAG TTGTTGGAGA   
  
  
- GGCCATAAAA GAAGGCGGTG GATGAGCCTC AACTTATGAC TAACAGTTTG AGTTGACGGG TACAGATTGG   
  
  
- AGGGGCCTTT CTAGCAAGCC TTCGCCTCAC GACGACTTTA CCTCGTCGCG AGGCGAGGCT AACGCCCACT   
  
  
- AATGGGCGGT TGAGTAGTCG CCCATTAGTG GGCGGTGTTA CTCCGCCGGA GTAGTCACAG AAAGTAACTG   
  
  
- AGGAGTGAGA GCGGTGAAGT GGAGAAGTTG CCGAGGTTAA GCTGCTGGGG TAATCGGGGG CTGGCAGGAT   
  
  
- TACTAGGCCC AGACTCAGTC CCAGGCCTAG ACGGAGAACA CACGCCTAAG AGGCCTGAGG GAAATAAGGG   
  
  
- TGGCCACCTC TCTTCTGGAA GGTAGGGGCG GTGCTGGTGA CGACGGCGCC GCTAGCGCCA CCAAGGACAC   
  
  
- CTCCTCCTAC TAGGCTGCAC CTACCTATCG TAGTATTTTC TCGACTAAGT TTCGAGTTTG AGCTAGAGTT   
  
  
- AGGGTGTCAA CTAAGTCTTG CAATCTCTAT AGTAGATGGG TACATTAGGA TTAGACCCCA GGCGGTAACT   
  
  
- CAAGGCGGAG GCGAGCGAGC GGCGGCTGGG GGAGTAACGA GGCGGCGGTG AAGGCGGGAA GGTGGTGGTG   
  
  
- GTTGTGGTGG AGTTAGTTGT TTAGTGAGAA GGAGCTTAGT CGTTGTGATT GTTGTTGTTA GTACACTCGC   
  
  
- ACATGCGGTT GTTTCCTGGG CCTATGAAGT TGAACCCAGG CCCAGTCCCA GTCCCAGGCT AATTGTAGCT   
  
  
- GGTTCGATTG GAAAGGAAGG GAGGACTAAG GTGGCGGACA ACCCCACAGT CACAGTGTGG CGGTGGCAGA   
  
  
- AGGCGGCGGC CAAGGCCGAG GCCACCTTCG CCGTCATCAT CACCATTTAG TTTGGGATTG GGATTAGGGT   
  
  
- TGGGCTTGGG TTTGGGTTTG GGTTTGGGCT TGTTGTTTCG AGTCCTACAA GTTGAGGTTT GAGGTGTTGT   
  
  
- TGTCGTTGTC GTTGTCGTTG TTTAGGGGCA CCTAGTCCTC GTTCTGGGCC GCCGAGGTGG CCGCTGCCTT   
  
  
- CGTAGCGGTG GTAGAGGTTC TCGCCGCCGT GGTGGCGGCC GCCGTCACTT TCGTTCTCTT TCTCTCCTCT   
  
  
- ACGCCGTTTC CTTCGCGCTG CTTCTCCCAG AGGTGGAGGA TTGGGAGGAG GAGGTTACGC GTCTTCGTCA   
  
  
- TAGGCCGCTG TTTATGCTTC TTCGCTTGTT CTACGAAGAT CTTTAGAGCC TCACCCGGTG GGGTAAGCCG   
  
  
- TGGAGGCGGG TTGCGCAGCG GCGCATGAAG AGCCTTCGGT ACAGTCGGGC AGAGCATAGG AGGACGGAGC   
  
  
- CGTAGATGCG GCGGGAGGGG TGGCAGGGTG TGATGCAGTT CGAGGAGAGG CGGAAGGTTC AGAAGTTACC   
  
  
- GTAGTCGGGT AAGCAGTTTA AGAGAGTGAA GTGACGTTTG GTTCGTTAGG TTCTCCGGAA GGTCTCCCTT   
  
  
- CTGTCCCAGG TGTAGTAGCT GGAGCTATAG TACGTCCCCG AGGTCACCGG GCCCGACAAG GTGTAGGAGC   
  
  
- GCAGGGCCGG TCCACCCGGA GGGAAGCATT CCGAGTGGCC CGAGCCCTGG AGGTACCTCC GCGAGCTCCG   
  
  
- GTGGCCTTTT TCCGAGAGTC TGAAGCGGCT CTTCAACCCC AACGGGAAAC TCAAATATGG GCACCGCCTC   
  
  
- TTCTAACCTT TAAACCTGAA CCTTTCCAAC GTACAATCAT TTTCCCTTCG AGAACGACAC GTAACCAATG   
  
  
- TCGTGAGAAA CATACTACAA TGACCGAGAC TATGATTATG TGAACCGGAA GAAGTTTCCA ACCGCGGTTT   
  
  
- TCACCACTGC CACCACCTCG TTCTGGATTC GGCTTGTCCG AGAAAGGATC CTTCCAAACA TCTCCGCTAG   
  
  
- GTGATGATAA GTCGGGATAA ACTGAGGAAT CCTCGCTCAA TACCTCTCCT GTCACTCCTC TCCGTACACC   
  
  
- AACTCGTTGT CGAGGAAAGA TCCCTCTAAG CTTTGTAAGA CCGGCAACCA CCCGGGAGTT CCTGGCCCCT   
  
  
- CGGGTTCAAA CGCTCGACCT CCCTCTTCGA TGTTGTCAGG CCGAAATCCC CTTAGAGTAA CCGTCCGTTG   
  
  
- CGGCGACGGG TCCGGTGGAA CGAGGAGCCG TACAAGGGAA GACTACCCAT ATGAAATTAG CTCCTGTCAC   
  
  
- CGTGTGAATT CGAGCCCACC TTCCTGAACA CGGAGGACTG ACGAAGCCGG ACCTCCGGAA GGGTACGAGT   
  
  
- ATGATACTCG TGGCAGACAT GAGCCTCGGT TAT

+     DRE core

| Site Name | Organism | Position | Strand | Matrix score. | sequence | function |
| --- | --- | --- | --- | --- | --- | --- |
| DRE core | Arabidopsis thaliana | 2757 | + | 6 | GCCGAC |  |

>HU01G00472.1   
+ -Up\_Stream \_Len000GTCTCT GTAACACAAT TCATTAAATC CATCCAAAGG GGATCATAAA TAACCAAACT   
  
  
+ TTGTTGGAAA CTGCTTTGAT GTGGGCTTTC TCTTTCAGCA TTTATGTTAT ACATATATAT GGTTATAAAG   
  
  
+ TTACAAAAGT ATGACCCGAC ACGACAATTC AATCCGAATC CGACTCGAAG TTACTGAAAG TAATCTGAAT   
  
  
+ ACGATTTGAC AGCTTAGTTT AAAAACCTAT TACTTCTCAA TTTTGAGTAA ATCTAAGCTA TTTTTAATGC   
  
  
+ TGAATAGATC GACCCGATTC GACCCGTTTT CCGACTCTAT ATGGTTATGG TGCCCCTAAA AAAAATTAAA   
  
  
+ AAAGAAAAAG AATAGAAAAA TATAAAATAA AACTGAGATT TCTGAACACT CTCTCCCCTA GTCTTGTGGG   
  
  
+ CATGGAAAGT GGTTGGAGAT GGAGATGTTT GAGTTTCATT TTCTGATATT ATTTTTTAGT CATTAGAAGT   
  
  
+ ACAAAGTCTA AACTCAACAA GAGCATGAGG GGAGAATGGT TGTTGTAGCC AATGGAAAAT AAAAAGAACA   
  
  
+ TTCACTCTTC TTGTGCTTTA TTTATGGTGA TAATTTGTTA CTTGTATTTT TAAAAATGAT TAAATATATT   
  
  
+ GATCCTTTGA TATTATGTTG AAATAATAAA AATATAAAAA TTAGAAAATA TTTTACCTCG TGAAATTTTT   
  
  
+ TTTCTGATTA ATCATTGAAA TTATGGTGAA AAATAAAGGA ACATCTCTTG TAAACACTCT ATCCGTTTAC   
  
  
+ GTTTGACCAC AAACTTGCTA TCCACAAGCA TCTCAACCTT TGCCTTTAAC CCAATTGTGG GTTGATGAAA   
  
  
+ TCTAACCATT ATAGGATTAG ATGGTAGTCG TTAATCCTGT GAAACAAGTT TTTGGTTATG ATGTATCCAA   
  
  
+ TTATGAGGGC CTGATAACTC TTGCCTCGGC ATTTGCGTGA TAATGACGTG GTACTAATAG CAATTATTTT   
  
  
+ TCGCAAATAT TCGAAATTTT TTAAGAATAA ACGCAAAATT ATTGATCTTG ACAAATGATT TTATTTTTTT   
  
  
+ GTTGGGAAAA GGATAAACAT CTAGTGTCAA TTGTTGATGA CCTGATAGCT CTCGATTTCT TATTTATGAG   
  
  
+ TTATGTTGAT ATGACACTAA TTGTAATTAT TTGTTGTAAA CTTAAACATT TTTAAAAATC AAAGCAAAAT   
  
  
+ TTGTTAATTA CAATAAATCA ATCTACTTTT ACTATGATCT TATTTGGTGT AATTATTGAT CTTGAATTTT   
  
  
+ TTTTTTTCTT TTTGAAAAAG ATAAGCATCG AATTTTTTCA AAAATAAAAA GGGTGAAGGA AGAAAAAATG   
  
  
+ GAAACAGTTA CCGAGTAAGG AAGGACAGAG CGCGAGAGGA CAAGACACAA AGAGAGGGGA GAGAGAGAGA   
  
  
+ GAAGGGGGCA ATGGCAGTGG GGAAGGTCGA GGAGCACCTT TCAACTTCAA GGCTCAACAC CCCCCCCCCC   
  
  
+ CCCCTCTTTC CTCATTTTGC GACGTTAGCA TTTGACATTT TGCTTTCAAC CGTCCCTCGG CATCATCAGC   
  
  
+ CCTGCCCTTG CTCAGCTTTT CCCATCTTTT TTTGGCTTTT TGTTTGACTC CCTCTATAAC TCTATTCACG   
  
  
+ TCGAGCCTTG TAAAAATTTT ACATATTTTA TTTTATTTTA CTTTTTTAGG TTTATCCCTC TCTATTCTTG   
  
  
+ ACCTACGGGC ATTTTTTATT TTCTTTGGGT TTGAGACTTG TGAACTTTTT GCACATTTTC TCACAAAAAT   
  
  
+ TTATCTTGTC TTTTTCACTT TCAACGTTCC TTCTCTCTCT CTCTCTCTCT CTCTCTCTCT CTCACTCACA   
  
  
+ TCCTAGACAG ACGTCCTTTT TAAAGAATTT ATTTAACAAG TTAGAAGAAA CCGTTGTTTA AGTAGAACCA   
  
  
+ CCCTGTTTGG ATGCCAAAGG AAATACCAGT CAAGAAAAAT CACGACAATC ACAACACATC CATTTCGTTT   
  
  
+ ACATCTCATC CTCATCCTCA TCTTCCTCAT CTCTACCCAT CTCCCTCTTT CTGTATGGCT GCTTCTTCTA   
  
  
+ CTTTGTTCCC TGACCCTAAC AATACTAGTT TAGTCCTCCC TTATTATTAT TATCCTCCTC CTTCCTCCTC   
  
  
+ TTACTATAAT GGTAACCCTT ACCTTCCTCT TCCTAACACC ACTGCTATTC CTACTCCTTC AACAACCTCT   
  
  
+ CCGGTATTTT CTTCCGCCAC CTACTCGGAG TTGAATACTG ATTGTCAAAC TCAACTGCCC ATGTCTAACC   
  
  
+ TCCCCGGAAA GATCGTTCGG AAGCGGAGTG CTGCTGAAAT GGAGCAGCGC TCCGCTCCGA TTGCGGGTGA   
  
  
+ TTACCCGCCA ACTCATCAGC GGGTAATCAC CCGCCACAAT GAGGCGGCCT CATCAGTGTC TTTCATTGAC   
  
  
+ TCCTCACTCT CGCCACTTCA CCTCTTCAAC GGCTCCAATT CGACGACCCC ATTAGCCCCC GACCGTCCTA   
  
  
+ ATGATCCGGG TCTGAGTCAG GGTCCGGATC TGCCTCTTGT GTGCGGATTC TCCGGACTCC CTTTATTCCC   
  
  
+ ACCGGTGGAG AGAAGACCTT CCATCCCCGC CACGACCACT GCTGCCGCGG CGATCGCGGT GGTTCCTGTG   
  
  
+ GAGGAGGATG ATCCGACGTG GATGGATAGC ATCATAAAAG AGCTGATTCA AAGCTCAAAC TCGATCTCAA   
  
  
+ TCCCACAGTT GATTCAGAAC GTTAGAGATA TCATCTACCC ATGTAATCCT AATCTGGGGT CCGCCATTGA   
  
  
+ GTTCCGCCTC CGCTCGCTCG CCGCCGACCC CCTCATTGCT CCGCCGCCAC TTCCGCCCTT CCACCACCAC   
  
  
+ CAACACCACC TCAATCAACA AATCACTCTT CCTCGAATCA GCAACACTAA CAACAACAAT CATGTGAGCG   
  
  
+ TGTACGCCAA CAAAGGACCC GGATACTTCA ACTTGGGTCC GGGTCAGGGT CAGGGTCCGA TTAACATCGA   
  
  
+ CCAAGCTAAC CTTTCCTTCC CTCCTGATTC CACCGCCTGT TGGGGTGTCA GTGTCACACC GCCACCGTCT   
  
  
+ TCCGCCGCCG GTTCCGGCTC CGGTGGAAGC GGCAGTAGTA GTGGTAAATC AAACCCTAAC CCTAATCCCA   
  
  
+ ACCCGAACCC AAACCCAAAC CCAAACCCGA ACAACAAAGC TCAGGATGTT CAACTCCAAA CTCCACAACA   
  
  
+ ACAGCAACAG CAACAGCAAC AAATCCCCGT GGATCAGGAG CAAGACCCGG CGGCTCCACC GGCGACGGAA   
  
  
+ GCATCGCCAC CATCTCCAAG AGCGGCGGCA CCACCGCCGG CGGCAGTGAA AGCAAGAGAA AGAGAGGAGA   
  
  
+ TGCGGCAAAG GAAGCGCGAC GAAGAGGGTC TCCACCTCCT AACCCTCCTC CTCCAATGCG CAGAAGCAGT   
  
  
+ ATCCGGCGAC AAATACGAAG AAGCGAACAA GATGCTTCTA GAAATCTCGG AGTGGGCCAC CCCATTCGGC   
  
  
+ ACCTCCGCCC AACGCGTCGC CGCGTACTTC TCGGAAGCCA TGTCAGCCCG TCTCGTATCC TCCTGCCTCG   
  
  
+ GCATCTACGC CGCCCTCCCC ACCGTCCCAC ACTACGTCAA GCTCCTCTCC GCCTTCCAAG TCTTCAATGG   
  
  
+ CATCAGCCCA TTCGTCAAAT TCTCTCACTT CACTGCAAAC CAAGCAATCC AAGAGGCCTT CCAGAGGGAA   
  
  
+ GACAGGGTCC ACATCATCGA CCTCGATATC ATGCAGGGGC TCCAGTGGCC CGGGCTGTTC CACATCCTCG   
  
  
+ CGTCCCGGCC AGGTGGGCCT CCCTTCGTAA GGCTCACCGG GCTCGGGACC TCCATGGAGG CGCTCGAGGC   
  
  
+ CACCGGAAAA AGGCTCTCAG ACTTCGCCGA GAAGTTGGGG TTGCCCTTTG AGTTTATACC CGTGGCGGAG   
  
  
+ AAGATTGGAA ATTTGGACTT GGAAAGGTTG CATGTTAGTA AAAGGGAAGC TCTTGCTGTG CATTGGTTAC   
  
  
+ AGCACTCTTT GTATGATGTT ACTGGCTCTG ATACTAATAC ACTTGGCCTT CTTCAAAGGT TGGCGCCAAA   
  
  
+ AGTGGTGACG GTGGTGGAGC AAGACCTAAG CCGAACAGGC TCTTTCCTAG GAAGGTTTGT AGAGGCGATC   
  
  
+ CACTACTATT CAGCCCTATT TGACTCCTTA GGAGCGAGTT ATGGAGAGGA CAGTGAGGAG AGGCATGTGG   
  
  
+ TTGAGCAACA GCTCCTTTCT AGGGAGATTC GAAACATTCT GGCCGTTGGT GGGCCCTCAA GGACCGGGGA   
  
  
+ GCCCAAGTTT GCGAGCTGGA GGGAGAAGCT ACAACAGTCC GGCTTTAGGG GAATCTCATT GGCAGGCAAC   
  
  
+ GCCGCTGCCC AGGCCACCTT GCTCCTCGGC ATGTTCCCTT CTGATGGGTA TACTTTAATC GAGGACAGTG   
  
  
+ GCACACTTAA GCTCGGGTGG AAGGACTTGT GCCTCCTGAC TGCTTCGGCC TGGAGGCCTT CCCATGCTCA   
  
  
+ TACTATGAGC ACCGTCTGTA CTCGGAGCCA ATA  

- -Up\_Stream \_Len000CAGAGA CATTGTGTTA AGTAATTTAG GTAGGTTTCC CCTAGTATTT ATTGGTTTGA   
  
  
- AACAACCTTT GACGAAACTA CACCCGAAAG AGAAAGTCGT AAATACAATA TGTATATATA CCAATATTTC   
  
  
- AATGTTTTCA TACTGGGCTG TGCTGTTAAG TTAGGCTTAG GCTGAGCTTC AATGACTTTC ATTAGACTTA   
  
  
- TGCTAAACTG TCGAATCAAA TTTTTGGATA ATGAAGAGTT AAAACTCATT TAGATTCGAT AAAAATTACG   
  
  
- ACTTATCTAG CTGGGCTAAG CTGGGCAAAA GGCTGAGATA TACCAATACC ACGGGGATTT TTTTTAATTT   
  
  
- TTTCTTTTTC TTATCTTTTT ATATTTTATT TTGACTCTAA AGACTTGTGA GAGAGGGGAT CAGAACACCC   
  
  
- GTACCTTTCA CCAACCTCTA CCTCTACAAA CTCAAAGTAA AAGACTATAA TAAAAAATCA GTAATCTTCA   
  
  
- TGTTTCAGAT TTGAGTTGTT CTCGTACTCC CCTCTTACCA ACAACATCGG TTACCTTTTA TTTTTCTTGT   
  
  
- AAGTGAGAAG AACACGAAAT AAATACCACT ATTAAACAAT GAACATAAAA ATTTTTACTA ATTTATATAA   
  
  
- CTAGGAAACT ATAATACAAC TTTATTATTT TTATATTTTT AATCTTTTAT AAAATGGAGC ACTTTAAAAA   
  
  
- AAAGACTAAT TAGTAACTTT AATACCACTT TTTATTTCCT TGTAGAGAAC ATTTGTGAGA TAGGCAAATG   
  
  
- CAAACTGGTG TTTGAACGAT AGGTGTTCGT AGAGTTGGAA ACGGAAATTG GGTTAACACC CAACTACTTT   
  
  
- AGATTGGTAA TATCCTAATC TACCATCAGC AATTAGGACA CTTTGTTCAA AAACCAATAC TACATAGGTT   
  
  
- AATACTCCCG GACTATTGAG AACGGAGCCG TAAACGCACT ATTACTGCAC CATGATTATC GTTAATAAAA   
  
  
- AGCGTTTATA AGCTTTAAAA AATTCTTATT TGCGTTTTAA TAACTAGAAC TGTTTACTAA AATAAAAAAA   
  
  
- CAACCCTTTT CCTATTTGTA GATCACAGTT AACAACTACT GGACTATCGA GAGCTAAAGA ATAAATACTC   
  
  
- AATACAACTA TACTGTGATT AACATTAATA AACAACATTT GAATTTGTAA AAATTTTTAG TTTCGTTTTA   
  
  
- AACAATTAAT GTTATTTAGT TAGATGAAAA TGATACTAGA ATAAACCACA TTAATAACTA GAACTTAAAA   
  
  
- AAAAAAAGAA AAACTTTTTC TATTCGTAGC TTAAAAAAGT TTTTATTTTT CCCACTTCCT TCTTTTTTAC   
  
  
- CTTTGTCAAT GGCTCATTCC TTCCTGTCTC GCGCTCTCCT GTTCTGTGTT TCTCTCCCCT CTCTCTCTCT   
  
  
- CTTCCCCCGT TACCGTCACC CCTTCCAGCT CCTCGTGGAA AGTTGAAGTT CCGAGTTGTG GGGGGGGGGG   
  
  
- GGGGAGAAAG GAGTAAAACG CTGCAATCGT AAACTGTAAA ACGAAAGTTG GCAGGGAGCC GTAGTAGTCG   
  
  
- GGACGGGAAC GAGTCGAAAA GGGTAGAAAA AAACCGAAAA ACAAACTGAG GGAGATATTG AGATAAGTGC   
  
  
- AGCTCGGAAC ATTTTTAAAA TGTATAAAAT AAAATAAAAT GAAAAAATCC AAATAGGGAG AGATAAGAAC   
  
  
- TGGATGCCCG TAAAAAATAA AAGAAACCCA AACTCTGAAC ACTTGAAAAA CGTGTAAAAG AGTGTTTTTA   
  
  
- AATAGAACAG AAAAAGTGAA AGTTGCAAGG AAGAGAGAGA GAGAGAGAGA GAGAGAGAGA GAGTGAGTGT   
  
  
- AGGATCTGTC TGCAGGAAAA ATTTCTTAAA TAAATTGTTC AATCTTCTTT GGCAACAAAT TCATCTTGGT   
  
  
- GGGACAAACC TACGGTTTCC TTTATGGTCA GTTCTTTTTA GTGCTGTTAG TGTTGTGTAG GTAAAGCAAA   
  
  
- TGTAGAGTAG GAGTAGGAGT AGAAGGAGTA GAGATGGGTA GAGGGAGAAA GACATACCGA CGAAGAAGAT   
  
  
- GAAACAAGGG ACTGGGATTG TTATGATCAA ATCAGGAGGG AATAATAATA ATAGGAGGAG GAAGGAGGAG   
  
  
- AATGATATTA CCATTGGGAA TGGAAGGAGA AGGATTGTGG TGACGATAAG GATGAGGAAG TTGTTGGAGA   
  
  
- GGCCATAAAA GAAGGCGGTG GATGAGCCTC AACTTATGAC TAACAGTTTG AGTTGACGGG TACAGATTGG   
  
  
- AGGGGCCTTT CTAGCAAGCC TTCGCCTCAC GACGACTTTA CCTCGTCGCG AGGCGAGGCT AACGCCCACT   
  
  
- AATGGGCGGT TGAGTAGTCG CCCATTAGTG GGCGGTGTTA CTCCGCCGGA GTAGTCACAG AAAGTAACTG   
  
  
- AGGAGTGAGA GCGGTGAAGT GGAGAAGTTG CCGAGGTTAA GCTGCTGGGG TAATCGGGGG CTGGCAGGAT   
  
  
- TACTAGGCCC AGACTCAGTC CCAGGCCTAG ACGGAGAACA CACGCCTAAG AGGCCTGAGG GAAATAAGGG   
  
  
- TGGCCACCTC TCTTCTGGAA GGTAGGGGCG GTGCTGGTGA CGACGGCGCC GCTAGCGCCA CCAAGGACAC   
  
  
- CTCCTCCTAC TAGGCTGCAC CTACCTATCG TAGTATTTTC TCGACTAAGT TTCGAGTTTG AGCTAGAGTT   
  
  
- AGGGTGTCAA CTAAGTCTTG CAATCTCTAT AGTAGATGGG TACATTAGGA TTAGACCCCA GGCGGTAACT   
  
  
- CAAGGCGGAG GCGAGCGAGC GGCGGCTGGG GGAGTAACGA GGCGGCGGTG AAGGCGGGAA GGTGGTGGTG   
  
  
- GTTGTGGTGG AGTTAGTTGT TTAGTGAGAA GGAGCTTAGT CGTTGTGATT GTTGTTGTTA GTACACTCGC   
  
  
- ACATGCGGTT GTTTCCTGGG CCTATGAAGT TGAACCCAGG CCCAGTCCCA GTCCCAGGCT AATTGTAGCT   
  
  
- GGTTCGATTG GAAAGGAAGG GAGGACTAAG GTGGCGGACA ACCCCACAGT CACAGTGTGG CGGTGGCAGA   
  
  
- AGGCGGCGGC CAAGGCCGAG GCCACCTTCG CCGTCATCAT CACCATTTAG TTTGGGATTG GGATTAGGGT   
  
  
- TGGGCTTGGG TTTGGGTTTG GGTTTGGGCT TGTTGTTTCG AGTCCTACAA GTTGAGGTTT GAGGTGTTGT   
  
  
- TGTCGTTGTC GTTGTCGTTG TTTAGGGGCA CCTAGTCCTC GTTCTGGGCC GCCGAGGTGG CCGCTGCCTT   
  
  
- CGTAGCGGTG GTAGAGGTTC TCGCCGCCGT GGTGGCGGCC GCCGTCACTT TCGTTCTCTT TCTCTCCTCT   
  
  
- ACGCCGTTTC CTTCGCGCTG CTTCTCCCAG AGGTGGAGGA TTGGGAGGAG GAGGTTACGC GTCTTCGTCA   
  
  
- TAGGCCGCTG TTTATGCTTC TTCGCTTGTT CTACGAAGAT CTTTAGAGCC TCACCCGGTG GGGTAAGCCG   
  
  
- TGGAGGCGGG TTGCGCAGCG GCGCATGAAG AGCCTTCGGT ACAGTCGGGC AGAGCATAGG AGGACGGAGC   
  
  
- CGTAGATGCG GCGGGAGGGG TGGCAGGGTG TGATGCAGTT CGAGGAGAGG CGGAAGGTTC AGAAGTTACC   
  
  
- GTAGTCGGGT AAGCAGTTTA AGAGAGTGAA GTGACGTTTG GTTCGTTAGG TTCTCCGGAA GGTCTCCCTT   
  
  
- CTGTCCCAGG TGTAGTAGCT GGAGCTATAG TACGTCCCCG AGGTCACCGG GCCCGACAAG GTGTAGGAGC   
  
  
- GCAGGGCCGG TCCACCCGGA GGGAAGCATT CCGAGTGGCC CGAGCCCTGG AGGTACCTCC GCGAGCTCCG   
  
  
- GTGGCCTTTT TCCGAGAGTC TGAAGCGGCT CTTCAACCCC AACGGGAAAC TCAAATATGG GCACCGCCTC   
  
  
- TTCTAACCTT TAAACCTGAA CCTTTCCAAC GTACAATCAT TTTCCCTTCG AGAACGACAC GTAACCAATG   
  
  
- TCGTGAGAAA CATACTACAA TGACCGAGAC TATGATTATG TGAACCGGAA GAAGTTTCCA ACCGCGGTTT   
  
  
- TCACCACTGC CACCACCTCG TTCTGGATTC GGCTTGTCCG AGAAAGGATC CTTCCAAACA TCTCCGCTAG   
  
  
- GTGATGATAA GTCGGGATAA ACTGAGGAAT CCTCGCTCAA TACCTCTCCT GTCACTCCTC TCCGTACACC   
  
  
- AACTCGTTGT CGAGGAAAGA TCCCTCTAAG CTTTGTAAGA CCGGCAACCA CCCGGGAGTT CCTGGCCCCT   
  
  
- CGGGTTCAAA CGCTCGACCT CCCTCTTCGA TGTTGTCAGG CCGAAATCCC CTTAGAGTAA CCGTCCGTTG   
  
  
- CGGCGACGGG TCCGGTGGAA CGAGGAGCCG TACAAGGGAA GACTACCCAT ATGAAATTAG CTCCTGTCAC   
  
  
- CGTGTGAATT CGAGCCCACC TTCCTGAACA CGGAGGACTG ACGAAGCCGG ACCTCCGGAA GGGTACGAGT   
  
  
- ATGATACTCG TGGCAGACAT GAGCCTCGGT TAT

+     E2Fb

| Site Name | Organism | Position | Strand | Matrix score. | sequence | function |
| --- | --- | --- | --- | --- | --- | --- |
| E2Fb | Nicotiana tabacum | 3296 | - | 8 | TTTGCCGC |  |

>HU01G00472.1   
+ -Up\_Stream \_Len000GTCTCT GTAACACAAT TCATTAAATC CATCCAAAGG GGATCATAAA TAACCAAACT   
  
  
+ TTGTTGGAAA CTGCTTTGAT GTGGGCTTTC TCTTTCAGCA TTTATGTTAT ACATATATAT GGTTATAAAG   
  
  
+ TTACAAAAGT ATGACCCGAC ACGACAATTC AATCCGAATC CGACTCGAAG TTACTGAAAG TAATCTGAAT   
  
  
+ ACGATTTGAC AGCTTAGTTT AAAAACCTAT TACTTCTCAA TTTTGAGTAA ATCTAAGCTA TTTTTAATGC   
  
  
+ TGAATAGATC GACCCGATTC GACCCGTTTT CCGACTCTAT ATGGTTATGG TGCCCCTAAA AAAAATTAAA   
  
  
+ AAAGAAAAAG AATAGAAAAA TATAAAATAA AACTGAGATT TCTGAACACT CTCTCCCCTA GTCTTGTGGG   
  
  
+ CATGGAAAGT GGTTGGAGAT GGAGATGTTT GAGTTTCATT TTCTGATATT ATTTTTTAGT CATTAGAAGT   
  
  
+ ACAAAGTCTA AACTCAACAA GAGCATGAGG GGAGAATGGT TGTTGTAGCC AATGGAAAAT AAAAAGAACA   
  
  
+ TTCACTCTTC TTGTGCTTTA TTTATGGTGA TAATTTGTTA CTTGTATTTT TAAAAATGAT TAAATATATT   
  
  
+ GATCCTTTGA TATTATGTTG AAATAATAAA AATATAAAAA TTAGAAAATA TTTTACCTCG TGAAATTTTT   
  
  
+ TTTCTGATTA ATCATTGAAA TTATGGTGAA AAATAAAGGA ACATCTCTTG TAAACACTCT ATCCGTTTAC   
  
  
+ GTTTGACCAC AAACTTGCTA TCCACAAGCA TCTCAACCTT TGCCTTTAAC CCAATTGTGG GTTGATGAAA   
  
  
+ TCTAACCATT ATAGGATTAG ATGGTAGTCG TTAATCCTGT GAAACAAGTT TTTGGTTATG ATGTATCCAA   
  
  
+ TTATGAGGGC CTGATAACTC TTGCCTCGGC ATTTGCGTGA TAATGACGTG GTACTAATAG CAATTATTTT   
  
  
+ TCGCAAATAT TCGAAATTTT TTAAGAATAA ACGCAAAATT ATTGATCTTG ACAAATGATT TTATTTTTTT   
  
  
+ GTTGGGAAAA GGATAAACAT CTAGTGTCAA TTGTTGATGA CCTGATAGCT CTCGATTTCT TATTTATGAG   
  
  
+ TTATGTTGAT ATGACACTAA TTGTAATTAT TTGTTGTAAA CTTAAACATT TTTAAAAATC AAAGCAAAAT   
  
  
+ TTGTTAATTA CAATAAATCA ATCTACTTTT ACTATGATCT TATTTGGTGT AATTATTGAT CTTGAATTTT   
  
  
+ TTTTTTTCTT TTTGAAAAAG ATAAGCATCG AATTTTTTCA AAAATAAAAA GGGTGAAGGA AGAAAAAATG   
  
  
+ GAAACAGTTA CCGAGTAAGG AAGGACAGAG CGCGAGAGGA CAAGACACAA AGAGAGGGGA GAGAGAGAGA   
  
  
+ GAAGGGGGCA ATGGCAGTGG GGAAGGTCGA GGAGCACCTT TCAACTTCAA GGCTCAACAC CCCCCCCCCC   
  
  
+ CCCCTCTTTC CTCATTTTGC GACGTTAGCA TTTGACATTT TGCTTTCAAC CGTCCCTCGG CATCATCAGC   
  
  
+ CCTGCCCTTG CTCAGCTTTT CCCATCTTTT TTTGGCTTTT TGTTTGACTC CCTCTATAAC TCTATTCACG   
  
  
+ TCGAGCCTTG TAAAAATTTT ACATATTTTA TTTTATTTTA CTTTTTTAGG TTTATCCCTC TCTATTCTTG   
  
  
+ ACCTACGGGC ATTTTTTATT TTCTTTGGGT TTGAGACTTG TGAACTTTTT GCACATTTTC TCACAAAAAT   
  
  
+ TTATCTTGTC TTTTTCACTT TCAACGTTCC TTCTCTCTCT CTCTCTCTCT CTCTCTCTCT CTCACTCACA   
  
  
+ TCCTAGACAG ACGTCCTTTT TAAAGAATTT ATTTAACAAG TTAGAAGAAA CCGTTGTTTA AGTAGAACCA   
  
  
+ CCCTGTTTGG ATGCCAAAGG AAATACCAGT CAAGAAAAAT CACGACAATC ACAACACATC CATTTCGTTT   
  
  
+ ACATCTCATC CTCATCCTCA TCTTCCTCAT CTCTACCCAT CTCCCTCTTT CTGTATGGCT GCTTCTTCTA   
  
  
+ CTTTGTTCCC TGACCCTAAC AATACTAGTT TAGTCCTCCC TTATTATTAT TATCCTCCTC CTTCCTCCTC   
  
  
+ TTACTATAAT GGTAACCCTT ACCTTCCTCT TCCTAACACC ACTGCTATTC CTACTCCTTC AACAACCTCT   
  
  
+ CCGGTATTTT CTTCCGCCAC CTACTCGGAG TTGAATACTG ATTGTCAAAC TCAACTGCCC ATGTCTAACC   
  
  
+ TCCCCGGAAA GATCGTTCGG AAGCGGAGTG CTGCTGAAAT GGAGCAGCGC TCCGCTCCGA TTGCGGGTGA   
  
  
+ TTACCCGCCA ACTCATCAGC GGGTAATCAC CCGCCACAAT GAGGCGGCCT CATCAGTGTC TTTCATTGAC   
  
  
+ TCCTCACTCT CGCCACTTCA CCTCTTCAAC GGCTCCAATT CGACGACCCC ATTAGCCCCC GACCGTCCTA   
  
  
+ ATGATCCGGG TCTGAGTCAG GGTCCGGATC TGCCTCTTGT GTGCGGATTC TCCGGACTCC CTTTATTCCC   
  
  
+ ACCGGTGGAG AGAAGACCTT CCATCCCCGC CACGACCACT GCTGCCGCGG CGATCGCGGT GGTTCCTGTG   
  
  
+ GAGGAGGATG ATCCGACGTG GATGGATAGC ATCATAAAAG AGCTGATTCA AAGCTCAAAC TCGATCTCAA   
  
  
+ TCCCACAGTT GATTCAGAAC GTTAGAGATA TCATCTACCC ATGTAATCCT AATCTGGGGT CCGCCATTGA   
  
  
+ GTTCCGCCTC CGCTCGCTCG CCGCCGACCC CCTCATTGCT CCGCCGCCAC TTCCGCCCTT CCACCACCAC   
  
  
+ CAACACCACC TCAATCAACA AATCACTCTT CCTCGAATCA GCAACACTAA CAACAACAAT CATGTGAGCG   
  
  
+ TGTACGCCAA CAAAGGACCC GGATACTTCA ACTTGGGTCC GGGTCAGGGT CAGGGTCCGA TTAACATCGA   
  
  
+ CCAAGCTAAC CTTTCCTTCC CTCCTGATTC CACCGCCTGT TGGGGTGTCA GTGTCACACC GCCACCGTCT   
  
  
+ TCCGCCGCCG GTTCCGGCTC CGGTGGAAGC GGCAGTAGTA GTGGTAAATC AAACCCTAAC CCTAATCCCA   
  
  
+ ACCCGAACCC AAACCCAAAC CCAAACCCGA ACAACAAAGC TCAGGATGTT CAACTCCAAA CTCCACAACA   
  
  
+ ACAGCAACAG CAACAGCAAC AAATCCCCGT GGATCAGGAG CAAGACCCGG CGGCTCCACC GGCGACGGAA   
  
  
+ GCATCGCCAC CATCTCCAAG AGCGGCGGCA CCACCGCCGG CGGCAGTGAA AGCAAGAGAA AGAGAGGAGA   
  
  
+ TGCGGCAAAG GAAGCGCGAC GAAGAGGGTC TCCACCTCCT AACCCTCCTC CTCCAATGCG CAGAAGCAGT   
  
  
+ ATCCGGCGAC AAATACGAAG AAGCGAACAA GATGCTTCTA GAAATCTCGG AGTGGGCCAC CCCATTCGGC   
  
  
+ ACCTCCGCCC AACGCGTCGC CGCGTACTTC TCGGAAGCCA TGTCAGCCCG TCTCGTATCC TCCTGCCTCG   
  
  
+ GCATCTACGC CGCCCTCCCC ACCGTCCCAC ACTACGTCAA GCTCCTCTCC GCCTTCCAAG TCTTCAATGG   
  
  
+ CATCAGCCCA TTCGTCAAAT TCTCTCACTT CACTGCAAAC CAAGCAATCC AAGAGGCCTT CCAGAGGGAA   
  
  
+ GACAGGGTCC ACATCATCGA CCTCGATATC ATGCAGGGGC TCCAGTGGCC CGGGCTGTTC CACATCCTCG   
  
  
+ CGTCCCGGCC AGGTGGGCCT CCCTTCGTAA GGCTCACCGG GCTCGGGACC TCCATGGAGG CGCTCGAGGC   
  
  
+ CACCGGAAAA AGGCTCTCAG ACTTCGCCGA GAAGTTGGGG TTGCCCTTTG AGTTTATACC CGTGGCGGAG   
  
  
+ AAGATTGGAA ATTTGGACTT GGAAAGGTTG CATGTTAGTA AAAGGGAAGC TCTTGCTGTG CATTGGTTAC   
  
  
+ AGCACTCTTT GTATGATGTT ACTGGCTCTG ATACTAATAC ACTTGGCCTT CTTCAAAGGT TGGCGCCAAA   
  
  
+ AGTGGTGACG GTGGTGGAGC AAGACCTAAG CCGAACAGGC TCTTTCCTAG GAAGGTTTGT AGAGGCGATC   
  
  
+ CACTACTATT CAGCCCTATT TGACTCCTTA GGAGCGAGTT ATGGAGAGGA CAGTGAGGAG AGGCATGTGG   
  
  
+ TTGAGCAACA GCTCCTTTCT AGGGAGATTC GAAACATTCT GGCCGTTGGT GGGCCCTCAA GGACCGGGGA   
  
  
+ GCCCAAGTTT GCGAGCTGGA GGGAGAAGCT ACAACAGTCC GGCTTTAGGG GAATCTCATT GGCAGGCAAC   
  
  
+ GCCGCTGCCC AGGCCACCTT GCTCCTCGGC ATGTTCCCTT CTGATGGGTA TACTTTAATC GAGGACAGTG   
  
  
+ GCACACTTAA GCTCGGGTGG AAGGACTTGT GCCTCCTGAC TGCTTCGGCC TGGAGGCCTT CCCATGCTCA   
  
  
+ TACTATGAGC ACCGTCTGTA CTCGGAGCCA ATA  

- -Up\_Stream \_Len000CAGAGA CATTGTGTTA AGTAATTTAG GTAGGTTTCC CCTAGTATTT ATTGGTTTGA   
  
  
- AACAACCTTT GACGAAACTA CACCCGAAAG AGAAAGTCGT AAATACAATA TGTATATATA CCAATATTTC   
  
  
- AATGTTTTCA TACTGGGCTG TGCTGTTAAG TTAGGCTTAG GCTGAGCTTC AATGACTTTC ATTAGACTTA   
  
  
- TGCTAAACTG TCGAATCAAA TTTTTGGATA ATGAAGAGTT AAAACTCATT TAGATTCGAT AAAAATTACG   
  
  
- ACTTATCTAG CTGGGCTAAG CTGGGCAAAA GGCTGAGATA TACCAATACC ACGGGGATTT TTTTTAATTT   
  
  
- TTTCTTTTTC TTATCTTTTT ATATTTTATT TTGACTCTAA AGACTTGTGA GAGAGGGGAT CAGAACACCC   
  
  
- GTACCTTTCA CCAACCTCTA CCTCTACAAA CTCAAAGTAA AAGACTATAA TAAAAAATCA GTAATCTTCA   
  
  
- TGTTTCAGAT TTGAGTTGTT CTCGTACTCC CCTCTTACCA ACAACATCGG TTACCTTTTA TTTTTCTTGT   
  
  
- AAGTGAGAAG AACACGAAAT AAATACCACT ATTAAACAAT GAACATAAAA ATTTTTACTA ATTTATATAA   
  
  
- CTAGGAAACT ATAATACAAC TTTATTATTT TTATATTTTT AATCTTTTAT AAAATGGAGC ACTTTAAAAA   
  
  
- AAAGACTAAT TAGTAACTTT AATACCACTT TTTATTTCCT TGTAGAGAAC ATTTGTGAGA TAGGCAAATG   
  
  
- CAAACTGGTG TTTGAACGAT AGGTGTTCGT AGAGTTGGAA ACGGAAATTG GGTTAACACC CAACTACTTT   
  
  
- AGATTGGTAA TATCCTAATC TACCATCAGC AATTAGGACA CTTTGTTCAA AAACCAATAC TACATAGGTT   
  
  
- AATACTCCCG GACTATTGAG AACGGAGCCG TAAACGCACT ATTACTGCAC CATGATTATC GTTAATAAAA   
  
  
- AGCGTTTATA AGCTTTAAAA AATTCTTATT TGCGTTTTAA TAACTAGAAC TGTTTACTAA AATAAAAAAA   
  
  
- CAACCCTTTT CCTATTTGTA GATCACAGTT AACAACTACT GGACTATCGA GAGCTAAAGA ATAAATACTC   
  
  
- AATACAACTA TACTGTGATT AACATTAATA AACAACATTT GAATTTGTAA AAATTTTTAG TTTCGTTTTA   
  
  
- AACAATTAAT GTTATTTAGT TAGATGAAAA TGATACTAGA ATAAACCACA TTAATAACTA GAACTTAAAA   
  
  
- AAAAAAAGAA AAACTTTTTC TATTCGTAGC TTAAAAAAGT TTTTATTTTT CCCACTTCCT TCTTTTTTAC   
  
  
- CTTTGTCAAT GGCTCATTCC TTCCTGTCTC GCGCTCTCCT GTTCTGTGTT TCTCTCCCCT CTCTCTCTCT   
  
  
- CTTCCCCCGT TACCGTCACC CCTTCCAGCT CCTCGTGGAA AGTTGAAGTT CCGAGTTGTG GGGGGGGGGG   
  
  
- GGGGAGAAAG GAGTAAAACG CTGCAATCGT AAACTGTAAA ACGAAAGTTG GCAGGGAGCC GTAGTAGTCG   
  
  
- GGACGGGAAC GAGTCGAAAA GGGTAGAAAA AAACCGAAAA ACAAACTGAG GGAGATATTG AGATAAGTGC   
  
  
- AGCTCGGAAC ATTTTTAAAA TGTATAAAAT AAAATAAAAT GAAAAAATCC AAATAGGGAG AGATAAGAAC   
  
  
- TGGATGCCCG TAAAAAATAA AAGAAACCCA AACTCTGAAC ACTTGAAAAA CGTGTAAAAG AGTGTTTTTA   
  
  
- AATAGAACAG AAAAAGTGAA AGTTGCAAGG AAGAGAGAGA GAGAGAGAGA GAGAGAGAGA GAGTGAGTGT   
  
  
- AGGATCTGTC TGCAGGAAAA ATTTCTTAAA TAAATTGTTC AATCTTCTTT GGCAACAAAT TCATCTTGGT   
  
  
- GGGACAAACC TACGGTTTCC TTTATGGTCA GTTCTTTTTA GTGCTGTTAG TGTTGTGTAG GTAAAGCAAA   
  
  
- TGTAGAGTAG GAGTAGGAGT AGAAGGAGTA GAGATGGGTA GAGGGAGAAA GACATACCGA CGAAGAAGAT   
  
  
- GAAACAAGGG ACTGGGATTG TTATGATCAA ATCAGGAGGG AATAATAATA ATAGGAGGAG GAAGGAGGAG   
  
  
- AATGATATTA CCATTGGGAA TGGAAGGAGA AGGATTGTGG TGACGATAAG GATGAGGAAG TTGTTGGAGA   
  
  
- GGCCATAAAA GAAGGCGGTG GATGAGCCTC AACTTATGAC TAACAGTTTG AGTTGACGGG TACAGATTGG   
  
  
- AGGGGCCTTT CTAGCAAGCC TTCGCCTCAC GACGACTTTA CCTCGTCGCG AGGCGAGGCT AACGCCCACT   
  
  
- AATGGGCGGT TGAGTAGTCG CCCATTAGTG GGCGGTGTTA CTCCGCCGGA GTAGTCACAG AAAGTAACTG   
  
  
- AGGAGTGAGA GCGGTGAAGT GGAGAAGTTG CCGAGGTTAA GCTGCTGGGG TAATCGGGGG CTGGCAGGAT   
  
  
- TACTAGGCCC AGACTCAGTC CCAGGCCTAG ACGGAGAACA CACGCCTAAG AGGCCTGAGG GAAATAAGGG   
  
  
- TGGCCACCTC TCTTCTGGAA GGTAGGGGCG GTGCTGGTGA CGACGGCGCC GCTAGCGCCA CCAAGGACAC   
  
  
- CTCCTCCTAC TAGGCTGCAC CTACCTATCG TAGTATTTTC TCGACTAAGT TTCGAGTTTG AGCTAGAGTT   
  
  
- AGGGTGTCAA CTAAGTCTTG CAATCTCTAT AGTAGATGGG TACATTAGGA TTAGACCCCA GGCGGTAACT   
  
  
- CAAGGCGGAG GCGAGCGAGC GGCGGCTGGG GGAGTAACGA GGCGGCGGTG AAGGCGGGAA GGTGGTGGTG   
  
  
- GTTGTGGTGG AGTTAGTTGT TTAGTGAGAA GGAGCTTAGT CGTTGTGATT GTTGTTGTTA GTACACTCGC   
  
  
- ACATGCGGTT GTTTCCTGGG CCTATGAAGT TGAACCCAGG CCCAGTCCCA GTCCCAGGCT AATTGTAGCT   
  
  
- GGTTCGATTG GAAAGGAAGG GAGGACTAAG GTGGCGGACA ACCCCACAGT CACAGTGTGG CGGTGGCAGA   
  
  
- AGGCGGCGGC CAAGGCCGAG GCCACCTTCG CCGTCATCAT CACCATTTAG TTTGGGATTG GGATTAGGGT   
  
  
- TGGGCTTGGG TTTGGGTTTG GGTTTGGGCT TGTTGTTTCG AGTCCTACAA GTTGAGGTTT GAGGTGTTGT   
  
  
- TGTCGTTGTC GTTGTCGTTG TTTAGGGGCA CCTAGTCCTC GTTCTGGGCC GCCGAGGTGG CCGCTGCCTT   
  
  
- CGTAGCGGTG GTAGAGGTTC TCGCCGCCGT GGTGGCGGCC GCCGTCACTT TCGTTCTCTT TCTCTCCTCT   
  
  
- ACGCCGTTTC CTTCGCGCTG CTTCTCCCAG AGGTGGAGGA TTGGGAGGAG GAGGTTACGC GTCTTCGTCA   
  
  
- TAGGCCGCTG TTTATGCTTC TTCGCTTGTT CTACGAAGAT CTTTAGAGCC TCACCCGGTG GGGTAAGCCG   
  
  
- TGGAGGCGGG TTGCGCAGCG GCGCATGAAG AGCCTTCGGT ACAGTCGGGC AGAGCATAGG AGGACGGAGC   
  
  
- CGTAGATGCG GCGGGAGGGG TGGCAGGGTG TGATGCAGTT CGAGGAGAGG CGGAAGGTTC AGAAGTTACC   
  
  
- GTAGTCGGGT AAGCAGTTTA AGAGAGTGAA GTGACGTTTG GTTCGTTAGG TTCTCCGGAA GGTCTCCCTT   
  
  
- CTGTCCCAGG TGTAGTAGCT GGAGCTATAG TACGTCCCCG AGGTCACCGG GCCCGACAAG GTGTAGGAGC   
  
  
- GCAGGGCCGG TCCACCCGGA GGGAAGCATT CCGAGTGGCC CGAGCCCTGG AGGTACCTCC GCGAGCTCCG   
  
  
- GTGGCCTTTT TCCGAGAGTC TGAAGCGGCT CTTCAACCCC AACGGGAAAC TCAAATATGG GCACCGCCTC   
  
  
- TTCTAACCTT TAAACCTGAA CCTTTCCAAC GTACAATCAT TTTCCCTTCG AGAACGACAC GTAACCAATG   
  
  
- TCGTGAGAAA CATACTACAA TGACCGAGAC TATGATTATG TGAACCGGAA GAAGTTTCCA ACCGCGGTTT   
  
  
- TCACCACTGC CACCACCTCG TTCTGGATTC GGCTTGTCCG AGAAAGGATC CTTCCAAACA TCTCCGCTAG   
  
  
- GTGATGATAA GTCGGGATAA ACTGAGGAAT CCTCGCTCAA TACCTCTCCT GTCACTCCTC TCCGTACACC   
  
  
- AACTCGTTGT CGAGGAAAGA TCCCTCTAAG CTTTGTAAGA CCGGCAACCA CCCGGGAGTT CCTGGCCCCT   
  
  
- CGGGTTCAAA CGCTCGACCT CCCTCTTCGA TGTTGTCAGG CCGAAATCCC CTTAGAGTAA CCGTCCGTTG   
  
  
- CGGCGACGGG TCCGGTGGAA CGAGGAGCCG TACAAGGGAA GACTACCCAT ATGAAATTAG CTCCTGTCAC   
  
  
- CGTGTGAATT CGAGCCCACC TTCCTGAACA CGGAGGACTG ACGAAGCCGG ACCTCCGGAA GGGTACGAGT   
  
  
- ATGATACTCG TGGCAGACAT GAGCCTCGGT TAT

+     G-box

| Site Name | Organism | Position | Strand | Matrix score. | sequence | function |
| --- | --- | --- | --- | --- | --- | --- |
| G-box | Zea mays | 164 | + | 6 | CACGAC | cis-acting regulatory element involved in light responsiveness |
| G-box | Zea mays | 2555 | + | 6 | CACGAC | cis-acting regulatory element involved in light responsiveness |
| G-box | Zea mays | 1611 | + | 6 | CACGTC | cis-acting regulatory element involved in light responsiveness |
| G-box | Zea mays | 2609 | - | 6 | CACGTC | cis-acting regulatory element involved in light responsiveness |
| G-box | Zea mays | 959 | - | 6 | CACGTC | cis-acting regulatory element involved in light responsiveness |
| G-box | Zea mays | 1935 | + | 6 | CACGAC | cis-acting regulatory element involved in light responsiveness |

>HU01G00472.1   
+ -Up\_Stream \_Len000GTCTCT GTAACACAAT TCATTAAATC CATCCAAAGG GGATCATAAA TAACCAAACT   
  
  
+ TTGTTGGAAA CTGCTTTGAT GTGGGCTTTC TCTTTCAGCA TTTATGTTAT ACATATATAT GGTTATAAAG   
  
  
+ TTACAAAAGT ATGACCCGAC ACGACAATTC AATCCGAATC CGACTCGAAG TTACTGAAAG TAATCTGAAT   
  
  
+ ACGATTTGAC AGCTTAGTTT AAAAACCTAT TACTTCTCAA TTTTGAGTAA ATCTAAGCTA TTTTTAATGC   
  
  
+ TGAATAGATC GACCCGATTC GACCCGTTTT CCGACTCTAT ATGGTTATGG TGCCCCTAAA AAAAATTAAA   
  
  
+ AAAGAAAAAG AATAGAAAAA TATAAAATAA AACTGAGATT TCTGAACACT CTCTCCCCTA GTCTTGTGGG   
  
  
+ CATGGAAAGT GGTTGGAGAT GGAGATGTTT GAGTTTCATT TTCTGATATT ATTTTTTAGT CATTAGAAGT   
  
  
+ ACAAAGTCTA AACTCAACAA GAGCATGAGG GGAGAATGGT TGTTGTAGCC AATGGAAAAT AAAAAGAACA   
  
  
+ TTCACTCTTC TTGTGCTTTA TTTATGGTGA TAATTTGTTA CTTGTATTTT TAAAAATGAT TAAATATATT   
  
  
+ GATCCTTTGA TATTATGTTG AAATAATAAA AATATAAAAA TTAGAAAATA TTTTACCTCG TGAAATTTTT   
  
  
+ TTTCTGATTA ATCATTGAAA TTATGGTGAA AAATAAAGGA ACATCTCTTG TAAACACTCT ATCCGTTTAC   
  
  
+ GTTTGACCAC AAACTTGCTA TCCACAAGCA TCTCAACCTT TGCCTTTAAC CCAATTGTGG GTTGATGAAA   
  
  
+ TCTAACCATT ATAGGATTAG ATGGTAGTCG TTAATCCTGT GAAACAAGTT TTTGGTTATG ATGTATCCAA   
  
  
+ TTATGAGGGC CTGATAACTC TTGCCTCGGC ATTTGCGTGA TAATGACGTG GTACTAATAG CAATTATTTT   
  
  
+ TCGCAAATAT TCGAAATTTT TTAAGAATAA ACGCAAAATT ATTGATCTTG ACAAATGATT TTATTTTTTT   
  
  
+ GTTGGGAAAA GGATAAACAT CTAGTGTCAA TTGTTGATGA CCTGATAGCT CTCGATTTCT TATTTATGAG   
  
  
+ TTATGTTGAT ATGACACTAA TTGTAATTAT TTGTTGTAAA CTTAAACATT TTTAAAAATC AAAGCAAAAT   
  
  
+ TTGTTAATTA CAATAAATCA ATCTACTTTT ACTATGATCT TATTTGGTGT AATTATTGAT CTTGAATTTT   
  
  
+ TTTTTTTCTT TTTGAAAAAG ATAAGCATCG AATTTTTTCA AAAATAAAAA GGGTGAAGGA AGAAAAAATG   
  
  
+ GAAACAGTTA CCGAGTAAGG AAGGACAGAG CGCGAGAGGA CAAGACACAA AGAGAGGGGA GAGAGAGAGA   
  
  
+ GAAGGGGGCA ATGGCAGTGG GGAAGGTCGA GGAGCACCTT TCAACTTCAA GGCTCAACAC CCCCCCCCCC   
  
  
+ CCCCTCTTTC CTCATTTTGC GACGTTAGCA TTTGACATTT TGCTTTCAAC CGTCCCTCGG CATCATCAGC   
  
  
+ CCTGCCCTTG CTCAGCTTTT CCCATCTTTT TTTGGCTTTT TGTTTGACTC CCTCTATAAC TCTATTCACG   
  
  
+ TCGAGCCTTG TAAAAATTTT ACATATTTTA TTTTATTTTA CTTTTTTAGG TTTATCCCTC TCTATTCTTG   
  
  
+ ACCTACGGGC ATTTTTTATT TTCTTTGGGT TTGAGACTTG TGAACTTTTT GCACATTTTC TCACAAAAAT   
  
  
+ TTATCTTGTC TTTTTCACTT TCAACGTTCC TTCTCTCTCT CTCTCTCTCT CTCTCTCTCT CTCACTCACA   
  
  
+ TCCTAGACAG ACGTCCTTTT TAAAGAATTT ATTTAACAAG TTAGAAGAAA CCGTTGTTTA AGTAGAACCA   
  
  
+ CCCTGTTTGG ATGCCAAAGG AAATACCAGT CAAGAAAAAT CACGACAATC ACAACACATC CATTTCGTTT   
  
  
+ ACATCTCATC CTCATCCTCA TCTTCCTCAT CTCTACCCAT CTCCCTCTTT CTGTATGGCT GCTTCTTCTA   
  
  
+ CTTTGTTCCC TGACCCTAAC AATACTAGTT TAGTCCTCCC TTATTATTAT TATCCTCCTC CTTCCTCCTC   
  
  
+ TTACTATAAT GGTAACCCTT ACCTTCCTCT TCCTAACACC ACTGCTATTC CTACTCCTTC AACAACCTCT   
  
  
+ CCGGTATTTT CTTCCGCCAC CTACTCGGAG TTGAATACTG ATTGTCAAAC TCAACTGCCC ATGTCTAACC   
  
  
+ TCCCCGGAAA GATCGTTCGG AAGCGGAGTG CTGCTGAAAT GGAGCAGCGC TCCGCTCCGA TTGCGGGTGA   
  
  
+ TTACCCGCCA ACTCATCAGC GGGTAATCAC CCGCCACAAT GAGGCGGCCT CATCAGTGTC TTTCATTGAC   
  
  
+ TCCTCACTCT CGCCACTTCA CCTCTTCAAC GGCTCCAATT CGACGACCCC ATTAGCCCCC GACCGTCCTA   
  
  
+ ATGATCCGGG TCTGAGTCAG GGTCCGGATC TGCCTCTTGT GTGCGGATTC TCCGGACTCC CTTTATTCCC   
  
  
+ ACCGGTGGAG AGAAGACCTT CCATCCCCGC CACGACCACT GCTGCCGCGG CGATCGCGGT GGTTCCTGTG   
  
  
+ GAGGAGGATG ATCCGACGTG GATGGATAGC ATCATAAAAG AGCTGATTCA AAGCTCAAAC TCGATCTCAA   
  
  
+ TCCCACAGTT GATTCAGAAC GTTAGAGATA TCATCTACCC ATGTAATCCT AATCTGGGGT CCGCCATTGA   
  
  
+ GTTCCGCCTC CGCTCGCTCG CCGCCGACCC CCTCATTGCT CCGCCGCCAC TTCCGCCCTT CCACCACCAC   
  
  
+ CAACACCACC TCAATCAACA AATCACTCTT CCTCGAATCA GCAACACTAA CAACAACAAT CATGTGAGCG   
  
  
+ TGTACGCCAA CAAAGGACCC GGATACTTCA ACTTGGGTCC GGGTCAGGGT CAGGGTCCGA TTAACATCGA   
  
  
+ CCAAGCTAAC CTTTCCTTCC CTCCTGATTC CACCGCCTGT TGGGGTGTCA GTGTCACACC GCCACCGTCT   
  
  
+ TCCGCCGCCG GTTCCGGCTC CGGTGGAAGC GGCAGTAGTA GTGGTAAATC AAACCCTAAC CCTAATCCCA   
  
  
+ ACCCGAACCC AAACCCAAAC CCAAACCCGA ACAACAAAGC TCAGGATGTT CAACTCCAAA CTCCACAACA   
  
  
+ ACAGCAACAG CAACAGCAAC AAATCCCCGT GGATCAGGAG CAAGACCCGG CGGCTCCACC GGCGACGGAA   
  
  
+ GCATCGCCAC CATCTCCAAG AGCGGCGGCA CCACCGCCGG CGGCAGTGAA AGCAAGAGAA AGAGAGGAGA   
  
  
+ TGCGGCAAAG GAAGCGCGAC GAAGAGGGTC TCCACCTCCT AACCCTCCTC CTCCAATGCG CAGAAGCAGT   
  
  
+ ATCCGGCGAC AAATACGAAG AAGCGAACAA GATGCTTCTA GAAATCTCGG AGTGGGCCAC CCCATTCGGC   
  
  
+ ACCTCCGCCC AACGCGTCGC CGCGTACTTC TCGGAAGCCA TGTCAGCCCG TCTCGTATCC TCCTGCCTCG   
  
  
+ GCATCTACGC CGCCCTCCCC ACCGTCCCAC ACTACGTCAA GCTCCTCTCC GCCTTCCAAG TCTTCAATGG   
  
  
+ CATCAGCCCA TTCGTCAAAT TCTCTCACTT CACTGCAAAC CAAGCAATCC AAGAGGCCTT CCAGAGGGAA   
  
  
+ GACAGGGTCC ACATCATCGA CCTCGATATC ATGCAGGGGC TCCAGTGGCC CGGGCTGTTC CACATCCTCG   
  
  
+ CGTCCCGGCC AGGTGGGCCT CCCTTCGTAA GGCTCACCGG GCTCGGGACC TCCATGGAGG CGCTCGAGGC   
  
  
+ CACCGGAAAA AGGCTCTCAG ACTTCGCCGA GAAGTTGGGG TTGCCCTTTG AGTTTATACC CGTGGCGGAG   
  
  
+ AAGATTGGAA ATTTGGACTT GGAAAGGTTG CATGTTAGTA AAAGGGAAGC TCTTGCTGTG CATTGGTTAC   
  
  
+ AGCACTCTTT GTATGATGTT ACTGGCTCTG ATACTAATAC ACTTGGCCTT CTTCAAAGGT TGGCGCCAAA   
  
  
+ AGTGGTGACG GTGGTGGAGC AAGACCTAAG CCGAACAGGC TCTTTCCTAG GAAGGTTTGT AGAGGCGATC   
  
  
+ CACTACTATT CAGCCCTATT TGACTCCTTA GGAGCGAGTT ATGGAGAGGA CAGTGAGGAG AGGCATGTGG   
  
  
+ TTGAGCAACA GCTCCTTTCT AGGGAGATTC GAAACATTCT GGCCGTTGGT GGGCCCTCAA GGACCGGGGA   
  
  
+ GCCCAAGTTT GCGAGCTGGA GGGAGAAGCT ACAACAGTCC GGCTTTAGGG GAATCTCATT GGCAGGCAAC   
  
  
+ GCCGCTGCCC AGGCCACCTT GCTCCTCGGC ATGTTCCCTT CTGATGGGTA TACTTTAATC GAGGACAGTG   
  
  
+ GCACACTTAA GCTCGGGTGG AAGGACTTGT GCCTCCTGAC TGCTTCGGCC TGGAGGCCTT CCCATGCTCA   
  
  
+ TACTATGAGC ACCGTCTGTA CTCGGAGCCA ATA  

- -Up\_Stream \_Len000CAGAGA CATTGTGTTA AGTAATTTAG GTAGGTTTCC CCTAGTATTT ATTGGTTTGA   
  
  
- AACAACCTTT GACGAAACTA CACCCGAAAG AGAAAGTCGT AAATACAATA TGTATATATA CCAATATTTC   
  
  
- AATGTTTTCA TACTGGGCTG TGCTGTTAAG TTAGGCTTAG GCTGAGCTTC AATGACTTTC ATTAGACTTA   
  
  
- TGCTAAACTG TCGAATCAAA TTTTTGGATA ATGAAGAGTT AAAACTCATT TAGATTCGAT AAAAATTACG   
  
  
- ACTTATCTAG CTGGGCTAAG CTGGGCAAAA GGCTGAGATA TACCAATACC ACGGGGATTT TTTTTAATTT   
  
  
- TTTCTTTTTC TTATCTTTTT ATATTTTATT TTGACTCTAA AGACTTGTGA GAGAGGGGAT CAGAACACCC   
  
  
- GTACCTTTCA CCAACCTCTA CCTCTACAAA CTCAAAGTAA AAGACTATAA TAAAAAATCA GTAATCTTCA   
  
  
- TGTTTCAGAT TTGAGTTGTT CTCGTACTCC CCTCTTACCA ACAACATCGG TTACCTTTTA TTTTTCTTGT   
  
  
- AAGTGAGAAG AACACGAAAT AAATACCACT ATTAAACAAT GAACATAAAA ATTTTTACTA ATTTATATAA   
  
  
- CTAGGAAACT ATAATACAAC TTTATTATTT TTATATTTTT AATCTTTTAT AAAATGGAGC ACTTTAAAAA   
  
  
- AAAGACTAAT TAGTAACTTT AATACCACTT TTTATTTCCT TGTAGAGAAC ATTTGTGAGA TAGGCAAATG   
  
  
- CAAACTGGTG TTTGAACGAT AGGTGTTCGT AGAGTTGGAA ACGGAAATTG GGTTAACACC CAACTACTTT   
  
  
- AGATTGGTAA TATCCTAATC TACCATCAGC AATTAGGACA CTTTGTTCAA AAACCAATAC TACATAGGTT   
  
  
- AATACTCCCG GACTATTGAG AACGGAGCCG TAAACGCACT ATTACTGCAC CATGATTATC GTTAATAAAA   
  
  
- AGCGTTTATA AGCTTTAAAA AATTCTTATT TGCGTTTTAA TAACTAGAAC TGTTTACTAA AATAAAAAAA   
  
  
- CAACCCTTTT CCTATTTGTA GATCACAGTT AACAACTACT GGACTATCGA GAGCTAAAGA ATAAATACTC   
  
  
- AATACAACTA TACTGTGATT AACATTAATA AACAACATTT GAATTTGTAA AAATTTTTAG TTTCGTTTTA   
  
  
- AACAATTAAT GTTATTTAGT TAGATGAAAA TGATACTAGA ATAAACCACA TTAATAACTA GAACTTAAAA   
  
  
- AAAAAAAGAA AAACTTTTTC TATTCGTAGC TTAAAAAAGT TTTTATTTTT CCCACTTCCT TCTTTTTTAC   
  
  
- CTTTGTCAAT GGCTCATTCC TTCCTGTCTC GCGCTCTCCT GTTCTGTGTT TCTCTCCCCT CTCTCTCTCT   
  
  
- CTTCCCCCGT TACCGTCACC CCTTCCAGCT CCTCGTGGAA AGTTGAAGTT CCGAGTTGTG GGGGGGGGGG   
  
  
- GGGGAGAAAG GAGTAAAACG CTGCAATCGT AAACTGTAAA ACGAAAGTTG GCAGGGAGCC GTAGTAGTCG   
  
  
- GGACGGGAAC GAGTCGAAAA GGGTAGAAAA AAACCGAAAA ACAAACTGAG GGAGATATTG AGATAAGTGC   
  
  
- AGCTCGGAAC ATTTTTAAAA TGTATAAAAT AAAATAAAAT GAAAAAATCC AAATAGGGAG AGATAAGAAC   
  
  
- TGGATGCCCG TAAAAAATAA AAGAAACCCA AACTCTGAAC ACTTGAAAAA CGTGTAAAAG AGTGTTTTTA   
  
  
- AATAGAACAG AAAAAGTGAA AGTTGCAAGG AAGAGAGAGA GAGAGAGAGA GAGAGAGAGA GAGTGAGTGT   
  
  
- AGGATCTGTC TGCAGGAAAA ATTTCTTAAA TAAATTGTTC AATCTTCTTT GGCAACAAAT TCATCTTGGT   
  
  
- GGGACAAACC TACGGTTTCC TTTATGGTCA GTTCTTTTTA GTGCTGTTAG TGTTGTGTAG GTAAAGCAAA   
  
  
- TGTAGAGTAG GAGTAGGAGT AGAAGGAGTA GAGATGGGTA GAGGGAGAAA GACATACCGA CGAAGAAGAT   
  
  
- GAAACAAGGG ACTGGGATTG TTATGATCAA ATCAGGAGGG AATAATAATA ATAGGAGGAG GAAGGAGGAG   
  
  
- AATGATATTA CCATTGGGAA TGGAAGGAGA AGGATTGTGG TGACGATAAG GATGAGGAAG TTGTTGGAGA   
  
  
- GGCCATAAAA GAAGGCGGTG GATGAGCCTC AACTTATGAC TAACAGTTTG AGTTGACGGG TACAGATTGG   
  
  
- AGGGGCCTTT CTAGCAAGCC TTCGCCTCAC GACGACTTTA CCTCGTCGCG AGGCGAGGCT AACGCCCACT   
  
  
- AATGGGCGGT TGAGTAGTCG CCCATTAGTG GGCGGTGTTA CTCCGCCGGA GTAGTCACAG AAAGTAACTG   
  
  
- AGGAGTGAGA GCGGTGAAGT GGAGAAGTTG CCGAGGTTAA GCTGCTGGGG TAATCGGGGG CTGGCAGGAT   
  
  
- TACTAGGCCC AGACTCAGTC CCAGGCCTAG ACGGAGAACA CACGCCTAAG AGGCCTGAGG GAAATAAGGG   
  
  
- TGGCCACCTC TCTTCTGGAA GGTAGGGGCG GTGCTGGTGA CGACGGCGCC GCTAGCGCCA CCAAGGACAC   
  
  
- CTCCTCCTAC TAGGCTGCAC CTACCTATCG TAGTATTTTC TCGACTAAGT TTCGAGTTTG AGCTAGAGTT   
  
  
- AGGGTGTCAA CTAAGTCTTG CAATCTCTAT AGTAGATGGG TACATTAGGA TTAGACCCCA GGCGGTAACT   
  
  
- CAAGGCGGAG GCGAGCGAGC GGCGGCTGGG GGAGTAACGA GGCGGCGGTG AAGGCGGGAA GGTGGTGGTG   
  
  
- GTTGTGGTGG AGTTAGTTGT TTAGTGAGAA GGAGCTTAGT CGTTGTGATT GTTGTTGTTA GTACACTCGC   
  
  
- ACATGCGGTT GTTTCCTGGG CCTATGAAGT TGAACCCAGG CCCAGTCCCA GTCCCAGGCT AATTGTAGCT   
  
  
- GGTTCGATTG GAAAGGAAGG GAGGACTAAG GTGGCGGACA ACCCCACAGT CACAGTGTGG CGGTGGCAGA   
  
  
- AGGCGGCGGC CAAGGCCGAG GCCACCTTCG CCGTCATCAT CACCATTTAG TTTGGGATTG GGATTAGGGT   
  
  
- TGGGCTTGGG TTTGGGTTTG GGTTTGGGCT TGTTGTTTCG AGTCCTACAA GTTGAGGTTT GAGGTGTTGT   
  
  
- TGTCGTTGTC GTTGTCGTTG TTTAGGGGCA CCTAGTCCTC GTTCTGGGCC GCCGAGGTGG CCGCTGCCTT   
  
  
- CGTAGCGGTG GTAGAGGTTC TCGCCGCCGT GGTGGCGGCC GCCGTCACTT TCGTTCTCTT TCTCTCCTCT   
  
  
- ACGCCGTTTC CTTCGCGCTG CTTCTCCCAG AGGTGGAGGA TTGGGAGGAG GAGGTTACGC GTCTTCGTCA   
  
  
- TAGGCCGCTG TTTATGCTTC TTCGCTTGTT CTACGAAGAT CTTTAGAGCC TCACCCGGTG GGGTAAGCCG   
  
  
- TGGAGGCGGG TTGCGCAGCG GCGCATGAAG AGCCTTCGGT ACAGTCGGGC AGAGCATAGG AGGACGGAGC   
  
  
- CGTAGATGCG GCGGGAGGGG TGGCAGGGTG TGATGCAGTT CGAGGAGAGG CGGAAGGTTC AGAAGTTACC   
  
  
- GTAGTCGGGT AAGCAGTTTA AGAGAGTGAA GTGACGTTTG GTTCGTTAGG TTCTCCGGAA GGTCTCCCTT   
  
  
- CTGTCCCAGG TGTAGTAGCT GGAGCTATAG TACGTCCCCG AGGTCACCGG GCCCGACAAG GTGTAGGAGC   
  
  
- GCAGGGCCGG TCCACCCGGA GGGAAGCATT CCGAGTGGCC CGAGCCCTGG AGGTACCTCC GCGAGCTCCG   
  
  
- GTGGCCTTTT TCCGAGAGTC TGAAGCGGCT CTTCAACCCC AACGGGAAAC TCAAATATGG GCACCGCCTC   
  
  
- TTCTAACCTT TAAACCTGAA CCTTTCCAAC GTACAATCAT TTTCCCTTCG AGAACGACAC GTAACCAATG   
  
  
- TCGTGAGAAA CATACTACAA TGACCGAGAC TATGATTATG TGAACCGGAA GAAGTTTCCA ACCGCGGTTT   
  
  
- TCACCACTGC CACCACCTCG TTCTGGATTC GGCTTGTCCG AGAAAGGATC CTTCCAAACA TCTCCGCTAG   
  
  
- GTGATGATAA GTCGGGATAA ACTGAGGAAT CCTCGCTCAA TACCTCTCCT GTCACTCCTC TCCGTACACC   
  
  
- AACTCGTTGT CGAGGAAAGA TCCCTCTAAG CTTTGTAAGA CCGGCAACCA CCCGGGAGTT CCTGGCCCCT   
  
  
- CGGGTTCAAA CGCTCGACCT CCCTCTTCGA TGTTGTCAGG CCGAAATCCC CTTAGAGTAA CCGTCCGTTG   
  
  
- CGGCGACGGG TCCGGTGGAA CGAGGAGCCG TACAAGGGAA GACTACCCAT ATGAAATTAG CTCCTGTCAC   
  
  
- CGTGTGAATT CGAGCCCACC TTCCTGAACA CGGAGGACTG ACGAAGCCGG ACCTCCGGAA GGGTACGAGT   
  
  
- ATGATACTCG TGGCAGACAT GAGCCTCGGT TAT

+     GC-motif

| Site Name | Organism | Position | Strand | Matrix score. | sequence | function |
| --- | --- | --- | --- | --- | --- | --- |
| GC-motif | Zea mays | 2440 | + | 6 | CCCCCG | enhancer-like element involved in anoxic specific inducibility |

>HU01G00472.1   
+ -Up\_Stream \_Len000GTCTCT GTAACACAAT TCATTAAATC CATCCAAAGG GGATCATAAA TAACCAAACT   
  
  
+ TTGTTGGAAA CTGCTTTGAT GTGGGCTTTC TCTTTCAGCA TTTATGTTAT ACATATATAT GGTTATAAAG   
  
  
+ TTACAAAAGT ATGACCCGAC ACGACAATTC AATCCGAATC CGACTCGAAG TTACTGAAAG TAATCTGAAT   
  
  
+ ACGATTTGAC AGCTTAGTTT AAAAACCTAT TACTTCTCAA TTTTGAGTAA ATCTAAGCTA TTTTTAATGC   
  
  
+ TGAATAGATC GACCCGATTC GACCCGTTTT CCGACTCTAT ATGGTTATGG TGCCCCTAAA AAAAATTAAA   
  
  
+ AAAGAAAAAG AATAGAAAAA TATAAAATAA AACTGAGATT TCTGAACACT CTCTCCCCTA GTCTTGTGGG   
  
  
+ CATGGAAAGT GGTTGGAGAT GGAGATGTTT GAGTTTCATT TTCTGATATT ATTTTTTAGT CATTAGAAGT   
  
  
+ ACAAAGTCTA AACTCAACAA GAGCATGAGG GGAGAATGGT TGTTGTAGCC AATGGAAAAT AAAAAGAACA   
  
  
+ TTCACTCTTC TTGTGCTTTA TTTATGGTGA TAATTTGTTA CTTGTATTTT TAAAAATGAT TAAATATATT   
  
  
+ GATCCTTTGA TATTATGTTG AAATAATAAA AATATAAAAA TTAGAAAATA TTTTACCTCG TGAAATTTTT   
  
  
+ TTTCTGATTA ATCATTGAAA TTATGGTGAA AAATAAAGGA ACATCTCTTG TAAACACTCT ATCCGTTTAC   
  
  
+ GTTTGACCAC AAACTTGCTA TCCACAAGCA TCTCAACCTT TGCCTTTAAC CCAATTGTGG GTTGATGAAA   
  
  
+ TCTAACCATT ATAGGATTAG ATGGTAGTCG TTAATCCTGT GAAACAAGTT TTTGGTTATG ATGTATCCAA   
  
  
+ TTATGAGGGC CTGATAACTC TTGCCTCGGC ATTTGCGTGA TAATGACGTG GTACTAATAG CAATTATTTT   
  
  
+ TCGCAAATAT TCGAAATTTT TTAAGAATAA ACGCAAAATT ATTGATCTTG ACAAATGATT TTATTTTTTT   
  
  
+ GTTGGGAAAA GGATAAACAT CTAGTGTCAA TTGTTGATGA CCTGATAGCT CTCGATTTCT TATTTATGAG   
  
  
+ TTATGTTGAT ATGACACTAA TTGTAATTAT TTGTTGTAAA CTTAAACATT TTTAAAAATC AAAGCAAAAT   
  
  
+ TTGTTAATTA CAATAAATCA ATCTACTTTT ACTATGATCT TATTTGGTGT AATTATTGAT CTTGAATTTT   
  
  
+ TTTTTTTCTT TTTGAAAAAG ATAAGCATCG AATTTTTTCA AAAATAAAAA GGGTGAAGGA AGAAAAAATG   
  
  
+ GAAACAGTTA CCGAGTAAGG AAGGACAGAG CGCGAGAGGA CAAGACACAA AGAGAGGGGA GAGAGAGAGA   
  
  
+ GAAGGGGGCA ATGGCAGTGG GGAAGGTCGA GGAGCACCTT TCAACTTCAA GGCTCAACAC CCCCCCCCCC   
  
  
+ CCCCTCTTTC CTCATTTTGC GACGTTAGCA TTTGACATTT TGCTTTCAAC CGTCCCTCGG CATCATCAGC   
  
  
+ CCTGCCCTTG CTCAGCTTTT CCCATCTTTT TTTGGCTTTT TGTTTGACTC CCTCTATAAC TCTATTCACG   
  
  
+ TCGAGCCTTG TAAAAATTTT ACATATTTTA TTTTATTTTA CTTTTTTAGG TTTATCCCTC TCTATTCTTG   
  
  
+ ACCTACGGGC ATTTTTTATT TTCTTTGGGT TTGAGACTTG TGAACTTTTT GCACATTTTC TCACAAAAAT   
  
  
+ TTATCTTGTC TTTTTCACTT TCAACGTTCC TTCTCTCTCT CTCTCTCTCT CTCTCTCTCT CTCACTCACA   
  
  
+ TCCTAGACAG ACGTCCTTTT TAAAGAATTT ATTTAACAAG TTAGAAGAAA CCGTTGTTTA AGTAGAACCA   
  
  
+ CCCTGTTTGG ATGCCAAAGG AAATACCAGT CAAGAAAAAT CACGACAATC ACAACACATC CATTTCGTTT   
  
  
+ ACATCTCATC CTCATCCTCA TCTTCCTCAT CTCTACCCAT CTCCCTCTTT CTGTATGGCT GCTTCTTCTA   
  
  
+ CTTTGTTCCC TGACCCTAAC AATACTAGTT TAGTCCTCCC TTATTATTAT TATCCTCCTC CTTCCTCCTC   
  
  
+ TTACTATAAT GGTAACCCTT ACCTTCCTCT TCCTAACACC ACTGCTATTC CTACTCCTTC AACAACCTCT   
  
  
+ CCGGTATTTT CTTCCGCCAC CTACTCGGAG TTGAATACTG ATTGTCAAAC TCAACTGCCC ATGTCTAACC   
  
  
+ TCCCCGGAAA GATCGTTCGG AAGCGGAGTG CTGCTGAAAT GGAGCAGCGC TCCGCTCCGA TTGCGGGTGA   
  
  
+ TTACCCGCCA ACTCATCAGC GGGTAATCAC CCGCCACAAT GAGGCGGCCT CATCAGTGTC TTTCATTGAC   
  
  
+ TCCTCACTCT CGCCACTTCA CCTCTTCAAC GGCTCCAATT CGACGACCCC ATTAGCCCCC GACCGTCCTA   
  
  
+ ATGATCCGGG TCTGAGTCAG GGTCCGGATC TGCCTCTTGT GTGCGGATTC TCCGGACTCC CTTTATTCCC   
  
  
+ ACCGGTGGAG AGAAGACCTT CCATCCCCGC CACGACCACT GCTGCCGCGG CGATCGCGGT GGTTCCTGTG   
  
  
+ GAGGAGGATG ATCCGACGTG GATGGATAGC ATCATAAAAG AGCTGATTCA AAGCTCAAAC TCGATCTCAA   
  
  
+ TCCCACAGTT GATTCAGAAC GTTAGAGATA TCATCTACCC ATGTAATCCT AATCTGGGGT CCGCCATTGA   
  
  
+ GTTCCGCCTC CGCTCGCTCG CCGCCGACCC CCTCATTGCT CCGCCGCCAC TTCCGCCCTT CCACCACCAC   
  
  
+ CAACACCACC TCAATCAACA AATCACTCTT CCTCGAATCA GCAACACTAA CAACAACAAT CATGTGAGCG   
  
  
+ TGTACGCCAA CAAAGGACCC GGATACTTCA ACTTGGGTCC GGGTCAGGGT CAGGGTCCGA TTAACATCGA   
  
  
+ CCAAGCTAAC CTTTCCTTCC CTCCTGATTC CACCGCCTGT TGGGGTGTCA GTGTCACACC GCCACCGTCT   
  
  
+ TCCGCCGCCG GTTCCGGCTC CGGTGGAAGC GGCAGTAGTA GTGGTAAATC AAACCCTAAC CCTAATCCCA   
  
  
+ ACCCGAACCC AAACCCAAAC CCAAACCCGA ACAACAAAGC TCAGGATGTT CAACTCCAAA CTCCACAACA   
  
  
+ ACAGCAACAG CAACAGCAAC AAATCCCCGT GGATCAGGAG CAAGACCCGG CGGCTCCACC GGCGACGGAA   
  
  
+ GCATCGCCAC CATCTCCAAG AGCGGCGGCA CCACCGCCGG CGGCAGTGAA AGCAAGAGAA AGAGAGGAGA   
  
  
+ TGCGGCAAAG GAAGCGCGAC GAAGAGGGTC TCCACCTCCT AACCCTCCTC CTCCAATGCG CAGAAGCAGT   
  
  
+ ATCCGGCGAC AAATACGAAG AAGCGAACAA GATGCTTCTA GAAATCTCGG AGTGGGCCAC CCCATTCGGC   
  
  
+ ACCTCCGCCC AACGCGTCGC CGCGTACTTC TCGGAAGCCA TGTCAGCCCG TCTCGTATCC TCCTGCCTCG   
  
  
+ GCATCTACGC CGCCCTCCCC ACCGTCCCAC ACTACGTCAA GCTCCTCTCC GCCTTCCAAG TCTTCAATGG   
  
  
+ CATCAGCCCA TTCGTCAAAT TCTCTCACTT CACTGCAAAC CAAGCAATCC AAGAGGCCTT CCAGAGGGAA   
  
  
+ GACAGGGTCC ACATCATCGA CCTCGATATC ATGCAGGGGC TCCAGTGGCC CGGGCTGTTC CACATCCTCG   
  
  
+ CGTCCCGGCC AGGTGGGCCT CCCTTCGTAA GGCTCACCGG GCTCGGGACC TCCATGGAGG CGCTCGAGGC   
  
  
+ CACCGGAAAA AGGCTCTCAG ACTTCGCCGA GAAGTTGGGG TTGCCCTTTG AGTTTATACC CGTGGCGGAG   
  
  
+ AAGATTGGAA ATTTGGACTT GGAAAGGTTG CATGTTAGTA AAAGGGAAGC TCTTGCTGTG CATTGGTTAC   
  
  
+ AGCACTCTTT GTATGATGTT ACTGGCTCTG ATACTAATAC ACTTGGCCTT CTTCAAAGGT TGGCGCCAAA   
  
  
+ AGTGGTGACG GTGGTGGAGC AAGACCTAAG CCGAACAGGC TCTTTCCTAG GAAGGTTTGT AGAGGCGATC   
  
  
+ CACTACTATT CAGCCCTATT TGACTCCTTA GGAGCGAGTT ATGGAGAGGA CAGTGAGGAG AGGCATGTGG   
  
  
+ TTGAGCAACA GCTCCTTTCT AGGGAGATTC GAAACATTCT GGCCGTTGGT GGGCCCTCAA GGACCGGGGA   
  
  
+ GCCCAAGTTT GCGAGCTGGA GGGAGAAGCT ACAACAGTCC GGCTTTAGGG GAATCTCATT GGCAGGCAAC   
  
  
+ GCCGCTGCCC AGGCCACCTT GCTCCTCGGC ATGTTCCCTT CTGATGGGTA TACTTTAATC GAGGACAGTG   
  
  
+ GCACACTTAA GCTCGGGTGG AAGGACTTGT GCCTCCTGAC TGCTTCGGCC TGGAGGCCTT CCCATGCTCA   
  
  
+ TACTATGAGC ACCGTCTGTA CTCGGAGCCA ATA  

- -Up\_Stream \_Len000CAGAGA CATTGTGTTA AGTAATTTAG GTAGGTTTCC CCTAGTATTT ATTGGTTTGA   
  
  
- AACAACCTTT GACGAAACTA CACCCGAAAG AGAAAGTCGT AAATACAATA TGTATATATA CCAATATTTC   
  
  
- AATGTTTTCA TACTGGGCTG TGCTGTTAAG TTAGGCTTAG GCTGAGCTTC AATGACTTTC ATTAGACTTA   
  
  
- TGCTAAACTG TCGAATCAAA TTTTTGGATA ATGAAGAGTT AAAACTCATT TAGATTCGAT AAAAATTACG   
  
  
- ACTTATCTAG CTGGGCTAAG CTGGGCAAAA GGCTGAGATA TACCAATACC ACGGGGATTT TTTTTAATTT   
  
  
- TTTCTTTTTC TTATCTTTTT ATATTTTATT TTGACTCTAA AGACTTGTGA GAGAGGGGAT CAGAACACCC   
  
  
- GTACCTTTCA CCAACCTCTA CCTCTACAAA CTCAAAGTAA AAGACTATAA TAAAAAATCA GTAATCTTCA   
  
  
- TGTTTCAGAT TTGAGTTGTT CTCGTACTCC CCTCTTACCA ACAACATCGG TTACCTTTTA TTTTTCTTGT   
  
  
- AAGTGAGAAG AACACGAAAT AAATACCACT ATTAAACAAT GAACATAAAA ATTTTTACTA ATTTATATAA   
  
  
- CTAGGAAACT ATAATACAAC TTTATTATTT TTATATTTTT AATCTTTTAT AAAATGGAGC ACTTTAAAAA   
  
  
- AAAGACTAAT TAGTAACTTT AATACCACTT TTTATTTCCT TGTAGAGAAC ATTTGTGAGA TAGGCAAATG   
  
  
- CAAACTGGTG TTTGAACGAT AGGTGTTCGT AGAGTTGGAA ACGGAAATTG GGTTAACACC CAACTACTTT   
  
  
- AGATTGGTAA TATCCTAATC TACCATCAGC AATTAGGACA CTTTGTTCAA AAACCAATAC TACATAGGTT   
  
  
- AATACTCCCG GACTATTGAG AACGGAGCCG TAAACGCACT ATTACTGCAC CATGATTATC GTTAATAAAA   
  
  
- AGCGTTTATA AGCTTTAAAA AATTCTTATT TGCGTTTTAA TAACTAGAAC TGTTTACTAA AATAAAAAAA   
  
  
- CAACCCTTTT CCTATTTGTA GATCACAGTT AACAACTACT GGACTATCGA GAGCTAAAGA ATAAATACTC   
  
  
- AATACAACTA TACTGTGATT AACATTAATA AACAACATTT GAATTTGTAA AAATTTTTAG TTTCGTTTTA   
  
  
- AACAATTAAT GTTATTTAGT TAGATGAAAA TGATACTAGA ATAAACCACA TTAATAACTA GAACTTAAAA   
  
  
- AAAAAAAGAA AAACTTTTTC TATTCGTAGC TTAAAAAAGT TTTTATTTTT CCCACTTCCT TCTTTTTTAC   
  
  
- CTTTGTCAAT GGCTCATTCC TTCCTGTCTC GCGCTCTCCT GTTCTGTGTT TCTCTCCCCT CTCTCTCTCT   
  
  
- CTTCCCCCGT TACCGTCACC CCTTCCAGCT CCTCGTGGAA AGTTGAAGTT CCGAGTTGTG GGGGGGGGGG   
  
  
- GGGGAGAAAG GAGTAAAACG CTGCAATCGT AAACTGTAAA ACGAAAGTTG GCAGGGAGCC GTAGTAGTCG   
  
  
- GGACGGGAAC GAGTCGAAAA GGGTAGAAAA AAACCGAAAA ACAAACTGAG GGAGATATTG AGATAAGTGC   
  
  
- AGCTCGGAAC ATTTTTAAAA TGTATAAAAT AAAATAAAAT GAAAAAATCC AAATAGGGAG AGATAAGAAC   
  
  
- TGGATGCCCG TAAAAAATAA AAGAAACCCA AACTCTGAAC ACTTGAAAAA CGTGTAAAAG AGTGTTTTTA   
  
  
- AATAGAACAG AAAAAGTGAA AGTTGCAAGG AAGAGAGAGA GAGAGAGAGA GAGAGAGAGA GAGTGAGTGT   
  
  
- AGGATCTGTC TGCAGGAAAA ATTTCTTAAA TAAATTGTTC AATCTTCTTT GGCAACAAAT TCATCTTGGT   
  
  
- GGGACAAACC TACGGTTTCC TTTATGGTCA GTTCTTTTTA GTGCTGTTAG TGTTGTGTAG GTAAAGCAAA   
  
  
- TGTAGAGTAG GAGTAGGAGT AGAAGGAGTA GAGATGGGTA GAGGGAGAAA GACATACCGA CGAAGAAGAT   
  
  
- GAAACAAGGG ACTGGGATTG TTATGATCAA ATCAGGAGGG AATAATAATA ATAGGAGGAG GAAGGAGGAG   
  
  
- AATGATATTA CCATTGGGAA TGGAAGGAGA AGGATTGTGG TGACGATAAG GATGAGGAAG TTGTTGGAGA   
  
  
- GGCCATAAAA GAAGGCGGTG GATGAGCCTC AACTTATGAC TAACAGTTTG AGTTGACGGG TACAGATTGG   
  
  
- AGGGGCCTTT CTAGCAAGCC TTCGCCTCAC GACGACTTTA CCTCGTCGCG AGGCGAGGCT AACGCCCACT   
  
  
- AATGGGCGGT TGAGTAGTCG CCCATTAGTG GGCGGTGTTA CTCCGCCGGA GTAGTCACAG AAAGTAACTG   
  
  
- AGGAGTGAGA GCGGTGAAGT GGAGAAGTTG CCGAGGTTAA GCTGCTGGGG TAATCGGGGG CTGGCAGGAT   
  
  
- TACTAGGCCC AGACTCAGTC CCAGGCCTAG ACGGAGAACA CACGCCTAAG AGGCCTGAGG GAAATAAGGG   
  
  
- TGGCCACCTC TCTTCTGGAA GGTAGGGGCG GTGCTGGTGA CGACGGCGCC GCTAGCGCCA CCAAGGACAC   
  
  
- CTCCTCCTAC TAGGCTGCAC CTACCTATCG TAGTATTTTC TCGACTAAGT TTCGAGTTTG AGCTAGAGTT   
  
  
- AGGGTGTCAA CTAAGTCTTG CAATCTCTAT AGTAGATGGG TACATTAGGA TTAGACCCCA GGCGGTAACT   
  
  
- CAAGGCGGAG GCGAGCGAGC GGCGGCTGGG GGAGTAACGA GGCGGCGGTG AAGGCGGGAA GGTGGTGGTG   
  
  
- GTTGTGGTGG AGTTAGTTGT TTAGTGAGAA GGAGCTTAGT CGTTGTGATT GTTGTTGTTA GTACACTCGC   
  
  
- ACATGCGGTT GTTTCCTGGG CCTATGAAGT TGAACCCAGG CCCAGTCCCA GTCCCAGGCT AATTGTAGCT   
  
  
- GGTTCGATTG GAAAGGAAGG GAGGACTAAG GTGGCGGACA ACCCCACAGT CACAGTGTGG CGGTGGCAGA   
  
  
- AGGCGGCGGC CAAGGCCGAG GCCACCTTCG CCGTCATCAT CACCATTTAG TTTGGGATTG GGATTAGGGT   
  
  
- TGGGCTTGGG TTTGGGTTTG GGTTTGGGCT TGTTGTTTCG AGTCCTACAA GTTGAGGTTT GAGGTGTTGT   
  
  
- TGTCGTTGTC GTTGTCGTTG TTTAGGGGCA CCTAGTCCTC GTTCTGGGCC GCCGAGGTGG CCGCTGCCTT   
  
  
- CGTAGCGGTG GTAGAGGTTC TCGCCGCCGT GGTGGCGGCC GCCGTCACTT TCGTTCTCTT TCTCTCCTCT   
  
  
- ACGCCGTTTC CTTCGCGCTG CTTCTCCCAG AGGTGGAGGA TTGGGAGGAG GAGGTTACGC GTCTTCGTCA   
  
  
- TAGGCCGCTG TTTATGCTTC TTCGCTTGTT CTACGAAGAT CTTTAGAGCC TCACCCGGTG GGGTAAGCCG   
  
  
- TGGAGGCGGG TTGCGCAGCG GCGCATGAAG AGCCTTCGGT ACAGTCGGGC AGAGCATAGG AGGACGGAGC   
  
  
- CGTAGATGCG GCGGGAGGGG TGGCAGGGTG TGATGCAGTT CGAGGAGAGG CGGAAGGTTC AGAAGTTACC   
  
  
- GTAGTCGGGT AAGCAGTTTA AGAGAGTGAA GTGACGTTTG GTTCGTTAGG TTCTCCGGAA GGTCTCCCTT   
  
  
- CTGTCCCAGG TGTAGTAGCT GGAGCTATAG TACGTCCCCG AGGTCACCGG GCCCGACAAG GTGTAGGAGC   
  
  
- GCAGGGCCGG TCCACCCGGA GGGAAGCATT CCGAGTGGCC CGAGCCCTGG AGGTACCTCC GCGAGCTCCG   
  
  
- GTGGCCTTTT TCCGAGAGTC TGAAGCGGCT CTTCAACCCC AACGGGAAAC TCAAATATGG GCACCGCCTC   
  
  
- TTCTAACCTT TAAACCTGAA CCTTTCCAAC GTACAATCAT TTTCCCTTCG AGAACGACAC GTAACCAATG   
  
  
- TCGTGAGAAA CATACTACAA TGACCGAGAC TATGATTATG TGAACCGGAA GAAGTTTCCA ACCGCGGTTT   
  
  
- TCACCACTGC CACCACCTCG TTCTGGATTC GGCTTGTCCG AGAAAGGATC CTTCCAAACA TCTCCGCTAG   
  
  
- GTGATGATAA GTCGGGATAA ACTGAGGAAT CCTCGCTCAA TACCTCTCCT GTCACTCCTC TCCGTACACC   
  
  
- AACTCGTTGT CGAGGAAAGA TCCCTCTAAG CTTTGTAAGA CCGGCAACCA CCCGGGAGTT CCTGGCCCCT   
  
  
- CGGGTTCAAA CGCTCGACCT CCCTCTTCGA TGTTGTCAGG CCGAAATCCC CTTAGAGTAA CCGTCCGTTG   
  
  
- CGGCGACGGG TCCGGTGGAA CGAGGAGCCG TACAAGGGAA GACTACCCAT ATGAAATTAG CTCCTGTCAC   
  
  
- CGTGTGAATT CGAGCCCACC TTCCTGAACA CGGAGGACTG ACGAAGCCGG ACCTCCGGAA GGGTACGAGT   
  
  
- ATGATACTCG TGGCAGACAT GAGCCTCGGT TAT

+     GCN4\_motif

| Site Name | Organism | Position | Strand | Matrix score. | sequence | function |
| --- | --- | --- | --- | --- | --- | --- |
| GCN4\_motif | Oryza sativa | 2467 | + | 7 | TGAGTCA | cis-regulatory element involved in endosperm expression |

>HU01G00472.1   
+ -Up\_Stream \_Len000GTCTCT GTAACACAAT TCATTAAATC CATCCAAAGG GGATCATAAA TAACCAAACT   
  
  
+ TTGTTGGAAA CTGCTTTGAT GTGGGCTTTC TCTTTCAGCA TTTATGTTAT ACATATATAT GGTTATAAAG   
  
  
+ TTACAAAAGT ATGACCCGAC ACGACAATTC AATCCGAATC CGACTCGAAG TTACTGAAAG TAATCTGAAT   
  
  
+ ACGATTTGAC AGCTTAGTTT AAAAACCTAT TACTTCTCAA TTTTGAGTAA ATCTAAGCTA TTTTTAATGC   
  
  
+ TGAATAGATC GACCCGATTC GACCCGTTTT CCGACTCTAT ATGGTTATGG TGCCCCTAAA AAAAATTAAA   
  
  
+ AAAGAAAAAG AATAGAAAAA TATAAAATAA AACTGAGATT TCTGAACACT CTCTCCCCTA GTCTTGTGGG   
  
  
+ CATGGAAAGT GGTTGGAGAT GGAGATGTTT GAGTTTCATT TTCTGATATT ATTTTTTAGT CATTAGAAGT   
  
  
+ ACAAAGTCTA AACTCAACAA GAGCATGAGG GGAGAATGGT TGTTGTAGCC AATGGAAAAT AAAAAGAACA   
  
  
+ TTCACTCTTC TTGTGCTTTA TTTATGGTGA TAATTTGTTA CTTGTATTTT TAAAAATGAT TAAATATATT   
  
  
+ GATCCTTTGA TATTATGTTG AAATAATAAA AATATAAAAA TTAGAAAATA TTTTACCTCG TGAAATTTTT   
  
  
+ TTTCTGATTA ATCATTGAAA TTATGGTGAA AAATAAAGGA ACATCTCTTG TAAACACTCT ATCCGTTTAC   
  
  
+ GTTTGACCAC AAACTTGCTA TCCACAAGCA TCTCAACCTT TGCCTTTAAC CCAATTGTGG GTTGATGAAA   
  
  
+ TCTAACCATT ATAGGATTAG ATGGTAGTCG TTAATCCTGT GAAACAAGTT TTTGGTTATG ATGTATCCAA   
  
  
+ TTATGAGGGC CTGATAACTC TTGCCTCGGC ATTTGCGTGA TAATGACGTG GTACTAATAG CAATTATTTT   
  
  
+ TCGCAAATAT TCGAAATTTT TTAAGAATAA ACGCAAAATT ATTGATCTTG ACAAATGATT TTATTTTTTT   
  
  
+ GTTGGGAAAA GGATAAACAT CTAGTGTCAA TTGTTGATGA CCTGATAGCT CTCGATTTCT TATTTATGAG   
  
  
+ TTATGTTGAT ATGACACTAA TTGTAATTAT TTGTTGTAAA CTTAAACATT TTTAAAAATC AAAGCAAAAT   
  
  
+ TTGTTAATTA CAATAAATCA ATCTACTTTT ACTATGATCT TATTTGGTGT AATTATTGAT CTTGAATTTT   
  
  
+ TTTTTTTCTT TTTGAAAAAG ATAAGCATCG AATTTTTTCA AAAATAAAAA GGGTGAAGGA AGAAAAAATG   
  
  
+ GAAACAGTTA CCGAGTAAGG AAGGACAGAG CGCGAGAGGA CAAGACACAA AGAGAGGGGA GAGAGAGAGA   
  
  
+ GAAGGGGGCA ATGGCAGTGG GGAAGGTCGA GGAGCACCTT TCAACTTCAA GGCTCAACAC CCCCCCCCCC   
  
  
+ CCCCTCTTTC CTCATTTTGC GACGTTAGCA TTTGACATTT TGCTTTCAAC CGTCCCTCGG CATCATCAGC   
  
  
+ CCTGCCCTTG CTCAGCTTTT CCCATCTTTT TTTGGCTTTT TGTTTGACTC CCTCTATAAC TCTATTCACG   
  
  
+ TCGAGCCTTG TAAAAATTTT ACATATTTTA TTTTATTTTA CTTTTTTAGG TTTATCCCTC TCTATTCTTG   
  
  
+ ACCTACGGGC ATTTTTTATT TTCTTTGGGT TTGAGACTTG TGAACTTTTT GCACATTTTC TCACAAAAAT   
  
  
+ TTATCTTGTC TTTTTCACTT TCAACGTTCC TTCTCTCTCT CTCTCTCTCT CTCTCTCTCT CTCACTCACA   
  
  
+ TCCTAGACAG ACGTCCTTTT TAAAGAATTT ATTTAACAAG TTAGAAGAAA CCGTTGTTTA AGTAGAACCA   
  
  
+ CCCTGTTTGG ATGCCAAAGG AAATACCAGT CAAGAAAAAT CACGACAATC ACAACACATC CATTTCGTTT   
  
  
+ ACATCTCATC CTCATCCTCA TCTTCCTCAT CTCTACCCAT CTCCCTCTTT CTGTATGGCT GCTTCTTCTA   
  
  
+ CTTTGTTCCC TGACCCTAAC AATACTAGTT TAGTCCTCCC TTATTATTAT TATCCTCCTC CTTCCTCCTC   
  
  
+ TTACTATAAT GGTAACCCTT ACCTTCCTCT TCCTAACACC ACTGCTATTC CTACTCCTTC AACAACCTCT   
  
  
+ CCGGTATTTT CTTCCGCCAC CTACTCGGAG TTGAATACTG ATTGTCAAAC TCAACTGCCC ATGTCTAACC   
  
  
+ TCCCCGGAAA GATCGTTCGG AAGCGGAGTG CTGCTGAAAT GGAGCAGCGC TCCGCTCCGA TTGCGGGTGA   
  
  
+ TTACCCGCCA ACTCATCAGC GGGTAATCAC CCGCCACAAT GAGGCGGCCT CATCAGTGTC TTTCATTGAC   
  
  
+ TCCTCACTCT CGCCACTTCA CCTCTTCAAC GGCTCCAATT CGACGACCCC ATTAGCCCCC GACCGTCCTA   
  
  
+ ATGATCCGGG TCTGAGTCAG GGTCCGGATC TGCCTCTTGT GTGCGGATTC TCCGGACTCC CTTTATTCCC   
  
  
+ ACCGGTGGAG AGAAGACCTT CCATCCCCGC CACGACCACT GCTGCCGCGG CGATCGCGGT GGTTCCTGTG   
  
  
+ GAGGAGGATG ATCCGACGTG GATGGATAGC ATCATAAAAG AGCTGATTCA AAGCTCAAAC TCGATCTCAA   
  
  
+ TCCCACAGTT GATTCAGAAC GTTAGAGATA TCATCTACCC ATGTAATCCT AATCTGGGGT CCGCCATTGA   
  
  
+ GTTCCGCCTC CGCTCGCTCG CCGCCGACCC CCTCATTGCT CCGCCGCCAC TTCCGCCCTT CCACCACCAC   
  
  
+ CAACACCACC TCAATCAACA AATCACTCTT CCTCGAATCA GCAACACTAA CAACAACAAT CATGTGAGCG   
  
  
+ TGTACGCCAA CAAAGGACCC GGATACTTCA ACTTGGGTCC GGGTCAGGGT CAGGGTCCGA TTAACATCGA   
  
  
+ CCAAGCTAAC CTTTCCTTCC CTCCTGATTC CACCGCCTGT TGGGGTGTCA GTGTCACACC GCCACCGTCT   
  
  
+ TCCGCCGCCG GTTCCGGCTC CGGTGGAAGC GGCAGTAGTA GTGGTAAATC AAACCCTAAC CCTAATCCCA   
  
  
+ ACCCGAACCC AAACCCAAAC CCAAACCCGA ACAACAAAGC TCAGGATGTT CAACTCCAAA CTCCACAACA   
  
  
+ ACAGCAACAG CAACAGCAAC AAATCCCCGT GGATCAGGAG CAAGACCCGG CGGCTCCACC GGCGACGGAA   
  
  
+ GCATCGCCAC CATCTCCAAG AGCGGCGGCA CCACCGCCGG CGGCAGTGAA AGCAAGAGAA AGAGAGGAGA   
  
  
+ TGCGGCAAAG GAAGCGCGAC GAAGAGGGTC TCCACCTCCT AACCCTCCTC CTCCAATGCG CAGAAGCAGT   
  
  
+ ATCCGGCGAC AAATACGAAG AAGCGAACAA GATGCTTCTA GAAATCTCGG AGTGGGCCAC CCCATTCGGC   
  
  
+ ACCTCCGCCC AACGCGTCGC CGCGTACTTC TCGGAAGCCA TGTCAGCCCG TCTCGTATCC TCCTGCCTCG   
  
  
+ GCATCTACGC CGCCCTCCCC ACCGTCCCAC ACTACGTCAA GCTCCTCTCC GCCTTCCAAG TCTTCAATGG   
  
  
+ CATCAGCCCA TTCGTCAAAT TCTCTCACTT CACTGCAAAC CAAGCAATCC AAGAGGCCTT CCAGAGGGAA   
  
  
+ GACAGGGTCC ACATCATCGA CCTCGATATC ATGCAGGGGC TCCAGTGGCC CGGGCTGTTC CACATCCTCG   
  
  
+ CGTCCCGGCC AGGTGGGCCT CCCTTCGTAA GGCTCACCGG GCTCGGGACC TCCATGGAGG CGCTCGAGGC   
  
  
+ CACCGGAAAA AGGCTCTCAG ACTTCGCCGA GAAGTTGGGG TTGCCCTTTG AGTTTATACC CGTGGCGGAG   
  
  
+ AAGATTGGAA ATTTGGACTT GGAAAGGTTG CATGTTAGTA AAAGGGAAGC TCTTGCTGTG CATTGGTTAC   
  
  
+ AGCACTCTTT GTATGATGTT ACTGGCTCTG ATACTAATAC ACTTGGCCTT CTTCAAAGGT TGGCGCCAAA   
  
  
+ AGTGGTGACG GTGGTGGAGC AAGACCTAAG CCGAACAGGC TCTTTCCTAG GAAGGTTTGT AGAGGCGATC   
  
  
+ CACTACTATT CAGCCCTATT TGACTCCTTA GGAGCGAGTT ATGGAGAGGA CAGTGAGGAG AGGCATGTGG   
  
  
+ TTGAGCAACA GCTCCTTTCT AGGGAGATTC GAAACATTCT GGCCGTTGGT GGGCCCTCAA GGACCGGGGA   
  
  
+ GCCCAAGTTT GCGAGCTGGA GGGAGAAGCT ACAACAGTCC GGCTTTAGGG GAATCTCATT GGCAGGCAAC   
  
  
+ GCCGCTGCCC AGGCCACCTT GCTCCTCGGC ATGTTCCCTT CTGATGGGTA TACTTTAATC GAGGACAGTG   
  
  
+ GCACACTTAA GCTCGGGTGG AAGGACTTGT GCCTCCTGAC TGCTTCGGCC TGGAGGCCTT CCCATGCTCA   
  
  
+ TACTATGAGC ACCGTCTGTA CTCGGAGCCA ATA  

- -Up\_Stream \_Len000CAGAGA CATTGTGTTA AGTAATTTAG GTAGGTTTCC CCTAGTATTT ATTGGTTTGA   
  
  
- AACAACCTTT GACGAAACTA CACCCGAAAG AGAAAGTCGT AAATACAATA TGTATATATA CCAATATTTC   
  
  
- AATGTTTTCA TACTGGGCTG TGCTGTTAAG TTAGGCTTAG GCTGAGCTTC AATGACTTTC ATTAGACTTA   
  
  
- TGCTAAACTG TCGAATCAAA TTTTTGGATA ATGAAGAGTT AAAACTCATT TAGATTCGAT AAAAATTACG   
  
  
- ACTTATCTAG CTGGGCTAAG CTGGGCAAAA GGCTGAGATA TACCAATACC ACGGGGATTT TTTTTAATTT   
  
  
- TTTCTTTTTC TTATCTTTTT ATATTTTATT TTGACTCTAA AGACTTGTGA GAGAGGGGAT CAGAACACCC   
  
  
- GTACCTTTCA CCAACCTCTA CCTCTACAAA CTCAAAGTAA AAGACTATAA TAAAAAATCA GTAATCTTCA   
  
  
- TGTTTCAGAT TTGAGTTGTT CTCGTACTCC CCTCTTACCA ACAACATCGG TTACCTTTTA TTTTTCTTGT   
  
  
- AAGTGAGAAG AACACGAAAT AAATACCACT ATTAAACAAT GAACATAAAA ATTTTTACTA ATTTATATAA   
  
  
- CTAGGAAACT ATAATACAAC TTTATTATTT TTATATTTTT AATCTTTTAT AAAATGGAGC ACTTTAAAAA   
  
  
- AAAGACTAAT TAGTAACTTT AATACCACTT TTTATTTCCT TGTAGAGAAC ATTTGTGAGA TAGGCAAATG   
  
  
- CAAACTGGTG TTTGAACGAT AGGTGTTCGT AGAGTTGGAA ACGGAAATTG GGTTAACACC CAACTACTTT   
  
  
- AGATTGGTAA TATCCTAATC TACCATCAGC AATTAGGACA CTTTGTTCAA AAACCAATAC TACATAGGTT   
  
  
- AATACTCCCG GACTATTGAG AACGGAGCCG TAAACGCACT ATTACTGCAC CATGATTATC GTTAATAAAA   
  
  
- AGCGTTTATA AGCTTTAAAA AATTCTTATT TGCGTTTTAA TAACTAGAAC TGTTTACTAA AATAAAAAAA   
  
  
- CAACCCTTTT CCTATTTGTA GATCACAGTT AACAACTACT GGACTATCGA GAGCTAAAGA ATAAATACTC   
  
  
- AATACAACTA TACTGTGATT AACATTAATA AACAACATTT GAATTTGTAA AAATTTTTAG TTTCGTTTTA   
  
  
- AACAATTAAT GTTATTTAGT TAGATGAAAA TGATACTAGA ATAAACCACA TTAATAACTA GAACTTAAAA   
  
  
- AAAAAAAGAA AAACTTTTTC TATTCGTAGC TTAAAAAAGT TTTTATTTTT CCCACTTCCT TCTTTTTTAC   
  
  
- CTTTGTCAAT GGCTCATTCC TTCCTGTCTC GCGCTCTCCT GTTCTGTGTT TCTCTCCCCT CTCTCTCTCT   
  
  
- CTTCCCCCGT TACCGTCACC CCTTCCAGCT CCTCGTGGAA AGTTGAAGTT CCGAGTTGTG GGGGGGGGGG   
  
  
- GGGGAGAAAG GAGTAAAACG CTGCAATCGT AAACTGTAAA ACGAAAGTTG GCAGGGAGCC GTAGTAGTCG   
  
  
- GGACGGGAAC GAGTCGAAAA GGGTAGAAAA AAACCGAAAA ACAAACTGAG GGAGATATTG AGATAAGTGC   
  
  
- AGCTCGGAAC ATTTTTAAAA TGTATAAAAT AAAATAAAAT GAAAAAATCC AAATAGGGAG AGATAAGAAC   
  
  
- TGGATGCCCG TAAAAAATAA AAGAAACCCA AACTCTGAAC ACTTGAAAAA CGTGTAAAAG AGTGTTTTTA   
  
  
- AATAGAACAG AAAAAGTGAA AGTTGCAAGG AAGAGAGAGA GAGAGAGAGA GAGAGAGAGA GAGTGAGTGT   
  
  
- AGGATCTGTC TGCAGGAAAA ATTTCTTAAA TAAATTGTTC AATCTTCTTT GGCAACAAAT TCATCTTGGT   
  
  
- GGGACAAACC TACGGTTTCC TTTATGGTCA GTTCTTTTTA GTGCTGTTAG TGTTGTGTAG GTAAAGCAAA   
  
  
- TGTAGAGTAG GAGTAGGAGT AGAAGGAGTA GAGATGGGTA GAGGGAGAAA GACATACCGA CGAAGAAGAT   
  
  
- GAAACAAGGG ACTGGGATTG TTATGATCAA ATCAGGAGGG AATAATAATA ATAGGAGGAG GAAGGAGGAG   
  
  
- AATGATATTA CCATTGGGAA TGGAAGGAGA AGGATTGTGG TGACGATAAG GATGAGGAAG TTGTTGGAGA   
  
  
- GGCCATAAAA GAAGGCGGTG GATGAGCCTC AACTTATGAC TAACAGTTTG AGTTGACGGG TACAGATTGG   
  
  
- AGGGGCCTTT CTAGCAAGCC TTCGCCTCAC GACGACTTTA CCTCGTCGCG AGGCGAGGCT AACGCCCACT   
  
  
- AATGGGCGGT TGAGTAGTCG CCCATTAGTG GGCGGTGTTA CTCCGCCGGA GTAGTCACAG AAAGTAACTG   
  
  
- AGGAGTGAGA GCGGTGAAGT GGAGAAGTTG CCGAGGTTAA GCTGCTGGGG TAATCGGGGG CTGGCAGGAT   
  
  
- TACTAGGCCC AGACTCAGTC CCAGGCCTAG ACGGAGAACA CACGCCTAAG AGGCCTGAGG GAAATAAGGG   
  
  
- TGGCCACCTC TCTTCTGGAA GGTAGGGGCG GTGCTGGTGA CGACGGCGCC GCTAGCGCCA CCAAGGACAC   
  
  
- CTCCTCCTAC TAGGCTGCAC CTACCTATCG TAGTATTTTC TCGACTAAGT TTCGAGTTTG AGCTAGAGTT   
  
  
- AGGGTGTCAA CTAAGTCTTG CAATCTCTAT AGTAGATGGG TACATTAGGA TTAGACCCCA GGCGGTAACT   
  
  
- CAAGGCGGAG GCGAGCGAGC GGCGGCTGGG GGAGTAACGA GGCGGCGGTG AAGGCGGGAA GGTGGTGGTG   
  
  
- GTTGTGGTGG AGTTAGTTGT TTAGTGAGAA GGAGCTTAGT CGTTGTGATT GTTGTTGTTA GTACACTCGC   
  
  
- ACATGCGGTT GTTTCCTGGG CCTATGAAGT TGAACCCAGG CCCAGTCCCA GTCCCAGGCT AATTGTAGCT   
  
  
- GGTTCGATTG GAAAGGAAGG GAGGACTAAG GTGGCGGACA ACCCCACAGT CACAGTGTGG CGGTGGCAGA   
  
  
- AGGCGGCGGC CAAGGCCGAG GCCACCTTCG CCGTCATCAT CACCATTTAG TTTGGGATTG GGATTAGGGT   
  
  
- TGGGCTTGGG TTTGGGTTTG GGTTTGGGCT TGTTGTTTCG AGTCCTACAA GTTGAGGTTT GAGGTGTTGT   
  
  
- TGTCGTTGTC GTTGTCGTTG TTTAGGGGCA CCTAGTCCTC GTTCTGGGCC GCCGAGGTGG CCGCTGCCTT   
  
  
- CGTAGCGGTG GTAGAGGTTC TCGCCGCCGT GGTGGCGGCC GCCGTCACTT TCGTTCTCTT TCTCTCCTCT   
  
  
- ACGCCGTTTC CTTCGCGCTG CTTCTCCCAG AGGTGGAGGA TTGGGAGGAG GAGGTTACGC GTCTTCGTCA   
  
  
- TAGGCCGCTG TTTATGCTTC TTCGCTTGTT CTACGAAGAT CTTTAGAGCC TCACCCGGTG GGGTAAGCCG   
  
  
- TGGAGGCGGG TTGCGCAGCG GCGCATGAAG AGCCTTCGGT ACAGTCGGGC AGAGCATAGG AGGACGGAGC   
  
  
- CGTAGATGCG GCGGGAGGGG TGGCAGGGTG TGATGCAGTT CGAGGAGAGG CGGAAGGTTC AGAAGTTACC   
  
  
- GTAGTCGGGT AAGCAGTTTA AGAGAGTGAA GTGACGTTTG GTTCGTTAGG TTCTCCGGAA GGTCTCCCTT   
  
  
- CTGTCCCAGG TGTAGTAGCT GGAGCTATAG TACGTCCCCG AGGTCACCGG GCCCGACAAG GTGTAGGAGC   
  
  
- GCAGGGCCGG TCCACCCGGA GGGAAGCATT CCGAGTGGCC CGAGCCCTGG AGGTACCTCC GCGAGCTCCG   
  
  
- GTGGCCTTTT TCCGAGAGTC TGAAGCGGCT CTTCAACCCC AACGGGAAAC TCAAATATGG GCACCGCCTC   
  
  
- TTCTAACCTT TAAACCTGAA CCTTTCCAAC GTACAATCAT TTTCCCTTCG AGAACGACAC GTAACCAATG   
  
  
- TCGTGAGAAA CATACTACAA TGACCGAGAC TATGATTATG TGAACCGGAA GAAGTTTCCA ACCGCGGTTT   
  
  
- TCACCACTGC CACCACCTCG TTCTGGATTC GGCTTGTCCG AGAAAGGATC CTTCCAAACA TCTCCGCTAG   
  
  
- GTGATGATAA GTCGGGATAA ACTGAGGAAT CCTCGCTCAA TACCTCTCCT GTCACTCCTC TCCGTACACC   
  
  
- AACTCGTTGT CGAGGAAAGA TCCCTCTAAG CTTTGTAAGA CCGGCAACCA CCCGGGAGTT CCTGGCCCCT   
  
  
- CGGGTTCAAA CGCTCGACCT CCCTCTTCGA TGTTGTCAGG CCGAAATCCC CTTAGAGTAA CCGTCCGTTG   
  
  
- CGGCGACGGG TCCGGTGGAA CGAGGAGCCG TACAAGGGAA GACTACCCAT ATGAAATTAG CTCCTGTCAC   
  
  
- CGTGTGAATT CGAGCCCACC TTCCTGAACA CGGAGGACTG ACGAAGCCGG ACCTCCGGAA GGGTACGAGT   
  
  
- ATGATACTCG TGGCAGACAT GAGCCTCGGT TAT

+     GT1-motif

| Site Name | Organism | Position | Strand | Matrix score. | sequence | function |
| --- | --- | --- | --- | --- | --- | --- |
| GT1-motif | Arabidopsis thaliana | 820 | - | 6 | GGTTAA | light responsive element |

>HU01G00472.1   
+ -Up\_Stream \_Len000GTCTCT GTAACACAAT TCATTAAATC CATCCAAAGG GGATCATAAA TAACCAAACT   
  
  
+ TTGTTGGAAA CTGCTTTGAT GTGGGCTTTC TCTTTCAGCA TTTATGTTAT ACATATATAT GGTTATAAAG   
  
  
+ TTACAAAAGT ATGACCCGAC ACGACAATTC AATCCGAATC CGACTCGAAG TTACTGAAAG TAATCTGAAT   
  
  
+ ACGATTTGAC AGCTTAGTTT AAAAACCTAT TACTTCTCAA TTTTGAGTAA ATCTAAGCTA TTTTTAATGC   
  
  
+ TGAATAGATC GACCCGATTC GACCCGTTTT CCGACTCTAT ATGGTTATGG TGCCCCTAAA AAAAATTAAA   
  
  
+ AAAGAAAAAG AATAGAAAAA TATAAAATAA AACTGAGATT TCTGAACACT CTCTCCCCTA GTCTTGTGGG   
  
  
+ CATGGAAAGT GGTTGGAGAT GGAGATGTTT GAGTTTCATT TTCTGATATT ATTTTTTAGT CATTAGAAGT   
  
  
+ ACAAAGTCTA AACTCAACAA GAGCATGAGG GGAGAATGGT TGTTGTAGCC AATGGAAAAT AAAAAGAACA   
  
  
+ TTCACTCTTC TTGTGCTTTA TTTATGGTGA TAATTTGTTA CTTGTATTTT TAAAAATGAT TAAATATATT   
  
  
+ GATCCTTTGA TATTATGTTG AAATAATAAA AATATAAAAA TTAGAAAATA TTTTACCTCG TGAAATTTTT   
  
  
+ TTTCTGATTA ATCATTGAAA TTATGGTGAA AAATAAAGGA ACATCTCTTG TAAACACTCT ATCCGTTTAC   
  
  
+ GTTTGACCAC AAACTTGCTA TCCACAAGCA TCTCAACCTT TGCCTTTAAC CCAATTGTGG GTTGATGAAA   
  
  
+ TCTAACCATT ATAGGATTAG ATGGTAGTCG TTAATCCTGT GAAACAAGTT TTTGGTTATG ATGTATCCAA   
  
  
+ TTATGAGGGC CTGATAACTC TTGCCTCGGC ATTTGCGTGA TAATGACGTG GTACTAATAG CAATTATTTT   
  
  
+ TCGCAAATAT TCGAAATTTT TTAAGAATAA ACGCAAAATT ATTGATCTTG ACAAATGATT TTATTTTTTT   
  
  
+ GTTGGGAAAA GGATAAACAT CTAGTGTCAA TTGTTGATGA CCTGATAGCT CTCGATTTCT TATTTATGAG   
  
  
+ TTATGTTGAT ATGACACTAA TTGTAATTAT TTGTTGTAAA CTTAAACATT TTTAAAAATC AAAGCAAAAT   
  
  
+ TTGTTAATTA CAATAAATCA ATCTACTTTT ACTATGATCT TATTTGGTGT AATTATTGAT CTTGAATTTT   
  
  
+ TTTTTTTCTT TTTGAAAAAG ATAAGCATCG AATTTTTTCA AAAATAAAAA GGGTGAAGGA AGAAAAAATG   
  
  
+ GAAACAGTTA CCGAGTAAGG AAGGACAGAG CGCGAGAGGA CAAGACACAA AGAGAGGGGA GAGAGAGAGA   
  
  
+ GAAGGGGGCA ATGGCAGTGG GGAAGGTCGA GGAGCACCTT TCAACTTCAA GGCTCAACAC CCCCCCCCCC   
  
  
+ CCCCTCTTTC CTCATTTTGC GACGTTAGCA TTTGACATTT TGCTTTCAAC CGTCCCTCGG CATCATCAGC   
  
  
+ CCTGCCCTTG CTCAGCTTTT CCCATCTTTT TTTGGCTTTT TGTTTGACTC CCTCTATAAC TCTATTCACG   
  
  
+ TCGAGCCTTG TAAAAATTTT ACATATTTTA TTTTATTTTA CTTTTTTAGG TTTATCCCTC TCTATTCTTG   
  
  
+ ACCTACGGGC ATTTTTTATT TTCTTTGGGT TTGAGACTTG TGAACTTTTT GCACATTTTC TCACAAAAAT   
  
  
+ TTATCTTGTC TTTTTCACTT TCAACGTTCC TTCTCTCTCT CTCTCTCTCT CTCTCTCTCT CTCACTCACA   
  
  
+ TCCTAGACAG ACGTCCTTTT TAAAGAATTT ATTTAACAAG TTAGAAGAAA CCGTTGTTTA AGTAGAACCA   
  
  
+ CCCTGTTTGG ATGCCAAAGG AAATACCAGT CAAGAAAAAT CACGACAATC ACAACACATC CATTTCGTTT   
  
  
+ ACATCTCATC CTCATCCTCA TCTTCCTCAT CTCTACCCAT CTCCCTCTTT CTGTATGGCT GCTTCTTCTA   
  
  
+ CTTTGTTCCC TGACCCTAAC AATACTAGTT TAGTCCTCCC TTATTATTAT TATCCTCCTC CTTCCTCCTC   
  
  
+ TTACTATAAT GGTAACCCTT ACCTTCCTCT TCCTAACACC ACTGCTATTC CTACTCCTTC AACAACCTCT   
  
  
+ CCGGTATTTT CTTCCGCCAC CTACTCGGAG TTGAATACTG ATTGTCAAAC TCAACTGCCC ATGTCTAACC   
  
  
+ TCCCCGGAAA GATCGTTCGG AAGCGGAGTG CTGCTGAAAT GGAGCAGCGC TCCGCTCCGA TTGCGGGTGA   
  
  
+ TTACCCGCCA ACTCATCAGC GGGTAATCAC CCGCCACAAT GAGGCGGCCT CATCAGTGTC TTTCATTGAC   
  
  
+ TCCTCACTCT CGCCACTTCA CCTCTTCAAC GGCTCCAATT CGACGACCCC ATTAGCCCCC GACCGTCCTA   
  
  
+ ATGATCCGGG TCTGAGTCAG GGTCCGGATC TGCCTCTTGT GTGCGGATTC TCCGGACTCC CTTTATTCCC   
  
  
+ ACCGGTGGAG AGAAGACCTT CCATCCCCGC CACGACCACT GCTGCCGCGG CGATCGCGGT GGTTCCTGTG   
  
  
+ GAGGAGGATG ATCCGACGTG GATGGATAGC ATCATAAAAG AGCTGATTCA AAGCTCAAAC TCGATCTCAA   
  
  
+ TCCCACAGTT GATTCAGAAC GTTAGAGATA TCATCTACCC ATGTAATCCT AATCTGGGGT CCGCCATTGA   
  
  
+ GTTCCGCCTC CGCTCGCTCG CCGCCGACCC CCTCATTGCT CCGCCGCCAC TTCCGCCCTT CCACCACCAC   
  
  
+ CAACACCACC TCAATCAACA AATCACTCTT CCTCGAATCA GCAACACTAA CAACAACAAT CATGTGAGCG   
  
  
+ TGTACGCCAA CAAAGGACCC GGATACTTCA ACTTGGGTCC GGGTCAGGGT CAGGGTCCGA TTAACATCGA   
  
  
+ CCAAGCTAAC CTTTCCTTCC CTCCTGATTC CACCGCCTGT TGGGGTGTCA GTGTCACACC GCCACCGTCT   
  
  
+ TCCGCCGCCG GTTCCGGCTC CGGTGGAAGC GGCAGTAGTA GTGGTAAATC AAACCCTAAC CCTAATCCCA   
  
  
+ ACCCGAACCC AAACCCAAAC CCAAACCCGA ACAACAAAGC TCAGGATGTT CAACTCCAAA CTCCACAACA   
  
  
+ ACAGCAACAG CAACAGCAAC AAATCCCCGT GGATCAGGAG CAAGACCCGG CGGCTCCACC GGCGACGGAA   
  
  
+ GCATCGCCAC CATCTCCAAG AGCGGCGGCA CCACCGCCGG CGGCAGTGAA AGCAAGAGAA AGAGAGGAGA   
  
  
+ TGCGGCAAAG GAAGCGCGAC GAAGAGGGTC TCCACCTCCT AACCCTCCTC CTCCAATGCG CAGAAGCAGT   
  
  
+ ATCCGGCGAC AAATACGAAG AAGCGAACAA GATGCTTCTA GAAATCTCGG AGTGGGCCAC CCCATTCGGC   
  
  
+ ACCTCCGCCC AACGCGTCGC CGCGTACTTC TCGGAAGCCA TGTCAGCCCG TCTCGTATCC TCCTGCCTCG   
  
  
+ GCATCTACGC CGCCCTCCCC ACCGTCCCAC ACTACGTCAA GCTCCTCTCC GCCTTCCAAG TCTTCAATGG   
  
  
+ CATCAGCCCA TTCGTCAAAT TCTCTCACTT CACTGCAAAC CAAGCAATCC AAGAGGCCTT CCAGAGGGAA   
  
  
+ GACAGGGTCC ACATCATCGA CCTCGATATC ATGCAGGGGC TCCAGTGGCC CGGGCTGTTC CACATCCTCG   
  
  
+ CGTCCCGGCC AGGTGGGCCT CCCTTCGTAA GGCTCACCGG GCTCGGGACC TCCATGGAGG CGCTCGAGGC   
  
  
+ CACCGGAAAA AGGCTCTCAG ACTTCGCCGA GAAGTTGGGG TTGCCCTTTG AGTTTATACC CGTGGCGGAG   
  
  
+ AAGATTGGAA ATTTGGACTT GGAAAGGTTG CATGTTAGTA AAAGGGAAGC TCTTGCTGTG CATTGGTTAC   
  
  
+ AGCACTCTTT GTATGATGTT ACTGGCTCTG ATACTAATAC ACTTGGCCTT CTTCAAAGGT TGGCGCCAAA   
  
  
+ AGTGGTGACG GTGGTGGAGC AAGACCTAAG CCGAACAGGC TCTTTCCTAG GAAGGTTTGT AGAGGCGATC   
  
  
+ CACTACTATT CAGCCCTATT TGACTCCTTA GGAGCGAGTT ATGGAGAGGA CAGTGAGGAG AGGCATGTGG   
  
  
+ TTGAGCAACA GCTCCTTTCT AGGGAGATTC GAAACATTCT GGCCGTTGGT GGGCCCTCAA GGACCGGGGA   
  
  
+ GCCCAAGTTT GCGAGCTGGA GGGAGAAGCT ACAACAGTCC GGCTTTAGGG GAATCTCATT GGCAGGCAAC   
  
  
+ GCCGCTGCCC AGGCCACCTT GCTCCTCGGC ATGTTCCCTT CTGATGGGTA TACTTTAATC GAGGACAGTG   
  
  
+ GCACACTTAA GCTCGGGTGG AAGGACTTGT GCCTCCTGAC TGCTTCGGCC TGGAGGCCTT CCCATGCTCA   
  
  
+ TACTATGAGC ACCGTCTGTA CTCGGAGCCA ATA  

- -Up\_Stream \_Len000CAGAGA CATTGTGTTA AGTAATTTAG GTAGGTTTCC CCTAGTATTT ATTGGTTTGA   
  
  
- AACAACCTTT GACGAAACTA CACCCGAAAG AGAAAGTCGT AAATACAATA TGTATATATA CCAATATTTC   
  
  
- AATGTTTTCA TACTGGGCTG TGCTGTTAAG TTAGGCTTAG GCTGAGCTTC AATGACTTTC ATTAGACTTA   
  
  
- TGCTAAACTG TCGAATCAAA TTTTTGGATA ATGAAGAGTT AAAACTCATT TAGATTCGAT AAAAATTACG   
  
  
- ACTTATCTAG CTGGGCTAAG CTGGGCAAAA GGCTGAGATA TACCAATACC ACGGGGATTT TTTTTAATTT   
  
  
- TTTCTTTTTC TTATCTTTTT ATATTTTATT TTGACTCTAA AGACTTGTGA GAGAGGGGAT CAGAACACCC   
  
  
- GTACCTTTCA CCAACCTCTA CCTCTACAAA CTCAAAGTAA AAGACTATAA TAAAAAATCA GTAATCTTCA   
  
  
- TGTTTCAGAT TTGAGTTGTT CTCGTACTCC CCTCTTACCA ACAACATCGG TTACCTTTTA TTTTTCTTGT   
  
  
- AAGTGAGAAG AACACGAAAT AAATACCACT ATTAAACAAT GAACATAAAA ATTTTTACTA ATTTATATAA   
  
  
- CTAGGAAACT ATAATACAAC TTTATTATTT TTATATTTTT AATCTTTTAT AAAATGGAGC ACTTTAAAAA   
  
  
- AAAGACTAAT TAGTAACTTT AATACCACTT TTTATTTCCT TGTAGAGAAC ATTTGTGAGA TAGGCAAATG   
  
  
- CAAACTGGTG TTTGAACGAT AGGTGTTCGT AGAGTTGGAA ACGGAAATTG GGTTAACACC CAACTACTTT   
  
  
- AGATTGGTAA TATCCTAATC TACCATCAGC AATTAGGACA CTTTGTTCAA AAACCAATAC TACATAGGTT   
  
  
- AATACTCCCG GACTATTGAG AACGGAGCCG TAAACGCACT ATTACTGCAC CATGATTATC GTTAATAAAA   
  
  
- AGCGTTTATA AGCTTTAAAA AATTCTTATT TGCGTTTTAA TAACTAGAAC TGTTTACTAA AATAAAAAAA   
  
  
- CAACCCTTTT CCTATTTGTA GATCACAGTT AACAACTACT GGACTATCGA GAGCTAAAGA ATAAATACTC   
  
  
- AATACAACTA TACTGTGATT AACATTAATA AACAACATTT GAATTTGTAA AAATTTTTAG TTTCGTTTTA   
  
  
- AACAATTAAT GTTATTTAGT TAGATGAAAA TGATACTAGA ATAAACCACA TTAATAACTA GAACTTAAAA   
  
  
- AAAAAAAGAA AAACTTTTTC TATTCGTAGC TTAAAAAAGT TTTTATTTTT CCCACTTCCT TCTTTTTTAC   
  
  
- CTTTGTCAAT GGCTCATTCC TTCCTGTCTC GCGCTCTCCT GTTCTGTGTT TCTCTCCCCT CTCTCTCTCT   
  
  
- CTTCCCCCGT TACCGTCACC CCTTCCAGCT CCTCGTGGAA AGTTGAAGTT CCGAGTTGTG GGGGGGGGGG   
  
  
- GGGGAGAAAG GAGTAAAACG CTGCAATCGT AAACTGTAAA ACGAAAGTTG GCAGGGAGCC GTAGTAGTCG   
  
  
- GGACGGGAAC GAGTCGAAAA GGGTAGAAAA AAACCGAAAA ACAAACTGAG GGAGATATTG AGATAAGTGC   
  
  
- AGCTCGGAAC ATTTTTAAAA TGTATAAAAT AAAATAAAAT GAAAAAATCC AAATAGGGAG AGATAAGAAC   
  
  
- TGGATGCCCG TAAAAAATAA AAGAAACCCA AACTCTGAAC ACTTGAAAAA CGTGTAAAAG AGTGTTTTTA   
  
  
- AATAGAACAG AAAAAGTGAA AGTTGCAAGG AAGAGAGAGA GAGAGAGAGA GAGAGAGAGA GAGTGAGTGT   
  
  
- AGGATCTGTC TGCAGGAAAA ATTTCTTAAA TAAATTGTTC AATCTTCTTT GGCAACAAAT TCATCTTGGT   
  
  
- GGGACAAACC TACGGTTTCC TTTATGGTCA GTTCTTTTTA GTGCTGTTAG TGTTGTGTAG GTAAAGCAAA   
  
  
- TGTAGAGTAG GAGTAGGAGT AGAAGGAGTA GAGATGGGTA GAGGGAGAAA GACATACCGA CGAAGAAGAT   
  
  
- GAAACAAGGG ACTGGGATTG TTATGATCAA ATCAGGAGGG AATAATAATA ATAGGAGGAG GAAGGAGGAG   
  
  
- AATGATATTA CCATTGGGAA TGGAAGGAGA AGGATTGTGG TGACGATAAG GATGAGGAAG TTGTTGGAGA   
  
  
- GGCCATAAAA GAAGGCGGTG GATGAGCCTC AACTTATGAC TAACAGTTTG AGTTGACGGG TACAGATTGG   
  
  
- AGGGGCCTTT CTAGCAAGCC TTCGCCTCAC GACGACTTTA CCTCGTCGCG AGGCGAGGCT AACGCCCACT   
  
  
- AATGGGCGGT TGAGTAGTCG CCCATTAGTG GGCGGTGTTA CTCCGCCGGA GTAGTCACAG AAAGTAACTG   
  
  
- AGGAGTGAGA GCGGTGAAGT GGAGAAGTTG CCGAGGTTAA GCTGCTGGGG TAATCGGGGG CTGGCAGGAT   
  
  
- TACTAGGCCC AGACTCAGTC CCAGGCCTAG ACGGAGAACA CACGCCTAAG AGGCCTGAGG GAAATAAGGG   
  
  
- TGGCCACCTC TCTTCTGGAA GGTAGGGGCG GTGCTGGTGA CGACGGCGCC GCTAGCGCCA CCAAGGACAC   
  
  
- CTCCTCCTAC TAGGCTGCAC CTACCTATCG TAGTATTTTC TCGACTAAGT TTCGAGTTTG AGCTAGAGTT   
  
  
- AGGGTGTCAA CTAAGTCTTG CAATCTCTAT AGTAGATGGG TACATTAGGA TTAGACCCCA GGCGGTAACT   
  
  
- CAAGGCGGAG GCGAGCGAGC GGCGGCTGGG GGAGTAACGA GGCGGCGGTG AAGGCGGGAA GGTGGTGGTG   
  
  
- GTTGTGGTGG AGTTAGTTGT TTAGTGAGAA GGAGCTTAGT CGTTGTGATT GTTGTTGTTA GTACACTCGC   
  
  
- ACATGCGGTT GTTTCCTGGG CCTATGAAGT TGAACCCAGG CCCAGTCCCA GTCCCAGGCT AATTGTAGCT   
  
  
- GGTTCGATTG GAAAGGAAGG GAGGACTAAG GTGGCGGACA ACCCCACAGT CACAGTGTGG CGGTGGCAGA   
  
  
- AGGCGGCGGC CAAGGCCGAG GCCACCTTCG CCGTCATCAT CACCATTTAG TTTGGGATTG GGATTAGGGT   
  
  
- TGGGCTTGGG TTTGGGTTTG GGTTTGGGCT TGTTGTTTCG AGTCCTACAA GTTGAGGTTT GAGGTGTTGT   
  
  
- TGTCGTTGTC GTTGTCGTTG TTTAGGGGCA CCTAGTCCTC GTTCTGGGCC GCCGAGGTGG CCGCTGCCTT   
  
  
- CGTAGCGGTG GTAGAGGTTC TCGCCGCCGT GGTGGCGGCC GCCGTCACTT TCGTTCTCTT TCTCTCCTCT   
  
  
- ACGCCGTTTC CTTCGCGCTG CTTCTCCCAG AGGTGGAGGA TTGGGAGGAG GAGGTTACGC GTCTTCGTCA   
  
  
- TAGGCCGCTG TTTATGCTTC TTCGCTTGTT CTACGAAGAT CTTTAGAGCC TCACCCGGTG GGGTAAGCCG   
  
  
- TGGAGGCGGG TTGCGCAGCG GCGCATGAAG AGCCTTCGGT ACAGTCGGGC AGAGCATAGG AGGACGGAGC   
  
  
- CGTAGATGCG GCGGGAGGGG TGGCAGGGTG TGATGCAGTT CGAGGAGAGG CGGAAGGTTC AGAAGTTACC   
  
  
- GTAGTCGGGT AAGCAGTTTA AGAGAGTGAA GTGACGTTTG GTTCGTTAGG TTCTCCGGAA GGTCTCCCTT   
  
  
- CTGTCCCAGG TGTAGTAGCT GGAGCTATAG TACGTCCCCG AGGTCACCGG GCCCGACAAG GTGTAGGAGC   
  
  
- GCAGGGCCGG TCCACCCGGA GGGAAGCATT CCGAGTGGCC CGAGCCCTGG AGGTACCTCC GCGAGCTCCG   
  
  
- GTGGCCTTTT TCCGAGAGTC TGAAGCGGCT CTTCAACCCC AACGGGAAAC TCAAATATGG GCACCGCCTC   
  
  
- TTCTAACCTT TAAACCTGAA CCTTTCCAAC GTACAATCAT TTTCCCTTCG AGAACGACAC GTAACCAATG   
  
  
- TCGTGAGAAA CATACTACAA TGACCGAGAC TATGATTATG TGAACCGGAA GAAGTTTCCA ACCGCGGTTT   
  
  
- TCACCACTGC CACCACCTCG TTCTGGATTC GGCTTGTCCG AGAAAGGATC CTTCCAAACA TCTCCGCTAG   
  
  
- GTGATGATAA GTCGGGATAA ACTGAGGAAT CCTCGCTCAA TACCTCTCCT GTCACTCCTC TCCGTACACC   
  
  
- AACTCGTTGT CGAGGAAAGA TCCCTCTAAG CTTTGTAAGA CCGGCAACCA CCCGGGAGTT CCTGGCCCCT   
  
  
- CGGGTTCAAA CGCTCGACCT CCCTCTTCGA TGTTGTCAGG CCGAAATCCC CTTAGAGTAA CCGTCCGTTG   
  
  
- CGGCGACGGG TCCGGTGGAA CGAGGAGCCG TACAAGGGAA GACTACCCAT ATGAAATTAG CTCCTGTCAC   
  
  
- CGTGTGAATT CGAGCCCACC TTCCTGAACA CGGAGGACTG ACGAAGCCGG ACCTCCGGAA GGGTACGAGT   
  
  
- ATGATACTCG TGGCAGACAT GAGCCTCGGT TAT

+     MBS

| Site Name | Organism | Position | Strand | Matrix score. | sequence | function |
| --- | --- | --- | --- | --- | --- | --- |
| MBS | Arabidopsis thaliana | 2226 | + | 6 | CAACTG | MYB binding site involved in drought-inducibility |
| MBS | Arabidopsis thaliana | 2670 | - | 6 | CAACTG | MYB binding site involved in drought-inducibility |

>HU01G00472.1   
+ -Up\_Stream \_Len000GTCTCT GTAACACAAT TCATTAAATC CATCCAAAGG GGATCATAAA TAACCAAACT   
  
  
+ TTGTTGGAAA CTGCTTTGAT GTGGGCTTTC TCTTTCAGCA TTTATGTTAT ACATATATAT GGTTATAAAG   
  
  
+ TTACAAAAGT ATGACCCGAC ACGACAATTC AATCCGAATC CGACTCGAAG TTACTGAAAG TAATCTGAAT   
  
  
+ ACGATTTGAC AGCTTAGTTT AAAAACCTAT TACTTCTCAA TTTTGAGTAA ATCTAAGCTA TTTTTAATGC   
  
  
+ TGAATAGATC GACCCGATTC GACCCGTTTT CCGACTCTAT ATGGTTATGG TGCCCCTAAA AAAAATTAAA   
  
  
+ AAAGAAAAAG AATAGAAAAA TATAAAATAA AACTGAGATT TCTGAACACT CTCTCCCCTA GTCTTGTGGG   
  
  
+ CATGGAAAGT GGTTGGAGAT GGAGATGTTT GAGTTTCATT TTCTGATATT ATTTTTTAGT CATTAGAAGT   
  
  
+ ACAAAGTCTA AACTCAACAA GAGCATGAGG GGAGAATGGT TGTTGTAGCC AATGGAAAAT AAAAAGAACA   
  
  
+ TTCACTCTTC TTGTGCTTTA TTTATGGTGA TAATTTGTTA CTTGTATTTT TAAAAATGAT TAAATATATT   
  
  
+ GATCCTTTGA TATTATGTTG AAATAATAAA AATATAAAAA TTAGAAAATA TTTTACCTCG TGAAATTTTT   
  
  
+ TTTCTGATTA ATCATTGAAA TTATGGTGAA AAATAAAGGA ACATCTCTTG TAAACACTCT ATCCGTTTAC   
  
  
+ GTTTGACCAC AAACTTGCTA TCCACAAGCA TCTCAACCTT TGCCTTTAAC CCAATTGTGG GTTGATGAAA   
  
  
+ TCTAACCATT ATAGGATTAG ATGGTAGTCG TTAATCCTGT GAAACAAGTT TTTGGTTATG ATGTATCCAA   
  
  
+ TTATGAGGGC CTGATAACTC TTGCCTCGGC ATTTGCGTGA TAATGACGTG GTACTAATAG CAATTATTTT   
  
  
+ TCGCAAATAT TCGAAATTTT TTAAGAATAA ACGCAAAATT ATTGATCTTG ACAAATGATT TTATTTTTTT   
  
  
+ GTTGGGAAAA GGATAAACAT CTAGTGTCAA TTGTTGATGA CCTGATAGCT CTCGATTTCT TATTTATGAG   
  
  
+ TTATGTTGAT ATGACACTAA TTGTAATTAT TTGTTGTAAA CTTAAACATT TTTAAAAATC AAAGCAAAAT   
  
  
+ TTGTTAATTA CAATAAATCA ATCTACTTTT ACTATGATCT TATTTGGTGT AATTATTGAT CTTGAATTTT   
  
  
+ TTTTTTTCTT TTTGAAAAAG ATAAGCATCG AATTTTTTCA AAAATAAAAA GGGTGAAGGA AGAAAAAATG   
  
  
+ GAAACAGTTA CCGAGTAAGG AAGGACAGAG CGCGAGAGGA CAAGACACAA AGAGAGGGGA GAGAGAGAGA   
  
  
+ GAAGGGGGCA ATGGCAGTGG GGAAGGTCGA GGAGCACCTT TCAACTTCAA GGCTCAACAC CCCCCCCCCC   
  
  
+ CCCCTCTTTC CTCATTTTGC GACGTTAGCA TTTGACATTT TGCTTTCAAC CGTCCCTCGG CATCATCAGC   
  
  
+ CCTGCCCTTG CTCAGCTTTT CCCATCTTTT TTTGGCTTTT TGTTTGACTC CCTCTATAAC TCTATTCACG   
  
  
+ TCGAGCCTTG TAAAAATTTT ACATATTTTA TTTTATTTTA CTTTTTTAGG TTTATCCCTC TCTATTCTTG   
  
  
+ ACCTACGGGC ATTTTTTATT TTCTTTGGGT TTGAGACTTG TGAACTTTTT GCACATTTTC TCACAAAAAT   
  
  
+ TTATCTTGTC TTTTTCACTT TCAACGTTCC TTCTCTCTCT CTCTCTCTCT CTCTCTCTCT CTCACTCACA   
  
  
+ TCCTAGACAG ACGTCCTTTT TAAAGAATTT ATTTAACAAG TTAGAAGAAA CCGTTGTTTA AGTAGAACCA   
  
  
+ CCCTGTTTGG ATGCCAAAGG AAATACCAGT CAAGAAAAAT CACGACAATC ACAACACATC CATTTCGTTT   
  
  
+ ACATCTCATC CTCATCCTCA TCTTCCTCAT CTCTACCCAT CTCCCTCTTT CTGTATGGCT GCTTCTTCTA   
  
  
+ CTTTGTTCCC TGACCCTAAC AATACTAGTT TAGTCCTCCC TTATTATTAT TATCCTCCTC CTTCCTCCTC   
  
  
+ TTACTATAAT GGTAACCCTT ACCTTCCTCT TCCTAACACC ACTGCTATTC CTACTCCTTC AACAACCTCT   
  
  
+ CCGGTATTTT CTTCCGCCAC CTACTCGGAG TTGAATACTG ATTGTCAAAC TCAACTGCCC ATGTCTAACC   
  
  
+ TCCCCGGAAA GATCGTTCGG AAGCGGAGTG CTGCTGAAAT GGAGCAGCGC TCCGCTCCGA TTGCGGGTGA   
  
  
+ TTACCCGCCA ACTCATCAGC GGGTAATCAC CCGCCACAAT GAGGCGGCCT CATCAGTGTC TTTCATTGAC   
  
  
+ TCCTCACTCT CGCCACTTCA CCTCTTCAAC GGCTCCAATT CGACGACCCC ATTAGCCCCC GACCGTCCTA   
  
  
+ ATGATCCGGG TCTGAGTCAG GGTCCGGATC TGCCTCTTGT GTGCGGATTC TCCGGACTCC CTTTATTCCC   
  
  
+ ACCGGTGGAG AGAAGACCTT CCATCCCCGC CACGACCACT GCTGCCGCGG CGATCGCGGT GGTTCCTGTG   
  
  
+ GAGGAGGATG ATCCGACGTG GATGGATAGC ATCATAAAAG AGCTGATTCA AAGCTCAAAC TCGATCTCAA   
  
  
+ TCCCACAGTT GATTCAGAAC GTTAGAGATA TCATCTACCC ATGTAATCCT AATCTGGGGT CCGCCATTGA   
  
  
+ GTTCCGCCTC CGCTCGCTCG CCGCCGACCC CCTCATTGCT CCGCCGCCAC TTCCGCCCTT CCACCACCAC   
  
  
+ CAACACCACC TCAATCAACA AATCACTCTT CCTCGAATCA GCAACACTAA CAACAACAAT CATGTGAGCG   
  
  
+ TGTACGCCAA CAAAGGACCC GGATACTTCA ACTTGGGTCC GGGTCAGGGT CAGGGTCCGA TTAACATCGA   
  
  
+ CCAAGCTAAC CTTTCCTTCC CTCCTGATTC CACCGCCTGT TGGGGTGTCA GTGTCACACC GCCACCGTCT   
  
  
+ TCCGCCGCCG GTTCCGGCTC CGGTGGAAGC GGCAGTAGTA GTGGTAAATC AAACCCTAAC CCTAATCCCA   
  
  
+ ACCCGAACCC AAACCCAAAC CCAAACCCGA ACAACAAAGC TCAGGATGTT CAACTCCAAA CTCCACAACA   
  
  
+ ACAGCAACAG CAACAGCAAC AAATCCCCGT GGATCAGGAG CAAGACCCGG CGGCTCCACC GGCGACGGAA   
  
  
+ GCATCGCCAC CATCTCCAAG AGCGGCGGCA CCACCGCCGG CGGCAGTGAA AGCAAGAGAA AGAGAGGAGA   
  
  
+ TGCGGCAAAG GAAGCGCGAC GAAGAGGGTC TCCACCTCCT AACCCTCCTC CTCCAATGCG CAGAAGCAGT   
  
  
+ ATCCGGCGAC AAATACGAAG AAGCGAACAA GATGCTTCTA GAAATCTCGG AGTGGGCCAC CCCATTCGGC   
  
  
+ ACCTCCGCCC AACGCGTCGC CGCGTACTTC TCGGAAGCCA TGTCAGCCCG TCTCGTATCC TCCTGCCTCG   
  
  
+ GCATCTACGC CGCCCTCCCC ACCGTCCCAC ACTACGTCAA GCTCCTCTCC GCCTTCCAAG TCTTCAATGG   
  
  
+ CATCAGCCCA TTCGTCAAAT TCTCTCACTT CACTGCAAAC CAAGCAATCC AAGAGGCCTT CCAGAGGGAA   
  
  
+ GACAGGGTCC ACATCATCGA CCTCGATATC ATGCAGGGGC TCCAGTGGCC CGGGCTGTTC CACATCCTCG   
  
  
+ CGTCCCGGCC AGGTGGGCCT CCCTTCGTAA GGCTCACCGG GCTCGGGACC TCCATGGAGG CGCTCGAGGC   
  
  
+ CACCGGAAAA AGGCTCTCAG ACTTCGCCGA GAAGTTGGGG TTGCCCTTTG AGTTTATACC CGTGGCGGAG   
  
  
+ AAGATTGGAA ATTTGGACTT GGAAAGGTTG CATGTTAGTA AAAGGGAAGC TCTTGCTGTG CATTGGTTAC   
  
  
+ AGCACTCTTT GTATGATGTT ACTGGCTCTG ATACTAATAC ACTTGGCCTT CTTCAAAGGT TGGCGCCAAA   
  
  
+ AGTGGTGACG GTGGTGGAGC AAGACCTAAG CCGAACAGGC TCTTTCCTAG GAAGGTTTGT AGAGGCGATC   
  
  
+ CACTACTATT CAGCCCTATT TGACTCCTTA GGAGCGAGTT ATGGAGAGGA CAGTGAGGAG AGGCATGTGG   
  
  
+ TTGAGCAACA GCTCCTTTCT AGGGAGATTC GAAACATTCT GGCCGTTGGT GGGCCCTCAA GGACCGGGGA   
  
  
+ GCCCAAGTTT GCGAGCTGGA GGGAGAAGCT ACAACAGTCC GGCTTTAGGG GAATCTCATT GGCAGGCAAC   
  
  
+ GCCGCTGCCC AGGCCACCTT GCTCCTCGGC ATGTTCCCTT CTGATGGGTA TACTTTAATC GAGGACAGTG   
  
  
+ GCACACTTAA GCTCGGGTGG AAGGACTTGT GCCTCCTGAC TGCTTCGGCC TGGAGGCCTT CCCATGCTCA   
  
  
+ TACTATGAGC ACCGTCTGTA CTCGGAGCCA ATA  

- -Up\_Stream \_Len000CAGAGA CATTGTGTTA AGTAATTTAG GTAGGTTTCC CCTAGTATTT ATTGGTTTGA   
  
  
- AACAACCTTT GACGAAACTA CACCCGAAAG AGAAAGTCGT AAATACAATA TGTATATATA CCAATATTTC   
  
  
- AATGTTTTCA TACTGGGCTG TGCTGTTAAG TTAGGCTTAG GCTGAGCTTC AATGACTTTC ATTAGACTTA   
  
  
- TGCTAAACTG TCGAATCAAA TTTTTGGATA ATGAAGAGTT AAAACTCATT TAGATTCGAT AAAAATTACG   
  
  
- ACTTATCTAG CTGGGCTAAG CTGGGCAAAA GGCTGAGATA TACCAATACC ACGGGGATTT TTTTTAATTT   
  
  
- TTTCTTTTTC TTATCTTTTT ATATTTTATT TTGACTCTAA AGACTTGTGA GAGAGGGGAT CAGAACACCC   
  
  
- GTACCTTTCA CCAACCTCTA CCTCTACAAA CTCAAAGTAA AAGACTATAA TAAAAAATCA GTAATCTTCA   
  
  
- TGTTTCAGAT TTGAGTTGTT CTCGTACTCC CCTCTTACCA ACAACATCGG TTACCTTTTA TTTTTCTTGT   
  
  
- AAGTGAGAAG AACACGAAAT AAATACCACT ATTAAACAAT GAACATAAAA ATTTTTACTA ATTTATATAA   
  
  
- CTAGGAAACT ATAATACAAC TTTATTATTT TTATATTTTT AATCTTTTAT AAAATGGAGC ACTTTAAAAA   
  
  
- AAAGACTAAT TAGTAACTTT AATACCACTT TTTATTTCCT TGTAGAGAAC ATTTGTGAGA TAGGCAAATG   
  
  
- CAAACTGGTG TTTGAACGAT AGGTGTTCGT AGAGTTGGAA ACGGAAATTG GGTTAACACC CAACTACTTT   
  
  
- AGATTGGTAA TATCCTAATC TACCATCAGC AATTAGGACA CTTTGTTCAA AAACCAATAC TACATAGGTT   
  
  
- AATACTCCCG GACTATTGAG AACGGAGCCG TAAACGCACT ATTACTGCAC CATGATTATC GTTAATAAAA   
  
  
- AGCGTTTATA AGCTTTAAAA AATTCTTATT TGCGTTTTAA TAACTAGAAC TGTTTACTAA AATAAAAAAA   
  
  
- CAACCCTTTT CCTATTTGTA GATCACAGTT AACAACTACT GGACTATCGA GAGCTAAAGA ATAAATACTC   
  
  
- AATACAACTA TACTGTGATT AACATTAATA AACAACATTT GAATTTGTAA AAATTTTTAG TTTCGTTTTA   
  
  
- AACAATTAAT GTTATTTAGT TAGATGAAAA TGATACTAGA ATAAACCACA TTAATAACTA GAACTTAAAA   
  
  
- AAAAAAAGAA AAACTTTTTC TATTCGTAGC TTAAAAAAGT TTTTATTTTT CCCACTTCCT TCTTTTTTAC   
  
  
- CTTTGTCAAT GGCTCATTCC TTCCTGTCTC GCGCTCTCCT GTTCTGTGTT TCTCTCCCCT CTCTCTCTCT   
  
  
- CTTCCCCCGT TACCGTCACC CCTTCCAGCT CCTCGTGGAA AGTTGAAGTT CCGAGTTGTG GGGGGGGGGG   
  
  
- GGGGAGAAAG GAGTAAAACG CTGCAATCGT AAACTGTAAA ACGAAAGTTG GCAGGGAGCC GTAGTAGTCG   
  
  
- GGACGGGAAC GAGTCGAAAA GGGTAGAAAA AAACCGAAAA ACAAACTGAG GGAGATATTG AGATAAGTGC   
  
  
- AGCTCGGAAC ATTTTTAAAA TGTATAAAAT AAAATAAAAT GAAAAAATCC AAATAGGGAG AGATAAGAAC   
  
  
- TGGATGCCCG TAAAAAATAA AAGAAACCCA AACTCTGAAC ACTTGAAAAA CGTGTAAAAG AGTGTTTTTA   
  
  
- AATAGAACAG AAAAAGTGAA AGTTGCAAGG AAGAGAGAGA GAGAGAGAGA GAGAGAGAGA GAGTGAGTGT   
  
  
- AGGATCTGTC TGCAGGAAAA ATTTCTTAAA TAAATTGTTC AATCTTCTTT GGCAACAAAT TCATCTTGGT   
  
  
- GGGACAAACC TACGGTTTCC TTTATGGTCA GTTCTTTTTA GTGCTGTTAG TGTTGTGTAG GTAAAGCAAA   
  
  
- TGTAGAGTAG GAGTAGGAGT AGAAGGAGTA GAGATGGGTA GAGGGAGAAA GACATACCGA CGAAGAAGAT   
  
  
- GAAACAAGGG ACTGGGATTG TTATGATCAA ATCAGGAGGG AATAATAATA ATAGGAGGAG GAAGGAGGAG   
  
  
- AATGATATTA CCATTGGGAA TGGAAGGAGA AGGATTGTGG TGACGATAAG GATGAGGAAG TTGTTGGAGA   
  
  
- GGCCATAAAA GAAGGCGGTG GATGAGCCTC AACTTATGAC TAACAGTTTG AGTTGACGGG TACAGATTGG   
  
  
- AGGGGCCTTT CTAGCAAGCC TTCGCCTCAC GACGACTTTA CCTCGTCGCG AGGCGAGGCT AACGCCCACT   
  
  
- AATGGGCGGT TGAGTAGTCG CCCATTAGTG GGCGGTGTTA CTCCGCCGGA GTAGTCACAG AAAGTAACTG   
  
  
- AGGAGTGAGA GCGGTGAAGT GGAGAAGTTG CCGAGGTTAA GCTGCTGGGG TAATCGGGGG CTGGCAGGAT   
  
  
- TACTAGGCCC AGACTCAGTC CCAGGCCTAG ACGGAGAACA CACGCCTAAG AGGCCTGAGG GAAATAAGGG   
  
  
- TGGCCACCTC TCTTCTGGAA GGTAGGGGCG GTGCTGGTGA CGACGGCGCC GCTAGCGCCA CCAAGGACAC   
  
  
- CTCCTCCTAC TAGGCTGCAC CTACCTATCG TAGTATTTTC TCGACTAAGT TTCGAGTTTG AGCTAGAGTT   
  
  
- AGGGTGTCAA CTAAGTCTTG CAATCTCTAT AGTAGATGGG TACATTAGGA TTAGACCCCA GGCGGTAACT   
  
  
- CAAGGCGGAG GCGAGCGAGC GGCGGCTGGG GGAGTAACGA GGCGGCGGTG AAGGCGGGAA GGTGGTGGTG   
  
  
- GTTGTGGTGG AGTTAGTTGT TTAGTGAGAA GGAGCTTAGT CGTTGTGATT GTTGTTGTTA GTACACTCGC   
  
  
- ACATGCGGTT GTTTCCTGGG CCTATGAAGT TGAACCCAGG CCCAGTCCCA GTCCCAGGCT AATTGTAGCT   
  
  
- GGTTCGATTG GAAAGGAAGG GAGGACTAAG GTGGCGGACA ACCCCACAGT CACAGTGTGG CGGTGGCAGA   
  
  
- AGGCGGCGGC CAAGGCCGAG GCCACCTTCG CCGTCATCAT CACCATTTAG TTTGGGATTG GGATTAGGGT   
  
  
- TGGGCTTGGG TTTGGGTTTG GGTTTGGGCT TGTTGTTTCG AGTCCTACAA GTTGAGGTTT GAGGTGTTGT   
  
  
- TGTCGTTGTC GTTGTCGTTG TTTAGGGGCA CCTAGTCCTC GTTCTGGGCC GCCGAGGTGG CCGCTGCCTT   
  
  
- CGTAGCGGTG GTAGAGGTTC TCGCCGCCGT GGTGGCGGCC GCCGTCACTT TCGTTCTCTT TCTCTCCTCT   
  
  
- ACGCCGTTTC CTTCGCGCTG CTTCTCCCAG AGGTGGAGGA TTGGGAGGAG GAGGTTACGC GTCTTCGTCA   
  
  
- TAGGCCGCTG TTTATGCTTC TTCGCTTGTT CTACGAAGAT CTTTAGAGCC TCACCCGGTG GGGTAAGCCG   
  
  
- TGGAGGCGGG TTGCGCAGCG GCGCATGAAG AGCCTTCGGT ACAGTCGGGC AGAGCATAGG AGGACGGAGC   
  
  
- CGTAGATGCG GCGGGAGGGG TGGCAGGGTG TGATGCAGTT CGAGGAGAGG CGGAAGGTTC AGAAGTTACC   
  
  
- GTAGTCGGGT AAGCAGTTTA AGAGAGTGAA GTGACGTTTG GTTCGTTAGG TTCTCCGGAA GGTCTCCCTT   
  
  
- CTGTCCCAGG TGTAGTAGCT GGAGCTATAG TACGTCCCCG AGGTCACCGG GCCCGACAAG GTGTAGGAGC   
  
  
- GCAGGGCCGG TCCACCCGGA GGGAAGCATT CCGAGTGGCC CGAGCCCTGG AGGTACCTCC GCGAGCTCCG   
  
  
- GTGGCCTTTT TCCGAGAGTC TGAAGCGGCT CTTCAACCCC AACGGGAAAC TCAAATATGG GCACCGCCTC   
  
  
- TTCTAACCTT TAAACCTGAA CCTTTCCAAC GTACAATCAT TTTCCCTTCG AGAACGACAC GTAACCAATG   
  
  
- TCGTGAGAAA CATACTACAA TGACCGAGAC TATGATTATG TGAACCGGAA GAAGTTTCCA ACCGCGGTTT   
  
  
- TCACCACTGC CACCACCTCG TTCTGGATTC GGCTTGTCCG AGAAAGGATC CTTCCAAACA TCTCCGCTAG   
  
  
- GTGATGATAA GTCGGGATAA ACTGAGGAAT CCTCGCTCAA TACCTCTCCT GTCACTCCTC TCCGTACACC   
  
  
- AACTCGTTGT CGAGGAAAGA TCCCTCTAAG CTTTGTAAGA CCGGCAACCA CCCGGGAGTT CCTGGCCCCT   
  
  
- CGGGTTCAAA CGCTCGACCT CCCTCTTCGA TGTTGTCAGG CCGAAATCCC CTTAGAGTAA CCGTCCGTTG   
  
  
- CGGCGACGGG TCCGGTGGAA CGAGGAGCCG TACAAGGGAA GACTACCCAT ATGAAATTAG CTCCTGTCAC   
  
  
- CGTGTGAATT CGAGCCCACC TTCCTGAACA CGGAGGACTG ACGAAGCCGG ACCTCCGGAA GGGTACGAGT   
  
  
- ATGATACTCG TGGCAGACAT GAGCCTCGGT TAT

+     MRE

| Site Name | Organism | Position | Strand | Matrix score. | sequence | function |
| --- | --- | --- | --- | --- | --- | --- |
| MRE | Petroselinum crispum | 1660 | - | 7 | AACCTAA | MYB binding site involved in light responsiveness |

>HU01G00472.1   
+ -Up\_Stream \_Len000GTCTCT GTAACACAAT TCATTAAATC CATCCAAAGG GGATCATAAA TAACCAAACT   
  
  
+ TTGTTGGAAA CTGCTTTGAT GTGGGCTTTC TCTTTCAGCA TTTATGTTAT ACATATATAT GGTTATAAAG   
  
  
+ TTACAAAAGT ATGACCCGAC ACGACAATTC AATCCGAATC CGACTCGAAG TTACTGAAAG TAATCTGAAT   
  
  
+ ACGATTTGAC AGCTTAGTTT AAAAACCTAT TACTTCTCAA TTTTGAGTAA ATCTAAGCTA TTTTTAATGC   
  
  
+ TGAATAGATC GACCCGATTC GACCCGTTTT CCGACTCTAT ATGGTTATGG TGCCCCTAAA AAAAATTAAA   
  
  
+ AAAGAAAAAG AATAGAAAAA TATAAAATAA AACTGAGATT TCTGAACACT CTCTCCCCTA GTCTTGTGGG   
  
  
+ CATGGAAAGT GGTTGGAGAT GGAGATGTTT GAGTTTCATT TTCTGATATT ATTTTTTAGT CATTAGAAGT   
  
  
+ ACAAAGTCTA AACTCAACAA GAGCATGAGG GGAGAATGGT TGTTGTAGCC AATGGAAAAT AAAAAGAACA   
  
  
+ TTCACTCTTC TTGTGCTTTA TTTATGGTGA TAATTTGTTA CTTGTATTTT TAAAAATGAT TAAATATATT   
  
  
+ GATCCTTTGA TATTATGTTG AAATAATAAA AATATAAAAA TTAGAAAATA TTTTACCTCG TGAAATTTTT   
  
  
+ TTTCTGATTA ATCATTGAAA TTATGGTGAA AAATAAAGGA ACATCTCTTG TAAACACTCT ATCCGTTTAC   
  
  
+ GTTTGACCAC AAACTTGCTA TCCACAAGCA TCTCAACCTT TGCCTTTAAC CCAATTGTGG GTTGATGAAA   
  
  
+ TCTAACCATT ATAGGATTAG ATGGTAGTCG TTAATCCTGT GAAACAAGTT TTTGGTTATG ATGTATCCAA   
  
  
+ TTATGAGGGC CTGATAACTC TTGCCTCGGC ATTTGCGTGA TAATGACGTG GTACTAATAG CAATTATTTT   
  
  
+ TCGCAAATAT TCGAAATTTT TTAAGAATAA ACGCAAAATT ATTGATCTTG ACAAATGATT TTATTTTTTT   
  
  
+ GTTGGGAAAA GGATAAACAT CTAGTGTCAA TTGTTGATGA CCTGATAGCT CTCGATTTCT TATTTATGAG   
  
  
+ TTATGTTGAT ATGACACTAA TTGTAATTAT TTGTTGTAAA CTTAAACATT TTTAAAAATC AAAGCAAAAT   
  
  
+ TTGTTAATTA CAATAAATCA ATCTACTTTT ACTATGATCT TATTTGGTGT AATTATTGAT CTTGAATTTT   
  
  
+ TTTTTTTCTT TTTGAAAAAG ATAAGCATCG AATTTTTTCA AAAATAAAAA GGGTGAAGGA AGAAAAAATG   
  
  
+ GAAACAGTTA CCGAGTAAGG AAGGACAGAG CGCGAGAGGA CAAGACACAA AGAGAGGGGA GAGAGAGAGA   
  
  
+ GAAGGGGGCA ATGGCAGTGG GGAAGGTCGA GGAGCACCTT TCAACTTCAA GGCTCAACAC CCCCCCCCCC   
  
  
+ CCCCTCTTTC CTCATTTTGC GACGTTAGCA TTTGACATTT TGCTTTCAAC CGTCCCTCGG CATCATCAGC   
  
  
+ CCTGCCCTTG CTCAGCTTTT CCCATCTTTT TTTGGCTTTT TGTTTGACTC CCTCTATAAC TCTATTCACG   
  
  
+ TCGAGCCTTG TAAAAATTTT ACATATTTTA TTTTATTTTA CTTTTTTAGG TTTATCCCTC TCTATTCTTG   
  
  
+ ACCTACGGGC ATTTTTTATT TTCTTTGGGT TTGAGACTTG TGAACTTTTT GCACATTTTC TCACAAAAAT   
  
  
+ TTATCTTGTC TTTTTCACTT TCAACGTTCC TTCTCTCTCT CTCTCTCTCT CTCTCTCTCT CTCACTCACA   
  
  
+ TCCTAGACAG ACGTCCTTTT TAAAGAATTT ATTTAACAAG TTAGAAGAAA CCGTTGTTTA AGTAGAACCA   
  
  
+ CCCTGTTTGG ATGCCAAAGG AAATACCAGT CAAGAAAAAT CACGACAATC ACAACACATC CATTTCGTTT   
  
  
+ ACATCTCATC CTCATCCTCA TCTTCCTCAT CTCTACCCAT CTCCCTCTTT CTGTATGGCT GCTTCTTCTA   
  
  
+ CTTTGTTCCC TGACCCTAAC AATACTAGTT TAGTCCTCCC TTATTATTAT TATCCTCCTC CTTCCTCCTC   
  
  
+ TTACTATAAT GGTAACCCTT ACCTTCCTCT TCCTAACACC ACTGCTATTC CTACTCCTTC AACAACCTCT   
  
  
+ CCGGTATTTT CTTCCGCCAC CTACTCGGAG TTGAATACTG ATTGTCAAAC TCAACTGCCC ATGTCTAACC   
  
  
+ TCCCCGGAAA GATCGTTCGG AAGCGGAGTG CTGCTGAAAT GGAGCAGCGC TCCGCTCCGA TTGCGGGTGA   
  
  
+ TTACCCGCCA ACTCATCAGC GGGTAATCAC CCGCCACAAT GAGGCGGCCT CATCAGTGTC TTTCATTGAC   
  
  
+ TCCTCACTCT CGCCACTTCA CCTCTTCAAC GGCTCCAATT CGACGACCCC ATTAGCCCCC GACCGTCCTA   
  
  
+ ATGATCCGGG TCTGAGTCAG GGTCCGGATC TGCCTCTTGT GTGCGGATTC TCCGGACTCC CTTTATTCCC   
  
  
+ ACCGGTGGAG AGAAGACCTT CCATCCCCGC CACGACCACT GCTGCCGCGG CGATCGCGGT GGTTCCTGTG   
  
  
+ GAGGAGGATG ATCCGACGTG GATGGATAGC ATCATAAAAG AGCTGATTCA AAGCTCAAAC TCGATCTCAA   
  
  
+ TCCCACAGTT GATTCAGAAC GTTAGAGATA TCATCTACCC ATGTAATCCT AATCTGGGGT CCGCCATTGA   
  
  
+ GTTCCGCCTC CGCTCGCTCG CCGCCGACCC CCTCATTGCT CCGCCGCCAC TTCCGCCCTT CCACCACCAC   
  
  
+ CAACACCACC TCAATCAACA AATCACTCTT CCTCGAATCA GCAACACTAA CAACAACAAT CATGTGAGCG   
  
  
+ TGTACGCCAA CAAAGGACCC GGATACTTCA ACTTGGGTCC GGGTCAGGGT CAGGGTCCGA TTAACATCGA   
  
  
+ CCAAGCTAAC CTTTCCTTCC CTCCTGATTC CACCGCCTGT TGGGGTGTCA GTGTCACACC GCCACCGTCT   
  
  
+ TCCGCCGCCG GTTCCGGCTC CGGTGGAAGC GGCAGTAGTA GTGGTAAATC AAACCCTAAC CCTAATCCCA   
  
  
+ ACCCGAACCC AAACCCAAAC CCAAACCCGA ACAACAAAGC TCAGGATGTT CAACTCCAAA CTCCACAACA   
  
  
+ ACAGCAACAG CAACAGCAAC AAATCCCCGT GGATCAGGAG CAAGACCCGG CGGCTCCACC GGCGACGGAA   
  
  
+ GCATCGCCAC CATCTCCAAG AGCGGCGGCA CCACCGCCGG CGGCAGTGAA AGCAAGAGAA AGAGAGGAGA   
  
  
+ TGCGGCAAAG GAAGCGCGAC GAAGAGGGTC TCCACCTCCT AACCCTCCTC CTCCAATGCG CAGAAGCAGT   
  
  
+ ATCCGGCGAC AAATACGAAG AAGCGAACAA GATGCTTCTA GAAATCTCGG AGTGGGCCAC CCCATTCGGC   
  
  
+ ACCTCCGCCC AACGCGTCGC CGCGTACTTC TCGGAAGCCA TGTCAGCCCG TCTCGTATCC TCCTGCCTCG   
  
  
+ GCATCTACGC CGCCCTCCCC ACCGTCCCAC ACTACGTCAA GCTCCTCTCC GCCTTCCAAG TCTTCAATGG   
  
  
+ CATCAGCCCA TTCGTCAAAT TCTCTCACTT CACTGCAAAC CAAGCAATCC AAGAGGCCTT CCAGAGGGAA   
  
  
+ GACAGGGTCC ACATCATCGA CCTCGATATC ATGCAGGGGC TCCAGTGGCC CGGGCTGTTC CACATCCTCG   
  
  
+ CGTCCCGGCC AGGTGGGCCT CCCTTCGTAA GGCTCACCGG GCTCGGGACC TCCATGGAGG CGCTCGAGGC   
  
  
+ CACCGGAAAA AGGCTCTCAG ACTTCGCCGA GAAGTTGGGG TTGCCCTTTG AGTTTATACC CGTGGCGGAG   
  
  
+ AAGATTGGAA ATTTGGACTT GGAAAGGTTG CATGTTAGTA AAAGGGAAGC TCTTGCTGTG CATTGGTTAC   
  
  
+ AGCACTCTTT GTATGATGTT ACTGGCTCTG ATACTAATAC ACTTGGCCTT CTTCAAAGGT TGGCGCCAAA   
  
  
+ AGTGGTGACG GTGGTGGAGC AAGACCTAAG CCGAACAGGC TCTTTCCTAG GAAGGTTTGT AGAGGCGATC   
  
  
+ CACTACTATT CAGCCCTATT TGACTCCTTA GGAGCGAGTT ATGGAGAGGA CAGTGAGGAG AGGCATGTGG   
  
  
+ TTGAGCAACA GCTCCTTTCT AGGGAGATTC GAAACATTCT GGCCGTTGGT GGGCCCTCAA GGACCGGGGA   
  
  
+ GCCCAAGTTT GCGAGCTGGA GGGAGAAGCT ACAACAGTCC GGCTTTAGGG GAATCTCATT GGCAGGCAAC   
  
  
+ GCCGCTGCCC AGGCCACCTT GCTCCTCGGC ATGTTCCCTT CTGATGGGTA TACTTTAATC GAGGACAGTG   
  
  
+ GCACACTTAA GCTCGGGTGG AAGGACTTGT GCCTCCTGAC TGCTTCGGCC TGGAGGCCTT CCCATGCTCA   
  
  
+ TACTATGAGC ACCGTCTGTA CTCGGAGCCA ATA  

- -Up\_Stream \_Len000CAGAGA CATTGTGTTA AGTAATTTAG GTAGGTTTCC CCTAGTATTT ATTGGTTTGA   
  
  
- AACAACCTTT GACGAAACTA CACCCGAAAG AGAAAGTCGT AAATACAATA TGTATATATA CCAATATTTC   
  
  
- AATGTTTTCA TACTGGGCTG TGCTGTTAAG TTAGGCTTAG GCTGAGCTTC AATGACTTTC ATTAGACTTA   
  
  
- TGCTAAACTG TCGAATCAAA TTTTTGGATA ATGAAGAGTT AAAACTCATT TAGATTCGAT AAAAATTACG   
  
  
- ACTTATCTAG CTGGGCTAAG CTGGGCAAAA GGCTGAGATA TACCAATACC ACGGGGATTT TTTTTAATTT   
  
  
- TTTCTTTTTC TTATCTTTTT ATATTTTATT TTGACTCTAA AGACTTGTGA GAGAGGGGAT CAGAACACCC   
  
  
- GTACCTTTCA CCAACCTCTA CCTCTACAAA CTCAAAGTAA AAGACTATAA TAAAAAATCA GTAATCTTCA   
  
  
- TGTTTCAGAT TTGAGTTGTT CTCGTACTCC CCTCTTACCA ACAACATCGG TTACCTTTTA TTTTTCTTGT   
  
  
- AAGTGAGAAG AACACGAAAT AAATACCACT ATTAAACAAT GAACATAAAA ATTTTTACTA ATTTATATAA   
  
  
- CTAGGAAACT ATAATACAAC TTTATTATTT TTATATTTTT AATCTTTTAT AAAATGGAGC ACTTTAAAAA   
  
  
- AAAGACTAAT TAGTAACTTT AATACCACTT TTTATTTCCT TGTAGAGAAC ATTTGTGAGA TAGGCAAATG   
  
  
- CAAACTGGTG TTTGAACGAT AGGTGTTCGT AGAGTTGGAA ACGGAAATTG GGTTAACACC CAACTACTTT   
  
  
- AGATTGGTAA TATCCTAATC TACCATCAGC AATTAGGACA CTTTGTTCAA AAACCAATAC TACATAGGTT   
  
  
- AATACTCCCG GACTATTGAG AACGGAGCCG TAAACGCACT ATTACTGCAC CATGATTATC GTTAATAAAA   
  
  
- AGCGTTTATA AGCTTTAAAA AATTCTTATT TGCGTTTTAA TAACTAGAAC TGTTTACTAA AATAAAAAAA   
  
  
- CAACCCTTTT CCTATTTGTA GATCACAGTT AACAACTACT GGACTATCGA GAGCTAAAGA ATAAATACTC   
  
  
- AATACAACTA TACTGTGATT AACATTAATA AACAACATTT GAATTTGTAA AAATTTTTAG TTTCGTTTTA   
  
  
- AACAATTAAT GTTATTTAGT TAGATGAAAA TGATACTAGA ATAAACCACA TTAATAACTA GAACTTAAAA   
  
  
- AAAAAAAGAA AAACTTTTTC TATTCGTAGC TTAAAAAAGT TTTTATTTTT CCCACTTCCT TCTTTTTTAC   
  
  
- CTTTGTCAAT GGCTCATTCC TTCCTGTCTC GCGCTCTCCT GTTCTGTGTT TCTCTCCCCT CTCTCTCTCT   
  
  
- CTTCCCCCGT TACCGTCACC CCTTCCAGCT CCTCGTGGAA AGTTGAAGTT CCGAGTTGTG GGGGGGGGGG   
  
  
- GGGGAGAAAG GAGTAAAACG CTGCAATCGT AAACTGTAAA ACGAAAGTTG GCAGGGAGCC GTAGTAGTCG   
  
  
- GGACGGGAAC GAGTCGAAAA GGGTAGAAAA AAACCGAAAA ACAAACTGAG GGAGATATTG AGATAAGTGC   
  
  
- AGCTCGGAAC ATTTTTAAAA TGTATAAAAT AAAATAAAAT GAAAAAATCC AAATAGGGAG AGATAAGAAC   
  
  
- TGGATGCCCG TAAAAAATAA AAGAAACCCA AACTCTGAAC ACTTGAAAAA CGTGTAAAAG AGTGTTTTTA   
  
  
- AATAGAACAG AAAAAGTGAA AGTTGCAAGG AAGAGAGAGA GAGAGAGAGA GAGAGAGAGA GAGTGAGTGT   
  
  
- AGGATCTGTC TGCAGGAAAA ATTTCTTAAA TAAATTGTTC AATCTTCTTT GGCAACAAAT TCATCTTGGT   
  
  
- GGGACAAACC TACGGTTTCC TTTATGGTCA GTTCTTTTTA GTGCTGTTAG TGTTGTGTAG GTAAAGCAAA   
  
  
- TGTAGAGTAG GAGTAGGAGT AGAAGGAGTA GAGATGGGTA GAGGGAGAAA GACATACCGA CGAAGAAGAT   
  
  
- GAAACAAGGG ACTGGGATTG TTATGATCAA ATCAGGAGGG AATAATAATA ATAGGAGGAG GAAGGAGGAG   
  
  
- AATGATATTA CCATTGGGAA TGGAAGGAGA AGGATTGTGG TGACGATAAG GATGAGGAAG TTGTTGGAGA   
  
  
- GGCCATAAAA GAAGGCGGTG GATGAGCCTC AACTTATGAC TAACAGTTTG AGTTGACGGG TACAGATTGG   
  
  
- AGGGGCCTTT CTAGCAAGCC TTCGCCTCAC GACGACTTTA CCTCGTCGCG AGGCGAGGCT AACGCCCACT   
  
  
- AATGGGCGGT TGAGTAGTCG CCCATTAGTG GGCGGTGTTA CTCCGCCGGA GTAGTCACAG AAAGTAACTG   
  
  
- AGGAGTGAGA GCGGTGAAGT GGAGAAGTTG CCGAGGTTAA GCTGCTGGGG TAATCGGGGG CTGGCAGGAT   
  
  
- TACTAGGCCC AGACTCAGTC CCAGGCCTAG ACGGAGAACA CACGCCTAAG AGGCCTGAGG GAAATAAGGG   
  
  
- TGGCCACCTC TCTTCTGGAA GGTAGGGGCG GTGCTGGTGA CGACGGCGCC GCTAGCGCCA CCAAGGACAC   
  
  
- CTCCTCCTAC TAGGCTGCAC CTACCTATCG TAGTATTTTC TCGACTAAGT TTCGAGTTTG AGCTAGAGTT   
  
  
- AGGGTGTCAA CTAAGTCTTG CAATCTCTAT AGTAGATGGG TACATTAGGA TTAGACCCCA GGCGGTAACT   
  
  
- CAAGGCGGAG GCGAGCGAGC GGCGGCTGGG GGAGTAACGA GGCGGCGGTG AAGGCGGGAA GGTGGTGGTG   
  
  
- GTTGTGGTGG AGTTAGTTGT TTAGTGAGAA GGAGCTTAGT CGTTGTGATT GTTGTTGTTA GTACACTCGC   
  
  
- ACATGCGGTT GTTTCCTGGG CCTATGAAGT TGAACCCAGG CCCAGTCCCA GTCCCAGGCT AATTGTAGCT   
  
  
- GGTTCGATTG GAAAGGAAGG GAGGACTAAG GTGGCGGACA ACCCCACAGT CACAGTGTGG CGGTGGCAGA   
  
  
- AGGCGGCGGC CAAGGCCGAG GCCACCTTCG CCGTCATCAT CACCATTTAG TTTGGGATTG GGATTAGGGT   
  
  
- TGGGCTTGGG TTTGGGTTTG GGTTTGGGCT TGTTGTTTCG AGTCCTACAA GTTGAGGTTT GAGGTGTTGT   
  
  
- TGTCGTTGTC GTTGTCGTTG TTTAGGGGCA CCTAGTCCTC GTTCTGGGCC GCCGAGGTGG CCGCTGCCTT   
  
  
- CGTAGCGGTG GTAGAGGTTC TCGCCGCCGT GGTGGCGGCC GCCGTCACTT TCGTTCTCTT TCTCTCCTCT   
  
  
- ACGCCGTTTC CTTCGCGCTG CTTCTCCCAG AGGTGGAGGA TTGGGAGGAG GAGGTTACGC GTCTTCGTCA   
  
  
- TAGGCCGCTG TTTATGCTTC TTCGCTTGTT CTACGAAGAT CTTTAGAGCC TCACCCGGTG GGGTAAGCCG   
  
  
- TGGAGGCGGG TTGCGCAGCG GCGCATGAAG AGCCTTCGGT ACAGTCGGGC AGAGCATAGG AGGACGGAGC   
  
  
- CGTAGATGCG GCGGGAGGGG TGGCAGGGTG TGATGCAGTT CGAGGAGAGG CGGAAGGTTC AGAAGTTACC   
  
  
- GTAGTCGGGT AAGCAGTTTA AGAGAGTGAA GTGACGTTTG GTTCGTTAGG TTCTCCGGAA GGTCTCCCTT   
  
  
- CTGTCCCAGG TGTAGTAGCT GGAGCTATAG TACGTCCCCG AGGTCACCGG GCCCGACAAG GTGTAGGAGC   
  
  
- GCAGGGCCGG TCCACCCGGA GGGAAGCATT CCGAGTGGCC CGAGCCCTGG AGGTACCTCC GCGAGCTCCG   
  
  
- GTGGCCTTTT TCCGAGAGTC TGAAGCGGCT CTTCAACCCC AACGGGAAAC TCAAATATGG GCACCGCCTC   
  
  
- TTCTAACCTT TAAACCTGAA CCTTTCCAAC GTACAATCAT TTTCCCTTCG AGAACGACAC GTAACCAATG   
  
  
- TCGTGAGAAA CATACTACAA TGACCGAGAC TATGATTATG TGAACCGGAA GAAGTTTCCA ACCGCGGTTT   
  
  
- TCACCACTGC CACCACCTCG TTCTGGATTC GGCTTGTCCG AGAAAGGATC CTTCCAAACA TCTCCGCTAG   
  
  
- GTGATGATAA GTCGGGATAA ACTGAGGAAT CCTCGCTCAA TACCTCTCCT GTCACTCCTC TCCGTACACC   
  
  
- AACTCGTTGT CGAGGAAAGA TCCCTCTAAG CTTTGTAAGA CCGGCAACCA CCCGGGAGTT CCTGGCCCCT   
  
  
- CGGGTTCAAA CGCTCGACCT CCCTCTTCGA TGTTGTCAGG CCGAAATCCC CTTAGAGTAA CCGTCCGTTG   
  
  
- CGGCGACGGG TCCGGTGGAA CGAGGAGCCG TACAAGGGAA GACTACCCAT ATGAAATTAG CTCCTGTCAC   
  
  
- CGTGTGAATT CGAGCCCACC TTCCTGAACA CGGAGGACTG ACGAAGCCGG ACCTCCGGAA GGGTACGAGT   
  
  
- ATGATACTCG TGGCAGACAT GAGCCTCGGT TAT

+     MSA-like

| Site Name | Organism | Position | Strand | Matrix score. | sequence | function |
| --- | --- | --- | --- | --- | --- | --- |
| MSA-like | Catharanthus roseus | 4174 | - | 8.5 | (T/C)C(T/C)AACGG(T/C)(T/C)A | cis-acting element involved in cell cycle regulation |

>HU01G00472.1   
+ -Up\_Stream \_Len000GTCTCT GTAACACAAT TCATTAAATC CATCCAAAGG GGATCATAAA TAACCAAACT   
  
  
+ TTGTTGGAAA CTGCTTTGAT GTGGGCTTTC TCTTTCAGCA TTTATGTTAT ACATATATAT GGTTATAAAG   
  
  
+ TTACAAAAGT ATGACCCGAC ACGACAATTC AATCCGAATC CGACTCGAAG TTACTGAAAG TAATCTGAAT   
  
  
+ ACGATTTGAC AGCTTAGTTT AAAAACCTAT TACTTCTCAA TTTTGAGTAA ATCTAAGCTA TTTTTAATGC   
  
  
+ TGAATAGATC GACCCGATTC GACCCGTTTT CCGACTCTAT ATGGTTATGG TGCCCCTAAA AAAAATTAAA   
  
  
+ AAAGAAAAAG AATAGAAAAA TATAAAATAA AACTGAGATT TCTGAACACT CTCTCCCCTA GTCTTGTGGG   
  
  
+ CATGGAAAGT GGTTGGAGAT GGAGATGTTT GAGTTTCATT TTCTGATATT ATTTTTTAGT CATTAGAAGT   
  
  
+ ACAAAGTCTA AACTCAACAA GAGCATGAGG GGAGAATGGT TGTTGTAGCC AATGGAAAAT AAAAAGAACA   
  
  
+ TTCACTCTTC TTGTGCTTTA TTTATGGTGA TAATTTGTTA CTTGTATTTT TAAAAATGAT TAAATATATT   
  
  
+ GATCCTTTGA TATTATGTTG AAATAATAAA AATATAAAAA TTAGAAAATA TTTTACCTCG TGAAATTTTT   
  
  
+ TTTCTGATTA ATCATTGAAA TTATGGTGAA AAATAAAGGA ACATCTCTTG TAAACACTCT ATCCGTTTAC   
  
  
+ GTTTGACCAC AAACTTGCTA TCCACAAGCA TCTCAACCTT TGCCTTTAAC CCAATTGTGG GTTGATGAAA   
  
  
+ TCTAACCATT ATAGGATTAG ATGGTAGTCG TTAATCCTGT GAAACAAGTT TTTGGTTATG ATGTATCCAA   
  
  
+ TTATGAGGGC CTGATAACTC TTGCCTCGGC ATTTGCGTGA TAATGACGTG GTACTAATAG CAATTATTTT   
  
  
+ TCGCAAATAT TCGAAATTTT TTAAGAATAA ACGCAAAATT ATTGATCTTG ACAAATGATT TTATTTTTTT   
  
  
+ GTTGGGAAAA GGATAAACAT CTAGTGTCAA TTGTTGATGA CCTGATAGCT CTCGATTTCT TATTTATGAG   
  
  
+ TTATGTTGAT ATGACACTAA TTGTAATTAT TTGTTGTAAA CTTAAACATT TTTAAAAATC AAAGCAAAAT   
  
  
+ TTGTTAATTA CAATAAATCA ATCTACTTTT ACTATGATCT TATTTGGTGT AATTATTGAT CTTGAATTTT   
  
  
+ TTTTTTTCTT TTTGAAAAAG ATAAGCATCG AATTTTTTCA AAAATAAAAA GGGTGAAGGA AGAAAAAATG   
  
  
+ GAAACAGTTA CCGAGTAAGG AAGGACAGAG CGCGAGAGGA CAAGACACAA AGAGAGGGGA GAGAGAGAGA   
  
  
+ GAAGGGGGCA ATGGCAGTGG GGAAGGTCGA GGAGCACCTT TCAACTTCAA GGCTCAACAC CCCCCCCCCC   
  
  
+ CCCCTCTTTC CTCATTTTGC GACGTTAGCA TTTGACATTT TGCTTTCAAC CGTCCCTCGG CATCATCAGC   
  
  
+ CCTGCCCTTG CTCAGCTTTT CCCATCTTTT TTTGGCTTTT TGTTTGACTC CCTCTATAAC TCTATTCACG   
  
  
+ TCGAGCCTTG TAAAAATTTT ACATATTTTA TTTTATTTTA CTTTTTTAGG TTTATCCCTC TCTATTCTTG   
  
  
+ ACCTACGGGC ATTTTTTATT TTCTTTGGGT TTGAGACTTG TGAACTTTTT GCACATTTTC TCACAAAAAT   
  
  
+ TTATCTTGTC TTTTTCACTT TCAACGTTCC TTCTCTCTCT CTCTCTCTCT CTCTCTCTCT CTCACTCACA   
  
  
+ TCCTAGACAG ACGTCCTTTT TAAAGAATTT ATTTAACAAG TTAGAAGAAA CCGTTGTTTA AGTAGAACCA   
  
  
+ CCCTGTTTGG ATGCCAAAGG AAATACCAGT CAAGAAAAAT CACGACAATC ACAACACATC CATTTCGTTT   
  
  
+ ACATCTCATC CTCATCCTCA TCTTCCTCAT CTCTACCCAT CTCCCTCTTT CTGTATGGCT GCTTCTTCTA   
  
  
+ CTTTGTTCCC TGACCCTAAC AATACTAGTT TAGTCCTCCC TTATTATTAT TATCCTCCTC CTTCCTCCTC   
  
  
+ TTACTATAAT GGTAACCCTT ACCTTCCTCT TCCTAACACC ACTGCTATTC CTACTCCTTC AACAACCTCT   
  
  
+ CCGGTATTTT CTTCCGCCAC CTACTCGGAG TTGAATACTG ATTGTCAAAC TCAACTGCCC ATGTCTAACC   
  
  
+ TCCCCGGAAA GATCGTTCGG AAGCGGAGTG CTGCTGAAAT GGAGCAGCGC TCCGCTCCGA TTGCGGGTGA   
  
  
+ TTACCCGCCA ACTCATCAGC GGGTAATCAC CCGCCACAAT GAGGCGGCCT CATCAGTGTC TTTCATTGAC   
  
  
+ TCCTCACTCT CGCCACTTCA CCTCTTCAAC GGCTCCAATT CGACGACCCC ATTAGCCCCC GACCGTCCTA   
  
  
+ ATGATCCGGG TCTGAGTCAG GGTCCGGATC TGCCTCTTGT GTGCGGATTC TCCGGACTCC CTTTATTCCC   
  
  
+ ACCGGTGGAG AGAAGACCTT CCATCCCCGC CACGACCACT GCTGCCGCGG CGATCGCGGT GGTTCCTGTG   
  
  
+ GAGGAGGATG ATCCGACGTG GATGGATAGC ATCATAAAAG AGCTGATTCA AAGCTCAAAC TCGATCTCAA   
  
  
+ TCCCACAGTT GATTCAGAAC GTTAGAGATA TCATCTACCC ATGTAATCCT AATCTGGGGT CCGCCATTGA   
  
  
+ GTTCCGCCTC CGCTCGCTCG CCGCCGACCC CCTCATTGCT CCGCCGCCAC TTCCGCCCTT CCACCACCAC   
  
  
+ CAACACCACC TCAATCAACA AATCACTCTT CCTCGAATCA GCAACACTAA CAACAACAAT CATGTGAGCG   
  
  
+ TGTACGCCAA CAAAGGACCC GGATACTTCA ACTTGGGTCC GGGTCAGGGT CAGGGTCCGA TTAACATCGA   
  
  
+ CCAAGCTAAC CTTTCCTTCC CTCCTGATTC CACCGCCTGT TGGGGTGTCA GTGTCACACC GCCACCGTCT   
  
  
+ TCCGCCGCCG GTTCCGGCTC CGGTGGAAGC GGCAGTAGTA GTGGTAAATC AAACCCTAAC CCTAATCCCA   
  
  
+ ACCCGAACCC AAACCCAAAC CCAAACCCGA ACAACAAAGC TCAGGATGTT CAACTCCAAA CTCCACAACA   
  
  
+ ACAGCAACAG CAACAGCAAC AAATCCCCGT GGATCAGGAG CAAGACCCGG CGGCTCCACC GGCGACGGAA   
  
  
+ GCATCGCCAC CATCTCCAAG AGCGGCGGCA CCACCGCCGG CGGCAGTGAA AGCAAGAGAA AGAGAGGAGA   
  
  
+ TGCGGCAAAG GAAGCGCGAC GAAGAGGGTC TCCACCTCCT AACCCTCCTC CTCCAATGCG CAGAAGCAGT   
  
  
+ ATCCGGCGAC AAATACGAAG AAGCGAACAA GATGCTTCTA GAAATCTCGG AGTGGGCCAC CCCATTCGGC   
  
  
+ ACCTCCGCCC AACGCGTCGC CGCGTACTTC TCGGAAGCCA TGTCAGCCCG TCTCGTATCC TCCTGCCTCG   
  
  
+ GCATCTACGC CGCCCTCCCC ACCGTCCCAC ACTACGTCAA GCTCCTCTCC GCCTTCCAAG TCTTCAATGG   
  
  
+ CATCAGCCCA TTCGTCAAAT TCTCTCACTT CACTGCAAAC CAAGCAATCC AAGAGGCCTT CCAGAGGGAA   
  
  
+ GACAGGGTCC ACATCATCGA CCTCGATATC ATGCAGGGGC TCCAGTGGCC CGGGCTGTTC CACATCCTCG   
  
  
+ CGTCCCGGCC AGGTGGGCCT CCCTTCGTAA GGCTCACCGG GCTCGGGACC TCCATGGAGG CGCTCGAGGC   
  
  
+ CACCGGAAAA AGGCTCTCAG ACTTCGCCGA GAAGTTGGGG TTGCCCTTTG AGTTTATACC CGTGGCGGAG   
  
  
+ AAGATTGGAA ATTTGGACTT GGAAAGGTTG CATGTTAGTA AAAGGGAAGC TCTTGCTGTG CATTGGTTAC   
  
  
+ AGCACTCTTT GTATGATGTT ACTGGCTCTG ATACTAATAC ACTTGGCCTT CTTCAAAGGT TGGCGCCAAA   
  
  
+ AGTGGTGACG GTGGTGGAGC AAGACCTAAG CCGAACAGGC TCTTTCCTAG GAAGGTTTGT AGAGGCGATC   
  
  
+ CACTACTATT CAGCCCTATT TGACTCCTTA GGAGCGAGTT ATGGAGAGGA CAGTGAGGAG AGGCATGTGG   
  
  
+ TTGAGCAACA GCTCCTTTCT AGGGAGATTC GAAACATTCT GGCCGTTGGT GGGCCCTCAA GGACCGGGGA   
  
  
+ GCCCAAGTTT GCGAGCTGGA GGGAGAAGCT ACAACAGTCC GGCTTTAGGG GAATCTCATT GGCAGGCAAC   
  
  
+ GCCGCTGCCC AGGCCACCTT GCTCCTCGGC ATGTTCCCTT CTGATGGGTA TACTTTAATC GAGGACAGTG   
  
  
+ GCACACTTAA GCTCGGGTGG AAGGACTTGT GCCTCCTGAC TGCTTCGGCC TGGAGGCCTT CCCATGCTCA   
  
  
+ TACTATGAGC ACCGTCTGTA CTCGGAGCCA ATA  

- -Up\_Stream \_Len000CAGAGA CATTGTGTTA AGTAATTTAG GTAGGTTTCC CCTAGTATTT ATTGGTTTGA   
  
  
- AACAACCTTT GACGAAACTA CACCCGAAAG AGAAAGTCGT AAATACAATA TGTATATATA CCAATATTTC   
  
  
- AATGTTTTCA TACTGGGCTG TGCTGTTAAG TTAGGCTTAG GCTGAGCTTC AATGACTTTC ATTAGACTTA   
  
  
- TGCTAAACTG TCGAATCAAA TTTTTGGATA ATGAAGAGTT AAAACTCATT TAGATTCGAT AAAAATTACG   
  
  
- ACTTATCTAG CTGGGCTAAG CTGGGCAAAA GGCTGAGATA TACCAATACC ACGGGGATTT TTTTTAATTT   
  
  
- TTTCTTTTTC TTATCTTTTT ATATTTTATT TTGACTCTAA AGACTTGTGA GAGAGGGGAT CAGAACACCC   
  
  
- GTACCTTTCA CCAACCTCTA CCTCTACAAA CTCAAAGTAA AAGACTATAA TAAAAAATCA GTAATCTTCA   
  
  
- TGTTTCAGAT TTGAGTTGTT CTCGTACTCC CCTCTTACCA ACAACATCGG TTACCTTTTA TTTTTCTTGT   
  
  
- AAGTGAGAAG AACACGAAAT AAATACCACT ATTAAACAAT GAACATAAAA ATTTTTACTA ATTTATATAA   
  
  
- CTAGGAAACT ATAATACAAC TTTATTATTT TTATATTTTT AATCTTTTAT AAAATGGAGC ACTTTAAAAA   
  
  
- AAAGACTAAT TAGTAACTTT AATACCACTT TTTATTTCCT TGTAGAGAAC ATTTGTGAGA TAGGCAAATG   
  
  
- CAAACTGGTG TTTGAACGAT AGGTGTTCGT AGAGTTGGAA ACGGAAATTG GGTTAACACC CAACTACTTT   
  
  
- AGATTGGTAA TATCCTAATC TACCATCAGC AATTAGGACA CTTTGTTCAA AAACCAATAC TACATAGGTT   
  
  
- AATACTCCCG GACTATTGAG AACGGAGCCG TAAACGCACT ATTACTGCAC CATGATTATC GTTAATAAAA   
  
  
- AGCGTTTATA AGCTTTAAAA AATTCTTATT TGCGTTTTAA TAACTAGAAC TGTTTACTAA AATAAAAAAA   
  
  
- CAACCCTTTT CCTATTTGTA GATCACAGTT AACAACTACT GGACTATCGA GAGCTAAAGA ATAAATACTC   
  
  
- AATACAACTA TACTGTGATT AACATTAATA AACAACATTT GAATTTGTAA AAATTTTTAG TTTCGTTTTA   
  
  
- AACAATTAAT GTTATTTAGT TAGATGAAAA TGATACTAGA ATAAACCACA TTAATAACTA GAACTTAAAA   
  
  
- AAAAAAAGAA AAACTTTTTC TATTCGTAGC TTAAAAAAGT TTTTATTTTT CCCACTTCCT TCTTTTTTAC   
  
  
- CTTTGTCAAT GGCTCATTCC TTCCTGTCTC GCGCTCTCCT GTTCTGTGTT TCTCTCCCCT CTCTCTCTCT   
  
  
- CTTCCCCCGT TACCGTCACC CCTTCCAGCT CCTCGTGGAA AGTTGAAGTT CCGAGTTGTG GGGGGGGGGG   
  
  
- GGGGAGAAAG GAGTAAAACG CTGCAATCGT AAACTGTAAA ACGAAAGTTG GCAGGGAGCC GTAGTAGTCG   
  
  
- GGACGGGAAC GAGTCGAAAA GGGTAGAAAA AAACCGAAAA ACAAACTGAG GGAGATATTG AGATAAGTGC   
  
  
- AGCTCGGAAC ATTTTTAAAA TGTATAAAAT AAAATAAAAT GAAAAAATCC AAATAGGGAG AGATAAGAAC   
  
  
- TGGATGCCCG TAAAAAATAA AAGAAACCCA AACTCTGAAC ACTTGAAAAA CGTGTAAAAG AGTGTTTTTA   
  
  
- AATAGAACAG AAAAAGTGAA AGTTGCAAGG AAGAGAGAGA GAGAGAGAGA GAGAGAGAGA GAGTGAGTGT   
  
  
- AGGATCTGTC TGCAGGAAAA ATTTCTTAAA TAAATTGTTC AATCTTCTTT GGCAACAAAT TCATCTTGGT   
  
  
- GGGACAAACC TACGGTTTCC TTTATGGTCA GTTCTTTTTA GTGCTGTTAG TGTTGTGTAG GTAAAGCAAA   
  
  
- TGTAGAGTAG GAGTAGGAGT AGAAGGAGTA GAGATGGGTA GAGGGAGAAA GACATACCGA CGAAGAAGAT   
  
  
- GAAACAAGGG ACTGGGATTG TTATGATCAA ATCAGGAGGG AATAATAATA ATAGGAGGAG GAAGGAGGAG   
  
  
- AATGATATTA CCATTGGGAA TGGAAGGAGA AGGATTGTGG TGACGATAAG GATGAGGAAG TTGTTGGAGA   
  
  
- GGCCATAAAA GAAGGCGGTG GATGAGCCTC AACTTATGAC TAACAGTTTG AGTTGACGGG TACAGATTGG   
  
  
- AGGGGCCTTT CTAGCAAGCC TTCGCCTCAC GACGACTTTA CCTCGTCGCG AGGCGAGGCT AACGCCCACT   
  
  
- AATGGGCGGT TGAGTAGTCG CCCATTAGTG GGCGGTGTTA CTCCGCCGGA GTAGTCACAG AAAGTAACTG   
  
  
- AGGAGTGAGA GCGGTGAAGT GGAGAAGTTG CCGAGGTTAA GCTGCTGGGG TAATCGGGGG CTGGCAGGAT   
  
  
- TACTAGGCCC AGACTCAGTC CCAGGCCTAG ACGGAGAACA CACGCCTAAG AGGCCTGAGG GAAATAAGGG   
  
  
- TGGCCACCTC TCTTCTGGAA GGTAGGGGCG GTGCTGGTGA CGACGGCGCC GCTAGCGCCA CCAAGGACAC   
  
  
- CTCCTCCTAC TAGGCTGCAC CTACCTATCG TAGTATTTTC TCGACTAAGT TTCGAGTTTG AGCTAGAGTT   
  
  
- AGGGTGTCAA CTAAGTCTTG CAATCTCTAT AGTAGATGGG TACATTAGGA TTAGACCCCA GGCGGTAACT   
  
  
- CAAGGCGGAG GCGAGCGAGC GGCGGCTGGG GGAGTAACGA GGCGGCGGTG AAGGCGGGAA GGTGGTGGTG   
  
  
- GTTGTGGTGG AGTTAGTTGT TTAGTGAGAA GGAGCTTAGT CGTTGTGATT GTTGTTGTTA GTACACTCGC   
  
  
- ACATGCGGTT GTTTCCTGGG CCTATGAAGT TGAACCCAGG CCCAGTCCCA GTCCCAGGCT AATTGTAGCT   
  
  
- GGTTCGATTG GAAAGGAAGG GAGGACTAAG GTGGCGGACA ACCCCACAGT CACAGTGTGG CGGTGGCAGA   
  
  
- AGGCGGCGGC CAAGGCCGAG GCCACCTTCG CCGTCATCAT CACCATTTAG TTTGGGATTG GGATTAGGGT   
  
  
- TGGGCTTGGG TTTGGGTTTG GGTTTGGGCT TGTTGTTTCG AGTCCTACAA GTTGAGGTTT GAGGTGTTGT   
  
  
- TGTCGTTGTC GTTGTCGTTG TTTAGGGGCA CCTAGTCCTC GTTCTGGGCC GCCGAGGTGG CCGCTGCCTT   
  
  
- CGTAGCGGTG GTAGAGGTTC TCGCCGCCGT GGTGGCGGCC GCCGTCACTT TCGTTCTCTT TCTCTCCTCT   
  
  
- ACGCCGTTTC CTTCGCGCTG CTTCTCCCAG AGGTGGAGGA TTGGGAGGAG GAGGTTACGC GTCTTCGTCA   
  
  
- TAGGCCGCTG TTTATGCTTC TTCGCTTGTT CTACGAAGAT CTTTAGAGCC TCACCCGGTG GGGTAAGCCG   
  
  
- TGGAGGCGGG TTGCGCAGCG GCGCATGAAG AGCCTTCGGT ACAGTCGGGC AGAGCATAGG AGGACGGAGC   
  
  
- CGTAGATGCG GCGGGAGGGG TGGCAGGGTG TGATGCAGTT CGAGGAGAGG CGGAAGGTTC AGAAGTTACC   
  
  
- GTAGTCGGGT AAGCAGTTTA AGAGAGTGAA GTGACGTTTG GTTCGTTAGG TTCTCCGGAA GGTCTCCCTT   
  
  
- CTGTCCCAGG TGTAGTAGCT GGAGCTATAG TACGTCCCCG AGGTCACCGG GCCCGACAAG GTGTAGGAGC   
  
  
- GCAGGGCCGG TCCACCCGGA GGGAAGCATT CCGAGTGGCC CGAGCCCTGG AGGTACCTCC GCGAGCTCCG   
  
  
- GTGGCCTTTT TCCGAGAGTC TGAAGCGGCT CTTCAACCCC AACGGGAAAC TCAAATATGG GCACCGCCTC   
  
  
- TTCTAACCTT TAAACCTGAA CCTTTCCAAC GTACAATCAT TTTCCCTTCG AGAACGACAC GTAACCAATG   
  
  
- TCGTGAGAAA CATACTACAA TGACCGAGAC TATGATTATG TGAACCGGAA GAAGTTTCCA ACCGCGGTTT   
  
  
- TCACCACTGC CACCACCTCG TTCTGGATTC GGCTTGTCCG AGAAAGGATC CTTCCAAACA TCTCCGCTAG   
  
  
- GTGATGATAA GTCGGGATAA ACTGAGGAAT CCTCGCTCAA TACCTCTCCT GTCACTCCTC TCCGTACACC   
  
  
- AACTCGTTGT CGAGGAAAGA TCCCTCTAAG CTTTGTAAGA CCGGCAACCA CCCGGGAGTT CCTGGCCCCT   
  
  
- CGGGTTCAAA CGCTCGACCT CCCTCTTCGA TGTTGTCAGG CCGAAATCCC CTTAGAGTAA CCGTCCGTTG   
  
  
- CGGCGACGGG TCCGGTGGAA CGAGGAGCCG TACAAGGGAA GACTACCCAT ATGAAATTAG CTCCTGTCAC   
  
  
- CGTGTGAATT CGAGCCCACC TTCCTGAACA CGGAGGACTG ACGAAGCCGG ACCTCCGGAA GGGTACGAGT   
  
  
- ATGATACTCG TGGCAGACAT GAGCCTCGGT TAT

+     MYB

| Site Name | Organism | Position | Strand | Matrix score. | sequence | function |
| --- | --- | --- | --- | --- | --- | --- |
| MYB | Arabidopsis thaliana | 65 | + | 6 | TAACCA |  |
| MYB | Arabidopsis thaliana | 3165 | + | 6 | CAACAG |  |
| MYB | Arabidopsis thaliana | 531 | - | 6 | CAACCA |  |
| MYB | Arabidopsis thaliana | 4132 | - | 6 | CAACCA |  |
| MYB | Arabidopsis thaliana | 326 | - | 6 | TAACCA |  |
| MYB | Arabidopsis thaliana | 3153 | + | 6 | CAACAG |  |
| MYB | Arabidopsis thaliana | 897 | - | 6 | TAACCA |  |
| MYB | Arabidopsis thaliana | 4236 | + | 6 | CAACAG |  |
| MYB | Arabidopsis thaliana | 134 | - | 6 | TAACCA |  |
| MYB | Arabidopsis thaliana | 3159 | + | 6 | CAACAG |  |
| MYB | Arabidopsis thaliana | 434 | - | 6 | CAACCA |  |
| MYB | Arabidopsis thaliana | 4140 | + | 6 | CAACAG |  |
| MYB | Arabidopsis thaliana | 847 | + | 6 | TAACCA |  |
| MYB | Arabidopsis thaliana | 3918 | - | 6 | TAACCA |  |
| MYB | Arabidopsis thaliana | 2981 | - | 6 | CAACAG |  |

>HU01G00472.1   
+ -Up\_Stream \_Len000GTCTCT GTAACACAAT TCATTAAATC CATCCAAAGG GGATCATAAA TAACCAAACT   
  
  
+ TTGTTGGAAA CTGCTTTGAT GTGGGCTTTC TCTTTCAGCA TTTATGTTAT ACATATATAT GGTTATAAAG   
  
  
+ TTACAAAAGT ATGACCCGAC ACGACAATTC AATCCGAATC CGACTCGAAG TTACTGAAAG TAATCTGAAT   
  
  
+ ACGATTTGAC AGCTTAGTTT AAAAACCTAT TACTTCTCAA TTTTGAGTAA ATCTAAGCTA TTTTTAATGC   
  
  
+ TGAATAGATC GACCCGATTC GACCCGTTTT CCGACTCTAT ATGGTTATGG TGCCCCTAAA AAAAATTAAA   
  
  
+ AAAGAAAAAG AATAGAAAAA TATAAAATAA AACTGAGATT TCTGAACACT CTCTCCCCTA GTCTTGTGGG   
  
  
+ CATGGAAAGT GGTTGGAGAT GGAGATGTTT GAGTTTCATT TTCTGATATT ATTTTTTAGT CATTAGAAGT   
  
  
+ ACAAAGTCTA AACTCAACAA GAGCATGAGG GGAGAATGGT TGTTGTAGCC AATGGAAAAT AAAAAGAACA   
  
  
+ TTCACTCTTC TTGTGCTTTA TTTATGGTGA TAATTTGTTA CTTGTATTTT TAAAAATGAT TAAATATATT   
  
  
+ GATCCTTTGA TATTATGTTG AAATAATAAA AATATAAAAA TTAGAAAATA TTTTACCTCG TGAAATTTTT   
  
  
+ TTTCTGATTA ATCATTGAAA TTATGGTGAA AAATAAAGGA ACATCTCTTG TAAACACTCT ATCCGTTTAC   
  
  
+ GTTTGACCAC AAACTTGCTA TCCACAAGCA TCTCAACCTT TGCCTTTAAC CCAATTGTGG GTTGATGAAA   
  
  
+ TCTAACCATT ATAGGATTAG ATGGTAGTCG TTAATCCTGT GAAACAAGTT TTTGGTTATG ATGTATCCAA   
  
  
+ TTATGAGGGC CTGATAACTC TTGCCTCGGC ATTTGCGTGA TAATGACGTG GTACTAATAG CAATTATTTT   
  
  
+ TCGCAAATAT TCGAAATTTT TTAAGAATAA ACGCAAAATT ATTGATCTTG ACAAATGATT TTATTTTTTT   
  
  
+ GTTGGGAAAA GGATAAACAT CTAGTGTCAA TTGTTGATGA CCTGATAGCT CTCGATTTCT TATTTATGAG   
  
  
+ TTATGTTGAT ATGACACTAA TTGTAATTAT TTGTTGTAAA CTTAAACATT TTTAAAAATC AAAGCAAAAT   
  
  
+ TTGTTAATTA CAATAAATCA ATCTACTTTT ACTATGATCT TATTTGGTGT AATTATTGAT CTTGAATTTT   
  
  
+ TTTTTTTCTT TTTGAAAAAG ATAAGCATCG AATTTTTTCA AAAATAAAAA GGGTGAAGGA AGAAAAAATG   
  
  
+ GAAACAGTTA CCGAGTAAGG AAGGACAGAG CGCGAGAGGA CAAGACACAA AGAGAGGGGA GAGAGAGAGA   
  
  
+ GAAGGGGGCA ATGGCAGTGG GGAAGGTCGA GGAGCACCTT TCAACTTCAA GGCTCAACAC CCCCCCCCCC   
  
  
+ CCCCTCTTTC CTCATTTTGC GACGTTAGCA TTTGACATTT TGCTTTCAAC CGTCCCTCGG CATCATCAGC   
  
  
+ CCTGCCCTTG CTCAGCTTTT CCCATCTTTT TTTGGCTTTT TGTTTGACTC CCTCTATAAC TCTATTCACG   
  
  
+ TCGAGCCTTG TAAAAATTTT ACATATTTTA TTTTATTTTA CTTTTTTAGG TTTATCCCTC TCTATTCTTG   
  
  
+ ACCTACGGGC ATTTTTTATT TTCTTTGGGT TTGAGACTTG TGAACTTTTT GCACATTTTC TCACAAAAAT   
  
  
+ TTATCTTGTC TTTTTCACTT TCAACGTTCC TTCTCTCTCT CTCTCTCTCT CTCTCTCTCT CTCACTCACA   
  
  
+ TCCTAGACAG ACGTCCTTTT TAAAGAATTT ATTTAACAAG TTAGAAGAAA CCGTTGTTTA AGTAGAACCA   
  
  
+ CCCTGTTTGG ATGCCAAAGG AAATACCAGT CAAGAAAAAT CACGACAATC ACAACACATC CATTTCGTTT   
  
  
+ ACATCTCATC CTCATCCTCA TCTTCCTCAT CTCTACCCAT CTCCCTCTTT CTGTATGGCT GCTTCTTCTA   
  
  
+ CTTTGTTCCC TGACCCTAAC AATACTAGTT TAGTCCTCCC TTATTATTAT TATCCTCCTC CTTCCTCCTC   
  
  
+ TTACTATAAT GGTAACCCTT ACCTTCCTCT TCCTAACACC ACTGCTATTC CTACTCCTTC AACAACCTCT   
  
  
+ CCGGTATTTT CTTCCGCCAC CTACTCGGAG TTGAATACTG ATTGTCAAAC TCAACTGCCC ATGTCTAACC   
  
  
+ TCCCCGGAAA GATCGTTCGG AAGCGGAGTG CTGCTGAAAT GGAGCAGCGC TCCGCTCCGA TTGCGGGTGA   
  
  
+ TTACCCGCCA ACTCATCAGC GGGTAATCAC CCGCCACAAT GAGGCGGCCT CATCAGTGTC TTTCATTGAC   
  
  
+ TCCTCACTCT CGCCACTTCA CCTCTTCAAC GGCTCCAATT CGACGACCCC ATTAGCCCCC GACCGTCCTA   
  
  
+ ATGATCCGGG TCTGAGTCAG GGTCCGGATC TGCCTCTTGT GTGCGGATTC TCCGGACTCC CTTTATTCCC   
  
  
+ ACCGGTGGAG AGAAGACCTT CCATCCCCGC CACGACCACT GCTGCCGCGG CGATCGCGGT GGTTCCTGTG   
  
  
+ GAGGAGGATG ATCCGACGTG GATGGATAGC ATCATAAAAG AGCTGATTCA AAGCTCAAAC TCGATCTCAA   
  
  
+ TCCCACAGTT GATTCAGAAC GTTAGAGATA TCATCTACCC ATGTAATCCT AATCTGGGGT CCGCCATTGA   
  
  
+ GTTCCGCCTC CGCTCGCTCG CCGCCGACCC CCTCATTGCT CCGCCGCCAC TTCCGCCCTT CCACCACCAC   
  
  
+ CAACACCACC TCAATCAACA AATCACTCTT CCTCGAATCA GCAACACTAA CAACAACAAT CATGTGAGCG   
  
  
+ TGTACGCCAA CAAAGGACCC GGATACTTCA ACTTGGGTCC GGGTCAGGGT CAGGGTCCGA TTAACATCGA   
  
  
+ CCAAGCTAAC CTTTCCTTCC CTCCTGATTC CACCGCCTGT TGGGGTGTCA GTGTCACACC GCCACCGTCT   
  
  
+ TCCGCCGCCG GTTCCGGCTC CGGTGGAAGC GGCAGTAGTA GTGGTAAATC AAACCCTAAC CCTAATCCCA   
  
  
+ ACCCGAACCC AAACCCAAAC CCAAACCCGA ACAACAAAGC TCAGGATGTT CAACTCCAAA CTCCACAACA   
  
  
+ ACAGCAACAG CAACAGCAAC AAATCCCCGT GGATCAGGAG CAAGACCCGG CGGCTCCACC GGCGACGGAA   
  
  
+ GCATCGCCAC CATCTCCAAG AGCGGCGGCA CCACCGCCGG CGGCAGTGAA AGCAAGAGAA AGAGAGGAGA   
  
  
+ TGCGGCAAAG GAAGCGCGAC GAAGAGGGTC TCCACCTCCT AACCCTCCTC CTCCAATGCG CAGAAGCAGT   
  
  
+ ATCCGGCGAC AAATACGAAG AAGCGAACAA GATGCTTCTA GAAATCTCGG AGTGGGCCAC CCCATTCGGC   
  
  
+ ACCTCCGCCC AACGCGTCGC CGCGTACTTC TCGGAAGCCA TGTCAGCCCG TCTCGTATCC TCCTGCCTCG   
  
  
+ GCATCTACGC CGCCCTCCCC ACCGTCCCAC ACTACGTCAA GCTCCTCTCC GCCTTCCAAG TCTTCAATGG   
  
  
+ CATCAGCCCA TTCGTCAAAT TCTCTCACTT CACTGCAAAC CAAGCAATCC AAGAGGCCTT CCAGAGGGAA   
  
  
+ GACAGGGTCC ACATCATCGA CCTCGATATC ATGCAGGGGC TCCAGTGGCC CGGGCTGTTC CACATCCTCG   
  
  
+ CGTCCCGGCC AGGTGGGCCT CCCTTCGTAA GGCTCACCGG GCTCGGGACC TCCATGGAGG CGCTCGAGGC   
  
  
+ CACCGGAAAA AGGCTCTCAG ACTTCGCCGA GAAGTTGGGG TTGCCCTTTG AGTTTATACC CGTGGCGGAG   
  
  
+ AAGATTGGAA ATTTGGACTT GGAAAGGTTG CATGTTAGTA AAAGGGAAGC TCTTGCTGTG CATTGGTTAC   
  
  
+ AGCACTCTTT GTATGATGTT ACTGGCTCTG ATACTAATAC ACTTGGCCTT CTTCAAAGGT TGGCGCCAAA   
  
  
+ AGTGGTGACG GTGGTGGAGC AAGACCTAAG CCGAACAGGC TCTTTCCTAG GAAGGTTTGT AGAGGCGATC   
  
  
+ CACTACTATT CAGCCCTATT TGACTCCTTA GGAGCGAGTT ATGGAGAGGA CAGTGAGGAG AGGCATGTGG   
  
  
+ TTGAGCAACA GCTCCTTTCT AGGGAGATTC GAAACATTCT GGCCGTTGGT GGGCCCTCAA GGACCGGGGA   
  
  
+ GCCCAAGTTT GCGAGCTGGA GGGAGAAGCT ACAACAGTCC GGCTTTAGGG GAATCTCATT GGCAGGCAAC   
  
  
+ GCCGCTGCCC AGGCCACCTT GCTCCTCGGC ATGTTCCCTT CTGATGGGTA TACTTTAATC GAGGACAGTG   
  
  
+ GCACACTTAA GCTCGGGTGG AAGGACTTGT GCCTCCTGAC TGCTTCGGCC TGGAGGCCTT CCCATGCTCA   
  
  
+ TACTATGAGC ACCGTCTGTA CTCGGAGCCA ATA  

- -Up\_Stream \_Len000CAGAGA CATTGTGTTA AGTAATTTAG GTAGGTTTCC CCTAGTATTT ATTGGTTTGA   
  
  
- AACAACCTTT GACGAAACTA CACCCGAAAG AGAAAGTCGT AAATACAATA TGTATATATA CCAATATTTC   
  
  
- AATGTTTTCA TACTGGGCTG TGCTGTTAAG TTAGGCTTAG GCTGAGCTTC AATGACTTTC ATTAGACTTA   
  
  
- TGCTAAACTG TCGAATCAAA TTTTTGGATA ATGAAGAGTT AAAACTCATT TAGATTCGAT AAAAATTACG   
  
  
- ACTTATCTAG CTGGGCTAAG CTGGGCAAAA GGCTGAGATA TACCAATACC ACGGGGATTT TTTTTAATTT   
  
  
- TTTCTTTTTC TTATCTTTTT ATATTTTATT TTGACTCTAA AGACTTGTGA GAGAGGGGAT CAGAACACCC   
  
  
- GTACCTTTCA CCAACCTCTA CCTCTACAAA CTCAAAGTAA AAGACTATAA TAAAAAATCA GTAATCTTCA   
  
  
- TGTTTCAGAT TTGAGTTGTT CTCGTACTCC CCTCTTACCA ACAACATCGG TTACCTTTTA TTTTTCTTGT   
  
  
- AAGTGAGAAG AACACGAAAT AAATACCACT ATTAAACAAT GAACATAAAA ATTTTTACTA ATTTATATAA   
  
  
- CTAGGAAACT ATAATACAAC TTTATTATTT TTATATTTTT AATCTTTTAT AAAATGGAGC ACTTTAAAAA   
  
  
- AAAGACTAAT TAGTAACTTT AATACCACTT TTTATTTCCT TGTAGAGAAC ATTTGTGAGA TAGGCAAATG   
  
  
- CAAACTGGTG TTTGAACGAT AGGTGTTCGT AGAGTTGGAA ACGGAAATTG GGTTAACACC CAACTACTTT   
  
  
- AGATTGGTAA TATCCTAATC TACCATCAGC AATTAGGACA CTTTGTTCAA AAACCAATAC TACATAGGTT   
  
  
- AATACTCCCG GACTATTGAG AACGGAGCCG TAAACGCACT ATTACTGCAC CATGATTATC GTTAATAAAA   
  
  
- AGCGTTTATA AGCTTTAAAA AATTCTTATT TGCGTTTTAA TAACTAGAAC TGTTTACTAA AATAAAAAAA   
  
  
- CAACCCTTTT CCTATTTGTA GATCACAGTT AACAACTACT GGACTATCGA GAGCTAAAGA ATAAATACTC   
  
  
- AATACAACTA TACTGTGATT AACATTAATA AACAACATTT GAATTTGTAA AAATTTTTAG TTTCGTTTTA   
  
  
- AACAATTAAT GTTATTTAGT TAGATGAAAA TGATACTAGA ATAAACCACA TTAATAACTA GAACTTAAAA   
  
  
- AAAAAAAGAA AAACTTTTTC TATTCGTAGC TTAAAAAAGT TTTTATTTTT CCCACTTCCT TCTTTTTTAC   
  
  
- CTTTGTCAAT GGCTCATTCC TTCCTGTCTC GCGCTCTCCT GTTCTGTGTT TCTCTCCCCT CTCTCTCTCT   
  
  
- CTTCCCCCGT TACCGTCACC CCTTCCAGCT CCTCGTGGAA AGTTGAAGTT CCGAGTTGTG GGGGGGGGGG   
  
  
- GGGGAGAAAG GAGTAAAACG CTGCAATCGT AAACTGTAAA ACGAAAGTTG GCAGGGAGCC GTAGTAGTCG   
  
  
- GGACGGGAAC GAGTCGAAAA GGGTAGAAAA AAACCGAAAA ACAAACTGAG GGAGATATTG AGATAAGTGC   
  
  
- AGCTCGGAAC ATTTTTAAAA TGTATAAAAT AAAATAAAAT GAAAAAATCC AAATAGGGAG AGATAAGAAC   
  
  
- TGGATGCCCG TAAAAAATAA AAGAAACCCA AACTCTGAAC ACTTGAAAAA CGTGTAAAAG AGTGTTTTTA   
  
  
- AATAGAACAG AAAAAGTGAA AGTTGCAAGG AAGAGAGAGA GAGAGAGAGA GAGAGAGAGA GAGTGAGTGT   
  
  
- AGGATCTGTC TGCAGGAAAA ATTTCTTAAA TAAATTGTTC AATCTTCTTT GGCAACAAAT TCATCTTGGT   
  
  
- GGGACAAACC TACGGTTTCC TTTATGGTCA GTTCTTTTTA GTGCTGTTAG TGTTGTGTAG GTAAAGCAAA   
  
  
- TGTAGAGTAG GAGTAGGAGT AGAAGGAGTA GAGATGGGTA GAGGGAGAAA GACATACCGA CGAAGAAGAT   
  
  
- GAAACAAGGG ACTGGGATTG TTATGATCAA ATCAGGAGGG AATAATAATA ATAGGAGGAG GAAGGAGGAG   
  
  
- AATGATATTA CCATTGGGAA TGGAAGGAGA AGGATTGTGG TGACGATAAG GATGAGGAAG TTGTTGGAGA   
  
  
- GGCCATAAAA GAAGGCGGTG GATGAGCCTC AACTTATGAC TAACAGTTTG AGTTGACGGG TACAGATTGG   
  
  
- AGGGGCCTTT CTAGCAAGCC TTCGCCTCAC GACGACTTTA CCTCGTCGCG AGGCGAGGCT AACGCCCACT   
  
  
- AATGGGCGGT TGAGTAGTCG CCCATTAGTG GGCGGTGTTA CTCCGCCGGA GTAGTCACAG AAAGTAACTG   
  
  
- AGGAGTGAGA GCGGTGAAGT GGAGAAGTTG CCGAGGTTAA GCTGCTGGGG TAATCGGGGG CTGGCAGGAT   
  
  
- TACTAGGCCC AGACTCAGTC CCAGGCCTAG ACGGAGAACA CACGCCTAAG AGGCCTGAGG GAAATAAGGG   
  
  
- TGGCCACCTC TCTTCTGGAA GGTAGGGGCG GTGCTGGTGA CGACGGCGCC GCTAGCGCCA CCAAGGACAC   
  
  
- CTCCTCCTAC TAGGCTGCAC CTACCTATCG TAGTATTTTC TCGACTAAGT TTCGAGTTTG AGCTAGAGTT   
  
  
- AGGGTGTCAA CTAAGTCTTG CAATCTCTAT AGTAGATGGG TACATTAGGA TTAGACCCCA GGCGGTAACT   
  
  
- CAAGGCGGAG GCGAGCGAGC GGCGGCTGGG GGAGTAACGA GGCGGCGGTG AAGGCGGGAA GGTGGTGGTG   
  
  
- GTTGTGGTGG AGTTAGTTGT TTAGTGAGAA GGAGCTTAGT CGTTGTGATT GTTGTTGTTA GTACACTCGC   
  
  
- ACATGCGGTT GTTTCCTGGG CCTATGAAGT TGAACCCAGG CCCAGTCCCA GTCCCAGGCT AATTGTAGCT   
  
  
- GGTTCGATTG GAAAGGAAGG GAGGACTAAG GTGGCGGACA ACCCCACAGT CACAGTGTGG CGGTGGCAGA   
  
  
- AGGCGGCGGC CAAGGCCGAG GCCACCTTCG CCGTCATCAT CACCATTTAG TTTGGGATTG GGATTAGGGT   
  
  
- TGGGCTTGGG TTTGGGTTTG GGTTTGGGCT TGTTGTTTCG AGTCCTACAA GTTGAGGTTT GAGGTGTTGT   
  
  
- TGTCGTTGTC GTTGTCGTTG TTTAGGGGCA CCTAGTCCTC GTTCTGGGCC GCCGAGGTGG CCGCTGCCTT   
  
  
- CGTAGCGGTG GTAGAGGTTC TCGCCGCCGT GGTGGCGGCC GCCGTCACTT TCGTTCTCTT TCTCTCCTCT   
  
  
- ACGCCGTTTC CTTCGCGCTG CTTCTCCCAG AGGTGGAGGA TTGGGAGGAG GAGGTTACGC GTCTTCGTCA   
  
  
- TAGGCCGCTG TTTATGCTTC TTCGCTTGTT CTACGAAGAT CTTTAGAGCC TCACCCGGTG GGGTAAGCCG   
  
  
- TGGAGGCGGG TTGCGCAGCG GCGCATGAAG AGCCTTCGGT ACAGTCGGGC AGAGCATAGG AGGACGGAGC   
  
  
- CGTAGATGCG GCGGGAGGGG TGGCAGGGTG TGATGCAGTT CGAGGAGAGG CGGAAGGTTC AGAAGTTACC   
  
  
- GTAGTCGGGT AAGCAGTTTA AGAGAGTGAA GTGACGTTTG GTTCGTTAGG TTCTCCGGAA GGTCTCCCTT   
  
  
- CTGTCCCAGG TGTAGTAGCT GGAGCTATAG TACGTCCCCG AGGTCACCGG GCCCGACAAG GTGTAGGAGC   
  
  
- GCAGGGCCGG TCCACCCGGA GGGAAGCATT CCGAGTGGCC CGAGCCCTGG AGGTACCTCC GCGAGCTCCG   
  
  
- GTGGCCTTTT TCCGAGAGTC TGAAGCGGCT CTTCAACCCC AACGGGAAAC TCAAATATGG GCACCGCCTC   
  
  
- TTCTAACCTT TAAACCTGAA CCTTTCCAAC GTACAATCAT TTTCCCTTCG AGAACGACAC GTAACCAATG   
  
  
- TCGTGAGAAA CATACTACAA TGACCGAGAC TATGATTATG TGAACCGGAA GAAGTTTCCA ACCGCGGTTT   
  
  
- TCACCACTGC CACCACCTCG TTCTGGATTC GGCTTGTCCG AGAAAGGATC CTTCCAAACA TCTCCGCTAG   
  
  
- GTGATGATAA GTCGGGATAA ACTGAGGAAT CCTCGCTCAA TACCTCTCCT GTCACTCCTC TCCGTACACC   
  
  
- AACTCGTTGT CGAGGAAAGA TCCCTCTAAG CTTTGTAAGA CCGGCAACCA CCCGGGAGTT CCTGGCCCCT   
  
  
- CGGGTTCAAA CGCTCGACCT CCCTCTTCGA TGTTGTCAGG CCGAAATCCC CTTAGAGTAA CCGTCCGTTG   
  
  
- CGGCGACGGG TCCGGTGGAA CGAGGAGCCG TACAAGGGAA GACTACCCAT ATGAAATTAG CTCCTGTCAC   
  
  
- CGTGTGAATT CGAGCCCACC TTCCTGAACA CGGAGGACTG ACGAAGCCGG ACCTCCGGAA GGGTACGAGT   
  
  
- ATGATACTCG TGGCAGACAT GAGCCTCGGT TAT

+     MYB recognition site

| Site Name | Organism | Position | Strand | Matrix score. | sequence | function |
| --- | --- | --- | --- | --- | --- | --- |
| MYB recognition site | Arabidopsis thaliana | 1875 | + | 6 | CCGTTG |  |
| MYB recognition site | Arabidopsis thaliana | 4177 | + | 6 | CCGTTG |  |
| MYB recognition site | Arabidopsis thaliana | 2411 | - | 6 | CCGTTG |  |

>HU01G00472.1   
+ -Up\_Stream \_Len000GTCTCT GTAACACAAT TCATTAAATC CATCCAAAGG GGATCATAAA TAACCAAACT   
  
  
+ TTGTTGGAAA CTGCTTTGAT GTGGGCTTTC TCTTTCAGCA TTTATGTTAT ACATATATAT GGTTATAAAG   
  
  
+ TTACAAAAGT ATGACCCGAC ACGACAATTC AATCCGAATC CGACTCGAAG TTACTGAAAG TAATCTGAAT   
  
  
+ ACGATTTGAC AGCTTAGTTT AAAAACCTAT TACTTCTCAA TTTTGAGTAA ATCTAAGCTA TTTTTAATGC   
  
  
+ TGAATAGATC GACCCGATTC GACCCGTTTT CCGACTCTAT ATGGTTATGG TGCCCCTAAA AAAAATTAAA   
  
  
+ AAAGAAAAAG AATAGAAAAA TATAAAATAA AACTGAGATT TCTGAACACT CTCTCCCCTA GTCTTGTGGG   
  
  
+ CATGGAAAGT GGTTGGAGAT GGAGATGTTT GAGTTTCATT TTCTGATATT ATTTTTTAGT CATTAGAAGT   
  
  
+ ACAAAGTCTA AACTCAACAA GAGCATGAGG GGAGAATGGT TGTTGTAGCC AATGGAAAAT AAAAAGAACA   
  
  
+ TTCACTCTTC TTGTGCTTTA TTTATGGTGA TAATTTGTTA CTTGTATTTT TAAAAATGAT TAAATATATT   
  
  
+ GATCCTTTGA TATTATGTTG AAATAATAAA AATATAAAAA TTAGAAAATA TTTTACCTCG TGAAATTTTT   
  
  
+ TTTCTGATTA ATCATTGAAA TTATGGTGAA AAATAAAGGA ACATCTCTTG TAAACACTCT ATCCGTTTAC   
  
  
+ GTTTGACCAC AAACTTGCTA TCCACAAGCA TCTCAACCTT TGCCTTTAAC CCAATTGTGG GTTGATGAAA   
  
  
+ TCTAACCATT ATAGGATTAG ATGGTAGTCG TTAATCCTGT GAAACAAGTT TTTGGTTATG ATGTATCCAA   
  
  
+ TTATGAGGGC CTGATAACTC TTGCCTCGGC ATTTGCGTGA TAATGACGTG GTACTAATAG CAATTATTTT   
  
  
+ TCGCAAATAT TCGAAATTTT TTAAGAATAA ACGCAAAATT ATTGATCTTG ACAAATGATT TTATTTTTTT   
  
  
+ GTTGGGAAAA GGATAAACAT CTAGTGTCAA TTGTTGATGA CCTGATAGCT CTCGATTTCT TATTTATGAG   
  
  
+ TTATGTTGAT ATGACACTAA TTGTAATTAT TTGTTGTAAA CTTAAACATT TTTAAAAATC AAAGCAAAAT   
  
  
+ TTGTTAATTA CAATAAATCA ATCTACTTTT ACTATGATCT TATTTGGTGT AATTATTGAT CTTGAATTTT   
  
  
+ TTTTTTTCTT TTTGAAAAAG ATAAGCATCG AATTTTTTCA AAAATAAAAA GGGTGAAGGA AGAAAAAATG   
  
  
+ GAAACAGTTA CCGAGTAAGG AAGGACAGAG CGCGAGAGGA CAAGACACAA AGAGAGGGGA GAGAGAGAGA   
  
  
+ GAAGGGGGCA ATGGCAGTGG GGAAGGTCGA GGAGCACCTT TCAACTTCAA GGCTCAACAC CCCCCCCCCC   
  
  
+ CCCCTCTTTC CTCATTTTGC GACGTTAGCA TTTGACATTT TGCTTTCAAC CGTCCCTCGG CATCATCAGC   
  
  
+ CCTGCCCTTG CTCAGCTTTT CCCATCTTTT TTTGGCTTTT TGTTTGACTC CCTCTATAAC TCTATTCACG   
  
  
+ TCGAGCCTTG TAAAAATTTT ACATATTTTA TTTTATTTTA CTTTTTTAGG TTTATCCCTC TCTATTCTTG   
  
  
+ ACCTACGGGC ATTTTTTATT TTCTTTGGGT TTGAGACTTG TGAACTTTTT GCACATTTTC TCACAAAAAT   
  
  
+ TTATCTTGTC TTTTTCACTT TCAACGTTCC TTCTCTCTCT CTCTCTCTCT CTCTCTCTCT CTCACTCACA   
  
  
+ TCCTAGACAG ACGTCCTTTT TAAAGAATTT ATTTAACAAG TTAGAAGAAA CCGTTGTTTA AGTAGAACCA   
  
  
+ CCCTGTTTGG ATGCCAAAGG AAATACCAGT CAAGAAAAAT CACGACAATC ACAACACATC CATTTCGTTT   
  
  
+ ACATCTCATC CTCATCCTCA TCTTCCTCAT CTCTACCCAT CTCCCTCTTT CTGTATGGCT GCTTCTTCTA   
  
  
+ CTTTGTTCCC TGACCCTAAC AATACTAGTT TAGTCCTCCC TTATTATTAT TATCCTCCTC CTTCCTCCTC   
  
  
+ TTACTATAAT GGTAACCCTT ACCTTCCTCT TCCTAACACC ACTGCTATTC CTACTCCTTC AACAACCTCT   
  
  
+ CCGGTATTTT CTTCCGCCAC CTACTCGGAG TTGAATACTG ATTGTCAAAC TCAACTGCCC ATGTCTAACC   
  
  
+ TCCCCGGAAA GATCGTTCGG AAGCGGAGTG CTGCTGAAAT GGAGCAGCGC TCCGCTCCGA TTGCGGGTGA   
  
  
+ TTACCCGCCA ACTCATCAGC GGGTAATCAC CCGCCACAAT GAGGCGGCCT CATCAGTGTC TTTCATTGAC   
  
  
+ TCCTCACTCT CGCCACTTCA CCTCTTCAAC GGCTCCAATT CGACGACCCC ATTAGCCCCC GACCGTCCTA   
  
  
+ ATGATCCGGG TCTGAGTCAG GGTCCGGATC TGCCTCTTGT GTGCGGATTC TCCGGACTCC CTTTATTCCC   
  
  
+ ACCGGTGGAG AGAAGACCTT CCATCCCCGC CACGACCACT GCTGCCGCGG CGATCGCGGT GGTTCCTGTG   
  
  
+ GAGGAGGATG ATCCGACGTG GATGGATAGC ATCATAAAAG AGCTGATTCA AAGCTCAAAC TCGATCTCAA   
  
  
+ TCCCACAGTT GATTCAGAAC GTTAGAGATA TCATCTACCC ATGTAATCCT AATCTGGGGT CCGCCATTGA   
  
  
+ GTTCCGCCTC CGCTCGCTCG CCGCCGACCC CCTCATTGCT CCGCCGCCAC TTCCGCCCTT CCACCACCAC   
  
  
+ CAACACCACC TCAATCAACA AATCACTCTT CCTCGAATCA GCAACACTAA CAACAACAAT CATGTGAGCG   
  
  
+ TGTACGCCAA CAAAGGACCC GGATACTTCA ACTTGGGTCC GGGTCAGGGT CAGGGTCCGA TTAACATCGA   
  
  
+ CCAAGCTAAC CTTTCCTTCC CTCCTGATTC CACCGCCTGT TGGGGTGTCA GTGTCACACC GCCACCGTCT   
  
  
+ TCCGCCGCCG GTTCCGGCTC CGGTGGAAGC GGCAGTAGTA GTGGTAAATC AAACCCTAAC CCTAATCCCA   
  
  
+ ACCCGAACCC AAACCCAAAC CCAAACCCGA ACAACAAAGC TCAGGATGTT CAACTCCAAA CTCCACAACA   
  
  
+ ACAGCAACAG CAACAGCAAC AAATCCCCGT GGATCAGGAG CAAGACCCGG CGGCTCCACC GGCGACGGAA   
  
  
+ GCATCGCCAC CATCTCCAAG AGCGGCGGCA CCACCGCCGG CGGCAGTGAA AGCAAGAGAA AGAGAGGAGA   
  
  
+ TGCGGCAAAG GAAGCGCGAC GAAGAGGGTC TCCACCTCCT AACCCTCCTC CTCCAATGCG CAGAAGCAGT   
  
  
+ ATCCGGCGAC AAATACGAAG AAGCGAACAA GATGCTTCTA GAAATCTCGG AGTGGGCCAC CCCATTCGGC   
  
  
+ ACCTCCGCCC AACGCGTCGC CGCGTACTTC TCGGAAGCCA TGTCAGCCCG TCTCGTATCC TCCTGCCTCG   
  
  
+ GCATCTACGC CGCCCTCCCC ACCGTCCCAC ACTACGTCAA GCTCCTCTCC GCCTTCCAAG TCTTCAATGG   
  
  
+ CATCAGCCCA TTCGTCAAAT TCTCTCACTT CACTGCAAAC CAAGCAATCC AAGAGGCCTT CCAGAGGGAA   
  
  
+ GACAGGGTCC ACATCATCGA CCTCGATATC ATGCAGGGGC TCCAGTGGCC CGGGCTGTTC CACATCCTCG   
  
  
+ CGTCCCGGCC AGGTGGGCCT CCCTTCGTAA GGCTCACCGG GCTCGGGACC TCCATGGAGG CGCTCGAGGC   
  
  
+ CACCGGAAAA AGGCTCTCAG ACTTCGCCGA GAAGTTGGGG TTGCCCTTTG AGTTTATACC CGTGGCGGAG   
  
  
+ AAGATTGGAA ATTTGGACTT GGAAAGGTTG CATGTTAGTA AAAGGGAAGC TCTTGCTGTG CATTGGTTAC   
  
  
+ AGCACTCTTT GTATGATGTT ACTGGCTCTG ATACTAATAC ACTTGGCCTT CTTCAAAGGT TGGCGCCAAA   
  
  
+ AGTGGTGACG GTGGTGGAGC AAGACCTAAG CCGAACAGGC TCTTTCCTAG GAAGGTTTGT AGAGGCGATC   
  
  
+ CACTACTATT CAGCCCTATT TGACTCCTTA GGAGCGAGTT ATGGAGAGGA CAGTGAGGAG AGGCATGTGG   
  
  
+ TTGAGCAACA GCTCCTTTCT AGGGAGATTC GAAACATTCT GGCCGTTGGT GGGCCCTCAA GGACCGGGGA   
  
  
+ GCCCAAGTTT GCGAGCTGGA GGGAGAAGCT ACAACAGTCC GGCTTTAGGG GAATCTCATT GGCAGGCAAC   
  
  
+ GCCGCTGCCC AGGCCACCTT GCTCCTCGGC ATGTTCCCTT CTGATGGGTA TACTTTAATC GAGGACAGTG   
  
  
+ GCACACTTAA GCTCGGGTGG AAGGACTTGT GCCTCCTGAC TGCTTCGGCC TGGAGGCCTT CCCATGCTCA   
  
  
+ TACTATGAGC ACCGTCTGTA CTCGGAGCCA ATA  

- -Up\_Stream \_Len000CAGAGA CATTGTGTTA AGTAATTTAG GTAGGTTTCC CCTAGTATTT ATTGGTTTGA   
  
  
- AACAACCTTT GACGAAACTA CACCCGAAAG AGAAAGTCGT AAATACAATA TGTATATATA CCAATATTTC   
  
  
- AATGTTTTCA TACTGGGCTG TGCTGTTAAG TTAGGCTTAG GCTGAGCTTC AATGACTTTC ATTAGACTTA   
  
  
- TGCTAAACTG TCGAATCAAA TTTTTGGATA ATGAAGAGTT AAAACTCATT TAGATTCGAT AAAAATTACG   
  
  
- ACTTATCTAG CTGGGCTAAG CTGGGCAAAA GGCTGAGATA TACCAATACC ACGGGGATTT TTTTTAATTT   
  
  
- TTTCTTTTTC TTATCTTTTT ATATTTTATT TTGACTCTAA AGACTTGTGA GAGAGGGGAT CAGAACACCC   
  
  
- GTACCTTTCA CCAACCTCTA CCTCTACAAA CTCAAAGTAA AAGACTATAA TAAAAAATCA GTAATCTTCA   
  
  
- TGTTTCAGAT TTGAGTTGTT CTCGTACTCC CCTCTTACCA ACAACATCGG TTACCTTTTA TTTTTCTTGT   
  
  
- AAGTGAGAAG AACACGAAAT AAATACCACT ATTAAACAAT GAACATAAAA ATTTTTACTA ATTTATATAA   
  
  
- CTAGGAAACT ATAATACAAC TTTATTATTT TTATATTTTT AATCTTTTAT AAAATGGAGC ACTTTAAAAA   
  
  
- AAAGACTAAT TAGTAACTTT AATACCACTT TTTATTTCCT TGTAGAGAAC ATTTGTGAGA TAGGCAAATG   
  
  
- CAAACTGGTG TTTGAACGAT AGGTGTTCGT AGAGTTGGAA ACGGAAATTG GGTTAACACC CAACTACTTT   
  
  
- AGATTGGTAA TATCCTAATC TACCATCAGC AATTAGGACA CTTTGTTCAA AAACCAATAC TACATAGGTT   
  
  
- AATACTCCCG GACTATTGAG AACGGAGCCG TAAACGCACT ATTACTGCAC CATGATTATC GTTAATAAAA   
  
  
- AGCGTTTATA AGCTTTAAAA AATTCTTATT TGCGTTTTAA TAACTAGAAC TGTTTACTAA AATAAAAAAA   
  
  
- CAACCCTTTT CCTATTTGTA GATCACAGTT AACAACTACT GGACTATCGA GAGCTAAAGA ATAAATACTC   
  
  
- AATACAACTA TACTGTGATT AACATTAATA AACAACATTT GAATTTGTAA AAATTTTTAG TTTCGTTTTA   
  
  
- AACAATTAAT GTTATTTAGT TAGATGAAAA TGATACTAGA ATAAACCACA TTAATAACTA GAACTTAAAA   
  
  
- AAAAAAAGAA AAACTTTTTC TATTCGTAGC TTAAAAAAGT TTTTATTTTT CCCACTTCCT TCTTTTTTAC   
  
  
- CTTTGTCAAT GGCTCATTCC TTCCTGTCTC GCGCTCTCCT GTTCTGTGTT TCTCTCCCCT CTCTCTCTCT   
  
  
- CTTCCCCCGT TACCGTCACC CCTTCCAGCT CCTCGTGGAA AGTTGAAGTT CCGAGTTGTG GGGGGGGGGG   
  
  
- GGGGAGAAAG GAGTAAAACG CTGCAATCGT AAACTGTAAA ACGAAAGTTG GCAGGGAGCC GTAGTAGTCG   
  
  
- GGACGGGAAC GAGTCGAAAA GGGTAGAAAA AAACCGAAAA ACAAACTGAG GGAGATATTG AGATAAGTGC   
  
  
- AGCTCGGAAC ATTTTTAAAA TGTATAAAAT AAAATAAAAT GAAAAAATCC AAATAGGGAG AGATAAGAAC   
  
  
- TGGATGCCCG TAAAAAATAA AAGAAACCCA AACTCTGAAC ACTTGAAAAA CGTGTAAAAG AGTGTTTTTA   
  
  
- AATAGAACAG AAAAAGTGAA AGTTGCAAGG AAGAGAGAGA GAGAGAGAGA GAGAGAGAGA GAGTGAGTGT   
  
  
- AGGATCTGTC TGCAGGAAAA ATTTCTTAAA TAAATTGTTC AATCTTCTTT GGCAACAAAT TCATCTTGGT   
  
  
- GGGACAAACC TACGGTTTCC TTTATGGTCA GTTCTTTTTA GTGCTGTTAG TGTTGTGTAG GTAAAGCAAA   
  
  
- TGTAGAGTAG GAGTAGGAGT AGAAGGAGTA GAGATGGGTA GAGGGAGAAA GACATACCGA CGAAGAAGAT   
  
  
- GAAACAAGGG ACTGGGATTG TTATGATCAA ATCAGGAGGG AATAATAATA ATAGGAGGAG GAAGGAGGAG   
  
  
- AATGATATTA CCATTGGGAA TGGAAGGAGA AGGATTGTGG TGACGATAAG GATGAGGAAG TTGTTGGAGA   
  
  
- GGCCATAAAA GAAGGCGGTG GATGAGCCTC AACTTATGAC TAACAGTTTG AGTTGACGGG TACAGATTGG   
  
  
- AGGGGCCTTT CTAGCAAGCC TTCGCCTCAC GACGACTTTA CCTCGTCGCG AGGCGAGGCT AACGCCCACT   
  
  
- AATGGGCGGT TGAGTAGTCG CCCATTAGTG GGCGGTGTTA CTCCGCCGGA GTAGTCACAG AAAGTAACTG   
  
  
- AGGAGTGAGA GCGGTGAAGT GGAGAAGTTG CCGAGGTTAA GCTGCTGGGG TAATCGGGGG CTGGCAGGAT   
  
  
- TACTAGGCCC AGACTCAGTC CCAGGCCTAG ACGGAGAACA CACGCCTAAG AGGCCTGAGG GAAATAAGGG   
  
  
- TGGCCACCTC TCTTCTGGAA GGTAGGGGCG GTGCTGGTGA CGACGGCGCC GCTAGCGCCA CCAAGGACAC   
  
  
- CTCCTCCTAC TAGGCTGCAC CTACCTATCG TAGTATTTTC TCGACTAAGT TTCGAGTTTG AGCTAGAGTT   
  
  
- AGGGTGTCAA CTAAGTCTTG CAATCTCTAT AGTAGATGGG TACATTAGGA TTAGACCCCA GGCGGTAACT   
  
  
- CAAGGCGGAG GCGAGCGAGC GGCGGCTGGG GGAGTAACGA GGCGGCGGTG AAGGCGGGAA GGTGGTGGTG   
  
  
- GTTGTGGTGG AGTTAGTTGT TTAGTGAGAA GGAGCTTAGT CGTTGTGATT GTTGTTGTTA GTACACTCGC   
  
  
- ACATGCGGTT GTTTCCTGGG CCTATGAAGT TGAACCCAGG CCCAGTCCCA GTCCCAGGCT AATTGTAGCT   
  
  
- GGTTCGATTG GAAAGGAAGG GAGGACTAAG GTGGCGGACA ACCCCACAGT CACAGTGTGG CGGTGGCAGA   
  
  
- AGGCGGCGGC CAAGGCCGAG GCCACCTTCG CCGTCATCAT CACCATTTAG TTTGGGATTG GGATTAGGGT   
  
  
- TGGGCTTGGG TTTGGGTTTG GGTTTGGGCT TGTTGTTTCG AGTCCTACAA GTTGAGGTTT GAGGTGTTGT   
  
  
- TGTCGTTGTC GTTGTCGTTG TTTAGGGGCA CCTAGTCCTC GTTCTGGGCC GCCGAGGTGG CCGCTGCCTT   
  
  
- CGTAGCGGTG GTAGAGGTTC TCGCCGCCGT GGTGGCGGCC GCCGTCACTT TCGTTCTCTT TCTCTCCTCT   
  
  
- ACGCCGTTTC CTTCGCGCTG CTTCTCCCAG AGGTGGAGGA TTGGGAGGAG GAGGTTACGC GTCTTCGTCA   
  
  
- TAGGCCGCTG TTTATGCTTC TTCGCTTGTT CTACGAAGAT CTTTAGAGCC TCACCCGGTG GGGTAAGCCG   
  
  
- TGGAGGCGGG TTGCGCAGCG GCGCATGAAG AGCCTTCGGT ACAGTCGGGC AGAGCATAGG AGGACGGAGC   
  
  
- CGTAGATGCG GCGGGAGGGG TGGCAGGGTG TGATGCAGTT CGAGGAGAGG CGGAAGGTTC AGAAGTTACC   
  
  
- GTAGTCGGGT AAGCAGTTTA AGAGAGTGAA GTGACGTTTG GTTCGTTAGG TTCTCCGGAA GGTCTCCCTT   
  
  
- CTGTCCCAGG TGTAGTAGCT GGAGCTATAG TACGTCCCCG AGGTCACCGG GCCCGACAAG GTGTAGGAGC   
  
  
- GCAGGGCCGG TCCACCCGGA GGGAAGCATT CCGAGTGGCC CGAGCCCTGG AGGTACCTCC GCGAGCTCCG   
  
  
- GTGGCCTTTT TCCGAGAGTC TGAAGCGGCT CTTCAACCCC AACGGGAAAC TCAAATATGG GCACCGCCTC   
  
  
- TTCTAACCTT TAAACCTGAA CCTTTCCAAC GTACAATCAT TTTCCCTTCG AGAACGACAC GTAACCAATG   
  
  
- TCGTGAGAAA CATACTACAA TGACCGAGAC TATGATTATG TGAACCGGAA GAAGTTTCCA ACCGCGGTTT   
  
  
- TCACCACTGC CACCACCTCG TTCTGGATTC GGCTTGTCCG AGAAAGGATC CTTCCAAACA TCTCCGCTAG   
  
  
- GTGATGATAA GTCGGGATAA ACTGAGGAAT CCTCGCTCAA TACCTCTCCT GTCACTCCTC TCCGTACACC   
  
  
- AACTCGTTGT CGAGGAAAGA TCCCTCTAAG CTTTGTAAGA CCGGCAACCA CCCGGGAGTT CCTGGCCCCT   
  
  
- CGGGTTCAAA CGCTCGACCT CCCTCTTCGA TGTTGTCAGG CCGAAATCCC CTTAGAGTAA CCGTCCGTTG   
  
  
- CGGCGACGGG TCCGGTGGAA CGAGGAGCCG TACAAGGGAA GACTACCCAT ATGAAATTAG CTCCTGTCAC   
  
  
- CGTGTGAATT CGAGCCCACC TTCCTGAACA CGGAGGACTG ACGAAGCCGG ACCTCCGGAA GGGTACGAGT   
  
  
- ATGATACTCG TGGCAGACAT GAGCCTCGGT TAT

+     MYB-like sequence

| Site Name | Organism | Position | Strand | Matrix score. | sequence | function |
| --- | --- | --- | --- | --- | --- | --- |
| MYB-like sequence | Arabidopsis thaliana | 65 | + | 6 | TAACCA |  |
| MYB-like sequence | Arabidopsis thaliana | 897 | - | 6 | TAACCA |  |
| MYB-like sequence | Arabidopsis thaliana | 326 | - | 6 | TAACCA |  |
| MYB-like sequence | Arabidopsis thaliana | 3918 | - | 6 | TAACCA |  |
| MYB-like sequence | Arabidopsis thaliana | 134 | - | 6 | TAACCA |  |
| MYB-like sequence | Arabidopsis thaliana | 847 | + | 6 | TAACCA |  |

>HU01G00472.1   
+ -Up\_Stream \_Len000GTCTCT GTAACACAAT TCATTAAATC CATCCAAAGG GGATCATAAA TAACCAAACT   
  
  
+ TTGTTGGAAA CTGCTTTGAT GTGGGCTTTC TCTTTCAGCA TTTATGTTAT ACATATATAT GGTTATAAAG   
  
  
+ TTACAAAAGT ATGACCCGAC ACGACAATTC AATCCGAATC CGACTCGAAG TTACTGAAAG TAATCTGAAT   
  
  
+ ACGATTTGAC AGCTTAGTTT AAAAACCTAT TACTTCTCAA TTTTGAGTAA ATCTAAGCTA TTTTTAATGC   
  
  
+ TGAATAGATC GACCCGATTC GACCCGTTTT CCGACTCTAT ATGGTTATGG TGCCCCTAAA AAAAATTAAA   
  
  
+ AAAGAAAAAG AATAGAAAAA TATAAAATAA AACTGAGATT TCTGAACACT CTCTCCCCTA GTCTTGTGGG   
  
  
+ CATGGAAAGT GGTTGGAGAT GGAGATGTTT GAGTTTCATT TTCTGATATT ATTTTTTAGT CATTAGAAGT   
  
  
+ ACAAAGTCTA AACTCAACAA GAGCATGAGG GGAGAATGGT TGTTGTAGCC AATGGAAAAT AAAAAGAACA   
  
  
+ TTCACTCTTC TTGTGCTTTA TTTATGGTGA TAATTTGTTA CTTGTATTTT TAAAAATGAT TAAATATATT   
  
  
+ GATCCTTTGA TATTATGTTG AAATAATAAA AATATAAAAA TTAGAAAATA TTTTACCTCG TGAAATTTTT   
  
  
+ TTTCTGATTA ATCATTGAAA TTATGGTGAA AAATAAAGGA ACATCTCTTG TAAACACTCT ATCCGTTTAC   
  
  
+ GTTTGACCAC AAACTTGCTA TCCACAAGCA TCTCAACCTT TGCCTTTAAC CCAATTGTGG GTTGATGAAA   
  
  
+ TCTAACCATT ATAGGATTAG ATGGTAGTCG TTAATCCTGT GAAACAAGTT TTTGGTTATG ATGTATCCAA   
  
  
+ TTATGAGGGC CTGATAACTC TTGCCTCGGC ATTTGCGTGA TAATGACGTG GTACTAATAG CAATTATTTT   
  
  
+ TCGCAAATAT TCGAAATTTT TTAAGAATAA ACGCAAAATT ATTGATCTTG ACAAATGATT TTATTTTTTT   
  
  
+ GTTGGGAAAA GGATAAACAT CTAGTGTCAA TTGTTGATGA CCTGATAGCT CTCGATTTCT TATTTATGAG   
  
  
+ TTATGTTGAT ATGACACTAA TTGTAATTAT TTGTTGTAAA CTTAAACATT TTTAAAAATC AAAGCAAAAT   
  
  
+ TTGTTAATTA CAATAAATCA ATCTACTTTT ACTATGATCT TATTTGGTGT AATTATTGAT CTTGAATTTT   
  
  
+ TTTTTTTCTT TTTGAAAAAG ATAAGCATCG AATTTTTTCA AAAATAAAAA GGGTGAAGGA AGAAAAAATG   
  
  
+ GAAACAGTTA CCGAGTAAGG AAGGACAGAG CGCGAGAGGA CAAGACACAA AGAGAGGGGA GAGAGAGAGA   
  
  
+ GAAGGGGGCA ATGGCAGTGG GGAAGGTCGA GGAGCACCTT TCAACTTCAA GGCTCAACAC CCCCCCCCCC   
  
  
+ CCCCTCTTTC CTCATTTTGC GACGTTAGCA TTTGACATTT TGCTTTCAAC CGTCCCTCGG CATCATCAGC   
  
  
+ CCTGCCCTTG CTCAGCTTTT CCCATCTTTT TTTGGCTTTT TGTTTGACTC CCTCTATAAC TCTATTCACG   
  
  
+ TCGAGCCTTG TAAAAATTTT ACATATTTTA TTTTATTTTA CTTTTTTAGG TTTATCCCTC TCTATTCTTG   
  
  
+ ACCTACGGGC ATTTTTTATT TTCTTTGGGT TTGAGACTTG TGAACTTTTT GCACATTTTC TCACAAAAAT   
  
  
+ TTATCTTGTC TTTTTCACTT TCAACGTTCC TTCTCTCTCT CTCTCTCTCT CTCTCTCTCT CTCACTCACA   
  
  
+ TCCTAGACAG ACGTCCTTTT TAAAGAATTT ATTTAACAAG TTAGAAGAAA CCGTTGTTTA AGTAGAACCA   
  
  
+ CCCTGTTTGG ATGCCAAAGG AAATACCAGT CAAGAAAAAT CACGACAATC ACAACACATC CATTTCGTTT   
  
  
+ ACATCTCATC CTCATCCTCA TCTTCCTCAT CTCTACCCAT CTCCCTCTTT CTGTATGGCT GCTTCTTCTA   
  
  
+ CTTTGTTCCC TGACCCTAAC AATACTAGTT TAGTCCTCCC TTATTATTAT TATCCTCCTC CTTCCTCCTC   
  
  
+ TTACTATAAT GGTAACCCTT ACCTTCCTCT TCCTAACACC ACTGCTATTC CTACTCCTTC AACAACCTCT   
  
  
+ CCGGTATTTT CTTCCGCCAC CTACTCGGAG TTGAATACTG ATTGTCAAAC TCAACTGCCC ATGTCTAACC   
  
  
+ TCCCCGGAAA GATCGTTCGG AAGCGGAGTG CTGCTGAAAT GGAGCAGCGC TCCGCTCCGA TTGCGGGTGA   
  
  
+ TTACCCGCCA ACTCATCAGC GGGTAATCAC CCGCCACAAT GAGGCGGCCT CATCAGTGTC TTTCATTGAC   
  
  
+ TCCTCACTCT CGCCACTTCA CCTCTTCAAC GGCTCCAATT CGACGACCCC ATTAGCCCCC GACCGTCCTA   
  
  
+ ATGATCCGGG TCTGAGTCAG GGTCCGGATC TGCCTCTTGT GTGCGGATTC TCCGGACTCC CTTTATTCCC   
  
  
+ ACCGGTGGAG AGAAGACCTT CCATCCCCGC CACGACCACT GCTGCCGCGG CGATCGCGGT GGTTCCTGTG   
  
  
+ GAGGAGGATG ATCCGACGTG GATGGATAGC ATCATAAAAG AGCTGATTCA AAGCTCAAAC TCGATCTCAA   
  
  
+ TCCCACAGTT GATTCAGAAC GTTAGAGATA TCATCTACCC ATGTAATCCT AATCTGGGGT CCGCCATTGA   
  
  
+ GTTCCGCCTC CGCTCGCTCG CCGCCGACCC CCTCATTGCT CCGCCGCCAC TTCCGCCCTT CCACCACCAC   
  
  
+ CAACACCACC TCAATCAACA AATCACTCTT CCTCGAATCA GCAACACTAA CAACAACAAT CATGTGAGCG   
  
  
+ TGTACGCCAA CAAAGGACCC GGATACTTCA ACTTGGGTCC GGGTCAGGGT CAGGGTCCGA TTAACATCGA   
  
  
+ CCAAGCTAAC CTTTCCTTCC CTCCTGATTC CACCGCCTGT TGGGGTGTCA GTGTCACACC GCCACCGTCT   
  
  
+ TCCGCCGCCG GTTCCGGCTC CGGTGGAAGC GGCAGTAGTA GTGGTAAATC AAACCCTAAC CCTAATCCCA   
  
  
+ ACCCGAACCC AAACCCAAAC CCAAACCCGA ACAACAAAGC TCAGGATGTT CAACTCCAAA CTCCACAACA   
  
  
+ ACAGCAACAG CAACAGCAAC AAATCCCCGT GGATCAGGAG CAAGACCCGG CGGCTCCACC GGCGACGGAA   
  
  
+ GCATCGCCAC CATCTCCAAG AGCGGCGGCA CCACCGCCGG CGGCAGTGAA AGCAAGAGAA AGAGAGGAGA   
  
  
+ TGCGGCAAAG GAAGCGCGAC GAAGAGGGTC TCCACCTCCT AACCCTCCTC CTCCAATGCG CAGAAGCAGT   
  
  
+ ATCCGGCGAC AAATACGAAG AAGCGAACAA GATGCTTCTA GAAATCTCGG AGTGGGCCAC CCCATTCGGC   
  
  
+ ACCTCCGCCC AACGCGTCGC CGCGTACTTC TCGGAAGCCA TGTCAGCCCG TCTCGTATCC TCCTGCCTCG   
  
  
+ GCATCTACGC CGCCCTCCCC ACCGTCCCAC ACTACGTCAA GCTCCTCTCC GCCTTCCAAG TCTTCAATGG   
  
  
+ CATCAGCCCA TTCGTCAAAT TCTCTCACTT CACTGCAAAC CAAGCAATCC AAGAGGCCTT CCAGAGGGAA   
  
  
+ GACAGGGTCC ACATCATCGA CCTCGATATC ATGCAGGGGC TCCAGTGGCC CGGGCTGTTC CACATCCTCG   
  
  
+ CGTCCCGGCC AGGTGGGCCT CCCTTCGTAA GGCTCACCGG GCTCGGGACC TCCATGGAGG CGCTCGAGGC   
  
  
+ CACCGGAAAA AGGCTCTCAG ACTTCGCCGA GAAGTTGGGG TTGCCCTTTG AGTTTATACC CGTGGCGGAG   
  
  
+ AAGATTGGAA ATTTGGACTT GGAAAGGTTG CATGTTAGTA AAAGGGAAGC TCTTGCTGTG CATTGGTTAC   
  
  
+ AGCACTCTTT GTATGATGTT ACTGGCTCTG ATACTAATAC ACTTGGCCTT CTTCAAAGGT TGGCGCCAAA   
  
  
+ AGTGGTGACG GTGGTGGAGC AAGACCTAAG CCGAACAGGC TCTTTCCTAG GAAGGTTTGT AGAGGCGATC   
  
  
+ CACTACTATT CAGCCCTATT TGACTCCTTA GGAGCGAGTT ATGGAGAGGA CAGTGAGGAG AGGCATGTGG   
  
  
+ TTGAGCAACA GCTCCTTTCT AGGGAGATTC GAAACATTCT GGCCGTTGGT GGGCCCTCAA GGACCGGGGA   
  
  
+ GCCCAAGTTT GCGAGCTGGA GGGAGAAGCT ACAACAGTCC GGCTTTAGGG GAATCTCATT GGCAGGCAAC   
  
  
+ GCCGCTGCCC AGGCCACCTT GCTCCTCGGC ATGTTCCCTT CTGATGGGTA TACTTTAATC GAGGACAGTG   
  
  
+ GCACACTTAA GCTCGGGTGG AAGGACTTGT GCCTCCTGAC TGCTTCGGCC TGGAGGCCTT CCCATGCTCA   
  
  
+ TACTATGAGC ACCGTCTGTA CTCGGAGCCA ATA  

- -Up\_Stream \_Len000CAGAGA CATTGTGTTA AGTAATTTAG GTAGGTTTCC CCTAGTATTT ATTGGTTTGA   
  
  
- AACAACCTTT GACGAAACTA CACCCGAAAG AGAAAGTCGT AAATACAATA TGTATATATA CCAATATTTC   
  
  
- AATGTTTTCA TACTGGGCTG TGCTGTTAAG TTAGGCTTAG GCTGAGCTTC AATGACTTTC ATTAGACTTA   
  
  
- TGCTAAACTG TCGAATCAAA TTTTTGGATA ATGAAGAGTT AAAACTCATT TAGATTCGAT AAAAATTACG   
  
  
- ACTTATCTAG CTGGGCTAAG CTGGGCAAAA GGCTGAGATA TACCAATACC ACGGGGATTT TTTTTAATTT   
  
  
- TTTCTTTTTC TTATCTTTTT ATATTTTATT TTGACTCTAA AGACTTGTGA GAGAGGGGAT CAGAACACCC   
  
  
- GTACCTTTCA CCAACCTCTA CCTCTACAAA CTCAAAGTAA AAGACTATAA TAAAAAATCA GTAATCTTCA   
  
  
- TGTTTCAGAT TTGAGTTGTT CTCGTACTCC CCTCTTACCA ACAACATCGG TTACCTTTTA TTTTTCTTGT   
  
  
- AAGTGAGAAG AACACGAAAT AAATACCACT ATTAAACAAT GAACATAAAA ATTTTTACTA ATTTATATAA   
  
  
- CTAGGAAACT ATAATACAAC TTTATTATTT TTATATTTTT AATCTTTTAT AAAATGGAGC ACTTTAAAAA   
  
  
- AAAGACTAAT TAGTAACTTT AATACCACTT TTTATTTCCT TGTAGAGAAC ATTTGTGAGA TAGGCAAATG   
  
  
- CAAACTGGTG TTTGAACGAT AGGTGTTCGT AGAGTTGGAA ACGGAAATTG GGTTAACACC CAACTACTTT   
  
  
- AGATTGGTAA TATCCTAATC TACCATCAGC AATTAGGACA CTTTGTTCAA AAACCAATAC TACATAGGTT   
  
  
- AATACTCCCG GACTATTGAG AACGGAGCCG TAAACGCACT ATTACTGCAC CATGATTATC GTTAATAAAA   
  
  
- AGCGTTTATA AGCTTTAAAA AATTCTTATT TGCGTTTTAA TAACTAGAAC TGTTTACTAA AATAAAAAAA   
  
  
- CAACCCTTTT CCTATTTGTA GATCACAGTT AACAACTACT GGACTATCGA GAGCTAAAGA ATAAATACTC   
  
  
- AATACAACTA TACTGTGATT AACATTAATA AACAACATTT GAATTTGTAA AAATTTTTAG TTTCGTTTTA   
  
  
- AACAATTAAT GTTATTTAGT TAGATGAAAA TGATACTAGA ATAAACCACA TTAATAACTA GAACTTAAAA   
  
  
- AAAAAAAGAA AAACTTTTTC TATTCGTAGC TTAAAAAAGT TTTTATTTTT CCCACTTCCT TCTTTTTTAC   
  
  
- CTTTGTCAAT GGCTCATTCC TTCCTGTCTC GCGCTCTCCT GTTCTGTGTT TCTCTCCCCT CTCTCTCTCT   
  
  
- CTTCCCCCGT TACCGTCACC CCTTCCAGCT CCTCGTGGAA AGTTGAAGTT CCGAGTTGTG GGGGGGGGGG   
  
  
- GGGGAGAAAG GAGTAAAACG CTGCAATCGT AAACTGTAAA ACGAAAGTTG GCAGGGAGCC GTAGTAGTCG   
  
  
- GGACGGGAAC GAGTCGAAAA GGGTAGAAAA AAACCGAAAA ACAAACTGAG GGAGATATTG AGATAAGTGC   
  
  
- AGCTCGGAAC ATTTTTAAAA TGTATAAAAT AAAATAAAAT GAAAAAATCC AAATAGGGAG AGATAAGAAC   
  
  
- TGGATGCCCG TAAAAAATAA AAGAAACCCA AACTCTGAAC ACTTGAAAAA CGTGTAAAAG AGTGTTTTTA   
  
  
- AATAGAACAG AAAAAGTGAA AGTTGCAAGG AAGAGAGAGA GAGAGAGAGA GAGAGAGAGA GAGTGAGTGT   
  
  
- AGGATCTGTC TGCAGGAAAA ATTTCTTAAA TAAATTGTTC AATCTTCTTT GGCAACAAAT TCATCTTGGT   
  
  
- GGGACAAACC TACGGTTTCC TTTATGGTCA GTTCTTTTTA GTGCTGTTAG TGTTGTGTAG GTAAAGCAAA   
  
  
- TGTAGAGTAG GAGTAGGAGT AGAAGGAGTA GAGATGGGTA GAGGGAGAAA GACATACCGA CGAAGAAGAT   
  
  
- GAAACAAGGG ACTGGGATTG TTATGATCAA ATCAGGAGGG AATAATAATA ATAGGAGGAG GAAGGAGGAG   
  
  
- AATGATATTA CCATTGGGAA TGGAAGGAGA AGGATTGTGG TGACGATAAG GATGAGGAAG TTGTTGGAGA   
  
  
- GGCCATAAAA GAAGGCGGTG GATGAGCCTC AACTTATGAC TAACAGTTTG AGTTGACGGG TACAGATTGG   
  
  
- AGGGGCCTTT CTAGCAAGCC TTCGCCTCAC GACGACTTTA CCTCGTCGCG AGGCGAGGCT AACGCCCACT   
  
  
- AATGGGCGGT TGAGTAGTCG CCCATTAGTG GGCGGTGTTA CTCCGCCGGA GTAGTCACAG AAAGTAACTG   
  
  
- AGGAGTGAGA GCGGTGAAGT GGAGAAGTTG CCGAGGTTAA GCTGCTGGGG TAATCGGGGG CTGGCAGGAT   
  
  
- TACTAGGCCC AGACTCAGTC CCAGGCCTAG ACGGAGAACA CACGCCTAAG AGGCCTGAGG GAAATAAGGG   
  
  
- TGGCCACCTC TCTTCTGGAA GGTAGGGGCG GTGCTGGTGA CGACGGCGCC GCTAGCGCCA CCAAGGACAC   
  
  
- CTCCTCCTAC TAGGCTGCAC CTACCTATCG TAGTATTTTC TCGACTAAGT TTCGAGTTTG AGCTAGAGTT   
  
  
- AGGGTGTCAA CTAAGTCTTG CAATCTCTAT AGTAGATGGG TACATTAGGA TTAGACCCCA GGCGGTAACT   
  
  
- CAAGGCGGAG GCGAGCGAGC GGCGGCTGGG GGAGTAACGA GGCGGCGGTG AAGGCGGGAA GGTGGTGGTG   
  
  
- GTTGTGGTGG AGTTAGTTGT TTAGTGAGAA GGAGCTTAGT CGTTGTGATT GTTGTTGTTA GTACACTCGC   
  
  
- ACATGCGGTT GTTTCCTGGG CCTATGAAGT TGAACCCAGG CCCAGTCCCA GTCCCAGGCT AATTGTAGCT   
  
  
- GGTTCGATTG GAAAGGAAGG GAGGACTAAG GTGGCGGACA ACCCCACAGT CACAGTGTGG CGGTGGCAGA   
  
  
- AGGCGGCGGC CAAGGCCGAG GCCACCTTCG CCGTCATCAT CACCATTTAG TTTGGGATTG GGATTAGGGT   
  
  
- TGGGCTTGGG TTTGGGTTTG GGTTTGGGCT TGTTGTTTCG AGTCCTACAA GTTGAGGTTT GAGGTGTTGT   
  
  
- TGTCGTTGTC GTTGTCGTTG TTTAGGGGCA CCTAGTCCTC GTTCTGGGCC GCCGAGGTGG CCGCTGCCTT   
  
  
- CGTAGCGGTG GTAGAGGTTC TCGCCGCCGT GGTGGCGGCC GCCGTCACTT TCGTTCTCTT TCTCTCCTCT   
  
  
- ACGCCGTTTC CTTCGCGCTG CTTCTCCCAG AGGTGGAGGA TTGGGAGGAG GAGGTTACGC GTCTTCGTCA   
  
  
- TAGGCCGCTG TTTATGCTTC TTCGCTTGTT CTACGAAGAT CTTTAGAGCC TCACCCGGTG GGGTAAGCCG   
  
  
- TGGAGGCGGG TTGCGCAGCG GCGCATGAAG AGCCTTCGGT ACAGTCGGGC AGAGCATAGG AGGACGGAGC   
  
  
- CGTAGATGCG GCGGGAGGGG TGGCAGGGTG TGATGCAGTT CGAGGAGAGG CGGAAGGTTC AGAAGTTACC   
  
  
- GTAGTCGGGT AAGCAGTTTA AGAGAGTGAA GTGACGTTTG GTTCGTTAGG TTCTCCGGAA GGTCTCCCTT   
  
  
- CTGTCCCAGG TGTAGTAGCT GGAGCTATAG TACGTCCCCG AGGTCACCGG GCCCGACAAG GTGTAGGAGC   
  
  
- GCAGGGCCGG TCCACCCGGA GGGAAGCATT CCGAGTGGCC CGAGCCCTGG AGGTACCTCC GCGAGCTCCG   
  
  
- GTGGCCTTTT TCCGAGAGTC TGAAGCGGCT CTTCAACCCC AACGGGAAAC TCAAATATGG GCACCGCCTC   
  
  
- TTCTAACCTT TAAACCTGAA CCTTTCCAAC GTACAATCAT TTTCCCTTCG AGAACGACAC GTAACCAATG   
  
  
- TCGTGAGAAA CATACTACAA TGACCGAGAC TATGATTATG TGAACCGGAA GAAGTTTCCA ACCGCGGTTT   
  
  
- TCACCACTGC CACCACCTCG TTCTGGATTC GGCTTGTCCG AGAAAGGATC CTTCCAAACA TCTCCGCTAG   
  
  
- GTGATGATAA GTCGGGATAA ACTGAGGAAT CCTCGCTCAA TACCTCTCCT GTCACTCCTC TCCGTACACC   
  
  
- AACTCGTTGT CGAGGAAAGA TCCCTCTAAG CTTTGTAAGA CCGGCAACCA CCCGGGAGTT CCTGGCCCCT   
  
  
- CGGGTTCAAA CGCTCGACCT CCCTCTTCGA TGTTGTCAGG CCGAAATCCC CTTAGAGTAA CCGTCCGTTG   
  
  
- CGGCGACGGG TCCGGTGGAA CGAGGAGCCG TACAAGGGAA GACTACCCAT ATGAAATTAG CTCCTGTCAC   
  
  
- CGTGTGAATT CGAGCCCACC TTCCTGAACA CGGAGGACTG ACGAAGCCGG ACCTCCGGAA GGGTACGAGT   
  
  
- ATGATACTCG TGGCAGACAT GAGCCTCGGT TAT

+     MYC

| Site Name | Organism | Position | Strand | Matrix score. | sequence | function |
| --- | --- | --- | --- | --- | --- | --- |
| MYC | Arabidopsis thaliana | 826 | + | 6 | CAATTG |  |
| MYC | Arabidopsis thaliana | 1503 | + | 6 | CATTTG |  |
| MYC | Arabidopsis thaliana | 1036 | - | 6 | CATTTG |  |
| MYC | Arabidopsis thaliana | 4128 | + | 6 | CATGTG |  |
| MYC | Arabidopsis thaliana | 944 | + | 6 | CATTTG |  |
| MYC | Arabidopsis thaliana | 2865 | + | 6 | CATGTG |  |
| MYC | Arabidopsis thaliana | 1082 | + | 6 | CAATTG |  |

>HU01G00472.1   
+ -Up\_Stream \_Len000GTCTCT GTAACACAAT TCATTAAATC CATCCAAAGG GGATCATAAA TAACCAAACT   
  
  
+ TTGTTGGAAA CTGCTTTGAT GTGGGCTTTC TCTTTCAGCA TTTATGTTAT ACATATATAT GGTTATAAAG   
  
  
+ TTACAAAAGT ATGACCCGAC ACGACAATTC AATCCGAATC CGACTCGAAG TTACTGAAAG TAATCTGAAT   
  
  
+ ACGATTTGAC AGCTTAGTTT AAAAACCTAT TACTTCTCAA TTTTGAGTAA ATCTAAGCTA TTTTTAATGC   
  
  
+ TGAATAGATC GACCCGATTC GACCCGTTTT CCGACTCTAT ATGGTTATGG TGCCCCTAAA AAAAATTAAA   
  
  
+ AAAGAAAAAG AATAGAAAAA TATAAAATAA AACTGAGATT TCTGAACACT CTCTCCCCTA GTCTTGTGGG   
  
  
+ CATGGAAAGT GGTTGGAGAT GGAGATGTTT GAGTTTCATT TTCTGATATT ATTTTTTAGT CATTAGAAGT   
  
  
+ ACAAAGTCTA AACTCAACAA GAGCATGAGG GGAGAATGGT TGTTGTAGCC AATGGAAAAT AAAAAGAACA   
  
  
+ TTCACTCTTC TTGTGCTTTA TTTATGGTGA TAATTTGTTA CTTGTATTTT TAAAAATGAT TAAATATATT   
  
  
+ GATCCTTTGA TATTATGTTG AAATAATAAA AATATAAAAA TTAGAAAATA TTTTACCTCG TGAAATTTTT   
  
  
+ TTTCTGATTA ATCATTGAAA TTATGGTGAA AAATAAAGGA ACATCTCTTG TAAACACTCT ATCCGTTTAC   
  
  
+ GTTTGACCAC AAACTTGCTA TCCACAAGCA TCTCAACCTT TGCCTTTAAC CCAATTGTGG GTTGATGAAA   
  
  
+ TCTAACCATT ATAGGATTAG ATGGTAGTCG TTAATCCTGT GAAACAAGTT TTTGGTTATG ATGTATCCAA   
  
  
+ TTATGAGGGC CTGATAACTC TTGCCTCGGC ATTTGCGTGA TAATGACGTG GTACTAATAG CAATTATTTT   
  
  
+ TCGCAAATAT TCGAAATTTT TTAAGAATAA ACGCAAAATT ATTGATCTTG ACAAATGATT TTATTTTTTT   
  
  
+ GTTGGGAAAA GGATAAACAT CTAGTGTCAA TTGTTGATGA CCTGATAGCT CTCGATTTCT TATTTATGAG   
  
  
+ TTATGTTGAT ATGACACTAA TTGTAATTAT TTGTTGTAAA CTTAAACATT TTTAAAAATC AAAGCAAAAT   
  
  
+ TTGTTAATTA CAATAAATCA ATCTACTTTT ACTATGATCT TATTTGGTGT AATTATTGAT CTTGAATTTT   
  
  
+ TTTTTTTCTT TTTGAAAAAG ATAAGCATCG AATTTTTTCA AAAATAAAAA GGGTGAAGGA AGAAAAAATG   
  
  
+ GAAACAGTTA CCGAGTAAGG AAGGACAGAG CGCGAGAGGA CAAGACACAA AGAGAGGGGA GAGAGAGAGA   
  
  
+ GAAGGGGGCA ATGGCAGTGG GGAAGGTCGA GGAGCACCTT TCAACTTCAA GGCTCAACAC CCCCCCCCCC   
  
  
+ CCCCTCTTTC CTCATTTTGC GACGTTAGCA TTTGACATTT TGCTTTCAAC CGTCCCTCGG CATCATCAGC   
  
  
+ CCTGCCCTTG CTCAGCTTTT CCCATCTTTT TTTGGCTTTT TGTTTGACTC CCTCTATAAC TCTATTCACG   
  
  
+ TCGAGCCTTG TAAAAATTTT ACATATTTTA TTTTATTTTA CTTTTTTAGG TTTATCCCTC TCTATTCTTG   
  
  
+ ACCTACGGGC ATTTTTTATT TTCTTTGGGT TTGAGACTTG TGAACTTTTT GCACATTTTC TCACAAAAAT   
  
  
+ TTATCTTGTC TTTTTCACTT TCAACGTTCC TTCTCTCTCT CTCTCTCTCT CTCTCTCTCT CTCACTCACA   
  
  
+ TCCTAGACAG ACGTCCTTTT TAAAGAATTT ATTTAACAAG TTAGAAGAAA CCGTTGTTTA AGTAGAACCA   
  
  
+ CCCTGTTTGG ATGCCAAAGG AAATACCAGT CAAGAAAAAT CACGACAATC ACAACACATC CATTTCGTTT   
  
  
+ ACATCTCATC CTCATCCTCA TCTTCCTCAT CTCTACCCAT CTCCCTCTTT CTGTATGGCT GCTTCTTCTA   
  
  
+ CTTTGTTCCC TGACCCTAAC AATACTAGTT TAGTCCTCCC TTATTATTAT TATCCTCCTC CTTCCTCCTC   
  
  
+ TTACTATAAT GGTAACCCTT ACCTTCCTCT TCCTAACACC ACTGCTATTC CTACTCCTTC AACAACCTCT   
  
  
+ CCGGTATTTT CTTCCGCCAC CTACTCGGAG TTGAATACTG ATTGTCAAAC TCAACTGCCC ATGTCTAACC   
  
  
+ TCCCCGGAAA GATCGTTCGG AAGCGGAGTG CTGCTGAAAT GGAGCAGCGC TCCGCTCCGA TTGCGGGTGA   
  
  
+ TTACCCGCCA ACTCATCAGC GGGTAATCAC CCGCCACAAT GAGGCGGCCT CATCAGTGTC TTTCATTGAC   
  
  
+ TCCTCACTCT CGCCACTTCA CCTCTTCAAC GGCTCCAATT CGACGACCCC ATTAGCCCCC GACCGTCCTA   
  
  
+ ATGATCCGGG TCTGAGTCAG GGTCCGGATC TGCCTCTTGT GTGCGGATTC TCCGGACTCC CTTTATTCCC   
  
  
+ ACCGGTGGAG AGAAGACCTT CCATCCCCGC CACGACCACT GCTGCCGCGG CGATCGCGGT GGTTCCTGTG   
  
  
+ GAGGAGGATG ATCCGACGTG GATGGATAGC ATCATAAAAG AGCTGATTCA AAGCTCAAAC TCGATCTCAA   
  
  
+ TCCCACAGTT GATTCAGAAC GTTAGAGATA TCATCTACCC ATGTAATCCT AATCTGGGGT CCGCCATTGA   
  
  
+ GTTCCGCCTC CGCTCGCTCG CCGCCGACCC CCTCATTGCT CCGCCGCCAC TTCCGCCCTT CCACCACCAC   
  
  
+ CAACACCACC TCAATCAACA AATCACTCTT CCTCGAATCA GCAACACTAA CAACAACAAT CATGTGAGCG   
  
  
+ TGTACGCCAA CAAAGGACCC GGATACTTCA ACTTGGGTCC GGGTCAGGGT CAGGGTCCGA TTAACATCGA   
  
  
+ CCAAGCTAAC CTTTCCTTCC CTCCTGATTC CACCGCCTGT TGGGGTGTCA GTGTCACACC GCCACCGTCT   
  
  
+ TCCGCCGCCG GTTCCGGCTC CGGTGGAAGC GGCAGTAGTA GTGGTAAATC AAACCCTAAC CCTAATCCCA   
  
  
+ ACCCGAACCC AAACCCAAAC CCAAACCCGA ACAACAAAGC TCAGGATGTT CAACTCCAAA CTCCACAACA   
  
  
+ ACAGCAACAG CAACAGCAAC AAATCCCCGT GGATCAGGAG CAAGACCCGG CGGCTCCACC GGCGACGGAA   
  
  
+ GCATCGCCAC CATCTCCAAG AGCGGCGGCA CCACCGCCGG CGGCAGTGAA AGCAAGAGAA AGAGAGGAGA   
  
  
+ TGCGGCAAAG GAAGCGCGAC GAAGAGGGTC TCCACCTCCT AACCCTCCTC CTCCAATGCG CAGAAGCAGT   
  
  
+ ATCCGGCGAC AAATACGAAG AAGCGAACAA GATGCTTCTA GAAATCTCGG AGTGGGCCAC CCCATTCGGC   
  
  
+ ACCTCCGCCC AACGCGTCGC CGCGTACTTC TCGGAAGCCA TGTCAGCCCG TCTCGTATCC TCCTGCCTCG   
  
  
+ GCATCTACGC CGCCCTCCCC ACCGTCCCAC ACTACGTCAA GCTCCTCTCC GCCTTCCAAG TCTTCAATGG   
  
  
+ CATCAGCCCA TTCGTCAAAT TCTCTCACTT CACTGCAAAC CAAGCAATCC AAGAGGCCTT CCAGAGGGAA   
  
  
+ GACAGGGTCC ACATCATCGA CCTCGATATC ATGCAGGGGC TCCAGTGGCC CGGGCTGTTC CACATCCTCG   
  
  
+ CGTCCCGGCC AGGTGGGCCT CCCTTCGTAA GGCTCACCGG GCTCGGGACC TCCATGGAGG CGCTCGAGGC   
  
  
+ CACCGGAAAA AGGCTCTCAG ACTTCGCCGA GAAGTTGGGG TTGCCCTTTG AGTTTATACC CGTGGCGGAG   
  
  
+ AAGATTGGAA ATTTGGACTT GGAAAGGTTG CATGTTAGTA AAAGGGAAGC TCTTGCTGTG CATTGGTTAC   
  
  
+ AGCACTCTTT GTATGATGTT ACTGGCTCTG ATACTAATAC ACTTGGCCTT CTTCAAAGGT TGGCGCCAAA   
  
  
+ AGTGGTGACG GTGGTGGAGC AAGACCTAAG CCGAACAGGC TCTTTCCTAG GAAGGTTTGT AGAGGCGATC   
  
  
+ CACTACTATT CAGCCCTATT TGACTCCTTA GGAGCGAGTT ATGGAGAGGA CAGTGAGGAG AGGCATGTGG   
  
  
+ TTGAGCAACA GCTCCTTTCT AGGGAGATTC GAAACATTCT GGCCGTTGGT GGGCCCTCAA GGACCGGGGA   
  
  
+ GCCCAAGTTT GCGAGCTGGA GGGAGAAGCT ACAACAGTCC GGCTTTAGGG GAATCTCATT GGCAGGCAAC   
  
  
+ GCCGCTGCCC AGGCCACCTT GCTCCTCGGC ATGTTCCCTT CTGATGGGTA TACTTTAATC GAGGACAGTG   
  
  
+ GCACACTTAA GCTCGGGTGG AAGGACTTGT GCCTCCTGAC TGCTTCGGCC TGGAGGCCTT CCCATGCTCA   
  
  
+ TACTATGAGC ACCGTCTGTA CTCGGAGCCA ATA  

- -Up\_Stream \_Len000CAGAGA CATTGTGTTA AGTAATTTAG GTAGGTTTCC CCTAGTATTT ATTGGTTTGA   
  
  
- AACAACCTTT GACGAAACTA CACCCGAAAG AGAAAGTCGT AAATACAATA TGTATATATA CCAATATTTC   
  
  
- AATGTTTTCA TACTGGGCTG TGCTGTTAAG TTAGGCTTAG GCTGAGCTTC AATGACTTTC ATTAGACTTA   
  
  
- TGCTAAACTG TCGAATCAAA TTTTTGGATA ATGAAGAGTT AAAACTCATT TAGATTCGAT AAAAATTACG   
  
  
- ACTTATCTAG CTGGGCTAAG CTGGGCAAAA GGCTGAGATA TACCAATACC ACGGGGATTT TTTTTAATTT   
  
  
- TTTCTTTTTC TTATCTTTTT ATATTTTATT TTGACTCTAA AGACTTGTGA GAGAGGGGAT CAGAACACCC   
  
  
- GTACCTTTCA CCAACCTCTA CCTCTACAAA CTCAAAGTAA AAGACTATAA TAAAAAATCA GTAATCTTCA   
  
  
- TGTTTCAGAT TTGAGTTGTT CTCGTACTCC CCTCTTACCA ACAACATCGG TTACCTTTTA TTTTTCTTGT   
  
  
- AAGTGAGAAG AACACGAAAT AAATACCACT ATTAAACAAT GAACATAAAA ATTTTTACTA ATTTATATAA   
  
  
- CTAGGAAACT ATAATACAAC TTTATTATTT TTATATTTTT AATCTTTTAT AAAATGGAGC ACTTTAAAAA   
  
  
- AAAGACTAAT TAGTAACTTT AATACCACTT TTTATTTCCT TGTAGAGAAC ATTTGTGAGA TAGGCAAATG   
  
  
- CAAACTGGTG TTTGAACGAT AGGTGTTCGT AGAGTTGGAA ACGGAAATTG GGTTAACACC CAACTACTTT   
  
  
- AGATTGGTAA TATCCTAATC TACCATCAGC AATTAGGACA CTTTGTTCAA AAACCAATAC TACATAGGTT   
  
  
- AATACTCCCG GACTATTGAG AACGGAGCCG TAAACGCACT ATTACTGCAC CATGATTATC GTTAATAAAA   
  
  
- AGCGTTTATA AGCTTTAAAA AATTCTTATT TGCGTTTTAA TAACTAGAAC TGTTTACTAA AATAAAAAAA   
  
  
- CAACCCTTTT CCTATTTGTA GATCACAGTT AACAACTACT GGACTATCGA GAGCTAAAGA ATAAATACTC   
  
  
- AATACAACTA TACTGTGATT AACATTAATA AACAACATTT GAATTTGTAA AAATTTTTAG TTTCGTTTTA   
  
  
- AACAATTAAT GTTATTTAGT TAGATGAAAA TGATACTAGA ATAAACCACA TTAATAACTA GAACTTAAAA   
  
  
- AAAAAAAGAA AAACTTTTTC TATTCGTAGC TTAAAAAAGT TTTTATTTTT CCCACTTCCT TCTTTTTTAC   
  
  
- CTTTGTCAAT GGCTCATTCC TTCCTGTCTC GCGCTCTCCT GTTCTGTGTT TCTCTCCCCT CTCTCTCTCT   
  
  
- CTTCCCCCGT TACCGTCACC CCTTCCAGCT CCTCGTGGAA AGTTGAAGTT CCGAGTTGTG GGGGGGGGGG   
  
  
- GGGGAGAAAG GAGTAAAACG CTGCAATCGT AAACTGTAAA ACGAAAGTTG GCAGGGAGCC GTAGTAGTCG   
  
  
- GGACGGGAAC GAGTCGAAAA GGGTAGAAAA AAACCGAAAA ACAAACTGAG GGAGATATTG AGATAAGTGC   
  
  
- AGCTCGGAAC ATTTTTAAAA TGTATAAAAT AAAATAAAAT GAAAAAATCC AAATAGGGAG AGATAAGAAC   
  
  
- TGGATGCCCG TAAAAAATAA AAGAAACCCA AACTCTGAAC ACTTGAAAAA CGTGTAAAAG AGTGTTTTTA   
  
  
- AATAGAACAG AAAAAGTGAA AGTTGCAAGG AAGAGAGAGA GAGAGAGAGA GAGAGAGAGA GAGTGAGTGT   
  
  
- AGGATCTGTC TGCAGGAAAA ATTTCTTAAA TAAATTGTTC AATCTTCTTT GGCAACAAAT TCATCTTGGT   
  
  
- GGGACAAACC TACGGTTTCC TTTATGGTCA GTTCTTTTTA GTGCTGTTAG TGTTGTGTAG GTAAAGCAAA   
  
  
- TGTAGAGTAG GAGTAGGAGT AGAAGGAGTA GAGATGGGTA GAGGGAGAAA GACATACCGA CGAAGAAGAT   
  
  
- GAAACAAGGG ACTGGGATTG TTATGATCAA ATCAGGAGGG AATAATAATA ATAGGAGGAG GAAGGAGGAG   
  
  
- AATGATATTA CCATTGGGAA TGGAAGGAGA AGGATTGTGG TGACGATAAG GATGAGGAAG TTGTTGGAGA   
  
  
- GGCCATAAAA GAAGGCGGTG GATGAGCCTC AACTTATGAC TAACAGTTTG AGTTGACGGG TACAGATTGG   
  
  
- AGGGGCCTTT CTAGCAAGCC TTCGCCTCAC GACGACTTTA CCTCGTCGCG AGGCGAGGCT AACGCCCACT   
  
  
- AATGGGCGGT TGAGTAGTCG CCCATTAGTG GGCGGTGTTA CTCCGCCGGA GTAGTCACAG AAAGTAACTG   
  
  
- AGGAGTGAGA GCGGTGAAGT GGAGAAGTTG CCGAGGTTAA GCTGCTGGGG TAATCGGGGG CTGGCAGGAT   
  
  
- TACTAGGCCC AGACTCAGTC CCAGGCCTAG ACGGAGAACA CACGCCTAAG AGGCCTGAGG GAAATAAGGG   
  
  
- TGGCCACCTC TCTTCTGGAA GGTAGGGGCG GTGCTGGTGA CGACGGCGCC GCTAGCGCCA CCAAGGACAC   
  
  
- CTCCTCCTAC TAGGCTGCAC CTACCTATCG TAGTATTTTC TCGACTAAGT TTCGAGTTTG AGCTAGAGTT   
  
  
- AGGGTGTCAA CTAAGTCTTG CAATCTCTAT AGTAGATGGG TACATTAGGA TTAGACCCCA GGCGGTAACT   
  
  
- CAAGGCGGAG GCGAGCGAGC GGCGGCTGGG GGAGTAACGA GGCGGCGGTG AAGGCGGGAA GGTGGTGGTG   
  
  
- GTTGTGGTGG AGTTAGTTGT TTAGTGAGAA GGAGCTTAGT CGTTGTGATT GTTGTTGTTA GTACACTCGC   
  
  
- ACATGCGGTT GTTTCCTGGG CCTATGAAGT TGAACCCAGG CCCAGTCCCA GTCCCAGGCT AATTGTAGCT   
  
  
- GGTTCGATTG GAAAGGAAGG GAGGACTAAG GTGGCGGACA ACCCCACAGT CACAGTGTGG CGGTGGCAGA   
  
  
- AGGCGGCGGC CAAGGCCGAG GCCACCTTCG CCGTCATCAT CACCATTTAG TTTGGGATTG GGATTAGGGT   
  
  
- TGGGCTTGGG TTTGGGTTTG GGTTTGGGCT TGTTGTTTCG AGTCCTACAA GTTGAGGTTT GAGGTGTTGT   
  
  
- TGTCGTTGTC GTTGTCGTTG TTTAGGGGCA CCTAGTCCTC GTTCTGGGCC GCCGAGGTGG CCGCTGCCTT   
  
  
- CGTAGCGGTG GTAGAGGTTC TCGCCGCCGT GGTGGCGGCC GCCGTCACTT TCGTTCTCTT TCTCTCCTCT   
  
  
- ACGCCGTTTC CTTCGCGCTG CTTCTCCCAG AGGTGGAGGA TTGGGAGGAG GAGGTTACGC GTCTTCGTCA   
  
  
- TAGGCCGCTG TTTATGCTTC TTCGCTTGTT CTACGAAGAT CTTTAGAGCC TCACCCGGTG GGGTAAGCCG   
  
  
- TGGAGGCGGG TTGCGCAGCG GCGCATGAAG AGCCTTCGGT ACAGTCGGGC AGAGCATAGG AGGACGGAGC   
  
  
- CGTAGATGCG GCGGGAGGGG TGGCAGGGTG TGATGCAGTT CGAGGAGAGG CGGAAGGTTC AGAAGTTACC   
  
  
- GTAGTCGGGT AAGCAGTTTA AGAGAGTGAA GTGACGTTTG GTTCGTTAGG TTCTCCGGAA GGTCTCCCTT   
  
  
- CTGTCCCAGG TGTAGTAGCT GGAGCTATAG TACGTCCCCG AGGTCACCGG GCCCGACAAG GTGTAGGAGC   
  
  
- GCAGGGCCGG TCCACCCGGA GGGAAGCATT CCGAGTGGCC CGAGCCCTGG AGGTACCTCC GCGAGCTCCG   
  
  
- GTGGCCTTTT TCCGAGAGTC TGAAGCGGCT CTTCAACCCC AACGGGAAAC TCAAATATGG GCACCGCCTC   
  
  
- TTCTAACCTT TAAACCTGAA CCTTTCCAAC GTACAATCAT TTTCCCTTCG AGAACGACAC GTAACCAATG   
  
  
- TCGTGAGAAA CATACTACAA TGACCGAGAC TATGATTATG TGAACCGGAA GAAGTTTCCA ACCGCGGTTT   
  
  
- TCACCACTGC CACCACCTCG TTCTGGATTC GGCTTGTCCG AGAAAGGATC CTTCCAAACA TCTCCGCTAG   
  
  
- GTGATGATAA GTCGGGATAA ACTGAGGAAT CCTCGCTCAA TACCTCTCCT GTCACTCCTC TCCGTACACC   
  
  
- AACTCGTTGT CGAGGAAAGA TCCCTCTAAG CTTTGTAAGA CCGGCAACCA CCCGGGAGTT CCTGGCCCCT   
  
  
- CGGGTTCAAA CGCTCGACCT CCCTCTTCGA TGTTGTCAGG CCGAAATCCC CTTAGAGTAA CCGTCCGTTG   
  
  
- CGGCGACGGG TCCGGTGGAA CGAGGAGCCG TACAAGGGAA GACTACCCAT ATGAAATTAG CTCCTGTCAC   
  
  
- CGTGTGAATT CGAGCCCACC TTCCTGAACA CGGAGGACTG ACGAAGCCGG ACCTCCGGAA GGGTACGAGT   
  
  
- ATGATACTCG TGGCAGACAT GAGCCTCGGT TAT

+     Myb

| Site Name | Organism | Position | Strand | Matrix score. | sequence | function |
| --- | --- | --- | --- | --- | --- | --- |
| Myb | Arabidopsis thaliana | 1339 | - | 6 | TAACTG |  |
| Myb | Arabidopsis thaliana | 2670 | - | 6 | CAACTG |  |
| Myb | Arabidopsis thaliana | 2226 | + | 6 | CAACTG |  |

>HU01G00472.1   
+ -Up\_Stream \_Len000GTCTCT GTAACACAAT TCATTAAATC CATCCAAAGG GGATCATAAA TAACCAAACT   
  
  
+ TTGTTGGAAA CTGCTTTGAT GTGGGCTTTC TCTTTCAGCA TTTATGTTAT ACATATATAT GGTTATAAAG   
  
  
+ TTACAAAAGT ATGACCCGAC ACGACAATTC AATCCGAATC CGACTCGAAG TTACTGAAAG TAATCTGAAT   
  
  
+ ACGATTTGAC AGCTTAGTTT AAAAACCTAT TACTTCTCAA TTTTGAGTAA ATCTAAGCTA TTTTTAATGC   
  
  
+ TGAATAGATC GACCCGATTC GACCCGTTTT CCGACTCTAT ATGGTTATGG TGCCCCTAAA AAAAATTAAA   
  
  
+ AAAGAAAAAG AATAGAAAAA TATAAAATAA AACTGAGATT TCTGAACACT CTCTCCCCTA GTCTTGTGGG   
  
  
+ CATGGAAAGT GGTTGGAGAT GGAGATGTTT GAGTTTCATT TTCTGATATT ATTTTTTAGT CATTAGAAGT   
  
  
+ ACAAAGTCTA AACTCAACAA GAGCATGAGG GGAGAATGGT TGTTGTAGCC AATGGAAAAT AAAAAGAACA   
  
  
+ TTCACTCTTC TTGTGCTTTA TTTATGGTGA TAATTTGTTA CTTGTATTTT TAAAAATGAT TAAATATATT   
  
  
+ GATCCTTTGA TATTATGTTG AAATAATAAA AATATAAAAA TTAGAAAATA TTTTACCTCG TGAAATTTTT   
  
  
+ TTTCTGATTA ATCATTGAAA TTATGGTGAA AAATAAAGGA ACATCTCTTG TAAACACTCT ATCCGTTTAC   
  
  
+ GTTTGACCAC AAACTTGCTA TCCACAAGCA TCTCAACCTT TGCCTTTAAC CCAATTGTGG GTTGATGAAA   
  
  
+ TCTAACCATT ATAGGATTAG ATGGTAGTCG TTAATCCTGT GAAACAAGTT TTTGGTTATG ATGTATCCAA   
  
  
+ TTATGAGGGC CTGATAACTC TTGCCTCGGC ATTTGCGTGA TAATGACGTG GTACTAATAG CAATTATTTT   
  
  
+ TCGCAAATAT TCGAAATTTT TTAAGAATAA ACGCAAAATT ATTGATCTTG ACAAATGATT TTATTTTTTT   
  
  
+ GTTGGGAAAA GGATAAACAT CTAGTGTCAA TTGTTGATGA CCTGATAGCT CTCGATTTCT TATTTATGAG   
  
  
+ TTATGTTGAT ATGACACTAA TTGTAATTAT TTGTTGTAAA CTTAAACATT TTTAAAAATC AAAGCAAAAT   
  
  
+ TTGTTAATTA CAATAAATCA ATCTACTTTT ACTATGATCT TATTTGGTGT AATTATTGAT CTTGAATTTT   
  
  
+ TTTTTTTCTT TTTGAAAAAG ATAAGCATCG AATTTTTTCA AAAATAAAAA GGGTGAAGGA AGAAAAAATG   
  
  
+ GAAACAGTTA CCGAGTAAGG AAGGACAGAG CGCGAGAGGA CAAGACACAA AGAGAGGGGA GAGAGAGAGA   
  
  
+ GAAGGGGGCA ATGGCAGTGG GGAAGGTCGA GGAGCACCTT TCAACTTCAA GGCTCAACAC CCCCCCCCCC   
  
  
+ CCCCTCTTTC CTCATTTTGC GACGTTAGCA TTTGACATTT TGCTTTCAAC CGTCCCTCGG CATCATCAGC   
  
  
+ CCTGCCCTTG CTCAGCTTTT CCCATCTTTT TTTGGCTTTT TGTTTGACTC CCTCTATAAC TCTATTCACG   
  
  
+ TCGAGCCTTG TAAAAATTTT ACATATTTTA TTTTATTTTA CTTTTTTAGG TTTATCCCTC TCTATTCTTG   
  
  
+ ACCTACGGGC ATTTTTTATT TTCTTTGGGT TTGAGACTTG TGAACTTTTT GCACATTTTC TCACAAAAAT   
  
  
+ TTATCTTGTC TTTTTCACTT TCAACGTTCC TTCTCTCTCT CTCTCTCTCT CTCTCTCTCT CTCACTCACA   
  
  
+ TCCTAGACAG ACGTCCTTTT TAAAGAATTT ATTTAACAAG TTAGAAGAAA CCGTTGTTTA AGTAGAACCA   
  
  
+ CCCTGTTTGG ATGCCAAAGG AAATACCAGT CAAGAAAAAT CACGACAATC ACAACACATC CATTTCGTTT   
  
  
+ ACATCTCATC CTCATCCTCA TCTTCCTCAT CTCTACCCAT CTCCCTCTTT CTGTATGGCT GCTTCTTCTA   
  
  
+ CTTTGTTCCC TGACCCTAAC AATACTAGTT TAGTCCTCCC TTATTATTAT TATCCTCCTC CTTCCTCCTC   
  
  
+ TTACTATAAT GGTAACCCTT ACCTTCCTCT TCCTAACACC ACTGCTATTC CTACTCCTTC AACAACCTCT   
  
  
+ CCGGTATTTT CTTCCGCCAC CTACTCGGAG TTGAATACTG ATTGTCAAAC TCAACTGCCC ATGTCTAACC   
  
  
+ TCCCCGGAAA GATCGTTCGG AAGCGGAGTG CTGCTGAAAT GGAGCAGCGC TCCGCTCCGA TTGCGGGTGA   
  
  
+ TTACCCGCCA ACTCATCAGC GGGTAATCAC CCGCCACAAT GAGGCGGCCT CATCAGTGTC TTTCATTGAC   
  
  
+ TCCTCACTCT CGCCACTTCA CCTCTTCAAC GGCTCCAATT CGACGACCCC ATTAGCCCCC GACCGTCCTA   
  
  
+ ATGATCCGGG TCTGAGTCAG GGTCCGGATC TGCCTCTTGT GTGCGGATTC TCCGGACTCC CTTTATTCCC   
  
  
+ ACCGGTGGAG AGAAGACCTT CCATCCCCGC CACGACCACT GCTGCCGCGG CGATCGCGGT GGTTCCTGTG   
  
  
+ GAGGAGGATG ATCCGACGTG GATGGATAGC ATCATAAAAG AGCTGATTCA AAGCTCAAAC TCGATCTCAA   
  
  
+ TCCCACAGTT GATTCAGAAC GTTAGAGATA TCATCTACCC ATGTAATCCT AATCTGGGGT CCGCCATTGA   
  
  
+ GTTCCGCCTC CGCTCGCTCG CCGCCGACCC CCTCATTGCT CCGCCGCCAC TTCCGCCCTT CCACCACCAC   
  
  
+ CAACACCACC TCAATCAACA AATCACTCTT CCTCGAATCA GCAACACTAA CAACAACAAT CATGTGAGCG   
  
  
+ TGTACGCCAA CAAAGGACCC GGATACTTCA ACTTGGGTCC GGGTCAGGGT CAGGGTCCGA TTAACATCGA   
  
  
+ CCAAGCTAAC CTTTCCTTCC CTCCTGATTC CACCGCCTGT TGGGGTGTCA GTGTCACACC GCCACCGTCT   
  
  
+ TCCGCCGCCG GTTCCGGCTC CGGTGGAAGC GGCAGTAGTA GTGGTAAATC AAACCCTAAC CCTAATCCCA   
  
  
+ ACCCGAACCC AAACCCAAAC CCAAACCCGA ACAACAAAGC TCAGGATGTT CAACTCCAAA CTCCACAACA   
  
  
+ ACAGCAACAG CAACAGCAAC AAATCCCCGT GGATCAGGAG CAAGACCCGG CGGCTCCACC GGCGACGGAA   
  
  
+ GCATCGCCAC CATCTCCAAG AGCGGCGGCA CCACCGCCGG CGGCAGTGAA AGCAAGAGAA AGAGAGGAGA   
  
  
+ TGCGGCAAAG GAAGCGCGAC GAAGAGGGTC TCCACCTCCT AACCCTCCTC CTCCAATGCG CAGAAGCAGT   
  
  
+ ATCCGGCGAC AAATACGAAG AAGCGAACAA GATGCTTCTA GAAATCTCGG AGTGGGCCAC CCCATTCGGC   
  
  
+ ACCTCCGCCC AACGCGTCGC CGCGTACTTC TCGGAAGCCA TGTCAGCCCG TCTCGTATCC TCCTGCCTCG   
  
  
+ GCATCTACGC CGCCCTCCCC ACCGTCCCAC ACTACGTCAA GCTCCTCTCC GCCTTCCAAG TCTTCAATGG   
  
  
+ CATCAGCCCA TTCGTCAAAT TCTCTCACTT CACTGCAAAC CAAGCAATCC AAGAGGCCTT CCAGAGGGAA   
  
  
+ GACAGGGTCC ACATCATCGA CCTCGATATC ATGCAGGGGC TCCAGTGGCC CGGGCTGTTC CACATCCTCG   
  
  
+ CGTCCCGGCC AGGTGGGCCT CCCTTCGTAA GGCTCACCGG GCTCGGGACC TCCATGGAGG CGCTCGAGGC   
  
  
+ CACCGGAAAA AGGCTCTCAG ACTTCGCCGA GAAGTTGGGG TTGCCCTTTG AGTTTATACC CGTGGCGGAG   
  
  
+ AAGATTGGAA ATTTGGACTT GGAAAGGTTG CATGTTAGTA AAAGGGAAGC TCTTGCTGTG CATTGGTTAC   
  
  
+ AGCACTCTTT GTATGATGTT ACTGGCTCTG ATACTAATAC ACTTGGCCTT CTTCAAAGGT TGGCGCCAAA   
  
  
+ AGTGGTGACG GTGGTGGAGC AAGACCTAAG CCGAACAGGC TCTTTCCTAG GAAGGTTTGT AGAGGCGATC   
  
  
+ CACTACTATT CAGCCCTATT TGACTCCTTA GGAGCGAGTT ATGGAGAGGA CAGTGAGGAG AGGCATGTGG   
  
  
+ TTGAGCAACA GCTCCTTTCT AGGGAGATTC GAAACATTCT GGCCGTTGGT GGGCCCTCAA GGACCGGGGA   
  
  
+ GCCCAAGTTT GCGAGCTGGA GGGAGAAGCT ACAACAGTCC GGCTTTAGGG GAATCTCATT GGCAGGCAAC   
  
  
+ GCCGCTGCCC AGGCCACCTT GCTCCTCGGC ATGTTCCCTT CTGATGGGTA TACTTTAATC GAGGACAGTG   
  
  
+ GCACACTTAA GCTCGGGTGG AAGGACTTGT GCCTCCTGAC TGCTTCGGCC TGGAGGCCTT CCCATGCTCA   
  
  
+ TACTATGAGC ACCGTCTGTA CTCGGAGCCA ATA  

- -Up\_Stream \_Len000CAGAGA CATTGTGTTA AGTAATTTAG GTAGGTTTCC CCTAGTATTT ATTGGTTTGA   
  
  
- AACAACCTTT GACGAAACTA CACCCGAAAG AGAAAGTCGT AAATACAATA TGTATATATA CCAATATTTC   
  
  
- AATGTTTTCA TACTGGGCTG TGCTGTTAAG TTAGGCTTAG GCTGAGCTTC AATGACTTTC ATTAGACTTA   
  
  
- TGCTAAACTG TCGAATCAAA TTTTTGGATA ATGAAGAGTT AAAACTCATT TAGATTCGAT AAAAATTACG   
  
  
- ACTTATCTAG CTGGGCTAAG CTGGGCAAAA GGCTGAGATA TACCAATACC ACGGGGATTT TTTTTAATTT   
  
  
- TTTCTTTTTC TTATCTTTTT ATATTTTATT TTGACTCTAA AGACTTGTGA GAGAGGGGAT CAGAACACCC   
  
  
- GTACCTTTCA CCAACCTCTA CCTCTACAAA CTCAAAGTAA AAGACTATAA TAAAAAATCA GTAATCTTCA   
  
  
- TGTTTCAGAT TTGAGTTGTT CTCGTACTCC CCTCTTACCA ACAACATCGG TTACCTTTTA TTTTTCTTGT   
  
  
- AAGTGAGAAG AACACGAAAT AAATACCACT ATTAAACAAT GAACATAAAA ATTTTTACTA ATTTATATAA   
  
  
- CTAGGAAACT ATAATACAAC TTTATTATTT TTATATTTTT AATCTTTTAT AAAATGGAGC ACTTTAAAAA   
  
  
- AAAGACTAAT TAGTAACTTT AATACCACTT TTTATTTCCT TGTAGAGAAC ATTTGTGAGA TAGGCAAATG   
  
  
- CAAACTGGTG TTTGAACGAT AGGTGTTCGT AGAGTTGGAA ACGGAAATTG GGTTAACACC CAACTACTTT   
  
  
- AGATTGGTAA TATCCTAATC TACCATCAGC AATTAGGACA CTTTGTTCAA AAACCAATAC TACATAGGTT   
  
  
- AATACTCCCG GACTATTGAG AACGGAGCCG TAAACGCACT ATTACTGCAC CATGATTATC GTTAATAAAA   
  
  
- AGCGTTTATA AGCTTTAAAA AATTCTTATT TGCGTTTTAA TAACTAGAAC TGTTTACTAA AATAAAAAAA   
  
  
- CAACCCTTTT CCTATTTGTA GATCACAGTT AACAACTACT GGACTATCGA GAGCTAAAGA ATAAATACTC   
  
  
- AATACAACTA TACTGTGATT AACATTAATA AACAACATTT GAATTTGTAA AAATTTTTAG TTTCGTTTTA   
  
  
- AACAATTAAT GTTATTTAGT TAGATGAAAA TGATACTAGA ATAAACCACA TTAATAACTA GAACTTAAAA   
  
  
- AAAAAAAGAA AAACTTTTTC TATTCGTAGC TTAAAAAAGT TTTTATTTTT CCCACTTCCT TCTTTTTTAC   
  
  
- CTTTGTCAAT GGCTCATTCC TTCCTGTCTC GCGCTCTCCT GTTCTGTGTT TCTCTCCCCT CTCTCTCTCT   
  
  
- CTTCCCCCGT TACCGTCACC CCTTCCAGCT CCTCGTGGAA AGTTGAAGTT CCGAGTTGTG GGGGGGGGGG   
  
  
- GGGGAGAAAG GAGTAAAACG CTGCAATCGT AAACTGTAAA ACGAAAGTTG GCAGGGAGCC GTAGTAGTCG   
  
  
- GGACGGGAAC GAGTCGAAAA GGGTAGAAAA AAACCGAAAA ACAAACTGAG GGAGATATTG AGATAAGTGC   
  
  
- AGCTCGGAAC ATTTTTAAAA TGTATAAAAT AAAATAAAAT GAAAAAATCC AAATAGGGAG AGATAAGAAC   
  
  
- TGGATGCCCG TAAAAAATAA AAGAAACCCA AACTCTGAAC ACTTGAAAAA CGTGTAAAAG AGTGTTTTTA   
  
  
- AATAGAACAG AAAAAGTGAA AGTTGCAAGG AAGAGAGAGA GAGAGAGAGA GAGAGAGAGA GAGTGAGTGT   
  
  
- AGGATCTGTC TGCAGGAAAA ATTTCTTAAA TAAATTGTTC AATCTTCTTT GGCAACAAAT TCATCTTGGT   
  
  
- GGGACAAACC TACGGTTTCC TTTATGGTCA GTTCTTTTTA GTGCTGTTAG TGTTGTGTAG GTAAAGCAAA   
  
  
- TGTAGAGTAG GAGTAGGAGT AGAAGGAGTA GAGATGGGTA GAGGGAGAAA GACATACCGA CGAAGAAGAT   
  
  
- GAAACAAGGG ACTGGGATTG TTATGATCAA ATCAGGAGGG AATAATAATA ATAGGAGGAG GAAGGAGGAG   
  
  
- AATGATATTA CCATTGGGAA TGGAAGGAGA AGGATTGTGG TGACGATAAG GATGAGGAAG TTGTTGGAGA   
  
  
- GGCCATAAAA GAAGGCGGTG GATGAGCCTC AACTTATGAC TAACAGTTTG AGTTGACGGG TACAGATTGG   
  
  
- AGGGGCCTTT CTAGCAAGCC TTCGCCTCAC GACGACTTTA CCTCGTCGCG AGGCGAGGCT AACGCCCACT   
  
  
- AATGGGCGGT TGAGTAGTCG CCCATTAGTG GGCGGTGTTA CTCCGCCGGA GTAGTCACAG AAAGTAACTG   
  
  
- AGGAGTGAGA GCGGTGAAGT GGAGAAGTTG CCGAGGTTAA GCTGCTGGGG TAATCGGGGG CTGGCAGGAT   
  
  
- TACTAGGCCC AGACTCAGTC CCAGGCCTAG ACGGAGAACA CACGCCTAAG AGGCCTGAGG GAAATAAGGG   
  
  
- TGGCCACCTC TCTTCTGGAA GGTAGGGGCG GTGCTGGTGA CGACGGCGCC GCTAGCGCCA CCAAGGACAC   
  
  
- CTCCTCCTAC TAGGCTGCAC CTACCTATCG TAGTATTTTC TCGACTAAGT TTCGAGTTTG AGCTAGAGTT   
  
  
- AGGGTGTCAA CTAAGTCTTG CAATCTCTAT AGTAGATGGG TACATTAGGA TTAGACCCCA GGCGGTAACT   
  
  
- CAAGGCGGAG GCGAGCGAGC GGCGGCTGGG GGAGTAACGA GGCGGCGGTG AAGGCGGGAA GGTGGTGGTG   
  
  
- GTTGTGGTGG AGTTAGTTGT TTAGTGAGAA GGAGCTTAGT CGTTGTGATT GTTGTTGTTA GTACACTCGC   
  
  
- ACATGCGGTT GTTTCCTGGG CCTATGAAGT TGAACCCAGG CCCAGTCCCA GTCCCAGGCT AATTGTAGCT   
  
  
- GGTTCGATTG GAAAGGAAGG GAGGACTAAG GTGGCGGACA ACCCCACAGT CACAGTGTGG CGGTGGCAGA   
  
  
- AGGCGGCGGC CAAGGCCGAG GCCACCTTCG CCGTCATCAT CACCATTTAG TTTGGGATTG GGATTAGGGT   
  
  
- TGGGCTTGGG TTTGGGTTTG GGTTTGGGCT TGTTGTTTCG AGTCCTACAA GTTGAGGTTT GAGGTGTTGT   
  
  
- TGTCGTTGTC GTTGTCGTTG TTTAGGGGCA CCTAGTCCTC GTTCTGGGCC GCCGAGGTGG CCGCTGCCTT   
  
  
- CGTAGCGGTG GTAGAGGTTC TCGCCGCCGT GGTGGCGGCC GCCGTCACTT TCGTTCTCTT TCTCTCCTCT   
  
  
- ACGCCGTTTC CTTCGCGCTG CTTCTCCCAG AGGTGGAGGA TTGGGAGGAG GAGGTTACGC GTCTTCGTCA   
  
  
- TAGGCCGCTG TTTATGCTTC TTCGCTTGTT CTACGAAGAT CTTTAGAGCC TCACCCGGTG GGGTAAGCCG   
  
  
- TGGAGGCGGG TTGCGCAGCG GCGCATGAAG AGCCTTCGGT ACAGTCGGGC AGAGCATAGG AGGACGGAGC   
  
  
- CGTAGATGCG GCGGGAGGGG TGGCAGGGTG TGATGCAGTT CGAGGAGAGG CGGAAGGTTC AGAAGTTACC   
  
  
- GTAGTCGGGT AAGCAGTTTA AGAGAGTGAA GTGACGTTTG GTTCGTTAGG TTCTCCGGAA GGTCTCCCTT   
  
  
- CTGTCCCAGG TGTAGTAGCT GGAGCTATAG TACGTCCCCG AGGTCACCGG GCCCGACAAG GTGTAGGAGC   
  
  
- GCAGGGCCGG TCCACCCGGA GGGAAGCATT CCGAGTGGCC CGAGCCCTGG AGGTACCTCC GCGAGCTCCG   
  
  
- GTGGCCTTTT TCCGAGAGTC TGAAGCGGCT CTTCAACCCC AACGGGAAAC TCAAATATGG GCACCGCCTC   
  
  
- TTCTAACCTT TAAACCTGAA CCTTTCCAAC GTACAATCAT TTTCCCTTCG AGAACGACAC GTAACCAATG   
  
  
- TCGTGAGAAA CATACTACAA TGACCGAGAC TATGATTATG TGAACCGGAA GAAGTTTCCA ACCGCGGTTT   
  
  
- TCACCACTGC CACCACCTCG TTCTGGATTC GGCTTGTCCG AGAAAGGATC CTTCCAAACA TCTCCGCTAG   
  
  
- GTGATGATAA GTCGGGATAA ACTGAGGAAT CCTCGCTCAA TACCTCTCCT GTCACTCCTC TCCGTACACC   
  
  
- AACTCGTTGT CGAGGAAAGA TCCCTCTAAG CTTTGTAAGA CCGGCAACCA CCCGGGAGTT CCTGGCCCCT   
  
  
- CGGGTTCAAA CGCTCGACCT CCCTCTTCGA TGTTGTCAGG CCGAAATCCC CTTAGAGTAA CCGTCCGTTG   
  
  
- CGGCGACGGG TCCGGTGGAA CGAGGAGCCG TACAAGGGAA GACTACCCAT ATGAAATTAG CTCCTGTCAC   
  
  
- CGTGTGAATT CGAGCCCACC TTCCTGAACA CGGAGGACTG ACGAAGCCGG ACCTCCGGAA GGGTACGAGT   
  
  
- ATGATACTCG TGGCAGACAT GAGCCTCGGT TAT

+     Myb-binding site

| Site Name | Organism | Position | Strand | Matrix score. | sequence | function |
| --- | --- | --- | --- | --- | --- | --- |
| Myb-binding site | Nicotiana tabacum | 2981 | - | 6 | CAACAG |  |
| Myb-binding site | Nicotiana tabacum | 4140 | + | 6 | CAACAG |  |
| Myb-binding site | Nicotiana tabacum | 3159 | + | 6 | CAACAG |  |
| Myb-binding site | Nicotiana tabacum | 4236 | + | 6 | CAACAG |  |
| Myb-binding site | Nicotiana tabacum | 3153 | + | 6 | CAACAG |  |
| Myb-binding site | Nicotiana tabacum | 3165 | + | 6 | CAACAG |  |

>HU01G00472.1   
+ -Up\_Stream \_Len000GTCTCT GTAACACAAT TCATTAAATC CATCCAAAGG GGATCATAAA TAACCAAACT   
  
  
+ TTGTTGGAAA CTGCTTTGAT GTGGGCTTTC TCTTTCAGCA TTTATGTTAT ACATATATAT GGTTATAAAG   
  
  
+ TTACAAAAGT ATGACCCGAC ACGACAATTC AATCCGAATC CGACTCGAAG TTACTGAAAG TAATCTGAAT   
  
  
+ ACGATTTGAC AGCTTAGTTT AAAAACCTAT TACTTCTCAA TTTTGAGTAA ATCTAAGCTA TTTTTAATGC   
  
  
+ TGAATAGATC GACCCGATTC GACCCGTTTT CCGACTCTAT ATGGTTATGG TGCCCCTAAA AAAAATTAAA   
  
  
+ AAAGAAAAAG AATAGAAAAA TATAAAATAA AACTGAGATT TCTGAACACT CTCTCCCCTA GTCTTGTGGG   
  
  
+ CATGGAAAGT GGTTGGAGAT GGAGATGTTT GAGTTTCATT TTCTGATATT ATTTTTTAGT CATTAGAAGT   
  
  
+ ACAAAGTCTA AACTCAACAA GAGCATGAGG GGAGAATGGT TGTTGTAGCC AATGGAAAAT AAAAAGAACA   
  
  
+ TTCACTCTTC TTGTGCTTTA TTTATGGTGA TAATTTGTTA CTTGTATTTT TAAAAATGAT TAAATATATT   
  
  
+ GATCCTTTGA TATTATGTTG AAATAATAAA AATATAAAAA TTAGAAAATA TTTTACCTCG TGAAATTTTT   
  
  
+ TTTCTGATTA ATCATTGAAA TTATGGTGAA AAATAAAGGA ACATCTCTTG TAAACACTCT ATCCGTTTAC   
  
  
+ GTTTGACCAC AAACTTGCTA TCCACAAGCA TCTCAACCTT TGCCTTTAAC CCAATTGTGG GTTGATGAAA   
  
  
+ TCTAACCATT ATAGGATTAG ATGGTAGTCG TTAATCCTGT GAAACAAGTT TTTGGTTATG ATGTATCCAA   
  
  
+ TTATGAGGGC CTGATAACTC TTGCCTCGGC ATTTGCGTGA TAATGACGTG GTACTAATAG CAATTATTTT   
  
  
+ TCGCAAATAT TCGAAATTTT TTAAGAATAA ACGCAAAATT ATTGATCTTG ACAAATGATT TTATTTTTTT   
  
  
+ GTTGGGAAAA GGATAAACAT CTAGTGTCAA TTGTTGATGA CCTGATAGCT CTCGATTTCT TATTTATGAG   
  
  
+ TTATGTTGAT ATGACACTAA TTGTAATTAT TTGTTGTAAA CTTAAACATT TTTAAAAATC AAAGCAAAAT   
  
  
+ TTGTTAATTA CAATAAATCA ATCTACTTTT ACTATGATCT TATTTGGTGT AATTATTGAT CTTGAATTTT   
  
  
+ TTTTTTTCTT TTTGAAAAAG ATAAGCATCG AATTTTTTCA AAAATAAAAA GGGTGAAGGA AGAAAAAATG   
  
  
+ GAAACAGTTA CCGAGTAAGG AAGGACAGAG CGCGAGAGGA CAAGACACAA AGAGAGGGGA GAGAGAGAGA   
  
  
+ GAAGGGGGCA ATGGCAGTGG GGAAGGTCGA GGAGCACCTT TCAACTTCAA GGCTCAACAC CCCCCCCCCC   
  
  
+ CCCCTCTTTC CTCATTTTGC GACGTTAGCA TTTGACATTT TGCTTTCAAC CGTCCCTCGG CATCATCAGC   
  
  
+ CCTGCCCTTG CTCAGCTTTT CCCATCTTTT TTTGGCTTTT TGTTTGACTC CCTCTATAAC TCTATTCACG   
  
  
+ TCGAGCCTTG TAAAAATTTT ACATATTTTA TTTTATTTTA CTTTTTTAGG TTTATCCCTC TCTATTCTTG   
  
  
+ ACCTACGGGC ATTTTTTATT TTCTTTGGGT TTGAGACTTG TGAACTTTTT GCACATTTTC TCACAAAAAT   
  
  
+ TTATCTTGTC TTTTTCACTT TCAACGTTCC TTCTCTCTCT CTCTCTCTCT CTCTCTCTCT CTCACTCACA   
  
  
+ TCCTAGACAG ACGTCCTTTT TAAAGAATTT ATTTAACAAG TTAGAAGAAA CCGTTGTTTA AGTAGAACCA   
  
  
+ CCCTGTTTGG ATGCCAAAGG AAATACCAGT CAAGAAAAAT CACGACAATC ACAACACATC CATTTCGTTT   
  
  
+ ACATCTCATC CTCATCCTCA TCTTCCTCAT CTCTACCCAT CTCCCTCTTT CTGTATGGCT GCTTCTTCTA   
  
  
+ CTTTGTTCCC TGACCCTAAC AATACTAGTT TAGTCCTCCC TTATTATTAT TATCCTCCTC CTTCCTCCTC   
  
  
+ TTACTATAAT GGTAACCCTT ACCTTCCTCT TCCTAACACC ACTGCTATTC CTACTCCTTC AACAACCTCT   
  
  
+ CCGGTATTTT CTTCCGCCAC CTACTCGGAG TTGAATACTG ATTGTCAAAC TCAACTGCCC ATGTCTAACC   
  
  
+ TCCCCGGAAA GATCGTTCGG AAGCGGAGTG CTGCTGAAAT GGAGCAGCGC TCCGCTCCGA TTGCGGGTGA   
  
  
+ TTACCCGCCA ACTCATCAGC GGGTAATCAC CCGCCACAAT GAGGCGGCCT CATCAGTGTC TTTCATTGAC   
  
  
+ TCCTCACTCT CGCCACTTCA CCTCTTCAAC GGCTCCAATT CGACGACCCC ATTAGCCCCC GACCGTCCTA   
  
  
+ ATGATCCGGG TCTGAGTCAG GGTCCGGATC TGCCTCTTGT GTGCGGATTC TCCGGACTCC CTTTATTCCC   
  
  
+ ACCGGTGGAG AGAAGACCTT CCATCCCCGC CACGACCACT GCTGCCGCGG CGATCGCGGT GGTTCCTGTG   
  
  
+ GAGGAGGATG ATCCGACGTG GATGGATAGC ATCATAAAAG AGCTGATTCA AAGCTCAAAC TCGATCTCAA   
  
  
+ TCCCACAGTT GATTCAGAAC GTTAGAGATA TCATCTACCC ATGTAATCCT AATCTGGGGT CCGCCATTGA   
  
  
+ GTTCCGCCTC CGCTCGCTCG CCGCCGACCC CCTCATTGCT CCGCCGCCAC TTCCGCCCTT CCACCACCAC   
  
  
+ CAACACCACC TCAATCAACA AATCACTCTT CCTCGAATCA GCAACACTAA CAACAACAAT CATGTGAGCG   
  
  
+ TGTACGCCAA CAAAGGACCC GGATACTTCA ACTTGGGTCC GGGTCAGGGT CAGGGTCCGA TTAACATCGA   
  
  
+ CCAAGCTAAC CTTTCCTTCC CTCCTGATTC CACCGCCTGT TGGGGTGTCA GTGTCACACC GCCACCGTCT   
  
  
+ TCCGCCGCCG GTTCCGGCTC CGGTGGAAGC GGCAGTAGTA GTGGTAAATC AAACCCTAAC CCTAATCCCA   
  
  
+ ACCCGAACCC AAACCCAAAC CCAAACCCGA ACAACAAAGC TCAGGATGTT CAACTCCAAA CTCCACAACA   
  
  
+ ACAGCAACAG CAACAGCAAC AAATCCCCGT GGATCAGGAG CAAGACCCGG CGGCTCCACC GGCGACGGAA   
  
  
+ GCATCGCCAC CATCTCCAAG AGCGGCGGCA CCACCGCCGG CGGCAGTGAA AGCAAGAGAA AGAGAGGAGA   
  
  
+ TGCGGCAAAG GAAGCGCGAC GAAGAGGGTC TCCACCTCCT AACCCTCCTC CTCCAATGCG CAGAAGCAGT   
  
  
+ ATCCGGCGAC AAATACGAAG AAGCGAACAA GATGCTTCTA GAAATCTCGG AGTGGGCCAC CCCATTCGGC   
  
  
+ ACCTCCGCCC AACGCGTCGC CGCGTACTTC TCGGAAGCCA TGTCAGCCCG TCTCGTATCC TCCTGCCTCG   
  
  
+ GCATCTACGC CGCCCTCCCC ACCGTCCCAC ACTACGTCAA GCTCCTCTCC GCCTTCCAAG TCTTCAATGG   
  
  
+ CATCAGCCCA TTCGTCAAAT TCTCTCACTT CACTGCAAAC CAAGCAATCC AAGAGGCCTT CCAGAGGGAA   
  
  
+ GACAGGGTCC ACATCATCGA CCTCGATATC ATGCAGGGGC TCCAGTGGCC CGGGCTGTTC CACATCCTCG   
  
  
+ CGTCCCGGCC AGGTGGGCCT CCCTTCGTAA GGCTCACCGG GCTCGGGACC TCCATGGAGG CGCTCGAGGC   
  
  
+ CACCGGAAAA AGGCTCTCAG ACTTCGCCGA GAAGTTGGGG TTGCCCTTTG AGTTTATACC CGTGGCGGAG   
  
  
+ AAGATTGGAA ATTTGGACTT GGAAAGGTTG CATGTTAGTA AAAGGGAAGC TCTTGCTGTG CATTGGTTAC   
  
  
+ AGCACTCTTT GTATGATGTT ACTGGCTCTG ATACTAATAC ACTTGGCCTT CTTCAAAGGT TGGCGCCAAA   
  
  
+ AGTGGTGACG GTGGTGGAGC AAGACCTAAG CCGAACAGGC TCTTTCCTAG GAAGGTTTGT AGAGGCGATC   
  
  
+ CACTACTATT CAGCCCTATT TGACTCCTTA GGAGCGAGTT ATGGAGAGGA CAGTGAGGAG AGGCATGTGG   
  
  
+ TTGAGCAACA GCTCCTTTCT AGGGAGATTC GAAACATTCT GGCCGTTGGT GGGCCCTCAA GGACCGGGGA   
  
  
+ GCCCAAGTTT GCGAGCTGGA GGGAGAAGCT ACAACAGTCC GGCTTTAGGG GAATCTCATT GGCAGGCAAC   
  
  
+ GCCGCTGCCC AGGCCACCTT GCTCCTCGGC ATGTTCCCTT CTGATGGGTA TACTTTAATC GAGGACAGTG   
  
  
+ GCACACTTAA GCTCGGGTGG AAGGACTTGT GCCTCCTGAC TGCTTCGGCC TGGAGGCCTT CCCATGCTCA   
  
  
+ TACTATGAGC ACCGTCTGTA CTCGGAGCCA ATA  

- -Up\_Stream \_Len000CAGAGA CATTGTGTTA AGTAATTTAG GTAGGTTTCC CCTAGTATTT ATTGGTTTGA   
  
  
- AACAACCTTT GACGAAACTA CACCCGAAAG AGAAAGTCGT AAATACAATA TGTATATATA CCAATATTTC   
  
  
- AATGTTTTCA TACTGGGCTG TGCTGTTAAG TTAGGCTTAG GCTGAGCTTC AATGACTTTC ATTAGACTTA   
  
  
- TGCTAAACTG TCGAATCAAA TTTTTGGATA ATGAAGAGTT AAAACTCATT TAGATTCGAT AAAAATTACG   
  
  
- ACTTATCTAG CTGGGCTAAG CTGGGCAAAA GGCTGAGATA TACCAATACC ACGGGGATTT TTTTTAATTT   
  
  
- TTTCTTTTTC TTATCTTTTT ATATTTTATT TTGACTCTAA AGACTTGTGA GAGAGGGGAT CAGAACACCC   
  
  
- GTACCTTTCA CCAACCTCTA CCTCTACAAA CTCAAAGTAA AAGACTATAA TAAAAAATCA GTAATCTTCA   
  
  
- TGTTTCAGAT TTGAGTTGTT CTCGTACTCC CCTCTTACCA ACAACATCGG TTACCTTTTA TTTTTCTTGT   
  
  
- AAGTGAGAAG AACACGAAAT AAATACCACT ATTAAACAAT GAACATAAAA ATTTTTACTA ATTTATATAA   
  
  
- CTAGGAAACT ATAATACAAC TTTATTATTT TTATATTTTT AATCTTTTAT AAAATGGAGC ACTTTAAAAA   
  
  
- AAAGACTAAT TAGTAACTTT AATACCACTT TTTATTTCCT TGTAGAGAAC ATTTGTGAGA TAGGCAAATG   
  
  
- CAAACTGGTG TTTGAACGAT AGGTGTTCGT AGAGTTGGAA ACGGAAATTG GGTTAACACC CAACTACTTT   
  
  
- AGATTGGTAA TATCCTAATC TACCATCAGC AATTAGGACA CTTTGTTCAA AAACCAATAC TACATAGGTT   
  
  
- AATACTCCCG GACTATTGAG AACGGAGCCG TAAACGCACT ATTACTGCAC CATGATTATC GTTAATAAAA   
  
  
- AGCGTTTATA AGCTTTAAAA AATTCTTATT TGCGTTTTAA TAACTAGAAC TGTTTACTAA AATAAAAAAA   
  
  
- CAACCCTTTT CCTATTTGTA GATCACAGTT AACAACTACT GGACTATCGA GAGCTAAAGA ATAAATACTC   
  
  
- AATACAACTA TACTGTGATT AACATTAATA AACAACATTT GAATTTGTAA AAATTTTTAG TTTCGTTTTA   
  
  
- AACAATTAAT GTTATTTAGT TAGATGAAAA TGATACTAGA ATAAACCACA TTAATAACTA GAACTTAAAA   
  
  
- AAAAAAAGAA AAACTTTTTC TATTCGTAGC TTAAAAAAGT TTTTATTTTT CCCACTTCCT TCTTTTTTAC   
  
  
- CTTTGTCAAT GGCTCATTCC TTCCTGTCTC GCGCTCTCCT GTTCTGTGTT TCTCTCCCCT CTCTCTCTCT   
  
  
- CTTCCCCCGT TACCGTCACC CCTTCCAGCT CCTCGTGGAA AGTTGAAGTT CCGAGTTGTG GGGGGGGGGG   
  
  
- GGGGAGAAAG GAGTAAAACG CTGCAATCGT AAACTGTAAA ACGAAAGTTG GCAGGGAGCC GTAGTAGTCG   
  
  
- GGACGGGAAC GAGTCGAAAA GGGTAGAAAA AAACCGAAAA ACAAACTGAG GGAGATATTG AGATAAGTGC   
  
  
- AGCTCGGAAC ATTTTTAAAA TGTATAAAAT AAAATAAAAT GAAAAAATCC AAATAGGGAG AGATAAGAAC   
  
  
- TGGATGCCCG TAAAAAATAA AAGAAACCCA AACTCTGAAC ACTTGAAAAA CGTGTAAAAG AGTGTTTTTA   
  
  
- AATAGAACAG AAAAAGTGAA AGTTGCAAGG AAGAGAGAGA GAGAGAGAGA GAGAGAGAGA GAGTGAGTGT   
  
  
- AGGATCTGTC TGCAGGAAAA ATTTCTTAAA TAAATTGTTC AATCTTCTTT GGCAACAAAT TCATCTTGGT   
  
  
- GGGACAAACC TACGGTTTCC TTTATGGTCA GTTCTTTTTA GTGCTGTTAG TGTTGTGTAG GTAAAGCAAA   
  
  
- TGTAGAGTAG GAGTAGGAGT AGAAGGAGTA GAGATGGGTA GAGGGAGAAA GACATACCGA CGAAGAAGAT   
  
  
- GAAACAAGGG ACTGGGATTG TTATGATCAA ATCAGGAGGG AATAATAATA ATAGGAGGAG GAAGGAGGAG   
  
  
- AATGATATTA CCATTGGGAA TGGAAGGAGA AGGATTGTGG TGACGATAAG GATGAGGAAG TTGTTGGAGA   
  
  
- GGCCATAAAA GAAGGCGGTG GATGAGCCTC AACTTATGAC TAACAGTTTG AGTTGACGGG TACAGATTGG   
  
  
- AGGGGCCTTT CTAGCAAGCC TTCGCCTCAC GACGACTTTA CCTCGTCGCG AGGCGAGGCT AACGCCCACT   
  
  
- AATGGGCGGT TGAGTAGTCG CCCATTAGTG GGCGGTGTTA CTCCGCCGGA GTAGTCACAG AAAGTAACTG   
  
  
- AGGAGTGAGA GCGGTGAAGT GGAGAAGTTG CCGAGGTTAA GCTGCTGGGG TAATCGGGGG CTGGCAGGAT   
  
  
- TACTAGGCCC AGACTCAGTC CCAGGCCTAG ACGGAGAACA CACGCCTAAG AGGCCTGAGG GAAATAAGGG   
  
  
- TGGCCACCTC TCTTCTGGAA GGTAGGGGCG GTGCTGGTGA CGACGGCGCC GCTAGCGCCA CCAAGGACAC   
  
  
- CTCCTCCTAC TAGGCTGCAC CTACCTATCG TAGTATTTTC TCGACTAAGT TTCGAGTTTG AGCTAGAGTT   
  
  
- AGGGTGTCAA CTAAGTCTTG CAATCTCTAT AGTAGATGGG TACATTAGGA TTAGACCCCA GGCGGTAACT   
  
  
- CAAGGCGGAG GCGAGCGAGC GGCGGCTGGG GGAGTAACGA GGCGGCGGTG AAGGCGGGAA GGTGGTGGTG   
  
  
- GTTGTGGTGG AGTTAGTTGT TTAGTGAGAA GGAGCTTAGT CGTTGTGATT GTTGTTGTTA GTACACTCGC   
  
  
- ACATGCGGTT GTTTCCTGGG CCTATGAAGT TGAACCCAGG CCCAGTCCCA GTCCCAGGCT AATTGTAGCT   
  
  
- GGTTCGATTG GAAAGGAAGG GAGGACTAAG GTGGCGGACA ACCCCACAGT CACAGTGTGG CGGTGGCAGA   
  
  
- AGGCGGCGGC CAAGGCCGAG GCCACCTTCG CCGTCATCAT CACCATTTAG TTTGGGATTG GGATTAGGGT   
  
  
- TGGGCTTGGG TTTGGGTTTG GGTTTGGGCT TGTTGTTTCG AGTCCTACAA GTTGAGGTTT GAGGTGTTGT   
  
  
- TGTCGTTGTC GTTGTCGTTG TTTAGGGGCA CCTAGTCCTC GTTCTGGGCC GCCGAGGTGG CCGCTGCCTT   
  
  
- CGTAGCGGTG GTAGAGGTTC TCGCCGCCGT GGTGGCGGCC GCCGTCACTT TCGTTCTCTT TCTCTCCTCT   
  
  
- ACGCCGTTTC CTTCGCGCTG CTTCTCCCAG AGGTGGAGGA TTGGGAGGAG GAGGTTACGC GTCTTCGTCA   
  
  
- TAGGCCGCTG TTTATGCTTC TTCGCTTGTT CTACGAAGAT CTTTAGAGCC TCACCCGGTG GGGTAAGCCG   
  
  
- TGGAGGCGGG TTGCGCAGCG GCGCATGAAG AGCCTTCGGT ACAGTCGGGC AGAGCATAGG AGGACGGAGC   
  
  
- CGTAGATGCG GCGGGAGGGG TGGCAGGGTG TGATGCAGTT CGAGGAGAGG CGGAAGGTTC AGAAGTTACC   
  
  
- GTAGTCGGGT AAGCAGTTTA AGAGAGTGAA GTGACGTTTG GTTCGTTAGG TTCTCCGGAA GGTCTCCCTT   
  
  
- CTGTCCCAGG TGTAGTAGCT GGAGCTATAG TACGTCCCCG AGGTCACCGG GCCCGACAAG GTGTAGGAGC   
  
  
- GCAGGGCCGG TCCACCCGGA GGGAAGCATT CCGAGTGGCC CGAGCCCTGG AGGTACCTCC GCGAGCTCCG   
  
  
- GTGGCCTTTT TCCGAGAGTC TGAAGCGGCT CTTCAACCCC AACGGGAAAC TCAAATATGG GCACCGCCTC   
  
  
- TTCTAACCTT TAAACCTGAA CCTTTCCAAC GTACAATCAT TTTCCCTTCG AGAACGACAC GTAACCAATG   
  
  
- TCGTGAGAAA CATACTACAA TGACCGAGAC TATGATTATG TGAACCGGAA GAAGTTTCCA ACCGCGGTTT   
  
  
- TCACCACTGC CACCACCTCG TTCTGGATTC GGCTTGTCCG AGAAAGGATC CTTCCAAACA TCTCCGCTAG   
  
  
- GTGATGATAA GTCGGGATAA ACTGAGGAAT CCTCGCTCAA TACCTCTCCT GTCACTCCTC TCCGTACACC   
  
  
- AACTCGTTGT CGAGGAAAGA TCCCTCTAAG CTTTGTAAGA CCGGCAACCA CCCGGGAGTT CCTGGCCCCT   
  
  
- CGGGTTCAAA CGCTCGACCT CCCTCTTCGA TGTTGTCAGG CCGAAATCCC CTTAGAGTAA CCGTCCGTTG   
  
  
- CGGCGACGGG TCCGGTGGAA CGAGGAGCCG TACAAGGGAA GACTACCCAT ATGAAATTAG CTCCTGTCAC   
  
  
- CGTGTGAATT CGAGCCCACC TTCCTGAACA CGGAGGACTG ACGAAGCCGG ACCTCCGGAA GGGTACGAGT   
  
  
- ATGATACTCG TGGCAGACAT GAGCCTCGGT TAT

+     O2-site

| Site Name | Organism | Position | Strand | Matrix score. | sequence | function |
| --- | --- | --- | --- | --- | --- | --- |
| O2-site | Zea mays | 1090 | + | 8 | GATGA(C/T)(A/G)TG(A/G) | cis-acting regulatory element involved in zein metabolism regulation |
| O2-site | Zea mays | 3704 | - | 9 | GATGATGTGG | cis-acting regulatory element involved in zein metabolism regulation |
| O2-site | Zea mays | 3472 | - | 9 | GATGACATGG | cis-acting regulatory element involved in zein metabolism regulation |
| O2-site | Zea mays | 2703 | - | 9 | GATGACATGG | cis-acting regulatory element involved in zein metabolism regulation |
| O2-site | Zea mays | 3653 | - | 10 | GATGATGTGG | cis-acting regulatory element involved in zein metabolism regulation |

>HU01G00472.1   
+ -Up\_Stream \_Len000GTCTCT GTAACACAAT TCATTAAATC CATCCAAAGG GGATCATAAA TAACCAAACT   
  
  
+ TTGTTGGAAA CTGCTTTGAT GTGGGCTTTC TCTTTCAGCA TTTATGTTAT ACATATATAT GGTTATAAAG   
  
  
+ TTACAAAAGT ATGACCCGAC ACGACAATTC AATCCGAATC CGACTCGAAG TTACTGAAAG TAATCTGAAT   
  
  
+ ACGATTTGAC AGCTTAGTTT AAAAACCTAT TACTTCTCAA TTTTGAGTAA ATCTAAGCTA TTTTTAATGC   
  
  
+ TGAATAGATC GACCCGATTC GACCCGTTTT CCGACTCTAT ATGGTTATGG TGCCCCTAAA AAAAATTAAA   
  
  
+ AAAGAAAAAG AATAGAAAAA TATAAAATAA AACTGAGATT TCTGAACACT CTCTCCCCTA GTCTTGTGGG   
  
  
+ CATGGAAAGT GGTTGGAGAT GGAGATGTTT GAGTTTCATT TTCTGATATT ATTTTTTAGT CATTAGAAGT   
  
  
+ ACAAAGTCTA AACTCAACAA GAGCATGAGG GGAGAATGGT TGTTGTAGCC AATGGAAAAT AAAAAGAACA   
  
  
+ TTCACTCTTC TTGTGCTTTA TTTATGGTGA TAATTTGTTA CTTGTATTTT TAAAAATGAT TAAATATATT   
  
  
+ GATCCTTTGA TATTATGTTG AAATAATAAA AATATAAAAA TTAGAAAATA TTTTACCTCG TGAAATTTTT   
  
  
+ TTTCTGATTA ATCATTGAAA TTATGGTGAA AAATAAAGGA ACATCTCTTG TAAACACTCT ATCCGTTTAC   
  
  
+ GTTTGACCAC AAACTTGCTA TCCACAAGCA TCTCAACCTT TGCCTTTAAC CCAATTGTGG GTTGATGAAA   
  
  
+ TCTAACCATT ATAGGATTAG ATGGTAGTCG TTAATCCTGT GAAACAAGTT TTTGGTTATG ATGTATCCAA   
  
  
+ TTATGAGGGC CTGATAACTC TTGCCTCGGC ATTTGCGTGA TAATGACGTG GTACTAATAG CAATTATTTT   
  
  
+ TCGCAAATAT TCGAAATTTT TTAAGAATAA ACGCAAAATT ATTGATCTTG ACAAATGATT TTATTTTTTT   
  
  
+ GTTGGGAAAA GGATAAACAT CTAGTGTCAA TTGTTGATGA CCTGATAGCT CTCGATTTCT TATTTATGAG   
  
  
+ TTATGTTGAT ATGACACTAA TTGTAATTAT TTGTTGTAAA CTTAAACATT TTTAAAAATC AAAGCAAAAT   
  
  
+ TTGTTAATTA CAATAAATCA ATCTACTTTT ACTATGATCT TATTTGGTGT AATTATTGAT CTTGAATTTT   
  
  
+ TTTTTTTCTT TTTGAAAAAG ATAAGCATCG AATTTTTTCA AAAATAAAAA GGGTGAAGGA AGAAAAAATG   
  
  
+ GAAACAGTTA CCGAGTAAGG AAGGACAGAG CGCGAGAGGA CAAGACACAA AGAGAGGGGA GAGAGAGAGA   
  
  
+ GAAGGGGGCA ATGGCAGTGG GGAAGGTCGA GGAGCACCTT TCAACTTCAA GGCTCAACAC CCCCCCCCCC   
  
  
+ CCCCTCTTTC CTCATTTTGC GACGTTAGCA TTTGACATTT TGCTTTCAAC CGTCCCTCGG CATCATCAGC   
  
  
+ CCTGCCCTTG CTCAGCTTTT CCCATCTTTT TTTGGCTTTT TGTTTGACTC CCTCTATAAC TCTATTCACG   
  
  
+ TCGAGCCTTG TAAAAATTTT ACATATTTTA TTTTATTTTA CTTTTTTAGG TTTATCCCTC TCTATTCTTG   
  
  
+ ACCTACGGGC ATTTTTTATT TTCTTTGGGT TTGAGACTTG TGAACTTTTT GCACATTTTC TCACAAAAAT   
  
  
+ TTATCTTGTC TTTTTCACTT TCAACGTTCC TTCTCTCTCT CTCTCTCTCT CTCTCTCTCT CTCACTCACA   
  
  
+ TCCTAGACAG ACGTCCTTTT TAAAGAATTT ATTTAACAAG TTAGAAGAAA CCGTTGTTTA AGTAGAACCA   
  
  
+ CCCTGTTTGG ATGCCAAAGG AAATACCAGT CAAGAAAAAT CACGACAATC ACAACACATC CATTTCGTTT   
  
  
+ ACATCTCATC CTCATCCTCA TCTTCCTCAT CTCTACCCAT CTCCCTCTTT CTGTATGGCT GCTTCTTCTA   
  
  
+ CTTTGTTCCC TGACCCTAAC AATACTAGTT TAGTCCTCCC TTATTATTAT TATCCTCCTC CTTCCTCCTC   
  
  
+ TTACTATAAT GGTAACCCTT ACCTTCCTCT TCCTAACACC ACTGCTATTC CTACTCCTTC AACAACCTCT   
  
  
+ CCGGTATTTT CTTCCGCCAC CTACTCGGAG TTGAATACTG ATTGTCAAAC TCAACTGCCC ATGTCTAACC   
  
  
+ TCCCCGGAAA GATCGTTCGG AAGCGGAGTG CTGCTGAAAT GGAGCAGCGC TCCGCTCCGA TTGCGGGTGA   
  
  
+ TTACCCGCCA ACTCATCAGC GGGTAATCAC CCGCCACAAT GAGGCGGCCT CATCAGTGTC TTTCATTGAC   
  
  
+ TCCTCACTCT CGCCACTTCA CCTCTTCAAC GGCTCCAATT CGACGACCCC ATTAGCCCCC GACCGTCCTA   
  
  
+ ATGATCCGGG TCTGAGTCAG GGTCCGGATC TGCCTCTTGT GTGCGGATTC TCCGGACTCC CTTTATTCCC   
  
  
+ ACCGGTGGAG AGAAGACCTT CCATCCCCGC CACGACCACT GCTGCCGCGG CGATCGCGGT GGTTCCTGTG   
  
  
+ GAGGAGGATG ATCCGACGTG GATGGATAGC ATCATAAAAG AGCTGATTCA AAGCTCAAAC TCGATCTCAA   
  
  
+ TCCCACAGTT GATTCAGAAC GTTAGAGATA TCATCTACCC ATGTAATCCT AATCTGGGGT CCGCCATTGA   
  
  
+ GTTCCGCCTC CGCTCGCTCG CCGCCGACCC CCTCATTGCT CCGCCGCCAC TTCCGCCCTT CCACCACCAC   
  
  
+ CAACACCACC TCAATCAACA AATCACTCTT CCTCGAATCA GCAACACTAA CAACAACAAT CATGTGAGCG   
  
  
+ TGTACGCCAA CAAAGGACCC GGATACTTCA ACTTGGGTCC GGGTCAGGGT CAGGGTCCGA TTAACATCGA   
  
  
+ CCAAGCTAAC CTTTCCTTCC CTCCTGATTC CACCGCCTGT TGGGGTGTCA GTGTCACACC GCCACCGTCT   
  
  
+ TCCGCCGCCG GTTCCGGCTC CGGTGGAAGC GGCAGTAGTA GTGGTAAATC AAACCCTAAC CCTAATCCCA   
  
  
+ ACCCGAACCC AAACCCAAAC CCAAACCCGA ACAACAAAGC TCAGGATGTT CAACTCCAAA CTCCACAACA   
  
  
+ ACAGCAACAG CAACAGCAAC AAATCCCCGT GGATCAGGAG CAAGACCCGG CGGCTCCACC GGCGACGGAA   
  
  
+ GCATCGCCAC CATCTCCAAG AGCGGCGGCA CCACCGCCGG CGGCAGTGAA AGCAAGAGAA AGAGAGGAGA   
  
  
+ TGCGGCAAAG GAAGCGCGAC GAAGAGGGTC TCCACCTCCT AACCCTCCTC CTCCAATGCG CAGAAGCAGT   
  
  
+ ATCCGGCGAC AAATACGAAG AAGCGAACAA GATGCTTCTA GAAATCTCGG AGTGGGCCAC CCCATTCGGC   
  
  
+ ACCTCCGCCC AACGCGTCGC CGCGTACTTC TCGGAAGCCA TGTCAGCCCG TCTCGTATCC TCCTGCCTCG   
  
  
+ GCATCTACGC CGCCCTCCCC ACCGTCCCAC ACTACGTCAA GCTCCTCTCC GCCTTCCAAG TCTTCAATGG   
  
  
+ CATCAGCCCA TTCGTCAAAT TCTCTCACTT CACTGCAAAC CAAGCAATCC AAGAGGCCTT CCAGAGGGAA   
  
  
+ GACAGGGTCC ACATCATCGA CCTCGATATC ATGCAGGGGC TCCAGTGGCC CGGGCTGTTC CACATCCTCG   
  
  
+ CGTCCCGGCC AGGTGGGCCT CCCTTCGTAA GGCTCACCGG GCTCGGGACC TCCATGGAGG CGCTCGAGGC   
  
  
+ CACCGGAAAA AGGCTCTCAG ACTTCGCCGA GAAGTTGGGG TTGCCCTTTG AGTTTATACC CGTGGCGGAG   
  
  
+ AAGATTGGAA ATTTGGACTT GGAAAGGTTG CATGTTAGTA AAAGGGAAGC TCTTGCTGTG CATTGGTTAC   
  
  
+ AGCACTCTTT GTATGATGTT ACTGGCTCTG ATACTAATAC ACTTGGCCTT CTTCAAAGGT TGGCGCCAAA   
  
  
+ AGTGGTGACG GTGGTGGAGC AAGACCTAAG CCGAACAGGC TCTTTCCTAG GAAGGTTTGT AGAGGCGATC   
  
  
+ CACTACTATT CAGCCCTATT TGACTCCTTA GGAGCGAGTT ATGGAGAGGA CAGTGAGGAG AGGCATGTGG   
  
  
+ TTGAGCAACA GCTCCTTTCT AGGGAGATTC GAAACATTCT GGCCGTTGGT GGGCCCTCAA GGACCGGGGA   
  
  
+ GCCCAAGTTT GCGAGCTGGA GGGAGAAGCT ACAACAGTCC GGCTTTAGGG GAATCTCATT GGCAGGCAAC   
  
  
+ GCCGCTGCCC AGGCCACCTT GCTCCTCGGC ATGTTCCCTT CTGATGGGTA TACTTTAATC GAGGACAGTG   
  
  
+ GCACACTTAA GCTCGGGTGG AAGGACTTGT GCCTCCTGAC TGCTTCGGCC TGGAGGCCTT CCCATGCTCA   
  
  
+ TACTATGAGC ACCGTCTGTA CTCGGAGCCA ATA  

- -Up\_Stream \_Len000CAGAGA CATTGTGTTA AGTAATTTAG GTAGGTTTCC CCTAGTATTT ATTGGTTTGA   
  
  
- AACAACCTTT GACGAAACTA CACCCGAAAG AGAAAGTCGT AAATACAATA TGTATATATA CCAATATTTC   
  
  
- AATGTTTTCA TACTGGGCTG TGCTGTTAAG TTAGGCTTAG GCTGAGCTTC AATGACTTTC ATTAGACTTA   
  
  
- TGCTAAACTG TCGAATCAAA TTTTTGGATA ATGAAGAGTT AAAACTCATT TAGATTCGAT AAAAATTACG   
  
  
- ACTTATCTAG CTGGGCTAAG CTGGGCAAAA GGCTGAGATA TACCAATACC ACGGGGATTT TTTTTAATTT   
  
  
- TTTCTTTTTC TTATCTTTTT ATATTTTATT TTGACTCTAA AGACTTGTGA GAGAGGGGAT CAGAACACCC   
  
  
- GTACCTTTCA CCAACCTCTA CCTCTACAAA CTCAAAGTAA AAGACTATAA TAAAAAATCA GTAATCTTCA   
  
  
- TGTTTCAGAT TTGAGTTGTT CTCGTACTCC CCTCTTACCA ACAACATCGG TTACCTTTTA TTTTTCTTGT   
  
  
- AAGTGAGAAG AACACGAAAT AAATACCACT ATTAAACAAT GAACATAAAA ATTTTTACTA ATTTATATAA   
  
  
- CTAGGAAACT ATAATACAAC TTTATTATTT TTATATTTTT AATCTTTTAT AAAATGGAGC ACTTTAAAAA   
  
  
- AAAGACTAAT TAGTAACTTT AATACCACTT TTTATTTCCT TGTAGAGAAC ATTTGTGAGA TAGGCAAATG   
  
  
- CAAACTGGTG TTTGAACGAT AGGTGTTCGT AGAGTTGGAA ACGGAAATTG GGTTAACACC CAACTACTTT   
  
  
- AGATTGGTAA TATCCTAATC TACCATCAGC AATTAGGACA CTTTGTTCAA AAACCAATAC TACATAGGTT   
  
  
- AATACTCCCG GACTATTGAG AACGGAGCCG TAAACGCACT ATTACTGCAC CATGATTATC GTTAATAAAA   
  
  
- AGCGTTTATA AGCTTTAAAA AATTCTTATT TGCGTTTTAA TAACTAGAAC TGTTTACTAA AATAAAAAAA   
  
  
- CAACCCTTTT CCTATTTGTA GATCACAGTT AACAACTACT GGACTATCGA GAGCTAAAGA ATAAATACTC   
  
  
- AATACAACTA TACTGTGATT AACATTAATA AACAACATTT GAATTTGTAA AAATTTTTAG TTTCGTTTTA   
  
  
- AACAATTAAT GTTATTTAGT TAGATGAAAA TGATACTAGA ATAAACCACA TTAATAACTA GAACTTAAAA   
  
  
- AAAAAAAGAA AAACTTTTTC TATTCGTAGC TTAAAAAAGT TTTTATTTTT CCCACTTCCT TCTTTTTTAC   
  
  
- CTTTGTCAAT GGCTCATTCC TTCCTGTCTC GCGCTCTCCT GTTCTGTGTT TCTCTCCCCT CTCTCTCTCT   
  
  
- CTTCCCCCGT TACCGTCACC CCTTCCAGCT CCTCGTGGAA AGTTGAAGTT CCGAGTTGTG GGGGGGGGGG   
  
  
- GGGGAGAAAG GAGTAAAACG CTGCAATCGT AAACTGTAAA ACGAAAGTTG GCAGGGAGCC GTAGTAGTCG   
  
  
- GGACGGGAAC GAGTCGAAAA GGGTAGAAAA AAACCGAAAA ACAAACTGAG GGAGATATTG AGATAAGTGC   
  
  
- AGCTCGGAAC ATTTTTAAAA TGTATAAAAT AAAATAAAAT GAAAAAATCC AAATAGGGAG AGATAAGAAC   
  
  
- TGGATGCCCG TAAAAAATAA AAGAAACCCA AACTCTGAAC ACTTGAAAAA CGTGTAAAAG AGTGTTTTTA   
  
  
- AATAGAACAG AAAAAGTGAA AGTTGCAAGG AAGAGAGAGA GAGAGAGAGA GAGAGAGAGA GAGTGAGTGT   
  
  
- AGGATCTGTC TGCAGGAAAA ATTTCTTAAA TAAATTGTTC AATCTTCTTT GGCAACAAAT TCATCTTGGT   
  
  
- GGGACAAACC TACGGTTTCC TTTATGGTCA GTTCTTTTTA GTGCTGTTAG TGTTGTGTAG GTAAAGCAAA   
  
  
- TGTAGAGTAG GAGTAGGAGT AGAAGGAGTA GAGATGGGTA GAGGGAGAAA GACATACCGA CGAAGAAGAT   
  
  
- GAAACAAGGG ACTGGGATTG TTATGATCAA ATCAGGAGGG AATAATAATA ATAGGAGGAG GAAGGAGGAG   
  
  
- AATGATATTA CCATTGGGAA TGGAAGGAGA AGGATTGTGG TGACGATAAG GATGAGGAAG TTGTTGGAGA   
  
  
- GGCCATAAAA GAAGGCGGTG GATGAGCCTC AACTTATGAC TAACAGTTTG AGTTGACGGG TACAGATTGG   
  
  
- AGGGGCCTTT CTAGCAAGCC TTCGCCTCAC GACGACTTTA CCTCGTCGCG AGGCGAGGCT AACGCCCACT   
  
  
- AATGGGCGGT TGAGTAGTCG CCCATTAGTG GGCGGTGTTA CTCCGCCGGA GTAGTCACAG AAAGTAACTG   
  
  
- AGGAGTGAGA GCGGTGAAGT GGAGAAGTTG CCGAGGTTAA GCTGCTGGGG TAATCGGGGG CTGGCAGGAT   
  
  
- TACTAGGCCC AGACTCAGTC CCAGGCCTAG ACGGAGAACA CACGCCTAAG AGGCCTGAGG GAAATAAGGG   
  
  
- TGGCCACCTC TCTTCTGGAA GGTAGGGGCG GTGCTGGTGA CGACGGCGCC GCTAGCGCCA CCAAGGACAC   
  
  
- CTCCTCCTAC TAGGCTGCAC CTACCTATCG TAGTATTTTC TCGACTAAGT TTCGAGTTTG AGCTAGAGTT   
  
  
- AGGGTGTCAA CTAAGTCTTG CAATCTCTAT AGTAGATGGG TACATTAGGA TTAGACCCCA GGCGGTAACT   
  
  
- CAAGGCGGAG GCGAGCGAGC GGCGGCTGGG GGAGTAACGA GGCGGCGGTG AAGGCGGGAA GGTGGTGGTG   
  
  
- GTTGTGGTGG AGTTAGTTGT TTAGTGAGAA GGAGCTTAGT CGTTGTGATT GTTGTTGTTA GTACACTCGC   
  
  
- ACATGCGGTT GTTTCCTGGG CCTATGAAGT TGAACCCAGG CCCAGTCCCA GTCCCAGGCT AATTGTAGCT   
  
  
- GGTTCGATTG GAAAGGAAGG GAGGACTAAG GTGGCGGACA ACCCCACAGT CACAGTGTGG CGGTGGCAGA   
  
  
- AGGCGGCGGC CAAGGCCGAG GCCACCTTCG CCGTCATCAT CACCATTTAG TTTGGGATTG GGATTAGGGT   
  
  
- TGGGCTTGGG TTTGGGTTTG GGTTTGGGCT TGTTGTTTCG AGTCCTACAA GTTGAGGTTT GAGGTGTTGT   
  
  
- TGTCGTTGTC GTTGTCGTTG TTTAGGGGCA CCTAGTCCTC GTTCTGGGCC GCCGAGGTGG CCGCTGCCTT   
  
  
- CGTAGCGGTG GTAGAGGTTC TCGCCGCCGT GGTGGCGGCC GCCGTCACTT TCGTTCTCTT TCTCTCCTCT   
  
  
- ACGCCGTTTC CTTCGCGCTG CTTCTCCCAG AGGTGGAGGA TTGGGAGGAG GAGGTTACGC GTCTTCGTCA   
  
  
- TAGGCCGCTG TTTATGCTTC TTCGCTTGTT CTACGAAGAT CTTTAGAGCC TCACCCGGTG GGGTAAGCCG   
  
  
- TGGAGGCGGG TTGCGCAGCG GCGCATGAAG AGCCTTCGGT ACAGTCGGGC AGAGCATAGG AGGACGGAGC   
  
  
- CGTAGATGCG GCGGGAGGGG TGGCAGGGTG TGATGCAGTT CGAGGAGAGG CGGAAGGTTC AGAAGTTACC   
  
  
- GTAGTCGGGT AAGCAGTTTA AGAGAGTGAA GTGACGTTTG GTTCGTTAGG TTCTCCGGAA GGTCTCCCTT   
  
  
- CTGTCCCAGG TGTAGTAGCT GGAGCTATAG TACGTCCCCG AGGTCACCGG GCCCGACAAG GTGTAGGAGC   
  
  
- GCAGGGCCGG TCCACCCGGA GGGAAGCATT CCGAGTGGCC CGAGCCCTGG AGGTACCTCC GCGAGCTCCG   
  
  
- GTGGCCTTTT TCCGAGAGTC TGAAGCGGCT CTTCAACCCC AACGGGAAAC TCAAATATGG GCACCGCCTC   
  
  
- TTCTAACCTT TAAACCTGAA CCTTTCCAAC GTACAATCAT TTTCCCTTCG AGAACGACAC GTAACCAATG   
  
  
- TCGTGAGAAA CATACTACAA TGACCGAGAC TATGATTATG TGAACCGGAA GAAGTTTCCA ACCGCGGTTT   
  
  
- TCACCACTGC CACCACCTCG TTCTGGATTC GGCTTGTCCG AGAAAGGATC CTTCCAAACA TCTCCGCTAG   
  
  
- GTGATGATAA GTCGGGATAA ACTGAGGAAT CCTCGCTCAA TACCTCTCCT GTCACTCCTC TCCGTACACC   
  
  
- AACTCGTTGT CGAGGAAAGA TCCCTCTAAG CTTTGTAAGA CCGGCAACCA CCCGGGAGTT CCTGGCCCCT   
  
  
- CGGGTTCAAA CGCTCGACCT CCCTCTTCGA TGTTGTCAGG CCGAAATCCC CTTAGAGTAA CCGTCCGTTG   
  
  
- CGGCGACGGG TCCGGTGGAA CGAGGAGCCG TACAAGGGAA GACTACCCAT ATGAAATTAG CTCCTGTCAC   
  
  
- CGTGTGAATT CGAGCCCACC TTCCTGAACA CGGAGGACTG ACGAAGCCGG ACCTCCGGAA GGGTACGAGT   
  
  
- ATGATACTCG TGGCAGACAT GAGCCTCGGT TAT

+     STRE

| Site Name | Organism | Position | Strand | Matrix score. | sequence | function |
| --- | --- | --- | --- | --- | --- | --- |
| STRE | Arabidopsis thaliana | 52 | + | 5 | AGGGG |  |
| STRE | Arabidopsis thaliana | 1475 | - | 5 | AGGGG |  |
| STRE | Arabidopsis thaliana | 1389 | + | 5 | AGGGG |  |
| STRE | Arabidopsis thaliana | 3679 | + | 5 | AGGGG |  |
| STRE | Arabidopsis thaliana | 409 | - | 5 | AGGGG |  |
| STRE | Arabidopsis thaliana | 1407 | + | 5 | AGGGG |  |
| STRE | Arabidopsis thaliana | 337 | - | 5 | AGGGG |  |
| STRE | Arabidopsis thaliana | 2763 | - | 5 | AGGGG |  |
| STRE | Arabidopsis thaliana | 522 | + | 5 | AGGGG |  |
| STRE | Arabidopsis thaliana | 4251 | + | 5 | AGGGG |  |

>HU01G00472.1   
+ -Up\_Stream \_Len000GTCTCT GTAACACAAT TCATTAAATC CATCCAAAGG GGATCATAAA TAACCAAACT   
  
  
+ TTGTTGGAAA CTGCTTTGAT GTGGGCTTTC TCTTTCAGCA TTTATGTTAT ACATATATAT GGTTATAAAG   
  
  
+ TTACAAAAGT ATGACCCGAC ACGACAATTC AATCCGAATC CGACTCGAAG TTACTGAAAG TAATCTGAAT   
  
  
+ ACGATTTGAC AGCTTAGTTT AAAAACCTAT TACTTCTCAA TTTTGAGTAA ATCTAAGCTA TTTTTAATGC   
  
  
+ TGAATAGATC GACCCGATTC GACCCGTTTT CCGACTCTAT ATGGTTATGG TGCCCCTAAA AAAAATTAAA   
  
  
+ AAAGAAAAAG AATAGAAAAA TATAAAATAA AACTGAGATT TCTGAACACT CTCTCCCCTA GTCTTGTGGG   
  
  
+ CATGGAAAGT GGTTGGAGAT GGAGATGTTT GAGTTTCATT TTCTGATATT ATTTTTTAGT CATTAGAAGT   
  
  
+ ACAAAGTCTA AACTCAACAA GAGCATGAGG GGAGAATGGT TGTTGTAGCC AATGGAAAAT AAAAAGAACA   
  
  
+ TTCACTCTTC TTGTGCTTTA TTTATGGTGA TAATTTGTTA CTTGTATTTT TAAAAATGAT TAAATATATT   
  
  
+ GATCCTTTGA TATTATGTTG AAATAATAAA AATATAAAAA TTAGAAAATA TTTTACCTCG TGAAATTTTT   
  
  
+ TTTCTGATTA ATCATTGAAA TTATGGTGAA AAATAAAGGA ACATCTCTTG TAAACACTCT ATCCGTTTAC   
  
  
+ GTTTGACCAC AAACTTGCTA TCCACAAGCA TCTCAACCTT TGCCTTTAAC CCAATTGTGG GTTGATGAAA   
  
  
+ TCTAACCATT ATAGGATTAG ATGGTAGTCG TTAATCCTGT GAAACAAGTT TTTGGTTATG ATGTATCCAA   
  
  
+ TTATGAGGGC CTGATAACTC TTGCCTCGGC ATTTGCGTGA TAATGACGTG GTACTAATAG CAATTATTTT   
  
  
+ TCGCAAATAT TCGAAATTTT TTAAGAATAA ACGCAAAATT ATTGATCTTG ACAAATGATT TTATTTTTTT   
  
  
+ GTTGGGAAAA GGATAAACAT CTAGTGTCAA TTGTTGATGA CCTGATAGCT CTCGATTTCT TATTTATGAG   
  
  
+ TTATGTTGAT ATGACACTAA TTGTAATTAT TTGTTGTAAA CTTAAACATT TTTAAAAATC AAAGCAAAAT   
  
  
+ TTGTTAATTA CAATAAATCA ATCTACTTTT ACTATGATCT TATTTGGTGT AATTATTGAT CTTGAATTTT   
  
  
+ TTTTTTTCTT TTTGAAAAAG ATAAGCATCG AATTTTTTCA AAAATAAAAA GGGTGAAGGA AGAAAAAATG   
  
  
+ GAAACAGTTA CCGAGTAAGG AAGGACAGAG CGCGAGAGGA CAAGACACAA AGAGAGGGGA GAGAGAGAGA   
  
  
+ GAAGGGGGCA ATGGCAGTGG GGAAGGTCGA GGAGCACCTT TCAACTTCAA GGCTCAACAC CCCCCCCCCC   
  
  
+ CCCCTCTTTC CTCATTTTGC GACGTTAGCA TTTGACATTT TGCTTTCAAC CGTCCCTCGG CATCATCAGC   
  
  
+ CCTGCCCTTG CTCAGCTTTT CCCATCTTTT TTTGGCTTTT TGTTTGACTC CCTCTATAAC TCTATTCACG   
  
  
+ TCGAGCCTTG TAAAAATTTT ACATATTTTA TTTTATTTTA CTTTTTTAGG TTTATCCCTC TCTATTCTTG   
  
  
+ ACCTACGGGC ATTTTTTATT TTCTTTGGGT TTGAGACTTG TGAACTTTTT GCACATTTTC TCACAAAAAT   
  
  
+ TTATCTTGTC TTTTTCACTT TCAACGTTCC TTCTCTCTCT CTCTCTCTCT CTCTCTCTCT CTCACTCACA   
  
  
+ TCCTAGACAG ACGTCCTTTT TAAAGAATTT ATTTAACAAG TTAGAAGAAA CCGTTGTTTA AGTAGAACCA   
  
  
+ CCCTGTTTGG ATGCCAAAGG AAATACCAGT CAAGAAAAAT CACGACAATC ACAACACATC CATTTCGTTT   
  
  
+ ACATCTCATC CTCATCCTCA TCTTCCTCAT CTCTACCCAT CTCCCTCTTT CTGTATGGCT GCTTCTTCTA   
  
  
+ CTTTGTTCCC TGACCCTAAC AATACTAGTT TAGTCCTCCC TTATTATTAT TATCCTCCTC CTTCCTCCTC   
  
  
+ TTACTATAAT GGTAACCCTT ACCTTCCTCT TCCTAACACC ACTGCTATTC CTACTCCTTC AACAACCTCT   
  
  
+ CCGGTATTTT CTTCCGCCAC CTACTCGGAG TTGAATACTG ATTGTCAAAC TCAACTGCCC ATGTCTAACC   
  
  
+ TCCCCGGAAA GATCGTTCGG AAGCGGAGTG CTGCTGAAAT GGAGCAGCGC TCCGCTCCGA TTGCGGGTGA   
  
  
+ TTACCCGCCA ACTCATCAGC GGGTAATCAC CCGCCACAAT GAGGCGGCCT CATCAGTGTC TTTCATTGAC   
  
  
+ TCCTCACTCT CGCCACTTCA CCTCTTCAAC GGCTCCAATT CGACGACCCC ATTAGCCCCC GACCGTCCTA   
  
  
+ ATGATCCGGG TCTGAGTCAG GGTCCGGATC TGCCTCTTGT GTGCGGATTC TCCGGACTCC CTTTATTCCC   
  
  
+ ACCGGTGGAG AGAAGACCTT CCATCCCCGC CACGACCACT GCTGCCGCGG CGATCGCGGT GGTTCCTGTG   
  
  
+ GAGGAGGATG ATCCGACGTG GATGGATAGC ATCATAAAAG AGCTGATTCA AAGCTCAAAC TCGATCTCAA   
  
  
+ TCCCACAGTT GATTCAGAAC GTTAGAGATA TCATCTACCC ATGTAATCCT AATCTGGGGT CCGCCATTGA   
  
  
+ GTTCCGCCTC CGCTCGCTCG CCGCCGACCC CCTCATTGCT CCGCCGCCAC TTCCGCCCTT CCACCACCAC   
  
  
+ CAACACCACC TCAATCAACA AATCACTCTT CCTCGAATCA GCAACACTAA CAACAACAAT CATGTGAGCG   
  
  
+ TGTACGCCAA CAAAGGACCC GGATACTTCA ACTTGGGTCC GGGTCAGGGT CAGGGTCCGA TTAACATCGA   
  
  
+ CCAAGCTAAC CTTTCCTTCC CTCCTGATTC CACCGCCTGT TGGGGTGTCA GTGTCACACC GCCACCGTCT   
  
  
+ TCCGCCGCCG GTTCCGGCTC CGGTGGAAGC GGCAGTAGTA GTGGTAAATC AAACCCTAAC CCTAATCCCA   
  
  
+ ACCCGAACCC AAACCCAAAC CCAAACCCGA ACAACAAAGC TCAGGATGTT CAACTCCAAA CTCCACAACA   
  
  
+ ACAGCAACAG CAACAGCAAC AAATCCCCGT GGATCAGGAG CAAGACCCGG CGGCTCCACC GGCGACGGAA   
  
  
+ GCATCGCCAC CATCTCCAAG AGCGGCGGCA CCACCGCCGG CGGCAGTGAA AGCAAGAGAA AGAGAGGAGA   
  
  
+ TGCGGCAAAG GAAGCGCGAC GAAGAGGGTC TCCACCTCCT AACCCTCCTC CTCCAATGCG CAGAAGCAGT   
  
  
+ ATCCGGCGAC AAATACGAAG AAGCGAACAA GATGCTTCTA GAAATCTCGG AGTGGGCCAC CCCATTCGGC   
  
  
+ ACCTCCGCCC AACGCGTCGC CGCGTACTTC TCGGAAGCCA TGTCAGCCCG TCTCGTATCC TCCTGCCTCG   
  
  
+ GCATCTACGC CGCCCTCCCC ACCGTCCCAC ACTACGTCAA GCTCCTCTCC GCCTTCCAAG TCTTCAATGG   
  
  
+ CATCAGCCCA TTCGTCAAAT TCTCTCACTT CACTGCAAAC CAAGCAATCC AAGAGGCCTT CCAGAGGGAA   
  
  
+ GACAGGGTCC ACATCATCGA CCTCGATATC ATGCAGGGGC TCCAGTGGCC CGGGCTGTTC CACATCCTCG   
  
  
+ CGTCCCGGCC AGGTGGGCCT CCCTTCGTAA GGCTCACCGG GCTCGGGACC TCCATGGAGG CGCTCGAGGC   
  
  
+ CACCGGAAAA AGGCTCTCAG ACTTCGCCGA GAAGTTGGGG TTGCCCTTTG AGTTTATACC CGTGGCGGAG   
  
  
+ AAGATTGGAA ATTTGGACTT GGAAAGGTTG CATGTTAGTA AAAGGGAAGC TCTTGCTGTG CATTGGTTAC   
  
  
+ AGCACTCTTT GTATGATGTT ACTGGCTCTG ATACTAATAC ACTTGGCCTT CTTCAAAGGT TGGCGCCAAA   
  
  
+ AGTGGTGACG GTGGTGGAGC AAGACCTAAG CCGAACAGGC TCTTTCCTAG GAAGGTTTGT AGAGGCGATC   
  
  
+ CACTACTATT CAGCCCTATT TGACTCCTTA GGAGCGAGTT ATGGAGAGGA CAGTGAGGAG AGGCATGTGG   
  
  
+ TTGAGCAACA GCTCCTTTCT AGGGAGATTC GAAACATTCT GGCCGTTGGT GGGCCCTCAA GGACCGGGGA   
  
  
+ GCCCAAGTTT GCGAGCTGGA GGGAGAAGCT ACAACAGTCC GGCTTTAGGG GAATCTCATT GGCAGGCAAC   
  
  
+ GCCGCTGCCC AGGCCACCTT GCTCCTCGGC ATGTTCCCTT CTGATGGGTA TACTTTAATC GAGGACAGTG   
  
  
+ GCACACTTAA GCTCGGGTGG AAGGACTTGT GCCTCCTGAC TGCTTCGGCC TGGAGGCCTT CCCATGCTCA   
  
  
+ TACTATGAGC ACCGTCTGTA CTCGGAGCCA ATA  

- -Up\_Stream \_Len000CAGAGA CATTGTGTTA AGTAATTTAG GTAGGTTTCC CCTAGTATTT ATTGGTTTGA   
  
  
- AACAACCTTT GACGAAACTA CACCCGAAAG AGAAAGTCGT AAATACAATA TGTATATATA CCAATATTTC   
  
  
- AATGTTTTCA TACTGGGCTG TGCTGTTAAG TTAGGCTTAG GCTGAGCTTC AATGACTTTC ATTAGACTTA   
  
  
- TGCTAAACTG TCGAATCAAA TTTTTGGATA ATGAAGAGTT AAAACTCATT TAGATTCGAT AAAAATTACG   
  
  
- ACTTATCTAG CTGGGCTAAG CTGGGCAAAA GGCTGAGATA TACCAATACC ACGGGGATTT TTTTTAATTT   
  
  
- TTTCTTTTTC TTATCTTTTT ATATTTTATT TTGACTCTAA AGACTTGTGA GAGAGGGGAT CAGAACACCC   
  
  
- GTACCTTTCA CCAACCTCTA CCTCTACAAA CTCAAAGTAA AAGACTATAA TAAAAAATCA GTAATCTTCA   
  
  
- TGTTTCAGAT TTGAGTTGTT CTCGTACTCC CCTCTTACCA ACAACATCGG TTACCTTTTA TTTTTCTTGT   
  
  
- AAGTGAGAAG AACACGAAAT AAATACCACT ATTAAACAAT GAACATAAAA ATTTTTACTA ATTTATATAA   
  
  
- CTAGGAAACT ATAATACAAC TTTATTATTT TTATATTTTT AATCTTTTAT AAAATGGAGC ACTTTAAAAA   
  
  
- AAAGACTAAT TAGTAACTTT AATACCACTT TTTATTTCCT TGTAGAGAAC ATTTGTGAGA TAGGCAAATG   
  
  
- CAAACTGGTG TTTGAACGAT AGGTGTTCGT AGAGTTGGAA ACGGAAATTG GGTTAACACC CAACTACTTT   
  
  
- AGATTGGTAA TATCCTAATC TACCATCAGC AATTAGGACA CTTTGTTCAA AAACCAATAC TACATAGGTT   
  
  
- AATACTCCCG GACTATTGAG AACGGAGCCG TAAACGCACT ATTACTGCAC CATGATTATC GTTAATAAAA   
  
  
- AGCGTTTATA AGCTTTAAAA AATTCTTATT TGCGTTTTAA TAACTAGAAC TGTTTACTAA AATAAAAAAA   
  
  
- CAACCCTTTT CCTATTTGTA GATCACAGTT AACAACTACT GGACTATCGA GAGCTAAAGA ATAAATACTC   
  
  
- AATACAACTA TACTGTGATT AACATTAATA AACAACATTT GAATTTGTAA AAATTTTTAG TTTCGTTTTA   
  
  
- AACAATTAAT GTTATTTAGT TAGATGAAAA TGATACTAGA ATAAACCACA TTAATAACTA GAACTTAAAA   
  
  
- AAAAAAAGAA AAACTTTTTC TATTCGTAGC TTAAAAAAGT TTTTATTTTT CCCACTTCCT TCTTTTTTAC   
  
  
- CTTTGTCAAT GGCTCATTCC TTCCTGTCTC GCGCTCTCCT GTTCTGTGTT TCTCTCCCCT CTCTCTCTCT   
  
  
- CTTCCCCCGT TACCGTCACC CCTTCCAGCT CCTCGTGGAA AGTTGAAGTT CCGAGTTGTG GGGGGGGGGG   
  
  
- GGGGAGAAAG GAGTAAAACG CTGCAATCGT AAACTGTAAA ACGAAAGTTG GCAGGGAGCC GTAGTAGTCG   
  
  
- GGACGGGAAC GAGTCGAAAA GGGTAGAAAA AAACCGAAAA ACAAACTGAG GGAGATATTG AGATAAGTGC   
  
  
- AGCTCGGAAC ATTTTTAAAA TGTATAAAAT AAAATAAAAT GAAAAAATCC AAATAGGGAG AGATAAGAAC   
  
  
- TGGATGCCCG TAAAAAATAA AAGAAACCCA AACTCTGAAC ACTTGAAAAA CGTGTAAAAG AGTGTTTTTA   
  
  
- AATAGAACAG AAAAAGTGAA AGTTGCAAGG AAGAGAGAGA GAGAGAGAGA GAGAGAGAGA GAGTGAGTGT   
  
  
- AGGATCTGTC TGCAGGAAAA ATTTCTTAAA TAAATTGTTC AATCTTCTTT GGCAACAAAT TCATCTTGGT   
  
  
- GGGACAAACC TACGGTTTCC TTTATGGTCA GTTCTTTTTA GTGCTGTTAG TGTTGTGTAG GTAAAGCAAA   
  
  
- TGTAGAGTAG GAGTAGGAGT AGAAGGAGTA GAGATGGGTA GAGGGAGAAA GACATACCGA CGAAGAAGAT   
  
  
- GAAACAAGGG ACTGGGATTG TTATGATCAA ATCAGGAGGG AATAATAATA ATAGGAGGAG GAAGGAGGAG   
  
  
- AATGATATTA CCATTGGGAA TGGAAGGAGA AGGATTGTGG TGACGATAAG GATGAGGAAG TTGTTGGAGA   
  
  
- GGCCATAAAA GAAGGCGGTG GATGAGCCTC AACTTATGAC TAACAGTTTG AGTTGACGGG TACAGATTGG   
  
  
- AGGGGCCTTT CTAGCAAGCC TTCGCCTCAC GACGACTTTA CCTCGTCGCG AGGCGAGGCT AACGCCCACT   
  
  
- AATGGGCGGT TGAGTAGTCG CCCATTAGTG GGCGGTGTTA CTCCGCCGGA GTAGTCACAG AAAGTAACTG   
  
  
- AGGAGTGAGA GCGGTGAAGT GGAGAAGTTG CCGAGGTTAA GCTGCTGGGG TAATCGGGGG CTGGCAGGAT   
  
  
- TACTAGGCCC AGACTCAGTC CCAGGCCTAG ACGGAGAACA CACGCCTAAG AGGCCTGAGG GAAATAAGGG   
  
  
- TGGCCACCTC TCTTCTGGAA GGTAGGGGCG GTGCTGGTGA CGACGGCGCC GCTAGCGCCA CCAAGGACAC   
  
  
- CTCCTCCTAC TAGGCTGCAC CTACCTATCG TAGTATTTTC TCGACTAAGT TTCGAGTTTG AGCTAGAGTT   
  
  
- AGGGTGTCAA CTAAGTCTTG CAATCTCTAT AGTAGATGGG TACATTAGGA TTAGACCCCA GGCGGTAACT   
  
  
- CAAGGCGGAG GCGAGCGAGC GGCGGCTGGG GGAGTAACGA GGCGGCGGTG AAGGCGGGAA GGTGGTGGTG   
  
  
- GTTGTGGTGG AGTTAGTTGT TTAGTGAGAA GGAGCTTAGT CGTTGTGATT GTTGTTGTTA GTACACTCGC   
  
  
- ACATGCGGTT GTTTCCTGGG CCTATGAAGT TGAACCCAGG CCCAGTCCCA GTCCCAGGCT AATTGTAGCT   
  
  
- GGTTCGATTG GAAAGGAAGG GAGGACTAAG GTGGCGGACA ACCCCACAGT CACAGTGTGG CGGTGGCAGA   
  
  
- AGGCGGCGGC CAAGGCCGAG GCCACCTTCG CCGTCATCAT CACCATTTAG TTTGGGATTG GGATTAGGGT   
  
  
- TGGGCTTGGG TTTGGGTTTG GGTTTGGGCT TGTTGTTTCG AGTCCTACAA GTTGAGGTTT GAGGTGTTGT   
  
  
- TGTCGTTGTC GTTGTCGTTG TTTAGGGGCA CCTAGTCCTC GTTCTGGGCC GCCGAGGTGG CCGCTGCCTT   
  
  
- CGTAGCGGTG GTAGAGGTTC TCGCCGCCGT GGTGGCGGCC GCCGTCACTT TCGTTCTCTT TCTCTCCTCT   
  
  
- ACGCCGTTTC CTTCGCGCTG CTTCTCCCAG AGGTGGAGGA TTGGGAGGAG GAGGTTACGC GTCTTCGTCA   
  
  
- TAGGCCGCTG TTTATGCTTC TTCGCTTGTT CTACGAAGAT CTTTAGAGCC TCACCCGGTG GGGTAAGCCG   
  
  
- TGGAGGCGGG TTGCGCAGCG GCGCATGAAG AGCCTTCGGT ACAGTCGGGC AGAGCATAGG AGGACGGAGC   
  
  
- CGTAGATGCG GCGGGAGGGG TGGCAGGGTG TGATGCAGTT CGAGGAGAGG CGGAAGGTTC AGAAGTTACC   
  
  
- GTAGTCGGGT AAGCAGTTTA AGAGAGTGAA GTGACGTTTG GTTCGTTAGG TTCTCCGGAA GGTCTCCCTT   
  
  
- CTGTCCCAGG TGTAGTAGCT GGAGCTATAG TACGTCCCCG AGGTCACCGG GCCCGACAAG GTGTAGGAGC   
  
  
- GCAGGGCCGG TCCACCCGGA GGGAAGCATT CCGAGTGGCC CGAGCCCTGG AGGTACCTCC GCGAGCTCCG   
  
  
- GTGGCCTTTT TCCGAGAGTC TGAAGCGGCT CTTCAACCCC AACGGGAAAC TCAAATATGG GCACCGCCTC   
  
  
- TTCTAACCTT TAAACCTGAA CCTTTCCAAC GTACAATCAT TTTCCCTTCG AGAACGACAC GTAACCAATG   
  
  
- TCGTGAGAAA CATACTACAA TGACCGAGAC TATGATTATG TGAACCGGAA GAAGTTTCCA ACCGCGGTTT   
  
  
- TCACCACTGC CACCACCTCG TTCTGGATTC GGCTTGTCCG AGAAAGGATC CTTCCAAACA TCTCCGCTAG   
  
  
- GTGATGATAA GTCGGGATAA ACTGAGGAAT CCTCGCTCAA TACCTCTCCT GTCACTCCTC TCCGTACACC   
  
  
- AACTCGTTGT CGAGGAAAGA TCCCTCTAAG CTTTGTAAGA CCGGCAACCA CCCGGGAGTT CCTGGCCCCT   
  
  
- CGGGTTCAAA CGCTCGACCT CCCTCTTCGA TGTTGTCAGG CCGAAATCCC CTTAGAGTAA CCGTCCGTTG   
  
  
- CGGCGACGGG TCCGGTGGAA CGAGGAGCCG TACAAGGGAA GACTACCCAT ATGAAATTAG CTCCTGTCAC   
  
  
- CGTGTGAATT CGAGCCCACC TTCCTGAACA CGGAGGACTG ACGAAGCCGG ACCTCCGGAA GGGTACGAGT   
  
  
- ATGATACTCG TGGCAGACAT GAGCCTCGGT TAT

+     Sp1

| Site Name | Organism | Position | Strand | Matrix score. | sequence | function |
| --- | --- | --- | --- | --- | --- | --- |
| Sp1 | Oryza sativa | 2787 | - | 6 | GGGCGG | light responsive element |
| Sp1 | Oryza sativa | 3514 | - | 6 | GGGCGG | light responsive element |
| Sp1 | Oryza sativa | 3439 | - | 6 | GGGCGG | light responsive element |

>HU01G00472.1   
+ -Up\_Stream \_Len000GTCTCT GTAACACAAT TCATTAAATC CATCCAAAGG GGATCATAAA TAACCAAACT   
  
  
+ TTGTTGGAAA CTGCTTTGAT GTGGGCTTTC TCTTTCAGCA TTTATGTTAT ACATATATAT GGTTATAAAG   
  
  
+ TTACAAAAGT ATGACCCGAC ACGACAATTC AATCCGAATC CGACTCGAAG TTACTGAAAG TAATCTGAAT   
  
  
+ ACGATTTGAC AGCTTAGTTT AAAAACCTAT TACTTCTCAA TTTTGAGTAA ATCTAAGCTA TTTTTAATGC   
  
  
+ TGAATAGATC GACCCGATTC GACCCGTTTT CCGACTCTAT ATGGTTATGG TGCCCCTAAA AAAAATTAAA   
  
  
+ AAAGAAAAAG AATAGAAAAA TATAAAATAA AACTGAGATT TCTGAACACT CTCTCCCCTA GTCTTGTGGG   
  
  
+ CATGGAAAGT GGTTGGAGAT GGAGATGTTT GAGTTTCATT TTCTGATATT ATTTTTTAGT CATTAGAAGT   
  
  
+ ACAAAGTCTA AACTCAACAA GAGCATGAGG GGAGAATGGT TGTTGTAGCC AATGGAAAAT AAAAAGAACA   
  
  
+ TTCACTCTTC TTGTGCTTTA TTTATGGTGA TAATTTGTTA CTTGTATTTT TAAAAATGAT TAAATATATT   
  
  
+ GATCCTTTGA TATTATGTTG AAATAATAAA AATATAAAAA TTAGAAAATA TTTTACCTCG TGAAATTTTT   
  
  
+ TTTCTGATTA ATCATTGAAA TTATGGTGAA AAATAAAGGA ACATCTCTTG TAAACACTCT ATCCGTTTAC   
  
  
+ GTTTGACCAC AAACTTGCTA TCCACAAGCA TCTCAACCTT TGCCTTTAAC CCAATTGTGG GTTGATGAAA   
  
  
+ TCTAACCATT ATAGGATTAG ATGGTAGTCG TTAATCCTGT GAAACAAGTT TTTGGTTATG ATGTATCCAA   
  
  
+ TTATGAGGGC CTGATAACTC TTGCCTCGGC ATTTGCGTGA TAATGACGTG GTACTAATAG CAATTATTTT   
  
  
+ TCGCAAATAT TCGAAATTTT TTAAGAATAA ACGCAAAATT ATTGATCTTG ACAAATGATT TTATTTTTTT   
  
  
+ GTTGGGAAAA GGATAAACAT CTAGTGTCAA TTGTTGATGA CCTGATAGCT CTCGATTTCT TATTTATGAG   
  
  
+ TTATGTTGAT ATGACACTAA TTGTAATTAT TTGTTGTAAA CTTAAACATT TTTAAAAATC AAAGCAAAAT   
  
  
+ TTGTTAATTA CAATAAATCA ATCTACTTTT ACTATGATCT TATTTGGTGT AATTATTGAT CTTGAATTTT   
  
  
+ TTTTTTTCTT TTTGAAAAAG ATAAGCATCG AATTTTTTCA AAAATAAAAA GGGTGAAGGA AGAAAAAATG   
  
  
+ GAAACAGTTA CCGAGTAAGG AAGGACAGAG CGCGAGAGGA CAAGACACAA AGAGAGGGGA GAGAGAGAGA   
  
  
+ GAAGGGGGCA ATGGCAGTGG GGAAGGTCGA GGAGCACCTT TCAACTTCAA GGCTCAACAC CCCCCCCCCC   
  
  
+ CCCCTCTTTC CTCATTTTGC GACGTTAGCA TTTGACATTT TGCTTTCAAC CGTCCCTCGG CATCATCAGC   
  
  
+ CCTGCCCTTG CTCAGCTTTT CCCATCTTTT TTTGGCTTTT TGTTTGACTC CCTCTATAAC TCTATTCACG   
  
  
+ TCGAGCCTTG TAAAAATTTT ACATATTTTA TTTTATTTTA CTTTTTTAGG TTTATCCCTC TCTATTCTTG   
  
  
+ ACCTACGGGC ATTTTTTATT TTCTTTGGGT TTGAGACTTG TGAACTTTTT GCACATTTTC TCACAAAAAT   
  
  
+ TTATCTTGTC TTTTTCACTT TCAACGTTCC TTCTCTCTCT CTCTCTCTCT CTCTCTCTCT CTCACTCACA   
  
  
+ TCCTAGACAG ACGTCCTTTT TAAAGAATTT ATTTAACAAG TTAGAAGAAA CCGTTGTTTA AGTAGAACCA   
  
  
+ CCCTGTTTGG ATGCCAAAGG AAATACCAGT CAAGAAAAAT CACGACAATC ACAACACATC CATTTCGTTT   
  
  
+ ACATCTCATC CTCATCCTCA TCTTCCTCAT CTCTACCCAT CTCCCTCTTT CTGTATGGCT GCTTCTTCTA   
  
  
+ CTTTGTTCCC TGACCCTAAC AATACTAGTT TAGTCCTCCC TTATTATTAT TATCCTCCTC CTTCCTCCTC   
  
  
+ TTACTATAAT GGTAACCCTT ACCTTCCTCT TCCTAACACC ACTGCTATTC CTACTCCTTC AACAACCTCT   
  
  
+ CCGGTATTTT CTTCCGCCAC CTACTCGGAG TTGAATACTG ATTGTCAAAC TCAACTGCCC ATGTCTAACC   
  
  
+ TCCCCGGAAA GATCGTTCGG AAGCGGAGTG CTGCTGAAAT GGAGCAGCGC TCCGCTCCGA TTGCGGGTGA   
  
  
+ TTACCCGCCA ACTCATCAGC GGGTAATCAC CCGCCACAAT GAGGCGGCCT CATCAGTGTC TTTCATTGAC   
  
  
+ TCCTCACTCT CGCCACTTCA CCTCTTCAAC GGCTCCAATT CGACGACCCC ATTAGCCCCC GACCGTCCTA   
  
  
+ ATGATCCGGG TCTGAGTCAG GGTCCGGATC TGCCTCTTGT GTGCGGATTC TCCGGACTCC CTTTATTCCC   
  
  
+ ACCGGTGGAG AGAAGACCTT CCATCCCCGC CACGACCACT GCTGCCGCGG CGATCGCGGT GGTTCCTGTG   
  
  
+ GAGGAGGATG ATCCGACGTG GATGGATAGC ATCATAAAAG AGCTGATTCA AAGCTCAAAC TCGATCTCAA   
  
  
+ TCCCACAGTT GATTCAGAAC GTTAGAGATA TCATCTACCC ATGTAATCCT AATCTGGGGT CCGCCATTGA   
  
  
+ GTTCCGCCTC CGCTCGCTCG CCGCCGACCC CCTCATTGCT CCGCCGCCAC TTCCGCCCTT CCACCACCAC   
  
  
+ CAACACCACC TCAATCAACA AATCACTCTT CCTCGAATCA GCAACACTAA CAACAACAAT CATGTGAGCG   
  
  
+ TGTACGCCAA CAAAGGACCC GGATACTTCA ACTTGGGTCC GGGTCAGGGT CAGGGTCCGA TTAACATCGA   
  
  
+ CCAAGCTAAC CTTTCCTTCC CTCCTGATTC CACCGCCTGT TGGGGTGTCA GTGTCACACC GCCACCGTCT   
  
  
+ TCCGCCGCCG GTTCCGGCTC CGGTGGAAGC GGCAGTAGTA GTGGTAAATC AAACCCTAAC CCTAATCCCA   
  
  
+ ACCCGAACCC AAACCCAAAC CCAAACCCGA ACAACAAAGC TCAGGATGTT CAACTCCAAA CTCCACAACA   
  
  
+ ACAGCAACAG CAACAGCAAC AAATCCCCGT GGATCAGGAG CAAGACCCGG CGGCTCCACC GGCGACGGAA   
  
  
+ GCATCGCCAC CATCTCCAAG AGCGGCGGCA CCACCGCCGG CGGCAGTGAA AGCAAGAGAA AGAGAGGAGA   
  
  
+ TGCGGCAAAG GAAGCGCGAC GAAGAGGGTC TCCACCTCCT AACCCTCCTC CTCCAATGCG CAGAAGCAGT   
  
  
+ ATCCGGCGAC AAATACGAAG AAGCGAACAA GATGCTTCTA GAAATCTCGG AGTGGGCCAC CCCATTCGGC   
  
  
+ ACCTCCGCCC AACGCGTCGC CGCGTACTTC TCGGAAGCCA TGTCAGCCCG TCTCGTATCC TCCTGCCTCG   
  
  
+ GCATCTACGC CGCCCTCCCC ACCGTCCCAC ACTACGTCAA GCTCCTCTCC GCCTTCCAAG TCTTCAATGG   
  
  
+ CATCAGCCCA TTCGTCAAAT TCTCTCACTT CACTGCAAAC CAAGCAATCC AAGAGGCCTT CCAGAGGGAA   
  
  
+ GACAGGGTCC ACATCATCGA CCTCGATATC ATGCAGGGGC TCCAGTGGCC CGGGCTGTTC CACATCCTCG   
  
  
+ CGTCCCGGCC AGGTGGGCCT CCCTTCGTAA GGCTCACCGG GCTCGGGACC TCCATGGAGG CGCTCGAGGC   
  
  
+ CACCGGAAAA AGGCTCTCAG ACTTCGCCGA GAAGTTGGGG TTGCCCTTTG AGTTTATACC CGTGGCGGAG   
  
  
+ AAGATTGGAA ATTTGGACTT GGAAAGGTTG CATGTTAGTA AAAGGGAAGC TCTTGCTGTG CATTGGTTAC   
  
  
+ AGCACTCTTT GTATGATGTT ACTGGCTCTG ATACTAATAC ACTTGGCCTT CTTCAAAGGT TGGCGCCAAA   
  
  
+ AGTGGTGACG GTGGTGGAGC AAGACCTAAG CCGAACAGGC TCTTTCCTAG GAAGGTTTGT AGAGGCGATC   
  
  
+ CACTACTATT CAGCCCTATT TGACTCCTTA GGAGCGAGTT ATGGAGAGGA CAGTGAGGAG AGGCATGTGG   
  
  
+ TTGAGCAACA GCTCCTTTCT AGGGAGATTC GAAACATTCT GGCCGTTGGT GGGCCCTCAA GGACCGGGGA   
  
  
+ GCCCAAGTTT GCGAGCTGGA GGGAGAAGCT ACAACAGTCC GGCTTTAGGG GAATCTCATT GGCAGGCAAC   
  
  
+ GCCGCTGCCC AGGCCACCTT GCTCCTCGGC ATGTTCCCTT CTGATGGGTA TACTTTAATC GAGGACAGTG   
  
  
+ GCACACTTAA GCTCGGGTGG AAGGACTTGT GCCTCCTGAC TGCTTCGGCC TGGAGGCCTT CCCATGCTCA   
  
  
+ TACTATGAGC ACCGTCTGTA CTCGGAGCCA ATA  

- -Up\_Stream \_Len000CAGAGA CATTGTGTTA AGTAATTTAG GTAGGTTTCC CCTAGTATTT ATTGGTTTGA   
  
  
- AACAACCTTT GACGAAACTA CACCCGAAAG AGAAAGTCGT AAATACAATA TGTATATATA CCAATATTTC   
  
  
- AATGTTTTCA TACTGGGCTG TGCTGTTAAG TTAGGCTTAG GCTGAGCTTC AATGACTTTC ATTAGACTTA   
  
  
- TGCTAAACTG TCGAATCAAA TTTTTGGATA ATGAAGAGTT AAAACTCATT TAGATTCGAT AAAAATTACG   
  
  
- ACTTATCTAG CTGGGCTAAG CTGGGCAAAA GGCTGAGATA TACCAATACC ACGGGGATTT TTTTTAATTT   
  
  
- TTTCTTTTTC TTATCTTTTT ATATTTTATT TTGACTCTAA AGACTTGTGA GAGAGGGGAT CAGAACACCC   
  
  
- GTACCTTTCA CCAACCTCTA CCTCTACAAA CTCAAAGTAA AAGACTATAA TAAAAAATCA GTAATCTTCA   
  
  
- TGTTTCAGAT TTGAGTTGTT CTCGTACTCC CCTCTTACCA ACAACATCGG TTACCTTTTA TTTTTCTTGT   
  
  
- AAGTGAGAAG AACACGAAAT AAATACCACT ATTAAACAAT GAACATAAAA ATTTTTACTA ATTTATATAA   
  
  
- CTAGGAAACT ATAATACAAC TTTATTATTT TTATATTTTT AATCTTTTAT AAAATGGAGC ACTTTAAAAA   
  
  
- AAAGACTAAT TAGTAACTTT AATACCACTT TTTATTTCCT TGTAGAGAAC ATTTGTGAGA TAGGCAAATG   
  
  
- CAAACTGGTG TTTGAACGAT AGGTGTTCGT AGAGTTGGAA ACGGAAATTG GGTTAACACC CAACTACTTT   
  
  
- AGATTGGTAA TATCCTAATC TACCATCAGC AATTAGGACA CTTTGTTCAA AAACCAATAC TACATAGGTT   
  
  
- AATACTCCCG GACTATTGAG AACGGAGCCG TAAACGCACT ATTACTGCAC CATGATTATC GTTAATAAAA   
  
  
- AGCGTTTATA AGCTTTAAAA AATTCTTATT TGCGTTTTAA TAACTAGAAC TGTTTACTAA AATAAAAAAA   
  
  
- CAACCCTTTT CCTATTTGTA GATCACAGTT AACAACTACT GGACTATCGA GAGCTAAAGA ATAAATACTC   
  
  
- AATACAACTA TACTGTGATT AACATTAATA AACAACATTT GAATTTGTAA AAATTTTTAG TTTCGTTTTA   
  
  
- AACAATTAAT GTTATTTAGT TAGATGAAAA TGATACTAGA ATAAACCACA TTAATAACTA GAACTTAAAA   
  
  
- AAAAAAAGAA AAACTTTTTC TATTCGTAGC TTAAAAAAGT TTTTATTTTT CCCACTTCCT TCTTTTTTAC   
  
  
- CTTTGTCAAT GGCTCATTCC TTCCTGTCTC GCGCTCTCCT GTTCTGTGTT TCTCTCCCCT CTCTCTCTCT   
  
  
- CTTCCCCCGT TACCGTCACC CCTTCCAGCT CCTCGTGGAA AGTTGAAGTT CCGAGTTGTG GGGGGGGGGG   
  
  
- GGGGAGAAAG GAGTAAAACG CTGCAATCGT AAACTGTAAA ACGAAAGTTG GCAGGGAGCC GTAGTAGTCG   
  
  
- GGACGGGAAC GAGTCGAAAA GGGTAGAAAA AAACCGAAAA ACAAACTGAG GGAGATATTG AGATAAGTGC   
  
  
- AGCTCGGAAC ATTTTTAAAA TGTATAAAAT AAAATAAAAT GAAAAAATCC AAATAGGGAG AGATAAGAAC   
  
  
- TGGATGCCCG TAAAAAATAA AAGAAACCCA AACTCTGAAC ACTTGAAAAA CGTGTAAAAG AGTGTTTTTA   
  
  
- AATAGAACAG AAAAAGTGAA AGTTGCAAGG AAGAGAGAGA GAGAGAGAGA GAGAGAGAGA GAGTGAGTGT   
  
  
- AGGATCTGTC TGCAGGAAAA ATTTCTTAAA TAAATTGTTC AATCTTCTTT GGCAACAAAT TCATCTTGGT   
  
  
- GGGACAAACC TACGGTTTCC TTTATGGTCA GTTCTTTTTA GTGCTGTTAG TGTTGTGTAG GTAAAGCAAA   
  
  
- TGTAGAGTAG GAGTAGGAGT AGAAGGAGTA GAGATGGGTA GAGGGAGAAA GACATACCGA CGAAGAAGAT   
  
  
- GAAACAAGGG ACTGGGATTG TTATGATCAA ATCAGGAGGG AATAATAATA ATAGGAGGAG GAAGGAGGAG   
  
  
- AATGATATTA CCATTGGGAA TGGAAGGAGA AGGATTGTGG TGACGATAAG GATGAGGAAG TTGTTGGAGA   
  
  
- GGCCATAAAA GAAGGCGGTG GATGAGCCTC AACTTATGAC TAACAGTTTG AGTTGACGGG TACAGATTGG   
  
  
- AGGGGCCTTT CTAGCAAGCC TTCGCCTCAC GACGACTTTA CCTCGTCGCG AGGCGAGGCT AACGCCCACT   
  
  
- AATGGGCGGT TGAGTAGTCG CCCATTAGTG GGCGGTGTTA CTCCGCCGGA GTAGTCACAG AAAGTAACTG   
  
  
- AGGAGTGAGA GCGGTGAAGT GGAGAAGTTG CCGAGGTTAA GCTGCTGGGG TAATCGGGGG CTGGCAGGAT   
  
  
- TACTAGGCCC AGACTCAGTC CCAGGCCTAG ACGGAGAACA CACGCCTAAG AGGCCTGAGG GAAATAAGGG   
  
  
- TGGCCACCTC TCTTCTGGAA GGTAGGGGCG GTGCTGGTGA CGACGGCGCC GCTAGCGCCA CCAAGGACAC   
  
  
- CTCCTCCTAC TAGGCTGCAC CTACCTATCG TAGTATTTTC TCGACTAAGT TTCGAGTTTG AGCTAGAGTT   
  
  
- AGGGTGTCAA CTAAGTCTTG CAATCTCTAT AGTAGATGGG TACATTAGGA TTAGACCCCA GGCGGTAACT   
  
  
- CAAGGCGGAG GCGAGCGAGC GGCGGCTGGG GGAGTAACGA GGCGGCGGTG AAGGCGGGAA GGTGGTGGTG   
  
  
- GTTGTGGTGG AGTTAGTTGT TTAGTGAGAA GGAGCTTAGT CGTTGTGATT GTTGTTGTTA GTACACTCGC   
  
  
- ACATGCGGTT GTTTCCTGGG CCTATGAAGT TGAACCCAGG CCCAGTCCCA GTCCCAGGCT AATTGTAGCT   
  
  
- GGTTCGATTG GAAAGGAAGG GAGGACTAAG GTGGCGGACA ACCCCACAGT CACAGTGTGG CGGTGGCAGA   
  
  
- AGGCGGCGGC CAAGGCCGAG GCCACCTTCG CCGTCATCAT CACCATTTAG TTTGGGATTG GGATTAGGGT   
  
  
- TGGGCTTGGG TTTGGGTTTG GGTTTGGGCT TGTTGTTTCG AGTCCTACAA GTTGAGGTTT GAGGTGTTGT   
  
  
- TGTCGTTGTC GTTGTCGTTG TTTAGGGGCA CCTAGTCCTC GTTCTGGGCC GCCGAGGTGG CCGCTGCCTT   
  
  
- CGTAGCGGTG GTAGAGGTTC TCGCCGCCGT GGTGGCGGCC GCCGTCACTT TCGTTCTCTT TCTCTCCTCT   
  
  
- ACGCCGTTTC CTTCGCGCTG CTTCTCCCAG AGGTGGAGGA TTGGGAGGAG GAGGTTACGC GTCTTCGTCA   
  
  
- TAGGCCGCTG TTTATGCTTC TTCGCTTGTT CTACGAAGAT CTTTAGAGCC TCACCCGGTG GGGTAAGCCG   
  
  
- TGGAGGCGGG TTGCGCAGCG GCGCATGAAG AGCCTTCGGT ACAGTCGGGC AGAGCATAGG AGGACGGAGC   
  
  
- CGTAGATGCG GCGGGAGGGG TGGCAGGGTG TGATGCAGTT CGAGGAGAGG CGGAAGGTTC AGAAGTTACC   
  
  
- GTAGTCGGGT AAGCAGTTTA AGAGAGTGAA GTGACGTTTG GTTCGTTAGG TTCTCCGGAA GGTCTCCCTT   
  
  
- CTGTCCCAGG TGTAGTAGCT GGAGCTATAG TACGTCCCCG AGGTCACCGG GCCCGACAAG GTGTAGGAGC   
  
  
- GCAGGGCCGG TCCACCCGGA GGGAAGCATT CCGAGTGGCC CGAGCCCTGG AGGTACCTCC GCGAGCTCCG   
  
  
- GTGGCCTTTT TCCGAGAGTC TGAAGCGGCT CTTCAACCCC AACGGGAAAC TCAAATATGG GCACCGCCTC   
  
  
- TTCTAACCTT TAAACCTGAA CCTTTCCAAC GTACAATCAT TTTCCCTTCG AGAACGACAC GTAACCAATG   
  
  
- TCGTGAGAAA CATACTACAA TGACCGAGAC TATGATTATG TGAACCGGAA GAAGTTTCCA ACCGCGGTTT   
  
  
- TCACCACTGC CACCACCTCG TTCTGGATTC GGCTTGTCCG AGAAAGGATC CTTCCAAACA TCTCCGCTAG   
  
  
- GTGATGATAA GTCGGGATAA ACTGAGGAAT CCTCGCTCAA TACCTCTCCT GTCACTCCTC TCCGTACACC   
  
  
- AACTCGTTGT CGAGGAAAGA TCCCTCTAAG CTTTGTAAGA CCGGCAACCA CCCGGGAGTT CCTGGCCCCT   
  
  
- CGGGTTCAAA CGCTCGACCT CCCTCTTCGA TGTTGTCAGG CCGAAATCCC CTTAGAGTAA CCGTCCGTTG   
  
  
- CGGCGACGGG TCCGGTGGAA CGAGGAGCCG TACAAGGGAA GACTACCCAT ATGAAATTAG CTCCTGTCAC   
  
  
- CGTGTGAATT CGAGCCCACC TTCCTGAACA CGGAGGACTG ACGAAGCCGG ACCTCCGGAA GGGTACGAGT   
  
  
- ATGATACTCG TGGCAGACAT GAGCCTCGGT TAT

+     TATA

| Site Name | Organism | Position | Strand | Matrix score. | sequence | function |
| --- | --- | --- | --- | --- | --- | --- |
| TATA | Arabidopsis thaliana | 375 | + | 8 | TATAAAAT |  |

>HU01G00472.1   
+ -Up\_Stream \_Len000GTCTCT GTAACACAAT TCATTAAATC CATCCAAAGG GGATCATAAA TAACCAAACT   
  
  
+ TTGTTGGAAA CTGCTTTGAT GTGGGCTTTC TCTTTCAGCA TTTATGTTAT ACATATATAT GGTTATAAAG   
  
  
+ TTACAAAAGT ATGACCCGAC ACGACAATTC AATCCGAATC CGACTCGAAG TTACTGAAAG TAATCTGAAT   
  
  
+ ACGATTTGAC AGCTTAGTTT AAAAACCTAT TACTTCTCAA TTTTGAGTAA ATCTAAGCTA TTTTTAATGC   
  
  
+ TGAATAGATC GACCCGATTC GACCCGTTTT CCGACTCTAT ATGGTTATGG TGCCCCTAAA AAAAATTAAA   
  
  
+ AAAGAAAAAG AATAGAAAAA TATAAAATAA AACTGAGATT TCTGAACACT CTCTCCCCTA GTCTTGTGGG   
  
  
+ CATGGAAAGT GGTTGGAGAT GGAGATGTTT GAGTTTCATT TTCTGATATT ATTTTTTAGT CATTAGAAGT   
  
  
+ ACAAAGTCTA AACTCAACAA GAGCATGAGG GGAGAATGGT TGTTGTAGCC AATGGAAAAT AAAAAGAACA   
  
  
+ TTCACTCTTC TTGTGCTTTA TTTATGGTGA TAATTTGTTA CTTGTATTTT TAAAAATGAT TAAATATATT   
  
  
+ GATCCTTTGA TATTATGTTG AAATAATAAA AATATAAAAA TTAGAAAATA TTTTACCTCG TGAAATTTTT   
  
  
+ TTTCTGATTA ATCATTGAAA TTATGGTGAA AAATAAAGGA ACATCTCTTG TAAACACTCT ATCCGTTTAC   
  
  
+ GTTTGACCAC AAACTTGCTA TCCACAAGCA TCTCAACCTT TGCCTTTAAC CCAATTGTGG GTTGATGAAA   
  
  
+ TCTAACCATT ATAGGATTAG ATGGTAGTCG TTAATCCTGT GAAACAAGTT TTTGGTTATG ATGTATCCAA   
  
  
+ TTATGAGGGC CTGATAACTC TTGCCTCGGC ATTTGCGTGA TAATGACGTG GTACTAATAG CAATTATTTT   
  
  
+ TCGCAAATAT TCGAAATTTT TTAAGAATAA ACGCAAAATT ATTGATCTTG ACAAATGATT TTATTTTTTT   
  
  
+ GTTGGGAAAA GGATAAACAT CTAGTGTCAA TTGTTGATGA CCTGATAGCT CTCGATTTCT TATTTATGAG   
  
  
+ TTATGTTGAT ATGACACTAA TTGTAATTAT TTGTTGTAAA CTTAAACATT TTTAAAAATC AAAGCAAAAT   
  
  
+ TTGTTAATTA CAATAAATCA ATCTACTTTT ACTATGATCT TATTTGGTGT AATTATTGAT CTTGAATTTT   
  
  
+ TTTTTTTCTT TTTGAAAAAG ATAAGCATCG AATTTTTTCA AAAATAAAAA GGGTGAAGGA AGAAAAAATG   
  
  
+ GAAACAGTTA CCGAGTAAGG AAGGACAGAG CGCGAGAGGA CAAGACACAA AGAGAGGGGA GAGAGAGAGA   
  
  
+ GAAGGGGGCA ATGGCAGTGG GGAAGGTCGA GGAGCACCTT TCAACTTCAA GGCTCAACAC CCCCCCCCCC   
  
  
+ CCCCTCTTTC CTCATTTTGC GACGTTAGCA TTTGACATTT TGCTTTCAAC CGTCCCTCGG CATCATCAGC   
  
  
+ CCTGCCCTTG CTCAGCTTTT CCCATCTTTT TTTGGCTTTT TGTTTGACTC CCTCTATAAC TCTATTCACG   
  
  
+ TCGAGCCTTG TAAAAATTTT ACATATTTTA TTTTATTTTA CTTTTTTAGG TTTATCCCTC TCTATTCTTG   
  
  
+ ACCTACGGGC ATTTTTTATT TTCTTTGGGT TTGAGACTTG TGAACTTTTT GCACATTTTC TCACAAAAAT   
  
  
+ TTATCTTGTC TTTTTCACTT TCAACGTTCC TTCTCTCTCT CTCTCTCTCT CTCTCTCTCT CTCACTCACA   
  
  
+ TCCTAGACAG ACGTCCTTTT TAAAGAATTT ATTTAACAAG TTAGAAGAAA CCGTTGTTTA AGTAGAACCA   
  
  
+ CCCTGTTTGG ATGCCAAAGG AAATACCAGT CAAGAAAAAT CACGACAATC ACAACACATC CATTTCGTTT   
  
  
+ ACATCTCATC CTCATCCTCA TCTTCCTCAT CTCTACCCAT CTCCCTCTTT CTGTATGGCT GCTTCTTCTA   
  
  
+ CTTTGTTCCC TGACCCTAAC AATACTAGTT TAGTCCTCCC TTATTATTAT TATCCTCCTC CTTCCTCCTC   
  
  
+ TTACTATAAT GGTAACCCTT ACCTTCCTCT TCCTAACACC ACTGCTATTC CTACTCCTTC AACAACCTCT   
  
  
+ CCGGTATTTT CTTCCGCCAC CTACTCGGAG TTGAATACTG ATTGTCAAAC TCAACTGCCC ATGTCTAACC   
  
  
+ TCCCCGGAAA GATCGTTCGG AAGCGGAGTG CTGCTGAAAT GGAGCAGCGC TCCGCTCCGA TTGCGGGTGA   
  
  
+ TTACCCGCCA ACTCATCAGC GGGTAATCAC CCGCCACAAT GAGGCGGCCT CATCAGTGTC TTTCATTGAC   
  
  
+ TCCTCACTCT CGCCACTTCA CCTCTTCAAC GGCTCCAATT CGACGACCCC ATTAGCCCCC GACCGTCCTA   
  
  
+ ATGATCCGGG TCTGAGTCAG GGTCCGGATC TGCCTCTTGT GTGCGGATTC TCCGGACTCC CTTTATTCCC   
  
  
+ ACCGGTGGAG AGAAGACCTT CCATCCCCGC CACGACCACT GCTGCCGCGG CGATCGCGGT GGTTCCTGTG   
  
  
+ GAGGAGGATG ATCCGACGTG GATGGATAGC ATCATAAAAG AGCTGATTCA AAGCTCAAAC TCGATCTCAA   
  
  
+ TCCCACAGTT GATTCAGAAC GTTAGAGATA TCATCTACCC ATGTAATCCT AATCTGGGGT CCGCCATTGA   
  
  
+ GTTCCGCCTC CGCTCGCTCG CCGCCGACCC CCTCATTGCT CCGCCGCCAC TTCCGCCCTT CCACCACCAC   
  
  
+ CAACACCACC TCAATCAACA AATCACTCTT CCTCGAATCA GCAACACTAA CAACAACAAT CATGTGAGCG   
  
  
+ TGTACGCCAA CAAAGGACCC GGATACTTCA ACTTGGGTCC GGGTCAGGGT CAGGGTCCGA TTAACATCGA   
  
  
+ CCAAGCTAAC CTTTCCTTCC CTCCTGATTC CACCGCCTGT TGGGGTGTCA GTGTCACACC GCCACCGTCT   
  
  
+ TCCGCCGCCG GTTCCGGCTC CGGTGGAAGC GGCAGTAGTA GTGGTAAATC AAACCCTAAC CCTAATCCCA   
  
  
+ ACCCGAACCC AAACCCAAAC CCAAACCCGA ACAACAAAGC TCAGGATGTT CAACTCCAAA CTCCACAACA   
  
  
+ ACAGCAACAG CAACAGCAAC AAATCCCCGT GGATCAGGAG CAAGACCCGG CGGCTCCACC GGCGACGGAA   
  
  
+ GCATCGCCAC CATCTCCAAG AGCGGCGGCA CCACCGCCGG CGGCAGTGAA AGCAAGAGAA AGAGAGGAGA   
  
  
+ TGCGGCAAAG GAAGCGCGAC GAAGAGGGTC TCCACCTCCT AACCCTCCTC CTCCAATGCG CAGAAGCAGT   
  
  
+ ATCCGGCGAC AAATACGAAG AAGCGAACAA GATGCTTCTA GAAATCTCGG AGTGGGCCAC CCCATTCGGC   
  
  
+ ACCTCCGCCC AACGCGTCGC CGCGTACTTC TCGGAAGCCA TGTCAGCCCG TCTCGTATCC TCCTGCCTCG   
  
  
+ GCATCTACGC CGCCCTCCCC ACCGTCCCAC ACTACGTCAA GCTCCTCTCC GCCTTCCAAG TCTTCAATGG   
  
  
+ CATCAGCCCA TTCGTCAAAT TCTCTCACTT CACTGCAAAC CAAGCAATCC AAGAGGCCTT CCAGAGGGAA   
  
  
+ GACAGGGTCC ACATCATCGA CCTCGATATC ATGCAGGGGC TCCAGTGGCC CGGGCTGTTC CACATCCTCG   
  
  
+ CGTCCCGGCC AGGTGGGCCT CCCTTCGTAA GGCTCACCGG GCTCGGGACC TCCATGGAGG CGCTCGAGGC   
  
  
+ CACCGGAAAA AGGCTCTCAG ACTTCGCCGA GAAGTTGGGG TTGCCCTTTG AGTTTATACC CGTGGCGGAG   
  
  
+ AAGATTGGAA ATTTGGACTT GGAAAGGTTG CATGTTAGTA AAAGGGAAGC TCTTGCTGTG CATTGGTTAC   
  
  
+ AGCACTCTTT GTATGATGTT ACTGGCTCTG ATACTAATAC ACTTGGCCTT CTTCAAAGGT TGGCGCCAAA   
  
  
+ AGTGGTGACG GTGGTGGAGC AAGACCTAAG CCGAACAGGC TCTTTCCTAG GAAGGTTTGT AGAGGCGATC   
  
  
+ CACTACTATT CAGCCCTATT TGACTCCTTA GGAGCGAGTT ATGGAGAGGA CAGTGAGGAG AGGCATGTGG   
  
  
+ TTGAGCAACA GCTCCTTTCT AGGGAGATTC GAAACATTCT GGCCGTTGGT GGGCCCTCAA GGACCGGGGA   
  
  
+ GCCCAAGTTT GCGAGCTGGA GGGAGAAGCT ACAACAGTCC GGCTTTAGGG GAATCTCATT GGCAGGCAAC   
  
  
+ GCCGCTGCCC AGGCCACCTT GCTCCTCGGC ATGTTCCCTT CTGATGGGTA TACTTTAATC GAGGACAGTG   
  
  
+ GCACACTTAA GCTCGGGTGG AAGGACTTGT GCCTCCTGAC TGCTTCGGCC TGGAGGCCTT CCCATGCTCA   
  
  
+ TACTATGAGC ACCGTCTGTA CTCGGAGCCA ATA  

- -Up\_Stream \_Len000CAGAGA CATTGTGTTA AGTAATTTAG GTAGGTTTCC CCTAGTATTT ATTGGTTTGA   
  
  
- AACAACCTTT GACGAAACTA CACCCGAAAG AGAAAGTCGT AAATACAATA TGTATATATA CCAATATTTC   
  
  
- AATGTTTTCA TACTGGGCTG TGCTGTTAAG TTAGGCTTAG GCTGAGCTTC AATGACTTTC ATTAGACTTA   
  
  
- TGCTAAACTG TCGAATCAAA TTTTTGGATA ATGAAGAGTT AAAACTCATT TAGATTCGAT AAAAATTACG   
  
  
- ACTTATCTAG CTGGGCTAAG CTGGGCAAAA GGCTGAGATA TACCAATACC ACGGGGATTT TTTTTAATTT   
  
  
- TTTCTTTTTC TTATCTTTTT ATATTTTATT TTGACTCTAA AGACTTGTGA GAGAGGGGAT CAGAACACCC   
  
  
- GTACCTTTCA CCAACCTCTA CCTCTACAAA CTCAAAGTAA AAGACTATAA TAAAAAATCA GTAATCTTCA   
  
  
- TGTTTCAGAT TTGAGTTGTT CTCGTACTCC CCTCTTACCA ACAACATCGG TTACCTTTTA TTTTTCTTGT   
  
  
- AAGTGAGAAG AACACGAAAT AAATACCACT ATTAAACAAT GAACATAAAA ATTTTTACTA ATTTATATAA   
  
  
- CTAGGAAACT ATAATACAAC TTTATTATTT TTATATTTTT AATCTTTTAT AAAATGGAGC ACTTTAAAAA   
  
  
- AAAGACTAAT TAGTAACTTT AATACCACTT TTTATTTCCT TGTAGAGAAC ATTTGTGAGA TAGGCAAATG   
  
  
- CAAACTGGTG TTTGAACGAT AGGTGTTCGT AGAGTTGGAA ACGGAAATTG GGTTAACACC CAACTACTTT   
  
  
- AGATTGGTAA TATCCTAATC TACCATCAGC AATTAGGACA CTTTGTTCAA AAACCAATAC TACATAGGTT   
  
  
- AATACTCCCG GACTATTGAG AACGGAGCCG TAAACGCACT ATTACTGCAC CATGATTATC GTTAATAAAA   
  
  
- AGCGTTTATA AGCTTTAAAA AATTCTTATT TGCGTTTTAA TAACTAGAAC TGTTTACTAA AATAAAAAAA   
  
  
- CAACCCTTTT CCTATTTGTA GATCACAGTT AACAACTACT GGACTATCGA GAGCTAAAGA ATAAATACTC   
  
  
- AATACAACTA TACTGTGATT AACATTAATA AACAACATTT GAATTTGTAA AAATTTTTAG TTTCGTTTTA   
  
  
- AACAATTAAT GTTATTTAGT TAGATGAAAA TGATACTAGA ATAAACCACA TTAATAACTA GAACTTAAAA   
  
  
- AAAAAAAGAA AAACTTTTTC TATTCGTAGC TTAAAAAAGT TTTTATTTTT CCCACTTCCT TCTTTTTTAC   
  
  
- CTTTGTCAAT GGCTCATTCC TTCCTGTCTC GCGCTCTCCT GTTCTGTGTT TCTCTCCCCT CTCTCTCTCT   
  
  
- CTTCCCCCGT TACCGTCACC CCTTCCAGCT CCTCGTGGAA AGTTGAAGTT CCGAGTTGTG GGGGGGGGGG   
  
  
- GGGGAGAAAG GAGTAAAACG CTGCAATCGT AAACTGTAAA ACGAAAGTTG GCAGGGAGCC GTAGTAGTCG   
  
  
- GGACGGGAAC GAGTCGAAAA GGGTAGAAAA AAACCGAAAA ACAAACTGAG GGAGATATTG AGATAAGTGC   
  
  
- AGCTCGGAAC ATTTTTAAAA TGTATAAAAT AAAATAAAAT GAAAAAATCC AAATAGGGAG AGATAAGAAC   
  
  
- TGGATGCCCG TAAAAAATAA AAGAAACCCA AACTCTGAAC ACTTGAAAAA CGTGTAAAAG AGTGTTTTTA   
  
  
- AATAGAACAG AAAAAGTGAA AGTTGCAAGG AAGAGAGAGA GAGAGAGAGA GAGAGAGAGA GAGTGAGTGT   
  
  
- AGGATCTGTC TGCAGGAAAA ATTTCTTAAA TAAATTGTTC AATCTTCTTT GGCAACAAAT TCATCTTGGT   
  
  
- GGGACAAACC TACGGTTTCC TTTATGGTCA GTTCTTTTTA GTGCTGTTAG TGTTGTGTAG GTAAAGCAAA   
  
  
- TGTAGAGTAG GAGTAGGAGT AGAAGGAGTA GAGATGGGTA GAGGGAGAAA GACATACCGA CGAAGAAGAT   
  
  
- GAAACAAGGG ACTGGGATTG TTATGATCAA ATCAGGAGGG AATAATAATA ATAGGAGGAG GAAGGAGGAG   
  
  
- AATGATATTA CCATTGGGAA TGGAAGGAGA AGGATTGTGG TGACGATAAG GATGAGGAAG TTGTTGGAGA   
  
  
- GGCCATAAAA GAAGGCGGTG GATGAGCCTC AACTTATGAC TAACAGTTTG AGTTGACGGG TACAGATTGG   
  
  
- AGGGGCCTTT CTAGCAAGCC TTCGCCTCAC GACGACTTTA CCTCGTCGCG AGGCGAGGCT AACGCCCACT   
  
  
- AATGGGCGGT TGAGTAGTCG CCCATTAGTG GGCGGTGTTA CTCCGCCGGA GTAGTCACAG AAAGTAACTG   
  
  
- AGGAGTGAGA GCGGTGAAGT GGAGAAGTTG CCGAGGTTAA GCTGCTGGGG TAATCGGGGG CTGGCAGGAT   
  
  
- TACTAGGCCC AGACTCAGTC CCAGGCCTAG ACGGAGAACA CACGCCTAAG AGGCCTGAGG GAAATAAGGG   
  
  
- TGGCCACCTC TCTTCTGGAA GGTAGGGGCG GTGCTGGTGA CGACGGCGCC GCTAGCGCCA CCAAGGACAC   
  
  
- CTCCTCCTAC TAGGCTGCAC CTACCTATCG TAGTATTTTC TCGACTAAGT TTCGAGTTTG AGCTAGAGTT   
  
  
- AGGGTGTCAA CTAAGTCTTG CAATCTCTAT AGTAGATGGG TACATTAGGA TTAGACCCCA GGCGGTAACT   
  
  
- CAAGGCGGAG GCGAGCGAGC GGCGGCTGGG GGAGTAACGA GGCGGCGGTG AAGGCGGGAA GGTGGTGGTG   
  
  
- GTTGTGGTGG AGTTAGTTGT TTAGTGAGAA GGAGCTTAGT CGTTGTGATT GTTGTTGTTA GTACACTCGC
[truncated: 193,526 more chars]
