# Supplementary material for: Genome-Wide Identification and Expression Pattern of the GRAS Gene Family in Pitaya (Selenicereus undatus L.)
Source: Biology (Basel). 2022 Dec 21;12(1):11. doi: 10.3390/biology12010011 (PMC9854919; doi:10.3390/biology12010011)
Supplement: Supplementary file 1 [file biology-12-00011-s001.zip › Supplementary file S5/HU01G00833.1_plantcare.html]

Content-Type: text/html; charset=ISO-8859-1


PlantCARE


Webmaster Firefox specific output  
To save the result:
click on the frame with the right mouse button and save the source code as a text file with extension .html  
REFERENCE:PlantCARE: a database of plant cis-acting regulatory elements and a portal to tools for in silico analysis of promoter sequences.  
Lescot, M., Déhais, P., Moreau, Y., De Moor, B., Rouzé ,P.,and Rombauts, S.  
Nucleic Acids Res., Database issue(2002), 30(1):325-327.   


---

>HU01G00833.1   
+ -Up\_Stream \_Len000AAAATA TTTTTACAAA AGCATTTCTC TATAAAATAT TTTACAAAAT TTCATTTTGT   
  
  
+ GGGTCTTTTA GAATCAATCT CTCTACAAGT AGGAATGCCG GTTGAGGTAA GGCTACCTAC ATCTAGATCT   
  
  
+ CTCCAGACCC CACAAAAATT AAACGAGACC CACACTGAAA GGTGGTGGTG GTGGTGTTGT TGTTGTATTG   
  
  
+ TCCTACCACG AGAGAGCTTC AAGTGCATCA CTTATTTCAA CAGGTCAATC ACATAGATCT CTAGTGTACT   
  
  
+ GTAAATTTTT TTTCCGTCTT ATTTTAATTT TCTTTTCCAT CCTAAAAGAT TATCATGTAG ATATTCCTAG   
  
  
+ TTTGATATAT TGACTTCAAC TTCAAAAATT ACACATTAAG ATCCACGAAT ATGACAGTTT AGGTTTCATT   
  
  
+ TGAAGGAGAA AGAAATGTTA CGGTGTTTTG GTGTTGTATT CGTAGACATT TTTTGCGCTT AACTGGCTCT   
  
  
+ TCTCATGGGG ACGAGACAGT GAAGGTAATT GTACTTGTTG ATTGATGACG TCATCTGTAG CTTGGATTTG   
  
  
+ TATAGGAATG AACATCTTGG TGTCTACGTG GAAAGTGTGA TTTGTGTTCT TTCATTGATC CTCCTCCTCA   
  
  
+ AGAAGAGGTG ATCTTTCTCC CTTGTTTTTT TTTTTTTTTG GCTAATATGT TGGCAGAACC GGCAAAAAAT   
  
  
+ ACATAATTTG AATAGTGCAT GCATATGAGT ACATGACAGT GAACCAACAA AACGAACCCC TAAAACACAA   
  
  
+ GAGAATTAAC ATGTTAAAAC ACTTAAACTA AATGCAACCA TCTCGATGAC CACTAAATAG CCAAGAAACC   
  
  
+ TAACGTAATT AACAAAAATA AAAAATAACT GAAAATAAAG TAGAGAAAGT CTAACCGAAT ACGATAACAT   
  
  
+ AACCTAAAAC TAACTATACT TGAACTTACT AAGAAATATG AATCAACCAC AAACCGAATT GATTTTGGTT   
  
  
+ TGCCATAATT TGACTATACA TTTAACTTAA AAAATATTTT TTTTTAATTT CAAATAAATC TAAATTATTT   
  
  
+ TTCAATATTC ATCCAAAATA GATTCAATTA TTAAACAAGT CAACCTATAC CGAGTTATTT TCTAGCTCTG   
  
  
+ ACTAAGACAT ATATATATTT TTTGTAAAAA AGATAAGGAT TATAATTTAA ATAGCCTTTC CCTTAAAAAA   
  
  
+ ATTTTTAAAA TTATCCTGAT AGAGAATAAA AAGAAATCCT TATACAAAAC TCTGATTGAC GCAGATGTGT   
  
  
+ ATATGGACAT ATAGAAGTAA TAAGAGGGGT AGGCTAGGAA GGGGGTGGAA ATATAGGGCA TAAATAGGGG   
  
  
+ GCGGGCATAG GGACATGTCT GTCTGAGGTA CGGTGGATGG GACCTTGCCA TGGAAGCAAC AATGTCACAA   
  
  
+ TAATAATATC AACGACTGAA GTTTCTTCAC CAGCCCACTC TTTGCCATAA TGCCATTAAT TATTTATTGC   
  
  
+ TACTATCTCT TTTGATGTGA AACGGTGGAC CTTATCTCTC CCCTCCACTT CCAATTGATG TGAGCAACTC   
  
  
+ AGAAACGCAA ACAAAACTTG TGCCACCTTC TTTACTTGCA TCCTCATTTT CCTCTGTCTC TCTCCGCCCC   
  
  
+ TGTTTCTCTC TCTCTCCCTC ACTCACTCAG AGTACTGATC TGAGCATCAG AAGTAGCAGA CCCAGAAACT   
  
  
+ CAGATTTAAA AACAACCCGC TCTTTTGTTC CCTCTACGTC TAATAATTTT GTCATCGCAT TTAATGTCAG   
  
  
+ ATAGAGAGAT AGAGCTCTAC TGTATTAATT TTCTCCCACC TCACTTGATT CTGTTGCCCT AAACCACCCA   
  
  
+ AAAAAAGAAA TACGAAGAAG CCACTCTCTC TCTCTCTCTC TCTCACACAC ACACACACCC CTCCTAGTGG   
  
  
+ ATTTGATAGA GTATAAATGA ACAGTAATCA GACGAGATAT ATAGTAGTGC TTAATTGACC GAGTAGCAAA   
  
  
+ AGGGGGAAGT TGAGTGAGGT ATCAAGAGAG TAGCGTAGCT AGCTAGGGCA AGATATGCTG GGGTCTTCCT   
  
  
+ CATACAACTC CGAGGAAGAC CACCAAGATG ACAACCATTC CTCACCGTCT GATTTGTCTA GGCACCCACC   
  
  
+ TAGATTCACC ACTACTACTT ACCAATACCA ACAACATATT AGCTCTCCAT CTTCACAATC AATACTAGCT   
  
  
+ GTGCCACCTC GCCAATTGTT AATCAATTGT GCGGAGTTAA TTTGCCGGTT CGACTTCTCC TCCGCCAACC   
  
  
+ GGCTTCTCTC TCTCCTCGCC TCCCACTTTG CCTCCCCTTC TGGGGACTCC ACCGAGAGAC TTGTGTTTTA   
  
  
+ CTTCTGCAAG GCTTTGGATC TCCGCCTCAA TAACAGTTAT TTATCCACTC GTAGTTTGAC TGGTTGTAAC   
  
  
+ CCTAGTACTG GTTCTGGTAT TGGGGCTCCT TTAGCTTCCA ATTTTCTATT TCTTCAACCA TTAGTGCATT   
  
  
+ CACAGGTAAC AAGTAATGCC CGTGTTGGGT ACTTGACATA TTTGACTCTG AACCAGGTCA CCCCATTCAT   
  
  
+ CCGATTCACC CACTTAACTG CCAACCAAGC CATCCTGGAA GCTTTGGAGG GGTACAAGGC GGTCCATATC   
  
  
+ CTTGACATGG ACATCATGCA TGGGGTCCAG TGGCCACCAC TTCTTCAAGC AATTGCAGAA AGATCTGCAA   
  
  
+ CCCTTGGTCA CCCTGCTCCC ACTGTTCGCA TCACAGGAGG GGGCAGGGAT CTAGAACTCC TGAACCGAAC   
  
  
+ TGGAGATCGG ATCAGGAAGT TCGCACAATC CTTGGGACTC GAGTTTCAAT TCCACCATCT TATAACGGTG   
  
  
+ GACTCGAACC TTACTGAGGC AGTGGAGGCC ATCAATGCCC TACAGCTACA CCTCCATGTC CAAGAGGAGG   
  
  
+ AGGTCTTCGC AGTGAATTGT GGAGATTTCC TCCACCGCCT CCTAACGGAG TATGATAGTA CATTCCTTAG   
  
  
+ GATGTTTCTC TACAAGGTGA AGACCCTAAA CCCTAGGGTC TTCACCGTGG GAGAAAGGGA GGCCGACCAC   
  
  
+ AACCACCCTC TCTTCTGGCA GCGATTCATT GAGGCACTTG ACCACTATGG GGCAGTGTTT GACTCTCTAG   
  
  
+ AAGCAACCCT ACCATCAACA AGTCAAGAGA GGGTGGCGGT GGAAGAGGGG TGGTTCGGGG AGGAGATTAA   
  
  
+ AGATGTGGTT GGACGAGAAG GAGGGTTGAG GAGACAGAGG CACCAGAAAT ACGAGTCTTG GGAGGTGTTG   
  
  
+ ATGAGGAGCT CTGGGTTTAA GAGCTTGCCC TTGAGCCCCT TCTCTGTGTC ACAAGCCAAG CTGCTACTTC   
  
  
+ GCCTTCATTA CCCTTCTGAA GGGTACCACC TTAAGGTCTT CCATAGTAAT TGTCTGTTGC TTGGGTGGAA   
  
  
+ GAATCGCCCT CTTTTCTCTG TTTCTTCTTG GCAATA  

- -Up\_Stream \_Len000TTTTAT AAAAATGTTT TCGTAAAGAG ATATTTTATA AAATGTTTTA AAGTAAAACA   
  
  
- CCCAGAAAAT CTTAGTTAGA GAGATGTTCA TCCTTACGGC CAACTCCATT CCGATGGATG TAGATCTAGA   
  
  
- GAGGTCTGGG GTGTTTTTAA TTTGCTCTGG GTGTGACTTT CCACCACCAC CACCACAACA ACAACATAAC   
  
  
- AGGATGGTGC TCTCTCGAAG TTCACGTAGT GAATAAAGTT GTCCAGTTAG TGTATCTAGA GATCACATGA   
  
  
- CATTTAAAAA AAAGGCAGAA TAAAATTAAA AGAAAAGGTA GGATTTTCTA ATAGTACATC TATAAGGATC   
  
  
- AAACTATATA ACTGAAGTTG AAGTTTTTAA TGTGTAATTC TAGGTGCTTA TACTGTCAAA TCCAAAGTAA   
  
  
- ACTTCCTCTT TCTTTACAAT GCCACAAAAC CACAACATAA GCATCTGTAA AAAACGCGAA TTGACCGAGA   
  
  
- AGAGTACCCC TGCTCTGTCA CTTCCATTAA CATGAACAAC TAACTACTGC AGTAGACATC GAACCTAAAC   
  
  
- ATATCCTTAC TTGTAGAACC ACAGATGCAC CTTTCACACT AAACACAAGA AAGTAACTAG GAGGAGGAGT   
  
  
- TCTTCTCCAC TAGAAAGAGG GAACAAAAAA AAAAAAAAAC CGATTATACA ACCGTCTTGG CCGTTTTTTA   
  
  
- TGTATTAAAC TTATCACGTA CGTATACTCA TGTACTGTCA CTTGGTTGTT TTGCTTGGGG ATTTTGTGTT   
  
  
- CTCTTAATTG TACAATTTTG TGAATTTGAT TTACGTTGGT AGAGCTACTG GTGATTTATC GGTTCTTTGG   
  
  
- ATTGCATTAA TTGTTTTTAT TTTTTATTGA CTTTTATTTC ATCTCTTTCA GATTGGCTTA TGCTATTGTA   
  
  
- TTGGATTTTG ATTGATATGA ACTTGAATGA TTCTTTATAC TTAGTTGGTG TTTGGCTTAA CTAAAACCAA   
  
  
- ACGGTATTAA ACTGATATGT AAATTGAATT TTTTATAAAA AAAAATTAAA GTTTATTTAG ATTTAATAAA   
  
  
- AAGTTATAAG TAGGTTTTAT CTAAGTTAAT AATTTGTTCA GTTGGATATG GCTCAATAAA AGATCGAGAC   
  
  
- TGATTCTGTA TATATATAAA AAACATTTTT TCTATTCCTA ATATTAAATT TATCGGAAAG GGAATTTTTT   
  
  
- TAAAAATTTT AATAGGACTA TCTCTTATTT TTCTTTAGGA ATATGTTTTG AGACTAACTG CGTCTACACA   
  
  
- TATACCTGTA TATCTTCATT ATTCTCCCCA TCCGATCCTT CCCCCACCTT TATATCCCGT ATTTATCCCC   
  
  
- CGCCCGTATC CCTGTACAGA CAGACTCCAT GCCACCTACC CTGGAACGGT ACCTTCGTTG TTACAGTGTT   
  
  
- ATTATTATAG TTGCTGACTT CAAAGAAGTG GTCGGGTGAG AAACGGTATT ACGGTAATTA ATAAATAACG   
  
  
- ATGATAGAGA AAACTACACT TTGCCACCTG GAATAGAGAG GGGAGGTGAA GGTTAACTAC ACTCGTTGAG   
  
  
- TCTTTGCGTT TGTTTTGAAC ACGGTGGAAG AAATGAACGT AGGAGTAAAA GGAGACAGAG AGAGGCGGGG   
  
  
- ACAAAGAGAG AGAGAGGGAG TGAGTGAGTC TCATGACTAG ACTCGTAGTC TTCATCGTCT GGGTCTTTGA   
  
  
- GTCTAAATTT TTGTTGGGCG AGAAAACAAG GGAGATGCAG ATTATTAAAA CAGTAGCGTA AATTACAGTC   
  
  
- TATCTCTCTA TCTCGAGATG ACATAATTAA AAGAGGGTGG AGTGAACTAA GACAACGGGA TTTGGTGGGT   
  
  
- TTTTTTCTTT ATGCTTCTTC GGTGAGAGAG AGAGAGAGAG AGAGTGTGTG TGTGTGTGGG GAGGATCACC   
  
  
- TAAACTATCT CATATTTACT TGTCATTAGT CTGCTCTATA TATCATCACG AATTAACTGG CTCATCGTTT   
  
  
- TCCCCCTTCA ACTCACTCCA TAGTTCTCTC ATCGCATCGA TCGATCCCGT TCTATACGAC CCCAGAAGGA   
  
  
- GTATGTTGAG GCTCCTTCTG GTGGTTCTAC TGTTGGTAAG GAGTGGCAGA CTAAACAGAT CCGTGGGTGG   
  
  
- ATCTAAGTGG TGATGATGAA TGGTTATGGT TGTTGTATAA TCGAGAGGTA GAAGTGTTAG TTATGATCGA   
  
  
- CACGGTGGAG CGGTTAACAA TTAGTTAACA CGCCTCAATT AAACGGCCAA GCTGAAGAGG AGGCGGTTGG   
  
  
- CCGAAGAGAG AGAGGAGCGG AGGGTGAAAC GGAGGGGAAG ACCCCTGAGG TGGCTCTCTG AACACAAAAT   
  
  
- GAAGACGTTC CGAAACCTAG AGGCGGAGTT ATTGTCAATA AATAGGTGAG CATCAAACTG ACCAACATTG   
  
  
- GGATCATGAC CAAGACCATA ACCCCGAGGA AATCGAAGGT TAAAAGATAA AGAAGTTGGT AATCACGTAA   
  
  
- GTGTCCATTG TTCATTACGG GCACAACCCA TGAACTGTAT AAACTGAGAC TTGGTCCAGT GGGGTAAGTA   
  
  
- GGCTAAGTGG GTGAATTGAC GGTTGGTTCG GTAGGACCTT CGAAACCTCC CCATGTTCCG CCAGGTATAG   
  
  
- GAACTGTACC TGTAGTACGT ACCCCAGGTC ACCGGTGGTG AAGAAGTTCG TTAACGTCTT TCTAGACGTT   
  
  
- GGGAACCAGT GGGACGAGGG TGACAAGCGT AGTGTCCTCC CCCGTCCCTA GATCTTGAGG ACTTGGCTTG   
  
  
- ACCTCTAGCC TAGTCCTTCA AGCGTGTTAG GAACCCTGAG CTCAAAGTTA AGGTGGTAGA ATATTGCCAC   
  
  
- CTGAGCTTGG AATGACTCCG TCACCTCCGG TAGTTACGGG ATGTCGATGT GGAGGTACAG GTTCTCCTCC   
  
  
- TCCAGAAGCG TCACTTAACA CCTCTAAAGG AGGTGGCGGA GGATTGCCTC ATACTATCAT GTAAGGAATC   
  
  
- CTACAAAGAG ATGTTCCACT TCTGGGATTT GGGATCCCAG AAGTGGCACC CTCTTTCCCT CCGGCTGGTG   
  
  
- TTGGTGGGAG AGAAGACCGT CGCTAAGTAA CTCCGTGAAC TGGTGATACC CCGTCACAAA CTGAGAGATC   
  
  
- TTCGTTGGGA TGGTAGTTGT TCAGTTCTCT CCCACCGCCA CCTTCTCCCC ACCAAGCCCC TCCTCTAATT   
  
  
- TCTACACCAA CCTGCTCTTC CTCCCAACTC CTCTGTCTCC GTGGTCTTTA TGCTCAGAAC CCTCCACAAC   
  
  
- TACTCCTCGA GACCCAAATT CTCGAACGGG AACTCGGGGA AGAGACACAG TGTTCGGTTC GACGATGAAG   
  
  
- CGGAAGTAAT GGGAAGACTT CCCATGGTGG AATTCCAGAA GGTATCATTA ACAGACAACG AACCCACCTT   
  
  
- CTTAGCGGGA GAAAAGAGAC AAAGAAGAAC CGTTAT

  
  
Motifs Found  

+   

| Site Name | Organism | Position | Strand | Matrix score. | sequence | function |
| --- | --- | --- | --- | --- | --- | --- |
|  | organism | 3146 | - | 4 | motif\_sequence | short\_function |
|  | organism | 3024 | + | 4 | motif\_sequence | short\_function |
|  | organism | 2147 | + | 4 | motif\_sequence | short\_function |
|  | organism | 2736 | - | 4 | motif\_sequence | short\_function |
|  | organism | 3126 | - | 4 | motif\_sequence | short\_function |
|  | organism | 2895 | - | 4 | motif\_sequence | short\_function |
|  | organism | 2860 | + | 4 | motif\_sequence | short\_function |
|  | organism | 2932 | - | 4 | motif\_sequence | short\_function |
|  | organism | 2952 | + | 4 | motif\_sequence | short\_function |
|  | organism | 1769 | + | 4 | motif\_sequence | short\_function |
|  | organism | 1716 | + | 4 | motif\_sequence | short\_function |
|  | organism | 884 | - | 4 | motif\_sequence | short\_function |
|  | organism | 1913 | - | 4 | motif\_sequence | short\_function |
|  | organism | 636 | - | 4 | motif\_sequence | short\_function |
|  | organism | 1626 | + | 4 | motif\_sequence | short\_function |
|  | organism | 1604 | + | 4 | motif\_sequence | short\_function |
|  | organism | 1510 | + | 4 | motif\_sequence | short\_function |
|  | organism | 1345 | - | 4 | motif\_sequence | short\_function |
|  | organism | 574 | - | 4 | motif\_sequence | short\_function |
|  | organism | 1255 | - | 4 | motif\_sequence | short\_function |
|  | organism | 733 | - | 4 | motif\_sequence | short\_function |
|  | organism | 2686 | + | 4 | motif\_sequence | short\_function |
|  | organism | 2657 | + | 4 | motif\_sequence | short\_function |
|  | organism | 2603 | - | 4 | motif\_sequence | short\_function |
|  | organism | 2153 | + | 4 | motif\_sequence | short\_function |
|  | organism | 2332 | + | 4 | motif\_sequence | short\_function |
|  | organism | 2254 | + | 4 | motif\_sequence | short\_function |
|  | organism | 2173 | + | 4 | motif\_sequence | short\_function |
|  | organism | 143 | + | 4 | motif\_sequence | short\_function |
|  | organism | 491 | + | 4 | motif\_sequence | short\_function |
|  | organism | 1269 | - | 4 | motif\_sequence | short\_function |
|  | organism | 95 | + | 4 | motif\_sequence | short\_function |
|  | organism | 341 | - | 4 | motif\_sequence | short\_function |

>HU01G00833.1   
+ -Up\_Stream \_Len000AAAATA TTTTTACAAA AGCATTTCTC TATAAAATAT TTTACAAAAT TTCATTTTGT   
  
  
+ GGGTCTTTTA GAATCAATCT CTCTACAAGT AGGAATGCCG GTTGAGGTAA GGCTACCTAC ATCTAGATCT   
  
  
+ CTCCAGACCC CACAAAAATT AAACGAGACC CACACTGAAA GGTGGTGGTG GTGGTGTTGT TGTTGTATTG   
  
  
+ TCCTACCACG AGAGAGCTTC AAGTGCATCA CTTATTTCAA CAGGTCAATC ACATAGATCT CTAGTGTACT   
  
  
+ GTAAATTTTT TTTCCGTCTT ATTTTAATTT TCTTTTCCAT CCTAAAAGAT TATCATGTAG ATATTCCTAG   
  
  
+ TTTGATATAT TGACTTCAAC TTCAAAAATT ACACATTAAG ATCCACGAAT ATGACAGTTT AGGTTTCATT   
  
  
+ TGAAGGAGAA AGAAATGTTA CGGTGTTTTG GTGTTGTATT CGTAGACATT TTTTGCGCTT AACTGGCTCT   
  
  
+ TCTCATGGGG ACGAGACAGT GAAGGTAATT GTACTTGTTG ATTGATGACG TCATCTGTAG CTTGGATTTG   
  
  
+ TATAGGAATG AACATCTTGG TGTCTACGTG GAAAGTGTGA TTTGTGTTCT TTCATTGATC CTCCTCCTCA   
  
  
+ AGAAGAGGTG ATCTTTCTCC CTTGTTTTTT TTTTTTTTTG GCTAATATGT TGGCAGAACC GGCAAAAAAT   
  
  
+ ACATAATTTG AATAGTGCAT GCATATGAGT ACATGACAGT GAACCAACAA AACGAACCCC TAAAACACAA   
  
  
+ GAGAATTAAC ATGTTAAAAC ACTTAAACTA AATGCAACCA TCTCGATGAC CACTAAATAG CCAAGAAACC   
  
  
+ TAACGTAATT AACAAAAATA AAAAATAACT GAAAATAAAG TAGAGAAAGT CTAACCGAAT ACGATAACAT   
  
  
+ AACCTAAAAC TAACTATACT TGAACTTACT AAGAAATATG AATCAACCAC AAACCGAATT GATTTTGGTT   
  
  
+ TGCCATAATT TGACTATACA TTTAACTTAA AAAATATTTT TTTTTAATTT CAAATAAATC TAAATTATTT   
  
  
+ TTCAATATTC ATCCAAAATA GATTCAATTA TTAAACAAGT CAACCTATAC CGAGTTATTT TCTAGCTCTG   
  
  
+ ACTAAGACAT ATATATATTT TTTGTAAAAA AGATAAGGAT TATAATTTAA ATAGCCTTTC CCTTAAAAAA   
  
  
+ ATTTTTAAAA TTATCCTGAT AGAGAATAAA AAGAAATCCT TATACAAAAC TCTGATTGAC GCAGATGTGT   
  
  
+ ATATGGACAT ATAGAAGTAA TAAGAGGGGT AGGCTAGGAA GGGGGTGGAA ATATAGGGCA TAAATAGGGG   
  
  
+ GCGGGCATAG GGACATGTCT GTCTGAGGTA CGGTGGATGG GACCTTGCCA TGGAAGCAAC AATGTCACAA   
  
  
+ TAATAATATC AACGACTGAA GTTTCTTCAC CAGCCCACTC TTTGCCATAA TGCCATTAAT TATTTATTGC   
  
  
+ TACTATCTCT TTTGATGTGA AACGGTGGAC CTTATCTCTC CCCTCCACTT CCAATTGATG TGAGCAACTC   
  
  
+ AGAAACGCAA ACAAAACTTG TGCCACCTTC TTTACTTGCA TCCTCATTTT CCTCTGTCTC TCTCCGCCCC   
  
  
+ TGTTTCTCTC TCTCTCCCTC ACTCACTCAG AGTACTGATC TGAGCATCAG AAGTAGCAGA CCCAGAAACT   
  
  
+ CAGATTTAAA AACAACCCGC TCTTTTGTTC CCTCTACGTC TAATAATTTT GTCATCGCAT TTAATGTCAG   
  
  
+ ATAGAGAGAT AGAGCTCTAC TGTATTAATT TTCTCCCACC TCACTTGATT CTGTTGCCCT AAACCACCCA   
  
  
+ AAAAAAGAAA TACGAAGAAG CCACTCTCTC TCTCTCTCTC TCTCACACAC ACACACACCC CTCCTAGTGG   
  
  
+ ATTTGATAGA GTATAAATGA ACAGTAATCA GACGAGATAT ATAGTAGTGC TTAATTGACC GAGTAGCAAA   
  
  
+ AGGGGGAAGT TGAGTGAGGT ATCAAGAGAG TAGCGTAGCT AGCTAGGGCA AGATATGCTG GGGTCTTCCT   
  
  
+ CATACAACTC CGAGGAAGAC CACCAAGATG ACAACCATTC CTCACCGTCT GATTTGTCTA GGCACCCACC   
  
  
+ TAGATTCACC ACTACTACTT ACCAATACCA ACAACATATT AGCTCTCCAT CTTCACAATC AATACTAGCT   
  
  
+ GTGCCACCTC GCCAATTGTT AATCAATTGT GCGGAGTTAA TTTGCCGGTT CGACTTCTCC TCCGCCAACC   
  
  
+ GGCTTCTCTC TCTCCTCGCC TCCCACTTTG CCTCCCCTTC TGGGGACTCC ACCGAGAGAC TTGTGTTTTA   
  
  
+ CTTCTGCAAG GCTTTGGATC TCCGCCTCAA TAACAGTTAT TTATCCACTC GTAGTTTGAC TGGTTGTAAC   
  
  
+ CCTAGTACTG GTTCTGGTAT TGGGGCTCCT TTAGCTTCCA ATTTTCTATT TCTTCAACCA TTAGTGCATT   
  
  
+ CACAGGTAAC AAGTAATGCC CGTGTTGGGT ACTTGACATA TTTGACTCTG AACCAGGTCA CCCCATTCAT   
  
  
+ CCGATTCACC CACTTAACTG CCAACCAAGC CATCCTGGAA GCTTTGGAGG GGTACAAGGC GGTCCATATC   
  
  
+ CTTGACATGG ACATCATGCA TGGGGTCCAG TGGCCACCAC TTCTTCAAGC AATTGCAGAA AGATCTGCAA   
  
  
+ CCCTTGGTCA CCCTGCTCCC ACTGTTCGCA TCACAGGAGG GGGCAGGGAT CTAGAACTCC TGAACCGAAC   
  
  
+ TGGAGATCGG ATCAGGAAGT TCGCACAATC CTTGGGACTC GAGTTTCAAT TCCACCATCT TATAACGGTG   
  
  
+ GACTCGAACC TTACTGAGGC AGTGGAGGCC ATCAATGCCC TACAGCTACA CCTCCATGTC CAAGAGGAGG   
  
  
+ AGGTCTTCGC AGTGAATTGT GGAGATTTCC TCCACCGCCT CCTAACGGAG TATGATAGTA CATTCCTTAG   
  
  
+ GATGTTTCTC TACAAGGTGA AGACCCTAAA CCCTAGGGTC TTCACCGTGG GAGAAAGGGA GGCCGACCAC   
  
  
+ AACCACCCTC TCTTCTGGCA GCGATTCATT GAGGCACTTG ACCACTATGG GGCAGTGTTT GACTCTCTAG   
  
  
+ AAGCAACCCT ACCATCAACA AGTCAAGAGA GGGTGGCGGT GGAAGAGGGG TGGTTCGGGG AGGAGATTAA   
  
  
+ AGATGTGGTT GGACGAGAAG GAGGGTTGAG GAGACAGAGG CACCAGAAAT ACGAGTCTTG GGAGGTGTTG   
  
  
+ ATGAGGAGCT CTGGGTTTAA GAGCTTGCCC TTGAGCCCCT TCTCTGTGTC ACAAGCCAAG CTGCTACTTC   
  
  
+ GCCTTCATTA CCCTTCTGAA GGGTACCACC TTAAGGTCTT CCATAGTAAT TGTCTGTTGC TTGGGTGGAA   
  
  
+ GAATCGCCCT CTTTTCTCTG TTTCTTCTTG GCAATA  

- -Up\_Stream \_Len000TTTTAT AAAAATGTTT TCGTAAAGAG ATATTTTATA AAATGTTTTA AAGTAAAACA   
  
  
- CCCAGAAAAT CTTAGTTAGA GAGATGTTCA TCCTTACGGC CAACTCCATT CCGATGGATG TAGATCTAGA   
  
  
- GAGGTCTGGG GTGTTTTTAA TTTGCTCTGG GTGTGACTTT CCACCACCAC CACCACAACA ACAACATAAC   
  
  
- AGGATGGTGC TCTCTCGAAG TTCACGTAGT GAATAAAGTT GTCCAGTTAG TGTATCTAGA GATCACATGA   
  
  
- CATTTAAAAA AAAGGCAGAA TAAAATTAAA AGAAAAGGTA GGATTTTCTA ATAGTACATC TATAAGGATC   
  
  
- AAACTATATA ACTGAAGTTG AAGTTTTTAA TGTGTAATTC TAGGTGCTTA TACTGTCAAA TCCAAAGTAA   
  
  
- ACTTCCTCTT TCTTTACAAT GCCACAAAAC CACAACATAA GCATCTGTAA AAAACGCGAA TTGACCGAGA   
  
  
- AGAGTACCCC TGCTCTGTCA CTTCCATTAA CATGAACAAC TAACTACTGC AGTAGACATC GAACCTAAAC   
  
  
- ATATCCTTAC TTGTAGAACC ACAGATGCAC CTTTCACACT AAACACAAGA AAGTAACTAG GAGGAGGAGT   
  
  
- TCTTCTCCAC TAGAAAGAGG GAACAAAAAA AAAAAAAAAC CGATTATACA ACCGTCTTGG CCGTTTTTTA   
  
  
- TGTATTAAAC TTATCACGTA CGTATACTCA TGTACTGTCA CTTGGTTGTT TTGCTTGGGG ATTTTGTGTT   
  
  
- CTCTTAATTG TACAATTTTG TGAATTTGAT TTACGTTGGT AGAGCTACTG GTGATTTATC GGTTCTTTGG   
  
  
- ATTGCATTAA TTGTTTTTAT TTTTTATTGA CTTTTATTTC ATCTCTTTCA GATTGGCTTA TGCTATTGTA   
  
  
- TTGGATTTTG ATTGATATGA ACTTGAATGA TTCTTTATAC TTAGTTGGTG TTTGGCTTAA CTAAAACCAA   
  
  
- ACGGTATTAA ACTGATATGT AAATTGAATT TTTTATAAAA AAAAATTAAA GTTTATTTAG ATTTAATAAA   
  
  
- AAGTTATAAG TAGGTTTTAT CTAAGTTAAT AATTTGTTCA GTTGGATATG GCTCAATAAA AGATCGAGAC   
  
  
- TGATTCTGTA TATATATAAA AAACATTTTT TCTATTCCTA ATATTAAATT TATCGGAAAG GGAATTTTTT   
  
  
- TAAAAATTTT AATAGGACTA TCTCTTATTT TTCTTTAGGA ATATGTTTTG AGACTAACTG CGTCTACACA   
  
  
- TATACCTGTA TATCTTCATT ATTCTCCCCA TCCGATCCTT CCCCCACCTT TATATCCCGT ATTTATCCCC   
  
  
- CGCCCGTATC CCTGTACAGA CAGACTCCAT GCCACCTACC CTGGAACGGT ACCTTCGTTG TTACAGTGTT   
  
  
- ATTATTATAG TTGCTGACTT CAAAGAAGTG GTCGGGTGAG AAACGGTATT ACGGTAATTA ATAAATAACG   
  
  
- ATGATAGAGA AAACTACACT TTGCCACCTG GAATAGAGAG GGGAGGTGAA GGTTAACTAC ACTCGTTGAG   
  
  
- TCTTTGCGTT TGTTTTGAAC ACGGTGGAAG AAATGAACGT AGGAGTAAAA GGAGACAGAG AGAGGCGGGG   
  
  
- ACAAAGAGAG AGAGAGGGAG TGAGTGAGTC TCATGACTAG ACTCGTAGTC TTCATCGTCT GGGTCTTTGA   
  
  
- GTCTAAATTT TTGTTGGGCG AGAAAACAAG GGAGATGCAG ATTATTAAAA CAGTAGCGTA AATTACAGTC   
  
  
- TATCTCTCTA TCTCGAGATG ACATAATTAA AAGAGGGTGG AGTGAACTAA GACAACGGGA TTTGGTGGGT   
  
  
- TTTTTTCTTT ATGCTTCTTC GGTGAGAGAG AGAGAGAGAG AGAGTGTGTG TGTGTGTGGG GAGGATCACC   
  
  
- TAAACTATCT CATATTTACT TGTCATTAGT CTGCTCTATA TATCATCACG AATTAACTGG CTCATCGTTT   
  
  
- TCCCCCTTCA ACTCACTCCA TAGTTCTCTC ATCGCATCGA TCGATCCCGT TCTATACGAC CCCAGAAGGA   
  
  
- GTATGTTGAG GCTCCTTCTG GTGGTTCTAC TGTTGGTAAG GAGTGGCAGA CTAAACAGAT CCGTGGGTGG   
  
  
- ATCTAAGTGG TGATGATGAA TGGTTATGGT TGTTGTATAA TCGAGAGGTA GAAGTGTTAG TTATGATCGA   
  
  
- CACGGTGGAG CGGTTAACAA TTAGTTAACA CGCCTCAATT AAACGGCCAA GCTGAAGAGG AGGCGGTTGG   
  
  
- CCGAAGAGAG AGAGGAGCGG AGGGTGAAAC GGAGGGGAAG ACCCCTGAGG TGGCTCTCTG AACACAAAAT   
  
  
- GAAGACGTTC CGAAACCTAG AGGCGGAGTT ATTGTCAATA AATAGGTGAG CATCAAACTG ACCAACATTG   
  
  
- GGATCATGAC CAAGACCATA ACCCCGAGGA AATCGAAGGT TAAAAGATAA AGAAGTTGGT AATCACGTAA   
  
  
- GTGTCCATTG TTCATTACGG GCACAACCCA TGAACTGTAT AAACTGAGAC TTGGTCCAGT GGGGTAAGTA   
  
  
- GGCTAAGTGG GTGAATTGAC GGTTGGTTCG GTAGGACCTT CGAAACCTCC CCATGTTCCG CCAGGTATAG   
  
  
- GAACTGTACC TGTAGTACGT ACCCCAGGTC ACCGGTGGTG AAGAAGTTCG TTAACGTCTT TCTAGACGTT   
  
  
- GGGAACCAGT GGGACGAGGG TGACAAGCGT AGTGTCCTCC CCCGTCCCTA GATCTTGAGG ACTTGGCTTG   
  
  
- ACCTCTAGCC TAGTCCTTCA AGCGTGTTAG GAACCCTGAG CTCAAAGTTA AGGTGGTAGA ATATTGCCAC   
  
  
- CTGAGCTTGG AATGACTCCG TCACCTCCGG TAGTTACGGG ATGTCGATGT GGAGGTACAG GTTCTCCTCC   
  
  
- TCCAGAAGCG TCACTTAACA CCTCTAAAGG AGGTGGCGGA GGATTGCCTC ATACTATCAT GTAAGGAATC   
  
  
- CTACAAAGAG ATGTTCCACT TCTGGGATTT GGGATCCCAG AAGTGGCACC CTCTTTCCCT CCGGCTGGTG   
  
  
- TTGGTGGGAG AGAAGACCGT CGCTAAGTAA CTCCGTGAAC TGGTGATACC CCGTCACAAA CTGAGAGATC   
  
  
- TTCGTTGGGA TGGTAGTTGT TCAGTTCTCT CCCACCGCCA CCTTCTCCCC ACCAAGCCCC TCCTCTAATT   
  
  
- TCTACACCAA CCTGCTCTTC CTCCCAACTC CTCTGTCTCC GTGGTCTTTA TGCTCAGAAC CCTCCACAAC   
  
  
- TACTCCTCGA GACCCAAATT CTCGAACGGG AACTCGGGGA AGAGACACAG TGTTCGGTTC GACGATGAAG   
  
  
- CGGAAGTAAT GGGAAGACTT CCCATGGTGG AATTCCAGAA GGTATCATTA ACAGACAACG AACCCACCTT   
  
  
- CTTAGCGGGA GAAAAGAGAC AAAGAAGAAC CGTTAT

+     AAGAA-motif

| Site Name | Organism | Position | Strand | Matrix score. | sequence | function |
| --- | --- | --- | --- | --- | --- | --- |
| AAGAA-motif | Avena sativa | 432 | + | 7 | GAAAGAA |  |
| AAGAA-motif | Avena sativa | 313 | - | 9 | gGTAAAGAAA |  |
| AAGAA-motif | Avena sativa | 1426 | - | 9 | gGTAAAGAAA |  |
| AAGAA-motif | Avena sativa | 611 | - | 7 | GAAAGAA |  |

>HU01G00833.1   
+ -Up\_Stream \_Len000AAAATA TTTTTACAAA AGCATTTCTC TATAAAATAT TTTACAAAAT TTCATTTTGT   
  
  
+ GGGTCTTTTA GAATCAATCT CTCTACAAGT AGGAATGCCG GTTGAGGTAA GGCTACCTAC ATCTAGATCT   
  
  
+ CTCCAGACCC CACAAAAATT AAACGAGACC CACACTGAAA GGTGGTGGTG GTGGTGTTGT TGTTGTATTG   
  
  
+ TCCTACCACG AGAGAGCTTC AAGTGCATCA CTTATTTCAA CAGGTCAATC ACATAGATCT CTAGTGTACT   
  
  
+ GTAAATTTTT TTTCCGTCTT ATTTTAATTT TCTTTTCCAT CCTAAAAGAT TATCATGTAG ATATTCCTAG   
  
  
+ TTTGATATAT TGACTTCAAC TTCAAAAATT ACACATTAAG ATCCACGAAT ATGACAGTTT AGGTTTCATT   
  
  
+ TGAAGGAGAA AGAAATGTTA CGGTGTTTTG GTGTTGTATT CGTAGACATT TTTTGCGCTT AACTGGCTCT   
  
  
+ TCTCATGGGG ACGAGACAGT GAAGGTAATT GTACTTGTTG ATTGATGACG TCATCTGTAG CTTGGATTTG   
  
  
+ TATAGGAATG AACATCTTGG TGTCTACGTG GAAAGTGTGA TTTGTGTTCT TTCATTGATC CTCCTCCTCA   
  
  
+ AGAAGAGGTG ATCTTTCTCC CTTGTTTTTT TTTTTTTTTG GCTAATATGT TGGCAGAACC GGCAAAAAAT   
  
  
+ ACATAATTTG AATAGTGCAT GCATATGAGT ACATGACAGT GAACCAACAA AACGAACCCC TAAAACACAA   
  
  
+ GAGAATTAAC ATGTTAAAAC ACTTAAACTA AATGCAACCA TCTCGATGAC CACTAAATAG CCAAGAAACC   
  
  
+ TAACGTAATT AACAAAAATA AAAAATAACT GAAAATAAAG TAGAGAAAGT CTAACCGAAT ACGATAACAT   
  
  
+ AACCTAAAAC TAACTATACT TGAACTTACT AAGAAATATG AATCAACCAC AAACCGAATT GATTTTGGTT   
  
  
+ TGCCATAATT TGACTATACA TTTAACTTAA AAAATATTTT TTTTTAATTT CAAATAAATC TAAATTATTT   
  
  
+ TTCAATATTC ATCCAAAATA GATTCAATTA TTAAACAAGT CAACCTATAC CGAGTTATTT TCTAGCTCTG   
  
  
+ ACTAAGACAT ATATATATTT TTTGTAAAAA AGATAAGGAT TATAATTTAA ATAGCCTTTC CCTTAAAAAA   
  
  
+ ATTTTTAAAA TTATCCTGAT AGAGAATAAA AAGAAATCCT TATACAAAAC TCTGATTGAC GCAGATGTGT   
  
  
+ ATATGGACAT ATAGAAGTAA TAAGAGGGGT AGGCTAGGAA GGGGGTGGAA ATATAGGGCA TAAATAGGGG   
  
  
+ GCGGGCATAG GGACATGTCT GTCTGAGGTA CGGTGGATGG GACCTTGCCA TGGAAGCAAC AATGTCACAA   
  
  
+ TAATAATATC AACGACTGAA GTTTCTTCAC CAGCCCACTC TTTGCCATAA TGCCATTAAT TATTTATTGC   
  
  
+ TACTATCTCT TTTGATGTGA AACGGTGGAC CTTATCTCTC CCCTCCACTT CCAATTGATG TGAGCAACTC   
  
  
+ AGAAACGCAA ACAAAACTTG TGCCACCTTC TTTACTTGCA TCCTCATTTT CCTCTGTCTC TCTCCGCCCC   
  
  
+ TGTTTCTCTC TCTCTCCCTC ACTCACTCAG AGTACTGATC TGAGCATCAG AAGTAGCAGA CCCAGAAACT   
  
  
+ CAGATTTAAA AACAACCCGC TCTTTTGTTC CCTCTACGTC TAATAATTTT GTCATCGCAT TTAATGTCAG   
  
  
+ ATAGAGAGAT AGAGCTCTAC TGTATTAATT TTCTCCCACC TCACTTGATT CTGTTGCCCT AAACCACCCA   
  
  
+ AAAAAAGAAA TACGAAGAAG CCACTCTCTC TCTCTCTCTC TCTCACACAC ACACACACCC CTCCTAGTGG   
  
  
+ ATTTGATAGA GTATAAATGA ACAGTAATCA GACGAGATAT ATAGTAGTGC TTAATTGACC GAGTAGCAAA   
  
  
+ AGGGGGAAGT TGAGTGAGGT ATCAAGAGAG TAGCGTAGCT AGCTAGGGCA AGATATGCTG GGGTCTTCCT   
  
  
+ CATACAACTC CGAGGAAGAC CACCAAGATG ACAACCATTC CTCACCGTCT GATTTGTCTA GGCACCCACC   
  
  
+ TAGATTCACC ACTACTACTT ACCAATACCA ACAACATATT AGCTCTCCAT CTTCACAATC AATACTAGCT   
  
  
+ GTGCCACCTC GCCAATTGTT AATCAATTGT GCGGAGTTAA TTTGCCGGTT CGACTTCTCC TCCGCCAACC   
  
  
+ GGCTTCTCTC TCTCCTCGCC TCCCACTTTG CCTCCCCTTC TGGGGACTCC ACCGAGAGAC TTGTGTTTTA   
  
  
+ CTTCTGCAAG GCTTTGGATC TCCGCCTCAA TAACAGTTAT TTATCCACTC GTAGTTTGAC TGGTTGTAAC   
  
  
+ CCTAGTACTG GTTCTGGTAT TGGGGCTCCT TTAGCTTCCA ATTTTCTATT TCTTCAACCA TTAGTGCATT   
  
  
+ CACAGGTAAC AAGTAATGCC CGTGTTGGGT ACTTGACATA TTTGACTCTG AACCAGGTCA CCCCATTCAT   
  
  
+ CCGATTCACC CACTTAACTG CCAACCAAGC CATCCTGGAA GCTTTGGAGG GGTACAAGGC GGTCCATATC   
  
  
+ CTTGACATGG ACATCATGCA TGGGGTCCAG TGGCCACCAC TTCTTCAAGC AATTGCAGAA AGATCTGCAA   
  
  
+ CCCTTGGTCA CCCTGCTCCC ACTGTTCGCA TCACAGGAGG GGGCAGGGAT CTAGAACTCC TGAACCGAAC   
  
  
+ TGGAGATCGG ATCAGGAAGT TCGCACAATC CTTGGGACTC GAGTTTCAAT TCCACCATCT TATAACGGTG   
  
  
+ GACTCGAACC TTACTGAGGC AGTGGAGGCC ATCAATGCCC TACAGCTACA CCTCCATGTC CAAGAGGAGG   
  
  
+ AGGTCTTCGC AGTGAATTGT GGAGATTTCC TCCACCGCCT CCTAACGGAG TATGATAGTA CATTCCTTAG   
  
  
+ GATGTTTCTC TACAAGGTGA AGACCCTAAA CCCTAGGGTC TTCACCGTGG GAGAAAGGGA GGCCGACCAC   
  
  
+ AACCACCCTC TCTTCTGGCA GCGATTCATT GAGGCACTTG ACCACTATGG GGCAGTGTTT GACTCTCTAG   
  
  
+ AAGCAACCCT ACCATCAACA AGTCAAGAGA GGGTGGCGGT GGAAGAGGGG TGGTTCGGGG AGGAGATTAA   
  
  
+ AGATGTGGTT GGACGAGAAG GAGGGTTGAG GAGACAGAGG CACCAGAAAT ACGAGTCTTG GGAGGTGTTG   
  
  
+ ATGAGGAGCT CTGGGTTTAA GAGCTTGCCC TTGAGCCCCT TCTCTGTGTC ACAAGCCAAG CTGCTACTTC   
  
  
+ GCCTTCATTA CCCTTCTGAA GGGTACCACC TTAAGGTCTT CCATAGTAAT TGTCTGTTGC TTGGGTGGAA   
  
  
+ GAATCGCCCT CTTTTCTCTG TTTCTTCTTG GCAATA  

- -Up\_Stream \_Len000TTTTAT AAAAATGTTT TCGTAAAGAG ATATTTTATA AAATGTTTTA AAGTAAAACA   
  
  
- CCCAGAAAAT CTTAGTTAGA GAGATGTTCA TCCTTACGGC CAACTCCATT CCGATGGATG TAGATCTAGA   
  
  
- GAGGTCTGGG GTGTTTTTAA TTTGCTCTGG GTGTGACTTT CCACCACCAC CACCACAACA ACAACATAAC   
  
  
- AGGATGGTGC TCTCTCGAAG TTCACGTAGT GAATAAAGTT GTCCAGTTAG TGTATCTAGA GATCACATGA   
  
  
- CATTTAAAAA AAAGGCAGAA TAAAATTAAA AGAAAAGGTA GGATTTTCTA ATAGTACATC TATAAGGATC   
  
  
- AAACTATATA ACTGAAGTTG AAGTTTTTAA TGTGTAATTC TAGGTGCTTA TACTGTCAAA TCCAAAGTAA   
  
  
- ACTTCCTCTT TCTTTACAAT GCCACAAAAC CACAACATAA GCATCTGTAA AAAACGCGAA TTGACCGAGA   
  
  
- AGAGTACCCC TGCTCTGTCA CTTCCATTAA CATGAACAAC TAACTACTGC AGTAGACATC GAACCTAAAC   
  
  
- ATATCCTTAC TTGTAGAACC ACAGATGCAC CTTTCACACT AAACACAAGA AAGTAACTAG GAGGAGGAGT   
  
  
- TCTTCTCCAC TAGAAAGAGG GAACAAAAAA AAAAAAAAAC CGATTATACA ACCGTCTTGG CCGTTTTTTA   
  
  
- TGTATTAAAC TTATCACGTA CGTATACTCA TGTACTGTCA CTTGGTTGTT TTGCTTGGGG ATTTTGTGTT   
  
  
- CTCTTAATTG TACAATTTTG TGAATTTGAT TTACGTTGGT AGAGCTACTG GTGATTTATC GGTTCTTTGG   
  
  
- ATTGCATTAA TTGTTTTTAT TTTTTATTGA CTTTTATTTC ATCTCTTTCA GATTGGCTTA TGCTATTGTA   
  
  
- TTGGATTTTG ATTGATATGA ACTTGAATGA TTCTTTATAC TTAGTTGGTG TTTGGCTTAA CTAAAACCAA   
  
  
- ACGGTATTAA ACTGATATGT AAATTGAATT TTTTATAAAA AAAAATTAAA GTTTATTTAG ATTTAATAAA   
  
  
- AAGTTATAAG TAGGTTTTAT CTAAGTTAAT AATTTGTTCA GTTGGATATG GCTCAATAAA AGATCGAGAC   
  
  
- TGATTCTGTA TATATATAAA AAACATTTTT TCTATTCCTA ATATTAAATT TATCGGAAAG GGAATTTTTT   
  
  
- TAAAAATTTT AATAGGACTA TCTCTTATTT TTCTTTAGGA ATATGTTTTG AGACTAACTG CGTCTACACA   
  
  
- TATACCTGTA TATCTTCATT ATTCTCCCCA TCCGATCCTT CCCCCACCTT TATATCCCGT ATTTATCCCC   
  
  
- CGCCCGTATC CCTGTACAGA CAGACTCCAT GCCACCTACC CTGGAACGGT ACCTTCGTTG TTACAGTGTT   
  
  
- ATTATTATAG TTGCTGACTT CAAAGAAGTG GTCGGGTGAG AAACGGTATT ACGGTAATTA ATAAATAACG   
  
  
- ATGATAGAGA AAACTACACT TTGCCACCTG GAATAGAGAG GGGAGGTGAA GGTTAACTAC ACTCGTTGAG   
  
  
- TCTTTGCGTT TGTTTTGAAC ACGGTGGAAG AAATGAACGT AGGAGTAAAA GGAGACAGAG AGAGGCGGGG   
  
  
- ACAAAGAGAG AGAGAGGGAG TGAGTGAGTC TCATGACTAG ACTCGTAGTC TTCATCGTCT GGGTCTTTGA   
  
  
- GTCTAAATTT TTGTTGGGCG AGAAAACAAG GGAGATGCAG ATTATTAAAA CAGTAGCGTA AATTACAGTC   
  
  
- TATCTCTCTA TCTCGAGATG ACATAATTAA AAGAGGGTGG AGTGAACTAA GACAACGGGA TTTGGTGGGT   
  
  
- TTTTTTCTTT ATGCTTCTTC GGTGAGAGAG AGAGAGAGAG AGAGTGTGTG TGTGTGTGGG GAGGATCACC   
  
  
- TAAACTATCT CATATTTACT TGTCATTAGT CTGCTCTATA TATCATCACG AATTAACTGG CTCATCGTTT   
  
  
- TCCCCCTTCA ACTCACTCCA TAGTTCTCTC ATCGCATCGA TCGATCCCGT TCTATACGAC CCCAGAAGGA   
  
  
- GTATGTTGAG GCTCCTTCTG GTGGTTCTAC TGTTGGTAAG GAGTGGCAGA CTAAACAGAT CCGTGGGTGG   
  
  
- ATCTAAGTGG TGATGATGAA TGGTTATGGT TGTTGTATAA TCGAGAGGTA GAAGTGTTAG TTATGATCGA   
  
  
- CACGGTGGAG CGGTTAACAA TTAGTTAACA CGCCTCAATT AAACGGCCAA GCTGAAGAGG AGGCGGTTGG   
  
  
- CCGAAGAGAG AGAGGAGCGG AGGGTGAAAC GGAGGGGAAG ACCCCTGAGG TGGCTCTCTG AACACAAAAT   
  
  
- GAAGACGTTC CGAAACCTAG AGGCGGAGTT ATTGTCAATA AATAGGTGAG CATCAAACTG ACCAACATTG   
  
  
- GGATCATGAC CAAGACCATA ACCCCGAGGA AATCGAAGGT TAAAAGATAA AGAAGTTGGT AATCACGTAA   
  
  
- GTGTCCATTG TTCATTACGG GCACAACCCA TGAACTGTAT AAACTGAGAC TTGGTCCAGT GGGGTAAGTA   
  
  
- GGCTAAGTGG GTGAATTGAC GGTTGGTTCG GTAGGACCTT CGAAACCTCC CCATGTTCCG CCAGGTATAG   
  
  
- GAACTGTACC TGTAGTACGT ACCCCAGGTC ACCGGTGGTG AAGAAGTTCG TTAACGTCTT TCTAGACGTT   
  
  
- GGGAACCAGT GGGACGAGGG TGACAAGCGT AGTGTCCTCC CCCGTCCCTA GATCTTGAGG ACTTGGCTTG   
  
  
- ACCTCTAGCC TAGTCCTTCA AGCGTGTTAG GAACCCTGAG CTCAAAGTTA AGGTGGTAGA ATATTGCCAC   
  
  
- CTGAGCTTGG AATGACTCCG TCACCTCCGG TAGTTACGGG ATGTCGATGT GGAGGTACAG GTTCTCCTCC   
  
  
- TCCAGAAGCG TCACTTAACA CCTCTAAAGG AGGTGGCGGA GGATTGCCTC ATACTATCAT GTAAGGAATC   
  
  
- CTACAAAGAG ATGTTCCACT TCTGGGATTT GGGATCCCAG AAGTGGCACC CTCTTTCCCT CCGGCTGGTG   
  
  
- TTGGTGGGAG AGAAGACCGT CGCTAAGTAA CTCCGTGAAC TGGTGATACC CCGTCACAAA CTGAGAGATC   
  
  
- TTCGTTGGGA TGGTAGTTGT TCAGTTCTCT CCCACCGCCA CCTTCTCCCC ACCAAGCCCC TCCTCTAATT   
  
  
- TCTACACCAA CCTGCTCTTC CTCCCAACTC CTCTGTCTCC GTGGTCTTTA TGCTCAGAAC CCTCCACAAC   
  
  
- TACTCCTCGA GACCCAAATT CTCGAACGGG AACTCGGGGA AGAGACACAG TGTTCGGTTC GACGATGAAG   
  
  
- CGGAAGTAAT GGGAAGACTT CCCATGGTGG AATTCCAGAA GGTATCATTA ACAGACAACG AACCCACCTT   
  
  
- CTTAGCGGGA GAAAAGAGAC AAAGAAGAAC CGTTAT

+     ABRE

| Site Name | Organism | Position | Strand | Matrix score. | sequence | function |
| --- | --- | --- | --- | --- | --- | --- |
| ABRE | Arabidopsis thaliana | 590 | + | 5 | ACGTG | cis-acting element involved in the abscisic acid responsiveness |

>HU01G00833.1   
+ -Up\_Stream \_Len000AAAATA TTTTTACAAA AGCATTTCTC TATAAAATAT TTTACAAAAT TTCATTTTGT   
  
  
+ GGGTCTTTTA GAATCAATCT CTCTACAAGT AGGAATGCCG GTTGAGGTAA GGCTACCTAC ATCTAGATCT   
  
  
+ CTCCAGACCC CACAAAAATT AAACGAGACC CACACTGAAA GGTGGTGGTG GTGGTGTTGT TGTTGTATTG   
  
  
+ TCCTACCACG AGAGAGCTTC AAGTGCATCA CTTATTTCAA CAGGTCAATC ACATAGATCT CTAGTGTACT   
  
  
+ GTAAATTTTT TTTCCGTCTT ATTTTAATTT TCTTTTCCAT CCTAAAAGAT TATCATGTAG ATATTCCTAG   
  
  
+ TTTGATATAT TGACTTCAAC TTCAAAAATT ACACATTAAG ATCCACGAAT ATGACAGTTT AGGTTTCATT   
  
  
+ TGAAGGAGAA AGAAATGTTA CGGTGTTTTG GTGTTGTATT CGTAGACATT TTTTGCGCTT AACTGGCTCT   
  
  
+ TCTCATGGGG ACGAGACAGT GAAGGTAATT GTACTTGTTG ATTGATGACG TCATCTGTAG CTTGGATTTG   
  
  
+ TATAGGAATG AACATCTTGG TGTCTACGTG GAAAGTGTGA TTTGTGTTCT TTCATTGATC CTCCTCCTCA   
  
  
+ AGAAGAGGTG ATCTTTCTCC CTTGTTTTTT TTTTTTTTTG GCTAATATGT TGGCAGAACC GGCAAAAAAT   
  
  
+ ACATAATTTG AATAGTGCAT GCATATGAGT ACATGACAGT GAACCAACAA AACGAACCCC TAAAACACAA   
  
  
+ GAGAATTAAC ATGTTAAAAC ACTTAAACTA AATGCAACCA TCTCGATGAC CACTAAATAG CCAAGAAACC   
  
  
+ TAACGTAATT AACAAAAATA AAAAATAACT GAAAATAAAG TAGAGAAAGT CTAACCGAAT ACGATAACAT   
  
  
+ AACCTAAAAC TAACTATACT TGAACTTACT AAGAAATATG AATCAACCAC AAACCGAATT GATTTTGGTT   
  
  
+ TGCCATAATT TGACTATACA TTTAACTTAA AAAATATTTT TTTTTAATTT CAAATAAATC TAAATTATTT   
  
  
+ TTCAATATTC ATCCAAAATA GATTCAATTA TTAAACAAGT CAACCTATAC CGAGTTATTT TCTAGCTCTG   
  
  
+ ACTAAGACAT ATATATATTT TTTGTAAAAA AGATAAGGAT TATAATTTAA ATAGCCTTTC CCTTAAAAAA   
  
  
+ ATTTTTAAAA TTATCCTGAT AGAGAATAAA AAGAAATCCT TATACAAAAC TCTGATTGAC GCAGATGTGT   
  
  
+ ATATGGACAT ATAGAAGTAA TAAGAGGGGT AGGCTAGGAA GGGGGTGGAA ATATAGGGCA TAAATAGGGG   
  
  
+ GCGGGCATAG GGACATGTCT GTCTGAGGTA CGGTGGATGG GACCTTGCCA TGGAAGCAAC AATGTCACAA   
  
  
+ TAATAATATC AACGACTGAA GTTTCTTCAC CAGCCCACTC TTTGCCATAA TGCCATTAAT TATTTATTGC   
  
  
+ TACTATCTCT TTTGATGTGA AACGGTGGAC CTTATCTCTC CCCTCCACTT CCAATTGATG TGAGCAACTC   
  
  
+ AGAAACGCAA ACAAAACTTG TGCCACCTTC TTTACTTGCA TCCTCATTTT CCTCTGTCTC TCTCCGCCCC   
  
  
+ TGTTTCTCTC TCTCTCCCTC ACTCACTCAG AGTACTGATC TGAGCATCAG AAGTAGCAGA CCCAGAAACT   
  
  
+ CAGATTTAAA AACAACCCGC TCTTTTGTTC CCTCTACGTC TAATAATTTT GTCATCGCAT TTAATGTCAG   
  
  
+ ATAGAGAGAT AGAGCTCTAC TGTATTAATT TTCTCCCACC TCACTTGATT CTGTTGCCCT AAACCACCCA   
  
  
+ AAAAAAGAAA TACGAAGAAG CCACTCTCTC TCTCTCTCTC TCTCACACAC ACACACACCC CTCCTAGTGG   
  
  
+ ATTTGATAGA GTATAAATGA ACAGTAATCA GACGAGATAT ATAGTAGTGC TTAATTGACC GAGTAGCAAA   
  
  
+ AGGGGGAAGT TGAGTGAGGT ATCAAGAGAG TAGCGTAGCT AGCTAGGGCA AGATATGCTG GGGTCTTCCT   
  
  
+ CATACAACTC CGAGGAAGAC CACCAAGATG ACAACCATTC CTCACCGTCT GATTTGTCTA GGCACCCACC   
  
  
+ TAGATTCACC ACTACTACTT ACCAATACCA ACAACATATT AGCTCTCCAT CTTCACAATC AATACTAGCT   
  
  
+ GTGCCACCTC GCCAATTGTT AATCAATTGT GCGGAGTTAA TTTGCCGGTT CGACTTCTCC TCCGCCAACC   
  
  
+ GGCTTCTCTC TCTCCTCGCC TCCCACTTTG CCTCCCCTTC TGGGGACTCC ACCGAGAGAC TTGTGTTTTA   
  
  
+ CTTCTGCAAG GCTTTGGATC TCCGCCTCAA TAACAGTTAT TTATCCACTC GTAGTTTGAC TGGTTGTAAC   
  
  
+ CCTAGTACTG GTTCTGGTAT TGGGGCTCCT TTAGCTTCCA ATTTTCTATT TCTTCAACCA TTAGTGCATT   
  
  
+ CACAGGTAAC AAGTAATGCC CGTGTTGGGT ACTTGACATA TTTGACTCTG AACCAGGTCA CCCCATTCAT   
  
  
+ CCGATTCACC CACTTAACTG CCAACCAAGC CATCCTGGAA GCTTTGGAGG GGTACAAGGC GGTCCATATC   
  
  
+ CTTGACATGG ACATCATGCA TGGGGTCCAG TGGCCACCAC TTCTTCAAGC AATTGCAGAA AGATCTGCAA   
  
  
+ CCCTTGGTCA CCCTGCTCCC ACTGTTCGCA TCACAGGAGG GGGCAGGGAT CTAGAACTCC TGAACCGAAC   
  
  
+ TGGAGATCGG ATCAGGAAGT TCGCACAATC CTTGGGACTC GAGTTTCAAT TCCACCATCT TATAACGGTG   
  
  
+ GACTCGAACC TTACTGAGGC AGTGGAGGCC ATCAATGCCC TACAGCTACA CCTCCATGTC CAAGAGGAGG   
  
  
+ AGGTCTTCGC AGTGAATTGT GGAGATTTCC TCCACCGCCT CCTAACGGAG TATGATAGTA CATTCCTTAG   
  
  
+ GATGTTTCTC TACAAGGTGA AGACCCTAAA CCCTAGGGTC TTCACCGTGG GAGAAAGGGA GGCCGACCAC   
  
  
+ AACCACCCTC TCTTCTGGCA GCGATTCATT GAGGCACTTG ACCACTATGG GGCAGTGTTT GACTCTCTAG   
  
  
+ AAGCAACCCT ACCATCAACA AGTCAAGAGA GGGTGGCGGT GGAAGAGGGG TGGTTCGGGG AGGAGATTAA   
  
  
+ AGATGTGGTT GGACGAGAAG GAGGGTTGAG GAGACAGAGG CACCAGAAAT ACGAGTCTTG GGAGGTGTTG   
  
  
+ ATGAGGAGCT CTGGGTTTAA GAGCTTGCCC TTGAGCCCCT TCTCTGTGTC ACAAGCCAAG CTGCTACTTC   
  
  
+ GCCTTCATTA CCCTTCTGAA GGGTACCACC TTAAGGTCTT CCATAGTAAT TGTCTGTTGC TTGGGTGGAA   
  
  
+ GAATCGCCCT CTTTTCTCTG TTTCTTCTTG GCAATA  

- -Up\_Stream \_Len000TTTTAT AAAAATGTTT TCGTAAAGAG ATATTTTATA AAATGTTTTA AAGTAAAACA   
  
  
- CCCAGAAAAT CTTAGTTAGA GAGATGTTCA TCCTTACGGC CAACTCCATT CCGATGGATG TAGATCTAGA   
  
  
- GAGGTCTGGG GTGTTTTTAA TTTGCTCTGG GTGTGACTTT CCACCACCAC CACCACAACA ACAACATAAC   
  
  
- AGGATGGTGC TCTCTCGAAG TTCACGTAGT GAATAAAGTT GTCCAGTTAG TGTATCTAGA GATCACATGA   
  
  
- CATTTAAAAA AAAGGCAGAA TAAAATTAAA AGAAAAGGTA GGATTTTCTA ATAGTACATC TATAAGGATC   
  
  
- AAACTATATA ACTGAAGTTG AAGTTTTTAA TGTGTAATTC TAGGTGCTTA TACTGTCAAA TCCAAAGTAA   
  
  
- ACTTCCTCTT TCTTTACAAT GCCACAAAAC CACAACATAA GCATCTGTAA AAAACGCGAA TTGACCGAGA   
  
  
- AGAGTACCCC TGCTCTGTCA CTTCCATTAA CATGAACAAC TAACTACTGC AGTAGACATC GAACCTAAAC   
  
  
- ATATCCTTAC TTGTAGAACC ACAGATGCAC CTTTCACACT AAACACAAGA AAGTAACTAG GAGGAGGAGT   
  
  
- TCTTCTCCAC TAGAAAGAGG GAACAAAAAA AAAAAAAAAC CGATTATACA ACCGTCTTGG CCGTTTTTTA   
  
  
- TGTATTAAAC TTATCACGTA CGTATACTCA TGTACTGTCA CTTGGTTGTT TTGCTTGGGG ATTTTGTGTT   
  
  
- CTCTTAATTG TACAATTTTG TGAATTTGAT TTACGTTGGT AGAGCTACTG GTGATTTATC GGTTCTTTGG   
  
  
- ATTGCATTAA TTGTTTTTAT TTTTTATTGA CTTTTATTTC ATCTCTTTCA GATTGGCTTA TGCTATTGTA   
  
  
- TTGGATTTTG ATTGATATGA ACTTGAATGA TTCTTTATAC TTAGTTGGTG TTTGGCTTAA CTAAAACCAA   
  
  
- ACGGTATTAA ACTGATATGT AAATTGAATT TTTTATAAAA AAAAATTAAA GTTTATTTAG ATTTAATAAA   
  
  
- AAGTTATAAG TAGGTTTTAT CTAAGTTAAT AATTTGTTCA GTTGGATATG GCTCAATAAA AGATCGAGAC   
  
  
- TGATTCTGTA TATATATAAA AAACATTTTT TCTATTCCTA ATATTAAATT TATCGGAAAG GGAATTTTTT   
  
  
- TAAAAATTTT AATAGGACTA TCTCTTATTT TTCTTTAGGA ATATGTTTTG AGACTAACTG CGTCTACACA   
  
  
- TATACCTGTA TATCTTCATT ATTCTCCCCA TCCGATCCTT CCCCCACCTT TATATCCCGT ATTTATCCCC   
  
  
- CGCCCGTATC CCTGTACAGA CAGACTCCAT GCCACCTACC CTGGAACGGT ACCTTCGTTG TTACAGTGTT   
  
  
- ATTATTATAG TTGCTGACTT CAAAGAAGTG GTCGGGTGAG AAACGGTATT ACGGTAATTA ATAAATAACG   
  
  
- ATGATAGAGA AAACTACACT TTGCCACCTG GAATAGAGAG GGGAGGTGAA GGTTAACTAC ACTCGTTGAG   
  
  
- TCTTTGCGTT TGTTTTGAAC ACGGTGGAAG AAATGAACGT AGGAGTAAAA GGAGACAGAG AGAGGCGGGG   
  
  
- ACAAAGAGAG AGAGAGGGAG TGAGTGAGTC TCATGACTAG ACTCGTAGTC TTCATCGTCT GGGTCTTTGA   
  
  
- GTCTAAATTT TTGTTGGGCG AGAAAACAAG GGAGATGCAG ATTATTAAAA CAGTAGCGTA AATTACAGTC   
  
  
- TATCTCTCTA TCTCGAGATG ACATAATTAA AAGAGGGTGG AGTGAACTAA GACAACGGGA TTTGGTGGGT   
  
  
- TTTTTTCTTT ATGCTTCTTC GGTGAGAGAG AGAGAGAGAG AGAGTGTGTG TGTGTGTGGG GAGGATCACC   
  
  
- TAAACTATCT CATATTTACT TGTCATTAGT CTGCTCTATA TATCATCACG AATTAACTGG CTCATCGTTT   
  
  
- TCCCCCTTCA ACTCACTCCA TAGTTCTCTC ATCGCATCGA TCGATCCCGT TCTATACGAC CCCAGAAGGA   
  
  
- GTATGTTGAG GCTCCTTCTG GTGGTTCTAC TGTTGGTAAG GAGTGGCAGA CTAAACAGAT CCGTGGGTGG   
  
  
- ATCTAAGTGG TGATGATGAA TGGTTATGGT TGTTGTATAA TCGAGAGGTA GAAGTGTTAG TTATGATCGA   
  
  
- CACGGTGGAG CGGTTAACAA TTAGTTAACA CGCCTCAATT AAACGGCCAA GCTGAAGAGG AGGCGGTTGG   
  
  
- CCGAAGAGAG AGAGGAGCGG AGGGTGAAAC GGAGGGGAAG ACCCCTGAGG TGGCTCTCTG AACACAAAAT   
  
  
- GAAGACGTTC CGAAACCTAG AGGCGGAGTT ATTGTCAATA AATAGGTGAG CATCAAACTG ACCAACATTG   
  
  
- GGATCATGAC CAAGACCATA ACCCCGAGGA AATCGAAGGT TAAAAGATAA AGAAGTTGGT AATCACGTAA   
  
  
- GTGTCCATTG TTCATTACGG GCACAACCCA TGAACTGTAT AAACTGAGAC TTGGTCCAGT GGGGTAAGTA   
  
  
- GGCTAAGTGG GTGAATTGAC GGTTGGTTCG GTAGGACCTT CGAAACCTCC CCATGTTCCG CCAGGTATAG   
  
  
- GAACTGTACC TGTAGTACGT ACCCCAGGTC ACCGGTGGTG AAGAAGTTCG TTAACGTCTT TCTAGACGTT   
  
  
- GGGAACCAGT GGGACGAGGG TGACAAGCGT AGTGTCCTCC CCCGTCCCTA GATCTTGAGG ACTTGGCTTG   
  
  
- ACCTCTAGCC TAGTCCTTCA AGCGTGTTAG GAACCCTGAG CTCAAAGTTA AGGTGGTAGA ATATTGCCAC   
  
  
- CTGAGCTTGG AATGACTCCG TCACCTCCGG TAGTTACGGG ATGTCGATGT GGAGGTACAG GTTCTCCTCC   
  
  
- TCCAGAAGCG TCACTTAACA CCTCTAAAGG AGGTGGCGGA GGATTGCCTC ATACTATCAT GTAAGGAATC   
  
  
- CTACAAAGAG ATGTTCCACT TCTGGGATTT GGGATCCCAG AAGTGGCACC CTCTTTCCCT CCGGCTGGTG   
  
  
- TTGGTGGGAG AGAAGACCGT CGCTAAGTAA CTCCGTGAAC TGGTGATACC CCGTCACAAA CTGAGAGATC   
  
  
- TTCGTTGGGA TGGTAGTTGT TCAGTTCTCT CCCACCGCCA CCTTCTCCCC ACCAAGCCCC TCCTCTAATT   
  
  
- TCTACACCAA CCTGCTCTTC CTCCCAACTC CTCTGTCTCC GTGGTCTTTA TGCTCAGAAC CCTCCACAAC   
  
  
- TACTCCTCGA GACCCAAATT CTCGAACGGG AACTCGGGGA AGAGACACAG TGTTCGGTTC GACGATGAAG   
  
  
- CGGAAGTAAT GGGAAGACTT CCCATGGTGG AATTCCAGAA GGTATCATTA ACAGACAACG AACCCACCTT   
  
  
- CTTAGCGGGA GAAAAGAGAC AAAGAAGAAC CGTTAT

+     ABRE3a

| Site Name | Organism | Position | Strand | Matrix score. | sequence | function |
| --- | --- | --- | --- | --- | --- | --- |
| ABRE3a | Zea mays | 589 | + | 6 | TACGTG |  |

>HU01G00833.1   
+ -Up\_Stream \_Len000AAAATA TTTTTACAAA AGCATTTCTC TATAAAATAT TTTACAAAAT TTCATTTTGT   
  
  
+ GGGTCTTTTA GAATCAATCT CTCTACAAGT AGGAATGCCG GTTGAGGTAA GGCTACCTAC ATCTAGATCT   
  
  
+ CTCCAGACCC CACAAAAATT AAACGAGACC CACACTGAAA GGTGGTGGTG GTGGTGTTGT TGTTGTATTG   
  
  
+ TCCTACCACG AGAGAGCTTC AAGTGCATCA CTTATTTCAA CAGGTCAATC ACATAGATCT CTAGTGTACT   
  
  
+ GTAAATTTTT TTTCCGTCTT ATTTTAATTT TCTTTTCCAT CCTAAAAGAT TATCATGTAG ATATTCCTAG   
  
  
+ TTTGATATAT TGACTTCAAC TTCAAAAATT ACACATTAAG ATCCACGAAT ATGACAGTTT AGGTTTCATT   
  
  
+ TGAAGGAGAA AGAAATGTTA CGGTGTTTTG GTGTTGTATT CGTAGACATT TTTTGCGCTT AACTGGCTCT   
  
  
+ TCTCATGGGG ACGAGACAGT GAAGGTAATT GTACTTGTTG ATTGATGACG TCATCTGTAG CTTGGATTTG   
  
  
+ TATAGGAATG AACATCTTGG TGTCTACGTG GAAAGTGTGA TTTGTGTTCT TTCATTGATC CTCCTCCTCA   
  
  
+ AGAAGAGGTG ATCTTTCTCC CTTGTTTTTT TTTTTTTTTG GCTAATATGT TGGCAGAACC GGCAAAAAAT   
  
  
+ ACATAATTTG AATAGTGCAT GCATATGAGT ACATGACAGT GAACCAACAA AACGAACCCC TAAAACACAA   
  
  
+ GAGAATTAAC ATGTTAAAAC ACTTAAACTA AATGCAACCA TCTCGATGAC CACTAAATAG CCAAGAAACC   
  
  
+ TAACGTAATT AACAAAAATA AAAAATAACT GAAAATAAAG TAGAGAAAGT CTAACCGAAT ACGATAACAT   
  
  
+ AACCTAAAAC TAACTATACT TGAACTTACT AAGAAATATG AATCAACCAC AAACCGAATT GATTTTGGTT   
  
  
+ TGCCATAATT TGACTATACA TTTAACTTAA AAAATATTTT TTTTTAATTT CAAATAAATC TAAATTATTT   
  
  
+ TTCAATATTC ATCCAAAATA GATTCAATTA TTAAACAAGT CAACCTATAC CGAGTTATTT TCTAGCTCTG   
  
  
+ ACTAAGACAT ATATATATTT TTTGTAAAAA AGATAAGGAT TATAATTTAA ATAGCCTTTC CCTTAAAAAA   
  
  
+ ATTTTTAAAA TTATCCTGAT AGAGAATAAA AAGAAATCCT TATACAAAAC TCTGATTGAC GCAGATGTGT   
  
  
+ ATATGGACAT ATAGAAGTAA TAAGAGGGGT AGGCTAGGAA GGGGGTGGAA ATATAGGGCA TAAATAGGGG   
  
  
+ GCGGGCATAG GGACATGTCT GTCTGAGGTA CGGTGGATGG GACCTTGCCA TGGAAGCAAC AATGTCACAA   
  
  
+ TAATAATATC AACGACTGAA GTTTCTTCAC CAGCCCACTC TTTGCCATAA TGCCATTAAT TATTTATTGC   
  
  
+ TACTATCTCT TTTGATGTGA AACGGTGGAC CTTATCTCTC CCCTCCACTT CCAATTGATG TGAGCAACTC   
  
  
+ AGAAACGCAA ACAAAACTTG TGCCACCTTC TTTACTTGCA TCCTCATTTT CCTCTGTCTC TCTCCGCCCC   
  
  
+ TGTTTCTCTC TCTCTCCCTC ACTCACTCAG AGTACTGATC TGAGCATCAG AAGTAGCAGA CCCAGAAACT   
  
  
+ CAGATTTAAA AACAACCCGC TCTTTTGTTC CCTCTACGTC TAATAATTTT GTCATCGCAT TTAATGTCAG   
  
  
+ ATAGAGAGAT AGAGCTCTAC TGTATTAATT TTCTCCCACC TCACTTGATT CTGTTGCCCT AAACCACCCA   
  
  
+ AAAAAAGAAA TACGAAGAAG CCACTCTCTC TCTCTCTCTC TCTCACACAC ACACACACCC CTCCTAGTGG   
  
  
+ ATTTGATAGA GTATAAATGA ACAGTAATCA GACGAGATAT ATAGTAGTGC TTAATTGACC GAGTAGCAAA   
  
  
+ AGGGGGAAGT TGAGTGAGGT ATCAAGAGAG TAGCGTAGCT AGCTAGGGCA AGATATGCTG GGGTCTTCCT   
  
  
+ CATACAACTC CGAGGAAGAC CACCAAGATG ACAACCATTC CTCACCGTCT GATTTGTCTA GGCACCCACC   
  
  
+ TAGATTCACC ACTACTACTT ACCAATACCA ACAACATATT AGCTCTCCAT CTTCACAATC AATACTAGCT   
  
  
+ GTGCCACCTC GCCAATTGTT AATCAATTGT GCGGAGTTAA TTTGCCGGTT CGACTTCTCC TCCGCCAACC   
  
  
+ GGCTTCTCTC TCTCCTCGCC TCCCACTTTG CCTCCCCTTC TGGGGACTCC ACCGAGAGAC TTGTGTTTTA   
  
  
+ CTTCTGCAAG GCTTTGGATC TCCGCCTCAA TAACAGTTAT TTATCCACTC GTAGTTTGAC TGGTTGTAAC   
  
  
+ CCTAGTACTG GTTCTGGTAT TGGGGCTCCT TTAGCTTCCA ATTTTCTATT TCTTCAACCA TTAGTGCATT   
  
  
+ CACAGGTAAC AAGTAATGCC CGTGTTGGGT ACTTGACATA TTTGACTCTG AACCAGGTCA CCCCATTCAT   
  
  
+ CCGATTCACC CACTTAACTG CCAACCAAGC CATCCTGGAA GCTTTGGAGG GGTACAAGGC GGTCCATATC   
  
  
+ CTTGACATGG ACATCATGCA TGGGGTCCAG TGGCCACCAC TTCTTCAAGC AATTGCAGAA AGATCTGCAA   
  
  
+ CCCTTGGTCA CCCTGCTCCC ACTGTTCGCA TCACAGGAGG GGGCAGGGAT CTAGAACTCC TGAACCGAAC   
  
  
+ TGGAGATCGG ATCAGGAAGT TCGCACAATC CTTGGGACTC GAGTTTCAAT TCCACCATCT TATAACGGTG   
  
  
+ GACTCGAACC TTACTGAGGC AGTGGAGGCC ATCAATGCCC TACAGCTACA CCTCCATGTC CAAGAGGAGG   
  
  
+ AGGTCTTCGC AGTGAATTGT GGAGATTTCC TCCACCGCCT CCTAACGGAG TATGATAGTA CATTCCTTAG   
  
  
+ GATGTTTCTC TACAAGGTGA AGACCCTAAA CCCTAGGGTC TTCACCGTGG GAGAAAGGGA GGCCGACCAC   
  
  
+ AACCACCCTC TCTTCTGGCA GCGATTCATT GAGGCACTTG ACCACTATGG GGCAGTGTTT GACTCTCTAG   
  
  
+ AAGCAACCCT ACCATCAACA AGTCAAGAGA GGGTGGCGGT GGAAGAGGGG TGGTTCGGGG AGGAGATTAA   
  
  
+ AGATGTGGTT GGACGAGAAG GAGGGTTGAG GAGACAGAGG CACCAGAAAT ACGAGTCTTG GGAGGTGTTG   
  
  
+ ATGAGGAGCT CTGGGTTTAA GAGCTTGCCC TTGAGCCCCT TCTCTGTGTC ACAAGCCAAG CTGCTACTTC   
  
  
+ GCCTTCATTA CCCTTCTGAA GGGTACCACC TTAAGGTCTT CCATAGTAAT TGTCTGTTGC TTGGGTGGAA   
  
  
+ GAATCGCCCT CTTTTCTCTG TTTCTTCTTG GCAATA  

- -Up\_Stream \_Len000TTTTAT AAAAATGTTT TCGTAAAGAG ATATTTTATA AAATGTTTTA AAGTAAAACA   
  
  
- CCCAGAAAAT CTTAGTTAGA GAGATGTTCA TCCTTACGGC CAACTCCATT CCGATGGATG TAGATCTAGA   
  
  
- GAGGTCTGGG GTGTTTTTAA TTTGCTCTGG GTGTGACTTT CCACCACCAC CACCACAACA ACAACATAAC   
  
  
- AGGATGGTGC TCTCTCGAAG TTCACGTAGT GAATAAAGTT GTCCAGTTAG TGTATCTAGA GATCACATGA   
  
  
- CATTTAAAAA AAAGGCAGAA TAAAATTAAA AGAAAAGGTA GGATTTTCTA ATAGTACATC TATAAGGATC   
  
  
- AAACTATATA ACTGAAGTTG AAGTTTTTAA TGTGTAATTC TAGGTGCTTA TACTGTCAAA TCCAAAGTAA   
  
  
- ACTTCCTCTT TCTTTACAAT GCCACAAAAC CACAACATAA GCATCTGTAA AAAACGCGAA TTGACCGAGA   
  
  
- AGAGTACCCC TGCTCTGTCA CTTCCATTAA CATGAACAAC TAACTACTGC AGTAGACATC GAACCTAAAC   
  
  
- ATATCCTTAC TTGTAGAACC ACAGATGCAC CTTTCACACT AAACACAAGA AAGTAACTAG GAGGAGGAGT   
  
  
- TCTTCTCCAC TAGAAAGAGG GAACAAAAAA AAAAAAAAAC CGATTATACA ACCGTCTTGG CCGTTTTTTA   
  
  
- TGTATTAAAC TTATCACGTA CGTATACTCA TGTACTGTCA CTTGGTTGTT TTGCTTGGGG ATTTTGTGTT   
  
  
- CTCTTAATTG TACAATTTTG TGAATTTGAT TTACGTTGGT AGAGCTACTG GTGATTTATC GGTTCTTTGG   
  
  
- ATTGCATTAA TTGTTTTTAT TTTTTATTGA CTTTTATTTC ATCTCTTTCA GATTGGCTTA TGCTATTGTA   
  
  
- TTGGATTTTG ATTGATATGA ACTTGAATGA TTCTTTATAC TTAGTTGGTG TTTGGCTTAA CTAAAACCAA   
  
  
- ACGGTATTAA ACTGATATGT AAATTGAATT TTTTATAAAA AAAAATTAAA GTTTATTTAG ATTTAATAAA   
  
  
- AAGTTATAAG TAGGTTTTAT CTAAGTTAAT AATTTGTTCA GTTGGATATG GCTCAATAAA AGATCGAGAC   
  
  
- TGATTCTGTA TATATATAAA AAACATTTTT TCTATTCCTA ATATTAAATT TATCGGAAAG GGAATTTTTT   
  
  
- TAAAAATTTT AATAGGACTA TCTCTTATTT TTCTTTAGGA ATATGTTTTG AGACTAACTG CGTCTACACA   
  
  
- TATACCTGTA TATCTTCATT ATTCTCCCCA TCCGATCCTT CCCCCACCTT TATATCCCGT ATTTATCCCC   
  
  
- CGCCCGTATC CCTGTACAGA CAGACTCCAT GCCACCTACC CTGGAACGGT ACCTTCGTTG TTACAGTGTT   
  
  
- ATTATTATAG TTGCTGACTT CAAAGAAGTG GTCGGGTGAG AAACGGTATT ACGGTAATTA ATAAATAACG   
  
  
- ATGATAGAGA AAACTACACT TTGCCACCTG GAATAGAGAG GGGAGGTGAA GGTTAACTAC ACTCGTTGAG   
  
  
- TCTTTGCGTT TGTTTTGAAC ACGGTGGAAG AAATGAACGT AGGAGTAAAA GGAGACAGAG AGAGGCGGGG   
  
  
- ACAAAGAGAG AGAGAGGGAG TGAGTGAGTC TCATGACTAG ACTCGTAGTC TTCATCGTCT GGGTCTTTGA   
  
  
- GTCTAAATTT TTGTTGGGCG AGAAAACAAG GGAGATGCAG ATTATTAAAA CAGTAGCGTA AATTACAGTC   
  
  
- TATCTCTCTA TCTCGAGATG ACATAATTAA AAGAGGGTGG AGTGAACTAA GACAACGGGA TTTGGTGGGT   
  
  
- TTTTTTCTTT ATGCTTCTTC GGTGAGAGAG AGAGAGAGAG AGAGTGTGTG TGTGTGTGGG GAGGATCACC   
  
  
- TAAACTATCT CATATTTACT TGTCATTAGT CTGCTCTATA TATCATCACG AATTAACTGG CTCATCGTTT   
  
  
- TCCCCCTTCA ACTCACTCCA TAGTTCTCTC ATCGCATCGA TCGATCCCGT TCTATACGAC CCCAGAAGGA   
  
  
- GTATGTTGAG GCTCCTTCTG GTGGTTCTAC TGTTGGTAAG GAGTGGCAGA CTAAACAGAT CCGTGGGTGG   
  
  
- ATCTAAGTGG TGATGATGAA TGGTTATGGT TGTTGTATAA TCGAGAGGTA GAAGTGTTAG TTATGATCGA   
  
  
- CACGGTGGAG CGGTTAACAA TTAGTTAACA CGCCTCAATT AAACGGCCAA GCTGAAGAGG AGGCGGTTGG   
  
  
- CCGAAGAGAG AGAGGAGCGG AGGGTGAAAC GGAGGGGAAG ACCCCTGAGG TGGCTCTCTG AACACAAAAT   
  
  
- GAAGACGTTC CGAAACCTAG AGGCGGAGTT ATTGTCAATA AATAGGTGAG CATCAAACTG ACCAACATTG   
  
  
- GGATCATGAC CAAGACCATA ACCCCGAGGA AATCGAAGGT TAAAAGATAA AGAAGTTGGT AATCACGTAA   
  
  
- GTGTCCATTG TTCATTACGG GCACAACCCA TGAACTGTAT AAACTGAGAC TTGGTCCAGT GGGGTAAGTA   
  
  
- GGCTAAGTGG GTGAATTGAC GGTTGGTTCG GTAGGACCTT CGAAACCTCC CCATGTTCCG CCAGGTATAG   
  
  
- GAACTGTACC TGTAGTACGT ACCCCAGGTC ACCGGTGGTG AAGAAGTTCG TTAACGTCTT TCTAGACGTT   
  
  
- GGGAACCAGT GGGACGAGGG TGACAAGCGT AGTGTCCTCC CCCGTCCCTA GATCTTGAGG ACTTGGCTTG   
  
  
- ACCTCTAGCC TAGTCCTTCA AGCGTGTTAG GAACCCTGAG CTCAAAGTTA AGGTGGTAGA ATATTGCCAC   
  
  
- CTGAGCTTGG AATGACTCCG TCACCTCCGG TAGTTACGGG ATGTCGATGT GGAGGTACAG GTTCTCCTCC   
  
  
- TCCAGAAGCG TCACTTAACA CCTCTAAAGG AGGTGGCGGA GGATTGCCTC ATACTATCAT GTAAGGAATC   
  
  
- CTACAAAGAG ATGTTCCACT TCTGGGATTT GGGATCCCAG AAGTGGCACC CTCTTTCCCT CCGGCTGGTG   
  
  
- TTGGTGGGAG AGAAGACCGT CGCTAAGTAA CTCCGTGAAC TGGTGATACC CCGTCACAAA CTGAGAGATC   
  
  
- TTCGTTGGGA TGGTAGTTGT TCAGTTCTCT CCCACCGCCA CCTTCTCCCC ACCAAGCCCC TCCTCTAATT   
  
  
- TCTACACCAA CCTGCTCTTC CTCCCAACTC CTCTGTCTCC GTGGTCTTTA TGCTCAGAAC CCTCCACAAC   
  
  
- TACTCCTCGA GACCCAAATT CTCGAACGGG AACTCGGGGA AGAGACACAG TGTTCGGTTC GACGATGAAG   
  
  
- CGGAAGTAAT GGGAAGACTT CCCATGGTGG AATTCCAGAA GGTATCATTA ACAGACAACG AACCCACCTT   
  
  
- CTTAGCGGGA GAAAAGAGAC AAAGAAGAAC CGTTAT

+     ABRE4

| Site Name | Organism | Position | Strand | Matrix score. | sequence | function |
| --- | --- | --- | --- | --- | --- | --- |
| ABRE4 | Zea mays | 589 | - | 6 | CACGTA |  |

>HU01G00833.1   
+ -Up\_Stream \_Len000AAAATA TTTTTACAAA AGCATTTCTC TATAAAATAT TTTACAAAAT TTCATTTTGT   
  
  
+ GGGTCTTTTA GAATCAATCT CTCTACAAGT AGGAATGCCG GTTGAGGTAA GGCTACCTAC ATCTAGATCT   
  
  
+ CTCCAGACCC CACAAAAATT AAACGAGACC CACACTGAAA GGTGGTGGTG GTGGTGTTGT TGTTGTATTG   
  
  
+ TCCTACCACG AGAGAGCTTC AAGTGCATCA CTTATTTCAA CAGGTCAATC ACATAGATCT CTAGTGTACT   
  
  
+ GTAAATTTTT TTTCCGTCTT ATTTTAATTT TCTTTTCCAT CCTAAAAGAT TATCATGTAG ATATTCCTAG   
  
  
+ TTTGATATAT TGACTTCAAC TTCAAAAATT ACACATTAAG ATCCACGAAT ATGACAGTTT AGGTTTCATT   
  
  
+ TGAAGGAGAA AGAAATGTTA CGGTGTTTTG GTGTTGTATT CGTAGACATT TTTTGCGCTT AACTGGCTCT   
  
  
+ TCTCATGGGG ACGAGACAGT GAAGGTAATT GTACTTGTTG ATTGATGACG TCATCTGTAG CTTGGATTTG   
  
  
+ TATAGGAATG AACATCTTGG TGTCTACGTG GAAAGTGTGA TTTGTGTTCT TTCATTGATC CTCCTCCTCA   
  
  
+ AGAAGAGGTG ATCTTTCTCC CTTGTTTTTT TTTTTTTTTG GCTAATATGT TGGCAGAACC GGCAAAAAAT   
  
  
+ ACATAATTTG AATAGTGCAT GCATATGAGT ACATGACAGT GAACCAACAA AACGAACCCC TAAAACACAA   
  
  
+ GAGAATTAAC ATGTTAAAAC ACTTAAACTA AATGCAACCA TCTCGATGAC CACTAAATAG CCAAGAAACC   
  
  
+ TAACGTAATT AACAAAAATA AAAAATAACT GAAAATAAAG TAGAGAAAGT CTAACCGAAT ACGATAACAT   
  
  
+ AACCTAAAAC TAACTATACT TGAACTTACT AAGAAATATG AATCAACCAC AAACCGAATT GATTTTGGTT   
  
  
+ TGCCATAATT TGACTATACA TTTAACTTAA AAAATATTTT TTTTTAATTT CAAATAAATC TAAATTATTT   
  
  
+ TTCAATATTC ATCCAAAATA GATTCAATTA TTAAACAAGT CAACCTATAC CGAGTTATTT TCTAGCTCTG   
  
  
+ ACTAAGACAT ATATATATTT TTTGTAAAAA AGATAAGGAT TATAATTTAA ATAGCCTTTC CCTTAAAAAA   
  
  
+ ATTTTTAAAA TTATCCTGAT AGAGAATAAA AAGAAATCCT TATACAAAAC TCTGATTGAC GCAGATGTGT   
  
  
+ ATATGGACAT ATAGAAGTAA TAAGAGGGGT AGGCTAGGAA GGGGGTGGAA ATATAGGGCA TAAATAGGGG   
  
  
+ GCGGGCATAG GGACATGTCT GTCTGAGGTA CGGTGGATGG GACCTTGCCA TGGAAGCAAC AATGTCACAA   
  
  
+ TAATAATATC AACGACTGAA GTTTCTTCAC CAGCCCACTC TTTGCCATAA TGCCATTAAT TATTTATTGC   
  
  
+ TACTATCTCT TTTGATGTGA AACGGTGGAC CTTATCTCTC CCCTCCACTT CCAATTGATG TGAGCAACTC   
  
  
+ AGAAACGCAA ACAAAACTTG TGCCACCTTC TTTACTTGCA TCCTCATTTT CCTCTGTCTC TCTCCGCCCC   
  
  
+ TGTTTCTCTC TCTCTCCCTC ACTCACTCAG AGTACTGATC TGAGCATCAG AAGTAGCAGA CCCAGAAACT   
  
  
+ CAGATTTAAA AACAACCCGC TCTTTTGTTC CCTCTACGTC TAATAATTTT GTCATCGCAT TTAATGTCAG   
  
  
+ ATAGAGAGAT AGAGCTCTAC TGTATTAATT TTCTCCCACC TCACTTGATT CTGTTGCCCT AAACCACCCA   
  
  
+ AAAAAAGAAA TACGAAGAAG CCACTCTCTC TCTCTCTCTC TCTCACACAC ACACACACCC CTCCTAGTGG   
  
  
+ ATTTGATAGA GTATAAATGA ACAGTAATCA GACGAGATAT ATAGTAGTGC TTAATTGACC GAGTAGCAAA   
  
  
+ AGGGGGAAGT TGAGTGAGGT ATCAAGAGAG TAGCGTAGCT AGCTAGGGCA AGATATGCTG GGGTCTTCCT   
  
  
+ CATACAACTC CGAGGAAGAC CACCAAGATG ACAACCATTC CTCACCGTCT GATTTGTCTA GGCACCCACC   
  
  
+ TAGATTCACC ACTACTACTT ACCAATACCA ACAACATATT AGCTCTCCAT CTTCACAATC AATACTAGCT   
  
  
+ GTGCCACCTC GCCAATTGTT AATCAATTGT GCGGAGTTAA TTTGCCGGTT CGACTTCTCC TCCGCCAACC   
  
  
+ GGCTTCTCTC TCTCCTCGCC TCCCACTTTG CCTCCCCTTC TGGGGACTCC ACCGAGAGAC TTGTGTTTTA   
  
  
+ CTTCTGCAAG GCTTTGGATC TCCGCCTCAA TAACAGTTAT TTATCCACTC GTAGTTTGAC TGGTTGTAAC   
  
  
+ CCTAGTACTG GTTCTGGTAT TGGGGCTCCT TTAGCTTCCA ATTTTCTATT TCTTCAACCA TTAGTGCATT   
  
  
+ CACAGGTAAC AAGTAATGCC CGTGTTGGGT ACTTGACATA TTTGACTCTG AACCAGGTCA CCCCATTCAT   
  
  
+ CCGATTCACC CACTTAACTG CCAACCAAGC CATCCTGGAA GCTTTGGAGG GGTACAAGGC GGTCCATATC   
  
  
+ CTTGACATGG ACATCATGCA TGGGGTCCAG TGGCCACCAC TTCTTCAAGC AATTGCAGAA AGATCTGCAA   
  
  
+ CCCTTGGTCA CCCTGCTCCC ACTGTTCGCA TCACAGGAGG GGGCAGGGAT CTAGAACTCC TGAACCGAAC   
  
  
+ TGGAGATCGG ATCAGGAAGT TCGCACAATC CTTGGGACTC GAGTTTCAAT TCCACCATCT TATAACGGTG   
  
  
+ GACTCGAACC TTACTGAGGC AGTGGAGGCC ATCAATGCCC TACAGCTACA CCTCCATGTC CAAGAGGAGG   
  
  
+ AGGTCTTCGC AGTGAATTGT GGAGATTTCC TCCACCGCCT CCTAACGGAG TATGATAGTA CATTCCTTAG   
  
  
+ GATGTTTCTC TACAAGGTGA AGACCCTAAA CCCTAGGGTC TTCACCGTGG GAGAAAGGGA GGCCGACCAC   
  
  
+ AACCACCCTC TCTTCTGGCA GCGATTCATT GAGGCACTTG ACCACTATGG GGCAGTGTTT GACTCTCTAG   
  
  
+ AAGCAACCCT ACCATCAACA AGTCAAGAGA GGGTGGCGGT GGAAGAGGGG TGGTTCGGGG AGGAGATTAA   
  
  
+ AGATGTGGTT GGACGAGAAG GAGGGTTGAG GAGACAGAGG CACCAGAAAT ACGAGTCTTG GGAGGTGTTG   
  
  
+ ATGAGGAGCT CTGGGTTTAA GAGCTTGCCC TTGAGCCCCT TCTCTGTGTC ACAAGCCAAG CTGCTACTTC   
  
  
+ GCCTTCATTA CCCTTCTGAA GGGTACCACC TTAAGGTCTT CCATAGTAAT TGTCTGTTGC TTGGGTGGAA   
  
  
+ GAATCGCCCT CTTTTCTCTG TTTCTTCTTG GCAATA  

- -Up\_Stream \_Len000TTTTAT AAAAATGTTT TCGTAAAGAG ATATTTTATA AAATGTTTTA AAGTAAAACA   
  
  
- CCCAGAAAAT CTTAGTTAGA GAGATGTTCA TCCTTACGGC CAACTCCATT CCGATGGATG TAGATCTAGA   
  
  
- GAGGTCTGGG GTGTTTTTAA TTTGCTCTGG GTGTGACTTT CCACCACCAC CACCACAACA ACAACATAAC   
  
  
- AGGATGGTGC TCTCTCGAAG TTCACGTAGT GAATAAAGTT GTCCAGTTAG TGTATCTAGA GATCACATGA   
  
  
- CATTTAAAAA AAAGGCAGAA TAAAATTAAA AGAAAAGGTA GGATTTTCTA ATAGTACATC TATAAGGATC   
  
  
- AAACTATATA ACTGAAGTTG AAGTTTTTAA TGTGTAATTC TAGGTGCTTA TACTGTCAAA TCCAAAGTAA   
  
  
- ACTTCCTCTT TCTTTACAAT GCCACAAAAC CACAACATAA GCATCTGTAA AAAACGCGAA TTGACCGAGA   
  
  
- AGAGTACCCC TGCTCTGTCA CTTCCATTAA CATGAACAAC TAACTACTGC AGTAGACATC GAACCTAAAC   
  
  
- ATATCCTTAC TTGTAGAACC ACAGATGCAC CTTTCACACT AAACACAAGA AAGTAACTAG GAGGAGGAGT   
  
  
- TCTTCTCCAC TAGAAAGAGG GAACAAAAAA AAAAAAAAAC CGATTATACA ACCGTCTTGG CCGTTTTTTA   
  
  
- TGTATTAAAC TTATCACGTA CGTATACTCA TGTACTGTCA CTTGGTTGTT TTGCTTGGGG ATTTTGTGTT   
  
  
- CTCTTAATTG TACAATTTTG TGAATTTGAT TTACGTTGGT AGAGCTACTG GTGATTTATC GGTTCTTTGG   
  
  
- ATTGCATTAA TTGTTTTTAT TTTTTATTGA CTTTTATTTC ATCTCTTTCA GATTGGCTTA TGCTATTGTA   
  
  
- TTGGATTTTG ATTGATATGA ACTTGAATGA TTCTTTATAC TTAGTTGGTG TTTGGCTTAA CTAAAACCAA   
  
  
- ACGGTATTAA ACTGATATGT AAATTGAATT TTTTATAAAA AAAAATTAAA GTTTATTTAG ATTTAATAAA   
  
  
- AAGTTATAAG TAGGTTTTAT CTAAGTTAAT AATTTGTTCA GTTGGATATG GCTCAATAAA AGATCGAGAC   
  
  
- TGATTCTGTA TATATATAAA AAACATTTTT TCTATTCCTA ATATTAAATT TATCGGAAAG GGAATTTTTT   
  
  
- TAAAAATTTT AATAGGACTA TCTCTTATTT TTCTTTAGGA ATATGTTTTG AGACTAACTG CGTCTACACA   
  
  
- TATACCTGTA TATCTTCATT ATTCTCCCCA TCCGATCCTT CCCCCACCTT TATATCCCGT ATTTATCCCC   
  
  
- CGCCCGTATC CCTGTACAGA CAGACTCCAT GCCACCTACC CTGGAACGGT ACCTTCGTTG TTACAGTGTT   
  
  
- ATTATTATAG TTGCTGACTT CAAAGAAGTG GTCGGGTGAG AAACGGTATT ACGGTAATTA ATAAATAACG   
  
  
- ATGATAGAGA AAACTACACT TTGCCACCTG GAATAGAGAG GGGAGGTGAA GGTTAACTAC ACTCGTTGAG   
  
  
- TCTTTGCGTT TGTTTTGAAC ACGGTGGAAG AAATGAACGT AGGAGTAAAA GGAGACAGAG AGAGGCGGGG   
  
  
- ACAAAGAGAG AGAGAGGGAG TGAGTGAGTC TCATGACTAG ACTCGTAGTC TTCATCGTCT GGGTCTTTGA   
  
  
- GTCTAAATTT TTGTTGGGCG AGAAAACAAG GGAGATGCAG ATTATTAAAA CAGTAGCGTA AATTACAGTC   
  
  
- TATCTCTCTA TCTCGAGATG ACATAATTAA AAGAGGGTGG AGTGAACTAA GACAACGGGA TTTGGTGGGT   
  
  
- TTTTTTCTTT ATGCTTCTTC GGTGAGAGAG AGAGAGAGAG AGAGTGTGTG TGTGTGTGGG GAGGATCACC   
  
  
- TAAACTATCT CATATTTACT TGTCATTAGT CTGCTCTATA TATCATCACG AATTAACTGG CTCATCGTTT   
  
  
- TCCCCCTTCA ACTCACTCCA TAGTTCTCTC ATCGCATCGA TCGATCCCGT TCTATACGAC CCCAGAAGGA   
  
  
- GTATGTTGAG GCTCCTTCTG GTGGTTCTAC TGTTGGTAAG GAGTGGCAGA CTAAACAGAT CCGTGGGTGG   
  
  
- ATCTAAGTGG TGATGATGAA TGGTTATGGT TGTTGTATAA TCGAGAGGTA GAAGTGTTAG TTATGATCGA   
  
  
- CACGGTGGAG CGGTTAACAA TTAGTTAACA CGCCTCAATT AAACGGCCAA GCTGAAGAGG AGGCGGTTGG   
  
  
- CCGAAGAGAG AGAGGAGCGG AGGGTGAAAC GGAGGGGAAG ACCCCTGAGG TGGCTCTCTG AACACAAAAT   
  
  
- GAAGACGTTC CGAAACCTAG AGGCGGAGTT ATTGTCAATA AATAGGTGAG CATCAAACTG ACCAACATTG   
  
  
- GGATCATGAC CAAGACCATA ACCCCGAGGA AATCGAAGGT TAAAAGATAA AGAAGTTGGT AATCACGTAA   
  
  
- GTGTCCATTG TTCATTACGG GCACAACCCA TGAACTGTAT AAACTGAGAC TTGGTCCAGT GGGGTAAGTA   
  
  
- GGCTAAGTGG GTGAATTGAC GGTTGGTTCG GTAGGACCTT CGAAACCTCC CCATGTTCCG CCAGGTATAG   
  
  
- GAACTGTACC TGTAGTACGT ACCCCAGGTC ACCGGTGGTG AAGAAGTTCG TTAACGTCTT TCTAGACGTT   
  
  
- GGGAACCAGT GGGACGAGGG TGACAAGCGT AGTGTCCTCC CCCGTCCCTA GATCTTGAGG ACTTGGCTTG   
  
  
- ACCTCTAGCC TAGTCCTTCA AGCGTGTTAG GAACCCTGAG CTCAAAGTTA AGGTGGTAGA ATATTGCCAC   
  
  
- CTGAGCTTGG AATGACTCCG TCACCTCCGG TAGTTACGGG ATGTCGATGT GGAGGTACAG GTTCTCCTCC   
  
  
- TCCAGAAGCG TCACTTAACA CCTCTAAAGG AGGTGGCGGA GGATTGCCTC ATACTATCAT GTAAGGAATC   
  
  
- CTACAAAGAG ATGTTCCACT TCTGGGATTT GGGATCCCAG AAGTGGCACC CTCTTTCCCT CCGGCTGGTG   
  
  
- TTGGTGGGAG AGAAGACCGT CGCTAAGTAA CTCCGTGAAC TGGTGATACC CCGTCACAAA CTGAGAGATC   
  
  
- TTCGTTGGGA TGGTAGTTGT TCAGTTCTCT CCCACCGCCA CCTTCTCCCC ACCAAGCCCC TCCTCTAATT   
  
  
- TCTACACCAA CCTGCTCTTC CTCCCAACTC CTCTGTCTCC GTGGTCTTTA TGCTCAGAAC CCTCCACAAC   
  
  
- TACTCCTCGA GACCCAAATT CTCGAACGGG AACTCGGGGA AGAGACACAG TGTTCGGTTC GACGATGAAG   
  
  
- CGGAAGTAAT GGGAAGACTT CCCATGGTGG AATTCCAGAA GGTATCATTA ACAGACAACG AACCCACCTT   
  
  
- CTTAGCGGGA GAAAAGAGAC AAAGAAGAAC CGTTAT

+     ACE

| Site Name | Organism | Position | Strand | Matrix score. | sequence | function |
| --- | --- | --- | --- | --- | --- | --- |
| ACE | Petroselinum crispum | 844 | + | 9 | CTAACGTATT | cis-acting element involved in light responsiveness |

>HU01G00833.1   
+ -Up\_Stream \_Len000AAAATA TTTTTACAAA AGCATTTCTC TATAAAATAT TTTACAAAAT TTCATTTTGT   
  
  
+ GGGTCTTTTA GAATCAATCT CTCTACAAGT AGGAATGCCG GTTGAGGTAA GGCTACCTAC ATCTAGATCT   
  
  
+ CTCCAGACCC CACAAAAATT AAACGAGACC CACACTGAAA GGTGGTGGTG GTGGTGTTGT TGTTGTATTG   
  
  
+ TCCTACCACG AGAGAGCTTC AAGTGCATCA CTTATTTCAA CAGGTCAATC ACATAGATCT CTAGTGTACT   
  
  
+ GTAAATTTTT TTTCCGTCTT ATTTTAATTT TCTTTTCCAT CCTAAAAGAT TATCATGTAG ATATTCCTAG   
  
  
+ TTTGATATAT TGACTTCAAC TTCAAAAATT ACACATTAAG ATCCACGAAT ATGACAGTTT AGGTTTCATT   
  
  
+ TGAAGGAGAA AGAAATGTTA CGGTGTTTTG GTGTTGTATT CGTAGACATT TTTTGCGCTT AACTGGCTCT   
  
  
+ TCTCATGGGG ACGAGACAGT GAAGGTAATT GTACTTGTTG ATTGATGACG TCATCTGTAG CTTGGATTTG   
  
  
+ TATAGGAATG AACATCTTGG TGTCTACGTG GAAAGTGTGA TTTGTGTTCT TTCATTGATC CTCCTCCTCA   
  
  
+ AGAAGAGGTG ATCTTTCTCC CTTGTTTTTT TTTTTTTTTG GCTAATATGT TGGCAGAACC GGCAAAAAAT   
  
  
+ ACATAATTTG AATAGTGCAT GCATATGAGT ACATGACAGT GAACCAACAA AACGAACCCC TAAAACACAA   
  
  
+ GAGAATTAAC ATGTTAAAAC ACTTAAACTA AATGCAACCA TCTCGATGAC CACTAAATAG CCAAGAAACC   
  
  
+ TAACGTAATT AACAAAAATA AAAAATAACT GAAAATAAAG TAGAGAAAGT CTAACCGAAT ACGATAACAT   
  
  
+ AACCTAAAAC TAACTATACT TGAACTTACT AAGAAATATG AATCAACCAC AAACCGAATT GATTTTGGTT   
  
  
+ TGCCATAATT TGACTATACA TTTAACTTAA AAAATATTTT TTTTTAATTT CAAATAAATC TAAATTATTT   
  
  
+ TTCAATATTC ATCCAAAATA GATTCAATTA TTAAACAAGT CAACCTATAC CGAGTTATTT TCTAGCTCTG   
  
  
+ ACTAAGACAT ATATATATTT TTTGTAAAAA AGATAAGGAT TATAATTTAA ATAGCCTTTC CCTTAAAAAA   
  
  
+ ATTTTTAAAA TTATCCTGAT AGAGAATAAA AAGAAATCCT TATACAAAAC TCTGATTGAC GCAGATGTGT   
  
  
+ ATATGGACAT ATAGAAGTAA TAAGAGGGGT AGGCTAGGAA GGGGGTGGAA ATATAGGGCA TAAATAGGGG   
  
  
+ GCGGGCATAG GGACATGTCT GTCTGAGGTA CGGTGGATGG GACCTTGCCA TGGAAGCAAC AATGTCACAA   
  
  
+ TAATAATATC AACGACTGAA GTTTCTTCAC CAGCCCACTC TTTGCCATAA TGCCATTAAT TATTTATTGC   
  
  
+ TACTATCTCT TTTGATGTGA AACGGTGGAC CTTATCTCTC CCCTCCACTT CCAATTGATG TGAGCAACTC   
  
  
+ AGAAACGCAA ACAAAACTTG TGCCACCTTC TTTACTTGCA TCCTCATTTT CCTCTGTCTC TCTCCGCCCC   
  
  
+ TGTTTCTCTC TCTCTCCCTC ACTCACTCAG AGTACTGATC TGAGCATCAG AAGTAGCAGA CCCAGAAACT   
  
  
+ CAGATTTAAA AACAACCCGC TCTTTTGTTC CCTCTACGTC TAATAATTTT GTCATCGCAT TTAATGTCAG   
  
  
+ ATAGAGAGAT AGAGCTCTAC TGTATTAATT TTCTCCCACC TCACTTGATT CTGTTGCCCT AAACCACCCA   
  
  
+ AAAAAAGAAA TACGAAGAAG CCACTCTCTC TCTCTCTCTC TCTCACACAC ACACACACCC CTCCTAGTGG   
  
  
+ ATTTGATAGA GTATAAATGA ACAGTAATCA GACGAGATAT ATAGTAGTGC TTAATTGACC GAGTAGCAAA   
  
  
+ AGGGGGAAGT TGAGTGAGGT ATCAAGAGAG TAGCGTAGCT AGCTAGGGCA AGATATGCTG GGGTCTTCCT   
  
  
+ CATACAACTC CGAGGAAGAC CACCAAGATG ACAACCATTC CTCACCGTCT GATTTGTCTA GGCACCCACC   
  
  
+ TAGATTCACC ACTACTACTT ACCAATACCA ACAACATATT AGCTCTCCAT CTTCACAATC AATACTAGCT   
  
  
+ GTGCCACCTC GCCAATTGTT AATCAATTGT GCGGAGTTAA TTTGCCGGTT CGACTTCTCC TCCGCCAACC   
  
  
+ GGCTTCTCTC TCTCCTCGCC TCCCACTTTG CCTCCCCTTC TGGGGACTCC ACCGAGAGAC TTGTGTTTTA   
  
  
+ CTTCTGCAAG GCTTTGGATC TCCGCCTCAA TAACAGTTAT TTATCCACTC GTAGTTTGAC TGGTTGTAAC   
  
  
+ CCTAGTACTG GTTCTGGTAT TGGGGCTCCT TTAGCTTCCA ATTTTCTATT TCTTCAACCA TTAGTGCATT   
  
  
+ CACAGGTAAC AAGTAATGCC CGTGTTGGGT ACTTGACATA TTTGACTCTG AACCAGGTCA CCCCATTCAT   
  
  
+ CCGATTCACC CACTTAACTG CCAACCAAGC CATCCTGGAA GCTTTGGAGG GGTACAAGGC GGTCCATATC   
  
  
+ CTTGACATGG ACATCATGCA TGGGGTCCAG TGGCCACCAC TTCTTCAAGC AATTGCAGAA AGATCTGCAA   
  
  
+ CCCTTGGTCA CCCTGCTCCC ACTGTTCGCA TCACAGGAGG GGGCAGGGAT CTAGAACTCC TGAACCGAAC   
  
  
+ TGGAGATCGG ATCAGGAAGT TCGCACAATC CTTGGGACTC GAGTTTCAAT TCCACCATCT TATAACGGTG   
  
  
+ GACTCGAACC TTACTGAGGC AGTGGAGGCC ATCAATGCCC TACAGCTACA CCTCCATGTC CAAGAGGAGG   
  
  
+ AGGTCTTCGC AGTGAATTGT GGAGATTTCC TCCACCGCCT CCTAACGGAG TATGATAGTA CATTCCTTAG   
  
  
+ GATGTTTCTC TACAAGGTGA AGACCCTAAA CCCTAGGGTC TTCACCGTGG GAGAAAGGGA GGCCGACCAC   
  
  
+ AACCACCCTC TCTTCTGGCA GCGATTCATT GAGGCACTTG ACCACTATGG GGCAGTGTTT GACTCTCTAG   
  
  
+ AAGCAACCCT ACCATCAACA AGTCAAGAGA GGGTGGCGGT GGAAGAGGGG TGGTTCGGGG AGGAGATTAA   
  
  
+ AGATGTGGTT GGACGAGAAG GAGGGTTGAG GAGACAGAGG CACCAGAAAT ACGAGTCTTG GGAGGTGTTG   
  
  
+ ATGAGGAGCT CTGGGTTTAA GAGCTTGCCC TTGAGCCCCT TCTCTGTGTC ACAAGCCAAG CTGCTACTTC   
  
  
+ GCCTTCATTA CCCTTCTGAA GGGTACCACC TTAAGGTCTT CCATAGTAAT TGTCTGTTGC TTGGGTGGAA   
  
  
+ GAATCGCCCT CTTTTCTCTG TTTCTTCTTG GCAATA  

- -Up\_Stream \_Len000TTTTAT AAAAATGTTT TCGTAAAGAG ATATTTTATA AAATGTTTTA AAGTAAAACA   
  
  
- CCCAGAAAAT CTTAGTTAGA GAGATGTTCA TCCTTACGGC CAACTCCATT CCGATGGATG TAGATCTAGA   
  
  
- GAGGTCTGGG GTGTTTTTAA TTTGCTCTGG GTGTGACTTT CCACCACCAC CACCACAACA ACAACATAAC   
  
  
- AGGATGGTGC TCTCTCGAAG TTCACGTAGT GAATAAAGTT GTCCAGTTAG TGTATCTAGA GATCACATGA   
  
  
- CATTTAAAAA AAAGGCAGAA TAAAATTAAA AGAAAAGGTA GGATTTTCTA ATAGTACATC TATAAGGATC   
  
  
- AAACTATATA ACTGAAGTTG AAGTTTTTAA TGTGTAATTC TAGGTGCTTA TACTGTCAAA TCCAAAGTAA   
  
  
- ACTTCCTCTT TCTTTACAAT GCCACAAAAC CACAACATAA GCATCTGTAA AAAACGCGAA TTGACCGAGA   
  
  
- AGAGTACCCC TGCTCTGTCA CTTCCATTAA CATGAACAAC TAACTACTGC AGTAGACATC GAACCTAAAC   
  
  
- ATATCCTTAC TTGTAGAACC ACAGATGCAC CTTTCACACT AAACACAAGA AAGTAACTAG GAGGAGGAGT   
  
  
- TCTTCTCCAC TAGAAAGAGG GAACAAAAAA AAAAAAAAAC CGATTATACA ACCGTCTTGG CCGTTTTTTA   
  
  
- TGTATTAAAC TTATCACGTA CGTATACTCA TGTACTGTCA CTTGGTTGTT TTGCTTGGGG ATTTTGTGTT   
  
  
- CTCTTAATTG TACAATTTTG TGAATTTGAT TTACGTTGGT AGAGCTACTG GTGATTTATC GGTTCTTTGG   
  
  
- ATTGCATTAA TTGTTTTTAT TTTTTATTGA CTTTTATTTC ATCTCTTTCA GATTGGCTTA TGCTATTGTA   
  
  
- TTGGATTTTG ATTGATATGA ACTTGAATGA TTCTTTATAC TTAGTTGGTG TTTGGCTTAA CTAAAACCAA   
  
  
- ACGGTATTAA ACTGATATGT AAATTGAATT TTTTATAAAA AAAAATTAAA GTTTATTTAG ATTTAATAAA   
  
  
- AAGTTATAAG TAGGTTTTAT CTAAGTTAAT AATTTGTTCA GTTGGATATG GCTCAATAAA AGATCGAGAC   
  
  
- TGATTCTGTA TATATATAAA AAACATTTTT TCTATTCCTA ATATTAAATT TATCGGAAAG GGAATTTTTT   
  
  
- TAAAAATTTT AATAGGACTA TCTCTTATTT TTCTTTAGGA ATATGTTTTG AGACTAACTG CGTCTACACA   
  
  
- TATACCTGTA TATCTTCATT ATTCTCCCCA TCCGATCCTT CCCCCACCTT TATATCCCGT ATTTATCCCC   
  
  
- CGCCCGTATC CCTGTACAGA CAGACTCCAT GCCACCTACC CTGGAACGGT ACCTTCGTTG TTACAGTGTT   
  
  
- ATTATTATAG TTGCTGACTT CAAAGAAGTG GTCGGGTGAG AAACGGTATT ACGGTAATTA ATAAATAACG   
  
  
- ATGATAGAGA AAACTACACT TTGCCACCTG GAATAGAGAG GGGAGGTGAA GGTTAACTAC ACTCGTTGAG   
  
  
- TCTTTGCGTT TGTTTTGAAC ACGGTGGAAG AAATGAACGT AGGAGTAAAA GGAGACAGAG AGAGGCGGGG   
  
  
- ACAAAGAGAG AGAGAGGGAG TGAGTGAGTC TCATGACTAG ACTCGTAGTC TTCATCGTCT GGGTCTTTGA   
  
  
- GTCTAAATTT TTGTTGGGCG AGAAAACAAG GGAGATGCAG ATTATTAAAA CAGTAGCGTA AATTACAGTC   
  
  
- TATCTCTCTA TCTCGAGATG ACATAATTAA AAGAGGGTGG AGTGAACTAA GACAACGGGA TTTGGTGGGT   
  
  
- TTTTTTCTTT ATGCTTCTTC GGTGAGAGAG AGAGAGAGAG AGAGTGTGTG TGTGTGTGGG GAGGATCACC   
  
  
- TAAACTATCT CATATTTACT TGTCATTAGT CTGCTCTATA TATCATCACG AATTAACTGG CTCATCGTTT   
  
  
- TCCCCCTTCA ACTCACTCCA TAGTTCTCTC ATCGCATCGA TCGATCCCGT TCTATACGAC CCCAGAAGGA   
  
  
- GTATGTTGAG GCTCCTTCTG GTGGTTCTAC TGTTGGTAAG GAGTGGCAGA CTAAACAGAT CCGTGGGTGG   
  
  
- ATCTAAGTGG TGATGATGAA TGGTTATGGT TGTTGTATAA TCGAGAGGTA GAAGTGTTAG TTATGATCGA   
  
  
- CACGGTGGAG CGGTTAACAA TTAGTTAACA CGCCTCAATT AAACGGCCAA GCTGAAGAGG AGGCGGTTGG   
  
  
- CCGAAGAGAG AGAGGAGCGG AGGGTGAAAC GGAGGGGAAG ACCCCTGAGG TGGCTCTCTG AACACAAAAT   
  
  
- GAAGACGTTC CGAAACCTAG AGGCGGAGTT ATTGTCAATA AATAGGTGAG CATCAAACTG ACCAACATTG   
  
  
- GGATCATGAC CAAGACCATA ACCCCGAGGA AATCGAAGGT TAAAAGATAA AGAAGTTGGT AATCACGTAA   
  
  
- GTGTCCATTG TTCATTACGG GCACAACCCA TGAACTGTAT AAACTGAGAC TTGGTCCAGT GGGGTAAGTA   
  
  
- GGCTAAGTGG GTGAATTGAC GGTTGGTTCG GTAGGACCTT CGAAACCTCC CCATGTTCCG CCAGGTATAG   
  
  
- GAACTGTACC TGTAGTACGT ACCCCAGGTC ACCGGTGGTG AAGAAGTTCG TTAACGTCTT TCTAGACGTT   
  
  
- GGGAACCAGT GGGACGAGGG TGACAAGCGT AGTGTCCTCC CCCGTCCCTA GATCTTGAGG ACTTGGCTTG   
  
  
- ACCTCTAGCC TAGTCCTTCA AGCGTGTTAG GAACCCTGAG CTCAAAGTTA AGGTGGTAGA ATATTGCCAC   
  
  
- CTGAGCTTGG AATGACTCCG TCACCTCCGG TAGTTACGGG ATGTCGATGT GGAGGTACAG GTTCTCCTCC   
  
  
- TCCAGAAGCG TCACTTAACA CCTCTAAAGG AGGTGGCGGA GGATTGCCTC ATACTATCAT GTAAGGAATC   
  
  
- CTACAAAGAG ATGTTCCACT TCTGGGATTT GGGATCCCAG AAGTGGCACC CTCTTTCCCT CCGGCTGGTG   
  
  
- TTGGTGGGAG AGAAGACCGT CGCTAAGTAA CTCCGTGAAC TGGTGATACC CCGTCACAAA CTGAGAGATC   
  
  
- TTCGTTGGGA TGGTAGTTGT TCAGTTCTCT CCCACCGCCA CCTTCTCCCC ACCAAGCCCC TCCTCTAATT   
  
  
- TCTACACCAA CCTGCTCTTC CTCCCAACTC CTCTGTCTCC GTGGTCTTTA TGCTCAGAAC CCTCCACAAC   
  
  
- TACTCCTCGA GACCCAAATT CTCGAACGGG AACTCGGGGA AGAGACACAG TGTTCGGTTC GACGATGAAG   
  
  
- CGGAAGTAAT GGGAAGACTT CCCATGGTGG AATTCCAGAA GGTATCATTA ACAGACAACG AACCCACCTT   
  
  
- CTTAGCGGGA GAAAAGAGAC AAAGAAGAAC CGTTAT

+     AE-box

| Site Name | Organism | Position | Strand | Matrix score. | sequence | function |
| --- | --- | --- | --- | --- | --- | --- |
| AE-box | Arabidopsis thaliana | 1423 | - | 8 | AGAAACTT | part of a module for light response |

>HU01G00833.1   
+ -Up\_Stream \_Len000AAAATA TTTTTACAAA AGCATTTCTC TATAAAATAT TTTACAAAAT TTCATTTTGT   
  
  
+ GGGTCTTTTA GAATCAATCT CTCTACAAGT AGGAATGCCG GTTGAGGTAA GGCTACCTAC ATCTAGATCT   
  
  
+ CTCCAGACCC CACAAAAATT AAACGAGACC CACACTGAAA GGTGGTGGTG GTGGTGTTGT TGTTGTATTG   
  
  
+ TCCTACCACG AGAGAGCTTC AAGTGCATCA CTTATTTCAA CAGGTCAATC ACATAGATCT CTAGTGTACT   
  
  
+ GTAAATTTTT TTTCCGTCTT ATTTTAATTT TCTTTTCCAT CCTAAAAGAT TATCATGTAG ATATTCCTAG   
  
  
+ TTTGATATAT TGACTTCAAC TTCAAAAATT ACACATTAAG ATCCACGAAT ATGACAGTTT AGGTTTCATT   
  
  
+ TGAAGGAGAA AGAAATGTTA CGGTGTTTTG GTGTTGTATT CGTAGACATT TTTTGCGCTT AACTGGCTCT   
  
  
+ TCTCATGGGG ACGAGACAGT GAAGGTAATT GTACTTGTTG ATTGATGACG TCATCTGTAG CTTGGATTTG   
  
  
+ TATAGGAATG AACATCTTGG TGTCTACGTG GAAAGTGTGA TTTGTGTTCT TTCATTGATC CTCCTCCTCA   
  
  
+ AGAAGAGGTG ATCTTTCTCC CTTGTTTTTT TTTTTTTTTG GCTAATATGT TGGCAGAACC GGCAAAAAAT   
  
  
+ ACATAATTTG AATAGTGCAT GCATATGAGT ACATGACAGT GAACCAACAA AACGAACCCC TAAAACACAA   
  
  
+ GAGAATTAAC ATGTTAAAAC ACTTAAACTA AATGCAACCA TCTCGATGAC CACTAAATAG CCAAGAAACC   
  
  
+ TAACGTAATT AACAAAAATA AAAAATAACT GAAAATAAAG TAGAGAAAGT CTAACCGAAT ACGATAACAT   
  
  
+ AACCTAAAAC TAACTATACT TGAACTTACT AAGAAATATG AATCAACCAC AAACCGAATT GATTTTGGTT   
  
  
+ TGCCATAATT TGACTATACA TTTAACTTAA AAAATATTTT TTTTTAATTT CAAATAAATC TAAATTATTT   
  
  
+ TTCAATATTC ATCCAAAATA GATTCAATTA TTAAACAAGT CAACCTATAC CGAGTTATTT TCTAGCTCTG   
  
  
+ ACTAAGACAT ATATATATTT TTTGTAAAAA AGATAAGGAT TATAATTTAA ATAGCCTTTC CCTTAAAAAA   
  
  
+ ATTTTTAAAA TTATCCTGAT AGAGAATAAA AAGAAATCCT TATACAAAAC TCTGATTGAC GCAGATGTGT   
  
  
+ ATATGGACAT ATAGAAGTAA TAAGAGGGGT AGGCTAGGAA GGGGGTGGAA ATATAGGGCA TAAATAGGGG   
  
  
+ GCGGGCATAG GGACATGTCT GTCTGAGGTA CGGTGGATGG GACCTTGCCA TGGAAGCAAC AATGTCACAA   
  
  
+ TAATAATATC AACGACTGAA GTTTCTTCAC CAGCCCACTC TTTGCCATAA TGCCATTAAT TATTTATTGC   
  
  
+ TACTATCTCT TTTGATGTGA AACGGTGGAC CTTATCTCTC CCCTCCACTT CCAATTGATG TGAGCAACTC   
  
  
+ AGAAACGCAA ACAAAACTTG TGCCACCTTC TTTACTTGCA TCCTCATTTT CCTCTGTCTC TCTCCGCCCC   
  
  
+ TGTTTCTCTC TCTCTCCCTC ACTCACTCAG AGTACTGATC TGAGCATCAG AAGTAGCAGA CCCAGAAACT   
  
  
+ CAGATTTAAA AACAACCCGC TCTTTTGTTC CCTCTACGTC TAATAATTTT GTCATCGCAT TTAATGTCAG   
  
  
+ ATAGAGAGAT AGAGCTCTAC TGTATTAATT TTCTCCCACC TCACTTGATT CTGTTGCCCT AAACCACCCA   
  
  
+ AAAAAAGAAA TACGAAGAAG CCACTCTCTC TCTCTCTCTC TCTCACACAC ACACACACCC CTCCTAGTGG   
  
  
+ ATTTGATAGA GTATAAATGA ACAGTAATCA GACGAGATAT ATAGTAGTGC TTAATTGACC GAGTAGCAAA   
  
  
+ AGGGGGAAGT TGAGTGAGGT ATCAAGAGAG TAGCGTAGCT AGCTAGGGCA AGATATGCTG GGGTCTTCCT   
  
  
+ CATACAACTC CGAGGAAGAC CACCAAGATG ACAACCATTC CTCACCGTCT GATTTGTCTA GGCACCCACC   
  
  
+ TAGATTCACC ACTACTACTT ACCAATACCA ACAACATATT AGCTCTCCAT CTTCACAATC AATACTAGCT   
  
  
+ GTGCCACCTC GCCAATTGTT AATCAATTGT GCGGAGTTAA TTTGCCGGTT CGACTTCTCC TCCGCCAACC   
  
  
+ GGCTTCTCTC TCTCCTCGCC TCCCACTTTG CCTCCCCTTC TGGGGACTCC ACCGAGAGAC TTGTGTTTTA   
  
  
+ CTTCTGCAAG GCTTTGGATC TCCGCCTCAA TAACAGTTAT TTATCCACTC GTAGTTTGAC TGGTTGTAAC   
  
  
+ CCTAGTACTG GTTCTGGTAT TGGGGCTCCT TTAGCTTCCA ATTTTCTATT TCTTCAACCA TTAGTGCATT   
  
  
+ CACAGGTAAC AAGTAATGCC CGTGTTGGGT ACTTGACATA TTTGACTCTG AACCAGGTCA CCCCATTCAT   
  
  
+ CCGATTCACC CACTTAACTG CCAACCAAGC CATCCTGGAA GCTTTGGAGG GGTACAAGGC GGTCCATATC   
  
  
+ CTTGACATGG ACATCATGCA TGGGGTCCAG TGGCCACCAC TTCTTCAAGC AATTGCAGAA AGATCTGCAA   
  
  
+ CCCTTGGTCA CCCTGCTCCC ACTGTTCGCA TCACAGGAGG GGGCAGGGAT CTAGAACTCC TGAACCGAAC   
  
  
+ TGGAGATCGG ATCAGGAAGT TCGCACAATC CTTGGGACTC GAGTTTCAAT TCCACCATCT TATAACGGTG   
  
  
+ GACTCGAACC TTACTGAGGC AGTGGAGGCC ATCAATGCCC TACAGCTACA CCTCCATGTC CAAGAGGAGG   
  
  
+ AGGTCTTCGC AGTGAATTGT GGAGATTTCC TCCACCGCCT CCTAACGGAG TATGATAGTA CATTCCTTAG   
  
  
+ GATGTTTCTC TACAAGGTGA AGACCCTAAA CCCTAGGGTC TTCACCGTGG GAGAAAGGGA GGCCGACCAC   
  
  
+ AACCACCCTC TCTTCTGGCA GCGATTCATT GAGGCACTTG ACCACTATGG GGCAGTGTTT GACTCTCTAG   
  
  
+ AAGCAACCCT ACCATCAACA AGTCAAGAGA GGGTGGCGGT GGAAGAGGGG TGGTTCGGGG AGGAGATTAA   
  
  
+ AGATGTGGTT GGACGAGAAG GAGGGTTGAG GAGACAGAGG CACCAGAAAT ACGAGTCTTG GGAGGTGTTG   
  
  
+ ATGAGGAGCT CTGGGTTTAA GAGCTTGCCC TTGAGCCCCT TCTCTGTGTC ACAAGCCAAG CTGCTACTTC   
  
  
+ GCCTTCATTA CCCTTCTGAA GGGTACCACC TTAAGGTCTT CCATAGTAAT TGTCTGTTGC TTGGGTGGAA   
  
  
+ GAATCGCCCT CTTTTCTCTG TTTCTTCTTG GCAATA  

- -Up\_Stream \_Len000TTTTAT AAAAATGTTT TCGTAAAGAG ATATTTTATA AAATGTTTTA AAGTAAAACA   
  
  
- CCCAGAAAAT CTTAGTTAGA GAGATGTTCA TCCTTACGGC CAACTCCATT CCGATGGATG TAGATCTAGA   
  
  
- GAGGTCTGGG GTGTTTTTAA TTTGCTCTGG GTGTGACTTT CCACCACCAC CACCACAACA ACAACATAAC   
  
  
- AGGATGGTGC TCTCTCGAAG TTCACGTAGT GAATAAAGTT GTCCAGTTAG TGTATCTAGA GATCACATGA   
  
  
- CATTTAAAAA AAAGGCAGAA TAAAATTAAA AGAAAAGGTA GGATTTTCTA ATAGTACATC TATAAGGATC   
  
  
- AAACTATATA ACTGAAGTTG AAGTTTTTAA TGTGTAATTC TAGGTGCTTA TACTGTCAAA TCCAAAGTAA   
  
  
- ACTTCCTCTT TCTTTACAAT GCCACAAAAC CACAACATAA GCATCTGTAA AAAACGCGAA TTGACCGAGA   
  
  
- AGAGTACCCC TGCTCTGTCA CTTCCATTAA CATGAACAAC TAACTACTGC AGTAGACATC GAACCTAAAC   
  
  
- ATATCCTTAC TTGTAGAACC ACAGATGCAC CTTTCACACT AAACACAAGA AAGTAACTAG GAGGAGGAGT   
  
  
- TCTTCTCCAC TAGAAAGAGG GAACAAAAAA AAAAAAAAAC CGATTATACA ACCGTCTTGG CCGTTTTTTA   
  
  
- TGTATTAAAC TTATCACGTA CGTATACTCA TGTACTGTCA CTTGGTTGTT TTGCTTGGGG ATTTTGTGTT   
  
  
- CTCTTAATTG TACAATTTTG TGAATTTGAT TTACGTTGGT AGAGCTACTG GTGATTTATC GGTTCTTTGG   
  
  
- ATTGCATTAA TTGTTTTTAT TTTTTATTGA CTTTTATTTC ATCTCTTTCA GATTGGCTTA TGCTATTGTA   
  
  
- TTGGATTTTG ATTGATATGA ACTTGAATGA TTCTTTATAC TTAGTTGGTG TTTGGCTTAA CTAAAACCAA   
  
  
- ACGGTATTAA ACTGATATGT AAATTGAATT TTTTATAAAA AAAAATTAAA GTTTATTTAG ATTTAATAAA   
  
  
- AAGTTATAAG TAGGTTTTAT CTAAGTTAAT AATTTGTTCA GTTGGATATG GCTCAATAAA AGATCGAGAC   
  
  
- TGATTCTGTA TATATATAAA AAACATTTTT TCTATTCCTA ATATTAAATT TATCGGAAAG GGAATTTTTT   
  
  
- TAAAAATTTT AATAGGACTA TCTCTTATTT TTCTTTAGGA ATATGTTTTG AGACTAACTG CGTCTACACA   
  
  
- TATACCTGTA TATCTTCATT ATTCTCCCCA TCCGATCCTT CCCCCACCTT TATATCCCGT ATTTATCCCC   
  
  
- CGCCCGTATC CCTGTACAGA CAGACTCCAT GCCACCTACC CTGGAACGGT ACCTTCGTTG TTACAGTGTT   
  
  
- ATTATTATAG TTGCTGACTT CAAAGAAGTG GTCGGGTGAG AAACGGTATT ACGGTAATTA ATAAATAACG   
  
  
- ATGATAGAGA AAACTACACT TTGCCACCTG GAATAGAGAG GGGAGGTGAA GGTTAACTAC ACTCGTTGAG   
  
  
- TCTTTGCGTT TGTTTTGAAC ACGGTGGAAG AAATGAACGT AGGAGTAAAA GGAGACAGAG AGAGGCGGGG   
  
  
- ACAAAGAGAG AGAGAGGGAG TGAGTGAGTC TCATGACTAG ACTCGTAGTC TTCATCGTCT GGGTCTTTGA   
  
  
- GTCTAAATTT TTGTTGGGCG AGAAAACAAG GGAGATGCAG ATTATTAAAA CAGTAGCGTA AATTACAGTC   
  
  
- TATCTCTCTA TCTCGAGATG ACATAATTAA AAGAGGGTGG AGTGAACTAA GACAACGGGA TTTGGTGGGT   
  
  
- TTTTTTCTTT ATGCTTCTTC GGTGAGAGAG AGAGAGAGAG AGAGTGTGTG TGTGTGTGGG GAGGATCACC   
  
  
- TAAACTATCT CATATTTACT TGTCATTAGT CTGCTCTATA TATCATCACG AATTAACTGG CTCATCGTTT   
  
  
- TCCCCCTTCA ACTCACTCCA TAGTTCTCTC ATCGCATCGA TCGATCCCGT TCTATACGAC CCCAGAAGGA   
  
  
- GTATGTTGAG GCTCCTTCTG GTGGTTCTAC TGTTGGTAAG GAGTGGCAGA CTAAACAGAT CCGTGGGTGG   
  
  
- ATCTAAGTGG TGATGATGAA TGGTTATGGT TGTTGTATAA TCGAGAGGTA GAAGTGTTAG TTATGATCGA   
  
  
- CACGGTGGAG CGGTTAACAA TTAGTTAACA CGCCTCAATT AAACGGCCAA GCTGAAGAGG AGGCGGTTGG   
  
  
- CCGAAGAGAG AGAGGAGCGG AGGGTGAAAC GGAGGGGAAG ACCCCTGAGG TGGCTCTCTG AACACAAAAT   
  
  
- GAAGACGTTC CGAAACCTAG AGGCGGAGTT ATTGTCAATA AATAGGTGAG CATCAAACTG ACCAACATTG   
  
  
- GGATCATGAC CAAGACCATA ACCCCGAGGA AATCGAAGGT TAAAAGATAA AGAAGTTGGT AATCACGTAA   
  
  
- GTGTCCATTG TTCATTACGG GCACAACCCA TGAACTGTAT AAACTGAGAC TTGGTCCAGT GGGGTAAGTA   
  
  
- GGCTAAGTGG GTGAATTGAC GGTTGGTTCG GTAGGACCTT CGAAACCTCC CCATGTTCCG CCAGGTATAG   
  
  
- GAACTGTACC TGTAGTACGT ACCCCAGGTC ACCGGTGGTG AAGAAGTTCG TTAACGTCTT TCTAGACGTT   
  
  
- GGGAACCAGT GGGACGAGGG TGACAAGCGT AGTGTCCTCC CCCGTCCCTA GATCTTGAGG ACTTGGCTTG   
  
  
- ACCTCTAGCC TAGTCCTTCA AGCGTGTTAG GAACCCTGAG CTCAAAGTTA AGGTGGTAGA ATATTGCCAC   
  
  
- CTGAGCTTGG AATGACTCCG TCACCTCCGG TAGTTACGGG ATGTCGATGT GGAGGTACAG GTTCTCCTCC   
  
  
- TCCAGAAGCG TCACTTAACA CCTCTAAAGG AGGTGGCGGA GGATTGCCTC ATACTATCAT GTAAGGAATC   
  
  
- CTACAAAGAG ATGTTCCACT TCTGGGATTT GGGATCCCAG AAGTGGCACC CTCTTTCCCT CCGGCTGGTG   
  
  
- TTGGTGGGAG AGAAGACCGT CGCTAAGTAA CTCCGTGAAC TGGTGATACC CCGTCACAAA CTGAGAGATC   
  
  
- TTCGTTGGGA TGGTAGTTGT TCAGTTCTCT CCCACCGCCA CCTTCTCCCC ACCAAGCCCC TCCTCTAATT   
  
  
- TCTACACCAA CCTGCTCTTC CTCCCAACTC CTCTGTCTCC GTGGTCTTTA TGCTCAGAAC CCTCCACAAC   
  
  
- TACTCCTCGA GACCCAAATT CTCGAACGGG AACTCGGGGA AGAGACACAG TGTTCGGTTC GACGATGAAG   
  
  
- CGGAAGTAAT GGGAAGACTT CCCATGGTGG AATTCCAGAA GGTATCATTA ACAGACAACG AACCCACCTT   
  
  
- CTTAGCGGGA GAAAAGAGAC AAAGAAGAAC CGTTAT

+     ARE

| Site Name | Organism | Position | Strand | Matrix score. | sequence | function |
| --- | --- | --- | --- | --- | --- | --- |
| ARE | Zea mays | 1815 | + | 6 | AAACCA | cis-acting regulatory element essential for the anaerobic induction |
| ARE | Zea mays | 980 | - | 6 | AAACCA | cis-acting regulatory element essential for the anaerobic induction |

>HU01G00833.1   
+ -Up\_Stream \_Len000AAAATA TTTTTACAAA AGCATTTCTC TATAAAATAT TTTACAAAAT TTCATTTTGT   
  
  
+ GGGTCTTTTA GAATCAATCT CTCTACAAGT AGGAATGCCG GTTGAGGTAA GGCTACCTAC ATCTAGATCT   
  
  
+ CTCCAGACCC CACAAAAATT AAACGAGACC CACACTGAAA GGTGGTGGTG GTGGTGTTGT TGTTGTATTG   
  
  
+ TCCTACCACG AGAGAGCTTC AAGTGCATCA CTTATTTCAA CAGGTCAATC ACATAGATCT CTAGTGTACT   
  
  
+ GTAAATTTTT TTTCCGTCTT ATTTTAATTT TCTTTTCCAT CCTAAAAGAT TATCATGTAG ATATTCCTAG   
  
  
+ TTTGATATAT TGACTTCAAC TTCAAAAATT ACACATTAAG ATCCACGAAT ATGACAGTTT AGGTTTCATT   
  
  
+ TGAAGGAGAA AGAAATGTTA CGGTGTTTTG GTGTTGTATT CGTAGACATT TTTTGCGCTT AACTGGCTCT   
  
  
+ TCTCATGGGG ACGAGACAGT GAAGGTAATT GTACTTGTTG ATTGATGACG TCATCTGTAG CTTGGATTTG   
  
  
+ TATAGGAATG AACATCTTGG TGTCTACGTG GAAAGTGTGA TTTGTGTTCT TTCATTGATC CTCCTCCTCA   
  
  
+ AGAAGAGGTG ATCTTTCTCC CTTGTTTTTT TTTTTTTTTG GCTAATATGT TGGCAGAACC GGCAAAAAAT   
  
  
+ ACATAATTTG AATAGTGCAT GCATATGAGT ACATGACAGT GAACCAACAA AACGAACCCC TAAAACACAA   
  
  
+ GAGAATTAAC ATGTTAAAAC ACTTAAACTA AATGCAACCA TCTCGATGAC CACTAAATAG CCAAGAAACC   
  
  
+ TAACGTAATT AACAAAAATA AAAAATAACT GAAAATAAAG TAGAGAAAGT CTAACCGAAT ACGATAACAT   
  
  
+ AACCTAAAAC TAACTATACT TGAACTTACT AAGAAATATG AATCAACCAC AAACCGAATT GATTTTGGTT   
  
  
+ TGCCATAATT TGACTATACA TTTAACTTAA AAAATATTTT TTTTTAATTT CAAATAAATC TAAATTATTT   
  
  
+ TTCAATATTC ATCCAAAATA GATTCAATTA TTAAACAAGT CAACCTATAC CGAGTTATTT TCTAGCTCTG   
  
  
+ ACTAAGACAT ATATATATTT TTTGTAAAAA AGATAAGGAT TATAATTTAA ATAGCCTTTC CCTTAAAAAA   
  
  
+ ATTTTTAAAA TTATCCTGAT AGAGAATAAA AAGAAATCCT TATACAAAAC TCTGATTGAC GCAGATGTGT   
  
  
+ ATATGGACAT ATAGAAGTAA TAAGAGGGGT AGGCTAGGAA GGGGGTGGAA ATATAGGGCA TAAATAGGGG   
  
  
+ GCGGGCATAG GGACATGTCT GTCTGAGGTA CGGTGGATGG GACCTTGCCA TGGAAGCAAC AATGTCACAA   
  
  
+ TAATAATATC AACGACTGAA GTTTCTTCAC CAGCCCACTC TTTGCCATAA TGCCATTAAT TATTTATTGC   
  
  
+ TACTATCTCT TTTGATGTGA AACGGTGGAC CTTATCTCTC CCCTCCACTT CCAATTGATG TGAGCAACTC   
  
  
+ AGAAACGCAA ACAAAACTTG TGCCACCTTC TTTACTTGCA TCCTCATTTT CCTCTGTCTC TCTCCGCCCC   
  
  
+ TGTTTCTCTC TCTCTCCCTC ACTCACTCAG AGTACTGATC TGAGCATCAG AAGTAGCAGA CCCAGAAACT   
  
  
+ CAGATTTAAA AACAACCCGC TCTTTTGTTC CCTCTACGTC TAATAATTTT GTCATCGCAT TTAATGTCAG   
  
  
+ ATAGAGAGAT AGAGCTCTAC TGTATTAATT TTCTCCCACC TCACTTGATT CTGTTGCCCT AAACCACCCA   
  
  
+ AAAAAAGAAA TACGAAGAAG CCACTCTCTC TCTCTCTCTC TCTCACACAC ACACACACCC CTCCTAGTGG   
  
  
+ ATTTGATAGA GTATAAATGA ACAGTAATCA GACGAGATAT ATAGTAGTGC TTAATTGACC GAGTAGCAAA   
  
  
+ AGGGGGAAGT TGAGTGAGGT ATCAAGAGAG TAGCGTAGCT AGCTAGGGCA AGATATGCTG GGGTCTTCCT   
  
  
+ CATACAACTC CGAGGAAGAC CACCAAGATG ACAACCATTC CTCACCGTCT GATTTGTCTA GGCACCCACC   
  
  
+ TAGATTCACC ACTACTACTT ACCAATACCA ACAACATATT AGCTCTCCAT CTTCACAATC AATACTAGCT   
  
  
+ GTGCCACCTC GCCAATTGTT AATCAATTGT GCGGAGTTAA TTTGCCGGTT CGACTTCTCC TCCGCCAACC   
  
  
+ GGCTTCTCTC TCTCCTCGCC TCCCACTTTG CCTCCCCTTC TGGGGACTCC ACCGAGAGAC TTGTGTTTTA   
  
  
+ CTTCTGCAAG GCTTTGGATC TCCGCCTCAA TAACAGTTAT TTATCCACTC GTAGTTTGAC TGGTTGTAAC   
  
  
+ CCTAGTACTG GTTCTGGTAT TGGGGCTCCT TTAGCTTCCA ATTTTCTATT TCTTCAACCA TTAGTGCATT   
  
  
+ CACAGGTAAC AAGTAATGCC CGTGTTGGGT ACTTGACATA TTTGACTCTG AACCAGGTCA CCCCATTCAT   
  
  
+ CCGATTCACC CACTTAACTG CCAACCAAGC CATCCTGGAA GCTTTGGAGG GGTACAAGGC GGTCCATATC   
  
  
+ CTTGACATGG ACATCATGCA TGGGGTCCAG TGGCCACCAC TTCTTCAAGC AATTGCAGAA AGATCTGCAA   
  
  
+ CCCTTGGTCA CCCTGCTCCC ACTGTTCGCA TCACAGGAGG GGGCAGGGAT CTAGAACTCC TGAACCGAAC   
  
  
+ TGGAGATCGG ATCAGGAAGT TCGCACAATC CTTGGGACTC GAGTTTCAAT TCCACCATCT TATAACGGTG   
  
  
+ GACTCGAACC TTACTGAGGC AGTGGAGGCC ATCAATGCCC TACAGCTACA CCTCCATGTC CAAGAGGAGG   
  
  
+ AGGTCTTCGC AGTGAATTGT GGAGATTTCC TCCACCGCCT CCTAACGGAG TATGATAGTA CATTCCTTAG   
  
  
+ GATGTTTCTC TACAAGGTGA AGACCCTAAA CCCTAGGGTC TTCACCGTGG GAGAAAGGGA GGCCGACCAC   
  
  
+ AACCACCCTC TCTTCTGGCA GCGATTCATT GAGGCACTTG ACCACTATGG GGCAGTGTTT GACTCTCTAG   
  
  
+ AAGCAACCCT ACCATCAACA AGTCAAGAGA GGGTGGCGGT GGAAGAGGGG TGGTTCGGGG AGGAGATTAA   
  
  
+ AGATGTGGTT GGACGAGAAG GAGGGTTGAG GAGACAGAGG CACCAGAAAT ACGAGTCTTG GGAGGTGTTG   
  
  
+ ATGAGGAGCT CTGGGTTTAA GAGCTTGCCC TTGAGCCCCT TCTCTGTGTC ACAAGCCAAG CTGCTACTTC   
  
  
+ GCCTTCATTA CCCTTCTGAA GGGTACCACC TTAAGGTCTT CCATAGTAAT TGTCTGTTGC TTGGGTGGAA   
  
  
+ GAATCGCCCT CTTTTCTCTG TTTCTTCTTG GCAATA  

- -Up\_Stream \_Len000TTTTAT AAAAATGTTT TCGTAAAGAG ATATTTTATA AAATGTTTTA AAGTAAAACA   
  
  
- CCCAGAAAAT CTTAGTTAGA GAGATGTTCA TCCTTACGGC CAACTCCATT CCGATGGATG TAGATCTAGA   
  
  
- GAGGTCTGGG GTGTTTTTAA TTTGCTCTGG GTGTGACTTT CCACCACCAC CACCACAACA ACAACATAAC   
  
  
- AGGATGGTGC TCTCTCGAAG TTCACGTAGT GAATAAAGTT GTCCAGTTAG TGTATCTAGA GATCACATGA   
  
  
- CATTTAAAAA AAAGGCAGAA TAAAATTAAA AGAAAAGGTA GGATTTTCTA ATAGTACATC TATAAGGATC   
  
  
- AAACTATATA ACTGAAGTTG AAGTTTTTAA TGTGTAATTC TAGGTGCTTA TACTGTCAAA TCCAAAGTAA   
  
  
- ACTTCCTCTT TCTTTACAAT GCCACAAAAC CACAACATAA GCATCTGTAA AAAACGCGAA TTGACCGAGA   
  
  
- AGAGTACCCC TGCTCTGTCA CTTCCATTAA CATGAACAAC TAACTACTGC AGTAGACATC GAACCTAAAC   
  
  
- ATATCCTTAC TTGTAGAACC ACAGATGCAC CTTTCACACT AAACACAAGA AAGTAACTAG GAGGAGGAGT   
  
  
- TCTTCTCCAC TAGAAAGAGG GAACAAAAAA AAAAAAAAAC CGATTATACA ACCGTCTTGG CCGTTTTTTA   
  
  
- TGTATTAAAC TTATCACGTA CGTATACTCA TGTACTGTCA CTTGGTTGTT TTGCTTGGGG ATTTTGTGTT   
  
  
- CTCTTAATTG TACAATTTTG TGAATTTGAT TTACGTTGGT AGAGCTACTG GTGATTTATC GGTTCTTTGG   
  
  
- ATTGCATTAA TTGTTTTTAT TTTTTATTGA CTTTTATTTC ATCTCTTTCA GATTGGCTTA TGCTATTGTA   
  
  
- TTGGATTTTG ATTGATATGA ACTTGAATGA TTCTTTATAC TTAGTTGGTG TTTGGCTTAA CTAAAACCAA   
  
  
- ACGGTATTAA ACTGATATGT AAATTGAATT TTTTATAAAA AAAAATTAAA GTTTATTTAG ATTTAATAAA   
  
  
- AAGTTATAAG TAGGTTTTAT CTAAGTTAAT AATTTGTTCA GTTGGATATG GCTCAATAAA AGATCGAGAC   
  
  
- TGATTCTGTA TATATATAAA AAACATTTTT TCTATTCCTA ATATTAAATT TATCGGAAAG GGAATTTTTT   
  
  
- TAAAAATTTT AATAGGACTA TCTCTTATTT TTCTTTAGGA ATATGTTTTG AGACTAACTG CGTCTACACA   
  
  
- TATACCTGTA TATCTTCATT ATTCTCCCCA TCCGATCCTT CCCCCACCTT TATATCCCGT ATTTATCCCC   
  
  
- CGCCCGTATC CCTGTACAGA CAGACTCCAT GCCACCTACC CTGGAACGGT ACCTTCGTTG TTACAGTGTT   
  
  
- ATTATTATAG TTGCTGACTT CAAAGAAGTG GTCGGGTGAG AAACGGTATT ACGGTAATTA ATAAATAACG   
  
  
- ATGATAGAGA AAACTACACT TTGCCACCTG GAATAGAGAG GGGAGGTGAA GGTTAACTAC ACTCGTTGAG   
  
  
- TCTTTGCGTT TGTTTTGAAC ACGGTGGAAG AAATGAACGT AGGAGTAAAA GGAGACAGAG AGAGGCGGGG   
  
  
- ACAAAGAGAG AGAGAGGGAG TGAGTGAGTC TCATGACTAG ACTCGTAGTC TTCATCGTCT GGGTCTTTGA   
  
  
- GTCTAAATTT TTGTTGGGCG AGAAAACAAG GGAGATGCAG ATTATTAAAA CAGTAGCGTA AATTACAGTC   
  
  
- TATCTCTCTA TCTCGAGATG ACATAATTAA AAGAGGGTGG AGTGAACTAA GACAACGGGA TTTGGTGGGT   
  
  
- TTTTTTCTTT ATGCTTCTTC GGTGAGAGAG AGAGAGAGAG AGAGTGTGTG TGTGTGTGGG GAGGATCACC   
  
  
- TAAACTATCT CATATTTACT TGTCATTAGT CTGCTCTATA TATCATCACG AATTAACTGG CTCATCGTTT   
  
  
- TCCCCCTTCA ACTCACTCCA TAGTTCTCTC ATCGCATCGA TCGATCCCGT TCTATACGAC CCCAGAAGGA   
  
  
- GTATGTTGAG GCTCCTTCTG GTGGTTCTAC TGTTGGTAAG GAGTGGCAGA CTAAACAGAT CCGTGGGTGG   
  
  
- ATCTAAGTGG TGATGATGAA TGGTTATGGT TGTTGTATAA TCGAGAGGTA GAAGTGTTAG TTATGATCGA   
  
  
- CACGGTGGAG CGGTTAACAA TTAGTTAACA CGCCTCAATT AAACGGCCAA GCTGAAGAGG AGGCGGTTGG   
  
  
- CCGAAGAGAG AGAGGAGCGG AGGGTGAAAC GGAGGGGAAG ACCCCTGAGG TGGCTCTCTG AACACAAAAT   
  
  
- GAAGACGTTC CGAAACCTAG AGGCGGAGTT ATTGTCAATA AATAGGTGAG CATCAAACTG ACCAACATTG   
  
  
- GGATCATGAC CAAGACCATA ACCCCGAGGA AATCGAAGGT TAAAAGATAA AGAAGTTGGT AATCACGTAA   
  
  
- GTGTCCATTG TTCATTACGG GCACAACCCA TGAACTGTAT AAACTGAGAC TTGGTCCAGT GGGGTAAGTA   
  
  
- GGCTAAGTGG GTGAATTGAC GGTTGGTTCG GTAGGACCTT CGAAACCTCC CCATGTTCCG CCAGGTATAG   
  
  
- GAACTGTACC TGTAGTACGT ACCCCAGGTC ACCGGTGGTG AAGAAGTTCG TTAACGTCTT TCTAGACGTT   
  
  
- GGGAACCAGT GGGACGAGGG TGACAAGCGT AGTGTCCTCC CCCGTCCCTA GATCTTGAGG ACTTGGCTTG   
  
  
- ACCTCTAGCC TAGTCCTTCA AGCGTGTTAG GAACCCTGAG CTCAAAGTTA AGGTGGTAGA ATATTGCCAC   
  
  
- CTGAGCTTGG AATGACTCCG TCACCTCCGG TAGTTACGGG ATGTCGATGT GGAGGTACAG GTTCTCCTCC   
  
  
- TCCAGAAGCG TCACTTAACA CCTCTAAAGG AGGTGGCGGA GGATTGCCTC ATACTATCAT GTAAGGAATC   
  
  
- CTACAAAGAG ATGTTCCACT TCTGGGATTT GGGATCCCAG AAGTGGCACC CTCTTTCCCT CCGGCTGGTG   
  
  
- TTGGTGGGAG AGAAGACCGT CGCTAAGTAA CTCCGTGAAC TGGTGATACC CCGTCACAAA CTGAGAGATC   
  
  
- TTCGTTGGGA TGGTAGTTGT TCAGTTCTCT CCCACCGCCA CCTTCTCCCC ACCAAGCCCC TCCTCTAATT   
  
  
- TCTACACCAA CCTGCTCTTC CTCCCAACTC CTCTGTCTCC GTGGTCTTTA TGCTCAGAAC CCTCCACAAC   
  
  
- TACTCCTCGA GACCCAAATT CTCGAACGGG AACTCGGGGA AGAGACACAG TGTTCGGTTC GACGATGAAG   
  
  
- CGGAAGTAAT GGGAAGACTT CCCATGGTGG AATTCCAGAA GGTATCATTA ACAGACAACG AACCCACCTT   
  
  
- CTTAGCGGGA GAAAAGAGAC AAAGAAGAAC CGTTAT

+     AT1-motif

| Site Name | Organism | Position | Strand | Matrix score. | sequence | function |
| --- | --- | --- | --- | --- | --- | --- |
| AT1-motif | Solanum tuberosum | 861 | - | 13 | AATTATTTTTTATT | part of a light responsive module |

>HU01G00833.1   
+ -Up\_Stream \_Len000AAAATA TTTTTACAAA AGCATTTCTC TATAAAATAT TTTACAAAAT TTCATTTTGT   
  
  
+ GGGTCTTTTA GAATCAATCT CTCTACAAGT AGGAATGCCG GTTGAGGTAA GGCTACCTAC ATCTAGATCT   
  
  
+ CTCCAGACCC CACAAAAATT AAACGAGACC CACACTGAAA GGTGGTGGTG GTGGTGTTGT TGTTGTATTG   
  
  
+ TCCTACCACG AGAGAGCTTC AAGTGCATCA CTTATTTCAA CAGGTCAATC ACATAGATCT CTAGTGTACT   
  
  
+ GTAAATTTTT TTTCCGTCTT ATTTTAATTT TCTTTTCCAT CCTAAAAGAT TATCATGTAG ATATTCCTAG   
  
  
+ TTTGATATAT TGACTTCAAC TTCAAAAATT ACACATTAAG ATCCACGAAT ATGACAGTTT AGGTTTCATT   
  
  
+ TGAAGGAGAA AGAAATGTTA CGGTGTTTTG GTGTTGTATT CGTAGACATT TTTTGCGCTT AACTGGCTCT   
  
  
+ TCTCATGGGG ACGAGACAGT GAAGGTAATT GTACTTGTTG ATTGATGACG TCATCTGTAG CTTGGATTTG   
  
  
+ TATAGGAATG AACATCTTGG TGTCTACGTG GAAAGTGTGA TTTGTGTTCT TTCATTGATC CTCCTCCTCA   
  
  
+ AGAAGAGGTG ATCTTTCTCC CTTGTTTTTT TTTTTTTTTG GCTAATATGT TGGCAGAACC GGCAAAAAAT   
  
  
+ ACATAATTTG AATAGTGCAT GCATATGAGT ACATGACAGT GAACCAACAA AACGAACCCC TAAAACACAA   
  
  
+ GAGAATTAAC ATGTTAAAAC ACTTAAACTA AATGCAACCA TCTCGATGAC CACTAAATAG CCAAGAAACC   
  
  
+ TAACGTAATT AACAAAAATA AAAAATAACT GAAAATAAAG TAGAGAAAGT CTAACCGAAT ACGATAACAT   
  
  
+ AACCTAAAAC TAACTATACT TGAACTTACT AAGAAATATG AATCAACCAC AAACCGAATT GATTTTGGTT   
  
  
+ TGCCATAATT TGACTATACA TTTAACTTAA AAAATATTTT TTTTTAATTT CAAATAAATC TAAATTATTT   
  
  
+ TTCAATATTC ATCCAAAATA GATTCAATTA TTAAACAAGT CAACCTATAC CGAGTTATTT TCTAGCTCTG   
  
  
+ ACTAAGACAT ATATATATTT TTTGTAAAAA AGATAAGGAT TATAATTTAA ATAGCCTTTC CCTTAAAAAA   
  
  
+ ATTTTTAAAA TTATCCTGAT AGAGAATAAA AAGAAATCCT TATACAAAAC TCTGATTGAC GCAGATGTGT   
  
  
+ ATATGGACAT ATAGAAGTAA TAAGAGGGGT AGGCTAGGAA GGGGGTGGAA ATATAGGGCA TAAATAGGGG   
  
  
+ GCGGGCATAG GGACATGTCT GTCTGAGGTA CGGTGGATGG GACCTTGCCA TGGAAGCAAC AATGTCACAA   
  
  
+ TAATAATATC AACGACTGAA GTTTCTTCAC CAGCCCACTC TTTGCCATAA TGCCATTAAT TATTTATTGC   
  
  
+ TACTATCTCT TTTGATGTGA AACGGTGGAC CTTATCTCTC CCCTCCACTT CCAATTGATG TGAGCAACTC   
  
  
+ AGAAACGCAA ACAAAACTTG TGCCACCTTC TTTACTTGCA TCCTCATTTT CCTCTGTCTC TCTCCGCCCC   
  
  
+ TGTTTCTCTC TCTCTCCCTC ACTCACTCAG AGTACTGATC TGAGCATCAG AAGTAGCAGA CCCAGAAACT   
  
  
+ CAGATTTAAA AACAACCCGC TCTTTTGTTC CCTCTACGTC TAATAATTTT GTCATCGCAT TTAATGTCAG   
  
  
+ ATAGAGAGAT AGAGCTCTAC TGTATTAATT TTCTCCCACC TCACTTGATT CTGTTGCCCT AAACCACCCA   
  
  
+ AAAAAAGAAA TACGAAGAAG CCACTCTCTC TCTCTCTCTC TCTCACACAC ACACACACCC CTCCTAGTGG   
  
  
+ ATTTGATAGA GTATAAATGA ACAGTAATCA GACGAGATAT ATAGTAGTGC TTAATTGACC GAGTAGCAAA   
  
  
+ AGGGGGAAGT TGAGTGAGGT ATCAAGAGAG TAGCGTAGCT AGCTAGGGCA AGATATGCTG GGGTCTTCCT   
  
  
+ CATACAACTC CGAGGAAGAC CACCAAGATG ACAACCATTC CTCACCGTCT GATTTGTCTA GGCACCCACC   
  
  
+ TAGATTCACC ACTACTACTT ACCAATACCA ACAACATATT AGCTCTCCAT CTTCACAATC AATACTAGCT   
  
  
+ GTGCCACCTC GCCAATTGTT AATCAATTGT GCGGAGTTAA TTTGCCGGTT CGACTTCTCC TCCGCCAACC   
  
  
+ GGCTTCTCTC TCTCCTCGCC TCCCACTTTG CCTCCCCTTC TGGGGACTCC ACCGAGAGAC TTGTGTTTTA   
  
  
+ CTTCTGCAAG GCTTTGGATC TCCGCCTCAA TAACAGTTAT TTATCCACTC GTAGTTTGAC TGGTTGTAAC   
  
  
+ CCTAGTACTG GTTCTGGTAT TGGGGCTCCT TTAGCTTCCA ATTTTCTATT TCTTCAACCA TTAGTGCATT   
  
  
+ CACAGGTAAC AAGTAATGCC CGTGTTGGGT ACTTGACATA TTTGACTCTG AACCAGGTCA CCCCATTCAT   
  
  
+ CCGATTCACC CACTTAACTG CCAACCAAGC CATCCTGGAA GCTTTGGAGG GGTACAAGGC GGTCCATATC   
  
  
+ CTTGACATGG ACATCATGCA TGGGGTCCAG TGGCCACCAC TTCTTCAAGC AATTGCAGAA AGATCTGCAA   
  
  
+ CCCTTGGTCA CCCTGCTCCC ACTGTTCGCA TCACAGGAGG GGGCAGGGAT CTAGAACTCC TGAACCGAAC   
  
  
+ TGGAGATCGG ATCAGGAAGT TCGCACAATC CTTGGGACTC GAGTTTCAAT TCCACCATCT TATAACGGTG   
  
  
+ GACTCGAACC TTACTGAGGC AGTGGAGGCC ATCAATGCCC TACAGCTACA CCTCCATGTC CAAGAGGAGG   
  
  
+ AGGTCTTCGC AGTGAATTGT GGAGATTTCC TCCACCGCCT CCTAACGGAG TATGATAGTA CATTCCTTAG   
  
  
+ GATGTTTCTC TACAAGGTGA AGACCCTAAA CCCTAGGGTC TTCACCGTGG GAGAAAGGGA GGCCGACCAC   
  
  
+ AACCACCCTC TCTTCTGGCA GCGATTCATT GAGGCACTTG ACCACTATGG GGCAGTGTTT GACTCTCTAG   
  
  
+ AAGCAACCCT ACCATCAACA AGTCAAGAGA GGGTGGCGGT GGAAGAGGGG TGGTTCGGGG AGGAGATTAA   
  
  
+ AGATGTGGTT GGACGAGAAG GAGGGTTGAG GAGACAGAGG CACCAGAAAT ACGAGTCTTG GGAGGTGTTG   
  
  
+ ATGAGGAGCT CTGGGTTTAA GAGCTTGCCC TTGAGCCCCT TCTCTGTGTC ACAAGCCAAG CTGCTACTTC   
  
  
+ GCCTTCATTA CCCTTCTGAA GGGTACCACC TTAAGGTCTT CCATAGTAAT TGTCTGTTGC TTGGGTGGAA   
  
  
+ GAATCGCCCT CTTTTCTCTG TTTCTTCTTG GCAATA  

- -Up\_Stream \_Len000TTTTAT AAAAATGTTT TCGTAAAGAG ATATTTTATA AAATGTTTTA AAGTAAAACA   
  
  
- CCCAGAAAAT CTTAGTTAGA GAGATGTTCA TCCTTACGGC CAACTCCATT CCGATGGATG TAGATCTAGA   
  
  
- GAGGTCTGGG GTGTTTTTAA TTTGCTCTGG GTGTGACTTT CCACCACCAC CACCACAACA ACAACATAAC   
  
  
- AGGATGGTGC TCTCTCGAAG TTCACGTAGT GAATAAAGTT GTCCAGTTAG TGTATCTAGA GATCACATGA   
  
  
- CATTTAAAAA AAAGGCAGAA TAAAATTAAA AGAAAAGGTA GGATTTTCTA ATAGTACATC TATAAGGATC   
  
  
- AAACTATATA ACTGAAGTTG AAGTTTTTAA TGTGTAATTC TAGGTGCTTA TACTGTCAAA TCCAAAGTAA   
  
  
- ACTTCCTCTT TCTTTACAAT GCCACAAAAC CACAACATAA GCATCTGTAA AAAACGCGAA TTGACCGAGA   
  
  
- AGAGTACCCC TGCTCTGTCA CTTCCATTAA CATGAACAAC TAACTACTGC AGTAGACATC GAACCTAAAC   
  
  
- ATATCCTTAC TTGTAGAACC ACAGATGCAC CTTTCACACT AAACACAAGA AAGTAACTAG GAGGAGGAGT   
  
  
- TCTTCTCCAC TAGAAAGAGG GAACAAAAAA AAAAAAAAAC CGATTATACA ACCGTCTTGG CCGTTTTTTA   
  
  
- TGTATTAAAC TTATCACGTA CGTATACTCA TGTACTGTCA CTTGGTTGTT TTGCTTGGGG ATTTTGTGTT   
  
  
- CTCTTAATTG TACAATTTTG TGAATTTGAT TTACGTTGGT AGAGCTACTG GTGATTTATC GGTTCTTTGG   
  
  
- ATTGCATTAA TTGTTTTTAT TTTTTATTGA CTTTTATTTC ATCTCTTTCA GATTGGCTTA TGCTATTGTA   
  
  
- TTGGATTTTG ATTGATATGA ACTTGAATGA TTCTTTATAC TTAGTTGGTG TTTGGCTTAA CTAAAACCAA   
  
  
- ACGGTATTAA ACTGATATGT AAATTGAATT TTTTATAAAA AAAAATTAAA GTTTATTTAG ATTTAATAAA   
  
  
- AAGTTATAAG TAGGTTTTAT CTAAGTTAAT AATTTGTTCA GTTGGATATG GCTCAATAAA AGATCGAGAC   
  
  
- TGATTCTGTA TATATATAAA AAACATTTTT TCTATTCCTA ATATTAAATT TATCGGAAAG GGAATTTTTT   
  
  
- TAAAAATTTT AATAGGACTA TCTCTTATTT TTCTTTAGGA ATATGTTTTG AGACTAACTG CGTCTACACA   
  
  
- TATACCTGTA TATCTTCATT ATTCTCCCCA TCCGATCCTT CCCCCACCTT TATATCCCGT ATTTATCCCC   
  
  
- CGCCCGTATC CCTGTACAGA CAGACTCCAT GCCACCTACC CTGGAACGGT ACCTTCGTTG TTACAGTGTT   
  
  
- ATTATTATAG TTGCTGACTT CAAAGAAGTG GTCGGGTGAG AAACGGTATT ACGGTAATTA ATAAATAACG   
  
  
- ATGATAGAGA AAACTACACT TTGCCACCTG GAATAGAGAG GGGAGGTGAA GGTTAACTAC ACTCGTTGAG   
  
  
- TCTTTGCGTT TGTTTTGAAC ACGGTGGAAG AAATGAACGT AGGAGTAAAA GGAGACAGAG AGAGGCGGGG   
  
  
- ACAAAGAGAG AGAGAGGGAG TGAGTGAGTC TCATGACTAG ACTCGTAGTC TTCATCGTCT GGGTCTTTGA   
  
  
- GTCTAAATTT TTGTTGGGCG AGAAAACAAG GGAGATGCAG ATTATTAAAA CAGTAGCGTA AATTACAGTC   
  
  
- TATCTCTCTA TCTCGAGATG ACATAATTAA AAGAGGGTGG AGTGAACTAA GACAACGGGA TTTGGTGGGT   
  
  
- TTTTTTCTTT ATGCTTCTTC GGTGAGAGAG AGAGAGAGAG AGAGTGTGTG TGTGTGTGGG GAGGATCACC   
  
  
- TAAACTATCT CATATTTACT TGTCATTAGT CTGCTCTATA TATCATCACG AATTAACTGG CTCATCGTTT   
  
  
- TCCCCCTTCA ACTCACTCCA TAGTTCTCTC ATCGCATCGA TCGATCCCGT TCTATACGAC CCCAGAAGGA   
  
  
- GTATGTTGAG GCTCCTTCTG GTGGTTCTAC TGTTGGTAAG GAGTGGCAGA CTAAACAGAT CCGTGGGTGG   
  
  
- ATCTAAGTGG TGATGATGAA TGGTTATGGT TGTTGTATAA TCGAGAGGTA GAAGTGTTAG TTATGATCGA   
  
  
- CACGGTGGAG CGGTTAACAA TTAGTTAACA CGCCTCAATT AAACGGCCAA GCTGAAGAGG AGGCGGTTGG   
  
  
- CCGAAGAGAG AGAGGAGCGG AGGGTGAAAC GGAGGGGAAG ACCCCTGAGG TGGCTCTCTG AACACAAAAT   
  
  
- GAAGACGTTC CGAAACCTAG AGGCGGAGTT ATTGTCAATA AATAGGTGAG CATCAAACTG ACCAACATTG   
  
  
- GGATCATGAC CAAGACCATA ACCCCGAGGA AATCGAAGGT TAAAAGATAA AGAAGTTGGT AATCACGTAA   
  
  
- GTGTCCATTG TTCATTACGG GCACAACCCA TGAACTGTAT AAACTGAGAC TTGGTCCAGT GGGGTAAGTA   
  
  
- GGCTAAGTGG GTGAATTGAC GGTTGGTTCG GTAGGACCTT CGAAACCTCC CCATGTTCCG CCAGGTATAG   
  
  
- GAACTGTACC TGTAGTACGT ACCCCAGGTC ACCGGTGGTG AAGAAGTTCG TTAACGTCTT TCTAGACGTT   
  
  
- GGGAACCAGT GGGACGAGGG TGACAAGCGT AGTGTCCTCC CCCGTCCCTA GATCTTGAGG ACTTGGCTTG   
  
  
- ACCTCTAGCC TAGTCCTTCA AGCGTGTTAG GAACCCTGAG CTCAAAGTTA AGGTGGTAGA ATATTGCCAC   
  
  
- CTGAGCTTGG AATGACTCCG TCACCTCCGG TAGTTACGGG ATGTCGATGT GGAGGTACAG GTTCTCCTCC   
  
  
- TCCAGAAGCG TCACTTAACA CCTCTAAAGG AGGTGGCGGA GGATTGCCTC ATACTATCAT GTAAGGAATC   
  
  
- CTACAAAGAG ATGTTCCACT TCTGGGATTT GGGATCCCAG AAGTGGCACC CTCTTTCCCT CCGGCTGGTG   
  
  
- TTGGTGGGAG AGAAGACCGT CGCTAAGTAA CTCCGTGAAC TGGTGATACC CCGTCACAAA CTGAGAGATC   
  
  
- TTCGTTGGGA TGGTAGTTGT TCAGTTCTCT CCCACCGCCA CCTTCTCCCC ACCAAGCCCC TCCTCTAATT   
  
  
- TCTACACCAA CCTGCTCTTC CTCCCAACTC CTCTGTCTCC GTGGTCTTTA TGCTCAGAAC CCTCCACAAC   
  
  
- TACTCCTCGA GACCCAAATT CTCGAACGGG AACTCGGGGA AGAGACACAG TGTTCGGTTC GACGATGAAG   
  
  
- CGGAAGTAAT GGGAAGACTT CCCATGGTGG AATTCCAGAA GGTATCATTA ACAGACAACG AACCCACCTT   
  
  
- CTTAGCGGGA GAAAAGAGAC AAAGAAGAAC CGTTAT

+     AT~TATA-box

| Site Name | Organism | Position | Strand | Matrix score. | sequence | function |
| --- | --- | --- | --- | --- | --- | --- |
| AT~TATA-box | Arabidopsis thaliana | 1932 | - | 6 | TATATA |  |
| AT~TATA-box | Arabidopsis thaliana | 1136 | + | 6 | TATATA |  |
| AT~TATA-box | Arabidopsis thaliana | 1134 | + | 6 | TATATA |  |

>HU01G00833.1   
+ -Up\_Stream \_Len000AAAATA TTTTTACAAA AGCATTTCTC TATAAAATAT TTTACAAAAT TTCATTTTGT   
  
  
+ GGGTCTTTTA GAATCAATCT CTCTACAAGT AGGAATGCCG GTTGAGGTAA GGCTACCTAC ATCTAGATCT   
  
  
+ CTCCAGACCC CACAAAAATT AAACGAGACC CACACTGAAA GGTGGTGGTG GTGGTGTTGT TGTTGTATTG   
  
  
+ TCCTACCACG AGAGAGCTTC AAGTGCATCA CTTATTTCAA CAGGTCAATC ACATAGATCT CTAGTGTACT   
  
  
+ GTAAATTTTT TTTCCGTCTT ATTTTAATTT TCTTTTCCAT CCTAAAAGAT TATCATGTAG ATATTCCTAG   
  
  
+ TTTGATATAT TGACTTCAAC TTCAAAAATT ACACATTAAG ATCCACGAAT ATGACAGTTT AGGTTTCATT   
  
  
+ TGAAGGAGAA AGAAATGTTA CGGTGTTTTG GTGTTGTATT CGTAGACATT TTTTGCGCTT AACTGGCTCT   
  
  
+ TCTCATGGGG ACGAGACAGT GAAGGTAATT GTACTTGTTG ATTGATGACG TCATCTGTAG CTTGGATTTG   
  
  
+ TATAGGAATG AACATCTTGG TGTCTACGTG GAAAGTGTGA TTTGTGTTCT TTCATTGATC CTCCTCCTCA   
  
  
+ AGAAGAGGTG ATCTTTCTCC CTTGTTTTTT TTTTTTTTTG GCTAATATGT TGGCAGAACC GGCAAAAAAT   
  
  
+ ACATAATTTG AATAGTGCAT GCATATGAGT ACATGACAGT GAACCAACAA AACGAACCCC TAAAACACAA   
  
  
+ GAGAATTAAC ATGTTAAAAC ACTTAAACTA AATGCAACCA TCTCGATGAC CACTAAATAG CCAAGAAACC   
  
  
+ TAACGTAATT AACAAAAATA AAAAATAACT GAAAATAAAG TAGAGAAAGT CTAACCGAAT ACGATAACAT   
  
  
+ AACCTAAAAC TAACTATACT TGAACTTACT AAGAAATATG AATCAACCAC AAACCGAATT GATTTTGGTT   
  
  
+ TGCCATAATT TGACTATACA TTTAACTTAA AAAATATTTT TTTTTAATTT CAAATAAATC TAAATTATTT   
  
  
+ TTCAATATTC ATCCAAAATA GATTCAATTA TTAAACAAGT CAACCTATAC CGAGTTATTT TCTAGCTCTG   
  
  
+ ACTAAGACAT ATATATATTT TTTGTAAAAA AGATAAGGAT TATAATTTAA ATAGCCTTTC CCTTAAAAAA   
  
  
+ ATTTTTAAAA TTATCCTGAT AGAGAATAAA AAGAAATCCT TATACAAAAC TCTGATTGAC GCAGATGTGT   
  
  
+ ATATGGACAT ATAGAAGTAA TAAGAGGGGT AGGCTAGGAA GGGGGTGGAA ATATAGGGCA TAAATAGGGG   
  
  
+ GCGGGCATAG GGACATGTCT GTCTGAGGTA CGGTGGATGG GACCTTGCCA TGGAAGCAAC AATGTCACAA   
  
  
+ TAATAATATC AACGACTGAA GTTTCTTCAC CAGCCCACTC TTTGCCATAA TGCCATTAAT TATTTATTGC   
  
  
+ TACTATCTCT TTTGATGTGA AACGGTGGAC CTTATCTCTC CCCTCCACTT CCAATTGATG TGAGCAACTC   
  
  
+ AGAAACGCAA ACAAAACTTG TGCCACCTTC TTTACTTGCA TCCTCATTTT CCTCTGTCTC TCTCCGCCCC   
  
  
+ TGTTTCTCTC TCTCTCCCTC ACTCACTCAG AGTACTGATC TGAGCATCAG AAGTAGCAGA CCCAGAAACT   
  
  
+ CAGATTTAAA AACAACCCGC TCTTTTGTTC CCTCTACGTC TAATAATTTT GTCATCGCAT TTAATGTCAG   
  
  
+ ATAGAGAGAT AGAGCTCTAC TGTATTAATT TTCTCCCACC TCACTTGATT CTGTTGCCCT AAACCACCCA   
  
  
+ AAAAAAGAAA TACGAAGAAG CCACTCTCTC TCTCTCTCTC TCTCACACAC ACACACACCC CTCCTAGTGG   
  
  
+ ATTTGATAGA GTATAAATGA ACAGTAATCA GACGAGATAT ATAGTAGTGC TTAATTGACC GAGTAGCAAA   
  
  
+ AGGGGGAAGT TGAGTGAGGT ATCAAGAGAG TAGCGTAGCT AGCTAGGGCA AGATATGCTG GGGTCTTCCT   
  
  
+ CATACAACTC CGAGGAAGAC CACCAAGATG ACAACCATTC CTCACCGTCT GATTTGTCTA GGCACCCACC   
  
  
+ TAGATTCACC ACTACTACTT ACCAATACCA ACAACATATT AGCTCTCCAT CTTCACAATC AATACTAGCT   
  
  
+ GTGCCACCTC GCCAATTGTT AATCAATTGT GCGGAGTTAA TTTGCCGGTT CGACTTCTCC TCCGCCAACC   
  
  
+ GGCTTCTCTC TCTCCTCGCC TCCCACTTTG CCTCCCCTTC TGGGGACTCC ACCGAGAGAC TTGTGTTTTA   
  
  
+ CTTCTGCAAG GCTTTGGATC TCCGCCTCAA TAACAGTTAT TTATCCACTC GTAGTTTGAC TGGTTGTAAC   
  
  
+ CCTAGTACTG GTTCTGGTAT TGGGGCTCCT TTAGCTTCCA ATTTTCTATT TCTTCAACCA TTAGTGCATT   
  
  
+ CACAGGTAAC AAGTAATGCC CGTGTTGGGT ACTTGACATA TTTGACTCTG AACCAGGTCA CCCCATTCAT   
  
  
+ CCGATTCACC CACTTAACTG CCAACCAAGC CATCCTGGAA GCTTTGGAGG GGTACAAGGC GGTCCATATC   
  
  
+ CTTGACATGG ACATCATGCA TGGGGTCCAG TGGCCACCAC TTCTTCAAGC AATTGCAGAA AGATCTGCAA   
  
  
+ CCCTTGGTCA CCCTGCTCCC ACTGTTCGCA TCACAGGAGG GGGCAGGGAT CTAGAACTCC TGAACCGAAC   
  
  
+ TGGAGATCGG ATCAGGAAGT TCGCACAATC CTTGGGACTC GAGTTTCAAT TCCACCATCT TATAACGGTG   
  
  
+ GACTCGAACC TTACTGAGGC AGTGGAGGCC ATCAATGCCC TACAGCTACA CCTCCATGTC CAAGAGGAGG   
  
  
+ AGGTCTTCGC AGTGAATTGT GGAGATTTCC TCCACCGCCT CCTAACGGAG TATGATAGTA CATTCCTTAG   
  
  
+ GATGTTTCTC TACAAGGTGA AGACCCTAAA CCCTAGGGTC TTCACCGTGG GAGAAAGGGA GGCCGACCAC   
  
  
+ AACCACCCTC TCTTCTGGCA GCGATTCATT GAGGCACTTG ACCACTATGG GGCAGTGTTT GACTCTCTAG   
  
  
+ AAGCAACCCT ACCATCAACA AGTCAAGAGA GGGTGGCGGT GGAAGAGGGG TGGTTCGGGG AGGAGATTAA   
  
  
+ AGATGTGGTT GGACGAGAAG GAGGGTTGAG GAGACAGAGG CACCAGAAAT ACGAGTCTTG GGAGGTGTTG   
  
  
+ ATGAGGAGCT CTGGGTTTAA GAGCTTGCCC TTGAGCCCCT TCTCTGTGTC ACAAGCCAAG CTGCTACTTC   
  
  
+ GCCTTCATTA CCCTTCTGAA GGGTACCACC TTAAGGTCTT CCATAGTAAT TGTCTGTTGC TTGGGTGGAA   
  
  
+ GAATCGCCCT CTTTTCTCTG TTTCTTCTTG GCAATA  

- -Up\_Stream \_Len000TTTTAT AAAAATGTTT TCGTAAAGAG ATATTTTATA AAATGTTTTA AAGTAAAACA   
  
  
- CCCAGAAAAT CTTAGTTAGA GAGATGTTCA TCCTTACGGC CAACTCCATT CCGATGGATG TAGATCTAGA   
  
  
- GAGGTCTGGG GTGTTTTTAA TTTGCTCTGG GTGTGACTTT CCACCACCAC CACCACAACA ACAACATAAC   
  
  
- AGGATGGTGC TCTCTCGAAG TTCACGTAGT GAATAAAGTT GTCCAGTTAG TGTATCTAGA GATCACATGA   
  
  
- CATTTAAAAA AAAGGCAGAA TAAAATTAAA AGAAAAGGTA GGATTTTCTA ATAGTACATC TATAAGGATC   
  
  
- AAACTATATA ACTGAAGTTG AAGTTTTTAA TGTGTAATTC TAGGTGCTTA TACTGTCAAA TCCAAAGTAA   
  
  
- ACTTCCTCTT TCTTTACAAT GCCACAAAAC CACAACATAA GCATCTGTAA AAAACGCGAA TTGACCGAGA   
  
  
- AGAGTACCCC TGCTCTGTCA CTTCCATTAA CATGAACAAC TAACTACTGC AGTAGACATC GAACCTAAAC   
  
  
- ATATCCTTAC TTGTAGAACC ACAGATGCAC CTTTCACACT AAACACAAGA AAGTAACTAG GAGGAGGAGT   
  
  
- TCTTCTCCAC TAGAAAGAGG GAACAAAAAA AAAAAAAAAC CGATTATACA ACCGTCTTGG CCGTTTTTTA   
  
  
- TGTATTAAAC TTATCACGTA CGTATACTCA TGTACTGTCA CTTGGTTGTT TTGCTTGGGG ATTTTGTGTT   
  
  
- CTCTTAATTG TACAATTTTG TGAATTTGAT TTACGTTGGT AGAGCTACTG GTGATTTATC GGTTCTTTGG   
  
  
- ATTGCATTAA TTGTTTTTAT TTTTTATTGA CTTTTATTTC ATCTCTTTCA GATTGGCTTA TGCTATTGTA   
  
  
- TTGGATTTTG ATTGATATGA ACTTGAATGA TTCTTTATAC TTAGTTGGTG TTTGGCTTAA CTAAAACCAA   
  
  
- ACGGTATTAA ACTGATATGT AAATTGAATT TTTTATAAAA AAAAATTAAA GTTTATTTAG ATTTAATAAA   
  
  
- AAGTTATAAG TAGGTTTTAT CTAAGTTAAT AATTTGTTCA GTTGGATATG GCTCAATAAA AGATCGAGAC   
  
  
- TGATTCTGTA TATATATAAA AAACATTTTT TCTATTCCTA ATATTAAATT TATCGGAAAG GGAATTTTTT   
  
  
- TAAAAATTTT AATAGGACTA TCTCTTATTT TTCTTTAGGA ATATGTTTTG AGACTAACTG CGTCTACACA   
  
  
- TATACCTGTA TATCTTCATT ATTCTCCCCA TCCGATCCTT CCCCCACCTT TATATCCCGT ATTTATCCCC   
  
  
- CGCCCGTATC CCTGTACAGA CAGACTCCAT GCCACCTACC CTGGAACGGT ACCTTCGTTG TTACAGTGTT   
  
  
- ATTATTATAG TTGCTGACTT CAAAGAAGTG GTCGGGTGAG AAACGGTATT ACGGTAATTA ATAAATAACG   
  
  
- ATGATAGAGA AAACTACACT TTGCCACCTG GAATAGAGAG GGGAGGTGAA GGTTAACTAC ACTCGTTGAG   
  
  
- TCTTTGCGTT TGTTTTGAAC ACGGTGGAAG AAATGAACGT AGGAGTAAAA GGAGACAGAG AGAGGCGGGG   
  
  
- ACAAAGAGAG AGAGAGGGAG TGAGTGAGTC TCATGACTAG ACTCGTAGTC TTCATCGTCT GGGTCTTTGA   
  
  
- GTCTAAATTT TTGTTGGGCG AGAAAACAAG GGAGATGCAG ATTATTAAAA CAGTAGCGTA AATTACAGTC   
  
  
- TATCTCTCTA TCTCGAGATG ACATAATTAA AAGAGGGTGG AGTGAACTAA GACAACGGGA TTTGGTGGGT   
  
  
- TTTTTTCTTT ATGCTTCTTC GGTGAGAGAG AGAGAGAGAG AGAGTGTGTG TGTGTGTGGG GAGGATCACC   
  
  
- TAAACTATCT CATATTTACT TGTCATTAGT CTGCTCTATA TATCATCACG AATTAACTGG CTCATCGTTT   
  
  
- TCCCCCTTCA ACTCACTCCA TAGTTCTCTC ATCGCATCGA TCGATCCCGT TCTATACGAC CCCAGAAGGA   
  
  
- GTATGTTGAG GCTCCTTCTG GTGGTTCTAC TGTTGGTAAG GAGTGGCAGA CTAAACAGAT CCGTGGGTGG   
  
  
- ATCTAAGTGG TGATGATGAA TGGTTATGGT TGTTGTATAA TCGAGAGGTA GAAGTGTTAG TTATGATCGA   
  
  
- CACGGTGGAG CGGTTAACAA TTAGTTAACA CGCCTCAATT AAACGGCCAA GCTGAAGAGG AGGCGGTTGG   
  
  
- CCGAAGAGAG AGAGGAGCGG AGGGTGAAAC GGAGGGGAAG ACCCCTGAGG TGGCTCTCTG AACACAAAAT   
  
  
- GAAGACGTTC CGAAACCTAG AGGCGGAGTT ATTGTCAATA AATAGGTGAG CATCAAACTG ACCAACATTG   
  
  
- GGATCATGAC CAAGACCATA ACCCCGAGGA AATCGAAGGT TAAAAGATAA AGAAGTTGGT AATCACGTAA   
  
  
- GTGTCCATTG TTCATTACGG GCACAACCCA TGAACTGTAT AAACTGAGAC TTGGTCCAGT GGGGTAAGTA   
  
  
- GGCTAAGTGG GTGAATTGAC GGTTGGTTCG GTAGGACCTT CGAAACCTCC CCATGTTCCG CCAGGTATAG   
  
  
- GAACTGTACC TGTAGTACGT ACCCCAGGTC ACCGGTGGTG AAGAAGTTCG TTAACGTCTT TCTAGACGTT   
  
  
- GGGAACCAGT GGGACGAGGG TGACAAGCGT AGTGTCCTCC CCCGTCCCTA GATCTTGAGG ACTTGGCTTG   
  
  
- ACCTCTAGCC TAGTCCTTCA AGCGTGTTAG GAACCCTGAG CTCAAAGTTA AGGTGGTAGA ATATTGCCAC   
  
  
- CTGAGCTTGG AATGACTCCG TCACCTCCGG TAGTTACGGG ATGTCGATGT GGAGGTACAG GTTCTCCTCC   
  
  
- TCCAGAAGCG TCACTTAACA CCTCTAAAGG AGGTGGCGGA GGATTGCCTC ATACTATCAT GTAAGGAATC   
  
  
- CTACAAAGAG ATGTTCCACT TCTGGGATTT GGGATCCCAG AAGTGGCACC CTCTTTCCCT CCGGCTGGTG   
  
  
- TTGGTGGGAG AGAAGACCGT CGCTAAGTAA CTCCGTGAAC TGGTGATACC CCGTCACAAA CTGAGAGATC   
  
  
- TTCGTTGGGA TGGTAGTTGT TCAGTTCTCT CCCACCGCCA CCTTCTCCCC ACCAAGCCCC TCCTCTAATT   
  
  
- TCTACACCAA CCTGCTCTTC CTCCCAACTC CTCTGTCTCC GTGGTCTTTA TGCTCAGAAC CCTCCACAAC   
  
  
- TACTCCTCGA GACCCAAATT CTCGAACGGG AACTCGGGGA AGAGACACAG TGTTCGGTTC GACGATGAAG   
  
  
- CGGAAGTAAT GGGAAGACTT CCCATGGTGG AATTCCAGAA GGTATCATTA ACAGACAACG AACCCACCTT   
  
  
- CTTAGCGGGA GAAAAGAGAC AAAGAAGAAC CGTTAT

+     AuxRR-core

| Site Name | Organism | Position | Strand | Matrix score. | sequence | function |
| --- | --- | --- | --- | --- | --- | --- |
| AuxRR-core | Nicotiana tabacum | 2585 | + | 7 | GGTCCAT | cis-acting regulatory element involved in auxin responsiveness |

>HU01G00833.1   
+ -Up\_Stream \_Len000AAAATA TTTTTACAAA AGCATTTCTC TATAAAATAT TTTACAAAAT TTCATTTTGT   
  
  
+ GGGTCTTTTA GAATCAATCT CTCTACAAGT AGGAATGCCG GTTGAGGTAA GGCTACCTAC ATCTAGATCT   
  
  
+ CTCCAGACCC CACAAAAATT AAACGAGACC CACACTGAAA GGTGGTGGTG GTGGTGTTGT TGTTGTATTG   
  
  
+ TCCTACCACG AGAGAGCTTC AAGTGCATCA CTTATTTCAA CAGGTCAATC ACATAGATCT CTAGTGTACT   
  
  
+ GTAAATTTTT TTTCCGTCTT ATTTTAATTT TCTTTTCCAT CCTAAAAGAT TATCATGTAG ATATTCCTAG   
  
  
+ TTTGATATAT TGACTTCAAC TTCAAAAATT ACACATTAAG ATCCACGAAT ATGACAGTTT AGGTTTCATT   
  
  
+ TGAAGGAGAA AGAAATGTTA CGGTGTTTTG GTGTTGTATT CGTAGACATT TTTTGCGCTT AACTGGCTCT   
  
  
+ TCTCATGGGG ACGAGACAGT GAAGGTAATT GTACTTGTTG ATTGATGACG TCATCTGTAG CTTGGATTTG   
  
  
+ TATAGGAATG AACATCTTGG TGTCTACGTG GAAAGTGTGA TTTGTGTTCT TTCATTGATC CTCCTCCTCA   
  
  
+ AGAAGAGGTG ATCTTTCTCC CTTGTTTTTT TTTTTTTTTG GCTAATATGT TGGCAGAACC GGCAAAAAAT   
  
  
+ ACATAATTTG AATAGTGCAT GCATATGAGT ACATGACAGT GAACCAACAA AACGAACCCC TAAAACACAA   
  
  
+ GAGAATTAAC ATGTTAAAAC ACTTAAACTA AATGCAACCA TCTCGATGAC CACTAAATAG CCAAGAAACC   
  
  
+ TAACGTAATT AACAAAAATA AAAAATAACT GAAAATAAAG TAGAGAAAGT CTAACCGAAT ACGATAACAT   
  
  
+ AACCTAAAAC TAACTATACT TGAACTTACT AAGAAATATG AATCAACCAC AAACCGAATT GATTTTGGTT   
  
  
+ TGCCATAATT TGACTATACA TTTAACTTAA AAAATATTTT TTTTTAATTT CAAATAAATC TAAATTATTT   
  
  
+ TTCAATATTC ATCCAAAATA GATTCAATTA TTAAACAAGT CAACCTATAC CGAGTTATTT TCTAGCTCTG   
  
  
+ ACTAAGACAT ATATATATTT TTTGTAAAAA AGATAAGGAT TATAATTTAA ATAGCCTTTC CCTTAAAAAA   
  
  
+ ATTTTTAAAA TTATCCTGAT AGAGAATAAA AAGAAATCCT TATACAAAAC TCTGATTGAC GCAGATGTGT   
  
  
+ ATATGGACAT ATAGAAGTAA TAAGAGGGGT AGGCTAGGAA GGGGGTGGAA ATATAGGGCA TAAATAGGGG   
  
  
+ GCGGGCATAG GGACATGTCT GTCTGAGGTA CGGTGGATGG GACCTTGCCA TGGAAGCAAC AATGTCACAA   
  
  
+ TAATAATATC AACGACTGAA GTTTCTTCAC CAGCCCACTC TTTGCCATAA TGCCATTAAT TATTTATTGC   
  
  
+ TACTATCTCT TTTGATGTGA AACGGTGGAC CTTATCTCTC CCCTCCACTT CCAATTGATG TGAGCAACTC   
  
  
+ AGAAACGCAA ACAAAACTTG TGCCACCTTC TTTACTTGCA TCCTCATTTT CCTCTGTCTC TCTCCGCCCC   
  
  
+ TGTTTCTCTC TCTCTCCCTC ACTCACTCAG AGTACTGATC TGAGCATCAG AAGTAGCAGA CCCAGAAACT   
  
  
+ CAGATTTAAA AACAACCCGC TCTTTTGTTC CCTCTACGTC TAATAATTTT GTCATCGCAT TTAATGTCAG   
  
  
+ ATAGAGAGAT AGAGCTCTAC TGTATTAATT TTCTCCCACC TCACTTGATT CTGTTGCCCT AAACCACCCA   
  
  
+ AAAAAAGAAA TACGAAGAAG CCACTCTCTC TCTCTCTCTC TCTCACACAC ACACACACCC CTCCTAGTGG   
  
  
+ ATTTGATAGA GTATAAATGA ACAGTAATCA GACGAGATAT ATAGTAGTGC TTAATTGACC GAGTAGCAAA   
  
  
+ AGGGGGAAGT TGAGTGAGGT ATCAAGAGAG TAGCGTAGCT AGCTAGGGCA AGATATGCTG GGGTCTTCCT   
  
  
+ CATACAACTC CGAGGAAGAC CACCAAGATG ACAACCATTC CTCACCGTCT GATTTGTCTA GGCACCCACC   
  
  
+ TAGATTCACC ACTACTACTT ACCAATACCA ACAACATATT AGCTCTCCAT CTTCACAATC AATACTAGCT   
  
  
+ GTGCCACCTC GCCAATTGTT AATCAATTGT GCGGAGTTAA TTTGCCGGTT CGACTTCTCC TCCGCCAACC   
  
  
+ GGCTTCTCTC TCTCCTCGCC TCCCACTTTG CCTCCCCTTC TGGGGACTCC ACCGAGAGAC TTGTGTTTTA   
  
  
+ CTTCTGCAAG GCTTTGGATC TCCGCCTCAA TAACAGTTAT TTATCCACTC GTAGTTTGAC TGGTTGTAAC   
  
  
+ CCTAGTACTG GTTCTGGTAT TGGGGCTCCT TTAGCTTCCA ATTTTCTATT TCTTCAACCA TTAGTGCATT   
  
  
+ CACAGGTAAC AAGTAATGCC CGTGTTGGGT ACTTGACATA TTTGACTCTG AACCAGGTCA CCCCATTCAT   
  
  
+ CCGATTCACC CACTTAACTG CCAACCAAGC CATCCTGGAA GCTTTGGAGG GGTACAAGGC GGTCCATATC   
  
  
+ CTTGACATGG ACATCATGCA TGGGGTCCAG TGGCCACCAC TTCTTCAAGC AATTGCAGAA AGATCTGCAA   
  
  
+ CCCTTGGTCA CCCTGCTCCC ACTGTTCGCA TCACAGGAGG GGGCAGGGAT CTAGAACTCC TGAACCGAAC   
  
  
+ TGGAGATCGG ATCAGGAAGT TCGCACAATC CTTGGGACTC GAGTTTCAAT TCCACCATCT TATAACGGTG   
  
  
+ GACTCGAACC TTACTGAGGC AGTGGAGGCC ATCAATGCCC TACAGCTACA CCTCCATGTC CAAGAGGAGG   
  
  
+ AGGTCTTCGC AGTGAATTGT GGAGATTTCC TCCACCGCCT CCTAACGGAG TATGATAGTA CATTCCTTAG   
  
  
+ GATGTTTCTC TACAAGGTGA AGACCCTAAA CCCTAGGGTC TTCACCGTGG GAGAAAGGGA GGCCGACCAC   
  
  
+ AACCACCCTC TCTTCTGGCA GCGATTCATT GAGGCACTTG ACCACTATGG GGCAGTGTTT GACTCTCTAG   
  
  
+ AAGCAACCCT ACCATCAACA AGTCAAGAGA GGGTGGCGGT GGAAGAGGGG TGGTTCGGGG AGGAGATTAA   
  
  
+ AGATGTGGTT GGACGAGAAG GAGGGTTGAG GAGACAGAGG CACCAGAAAT ACGAGTCTTG GGAGGTGTTG   
  
  
+ ATGAGGAGCT CTGGGTTTAA GAGCTTGCCC TTGAGCCCCT TCTCTGTGTC ACAAGCCAAG CTGCTACTTC   
  
  
+ GCCTTCATTA CCCTTCTGAA GGGTACCACC TTAAGGTCTT CCATAGTAAT TGTCTGTTGC TTGGGTGGAA   
  
  
+ GAATCGCCCT CTTTTCTCTG TTTCTTCTTG GCAATA  

- -Up\_Stream \_Len000TTTTAT AAAAATGTTT TCGTAAAGAG ATATTTTATA AAATGTTTTA AAGTAAAACA   
  
  
- CCCAGAAAAT CTTAGTTAGA GAGATGTTCA TCCTTACGGC CAACTCCATT CCGATGGATG TAGATCTAGA   
  
  
- GAGGTCTGGG GTGTTTTTAA TTTGCTCTGG GTGTGACTTT CCACCACCAC CACCACAACA ACAACATAAC   
  
  
- AGGATGGTGC TCTCTCGAAG TTCACGTAGT GAATAAAGTT GTCCAGTTAG TGTATCTAGA GATCACATGA   
  
  
- CATTTAAAAA AAAGGCAGAA TAAAATTAAA AGAAAAGGTA GGATTTTCTA ATAGTACATC TATAAGGATC   
  
  
- AAACTATATA ACTGAAGTTG AAGTTTTTAA TGTGTAATTC TAGGTGCTTA TACTGTCAAA TCCAAAGTAA   
  
  
- ACTTCCTCTT TCTTTACAAT GCCACAAAAC CACAACATAA GCATCTGTAA AAAACGCGAA TTGACCGAGA   
  
  
- AGAGTACCCC TGCTCTGTCA CTTCCATTAA CATGAACAAC TAACTACTGC AGTAGACATC GAACCTAAAC   
  
  
- ATATCCTTAC TTGTAGAACC ACAGATGCAC CTTTCACACT AAACACAAGA AAGTAACTAG GAGGAGGAGT   
  
  
- TCTTCTCCAC TAGAAAGAGG GAACAAAAAA AAAAAAAAAC CGATTATACA ACCGTCTTGG CCGTTTTTTA   
  
  
- TGTATTAAAC TTATCACGTA CGTATACTCA TGTACTGTCA CTTGGTTGTT TTGCTTGGGG ATTTTGTGTT   
  
  
- CTCTTAATTG TACAATTTTG TGAATTTGAT TTACGTTGGT AGAGCTACTG GTGATTTATC GGTTCTTTGG   
  
  
- ATTGCATTAA TTGTTTTTAT TTTTTATTGA CTTTTATTTC ATCTCTTTCA GATTGGCTTA TGCTATTGTA   
  
  
- TTGGATTTTG ATTGATATGA ACTTGAATGA TTCTTTATAC TTAGTTGGTG TTTGGCTTAA CTAAAACCAA   
  
  
- ACGGTATTAA ACTGATATGT AAATTGAATT TTTTATAAAA AAAAATTAAA GTTTATTTAG ATTTAATAAA   
  
  
- AAGTTATAAG TAGGTTTTAT CTAAGTTAAT AATTTGTTCA GTTGGATATG GCTCAATAAA AGATCGAGAC   
  
  
- TGATTCTGTA TATATATAAA AAACATTTTT TCTATTCCTA ATATTAAATT TATCGGAAAG GGAATTTTTT   
  
  
- TAAAAATTTT AATAGGACTA TCTCTTATTT TTCTTTAGGA ATATGTTTTG AGACTAACTG CGTCTACACA   
  
  
- TATACCTGTA TATCTTCATT ATTCTCCCCA TCCGATCCTT CCCCCACCTT TATATCCCGT ATTTATCCCC   
  
  
- CGCCCGTATC CCTGTACAGA CAGACTCCAT GCCACCTACC CTGGAACGGT ACCTTCGTTG TTACAGTGTT   
  
  
- ATTATTATAG TTGCTGACTT CAAAGAAGTG GTCGGGTGAG AAACGGTATT ACGGTAATTA ATAAATAACG   
  
  
- ATGATAGAGA AAACTACACT TTGCCACCTG GAATAGAGAG GGGAGGTGAA GGTTAACTAC ACTCGTTGAG   
  
  
- TCTTTGCGTT TGTTTTGAAC ACGGTGGAAG AAATGAACGT AGGAGTAAAA GGAGACAGAG AGAGGCGGGG   
  
  
- ACAAAGAGAG AGAGAGGGAG TGAGTGAGTC TCATGACTAG ACTCGTAGTC TTCATCGTCT GGGTCTTTGA   
  
  
- GTCTAAATTT TTGTTGGGCG AGAAAACAAG GGAGATGCAG ATTATTAAAA CAGTAGCGTA AATTACAGTC   
  
  
- TATCTCTCTA TCTCGAGATG ACATAATTAA AAGAGGGTGG AGTGAACTAA GACAACGGGA TTTGGTGGGT   
  
  
- TTTTTTCTTT ATGCTTCTTC GGTGAGAGAG AGAGAGAGAG AGAGTGTGTG TGTGTGTGGG GAGGATCACC   
  
  
- TAAACTATCT CATATTTACT TGTCATTAGT CTGCTCTATA TATCATCACG AATTAACTGG CTCATCGTTT   
  
  
- TCCCCCTTCA ACTCACTCCA TAGTTCTCTC ATCGCATCGA TCGATCCCGT TCTATACGAC CCCAGAAGGA   
  
  
- GTATGTTGAG GCTCCTTCTG GTGGTTCTAC TGTTGGTAAG GAGTGGCAGA CTAAACAGAT CCGTGGGTGG   
  
  
- ATCTAAGTGG TGATGATGAA TGGTTATGGT TGTTGTATAA TCGAGAGGTA GAAGTGTTAG TTATGATCGA   
  
  
- CACGGTGGAG CGGTTAACAA TTAGTTAACA CGCCTCAATT AAACGGCCAA GCTGAAGAGG AGGCGGTTGG   
  
  
- CCGAAGAGAG AGAGGAGCGG AGGGTGAAAC GGAGGGGAAG ACCCCTGAGG TGGCTCTCTG AACACAAAAT   
  
  
- GAAGACGTTC CGAAACCTAG AGGCGGAGTT ATTGTCAATA AATAGGTGAG CATCAAACTG ACCAACATTG   
  
  
- GGATCATGAC CAAGACCATA ACCCCGAGGA AATCGAAGGT TAAAAGATAA AGAAGTTGGT AATCACGTAA   
  
  
- GTGTCCATTG TTCATTACGG GCACAACCCA TGAACTGTAT AAACTGAGAC TTGGTCCAGT GGGGTAAGTA   
  
  
- GGCTAAGTGG GTGAATTGAC GGTTGGTTCG GTAGGACCTT CGAAACCTCC CCATGTTCCG CCAGGTATAG   
  
  
- GAACTGTACC TGTAGTACGT ACCCCAGGTC ACCGGTGGTG AAGAAGTTCG TTAACGTCTT TCTAGACGTT   
  
  
- GGGAACCAGT GGGACGAGGG TGACAAGCGT AGTGTCCTCC CCCGTCCCTA GATCTTGAGG ACTTGGCTTG   
  
  
- ACCTCTAGCC TAGTCCTTCA AGCGTGTTAG GAACCCTGAG CTCAAAGTTA AGGTGGTAGA ATATTGCCAC   
  
  
- CTGAGCTTGG AATGACTCCG TCACCTCCGG TAGTTACGGG ATGTCGATGT GGAGGTACAG GTTCTCCTCC   
  
  
- TCCAGAAGCG TCACTTAACA CCTCTAAAGG AGGTGGCGGA GGATTGCCTC ATACTATCAT GTAAGGAATC   
  
  
- CTACAAAGAG ATGTTCCACT TCTGGGATTT GGGATCCCAG AAGTGGCACC CTCTTTCCCT CCGGCTGGTG   
  
  
- TTGGTGGGAG AGAAGACCGT CGCTAAGTAA CTCCGTGAAC TGGTGATACC CCGTCACAAA CTGAGAGATC   
  
  
- TTCGTTGGGA TGGTAGTTGT TCAGTTCTCT CCCACCGCCA CCTTCTCCCC ACCAAGCCCC TCCTCTAATT   
  
  
- TCTACACCAA CCTGCTCTTC CTCCCAACTC CTCTGTCTCC GTGGTCTTTA TGCTCAGAAC CCTCCACAAC   
  
  
- TACTCCTCGA GACCCAAATT CTCGAACGGG AACTCGGGGA AGAGACACAG TGTTCGGTTC GACGATGAAG   
  
  
- CGGAAGTAAT GGGAAGACTT CCCATGGTGG AATTCCAGAA GGTATCATTA ACAGACAACG AACCCACCTT   
  
  
- CTTAGCGGGA GAAAAGAGAC AAAGAAGAAC CGTTAT

+     Box 4

| Site Name | Organism | Position | Strand | Matrix score. | sequence | function |
| --- | --- | --- | --- | --- | --- | --- |
| Box 4 | Petroselinum crispum | 1778 | - | 6 | ATTAAT | part of a conserved DNA module involved in light responsiveness |
| Box 4 | Petroselinum crispum | 1459 | + | 6 | ATTAAT | part of a conserved DNA module involved in light responsiveness |

>HU01G00833.1   
+ -Up\_Stream \_Len000AAAATA TTTTTACAAA AGCATTTCTC TATAAAATAT TTTACAAAAT TTCATTTTGT   
  
  
+ GGGTCTTTTA GAATCAATCT CTCTACAAGT AGGAATGCCG GTTGAGGTAA GGCTACCTAC ATCTAGATCT   
  
  
+ CTCCAGACCC CACAAAAATT AAACGAGACC CACACTGAAA GGTGGTGGTG GTGGTGTTGT TGTTGTATTG   
  
  
+ TCCTACCACG AGAGAGCTTC AAGTGCATCA CTTATTTCAA CAGGTCAATC ACATAGATCT CTAGTGTACT   
  
  
+ GTAAATTTTT TTTCCGTCTT ATTTTAATTT TCTTTTCCAT CCTAAAAGAT TATCATGTAG ATATTCCTAG   
  
  
+ TTTGATATAT TGACTTCAAC TTCAAAAATT ACACATTAAG ATCCACGAAT ATGACAGTTT AGGTTTCATT   
  
  
+ TGAAGGAGAA AGAAATGTTA CGGTGTTTTG GTGTTGTATT CGTAGACATT TTTTGCGCTT AACTGGCTCT   
  
  
+ TCTCATGGGG ACGAGACAGT GAAGGTAATT GTACTTGTTG ATTGATGACG TCATCTGTAG CTTGGATTTG   
  
  
+ TATAGGAATG AACATCTTGG TGTCTACGTG GAAAGTGTGA TTTGTGTTCT TTCATTGATC CTCCTCCTCA   
  
  
+ AGAAGAGGTG ATCTTTCTCC CTTGTTTTTT TTTTTTTTTG GCTAATATGT TGGCAGAACC GGCAAAAAAT   
  
  
+ ACATAATTTG AATAGTGCAT GCATATGAGT ACATGACAGT GAACCAACAA AACGAACCCC TAAAACACAA   
  
  
+ GAGAATTAAC ATGTTAAAAC ACTTAAACTA AATGCAACCA TCTCGATGAC CACTAAATAG CCAAGAAACC   
  
  
+ TAACGTAATT AACAAAAATA AAAAATAACT GAAAATAAAG TAGAGAAAGT CTAACCGAAT ACGATAACAT   
  
  
+ AACCTAAAAC TAACTATACT TGAACTTACT AAGAAATATG AATCAACCAC AAACCGAATT GATTTTGGTT   
  
  
+ TGCCATAATT TGACTATACA TTTAACTTAA AAAATATTTT TTTTTAATTT CAAATAAATC TAAATTATTT   
  
  
+ TTCAATATTC ATCCAAAATA GATTCAATTA TTAAACAAGT CAACCTATAC CGAGTTATTT TCTAGCTCTG   
  
  
+ ACTAAGACAT ATATATATTT TTTGTAAAAA AGATAAGGAT TATAATTTAA ATAGCCTTTC CCTTAAAAAA   
  
  
+ ATTTTTAAAA TTATCCTGAT AGAGAATAAA AAGAAATCCT TATACAAAAC TCTGATTGAC GCAGATGTGT   
  
  
+ ATATGGACAT ATAGAAGTAA TAAGAGGGGT AGGCTAGGAA GGGGGTGGAA ATATAGGGCA TAAATAGGGG   
  
  
+ GCGGGCATAG GGACATGTCT GTCTGAGGTA CGGTGGATGG GACCTTGCCA TGGAAGCAAC AATGTCACAA   
  
  
+ TAATAATATC AACGACTGAA GTTTCTTCAC CAGCCCACTC TTTGCCATAA TGCCATTAAT TATTTATTGC   
  
  
+ TACTATCTCT TTTGATGTGA AACGGTGGAC CTTATCTCTC CCCTCCACTT CCAATTGATG TGAGCAACTC   
  
  
+ AGAAACGCAA ACAAAACTTG TGCCACCTTC TTTACTTGCA TCCTCATTTT CCTCTGTCTC TCTCCGCCCC   
  
  
+ TGTTTCTCTC TCTCTCCCTC ACTCACTCAG AGTACTGATC TGAGCATCAG AAGTAGCAGA CCCAGAAACT   
  
  
+ CAGATTTAAA AACAACCCGC TCTTTTGTTC CCTCTACGTC TAATAATTTT GTCATCGCAT TTAATGTCAG   
  
  
+ ATAGAGAGAT AGAGCTCTAC TGTATTAATT TTCTCCCACC TCACTTGATT CTGTTGCCCT AAACCACCCA   
  
  
+ AAAAAAGAAA TACGAAGAAG CCACTCTCTC TCTCTCTCTC TCTCACACAC ACACACACCC CTCCTAGTGG   
  
  
+ ATTTGATAGA GTATAAATGA ACAGTAATCA GACGAGATAT ATAGTAGTGC TTAATTGACC GAGTAGCAAA   
  
  
+ AGGGGGAAGT TGAGTGAGGT ATCAAGAGAG TAGCGTAGCT AGCTAGGGCA AGATATGCTG GGGTCTTCCT   
  
  
+ CATACAACTC CGAGGAAGAC CACCAAGATG ACAACCATTC CTCACCGTCT GATTTGTCTA GGCACCCACC   
  
  
+ TAGATTCACC ACTACTACTT ACCAATACCA ACAACATATT AGCTCTCCAT CTTCACAATC AATACTAGCT   
  
  
+ GTGCCACCTC GCCAATTGTT AATCAATTGT GCGGAGTTAA TTTGCCGGTT CGACTTCTCC TCCGCCAACC   
  
  
+ GGCTTCTCTC TCTCCTCGCC TCCCACTTTG CCTCCCCTTC TGGGGACTCC ACCGAGAGAC TTGTGTTTTA   
  
  
+ CTTCTGCAAG GCTTTGGATC TCCGCCTCAA TAACAGTTAT TTATCCACTC GTAGTTTGAC TGGTTGTAAC   
  
  
+ CCTAGTACTG GTTCTGGTAT TGGGGCTCCT TTAGCTTCCA ATTTTCTATT TCTTCAACCA TTAGTGCATT   
  
  
+ CACAGGTAAC AAGTAATGCC CGTGTTGGGT ACTTGACATA TTTGACTCTG AACCAGGTCA CCCCATTCAT   
  
  
+ CCGATTCACC CACTTAACTG CCAACCAAGC CATCCTGGAA GCTTTGGAGG GGTACAAGGC GGTCCATATC   
  
  
+ CTTGACATGG ACATCATGCA TGGGGTCCAG TGGCCACCAC TTCTTCAAGC AATTGCAGAA AGATCTGCAA   
  
  
+ CCCTTGGTCA CCCTGCTCCC ACTGTTCGCA TCACAGGAGG GGGCAGGGAT CTAGAACTCC TGAACCGAAC   
  
  
+ TGGAGATCGG ATCAGGAAGT TCGCACAATC CTTGGGACTC GAGTTTCAAT TCCACCATCT TATAACGGTG   
  
  
+ GACTCGAACC TTACTGAGGC AGTGGAGGCC ATCAATGCCC TACAGCTACA CCTCCATGTC CAAGAGGAGG   
  
  
+ AGGTCTTCGC AGTGAATTGT GGAGATTTCC TCCACCGCCT CCTAACGGAG TATGATAGTA CATTCCTTAG   
  
  
+ GATGTTTCTC TACAAGGTGA AGACCCTAAA CCCTAGGGTC TTCACCGTGG GAGAAAGGGA GGCCGACCAC   
  
  
+ AACCACCCTC TCTTCTGGCA GCGATTCATT GAGGCACTTG ACCACTATGG GGCAGTGTTT GACTCTCTAG   
  
  
+ AAGCAACCCT ACCATCAACA AGTCAAGAGA GGGTGGCGGT GGAAGAGGGG TGGTTCGGGG AGGAGATTAA   
  
  
+ AGATGTGGTT GGACGAGAAG GAGGGTTGAG GAGACAGAGG CACCAGAAAT ACGAGTCTTG GGAGGTGTTG   
  
  
+ ATGAGGAGCT CTGGGTTTAA GAGCTTGCCC TTGAGCCCCT TCTCTGTGTC ACAAGCCAAG CTGCTACTTC   
  
  
+ GCCTTCATTA CCCTTCTGAA GGGTACCACC TTAAGGTCTT CCATAGTAAT TGTCTGTTGC TTGGGTGGAA   
  
  
+ GAATCGCCCT CTTTTCTCTG TTTCTTCTTG GCAATA  

- -Up\_Stream \_Len000TTTTAT AAAAATGTTT TCGTAAAGAG ATATTTTATA AAATGTTTTA AAGTAAAACA   
  
  
- CCCAGAAAAT CTTAGTTAGA GAGATGTTCA TCCTTACGGC CAACTCCATT CCGATGGATG TAGATCTAGA   
  
  
- GAGGTCTGGG GTGTTTTTAA TTTGCTCTGG GTGTGACTTT CCACCACCAC CACCACAACA ACAACATAAC   
  
  
- AGGATGGTGC TCTCTCGAAG TTCACGTAGT GAATAAAGTT GTCCAGTTAG TGTATCTAGA GATCACATGA   
  
  
- CATTTAAAAA AAAGGCAGAA TAAAATTAAA AGAAAAGGTA GGATTTTCTA ATAGTACATC TATAAGGATC   
  
  
- AAACTATATA ACTGAAGTTG AAGTTTTTAA TGTGTAATTC TAGGTGCTTA TACTGTCAAA TCCAAAGTAA   
  
  
- ACTTCCTCTT TCTTTACAAT GCCACAAAAC CACAACATAA GCATCTGTAA AAAACGCGAA TTGACCGAGA   
  
  
- AGAGTACCCC TGCTCTGTCA CTTCCATTAA CATGAACAAC TAACTACTGC AGTAGACATC GAACCTAAAC   
  
  
- ATATCCTTAC TTGTAGAACC ACAGATGCAC CTTTCACACT AAACACAAGA AAGTAACTAG GAGGAGGAGT   
  
  
- TCTTCTCCAC TAGAAAGAGG GAACAAAAAA AAAAAAAAAC CGATTATACA ACCGTCTTGG CCGTTTTTTA   
  
  
- TGTATTAAAC TTATCACGTA CGTATACTCA TGTACTGTCA CTTGGTTGTT TTGCTTGGGG ATTTTGTGTT   
  
  
- CTCTTAATTG TACAATTTTG TGAATTTGAT TTACGTTGGT AGAGCTACTG GTGATTTATC GGTTCTTTGG   
  
  
- ATTGCATTAA TTGTTTTTAT TTTTTATTGA CTTTTATTTC ATCTCTTTCA GATTGGCTTA TGCTATTGTA   
  
  
- TTGGATTTTG ATTGATATGA ACTTGAATGA TTCTTTATAC TTAGTTGGTG TTTGGCTTAA CTAAAACCAA   
  
  
- ACGGTATTAA ACTGATATGT AAATTGAATT TTTTATAAAA AAAAATTAAA GTTTATTTAG ATTTAATAAA   
  
  
- AAGTTATAAG TAGGTTTTAT CTAAGTTAAT AATTTGTTCA GTTGGATATG GCTCAATAAA AGATCGAGAC   
  
  
- TGATTCTGTA TATATATAAA AAACATTTTT TCTATTCCTA ATATTAAATT TATCGGAAAG GGAATTTTTT   
  
  
- TAAAAATTTT AATAGGACTA TCTCTTATTT TTCTTTAGGA ATATGTTTTG AGACTAACTG CGTCTACACA   
  
  
- TATACCTGTA TATCTTCATT ATTCTCCCCA TCCGATCCTT CCCCCACCTT TATATCCCGT ATTTATCCCC   
  
  
- CGCCCGTATC CCTGTACAGA CAGACTCCAT GCCACCTACC CTGGAACGGT ACCTTCGTTG TTACAGTGTT   
  
  
- ATTATTATAG TTGCTGACTT CAAAGAAGTG GTCGGGTGAG AAACGGTATT ACGGTAATTA ATAAATAACG   
  
  
- ATGATAGAGA AAACTACACT TTGCCACCTG GAATAGAGAG GGGAGGTGAA GGTTAACTAC ACTCGTTGAG   
  
  
- TCTTTGCGTT TGTTTTGAAC ACGGTGGAAG AAATGAACGT AGGAGTAAAA GGAGACAGAG AGAGGCGGGG   
  
  
- ACAAAGAGAG AGAGAGGGAG TGAGTGAGTC TCATGACTAG ACTCGTAGTC TTCATCGTCT GGGTCTTTGA   
  
  
- GTCTAAATTT TTGTTGGGCG AGAAAACAAG GGAGATGCAG ATTATTAAAA CAGTAGCGTA AATTACAGTC   
  
  
- TATCTCTCTA TCTCGAGATG ACATAATTAA AAGAGGGTGG AGTGAACTAA GACAACGGGA TTTGGTGGGT   
  
  
- TTTTTTCTTT ATGCTTCTTC GGTGAGAGAG AGAGAGAGAG AGAGTGTGTG TGTGTGTGGG GAGGATCACC   
  
  
- TAAACTATCT CATATTTACT TGTCATTAGT CTGCTCTATA TATCATCACG AATTAACTGG CTCATCGTTT   
  
  
- TCCCCCTTCA ACTCACTCCA TAGTTCTCTC ATCGCATCGA TCGATCCCGT TCTATACGAC CCCAGAAGGA   
  
  
- GTATGTTGAG GCTCCTTCTG GTGGTTCTAC TGTTGGTAAG GAGTGGCAGA CTAAACAGAT CCGTGGGTGG   
  
  
- ATCTAAGTGG TGATGATGAA TGGTTATGGT TGTTGTATAA TCGAGAGGTA GAAGTGTTAG TTATGATCGA   
  
  
- CACGGTGGAG CGGTTAACAA TTAGTTAACA CGCCTCAATT AAACGGCCAA GCTGAAGAGG AGGCGGTTGG   
  
  
- CCGAAGAGAG AGAGGAGCGG AGGGTGAAAC GGAGGGGAAG ACCCCTGAGG TGGCTCTCTG AACACAAAAT   
  
  
- GAAGACGTTC CGAAACCTAG AGGCGGAGTT ATTGTCAATA AATAGGTGAG CATCAAACTG ACCAACATTG   
  
  
- GGATCATGAC CAAGACCATA ACCCCGAGGA AATCGAAGGT TAAAAGATAA AGAAGTTGGT AATCACGTAA   
  
  
- GTGTCCATTG TTCATTACGG GCACAACCCA TGAACTGTAT AAACTGAGAC TTGGTCCAGT GGGGTAAGTA   
  
  
- GGCTAAGTGG GTGAATTGAC GGTTGGTTCG GTAGGACCTT CGAAACCTCC CCATGTTCCG CCAGGTATAG   
  
  
- GAACTGTACC TGTAGTACGT ACCCCAGGTC ACCGGTGGTG AAGAAGTTCG TTAACGTCTT TCTAGACGTT   
  
  
- GGGAACCAGT GGGACGAGGG TGACAAGCGT AGTGTCCTCC CCCGTCCCTA GATCTTGAGG ACTTGGCTTG   
  
  
- ACCTCTAGCC TAGTCCTTCA AGCGTGTTAG GAACCCTGAG CTCAAAGTTA AGGTGGTAGA ATATTGCCAC   
  
  
- CTGAGCTTGG AATGACTCCG TCACCTCCGG TAGTTACGGG ATGTCGATGT GGAGGTACAG GTTCTCCTCC   
  
  
- TCCAGAAGCG TCACTTAACA CCTCTAAAGG AGGTGGCGGA GGATTGCCTC ATACTATCAT GTAAGGAATC   
  
  
- CTACAAAGAG ATGTTCCACT TCTGGGATTT GGGATCCCAG AAGTGGCACC CTCTTTCCCT CCGGCTGGTG   
  
  
- TTGGTGGGAG AGAAGACCGT CGCTAAGTAA CTCCGTGAAC TGGTGATACC CCGTCACAAA CTGAGAGATC   
  
  
- TTCGTTGGGA TGGTAGTTGT TCAGTTCTCT CCCACCGCCA CCTTCTCCCC ACCAAGCCCC TCCTCTAATT   
  
  
- TCTACACCAA CCTGCTCTTC CTCCCAACTC CTCTGTCTCC GTGGTCTTTA TGCTCAGAAC CCTCCACAAC   
  
  
- TACTCCTCGA GACCCAAATT CTCGAACGGG AACTCGGGGA AGAGACACAG TGTTCGGTTC GACGATGAAG   
  
  
- CGGAAGTAAT GGGAAGACTT CCCATGGTGG AATTCCAGAA GGTATCATTA ACAGACAACG AACCCACCTT   
  
  
- CTTAGCGGGA GAAAAGAGAC AAAGAAGAAC CGTTAT

+     CAAT-box

| Site Name | Organism | Position | Strand | Matrix score. | sequence | function |
| --- | --- | --- | --- | --- | --- | --- |
| CAAT-box | Arabidopsis thaliana | 2422 | + | 5 | CCAAT | common cis-acting element in promoter and enhancer regions |
| CAAT-box | Nicotiana glutinosa | 1948 | - | 4 | CAAT |  |
| CAAT-box | Arabidopsis thaliana | 2403 | - | 5 | CCAAT | common cis-acting element in promoter and enhancer regions |
| CAAT-box | Nicotiana glutinosa | 3343 | - | 4 | CAAT |  |
| CAAT-box | Nicotiana glutinosa | 2890 | - | 4 | CAAT |  |
| CAAT-box | Nicotiana glutinosa | 2198 | + | 4 | CAAT |  |
| CAAT-box | Nicotiana glutinosa | 3396 | + | 4 | CAAT |  |
| CAAT-box | Nicotiana glutinosa | 2200 | - | 4 | CAAT |  |
| CAAT-box | Nicotiana glutinosa | 3042 | - | 4 | CAAT |  |
| CAAT-box | Nicotiana glutinosa | 2781 | + | 4 | CAAT |  |
| CAAT-box | Nicotiana glutinosa | 2646 | - | 4 | CAAT |  |
| CAAT-box | Nicotiana glutinosa | 2837 | + | 4 | CAAT |  |
| CAAT-box | Nicotiana glutinosa | 2760 | + | 4 | CAAT |  |
| CAAT-box | Nicotiana glutinosa | 2423 | + | 4 | CAAT |  |
| CAAT-box | Pisum sativum | 2494 | - | 5 | CAAAT | common cis-acting element in promoter and enhancer regions |
| CAAT-box | Petunia hybrida | 2543 | + | 7 | TGCCAAC | common cis-acting element in promoter and enhancer regions |
| CAAT-box | Nicotiana glutinosa | 2644 | + | 4 | CAAT |  |
| CAAT-box | Pisum sativum | 2214 | - | 5 | CAAAT | common cis-acting element in promoter and enhancer regions |
| CAAT-box | Nicotiana glutinosa | 2342 | + | 4 | CAAT |  |
| CAAT-box | Nicotiana glutinosa | 2187 | + | 4 | CAAT |  |
| CAAT-box | Nicotiana glutinosa | 2189 | - | 4 | CAAT |  |
| CAAT-box | Arabidopsis thaliana | 2186 | + | 5 | CCAAT | common cis-acting element in promoter and enhancer regions |
| CAAT-box | Nicotiana glutinosa | 2164 | + | 4 | CAAT |  |
| CAAT-box | Arabidopsis thaliana | 2126 | + | 5 | CCAAT | common cis-acting element in promoter and enhancer regions |
| CAAT-box | Pisum sativum | 2086 | - | 5 | CAAAT | common cis-acting element in promoter and enhancer regions |
| CAAT-box | Nicotiana glutinosa | 2160 | + | 4 | CAAT |  |
| CAAT-box | Nicotiana glutinosa | 2127 | + | 4 | CAAT |  |
| CAAT-box | Pisum sativum | 1895 | - | 5 | CAAAT | common cis-acting element in promoter and enhancer regions |
| CAAT-box | Arabidopsis thaliana | 1525 | + | 5 | CCAAT | common cis-acting element in promoter and enhancer regions |
| CAAT-box | Nicotiana glutinosa | 1470 | - | 4 | CAAT |  |
| CAAT-box | Nicotiana glutinosa | 1402 | + | 4 | CAAT |  |
| CAAT-box | Nicotiana glutinosa | 211 | - | 4 | CAAT |  |
| CAAT-box | Nicotiana glutinosa | 1528 | - | 4 | CAAT |  |
| CAAT-box | Nicotiana glutinosa | 1526 | + | 4 | CAAT |  |
| CAAT-box | Nicotiana glutinosa | 1394 | + | 4 | CAAT |  |
| CAAT-box | Nicotiana glutinosa | 1057 | + | 4 | CAAT |  |
| CAAT-box | Pisum sativum | 1035 | + | 5 | CAAAT | common cis-acting element in promoter and enhancer regions |
| CAAT-box | Nicotiana glutinosa | 1249 | - | 4 | CAAT |  |
| CAAT-box | Nicotiana glutinosa | 1079 | + | 4 | CAAT |  |
| CAAT-box | Nicotiana glutinosa | 618 | - | 4 | CAAT |  |
| CAAT-box | Pisum sativum | 710 | - | 5 | CAAAT | common cis-acting element in promoter and enhancer regions |
| CAAT-box | Petunia hybrida | 683 | - | 7 | TGCCAAC | common cis-acting element in promoter and enhancer regions |
| CAAT-box | Nicotiana glutinosa | 363 | - | 4 | CAAT |  |
| CAAT-box | Nicotiana glutinosa | 535 | - | 4 | CAAT |  |
| CAAT-box | Nicotiana glutinosa | 89 | + | 4 | CAAT |  |
| CAAT-box | Pisum sativum | 992 | - | 5 | CAAAT | common cis-acting element in promoter and enhancer regions |
| CAAT-box | Pisum sativum | 560 | - | 5 | CAAAT | common cis-acting element in promoter and enhancer regions |
| CAAT-box | Pisum sativum | 604 | - | 5 | CAAAT | common cis-acting element in promoter and enhancer regions |
| CAAT-box | Nicotiana glutinosa | 260 | + | 4 | CAAT |  |
| CAAT-box | Nicotiana glutinosa | 972 | - | 4 | CAAT |  |
| CAAT-box | Nicotiana glutinosa | 522 | - | 4 | CAAT |  |
| CAAT-box | Pisum sativum | 422 | - | 5 | CAAAT | common cis-acting element in promoter and enhancer regions |

>HU01G00833.1   
+ -Up\_Stream \_Len000AAAATA TTTTTACAAA AGCATTTCTC TATAAAATAT TTTACAAAAT TTCATTTTGT   
  
  
+ GGGTCTTTTA GAATCAATCT CTCTACAAGT AGGAATGCCG GTTGAGGTAA GGCTACCTAC ATCTAGATCT   
  
  
+ CTCCAGACCC CACAAAAATT AAACGAGACC CACACTGAAA GGTGGTGGTG GTGGTGTTGT TGTTGTATTG   
  
  
+ TCCTACCACG AGAGAGCTTC AAGTGCATCA CTTATTTCAA CAGGTCAATC ACATAGATCT CTAGTGTACT   
  
  
+ GTAAATTTTT TTTCCGTCTT ATTTTAATTT TCTTTTCCAT CCTAAAAGAT TATCATGTAG ATATTCCTAG   
  
  
+ TTTGATATAT TGACTTCAAC TTCAAAAATT ACACATTAAG ATCCACGAAT ATGACAGTTT AGGTTTCATT   
  
  
+ TGAAGGAGAA AGAAATGTTA CGGTGTTTTG GTGTTGTATT CGTAGACATT TTTTGCGCTT AACTGGCTCT   
  
  
+ TCTCATGGGG ACGAGACAGT GAAGGTAATT GTACTTGTTG ATTGATGACG TCATCTGTAG CTTGGATTTG   
  
  
+ TATAGGAATG AACATCTTGG TGTCTACGTG GAAAGTGTGA TTTGTGTTCT TTCATTGATC CTCCTCCTCA   
  
  
+ AGAAGAGGTG ATCTTTCTCC CTTGTTTTTT TTTTTTTTTG GCTAATATGT TGGCAGAACC GGCAAAAAAT   
  
  
+ ACATAATTTG AATAGTGCAT GCATATGAGT ACATGACAGT GAACCAACAA AACGAACCCC TAAAACACAA   
  
  
+ GAGAATTAAC ATGTTAAAAC ACTTAAACTA AATGCAACCA TCTCGATGAC CACTAAATAG CCAAGAAACC   
  
  
+ TAACGTAATT AACAAAAATA AAAAATAACT GAAAATAAAG TAGAGAAAGT CTAACCGAAT ACGATAACAT   
  
  
+ AACCTAAAAC TAACTATACT TGAACTTACT AAGAAATATG AATCAACCAC AAACCGAATT GATTTTGGTT   
  
  
+ TGCCATAATT TGACTATACA TTTAACTTAA AAAATATTTT TTTTTAATTT CAAATAAATC TAAATTATTT   
  
  
+ TTCAATATTC ATCCAAAATA GATTCAATTA TTAAACAAGT CAACCTATAC CGAGTTATTT TCTAGCTCTG   
  
  
+ ACTAAGACAT ATATATATTT TTTGTAAAAA AGATAAGGAT TATAATTTAA ATAGCCTTTC CCTTAAAAAA   
  
  
+ ATTTTTAAAA TTATCCTGAT AGAGAATAAA AAGAAATCCT TATACAAAAC TCTGATTGAC GCAGATGTGT   
  
  
+ ATATGGACAT ATAGAAGTAA TAAGAGGGGT AGGCTAGGAA GGGGGTGGAA ATATAGGGCA TAAATAGGGG   
  
  
+ GCGGGCATAG GGACATGTCT GTCTGAGGTA CGGTGGATGG GACCTTGCCA TGGAAGCAAC AATGTCACAA   
  
  
+ TAATAATATC AACGACTGAA GTTTCTTCAC CAGCCCACTC TTTGCCATAA TGCCATTAAT TATTTATTGC   
  
  
+ TACTATCTCT TTTGATGTGA AACGGTGGAC CTTATCTCTC CCCTCCACTT CCAATTGATG TGAGCAACTC   
  
  
+ AGAAACGCAA ACAAAACTTG TGCCACCTTC TTTACTTGCA TCCTCATTTT CCTCTGTCTC TCTCCGCCCC   
  
  
+ TGTTTCTCTC TCTCTCCCTC ACTCACTCAG AGTACTGATC TGAGCATCAG AAGTAGCAGA CCCAGAAACT   
  
  
+ CAGATTTAAA AACAACCCGC TCTTTTGTTC CCTCTACGTC TAATAATTTT GTCATCGCAT TTAATGTCAG   
  
  
+ ATAGAGAGAT AGAGCTCTAC TGTATTAATT TTCTCCCACC TCACTTGATT CTGTTGCCCT AAACCACCCA   
  
  
+ AAAAAAGAAA TACGAAGAAG CCACTCTCTC TCTCTCTCTC TCTCACACAC ACACACACCC CTCCTAGTGG   
  
  
+ ATTTGATAGA GTATAAATGA ACAGTAATCA GACGAGATAT ATAGTAGTGC TTAATTGACC GAGTAGCAAA   
  
  
+ AGGGGGAAGT TGAGTGAGGT ATCAAGAGAG TAGCGTAGCT AGCTAGGGCA AGATATGCTG GGGTCTTCCT   
  
  
+ CATACAACTC CGAGGAAGAC CACCAAGATG ACAACCATTC CTCACCGTCT GATTTGTCTA GGCACCCACC   
  
  
+ TAGATTCACC ACTACTACTT ACCAATACCA ACAACATATT AGCTCTCCAT CTTCACAATC AATACTAGCT   
  
  
+ GTGCCACCTC GCCAATTGTT AATCAATTGT GCGGAGTTAA TTTGCCGGTT CGACTTCTCC TCCGCCAACC   
  
  
+ GGCTTCTCTC TCTCCTCGCC TCCCACTTTG CCTCCCCTTC TGGGGACTCC ACCGAGAGAC TTGTGTTTTA   
  
  
+ CTTCTGCAAG GCTTTGGATC TCCGCCTCAA TAACAGTTAT TTATCCACTC GTAGTTTGAC TGGTTGTAAC   
  
  
+ CCTAGTACTG GTTCTGGTAT TGGGGCTCCT TTAGCTTCCA ATTTTCTATT TCTTCAACCA TTAGTGCATT   
  
  
+ CACAGGTAAC AAGTAATGCC CGTGTTGGGT ACTTGACATA TTTGACTCTG AACCAGGTCA CCCCATTCAT   
  
  
+ CCGATTCACC CACTTAACTG CCAACCAAGC CATCCTGGAA GCTTTGGAGG GGTACAAGGC GGTCCATATC   
  
  
+ CTTGACATGG ACATCATGCA TGGGGTCCAG TGGCCACCAC TTCTTCAAGC AATTGCAGAA AGATCTGCAA   
  
  
+ CCCTTGGTCA CCCTGCTCCC ACTGTTCGCA TCACAGGAGG GGGCAGGGAT CTAGAACTCC TGAACCGAAC   
  
  
+ TGGAGATCGG ATCAGGAAGT TCGCACAATC CTTGGGACTC GAGTTTCAAT TCCACCATCT TATAACGGTG   
  
  
+ GACTCGAACC TTACTGAGGC AGTGGAGGCC ATCAATGCCC TACAGCTACA CCTCCATGTC CAAGAGGAGG   
  
  
+ AGGTCTTCGC AGTGAATTGT GGAGATTTCC TCCACCGCCT CCTAACGGAG TATGATAGTA CATTCCTTAG   
  
  
+ GATGTTTCTC TACAAGGTGA AGACCCTAAA CCCTAGGGTC TTCACCGTGG GAGAAAGGGA GGCCGACCAC   
  
  
+ AACCACCCTC TCTTCTGGCA GCGATTCATT GAGGCACTTG ACCACTATGG GGCAGTGTTT GACTCTCTAG   
  
  
+ AAGCAACCCT ACCATCAACA AGTCAAGAGA GGGTGGCGGT GGAAGAGGGG TGGTTCGGGG AGGAGATTAA   
  
  
+ AGATGTGGTT GGACGAGAAG GAGGGTTGAG GAGACAGAGG CACCAGAAAT ACGAGTCTTG GGAGGTGTTG   
  
  
+ ATGAGGAGCT CTGGGTTTAA GAGCTTGCCC TTGAGCCCCT TCTCTGTGTC ACAAGCCAAG CTGCTACTTC   
  
  
+ GCCTTCATTA CCCTTCTGAA GGGTACCACC TTAAGGTCTT CCATAGTAAT TGTCTGTTGC TTGGGTGGAA   
  
  
+ GAATCGCCCT CTTTTCTCTG TTTCTTCTTG GCAATA  

- -Up\_Stream \_Len000TTTTAT AAAAATGTTT TCGTAAAGAG ATATTTTATA AAATGTTTTA AAGTAAAACA   
  
  
- CCCAGAAAAT CTTAGTTAGA GAGATGTTCA TCCTTACGGC CAACTCCATT CCGATGGATG TAGATCTAGA   
  
  
- GAGGTCTGGG GTGTTTTTAA TTTGCTCTGG GTGTGACTTT CCACCACCAC CACCACAACA ACAACATAAC   
  
  
- AGGATGGTGC TCTCTCGAAG TTCACGTAGT GAATAAAGTT GTCCAGTTAG TGTATCTAGA GATCACATGA   
  
  
- CATTTAAAAA AAAGGCAGAA TAAAATTAAA AGAAAAGGTA GGATTTTCTA ATAGTACATC TATAAGGATC   
  
  
- AAACTATATA ACTGAAGTTG AAGTTTTTAA TGTGTAATTC TAGGTGCTTA TACTGTCAAA TCCAAAGTAA   
  
  
- ACTTCCTCTT TCTTTACAAT GCCACAAAAC CACAACATAA GCATCTGTAA AAAACGCGAA TTGACCGAGA   
  
  
- AGAGTACCCC TGCTCTGTCA CTTCCATTAA CATGAACAAC TAACTACTGC AGTAGACATC GAACCTAAAC   
  
  
- ATATCCTTAC TTGTAGAACC ACAGATGCAC CTTTCACACT AAACACAAGA AAGTAACTAG GAGGAGGAGT   
  
  
- TCTTCTCCAC TAGAAAGAGG GAACAAAAAA AAAAAAAAAC CGATTATACA ACCGTCTTGG CCGTTTTTTA   
  
  
- TGTATTAAAC TTATCACGTA CGTATACTCA TGTACTGTCA CTTGGTTGTT TTGCTTGGGG ATTTTGTGTT   
  
  
- CTCTTAATTG TACAATTTTG TGAATTTGAT TTACGTTGGT AGAGCTACTG GTGATTTATC GGTTCTTTGG   
  
  
- ATTGCATTAA TTGTTTTTAT TTTTTATTGA CTTTTATTTC ATCTCTTTCA GATTGGCTTA TGCTATTGTA   
  
  
- TTGGATTTTG ATTGATATGA ACTTGAATGA TTCTTTATAC TTAGTTGGTG TTTGGCTTAA CTAAAACCAA   
  
  
- ACGGTATTAA ACTGATATGT AAATTGAATT TTTTATAAAA AAAAATTAAA GTTTATTTAG ATTTAATAAA   
  
  
- AAGTTATAAG TAGGTTTTAT CTAAGTTAAT AATTTGTTCA GTTGGATATG GCTCAATAAA AGATCGAGAC   
  
  
- TGATTCTGTA TATATATAAA AAACATTTTT TCTATTCCTA ATATTAAATT TATCGGAAAG GGAATTTTTT   
  
  
- TAAAAATTTT AATAGGACTA TCTCTTATTT TTCTTTAGGA ATATGTTTTG AGACTAACTG CGTCTACACA   
  
  
- TATACCTGTA TATCTTCATT ATTCTCCCCA TCCGATCCTT CCCCCACCTT TATATCCCGT ATTTATCCCC   
  
  
- CGCCCGTATC CCTGTACAGA CAGACTCCAT GCCACCTACC CTGGAACGGT ACCTTCGTTG TTACAGTGTT   
  
  
- ATTATTATAG TTGCTGACTT CAAAGAAGTG GTCGGGTGAG AAACGGTATT ACGGTAATTA ATAAATAACG   
  
  
- ATGATAGAGA AAACTACACT TTGCCACCTG GAATAGAGAG GGGAGGTGAA GGTTAACTAC ACTCGTTGAG   
  
  
- TCTTTGCGTT TGTTTTGAAC ACGGTGGAAG AAATGAACGT AGGAGTAAAA GGAGACAGAG AGAGGCGGGG   
  
  
- ACAAAGAGAG AGAGAGGGAG TGAGTGAGTC TCATGACTAG ACTCGTAGTC TTCATCGTCT GGGTCTTTGA   
  
  
- GTCTAAATTT TTGTTGGGCG AGAAAACAAG GGAGATGCAG ATTATTAAAA CAGTAGCGTA AATTACAGTC   
  
  
- TATCTCTCTA TCTCGAGATG ACATAATTAA AAGAGGGTGG AGTGAACTAA GACAACGGGA TTTGGTGGGT   
  
  
- TTTTTTCTTT ATGCTTCTTC GGTGAGAGAG AGAGAGAGAG AGAGTGTGTG TGTGTGTGGG GAGGATCACC   
  
  
- TAAACTATCT CATATTTACT TGTCATTAGT CTGCTCTATA TATCATCACG AATTAACTGG CTCATCGTTT   
  
  
- TCCCCCTTCA ACTCACTCCA TAGTTCTCTC ATCGCATCGA TCGATCCCGT TCTATACGAC CCCAGAAGGA   
  
  
- GTATGTTGAG GCTCCTTCTG GTGGTTCTAC TGTTGGTAAG GAGTGGCAGA CTAAACAGAT CCGTGGGTGG   
  
  
- ATCTAAGTGG TGATGATGAA TGGTTATGGT TGTTGTATAA TCGAGAGGTA GAAGTGTTAG TTATGATCGA   
  
  
- CACGGTGGAG CGGTTAACAA TTAGTTAACA CGCCTCAATT AAACGGCCAA GCTGAAGAGG AGGCGGTTGG   
  
  
- CCGAAGAGAG AGAGGAGCGG AGGGTGAAAC GGAGGGGAAG ACCCCTGAGG TGGCTCTCTG AACACAAAAT   
  
  
- GAAGACGTTC CGAAACCTAG AGGCGGAGTT ATTGTCAATA AATAGGTGAG CATCAAACTG ACCAACATTG   
  
  
- GGATCATGAC CAAGACCATA ACCCCGAGGA AATCGAAGGT TAAAAGATAA AGAAGTTGGT AATCACGTAA   
  
  
- GTGTCCATTG TTCATTACGG GCACAACCCA TGAACTGTAT AAACTGAGAC TTGGTCCAGT GGGGTAAGTA   
  
  
- GGCTAAGTGG GTGAATTGAC GGTTGGTTCG GTAGGACCTT CGAAACCTCC CCATGTTCCG CCAGGTATAG   
  
  
- GAACTGTACC TGTAGTACGT ACCCCAGGTC ACCGGTGGTG AAGAAGTTCG TTAACGTCTT TCTAGACGTT   
  
  
- GGGAACCAGT GGGACGAGGG TGACAAGCGT AGTGTCCTCC CCCGTCCCTA GATCTTGAGG ACTTGGCTTG   
  
  
- ACCTCTAGCC TAGTCCTTCA AGCGTGTTAG GAACCCTGAG CTCAAAGTTA AGGTGGTAGA ATATTGCCAC   
  
  
- CTGAGCTTGG AATGACTCCG TCACCTCCGG TAGTTACGGG ATGTCGATGT GGAGGTACAG GTTCTCCTCC   
  
  
- TCCAGAAGCG TCACTTAACA CCTCTAAAGG AGGTGGCGGA GGATTGCCTC ATACTATCAT GTAAGGAATC   
  
  
- CTACAAAGAG ATGTTCCACT TCTGGGATTT GGGATCCCAG AAGTGGCACC CTCTTTCCCT CCGGCTGGTG   
  
  
- TTGGTGGGAG AGAAGACCGT CGCTAAGTAA CTCCGTGAAC TGGTGATACC CCGTCACAAA CTGAGAGATC   
  
  
- TTCGTTGGGA TGGTAGTTGT TCAGTTCTCT CCCACCGCCA CCTTCTCCCC ACCAAGCCCC TCCTCTAATT   
  
  
- TCTACACCAA CCTGCTCTTC CTCCCAACTC CTCTGTCTCC GTGGTCTTTA TGCTCAGAAC CCTCCACAAC   
  
  
- TACTCCTCGA GACCCAAATT CTCGAACGGG AACTCGGGGA AGAGACACAG TGTTCGGTTC GACGATGAAG   
  
  
- CGGAAGTAAT GGGAAGACTT CCCATGGTGG AATTCCAGAA GGTATCATTA ACAGACAACG AACCCACCTT   
  
  
- CTTAGCGGGA GAAAAGAGAC AAAGAAGAAC CGTTAT

+     CAT-box

| Site Name | Organism | Position | Strand | Matrix score. | sequence | function |
| --- | --- | --- | --- | --- | --- | --- |
| CAT-box | Arabidopsis thaliana | 2623 | - | 6 | GCCACT | cis-acting regulatory element related to meristem expression |
| CAT-box | Arabidopsis thaliana | 1844 | + | 6 | GCCACT | cis-acting regulatory element related to meristem expression |

>HU01G00833.1   
+ -Up\_Stream \_Len000AAAATA TTTTTACAAA AGCATTTCTC TATAAAATAT TTTACAAAAT TTCATTTTGT   
  
  
+ GGGTCTTTTA GAATCAATCT CTCTACAAGT AGGAATGCCG GTTGAGGTAA GGCTACCTAC ATCTAGATCT   
  
  
+ CTCCAGACCC CACAAAAATT AAACGAGACC CACACTGAAA GGTGGTGGTG GTGGTGTTGT TGTTGTATTG   
  
  
+ TCCTACCACG AGAGAGCTTC AAGTGCATCA CTTATTTCAA CAGGTCAATC ACATAGATCT CTAGTGTACT   
  
  
+ GTAAATTTTT TTTCCGTCTT ATTTTAATTT TCTTTTCCAT CCTAAAAGAT TATCATGTAG ATATTCCTAG   
  
  
+ TTTGATATAT TGACTTCAAC TTCAAAAATT ACACATTAAG ATCCACGAAT ATGACAGTTT AGGTTTCATT   
  
  
+ TGAAGGAGAA AGAAATGTTA CGGTGTTTTG GTGTTGTATT CGTAGACATT TTTTGCGCTT AACTGGCTCT   
  
  
+ TCTCATGGGG ACGAGACAGT GAAGGTAATT GTACTTGTTG ATTGATGACG TCATCTGTAG CTTGGATTTG   
  
  
+ TATAGGAATG AACATCTTGG TGTCTACGTG GAAAGTGTGA TTTGTGTTCT TTCATTGATC CTCCTCCTCA   
  
  
+ AGAAGAGGTG ATCTTTCTCC CTTGTTTTTT TTTTTTTTTG GCTAATATGT TGGCAGAACC GGCAAAAAAT   
  
  
+ ACATAATTTG AATAGTGCAT GCATATGAGT ACATGACAGT GAACCAACAA AACGAACCCC TAAAACACAA   
  
  
+ GAGAATTAAC ATGTTAAAAC ACTTAAACTA AATGCAACCA TCTCGATGAC CACTAAATAG CCAAGAAACC   
  
  
+ TAACGTAATT AACAAAAATA AAAAATAACT GAAAATAAAG TAGAGAAAGT CTAACCGAAT ACGATAACAT   
  
  
+ AACCTAAAAC TAACTATACT TGAACTTACT AAGAAATATG AATCAACCAC AAACCGAATT GATTTTGGTT   
  
  
+ TGCCATAATT TGACTATACA TTTAACTTAA AAAATATTTT TTTTTAATTT CAAATAAATC TAAATTATTT   
  
  
+ TTCAATATTC ATCCAAAATA GATTCAATTA TTAAACAAGT CAACCTATAC CGAGTTATTT TCTAGCTCTG   
  
  
+ ACTAAGACAT ATATATATTT TTTGTAAAAA AGATAAGGAT TATAATTTAA ATAGCCTTTC CCTTAAAAAA   
  
  
+ ATTTTTAAAA TTATCCTGAT AGAGAATAAA AAGAAATCCT TATACAAAAC TCTGATTGAC GCAGATGTGT   
  
  
+ ATATGGACAT ATAGAAGTAA TAAGAGGGGT AGGCTAGGAA GGGGGTGGAA ATATAGGGCA TAAATAGGGG   
  
  
+ GCGGGCATAG GGACATGTCT GTCTGAGGTA CGGTGGATGG GACCTTGCCA TGGAAGCAAC AATGTCACAA   
  
  
+ TAATAATATC AACGACTGAA GTTTCTTCAC CAGCCCACTC TTTGCCATAA TGCCATTAAT TATTTATTGC   
  
  
+ TACTATCTCT TTTGATGTGA AACGGTGGAC CTTATCTCTC CCCTCCACTT CCAATTGATG TGAGCAACTC   
  
  
+ AGAAACGCAA ACAAAACTTG TGCCACCTTC TTTACTTGCA TCCTCATTTT CCTCTGTCTC TCTCCGCCCC   
  
  
+ TGTTTCTCTC TCTCTCCCTC ACTCACTCAG AGTACTGATC TGAGCATCAG AAGTAGCAGA CCCAGAAACT   
  
  
+ CAGATTTAAA AACAACCCGC TCTTTTGTTC CCTCTACGTC TAATAATTTT GTCATCGCAT TTAATGTCAG   
  
  
+ ATAGAGAGAT AGAGCTCTAC TGTATTAATT TTCTCCCACC TCACTTGATT CTGTTGCCCT AAACCACCCA   
  
  
+ AAAAAAGAAA TACGAAGAAG CCACTCTCTC TCTCTCTCTC TCTCACACAC ACACACACCC CTCCTAGTGG   
  
  
+ ATTTGATAGA GTATAAATGA ACAGTAATCA GACGAGATAT ATAGTAGTGC TTAATTGACC GAGTAGCAAA   
  
  
+ AGGGGGAAGT TGAGTGAGGT ATCAAGAGAG TAGCGTAGCT AGCTAGGGCA AGATATGCTG GGGTCTTCCT   
  
  
+ CATACAACTC CGAGGAAGAC CACCAAGATG ACAACCATTC CTCACCGTCT GATTTGTCTA GGCACCCACC   
  
  
+ TAGATTCACC ACTACTACTT ACCAATACCA ACAACATATT AGCTCTCCAT CTTCACAATC AATACTAGCT   
  
  
+ GTGCCACCTC GCCAATTGTT AATCAATTGT GCGGAGTTAA TTTGCCGGTT CGACTTCTCC TCCGCCAACC   
  
  
+ GGCTTCTCTC TCTCCTCGCC TCCCACTTTG CCTCCCCTTC TGGGGACTCC ACCGAGAGAC TTGTGTTTTA   
  
  
+ CTTCTGCAAG GCTTTGGATC TCCGCCTCAA TAACAGTTAT TTATCCACTC GTAGTTTGAC TGGTTGTAAC   
  
  
+ CCTAGTACTG GTTCTGGTAT TGGGGCTCCT TTAGCTTCCA ATTTTCTATT TCTTCAACCA TTAGTGCATT   
  
  
+ CACAGGTAAC AAGTAATGCC CGTGTTGGGT ACTTGACATA TTTGACTCTG AACCAGGTCA CCCCATTCAT   
  
  
+ CCGATTCACC CACTTAACTG CCAACCAAGC CATCCTGGAA GCTTTGGAGG GGTACAAGGC GGTCCATATC   
  
  
+ CTTGACATGG ACATCATGCA TGGGGTCCAG TGGCCACCAC TTCTTCAAGC AATTGCAGAA AGATCTGCAA   
  
  
+ CCCTTGGTCA CCCTGCTCCC ACTGTTCGCA TCACAGGAGG GGGCAGGGAT CTAGAACTCC TGAACCGAAC   
  
  
+ TGGAGATCGG ATCAGGAAGT TCGCACAATC CTTGGGACTC GAGTTTCAAT TCCACCATCT TATAACGGTG   
  
  
+ GACTCGAACC TTACTGAGGC AGTGGAGGCC ATCAATGCCC TACAGCTACA CCTCCATGTC CAAGAGGAGG   
  
  
+ AGGTCTTCGC AGTGAATTGT GGAGATTTCC TCCACCGCCT CCTAACGGAG TATGATAGTA CATTCCTTAG   
  
  
+ GATGTTTCTC TACAAGGTGA AGACCCTAAA CCCTAGGGTC TTCACCGTGG GAGAAAGGGA GGCCGACCAC   
  
  
+ AACCACCCTC TCTTCTGGCA GCGATTCATT GAGGCACTTG ACCACTATGG GGCAGTGTTT GACTCTCTAG   
  
  
+ AAGCAACCCT ACCATCAACA AGTCAAGAGA GGGTGGCGGT GGAAGAGGGG TGGTTCGGGG AGGAGATTAA   
  
  
+ AGATGTGGTT GGACGAGAAG GAGGGTTGAG GAGACAGAGG CACCAGAAAT ACGAGTCTTG GGAGGTGTTG   
  
  
+ ATGAGGAGCT CTGGGTTTAA GAGCTTGCCC TTGAGCCCCT TCTCTGTGTC ACAAGCCAAG CTGCTACTTC   
  
  
+ GCCTTCATTA CCCTTCTGAA GGGTACCACC TTAAGGTCTT CCATAGTAAT TGTCTGTTGC TTGGGTGGAA   
  
  
+ GAATCGCCCT CTTTTCTCTG TTTCTTCTTG GCAATA  

- -Up\_Stream \_Len000TTTTAT AAAAATGTTT TCGTAAAGAG ATATTTTATA AAATGTTTTA AAGTAAAACA   
  
  
- CCCAGAAAAT CTTAGTTAGA GAGATGTTCA TCCTTACGGC CAACTCCATT CCGATGGATG TAGATCTAGA   
  
  
- GAGGTCTGGG GTGTTTTTAA TTTGCTCTGG GTGTGACTTT CCACCACCAC CACCACAACA ACAACATAAC   
  
  
- AGGATGGTGC TCTCTCGAAG TTCACGTAGT GAATAAAGTT GTCCAGTTAG TGTATCTAGA GATCACATGA   
  
  
- CATTTAAAAA AAAGGCAGAA TAAAATTAAA AGAAAAGGTA GGATTTTCTA ATAGTACATC TATAAGGATC   
  
  
- AAACTATATA ACTGAAGTTG AAGTTTTTAA TGTGTAATTC TAGGTGCTTA TACTGTCAAA TCCAAAGTAA   
  
  
- ACTTCCTCTT TCTTTACAAT GCCACAAAAC CACAACATAA GCATCTGTAA AAAACGCGAA TTGACCGAGA   
  
  
- AGAGTACCCC TGCTCTGTCA CTTCCATTAA CATGAACAAC TAACTACTGC AGTAGACATC GAACCTAAAC   
  
  
- ATATCCTTAC TTGTAGAACC ACAGATGCAC CTTTCACACT AAACACAAGA AAGTAACTAG GAGGAGGAGT   
  
  
- TCTTCTCCAC TAGAAAGAGG GAACAAAAAA AAAAAAAAAC CGATTATACA ACCGTCTTGG CCGTTTTTTA   
  
  
- TGTATTAAAC TTATCACGTA CGTATACTCA TGTACTGTCA CTTGGTTGTT TTGCTTGGGG ATTTTGTGTT   
  
  
- CTCTTAATTG TACAATTTTG TGAATTTGAT TTACGTTGGT AGAGCTACTG GTGATTTATC GGTTCTTTGG   
  
  
- ATTGCATTAA TTGTTTTTAT TTTTTATTGA CTTTTATTTC ATCTCTTTCA GATTGGCTTA TGCTATTGTA   
  
  
- TTGGATTTTG ATTGATATGA ACTTGAATGA TTCTTTATAC TTAGTTGGTG TTTGGCTTAA CTAAAACCAA   
  
  
- ACGGTATTAA ACTGATATGT AAATTGAATT TTTTATAAAA AAAAATTAAA GTTTATTTAG ATTTAATAAA   
  
  
- AAGTTATAAG TAGGTTTTAT CTAAGTTAAT AATTTGTTCA GTTGGATATG GCTCAATAAA AGATCGAGAC   
  
  
- TGATTCTGTA TATATATAAA AAACATTTTT TCTATTCCTA ATATTAAATT TATCGGAAAG GGAATTTTTT   
  
  
- TAAAAATTTT AATAGGACTA TCTCTTATTT TTCTTTAGGA ATATGTTTTG AGACTAACTG CGTCTACACA   
  
  
- TATACCTGTA TATCTTCATT ATTCTCCCCA TCCGATCCTT CCCCCACCTT TATATCCCGT ATTTATCCCC   
  
  
- CGCCCGTATC CCTGTACAGA CAGACTCCAT GCCACCTACC CTGGAACGGT ACCTTCGTTG TTACAGTGTT   
  
  
- ATTATTATAG TTGCTGACTT CAAAGAAGTG GTCGGGTGAG AAACGGTATT ACGGTAATTA ATAAATAACG   
  
  
- ATGATAGAGA AAACTACACT TTGCCACCTG GAATAGAGAG GGGAGGTGAA GGTTAACTAC ACTCGTTGAG   
  
  
- TCTTTGCGTT TGTTTTGAAC ACGGTGGAAG AAATGAACGT AGGAGTAAAA GGAGACAGAG AGAGGCGGGG   
  
  
- ACAAAGAGAG AGAGAGGGAG TGAGTGAGTC TCATGACTAG ACTCGTAGTC TTCATCGTCT GGGTCTTTGA   
  
  
- GTCTAAATTT TTGTTGGGCG AGAAAACAAG GGAGATGCAG ATTATTAAAA CAGTAGCGTA AATTACAGTC   
  
  
- TATCTCTCTA TCTCGAGATG ACATAATTAA AAGAGGGTGG AGTGAACTAA GACAACGGGA TTTGGTGGGT   
  
  
- TTTTTTCTTT ATGCTTCTTC GGTGAGAGAG AGAGAGAGAG AGAGTGTGTG TGTGTGTGGG GAGGATCACC   
  
  
- TAAACTATCT CATATTTACT TGTCATTAGT CTGCTCTATA TATCATCACG AATTAACTGG CTCATCGTTT   
  
  
- TCCCCCTTCA ACTCACTCCA TAGTTCTCTC ATCGCATCGA TCGATCCCGT TCTATACGAC CCCAGAAGGA   
  
  
- GTATGTTGAG GCTCCTTCTG GTGGTTCTAC TGTTGGTAAG GAGTGGCAGA CTAAACAGAT CCGTGGGTGG   
  
  
- ATCTAAGTGG TGATGATGAA TGGTTATGGT TGTTGTATAA TCGAGAGGTA GAAGTGTTAG TTATGATCGA   
  
  
- CACGGTGGAG CGGTTAACAA TTAGTTAACA CGCCTCAATT AAACGGCCAA GCTGAAGAGG AGGCGGTTGG   
  
  
- CCGAAGAGAG AGAGGAGCGG AGGGTGAAAC GGAGGGGAAG ACCCCTGAGG TGGCTCTCTG AACACAAAAT   
  
  
- GAAGACGTTC CGAAACCTAG AGGCGGAGTT ATTGTCAATA AATAGGTGAG CATCAAACTG ACCAACATTG   
  
  
- GGATCATGAC CAAGACCATA ACCCCGAGGA AATCGAAGGT TAAAAGATAA AGAAGTTGGT AATCACGTAA   
  
  
- GTGTCCATTG TTCATTACGG GCACAACCCA TGAACTGTAT AAACTGAGAC TTGGTCCAGT GGGGTAAGTA   
  
  
- GGCTAAGTGG GTGAATTGAC GGTTGGTTCG GTAGGACCTT CGAAACCTCC CCATGTTCCG CCAGGTATAG   
  
  
- GAACTGTACC TGTAGTACGT ACCCCAGGTC ACCGGTGGTG AAGAAGTTCG TTAACGTCTT TCTAGACGTT   
  
  
- GGGAACCAGT GGGACGAGGG TGACAAGCGT AGTGTCCTCC CCCGTCCCTA GATCTTGAGG ACTTGGCTTG   
  
  
- ACCTCTAGCC TAGTCCTTCA AGCGTGTTAG GAACCCTGAG CTCAAAGTTA AGGTGGTAGA ATATTGCCAC   
  
  
- CTGAGCTTGG AATGACTCCG TCACCTCCGG TAGTTACGGG ATGTCGATGT GGAGGTACAG GTTCTCCTCC   
  
  
- TCCAGAAGCG TCACTTAACA CCTCTAAAGG AGGTGGCGGA GGATTGCCTC ATACTATCAT GTAAGGAATC   
  
  
- CTACAAAGAG ATGTTCCACT TCTGGGATTT GGGATCCCAG AAGTGGCACC CTCTTTCCCT CCGGCTGGTG   
  
  
- TTGGTGGGAG AGAAGACCGT CGCTAAGTAA CTCCGTGAAC TGGTGATACC CCGTCACAAA CTGAGAGATC   
  
  
- TTCGTTGGGA TGGTAGTTGT TCAGTTCTCT CCCACCGCCA CCTTCTCCCC ACCAAGCCCC TCCTCTAATT   
  
  
- TCTACACCAA CCTGCTCTTC CTCCCAACTC CTCTGTCTCC GTGGTCTTTA TGCTCAGAAC CCTCCACAAC   
  
  
- TACTCCTCGA GACCCAAATT CTCGAACGGG AACTCGGGGA AGAGACACAG TGTTCGGTTC GACGATGAAG   
  
  
- CGGAAGTAAT GGGAAGACTT CCCATGGTGG AATTCCAGAA GGTATCATTA ACAGACAACG AACCCACCTT   
  
  
- CTTAGCGGGA GAAAAGAGAC AAAGAAGAAC CGTTAT

+     CGTCA-motif

| Site Name | Organism | Position | Strand | Matrix score. | sequence | function |
| --- | --- | --- | --- | --- | --- | --- |
| CGTCA-motif | Hordeum vulgare | 543 | + | 5 | CGTCA | cis-acting regulatory element involved in the MeJA-responsiveness |
| CGTCA-motif | Hordeum vulgare | 540 | - | 5 | CGTCA | cis-acting regulatory element involved in the MeJA-responsiveness |
| CGTCA-motif | Hordeum vulgare | 1251 | - | 5 | CGTCA | cis-acting regulatory element involved in the MeJA-responsiveness |

>HU01G00833.1   
+ -Up\_Stream \_Len000AAAATA TTTTTACAAA AGCATTTCTC TATAAAATAT TTTACAAAAT TTCATTTTGT   
  
  
+ GGGTCTTTTA GAATCAATCT CTCTACAAGT AGGAATGCCG GTTGAGGTAA GGCTACCTAC ATCTAGATCT   
  
  
+ CTCCAGACCC CACAAAAATT AAACGAGACC CACACTGAAA GGTGGTGGTG GTGGTGTTGT TGTTGTATTG   
  
  
+ TCCTACCACG AGAGAGCTTC AAGTGCATCA CTTATTTCAA CAGGTCAATC ACATAGATCT CTAGTGTACT   
  
  
+ GTAAATTTTT TTTCCGTCTT ATTTTAATTT TCTTTTCCAT CCTAAAAGAT TATCATGTAG ATATTCCTAG   
  
  
+ TTTGATATAT TGACTTCAAC TTCAAAAATT ACACATTAAG ATCCACGAAT ATGACAGTTT AGGTTTCATT   
  
  
+ TGAAGGAGAA AGAAATGTTA CGGTGTTTTG GTGTTGTATT CGTAGACATT TTTTGCGCTT AACTGGCTCT   
  
  
+ TCTCATGGGG ACGAGACAGT GAAGGTAATT GTACTTGTTG ATTGATGACG TCATCTGTAG CTTGGATTTG   
  
  
+ TATAGGAATG AACATCTTGG TGTCTACGTG GAAAGTGTGA TTTGTGTTCT TTCATTGATC CTCCTCCTCA   
  
  
+ AGAAGAGGTG ATCTTTCTCC CTTGTTTTTT TTTTTTTTTG GCTAATATGT TGGCAGAACC GGCAAAAAAT   
  
  
+ ACATAATTTG AATAGTGCAT GCATATGAGT ACATGACAGT GAACCAACAA AACGAACCCC TAAAACACAA   
  
  
+ GAGAATTAAC ATGTTAAAAC ACTTAAACTA AATGCAACCA TCTCGATGAC CACTAAATAG CCAAGAAACC   
  
  
+ TAACGTAATT AACAAAAATA AAAAATAACT GAAAATAAAG TAGAGAAAGT CTAACCGAAT ACGATAACAT   
  
  
+ AACCTAAAAC TAACTATACT TGAACTTACT AAGAAATATG AATCAACCAC AAACCGAATT GATTTTGGTT   
  
  
+ TGCCATAATT TGACTATACA TTTAACTTAA AAAATATTTT TTTTTAATTT CAAATAAATC TAAATTATTT   
  
  
+ TTCAATATTC ATCCAAAATA GATTCAATTA TTAAACAAGT CAACCTATAC CGAGTTATTT TCTAGCTCTG   
  
  
+ ACTAAGACAT ATATATATTT TTTGTAAAAA AGATAAGGAT TATAATTTAA ATAGCCTTTC CCTTAAAAAA   
  
  
+ ATTTTTAAAA TTATCCTGAT AGAGAATAAA AAGAAATCCT TATACAAAAC TCTGATTGAC GCAGATGTGT   
  
  
+ ATATGGACAT ATAGAAGTAA TAAGAGGGGT AGGCTAGGAA GGGGGTGGAA ATATAGGGCA TAAATAGGGG   
  
  
+ GCGGGCATAG GGACATGTCT GTCTGAGGTA CGGTGGATGG GACCTTGCCA TGGAAGCAAC AATGTCACAA   
  
  
+ TAATAATATC AACGACTGAA GTTTCTTCAC CAGCCCACTC TTTGCCATAA TGCCATTAAT TATTTATTGC   
  
  
+ TACTATCTCT TTTGATGTGA AACGGTGGAC CTTATCTCTC CCCTCCACTT CCAATTGATG TGAGCAACTC   
  
  
+ AGAAACGCAA ACAAAACTTG TGCCACCTTC TTTACTTGCA TCCTCATTTT CCTCTGTCTC TCTCCGCCCC   
  
  
+ TGTTTCTCTC TCTCTCCCTC ACTCACTCAG AGTACTGATC TGAGCATCAG AAGTAGCAGA CCCAGAAACT   
  
  
+ CAGATTTAAA AACAACCCGC TCTTTTGTTC CCTCTACGTC TAATAATTTT GTCATCGCAT TTAATGTCAG   
  
  
+ ATAGAGAGAT AGAGCTCTAC TGTATTAATT TTCTCCCACC TCACTTGATT CTGTTGCCCT AAACCACCCA   
  
  
+ AAAAAAGAAA TACGAAGAAG CCACTCTCTC TCTCTCTCTC TCTCACACAC ACACACACCC CTCCTAGTGG   
  
  
+ ATTTGATAGA GTATAAATGA ACAGTAATCA GACGAGATAT ATAGTAGTGC TTAATTGACC GAGTAGCAAA   
  
  
+ AGGGGGAAGT TGAGTGAGGT ATCAAGAGAG TAGCGTAGCT AGCTAGGGCA AGATATGCTG GGGTCTTCCT   
  
  
+ CATACAACTC CGAGGAAGAC CACCAAGATG ACAACCATTC CTCACCGTCT GATTTGTCTA GGCACCCACC   
  
  
+ TAGATTCACC ACTACTACTT ACCAATACCA ACAACATATT AGCTCTCCAT CTTCACAATC AATACTAGCT   
  
  
+ GTGCCACCTC GCCAATTGTT AATCAATTGT GCGGAGTTAA TTTGCCGGTT CGACTTCTCC TCCGCCAACC   
  
  
+ GGCTTCTCTC TCTCCTCGCC TCCCACTTTG CCTCCCCTTC TGGGGACTCC ACCGAGAGAC TTGTGTTTTA   
  
  
+ CTTCTGCAAG GCTTTGGATC TCCGCCTCAA TAACAGTTAT TTATCCACTC GTAGTTTGAC TGGTTGTAAC   
  
  
+ CCTAGTACTG GTTCTGGTAT TGGGGCTCCT TTAGCTTCCA ATTTTCTATT TCTTCAACCA TTAGTGCATT   
  
  
+ CACAGGTAAC AAGTAATGCC CGTGTTGGGT ACTTGACATA TTTGACTCTG AACCAGGTCA CCCCATTCAT   
  
  
+ CCGATTCACC CACTTAACTG CCAACCAAGC CATCCTGGAA GCTTTGGAGG GGTACAAGGC GGTCCATATC   
  
  
+ CTTGACATGG ACATCATGCA TGGGGTCCAG TGGCCACCAC TTCTTCAAGC AATTGCAGAA AGATCTGCAA   
  
  
+ CCCTTGGTCA CCCTGCTCCC ACTGTTCGCA TCACAGGAGG GGGCAGGGAT CTAGAACTCC TGAACCGAAC   
  
  
+ TGGAGATCGG ATCAGGAAGT TCGCACAATC CTTGGGACTC GAGTTTCAAT TCCACCATCT TATAACGGTG   
  
  
+ GACTCGAACC TTACTGAGGC AGTGGAGGCC ATCAATGCCC TACAGCTACA CCTCCATGTC CAAGAGGAGG   
  
  
+ AGGTCTTCGC AGTGAATTGT GGAGATTTCC TCCACCGCCT CCTAACGGAG TATGATAGTA CATTCCTTAG   
  
  
+ GATGTTTCTC TACAAGGTGA AGACCCTAAA CCCTAGGGTC TTCACCGTGG GAGAAAGGGA GGCCGACCAC   
  
  
+ AACCACCCTC TCTTCTGGCA GCGATTCATT GAGGCACTTG ACCACTATGG GGCAGTGTTT GACTCTCTAG   
  
  
+ AAGCAACCCT ACCATCAACA AGTCAAGAGA GGGTGGCGGT GGAAGAGGGG TGGTTCGGGG AGGAGATTAA   
  
  
+ AGATGTGGTT GGACGAGAAG GAGGGTTGAG GAGACAGAGG CACCAGAAAT ACGAGTCTTG GGAGGTGTTG   
  
  
+ ATGAGGAGCT CTGGGTTTAA GAGCTTGCCC TTGAGCCCCT TCTCTGTGTC ACAAGCCAAG CTGCTACTTC   
  
  
+ GCCTTCATTA CCCTTCTGAA GGGTACCACC TTAAGGTCTT CCATAGTAAT TGTCTGTTGC TTGGGTGGAA   
  
  
+ GAATCGCCCT CTTTTCTCTG TTTCTTCTTG GCAATA  

- -Up\_Stream \_Len000TTTTAT AAAAATGTTT TCGTAAAGAG ATATTTTATA AAATGTTTTA AAGTAAAACA   
  
  
- CCCAGAAAAT CTTAGTTAGA GAGATGTTCA TCCTTACGGC CAACTCCATT CCGATGGATG TAGATCTAGA   
  
  
- GAGGTCTGGG GTGTTTTTAA TTTGCTCTGG GTGTGACTTT CCACCACCAC CACCACAACA ACAACATAAC   
  
  
- AGGATGGTGC TCTCTCGAAG TTCACGTAGT GAATAAAGTT GTCCAGTTAG TGTATCTAGA GATCACATGA   
  
  
- CATTTAAAAA AAAGGCAGAA TAAAATTAAA AGAAAAGGTA GGATTTTCTA ATAGTACATC TATAAGGATC   
  
  
- AAACTATATA ACTGAAGTTG AAGTTTTTAA TGTGTAATTC TAGGTGCTTA TACTGTCAAA TCCAAAGTAA   
  
  
- ACTTCCTCTT TCTTTACAAT GCCACAAAAC CACAACATAA GCATCTGTAA AAAACGCGAA TTGACCGAGA   
  
  
- AGAGTACCCC TGCTCTGTCA CTTCCATTAA CATGAACAAC TAACTACTGC AGTAGACATC GAACCTAAAC   
  
  
- ATATCCTTAC TTGTAGAACC ACAGATGCAC CTTTCACACT AAACACAAGA AAGTAACTAG GAGGAGGAGT   
  
  
- TCTTCTCCAC TAGAAAGAGG GAACAAAAAA AAAAAAAAAC CGATTATACA ACCGTCTTGG CCGTTTTTTA   
  
  
- TGTATTAAAC TTATCACGTA CGTATACTCA TGTACTGTCA CTTGGTTGTT TTGCTTGGGG ATTTTGTGTT   
  
  
- CTCTTAATTG TACAATTTTG TGAATTTGAT TTACGTTGGT AGAGCTACTG GTGATTTATC GGTTCTTTGG   
  
  
- ATTGCATTAA TTGTTTTTAT TTTTTATTGA CTTTTATTTC ATCTCTTTCA GATTGGCTTA TGCTATTGTA   
  
  
- TTGGATTTTG ATTGATATGA ACTTGAATGA TTCTTTATAC TTAGTTGGTG TTTGGCTTAA CTAAAACCAA   
  
  
- ACGGTATTAA ACTGATATGT AAATTGAATT TTTTATAAAA AAAAATTAAA GTTTATTTAG ATTTAATAAA   
  
  
- AAGTTATAAG TAGGTTTTAT CTAAGTTAAT AATTTGTTCA GTTGGATATG GCTCAATAAA AGATCGAGAC   
  
  
- TGATTCTGTA TATATATAAA AAACATTTTT TCTATTCCTA ATATTAAATT TATCGGAAAG GGAATTTTTT   
  
  
- TAAAAATTTT AATAGGACTA TCTCTTATTT TTCTTTAGGA ATATGTTTTG AGACTAACTG CGTCTACACA   
  
  
- TATACCTGTA TATCTTCATT ATTCTCCCCA TCCGATCCTT CCCCCACCTT TATATCCCGT ATTTATCCCC   
  
  
- CGCCCGTATC CCTGTACAGA CAGACTCCAT GCCACCTACC CTGGAACGGT ACCTTCGTTG TTACAGTGTT   
  
  
- ATTATTATAG TTGCTGACTT CAAAGAAGTG GTCGGGTGAG AAACGGTATT ACGGTAATTA ATAAATAACG   
  
  
- ATGATAGAGA AAACTACACT TTGCCACCTG GAATAGAGAG GGGAGGTGAA GGTTAACTAC ACTCGTTGAG   
  
  
- TCTTTGCGTT TGTTTTGAAC ACGGTGGAAG AAATGAACGT AGGAGTAAAA GGAGACAGAG AGAGGCGGGG   
  
  
- ACAAAGAGAG AGAGAGGGAG TGAGTGAGTC TCATGACTAG ACTCGTAGTC TTCATCGTCT GGGTCTTTGA   
  
  
- GTCTAAATTT TTGTTGGGCG AGAAAACAAG GGAGATGCAG ATTATTAAAA CAGTAGCGTA AATTACAGTC   
  
  
- TATCTCTCTA TCTCGAGATG ACATAATTAA AAGAGGGTGG AGTGAACTAA GACAACGGGA TTTGGTGGGT   
  
  
- TTTTTTCTTT ATGCTTCTTC GGTGAGAGAG AGAGAGAGAG AGAGTGTGTG TGTGTGTGGG GAGGATCACC   
  
  
- TAAACTATCT CATATTTACT TGTCATTAGT CTGCTCTATA TATCATCACG AATTAACTGG CTCATCGTTT   
  
  
- TCCCCCTTCA ACTCACTCCA TAGTTCTCTC ATCGCATCGA TCGATCCCGT TCTATACGAC CCCAGAAGGA   
  
  
- GTATGTTGAG GCTCCTTCTG GTGGTTCTAC TGTTGGTAAG GAGTGGCAGA CTAAACAGAT CCGTGGGTGG   
  
  
- ATCTAAGTGG TGATGATGAA TGGTTATGGT TGTTGTATAA TCGAGAGGTA GAAGTGTTAG TTATGATCGA   
  
  
- CACGGTGGAG CGGTTAACAA TTAGTTAACA CGCCTCAATT AAACGGCCAA GCTGAAGAGG AGGCGGTTGG   
  
  
- CCGAAGAGAG AGAGGAGCGG AGGGTGAAAC GGAGGGGAAG ACCCCTGAGG TGGCTCTCTG AACACAAAAT   
  
  
- GAAGACGTTC CGAAACCTAG AGGCGGAGTT ATTGTCAATA AATAGGTGAG CATCAAACTG ACCAACATTG   
  
  
- GGATCATGAC CAAGACCATA ACCCCGAGGA AATCGAAGGT TAAAAGATAA AGAAGTTGGT AATCACGTAA   
  
  
- GTGTCCATTG TTCATTACGG GCACAACCCA TGAACTGTAT AAACTGAGAC TTGGTCCAGT GGGGTAAGTA   
  
  
- GGCTAAGTGG GTGAATTGAC GGTTGGTTCG GTAGGACCTT CGAAACCTCC CCATGTTCCG CCAGGTATAG   
  
  
- GAACTGTACC TGTAGTACGT ACCCCAGGTC ACCGGTGGTG AAGAAGTTCG TTAACGTCTT TCTAGACGTT   
  
  
- GGGAACCAGT GGGACGAGGG TGACAAGCGT AGTGTCCTCC CCCGTCCCTA GATCTTGAGG ACTTGGCTTG   
  
  
- ACCTCTAGCC TAGTCCTTCA AGCGTGTTAG GAACCCTGAG CTCAAAGTTA AGGTGGTAGA ATATTGCCAC   
  
  
- CTGAGCTTGG AATGACTCCG TCACCTCCGG TAGTTACGGG ATGTCGATGT GGAGGTACAG GTTCTCCTCC   
  
  
- TCCAGAAGCG TCACTTAACA CCTCTAAAGG AGGTGGCGGA GGATTGCCTC ATACTATCAT GTAAGGAATC   
  
  
- CTACAAAGAG ATGTTCCACT TCTGGGATTT GGGATCCCAG AAGTGGCACC CTCTTTCCCT CCGGCTGGTG   
  
  
- TTGGTGGGAG AGAAGACCGT CGCTAAGTAA CTCCGTGAAC TGGTGATACC CCGTCACAAA CTGAGAGATC   
  
  
- TTCGTTGGGA TGGTAGTTGT TCAGTTCTCT CCCACCGCCA CCTTCTCCCC ACCAAGCCCC TCCTCTAATT   
  
  
- TCTACACCAA CCTGCTCTTC CTCCCAACTC CTCTGTCTCC GTGGTCTTTA TGCTCAGAAC CCTCCACAAC   
  
  
- TACTCCTCGA GACCCAAATT CTCGAACGGG AACTCGGGGA AGAGACACAG TGTTCGGTTC GACGATGAAG   
  
  
- CGGAAGTAAT GGGAAGACTT CCCATGGTGG AATTCCAGAA GGTATCATTA ACAGACAACG AACCCACCTT   
  
  
- CTTAGCGGGA GAAAAGAGAC AAAGAAGAAC CGTTAT

+     DRE core

| Site Name | Organism | Position | Strand | Matrix score. | sequence | function |
| --- | --- | --- | --- | --- | --- | --- |
| DRE core | Arabidopsis thaliana | 3006 | + | 6 | GCCGAC |  |

>HU01G00833.1   
+ -Up\_Stream \_Len000AAAATA TTTTTACAAA AGCATTTCTC TATAAAATAT TTTACAAAAT TTCATTTTGT   
  
  
+ GGGTCTTTTA GAATCAATCT CTCTACAAGT AGGAATGCCG GTTGAGGTAA GGCTACCTAC ATCTAGATCT   
  
  
+ CTCCAGACCC CACAAAAATT AAACGAGACC CACACTGAAA GGTGGTGGTG GTGGTGTTGT TGTTGTATTG   
  
  
+ TCCTACCACG AGAGAGCTTC AAGTGCATCA CTTATTTCAA CAGGTCAATC ACATAGATCT CTAGTGTACT   
  
  
+ GTAAATTTTT TTTCCGTCTT ATTTTAATTT TCTTTTCCAT CCTAAAAGAT TATCATGTAG ATATTCCTAG   
  
  
+ TTTGATATAT TGACTTCAAC TTCAAAAATT ACACATTAAG ATCCACGAAT ATGACAGTTT AGGTTTCATT   
  
  
+ TGAAGGAGAA AGAAATGTTA CGGTGTTTTG GTGTTGTATT CGTAGACATT TTTTGCGCTT AACTGGCTCT   
  
  
+ TCTCATGGGG ACGAGACAGT GAAGGTAATT GTACTTGTTG ATTGATGACG TCATCTGTAG CTTGGATTTG   
  
  
+ TATAGGAATG AACATCTTGG TGTCTACGTG GAAAGTGTGA TTTGTGTTCT TTCATTGATC CTCCTCCTCA   
  
  
+ AGAAGAGGTG ATCTTTCTCC CTTGTTTTTT TTTTTTTTTG GCTAATATGT TGGCAGAACC GGCAAAAAAT   
  
  
+ ACATAATTTG AATAGTGCAT GCATATGAGT ACATGACAGT GAACCAACAA AACGAACCCC TAAAACACAA   
  
  
+ GAGAATTAAC ATGTTAAAAC ACTTAAACTA AATGCAACCA TCTCGATGAC CACTAAATAG CCAAGAAACC   
  
  
+ TAACGTAATT AACAAAAATA AAAAATAACT GAAAATAAAG TAGAGAAAGT CTAACCGAAT ACGATAACAT   
  
  
+ AACCTAAAAC TAACTATACT TGAACTTACT AAGAAATATG AATCAACCAC AAACCGAATT GATTTTGGTT   
  
  
+ TGCCATAATT TGACTATACA TTTAACTTAA AAAATATTTT TTTTTAATTT CAAATAAATC TAAATTATTT   
  
  
+ TTCAATATTC ATCCAAAATA GATTCAATTA TTAAACAAGT CAACCTATAC CGAGTTATTT TCTAGCTCTG   
  
  
+ ACTAAGACAT ATATATATTT TTTGTAAAAA AGATAAGGAT TATAATTTAA ATAGCCTTTC CCTTAAAAAA   
  
  
+ ATTTTTAAAA TTATCCTGAT AGAGAATAAA AAGAAATCCT TATACAAAAC TCTGATTGAC GCAGATGTGT   
  
  
+ ATATGGACAT ATAGAAGTAA TAAGAGGGGT AGGCTAGGAA GGGGGTGGAA ATATAGGGCA TAAATAGGGG   
  
  
+ GCGGGCATAG GGACATGTCT GTCTGAGGTA CGGTGGATGG GACCTTGCCA TGGAAGCAAC AATGTCACAA   
  
  
+ TAATAATATC AACGACTGAA GTTTCTTCAC CAGCCCACTC TTTGCCATAA TGCCATTAAT TATTTATTGC   
  
  
+ TACTATCTCT TTTGATGTGA AACGGTGGAC CTTATCTCTC CCCTCCACTT CCAATTGATG TGAGCAACTC   
  
  
+ AGAAACGCAA ACAAAACTTG TGCCACCTTC TTTACTTGCA TCCTCATTTT CCTCTGTCTC TCTCCGCCCC   
  
  
+ TGTTTCTCTC TCTCTCCCTC ACTCACTCAG AGTACTGATC TGAGCATCAG AAGTAGCAGA CCCAGAAACT   
  
  
+ CAGATTTAAA AACAACCCGC TCTTTTGTTC CCTCTACGTC TAATAATTTT GTCATCGCAT TTAATGTCAG   
  
  
+ ATAGAGAGAT AGAGCTCTAC TGTATTAATT TTCTCCCACC TCACTTGATT CTGTTGCCCT AAACCACCCA   
  
  
+ AAAAAAGAAA TACGAAGAAG CCACTCTCTC TCTCTCTCTC TCTCACACAC ACACACACCC CTCCTAGTGG   
  
  
+ ATTTGATAGA GTATAAATGA ACAGTAATCA GACGAGATAT ATAGTAGTGC TTAATTGACC GAGTAGCAAA   
  
  
+ AGGGGGAAGT TGAGTGAGGT ATCAAGAGAG TAGCGTAGCT AGCTAGGGCA AGATATGCTG GGGTCTTCCT   
  
  
+ CATACAACTC CGAGGAAGAC CACCAAGATG ACAACCATTC CTCACCGTCT GATTTGTCTA GGCACCCACC   
  
  
+ TAGATTCACC ACTACTACTT ACCAATACCA ACAACATATT AGCTCTCCAT CTTCACAATC AATACTAGCT   
  
  
+ GTGCCACCTC GCCAATTGTT AATCAATTGT GCGGAGTTAA TTTGCCGGTT CGACTTCTCC TCCGCCAACC   
  
  
+ GGCTTCTCTC TCTCCTCGCC TCCCACTTTG CCTCCCCTTC TGGGGACTCC ACCGAGAGAC TTGTGTTTTA   
  
  
+ CTTCTGCAAG GCTTTGGATC TCCGCCTCAA TAACAGTTAT TTATCCACTC GTAGTTTGAC TGGTTGTAAC   
  
  
+ CCTAGTACTG GTTCTGGTAT TGGGGCTCCT TTAGCTTCCA ATTTTCTATT TCTTCAACCA TTAGTGCATT   
  
  
+ CACAGGTAAC AAGTAATGCC CGTGTTGGGT ACTTGACATA TTTGACTCTG AACCAGGTCA CCCCATTCAT   
  
  
+ CCGATTCACC CACTTAACTG CCAACCAAGC CATCCTGGAA GCTTTGGAGG GGTACAAGGC GGTCCATATC   
  
  
+ CTTGACATGG ACATCATGCA TGGGGTCCAG TGGCCACCAC TTCTTCAAGC AATTGCAGAA AGATCTGCAA   
  
  
+ CCCTTGGTCA CCCTGCTCCC ACTGTTCGCA TCACAGGAGG GGGCAGGGAT CTAGAACTCC TGAACCGAAC   
  
  
+ TGGAGATCGG ATCAGGAAGT TCGCACAATC CTTGGGACTC GAGTTTCAAT TCCACCATCT TATAACGGTG   
  
  
+ GACTCGAACC TTACTGAGGC AGTGGAGGCC ATCAATGCCC TACAGCTACA CCTCCATGTC CAAGAGGAGG   
  
  
+ AGGTCTTCGC AGTGAATTGT GGAGATTTCC TCCACCGCCT CCTAACGGAG TATGATAGTA CATTCCTTAG   
  
  
+ GATGTTTCTC TACAAGGTGA AGACCCTAAA CCCTAGGGTC TTCACCGTGG GAGAAAGGGA GGCCGACCAC   
  
  
+ AACCACCCTC TCTTCTGGCA GCGATTCATT GAGGCACTTG ACCACTATGG GGCAGTGTTT GACTCTCTAG   
  
  
+ AAGCAACCCT ACCATCAACA AGTCAAGAGA GGGTGGCGGT GGAAGAGGGG TGGTTCGGGG AGGAGATTAA   
  
  
+ AGATGTGGTT GGACGAGAAG GAGGGTTGAG GAGACAGAGG CACCAGAAAT ACGAGTCTTG GGAGGTGTTG   
  
  
+ ATGAGGAGCT CTGGGTTTAA GAGCTTGCCC TTGAGCCCCT TCTCTGTGTC ACAAGCCAAG CTGCTACTTC   
  
  
+ GCCTTCATTA CCCTTCTGAA GGGTACCACC TTAAGGTCTT CCATAGTAAT TGTCTGTTGC TTGGGTGGAA   
  
  
+ GAATCGCCCT CTTTTCTCTG TTTCTTCTTG GCAATA  

- -Up\_Stream \_Len000TTTTAT AAAAATGTTT TCGTAAAGAG ATATTTTATA AAATGTTTTA AAGTAAAACA   
  
  
- CCCAGAAAAT CTTAGTTAGA GAGATGTTCA TCCTTACGGC CAACTCCATT CCGATGGATG TAGATCTAGA   
  
  
- GAGGTCTGGG GTGTTTTTAA TTTGCTCTGG GTGTGACTTT CCACCACCAC CACCACAACA ACAACATAAC   
  
  
- AGGATGGTGC TCTCTCGAAG TTCACGTAGT GAATAAAGTT GTCCAGTTAG TGTATCTAGA GATCACATGA   
  
  
- CATTTAAAAA AAAGGCAGAA TAAAATTAAA AGAAAAGGTA GGATTTTCTA ATAGTACATC TATAAGGATC   
  
  
- AAACTATATA ACTGAAGTTG AAGTTTTTAA TGTGTAATTC TAGGTGCTTA TACTGTCAAA TCCAAAGTAA   
  
  
- ACTTCCTCTT TCTTTACAAT GCCACAAAAC CACAACATAA GCATCTGTAA AAAACGCGAA TTGACCGAGA   
  
  
- AGAGTACCCC TGCTCTGTCA CTTCCATTAA CATGAACAAC TAACTACTGC AGTAGACATC GAACCTAAAC   
  
  
- ATATCCTTAC TTGTAGAACC ACAGATGCAC CTTTCACACT AAACACAAGA AAGTAACTAG GAGGAGGAGT   
  
  
- TCTTCTCCAC TAGAAAGAGG GAACAAAAAA AAAAAAAAAC CGATTATACA ACCGTCTTGG CCGTTTTTTA   
  
  
- TGTATTAAAC TTATCACGTA CGTATACTCA TGTACTGTCA CTTGGTTGTT TTGCTTGGGG ATTTTGTGTT   
  
  
- CTCTTAATTG TACAATTTTG TGAATTTGAT TTACGTTGGT AGAGCTACTG GTGATTTATC GGTTCTTTGG   
  
  
- ATTGCATTAA TTGTTTTTAT TTTTTATTGA CTTTTATTTC ATCTCTTTCA GATTGGCTTA TGCTATTGTA   
  
  
- TTGGATTTTG ATTGATATGA ACTTGAATGA TTCTTTATAC TTAGTTGGTG TTTGGCTTAA CTAAAACCAA   
  
  
- ACGGTATTAA ACTGATATGT AAATTGAATT TTTTATAAAA AAAAATTAAA GTTTATTTAG ATTTAATAAA   
  
  
- AAGTTATAAG TAGGTTTTAT CTAAGTTAAT AATTTGTTCA GTTGGATATG GCTCAATAAA AGATCGAGAC   
  
  
- TGATTCTGTA TATATATAAA AAACATTTTT TCTATTCCTA ATATTAAATT TATCGGAAAG GGAATTTTTT   
  
  
- TAAAAATTTT AATAGGACTA TCTCTTATTT TTCTTTAGGA ATATGTTTTG AGACTAACTG CGTCTACACA   
  
  
- TATACCTGTA TATCTTCATT ATTCTCCCCA TCCGATCCTT CCCCCACCTT TATATCCCGT ATTTATCCCC   
  
  
- CGCCCGTATC CCTGTACAGA CAGACTCCAT GCCACCTACC CTGGAACGGT ACCTTCGTTG TTACAGTGTT   
  
  
- ATTATTATAG TTGCTGACTT CAAAGAAGTG GTCGGGTGAG AAACGGTATT ACGGTAATTA ATAAATAACG   
  
  
- ATGATAGAGA AAACTACACT TTGCCACCTG GAATAGAGAG GGGAGGTGAA GGTTAACTAC ACTCGTTGAG   
  
  
- TCTTTGCGTT TGTTTTGAAC ACGGTGGAAG AAATGAACGT AGGAGTAAAA GGAGACAGAG AGAGGCGGGG   
  
  
- ACAAAGAGAG AGAGAGGGAG TGAGTGAGTC TCATGACTAG ACTCGTAGTC TTCATCGTCT GGGTCTTTGA   
  
  
- GTCTAAATTT TTGTTGGGCG AGAAAACAAG GGAGATGCAG ATTATTAAAA CAGTAGCGTA AATTACAGTC   
  
  
- TATCTCTCTA TCTCGAGATG ACATAATTAA AAGAGGGTGG AGTGAACTAA GACAACGGGA TTTGGTGGGT   
  
  
- TTTTTTCTTT ATGCTTCTTC GGTGAGAGAG AGAGAGAGAG AGAGTGTGTG TGTGTGTGGG GAGGATCACC   
  
  
- TAAACTATCT CATATTTACT TGTCATTAGT CTGCTCTATA TATCATCACG AATTAACTGG CTCATCGTTT   
  
  
- TCCCCCTTCA ACTCACTCCA TAGTTCTCTC ATCGCATCGA TCGATCCCGT TCTATACGAC CCCAGAAGGA   
  
  
- GTATGTTGAG GCTCCTTCTG GTGGTTCTAC TGTTGGTAAG GAGTGGCAGA CTAAACAGAT CCGTGGGTGG   
  
  
- ATCTAAGTGG TGATGATGAA TGGTTATGGT TGTTGTATAA TCGAGAGGTA GAAGTGTTAG TTATGATCGA   
  
  
- CACGGTGGAG CGGTTAACAA TTAGTTAACA CGCCTCAATT AAACGGCCAA GCTGAAGAGG AGGCGGTTGG   
  
  
- CCGAAGAGAG AGAGGAGCGG AGGGTGAAAC GGAGGGGAAG ACCCCTGAGG TGGCTCTCTG AACACAAAAT   
  
  
- GAAGACGTTC CGAAACCTAG AGGCGGAGTT ATTGTCAATA AATAGGTGAG CATCAAACTG ACCAACATTG   
  
  
- GGATCATGAC CAAGACCATA ACCCCGAGGA AATCGAAGGT TAAAAGATAA AGAAGTTGGT AATCACGTAA   
  
  
- GTGTCCATTG TTCATTACGG GCACAACCCA TGAACTGTAT AAACTGAGAC TTGGTCCAGT GGGGTAAGTA   
  
  
- GGCTAAGTGG GTGAATTGAC GGTTGGTTCG GTAGGACCTT CGAAACCTCC CCATGTTCCG CCAGGTATAG   
  
  
- GAACTGTACC TGTAGTACGT ACCCCAGGTC ACCGGTGGTG AAGAAGTTCG TTAACGTCTT TCTAGACGTT   
  
  
- GGGAACCAGT GGGACGAGGG TGACAAGCGT AGTGTCCTCC CCCGTCCCTA GATCTTGAGG ACTTGGCTTG   
  
  
- ACCTCTAGCC TAGTCCTTCA AGCGTGTTAG GAACCCTGAG CTCAAAGTTA AGGTGGTAGA ATATTGCCAC   
  
  
- CTGAGCTTGG AATGACTCCG TCACCTCCGG TAGTTACGGG ATGTCGATGT GGAGGTACAG GTTCTCCTCC   
  
  
- TCCAGAAGCG TCACTTAACA CCTCTAAAGG AGGTGGCGGA GGATTGCCTC ATACTATCAT GTAAGGAATC   
  
  
- CTACAAAGAG ATGTTCCACT TCTGGGATTT GGGATCCCAG AAGTGGCACC CTCTTTCCCT CCGGCTGGTG   
  
  
- TTGGTGGGAG AGAAGACCGT CGCTAAGTAA CTCCGTGAAC TGGTGATACC CCGTCACAAA CTGAGAGATC   
  
  
- TTCGTTGGGA TGGTAGTTGT TCAGTTCTCT CCCACCGCCA CCTTCTCCCC ACCAAGCCCC TCCTCTAATT   
  
  
- TCTACACCAA CCTGCTCTTC CTCCCAACTC CTCTGTCTCC GTGGTCTTTA TGCTCAGAAC CCTCCACAAC   
  
  
- TACTCCTCGA GACCCAAATT CTCGAACGGG AACTCGGGGA AGAGACACAG TGTTCGGTTC GACGATGAAG   
  
  
- CGGAAGTAAT GGGAAGACTT CCCATGGTGG AATTCCAGAA GGTATCATTA ACAGACAACG AACCCACCTT   
  
  
- CTTAGCGGGA GAAAAGAGAC AAAGAAGAAC CGTTAT

+     DRE1

| Site Name | Organism | Position | Strand | Matrix score. | sequence | function |
| --- | --- | --- | --- | --- | --- | --- |
| DRE1 | Zea mays | 2295 | + | 7 | ACCGAGA |  |

>HU01G00833.1   
+ -Up\_Stream \_Len000AAAATA TTTTTACAAA AGCATTTCTC TATAAAATAT TTTACAAAAT TTCATTTTGT   
  
  
+ GGGTCTTTTA GAATCAATCT CTCTACAAGT AGGAATGCCG GTTGAGGTAA GGCTACCTAC ATCTAGATCT   
  
  
+ CTCCAGACCC CACAAAAATT AAACGAGACC CACACTGAAA GGTGGTGGTG GTGGTGTTGT TGTTGTATTG   
  
  
+ TCCTACCACG AGAGAGCTTC AAGTGCATCA CTTATTTCAA CAGGTCAATC ACATAGATCT CTAGTGTACT   
  
  
+ GTAAATTTTT TTTCCGTCTT ATTTTAATTT TCTTTTCCAT CCTAAAAGAT TATCATGTAG ATATTCCTAG   
  
  
+ TTTGATATAT TGACTTCAAC TTCAAAAATT ACACATTAAG ATCCACGAAT ATGACAGTTT AGGTTTCATT   
  
  
+ TGAAGGAGAA AGAAATGTTA CGGTGTTTTG GTGTTGTATT CGTAGACATT TTTTGCGCTT AACTGGCTCT   
  
  
+ TCTCATGGGG ACGAGACAGT GAAGGTAATT GTACTTGTTG ATTGATGACG TCATCTGTAG CTTGGATTTG   
  
  
+ TATAGGAATG AACATCTTGG TGTCTACGTG GAAAGTGTGA TTTGTGTTCT TTCATTGATC CTCCTCCTCA   
  
  
+ AGAAGAGGTG ATCTTTCTCC CTTGTTTTTT TTTTTTTTTG GCTAATATGT TGGCAGAACC GGCAAAAAAT   
  
  
+ ACATAATTTG AATAGTGCAT GCATATGAGT ACATGACAGT GAACCAACAA AACGAACCCC TAAAACACAA   
  
  
+ GAGAATTAAC ATGTTAAAAC ACTTAAACTA AATGCAACCA TCTCGATGAC CACTAAATAG CCAAGAAACC   
  
  
+ TAACGTAATT AACAAAAATA AAAAATAACT GAAAATAAAG TAGAGAAAGT CTAACCGAAT ACGATAACAT   
  
  
+ AACCTAAAAC TAACTATACT TGAACTTACT AAGAAATATG AATCAACCAC AAACCGAATT GATTTTGGTT   
  
  
+ TGCCATAATT TGACTATACA TTTAACTTAA AAAATATTTT TTTTTAATTT CAAATAAATC TAAATTATTT   
  
  
+ TTCAATATTC ATCCAAAATA GATTCAATTA TTAAACAAGT CAACCTATAC CGAGTTATTT TCTAGCTCTG   
  
  
+ ACTAAGACAT ATATATATTT TTTGTAAAAA AGATAAGGAT TATAATTTAA ATAGCCTTTC CCTTAAAAAA   
  
  
+ ATTTTTAAAA TTATCCTGAT AGAGAATAAA AAGAAATCCT TATACAAAAC TCTGATTGAC GCAGATGTGT   
  
  
+ ATATGGACAT ATAGAAGTAA TAAGAGGGGT AGGCTAGGAA GGGGGTGGAA ATATAGGGCA TAAATAGGGG   
  
  
+ GCGGGCATAG GGACATGTCT GTCTGAGGTA CGGTGGATGG GACCTTGCCA TGGAAGCAAC AATGTCACAA   
  
  
+ TAATAATATC AACGACTGAA GTTTCTTCAC CAGCCCACTC TTTGCCATAA TGCCATTAAT TATTTATTGC   
  
  
+ TACTATCTCT TTTGATGTGA AACGGTGGAC CTTATCTCTC CCCTCCACTT CCAATTGATG TGAGCAACTC   
  
  
+ AGAAACGCAA ACAAAACTTG TGCCACCTTC TTTACTTGCA TCCTCATTTT CCTCTGTCTC TCTCCGCCCC   
  
  
+ TGTTTCTCTC TCTCTCCCTC ACTCACTCAG AGTACTGATC TGAGCATCAG AAGTAGCAGA CCCAGAAACT   
  
  
+ CAGATTTAAA AACAACCCGC TCTTTTGTTC CCTCTACGTC TAATAATTTT GTCATCGCAT TTAATGTCAG   
  
  
+ ATAGAGAGAT AGAGCTCTAC TGTATTAATT TTCTCCCACC TCACTTGATT CTGTTGCCCT AAACCACCCA   
  
  
+ AAAAAAGAAA TACGAAGAAG CCACTCTCTC TCTCTCTCTC TCTCACACAC ACACACACCC CTCCTAGTGG   
  
  
+ ATTTGATAGA GTATAAATGA ACAGTAATCA GACGAGATAT ATAGTAGTGC TTAATTGACC GAGTAGCAAA   
  
  
+ AGGGGGAAGT TGAGTGAGGT ATCAAGAGAG TAGCGTAGCT AGCTAGGGCA AGATATGCTG GGGTCTTCCT   
  
  
+ CATACAACTC CGAGGAAGAC CACCAAGATG ACAACCATTC CTCACCGTCT GATTTGTCTA GGCACCCACC   
  
  
+ TAGATTCACC ACTACTACTT ACCAATACCA ACAACATATT AGCTCTCCAT CTTCACAATC AATACTAGCT   
  
  
+ GTGCCACCTC GCCAATTGTT AATCAATTGT GCGGAGTTAA TTTGCCGGTT CGACTTCTCC TCCGCCAACC   
  
  
+ GGCTTCTCTC TCTCCTCGCC TCCCACTTTG CCTCCCCTTC TGGGGACTCC ACCGAGAGAC TTGTGTTTTA   
  
  
+ CTTCTGCAAG GCTTTGGATC TCCGCCTCAA TAACAGTTAT TTATCCACTC GTAGTTTGAC TGGTTGTAAC   
  
  
+ CCTAGTACTG GTTCTGGTAT TGGGGCTCCT TTAGCTTCCA ATTTTCTATT TCTTCAACCA TTAGTGCATT   
  
  
+ CACAGGTAAC AAGTAATGCC CGTGTTGGGT ACTTGACATA TTTGACTCTG AACCAGGTCA CCCCATTCAT   
  
  
+ CCGATTCACC CACTTAACTG CCAACCAAGC CATCCTGGAA GCTTTGGAGG GGTACAAGGC GGTCCATATC   
  
  
+ CTTGACATGG ACATCATGCA TGGGGTCCAG TGGCCACCAC TTCTTCAAGC AATTGCAGAA AGATCTGCAA   
  
  
+ CCCTTGGTCA CCCTGCTCCC ACTGTTCGCA TCACAGGAGG GGGCAGGGAT CTAGAACTCC TGAACCGAAC   
  
  
+ TGGAGATCGG ATCAGGAAGT TCGCACAATC CTTGGGACTC GAGTTTCAAT TCCACCATCT TATAACGGTG   
  
  
+ GACTCGAACC TTACTGAGGC AGTGGAGGCC ATCAATGCCC TACAGCTACA CCTCCATGTC CAAGAGGAGG   
  
  
+ AGGTCTTCGC AGTGAATTGT GGAGATTTCC TCCACCGCCT CCTAACGGAG TATGATAGTA CATTCCTTAG   
  
  
+ GATGTTTCTC TACAAGGTGA AGACCCTAAA CCCTAGGGTC TTCACCGTGG GAGAAAGGGA GGCCGACCAC   
  
  
+ AACCACCCTC TCTTCTGGCA GCGATTCATT GAGGCACTTG ACCACTATGG GGCAGTGTTT GACTCTCTAG   
  
  
+ AAGCAACCCT ACCATCAACA AGTCAAGAGA GGGTGGCGGT GGAAGAGGGG TGGTTCGGGG AGGAGATTAA   
  
  
+ AGATGTGGTT GGACGAGAAG GAGGGTTGAG GAGACAGAGG CACCAGAAAT ACGAGTCTTG GGAGGTGTTG   
  
  
+ ATGAGGAGCT CTGGGTTTAA GAGCTTGCCC TTGAGCCCCT TCTCTGTGTC ACAAGCCAAG CTGCTACTTC   
  
  
+ GCCTTCATTA CCCTTCTGAA GGGTACCACC TTAAGGTCTT CCATAGTAAT TGTCTGTTGC TTGGGTGGAA   
  
  
+ GAATCGCCCT CTTTTCTCTG TTTCTTCTTG GCAATA  

- -Up\_Stream \_Len000TTTTAT AAAAATGTTT TCGTAAAGAG ATATTTTATA AAATGTTTTA AAGTAAAACA   
  
  
- CCCAGAAAAT CTTAGTTAGA GAGATGTTCA TCCTTACGGC CAACTCCATT CCGATGGATG TAGATCTAGA   
  
  
- GAGGTCTGGG GTGTTTTTAA TTTGCTCTGG GTGTGACTTT CCACCACCAC CACCACAACA ACAACATAAC   
  
  
- AGGATGGTGC TCTCTCGAAG TTCACGTAGT GAATAAAGTT GTCCAGTTAG TGTATCTAGA GATCACATGA   
  
  
- CATTTAAAAA AAAGGCAGAA TAAAATTAAA AGAAAAGGTA GGATTTTCTA ATAGTACATC TATAAGGATC   
  
  
- AAACTATATA ACTGAAGTTG AAGTTTTTAA TGTGTAATTC TAGGTGCTTA TACTGTCAAA TCCAAAGTAA   
  
  
- ACTTCCTCTT TCTTTACAAT GCCACAAAAC CACAACATAA GCATCTGTAA AAAACGCGAA TTGACCGAGA   
  
  
- AGAGTACCCC TGCTCTGTCA CTTCCATTAA CATGAACAAC TAACTACTGC AGTAGACATC GAACCTAAAC   
  
  
- ATATCCTTAC TTGTAGAACC ACAGATGCAC CTTTCACACT AAACACAAGA AAGTAACTAG GAGGAGGAGT   
  
  
- TCTTCTCCAC TAGAAAGAGG GAACAAAAAA AAAAAAAAAC CGATTATACA ACCGTCTTGG CCGTTTTTTA   
  
  
- TGTATTAAAC TTATCACGTA CGTATACTCA TGTACTGTCA CTTGGTTGTT TTGCTTGGGG ATTTTGTGTT   
  
  
- CTCTTAATTG TACAATTTTG TGAATTTGAT TTACGTTGGT AGAGCTACTG GTGATTTATC GGTTCTTTGG   
  
  
- ATTGCATTAA TTGTTTTTAT TTTTTATTGA CTTTTATTTC ATCTCTTTCA GATTGGCTTA TGCTATTGTA   
  
  
- TTGGATTTTG ATTGATATGA ACTTGAATGA TTCTTTATAC TTAGTTGGTG TTTGGCTTAA CTAAAACCAA   
  
  
- ACGGTATTAA ACTGATATGT AAATTGAATT TTTTATAAAA AAAAATTAAA GTTTATTTAG ATTTAATAAA   
  
  
- AAGTTATAAG TAGGTTTTAT CTAAGTTAAT AATTTGTTCA GTTGGATATG GCTCAATAAA AGATCGAGAC   
  
  
- TGATTCTGTA TATATATAAA AAACATTTTT TCTATTCCTA ATATTAAATT TATCGGAAAG GGAATTTTTT   
  
  
- TAAAAATTTT AATAGGACTA TCTCTTATTT TTCTTTAGGA ATATGTTTTG AGACTAACTG CGTCTACACA   
  
  
- TATACCTGTA TATCTTCATT ATTCTCCCCA TCCGATCCTT CCCCCACCTT TATATCCCGT ATTTATCCCC   
  
  
- CGCCCGTATC CCTGTACAGA CAGACTCCAT GCCACCTACC CTGGAACGGT ACCTTCGTTG TTACAGTGTT   
  
  
- ATTATTATAG TTGCTGACTT CAAAGAAGTG GTCGGGTGAG AAACGGTATT ACGGTAATTA ATAAATAACG   
  
  
- ATGATAGAGA AAACTACACT TTGCCACCTG GAATAGAGAG GGGAGGTGAA GGTTAACTAC ACTCGTTGAG   
  
  
- TCTTTGCGTT TGTTTTGAAC ACGGTGGAAG AAATGAACGT AGGAGTAAAA GGAGACAGAG AGAGGCGGGG   
  
  
- ACAAAGAGAG AGAGAGGGAG TGAGTGAGTC TCATGACTAG ACTCGTAGTC TTCATCGTCT GGGTCTTTGA   
  
  
- GTCTAAATTT TTGTTGGGCG AGAAAACAAG GGAGATGCAG ATTATTAAAA CAGTAGCGTA AATTACAGTC   
  
  
- TATCTCTCTA TCTCGAGATG ACATAATTAA AAGAGGGTGG AGTGAACTAA GACAACGGGA TTTGGTGGGT   
  
  
- TTTTTTCTTT ATGCTTCTTC GGTGAGAGAG AGAGAGAGAG AGAGTGTGTG TGTGTGTGGG GAGGATCACC   
  
  
- TAAACTATCT CATATTTACT TGTCATTAGT CTGCTCTATA TATCATCACG AATTAACTGG CTCATCGTTT   
  
  
- TCCCCCTTCA ACTCACTCCA TAGTTCTCTC ATCGCATCGA TCGATCCCGT TCTATACGAC CCCAGAAGGA   
  
  
- GTATGTTGAG GCTCCTTCTG GTGGTTCTAC TGTTGGTAAG GAGTGGCAGA CTAAACAGAT CCGTGGGTGG   
  
  
- ATCTAAGTGG TGATGATGAA TGGTTATGGT TGTTGTATAA TCGAGAGGTA GAAGTGTTAG TTATGATCGA   
  
  
- CACGGTGGAG CGGTTAACAA TTAGTTAACA CGCCTCAATT AAACGGCCAA GCTGAAGAGG AGGCGGTTGG   
  
  
- CCGAAGAGAG AGAGGAGCGG AGGGTGAAAC GGAGGGGAAG ACCCCTGAGG TGGCTCTCTG AACACAAAAT   
  
  
- GAAGACGTTC CGAAACCTAG AGGCGGAGTT ATTGTCAATA AATAGGTGAG CATCAAACTG ACCAACATTG   
  
  
- GGATCATGAC CAAGACCATA ACCCCGAGGA AATCGAAGGT TAAAAGATAA AGAAGTTGGT AATCACGTAA   
  
  
- GTGTCCATTG TTCATTACGG GCACAACCCA TGAACTGTAT AAACTGAGAC TTGGTCCAGT GGGGTAAGTA   
  
  
- GGCTAAGTGG GTGAATTGAC GGTTGGTTCG GTAGGACCTT CGAAACCTCC CCATGTTCCG CCAGGTATAG   
  
  
- GAACTGTACC TGTAGTACGT ACCCCAGGTC ACCGGTGGTG AAGAAGTTCG TTAACGTCTT TCTAGACGTT   
  
  
- GGGAACCAGT GGGACGAGGG TGACAAGCGT AGTGTCCTCC CCCGTCCCTA GATCTTGAGG ACTTGGCTTG   
  
  
- ACCTCTAGCC TAGTCCTTCA AGCGTGTTAG GAACCCTGAG CTCAAAGTTA AGGTGGTAGA ATATTGCCAC   
  
  
- CTGAGCTTGG AATGACTCCG TCACCTCCGG TAGTTACGGG ATGTCGATGT GGAGGTACAG GTTCTCCTCC   
  
  
- TCCAGAAGCG TCACTTAACA CCTCTAAAGG AGGTGGCGGA GGATTGCCTC ATACTATCAT GTAAGGAATC   
  
  
- CTACAAAGAG ATGTTCCACT TCTGGGATTT GGGATCCCAG AAGTGGCACC CTCTTTCCCT CCGGCTGGTG   
  
  
- TTGGTGGGAG AGAAGACCGT CGCTAAGTAA CTCCGTGAAC TGGTGATACC CCGTCACAAA CTGAGAGATC   
  
  
- TTCGTTGGGA TGGTAGTTGT TCAGTTCTCT CCCACCGCCA CCTTCTCCCC ACCAAGCCCC TCCTCTAATT   
  
  
- TCTACACCAA CCTGCTCTTC CTCCCAACTC CTCTGTCTCC GTGGTCTTTA TGCTCAGAAC CCTCCACAAC   
  
  
- TACTCCTCGA GACCCAAATT CTCGAACGGG AACTCGGGGA AGAGACACAG TGTTCGGTTC GACGATGAAG   
  
  
- CGGAAGTAAT GGGAAGACTT CCCATGGTGG AATTCCAGAA GGTATCATTA ACAGACAACG AACCCACCTT   
  
  
- CTTAGCGGGA GAAAAGAGAC AAAGAAGAAC CGTTAT

+     ERE

| Site Name | Organism | Position | Strand | Matrix score. | sequence | function |
| --- | --- | --- | --- | --- | --- | --- |
| ERE | Nicotiana glutinos | 1198 | - | 8 | ATTTTAAA |  |

>HU01G00833.1   
+ -Up\_Stream \_Len000AAAATA TTTTTACAAA AGCATTTCTC TATAAAATAT TTTACAAAAT TTCATTTTGT   
  
  
+ GGGTCTTTTA GAATCAATCT CTCTACAAGT AGGAATGCCG GTTGAGGTAA GGCTACCTAC ATCTAGATCT   
  
  
+ CTCCAGACCC CACAAAAATT AAACGAGACC CACACTGAAA GGTGGTGGTG GTGGTGTTGT TGTTGTATTG   
  
  
+ TCCTACCACG AGAGAGCTTC AAGTGCATCA CTTATTTCAA CAGGTCAATC ACATAGATCT CTAGTGTACT   
  
  
+ GTAAATTTTT TTTCCGTCTT ATTTTAATTT TCTTTTCCAT CCTAAAAGAT TATCATGTAG ATATTCCTAG   
  
  
+ TTTGATATAT TGACTTCAAC TTCAAAAATT ACACATTAAG ATCCACGAAT ATGACAGTTT AGGTTTCATT   
  
  
+ TGAAGGAGAA AGAAATGTTA CGGTGTTTTG GTGTTGTATT CGTAGACATT TTTTGCGCTT AACTGGCTCT   
  
  
+ TCTCATGGGG ACGAGACAGT GAAGGTAATT GTACTTGTTG ATTGATGACG TCATCTGTAG CTTGGATTTG   
  
  
+ TATAGGAATG AACATCTTGG TGTCTACGTG GAAAGTGTGA TTTGTGTTCT TTCATTGATC CTCCTCCTCA   
  
  
+ AGAAGAGGTG ATCTTTCTCC CTTGTTTTTT TTTTTTTTTG GCTAATATGT TGGCAGAACC GGCAAAAAAT   
  
  
+ ACATAATTTG AATAGTGCAT GCATATGAGT ACATGACAGT GAACCAACAA AACGAACCCC TAAAACACAA   
  
  
+ GAGAATTAAC ATGTTAAAAC ACTTAAACTA AATGCAACCA TCTCGATGAC CACTAAATAG CCAAGAAACC   
  
  
+ TAACGTAATT AACAAAAATA AAAAATAACT GAAAATAAAG TAGAGAAAGT CTAACCGAAT ACGATAACAT   
  
  
+ AACCTAAAAC TAACTATACT TGAACTTACT AAGAAATATG AATCAACCAC AAACCGAATT GATTTTGGTT   
  
  
+ TGCCATAATT TGACTATACA TTTAACTTAA AAAATATTTT TTTTTAATTT CAAATAAATC TAAATTATTT   
  
  
+ TTCAATATTC ATCCAAAATA GATTCAATTA TTAAACAAGT CAACCTATAC CGAGTTATTT TCTAGCTCTG   
  
  
+ ACTAAGACAT ATATATATTT TTTGTAAAAA AGATAAGGAT TATAATTTAA ATAGCCTTTC CCTTAAAAAA   
  
  
+ ATTTTTAAAA TTATCCTGAT AGAGAATAAA AAGAAATCCT TATACAAAAC TCTGATTGAC GCAGATGTGT   
  
  
+ ATATGGACAT ATAGAAGTAA TAAGAGGGGT AGGCTAGGAA GGGGGTGGAA ATATAGGGCA TAAATAGGGG   
  
  
+ GCGGGCATAG GGACATGTCT GTCTGAGGTA CGGTGGATGG GACCTTGCCA TGGAAGCAAC AATGTCACAA   
  
  
+ TAATAATATC AACGACTGAA GTTTCTTCAC CAGCCCACTC TTTGCCATAA TGCCATTAAT TATTTATTGC   
  
  
+ TACTATCTCT TTTGATGTGA AACGGTGGAC CTTATCTCTC CCCTCCACTT CCAATTGATG TGAGCAACTC   
  
  
+ AGAAACGCAA ACAAAACTTG TGCCACCTTC TTTACTTGCA TCCTCATTTT CCTCTGTCTC TCTCCGCCCC   
  
  
+ TGTTTCTCTC TCTCTCCCTC ACTCACTCAG AGTACTGATC TGAGCATCAG AAGTAGCAGA CCCAGAAACT   
  
  
+ CAGATTTAAA AACAACCCGC TCTTTTGTTC CCTCTACGTC TAATAATTTT GTCATCGCAT TTAATGTCAG   
  
  
+ ATAGAGAGAT AGAGCTCTAC TGTATTAATT TTCTCCCACC TCACTTGATT CTGTTGCCCT AAACCACCCA   
  
  
+ AAAAAAGAAA TACGAAGAAG CCACTCTCTC TCTCTCTCTC TCTCACACAC ACACACACCC CTCCTAGTGG   
  
  
+ ATTTGATAGA GTATAAATGA ACAGTAATCA GACGAGATAT ATAGTAGTGC TTAATTGACC GAGTAGCAAA   
  
  
+ AGGGGGAAGT TGAGTGAGGT ATCAAGAGAG TAGCGTAGCT AGCTAGGGCA AGATATGCTG GGGTCTTCCT   
  
  
+ CATACAACTC CGAGGAAGAC CACCAAGATG ACAACCATTC CTCACCGTCT GATTTGTCTA GGCACCCACC   
  
  
+ TAGATTCACC ACTACTACTT ACCAATACCA ACAACATATT AGCTCTCCAT CTTCACAATC AATACTAGCT   
  
  
+ GTGCCACCTC GCCAATTGTT AATCAATTGT GCGGAGTTAA TTTGCCGGTT CGACTTCTCC TCCGCCAACC   
  
  
+ GGCTTCTCTC TCTCCTCGCC TCCCACTTTG CCTCCCCTTC TGGGGACTCC ACCGAGAGAC TTGTGTTTTA   
  
  
+ CTTCTGCAAG GCTTTGGATC TCCGCCTCAA TAACAGTTAT TTATCCACTC GTAGTTTGAC TGGTTGTAAC   
  
  
+ CCTAGTACTG GTTCTGGTAT TGGGGCTCCT TTAGCTTCCA ATTTTCTATT TCTTCAACCA TTAGTGCATT   
  
  
+ CACAGGTAAC AAGTAATGCC CGTGTTGGGT ACTTGACATA TTTGACTCTG AACCAGGTCA CCCCATTCAT   
  
  
+ CCGATTCACC CACTTAACTG CCAACCAAGC CATCCTGGAA GCTTTGGAGG GGTACAAGGC GGTCCATATC   
  
  
+ CTTGACATGG ACATCATGCA TGGGGTCCAG TGGCCACCAC TTCTTCAAGC AATTGCAGAA AGATCTGCAA   
  
  
+ CCCTTGGTCA CCCTGCTCCC ACTGTTCGCA TCACAGGAGG GGGCAGGGAT CTAGAACTCC TGAACCGAAC   
  
  
+ TGGAGATCGG ATCAGGAAGT TCGCACAATC CTTGGGACTC GAGTTTCAAT TCCACCATCT TATAACGGTG   
  
  
+ GACTCGAACC TTACTGAGGC AGTGGAGGCC ATCAATGCCC TACAGCTACA CCTCCATGTC CAAGAGGAGG   
  
  
+ AGGTCTTCGC AGTGAATTGT GGAGATTTCC TCCACCGCCT CCTAACGGAG TATGATAGTA CATTCCTTAG   
  
  
+ GATGTTTCTC TACAAGGTGA AGACCCTAAA CCCTAGGGTC TTCACCGTGG GAGAAAGGGA GGCCGACCAC   
  
  
+ AACCACCCTC TCTTCTGGCA GCGATTCATT GAGGCACTTG ACCACTATGG GGCAGTGTTT GACTCTCTAG   
  
  
+ AAGCAACCCT ACCATCAACA AGTCAAGAGA GGGTGGCGGT GGAAGAGGGG TGGTTCGGGG AGGAGATTAA   
  
  
+ AGATGTGGTT GGACGAGAAG GAGGGTTGAG GAGACAGAGG CACCAGAAAT ACGAGTCTTG GGAGGTGTTG   
  
  
+ ATGAGGAGCT CTGGGTTTAA GAGCTTGCCC TTGAGCCCCT TCTCTGTGTC ACAAGCCAAG CTGCTACTTC   
  
  
+ GCCTTCATTA CCCTTCTGAA GGGTACCACC TTAAGGTCTT CCATAGTAAT TGTCTGTTGC TTGGGTGGAA   
  
  
+ GAATCGCCCT CTTTTCTCTG TTTCTTCTTG GCAATA  

- -Up\_Stream \_Len000TTTTAT AAAAATGTTT TCGTAAAGAG ATATTTTATA AAATGTTTTA AAGTAAAACA   
  
  
- CCCAGAAAAT CTTAGTTAGA GAGATGTTCA TCCTTACGGC CAACTCCATT CCGATGGATG TAGATCTAGA   
  
  
- GAGGTCTGGG GTGTTTTTAA TTTGCTCTGG GTGTGACTTT CCACCACCAC CACCACAACA ACAACATAAC   
  
  
- AGGATGGTGC TCTCTCGAAG TTCACGTAGT GAATAAAGTT GTCCAGTTAG TGTATCTAGA GATCACATGA   
  
  
- CATTTAAAAA AAAGGCAGAA TAAAATTAAA AGAAAAGGTA GGATTTTCTA ATAGTACATC TATAAGGATC   
  
  
- AAACTATATA ACTGAAGTTG AAGTTTTTAA TGTGTAATTC TAGGTGCTTA TACTGTCAAA TCCAAAGTAA   
  
  
- ACTTCCTCTT TCTTTACAAT GCCACAAAAC CACAACATAA GCATCTGTAA AAAACGCGAA TTGACCGAGA   
  
  
- AGAGTACCCC TGCTCTGTCA CTTCCATTAA CATGAACAAC TAACTACTGC AGTAGACATC GAACCTAAAC   
  
  
- ATATCCTTAC TTGTAGAACC ACAGATGCAC CTTTCACACT AAACACAAGA AAGTAACTAG GAGGAGGAGT   
  
  
- TCTTCTCCAC TAGAAAGAGG GAACAAAAAA AAAAAAAAAC CGATTATACA ACCGTCTTGG CCGTTTTTTA   
  
  
- TGTATTAAAC TTATCACGTA CGTATACTCA TGTACTGTCA CTTGGTTGTT TTGCTTGGGG ATTTTGTGTT   
  
  
- CTCTTAATTG TACAATTTTG TGAATTTGAT TTACGTTGGT AGAGCTACTG GTGATTTATC GGTTCTTTGG   
  
  
- ATTGCATTAA TTGTTTTTAT TTTTTATTGA CTTTTATTTC ATCTCTTTCA GATTGGCTTA TGCTATTGTA   
  
  
- TTGGATTTTG ATTGATATGA ACTTGAATGA TTCTTTATAC TTAGTTGGTG TTTGGCTTAA CTAAAACCAA   
  
  
- ACGGTATTAA ACTGATATGT AAATTGAATT TTTTATAAAA AAAAATTAAA GTTTATTTAG ATTTAATAAA   
  
  
- AAGTTATAAG TAGGTTTTAT CTAAGTTAAT AATTTGTTCA GTTGGATATG GCTCAATAAA AGATCGAGAC   
  
  
- TGATTCTGTA TATATATAAA AAACATTTTT TCTATTCCTA ATATTAAATT TATCGGAAAG GGAATTTTTT   
  
  
- TAAAAATTTT AATAGGACTA TCTCTTATTT TTCTTTAGGA ATATGTTTTG AGACTAACTG CGTCTACACA   
  
  
- TATACCTGTA TATCTTCATT ATTCTCCCCA TCCGATCCTT CCCCCACCTT TATATCCCGT ATTTATCCCC   
  
  
- CGCCCGTATC CCTGTACAGA CAGACTCCAT GCCACCTACC CTGGAACGGT ACCTTCGTTG TTACAGTGTT   
  
  
- ATTATTATAG TTGCTGACTT CAAAGAAGTG GTCGGGTGAG AAACGGTATT ACGGTAATTA ATAAATAACG   
  
  
- ATGATAGAGA AAACTACACT TTGCCACCTG GAATAGAGAG GGGAGGTGAA GGTTAACTAC ACTCGTTGAG   
  
  
- TCTTTGCGTT TGTTTTGAAC ACGGTGGAAG AAATGAACGT AGGAGTAAAA GGAGACAGAG AGAGGCGGGG   
  
  
- ACAAAGAGAG AGAGAGGGAG TGAGTGAGTC TCATGACTAG ACTCGTAGTC TTCATCGTCT GGGTCTTTGA   
  
  
- GTCTAAATTT TTGTTGGGCG AGAAAACAAG GGAGATGCAG ATTATTAAAA CAGTAGCGTA AATTACAGTC   
  
  
- TATCTCTCTA TCTCGAGATG ACATAATTAA AAGAGGGTGG AGTGAACTAA GACAACGGGA TTTGGTGGGT   
  
  
- TTTTTTCTTT ATGCTTCTTC GGTGAGAGAG AGAGAGAGAG AGAGTGTGTG TGTGTGTGGG GAGGATCACC   
  
  
- TAAACTATCT CATATTTACT TGTCATTAGT CTGCTCTATA TATCATCACG AATTAACTGG CTCATCGTTT   
  
  
- TCCCCCTTCA ACTCACTCCA TAGTTCTCTC ATCGCATCGA TCGATCCCGT TCTATACGAC CCCAGAAGGA   
  
  
- GTATGTTGAG GCTCCTTCTG GTGGTTCTAC TGTTGGTAAG GAGTGGCAGA CTAAACAGAT CCGTGGGTGG   
  
  
- ATCTAAGTGG TGATGATGAA TGGTTATGGT TGTTGTATAA TCGAGAGGTA GAAGTGTTAG TTATGATCGA   
  
  
- CACGGTGGAG CGGTTAACAA TTAGTTAACA CGCCTCAATT AAACGGCCAA GCTGAAGAGG AGGCGGTTGG   
  
  
- CCGAAGAGAG AGAGGAGCGG AGGGTGAAAC GGAGGGGAAG ACCCCTGAGG TGGCTCTCTG AACACAAAAT   
  
  
- GAAGACGTTC CGAAACCTAG AGGCGGAGTT ATTGTCAATA AATAGGTGAG CATCAAACTG ACCAACATTG   
  
  
- GGATCATGAC CAAGACCATA ACCCCGAGGA AATCGAAGGT TAAAAGATAA AGAAGTTGGT AATCACGTAA   
  
  
- GTGTCCATTG TTCATTACGG GCACAACCCA TGAACTGTAT AAACTGAGAC TTGGTCCAGT GGGGTAAGTA   
  
  
- GGCTAAGTGG GTGAATTGAC GGTTGGTTCG GTAGGACCTT CGAAACCTCC CCATGTTCCG CCAGGTATAG   
  
  
- GAACTGTACC TGTAGTACGT ACCCCAGGTC ACCGGTGGTG AAGAAGTTCG TTAACGTCTT TCTAGACGTT   
  
  
- GGGAACCAGT GGGACGAGGG TGACAAGCGT AGTGTCCTCC CCCGTCCCTA GATCTTGAGG ACTTGGCTTG   
  
  
- ACCTCTAGCC TAGTCCTTCA AGCGTGTTAG GAACCCTGAG CTCAAAGTTA AGGTGGTAGA ATATTGCCAC   
  
  
- CTGAGCTTGG AATGACTCCG TCACCTCCGG TAGTTACGGG ATGTCGATGT GGAGGTACAG GTTCTCCTCC   
  
  
- TCCAGAAGCG TCACTTAACA CCTCTAAAGG AGGTGGCGGA GGATTGCCTC ATACTATCAT GTAAGGAATC   
  
  
- CTACAAAGAG ATGTTCCACT TCTGGGATTT GGGATCCCAG AAGTGGCACC CTCTTTCCCT CCGGCTGGTG   
  
  
- TTGGTGGGAG AGAAGACCGT CGCTAAGTAA CTCCGTGAAC TGGTGATACC CCGTCACAAA CTGAGAGATC   
  
  
- TTCGTTGGGA TGGTAGTTGT TCAGTTCTCT CCCACCGCCA CCTTCTCCCC ACCAAGCCCC TCCTCTAATT   
  
  
- TCTACACCAA CCTGCTCTTC CTCCCAACTC CTCTGTCTCC GTGGTCTTTA TGCTCAGAAC CCTCCACAAC   
  
  
- TACTCCTCGA GACCCAAATT CTCGAACGGG AACTCGGGGA AGAGACACAG TGTTCGGTTC GACGATGAAG   
  
  
- CGGAAGTAAT GGGAAGACTT CCCATGGTGG AATTCCAGAA GGTATCATTA ACAGACAACG AACCCACCTT   
  
  
- CTTAGCGGGA GAAAAGAGAC AAAGAAGAAC CGTTAT

+     G-box

| Site Name | Organism | Position | Strand | Matrix score. | sequence | function |
| --- | --- | --- | --- | --- | --- | --- |
| G-box | Arabidopsis thaliana | 589 | + | 6 | TACGTG | cis-acting regulatory element involved in light responsiveness |

>HU01G00833.1   
+ -Up\_Stream \_Len000AAAATA TTTTTACAAA AGCATTTCTC TATAAAATAT TTTACAAAAT TTCATTTTGT   
  
  
+ GGGTCTTTTA GAATCAATCT CTCTACAAGT AGGAATGCCG GTTGAGGTAA GGCTACCTAC ATCTAGATCT   
  
  
+ CTCCAGACCC CACAAAAATT AAACGAGACC CACACTGAAA GGTGGTGGTG GTGGTGTTGT TGTTGTATTG   
  
  
+ TCCTACCACG AGAGAGCTTC AAGTGCATCA CTTATTTCAA CAGGTCAATC ACATAGATCT CTAGTGTACT   
  
  
+ GTAAATTTTT TTTCCGTCTT ATTTTAATTT TCTTTTCCAT CCTAAAAGAT TATCATGTAG ATATTCCTAG   
  
  
+ TTTGATATAT TGACTTCAAC TTCAAAAATT ACACATTAAG ATCCACGAAT ATGACAGTTT AGGTTTCATT   
  
  
+ TGAAGGAGAA AGAAATGTTA CGGTGTTTTG GTGTTGTATT CGTAGACATT TTTTGCGCTT AACTGGCTCT   
  
  
+ TCTCATGGGG ACGAGACAGT GAAGGTAATT GTACTTGTTG ATTGATGACG TCATCTGTAG CTTGGATTTG   
  
  
+ TATAGGAATG AACATCTTGG TGTCTACGTG GAAAGTGTGA TTTGTGTTCT TTCATTGATC CTCCTCCTCA   
  
  
+ AGAAGAGGTG ATCTTTCTCC CTTGTTTTTT TTTTTTTTTG GCTAATATGT TGGCAGAACC GGCAAAAAAT   
  
  
+ ACATAATTTG AATAGTGCAT GCATATGAGT ACATGACAGT GAACCAACAA AACGAACCCC TAAAACACAA   
  
  
+ GAGAATTAAC ATGTTAAAAC ACTTAAACTA AATGCAACCA TCTCGATGAC CACTAAATAG CCAAGAAACC   
  
  
+ TAACGTAATT AACAAAAATA AAAAATAACT GAAAATAAAG TAGAGAAAGT CTAACCGAAT ACGATAACAT   
  
  
+ AACCTAAAAC TAACTATACT TGAACTTACT AAGAAATATG AATCAACCAC AAACCGAATT GATTTTGGTT   
  
  
+ TGCCATAATT TGACTATACA TTTAACTTAA AAAATATTTT TTTTTAATTT CAAATAAATC TAAATTATTT   
  
  
+ TTCAATATTC ATCCAAAATA GATTCAATTA TTAAACAAGT CAACCTATAC CGAGTTATTT TCTAGCTCTG   
  
  
+ ACTAAGACAT ATATATATTT TTTGTAAAAA AGATAAGGAT TATAATTTAA ATAGCCTTTC CCTTAAAAAA   
  
  
+ ATTTTTAAAA TTATCCTGAT AGAGAATAAA AAGAAATCCT TATACAAAAC TCTGATTGAC GCAGATGTGT   
  
  
+ ATATGGACAT ATAGAAGTAA TAAGAGGGGT AGGCTAGGAA GGGGGTGGAA ATATAGGGCA TAAATAGGGG   
  
  
+ GCGGGCATAG GGACATGTCT GTCTGAGGTA CGGTGGATGG GACCTTGCCA TGGAAGCAAC AATGTCACAA   
  
  
+ TAATAATATC AACGACTGAA GTTTCTTCAC CAGCCCACTC TTTGCCATAA TGCCATTAAT TATTTATTGC   
  
  
+ TACTATCTCT TTTGATGTGA AACGGTGGAC CTTATCTCTC CCCTCCACTT CCAATTGATG TGAGCAACTC   
  
  
+ AGAAACGCAA ACAAAACTTG TGCCACCTTC TTTACTTGCA TCCTCATTTT CCTCTGTCTC TCTCCGCCCC   
  
  
+ TGTTTCTCTC TCTCTCCCTC ACTCACTCAG AGTACTGATC TGAGCATCAG AAGTAGCAGA CCCAGAAACT   
  
  
+ CAGATTTAAA AACAACCCGC TCTTTTGTTC CCTCTACGTC TAATAATTTT GTCATCGCAT TTAATGTCAG   
  
  
+ ATAGAGAGAT AGAGCTCTAC TGTATTAATT TTCTCCCACC TCACTTGATT CTGTTGCCCT AAACCACCCA   
  
  
+ AAAAAAGAAA TACGAAGAAG CCACTCTCTC TCTCTCTCTC TCTCACACAC ACACACACCC CTCCTAGTGG   
  
  
+ ATTTGATAGA GTATAAATGA ACAGTAATCA GACGAGATAT ATAGTAGTGC TTAATTGACC GAGTAGCAAA   
  
  
+ AGGGGGAAGT TGAGTGAGGT ATCAAGAGAG TAGCGTAGCT AGCTAGGGCA AGATATGCTG GGGTCTTCCT   
  
  
+ CATACAACTC CGAGGAAGAC CACCAAGATG ACAACCATTC CTCACCGTCT GATTTGTCTA GGCACCCACC   
  
  
+ TAGATTCACC ACTACTACTT ACCAATACCA ACAACATATT AGCTCTCCAT CTTCACAATC AATACTAGCT   
  
  
+ GTGCCACCTC GCCAATTGTT AATCAATTGT GCGGAGTTAA TTTGCCGGTT CGACTTCTCC TCCGCCAACC   
  
  
+ GGCTTCTCTC TCTCCTCGCC TCCCACTTTG CCTCCCCTTC TGGGGACTCC ACCGAGAGAC TTGTGTTTTA   
  
  
+ CTTCTGCAAG GCTTTGGATC TCCGCCTCAA TAACAGTTAT TTATCCACTC GTAGTTTGAC TGGTTGTAAC   
  
  
+ CCTAGTACTG GTTCTGGTAT TGGGGCTCCT TTAGCTTCCA ATTTTCTATT TCTTCAACCA TTAGTGCATT   
  
  
+ CACAGGTAAC AAGTAATGCC CGTGTTGGGT ACTTGACATA TTTGACTCTG AACCAGGTCA CCCCATTCAT   
  
  
+ CCGATTCACC CACTTAACTG CCAACCAAGC CATCCTGGAA GCTTTGGAGG GGTACAAGGC GGTCCATATC   
  
  
+ CTTGACATGG ACATCATGCA TGGGGTCCAG TGGCCACCAC TTCTTCAAGC AATTGCAGAA AGATCTGCAA   
  
  
+ CCCTTGGTCA CCCTGCTCCC ACTGTTCGCA TCACAGGAGG GGGCAGGGAT CTAGAACTCC TGAACCGAAC   
  
  
+ TGGAGATCGG ATCAGGAAGT TCGCACAATC CTTGGGACTC GAGTTTCAAT TCCACCATCT TATAACGGTG   
  
  
+ GACTCGAACC TTACTGAGGC AGTGGAGGCC ATCAATGCCC TACAGCTACA CCTCCATGTC CAAGAGGAGG   
  
  
+ AGGTCTTCGC AGTGAATTGT GGAGATTTCC TCCACCGCCT CCTAACGGAG TATGATAGTA CATTCCTTAG   
  
  
+ GATGTTTCTC TACAAGGTGA AGACCCTAAA CCCTAGGGTC TTCACCGTGG GAGAAAGGGA GGCCGACCAC   
  
  
+ AACCACCCTC TCTTCTGGCA GCGATTCATT GAGGCACTTG ACCACTATGG GGCAGTGTTT GACTCTCTAG   
  
  
+ AAGCAACCCT ACCATCAACA AGTCAAGAGA GGGTGGCGGT GGAAGAGGGG TGGTTCGGGG AGGAGATTAA   
  
  
+ AGATGTGGTT GGACGAGAAG GAGGGTTGAG GAGACAGAGG CACCAGAAAT ACGAGTCTTG GGAGGTGTTG   
  
  
+ ATGAGGAGCT CTGGGTTTAA GAGCTTGCCC TTGAGCCCCT TCTCTGTGTC ACAAGCCAAG CTGCTACTTC   
  
  
+ GCCTTCATTA CCCTTCTGAA GGGTACCACC TTAAGGTCTT CCATAGTAAT TGTCTGTTGC TTGGGTGGAA   
  
  
+ GAATCGCCCT CTTTTCTCTG TTTCTTCTTG GCAATA  

- -Up\_Stream \_Len000TTTTAT AAAAATGTTT TCGTAAAGAG ATATTTTATA AAATGTTTTA AAGTAAAACA   
  
  
- CCCAGAAAAT CTTAGTTAGA GAGATGTTCA TCCTTACGGC CAACTCCATT CCGATGGATG TAGATCTAGA   
  
  
- GAGGTCTGGG GTGTTTTTAA TTTGCTCTGG GTGTGACTTT CCACCACCAC CACCACAACA ACAACATAAC   
  
  
- AGGATGGTGC TCTCTCGAAG TTCACGTAGT GAATAAAGTT GTCCAGTTAG TGTATCTAGA GATCACATGA   
  
  
- CATTTAAAAA AAAGGCAGAA TAAAATTAAA AGAAAAGGTA GGATTTTCTA ATAGTACATC TATAAGGATC   
  
  
- AAACTATATA ACTGAAGTTG AAGTTTTTAA TGTGTAATTC TAGGTGCTTA TACTGTCAAA TCCAAAGTAA   
  
  
- ACTTCCTCTT TCTTTACAAT GCCACAAAAC CACAACATAA GCATCTGTAA AAAACGCGAA TTGACCGAGA   
  
  
- AGAGTACCCC TGCTCTGTCA CTTCCATTAA CATGAACAAC TAACTACTGC AGTAGACATC GAACCTAAAC   
  
  
- ATATCCTTAC TTGTAGAACC ACAGATGCAC CTTTCACACT AAACACAAGA AAGTAACTAG GAGGAGGAGT   
  
  
- TCTTCTCCAC TAGAAAGAGG GAACAAAAAA AAAAAAAAAC CGATTATACA ACCGTCTTGG CCGTTTTTTA   
  
  
- TGTATTAAAC TTATCACGTA CGTATACTCA TGTACTGTCA CTTGGTTGTT TTGCTTGGGG ATTTTGTGTT   
  
  
- CTCTTAATTG TACAATTTTG TGAATTTGAT TTACGTTGGT AGAGCTACTG GTGATTTATC GGTTCTTTGG   
  
  
- ATTGCATTAA TTGTTTTTAT TTTTTATTGA CTTTTATTTC ATCTCTTTCA GATTGGCTTA TGCTATTGTA   
  
  
- TTGGATTTTG ATTGATATGA ACTTGAATGA TTCTTTATAC TTAGTTGGTG TTTGGCTTAA CTAAAACCAA   
  
  
- ACGGTATTAA ACTGATATGT AAATTGAATT TTTTATAAAA AAAAATTAAA GTTTATTTAG ATTTAATAAA   
  
  
- AAGTTATAAG TAGGTTTTAT CTAAGTTAAT AATTTGTTCA GTTGGATATG GCTCAATAAA AGATCGAGAC   
  
  
- TGATTCTGTA TATATATAAA AAACATTTTT TCTATTCCTA ATATTAAATT TATCGGAAAG GGAATTTTTT   
  
  
- TAAAAATTTT AATAGGACTA TCTCTTATTT TTCTTTAGGA ATATGTTTTG AGACTAACTG CGTCTACACA   
  
  
- TATACCTGTA TATCTTCATT ATTCTCCCCA TCCGATCCTT CCCCCACCTT TATATCCCGT ATTTATCCCC   
  
  
- CGCCCGTATC CCTGTACAGA CAGACTCCAT GCCACCTACC CTGGAACGGT ACCTTCGTTG TTACAGTGTT   
  
  
- ATTATTATAG TTGCTGACTT CAAAGAAGTG GTCGGGTGAG AAACGGTATT ACGGTAATTA ATAAATAACG   
  
  
- ATGATAGAGA AAACTACACT TTGCCACCTG GAATAGAGAG GGGAGGTGAA GGTTAACTAC ACTCGTTGAG   
  
  
- TCTTTGCGTT TGTTTTGAAC ACGGTGGAAG AAATGAACGT AGGAGTAAAA GGAGACAGAG AGAGGCGGGG   
  
  
- ACAAAGAGAG AGAGAGGGAG TGAGTGAGTC TCATGACTAG ACTCGTAGTC TTCATCGTCT GGGTCTTTGA   
  
  
- GTCTAAATTT TTGTTGGGCG AGAAAACAAG GGAGATGCAG ATTATTAAAA CAGTAGCGTA AATTACAGTC   
  
  
- TATCTCTCTA TCTCGAGATG ACATAATTAA AAGAGGGTGG AGTGAACTAA GACAACGGGA TTTGGTGGGT   
  
  
- TTTTTTCTTT ATGCTTCTTC GGTGAGAGAG AGAGAGAGAG AGAGTGTGTG TGTGTGTGGG GAGGATCACC   
  
  
- TAAACTATCT CATATTTACT TGTCATTAGT CTGCTCTATA TATCATCACG AATTAACTGG CTCATCGTTT   
  
  
- TCCCCCTTCA ACTCACTCCA TAGTTCTCTC ATCGCATCGA TCGATCCCGT TCTATACGAC CCCAGAAGGA   
  
  
- GTATGTTGAG GCTCCTTCTG GTGGTTCTAC TGTTGGTAAG GAGTGGCAGA CTAAACAGAT CCGTGGGTGG   
  
  
- ATCTAAGTGG TGATGATGAA TGGTTATGGT TGTTGTATAA TCGAGAGGTA GAAGTGTTAG TTATGATCGA   
  
  
- CACGGTGGAG CGGTTAACAA TTAGTTAACA CGCCTCAATT AAACGGCCAA GCTGAAGAGG AGGCGGTTGG   
  
  
- CCGAAGAGAG AGAGGAGCGG AGGGTGAAAC GGAGGGGAAG ACCCCTGAGG TGGCTCTCTG AACACAAAAT   
  
  
- GAAGACGTTC CGAAACCTAG AGGCGGAGTT ATTGTCAATA AATAGGTGAG CATCAAACTG ACCAACATTG   
  
  
- GGATCATGAC CAAGACCATA ACCCCGAGGA AATCGAAGGT TAAAAGATAA AGAAGTTGGT AATCACGTAA   
  
  
- GTGTCCATTG TTCATTACGG GCACAACCCA TGAACTGTAT AAACTGAGAC TTGGTCCAGT GGGGTAAGTA   
  
  
- GGCTAAGTGG GTGAATTGAC GGTTGGTTCG GTAGGACCTT CGAAACCTCC CCATGTTCCG CCAGGTATAG   
  
  
- GAACTGTACC TGTAGTACGT ACCCCAGGTC ACCGGTGGTG AAGAAGTTCG TTAACGTCTT TCTAGACGTT   
  
  
- GGGAACCAGT GGGACGAGGG TGACAAGCGT AGTGTCCTCC CCCGTCCCTA GATCTTGAGG ACTTGGCTTG   
  
  
- ACCTCTAGCC TAGTCCTTCA AGCGTGTTAG GAACCCTGAG CTCAAAGTTA AGGTGGTAGA ATATTGCCAC   
  
  
- CTGAGCTTGG AATGACTCCG TCACCTCCGG TAGTTACGGG ATGTCGATGT GGAGGTACAG GTTCTCCTCC   
  
  
- TCCAGAAGCG TCACTTAACA CCTCTAAAGG AGGTGGCGGA GGATTGCCTC ATACTATCAT GTAAGGAATC   
  
  
- CTACAAAGAG ATGTTCCACT TCTGGGATTT GGGATCCCAG AAGTGGCACC CTCTTTCCCT CCGGCTGGTG   
  
  
- TTGGTGGGAG AGAAGACCGT CGCTAAGTAA CTCCGTGAAC TGGTGATACC CCGTCACAAA CTGAGAGATC   
  
  
- TTCGTTGGGA TGGTAGTTGT TCAGTTCTCT CCCACCGCCA CCTTCTCCCC ACCAAGCCCC TCCTCTAATT   
  
  
- TCTACACCAA CCTGCTCTTC CTCCCAACTC CTCTGTCTCC GTGGTCTTTA TGCTCAGAAC CCTCCACAAC   
  
  
- TACTCCTCGA GACCCAAATT CTCGAACGGG AACTCGGGGA AGAGACACAG TGTTCGGTTC GACGATGAAG   
  
  
- CGGAAGTAAT GGGAAGACTT CCCATGGTGG AATTCCAGAA GGTATCATTA ACAGACAACG AACCCACCTT   
  
  
- CTTAGCGGGA GAAAAGAGAC AAAGAAGAAC CGTTAT

+     GARE-motif

| Site Name | Organism | Position | Strand | Matrix score. | sequence | function |
| --- | --- | --- | --- | --- | --- | --- |
| GARE-motif | Brassica oleracea | 3347 | + | 7 | TCTGTTG | gibberellin-responsive element |
| GARE-motif | Brassica oleracea | 1804 | + | 7 | TCTGTTG | gibberellin-responsive element |

>HU01G00833.1   
+ -Up\_Stream \_Len000AAAATA TTTTTACAAA AGCATTTCTC TATAAAATAT TTTACAAAAT TTCATTTTGT   
  
  
+ GGGTCTTTTA GAATCAATCT CTCTACAAGT AGGAATGCCG GTTGAGGTAA GGCTACCTAC ATCTAGATCT   
  
  
+ CTCCAGACCC CACAAAAATT AAACGAGACC CACACTGAAA GGTGGTGGTG GTGGTGTTGT TGTTGTATTG   
  
  
+ TCCTACCACG AGAGAGCTTC AAGTGCATCA CTTATTTCAA CAGGTCAATC ACATAGATCT CTAGTGTACT   
  
  
+ GTAAATTTTT TTTCCGTCTT ATTTTAATTT TCTTTTCCAT CCTAAAAGAT TATCATGTAG ATATTCCTAG   
  
  
+ TTTGATATAT TGACTTCAAC TTCAAAAATT ACACATTAAG ATCCACGAAT ATGACAGTTT AGGTTTCATT   
  
  
+ TGAAGGAGAA AGAAATGTTA CGGTGTTTTG GTGTTGTATT CGTAGACATT TTTTGCGCTT AACTGGCTCT   
  
  
+ TCTCATGGGG ACGAGACAGT GAAGGTAATT GTACTTGTTG ATTGATGACG TCATCTGTAG CTTGGATTTG   
  
  
+ TATAGGAATG AACATCTTGG TGTCTACGTG GAAAGTGTGA TTTGTGTTCT TTCATTGATC CTCCTCCTCA   
  
  
+ AGAAGAGGTG ATCTTTCTCC CTTGTTTTTT TTTTTTTTTG GCTAATATGT TGGCAGAACC GGCAAAAAAT   
  
  
+ ACATAATTTG AATAGTGCAT GCATATGAGT ACATGACAGT GAACCAACAA AACGAACCCC TAAAACACAA   
  
  
+ GAGAATTAAC ATGTTAAAAC ACTTAAACTA AATGCAACCA TCTCGATGAC CACTAAATAG CCAAGAAACC   
  
  
+ TAACGTAATT AACAAAAATA AAAAATAACT GAAAATAAAG TAGAGAAAGT CTAACCGAAT ACGATAACAT   
  
  
+ AACCTAAAAC TAACTATACT TGAACTTACT AAGAAATATG AATCAACCAC AAACCGAATT GATTTTGGTT   
  
  
+ TGCCATAATT TGACTATACA TTTAACTTAA AAAATATTTT TTTTTAATTT CAAATAAATC TAAATTATTT   
  
  
+ TTCAATATTC ATCCAAAATA GATTCAATTA TTAAACAAGT CAACCTATAC CGAGTTATTT TCTAGCTCTG   
  
  
+ ACTAAGACAT ATATATATTT TTTGTAAAAA AGATAAGGAT TATAATTTAA ATAGCCTTTC CCTTAAAAAA   
  
  
+ ATTTTTAAAA TTATCCTGAT AGAGAATAAA AAGAAATCCT TATACAAAAC TCTGATTGAC GCAGATGTGT   
  
  
+ ATATGGACAT ATAGAAGTAA TAAGAGGGGT AGGCTAGGAA GGGGGTGGAA ATATAGGGCA TAAATAGGGG   
  
  
+ GCGGGCATAG GGACATGTCT GTCTGAGGTA CGGTGGATGG GACCTTGCCA TGGAAGCAAC AATGTCACAA   
  
  
+ TAATAATATC AACGACTGAA GTTTCTTCAC CAGCCCACTC TTTGCCATAA TGCCATTAAT TATTTATTGC   
  
  
+ TACTATCTCT TTTGATGTGA AACGGTGGAC CTTATCTCTC CCCTCCACTT CCAATTGATG TGAGCAACTC   
  
  
+ AGAAACGCAA ACAAAACTTG TGCCACCTTC TTTACTTGCA TCCTCATTTT CCTCTGTCTC TCTCCGCCCC   
  
  
+ TGTTTCTCTC TCTCTCCCTC ACTCACTCAG AGTACTGATC TGAGCATCAG AAGTAGCAGA CCCAGAAACT   
  
  
+ CAGATTTAAA AACAACCCGC TCTTTTGTTC CCTCTACGTC TAATAATTTT GTCATCGCAT TTAATGTCAG   
  
  
+ ATAGAGAGAT AGAGCTCTAC TGTATTAATT TTCTCCCACC TCACTTGATT CTGTTGCCCT AAACCACCCA   
  
  
+ AAAAAAGAAA TACGAAGAAG CCACTCTCTC TCTCTCTCTC TCTCACACAC ACACACACCC CTCCTAGTGG   
  
  
+ ATTTGATAGA GTATAAATGA ACAGTAATCA GACGAGATAT ATAGTAGTGC TTAATTGACC GAGTAGCAAA   
  
  
+ AGGGGGAAGT TGAGTGAGGT ATCAAGAGAG TAGCGTAGCT AGCTAGGGCA AGATATGCTG GGGTCTTCCT   
  
  
+ CATACAACTC CGAGGAAGAC CACCAAGATG ACAACCATTC CTCACCGTCT GATTTGTCTA GGCACCCACC   
  
  
+ TAGATTCACC ACTACTACTT ACCAATACCA ACAACATATT AGCTCTCCAT CTTCACAATC AATACTAGCT   
  
  
+ GTGCCACCTC GCCAATTGTT AATCAATTGT GCGGAGTTAA TTTGCCGGTT CGACTTCTCC TCCGCCAACC   
  
  
+ GGCTTCTCTC TCTCCTCGCC TCCCACTTTG CCTCCCCTTC TGGGGACTCC ACCGAGAGAC TTGTGTTTTA   
  
  
+ CTTCTGCAAG GCTTTGGATC TCCGCCTCAA TAACAGTTAT TTATCCACTC GTAGTTTGAC TGGTTGTAAC   
  
  
+ CCTAGTACTG GTTCTGGTAT TGGGGCTCCT TTAGCTTCCA ATTTTCTATT TCTTCAACCA TTAGTGCATT   
  
  
+ CACAGGTAAC AAGTAATGCC CGTGTTGGGT ACTTGACATA TTTGACTCTG AACCAGGTCA CCCCATTCAT   
  
  
+ CCGATTCACC CACTTAACTG CCAACCAAGC CATCCTGGAA GCTTTGGAGG GGTACAAGGC GGTCCATATC   
  
  
+ CTTGACATGG ACATCATGCA TGGGGTCCAG TGGCCACCAC TTCTTCAAGC AATTGCAGAA AGATCTGCAA   
  
  
+ CCCTTGGTCA CCCTGCTCCC ACTGTTCGCA TCACAGGAGG GGGCAGGGAT CTAGAACTCC TGAACCGAAC   
  
  
+ TGGAGATCGG ATCAGGAAGT TCGCACAATC CTTGGGACTC GAGTTTCAAT TCCACCATCT TATAACGGTG   
  
  
+ GACTCGAACC TTACTGAGGC AGTGGAGGCC ATCAATGCCC TACAGCTACA CCTCCATGTC CAAGAGGAGG   
  
  
+ AGGTCTTCGC AGTGAATTGT GGAGATTTCC TCCACCGCCT CCTAACGGAG TATGATAGTA CATTCCTTAG   
  
  
+ GATGTTTCTC TACAAGGTGA AGACCCTAAA CCCTAGGGTC TTCACCGTGG GAGAAAGGGA GGCCGACCAC   
  
  
+ AACCACCCTC TCTTCTGGCA GCGATTCATT GAGGCACTTG ACCACTATGG GGCAGTGTTT GACTCTCTAG   
  
  
+ AAGCAACCCT ACCATCAACA AGTCAAGAGA GGGTGGCGGT GGAAGAGGGG TGGTTCGGGG AGGAGATTAA   
  
  
+ AGATGTGGTT GGACGAGAAG GAGGGTTGAG GAGACAGAGG CACCAGAAAT ACGAGTCTTG GGAGGTGTTG   
  
  
+ ATGAGGAGCT CTGGGTTTAA GAGCTTGCCC TTGAGCCCCT TCTCTGTGTC ACAAGCCAAG CTGCTACTTC   
  
  
+ GCCTTCATTA CCCTTCTGAA GGGTACCACC TTAAGGTCTT CCATAGTAAT TGTCTGTTGC TTGGGTGGAA   
  
  
+ GAATCGCCCT CTTTTCTCTG TTTCTTCTTG GCAATA  

- -Up\_Stream \_Len000TTTTAT AAAAATGTTT TCGTAAAGAG ATATTTTATA AAATGTTTTA AAGTAAAACA   
  
  
- CCCAGAAAAT CTTAGTTAGA GAGATGTTCA TCCTTACGGC CAACTCCATT CCGATGGATG TAGATCTAGA   
  
  
- GAGGTCTGGG GTGTTTTTAA TTTGCTCTGG GTGTGACTTT CCACCACCAC CACCACAACA ACAACATAAC   
  
  
- AGGATGGTGC TCTCTCGAAG TTCACGTAGT GAATAAAGTT GTCCAGTTAG TGTATCTAGA GATCACATGA   
  
  
- CATTTAAAAA AAAGGCAGAA TAAAATTAAA AGAAAAGGTA GGATTTTCTA ATAGTACATC TATAAGGATC   
  
  
- AAACTATATA ACTGAAGTTG AAGTTTTTAA TGTGTAATTC TAGGTGCTTA TACTGTCAAA TCCAAAGTAA   
  
  
- ACTTCCTCTT TCTTTACAAT GCCACAAAAC CACAACATAA GCATCTGTAA AAAACGCGAA TTGACCGAGA   
  
  
- AGAGTACCCC TGCTCTGTCA CTTCCATTAA CATGAACAAC TAACTACTGC AGTAGACATC GAACCTAAAC   
  
  
- ATATCCTTAC TTGTAGAACC ACAGATGCAC CTTTCACACT AAACACAAGA AAGTAACTAG GAGGAGGAGT   
  
  
- TCTTCTCCAC TAGAAAGAGG GAACAAAAAA AAAAAAAAAC CGATTATACA ACCGTCTTGG CCGTTTTTTA   
  
  
- TGTATTAAAC TTATCACGTA CGTATACTCA TGTACTGTCA CTTGGTTGTT TTGCTTGGGG ATTTTGTGTT   
  
  
- CTCTTAATTG TACAATTTTG TGAATTTGAT TTACGTTGGT AGAGCTACTG GTGATTTATC GGTTCTTTGG   
  
  
- ATTGCATTAA TTGTTTTTAT TTTTTATTGA CTTTTATTTC ATCTCTTTCA GATTGGCTTA TGCTATTGTA   
  
  
- TTGGATTTTG ATTGATATGA ACTTGAATGA TTCTTTATAC TTAGTTGGTG TTTGGCTTAA CTAAAACCAA   
  
  
- ACGGTATTAA ACTGATATGT AAATTGAATT TTTTATAAAA AAAAATTAAA GTTTATTTAG ATTTAATAAA   
  
  
- AAGTTATAAG TAGGTTTTAT CTAAGTTAAT AATTTGTTCA GTTGGATATG GCTCAATAAA AGATCGAGAC   
  
  
- TGATTCTGTA TATATATAAA AAACATTTTT TCTATTCCTA ATATTAAATT TATCGGAAAG GGAATTTTTT   
  
  
- TAAAAATTTT AATAGGACTA TCTCTTATTT TTCTTTAGGA ATATGTTTTG AGACTAACTG CGTCTACACA   
  
  
- TATACCTGTA TATCTTCATT ATTCTCCCCA TCCGATCCTT CCCCCACCTT TATATCCCGT ATTTATCCCC   
  
  
- CGCCCGTATC CCTGTACAGA CAGACTCCAT GCCACCTACC CTGGAACGGT ACCTTCGTTG TTACAGTGTT   
  
  
- ATTATTATAG TTGCTGACTT CAAAGAAGTG GTCGGGTGAG AAACGGTATT ACGGTAATTA ATAAATAACG   
  
  
- ATGATAGAGA AAACTACACT TTGCCACCTG GAATAGAGAG GGGAGGTGAA GGTTAACTAC ACTCGTTGAG   
  
  
- TCTTTGCGTT TGTTTTGAAC ACGGTGGAAG AAATGAACGT AGGAGTAAAA GGAGACAGAG AGAGGCGGGG   
  
  
- ACAAAGAGAG AGAGAGGGAG TGAGTGAGTC TCATGACTAG ACTCGTAGTC TTCATCGTCT GGGTCTTTGA   
  
  
- GTCTAAATTT TTGTTGGGCG AGAAAACAAG GGAGATGCAG ATTATTAAAA CAGTAGCGTA AATTACAGTC   
  
  
- TATCTCTCTA TCTCGAGATG ACATAATTAA AAGAGGGTGG AGTGAACTAA GACAACGGGA TTTGGTGGGT   
  
  
- TTTTTTCTTT ATGCTTCTTC GGTGAGAGAG AGAGAGAGAG AGAGTGTGTG TGTGTGTGGG GAGGATCACC   
  
  
- TAAACTATCT CATATTTACT TGTCATTAGT CTGCTCTATA TATCATCACG AATTAACTGG CTCATCGTTT   
  
  
- TCCCCCTTCA ACTCACTCCA TAGTTCTCTC ATCGCATCGA TCGATCCCGT TCTATACGAC CCCAGAAGGA   
  
  
- GTATGTTGAG GCTCCTTCTG GTGGTTCTAC TGTTGGTAAG GAGTGGCAGA CTAAACAGAT CCGTGGGTGG   
  
  
- ATCTAAGTGG TGATGATGAA TGGTTATGGT TGTTGTATAA TCGAGAGGTA GAAGTGTTAG TTATGATCGA   
  
  
- CACGGTGGAG CGGTTAACAA TTAGTTAACA CGCCTCAATT AAACGGCCAA GCTGAAGAGG AGGCGGTTGG   
  
  
- CCGAAGAGAG AGAGGAGCGG AGGGTGAAAC GGAGGGGAAG ACCCCTGAGG TGGCTCTCTG AACACAAAAT   
  
  
- GAAGACGTTC CGAAACCTAG AGGCGGAGTT ATTGTCAATA AATAGGTGAG CATCAAACTG ACCAACATTG   
  
  
- GGATCATGAC CAAGACCATA ACCCCGAGGA AATCGAAGGT TAAAAGATAA AGAAGTTGGT AATCACGTAA   
  
  
- GTGTCCATTG TTCATTACGG GCACAACCCA TGAACTGTAT AAACTGAGAC TTGGTCCAGT GGGGTAAGTA   
  
  
- GGCTAAGTGG GTGAATTGAC GGTTGGTTCG GTAGGACCTT CGAAACCTCC CCATGTTCCG CCAGGTATAG   
  
  
- GAACTGTACC TGTAGTACGT ACCCCAGGTC ACCGGTGGTG AAGAAGTTCG TTAACGTCTT TCTAGACGTT   
  
  
- GGGAACCAGT GGGACGAGGG TGACAAGCGT AGTGTCCTCC CCCGTCCCTA GATCTTGAGG ACTTGGCTTG   
  
  
- ACCTCTAGCC TAGTCCTTCA AGCGTGTTAG GAACCCTGAG CTCAAAGTTA AGGTGGTAGA ATATTGCCAC   
  
  
- CTGAGCTTGG AATGACTCCG TCACCTCCGG TAGTTACGGG ATGTCGATGT GGAGGTACAG GTTCTCCTCC   
  
  
- TCCAGAAGCG TCACTTAACA CCTCTAAAGG AGGTGGCGGA GGATTGCCTC ATACTATCAT GTAAGGAATC   
  
  
- CTACAAAGAG ATGTTCCACT TCTGGGATTT GGGATCCCAG AAGTGGCACC CTCTTTCCCT CCGGCTGGTG   
  
  
- TTGGTGGGAG AGAAGACCGT CGCTAAGTAA CTCCGTGAAC TGGTGATACC CCGTCACAAA CTGAGAGATC   
  
  
- TTCGTTGGGA TGGTAGTTGT TCAGTTCTCT CCCACCGCCA CCTTCTCCCC ACCAAGCCCC TCCTCTAATT   
  
  
- TCTACACCAA CCTGCTCTTC CTCCCAACTC CTCTGTCTCC GTGGTCTTTA TGCTCAGAAC CCTCCACAAC   
  
  
- TACTCCTCGA GACCCAAATT CTCGAACGGG AACTCGGGGA AGAGACACAG TGTTCGGTTC GACGATGAAG   
  
  
- CGGAAGTAAT GGGAAGACTT CCCATGGTGG AATTCCAGAA GGTATCATTA ACAGACAACG AACCCACCTT   
  
  
- CTTAGCGGGA GAAAAGAGAC AAAGAAGAAC CGTTAT

+     GATA-motif

| Site Name | Organism | Position | Strand | Matrix score. | sequence | function |
| --- | --- | --- | --- | --- | --- | --- |
| GATA-motif | Solanum tuberosum | 2588 | - | 9 | AAGGATAAGG | part of a light responsive element |
| GATA-motif | Solanum tuberosum | 1153 | + | 9 | AAGGATAAGG | part of a light responsive element |

>HU01G00833.1   
+ -Up\_Stream \_Len000AAAATA TTTTTACAAA AGCATTTCTC TATAAAATAT TTTACAAAAT TTCATTTTGT   
  
  
+ GGGTCTTTTA GAATCAATCT CTCTACAAGT AGGAATGCCG GTTGAGGTAA GGCTACCTAC ATCTAGATCT   
  
  
+ CTCCAGACCC CACAAAAATT AAACGAGACC CACACTGAAA GGTGGTGGTG GTGGTGTTGT TGTTGTATTG   
  
  
+ TCCTACCACG AGAGAGCTTC AAGTGCATCA CTTATTTCAA CAGGTCAATC ACATAGATCT CTAGTGTACT   
  
  
+ GTAAATTTTT TTTCCGTCTT ATTTTAATTT TCTTTTCCAT CCTAAAAGAT TATCATGTAG ATATTCCTAG   
  
  
+ TTTGATATAT TGACTTCAAC TTCAAAAATT ACACATTAAG ATCCACGAAT ATGACAGTTT AGGTTTCATT   
  
  
+ TGAAGGAGAA AGAAATGTTA CGGTGTTTTG GTGTTGTATT CGTAGACATT TTTTGCGCTT AACTGGCTCT   
  
  
+ TCTCATGGGG ACGAGACAGT GAAGGTAATT GTACTTGTTG ATTGATGACG TCATCTGTAG CTTGGATTTG   
  
  
+ TATAGGAATG AACATCTTGG TGTCTACGTG GAAAGTGTGA TTTGTGTTCT TTCATTGATC CTCCTCCTCA   
  
  
+ AGAAGAGGTG ATCTTTCTCC CTTGTTTTTT TTTTTTTTTG GCTAATATGT TGGCAGAACC GGCAAAAAAT   
  
  
+ ACATAATTTG AATAGTGCAT GCATATGAGT ACATGACAGT GAACCAACAA AACGAACCCC TAAAACACAA   
  
  
+ GAGAATTAAC ATGTTAAAAC ACTTAAACTA AATGCAACCA TCTCGATGAC CACTAAATAG CCAAGAAACC   
  
  
+ TAACGTAATT AACAAAAATA AAAAATAACT GAAAATAAAG TAGAGAAAGT CTAACCGAAT ACGATAACAT   
  
  
+ AACCTAAAAC TAACTATACT TGAACTTACT AAGAAATATG AATCAACCAC AAACCGAATT GATTTTGGTT   
  
  
+ TGCCATAATT TGACTATACA TTTAACTTAA AAAATATTTT TTTTTAATTT CAAATAAATC TAAATTATTT   
  
  
+ TTCAATATTC ATCCAAAATA GATTCAATTA TTAAACAAGT CAACCTATAC CGAGTTATTT TCTAGCTCTG   
  
  
+ ACTAAGACAT ATATATATTT TTTGTAAAAA AGATAAGGAT TATAATTTAA ATAGCCTTTC CCTTAAAAAA   
  
  
+ ATTTTTAAAA TTATCCTGAT AGAGAATAAA AAGAAATCCT TATACAAAAC TCTGATTGAC GCAGATGTGT   
  
  
+ ATATGGACAT ATAGAAGTAA TAAGAGGGGT AGGCTAGGAA GGGGGTGGAA ATATAGGGCA TAAATAGGGG   
  
  
+ GCGGGCATAG GGACATGTCT GTCTGAGGTA CGGTGGATGG GACCTTGCCA TGGAAGCAAC AATGTCACAA   
  
  
+ TAATAATATC AACGACTGAA GTTTCTTCAC CAGCCCACTC TTTGCCATAA TGCCATTAAT TATTTATTGC   
  
  
+ TACTATCTCT TTTGATGTGA AACGGTGGAC CTTATCTCTC CCCTCCACTT CCAATTGATG TGAGCAACTC   
  
  
+ AGAAACGCAA ACAAAACTTG TGCCACCTTC TTTACTTGCA TCCTCATTTT CCTCTGTCTC TCTCCGCCCC   
  
  
+ TGTTTCTCTC TCTCTCCCTC ACTCACTCAG AGTACTGATC TGAGCATCAG AAGTAGCAGA CCCAGAAACT   
  
  
+ CAGATTTAAA AACAACCCGC TCTTTTGTTC CCTCTACGTC TAATAATTTT GTCATCGCAT TTAATGTCAG   
  
  
+ ATAGAGAGAT AGAGCTCTAC TGTATTAATT TTCTCCCACC TCACTTGATT CTGTTGCCCT AAACCACCCA   
  
  
+ AAAAAAGAAA TACGAAGAAG CCACTCTCTC TCTCTCTCTC TCTCACACAC ACACACACCC CTCCTAGTGG   
  
  
+ ATTTGATAGA GTATAAATGA ACAGTAATCA GACGAGATAT ATAGTAGTGC TTAATTGACC GAGTAGCAAA   
  
  
+ AGGGGGAAGT TGAGTGAGGT ATCAAGAGAG TAGCGTAGCT AGCTAGGGCA AGATATGCTG GGGTCTTCCT   
  
  
+ CATACAACTC CGAGGAAGAC CACCAAGATG ACAACCATTC CTCACCGTCT GATTTGTCTA GGCACCCACC   
  
  
+ TAGATTCACC ACTACTACTT ACCAATACCA ACAACATATT AGCTCTCCAT CTTCACAATC AATACTAGCT   
  
  
+ GTGCCACCTC GCCAATTGTT AATCAATTGT GCGGAGTTAA TTTGCCGGTT CGACTTCTCC TCCGCCAACC   
  
  
+ GGCTTCTCTC TCTCCTCGCC TCCCACTTTG CCTCCCCTTC TGGGGACTCC ACCGAGAGAC TTGTGTTTTA   
  
  
+ CTTCTGCAAG GCTTTGGATC TCCGCCTCAA TAACAGTTAT TTATCCACTC GTAGTTTGAC TGGTTGTAAC   
  
  
+ CCTAGTACTG GTTCTGGTAT TGGGGCTCCT TTAGCTTCCA ATTTTCTATT TCTTCAACCA TTAGTGCATT   
  
  
+ CACAGGTAAC AAGTAATGCC CGTGTTGGGT ACTTGACATA TTTGACTCTG AACCAGGTCA CCCCATTCAT   
  
  
+ CCGATTCACC CACTTAACTG CCAACCAAGC CATCCTGGAA GCTTTGGAGG GGTACAAGGC GGTCCATATC   
  
  
+ CTTGACATGG ACATCATGCA TGGGGTCCAG TGGCCACCAC TTCTTCAAGC AATTGCAGAA AGATCTGCAA   
  
  
+ CCCTTGGTCA CCCTGCTCCC ACTGTTCGCA TCACAGGAGG GGGCAGGGAT CTAGAACTCC TGAACCGAAC   
  
  
+ TGGAGATCGG ATCAGGAAGT TCGCACAATC CTTGGGACTC GAGTTTCAAT TCCACCATCT TATAACGGTG   
  
  
+ GACTCGAACC TTACTGAGGC AGTGGAGGCC ATCAATGCCC TACAGCTACA CCTCCATGTC CAAGAGGAGG   
  
  
+ AGGTCTTCGC AGTGAATTGT GGAGATTTCC TCCACCGCCT CCTAACGGAG TATGATAGTA CATTCCTTAG   
  
  
+ GATGTTTCTC TACAAGGTGA AGACCCTAAA CCCTAGGGTC TTCACCGTGG GAGAAAGGGA GGCCGACCAC   
  
  
+ AACCACCCTC TCTTCTGGCA GCGATTCATT GAGGCACTTG ACCACTATGG GGCAGTGTTT GACTCTCTAG   
  
  
+ AAGCAACCCT ACCATCAACA AGTCAAGAGA GGGTGGCGGT GGAAGAGGGG TGGTTCGGGG AGGAGATTAA   
  
  
+ AGATGTGGTT GGACGAGAAG GAGGGTTGAG GAGACAGAGG CACCAGAAAT ACGAGTCTTG GGAGGTGTTG   
  
  
+ ATGAGGAGCT CTGGGTTTAA GAGCTTGCCC TTGAGCCCCT TCTCTGTGTC ACAAGCCAAG CTGCTACTTC   
  
  
+ GCCTTCATTA CCCTTCTGAA GGGTACCACC TTAAGGTCTT CCATAGTAAT TGTCTGTTGC TTGGGTGGAA   
  
  
+ GAATCGCCCT CTTTTCTCTG TTTCTTCTTG GCAATA  

- -Up\_Stream \_Len000TTTTAT AAAAATGTTT TCGTAAAGAG ATATTTTATA AAATGTTTTA AAGTAAAACA   
  
  
- CCCAGAAAAT CTTAGTTAGA GAGATGTTCA TCCTTACGGC CAACTCCATT CCGATGGATG TAGATCTAGA   
  
  
- GAGGTCTGGG GTGTTTTTAA TTTGCTCTGG GTGTGACTTT CCACCACCAC CACCACAACA ACAACATAAC   
  
  
- AGGATGGTGC TCTCTCGAAG TTCACGTAGT GAATAAAGTT GTCCAGTTAG TGTATCTAGA GATCACATGA   
  
  
- CATTTAAAAA AAAGGCAGAA TAAAATTAAA AGAAAAGGTA GGATTTTCTA ATAGTACATC TATAAGGATC   
  
  
- AAACTATATA ACTGAAGTTG AAGTTTTTAA TGTGTAATTC TAGGTGCTTA TACTGTCAAA TCCAAAGTAA   
  
  
- ACTTCCTCTT TCTTTACAAT GCCACAAAAC CACAACATAA GCATCTGTAA AAAACGCGAA TTGACCGAGA   
  
  
- AGAGTACCCC TGCTCTGTCA CTTCCATTAA CATGAACAAC TAACTACTGC AGTAGACATC GAACCTAAAC   
  
  
- ATATCCTTAC TTGTAGAACC ACAGATGCAC CTTTCACACT AAACACAAGA AAGTAACTAG GAGGAGGAGT   
  
  
- TCTTCTCCAC TAGAAAGAGG GAACAAAAAA AAAAAAAAAC CGATTATACA ACCGTCTTGG CCGTTTTTTA   
  
  
- TGTATTAAAC TTATCACGTA CGTATACTCA TGTACTGTCA CTTGGTTGTT TTGCTTGGGG ATTTTGTGTT   
  
  
- CTCTTAATTG TACAATTTTG TGAATTTGAT TTACGTTGGT AGAGCTACTG GTGATTTATC GGTTCTTTGG   
  
  
- ATTGCATTAA TTGTTTTTAT TTTTTATTGA CTTTTATTTC ATCTCTTTCA GATTGGCTTA TGCTATTGTA   
  
  
- TTGGATTTTG ATTGATATGA ACTTGAATGA TTCTTTATAC TTAGTTGGTG TTTGGCTTAA CTAAAACCAA   
  
  
- ACGGTATTAA ACTGATATGT AAATTGAATT TTTTATAAAA AAAAATTAAA GTTTATTTAG ATTTAATAAA   
  
  
- AAGTTATAAG TAGGTTTTAT CTAAGTTAAT AATTTGTTCA GTTGGATATG GCTCAATAAA AGATCGAGAC   
  
  
- TGATTCTGTA TATATATAAA AAACATTTTT TCTATTCCTA ATATTAAATT TATCGGAAAG GGAATTTTTT   
  
  
- TAAAAATTTT AATAGGACTA TCTCTTATTT TTCTTTAGGA ATATGTTTTG AGACTAACTG CGTCTACACA   
  
  
- TATACCTGTA TATCTTCATT ATTCTCCCCA TCCGATCCTT CCCCCACCTT TATATCCCGT ATTTATCCCC   
  
  
- CGCCCGTATC CCTGTACAGA CAGACTCCAT GCCACCTACC CTGGAACGGT ACCTTCGTTG TTACAGTGTT   
  
  
- ATTATTATAG TTGCTGACTT CAAAGAAGTG GTCGGGTGAG AAACGGTATT ACGGTAATTA ATAAATAACG   
  
  
- ATGATAGAGA AAACTACACT TTGCCACCTG GAATAGAGAG GGGAGGTGAA GGTTAACTAC ACTCGTTGAG   
  
  
- TCTTTGCGTT TGTTTTGAAC ACGGTGGAAG AAATGAACGT AGGAGTAAAA GGAGACAGAG AGAGGCGGGG   
  
  
- ACAAAGAGAG AGAGAGGGAG TGAGTGAGTC TCATGACTAG ACTCGTAGTC TTCATCGTCT GGGTCTTTGA   
  
  
- GTCTAAATTT TTGTTGGGCG AGAAAACAAG GGAGATGCAG ATTATTAAAA CAGTAGCGTA AATTACAGTC   
  
  
- TATCTCTCTA TCTCGAGATG ACATAATTAA AAGAGGGTGG AGTGAACTAA GACAACGGGA TTTGGTGGGT   
  
  
- TTTTTTCTTT ATGCTTCTTC GGTGAGAGAG AGAGAGAGAG AGAGTGTGTG TGTGTGTGGG GAGGATCACC   
  
  
- TAAACTATCT CATATTTACT TGTCATTAGT CTGCTCTATA TATCATCACG AATTAACTGG CTCATCGTTT   
  
  
- TCCCCCTTCA ACTCACTCCA TAGTTCTCTC ATCGCATCGA TCGATCCCGT TCTATACGAC CCCAGAAGGA   
  
  
- GTATGTTGAG GCTCCTTCTG GTGGTTCTAC TGTTGGTAAG GAGTGGCAGA CTAAACAGAT CCGTGGGTGG   
  
  
- ATCTAAGTGG TGATGATGAA TGGTTATGGT TGTTGTATAA TCGAGAGGTA GAAGTGTTAG TTATGATCGA   
  
  
- CACGGTGGAG CGGTTAACAA TTAGTTAACA CGCCTCAATT AAACGGCCAA GCTGAAGAGG AGGCGGTTGG   
  
  
- CCGAAGAGAG AGAGGAGCGG AGGGTGAAAC GGAGGGGAAG ACCCCTGAGG TGGCTCTCTG AACACAAAAT   
  
  
- GAAGACGTTC CGAAACCTAG AGGCGGAGTT ATTGTCAATA AATAGGTGAG CATCAAACTG ACCAACATTG   
  
  
- GGATCATGAC CAAGACCATA ACCCCGAGGA AATCGAAGGT TAAAAGATAA AGAAGTTGGT AATCACGTAA   
  
  
- GTGTCCATTG TTCATTACGG GCACAACCCA TGAACTGTAT AAACTGAGAC TTGGTCCAGT GGGGTAAGTA   
  
  
- GGCTAAGTGG GTGAATTGAC GGTTGGTTCG GTAGGACCTT CGAAACCTCC CCATGTTCCG CCAGGTATAG   
  
  
- GAACTGTACC TGTAGTACGT ACCCCAGGTC ACCGGTGGTG AAGAAGTTCG TTAACGTCTT TCTAGACGTT   
  
  
- GGGAACCAGT GGGACGAGGG TGACAAGCGT AGTGTCCTCC CCCGTCCCTA GATCTTGAGG ACTTGGCTTG   
  
  
- ACCTCTAGCC TAGTCCTTCA AGCGTGTTAG GAACCCTGAG CTCAAAGTTA AGGTGGTAGA ATATTGCCAC   
  
  
- CTGAGCTTGG AATGACTCCG TCACCTCCGG TAGTTACGGG ATGTCGATGT GGAGGTACAG GTTCTCCTCC   
  
  
- TCCAGAAGCG TCACTTAACA CCTCTAAAGG AGGTGGCGGA GGATTGCCTC ATACTATCAT GTAAGGAATC   
  
  
- CTACAAAGAG ATGTTCCACT TCTGGGATTT GGGATCCCAG AAGTGGCACC CTCTTTCCCT CCGGCTGGTG   
  
  
- TTGGTGGGAG AGAAGACCGT CGCTAAGTAA CTCCGTGAAC TGGTGATACC CCGTCACAAA CTGAGAGATC   
  
  
- TTCGTTGGGA TGGTAGTTGT TCAGTTCTCT CCCACCGCCA CCTTCTCCCC ACCAAGCCCC TCCTCTAATT   
  
  
- TCTACACCAA CCTGCTCTTC CTCCCAACTC CTCTGTCTCC GTGGTCTTTA TGCTCAGAAC CCTCCACAAC   
  
  
- TACTCCTCGA GACCCAAATT CTCGAACGGG AACTCGGGGA AGAGACACAG TGTTCGGTTC GACGATGAAG   
  
  
- CGGAAGTAAT GGGAAGACTT CCCATGGTGG AATTCCAGAA GGTATCATTA ACAGACAACG AACCCACCTT   
  
  
- CTTAGCGGGA GAAAAGAGAC AAAGAAGAAC CGTTAT

+     GTGGC-motif

| Site Name | Organism | Position | Strand | Matrix score. | sequence | function |
| --- | --- | --- | --- | --- | --- | --- |
| GTGGC-motif | Spinacia oleracea | 1801 | + | 10 | GATTCTGTGGC | part of a light responsive element |

>HU01G00833.1   
+ -Up\_Stream \_Len000AAAATA TTTTTACAAA AGCATTTCTC TATAAAATAT TTTACAAAAT TTCATTTTGT   
  
  
+ GGGTCTTTTA GAATCAATCT CTCTACAAGT AGGAATGCCG GTTGAGGTAA GGCTACCTAC ATCTAGATCT   
  
  
+ CTCCAGACCC CACAAAAATT AAACGAGACC CACACTGAAA GGTGGTGGTG GTGGTGTTGT TGTTGTATTG   
  
  
+ TCCTACCACG AGAGAGCTTC AAGTGCATCA CTTATTTCAA CAGGTCAATC ACATAGATCT CTAGTGTACT   
  
  
+ GTAAATTTTT TTTCCGTCTT ATTTTAATTT TCTTTTCCAT CCTAAAAGAT TATCATGTAG ATATTCCTAG   
  
  
+ TTTGATATAT TGACTTCAAC TTCAAAAATT ACACATTAAG ATCCACGAAT ATGACAGTTT AGGTTTCATT   
  
  
+ TGAAGGAGAA AGAAATGTTA CGGTGTTTTG GTGTTGTATT CGTAGACATT TTTTGCGCTT AACTGGCTCT   
  
  
+ TCTCATGGGG ACGAGACAGT GAAGGTAATT GTACTTGTTG ATTGATGACG TCATCTGTAG CTTGGATTTG   
  
  
+ TATAGGAATG AACATCTTGG TGTCTACGTG GAAAGTGTGA TTTGTGTTCT TTCATTGATC CTCCTCCTCA   
  
  
+ AGAAGAGGTG ATCTTTCTCC CTTGTTTTTT TTTTTTTTTG GCTAATATGT TGGCAGAACC GGCAAAAAAT   
  
  
+ ACATAATTTG AATAGTGCAT GCATATGAGT ACATGACAGT GAACCAACAA AACGAACCCC TAAAACACAA   
  
  
+ GAGAATTAAC ATGTTAAAAC ACTTAAACTA AATGCAACCA TCTCGATGAC CACTAAATAG CCAAGAAACC   
  
  
+ TAACGTAATT AACAAAAATA AAAAATAACT GAAAATAAAG TAGAGAAAGT CTAACCGAAT ACGATAACAT   
  
  
+ AACCTAAAAC TAACTATACT TGAACTTACT AAGAAATATG AATCAACCAC AAACCGAATT GATTTTGGTT   
  
  
+ TGCCATAATT TGACTATACA TTTAACTTAA AAAATATTTT TTTTTAATTT CAAATAAATC TAAATTATTT   
  
  
+ TTCAATATTC ATCCAAAATA GATTCAATTA TTAAACAAGT CAACCTATAC CGAGTTATTT TCTAGCTCTG   
  
  
+ ACTAAGACAT ATATATATTT TTTGTAAAAA AGATAAGGAT TATAATTTAA ATAGCCTTTC CCTTAAAAAA   
  
  
+ ATTTTTAAAA TTATCCTGAT AGAGAATAAA AAGAAATCCT TATACAAAAC TCTGATTGAC GCAGATGTGT   
  
  
+ ATATGGACAT ATAGAAGTAA TAAGAGGGGT AGGCTAGGAA GGGGGTGGAA ATATAGGGCA TAAATAGGGG   
  
  
+ GCGGGCATAG GGACATGTCT GTCTGAGGTA CGGTGGATGG GACCTTGCCA TGGAAGCAAC AATGTCACAA   
  
  
+ TAATAATATC AACGACTGAA GTTTCTTCAC CAGCCCACTC TTTGCCATAA TGCCATTAAT TATTTATTGC   
  
  
+ TACTATCTCT TTTGATGTGA AACGGTGGAC CTTATCTCTC CCCTCCACTT CCAATTGATG TGAGCAACTC   
  
  
+ AGAAACGCAA ACAAAACTTG TGCCACCTTC TTTACTTGCA TCCTCATTTT CCTCTGTCTC TCTCCGCCCC   
  
  
+ TGTTTCTCTC TCTCTCCCTC ACTCACTCAG AGTACTGATC TGAGCATCAG AAGTAGCAGA CCCAGAAACT   
  
  
+ CAGATTTAAA AACAACCCGC TCTTTTGTTC CCTCTACGTC TAATAATTTT GTCATCGCAT TTAATGTCAG   
  
  
+ ATAGAGAGAT AGAGCTCTAC TGTATTAATT TTCTCCCACC TCACTTGATT CTGTTGCCCT AAACCACCCA   
  
  
+ AAAAAAGAAA TACGAAGAAG CCACTCTCTC TCTCTCTCTC TCTCACACAC ACACACACCC CTCCTAGTGG   
  
  
+ ATTTGATAGA GTATAAATGA ACAGTAATCA GACGAGATAT ATAGTAGTGC TTAATTGACC GAGTAGCAAA   
  
  
+ AGGGGGAAGT TGAGTGAGGT ATCAAGAGAG TAGCGTAGCT AGCTAGGGCA AGATATGCTG GGGTCTTCCT   
  
  
+ CATACAACTC CGAGGAAGAC CACCAAGATG ACAACCATTC CTCACCGTCT GATTTGTCTA GGCACCCACC   
  
  
+ TAGATTCACC ACTACTACTT ACCAATACCA ACAACATATT AGCTCTCCAT CTTCACAATC AATACTAGCT   
  
  
+ GTGCCACCTC GCCAATTGTT AATCAATTGT GCGGAGTTAA TTTGCCGGTT CGACTTCTCC TCCGCCAACC   
  
  
+ GGCTTCTCTC TCTCCTCGCC TCCCACTTTG CCTCCCCTTC TGGGGACTCC ACCGAGAGAC TTGTGTTTTA   
  
  
+ CTTCTGCAAG GCTTTGGATC TCCGCCTCAA TAACAGTTAT TTATCCACTC GTAGTTTGAC TGGTTGTAAC   
  
  
+ CCTAGTACTG GTTCTGGTAT TGGGGCTCCT TTAGCTTCCA ATTTTCTATT TCTTCAACCA TTAGTGCATT   
  
  
+ CACAGGTAAC AAGTAATGCC CGTGTTGGGT ACTTGACATA TTTGACTCTG AACCAGGTCA CCCCATTCAT   
  
  
+ CCGATTCACC CACTTAACTG CCAACCAAGC CATCCTGGAA GCTTTGGAGG GGTACAAGGC GGTCCATATC   
  
  
+ CTTGACATGG ACATCATGCA TGGGGTCCAG TGGCCACCAC TTCTTCAAGC AATTGCAGAA AGATCTGCAA   
  
  
+ CCCTTGGTCA CCCTGCTCCC ACTGTTCGCA TCACAGGAGG GGGCAGGGAT CTAGAACTCC TGAACCGAAC   
  
  
+ TGGAGATCGG ATCAGGAAGT TCGCACAATC CTTGGGACTC GAGTTTCAAT TCCACCATCT TATAACGGTG   
  
  
+ GACTCGAACC TTACTGAGGC AGTGGAGGCC ATCAATGCCC TACAGCTACA CCTCCATGTC CAAGAGGAGG   
  
  
+ AGGTCTTCGC AGTGAATTGT GGAGATTTCC TCCACCGCCT CCTAACGGAG TATGATAGTA CATTCCTTAG   
  
  
+ GATGTTTCTC TACAAGGTGA AGACCCTAAA CCCTAGGGTC TTCACCGTGG GAGAAAGGGA GGCCGACCAC   
  
  
+ AACCACCCTC TCTTCTGGCA GCGATTCATT GAGGCACTTG ACCACTATGG GGCAGTGTTT GACTCTCTAG   
  
  
+ AAGCAACCCT ACCATCAACA AGTCAAGAGA GGGTGGCGGT GGAAGAGGGG TGGTTCGGGG AGGAGATTAA   
  
  
+ AGATGTGGTT GGACGAGAAG GAGGGTTGAG GAGACAGAGG CACCAGAAAT ACGAGTCTTG GGAGGTGTTG   
  
  
+ ATGAGGAGCT CTGGGTTTAA GAGCTTGCCC TTGAGCCCCT TCTCTGTGTC ACAAGCCAAG CTGCTACTTC   
  
  
+ GCCTTCATTA CCCTTCTGAA GGGTACCACC TTAAGGTCTT CCATAGTAAT TGTCTGTTGC TTGGGTGGAA   
  
  
+ GAATCGCCCT CTTTTCTCTG TTTCTTCTTG GCAATA  

- -Up\_Stream \_Len000TTTTAT AAAAATGTTT TCGTAAAGAG ATATTTTATA AAATGTTTTA AAGTAAAACA   
  
  
- CCCAGAAAAT CTTAGTTAGA GAGATGTTCA TCCTTACGGC CAACTCCATT CCGATGGATG TAGATCTAGA   
  
  
- GAGGTCTGGG GTGTTTTTAA TTTGCTCTGG GTGTGACTTT CCACCACCAC CACCACAACA ACAACATAAC   
  
  
- AGGATGGTGC TCTCTCGAAG TTCACGTAGT GAATAAAGTT GTCCAGTTAG TGTATCTAGA GATCACATGA   
  
  
- CATTTAAAAA AAAGGCAGAA TAAAATTAAA AGAAAAGGTA GGATTTTCTA ATAGTACATC TATAAGGATC   
  
  
- AAACTATATA ACTGAAGTTG AAGTTTTTAA TGTGTAATTC TAGGTGCTTA TACTGTCAAA TCCAAAGTAA   
  
  
- ACTTCCTCTT TCTTTACAAT GCCACAAAAC CACAACATAA GCATCTGTAA AAAACGCGAA TTGACCGAGA   
  
  
- AGAGTACCCC TGCTCTGTCA CTTCCATTAA CATGAACAAC TAACTACTGC AGTAGACATC GAACCTAAAC   
  
  
- ATATCCTTAC TTGTAGAACC ACAGATGCAC CTTTCACACT AAACACAAGA AAGTAACTAG GAGGAGGAGT   
  
  
- TCTTCTCCAC TAGAAAGAGG GAACAAAAAA AAAAAAAAAC CGATTATACA ACCGTCTTGG CCGTTTTTTA   
  
  
- TGTATTAAAC TTATCACGTA CGTATACTCA TGTACTGTCA CTTGGTTGTT TTGCTTGGGG ATTTTGTGTT   
  
  
- CTCTTAATTG TACAATTTTG TGAATTTGAT TTACGTTGGT AGAGCTACTG GTGATTTATC GGTTCTTTGG   
  
  
- ATTGCATTAA TTGTTTTTAT TTTTTATTGA CTTTTATTTC ATCTCTTTCA GATTGGCTTA TGCTATTGTA   
  
  
- TTGGATTTTG ATTGATATGA ACTTGAATGA TTCTTTATAC TTAGTTGGTG TTTGGCTTAA CTAAAACCAA   
  
  
- ACGGTATTAA ACTGATATGT AAATTGAATT TTTTATAAAA AAAAATTAAA GTTTATTTAG ATTTAATAAA   
  
  
- AAGTTATAAG TAGGTTTTAT CTAAGTTAAT AATTTGTTCA GTTGGATATG GCTCAATAAA AGATCGAGAC   
  
  
- TGATTCTGTA TATATATAAA AAACATTTTT TCTATTCCTA ATATTAAATT TATCGGAAAG GGAATTTTTT   
  
  
- TAAAAATTTT AATAGGACTA TCTCTTATTT TTCTTTAGGA ATATGTTTTG AGACTAACTG CGTCTACACA   
  
  
- TATACCTGTA TATCTTCATT ATTCTCCCCA TCCGATCCTT CCCCCACCTT TATATCCCGT ATTTATCCCC   
  
  
- CGCCCGTATC CCTGTACAGA CAGACTCCAT GCCACCTACC CTGGAACGGT ACCTTCGTTG TTACAGTGTT   
  
  
- ATTATTATAG TTGCTGACTT CAAAGAAGTG GTCGGGTGAG AAACGGTATT ACGGTAATTA ATAAATAACG   
  
  
- ATGATAGAGA AAACTACACT TTGCCACCTG GAATAGAGAG GGGAGGTGAA GGTTAACTAC ACTCGTTGAG   
  
  
- TCTTTGCGTT TGTTTTGAAC ACGGTGGAAG AAATGAACGT AGGAGTAAAA GGAGACAGAG AGAGGCGGGG   
  
  
- ACAAAGAGAG AGAGAGGGAG TGAGTGAGTC TCATGACTAG ACTCGTAGTC TTCATCGTCT GGGTCTTTGA   
  
  
- GTCTAAATTT TTGTTGGGCG AGAAAACAAG GGAGATGCAG ATTATTAAAA CAGTAGCGTA AATTACAGTC   
  
  
- TATCTCTCTA TCTCGAGATG ACATAATTAA AAGAGGGTGG AGTGAACTAA GACAACGGGA TTTGGTGGGT   
  
  
- TTTTTTCTTT ATGCTTCTTC GGTGAGAGAG AGAGAGAGAG AGAGTGTGTG TGTGTGTGGG GAGGATCACC   
  
  
- TAAACTATCT CATATTTACT TGTCATTAGT CTGCTCTATA TATCATCACG AATTAACTGG CTCATCGTTT   
  
  
- TCCCCCTTCA ACTCACTCCA TAGTTCTCTC ATCGCATCGA TCGATCCCGT TCTATACGAC CCCAGAAGGA   
  
  
- GTATGTTGAG GCTCCTTCTG GTGGTTCTAC TGTTGGTAAG GAGTGGCAGA CTAAACAGAT CCGTGGGTGG   
  
  
- ATCTAAGTGG TGATGATGAA TGGTTATGGT TGTTGTATAA TCGAGAGGTA GAAGTGTTAG TTATGATCGA   
  
  
- CACGGTGGAG CGGTTAACAA TTAGTTAACA CGCCTCAATT AAACGGCCAA GCTGAAGAGG AGGCGGTTGG   
  
  
- CCGAAGAGAG AGAGGAGCGG AGGGTGAAAC GGAGGGGAAG ACCCCTGAGG TGGCTCTCTG AACACAAAAT   
  
  
- GAAGACGTTC CGAAACCTAG AGGCGGAGTT ATTGTCAATA AATAGGTGAG CATCAAACTG ACCAACATTG   
  
  
- GGATCATGAC CAAGACCATA ACCCCGAGGA AATCGAAGGT TAAAAGATAA AGAAGTTGGT AATCACGTAA   
  
  
- GTGTCCATTG TTCATTACGG GCACAACCCA TGAACTGTAT AAACTGAGAC TTGGTCCAGT GGGGTAAGTA   
  
  
- GGCTAAGTGG GTGAATTGAC GGTTGGTTCG GTAGGACCTT CGAAACCTCC CCATGTTCCG CCAGGTATAG   
  
  
- GAACTGTACC TGTAGTACGT ACCCCAGGTC ACCGGTGGTG AAGAAGTTCG TTAACGTCTT TCTAGACGTT   
  
  
- GGGAACCAGT GGGACGAGGG TGACAAGCGT AGTGTCCTCC CCCGTCCCTA GATCTTGAGG ACTTGGCTTG   
  
  
- ACCTCTAGCC TAGTCCTTCA AGCGTGTTAG GAACCCTGAG CTCAAAGTTA AGGTGGTAGA ATATTGCCAC   
  
  
- CTGAGCTTGG AATGACTCCG TCACCTCCGG TAGTTACGGG ATGTCGATGT GGAGGTACAG GTTCTCCTCC   
  
  
- TCCAGAAGCG TCACTTAACA CCTCTAAAGG AGGTGGCGGA GGATTGCCTC ATACTATCAT GTAAGGAATC   
  
  
- CTACAAAGAG ATGTTCCACT TCTGGGATTT GGGATCCCAG AAGTGGCACC CTCTTTCCCT CCGGCTGGTG   
  
  
- TTGGTGGGAG AGAAGACCGT CGCTAAGTAA CTCCGTGAAC TGGTGATACC CCGTCACAAA CTGAGAGATC   
  
  
- TTCGTTGGGA TGGTAGTTGT TCAGTTCTCT CCCACCGCCA CCTTCTCCCC ACCAAGCCCC TCCTCTAATT   
  
  
- TCTACACCAA CCTGCTCTTC CTCCCAACTC CTCTGTCTCC GTGGTCTTTA TGCTCAGAAC CCTCCACAAC   
  
  
- TACTCCTCGA GACCCAAATT CTCGAACGGG AACTCGGGGA AGAGACACAG TGTTCGGTTC GACGATGAAG   
  
  
- CGGAAGTAAT GGGAAGACTT CCCATGGTGG AATTCCAGAA GGTATCATTA ACAGACAACG AACCCACCTT   
  
  
- CTTAGCGGGA GAAAAGAGAC AAAGAAGAAC CGTTAT

+     I-box

| Site Name | Organism | Position | Strand | Matrix score. | sequence | function |
| --- | --- | --- | --- | --- | --- | --- |
| I-box | Triticum aestivum | 1504 | - | 8 | AGATAAGG | part of a light responsive element |
| I-box | Triticum aestivum | 1155 | + | 8 | AGATAAGG | part of a light responsive element |
| I-box | Gossypium hirsutum | 1154 | + | 10 | AAGATAAGGCT | part of a light responsive element |

>HU01G00833.1   
+ -Up\_Stream \_Len000AAAATA TTTTTACAAA AGCATTTCTC TATAAAATAT TTTACAAAAT TTCATTTTGT   
  
  
+ GGGTCTTTTA GAATCAATCT CTCTACAAGT AGGAATGCCG GTTGAGGTAA GGCTACCTAC ATCTAGATCT   
  
  
+ CTCCAGACCC CACAAAAATT AAACGAGACC CACACTGAAA GGTGGTGGTG GTGGTGTTGT TGTTGTATTG   
  
  
+ TCCTACCACG AGAGAGCTTC AAGTGCATCA CTTATTTCAA CAGGTCAATC ACATAGATCT CTAGTGTACT   
  
  
+ GTAAATTTTT TTTCCGTCTT ATTTTAATTT TCTTTTCCAT CCTAAAAGAT TATCATGTAG ATATTCCTAG   
  
  
+ TTTGATATAT TGACTTCAAC TTCAAAAATT ACACATTAAG ATCCACGAAT ATGACAGTTT AGGTTTCATT   
  
  
+ TGAAGGAGAA AGAAATGTTA CGGTGTTTTG GTGTTGTATT CGTAGACATT TTTTGCGCTT AACTGGCTCT   
  
  
+ TCTCATGGGG ACGAGACAGT GAAGGTAATT GTACTTGTTG ATTGATGACG TCATCTGTAG CTTGGATTTG   
  
  
+ TATAGGAATG AACATCTTGG TGTCTACGTG GAAAGTGTGA TTTGTGTTCT TTCATTGATC CTCCTCCTCA   
  
  
+ AGAAGAGGTG ATCTTTCTCC CTTGTTTTTT TTTTTTTTTG GCTAATATGT TGGCAGAACC GGCAAAAAAT   
  
  
+ ACATAATTTG AATAGTGCAT GCATATGAGT ACATGACAGT GAACCAACAA AACGAACCCC TAAAACACAA   
  
  
+ GAGAATTAAC ATGTTAAAAC ACTTAAACTA AATGCAACCA TCTCGATGAC CACTAAATAG CCAAGAAACC   
  
  
+ TAACGTAATT AACAAAAATA AAAAATAACT GAAAATAAAG TAGAGAAAGT CTAACCGAAT ACGATAACAT   
  
  
+ AACCTAAAAC TAACTATACT TGAACTTACT AAGAAATATG AATCAACCAC AAACCGAATT GATTTTGGTT   
  
  
+ TGCCATAATT TGACTATACA TTTAACTTAA AAAATATTTT TTTTTAATTT CAAATAAATC TAAATTATTT   
  
  
+ TTCAATATTC ATCCAAAATA GATTCAATTA TTAAACAAGT CAACCTATAC CGAGTTATTT TCTAGCTCTG   
  
  
+ ACTAAGACAT ATATATATTT TTTGTAAAAA AGATAAGGAT TATAATTTAA ATAGCCTTTC CCTTAAAAAA   
  
  
+ ATTTTTAAAA TTATCCTGAT AGAGAATAAA AAGAAATCCT TATACAAAAC TCTGATTGAC GCAGATGTGT   
  
  
+ ATATGGACAT ATAGAAGTAA TAAGAGGGGT AGGCTAGGAA GGGGGTGGAA ATATAGGGCA TAAATAGGGG   
  
  
+ GCGGGCATAG GGACATGTCT GTCTGAGGTA CGGTGGATGG GACCTTGCCA TGGAAGCAAC AATGTCACAA   
  
  
+ TAATAATATC AACGACTGAA GTTTCTTCAC CAGCCCACTC TTTGCCATAA TGCCATTAAT TATTTATTGC   
  
  
+ TACTATCTCT TTTGATGTGA AACGGTGGAC CTTATCTCTC CCCTCCACTT CCAATTGATG TGAGCAACTC   
  
  
+ AGAAACGCAA ACAAAACTTG TGCCACCTTC TTTACTTGCA TCCTCATTTT CCTCTGTCTC TCTCCGCCCC   
  
  
+ TGTTTCTCTC TCTCTCCCTC ACTCACTCAG AGTACTGATC TGAGCATCAG AAGTAGCAGA CCCAGAAACT   
  
  
+ CAGATTTAAA AACAACCCGC TCTTTTGTTC CCTCTACGTC TAATAATTTT GTCATCGCAT TTAATGTCAG   
  
  
+ ATAGAGAGAT AGAGCTCTAC TGTATTAATT TTCTCCCACC TCACTTGATT CTGTTGCCCT AAACCACCCA   
  
  
+ AAAAAAGAAA TACGAAGAAG CCACTCTCTC TCTCTCTCTC TCTCACACAC ACACACACCC CTCCTAGTGG   
  
  
+ ATTTGATAGA GTATAAATGA ACAGTAATCA GACGAGATAT ATAGTAGTGC TTAATTGACC GAGTAGCAAA   
  
  
+ AGGGGGAAGT TGAGTGAGGT ATCAAGAGAG TAGCGTAGCT AGCTAGGGCA AGATATGCTG GGGTCTTCCT   
  
  
+ CATACAACTC CGAGGAAGAC CACCAAGATG ACAACCATTC CTCACCGTCT GATTTGTCTA GGCACCCACC   
  
  
+ TAGATTCACC ACTACTACTT ACCAATACCA ACAACATATT AGCTCTCCAT CTTCACAATC AATACTAGCT   
  
  
+ GTGCCACCTC GCCAATTGTT AATCAATTGT GCGGAGTTAA TTTGCCGGTT CGACTTCTCC TCCGCCAACC   
  
  
+ GGCTTCTCTC TCTCCTCGCC TCCCACTTTG CCTCCCCTTC TGGGGACTCC ACCGAGAGAC TTGTGTTTTA   
  
  
+ CTTCTGCAAG GCTTTGGATC TCCGCCTCAA TAACAGTTAT TTATCCACTC GTAGTTTGAC TGGTTGTAAC   
  
  
+ CCTAGTACTG GTTCTGGTAT TGGGGCTCCT TTAGCTTCCA ATTTTCTATT TCTTCAACCA TTAGTGCATT   
  
  
+ CACAGGTAAC AAGTAATGCC CGTGTTGGGT ACTTGACATA TTTGACTCTG AACCAGGTCA CCCCATTCAT   
  
  
+ CCGATTCACC CACTTAACTG CCAACCAAGC CATCCTGGAA GCTTTGGAGG GGTACAAGGC GGTCCATATC   
  
  
+ CTTGACATGG ACATCATGCA TGGGGTCCAG TGGCCACCAC TTCTTCAAGC AATTGCAGAA AGATCTGCAA   
  
  
+ CCCTTGGTCA CCCTGCTCCC ACTGTTCGCA TCACAGGAGG GGGCAGGGAT CTAGAACTCC TGAACCGAAC   
  
  
+ TGGAGATCGG ATCAGGAAGT TCGCACAATC CTTGGGACTC GAGTTTCAAT TCCACCATCT TATAACGGTG   
  
  
+ GACTCGAACC TTACTGAGGC AGTGGAGGCC ATCAATGCCC TACAGCTACA CCTCCATGTC CAAGAGGAGG   
  
  
+ AGGTCTTCGC AGTGAATTGT GGAGATTTCC TCCACCGCCT CCTAACGGAG TATGATAGTA CATTCCTTAG   
  
  
+ GATGTTTCTC TACAAGGTGA AGACCCTAAA CCCTAGGGTC TTCACCGTGG GAGAAAGGGA GGCCGACCAC   
  
  
+ AACCACCCTC TCTTCTGGCA GCGATTCATT GAGGCACTTG ACCACTATGG GGCAGTGTTT GACTCTCTAG   
  
  
+ AAGCAACCCT ACCATCAACA AGTCAAGAGA GGGTGGCGGT GGAAGAGGGG TGGTTCGGGG AGGAGATTAA   
  
  
+ AGATGTGGTT GGACGAGAAG GAGGGTTGAG GAGACAGAGG CACCAGAAAT ACGAGTCTTG GGAGGTGTTG   
  
  
+ ATGAGGAGCT CTGGGTTTAA GAGCTTGCCC TTGAGCCCCT TCTCTGTGTC ACAAGCCAAG CTGCTACTTC   
  
  
+ GCCTTCATTA CCCTTCTGAA GGGTACCACC TTAAGGTCTT CCATAGTAAT TGTCTGTTGC TTGGGTGGAA   
  
  
+ GAATCGCCCT CTTTTCTCTG TTTCTTCTTG GCAATA  

- -Up\_Stream \_Len000TTTTAT AAAAATGTTT TCGTAAAGAG ATATTTTATA AAATGTTTTA AAGTAAAACA   
  
  
- CCCAGAAAAT CTTAGTTAGA GAGATGTTCA TCCTTACGGC CAACTCCATT CCGATGGATG TAGATCTAGA   
  
  
- GAGGTCTGGG GTGTTTTTAA TTTGCTCTGG GTGTGACTTT CCACCACCAC CACCACAACA ACAACATAAC   
  
  
- AGGATGGTGC TCTCTCGAAG TTCACGTAGT GAATAAAGTT GTCCAGTTAG TGTATCTAGA GATCACATGA   
  
  
- CATTTAAAAA AAAGGCAGAA TAAAATTAAA AGAAAAGGTA GGATTTTCTA ATAGTACATC TATAAGGATC   
  
  
- AAACTATATA ACTGAAGTTG AAGTTTTTAA TGTGTAATTC TAGGTGCTTA TACTGTCAAA TCCAAAGTAA   
  
  
- ACTTCCTCTT TCTTTACAAT GCCACAAAAC CACAACATAA GCATCTGTAA AAAACGCGAA TTGACCGAGA   
  
  
- AGAGTACCCC TGCTCTGTCA CTTCCATTAA CATGAACAAC TAACTACTGC AGTAGACATC GAACCTAAAC   
  
  
- ATATCCTTAC TTGTAGAACC ACAGATGCAC CTTTCACACT AAACACAAGA AAGTAACTAG GAGGAGGAGT   
  
  
- TCTTCTCCAC TAGAAAGAGG GAACAAAAAA AAAAAAAAAC CGATTATACA ACCGTCTTGG CCGTTTTTTA   
  
  
- TGTATTAAAC TTATCACGTA CGTATACTCA TGTACTGTCA CTTGGTTGTT TTGCTTGGGG ATTTTGTGTT   
  
  
- CTCTTAATTG TACAATTTTG TGAATTTGAT TTACGTTGGT AGAGCTACTG GTGATTTATC GGTTCTTTGG   
  
  
- ATTGCATTAA TTGTTTTTAT TTTTTATTGA CTTTTATTTC ATCTCTTTCA GATTGGCTTA TGCTATTGTA   
  
  
- TTGGATTTTG ATTGATATGA ACTTGAATGA TTCTTTATAC TTAGTTGGTG TTTGGCTTAA CTAAAACCAA   
  
  
- ACGGTATTAA ACTGATATGT AAATTGAATT TTTTATAAAA AAAAATTAAA GTTTATTTAG ATTTAATAAA   
  
  
- AAGTTATAAG TAGGTTTTAT CTAAGTTAAT AATTTGTTCA GTTGGATATG GCTCAATAAA AGATCGAGAC   
  
  
- TGATTCTGTA TATATATAAA AAACATTTTT TCTATTCCTA ATATTAAATT TATCGGAAAG GGAATTTTTT   
  
  
- TAAAAATTTT AATAGGACTA TCTCTTATTT TTCTTTAGGA ATATGTTTTG AGACTAACTG CGTCTACACA   
  
  
- TATACCTGTA TATCTTCATT ATTCTCCCCA TCCGATCCTT CCCCCACCTT TATATCCCGT ATTTATCCCC   
  
  
- CGCCCGTATC CCTGTACAGA CAGACTCCAT GCCACCTACC CTGGAACGGT ACCTTCGTTG TTACAGTGTT   
  
  
- ATTATTATAG TTGCTGACTT CAAAGAAGTG GTCGGGTGAG AAACGGTATT ACGGTAATTA ATAAATAACG   
  
  
- ATGATAGAGA AAACTACACT TTGCCACCTG GAATAGAGAG GGGAGGTGAA GGTTAACTAC ACTCGTTGAG   
  
  
- TCTTTGCGTT TGTTTTGAAC ACGGTGGAAG AAATGAACGT AGGAGTAAAA GGAGACAGAG AGAGGCGGGG   
  
  
- ACAAAGAGAG AGAGAGGGAG TGAGTGAGTC TCATGACTAG ACTCGTAGTC TTCATCGTCT GGGTCTTTGA   
  
  
- GTCTAAATTT TTGTTGGGCG AGAAAACAAG GGAGATGCAG ATTATTAAAA CAGTAGCGTA AATTACAGTC   
  
  
- TATCTCTCTA TCTCGAGATG ACATAATTAA AAGAGGGTGG AGTGAACTAA GACAACGGGA TTTGGTGGGT   
  
  
- TTTTTTCTTT ATGCTTCTTC GGTGAGAGAG AGAGAGAGAG AGAGTGTGTG TGTGTGTGGG GAGGATCACC   
  
  
- TAAACTATCT CATATTTACT TGTCATTAGT CTGCTCTATA TATCATCACG AATTAACTGG CTCATCGTTT   
  
  
- TCCCCCTTCA ACTCACTCCA TAGTTCTCTC ATCGCATCGA TCGATCCCGT TCTATACGAC CCCAGAAGGA   
  
  
- GTATGTTGAG GCTCCTTCTG GTGGTTCTAC TGTTGGTAAG GAGTGGCAGA CTAAACAGAT CCGTGGGTGG   
  
  
- ATCTAAGTGG TGATGATGAA TGGTTATGGT TGTTGTATAA TCGAGAGGTA GAAGTGTTAG TTATGATCGA   
  
  
- CACGGTGGAG CGGTTAACAA TTAGTTAACA CGCCTCAATT AAACGGCCAA GCTGAAGAGG AGGCGGTTGG   
  
  
- CCGAAGAGAG AGAGGAGCGG AGGGTGAAAC GGAGGGGAAG ACCCCTGAGG TGGCTCTCTG AACACAAAAT   
  
  
- GAAGACGTTC CGAAACCTAG AGGCGGAGTT ATTGTCAATA AATAGGTGAG CATCAAACTG ACCAACATTG   
  
  
- GGATCATGAC CAAGACCATA ACCCCGAGGA AATCGAAGGT TAAAAGATAA AGAAGTTGGT AATCACGTAA   
  
  
- GTGTCCATTG TTCATTACGG GCACAACCCA TGAACTGTAT AAACTGAGAC TTGGTCCAGT GGGGTAAGTA   
  
  
- GGCTAAGTGG GTGAATTGAC GGTTGGTTCG GTAGGACCTT CGAAACCTCC CCATGTTCCG CCAGGTATAG   
  
  
- GAACTGTACC TGTAGTACGT ACCCCAGGTC ACCGGTGGTG AAGAAGTTCG TTAACGTCTT TCTAGACGTT   
  
  
- GGGAACCAGT GGGACGAGGG TGACAAGCGT AGTGTCCTCC CCCGTCCCTA GATCTTGAGG ACTTGGCTTG   
  
  
- ACCTCTAGCC TAGTCCTTCA AGCGTGTTAG GAACCCTGAG CTCAAAGTTA AGGTGGTAGA ATATTGCCAC   
  
  
- CTGAGCTTGG AATGACTCCG TCACCTCCGG TAGTTACGGG ATGTCGATGT GGAGGTACAG GTTCTCCTCC   
  
  
- TCCAGAAGCG TCACTTAACA CCTCTAAAGG AGGTGGCGGA GGATTGCCTC ATACTATCAT GTAAGGAATC   
  
  
- CTACAAAGAG ATGTTCCACT TCTGGGATTT GGGATCCCAG AAGTGGCACC CTCTTTCCCT CCGGCTGGTG   
  
  
- TTGGTGGGAG AGAAGACCGT CGCTAAGTAA CTCCGTGAAC TGGTGATACC CCGTCACAAA CTGAGAGATC   
  
  
- TTCGTTGGGA TGGTAGTTGT TCAGTTCTCT CCCACCGCCA CCTTCTCCCC ACCAAGCCCC TCCTCTAATT   
  
  
- TCTACACCAA CCTGCTCTTC CTCCCAACTC CTCTGTCTCC GTGGTCTTTA TGCTCAGAAC CCTCCACAAC   
  
  
- TACTCCTCGA GACCCAAATT CTCGAACGGG AACTCGGGGA AGAGACACAG TGTTCGGTTC GACGATGAAG   
  
  
- CGGAAGTAAT GGGAAGACTT CCCATGGTGG AATTCCAGAA GGTATCATTA ACAGACAACG AACCCACCTT   
  
  
- CTTAGCGGGA GAAAAGAGAC AAAGAAGAAC CGTTAT

+     MRE

| Site Name | Organism | Position | Strand | Matrix score. | sequence | function |
| --- | --- | --- | --- | --- | --- | --- |
| MRE | Petroselinum crispum | 915 | + | 7 | AACCTAA | MYB binding site involved in light responsiveness |
| MRE | Petroselinum crispum | 841 | + | 7 | AACCTAA | MYB binding site involved in light responsiveness |
| MRE | Petroselinum crispum | 413 | - | 7 | AACCTAA | MYB binding site involved in light responsiveness |

>HU01G00833.1   
+ -Up\_Stream \_Len000AAAATA TTTTTACAAA AGCATTTCTC TATAAAATAT TTTACAAAAT TTCATTTTGT   
  
  
+ GGGTCTTTTA GAATCAATCT CTCTACAAGT AGGAATGCCG GTTGAGGTAA GGCTACCTAC ATCTAGATCT   
  
  
+ CTCCAGACCC CACAAAAATT AAACGAGACC CACACTGAAA GGTGGTGGTG GTGGTGTTGT TGTTGTATTG   
  
  
+ TCCTACCACG AGAGAGCTTC AAGTGCATCA CTTATTTCAA CAGGTCAATC ACATAGATCT CTAGTGTACT   
  
  
+ GTAAATTTTT TTTCCGTCTT ATTTTAATTT TCTTTTCCAT CCTAAAAGAT TATCATGTAG ATATTCCTAG   
  
  
+ TTTGATATAT TGACTTCAAC TTCAAAAATT ACACATTAAG ATCCACGAAT ATGACAGTTT AGGTTTCATT   
  
  
+ TGAAGGAGAA AGAAATGTTA CGGTGTTTTG GTGTTGTATT CGTAGACATT TTTTGCGCTT AACTGGCTCT   
  
  
+ TCTCATGGGG ACGAGACAGT GAAGGTAATT GTACTTGTTG ATTGATGACG TCATCTGTAG CTTGGATTTG   
  
  
+ TATAGGAATG AACATCTTGG TGTCTACGTG GAAAGTGTGA TTTGTGTTCT TTCATTGATC CTCCTCCTCA   
  
  
+ AGAAGAGGTG ATCTTTCTCC CTTGTTTTTT TTTTTTTTTG GCTAATATGT TGGCAGAACC GGCAAAAAAT   
  
  
+ ACATAATTTG AATAGTGCAT GCATATGAGT ACATGACAGT GAACCAACAA AACGAACCCC TAAAACACAA   
  
  
+ GAGAATTAAC ATGTTAAAAC ACTTAAACTA AATGCAACCA TCTCGATGAC CACTAAATAG CCAAGAAACC   
  
  
+ TAACGTAATT AACAAAAATA AAAAATAACT GAAAATAAAG TAGAGAAAGT CTAACCGAAT ACGATAACAT   
  
  
+ AACCTAAAAC TAACTATACT TGAACTTACT AAGAAATATG AATCAACCAC AAACCGAATT GATTTTGGTT   
  
  
+ TGCCATAATT TGACTATACA TTTAACTTAA AAAATATTTT TTTTTAATTT CAAATAAATC TAAATTATTT   
  
  
+ TTCAATATTC ATCCAAAATA GATTCAATTA TTAAACAAGT CAACCTATAC CGAGTTATTT TCTAGCTCTG   
  
  
+ ACTAAGACAT ATATATATTT TTTGTAAAAA AGATAAGGAT TATAATTTAA ATAGCCTTTC CCTTAAAAAA   
  
  
+ ATTTTTAAAA TTATCCTGAT AGAGAATAAA AAGAAATCCT TATACAAAAC TCTGATTGAC GCAGATGTGT   
  
  
+ ATATGGACAT ATAGAAGTAA TAAGAGGGGT AGGCTAGGAA GGGGGTGGAA ATATAGGGCA TAAATAGGGG   
  
  
+ GCGGGCATAG GGACATGTCT GTCTGAGGTA CGGTGGATGG GACCTTGCCA TGGAAGCAAC AATGTCACAA   
  
  
+ TAATAATATC AACGACTGAA GTTTCTTCAC CAGCCCACTC TTTGCCATAA TGCCATTAAT TATTTATTGC   
  
  
+ TACTATCTCT TTTGATGTGA AACGGTGGAC CTTATCTCTC CCCTCCACTT CCAATTGATG TGAGCAACTC   
  
  
+ AGAAACGCAA ACAAAACTTG TGCCACCTTC TTTACTTGCA TCCTCATTTT CCTCTGTCTC TCTCCGCCCC   
  
  
+ TGTTTCTCTC TCTCTCCCTC ACTCACTCAG AGTACTGATC TGAGCATCAG AAGTAGCAGA CCCAGAAACT   
  
  
+ CAGATTTAAA AACAACCCGC TCTTTTGTTC CCTCTACGTC TAATAATTTT GTCATCGCAT TTAATGTCAG   
  
  
+ ATAGAGAGAT AGAGCTCTAC TGTATTAATT TTCTCCCACC TCACTTGATT CTGTTGCCCT AAACCACCCA   
  
  
+ AAAAAAGAAA TACGAAGAAG CCACTCTCTC TCTCTCTCTC TCTCACACAC ACACACACCC CTCCTAGTGG   
  
  
+ ATTTGATAGA GTATAAATGA ACAGTAATCA GACGAGATAT ATAGTAGTGC TTAATTGACC GAGTAGCAAA   
  
  
+ AGGGGGAAGT TGAGTGAGGT ATCAAGAGAG TAGCGTAGCT AGCTAGGGCA AGATATGCTG GGGTCTTCCT   
  
  
+ CATACAACTC CGAGGAAGAC CACCAAGATG ACAACCATTC CTCACCGTCT GATTTGTCTA GGCACCCACC   
  
  
+ TAGATTCACC ACTACTACTT ACCAATACCA ACAACATATT AGCTCTCCAT CTTCACAATC AATACTAGCT   
  
  
+ GTGCCACCTC GCCAATTGTT AATCAATTGT GCGGAGTTAA TTTGCCGGTT CGACTTCTCC TCCGCCAACC   
  
  
+ GGCTTCTCTC TCTCCTCGCC TCCCACTTTG CCTCCCCTTC TGGGGACTCC ACCGAGAGAC TTGTGTTTTA   
  
  
+ CTTCTGCAAG GCTTTGGATC TCCGCCTCAA TAACAGTTAT TTATCCACTC GTAGTTTGAC TGGTTGTAAC   
  
  
+ CCTAGTACTG GTTCTGGTAT TGGGGCTCCT TTAGCTTCCA ATTTTCTATT TCTTCAACCA TTAGTGCATT   
  
  
+ CACAGGTAAC AAGTAATGCC CGTGTTGGGT ACTTGACATA TTTGACTCTG AACCAGGTCA CCCCATTCAT   
  
  
+ CCGATTCACC CACTTAACTG CCAACCAAGC CATCCTGGAA GCTTTGGAGG GGTACAAGGC GGTCCATATC   
  
  
+ CTTGACATGG ACATCATGCA TGGGGTCCAG TGGCCACCAC TTCTTCAAGC AATTGCAGAA AGATCTGCAA   
  
  
+ CCCTTGGTCA CCCTGCTCCC ACTGTTCGCA TCACAGGAGG GGGCAGGGAT CTAGAACTCC TGAACCGAAC   
  
  
+ TGGAGATCGG ATCAGGAAGT TCGCACAATC CTTGGGACTC GAGTTTCAAT TCCACCATCT TATAACGGTG   
  
  
+ GACTCGAACC TTACTGAGGC AGTGGAGGCC ATCAATGCCC TACAGCTACA CCTCCATGTC CAAGAGGAGG   
  
  
+ AGGTCTTCGC AGTGAATTGT GGAGATTTCC TCCACCGCCT CCTAACGGAG TATGATAGTA CATTCCTTAG   
  
  
+ GATGTTTCTC TACAAGGTGA AGACCCTAAA CCCTAGGGTC TTCACCGTGG GAGAAAGGGA GGCCGACCAC   
  
  
+ AACCACCCTC TCTTCTGGCA GCGATTCATT GAGGCACTTG ACCACTATGG GGCAGTGTTT GACTCTCTAG   
  
  
+ AAGCAACCCT ACCATCAACA AGTCAAGAGA GGGTGGCGGT GGAAGAGGGG TGGTTCGGGG AGGAGATTAA   
  
  
+ AGATGTGGTT GGACGAGAAG GAGGGTTGAG GAGACAGAGG CACCAGAAAT ACGAGTCTTG GGAGGTGTTG   
  
  
+ ATGAGGAGCT CTGGGTTTAA GAGCTTGCCC TTGAGCCCCT TCTCTGTGTC ACAAGCCAAG CTGCTACTTC   
  
  
+ GCCTTCATTA CCCTTCTGAA GGGTACCACC TTAAGGTCTT CCATAGTAAT TGTCTGTTGC TTGGGTGGAA   
  
  
+ GAATCGCCCT CTTTTCTCTG TTTCTTCTTG GCAATA  

- -Up\_Stream \_Len000TTTTAT AAAAATGTTT TCGTAAAGAG ATATTTTATA AAATGTTTTA AAGTAAAACA   
  
  
- CCCAGAAAAT CTTAGTTAGA GAGATGTTCA TCCTTACGGC CAACTCCATT CCGATGGATG TAGATCTAGA   
  
  
- GAGGTCTGGG GTGTTTTTAA TTTGCTCTGG GTGTGACTTT CCACCACCAC CACCACAACA ACAACATAAC   
  
  
- AGGATGGTGC TCTCTCGAAG TTCACGTAGT GAATAAAGTT GTCCAGTTAG TGTATCTAGA GATCACATGA   
  
  
- CATTTAAAAA AAAGGCAGAA TAAAATTAAA AGAAAAGGTA GGATTTTCTA ATAGTACATC TATAAGGATC   
  
  
- AAACTATATA ACTGAAGTTG AAGTTTTTAA TGTGTAATTC TAGGTGCTTA TACTGTCAAA TCCAAAGTAA   
  
  
- ACTTCCTCTT TCTTTACAAT GCCACAAAAC CACAACATAA GCATCTGTAA AAAACGCGAA TTGACCGAGA   
  
  
- AGAGTACCCC TGCTCTGTCA CTTCCATTAA CATGAACAAC TAACTACTGC AGTAGACATC GAACCTAAAC   
  
  
- ATATCCTTAC TTGTAGAACC ACAGATGCAC CTTTCACACT AAACACAAGA AAGTAACTAG GAGGAGGAGT   
  
  
- TCTTCTCCAC TAGAAAGAGG GAACAAAAAA AAAAAAAAAC CGATTATACA ACCGTCTTGG CCGTTTTTTA   
  
  
- TGTATTAAAC TTATCACGTA CGTATACTCA TGTACTGTCA CTTGGTTGTT TTGCTTGGGG ATTTTGTGTT   
  
  
- CTCTTAATTG TACAATTTTG TGAATTTGAT TTACGTTGGT AGAGCTACTG GTGATTTATC GGTTCTTTGG   
  
  
- ATTGCATTAA TTGTTTTTAT TTTTTATTGA CTTTTATTTC ATCTCTTTCA GATTGGCTTA TGCTATTGTA   
  
  
- TTGGATTTTG ATTGATATGA ACTTGAATGA TTCTTTATAC TTAGTTGGTG TTTGGCTTAA CTAAAACCAA   
  
  
- ACGGTATTAA ACTGATATGT AAATTGAATT TTTTATAAAA AAAAATTAAA GTTTATTTAG ATTTAATAAA   
  
  
- AAGTTATAAG TAGGTTTTAT CTAAGTTAAT AATTTGTTCA GTTGGATATG GCTCAATAAA AGATCGAGAC   
  
  
- TGATTCTGTA TATATATAAA AAACATTTTT TCTATTCCTA ATATTAAATT TATCGGAAAG GGAATTTTTT   
  
  
- TAAAAATTTT AATAGGACTA TCTCTTATTT TTCTTTAGGA ATATGTTTTG AGACTAACTG CGTCTACACA   
  
  
- TATACCTGTA TATCTTCATT ATTCTCCCCA TCCGATCCTT CCCCCACCTT TATATCCCGT ATTTATCCCC   
  
  
- CGCCCGTATC CCTGTACAGA CAGACTCCAT GCCACCTACC CTGGAACGGT ACCTTCGTTG TTACAGTGTT   
  
  
- ATTATTATAG TTGCTGACTT CAAAGAAGTG GTCGGGTGAG AAACGGTATT ACGGTAATTA ATAAATAACG   
  
  
- ATGATAGAGA AAACTACACT TTGCCACCTG GAATAGAGAG GGGAGGTGAA GGTTAACTAC ACTCGTTGAG   
  
  
- TCTTTGCGTT TGTTTTGAAC ACGGTGGAAG AAATGAACGT AGGAGTAAAA GGAGACAGAG AGAGGCGGGG   
  
  
- ACAAAGAGAG AGAGAGGGAG TGAGTGAGTC TCATGACTAG ACTCGTAGTC TTCATCGTCT GGGTCTTTGA   
  
  
- GTCTAAATTT TTGTTGGGCG AGAAAACAAG GGAGATGCAG ATTATTAAAA CAGTAGCGTA AATTACAGTC   
  
  
- TATCTCTCTA TCTCGAGATG ACATAATTAA AAGAGGGTGG AGTGAACTAA GACAACGGGA TTTGGTGGGT   
  
  
- TTTTTTCTTT ATGCTTCTTC GGTGAGAGAG AGAGAGAGAG AGAGTGTGTG TGTGTGTGGG GAGGATCACC   
  
  
- TAAACTATCT CATATTTACT TGTCATTAGT CTGCTCTATA TATCATCACG AATTAACTGG CTCATCGTTT   
  
  
- TCCCCCTTCA ACTCACTCCA TAGTTCTCTC ATCGCATCGA TCGATCCCGT TCTATACGAC CCCAGAAGGA   
  
  
- GTATGTTGAG GCTCCTTCTG GTGGTTCTAC TGTTGGTAAG GAGTGGCAGA CTAAACAGAT CCGTGGGTGG   
  
  
- ATCTAAGTGG TGATGATGAA TGGTTATGGT TGTTGTATAA TCGAGAGGTA GAAGTGTTAG TTATGATCGA   
  
  
- CACGGTGGAG CGGTTAACAA TTAGTTAACA CGCCTCAATT AAACGGCCAA GCTGAAGAGG AGGCGGTTGG   
  
  
- CCGAAGAGAG AGAGGAGCGG AGGGTGAAAC GGAGGGGAAG ACCCCTGAGG TGGCTCTCTG AACACAAAAT   
  
  
- GAAGACGTTC CGAAACCTAG AGGCGGAGTT ATTGTCAATA AATAGGTGAG CATCAAACTG ACCAACATTG   
  
  
- GGATCATGAC CAAGACCATA ACCCCGAGGA AATCGAAGGT TAAAAGATAA AGAAGTTGGT AATCACGTAA   
  
  
- GTGTCCATTG TTCATTACGG GCACAACCCA TGAACTGTAT AAACTGAGAC TTGGTCCAGT GGGGTAAGTA   
  
  
- GGCTAAGTGG GTGAATTGAC GGTTGGTTCG GTAGGACCTT CGAAACCTCC CCATGTTCCG CCAGGTATAG   
  
  
- GAACTGTACC TGTAGTACGT ACCCCAGGTC ACCGGTGGTG AAGAAGTTCG TTAACGTCTT TCTAGACGTT   
  
  
- GGGAACCAGT GGGACGAGGG TGACAAGCGT AGTGTCCTCC CCCGTCCCTA GATCTTGAGG ACTTGGCTTG   
  
  
- ACCTCTAGCC TAGTCCTTCA AGCGTGTTAG GAACCCTGAG CTCAAAGTTA AGGTGGTAGA ATATTGCCAC   
  
  
- CTGAGCTTGG AATGACTCCG TCACCTCCGG TAGTTACGGG ATGTCGATGT GGAGGTACAG GTTCTCCTCC   
  
  
- TCCAGAAGCG TCACTTAACA CCTCTAAAGG AGGTGGCGGA GGATTGCCTC ATACTATCAT GTAAGGAATC   
  
  
- CTACAAAGAG ATGTTCCACT TCTGGGATTT GGGATCCCAG AAGTGGCACC CTCTTTCCCT CCGGCTGGTG   
  
  
- TTGGTGGGAG AGAAGACCGT CGCTAAGTAA CTCCGTGAAC TGGTGATACC CCGTCACAAA CTGAGAGATC   
  
  
- TTCGTTGGGA TGGTAGTTGT TCAGTTCTCT CCCACCGCCA CCTTCTCCCC ACCAAGCCCC TCCTCTAATT   
  
  
- TCTACACCAA CCTGCTCTTC CTCCCAACTC CTCTGTCTCC GTGGTCTTTA TGCTCAGAAC CCTCCACAAC   
  
  
- TACTCCTCGA GACCCAAATT CTCGAACGGG AACTCGGGGA AGAGACACAG TGTTCGGTTC GACGATGAAG   
  
  
- CGGAAGTAAT GGGAAGACTT CCCATGGTGG AATTCCAGAA GGTATCATTA ACAGACAACG AACCCACCTT   
  
  
- CTTAGCGGGA GAAAAGAGAC AAAGAAGAAC CGTTAT

+     MYB

| Site Name | Organism | Position | Strand | Matrix score. | sequence | function |
| --- | --- | --- | --- | --- | --- | --- |
| MYB | Arabidopsis thaliana | 2375 | - | 6 | CAACCA |  |
| MYB | Arabidopsis thaliana | 3160 | - | 6 | CAACCA |  |
| MYB | Arabidopsis thaliana | 3014 | + | 6 | CAACCA |  |
| MYB | Arabidopsis thaliana | 2066 | + | 6 | CAACCA |  |
| MYB | Arabidopsis thaliana | 2439 | + | 6 | CAACCA |  |
| MYB | Arabidopsis thaliana | 2546 | + | 6 | CAACCA |  |
| MYB | Arabidopsis thaliana | 3348 | - | 6 | CAACAG |  |
| MYB | Arabidopsis thaliana | 958 | + | 6 | CAACCA |  |
| MYB | Arabidopsis thaliana | 1805 | - | 6 | CAACAG |  |
| MYB | Arabidopsis thaliana | 809 | + | 6 | CAACCA |  |
| MYB | Arabidopsis thaliana | 252 | + | 6 | CAACAG |  |

>HU01G00833.1   
+ -Up\_Stream \_Len000AAAATA TTTTTACAAA AGCATTTCTC TATAAAATAT TTTACAAAAT TTCATTTTGT   
  
  
+ GGGTCTTTTA GAATCAATCT CTCTACAAGT AGGAATGCCG GTTGAGGTAA GGCTACCTAC ATCTAGATCT   
  
  
+ CTCCAGACCC CACAAAAATT AAACGAGACC CACACTGAAA GGTGGTGGTG GTGGTGTTGT TGTTGTATTG   
  
  
+ TCCTACCACG AGAGAGCTTC AAGTGCATCA CTTATTTCAA CAGGTCAATC ACATAGATCT CTAGTGTACT   
  
  
+ GTAAATTTTT TTTCCGTCTT ATTTTAATTT TCTTTTCCAT CCTAAAAGAT TATCATGTAG ATATTCCTAG   
  
  
+ TTTGATATAT TGACTTCAAC TTCAAAAATT ACACATTAAG ATCCACGAAT ATGACAGTTT AGGTTTCATT   
  
  
+ TGAAGGAGAA AGAAATGTTA CGGTGTTTTG GTGTTGTATT CGTAGACATT TTTTGCGCTT AACTGGCTCT   
  
  
+ TCTCATGGGG ACGAGACAGT GAAGGTAATT GTACTTGTTG ATTGATGACG TCATCTGTAG CTTGGATTTG   
  
  
+ TATAGGAATG AACATCTTGG TGTCTACGTG GAAAGTGTGA TTTGTGTTCT TTCATTGATC CTCCTCCTCA   
  
  
+ AGAAGAGGTG ATCTTTCTCC CTTGTTTTTT TTTTTTTTTG GCTAATATGT TGGCAGAACC GGCAAAAAAT   
  
  
+ ACATAATTTG AATAGTGCAT GCATATGAGT ACATGACAGT GAACCAACAA AACGAACCCC TAAAACACAA   
  
  
+ GAGAATTAAC ATGTTAAAAC ACTTAAACTA AATGCAACCA TCTCGATGAC CACTAAATAG CCAAGAAACC   
  
  
+ TAACGTAATT AACAAAAATA AAAAATAACT GAAAATAAAG TAGAGAAAGT CTAACCGAAT ACGATAACAT   
  
  
+ AACCTAAAAC TAACTATACT TGAACTTACT AAGAAATATG AATCAACCAC AAACCGAATT GATTTTGGTT   
  
  
+ TGCCATAATT TGACTATACA TTTAACTTAA AAAATATTTT TTTTTAATTT CAAATAAATC TAAATTATTT   
  
  
+ TTCAATATTC ATCCAAAATA GATTCAATTA TTAAACAAGT CAACCTATAC CGAGTTATTT TCTAGCTCTG   
  
  
+ ACTAAGACAT ATATATATTT TTTGTAAAAA AGATAAGGAT TATAATTTAA ATAGCCTTTC CCTTAAAAAA   
  
  
+ ATTTTTAAAA TTATCCTGAT AGAGAATAAA AAGAAATCCT TATACAAAAC TCTGATTGAC GCAGATGTGT   
  
  
+ ATATGGACAT ATAGAAGTAA TAAGAGGGGT AGGCTAGGAA GGGGGTGGAA ATATAGGGCA TAAATAGGGG   
  
  
+ GCGGGCATAG GGACATGTCT GTCTGAGGTA CGGTGGATGG GACCTTGCCA TGGAAGCAAC AATGTCACAA   
  
  
+ TAATAATATC AACGACTGAA GTTTCTTCAC CAGCCCACTC TTTGCCATAA TGCCATTAAT TATTTATTGC   
  
  
+ TACTATCTCT TTTGATGTGA AACGGTGGAC CTTATCTCTC CCCTCCACTT CCAATTGATG TGAGCAACTC   
  
  
+ AGAAACGCAA ACAAAACTTG TGCCACCTTC TTTACTTGCA TCCTCATTTT CCTCTGTCTC TCTCCGCCCC   
  
  
+ TGTTTCTCTC TCTCTCCCTC ACTCACTCAG AGTACTGATC TGAGCATCAG AAGTAGCAGA CCCAGAAACT   
  
  
+ CAGATTTAAA AACAACCCGC TCTTTTGTTC CCTCTACGTC TAATAATTTT GTCATCGCAT TTAATGTCAG   
  
  
+ ATAGAGAGAT AGAGCTCTAC TGTATTAATT TTCTCCCACC TCACTTGATT CTGTTGCCCT AAACCACCCA   
  
  
+ AAAAAAGAAA TACGAAGAAG CCACTCTCTC TCTCTCTCTC TCTCACACAC ACACACACCC CTCCTAGTGG   
  
  
+ ATTTGATAGA GTATAAATGA ACAGTAATCA GACGAGATAT ATAGTAGTGC TTAATTGACC GAGTAGCAAA   
  
  
+ AGGGGGAAGT TGAGTGAGGT ATCAAGAGAG TAGCGTAGCT AGCTAGGGCA AGATATGCTG GGGTCTTCCT   
  
  
+ CATACAACTC CGAGGAAGAC CACCAAGATG ACAACCATTC CTCACCGTCT GATTTGTCTA GGCACCCACC   
  
  
+ TAGATTCACC ACTACTACTT ACCAATACCA ACAACATATT AGCTCTCCAT CTTCACAATC AATACTAGCT   
  
  
+ GTGCCACCTC GCCAATTGTT AATCAATTGT GCGGAGTTAA TTTGCCGGTT CGACTTCTCC TCCGCCAACC   
  
  
+ GGCTTCTCTC TCTCCTCGCC TCCCACTTTG CCTCCCCTTC TGGGGACTCC ACCGAGAGAC TTGTGTTTTA   
  
  
+ CTTCTGCAAG GCTTTGGATC TCCGCCTCAA TAACAGTTAT TTATCCACTC GTAGTTTGAC TGGTTGTAAC   
  
  
+ CCTAGTACTG GTTCTGGTAT TGGGGCTCCT TTAGCTTCCA ATTTTCTATT TCTTCAACCA TTAGTGCATT   
  
  
+ CACAGGTAAC AAGTAATGCC CGTGTTGGGT ACTTGACATA TTTGACTCTG AACCAGGTCA CCCCATTCAT   
  
  
+ CCGATTCACC CACTTAACTG CCAACCAAGC CATCCTGGAA GCTTTGGAGG GGTACAAGGC GGTCCATATC   
  
  
+ CTTGACATGG ACATCATGCA TGGGGTCCAG TGGCCACCAC TTCTTCAAGC AATTGCAGAA AGATCTGCAA   
  
  
+ CCCTTGGTCA CCCTGCTCCC ACTGTTCGCA TCACAGGAGG GGGCAGGGAT CTAGAACTCC TGAACCGAAC   
  
  
+ TGGAGATCGG ATCAGGAAGT TCGCACAATC CTTGGGACTC GAGTTTCAAT TCCACCATCT TATAACGGTG   
  
  
+ GACTCGAACC TTACTGAGGC AGTGGAGGCC ATCAATGCCC TACAGCTACA CCTCCATGTC CAAGAGGAGG   
  
  
+ AGGTCTTCGC AGTGAATTGT GGAGATTTCC TCCACCGCCT CCTAACGGAG TATGATAGTA CATTCCTTAG   
  
  
+ GATGTTTCTC TACAAGGTGA AGACCCTAAA CCCTAGGGTC TTCACCGTGG GAGAAAGGGA GGCCGACCAC   
  
  
+ AACCACCCTC TCTTCTGGCA GCGATTCATT GAGGCACTTG ACCACTATGG GGCAGTGTTT GACTCTCTAG   
  
  
+ AAGCAACCCT ACCATCAACA AGTCAAGAGA GGGTGGCGGT GGAAGAGGGG TGGTTCGGGG AGGAGATTAA   
  
  
+ AGATGTGGTT GGACGAGAAG GAGGGTTGAG GAGACAGAGG CACCAGAAAT ACGAGTCTTG GGAGGTGTTG   
  
  
+ ATGAGGAGCT CTGGGTTTAA GAGCTTGCCC TTGAGCCCCT TCTCTGTGTC ACAAGCCAAG CTGCTACTTC   
  
  
+ GCCTTCATTA CCCTTCTGAA GGGTACCACC TTAAGGTCTT CCATAGTAAT TGTCTGTTGC TTGGGTGGAA   
  
  
+ GAATCGCCCT CTTTTCTCTG TTTCTTCTTG GCAATA  

- -Up\_Stream \_Len000TTTTAT AAAAATGTTT TCGTAAAGAG ATATTTTATA AAATGTTTTA AAGTAAAACA   
  
  
- CCCAGAAAAT CTTAGTTAGA GAGATGTTCA TCCTTACGGC CAACTCCATT CCGATGGATG TAGATCTAGA   
  
  
- GAGGTCTGGG GTGTTTTTAA TTTGCTCTGG GTGTGACTTT CCACCACCAC CACCACAACA ACAACATAAC   
  
  
- AGGATGGTGC TCTCTCGAAG TTCACGTAGT GAATAAAGTT GTCCAGTTAG TGTATCTAGA GATCACATGA   
  
  
- CATTTAAAAA AAAGGCAGAA TAAAATTAAA AGAAAAGGTA GGATTTTCTA ATAGTACATC TATAAGGATC   
  
  
- AAACTATATA ACTGAAGTTG AAGTTTTTAA TGTGTAATTC TAGGTGCTTA TACTGTCAAA TCCAAAGTAA   
  
  
- ACTTCCTCTT TCTTTACAAT GCCACAAAAC CACAACATAA GCATCTGTAA AAAACGCGAA TTGACCGAGA   
  
  
- AGAGTACCCC TGCTCTGTCA CTTCCATTAA CATGAACAAC TAACTACTGC AGTAGACATC GAACCTAAAC   
  
  
- ATATCCTTAC TTGTAGAACC ACAGATGCAC CTTTCACACT AAACACAAGA AAGTAACTAG GAGGAGGAGT   
  
  
- TCTTCTCCAC TAGAAAGAGG GAACAAAAAA AAAAAAAAAC CGATTATACA ACCGTCTTGG CCGTTTTTTA   
  
  
- TGTATTAAAC TTATCACGTA CGTATACTCA TGTACTGTCA CTTGGTTGTT TTGCTTGGGG ATTTTGTGTT   
  
  
- CTCTTAATTG TACAATTTTG TGAATTTGAT TTACGTTGGT AGAGCTACTG GTGATTTATC GGTTCTTTGG   
  
  
- ATTGCATTAA TTGTTTTTAT TTTTTATTGA CTTTTATTTC ATCTCTTTCA GATTGGCTTA TGCTATTGTA   
  
  
- TTGGATTTTG ATTGATATGA ACTTGAATGA TTCTTTATAC TTAGTTGGTG TTTGGCTTAA CTAAAACCAA   
  
  
- ACGGTATTAA ACTGATATGT AAATTGAATT TTTTATAAAA AAAAATTAAA GTTTATTTAG ATTTAATAAA   
  
  
- AAGTTATAAG TAGGTTTTAT CTAAGTTAAT AATTTGTTCA GTTGGATATG GCTCAATAAA AGATCGAGAC   
  
  
- TGATTCTGTA TATATATAAA AAACATTTTT TCTATTCCTA ATATTAAATT TATCGGAAAG GGAATTTTTT   
  
  
- TAAAAATTTT AATAGGACTA TCTCTTATTT TTCTTTAGGA ATATGTTTTG AGACTAACTG CGTCTACACA   
  
  
- TATACCTGTA TATCTTCATT ATTCTCCCCA TCCGATCCTT CCCCCACCTT TATATCCCGT ATTTATCCCC   
  
  
- CGCCCGTATC CCTGTACAGA CAGACTCCAT GCCACCTACC CTGGAACGGT ACCTTCGTTG TTACAGTGTT   
  
  
- ATTATTATAG TTGCTGACTT CAAAGAAGTG GTCGGGTGAG AAACGGTATT ACGGTAATTA ATAAATAACG   
  
  
- ATGATAGAGA AAACTACACT TTGCCACCTG GAATAGAGAG GGGAGGTGAA GGTTAACTAC ACTCGTTGAG   
  
  
- TCTTTGCGTT TGTTTTGAAC ACGGTGGAAG AAATGAACGT AGGAGTAAAA GGAGACAGAG AGAGGCGGGG   
  
  
- ACAAAGAGAG AGAGAGGGAG TGAGTGAGTC TCATGACTAG ACTCGTAGTC TTCATCGTCT GGGTCTTTGA   
  
  
- GTCTAAATTT TTGTTGGGCG AGAAAACAAG GGAGATGCAG ATTATTAAAA CAGTAGCGTA AATTACAGTC   
  
  
- TATCTCTCTA TCTCGAGATG ACATAATTAA AAGAGGGTGG AGTGAACTAA GACAACGGGA TTTGGTGGGT   
  
  
- TTTTTTCTTT ATGCTTCTTC GGTGAGAGAG AGAGAGAGAG AGAGTGTGTG TGTGTGTGGG GAGGATCACC   
  
  
- TAAACTATCT CATATTTACT TGTCATTAGT CTGCTCTATA TATCATCACG AATTAACTGG CTCATCGTTT   
  
  
- TCCCCCTTCA ACTCACTCCA TAGTTCTCTC ATCGCATCGA TCGATCCCGT TCTATACGAC CCCAGAAGGA   
  
  
- GTATGTTGAG GCTCCTTCTG GTGGTTCTAC TGTTGGTAAG GAGTGGCAGA CTAAACAGAT CCGTGGGTGG   
  
  
- ATCTAAGTGG TGATGATGAA TGGTTATGGT TGTTGTATAA TCGAGAGGTA GAAGTGTTAG TTATGATCGA   
  
  
- CACGGTGGAG CGGTTAACAA TTAGTTAACA CGCCTCAATT AAACGGCCAA GCTGAAGAGG AGGCGGTTGG   
  
  
- CCGAAGAGAG AGAGGAGCGG AGGGTGAAAC GGAGGGGAAG ACCCCTGAGG TGGCTCTCTG AACACAAAAT   
  
  
- GAAGACGTTC CGAAACCTAG AGGCGGAGTT ATTGTCAATA AATAGGTGAG CATCAAACTG ACCAACATTG   
  
  
- GGATCATGAC CAAGACCATA ACCCCGAGGA AATCGAAGGT TAAAAGATAA AGAAGTTGGT AATCACGTAA   
  
  
- GTGTCCATTG TTCATTACGG GCACAACCCA TGAACTGTAT AAACTGAGAC TTGGTCCAGT GGGGTAAGTA   
  
  
- GGCTAAGTGG GTGAATTGAC GGTTGGTTCG GTAGGACCTT CGAAACCTCC CCATGTTCCG CCAGGTATAG   
  
  
- GAACTGTACC TGTAGTACGT ACCCCAGGTC ACCGGTGGTG AAGAAGTTCG TTAACGTCTT TCTAGACGTT   
  
  
- GGGAACCAGT GGGACGAGGG TGACAAGCGT AGTGTCCTCC CCCGTCCCTA GATCTTGAGG ACTTGGCTTG   
  
  
- ACCTCTAGCC TAGTCCTTCA AGCGTGTTAG GAACCCTGAG CTCAAAGTTA AGGTGGTAGA ATATTGCCAC   
  
  
- CTGAGCTTGG AATGACTCCG TCACCTCCGG TAGTTACGGG ATGTCGATGT GGAGGTACAG GTTCTCCTCC   
  
  
- TCCAGAAGCG TCACTTAACA CCTCTAAAGG AGGTGGCGGA GGATTGCCTC ATACTATCAT GTAAGGAATC   
  
  
- CTACAAAGAG ATGTTCCACT TCTGGGATTT GGGATCCCAG AAGTGGCACC CTCTTTCCCT CCGGCTGGTG   
  
  
- TTGGTGGGAG AGAAGACCGT CGCTAAGTAA CTCCGTGAAC TGGTGATACC CCGTCACAAA CTGAGAGATC   
  
  
- TTCGTTGGGA TGGTAGTTGT TCAGTTCTCT CCCACCGCCA CCTTCTCCCC ACCAAGCCCC TCCTCTAATT   
  
  
- TCTACACCAA CCTGCTCTTC CTCCCAACTC CTCTGTCTCC GTGGTCTTTA TGCTCAGAAC CCTCCACAAC   
  
  
- TACTCCTCGA GACCCAAATT CTCGAACGGG AACTCGGGGA AGAGACACAG TGTTCGGTTC GACGATGAAG   
  
  
- CGGAAGTAAT GGGAAGACTT CCCATGGTGG AATTCCAGAA GGTATCATTA ACAGACAACG AACCCACCTT   
  
  
- CTTAGCGGGA GAAAAGAGAC AAAGAAGAAC CGTTAT

+     MYC

| Site Name | Organism | Position | Strand | Matrix score. | sequence | function |
| --- | --- | --- | --- | --- | --- | --- |
| MYC | Arabidopsis thaliana | 2644 | - | 6 | CAATTG |  |
| MYC | Arabidopsis thaliana | 2198 | - | 6 | CAATTG |  |
| MYC | Arabidopsis thaliana | 2187 | - | 6 | CAATTG |  |
| MYC | Arabidopsis thaliana | 1526 | + | 6 | CAATTG |  |
| MYC | Arabidopsis thaliana | 421 | + | 6 | CATTTG |  |

>HU01G00833.1   
+ -Up\_Stream \_Len000AAAATA TTTTTACAAA AGCATTTCTC TATAAAATAT TTTACAAAAT TTCATTTTGT   
  
  
+ GGGTCTTTTA GAATCAATCT CTCTACAAGT AGGAATGCCG GTTGAGGTAA GGCTACCTAC ATCTAGATCT   
  
  
+ CTCCAGACCC CACAAAAATT AAACGAGACC CACACTGAAA GGTGGTGGTG GTGGTGTTGT TGTTGTATTG   
  
  
+ TCCTACCACG AGAGAGCTTC AAGTGCATCA CTTATTTCAA CAGGTCAATC ACATAGATCT CTAGTGTACT   
  
  
+ GTAAATTTTT TTTCCGTCTT ATTTTAATTT TCTTTTCCAT CCTAAAAGAT TATCATGTAG ATATTCCTAG   
  
  
+ TTTGATATAT TGACTTCAAC TTCAAAAATT ACACATTAAG ATCCACGAAT ATGACAGTTT AGGTTTCATT   
  
  
+ TGAAGGAGAA AGAAATGTTA CGGTGTTTTG GTGTTGTATT CGTAGACATT TTTTGCGCTT AACTGGCTCT   
  
  
+ TCTCATGGGG ACGAGACAGT GAAGGTAATT GTACTTGTTG ATTGATGACG TCATCTGTAG CTTGGATTTG   
  
  
+ TATAGGAATG AACATCTTGG TGTCTACGTG GAAAGTGTGA TTTGTGTTCT TTCATTGATC CTCCTCCTCA   
  
  
+ AGAAGAGGTG ATCTTTCTCC CTTGTTTTTT TTTTTTTTTG GCTAATATGT TGGCAGAACC GGCAAAAAAT   
  
  
+ ACATAATTTG AATAGTGCAT GCATATGAGT ACATGACAGT GAACCAACAA AACGAACCCC TAAAACACAA   
  
  
+ GAGAATTAAC ATGTTAAAAC ACTTAAACTA AATGCAACCA TCTCGATGAC CACTAAATAG CCAAGAAACC   
  
  
+ TAACGTAATT AACAAAAATA AAAAATAACT GAAAATAAAG TAGAGAAAGT CTAACCGAAT ACGATAACAT   
  
  
+ AACCTAAAAC TAACTATACT TGAACTTACT AAGAAATATG AATCAACCAC AAACCGAATT GATTTTGGTT   
  
  
+ TGCCATAATT TGACTATACA TTTAACTTAA AAAATATTTT TTTTTAATTT CAAATAAATC TAAATTATTT   
  
  
+ TTCAATATTC ATCCAAAATA GATTCAATTA TTAAACAAGT CAACCTATAC CGAGTTATTT TCTAGCTCTG   
  
  
+ ACTAAGACAT ATATATATTT TTTGTAAAAA AGATAAGGAT TATAATTTAA ATAGCCTTTC CCTTAAAAAA   
  
  
+ ATTTTTAAAA TTATCCTGAT AGAGAATAAA AAGAAATCCT TATACAAAAC TCTGATTGAC GCAGATGTGT   
  
  
+ ATATGGACAT ATAGAAGTAA TAAGAGGGGT AGGCTAGGAA GGGGGTGGAA ATATAGGGCA TAAATAGGGG   
  
  
+ GCGGGCATAG GGACATGTCT GTCTGAGGTA CGGTGGATGG GACCTTGCCA TGGAAGCAAC AATGTCACAA   
  
  
+ TAATAATATC AACGACTGAA GTTTCTTCAC CAGCCCACTC TTTGCCATAA TGCCATTAAT TATTTATTGC   
  
  
+ TACTATCTCT TTTGATGTGA AACGGTGGAC CTTATCTCTC CCCTCCACTT CCAATTGATG TGAGCAACTC   
  
  
+ AGAAACGCAA ACAAAACTTG TGCCACCTTC TTTACTTGCA TCCTCATTTT CCTCTGTCTC TCTCCGCCCC   
  
  
+ TGTTTCTCTC TCTCTCCCTC ACTCACTCAG AGTACTGATC TGAGCATCAG AAGTAGCAGA CCCAGAAACT   
  
  
+ CAGATTTAAA AACAACCCGC TCTTTTGTTC CCTCTACGTC TAATAATTTT GTCATCGCAT TTAATGTCAG   
  
  
+ ATAGAGAGAT AGAGCTCTAC TGTATTAATT TTCTCCCACC TCACTTGATT CTGTTGCCCT AAACCACCCA   
  
  
+ AAAAAAGAAA TACGAAGAAG CCACTCTCTC TCTCTCTCTC TCTCACACAC ACACACACCC CTCCTAGTGG   
  
  
+ ATTTGATAGA GTATAAATGA ACAGTAATCA GACGAGATAT ATAGTAGTGC TTAATTGACC GAGTAGCAAA   
  
  
+ AGGGGGAAGT TGAGTGAGGT ATCAAGAGAG TAGCGTAGCT AGCTAGGGCA AGATATGCTG GGGTCTTCCT   
  
  
+ CATACAACTC CGAGGAAGAC CACCAAGATG ACAACCATTC CTCACCGTCT GATTTGTCTA GGCACCCACC   
  
  
+ TAGATTCACC ACTACTACTT ACCAATACCA ACAACATATT AGCTCTCCAT CTTCACAATC AATACTAGCT   
  
  
+ GTGCCACCTC GCCAATTGTT AATCAATTGT GCGGAGTTAA TTTGCCGGTT CGACTTCTCC TCCGCCAACC   
  
  
+ GGCTTCTCTC TCTCCTCGCC TCCCACTTTG CCTCCCCTTC TGGGGACTCC ACCGAGAGAC TTGTGTTTTA   
  
  
+ CTTCTGCAAG GCTTTGGATC TCCGCCTCAA TAACAGTTAT TTATCCACTC GTAGTTTGAC TGGTTGTAAC   
  
  
+ CCTAGTACTG GTTCTGGTAT TGGGGCTCCT TTAGCTTCCA ATTTTCTATT TCTTCAACCA TTAGTGCATT   
  
  
+ CACAGGTAAC AAGTAATGCC CGTGTTGGGT ACTTGACATA TTTGACTCTG AACCAGGTCA CCCCATTCAT   
  
  
+ CCGATTCACC CACTTAACTG CCAACCAAGC CATCCTGGAA GCTTTGGAGG GGTACAAGGC GGTCCATATC   
  
  
+ CTTGACATGG ACATCATGCA TGGGGTCCAG TGGCCACCAC TTCTTCAAGC AATTGCAGAA AGATCTGCAA   
  
  
+ CCCTTGGTCA CCCTGCTCCC ACTGTTCGCA TCACAGGAGG GGGCAGGGAT CTAGAACTCC TGAACCGAAC   
  
  
+ TGGAGATCGG ATCAGGAAGT TCGCACAATC CTTGGGACTC GAGTTTCAAT TCCACCATCT TATAACGGTG   
  
  
+ GACTCGAACC TTACTGAGGC AGTGGAGGCC ATCAATGCCC TACAGCTACA CCTCCATGTC CAAGAGGAGG   
  
  
+ AGGTCTTCGC AGTGAATTGT GGAGATTTCC TCCACCGCCT CCTAACGGAG TATGATAGTA CATTCCTTAG   
  
  
+ GATGTTTCTC TACAAGGTGA AGACCCTAAA CCCTAGGGTC TTCACCGTGG GAGAAAGGGA GGCCGACCAC   
  
  
+ AACCACCCTC TCTTCTGGCA GCGATTCATT GAGGCACTTG ACCACTATGG GGCAGTGTTT GACTCTCTAG   
  
  
+ AAGCAACCCT ACCATCAACA AGTCAAGAGA GGGTGGCGGT GGAAGAGGGG TGGTTCGGGG AGGAGATTAA   
  
  
+ AGATGTGGTT GGACGAGAAG GAGGGTTGAG GAGACAGAGG CACCAGAAAT ACGAGTCTTG GGAGGTGTTG   
  
  
+ ATGAGGAGCT CTGGGTTTAA GAGCTTGCCC TTGAGCCCCT TCTCTGTGTC ACAAGCCAAG CTGCTACTTC   
  
  
+ GCCTTCATTA CCCTTCTGAA GGGTACCACC TTAAGGTCTT CCATAGTAAT TGTCTGTTGC TTGGGTGGAA   
  
  
+ GAATCGCCCT CTTTTCTCTG TTTCTTCTTG GCAATA  

- -Up\_Stream \_Len000TTTTAT AAAAATGTTT TCGTAAAGAG ATATTTTATA AAATGTTTTA AAGTAAAACA   
  
  
- CCCAGAAAAT CTTAGTTAGA GAGATGTTCA TCCTTACGGC CAACTCCATT CCGATGGATG TAGATCTAGA   
  
  
- GAGGTCTGGG GTGTTTTTAA TTTGCTCTGG GTGTGACTTT CCACCACCAC CACCACAACA ACAACATAAC   
  
  
- AGGATGGTGC TCTCTCGAAG TTCACGTAGT GAATAAAGTT GTCCAGTTAG TGTATCTAGA GATCACATGA   
  
  
- CATTTAAAAA AAAGGCAGAA TAAAATTAAA AGAAAAGGTA GGATTTTCTA ATAGTACATC TATAAGGATC   
  
  
- AAACTATATA ACTGAAGTTG AAGTTTTTAA TGTGTAATTC TAGGTGCTTA TACTGTCAAA TCCAAAGTAA   
  
  
- ACTTCCTCTT TCTTTACAAT GCCACAAAAC CACAACATAA GCATCTGTAA AAAACGCGAA TTGACCGAGA   
  
  
- AGAGTACCCC TGCTCTGTCA CTTCCATTAA CATGAACAAC TAACTACTGC AGTAGACATC GAACCTAAAC   
  
  
- ATATCCTTAC TTGTAGAACC ACAGATGCAC CTTTCACACT AAACACAAGA AAGTAACTAG GAGGAGGAGT   
  
  
- TCTTCTCCAC TAGAAAGAGG GAACAAAAAA AAAAAAAAAC CGATTATACA ACCGTCTTGG CCGTTTTTTA   
  
  
- TGTATTAAAC TTATCACGTA CGTATACTCA TGTACTGTCA CTTGGTTGTT TTGCTTGGGG ATTTTGTGTT   
  
  
- CTCTTAATTG TACAATTTTG TGAATTTGAT TTACGTTGGT AGAGCTACTG GTGATTTATC GGTTCTTTGG   
  
  
- ATTGCATTAA TTGTTTTTAT TTTTTATTGA CTTTTATTTC ATCTCTTTCA GATTGGCTTA TGCTATTGTA   
  
  
- TTGGATTTTG ATTGATATGA ACTTGAATGA TTCTTTATAC TTAGTTGGTG TTTGGCTTAA CTAAAACCAA   
  
  
- ACGGTATTAA ACTGATATGT AAATTGAATT TTTTATAAAA AAAAATTAAA GTTTATTTAG ATTTAATAAA   
  
  
- AAGTTATAAG TAGGTTTTAT CTAAGTTAAT AATTTGTTCA GTTGGATATG GCTCAATAAA AGATCGAGAC   
  
  
- TGATTCTGTA TATATATAAA AAACATTTTT TCTATTCCTA ATATTAAATT TATCGGAAAG GGAATTTTTT   
  
  
- TAAAAATTTT AATAGGACTA TCTCTTATTT TTCTTTAGGA ATATGTTTTG AGACTAACTG CGTCTACACA   
  
  
- TATACCTGTA TATCTTCATT ATTCTCCCCA TCCGATCCTT CCCCCACCTT TATATCCCGT ATTTATCCCC   
  
  
- CGCCCGTATC CCTGTACAGA CAGACTCCAT GCCACCTACC CTGGAACGGT ACCTTCGTTG TTACAGTGTT   
  
  
- ATTATTATAG TTGCTGACTT CAAAGAAGTG GTCGGGTGAG AAACGGTATT ACGGTAATTA ATAAATAACG   
  
  
- ATGATAGAGA AAACTACACT TTGCCACCTG GAATAGAGAG GGGAGGTGAA GGTTAACTAC ACTCGTTGAG   
  
  
- TCTTTGCGTT TGTTTTGAAC ACGGTGGAAG AAATGAACGT AGGAGTAAAA GGAGACAGAG AGAGGCGGGG   
  
  
- ACAAAGAGAG AGAGAGGGAG TGAGTGAGTC TCATGACTAG ACTCGTAGTC TTCATCGTCT GGGTCTTTGA   
  
  
- GTCTAAATTT TTGTTGGGCG AGAAAACAAG GGAGATGCAG ATTATTAAAA CAGTAGCGTA AATTACAGTC   
  
  
- TATCTCTCTA TCTCGAGATG ACATAATTAA AAGAGGGTGG AGTGAACTAA GACAACGGGA TTTGGTGGGT   
  
  
- TTTTTTCTTT ATGCTTCTTC GGTGAGAGAG AGAGAGAGAG AGAGTGTGTG TGTGTGTGGG GAGGATCACC   
  
  
- TAAACTATCT CATATTTACT TGTCATTAGT CTGCTCTATA TATCATCACG AATTAACTGG CTCATCGTTT   
  
  
- TCCCCCTTCA ACTCACTCCA TAGTTCTCTC ATCGCATCGA TCGATCCCGT TCTATACGAC CCCAGAAGGA   
  
  
- GTATGTTGAG GCTCCTTCTG GTGGTTCTAC TGTTGGTAAG GAGTGGCAGA CTAAACAGAT CCGTGGGTGG   
  
  
- ATCTAAGTGG TGATGATGAA TGGTTATGGT TGTTGTATAA TCGAGAGGTA GAAGTGTTAG TTATGATCGA   
  
  
- CACGGTGGAG CGGTTAACAA TTAGTTAACA CGCCTCAATT AAACGGCCAA GCTGAAGAGG AGGCGGTTGG   
  
  
- CCGAAGAGAG AGAGGAGCGG AGGGTGAAAC GGAGGGGAAG ACCCCTGAGG TGGCTCTCTG AACACAAAAT   
  
  
- GAAGACGTTC CGAAACCTAG AGGCGGAGTT ATTGTCAATA AATAGGTGAG CATCAAACTG ACCAACATTG   
  
  
- GGATCATGAC CAAGACCATA ACCCCGAGGA AATCGAAGGT TAAAAGATAA AGAAGTTGGT AATCACGTAA   
  
  
- GTGTCCATTG TTCATTACGG GCACAACCCA TGAACTGTAT AAACTGAGAC TTGGTCCAGT GGGGTAAGTA   
  
  
- GGCTAAGTGG GTGAATTGAC GGTTGGTTCG GTAGGACCTT CGAAACCTCC CCATGTTCCG CCAGGTATAG   
  
  
- GAACTGTACC TGTAGTACGT ACCCCAGGTC ACCGGTGGTG AAGAAGTTCG TTAACGTCTT TCTAGACGTT   
  
  
- GGGAACCAGT GGGACGAGGG TGACAAGCGT AGTGTCCTCC CCCGTCCCTA GATCTTGAGG ACTTGGCTTG   
  
  
- ACCTCTAGCC TAGTCCTTCA AGCGTGTTAG GAACCCTGAG CTCAAAGTTA AGGTGGTAGA ATATTGCCAC   
  
  
- CTGAGCTTGG AATGACTCCG TCACCTCCGG TAGTTACGGG ATGTCGATGT GGAGGTACAG GTTCTCCTCC   
  
  
- TCCAGAAGCG TCACTTAACA CCTCTAAAGG AGGTGGCGGA GGATTGCCTC ATACTATCAT GTAAGGAATC   
  
  
- CTACAAAGAG ATGTTCCACT TCTGGGATTT GGGATCCCAG AAGTGGCACC CTCTTTCCCT CCGGCTGGTG   
  
  
- TTGGTGGGAG AGAAGACCGT CGCTAAGTAA CTCCGTGAAC TGGTGATACC CCGTCACAAA CTGAGAGATC   
  
  
- TTCGTTGGGA TGGTAGTTGT TCAGTTCTCT CCCACCGCCA CCTTCTCCCC ACCAAGCCCC TCCTCTAATT   
  
  
- TCTACACCAA CCTGCTCTTC CTCCCAACTC CTCTGTCTCC GTGGTCTTTA TGCTCAGAAC CCTCCACAAC   
  
  
- TACTCCTCGA GACCCAAATT CTCGAACGGG AACTCGGGGA AGAGACACAG TGTTCGGTTC GACGATGAAG   
  
  
- CGGAAGTAAT GGGAAGACTT CCCATGGTGG AATTCCAGAA GGTATCATTA ACAGACAACG AACCCACCTT   
  
  
- CTTAGCGGGA GAAAAGAGAC AAAGAAGAAC CGTTAT

+     Myb

| Site Name | Organism | Position | Strand | Matrix score. | sequence | function |
| --- | --- | --- | --- | --- | --- | --- |
| Myb | Arabidopsis thaliana | 2348 | - | 6 | TAACTG |  |
| Myb | Arabidopsis thaliana | 2539 | + | 6 | TAACTG |  |
| Myb | Arabidopsis thaliana | 870 | + | 6 | TAACTG |  |
| Myb | Arabidopsis thaliana | 484 | + | 6 | TAACTG |  |

>HU01G00833.1   
+ -Up\_Stream \_Len000AAAATA TTTTTACAAA AGCATTTCTC TATAAAATAT TTTACAAAAT TTCATTTTGT   
  
  
+ GGGTCTTTTA GAATCAATCT CTCTACAAGT AGGAATGCCG GTTGAGGTAA GGCTACCTAC ATCTAGATCT   
  
  
+ CTCCAGACCC CACAAAAATT AAACGAGACC CACACTGAAA GGTGGTGGTG GTGGTGTTGT TGTTGTATTG   
  
  
+ TCCTACCACG AGAGAGCTTC AAGTGCATCA CTTATTTCAA CAGGTCAATC ACATAGATCT CTAGTGTACT   
  
  
+ GTAAATTTTT TTTCCGTCTT ATTTTAATTT TCTTTTCCAT CCTAAAAGAT TATCATGTAG ATATTCCTAG   
  
  
+ TTTGATATAT TGACTTCAAC TTCAAAAATT ACACATTAAG ATCCACGAAT ATGACAGTTT AGGTTTCATT   
  
  
+ TGAAGGAGAA AGAAATGTTA CGGTGTTTTG GTGTTGTATT CGTAGACATT TTTTGCGCTT AACTGGCTCT   
  
  
+ TCTCATGGGG ACGAGACAGT GAAGGTAATT GTACTTGTTG ATTGATGACG TCATCTGTAG CTTGGATTTG   
  
  
+ TATAGGAATG AACATCTTGG TGTCTACGTG GAAAGTGTGA TTTGTGTTCT TTCATTGATC CTCCTCCTCA   
  
  
+ AGAAGAGGTG ATCTTTCTCC CTTGTTTTTT TTTTTTTTTG GCTAATATGT TGGCAGAACC GGCAAAAAAT   
  
  
+ ACATAATTTG AATAGTGCAT GCATATGAGT ACATGACAGT GAACCAACAA AACGAACCCC TAAAACACAA   
  
  
+ GAGAATTAAC ATGTTAAAAC ACTTAAACTA AATGCAACCA TCTCGATGAC CACTAAATAG CCAAGAAACC   
  
  
+ TAACGTAATT AACAAAAATA AAAAATAACT GAAAATAAAG TAGAGAAAGT CTAACCGAAT ACGATAACAT   
  
  
+ AACCTAAAAC TAACTATACT TGAACTTACT AAGAAATATG AATCAACCAC AAACCGAATT GATTTTGGTT   
  
  
+ TGCCATAATT TGACTATACA TTTAACTTAA AAAATATTTT TTTTTAATTT CAAATAAATC TAAATTATTT   
  
  
+ TTCAATATTC ATCCAAAATA GATTCAATTA TTAAACAAGT CAACCTATAC CGAGTTATTT TCTAGCTCTG   
  
  
+ ACTAAGACAT ATATATATTT TTTGTAAAAA AGATAAGGAT TATAATTTAA ATAGCCTTTC CCTTAAAAAA   
  
  
+ ATTTTTAAAA TTATCCTGAT AGAGAATAAA AAGAAATCCT TATACAAAAC TCTGATTGAC GCAGATGTGT   
  
  
+ ATATGGACAT ATAGAAGTAA TAAGAGGGGT AGGCTAGGAA GGGGGTGGAA ATATAGGGCA TAAATAGGGG   
  
  
+ GCGGGCATAG GGACATGTCT GTCTGAGGTA CGGTGGATGG GACCTTGCCA TGGAAGCAAC AATGTCACAA   
  
  
+ TAATAATATC AACGACTGAA GTTTCTTCAC CAGCCCACTC TTTGCCATAA TGCCATTAAT TATTTATTGC   
  
  
+ TACTATCTCT TTTGATGTGA AACGGTGGAC CTTATCTCTC CCCTCCACTT CCAATTGATG TGAGCAACTC   
  
  
+ AGAAACGCAA ACAAAACTTG TGCCACCTTC TTTACTTGCA TCCTCATTTT CCTCTGTCTC TCTCCGCCCC   
  
  
+ TGTTTCTCTC TCTCTCCCTC ACTCACTCAG AGTACTGATC TGAGCATCAG AAGTAGCAGA CCCAGAAACT   
  
  
+ CAGATTTAAA AACAACCCGC TCTTTTGTTC CCTCTACGTC TAATAATTTT GTCATCGCAT TTAATGTCAG   
  
  
+ ATAGAGAGAT AGAGCTCTAC TGTATTAATT TTCTCCCACC TCACTTGATT CTGTTGCCCT AAACCACCCA   
  
  
+ AAAAAAGAAA TACGAAGAAG CCACTCTCTC TCTCTCTCTC TCTCACACAC ACACACACCC CTCCTAGTGG   
  
  
+ ATTTGATAGA GTATAAATGA ACAGTAATCA GACGAGATAT ATAGTAGTGC TTAATTGACC GAGTAGCAAA   
  
  
+ AGGGGGAAGT TGAGTGAGGT ATCAAGAGAG TAGCGTAGCT AGCTAGGGCA AGATATGCTG GGGTCTTCCT   
  
  
+ CATACAACTC CGAGGAAGAC CACCAAGATG ACAACCATTC CTCACCGTCT GATTTGTCTA GGCACCCACC   
  
  
+ TAGATTCACC ACTACTACTT ACCAATACCA ACAACATATT AGCTCTCCAT CTTCACAATC AATACTAGCT   
  
  
+ GTGCCACCTC GCCAATTGTT AATCAATTGT GCGGAGTTAA TTTGCCGGTT CGACTTCTCC TCCGCCAACC   
  
  
+ GGCTTCTCTC TCTCCTCGCC TCCCACTTTG CCTCCCCTTC TGGGGACTCC ACCGAGAGAC TTGTGTTTTA   
  
  
+ CTTCTGCAAG GCTTTGGATC TCCGCCTCAA TAACAGTTAT TTATCCACTC GTAGTTTGAC TGGTTGTAAC   
  
  
+ CCTAGTACTG GTTCTGGTAT TGGGGCTCCT TTAGCTTCCA ATTTTCTATT TCTTCAACCA TTAGTGCATT   
  
  
+ CACAGGTAAC AAGTAATGCC CGTGTTGGGT ACTTGACATA TTTGACTCTG AACCAGGTCA CCCCATTCAT   
  
  
+ CCGATTCACC CACTTAACTG CCAACCAAGC CATCCTGGAA GCTTTGGAGG GGTACAAGGC GGTCCATATC   
  
  
+ CTTGACATGG ACATCATGCA TGGGGTCCAG TGGCCACCAC TTCTTCAAGC AATTGCAGAA AGATCTGCAA   
  
  
+ CCCTTGGTCA CCCTGCTCCC ACTGTTCGCA TCACAGGAGG GGGCAGGGAT CTAGAACTCC TGAACCGAAC   
  
  
+ TGGAGATCGG ATCAGGAAGT TCGCACAATC CTTGGGACTC GAGTTTCAAT TCCACCATCT TATAACGGTG   
  
  
+ GACTCGAACC TTACTGAGGC AGTGGAGGCC ATCAATGCCC TACAGCTACA CCTCCATGTC CAAGAGGAGG   
  
  
+ AGGTCTTCGC AGTGAATTGT GGAGATTTCC TCCACCGCCT CCTAACGGAG TATGATAGTA CATTCCTTAG   
  
  
+ GATGTTTCTC TACAAGGTGA AGACCCTAAA CCCTAGGGTC TTCACCGTGG GAGAAAGGGA GGCCGACCAC   
  
  
+ AACCACCCTC TCTTCTGGCA GCGATTCATT GAGGCACTTG ACCACTATGG GGCAGTGTTT GACTCTCTAG   
  
  
+ AAGCAACCCT ACCATCAACA AGTCAAGAGA GGGTGGCGGT GGAAGAGGGG TGGTTCGGGG AGGAGATTAA   
  
  
+ AGATGTGGTT GGACGAGAAG GAGGGTTGAG GAGACAGAGG CACCAGAAAT ACGAGTCTTG GGAGGTGTTG   
  
  
+ ATGAGGAGCT CTGGGTTTAA GAGCTTGCCC TTGAGCCCCT TCTCTGTGTC ACAAGCCAAG CTGCTACTTC   
  
  
+ GCCTTCATTA CCCTTCTGAA GGGTACCACC TTAAGGTCTT CCATAGTAAT TGTCTGTTGC TTGGGTGGAA   
  
  
+ GAATCGCCCT CTTTTCTCTG TTTCTTCTTG GCAATA  

- -Up\_Stream \_Len000TTTTAT AAAAATGTTT TCGTAAAGAG ATATTTTATA AAATGTTTTA AAGTAAAACA   
  
  
- CCCAGAAAAT CTTAGTTAGA GAGATGTTCA TCCTTACGGC CAACTCCATT CCGATGGATG TAGATCTAGA   
  
  
- GAGGTCTGGG GTGTTTTTAA TTTGCTCTGG GTGTGACTTT CCACCACCAC CACCACAACA ACAACATAAC   
  
  
- AGGATGGTGC TCTCTCGAAG TTCACGTAGT GAATAAAGTT GTCCAGTTAG TGTATCTAGA GATCACATGA   
  
  
- CATTTAAAAA AAAGGCAGAA TAAAATTAAA AGAAAAGGTA GGATTTTCTA ATAGTACATC TATAAGGATC   
  
  
- AAACTATATA ACTGAAGTTG AAGTTTTTAA TGTGTAATTC TAGGTGCTTA TACTGTCAAA TCCAAAGTAA   
  
  
- ACTTCCTCTT TCTTTACAAT GCCACAAAAC CACAACATAA GCATCTGTAA AAAACGCGAA TTGACCGAGA   
  
  
- AGAGTACCCC TGCTCTGTCA CTTCCATTAA CATGAACAAC TAACTACTGC AGTAGACATC GAACCTAAAC   
  
  
- ATATCCTTAC TTGTAGAACC ACAGATGCAC CTTTCACACT AAACACAAGA AAGTAACTAG GAGGAGGAGT   
  
  
- TCTTCTCCAC TAGAAAGAGG GAACAAAAAA AAAAAAAAAC CGATTATACA ACCGTCTTGG CCGTTTTTTA   
  
  
- TGTATTAAAC TTATCACGTA CGTATACTCA TGTACTGTCA CTTGGTTGTT TTGCTTGGGG ATTTTGTGTT   
  
  
- CTCTTAATTG TACAATTTTG TGAATTTGAT TTACGTTGGT AGAGCTACTG GTGATTTATC GGTTCTTTGG   
  
  
- ATTGCATTAA TTGTTTTTAT TTTTTATTGA CTTTTATTTC ATCTCTTTCA GATTGGCTTA TGCTATTGTA   
  
  
- TTGGATTTTG ATTGATATGA ACTTGAATGA TTCTTTATAC TTAGTTGGTG TTTGGCTTAA CTAAAACCAA   
  
  
- ACGGTATTAA ACTGATATGT AAATTGAATT TTTTATAAAA AAAAATTAAA GTTTATTTAG ATTTAATAAA   
  
  
- AAGTTATAAG TAGGTTTTAT CTAAGTTAAT AATTTGTTCA GTTGGATATG GCTCAATAAA AGATCGAGAC   
  
  
- TGATTCTGTA TATATATAAA AAACATTTTT TCTATTCCTA ATATTAAATT TATCGGAAAG GGAATTTTTT   
  
  
- TAAAAATTTT AATAGGACTA TCTCTTATTT TTCTTTAGGA ATATGTTTTG AGACTAACTG CGTCTACACA   
  
  
- TATACCTGTA TATCTTCATT ATTCTCCCCA TCCGATCCTT CCCCCACCTT TATATCCCGT ATTTATCCCC   
  
  
- CGCCCGTATC CCTGTACAGA CAGACTCCAT GCCACCTACC CTGGAACGGT ACCTTCGTTG TTACAGTGTT   
  
  
- ATTATTATAG TTGCTGACTT CAAAGAAGTG GTCGGGTGAG AAACGGTATT ACGGTAATTA ATAAATAACG   
  
  
- ATGATAGAGA AAACTACACT TTGCCACCTG GAATAGAGAG GGGAGGTGAA GGTTAACTAC ACTCGTTGAG   
  
  
- TCTTTGCGTT TGTTTTGAAC ACGGTGGAAG AAATGAACGT AGGAGTAAAA GGAGACAGAG AGAGGCGGGG   
  
  
- ACAAAGAGAG AGAGAGGGAG TGAGTGAGTC TCATGACTAG ACTCGTAGTC TTCATCGTCT GGGTCTTTGA   
  
  
- GTCTAAATTT TTGTTGGGCG AGAAAACAAG GGAGATGCAG ATTATTAAAA CAGTAGCGTA AATTACAGTC   
  
  
- TATCTCTCTA TCTCGAGATG ACATAATTAA AAGAGGGTGG AGTGAACTAA GACAACGGGA TTTGGTGGGT   
  
  
- TTTTTTCTTT ATGCTTCTTC GGTGAGAGAG AGAGAGAGAG AGAGTGTGTG TGTGTGTGGG GAGGATCACC   
  
  
- TAAACTATCT CATATTTACT TGTCATTAGT CTGCTCTATA TATCATCACG AATTAACTGG CTCATCGTTT   
  
  
- TCCCCCTTCA ACTCACTCCA TAGTTCTCTC ATCGCATCGA TCGATCCCGT TCTATACGAC CCCAGAAGGA   
  
  
- GTATGTTGAG GCTCCTTCTG GTGGTTCTAC TGTTGGTAAG GAGTGGCAGA CTAAACAGAT CCGTGGGTGG   
  
  
- ATCTAAGTGG TGATGATGAA TGGTTATGGT TGTTGTATAA TCGAGAGGTA GAAGTGTTAG TTATGATCGA   
  
  
- CACGGTGGAG CGGTTAACAA TTAGTTAACA CGCCTCAATT AAACGGCCAA GCTGAAGAGG AGGCGGTTGG   
  
  
- CCGAAGAGAG AGAGGAGCGG AGGGTGAAAC GGAGGGGAAG ACCCCTGAGG TGGCTCTCTG AACACAAAAT   
  
  
- GAAGACGTTC CGAAACCTAG AGGCGGAGTT ATTGTCAATA AATAGGTGAG CATCAAACTG ACCAACATTG   
  
  
- GGATCATGAC CAAGACCATA ACCCCGAGGA AATCGAAGGT TAAAAGATAA AGAAGTTGGT AATCACGTAA   
  
  
- GTGTCCATTG TTCATTACGG GCACAACCCA TGAACTGTAT AAACTGAGAC TTGGTCCAGT GGGGTAAGTA   
  
  
- GGCTAAGTGG GTGAATTGAC GGTTGGTTCG GTAGGACCTT CGAAACCTCC CCATGTTCCG CCAGGTATAG   
  
  
- GAACTGTACC TGTAGTACGT ACCCCAGGTC ACCGGTGGTG AAGAAGTTCG TTAACGTCTT TCTAGACGTT   
  
  
- GGGAACCAGT GGGACGAGGG TGACAAGCGT AGTGTCCTCC CCCGTCCCTA GATCTTGAGG ACTTGGCTTG   
  
  
- ACCTCTAGCC TAGTCCTTCA AGCGTGTTAG GAACCCTGAG CTCAAAGTTA AGGTGGTAGA ATATTGCCAC   
  
  
- CTGAGCTTGG AATGACTCCG TCACCTCCGG TAGTTACGGG ATGTCGATGT GGAGGTACAG GTTCTCCTCC   
  
  
- TCCAGAAGCG TCACTTAACA CCTCTAAAGG AGGTGGCGGA GGATTGCCTC ATACTATCAT GTAAGGAATC   
  
  
- CTACAAAGAG ATGTTCCACT TCTGGGATTT GGGATCCCAG AAGTGGCACC CTCTTTCCCT CCGGCTGGTG   
  
  
- TTGGTGGGAG AGAAGACCGT CGCTAAGTAA CTCCGTGAAC TGGTGATACC CCGTCACAAA CTGAGAGATC   
  
  
- TTCGTTGGGA TGGTAGTTGT TCAGTTCTCT CCCACCGCCA CCTTCTCCCC ACCAAGCCCC TCCTCTAATT   
  
  
- TCTACACCAA CCTGCTCTTC CTCCCAACTC CTCTGTCTCC GTGGTCTTTA TGCTCAGAAC CCTCCACAAC   
  
  
- TACTCCTCGA GACCCAAATT CTCGAACGGG AACTCGGGGA AGAGACACAG TGTTCGGTTC GACGATGAAG   
  
  
- CGGAAGTAAT GGGAAGACTT CCCATGGTGG AATTCCAGAA GGTATCATTA ACAGACAACG AACCCACCTT   
  
  
- CTTAGCGGGA GAAAAGAGAC AAAGAAGAAC CGTTAT

+     Myb-binding site

| Site Name | Organism | Position | Strand | Matrix score. | sequence | function |
| --- | --- | --- | --- | --- | --- | --- |
| Myb-binding site | Nicotiana tabacum | 3348 | - | 6 | CAACAG |  |
| Myb-binding site | Nicotiana tabacum | 1805 | - | 6 | CAACAG |  |
| Myb-binding site | Nicotiana tabacum | 252 | + | 6 | CAACAG |  |

>HU01G00833.1   
+ -Up\_Stream \_Len000AAAATA TTTTTACAAA AGCATTTCTC TATAAAATAT TTTACAAAAT TTCATTTTGT   
  
  
+ GGGTCTTTTA GAATCAATCT CTCTACAAGT AGGAATGCCG GTTGAGGTAA GGCTACCTAC ATCTAGATCT   
  
  
+ CTCCAGACCC CACAAAAATT AAACGAGACC CACACTGAAA GGTGGTGGTG GTGGTGTTGT TGTTGTATTG   
  
  
+ TCCTACCACG AGAGAGCTTC AAGTGCATCA CTTATTTCAA CAGGTCAATC ACATAGATCT CTAGTGTACT   
  
  
+ GTAAATTTTT TTTCCGTCTT ATTTTAATTT TCTTTTCCAT CCTAAAAGAT TATCATGTAG ATATTCCTAG   
  
  
+ TTTGATATAT TGACTTCAAC TTCAAAAATT ACACATTAAG ATCCACGAAT ATGACAGTTT AGGTTTCATT   
  
  
+ TGAAGGAGAA AGAAATGTTA CGGTGTTTTG GTGTTGTATT CGTAGACATT TTTTGCGCTT AACTGGCTCT   
  
  
+ TCTCATGGGG ACGAGACAGT GAAGGTAATT GTACTTGTTG ATTGATGACG TCATCTGTAG CTTGGATTTG   
  
  
+ TATAGGAATG AACATCTTGG TGTCTACGTG GAAAGTGTGA TTTGTGTTCT TTCATTGATC CTCCTCCTCA   
  
  
+ AGAAGAGGTG ATCTTTCTCC CTTGTTTTTT TTTTTTTTTG GCTAATATGT TGGCAGAACC GGCAAAAAAT   
  
  
+ ACATAATTTG AATAGTGCAT GCATATGAGT ACATGACAGT GAACCAACAA AACGAACCCC TAAAACACAA   
  
  
+ GAGAATTAAC ATGTTAAAAC ACTTAAACTA AATGCAACCA TCTCGATGAC CACTAAATAG CCAAGAAACC   
  
  
+ TAACGTAATT AACAAAAATA AAAAATAACT GAAAATAAAG TAGAGAAAGT CTAACCGAAT ACGATAACAT   
  
  
+ AACCTAAAAC TAACTATACT TGAACTTACT AAGAAATATG AATCAACCAC AAACCGAATT GATTTTGGTT   
  
  
+ TGCCATAATT TGACTATACA TTTAACTTAA AAAATATTTT TTTTTAATTT CAAATAAATC TAAATTATTT   
  
  
+ TTCAATATTC ATCCAAAATA GATTCAATTA TTAAACAAGT CAACCTATAC CGAGTTATTT TCTAGCTCTG   
  
  
+ ACTAAGACAT ATATATATTT TTTGTAAAAA AGATAAGGAT TATAATTTAA ATAGCCTTTC CCTTAAAAAA   
  
  
+ ATTTTTAAAA TTATCCTGAT AGAGAATAAA AAGAAATCCT TATACAAAAC TCTGATTGAC GCAGATGTGT   
  
  
+ ATATGGACAT ATAGAAGTAA TAAGAGGGGT AGGCTAGGAA GGGGGTGGAA ATATAGGGCA TAAATAGGGG   
  
  
+ GCGGGCATAG GGACATGTCT GTCTGAGGTA CGGTGGATGG GACCTTGCCA TGGAAGCAAC AATGTCACAA   
  
  
+ TAATAATATC AACGACTGAA GTTTCTTCAC CAGCCCACTC TTTGCCATAA TGCCATTAAT TATTTATTGC   
  
  
+ TACTATCTCT TTTGATGTGA AACGGTGGAC CTTATCTCTC CCCTCCACTT CCAATTGATG TGAGCAACTC   
  
  
+ AGAAACGCAA ACAAAACTTG TGCCACCTTC TTTACTTGCA TCCTCATTTT CCTCTGTCTC TCTCCGCCCC   
  
  
+ TGTTTCTCTC TCTCTCCCTC ACTCACTCAG AGTACTGATC TGAGCATCAG AAGTAGCAGA CCCAGAAACT   
  
  
+ CAGATTTAAA AACAACCCGC TCTTTTGTTC CCTCTACGTC TAATAATTTT GTCATCGCAT TTAATGTCAG   
  
  
+ ATAGAGAGAT AGAGCTCTAC TGTATTAATT TTCTCCCACC TCACTTGATT CTGTTGCCCT AAACCACCCA   
  
  
+ AAAAAAGAAA TACGAAGAAG CCACTCTCTC TCTCTCTCTC TCTCACACAC ACACACACCC CTCCTAGTGG   
  
  
+ ATTTGATAGA GTATAAATGA ACAGTAATCA GACGAGATAT ATAGTAGTGC TTAATTGACC GAGTAGCAAA   
  
  
+ AGGGGGAAGT TGAGTGAGGT ATCAAGAGAG TAGCGTAGCT AGCTAGGGCA AGATATGCTG GGGTCTTCCT   
  
  
+ CATACAACTC CGAGGAAGAC CACCAAGATG ACAACCATTC CTCACCGTCT GATTTGTCTA GGCACCCACC   
  
  
+ TAGATTCACC ACTACTACTT ACCAATACCA ACAACATATT AGCTCTCCAT CTTCACAATC AATACTAGCT   
  
  
+ GTGCCACCTC GCCAATTGTT AATCAATTGT GCGGAGTTAA TTTGCCGGTT CGACTTCTCC TCCGCCAACC   
  
  
+ GGCTTCTCTC TCTCCTCGCC TCCCACTTTG CCTCCCCTTC TGGGGACTCC ACCGAGAGAC TTGTGTTTTA   
  
  
+ CTTCTGCAAG GCTTTGGATC TCCGCCTCAA TAACAGTTAT TTATCCACTC GTAGTTTGAC TGGTTGTAAC   
  
  
+ CCTAGTACTG GTTCTGGTAT TGGGGCTCCT TTAGCTTCCA ATTTTCTATT TCTTCAACCA TTAGTGCATT   
  
  
+ CACAGGTAAC AAGTAATGCC CGTGTTGGGT ACTTGACATA TTTGACTCTG AACCAGGTCA CCCCATTCAT   
  
  
+ CCGATTCACC CACTTAACTG CCAACCAAGC CATCCTGGAA GCTTTGGAGG GGTACAAGGC GGTCCATATC   
  
  
+ CTTGACATGG ACATCATGCA TGGGGTCCAG TGGCCACCAC TTCTTCAAGC AATTGCAGAA AGATCTGCAA   
  
  
+ CCCTTGGTCA CCCTGCTCCC ACTGTTCGCA TCACAGGAGG GGGCAGGGAT CTAGAACTCC TGAACCGAAC   
  
  
+ TGGAGATCGG ATCAGGAAGT TCGCACAATC CTTGGGACTC GAGTTTCAAT TCCACCATCT TATAACGGTG   
  
  
+ GACTCGAACC TTACTGAGGC AGTGGAGGCC ATCAATGCCC TACAGCTACA CCTCCATGTC CAAGAGGAGG   
  
  
+ AGGTCTTCGC AGTGAATTGT GGAGATTTCC TCCACCGCCT CCTAACGGAG TATGATAGTA CATTCCTTAG   
  
  
+ GATGTTTCTC TACAAGGTGA AGACCCTAAA CCCTAGGGTC TTCACCGTGG GAGAAAGGGA GGCCGACCAC   
  
  
+ AACCACCCTC TCTTCTGGCA GCGATTCATT GAGGCACTTG ACCACTATGG GGCAGTGTTT GACTCTCTAG   
  
  
+ AAGCAACCCT ACCATCAACA AGTCAAGAGA GGGTGGCGGT GGAAGAGGGG TGGTTCGGGG AGGAGATTAA   
  
  
+ AGATGTGGTT GGACGAGAAG GAGGGTTGAG GAGACAGAGG CACCAGAAAT ACGAGTCTTG GGAGGTGTTG   
  
  
+ ATGAGGAGCT CTGGGTTTAA GAGCTTGCCC TTGAGCCCCT TCTCTGTGTC ACAAGCCAAG CTGCTACTTC   
  
  
+ GCCTTCATTA CCCTTCTGAA GGGTACCACC TTAAGGTCTT CCATAGTAAT TGTCTGTTGC TTGGGTGGAA   
  
  
+ GAATCGCCCT CTTTTCTCTG TTTCTTCTTG GCAATA  

- -Up\_Stream \_Len000TTTTAT AAAAATGTTT TCGTAAAGAG ATATTTTATA AAATGTTTTA AAGTAAAACA   
  
  
- CCCAGAAAAT CTTAGTTAGA GAGATGTTCA TCCTTACGGC CAACTCCATT CCGATGGATG TAGATCTAGA   
  
  
- GAGGTCTGGG GTGTTTTTAA TTTGCTCTGG GTGTGACTTT CCACCACCAC CACCACAACA ACAACATAAC   
  
  
- AGGATGGTGC TCTCTCGAAG TTCACGTAGT GAATAAAGTT GTCCAGTTAG TGTATCTAGA GATCACATGA   
  
  
- CATTTAAAAA AAAGGCAGAA TAAAATTAAA AGAAAAGGTA GGATTTTCTA ATAGTACATC TATAAGGATC   
  
  
- AAACTATATA ACTGAAGTTG AAGTTTTTAA TGTGTAATTC TAGGTGCTTA TACTGTCAAA TCCAAAGTAA   
  
  
- ACTTCCTCTT TCTTTACAAT GCCACAAAAC CACAACATAA GCATCTGTAA AAAACGCGAA TTGACCGAGA   
  
  
- AGAGTACCCC TGCTCTGTCA CTTCCATTAA CATGAACAAC TAACTACTGC AGTAGACATC GAACCTAAAC   
  
  
- ATATCCTTAC TTGTAGAACC ACAGATGCAC CTTTCACACT AAACACAAGA AAGTAACTAG GAGGAGGAGT   
  
  
- TCTTCTCCAC TAGAAAGAGG GAACAAAAAA AAAAAAAAAC CGATTATACA ACCGTCTTGG CCGTTTTTTA   
  
  
- TGTATTAAAC TTATCACGTA CGTATACTCA TGTACTGTCA CTTGGTTGTT TTGCTTGGGG ATTTTGTGTT   
  
  
- CTCTTAATTG TACAATTTTG TGAATTTGAT TTACGTTGGT AGAGCTACTG GTGATTTATC GGTTCTTTGG   
  
  
- ATTGCATTAA TTGTTTTTAT TTTTTATTGA CTTTTATTTC ATCTCTTTCA GATTGGCTTA TGCTATTGTA   
  
  
- TTGGATTTTG ATTGATATGA ACTTGAATGA TTCTTTATAC TTAGTTGGTG TTTGGCTTAA CTAAAACCAA   
  
  
- ACGGTATTAA ACTGATATGT AAATTGAATT TTTTATAAAA AAAAATTAAA GTTTATTTAG ATTTAATAAA   
  
  
- AAGTTATAAG TAGGTTTTAT CTAAGTTAAT AATTTGTTCA GTTGGATATG GCTCAATAAA AGATCGAGAC   
  
  
- TGATTCTGTA TATATATAAA AAACATTTTT TCTATTCCTA ATATTAAATT TATCGGAAAG GGAATTTTTT   
  
  
- TAAAAATTTT AATAGGACTA TCTCTTATTT TTCTTTAGGA ATATGTTTTG AGACTAACTG CGTCTACACA   
  
  
- TATACCTGTA TATCTTCATT ATTCTCCCCA TCCGATCCTT CCCCCACCTT TATATCCCGT ATTTATCCCC   
  
  
- CGCCCGTATC CCTGTACAGA CAGACTCCAT GCCACCTACC CTGGAACGGT ACCTTCGTTG TTACAGTGTT   
  
  
- ATTATTATAG TTGCTGACTT CAAAGAAGTG GTCGGGTGAG AAACGGTATT ACGGTAATTA ATAAATAACG   
  
  
- ATGATAGAGA AAACTACACT TTGCCACCTG GAATAGAGAG GGGAGGTGAA GGTTAACTAC ACTCGTTGAG   
  
  
- TCTTTGCGTT TGTTTTGAAC ACGGTGGAAG AAATGAACGT AGGAGTAAAA GGAGACAGAG AGAGGCGGGG   
  
  
- ACAAAGAGAG AGAGAGGGAG TGAGTGAGTC TCATGACTAG ACTCGTAGTC TTCATCGTCT GGGTCTTTGA   
  
  
- GTCTAAATTT TTGTTGGGCG AGAAAACAAG GGAGATGCAG ATTATTAAAA CAGTAGCGTA AATTACAGTC   
  
  
- TATCTCTCTA TCTCGAGATG ACATAATTAA AAGAGGGTGG AGTGAACTAA GACAACGGGA TTTGGTGGGT   
  
  
- TTTTTTCTTT ATGCTTCTTC GGTGAGAGAG AGAGAGAGAG AGAGTGTGTG TGTGTGTGGG GAGGATCACC   
  
  
- TAAACTATCT CATATTTACT TGTCATTAGT CTGCTCTATA TATCATCACG AATTAACTGG CTCATCGTTT   
  
  
- TCCCCCTTCA ACTCACTCCA TAGTTCTCTC ATCGCATCGA TCGATCCCGT TCTATACGAC CCCAGAAGGA   
  
  
- GTATGTTGAG GCTCCTTCTG GTGGTTCTAC TGTTGGTAAG GAGTGGCAGA CTAAACAGAT CCGTGGGTGG   
  
  
- ATCTAAGTGG TGATGATGAA TGGTTATGGT TGTTGTATAA TCGAGAGGTA GAAGTGTTAG TTATGATCGA   
  
  
- CACGGTGGAG CGGTTAACAA TTAGTTAACA CGCCTCAATT AAACGGCCAA GCTGAAGAGG AGGCGGTTGG   
  
  
- CCGAAGAGAG AGAGGAGCGG AGGGTGAAAC GGAGGGGAAG ACCCCTGAGG TGGCTCTCTG AACACAAAAT   
  
  
- GAAGACGTTC CGAAACCTAG AGGCGGAGTT ATTGTCAATA AATAGGTGAG CATCAAACTG ACCAACATTG   
  
  
- GGATCATGAC CAAGACCATA ACCCCGAGGA AATCGAAGGT TAAAAGATAA AGAAGTTGGT AATCACGTAA   
  
  
- GTGTCCATTG TTCATTACGG GCACAACCCA TGAACTGTAT AAACTGAGAC TTGGTCCAGT GGGGTAAGTA   
  
  
- GGCTAAGTGG GTGAATTGAC GGTTGGTTCG GTAGGACCTT CGAAACCTCC CCATGTTCCG CCAGGTATAG   
  
  
- GAACTGTACC TGTAGTACGT ACCCCAGGTC ACCGGTGGTG AAGAAGTTCG TTAACGTCTT TCTAGACGTT   
  
  
- GGGAACCAGT GGGACGAGGG TGACAAGCGT AGTGTCCTCC CCCGTCCCTA GATCTTGAGG ACTTGGCTTG   
  
  
- ACCTCTAGCC TAGTCCTTCA AGCGTGTTAG GAACCCTGAG CTCAAAGTTA AGGTGGTAGA ATATTGCCAC   
  
  
- CTGAGCTTGG AATGACTCCG TCACCTCCGG TAGTTACGGG ATGTCGATGT GGAGGTACAG GTTCTCCTCC   
  
  
- TCCAGAAGCG TCACTTAACA CCTCTAAAGG AGGTGGCGGA GGATTGCCTC ATACTATCAT GTAAGGAATC   
  
  
- CTACAAAGAG ATGTTCCACT TCTGGGATTT GGGATCCCAG AAGTGGCACC CTCTTTCCCT CCGGCTGGTG   
  
  
- TTGGTGGGAG AGAAGACCGT CGCTAAGTAA CTCCGTGAAC TGGTGATACC CCGTCACAAA CTGAGAGATC   
  
  
- TTCGTTGGGA TGGTAGTTGT TCAGTTCTCT CCCACCGCCA CCTTCTCCCC ACCAAGCCCC TCCTCTAATT   
  
  
- TCTACACCAA CCTGCTCTTC CTCCCAACTC CTCTGTCTCC GTGGTCTTTA TGCTCAGAAC CCTCCACAAC   
  
  
- TACTCCTCGA GACCCAAATT CTCGAACGGG AACTCGGGGA AGAGACACAG TGTTCGGTTC GACGATGAAG   
  
  
- CGGAAGTAAT GGGAAGACTT CCCATGGTGG AATTCCAGAA GGTATCATTA ACAGACAACG AACCCACCTT   
  
  
- CTTAGCGGGA GAAAAGAGAC AAAGAAGAAC CGTTAT

+     P-box

| Site Name | Organism | Position | Strand | Matrix score. | sequence | function |
| --- | --- | --- | --- | --- | --- | --- |
| P-box | Oryza sativa | 1961 | - | 7 | CCTTTTG | gibberellin-responsive element |

>HU01G00833.1   
+ -Up\_Stream \_Len000AAAATA TTTTTACAAA AGCATTTCTC TATAAAATAT TTTACAAAAT TTCATTTTGT   
  
  
+ GGGTCTTTTA GAATCAATCT CTCTACAAGT AGGAATGCCG GTTGAGGTAA GGCTACCTAC ATCTAGATCT   
  
  
+ CTCCAGACCC CACAAAAATT AAACGAGACC CACACTGAAA GGTGGTGGTG GTGGTGTTGT TGTTGTATTG   
  
  
+ TCCTACCACG AGAGAGCTTC AAGTGCATCA CTTATTTCAA CAGGTCAATC ACATAGATCT CTAGTGTACT   
  
  
+ GTAAATTTTT TTTCCGTCTT ATTTTAATTT TCTTTTCCAT CCTAAAAGAT TATCATGTAG ATATTCCTAG   
  
  
+ TTTGATATAT TGACTTCAAC TTCAAAAATT ACACATTAAG ATCCACGAAT ATGACAGTTT AGGTTTCATT   
  
  
+ TGAAGGAGAA AGAAATGTTA CGGTGTTTTG GTGTTGTATT CGTAGACATT TTTTGCGCTT AACTGGCTCT   
  
  
+ TCTCATGGGG ACGAGACAGT GAAGGTAATT GTACTTGTTG ATTGATGACG TCATCTGTAG CTTGGATTTG   
  
  
+ TATAGGAATG AACATCTTGG TGTCTACGTG GAAAGTGTGA TTTGTGTTCT TTCATTGATC CTCCTCCTCA   
  
  
+ AGAAGAGGTG ATCTTTCTCC CTTGTTTTTT TTTTTTTTTG GCTAATATGT TGGCAGAACC GGCAAAAAAT   
  
  
+ ACATAATTTG AATAGTGCAT GCATATGAGT ACATGACAGT GAACCAACAA AACGAACCCC TAAAACACAA   
  
  
+ GAGAATTAAC ATGTTAAAAC ACTTAAACTA AATGCAACCA TCTCGATGAC CACTAAATAG CCAAGAAACC   
  
  
+ TAACGTAATT AACAAAAATA AAAAATAACT GAAAATAAAG TAGAGAAAGT CTAACCGAAT ACGATAACAT   
  
  
+ AACCTAAAAC TAACTATACT TGAACTTACT AAGAAATATG AATCAACCAC AAACCGAATT GATTTTGGTT   
  
  
+ TGCCATAATT TGACTATACA TTTAACTTAA AAAATATTTT TTTTTAATTT CAAATAAATC TAAATTATTT   
  
  
+ TTCAATATTC ATCCAAAATA GATTCAATTA TTAAACAAGT CAACCTATAC CGAGTTATTT TCTAGCTCTG   
  
  
+ ACTAAGACAT ATATATATTT TTTGTAAAAA AGATAAGGAT TATAATTTAA ATAGCCTTTC CCTTAAAAAA   
  
  
+ ATTTTTAAAA TTATCCTGAT AGAGAATAAA AAGAAATCCT TATACAAAAC TCTGATTGAC GCAGATGTGT   
  
  
+ ATATGGACAT ATAGAAGTAA TAAGAGGGGT AGGCTAGGAA GGGGGTGGAA ATATAGGGCA TAAATAGGGG   
  
  
+ GCGGGCATAG GGACATGTCT GTCTGAGGTA CGGTGGATGG GACCTTGCCA TGGAAGCAAC AATGTCACAA   
  
  
+ TAATAATATC AACGACTGAA GTTTCTTCAC CAGCCCACTC TTTGCCATAA TGCCATTAAT TATTTATTGC   
  
  
+ TACTATCTCT TTTGATGTGA AACGGTGGAC CTTATCTCTC CCCTCCACTT CCAATTGATG TGAGCAACTC   
  
  
+ AGAAACGCAA ACAAAACTTG TGCCACCTTC TTTACTTGCA TCCTCATTTT CCTCTGTCTC TCTCCGCCCC   
  
  
+ TGTTTCTCTC TCTCTCCCTC ACTCACTCAG AGTACTGATC TGAGCATCAG AAGTAGCAGA CCCAGAAACT   
  
  
+ CAGATTTAAA AACAACCCGC TCTTTTGTTC CCTCTACGTC TAATAATTTT GTCATCGCAT TTAATGTCAG   
  
  
+ ATAGAGAGAT AGAGCTCTAC TGTATTAATT TTCTCCCACC TCACTTGATT CTGTTGCCCT AAACCACCCA   
  
  
+ AAAAAAGAAA TACGAAGAAG CCACTCTCTC TCTCTCTCTC TCTCACACAC ACACACACCC CTCCTAGTGG   
  
  
+ ATTTGATAGA GTATAAATGA ACAGTAATCA GACGAGATAT ATAGTAGTGC TTAATTGACC GAGTAGCAAA   
  
  
+ AGGGGGAAGT TGAGTGAGGT ATCAAGAGAG TAGCGTAGCT AGCTAGGGCA AGATATGCTG GGGTCTTCCT   
  
  
+ CATACAACTC CGAGGAAGAC CACCAAGATG ACAACCATTC CTCACCGTCT GATTTGTCTA GGCACCCACC   
  
  
+ TAGATTCACC ACTACTACTT ACCAATACCA ACAACATATT AGCTCTCCAT CTTCACAATC AATACTAGCT   
  
  
+ GTGCCACCTC GCCAATTGTT AATCAATTGT GCGGAGTTAA TTTGCCGGTT CGACTTCTCC TCCGCCAACC   
  
  
+ GGCTTCTCTC TCTCCTCGCC TCCCACTTTG CCTCCCCTTC TGGGGACTCC ACCGAGAGAC TTGTGTTTTA   
  
  
+ CTTCTGCAAG GCTTTGGATC TCCGCCTCAA TAACAGTTAT TTATCCACTC GTAGTTTGAC TGGTTGTAAC   
  
  
+ CCTAGTACTG GTTCTGGTAT TGGGGCTCCT TTAGCTTCCA ATTTTCTATT TCTTCAACCA TTAGTGCATT   
  
  
+ CACAGGTAAC AAGTAATGCC CGTGTTGGGT ACTTGACATA TTTGACTCTG AACCAGGTCA CCCCATTCAT   
  
  
+ CCGATTCACC CACTTAACTG CCAACCAAGC CATCCTGGAA GCTTTGGAGG GGTACAAGGC GGTCCATATC   
  
  
+ CTTGACATGG ACATCATGCA TGGGGTCCAG TGGCCACCAC TTCTTCAAGC AATTGCAGAA AGATCTGCAA   
  
  
+ CCCTTGGTCA CCCTGCTCCC ACTGTTCGCA TCACAGGAGG GGGCAGGGAT CTAGAACTCC TGAACCGAAC   
  
  
+ TGGAGATCGG ATCAGGAAGT TCGCACAATC CTTGGGACTC GAGTTTCAAT TCCACCATCT TATAACGGTG   
  
  
+ GACTCGAACC TTACTGAGGC AGTGGAGGCC ATCAATGCCC TACAGCTACA CCTCCATGTC CAAGAGGAGG   
  
  
+ AGGTCTTCGC AGTGAATTGT GGAGATTTCC TCCACCGCCT CCTAACGGAG TATGATAGTA CATTCCTTAG   
  
  
+ GATGTTTCTC TACAAGGTGA AGACCCTAAA CCCTAGGGTC TTCACCGTGG GAGAAAGGGA GGCCGACCAC   
  
  
+ AACCACCCTC TCTTCTGGCA GCGATTCATT GAGGCACTTG ACCACTATGG GGCAGTGTTT GACTCTCTAG   
  
  
+ AAGCAACCCT ACCATCAACA AGTCAAGAGA GGGTGGCGGT GGAAGAGGGG TGGTTCGGGG AGGAGATTAA   
  
  
+ AGATGTGGTT GGACGAGAAG GAGGGTTGAG GAGACAGAGG CACCAGAAAT ACGAGTCTTG GGAGGTGTTG   
  
  
+ ATGAGGAGCT CTGGGTTTAA GAGCTTGCCC TTGAGCCCCT TCTCTGTGTC ACAAGCCAAG CTGCTACTTC   
  
  
+ GCCTTCATTA CCCTTCTGAA GGGTACCACC TTAAGGTCTT CCATAGTAAT TGTCTGTTGC TTGGGTGGAA   
  
  
+ GAATCGCCCT CTTTTCTCTG TTTCTTCTTG GCAATA  

- -Up\_Stream \_Len000TTTTAT AAAAATGTTT TCGTAAAGAG ATATTTTATA AAATGTTTTA AAGTAAAACA   
  
  
- CCCAGAAAAT CTTAGTTAGA GAGATGTTCA TCCTTACGGC CAACTCCATT CCGATGGATG TAGATCTAGA   
  
  
- GAGGTCTGGG GTGTTTTTAA TTTGCTCTGG GTGTGACTTT CCACCACCAC CACCACAACA ACAACATAAC   
  
  
- AGGATGGTGC TCTCTCGAAG TTCACGTAGT GAATAAAGTT GTCCAGTTAG TGTATCTAGA GATCACATGA   
  
  
- CATTTAAAAA AAAGGCAGAA TAAAATTAAA AGAAAAGGTA GGATTTTCTA ATAGTACATC TATAAGGATC   
  
  
- AAACTATATA ACTGAAGTTG AAGTTTTTAA TGTGTAATTC TAGGTGCTTA TACTGTCAAA TCCAAAGTAA   
  
  
- ACTTCCTCTT TCTTTACAAT GCCACAAAAC CACAACATAA GCATCTGTAA AAAACGCGAA TTGACCGAGA   
  
  
- AGAGTACCCC TGCTCTGTCA CTTCCATTAA CATGAACAAC TAACTACTGC AGTAGACATC GAACCTAAAC   
  
  
- ATATCCTTAC TTGTAGAACC ACAGATGCAC CTTTCACACT AAACACAAGA AAGTAACTAG GAGGAGGAGT   
  
  
- TCTTCTCCAC TAGAAAGAGG GAACAAAAAA AAAAAAAAAC CGATTATACA ACCGTCTTGG CCGTTTTTTA   
  
  
- TGTATTAAAC TTATCACGTA CGTATACTCA TGTACTGTCA CTTGGTTGTT TTGCTTGGGG ATTTTGTGTT   
  
  
- CTCTTAATTG TACAATTTTG TGAATTTGAT TTACGTTGGT AGAGCTACTG GTGATTTATC GGTTCTTTGG   
  
  
- ATTGCATTAA TTGTTTTTAT TTTTTATTGA CTTTTATTTC ATCTCTTTCA GATTGGCTTA TGCTATTGTA   
  
  
- TTGGATTTTG ATTGATATGA ACTTGAATGA TTCTTTATAC TTAGTTGGTG TTTGGCTTAA CTAAAACCAA   
  
  
- ACGGTATTAA ACTGATATGT AAATTGAATT TTTTATAAAA AAAAATTAAA GTTTATTTAG ATTTAATAAA   
  
  
- AAGTTATAAG TAGGTTTTAT CTAAGTTAAT AATTTGTTCA GTTGGATATG GCTCAATAAA AGATCGAGAC   
  
  
- TGATTCTGTA TATATATAAA AAACATTTTT TCTATTCCTA ATATTAAATT TATCGGAAAG GGAATTTTTT   
  
  
- TAAAAATTTT AATAGGACTA TCTCTTATTT TTCTTTAGGA ATATGTTTTG AGACTAACTG CGTCTACACA   
  
  
- TATACCTGTA TATCTTCATT ATTCTCCCCA TCCGATCCTT CCCCCACCTT TATATCCCGT ATTTATCCCC   
  
  
- CGCCCGTATC CCTGTACAGA CAGACTCCAT GCCACCTACC CTGGAACGGT ACCTTCGTTG TTACAGTGTT   
  
  
- ATTATTATAG TTGCTGACTT CAAAGAAGTG GTCGGGTGAG AAACGGTATT ACGGTAATTA ATAAATAACG   
  
  
- ATGATAGAGA AAACTACACT TTGCCACCTG GAATAGAGAG GGGAGGTGAA GGTTAACTAC ACTCGTTGAG   
  
  
- TCTTTGCGTT TGTTTTGAAC ACGGTGGAAG AAATGAACGT AGGAGTAAAA GGAGACAGAG AGAGGCGGGG   
  
  
- ACAAAGAGAG AGAGAGGGAG TGAGTGAGTC TCATGACTAG ACTCGTAGTC TTCATCGTCT GGGTCTTTGA   
  
  
- GTCTAAATTT TTGTTGGGCG AGAAAACAAG GGAGATGCAG ATTATTAAAA CAGTAGCGTA AATTACAGTC   
  
  
- TATCTCTCTA TCTCGAGATG ACATAATTAA AAGAGGGTGG AGTGAACTAA GACAACGGGA TTTGGTGGGT   
  
  
- TTTTTTCTTT ATGCTTCTTC GGTGAGAGAG AGAGAGAGAG AGAGTGTGTG TGTGTGTGGG GAGGATCACC   
  
  
- TAAACTATCT CATATTTACT TGTCATTAGT CTGCTCTATA TATCATCACG AATTAACTGG CTCATCGTTT   
  
  
- TCCCCCTTCA ACTCACTCCA TAGTTCTCTC ATCGCATCGA TCGATCCCGT TCTATACGAC CCCAGAAGGA   
  
  
- GTATGTTGAG GCTCCTTCTG GTGGTTCTAC TGTTGGTAAG GAGTGGCAGA CTAAACAGAT CCGTGGGTGG   
  
  
- ATCTAAGTGG TGATGATGAA TGGTTATGGT TGTTGTATAA TCGAGAGGTA GAAGTGTTAG TTATGATCGA   
  
  
- CACGGTGGAG CGGTTAACAA TTAGTTAACA CGCCTCAATT AAACGGCCAA GCTGAAGAGG AGGCGGTTGG   
  
  
- CCGAAGAGAG AGAGGAGCGG AGGGTGAAAC GGAGGGGAAG ACCCCTGAGG TGGCTCTCTG AACACAAAAT   
  
  
- GAAGACGTTC CGAAACCTAG AGGCGGAGTT ATTGTCAATA AATAGGTGAG CATCAAACTG ACCAACATTG   
  
  
- GGATCATGAC CAAGACCATA ACCCCGAGGA AATCGAAGGT TAAAAGATAA AGAAGTTGGT AATCACGTAA   
  
  
- GTGTCCATTG TTCATTACGG GCACAACCCA TGAACTGTAT AAACTGAGAC TTGGTCCAGT GGGGTAAGTA   
  
  
- GGCTAAGTGG GTGAATTGAC GGTTGGTTCG GTAGGACCTT CGAAACCTCC CCATGTTCCG CCAGGTATAG   
  
  
- GAACTGTACC TGTAGTACGT ACCCCAGGTC ACCGGTGGTG AAGAAGTTCG TTAACGTCTT TCTAGACGTT   
  
  
- GGGAACCAGT GGGACGAGGG TGACAAGCGT AGTGTCCTCC CCCGTCCCTA GATCTTGAGG ACTTGGCTTG   
  
  
- ACCTCTAGCC TAGTCCTTCA AGCGTGTTAG GAACCCTGAG CTCAAAGTTA AGGTGGTAGA ATATTGCCAC   
  
  
- CTGAGCTTGG AATGACTCCG TCACCTCCGG TAGTTACGGG ATGTCGATGT GGAGGTACAG GTTCTCCTCC   
  
  
- TCCAGAAGCG TCACTTAACA CCTCTAAAGG AGGTGGCGGA GGATTGCCTC ATACTATCAT GTAAGGAATC   
  
  
- CTACAAAGAG ATGTTCCACT TCTGGGATTT GGGATCCCAG AAGTGGCACC CTCTTTCCCT CCGGCTGGTG   
  
  
- TTGGTGGGAG AGAAGACCGT CGCTAAGTAA CTCCGTGAAC TGGTGATACC CCGTCACAAA CTGAGAGATC   
  
  
- TTCGTTGGGA TGGTAGTTGT TCAGTTCTCT CCCACCGCCA CCTTCTCCCC ACCAAGCCCC TCCTCTAATT   
  
  
- TCTACACCAA CCTGCTCTTC CTCCCAACTC CTCTGTCTCC GTGGTCTTTA TGCTCAGAAC CCTCCACAAC   
  
  
- TACTCCTCGA GACCCAAATT CTCGAACGGG AACTCGGGGA AGAGACACAG TGTTCGGTTC GACGATGAAG   
  
  
- CGGAAGTAAT GGGAAGACTT CCCATGGTGG AATTCCAGAA GGTATCATTA ACAGACAACG AACCCACCTT   
  
  
- CTTAGCGGGA GAAAAGAGAC AAAGAAGAAC CGTTAT

+     RY-element

| Site Name | Organism | Position | Strand | Matrix score. | sequence | function |
| --- | --- | --- | --- | --- | --- | --- |
| RY-element | Helianthus annuus | 2609 | - | 8 | CATGCATG | cis-acting regulatory element involved in seed-specific regulation |

>HU01G00833.1   
+ -Up\_Stream \_Len000AAAATA TTTTTACAAA AGCATTTCTC TATAAAATAT TTTACAAAAT TTCATTTTGT   
  
  
+ GGGTCTTTTA GAATCAATCT CTCTACAAGT AGGAATGCCG GTTGAGGTAA GGCTACCTAC ATCTAGATCT   
  
  
+ CTCCAGACCC CACAAAAATT AAACGAGACC CACACTGAAA GGTGGTGGTG GTGGTGTTGT TGTTGTATTG   
  
  
+ TCCTACCACG AGAGAGCTTC AAGTGCATCA CTTATTTCAA CAGGTCAATC ACATAGATCT CTAGTGTACT   
  
  
+ GTAAATTTTT TTTCCGTCTT ATTTTAATTT TCTTTTCCAT CCTAAAAGAT TATCATGTAG ATATTCCTAG   
  
  
+ TTTGATATAT TGACTTCAAC TTCAAAAATT ACACATTAAG ATCCACGAAT ATGACAGTTT AGGTTTCATT   
  
  
+ TGAAGGAGAA AGAAATGTTA CGGTGTTTTG GTGTTGTATT CGTAGACATT TTTTGCGCTT AACTGGCTCT   
  
  
+ TCTCATGGGG ACGAGACAGT GAAGGTAATT GTACTTGTTG ATTGATGACG TCATCTGTAG CTTGGATTTG   
  
  
+ TATAGGAATG AACATCTTGG TGTCTACGTG GAAAGTGTGA TTTGTGTTCT TTCATTGATC CTCCTCCTCA   
  
  
+ AGAAGAGGTG ATCTTTCTCC CTTGTTTTTT TTTTTTTTTG GCTAATATGT TGGCAGAACC GGCAAAAAAT   
  
  
+ ACATAATTTG AATAGTGCAT GCATATGAGT ACATGACAGT GAACCAACAA AACGAACCCC TAAAACACAA   
  
  
+ GAGAATTAAC ATGTTAAAAC ACTTAAACTA AATGCAACCA TCTCGATGAC CACTAAATAG CCAAGAAACC   
  
  
+ TAACGTAATT AACAAAAATA AAAAATAACT GAAAATAAAG TAGAGAAAGT CTAACCGAAT ACGATAACAT   
  
  
+ AACCTAAAAC TAACTATACT TGAACTTACT AAGAAATATG AATCAACCAC AAACCGAATT GATTTTGGTT   
  
  
+ TGCCATAATT TGACTATACA TTTAACTTAA AAAATATTTT TTTTTAATTT CAAATAAATC TAAATTATTT   
  
  
+ TTCAATATTC ATCCAAAATA GATTCAATTA TTAAACAAGT CAACCTATAC CGAGTTATTT TCTAGCTCTG   
  
  
+ ACTAAGACAT ATATATATTT TTTGTAAAAA AGATAAGGAT TATAATTTAA ATAGCCTTTC CCTTAAAAAA   
  
  
+ ATTTTTAAAA TTATCCTGAT AGAGAATAAA AAGAAATCCT TATACAAAAC TCTGATTGAC GCAGATGTGT   
  
  
+ ATATGGACAT ATAGAAGTAA TAAGAGGGGT AGGCTAGGAA GGGGGTGGAA ATATAGGGCA TAAATAGGGG   
  
  
+ GCGGGCATAG GGACATGTCT GTCTGAGGTA CGGTGGATGG GACCTTGCCA TGGAAGCAAC AATGTCACAA   
  
  
+ TAATAATATC AACGACTGAA GTTTCTTCAC CAGCCCACTC TTTGCCATAA TGCCATTAAT TATTTATTGC   
  
  
+ TACTATCTCT TTTGATGTGA AACGGTGGAC CTTATCTCTC CCCTCCACTT CCAATTGATG TGAGCAACTC   
  
  
+ AGAAACGCAA ACAAAACTTG TGCCACCTTC TTTACTTGCA TCCTCATTTT CCTCTGTCTC TCTCCGCCCC   
  
  
+ TGTTTCTCTC TCTCTCCCTC ACTCACTCAG AGTACTGATC TGAGCATCAG AAGTAGCAGA CCCAGAAACT   
  
  
+ CAGATTTAAA AACAACCCGC TCTTTTGTTC CCTCTACGTC TAATAATTTT GTCATCGCAT TTAATGTCAG   
  
  
+ ATAGAGAGAT AGAGCTCTAC TGTATTAATT TTCTCCCACC TCACTTGATT CTGTTGCCCT AAACCACCCA   
  
  
+ AAAAAAGAAA TACGAAGAAG CCACTCTCTC TCTCTCTCTC TCTCACACAC ACACACACCC CTCCTAGTGG   
  
  
+ ATTTGATAGA GTATAAATGA ACAGTAATCA GACGAGATAT ATAGTAGTGC TTAATTGACC GAGTAGCAAA   
  
  
+ AGGGGGAAGT TGAGTGAGGT ATCAAGAGAG TAGCGTAGCT AGCTAGGGCA AGATATGCTG GGGTCTTCCT   
  
  
+ CATACAACTC CGAGGAAGAC CACCAAGATG ACAACCATTC CTCACCGTCT GATTTGTCTA GGCACCCACC   
  
  
+ TAGATTCACC ACTACTACTT ACCAATACCA ACAACATATT AGCTCTCCAT CTTCACAATC AATACTAGCT   
  
  
+ GTGCCACCTC GCCAATTGTT AATCAATTGT GCGGAGTTAA TTTGCCGGTT CGACTTCTCC TCCGCCAACC   
  
  
+ GGCTTCTCTC TCTCCTCGCC TCCCACTTTG CCTCCCCTTC TGGGGACTCC ACCGAGAGAC TTGTGTTTTA   
  
  
+ CTTCTGCAAG GCTTTGGATC TCCGCCTCAA TAACAGTTAT TTATCCACTC GTAGTTTGAC TGGTTGTAAC   
  
  
+ CCTAGTACTG GTTCTGGTAT TGGGGCTCCT TTAGCTTCCA ATTTTCTATT TCTTCAACCA TTAGTGCATT   
  
  
+ CACAGGTAAC AAGTAATGCC CGTGTTGGGT ACTTGACATA TTTGACTCTG AACCAGGTCA CCCCATTCAT   
  
  
+ CCGATTCACC CACTTAACTG CCAACCAAGC CATCCTGGAA GCTTTGGAGG GGTACAAGGC GGTCCATATC   
  
  
+ CTTGACATGG ACATCATGCA TGGGGTCCAG TGGCCACCAC TTCTTCAAGC AATTGCAGAA AGATCTGCAA   
  
  
+ CCCTTGGTCA CCCTGCTCCC ACTGTTCGCA TCACAGGAGG GGGCAGGGAT CTAGAACTCC TGAACCGAAC   
  
  
+ TGGAGATCGG ATCAGGAAGT TCGCACAATC CTTGGGACTC GAGTTTCAAT TCCACCATCT TATAACGGTG   
  
  
+ GACTCGAACC TTACTGAGGC AGTGGAGGCC ATCAATGCCC TACAGCTACA CCTCCATGTC CAAGAGGAGG   
  
  
+ AGGTCTTCGC AGTGAATTGT GGAGATTTCC TCCACCGCCT CCTAACGGAG TATGATAGTA CATTCCTTAG   
  
  
+ GATGTTTCTC TACAAGGTGA AGACCCTAAA CCCTAGGGTC TTCACCGTGG GAGAAAGGGA GGCCGACCAC   
  
  
+ AACCACCCTC TCTTCTGGCA GCGATTCATT GAGGCACTTG ACCACTATGG GGCAGTGTTT GACTCTCTAG   
  
  
+ AAGCAACCCT ACCATCAACA AGTCAAGAGA GGGTGGCGGT GGAAGAGGGG TGGTTCGGGG AGGAGATTAA   
  
  
+ AGATGTGGTT GGACGAGAAG GAGGGTTGAG GAGACAGAGG CACCAGAAAT ACGAGTCTTG GGAGGTGTTG   
  
  
+ ATGAGGAGCT CTGGGTTTAA GAGCTTGCCC TTGAGCCCCT TCTCTGTGTC ACAAGCCAAG CTGCTACTTC   
  
  
+ GCCTTCATTA CCCTTCTGAA GGGTACCACC TTAAGGTCTT CCATAGTAAT TGTCTGTTGC TTGGGTGGAA   
  
  
+ GAATCGCCCT CTTTTCTCTG TTTCTTCTTG GCAATA  

- -Up\_Stream \_Len000TTTTAT AAAAATGTTT TCGTAAAGAG ATATTTTATA AAATGTTTTA AAGTAAAACA   
  
  
- CCCAGAAAAT CTTAGTTAGA GAGATGTTCA TCCTTACGGC CAACTCCATT CCGATGGATG TAGATCTAGA   
  
  
- GAGGTCTGGG GTGTTTTTAA TTTGCTCTGG GTGTGACTTT CCACCACCAC CACCACAACA ACAACATAAC   
  
  
- AGGATGGTGC TCTCTCGAAG TTCACGTAGT GAATAAAGTT GTCCAGTTAG TGTATCTAGA GATCACATGA   
  
  
- CATTTAAAAA AAAGGCAGAA TAAAATTAAA AGAAAAGGTA GGATTTTCTA ATAGTACATC TATAAGGATC   
  
  
- AAACTATATA ACTGAAGTTG AAGTTTTTAA TGTGTAATTC TAGGTGCTTA TACTGTCAAA TCCAAAGTAA   
  
  
- ACTTCCTCTT TCTTTACAAT GCCACAAAAC CACAACATAA GCATCTGTAA AAAACGCGAA TTGACCGAGA   
  
  
- AGAGTACCCC TGCTCTGTCA CTTCCATTAA CATGAACAAC TAACTACTGC AGTAGACATC GAACCTAAAC   
  
  
- ATATCCTTAC TTGTAGAACC ACAGATGCAC CTTTCACACT AAACACAAGA AAGTAACTAG GAGGAGGAGT   
  
  
- TCTTCTCCAC TAGAAAGAGG GAACAAAAAA AAAAAAAAAC CGATTATACA ACCGTCTTGG CCGTTTTTTA   
  
  
- TGTATTAAAC TTATCACGTA CGTATACTCA TGTACTGTCA CTTGGTTGTT TTGCTTGGGG ATTTTGTGTT   
  
  
- CTCTTAATTG TACAATTTTG TGAATTTGAT TTACGTTGGT AGAGCTACTG GTGATTTATC GGTTCTTTGG   
  
  
- ATTGCATTAA TTGTTTTTAT TTTTTATTGA CTTTTATTTC ATCTCTTTCA GATTGGCTTA TGCTATTGTA   
  
  
- TTGGATTTTG ATTGATATGA ACTTGAATGA TTCTTTATAC TTAGTTGGTG TTTGGCTTAA CTAAAACCAA   
  
  
- ACGGTATTAA ACTGATATGT AAATTGAATT TTTTATAAAA AAAAATTAAA GTTTATTTAG ATTTAATAAA   
  
  
- AAGTTATAAG TAGGTTTTAT CTAAGTTAAT AATTTGTTCA GTTGGATATG GCTCAATAAA AGATCGAGAC   
  
  
- TGATTCTGTA TATATATAAA AAACATTTTT TCTATTCCTA ATATTAAATT TATCGGAAAG GGAATTTTTT   
  
  
- TAAAAATTTT AATAGGACTA TCTCTTATTT TTCTTTAGGA ATATGTTTTG AGACTAACTG CGTCTACACA   
  
  
- TATACCTGTA TATCTTCATT ATTCTCCCCA TCCGATCCTT CCCCCACCTT TATATCCCGT ATTTATCCCC   
  
  
- CGCCCGTATC CCTGTACAGA CAGACTCCAT GCCACCTACC CTGGAACGGT ACCTTCGTTG TTACAGTGTT   
  
  
- ATTATTATAG TTGCTGACTT CAAAGAAGTG GTCGGGTGAG AAACGGTATT ACGGTAATTA ATAAATAACG   
  
  
- ATGATAGAGA AAACTACACT TTGCCACCTG GAATAGAGAG GGGAGGTGAA GGTTAACTAC ACTCGTTGAG   
  
  
- TCTTTGCGTT TGTTTTGAAC ACGGTGGAAG AAATGAACGT AGGAGTAAAA GGAGACAGAG AGAGGCGGGG   
  
  
- ACAAAGAGAG AGAGAGGGAG TGAGTGAGTC TCATGACTAG ACTCGTAGTC TTCATCGTCT GGGTCTTTGA   
  
  
- GTCTAAATTT TTGTTGGGCG AGAAAACAAG GGAGATGCAG ATTATTAAAA CAGTAGCGTA AATTACAGTC   
  
  
- TATCTCTCTA TCTCGAGATG ACATAATTAA AAGAGGGTGG AGTGAACTAA GACAACGGGA TTTGGTGGGT   
  
  
- TTTTTTCTTT ATGCTTCTTC GGTGAGAGAG AGAGAGAGAG AGAGTGTGTG TGTGTGTGGG GAGGATCACC   
  
  
- TAAACTATCT CATATTTACT TGTCATTAGT CTGCTCTATA TATCATCACG AATTAACTGG CTCATCGTTT   
  
  
- TCCCCCTTCA ACTCACTCCA TAGTTCTCTC ATCGCATCGA TCGATCCCGT TCTATACGAC CCCAGAAGGA   
  
  
- GTATGTTGAG GCTCCTTCTG GTGGTTCTAC TGTTGGTAAG GAGTGGCAGA CTAAACAGAT CCGTGGGTGG   
  
  
- ATCTAAGTGG TGATGATGAA TGGTTATGGT TGTTGTATAA TCGAGAGGTA GAAGTGTTAG TTATGATCGA   
  
  
- CACGGTGGAG CGGTTAACAA TTAGTTAACA CGCCTCAATT AAACGGCCAA GCTGAAGAGG AGGCGGTTGG   
  
  
- CCGAAGAGAG AGAGGAGCGG AGGGTGAAAC GGAGGGGAAG ACCCCTGAGG TGGCTCTCTG AACACAAAAT   
  
  
- GAAGACGTTC CGAAACCTAG AGGCGGAGTT ATTGTCAATA AATAGGTGAG CATCAAACTG ACCAACATTG   
  
  
- GGATCATGAC CAAGACCATA ACCCCGAGGA AATCGAAGGT TAAAAGATAA AGAAGTTGGT AATCACGTAA   
  
  
- GTGTCCATTG TTCATTACGG GCACAACCCA TGAACTGTAT AAACTGAGAC TTGGTCCAGT GGGGTAAGTA   
  
  
- GGCTAAGTGG GTGAATTGAC GGTTGGTTCG GTAGGACCTT CGAAACCTCC CCATGTTCCG CCAGGTATAG   
  
  
- GAACTGTACC TGTAGTACGT ACCCCAGGTC ACCGGTGGTG AAGAAGTTCG TTAACGTCTT TCTAGACGTT   
  
  
- GGGAACCAGT GGGACGAGGG TGACAAGCGT AGTGTCCTCC CCCGTCCCTA GATCTTGAGG ACTTGGCTTG   
  
  
- ACCTCTAGCC TAGTCCTTCA AGCGTGTTAG GAACCCTGAG CTCAAAGTTA AGGTGGTAGA ATATTGCCAC   
  
  
- CTGAGCTTGG AATGACTCCG TCACCTCCGG TAGTTACGGG ATGTCGATGT GGAGGTACAG GTTCTCCTCC   
  
  
- TCCAGAAGCG TCACTTAACA CCTCTAAAGG AGGTGGCGGA GGATTGCCTC ATACTATCAT GTAAGGAATC   
  
  
- CTACAAAGAG ATGTTCCACT TCTGGGATTT GGGATCCCAG AAGTGGCACC CTCTTTCCCT CCGGCTGGTG   
  
  
- TTGGTGGGAG AGAAGACCGT CGCTAAGTAA CTCCGTGAAC TGGTGATACC CCGTCACAAA CTGAGAGATC   
  
  
- TTCGTTGGGA TGGTAGTTGT TCAGTTCTCT CCCACCGCCA CCTTCTCCCC ACCAAGCCCC TCCTCTAATT   
  
  
- TCTACACCAA CCTGCTCTTC CTCCCAACTC CTCTGTCTCC GTGGTCTTTA TGCTCAGAAC CCTCCACAAC   
  
  
- TACTCCTCGA GACCCAAATT CTCGAACGGG AACTCGGGGA AGAGACACAG TGTTCGGTTC GACGATGAAG   
  
  
- CGGAAGTAAT GGGAAGACTT CCCATGGTGG AATTCCAGAA GGTATCATTA ACAGACAACG AACCCACCTT   
  
  
- CTTAGCGGGA GAAAAGAGAC AAAGAAGAAC CGTTAT

+     SARE

| Site Name | Organism | Position | Strand | Matrix score. | sequence | function |
| --- | --- | --- | --- | --- | --- | --- |
| SARE | Nicotiana tabacum | 2784 | + | 11 | TTCGACCATCTT | cis-acting element involved in salicylic acid responsiveness |

>HU01G00833.1   
+ -Up\_Stream \_Len000AAAATA TTTTTACAAA AGCATTTCTC TATAAAATAT TTTACAAAAT TTCATTTTGT   
  
  
+ GGGTCTTTTA GAATCAATCT CTCTACAAGT AGGAATGCCG GTTGAGGTAA GGCTACCTAC ATCTAGATCT   
  
  
+ CTCCAGACCC CACAAAAATT AAACGAGACC CACACTGAAA GGTGGTGGTG GTGGTGTTGT TGTTGTATTG   
  
  
+ TCCTACCACG AGAGAGCTTC AAGTGCATCA CTTATTTCAA CAGGTCAATC ACATAGATCT CTAGTGTACT   
  
  
+ GTAAATTTTT TTTCCGTCTT ATTTTAATTT TCTTTTCCAT CCTAAAAGAT TATCATGTAG ATATTCCTAG   
  
  
+ TTTGATATAT TGACTTCAAC TTCAAAAATT ACACATTAAG ATCCACGAAT ATGACAGTTT AGGTTTCATT   
  
  
+ TGAAGGAGAA AGAAATGTTA CGGTGTTTTG GTGTTGTATT CGTAGACATT TTTTGCGCTT AACTGGCTCT   
  
  
+ TCTCATGGGG ACGAGACAGT GAAGGTAATT GTACTTGTTG ATTGATGACG TCATCTGTAG CTTGGATTTG   
  
  
+ TATAGGAATG AACATCTTGG TGTCTACGTG GAAAGTGTGA TTTGTGTTCT TTCATTGATC CTCCTCCTCA   
  
  
+ AGAAGAGGTG ATCTTTCTCC CTTGTTTTTT TTTTTTTTTG GCTAATATGT TGGCAGAACC GGCAAAAAAT   
  
  
+ ACATAATTTG AATAGTGCAT GCATATGAGT ACATGACAGT GAACCAACAA AACGAACCCC TAAAACACAA   
  
  
+ GAGAATTAAC ATGTTAAAAC ACTTAAACTA AATGCAACCA TCTCGATGAC CACTAAATAG CCAAGAAACC   
  
  
+ TAACGTAATT AACAAAAATA AAAAATAACT GAAAATAAAG TAGAGAAAGT CTAACCGAAT ACGATAACAT   
  
  
+ AACCTAAAAC TAACTATACT TGAACTTACT AAGAAATATG AATCAACCAC AAACCGAATT GATTTTGGTT   
  
  
+ TGCCATAATT TGACTATACA TTTAACTTAA AAAATATTTT TTTTTAATTT CAAATAAATC TAAATTATTT   
  
  
+ TTCAATATTC ATCCAAAATA GATTCAATTA TTAAACAAGT CAACCTATAC CGAGTTATTT TCTAGCTCTG   
  
  
+ ACTAAGACAT ATATATATTT TTTGTAAAAA AGATAAGGAT TATAATTTAA ATAGCCTTTC CCTTAAAAAA   
  
  
+ ATTTTTAAAA TTATCCTGAT AGAGAATAAA AAGAAATCCT TATACAAAAC TCTGATTGAC GCAGATGTGT   
  
  
+ ATATGGACAT ATAGAAGTAA TAAGAGGGGT AGGCTAGGAA GGGGGTGGAA ATATAGGGCA TAAATAGGGG   
  
  
+ GCGGGCATAG GGACATGTCT GTCTGAGGTA CGGTGGATGG GACCTTGCCA TGGAAGCAAC AATGTCACAA   
  
  
+ TAATAATATC AACGACTGAA GTTTCTTCAC CAGCCCACTC TTTGCCATAA TGCCATTAAT TATTTATTGC   
  
  
+ TACTATCTCT TTTGATGTGA AACGGTGGAC CTTATCTCTC CCCTCCACTT CCAATTGATG TGAGCAACTC   
  
  
+ AGAAACGCAA ACAAAACTTG TGCCACCTTC TTTACTTGCA TCCTCATTTT CCTCTGTCTC TCTCCGCCCC   
  
  
+ TGTTTCTCTC TCTCTCCCTC ACTCACTCAG AGTACTGATC TGAGCATCAG AAGTAGCAGA CCCAGAAACT   
  
  
+ CAGATTTAAA AACAACCCGC TCTTTTGTTC CCTCTACGTC TAATAATTTT GTCATCGCAT TTAATGTCAG   
  
  
+ ATAGAGAGAT AGAGCTCTAC TGTATTAATT TTCTCCCACC TCACTTGATT CTGTTGCCCT AAACCACCCA   
  
  
+ AAAAAAGAAA TACGAAGAAG CCACTCTCTC TCTCTCTCTC TCTCACACAC ACACACACCC CTCCTAGTGG   
  
  
+ ATTTGATAGA GTATAAATGA ACAGTAATCA GACGAGATAT ATAGTAGTGC TTAATTGACC GAGTAGCAAA   
  
  
+ AGGGGGAAGT TGAGTGAGGT ATCAAGAGAG TAGCGTAGCT AGCTAGGGCA AGATATGCTG GGGTCTTCCT   
  
  
+ CATACAACTC CGAGGAAGAC CACCAAGATG ACAACCATTC CTCACCGTCT GATTTGTCTA GGCACCCACC   
  
  
+ TAGATTCACC ACTACTACTT ACCAATACCA ACAACATATT AGCTCTCCAT CTTCACAATC AATACTAGCT   
  
  
+ GTGCCACCTC GCCAATTGTT AATCAATTGT GCGGAGTTAA TTTGCCGGTT CGACTTCTCC TCCGCCAACC   
  
  
+ GGCTTCTCTC TCTCCTCGCC TCCCACTTTG CCTCCCCTTC TGGGGACTCC ACCGAGAGAC TTGTGTTTTA   
  
  
+ CTTCTGCAAG GCTTTGGATC TCCGCCTCAA TAACAGTTAT TTATCCACTC GTAGTTTGAC TGGTTGTAAC   
  
  
+ CCTAGTACTG GTTCTGGTAT TGGGGCTCCT TTAGCTTCCA ATTTTCTATT TCTTCAACCA TTAGTGCATT   
  
  
+ CACAGGTAAC AAGTAATGCC CGTGTTGGGT ACTTGACATA TTTGACTCTG AACCAGGTCA CCCCATTCAT   
  
  
+ CCGATTCACC CACTTAACTG CCAACCAAGC CATCCTGGAA GCTTTGGAGG GGTACAAGGC GGTCCATATC   
  
  
+ CTTGACATGG ACATCATGCA TGGGGTCCAG TGGCCACCAC TTCTTCAAGC AATTGCAGAA AGATCTGCAA   
  
  
+ CCCTTGGTCA CCCTGCTCCC ACTGTTCGCA TCACAGGAGG GGGCAGGGAT CTAGAACTCC TGAACCGAAC   
  
  
+ TGGAGATCGG ATCAGGAAGT TCGCACAATC CTTGGGACTC GAGTTTCAAT TCCACCATCT TATAACGGTG   
  
  
+ GACTCGAACC TTACTGAGGC AGTGGAGGCC ATCAATGCCC TACAGCTACA CCTCCATGTC CAAGAGGAGG   
  
  
+ AGGTCTTCGC AGTGAATTGT GGAGATTTCC TCCACCGCCT CCTAACGGAG TATGATAGTA CATTCCTTAG   
  
  
+ GATGTTTCTC TACAAGGTGA AGACCCTAAA CCCTAGGGTC TTCACCGTGG GAGAAAGGGA GGCCGACCAC   
  
  
+ AACCACCCTC TCTTCTGGCA GCGATTCATT GAGGCACTTG ACCACTATGG GGCAGTGTTT GACTCTCTAG   
  
  
+ AAGCAACCCT ACCATCAACA AGTCAAGAGA GGGTGGCGGT GGAAGAGGGG TGGTTCGGGG AGGAGATTAA   
  
  
+ AGATGTGGTT GGACGAGAAG GAGGGTTGAG GAGACAGAGG CACCAGAAAT ACGAGTCTTG GGAGGTGTTG   
  
  
+ ATGAGGAGCT CTGGGTTTAA GAGCTTGCCC TTGAGCCCCT TCTCTGTGTC ACAAGCCAAG CTGCTACTTC   
  
  
+ GCCTTCATTA CCCTTCTGAA GGGTACCACC TTAAGGTCTT CCATAGTAAT TGTCTGTTGC TTGGGTGGAA   
  
  
+ GAATCGCCCT CTTTTCTCTG TTTCTTCTTG GCAATA  

- -Up\_Stream \_Len000TTTTAT AAAAATGTTT TCGTAAAGAG ATATTTTATA AAATGTTTTA AAGTAAAACA   
  
  
- CCCAGAAAAT CTTAGTTAGA GAGATGTTCA TCCTTACGGC CAACTCCATT CCGATGGATG TAGATCTAGA   
  
  
- GAGGTCTGGG GTGTTTTTAA TTTGCTCTGG GTGTGACTTT CCACCACCAC CACCACAACA ACAACATAAC   
  
  
- AGGATGGTGC TCTCTCGAAG TTCACGTAGT GAATAAAGTT GTCCAGTTAG TGTATCTAGA GATCACATGA   
  
  
- CATTTAAAAA AAAGGCAGAA TAAAATTAAA AGAAAAGGTA GGATTTTCTA ATAGTACATC TATAAGGATC   
  
  
- AAACTATATA ACTGAAGTTG AAGTTTTTAA TGTGTAATTC TAGGTGCTTA TACTGTCAAA TCCAAAGTAA   
  
  
- ACTTCCTCTT TCTTTACAAT GCCACAAAAC CACAACATAA GCATCTGTAA AAAACGCGAA TTGACCGAGA   
  
  
- AGAGTACCCC TGCTCTGTCA CTTCCATTAA CATGAACAAC TAACTACTGC AGTAGACATC GAACCTAAAC   
  
  
- ATATCCTTAC TTGTAGAACC ACAGATGCAC CTTTCACACT AAACACAAGA AAGTAACTAG GAGGAGGAGT   
  
  
- TCTTCTCCAC TAGAAAGAGG GAACAAAAAA AAAAAAAAAC CGATTATACA ACCGTCTTGG CCGTTTTTTA   
  
  
- TGTATTAAAC TTATCACGTA CGTATACTCA TGTACTGTCA CTTGGTTGTT TTGCTTGGGG ATTTTGTGTT   
  
  
- CTCTTAATTG TACAATTTTG TGAATTTGAT TTACGTTGGT AGAGCTACTG GTGATTTATC GGTTCTTTGG   
  
  
- ATTGCATTAA TTGTTTTTAT TTTTTATTGA CTTTTATTTC ATCTCTTTCA GATTGGCTTA TGCTATTGTA   
  
  
- TTGGATTTTG ATTGATATGA ACTTGAATGA TTCTTTATAC TTAGTTGGTG TTTGGCTTAA CTAAAACCAA   
  
  
- ACGGTATTAA ACTGATATGT AAATTGAATT TTTTATAAAA AAAAATTAAA GTTTATTTAG ATTTAATAAA   
  
  
- AAGTTATAAG TAGGTTTTAT CTAAGTTAAT AATTTGTTCA GTTGGATATG GCTCAATAAA AGATCGAGAC   
  
  
- TGATTCTGTA TATATATAAA AAACATTTTT TCTATTCCTA ATATTAAATT TATCGGAAAG GGAATTTTTT   
  
  
- TAAAAATTTT AATAGGACTA TCTCTTATTT TTCTTTAGGA ATATGTTTTG AGACTAACTG CGTCTACACA   
  
  
- TATACCTGTA TATCTTCATT ATTCTCCCCA TCCGATCCTT CCCCCACCTT TATATCCCGT ATTTATCCCC   
  
  
- CGCCCGTATC CCTGTACAGA CAGACTCCAT GCCACCTACC CTGGAACGGT ACCTTCGTTG TTACAGTGTT   
  
  
- ATTATTATAG TTGCTGACTT CAAAGAAGTG GTCGGGTGAG AAACGGTATT ACGGTAATTA ATAAATAACG   
  
  
- ATGATAGAGA AAACTACACT TTGCCACCTG GAATAGAGAG GGGAGGTGAA GGTTAACTAC ACTCGTTGAG   
  
  
- TCTTTGCGTT TGTTTTGAAC ACGGTGGAAG AAATGAACGT AGGAGTAAAA GGAGACAGAG AGAGGCGGGG   
  
  
- ACAAAGAGAG AGAGAGGGAG TGAGTGAGTC TCATGACTAG ACTCGTAGTC TTCATCGTCT GGGTCTTTGA   
  
  
- GTCTAAATTT TTGTTGGGCG AGAAAACAAG GGAGATGCAG ATTATTAAAA CAGTAGCGTA AATTACAGTC   
  
  
- TATCTCTCTA TCTCGAGATG ACATAATTAA AAGAGGGTGG AGTGAACTAA GACAACGGGA TTTGGTGGGT   
  
  
- TTTTTTCTTT ATGCTTCTTC GGTGAGAGAG AGAGAGAGAG AGAGTGTGTG TGTGTGTGGG GAGGATCACC   
  
  
- TAAACTATCT CATATTTACT TGTCATTAGT CTGCTCTATA TATCATCACG AATTAACTGG CTCATCGTTT   
  
  
- TCCCCCTTCA ACTCACTCCA TAGTTCTCTC ATCGCATCGA TCGATCCCGT TCTATACGAC CCCAGAAGGA   
  
  
- GTATGTTGAG GCTCCTTCTG GTGGTTCTAC TGTTGGTAAG GAGTGGCAGA CTAAACAGAT CCGTGGGTGG   
  
  
- ATCTAAGTGG TGATGATGAA TGGTTATGGT TGTTGTATAA TCGAGAGGTA GAAGTGTTAG TTATGATCGA   
  
  
- CACGGTGGAG CGGTTAACAA TTAGTTAACA CGCCTCAATT AAACGGCCAA GCTGAAGAGG AGGCGGTTGG   
  
  
- CCGAAGAGAG AGAGGAGCGG AGGGTGAAAC GGAGGGGAAG ACCCCTGAGG TGGCTCTCTG AACACAAAAT   
  
  
- GAAGACGTTC CGAAACCTAG AGGCGGAGTT ATTGTCAATA AATAGGTGAG CATCAAACTG ACCAACATTG   
  
  
- GGATCATGAC CAAGACCATA ACCCCGAGGA AATCGAAGGT TAAAAGATAA AGAAGTTGGT AATCACGTAA   
  
  
- GTGTCCATTG TTCATTACGG GCACAACCCA TGAACTGTAT AAACTGAGAC TTGGTCCAGT GGGGTAAGTA   
  
  
- GGCTAAGTGG GTGAATTGAC GGTTGGTTCG GTAGGACCTT CGAAACCTCC CCATGTTCCG CCAGGTATAG   
  
  
- GAACTGTACC TGTAGTACGT ACCCCAGGTC ACCGGTGGTG AAGAAGTTCG TTAACGTCTT TCTAGACGTT   
  
  
- GGGAACCAGT GGGACGAGGG TGACAAGCGT AGTGTCCTCC CCCGTCCCTA GATCTTGAGG ACTTGGCTTG   
  
  
- ACCTCTAGCC TAGTCCTTCA AGCGTGTTAG GAACCCTGAG CTCAAAGTTA AGGTGGTAGA ATATTGCCAC   
  
  
- CTGAGCTTGG AATGACTCCG TCACCTCCGG TAGTTACGGG ATGTCGATGT GGAGGTACAG GTTCTCCTCC   
  
  
- TCCAGAAGCG TCACTTAACA CCTCTAAAGG AGGTGGCGGA GGATTGCCTC ATACTATCAT GTAAGGAATC   
  
  
- CTACAAAGAG ATGTTCCACT TCTGGGATTT GGGATCCCAG AAGTGGCACC CTCTTTCCCT CCGGCTGGTG   
  
  
- TTGGTGGGAG AGAAGACCGT CGCTAAGTAA CTCCGTGAAC TGGTGATACC CCGTCACAAA CTGAGAGATC   
  
  
- TTCGTTGGGA TGGTAGTTGT TCAGTTCTCT CCCACCGCCA CCTTCTCCCC ACCAAGCCCC TCCTCTAATT   
  
  
- TCTACACCAA CCTGCTCTTC CTCCCAACTC CTCTGTCTCC GTGGTCTTTA TGCTCAGAAC CCTCCACAAC   
  
  
- TACTCCTCGA GACCCAAATT CTCGAACGGG AACTCGGGGA AGAGACACAG TGTTCGGTTC GACGATGAAG   
  
  
- CGGAAGTAAT GGGAAGACTT CCCATGGTGG AATTCCAGAA GGTATCATTA ACAGACAACG AACCCACCTT   
  
  
- CTTAGCGGGA GAAAAGAGAC AAAGAAGAAC CGTTAT

+     STRE

| Site Name | Organism | Position | Strand | Matrix score. | sequence | function |
| --- | --- | --- | --- | --- | --- | --- |
| STRE | Arabidopsis thaliana | 3260 | - | 5 | AGGGG |  |
| STRE | Arabidopsis thaliana | 3130 | + | 5 | AGGGG |  |
| STRE | Arabidopsis thaliana | 2702 | + | 5 | AGGGG |  |
| STRE | Arabidopsis thaliana | 2572 | + | 5 | AGGGG |  |
| STRE | Arabidopsis thaliana | 2278 | - | 5 | AGGGG |  |
| STRE | Arabidopsis thaliana | 1330 | + | 5 | AGGGG |  |
| STRE | Arabidopsis thaliana | 1304 | + | 5 | AGGGG |  |
| STRE | Arabidopsis thaliana | 761 | - | 5 | AGGGG |  |
| STRE | Arabidopsis thaliana | 1289 | + | 5 | AGGGG |  |
| STRE | Arabidopsis thaliana | 1965 | + | 5 | AGGGG |  |
| STRE | Arabidopsis thaliana | 1882 | - | 5 | AGGGG |  |
| STRE | Arabidopsis thaliana | 1611 | - | 5 | AGGGG |  |
| STRE | Arabidopsis thaliana | 1514 | - | 5 | AGGGG |  |

>HU01G00833.1   
+ -Up\_Stream \_Len000AAAATA TTTTTACAAA AGCATTTCTC TATAAAATAT TTTACAAAAT TTCATTTTGT   
  
  
+ GGGTCTTTTA GAATCAATCT CTCTACAAGT AGGAATGCCG GTTGAGGTAA GGCTACCTAC ATCTAGATCT   
  
  
+ CTCCAGACCC CACAAAAATT AAACGAGACC CACACTGAAA GGTGGTGGTG GTGGTGTTGT TGTTGTATTG   
  
  
+ TCCTACCACG AGAGAGCTTC AAGTGCATCA CTTATTTCAA CAGGTCAATC ACATAGATCT CTAGTGTACT   
  
  
+ GTAAATTTTT TTTCCGTCTT ATTTTAATTT TCTTTTCCAT CCTAAAAGAT TATCATGTAG ATATTCCTAG   
  
  
+ TTTGATATAT TGACTTCAAC TTCAAAAATT ACACATTAAG ATCCACGAAT ATGACAGTTT AGGTTTCATT   
  
  
+ TGAAGGAGAA AGAAATGTTA CGGTGTTTTG GTGTTGTATT CGTAGACATT TTTTGCGCTT AACTGGCTCT   
  
  
+ TCTCATGGGG ACGAGACAGT GAAGGTAATT GTACTTGTTG ATTGATGACG TCATCTGTAG CTTGGATTTG   
  
  
+ TATAGGAATG AACATCTTGG TGTCTACGTG GAAAGTGTGA TTTGTGTTCT TTCATTGATC CTCCTCCTCA   
  
  
+ AGAAGAGGTG ATCTTTCTCC CTTGTTTTTT TTTTTTTTTG GCTAATATGT TGGCAGAACC GGCAAAAAAT   
  
  
+ ACATAATTTG AATAGTGCAT GCATATGAGT ACATGACAGT GAACCAACAA AACGAACCCC TAAAACACAA   
  
  
+ GAGAATTAAC ATGTTAAAAC ACTTAAACTA AATGCAACCA TCTCGATGAC CACTAAATAG CCAAGAAACC   
  
  
+ TAACGTAATT AACAAAAATA AAAAATAACT GAAAATAAAG TAGAGAAAGT CTAACCGAAT ACGATAACAT   
  
  
+ AACCTAAAAC TAACTATACT TGAACTTACT AAGAAATATG AATCAACCAC AAACCGAATT GATTTTGGTT   
  
  
+ TGCCATAATT TGACTATACA TTTAACTTAA AAAATATTTT TTTTTAATTT CAAATAAATC TAAATTATTT   
  
  
+ TTCAATATTC ATCCAAAATA GATTCAATTA TTAAACAAGT CAACCTATAC CGAGTTATTT TCTAGCTCTG   
  
  
+ ACTAAGACAT ATATATATTT TTTGTAAAAA AGATAAGGAT TATAATTTAA ATAGCCTTTC CCTTAAAAAA   
  
  
+ ATTTTTAAAA TTATCCTGAT AGAGAATAAA AAGAAATCCT TATACAAAAC TCTGATTGAC GCAGATGTGT   
  
  
+ ATATGGACAT ATAGAAGTAA TAAGAGGGGT AGGCTAGGAA GGGGGTGGAA ATATAGGGCA TAAATAGGGG   
  
  
+ GCGGGCATAG GGACATGTCT GTCTGAGGTA CGGTGGATGG GACCTTGCCA TGGAAGCAAC AATGTCACAA   
  
  
+ TAATAATATC AACGACTGAA GTTTCTTCAC CAGCCCACTC TTTGCCATAA TGCCATTAAT TATTTATTGC   
  
  
+ TACTATCTCT TTTGATGTGA AACGGTGGAC CTTATCTCTC CCCTCCACTT CCAATTGATG TGAGCAACTC   
  
  
+ AGAAACGCAA ACAAAACTTG TGCCACCTTC TTTACTTGCA TCCTCATTTT CCTCTGTCTC TCTCCGCCCC   
  
  
+ TGTTTCTCTC TCTCTCCCTC ACTCACTCAG AGTACTGATC TGAGCATCAG AAGTAGCAGA CCCAGAAACT   
  
  
+ CAGATTTAAA AACAACCCGC TCTTTTGTTC CCTCTACGTC TAATAATTTT GTCATCGCAT TTAATGTCAG   
  
  
+ ATAGAGAGAT AGAGCTCTAC TGTATTAATT TTCTCCCACC TCACTTGATT CTGTTGCCCT AAACCACCCA   
  
  
+ AAAAAAGAAA TACGAAGAAG CCACTCTCTC TCTCTCTCTC TCTCACACAC ACACACACCC CTCCTAGTGG   
  
  
+ ATTTGATAGA GTATAAATGA ACAGTAATCA GACGAGATAT ATAGTAGTGC TTAATTGACC GAGTAGCAAA   
  
  
+ AGGGGGAAGT TGAGTGAGGT ATCAAGAGAG TAGCGTAGCT AGCTAGGGCA AGATATGCTG GGGTCTTCCT   
  
  
+ CATACAACTC CGAGGAAGAC CACCAAGATG ACAACCATTC CTCACCGTCT GATTTGTCTA GGCACCCACC   
  
  
+ TAGATTCACC ACTACTACTT ACCAATACCA ACAACATATT AGCTCTCCAT CTTCACAATC AATACTAGCT   
  
  
+ GTGCCACCTC GCCAATTGTT AATCAATTGT GCGGAGTTAA TTTGCCGGTT CGACTTCTCC TCCGCCAACC   
  
  
+ GGCTTCTCTC TCTCCTCGCC TCCCACTTTG CCTCCCCTTC TGGGGACTCC ACCGAGAGAC TTGTGTTTTA   
  
  
+ CTTCTGCAAG GCTTTGGATC TCCGCCTCAA TAACAGTTAT TTATCCACTC GTAGTTTGAC TGGTTGTAAC   
  
  
+ CCTAGTACTG GTTCTGGTAT TGGGGCTCCT TTAGCTTCCA ATTTTCTATT TCTTCAACCA TTAGTGCATT   
  
  
+ CACAGGTAAC AAGTAATGCC CGTGTTGGGT ACTTGACATA TTTGACTCTG AACCAGGTCA CCCCATTCAT   
  
  
+ CCGATTCACC CACTTAACTG CCAACCAAGC CATCCTGGAA GCTTTGGAGG GGTACAAGGC GGTCCATATC   
  
  
+ CTTGACATGG ACATCATGCA TGGGGTCCAG TGGCCACCAC TTCTTCAAGC AATTGCAGAA AGATCTGCAA   
  
  
+ CCCTTGGTCA CCCTGCTCCC ACTGTTCGCA TCACAGGAGG GGGCAGGGAT CTAGAACTCC TGAACCGAAC   
  
  
+ TGGAGATCGG ATCAGGAAGT TCGCACAATC CTTGGGACTC GAGTTTCAAT TCCACCATCT TATAACGGTG   
  
  
+ GACTCGAACC TTACTGAGGC AGTGGAGGCC ATCAATGCCC TACAGCTACA CCTCCATGTC CAAGAGGAGG   
  
  
+ AGGTCTTCGC AGTGAATTGT GGAGATTTCC TCCACCGCCT CCTAACGGAG TATGATAGTA CATTCCTTAG   
  
  
+ GATGTTTCTC TACAAGGTGA AGACCCTAAA CCCTAGGGTC TTCACCGTGG GAGAAAGGGA GGCCGACCAC   
  
  
+ AACCACCCTC TCTTCTGGCA GCGATTCATT GAGGCACTTG ACCACTATGG GGCAGTGTTT GACTCTCTAG   
  
  
+ AAGCAACCCT ACCATCAACA AGTCAAGAGA GGGTGGCGGT GGAAGAGGGG TGGTTCGGGG AGGAGATTAA   
  
  
+ AGATGTGGTT GGACGAGAAG GAGGGTTGAG GAGACAGAGG CACCAGAAAT ACGAGTCTTG GGAGGTGTTG   
  
  
+ ATGAGGAGCT CTGGGTTTAA GAGCTTGCCC TTGAGCCCCT TCTCTGTGTC ACAAGCCAAG CTGCTACTTC   
  
  
+ GCCTTCATTA CCCTTCTGAA GGGTACCACC TTAAGGTCTT CCATAGTAAT TGTCTGTTGC TTGGGTGGAA   
  
  
+ GAATCGCCCT CTTTTCTCTG TTTCTTCTTG GCAATA  

- -Up\_Stream \_Len000TTTTAT AAAAATGTTT TCGTAAAGAG ATATTTTATA AAATGTTTTA AAGTAAAACA   
  
  
- CCCAGAAAAT CTTAGTTAGA GAGATGTTCA TCCTTACGGC CAACTCCATT CCGATGGATG TAGATCTAGA   
  
  
- GAGGTCTGGG GTGTTTTTAA TTTGCTCTGG GTGTGACTTT CCACCACCAC CACCACAACA ACAACATAAC   
  
  
- AGGATGGTGC TCTCTCGAAG TTCACGTAGT GAATAAAGTT GTCCAGTTAG TGTATCTAGA GATCACATGA   
  
  
- CATTTAAAAA AAAGGCAGAA TAAAATTAAA AGAAAAGGTA GGATTTTCTA ATAGTACATC TATAAGGATC   
  
  
- AAACTATATA ACTGAAGTTG AAGTTTTTAA TGTGTAATTC TAGGTGCTTA TACTGTCAAA TCCAAAGTAA   
  
  
- ACTTCCTCTT TCTTTACAAT GCCACAAAAC CACAACATAA GCATCTGTAA AAAACGCGAA TTGACCGAGA   
  
  
- AGAGTACCCC TGCTCTGTCA CTTCCATTAA CATGAACAAC TAACTACTGC AGTAGACATC GAACCTAAAC   
  
  
- ATATCCTTAC TTGTAGAACC ACAGATGCAC CTTTCACACT AAACACAAGA AAGTAACTAG GAGGAGGAGT   
  
  
- TCTTCTCCAC TAGAAAGAGG GAACAAAAAA AAAAAAAAAC CGATTATACA ACCGTCTTGG CCGTTTTTTA   
  
  
- TGTATTAAAC TTATCACGTA CGTATACTCA TGTACTGTCA CTTGGTTGTT TTGCTTGGGG ATTTTGTGTT   
  
  
- CTCTTAATTG TACAATTTTG TGAATTTGAT TTACGTTGGT AGAGCTACTG GTGATTTATC GGTTCTTTGG   
  
  
- ATTGCATTAA TTGTTTTTAT TTTTTATTGA CTTTTATTTC ATCTCTTTCA GATTGGCTTA TGCTATTGTA   
  
  
- TTGGATTTTG ATTGATATGA ACTTGAATGA TTCTTTATAC TTAGTTGGTG TTTGGCTTAA CTAAAACCAA   
  
  
- ACGGTATTAA ACTGATATGT AAATTGAATT TTTTATAAAA AAAAATTAAA GTTTATTTAG ATTTAATAAA   
  
  
- AAGTTATAAG TAGGTTTTAT CTAAGTTAAT AATTTGTTCA GTTGGATATG GCTCAATAAA AGATCGAGAC   
  
  
- TGATTCTGTA TATATATAAA AAACATTTTT TCTATTCCTA ATATTAAATT TATCGGAAAG GGAATTTTTT   
  
  
- TAAAAATTTT AATAGGACTA TCTCTTATTT TTCTTTAGGA ATATGTTTTG AGACTAACTG CGTCTACACA   
  
  
- TATACCTGTA TATCTTCATT ATTCTCCCCA TCCGATCCTT CCCCCACCTT TATATCCCGT ATTTATCCCC   
  
  
- CGCCCGTATC CCTGTACAGA CAGACTCCAT GCCACCTACC CTGGAACGGT ACCTTCGTTG TTACAGTGTT   
  
  
- ATTATTATAG TTGCTGACTT CAAAGAAGTG GTCGGGTGAG AAACGGTATT ACGGTAATTA ATAAATAACG   
  
  
- ATGATAGAGA AAACTACACT TTGCCACCTG GAATAGAGAG GGGAGGTGAA GGTTAACTAC ACTCGTTGAG   
  
  
- TCTTTGCGTT TGTTTTGAAC ACGGTGGAAG AAATGAACGT AGGAGTAAAA GGAGACAGAG AGAGGCGGGG   
  
  
- ACAAAGAGAG AGAGAGGGAG TGAGTGAGTC TCATGACTAG ACTCGTAGTC TTCATCGTCT GGGTCTTTGA   
  
  
- GTCTAAATTT TTGTTGGGCG AGAAAACAAG GGAGATGCAG ATTATTAAAA CAGTAGCGTA AATTACAGTC   
  
  
- TATCTCTCTA TCTCGAGATG ACATAATTAA AAGAGGGTGG AGTGAACTAA GACAACGGGA TTTGGTGGGT   
  
  
- TTTTTTCTTT ATGCTTCTTC GGTGAGAGAG AGAGAGAGAG AGAGTGTGTG TGTGTGTGGG GAGGATCACC   
  
  
- TAAACTATCT CATATTTACT TGTCATTAGT CTGCTCTATA TATCATCACG AATTAACTGG CTCATCGTTT   
  
  
- TCCCCCTTCA ACTCACTCCA TAGTTCTCTC ATCGCATCGA TCGATCCCGT TCTATACGAC CCCAGAAGGA   
  
  
- GTATGTTGAG GCTCCTTCTG GTGGTTCTAC TGTTGGTAAG GAGTGGCAGA CTAAACAGAT CCGTGGGTGG   
  
  
- ATCTAAGTGG TGATGATGAA TGGTTATGGT TGTTGTATAA TCGAGAGGTA GAAGTGTTAG TTATGATCGA   
  
  
- CACGGTGGAG CGGTTAACAA TTAGTTAACA CGCCTCAATT AAACGGCCAA GCTGAAGAGG AGGCGGTTGG   
  
  
- CCGAAGAGAG AGAGGAGCGG AGGGTGAAAC GGAGGGGAAG ACCCCTGAGG TGGCTCTCTG AACACAAAAT   
  
  
- GAAGACGTTC CGAAACCTAG AGGCGGAGTT ATTGTCAATA AATAGGTGAG CATCAAACTG ACCAACATTG   
  
  
- GGATCATGAC CAAGACCATA ACCCCGAGGA AATCGAAGGT TAAAAGATAA AGAAGTTGGT AATCACGTAA   
  
  
- GTGTCCATTG TTCATTACGG GCACAACCCA TGAACTGTAT AAACTGAGAC TTGGTCCAGT GGGGTAAGTA   
  
  
- GGCTAAGTGG GTGAATTGAC GGTTGGTTCG GTAGGACCTT CGAAACCTCC CCATGTTCCG CCAGGTATAG   
  
  
- GAACTGTACC TGTAGTACGT ACCCCAGGTC ACCGGTGGTG AAGAAGTTCG TTAACGTCTT TCTAGACGTT   
  
  
- GGGAACCAGT GGGACGAGGG TGACAAGCGT AGTGTCCTCC CCCGTCCCTA GATCTTGAGG ACTTGGCTTG   
  
  
- ACCTCTAGCC TAGTCCTTCA AGCGTGTTAG GAACCCTGAG CTCAAAGTTA AGGTGGTAGA ATATTGCCAC   
  
  
- CTGAGCTTGG AATGACTCCG TCACCTCCGG TAGTTACGGG ATGTCGATGT GGAGGTACAG GTTCTCCTCC   
  
  
- TCCAGAAGCG TCACTTAACA CCTCTAAAGG AGGTGGCGGA GGATTGCCTC ATACTATCAT GTAAGGAATC   
  
  
- CTACAAAGAG ATGTTCCACT TCTGGGATTT GGGATCCCAG AAGTGGCACC CTCTTTCCCT CCGGCTGGTG   
  
  
- TTGGTGGGAG AGAAGACCGT CGCTAAGTAA CTCCGTGAAC TGGTGATACC CCGTCACAAA CTGAGAGATC   
  
  
- TTCGTTGGGA TGGTAGTTGT TCAGTTCTCT CCCACCGCCA CCTTCTCCCC ACCAAGCCCC TCCTCTAATT   
  
  
- TCTACACCAA CCTGCTCTTC CTCCCAACTC CTCTGTCTCC GTGGTCTTTA TGCTCAGAAC CCTCCACAAC   
  
  
- TACTCCTCGA GACCCAAATT CTCGAACGGG AACTCGGGGA AGAGACACAG TGTTCGGTTC GACGATGAAG   
  
  
- CGGAAGTAAT GGGAAGACTT CCCATGGTGG AATTCCAGAA GGTATCATTA ACAGACAACG AACCCACCTT   
  
  
- CTTAGCGGGA GAAAAGAGAC AAAGAAGAAC CGTTAT

+     Sp1

| Site Name | Organism | Position | Strand | Matrix score. | sequence | function |
| --- | --- | --- | --- | --- | --- | --- |
| Sp1 | Oryza sativa | 1608 | - | 6 | GGGCGG | light responsive element |
| Sp1 | Oryza sativa | 1333 | + | 6 | GGGCGG | light responsive element |

>HU01G00833.1   
+ -Up\_Stream \_Len000AAAATA TTTTTACAAA AGCATTTCTC TATAAAATAT TTTACAAAAT TTCATTTTGT   
  
  
+ GGGTCTTTTA GAATCAATCT CTCTACAAGT AGGAATGCCG GTTGAGGTAA GGCTACCTAC ATCTAGATCT   
  
  
+ CTCCAGACCC CACAAAAATT AAACGAGACC CACACTGAAA GGTGGTGGTG GTGGTGTTGT TGTTGTATTG   
  
  
+ TCCTACCACG AGAGAGCTTC AAGTGCATCA CTTATTTCAA CAGGTCAATC ACATAGATCT CTAGTGTACT   
  
  
+ GTAAATTTTT TTTCCGTCTT ATTTTAATTT TCTTTTCCAT CCTAAAAGAT TATCATGTAG ATATTCCTAG   
  
  
+ TTTGATATAT TGACTTCAAC TTCAAAAATT ACACATTAAG ATCCACGAAT ATGACAGTTT AGGTTTCATT   
  
  
+ TGAAGGAGAA AGAAATGTTA CGGTGTTTTG GTGTTGTATT CGTAGACATT TTTTGCGCTT AACTGGCTCT   
  
  
+ TCTCATGGGG ACGAGACAGT GAAGGTAATT GTACTTGTTG ATTGATGACG TCATCTGTAG CTTGGATTTG   
  
  
+ TATAGGAATG AACATCTTGG TGTCTACGTG GAAAGTGTGA TTTGTGTTCT TTCATTGATC CTCCTCCTCA   
  
  
+ AGAAGAGGTG ATCTTTCTCC CTTGTTTTTT TTTTTTTTTG GCTAATATGT TGGCAGAACC GGCAAAAAAT   
  
  
+ ACATAATTTG AATAGTGCAT GCATATGAGT ACATGACAGT GAACCAACAA AACGAACCCC TAAAACACAA   
  
  
+ GAGAATTAAC ATGTTAAAAC ACTTAAACTA AATGCAACCA TCTCGATGAC CACTAAATAG CCAAGAAACC   
  
  
+ TAACGTAATT AACAAAAATA AAAAATAACT GAAAATAAAG TAGAGAAAGT CTAACCGAAT ACGATAACAT   
  
  
+ AACCTAAAAC TAACTATACT TGAACTTACT AAGAAATATG AATCAACCAC AAACCGAATT GATTTTGGTT   
  
  
+ TGCCATAATT TGACTATACA TTTAACTTAA AAAATATTTT TTTTTAATTT CAAATAAATC TAAATTATTT   
  
  
+ TTCAATATTC ATCCAAAATA GATTCAATTA TTAAACAAGT CAACCTATAC CGAGTTATTT TCTAGCTCTG   
  
  
+ ACTAAGACAT ATATATATTT TTTGTAAAAA AGATAAGGAT TATAATTTAA ATAGCCTTTC CCTTAAAAAA   
  
  
+ ATTTTTAAAA TTATCCTGAT AGAGAATAAA AAGAAATCCT TATACAAAAC TCTGATTGAC GCAGATGTGT   
  
  
+ ATATGGACAT ATAGAAGTAA TAAGAGGGGT AGGCTAGGAA GGGGGTGGAA ATATAGGGCA TAAATAGGGG   
  
  
+ GCGGGCATAG GGACATGTCT GTCTGAGGTA CGGTGGATGG GACCTTGCCA TGGAAGCAAC AATGTCACAA   
  
  
+ TAATAATATC AACGACTGAA GTTTCTTCAC CAGCCCACTC TTTGCCATAA TGCCATTAAT TATTTATTGC   
  
  
+ TACTATCTCT TTTGATGTGA AACGGTGGAC CTTATCTCTC CCCTCCACTT CCAATTGATG TGAGCAACTC   
  
  
+ AGAAACGCAA ACAAAACTTG TGCCACCTTC TTTACTTGCA TCCTCATTTT CCTCTGTCTC TCTCCGCCCC   
  
  
+ TGTTTCTCTC TCTCTCCCTC ACTCACTCAG AGTACTGATC TGAGCATCAG AAGTAGCAGA CCCAGAAACT   
  
  
+ CAGATTTAAA AACAACCCGC TCTTTTGTTC CCTCTACGTC TAATAATTTT GTCATCGCAT TTAATGTCAG   
  
  
+ ATAGAGAGAT AGAGCTCTAC TGTATTAATT TTCTCCCACC TCACTTGATT CTGTTGCCCT AAACCACCCA   
  
  
+ AAAAAAGAAA TACGAAGAAG CCACTCTCTC TCTCTCTCTC TCTCACACAC ACACACACCC CTCCTAGTGG   
  
  
+ ATTTGATAGA GTATAAATGA ACAGTAATCA GACGAGATAT ATAGTAGTGC TTAATTGACC GAGTAGCAAA   
  
  
+ AGGGGGAAGT TGAGTGAGGT ATCAAGAGAG TAGCGTAGCT AGCTAGGGCA AGATATGCTG GGGTCTTCCT   
  
  
+ CATACAACTC CGAGGAAGAC CACCAAGATG ACAACCATTC CTCACCGTCT GATTTGTCTA GGCACCCACC   
  
  
+ TAGATTCACC ACTACTACTT ACCAATACCA ACAACATATT AGCTCTCCAT CTTCACAATC AATACTAGCT   
  
  
+ GTGCCACCTC GCCAATTGTT AATCAATTGT GCGGAGTTAA TTTGCCGGTT CGACTTCTCC TCCGCCAACC   
  
  
+ GGCTTCTCTC TCTCCTCGCC TCCCACTTTG CCTCCCCTTC TGGGGACTCC ACCGAGAGAC TTGTGTTTTA   
  
  
+ CTTCTGCAAG GCTTTGGATC TCCGCCTCAA TAACAGTTAT TTATCCACTC GTAGTTTGAC TGGTTGTAAC   
  
  
+ CCTAGTACTG GTTCTGGTAT TGGGGCTCCT TTAGCTTCCA ATTTTCTATT TCTTCAACCA TTAGTGCATT   
  
  
+ CACAGGTAAC AAGTAATGCC CGTGTTGGGT ACTTGACATA TTTGACTCTG AACCAGGTCA CCCCATTCAT   
  
  
+ CCGATTCACC CACTTAACTG CCAACCAAGC CATCCTGGAA GCTTTGGAGG GGTACAAGGC GGTCCATATC   
  
  
+ CTTGACATGG ACATCATGCA TGGGGTCCAG TGGCCACCAC TTCTTCAAGC AATTGCAGAA AGATCTGCAA   
  
  
+ CCCTTGGTCA CCCTGCTCCC ACTGTTCGCA TCACAGGAGG GGGCAGGGAT CTAGAACTCC TGAACCGAAC   
  
  
+ TGGAGATCGG ATCAGGAAGT TCGCACAATC CTTGGGACTC GAGTTTCAAT TCCACCATCT TATAACGGTG   
  
  
+ GACTCGAACC TTACTGAGGC AGTGGAGGCC ATCAATGCCC TACAGCTACA CCTCCATGTC CAAGAGGAGG   
  
  
+ AGGTCTTCGC AGTGAATTGT GGAGATTTCC TCCACCGCCT CCTAACGGAG TATGATAGTA CATTCCTTAG   
  
  
+ GATGTTTCTC TACAAGGTGA AGACCCTAAA CCCTAGGGTC TTCACCGTGG GAGAAAGGGA GGCCGACCAC   
  
  
+ AACCACCCTC TCTTCTGGCA GCGATTCATT GAGGCACTTG ACCACTATGG GGCAGTGTTT GACTCTCTAG   
  
  
+ AAGCAACCCT ACCATCAACA AGTCAAGAGA GGGTGGCGGT GGAAGAGGGG TGGTTCGGGG AGGAGATTAA   
  
  
+ AGATGTGGTT GGACGAGAAG GAGGGTTGAG GAGACAGAGG CACCAGAAAT ACGAGTCTTG GGAGGTGTTG   
  
  
+ ATGAGGAGCT CTGGGTTTAA GAGCTTGCCC TTGAGCCCCT TCTCTGTGTC ACAAGCCAAG CTGCTACTTC   
  
  
+ GCCTTCATTA CCCTTCTGAA GGGTACCACC TTAAGGTCTT CCATAGTAAT TGTCTGTTGC TTGGGTGGAA   
  
  
+ GAATCGCCCT CTTTTCTCTG TTTCTTCTTG GCAATA  

- -Up\_Stream \_Len000TTTTAT AAAAATGTTT TCGTAAAGAG ATATTTTATA AAATGTTTTA AAGTAAAACA   
  
  
- CCCAGAAAAT CTTAGTTAGA GAGATGTTCA TCCTTACGGC CAACTCCATT CCGATGGATG TAGATCTAGA   
  
  
- GAGGTCTGGG GTGTTTTTAA TTTGCTCTGG GTGTGACTTT CCACCACCAC CACCACAACA ACAACATAAC   
  
  
- AGGATGGTGC TCTCTCGAAG TTCACGTAGT GAATAAAGTT GTCCAGTTAG TGTATCTAGA GATCACATGA   
  
  
- CATTTAAAAA AAAGGCAGAA TAAAATTAAA AGAAAAGGTA GGATTTTCTA ATAGTACATC TATAAGGATC   
  
  
- AAACTATATA ACTGAAGTTG AAGTTTTTAA TGTGTAATTC TAGGTGCTTA TACTGTCAAA TCCAAAGTAA   
  
  
- ACTTCCTCTT TCTTTACAAT GCCACAAAAC CACAACATAA GCATCTGTAA AAAACGCGAA TTGACCGAGA   
  
  
- AGAGTACCCC TGCTCTGTCA CTTCCATTAA CATGAACAAC TAACTACTGC AGTAGACATC GAACCTAAAC   
  
  
- ATATCCTTAC TTGTAGAACC ACAGATGCAC CTTTCACACT AAACACAAGA AAGTAACTAG GAGGAGGAGT   
  
  
- TCTTCTCCAC TAGAAAGAGG GAACAAAAAA AAAAAAAAAC CGATTATACA ACCGTCTTGG CCGTTTTTTA   
  
  
- TGTATTAAAC TTATCACGTA CGTATACTCA TGTACTGTCA CTTGGTTGTT TTGCTTGGGG ATTTTGTGTT   
  
  
- CTCTTAATTG TACAATTTTG TGAATTTGAT TTACGTTGGT AGAGCTACTG GTGATTTATC GGTTCTTTGG   
  
  
- ATTGCATTAA TTGTTTTTAT TTTTTATTGA CTTTTATTTC ATCTCTTTCA GATTGGCTTA TGCTATTGTA   
  
  
- TTGGATTTTG ATTGATATGA ACTTGAATGA TTCTTTATAC TTAGTTGGTG TTTGGCTTAA CTAAAACCAA   
  
  
- ACGGTATTAA ACTGATATGT AAATTGAATT TTTTATAAAA AAAAATTAAA GTTTATTTAG ATTTAATAAA   
  
  
- AAGTTATAAG TAGGTTTTAT CTAAGTTAAT AATTTGTTCA GTTGGATATG GCTCAATAAA AGATCGAGAC   
  
  
- TGATTCTGTA TATATATAAA AAACATTTTT TCTATTCCTA ATATTAAATT TATCGGAAAG GGAATTTTTT   
  
  
- TAAAAATTTT AATAGGACTA TCTCTTATTT TTCTTTAGGA ATATGTTTTG AGACTAACTG CGTCTACACA   
  
  
- TATACCTGTA TATCTTCATT ATTCTCCCCA TCCGATCCTT CCCCCACCTT TATATCCCGT ATTTATCCCC   
  
  
- CGCCCGTATC CCTGTACAGA CAGACTCCAT GCCACCTACC CTGGAACGGT ACCTTCGTTG TTACAGTGTT   
  
  
- ATTATTATAG TTGCTGACTT CAAAGAAGTG GTCGGGTGAG AAACGGTATT ACGGTAATTA ATAAATAACG   
  
  
- ATGATAGAGA AAACTACACT TTGCCACCTG GAATAGAGAG GGGAGGTGAA GGTTAACTAC ACTCGTTGAG   
  
  
- TCTTTGCGTT TGTTTTGAAC ACGGTGGAAG AAATGAACGT AGGAGTAAAA GGAGACAGAG AGAGGCGGGG   
  
  
- ACAAAGAGAG AGAGAGGGAG TGAGTGAGTC TCATGACTAG ACTCGTAGTC TTCATCGTCT GGGTCTTTGA   
  
  
- GTCTAAATTT TTGTTGGGCG AGAAAACAAG GGAGATGCAG ATTATTAAAA CAGTAGCGTA AATTACAGTC   
  
  
- TATCTCTCTA TCTCGAGATG ACATAATTAA AAGAGGGTGG AGTGAACTAA GACAACGGGA TTTGGTGGGT   
  
  
- TTTTTTCTTT ATGCTTCTTC GGTGAGAGAG AGAGAGAGAG AGAGTGTGTG TGTGTGTGGG GAGGATCACC   
  
  
- TAAACTATCT CATATTTACT TGTCATTAGT CTGCTCTATA TATCATCACG AATTAACTGG CTCATCGTTT   
  
  
- TCCCCCTTCA ACTCACTCCA TAGTTCTCTC ATCGCATCGA TCGATCCCGT TCTATACGAC CCCAGAAGGA   
  
  
- GTATGTTGAG GCTCCTTCTG GTGGTTCTAC TGTTGGTAAG GAGTGGCAGA CTAAACAGAT CCGTGGGTGG   
  
  
- ATCTAAGTGG TGATGATGAA TGGTTATGGT TGTTGTATAA TCGAGAGGTA GAAGTGTTAG TTATGATCGA   
  
  
- CACGGTGGAG CGGTTAACAA TTAGTTAACA CGCCTCAATT AAACGGCCAA GCTGAAGAGG AGGCGGTTGG   
  
  
- CCGAAGAGAG AGAGGAGCGG AGGGTGAAAC GGAGGGGAAG ACCCCTGAGG TGGCTCTCTG AACACAAAAT   
  
  
- GAAGACGTTC CGAAACCTAG AGGCGGAGTT ATTGTCAATA AATAGGTGAG CATCAAACTG ACCAACATTG   
  
  
- GGATCATGAC CAAGACCATA ACCCCGAGGA AATCGAAGGT TAAAAGATAA AGAAGTTGGT AATCACGTAA   
  
  
- GTGTCCATTG TTCATTACGG GCACAACCCA TGAACTGTAT AAACTGAGAC TTGGTCCAGT GGGGTAAGTA   
  
  
- GGCTAAGTGG GTGAATTGAC GGTTGGTTCG GTAGGACCTT CGAAACCTCC CCATGTTCCG CCAGGTATAG   
  
  
- GAACTGTACC TGTAGTACGT ACCCCAGGTC ACCGGTGGTG AAGAAGTTCG TTAACGTCTT TCTAGACGTT   
  
  
- GGGAACCAGT GGGACGAGGG TGACAAGCGT AGTGTCCTCC CCCGTCCCTA GATCTTGAGG ACTTGGCTTG   
  
  
- ACCTCTAGCC TAGTCCTTCA AGCGTGTTAG GAACCCTGAG CTCAAAGTTA AGGTGGTAGA ATATTGCCAC   
  
  
- CTGAGCTTGG AATGACTCCG TCACCTCCGG TAGTTACGGG ATGTCGATGT GGAGGTACAG GTTCTCCTCC   
  
  
- TCCAGAAGCG TCACTTAACA CCTCTAAAGG AGGTGGCGGA GGATTGCCTC ATACTATCAT GTAAGGAATC   
  
  
- CTACAAAGAG ATGTTCCACT TCTGGGATTT GGGATCCCAG AAGTGGCACC CTCTTTCCCT CCGGCTGGTG   
  
  
- TTGGTGGGAG AGAAGACCGT CGCTAAGTAA CTCCGTGAAC TGGTGATACC CCGTCACAAA CTGAGAGATC   
  
  
- TTCGTTGGGA TGGTAGTTGT TCAGTTCTCT CCCACCGCCA CCTTCTCCCC ACCAAGCCCC TCCTCTAATT   
  
  
- TCTACACCAA CCTGCTCTTC CTCCCAACTC CTCTGTCTCC GTGGTCTTTA TGCTCAGAAC CCTCCACAAC   
  
  
- TACTCCTCGA GACCCAAATT CTCGAACGGG AACTCGGGGA AGAGACACAG TGTTCGGTTC GACGATGAAG   
  
  
- CGGAAGTAAT GGGAAGACTT CCCATGGTGG AATTCCAGAA GGTATCATTA ACAGACAACG AACCCACCTT   
  
  
- CTTAGCGGGA GAAAAGAGAC AAAGAAGAAC CGTTAT

+     TATA

| Site Name | Organism | Position | Strand | Matrix score. | sequence | function |
| --- | --- | --- | --- | --- | --- | --- |
| TATA | Arabidopsis thaliana | 45 | + | 8 | TATAAAAT |  |

>HU01G00833.1   
+ -Up\_Stream \_Len000AAAATA TTTTTACAAA AGCATTTCTC TATAAAATAT TTTACAAAAT TTCATTTTGT   
  
  
+ GGGTCTTTTA GAATCAATCT CTCTACAAGT AGGAATGCCG GTTGAGGTAA GGCTACCTAC ATCTAGATCT   
  
  
+ CTCCAGACCC CACAAAAATT AAACGAGACC CACACTGAAA GGTGGTGGTG GTGGTGTTGT TGTTGTATTG   
  
  
+ TCCTACCACG AGAGAGCTTC AAGTGCATCA CTTATTTCAA CAGGTCAATC ACATAGATCT CTAGTGTACT   
  
  
+ GTAAATTTTT TTTCCGTCTT ATTTTAATTT TCTTTTCCAT CCTAAAAGAT TATCATGTAG ATATTCCTAG   
  
  
+ TTTGATATAT TGACTTCAAC TTCAAAAATT ACACATTAAG ATCCACGAAT ATGACAGTTT AGGTTTCATT   
  
  
+ TGAAGGAGAA AGAAATGTTA CGGTGTTTTG GTGTTGTATT CGTAGACATT TTTTGCGCTT AACTGGCTCT   
  
  
+ TCTCATGGGG ACGAGACAGT GAAGGTAATT GTACTTGTTG ATTGATGACG TCATCTGTAG CTTGGATTTG   
  
  
+ TATAGGAATG AACATCTTGG TGTCTACGTG GAAAGTGTGA TTTGTGTTCT TTCATTGATC CTCCTCCTCA   
  
  
+ AGAAGAGGTG ATCTTTCTCC CTTGTTTTTT TTTTTTTTTG GCTAATATGT TGGCAGAACC GGCAAAAAAT   
  
  
+ ACATAATTTG AATAGTGCAT GCATATGAGT ACATGACAGT GAACCAACAA AACGAACCCC TAAAACACAA   
  
  
+ GAGAATTAAC ATGTTAAAAC ACTTAAACTA AATGCAACCA TCTCGATGAC CACTAAATAG CCAAGAAACC   
  
  
+ TAACGTAATT AACAAAAATA AAAAATAACT GAAAATAAAG TAGAGAAAGT CTAACCGAAT ACGATAACAT   
  
  
+ AACCTAAAAC TAACTATACT TGAACTTACT AAGAAATATG AATCAACCAC AAACCGAATT GATTTTGGTT   
  
  
+ TGCCATAATT TGACTATACA TTTAACTTAA AAAATATTTT TTTTTAATTT CAAATAAATC TAAATTATTT   
  
  
+ TTCAATATTC ATCCAAAATA GATTCAATTA TTAAACAAGT CAACCTATAC CGAGTTATTT TCTAGCTCTG   
  
  
+ ACTAAGACAT ATATATATTT TTTGTAAAAA AGATAAGGAT TATAATTTAA ATAGCCTTTC CCTTAAAAAA   
  
  
+ ATTTTTAAAA TTATCCTGAT AGAGAATAAA AAGAAATCCT TATACAAAAC TCTGATTGAC GCAGATGTGT   
  
  
+ ATATGGACAT ATAGAAGTAA TAAGAGGGGT AGGCTAGGAA GGGGGTGGAA ATATAGGGCA TAAATAGGGG   
  
  
+ GCGGGCATAG GGACATGTCT GTCTGAGGTA CGGTGGATGG GACCTTGCCA TGGAAGCAAC AATGTCACAA   
  
  
+ TAATAATATC AACGACTGAA GTTTCTTCAC CAGCCCACTC TTTGCCATAA TGCCATTAAT TATTTATTGC   
  
  
+ TACTATCTCT TTTGATGTGA AACGGTGGAC CTTATCTCTC CCCTCCACTT CCAATTGATG TGAGCAACTC   
  
  
+ AGAAACGCAA ACAAAACTTG TGCCACCTTC TTTACTTGCA TCCTCATTTT CCTCTGTCTC TCTCCGCCCC   
  
  
+ TGTTTCTCTC TCTCTCCCTC ACTCACTCAG AGTACTGATC TGAGCATCAG AAGTAGCAGA CCCAGAAACT   
  
  
+ CAGATTTAAA AACAACCCGC TCTTTTGTTC CCTCTACGTC TAATAATTTT GTCATCGCAT TTAATGTCAG   
  
  
+ ATAGAGAGAT AGAGCTCTAC TGTATTAATT TTCTCCCACC TCACTTGATT CTGTTGCCCT AAACCACCCA   
  
  
+ AAAAAAGAAA TACGAAGAAG CCACTCTCTC TCTCTCTCTC TCTCACACAC ACACACACCC CTCCTAGTGG   
  
  
+ ATTTGATAGA GTATAAATGA ACAGTAATCA GACGAGATAT ATAGTAGTGC TTAATTGACC GAGTAGCAAA   
  
  
+ AGGGGGAAGT TGAGTGAGGT ATCAAGAGAG TAGCGTAGCT AGCTAGGGCA AGATATGCTG GGGTCTTCCT   
  
  
+ CATACAACTC CGAGGAAGAC CACCAAGATG ACAACCATTC CTCACCGTCT GATTTGTCTA GGCACCCACC   
  
  
+ TAGATTCACC ACTACTACTT ACCAATACCA ACAACATATT AGCTCTCCAT CTTCACAATC AATACTAGCT   
  
  
+ GTGCCACCTC GCCAATTGTT AATCAATTGT GCGGAGTTAA TTTGCCGGTT CGACTTCTCC TCCGCCAACC   
  
  
+ GGCTTCTCTC TCTCCTCGCC TCCCACTTTG CCTCCCCTTC TGGGGACTCC ACCGAGAGAC TTGTGTTTTA   
  
  
+ CTTCTGCAAG GCTTTGGATC TCCGCCTCAA TAACAGTTAT TTATCCACTC GTAGTTTGAC TGGTTGTAAC   
  
  
+ CCTAGTACTG GTTCTGGTAT TGGGGCTCCT TTAGCTTCCA ATTTTCTATT TCTTCAACCA TTAGTGCATT   
  
  
+ CACAGGTAAC AAGTAATGCC CGTGTTGGGT ACTTGACATA TTTGACTCTG AACCAGGTCA CCCCATTCAT   
  
  
+ CCGATTCACC CACTTAACTG CCAACCAAGC CATCCTGGAA GCTTTGGAGG GGTACAAGGC GGTCCATATC   
  
  
+ CTTGACATGG ACATCATGCA TGGGGTCCAG TGGCCACCAC TTCTTCAAGC AATTGCAGAA AGATCTGCAA   
  
  
+ CCCTTGGTCA CCCTGCTCCC ACTGTTCGCA TCACAGGAGG GGGCAGGGAT CTAGAACTCC TGAACCGAAC   
  
  
+ TGGAGATCGG ATCAGGAAGT TCGCACAATC CTTGGGACTC GAGTTTCAAT TCCACCATCT TATAACGGTG   
  
  
+ GACTCGAACC TTACTGAGGC AGTGGAGGCC ATCAATGCCC TACAGCTACA CCTCCATGTC CAAGAGGAGG   
  
  
+ AGGTCTTCGC AGTGAATTGT GGAGATTTCC TCCACCGCCT CCTAACGGAG TATGATAGTA CATTCCTTAG   
  
  
+ GATGTTTCTC TACAAGGTGA AGACCCTAAA CCCTAGGGTC TTCACCGTGG GAGAAAGGGA GGCCGACCAC   
  
  
+ AACCACCCTC TCTTCTGGCA GCGATTCATT GAGGCACTTG ACCACTATGG GGCAGTGTTT GACTCTCTAG   
  
  
+ AAGCAACCCT ACCATCAACA AGTCAAGAGA GGGTGGCGGT GGAAGAGGGG TGGTTCGGGG AGGAGATTAA   
  
  
+ AGATGTGGTT GGACGAGAAG GAGGGTTGAG GAGACAGAGG CACCAGAAAT ACGAGTCTTG GGAGGTGTTG   
  
  
+ ATGAGGAGCT CTGGGTTTAA GAGCTTGCCC TTGAGCCCCT TCTCTGTGTC ACAAGCCAAG CTGCTACTTC   
  
  
+ GCCTTCATTA CCCTTCTGAA GGGTACCACC TTAAGGTCTT CCATAGTAAT TGTCTGTTGC TTGGGTGGAA   
  
  
+ GAATCGCCCT CTTTTCTCTG TTTCTTCTTG GCAATA  

- -Up\_Stream \_Len000TTTTAT AAAAATGTTT TCGTAAAGAG ATATTTTATA AAATGTTTTA AAGTAAAACA   
  
  
- CCCAGAAAAT CTTAGTTAGA GAGATGTTCA TCCTTACGGC CAACTCCATT CCGATGGATG TAGATCTAGA   
  
  
- GAGGTCTGGG GTGTTTTTAA TTTGCTCTGG GTGTGACTTT CCACCACCAC CACCACAACA ACAACATAAC   
  
  
- AGGATGGTGC TCTCTCGAAG TTCACGTAGT GAATAAAGTT GTCCAGTTAG TGTATCTAGA GATCACATGA   
  
  
- CATTTAAAAA AAAGGCAGAA TAAAATTAAA AGAAAAGGTA GGATTTTCTA ATAGTACATC TATAAGGATC   
  
  
- AAACTATATA ACTGAAGTTG AAGTTTTTAA TGTGTAATTC TAGGTGCTTA TACTGTCAAA TCCAAAGTAA   
  
  
- ACTTCCTCTT TCTTTACAAT GCCACAAAAC CACAACATAA GCATCTGTAA AAAACGCGAA TTGACCGAGA   
  
  
- AGAGTACCCC TGCTCTGTCA CTTCCATTAA CATGAACAAC TAACTACTGC AGTAGACATC GAACCTAAAC   
  
  
- ATATCCTTAC TTGTAGAACC ACAGATGCAC CTTTCACACT AAACACAAGA AAGTAACTAG GAGGAGGAGT   
  
  
- TCTTCTCCAC TAGAAAGAGG GAACAAAAAA AAAAAAAAAC CGATTATACA ACCGTCTTGG CCGTTTTTTA   
  
  
- TGTATTAAAC TTATCACGTA CGTATACTCA TGTACTGTCA CTTGGTTGTT TTGCTTGGGG ATTTTGTGTT   
  
  
- CTCTTAATTG TACAATTTTG TGAATTTGAT TTACGTTGGT AGAGCTACTG GTGATTTATC GGTTCTTTGG   
  
  
- ATTGCATTAA TTGTTTTTAT TTTTTATTGA CTTTTATTTC ATCTCTTTCA GATTGGCTTA TGCTATTGTA   
  
  
- TTGGATTTTG ATTGATATGA ACTTGAATGA TTCTTTATAC TTAGTTGGTG TTTGGCTTAA CTAAAACCAA   
  
  
- ACGGTATTAA ACTGATATGT AAATTGAATT TTTTATAAAA AAAAATTAAA GTTTATTTAG ATTTAATAAA   
  
  
- AAGTTATAAG TAGGTTTTAT CTAAGTTAAT AATTTGTTCA GTTGGATATG GCTCAATAAA AGATCGAGAC   
  
  
- TGATTCTGTA TATATATAAA AAACATTTTT TCTATTCCTA ATATTAAATT TATCGGAAAG GGAATTTTTT   
  
  
- TAAAAATTTT AATAGGACTA TCTCTTATTT TTCTTTAGGA ATATGTTTTG AGACTAACTG CGTCTACACA   
  
  
- TATACCTGTA TATCTTCATT ATTCTCCCCA TCCGATCCTT CCCCCACCTT TATATCCCGT ATTTATCCCC   
  
  
- CGCCCGTATC CCTGTACAGA CAGACTCCAT GCCACCTACC CTGGAACGGT ACCTTCGTTG TTACAGTGTT   
  
  
- ATTATTATAG TTGCTGACTT CAAAGAAGTG GTCGGGTGAG AAACGGTATT ACGGTAATTA ATAAATAACG   
  
  
- ATGATAGAGA AAACTACACT TTGCCACCTG GAATAGAGAG GGGAGGTGAA GGTTAACTAC ACTCGTTGAG   
  
  
- TCTTTGCGTT TGTTTTGAAC ACGGTGGAAG AAATGAACGT AGGAGTAAAA GGAGACAGAG AGAGGCGGGG   
  
  
- ACAAAGAGAG AGAGAGGGAG TGAGTGAGTC TCATGACTAG ACTCGTAGTC TTCATCGTCT GGGTCTTTGA   
  
  
- GTCTAAATTT TTGTTGGGCG AGAAAACAAG GGAGATGCAG ATTATTAAAA CAGTAGCGTA AATTACAGTC   
  
  
- TATCTCTCTA TCTCGAGATG ACATAATTAA AAGAGGGTGG AGTGAACTAA GACAACGGGA TTTGGTGGGT   
  
  
- TTTTTTCTTT ATGCTTCTTC GGTGAGAGAG AGAGAGAGAG AGAGTGTGTG TGTGTGTGGG GAGGATCACC   
  
  
- TAAACTATCT CATATTTACT TGTCATTAGT CTGCTCTATA TATCATCACG AATTAACTGG CTCATCGTTT   
  
  
- TCCCCCTTCA ACTCACTCCA TAGTTCTCTC ATCGCATCGA TCGATCCCGT TCTATACGAC CCCAGAAGGA   
  
  
- GTATGTTGAG GCTCCTTCTG GTGGTTCTAC TGTTGGTAAG GAGTGGCAGA CTAAACAGAT CCGTGGGTGG   
  
  
- ATCTAAGTGG TGATGATGAA TGGTTATGGT TGTTGTATAA TCGAGAGGTA GAAGTGTTAG TTATGATCGA   
  
  
- CACGGTGGAG CGGTTAACAA TTAGTTAACA CGCCTCAATT AAACGGCCAA GCTGAAGAGG AGGCGGTTGG   
  
  
- CCGAAGAGAG AGAGGAGCGG AGGGTGAAAC GGAGGGGAAG ACCCCTGAGG TGGCTCTCTG AACACAAAAT   
  
  
- GAAGACGTTC CGAAACCTAG AGGCGGAGTT ATTGTCAATA AATAGGTGAG CATCAAACTG ACCAACATTG   
  
  
- GGATCATGAC CAAGACCATA ACCCCGAGGA AATCGAAGGT TAAAAGATAA AGAAGTTGGT AATCACGTAA   
  
  
- GTGTCCATTG TTCATTACGG GCACAACCCA TGAACTGTAT AAACTGAGAC TTGGTCCAGT GGGGTAAGTA   
  
  
- GGCTAAGTGG GTGAATTGAC GGTTGGTTCG GTAGGACCTT CGAAACCTCC CCATGTTCCG CCAGGTATAG   
  
  
- GAACTGTACC TGTAGTACGT ACCCCAGGTC ACCGGTGGTG AAGAAGTTCG TTAACGTCTT TCTAGACGTT   
  
  
- GGGAACCAGT GGGACGAGGG TGACAAGCGT AGTGTCCTCC CCCGTCCCTA GATCTTGAGG ACTTGGCTTG   
  
  
- ACCTCTAGCC TAGTCCTTCA AGCGTGTTAG GAACCCTGAG CTCAAAGTTA AGGTGGTAGA ATATTGCCAC   
  
  
- CTGAGCTTGG AATGACTCCG TCACCTCCGG TAGTTACGGG ATGTCGATGT GGAGGTACAG GTTCTCCTCC   
  
  
- TCCAGAAGCG TCACTTAACA CCTCTAAAGG AGGTGGCGGA GGATTGCCTC ATACTATCAT GTAAGGAATC   
  
  
- CTACAAAGAG ATGTTCCACT TCTGGGATTT GGGATCCCAG AAGTGGCACC CTCTTTCCCT CCGGCTGGTG   
  
  
- TTGGTGGGAG AGAAGACCGT CGCTAAGTAA CTCCGTGAAC TGGTGATACC CCGTCACAAA CTGAGAGATC   
  
  
- TTCGTTGGGA TGGTAGTTGT TCAGTTCTCT CCCACCGCCA CCTTCTCCCC ACCAAGCCCC TCCTCTAATT   
  
  
- TCTACACCAA CCTGCTCTTC CTCCCAACTC CTCTGTCTCC GTGGTCTTTA TGCTCAGAAC CCTCCACAAC   
  
  
- TACTCCTCGA GACCCAAATT CTCGAACGGG AACTCGGGGA AGAGACACAG TGTTCGGTTC GACGATGAAG   
  
  
- CGGAAGTAAT GGGAAGACTT CCCATGGTGG AATTCCAGAA GGTATCATTA ACAGACAACG AACCCACCTT   
  
  
- CTTAGCGGGA GAAAAGAGAC AAAGAAGAAC CGTTAT

+     TATA-box

| Site Name | Organism | Position | Strand | Matrix score. | sequence | function |
| --- | --- | --- | --- | --- | --- | --- |
| TATA-box | Arabidopsis thaliana | 2795 | - | 4 | TATA | core promoter element around -30 of transcription start |
| TATA-box | Arabidopsis thaliana | 2794 | - | 5 | TATAA | core promoter element around -30 of transcription start |
| TATA-box | Brassica napus | 1931 | - | 6 | ATATAT | core promoter element around -30 of transcription start |
| TATA-box | Arabidopsis thaliana | 1934 | - | 4 | TATA | core promoter element around -30 of transcription start |
| TATA-box | Arabidopsis thaliana | 1138 | + | 4 | TATA | core promoter element around -30 of transcription start |
| TATA-box | Arabidopsis thaliana | 1932 | - | 6 | TATATA | core promoter element around -30 of transcription start |
| TATA-box | Arabidopsis thaliana | 1264 | + | 4 | TATA | core promoter element around -30 of transcription start |
| TATA-box | Arabidopsis thaliana | 1906 | - | 4 | TATA | core promoter element around -30 of transcription start |
| TATA-box | Arabidopsis thaliana | 1274 | + | 4 | TATA | core promoter element around -30 of transcription start |
| TATA-box | Arabidopsis thaliana | 1136 | + | 6 | TATATA | core promoter element around -30 of transcription start |
| TATA-box | Arabidopsis thaliana | 1316 | + | 4 | TATA | core promoter element around -30 of transcription start |
| TATA-box | Arabidopsis thaliana | 1165 | + | 4 | TATA | core promoter element around -30 of transcription start |
| TATA-box | Arabidopsis thaliana | 565 | + | 4 | TATA | core promoter element around -30 of transcription start |
| TATA-box | Oryza sativa | 1144 | - | 7 | TACAAAA | core promoter element around -30 of transcription start |
| TATA-box | Arabidopsis thaliana | 1164 | - | 5 | TATAA | core promoter element around -30 of transcription start |
| TATA-box | Helianthus annuus | 1262 | - | 6 | TATACA | core promoter element around -30 of transcription start |
| TATA-box | Brassica napus | 1163 | + | 6 | ATTATA | core promoter element around -30 of transcription start |
| TATA-box | Arabidopsis thaliana | 1234 | - | 5 | TATAA | core promoter element around -30 of transcription start |
| TATA-box | Arabidopsis thaliana | 1170 | - | 8 | TATTTAAA | core promoter element around -30 of transcription start |
| TATA-box | Oryza sativa | 1237 | + | 7 | TACAAAA | core promoter element around -30 of transcription start |
| TATA-box | Arabidopsis thaliana | 1134 | + | 6 | TATATA | core promoter element around -30 of transcription start |
| TATA-box | Arabidopsis thaliana | 1235 | + | 4 | TATA | core promoter element around -30 of transcription start |
| TATA-box | Brassica napus | 1135 | + | 6 | ATATAT | core promoter element around -30 of transcription start |
| TATA-box | Arabidopsis thaliana | 559 | - | 9 | taTATAAAtc | core promoter element around -30 of transcription start |
| TATA-box | Arabidopsis thaliana | 929 | + | 4 | TATA | core promoter element around -30 of transcription start |
| TATA-box | Brassica napus | 1137 | + | 6 | ATATAT | core promoter element around -30 of transcription start |
| TATA-box | Arabidopsis thaliana | 1100 | + | 4 | TATA | core promoter element around -30 of transcription start |
| TATA-box | Arabidopsis thaliana | 999 | + | 4 | TATA | core promoter element around -30 of transcription start |
| TATA-box | Brassica napus | 1133 | + | 6 | ATATAT | core promoter element around -30 of transcription start |
| TATA-box | Helianthus annuus | 563 | - | 6 | TATACA | core promoter element around -30 of transcription start |
| TATA-box | Arabidopsis thaliana | 561 | - | 9 | ccTATAAAaa | core promoter element around -30 of transcription start |
| TATA-box | Arabidopsis thaliana | 360 | + | 4 | TATA | core promoter element around -30 of transcription start |
| TATA-box | Brassica napus | 359 | + | 6 | ATATAT | core promoter element around -30 of transcription start |
| TATA-box | Arabidopsis thaliana | 45 | + | 4 | TATA | core promoter element around -30 of transcription start |
| TATA-box | Oryza sativa | 57 | + | 7 | TACAAAA | core promoter element around -30 of transcription start |
| TATA-box | Oryza sativa | 29 | + | 7 | TACAAAA | core promoter element around -30 of transcription start |

>HU01G00833.1   
+ -Up\_Stream \_Len000AAAATA TTTTTACAAA AGCATTTCTC TATAAAATAT TTTACAAAAT TTCATTTTGT   
  
  
+ GGGTCTTTTA GAATCAATCT CTCTACAAGT AGGAATGCCG GTTGAGGTAA GGCTACCTAC ATCTAGATCT   
  
  
+ CTCCAGACCC CACAAAAATT AAACGAGACC CACACTGAAA GGTGGTGGTG GTGGTGTTGT TGTTGTATTG   
  
  
+ TCCTACCACG AGAGAGCTTC AAGTGCATCA CTTATTTCAA CAGGTCAATC ACATAGATCT CTAGTGTACT   
  
  
+ GTAAATTTTT TTTCCGTCTT ATTTTAATTT TCTTTTCCAT CCTAAAAGAT TATCATGTAG ATATTCCTAG   
  
  
+ TTTGATATAT TGACTTCAAC TTCAAAAATT ACACATTAAG ATCCACGAAT ATGACAGTTT AGGTTTCATT   
  
  
+ TGAAGGAGAA AGAAATGTTA CGGTGTTTTG GTGTTGTATT CGTAGACATT TTTTGCGCTT AACTGGCTCT   
  
  
+ TCTCATGGGG ACGAGACAGT GAAGGTAATT GTACTTGTTG ATTGATGACG TCATCTGTAG CTTGGATTTG   
  
  
+ TATAGGAATG AACATCTTGG TGTCTACGTG GAAAGTGTGA TTTGTGTTCT TTCATTGATC CTCCTCCTCA   
  
  
+ AGAAGAGGTG ATCTTTCTCC CTTGTTTTTT TTTTTTTTTG GCTAATATGT TGGCAGAACC GGCAAAAAAT   
  
  
+ ACATAATTTG AATAGTGCAT GCATATGAGT ACATGACAGT GAACCAACAA AACGAACCCC TAAAACACAA   
  
  
+ GAGAATTAAC ATGTTAAAAC ACTTAAACTA AATGCAACCA TCTCGATGAC CACTAAATAG CCAAGAAACC   
  
  
+ TAACGTAATT AACAAAAATA AAAAATAACT GAAAATAAAG TAGAGAAAGT CTAACCGAAT ACGATAACAT   
  
  
+ AACCTAAAAC TAACTATACT TGAACTTACT AAGAAATATG AATCAACCAC AAACCGAATT GATTTTGGTT   
  
  
+ TGCCATAATT TGACTATACA TTTAACTTAA AAAATATTTT TTTTTAATTT CAAATAAATC TAAATTATTT   
  
  
+ TTCAATATTC ATCCAAAATA GATTCAATTA TTAAACAAGT CAACCTATAC CGAGTTATTT TCTAGCTCTG   
  
  
+ ACTAAGACAT ATATATATTT TTTGTAAAAA AGATAAGGAT TATAATTTAA ATAGCCTTTC CCTTAAAAAA   
  
  
+ ATTTTTAAAA TTATCCTGAT AGAGAATAAA AAGAAATCCT TATACAAAAC TCTGATTGAC GCAGATGTGT   
  
  
+ ATATGGACAT ATAGAAGTAA TAAGAGGGGT AGGCTAGGAA GGGGGTGGAA ATATAGGGCA TAAATAGGGG   
  
  
+ GCGGGCATAG GGACATGTCT GTCTGAGGTA CGGTGGATGG GACCTTGCCA TGGAAGCAAC AATGTCACAA   
  
  
+ TAATAATATC AACGACTGAA GTTTCTTCAC CAGCCCACTC TTTGCCATAA TGCCATTAAT TATTTATTGC   
  
  
+ TACTATCTCT TTTGATGTGA AACGGTGGAC CTTATCTCTC CCCTCCACTT CCAATTGATG TGAGCAACTC   
  
  
+ AGAAACGCAA ACAAAACTTG TGCCACCTTC TTTACTTGCA TCCTCATTTT CCTCTGTCTC TCTCCGCCCC   
  
  
+ TGTTTCTCTC TCTCTCCCTC ACTCACTCAG AGTACTGATC TGAGCATCAG AAGTAGCAGA CCCAGAAACT   
  
  
+ CAGATTTAAA AACAACCCGC TCTTTTGTTC CCTCTACGTC TAATAATTTT GTCATCGCAT TTAATGTCAG   
  
  
+ ATAGAGAGAT AGAGCTCTAC TGTATTAATT TTCTCCCACC TCACTTGATT CTGTTGCCCT AAACCACCCA   
  
  
+ AAAAAAGAAA TACGAAGAAG CCACTCTCTC TCTCTCTCTC TCTCACACAC ACACACACCC CTCCTAGTGG   
  
  
+ ATTTGATAGA GTATAAATGA ACAGTAATCA GACGAGATAT ATAGTAGTGC TTAATTGACC GAGTAGCAAA   
  
  
+ AGGGGGAAGT TGAGTGAGGT ATCAAGAGAG TAGCGTAGCT AGCTAGGGCA AGATATGCTG GGGTCTTCCT   
  
  
+ CATACAACTC CGAGGAAGAC CACCAAGATG ACAACCATTC CTCACCGTCT GATTTGTCTA GGCACCCACC   
  
  
+ TAGATTCACC ACTACTACTT ACCAATACCA ACAACATATT AGCTCTCCAT CTTCACAATC AATACTAGCT   
  
  
+ GTGCCACCTC GCCAATTGTT AATCAATTGT GCGGAGTTAA TTTGCCGGTT CGACTTCTCC TCCGCCAACC   
  
  
+ GGCTTCTCTC TCTCCTCGCC TCCCACTTTG CCTCCCCTTC TGGGGACTCC ACCGAGAGAC TTGTGTTTTA   
  
  
+ CTTCTGCAAG GCTTTGGATC TCCGCCTCAA TAACAGTTAT TTATCCACTC GTAGTTTGAC TGGTTGTAAC   
  
  
+ CCTAGTACTG GTTCTGGTAT TGGGGCTCCT TTAGCTTCCA ATTTTCTATT TCTTCAACCA TTAGTGCATT   
  
  
+ CACAGGTAAC AAGTAATGCC CGTGTTGGGT ACTTGACATA TTTGACTCTG AACCAGGTCA CCCCATTCAT   
  
  
+ CCGATTCACC CACTTAACTG CCAACCAAGC CATCCTGGAA GCTTTGGAGG GGTACAAGGC GGTCCATATC   
  
  
+ CTTGACATGG ACATCATGCA TGGGGTCCAG TGGCCACCAC TTCTTCAAGC AATTGCAGAA AGATCTGCAA   
  
  
+ CCCTTGGTCA CCCTGCTCCC ACTGTTCGCA TCACAGGAGG GGGCAGGGAT CTAGAACTCC TGAACCGAAC   
  
  
+ TGGAGATCGG ATCAGGAAGT TCGCACAATC CTTGGGACTC GAGTTTCAAT TCCACCATCT TATAACGGTG   
  
  
+ GACTCGAACC TTACTGAGGC AGTGGAGGCC ATCAATGCCC TACAGCTACA CCTCCATGTC CAAGAGGAGG   
  
  
+ AGGTCTTCGC AGTGAATTGT GGAGATTTCC TCCACCGCCT CCTAACGGAG TATGATAGTA CATTCCTTAG   
  
  
+ GATGTTTCTC TACAAGGTGA AGACCCTAAA CCCTAGGGTC TTCACCGTGG GAGAAAGGGA GGCCGACCAC   
  
  
+ AACCACCCTC TCTTCTGGCA GCGATTCATT GAGGCACTTG ACCACTATGG GGCAGTGTTT GACTCTCTAG   
  
  
+ AAGCAACCCT ACCATCAACA AGTCAAGAGA GGGTGGCGGT GGAAGAGGGG TGGTTCGGGG AGGAGATTAA   
  
  
+ AGATGTGGTT GGACGAGAAG GAGGGTTGAG GAGACAGAGG CACCAGAAAT ACGAGTCTTG GGAGGTGTTG   
  
  
+ ATGAGGAGCT CTGGGTTTAA GAGCTTGCCC TTGAGCCCCT TCTCTGTGTC ACAAGCCAAG CTGCTACTTC   
  
  
+ GCCTTCATTA CCCTTCTGAA GGGTACCACC TTAAGGTCTT CCATAGTAAT TGTCTGTTGC TTGGGTGGAA   
  
  
+ GAATCGCCCT CTTTTCTCTG TTTCTTCTTG GCAATA  

- -Up\_Stream \_Len000TTTTAT AAAAATGTTT TCGTAAAGAG ATATTTTATA AAATGTTTTA AAGTAAAACA   
  
  
- CCCAGAAAAT CTTAGTTAGA GAGATGTTCA TCCTTACGGC CAACTCCATT CCGATGGATG TAGATCTAGA   
  
  
- GAGGTCTGGG GTGTTTTTAA TTTGCTCTGG GTGTGACTTT CCACCACCAC CACCACAACA ACAACATAAC   
  
  
- AGGATGGTGC TCTCTCGAAG TTCACGTAGT GAATAAAGTT GTCCAGTTAG TGTATCTAGA GATCACATGA   
  
  
- CATTTAAAAA AAAGGCAGAA TAAAATTAAA AGAAAAGGTA GGATTTTCTA ATAGTACATC TATAAGGATC   
  
  
- AAACTATATA ACTGAAGTTG AAGTTTTTAA TGTGTAATTC TAGGTGCTTA TACTGTCAAA TCCAAAGTAA   
  
  
- ACTTCCTCTT TCTTTACAAT GCCACAAAAC CACAACATAA GCATCTGTAA AAAACGCGAA TTGACCGAGA   
  
  
- AGAGTACCCC TGCTCTGTCA CTTCCATTAA CATGAACAAC TAACTACTGC AGTAGACATC GAACCTAAAC   
  
  
- ATATCCTTAC TTGTAGAACC ACAGATGCAC CTTTCACACT AAACACAAGA AAGTAACTAG GAGGAGGAGT   
  
  
- TCTTCTCCAC TAGAAAGAGG GAACAAAAAA AAAAAAAAAC CGATTATACA ACCGTCTTGG CCGTTTTTTA   
  
  
- TGTATTAAAC TTATCACGTA CGTATACTCA TGTACTGTCA CTTGGTTGTT TTGCTTGGGG ATTTTGTGTT   
  
  
- CTCTTAATTG TACAATTTTG TGAATTTGAT TTACGTTGGT AGAGCTACTG GTGATTTATC GGTTCTTTGG   
  
  
- ATTGCATTAA TTGTTTTTAT TTTTTATTGA CTTTTATTTC ATCTCTTTCA GATTGGCTTA TGCTATTGTA   
  
  
- TTGGATTTTG ATTGATATGA ACTTGAATGA TTCTTTATAC TTAGTTGGTG TTTGGCTTAA CTAAAACCAA   
  
  
- ACGGTATTAA ACTGATATGT AAATTGAATT TTTTATAAAA AAAAATTAAA GTTTATTTAG ATTTAATAAA   
  
  
- AAGTTATAAG TAGGTTTTAT CTAAGTTAAT AATTTGTTCA GTTGGATATG GCTCAATAAA AGATCGAGAC   
  
  
- TGATTCTGTA TATATATAAA AAACATTTTT TCTATTCCTA ATATTAAATT TATCGGAAAG GGAATTTTTT   
  
  
- TAAAAATTTT AATAGGACTA TCTCTTATTT TTCTTTAGGA ATATGTTTTG AGACTAACTG CGTCTACACA   
  
  
- TATACCTGTA TATCTTCATT ATTCTCCCCA TCCGATCCTT CCCCCACCTT TATATCCCGT ATTTATCCCC   
  
  
- CGCCCGTATC CCTGTACAGA CAGACTCCAT GCCACCTACC CTGGAACGGT ACCTTCGTTG TTACAGTGTT   
  
  
- ATTATTATAG TTGCTGACTT CAAAGAAGTG GTCGGGTGAG AAACGGTATT ACGGTAATTA ATAAATAACG   
  
  
- ATGATAGAGA AAACTACACT TTGCCACCTG GAATAGAGAG GGGAGGTGAA GGTTAACTAC ACTCGTTGAG   
  
  
- TCTTTGCGTT TGTTTTGAAC ACGGTGGAAG AAATGAACGT AGGAGTAAAA GGAGACAGAG AGAGGCGGGG   
  
  
- ACAAAGAGAG AGAGAGGGAG TGAGTGAGTC TCATGACTAG ACTCGTAGTC TTCATCGTCT GGGTCTTTGA   
  
  
- GTCTAAATTT TTGTTGGGCG AGAAAACAAG GGAGATGCAG ATTATTAAAA CAGTAGCGTA AATTACAGTC   
  
  
- TATCTCTCTA TCTCGAGATG ACATAATTAA AAGAGGGTGG AGTGAACTAA GACAACGGGA TTTGGTGGGT   
  
  
- TTTTTTCTTT ATGCTTCTTC GGTGAGAGAG AGAGAGAGAG AGAGTGTGTG TGTGTGTGGG GAGGATCACC   
  
  
- TAAACTATCT CATATTTACT TGTCATTAGT CTGCTCTATA TATCATCACG AATTAACTGG CTCATCGTTT   
  
  
- TCCCCCTTCA ACTCACTCCA TAGTTCTCTC ATCGCATCGA TCGATCCCGT TCTATACGAC CCCAGAAGGA   
  
  
- GTATGTTGAG GCTCCTTCTG GTGGTTCTAC TGTTGGTAAG GAGTGGCAGA CTAAACAGAT CCGTGGGTGG   
  
  
- ATCTAAGTGG TGATGATGAA TGGTTATGGT TGTTGTATAA TCGAGAGGTA GAAGTGTTAG TTATGATCGA   
  
  
- CACGGTGGAG CGGTTAACAA TTAGTTAACA CGCCTCAATT AAACGGCCAA GCTGAAGAGG AGGCGGTTGG   
  
  
- CCGAAGAGAG AGAGGAGCGG AGGGTGAAAC GGAGGGGAAG ACCCCTGAGG TGGCTCTCTG AACACAAAAT   
  
  
- GAAGACGTTC CGAAACCTAG AGGCGGAGTT ATTGTCAATA AATAGGTGAG CATCAAACTG ACCAACATTG   
  
  
- GGATCATGAC CAAGACCATA ACCCCGAGGA AATCGAAGGT TAAAAGATAA AGAAGTTGGT AATCACGTAA   
  
  
- GTGTCCATTG TTCATTACGG GCACAACCCA TGAACTGTAT AAACTGAGAC TTGGTCCAGT GGGGTAAGTA   
  
  
- GGCTAAGTGG GTGAATTGAC GGTTGGTTCG GTAGGACCTT CGAAACCTCC CCATGTTCCG CCAGGTATAG   
  
  
- GAACTGTACC TGTAGTACGT ACCCCAGGTC ACCGGTGGTG AAGAAGTTCG TTAACGTCTT TCTAGACGTT   
  
  
- GGGAACCAGT GGGACGAGGG TGACAAGCGT AGTGTCCTCC CCCGTCCCTA GATCTTGAGG ACTTGGCTTG   
  
  
- ACCTCTAGCC TAGTCCTTCA AGCGTGTTAG GAACCCTGAG CTCAAAGTTA AGGTGGTAGA ATATTGCCAC   
  
  
- CTGAGCTTGG AATGACTCCG TCACCTCCGG TAGTTACGGG ATGTCGATGT GGAGGTACAG GTTCTCCTCC   
  
  
- TCCAGAAGCG TCACTTAACA CCTCTAAAGG AGGTGGCGGA GGATTGCCTC ATACTATCAT GTAAGGAATC   
  
  
- CTACAAAGAG ATGTTCCACT TCTGGGATTT GGGATCCCAG AAGTGGCACC CTCTTTCCCT CCGGCTGGTG   
  
  
- TTGGTGGGAG AGAAGACCGT CGCTAAGTAA CTCCGTGAAC TGGTGATACC CCGTCACAAA CTGAGAGATC   
  
  
- TTCGTTGGGA TGGTAGTTGT TCAGTTCTCT CCCACCGCCA CCTTCTCCCC ACCAAGCCCC TCCTCTAATT   
  
  
- TCTACACCAA CCTGCTCTTC CTCCCAACTC CTCTGTCTCC GTGGTCTTTA TGCTCAGAAC CCTCCACAAC   
  
  
- TACTCCTCGA GACCCAAATT CTCGAACGGG AACTCGGGGA AGAGACACAG TGTTCGGTTC GACGATGAAG   
  
  
- CGGAAGTAAT GGGAAGACTT CCCATGGTGG AATTCCAGAA GGTATCATTA ACAGACAACG AACCCACCTT   
  
  
- CTTAGCGGGA GAAAAGAGAC AAAGAAGAAC CGTTAT

+     TC-rich repeats

| Site Name | Organism | Position | Strand | Matrix score. | sequence | function |
| --- | --- | --- | --- | --- | --- | --- |
| TC-rich repeats | Nicotiana tabacum | 1212 | - | 9 | ATTCTCTAAC | cis-acting element involved in defense and stress responsiveness |

>HU01G00833.1   
+ -Up\_Stream \_Len000AAAATA TTTTTACAAA AGCATTTCTC TATAAAATAT TTTACAAAAT TTCATTTTGT   
  
  
+ GGGTCTTTTA GAATCAATCT CTCTACAAGT AGGAATGCCG GTTGAGGTAA GGCTACCTAC ATCTAGATCT   
  
  
+ CTCCAGACCC CACAAAAATT AAACGAGACC CACACTGAAA GGTGGTGGTG GTGGTGTTGT TGTTGTATTG   
  
  
+ TCCTACCACG AGAGAGCTTC AAGTGCATCA CTTATTTCAA CAGGTCAATC ACATAGATCT CTAGTGTACT   
  
  
+ GTAAATTTTT TTTCCGTCTT ATTTTAATTT TCTTTTCCAT CCTAAAAGAT TATCATGTAG ATATTCCTAG   
  
  
+ TTTGATATAT TGACTTCAAC TTCAAAAATT ACACATTAAG ATCCACGAAT ATGACAGTTT AGGTTTCATT   
  
  
+ TGAAGGAGAA AGAAATGTTA CGGTGTTTTG GTGTTGTATT CGTAGACATT TTTTGCGCTT AACTGGCTCT   
  
  
+ TCTCATGGGG ACGAGACAGT GAAGGTAATT GTACTTGTTG ATTGATGACG TCATCTGTAG CTTGGATTTG   
  
  
+ TATAGGAATG AACATCTTGG TGTCTACGTG GAAAGTGTGA TTTGTGTTCT TTCATTGATC CTCCTCCTCA   
  
  
+ AGAAGAGGTG ATCTTTCTCC CTTGTTTTTT TTTTTTTTTG GCTAATATGT TGGCAGAACC GGCAAAAAAT   
  
  
+ ACATAATTTG AATAGTGCAT GCATATGAGT ACATGACAGT GAACCAACAA AACGAACCCC TAAAACACAA   
  
  
+ GAGAATTAAC ATGTTAAAAC ACTTAAACTA AATGCAACCA TCTCGATGAC CACTAAATAG CCAAGAAACC   
  
  
+ TAACGTAATT AACAAAAATA AAAAATAACT GAAAATAAAG TAGAGAAAGT CTAACCGAAT ACGATAACAT   
  
  
+ AACCTAAAAC TAACTATACT TGAACTTACT AAGAAATATG AATCAACCAC AAACCGAATT GATTTTGGTT   
  
  
+ TGCCATAATT TGACTATACA TTTAACTTAA AAAATATTTT TTTTTAATTT CAAATAAATC TAAATTATTT   
  
  
+ TTCAATATTC ATCCAAAATA GATTCAATTA TTAAACAAGT CAACCTATAC CGAGTTATTT TCTAGCTCTG   
  
  
+ ACTAAGACAT ATATATATTT TTTGTAAAAA AGATAAGGAT TATAATTTAA ATAGCCTTTC CCTTAAAAAA   
  
  
+ ATTTTTAAAA TTATCCTGAT AGAGAATAAA AAGAAATCCT TATACAAAAC TCTGATTGAC GCAGATGTGT   
  
  
+ ATATGGACAT ATAGAAGTAA TAAGAGGGGT AGGCTAGGAA GGGGGTGGAA ATATAGGGCA TAAATAGGGG   
  
  
+ GCGGGCATAG GGACATGTCT GTCTGAGGTA CGGTGGATGG GACCTTGCCA TGGAAGCAAC AATGTCACAA   
  
  
+ TAATAATATC AACGACTGAA GTTTCTTCAC CAGCCCACTC TTTGCCATAA TGCCATTAAT TATTTATTGC   
  
  
+ TACTATCTCT TTTGATGTGA AACGGTGGAC CTTATCTCTC CCCTCCACTT CCAATTGATG TGAGCAACTC   
  
  
+ AGAAACGCAA ACAAAACTTG TGCCACCTTC TTTACTTGCA TCCTCATTTT CCTCTGTCTC TCTCCGCCCC   
  
  
+ TGTTTCTCTC TCTCTCCCTC ACTCACTCAG AGTACTGATC TGAGCATCAG AAGTAGCAGA CCCAGAAACT   
  
  
+ CAGATTTAAA AACAACCCGC TCTTTTGTTC CCTCTACGTC TAATAATTTT GTCATCGCAT TTAATGTCAG   
  
  
+ ATAGAGAGAT AGAGCTCTAC TGTATTAATT TTCTCCCACC TCACTTGATT CTGTTGCCCT AAACCACCCA   
  
  
+ AAAAAAGAAA TACGAAGAAG CCACTCTCTC TCTCTCTCTC TCTCACACAC ACACACACCC CTCCTAGTGG   
  
  
+ ATTTGATAGA GTATAAATGA ACAGTAATCA GACGAGATAT ATAGTAGTGC TTAATTGACC GAGTAGCAAA   
  
  
+ AGGGGGAAGT TGAGTGAGGT ATCAAGAGAG TAGCGTAGCT AGCTAGGGCA AGATATGCTG GGGTCTTCCT   
  
  
+ CATACAACTC CGAGGAAGAC CACCAAGATG ACAACCATTC CTCACCGTCT GATTTGTCTA GGCACCCACC   
  
  
+ TAGATTCACC ACTACTACTT ACCAATACCA ACAACATATT AGCTCTCCAT CTTCACAATC AATACTAGCT   
  
  
+ GTGCCACCTC GCCAATTGTT AATCAATTGT GCGGAGTTAA TTTGCCGGTT CGACTTCTCC TCCGCCAACC   
  
  
+ GGCTTCTCTC TCTCCTCGCC TCCCACTTTG CCTCCCCTTC TGGGGACTCC ACCGAGAGAC TTGTGTTTTA   
  
  
+ CTTCTGCAAG GCTTTGGATC TCCGCCTCAA TAACAGTTAT TTATCCACTC GTAGTTTGAC TGGTTGTAAC   
  
  
+ CCTAGTACTG GTTCTGGTAT TGGGGCTCCT TTAGCTTCCA ATTTTCTATT TCTTCAACCA TTAGTGCATT   
  
  
+ CACAGGTAAC AAGTAATGCC CGTGTTGGGT ACTTGACATA TTTGACTCTG AACCAGGTCA CCCCATTCAT   
  
  
+ CCGATTCACC CACTTAACTG CCAACCAAGC CATCCTGGAA GCTTTGGAGG GGTACAAGGC GGTCCATATC   
  
  
+ CTTGACATGG ACATCATGCA TGGGGTCCAG TGGCCACCAC TTCTTCAAGC AATTGCAGAA AGATCTGCAA   
  
  
+ CCCTTGGTCA CCCTGCTCCC ACTGTTCGCA TCACAGGAGG GGGCAGGGAT CTAGAACTCC TGAACCGAAC   
  
  
+ TGGAGATCGG ATCAGGAAGT TCGCACAATC CTTGGGACTC GAGTTTCAAT TCCACCATCT TATAACGGTG   
  
  
+ GACTCGAACC TTACTGAGGC AGTGGAGGCC ATCAATGCCC TACAGCTACA CCTCCATGTC CAAGAGGAGG   
  
  
+ AGGTCTTCGC AGTGAATTGT GGAGATTTCC TCCACCGCCT CCTAACGGAG TATGATAGTA CATTCCTTAG   
  
  
+ GATGTTTCTC TACAAGGTGA AGACCCTAAA CCCTAGGGTC TTCACCGTGG GAGAAAGGGA GGCCGACCAC   
  
  
+ AACCACCCTC TCTTCTGGCA GCGATTCATT GAGGCACTTG ACCACTATGG GGCAGTGTTT GACTCTCTAG   
  
  
+ AAGCAACCCT ACCATCAACA AGTCAAGAGA GGGTGGCGGT GGAAGAGGGG TGGTTCGGGG AGGAGATTAA   
  
  
+ AGATGTGGTT GGACGAGAAG GAGGGTTGAG GAGACAGAGG CACCAGAAAT ACGAGTCTTG GGAGGTGTTG   
  
  
+ ATGAGGAGCT CTGGGTTTAA GAGCTTGCCC TTGAGCCCCT TCTCTGTGTC ACAAGCCAAG CTGCTACTTC   
  
  
+ GCCTTCATTA CCCTTCTGAA GGGTACCACC TTAAGGTCTT CCATAGTAAT TGTCTGTTGC TTGGGTGGAA   
  
  
+ GAATCGCCCT CTTTTCTCTG TTTCTTCTTG GCAATA  

- -Up\_Stream \_Len000TTTTAT AAAAATGTTT TCGTAAAGAG ATATTTTATA AAATGTTTTA AAGTAAAACA   
  
  
- CCCAGAAAAT CTTAGTTAGA GAGATGTTCA TCCTTACGGC CAACTCCATT CCGATGGATG TAGATCTAGA   
  
  
- GAGGTCTGGG GTGTTTTTAA TTTGCTCTGG GTGTGACTTT CCACCACCAC CACCACAACA ACAACATAAC   
  
  
- AGGATGGTGC TCTCTCGAAG TTCACGTAGT GAATAAAGTT GTCCAGTTAG TGTATCTAGA GATCACATGA   
  
  
- CATTTAAAAA AAAGGCAGAA TAAAATTAAA AGAAAAGGTA GGATTTTCTA ATAGTACATC TATAAGGATC   
  
  
- AAACTATATA ACTGAAGTTG AAGTTTTTAA TGTGTAATTC TAGGTGCTTA TACTGTCAAA TCCAAAGTAA   
  
  
- ACTTCCTCTT TCTTTACAAT GCCACAAAAC CACAACATAA GCATCTGTAA AAAACGCGAA TTGACCGAGA   
  
  
- AGAGTACCCC TGCTCTGTCA CTTCCATTAA CATGAACAAC TAACTACTGC AGTAGACATC GAACCTAAAC   
  
  
- ATATCCTTAC TTGTAGAACC ACAGATGCAC CTTTCACACT AAACACAAGA AAGTAACTAG GAGGAGGAGT   
  
  
- TCTTCTCCAC TAGAAAGAGG GAACAAAAAA AAAAAAAAAC CGATTATACA ACCGTCTTGG CCGTTTTTTA   
  
  
- TGTATTAAAC TTATCACGTA CGTATACTCA TGTACTGTCA CTTGGTTGTT TTGCTTGGGG ATTTTGTGTT   
  
  
- CTCTTAATTG TACAATTTTG TGAATTTGAT TTACGTTGGT AGAGCTACTG GTGATTTATC GGTTCTTTGG   
  
  
- ATTGCATTAA TTGTTTTTAT TTTTTATTGA CTTTTATTTC ATCTCTTTCA GATTGGCTTA TGCTATTGTA   
  
  
- TTGGATTTTG ATTGATATGA ACTTGAATGA TTCTTTATAC TTAGTTGGTG TTTGGCTTAA CTAAAACCAA   
  
  
- ACGGTATTAA ACTGATATGT AAATTGAATT TTTTATAAAA AAAAATTAAA GTTTATTTAG ATTTAATAAA   
  
  
- AAGTTATAAG TAGGTTTTAT CTAAGTTAAT AATTTGTTCA GTTGGATATG GCTCAATAAA AGATCGAGAC   
  
  
- TGATTCTGTA TATATATAAA AAACATTTTT TCTATTCCTA ATATTAAATT TATCGGAAAG GGAATTTTTT   
  
  
- TAAAAATTTT AATAGGACTA TCTCTTATTT TTCTTTAGGA ATATGTTTTG AGACTAACTG CGTCTACACA   
  
  
- TATACCTGTA TATCTTCATT ATTCTCCCCA TCCGATCCTT CCCCCACCTT TATATCCCGT ATTTATCCCC   
  
  
- CGCCCGTATC CCTGTACAGA CAGACTCCAT GCCACCTACC CTGGAACGGT ACCTTCGTTG TTACAGTGTT   
  
  
- ATTATTATAG TTGCTGACTT CAAAGAAGTG GTCGGGTGAG AAACGGTATT ACGGTAATTA ATAAATAACG   
  
  
- ATGATAGAGA AAACTACACT TTGCCACCTG GAATAGAGAG GGGAGGTGAA GGTTAACTAC ACTCGTTGAG   
  
  
- TCTTTGCGTT TGTTTTGAAC ACGGTGGAAG AAATGAACGT AGGAGTAAAA GGAGACAGAG AGAGGCGGGG   
  
  
- ACAAAGAGAG AGAGAGGGAG TGAGTGAGTC TCATGACTAG ACTCGTAGTC TTCATCGTCT GGGTCTTTGA   
  
  
- GTCTAAATTT TTGTTGGGCG AGAAAACAAG GGAGATGCAG ATTATTAAAA CAGTAGCGTA AATTACAGTC   
  
  
- TATCTCTCTA TCTCGAGATG ACATAATTAA AAGAGGGTGG AGTGAACTAA GACAACGGGA TTTGGTGGGT   
  
  
- TTTTTTCTTT ATGCTTCTTC GGTGAGAGAG AGAGAGAGAG AGAGTGTGTG TGTGTGTGGG GAGGATCACC   
  
  
- TAAACTATCT CATATTTACT TGTCATTAGT CTGCTCTATA TATCATCACG AATTAACTGG CTCATCGTTT   
  
  
- TCCCCCTTCA ACTCACTCCA TAGTTCTCTC ATCGCATCGA TCGATCCCGT TCTATACGAC CCCAGAAGGA   
  
  
- GTATGTTGAG GCTCCTTCTG GTGGTTCTAC TGTTGGTAAG GAGTGGCAGA CTAAACAGAT CCGTGGGTGG   
  
  
- ATCTAAGTGG TGATGATGAA TGGTTATGGT TGTTGTATAA TCGAGAGGTA GAAGTGTTAG TTATGATCGA   
  
  
- CACGGTGGAG CGGTTAACAA TTAGTTAACA CGCCTCAATT AAACGGCCAA GCTGAAGAGG AGGCGGTTGG   
  
  
- CCGAAGAGAG AGAGGAGCGG AGGGTGAAAC GGAGGGGAAG ACCCCTGAGG TGGCTCTCTG AACACAAAAT   
  
  
- GAAGACGTTC CGAAACCTAG AGGCGGAGTT ATTGTCAATA AATAGGTGAG CATCAAACTG ACCAACATTG   
  
  
- GGATCATGAC CAAGACCATA ACCCCGAGGA AATCGAAGGT TAAAAGATAA AGAAGTTGGT AATCACGTAA   
  
  
- GTGTCCATTG TTCATTACGG GCACAACCCA TGAACTGTAT AAACTGAGAC TTGGTCCAGT GGGGTAAGTA   
  
  
- GGCTAAGTGG GTGAATTGAC GGTTGGTTCG GTAGGACCTT CGAAACCTCC CCATGTTCCG CCAGGTATAG   
  
  
- GAACTGTACC TGTAGTACGT ACCCCAGGTC ACCGGTGGTG AAGAAGTTCG TTAACGTCTT TCTAGACGTT   
  
  
- GGGAACCAGT GGGACGAGGG TGACAAGCGT AGTGTCCTCC CCCGTCCCTA GATCTTGAGG ACTTGGCTTG   
  
  
- ACCTCTAGCC TAGTCCTTCA AGCGTGTTAG GAACCCTGAG CTCAAAGTTA AGGTGGTAGA ATATTGCCAC   
  
  
- CTGAGCTTGG AATGACTCCG TCACCTCCGG TAGTTACGGG ATGTCGATGT GGAGGTACAG GTTCTCCTCC   
  
  
- TCCAGAAGCG TCACTTAACA CCTCTAAAGG AGGTGGCGGA GGATTGCCTC ATACTATCAT GTAAGGAATC   
  
  
- CTACAAAGAG ATGTTCCACT TCTGGGATTT GGGATCCCAG AAGTGGCACC CTCTTTCCCT CCGGCTGGTG   
  
  
- TTGGTGGGAG AGAAGACCGT CGCTAAGTAA CTCCGTGAAC TGGTGATACC CCGTCACAAA CTGAGAGATC   
  
  
- TTCGTTGGGA TGGTAGTTGT TCAGTTCTCT CCCACCGCCA CCTTCTCCCC ACCAAGCCCC TCCTCTAATT   
  
  
- TCTACACCAA CCTGCTCTTC CTCCCAACTC CTCTGTCTCC GTGGTCTTTA TGCTCAGAAC CCTCCACAAC   
  
  
- TACTCCTCGA GACCCAAATT CTCGAACGGG AACTCGGGGA AGAGACACAG TGTTCGGTTC GACGATGAAG   
  
  
- CGGAAGTAAT GGGAAGACTT CCCATGGTGG AATTCCAGAA GGTATCATTA ACAGACAACG AACCCACCTT   
  
  
- CTTAGCGGGA GAAAAGAGAC AAAGAAGAAC CGTTAT

+     TCCC-motif

| Site Name | Organism | Position | Strand | Matrix score. | sequence | function |
| --- | --- | --- | --- | --- | --- | --- |
| TCCC-motif | Spinacia oleracea | 1627 | + | 7 | TCTCCCT | part of a light responsive element |
| TCCC-motif | Spinacia oleracea | 650 | + | 7 | TCTCCCT | part of a light responsive element |

>HU01G00833.1   
+ -Up\_Stream \_Len000AAAATA TTTTTACAAA AGCATTTCTC TATAAAATAT TTTACAAAAT TTCATTTTGT   
  
  
+ GGGTCTTTTA GAATCAATCT CTCTACAAGT AGGAATGCCG GTTGAGGTAA GGCTACCTAC ATCTAGATCT   
  
  
+ CTCCAGACCC CACAAAAATT AAACGAGACC CACACTGAAA GGTGGTGGTG GTGGTGTTGT TGTTGTATTG   
  
  
+ TCCTACCACG AGAGAGCTTC AAGTGCATCA CTTATTTCAA CAGGTCAATC ACATAGATCT CTAGTGTACT   
  
  
+ GTAAATTTTT TTTCCGTCTT ATTTTAATTT TCTTTTCCAT CCTAAAAGAT TATCATGTAG ATATTCCTAG   
  
  
+ TTTGATATAT TGACTTCAAC TTCAAAAATT ACACATTAAG ATCCACGAAT ATGACAGTTT AGGTTTCATT   
  
  
+ TGAAGGAGAA AGAAATGTTA CGGTGTTTTG GTGTTGTATT CGTAGACATT TTTTGCGCTT AACTGGCTCT   
  
  
+ TCTCATGGGG ACGAGACAGT GAAGGTAATT GTACTTGTTG ATTGATGACG TCATCTGTAG CTTGGATTTG   
  
  
+ TATAGGAATG AACATCTTGG TGTCTACGTG GAAAGTGTGA TTTGTGTTCT TTCATTGATC CTCCTCCTCA   
  
  
+ AGAAGAGGTG ATCTTTCTCC CTTGTTTTTT TTTTTTTTTG GCTAATATGT TGGCAGAACC GGCAAAAAAT   
  
  
+ ACATAATTTG AATAGTGCAT GCATATGAGT ACATGACAGT GAACCAACAA AACGAACCCC TAAAACACAA   
  
  
+ GAGAATTAAC ATGTTAAAAC ACTTAAACTA AATGCAACCA TCTCGATGAC CACTAAATAG CCAAGAAACC   
  
  
+ TAACGTAATT AACAAAAATA AAAAATAACT GAAAATAAAG TAGAGAAAGT CTAACCGAAT ACGATAACAT   
  
  
+ AACCTAAAAC TAACTATACT TGAACTTACT AAGAAATATG AATCAACCAC AAACCGAATT GATTTTGGTT   
  
  
+ TGCCATAATT TGACTATACA TTTAACTTAA AAAATATTTT TTTTTAATTT CAAATAAATC TAAATTATTT   
  
  
+ TTCAATATTC ATCCAAAATA GATTCAATTA TTAAACAAGT CAACCTATAC CGAGTTATTT TCTAGCTCTG   
  
  
+ ACTAAGACAT ATATATATTT TTTGTAAAAA AGATAAGGAT TATAATTTAA ATAGCCTTTC CCTTAAAAAA   
  
  
+ ATTTTTAAAA TTATCCTGAT AGAGAATAAA AAGAAATCCT TATACAAAAC TCTGATTGAC GCAGATGTGT   
  
  
+ ATATGGACAT ATAGAAGTAA TAAGAGGGGT AGGCTAGGAA GGGGGTGGAA ATATAGGGCA TAAATAGGGG   
  
  
+ GCGGGCATAG GGACATGTCT GTCTGAGGTA CGGTGGATGG GACCTTGCCA TGGAAGCAAC AATGTCACAA   
  
  
+ TAATAATATC AACGACTGAA GTTTCTTCAC CAGCCCACTC TTTGCCATAA TGCCATTAAT TATTTATTGC   
  
  
+ TACTATCTCT TTTGATGTGA AACGGTGGAC CTTATCTCTC CCCTCCACTT CCAATTGATG TGAGCAACTC   
  
  
+ AGAAACGCAA ACAAAACTTG TGCCACCTTC TTTACTTGCA TCCTCATTTT CCTCTGTCTC TCTCCGCCCC   
  
  
+ TGTTTCTCTC TCTCTCCCTC ACTCACTCAG AGTACTGATC TGAGCATCAG AAGTAGCAGA CCCAGAAACT   
  
  
+ CAGATTTAAA AACAACCCGC TCTTTTGTTC CCTCTACGTC TAATAATTTT GTCATCGCAT TTAATGTCAG   
  
  
+ ATAGAGAGAT AGAGCTCTAC TGTATTAATT TTCTCCCACC TCACTTGATT CTGTTGCCCT AAACCACCCA   
  
  
+ AAAAAAGAAA TACGAAGAAG CCACTCTCTC TCTCTCTCTC TCTCACACAC ACACACACCC CTCCTAGTGG   
  
  
+ ATTTGATAGA GTATAAATGA ACAGTAATCA GACGAGATAT ATAGTAGTGC TTAATTGACC GAGTAGCAAA   
  
  
+ AGGGGGAAGT TGAGTGAGGT ATCAAGAGAG TAGCGTAGCT AGCTAGGGCA AGATATGCTG GGGTCTTCCT   
  
  
+ CATACAACTC CGAGGAAGAC CACCAAGATG ACAACCATTC CTCACCGTCT GATTTGTCTA GGCACCCACC   
  
  
+ TAGATTCACC ACTACTACTT ACCAATACCA ACAACATATT AGCTCTCCAT CTTCACAATC AATACTAGCT   
  
  
+ GTGCCACCTC GCCAATTGTT AATCAATTGT GCGGAGTTAA TTTGCCGGTT CGACTTCTCC TCCGCCAACC   
  
  
+ GGCTTCTCTC TCTCCTCGCC TCCCACTTTG CCTCCCCTTC TGGGGACTCC ACCGAGAGAC TTGTGTTTTA   
  
  
+ CTTCTGCAAG GCTTTGGATC TCCGCCTCAA TAACAGTTAT TTATCCACTC GTAGTTTGAC TGGTTGTAAC   
  
  
+ CCTAGTACTG GTTCTGGTAT TGGGGCTCCT TTAGCTTCCA ATTTTCTATT TCTTCAACCA TTAGTGCATT   
  
  
+ CACAGGTAAC AAGTAATGCC CGTGTTGGGT ACTTGACATA TTTGACTCTG AACCAGGTCA CCCCATTCAT   
  
  
+ CCGATTCACC CACTTAACTG CCAACCAAGC CATCCTGGAA GCTTTGGAGG GGTACAAGGC GGTCCATATC   
  
  
+ CTTGACATGG ACATCATGCA TGGGGTCCAG TGGCCACCAC TTCTTCAAGC AATTGCAGAA AGATCTGCAA   
  
  
+ CCCTTGGTCA CCCTGCTCCC ACTGTTCGCA TCACAGGAGG GGGCAGGGAT CTAGAACTCC TGAACCGAAC   
  
  
+ TGGAGATCGG ATCAGGAAGT TCGCACAATC CTTGGGACTC GAGTTTCAAT TCCACCATCT TATAACGGTG   
  
  
+ GACTCGAACC TTACTGAGGC AGTGGAGGCC ATCAATGCCC TACAGCTACA CCTCCATGTC CAAGAGGAGG   
  
  
+ AGGTCTTCGC AGTGAATTGT GGAGATTTCC TCCACCGCCT CCTAACGGAG TATGATAGTA CATTCCTTAG   
  
  
+ GATGTTTCTC TACAAGGTGA AGACCCTAAA CCCTAGGGTC TTCACCGTGG GAGAAAGGGA GGCCGACCAC   
  
  
+ AACCACCCTC TCTTCTGGCA GCGATTCATT GAGGCACTTG ACCACTATGG GGCAGTGTTT GACTCTCTAG   
  
  
+ AAGCAACCCT ACCATCAACA AGTCAAGAGA GGGTGGCGGT GGAAGAGGGG TGGTTCGGGG AGGAGATTAA   
  
  
+ AGATGTGGTT GGACGAGAAG GAGGGTTGAG GAGACAGAGG CACCAGAAAT ACGAGTCTTG GGAGGTGTTG   
  
  
+ ATGAGGAGCT CTGGGTTTAA GAGCTTGCCC TTGAGCCCCT TCTCTGTGTC ACAAGCCAAG CTGCTACTTC   
  
  
+ GCCTTCATTA CCCTTCTGAA GGGTACCACC TTAAGGTCTT CCATAGTAAT TGTCTGTTGC TTGGGTGGAA   
  
  
+ GAATCGCCCT CTTTTCTCTG TTTCTTCTTG GCAATA  

- -Up\_Stream \_Len000TTTTAT AAAAATGTTT TCGTAAAGAG ATATTTTATA AAATGTTTTA AAGTAAAACA   
  
  
- CCCAGAAAAT CTTAGTTAGA GAGATGTTCA TCCTTACGGC CAACTCCATT CCGATGGATG TAGATCTAGA   
  
  
- GAGGTCTGGG GTGTTTTTAA TTTGCTCTGG GTGTGACTTT CCACCACCAC CACCACAACA ACAACATAAC   
  
  
- AGGATGGTGC TCTCTCGAAG TTCACGTAGT GAATAAAGTT GTCCAGTTAG TGTATCTAGA GATCACATGA   
  
  
- CATTTAAAAA AAAGGCAGAA TAAAATTAAA AGAAAAGGTA GGATTTTCTA ATAGTACATC TATAAGGATC   
  
  
- AAACTATATA ACTGAAGTTG AAGTTTTTAA TGTGTAATTC TAGGTGCTTA TACTGTCAAA TCCAAAGTAA   
  
  
- ACTTCCTCTT TCTTTACAAT GCCACAAAAC CACAACATAA GCATCTGTAA AAAACGCGAA TTGACCGAGA   
  
  
- AGAGTACCCC TGCTCTGTCA CTTCCATTAA CATGAACAAC TAACTACTGC AGTAGACATC GAACCTAAAC   
  
  
- ATATCCTTAC TTGTAGAACC ACAGATGCAC CTTTCACACT AAACACAAGA AAGTAACTAG GAGGAGGAGT   
  
  
- TCTTCTCCAC TAGAAAGAGG GAACAAAAAA AAAAAAAAAC CGATTATACA ACCGTCTTGG CCGTTTTTTA   
  
  
- TGTATTAAAC TTATCACGTA CGTATACTCA TGTACTGTCA CTTGGTTGTT TTGCTTGGGG ATTTTGTGTT   
  
  
- CTCTTAATTG TACAATTTTG TGAATTTGAT TTACGTTGGT AGAGCTACTG GTGATTTATC GGTTCTTTGG   
  
  
- ATTGCATTAA TTGTTTTTAT TTTTTATTGA CTTTTATTTC ATCTCTTTCA GATTGGCTTA TGCTATTGTA   
  
  
- TTGGATTTTG ATTGATATGA ACTTGAATGA TTCTTTATAC TTAGTTGGTG TTTGGCTTAA CTAAAACCAA   
  
  
- ACGGTATTAA ACTGATATGT AAATTGAATT TTTTATAAAA AAAAATTAAA GTTTATTTAG ATTTAATAAA   
  
  
- AAGTTATAAG TAGGTTTTAT CTAAGTTAAT AATTTGTTCA GTTGGATATG GCTCAATAAA AGATCGAGAC   
  
  
- TGATTCTGTA TATATATAAA AAACATTTTT TCTATTCCTA ATATTAAATT TATCGGAAAG GGAATTTTTT   
  
  
- TAAAAATTTT AATAGGACTA TCTCTTATTT TTCTTTAGGA ATATGTTTTG AGACTAACTG CGTCTACACA   
  
  
- TATACCTGTA TATCTTCATT ATTCTCCCCA TCCGATCCTT CCCCCACCTT TATATCCCGT ATTTATCCCC   
  
  
- CGCCCGTATC CCTGTACAGA CAGACTCCAT GCCACCTACC CTGGAACGGT ACCTTCGTTG TTACAGTGTT   
  
  
- ATTATTATAG TTGCTGACTT CAAAGAAGTG GTCGGGTGAG AAACGGTATT ACGGTAATTA ATAAATAACG   
  
  
- ATGATAGAGA AAACTACACT TTGCCACCTG GAATAGAGAG GGGAGGTGAA GGTTAACTAC ACTCGTTGAG   
  
  
- TCTTTGCGTT TGTTTTGAAC ACGGTGGAAG AAATGAACGT AGGAGTAAAA GGAGACAGAG AGAGGCGGGG   
  
  
- ACAAAGAGAG AGAGAGGGAG TGAGTGAGTC TCATGACTAG ACTCGTAGTC TTCATCGTCT GGGTCTTTGA   
  
  
- GTCTAAATTT TTGTTGGGCG AGAAAACAAG GGAGATGCAG ATTATTAAAA CAGTAGCGTA AATTACAGTC   
  
  
- TATCTCTCTA TCTCGAGATG ACATAATTAA AAGAGGGTGG AGTGAACTAA GACAACGGGA TTTGGTGGGT   
  
  
- TTTTTTCTTT ATGCTTCTTC GGTGAGAGAG AGAGAGAGAG AGAGTGTGTG TGTGTGTGGG GAGGATCACC   
  
  
- TAAACTATCT CATATTTACT TGTCATTAGT CTGCTCTATA TATCATCACG AATTAACTGG CTCATCGTTT   
  
  
- TCCCCCTTCA ACTCACTCCA TAGTTCTCTC ATCGCATCGA TCGATCCCGT TCTATACGAC CCCAGAAGGA   
  
  
- GTATGTTGAG GCTCCTTCTG GTGGTTCTAC TGTTGGTAAG GAGTGGCAGA CTAAACAGAT CCGTGGGTGG   
  
  
- ATCTAAGTGG TGATGATGAA TGGTTATGGT TGTTGTATAA TCGAGAGGTA GAAGTGTTAG TTATGATCGA   
  
  
- CACGGTGGAG CGGTTAACAA TTAGTTAACA CGCCTCAATT AAACGGCCAA GCTGAAGAGG AGGCGGTTGG   
  
  
- CCGAAGAGAG AGAGGAGCGG AGGGTGAAAC GGAGGGGAAG ACCCCTGAGG TGGCTCTCTG AACACAAAAT   
  
  
- GAAGACGTTC CGAAACCTAG AGGCGGAGTT ATTGTCAATA AATAGGTGAG CATCAAACTG ACCAACATTG   
  
  
- GGATCATGAC CAAGACCATA ACCCCGAGGA AATCGAAGGT TAAAAGATAA AGAAGTTGGT AATCACGTAA   
  
  
- GTGTCCATTG TTCATTACGG GCACAACCCA TGAACTGTAT AAACTGAGAC TTGGTCCAGT GGGGTAAGTA   
  
  
- GGCTAAGTGG GTGAATTGAC GGTTGGTTCG GTAGGACCTT CGAAACCTCC CCATGTTCCG CCAGGTATAG   
  
  
- GAACTGTACC TGTAGTACGT ACCCCAGGTC ACCGGTGGTG AAGAAGTTCG TTAACGTCTT TCTAGACGTT   
  
  
- GGGAACCAGT GGGACGAGGG TGACAAGCGT AGTGTCCTCC CCCGTCCCTA GATCTTGAGG ACTTGGCTTG   
  
  
- ACCTCTAGCC TAGTCCTTCA AGCGTGTTAG GAACCCTGAG CTCAAAGTTA AGGTGGTAGA ATATTGCCAC   
  
  
- CTGAGCTTGG AATGACTCCG TCACCTCCGG TAGTTACGGG ATGTCGATGT GGAGGTACAG GTTCTCCTCC   
  
  
- TCCAGAAGCG TCACTTAACA CCTCTAAAGG AGGTGGCGGA GGATTGCCTC ATACTATCAT GTAAGGAATC   
  
  
- CTACAAAGAG ATGTTCCACT TCTGGGATTT GGGATCCCAG AAGTGGCACC CTCTTTCCCT CCGGCTGGTG   
  
  
- TTGGTGGGAG AGAAGACCGT CGCTAAGTAA CTCCGTGAAC TGGTGATACC CCGTCACAAA CTGAGAGATC   
  
  
- TTCGTTGGGA TGGTAGTTGT TCAGTTCTCT CCCACCGCCA CCTTCTCCCC ACCAAGCCCC TCCTCTAATT   
  
  
- TCTACACCAA CCTGCTCTTC CTCCCAACTC CTCTGTCTCC GTGGTCTTTA TGCTCAGAAC CCTCCACAAC   
  
  
- TACTCCTCGA GACCCAAATT CTCGAACGGG AACTCGGGGA AGAGACACAG TGTTCGGTTC GACGATGAAG   
  
  
- CGGAAGTAAT GGGAAGACTT CCCATGGTGG AATTCCAGAA GGTATCATTA ACAGACAACG AACCCACCTT   
  
  
- CTTAGCGGGA GAAAAGAGAC AAAGAAGAAC CGTTAT

+     TGA-element

| Site Name | Organism | Position | Strand | Matrix score. | sequence | function |
| --- | --- | --- | --- | --- | --- | --- |
| TGA-element | Brassica oleracea | 1415 | + | 6 | AACGAC | auxin-responsive element |

>HU01G00833.1   
+ -Up\_Stream \_Len000AAAATA TTTTTACAAA AGCATTTCTC TATAAAATAT TTTACAAAAT TTCATTTTGT   
  
  
+ GGGTCTTTTA GAATCAATCT CTCTACAAGT AGGAATGCCG GTTGAGGTAA GGCTACCTAC ATCTAGATCT   
  
  
+ CTCCAGACCC CACAAAAATT AAACGAGACC CACACTGAAA GGTGGTGGTG GTGGTGTTGT TGTTGTATTG   
  
  
+ TCCTACCACG AGAGAGCTTC AAGTGCATCA CTTATTTCAA CAGGTCAATC ACATAGATCT CTAGTGTACT   
  
  
+ GTAAATTTTT TTTCCGTCTT ATTTTAATTT TCTTTTCCAT CCTAAAAGAT TATCATGTAG ATATTCCTAG   
  
  
+ TTTGATATAT TGACTTCAAC TTCAAAAATT ACACATTAAG ATCCACGAAT ATGACAGTTT AGGTTTCATT   
  
  
+ TGAAGGAGAA AGAAATGTTA CGGTGTTTTG GTGTTGTATT CGTAGACATT TTTTGCGCTT AACTGGCTCT   
  
  
+ TCTCATGGGG ACGAGACAGT GAAGGTAATT GTACTTGTTG ATTGATGACG TCATCTGTAG CTTGGATTTG   
  
  
+ TATAGGAATG AACATCTTGG TGTCTACGTG GAAAGTGTGA TTTGTGTTCT TTCATTGATC CTCCTCCTCA   
  
  
+ AGAAGAGGTG ATCTTTCTCC CTTGTTTTTT TTTTTTTTTG GCTAATATGT TGGCAGAACC GGCAAAAAAT   
  
  
+ ACATAATTTG AATAGTGCAT GCATATGAGT ACATGACAGT GAACCAACAA AACGAACCCC TAAAACACAA   
  
  
+ GAGAATTAAC ATGTTAAAAC ACTTAAACTA AATGCAACCA TCTCGATGAC CACTAAATAG CCAAGAAACC   
  
  
+ TAACGTAATT AACAAAAATA AAAAATAACT GAAAATAAAG TAGAGAAAGT CTAACCGAAT ACGATAACAT   
  
  
+ AACCTAAAAC TAACTATACT TGAACTTACT AAGAAATATG AATCAACCAC AAACCGAATT GATTTTGGTT   
  
  
+ TGCCATAATT TGACTATACA TTTAACTTAA AAAATATTTT TTTTTAATTT CAAATAAATC TAAATTATTT   
  
  
+ TTCAATATTC ATCCAAAATA GATTCAATTA TTAAACAAGT CAACCTATAC CGAGTTATTT TCTAGCTCTG   
  
  
+ ACTAAGACAT ATATATATTT TTTGTAAAAA AGATAAGGAT TATAATTTAA ATAGCCTTTC CCTTAAAAAA   
  
  
+ ATTTTTAAAA TTATCCTGAT AGAGAATAAA AAGAAATCCT TATACAAAAC TCTGATTGAC GCAGATGTGT   
  
  
+ ATATGGACAT ATAGAAGTAA TAAGAGGGGT AGGCTAGGAA GGGGGTGGAA ATATAGGGCA TAAATAGGGG   
  
  
+ GCGGGCATAG GGACATGTCT GTCTGAGGTA CGGTGGATGG GACCTTGCCA TGGAAGCAAC AATGTCACAA   
  
  
+ TAATAATATC AACGACTGAA GTTTCTTCAC CAGCCCACTC TTTGCCATAA TGCCATTAAT TATTTATTGC   
  
  
+ TACTATCTCT TTTGATGTGA AACGGTGGAC CTTATCTCTC CCCTCCACTT CCAATTGATG TGAGCAACTC   
  
  
+ AGAAACGCAA ACAAAACTTG TGCCACCTTC TTTACTTGCA TCCTCATTTT CCTCTGTCTC TCTCCGCCCC   
  
  
+ TGTTTCTCTC TCTCTCCCTC ACTCACTCAG AGTACTGATC TGAGCATCAG AAGTAGCAGA CCCAGAAACT   
  
  
+ CAGATTTAAA AACAACCCGC TCTTTTGTTC CCTCTACGTC TAATAATTTT GTCATCGCAT TTAATGTCAG   
  
  
+ ATAGAGAGAT AGAGCTCTAC TGTATTAATT TTCTCCCACC TCACTTGATT CTGTTGCCCT AAACCACCCA   
  
  
+ AAAAAAGAAA TACGAAGAAG CCACTCTCTC TCTCTCTCTC TCTCACACAC ACACACACCC CTCCTAGTGG   
  
  
+ ATTTGATAGA GTATAAATGA ACAGTAATCA GACGAGATAT ATAGTAGTGC TTAATTGACC GAGTAGCAAA   
  
  
+ AGGGGGAAGT TGAGTGAGGT ATCAAGAGAG TAGCGTAGCT AGCTAGGGCA AGATATGCTG GGGTCTTCCT   
  
  
+ CATACAACTC CGAGGAAGAC CACCAAGATG ACAACCATTC CTCACCGTCT GATTTGTCTA GGCACCCACC   
  
  
+ TAGATTCACC ACTACTACTT ACCAATACCA ACAACATATT AGCTCTCCAT CTTCACAATC AATACTAGCT   
  
  
+ GTGCCACCTC GCCAATTGTT AATCAATTGT GCGGAGTTAA TTTGCCGGTT CGACTTCTCC TCCGCCAACC   
  
  
+ GGCTTCTCTC TCTCCTCGCC TCCCACTTTG CCTCCCCTTC TGGGGACTCC ACCGAGAGAC TTGTGTTTTA   
  
  
+ CTTCTGCAAG GCTTTGGATC TCCGCCTCAA TAACAGTTAT TTATCCACTC GTAGTTTGAC TGGTTGTAAC   
  
  
+ CCTAGTACTG GTTCTGGTAT TGGGGCTCCT TTAGCTTCCA ATTTTCTATT TCTTCAACCA TTAGTGCATT   
  
  
+ CACAGGTAAC AAGTAATGCC CGTGTTGGGT ACTTGACATA TTTGACTCTG AACCAGGTCA CCCCATTCAT   
  
  
+ CCGATTCACC CACTTAACTG CCAACCAAGC CATCCTGGAA GCTTTGGAGG GGTACAAGGC GGTCCATATC   
  
  
+ CTTGACATGG ACATCATGCA TGGGGTCCAG TGGCCACCAC TTCTTCAAGC AATTGCAGAA AGATCTGCAA   
  
  
+ CCCTTGGTCA CCCTGCTCCC ACTGTTCGCA TCACAGGAGG GGGCAGGGAT CTAGAACTCC TGAACCGAAC   
  
  
+ TGGAGATCGG ATCAGGAAGT TCGCACAATC CTTGGGACTC GAGTTTCAAT TCCACCATCT TATAACGGTG   
  
  
+ GACTCGAACC TTACTGAGGC AGTGGAGGCC ATCAATGCCC TACAGCTACA CCTCCATGTC CAAGAGGAGG   
  
  
+ AGGTCTTCGC AGTGAATTGT GGAGATTTCC TCCACCGCCT CCTAACGGAG TATGATAGTA CATTCCTTAG   
  
  
+ GATGTTTCTC TACAAGGTGA AGACCCTAAA CCCTAGGGTC TTCACCGTGG GAGAAAGGGA GGCCGACCAC   
  
  
+ AACCACCCTC TCTTCTGGCA GCGATTCATT GAGGCACTTG ACCACTATGG GGCAGTGTTT GACTCTCTAG   
  
  
+ AAGCAACCCT ACCATCAACA AGTCAAGAGA GGGTGGCGGT GGAAGAGGGG TGGTTCGGGG AGGAGATTAA   
  
  
+ AGATGTGGTT GGACGAGAAG GAGGGTTGAG GAGACAGAGG CACCAGAAAT ACGAGTCTTG GGAGGTGTTG   
  
  
+ ATGAGGAGCT CTGGGTTTAA GAGCTTGCCC TTGAGCCCCT TCTCTGTGTC ACAAGCCAAG CTGCTACTTC   
  
  
+ GCCTTCATTA CCCTTCTGAA GGGTACCACC TTAAGGTCTT CCATAGTAAT TGTCTGTTGC TTGGGTGGAA   
  
  
+ GAATCGCCCT CTTTTCTCTG TTTCTTCTTG GCAATA  

- -Up\_Stream \_Len000TTTTAT AAAAATGTTT TCGTAAAGAG ATATTTTATA AAATGTTTTA AAGTAAAACA   
  
  
- CCCAGAAAAT CTTAGTTAGA GAGATGTTCA TCCTTACGGC CAACTCCATT CCGATGGATG TAGATCTAGA   
  
  
- GAGGTCTGGG GTGTTTTTAA TTTGCTCTGG GTGTGACTTT CCACCACCAC CACCACAACA ACAACATAAC   
  
  
- AGGATGGTGC TCTCTCGAAG TTCACGTAGT GAATAAAGTT GTCCAGTTAG TGTATCTAGA GATCACATGA   
  
  
- CATTTAAAAA AAAGGCAGAA TAAAATTAAA AGAAAAGGTA GGATTTTCTA ATAGTACATC TATAAGGATC   
  
  
- AAACTATATA ACTGAAGTTG AAGTTTTTAA TGTGTAATTC TAGGTGCTTA TACTGTCAAA TCCAAAGTAA   
  
  
- ACTTCCTCTT TCTTTACAAT GCCACAAAAC CACAACATAA GCATCTGTAA AAAACGCGAA TTGACCGAGA   
  
  
- AGAGTACCCC TGCTCTGTCA CTTCCATTAA CATGAACAAC TAACTACTGC AGTAGACATC GAACCTAAAC   
  
  
- ATATCCTTAC TTGTAGAACC ACAGATGCAC CTTTCACACT AAACACAAGA AAGTAACTAG GAGGAGGAGT   
  
  
- TCTTCTCCAC TAGAAAGAGG GAACAAAAAA AAAAAAAAAC CGATTATACA ACCGTCTTGG CCGTTTTTTA   
  
  
- TGTATTAAAC TTATCACGTA CGTATACTCA TGTACTGTCA CTTGGTTGTT TTGCTTGGGG ATTTTGTGTT   
  
  
- CTCTTAATTG TACAATTTTG TGAATTTGAT TTACGTTGGT AGAGCTACTG GTGATTTATC GGTTCTTTGG   
  
  
- ATTGCATTAA TTGTTTTTAT TTTTTATTGA CTTTTATTTC ATCTCTTTCA GATTGGCTTA TGCTATTGTA   
  
  
- TTGGATTTTG ATTGATATGA ACTTGAATGA TTCTTTATAC TTAGTTGGTG TTTGGCTTAA CTAAAACCAA   
  
  
- ACGGTATTAA ACTGATATGT AAATTGAATT TTTTATAAAA AAAAATTAAA GTTTATTTAG ATTTAATAAA   
  
  
- AAGTTATAAG TAGGTTTTAT CTAAGTTAAT AATTTGTTCA GTTGGATATG GCTCAATAAA AGATCGAGAC   
  
  
- TGATTCTGTA TATATATAAA AAACATTTTT TCTATTCCTA ATATTAAATT TATCGGAAAG GGAATTTTTT   
  
  
- TAAAAATTTT AATAGGACTA TCTCTTATTT TTCTTTAGGA ATATGTTTTG AGACTAACTG CGTCTACACA   
  
  
- TATACCTGTA TATCTTCATT ATTCTCCCCA TCCGATCCTT CCCCCACCTT TATATCCCGT ATTTATCCCC   
  
  
- CGCCCGTATC CCTGTACAGA CAGACTCCAT GCCACCTACC CTGGAACGGT ACCTTCGTTG TTACAGTGTT   
  
  
- ATTATTATAG TTGCTGACTT CAAAGAAGTG GTCGGGTGAG AAACGGTATT ACGGTAATTA ATAAATAACG   
  
  
- ATGATAGAGA AAACTACACT TTGCCACCTG GAATAGAGAG GGGAGGTGAA GGTTAACTAC ACTCGTTGAG   
  
  
- TCTTTGCGTT TGTTTTGAAC ACGGTGGAAG AAATGAACGT AGGAGTAAAA GGAGACAGAG AGAGGCGGGG   
  
  
- ACAAAGAGAG AGAGAGGGAG TGAGTGAGTC TCATGACTAG ACTCGTAGTC TTCATCGTCT GGGTCTTTGA   
  
  
- GTCTAAATTT TTGTTGGGCG AGAAAACAAG GGAGATGCAG ATTATTAAAA CAGTAGCGTA AATTACAGTC   
  
  
- TATCTCTCTA TCTCGAGATG ACATAATTAA AAGAGGGTGG AGTGAACTAA GACAACGGGA TTTGGTGGGT   
  
  
- TTTTTTCTTT ATGCTTCTTC GGTGAGAGAG AGAGAGAGAG AGAGTGTGTG TGTGTGTGGG GAGGATCACC   
  
  
- TAAACTATCT CATATTTACT TGTCATTAGT CTGCTCTATA TATCATCACG AATTAACTGG CTCATCGTTT   
  
  
- TCCCCCTTCA ACTCACTCCA TAGTTCTCTC ATCGCATCGA TCGATCCCGT TCTATACGAC CCCAGAAGGA   
  
  
- GTATGTTGAG GCTCCTTCTG GTGGTTCTAC TGTTGGTAAG GAGTGGCAGA CTAAACAGAT CCGTGGGTGG   
  
  
- ATCTAAGTGG TGATGATGAA TGGTTATGGT TGTTGTATAA TCGAGAGGTA GAAGTGTTAG TTATGATCGA   
  
  
- CACGGTGGAG CGGTTAACAA TTAGTTAACA CGCCTCAATT AAACGGCCAA GCTGAAGAGG AGGCGGTTGG   
  
  
- CCGAAGAGAG AGAGGAGCGG AGGGTGAAAC GGAGGGGAAG ACCCCTGAGG TGGCTCTCTG AACACAAAAT   
  
  
- GAAGACGTTC CGAAACCTAG AGGCGGAGTT ATTGTCAATA AATAGGTGAG CATCAAACTG ACCAACATTG   
  
  
- GGATCATGAC CAAGACCATA ACCCCGAGGA AATCGAAGGT TAAAAGATAA AGAAGTTGGT AATCACGTAA   
  
  
- GTGTCCATTG TTCATTACGG GCACAACCCA TGAACTGTAT AAACTGAGAC TTGGTCCAGT GGGGTAAGTA   
  
  
- GGCTAAGTGG GTGAATTGAC GGTTGGTTCG GTAGGACCTT CGAAACCTCC CCATGTTCCG CCAGGTATAG   
  
  
- GAACTGTACC TGTAGTACGT ACCCCAGGTC ACCGGTGGTG AAGAAGTTCG TTAACGTCTT TCTAGACGTT   
  
  
- GGGAACCAGT GGGACGAGGG TGACAAGCGT AGTGTCCTCC CCCGTCCCTA GATCTTGAGG ACTTGGCTTG   
  
  
- ACCTCTAGCC TAGTCCTTCA AGCGTGTTAG GAACCCTGAG CTCAAAGTTA AGGTGGTAGA ATATTGCCAC   
  
  
- CTGAGCTTGG AATGACTCCG TCACCTCCGG TAGTTACGGG ATGTCGATGT GGAGGTACAG GTTCTCCTCC   
  
  
- TCCAGAAGCG TCACTTAACA CCTCTAAAGG AGGTGGCGGA GGATTGCCTC ATACTATCAT GTAAGGAATC   
  
  
- CTACAAAGAG ATGTTCCACT TCTGGGATTT GGGATCCCAG AAGTGGCACC CTCTTTCCCT CCGGCTGGTG   
  
  
- TTGGTGGGAG AGAAGACCGT CGCTAAGTAA CTCCGTGAAC TGGTGATACC CCGTCACAAA CTGAGAGATC   
  
  
- TTCGTTGGGA TGGTAGTTGT TCAGTTCTCT CCCACCGCCA CCTTCTCCCC ACCAAGCCCC TCCTCTAATT   
  
  
- TCTACACCAA CCTGCTCTTC CTCCCAACTC CTCTGTCTCC GTGGTCTTTA TGCTCAGAAC CCTCCACAAC   
  
  
- TACTCCTCGA GACCCAAATT CTCGAACGGG AACTCGGGGA AGAGACACAG TGTTCGGTTC GACGATGAAG   
  
  
- CGGAAGTAAT GGGAAGACTT CCCATGGTGG AATTCCAGAA GGTATCATTA ACAGACAACG AACCCACCTT   
  
  
- CTTAGCGGGA GAAAAGAGAC AAAGAAGAAC CGTTAT

+     TGACG-motif

| Site Name | Organism | Position | Strand | Matrix score. | sequence | function |
| --- | --- | --- | --- | --- | --- | --- |
| TGACG-motif | Hordeum vulgare | 1251 | + | 5 | TGACG | cis-acting regulatory element involved in the MeJA-responsiveness |
| TGACG-motif | Hordeum vulgare | 543 | - | 5 | TGACG | cis-acting regulatory element involved in the MeJA-responsiveness |
| TGACG-motif | Hordeum vulgare | 540 | + | 5 | TGACG | cis-acting regulatory element involved in the MeJA-responsiveness |

>HU01G00833.1   
+ -Up\_Stream \_Len000AAAATA TTTTTACAAA AGCATTTCTC TATAAAATAT TTTACAAAAT TTCATTTTGT   
  
  
+ GGGTCTTTTA GAATCAATCT CTCTACAAGT AGGAATGCCG GTTGAGGTAA GGCTACCTAC ATCTAGATCT   
  
  
+ CTCCAGACCC CACAAAAATT AAACGAGACC CACACTGAAA GGTGGTGGTG GTGGTGTTGT TGTTGTATTG   
  
  
+ TCCTACCACG AGAGAGCTTC AAGTGCATCA CTTATTTCAA CAGGTCAATC ACATAGATCT CTAGTGTACT   
  
  
+ GTAAATTTTT TTTCCGTCTT ATTTTAATTT TCTTTTCCAT CCTAAAAGAT TATCATGTAG ATATTCCTAG   
  
  
+ TTTGATATAT TGACTTCAAC TTCAAAAATT ACACATTAAG ATCCACGAAT ATGACAGTTT AGGTTTCATT   
  
  
+ TGAAGGAGAA AGAAATGTTA CGGTGTTTTG GTGTTGTATT CGTAGACATT TTTTGCGCTT AACTGGCTCT   
  
  
+ TCTCATGGGG ACGAGACAGT GAAGGTAATT GTACTTGTTG ATTGATGACG TCATCTGTAG CTTGGATTTG   
  
  
+ TATAGGAATG AACATCTTGG TGTCTACGTG GAAAGTGTGA TTTGTGTTCT TTCATTGATC CTCCTCCTCA   
  
  
+ AGAAGAGGTG ATCTTTCTCC CTTGTTTTTT TTTTTTTTTG GCTAATATGT TGGCAGAACC GGCAAAAAAT   
  
  
+ ACATAATTTG AATAGTGCAT GCATATGAGT ACATGACAGT GAACCAACAA AACGAACCCC TAAAACACAA   
  
  
+ GAGAATTAAC ATGTTAAAAC ACTTAAACTA AATGCAACCA TCTCGATGAC CACTAAATAG CCAAGAAACC   
  
  
+ TAACGTAATT AACAAAAATA AAAAATAACT GAAAATAAAG TAGAGAAAGT CTAACCGAAT ACGATAACAT   
  
  
+ AACCTAAAAC TAACTATACT TGAACTTACT AAGAAATATG AATCAACCAC AAACCGAATT GATTTTGGTT   
  
  
+ TGCCATAATT TGACTATACA TTTAACTTAA AAAATATTTT TTTTTAATTT CAAATAAATC TAAATTATTT   
  
  
+ TTCAATATTC ATCCAAAATA GATTCAATTA TTAAACAAGT CAACCTATAC CGAGTTATTT TCTAGCTCTG   
  
  
+ ACTAAGACAT ATATATATTT TTTGTAAAAA AGATAAGGAT TATAATTTAA ATAGCCTTTC CCTTAAAAAA   
  
  
+ ATTTTTAAAA TTATCCTGAT AGAGAATAAA AAGAAATCCT TATACAAAAC TCTGATTGAC GCAGATGTGT   
  
  
+ ATATGGACAT ATAGAAGTAA TAAGAGGGGT AGGCTAGGAA GGGGGTGGAA ATATAGGGCA TAAATAGGGG   
  
  
+ GCGGGCATAG GGACATGTCT GTCTGAGGTA CGGTGGATGG GACCTTGCCA TGGAAGCAAC AATGTCACAA   
  
  
+ TAATAATATC AACGACTGAA GTTTCTTCAC CAGCCCACTC TTTGCCATAA TGCCATTAAT TATTTATTGC   
  
  
+ TACTATCTCT TTTGATGTGA AACGGTGGAC CTTATCTCTC CCCTCCACTT CCAATTGATG TGAGCAACTC   
  
  
+ AGAAACGCAA ACAAAACTTG TGCCACCTTC TTTACTTGCA TCCTCATTTT CCTCTGTCTC TCTCCGCCCC   
  
  
+ TGTTTCTCTC TCTCTCCCTC ACTCACTCAG AGTACTGATC TGAGCATCAG AAGTAGCAGA CCCAGAAACT   
  
  
+ CAGATTTAAA AACAACCCGC TCTTTTGTTC CCTCTACGTC TAATAATTTT GTCATCGCAT TTAATGTCAG   
  
  
+ ATAGAGAGAT AGAGCTCTAC TGTATTAATT TTCTCCCACC TCACTTGATT CTGTTGCCCT AAACCACCCA   
  
  
+ AAAAAAGAAA TACGAAGAAG CCACTCTCTC TCTCTCTCTC TCTCACACAC ACACACACCC CTCCTAGTGG   
  
  
+ ATTTGATAGA GTATAAATGA ACAGTAATCA GACGAGATAT ATAGTAGTGC TTAATTGACC GAGTAGCAAA   
  
  
+ AGGGGGAAGT TGAGTGAGGT ATCAAGAGAG TAGCGTAGCT AGCTAGGGCA AGATATGCTG GGGTCTTCCT   
  
  
+ CATACAACTC CGAGGAAGAC CACCAAGATG ACAACCATTC CTCACCGTCT GATTTGTCTA GGCACCCACC   
  
  
+ TAGATTCACC ACTACTACTT ACCAATACCA ACAACATATT AGCTCTCCAT CTTCACAATC AATACTAGCT   
  
  
+ GTGCCACCTC GCCAATTGTT AATCAATTGT GCGGAGTTAA TTTGCCGGTT CGACTTCTCC TCCGCCAACC   
  
  
+ GGCTTCTCTC TCTCCTCGCC TCCCACTTTG CCTCCCCTTC TGGGGACTCC ACCGAGAGAC TTGTGTTTTA   
  
  
+ CTTCTGCAAG GCTTTGGATC TCCGCCTCAA TAACAGTTAT TTATCCACTC GTAGTTTGAC TGGTTGTAAC   
  
  
+ CCTAGTACTG GTTCTGGTAT TGGGGCTCCT TTAGCTTCCA ATTTTCTATT TCTTCAACCA TTAGTGCATT   
  
  
+ CACAGGTAAC AAGTAATGCC CGTGTTGGGT ACTTGACATA TTTGACTCTG AACCAGGTCA CCCCATTCAT   
  
  
+ CCGATTCACC CACTTAACTG CCAACCAAGC CATCCTGGAA GCTTTGGAGG GGTACAAGGC GGTCCATATC   
  
  
+ CTTGACATGG ACATCATGCA TGGGGTCCAG TGGCCACCAC TTCTTCAAGC AATTGCAGAA AGATCTGCAA   
  
  
+ CCCTTGGTCA CCCTGCTCCC ACTGTTCGCA TCACAGGAGG GGGCAGGGAT CTAGAACTCC TGAACCGAAC   
  
  
+ TGGAGATCGG ATCAGGAAGT TCGCACAATC CTTGGGACTC GAGTTTCAAT TCCACCATCT TATAACGGTG   
  
  
+ GACTCGAACC TTACTGAGGC AGTGGAGGCC ATCAATGCCC TACAGCTACA CCTCCATGTC CAAGAGGAGG   
  
  
+ AGGTCTTCGC AGTGAATTGT GGAGATTTCC TCCACCGCCT CCTAACGGAG TATGATAGTA CATTCCTTAG   
  
  
+ GATGTTTCTC TACAAGGTGA AGACCCTAAA CCCTAGGGTC TTCACCGTGG GAGAAAGGGA GGCCGACCAC   
  
  
+ AACCACCCTC TCTTCTGGCA GCGATTCATT GAGGCACTTG ACCACTATGG GGCAGTGTTT GACTCTCTAG   
  
  
+ AAGCAACCCT ACCATCAACA AGTCAAGAGA GGGTGGCGGT GGAAGAGGGG TGGTTCGGGG AGGAGATTAA   
  
  
+ AGATGTGGTT GGACGAGAAG GAGGGTTGAG GAGACAGAGG CACCAGAAAT ACGAGTCTTG GGAGGTGTTG   
  
  
+ ATGAGGAGCT CTGGGTTTAA GAGCTTGCCC TTGAGCCCCT TCTCTGTGTC ACAAGCCAAG CTGCTACTTC   
  
  
+ GCCTTCATTA CCCTTCTGAA GGGTACCACC TTAAGGTCTT CCATAGTAAT TGTCTGTTGC TTGGGTGGAA   
  
  
+ GAATCGCCCT CTTTTCTCTG TTTCTTCTTG GCAATA  

- -Up\_Stream \_Len000TTTTAT AAAAATGTTT TCGTAAAGAG ATATTTTATA AAATGTTTTA AAGTAAAACA   
  
  
- CCCAGAAAAT CTTAGTTAGA GAGATGTTCA TCCTTACGGC CAACTCCATT CCGATGGATG TAGATCTAGA   
  
  
- GAGGTCTGGG GTGTTTTTAA TTTGCTCTGG GTGTGACTTT CCACCACCAC CACCACAACA ACAACATAAC   
  
  
- AGGATGGTGC TCTCTCGAAG TTCACGTAGT GAATAAAGTT GTCCAGTTAG TGTATCTAGA GATCACATGA   
  
  
- CATTTAAAAA AAAGGCAGAA TAAAATTAAA AGAAAAGGTA GGATTTTCTA ATAGTACATC TATAAGGATC   
  
  
- AAACTATATA ACTGAAGTTG AAGTTTTTAA TGTGTAATTC TAGGTGCTTA TACTGTCAAA TCCAAAGTAA   
  
  
- ACTTCCTCTT TCTTTACAAT GCCACAAAAC CACAACATAA GCATCTGTAA AAAACGCGAA TTGACCGAGA   
  
  
- AGAGTACCCC TGCTCTGTCA CTTCCATTAA CATGAACAAC TAACTACTGC AGTAGACATC GAACCTAAAC   
  
  
- ATATCCTTAC TTGTAGAACC ACAGATGCAC CTTTCACACT AAACACAAGA AAGTAACTAG GAGGAGGAGT   
  
  
- TCTTCTCCAC TAGAAAGAGG GAACAAAAAA AAAAAAAAAC CGATTATACA ACCGTCTTGG CCGTTTTTTA   
  
  
- TGTATTAAAC TTATCACGTA CGTATACTCA TGTACTGTCA CTTGGTTGTT TTGCTTGGGG ATTTTGTGTT   
  
  
- CTCTTAATTG TACAATTTTG TGAATTTGAT TTACGTTGGT AGAGCTACTG GTGATTTATC GGTTCTTTGG   
  
  
- ATTGCATTAA TTGTTTTTAT TTTTTATTGA CTTTTATTTC ATCTCTTTCA GATTGGCTTA TGCTATTGTA   
  
  
- TTGGATTTTG ATTGATATGA ACTTGAATGA TTCTTTATAC TTAGTTGGTG TTTGGCTTAA CTAAAACCAA   
  
  
- ACGGTATTAA ACTGATATGT AAATTGAATT TTTTATAAAA AAAAATTAAA GTTTATTTAG ATTTAATAAA   
  
  
- AAGTTATAAG TAGGTTTTAT CTAAGTTAAT AATTTGTTCA GTTGGATATG GCTCAATAAA AGATCGAGAC   
  
  
- TGATTCTGTA TATATATAAA AAACATTTTT TCTATTCCTA ATATTAAATT TATCGGAAAG GGAATTTTTT   
  
  
- TAAAAATTTT AATAGGACTA TCTCTTATTT TTCTTTAGGA ATATGTTTTG AGACTAACTG CGTCTACACA   
  
  
- TATACCTGTA TATCTTCATT ATTCTCCCCA TCCGATCCTT CCCCCACCTT TATATCCCGT ATTTATCCCC   
  
  
- CGCCCGTATC CCTGTACAGA CAGACTCCAT GCCACCTACC CTGGAACGGT ACCTTCGTTG TTACAGTGTT   
  
  
- ATTATTATAG TTGCTGACTT CAAAGAAGTG GTCGGGTGAG AAACGGTATT ACGGTAATTA ATAAATAACG   
  
  
- ATGATAGAGA AAACTACACT TTGCCACCTG GAATAGAGAG GGGAGGTGAA GGTTAACTAC ACTCGTTGAG   
  
  
- TCTTTGCGTT TGTTTTGAAC ACGGTGGAAG AAATGAACGT AGGAGTAAAA GGAGACAGAG AGAGGCGGGG   
  
  
- ACAAAGAGAG AGAGAGGGAG TGAGTGAGTC TCATGACTAG ACTCGTAGTC TTCATCGTCT GGGTCTTTGA   
  
  
- GTCTAAATTT TTGTTGGGCG AGAAAACAAG GGAGATGCAG ATTATTAAAA CAGTAGCGTA AATTACAGTC   
  
  
- TATCTCTCTA TCTCGAGATG ACATAATTAA AAGAGGGTGG AGTGAACTAA GACAACGGGA TTTGGTGGGT   
  
  
- TTTTTTCTTT ATGCTTCTTC GGTGAGAGAG AGAGAGAGAG AGAGTGTGTG TGTGTGTGGG GAGGATCACC   
  
  
- TAAACTATCT CATATTTACT TGTCATTAGT CTGCTCTATA TATCATCACG AATTAACTGG CTCATCGTTT   
  
  
- TCCCCCTTCA ACTCACTCCA TAGTTCTCTC ATCGCATCGA TCGATCCCGT TCTATACGAC CCCAGAAGGA   
  
  
- GTATGTTGAG GCTCCTTCTG GTGGTTCTAC TGTTGGTAAG GAGTGGCAGA CTAAACAGAT CCGTGGGTGG   
  
  
- ATCTAAGTGG TGATGATGAA TGGTTATGGT TGTTGTATAA TCGAGAGGTA GAAGTGTTAG TTATGATCGA   
  
  
- CACGGTGGAG CGGTTAACAA TTAGTTAACA CGCCTCAATT AAACGGCCAA GCTGAAGAGG AGGCGGTTGG   
  
  
- CCGAAGAGAG AGAGGAGCGG AGGGTGAAAC GGAGGGGAAG ACCCCTGAGG TGGCTCTCTG AACACAAAAT   
  
  
- GAAGACGTTC CGAAACCTAG AGGCGGAGTT ATTGTCAATA AATAGGTGAG CATCAAACTG ACCAACATTG   
  
  
- GGATCATGAC CAAGACCATA ACCCCGAGGA AATCGAAGGT TAAAAGATAA AGAAGTTGGT AATCACGTAA   
  
  
- GTGTCCATTG TTCATTACGG GCACAACCCA TGAACTGTAT AAACTGAGAC TTGGTCCAGT GGGGTAAGTA   
  
  
- GGCTAAGTGG GTGAATTGAC GGTTGGTTCG GTAGGACCTT CGAAACCTCC CCATGTTCCG CCAGGTATAG   
  
  
- GAACTGTACC TGTAGTACGT ACCCCAGGTC ACCGGTGGTG AAGAAGTTCG TTAACGTCTT TCTAGACGTT   
  
  
- GGGAACCAGT GGGACGAGGG TGACAAGCGT AGTGTCCTCC CCCGTCCCTA GATCTTGAGG ACTTGGCTTG   
  
  
- ACCTCTAGCC TAGTCCTTCA AGCGTGTTAG GAACCCTGAG CTCAAAGTTA AGGTGGTAGA ATATTGCCAC   
  
  
- CTGAGCTTGG AATGACTCCG TCACCTCCGG TAGTTACGGG ATGTCGATGT GGAGGTACAG GTTCTCCTCC   
  
  
- TCCAGAAGCG TCACTTAACA CCTCTAAAGG AGGTGGCGGA GGATTGCCTC ATACTATCAT GTAAGGAATC   
  
  
- CTACAAAGAG ATGTTCCACT TCTGGGATTT GGGATCCCAG AAGTGGCACC CTCTTTCCCT CCGGCTGGTG   
  
  
- TTGGTGGGAG AGAAGACCGT CGCTAAGTAA CTCCGTGAAC TGGTGATACC CCGTCACAAA CTGAGAGATC   
  
  
- TTCGTTGGGA TGGTAGTTGT TCAGTTCTCT CCCACCGCCA CCTTCTCCCC ACCAAGCCCC TCCTCTAATT   
  
  
- TCTACACCAA CCTGCTCTTC CTCCCAACTC CTCTGTCTCC GTGGTCTTTA TGCTCAGAAC CCTCCACAAC   
  
  
- TACTCCTCGA GACCCAAATT CTCGAACGGG AACTCGGGGA AGAGACACAG TGTTCGGTTC GACGATGAAG   
  
  
- CGGAAGTAAT GGGAAGACTT CCCATGGTGG AATTCCAGAA GGTATCATTA ACAGACAACG AACCCACCTT   
  
  
- CTTAGCGGGA GAAAAGAGAC AAAGAAGAAC CGTTAT

+     Unnamed\_\_1

| Site Name | Organism | Position | Strand | Matrix score. | sequence | function |
| --- | --- | --- | --- | --- | --- | --- |
| Unnamed\_\_1 | Zea mays | 397 | - | 5 | CGTGG |  |
| Unnamed\_\_1 | Zea mays | 220 | - | 5 | CGTGG |  |
| Unnamed\_\_1 | Zea mays | 591 | + | 5 | CGTGG |  |
| Unnamed\_\_1 | Zea mays | 2990 | + | 5 | CGTGG |  |

>HU01G00833.1   
+ -Up\_Stream \_Len000AAAATA TTTTTACAAA AGCATTTCTC TATAAAATAT TTTACAAAAT TTCATTTTGT   
  
  
+ GGGTCTTTTA GAATCAATCT CTCTACAAGT AGGAATGCCG GTTGAGGTAA GGCTACCTAC ATCTAGATCT   
  
  
+ CTCCAGACCC CACAAAAATT AAACGAGACC CACACTGAAA GGTGGTGGTG GTGGTGTTGT TGTTGTATTG   
  
  
+ TCCTACCACG AGAGAGCTTC AAGTGCATCA CTTATTTCAA CAGGTCAATC ACATAGATCT CTAGTGTACT   
  
  
+ GTAAATTTTT TTTCCGTCTT ATTTTAATTT TCTTTTCCAT CCTAAAAGAT TATCATGTAG ATATTCCTAG   
  
  
+ TTTGATATAT TGACTTCAAC TTCAAAAATT ACACATTAAG ATCCACGAAT ATGACAGTTT AGGTTTCATT   
  
  
+ TGAAGGAGAA AGAAATGTTA CGGTGTTTTG GTGTTGTATT CGTAGACATT TTTTGCGCTT AACTGGCTCT   
  
  
+ TCTCATGGGG ACGAGACAGT GAAGGTAATT GTACTTGTTG ATTGATGACG TCATCTGTAG CTTGGATTTG   
  
  
+ TATAGGAATG AACATCTTGG TGTCTACGTG GAAAGTGTGA TTTGTGTTCT TTCATTGATC CTCCTCCTCA   
  
  
+ AGAAGAGGTG ATCTTTCTCC CTTGTTTTTT TTTTTTTTTG GCTAATATGT TGGCAGAACC GGCAAAAAAT   
  
  
+ ACATAATTTG AATAGTGCAT GCATATGAGT ACATGACAGT GAACCAACAA AACGAACCCC TAAAACACAA   
  
  
+ GAGAATTAAC ATGTTAAAAC ACTTAAACTA AATGCAACCA TCTCGATGAC CACTAAATAG CCAAGAAACC   
  
  
+ TAACGTAATT AACAAAAATA AAAAATAACT GAAAATAAAG TAGAGAAAGT CTAACCGAAT ACGATAACAT   
  
  
+ AACCTAAAAC TAACTATACT TGAACTTACT AAGAAATATG AATCAACCAC AAACCGAATT GATTTTGGTT   
  
  
+ TGCCATAATT TGACTATACA TTTAACTTAA AAAATATTTT TTTTTAATTT CAAATAAATC TAAATTATTT   
  
  
+ TTCAATATTC ATCCAAAATA GATTCAATTA TTAAACAAGT CAACCTATAC CGAGTTATTT TCTAGCTCTG   
  
  
+ ACTAAGACAT ATATATATTT TTTGTAAAAA AGATAAGGAT TATAATTTAA ATAGCCTTTC CCTTAAAAAA   
  
  
+ ATTTTTAAAA TTATCCTGAT AGAGAATAAA AAGAAATCCT TATACAAAAC TCTGATTGAC GCAGATGTGT   
  
  
+ ATATGGACAT ATAGAAGTAA TAAGAGGGGT AGGCTAGGAA GGGGGTGGAA ATATAGGGCA TAAATAGGGG   
  
  
+ GCGGGCATAG GGACATGTCT GTCTGAGGTA CGGTGGATGG GACCTTGCCA TGGAAGCAAC AATGTCACAA   
  
  
+ TAATAATATC AACGACTGAA GTTTCTTCAC CAGCCCACTC TTTGCCATAA TGCCATTAAT TATTTATTGC   
  
  
+ TACTATCTCT TTTGATGTGA AACGGTGGAC CTTATCTCTC CCCTCCACTT CCAATTGATG TGAGCAACTC   
  
  
+ AGAAACGCAA ACAAAACTTG TGCCACCTTC TTTACTTGCA TCCTCATTTT CCTCTGTCTC TCTCCGCCCC   
  
  
+ TGTTTCTCTC TCTCTCCCTC ACTCACTCAG AGTACTGATC TGAGCATCAG AAGTAGCAGA CCCAGAAACT   
  
  
+ CAGATTTAAA AACAACCCGC TCTTTTGTTC CCTCTACGTC TAATAATTTT GTCATCGCAT TTAATGTCAG   
  
  
+ ATAGAGAGAT AGAGCTCTAC TGTATTAATT TTCTCCCACC TCACTTGATT CTGTTGCCCT AAACCACCCA   
  
  
+ AAAAAAGAAA TACGAAGAAG CCACTCTCTC TCTCTCTCTC TCTCACACAC ACACACACCC CTCCTAGTGG   
  
  
+ ATTTGATAGA GTATAAATGA ACAGTAATCA GACGAGATAT ATAGTAGTGC TTAATTGACC GAGTAGCAAA   
  
  
+ AGGGGGAAGT TGAGTGAGGT ATCAAGAGAG TAGCGTAGCT AGCTAGGGCA AGATATGCTG GGGTCTTCCT   
  
  
+ CATACAACTC CGAGGAAGAC CACCAAGATG ACAACCATTC CTCACCGTCT GATTTGTCTA GGCACCCACC   
  
  
+ TAGATTCACC ACTACTACTT ACCAATACCA ACAACATATT AGCTCTCCAT CTTCACAATC AATACTAGCT   
  
  
+ GTGCCACCTC GCCAATTGTT AATCAATTGT GCGGAGTTAA TTTGCCGGTT CGACTTCTCC TCCGCCAACC   
  
  
+ GGCTTCTCTC TCTCCTCGCC TCCCACTTTG CCTCCCCTTC TGGGGACTCC ACCGAGAGAC TTGTGTTTTA   
  
  
+ CTTCTGCAAG GCTTTGGATC TCCGCCTCAA TAACAGTTAT TTATCCACTC GTAGTTTGAC TGGTTGTAAC   
  
  
+ CCTAGTACTG GTTCTGGTAT TGGGGCTCCT TTAGCTTCCA ATTTTCTATT TCTTCAACCA TTAGTGCATT   
  
  
+ CACAGGTAAC AAGTAATGCC CGTGTTGGGT ACTTGACATA TTTGACTCTG AACCAGGTCA CCCCATTCAT   
  
  
+ CCGATTCACC CACTTAACTG CCAACCAAGC CATCCTGGAA GCTTTGGAGG GGTACAAGGC GGTCCATATC   
  
  
+ CTTGACATGG ACATCATGCA TGGGGTCCAG TGGCCACCAC TTCTTCAAGC AATTGCAGAA AGATCTGCAA   
  
  
+ CCCTTGGTCA CCCTGCTCCC ACTGTTCGCA TCACAGGAGG GGGCAGGGAT CTAGAACTCC TGAACCGAAC   
  
  
+ TGGAGATCGG ATCAGGAAGT TCGCACAATC CTTGGGACTC GAGTTTCAAT TCCACCATCT TATAACGGTG   
  
  
+ GACTCGAACC TTACTGAGGC AGTGGAGGCC ATCAATGCCC TACAGCTACA CCTCCATGTC CAAGAGGAGG   
  
  
+ AGGTCTTCGC AGTGAATTGT GGAGATTTCC TCCACCGCCT CCTAACGGAG TATGATAGTA CATTCCTTAG   
  
  
+ GATGTTTCTC TACAAGGTGA AGACCCTAAA CCCTAGGGTC TTCACCGTGG GAGAAAGGGA GGCCGACCAC   
  
  
+ AACCACCCTC TCTTCTGGCA GCGATTCATT GAGGCACTTG ACCACTATGG GGCAGTGTTT GACTCTCTAG   
  
  
+ AAGCAACCCT ACCATCAACA AGTCAAGAGA GGGTGGCGGT GGAAGAGGGG TGGTTCGGGG AGGAGATTAA   
  
  
+ AGATGTGGTT GGACGAGAAG GAGGGTTGAG GAGACAGAGG CACCAGAAAT ACGAGTCTTG GGAGGTGTTG   
  
  
+ ATGAGGAGCT CTGGGTTTAA GAGCTTGCCC TTGAGCCCCT TCTCTGTGTC ACAAGCCAAG CTGCTACTTC   
  
  
+ GCCTTCATTA CCCTTCTGAA GGGTACCACC TTAAGGTCTT CCATAGTAAT TGTCTGTTGC TTGGGTGGAA   
  
  
+ GAATCGCCCT CTTTTCTCTG TTTCTTCTTG GCAATA  

- -Up\_Stream \_Len000TTTTAT AAAAATGTTT TCGTAAAGAG ATATTTTATA AAATGTTTTA AAGTAAAACA   
  
  
- CCCAGAAAAT CTTAGTTAGA GAGATGTTCA TCCTTACGGC CAACTCCATT CCGATGGATG TAGATCTAGA   
  
  
- GAGGTCTGGG GTGTTTTTAA TTTGCTCTGG GTGTGACTTT CCACCACCAC CACCACAACA ACAACATAAC   
  
  
- AGGATGGTGC TCTCTCGAAG TTCACGTAGT GAATAAAGTT GTCCAGTTAG TGTATCTAGA GATCACATGA   
  
  
- CATTTAAAAA AAAGGCAGAA TAAAATTAAA AGAAAAGGTA GGATTTTCTA ATAGTACATC TATAAGGATC   
  
  
- AAACTATATA ACTGAAGTTG AAGTTTTTAA TGTGTAATTC TAGGTGCTTA TACTGTCAAA TCCAAAGTAA   
  
  
- ACTTCCTCTT TCTTTACAAT GCCACAAAAC CACAACATAA GCATCTGTAA AAAACGCGAA TTGACCGAGA   
  
  
- AGAGTACCCC TGCTCTGTCA CTTCCATTAA CATGAACAAC TAACTACTGC AGTAGACATC GAACCTAAAC   
  
  
- ATATCCTTAC TTGTAGAACC ACAGATGCAC CTTTCACACT AAACACAAGA AAGTAACTAG GAGGAGGAGT   
  
  
- TCTTCTCCAC TAGAAAGAGG GAACAAAAAA AAAAAAAAAC CGATTATACA ACCGTCTTGG CCGTTTTTTA   
  
  
- TGTATTAAAC TTATCACGTA CGTATACTCA TGTACTGTCA CTTGGTTGTT TTGCTTGGGG ATTTTGTGTT   
  
  
- CTCTTAATTG TACAATTTTG TGAATTTGAT TTACGTTGGT AGAGCTACTG GTGATTTATC GGTTCTTTGG   
  
  
- ATTGCATTAA TTGTTTTTAT TTTTTATTGA CTTTTATTTC ATCTCTTTCA GATTGGCTTA TGCTATTGTA   
  
  
- TTGGATTTTG ATTGATATGA ACTTGAATGA TTCTTTATAC TTAGTTGGTG TTTGGCTTAA CTAAAACCAA   
  
  
- ACGGTATTAA ACTGATATGT AAATTGAATT TTTTATAAAA AAAAATTAAA GTTTATTTAG ATTTAATAAA   
  
  
- AAGTTATAAG TAGGTTTTAT CTAAGTTAAT AATTTGTTCA GTTGGATATG GCTCAATAAA AGATCGAGAC   
  
  
- TGATTCTGTA TATATATAAA AAACATTTTT TCTATTCCTA ATATTAAATT TATCGGAAAG GGAATTTTTT   
  
  
- TAAAAATTTT AATAGGACTA TCTCTTATTT TTCTTTAGGA ATATGTTTTG AGACTAACTG CGTCTACACA   
  
  
- TATACCTGTA TATCTTCATT ATTCTCCCCA TCCGATCCTT CCCCCACCTT TATATCCCGT ATTTATCCCC   
  
  
- CGCCCGTATC CCTGTACAGA CAGACTCCAT GCCACCTACC CTGGAACGGT ACCTTCGTTG TTACAGTGTT   
  
  
- ATTATTATAG TTGCTGACTT CAAAGAAGTG GTCGGGTGAG AAACGGTATT ACGGTAATTA ATAAATAACG   
  
  
- ATGATAGAGA AAACTACACT TTGCCACCTG GAATAGAGAG GGGAGGTGAA GGTTAACTAC ACTCGTTGAG   
  
  
- TCTTTGCGTT TGTTTTGAAC ACGGTGGAAG AAATGAACGT AGGAGTAAAA GGAGACAGAG AGAGGCGGGG   
  
  
- ACAAAGAGAG AGAGAGGGAG TGAGTGAGTC TCATGACTAG ACTCGTAGTC TTCATCGTCT GGGTCTTTGA   
  
  
- GTCTAAATTT TTGTTGGGCG AGAAAACAAG GGAGATGCAG ATTATTAAAA CAGTAGCGTA AATTACAGTC   
  
  
- TATCTCTCTA TCTCGAGATG ACATAATTAA AAGAGGGTGG AGTGAACTAA GACAACGGGA TTTGGTGGGT   
  
  
- TTTTTTCTTT ATGCTTCTTC GGTGAGAGAG AGAGAGAGAG AGAGTGTGTG TGTGTGTGGG GAGGATCACC   
  
  
- TAAACTATCT CATATTTACT TGTCATTAGT CTGCTCTATA TATCATCACG AATTAACTGG CTCATCGTTT   
  
  
- TCCCCCTTCA ACTCACTCCA TAGTTCTCTC ATCGCATCGA TCGATCCCGT TCTATACGAC CCCAGAAGGA   
  
  
- GTATGTTGAG GCTCCTTCTG GTGGTTCTAC TGTTGGTAAG GAGTGGCAGA CTAAACAGAT CCGTGGGTGG   
  
  
- ATCTAAGTGG TGATGATGAA TGGTTATGGT TGTTGTATAA TCGAGAGGTA GAAGTGTTAG TTATGATCGA   
  
  
- CACGGTGGAG CGGTTAACAA TTAGTTAACA CGCCTCAATT AAACGGCCAA GCTGAAGAGG AGGCGGTTGG   
  
  
- CCGAAGAGAG AGAGGAGCGG AGGGTGAAAC GGAGGGGAAG ACCCCTGAGG TGGCTCTCTG AACACAAAAT   
  
  
- GAAGACGTTC CGAAACCTAG AGGCGGAGTT ATTGTCAATA AATAGGTGAG CATCAAACTG ACCAACATTG   
  
  
- GGATCATGAC CAAGACCATA ACCCCGAGGA AATCGAAGGT TAAAAGATAA AGAAGTTGGT AATCACGTAA   
  
  
- GTGTCCATTG TTCATTACGG GCACAACCCA TGAACTGTAT AAACTGAGAC TTGGTCCAGT GGGGTAAGTA   
  
  
- GGCTAAGTGG GTGAATTGAC GGTTGGTTCG GTAGGACCTT CGAAACCTCC CCATGTTCCG CCAGGTATAG   
  
  
- GAACTGTACC TGTAGTACGT ACCCCAGGTC ACCGGTGGTG AAGAAGTTCG TTAACGTCTT TCTAGACGTT   
  
  
- GGGAACCAGT GGGACGAGGG TGACAAGCGT AGTGTCCTCC CCCGTCCCTA GATCTTGAGG ACTTGGCTTG   
  
  
- ACCTCTAGCC TAGTCCTTCA AGCGTGTTAG GAACCCTGAG CTCAAAGTTA AGGTGGTAGA ATATTGCCAC   
  
  
- CTGAGCTTGG AATGACTCCG TCACCTCCGG TAGTTACGGG ATGTCGATGT GGAGGTACAG GTTCTCCTCC   
  
  
- TCCAGAAGCG TCACTTAACA CCTCTAAAGG AGGTGGCGGA GGATTGCCTC ATACTATCAT GTAAGGAATC   
  
  
- CTACAAAGAG ATGTTCCACT TCTGGGATTT GGGATCCCAG AAGTGGCACC CTCTTTCCCT CCGGCTGGTG   
  
  
- TTGGTGGGAG AGAAGACCGT CGCTAAGTAA CTCCGTGAAC TGGTGATACC CCGTCACAAA CTGAGAGATC   
  
  
- TTCGTTGGGA TGGTAGTTGT TCAGTTCTCT CCCACCGCCA CCTTCTCCCC ACCAAGCCCC TCCTCTAATT   
  
  
- TCTACACCAA CCTGCTCTTC CTCCCAACTC CTCTGTCTCC GTGGTCTTTA TGCTCAGAAC CCTCCACAAC   
  
  
- TACTCCTCGA GACCCAAATT CTCGAACGGG AACTCGGGGA AGAGACACAG TGTTCGGTTC GACGATGAAG   
  
  
- CGGAAGTAAT GGGAAGACTT CCCATGGTGG AATTCCAGAA GGTATCATTA ACAGACAACG AACCCACCTT   
  
  
- CTTAGCGGGA GAAAAGAGAC AAAGAAGAAC CGTTAT

+     Unnamed\_\_10

| Site Name | Organism | Position | Strand | Matrix score. | sequence | function |
| --- | --- | --- | --- | --- | --- | --- |
| Unnamed\_\_10 | Zea mays | 587 | - | 10 | TCCACGTAGA |  |

>HU01G00833.1   
+ -Up\_Stream \_Len000AAAATA TTTTTACAAA AGCATTTCTC TATAAAATAT TTTACAAAAT TTCATTTTGT   
  
  
+ GGGTCTTTTA GAATCAATCT CTCTACAAGT AGGAATGCCG GTTGAGGTAA GGCTACCTAC ATCTAGATCT   
  
  
+ CTCCAGACCC CACAAAAATT AAACGAGACC CACACTGAAA GGTGGTGGTG GTGGTGTTGT TGTTGTATTG   
  
  
+ TCCTACCACG AGAGAGCTTC AAGTGCATCA CTTATTTCAA CAGGTCAATC ACATAGATCT CTAGTGTACT   
  
  
+ GTAAATTTTT TTTCCGTCTT ATTTTAATTT TCTTTTCCAT CCTAAAAGAT TATCATGTAG ATATTCCTAG   
  
  
+ TTTGATATAT TGACTTCAAC TTCAAAAATT ACACATTAAG ATCCACGAAT ATGACAGTTT AGGTTTCATT   
  
  
+ TGAAGGAGAA AGAAATGTTA CGGTGTTTTG GTGTTGTATT CGTAGACATT TTTTGCGCTT AACTGGCTCT   
  
  
+ TCTCATGGGG ACGAGACAGT GAAGGTAATT GTACTTGTTG ATTGATGACG TCATCTGTAG CTTGGATTTG   
  
  
+ TATAGGAATG AACATCTTGG TGTCTACGTG GAAAGTGTGA TTTGTGTTCT TTCATTGATC CTCCTCCTCA   
  
  
+ AGAAGAGGTG ATCTTTCTCC CTTGTTTTTT TTTTTTTTTG GCTAATATGT TGGCAGAACC GGCAAAAAAT   
  
  
+ ACATAATTTG AATAGTGCAT GCATATGAGT ACATGACAGT GAACCAACAA AACGAACCCC TAAAACACAA   
  
  
+ GAGAATTAAC ATGTTAAAAC ACTTAAACTA AATGCAACCA TCTCGATGAC CACTAAATAG CCAAGAAACC   
  
  
+ TAACGTAATT AACAAAAATA AAAAATAACT GAAAATAAAG TAGAGAAAGT CTAACCGAAT ACGATAACAT   
  
  
+ AACCTAAAAC TAACTATACT TGAACTTACT AAGAAATATG AATCAACCAC AAACCGAATT GATTTTGGTT   
  
  
+ TGCCATAATT TGACTATACA TTTAACTTAA AAAATATTTT TTTTTAATTT CAAATAAATC TAAATTATTT   
  
  
+ TTCAATATTC ATCCAAAATA GATTCAATTA TTAAACAAGT CAACCTATAC CGAGTTATTT TCTAGCTCTG   
  
  
+ ACTAAGACAT ATATATATTT TTTGTAAAAA AGATAAGGAT TATAATTTAA ATAGCCTTTC CCTTAAAAAA   
  
  
+ ATTTTTAAAA TTATCCTGAT AGAGAATAAA AAGAAATCCT TATACAAAAC TCTGATTGAC GCAGATGTGT   
  
  
+ ATATGGACAT ATAGAAGTAA TAAGAGGGGT AGGCTAGGAA GGGGGTGGAA ATATAGGGCA TAAATAGGGG   
  
  
+ GCGGGCATAG GGACATGTCT GTCTGAGGTA CGGTGGATGG GACCTTGCCA TGGAAGCAAC AATGTCACAA   
  
  
+ TAATAATATC AACGACTGAA GTTTCTTCAC CAGCCCACTC TTTGCCATAA TGCCATTAAT TATTTATTGC   
  
  
+ TACTATCTCT TTTGATGTGA AACGGTGGAC CTTATCTCTC CCCTCCACTT CCAATTGATG TGAGCAACTC   
  
  
+ AGAAACGCAA ACAAAACTTG TGCCACCTTC TTTACTTGCA TCCTCATTTT CCTCTGTCTC TCTCCGCCCC   
  
  
+ TGTTTCTCTC TCTCTCCCTC ACTCACTCAG AGTACTGATC TGAGCATCAG AAGTAGCAGA CCCAGAAACT   
  
  
+ CAGATTTAAA AACAACCCGC TCTTTTGTTC CCTCTACGTC TAATAATTTT GTCATCGCAT TTAATGTCAG   
  
  
+ ATAGAGAGAT AGAGCTCTAC TGTATTAATT TTCTCCCACC TCACTTGATT CTGTTGCCCT AAACCACCCA   
  
  
+ AAAAAAGAAA TACGAAGAAG CCACTCTCTC TCTCTCTCTC TCTCACACAC ACACACACCC CTCCTAGTGG   
  
  
+ ATTTGATAGA GTATAAATGA ACAGTAATCA GACGAGATAT ATAGTAGTGC TTAATTGACC GAGTAGCAAA   
  
  
+ AGGGGGAAGT TGAGTGAGGT ATCAAGAGAG TAGCGTAGCT AGCTAGGGCA AGATATGCTG GGGTCTTCCT   
  
  
+ CATACAACTC CGAGGAAGAC CACCAAGATG ACAACCATTC CTCACCGTCT GATTTGTCTA GGCACCCACC   
  
  
+ TAGATTCACC ACTACTACTT ACCAATACCA ACAACATATT AGCTCTCCAT CTTCACAATC AATACTAGCT   
  
  
+ GTGCCACCTC GCCAATTGTT AATCAATTGT GCGGAGTTAA TTTGCCGGTT CGACTTCTCC TCCGCCAACC   
  
  
+ GGCTTCTCTC TCTCCTCGCC TCCCACTTTG CCTCCCCTTC TGGGGACTCC ACCGAGAGAC TTGTGTTTTA   
  
  
+ CTTCTGCAAG GCTTTGGATC TCCGCCTCAA TAACAGTTAT TTATCCACTC GTAGTTTGAC TGGTTGTAAC   
  
  
+ CCTAGTACTG GTTCTGGTAT TGGGGCTCCT TTAGCTTCCA ATTTTCTATT TCTTCAACCA TTAGTGCATT   
  
  
+ CACAGGTAAC AAGTAATGCC CGTGTTGGGT ACTTGACATA TTTGACTCTG AACCAGGTCA CCCCATTCAT   
  
  
+ CCGATTCACC CACTTAACTG CCAACCAAGC CATCCTGGAA GCTTTGGAGG GGTACAAGGC GGTCCATATC   
  
  
+ CTTGACATGG ACATCATGCA TGGGGTCCAG TGGCCACCAC TTCTTCAAGC AATTGCAGAA AGATCTGCAA   
  
  
+ CCCTTGGTCA CCCTGCTCCC ACTGTTCGCA TCACAGGAGG GGGCAGGGAT CTAGAACTCC TGAACCGAAC   
  
  
+ TGGAGATCGG ATCAGGAAGT TCGCACAATC CTTGGGACTC GAGTTTCAAT TCCACCATCT TATAACGGTG   
  
  
+ GACTCGAACC TTACTGAGGC AGTGGAGGCC ATCAATGCCC TACAGCTACA CCTCCATGTC CAAGAGGAGG   
  
  
+ AGGTCTTCGC AGTGAATTGT GGAGATTTCC TCCACCGCCT CCTAACGGAG TATGATAGTA CATTCCTTAG   
  
  
+ GATGTTTCTC TACAAGGTGA AGACCCTAAA CCCTAGGGTC TTCACCGTGG GAGAAAGGGA GGCCGACCAC   
  
  
+ AACCACCCTC TCTTCTGGCA GCGATTCATT GAGGCACTTG ACCACTATGG GGCAGTGTTT GACTCTCTAG   
  
  
+ AAGCAACCCT ACCATCAACA AGTCAAGAGA GGGTGGCGGT GGAAGAGGGG TGGTTCGGGG AGGAGATTAA   
  
  
+ AGATGTGGTT GGACGAGAAG GAGGGTTGAG GAGACAGAGG CACCAGAAAT ACGAGTCTTG GGAGGTGTTG   
  
  
+ ATGAGGAGCT CTGGGTTTAA GAGCTTGCCC TTGAGCCCCT TCTCTGTGTC ACAAGCCAAG CTGCTACTTC   
  
  
+ GCCTTCATTA CCCTTCTGAA GGGTACCACC TTAAGGTCTT CCATAGTAAT TGTCTGTTGC TTGGGTGGAA   
  
  
+ GAATCGCCCT CTTTTCTCTG TTTCTTCTTG GCAATA  

- -Up\_Stream \_Len000TTTTAT AAAAATGTTT TCGTAAAGAG ATATTTTATA AAATGTTTTA AAGTAAAACA   
  
  
- CCCAGAAAAT CTTAGTTAGA GAGATGTTCA TCCTTACGGC CAACTCCATT CCGATGGATG TAGATCTAGA   
  
  
- GAGGTCTGGG GTGTTTTTAA TTTGCTCTGG GTGTGACTTT CCACCACCAC CACCACAACA ACAACATAAC   
  
  
- AGGATGGTGC TCTCTCGAAG TTCACGTAGT GAATAAAGTT GTCCAGTTAG TGTATCTAGA GATCACATGA   
  
  
- CATTTAAAAA AAAGGCAGAA TAAAATTAAA AGAAAAGGTA GGATTTTCTA ATAGTACATC TATAAGGATC   
  
  
- AAACTATATA ACTGAAGTTG AAGTTTTTAA TGTGTAATTC TAGGTGCTTA TACTGTCAAA TCCAAAGTAA   
  
  
- ACTTCCTCTT TCTTTACAAT GCCACAAAAC CACAACATAA GCATCTGTAA AAAACGCGAA TTGACCGAGA   
  
  
- AGAGTACCCC TGCTCTGTCA CTTCCATTAA CATGAACAAC TAACTACTGC AGTAGACATC GAACCTAAAC   
  
  
- ATATCCTTAC TTGTAGAACC ACAGATGCAC CTTTCACACT AAACACAAGA AAGTAACTAG GAGGAGGAGT   
  
  
- TCTTCTCCAC TAGAAAGAGG GAACAAAAAA AAAAAAAAAC CGATTATACA ACCGTCTTGG CCGTTTTTTA   
  
  
- TGTATTAAAC TTATCACGTA CGTATACTCA TGTACTGTCA CTTGGTTGTT TTGCTTGGGG ATTTTGTGTT   
  
  
- CTCTTAATTG TACAATTTTG TGAATTTGAT TTACGTTGGT AGAGCTACTG GTGATTTATC GGTTCTTTGG   
  
  
- ATTGCATTAA TTGTTTTTAT TTTTTATTGA CTTTTATTTC ATCTCTTTCA GATTGGCTTA TGCTATTGTA   
  
  
- TTGGATTTTG ATTGATATGA ACTTGAATGA TTCTTTATAC TTAGTTGGTG TTTGGCTTAA CTAAAACCAA   
  
  
- ACGGTATTAA ACTGATATGT AAATTGAATT TTTTATAAAA AAAAATTAAA GTTTATTTAG ATTTAATAAA   
  
  
- AAGTTATAAG TAGGTTTTAT CTAAGTTAAT AATTTGTTCA GTTGGATATG GCTCAATAAA AGATCGAGAC   
  
  
- TGATTCTGTA TATATATAAA AAACATTTTT TCTATTCCTA ATATTAAATT TATCGGAAAG GGAATTTTTT   
  
  
- TAAAAATTTT AATAGGACTA TCTCTTATTT TTCTTTAGGA ATATGTTTTG AGACTAACTG CGTCTACACA   
  
  
- TATACCTGTA TATCTTCATT ATTCTCCCCA TCCGATCCTT CCCCCACCTT TATATCCCGT ATTTATCCCC   
  
  
- CGCCCGTATC CCTGTACAGA CAGACTCCAT GCCACCTACC CTGGAACGGT ACCTTCGTTG TTACAGTGTT   
  
  
- ATTATTATAG TTGCTGACTT CAAAGAAGTG GTCGGGTGAG AAACGGTATT ACGGTAATTA ATAAATAACG   
  
  
- ATGATAGAGA AAACTACACT TTGCCACCTG GAATAGAGAG GGGAGGTGAA GGTTAACTAC ACTCGTTGAG   
  
  
- TCTTTGCGTT TGTTTTGAAC ACGGTGGAAG AAATGAACGT AGGAGTAAAA GGAGACAGAG AGAGGCGGGG   
  
  
- ACAAAGAGAG AGAGAGGGAG TGAGTGAGTC TCATGACTAG ACTCGTAGTC TTCATCGTCT GGGTCTTTGA   
  
  
- GTCTAAATTT TTGTTGGGCG AGAAAACAAG GGAGATGCAG ATTATTAAAA CAGTAGCGTA AATTACAGTC   
  
  
- TATCTCTCTA TCTCGAGATG ACATAATTAA AAGAGGGTGG AGTGAACTAA GACAACGGGA TTTGGTGGGT   
  
  
- TTTTTTCTTT ATGCTTCTTC GGTGAGAGAG AGAGAGAGAG AGAGTGTGTG TGTGTGTGGG GAGGATCACC   
  
  
- TAAACTATCT CATATTTACT TGTCATTAGT CTGCTCTATA TATCATCACG AATTAACTGG CTCATCGTTT   
  
  
- TCCCCCTTCA ACTCACTCCA TAGTTCTCTC ATCGCATCGA TCGATCCCGT TCTATACGAC CCCAGAAGGA   
  
  
- GTATGTTGAG GCTCCTTCTG GTGGTTCTAC TGTTGGTAAG GAGTGGCAGA CTAAACAGAT CCGTGGGTGG   
  
  
- ATCTAAGTGG TGATGATGAA TGGTTATGGT TGTTGTATAA TCGAGAGGTA GAAGTGTTAG TTATGATCGA   
  
  
- CACGGTGGAG CGGTTAACAA TTAGTTAACA CGCCTCAATT AAACGGCCAA GCTGAAGAGG AGGCGGTTGG   
  
  
- CCGAAGAGAG AGAGGAGCGG AGGGTGAAAC GGAGGGGAAG ACCCCTGAGG TGGCTCTCTG AACACAAAAT   
  
  
- GAAGACGTTC CGAAACCTAG AGGCGGAGTT ATTGTCAATA AATAGGTGAG CATCAAACTG ACCAACATTG   
  
  
- GGATCATGAC CAAGACCATA ACCCCGAGGA AATCGAAGGT TAAAAGATAA AGAAGTTGGT AATCACGTAA   
  
  
- GTGTCCATTG TTCATTACGG GCACAACCCA TGAACTGTAT AAACTGAGAC TTGGTCCAGT GGGGTAAGTA   
  
  
- GGCTAAGTGG GTGAATTGAC GGTTGGTTCG GTAGGACCTT CGAAACCTCC CCATGTTCCG CCAGGTATAG   
  
  
- GAACTGTACC TGTAGTACGT ACCCCAGGTC ACCGGTGGTG AAGAAGTTCG TTAACGTCTT TCTAGACGTT   
  
  
- GGGAACCAGT GGGACGAGGG TGACAAGCGT AGTGTCCTCC CCCGTCCCTA GATCTTGAGG ACTTGGCTTG   
  
  
- ACCTCTAGCC TAGTCCTTCA AGCGTGTTAG GAACCCTGAG CTCAAAGTTA AGGTGGTAGA ATATTGCCAC   
  
  
- CTGAGCTTGG AATGACTCCG TCACCTCCGG TAGTTACGGG ATGTCGATGT GGAGGTACAG GTTCTCCTCC   
  
  
- TCCAGAAGCG TCACTTAACA CCTCTAAAGG AGGTGGCGGA GGATTGCCTC ATACTATCAT GTAAGGAATC   
  
  
- CTACAAAGAG ATGTTCCACT TCTGGGATTT GGGATCCCAG AAGTGGCACC CTCTTTCCCT CCGGCTGGTG   
  
  
- TTGGTGGGAG AGAAGACCGT CGCTAAGTAA CTCCGTGAAC TGGTGATACC CCGTCACAAA CTGAGAGATC   
  
  
- TTCGTTGGGA TGGTAGTTGT TCAGTTCTCT CCCACCGCCA CCTTCTCCCC ACCAAGCCCC TCCTCTAATT   
  
  
- TCTACACCAA CCTGCTCTTC CTCCCAACTC CTCTGTCTCC GTGGTCTTTA TGCTCAGAAC CCTCCACAAC   
  
  
- TACTCCTCGA GACCCAAATT CTCGAACGGG AACTCGGGGA AGAGACACAG TGTTCGGTTC GACGATGAAG   
  
  
- CGGAAGTAAT GGGAAGACTT CCCATGGTGG AATTCCAGAA GGTATCATTA ACAGACAACG AACCCACCTT   
  
  
- CTTAGCGGGA GAAAAGAGAC AAAGAAGAAC CGTTAT

+     Unnamed\_\_12

| Site Name | Organism | Position | Strand | Matrix score. | sequence | function |
| --- | --- | --- | --- | --- | --- | --- |
| Unnamed\_\_12 | Zea mays | 587 | - | 10 | TCCACGTAGA |  |

>HU01G00833.1   
+ -Up\_Stream \_Len000AAAATA TTTTTACAAA AGCATTTCTC TATAAAATAT TTTACAAAAT TTCATTTTGT   
  
  
+ GGGTCTTTTA GAATCAATCT CTCTACAAGT AGGAATGCCG GTTGAGGTAA GGCTACCTAC ATCTAGATCT   
  
  
+ CTCCAGACCC CACAAAAATT AAACGAGACC CACACTGAAA GGTGGTGGTG GTGGTGTTGT TGTTGTATTG   
  
  
+ TCCTACCACG AGAGAGCTTC AAGTGCATCA CTTATTTCAA CAGGTCAATC ACATAGATCT CTAGTGTACT   
  
  
+ GTAAATTTTT TTTCCGTCTT ATTTTAATTT TCTTTTCCAT CCTAAAAGAT TATCATGTAG ATATTCCTAG   
  
  
+ TTTGATATAT TGACTTCAAC TTCAAAAATT ACACATTAAG ATCCACGAAT ATGACAGTTT AGGTTTCATT   
  
  
+ TGAAGGAGAA AGAAATGTTA CGGTGTTTTG GTGTTGTATT CGTAGACATT TTTTGCGCTT AACTGGCTCT   
  
  
+ TCTCATGGGG ACGAGACAGT GAAGGTAATT GTACTTGTTG ATTGATGACG TCATCTGTAG CTTGGATTTG   
  
  
+ TATAGGAATG AACATCTTGG TGTCTACGTG GAAAGTGTGA TTTGTGTTCT TTCATTGATC CTCCTCCTCA   
  
  
+ AGAAGAGGTG ATCTTTCTCC CTTGTTTTTT TTTTTTTTTG GCTAATATGT TGGCAGAACC GGCAAAAAAT   
  
  
+ ACATAATTTG AATAGTGCAT GCATATGAGT ACATGACAGT GAACCAACAA AACGAACCCC TAAAACACAA   
  
  
+ GAGAATTAAC ATGTTAAAAC ACTTAAACTA AATGCAACCA TCTCGATGAC CACTAAATAG CCAAGAAACC   
  
  
+ TAACGTAATT AACAAAAATA AAAAATAACT GAAAATAAAG TAGAGAAAGT CTAACCGAAT ACGATAACAT   
  
  
+ AACCTAAAAC TAACTATACT TGAACTTACT AAGAAATATG AATCAACCAC AAACCGAATT GATTTTGGTT   
  
  
+ TGCCATAATT TGACTATACA TTTAACTTAA AAAATATTTT TTTTTAATTT CAAATAAATC TAAATTATTT   
  
  
+ TTCAATATTC ATCCAAAATA GATTCAATTA TTAAACAAGT CAACCTATAC CGAGTTATTT TCTAGCTCTG   
  
  
+ ACTAAGACAT ATATATATTT TTTGTAAAAA AGATAAGGAT TATAATTTAA ATAGCCTTTC CCTTAAAAAA   
  
  
+ ATTTTTAAAA TTATCCTGAT AGAGAATAAA AAGAAATCCT TATACAAAAC TCTGATTGAC GCAGATGTGT   
  
  
+ ATATGGACAT ATAGAAGTAA TAAGAGGGGT AGGCTAGGAA GGGGGTGGAA ATATAGGGCA TAAATAGGGG   
  
  
+ GCGGGCATAG GGACATGTCT GTCTGAGGTA CGGTGGATGG GACCTTGCCA TGGAAGCAAC AATGTCACAA   
  
  
+ TAATAATATC AACGACTGAA GTTTCTTCAC CAGCCCACTC TTTGCCATAA TGCCATTAAT TATTTATTGC   
  
  
+ TACTATCTCT TTTGATGTGA AACGGTGGAC CTTATCTCTC CCCTCCACTT CCAATTGATG TGAGCAACTC   
  
  
+ AGAAACGCAA ACAAAACTTG TGCCACCTTC TTTACTTGCA TCCTCATTTT CCTCTGTCTC TCTCCGCCCC   
  
  
+ TGTTTCTCTC TCTCTCCCTC ACTCACTCAG AGTACTGATC TGAGCATCAG AAGTAGCAGA CCCAGAAACT   
  
  
+ CAGATTTAAA AACAACCCGC TCTTTTGTTC CCTCTACGTC TAATAATTTT GTCATCGCAT TTAATGTCAG   
  
  
+ ATAGAGAGAT AGAGCTCTAC TGTATTAATT TTCTCCCACC TCACTTGATT CTGTTGCCCT AAACCACCCA   
  
  
+ AAAAAAGAAA TACGAAGAAG CCACTCTCTC TCTCTCTCTC TCTCACACAC ACACACACCC CTCCTAGTGG   
  
  
+ ATTTGATAGA GTATAAATGA ACAGTAATCA GACGAGATAT ATAGTAGTGC TTAATTGACC GAGTAGCAAA   
  
  
+ AGGGGGAAGT TGAGTGAGGT ATCAAGAGAG TAGCGTAGCT AGCTAGGGCA AGATATGCTG GGGTCTTCCT   
  
  
+ CATACAACTC CGAGGAAGAC CACCAAGATG ACAACCATTC CTCACCGTCT GATTTGTCTA GGCACCCACC   
  
  
+ TAGATTCACC ACTACTACTT ACCAATACCA ACAACATATT AGCTCTCCAT CTTCACAATC AATACTAGCT   
  
  
+ GTGCCACCTC GCCAATTGTT AATCAATTGT GCGGAGTTAA TTTGCCGGTT CGACTTCTCC TCCGCCAACC   
  
  
+ GGCTTCTCTC TCTCCTCGCC TCCCACTTTG CCTCCCCTTC TGGGGACTCC ACCGAGAGAC TTGTGTTTTA   
  
  
+ CTTCTGCAAG GCTTTGGATC TCCGCCTCAA TAACAGTTAT TTATCCACTC GTAGTTTGAC TGGTTGTAAC   
  
  
+ CCTAGTACTG GTTCTGGTAT TGGGGCTCCT TTAGCTTCCA ATTTTCTATT TCTTCAACCA TTAGTGCATT   
  
  
+ CACAGGTAAC AAGTAATGCC CGTGTTGGGT ACTTGACATA TTTGACTCTG AACCAGGTCA CCCCATTCAT   
  
  
+ CCGATTCACC CACTTAACTG CCAACCAAGC CATCCTGGAA GCTTTGGAGG GGTACAAGGC GGTCCATATC   
  
  
+ CTTGACATGG ACATCATGCA TGGGGTCCAG TGGCCACCAC TTCTTCAAGC AATTGCAGAA AGATCTGCAA   
  
  
+ CCCTTGGTCA CCCTGCTCCC ACTGTTCGCA TCACAGGAGG GGGCAGGGAT CTAGAACTCC TGAACCGAAC   
  
  
+ TGGAGATCGG ATCAGGAAGT TCGCACAATC CTTGGGACTC GAGTTTCAAT TCCACCATCT TATAACGGTG   
  
  
+ GACTCGAACC TTACTGAGGC AGTGGAGGCC ATCAATGCCC TACAGCTACA CCTCCATGTC CAAGAGGAGG   
  
  
+ AGGTCTTCGC AGTGAATTGT GGAGATTTCC TCCACCGCCT CCTAACGGAG TATGATAGTA CATTCCTTAG   
  
  
+ GATGTTTCTC TACAAGGTGA AGACCCTAAA CCCTAGGGTC TTCACCGTGG GAGAAAGGGA GGCCGACCAC   
  
  
+ AACCACCCTC TCTTCTGGCA GCGATTCATT GAGGCACTTG ACCACTATGG GGCAGTGTTT GACTCTCTAG   
  
  
+ AAGCAACCCT ACCATCAACA AGTCAAGAGA GGGTGGCGGT GGAAGAGGGG TGGTTCGGGG AGGAGATTAA   
  
  
+ AGATGTGGTT GGACGAGAAG GAGGGTTGAG GAGACAGAGG CACCAGAAAT ACGAGTCTTG GGAGGTGTTG   
  
  
+ ATGAGGAGCT CTGGGTTTAA GAGCTTGCCC TTGAGCCCCT TCTCTGTGTC ACAAGCCAAG CTGCTACTTC   
  
  
+ GCCTTCATTA CCCTTCTGAA GGGTACCACC TTAAGGTCTT CCATAGTAAT TGTCTGTTGC TTGGGTGGAA   
  
  
+ GAATCGCCCT CTTTTCTCTG TTTCTTCTTG GCAATA  

- -Up\_Stream \_Len000TTTTAT AAAAATGTTT TCGTAAAGAG ATATTTTATA AAATGTTTTA AAGTAAAACA   
  
  
- CCCAGAAAAT CTTAGTTAGA GAGATGTTCA TCCTTACGGC CAACTCCATT CCGATGGATG TAGATCTAGA   
  
  
- GAGGTCTGGG GTGTTTTTAA TTTGCTCTGG GTGTGACTTT CCACCACCAC CACCACAACA ACAACATAAC   
  
  
- AGGATGGTGC TCTCTCGAAG TTCACGTAGT GAATAAAGTT GTCCAGTTAG TGTATCTAGA GATCACATGA   
  
  
- CATTTAAAAA AAAGGCAGAA TAAAATTAAA AGAAAAGGTA GGATTTTCTA ATAGTACATC TATAAGGATC   
  
  
- AAACTATATA ACTGAAGTTG AAGTTTTTAA TGTGTAATTC TAGGTGCTTA TACTGTCAAA TCCAAAGTAA   
  
  
- ACTTCCTCTT TCTTTACAAT GCCACAAAAC CACAACATAA GCATCTGTAA AAAACGCGAA TTGACCGAGA   
  
  
- AGAGTACCCC TGCTCTGTCA CTTCCATTAA CATGAACAAC TAACTACTGC AGTAGACATC GAACCTAAAC   
  
  
- ATATCCTTAC TTGTAGAACC ACAGATGCAC CTTTCACACT AAACACAAGA AAGTAACTAG GAGGAGGAGT   
  
  
- TCTTCTCCAC TAGAAAGAGG GAACAAAAAA AAAAAAAAAC CGATTATACA ACCGTCTTGG CCGTTTTTTA   
  
  
- TGTATTAAAC TTATCACGTA CGTATACTCA TGTACTGTCA CTTGGTTGTT TTGCTTGGGG ATTTTGTGTT   
  
  
- CTCTTAATTG TACAATTTTG TGAATTTGAT TTACGTTGGT AGAGCTACTG GTGATTTATC GGTTCTTTGG   
  
  
- ATTGCATTAA TTGTTTTTAT TTTTTATTGA CTTTTATTTC ATCTCTTTCA GATTGGCTTA TGCTATTGTA   
  
  
- TTGGATTTTG ATTGATATGA ACTTGAATGA TTCTTTATAC TTAGTTGGTG TTTGGCTTAA CTAAAACCAA   
  
  
- ACGGTATTAA ACTGATATGT AAATTGAATT TTTTATAAAA AAAAATTAAA GTTTATTTAG ATTTAATAAA   
  
  
- AAGTTATAAG TAGGTTTTAT CTAAGTTAAT AATTTGTTCA GTTGGATATG GCTCAATAAA AGATCGAGAC   
  
  
- TGATTCTGTA TATATATAAA AAACATTTTT TCTATTCCTA ATATTAAATT TATCGGAAAG GGAATTTTTT   
  
  
- TAAAAATTTT AATAGGACTA TCTCTTATTT TTCTTTAGGA ATATGTTTTG AGACTAACTG CGTCTACACA   
  
  
- TATACCTGTA TATCTTCATT ATTCTCCCCA TCCGATCCTT CCCCCACCTT TATATCCCGT ATTTATCCCC   
  
  
- CGCCCGTATC CCTGTACAGA CAGACTCCAT GCCACCTACC CTGGAACGGT ACCTTCGTTG TTACAGTGTT   
  
  
- ATTATTATAG TTGCTGACTT CAAAGAAGTG GTCGGGTGAG AAACGGTATT ACGGTAATTA ATAAATAACG   
  
  
- ATGATAGAGA AAACTACACT TTGCCACCTG GAATAGAGAG GGGAGGTGAA GGTTAACTAC ACTCGTTGAG   
  
  
- TCTTTGCGTT TGTTTTGAAC ACGGTGGAAG AAATGAACGT AGGAGTAAAA GGAGACAGAG AGAGGCGGGG   
  
  
- ACAAAGAGAG AGAGAGGGAG TGAGTGAGTC TCATGACTAG ACTCGTAGTC TTCATCGTCT GGGTCTTTGA   
  
  
- GTCTAAATTT TTGTTGGGCG AGAAAACAAG GGAGATGCAG ATTATTAAAA CAGTAGCGTA AATTACAGTC   
  
  
- TATCTCTCTA TCTCGAGATG ACATAATTAA AAGAGGGTGG AGTGAACTAA GACAACGGGA TTTGGTGGGT   
  
  
- TTTTTTCTTT ATGCTTCTTC GGTGAGAGAG AGAGAGAGAG AGAGTGTGTG TGTGTGTGGG GAGGATCACC   
  
  
- TAAACTATCT CATATTTACT TGTCATTAGT CTGCTCTATA TATCATCACG AATTAACTGG CTCATCGTTT   
  
  
- TCCCCCTTCA ACTCACTCCA TAGTTCTCTC ATCGCATCGA TCGATCCCGT TCTATACGAC CCCAGAAGGA   
  
  
- GTATGTTGAG GCTCCTTCTG GTGGTTCTAC TGTTGGTAAG GAGTGGCAGA CTAAACAGAT CCGTGGGTGG   
  
  
- ATCTAAGTGG TGATGATGAA TGGTTATGGT TGTTGTATAA TCGAGAGGTA GAAGTGTTAG TTATGATCGA   
  
  
- CACGGTGGAG CGGTTAACAA TTAGTTAACA CGCCTCAATT AAACGGCCAA GCTGAAGAGG AGGCGGTTGG   
  
  
- CCGAAGAGAG AGAGGAGCGG AGGGTGAAAC GGAGGGGAAG ACCCCTGAGG TGGCTCTCTG AACACAAAAT   
  
  
- GAAGACGTTC CGAAACCTAG AGGCGGAGTT ATTGTCAATA AATAGGTGAG CATCAAACTG ACCAACATTG   
  
  
- GGATCATGAC CAAGACCATA ACCCCGAGGA AATCGAAGGT TAAAAGATAA AGAAGTTGGT AATCACGTAA   
  
  
- GTGTCCATTG TTCATTACGG GCACAACCCA TGAACTGTAT AAACTGAGAC TTGGTCCAGT GGGGTAAGTA   
  
  
- GGCTAAGTGG GTGAATTGAC GGTTGGTTCG GTAGGACCTT CGAAACCTCC CCATGTTCCG CCAGGTATAG   
  
  
- GAACTGTACC TGTAGTACGT ACCCCAGGTC ACCGGTGGTG AAGAAGTTCG TTAACGTCTT TCTAGACGTT   
  
  
- GGGAACCAGT GGGACGAGGG TGACAAGCGT AGTGTCCTCC CCCGTCCCTA GATCTTGAGG ACTTGGCTTG   
  
  
- ACCTCTAGCC TAGTCCTTCA AGCGTGTTAG GAACCCTGAG CTCAAAGTTA AGGTGGTAGA ATATTGCCAC   
  
  
- CTGAGCTTGG AATGACTCCG TCACCTCCGG TAGTTACGGG ATGTCGATGT GGAGGTACAG GTTCTCCTCC   
  
  
- TCCAGAAGCG TCACTTAACA CCTCTAAAGG AGGTGGCGGA GGATTGCCTC ATACTATCAT GTAAGGAATC   
  
  
- CTACAAAGAG ATGTTCCACT TCTGGGATTT GGGATCCCAG AAGTGGCACC CTCTTTCCCT CCGGCTGGTG   
  
  
- TTGGTGGGAG AGAAGACCGT CGCTAAGTAA CTCCGTGAAC TGGTGATACC CCGTCACAAA CTGAGAGATC   
  
  
- TTCGTTGGGA TGGTAGTTGT TCAGTTCTCT CCCACCGCCA CCTTCTCCCC ACCAAGCCCC TCCTCTAATT   
  
  
- TCTACACCAA CCTGCTCTTC CTCCCAACTC CTCTGTCTCC GTGGTCTTTA TGCTCAGAAC CCTCCACAAC   
  
  
- TACTCCTCGA GACCCAAATT CTCGAACGGG AACTCGGGGA AGAGACACAG TGTTCGGTTC GACGATGAAG   
  
  
- CGGAAGTAAT GGGAAGACTT CCCATGGTGG AATTCCAGAA GGTATCATTA ACAGACAACG AACCCACCTT   
  
  
- CTTAGCGGGA GAAAAGAGAC AAAGAAGAAC CGTTAT

+     Unnamed\_\_14

| Site Name | Organism | Position | Strand | Matrix score. | sequence | function |
| --- | --- | --- | --- | --- | --- | --- |
| Unnamed\_\_14 | Zea mays | 587 | - | 10 | TCCACGTAGA |  |

>HU01G00833.1   
+ -Up\_Stream \_Len000AAAATA TTTTTACAAA AGCATTTCTC TATAAAATAT TTTACAAAAT TTCATTTTGT   
  
  
+ GGGTCTTTTA GAATCAATCT CTCTACAAGT AGGAATGCCG GTTGAGGTAA GGCTACCTAC ATCTAGATCT   
  
  
+ CTCCAGACCC CACAAAAATT AAACGAGACC CACACTGAAA GGTGGTGGTG GTGGTGTTGT TGTTGTATTG   
  
  
+ TCCTACCACG AGAGAGCTTC AAGTGCATCA CTTATTTCAA CAGGTCAATC ACATAGATCT CTAGTGTACT   
  
  
+ GTAAATTTTT TTTCCGTCTT ATTTTAATTT TCTTTTCCAT CCTAAAAGAT TATCATGTAG ATATTCCTAG   
  
  
+ TTTGATATAT TGACTTCAAC TTCAAAAATT ACACATTAAG ATCCACGAAT ATGACAGTTT AGGTTTCATT   
  
  
+ TGAAGGAGAA AGAAATGTTA CGGTGTTTTG GTGTTGTATT CGTAGACATT TTTTGCGCTT AACTGGCTCT   
  
  
+ TCTCATGGGG ACGAGACAGT GAAGGTAATT GTACTTGTTG ATTGATGACG TCATCTGTAG CTTGGATTTG   
  
  
+ TATAGGAATG AACATCTTGG TGTCTACGTG GAAAGTGTGA TTTGTGTTCT TTCATTGATC CTCCTCCTCA   
  
  
+ AGAAGAGGTG ATCTTTCTCC CTTGTTTTTT TTTTTTTTTG GCTAATATGT TGGCAGAACC GGCAAAAAAT   
  
  
+ ACATAATTTG AATAGTGCAT GCATATGAGT ACATGACAGT GAACCAACAA AACGAACCCC TAAAACACAA   
  
  
+ GAGAATTAAC ATGTTAAAAC ACTTAAACTA AATGCAACCA TCTCGATGAC CACTAAATAG CCAAGAAACC   
  
  
+ TAACGTAATT AACAAAAATA AAAAATAACT GAAAATAAAG TAGAGAAAGT CTAACCGAAT ACGATAACAT   
  
  
+ AACCTAAAAC TAACTATACT TGAACTTACT AAGAAATATG AATCAACCAC AAACCGAATT GATTTTGGTT   
  
  
+ TGCCATAATT TGACTATACA TTTAACTTAA AAAATATTTT TTTTTAATTT CAAATAAATC TAAATTATTT   
  
  
+ TTCAATATTC ATCCAAAATA GATTCAATTA TTAAACAAGT CAACCTATAC CGAGTTATTT TCTAGCTCTG   
  
  
+ ACTAAGACAT ATATATATTT TTTGTAAAAA AGATAAGGAT TATAATTTAA ATAGCCTTTC CCTTAAAAAA   
  
  
+ ATTTTTAAAA TTATCCTGAT AGAGAATAAA AAGAAATCCT TATACAAAAC TCTGATTGAC GCAGATGTGT   
  
  
+ ATATGGACAT ATAGAAGTAA TAAGAGGGGT AGGCTAGGAA GGGGGTGGAA ATATAGGGCA TAAATAGGGG   
  
  
+ GCGGGCATAG GGACATGTCT GTCTGAGGTA CGGTGGATGG GACCTTGCCA TGGAAGCAAC AATGTCACAA   
  
  
+ TAATAATATC AACGACTGAA GTTTCTTCAC CAGCCCACTC TTTGCCATAA TGCCATTAAT TATTTATTGC   
  
  
+ TACTATCTCT TTTGATGTGA AACGGTGGAC CTTATCTCTC CCCTCCACTT CCAATTGATG TGAGCAACTC   
  
  
+ AGAAACGCAA ACAAAACTTG TGCCACCTTC TTTACTTGCA TCCTCATTTT CCTCTGTCTC TCTCCGCCCC   
  
  
+ TGTTTCTCTC TCTCTCCCTC ACTCACTCAG AGTACTGATC TGAGCATCAG AAGTAGCAGA CCCAGAAACT   
  
  
+ CAGATTTAAA AACAACCCGC TCTTTTGTTC CCTCTACGTC TAATAATTTT GTCATCGCAT TTAATGTCAG   
  
  
+ ATAGAGAGAT AGAGCTCTAC TGTATTAATT TTCTCCCACC TCACTTGATT CTGTTGCCCT AAACCACCCA   
  
  
+ AAAAAAGAAA TACGAAGAAG CCACTCTCTC TCTCTCTCTC TCTCACACAC ACACACACCC CTCCTAGTGG   
  
  
+ ATTTGATAGA GTATAAATGA ACAGTAATCA GACGAGATAT ATAGTAGTGC TTAATTGACC GAGTAGCAAA   
  
  
+ AGGGGGAAGT TGAGTGAGGT ATCAAGAGAG TAGCGTAGCT AGCTAGGGCA AGATATGCTG GGGTCTTCCT   
  
  
+ CATACAACTC CGAGGAAGAC CACCAAGATG ACAACCATTC CTCACCGTCT GATTTGTCTA GGCACCCACC   
  
  
+ TAGATTCACC ACTACTACTT ACCAATACCA ACAACATATT AGCTCTCCAT CTTCACAATC AATACTAGCT   
  
  
+ GTGCCACCTC GCCAATTGTT AATCAATTGT GCGGAGTTAA TTTGCCGGTT CGACTTCTCC TCCGCCAACC   
  
  
+ GGCTTCTCTC TCTCCTCGCC TCCCACTTTG CCTCCCCTTC TGGGGACTCC ACCGAGAGAC TTGTGTTTTA   
  
  
+ CTTCTGCAAG GCTTTGGATC TCCGCCTCAA TAACAGTTAT TTATCCACTC GTAGTTTGAC TGGTTGTAAC   
  
  
+ CCTAGTACTG GTTCTGGTAT TGGGGCTCCT TTAGCTTCCA ATTTTCTATT TCTTCAACCA TTAGTGCATT   
  
  
+ CACAGGTAAC AAGTAATGCC CGTGTTGGGT ACTTGACATA TTTGACTCTG AACCAGGTCA CCCCATTCAT   
  
  
+ CCGATTCACC CACTTAACTG CCAACCAAGC CATCCTGGAA GCTTTGGAGG GGTACAAGGC GGTCCATATC   
  
  
+ CTTGACATGG ACATCATGCA TGGGGTCCAG TGGCCACCAC TTCTTCAAGC AATTGCAGAA AGATCTGCAA   
  
  
+ CCCTTGGTCA CCCTGCTCCC ACTGTTCGCA TCACAGGAGG GGGCAGGGAT CTAGAACTCC TGAACCGAAC   
  
  
+ TGGAGATCGG ATCAGGAAGT TCGCACAATC CTTGGGACTC GAGTTTCAAT TCCACCATCT TATAACGGTG   
  
  
+ GACTCGAACC TTACTGAGGC AGTGGAGGCC ATCAATGCCC TACAGCTACA CCTCCATGTC CAAGAGGAGG   
  
  
+ AGGTCTTCGC AGTGAATTGT GGAGATTTCC TCCACCGCCT CCTAACGGAG TATGATAGTA CATTCCTTAG   
  
  
+ GATGTTTCTC TACAAGGTGA AGACCCTAAA CCCTAGGGTC TTCACCGTGG GAGAAAGGGA GGCCGACCAC   
  
  
+ AACCACCCTC TCTTCTGGCA GCGATTCATT GAGGCACTTG ACCACTATGG GGCAGTGTTT GACTCTCTAG   
  
  
+ AAGCAACCCT ACCATCAACA AGTCAAGAGA GGGTGGCGGT GGAAGAGGGG TGGTTCGGGG AGGAGATTAA   
  
  
+ AGATGTGGTT GGACGAGAAG GAGGGTTGAG GAGACAGAGG CACCAGAAAT ACGAGTCTTG GGAGGTGTTG   
  
  
+ ATGAGGAGCT CTGGGTTTAA GAGCTTGCCC TTGAGCCCCT TCTCTGTGTC ACAAGCCAAG CTGCTACTTC   
  
  
+ GCCTTCATTA CCCTTCTGAA GGGTACCACC TTAAGGTCTT CCATAGTAAT TGTCTGTTGC TTGGGTGGAA   
  
  
+ GAATCGCCCT CTTTTCTCTG TTTCTTCTTG GCAATA  

- -Up\_Stream \_Len000TTTTAT AAAAATGTTT TCGTAAAGAG ATATTTTATA AAATGTTTTA AAGTAAAACA   
  
  
- CCCAGAAAAT CTTAGTTAGA GAGATGTTCA TCCTTACGGC CAACTCCATT CCGATGGATG TAGATCTAGA   
  
  
- GAGGTCTGGG GTGTTTTTAA TTTGCTCTGG GTGTGACTTT CCACCACCAC CACCACAACA ACAACATAAC   
  
  
- AGGATGGTGC TCTCTCGAAG TTCACGTAGT GAATAAAGTT GTCCAGTTAG TGTATCTAGA GATCACATGA   
  
  
- CATTTAAAAA AAAGGCAGAA TAAAATTAAA AGAAAAGGTA GGATTTTCTA ATAGTACATC TATAAGGATC   
  
  
- AAACTATATA ACTGAAGTTG AAGTTTTTAA TGTGTAATTC TAGGTGCTTA TACTGTCAAA TCCAAAGTAA   
  
  
- ACTTCCTCTT TCTTTACAAT GCCACAAAAC CACAACATAA GCATCTGTAA AAAACGCGAA TTGACCGAGA   
  
  
- AGAGTACCCC TGCTCTGTCA CTTCCATTAA CATGAACAAC TAACTACTGC AGTAGACATC GAACCTAAAC   
  
  
- ATATCCTTAC TTGTAGAACC ACAGATGCAC CTTTCACACT AAACACAAGA AAGTAACTAG GAGGAGGAGT   
  
  
- TCTTCTCCAC TAGAAAGAGG GAACAAAAAA AAAAAAAAAC CGATTATACA ACCGTCTTGG CCGTTTTTTA   
  
  
- TGTATTAAAC TTATCACGTA CGTATACTCA TGTACTGTCA CTTGGTTGTT TTGCTTGGGG ATTTTGTGTT   
  
  
- CTCTTAATTG TACAATTTTG TGAATTTGAT TTACGTTGGT AGAGCTACTG GTGATTTATC GGTTCTTTGG   
  
  
- ATTGCATTAA TTGTTTTTAT TTTTTATTGA CTTTTATTTC ATCTCTTTCA GATTGGCTTA TGCTATTGTA   
  
  
- TTGGATTTTG ATTGATATGA ACTTGAATGA TTCTTTATAC TTAGTTGGTG TTTGGCTTAA CTAAAACCAA   
  
  
- ACGGTATTAA ACTGATATGT AAATTGAATT TTTTATAAAA AAAAATTAAA GTTTATTTAG ATTTAATAAA   
  
  
- AAGTTATAAG TAGGTTTTAT CTAAGTTAAT AATTTGTTCA GTTGGATATG GCTCAATAAA AGATCGAGAC   
  
  
- TGATTCTGTA TATATATAAA AAACATTTTT TCTATTCCTA ATATTAAATT TATCGGAAAG GGAATTTTTT   
  
  
- TAAAAATTTT AATAGGACTA TCTCTTATTT TTCTTTAGGA ATATGTTTTG AGACTAACTG CGTCTACACA   
  
  
- TATACCTGTA TATCTTCATT ATTCTCCCCA TCCGATCCTT CCCCCACCTT TATATCCCGT ATTTATCCCC   
  
  
- CGCCCGTATC CCTGTACAGA CAGACTCCAT GCCACCTACC CTGGAACGGT ACCTTCGTTG TTACAGTGTT   
  
  
- ATTATTATAG TTGCTGACTT CAAAGAAGTG GTCGGGTGAG AAACGGTATT ACGGTAATTA ATAAATAACG   
  
  
- ATGATAGAGA AAACTACACT TTGCCACCTG GAATAGAGAG GGGAGGTGAA GGTTAACTAC ACTCGTTGAG   
  
  
- TCTTTGCGTT TGTTTTGAAC ACGGTGGAAG AAATGAACGT AGGAGTAAAA GGAGACAGAG AGAGGCGGGG   
  
  
- ACAAAGAGAG AGAGAGGGAG TGAGTGAGTC TCATGACTAG ACTCGTAGTC TTCATCGTCT GGGTCTTTGA   
  
  
- GTCTAAATTT TTGTTGGGCG AGAAAACAAG GGAGATGCAG ATTATTAAAA CAGTAGCGTA AATTACAGTC   
  
  
- TATCTCTCTA TCTCGAGATG ACATAATTAA AAGAGGGTGG AGTGAACTAA GACAACGGGA TTTGGTGGGT   
  
  
- TTTTTTCTTT ATGCTTCTTC GGTGAGAGAG AGAGAGAGAG AGAGTGTGTG TGTGTGTGGG GAGGATCACC   
  
  
- TAAACTATCT CATATTTACT TGTCATTAGT CTGCTCTATA TATCATCACG AATTAACTGG CTCATCGTTT   
  
  
- TCCCCCTTCA ACTCACTCCA TAGTTCTCTC ATCGCATCGA TCGATCCCGT TCTATACGAC CCCAGAAGGA   
  
  
- GTATGTTGAG GCTCCTTCTG GTGGTTCTAC TGTTGGTAAG GAGTGGCAGA CTAAACAGAT CCGTGGGTGG   
  
  
- ATCTAAGTGG TGATGATGAA TGGTTATGGT TGTTGTATAA TCGAGAGGTA GAAGTGTTAG TTATGATCGA   
  
  
- CACGGTGGAG CGGTTAACAA TTAGTTAACA CGCCTCAATT AAACGGCCAA GCTGAAGAGG AGGCGGTTGG   
  
  
- CCGAAGAGAG AGAGGAGCGG AGGGTGAAAC GGAGGGGAAG ACCCCTGAGG TGGCTCTCTG AACACAAAAT   
  
  
- GAAGACGTTC CGAAACCTAG AGGCGGAGTT ATTGTCAATA AATAGGTGAG CATCAAACTG ACCAACATTG
[truncated: 66,092 more chars]
