# Supplementary material for: Genome-Wide Identification and Expression Pattern of the GRAS Gene Family in Pitaya (Selenicereus undatus L.)
Source: Biology (Basel). 2022 Dec 21;12(1):11. doi: 10.3390/biology12010011 (PMC9854919; doi:10.3390/biology12010011)
Supplement: Supplementary file 1 [file biology-12-00011-s001.zip › Supplementary file S5/HU01G01391.1_plantcare.html]

Content-Type: text/html; charset=ISO-8859-1


PlantCARE


Webmaster Firefox specific output  
To save the result:
click on the frame with the right mouse button and save the source code as a text file with extension .html  
REFERENCE:PlantCARE: a database of plant cis-acting regulatory elements and a portal to tools for in silico analysis of promoter sequences.  
Lescot, M., Déhais, P., Moreau, Y., De Moor, B., Rouzé ,P.,and Rombauts, S.  
Nucleic Acids Res., Database issue(2002), 30(1):325-327.   


---

>HU01G01391.1   
+ +Up\_Stream \_Len000CATTTT AGTAATAAAT TCCCATGTGA GGACCACTGC CTACTGATTC CGAATGGCAT   
  
  
+ TTTAGTAATA AACTCCCATG TGAGGACCAC TTGATGAGGA ATTCAGTTTT ATAATCCACA GGTGTCAAAG   
  
  
+ CCTATTCCTA CTCTCACCAC TCTGCTATCT CTCCCCCCCC CCCCCCCCCG CCCCCCTCAA AATCTGCCTC   
  
  
+ CTCCGTTCTC TCCACAGCAG ATTCCTCAAT CAGGAAAGGT ATGTCAGAGC TCAAAACGAT TCAACATCAA   
  
  
+ AATGGGATAA TTTATTCTGC TTTCAGTTCT GGGTTATTGT CATATGTTCA TTCAGTTCAG TGTTTCTAGC   
  
  
+ ATGATTGCTT TTTGATGCCT TCTTTTTTGT TCTGGGTTGG ACGAACTCTG TCTTTTCTGT GCATAGATTT   
  
  
+ GAGCTCCGAG CTCAATTTTT GGGATTTTTT TAGCTGACTT TTCTGCTTGG TGTTGTGAAA TTGTGATGGG   
  
  
+ TTTTGGCTTT GTTAAACTTG TTTATAACTT TTTCTGTCAA AACTTTATAG TAAATTGTTG CAATTTGAGA   
  
  
+ ATTATGCACT CAGTGGCTCT GTGATTTATC GTCCGCTGGC AAATTTTCTG ATGAAAGGAG ATGATTTATT   
  
  
+ AATTTTTCAG GCATGGTCAA ATTTTCTAGC CTTTCATGTA CATTGGTTGG TGATAGGCTG ATAGCTAAAT   
  
  
+ TATGTCTCAT TTCTCTAAAT TTATTGCCAG ATATGAAATT GGTGGAACAG TTTAAGGGTT TTGGTCGTCT   
  
  
+ TTTTTGCCCT TTTTTTTGGG TCCCCCCCCC GCCCGGGGGG GAACCCGGAG GGCAGAAAAA GTAACATAAC   
  
  
+ TTAAAATTAT CATTGACGTA GTCCTCCAAC CATAATGAAA GCAACTGAAA TAAGAGCATG CTAAGCTAGA   
  
  
+ GAGTAGGCTG CCTTGTCAGC CTCCCTTTTG ACATGAGAAA GATGGGAGAA GTTTAAAATA AAGTTCCTCG   
  
  
+ TCTTTGCATC CCGGACCACA ATTCGGAACT GGGTGAGGAC CTCCTTAGGC TTGTAAACAC AAGTTTCGAA   
  
  
+ CTTAGATTTG CGTACATGTC AATGCTTTAC TGGATAAGCT TAGCTAACAC CCCTCATCCT TCAACCTGTA   
  
  
+ CCTTCATATA AAAAAAAGGA CAATCCGGTG CACAAGCATC CCGCATTCAC GCAAGGTCTG GGGAAGGGCC   
  
  
+ GCACCCCAAA GGGTGAATTG TAGGCAGCCT AACCTGACTT TGTCAGTGGC TAATTCCACG ATTCGAACCC   
  
  
+ ATGATTTTTT GGTTACACGG GAACAACCTT ACCATTGCTC AAAGGCTCCC CTTCTTCAAC CTCTACCTTC   
  
  
+ ATATATGAAT GAAAATCCTT TGGCTAAGAA TTGAAAAGGA AATATGTGGT TATTGGGTTT GGGTTCTTAT   
  
  
+ TCCGTTGGTT GCCCCTTCAC ATTGAATGCA TCCTCTCGCT AAGAACTGAA AAGGACATAT GGGGATGTAT   
  
  
+ AGGTCAAATT TGACTCAATG ATAACTGAGG ATACAAGAAT TCTAGAAGTC TTTATCAGGG GAATTTTTTC   
  
  
+ TTTTTTCCAC TTGTGACTTT TTGATGCCTT GCTTTTTACT CTTCATTGAA TGAGTACCCT TCTTGACAGA   
  
  
+ TTCTTCCTCC CTTTTTTCTT AATTTCTTCT TGTAGTGGAA TATAAGTATG CTTCTAGGTA GTTGGATGCC   
  
  
+ ATGGAATCTC ATCCCTGTGT GTGCATTCTT CCCCTCTCCC GCCGCCCCCC CCCCCCCAAA AAGAAACCAA   
  
  
+ AAAAAAAAAA AAAATGGATG ATTCTTAGAA CTGACCATTT TTCCTTCCTC CTCCCATTTT ATTTTTTTCA   
  
  
+ GGCTGTTTTT CTTCTACCTG TTGCTTTAGC ATCACTCATA AAAACAAGGT GCAAGCGCAT GACATCGGGT   
  
  
+ ACTGAGCTTC TGAGTTTACA TGCATCGTCA TGAAATTCGG TAGACTTTGA AGTAAAGTTT CCCCATCGTA   
  
  
+ ACTGGTGGTG CATACCTGTC TTCACGCCCA TCAGTAGAGT GGTGAAGTGA AGCAATGTCG AATAGTTTGT   
  
  
+ ATTACGAGCC CAAGAGAGAA ACTGATGCAT ATTTTATGCC TCAATGTCAA TCCTTGAACC CCCAGCTCGA   
  
  
+ TTACAACACC ATTGACTATG GAGCTTTTAT GTGCTCAAAA GTTTTCCTGG GTCAGTACTG CACTCTGGAA   
  
  
+ TCATCCTCAG GAACTGGGAC TTGTCCGGTG TCTAGCTCCA CATCAACTCT CAGCTTCTCA TCCAATGGTA   
  
  
+ GCCCTGGATC ACAGCTTGAT TCCAACTCTT ATCCTTCTGA TCAAAATTGC TCTCCTGATA ATGCCAATTC   
  
  
+ CTCATCTTTA AGTCATTCCT GCGTCACAGA TGATGTGGAT GACCTGAAAT ACAGGCTGAG AGAGTTGGAA   
  
  
+ ACAGTGATGT TGGGACCTGA TCCCGATTTT GTCTACGGCA ATAGCAACCA CACATTTGGG GTTGGGATCC   
  
  
+ ATGTAGTGTC ATCAGAGTTT GACAGCTGGG GGAAAGTGAT GGAGATGATC GCTCGAAAGG ATTTGAAACT   
  
  
+ GGTGCTAATT GCCTGTGCAA GAGCAGTTGC TGAAAATGAT CAGTTGTTGG CCCAGTGGCT GATGGATGAA   
  
  
+ TTGAGACAGA TGGTTTCAGT TTCTGGTGAA CCAATTCAAA GATTGGGTGC TTATATGTTG GAAGGGCTCG   
  
  
+ TAGCGAGGCA GGCCTCCTCA GGTAGCTCCA TTTATAAAGC ATTGAGATGC AAGGAACCCG CGAGTGCTGA   
  
  
+ CCTTTTATCT TACATGCACA TACTCTTTGA GGTTTGCCCA TACATCAAAT TTGGCTACAT GTCAGCAAAT   
  
  
+ GGTGCCATTG CAGAAGCAAT GAAGGATGAA AAGAGAGTCC ACATTGTCGA TTTCCAAATT GGGCAGGGAA   
  
  
+ GCCAATGGGT GACCCTAATC CAGGCATTTG CAGCCCGGCC AGGGGGTCCA CCCCACATTC GCATTACCGG   
  
  
+ TATCGATGAC TCCTACTCTG CATATGCTCG TGGAGGTGGG TTGAACATTG TGGGCCATAG GCTCTTGAGG   
  
  
+ TTGGCTCAGT CATTTAAGGT CCCATTTGAG TTCAACGCAG TTGACGTCCC GGCTTGTCAA GTCCTGCTTA   
  
  
+ AAGATCTCGG CATTCAACAT GGTGAGGCCT TGGCTGTGAA CTTTGCCTTC ATTCTTCACC ACATGCCCGA   
  
  
+ TGAGAGCGTG AGCACAGAGA ATCACCGGGA TCGTCTTCTG AGGGTGGTAA AAGGGCTGAA GCCAAAAGTG   
  
  
+ GTAACGTTAG TGGAGCAAGA GTGTAACACA AACTCTGCTG CTTTCTTGCC TCGCTTTGTT GAGACATTGG   
  
  
+ AATACTACAC AGCGATGTTT GAGTCCATGG ATGTGACTTT GCCTAGGGAT CATAAAGACC GGATCAATGT   
  
  
+ TGAGCAGCAT TGTCTTGCTC GAGATGTTGT GAACTTGGTT GCGTGTGAAG GAGCTGAGAG GGTGGAGCGG   
  
  
+ CATGAGCTCC TTGGGAAATG GAGGTCGCGG TTCACAATGG CAGGGTTTAA GCCATACCCT TTAAGCACTC   
  
  
+ TGGTAAACAA CACAATAAGA ACTTTGTTGA AGAACTACTG TGATAGGTAT GGGCTAGAGG AGAGGAATGG   
  
  
+ AGGTCTTTAT CTTGGGTGGA TGAATAGAGA TTTGGTTGCT TCGAGTGCCT GGCAGTGTAG TAATTA  

- +Up\_Stream \_Len000GTAAAA TCATTATTTA AGGGTACACT CCTGGTGACG GATGACTAAG GCTTACCGTA   
  
  
- AAATCATTAT TTGAGGGTAC ACTCCTGGTG AACTACTCCT TAAGTCAAAA TATTAGGTGT CCACAGTTTC   
  
  
- GGATAAGGAT GAGAGTGGTG AGACGATAGA GAGGGGGGGG GGGGGGGGGC GGGGGGAGTT TTAGACGGAG   
  
  
- GAGGCAAGAG AGGTGTCGTC TAAGGAGTTA GTCCTTTCCA TACAGTCTCG AGTTTTGCTA AGTTGTAGTT   
  
  
- TTACCCTATT AAATAAGACG AAAGTCAAGA CCCAATAACA GTATACAAGT AAGTCAAGTC ACAAAGATCG   
  
  
- TACTAACGAA AAACTACGGA AGAAAAAACA AGACCCAACC TGCTTGAGAC AGAAAAGACA CGTATCTAAA   
  
  
- CTCGAGGCTC GAGTTAAAAA CCCTAAAAAA ATCGACTGAA AAGACGAACC ACAACACTTT AACACTACCC   
  
  
- AAAACCGAAA CAATTTGAAC AAATATTGAA AAAGACAGTT TTGAAATATC ATTTAACAAC GTTAAACTCT   
  
  
- TAATACGTGA GTCACCGAGA CACTAAATAG CAGGCGACCG TTTAAAAGAC TACTTTCCTC TACTAAATAA   
  
  
- TTAAAAAGTC CGTACCAGTT TAAAAGATCG GAAAGTACAT GTAACCAACC ACTATCCGAC TATCGATTTA   
  
  
- ATACAGAGTA AAGAGATTTA AATAACGGTC TATACTTTAA CCACCTTGTC AAATTCCCAA AACCAGCAGA   
  
  
- AAAAACGGGA AAAAAAACCC AGGGGGGGGG CGGGCCCCCC CTTGGGCCTC CCGTCTTTTT CATTGTATTG   
  
  
- AATTTTAATA GTAACTGCAT CAGGAGGTTG GTATTACTTT CGTTGACTTT ATTCTCGTAC GATTCGATCT   
  
  
- CTCATCCGAC GGAACAGTCG GAGGGAAAAC TGTACTCTTT CTACCCTCTT CAAATTTTAT TTCAAGGAGC   
  
  
- AGAAACGTAG GGCCTGGTGT TAAGCCTTGA CCCACTCCTG GAGGAATCCG AACATTTGTG TTCAAAGCTT   
  
  
- GAATCTAAAC GCATGTACAG TTACGAAATG ACCTATTCGA ATCGATTGTG GGGAGTAGGA AGTTGGACAT   
  
  
- GGAAGTATAT TTTTTTTCCT GTTAGGCCAC GTGTTCGTAG GGCGTAAGTG CGTTCCAGAC CCCTTCCCGG   
  
  
- CGTGGGGTTT CCCACTTAAC ATCCGTCGGA TTGGACTGAA ACAGTCACCG ATTAAGGTGC TAAGCTTGGG   
  
  
- TACTAAAAAA CCAATGTGCC CTTGTTGGAA TGGTAACGAG TTTCCGAGGG GAAGAAGTTG GAGATGGAAG   
  
  
- TATATACTTA CTTTTAGGAA ACCGATTCTT AACTTTTCCT TTATACACCA ATAACCCAAA CCCAAGAATA   
  
  
- AGGCAACCAA CGGGGAAGTG TAACTTACGT AGGAGAGCGA TTCTTGACTT TTCCTGTATA CCCCTACATA   
  
  
- TCCAGTTTAA ACTGAGTTAC TATTGACTCC TATGTTCTTA AGATCTTCAG AAATAGTCCC CTTAAAAAAG   
  
  
- AAAAAAGGTG AACACTGAAA AACTACGGAA CGAAAAATGA GAAGTAACTT ACTCATGGGA AGAACTGTCT   
  
  
- AAGAAGGAGG GAAAAAAGAA TTAAAGAAGA ACATCACCTT ATATTCATAC GAAGATCCAT CAACCTACGG   
  
  
- TACCTTAGAG TAGGGACACA CACGTAAGAA GGGGAGAGGG CGGCGGGGGG GGGGGGGTTT TTCTTTGGTT   
  
  
- TTTTTTTTTT TTTTACCTAC TAAGAATCTT GACTGGTAAA AAGGAAGGAG GAGGGTAAAA TAAAAAAAGT   
  
  
- CCGACAAAAA GAAGATGGAC AACGAAATCG TAGTGAGTAT TTTTGTTCCA CGTTCGCGTA CTGTAGCCCA   
  
  
- TGACTCGAAG ACTCAAATGT ACGTAGCAGT ACTTTAAGCC ATCTGAAACT TCATTTCAAA GGGGTAGCAT   
  
  
- TGACCACCAC GTATGGACAG AAGTGCGGGT AGTCATCTCA CCACTTCACT TCGTTACAGC TTATCAAACA   
  
  
- TAATGCTCGG GTTCTCTCTT TGACTACGTA TAAAATACGG AGTTACAGTT AGGAACTTGG GGGTCGAGCT   
  
  
- AATGTTGTGG TAACTGATAC CTCGAAAATA CACGAGTTTT CAAAAGGACC CAGTCATGAC GTGAGACCTT   
  
  
- AGTAGGAGTC CTTGACCCTG AACAGGCCAC AGATCGAGGT GTAGTTGAGA GTCGAAGAGT AGGTTACCAT   
  
  
- CGGGACCTAG TGTCGAACTA AGGTTGAGAA TAGGAAGACT AGTTTTAACG AGAGGACTAT TACGGTTAAG   
  
  
- GAGTAGAAAT TCAGTAAGGA CGCAGTGTCT ACTACACCTA CTGGACTTTA TGTCCGACTC TCTCAACCTT   
  
  
- TGTCACTACA ACCCTGGACT AGGGCTAAAA CAGATGCCGT TATCGTTGGT GTGTAAACCC CAACCCTAGG   
  
  
- TACATCACAG TAGTCTCAAA CTGTCGACCC CCTTTCACTA CCTCTACTAG CGAGCTTTCC TAAACTTTGA   
  
  
- CCACGATTAA CGGACACGTT CTCGTCAACG ACTTTTACTA GTCAACAACC GGGTCACCGA CTACCTACTT   
  
  
- AACTCTGTCT ACCAAAGTCA AAGACCACTT GGTTAAGTTT CTAACCCACG AATATACAAC CTTCCCGAGC   
  
  
- ATCGCTCCGT CCGGAGGAGT CCATCGAGGT AAATATTTCG TAACTCTACG TTCCTTGGGC GCTCACGACT   
  
  
- GGAAAATAGA ATGTACGTGT ATGAGAAACT CCAAACGGGT ATGTAGTTTA AACCGATGTA CAGTCGTTTA   
  
  
- CCACGGTAAC GTCTTCGTTA CTTCCTACTT TTCTCTCAGG TGTAACAGCT AAAGGTTTAA CCCGTCCCTT   
  
  
- CGGTTACCCA CTGGGATTAG GTCCGTAAAC GTCGGGCCGG TCCCCCAGGT GGGGTGTAAG CGTAATGGCC   
  
  
- ATAGCTACTG AGGATGAGAC GTATACGAGC ACCTCCACCC AACTTGTAAC ACCCGGTATC CGAGAACTCC   
  
  
- AACCGAGTCA GTAAATTCCA GGGTAAACTC AAGTTGCGTC AACTGCAGGG CCGAACAGTT CAGGACGAAT   
  
  
- TTCTAGAGCC GTAAGTTGTA CCACTCCGGA ACCGACACTT GAAACGGAAG TAAGAAGTGG TGTACGGGCT   
  
  
- ACTCTCGCAC TCGTGTCTCT TAGTGGCCCT AGCAGAAGAC TCCCACCATT TTCCCGACTT CGGTTTTCAC   
  
  
- CATTGCAATC ACCTCGTTCT CACATTGTGT TTGAGACGAC GAAAGAACGG AGCGAAACAA CTCTGTAACC   
  
  
- TTATGATGTG TCGCTACAAA CTCAGGTACC TACACTGAAA CGGATCCCTA GTATTTCTGG CCTAGTTACA   
  
  
- ACTCGTCGTA ACAGAACGAG CTCTACAACA CTTGAACCAA CGCACACTTC CTCGACTCTC CCACCTCGCC   
  
  
- GTACTCGAGG AACCCTTTAC CTCCAGCGCC AAGTGTTACC GTCCCAAATT CGGTATGGGA AATTCGTGAG   
  
  
- ACCATTTGTT GTGTTATTCT TGAAACAACT TCTTGATGAC ACTATCCATA CCCGATCTCC TCTCCTTACC   
  
  
- TCCAGAAATA GAACCCACCT ACTTATCTCT AAACCAACGA AGCTCACGGA CCGTCACATC ATTAAT

  
  
Motifs Found  

+   

| Site Name | Organism | Position | Strand | Matrix score. | sequence | function |
| --- | --- | --- | --- | --- | --- | --- |
|  | organism | 3257 | + | 4 | motif\_sequence | short\_function |
|  | organism | 3563 | - | 4 | motif\_sequence | short\_function |
|  | organism | 2987 | - | 4 | motif\_sequence | short\_function |
|  | organism | 3166 | - | 4 | motif\_sequence | short\_function |
|  | organism | 2750 | - | 4 | motif\_sequence | short\_function |
|  | organism | 2960 | + | 4 | motif\_sequence | short\_function |
|  | organism | 2537 | + | 4 | motif\_sequence | short\_function |
|  | organism | 2495 | - | 4 | motif\_sequence | short\_function |
|  | organism | 2133 | + | 4 | motif\_sequence | short\_function |
|  | organism | 2294 | + | 4 | motif\_sequence | short\_function |
|  | organism | 1718 | + | 4 | motif\_sequence | short\_function |
|  | organism | 1998 | - | 4 | motif\_sequence | short\_function |
|  | organism | 1583 | + | 4 | motif\_sequence | short\_function |
|  | organism | 1457 | - | 4 | motif\_sequence | short\_function |
|  | organism | 749 | - | 4 | motif\_sequence | short\_function |
|  | organism | 1120 | + | 4 | motif\_sequence | short\_function |
|  | organism | 164 | + | 4 | motif\_sequence | short\_function |
|  | organism | 621 | - | 4 | motif\_sequence | short\_function |
|  | organism | 1325 | + | 4 | motif\_sequence | short\_function |
|  | organism | 672 | - | 4 | motif\_sequence | short\_function |
|  | organism | 670 | + | 4 | motif\_sequence | short\_function |
|  | organism | 1066 | - | 4 | motif\_sequence | short\_function |
|  | organism | 173 | + | 4 | motif\_sequence | short\_function |
|  | organism | 328 | + | 4 | motif\_sequence | short\_function |
|  | organism | 231 | - | 4 | motif\_sequence | short\_function |
|  | organism | 222 | + | 4 | motif\_sequence | short\_function |
|  | organism | 411 | + | 4 | motif\_sequence | short\_function |
|  | organism | 206 | + | 4 | motif\_sequence | short\_function |

>HU01G01391.1   
+ +Up\_Stream \_Len000CATTTT AGTAATAAAT TCCCATGTGA GGACCACTGC CTACTGATTC CGAATGGCAT   
  
  
+ TTTAGTAATA AACTCCCATG TGAGGACCAC TTGATGAGGA ATTCAGTTTT ATAATCCACA GGTGTCAAAG   
  
  
+ CCTATTCCTA CTCTCACCAC TCTGCTATCT CTCCCCCCCC CCCCCCCCCG CCCCCCTCAA AATCTGCCTC   
  
  
+ CTCCGTTCTC TCCACAGCAG ATTCCTCAAT CAGGAAAGGT ATGTCAGAGC TCAAAACGAT TCAACATCAA   
  
  
+ AATGGGATAA TTTATTCTGC TTTCAGTTCT GGGTTATTGT CATATGTTCA TTCAGTTCAG TGTTTCTAGC   
  
  
+ ATGATTGCTT TTTGATGCCT TCTTTTTTGT TCTGGGTTGG ACGAACTCTG TCTTTTCTGT GCATAGATTT   
  
  
+ GAGCTCCGAG CTCAATTTTT GGGATTTTTT TAGCTGACTT TTCTGCTTGG TGTTGTGAAA TTGTGATGGG   
  
  
+ TTTTGGCTTT GTTAAACTTG TTTATAACTT TTTCTGTCAA AACTTTATAG TAAATTGTTG CAATTTGAGA   
  
  
+ ATTATGCACT CAGTGGCTCT GTGATTTATC GTCCGCTGGC AAATTTTCTG ATGAAAGGAG ATGATTTATT   
  
  
+ AATTTTTCAG GCATGGTCAA ATTTTCTAGC CTTTCATGTA CATTGGTTGG TGATAGGCTG ATAGCTAAAT   
  
  
+ TATGTCTCAT TTCTCTAAAT TTATTGCCAG ATATGAAATT GGTGGAACAG TTTAAGGGTT TTGGTCGTCT   
  
  
+ TTTTTGCCCT TTTTTTTGGG TCCCCCCCCC GCCCGGGGGG GAACCCGGAG GGCAGAAAAA GTAACATAAC   
  
  
+ TTAAAATTAT CATTGACGTA GTCCTCCAAC CATAATGAAA GCAACTGAAA TAAGAGCATG CTAAGCTAGA   
  
  
+ GAGTAGGCTG CCTTGTCAGC CTCCCTTTTG ACATGAGAAA GATGGGAGAA GTTTAAAATA AAGTTCCTCG   
  
  
+ TCTTTGCATC CCGGACCACA ATTCGGAACT GGGTGAGGAC CTCCTTAGGC TTGTAAACAC AAGTTTCGAA   
  
  
+ CTTAGATTTG CGTACATGTC AATGCTTTAC TGGATAAGCT TAGCTAACAC CCCTCATCCT TCAACCTGTA   
  
  
+ CCTTCATATA AAAAAAAGGA CAATCCGGTG CACAAGCATC CCGCATTCAC GCAAGGTCTG GGGAAGGGCC   
  
  
+ GCACCCCAAA GGGTGAATTG TAGGCAGCCT AACCTGACTT TGTCAGTGGC TAATTCCACG ATTCGAACCC   
  
  
+ ATGATTTTTT GGTTACACGG GAACAACCTT ACCATTGCTC AAAGGCTCCC CTTCTTCAAC CTCTACCTTC   
  
  
+ ATATATGAAT GAAAATCCTT TGGCTAAGAA TTGAAAAGGA AATATGTGGT TATTGGGTTT GGGTTCTTAT   
  
  
+ TCCGTTGGTT GCCCCTTCAC ATTGAATGCA TCCTCTCGCT AAGAACTGAA AAGGACATAT GGGGATGTAT   
  
  
+ AGGTCAAATT TGACTCAATG ATAACTGAGG ATACAAGAAT TCTAGAAGTC TTTATCAGGG GAATTTTTTC   
  
  
+ TTTTTTCCAC TTGTGACTTT TTGATGCCTT GCTTTTTACT CTTCATTGAA TGAGTACCCT TCTTGACAGA   
  
  
+ TTCTTCCTCC CTTTTTTCTT AATTTCTTCT TGTAGTGGAA TATAAGTATG CTTCTAGGTA GTTGGATGCC   
  
  
+ ATGGAATCTC ATCCCTGTGT GTGCATTCTT CCCCTCTCCC GCCGCCCCCC CCCCCCCAAA AAGAAACCAA   
  
  
+ AAAAAAAAAA AAAATGGATG ATTCTTAGAA CTGACCATTT TTCCTTCCTC CTCCCATTTT ATTTTTTTCA   
  
  
+ GGCTGTTTTT CTTCTACCTG TTGCTTTAGC ATCACTCATA AAAACAAGGT GCAAGCGCAT GACATCGGGT   
  
  
+ ACTGAGCTTC TGAGTTTACA TGCATCGTCA TGAAATTCGG TAGACTTTGA AGTAAAGTTT CCCCATCGTA   
  
  
+ ACTGGTGGTG CATACCTGTC TTCACGCCCA TCAGTAGAGT GGTGAAGTGA AGCAATGTCG AATAGTTTGT   
  
  
+ ATTACGAGCC CAAGAGAGAA ACTGATGCAT ATTTTATGCC TCAATGTCAA TCCTTGAACC CCCAGCTCGA   
  
  
+ TTACAACACC ATTGACTATG GAGCTTTTAT GTGCTCAAAA GTTTTCCTGG GTCAGTACTG CACTCTGGAA   
  
  
+ TCATCCTCAG GAACTGGGAC TTGTCCGGTG TCTAGCTCCA CATCAACTCT CAGCTTCTCA TCCAATGGTA   
  
  
+ GCCCTGGATC ACAGCTTGAT TCCAACTCTT ATCCTTCTGA TCAAAATTGC TCTCCTGATA ATGCCAATTC   
  
  
+ CTCATCTTTA AGTCATTCCT GCGTCACAGA TGATGTGGAT GACCTGAAAT ACAGGCTGAG AGAGTTGGAA   
  
  
+ ACAGTGATGT TGGGACCTGA TCCCGATTTT GTCTACGGCA ATAGCAACCA CACATTTGGG GTTGGGATCC   
  
  
+ ATGTAGTGTC ATCAGAGTTT GACAGCTGGG GGAAAGTGAT GGAGATGATC GCTCGAAAGG ATTTGAAACT   
  
  
+ GGTGCTAATT GCCTGTGCAA GAGCAGTTGC TGAAAATGAT CAGTTGTTGG CCCAGTGGCT GATGGATGAA   
  
  
+ TTGAGACAGA TGGTTTCAGT TTCTGGTGAA CCAATTCAAA GATTGGGTGC TTATATGTTG GAAGGGCTCG   
  
  
+ TAGCGAGGCA GGCCTCCTCA GGTAGCTCCA TTTATAAAGC ATTGAGATGC AAGGAACCCG CGAGTGCTGA   
  
  
+ CCTTTTATCT TACATGCACA TACTCTTTGA GGTTTGCCCA TACATCAAAT TTGGCTACAT GTCAGCAAAT   
  
  
+ GGTGCCATTG CAGAAGCAAT GAAGGATGAA AAGAGAGTCC ACATTGTCGA TTTCCAAATT GGGCAGGGAA   
  
  
+ GCCAATGGGT GACCCTAATC CAGGCATTTG CAGCCCGGCC AGGGGGTCCA CCCCACATTC GCATTACCGG   
  
  
+ TATCGATGAC TCCTACTCTG CATATGCTCG TGGAGGTGGG TTGAACATTG TGGGCCATAG GCTCTTGAGG   
  
  
+ TTGGCTCAGT CATTTAAGGT CCCATTTGAG TTCAACGCAG TTGACGTCCC GGCTTGTCAA GTCCTGCTTA   
  
  
+ AAGATCTCGG CATTCAACAT GGTGAGGCCT TGGCTGTGAA CTTTGCCTTC ATTCTTCACC ACATGCCCGA   
  
  
+ TGAGAGCGTG AGCACAGAGA ATCACCGGGA TCGTCTTCTG AGGGTGGTAA AAGGGCTGAA GCCAAAAGTG   
  
  
+ GTAACGTTAG TGGAGCAAGA GTGTAACACA AACTCTGCTG CTTTCTTGCC TCGCTTTGTT GAGACATTGG   
  
  
+ AATACTACAC AGCGATGTTT GAGTCCATGG ATGTGACTTT GCCTAGGGAT CATAAAGACC GGATCAATGT   
  
  
+ TGAGCAGCAT TGTCTTGCTC GAGATGTTGT GAACTTGGTT GCGTGTGAAG GAGCTGAGAG GGTGGAGCGG   
  
  
+ CATGAGCTCC TTGGGAAATG GAGGTCGCGG TTCACAATGG CAGGGTTTAA GCCATACCCT TTAAGCACTC   
  
  
+ TGGTAAACAA CACAATAAGA ACTTTGTTGA AGAACTACTG TGATAGGTAT GGGCTAGAGG AGAGGAATGG   
  
  
+ AGGTCTTTAT CTTGGGTGGA TGAATAGAGA TTTGGTTGCT TCGAGTGCCT GGCAGTGTAG TAATTA  

- +Up\_Stream \_Len000GTAAAA TCATTATTTA AGGGTACACT CCTGGTGACG GATGACTAAG GCTTACCGTA   
  
  
- AAATCATTAT TTGAGGGTAC ACTCCTGGTG AACTACTCCT TAAGTCAAAA TATTAGGTGT CCACAGTTTC   
  
  
- GGATAAGGAT GAGAGTGGTG AGACGATAGA GAGGGGGGGG GGGGGGGGGC GGGGGGAGTT TTAGACGGAG   
  
  
- GAGGCAAGAG AGGTGTCGTC TAAGGAGTTA GTCCTTTCCA TACAGTCTCG AGTTTTGCTA AGTTGTAGTT   
  
  
- TTACCCTATT AAATAAGACG AAAGTCAAGA CCCAATAACA GTATACAAGT AAGTCAAGTC ACAAAGATCG   
  
  
- TACTAACGAA AAACTACGGA AGAAAAAACA AGACCCAACC TGCTTGAGAC AGAAAAGACA CGTATCTAAA   
  
  
- CTCGAGGCTC GAGTTAAAAA CCCTAAAAAA ATCGACTGAA AAGACGAACC ACAACACTTT AACACTACCC   
  
  
- AAAACCGAAA CAATTTGAAC AAATATTGAA AAAGACAGTT TTGAAATATC ATTTAACAAC GTTAAACTCT   
  
  
- TAATACGTGA GTCACCGAGA CACTAAATAG CAGGCGACCG TTTAAAAGAC TACTTTCCTC TACTAAATAA   
  
  
- TTAAAAAGTC CGTACCAGTT TAAAAGATCG GAAAGTACAT GTAACCAACC ACTATCCGAC TATCGATTTA   
  
  
- ATACAGAGTA AAGAGATTTA AATAACGGTC TATACTTTAA CCACCTTGTC AAATTCCCAA AACCAGCAGA   
  
  
- AAAAACGGGA AAAAAAACCC AGGGGGGGGG CGGGCCCCCC CTTGGGCCTC CCGTCTTTTT CATTGTATTG   
  
  
- AATTTTAATA GTAACTGCAT CAGGAGGTTG GTATTACTTT CGTTGACTTT ATTCTCGTAC GATTCGATCT   
  
  
- CTCATCCGAC GGAACAGTCG GAGGGAAAAC TGTACTCTTT CTACCCTCTT CAAATTTTAT TTCAAGGAGC   
  
  
- AGAAACGTAG GGCCTGGTGT TAAGCCTTGA CCCACTCCTG GAGGAATCCG AACATTTGTG TTCAAAGCTT   
  
  
- GAATCTAAAC GCATGTACAG TTACGAAATG ACCTATTCGA ATCGATTGTG GGGAGTAGGA AGTTGGACAT   
  
  
- GGAAGTATAT TTTTTTTCCT GTTAGGCCAC GTGTTCGTAG GGCGTAAGTG CGTTCCAGAC CCCTTCCCGG   
  
  
- CGTGGGGTTT CCCACTTAAC ATCCGTCGGA TTGGACTGAA ACAGTCACCG ATTAAGGTGC TAAGCTTGGG   
  
  
- TACTAAAAAA CCAATGTGCC CTTGTTGGAA TGGTAACGAG TTTCCGAGGG GAAGAAGTTG GAGATGGAAG   
  
  
- TATATACTTA CTTTTAGGAA ACCGATTCTT AACTTTTCCT TTATACACCA ATAACCCAAA CCCAAGAATA   
  
  
- AGGCAACCAA CGGGGAAGTG TAACTTACGT AGGAGAGCGA TTCTTGACTT TTCCTGTATA CCCCTACATA   
  
  
- TCCAGTTTAA ACTGAGTTAC TATTGACTCC TATGTTCTTA AGATCTTCAG AAATAGTCCC CTTAAAAAAG   
  
  
- AAAAAAGGTG AACACTGAAA AACTACGGAA CGAAAAATGA GAAGTAACTT ACTCATGGGA AGAACTGTCT   
  
  
- AAGAAGGAGG GAAAAAAGAA TTAAAGAAGA ACATCACCTT ATATTCATAC GAAGATCCAT CAACCTACGG   
  
  
- TACCTTAGAG TAGGGACACA CACGTAAGAA GGGGAGAGGG CGGCGGGGGG GGGGGGGTTT TTCTTTGGTT   
  
  
- TTTTTTTTTT TTTTACCTAC TAAGAATCTT GACTGGTAAA AAGGAAGGAG GAGGGTAAAA TAAAAAAAGT   
  
  
- CCGACAAAAA GAAGATGGAC AACGAAATCG TAGTGAGTAT TTTTGTTCCA CGTTCGCGTA CTGTAGCCCA   
  
  
- TGACTCGAAG ACTCAAATGT ACGTAGCAGT ACTTTAAGCC ATCTGAAACT TCATTTCAAA GGGGTAGCAT   
  
  
- TGACCACCAC GTATGGACAG AAGTGCGGGT AGTCATCTCA CCACTTCACT TCGTTACAGC TTATCAAACA   
  
  
- TAATGCTCGG GTTCTCTCTT TGACTACGTA TAAAATACGG AGTTACAGTT AGGAACTTGG GGGTCGAGCT   
  
  
- AATGTTGTGG TAACTGATAC CTCGAAAATA CACGAGTTTT CAAAAGGACC CAGTCATGAC GTGAGACCTT   
  
  
- AGTAGGAGTC CTTGACCCTG AACAGGCCAC AGATCGAGGT GTAGTTGAGA GTCGAAGAGT AGGTTACCAT   
  
  
- CGGGACCTAG TGTCGAACTA AGGTTGAGAA TAGGAAGACT AGTTTTAACG AGAGGACTAT TACGGTTAAG   
  
  
- GAGTAGAAAT TCAGTAAGGA CGCAGTGTCT ACTACACCTA CTGGACTTTA TGTCCGACTC TCTCAACCTT   
  
  
- TGTCACTACA ACCCTGGACT AGGGCTAAAA CAGATGCCGT TATCGTTGGT GTGTAAACCC CAACCCTAGG   
  
  
- TACATCACAG TAGTCTCAAA CTGTCGACCC CCTTTCACTA CCTCTACTAG CGAGCTTTCC TAAACTTTGA   
  
  
- CCACGATTAA CGGACACGTT CTCGTCAACG ACTTTTACTA GTCAACAACC GGGTCACCGA CTACCTACTT   
  
  
- AACTCTGTCT ACCAAAGTCA AAGACCACTT GGTTAAGTTT CTAACCCACG AATATACAAC CTTCCCGAGC   
  
  
- ATCGCTCCGT CCGGAGGAGT CCATCGAGGT AAATATTTCG TAACTCTACG TTCCTTGGGC GCTCACGACT   
  
  
- GGAAAATAGA ATGTACGTGT ATGAGAAACT CCAAACGGGT ATGTAGTTTA AACCGATGTA CAGTCGTTTA   
  
  
- CCACGGTAAC GTCTTCGTTA CTTCCTACTT TTCTCTCAGG TGTAACAGCT AAAGGTTTAA CCCGTCCCTT   
  
  
- CGGTTACCCA CTGGGATTAG GTCCGTAAAC GTCGGGCCGG TCCCCCAGGT GGGGTGTAAG CGTAATGGCC   
  
  
- ATAGCTACTG AGGATGAGAC GTATACGAGC ACCTCCACCC AACTTGTAAC ACCCGGTATC CGAGAACTCC   
  
  
- AACCGAGTCA GTAAATTCCA GGGTAAACTC AAGTTGCGTC AACTGCAGGG CCGAACAGTT CAGGACGAAT   
  
  
- TTCTAGAGCC GTAAGTTGTA CCACTCCGGA ACCGACACTT GAAACGGAAG TAAGAAGTGG TGTACGGGCT   
  
  
- ACTCTCGCAC TCGTGTCTCT TAGTGGCCCT AGCAGAAGAC TCCCACCATT TTCCCGACTT CGGTTTTCAC   
  
  
- CATTGCAATC ACCTCGTTCT CACATTGTGT TTGAGACGAC GAAAGAACGG AGCGAAACAA CTCTGTAACC   
  
  
- TTATGATGTG TCGCTACAAA CTCAGGTACC TACACTGAAA CGGATCCCTA GTATTTCTGG CCTAGTTACA   
  
  
- ACTCGTCGTA ACAGAACGAG CTCTACAACA CTTGAACCAA CGCACACTTC CTCGACTCTC CCACCTCGCC   
  
  
- GTACTCGAGG AACCCTTTAC CTCCAGCGCC AAGTGTTACC GTCCCAAATT CGGTATGGGA AATTCGTGAG   
  
  
- ACCATTTGTT GTGTTATTCT TGAAACAACT TCTTGATGAC ACTATCCATA CCCGATCTCC TCTCCTTACC   
  
  
- TCCAGAAATA GAACCCACCT ACTTATCTCT AAACCAACGA AGCTCACGGA CCGTCACATC ATTAAT

+     ABRE

| Site Name | Organism | Position | Strand | Matrix score. | sequence | function |
| --- | --- | --- | --- | --- | --- | --- |
| ABRE | Arabidopsis thaliana | 816 | + | 7 | AACCCGG | cis-acting element involved in the abscisic acid responsiveness |

>HU01G01391.1   
+ +Up\_Stream \_Len000CATTTT AGTAATAAAT TCCCATGTGA GGACCACTGC CTACTGATTC CGAATGGCAT   
  
  
+ TTTAGTAATA AACTCCCATG TGAGGACCAC TTGATGAGGA ATTCAGTTTT ATAATCCACA GGTGTCAAAG   
  
  
+ CCTATTCCTA CTCTCACCAC TCTGCTATCT CTCCCCCCCC CCCCCCCCCG CCCCCCTCAA AATCTGCCTC   
  
  
+ CTCCGTTCTC TCCACAGCAG ATTCCTCAAT CAGGAAAGGT ATGTCAGAGC TCAAAACGAT TCAACATCAA   
  
  
+ AATGGGATAA TTTATTCTGC TTTCAGTTCT GGGTTATTGT CATATGTTCA TTCAGTTCAG TGTTTCTAGC   
  
  
+ ATGATTGCTT TTTGATGCCT TCTTTTTTGT TCTGGGTTGG ACGAACTCTG TCTTTTCTGT GCATAGATTT   
  
  
+ GAGCTCCGAG CTCAATTTTT GGGATTTTTT TAGCTGACTT TTCTGCTTGG TGTTGTGAAA TTGTGATGGG   
  
  
+ TTTTGGCTTT GTTAAACTTG TTTATAACTT TTTCTGTCAA AACTTTATAG TAAATTGTTG CAATTTGAGA   
  
  
+ ATTATGCACT CAGTGGCTCT GTGATTTATC GTCCGCTGGC AAATTTTCTG ATGAAAGGAG ATGATTTATT   
  
  
+ AATTTTTCAG GCATGGTCAA ATTTTCTAGC CTTTCATGTA CATTGGTTGG TGATAGGCTG ATAGCTAAAT   
  
  
+ TATGTCTCAT TTCTCTAAAT TTATTGCCAG ATATGAAATT GGTGGAACAG TTTAAGGGTT TTGGTCGTCT   
  
  
+ TTTTTGCCCT TTTTTTTGGG TCCCCCCCCC GCCCGGGGGG GAACCCGGAG GGCAGAAAAA GTAACATAAC   
  
  
+ TTAAAATTAT CATTGACGTA GTCCTCCAAC CATAATGAAA GCAACTGAAA TAAGAGCATG CTAAGCTAGA   
  
  
+ GAGTAGGCTG CCTTGTCAGC CTCCCTTTTG ACATGAGAAA GATGGGAGAA GTTTAAAATA AAGTTCCTCG   
  
  
+ TCTTTGCATC CCGGACCACA ATTCGGAACT GGGTGAGGAC CTCCTTAGGC TTGTAAACAC AAGTTTCGAA   
  
  
+ CTTAGATTTG CGTACATGTC AATGCTTTAC TGGATAAGCT TAGCTAACAC CCCTCATCCT TCAACCTGTA   
  
  
+ CCTTCATATA AAAAAAAGGA CAATCCGGTG CACAAGCATC CCGCATTCAC GCAAGGTCTG GGGAAGGGCC   
  
  
+ GCACCCCAAA GGGTGAATTG TAGGCAGCCT AACCTGACTT TGTCAGTGGC TAATTCCACG ATTCGAACCC   
  
  
+ ATGATTTTTT GGTTACACGG GAACAACCTT ACCATTGCTC AAAGGCTCCC CTTCTTCAAC CTCTACCTTC   
  
  
+ ATATATGAAT GAAAATCCTT TGGCTAAGAA TTGAAAAGGA AATATGTGGT TATTGGGTTT GGGTTCTTAT   
  
  
+ TCCGTTGGTT GCCCCTTCAC ATTGAATGCA TCCTCTCGCT AAGAACTGAA AAGGACATAT GGGGATGTAT   
  
  
+ AGGTCAAATT TGACTCAATG ATAACTGAGG ATACAAGAAT TCTAGAAGTC TTTATCAGGG GAATTTTTTC   
  
  
+ TTTTTTCCAC TTGTGACTTT TTGATGCCTT GCTTTTTACT CTTCATTGAA TGAGTACCCT TCTTGACAGA   
  
  
+ TTCTTCCTCC CTTTTTTCTT AATTTCTTCT TGTAGTGGAA TATAAGTATG CTTCTAGGTA GTTGGATGCC   
  
  
+ ATGGAATCTC ATCCCTGTGT GTGCATTCTT CCCCTCTCCC GCCGCCCCCC CCCCCCCAAA AAGAAACCAA   
  
  
+ AAAAAAAAAA AAAATGGATG ATTCTTAGAA CTGACCATTT TTCCTTCCTC CTCCCATTTT ATTTTTTTCA   
  
  
+ GGCTGTTTTT CTTCTACCTG TTGCTTTAGC ATCACTCATA AAAACAAGGT GCAAGCGCAT GACATCGGGT   
  
  
+ ACTGAGCTTC TGAGTTTACA TGCATCGTCA TGAAATTCGG TAGACTTTGA AGTAAAGTTT CCCCATCGTA   
  
  
+ ACTGGTGGTG CATACCTGTC TTCACGCCCA TCAGTAGAGT GGTGAAGTGA AGCAATGTCG AATAGTTTGT   
  
  
+ ATTACGAGCC CAAGAGAGAA ACTGATGCAT ATTTTATGCC TCAATGTCAA TCCTTGAACC CCCAGCTCGA   
  
  
+ TTACAACACC ATTGACTATG GAGCTTTTAT GTGCTCAAAA GTTTTCCTGG GTCAGTACTG CACTCTGGAA   
  
  
+ TCATCCTCAG GAACTGGGAC TTGTCCGGTG TCTAGCTCCA CATCAACTCT CAGCTTCTCA TCCAATGGTA   
  
  
+ GCCCTGGATC ACAGCTTGAT TCCAACTCTT ATCCTTCTGA TCAAAATTGC TCTCCTGATA ATGCCAATTC   
  
  
+ CTCATCTTTA AGTCATTCCT GCGTCACAGA TGATGTGGAT GACCTGAAAT ACAGGCTGAG AGAGTTGGAA   
  
  
+ ACAGTGATGT TGGGACCTGA TCCCGATTTT GTCTACGGCA ATAGCAACCA CACATTTGGG GTTGGGATCC   
  
  
+ ATGTAGTGTC ATCAGAGTTT GACAGCTGGG GGAAAGTGAT GGAGATGATC GCTCGAAAGG ATTTGAAACT   
  
  
+ GGTGCTAATT GCCTGTGCAA GAGCAGTTGC TGAAAATGAT CAGTTGTTGG CCCAGTGGCT GATGGATGAA   
  
  
+ TTGAGACAGA TGGTTTCAGT TTCTGGTGAA CCAATTCAAA GATTGGGTGC TTATATGTTG GAAGGGCTCG   
  
  
+ TAGCGAGGCA GGCCTCCTCA GGTAGCTCCA TTTATAAAGC ATTGAGATGC AAGGAACCCG CGAGTGCTGA   
  
  
+ CCTTTTATCT TACATGCACA TACTCTTTGA GGTTTGCCCA TACATCAAAT TTGGCTACAT GTCAGCAAAT   
  
  
+ GGTGCCATTG CAGAAGCAAT GAAGGATGAA AAGAGAGTCC ACATTGTCGA TTTCCAAATT GGGCAGGGAA   
  
  
+ GCCAATGGGT GACCCTAATC CAGGCATTTG CAGCCCGGCC AGGGGGTCCA CCCCACATTC GCATTACCGG   
  
  
+ TATCGATGAC TCCTACTCTG CATATGCTCG TGGAGGTGGG TTGAACATTG TGGGCCATAG GCTCTTGAGG   
  
  
+ TTGGCTCAGT CATTTAAGGT CCCATTTGAG TTCAACGCAG TTGACGTCCC GGCTTGTCAA GTCCTGCTTA   
  
  
+ AAGATCTCGG CATTCAACAT GGTGAGGCCT TGGCTGTGAA CTTTGCCTTC ATTCTTCACC ACATGCCCGA   
  
  
+ TGAGAGCGTG AGCACAGAGA ATCACCGGGA TCGTCTTCTG AGGGTGGTAA AAGGGCTGAA GCCAAAAGTG   
  
  
+ GTAACGTTAG TGGAGCAAGA GTGTAACACA AACTCTGCTG CTTTCTTGCC TCGCTTTGTT GAGACATTGG   
  
  
+ AATACTACAC AGCGATGTTT GAGTCCATGG ATGTGACTTT GCCTAGGGAT CATAAAGACC GGATCAATGT   
  
  
+ TGAGCAGCAT TGTCTTGCTC GAGATGTTGT GAACTTGGTT GCGTGTGAAG GAGCTGAGAG GGTGGAGCGG   
  
  
+ CATGAGCTCC TTGGGAAATG GAGGTCGCGG TTCACAATGG CAGGGTTTAA GCCATACCCT TTAAGCACTC   
  
  
+ TGGTAAACAA CACAATAAGA ACTTTGTTGA AGAACTACTG TGATAGGTAT GGGCTAGAGG AGAGGAATGG   
  
  
+ AGGTCTTTAT CTTGGGTGGA TGAATAGAGA TTTGGTTGCT TCGAGTGCCT GGCAGTGTAG TAATTA  

- +Up\_Stream \_Len000GTAAAA TCATTATTTA AGGGTACACT CCTGGTGACG GATGACTAAG GCTTACCGTA   
  
  
- AAATCATTAT TTGAGGGTAC ACTCCTGGTG AACTACTCCT TAAGTCAAAA TATTAGGTGT CCACAGTTTC   
  
  
- GGATAAGGAT GAGAGTGGTG AGACGATAGA GAGGGGGGGG GGGGGGGGGC GGGGGGAGTT TTAGACGGAG   
  
  
- GAGGCAAGAG AGGTGTCGTC TAAGGAGTTA GTCCTTTCCA TACAGTCTCG AGTTTTGCTA AGTTGTAGTT   
  
  
- TTACCCTATT AAATAAGACG AAAGTCAAGA CCCAATAACA GTATACAAGT AAGTCAAGTC ACAAAGATCG   
  
  
- TACTAACGAA AAACTACGGA AGAAAAAACA AGACCCAACC TGCTTGAGAC AGAAAAGACA CGTATCTAAA   
  
  
- CTCGAGGCTC GAGTTAAAAA CCCTAAAAAA ATCGACTGAA AAGACGAACC ACAACACTTT AACACTACCC   
  
  
- AAAACCGAAA CAATTTGAAC AAATATTGAA AAAGACAGTT TTGAAATATC ATTTAACAAC GTTAAACTCT   
  
  
- TAATACGTGA GTCACCGAGA CACTAAATAG CAGGCGACCG TTTAAAAGAC TACTTTCCTC TACTAAATAA   
  
  
- TTAAAAAGTC CGTACCAGTT TAAAAGATCG GAAAGTACAT GTAACCAACC ACTATCCGAC TATCGATTTA   
  
  
- ATACAGAGTA AAGAGATTTA AATAACGGTC TATACTTTAA CCACCTTGTC AAATTCCCAA AACCAGCAGA   
  
  
- AAAAACGGGA AAAAAAACCC AGGGGGGGGG CGGGCCCCCC CTTGGGCCTC CCGTCTTTTT CATTGTATTG   
  
  
- AATTTTAATA GTAACTGCAT CAGGAGGTTG GTATTACTTT CGTTGACTTT ATTCTCGTAC GATTCGATCT   
  
  
- CTCATCCGAC GGAACAGTCG GAGGGAAAAC TGTACTCTTT CTACCCTCTT CAAATTTTAT TTCAAGGAGC   
  
  
- AGAAACGTAG GGCCTGGTGT TAAGCCTTGA CCCACTCCTG GAGGAATCCG AACATTTGTG TTCAAAGCTT   
  
  
- GAATCTAAAC GCATGTACAG TTACGAAATG ACCTATTCGA ATCGATTGTG GGGAGTAGGA AGTTGGACAT   
  
  
- GGAAGTATAT TTTTTTTCCT GTTAGGCCAC GTGTTCGTAG GGCGTAAGTG CGTTCCAGAC CCCTTCCCGG   
  
  
- CGTGGGGTTT CCCACTTAAC ATCCGTCGGA TTGGACTGAA ACAGTCACCG ATTAAGGTGC TAAGCTTGGG   
  
  
- TACTAAAAAA CCAATGTGCC CTTGTTGGAA TGGTAACGAG TTTCCGAGGG GAAGAAGTTG GAGATGGAAG   
  
  
- TATATACTTA CTTTTAGGAA ACCGATTCTT AACTTTTCCT TTATACACCA ATAACCCAAA CCCAAGAATA   
  
  
- AGGCAACCAA CGGGGAAGTG TAACTTACGT AGGAGAGCGA TTCTTGACTT TTCCTGTATA CCCCTACATA   
  
  
- TCCAGTTTAA ACTGAGTTAC TATTGACTCC TATGTTCTTA AGATCTTCAG AAATAGTCCC CTTAAAAAAG   
  
  
- AAAAAAGGTG AACACTGAAA AACTACGGAA CGAAAAATGA GAAGTAACTT ACTCATGGGA AGAACTGTCT   
  
  
- AAGAAGGAGG GAAAAAAGAA TTAAAGAAGA ACATCACCTT ATATTCATAC GAAGATCCAT CAACCTACGG   
  
  
- TACCTTAGAG TAGGGACACA CACGTAAGAA GGGGAGAGGG CGGCGGGGGG GGGGGGGTTT TTCTTTGGTT   
  
  
- TTTTTTTTTT TTTTACCTAC TAAGAATCTT GACTGGTAAA AAGGAAGGAG GAGGGTAAAA TAAAAAAAGT   
  
  
- CCGACAAAAA GAAGATGGAC AACGAAATCG TAGTGAGTAT TTTTGTTCCA CGTTCGCGTA CTGTAGCCCA   
  
  
- TGACTCGAAG ACTCAAATGT ACGTAGCAGT ACTTTAAGCC ATCTGAAACT TCATTTCAAA GGGGTAGCAT   
  
  
- TGACCACCAC GTATGGACAG AAGTGCGGGT AGTCATCTCA CCACTTCACT TCGTTACAGC TTATCAAACA   
  
  
- TAATGCTCGG GTTCTCTCTT TGACTACGTA TAAAATACGG AGTTACAGTT AGGAACTTGG GGGTCGAGCT   
  
  
- AATGTTGTGG TAACTGATAC CTCGAAAATA CACGAGTTTT CAAAAGGACC CAGTCATGAC GTGAGACCTT   
  
  
- AGTAGGAGTC CTTGACCCTG AACAGGCCAC AGATCGAGGT GTAGTTGAGA GTCGAAGAGT AGGTTACCAT   
  
  
- CGGGACCTAG TGTCGAACTA AGGTTGAGAA TAGGAAGACT AGTTTTAACG AGAGGACTAT TACGGTTAAG   
  
  
- GAGTAGAAAT TCAGTAAGGA CGCAGTGTCT ACTACACCTA CTGGACTTTA TGTCCGACTC TCTCAACCTT   
  
  
- TGTCACTACA ACCCTGGACT AGGGCTAAAA CAGATGCCGT TATCGTTGGT GTGTAAACCC CAACCCTAGG   
  
  
- TACATCACAG TAGTCTCAAA CTGTCGACCC CCTTTCACTA CCTCTACTAG CGAGCTTTCC TAAACTTTGA   
  
  
- CCACGATTAA CGGACACGTT CTCGTCAACG ACTTTTACTA GTCAACAACC GGGTCACCGA CTACCTACTT   
  
  
- AACTCTGTCT ACCAAAGTCA AAGACCACTT GGTTAAGTTT CTAACCCACG AATATACAAC CTTCCCGAGC   
  
  
- ATCGCTCCGT CCGGAGGAGT CCATCGAGGT AAATATTTCG TAACTCTACG TTCCTTGGGC GCTCACGACT   
  
  
- GGAAAATAGA ATGTACGTGT ATGAGAAACT CCAAACGGGT ATGTAGTTTA AACCGATGTA CAGTCGTTTA   
  
  
- CCACGGTAAC GTCTTCGTTA CTTCCTACTT TTCTCTCAGG TGTAACAGCT AAAGGTTTAA CCCGTCCCTT   
  
  
- CGGTTACCCA CTGGGATTAG GTCCGTAAAC GTCGGGCCGG TCCCCCAGGT GGGGTGTAAG CGTAATGGCC   
  
  
- ATAGCTACTG AGGATGAGAC GTATACGAGC ACCTCCACCC AACTTGTAAC ACCCGGTATC CGAGAACTCC   
  
  
- AACCGAGTCA GTAAATTCCA GGGTAAACTC AAGTTGCGTC AACTGCAGGG CCGAACAGTT CAGGACGAAT   
  
  
- TTCTAGAGCC GTAAGTTGTA CCACTCCGGA ACCGACACTT GAAACGGAAG TAAGAAGTGG TGTACGGGCT   
  
  
- ACTCTCGCAC TCGTGTCTCT TAGTGGCCCT AGCAGAAGAC TCCCACCATT TTCCCGACTT CGGTTTTCAC   
  
  
- CATTGCAATC ACCTCGTTCT CACATTGTGT TTGAGACGAC GAAAGAACGG AGCGAAACAA CTCTGTAACC   
  
  
- TTATGATGTG TCGCTACAAA CTCAGGTACC TACACTGAAA CGGATCCCTA GTATTTCTGG CCTAGTTACA   
  
  
- ACTCGTCGTA ACAGAACGAG CTCTACAACA CTTGAACCAA CGCACACTTC CTCGACTCTC CCACCTCGCC   
  
  
- GTACTCGAGG AACCCTTTAC CTCCAGCGCC AAGTGTTACC GTCCCAAATT CGGTATGGGA AATTCGTGAG   
  
  
- ACCATTTGTT GTGTTATTCT TGAAACAACT TCTTGATGAC ACTATCCATA CCCGATCTCC TCTCCTTACC   
  
  
- TCCAGAAATA GAACCCACCT ACTTATCTCT AAACCAACGA AGCTCACGGA CCGTCACATC ATTAAT

+     ACE

| Site Name | Organism | Position | Strand | Matrix score. | sequence | function |
| --- | --- | --- | --- | --- | --- | --- |
| ACE | Petroselinum crispum | 1975 | - | 9 | GACACGTATG | cis-acting element involved in light responsiveness |

>HU01G01391.1   
+ +Up\_Stream \_Len000CATTTT AGTAATAAAT TCCCATGTGA GGACCACTGC CTACTGATTC CGAATGGCAT   
  
  
+ TTTAGTAATA AACTCCCATG TGAGGACCAC TTGATGAGGA ATTCAGTTTT ATAATCCACA GGTGTCAAAG   
  
  
+ CCTATTCCTA CTCTCACCAC TCTGCTATCT CTCCCCCCCC CCCCCCCCCG CCCCCCTCAA AATCTGCCTC   
  
  
+ CTCCGTTCTC TCCACAGCAG ATTCCTCAAT CAGGAAAGGT ATGTCAGAGC TCAAAACGAT TCAACATCAA   
  
  
+ AATGGGATAA TTTATTCTGC TTTCAGTTCT GGGTTATTGT CATATGTTCA TTCAGTTCAG TGTTTCTAGC   
  
  
+ ATGATTGCTT TTTGATGCCT TCTTTTTTGT TCTGGGTTGG ACGAACTCTG TCTTTTCTGT GCATAGATTT   
  
  
+ GAGCTCCGAG CTCAATTTTT GGGATTTTTT TAGCTGACTT TTCTGCTTGG TGTTGTGAAA TTGTGATGGG   
  
  
+ TTTTGGCTTT GTTAAACTTG TTTATAACTT TTTCTGTCAA AACTTTATAG TAAATTGTTG CAATTTGAGA   
  
  
+ ATTATGCACT CAGTGGCTCT GTGATTTATC GTCCGCTGGC AAATTTTCTG ATGAAAGGAG ATGATTTATT   
  
  
+ AATTTTTCAG GCATGGTCAA ATTTTCTAGC CTTTCATGTA CATTGGTTGG TGATAGGCTG ATAGCTAAAT   
  
  
+ TATGTCTCAT TTCTCTAAAT TTATTGCCAG ATATGAAATT GGTGGAACAG TTTAAGGGTT TTGGTCGTCT   
  
  
+ TTTTTGCCCT TTTTTTTGGG TCCCCCCCCC GCCCGGGGGG GAACCCGGAG GGCAGAAAAA GTAACATAAC   
  
  
+ TTAAAATTAT CATTGACGTA GTCCTCCAAC CATAATGAAA GCAACTGAAA TAAGAGCATG CTAAGCTAGA   
  
  
+ GAGTAGGCTG CCTTGTCAGC CTCCCTTTTG ACATGAGAAA GATGGGAGAA GTTTAAAATA AAGTTCCTCG   
  
  
+ TCTTTGCATC CCGGACCACA ATTCGGAACT GGGTGAGGAC CTCCTTAGGC TTGTAAACAC AAGTTTCGAA   
  
  
+ CTTAGATTTG CGTACATGTC AATGCTTTAC TGGATAAGCT TAGCTAACAC CCCTCATCCT TCAACCTGTA   
  
  
+ CCTTCATATA AAAAAAAGGA CAATCCGGTG CACAAGCATC CCGCATTCAC GCAAGGTCTG GGGAAGGGCC   
  
  
+ GCACCCCAAA GGGTGAATTG TAGGCAGCCT AACCTGACTT TGTCAGTGGC TAATTCCACG ATTCGAACCC   
  
  
+ ATGATTTTTT GGTTACACGG GAACAACCTT ACCATTGCTC AAAGGCTCCC CTTCTTCAAC CTCTACCTTC   
  
  
+ ATATATGAAT GAAAATCCTT TGGCTAAGAA TTGAAAAGGA AATATGTGGT TATTGGGTTT GGGTTCTTAT   
  
  
+ TCCGTTGGTT GCCCCTTCAC ATTGAATGCA TCCTCTCGCT AAGAACTGAA AAGGACATAT GGGGATGTAT   
  
  
+ AGGTCAAATT TGACTCAATG ATAACTGAGG ATACAAGAAT TCTAGAAGTC TTTATCAGGG GAATTTTTTC   
  
  
+ TTTTTTCCAC TTGTGACTTT TTGATGCCTT GCTTTTTACT CTTCATTGAA TGAGTACCCT TCTTGACAGA   
  
  
+ TTCTTCCTCC CTTTTTTCTT AATTTCTTCT TGTAGTGGAA TATAAGTATG CTTCTAGGTA GTTGGATGCC   
  
  
+ ATGGAATCTC ATCCCTGTGT GTGCATTCTT CCCCTCTCCC GCCGCCCCCC CCCCCCCAAA AAGAAACCAA   
  
  
+ AAAAAAAAAA AAAATGGATG ATTCTTAGAA CTGACCATTT TTCCTTCCTC CTCCCATTTT ATTTTTTTCA   
  
  
+ GGCTGTTTTT CTTCTACCTG TTGCTTTAGC ATCACTCATA AAAACAAGGT GCAAGCGCAT GACATCGGGT   
  
  
+ ACTGAGCTTC TGAGTTTACA TGCATCGTCA TGAAATTCGG TAGACTTTGA AGTAAAGTTT CCCCATCGTA   
  
  
+ ACTGGTGGTG CATACCTGTC TTCACGCCCA TCAGTAGAGT GGTGAAGTGA AGCAATGTCG AATAGTTTGT   
  
  
+ ATTACGAGCC CAAGAGAGAA ACTGATGCAT ATTTTATGCC TCAATGTCAA TCCTTGAACC CCCAGCTCGA   
  
  
+ TTACAACACC ATTGACTATG GAGCTTTTAT GTGCTCAAAA GTTTTCCTGG GTCAGTACTG CACTCTGGAA   
  
  
+ TCATCCTCAG GAACTGGGAC TTGTCCGGTG TCTAGCTCCA CATCAACTCT CAGCTTCTCA TCCAATGGTA   
  
  
+ GCCCTGGATC ACAGCTTGAT TCCAACTCTT ATCCTTCTGA TCAAAATTGC TCTCCTGATA ATGCCAATTC   
  
  
+ CTCATCTTTA AGTCATTCCT GCGTCACAGA TGATGTGGAT GACCTGAAAT ACAGGCTGAG AGAGTTGGAA   
  
  
+ ACAGTGATGT TGGGACCTGA TCCCGATTTT GTCTACGGCA ATAGCAACCA CACATTTGGG GTTGGGATCC   
  
  
+ ATGTAGTGTC ATCAGAGTTT GACAGCTGGG GGAAAGTGAT GGAGATGATC GCTCGAAAGG ATTTGAAACT   
  
  
+ GGTGCTAATT GCCTGTGCAA GAGCAGTTGC TGAAAATGAT CAGTTGTTGG CCCAGTGGCT GATGGATGAA   
  
  
+ TTGAGACAGA TGGTTTCAGT TTCTGGTGAA CCAATTCAAA GATTGGGTGC TTATATGTTG GAAGGGCTCG   
  
  
+ TAGCGAGGCA GGCCTCCTCA GGTAGCTCCA TTTATAAAGC ATTGAGATGC AAGGAACCCG CGAGTGCTGA   
  
  
+ CCTTTTATCT TACATGCACA TACTCTTTGA GGTTTGCCCA TACATCAAAT TTGGCTACAT GTCAGCAAAT   
  
  
+ GGTGCCATTG CAGAAGCAAT GAAGGATGAA AAGAGAGTCC ACATTGTCGA TTTCCAAATT GGGCAGGGAA   
  
  
+ GCCAATGGGT GACCCTAATC CAGGCATTTG CAGCCCGGCC AGGGGGTCCA CCCCACATTC GCATTACCGG   
  
  
+ TATCGATGAC TCCTACTCTG CATATGCTCG TGGAGGTGGG TTGAACATTG TGGGCCATAG GCTCTTGAGG   
  
  
+ TTGGCTCAGT CATTTAAGGT CCCATTTGAG TTCAACGCAG TTGACGTCCC GGCTTGTCAA GTCCTGCTTA   
  
  
+ AAGATCTCGG CATTCAACAT GGTGAGGCCT TGGCTGTGAA CTTTGCCTTC ATTCTTCACC ACATGCCCGA   
  
  
+ TGAGAGCGTG AGCACAGAGA ATCACCGGGA TCGTCTTCTG AGGGTGGTAA AAGGGCTGAA GCCAAAAGTG   
  
  
+ GTAACGTTAG TGGAGCAAGA GTGTAACACA AACTCTGCTG CTTTCTTGCC TCGCTTTGTT GAGACATTGG   
  
  
+ AATACTACAC AGCGATGTTT GAGTCCATGG ATGTGACTTT GCCTAGGGAT CATAAAGACC GGATCAATGT   
  
  
+ TGAGCAGCAT TGTCTTGCTC GAGATGTTGT GAACTTGGTT GCGTGTGAAG GAGCTGAGAG GGTGGAGCGG   
  
  
+ CATGAGCTCC TTGGGAAATG GAGGTCGCGG TTCACAATGG CAGGGTTTAA GCCATACCCT TTAAGCACTC   
  
  
+ TGGTAAACAA CACAATAAGA ACTTTGTTGA AGAACTACTG TGATAGGTAT GGGCTAGAGG AGAGGAATGG   
  
  
+ AGGTCTTTAT CTTGGGTGGA TGAATAGAGA TTTGGTTGCT TCGAGTGCCT GGCAGTGTAG TAATTA  

- +Up\_Stream \_Len000GTAAAA TCATTATTTA AGGGTACACT CCTGGTGACG GATGACTAAG GCTTACCGTA   
  
  
- AAATCATTAT TTGAGGGTAC ACTCCTGGTG AACTACTCCT TAAGTCAAAA TATTAGGTGT CCACAGTTTC   
  
  
- GGATAAGGAT GAGAGTGGTG AGACGATAGA GAGGGGGGGG GGGGGGGGGC GGGGGGAGTT TTAGACGGAG   
  
  
- GAGGCAAGAG AGGTGTCGTC TAAGGAGTTA GTCCTTTCCA TACAGTCTCG AGTTTTGCTA AGTTGTAGTT   
  
  
- TTACCCTATT AAATAAGACG AAAGTCAAGA CCCAATAACA GTATACAAGT AAGTCAAGTC ACAAAGATCG   
  
  
- TACTAACGAA AAACTACGGA AGAAAAAACA AGACCCAACC TGCTTGAGAC AGAAAAGACA CGTATCTAAA   
  
  
- CTCGAGGCTC GAGTTAAAAA CCCTAAAAAA ATCGACTGAA AAGACGAACC ACAACACTTT AACACTACCC   
  
  
- AAAACCGAAA CAATTTGAAC AAATATTGAA AAAGACAGTT TTGAAATATC ATTTAACAAC GTTAAACTCT   
  
  
- TAATACGTGA GTCACCGAGA CACTAAATAG CAGGCGACCG TTTAAAAGAC TACTTTCCTC TACTAAATAA   
  
  
- TTAAAAAGTC CGTACCAGTT TAAAAGATCG GAAAGTACAT GTAACCAACC ACTATCCGAC TATCGATTTA   
  
  
- ATACAGAGTA AAGAGATTTA AATAACGGTC TATACTTTAA CCACCTTGTC AAATTCCCAA AACCAGCAGA   
  
  
- AAAAACGGGA AAAAAAACCC AGGGGGGGGG CGGGCCCCCC CTTGGGCCTC CCGTCTTTTT CATTGTATTG   
  
  
- AATTTTAATA GTAACTGCAT CAGGAGGTTG GTATTACTTT CGTTGACTTT ATTCTCGTAC GATTCGATCT   
  
  
- CTCATCCGAC GGAACAGTCG GAGGGAAAAC TGTACTCTTT CTACCCTCTT CAAATTTTAT TTCAAGGAGC   
  
  
- AGAAACGTAG GGCCTGGTGT TAAGCCTTGA CCCACTCCTG GAGGAATCCG AACATTTGTG TTCAAAGCTT   
  
  
- GAATCTAAAC GCATGTACAG TTACGAAATG ACCTATTCGA ATCGATTGTG GGGAGTAGGA AGTTGGACAT   
  
  
- GGAAGTATAT TTTTTTTCCT GTTAGGCCAC GTGTTCGTAG GGCGTAAGTG CGTTCCAGAC CCCTTCCCGG   
  
  
- CGTGGGGTTT CCCACTTAAC ATCCGTCGGA TTGGACTGAA ACAGTCACCG ATTAAGGTGC TAAGCTTGGG   
  
  
- TACTAAAAAA CCAATGTGCC CTTGTTGGAA TGGTAACGAG TTTCCGAGGG GAAGAAGTTG GAGATGGAAG   
  
  
- TATATACTTA CTTTTAGGAA ACCGATTCTT AACTTTTCCT TTATACACCA ATAACCCAAA CCCAAGAATA   
  
  
- AGGCAACCAA CGGGGAAGTG TAACTTACGT AGGAGAGCGA TTCTTGACTT TTCCTGTATA CCCCTACATA   
  
  
- TCCAGTTTAA ACTGAGTTAC TATTGACTCC TATGTTCTTA AGATCTTCAG AAATAGTCCC CTTAAAAAAG   
  
  
- AAAAAAGGTG AACACTGAAA AACTACGGAA CGAAAAATGA GAAGTAACTT ACTCATGGGA AGAACTGTCT   
  
  
- AAGAAGGAGG GAAAAAAGAA TTAAAGAAGA ACATCACCTT ATATTCATAC GAAGATCCAT CAACCTACGG   
  
  
- TACCTTAGAG TAGGGACACA CACGTAAGAA GGGGAGAGGG CGGCGGGGGG GGGGGGGTTT TTCTTTGGTT   
  
  
- TTTTTTTTTT TTTTACCTAC TAAGAATCTT GACTGGTAAA AAGGAAGGAG GAGGGTAAAA TAAAAAAAGT   
  
  
- CCGACAAAAA GAAGATGGAC AACGAAATCG TAGTGAGTAT TTTTGTTCCA CGTTCGCGTA CTGTAGCCCA   
  
  
- TGACTCGAAG ACTCAAATGT ACGTAGCAGT ACTTTAAGCC ATCTGAAACT TCATTTCAAA GGGGTAGCAT   
  
  
- TGACCACCAC GTATGGACAG AAGTGCGGGT AGTCATCTCA CCACTTCACT TCGTTACAGC TTATCAAACA   
  
  
- TAATGCTCGG GTTCTCTCTT TGACTACGTA TAAAATACGG AGTTACAGTT AGGAACTTGG GGGTCGAGCT   
  
  
- AATGTTGTGG TAACTGATAC CTCGAAAATA CACGAGTTTT CAAAAGGACC CAGTCATGAC GTGAGACCTT   
  
  
- AGTAGGAGTC CTTGACCCTG AACAGGCCAC AGATCGAGGT GTAGTTGAGA GTCGAAGAGT AGGTTACCAT   
  
  
- CGGGACCTAG TGTCGAACTA AGGTTGAGAA TAGGAAGACT AGTTTTAACG AGAGGACTAT TACGGTTAAG   
  
  
- GAGTAGAAAT TCAGTAAGGA CGCAGTGTCT ACTACACCTA CTGGACTTTA TGTCCGACTC TCTCAACCTT   
  
  
- TGTCACTACA ACCCTGGACT AGGGCTAAAA CAGATGCCGT TATCGTTGGT GTGTAAACCC CAACCCTAGG   
  
  
- TACATCACAG TAGTCTCAAA CTGTCGACCC CCTTTCACTA CCTCTACTAG CGAGCTTTCC TAAACTTTGA   
  
  
- CCACGATTAA CGGACACGTT CTCGTCAACG ACTTTTACTA GTCAACAACC GGGTCACCGA CTACCTACTT   
  
  
- AACTCTGTCT ACCAAAGTCA AAGACCACTT GGTTAAGTTT CTAACCCACG AATATACAAC CTTCCCGAGC   
  
  
- ATCGCTCCGT CCGGAGGAGT CCATCGAGGT AAATATTTCG TAACTCTACG TTCCTTGGGC GCTCACGACT   
  
  
- GGAAAATAGA ATGTACGTGT ATGAGAAACT CCAAACGGGT ATGTAGTTTA AACCGATGTA CAGTCGTTTA   
  
  
- CCACGGTAAC GTCTTCGTTA CTTCCTACTT TTCTCTCAGG TGTAACAGCT AAAGGTTTAA CCCGTCCCTT   
  
  
- CGGTTACCCA CTGGGATTAG GTCCGTAAAC GTCGGGCCGG TCCCCCAGGT GGGGTGTAAG CGTAATGGCC   
  
  
- ATAGCTACTG AGGATGAGAC GTATACGAGC ACCTCCACCC AACTTGTAAC ACCCGGTATC CGAGAACTCC   
  
  
- AACCGAGTCA GTAAATTCCA GGGTAAACTC AAGTTGCGTC AACTGCAGGG CCGAACAGTT CAGGACGAAT   
  
  
- TTCTAGAGCC GTAAGTTGTA CCACTCCGGA ACCGACACTT GAAACGGAAG TAAGAAGTGG TGTACGGGCT   
  
  
- ACTCTCGCAC TCGTGTCTCT TAGTGGCCCT AGCAGAAGAC TCCCACCATT TTCCCGACTT CGGTTTTCAC   
  
  
- CATTGCAATC ACCTCGTTCT CACATTGTGT TTGAGACGAC GAAAGAACGG AGCGAAACAA CTCTGTAACC   
  
  
- TTATGATGTG TCGCTACAAA CTCAGGTACC TACACTGAAA CGGATCCCTA GTATTTCTGG CCTAGTTACA   
  
  
- ACTCGTCGTA ACAGAACGAG CTCTACAACA CTTGAACCAA CGCACACTTC CTCGACTCTC CCACCTCGCC   
  
  
- GTACTCGAGG AACCCTTTAC CTCCAGCGCC AAGTGTTACC GTCCCAAATT CGGTATGGGA AATTCGTGAG   
  
  
- ACCATTTGTT GTGTTATTCT TGAAACAACT TCTTGATGAC ACTATCCATA CCCGATCTCC TCTCCTTACC   
  
  
- TCCAGAAATA GAACCCACCT ACTTATCTCT AAACCAACGA AGCTCACGGA CCGTCACATC ATTAAT

+     ARE

| Site Name | Organism | Position | Strand | Matrix score. | sequence | function |
| --- | --- | --- | --- | --- | --- | --- |
| ARE | Zea mays | 2605 | - | 6 | AAACCA | cis-acting regulatory element essential for the anaerobic induction |
| ARE | Zea mays | 1748 | + | 6 | AAACCA | cis-acting regulatory element essential for the anaerobic induction |

>HU01G01391.1   
+ +Up\_Stream \_Len000CATTTT AGTAATAAAT TCCCATGTGA GGACCACTGC CTACTGATTC CGAATGGCAT   
  
  
+ TTTAGTAATA AACTCCCATG TGAGGACCAC TTGATGAGGA ATTCAGTTTT ATAATCCACA GGTGTCAAAG   
  
  
+ CCTATTCCTA CTCTCACCAC TCTGCTATCT CTCCCCCCCC CCCCCCCCCG CCCCCCTCAA AATCTGCCTC   
  
  
+ CTCCGTTCTC TCCACAGCAG ATTCCTCAAT CAGGAAAGGT ATGTCAGAGC TCAAAACGAT TCAACATCAA   
  
  
+ AATGGGATAA TTTATTCTGC TTTCAGTTCT GGGTTATTGT CATATGTTCA TTCAGTTCAG TGTTTCTAGC   
  
  
+ ATGATTGCTT TTTGATGCCT TCTTTTTTGT TCTGGGTTGG ACGAACTCTG TCTTTTCTGT GCATAGATTT   
  
  
+ GAGCTCCGAG CTCAATTTTT GGGATTTTTT TAGCTGACTT TTCTGCTTGG TGTTGTGAAA TTGTGATGGG   
  
  
+ TTTTGGCTTT GTTAAACTTG TTTATAACTT TTTCTGTCAA AACTTTATAG TAAATTGTTG CAATTTGAGA   
  
  
+ ATTATGCACT CAGTGGCTCT GTGATTTATC GTCCGCTGGC AAATTTTCTG ATGAAAGGAG ATGATTTATT   
  
  
+ AATTTTTCAG GCATGGTCAA ATTTTCTAGC CTTTCATGTA CATTGGTTGG TGATAGGCTG ATAGCTAAAT   
  
  
+ TATGTCTCAT TTCTCTAAAT TTATTGCCAG ATATGAAATT GGTGGAACAG TTTAAGGGTT TTGGTCGTCT   
  
  
+ TTTTTGCCCT TTTTTTTGGG TCCCCCCCCC GCCCGGGGGG GAACCCGGAG GGCAGAAAAA GTAACATAAC   
  
  
+ TTAAAATTAT CATTGACGTA GTCCTCCAAC CATAATGAAA GCAACTGAAA TAAGAGCATG CTAAGCTAGA   
  
  
+ GAGTAGGCTG CCTTGTCAGC CTCCCTTTTG ACATGAGAAA GATGGGAGAA GTTTAAAATA AAGTTCCTCG   
  
  
+ TCTTTGCATC CCGGACCACA ATTCGGAACT GGGTGAGGAC CTCCTTAGGC TTGTAAACAC AAGTTTCGAA   
  
  
+ CTTAGATTTG CGTACATGTC AATGCTTTAC TGGATAAGCT TAGCTAACAC CCCTCATCCT TCAACCTGTA   
  
  
+ CCTTCATATA AAAAAAAGGA CAATCCGGTG CACAAGCATC CCGCATTCAC GCAAGGTCTG GGGAAGGGCC   
  
  
+ GCACCCCAAA GGGTGAATTG TAGGCAGCCT AACCTGACTT TGTCAGTGGC TAATTCCACG ATTCGAACCC   
  
  
+ ATGATTTTTT GGTTACACGG GAACAACCTT ACCATTGCTC AAAGGCTCCC CTTCTTCAAC CTCTACCTTC   
  
  
+ ATATATGAAT GAAAATCCTT TGGCTAAGAA TTGAAAAGGA AATATGTGGT TATTGGGTTT GGGTTCTTAT   
  
  
+ TCCGTTGGTT GCCCCTTCAC ATTGAATGCA TCCTCTCGCT AAGAACTGAA AAGGACATAT GGGGATGTAT   
  
  
+ AGGTCAAATT TGACTCAATG ATAACTGAGG ATACAAGAAT TCTAGAAGTC TTTATCAGGG GAATTTTTTC   
  
  
+ TTTTTTCCAC TTGTGACTTT TTGATGCCTT GCTTTTTACT CTTCATTGAA TGAGTACCCT TCTTGACAGA   
  
  
+ TTCTTCCTCC CTTTTTTCTT AATTTCTTCT TGTAGTGGAA TATAAGTATG CTTCTAGGTA GTTGGATGCC   
  
  
+ ATGGAATCTC ATCCCTGTGT GTGCATTCTT CCCCTCTCCC GCCGCCCCCC CCCCCCCAAA AAGAAACCAA   
  
  
+ AAAAAAAAAA AAAATGGATG ATTCTTAGAA CTGACCATTT TTCCTTCCTC CTCCCATTTT ATTTTTTTCA   
  
  
+ GGCTGTTTTT CTTCTACCTG TTGCTTTAGC ATCACTCATA AAAACAAGGT GCAAGCGCAT GACATCGGGT   
  
  
+ ACTGAGCTTC TGAGTTTACA TGCATCGTCA TGAAATTCGG TAGACTTTGA AGTAAAGTTT CCCCATCGTA   
  
  
+ ACTGGTGGTG CATACCTGTC TTCACGCCCA TCAGTAGAGT GGTGAAGTGA AGCAATGTCG AATAGTTTGT   
  
  
+ ATTACGAGCC CAAGAGAGAA ACTGATGCAT ATTTTATGCC TCAATGTCAA TCCTTGAACC CCCAGCTCGA   
  
  
+ TTACAACACC ATTGACTATG GAGCTTTTAT GTGCTCAAAA GTTTTCCTGG GTCAGTACTG CACTCTGGAA   
  
  
+ TCATCCTCAG GAACTGGGAC TTGTCCGGTG TCTAGCTCCA CATCAACTCT CAGCTTCTCA TCCAATGGTA   
  
  
+ GCCCTGGATC ACAGCTTGAT TCCAACTCTT ATCCTTCTGA TCAAAATTGC TCTCCTGATA ATGCCAATTC   
  
  
+ CTCATCTTTA AGTCATTCCT GCGTCACAGA TGATGTGGAT GACCTGAAAT ACAGGCTGAG AGAGTTGGAA   
  
  
+ ACAGTGATGT TGGGACCTGA TCCCGATTTT GTCTACGGCA ATAGCAACCA CACATTTGGG GTTGGGATCC   
  
  
+ ATGTAGTGTC ATCAGAGTTT GACAGCTGGG GGAAAGTGAT GGAGATGATC GCTCGAAAGG ATTTGAAACT   
  
  
+ GGTGCTAATT GCCTGTGCAA GAGCAGTTGC TGAAAATGAT CAGTTGTTGG CCCAGTGGCT GATGGATGAA   
  
  
+ TTGAGACAGA TGGTTTCAGT TTCTGGTGAA CCAATTCAAA GATTGGGTGC TTATATGTTG GAAGGGCTCG   
  
  
+ TAGCGAGGCA GGCCTCCTCA GGTAGCTCCA TTTATAAAGC ATTGAGATGC AAGGAACCCG CGAGTGCTGA   
  
  
+ CCTTTTATCT TACATGCACA TACTCTTTGA GGTTTGCCCA TACATCAAAT TTGGCTACAT GTCAGCAAAT   
  
  
+ GGTGCCATTG CAGAAGCAAT GAAGGATGAA AAGAGAGTCC ACATTGTCGA TTTCCAAATT GGGCAGGGAA   
  
  
+ GCCAATGGGT GACCCTAATC CAGGCATTTG CAGCCCGGCC AGGGGGTCCA CCCCACATTC GCATTACCGG   
  
  
+ TATCGATGAC TCCTACTCTG CATATGCTCG TGGAGGTGGG TTGAACATTG TGGGCCATAG GCTCTTGAGG   
  
  
+ TTGGCTCAGT CATTTAAGGT CCCATTTGAG TTCAACGCAG TTGACGTCCC GGCTTGTCAA GTCCTGCTTA   
  
  
+ AAGATCTCGG CATTCAACAT GGTGAGGCCT TGGCTGTGAA CTTTGCCTTC ATTCTTCACC ACATGCCCGA   
  
  
+ TGAGAGCGTG AGCACAGAGA ATCACCGGGA TCGTCTTCTG AGGGTGGTAA AAGGGCTGAA GCCAAAAGTG   
  
  
+ GTAACGTTAG TGGAGCAAGA GTGTAACACA AACTCTGCTG CTTTCTTGCC TCGCTTTGTT GAGACATTGG   
  
  
+ AATACTACAC AGCGATGTTT GAGTCCATGG ATGTGACTTT GCCTAGGGAT CATAAAGACC GGATCAATGT   
  
  
+ TGAGCAGCAT TGTCTTGCTC GAGATGTTGT GAACTTGGTT GCGTGTGAAG GAGCTGAGAG GGTGGAGCGG   
  
  
+ CATGAGCTCC TTGGGAAATG GAGGTCGCGG TTCACAATGG CAGGGTTTAA GCCATACCCT TTAAGCACTC   
  
  
+ TGGTAAACAA CACAATAAGA ACTTTGTTGA AGAACTACTG TGATAGGTAT GGGCTAGAGG AGAGGAATGG   
  
  
+ AGGTCTTTAT CTTGGGTGGA TGAATAGAGA TTTGGTTGCT TCGAGTGCCT GGCAGTGTAG TAATTA  

- +Up\_Stream \_Len000GTAAAA TCATTATTTA AGGGTACACT CCTGGTGACG GATGACTAAG GCTTACCGTA   
  
  
- AAATCATTAT TTGAGGGTAC ACTCCTGGTG AACTACTCCT TAAGTCAAAA TATTAGGTGT CCACAGTTTC   
  
  
- GGATAAGGAT GAGAGTGGTG AGACGATAGA GAGGGGGGGG GGGGGGGGGC GGGGGGAGTT TTAGACGGAG   
  
  
- GAGGCAAGAG AGGTGTCGTC TAAGGAGTTA GTCCTTTCCA TACAGTCTCG AGTTTTGCTA AGTTGTAGTT   
  
  
- TTACCCTATT AAATAAGACG AAAGTCAAGA CCCAATAACA GTATACAAGT AAGTCAAGTC ACAAAGATCG   
  
  
- TACTAACGAA AAACTACGGA AGAAAAAACA AGACCCAACC TGCTTGAGAC AGAAAAGACA CGTATCTAAA   
  
  
- CTCGAGGCTC GAGTTAAAAA CCCTAAAAAA ATCGACTGAA AAGACGAACC ACAACACTTT AACACTACCC   
  
  
- AAAACCGAAA CAATTTGAAC AAATATTGAA AAAGACAGTT TTGAAATATC ATTTAACAAC GTTAAACTCT   
  
  
- TAATACGTGA GTCACCGAGA CACTAAATAG CAGGCGACCG TTTAAAAGAC TACTTTCCTC TACTAAATAA   
  
  
- TTAAAAAGTC CGTACCAGTT TAAAAGATCG GAAAGTACAT GTAACCAACC ACTATCCGAC TATCGATTTA   
  
  
- ATACAGAGTA AAGAGATTTA AATAACGGTC TATACTTTAA CCACCTTGTC AAATTCCCAA AACCAGCAGA   
  
  
- AAAAACGGGA AAAAAAACCC AGGGGGGGGG CGGGCCCCCC CTTGGGCCTC CCGTCTTTTT CATTGTATTG   
  
  
- AATTTTAATA GTAACTGCAT CAGGAGGTTG GTATTACTTT CGTTGACTTT ATTCTCGTAC GATTCGATCT   
  
  
- CTCATCCGAC GGAACAGTCG GAGGGAAAAC TGTACTCTTT CTACCCTCTT CAAATTTTAT TTCAAGGAGC   
  
  
- AGAAACGTAG GGCCTGGTGT TAAGCCTTGA CCCACTCCTG GAGGAATCCG AACATTTGTG TTCAAAGCTT   
  
  
- GAATCTAAAC GCATGTACAG TTACGAAATG ACCTATTCGA ATCGATTGTG GGGAGTAGGA AGTTGGACAT   
  
  
- GGAAGTATAT TTTTTTTCCT GTTAGGCCAC GTGTTCGTAG GGCGTAAGTG CGTTCCAGAC CCCTTCCCGG   
  
  
- CGTGGGGTTT CCCACTTAAC ATCCGTCGGA TTGGACTGAA ACAGTCACCG ATTAAGGTGC TAAGCTTGGG   
  
  
- TACTAAAAAA CCAATGTGCC CTTGTTGGAA TGGTAACGAG TTTCCGAGGG GAAGAAGTTG GAGATGGAAG   
  
  
- TATATACTTA CTTTTAGGAA ACCGATTCTT AACTTTTCCT TTATACACCA ATAACCCAAA CCCAAGAATA   
  
  
- AGGCAACCAA CGGGGAAGTG TAACTTACGT AGGAGAGCGA TTCTTGACTT TTCCTGTATA CCCCTACATA   
  
  
- TCCAGTTTAA ACTGAGTTAC TATTGACTCC TATGTTCTTA AGATCTTCAG AAATAGTCCC CTTAAAAAAG   
  
  
- AAAAAAGGTG AACACTGAAA AACTACGGAA CGAAAAATGA GAAGTAACTT ACTCATGGGA AGAACTGTCT   
  
  
- AAGAAGGAGG GAAAAAAGAA TTAAAGAAGA ACATCACCTT ATATTCATAC GAAGATCCAT CAACCTACGG   
  
  
- TACCTTAGAG TAGGGACACA CACGTAAGAA GGGGAGAGGG CGGCGGGGGG GGGGGGGTTT TTCTTTGGTT   
  
  
- TTTTTTTTTT TTTTACCTAC TAAGAATCTT GACTGGTAAA AAGGAAGGAG GAGGGTAAAA TAAAAAAAGT   
  
  
- CCGACAAAAA GAAGATGGAC AACGAAATCG TAGTGAGTAT TTTTGTTCCA CGTTCGCGTA CTGTAGCCCA   
  
  
- TGACTCGAAG ACTCAAATGT ACGTAGCAGT ACTTTAAGCC ATCTGAAACT TCATTTCAAA GGGGTAGCAT   
  
  
- TGACCACCAC GTATGGACAG AAGTGCGGGT AGTCATCTCA CCACTTCACT TCGTTACAGC TTATCAAACA   
  
  
- TAATGCTCGG GTTCTCTCTT TGACTACGTA TAAAATACGG AGTTACAGTT AGGAACTTGG GGGTCGAGCT   
  
  
- AATGTTGTGG TAACTGATAC CTCGAAAATA CACGAGTTTT CAAAAGGACC CAGTCATGAC GTGAGACCTT   
  
  
- AGTAGGAGTC CTTGACCCTG AACAGGCCAC AGATCGAGGT GTAGTTGAGA GTCGAAGAGT AGGTTACCAT   
  
  
- CGGGACCTAG TGTCGAACTA AGGTTGAGAA TAGGAAGACT AGTTTTAACG AGAGGACTAT TACGGTTAAG   
  
  
- GAGTAGAAAT TCAGTAAGGA CGCAGTGTCT ACTACACCTA CTGGACTTTA TGTCCGACTC TCTCAACCTT   
  
  
- TGTCACTACA ACCCTGGACT AGGGCTAAAA CAGATGCCGT TATCGTTGGT GTGTAAACCC CAACCCTAGG   
  
  
- TACATCACAG TAGTCTCAAA CTGTCGACCC CCTTTCACTA CCTCTACTAG CGAGCTTTCC TAAACTTTGA   
  
  
- CCACGATTAA CGGACACGTT CTCGTCAACG ACTTTTACTA GTCAACAACC GGGTCACCGA CTACCTACTT   
  
  
- AACTCTGTCT ACCAAAGTCA AAGACCACTT GGTTAAGTTT CTAACCCACG AATATACAAC CTTCCCGAGC   
  
  
- ATCGCTCCGT CCGGAGGAGT CCATCGAGGT AAATATTTCG TAACTCTACG TTCCTTGGGC GCTCACGACT   
  
  
- GGAAAATAGA ATGTACGTGT ATGAGAAACT CCAAACGGGT ATGTAGTTTA AACCGATGTA CAGTCGTTTA   
  
  
- CCACGGTAAC GTCTTCGTTA CTTCCTACTT TTCTCTCAGG TGTAACAGCT AAAGGTTTAA CCCGTCCCTT   
  
  
- CGGTTACCCA CTGGGATTAG GTCCGTAAAC GTCGGGCCGG TCCCCCAGGT GGGGTGTAAG CGTAATGGCC   
  
  
- ATAGCTACTG AGGATGAGAC GTATACGAGC ACCTCCACCC AACTTGTAAC ACCCGGTATC CGAGAACTCC   
  
  
- AACCGAGTCA GTAAATTCCA GGGTAAACTC AAGTTGCGTC AACTGCAGGG CCGAACAGTT CAGGACGAAT   
  
  
- TTCTAGAGCC GTAAGTTGTA CCACTCCGGA ACCGACACTT GAAACGGAAG TAAGAAGTGG TGTACGGGCT   
  
  
- ACTCTCGCAC TCGTGTCTCT TAGTGGCCCT AGCAGAAGAC TCCCACCATT TTCCCGACTT CGGTTTTCAC   
  
  
- CATTGCAATC ACCTCGTTCT CACATTGTGT TTGAGACGAC GAAAGAACGG AGCGAAACAA CTCTGTAACC   
  
  
- TTATGATGTG TCGCTACAAA CTCAGGTACC TACACTGAAA CGGATCCCTA GTATTTCTGG CCTAGTTACA   
  
  
- ACTCGTCGTA ACAGAACGAG CTCTACAACA CTTGAACCAA CGCACACTTC CTCGACTCTC CCACCTCGCC   
  
  
- GTACTCGAGG AACCCTTTAC CTCCAGCGCC AAGTGTTACC GTCCCAAATT CGGTATGGGA AATTCGTGAG   
  
  
- ACCATTTGTT GTGTTATTCT TGAAACAACT TCTTGATGAC ACTATCCATA CCCGATCTCC TCTCCTTACC   
  
  
- TCCAGAAATA GAACCCACCT ACTTATCTCT AAACCAACGA AGCTCACGGA CCGTCACATC ATTAAT

+     ATCT-motif

| Site Name | Organism | Position | Strand | Matrix score. | sequence | function |
| --- | --- | --- | --- | --- | --- | --- |
| ATCT-motif | Pisum sativum | 1689 | + | 9 | AATCTAATCC | part of a conserved DNA module involved in light responsiveness |

>HU01G01391.1   
+ +Up\_Stream \_Len000CATTTT AGTAATAAAT TCCCATGTGA GGACCACTGC CTACTGATTC CGAATGGCAT   
  
  
+ TTTAGTAATA AACTCCCATG TGAGGACCAC TTGATGAGGA ATTCAGTTTT ATAATCCACA GGTGTCAAAG   
  
  
+ CCTATTCCTA CTCTCACCAC TCTGCTATCT CTCCCCCCCC CCCCCCCCCG CCCCCCTCAA AATCTGCCTC   
  
  
+ CTCCGTTCTC TCCACAGCAG ATTCCTCAAT CAGGAAAGGT ATGTCAGAGC TCAAAACGAT TCAACATCAA   
  
  
+ AATGGGATAA TTTATTCTGC TTTCAGTTCT GGGTTATTGT CATATGTTCA TTCAGTTCAG TGTTTCTAGC   
  
  
+ ATGATTGCTT TTTGATGCCT TCTTTTTTGT TCTGGGTTGG ACGAACTCTG TCTTTTCTGT GCATAGATTT   
  
  
+ GAGCTCCGAG CTCAATTTTT GGGATTTTTT TAGCTGACTT TTCTGCTTGG TGTTGTGAAA TTGTGATGGG   
  
  
+ TTTTGGCTTT GTTAAACTTG TTTATAACTT TTTCTGTCAA AACTTTATAG TAAATTGTTG CAATTTGAGA   
  
  
+ ATTATGCACT CAGTGGCTCT GTGATTTATC GTCCGCTGGC AAATTTTCTG ATGAAAGGAG ATGATTTATT   
  
  
+ AATTTTTCAG GCATGGTCAA ATTTTCTAGC CTTTCATGTA CATTGGTTGG TGATAGGCTG ATAGCTAAAT   
  
  
+ TATGTCTCAT TTCTCTAAAT TTATTGCCAG ATATGAAATT GGTGGAACAG TTTAAGGGTT TTGGTCGTCT   
  
  
+ TTTTTGCCCT TTTTTTTGGG TCCCCCCCCC GCCCGGGGGG GAACCCGGAG GGCAGAAAAA GTAACATAAC   
  
  
+ TTAAAATTAT CATTGACGTA GTCCTCCAAC CATAATGAAA GCAACTGAAA TAAGAGCATG CTAAGCTAGA   
  
  
+ GAGTAGGCTG CCTTGTCAGC CTCCCTTTTG ACATGAGAAA GATGGGAGAA GTTTAAAATA AAGTTCCTCG   
  
  
+ TCTTTGCATC CCGGACCACA ATTCGGAACT GGGTGAGGAC CTCCTTAGGC TTGTAAACAC AAGTTTCGAA   
  
  
+ CTTAGATTTG CGTACATGTC AATGCTTTAC TGGATAAGCT TAGCTAACAC CCCTCATCCT TCAACCTGTA   
  
  
+ CCTTCATATA AAAAAAAGGA CAATCCGGTG CACAAGCATC CCGCATTCAC GCAAGGTCTG GGGAAGGGCC   
  
  
+ GCACCCCAAA GGGTGAATTG TAGGCAGCCT AACCTGACTT TGTCAGTGGC TAATTCCACG ATTCGAACCC   
  
  
+ ATGATTTTTT GGTTACACGG GAACAACCTT ACCATTGCTC AAAGGCTCCC CTTCTTCAAC CTCTACCTTC   
  
  
+ ATATATGAAT GAAAATCCTT TGGCTAAGAA TTGAAAAGGA AATATGTGGT TATTGGGTTT GGGTTCTTAT   
  
  
+ TCCGTTGGTT GCCCCTTCAC ATTGAATGCA TCCTCTCGCT AAGAACTGAA AAGGACATAT GGGGATGTAT   
  
  
+ AGGTCAAATT TGACTCAATG ATAACTGAGG ATACAAGAAT TCTAGAAGTC TTTATCAGGG GAATTTTTTC   
  
  
+ TTTTTTCCAC TTGTGACTTT TTGATGCCTT GCTTTTTACT CTTCATTGAA TGAGTACCCT TCTTGACAGA   
  
  
+ TTCTTCCTCC CTTTTTTCTT AATTTCTTCT TGTAGTGGAA TATAAGTATG CTTCTAGGTA GTTGGATGCC   
  
  
+ ATGGAATCTC ATCCCTGTGT GTGCATTCTT CCCCTCTCCC GCCGCCCCCC CCCCCCCAAA AAGAAACCAA   
  
  
+ AAAAAAAAAA AAAATGGATG ATTCTTAGAA CTGACCATTT TTCCTTCCTC CTCCCATTTT ATTTTTTTCA   
  
  
+ GGCTGTTTTT CTTCTACCTG TTGCTTTAGC ATCACTCATA AAAACAAGGT GCAAGCGCAT GACATCGGGT   
  
  
+ ACTGAGCTTC TGAGTTTACA TGCATCGTCA TGAAATTCGG TAGACTTTGA AGTAAAGTTT CCCCATCGTA   
  
  
+ ACTGGTGGTG CATACCTGTC TTCACGCCCA TCAGTAGAGT GGTGAAGTGA AGCAATGTCG AATAGTTTGT   
  
  
+ ATTACGAGCC CAAGAGAGAA ACTGATGCAT ATTTTATGCC TCAATGTCAA TCCTTGAACC CCCAGCTCGA   
  
  
+ TTACAACACC ATTGACTATG GAGCTTTTAT GTGCTCAAAA GTTTTCCTGG GTCAGTACTG CACTCTGGAA   
  
  
+ TCATCCTCAG GAACTGGGAC TTGTCCGGTG TCTAGCTCCA CATCAACTCT CAGCTTCTCA TCCAATGGTA   
  
  
+ GCCCTGGATC ACAGCTTGAT TCCAACTCTT ATCCTTCTGA TCAAAATTGC TCTCCTGATA ATGCCAATTC   
  
  
+ CTCATCTTTA AGTCATTCCT GCGTCACAGA TGATGTGGAT GACCTGAAAT ACAGGCTGAG AGAGTTGGAA   
  
  
+ ACAGTGATGT TGGGACCTGA TCCCGATTTT GTCTACGGCA ATAGCAACCA CACATTTGGG GTTGGGATCC   
  
  
+ ATGTAGTGTC ATCAGAGTTT GACAGCTGGG GGAAAGTGAT GGAGATGATC GCTCGAAAGG ATTTGAAACT   
  
  
+ GGTGCTAATT GCCTGTGCAA GAGCAGTTGC TGAAAATGAT CAGTTGTTGG CCCAGTGGCT GATGGATGAA   
  
  
+ TTGAGACAGA TGGTTTCAGT TTCTGGTGAA CCAATTCAAA GATTGGGTGC TTATATGTTG GAAGGGCTCG   
  
  
+ TAGCGAGGCA GGCCTCCTCA GGTAGCTCCA TTTATAAAGC ATTGAGATGC AAGGAACCCG CGAGTGCTGA   
  
  
+ CCTTTTATCT TACATGCACA TACTCTTTGA GGTTTGCCCA TACATCAAAT TTGGCTACAT GTCAGCAAAT   
  
  
+ GGTGCCATTG CAGAAGCAAT GAAGGATGAA AAGAGAGTCC ACATTGTCGA TTTCCAAATT GGGCAGGGAA   
  
  
+ GCCAATGGGT GACCCTAATC CAGGCATTTG CAGCCCGGCC AGGGGGTCCA CCCCACATTC GCATTACCGG   
  
  
+ TATCGATGAC TCCTACTCTG CATATGCTCG TGGAGGTGGG TTGAACATTG TGGGCCATAG GCTCTTGAGG   
  
  
+ TTGGCTCAGT CATTTAAGGT CCCATTTGAG TTCAACGCAG TTGACGTCCC GGCTTGTCAA GTCCTGCTTA   
  
  
+ AAGATCTCGG CATTCAACAT GGTGAGGCCT TGGCTGTGAA CTTTGCCTTC ATTCTTCACC ACATGCCCGA   
  
  
+ TGAGAGCGTG AGCACAGAGA ATCACCGGGA TCGTCTTCTG AGGGTGGTAA AAGGGCTGAA GCCAAAAGTG   
  
  
+ GTAACGTTAG TGGAGCAAGA GTGTAACACA AACTCTGCTG CTTTCTTGCC TCGCTTTGTT GAGACATTGG   
  
  
+ AATACTACAC AGCGATGTTT GAGTCCATGG ATGTGACTTT GCCTAGGGAT CATAAAGACC GGATCAATGT   
  
  
+ TGAGCAGCAT TGTCTTGCTC GAGATGTTGT GAACTTGGTT GCGTGTGAAG GAGCTGAGAG GGTGGAGCGG   
  
  
+ CATGAGCTCC TTGGGAAATG GAGGTCGCGG TTCACAATGG CAGGGTTTAA GCCATACCCT TTAAGCACTC   
  
  
+ TGGTAAACAA CACAATAAGA ACTTTGTTGA AGAACTACTG TGATAGGTAT GGGCTAGAGG AGAGGAATGG   
  
  
+ AGGTCTTTAT CTTGGGTGGA TGAATAGAGA TTTGGTTGCT TCGAGTGCCT GGCAGTGTAG TAATTA  

- +Up\_Stream \_Len000GTAAAA TCATTATTTA AGGGTACACT CCTGGTGACG GATGACTAAG GCTTACCGTA   
  
  
- AAATCATTAT TTGAGGGTAC ACTCCTGGTG AACTACTCCT TAAGTCAAAA TATTAGGTGT CCACAGTTTC   
  
  
- GGATAAGGAT GAGAGTGGTG AGACGATAGA GAGGGGGGGG GGGGGGGGGC GGGGGGAGTT TTAGACGGAG   
  
  
- GAGGCAAGAG AGGTGTCGTC TAAGGAGTTA GTCCTTTCCA TACAGTCTCG AGTTTTGCTA AGTTGTAGTT   
  
  
- TTACCCTATT AAATAAGACG AAAGTCAAGA CCCAATAACA GTATACAAGT AAGTCAAGTC ACAAAGATCG   
  
  
- TACTAACGAA AAACTACGGA AGAAAAAACA AGACCCAACC TGCTTGAGAC AGAAAAGACA CGTATCTAAA   
  
  
- CTCGAGGCTC GAGTTAAAAA CCCTAAAAAA ATCGACTGAA AAGACGAACC ACAACACTTT AACACTACCC   
  
  
- AAAACCGAAA CAATTTGAAC AAATATTGAA AAAGACAGTT TTGAAATATC ATTTAACAAC GTTAAACTCT   
  
  
- TAATACGTGA GTCACCGAGA CACTAAATAG CAGGCGACCG TTTAAAAGAC TACTTTCCTC TACTAAATAA   
  
  
- TTAAAAAGTC CGTACCAGTT TAAAAGATCG GAAAGTACAT GTAACCAACC ACTATCCGAC TATCGATTTA   
  
  
- ATACAGAGTA AAGAGATTTA AATAACGGTC TATACTTTAA CCACCTTGTC AAATTCCCAA AACCAGCAGA   
  
  
- AAAAACGGGA AAAAAAACCC AGGGGGGGGG CGGGCCCCCC CTTGGGCCTC CCGTCTTTTT CATTGTATTG   
  
  
- AATTTTAATA GTAACTGCAT CAGGAGGTTG GTATTACTTT CGTTGACTTT ATTCTCGTAC GATTCGATCT   
  
  
- CTCATCCGAC GGAACAGTCG GAGGGAAAAC TGTACTCTTT CTACCCTCTT CAAATTTTAT TTCAAGGAGC   
  
  
- AGAAACGTAG GGCCTGGTGT TAAGCCTTGA CCCACTCCTG GAGGAATCCG AACATTTGTG TTCAAAGCTT   
  
  
- GAATCTAAAC GCATGTACAG TTACGAAATG ACCTATTCGA ATCGATTGTG GGGAGTAGGA AGTTGGACAT   
  
  
- GGAAGTATAT TTTTTTTCCT GTTAGGCCAC GTGTTCGTAG GGCGTAAGTG CGTTCCAGAC CCCTTCCCGG   
  
  
- CGTGGGGTTT CCCACTTAAC ATCCGTCGGA TTGGACTGAA ACAGTCACCG ATTAAGGTGC TAAGCTTGGG   
  
  
- TACTAAAAAA CCAATGTGCC CTTGTTGGAA TGGTAACGAG TTTCCGAGGG GAAGAAGTTG GAGATGGAAG   
  
  
- TATATACTTA CTTTTAGGAA ACCGATTCTT AACTTTTCCT TTATACACCA ATAACCCAAA CCCAAGAATA   
  
  
- AGGCAACCAA CGGGGAAGTG TAACTTACGT AGGAGAGCGA TTCTTGACTT TTCCTGTATA CCCCTACATA   
  
  
- TCCAGTTTAA ACTGAGTTAC TATTGACTCC TATGTTCTTA AGATCTTCAG AAATAGTCCC CTTAAAAAAG   
  
  
- AAAAAAGGTG AACACTGAAA AACTACGGAA CGAAAAATGA GAAGTAACTT ACTCATGGGA AGAACTGTCT   
  
  
- AAGAAGGAGG GAAAAAAGAA TTAAAGAAGA ACATCACCTT ATATTCATAC GAAGATCCAT CAACCTACGG   
  
  
- TACCTTAGAG TAGGGACACA CACGTAAGAA GGGGAGAGGG CGGCGGGGGG GGGGGGGTTT TTCTTTGGTT   
  
  
- TTTTTTTTTT TTTTACCTAC TAAGAATCTT GACTGGTAAA AAGGAAGGAG GAGGGTAAAA TAAAAAAAGT   
  
  
- CCGACAAAAA GAAGATGGAC AACGAAATCG TAGTGAGTAT TTTTGTTCCA CGTTCGCGTA CTGTAGCCCA   
  
  
- TGACTCGAAG ACTCAAATGT ACGTAGCAGT ACTTTAAGCC ATCTGAAACT TCATTTCAAA GGGGTAGCAT   
  
  
- TGACCACCAC GTATGGACAG AAGTGCGGGT AGTCATCTCA CCACTTCACT TCGTTACAGC TTATCAAACA   
  
  
- TAATGCTCGG GTTCTCTCTT TGACTACGTA TAAAATACGG AGTTACAGTT AGGAACTTGG GGGTCGAGCT   
  
  
- AATGTTGTGG TAACTGATAC CTCGAAAATA CACGAGTTTT CAAAAGGACC CAGTCATGAC GTGAGACCTT   
  
  
- AGTAGGAGTC CTTGACCCTG AACAGGCCAC AGATCGAGGT GTAGTTGAGA GTCGAAGAGT AGGTTACCAT   
  
  
- CGGGACCTAG TGTCGAACTA AGGTTGAGAA TAGGAAGACT AGTTTTAACG AGAGGACTAT TACGGTTAAG   
  
  
- GAGTAGAAAT TCAGTAAGGA CGCAGTGTCT ACTACACCTA CTGGACTTTA TGTCCGACTC TCTCAACCTT   
  
  
- TGTCACTACA ACCCTGGACT AGGGCTAAAA CAGATGCCGT TATCGTTGGT GTGTAAACCC CAACCCTAGG   
  
  
- TACATCACAG TAGTCTCAAA CTGTCGACCC CCTTTCACTA CCTCTACTAG CGAGCTTTCC TAAACTTTGA   
  
  
- CCACGATTAA CGGACACGTT CTCGTCAACG ACTTTTACTA GTCAACAACC GGGTCACCGA CTACCTACTT   
  
  
- AACTCTGTCT ACCAAAGTCA AAGACCACTT GGTTAAGTTT CTAACCCACG AATATACAAC CTTCCCGAGC   
  
  
- ATCGCTCCGT CCGGAGGAGT CCATCGAGGT AAATATTTCG TAACTCTACG TTCCTTGGGC GCTCACGACT   
  
  
- GGAAAATAGA ATGTACGTGT ATGAGAAACT CCAAACGGGT ATGTAGTTTA AACCGATGTA CAGTCGTTTA   
  
  
- CCACGGTAAC GTCTTCGTTA CTTCCTACTT TTCTCTCAGG TGTAACAGCT AAAGGTTTAA CCCGTCCCTT   
  
  
- CGGTTACCCA CTGGGATTAG GTCCGTAAAC GTCGGGCCGG TCCCCCAGGT GGGGTGTAAG CGTAATGGCC   
  
  
- ATAGCTACTG AGGATGAGAC GTATACGAGC ACCTCCACCC AACTTGTAAC ACCCGGTATC CGAGAACTCC   
  
  
- AACCGAGTCA GTAAATTCCA GGGTAAACTC AAGTTGCGTC AACTGCAGGG CCGAACAGTT CAGGACGAAT   
  
  
- TTCTAGAGCC GTAAGTTGTA CCACTCCGGA ACCGACACTT GAAACGGAAG TAAGAAGTGG TGTACGGGCT   
  
  
- ACTCTCGCAC TCGTGTCTCT TAGTGGCCCT AGCAGAAGAC TCCCACCATT TTCCCGACTT CGGTTTTCAC   
  
  
- CATTGCAATC ACCTCGTTCT CACATTGTGT TTGAGACGAC GAAAGAACGG AGCGAAACAA CTCTGTAACC   
  
  
- TTATGATGTG TCGCTACAAA CTCAGGTACC TACACTGAAA CGGATCCCTA GTATTTCTGG CCTAGTTACA   
  
  
- ACTCGTCGTA ACAGAACGAG CTCTACAACA CTTGAACCAA CGCACACTTC CTCGACTCTC CCACCTCGCC   
  
  
- GTACTCGAGG AACCCTTTAC CTCCAGCGCC AAGTGTTACC GTCCCAAATT CGGTATGGGA AATTCGTGAG   
  
  
- ACCATTTGTT GTGTTATTCT TGAAACAACT TCTTGATGAC ACTATCCATA CCCGATCTCC TCTCCTTACC   
  
  
- TCCAGAAATA GAACCCACCT ACTTATCTCT AAACCAACGA AGCTCACGGA CCGTCACATC ATTAAT

+     Box 4

| Site Name | Organism | Position | Strand | Matrix score. | sequence | function |
| --- | --- | --- | --- | --- | --- | --- |
| Box 4 | Petroselinum crispum | 632 | + | 6 | ATTAAT | part of a conserved DNA module involved in light responsiveness |

>HU01G01391.1   
+ +Up\_Stream \_Len000CATTTT AGTAATAAAT TCCCATGTGA GGACCACTGC CTACTGATTC CGAATGGCAT   
  
  
+ TTTAGTAATA AACTCCCATG TGAGGACCAC TTGATGAGGA ATTCAGTTTT ATAATCCACA GGTGTCAAAG   
  
  
+ CCTATTCCTA CTCTCACCAC TCTGCTATCT CTCCCCCCCC CCCCCCCCCG CCCCCCTCAA AATCTGCCTC   
  
  
+ CTCCGTTCTC TCCACAGCAG ATTCCTCAAT CAGGAAAGGT ATGTCAGAGC TCAAAACGAT TCAACATCAA   
  
  
+ AATGGGATAA TTTATTCTGC TTTCAGTTCT GGGTTATTGT CATATGTTCA TTCAGTTCAG TGTTTCTAGC   
  
  
+ ATGATTGCTT TTTGATGCCT TCTTTTTTGT TCTGGGTTGG ACGAACTCTG TCTTTTCTGT GCATAGATTT   
  
  
+ GAGCTCCGAG CTCAATTTTT GGGATTTTTT TAGCTGACTT TTCTGCTTGG TGTTGTGAAA TTGTGATGGG   
  
  
+ TTTTGGCTTT GTTAAACTTG TTTATAACTT TTTCTGTCAA AACTTTATAG TAAATTGTTG CAATTTGAGA   
  
  
+ ATTATGCACT CAGTGGCTCT GTGATTTATC GTCCGCTGGC AAATTTTCTG ATGAAAGGAG ATGATTTATT   
  
  
+ AATTTTTCAG GCATGGTCAA ATTTTCTAGC CTTTCATGTA CATTGGTTGG TGATAGGCTG ATAGCTAAAT   
  
  
+ TATGTCTCAT TTCTCTAAAT TTATTGCCAG ATATGAAATT GGTGGAACAG TTTAAGGGTT TTGGTCGTCT   
  
  
+ TTTTTGCCCT TTTTTTTGGG TCCCCCCCCC GCCCGGGGGG GAACCCGGAG GGCAGAAAAA GTAACATAAC   
  
  
+ TTAAAATTAT CATTGACGTA GTCCTCCAAC CATAATGAAA GCAACTGAAA TAAGAGCATG CTAAGCTAGA   
  
  
+ GAGTAGGCTG CCTTGTCAGC CTCCCTTTTG ACATGAGAAA GATGGGAGAA GTTTAAAATA AAGTTCCTCG   
  
  
+ TCTTTGCATC CCGGACCACA ATTCGGAACT GGGTGAGGAC CTCCTTAGGC TTGTAAACAC AAGTTTCGAA   
  
  
+ CTTAGATTTG CGTACATGTC AATGCTTTAC TGGATAAGCT TAGCTAACAC CCCTCATCCT TCAACCTGTA   
  
  
+ CCTTCATATA AAAAAAAGGA CAATCCGGTG CACAAGCATC CCGCATTCAC GCAAGGTCTG GGGAAGGGCC   
  
  
+ GCACCCCAAA GGGTGAATTG TAGGCAGCCT AACCTGACTT TGTCAGTGGC TAATTCCACG ATTCGAACCC   
  
  
+ ATGATTTTTT GGTTACACGG GAACAACCTT ACCATTGCTC AAAGGCTCCC CTTCTTCAAC CTCTACCTTC   
  
  
+ ATATATGAAT GAAAATCCTT TGGCTAAGAA TTGAAAAGGA AATATGTGGT TATTGGGTTT GGGTTCTTAT   
  
  
+ TCCGTTGGTT GCCCCTTCAC ATTGAATGCA TCCTCTCGCT AAGAACTGAA AAGGACATAT GGGGATGTAT   
  
  
+ AGGTCAAATT TGACTCAATG ATAACTGAGG ATACAAGAAT TCTAGAAGTC TTTATCAGGG GAATTTTTTC   
  
  
+ TTTTTTCCAC TTGTGACTTT TTGATGCCTT GCTTTTTACT CTTCATTGAA TGAGTACCCT TCTTGACAGA   
  
  
+ TTCTTCCTCC CTTTTTTCTT AATTTCTTCT TGTAGTGGAA TATAAGTATG CTTCTAGGTA GTTGGATGCC   
  
  
+ ATGGAATCTC ATCCCTGTGT GTGCATTCTT CCCCTCTCCC GCCGCCCCCC CCCCCCCAAA AAGAAACCAA   
  
  
+ AAAAAAAAAA AAAATGGATG ATTCTTAGAA CTGACCATTT TTCCTTCCTC CTCCCATTTT ATTTTTTTCA   
  
  
+ GGCTGTTTTT CTTCTACCTG TTGCTTTAGC ATCACTCATA AAAACAAGGT GCAAGCGCAT GACATCGGGT   
  
  
+ ACTGAGCTTC TGAGTTTACA TGCATCGTCA TGAAATTCGG TAGACTTTGA AGTAAAGTTT CCCCATCGTA   
  
  
+ ACTGGTGGTG CATACCTGTC TTCACGCCCA TCAGTAGAGT GGTGAAGTGA AGCAATGTCG AATAGTTTGT   
  
  
+ ATTACGAGCC CAAGAGAGAA ACTGATGCAT ATTTTATGCC TCAATGTCAA TCCTTGAACC CCCAGCTCGA   
  
  
+ TTACAACACC ATTGACTATG GAGCTTTTAT GTGCTCAAAA GTTTTCCTGG GTCAGTACTG CACTCTGGAA   
  
  
+ TCATCCTCAG GAACTGGGAC TTGTCCGGTG TCTAGCTCCA CATCAACTCT CAGCTTCTCA TCCAATGGTA   
  
  
+ GCCCTGGATC ACAGCTTGAT TCCAACTCTT ATCCTTCTGA TCAAAATTGC TCTCCTGATA ATGCCAATTC   
  
  
+ CTCATCTTTA AGTCATTCCT GCGTCACAGA TGATGTGGAT GACCTGAAAT ACAGGCTGAG AGAGTTGGAA   
  
  
+ ACAGTGATGT TGGGACCTGA TCCCGATTTT GTCTACGGCA ATAGCAACCA CACATTTGGG GTTGGGATCC   
  
  
+ ATGTAGTGTC ATCAGAGTTT GACAGCTGGG GGAAAGTGAT GGAGATGATC GCTCGAAAGG ATTTGAAACT   
  
  
+ GGTGCTAATT GCCTGTGCAA GAGCAGTTGC TGAAAATGAT CAGTTGTTGG CCCAGTGGCT GATGGATGAA   
  
  
+ TTGAGACAGA TGGTTTCAGT TTCTGGTGAA CCAATTCAAA GATTGGGTGC TTATATGTTG GAAGGGCTCG   
  
  
+ TAGCGAGGCA GGCCTCCTCA GGTAGCTCCA TTTATAAAGC ATTGAGATGC AAGGAACCCG CGAGTGCTGA   
  
  
+ CCTTTTATCT TACATGCACA TACTCTTTGA GGTTTGCCCA TACATCAAAT TTGGCTACAT GTCAGCAAAT   
  
  
+ GGTGCCATTG CAGAAGCAAT GAAGGATGAA AAGAGAGTCC ACATTGTCGA TTTCCAAATT GGGCAGGGAA   
  
  
+ GCCAATGGGT GACCCTAATC CAGGCATTTG CAGCCCGGCC AGGGGGTCCA CCCCACATTC GCATTACCGG   
  
  
+ TATCGATGAC TCCTACTCTG CATATGCTCG TGGAGGTGGG TTGAACATTG TGGGCCATAG GCTCTTGAGG   
  
  
+ TTGGCTCAGT CATTTAAGGT CCCATTTGAG TTCAACGCAG TTGACGTCCC GGCTTGTCAA GTCCTGCTTA   
  
  
+ AAGATCTCGG CATTCAACAT GGTGAGGCCT TGGCTGTGAA CTTTGCCTTC ATTCTTCACC ACATGCCCGA   
  
  
+ TGAGAGCGTG AGCACAGAGA ATCACCGGGA TCGTCTTCTG AGGGTGGTAA AAGGGCTGAA GCCAAAAGTG   
  
  
+ GTAACGTTAG TGGAGCAAGA GTGTAACACA AACTCTGCTG CTTTCTTGCC TCGCTTTGTT GAGACATTGG   
  
  
+ AATACTACAC AGCGATGTTT GAGTCCATGG ATGTGACTTT GCCTAGGGAT CATAAAGACC GGATCAATGT   
  
  
+ TGAGCAGCAT TGTCTTGCTC GAGATGTTGT GAACTTGGTT GCGTGTGAAG GAGCTGAGAG GGTGGAGCGG   
  
  
+ CATGAGCTCC TTGGGAAATG GAGGTCGCGG TTCACAATGG CAGGGTTTAA GCCATACCCT TTAAGCACTC   
  
  
+ TGGTAAACAA CACAATAAGA ACTTTGTTGA AGAACTACTG TGATAGGTAT GGGCTAGAGG AGAGGAATGG   
  
  
+ AGGTCTTTAT CTTGGGTGGA TGAATAGAGA TTTGGTTGCT TCGAGTGCCT GGCAGTGTAG TAATTA  

- +Up\_Stream \_Len000GTAAAA TCATTATTTA AGGGTACACT CCTGGTGACG GATGACTAAG GCTTACCGTA   
  
  
- AAATCATTAT TTGAGGGTAC ACTCCTGGTG AACTACTCCT TAAGTCAAAA TATTAGGTGT CCACAGTTTC   
  
  
- GGATAAGGAT GAGAGTGGTG AGACGATAGA GAGGGGGGGG GGGGGGGGGC GGGGGGAGTT TTAGACGGAG   
  
  
- GAGGCAAGAG AGGTGTCGTC TAAGGAGTTA GTCCTTTCCA TACAGTCTCG AGTTTTGCTA AGTTGTAGTT   
  
  
- TTACCCTATT AAATAAGACG AAAGTCAAGA CCCAATAACA GTATACAAGT AAGTCAAGTC ACAAAGATCG   
  
  
- TACTAACGAA AAACTACGGA AGAAAAAACA AGACCCAACC TGCTTGAGAC AGAAAAGACA CGTATCTAAA   
  
  
- CTCGAGGCTC GAGTTAAAAA CCCTAAAAAA ATCGACTGAA AAGACGAACC ACAACACTTT AACACTACCC   
  
  
- AAAACCGAAA CAATTTGAAC AAATATTGAA AAAGACAGTT TTGAAATATC ATTTAACAAC GTTAAACTCT   
  
  
- TAATACGTGA GTCACCGAGA CACTAAATAG CAGGCGACCG TTTAAAAGAC TACTTTCCTC TACTAAATAA   
  
  
- TTAAAAAGTC CGTACCAGTT TAAAAGATCG GAAAGTACAT GTAACCAACC ACTATCCGAC TATCGATTTA   
  
  
- ATACAGAGTA AAGAGATTTA AATAACGGTC TATACTTTAA CCACCTTGTC AAATTCCCAA AACCAGCAGA   
  
  
- AAAAACGGGA AAAAAAACCC AGGGGGGGGG CGGGCCCCCC CTTGGGCCTC CCGTCTTTTT CATTGTATTG   
  
  
- AATTTTAATA GTAACTGCAT CAGGAGGTTG GTATTACTTT CGTTGACTTT ATTCTCGTAC GATTCGATCT   
  
  
- CTCATCCGAC GGAACAGTCG GAGGGAAAAC TGTACTCTTT CTACCCTCTT CAAATTTTAT TTCAAGGAGC   
  
  
- AGAAACGTAG GGCCTGGTGT TAAGCCTTGA CCCACTCCTG GAGGAATCCG AACATTTGTG TTCAAAGCTT   
  
  
- GAATCTAAAC GCATGTACAG TTACGAAATG ACCTATTCGA ATCGATTGTG GGGAGTAGGA AGTTGGACAT   
  
  
- GGAAGTATAT TTTTTTTCCT GTTAGGCCAC GTGTTCGTAG GGCGTAAGTG CGTTCCAGAC CCCTTCCCGG   
  
  
- CGTGGGGTTT CCCACTTAAC ATCCGTCGGA TTGGACTGAA ACAGTCACCG ATTAAGGTGC TAAGCTTGGG   
  
  
- TACTAAAAAA CCAATGTGCC CTTGTTGGAA TGGTAACGAG TTTCCGAGGG GAAGAAGTTG GAGATGGAAG   
  
  
- TATATACTTA CTTTTAGGAA ACCGATTCTT AACTTTTCCT TTATACACCA ATAACCCAAA CCCAAGAATA   
  
  
- AGGCAACCAA CGGGGAAGTG TAACTTACGT AGGAGAGCGA TTCTTGACTT TTCCTGTATA CCCCTACATA   
  
  
- TCCAGTTTAA ACTGAGTTAC TATTGACTCC TATGTTCTTA AGATCTTCAG AAATAGTCCC CTTAAAAAAG   
  
  
- AAAAAAGGTG AACACTGAAA AACTACGGAA CGAAAAATGA GAAGTAACTT ACTCATGGGA AGAACTGTCT   
  
  
- AAGAAGGAGG GAAAAAAGAA TTAAAGAAGA ACATCACCTT ATATTCATAC GAAGATCCAT CAACCTACGG   
  
  
- TACCTTAGAG TAGGGACACA CACGTAAGAA GGGGAGAGGG CGGCGGGGGG GGGGGGGTTT TTCTTTGGTT   
  
  
- TTTTTTTTTT TTTTACCTAC TAAGAATCTT GACTGGTAAA AAGGAAGGAG GAGGGTAAAA TAAAAAAAGT   
  
  
- CCGACAAAAA GAAGATGGAC AACGAAATCG TAGTGAGTAT TTTTGTTCCA CGTTCGCGTA CTGTAGCCCA   
  
  
- TGACTCGAAG ACTCAAATGT ACGTAGCAGT ACTTTAAGCC ATCTGAAACT TCATTTCAAA GGGGTAGCAT   
  
  
- TGACCACCAC GTATGGACAG AAGTGCGGGT AGTCATCTCA CCACTTCACT TCGTTACAGC TTATCAAACA   
  
  
- TAATGCTCGG GTTCTCTCTT TGACTACGTA TAAAATACGG AGTTACAGTT AGGAACTTGG GGGTCGAGCT   
  
  
- AATGTTGTGG TAACTGATAC CTCGAAAATA CACGAGTTTT CAAAAGGACC CAGTCATGAC GTGAGACCTT   
  
  
- AGTAGGAGTC CTTGACCCTG AACAGGCCAC AGATCGAGGT GTAGTTGAGA GTCGAAGAGT AGGTTACCAT   
  
  
- CGGGACCTAG TGTCGAACTA AGGTTGAGAA TAGGAAGACT AGTTTTAACG AGAGGACTAT TACGGTTAAG   
  
  
- GAGTAGAAAT TCAGTAAGGA CGCAGTGTCT ACTACACCTA CTGGACTTTA TGTCCGACTC TCTCAACCTT   
  
  
- TGTCACTACA ACCCTGGACT AGGGCTAAAA CAGATGCCGT TATCGTTGGT GTGTAAACCC CAACCCTAGG   
  
  
- TACATCACAG TAGTCTCAAA CTGTCGACCC CCTTTCACTA CCTCTACTAG CGAGCTTTCC TAAACTTTGA   
  
  
- CCACGATTAA CGGACACGTT CTCGTCAACG ACTTTTACTA GTCAACAACC GGGTCACCGA CTACCTACTT   
  
  
- AACTCTGTCT ACCAAAGTCA AAGACCACTT GGTTAAGTTT CTAACCCACG AATATACAAC CTTCCCGAGC   
  
  
- ATCGCTCCGT CCGGAGGAGT CCATCGAGGT AAATATTTCG TAACTCTACG TTCCTTGGGC GCTCACGACT   
  
  
- GGAAAATAGA ATGTACGTGT ATGAGAAACT CCAAACGGGT ATGTAGTTTA AACCGATGTA CAGTCGTTTA   
  
  
- CCACGGTAAC GTCTTCGTTA CTTCCTACTT TTCTCTCAGG TGTAACAGCT AAAGGTTTAA CCCGTCCCTT   
  
  
- CGGTTACCCA CTGGGATTAG GTCCGTAAAC GTCGGGCCGG TCCCCCAGGT GGGGTGTAAG CGTAATGGCC   
  
  
- ATAGCTACTG AGGATGAGAC GTATACGAGC ACCTCCACCC AACTTGTAAC ACCCGGTATC CGAGAACTCC   
  
  
- AACCGAGTCA GTAAATTCCA GGGTAAACTC AAGTTGCGTC AACTGCAGGG CCGAACAGTT CAGGACGAAT   
  
  
- TTCTAGAGCC GTAAGTTGTA CCACTCCGGA ACCGACACTT GAAACGGAAG TAAGAAGTGG TGTACGGGCT   
  
  
- ACTCTCGCAC TCGTGTCTCT TAGTGGCCCT AGCAGAAGAC TCCCACCATT TTCCCGACTT CGGTTTTCAC   
  
  
- CATTGCAATC ACCTCGTTCT CACATTGTGT TTGAGACGAC GAAAGAACGG AGCGAAACAA CTCTGTAACC   
  
  
- TTATGATGTG TCGCTACAAA CTCAGGTACC TACACTGAAA CGGATCCCTA GTATTTCTGG CCTAGTTACA   
  
  
- ACTCGTCGTA ACAGAACGAG CTCTACAACA CTTGAACCAA CGCACACTTC CTCGACTCTC CCACCTCGCC   
  
  
- GTACTCGAGG AACCCTTTAC CTCCAGCGCC AAGTGTTACC GTCCCAAATT CGGTATGGGA AATTCGTGAG   
  
  
- ACCATTTGTT GTGTTATTCT TGAAACAACT TCTTGATGAC ACTATCCATA CCCGATCTCC TCTCCTTACC   
  
  
- TCCAGAAATA GAACCCACCT ACTTATCTCT AAACCAACGA AGCTCACGGA CCGTCACATC ATTAAT

+     CAAT-box

| Site Name | Organism | Position | Strand | Matrix score. | sequence | function |
| --- | --- | --- | --- | --- | --- | --- |
| CAAT-box | Nicotiana glutinosa | 3517 | + | 4 | CAAT |  |
| CAAT-box | Arabidopsis thaliana | 3290 | - | 5 | CCAAT | common cis-acting element in promoter and enhancer regions |
| CAAT-box | Nicotiana glutinosa | 3359 | + | 4 | CAAT |  |
| CAAT-box | Arabidopsis thaliana | 2876 | + | 5 | CCAAT | common cis-acting element in promoter and enhancer regions |
| CAAT-box | Arabidopsis thaliana | 2862 | - | 5 | CCAAT | common cis-acting element in promoter and enhancer regions |
| CAAT-box | Arabidopsis thaliana | 2860 | - | 8 | CCCAATTT | common cis-acting element in promoter and enhancer regions |
| CAAT-box | Nicotiana glutinosa | 2847 | - | 4 | CAAT |  |
| CAAT-box | Nicotiana glutinosa | 2821 | + | 4 | CAAT |  |
| CAAT-box | Nicotiana glutinosa | 2705 | - | 4 | CAAT |  |
| CAAT-box | Pisum sativum | 3038 | - | 5 | CAAAT | common cis-acting element in promoter and enhancer regions |
| CAAT-box | Nicotiana glutinosa | 2991 | - | 4 | CAAT |  |
| CAAT-box | Arabidopsis thaliana | 2636 | - | 5 | CCAAT | common cis-acting element in promoter and enhancer regions |
| CAAT-box | Arabidopsis thaliana | 2625 | + | 5 | CCAAT | common cis-acting element in promoter and enhancer regions |
| CAAT-box | Nicotiana glutinosa | 2626 | + | 4 | CAAT |  |
| CAAT-box | Nicotiana glutinosa | 2532 | - | 4 | CAAT |  |
| CAAT-box | Nicotiana glutinosa | 2594 | - | 4 | CAAT |  |
| CAAT-box | Nicotiana glutinosa | 2309 | + | 4 | CAAT |  |
| CAAT-box | Pisum sativum | 2438 | - | 5 | CAAAT | common cis-acting element in promoter and enhancer regions |
| CAAT-box | Nicotiana glutinosa | 2423 | + | 4 | CAAT |  |
| CAAT-box | Pisum sativum | 2515 | - | 5 | CAAAT | common cis-acting element in promoter and enhancer regions |
| CAAT-box | Arabidopsis thaliana | 2308 | + | 5 | CCAAT | common cis-acting element in promoter and enhancer regions |
| CAAT-box | Nicotiana glutinosa | 2290 | - | 4 | CAAT |  |
| CAAT-box | Pisum sativum | 2780 | + | 5 | CAAAT | common cis-acting element in promoter and enhancer regions |
| CAAT-box | Pisum sativum | 2800 | + | 5 | CAAAT | common cis-acting element in promoter and enhancer regions |
| CAAT-box | Nicotiana glutinosa | 2811 | - | 4 | CAAT |  |
| CAAT-box | Pisum sativum | 2859 | + | 5 | CAAAT | common cis-acting element in promoter and enhancer regions |
| CAAT-box | Pisum sativum | 2783 | - | 5 | CAAAT | common cis-acting element in promoter and enhancer regions |
| CAAT-box | Pisum sativum | 2900 | - | 5 | CAAAT | common cis-acting element in promoter and enhancer regions |
| CAAT-box | Nicotiana glutinosa | 2877 | + | 4 | CAAT |  |
| CAAT-box | Pisum sativum | 3604 | - | 5 | CAAAT | common cis-acting element in promoter and enhancer regions |
| CAAT-box | Nicotiana glutinosa | 3469 | + | 4 | CAAT |  |
| CAAT-box | Nicotiana glutinosa | 3373 | - | 4 | CAAT |  |
| CAAT-box | Arabidopsis thaliana | 2236 | + | 5 | CCAAT | common cis-acting element in promoter and enhancer regions |
| CAAT-box | Nicotiana glutinosa | 2237 | + | 4 | CAAT |  |
| CAAT-box | Nicotiana glutinosa | 2082 | + | 4 | CAAT |  |
| CAAT-box | Nicotiana glutinosa | 2115 | - | 4 | CAAT |  |
| CAAT-box | Nicotiana glutinosa | 2017 | + | 4 | CAAT |  |
| CAAT-box | Nicotiana glutinosa | 2076 | + | 4 | CAAT |  |
| CAAT-box | Nicotiana glutinosa | 1589 | - | 4 | CAAT |  |
| CAAT-box | Nicotiana glutinosa | 1425 | - | 4 | CAAT |  |
| CAAT-box | Nicotiana glutinosa | 1490 | + | 4 | CAAT |  |
| CAAT-box | Arabidopsis thaliana | 1386 | - | 5 | CCAAT | common cis-acting element in promoter and enhancer regions |
| CAAT-box | Nicotiana glutinosa | 1364 | - | 4 | CAAT |  |
| CAAT-box | Nicotiana glutinosa | 1298 | - | 4 | CAAT |  |
| CAAT-box | Nicotiana glutinosa | 1211 | - | 4 | CAAT |  |
| CAAT-box | Nicotiana glutinosa | 1074 | + | 4 | CAAT |  |
| CAAT-box | Nicotiana glutinosa | 1145 | + | 4 | CAAT |  |
| CAAT-box | Nicotiana glutinosa | 1003 | + | 4 | CAAT |  |
| CAAT-box | Nicotiana glutinosa | 856 | - | 4 | CAAT |  |
| CAAT-box | Arabidopsis thaliana | 742 | - | 5 | CCAAT | common cis-acting element in promoter and enhancer regions |
| CAAT-box | Nicotiana glutinosa | 727 | - | 4 | CAAT |  |
| CAAT-box | Arabidopsis thaliana | 676 | - | 5 | CCAAT | common cis-acting element in promoter and enhancer regions |
| CAAT-box | Nicotiana glutinosa | 320 | - | 4 | CAAT |  |
| CAAT-box | Pisum sativum | 652 | + | 5 | CAAAT | common cis-acting element in promoter and enhancer regions |
| CAAT-box | Nicotiana glutinosa | 484 | - | 4 | CAAT |  |
| CAAT-box | Nicotiana glutinosa | 437 | + | 4 | CAAT |  |
| CAAT-box | Nicotiana glutinosa | 358 | - | 4 | CAAT |  |
| CAAT-box | Nicotiana glutinosa | 241 | + | 4 | CAAT |  |
| CAAT-box | Pisum sativum | 1060 | - | 5 | CAAAT | common cis-acting element in promoter and enhancer regions |
| CAAT-box | Pisum sativum | 421 | - | 5 | CAAAT | common cis-acting element in promoter and enhancer regions |
| CAAT-box | Nicotiana glutinosa | 555 | + | 4 | CAAT |  |
| CAAT-box | Pisum sativum | 557 | - | 5 | CAAAT | common cis-acting element in promoter and enhancer regions |
| CAAT-box | Nicotiana glutinosa | 548 | - | 4 | CAAT |  |
| CAAT-box | Pisum sativum | 604 | + | 5 | CAAAT | common cis-acting element in promoter and enhancer regions |
| CAAT-box | Pisum sativum | 1479 | + | 5 | CAAAT | common cis-acting element in promoter and enhancer regions |
| CAAT-box | Pisum sativum | 1482 | - | 5 | CAAAT | common cis-acting element in promoter and enhancer regions |

>HU01G01391.1   
+ +Up\_Stream \_Len000CATTTT AGTAATAAAT TCCCATGTGA GGACCACTGC CTACTGATTC CGAATGGCAT   
  
  
+ TTTAGTAATA AACTCCCATG TGAGGACCAC TTGATGAGGA ATTCAGTTTT ATAATCCACA GGTGTCAAAG   
  
  
+ CCTATTCCTA CTCTCACCAC TCTGCTATCT CTCCCCCCCC CCCCCCCCCG CCCCCCTCAA AATCTGCCTC   
  
  
+ CTCCGTTCTC TCCACAGCAG ATTCCTCAAT CAGGAAAGGT ATGTCAGAGC TCAAAACGAT TCAACATCAA   
  
  
+ AATGGGATAA TTTATTCTGC TTTCAGTTCT GGGTTATTGT CATATGTTCA TTCAGTTCAG TGTTTCTAGC   
  
  
+ ATGATTGCTT TTTGATGCCT TCTTTTTTGT TCTGGGTTGG ACGAACTCTG TCTTTTCTGT GCATAGATTT   
  
  
+ GAGCTCCGAG CTCAATTTTT GGGATTTTTT TAGCTGACTT TTCTGCTTGG TGTTGTGAAA TTGTGATGGG   
  
  
+ TTTTGGCTTT GTTAAACTTG TTTATAACTT TTTCTGTCAA AACTTTATAG TAAATTGTTG CAATTTGAGA   
  
  
+ ATTATGCACT CAGTGGCTCT GTGATTTATC GTCCGCTGGC AAATTTTCTG ATGAAAGGAG ATGATTTATT   
  
  
+ AATTTTTCAG GCATGGTCAA ATTTTCTAGC CTTTCATGTA CATTGGTTGG TGATAGGCTG ATAGCTAAAT   
  
  
+ TATGTCTCAT TTCTCTAAAT TTATTGCCAG ATATGAAATT GGTGGAACAG TTTAAGGGTT TTGGTCGTCT   
  
  
+ TTTTTGCCCT TTTTTTTGGG TCCCCCCCCC GCCCGGGGGG GAACCCGGAG GGCAGAAAAA GTAACATAAC   
  
  
+ TTAAAATTAT CATTGACGTA GTCCTCCAAC CATAATGAAA GCAACTGAAA TAAGAGCATG CTAAGCTAGA   
  
  
+ GAGTAGGCTG CCTTGTCAGC CTCCCTTTTG ACATGAGAAA GATGGGAGAA GTTTAAAATA AAGTTCCTCG   
  
  
+ TCTTTGCATC CCGGACCACA ATTCGGAACT GGGTGAGGAC CTCCTTAGGC TTGTAAACAC AAGTTTCGAA   
  
  
+ CTTAGATTTG CGTACATGTC AATGCTTTAC TGGATAAGCT TAGCTAACAC CCCTCATCCT TCAACCTGTA   
  
  
+ CCTTCATATA AAAAAAAGGA CAATCCGGTG CACAAGCATC CCGCATTCAC GCAAGGTCTG GGGAAGGGCC   
  
  
+ GCACCCCAAA GGGTGAATTG TAGGCAGCCT AACCTGACTT TGTCAGTGGC TAATTCCACG ATTCGAACCC   
  
  
+ ATGATTTTTT GGTTACACGG GAACAACCTT ACCATTGCTC AAAGGCTCCC CTTCTTCAAC CTCTACCTTC   
  
  
+ ATATATGAAT GAAAATCCTT TGGCTAAGAA TTGAAAAGGA AATATGTGGT TATTGGGTTT GGGTTCTTAT   
  
  
+ TCCGTTGGTT GCCCCTTCAC ATTGAATGCA TCCTCTCGCT AAGAACTGAA AAGGACATAT GGGGATGTAT   
  
  
+ AGGTCAAATT TGACTCAATG ATAACTGAGG ATACAAGAAT TCTAGAAGTC TTTATCAGGG GAATTTTTTC   
  
  
+ TTTTTTCCAC TTGTGACTTT TTGATGCCTT GCTTTTTACT CTTCATTGAA TGAGTACCCT TCTTGACAGA   
  
  
+ TTCTTCCTCC CTTTTTTCTT AATTTCTTCT TGTAGTGGAA TATAAGTATG CTTCTAGGTA GTTGGATGCC   
  
  
+ ATGGAATCTC ATCCCTGTGT GTGCATTCTT CCCCTCTCCC GCCGCCCCCC CCCCCCCAAA AAGAAACCAA   
  
  
+ AAAAAAAAAA AAAATGGATG ATTCTTAGAA CTGACCATTT TTCCTTCCTC CTCCCATTTT ATTTTTTTCA   
  
  
+ GGCTGTTTTT CTTCTACCTG TTGCTTTAGC ATCACTCATA AAAACAAGGT GCAAGCGCAT GACATCGGGT   
  
  
+ ACTGAGCTTC TGAGTTTACA TGCATCGTCA TGAAATTCGG TAGACTTTGA AGTAAAGTTT CCCCATCGTA   
  
  
+ ACTGGTGGTG CATACCTGTC TTCACGCCCA TCAGTAGAGT GGTGAAGTGA AGCAATGTCG AATAGTTTGT   
  
  
+ ATTACGAGCC CAAGAGAGAA ACTGATGCAT ATTTTATGCC TCAATGTCAA TCCTTGAACC CCCAGCTCGA   
  
  
+ TTACAACACC ATTGACTATG GAGCTTTTAT GTGCTCAAAA GTTTTCCTGG GTCAGTACTG CACTCTGGAA   
  
  
+ TCATCCTCAG GAACTGGGAC TTGTCCGGTG TCTAGCTCCA CATCAACTCT CAGCTTCTCA TCCAATGGTA   
  
  
+ GCCCTGGATC ACAGCTTGAT TCCAACTCTT ATCCTTCTGA TCAAAATTGC TCTCCTGATA ATGCCAATTC   
  
  
+ CTCATCTTTA AGTCATTCCT GCGTCACAGA TGATGTGGAT GACCTGAAAT ACAGGCTGAG AGAGTTGGAA   
  
  
+ ACAGTGATGT TGGGACCTGA TCCCGATTTT GTCTACGGCA ATAGCAACCA CACATTTGGG GTTGGGATCC   
  
  
+ ATGTAGTGTC ATCAGAGTTT GACAGCTGGG GGAAAGTGAT GGAGATGATC GCTCGAAAGG ATTTGAAACT   
  
  
+ GGTGCTAATT GCCTGTGCAA GAGCAGTTGC TGAAAATGAT CAGTTGTTGG CCCAGTGGCT GATGGATGAA   
  
  
+ TTGAGACAGA TGGTTTCAGT TTCTGGTGAA CCAATTCAAA GATTGGGTGC TTATATGTTG GAAGGGCTCG   
  
  
+ TAGCGAGGCA GGCCTCCTCA GGTAGCTCCA TTTATAAAGC ATTGAGATGC AAGGAACCCG CGAGTGCTGA   
  
  
+ CCTTTTATCT TACATGCACA TACTCTTTGA GGTTTGCCCA TACATCAAAT TTGGCTACAT GTCAGCAAAT   
  
  
+ GGTGCCATTG CAGAAGCAAT GAAGGATGAA AAGAGAGTCC ACATTGTCGA TTTCCAAATT GGGCAGGGAA   
  
  
+ GCCAATGGGT GACCCTAATC CAGGCATTTG CAGCCCGGCC AGGGGGTCCA CCCCACATTC GCATTACCGG   
  
  
+ TATCGATGAC TCCTACTCTG CATATGCTCG TGGAGGTGGG TTGAACATTG TGGGCCATAG GCTCTTGAGG   
  
  
+ TTGGCTCAGT CATTTAAGGT CCCATTTGAG TTCAACGCAG TTGACGTCCC GGCTTGTCAA GTCCTGCTTA   
  
  
+ AAGATCTCGG CATTCAACAT GGTGAGGCCT TGGCTGTGAA CTTTGCCTTC ATTCTTCACC ACATGCCCGA   
  
  
+ TGAGAGCGTG AGCACAGAGA ATCACCGGGA TCGTCTTCTG AGGGTGGTAA AAGGGCTGAA GCCAAAAGTG   
  
  
+ GTAACGTTAG TGGAGCAAGA GTGTAACACA AACTCTGCTG CTTTCTTGCC TCGCTTTGTT GAGACATTGG   
  
  
+ AATACTACAC AGCGATGTTT GAGTCCATGG ATGTGACTTT GCCTAGGGAT CATAAAGACC GGATCAATGT   
  
  
+ TGAGCAGCAT TGTCTTGCTC GAGATGTTGT GAACTTGGTT GCGTGTGAAG GAGCTGAGAG GGTGGAGCGG   
  
  
+ CATGAGCTCC TTGGGAAATG GAGGTCGCGG TTCACAATGG CAGGGTTTAA GCCATACCCT TTAAGCACTC   
  
  
+ TGGTAAACAA CACAATAAGA ACTTTGTTGA AGAACTACTG TGATAGGTAT GGGCTAGAGG AGAGGAATGG   
  
  
+ AGGTCTTTAT CTTGGGTGGA TGAATAGAGA TTTGGTTGCT TCGAGTGCCT GGCAGTGTAG TAATTA  

- +Up\_Stream \_Len000GTAAAA TCATTATTTA AGGGTACACT CCTGGTGACG GATGACTAAG GCTTACCGTA   
  
  
- AAATCATTAT TTGAGGGTAC ACTCCTGGTG AACTACTCCT TAAGTCAAAA TATTAGGTGT CCACAGTTTC   
  
  
- GGATAAGGAT GAGAGTGGTG AGACGATAGA GAGGGGGGGG GGGGGGGGGC GGGGGGAGTT TTAGACGGAG   
  
  
- GAGGCAAGAG AGGTGTCGTC TAAGGAGTTA GTCCTTTCCA TACAGTCTCG AGTTTTGCTA AGTTGTAGTT   
  
  
- TTACCCTATT AAATAAGACG AAAGTCAAGA CCCAATAACA GTATACAAGT AAGTCAAGTC ACAAAGATCG   
  
  
- TACTAACGAA AAACTACGGA AGAAAAAACA AGACCCAACC TGCTTGAGAC AGAAAAGACA CGTATCTAAA   
  
  
- CTCGAGGCTC GAGTTAAAAA CCCTAAAAAA ATCGACTGAA AAGACGAACC ACAACACTTT AACACTACCC   
  
  
- AAAACCGAAA CAATTTGAAC AAATATTGAA AAAGACAGTT TTGAAATATC ATTTAACAAC GTTAAACTCT   
  
  
- TAATACGTGA GTCACCGAGA CACTAAATAG CAGGCGACCG TTTAAAAGAC TACTTTCCTC TACTAAATAA   
  
  
- TTAAAAAGTC CGTACCAGTT TAAAAGATCG GAAAGTACAT GTAACCAACC ACTATCCGAC TATCGATTTA   
  
  
- ATACAGAGTA AAGAGATTTA AATAACGGTC TATACTTTAA CCACCTTGTC AAATTCCCAA AACCAGCAGA   
  
  
- AAAAACGGGA AAAAAAACCC AGGGGGGGGG CGGGCCCCCC CTTGGGCCTC CCGTCTTTTT CATTGTATTG   
  
  
- AATTTTAATA GTAACTGCAT CAGGAGGTTG GTATTACTTT CGTTGACTTT ATTCTCGTAC GATTCGATCT   
  
  
- CTCATCCGAC GGAACAGTCG GAGGGAAAAC TGTACTCTTT CTACCCTCTT CAAATTTTAT TTCAAGGAGC   
  
  
- AGAAACGTAG GGCCTGGTGT TAAGCCTTGA CCCACTCCTG GAGGAATCCG AACATTTGTG TTCAAAGCTT   
  
  
- GAATCTAAAC GCATGTACAG TTACGAAATG ACCTATTCGA ATCGATTGTG GGGAGTAGGA AGTTGGACAT   
  
  
- GGAAGTATAT TTTTTTTCCT GTTAGGCCAC GTGTTCGTAG GGCGTAAGTG CGTTCCAGAC CCCTTCCCGG   
  
  
- CGTGGGGTTT CCCACTTAAC ATCCGTCGGA TTGGACTGAA ACAGTCACCG ATTAAGGTGC TAAGCTTGGG   
  
  
- TACTAAAAAA CCAATGTGCC CTTGTTGGAA TGGTAACGAG TTTCCGAGGG GAAGAAGTTG GAGATGGAAG   
  
  
- TATATACTTA CTTTTAGGAA ACCGATTCTT AACTTTTCCT TTATACACCA ATAACCCAAA CCCAAGAATA   
  
  
- AGGCAACCAA CGGGGAAGTG TAACTTACGT AGGAGAGCGA TTCTTGACTT TTCCTGTATA CCCCTACATA   
  
  
- TCCAGTTTAA ACTGAGTTAC TATTGACTCC TATGTTCTTA AGATCTTCAG AAATAGTCCC CTTAAAAAAG   
  
  
- AAAAAAGGTG AACACTGAAA AACTACGGAA CGAAAAATGA GAAGTAACTT ACTCATGGGA AGAACTGTCT   
  
  
- AAGAAGGAGG GAAAAAAGAA TTAAAGAAGA ACATCACCTT ATATTCATAC GAAGATCCAT CAACCTACGG   
  
  
- TACCTTAGAG TAGGGACACA CACGTAAGAA GGGGAGAGGG CGGCGGGGGG GGGGGGGTTT TTCTTTGGTT   
  
  
- TTTTTTTTTT TTTTACCTAC TAAGAATCTT GACTGGTAAA AAGGAAGGAG GAGGGTAAAA TAAAAAAAGT   
  
  
- CCGACAAAAA GAAGATGGAC AACGAAATCG TAGTGAGTAT TTTTGTTCCA CGTTCGCGTA CTGTAGCCCA   
  
  
- TGACTCGAAG ACTCAAATGT ACGTAGCAGT ACTTTAAGCC ATCTGAAACT TCATTTCAAA GGGGTAGCAT   
  
  
- TGACCACCAC GTATGGACAG AAGTGCGGGT AGTCATCTCA CCACTTCACT TCGTTACAGC TTATCAAACA   
  
  
- TAATGCTCGG GTTCTCTCTT TGACTACGTA TAAAATACGG AGTTACAGTT AGGAACTTGG GGGTCGAGCT   
  
  
- AATGTTGTGG TAACTGATAC CTCGAAAATA CACGAGTTTT CAAAAGGACC CAGTCATGAC GTGAGACCTT   
  
  
- AGTAGGAGTC CTTGACCCTG AACAGGCCAC AGATCGAGGT GTAGTTGAGA GTCGAAGAGT AGGTTACCAT   
  
  
- CGGGACCTAG TGTCGAACTA AGGTTGAGAA TAGGAAGACT AGTTTTAACG AGAGGACTAT TACGGTTAAG   
  
  
- GAGTAGAAAT TCAGTAAGGA CGCAGTGTCT ACTACACCTA CTGGACTTTA TGTCCGACTC TCTCAACCTT   
  
  
- TGTCACTACA ACCCTGGACT AGGGCTAAAA CAGATGCCGT TATCGTTGGT GTGTAAACCC CAACCCTAGG   
  
  
- TACATCACAG TAGTCTCAAA CTGTCGACCC CCTTTCACTA CCTCTACTAG CGAGCTTTCC TAAACTTTGA   
  
  
- CCACGATTAA CGGACACGTT CTCGTCAACG ACTTTTACTA GTCAACAACC GGGTCACCGA CTACCTACTT   
  
  
- AACTCTGTCT ACCAAAGTCA AAGACCACTT GGTTAAGTTT CTAACCCACG AATATACAAC CTTCCCGAGC   
  
  
- ATCGCTCCGT CCGGAGGAGT CCATCGAGGT AAATATTTCG TAACTCTACG TTCCTTGGGC GCTCACGACT   
  
  
- GGAAAATAGA ATGTACGTGT ATGAGAAACT CCAAACGGGT ATGTAGTTTA AACCGATGTA CAGTCGTTTA   
  
  
- CCACGGTAAC GTCTTCGTTA CTTCCTACTT TTCTCTCAGG TGTAACAGCT AAAGGTTTAA CCCGTCCCTT   
  
  
- CGGTTACCCA CTGGGATTAG GTCCGTAAAC GTCGGGCCGG TCCCCCAGGT GGGGTGTAAG CGTAATGGCC   
  
  
- ATAGCTACTG AGGATGAGAC GTATACGAGC ACCTCCACCC AACTTGTAAC ACCCGGTATC CGAGAACTCC   
  
  
- AACCGAGTCA GTAAATTCCA GGGTAAACTC AAGTTGCGTC AACTGCAGGG CCGAACAGTT CAGGACGAAT   
  
  
- TTCTAGAGCC GTAAGTTGTA CCACTCCGGA ACCGACACTT GAAACGGAAG TAAGAAGTGG TGTACGGGCT   
  
  
- ACTCTCGCAC TCGTGTCTCT TAGTGGCCCT AGCAGAAGAC TCCCACCATT TTCCCGACTT CGGTTTTCAC   
  
  
- CATTGCAATC ACCTCGTTCT CACATTGTGT TTGAGACGAC GAAAGAACGG AGCGAAACAA CTCTGTAACC   
  
  
- TTATGATGTG TCGCTACAAA CTCAGGTACC TACACTGAAA CGGATCCCTA GTATTTCTGG CCTAGTTACA   
  
  
- ACTCGTCGTA ACAGAACGAG CTCTACAACA CTTGAACCAA CGCACACTTC CTCGACTCTC CCACCTCGCC   
  
  
- GTACTCGAGG AACCCTTTAC CTCCAGCGCC AAGTGTTACC GTCCCAAATT CGGTATGGGA AATTCGTGAG   
  
  
- ACCATTTGTT GTGTTATTCT TGAAACAACT TCTTGATGAC ACTATCCATA CCCGATCTCC TCTCCTTACC   
  
  
- TCCAGAAATA GAACCCACCT ACTTATCTCT AAACCAACGA AGCTCACGGA CCGTCACATC ATTAAT

+     CAT-box

| Site Name | Organism | Position | Strand | Matrix score. | sequence | function |
| --- | --- | --- | --- | --- | --- | --- |
| CAT-box | Arabidopsis thaliana | 576 | - | 6 | GCCACT | cis-acting regulatory element related to meristem expression |
| CAT-box | Arabidopsis thaliana | 2578 | - | 6 | GCCACT | cis-acting regulatory element related to meristem expression |
| CAT-box | Arabidopsis thaliana | 1239 | - | 6 | GCCACT | cis-acting regulatory element related to meristem expression |

>HU01G01391.1   
+ +Up\_Stream \_Len000CATTTT AGTAATAAAT TCCCATGTGA GGACCACTGC CTACTGATTC CGAATGGCAT   
  
  
+ TTTAGTAATA AACTCCCATG TGAGGACCAC TTGATGAGGA ATTCAGTTTT ATAATCCACA GGTGTCAAAG   
  
  
+ CCTATTCCTA CTCTCACCAC TCTGCTATCT CTCCCCCCCC CCCCCCCCCG CCCCCCTCAA AATCTGCCTC   
  
  
+ CTCCGTTCTC TCCACAGCAG ATTCCTCAAT CAGGAAAGGT ATGTCAGAGC TCAAAACGAT TCAACATCAA   
  
  
+ AATGGGATAA TTTATTCTGC TTTCAGTTCT GGGTTATTGT CATATGTTCA TTCAGTTCAG TGTTTCTAGC   
  
  
+ ATGATTGCTT TTTGATGCCT TCTTTTTTGT TCTGGGTTGG ACGAACTCTG TCTTTTCTGT GCATAGATTT   
  
  
+ GAGCTCCGAG CTCAATTTTT GGGATTTTTT TAGCTGACTT TTCTGCTTGG TGTTGTGAAA TTGTGATGGG   
  
  
+ TTTTGGCTTT GTTAAACTTG TTTATAACTT TTTCTGTCAA AACTTTATAG TAAATTGTTG CAATTTGAGA   
  
  
+ ATTATGCACT CAGTGGCTCT GTGATTTATC GTCCGCTGGC AAATTTTCTG ATGAAAGGAG ATGATTTATT   
  
  
+ AATTTTTCAG GCATGGTCAA ATTTTCTAGC CTTTCATGTA CATTGGTTGG TGATAGGCTG ATAGCTAAAT   
  
  
+ TATGTCTCAT TTCTCTAAAT TTATTGCCAG ATATGAAATT GGTGGAACAG TTTAAGGGTT TTGGTCGTCT   
  
  
+ TTTTTGCCCT TTTTTTTGGG TCCCCCCCCC GCCCGGGGGG GAACCCGGAG GGCAGAAAAA GTAACATAAC   
  
  
+ TTAAAATTAT CATTGACGTA GTCCTCCAAC CATAATGAAA GCAACTGAAA TAAGAGCATG CTAAGCTAGA   
  
  
+ GAGTAGGCTG CCTTGTCAGC CTCCCTTTTG ACATGAGAAA GATGGGAGAA GTTTAAAATA AAGTTCCTCG   
  
  
+ TCTTTGCATC CCGGACCACA ATTCGGAACT GGGTGAGGAC CTCCTTAGGC TTGTAAACAC AAGTTTCGAA   
  
  
+ CTTAGATTTG CGTACATGTC AATGCTTTAC TGGATAAGCT TAGCTAACAC CCCTCATCCT TCAACCTGTA   
  
  
+ CCTTCATATA AAAAAAAGGA CAATCCGGTG CACAAGCATC CCGCATTCAC GCAAGGTCTG GGGAAGGGCC   
  
  
+ GCACCCCAAA GGGTGAATTG TAGGCAGCCT AACCTGACTT TGTCAGTGGC TAATTCCACG ATTCGAACCC   
  
  
+ ATGATTTTTT GGTTACACGG GAACAACCTT ACCATTGCTC AAAGGCTCCC CTTCTTCAAC CTCTACCTTC   
  
  
+ ATATATGAAT GAAAATCCTT TGGCTAAGAA TTGAAAAGGA AATATGTGGT TATTGGGTTT GGGTTCTTAT   
  
  
+ TCCGTTGGTT GCCCCTTCAC ATTGAATGCA TCCTCTCGCT AAGAACTGAA AAGGACATAT GGGGATGTAT   
  
  
+ AGGTCAAATT TGACTCAATG ATAACTGAGG ATACAAGAAT TCTAGAAGTC TTTATCAGGG GAATTTTTTC   
  
  
+ TTTTTTCCAC TTGTGACTTT TTGATGCCTT GCTTTTTACT CTTCATTGAA TGAGTACCCT TCTTGACAGA   
  
  
+ TTCTTCCTCC CTTTTTTCTT AATTTCTTCT TGTAGTGGAA TATAAGTATG CTTCTAGGTA GTTGGATGCC   
  
  
+ ATGGAATCTC ATCCCTGTGT GTGCATTCTT CCCCTCTCCC GCCGCCCCCC CCCCCCCAAA AAGAAACCAA   
  
  
+ AAAAAAAAAA AAAATGGATG ATTCTTAGAA CTGACCATTT TTCCTTCCTC CTCCCATTTT ATTTTTTTCA   
  
  
+ GGCTGTTTTT CTTCTACCTG TTGCTTTAGC ATCACTCATA AAAACAAGGT GCAAGCGCAT GACATCGGGT   
  
  
+ ACTGAGCTTC TGAGTTTACA TGCATCGTCA TGAAATTCGG TAGACTTTGA AGTAAAGTTT CCCCATCGTA   
  
  
+ ACTGGTGGTG CATACCTGTC TTCACGCCCA TCAGTAGAGT GGTGAAGTGA AGCAATGTCG AATAGTTTGT   
  
  
+ ATTACGAGCC CAAGAGAGAA ACTGATGCAT ATTTTATGCC TCAATGTCAA TCCTTGAACC CCCAGCTCGA   
  
  
+ TTACAACACC ATTGACTATG GAGCTTTTAT GTGCTCAAAA GTTTTCCTGG GTCAGTACTG CACTCTGGAA   
  
  
+ TCATCCTCAG GAACTGGGAC TTGTCCGGTG TCTAGCTCCA CATCAACTCT CAGCTTCTCA TCCAATGGTA   
  
  
+ GCCCTGGATC ACAGCTTGAT TCCAACTCTT ATCCTTCTGA TCAAAATTGC TCTCCTGATA ATGCCAATTC   
  
  
+ CTCATCTTTA AGTCATTCCT GCGTCACAGA TGATGTGGAT GACCTGAAAT ACAGGCTGAG AGAGTTGGAA   
  
  
+ ACAGTGATGT TGGGACCTGA TCCCGATTTT GTCTACGGCA ATAGCAACCA CACATTTGGG GTTGGGATCC   
  
  
+ ATGTAGTGTC ATCAGAGTTT GACAGCTGGG GGAAAGTGAT GGAGATGATC GCTCGAAAGG ATTTGAAACT   
  
  
+ GGTGCTAATT GCCTGTGCAA GAGCAGTTGC TGAAAATGAT CAGTTGTTGG CCCAGTGGCT GATGGATGAA   
  
  
+ TTGAGACAGA TGGTTTCAGT TTCTGGTGAA CCAATTCAAA GATTGGGTGC TTATATGTTG GAAGGGCTCG   
  
  
+ TAGCGAGGCA GGCCTCCTCA GGTAGCTCCA TTTATAAAGC ATTGAGATGC AAGGAACCCG CGAGTGCTGA   
  
  
+ CCTTTTATCT TACATGCACA TACTCTTTGA GGTTTGCCCA TACATCAAAT TTGGCTACAT GTCAGCAAAT   
  
  
+ GGTGCCATTG CAGAAGCAAT GAAGGATGAA AAGAGAGTCC ACATTGTCGA TTTCCAAATT GGGCAGGGAA   
  
  
+ GCCAATGGGT GACCCTAATC CAGGCATTTG CAGCCCGGCC AGGGGGTCCA CCCCACATTC GCATTACCGG   
  
  
+ TATCGATGAC TCCTACTCTG CATATGCTCG TGGAGGTGGG TTGAACATTG TGGGCCATAG GCTCTTGAGG   
  
  
+ TTGGCTCAGT CATTTAAGGT CCCATTTGAG TTCAACGCAG TTGACGTCCC GGCTTGTCAA GTCCTGCTTA   
  
  
+ AAGATCTCGG CATTCAACAT GGTGAGGCCT TGGCTGTGAA CTTTGCCTTC ATTCTTCACC ACATGCCCGA   
  
  
+ TGAGAGCGTG AGCACAGAGA ATCACCGGGA TCGTCTTCTG AGGGTGGTAA AAGGGCTGAA GCCAAAAGTG   
  
  
+ GTAACGTTAG TGGAGCAAGA GTGTAACACA AACTCTGCTG CTTTCTTGCC TCGCTTTGTT GAGACATTGG   
  
  
+ AATACTACAC AGCGATGTTT GAGTCCATGG ATGTGACTTT GCCTAGGGAT CATAAAGACC GGATCAATGT   
  
  
+ TGAGCAGCAT TGTCTTGCTC GAGATGTTGT GAACTTGGTT GCGTGTGAAG GAGCTGAGAG GGTGGAGCGG   
  
  
+ CATGAGCTCC TTGGGAAATG GAGGTCGCGG TTCACAATGG CAGGGTTTAA GCCATACCCT TTAAGCACTC   
  
  
+ TGGTAAACAA CACAATAAGA ACTTTGTTGA AGAACTACTG TGATAGGTAT GGGCTAGAGG AGAGGAATGG   
  
  
+ AGGTCTTTAT CTTGGGTGGA TGAATAGAGA TTTGGTTGCT TCGAGTGCCT GGCAGTGTAG TAATTA  

- +Up\_Stream \_Len000GTAAAA TCATTATTTA AGGGTACACT CCTGGTGACG GATGACTAAG GCTTACCGTA   
  
  
- AAATCATTAT TTGAGGGTAC ACTCCTGGTG AACTACTCCT TAAGTCAAAA TATTAGGTGT CCACAGTTTC   
  
  
- GGATAAGGAT GAGAGTGGTG AGACGATAGA GAGGGGGGGG GGGGGGGGGC GGGGGGAGTT TTAGACGGAG   
  
  
- GAGGCAAGAG AGGTGTCGTC TAAGGAGTTA GTCCTTTCCA TACAGTCTCG AGTTTTGCTA AGTTGTAGTT   
  
  
- TTACCCTATT AAATAAGACG AAAGTCAAGA CCCAATAACA GTATACAAGT AAGTCAAGTC ACAAAGATCG   
  
  
- TACTAACGAA AAACTACGGA AGAAAAAACA AGACCCAACC TGCTTGAGAC AGAAAAGACA CGTATCTAAA   
  
  
- CTCGAGGCTC GAGTTAAAAA CCCTAAAAAA ATCGACTGAA AAGACGAACC ACAACACTTT AACACTACCC   
  
  
- AAAACCGAAA CAATTTGAAC AAATATTGAA AAAGACAGTT TTGAAATATC ATTTAACAAC GTTAAACTCT   
  
  
- TAATACGTGA GTCACCGAGA CACTAAATAG CAGGCGACCG TTTAAAAGAC TACTTTCCTC TACTAAATAA   
  
  
- TTAAAAAGTC CGTACCAGTT TAAAAGATCG GAAAGTACAT GTAACCAACC ACTATCCGAC TATCGATTTA   
  
  
- ATACAGAGTA AAGAGATTTA AATAACGGTC TATACTTTAA CCACCTTGTC AAATTCCCAA AACCAGCAGA   
  
  
- AAAAACGGGA AAAAAAACCC AGGGGGGGGG CGGGCCCCCC CTTGGGCCTC CCGTCTTTTT CATTGTATTG   
  
  
- AATTTTAATA GTAACTGCAT CAGGAGGTTG GTATTACTTT CGTTGACTTT ATTCTCGTAC GATTCGATCT   
  
  
- CTCATCCGAC GGAACAGTCG GAGGGAAAAC TGTACTCTTT CTACCCTCTT CAAATTTTAT TTCAAGGAGC   
  
  
- AGAAACGTAG GGCCTGGTGT TAAGCCTTGA CCCACTCCTG GAGGAATCCG AACATTTGTG TTCAAAGCTT   
  
  
- GAATCTAAAC GCATGTACAG TTACGAAATG ACCTATTCGA ATCGATTGTG GGGAGTAGGA AGTTGGACAT   
  
  
- GGAAGTATAT TTTTTTTCCT GTTAGGCCAC GTGTTCGTAG GGCGTAAGTG CGTTCCAGAC CCCTTCCCGG   
  
  
- CGTGGGGTTT CCCACTTAAC ATCCGTCGGA TTGGACTGAA ACAGTCACCG ATTAAGGTGC TAAGCTTGGG   
  
  
- TACTAAAAAA CCAATGTGCC CTTGTTGGAA TGGTAACGAG TTTCCGAGGG GAAGAAGTTG GAGATGGAAG   
  
  
- TATATACTTA CTTTTAGGAA ACCGATTCTT AACTTTTCCT TTATACACCA ATAACCCAAA CCCAAGAATA   
  
  
- AGGCAACCAA CGGGGAAGTG TAACTTACGT AGGAGAGCGA TTCTTGACTT TTCCTGTATA CCCCTACATA   
  
  
- TCCAGTTTAA ACTGAGTTAC TATTGACTCC TATGTTCTTA AGATCTTCAG AAATAGTCCC CTTAAAAAAG   
  
  
- AAAAAAGGTG AACACTGAAA AACTACGGAA CGAAAAATGA GAAGTAACTT ACTCATGGGA AGAACTGTCT   
  
  
- AAGAAGGAGG GAAAAAAGAA TTAAAGAAGA ACATCACCTT ATATTCATAC GAAGATCCAT CAACCTACGG   
  
  
- TACCTTAGAG TAGGGACACA CACGTAAGAA GGGGAGAGGG CGGCGGGGGG GGGGGGGTTT TTCTTTGGTT   
  
  
- TTTTTTTTTT TTTTACCTAC TAAGAATCTT GACTGGTAAA AAGGAAGGAG GAGGGTAAAA TAAAAAAAGT   
  
  
- CCGACAAAAA GAAGATGGAC AACGAAATCG TAGTGAGTAT TTTTGTTCCA CGTTCGCGTA CTGTAGCCCA   
  
  
- TGACTCGAAG ACTCAAATGT ACGTAGCAGT ACTTTAAGCC ATCTGAAACT TCATTTCAAA GGGGTAGCAT   
  
  
- TGACCACCAC GTATGGACAG AAGTGCGGGT AGTCATCTCA CCACTTCACT TCGTTACAGC TTATCAAACA   
  
  
- TAATGCTCGG GTTCTCTCTT TGACTACGTA TAAAATACGG AGTTACAGTT AGGAACTTGG GGGTCGAGCT   
  
  
- AATGTTGTGG TAACTGATAC CTCGAAAATA CACGAGTTTT CAAAAGGACC CAGTCATGAC GTGAGACCTT   
  
  
- AGTAGGAGTC CTTGACCCTG AACAGGCCAC AGATCGAGGT GTAGTTGAGA GTCGAAGAGT AGGTTACCAT   
  
  
- CGGGACCTAG TGTCGAACTA AGGTTGAGAA TAGGAAGACT AGTTTTAACG AGAGGACTAT TACGGTTAAG   
  
  
- GAGTAGAAAT TCAGTAAGGA CGCAGTGTCT ACTACACCTA CTGGACTTTA TGTCCGACTC TCTCAACCTT   
  
  
- TGTCACTACA ACCCTGGACT AGGGCTAAAA CAGATGCCGT TATCGTTGGT GTGTAAACCC CAACCCTAGG   
  
  
- TACATCACAG TAGTCTCAAA CTGTCGACCC CCTTTCACTA CCTCTACTAG CGAGCTTTCC TAAACTTTGA   
  
  
- CCACGATTAA CGGACACGTT CTCGTCAACG ACTTTTACTA GTCAACAACC GGGTCACCGA CTACCTACTT   
  
  
- AACTCTGTCT ACCAAAGTCA AAGACCACTT GGTTAAGTTT CTAACCCACG AATATACAAC CTTCCCGAGC   
  
  
- ATCGCTCCGT CCGGAGGAGT CCATCGAGGT AAATATTTCG TAACTCTACG TTCCTTGGGC GCTCACGACT   
  
  
- GGAAAATAGA ATGTACGTGT ATGAGAAACT CCAAACGGGT ATGTAGTTTA AACCGATGTA CAGTCGTTTA   
  
  
- CCACGGTAAC GTCTTCGTTA CTTCCTACTT TTCTCTCAGG TGTAACAGCT AAAGGTTTAA CCCGTCCCTT   
  
  
- CGGTTACCCA CTGGGATTAG GTCCGTAAAC GTCGGGCCGG TCCCCCAGGT GGGGTGTAAG CGTAATGGCC   
  
  
- ATAGCTACTG AGGATGAGAC GTATACGAGC ACCTCCACCC AACTTGTAAC ACCCGGTATC CGAGAACTCC   
  
  
- AACCGAGTCA GTAAATTCCA GGGTAAACTC AAGTTGCGTC AACTGCAGGG CCGAACAGTT CAGGACGAAT   
  
  
- TTCTAGAGCC GTAAGTTGTA CCACTCCGGA ACCGACACTT GAAACGGAAG TAAGAAGTGG TGTACGGGCT   
  
  
- ACTCTCGCAC TCGTGTCTCT TAGTGGCCCT AGCAGAAGAC TCCCACCATT TTCCCGACTT CGGTTTTCAC   
  
  
- CATTGCAATC ACCTCGTTCT CACATTGTGT TTGAGACGAC GAAAGAACGG AGCGAAACAA CTCTGTAACC   
  
  
- TTATGATGTG TCGCTACAAA CTCAGGTACC TACACTGAAA CGGATCCCTA GTATTTCTGG CCTAGTTACA   
  
  
- ACTCGTCGTA ACAGAACGAG CTCTACAACA CTTGAACCAA CGCACACTTC CTCGACTCTC CCACCTCGCC   
  
  
- GTACTCGAGG AACCCTTTAC CTCCAGCGCC AAGTGTTACC GTCCCAAATT CGGTATGGGA AATTCGTGAG   
  
  
- ACCATTTGTT GTGTTATTCT TGAAACAACT TCTTGATGAC ACTATCCATA CCCGATCTCC TCTCCTTACC   
  
  
- TCCAGAAATA GAACCCACCT ACTTATCTCT AAACCAACGA AGCTCACGGA CCGTCACATC ATTAAT

+     CCAAT-box

| Site Name | Organism | Position | Strand | Matrix score. | sequence | function |
| --- | --- | --- | --- | --- | --- | --- |
| CCAAT-box | Hordeum vulgare | 1406 | - | 6 | CAACGG | MYBHv1 binding site |

>HU01G01391.1   
+ +Up\_Stream \_Len000CATTTT AGTAATAAAT TCCCATGTGA GGACCACTGC CTACTGATTC CGAATGGCAT   
  
  
+ TTTAGTAATA AACTCCCATG TGAGGACCAC TTGATGAGGA ATTCAGTTTT ATAATCCACA GGTGTCAAAG   
  
  
+ CCTATTCCTA CTCTCACCAC TCTGCTATCT CTCCCCCCCC CCCCCCCCCG CCCCCCTCAA AATCTGCCTC   
  
  
+ CTCCGTTCTC TCCACAGCAG ATTCCTCAAT CAGGAAAGGT ATGTCAGAGC TCAAAACGAT TCAACATCAA   
  
  
+ AATGGGATAA TTTATTCTGC TTTCAGTTCT GGGTTATTGT CATATGTTCA TTCAGTTCAG TGTTTCTAGC   
  
  
+ ATGATTGCTT TTTGATGCCT TCTTTTTTGT TCTGGGTTGG ACGAACTCTG TCTTTTCTGT GCATAGATTT   
  
  
+ GAGCTCCGAG CTCAATTTTT GGGATTTTTT TAGCTGACTT TTCTGCTTGG TGTTGTGAAA TTGTGATGGG   
  
  
+ TTTTGGCTTT GTTAAACTTG TTTATAACTT TTTCTGTCAA AACTTTATAG TAAATTGTTG CAATTTGAGA   
  
  
+ ATTATGCACT CAGTGGCTCT GTGATTTATC GTCCGCTGGC AAATTTTCTG ATGAAAGGAG ATGATTTATT   
  
  
+ AATTTTTCAG GCATGGTCAA ATTTTCTAGC CTTTCATGTA CATTGGTTGG TGATAGGCTG ATAGCTAAAT   
  
  
+ TATGTCTCAT TTCTCTAAAT TTATTGCCAG ATATGAAATT GGTGGAACAG TTTAAGGGTT TTGGTCGTCT   
  
  
+ TTTTTGCCCT TTTTTTTGGG TCCCCCCCCC GCCCGGGGGG GAACCCGGAG GGCAGAAAAA GTAACATAAC   
  
  
+ TTAAAATTAT CATTGACGTA GTCCTCCAAC CATAATGAAA GCAACTGAAA TAAGAGCATG CTAAGCTAGA   
  
  
+ GAGTAGGCTG CCTTGTCAGC CTCCCTTTTG ACATGAGAAA GATGGGAGAA GTTTAAAATA AAGTTCCTCG   
  
  
+ TCTTTGCATC CCGGACCACA ATTCGGAACT GGGTGAGGAC CTCCTTAGGC TTGTAAACAC AAGTTTCGAA   
  
  
+ CTTAGATTTG CGTACATGTC AATGCTTTAC TGGATAAGCT TAGCTAACAC CCCTCATCCT TCAACCTGTA   
  
  
+ CCTTCATATA AAAAAAAGGA CAATCCGGTG CACAAGCATC CCGCATTCAC GCAAGGTCTG GGGAAGGGCC   
  
  
+ GCACCCCAAA GGGTGAATTG TAGGCAGCCT AACCTGACTT TGTCAGTGGC TAATTCCACG ATTCGAACCC   
  
  
+ ATGATTTTTT GGTTACACGG GAACAACCTT ACCATTGCTC AAAGGCTCCC CTTCTTCAAC CTCTACCTTC   
  
  
+ ATATATGAAT GAAAATCCTT TGGCTAAGAA TTGAAAAGGA AATATGTGGT TATTGGGTTT GGGTTCTTAT   
  
  
+ TCCGTTGGTT GCCCCTTCAC ATTGAATGCA TCCTCTCGCT AAGAACTGAA AAGGACATAT GGGGATGTAT   
  
  
+ AGGTCAAATT TGACTCAATG ATAACTGAGG ATACAAGAAT TCTAGAAGTC TTTATCAGGG GAATTTTTTC   
  
  
+ TTTTTTCCAC TTGTGACTTT TTGATGCCTT GCTTTTTACT CTTCATTGAA TGAGTACCCT TCTTGACAGA   
  
  
+ TTCTTCCTCC CTTTTTTCTT AATTTCTTCT TGTAGTGGAA TATAAGTATG CTTCTAGGTA GTTGGATGCC   
  
  
+ ATGGAATCTC ATCCCTGTGT GTGCATTCTT CCCCTCTCCC GCCGCCCCCC CCCCCCCAAA AAGAAACCAA   
  
  
+ AAAAAAAAAA AAAATGGATG ATTCTTAGAA CTGACCATTT TTCCTTCCTC CTCCCATTTT ATTTTTTTCA   
  
  
+ GGCTGTTTTT CTTCTACCTG TTGCTTTAGC ATCACTCATA AAAACAAGGT GCAAGCGCAT GACATCGGGT   
  
  
+ ACTGAGCTTC TGAGTTTACA TGCATCGTCA TGAAATTCGG TAGACTTTGA AGTAAAGTTT CCCCATCGTA   
  
  
+ ACTGGTGGTG CATACCTGTC TTCACGCCCA TCAGTAGAGT GGTGAAGTGA AGCAATGTCG AATAGTTTGT   
  
  
+ ATTACGAGCC CAAGAGAGAA ACTGATGCAT ATTTTATGCC TCAATGTCAA TCCTTGAACC CCCAGCTCGA   
  
  
+ TTACAACACC ATTGACTATG GAGCTTTTAT GTGCTCAAAA GTTTTCCTGG GTCAGTACTG CACTCTGGAA   
  
  
+ TCATCCTCAG GAACTGGGAC TTGTCCGGTG TCTAGCTCCA CATCAACTCT CAGCTTCTCA TCCAATGGTA   
  
  
+ GCCCTGGATC ACAGCTTGAT TCCAACTCTT ATCCTTCTGA TCAAAATTGC TCTCCTGATA ATGCCAATTC   
  
  
+ CTCATCTTTA AGTCATTCCT GCGTCACAGA TGATGTGGAT GACCTGAAAT ACAGGCTGAG AGAGTTGGAA   
  
  
+ ACAGTGATGT TGGGACCTGA TCCCGATTTT GTCTACGGCA ATAGCAACCA CACATTTGGG GTTGGGATCC   
  
  
+ ATGTAGTGTC ATCAGAGTTT GACAGCTGGG GGAAAGTGAT GGAGATGATC GCTCGAAAGG ATTTGAAACT   
  
  
+ GGTGCTAATT GCCTGTGCAA GAGCAGTTGC TGAAAATGAT CAGTTGTTGG CCCAGTGGCT GATGGATGAA   
  
  
+ TTGAGACAGA TGGTTTCAGT TTCTGGTGAA CCAATTCAAA GATTGGGTGC TTATATGTTG GAAGGGCTCG   
  
  
+ TAGCGAGGCA GGCCTCCTCA GGTAGCTCCA TTTATAAAGC ATTGAGATGC AAGGAACCCG CGAGTGCTGA   
  
  
+ CCTTTTATCT TACATGCACA TACTCTTTGA GGTTTGCCCA TACATCAAAT TTGGCTACAT GTCAGCAAAT   
  
  
+ GGTGCCATTG CAGAAGCAAT GAAGGATGAA AAGAGAGTCC ACATTGTCGA TTTCCAAATT GGGCAGGGAA   
  
  
+ GCCAATGGGT GACCCTAATC CAGGCATTTG CAGCCCGGCC AGGGGGTCCA CCCCACATTC GCATTACCGG   
  
  
+ TATCGATGAC TCCTACTCTG CATATGCTCG TGGAGGTGGG TTGAACATTG TGGGCCATAG GCTCTTGAGG   
  
  
+ TTGGCTCAGT CATTTAAGGT CCCATTTGAG TTCAACGCAG TTGACGTCCC GGCTTGTCAA GTCCTGCTTA   
  
  
+ AAGATCTCGG CATTCAACAT GGTGAGGCCT TGGCTGTGAA CTTTGCCTTC ATTCTTCACC ACATGCCCGA   
  
  
+ TGAGAGCGTG AGCACAGAGA ATCACCGGGA TCGTCTTCTG AGGGTGGTAA AAGGGCTGAA GCCAAAAGTG   
  
  
+ GTAACGTTAG TGGAGCAAGA GTGTAACACA AACTCTGCTG CTTTCTTGCC TCGCTTTGTT GAGACATTGG   
  
  
+ AATACTACAC AGCGATGTTT GAGTCCATGG ATGTGACTTT GCCTAGGGAT CATAAAGACC GGATCAATGT   
  
  
+ TGAGCAGCAT TGTCTTGCTC GAGATGTTGT GAACTTGGTT GCGTGTGAAG GAGCTGAGAG GGTGGAGCGG   
  
  
+ CATGAGCTCC TTGGGAAATG GAGGTCGCGG TTCACAATGG CAGGGTTTAA GCCATACCCT TTAAGCACTC   
  
  
+ TGGTAAACAA CACAATAAGA ACTTTGTTGA AGAACTACTG TGATAGGTAT GGGCTAGAGG AGAGGAATGG   
  
  
+ AGGTCTTTAT CTTGGGTGGA TGAATAGAGA TTTGGTTGCT TCGAGTGCCT GGCAGTGTAG TAATTA  

- +Up\_Stream \_Len000GTAAAA TCATTATTTA AGGGTACACT CCTGGTGACG GATGACTAAG GCTTACCGTA   
  
  
- AAATCATTAT TTGAGGGTAC ACTCCTGGTG AACTACTCCT TAAGTCAAAA TATTAGGTGT CCACAGTTTC   
  
  
- GGATAAGGAT GAGAGTGGTG AGACGATAGA GAGGGGGGGG GGGGGGGGGC GGGGGGAGTT TTAGACGGAG   
  
  
- GAGGCAAGAG AGGTGTCGTC TAAGGAGTTA GTCCTTTCCA TACAGTCTCG AGTTTTGCTA AGTTGTAGTT   
  
  
- TTACCCTATT AAATAAGACG AAAGTCAAGA CCCAATAACA GTATACAAGT AAGTCAAGTC ACAAAGATCG   
  
  
- TACTAACGAA AAACTACGGA AGAAAAAACA AGACCCAACC TGCTTGAGAC AGAAAAGACA CGTATCTAAA   
  
  
- CTCGAGGCTC GAGTTAAAAA CCCTAAAAAA ATCGACTGAA AAGACGAACC ACAACACTTT AACACTACCC   
  
  
- AAAACCGAAA CAATTTGAAC AAATATTGAA AAAGACAGTT TTGAAATATC ATTTAACAAC GTTAAACTCT   
  
  
- TAATACGTGA GTCACCGAGA CACTAAATAG CAGGCGACCG TTTAAAAGAC TACTTTCCTC TACTAAATAA   
  
  
- TTAAAAAGTC CGTACCAGTT TAAAAGATCG GAAAGTACAT GTAACCAACC ACTATCCGAC TATCGATTTA   
  
  
- ATACAGAGTA AAGAGATTTA AATAACGGTC TATACTTTAA CCACCTTGTC AAATTCCCAA AACCAGCAGA   
  
  
- AAAAACGGGA AAAAAAACCC AGGGGGGGGG CGGGCCCCCC CTTGGGCCTC CCGTCTTTTT CATTGTATTG   
  
  
- AATTTTAATA GTAACTGCAT CAGGAGGTTG GTATTACTTT CGTTGACTTT ATTCTCGTAC GATTCGATCT   
  
  
- CTCATCCGAC GGAACAGTCG GAGGGAAAAC TGTACTCTTT CTACCCTCTT CAAATTTTAT TTCAAGGAGC   
  
  
- AGAAACGTAG GGCCTGGTGT TAAGCCTTGA CCCACTCCTG GAGGAATCCG AACATTTGTG TTCAAAGCTT   
  
  
- GAATCTAAAC GCATGTACAG TTACGAAATG ACCTATTCGA ATCGATTGTG GGGAGTAGGA AGTTGGACAT   
  
  
- GGAAGTATAT TTTTTTTCCT GTTAGGCCAC GTGTTCGTAG GGCGTAAGTG CGTTCCAGAC CCCTTCCCGG   
  
  
- CGTGGGGTTT CCCACTTAAC ATCCGTCGGA TTGGACTGAA ACAGTCACCG ATTAAGGTGC TAAGCTTGGG   
  
  
- TACTAAAAAA CCAATGTGCC CTTGTTGGAA TGGTAACGAG TTTCCGAGGG GAAGAAGTTG GAGATGGAAG   
  
  
- TATATACTTA CTTTTAGGAA ACCGATTCTT AACTTTTCCT TTATACACCA ATAACCCAAA CCCAAGAATA   
  
  
- AGGCAACCAA CGGGGAAGTG TAACTTACGT AGGAGAGCGA TTCTTGACTT TTCCTGTATA CCCCTACATA   
  
  
- TCCAGTTTAA ACTGAGTTAC TATTGACTCC TATGTTCTTA AGATCTTCAG AAATAGTCCC CTTAAAAAAG   
  
  
- AAAAAAGGTG AACACTGAAA AACTACGGAA CGAAAAATGA GAAGTAACTT ACTCATGGGA AGAACTGTCT   
  
  
- AAGAAGGAGG GAAAAAAGAA TTAAAGAAGA ACATCACCTT ATATTCATAC GAAGATCCAT CAACCTACGG   
  
  
- TACCTTAGAG TAGGGACACA CACGTAAGAA GGGGAGAGGG CGGCGGGGGG GGGGGGGTTT TTCTTTGGTT   
  
  
- TTTTTTTTTT TTTTACCTAC TAAGAATCTT GACTGGTAAA AAGGAAGGAG GAGGGTAAAA TAAAAAAAGT   
  
  
- CCGACAAAAA GAAGATGGAC AACGAAATCG TAGTGAGTAT TTTTGTTCCA CGTTCGCGTA CTGTAGCCCA   
  
  
- TGACTCGAAG ACTCAAATGT ACGTAGCAGT ACTTTAAGCC ATCTGAAACT TCATTTCAAA GGGGTAGCAT   
  
  
- TGACCACCAC GTATGGACAG AAGTGCGGGT AGTCATCTCA CCACTTCACT TCGTTACAGC TTATCAAACA   
  
  
- TAATGCTCGG GTTCTCTCTT TGACTACGTA TAAAATACGG AGTTACAGTT AGGAACTTGG GGGTCGAGCT   
  
  
- AATGTTGTGG TAACTGATAC CTCGAAAATA CACGAGTTTT CAAAAGGACC CAGTCATGAC GTGAGACCTT   
  
  
- AGTAGGAGTC CTTGACCCTG AACAGGCCAC AGATCGAGGT GTAGTTGAGA GTCGAAGAGT AGGTTACCAT   
  
  
- CGGGACCTAG TGTCGAACTA AGGTTGAGAA TAGGAAGACT AGTTTTAACG AGAGGACTAT TACGGTTAAG   
  
  
- GAGTAGAAAT TCAGTAAGGA CGCAGTGTCT ACTACACCTA CTGGACTTTA TGTCCGACTC TCTCAACCTT   
  
  
- TGTCACTACA ACCCTGGACT AGGGCTAAAA CAGATGCCGT TATCGTTGGT GTGTAAACCC CAACCCTAGG   
  
  
- TACATCACAG TAGTCTCAAA CTGTCGACCC CCTTTCACTA CCTCTACTAG CGAGCTTTCC TAAACTTTGA   
  
  
- CCACGATTAA CGGACACGTT CTCGTCAACG ACTTTTACTA GTCAACAACC GGGTCACCGA CTACCTACTT   
  
  
- AACTCTGTCT ACCAAAGTCA AAGACCACTT GGTTAAGTTT CTAACCCACG AATATACAAC CTTCCCGAGC   
  
  
- ATCGCTCCGT CCGGAGGAGT CCATCGAGGT AAATATTTCG TAACTCTACG TTCCTTGGGC GCTCACGACT   
  
  
- GGAAAATAGA ATGTACGTGT ATGAGAAACT CCAAACGGGT ATGTAGTTTA AACCGATGTA CAGTCGTTTA   
  
  
- CCACGGTAAC GTCTTCGTTA CTTCCTACTT TTCTCTCAGG TGTAACAGCT AAAGGTTTAA CCCGTCCCTT   
  
  
- CGGTTACCCA CTGGGATTAG GTCCGTAAAC GTCGGGCCGG TCCCCCAGGT GGGGTGTAAG CGTAATGGCC   
  
  
- ATAGCTACTG AGGATGAGAC GTATACGAGC ACCTCCACCC AACTTGTAAC ACCCGGTATC CGAGAACTCC   
  
  
- AACCGAGTCA GTAAATTCCA GGGTAAACTC AAGTTGCGTC AACTGCAGGG CCGAACAGTT CAGGACGAAT   
  
  
- TTCTAGAGCC GTAAGTTGTA CCACTCCGGA ACCGACACTT GAAACGGAAG TAAGAAGTGG TGTACGGGCT   
  
  
- ACTCTCGCAC TCGTGTCTCT TAGTGGCCCT AGCAGAAGAC TCCCACCATT TTCCCGACTT CGGTTTTCAC   
  
  
- CATTGCAATC ACCTCGTTCT CACATTGTGT TTGAGACGAC GAAAGAACGG AGCGAAACAA CTCTGTAACC   
  
  
- TTATGATGTG TCGCTACAAA CTCAGGTACC TACACTGAAA CGGATCCCTA GTATTTCTGG CCTAGTTACA   
  
  
- ACTCGTCGTA ACAGAACGAG CTCTACAACA CTTGAACCAA CGCACACTTC CTCGACTCTC CCACCTCGCC   
  
  
- GTACTCGAGG AACCCTTTAC CTCCAGCGCC AAGTGTTACC GTCCCAAATT CGGTATGGGA AATTCGTGAG   
  
  
- ACCATTTGTT GTGTTATTCT TGAAACAACT TCTTGATGAC ACTATCCATA CCCGATCTCC TCTCCTTACC   
  
  
- TCCAGAAATA GAACCCACCT ACTTATCTCT AAACCAACGA AGCTCACGGA CCGTCACATC ATTAAT

+     CGTCA-motif

| Site Name | Organism | Position | Strand | Matrix score. | sequence | function |
| --- | --- | --- | --- | --- | --- | --- |
| CGTCA-motif | Hordeum vulgare | 3056 | - | 5 | CGTCA | cis-acting regulatory element involved in the MeJA-responsiveness |
| CGTCA-motif | Hordeum vulgare | 2336 | + | 5 | CGTCA | cis-acting regulatory element involved in the MeJA-responsiveness |
| CGTCA-motif | Hordeum vulgare | 858 | - | 5 | CGTCA | cis-acting regulatory element involved in the MeJA-responsiveness |
| CGTCA-motif | Hordeum vulgare | 1920 | + | 5 | CGTCA | cis-acting regulatory element involved in the MeJA-responsiveness |

>HU01G01391.1   
+ +Up\_Stream \_Len000CATTTT AGTAATAAAT TCCCATGTGA GGACCACTGC CTACTGATTC CGAATGGCAT   
  
  
+ TTTAGTAATA AACTCCCATG TGAGGACCAC TTGATGAGGA ATTCAGTTTT ATAATCCACA GGTGTCAAAG   
  
  
+ CCTATTCCTA CTCTCACCAC TCTGCTATCT CTCCCCCCCC CCCCCCCCCG CCCCCCTCAA AATCTGCCTC   
  
  
+ CTCCGTTCTC TCCACAGCAG ATTCCTCAAT CAGGAAAGGT ATGTCAGAGC TCAAAACGAT TCAACATCAA   
  
  
+ AATGGGATAA TTTATTCTGC TTTCAGTTCT GGGTTATTGT CATATGTTCA TTCAGTTCAG TGTTTCTAGC   
  
  
+ ATGATTGCTT TTTGATGCCT TCTTTTTTGT TCTGGGTTGG ACGAACTCTG TCTTTTCTGT GCATAGATTT   
  
  
+ GAGCTCCGAG CTCAATTTTT GGGATTTTTT TAGCTGACTT TTCTGCTTGG TGTTGTGAAA TTGTGATGGG   
  
  
+ TTTTGGCTTT GTTAAACTTG TTTATAACTT TTTCTGTCAA AACTTTATAG TAAATTGTTG CAATTTGAGA   
  
  
+ ATTATGCACT CAGTGGCTCT GTGATTTATC GTCCGCTGGC AAATTTTCTG ATGAAAGGAG ATGATTTATT   
  
  
+ AATTTTTCAG GCATGGTCAA ATTTTCTAGC CTTTCATGTA CATTGGTTGG TGATAGGCTG ATAGCTAAAT   
  
  
+ TATGTCTCAT TTCTCTAAAT TTATTGCCAG ATATGAAATT GGTGGAACAG TTTAAGGGTT TTGGTCGTCT   
  
  
+ TTTTTGCCCT TTTTTTTGGG TCCCCCCCCC GCCCGGGGGG GAACCCGGAG GGCAGAAAAA GTAACATAAC   
  
  
+ TTAAAATTAT CATTGACGTA GTCCTCCAAC CATAATGAAA GCAACTGAAA TAAGAGCATG CTAAGCTAGA   
  
  
+ GAGTAGGCTG CCTTGTCAGC CTCCCTTTTG ACATGAGAAA GATGGGAGAA GTTTAAAATA AAGTTCCTCG   
  
  
+ TCTTTGCATC CCGGACCACA ATTCGGAACT GGGTGAGGAC CTCCTTAGGC TTGTAAACAC AAGTTTCGAA   
  
  
+ CTTAGATTTG CGTACATGTC AATGCTTTAC TGGATAAGCT TAGCTAACAC CCCTCATCCT TCAACCTGTA   
  
  
+ CCTTCATATA AAAAAAAGGA CAATCCGGTG CACAAGCATC CCGCATTCAC GCAAGGTCTG GGGAAGGGCC   
  
  
+ GCACCCCAAA GGGTGAATTG TAGGCAGCCT AACCTGACTT TGTCAGTGGC TAATTCCACG ATTCGAACCC   
  
  
+ ATGATTTTTT GGTTACACGG GAACAACCTT ACCATTGCTC AAAGGCTCCC CTTCTTCAAC CTCTACCTTC   
  
  
+ ATATATGAAT GAAAATCCTT TGGCTAAGAA TTGAAAAGGA AATATGTGGT TATTGGGTTT GGGTTCTTAT   
  
  
+ TCCGTTGGTT GCCCCTTCAC ATTGAATGCA TCCTCTCGCT AAGAACTGAA AAGGACATAT GGGGATGTAT   
  
  
+ AGGTCAAATT TGACTCAATG ATAACTGAGG ATACAAGAAT TCTAGAAGTC TTTATCAGGG GAATTTTTTC   
  
  
+ TTTTTTCCAC TTGTGACTTT TTGATGCCTT GCTTTTTACT CTTCATTGAA TGAGTACCCT TCTTGACAGA   
  
  
+ TTCTTCCTCC CTTTTTTCTT AATTTCTTCT TGTAGTGGAA TATAAGTATG CTTCTAGGTA GTTGGATGCC   
  
  
+ ATGGAATCTC ATCCCTGTGT GTGCATTCTT CCCCTCTCCC GCCGCCCCCC CCCCCCCAAA AAGAAACCAA   
  
  
+ AAAAAAAAAA AAAATGGATG ATTCTTAGAA CTGACCATTT TTCCTTCCTC CTCCCATTTT ATTTTTTTCA   
  
  
+ GGCTGTTTTT CTTCTACCTG TTGCTTTAGC ATCACTCATA AAAACAAGGT GCAAGCGCAT GACATCGGGT   
  
  
+ ACTGAGCTTC TGAGTTTACA TGCATCGTCA TGAAATTCGG TAGACTTTGA AGTAAAGTTT CCCCATCGTA   
  
  
+ ACTGGTGGTG CATACCTGTC TTCACGCCCA TCAGTAGAGT GGTGAAGTGA AGCAATGTCG AATAGTTTGT   
  
  
+ ATTACGAGCC CAAGAGAGAA ACTGATGCAT ATTTTATGCC TCAATGTCAA TCCTTGAACC CCCAGCTCGA   
  
  
+ TTACAACACC ATTGACTATG GAGCTTTTAT GTGCTCAAAA GTTTTCCTGG GTCAGTACTG CACTCTGGAA   
  
  
+ TCATCCTCAG GAACTGGGAC TTGTCCGGTG TCTAGCTCCA CATCAACTCT CAGCTTCTCA TCCAATGGTA   
  
  
+ GCCCTGGATC ACAGCTTGAT TCCAACTCTT ATCCTTCTGA TCAAAATTGC TCTCCTGATA ATGCCAATTC   
  
  
+ CTCATCTTTA AGTCATTCCT GCGTCACAGA TGATGTGGAT GACCTGAAAT ACAGGCTGAG AGAGTTGGAA   
  
  
+ ACAGTGATGT TGGGACCTGA TCCCGATTTT GTCTACGGCA ATAGCAACCA CACATTTGGG GTTGGGATCC   
  
  
+ ATGTAGTGTC ATCAGAGTTT GACAGCTGGG GGAAAGTGAT GGAGATGATC GCTCGAAAGG ATTTGAAACT   
  
  
+ GGTGCTAATT GCCTGTGCAA GAGCAGTTGC TGAAAATGAT CAGTTGTTGG CCCAGTGGCT GATGGATGAA   
  
  
+ TTGAGACAGA TGGTTTCAGT TTCTGGTGAA CCAATTCAAA GATTGGGTGC TTATATGTTG GAAGGGCTCG   
  
  
+ TAGCGAGGCA GGCCTCCTCA GGTAGCTCCA TTTATAAAGC ATTGAGATGC AAGGAACCCG CGAGTGCTGA   
  
  
+ CCTTTTATCT TACATGCACA TACTCTTTGA GGTTTGCCCA TACATCAAAT TTGGCTACAT GTCAGCAAAT   
  
  
+ GGTGCCATTG CAGAAGCAAT GAAGGATGAA AAGAGAGTCC ACATTGTCGA TTTCCAAATT GGGCAGGGAA   
  
  
+ GCCAATGGGT GACCCTAATC CAGGCATTTG CAGCCCGGCC AGGGGGTCCA CCCCACATTC GCATTACCGG   
  
  
+ TATCGATGAC TCCTACTCTG CATATGCTCG TGGAGGTGGG TTGAACATTG TGGGCCATAG GCTCTTGAGG   
  
  
+ TTGGCTCAGT CATTTAAGGT CCCATTTGAG TTCAACGCAG TTGACGTCCC GGCTTGTCAA GTCCTGCTTA   
  
  
+ AAGATCTCGG CATTCAACAT GGTGAGGCCT TGGCTGTGAA CTTTGCCTTC ATTCTTCACC ACATGCCCGA   
  
  
+ TGAGAGCGTG AGCACAGAGA ATCACCGGGA TCGTCTTCTG AGGGTGGTAA AAGGGCTGAA GCCAAAAGTG   
  
  
+ GTAACGTTAG TGGAGCAAGA GTGTAACACA AACTCTGCTG CTTTCTTGCC TCGCTTTGTT GAGACATTGG   
  
  
+ AATACTACAC AGCGATGTTT GAGTCCATGG ATGTGACTTT GCCTAGGGAT CATAAAGACC GGATCAATGT   
  
  
+ TGAGCAGCAT TGTCTTGCTC GAGATGTTGT GAACTTGGTT GCGTGTGAAG GAGCTGAGAG GGTGGAGCGG   
  
  
+ CATGAGCTCC TTGGGAAATG GAGGTCGCGG TTCACAATGG CAGGGTTTAA GCCATACCCT TTAAGCACTC   
  
  
+ TGGTAAACAA CACAATAAGA ACTTTGTTGA AGAACTACTG TGATAGGTAT GGGCTAGAGG AGAGGAATGG   
  
  
+ AGGTCTTTAT CTTGGGTGGA TGAATAGAGA TTTGGTTGCT TCGAGTGCCT GGCAGTGTAG TAATTA  

- +Up\_Stream \_Len000GTAAAA TCATTATTTA AGGGTACACT CCTGGTGACG GATGACTAAG GCTTACCGTA   
  
  
- AAATCATTAT TTGAGGGTAC ACTCCTGGTG AACTACTCCT TAAGTCAAAA TATTAGGTGT CCACAGTTTC   
  
  
- GGATAAGGAT GAGAGTGGTG AGACGATAGA GAGGGGGGGG GGGGGGGGGC GGGGGGAGTT TTAGACGGAG   
  
  
- GAGGCAAGAG AGGTGTCGTC TAAGGAGTTA GTCCTTTCCA TACAGTCTCG AGTTTTGCTA AGTTGTAGTT   
  
  
- TTACCCTATT AAATAAGACG AAAGTCAAGA CCCAATAACA GTATACAAGT AAGTCAAGTC ACAAAGATCG   
  
  
- TACTAACGAA AAACTACGGA AGAAAAAACA AGACCCAACC TGCTTGAGAC AGAAAAGACA CGTATCTAAA   
  
  
- CTCGAGGCTC GAGTTAAAAA CCCTAAAAAA ATCGACTGAA AAGACGAACC ACAACACTTT AACACTACCC   
  
  
- AAAACCGAAA CAATTTGAAC AAATATTGAA AAAGACAGTT TTGAAATATC ATTTAACAAC GTTAAACTCT   
  
  
- TAATACGTGA GTCACCGAGA CACTAAATAG CAGGCGACCG TTTAAAAGAC TACTTTCCTC TACTAAATAA   
  
  
- TTAAAAAGTC CGTACCAGTT TAAAAGATCG GAAAGTACAT GTAACCAACC ACTATCCGAC TATCGATTTA   
  
  
- ATACAGAGTA AAGAGATTTA AATAACGGTC TATACTTTAA CCACCTTGTC AAATTCCCAA AACCAGCAGA   
  
  
- AAAAACGGGA AAAAAAACCC AGGGGGGGGG CGGGCCCCCC CTTGGGCCTC CCGTCTTTTT CATTGTATTG   
  
  
- AATTTTAATA GTAACTGCAT CAGGAGGTTG GTATTACTTT CGTTGACTTT ATTCTCGTAC GATTCGATCT   
  
  
- CTCATCCGAC GGAACAGTCG GAGGGAAAAC TGTACTCTTT CTACCCTCTT CAAATTTTAT TTCAAGGAGC   
  
  
- AGAAACGTAG GGCCTGGTGT TAAGCCTTGA CCCACTCCTG GAGGAATCCG AACATTTGTG TTCAAAGCTT   
  
  
- GAATCTAAAC GCATGTACAG TTACGAAATG ACCTATTCGA ATCGATTGTG GGGAGTAGGA AGTTGGACAT   
  
  
- GGAAGTATAT TTTTTTTCCT GTTAGGCCAC GTGTTCGTAG GGCGTAAGTG CGTTCCAGAC CCCTTCCCGG   
  
  
- CGTGGGGTTT CCCACTTAAC ATCCGTCGGA TTGGACTGAA ACAGTCACCG ATTAAGGTGC TAAGCTTGGG   
  
  
- TACTAAAAAA CCAATGTGCC CTTGTTGGAA TGGTAACGAG TTTCCGAGGG GAAGAAGTTG GAGATGGAAG   
  
  
- TATATACTTA CTTTTAGGAA ACCGATTCTT AACTTTTCCT TTATACACCA ATAACCCAAA CCCAAGAATA   
  
  
- AGGCAACCAA CGGGGAAGTG TAACTTACGT AGGAGAGCGA TTCTTGACTT TTCCTGTATA CCCCTACATA   
  
  
- TCCAGTTTAA ACTGAGTTAC TATTGACTCC TATGTTCTTA AGATCTTCAG AAATAGTCCC CTTAAAAAAG   
  
  
- AAAAAAGGTG AACACTGAAA AACTACGGAA CGAAAAATGA GAAGTAACTT ACTCATGGGA AGAACTGTCT   
  
  
- AAGAAGGAGG GAAAAAAGAA TTAAAGAAGA ACATCACCTT ATATTCATAC GAAGATCCAT CAACCTACGG   
  
  
- TACCTTAGAG TAGGGACACA CACGTAAGAA GGGGAGAGGG CGGCGGGGGG GGGGGGGTTT TTCTTTGGTT   
  
  
- TTTTTTTTTT TTTTACCTAC TAAGAATCTT GACTGGTAAA AAGGAAGGAG GAGGGTAAAA TAAAAAAAGT   
  
  
- CCGACAAAAA GAAGATGGAC AACGAAATCG TAGTGAGTAT TTTTGTTCCA CGTTCGCGTA CTGTAGCCCA   
  
  
- TGACTCGAAG ACTCAAATGT ACGTAGCAGT ACTTTAAGCC ATCTGAAACT TCATTTCAAA GGGGTAGCAT   
  
  
- TGACCACCAC GTATGGACAG AAGTGCGGGT AGTCATCTCA CCACTTCACT TCGTTACAGC TTATCAAACA   
  
  
- TAATGCTCGG GTTCTCTCTT TGACTACGTA TAAAATACGG AGTTACAGTT AGGAACTTGG GGGTCGAGCT   
  
  
- AATGTTGTGG TAACTGATAC CTCGAAAATA CACGAGTTTT CAAAAGGACC CAGTCATGAC GTGAGACCTT   
  
  
- AGTAGGAGTC CTTGACCCTG AACAGGCCAC AGATCGAGGT GTAGTTGAGA GTCGAAGAGT AGGTTACCAT   
  
  
- CGGGACCTAG TGTCGAACTA AGGTTGAGAA TAGGAAGACT AGTTTTAACG AGAGGACTAT TACGGTTAAG   
  
  
- GAGTAGAAAT TCAGTAAGGA CGCAGTGTCT ACTACACCTA CTGGACTTTA TGTCCGACTC TCTCAACCTT   
  
  
- TGTCACTACA ACCCTGGACT AGGGCTAAAA CAGATGCCGT TATCGTTGGT GTGTAAACCC CAACCCTAGG   
  
  
- TACATCACAG TAGTCTCAAA CTGTCGACCC CCTTTCACTA CCTCTACTAG CGAGCTTTCC TAAACTTTGA   
  
  
- CCACGATTAA CGGACACGTT CTCGTCAACG ACTTTTACTA GTCAACAACC GGGTCACCGA CTACCTACTT   
  
  
- AACTCTGTCT ACCAAAGTCA AAGACCACTT GGTTAAGTTT CTAACCCACG AATATACAAC CTTCCCGAGC   
  
  
- ATCGCTCCGT CCGGAGGAGT CCATCGAGGT AAATATTTCG TAACTCTACG TTCCTTGGGC GCTCACGACT   
  
  
- GGAAAATAGA ATGTACGTGT ATGAGAAACT CCAAACGGGT ATGTAGTTTA AACCGATGTA CAGTCGTTTA   
  
  
- CCACGGTAAC GTCTTCGTTA CTTCCTACTT TTCTCTCAGG TGTAACAGCT AAAGGTTTAA CCCGTCCCTT   
  
  
- CGGTTACCCA CTGGGATTAG GTCCGTAAAC GTCGGGCCGG TCCCCCAGGT GGGGTGTAAG CGTAATGGCC   
  
  
- ATAGCTACTG AGGATGAGAC GTATACGAGC ACCTCCACCC AACTTGTAAC ACCCGGTATC CGAGAACTCC   
  
  
- AACCGAGTCA GTAAATTCCA GGGTAAACTC AAGTTGCGTC AACTGCAGGG CCGAACAGTT CAGGACGAAT   
  
  
- TTCTAGAGCC GTAAGTTGTA CCACTCCGGA ACCGACACTT GAAACGGAAG TAAGAAGTGG TGTACGGGCT   
  
  
- ACTCTCGCAC TCGTGTCTCT TAGTGGCCCT AGCAGAAGAC TCCCACCATT TTCCCGACTT CGGTTTTCAC   
  
  
- CATTGCAATC ACCTCGTTCT CACATTGTGT TTGAGACGAC GAAAGAACGG AGCGAAACAA CTCTGTAACC   
  
  
- TTATGATGTG TCGCTACAAA CTCAGGTACC TACACTGAAA CGGATCCCTA GTATTTCTGG CCTAGTTACA   
  
  
- ACTCGTCGTA ACAGAACGAG CTCTACAACA CTTGAACCAA CGCACACTTC CTCGACTCTC CCACCTCGCC   
  
  
- GTACTCGAGG AACCCTTTAC CTCCAGCGCC AAGTGTTACC GTCCCAAATT CGGTATGGGA AATTCGTGAG   
  
  
- ACCATTTGTT GTGTTATTCT TGAAACAACT TCTTGATGAC ACTATCCATA CCCGATCTCC TCTCCTTACC   
  
  
- TCCAGAAATA GAACCCACCT ACTTATCTCT AAACCAACGA AGCTCACGGA CCGTCACATC ATTAAT

+     ERE

| Site Name | Organism | Position | Strand | Matrix score. | sequence | function |
| --- | --- | --- | --- | --- | --- | --- |
| ERE | Nicotiana glutinos | 966 | - | 8 | ATTTTAAA |  |
| ERE | Nicotiana glutinos | 736 | - | 8 | ATTTCATA |  |

>HU01G01391.1   
+ +Up\_Stream \_Len000CATTTT AGTAATAAAT TCCCATGTGA GGACCACTGC CTACTGATTC CGAATGGCAT   
  
  
+ TTTAGTAATA AACTCCCATG TGAGGACCAC TTGATGAGGA ATTCAGTTTT ATAATCCACA GGTGTCAAAG   
  
  
+ CCTATTCCTA CTCTCACCAC TCTGCTATCT CTCCCCCCCC CCCCCCCCCG CCCCCCTCAA AATCTGCCTC   
  
  
+ CTCCGTTCTC TCCACAGCAG ATTCCTCAAT CAGGAAAGGT ATGTCAGAGC TCAAAACGAT TCAACATCAA   
  
  
+ AATGGGATAA TTTATTCTGC TTTCAGTTCT GGGTTATTGT CATATGTTCA TTCAGTTCAG TGTTTCTAGC   
  
  
+ ATGATTGCTT TTTGATGCCT TCTTTTTTGT TCTGGGTTGG ACGAACTCTG TCTTTTCTGT GCATAGATTT   
  
  
+ GAGCTCCGAG CTCAATTTTT GGGATTTTTT TAGCTGACTT TTCTGCTTGG TGTTGTGAAA TTGTGATGGG   
  
  
+ TTTTGGCTTT GTTAAACTTG TTTATAACTT TTTCTGTCAA AACTTTATAG TAAATTGTTG CAATTTGAGA   
  
  
+ ATTATGCACT CAGTGGCTCT GTGATTTATC GTCCGCTGGC AAATTTTCTG ATGAAAGGAG ATGATTTATT   
  
  
+ AATTTTTCAG GCATGGTCAA ATTTTCTAGC CTTTCATGTA CATTGGTTGG TGATAGGCTG ATAGCTAAAT   
  
  
+ TATGTCTCAT TTCTCTAAAT TTATTGCCAG ATATGAAATT GGTGGAACAG TTTAAGGGTT TTGGTCGTCT   
  
  
+ TTTTTGCCCT TTTTTTTGGG TCCCCCCCCC GCCCGGGGGG GAACCCGGAG GGCAGAAAAA GTAACATAAC   
  
  
+ TTAAAATTAT CATTGACGTA GTCCTCCAAC CATAATGAAA GCAACTGAAA TAAGAGCATG CTAAGCTAGA   
  
  
+ GAGTAGGCTG CCTTGTCAGC CTCCCTTTTG ACATGAGAAA GATGGGAGAA GTTTAAAATA AAGTTCCTCG   
  
  
+ TCTTTGCATC CCGGACCACA ATTCGGAACT GGGTGAGGAC CTCCTTAGGC TTGTAAACAC AAGTTTCGAA   
  
  
+ CTTAGATTTG CGTACATGTC AATGCTTTAC TGGATAAGCT TAGCTAACAC CCCTCATCCT TCAACCTGTA   
  
  
+ CCTTCATATA AAAAAAAGGA CAATCCGGTG CACAAGCATC CCGCATTCAC GCAAGGTCTG GGGAAGGGCC   
  
  
+ GCACCCCAAA GGGTGAATTG TAGGCAGCCT AACCTGACTT TGTCAGTGGC TAATTCCACG ATTCGAACCC   
  
  
+ ATGATTTTTT GGTTACACGG GAACAACCTT ACCATTGCTC AAAGGCTCCC CTTCTTCAAC CTCTACCTTC   
  
  
+ ATATATGAAT GAAAATCCTT TGGCTAAGAA TTGAAAAGGA AATATGTGGT TATTGGGTTT GGGTTCTTAT   
  
  
+ TCCGTTGGTT GCCCCTTCAC ATTGAATGCA TCCTCTCGCT AAGAACTGAA AAGGACATAT GGGGATGTAT   
  
  
+ AGGTCAAATT TGACTCAATG ATAACTGAGG ATACAAGAAT TCTAGAAGTC TTTATCAGGG GAATTTTTTC   
  
  
+ TTTTTTCCAC TTGTGACTTT TTGATGCCTT GCTTTTTACT CTTCATTGAA TGAGTACCCT TCTTGACAGA   
  
  
+ TTCTTCCTCC CTTTTTTCTT AATTTCTTCT TGTAGTGGAA TATAAGTATG CTTCTAGGTA GTTGGATGCC   
  
  
+ ATGGAATCTC ATCCCTGTGT GTGCATTCTT CCCCTCTCCC GCCGCCCCCC CCCCCCCAAA AAGAAACCAA   
  
  
+ AAAAAAAAAA AAAATGGATG ATTCTTAGAA CTGACCATTT TTCCTTCCTC CTCCCATTTT ATTTTTTTCA   
  
  
+ GGCTGTTTTT CTTCTACCTG TTGCTTTAGC ATCACTCATA AAAACAAGGT GCAAGCGCAT GACATCGGGT   
  
  
+ ACTGAGCTTC TGAGTTTACA TGCATCGTCA TGAAATTCGG TAGACTTTGA AGTAAAGTTT CCCCATCGTA   
  
  
+ ACTGGTGGTG CATACCTGTC TTCACGCCCA TCAGTAGAGT GGTGAAGTGA AGCAATGTCG AATAGTTTGT   
  
  
+ ATTACGAGCC CAAGAGAGAA ACTGATGCAT ATTTTATGCC TCAATGTCAA TCCTTGAACC CCCAGCTCGA   
  
  
+ TTACAACACC ATTGACTATG GAGCTTTTAT GTGCTCAAAA GTTTTCCTGG GTCAGTACTG CACTCTGGAA   
  
  
+ TCATCCTCAG GAACTGGGAC TTGTCCGGTG TCTAGCTCCA CATCAACTCT CAGCTTCTCA TCCAATGGTA   
  
  
+ GCCCTGGATC ACAGCTTGAT TCCAACTCTT ATCCTTCTGA TCAAAATTGC TCTCCTGATA ATGCCAATTC   
  
  
+ CTCATCTTTA AGTCATTCCT GCGTCACAGA TGATGTGGAT GACCTGAAAT ACAGGCTGAG AGAGTTGGAA   
  
  
+ ACAGTGATGT TGGGACCTGA TCCCGATTTT GTCTACGGCA ATAGCAACCA CACATTTGGG GTTGGGATCC   
  
  
+ ATGTAGTGTC ATCAGAGTTT GACAGCTGGG GGAAAGTGAT GGAGATGATC GCTCGAAAGG ATTTGAAACT   
  
  
+ GGTGCTAATT GCCTGTGCAA GAGCAGTTGC TGAAAATGAT CAGTTGTTGG CCCAGTGGCT GATGGATGAA   
  
  
+ TTGAGACAGA TGGTTTCAGT TTCTGGTGAA CCAATTCAAA GATTGGGTGC TTATATGTTG GAAGGGCTCG   
  
  
+ TAGCGAGGCA GGCCTCCTCA GGTAGCTCCA TTTATAAAGC ATTGAGATGC AAGGAACCCG CGAGTGCTGA   
  
  
+ CCTTTTATCT TACATGCACA TACTCTTTGA GGTTTGCCCA TACATCAAAT TTGGCTACAT GTCAGCAAAT   
  
  
+ GGTGCCATTG CAGAAGCAAT GAAGGATGAA AAGAGAGTCC ACATTGTCGA TTTCCAAATT GGGCAGGGAA   
  
  
+ GCCAATGGGT GACCCTAATC CAGGCATTTG CAGCCCGGCC AGGGGGTCCA CCCCACATTC GCATTACCGG   
  
  
+ TATCGATGAC TCCTACTCTG CATATGCTCG TGGAGGTGGG TTGAACATTG TGGGCCATAG GCTCTTGAGG   
  
  
+ TTGGCTCAGT CATTTAAGGT CCCATTTGAG TTCAACGCAG TTGACGTCCC GGCTTGTCAA GTCCTGCTTA   
  
  
+ AAGATCTCGG CATTCAACAT GGTGAGGCCT TGGCTGTGAA CTTTGCCTTC ATTCTTCACC ACATGCCCGA   
  
  
+ TGAGAGCGTG AGCACAGAGA ATCACCGGGA TCGTCTTCTG AGGGTGGTAA AAGGGCTGAA GCCAAAAGTG   
  
  
+ GTAACGTTAG TGGAGCAAGA GTGTAACACA AACTCTGCTG CTTTCTTGCC TCGCTTTGTT GAGACATTGG   
  
  
+ AATACTACAC AGCGATGTTT GAGTCCATGG ATGTGACTTT GCCTAGGGAT CATAAAGACC GGATCAATGT   
  
  
+ TGAGCAGCAT TGTCTTGCTC GAGATGTTGT GAACTTGGTT GCGTGTGAAG GAGCTGAGAG GGTGGAGCGG   
  
  
+ CATGAGCTCC TTGGGAAATG GAGGTCGCGG TTCACAATGG CAGGGTTTAA GCCATACCCT TTAAGCACTC   
  
  
+ TGGTAAACAA CACAATAAGA ACTTTGTTGA AGAACTACTG TGATAGGTAT GGGCTAGAGG AGAGGAATGG   
  
  
+ AGGTCTTTAT CTTGGGTGGA TGAATAGAGA TTTGGTTGCT TCGAGTGCCT GGCAGTGTAG TAATTA  

- +Up\_Stream \_Len000GTAAAA TCATTATTTA AGGGTACACT CCTGGTGACG GATGACTAAG GCTTACCGTA   
  
  
- AAATCATTAT TTGAGGGTAC ACTCCTGGTG AACTACTCCT TAAGTCAAAA TATTAGGTGT CCACAGTTTC   
  
  
- GGATAAGGAT GAGAGTGGTG AGACGATAGA GAGGGGGGGG GGGGGGGGGC GGGGGGAGTT TTAGACGGAG   
  
  
- GAGGCAAGAG AGGTGTCGTC TAAGGAGTTA GTCCTTTCCA TACAGTCTCG AGTTTTGCTA AGTTGTAGTT   
  
  
- TTACCCTATT AAATAAGACG AAAGTCAAGA CCCAATAACA GTATACAAGT AAGTCAAGTC ACAAAGATCG   
  
  
- TACTAACGAA AAACTACGGA AGAAAAAACA AGACCCAACC TGCTTGAGAC AGAAAAGACA CGTATCTAAA   
  
  
- CTCGAGGCTC GAGTTAAAAA CCCTAAAAAA ATCGACTGAA AAGACGAACC ACAACACTTT AACACTACCC   
  
  
- AAAACCGAAA CAATTTGAAC AAATATTGAA AAAGACAGTT TTGAAATATC ATTTAACAAC GTTAAACTCT   
  
  
- TAATACGTGA GTCACCGAGA CACTAAATAG CAGGCGACCG TTTAAAAGAC TACTTTCCTC TACTAAATAA   
  
  
- TTAAAAAGTC CGTACCAGTT TAAAAGATCG GAAAGTACAT GTAACCAACC ACTATCCGAC TATCGATTTA   
  
  
- ATACAGAGTA AAGAGATTTA AATAACGGTC TATACTTTAA CCACCTTGTC AAATTCCCAA AACCAGCAGA   
  
  
- AAAAACGGGA AAAAAAACCC AGGGGGGGGG CGGGCCCCCC CTTGGGCCTC CCGTCTTTTT CATTGTATTG   
  
  
- AATTTTAATA GTAACTGCAT CAGGAGGTTG GTATTACTTT CGTTGACTTT ATTCTCGTAC GATTCGATCT   
  
  
- CTCATCCGAC GGAACAGTCG GAGGGAAAAC TGTACTCTTT CTACCCTCTT CAAATTTTAT TTCAAGGAGC   
  
  
- AGAAACGTAG GGCCTGGTGT TAAGCCTTGA CCCACTCCTG GAGGAATCCG AACATTTGTG TTCAAAGCTT   
  
  
- GAATCTAAAC GCATGTACAG TTACGAAATG ACCTATTCGA ATCGATTGTG GGGAGTAGGA AGTTGGACAT   
  
  
- GGAAGTATAT TTTTTTTCCT GTTAGGCCAC GTGTTCGTAG GGCGTAAGTG CGTTCCAGAC CCCTTCCCGG   
  
  
- CGTGGGGTTT CCCACTTAAC ATCCGTCGGA TTGGACTGAA ACAGTCACCG ATTAAGGTGC TAAGCTTGGG   
  
  
- TACTAAAAAA CCAATGTGCC CTTGTTGGAA TGGTAACGAG TTTCCGAGGG GAAGAAGTTG GAGATGGAAG   
  
  
- TATATACTTA CTTTTAGGAA ACCGATTCTT AACTTTTCCT TTATACACCA ATAACCCAAA CCCAAGAATA   
  
  
- AGGCAACCAA CGGGGAAGTG TAACTTACGT AGGAGAGCGA TTCTTGACTT TTCCTGTATA CCCCTACATA   
  
  
- TCCAGTTTAA ACTGAGTTAC TATTGACTCC TATGTTCTTA AGATCTTCAG AAATAGTCCC CTTAAAAAAG   
  
  
- AAAAAAGGTG AACACTGAAA AACTACGGAA CGAAAAATGA GAAGTAACTT ACTCATGGGA AGAACTGTCT   
  
  
- AAGAAGGAGG GAAAAAAGAA TTAAAGAAGA ACATCACCTT ATATTCATAC GAAGATCCAT CAACCTACGG   
  
  
- TACCTTAGAG TAGGGACACA CACGTAAGAA GGGGAGAGGG CGGCGGGGGG GGGGGGGTTT TTCTTTGGTT   
  
  
- TTTTTTTTTT TTTTACCTAC TAAGAATCTT GACTGGTAAA AAGGAAGGAG GAGGGTAAAA TAAAAAAAGT   
  
  
- CCGACAAAAA GAAGATGGAC AACGAAATCG TAGTGAGTAT TTTTGTTCCA CGTTCGCGTA CTGTAGCCCA   
  
  
- TGACTCGAAG ACTCAAATGT ACGTAGCAGT ACTTTAAGCC ATCTGAAACT TCATTTCAAA GGGGTAGCAT   
  
  
- TGACCACCAC GTATGGACAG AAGTGCGGGT AGTCATCTCA CCACTTCACT TCGTTACAGC TTATCAAACA   
  
  
- TAATGCTCGG GTTCTCTCTT TGACTACGTA TAAAATACGG AGTTACAGTT AGGAACTTGG GGGTCGAGCT   
  
  
- AATGTTGTGG TAACTGATAC CTCGAAAATA CACGAGTTTT CAAAAGGACC CAGTCATGAC GTGAGACCTT   
  
  
- AGTAGGAGTC CTTGACCCTG AACAGGCCAC AGATCGAGGT GTAGTTGAGA GTCGAAGAGT AGGTTACCAT   
  
  
- CGGGACCTAG TGTCGAACTA AGGTTGAGAA TAGGAAGACT AGTTTTAACG AGAGGACTAT TACGGTTAAG   
  
  
- GAGTAGAAAT TCAGTAAGGA CGCAGTGTCT ACTACACCTA CTGGACTTTA TGTCCGACTC TCTCAACCTT   
  
  
- TGTCACTACA ACCCTGGACT AGGGCTAAAA CAGATGCCGT TATCGTTGGT GTGTAAACCC CAACCCTAGG   
  
  
- TACATCACAG TAGTCTCAAA CTGTCGACCC CCTTTCACTA CCTCTACTAG CGAGCTTTCC TAAACTTTGA   
  
  
- CCACGATTAA CGGACACGTT CTCGTCAACG ACTTTTACTA GTCAACAACC GGGTCACCGA CTACCTACTT   
  
  
- AACTCTGTCT ACCAAAGTCA AAGACCACTT GGTTAAGTTT CTAACCCACG AATATACAAC CTTCCCGAGC   
  
  
- ATCGCTCCGT CCGGAGGAGT CCATCGAGGT AAATATTTCG TAACTCTACG TTCCTTGGGC GCTCACGACT   
  
  
- GGAAAATAGA ATGTACGTGT ATGAGAAACT CCAAACGGGT ATGTAGTTTA AACCGATGTA CAGTCGTTTA   
  
  
- CCACGGTAAC GTCTTCGTTA CTTCCTACTT TTCTCTCAGG TGTAACAGCT AAAGGTTTAA CCCGTCCCTT   
  
  
- CGGTTACCCA CTGGGATTAG GTCCGTAAAC GTCGGGCCGG TCCCCCAGGT GGGGTGTAAG CGTAATGGCC   
  
  
- ATAGCTACTG AGGATGAGAC GTATACGAGC ACCTCCACCC AACTTGTAAC ACCCGGTATC CGAGAACTCC   
  
  
- AACCGAGTCA GTAAATTCCA GGGTAAACTC AAGTTGCGTC AACTGCAGGG CCGAACAGTT CAGGACGAAT   
  
  
- TTCTAGAGCC GTAAGTTGTA CCACTCCGGA ACCGACACTT GAAACGGAAG TAAGAAGTGG TGTACGGGCT   
  
  
- ACTCTCGCAC TCGTGTCTCT TAGTGGCCCT AGCAGAAGAC TCCCACCATT TTCCCGACTT CGGTTTTCAC   
  
  
- CATTGCAATC ACCTCGTTCT CACATTGTGT TTGAGACGAC GAAAGAACGG AGCGAAACAA CTCTGTAACC   
  
  
- TTATGATGTG TCGCTACAAA CTCAGGTACC TACACTGAAA CGGATCCCTA GTATTTCTGG CCTAGTTACA   
  
  
- ACTCGTCGTA ACAGAACGAG CTCTACAACA CTTGAACCAA CGCACACTTC CTCGACTCTC CCACCTCGCC   
  
  
- GTACTCGAGG AACCCTTTAC CTCCAGCGCC AAGTGTTACC GTCCCAAATT CGGTATGGGA AATTCGTGAG   
  
  
- ACCATTTGTT GTGTTATTCT TGAAACAACT TCTTGATGAC ACTATCCATA CCCGATCTCC TCTCCTTACC   
  
  
- TCCAGAAATA GAACCCACCT ACTTATCTCT AAACCAACGA AGCTCACGGA CCGTCACATC ATTAAT

+     GATA-motif

| Site Name | Organism | Position | Strand | Matrix score. | sequence | function |
| --- | --- | --- | --- | --- | --- | --- |
| GATA-motif | Solanum tuberosum | 1106 | - | 9 | AAGGATAAGG | part of a light responsive element |
| GATA-motif | Solanum tuberosum | 2271 | - | 9 | AAGGATAAGG | part of a light responsive element |

>HU01G01391.1   
+ +Up\_Stream \_Len000CATTTT AGTAATAAAT TCCCATGTGA GGACCACTGC CTACTGATTC CGAATGGCAT   
  
  
+ TTTAGTAATA AACTCCCATG TGAGGACCAC TTGATGAGGA ATTCAGTTTT ATAATCCACA GGTGTCAAAG   
  
  
+ CCTATTCCTA CTCTCACCAC TCTGCTATCT CTCCCCCCCC CCCCCCCCCG CCCCCCTCAA AATCTGCCTC   
  
  
+ CTCCGTTCTC TCCACAGCAG ATTCCTCAAT CAGGAAAGGT ATGTCAGAGC TCAAAACGAT TCAACATCAA   
  
  
+ AATGGGATAA TTTATTCTGC TTTCAGTTCT GGGTTATTGT CATATGTTCA TTCAGTTCAG TGTTTCTAGC   
  
  
+ ATGATTGCTT TTTGATGCCT TCTTTTTTGT TCTGGGTTGG ACGAACTCTG TCTTTTCTGT GCATAGATTT   
  
  
+ GAGCTCCGAG CTCAATTTTT GGGATTTTTT TAGCTGACTT TTCTGCTTGG TGTTGTGAAA TTGTGATGGG   
  
  
+ TTTTGGCTTT GTTAAACTTG TTTATAACTT TTTCTGTCAA AACTTTATAG TAAATTGTTG CAATTTGAGA   
  
  
+ ATTATGCACT CAGTGGCTCT GTGATTTATC GTCCGCTGGC AAATTTTCTG ATGAAAGGAG ATGATTTATT   
  
  
+ AATTTTTCAG GCATGGTCAA ATTTTCTAGC CTTTCATGTA CATTGGTTGG TGATAGGCTG ATAGCTAAAT   
  
  
+ TATGTCTCAT TTCTCTAAAT TTATTGCCAG ATATGAAATT GGTGGAACAG TTTAAGGGTT TTGGTCGTCT   
  
  
+ TTTTTGCCCT TTTTTTTGGG TCCCCCCCCC GCCCGGGGGG GAACCCGGAG GGCAGAAAAA GTAACATAAC   
  
  
+ TTAAAATTAT CATTGACGTA GTCCTCCAAC CATAATGAAA GCAACTGAAA TAAGAGCATG CTAAGCTAGA   
  
  
+ GAGTAGGCTG CCTTGTCAGC CTCCCTTTTG ACATGAGAAA GATGGGAGAA GTTTAAAATA AAGTTCCTCG   
  
  
+ TCTTTGCATC CCGGACCACA ATTCGGAACT GGGTGAGGAC CTCCTTAGGC TTGTAAACAC AAGTTTCGAA   
  
  
+ CTTAGATTTG CGTACATGTC AATGCTTTAC TGGATAAGCT TAGCTAACAC CCCTCATCCT TCAACCTGTA   
  
  
+ CCTTCATATA AAAAAAAGGA CAATCCGGTG CACAAGCATC CCGCATTCAC GCAAGGTCTG GGGAAGGGCC   
  
  
+ GCACCCCAAA GGGTGAATTG TAGGCAGCCT AACCTGACTT TGTCAGTGGC TAATTCCACG ATTCGAACCC   
  
  
+ ATGATTTTTT GGTTACACGG GAACAACCTT ACCATTGCTC AAAGGCTCCC CTTCTTCAAC CTCTACCTTC   
  
  
+ ATATATGAAT GAAAATCCTT TGGCTAAGAA TTGAAAAGGA AATATGTGGT TATTGGGTTT GGGTTCTTAT   
  
  
+ TCCGTTGGTT GCCCCTTCAC ATTGAATGCA TCCTCTCGCT AAGAACTGAA AAGGACATAT GGGGATGTAT   
  
  
+ AGGTCAAATT TGACTCAATG ATAACTGAGG ATACAAGAAT TCTAGAAGTC TTTATCAGGG GAATTTTTTC   
  
  
+ TTTTTTCCAC TTGTGACTTT TTGATGCCTT GCTTTTTACT CTTCATTGAA TGAGTACCCT TCTTGACAGA   
  
  
+ TTCTTCCTCC CTTTTTTCTT AATTTCTTCT TGTAGTGGAA TATAAGTATG CTTCTAGGTA GTTGGATGCC   
  
  
+ ATGGAATCTC ATCCCTGTGT GTGCATTCTT CCCCTCTCCC GCCGCCCCCC CCCCCCCAAA AAGAAACCAA   
  
  
+ AAAAAAAAAA AAAATGGATG ATTCTTAGAA CTGACCATTT TTCCTTCCTC CTCCCATTTT ATTTTTTTCA   
  
  
+ GGCTGTTTTT CTTCTACCTG TTGCTTTAGC ATCACTCATA AAAACAAGGT GCAAGCGCAT GACATCGGGT   
  
  
+ ACTGAGCTTC TGAGTTTACA TGCATCGTCA TGAAATTCGG TAGACTTTGA AGTAAAGTTT CCCCATCGTA   
  
  
+ ACTGGTGGTG CATACCTGTC TTCACGCCCA TCAGTAGAGT GGTGAAGTGA AGCAATGTCG AATAGTTTGT   
  
  
+ ATTACGAGCC CAAGAGAGAA ACTGATGCAT ATTTTATGCC TCAATGTCAA TCCTTGAACC CCCAGCTCGA   
  
  
+ TTACAACACC ATTGACTATG GAGCTTTTAT GTGCTCAAAA GTTTTCCTGG GTCAGTACTG CACTCTGGAA   
  
  
+ TCATCCTCAG GAACTGGGAC TTGTCCGGTG TCTAGCTCCA CATCAACTCT CAGCTTCTCA TCCAATGGTA   
  
  
+ GCCCTGGATC ACAGCTTGAT TCCAACTCTT ATCCTTCTGA TCAAAATTGC TCTCCTGATA ATGCCAATTC   
  
  
+ CTCATCTTTA AGTCATTCCT GCGTCACAGA TGATGTGGAT GACCTGAAAT ACAGGCTGAG AGAGTTGGAA   
  
  
+ ACAGTGATGT TGGGACCTGA TCCCGATTTT GTCTACGGCA ATAGCAACCA CACATTTGGG GTTGGGATCC   
  
  
+ ATGTAGTGTC ATCAGAGTTT GACAGCTGGG GGAAAGTGAT GGAGATGATC GCTCGAAAGG ATTTGAAACT   
  
  
+ GGTGCTAATT GCCTGTGCAA GAGCAGTTGC TGAAAATGAT CAGTTGTTGG CCCAGTGGCT GATGGATGAA   
  
  
+ TTGAGACAGA TGGTTTCAGT TTCTGGTGAA CCAATTCAAA GATTGGGTGC TTATATGTTG GAAGGGCTCG   
  
  
+ TAGCGAGGCA GGCCTCCTCA GGTAGCTCCA TTTATAAAGC ATTGAGATGC AAGGAACCCG CGAGTGCTGA   
  
  
+ CCTTTTATCT TACATGCACA TACTCTTTGA GGTTTGCCCA TACATCAAAT TTGGCTACAT GTCAGCAAAT   
  
  
+ GGTGCCATTG CAGAAGCAAT GAAGGATGAA AAGAGAGTCC ACATTGTCGA TTTCCAAATT GGGCAGGGAA   
  
  
+ GCCAATGGGT GACCCTAATC CAGGCATTTG CAGCCCGGCC AGGGGGTCCA CCCCACATTC GCATTACCGG   
  
  
+ TATCGATGAC TCCTACTCTG CATATGCTCG TGGAGGTGGG TTGAACATTG TGGGCCATAG GCTCTTGAGG   
  
  
+ TTGGCTCAGT CATTTAAGGT CCCATTTGAG TTCAACGCAG TTGACGTCCC GGCTTGTCAA GTCCTGCTTA   
  
  
+ AAGATCTCGG CATTCAACAT GGTGAGGCCT TGGCTGTGAA CTTTGCCTTC ATTCTTCACC ACATGCCCGA   
  
  
+ TGAGAGCGTG AGCACAGAGA ATCACCGGGA TCGTCTTCTG AGGGTGGTAA AAGGGCTGAA GCCAAAAGTG   
  
  
+ GTAACGTTAG TGGAGCAAGA GTGTAACACA AACTCTGCTG CTTTCTTGCC TCGCTTTGTT GAGACATTGG   
  
  
+ AATACTACAC AGCGATGTTT GAGTCCATGG ATGTGACTTT GCCTAGGGAT CATAAAGACC GGATCAATGT   
  
  
+ TGAGCAGCAT TGTCTTGCTC GAGATGTTGT GAACTTGGTT GCGTGTGAAG GAGCTGAGAG GGTGGAGCGG   
  
  
+ CATGAGCTCC TTGGGAAATG GAGGTCGCGG TTCACAATGG CAGGGTTTAA GCCATACCCT TTAAGCACTC   
  
  
+ TGGTAAACAA CACAATAAGA ACTTTGTTGA AGAACTACTG TGATAGGTAT GGGCTAGAGG AGAGGAATGG   
  
  
+ AGGTCTTTAT CTTGGGTGGA TGAATAGAGA TTTGGTTGCT TCGAGTGCCT GGCAGTGTAG TAATTA  

- +Up\_Stream \_Len000GTAAAA TCATTATTTA AGGGTACACT CCTGGTGACG GATGACTAAG GCTTACCGTA   
  
  
- AAATCATTAT TTGAGGGTAC ACTCCTGGTG AACTACTCCT TAAGTCAAAA TATTAGGTGT CCACAGTTTC   
  
  
- GGATAAGGAT GAGAGTGGTG AGACGATAGA GAGGGGGGGG GGGGGGGGGC GGGGGGAGTT TTAGACGGAG   
  
  
- GAGGCAAGAG AGGTGTCGTC TAAGGAGTTA GTCCTTTCCA TACAGTCTCG AGTTTTGCTA AGTTGTAGTT   
  
  
- TTACCCTATT AAATAAGACG AAAGTCAAGA CCCAATAACA GTATACAAGT AAGTCAAGTC ACAAAGATCG   
  
  
- TACTAACGAA AAACTACGGA AGAAAAAACA AGACCCAACC TGCTTGAGAC AGAAAAGACA CGTATCTAAA   
  
  
- CTCGAGGCTC GAGTTAAAAA CCCTAAAAAA ATCGACTGAA AAGACGAACC ACAACACTTT AACACTACCC   
  
  
- AAAACCGAAA CAATTTGAAC AAATATTGAA AAAGACAGTT TTGAAATATC ATTTAACAAC GTTAAACTCT   
  
  
- TAATACGTGA GTCACCGAGA CACTAAATAG CAGGCGACCG TTTAAAAGAC TACTTTCCTC TACTAAATAA   
  
  
- TTAAAAAGTC CGTACCAGTT TAAAAGATCG GAAAGTACAT GTAACCAACC ACTATCCGAC TATCGATTTA   
  
  
- ATACAGAGTA AAGAGATTTA AATAACGGTC TATACTTTAA CCACCTTGTC AAATTCCCAA AACCAGCAGA   
  
  
- AAAAACGGGA AAAAAAACCC AGGGGGGGGG CGGGCCCCCC CTTGGGCCTC CCGTCTTTTT CATTGTATTG   
  
  
- AATTTTAATA GTAACTGCAT CAGGAGGTTG GTATTACTTT CGTTGACTTT ATTCTCGTAC GATTCGATCT   
  
  
- CTCATCCGAC GGAACAGTCG GAGGGAAAAC TGTACTCTTT CTACCCTCTT CAAATTTTAT TTCAAGGAGC   
  
  
- AGAAACGTAG GGCCTGGTGT TAAGCCTTGA CCCACTCCTG GAGGAATCCG AACATTTGTG TTCAAAGCTT   
  
  
- GAATCTAAAC GCATGTACAG TTACGAAATG ACCTATTCGA ATCGATTGTG GGGAGTAGGA AGTTGGACAT   
  
  
- GGAAGTATAT TTTTTTTCCT GTTAGGCCAC GTGTTCGTAG GGCGTAAGTG CGTTCCAGAC CCCTTCCCGG   
  
  
- CGTGGGGTTT CCCACTTAAC ATCCGTCGGA TTGGACTGAA ACAGTCACCG ATTAAGGTGC TAAGCTTGGG   
  
  
- TACTAAAAAA CCAATGTGCC CTTGTTGGAA TGGTAACGAG TTTCCGAGGG GAAGAAGTTG GAGATGGAAG   
  
  
- TATATACTTA CTTTTAGGAA ACCGATTCTT AACTTTTCCT TTATACACCA ATAACCCAAA CCCAAGAATA   
  
  
- AGGCAACCAA CGGGGAAGTG TAACTTACGT AGGAGAGCGA TTCTTGACTT TTCCTGTATA CCCCTACATA   
  
  
- TCCAGTTTAA ACTGAGTTAC TATTGACTCC TATGTTCTTA AGATCTTCAG AAATAGTCCC CTTAAAAAAG   
  
  
- AAAAAAGGTG AACACTGAAA AACTACGGAA CGAAAAATGA GAAGTAACTT ACTCATGGGA AGAACTGTCT   
  
  
- AAGAAGGAGG GAAAAAAGAA TTAAAGAAGA ACATCACCTT ATATTCATAC GAAGATCCAT CAACCTACGG   
  
  
- TACCTTAGAG TAGGGACACA CACGTAAGAA GGGGAGAGGG CGGCGGGGGG GGGGGGGTTT TTCTTTGGTT   
  
  
- TTTTTTTTTT TTTTACCTAC TAAGAATCTT GACTGGTAAA AAGGAAGGAG GAGGGTAAAA TAAAAAAAGT   
  
  
- CCGACAAAAA GAAGATGGAC AACGAAATCG TAGTGAGTAT TTTTGTTCCA CGTTCGCGTA CTGTAGCCCA   
  
  
- TGACTCGAAG ACTCAAATGT ACGTAGCAGT ACTTTAAGCC ATCTGAAACT TCATTTCAAA GGGGTAGCAT   
  
  
- TGACCACCAC GTATGGACAG AAGTGCGGGT AGTCATCTCA CCACTTCACT TCGTTACAGC TTATCAAACA   
  
  
- TAATGCTCGG GTTCTCTCTT TGACTACGTA TAAAATACGG AGTTACAGTT AGGAACTTGG GGGTCGAGCT   
  
  
- AATGTTGTGG TAACTGATAC CTCGAAAATA CACGAGTTTT CAAAAGGACC CAGTCATGAC GTGAGACCTT   
  
  
- AGTAGGAGTC CTTGACCCTG AACAGGCCAC AGATCGAGGT GTAGTTGAGA GTCGAAGAGT AGGTTACCAT   
  
  
- CGGGACCTAG TGTCGAACTA AGGTTGAGAA TAGGAAGACT AGTTTTAACG AGAGGACTAT TACGGTTAAG   
  
  
- GAGTAGAAAT TCAGTAAGGA CGCAGTGTCT ACTACACCTA CTGGACTTTA TGTCCGACTC TCTCAACCTT   
  
  
- TGTCACTACA ACCCTGGACT AGGGCTAAAA CAGATGCCGT TATCGTTGGT GTGTAAACCC CAACCCTAGG   
  
  
- TACATCACAG TAGTCTCAAA CTGTCGACCC CCTTTCACTA CCTCTACTAG CGAGCTTTCC TAAACTTTGA   
  
  
- CCACGATTAA CGGACACGTT CTCGTCAACG ACTTTTACTA GTCAACAACC GGGTCACCGA CTACCTACTT   
  
  
- AACTCTGTCT ACCAAAGTCA AAGACCACTT GGTTAAGTTT CTAACCCACG AATATACAAC CTTCCCGAGC   
  
  
- ATCGCTCCGT CCGGAGGAGT CCATCGAGGT AAATATTTCG TAACTCTACG TTCCTTGGGC GCTCACGACT   
  
  
- GGAAAATAGA ATGTACGTGT ATGAGAAACT CCAAACGGGT ATGTAGTTTA AACCGATGTA CAGTCGTTTA   
  
  
- CCACGGTAAC GTCTTCGTTA CTTCCTACTT TTCTCTCAGG TGTAACAGCT AAAGGTTTAA CCCGTCCCTT   
  
  
- CGGTTACCCA CTGGGATTAG GTCCGTAAAC GTCGGGCCGG TCCCCCAGGT GGGGTGTAAG CGTAATGGCC   
  
  
- ATAGCTACTG AGGATGAGAC GTATACGAGC ACCTCCACCC AACTTGTAAC ACCCGGTATC CGAGAACTCC   
  
  
- AACCGAGTCA GTAAATTCCA GGGTAAACTC AAGTTGCGTC AACTGCAGGG CCGAACAGTT CAGGACGAAT   
  
  
- TTCTAGAGCC GTAAGTTGTA CCACTCCGGA ACCGACACTT GAAACGGAAG TAAGAAGTGG TGTACGGGCT   
  
  
- ACTCTCGCAC TCGTGTCTCT TAGTGGCCCT AGCAGAAGAC TCCCACCATT TTCCCGACTT CGGTTTTCAC   
  
  
- CATTGCAATC ACCTCGTTCT CACATTGTGT TTGAGACGAC GAAAGAACGG AGCGAAACAA CTCTGTAACC   
  
  
- TTATGATGTG TCGCTACAAA CTCAGGTACC TACACTGAAA CGGATCCCTA GTATTTCTGG CCTAGTTACA   
  
  
- ACTCGTCGTA ACAGAACGAG CTCTACAACA CTTGAACCAA CGCACACTTC CTCGACTCTC CCACCTCGCC   
  
  
- GTACTCGAGG AACCCTTTAC CTCCAGCGCC AAGTGTTACC GTCCCAAATT CGGTATGGGA AATTCGTGAG   
  
  
- ACCATTTGTT GTGTTATTCT TGAAACAACT TCTTGATGAC ACTATCCATA CCCGATCTCC TCTCCTTACC   
  
  
- TCCAGAAATA GAACCCACCT ACTTATCTCT AAACCAACGA AGCTCACGGA CCGTCACATC ATTAAT

+     GC-motif

| Site Name | Organism | Position | Strand | Matrix score. | sequence | function |
| --- | --- | --- | --- | --- | --- | --- |
| GC-motif | Zea mays | 808 | - | 6 | CCCCCG | enhancer-like element involved in anoxic specific inducibility |
| GC-motif | Zea mays | 189 | + | 6 | CCCCCG | enhancer-like element involved in anoxic specific inducibility |
| GC-motif | Zea mays | 800 | + | 6 | CCCCCG | enhancer-like element involved in anoxic specific inducibility |

>HU01G01391.1   
+ +Up\_Stream \_Len000CATTTT AGTAATAAAT TCCCATGTGA GGACCACTGC CTACTGATTC CGAATGGCAT   
  
  
+ TTTAGTAATA AACTCCCATG TGAGGACCAC TTGATGAGGA ATTCAGTTTT ATAATCCACA GGTGTCAAAG   
  
  
+ CCTATTCCTA CTCTCACCAC TCTGCTATCT CTCCCCCCCC CCCCCCCCCG CCCCCCTCAA AATCTGCCTC   
  
  
+ CTCCGTTCTC TCCACAGCAG ATTCCTCAAT CAGGAAAGGT ATGTCAGAGC TCAAAACGAT TCAACATCAA   
  
  
+ AATGGGATAA TTTATTCTGC TTTCAGTTCT GGGTTATTGT CATATGTTCA TTCAGTTCAG TGTTTCTAGC   
  
  
+ ATGATTGCTT TTTGATGCCT TCTTTTTTGT TCTGGGTTGG ACGAACTCTG TCTTTTCTGT GCATAGATTT   
  
  
+ GAGCTCCGAG CTCAATTTTT GGGATTTTTT TAGCTGACTT TTCTGCTTGG TGTTGTGAAA TTGTGATGGG   
  
  
+ TTTTGGCTTT GTTAAACTTG TTTATAACTT TTTCTGTCAA AACTTTATAG TAAATTGTTG CAATTTGAGA   
  
  
+ ATTATGCACT CAGTGGCTCT GTGATTTATC GTCCGCTGGC AAATTTTCTG ATGAAAGGAG ATGATTTATT   
  
  
+ AATTTTTCAG GCATGGTCAA ATTTTCTAGC CTTTCATGTA CATTGGTTGG TGATAGGCTG ATAGCTAAAT   
  
  
+ TATGTCTCAT TTCTCTAAAT TTATTGCCAG ATATGAAATT GGTGGAACAG TTTAAGGGTT TTGGTCGTCT   
  
  
+ TTTTTGCCCT TTTTTTTGGG TCCCCCCCCC GCCCGGGGGG GAACCCGGAG GGCAGAAAAA GTAACATAAC   
  
  
+ TTAAAATTAT CATTGACGTA GTCCTCCAAC CATAATGAAA GCAACTGAAA TAAGAGCATG CTAAGCTAGA   
  
  
+ GAGTAGGCTG CCTTGTCAGC CTCCCTTTTG ACATGAGAAA GATGGGAGAA GTTTAAAATA AAGTTCCTCG   
  
  
+ TCTTTGCATC CCGGACCACA ATTCGGAACT GGGTGAGGAC CTCCTTAGGC TTGTAAACAC AAGTTTCGAA   
  
  
+ CTTAGATTTG CGTACATGTC AATGCTTTAC TGGATAAGCT TAGCTAACAC CCCTCATCCT TCAACCTGTA   
  
  
+ CCTTCATATA AAAAAAAGGA CAATCCGGTG CACAAGCATC CCGCATTCAC GCAAGGTCTG GGGAAGGGCC   
  
  
+ GCACCCCAAA GGGTGAATTG TAGGCAGCCT AACCTGACTT TGTCAGTGGC TAATTCCACG ATTCGAACCC   
  
  
+ ATGATTTTTT GGTTACACGG GAACAACCTT ACCATTGCTC AAAGGCTCCC CTTCTTCAAC CTCTACCTTC   
  
  
+ ATATATGAAT GAAAATCCTT TGGCTAAGAA TTGAAAAGGA AATATGTGGT TATTGGGTTT GGGTTCTTAT   
  
  
+ TCCGTTGGTT GCCCCTTCAC ATTGAATGCA TCCTCTCGCT AAGAACTGAA AAGGACATAT GGGGATGTAT   
  
  
+ AGGTCAAATT TGACTCAATG ATAACTGAGG ATACAAGAAT TCTAGAAGTC TTTATCAGGG GAATTTTTTC   
  
  
+ TTTTTTCCAC TTGTGACTTT TTGATGCCTT GCTTTTTACT CTTCATTGAA TGAGTACCCT TCTTGACAGA   
  
  
+ TTCTTCCTCC CTTTTTTCTT AATTTCTTCT TGTAGTGGAA TATAAGTATG CTTCTAGGTA GTTGGATGCC   
  
  
+ ATGGAATCTC ATCCCTGTGT GTGCATTCTT CCCCTCTCCC GCCGCCCCCC CCCCCCCAAA AAGAAACCAA   
  
  
+ AAAAAAAAAA AAAATGGATG ATTCTTAGAA CTGACCATTT TTCCTTCCTC CTCCCATTTT ATTTTTTTCA   
  
  
+ GGCTGTTTTT CTTCTACCTG TTGCTTTAGC ATCACTCATA AAAACAAGGT GCAAGCGCAT GACATCGGGT   
  
  
+ ACTGAGCTTC TGAGTTTACA TGCATCGTCA TGAAATTCGG TAGACTTTGA AGTAAAGTTT CCCCATCGTA   
  
  
+ ACTGGTGGTG CATACCTGTC TTCACGCCCA TCAGTAGAGT GGTGAAGTGA AGCAATGTCG AATAGTTTGT   
  
  
+ ATTACGAGCC CAAGAGAGAA ACTGATGCAT ATTTTATGCC TCAATGTCAA TCCTTGAACC CCCAGCTCGA   
  
  
+ TTACAACACC ATTGACTATG GAGCTTTTAT GTGCTCAAAA GTTTTCCTGG GTCAGTACTG CACTCTGGAA   
  
  
+ TCATCCTCAG GAACTGGGAC TTGTCCGGTG TCTAGCTCCA CATCAACTCT CAGCTTCTCA TCCAATGGTA   
  
  
+ GCCCTGGATC ACAGCTTGAT TCCAACTCTT ATCCTTCTGA TCAAAATTGC TCTCCTGATA ATGCCAATTC   
  
  
+ CTCATCTTTA AGTCATTCCT GCGTCACAGA TGATGTGGAT GACCTGAAAT ACAGGCTGAG AGAGTTGGAA   
  
  
+ ACAGTGATGT TGGGACCTGA TCCCGATTTT GTCTACGGCA ATAGCAACCA CACATTTGGG GTTGGGATCC   
  
  
+ ATGTAGTGTC ATCAGAGTTT GACAGCTGGG GGAAAGTGAT GGAGATGATC GCTCGAAAGG ATTTGAAACT   
  
  
+ GGTGCTAATT GCCTGTGCAA GAGCAGTTGC TGAAAATGAT CAGTTGTTGG CCCAGTGGCT GATGGATGAA   
  
  
+ TTGAGACAGA TGGTTTCAGT TTCTGGTGAA CCAATTCAAA GATTGGGTGC TTATATGTTG GAAGGGCTCG   
  
  
+ TAGCGAGGCA GGCCTCCTCA GGTAGCTCCA TTTATAAAGC ATTGAGATGC AAGGAACCCG CGAGTGCTGA   
  
  
+ CCTTTTATCT TACATGCACA TACTCTTTGA GGTTTGCCCA TACATCAAAT TTGGCTACAT GTCAGCAAAT   
  
  
+ GGTGCCATTG CAGAAGCAAT GAAGGATGAA AAGAGAGTCC ACATTGTCGA TTTCCAAATT GGGCAGGGAA   
  
  
+ GCCAATGGGT GACCCTAATC CAGGCATTTG CAGCCCGGCC AGGGGGTCCA CCCCACATTC GCATTACCGG   
  
  
+ TATCGATGAC TCCTACTCTG CATATGCTCG TGGAGGTGGG TTGAACATTG TGGGCCATAG GCTCTTGAGG   
  
  
+ TTGGCTCAGT CATTTAAGGT CCCATTTGAG TTCAACGCAG TTGACGTCCC GGCTTGTCAA GTCCTGCTTA   
  
  
+ AAGATCTCGG CATTCAACAT GGTGAGGCCT TGGCTGTGAA CTTTGCCTTC ATTCTTCACC ACATGCCCGA   
  
  
+ TGAGAGCGTG AGCACAGAGA ATCACCGGGA TCGTCTTCTG AGGGTGGTAA AAGGGCTGAA GCCAAAAGTG   
  
  
+ GTAACGTTAG TGGAGCAAGA GTGTAACACA AACTCTGCTG CTTTCTTGCC TCGCTTTGTT GAGACATTGG   
  
  
+ AATACTACAC AGCGATGTTT GAGTCCATGG ATGTGACTTT GCCTAGGGAT CATAAAGACC GGATCAATGT   
  
  
+ TGAGCAGCAT TGTCTTGCTC GAGATGTTGT GAACTTGGTT GCGTGTGAAG GAGCTGAGAG GGTGGAGCGG   
  
  
+ CATGAGCTCC TTGGGAAATG GAGGTCGCGG TTCACAATGG CAGGGTTTAA GCCATACCCT TTAAGCACTC   
  
  
+ TGGTAAACAA CACAATAAGA ACTTTGTTGA AGAACTACTG TGATAGGTAT GGGCTAGAGG AGAGGAATGG   
  
  
+ AGGTCTTTAT CTTGGGTGGA TGAATAGAGA TTTGGTTGCT TCGAGTGCCT GGCAGTGTAG TAATTA  

- +Up\_Stream \_Len000GTAAAA TCATTATTTA AGGGTACACT CCTGGTGACG GATGACTAAG GCTTACCGTA   
  
  
- AAATCATTAT TTGAGGGTAC ACTCCTGGTG AACTACTCCT TAAGTCAAAA TATTAGGTGT CCACAGTTTC   
  
  
- GGATAAGGAT GAGAGTGGTG AGACGATAGA GAGGGGGGGG GGGGGGGGGC GGGGGGAGTT TTAGACGGAG   
  
  
- GAGGCAAGAG AGGTGTCGTC TAAGGAGTTA GTCCTTTCCA TACAGTCTCG AGTTTTGCTA AGTTGTAGTT   
  
  
- TTACCCTATT AAATAAGACG AAAGTCAAGA CCCAATAACA GTATACAAGT AAGTCAAGTC ACAAAGATCG   
  
  
- TACTAACGAA AAACTACGGA AGAAAAAACA AGACCCAACC TGCTTGAGAC AGAAAAGACA CGTATCTAAA   
  
  
- CTCGAGGCTC GAGTTAAAAA CCCTAAAAAA ATCGACTGAA AAGACGAACC ACAACACTTT AACACTACCC   
  
  
- AAAACCGAAA CAATTTGAAC AAATATTGAA AAAGACAGTT TTGAAATATC ATTTAACAAC GTTAAACTCT   
  
  
- TAATACGTGA GTCACCGAGA CACTAAATAG CAGGCGACCG TTTAAAAGAC TACTTTCCTC TACTAAATAA   
  
  
- TTAAAAAGTC CGTACCAGTT TAAAAGATCG GAAAGTACAT GTAACCAACC ACTATCCGAC TATCGATTTA   
  
  
- ATACAGAGTA AAGAGATTTA AATAACGGTC TATACTTTAA CCACCTTGTC AAATTCCCAA AACCAGCAGA   
  
  
- AAAAACGGGA AAAAAAACCC AGGGGGGGGG CGGGCCCCCC CTTGGGCCTC CCGTCTTTTT CATTGTATTG   
  
  
- AATTTTAATA GTAACTGCAT CAGGAGGTTG GTATTACTTT CGTTGACTTT ATTCTCGTAC GATTCGATCT   
  
  
- CTCATCCGAC GGAACAGTCG GAGGGAAAAC TGTACTCTTT CTACCCTCTT CAAATTTTAT TTCAAGGAGC   
  
  
- AGAAACGTAG GGCCTGGTGT TAAGCCTTGA CCCACTCCTG GAGGAATCCG AACATTTGTG TTCAAAGCTT   
  
  
- GAATCTAAAC GCATGTACAG TTACGAAATG ACCTATTCGA ATCGATTGTG GGGAGTAGGA AGTTGGACAT   
  
  
- GGAAGTATAT TTTTTTTCCT GTTAGGCCAC GTGTTCGTAG GGCGTAAGTG CGTTCCAGAC CCCTTCCCGG   
  
  
- CGTGGGGTTT CCCACTTAAC ATCCGTCGGA TTGGACTGAA ACAGTCACCG ATTAAGGTGC TAAGCTTGGG   
  
  
- TACTAAAAAA CCAATGTGCC CTTGTTGGAA TGGTAACGAG TTTCCGAGGG GAAGAAGTTG GAGATGGAAG   
  
  
- TATATACTTA CTTTTAGGAA ACCGATTCTT AACTTTTCCT TTATACACCA ATAACCCAAA CCCAAGAATA   
  
  
- AGGCAACCAA CGGGGAAGTG TAACTTACGT AGGAGAGCGA TTCTTGACTT TTCCTGTATA CCCCTACATA   
  
  
- TCCAGTTTAA ACTGAGTTAC TATTGACTCC TATGTTCTTA AGATCTTCAG AAATAGTCCC CTTAAAAAAG   
  
  
- AAAAAAGGTG AACACTGAAA AACTACGGAA CGAAAAATGA GAAGTAACTT ACTCATGGGA AGAACTGTCT   
  
  
- AAGAAGGAGG GAAAAAAGAA TTAAAGAAGA ACATCACCTT ATATTCATAC GAAGATCCAT CAACCTACGG   
  
  
- TACCTTAGAG TAGGGACACA CACGTAAGAA GGGGAGAGGG CGGCGGGGGG GGGGGGGTTT TTCTTTGGTT   
  
  
- TTTTTTTTTT TTTTACCTAC TAAGAATCTT GACTGGTAAA AAGGAAGGAG GAGGGTAAAA TAAAAAAAGT   
  
  
- CCGACAAAAA GAAGATGGAC AACGAAATCG TAGTGAGTAT TTTTGTTCCA CGTTCGCGTA CTGTAGCCCA   
  
  
- TGACTCGAAG ACTCAAATGT ACGTAGCAGT ACTTTAAGCC ATCTGAAACT TCATTTCAAA GGGGTAGCAT   
  
  
- TGACCACCAC GTATGGACAG AAGTGCGGGT AGTCATCTCA CCACTTCACT TCGTTACAGC TTATCAAACA   
  
  
- TAATGCTCGG GTTCTCTCTT TGACTACGTA TAAAATACGG AGTTACAGTT AGGAACTTGG GGGTCGAGCT   
  
  
- AATGTTGTGG TAACTGATAC CTCGAAAATA CACGAGTTTT CAAAAGGACC CAGTCATGAC GTGAGACCTT   
  
  
- AGTAGGAGTC CTTGACCCTG AACAGGCCAC AGATCGAGGT GTAGTTGAGA GTCGAAGAGT AGGTTACCAT   
  
  
- CGGGACCTAG TGTCGAACTA AGGTTGAGAA TAGGAAGACT AGTTTTAACG AGAGGACTAT TACGGTTAAG   
  
  
- GAGTAGAAAT TCAGTAAGGA CGCAGTGTCT ACTACACCTA CTGGACTTTA TGTCCGACTC TCTCAACCTT   
  
  
- TGTCACTACA ACCCTGGACT AGGGCTAAAA CAGATGCCGT TATCGTTGGT GTGTAAACCC CAACCCTAGG   
  
  
- TACATCACAG TAGTCTCAAA CTGTCGACCC CCTTTCACTA CCTCTACTAG CGAGCTTTCC TAAACTTTGA   
  
  
- CCACGATTAA CGGACACGTT CTCGTCAACG ACTTTTACTA GTCAACAACC GGGTCACCGA CTACCTACTT   
  
  
- AACTCTGTCT ACCAAAGTCA AAGACCACTT GGTTAAGTTT CTAACCCACG AATATACAAC CTTCCCGAGC   
  
  
- ATCGCTCCGT CCGGAGGAGT CCATCGAGGT AAATATTTCG TAACTCTACG TTCCTTGGGC GCTCACGACT   
  
  
- GGAAAATAGA ATGTACGTGT ATGAGAAACT CCAAACGGGT ATGTAGTTTA AACCGATGTA CAGTCGTTTA   
  
  
- CCACGGTAAC GTCTTCGTTA CTTCCTACTT TTCTCTCAGG TGTAACAGCT AAAGGTTTAA CCCGTCCCTT   
  
  
- CGGTTACCCA CTGGGATTAG GTCCGTAAAC GTCGGGCCGG TCCCCCAGGT GGGGTGTAAG CGTAATGGCC   
  
  
- ATAGCTACTG AGGATGAGAC GTATACGAGC ACCTCCACCC AACTTGTAAC ACCCGGTATC CGAGAACTCC   
  
  
- AACCGAGTCA GTAAATTCCA GGGTAAACTC AAGTTGCGTC AACTGCAGGG CCGAACAGTT CAGGACGAAT   
  
  
- TTCTAGAGCC GTAAGTTGTA CCACTCCGGA ACCGACACTT GAAACGGAAG TAAGAAGTGG TGTACGGGCT   
  
  
- ACTCTCGCAC TCGTGTCTCT TAGTGGCCCT AGCAGAAGAC TCCCACCATT TTCCCGACTT CGGTTTTCAC   
  
  
- CATTGCAATC ACCTCGTTCT CACATTGTGT TTGAGACGAC GAAAGAACGG AGCGAAACAA CTCTGTAACC   
  
  
- TTATGATGTG TCGCTACAAA CTCAGGTACC TACACTGAAA CGGATCCCTA GTATTTCTGG CCTAGTTACA   
  
  
- ACTCGTCGTA ACAGAACGAG CTCTACAACA CTTGAACCAA CGCACACTTC CTCGACTCTC CCACCTCGCC   
  
  
- GTACTCGAGG AACCCTTTAC CTCCAGCGCC AAGTGTTACC GTCCCAAATT CGGTATGGGA AATTCGTGAG   
  
  
- ACCATTTGTT GTGTTATTCT TGAAACAACT TCTTGATGAC ACTATCCATA CCCGATCTCC TCTCCTTACC   
  
  
- TCCAGAAATA GAACCCACCT ACTTATCTCT AAACCAACGA AGCTCACGGA CCGTCACATC ATTAAT

+     GCN4\_motif

| Site Name | Organism | Position | Strand | Matrix score. | sequence | function |
| --- | --- | --- | --- | --- | --- | --- |
| GCN4\_motif | Oryza sativa | 1485 | - | 7 | TGAGTCA | cis-regulatory element involved in endosperm expression |

>HU01G01391.1   
+ +Up\_Stream \_Len000CATTTT AGTAATAAAT TCCCATGTGA GGACCACTGC CTACTGATTC CGAATGGCAT   
  
  
+ TTTAGTAATA AACTCCCATG TGAGGACCAC TTGATGAGGA ATTCAGTTTT ATAATCCACA GGTGTCAAAG   
  
  
+ CCTATTCCTA CTCTCACCAC TCTGCTATCT CTCCCCCCCC CCCCCCCCCG CCCCCCTCAA AATCTGCCTC   
  
  
+ CTCCGTTCTC TCCACAGCAG ATTCCTCAAT CAGGAAAGGT ATGTCAGAGC TCAAAACGAT TCAACATCAA   
  
  
+ AATGGGATAA TTTATTCTGC TTTCAGTTCT GGGTTATTGT CATATGTTCA TTCAGTTCAG TGTTTCTAGC   
  
  
+ ATGATTGCTT TTTGATGCCT TCTTTTTTGT TCTGGGTTGG ACGAACTCTG TCTTTTCTGT GCATAGATTT   
  
  
+ GAGCTCCGAG CTCAATTTTT GGGATTTTTT TAGCTGACTT TTCTGCTTGG TGTTGTGAAA TTGTGATGGG   
  
  
+ TTTTGGCTTT GTTAAACTTG TTTATAACTT TTTCTGTCAA AACTTTATAG TAAATTGTTG CAATTTGAGA   
  
  
+ ATTATGCACT CAGTGGCTCT GTGATTTATC GTCCGCTGGC AAATTTTCTG ATGAAAGGAG ATGATTTATT   
  
  
+ AATTTTTCAG GCATGGTCAA ATTTTCTAGC CTTTCATGTA CATTGGTTGG TGATAGGCTG ATAGCTAAAT   
  
  
+ TATGTCTCAT TTCTCTAAAT TTATTGCCAG ATATGAAATT GGTGGAACAG TTTAAGGGTT TTGGTCGTCT   
  
  
+ TTTTTGCCCT TTTTTTTGGG TCCCCCCCCC GCCCGGGGGG GAACCCGGAG GGCAGAAAAA GTAACATAAC   
  
  
+ TTAAAATTAT CATTGACGTA GTCCTCCAAC CATAATGAAA GCAACTGAAA TAAGAGCATG CTAAGCTAGA   
  
  
+ GAGTAGGCTG CCTTGTCAGC CTCCCTTTTG ACATGAGAAA GATGGGAGAA GTTTAAAATA AAGTTCCTCG   
  
  
+ TCTTTGCATC CCGGACCACA ATTCGGAACT GGGTGAGGAC CTCCTTAGGC TTGTAAACAC AAGTTTCGAA   
  
  
+ CTTAGATTTG CGTACATGTC AATGCTTTAC TGGATAAGCT TAGCTAACAC CCCTCATCCT TCAACCTGTA   
  
  
+ CCTTCATATA AAAAAAAGGA CAATCCGGTG CACAAGCATC CCGCATTCAC GCAAGGTCTG GGGAAGGGCC   
  
  
+ GCACCCCAAA GGGTGAATTG TAGGCAGCCT AACCTGACTT TGTCAGTGGC TAATTCCACG ATTCGAACCC   
  
  
+ ATGATTTTTT GGTTACACGG GAACAACCTT ACCATTGCTC AAAGGCTCCC CTTCTTCAAC CTCTACCTTC   
  
  
+ ATATATGAAT GAAAATCCTT TGGCTAAGAA TTGAAAAGGA AATATGTGGT TATTGGGTTT GGGTTCTTAT   
  
  
+ TCCGTTGGTT GCCCCTTCAC ATTGAATGCA TCCTCTCGCT AAGAACTGAA AAGGACATAT GGGGATGTAT   
  
  
+ AGGTCAAATT TGACTCAATG ATAACTGAGG ATACAAGAAT TCTAGAAGTC TTTATCAGGG GAATTTTTTC   
  
  
+ TTTTTTCCAC TTGTGACTTT TTGATGCCTT GCTTTTTACT CTTCATTGAA TGAGTACCCT TCTTGACAGA   
  
  
+ TTCTTCCTCC CTTTTTTCTT AATTTCTTCT TGTAGTGGAA TATAAGTATG CTTCTAGGTA GTTGGATGCC   
  
  
+ ATGGAATCTC ATCCCTGTGT GTGCATTCTT CCCCTCTCCC GCCGCCCCCC CCCCCCCAAA AAGAAACCAA   
  
  
+ AAAAAAAAAA AAAATGGATG ATTCTTAGAA CTGACCATTT TTCCTTCCTC CTCCCATTTT ATTTTTTTCA   
  
  
+ GGCTGTTTTT CTTCTACCTG TTGCTTTAGC ATCACTCATA AAAACAAGGT GCAAGCGCAT GACATCGGGT   
  
  
+ ACTGAGCTTC TGAGTTTACA TGCATCGTCA TGAAATTCGG TAGACTTTGA AGTAAAGTTT CCCCATCGTA   
  
  
+ ACTGGTGGTG CATACCTGTC TTCACGCCCA TCAGTAGAGT GGTGAAGTGA AGCAATGTCG AATAGTTTGT   
  
  
+ ATTACGAGCC CAAGAGAGAA ACTGATGCAT ATTTTATGCC TCAATGTCAA TCCTTGAACC CCCAGCTCGA   
  
  
+ TTACAACACC ATTGACTATG GAGCTTTTAT GTGCTCAAAA GTTTTCCTGG GTCAGTACTG CACTCTGGAA   
  
  
+ TCATCCTCAG GAACTGGGAC TTGTCCGGTG TCTAGCTCCA CATCAACTCT CAGCTTCTCA TCCAATGGTA   
  
  
+ GCCCTGGATC ACAGCTTGAT TCCAACTCTT ATCCTTCTGA TCAAAATTGC TCTCCTGATA ATGCCAATTC   
  
  
+ CTCATCTTTA AGTCATTCCT GCGTCACAGA TGATGTGGAT GACCTGAAAT ACAGGCTGAG AGAGTTGGAA   
  
  
+ ACAGTGATGT TGGGACCTGA TCCCGATTTT GTCTACGGCA ATAGCAACCA CACATTTGGG GTTGGGATCC   
  
  
+ ATGTAGTGTC ATCAGAGTTT GACAGCTGGG GGAAAGTGAT GGAGATGATC GCTCGAAAGG ATTTGAAACT   
  
  
+ GGTGCTAATT GCCTGTGCAA GAGCAGTTGC TGAAAATGAT CAGTTGTTGG CCCAGTGGCT GATGGATGAA   
  
  
+ TTGAGACAGA TGGTTTCAGT TTCTGGTGAA CCAATTCAAA GATTGGGTGC TTATATGTTG GAAGGGCTCG   
  
  
+ TAGCGAGGCA GGCCTCCTCA GGTAGCTCCA TTTATAAAGC ATTGAGATGC AAGGAACCCG CGAGTGCTGA   
  
  
+ CCTTTTATCT TACATGCACA TACTCTTTGA GGTTTGCCCA TACATCAAAT TTGGCTACAT GTCAGCAAAT   
  
  
+ GGTGCCATTG CAGAAGCAAT GAAGGATGAA AAGAGAGTCC ACATTGTCGA TTTCCAAATT GGGCAGGGAA   
  
  
+ GCCAATGGGT GACCCTAATC CAGGCATTTG CAGCCCGGCC AGGGGGTCCA CCCCACATTC GCATTACCGG   
  
  
+ TATCGATGAC TCCTACTCTG CATATGCTCG TGGAGGTGGG TTGAACATTG TGGGCCATAG GCTCTTGAGG   
  
  
+ TTGGCTCAGT CATTTAAGGT CCCATTTGAG TTCAACGCAG TTGACGTCCC GGCTTGTCAA GTCCTGCTTA   
  
  
+ AAGATCTCGG CATTCAACAT GGTGAGGCCT TGGCTGTGAA CTTTGCCTTC ATTCTTCACC ACATGCCCGA   
  
  
+ TGAGAGCGTG AGCACAGAGA ATCACCGGGA TCGTCTTCTG AGGGTGGTAA AAGGGCTGAA GCCAAAAGTG   
  
  
+ GTAACGTTAG TGGAGCAAGA GTGTAACACA AACTCTGCTG CTTTCTTGCC TCGCTTTGTT GAGACATTGG   
  
  
+ AATACTACAC AGCGATGTTT GAGTCCATGG ATGTGACTTT GCCTAGGGAT CATAAAGACC GGATCAATGT   
  
  
+ TGAGCAGCAT TGTCTTGCTC GAGATGTTGT GAACTTGGTT GCGTGTGAAG GAGCTGAGAG GGTGGAGCGG   
  
  
+ CATGAGCTCC TTGGGAAATG GAGGTCGCGG TTCACAATGG CAGGGTTTAA GCCATACCCT TTAAGCACTC   
  
  
+ TGGTAAACAA CACAATAAGA ACTTTGTTGA AGAACTACTG TGATAGGTAT GGGCTAGAGG AGAGGAATGG   
  
  
+ AGGTCTTTAT CTTGGGTGGA TGAATAGAGA TTTGGTTGCT TCGAGTGCCT GGCAGTGTAG TAATTA  

- +Up\_Stream \_Len000GTAAAA TCATTATTTA AGGGTACACT CCTGGTGACG GATGACTAAG GCTTACCGTA   
  
  
- AAATCATTAT TTGAGGGTAC ACTCCTGGTG AACTACTCCT TAAGTCAAAA TATTAGGTGT CCACAGTTTC   
  
  
- GGATAAGGAT GAGAGTGGTG AGACGATAGA GAGGGGGGGG GGGGGGGGGC GGGGGGAGTT TTAGACGGAG   
  
  
- GAGGCAAGAG AGGTGTCGTC TAAGGAGTTA GTCCTTTCCA TACAGTCTCG AGTTTTGCTA AGTTGTAGTT   
  
  
- TTACCCTATT AAATAAGACG AAAGTCAAGA CCCAATAACA GTATACAAGT AAGTCAAGTC ACAAAGATCG   
  
  
- TACTAACGAA AAACTACGGA AGAAAAAACA AGACCCAACC TGCTTGAGAC AGAAAAGACA CGTATCTAAA   
  
  
- CTCGAGGCTC GAGTTAAAAA CCCTAAAAAA ATCGACTGAA AAGACGAACC ACAACACTTT AACACTACCC   
  
  
- AAAACCGAAA CAATTTGAAC AAATATTGAA AAAGACAGTT TTGAAATATC ATTTAACAAC GTTAAACTCT   
  
  
- TAATACGTGA GTCACCGAGA CACTAAATAG CAGGCGACCG TTTAAAAGAC TACTTTCCTC TACTAAATAA   
  
  
- TTAAAAAGTC CGTACCAGTT TAAAAGATCG GAAAGTACAT GTAACCAACC ACTATCCGAC TATCGATTTA   
  
  
- ATACAGAGTA AAGAGATTTA AATAACGGTC TATACTTTAA CCACCTTGTC AAATTCCCAA AACCAGCAGA   
  
  
- AAAAACGGGA AAAAAAACCC AGGGGGGGGG CGGGCCCCCC CTTGGGCCTC CCGTCTTTTT CATTGTATTG   
  
  
- AATTTTAATA GTAACTGCAT CAGGAGGTTG GTATTACTTT CGTTGACTTT ATTCTCGTAC GATTCGATCT   
  
  
- CTCATCCGAC GGAACAGTCG GAGGGAAAAC TGTACTCTTT CTACCCTCTT CAAATTTTAT TTCAAGGAGC   
  
  
- AGAAACGTAG GGCCTGGTGT TAAGCCTTGA CCCACTCCTG GAGGAATCCG AACATTTGTG TTCAAAGCTT   
  
  
- GAATCTAAAC GCATGTACAG TTACGAAATG ACCTATTCGA ATCGATTGTG GGGAGTAGGA AGTTGGACAT   
  
  
- GGAAGTATAT TTTTTTTCCT GTTAGGCCAC GTGTTCGTAG GGCGTAAGTG CGTTCCAGAC CCCTTCCCGG   
  
  
- CGTGGGGTTT CCCACTTAAC ATCCGTCGGA TTGGACTGAA ACAGTCACCG ATTAAGGTGC TAAGCTTGGG   
  
  
- TACTAAAAAA CCAATGTGCC CTTGTTGGAA TGGTAACGAG TTTCCGAGGG GAAGAAGTTG GAGATGGAAG   
  
  
- TATATACTTA CTTTTAGGAA ACCGATTCTT AACTTTTCCT TTATACACCA ATAACCCAAA CCCAAGAATA   
  
  
- AGGCAACCAA CGGGGAAGTG TAACTTACGT AGGAGAGCGA TTCTTGACTT TTCCTGTATA CCCCTACATA   
  
  
- TCCAGTTTAA ACTGAGTTAC TATTGACTCC TATGTTCTTA AGATCTTCAG AAATAGTCCC CTTAAAAAAG   
  
  
- AAAAAAGGTG AACACTGAAA AACTACGGAA CGAAAAATGA GAAGTAACTT ACTCATGGGA AGAACTGTCT   
  
  
- AAGAAGGAGG GAAAAAAGAA TTAAAGAAGA ACATCACCTT ATATTCATAC GAAGATCCAT CAACCTACGG   
  
  
- TACCTTAGAG TAGGGACACA CACGTAAGAA GGGGAGAGGG CGGCGGGGGG GGGGGGGTTT TTCTTTGGTT   
  
  
- TTTTTTTTTT TTTTACCTAC TAAGAATCTT GACTGGTAAA AAGGAAGGAG GAGGGTAAAA TAAAAAAAGT   
  
  
- CCGACAAAAA GAAGATGGAC AACGAAATCG TAGTGAGTAT TTTTGTTCCA CGTTCGCGTA CTGTAGCCCA   
  
  
- TGACTCGAAG ACTCAAATGT ACGTAGCAGT ACTTTAAGCC ATCTGAAACT TCATTTCAAA GGGGTAGCAT   
  
  
- TGACCACCAC GTATGGACAG AAGTGCGGGT AGTCATCTCA CCACTTCACT TCGTTACAGC TTATCAAACA   
  
  
- TAATGCTCGG GTTCTCTCTT TGACTACGTA TAAAATACGG AGTTACAGTT AGGAACTTGG GGGTCGAGCT   
  
  
- AATGTTGTGG TAACTGATAC CTCGAAAATA CACGAGTTTT CAAAAGGACC CAGTCATGAC GTGAGACCTT   
  
  
- AGTAGGAGTC CTTGACCCTG AACAGGCCAC AGATCGAGGT GTAGTTGAGA GTCGAAGAGT AGGTTACCAT   
  
  
- CGGGACCTAG TGTCGAACTA AGGTTGAGAA TAGGAAGACT AGTTTTAACG AGAGGACTAT TACGGTTAAG   
  
  
- GAGTAGAAAT TCAGTAAGGA CGCAGTGTCT ACTACACCTA CTGGACTTTA TGTCCGACTC TCTCAACCTT   
  
  
- TGTCACTACA ACCCTGGACT AGGGCTAAAA CAGATGCCGT TATCGTTGGT GTGTAAACCC CAACCCTAGG   
  
  
- TACATCACAG TAGTCTCAAA CTGTCGACCC CCTTTCACTA CCTCTACTAG CGAGCTTTCC TAAACTTTGA   
  
  
- CCACGATTAA CGGACACGTT CTCGTCAACG ACTTTTACTA GTCAACAACC GGGTCACCGA CTACCTACTT   
  
  
- AACTCTGTCT ACCAAAGTCA AAGACCACTT GGTTAAGTTT CTAACCCACG AATATACAAC CTTCCCGAGC   
  
  
- ATCGCTCCGT CCGGAGGAGT CCATCGAGGT AAATATTTCG TAACTCTACG TTCCTTGGGC GCTCACGACT   
  
  
- GGAAAATAGA ATGTACGTGT ATGAGAAACT CCAAACGGGT ATGTAGTTTA AACCGATGTA CAGTCGTTTA   
  
  
- CCACGGTAAC GTCTTCGTTA CTTCCTACTT TTCTCTCAGG TGTAACAGCT AAAGGTTTAA CCCGTCCCTT   
  
  
- CGGTTACCCA CTGGGATTAG GTCCGTAAAC GTCGGGCCGG TCCCCCAGGT GGGGTGTAAG CGTAATGGCC   
  
  
- ATAGCTACTG AGGATGAGAC GTATACGAGC ACCTCCACCC AACTTGTAAC ACCCGGTATC CGAGAACTCC   
  
  
- AACCGAGTCA GTAAATTCCA GGGTAAACTC AAGTTGCGTC AACTGCAGGG CCGAACAGTT CAGGACGAAT   
  
  
- TTCTAGAGCC GTAAGTTGTA CCACTCCGGA ACCGACACTT GAAACGGAAG TAAGAAGTGG TGTACGGGCT   
  
  
- ACTCTCGCAC TCGTGTCTCT TAGTGGCCCT AGCAGAAGAC TCCCACCATT TTCCCGACTT CGGTTTTCAC   
  
  
- CATTGCAATC ACCTCGTTCT CACATTGTGT TTGAGACGAC GAAAGAACGG AGCGAAACAA CTCTGTAACC   
  
  
- TTATGATGTG TCGCTACAAA CTCAGGTACC TACACTGAAA CGGATCCCTA GTATTTCTGG CCTAGTTACA   
  
  
- ACTCGTCGTA ACAGAACGAG CTCTACAACA CTTGAACCAA CGCACACTTC CTCGACTCTC CCACCTCGCC   
  
  
- GTACTCGAGG AACCCTTTAC CTCCAGCGCC AAGTGTTACC GTCCCAAATT CGGTATGGGA AATTCGTGAG   
  
  
- ACCATTTGTT GTGTTATTCT TGAAACAACT TCTTGATGAC ACTATCCATA CCCGATCTCC TCTCCTTACC   
  
  
- TCCAGAAATA GAACCCACCT ACTTATCTCT AAACCAACGA AGCTCACGGA CCGTCACATC ATTAAT

+     LAMP-element

| Site Name | Organism | Position | Strand | Matrix score. | sequence | function |
| --- | --- | --- | --- | --- | --- | --- |
| LAMP-element | Pisum sativum | 1524 | + | 8 | CTTTATCA | part of a light responsive element |

>HU01G01391.1   
+ +Up\_Stream \_Len000CATTTT AGTAATAAAT TCCCATGTGA GGACCACTGC CTACTGATTC CGAATGGCAT   
  
  
+ TTTAGTAATA AACTCCCATG TGAGGACCAC TTGATGAGGA ATTCAGTTTT ATAATCCACA GGTGTCAAAG   
  
  
+ CCTATTCCTA CTCTCACCAC TCTGCTATCT CTCCCCCCCC CCCCCCCCCG CCCCCCTCAA AATCTGCCTC   
  
  
+ CTCCGTTCTC TCCACAGCAG ATTCCTCAAT CAGGAAAGGT ATGTCAGAGC TCAAAACGAT TCAACATCAA   
  
  
+ AATGGGATAA TTTATTCTGC TTTCAGTTCT GGGTTATTGT CATATGTTCA TTCAGTTCAG TGTTTCTAGC   
  
  
+ ATGATTGCTT TTTGATGCCT TCTTTTTTGT TCTGGGTTGG ACGAACTCTG TCTTTTCTGT GCATAGATTT   
  
  
+ GAGCTCCGAG CTCAATTTTT GGGATTTTTT TAGCTGACTT TTCTGCTTGG TGTTGTGAAA TTGTGATGGG   
  
  
+ TTTTGGCTTT GTTAAACTTG TTTATAACTT TTTCTGTCAA AACTTTATAG TAAATTGTTG CAATTTGAGA   
  
  
+ ATTATGCACT CAGTGGCTCT GTGATTTATC GTCCGCTGGC AAATTTTCTG ATGAAAGGAG ATGATTTATT   
  
  
+ AATTTTTCAG GCATGGTCAA ATTTTCTAGC CTTTCATGTA CATTGGTTGG TGATAGGCTG ATAGCTAAAT   
  
  
+ TATGTCTCAT TTCTCTAAAT TTATTGCCAG ATATGAAATT GGTGGAACAG TTTAAGGGTT TTGGTCGTCT   
  
  
+ TTTTTGCCCT TTTTTTTGGG TCCCCCCCCC GCCCGGGGGG GAACCCGGAG GGCAGAAAAA GTAACATAAC   
  
  
+ TTAAAATTAT CATTGACGTA GTCCTCCAAC CATAATGAAA GCAACTGAAA TAAGAGCATG CTAAGCTAGA   
  
  
+ GAGTAGGCTG CCTTGTCAGC CTCCCTTTTG ACATGAGAAA GATGGGAGAA GTTTAAAATA AAGTTCCTCG   
  
  
+ TCTTTGCATC CCGGACCACA ATTCGGAACT GGGTGAGGAC CTCCTTAGGC TTGTAAACAC AAGTTTCGAA   
  
  
+ CTTAGATTTG CGTACATGTC AATGCTTTAC TGGATAAGCT TAGCTAACAC CCCTCATCCT TCAACCTGTA   
  
  
+ CCTTCATATA AAAAAAAGGA CAATCCGGTG CACAAGCATC CCGCATTCAC GCAAGGTCTG GGGAAGGGCC   
  
  
+ GCACCCCAAA GGGTGAATTG TAGGCAGCCT AACCTGACTT TGTCAGTGGC TAATTCCACG ATTCGAACCC   
  
  
+ ATGATTTTTT GGTTACACGG GAACAACCTT ACCATTGCTC AAAGGCTCCC CTTCTTCAAC CTCTACCTTC   
  
  
+ ATATATGAAT GAAAATCCTT TGGCTAAGAA TTGAAAAGGA AATATGTGGT TATTGGGTTT GGGTTCTTAT   
  
  
+ TCCGTTGGTT GCCCCTTCAC ATTGAATGCA TCCTCTCGCT AAGAACTGAA AAGGACATAT GGGGATGTAT   
  
  
+ AGGTCAAATT TGACTCAATG ATAACTGAGG ATACAAGAAT TCTAGAAGTC TTTATCAGGG GAATTTTTTC   
  
  
+ TTTTTTCCAC TTGTGACTTT TTGATGCCTT GCTTTTTACT CTTCATTGAA TGAGTACCCT TCTTGACAGA   
  
  
+ TTCTTCCTCC CTTTTTTCTT AATTTCTTCT TGTAGTGGAA TATAAGTATG CTTCTAGGTA GTTGGATGCC   
  
  
+ ATGGAATCTC ATCCCTGTGT GTGCATTCTT CCCCTCTCCC GCCGCCCCCC CCCCCCCAAA AAGAAACCAA   
  
  
+ AAAAAAAAAA AAAATGGATG ATTCTTAGAA CTGACCATTT TTCCTTCCTC CTCCCATTTT ATTTTTTTCA   
  
  
+ GGCTGTTTTT CTTCTACCTG TTGCTTTAGC ATCACTCATA AAAACAAGGT GCAAGCGCAT GACATCGGGT   
  
  
+ ACTGAGCTTC TGAGTTTACA TGCATCGTCA TGAAATTCGG TAGACTTTGA AGTAAAGTTT CCCCATCGTA   
  
  
+ ACTGGTGGTG CATACCTGTC TTCACGCCCA TCAGTAGAGT GGTGAAGTGA AGCAATGTCG AATAGTTTGT   
  
  
+ ATTACGAGCC CAAGAGAGAA ACTGATGCAT ATTTTATGCC TCAATGTCAA TCCTTGAACC CCCAGCTCGA   
  
  
+ TTACAACACC ATTGACTATG GAGCTTTTAT GTGCTCAAAA GTTTTCCTGG GTCAGTACTG CACTCTGGAA   
  
  
+ TCATCCTCAG GAACTGGGAC TTGTCCGGTG TCTAGCTCCA CATCAACTCT CAGCTTCTCA TCCAATGGTA   
  
  
+ GCCCTGGATC ACAGCTTGAT TCCAACTCTT ATCCTTCTGA TCAAAATTGC TCTCCTGATA ATGCCAATTC   
  
  
+ CTCATCTTTA AGTCATTCCT GCGTCACAGA TGATGTGGAT GACCTGAAAT ACAGGCTGAG AGAGTTGGAA   
  
  
+ ACAGTGATGT TGGGACCTGA TCCCGATTTT GTCTACGGCA ATAGCAACCA CACATTTGGG GTTGGGATCC   
  
  
+ ATGTAGTGTC ATCAGAGTTT GACAGCTGGG GGAAAGTGAT GGAGATGATC GCTCGAAAGG ATTTGAAACT   
  
  
+ GGTGCTAATT GCCTGTGCAA GAGCAGTTGC TGAAAATGAT CAGTTGTTGG CCCAGTGGCT GATGGATGAA   
  
  
+ TTGAGACAGA TGGTTTCAGT TTCTGGTGAA CCAATTCAAA GATTGGGTGC TTATATGTTG GAAGGGCTCG   
  
  
+ TAGCGAGGCA GGCCTCCTCA GGTAGCTCCA TTTATAAAGC ATTGAGATGC AAGGAACCCG CGAGTGCTGA   
  
  
+ CCTTTTATCT TACATGCACA TACTCTTTGA GGTTTGCCCA TACATCAAAT TTGGCTACAT GTCAGCAAAT   
  
  
+ GGTGCCATTG CAGAAGCAAT GAAGGATGAA AAGAGAGTCC ACATTGTCGA TTTCCAAATT GGGCAGGGAA   
  
  
+ GCCAATGGGT GACCCTAATC CAGGCATTTG CAGCCCGGCC AGGGGGTCCA CCCCACATTC GCATTACCGG   
  
  
+ TATCGATGAC TCCTACTCTG CATATGCTCG TGGAGGTGGG TTGAACATTG TGGGCCATAG GCTCTTGAGG   
  
  
+ TTGGCTCAGT CATTTAAGGT CCCATTTGAG TTCAACGCAG TTGACGTCCC GGCTTGTCAA GTCCTGCTTA   
  
  
+ AAGATCTCGG CATTCAACAT GGTGAGGCCT TGGCTGTGAA CTTTGCCTTC ATTCTTCACC ACATGCCCGA   
  
  
+ TGAGAGCGTG AGCACAGAGA ATCACCGGGA TCGTCTTCTG AGGGTGGTAA AAGGGCTGAA GCCAAAAGTG   
  
  
+ GTAACGTTAG TGGAGCAAGA GTGTAACACA AACTCTGCTG CTTTCTTGCC TCGCTTTGTT GAGACATTGG   
  
  
+ AATACTACAC AGCGATGTTT GAGTCCATGG ATGTGACTTT GCCTAGGGAT CATAAAGACC GGATCAATGT   
  
  
+ TGAGCAGCAT TGTCTTGCTC GAGATGTTGT GAACTTGGTT GCGTGTGAAG GAGCTGAGAG GGTGGAGCGG   
  
  
+ CATGAGCTCC TTGGGAAATG GAGGTCGCGG TTCACAATGG CAGGGTTTAA GCCATACCCT TTAAGCACTC   
  
  
+ TGGTAAACAA CACAATAAGA ACTTTGTTGA AGAACTACTG TGATAGGTAT GGGCTAGAGG AGAGGAATGG   
  
  
+ AGGTCTTTAT CTTGGGTGGA TGAATAGAGA TTTGGTTGCT TCGAGTGCCT GGCAGTGTAG TAATTA  

- +Up\_Stream \_Len000GTAAAA TCATTATTTA AGGGTACACT CCTGGTGACG GATGACTAAG GCTTACCGTA   
  
  
- AAATCATTAT TTGAGGGTAC ACTCCTGGTG AACTACTCCT TAAGTCAAAA TATTAGGTGT CCACAGTTTC   
  
  
- GGATAAGGAT GAGAGTGGTG AGACGATAGA GAGGGGGGGG GGGGGGGGGC GGGGGGAGTT TTAGACGGAG   
  
  
- GAGGCAAGAG AGGTGTCGTC TAAGGAGTTA GTCCTTTCCA TACAGTCTCG AGTTTTGCTA AGTTGTAGTT   
  
  
- TTACCCTATT AAATAAGACG AAAGTCAAGA CCCAATAACA GTATACAAGT AAGTCAAGTC ACAAAGATCG   
  
  
- TACTAACGAA AAACTACGGA AGAAAAAACA AGACCCAACC TGCTTGAGAC AGAAAAGACA CGTATCTAAA   
  
  
- CTCGAGGCTC GAGTTAAAAA CCCTAAAAAA ATCGACTGAA AAGACGAACC ACAACACTTT AACACTACCC   
  
  
- AAAACCGAAA CAATTTGAAC AAATATTGAA AAAGACAGTT TTGAAATATC ATTTAACAAC GTTAAACTCT   
  
  
- TAATACGTGA GTCACCGAGA CACTAAATAG CAGGCGACCG TTTAAAAGAC TACTTTCCTC TACTAAATAA   
  
  
- TTAAAAAGTC CGTACCAGTT TAAAAGATCG GAAAGTACAT GTAACCAACC ACTATCCGAC TATCGATTTA   
  
  
- ATACAGAGTA AAGAGATTTA AATAACGGTC TATACTTTAA CCACCTTGTC AAATTCCCAA AACCAGCAGA   
  
  
- AAAAACGGGA AAAAAAACCC AGGGGGGGGG CGGGCCCCCC CTTGGGCCTC CCGTCTTTTT CATTGTATTG   
  
  
- AATTTTAATA GTAACTGCAT CAGGAGGTTG GTATTACTTT CGTTGACTTT ATTCTCGTAC GATTCGATCT   
  
  
- CTCATCCGAC GGAACAGTCG GAGGGAAAAC TGTACTCTTT CTACCCTCTT CAAATTTTAT TTCAAGGAGC   
  
  
- AGAAACGTAG GGCCTGGTGT TAAGCCTTGA CCCACTCCTG GAGGAATCCG AACATTTGTG TTCAAAGCTT   
  
  
- GAATCTAAAC GCATGTACAG TTACGAAATG ACCTATTCGA ATCGATTGTG GGGAGTAGGA AGTTGGACAT   
  
  
- GGAAGTATAT TTTTTTTCCT GTTAGGCCAC GTGTTCGTAG GGCGTAAGTG CGTTCCAGAC CCCTTCCCGG   
  
  
- CGTGGGGTTT CCCACTTAAC ATCCGTCGGA TTGGACTGAA ACAGTCACCG ATTAAGGTGC TAAGCTTGGG   
  
  
- TACTAAAAAA CCAATGTGCC CTTGTTGGAA TGGTAACGAG TTTCCGAGGG GAAGAAGTTG GAGATGGAAG   
  
  
- TATATACTTA CTTTTAGGAA ACCGATTCTT AACTTTTCCT TTATACACCA ATAACCCAAA CCCAAGAATA   
  
  
- AGGCAACCAA CGGGGAAGTG TAACTTACGT AGGAGAGCGA TTCTTGACTT TTCCTGTATA CCCCTACATA   
  
  
- TCCAGTTTAA ACTGAGTTAC TATTGACTCC TATGTTCTTA AGATCTTCAG AAATAGTCCC CTTAAAAAAG   
  
  
- AAAAAAGGTG AACACTGAAA AACTACGGAA CGAAAAATGA GAAGTAACTT ACTCATGGGA AGAACTGTCT   
  
  
- AAGAAGGAGG GAAAAAAGAA TTAAAGAAGA ACATCACCTT ATATTCATAC GAAGATCCAT CAACCTACGG   
  
  
- TACCTTAGAG TAGGGACACA CACGTAAGAA GGGGAGAGGG CGGCGGGGGG GGGGGGGTTT TTCTTTGGTT   
  
  
- TTTTTTTTTT TTTTACCTAC TAAGAATCTT GACTGGTAAA AAGGAAGGAG GAGGGTAAAA TAAAAAAAGT   
  
  
- CCGACAAAAA GAAGATGGAC AACGAAATCG TAGTGAGTAT TTTTGTTCCA CGTTCGCGTA CTGTAGCCCA   
  
  
- TGACTCGAAG ACTCAAATGT ACGTAGCAGT ACTTTAAGCC ATCTGAAACT TCATTTCAAA GGGGTAGCAT   
  
  
- TGACCACCAC GTATGGACAG AAGTGCGGGT AGTCATCTCA CCACTTCACT TCGTTACAGC TTATCAAACA   
  
  
- TAATGCTCGG GTTCTCTCTT TGACTACGTA TAAAATACGG AGTTACAGTT AGGAACTTGG GGGTCGAGCT   
  
  
- AATGTTGTGG TAACTGATAC CTCGAAAATA CACGAGTTTT CAAAAGGACC CAGTCATGAC GTGAGACCTT   
  
  
- AGTAGGAGTC CTTGACCCTG AACAGGCCAC AGATCGAGGT GTAGTTGAGA GTCGAAGAGT AGGTTACCAT   
  
  
- CGGGACCTAG TGTCGAACTA AGGTTGAGAA TAGGAAGACT AGTTTTAACG AGAGGACTAT TACGGTTAAG   
  
  
- GAGTAGAAAT TCAGTAAGGA CGCAGTGTCT ACTACACCTA CTGGACTTTA TGTCCGACTC TCTCAACCTT   
  
  
- TGTCACTACA ACCCTGGACT AGGGCTAAAA CAGATGCCGT TATCGTTGGT GTGTAAACCC CAACCCTAGG   
  
  
- TACATCACAG TAGTCTCAAA CTGTCGACCC CCTTTCACTA CCTCTACTAG CGAGCTTTCC TAAACTTTGA   
  
  
- CCACGATTAA CGGACACGTT CTCGTCAACG ACTTTTACTA GTCAACAACC GGGTCACCGA CTACCTACTT   
  
  
- AACTCTGTCT ACCAAAGTCA AAGACCACTT GGTTAAGTTT CTAACCCACG AATATACAAC CTTCCCGAGC   
  
  
- ATCGCTCCGT CCGGAGGAGT CCATCGAGGT AAATATTTCG TAACTCTACG TTCCTTGGGC GCTCACGACT   
  
  
- GGAAAATAGA ATGTACGTGT ATGAGAAACT CCAAACGGGT ATGTAGTTTA AACCGATGTA CAGTCGTTTA   
  
  
- CCACGGTAAC GTCTTCGTTA CTTCCTACTT TTCTCTCAGG TGTAACAGCT AAAGGTTTAA CCCGTCCCTT   
  
  
- CGGTTACCCA CTGGGATTAG GTCCGTAAAC GTCGGGCCGG TCCCCCAGGT GGGGTGTAAG CGTAATGGCC   
  
  
- ATAGCTACTG AGGATGAGAC GTATACGAGC ACCTCCACCC AACTTGTAAC ACCCGGTATC CGAGAACTCC   
  
  
- AACCGAGTCA GTAAATTCCA GGGTAAACTC AAGTTGCGTC AACTGCAGGG CCGAACAGTT CAGGACGAAT   
  
  
- TTCTAGAGCC GTAAGTTGTA CCACTCCGGA ACCGACACTT GAAACGGAAG TAAGAAGTGG TGTACGGGCT   
  
  
- ACTCTCGCAC TCGTGTCTCT TAGTGGCCCT AGCAGAAGAC TCCCACCATT TTCCCGACTT CGGTTTTCAC   
  
  
- CATTGCAATC ACCTCGTTCT CACATTGTGT TTGAGACGAC GAAAGAACGG AGCGAAACAA CTCTGTAACC   
  
  
- TTATGATGTG TCGCTACAAA CTCAGGTACC TACACTGAAA CGGATCCCTA GTATTTCTGG CCTAGTTACA   
  
  
- ACTCGTCGTA ACAGAACGAG CTCTACAACA CTTGAACCAA CGCACACTTC CTCGACTCTC CCACCTCGCC   
  
  
- GTACTCGAGG AACCCTTTAC CTCCAGCGCC AAGTGTTACC GTCCCAAATT CGGTATGGGA AATTCGTGAG   
  
  
- ACCATTTGTT GTGTTATTCT TGAAACAACT TCTTGATGAC ACTATCCATA CCCGATCTCC TCTCCTTACC   
  
  
- TCCAGAAATA GAACCCACCT ACTTATCTCT AAACCAACGA AGCTCACGGA CCGTCACATC ATTAAT

+     MBS

| Site Name | Organism | Position | Strand | Matrix score. | sequence | function |
| --- | --- | --- | --- | --- | --- | --- |
| MBS | Arabidopsis thaliana | 3052 | - | 6 | CAACTG | MYB binding site involved in drought-inducibility |
| MBS | Arabidopsis thaliana | 2565 | - | 6 | CAACTG | MYB binding site involved in drought-inducibility |
| MBS | Arabidopsis thaliana | 2548 | - | 6 | CAACTG | MYB binding site involved in drought-inducibility |
| MBS | Arabidopsis thaliana | 886 | + | 6 | CAACTG | MYB binding site involved in drought-inducibility |

>HU01G01391.1   
+ +Up\_Stream \_Len000CATTTT AGTAATAAAT TCCCATGTGA GGACCACTGC CTACTGATTC CGAATGGCAT   
  
  
+ TTTAGTAATA AACTCCCATG TGAGGACCAC TTGATGAGGA ATTCAGTTTT ATAATCCACA GGTGTCAAAG   
  
  
+ CCTATTCCTA CTCTCACCAC TCTGCTATCT CTCCCCCCCC CCCCCCCCCG CCCCCCTCAA AATCTGCCTC   
  
  
+ CTCCGTTCTC TCCACAGCAG ATTCCTCAAT CAGGAAAGGT ATGTCAGAGC TCAAAACGAT TCAACATCAA   
  
  
+ AATGGGATAA TTTATTCTGC TTTCAGTTCT GGGTTATTGT CATATGTTCA TTCAGTTCAG TGTTTCTAGC   
  
  
+ ATGATTGCTT TTTGATGCCT TCTTTTTTGT TCTGGGTTGG ACGAACTCTG TCTTTTCTGT GCATAGATTT   
  
  
+ GAGCTCCGAG CTCAATTTTT GGGATTTTTT TAGCTGACTT TTCTGCTTGG TGTTGTGAAA TTGTGATGGG   
  
  
+ TTTTGGCTTT GTTAAACTTG TTTATAACTT TTTCTGTCAA AACTTTATAG TAAATTGTTG CAATTTGAGA   
  
  
+ ATTATGCACT CAGTGGCTCT GTGATTTATC GTCCGCTGGC AAATTTTCTG ATGAAAGGAG ATGATTTATT   
  
  
+ AATTTTTCAG GCATGGTCAA ATTTTCTAGC CTTTCATGTA CATTGGTTGG TGATAGGCTG ATAGCTAAAT   
  
  
+ TATGTCTCAT TTCTCTAAAT TTATTGCCAG ATATGAAATT GGTGGAACAG TTTAAGGGTT TTGGTCGTCT   
  
  
+ TTTTTGCCCT TTTTTTTGGG TCCCCCCCCC GCCCGGGGGG GAACCCGGAG GGCAGAAAAA GTAACATAAC   
  
  
+ TTAAAATTAT CATTGACGTA GTCCTCCAAC CATAATGAAA GCAACTGAAA TAAGAGCATG CTAAGCTAGA   
  
  
+ GAGTAGGCTG CCTTGTCAGC CTCCCTTTTG ACATGAGAAA GATGGGAGAA GTTTAAAATA AAGTTCCTCG   
  
  
+ TCTTTGCATC CCGGACCACA ATTCGGAACT GGGTGAGGAC CTCCTTAGGC TTGTAAACAC AAGTTTCGAA   
  
  
+ CTTAGATTTG CGTACATGTC AATGCTTTAC TGGATAAGCT TAGCTAACAC CCCTCATCCT TCAACCTGTA   
  
  
+ CCTTCATATA AAAAAAAGGA CAATCCGGTG CACAAGCATC CCGCATTCAC GCAAGGTCTG GGGAAGGGCC   
  
  
+ GCACCCCAAA GGGTGAATTG TAGGCAGCCT AACCTGACTT TGTCAGTGGC TAATTCCACG ATTCGAACCC   
  
  
+ ATGATTTTTT GGTTACACGG GAACAACCTT ACCATTGCTC AAAGGCTCCC CTTCTTCAAC CTCTACCTTC   
  
  
+ ATATATGAAT GAAAATCCTT TGGCTAAGAA TTGAAAAGGA AATATGTGGT TATTGGGTTT GGGTTCTTAT   
  
  
+ TCCGTTGGTT GCCCCTTCAC ATTGAATGCA TCCTCTCGCT AAGAACTGAA AAGGACATAT GGGGATGTAT   
  
  
+ AGGTCAAATT TGACTCAATG ATAACTGAGG ATACAAGAAT TCTAGAAGTC TTTATCAGGG GAATTTTTTC   
  
  
+ TTTTTTCCAC TTGTGACTTT TTGATGCCTT GCTTTTTACT CTTCATTGAA TGAGTACCCT TCTTGACAGA   
  
  
+ TTCTTCCTCC CTTTTTTCTT AATTTCTTCT TGTAGTGGAA TATAAGTATG CTTCTAGGTA GTTGGATGCC   
  
  
+ ATGGAATCTC ATCCCTGTGT GTGCATTCTT CCCCTCTCCC GCCGCCCCCC CCCCCCCAAA AAGAAACCAA   
  
  
+ AAAAAAAAAA AAAATGGATG ATTCTTAGAA CTGACCATTT TTCCTTCCTC CTCCCATTTT ATTTTTTTCA   
  
  
+ GGCTGTTTTT CTTCTACCTG TTGCTTTAGC ATCACTCATA AAAACAAGGT GCAAGCGCAT GACATCGGGT   
  
  
+ ACTGAGCTTC TGAGTTTACA TGCATCGTCA TGAAATTCGG TAGACTTTGA AGTAAAGTTT CCCCATCGTA   
  
  
+ ACTGGTGGTG CATACCTGTC TTCACGCCCA TCAGTAGAGT GGTGAAGTGA AGCAATGTCG AATAGTTTGT   
  
  
+ ATTACGAGCC CAAGAGAGAA ACTGATGCAT ATTTTATGCC TCAATGTCAA TCCTTGAACC CCCAGCTCGA   
  
  
+ TTACAACACC ATTGACTATG GAGCTTTTAT GTGCTCAAAA GTTTTCCTGG GTCAGTACTG CACTCTGGAA   
  
  
+ TCATCCTCAG GAACTGGGAC TTGTCCGGTG TCTAGCTCCA CATCAACTCT CAGCTTCTCA TCCAATGGTA   
  
  
+ GCCCTGGATC ACAGCTTGAT TCCAACTCTT ATCCTTCTGA TCAAAATTGC TCTCCTGATA ATGCCAATTC   
  
  
+ CTCATCTTTA AGTCATTCCT GCGTCACAGA TGATGTGGAT GACCTGAAAT ACAGGCTGAG AGAGTTGGAA   
  
  
+ ACAGTGATGT TGGGACCTGA TCCCGATTTT GTCTACGGCA ATAGCAACCA CACATTTGGG GTTGGGATCC   
  
  
+ ATGTAGTGTC ATCAGAGTTT GACAGCTGGG GGAAAGTGAT GGAGATGATC GCTCGAAAGG ATTTGAAACT   
  
  
+ GGTGCTAATT GCCTGTGCAA GAGCAGTTGC TGAAAATGAT CAGTTGTTGG CCCAGTGGCT GATGGATGAA   
  
  
+ TTGAGACAGA TGGTTTCAGT TTCTGGTGAA CCAATTCAAA GATTGGGTGC TTATATGTTG GAAGGGCTCG   
  
  
+ TAGCGAGGCA GGCCTCCTCA GGTAGCTCCA TTTATAAAGC ATTGAGATGC AAGGAACCCG CGAGTGCTGA   
  
  
+ CCTTTTATCT TACATGCACA TACTCTTTGA GGTTTGCCCA TACATCAAAT TTGGCTACAT GTCAGCAAAT   
  
  
+ GGTGCCATTG CAGAAGCAAT GAAGGATGAA AAGAGAGTCC ACATTGTCGA TTTCCAAATT GGGCAGGGAA   
  
  
+ GCCAATGGGT GACCCTAATC CAGGCATTTG CAGCCCGGCC AGGGGGTCCA CCCCACATTC GCATTACCGG   
  
  
+ TATCGATGAC TCCTACTCTG CATATGCTCG TGGAGGTGGG TTGAACATTG TGGGCCATAG GCTCTTGAGG   
  
  
+ TTGGCTCAGT CATTTAAGGT CCCATTTGAG TTCAACGCAG TTGACGTCCC GGCTTGTCAA GTCCTGCTTA   
  
  
+ AAGATCTCGG CATTCAACAT GGTGAGGCCT TGGCTGTGAA CTTTGCCTTC ATTCTTCACC ACATGCCCGA   
  
  
+ TGAGAGCGTG AGCACAGAGA ATCACCGGGA TCGTCTTCTG AGGGTGGTAA AAGGGCTGAA GCCAAAAGTG   
  
  
+ GTAACGTTAG TGGAGCAAGA GTGTAACACA AACTCTGCTG CTTTCTTGCC TCGCTTTGTT GAGACATTGG   
  
  
+ AATACTACAC AGCGATGTTT GAGTCCATGG ATGTGACTTT GCCTAGGGAT CATAAAGACC GGATCAATGT   
  
  
+ TGAGCAGCAT TGTCTTGCTC GAGATGTTGT GAACTTGGTT GCGTGTGAAG GAGCTGAGAG GGTGGAGCGG   
  
  
+ CATGAGCTCC TTGGGAAATG GAGGTCGCGG TTCACAATGG CAGGGTTTAA GCCATACCCT TTAAGCACTC   
  
  
+ TGGTAAACAA CACAATAAGA ACTTTGTTGA AGAACTACTG TGATAGGTAT GGGCTAGAGG AGAGGAATGG   
  
  
+ AGGTCTTTAT CTTGGGTGGA TGAATAGAGA TTTGGTTGCT TCGAGTGCCT GGCAGTGTAG TAATTA  

- +Up\_Stream \_Len000GTAAAA TCATTATTTA AGGGTACACT CCTGGTGACG GATGACTAAG GCTTACCGTA   
  
  
- AAATCATTAT TTGAGGGTAC ACTCCTGGTG AACTACTCCT TAAGTCAAAA TATTAGGTGT CCACAGTTTC   
  
  
- GGATAAGGAT GAGAGTGGTG AGACGATAGA GAGGGGGGGG GGGGGGGGGC GGGGGGAGTT TTAGACGGAG   
  
  
- GAGGCAAGAG AGGTGTCGTC TAAGGAGTTA GTCCTTTCCA TACAGTCTCG AGTTTTGCTA AGTTGTAGTT   
  
  
- TTACCCTATT AAATAAGACG AAAGTCAAGA CCCAATAACA GTATACAAGT AAGTCAAGTC ACAAAGATCG   
  
  
- TACTAACGAA AAACTACGGA AGAAAAAACA AGACCCAACC TGCTTGAGAC AGAAAAGACA CGTATCTAAA   
  
  
- CTCGAGGCTC GAGTTAAAAA CCCTAAAAAA ATCGACTGAA AAGACGAACC ACAACACTTT AACACTACCC   
  
  
- AAAACCGAAA CAATTTGAAC AAATATTGAA AAAGACAGTT TTGAAATATC ATTTAACAAC GTTAAACTCT   
  
  
- TAATACGTGA GTCACCGAGA CACTAAATAG CAGGCGACCG TTTAAAAGAC TACTTTCCTC TACTAAATAA   
  
  
- TTAAAAAGTC CGTACCAGTT TAAAAGATCG GAAAGTACAT GTAACCAACC ACTATCCGAC TATCGATTTA   
  
  
- ATACAGAGTA AAGAGATTTA AATAACGGTC TATACTTTAA CCACCTTGTC AAATTCCCAA AACCAGCAGA   
  
  
- AAAAACGGGA AAAAAAACCC AGGGGGGGGG CGGGCCCCCC CTTGGGCCTC CCGTCTTTTT CATTGTATTG   
  
  
- AATTTTAATA GTAACTGCAT CAGGAGGTTG GTATTACTTT CGTTGACTTT ATTCTCGTAC GATTCGATCT   
  
  
- CTCATCCGAC GGAACAGTCG GAGGGAAAAC TGTACTCTTT CTACCCTCTT CAAATTTTAT TTCAAGGAGC   
  
  
- AGAAACGTAG GGCCTGGTGT TAAGCCTTGA CCCACTCCTG GAGGAATCCG AACATTTGTG TTCAAAGCTT   
  
  
- GAATCTAAAC GCATGTACAG TTACGAAATG ACCTATTCGA ATCGATTGTG GGGAGTAGGA AGTTGGACAT   
  
  
- GGAAGTATAT TTTTTTTCCT GTTAGGCCAC GTGTTCGTAG GGCGTAAGTG CGTTCCAGAC CCCTTCCCGG   
  
  
- CGTGGGGTTT CCCACTTAAC ATCCGTCGGA TTGGACTGAA ACAGTCACCG ATTAAGGTGC TAAGCTTGGG   
  
  
- TACTAAAAAA CCAATGTGCC CTTGTTGGAA TGGTAACGAG TTTCCGAGGG GAAGAAGTTG GAGATGGAAG   
  
  
- TATATACTTA CTTTTAGGAA ACCGATTCTT AACTTTTCCT TTATACACCA ATAACCCAAA CCCAAGAATA   
  
  
- AGGCAACCAA CGGGGAAGTG TAACTTACGT AGGAGAGCGA TTCTTGACTT TTCCTGTATA CCCCTACATA   
  
  
- TCCAGTTTAA ACTGAGTTAC TATTGACTCC TATGTTCTTA AGATCTTCAG AAATAGTCCC CTTAAAAAAG   
  
  
- AAAAAAGGTG AACACTGAAA AACTACGGAA CGAAAAATGA GAAGTAACTT ACTCATGGGA AGAACTGTCT   
  
  
- AAGAAGGAGG GAAAAAAGAA TTAAAGAAGA ACATCACCTT ATATTCATAC GAAGATCCAT CAACCTACGG   
  
  
- TACCTTAGAG TAGGGACACA CACGTAAGAA GGGGAGAGGG CGGCGGGGGG GGGGGGGTTT TTCTTTGGTT   
  
  
- TTTTTTTTTT TTTTACCTAC TAAGAATCTT GACTGGTAAA AAGGAAGGAG GAGGGTAAAA TAAAAAAAGT   
  
  
- CCGACAAAAA GAAGATGGAC AACGAAATCG TAGTGAGTAT TTTTGTTCCA CGTTCGCGTA CTGTAGCCCA   
  
  
- TGACTCGAAG ACTCAAATGT ACGTAGCAGT ACTTTAAGCC ATCTGAAACT TCATTTCAAA GGGGTAGCAT   
  
  
- TGACCACCAC GTATGGACAG AAGTGCGGGT AGTCATCTCA CCACTTCACT TCGTTACAGC TTATCAAACA   
  
  
- TAATGCTCGG GTTCTCTCTT TGACTACGTA TAAAATACGG AGTTACAGTT AGGAACTTGG GGGTCGAGCT   
  
  
- AATGTTGTGG TAACTGATAC CTCGAAAATA CACGAGTTTT CAAAAGGACC CAGTCATGAC GTGAGACCTT   
  
  
- AGTAGGAGTC CTTGACCCTG AACAGGCCAC AGATCGAGGT GTAGTTGAGA GTCGAAGAGT AGGTTACCAT   
  
  
- CGGGACCTAG TGTCGAACTA AGGTTGAGAA TAGGAAGACT AGTTTTAACG AGAGGACTAT TACGGTTAAG   
  
  
- GAGTAGAAAT TCAGTAAGGA CGCAGTGTCT ACTACACCTA CTGGACTTTA TGTCCGACTC TCTCAACCTT   
  
  
- TGTCACTACA ACCCTGGACT AGGGCTAAAA CAGATGCCGT TATCGTTGGT GTGTAAACCC CAACCCTAGG   
  
  
- TACATCACAG TAGTCTCAAA CTGTCGACCC CCTTTCACTA CCTCTACTAG CGAGCTTTCC TAAACTTTGA   
  
  
- CCACGATTAA CGGACACGTT CTCGTCAACG ACTTTTACTA GTCAACAACC GGGTCACCGA CTACCTACTT   
  
  
- AACTCTGTCT ACCAAAGTCA AAGACCACTT GGTTAAGTTT CTAACCCACG AATATACAAC CTTCCCGAGC   
  
  
- ATCGCTCCGT CCGGAGGAGT CCATCGAGGT AAATATTTCG TAACTCTACG TTCCTTGGGC GCTCACGACT   
  
  
- GGAAAATAGA ATGTACGTGT ATGAGAAACT CCAAACGGGT ATGTAGTTTA AACCGATGTA CAGTCGTTTA   
  
  
- CCACGGTAAC GTCTTCGTTA CTTCCTACTT TTCTCTCAGG TGTAACAGCT AAAGGTTTAA CCCGTCCCTT   
  
  
- CGGTTACCCA CTGGGATTAG GTCCGTAAAC GTCGGGCCGG TCCCCCAGGT GGGGTGTAAG CGTAATGGCC   
  
  
- ATAGCTACTG AGGATGAGAC GTATACGAGC ACCTCCACCC AACTTGTAAC ACCCGGTATC CGAGAACTCC   
  
  
- AACCGAGTCA GTAAATTCCA GGGTAAACTC AAGTTGCGTC AACTGCAGGG CCGAACAGTT CAGGACGAAT   
  
  
- TTCTAGAGCC GTAAGTTGTA CCACTCCGGA ACCGACACTT GAAACGGAAG TAAGAAGTGG TGTACGGGCT   
  
  
- ACTCTCGCAC TCGTGTCTCT TAGTGGCCCT AGCAGAAGAC TCCCACCATT TTCCCGACTT CGGTTTTCAC   
  
  
- CATTGCAATC ACCTCGTTCT CACATTGTGT TTGAGACGAC GAAAGAACGG AGCGAAACAA CTCTGTAACC   
  
  
- TTATGATGTG TCGCTACAAA CTCAGGTACC TACACTGAAA CGGATCCCTA GTATTTCTGG CCTAGTTACA   
  
  
- ACTCGTCGTA ACAGAACGAG CTCTACAACA CTTGAACCAA CGCACACTTC CTCGACTCTC CCACCTCGCC   
  
  
- GTACTCGAGG AACCCTTTAC CTCCAGCGCC AAGTGTTACC GTCCCAAATT CGGTATGGGA AATTCGTGAG   
  
  
- ACCATTTGTT GTGTTATTCT TGAAACAACT TCTTGATGAC ACTATCCATA CCCGATCTCC TCTCCTTACC   
  
  
- TCCAGAAATA GAACCCACCT ACTTATCTCT AAACCAACGA AGCTCACGGA CCGTCACATC ATTAAT

+     MYB

| Site Name | Organism | Position | Strand | Matrix score. | sequence | function |
| --- | --- | --- | --- | --- | --- | --- |
| MYB | Arabidopsis thaliana | 3607 | - | 6 | CAACCA |  |
| MYB | Arabidopsis thaliana | 3400 | - | 6 | CAACCA |  |
| MYB | Arabidopsis thaliana | 2429 | + | 6 | CAACCA |  |
| MYB | Arabidopsis thaliana | 1842 | - | 6 | CAACAG |  |
| MYB | Arabidopsis thaliana | 1410 | - | 6 | CAACCA |  |
| MYB | Arabidopsis thaliana | 1381 | - | 6 | TAACCA |  |
| MYB | Arabidopsis thaliana | 1274 | - | 6 | TAACCA |  |
| MYB | Arabidopsis thaliana | 871 | + | 6 | CAACCA |  |
| MYB | Arabidopsis thaliana | 678 | - | 6 | CAACCA |  |

>HU01G01391.1   
+ +Up\_Stream \_Len000CATTTT AGTAATAAAT TCCCATGTGA GGACCACTGC CTACTGATTC CGAATGGCAT   
  
  
+ TTTAGTAATA AACTCCCATG TGAGGACCAC TTGATGAGGA ATTCAGTTTT ATAATCCACA GGTGTCAAAG   
  
  
+ CCTATTCCTA CTCTCACCAC TCTGCTATCT CTCCCCCCCC CCCCCCCCCG CCCCCCTCAA AATCTGCCTC   
  
  
+ CTCCGTTCTC TCCACAGCAG ATTCCTCAAT CAGGAAAGGT ATGTCAGAGC TCAAAACGAT TCAACATCAA   
  
  
+ AATGGGATAA TTTATTCTGC TTTCAGTTCT GGGTTATTGT CATATGTTCA TTCAGTTCAG TGTTTCTAGC   
  
  
+ ATGATTGCTT TTTGATGCCT TCTTTTTTGT TCTGGGTTGG ACGAACTCTG TCTTTTCTGT GCATAGATTT   
  
  
+ GAGCTCCGAG CTCAATTTTT GGGATTTTTT TAGCTGACTT TTCTGCTTGG TGTTGTGAAA TTGTGATGGG   
  
  
+ TTTTGGCTTT GTTAAACTTG TTTATAACTT TTTCTGTCAA AACTTTATAG TAAATTGTTG CAATTTGAGA   
  
  
+ ATTATGCACT CAGTGGCTCT GTGATTTATC GTCCGCTGGC AAATTTTCTG ATGAAAGGAG ATGATTTATT   
  
  
+ AATTTTTCAG GCATGGTCAA ATTTTCTAGC CTTTCATGTA CATTGGTTGG TGATAGGCTG ATAGCTAAAT   
  
  
+ TATGTCTCAT TTCTCTAAAT TTATTGCCAG ATATGAAATT GGTGGAACAG TTTAAGGGTT TTGGTCGTCT   
  
  
+ TTTTTGCCCT TTTTTTTGGG TCCCCCCCCC GCCCGGGGGG GAACCCGGAG GGCAGAAAAA GTAACATAAC   
  
  
+ TTAAAATTAT CATTGACGTA GTCCTCCAAC CATAATGAAA GCAACTGAAA TAAGAGCATG CTAAGCTAGA   
  
  
+ GAGTAGGCTG CCTTGTCAGC CTCCCTTTTG ACATGAGAAA GATGGGAGAA GTTTAAAATA AAGTTCCTCG   
  
  
+ TCTTTGCATC CCGGACCACA ATTCGGAACT GGGTGAGGAC CTCCTTAGGC TTGTAAACAC AAGTTTCGAA   
  
  
+ CTTAGATTTG CGTACATGTC AATGCTTTAC TGGATAAGCT TAGCTAACAC CCCTCATCCT TCAACCTGTA   
  
  
+ CCTTCATATA AAAAAAAGGA CAATCCGGTG CACAAGCATC CCGCATTCAC GCAAGGTCTG GGGAAGGGCC   
  
  
+ GCACCCCAAA GGGTGAATTG TAGGCAGCCT AACCTGACTT TGTCAGTGGC TAATTCCACG ATTCGAACCC   
  
  
+ ATGATTTTTT GGTTACACGG GAACAACCTT ACCATTGCTC AAAGGCTCCC CTTCTTCAAC CTCTACCTTC   
  
  
+ ATATATGAAT GAAAATCCTT TGGCTAAGAA TTGAAAAGGA AATATGTGGT TATTGGGTTT GGGTTCTTAT   
  
  
+ TCCGTTGGTT GCCCCTTCAC ATTGAATGCA TCCTCTCGCT AAGAACTGAA AAGGACATAT GGGGATGTAT   
  
  
+ AGGTCAAATT TGACTCAATG ATAACTGAGG ATACAAGAAT TCTAGAAGTC TTTATCAGGG GAATTTTTTC   
  
  
+ TTTTTTCCAC TTGTGACTTT TTGATGCCTT GCTTTTTACT CTTCATTGAA TGAGTACCCT TCTTGACAGA   
  
  
+ TTCTTCCTCC CTTTTTTCTT AATTTCTTCT TGTAGTGGAA TATAAGTATG CTTCTAGGTA GTTGGATGCC   
  
  
+ ATGGAATCTC ATCCCTGTGT GTGCATTCTT CCCCTCTCCC GCCGCCCCCC CCCCCCCAAA AAGAAACCAA   
  
  
+ AAAAAAAAAA AAAATGGATG ATTCTTAGAA CTGACCATTT TTCCTTCCTC CTCCCATTTT ATTTTTTTCA   
  
  
+ GGCTGTTTTT CTTCTACCTG TTGCTTTAGC ATCACTCATA AAAACAAGGT GCAAGCGCAT GACATCGGGT   
  
  
+ ACTGAGCTTC TGAGTTTACA TGCATCGTCA TGAAATTCGG TAGACTTTGA AGTAAAGTTT CCCCATCGTA   
  
  
+ ACTGGTGGTG CATACCTGTC TTCACGCCCA TCAGTAGAGT GGTGAAGTGA AGCAATGTCG AATAGTTTGT   
  
  
+ ATTACGAGCC CAAGAGAGAA ACTGATGCAT ATTTTATGCC TCAATGTCAA TCCTTGAACC CCCAGCTCGA   
  
  
+ TTACAACACC ATTGACTATG GAGCTTTTAT GTGCTCAAAA GTTTTCCTGG GTCAGTACTG CACTCTGGAA   
  
  
+ TCATCCTCAG GAACTGGGAC TTGTCCGGTG TCTAGCTCCA CATCAACTCT CAGCTTCTCA TCCAATGGTA   
  
  
+ GCCCTGGATC ACAGCTTGAT TCCAACTCTT ATCCTTCTGA TCAAAATTGC TCTCCTGATA ATGCCAATTC   
  
  
+ CTCATCTTTA AGTCATTCCT GCGTCACAGA TGATGTGGAT GACCTGAAAT ACAGGCTGAG AGAGTTGGAA   
  
  
+ ACAGTGATGT TGGGACCTGA TCCCGATTTT GTCTACGGCA ATAGCAACCA CACATTTGGG GTTGGGATCC   
  
  
+ ATGTAGTGTC ATCAGAGTTT GACAGCTGGG GGAAAGTGAT GGAGATGATC GCTCGAAAGG ATTTGAAACT   
  
  
+ GGTGCTAATT GCCTGTGCAA GAGCAGTTGC TGAAAATGAT CAGTTGTTGG CCCAGTGGCT GATGGATGAA   
  
  
+ TTGAGACAGA TGGTTTCAGT TTCTGGTGAA CCAATTCAAA GATTGGGTGC TTATATGTTG GAAGGGCTCG   
  
  
+ TAGCGAGGCA GGCCTCCTCA GGTAGCTCCA TTTATAAAGC ATTGAGATGC AAGGAACCCG CGAGTGCTGA   
  
  
+ CCTTTTATCT TACATGCACA TACTCTTTGA GGTTTGCCCA TACATCAAAT TTGGCTACAT GTCAGCAAAT   
  
  
+ GGTGCCATTG CAGAAGCAAT GAAGGATGAA AAGAGAGTCC ACATTGTCGA TTTCCAAATT GGGCAGGGAA   
  
  
+ GCCAATGGGT GACCCTAATC CAGGCATTTG CAGCCCGGCC AGGGGGTCCA CCCCACATTC GCATTACCGG   
  
  
+ TATCGATGAC TCCTACTCTG CATATGCTCG TGGAGGTGGG TTGAACATTG TGGGCCATAG GCTCTTGAGG   
  
  
+ TTGGCTCAGT CATTTAAGGT CCCATTTGAG TTCAACGCAG TTGACGTCCC GGCTTGTCAA GTCCTGCTTA   
  
  
+ AAGATCTCGG CATTCAACAT GGTGAGGCCT TGGCTGTGAA CTTTGCCTTC ATTCTTCACC ACATGCCCGA   
  
  
+ TGAGAGCGTG AGCACAGAGA ATCACCGGGA TCGTCTTCTG AGGGTGGTAA AAGGGCTGAA GCCAAAAGTG   
  
  
+ GTAACGTTAG TGGAGCAAGA GTGTAACACA AACTCTGCTG CTTTCTTGCC TCGCTTTGTT GAGACATTGG   
  
  
+ AATACTACAC AGCGATGTTT GAGTCCATGG ATGTGACTTT GCCTAGGGAT CATAAAGACC GGATCAATGT   
  
  
+ TGAGCAGCAT TGTCTTGCTC GAGATGTTGT GAACTTGGTT GCGTGTGAAG GAGCTGAGAG GGTGGAGCGG   
  
  
+ CATGAGCTCC TTGGGAAATG GAGGTCGCGG TTCACAATGG CAGGGTTTAA GCCATACCCT TTAAGCACTC   
  
  
+ TGGTAAACAA CACAATAAGA ACTTTGTTGA AGAACTACTG TGATAGGTAT GGGCTAGAGG AGAGGAATGG   
  
  
+ AGGTCTTTAT CTTGGGTGGA TGAATAGAGA TTTGGTTGCT TCGAGTGCCT GGCAGTGTAG TAATTA  

- +Up\_Stream \_Len000GTAAAA TCATTATTTA AGGGTACACT CCTGGTGACG GATGACTAAG GCTTACCGTA   
  
  
- AAATCATTAT TTGAGGGTAC ACTCCTGGTG AACTACTCCT TAAGTCAAAA TATTAGGTGT CCACAGTTTC   
  
  
- GGATAAGGAT GAGAGTGGTG AGACGATAGA GAGGGGGGGG GGGGGGGGGC GGGGGGAGTT TTAGACGGAG   
  
  
- GAGGCAAGAG AGGTGTCGTC TAAGGAGTTA GTCCTTTCCA TACAGTCTCG AGTTTTGCTA AGTTGTAGTT   
  
  
- TTACCCTATT AAATAAGACG AAAGTCAAGA CCCAATAACA GTATACAAGT AAGTCAAGTC ACAAAGATCG   
  
  
- TACTAACGAA AAACTACGGA AGAAAAAACA AGACCCAACC TGCTTGAGAC AGAAAAGACA CGTATCTAAA   
  
  
- CTCGAGGCTC GAGTTAAAAA CCCTAAAAAA ATCGACTGAA AAGACGAACC ACAACACTTT AACACTACCC   
  
  
- AAAACCGAAA CAATTTGAAC AAATATTGAA AAAGACAGTT TTGAAATATC ATTTAACAAC GTTAAACTCT   
  
  
- TAATACGTGA GTCACCGAGA CACTAAATAG CAGGCGACCG TTTAAAAGAC TACTTTCCTC TACTAAATAA   
  
  
- TTAAAAAGTC CGTACCAGTT TAAAAGATCG GAAAGTACAT GTAACCAACC ACTATCCGAC TATCGATTTA   
  
  
- ATACAGAGTA AAGAGATTTA AATAACGGTC TATACTTTAA CCACCTTGTC AAATTCCCAA AACCAGCAGA   
  
  
- AAAAACGGGA AAAAAAACCC AGGGGGGGGG CGGGCCCCCC CTTGGGCCTC CCGTCTTTTT CATTGTATTG   
  
  
- AATTTTAATA GTAACTGCAT CAGGAGGTTG GTATTACTTT CGTTGACTTT ATTCTCGTAC GATTCGATCT   
  
  
- CTCATCCGAC GGAACAGTCG GAGGGAAAAC TGTACTCTTT CTACCCTCTT CAAATTTTAT TTCAAGGAGC   
  
  
- AGAAACGTAG GGCCTGGTGT TAAGCCTTGA CCCACTCCTG GAGGAATCCG AACATTTGTG TTCAAAGCTT   
  
  
- GAATCTAAAC GCATGTACAG TTACGAAATG ACCTATTCGA ATCGATTGTG GGGAGTAGGA AGTTGGACAT   
  
  
- GGAAGTATAT TTTTTTTCCT GTTAGGCCAC GTGTTCGTAG GGCGTAAGTG CGTTCCAGAC CCCTTCCCGG   
  
  
- CGTGGGGTTT CCCACTTAAC ATCCGTCGGA TTGGACTGAA ACAGTCACCG ATTAAGGTGC TAAGCTTGGG   
  
  
- TACTAAAAAA CCAATGTGCC CTTGTTGGAA TGGTAACGAG TTTCCGAGGG GAAGAAGTTG GAGATGGAAG   
  
  
- TATATACTTA CTTTTAGGAA ACCGATTCTT AACTTTTCCT TTATACACCA ATAACCCAAA CCCAAGAATA   
  
  
- AGGCAACCAA CGGGGAAGTG TAACTTACGT AGGAGAGCGA TTCTTGACTT TTCCTGTATA CCCCTACATA   
  
  
- TCCAGTTTAA ACTGAGTTAC TATTGACTCC TATGTTCTTA AGATCTTCAG AAATAGTCCC CTTAAAAAAG   
  
  
- AAAAAAGGTG AACACTGAAA AACTACGGAA CGAAAAATGA GAAGTAACTT ACTCATGGGA AGAACTGTCT   
  
  
- AAGAAGGAGG GAAAAAAGAA TTAAAGAAGA ACATCACCTT ATATTCATAC GAAGATCCAT CAACCTACGG   
  
  
- TACCTTAGAG TAGGGACACA CACGTAAGAA GGGGAGAGGG CGGCGGGGGG GGGGGGGTTT TTCTTTGGTT   
  
  
- TTTTTTTTTT TTTTACCTAC TAAGAATCTT GACTGGTAAA AAGGAAGGAG GAGGGTAAAA TAAAAAAAGT   
  
  
- CCGACAAAAA GAAGATGGAC AACGAAATCG TAGTGAGTAT TTTTGTTCCA CGTTCGCGTA CTGTAGCCCA   
  
  
- TGACTCGAAG ACTCAAATGT ACGTAGCAGT ACTTTAAGCC ATCTGAAACT TCATTTCAAA GGGGTAGCAT   
  
  
- TGACCACCAC GTATGGACAG AAGTGCGGGT AGTCATCTCA CCACTTCACT TCGTTACAGC TTATCAAACA   
  
  
- TAATGCTCGG GTTCTCTCTT TGACTACGTA TAAAATACGG AGTTACAGTT AGGAACTTGG GGGTCGAGCT   
  
  
- AATGTTGTGG TAACTGATAC CTCGAAAATA CACGAGTTTT CAAAAGGACC CAGTCATGAC GTGAGACCTT   
  
  
- AGTAGGAGTC CTTGACCCTG AACAGGCCAC AGATCGAGGT GTAGTTGAGA GTCGAAGAGT AGGTTACCAT   
  
  
- CGGGACCTAG TGTCGAACTA AGGTTGAGAA TAGGAAGACT AGTTTTAACG AGAGGACTAT TACGGTTAAG   
  
  
- GAGTAGAAAT TCAGTAAGGA CGCAGTGTCT ACTACACCTA CTGGACTTTA TGTCCGACTC TCTCAACCTT   
  
  
- TGTCACTACA ACCCTGGACT AGGGCTAAAA CAGATGCCGT TATCGTTGGT GTGTAAACCC CAACCCTAGG   
  
  
- TACATCACAG TAGTCTCAAA CTGTCGACCC CCTTTCACTA CCTCTACTAG CGAGCTTTCC TAAACTTTGA   
  
  
- CCACGATTAA CGGACACGTT CTCGTCAACG ACTTTTACTA GTCAACAACC GGGTCACCGA CTACCTACTT   
  
  
- AACTCTGTCT ACCAAAGTCA AAGACCACTT GGTTAAGTTT CTAACCCACG AATATACAAC CTTCCCGAGC   
  
  
- ATCGCTCCGT CCGGAGGAGT CCATCGAGGT AAATATTTCG TAACTCTACG TTCCTTGGGC GCTCACGACT   
  
  
- GGAAAATAGA ATGTACGTGT ATGAGAAACT CCAAACGGGT ATGTAGTTTA AACCGATGTA CAGTCGTTTA   
  
  
- CCACGGTAAC GTCTTCGTTA CTTCCTACTT TTCTCTCAGG TGTAACAGCT AAAGGTTTAA CCCGTCCCTT   
  
  
- CGGTTACCCA CTGGGATTAG GTCCGTAAAC GTCGGGCCGG TCCCCCAGGT GGGGTGTAAG CGTAATGGCC   
  
  
- ATAGCTACTG AGGATGAGAC GTATACGAGC ACCTCCACCC AACTTGTAAC ACCCGGTATC CGAGAACTCC   
  
  
- AACCGAGTCA GTAAATTCCA GGGTAAACTC AAGTTGCGTC AACTGCAGGG CCGAACAGTT CAGGACGAAT   
  
  
- TTCTAGAGCC GTAAGTTGTA CCACTCCGGA ACCGACACTT GAAACGGAAG TAAGAAGTGG TGTACGGGCT   
  
  
- ACTCTCGCAC TCGTGTCTCT TAGTGGCCCT AGCAGAAGAC TCCCACCATT TTCCCGACTT CGGTTTTCAC   
  
  
- CATTGCAATC ACCTCGTTCT CACATTGTGT TTGAGACGAC GAAAGAACGG AGCGAAACAA CTCTGTAACC   
  
  
- TTATGATGTG TCGCTACAAA CTCAGGTACC TACACTGAAA CGGATCCCTA GTATTTCTGG CCTAGTTACA   
  
  
- ACTCGTCGTA ACAGAACGAG CTCTACAACA CTTGAACCAA CGCACACTTC CTCGACTCTC CCACCTCGCC   
  
  
- GTACTCGAGG AACCCTTTAC CTCCAGCGCC AAGTGTTACC GTCCCAAATT CGGTATGGGA AATTCGTGAG   
  
  
- ACCATTTGTT GTGTTATTCT TGAAACAACT TCTTGATGAC ACTATCCATA CCCGATCTCC TCTCCTTACC   
  
  
- TCCAGAAATA GAACCCACCT ACTTATCTCT AAACCAACGA AGCTCACGGA CCGTCACATC ATTAAT

+     MYB recognition site

| Site Name | Organism | Position | Strand | Matrix score. | sequence | function |
| --- | --- | --- | --- | --- | --- | --- |
| MYB recognition site | Arabidopsis thaliana | 1406 | + | 6 | CCGTTG |  |

>HU01G01391.1   
+ +Up\_Stream \_Len000CATTTT AGTAATAAAT TCCCATGTGA GGACCACTGC CTACTGATTC CGAATGGCAT   
  
  
+ TTTAGTAATA AACTCCCATG TGAGGACCAC TTGATGAGGA ATTCAGTTTT ATAATCCACA GGTGTCAAAG   
  
  
+ CCTATTCCTA CTCTCACCAC TCTGCTATCT CTCCCCCCCC CCCCCCCCCG CCCCCCTCAA AATCTGCCTC   
  
  
+ CTCCGTTCTC TCCACAGCAG ATTCCTCAAT CAGGAAAGGT ATGTCAGAGC TCAAAACGAT TCAACATCAA   
  
  
+ AATGGGATAA TTTATTCTGC TTTCAGTTCT GGGTTATTGT CATATGTTCA TTCAGTTCAG TGTTTCTAGC   
  
  
+ ATGATTGCTT TTTGATGCCT TCTTTTTTGT TCTGGGTTGG ACGAACTCTG TCTTTTCTGT GCATAGATTT   
  
  
+ GAGCTCCGAG CTCAATTTTT GGGATTTTTT TAGCTGACTT TTCTGCTTGG TGTTGTGAAA TTGTGATGGG   
  
  
+ TTTTGGCTTT GTTAAACTTG TTTATAACTT TTTCTGTCAA AACTTTATAG TAAATTGTTG CAATTTGAGA   
  
  
+ ATTATGCACT CAGTGGCTCT GTGATTTATC GTCCGCTGGC AAATTTTCTG ATGAAAGGAG ATGATTTATT   
  
  
+ AATTTTTCAG GCATGGTCAA ATTTTCTAGC CTTTCATGTA CATTGGTTGG TGATAGGCTG ATAGCTAAAT   
  
  
+ TATGTCTCAT TTCTCTAAAT TTATTGCCAG ATATGAAATT GGTGGAACAG TTTAAGGGTT TTGGTCGTCT   
  
  
+ TTTTTGCCCT TTTTTTTGGG TCCCCCCCCC GCCCGGGGGG GAACCCGGAG GGCAGAAAAA GTAACATAAC   
  
  
+ TTAAAATTAT CATTGACGTA GTCCTCCAAC CATAATGAAA GCAACTGAAA TAAGAGCATG CTAAGCTAGA   
  
  
+ GAGTAGGCTG CCTTGTCAGC CTCCCTTTTG ACATGAGAAA GATGGGAGAA GTTTAAAATA AAGTTCCTCG   
  
  
+ TCTTTGCATC CCGGACCACA ATTCGGAACT GGGTGAGGAC CTCCTTAGGC TTGTAAACAC AAGTTTCGAA   
  
  
+ CTTAGATTTG CGTACATGTC AATGCTTTAC TGGATAAGCT TAGCTAACAC CCCTCATCCT TCAACCTGTA   
  
  
+ CCTTCATATA AAAAAAAGGA CAATCCGGTG CACAAGCATC CCGCATTCAC GCAAGGTCTG GGGAAGGGCC   
  
  
+ GCACCCCAAA GGGTGAATTG TAGGCAGCCT AACCTGACTT TGTCAGTGGC TAATTCCACG ATTCGAACCC   
  
  
+ ATGATTTTTT GGTTACACGG GAACAACCTT ACCATTGCTC AAAGGCTCCC CTTCTTCAAC CTCTACCTTC   
  
  
+ ATATATGAAT GAAAATCCTT TGGCTAAGAA TTGAAAAGGA AATATGTGGT TATTGGGTTT GGGTTCTTAT   
  
  
+ TCCGTTGGTT GCCCCTTCAC ATTGAATGCA TCCTCTCGCT AAGAACTGAA AAGGACATAT GGGGATGTAT   
  
  
+ AGGTCAAATT TGACTCAATG ATAACTGAGG ATACAAGAAT TCTAGAAGTC TTTATCAGGG GAATTTTTTC   
  
  
+ TTTTTTCCAC TTGTGACTTT TTGATGCCTT GCTTTTTACT CTTCATTGAA TGAGTACCCT TCTTGACAGA   
  
  
+ TTCTTCCTCC CTTTTTTCTT AATTTCTTCT TGTAGTGGAA TATAAGTATG CTTCTAGGTA GTTGGATGCC   
  
  
+ ATGGAATCTC ATCCCTGTGT GTGCATTCTT CCCCTCTCCC GCCGCCCCCC CCCCCCCAAA AAGAAACCAA   
  
  
+ AAAAAAAAAA AAAATGGATG ATTCTTAGAA CTGACCATTT TTCCTTCCTC CTCCCATTTT ATTTTTTTCA   
  
  
+ GGCTGTTTTT CTTCTACCTG TTGCTTTAGC ATCACTCATA AAAACAAGGT GCAAGCGCAT GACATCGGGT   
  
  
+ ACTGAGCTTC TGAGTTTACA TGCATCGTCA TGAAATTCGG TAGACTTTGA AGTAAAGTTT CCCCATCGTA   
  
  
+ ACTGGTGGTG CATACCTGTC TTCACGCCCA TCAGTAGAGT GGTGAAGTGA AGCAATGTCG AATAGTTTGT   
  
  
+ ATTACGAGCC CAAGAGAGAA ACTGATGCAT ATTTTATGCC TCAATGTCAA TCCTTGAACC CCCAGCTCGA   
  
  
+ TTACAACACC ATTGACTATG GAGCTTTTAT GTGCTCAAAA GTTTTCCTGG GTCAGTACTG CACTCTGGAA   
  
  
+ TCATCCTCAG GAACTGGGAC TTGTCCGGTG TCTAGCTCCA CATCAACTCT CAGCTTCTCA TCCAATGGTA   
  
  
+ GCCCTGGATC ACAGCTTGAT TCCAACTCTT ATCCTTCTGA TCAAAATTGC TCTCCTGATA ATGCCAATTC   
  
  
+ CTCATCTTTA AGTCATTCCT GCGTCACAGA TGATGTGGAT GACCTGAAAT ACAGGCTGAG AGAGTTGGAA   
  
  
+ ACAGTGATGT TGGGACCTGA TCCCGATTTT GTCTACGGCA ATAGCAACCA CACATTTGGG GTTGGGATCC   
  
  
+ ATGTAGTGTC ATCAGAGTTT GACAGCTGGG GGAAAGTGAT GGAGATGATC GCTCGAAAGG ATTTGAAACT   
  
  
+ GGTGCTAATT GCCTGTGCAA GAGCAGTTGC TGAAAATGAT CAGTTGTTGG CCCAGTGGCT GATGGATGAA   
  
  
+ TTGAGACAGA TGGTTTCAGT TTCTGGTGAA CCAATTCAAA GATTGGGTGC TTATATGTTG GAAGGGCTCG   
  
  
+ TAGCGAGGCA GGCCTCCTCA GGTAGCTCCA TTTATAAAGC ATTGAGATGC AAGGAACCCG CGAGTGCTGA   
  
  
+ CCTTTTATCT TACATGCACA TACTCTTTGA GGTTTGCCCA TACATCAAAT TTGGCTACAT GTCAGCAAAT   
  
  
+ GGTGCCATTG CAGAAGCAAT GAAGGATGAA AAGAGAGTCC ACATTGTCGA TTTCCAAATT GGGCAGGGAA   
  
  
+ GCCAATGGGT GACCCTAATC CAGGCATTTG CAGCCCGGCC AGGGGGTCCA CCCCACATTC GCATTACCGG   
  
  
+ TATCGATGAC TCCTACTCTG CATATGCTCG TGGAGGTGGG TTGAACATTG TGGGCCATAG GCTCTTGAGG   
  
  
+ TTGGCTCAGT CATTTAAGGT CCCATTTGAG TTCAACGCAG TTGACGTCCC GGCTTGTCAA GTCCTGCTTA   
  
  
+ AAGATCTCGG CATTCAACAT GGTGAGGCCT TGGCTGTGAA CTTTGCCTTC ATTCTTCACC ACATGCCCGA   
  
  
+ TGAGAGCGTG AGCACAGAGA ATCACCGGGA TCGTCTTCTG AGGGTGGTAA AAGGGCTGAA GCCAAAAGTG   
  
  
+ GTAACGTTAG TGGAGCAAGA GTGTAACACA AACTCTGCTG CTTTCTTGCC TCGCTTTGTT GAGACATTGG   
  
  
+ AATACTACAC AGCGATGTTT GAGTCCATGG ATGTGACTTT GCCTAGGGAT CATAAAGACC GGATCAATGT   
  
  
+ TGAGCAGCAT TGTCTTGCTC GAGATGTTGT GAACTTGGTT GCGTGTGAAG GAGCTGAGAG GGTGGAGCGG   
  
  
+ CATGAGCTCC TTGGGAAATG GAGGTCGCGG TTCACAATGG CAGGGTTTAA GCCATACCCT TTAAGCACTC   
  
  
+ TGGTAAACAA CACAATAAGA ACTTTGTTGA AGAACTACTG TGATAGGTAT GGGCTAGAGG AGAGGAATGG   
  
  
+ AGGTCTTTAT CTTGGGTGGA TGAATAGAGA TTTGGTTGCT TCGAGTGCCT GGCAGTGTAG TAATTA  

- +Up\_Stream \_Len000GTAAAA TCATTATTTA AGGGTACACT CCTGGTGACG GATGACTAAG GCTTACCGTA   
  
  
- AAATCATTAT TTGAGGGTAC ACTCCTGGTG AACTACTCCT TAAGTCAAAA TATTAGGTGT CCACAGTTTC   
  
  
- GGATAAGGAT GAGAGTGGTG AGACGATAGA GAGGGGGGGG GGGGGGGGGC GGGGGGAGTT TTAGACGGAG   
  
  
- GAGGCAAGAG AGGTGTCGTC TAAGGAGTTA GTCCTTTCCA TACAGTCTCG AGTTTTGCTA AGTTGTAGTT   
  
  
- TTACCCTATT AAATAAGACG AAAGTCAAGA CCCAATAACA GTATACAAGT AAGTCAAGTC ACAAAGATCG   
  
  
- TACTAACGAA AAACTACGGA AGAAAAAACA AGACCCAACC TGCTTGAGAC AGAAAAGACA CGTATCTAAA   
  
  
- CTCGAGGCTC GAGTTAAAAA CCCTAAAAAA ATCGACTGAA AAGACGAACC ACAACACTTT AACACTACCC   
  
  
- AAAACCGAAA CAATTTGAAC AAATATTGAA AAAGACAGTT TTGAAATATC ATTTAACAAC GTTAAACTCT   
  
  
- TAATACGTGA GTCACCGAGA CACTAAATAG CAGGCGACCG TTTAAAAGAC TACTTTCCTC TACTAAATAA   
  
  
- TTAAAAAGTC CGTACCAGTT TAAAAGATCG GAAAGTACAT GTAACCAACC ACTATCCGAC TATCGATTTA   
  
  
- ATACAGAGTA AAGAGATTTA AATAACGGTC TATACTTTAA CCACCTTGTC AAATTCCCAA AACCAGCAGA   
  
  
- AAAAACGGGA AAAAAAACCC AGGGGGGGGG CGGGCCCCCC CTTGGGCCTC CCGTCTTTTT CATTGTATTG   
  
  
- AATTTTAATA GTAACTGCAT CAGGAGGTTG GTATTACTTT CGTTGACTTT ATTCTCGTAC GATTCGATCT   
  
  
- CTCATCCGAC GGAACAGTCG GAGGGAAAAC TGTACTCTTT CTACCCTCTT CAAATTTTAT TTCAAGGAGC   
  
  
- AGAAACGTAG GGCCTGGTGT TAAGCCTTGA CCCACTCCTG GAGGAATCCG AACATTTGTG TTCAAAGCTT   
  
  
- GAATCTAAAC GCATGTACAG TTACGAAATG ACCTATTCGA ATCGATTGTG GGGAGTAGGA AGTTGGACAT   
  
  
- GGAAGTATAT TTTTTTTCCT GTTAGGCCAC GTGTTCGTAG GGCGTAAGTG CGTTCCAGAC CCCTTCCCGG   
  
  
- CGTGGGGTTT CCCACTTAAC ATCCGTCGGA TTGGACTGAA ACAGTCACCG ATTAAGGTGC TAAGCTTGGG   
  
  
- TACTAAAAAA CCAATGTGCC CTTGTTGGAA TGGTAACGAG TTTCCGAGGG GAAGAAGTTG GAGATGGAAG   
  
  
- TATATACTTA CTTTTAGGAA ACCGATTCTT AACTTTTCCT TTATACACCA ATAACCCAAA CCCAAGAATA   
  
  
- AGGCAACCAA CGGGGAAGTG TAACTTACGT AGGAGAGCGA TTCTTGACTT TTCCTGTATA CCCCTACATA   
  
  
- TCCAGTTTAA ACTGAGTTAC TATTGACTCC TATGTTCTTA AGATCTTCAG AAATAGTCCC CTTAAAAAAG   
  
  
- AAAAAAGGTG AACACTGAAA AACTACGGAA CGAAAAATGA GAAGTAACTT ACTCATGGGA AGAACTGTCT   
  
  
- AAGAAGGAGG GAAAAAAGAA TTAAAGAAGA ACATCACCTT ATATTCATAC GAAGATCCAT CAACCTACGG   
  
  
- TACCTTAGAG TAGGGACACA CACGTAAGAA GGGGAGAGGG CGGCGGGGGG GGGGGGGTTT TTCTTTGGTT   
  
  
- TTTTTTTTTT TTTTACCTAC TAAGAATCTT GACTGGTAAA AAGGAAGGAG GAGGGTAAAA TAAAAAAAGT   
  
  
- CCGACAAAAA GAAGATGGAC AACGAAATCG TAGTGAGTAT TTTTGTTCCA CGTTCGCGTA CTGTAGCCCA   
  
  
- TGACTCGAAG ACTCAAATGT ACGTAGCAGT ACTTTAAGCC ATCTGAAACT TCATTTCAAA GGGGTAGCAT   
  
  
- TGACCACCAC GTATGGACAG AAGTGCGGGT AGTCATCTCA CCACTTCACT TCGTTACAGC TTATCAAACA   
  
  
- TAATGCTCGG GTTCTCTCTT TGACTACGTA TAAAATACGG AGTTACAGTT AGGAACTTGG GGGTCGAGCT   
  
  
- AATGTTGTGG TAACTGATAC CTCGAAAATA CACGAGTTTT CAAAAGGACC CAGTCATGAC GTGAGACCTT   
  
  
- AGTAGGAGTC CTTGACCCTG AACAGGCCAC AGATCGAGGT GTAGTTGAGA GTCGAAGAGT AGGTTACCAT   
  
  
- CGGGACCTAG TGTCGAACTA AGGTTGAGAA TAGGAAGACT AGTTTTAACG AGAGGACTAT TACGGTTAAG   
  
  
- GAGTAGAAAT TCAGTAAGGA CGCAGTGTCT ACTACACCTA CTGGACTTTA TGTCCGACTC TCTCAACCTT   
  
  
- TGTCACTACA ACCCTGGACT AGGGCTAAAA CAGATGCCGT TATCGTTGGT GTGTAAACCC CAACCCTAGG   
  
  
- TACATCACAG TAGTCTCAAA CTGTCGACCC CCTTTCACTA CCTCTACTAG CGAGCTTTCC TAAACTTTGA   
  
  
- CCACGATTAA CGGACACGTT CTCGTCAACG ACTTTTACTA GTCAACAACC GGGTCACCGA CTACCTACTT   
  
  
- AACTCTGTCT ACCAAAGTCA AAGACCACTT GGTTAAGTTT CTAACCCACG AATATACAAC CTTCCCGAGC   
  
  
- ATCGCTCCGT CCGGAGGAGT CCATCGAGGT AAATATTTCG TAACTCTACG TTCCTTGGGC GCTCACGACT   
  
  
- GGAAAATAGA ATGTACGTGT ATGAGAAACT CCAAACGGGT ATGTAGTTTA AACCGATGTA CAGTCGTTTA   
  
  
- CCACGGTAAC GTCTTCGTTA CTTCCTACTT TTCTCTCAGG TGTAACAGCT AAAGGTTTAA CCCGTCCCTT   
  
  
- CGGTTACCCA CTGGGATTAG GTCCGTAAAC GTCGGGCCGG TCCCCCAGGT GGGGTGTAAG CGTAATGGCC   
  
  
- ATAGCTACTG AGGATGAGAC GTATACGAGC ACCTCCACCC AACTTGTAAC ACCCGGTATC CGAGAACTCC   
  
  
- AACCGAGTCA GTAAATTCCA GGGTAAACTC AAGTTGCGTC AACTGCAGGG CCGAACAGTT CAGGACGAAT   
  
  
- TTCTAGAGCC GTAAGTTGTA CCACTCCGGA ACCGACACTT GAAACGGAAG TAAGAAGTGG TGTACGGGCT   
  
  
- ACTCTCGCAC TCGTGTCTCT TAGTGGCCCT AGCAGAAGAC TCCCACCATT TTCCCGACTT CGGTTTTCAC   
  
  
- CATTGCAATC ACCTCGTTCT CACATTGTGT TTGAGACGAC GAAAGAACGG AGCGAAACAA CTCTGTAACC   
  
  
- TTATGATGTG TCGCTACAAA CTCAGGTACC TACACTGAAA CGGATCCCTA GTATTTCTGG CCTAGTTACA   
  
  
- ACTCGTCGTA ACAGAACGAG CTCTACAACA CTTGAACCAA CGCACACTTC CTCGACTCTC CCACCTCGCC   
  
  
- GTACTCGAGG AACCCTTTAC CTCCAGCGCC AAGTGTTACC GTCCCAAATT CGGTATGGGA AATTCGTGAG   
  
  
- ACCATTTGTT GTGTTATTCT TGAAACAACT TCTTGATGAC ACTATCCATA CCCGATCTCC TCTCCTTACC   
  
  
- TCCAGAAATA GAACCCACCT ACTTATCTCT AAACCAACGA AGCTCACGGA CCGTCACATC ATTAAT

+     MYB-like sequence

| Site Name | Organism | Position | Strand | Matrix score. | sequence | function |
| --- | --- | --- | --- | --- | --- | --- |
| MYB-like sequence | Arabidopsis thaliana | 1381 | - | 6 | TAACCA |  |
| MYB-like sequence | Arabidopsis thaliana | 1274 | - | 6 | TAACCA |  |

>HU01G01391.1   
+ +Up\_Stream \_Len000CATTTT AGTAATAAAT TCCCATGTGA GGACCACTGC CTACTGATTC CGAATGGCAT   
  
  
+ TTTAGTAATA AACTCCCATG TGAGGACCAC TTGATGAGGA ATTCAGTTTT ATAATCCACA GGTGTCAAAG   
  
  
+ CCTATTCCTA CTCTCACCAC TCTGCTATCT CTCCCCCCCC CCCCCCCCCG CCCCCCTCAA AATCTGCCTC   
  
  
+ CTCCGTTCTC TCCACAGCAG ATTCCTCAAT CAGGAAAGGT ATGTCAGAGC TCAAAACGAT TCAACATCAA   
  
  
+ AATGGGATAA TTTATTCTGC TTTCAGTTCT GGGTTATTGT CATATGTTCA TTCAGTTCAG TGTTTCTAGC   
  
  
+ ATGATTGCTT TTTGATGCCT TCTTTTTTGT TCTGGGTTGG ACGAACTCTG TCTTTTCTGT GCATAGATTT   
  
  
+ GAGCTCCGAG CTCAATTTTT GGGATTTTTT TAGCTGACTT TTCTGCTTGG TGTTGTGAAA TTGTGATGGG   
  
  
+ TTTTGGCTTT GTTAAACTTG TTTATAACTT TTTCTGTCAA AACTTTATAG TAAATTGTTG CAATTTGAGA   
  
  
+ ATTATGCACT CAGTGGCTCT GTGATTTATC GTCCGCTGGC AAATTTTCTG ATGAAAGGAG ATGATTTATT   
  
  
+ AATTTTTCAG GCATGGTCAA ATTTTCTAGC CTTTCATGTA CATTGGTTGG TGATAGGCTG ATAGCTAAAT   
  
  
+ TATGTCTCAT TTCTCTAAAT TTATTGCCAG ATATGAAATT GGTGGAACAG TTTAAGGGTT TTGGTCGTCT   
  
  
+ TTTTTGCCCT TTTTTTTGGG TCCCCCCCCC GCCCGGGGGG GAACCCGGAG GGCAGAAAAA GTAACATAAC   
  
  
+ TTAAAATTAT CATTGACGTA GTCCTCCAAC CATAATGAAA GCAACTGAAA TAAGAGCATG CTAAGCTAGA   
  
  
+ GAGTAGGCTG CCTTGTCAGC CTCCCTTTTG ACATGAGAAA GATGGGAGAA GTTTAAAATA AAGTTCCTCG   
  
  
+ TCTTTGCATC CCGGACCACA ATTCGGAACT GGGTGAGGAC CTCCTTAGGC TTGTAAACAC AAGTTTCGAA   
  
  
+ CTTAGATTTG CGTACATGTC AATGCTTTAC TGGATAAGCT TAGCTAACAC CCCTCATCCT TCAACCTGTA   
  
  
+ CCTTCATATA AAAAAAAGGA CAATCCGGTG CACAAGCATC CCGCATTCAC GCAAGGTCTG GGGAAGGGCC   
  
  
+ GCACCCCAAA GGGTGAATTG TAGGCAGCCT AACCTGACTT TGTCAGTGGC TAATTCCACG ATTCGAACCC   
  
  
+ ATGATTTTTT GGTTACACGG GAACAACCTT ACCATTGCTC AAAGGCTCCC CTTCTTCAAC CTCTACCTTC   
  
  
+ ATATATGAAT GAAAATCCTT TGGCTAAGAA TTGAAAAGGA AATATGTGGT TATTGGGTTT GGGTTCTTAT   
  
  
+ TCCGTTGGTT GCCCCTTCAC ATTGAATGCA TCCTCTCGCT AAGAACTGAA AAGGACATAT GGGGATGTAT   
  
  
+ AGGTCAAATT TGACTCAATG ATAACTGAGG ATACAAGAAT TCTAGAAGTC TTTATCAGGG GAATTTTTTC   
  
  
+ TTTTTTCCAC TTGTGACTTT TTGATGCCTT GCTTTTTACT CTTCATTGAA TGAGTACCCT TCTTGACAGA   
  
  
+ TTCTTCCTCC CTTTTTTCTT AATTTCTTCT TGTAGTGGAA TATAAGTATG CTTCTAGGTA GTTGGATGCC   
  
  
+ ATGGAATCTC ATCCCTGTGT GTGCATTCTT CCCCTCTCCC GCCGCCCCCC CCCCCCCAAA AAGAAACCAA   
  
  
+ AAAAAAAAAA AAAATGGATG ATTCTTAGAA CTGACCATTT TTCCTTCCTC CTCCCATTTT ATTTTTTTCA   
  
  
+ GGCTGTTTTT CTTCTACCTG TTGCTTTAGC ATCACTCATA AAAACAAGGT GCAAGCGCAT GACATCGGGT   
  
  
+ ACTGAGCTTC TGAGTTTACA TGCATCGTCA TGAAATTCGG TAGACTTTGA AGTAAAGTTT CCCCATCGTA   
  
  
+ ACTGGTGGTG CATACCTGTC TTCACGCCCA TCAGTAGAGT GGTGAAGTGA AGCAATGTCG AATAGTTTGT   
  
  
+ ATTACGAGCC CAAGAGAGAA ACTGATGCAT ATTTTATGCC TCAATGTCAA TCCTTGAACC CCCAGCTCGA   
  
  
+ TTACAACACC ATTGACTATG GAGCTTTTAT GTGCTCAAAA GTTTTCCTGG GTCAGTACTG CACTCTGGAA   
  
  
+ TCATCCTCAG GAACTGGGAC TTGTCCGGTG TCTAGCTCCA CATCAACTCT CAGCTTCTCA TCCAATGGTA   
  
  
+ GCCCTGGATC ACAGCTTGAT TCCAACTCTT ATCCTTCTGA TCAAAATTGC TCTCCTGATA ATGCCAATTC   
  
  
+ CTCATCTTTA AGTCATTCCT GCGTCACAGA TGATGTGGAT GACCTGAAAT ACAGGCTGAG AGAGTTGGAA   
  
  
+ ACAGTGATGT TGGGACCTGA TCCCGATTTT GTCTACGGCA ATAGCAACCA CACATTTGGG GTTGGGATCC   
  
  
+ ATGTAGTGTC ATCAGAGTTT GACAGCTGGG GGAAAGTGAT GGAGATGATC GCTCGAAAGG ATTTGAAACT   
  
  
+ GGTGCTAATT GCCTGTGCAA GAGCAGTTGC TGAAAATGAT CAGTTGTTGG CCCAGTGGCT GATGGATGAA   
  
  
+ TTGAGACAGA TGGTTTCAGT TTCTGGTGAA CCAATTCAAA GATTGGGTGC TTATATGTTG GAAGGGCTCG   
  
  
+ TAGCGAGGCA GGCCTCCTCA GGTAGCTCCA TTTATAAAGC ATTGAGATGC AAGGAACCCG CGAGTGCTGA   
  
  
+ CCTTTTATCT TACATGCACA TACTCTTTGA GGTTTGCCCA TACATCAAAT TTGGCTACAT GTCAGCAAAT   
  
  
+ GGTGCCATTG CAGAAGCAAT GAAGGATGAA AAGAGAGTCC ACATTGTCGA TTTCCAAATT GGGCAGGGAA   
  
  
+ GCCAATGGGT GACCCTAATC CAGGCATTTG CAGCCCGGCC AGGGGGTCCA CCCCACATTC GCATTACCGG   
  
  
+ TATCGATGAC TCCTACTCTG CATATGCTCG TGGAGGTGGG TTGAACATTG TGGGCCATAG GCTCTTGAGG   
  
  
+ TTGGCTCAGT CATTTAAGGT CCCATTTGAG TTCAACGCAG TTGACGTCCC GGCTTGTCAA GTCCTGCTTA   
  
  
+ AAGATCTCGG CATTCAACAT GGTGAGGCCT TGGCTGTGAA CTTTGCCTTC ATTCTTCACC ACATGCCCGA   
  
  
+ TGAGAGCGTG AGCACAGAGA ATCACCGGGA TCGTCTTCTG AGGGTGGTAA AAGGGCTGAA GCCAAAAGTG   
  
  
+ GTAACGTTAG TGGAGCAAGA GTGTAACACA AACTCTGCTG CTTTCTTGCC TCGCTTTGTT GAGACATTGG   
  
  
+ AATACTACAC AGCGATGTTT GAGTCCATGG ATGTGACTTT GCCTAGGGAT CATAAAGACC GGATCAATGT   
  
  
+ TGAGCAGCAT TGTCTTGCTC GAGATGTTGT GAACTTGGTT GCGTGTGAAG GAGCTGAGAG GGTGGAGCGG   
  
  
+ CATGAGCTCC TTGGGAAATG GAGGTCGCGG TTCACAATGG CAGGGTTTAA GCCATACCCT TTAAGCACTC   
  
  
+ TGGTAAACAA CACAATAAGA ACTTTGTTGA AGAACTACTG TGATAGGTAT GGGCTAGAGG AGAGGAATGG   
  
  
+ AGGTCTTTAT CTTGGGTGGA TGAATAGAGA TTTGGTTGCT TCGAGTGCCT GGCAGTGTAG TAATTA  

- +Up\_Stream \_Len000GTAAAA TCATTATTTA AGGGTACACT CCTGGTGACG GATGACTAAG GCTTACCGTA   
  
  
- AAATCATTAT TTGAGGGTAC ACTCCTGGTG AACTACTCCT TAAGTCAAAA TATTAGGTGT CCACAGTTTC   
  
  
- GGATAAGGAT GAGAGTGGTG AGACGATAGA GAGGGGGGGG GGGGGGGGGC GGGGGGAGTT TTAGACGGAG   
  
  
- GAGGCAAGAG AGGTGTCGTC TAAGGAGTTA GTCCTTTCCA TACAGTCTCG AGTTTTGCTA AGTTGTAGTT   
  
  
- TTACCCTATT AAATAAGACG AAAGTCAAGA CCCAATAACA GTATACAAGT AAGTCAAGTC ACAAAGATCG   
  
  
- TACTAACGAA AAACTACGGA AGAAAAAACA AGACCCAACC TGCTTGAGAC AGAAAAGACA CGTATCTAAA   
  
  
- CTCGAGGCTC GAGTTAAAAA CCCTAAAAAA ATCGACTGAA AAGACGAACC ACAACACTTT AACACTACCC   
  
  
- AAAACCGAAA CAATTTGAAC AAATATTGAA AAAGACAGTT TTGAAATATC ATTTAACAAC GTTAAACTCT   
  
  
- TAATACGTGA GTCACCGAGA CACTAAATAG CAGGCGACCG TTTAAAAGAC TACTTTCCTC TACTAAATAA   
  
  
- TTAAAAAGTC CGTACCAGTT TAAAAGATCG GAAAGTACAT GTAACCAACC ACTATCCGAC TATCGATTTA   
  
  
- ATACAGAGTA AAGAGATTTA AATAACGGTC TATACTTTAA CCACCTTGTC AAATTCCCAA AACCAGCAGA   
  
  
- AAAAACGGGA AAAAAAACCC AGGGGGGGGG CGGGCCCCCC CTTGGGCCTC CCGTCTTTTT CATTGTATTG   
  
  
- AATTTTAATA GTAACTGCAT CAGGAGGTTG GTATTACTTT CGTTGACTTT ATTCTCGTAC GATTCGATCT   
  
  
- CTCATCCGAC GGAACAGTCG GAGGGAAAAC TGTACTCTTT CTACCCTCTT CAAATTTTAT TTCAAGGAGC   
  
  
- AGAAACGTAG GGCCTGGTGT TAAGCCTTGA CCCACTCCTG GAGGAATCCG AACATTTGTG TTCAAAGCTT   
  
  
- GAATCTAAAC GCATGTACAG TTACGAAATG ACCTATTCGA ATCGATTGTG GGGAGTAGGA AGTTGGACAT   
  
  
- GGAAGTATAT TTTTTTTCCT GTTAGGCCAC GTGTTCGTAG GGCGTAAGTG CGTTCCAGAC CCCTTCCCGG   
  
  
- CGTGGGGTTT CCCACTTAAC ATCCGTCGGA TTGGACTGAA ACAGTCACCG ATTAAGGTGC TAAGCTTGGG   
  
  
- TACTAAAAAA CCAATGTGCC CTTGTTGGAA TGGTAACGAG TTTCCGAGGG GAAGAAGTTG GAGATGGAAG   
  
  
- TATATACTTA CTTTTAGGAA ACCGATTCTT AACTTTTCCT TTATACACCA ATAACCCAAA CCCAAGAATA   
  
  
- AGGCAACCAA CGGGGAAGTG TAACTTACGT AGGAGAGCGA TTCTTGACTT TTCCTGTATA CCCCTACATA   
  
  
- TCCAGTTTAA ACTGAGTTAC TATTGACTCC TATGTTCTTA AGATCTTCAG AAATAGTCCC CTTAAAAAAG   
  
  
- AAAAAAGGTG AACACTGAAA AACTACGGAA CGAAAAATGA GAAGTAACTT ACTCATGGGA AGAACTGTCT   
  
  
- AAGAAGGAGG GAAAAAAGAA TTAAAGAAGA ACATCACCTT ATATTCATAC GAAGATCCAT CAACCTACGG   
  
  
- TACCTTAGAG TAGGGACACA CACGTAAGAA GGGGAGAGGG CGGCGGGGGG GGGGGGGTTT TTCTTTGGTT   
  
  
- TTTTTTTTTT TTTTACCTAC TAAGAATCTT GACTGGTAAA AAGGAAGGAG GAGGGTAAAA TAAAAAAAGT   
  
  
- CCGACAAAAA GAAGATGGAC AACGAAATCG TAGTGAGTAT TTTTGTTCCA CGTTCGCGTA CTGTAGCCCA   
  
  
- TGACTCGAAG ACTCAAATGT ACGTAGCAGT ACTTTAAGCC ATCTGAAACT TCATTTCAAA GGGGTAGCAT   
  
  
- TGACCACCAC GTATGGACAG AAGTGCGGGT AGTCATCTCA CCACTTCACT TCGTTACAGC TTATCAAACA   
  
  
- TAATGCTCGG GTTCTCTCTT TGACTACGTA TAAAATACGG AGTTACAGTT AGGAACTTGG GGGTCGAGCT   
  
  
- AATGTTGTGG TAACTGATAC CTCGAAAATA CACGAGTTTT CAAAAGGACC CAGTCATGAC GTGAGACCTT   
  
  
- AGTAGGAGTC CTTGACCCTG AACAGGCCAC AGATCGAGGT GTAGTTGAGA GTCGAAGAGT AGGTTACCAT   
  
  
- CGGGACCTAG TGTCGAACTA AGGTTGAGAA TAGGAAGACT AGTTTTAACG AGAGGACTAT TACGGTTAAG   
  
  
- GAGTAGAAAT TCAGTAAGGA CGCAGTGTCT ACTACACCTA CTGGACTTTA TGTCCGACTC TCTCAACCTT   
  
  
- TGTCACTACA ACCCTGGACT AGGGCTAAAA CAGATGCCGT TATCGTTGGT GTGTAAACCC CAACCCTAGG   
  
  
- TACATCACAG TAGTCTCAAA CTGTCGACCC CCTTTCACTA CCTCTACTAG CGAGCTTTCC TAAACTTTGA   
  
  
- CCACGATTAA CGGACACGTT CTCGTCAACG ACTTTTACTA GTCAACAACC GGGTCACCGA CTACCTACTT   
  
  
- AACTCTGTCT ACCAAAGTCA AAGACCACTT GGTTAAGTTT CTAACCCACG AATATACAAC CTTCCCGAGC   
  
  
- ATCGCTCCGT CCGGAGGAGT CCATCGAGGT AAATATTTCG TAACTCTACG TTCCTTGGGC GCTCACGACT   
  
  
- GGAAAATAGA ATGTACGTGT ATGAGAAACT CCAAACGGGT ATGTAGTTTA AACCGATGTA CAGTCGTTTA   
  
  
- CCACGGTAAC GTCTTCGTTA CTTCCTACTT TTCTCTCAGG TGTAACAGCT AAAGGTTTAA CCCGTCCCTT   
  
  
- CGGTTACCCA CTGGGATTAG GTCCGTAAAC GTCGGGCCGG TCCCCCAGGT GGGGTGTAAG CGTAATGGCC   
  
  
- ATAGCTACTG AGGATGAGAC GTATACGAGC ACCTCCACCC AACTTGTAAC ACCCGGTATC CGAGAACTCC   
  
  
- AACCGAGTCA GTAAATTCCA GGGTAAACTC AAGTTGCGTC AACTGCAGGG CCGAACAGTT CAGGACGAAT   
  
  
- TTCTAGAGCC GTAAGTTGTA CCACTCCGGA ACCGACACTT GAAACGGAAG TAAGAAGTGG TGTACGGGCT   
  
  
- ACTCTCGCAC TCGTGTCTCT TAGTGGCCCT AGCAGAAGAC TCCCACCATT TTCCCGACTT CGGTTTTCAC   
  
  
- CATTGCAATC ACCTCGTTCT CACATTGTGT TTGAGACGAC GAAAGAACGG AGCGAAACAA CTCTGTAACC   
  
  
- TTATGATGTG TCGCTACAAA CTCAGGTACC TACACTGAAA CGGATCCCTA GTATTTCTGG CCTAGTTACA   
  
  
- ACTCGTCGTA ACAGAACGAG CTCTACAACA CTTGAACCAA CGCACACTTC CTCGACTCTC CCACCTCGCC   
  
  
- GTACTCGAGG AACCCTTTAC CTCCAGCGCC AAGTGTTACC GTCCCAAATT CGGTATGGGA AATTCGTGAG   
  
  
- ACCATTTGTT GTGTTATTCT TGAAACAACT TCTTGATGAC ACTATCCATA CCCGATCTCC TCTCCTTACC   
  
  
- TCCAGAAATA GAACCCACCT ACTTATCTCT AAACCAACGA AGCTCACGGA CCGTCACATC ATTAAT

+     MYC

| Site Name | Organism | Position | Strand | Matrix score. | sequence | function |
| --- | --- | --- | --- | --- | --- | --- |
| MYC | Arabidopsis thaliana | 3144 | - | 6 | CATGTG |  |
| MYC | Arabidopsis thaliana | 3037 | + | 6 | CATTTG |  |
| MYC | Arabidopsis thaliana | 2899 | + | 6 | CATTTG |  |
| MYC | Arabidopsis thaliana | 2800 | - | 6 | CATTTG |  |
| MYC | Arabidopsis thaliana | 2437 | + | 6 | CATTTG |  |
| MYC | Arabidopsis thaliana | 91 | + | 6 | CATGTG |  |
| MYC | Arabidopsis thaliana | 38 | + | 6 | CATGTG |  |

>HU01G01391.1   
+ +Up\_Stream \_Len000CATTTT AGTAATAAAT TCCCATGTGA GGACCACTGC CTACTGATTC CGAATGGCAT   
  
  
+ TTTAGTAATA AACTCCCATG TGAGGACCAC TTGATGAGGA ATTCAGTTTT ATAATCCACA GGTGTCAAAG   
  
  
+ CCTATTCCTA CTCTCACCAC TCTGCTATCT CTCCCCCCCC CCCCCCCCCG CCCCCCTCAA AATCTGCCTC   
  
  
+ CTCCGTTCTC TCCACAGCAG ATTCCTCAAT CAGGAAAGGT ATGTCAGAGC TCAAAACGAT TCAACATCAA   
  
  
+ AATGGGATAA TTTATTCTGC TTTCAGTTCT GGGTTATTGT CATATGTTCA TTCAGTTCAG TGTTTCTAGC   
  
  
+ ATGATTGCTT TTTGATGCCT TCTTTTTTGT TCTGGGTTGG ACGAACTCTG TCTTTTCTGT GCATAGATTT   
  
  
+ GAGCTCCGAG CTCAATTTTT GGGATTTTTT TAGCTGACTT TTCTGCTTGG TGTTGTGAAA TTGTGATGGG   
  
  
+ TTTTGGCTTT GTTAAACTTG TTTATAACTT TTTCTGTCAA AACTTTATAG TAAATTGTTG CAATTTGAGA   
  
  
+ ATTATGCACT CAGTGGCTCT GTGATTTATC GTCCGCTGGC AAATTTTCTG ATGAAAGGAG ATGATTTATT   
  
  
+ AATTTTTCAG GCATGGTCAA ATTTTCTAGC CTTTCATGTA CATTGGTTGG TGATAGGCTG ATAGCTAAAT   
  
  
+ TATGTCTCAT TTCTCTAAAT TTATTGCCAG ATATGAAATT GGTGGAACAG TTTAAGGGTT TTGGTCGTCT   
  
  
+ TTTTTGCCCT TTTTTTTGGG TCCCCCCCCC GCCCGGGGGG GAACCCGGAG GGCAGAAAAA GTAACATAAC   
  
  
+ TTAAAATTAT CATTGACGTA GTCCTCCAAC CATAATGAAA GCAACTGAAA TAAGAGCATG CTAAGCTAGA   
  
  
+ GAGTAGGCTG CCTTGTCAGC CTCCCTTTTG ACATGAGAAA GATGGGAGAA GTTTAAAATA AAGTTCCTCG   
  
  
+ TCTTTGCATC CCGGACCACA ATTCGGAACT GGGTGAGGAC CTCCTTAGGC TTGTAAACAC AAGTTTCGAA   
  
  
+ CTTAGATTTG CGTACATGTC AATGCTTTAC TGGATAAGCT TAGCTAACAC CCCTCATCCT TCAACCTGTA   
  
  
+ CCTTCATATA AAAAAAAGGA CAATCCGGTG CACAAGCATC CCGCATTCAC GCAAGGTCTG GGGAAGGGCC   
  
  
+ GCACCCCAAA GGGTGAATTG TAGGCAGCCT AACCTGACTT TGTCAGTGGC TAATTCCACG ATTCGAACCC   
  
  
+ ATGATTTTTT GGTTACACGG GAACAACCTT ACCATTGCTC AAAGGCTCCC CTTCTTCAAC CTCTACCTTC   
  
  
+ ATATATGAAT GAAAATCCTT TGGCTAAGAA TTGAAAAGGA AATATGTGGT TATTGGGTTT GGGTTCTTAT   
  
  
+ TCCGTTGGTT GCCCCTTCAC ATTGAATGCA TCCTCTCGCT AAGAACTGAA AAGGACATAT GGGGATGTAT   
  
  
+ AGGTCAAATT TGACTCAATG ATAACTGAGG ATACAAGAAT TCTAGAAGTC TTTATCAGGG GAATTTTTTC   
  
  
+ TTTTTTCCAC TTGTGACTTT TTGATGCCTT GCTTTTTACT CTTCATTGAA TGAGTACCCT TCTTGACAGA   
  
  
+ TTCTTCCTCC CTTTTTTCTT AATTTCTTCT TGTAGTGGAA TATAAGTATG CTTCTAGGTA GTTGGATGCC   
  
  
+ ATGGAATCTC ATCCCTGTGT GTGCATTCTT CCCCTCTCCC GCCGCCCCCC CCCCCCCAAA AAGAAACCAA   
  
  
+ AAAAAAAAAA AAAATGGATG ATTCTTAGAA CTGACCATTT TTCCTTCCTC CTCCCATTTT ATTTTTTTCA   
  
  
+ GGCTGTTTTT CTTCTACCTG TTGCTTTAGC ATCACTCATA AAAACAAGGT GCAAGCGCAT GACATCGGGT   
  
  
+ ACTGAGCTTC TGAGTTTACA TGCATCGTCA TGAAATTCGG TAGACTTTGA AGTAAAGTTT CCCCATCGTA   
  
  
+ ACTGGTGGTG CATACCTGTC TTCACGCCCA TCAGTAGAGT GGTGAAGTGA AGCAATGTCG AATAGTTTGT   
  
  
+ ATTACGAGCC CAAGAGAGAA ACTGATGCAT ATTTTATGCC TCAATGTCAA TCCTTGAACC CCCAGCTCGA   
  
  
+ TTACAACACC ATTGACTATG GAGCTTTTAT GTGCTCAAAA GTTTTCCTGG GTCAGTACTG CACTCTGGAA   
  
  
+ TCATCCTCAG GAACTGGGAC TTGTCCGGTG TCTAGCTCCA CATCAACTCT CAGCTTCTCA TCCAATGGTA   
  
  
+ GCCCTGGATC ACAGCTTGAT TCCAACTCTT ATCCTTCTGA TCAAAATTGC TCTCCTGATA ATGCCAATTC   
  
  
+ CTCATCTTTA AGTCATTCCT GCGTCACAGA TGATGTGGAT GACCTGAAAT ACAGGCTGAG AGAGTTGGAA   
  
  
+ ACAGTGATGT TGGGACCTGA TCCCGATTTT GTCTACGGCA ATAGCAACCA CACATTTGGG GTTGGGATCC   
  
  
+ ATGTAGTGTC ATCAGAGTTT GACAGCTGGG GGAAAGTGAT GGAGATGATC GCTCGAAAGG ATTTGAAACT   
  
  
+ GGTGCTAATT GCCTGTGCAA GAGCAGTTGC TGAAAATGAT CAGTTGTTGG CCCAGTGGCT GATGGATGAA   
  
  
+ TTGAGACAGA TGGTTTCAGT TTCTGGTGAA CCAATTCAAA GATTGGGTGC TTATATGTTG GAAGGGCTCG   
  
  
+ TAGCGAGGCA GGCCTCCTCA GGTAGCTCCA TTTATAAAGC ATTGAGATGC AAGGAACCCG CGAGTGCTGA   
  
  
+ CCTTTTATCT TACATGCACA TACTCTTTGA GGTTTGCCCA TACATCAAAT TTGGCTACAT GTCAGCAAAT   
  
  
+ GGTGCCATTG CAGAAGCAAT GAAGGATGAA AAGAGAGTCC ACATTGTCGA TTTCCAAATT GGGCAGGGAA   
  
  
+ GCCAATGGGT GACCCTAATC CAGGCATTTG CAGCCCGGCC AGGGGGTCCA CCCCACATTC GCATTACCGG   
  
  
+ TATCGATGAC TCCTACTCTG CATATGCTCG TGGAGGTGGG TTGAACATTG TGGGCCATAG GCTCTTGAGG   
  
  
+ TTGGCTCAGT CATTTAAGGT CCCATTTGAG TTCAACGCAG TTGACGTCCC GGCTTGTCAA GTCCTGCTTA   
  
  
+ AAGATCTCGG CATTCAACAT GGTGAGGCCT TGGCTGTGAA CTTTGCCTTC ATTCTTCACC ACATGCCCGA   
  
  
+ TGAGAGCGTG AGCACAGAGA ATCACCGGGA TCGTCTTCTG AGGGTGGTAA AAGGGCTGAA GCCAAAAGTG   
  
  
+ GTAACGTTAG TGGAGCAAGA GTGTAACACA AACTCTGCTG CTTTCTTGCC TCGCTTTGTT GAGACATTGG   
  
  
+ AATACTACAC AGCGATGTTT GAGTCCATGG ATGTGACTTT GCCTAGGGAT CATAAAGACC GGATCAATGT   
  
  
+ TGAGCAGCAT TGTCTTGCTC GAGATGTTGT GAACTTGGTT GCGTGTGAAG GAGCTGAGAG GGTGGAGCGG   
  
  
+ CATGAGCTCC TTGGGAAATG GAGGTCGCGG TTCACAATGG CAGGGTTTAA GCCATACCCT TTAAGCACTC   
  
  
+ TGGTAAACAA CACAATAAGA ACTTTGTTGA AGAACTACTG TGATAGGTAT GGGCTAGAGG AGAGGAATGG   
  
  
+ AGGTCTTTAT CTTGGGTGGA TGAATAGAGA TTTGGTTGCT TCGAGTGCCT GGCAGTGTAG TAATTA  

- +Up\_Stream \_Len000GTAAAA TCATTATTTA AGGGTACACT CCTGGTGACG GATGACTAAG GCTTACCGTA   
  
  
- AAATCATTAT TTGAGGGTAC ACTCCTGGTG AACTACTCCT TAAGTCAAAA TATTAGGTGT CCACAGTTTC   
  
  
- GGATAAGGAT GAGAGTGGTG AGACGATAGA GAGGGGGGGG GGGGGGGGGC GGGGGGAGTT TTAGACGGAG   
  
  
- GAGGCAAGAG AGGTGTCGTC TAAGGAGTTA GTCCTTTCCA TACAGTCTCG AGTTTTGCTA AGTTGTAGTT   
  
  
- TTACCCTATT AAATAAGACG AAAGTCAAGA CCCAATAACA GTATACAAGT AAGTCAAGTC ACAAAGATCG   
  
  
- TACTAACGAA AAACTACGGA AGAAAAAACA AGACCCAACC TGCTTGAGAC AGAAAAGACA CGTATCTAAA   
  
  
- CTCGAGGCTC GAGTTAAAAA CCCTAAAAAA ATCGACTGAA AAGACGAACC ACAACACTTT AACACTACCC   
  
  
- AAAACCGAAA CAATTTGAAC AAATATTGAA AAAGACAGTT TTGAAATATC ATTTAACAAC GTTAAACTCT   
  
  
- TAATACGTGA GTCACCGAGA CACTAAATAG CAGGCGACCG TTTAAAAGAC TACTTTCCTC TACTAAATAA   
  
  
- TTAAAAAGTC CGTACCAGTT TAAAAGATCG GAAAGTACAT GTAACCAACC ACTATCCGAC TATCGATTTA   
  
  
- ATACAGAGTA AAGAGATTTA AATAACGGTC TATACTTTAA CCACCTTGTC AAATTCCCAA AACCAGCAGA   
  
  
- AAAAACGGGA AAAAAAACCC AGGGGGGGGG CGGGCCCCCC CTTGGGCCTC CCGTCTTTTT CATTGTATTG   
  
  
- AATTTTAATA GTAACTGCAT CAGGAGGTTG GTATTACTTT CGTTGACTTT ATTCTCGTAC GATTCGATCT   
  
  
- CTCATCCGAC GGAACAGTCG GAGGGAAAAC TGTACTCTTT CTACCCTCTT CAAATTTTAT TTCAAGGAGC   
  
  
- AGAAACGTAG GGCCTGGTGT TAAGCCTTGA CCCACTCCTG GAGGAATCCG AACATTTGTG TTCAAAGCTT   
  
  
- GAATCTAAAC GCATGTACAG TTACGAAATG ACCTATTCGA ATCGATTGTG GGGAGTAGGA AGTTGGACAT   
  
  
- GGAAGTATAT TTTTTTTCCT GTTAGGCCAC GTGTTCGTAG GGCGTAAGTG CGTTCCAGAC CCCTTCCCGG   
  
  
- CGTGGGGTTT CCCACTTAAC ATCCGTCGGA TTGGACTGAA ACAGTCACCG ATTAAGGTGC TAAGCTTGGG   
  
  
- TACTAAAAAA CCAATGTGCC CTTGTTGGAA TGGTAACGAG TTTCCGAGGG GAAGAAGTTG GAGATGGAAG   
  
  
- TATATACTTA CTTTTAGGAA ACCGATTCTT AACTTTTCCT TTATACACCA ATAACCCAAA CCCAAGAATA   
  
  
- AGGCAACCAA CGGGGAAGTG TAACTTACGT AGGAGAGCGA TTCTTGACTT TTCCTGTATA CCCCTACATA   
  
  
- TCCAGTTTAA ACTGAGTTAC TATTGACTCC TATGTTCTTA AGATCTTCAG AAATAGTCCC CTTAAAAAAG   
  
  
- AAAAAAGGTG AACACTGAAA AACTACGGAA CGAAAAATGA GAAGTAACTT ACTCATGGGA AGAACTGTCT   
  
  
- AAGAAGGAGG GAAAAAAGAA TTAAAGAAGA ACATCACCTT ATATTCATAC GAAGATCCAT CAACCTACGG   
  
  
- TACCTTAGAG TAGGGACACA CACGTAAGAA GGGGAGAGGG CGGCGGGGGG GGGGGGGTTT TTCTTTGGTT   
  
  
- TTTTTTTTTT TTTTACCTAC TAAGAATCTT GACTGGTAAA AAGGAAGGAG GAGGGTAAAA TAAAAAAAGT   
  
  
- CCGACAAAAA GAAGATGGAC AACGAAATCG TAGTGAGTAT TTTTGTTCCA CGTTCGCGTA CTGTAGCCCA   
  
  
- TGACTCGAAG ACTCAAATGT ACGTAGCAGT ACTTTAAGCC ATCTGAAACT TCATTTCAAA GGGGTAGCAT   
  
  
- TGACCACCAC GTATGGACAG AAGTGCGGGT AGTCATCTCA CCACTTCACT TCGTTACAGC TTATCAAACA   
  
  
- TAATGCTCGG GTTCTCTCTT TGACTACGTA TAAAATACGG AGTTACAGTT AGGAACTTGG GGGTCGAGCT   
  
  
- AATGTTGTGG TAACTGATAC CTCGAAAATA CACGAGTTTT CAAAAGGACC CAGTCATGAC GTGAGACCTT   
  
  
- AGTAGGAGTC CTTGACCCTG AACAGGCCAC AGATCGAGGT GTAGTTGAGA GTCGAAGAGT AGGTTACCAT   
  
  
- CGGGACCTAG TGTCGAACTA AGGTTGAGAA TAGGAAGACT AGTTTTAACG AGAGGACTAT TACGGTTAAG   
  
  
- GAGTAGAAAT TCAGTAAGGA CGCAGTGTCT ACTACACCTA CTGGACTTTA TGTCCGACTC TCTCAACCTT   
  
  
- TGTCACTACA ACCCTGGACT AGGGCTAAAA CAGATGCCGT TATCGTTGGT GTGTAAACCC CAACCCTAGG   
  
  
- TACATCACAG TAGTCTCAAA CTGTCGACCC CCTTTCACTA CCTCTACTAG CGAGCTTTCC TAAACTTTGA   
  
  
- CCACGATTAA CGGACACGTT CTCGTCAACG ACTTTTACTA GTCAACAACC GGGTCACCGA CTACCTACTT   
  
  
- AACTCTGTCT ACCAAAGTCA AAGACCACTT GGTTAAGTTT CTAACCCACG AATATACAAC CTTCCCGAGC   
  
  
- ATCGCTCCGT CCGGAGGAGT CCATCGAGGT AAATATTTCG TAACTCTACG TTCCTTGGGC GCTCACGACT   
  
  
- GGAAAATAGA ATGTACGTGT ATGAGAAACT CCAAACGGGT ATGTAGTTTA AACCGATGTA CAGTCGTTTA   
  
  
- CCACGGTAAC GTCTTCGTTA CTTCCTACTT TTCTCTCAGG TGTAACAGCT AAAGGTTTAA CCCGTCCCTT   
  
  
- CGGTTACCCA CTGGGATTAG GTCCGTAAAC GTCGGGCCGG TCCCCCAGGT GGGGTGTAAG CGTAATGGCC   
  
  
- ATAGCTACTG AGGATGAGAC GTATACGAGC ACCTCCACCC AACTTGTAAC ACCCGGTATC CGAGAACTCC   
  
  
- AACCGAGTCA GTAAATTCCA GGGTAAACTC AAGTTGCGTC AACTGCAGGG CCGAACAGTT CAGGACGAAT   
  
  
- TTCTAGAGCC GTAAGTTGTA CCACTCCGGA ACCGACACTT GAAACGGAAG TAAGAAGTGG TGTACGGGCT   
  
  
- ACTCTCGCAC TCGTGTCTCT TAGTGGCCCT AGCAGAAGAC TCCCACCATT TTCCCGACTT CGGTTTTCAC   
  
  
- CATTGCAATC ACCTCGTTCT CACATTGTGT TTGAGACGAC GAAAGAACGG AGCGAAACAA CTCTGTAACC   
  
  
- TTATGATGTG TCGCTACAAA CTCAGGTACC TACACTGAAA CGGATCCCTA GTATTTCTGG CCTAGTTACA   
  
  
- ACTCGTCGTA ACAGAACGAG CTCTACAACA CTTGAACCAA CGCACACTTC CTCGACTCTC CCACCTCGCC   
  
  
- GTACTCGAGG AACCCTTTAC CTCCAGCGCC AAGTGTTACC GTCCCAAATT CGGTATGGGA AATTCGTGAG   
  
  
- ACCATTTGTT GTGTTATTCT TGAAACAACT TCTTGATGAC ACTATCCATA CCCGATCTCC TCTCCTTACC   
  
  
- TCCAGAAATA GAACCCACCT ACTTATCTCT AAACCAACGA AGCTCACGGA CCGTCACATC ATTAAT

+     Myb

| Site Name | Organism | Position | Strand | Matrix score. | sequence | function |
| --- | --- | --- | --- | --- | --- | --- |
| Myb | Arabidopsis thaliana | 3052 | - | 6 | CAACTG |  |
| Myb | Arabidopsis thaliana | 2565 | - | 6 | CAACTG |  |
| Myb | Arabidopsis thaliana | 2548 | - | 6 | CAACTG |  |
| Myb | Arabidopsis thaliana | 1963 | + | 6 | TAACTG |  |
| Myb | Arabidopsis thaliana | 1496 | + | 6 | TAACTG |  |
| Myb | Arabidopsis thaliana | 886 | + | 6 | CAACTG |  |

>HU01G01391.1   
+ +Up\_Stream \_Len000CATTTT AGTAATAAAT TCCCATGTGA GGACCACTGC CTACTGATTC CGAATGGCAT   
  
  
+ TTTAGTAATA AACTCCCATG TGAGGACCAC TTGATGAGGA ATTCAGTTTT ATAATCCACA GGTGTCAAAG   
  
  
+ CCTATTCCTA CTCTCACCAC TCTGCTATCT CTCCCCCCCC CCCCCCCCCG CCCCCCTCAA AATCTGCCTC   
  
  
+ CTCCGTTCTC TCCACAGCAG ATTCCTCAAT CAGGAAAGGT ATGTCAGAGC TCAAAACGAT TCAACATCAA   
  
  
+ AATGGGATAA TTTATTCTGC TTTCAGTTCT GGGTTATTGT CATATGTTCA TTCAGTTCAG TGTTTCTAGC   
  
  
+ ATGATTGCTT TTTGATGCCT TCTTTTTTGT TCTGGGTTGG ACGAACTCTG TCTTTTCTGT GCATAGATTT   
  
  
+ GAGCTCCGAG CTCAATTTTT GGGATTTTTT TAGCTGACTT TTCTGCTTGG TGTTGTGAAA TTGTGATGGG   
  
  
+ TTTTGGCTTT GTTAAACTTG TTTATAACTT TTTCTGTCAA AACTTTATAG TAAATTGTTG CAATTTGAGA   
  
  
+ ATTATGCACT CAGTGGCTCT GTGATTTATC GTCCGCTGGC AAATTTTCTG ATGAAAGGAG ATGATTTATT   
  
  
+ AATTTTTCAG GCATGGTCAA ATTTTCTAGC CTTTCATGTA CATTGGTTGG TGATAGGCTG ATAGCTAAAT   
  
  
+ TATGTCTCAT TTCTCTAAAT TTATTGCCAG ATATGAAATT GGTGGAACAG TTTAAGGGTT TTGGTCGTCT   
  
  
+ TTTTTGCCCT TTTTTTTGGG TCCCCCCCCC GCCCGGGGGG GAACCCGGAG GGCAGAAAAA GTAACATAAC   
  
  
+ TTAAAATTAT CATTGACGTA GTCCTCCAAC CATAATGAAA GCAACTGAAA TAAGAGCATG CTAAGCTAGA   
  
  
+ GAGTAGGCTG CCTTGTCAGC CTCCCTTTTG ACATGAGAAA GATGGGAGAA GTTTAAAATA AAGTTCCTCG   
  
  
+ TCTTTGCATC CCGGACCACA ATTCGGAACT GGGTGAGGAC CTCCTTAGGC TTGTAAACAC AAGTTTCGAA   
  
  
+ CTTAGATTTG CGTACATGTC AATGCTTTAC TGGATAAGCT TAGCTAACAC CCCTCATCCT TCAACCTGTA   
  
  
+ CCTTCATATA AAAAAAAGGA CAATCCGGTG CACAAGCATC CCGCATTCAC GCAAGGTCTG GGGAAGGGCC   
  
  
+ GCACCCCAAA GGGTGAATTG TAGGCAGCCT AACCTGACTT TGTCAGTGGC TAATTCCACG ATTCGAACCC   
  
  
+ ATGATTTTTT GGTTACACGG GAACAACCTT ACCATTGCTC AAAGGCTCCC CTTCTTCAAC CTCTACCTTC   
  
  
+ ATATATGAAT GAAAATCCTT TGGCTAAGAA TTGAAAAGGA AATATGTGGT TATTGGGTTT GGGTTCTTAT   
  
  
+ TCCGTTGGTT GCCCCTTCAC ATTGAATGCA TCCTCTCGCT AAGAACTGAA AAGGACATAT GGGGATGTAT   
  
  
+ AGGTCAAATT TGACTCAATG ATAACTGAGG ATACAAGAAT TCTAGAAGTC TTTATCAGGG GAATTTTTTC   
  
  
+ TTTTTTCCAC TTGTGACTTT TTGATGCCTT GCTTTTTACT CTTCATTGAA TGAGTACCCT TCTTGACAGA   
  
  
+ TTCTTCCTCC CTTTTTTCTT AATTTCTTCT TGTAGTGGAA TATAAGTATG CTTCTAGGTA GTTGGATGCC   
  
  
+ ATGGAATCTC ATCCCTGTGT GTGCATTCTT CCCCTCTCCC GCCGCCCCCC CCCCCCCAAA AAGAAACCAA   
  
  
+ AAAAAAAAAA AAAATGGATG ATTCTTAGAA CTGACCATTT TTCCTTCCTC CTCCCATTTT ATTTTTTTCA   
  
  
+ GGCTGTTTTT CTTCTACCTG TTGCTTTAGC ATCACTCATA AAAACAAGGT GCAAGCGCAT GACATCGGGT   
  
  
+ ACTGAGCTTC TGAGTTTACA TGCATCGTCA TGAAATTCGG TAGACTTTGA AGTAAAGTTT CCCCATCGTA   
  
  
+ ACTGGTGGTG CATACCTGTC TTCACGCCCA TCAGTAGAGT GGTGAAGTGA AGCAATGTCG AATAGTTTGT   
  
  
+ ATTACGAGCC CAAGAGAGAA ACTGATGCAT ATTTTATGCC TCAATGTCAA TCCTTGAACC CCCAGCTCGA   
  
  
+ TTACAACACC ATTGACTATG GAGCTTTTAT GTGCTCAAAA GTTTTCCTGG GTCAGTACTG CACTCTGGAA   
  
  
+ TCATCCTCAG GAACTGGGAC TTGTCCGGTG TCTAGCTCCA CATCAACTCT CAGCTTCTCA TCCAATGGTA   
  
  
+ GCCCTGGATC ACAGCTTGAT TCCAACTCTT ATCCTTCTGA TCAAAATTGC TCTCCTGATA ATGCCAATTC   
  
  
+ CTCATCTTTA AGTCATTCCT GCGTCACAGA TGATGTGGAT GACCTGAAAT ACAGGCTGAG AGAGTTGGAA   
  
  
+ ACAGTGATGT TGGGACCTGA TCCCGATTTT GTCTACGGCA ATAGCAACCA CACATTTGGG GTTGGGATCC   
  
  
+ ATGTAGTGTC ATCAGAGTTT GACAGCTGGG GGAAAGTGAT GGAGATGATC GCTCGAAAGG ATTTGAAACT   
  
  
+ GGTGCTAATT GCCTGTGCAA GAGCAGTTGC TGAAAATGAT CAGTTGTTGG CCCAGTGGCT GATGGATGAA   
  
  
+ TTGAGACAGA TGGTTTCAGT TTCTGGTGAA CCAATTCAAA GATTGGGTGC TTATATGTTG GAAGGGCTCG   
  
  
+ TAGCGAGGCA GGCCTCCTCA GGTAGCTCCA TTTATAAAGC ATTGAGATGC AAGGAACCCG CGAGTGCTGA   
  
  
+ CCTTTTATCT TACATGCACA TACTCTTTGA GGTTTGCCCA TACATCAAAT TTGGCTACAT GTCAGCAAAT   
  
  
+ GGTGCCATTG CAGAAGCAAT GAAGGATGAA AAGAGAGTCC ACATTGTCGA TTTCCAAATT GGGCAGGGAA   
  
  
+ GCCAATGGGT GACCCTAATC CAGGCATTTG CAGCCCGGCC AGGGGGTCCA CCCCACATTC GCATTACCGG   
  
  
+ TATCGATGAC TCCTACTCTG CATATGCTCG TGGAGGTGGG TTGAACATTG TGGGCCATAG GCTCTTGAGG   
  
  
+ TTGGCTCAGT CATTTAAGGT CCCATTTGAG TTCAACGCAG TTGACGTCCC GGCTTGTCAA GTCCTGCTTA   
  
  
+ AAGATCTCGG CATTCAACAT GGTGAGGCCT TGGCTGTGAA CTTTGCCTTC ATTCTTCACC ACATGCCCGA   
  
  
+ TGAGAGCGTG AGCACAGAGA ATCACCGGGA TCGTCTTCTG AGGGTGGTAA AAGGGCTGAA GCCAAAAGTG   
  
  
+ GTAACGTTAG TGGAGCAAGA GTGTAACACA AACTCTGCTG CTTTCTTGCC TCGCTTTGTT GAGACATTGG   
  
  
+ AATACTACAC AGCGATGTTT GAGTCCATGG ATGTGACTTT GCCTAGGGAT CATAAAGACC GGATCAATGT   
  
  
+ TGAGCAGCAT TGTCTTGCTC GAGATGTTGT GAACTTGGTT GCGTGTGAAG GAGCTGAGAG GGTGGAGCGG   
  
  
+ CATGAGCTCC TTGGGAAATG GAGGTCGCGG TTCACAATGG CAGGGTTTAA GCCATACCCT TTAAGCACTC   
  
  
+ TGGTAAACAA CACAATAAGA ACTTTGTTGA AGAACTACTG TGATAGGTAT GGGCTAGAGG AGAGGAATGG   
  
  
+ AGGTCTTTAT CTTGGGTGGA TGAATAGAGA TTTGGTTGCT TCGAGTGCCT GGCAGTGTAG TAATTA  

- +Up\_Stream \_Len000GTAAAA TCATTATTTA AGGGTACACT CCTGGTGACG GATGACTAAG GCTTACCGTA   
  
  
- AAATCATTAT TTGAGGGTAC ACTCCTGGTG AACTACTCCT TAAGTCAAAA TATTAGGTGT CCACAGTTTC   
  
  
- GGATAAGGAT GAGAGTGGTG AGACGATAGA GAGGGGGGGG GGGGGGGGGC GGGGGGAGTT TTAGACGGAG   
  
  
- GAGGCAAGAG AGGTGTCGTC TAAGGAGTTA GTCCTTTCCA TACAGTCTCG AGTTTTGCTA AGTTGTAGTT   
  
  
- TTACCCTATT AAATAAGACG AAAGTCAAGA CCCAATAACA GTATACAAGT AAGTCAAGTC ACAAAGATCG   
  
  
- TACTAACGAA AAACTACGGA AGAAAAAACA AGACCCAACC TGCTTGAGAC AGAAAAGACA CGTATCTAAA   
  
  
- CTCGAGGCTC GAGTTAAAAA CCCTAAAAAA ATCGACTGAA AAGACGAACC ACAACACTTT AACACTACCC   
  
  
- AAAACCGAAA CAATTTGAAC AAATATTGAA AAAGACAGTT TTGAAATATC ATTTAACAAC GTTAAACTCT   
  
  
- TAATACGTGA GTCACCGAGA CACTAAATAG CAGGCGACCG TTTAAAAGAC TACTTTCCTC TACTAAATAA   
  
  
- TTAAAAAGTC CGTACCAGTT TAAAAGATCG GAAAGTACAT GTAACCAACC ACTATCCGAC TATCGATTTA   
  
  
- ATACAGAGTA AAGAGATTTA AATAACGGTC TATACTTTAA CCACCTTGTC AAATTCCCAA AACCAGCAGA   
  
  
- AAAAACGGGA AAAAAAACCC AGGGGGGGGG CGGGCCCCCC CTTGGGCCTC CCGTCTTTTT CATTGTATTG   
  
  
- AATTTTAATA GTAACTGCAT CAGGAGGTTG GTATTACTTT CGTTGACTTT ATTCTCGTAC GATTCGATCT   
  
  
- CTCATCCGAC GGAACAGTCG GAGGGAAAAC TGTACTCTTT CTACCCTCTT CAAATTTTAT TTCAAGGAGC   
  
  
- AGAAACGTAG GGCCTGGTGT TAAGCCTTGA CCCACTCCTG GAGGAATCCG AACATTTGTG TTCAAAGCTT   
  
  
- GAATCTAAAC GCATGTACAG TTACGAAATG ACCTATTCGA ATCGATTGTG GGGAGTAGGA AGTTGGACAT   
  
  
- GGAAGTATAT TTTTTTTCCT GTTAGGCCAC GTGTTCGTAG GGCGTAAGTG CGTTCCAGAC CCCTTCCCGG   
  
  
- CGTGGGGTTT CCCACTTAAC ATCCGTCGGA TTGGACTGAA ACAGTCACCG ATTAAGGTGC TAAGCTTGGG   
  
  
- TACTAAAAAA CCAATGTGCC CTTGTTGGAA TGGTAACGAG TTTCCGAGGG GAAGAAGTTG GAGATGGAAG   
  
  
- TATATACTTA CTTTTAGGAA ACCGATTCTT AACTTTTCCT TTATACACCA ATAACCCAAA CCCAAGAATA   
  
  
- AGGCAACCAA CGGGGAAGTG TAACTTACGT AGGAGAGCGA TTCTTGACTT TTCCTGTATA CCCCTACATA   
  
  
- TCCAGTTTAA ACTGAGTTAC TATTGACTCC TATGTTCTTA AGATCTTCAG AAATAGTCCC CTTAAAAAAG   
  
  
- AAAAAAGGTG AACACTGAAA AACTACGGAA CGAAAAATGA GAAGTAACTT ACTCATGGGA AGAACTGTCT   
  
  
- AAGAAGGAGG GAAAAAAGAA TTAAAGAAGA ACATCACCTT ATATTCATAC GAAGATCCAT CAACCTACGG   
  
  
- TACCTTAGAG TAGGGACACA CACGTAAGAA GGGGAGAGGG CGGCGGGGGG GGGGGGGTTT TTCTTTGGTT   
  
  
- TTTTTTTTTT TTTTACCTAC TAAGAATCTT GACTGGTAAA AAGGAAGGAG GAGGGTAAAA TAAAAAAAGT   
  
  
- CCGACAAAAA GAAGATGGAC AACGAAATCG TAGTGAGTAT TTTTGTTCCA CGTTCGCGTA CTGTAGCCCA   
  
  
- TGACTCGAAG ACTCAAATGT ACGTAGCAGT ACTTTAAGCC ATCTGAAACT TCATTTCAAA GGGGTAGCAT   
  
  
- TGACCACCAC GTATGGACAG AAGTGCGGGT AGTCATCTCA CCACTTCACT TCGTTACAGC TTATCAAACA   
  
  
- TAATGCTCGG GTTCTCTCTT TGACTACGTA TAAAATACGG AGTTACAGTT AGGAACTTGG GGGTCGAGCT   
  
  
- AATGTTGTGG TAACTGATAC CTCGAAAATA CACGAGTTTT CAAAAGGACC CAGTCATGAC GTGAGACCTT   
  
  
- AGTAGGAGTC CTTGACCCTG AACAGGCCAC AGATCGAGGT GTAGTTGAGA GTCGAAGAGT AGGTTACCAT   
  
  
- CGGGACCTAG TGTCGAACTA AGGTTGAGAA TAGGAAGACT AGTTTTAACG AGAGGACTAT TACGGTTAAG   
  
  
- GAGTAGAAAT TCAGTAAGGA CGCAGTGTCT ACTACACCTA CTGGACTTTA TGTCCGACTC TCTCAACCTT   
  
  
- TGTCACTACA ACCCTGGACT AGGGCTAAAA CAGATGCCGT TATCGTTGGT GTGTAAACCC CAACCCTAGG   
  
  
- TACATCACAG TAGTCTCAAA CTGTCGACCC CCTTTCACTA CCTCTACTAG CGAGCTTTCC TAAACTTTGA   
  
  
- CCACGATTAA CGGACACGTT CTCGTCAACG ACTTTTACTA GTCAACAACC GGGTCACCGA CTACCTACTT   
  
  
- AACTCTGTCT ACCAAAGTCA AAGACCACTT GGTTAAGTTT CTAACCCACG AATATACAAC CTTCCCGAGC   
  
  
- ATCGCTCCGT CCGGAGGAGT CCATCGAGGT AAATATTTCG TAACTCTACG TTCCTTGGGC GCTCACGACT   
  
  
- GGAAAATAGA ATGTACGTGT ATGAGAAACT CCAAACGGGT ATGTAGTTTA AACCGATGTA CAGTCGTTTA   
  
  
- CCACGGTAAC GTCTTCGTTA CTTCCTACTT TTCTCTCAGG TGTAACAGCT AAAGGTTTAA CCCGTCCCTT   
  
  
- CGGTTACCCA CTGGGATTAG GTCCGTAAAC GTCGGGCCGG TCCCCCAGGT GGGGTGTAAG CGTAATGGCC   
  
  
- ATAGCTACTG AGGATGAGAC GTATACGAGC ACCTCCACCC AACTTGTAAC ACCCGGTATC CGAGAACTCC   
  
  
- AACCGAGTCA GTAAATTCCA GGGTAAACTC AAGTTGCGTC AACTGCAGGG CCGAACAGTT CAGGACGAAT   
  
  
- TTCTAGAGCC GTAAGTTGTA CCACTCCGGA ACCGACACTT GAAACGGAAG TAAGAAGTGG TGTACGGGCT   
  
  
- ACTCTCGCAC TCGTGTCTCT TAGTGGCCCT AGCAGAAGAC TCCCACCATT TTCCCGACTT CGGTTTTCAC   
  
  
- CATTGCAATC ACCTCGTTCT CACATTGTGT TTGAGACGAC GAAAGAACGG AGCGAAACAA CTCTGTAACC   
  
  
- TTATGATGTG TCGCTACAAA CTCAGGTACC TACACTGAAA CGGATCCCTA GTATTTCTGG CCTAGTTACA   
  
  
- ACTCGTCGTA ACAGAACGAG CTCTACAACA CTTGAACCAA CGCACACTTC CTCGACTCTC CCACCTCGCC   
  
  
- GTACTCGAGG AACCCTTTAC CTCCAGCGCC AAGTGTTACC GTCCCAAATT CGGTATGGGA AATTCGTGAG   
  
  
- ACCATTTGTT GTGTTATTCT TGAAACAACT TCTTGATGAC ACTATCCATA CCCGATCTCC TCTCCTTACC   
  
  
- TCCAGAAATA GAACCCACCT ACTTATCTCT AAACCAACGA AGCTCACGGA CCGTCACATC ATTAAT

+     Myb-binding site

| Site Name | Organism | Position | Strand | Matrix score. | sequence | function |
| --- | --- | --- | --- | --- | --- | --- |
| Myb-binding site | Nicotiana tabacum | 1842 | - | 6 | CAACAG |  |

>HU01G01391.1   
+ +Up\_Stream \_Len000CATTTT AGTAATAAAT TCCCATGTGA GGACCACTGC CTACTGATTC CGAATGGCAT   
  
  
+ TTTAGTAATA AACTCCCATG TGAGGACCAC TTGATGAGGA ATTCAGTTTT ATAATCCACA GGTGTCAAAG   
  
  
+ CCTATTCCTA CTCTCACCAC TCTGCTATCT CTCCCCCCCC CCCCCCCCCG CCCCCCTCAA AATCTGCCTC   
  
  
+ CTCCGTTCTC TCCACAGCAG ATTCCTCAAT CAGGAAAGGT ATGTCAGAGC TCAAAACGAT TCAACATCAA   
  
  
+ AATGGGATAA TTTATTCTGC TTTCAGTTCT GGGTTATTGT CATATGTTCA TTCAGTTCAG TGTTTCTAGC   
  
  
+ ATGATTGCTT TTTGATGCCT TCTTTTTTGT TCTGGGTTGG ACGAACTCTG TCTTTTCTGT GCATAGATTT   
  
  
+ GAGCTCCGAG CTCAATTTTT GGGATTTTTT TAGCTGACTT TTCTGCTTGG TGTTGTGAAA TTGTGATGGG   
  
  
+ TTTTGGCTTT GTTAAACTTG TTTATAACTT TTTCTGTCAA AACTTTATAG TAAATTGTTG CAATTTGAGA   
  
  
+ ATTATGCACT CAGTGGCTCT GTGATTTATC GTCCGCTGGC AAATTTTCTG ATGAAAGGAG ATGATTTATT   
  
  
+ AATTTTTCAG GCATGGTCAA ATTTTCTAGC CTTTCATGTA CATTGGTTGG TGATAGGCTG ATAGCTAAAT   
  
  
+ TATGTCTCAT TTCTCTAAAT TTATTGCCAG ATATGAAATT GGTGGAACAG TTTAAGGGTT TTGGTCGTCT   
  
  
+ TTTTTGCCCT TTTTTTTGGG TCCCCCCCCC GCCCGGGGGG GAACCCGGAG GGCAGAAAAA GTAACATAAC   
  
  
+ TTAAAATTAT CATTGACGTA GTCCTCCAAC CATAATGAAA GCAACTGAAA TAAGAGCATG CTAAGCTAGA   
  
  
+ GAGTAGGCTG CCTTGTCAGC CTCCCTTTTG ACATGAGAAA GATGGGAGAA GTTTAAAATA AAGTTCCTCG   
  
  
+ TCTTTGCATC CCGGACCACA ATTCGGAACT GGGTGAGGAC CTCCTTAGGC TTGTAAACAC AAGTTTCGAA   
  
  
+ CTTAGATTTG CGTACATGTC AATGCTTTAC TGGATAAGCT TAGCTAACAC CCCTCATCCT TCAACCTGTA   
  
  
+ CCTTCATATA AAAAAAAGGA CAATCCGGTG CACAAGCATC CCGCATTCAC GCAAGGTCTG GGGAAGGGCC   
  
  
+ GCACCCCAAA GGGTGAATTG TAGGCAGCCT AACCTGACTT TGTCAGTGGC TAATTCCACG ATTCGAACCC   
  
  
+ ATGATTTTTT GGTTACACGG GAACAACCTT ACCATTGCTC AAAGGCTCCC CTTCTTCAAC CTCTACCTTC   
  
  
+ ATATATGAAT GAAAATCCTT TGGCTAAGAA TTGAAAAGGA AATATGTGGT TATTGGGTTT GGGTTCTTAT   
  
  
+ TCCGTTGGTT GCCCCTTCAC ATTGAATGCA TCCTCTCGCT AAGAACTGAA AAGGACATAT GGGGATGTAT   
  
  
+ AGGTCAAATT TGACTCAATG ATAACTGAGG ATACAAGAAT TCTAGAAGTC TTTATCAGGG GAATTTTTTC   
  
  
+ TTTTTTCCAC TTGTGACTTT TTGATGCCTT GCTTTTTACT CTTCATTGAA TGAGTACCCT TCTTGACAGA   
  
  
+ TTCTTCCTCC CTTTTTTCTT AATTTCTTCT TGTAGTGGAA TATAAGTATG CTTCTAGGTA GTTGGATGCC   
  
  
+ ATGGAATCTC ATCCCTGTGT GTGCATTCTT CCCCTCTCCC GCCGCCCCCC CCCCCCCAAA AAGAAACCAA   
  
  
+ AAAAAAAAAA AAAATGGATG ATTCTTAGAA CTGACCATTT TTCCTTCCTC CTCCCATTTT ATTTTTTTCA   
  
  
+ GGCTGTTTTT CTTCTACCTG TTGCTTTAGC ATCACTCATA AAAACAAGGT GCAAGCGCAT GACATCGGGT   
  
  
+ ACTGAGCTTC TGAGTTTACA TGCATCGTCA TGAAATTCGG TAGACTTTGA AGTAAAGTTT CCCCATCGTA   
  
  
+ ACTGGTGGTG CATACCTGTC TTCACGCCCA TCAGTAGAGT GGTGAAGTGA AGCAATGTCG AATAGTTTGT   
  
  
+ ATTACGAGCC CAAGAGAGAA ACTGATGCAT ATTTTATGCC TCAATGTCAA TCCTTGAACC CCCAGCTCGA   
  
  
+ TTACAACACC ATTGACTATG GAGCTTTTAT GTGCTCAAAA GTTTTCCTGG GTCAGTACTG CACTCTGGAA   
  
  
+ TCATCCTCAG GAACTGGGAC TTGTCCGGTG TCTAGCTCCA CATCAACTCT CAGCTTCTCA TCCAATGGTA   
  
  
+ GCCCTGGATC ACAGCTTGAT TCCAACTCTT ATCCTTCTGA TCAAAATTGC TCTCCTGATA ATGCCAATTC   
  
  
+ CTCATCTTTA AGTCATTCCT GCGTCACAGA TGATGTGGAT GACCTGAAAT ACAGGCTGAG AGAGTTGGAA   
  
  
+ ACAGTGATGT TGGGACCTGA TCCCGATTTT GTCTACGGCA ATAGCAACCA CACATTTGGG GTTGGGATCC   
  
  
+ ATGTAGTGTC ATCAGAGTTT GACAGCTGGG GGAAAGTGAT GGAGATGATC GCTCGAAAGG ATTTGAAACT   
  
  
+ GGTGCTAATT GCCTGTGCAA GAGCAGTTGC TGAAAATGAT CAGTTGTTGG CCCAGTGGCT GATGGATGAA   
  
  
+ TTGAGACAGA TGGTTTCAGT TTCTGGTGAA CCAATTCAAA GATTGGGTGC TTATATGTTG GAAGGGCTCG   
  
  
+ TAGCGAGGCA GGCCTCCTCA GGTAGCTCCA TTTATAAAGC ATTGAGATGC AAGGAACCCG CGAGTGCTGA   
  
  
+ CCTTTTATCT TACATGCACA TACTCTTTGA GGTTTGCCCA TACATCAAAT TTGGCTACAT GTCAGCAAAT   
  
  
+ GGTGCCATTG CAGAAGCAAT GAAGGATGAA AAGAGAGTCC ACATTGTCGA TTTCCAAATT GGGCAGGGAA   
  
  
+ GCCAATGGGT GACCCTAATC CAGGCATTTG CAGCCCGGCC AGGGGGTCCA CCCCACATTC GCATTACCGG   
  
  
+ TATCGATGAC TCCTACTCTG CATATGCTCG TGGAGGTGGG TTGAACATTG TGGGCCATAG GCTCTTGAGG   
  
  
+ TTGGCTCAGT CATTTAAGGT CCCATTTGAG TTCAACGCAG TTGACGTCCC GGCTTGTCAA GTCCTGCTTA   
  
  
+ AAGATCTCGG CATTCAACAT GGTGAGGCCT TGGCTGTGAA CTTTGCCTTC ATTCTTCACC ACATGCCCGA   
  
  
+ TGAGAGCGTG AGCACAGAGA ATCACCGGGA TCGTCTTCTG AGGGTGGTAA AAGGGCTGAA GCCAAAAGTG   
  
  
+ GTAACGTTAG TGGAGCAAGA GTGTAACACA AACTCTGCTG CTTTCTTGCC TCGCTTTGTT GAGACATTGG   
  
  
+ AATACTACAC AGCGATGTTT GAGTCCATGG ATGTGACTTT GCCTAGGGAT CATAAAGACC GGATCAATGT   
  
  
+ TGAGCAGCAT TGTCTTGCTC GAGATGTTGT GAACTTGGTT GCGTGTGAAG GAGCTGAGAG GGTGGAGCGG   
  
  
+ CATGAGCTCC TTGGGAAATG GAGGTCGCGG TTCACAATGG CAGGGTTTAA GCCATACCCT TTAAGCACTC   
  
  
+ TGGTAAACAA CACAATAAGA ACTTTGTTGA AGAACTACTG TGATAGGTAT GGGCTAGAGG AGAGGAATGG   
  
  
+ AGGTCTTTAT CTTGGGTGGA TGAATAGAGA TTTGGTTGCT TCGAGTGCCT GGCAGTGTAG TAATTA  

- +Up\_Stream \_Len000GTAAAA TCATTATTTA AGGGTACACT CCTGGTGACG GATGACTAAG GCTTACCGTA   
  
  
- AAATCATTAT TTGAGGGTAC ACTCCTGGTG AACTACTCCT TAAGTCAAAA TATTAGGTGT CCACAGTTTC   
  
  
- GGATAAGGAT GAGAGTGGTG AGACGATAGA GAGGGGGGGG GGGGGGGGGC GGGGGGAGTT TTAGACGGAG   
  
  
- GAGGCAAGAG AGGTGTCGTC TAAGGAGTTA GTCCTTTCCA TACAGTCTCG AGTTTTGCTA AGTTGTAGTT   
  
  
- TTACCCTATT AAATAAGACG AAAGTCAAGA CCCAATAACA GTATACAAGT AAGTCAAGTC ACAAAGATCG   
  
  
- TACTAACGAA AAACTACGGA AGAAAAAACA AGACCCAACC TGCTTGAGAC AGAAAAGACA CGTATCTAAA   
  
  
- CTCGAGGCTC GAGTTAAAAA CCCTAAAAAA ATCGACTGAA AAGACGAACC ACAACACTTT AACACTACCC   
  
  
- AAAACCGAAA CAATTTGAAC AAATATTGAA AAAGACAGTT TTGAAATATC ATTTAACAAC GTTAAACTCT   
  
  
- TAATACGTGA GTCACCGAGA CACTAAATAG CAGGCGACCG TTTAAAAGAC TACTTTCCTC TACTAAATAA   
  
  
- TTAAAAAGTC CGTACCAGTT TAAAAGATCG GAAAGTACAT GTAACCAACC ACTATCCGAC TATCGATTTA   
  
  
- ATACAGAGTA AAGAGATTTA AATAACGGTC TATACTTTAA CCACCTTGTC AAATTCCCAA AACCAGCAGA   
  
  
- AAAAACGGGA AAAAAAACCC AGGGGGGGGG CGGGCCCCCC CTTGGGCCTC CCGTCTTTTT CATTGTATTG   
  
  
- AATTTTAATA GTAACTGCAT CAGGAGGTTG GTATTACTTT CGTTGACTTT ATTCTCGTAC GATTCGATCT   
  
  
- CTCATCCGAC GGAACAGTCG GAGGGAAAAC TGTACTCTTT CTACCCTCTT CAAATTTTAT TTCAAGGAGC   
  
  
- AGAAACGTAG GGCCTGGTGT TAAGCCTTGA CCCACTCCTG GAGGAATCCG AACATTTGTG TTCAAAGCTT   
  
  
- GAATCTAAAC GCATGTACAG TTACGAAATG ACCTATTCGA ATCGATTGTG GGGAGTAGGA AGTTGGACAT   
  
  
- GGAAGTATAT TTTTTTTCCT GTTAGGCCAC GTGTTCGTAG GGCGTAAGTG CGTTCCAGAC CCCTTCCCGG   
  
  
- CGTGGGGTTT CCCACTTAAC ATCCGTCGGA TTGGACTGAA ACAGTCACCG ATTAAGGTGC TAAGCTTGGG   
  
  
- TACTAAAAAA CCAATGTGCC CTTGTTGGAA TGGTAACGAG TTTCCGAGGG GAAGAAGTTG GAGATGGAAG   
  
  
- TATATACTTA CTTTTAGGAA ACCGATTCTT AACTTTTCCT TTATACACCA ATAACCCAAA CCCAAGAATA   
  
  
- AGGCAACCAA CGGGGAAGTG TAACTTACGT AGGAGAGCGA TTCTTGACTT TTCCTGTATA CCCCTACATA   
  
  
- TCCAGTTTAA ACTGAGTTAC TATTGACTCC TATGTTCTTA AGATCTTCAG AAATAGTCCC CTTAAAAAAG   
  
  
- AAAAAAGGTG AACACTGAAA AACTACGGAA CGAAAAATGA GAAGTAACTT ACTCATGGGA AGAACTGTCT   
  
  
- AAGAAGGAGG GAAAAAAGAA TTAAAGAAGA ACATCACCTT ATATTCATAC GAAGATCCAT CAACCTACGG   
  
  
- TACCTTAGAG TAGGGACACA CACGTAAGAA GGGGAGAGGG CGGCGGGGGG GGGGGGGTTT TTCTTTGGTT   
  
  
- TTTTTTTTTT TTTTACCTAC TAAGAATCTT GACTGGTAAA AAGGAAGGAG GAGGGTAAAA TAAAAAAAGT   
  
  
- CCGACAAAAA GAAGATGGAC AACGAAATCG TAGTGAGTAT TTTTGTTCCA CGTTCGCGTA CTGTAGCCCA   
  
  
- TGACTCGAAG ACTCAAATGT ACGTAGCAGT ACTTTAAGCC ATCTGAAACT TCATTTCAAA GGGGTAGCAT   
  
  
- TGACCACCAC GTATGGACAG AAGTGCGGGT AGTCATCTCA CCACTTCACT TCGTTACAGC TTATCAAACA   
  
  
- TAATGCTCGG GTTCTCTCTT TGACTACGTA TAAAATACGG AGTTACAGTT AGGAACTTGG GGGTCGAGCT   
  
  
- AATGTTGTGG TAACTGATAC CTCGAAAATA CACGAGTTTT CAAAAGGACC CAGTCATGAC GTGAGACCTT   
  
  
- AGTAGGAGTC CTTGACCCTG AACAGGCCAC AGATCGAGGT GTAGTTGAGA GTCGAAGAGT AGGTTACCAT   
  
  
- CGGGACCTAG TGTCGAACTA AGGTTGAGAA TAGGAAGACT AGTTTTAACG AGAGGACTAT TACGGTTAAG   
  
  
- GAGTAGAAAT TCAGTAAGGA CGCAGTGTCT ACTACACCTA CTGGACTTTA TGTCCGACTC TCTCAACCTT   
  
  
- TGTCACTACA ACCCTGGACT AGGGCTAAAA CAGATGCCGT TATCGTTGGT GTGTAAACCC CAACCCTAGG   
  
  
- TACATCACAG TAGTCTCAAA CTGTCGACCC CCTTTCACTA CCTCTACTAG CGAGCTTTCC TAAACTTTGA   
  
  
- CCACGATTAA CGGACACGTT CTCGTCAACG ACTTTTACTA GTCAACAACC GGGTCACCGA CTACCTACTT   
  
  
- AACTCTGTCT ACCAAAGTCA AAGACCACTT GGTTAAGTTT CTAACCCACG AATATACAAC CTTCCCGAGC   
  
  
- ATCGCTCCGT CCGGAGGAGT CCATCGAGGT AAATATTTCG TAACTCTACG TTCCTTGGGC GCTCACGACT   
  
  
- GGAAAATAGA ATGTACGTGT ATGAGAAACT CCAAACGGGT ATGTAGTTTA AACCGATGTA CAGTCGTTTA   
  
  
- CCACGGTAAC GTCTTCGTTA CTTCCTACTT TTCTCTCAGG TGTAACAGCT AAAGGTTTAA CCCGTCCCTT   
  
  
- CGGTTACCCA CTGGGATTAG GTCCGTAAAC GTCGGGCCGG TCCCCCAGGT GGGGTGTAAG CGTAATGGCC   
  
  
- ATAGCTACTG AGGATGAGAC GTATACGAGC ACCTCCACCC AACTTGTAAC ACCCGGTATC CGAGAACTCC   
  
  
- AACCGAGTCA GTAAATTCCA GGGTAAACTC AAGTTGCGTC AACTGCAGGG CCGAACAGTT CAGGACGAAT   
  
  
- TTCTAGAGCC GTAAGTTGTA CCACTCCGGA ACCGACACTT GAAACGGAAG TAAGAAGTGG TGTACGGGCT   
  
  
- ACTCTCGCAC TCGTGTCTCT TAGTGGCCCT AGCAGAAGAC TCCCACCATT TTCCCGACTT CGGTTTTCAC   
  
  
- CATTGCAATC ACCTCGTTCT CACATTGTGT TTGAGACGAC GAAAGAACGG AGCGAAACAA CTCTGTAACC   
  
  
- TTATGATGTG TCGCTACAAA CTCAGGTACC TACACTGAAA CGGATCCCTA GTATTTCTGG CCTAGTTACA   
  
  
- ACTCGTCGTA ACAGAACGAG CTCTACAACA CTTGAACCAA CGCACACTTC CTCGACTCTC CCACCTCGCC   
  
  
- GTACTCGAGG AACCCTTTAC CTCCAGCGCC AAGTGTTACC GTCCCAAATT CGGTATGGGA AATTCGTGAG   
  
  
- ACCATTTGTT GTGTTATTCT TGAAACAACT TCTTGATGAC ACTATCCATA CCCGATCTCC TCTCCTTACC   
  
  
- TCCAGAAATA GAACCCACCT ACTTATCTCT AAACCAACGA AGCTCACGGA CCGTCACATC ATTAAT

+     O2-site

| Site Name | Organism | Position | Strand | Matrix score. | sequence | function |
| --- | --- | --- | --- | --- | --- | --- |
| O2-site | Zea mays | 1679 | + | 9 | GATGACATGG | cis-acting regulatory element involved in zein metabolism regulation |
| O2-site | Zea mays | 2352 | + | 8 | GATGA(C/T)(A/G)TG(A/G) | cis-acting regulatory element involved in zein metabolism regulation |
| O2-site | Zea mays | 2212 | - | 9 | GATGATGTGG | cis-acting regulatory element involved in zein metabolism regulation |
| O2-site | Zea mays | 2343 | + | 10 | GATGATGTGG | cis-acting regulatory element involved in zein metabolism regulation |

>HU01G01391.1   
+ +Up\_Stream \_Len000CATTTT AGTAATAAAT TCCCATGTGA GGACCACTGC CTACTGATTC CGAATGGCAT   
  
  
+ TTTAGTAATA AACTCCCATG TGAGGACCAC TTGATGAGGA ATTCAGTTTT ATAATCCACA GGTGTCAAAG   
  
  
+ CCTATTCCTA CTCTCACCAC TCTGCTATCT CTCCCCCCCC CCCCCCCCCG CCCCCCTCAA AATCTGCCTC   
  
  
+ CTCCGTTCTC TCCACAGCAG ATTCCTCAAT CAGGAAAGGT ATGTCAGAGC TCAAAACGAT TCAACATCAA   
  
  
+ AATGGGATAA TTTATTCTGC TTTCAGTTCT GGGTTATTGT CATATGTTCA TTCAGTTCAG TGTTTCTAGC   
  
  
+ ATGATTGCTT TTTGATGCCT TCTTTTTTGT TCTGGGTTGG ACGAACTCTG TCTTTTCTGT GCATAGATTT   
  
  
+ GAGCTCCGAG CTCAATTTTT GGGATTTTTT TAGCTGACTT TTCTGCTTGG TGTTGTGAAA TTGTGATGGG   
  
  
+ TTTTGGCTTT GTTAAACTTG TTTATAACTT TTTCTGTCAA AACTTTATAG TAAATTGTTG CAATTTGAGA   
  
  
+ ATTATGCACT CAGTGGCTCT GTGATTTATC GTCCGCTGGC AAATTTTCTG ATGAAAGGAG ATGATTTATT   
  
  
+ AATTTTTCAG GCATGGTCAA ATTTTCTAGC CTTTCATGTA CATTGGTTGG TGATAGGCTG ATAGCTAAAT   
  
  
+ TATGTCTCAT TTCTCTAAAT TTATTGCCAG ATATGAAATT GGTGGAACAG TTTAAGGGTT TTGGTCGTCT   
  
  
+ TTTTTGCCCT TTTTTTTGGG TCCCCCCCCC GCCCGGGGGG GAACCCGGAG GGCAGAAAAA GTAACATAAC   
  
  
+ TTAAAATTAT CATTGACGTA GTCCTCCAAC CATAATGAAA GCAACTGAAA TAAGAGCATG CTAAGCTAGA   
  
  
+ GAGTAGGCTG CCTTGTCAGC CTCCCTTTTG ACATGAGAAA GATGGGAGAA GTTTAAAATA AAGTTCCTCG   
  
  
+ TCTTTGCATC CCGGACCACA ATTCGGAACT GGGTGAGGAC CTCCTTAGGC TTGTAAACAC AAGTTTCGAA   
  
  
+ CTTAGATTTG CGTACATGTC AATGCTTTAC TGGATAAGCT TAGCTAACAC CCCTCATCCT TCAACCTGTA   
  
  
+ CCTTCATATA AAAAAAAGGA CAATCCGGTG CACAAGCATC CCGCATTCAC GCAAGGTCTG GGGAAGGGCC   
  
  
+ GCACCCCAAA GGGTGAATTG TAGGCAGCCT AACCTGACTT TGTCAGTGGC TAATTCCACG ATTCGAACCC   
  
  
+ ATGATTTTTT GGTTACACGG GAACAACCTT ACCATTGCTC AAAGGCTCCC CTTCTTCAAC CTCTACCTTC   
  
  
+ ATATATGAAT GAAAATCCTT TGGCTAAGAA TTGAAAAGGA AATATGTGGT TATTGGGTTT GGGTTCTTAT   
  
  
+ TCCGTTGGTT GCCCCTTCAC ATTGAATGCA TCCTCTCGCT AAGAACTGAA AAGGACATAT GGGGATGTAT   
  
  
+ AGGTCAAATT TGACTCAATG ATAACTGAGG ATACAAGAAT TCTAGAAGTC TTTATCAGGG GAATTTTTTC   
  
  
+ TTTTTTCCAC TTGTGACTTT TTGATGCCTT GCTTTTTACT CTTCATTGAA TGAGTACCCT TCTTGACAGA   
  
  
+ TTCTTCCTCC CTTTTTTCTT AATTTCTTCT TGTAGTGGAA TATAAGTATG CTTCTAGGTA GTTGGATGCC   
  
  
+ ATGGAATCTC ATCCCTGTGT GTGCATTCTT CCCCTCTCCC GCCGCCCCCC CCCCCCCAAA AAGAAACCAA   
  
  
+ AAAAAAAAAA AAAATGGATG ATTCTTAGAA CTGACCATTT TTCCTTCCTC CTCCCATTTT ATTTTTTTCA   
  
  
+ GGCTGTTTTT CTTCTACCTG TTGCTTTAGC ATCACTCATA AAAACAAGGT GCAAGCGCAT GACATCGGGT   
  
  
+ ACTGAGCTTC TGAGTTTACA TGCATCGTCA TGAAATTCGG TAGACTTTGA AGTAAAGTTT CCCCATCGTA   
  
  
+ ACTGGTGGTG CATACCTGTC TTCACGCCCA TCAGTAGAGT GGTGAAGTGA AGCAATGTCG AATAGTTTGT   
  
  
+ ATTACGAGCC CAAGAGAGAA ACTGATGCAT ATTTTATGCC TCAATGTCAA TCCTTGAACC CCCAGCTCGA   
  
  
+ TTACAACACC ATTGACTATG GAGCTTTTAT GTGCTCAAAA GTTTTCCTGG GTCAGTACTG CACTCTGGAA   
  
  
+ TCATCCTCAG GAACTGGGAC TTGTCCGGTG TCTAGCTCCA CATCAACTCT CAGCTTCTCA TCCAATGGTA   
  
  
+ GCCCTGGATC ACAGCTTGAT TCCAACTCTT ATCCTTCTGA TCAAAATTGC TCTCCTGATA ATGCCAATTC   
  
  
+ CTCATCTTTA AGTCATTCCT GCGTCACAGA TGATGTGGAT GACCTGAAAT ACAGGCTGAG AGAGTTGGAA   
  
  
+ ACAGTGATGT TGGGACCTGA TCCCGATTTT GTCTACGGCA ATAGCAACCA CACATTTGGG GTTGGGATCC   
  
  
+ ATGTAGTGTC ATCAGAGTTT GACAGCTGGG GGAAAGTGAT GGAGATGATC GCTCGAAAGG ATTTGAAACT   
  
  
+ GGTGCTAATT GCCTGTGCAA GAGCAGTTGC TGAAAATGAT CAGTTGTTGG CCCAGTGGCT GATGGATGAA   
  
  
+ TTGAGACAGA TGGTTTCAGT TTCTGGTGAA CCAATTCAAA GATTGGGTGC TTATATGTTG GAAGGGCTCG   
  
  
+ TAGCGAGGCA GGCCTCCTCA GGTAGCTCCA TTTATAAAGC ATTGAGATGC AAGGAACCCG CGAGTGCTGA   
  
  
+ CCTTTTATCT TACATGCACA TACTCTTTGA GGTTTGCCCA TACATCAAAT TTGGCTACAT GTCAGCAAAT   
  
  
+ GGTGCCATTG CAGAAGCAAT GAAGGATGAA AAGAGAGTCC ACATTGTCGA TTTCCAAATT GGGCAGGGAA   
  
  
+ GCCAATGGGT GACCCTAATC CAGGCATTTG CAGCCCGGCC AGGGGGTCCA CCCCACATTC GCATTACCGG   
  
  
+ TATCGATGAC TCCTACTCTG CATATGCTCG TGGAGGTGGG TTGAACATTG TGGGCCATAG GCTCTTGAGG   
  
  
+ TTGGCTCAGT CATTTAAGGT CCCATTTGAG TTCAACGCAG TTGACGTCCC GGCTTGTCAA GTCCTGCTTA   
  
  
+ AAGATCTCGG CATTCAACAT GGTGAGGCCT TGGCTGTGAA CTTTGCCTTC ATTCTTCACC ACATGCCCGA   
  
  
+ TGAGAGCGTG AGCACAGAGA ATCACCGGGA TCGTCTTCTG AGGGTGGTAA AAGGGCTGAA GCCAAAAGTG   
  
  
+ GTAACGTTAG TGGAGCAAGA GTGTAACACA AACTCTGCTG CTTTCTTGCC TCGCTTTGTT GAGACATTGG   
  
  
+ AATACTACAC AGCGATGTTT GAGTCCATGG ATGTGACTTT GCCTAGGGAT CATAAAGACC GGATCAATGT   
  
  
+ TGAGCAGCAT TGTCTTGCTC GAGATGTTGT GAACTTGGTT GCGTGTGAAG GAGCTGAGAG GGTGGAGCGG   
  
  
+ CATGAGCTCC TTGGGAAATG GAGGTCGCGG TTCACAATGG CAGGGTTTAA GCCATACCCT TTAAGCACTC   
  
  
+ TGGTAAACAA CACAATAAGA ACTTTGTTGA AGAACTACTG TGATAGGTAT GGGCTAGAGG AGAGGAATGG   
  
  
+ AGGTCTTTAT CTTGGGTGGA TGAATAGAGA TTTGGTTGCT TCGAGTGCCT GGCAGTGTAG TAATTA  

- +Up\_Stream \_Len000GTAAAA TCATTATTTA AGGGTACACT CCTGGTGACG GATGACTAAG GCTTACCGTA   
  
  
- AAATCATTAT TTGAGGGTAC ACTCCTGGTG AACTACTCCT TAAGTCAAAA TATTAGGTGT CCACAGTTTC   
  
  
- GGATAAGGAT GAGAGTGGTG AGACGATAGA GAGGGGGGGG GGGGGGGGGC GGGGGGAGTT TTAGACGGAG   
  
  
- GAGGCAAGAG AGGTGTCGTC TAAGGAGTTA GTCCTTTCCA TACAGTCTCG AGTTTTGCTA AGTTGTAGTT   
  
  
- TTACCCTATT AAATAAGACG AAAGTCAAGA CCCAATAACA GTATACAAGT AAGTCAAGTC ACAAAGATCG   
  
  
- TACTAACGAA AAACTACGGA AGAAAAAACA AGACCCAACC TGCTTGAGAC AGAAAAGACA CGTATCTAAA   
  
  
- CTCGAGGCTC GAGTTAAAAA CCCTAAAAAA ATCGACTGAA AAGACGAACC ACAACACTTT AACACTACCC   
  
  
- AAAACCGAAA CAATTTGAAC AAATATTGAA AAAGACAGTT TTGAAATATC ATTTAACAAC GTTAAACTCT   
  
  
- TAATACGTGA GTCACCGAGA CACTAAATAG CAGGCGACCG TTTAAAAGAC TACTTTCCTC TACTAAATAA   
  
  
- TTAAAAAGTC CGTACCAGTT TAAAAGATCG GAAAGTACAT GTAACCAACC ACTATCCGAC TATCGATTTA   
  
  
- ATACAGAGTA AAGAGATTTA AATAACGGTC TATACTTTAA CCACCTTGTC AAATTCCCAA AACCAGCAGA   
  
  
- AAAAACGGGA AAAAAAACCC AGGGGGGGGG CGGGCCCCCC CTTGGGCCTC CCGTCTTTTT CATTGTATTG   
  
  
- AATTTTAATA GTAACTGCAT CAGGAGGTTG GTATTACTTT CGTTGACTTT ATTCTCGTAC GATTCGATCT   
  
  
- CTCATCCGAC GGAACAGTCG GAGGGAAAAC TGTACTCTTT CTACCCTCTT CAAATTTTAT TTCAAGGAGC   
  
  
- AGAAACGTAG GGCCTGGTGT TAAGCCTTGA CCCACTCCTG GAGGAATCCG AACATTTGTG TTCAAAGCTT   
  
  
- GAATCTAAAC GCATGTACAG TTACGAAATG ACCTATTCGA ATCGATTGTG GGGAGTAGGA AGTTGGACAT   
  
  
- GGAAGTATAT TTTTTTTCCT GTTAGGCCAC GTGTTCGTAG GGCGTAAGTG CGTTCCAGAC CCCTTCCCGG   
  
  
- CGTGGGGTTT CCCACTTAAC ATCCGTCGGA TTGGACTGAA ACAGTCACCG ATTAAGGTGC TAAGCTTGGG   
  
  
- TACTAAAAAA CCAATGTGCC CTTGTTGGAA TGGTAACGAG TTTCCGAGGG GAAGAAGTTG GAGATGGAAG   
  
  
- TATATACTTA CTTTTAGGAA ACCGATTCTT AACTTTTCCT TTATACACCA ATAACCCAAA CCCAAGAATA   
  
  
- AGGCAACCAA CGGGGAAGTG TAACTTACGT AGGAGAGCGA TTCTTGACTT TTCCTGTATA CCCCTACATA   
  
  
- TCCAGTTTAA ACTGAGTTAC TATTGACTCC TATGTTCTTA AGATCTTCAG AAATAGTCCC CTTAAAAAAG   
  
  
- AAAAAAGGTG AACACTGAAA AACTACGGAA CGAAAAATGA GAAGTAACTT ACTCATGGGA AGAACTGTCT   
  
  
- AAGAAGGAGG GAAAAAAGAA TTAAAGAAGA ACATCACCTT ATATTCATAC GAAGATCCAT CAACCTACGG   
  
  
- TACCTTAGAG TAGGGACACA CACGTAAGAA GGGGAGAGGG CGGCGGGGGG GGGGGGGTTT TTCTTTGGTT   
  
  
- TTTTTTTTTT TTTTACCTAC TAAGAATCTT GACTGGTAAA AAGGAAGGAG GAGGGTAAAA TAAAAAAAGT   
  
  
- CCGACAAAAA GAAGATGGAC AACGAAATCG TAGTGAGTAT TTTTGTTCCA CGTTCGCGTA CTGTAGCCCA   
  
  
- TGACTCGAAG ACTCAAATGT ACGTAGCAGT ACTTTAAGCC ATCTGAAACT TCATTTCAAA GGGGTAGCAT   
  
  
- TGACCACCAC GTATGGACAG AAGTGCGGGT AGTCATCTCA CCACTTCACT TCGTTACAGC TTATCAAACA   
  
  
- TAATGCTCGG GTTCTCTCTT TGACTACGTA TAAAATACGG AGTTACAGTT AGGAACTTGG GGGTCGAGCT   
  
  
- AATGTTGTGG TAACTGATAC CTCGAAAATA CACGAGTTTT CAAAAGGACC CAGTCATGAC GTGAGACCTT   
  
  
- AGTAGGAGTC CTTGACCCTG AACAGGCCAC AGATCGAGGT GTAGTTGAGA GTCGAAGAGT AGGTTACCAT   
  
  
- CGGGACCTAG TGTCGAACTA AGGTTGAGAA TAGGAAGACT AGTTTTAACG AGAGGACTAT TACGGTTAAG   
  
  
- GAGTAGAAAT TCAGTAAGGA CGCAGTGTCT ACTACACCTA CTGGACTTTA TGTCCGACTC TCTCAACCTT   
  
  
- TGTCACTACA ACCCTGGACT AGGGCTAAAA CAGATGCCGT TATCGTTGGT GTGTAAACCC CAACCCTAGG   
  
  
- TACATCACAG TAGTCTCAAA CTGTCGACCC CCTTTCACTA CCTCTACTAG CGAGCTTTCC TAAACTTTGA   
  
  
- CCACGATTAA CGGACACGTT CTCGTCAACG ACTTTTACTA GTCAACAACC GGGTCACCGA CTACCTACTT   
  
  
- AACTCTGTCT ACCAAAGTCA AAGACCACTT GGTTAAGTTT CTAACCCACG AATATACAAC CTTCCCGAGC   
  
  
- ATCGCTCCGT CCGGAGGAGT CCATCGAGGT AAATATTTCG TAACTCTACG TTCCTTGGGC GCTCACGACT   
  
  
- GGAAAATAGA ATGTACGTGT ATGAGAAACT CCAAACGGGT ATGTAGTTTA AACCGATGTA CAGTCGTTTA   
  
  
- CCACGGTAAC GTCTTCGTTA CTTCCTACTT TTCTCTCAGG TGTAACAGCT AAAGGTTTAA CCCGTCCCTT   
  
  
- CGGTTACCCA CTGGGATTAG GTCCGTAAAC GTCGGGCCGG TCCCCCAGGT GGGGTGTAAG CGTAATGGCC   
  
  
- ATAGCTACTG AGGATGAGAC GTATACGAGC ACCTCCACCC AACTTGTAAC ACCCGGTATC CGAGAACTCC   
  
  
- AACCGAGTCA GTAAATTCCA GGGTAAACTC AAGTTGCGTC AACTGCAGGG CCGAACAGTT CAGGACGAAT   
  
  
- TTCTAGAGCC GTAAGTTGTA CCACTCCGGA ACCGACACTT GAAACGGAAG TAAGAAGTGG TGTACGGGCT   
  
  
- ACTCTCGCAC TCGTGTCTCT TAGTGGCCCT AGCAGAAGAC TCCCACCATT TTCCCGACTT CGGTTTTCAC   
  
  
- CATTGCAATC ACCTCGTTCT CACATTGTGT TTGAGACGAC GAAAGAACGG AGCGAAACAA CTCTGTAACC   
  
  
- TTATGATGTG TCGCTACAAA CTCAGGTACC TACACTGAAA CGGATCCCTA GTATTTCTGG CCTAGTTACA   
  
  
- ACTCGTCGTA ACAGAACGAG CTCTACAACA CTTGAACCAA CGCACACTTC CTCGACTCTC CCACCTCGCC   
  
  
- GTACTCGAGG AACCCTTTAC CTCCAGCGCC AAGTGTTACC GTCCCAAATT CGGTATGGGA AATTCGTGAG   
  
  
- ACCATTTGTT GTGTTATTCT TGAAACAACT TCTTGATGAC ACTATCCATA CCCGATCTCC TCTCCTTACC   
  
  
- TCCAGAAATA GAACCCACCT ACTTATCTCT AAACCAACGA AGCTCACGGA CCGTCACATC ATTAAT

+     P-box

| Site Name | Organism | Position | Strand | Matrix score. | sequence | function |
| --- | --- | --- | --- | --- | --- | --- |
| P-box | Oryza sativa | 938 | + | 7 | CCTTTTG | gibberellin-responsive element |

>HU01G01391.1   
+ +Up\_Stream \_Len000CATTTT AGTAATAAAT TCCCATGTGA GGACCACTGC CTACTGATTC CGAATGGCAT   
  
  
+ TTTAGTAATA AACTCCCATG TGAGGACCAC TTGATGAGGA ATTCAGTTTT ATAATCCACA GGTGTCAAAG   
  
  
+ CCTATTCCTA CTCTCACCAC TCTGCTATCT CTCCCCCCCC CCCCCCCCCG CCCCCCTCAA AATCTGCCTC   
  
  
+ CTCCGTTCTC TCCACAGCAG ATTCCTCAAT CAGGAAAGGT ATGTCAGAGC TCAAAACGAT TCAACATCAA   
  
  
+ AATGGGATAA TTTATTCTGC TTTCAGTTCT GGGTTATTGT CATATGTTCA TTCAGTTCAG TGTTTCTAGC   
  
  
+ ATGATTGCTT TTTGATGCCT TCTTTTTTGT TCTGGGTTGG ACGAACTCTG TCTTTTCTGT GCATAGATTT   
  
  
+ GAGCTCCGAG CTCAATTTTT GGGATTTTTT TAGCTGACTT TTCTGCTTGG TGTTGTGAAA TTGTGATGGG   
  
  
+ TTTTGGCTTT GTTAAACTTG TTTATAACTT TTTCTGTCAA AACTTTATAG TAAATTGTTG CAATTTGAGA   
  
  
+ ATTATGCACT CAGTGGCTCT GTGATTTATC GTCCGCTGGC AAATTTTCTG ATGAAAGGAG ATGATTTATT   
  
  
+ AATTTTTCAG GCATGGTCAA ATTTTCTAGC CTTTCATGTA CATTGGTTGG TGATAGGCTG ATAGCTAAAT   
  
  
+ TATGTCTCAT TTCTCTAAAT TTATTGCCAG ATATGAAATT GGTGGAACAG TTTAAGGGTT TTGGTCGTCT   
  
  
+ TTTTTGCCCT TTTTTTTGGG TCCCCCCCCC GCCCGGGGGG GAACCCGGAG GGCAGAAAAA GTAACATAAC   
  
  
+ TTAAAATTAT CATTGACGTA GTCCTCCAAC CATAATGAAA GCAACTGAAA TAAGAGCATG CTAAGCTAGA   
  
  
+ GAGTAGGCTG CCTTGTCAGC CTCCCTTTTG ACATGAGAAA GATGGGAGAA GTTTAAAATA AAGTTCCTCG   
  
  
+ TCTTTGCATC CCGGACCACA ATTCGGAACT GGGTGAGGAC CTCCTTAGGC TTGTAAACAC AAGTTTCGAA   
  
  
+ CTTAGATTTG CGTACATGTC AATGCTTTAC TGGATAAGCT TAGCTAACAC CCCTCATCCT TCAACCTGTA   
  
  
+ CCTTCATATA AAAAAAAGGA CAATCCGGTG CACAAGCATC CCGCATTCAC GCAAGGTCTG GGGAAGGGCC   
  
  
+ GCACCCCAAA GGGTGAATTG TAGGCAGCCT AACCTGACTT TGTCAGTGGC TAATTCCACG ATTCGAACCC   
  
  
+ ATGATTTTTT GGTTACACGG GAACAACCTT ACCATTGCTC AAAGGCTCCC CTTCTTCAAC CTCTACCTTC   
  
  
+ ATATATGAAT GAAAATCCTT TGGCTAAGAA TTGAAAAGGA AATATGTGGT TATTGGGTTT GGGTTCTTAT   
  
  
+ TCCGTTGGTT GCCCCTTCAC ATTGAATGCA TCCTCTCGCT AAGAACTGAA AAGGACATAT GGGGATGTAT   
  
  
+ AGGTCAAATT TGACTCAATG ATAACTGAGG ATACAAGAAT TCTAGAAGTC TTTATCAGGG GAATTTTTTC   
  
  
+ TTTTTTCCAC TTGTGACTTT TTGATGCCTT GCTTTTTACT CTTCATTGAA TGAGTACCCT TCTTGACAGA   
  
  
+ TTCTTCCTCC CTTTTTTCTT AATTTCTTCT TGTAGTGGAA TATAAGTATG CTTCTAGGTA GTTGGATGCC   
  
  
+ ATGGAATCTC ATCCCTGTGT GTGCATTCTT CCCCTCTCCC GCCGCCCCCC CCCCCCCAAA AAGAAACCAA   
  
  
+ AAAAAAAAAA AAAATGGATG ATTCTTAGAA CTGACCATTT TTCCTTCCTC CTCCCATTTT ATTTTTTTCA   
  
  
+ GGCTGTTTTT CTTCTACCTG TTGCTTTAGC ATCACTCATA AAAACAAGGT GCAAGCGCAT GACATCGGGT   
  
  
+ ACTGAGCTTC TGAGTTTACA TGCATCGTCA TGAAATTCGG TAGACTTTGA AGTAAAGTTT CCCCATCGTA   
  
  
+ ACTGGTGGTG CATACCTGTC TTCACGCCCA TCAGTAGAGT GGTGAAGTGA AGCAATGTCG AATAGTTTGT   
  
  
+ ATTACGAGCC CAAGAGAGAA ACTGATGCAT ATTTTATGCC TCAATGTCAA TCCTTGAACC CCCAGCTCGA   
  
  
+ TTACAACACC ATTGACTATG GAGCTTTTAT GTGCTCAAAA GTTTTCCTGG GTCAGTACTG CACTCTGGAA   
  
  
+ TCATCCTCAG GAACTGGGAC TTGTCCGGTG TCTAGCTCCA CATCAACTCT CAGCTTCTCA TCCAATGGTA   
  
  
+ GCCCTGGATC ACAGCTTGAT TCCAACTCTT ATCCTTCTGA TCAAAATTGC TCTCCTGATA ATGCCAATTC   
  
  
+ CTCATCTTTA AGTCATTCCT GCGTCACAGA TGATGTGGAT GACCTGAAAT ACAGGCTGAG AGAGTTGGAA   
  
  
+ ACAGTGATGT TGGGACCTGA TCCCGATTTT GTCTACGGCA ATAGCAACCA CACATTTGGG GTTGGGATCC   
  
  
+ ATGTAGTGTC ATCAGAGTTT GACAGCTGGG GGAAAGTGAT GGAGATGATC GCTCGAAAGG ATTTGAAACT   
  
  
+ GGTGCTAATT GCCTGTGCAA GAGCAGTTGC TGAAAATGAT CAGTTGTTGG CCCAGTGGCT GATGGATGAA   
  
  
+ TTGAGACAGA TGGTTTCAGT TTCTGGTGAA CCAATTCAAA GATTGGGTGC TTATATGTTG GAAGGGCTCG   
  
  
+ TAGCGAGGCA GGCCTCCTCA GGTAGCTCCA TTTATAAAGC ATTGAGATGC AAGGAACCCG CGAGTGCTGA   
  
  
+ CCTTTTATCT TACATGCACA TACTCTTTGA GGTTTGCCCA TACATCAAAT TTGGCTACAT GTCAGCAAAT   
  
  
+ GGTGCCATTG CAGAAGCAAT GAAGGATGAA AAGAGAGTCC ACATTGTCGA TTTCCAAATT GGGCAGGGAA   
  
  
+ GCCAATGGGT GACCCTAATC CAGGCATTTG CAGCCCGGCC AGGGGGTCCA CCCCACATTC GCATTACCGG   
  
  
+ TATCGATGAC TCCTACTCTG CATATGCTCG TGGAGGTGGG TTGAACATTG TGGGCCATAG GCTCTTGAGG   
  
  
+ TTGGCTCAGT CATTTAAGGT CCCATTTGAG TTCAACGCAG TTGACGTCCC GGCTTGTCAA GTCCTGCTTA   
  
  
+ AAGATCTCGG CATTCAACAT GGTGAGGCCT TGGCTGTGAA CTTTGCCTTC ATTCTTCACC ACATGCCCGA   
  
  
+ TGAGAGCGTG AGCACAGAGA ATCACCGGGA TCGTCTTCTG AGGGTGGTAA AAGGGCTGAA GCCAAAAGTG   
  
  
+ GTAACGTTAG TGGAGCAAGA GTGTAACACA AACTCTGCTG CTTTCTTGCC TCGCTTTGTT GAGACATTGG   
  
  
+ AATACTACAC AGCGATGTTT GAGTCCATGG ATGTGACTTT GCCTAGGGAT CATAAAGACC GGATCAATGT   
  
  
+ TGAGCAGCAT TGTCTTGCTC GAGATGTTGT GAACTTGGTT GCGTGTGAAG GAGCTGAGAG GGTGGAGCGG   
  
  
+ CATGAGCTCC TTGGGAAATG GAGGTCGCGG TTCACAATGG CAGGGTTTAA GCCATACCCT TTAAGCACTC   
  
  
+ TGGTAAACAA CACAATAAGA ACTTTGTTGA AGAACTACTG TGATAGGTAT GGGCTAGAGG AGAGGAATGG   
  
  
+ AGGTCTTTAT CTTGGGTGGA TGAATAGAGA TTTGGTTGCT TCGAGTGCCT GGCAGTGTAG TAATTA  

- +Up\_Stream \_Len000GTAAAA TCATTATTTA AGGGTACACT CCTGGTGACG GATGACTAAG GCTTACCGTA   
  
  
- AAATCATTAT TTGAGGGTAC ACTCCTGGTG AACTACTCCT TAAGTCAAAA TATTAGGTGT CCACAGTTTC   
  
  
- GGATAAGGAT GAGAGTGGTG AGACGATAGA GAGGGGGGGG GGGGGGGGGC GGGGGGAGTT TTAGACGGAG   
  
  
- GAGGCAAGAG AGGTGTCGTC TAAGGAGTTA GTCCTTTCCA TACAGTCTCG AGTTTTGCTA AGTTGTAGTT   
  
  
- TTACCCTATT AAATAAGACG AAAGTCAAGA CCCAATAACA GTATACAAGT AAGTCAAGTC ACAAAGATCG   
  
  
- TACTAACGAA AAACTACGGA AGAAAAAACA AGACCCAACC TGCTTGAGAC AGAAAAGACA CGTATCTAAA   
  
  
- CTCGAGGCTC GAGTTAAAAA CCCTAAAAAA ATCGACTGAA AAGACGAACC ACAACACTTT AACACTACCC   
  
  
- AAAACCGAAA CAATTTGAAC AAATATTGAA AAAGACAGTT TTGAAATATC ATTTAACAAC GTTAAACTCT   
  
  
- TAATACGTGA GTCACCGAGA CACTAAATAG CAGGCGACCG TTTAAAAGAC TACTTTCCTC TACTAAATAA   
  
  
- TTAAAAAGTC CGTACCAGTT TAAAAGATCG GAAAGTACAT GTAACCAACC ACTATCCGAC TATCGATTTA   
  
  
- ATACAGAGTA AAGAGATTTA AATAACGGTC TATACTTTAA CCACCTTGTC AAATTCCCAA AACCAGCAGA   
  
  
- AAAAACGGGA AAAAAAACCC AGGGGGGGGG CGGGCCCCCC CTTGGGCCTC CCGTCTTTTT CATTGTATTG   
  
  
- AATTTTAATA GTAACTGCAT CAGGAGGTTG GTATTACTTT CGTTGACTTT ATTCTCGTAC GATTCGATCT   
  
  
- CTCATCCGAC GGAACAGTCG GAGGGAAAAC TGTACTCTTT CTACCCTCTT CAAATTTTAT TTCAAGGAGC   
  
  
- AGAAACGTAG GGCCTGGTGT TAAGCCTTGA CCCACTCCTG GAGGAATCCG AACATTTGTG TTCAAAGCTT   
  
  
- GAATCTAAAC GCATGTACAG TTACGAAATG ACCTATTCGA ATCGATTGTG GGGAGTAGGA AGTTGGACAT   
  
  
- GGAAGTATAT TTTTTTTCCT GTTAGGCCAC GTGTTCGTAG GGCGTAAGTG CGTTCCAGAC CCCTTCCCGG   
  
  
- CGTGGGGTTT CCCACTTAAC ATCCGTCGGA TTGGACTGAA ACAGTCACCG ATTAAGGTGC TAAGCTTGGG   
  
  
- TACTAAAAAA CCAATGTGCC CTTGTTGGAA TGGTAACGAG TTTCCGAGGG GAAGAAGTTG GAGATGGAAG   
  
  
- TATATACTTA CTTTTAGGAA ACCGATTCTT AACTTTTCCT TTATACACCA ATAACCCAAA CCCAAGAATA   
  
  
- AGGCAACCAA CGGGGAAGTG TAACTTACGT AGGAGAGCGA TTCTTGACTT TTCCTGTATA CCCCTACATA   
  
  
- TCCAGTTTAA ACTGAGTTAC TATTGACTCC TATGTTCTTA AGATCTTCAG AAATAGTCCC CTTAAAAAAG   
  
  
- AAAAAAGGTG AACACTGAAA AACTACGGAA CGAAAAATGA GAAGTAACTT ACTCATGGGA AGAACTGTCT   
  
  
- AAGAAGGAGG GAAAAAAGAA TTAAAGAAGA ACATCACCTT ATATTCATAC GAAGATCCAT CAACCTACGG   
  
  
- TACCTTAGAG TAGGGACACA CACGTAAGAA GGGGAGAGGG CGGCGGGGGG GGGGGGGTTT TTCTTTGGTT   
  
  
- TTTTTTTTTT TTTTACCTAC TAAGAATCTT GACTGGTAAA AAGGAAGGAG GAGGGTAAAA TAAAAAAAGT   
  
  
- CCGACAAAAA GAAGATGGAC AACGAAATCG TAGTGAGTAT TTTTGTTCCA CGTTCGCGTA CTGTAGCCCA   
  
  
- TGACTCGAAG ACTCAAATGT ACGTAGCAGT ACTTTAAGCC ATCTGAAACT TCATTTCAAA GGGGTAGCAT   
  
  
- TGACCACCAC GTATGGACAG AAGTGCGGGT AGTCATCTCA CCACTTCACT TCGTTACAGC TTATCAAACA   
  
  
- TAATGCTCGG GTTCTCTCTT TGACTACGTA TAAAATACGG AGTTACAGTT AGGAACTTGG GGGTCGAGCT   
  
  
- AATGTTGTGG TAACTGATAC CTCGAAAATA CACGAGTTTT CAAAAGGACC CAGTCATGAC GTGAGACCTT   
  
  
- AGTAGGAGTC CTTGACCCTG AACAGGCCAC AGATCGAGGT GTAGTTGAGA GTCGAAGAGT AGGTTACCAT   
  
  
- CGGGACCTAG TGTCGAACTA AGGTTGAGAA TAGGAAGACT AGTTTTAACG AGAGGACTAT TACGGTTAAG   
  
  
- GAGTAGAAAT TCAGTAAGGA CGCAGTGTCT ACTACACCTA CTGGACTTTA TGTCCGACTC TCTCAACCTT   
  
  
- TGTCACTACA ACCCTGGACT AGGGCTAAAA CAGATGCCGT TATCGTTGGT GTGTAAACCC CAACCCTAGG   
  
  
- TACATCACAG TAGTCTCAAA CTGTCGACCC CCTTTCACTA CCTCTACTAG CGAGCTTTCC TAAACTTTGA   
  
  
- CCACGATTAA CGGACACGTT CTCGTCAACG ACTTTTACTA GTCAACAACC GGGTCACCGA CTACCTACTT   
  
  
- AACTCTGTCT ACCAAAGTCA AAGACCACTT GGTTAAGTTT CTAACCCACG AATATACAAC CTTCCCGAGC   
  
  
- ATCGCTCCGT CCGGAGGAGT CCATCGAGGT AAATATTTCG TAACTCTACG TTCCTTGGGC GCTCACGACT   
  
  
- GGAAAATAGA ATGTACGTGT ATGAGAAACT CCAAACGGGT ATGTAGTTTA AACCGATGTA CAGTCGTTTA   
  
  
- CCACGGTAAC GTCTTCGTTA CTTCCTACTT TTCTCTCAGG TGTAACAGCT AAAGGTTTAA CCCGTCCCTT   
  
  
- CGGTTACCCA CTGGGATTAG GTCCGTAAAC GTCGGGCCGG TCCCCCAGGT GGGGTGTAAG CGTAATGGCC   
  
  
- ATAGCTACTG AGGATGAGAC GTATACGAGC ACCTCCACCC AACTTGTAAC ACCCGGTATC CGAGAACTCC   
  
  
- AACCGAGTCA GTAAATTCCA GGGTAAACTC AAGTTGCGTC AACTGCAGGG CCGAACAGTT CAGGACGAAT   
  
  
- TTCTAGAGCC GTAAGTTGTA CCACTCCGGA ACCGACACTT GAAACGGAAG TAAGAAGTGG TGTACGGGCT   
  
  
- ACTCTCGCAC TCGTGTCTCT TAGTGGCCCT AGCAGAAGAC TCCCACCATT TTCCCGACTT CGGTTTTCAC   
  
  
- CATTGCAATC ACCTCGTTCT CACATTGTGT TTGAGACGAC GAAAGAACGG AGCGAAACAA CTCTGTAACC   
  
  
- TTATGATGTG TCGCTACAAA CTCAGGTACC TACACTGAAA CGGATCCCTA GTATTTCTGG CCTAGTTACA   
  
  
- ACTCGTCGTA ACAGAACGAG CTCTACAACA CTTGAACCAA CGCACACTTC CTCGACTCTC CCACCTCGCC   
  
  
- GTACTCGAGG AACCCTTTAC CTCCAGCGCC AAGTGTTACC GTCCCAAATT CGGTATGGGA AATTCGTGAG   
  
  
- ACCATTTGTT GTGTTATTCT TGAAACAACT TCTTGATGAC ACTATCCATA CCCGATCTCC TCTCCTTACC   
  
  
- TCCAGAAATA GAACCCACCT ACTTATCTCT AAACCAACGA AGCTCACGGA CCGTCACATC ATTAAT

+     STRE

| Site Name | Organism | Position | Strand | Matrix score. | sequence | function |
| --- | --- | --- | --- | --- | --- | --- |
| STRE | Arabidopsis thaliana | 2915 | + | 5 | AGGGG |  |
| STRE | Arabidopsis thaliana | 1715 | - | 5 | AGGGG |  |
| STRE | Arabidopsis thaliana | 1531 | + | 5 | AGGGG |  |
| STRE | Arabidopsis thaliana | 1416 | - | 5 | AGGGG |  |
| STRE | Arabidopsis thaliana | 1312 | - | 5 | AGGGG |  |
| STRE | Arabidopsis thaliana | 1104 | - | 5 | AGGGG |  |
| STRE | Arabidopsis thaliana | 197 | - | 5 | AGGGG |  |

>HU01G01391.1   
+ +Up\_Stream \_Len000CATTTT AGTAATAAAT TCCCATGTGA GGACCACTGC CTACTGATTC CGAATGGCAT   
  
  
+ TTTAGTAATA AACTCCCATG TGAGGACCAC TTGATGAGGA ATTCAGTTTT ATAATCCACA GGTGTCAAAG   
  
  
+ CCTATTCCTA CTCTCACCAC TCTGCTATCT CTCCCCCCCC CCCCCCCCCG CCCCCCTCAA AATCTGCCTC   
  
  
+ CTCCGTTCTC TCCACAGCAG ATTCCTCAAT CAGGAAAGGT ATGTCAGAGC TCAAAACGAT TCAACATCAA   
  
  
+ AATGGGATAA TTTATTCTGC TTTCAGTTCT GGGTTATTGT CATATGTTCA TTCAGTTCAG TGTTTCTAGC   
  
  
+ ATGATTGCTT TTTGATGCCT TCTTTTTTGT TCTGGGTTGG ACGAACTCTG TCTTTTCTGT GCATAGATTT   
  
  
+ GAGCTCCGAG CTCAATTTTT GGGATTTTTT TAGCTGACTT TTCTGCTTGG TGTTGTGAAA TTGTGATGGG   
  
  
+ TTTTGGCTTT GTTAAACTTG TTTATAACTT TTTCTGTCAA AACTTTATAG TAAATTGTTG CAATTTGAGA   
  
  
+ ATTATGCACT CAGTGGCTCT GTGATTTATC GTCCGCTGGC AAATTTTCTG ATGAAAGGAG ATGATTTATT   
  
  
+ AATTTTTCAG GCATGGTCAA ATTTTCTAGC CTTTCATGTA CATTGGTTGG TGATAGGCTG ATAGCTAAAT   
  
  
+ TATGTCTCAT TTCTCTAAAT TTATTGCCAG ATATGAAATT GGTGGAACAG TTTAAGGGTT TTGGTCGTCT   
  
  
+ TTTTTGCCCT TTTTTTTGGG TCCCCCCCCC GCCCGGGGGG GAACCCGGAG GGCAGAAAAA GTAACATAAC   
  
  
+ TTAAAATTAT CATTGACGTA GTCCTCCAAC CATAATGAAA GCAACTGAAA TAAGAGCATG CTAAGCTAGA   
  
  
+ GAGTAGGCTG CCTTGTCAGC CTCCCTTTTG ACATGAGAAA GATGGGAGAA GTTTAAAATA AAGTTCCTCG   
  
  
+ TCTTTGCATC CCGGACCACA ATTCGGAACT GGGTGAGGAC CTCCTTAGGC TTGTAAACAC AAGTTTCGAA   
  
  
+ CTTAGATTTG CGTACATGTC AATGCTTTAC TGGATAAGCT TAGCTAACAC CCCTCATCCT TCAACCTGTA   
  
  
+ CCTTCATATA AAAAAAAGGA CAATCCGGTG CACAAGCATC CCGCATTCAC GCAAGGTCTG GGGAAGGGCC   
  
  
+ GCACCCCAAA GGGTGAATTG TAGGCAGCCT AACCTGACTT TGTCAGTGGC TAATTCCACG ATTCGAACCC   
  
  
+ ATGATTTTTT GGTTACACGG GAACAACCTT ACCATTGCTC AAAGGCTCCC CTTCTTCAAC CTCTACCTTC   
  
  
+ ATATATGAAT GAAAATCCTT TGGCTAAGAA TTGAAAAGGA AATATGTGGT TATTGGGTTT GGGTTCTTAT   
  
  
+ TCCGTTGGTT GCCCCTTCAC ATTGAATGCA TCCTCTCGCT AAGAACTGAA AAGGACATAT GGGGATGTAT   
  
  
+ AGGTCAAATT TGACTCAATG ATAACTGAGG ATACAAGAAT TCTAGAAGTC TTTATCAGGG GAATTTTTTC   
  
  
+ TTTTTTCCAC TTGTGACTTT TTGATGCCTT GCTTTTTACT CTTCATTGAA TGAGTACCCT TCTTGACAGA   
  
  
+ TTCTTCCTCC CTTTTTTCTT AATTTCTTCT TGTAGTGGAA TATAAGTATG CTTCTAGGTA GTTGGATGCC   
  
  
+ ATGGAATCTC ATCCCTGTGT GTGCATTCTT CCCCTCTCCC GCCGCCCCCC CCCCCCCAAA AAGAAACCAA   
  
  
+ AAAAAAAAAA AAAATGGATG ATTCTTAGAA CTGACCATTT TTCCTTCCTC CTCCCATTTT ATTTTTTTCA   
  
  
+ GGCTGTTTTT CTTCTACCTG TTGCTTTAGC ATCACTCATA AAAACAAGGT GCAAGCGCAT GACATCGGGT   
  
  
+ ACTGAGCTTC TGAGTTTACA TGCATCGTCA TGAAATTCGG TAGACTTTGA AGTAAAGTTT CCCCATCGTA   
  
  
+ ACTGGTGGTG CATACCTGTC TTCACGCCCA TCAGTAGAGT GGTGAAGTGA AGCAATGTCG AATAGTTTGT   
  
  
+ ATTACGAGCC CAAGAGAGAA ACTGATGCAT ATTTTATGCC TCAATGTCAA TCCTTGAACC CCCAGCTCGA   
  
  
+ TTACAACACC ATTGACTATG GAGCTTTTAT GTGCTCAAAA GTTTTCCTGG GTCAGTACTG CACTCTGGAA   
  
  
+ TCATCCTCAG GAACTGGGAC TTGTCCGGTG TCTAGCTCCA CATCAACTCT CAGCTTCTCA TCCAATGGTA   
  
  
+ GCCCTGGATC ACAGCTTGAT TCCAACTCTT ATCCTTCTGA TCAAAATTGC TCTCCTGATA ATGCCAATTC   
  
  
+ CTCATCTTTA AGTCATTCCT GCGTCACAGA TGATGTGGAT GACCTGAAAT ACAGGCTGAG AGAGTTGGAA   
  
  
+ ACAGTGATGT TGGGACCTGA TCCCGATTTT GTCTACGGCA ATAGCAACCA CACATTTGGG GTTGGGATCC   
  
  
+ ATGTAGTGTC ATCAGAGTTT GACAGCTGGG GGAAAGTGAT GGAGATGATC GCTCGAAAGG ATTTGAAACT   
  
  
+ GGTGCTAATT GCCTGTGCAA GAGCAGTTGC TGAAAATGAT CAGTTGTTGG CCCAGTGGCT GATGGATGAA   
  
  
+ TTGAGACAGA TGGTTTCAGT TTCTGGTGAA CCAATTCAAA GATTGGGTGC TTATATGTTG GAAGGGCTCG   
  
  
+ TAGCGAGGCA GGCCTCCTCA GGTAGCTCCA TTTATAAAGC ATTGAGATGC AAGGAACCCG CGAGTGCTGA   
  
  
+ CCTTTTATCT TACATGCACA TACTCTTTGA GGTTTGCCCA TACATCAAAT TTGGCTACAT GTCAGCAAAT   
  
  
+ GGTGCCATTG CAGAAGCAAT GAAGGATGAA AAGAGAGTCC ACATTGTCGA TTTCCAAATT GGGCAGGGAA   
  
  
+ GCCAATGGGT GACCCTAATC CAGGCATTTG CAGCCCGGCC AGGGGGTCCA CCCCACATTC GCATTACCGG   
  
  
+ TATCGATGAC TCCTACTCTG CATATGCTCG TGGAGGTGGG TTGAACATTG TGGGCCATAG GCTCTTGAGG   
  
  
+ TTGGCTCAGT CATTTAAGGT CCCATTTGAG TTCAACGCAG TTGACGTCCC GGCTTGTCAA GTCCTGCTTA   
  
  
+ AAGATCTCGG CATTCAACAT GGTGAGGCCT TGGCTGTGAA CTTTGCCTTC ATTCTTCACC ACATGCCCGA   
  
  
+ TGAGAGCGTG AGCACAGAGA ATCACCGGGA TCGTCTTCTG AGGGTGGTAA AAGGGCTGAA GCCAAAAGTG   
  
  
+ GTAACGTTAG TGGAGCAAGA GTGTAACACA AACTCTGCTG CTTTCTTGCC TCGCTTTGTT GAGACATTGG   
  
  
+ AATACTACAC AGCGATGTTT GAGTCCATGG ATGTGACTTT GCCTAGGGAT CATAAAGACC GGATCAATGT   
  
  
+ TGAGCAGCAT TGTCTTGCTC GAGATGTTGT GAACTTGGTT GCGTGTGAAG GAGCTGAGAG GGTGGAGCGG   
  
  
+ CATGAGCTCC TTGGGAAATG GAGGTCGCGG TTCACAATGG CAGGGTTTAA GCCATACCCT TTAAGCACTC   
  
  
+ TGGTAAACAA CACAATAAGA ACTTTGTTGA AGAACTACTG TGATAGGTAT GGGCTAGAGG AGAGGAATGG   
  
  
+ AGGTCTTTAT CTTGGGTGGA TGAATAGAGA TTTGGTTGCT TCGAGTGCCT GGCAGTGTAG TAATTA  

- +Up\_Stream \_Len000GTAAAA TCATTATTTA AGGGTACACT CCTGGTGACG GATGACTAAG GCTTACCGTA   
  
  
- AAATCATTAT TTGAGGGTAC ACTCCTGGTG AACTACTCCT TAAGTCAAAA TATTAGGTGT CCACAGTTTC   
  
  
- GGATAAGGAT GAGAGTGGTG AGACGATAGA GAGGGGGGGG GGGGGGGGGC GGGGGGAGTT TTAGACGGAG   
  
  
- GAGGCAAGAG AGGTGTCGTC TAAGGAGTTA GTCCTTTCCA TACAGTCTCG AGTTTTGCTA AGTTGTAGTT   
  
  
- TTACCCTATT AAATAAGACG AAAGTCAAGA CCCAATAACA GTATACAAGT AAGTCAAGTC ACAAAGATCG   
  
  
- TACTAACGAA AAACTACGGA AGAAAAAACA AGACCCAACC TGCTTGAGAC AGAAAAGACA CGTATCTAAA   
  
  
- CTCGAGGCTC GAGTTAAAAA CCCTAAAAAA ATCGACTGAA AAGACGAACC ACAACACTTT AACACTACCC   
  
  
- AAAACCGAAA CAATTTGAAC AAATATTGAA AAAGACAGTT TTGAAATATC ATTTAACAAC GTTAAACTCT   
  
  
- TAATACGTGA GTCACCGAGA CACTAAATAG CAGGCGACCG TTTAAAAGAC TACTTTCCTC TACTAAATAA   
  
  
- TTAAAAAGTC CGTACCAGTT TAAAAGATCG GAAAGTACAT GTAACCAACC ACTATCCGAC TATCGATTTA   
  
  
- ATACAGAGTA AAGAGATTTA AATAACGGTC TATACTTTAA CCACCTTGTC AAATTCCCAA AACCAGCAGA   
  
  
- AAAAACGGGA AAAAAAACCC AGGGGGGGGG CGGGCCCCCC CTTGGGCCTC CCGTCTTTTT CATTGTATTG   
  
  
- AATTTTAATA GTAACTGCAT CAGGAGGTTG GTATTACTTT CGTTGACTTT ATTCTCGTAC GATTCGATCT   
  
  
- CTCATCCGAC GGAACAGTCG GAGGGAAAAC TGTACTCTTT CTACCCTCTT CAAATTTTAT TTCAAGGAGC   
  
  
- AGAAACGTAG GGCCTGGTGT TAAGCCTTGA CCCACTCCTG GAGGAATCCG AACATTTGTG TTCAAAGCTT   
  
  
- GAATCTAAAC GCATGTACAG TTACGAAATG ACCTATTCGA ATCGATTGTG GGGAGTAGGA AGTTGGACAT   
  
  
- GGAAGTATAT TTTTTTTCCT GTTAGGCCAC GTGTTCGTAG GGCGTAAGTG CGTTCCAGAC CCCTTCCCGG   
  
  
- CGTGGGGTTT CCCACTTAAC ATCCGTCGGA TTGGACTGAA ACAGTCACCG ATTAAGGTGC TAAGCTTGGG   
  
  
- TACTAAAAAA CCAATGTGCC CTTGTTGGAA TGGTAACGAG TTTCCGAGGG GAAGAAGTTG GAGATGGAAG   
  
  
- TATATACTTA CTTTTAGGAA ACCGATTCTT AACTTTTCCT TTATACACCA ATAACCCAAA CCCAAGAATA   
  
  
- AGGCAACCAA CGGGGAAGTG TAACTTACGT AGGAGAGCGA TTCTTGACTT TTCCTGTATA CCCCTACATA   
  
  
- TCCAGTTTAA ACTGAGTTAC TATTGACTCC TATGTTCTTA AGATCTTCAG AAATAGTCCC CTTAAAAAAG   
  
  
- AAAAAAGGTG AACACTGAAA AACTACGGAA CGAAAAATGA GAAGTAACTT ACTCATGGGA AGAACTGTCT   
  
  
- AAGAAGGAGG GAAAAAAGAA TTAAAGAAGA ACATCACCTT ATATTCATAC GAAGATCCAT CAACCTACGG   
  
  
- TACCTTAGAG TAGGGACACA CACGTAAGAA GGGGAGAGGG CGGCGGGGGG GGGGGGGTTT TTCTTTGGTT   
  
  
- TTTTTTTTTT TTTTACCTAC TAAGAATCTT GACTGGTAAA AAGGAAGGAG GAGGGTAAAA TAAAAAAAGT   
  
  
- CCGACAAAAA GAAGATGGAC AACGAAATCG TAGTGAGTAT TTTTGTTCCA CGTTCGCGTA CTGTAGCCCA   
  
  
- TGACTCGAAG ACTCAAATGT ACGTAGCAGT ACTTTAAGCC ATCTGAAACT TCATTTCAAA GGGGTAGCAT   
  
  
- TGACCACCAC GTATGGACAG AAGTGCGGGT AGTCATCTCA CCACTTCACT TCGTTACAGC TTATCAAACA   
  
  
- TAATGCTCGG GTTCTCTCTT TGACTACGTA TAAAATACGG AGTTACAGTT AGGAACTTGG GGGTCGAGCT   
  
  
- AATGTTGTGG TAACTGATAC CTCGAAAATA CACGAGTTTT CAAAAGGACC CAGTCATGAC GTGAGACCTT   
  
  
- AGTAGGAGTC CTTGACCCTG AACAGGCCAC AGATCGAGGT GTAGTTGAGA GTCGAAGAGT AGGTTACCAT   
  
  
- CGGGACCTAG TGTCGAACTA AGGTTGAGAA TAGGAAGACT AGTTTTAACG AGAGGACTAT TACGGTTAAG   
  
  
- GAGTAGAAAT TCAGTAAGGA CGCAGTGTCT ACTACACCTA CTGGACTTTA TGTCCGACTC TCTCAACCTT   
  
  
- TGTCACTACA ACCCTGGACT AGGGCTAAAA CAGATGCCGT TATCGTTGGT GTGTAAACCC CAACCCTAGG   
  
  
- TACATCACAG TAGTCTCAAA CTGTCGACCC CCTTTCACTA CCTCTACTAG CGAGCTTTCC TAAACTTTGA   
  
  
- CCACGATTAA CGGACACGTT CTCGTCAACG ACTTTTACTA GTCAACAACC GGGTCACCGA CTACCTACTT   
  
  
- AACTCTGTCT ACCAAAGTCA AAGACCACTT GGTTAAGTTT CTAACCCACG AATATACAAC CTTCCCGAGC   
  
  
- ATCGCTCCGT CCGGAGGAGT CCATCGAGGT AAATATTTCG TAACTCTACG TTCCTTGGGC GCTCACGACT   
  
  
- GGAAAATAGA ATGTACGTGT ATGAGAAACT CCAAACGGGT ATGTAGTTTA AACCGATGTA CAGTCGTTTA   
  
  
- CCACGGTAAC GTCTTCGTTA CTTCCTACTT TTCTCTCAGG TGTAACAGCT AAAGGTTTAA CCCGTCCCTT   
  
  
- CGGTTACCCA CTGGGATTAG GTCCGTAAAC GTCGGGCCGG TCCCCCAGGT GGGGTGTAAG CGTAATGGCC   
  
  
- ATAGCTACTG AGGATGAGAC GTATACGAGC ACCTCCACCC AACTTGTAAC ACCCGGTATC CGAGAACTCC   
  
  
- AACCGAGTCA GTAAATTCCA GGGTAAACTC AAGTTGCGTC AACTGCAGGG CCGAACAGTT CAGGACGAAT   
  
  
- TTCTAGAGCC GTAAGTTGTA CCACTCCGGA ACCGACACTT GAAACGGAAG TAAGAAGTGG TGTACGGGCT   
  
  
- ACTCTCGCAC TCGTGTCTCT TAGTGGCCCT AGCAGAAGAC TCCCACCATT TTCCCGACTT CGGTTTTCAC   
  
  
- CATTGCAATC ACCTCGTTCT CACATTGTGT TTGAGACGAC GAAAGAACGG AGCGAAACAA CTCTGTAACC   
  
  
- TTATGATGTG TCGCTACAAA CTCAGGTACC TACACTGAAA CGGATCCCTA GTATTTCTGG CCTAGTTACA   
  
  
- ACTCGTCGTA ACAGAACGAG CTCTACAACA CTTGAACCAA CGCACACTTC CTCGACTCTC CCACCTCGCC   
  
  
- GTACTCGAGG AACCCTTTAC CTCCAGCGCC AAGTGTTACC GTCCCAAATT CGGTATGGGA AATTCGTGAG   
  
  
- ACCATTTGTT GTGTTATTCT TGAAACAACT TCTTGATGAC ACTATCCATA CCCGATCTCC TCTCCTTACC   
  
  
- TCCAGAAATA GAACCCACCT ACTTATCTCT AAACCAACGA AGCTCACGGA CCGTCACATC ATTAAT

+     Sp1

| Site Name | Organism | Position | Strand | Matrix score. | sequence | function |
| --- | --- | --- | --- | --- | --- | --- |
| Sp1 | Oryza sativa | 1726 | - | 6 | GGGCGG | light responsive element |
| Sp1 | Oryza sativa | 803 | - | 6 | GGGCGG | light responsive element |
| Sp1 | Oryza sativa | 192 | - | 6 | GGGCGG | light responsive element |

>HU01G01391.1   
+ +Up\_Stream \_Len000CATTTT AGTAATAAAT TCCCATGTGA GGACCACTGC CTACTGATTC CGAATGGCAT   
  
  
+ TTTAGTAATA AACTCCCATG TGAGGACCAC TTGATGAGGA ATTCAGTTTT ATAATCCACA GGTGTCAAAG   
  
  
+ CCTATTCCTA CTCTCACCAC TCTGCTATCT CTCCCCCCCC CCCCCCCCCG CCCCCCTCAA AATCTGCCTC   
  
  
+ CTCCGTTCTC TCCACAGCAG ATTCCTCAAT CAGGAAAGGT ATGTCAGAGC TCAAAACGAT TCAACATCAA   
  
  
+ AATGGGATAA TTTATTCTGC TTTCAGTTCT GGGTTATTGT CATATGTTCA TTCAGTTCAG TGTTTCTAGC   
  
  
+ ATGATTGCTT TTTGATGCCT TCTTTTTTGT TCTGGGTTGG ACGAACTCTG TCTTTTCTGT GCATAGATTT   
  
  
+ GAGCTCCGAG CTCAATTTTT GGGATTTTTT TAGCTGACTT TTCTGCTTGG TGTTGTGAAA TTGTGATGGG   
  
  
+ TTTTGGCTTT GTTAAACTTG TTTATAACTT TTTCTGTCAA AACTTTATAG TAAATTGTTG CAATTTGAGA   
  
  
+ ATTATGCACT CAGTGGCTCT GTGATTTATC GTCCGCTGGC AAATTTTCTG ATGAAAGGAG ATGATTTATT   
  
  
+ AATTTTTCAG GCATGGTCAA ATTTTCTAGC CTTTCATGTA CATTGGTTGG TGATAGGCTG ATAGCTAAAT   
  
  
+ TATGTCTCAT TTCTCTAAAT TTATTGCCAG ATATGAAATT GGTGGAACAG TTTAAGGGTT TTGGTCGTCT   
  
  
+ TTTTTGCCCT TTTTTTTGGG TCCCCCCCCC GCCCGGGGGG GAACCCGGAG GGCAGAAAAA GTAACATAAC   
  
  
+ TTAAAATTAT CATTGACGTA GTCCTCCAAC CATAATGAAA GCAACTGAAA TAAGAGCATG CTAAGCTAGA   
  
  
+ GAGTAGGCTG CCTTGTCAGC CTCCCTTTTG ACATGAGAAA GATGGGAGAA GTTTAAAATA AAGTTCCTCG   
  
  
+ TCTTTGCATC CCGGACCACA ATTCGGAACT GGGTGAGGAC CTCCTTAGGC TTGTAAACAC AAGTTTCGAA   
  
  
+ CTTAGATTTG CGTACATGTC AATGCTTTAC TGGATAAGCT TAGCTAACAC CCCTCATCCT TCAACCTGTA   
  
  
+ CCTTCATATA AAAAAAAGGA CAATCCGGTG CACAAGCATC CCGCATTCAC GCAAGGTCTG GGGAAGGGCC   
  
  
+ GCACCCCAAA GGGTGAATTG TAGGCAGCCT AACCTGACTT TGTCAGTGGC TAATTCCACG ATTCGAACCC   
  
  
+ ATGATTTTTT GGTTACACGG GAACAACCTT ACCATTGCTC AAAGGCTCCC CTTCTTCAAC CTCTACCTTC   
  
  
+ ATATATGAAT GAAAATCCTT TGGCTAAGAA TTGAAAAGGA AATATGTGGT TATTGGGTTT GGGTTCTTAT   
  
  
+ TCCGTTGGTT GCCCCTTCAC ATTGAATGCA TCCTCTCGCT AAGAACTGAA AAGGACATAT GGGGATGTAT   
  
  
+ AGGTCAAATT TGACTCAATG ATAACTGAGG ATACAAGAAT TCTAGAAGTC TTTATCAGGG GAATTTTTTC   
  
  
+ TTTTTTCCAC TTGTGACTTT TTGATGCCTT GCTTTTTACT CTTCATTGAA TGAGTACCCT TCTTGACAGA   
  
  
+ TTCTTCCTCC CTTTTTTCTT AATTTCTTCT TGTAGTGGAA TATAAGTATG CTTCTAGGTA GTTGGATGCC   
  
  
+ ATGGAATCTC ATCCCTGTGT GTGCATTCTT CCCCTCTCCC GCCGCCCCCC CCCCCCCAAA AAGAAACCAA   
  
  
+ AAAAAAAAAA AAAATGGATG ATTCTTAGAA CTGACCATTT TTCCTTCCTC CTCCCATTTT ATTTTTTTCA   
  
  
+ GGCTGTTTTT CTTCTACCTG TTGCTTTAGC ATCACTCATA AAAACAAGGT GCAAGCGCAT GACATCGGGT   
  
  
+ ACTGAGCTTC TGAGTTTACA TGCATCGTCA TGAAATTCGG TAGACTTTGA AGTAAAGTTT CCCCATCGTA   
  
  
+ ACTGGTGGTG CATACCTGTC TTCACGCCCA TCAGTAGAGT GGTGAAGTGA AGCAATGTCG AATAGTTTGT   
  
  
+ ATTACGAGCC CAAGAGAGAA ACTGATGCAT ATTTTATGCC TCAATGTCAA TCCTTGAACC CCCAGCTCGA   
  
  
+ TTACAACACC ATTGACTATG GAGCTTTTAT GTGCTCAAAA GTTTTCCTGG GTCAGTACTG CACTCTGGAA   
  
  
+ TCATCCTCAG GAACTGGGAC TTGTCCGGTG TCTAGCTCCA CATCAACTCT CAGCTTCTCA TCCAATGGTA   
  
  
+ GCCCTGGATC ACAGCTTGAT TCCAACTCTT ATCCTTCTGA TCAAAATTGC TCTCCTGATA ATGCCAATTC   
  
  
+ CTCATCTTTA AGTCATTCCT GCGTCACAGA TGATGTGGAT GACCTGAAAT ACAGGCTGAG AGAGTTGGAA   
  
  
+ ACAGTGATGT TGGGACCTGA TCCCGATTTT GTCTACGGCA ATAGCAACCA CACATTTGGG GTTGGGATCC   
  
  
+ ATGTAGTGTC ATCAGAGTTT GACAGCTGGG GGAAAGTGAT GGAGATGATC GCTCGAAAGG ATTTGAAACT   
  
  
+ GGTGCTAATT GCCTGTGCAA GAGCAGTTGC TGAAAATGAT CAGTTGTTGG CCCAGTGGCT GATGGATGAA   
  
  
+ TTGAGACAGA TGGTTTCAGT TTCTGGTGAA CCAATTCAAA GATTGGGTGC TTATATGTTG GAAGGGCTCG   
  
  
+ TAGCGAGGCA GGCCTCCTCA GGTAGCTCCA TTTATAAAGC ATTGAGATGC AAGGAACCCG CGAGTGCTGA   
  
  
+ CCTTTTATCT TACATGCACA TACTCTTTGA GGTTTGCCCA TACATCAAAT TTGGCTACAT GTCAGCAAAT   
  
  
+ GGTGCCATTG CAGAAGCAAT GAAGGATGAA AAGAGAGTCC ACATTGTCGA TTTCCAAATT GGGCAGGGAA   
  
  
+ GCCAATGGGT GACCCTAATC CAGGCATTTG CAGCCCGGCC AGGGGGTCCA CCCCACATTC GCATTACCGG   
  
  
+ TATCGATGAC TCCTACTCTG CATATGCTCG TGGAGGTGGG TTGAACATTG TGGGCCATAG GCTCTTGAGG   
  
  
+ TTGGCTCAGT CATTTAAGGT CCCATTTGAG TTCAACGCAG TTGACGTCCC GGCTTGTCAA GTCCTGCTTA   
  
  
+ AAGATCTCGG CATTCAACAT GGTGAGGCCT TGGCTGTGAA CTTTGCCTTC ATTCTTCACC ACATGCCCGA   
  
  
+ TGAGAGCGTG AGCACAGAGA ATCACCGGGA TCGTCTTCTG AGGGTGGTAA AAGGGCTGAA GCCAAAAGTG   
  
  
+ GTAACGTTAG TGGAGCAAGA GTGTAACACA AACTCTGCTG CTTTCTTGCC TCGCTTTGTT GAGACATTGG   
  
  
+ AATACTACAC AGCGATGTTT GAGTCCATGG ATGTGACTTT GCCTAGGGAT CATAAAGACC GGATCAATGT   
  
  
+ TGAGCAGCAT TGTCTTGCTC GAGATGTTGT GAACTTGGTT GCGTGTGAAG GAGCTGAGAG GGTGGAGCGG   
  
  
+ CATGAGCTCC TTGGGAAATG GAGGTCGCGG TTCACAATGG CAGGGTTTAA GCCATACCCT TTAAGCACTC   
  
  
+ TGGTAAACAA CACAATAAGA ACTTTGTTGA AGAACTACTG TGATAGGTAT GGGCTAGAGG AGAGGAATGG   
  
  
+ AGGTCTTTAT CTTGGGTGGA TGAATAGAGA TTTGGTTGCT TCGAGTGCCT GGCAGTGTAG TAATTA  

- +Up\_Stream \_Len000GTAAAA TCATTATTTA AGGGTACACT CCTGGTGACG GATGACTAAG GCTTACCGTA   
  
  
- AAATCATTAT TTGAGGGTAC ACTCCTGGTG AACTACTCCT TAAGTCAAAA TATTAGGTGT CCACAGTTTC   
  
  
- GGATAAGGAT GAGAGTGGTG AGACGATAGA GAGGGGGGGG GGGGGGGGGC GGGGGGAGTT TTAGACGGAG   
  
  
- GAGGCAAGAG AGGTGTCGTC TAAGGAGTTA GTCCTTTCCA TACAGTCTCG AGTTTTGCTA AGTTGTAGTT   
  
  
- TTACCCTATT AAATAAGACG AAAGTCAAGA CCCAATAACA GTATACAAGT AAGTCAAGTC ACAAAGATCG   
  
  
- TACTAACGAA AAACTACGGA AGAAAAAACA AGACCCAACC TGCTTGAGAC AGAAAAGACA CGTATCTAAA   
  
  
- CTCGAGGCTC GAGTTAAAAA CCCTAAAAAA ATCGACTGAA AAGACGAACC ACAACACTTT AACACTACCC   
  
  
- AAAACCGAAA CAATTTGAAC AAATATTGAA AAAGACAGTT TTGAAATATC ATTTAACAAC GTTAAACTCT   
  
  
- TAATACGTGA GTCACCGAGA CACTAAATAG CAGGCGACCG TTTAAAAGAC TACTTTCCTC TACTAAATAA   
  
  
- TTAAAAAGTC CGTACCAGTT TAAAAGATCG GAAAGTACAT GTAACCAACC ACTATCCGAC TATCGATTTA   
  
  
- ATACAGAGTA AAGAGATTTA AATAACGGTC TATACTTTAA CCACCTTGTC AAATTCCCAA AACCAGCAGA   
  
  
- AAAAACGGGA AAAAAAACCC AGGGGGGGGG CGGGCCCCCC CTTGGGCCTC CCGTCTTTTT CATTGTATTG   
  
  
- AATTTTAATA GTAACTGCAT CAGGAGGTTG GTATTACTTT CGTTGACTTT ATTCTCGTAC GATTCGATCT   
  
  
- CTCATCCGAC GGAACAGTCG GAGGGAAAAC TGTACTCTTT CTACCCTCTT CAAATTTTAT TTCAAGGAGC   
  
  
- AGAAACGTAG GGCCTGGTGT TAAGCCTTGA CCCACTCCTG GAGGAATCCG AACATTTGTG TTCAAAGCTT   
  
  
- GAATCTAAAC GCATGTACAG TTACGAAATG ACCTATTCGA ATCGATTGTG GGGAGTAGGA AGTTGGACAT   
  
  
- GGAAGTATAT TTTTTTTCCT GTTAGGCCAC GTGTTCGTAG GGCGTAAGTG CGTTCCAGAC CCCTTCCCGG   
  
  
- CGTGGGGTTT CCCACTTAAC ATCCGTCGGA TTGGACTGAA ACAGTCACCG ATTAAGGTGC TAAGCTTGGG   
  
  
- TACTAAAAAA CCAATGTGCC CTTGTTGGAA TGGTAACGAG TTTCCGAGGG GAAGAAGTTG GAGATGGAAG   
  
  
- TATATACTTA CTTTTAGGAA ACCGATTCTT AACTTTTCCT TTATACACCA ATAACCCAAA CCCAAGAATA   
  
  
- AGGCAACCAA CGGGGAAGTG TAACTTACGT AGGAGAGCGA TTCTTGACTT TTCCTGTATA CCCCTACATA   
  
  
- TCCAGTTTAA ACTGAGTTAC TATTGACTCC TATGTTCTTA AGATCTTCAG AAATAGTCCC CTTAAAAAAG   
  
  
- AAAAAAGGTG AACACTGAAA AACTACGGAA CGAAAAATGA GAAGTAACTT ACTCATGGGA AGAACTGTCT   
  
  
- AAGAAGGAGG GAAAAAAGAA TTAAAGAAGA ACATCACCTT ATATTCATAC GAAGATCCAT CAACCTACGG   
  
  
- TACCTTAGAG TAGGGACACA CACGTAAGAA GGGGAGAGGG CGGCGGGGGG GGGGGGGTTT TTCTTTGGTT   
  
  
- TTTTTTTTTT TTTTACCTAC TAAGAATCTT GACTGGTAAA AAGGAAGGAG GAGGGTAAAA TAAAAAAAGT   
  
  
- CCGACAAAAA GAAGATGGAC AACGAAATCG TAGTGAGTAT TTTTGTTCCA CGTTCGCGTA CTGTAGCCCA   
  
  
- TGACTCGAAG ACTCAAATGT ACGTAGCAGT ACTTTAAGCC ATCTGAAACT TCATTTCAAA GGGGTAGCAT   
  
  
- TGACCACCAC GTATGGACAG AAGTGCGGGT AGTCATCTCA CCACTTCACT TCGTTACAGC TTATCAAACA   
  
  
- TAATGCTCGG GTTCTCTCTT TGACTACGTA TAAAATACGG AGTTACAGTT AGGAACTTGG GGGTCGAGCT   
  
  
- AATGTTGTGG TAACTGATAC CTCGAAAATA CACGAGTTTT CAAAAGGACC CAGTCATGAC GTGAGACCTT   
  
  
- AGTAGGAGTC CTTGACCCTG AACAGGCCAC AGATCGAGGT GTAGTTGAGA GTCGAAGAGT AGGTTACCAT   
  
  
- CGGGACCTAG TGTCGAACTA AGGTTGAGAA TAGGAAGACT AGTTTTAACG AGAGGACTAT TACGGTTAAG   
  
  
- GAGTAGAAAT TCAGTAAGGA CGCAGTGTCT ACTACACCTA CTGGACTTTA TGTCCGACTC TCTCAACCTT   
  
  
- TGTCACTACA ACCCTGGACT AGGGCTAAAA CAGATGCCGT TATCGTTGGT GTGTAAACCC CAACCCTAGG   
  
  
- TACATCACAG TAGTCTCAAA CTGTCGACCC CCTTTCACTA CCTCTACTAG CGAGCTTTCC TAAACTTTGA   
  
  
- CCACGATTAA CGGACACGTT CTCGTCAACG ACTTTTACTA GTCAACAACC GGGTCACCGA CTACCTACTT   
  
  
- AACTCTGTCT ACCAAAGTCA AAGACCACTT GGTTAAGTTT CTAACCCACG AATATACAAC CTTCCCGAGC   
  
  
- ATCGCTCCGT CCGGAGGAGT CCATCGAGGT AAATATTTCG TAACTCTACG TTCCTTGGGC GCTCACGACT   
  
  
- GGAAAATAGA ATGTACGTGT ATGAGAAACT CCAAACGGGT ATGTAGTTTA AACCGATGTA CAGTCGTTTA   
  
  
- CCACGGTAAC GTCTTCGTTA CTTCCTACTT TTCTCTCAGG TGTAACAGCT AAAGGTTTAA CCCGTCCCTT   
  
  
- CGGTTACCCA CTGGGATTAG GTCCGTAAAC GTCGGGCCGG TCCCCCAGGT GGGGTGTAAG CGTAATGGCC   
  
  
- ATAGCTACTG AGGATGAGAC GTATACGAGC ACCTCCACCC AACTTGTAAC ACCCGGTATC CGAGAACTCC   
  
  
- AACCGAGTCA GTAAATTCCA GGGTAAACTC AAGTTGCGTC AACTGCAGGG CCGAACAGTT CAGGACGAAT   
  
  
- TTCTAGAGCC GTAAGTTGTA CCACTCCGGA ACCGACACTT GAAACGGAAG TAAGAAGTGG TGTACGGGCT   
  
  
- ACTCTCGCAC TCGTGTCTCT TAGTGGCCCT AGCAGAAGAC TCCCACCATT TTCCCGACTT CGGTTTTCAC   
  
  
- CATTGCAATC ACCTCGTTCT CACATTGTGT TTGAGACGAC GAAAGAACGG AGCGAAACAA CTCTGTAACC   
  
  
- TTATGATGTG TCGCTACAAA CTCAGGTACC TACACTGAAA CGGATCCCTA GTATTTCTGG CCTAGTTACA   
  
  
- ACTCGTCGTA ACAGAACGAG CTCTACAACA CTTGAACCAA CGCACACTTC CTCGACTCTC CCACCTCGCC   
  
  
- GTACTCGAGG AACCCTTTAC CTCCAGCGCC AAGTGTTACC GTCCCAAATT CGGTATGGGA AATTCGTGAG   
  
  
- ACCATTTGTT GTGTTATTCT TGAAACAACT TCTTGATGAC ACTATCCATA CCCGATCTCC TCTCCTTACC   
  
  
- TCCAGAAATA GAACCCACCT ACTTATCTCT AAACCAACGA AGCTCACGGA CCGTCACATC ATTAAT

+     TATA-box

| Site Name | Organism | Position | Strand | Matrix score. | sequence | function |
| --- | --- | --- | --- | --- | --- | --- |
| TATA-box | Arabidopsis thaliana | 2697 | - | 4 | TATA | core promoter element around -30 of transcription start |
| TATA-box | Arabidopsis thaliana | 2696 | - | 5 | TATAA | core promoter element around -30 of transcription start |
| TATA-box | Helianthus annuus | 2695 | - | 6 | TATAAA | core promoter element around -30 of transcription start |
| TATA-box | Brassica juncea | 2694 | - | 7 | TATAAAT | core promoter element around -30 of transcription start |
| TATA-box | Arabidopsis thaliana | 2646 | - | 4 | TATA | core promoter element around -30 of transcription start |
| TATA-box | Arabidopsis thaliana | 2645 | - | 5 | TATAA | core promoter element around -30 of transcription start |
| TATA-box | Arabidopsis thaliana | 1655 | + | 4 | TATA | core promoter element around -30 of transcription start |
| TATA-box | Brassica oleracea | 1654 | + | 6 | ATATAA | core promoter element around -30 of transcription start |
| TATA-box | Arabidopsis thaliana | 1472 | + | 4 | TATA | core promoter element around -30 of transcription start |
| TATA-box | Helianthus annuus | 1470 | - | 6 | TATACA | core promoter element around -30 of transcription start |
| TATA-box | Arabidopsis thaliana | 1336 | + | 4 | TATA | core promoter element around -30 of transcription start |
| TATA-box | Brassica napus | 1335 | + | 6 | ATATAT | core promoter element around -30 of transcription start |
| TATA-box | Arabidopsis thaliana | 1131 | + | 4 | TATA | core promoter element around -30 of transcription start |
| TATA-box | Brassica oleracea | 1130 | + | 6 | ATATAA | core promoter element around -30 of transcription start |
| TATA-box | Arabidopsis thaliana | 1129 | + | 9 | ccTATAAAaa | core promoter element around -30 of transcription start |
| TATA-box | Arabidopsis thaliana | 540 | + | 4 | TATA | core promoter element around -30 of transcription start |
| TATA-box | Arabidopsis thaliana | 539 | - | 5 | TATAA | core promoter element around -30 of transcription start |
| TATA-box | Helianthus annuus | 538 | - | 6 | TATAAA | core promoter element around -30 of transcription start |
| TATA-box | Arabidopsis thaliana | 517 | + | 4 | TATA | core promoter element around -30 of transcription start |
| TATA-box | Arabidopsis thaliana | 516 | - | 5 | TATAA | core promoter element around -30 of transcription start |
| TATA-box | Helianthus annuus | 515 | - | 6 | TATAAA | core promoter element around -30 of transcription start |
| TATA-box | Arabidopsis thaliana | 124 | + | 4 | TATA | core promoter element around -30 of transcription start |
| TATA-box | Arabidopsis thaliana | 123 | - | 5 | TATAA | core promoter element around -30 of transcription start |
| TATA-box | Helianthus annuus | 122 | - | 6 | TATAAA | core promoter element around -30 of transcription start |
| TATA-box | Pisum sativum | 121 | - | 7 | TATAAAA | core promoter element around -30 of transcription start |

>HU01G01391.1   
+ +Up\_Stream \_Len000CATTTT AGTAATAAAT TCCCATGTGA GGACCACTGC CTACTGATTC CGAATGGCAT   
  
  
+ TTTAGTAATA AACTCCCATG TGAGGACCAC TTGATGAGGA ATTCAGTTTT ATAATCCACA GGTGTCAAAG   
  
  
+ CCTATTCCTA CTCTCACCAC TCTGCTATCT CTCCCCCCCC CCCCCCCCCG CCCCCCTCAA AATCTGCCTC   
  
  
+ CTCCGTTCTC TCCACAGCAG ATTCCTCAAT CAGGAAAGGT ATGTCAGAGC TCAAAACGAT TCAACATCAA   
  
  
+ AATGGGATAA TTTATTCTGC TTTCAGTTCT GGGTTATTGT CATATGTTCA TTCAGTTCAG TGTTTCTAGC   
  
  
+ ATGATTGCTT TTTGATGCCT TCTTTTTTGT TCTGGGTTGG ACGAACTCTG TCTTTTCTGT GCATAGATTT   
  
  
+ GAGCTCCGAG CTCAATTTTT GGGATTTTTT TAGCTGACTT TTCTGCTTGG TGTTGTGAAA TTGTGATGGG   
  
  
+ TTTTGGCTTT GTTAAACTTG TTTATAACTT TTTCTGTCAA AACTTTATAG TAAATTGTTG CAATTTGAGA   
  
  
+ ATTATGCACT CAGTGGCTCT GTGATTTATC GTCCGCTGGC AAATTTTCTG ATGAAAGGAG ATGATTTATT   
  
  
+ AATTTTTCAG GCATGGTCAA ATTTTCTAGC CTTTCATGTA CATTGGTTGG TGATAGGCTG ATAGCTAAAT   
  
  
+ TATGTCTCAT TTCTCTAAAT TTATTGCCAG ATATGAAATT GGTGGAACAG TTTAAGGGTT TTGGTCGTCT   
  
  
+ TTTTTGCCCT TTTTTTTGGG TCCCCCCCCC GCCCGGGGGG GAACCCGGAG GGCAGAAAAA GTAACATAAC   
  
  
+ TTAAAATTAT CATTGACGTA GTCCTCCAAC CATAATGAAA GCAACTGAAA TAAGAGCATG CTAAGCTAGA   
  
  
+ GAGTAGGCTG CCTTGTCAGC CTCCCTTTTG ACATGAGAAA GATGGGAGAA GTTTAAAATA AAGTTCCTCG   
  
  
+ TCTTTGCATC CCGGACCACA ATTCGGAACT GGGTGAGGAC CTCCTTAGGC TTGTAAACAC AAGTTTCGAA   
  
  
+ CTTAGATTTG CGTACATGTC AATGCTTTAC TGGATAAGCT TAGCTAACAC CCCTCATCCT TCAACCTGTA   
  
  
+ CCTTCATATA AAAAAAAGGA CAATCCGGTG CACAAGCATC CCGCATTCAC GCAAGGTCTG GGGAAGGGCC   
  
  
+ GCACCCCAAA GGGTGAATTG TAGGCAGCCT AACCTGACTT TGTCAGTGGC TAATTCCACG ATTCGAACCC   
  
  
+ ATGATTTTTT GGTTACACGG GAACAACCTT ACCATTGCTC AAAGGCTCCC CTTCTTCAAC CTCTACCTTC   
  
  
+ ATATATGAAT GAAAATCCTT TGGCTAAGAA TTGAAAAGGA AATATGTGGT TATTGGGTTT GGGTTCTTAT   
  
  
+ TCCGTTGGTT GCCCCTTCAC ATTGAATGCA TCCTCTCGCT AAGAACTGAA AAGGACATAT GGGGATGTAT   
  
  
+ AGGTCAAATT TGACTCAATG ATAACTGAGG ATACAAGAAT TCTAGAAGTC TTTATCAGGG GAATTTTTTC   
  
  
+ TTTTTTCCAC TTGTGACTTT TTGATGCCTT GCTTTTTACT CTTCATTGAA TGAGTACCCT TCTTGACAGA   
  
  
+ TTCTTCCTCC CTTTTTTCTT AATTTCTTCT TGTAGTGGAA TATAAGTATG CTTCTAGGTA GTTGGATGCC   
  
  
+ ATGGAATCTC ATCCCTGTGT GTGCATTCTT CCCCTCTCCC GCCGCCCCCC CCCCCCCAAA AAGAAACCAA   
  
  
+ AAAAAAAAAA AAAATGGATG ATTCTTAGAA CTGACCATTT TTCCTTCCTC CTCCCATTTT ATTTTTTTCA   
  
  
+ GGCTGTTTTT CTTCTACCTG TTGCTTTAGC ATCACTCATA AAAACAAGGT GCAAGCGCAT GACATCGGGT   
  
  
+ ACTGAGCTTC TGAGTTTACA TGCATCGTCA TGAAATTCGG TAGACTTTGA AGTAAAGTTT CCCCATCGTA   
  
  
+ ACTGGTGGTG CATACCTGTC TTCACGCCCA TCAGTAGAGT GGTGAAGTGA AGCAATGTCG AATAGTTTGT   
  
  
+ ATTACGAGCC CAAGAGAGAA ACTGATGCAT ATTTTATGCC TCAATGTCAA TCCTTGAACC CCCAGCTCGA   
  
  
+ TTACAACACC ATTGACTATG GAGCTTTTAT GTGCTCAAAA GTTTTCCTGG GTCAGTACTG CACTCTGGAA   
  
  
+ TCATCCTCAG GAACTGGGAC TTGTCCGGTG TCTAGCTCCA CATCAACTCT CAGCTTCTCA TCCAATGGTA   
  
  
+ GCCCTGGATC ACAGCTTGAT TCCAACTCTT ATCCTTCTGA TCAAAATTGC TCTCCTGATA ATGCCAATTC   
  
  
+ CTCATCTTTA AGTCATTCCT GCGTCACAGA TGATGTGGAT GACCTGAAAT ACAGGCTGAG AGAGTTGGAA   
  
  
+ ACAGTGATGT TGGGACCTGA TCCCGATTTT GTCTACGGCA ATAGCAACCA CACATTTGGG GTTGGGATCC   
  
  
+ ATGTAGTGTC ATCAGAGTTT GACAGCTGGG GGAAAGTGAT GGAGATGATC GCTCGAAAGG ATTTGAAACT   
  
  
+ GGTGCTAATT GCCTGTGCAA GAGCAGTTGC TGAAAATGAT CAGTTGTTGG CCCAGTGGCT GATGGATGAA   
  
  
+ TTGAGACAGA TGGTTTCAGT TTCTGGTGAA CCAATTCAAA GATTGGGTGC TTATATGTTG GAAGGGCTCG   
  
  
+ TAGCGAGGCA GGCCTCCTCA GGTAGCTCCA TTTATAAAGC ATTGAGATGC AAGGAACCCG CGAGTGCTGA   
  
  
+ CCTTTTATCT TACATGCACA TACTCTTTGA GGTTTGCCCA TACATCAAAT TTGGCTACAT GTCAGCAAAT   
  
  
+ GGTGCCATTG CAGAAGCAAT GAAGGATGAA AAGAGAGTCC ACATTGTCGA TTTCCAAATT GGGCAGGGAA   
  
  
+ GCCAATGGGT GACCCTAATC CAGGCATTTG CAGCCCGGCC AGGGGGTCCA CCCCACATTC GCATTACCGG   
  
  
+ TATCGATGAC TCCTACTCTG CATATGCTCG TGGAGGTGGG TTGAACATTG TGGGCCATAG GCTCTTGAGG   
  
  
+ TTGGCTCAGT CATTTAAGGT CCCATTTGAG TTCAACGCAG TTGACGTCCC GGCTTGTCAA GTCCTGCTTA   
  
  
+ AAGATCTCGG CATTCAACAT GGTGAGGCCT TGGCTGTGAA CTTTGCCTTC ATTCTTCACC ACATGCCCGA   
  
  
+ TGAGAGCGTG AGCACAGAGA ATCACCGGGA TCGTCTTCTG AGGGTGGTAA AAGGGCTGAA GCCAAAAGTG   
  
  
+ GTAACGTTAG TGGAGCAAGA GTGTAACACA AACTCTGCTG CTTTCTTGCC TCGCTTTGTT GAGACATTGG   
  
  
+ AATACTACAC AGCGATGTTT GAGTCCATGG ATGTGACTTT GCCTAGGGAT CATAAAGACC GGATCAATGT   
  
  
+ TGAGCAGCAT TGTCTTGCTC GAGATGTTGT GAACTTGGTT GCGTGTGAAG GAGCTGAGAG GGTGGAGCGG   
  
  
+ CATGAGCTCC TTGGGAAATG GAGGTCGCGG TTCACAATGG CAGGGTTTAA GCCATACCCT TTAAGCACTC   
  
  
+ TGGTAAACAA CACAATAAGA ACTTTGTTGA AGAACTACTG TGATAGGTAT GGGCTAGAGG AGAGGAATGG   
  
  
+ AGGTCTTTAT CTTGGGTGGA TGAATAGAGA TTTGGTTGCT TCGAGTGCCT GGCAGTGTAG TAATTA  

- +Up\_Stream \_Len000GTAAAA TCATTATTTA AGGGTACACT CCTGGTGACG GATGACTAAG GCTTACCGTA   
  
  
- AAATCATTAT TTGAGGGTAC ACTCCTGGTG AACTACTCCT TAAGTCAAAA TATTAGGTGT CCACAGTTTC   
  
  
- GGATAAGGAT GAGAGTGGTG AGACGATAGA GAGGGGGGGG GGGGGGGGGC GGGGGGAGTT TTAGACGGAG   
  
  
- GAGGCAAGAG AGGTGTCGTC TAAGGAGTTA GTCCTTTCCA TACAGTCTCG AGTTTTGCTA AGTTGTAGTT   
  
  
- TTACCCTATT AAATAAGACG AAAGTCAAGA CCCAATAACA GTATACAAGT AAGTCAAGTC ACAAAGATCG   
  
  
- TACTAACGAA AAACTACGGA AGAAAAAACA AGACCCAACC TGCTTGAGAC AGAAAAGACA CGTATCTAAA   
  
  
- CTCGAGGCTC GAGTTAAAAA CCCTAAAAAA ATCGACTGAA AAGACGAACC ACAACACTTT AACACTACCC   
  
  
- AAAACCGAAA CAATTTGAAC AAATATTGAA AAAGACAGTT TTGAAATATC ATTTAACAAC GTTAAACTCT   
  
  
- TAATACGTGA GTCACCGAGA CACTAAATAG CAGGCGACCG TTTAAAAGAC TACTTTCCTC TACTAAATAA   
  
  
- TTAAAAAGTC CGTACCAGTT TAAAAGATCG GAAAGTACAT GTAACCAACC ACTATCCGAC TATCGATTTA   
  
  
- ATACAGAGTA AAGAGATTTA AATAACGGTC TATACTTTAA CCACCTTGTC AAATTCCCAA AACCAGCAGA   
  
  
- AAAAACGGGA AAAAAAACCC AGGGGGGGGG CGGGCCCCCC CTTGGGCCTC CCGTCTTTTT CATTGTATTG   
  
  
- AATTTTAATA GTAACTGCAT CAGGAGGTTG GTATTACTTT CGTTGACTTT ATTCTCGTAC GATTCGATCT   
  
  
- CTCATCCGAC GGAACAGTCG GAGGGAAAAC TGTACTCTTT CTACCCTCTT CAAATTTTAT TTCAAGGAGC   
  
  
- AGAAACGTAG GGCCTGGTGT TAAGCCTTGA CCCACTCCTG GAGGAATCCG AACATTTGTG TTCAAAGCTT   
  
  
- GAATCTAAAC GCATGTACAG TTACGAAATG ACCTATTCGA ATCGATTGTG GGGAGTAGGA AGTTGGACAT   
  
  
- GGAAGTATAT TTTTTTTCCT GTTAGGCCAC GTGTTCGTAG GGCGTAAGTG CGTTCCAGAC CCCTTCCCGG   
  
  
- CGTGGGGTTT CCCACTTAAC ATCCGTCGGA TTGGACTGAA ACAGTCACCG ATTAAGGTGC TAAGCTTGGG   
  
  
- TACTAAAAAA CCAATGTGCC CTTGTTGGAA TGGTAACGAG TTTCCGAGGG GAAGAAGTTG GAGATGGAAG   
  
  
- TATATACTTA CTTTTAGGAA ACCGATTCTT AACTTTTCCT TTATACACCA ATAACCCAAA CCCAAGAATA   
  
  
- AGGCAACCAA CGGGGAAGTG TAACTTACGT AGGAGAGCGA TTCTTGACTT TTCCTGTATA CCCCTACATA   
  
  
- TCCAGTTTAA ACTGAGTTAC TATTGACTCC TATGTTCTTA AGATCTTCAG AAATAGTCCC CTTAAAAAAG   
  
  
- AAAAAAGGTG AACACTGAAA AACTACGGAA CGAAAAATGA GAAGTAACTT ACTCATGGGA AGAACTGTCT   
  
  
- AAGAAGGAGG GAAAAAAGAA TTAAAGAAGA ACATCACCTT ATATTCATAC GAAGATCCAT CAACCTACGG   
  
  
- TACCTTAGAG TAGGGACACA CACGTAAGAA GGGGAGAGGG CGGCGGGGGG GGGGGGGTTT TTCTTTGGTT   
  
  
- TTTTTTTTTT TTTTACCTAC TAAGAATCTT GACTGGTAAA AAGGAAGGAG GAGGGTAAAA TAAAAAAAGT   
  
  
- CCGACAAAAA GAAGATGGAC AACGAAATCG TAGTGAGTAT TTTTGTTCCA CGTTCGCGTA CTGTAGCCCA   
  
  
- TGACTCGAAG ACTCAAATGT ACGTAGCAGT ACTTTAAGCC ATCTGAAACT TCATTTCAAA GGGGTAGCAT   
  
  
- TGACCACCAC GTATGGACAG AAGTGCGGGT AGTCATCTCA CCACTTCACT TCGTTACAGC TTATCAAACA   
  
  
- TAATGCTCGG GTTCTCTCTT TGACTACGTA TAAAATACGG AGTTACAGTT AGGAACTTGG GGGTCGAGCT   
  
  
- AATGTTGTGG TAACTGATAC CTCGAAAATA CACGAGTTTT CAAAAGGACC CAGTCATGAC GTGAGACCTT   
  
  
- AGTAGGAGTC CTTGACCCTG AACAGGCCAC AGATCGAGGT GTAGTTGAGA GTCGAAGAGT AGGTTACCAT   
  
  
- CGGGACCTAG TGTCGAACTA AGGTTGAGAA TAGGAAGACT AGTTTTAACG AGAGGACTAT TACGGTTAAG   
  
  
- GAGTAGAAAT TCAGTAAGGA CGCAGTGTCT ACTACACCTA CTGGACTTTA TGTCCGACTC TCTCAACCTT   
  
  
- TGTCACTACA ACCCTGGACT AGGGCTAAAA CAGATGCCGT TATCGTTGGT GTGTAAACCC CAACCCTAGG   
  
  
- TACATCACAG TAGTCTCAAA CTGTCGACCC CCTTTCACTA CCTCTACTAG CGAGCTTTCC TAAACTTTGA   
  
  
- CCACGATTAA CGGACACGTT CTCGTCAACG ACTTTTACTA GTCAACAACC GGGTCACCGA CTACCTACTT   
  
  
- AACTCTGTCT ACCAAAGTCA AAGACCACTT GGTTAAGTTT CTAACCCACG AATATACAAC CTTCCCGAGC   
  
  
- ATCGCTCCGT CCGGAGGAGT CCATCGAGGT AAATATTTCG TAACTCTACG TTCCTTGGGC GCTCACGACT   
  
  
- GGAAAATAGA ATGTACGTGT ATGAGAAACT CCAAACGGGT ATGTAGTTTA AACCGATGTA CAGTCGTTTA   
  
  
- CCACGGTAAC GTCTTCGTTA CTTCCTACTT TTCTCTCAGG TGTAACAGCT AAAGGTTTAA CCCGTCCCTT   
  
  
- CGGTTACCCA CTGGGATTAG GTCCGTAAAC GTCGGGCCGG TCCCCCAGGT GGGGTGTAAG CGTAATGGCC   
  
  
- ATAGCTACTG AGGATGAGAC GTATACGAGC ACCTCCACCC AACTTGTAAC ACCCGGTATC CGAGAACTCC   
  
  
- AACCGAGTCA GTAAATTCCA GGGTAAACTC AAGTTGCGTC AACTGCAGGG CCGAACAGTT CAGGACGAAT   
  
  
- TTCTAGAGCC GTAAGTTGTA CCACTCCGGA ACCGACACTT GAAACGGAAG TAAGAAGTGG TGTACGGGCT   
  
  
- ACTCTCGCAC TCGTGTCTCT TAGTGGCCCT AGCAGAAGAC TCCCACCATT TTCCCGACTT CGGTTTTCAC   
  
  
- CATTGCAATC ACCTCGTTCT CACATTGTGT TTGAGACGAC GAAAGAACGG AGCGAAACAA CTCTGTAACC   
  
  
- TTATGATGTG TCGCTACAAA CTCAGGTACC TACACTGAAA CGGATCCCTA GTATTTCTGG CCTAGTTACA   
  
  
- ACTCGTCGTA ACAGAACGAG CTCTACAACA CTTGAACCAA CGCACACTTC CTCGACTCTC CCACCTCGCC   
  
  
- GTACTCGAGG AACCCTTTAC CTCCAGCGCC AAGTGTTACC GTCCCAAATT CGGTATGGGA AATTCGTGAG   
  
  
- ACCATTTGTT GTGTTATTCT TGAAACAACT TCTTGATGAC ACTATCCATA CCCGATCTCC TCTCCTTACC   
  
  
- TCCAGAAATA GAACCCACCT ACTTATCTCT AAACCAACGA AGCTCACGGA CCGTCACATC ATTAAT

+     TATC-box

| Site Name | Organism | Position | Strand | Matrix score. | sequence | function |
| --- | --- | --- | --- | --- | --- | --- |
| TATC-box | Oryza sativa | 287 | - | 7 | TATCCCA | cis-acting element involved in gibberellin-responsiveness |

>HU01G01391.1   
+ +Up\_Stream \_Len000CATTTT AGTAATAAAT TCCCATGTGA GGACCACTGC CTACTGATTC CGAATGGCAT   
  
  
+ TTTAGTAATA AACTCCCATG TGAGGACCAC TTGATGAGGA ATTCAGTTTT ATAATCCACA GGTGTCAAAG   
  
  
+ CCTATTCCTA CTCTCACCAC TCTGCTATCT CTCCCCCCCC CCCCCCCCCG CCCCCCTCAA AATCTGCCTC   
  
  
+ CTCCGTTCTC TCCACAGCAG ATTCCTCAAT CAGGAAAGGT ATGTCAGAGC TCAAAACGAT TCAACATCAA   
  
  
+ AATGGGATAA TTTATTCTGC TTTCAGTTCT GGGTTATTGT CATATGTTCA TTCAGTTCAG TGTTTCTAGC   
  
  
+ ATGATTGCTT TTTGATGCCT TCTTTTTTGT TCTGGGTTGG ACGAACTCTG TCTTTTCTGT GCATAGATTT   
  
  
+ GAGCTCCGAG CTCAATTTTT GGGATTTTTT TAGCTGACTT TTCTGCTTGG TGTTGTGAAA TTGTGATGGG   
  
  
+ TTTTGGCTTT GTTAAACTTG TTTATAACTT TTTCTGTCAA AACTTTATAG TAAATTGTTG CAATTTGAGA   
  
  
+ ATTATGCACT CAGTGGCTCT GTGATTTATC GTCCGCTGGC AAATTTTCTG ATGAAAGGAG ATGATTTATT   
  
  
+ AATTTTTCAG GCATGGTCAA ATTTTCTAGC CTTTCATGTA CATTGGTTGG TGATAGGCTG ATAGCTAAAT   
  
  
+ TATGTCTCAT TTCTCTAAAT TTATTGCCAG ATATGAAATT GGTGGAACAG TTTAAGGGTT TTGGTCGTCT   
  
  
+ TTTTTGCCCT TTTTTTTGGG TCCCCCCCCC GCCCGGGGGG GAACCCGGAG GGCAGAAAAA GTAACATAAC   
  
  
+ TTAAAATTAT CATTGACGTA GTCCTCCAAC CATAATGAAA GCAACTGAAA TAAGAGCATG CTAAGCTAGA   
  
  
+ GAGTAGGCTG CCTTGTCAGC CTCCCTTTTG ACATGAGAAA GATGGGAGAA GTTTAAAATA AAGTTCCTCG   
  
  
+ TCTTTGCATC CCGGACCACA ATTCGGAACT GGGTGAGGAC CTCCTTAGGC TTGTAAACAC AAGTTTCGAA   
  
  
+ CTTAGATTTG CGTACATGTC AATGCTTTAC TGGATAAGCT TAGCTAACAC CCCTCATCCT TCAACCTGTA   
  
  
+ CCTTCATATA AAAAAAAGGA CAATCCGGTG CACAAGCATC CCGCATTCAC GCAAGGTCTG GGGAAGGGCC   
  
  
+ GCACCCCAAA GGGTGAATTG TAGGCAGCCT AACCTGACTT TGTCAGTGGC TAATTCCACG ATTCGAACCC   
  
  
+ ATGATTTTTT GGTTACACGG GAACAACCTT ACCATTGCTC AAAGGCTCCC CTTCTTCAAC CTCTACCTTC   
  
  
+ ATATATGAAT GAAAATCCTT TGGCTAAGAA TTGAAAAGGA AATATGTGGT TATTGGGTTT GGGTTCTTAT   
  
  
+ TCCGTTGGTT GCCCCTTCAC ATTGAATGCA TCCTCTCGCT AAGAACTGAA AAGGACATAT GGGGATGTAT   
  
  
+ AGGTCAAATT TGACTCAATG ATAACTGAGG ATACAAGAAT TCTAGAAGTC TTTATCAGGG GAATTTTTTC   
  
  
+ TTTTTTCCAC TTGTGACTTT TTGATGCCTT GCTTTTTACT CTTCATTGAA TGAGTACCCT TCTTGACAGA   
  
  
+ TTCTTCCTCC CTTTTTTCTT AATTTCTTCT TGTAGTGGAA TATAAGTATG CTTCTAGGTA GTTGGATGCC   
  
  
+ ATGGAATCTC ATCCCTGTGT GTGCATTCTT CCCCTCTCCC GCCGCCCCCC CCCCCCCAAA AAGAAACCAA   
  
  
+ AAAAAAAAAA AAAATGGATG ATTCTTAGAA CTGACCATTT TTCCTTCCTC CTCCCATTTT ATTTTTTTCA   
  
  
+ GGCTGTTTTT CTTCTACCTG TTGCTTTAGC ATCACTCATA AAAACAAGGT GCAAGCGCAT GACATCGGGT   
  
  
+ ACTGAGCTTC TGAGTTTACA TGCATCGTCA TGAAATTCGG TAGACTTTGA AGTAAAGTTT CCCCATCGTA   
  
  
+ ACTGGTGGTG CATACCTGTC TTCACGCCCA TCAGTAGAGT GGTGAAGTGA AGCAATGTCG AATAGTTTGT   
  
  
+ ATTACGAGCC CAAGAGAGAA ACTGATGCAT ATTTTATGCC TCAATGTCAA TCCTTGAACC CCCAGCTCGA   
  
  
+ TTACAACACC ATTGACTATG GAGCTTTTAT GTGCTCAAAA GTTTTCCTGG GTCAGTACTG CACTCTGGAA   
  
  
+ TCATCCTCAG GAACTGGGAC TTGTCCGGTG TCTAGCTCCA CATCAACTCT CAGCTTCTCA TCCAATGGTA   
  
  
+ GCCCTGGATC ACAGCTTGAT TCCAACTCTT ATCCTTCTGA TCAAAATTGC TCTCCTGATA ATGCCAATTC   
  
  
+ CTCATCTTTA AGTCATTCCT GCGTCACAGA TGATGTGGAT GACCTGAAAT ACAGGCTGAG AGAGTTGGAA   
  
  
+ ACAGTGATGT TGGGACCTGA TCCCGATTTT GTCTACGGCA ATAGCAACCA CACATTTGGG GTTGGGATCC   
  
  
+ ATGTAGTGTC ATCAGAGTTT GACAGCTGGG GGAAAGTGAT GGAGATGATC GCTCGAAAGG ATTTGAAACT   
  
  
+ GGTGCTAATT GCCTGTGCAA GAGCAGTTGC TGAAAATGAT CAGTTGTTGG CCCAGTGGCT GATGGATGAA   
  
  
+ TTGAGACAGA TGGTTTCAGT TTCTGGTGAA CCAATTCAAA GATTGGGTGC TTATATGTTG GAAGGGCTCG   
  
  
+ TAGCGAGGCA GGCCTCCTCA GGTAGCTCCA TTTATAAAGC ATTGAGATGC AAGGAACCCG CGAGTGCTGA   
  
  
+ CCTTTTATCT TACATGCACA TACTCTTTGA GGTTTGCCCA TACATCAAAT TTGGCTACAT GTCAGCAAAT   
  
  
+ GGTGCCATTG CAGAAGCAAT GAAGGATGAA AAGAGAGTCC ACATTGTCGA TTTCCAAATT GGGCAGGGAA   
  
  
+ GCCAATGGGT GACCCTAATC CAGGCATTTG CAGCCCGGCC AGGGGGTCCA CCCCACATTC GCATTACCGG   
  
  
+ TATCGATGAC TCCTACTCTG CATATGCTCG TGGAGGTGGG TTGAACATTG TGGGCCATAG GCTCTTGAGG   
  
  
+ TTGGCTCAGT CATTTAAGGT CCCATTTGAG TTCAACGCAG TTGACGTCCC GGCTTGTCAA GTCCTGCTTA   
  
  
+ AAGATCTCGG CATTCAACAT GGTGAGGCCT TGGCTGTGAA CTTTGCCTTC ATTCTTCACC ACATGCCCGA   
  
  
+ TGAGAGCGTG AGCACAGAGA ATCACCGGGA TCGTCTTCTG AGGGTGGTAA AAGGGCTGAA GCCAAAAGTG   
  
  
+ GTAACGTTAG TGGAGCAAGA GTGTAACACA AACTCTGCTG CTTTCTTGCC TCGCTTTGTT GAGACATTGG   
  
  
+ AATACTACAC AGCGATGTTT GAGTCCATGG ATGTGACTTT GCCTAGGGAT CATAAAGACC GGATCAATGT   
  
  
+ TGAGCAGCAT TGTCTTGCTC GAGATGTTGT GAACTTGGTT GCGTGTGAAG GAGCTGAGAG GGTGGAGCGG   
  
  
+ CATGAGCTCC TTGGGAAATG GAGGTCGCGG TTCACAATGG CAGGGTTTAA GCCATACCCT TTAAGCACTC   
  
  
+ TGGTAAACAA CACAATAAGA ACTTTGTTGA AGAACTACTG TGATAGGTAT GGGCTAGAGG AGAGGAATGG   
  
  
+ AGGTCTTTAT CTTGGGTGGA TGAATAGAGA TTTGGTTGCT TCGAGTGCCT GGCAGTGTAG TAATTA  

- +Up\_Stream \_Len000GTAAAA TCATTATTTA AGGGTACACT CCTGGTGACG GATGACTAAG GCTTACCGTA   
  
  
- AAATCATTAT TTGAGGGTAC ACTCCTGGTG AACTACTCCT TAAGTCAAAA TATTAGGTGT CCACAGTTTC   
  
  
- GGATAAGGAT GAGAGTGGTG AGACGATAGA GAGGGGGGGG GGGGGGGGGC GGGGGGAGTT TTAGACGGAG   
  
  
- GAGGCAAGAG AGGTGTCGTC TAAGGAGTTA GTCCTTTCCA TACAGTCTCG AGTTTTGCTA AGTTGTAGTT   
  
  
- TTACCCTATT AAATAAGACG AAAGTCAAGA CCCAATAACA GTATACAAGT AAGTCAAGTC ACAAAGATCG   
  
  
- TACTAACGAA AAACTACGGA AGAAAAAACA AGACCCAACC TGCTTGAGAC AGAAAAGACA CGTATCTAAA   
  
  
- CTCGAGGCTC GAGTTAAAAA CCCTAAAAAA ATCGACTGAA AAGACGAACC ACAACACTTT AACACTACCC   
  
  
- AAAACCGAAA CAATTTGAAC AAATATTGAA AAAGACAGTT TTGAAATATC ATTTAACAAC GTTAAACTCT   
  
  
- TAATACGTGA GTCACCGAGA CACTAAATAG CAGGCGACCG TTTAAAAGAC TACTTTCCTC TACTAAATAA   
  
  
- TTAAAAAGTC CGTACCAGTT TAAAAGATCG GAAAGTACAT GTAACCAACC ACTATCCGAC TATCGATTTA   
  
  
- ATACAGAGTA AAGAGATTTA AATAACGGTC TATACTTTAA CCACCTTGTC AAATTCCCAA AACCAGCAGA   
  
  
- AAAAACGGGA AAAAAAACCC AGGGGGGGGG CGGGCCCCCC CTTGGGCCTC CCGTCTTTTT CATTGTATTG   
  
  
- AATTTTAATA GTAACTGCAT CAGGAGGTTG GTATTACTTT CGTTGACTTT ATTCTCGTAC GATTCGATCT   
  
  
- CTCATCCGAC GGAACAGTCG GAGGGAAAAC TGTACTCTTT CTACCCTCTT CAAATTTTAT TTCAAGGAGC   
  
  
- AGAAACGTAG GGCCTGGTGT TAAGCCTTGA CCCACTCCTG GAGGAATCCG AACATTTGTG TTCAAAGCTT   
  
  
- GAATCTAAAC GCATGTACAG TTACGAAATG ACCTATTCGA ATCGATTGTG GGGAGTAGGA AGTTGGACAT   
  
  
- GGAAGTATAT TTTTTTTCCT GTTAGGCCAC GTGTTCGTAG GGCGTAAGTG CGTTCCAGAC CCCTTCCCGG   
  
  
- CGTGGGGTTT CCCACTTAAC ATCCGTCGGA TTGGACTGAA ACAGTCACCG ATTAAGGTGC TAAGCTTGGG   
  
  
- TACTAAAAAA CCAATGTGCC CTTGTTGGAA TGGTAACGAG TTTCCGAGGG GAAGAAGTTG GAGATGGAAG   
  
  
- TATATACTTA CTTTTAGGAA ACCGATTCTT AACTTTTCCT TTATACACCA ATAACCCAAA CCCAAGAATA   
  
  
- AGGCAACCAA CGGGGAAGTG TAACTTACGT AGGAGAGCGA TTCTTGACTT TTCCTGTATA CCCCTACATA   
  
  
- TCCAGTTTAA ACTGAGTTAC TATTGACTCC TATGTTCTTA AGATCTTCAG AAATAGTCCC CTTAAAAAAG   
  
  
- AAAAAAGGTG AACACTGAAA AACTACGGAA CGAAAAATGA GAAGTAACTT ACTCATGGGA AGAACTGTCT   
  
  
- AAGAAGGAGG GAAAAAAGAA TTAAAGAAGA ACATCACCTT ATATTCATAC GAAGATCCAT CAACCTACGG   
  
  
- TACCTTAGAG TAGGGACACA CACGTAAGAA GGGGAGAGGG CGGCGGGGGG GGGGGGGTTT TTCTTTGGTT   
  
  
- TTTTTTTTTT TTTTACCTAC TAAGAATCTT GACTGGTAAA AAGGAAGGAG GAGGGTAAAA TAAAAAAAGT   
  
  
- CCGACAAAAA GAAGATGGAC AACGAAATCG TAGTGAGTAT TTTTGTTCCA CGTTCGCGTA CTGTAGCCCA   
  
  
- TGACTCGAAG ACTCAAATGT ACGTAGCAGT ACTTTAAGCC ATCTGAAACT TCATTTCAAA GGGGTAGCAT   
  
  
- TGACCACCAC GTATGGACAG AAGTGCGGGT AGTCATCTCA CCACTTCACT TCGTTACAGC TTATCAAACA   
  
  
- TAATGCTCGG GTTCTCTCTT TGACTACGTA TAAAATACGG AGTTACAGTT AGGAACTTGG GGGTCGAGCT   
  
  
- AATGTTGTGG TAACTGATAC CTCGAAAATA CACGAGTTTT CAAAAGGACC CAGTCATGAC GTGAGACCTT   
  
  
- AGTAGGAGTC CTTGACCCTG AACAGGCCAC AGATCGAGGT GTAGTTGAGA GTCGAAGAGT AGGTTACCAT   
  
  
- CGGGACCTAG TGTCGAACTA AGGTTGAGAA TAGGAAGACT AGTTTTAACG AGAGGACTAT TACGGTTAAG   
  
  
- GAGTAGAAAT TCAGTAAGGA CGCAGTGTCT ACTACACCTA CTGGACTTTA TGTCCGACTC TCTCAACCTT   
  
  
- TGTCACTACA ACCCTGGACT AGGGCTAAAA CAGATGCCGT TATCGTTGGT GTGTAAACCC CAACCCTAGG   
  
  
- TACATCACAG TAGTCTCAAA CTGTCGACCC CCTTTCACTA CCTCTACTAG CGAGCTTTCC TAAACTTTGA   
  
  
- CCACGATTAA CGGACACGTT CTCGTCAACG ACTTTTACTA GTCAACAACC GGGTCACCGA CTACCTACTT   
  
  
- AACTCTGTCT ACCAAAGTCA AAGACCACTT GGTTAAGTTT CTAACCCACG AATATACAAC CTTCCCGAGC   
  
  
- ATCGCTCCGT CCGGAGGAGT CCATCGAGGT AAATATTTCG TAACTCTACG TTCCTTGGGC GCTCACGACT   
  
  
- GGAAAATAGA ATGTACGTGT ATGAGAAACT CCAAACGGGT ATGTAGTTTA AACCGATGTA CAGTCGTTTA   
  
  
- CCACGGTAAC GTCTTCGTTA CTTCCTACTT TTCTCTCAGG TGTAACAGCT AAAGGTTTAA CCCGTCCCTT   
  
  
- CGGTTACCCA CTGGGATTAG GTCCGTAAAC GTCGGGCCGG TCCCCCAGGT GGGGTGTAAG CGTAATGGCC   
  
  
- ATAGCTACTG AGGATGAGAC GTATACGAGC ACCTCCACCC AACTTGTAAC ACCCGGTATC CGAGAACTCC   
  
  
- AACCGAGTCA GTAAATTCCA GGGTAAACTC AAGTTGCGTC AACTGCAGGG CCGAACAGTT CAGGACGAAT   
  
  
- TTCTAGAGCC GTAAGTTGTA CCACTCCGGA ACCGACACTT GAAACGGAAG TAAGAAGTGG TGTACGGGCT   
  
  
- ACTCTCGCAC TCGTGTCTCT TAGTGGCCCT AGCAGAAGAC TCCCACCATT TTCCCGACTT CGGTTTTCAC   
  
  
- CATTGCAATC ACCTCGTTCT CACATTGTGT TTGAGACGAC GAAAGAACGG AGCGAAACAA CTCTGTAACC   
  
  
- TTATGATGTG TCGCTACAAA CTCAGGTACC TACACTGAAA CGGATCCCTA GTATTTCTGG CCTAGTTACA   
  
  
- ACTCGTCGTA ACAGAACGAG CTCTACAACA CTTGAACCAA CGCACACTTC CTCGACTCTC CCACCTCGCC   
  
  
- GTACTCGAGG AACCCTTTAC CTCCAGCGCC AAGTGTTACC GTCCCAAATT CGGTATGGGA AATTCGTGAG   
  
  
- ACCATTTGTT GTGTTATTCT TGAAACAACT TCTTGATGAC ACTATCCATA CCCGATCTCC TCTCCTTACC   
  
  
- TCCAGAAATA GAACCCACCT ACTTATCTCT AAACCAACGA AGCTCACGGA CCGTCACATC ATTAAT

+     TCA

| Site Name | Organism | Position | Strand | Matrix score. | sequence | function |
| --- | --- | --- | --- | --- | --- | --- |
| TCA | Pisum sativum | 2493 | - | 9 | TCATCTTCAT |  |

>HU01G01391.1   
+ +Up\_Stream \_Len000CATTTT AGTAATAAAT TCCCATGTGA GGACCACTGC CTACTGATTC CGAATGGCAT   
  
  
+ TTTAGTAATA AACTCCCATG TGAGGACCAC TTGATGAGGA ATTCAGTTTT ATAATCCACA GGTGTCAAAG   
  
  
+ CCTATTCCTA CTCTCACCAC TCTGCTATCT CTCCCCCCCC CCCCCCCCCG CCCCCCTCAA AATCTGCCTC   
  
  
+ CTCCGTTCTC TCCACAGCAG ATTCCTCAAT CAGGAAAGGT ATGTCAGAGC TCAAAACGAT TCAACATCAA   
  
  
+ AATGGGATAA TTTATTCTGC TTTCAGTTCT GGGTTATTGT CATATGTTCA TTCAGTTCAG TGTTTCTAGC   
  
  
+ ATGATTGCTT TTTGATGCCT TCTTTTTTGT TCTGGGTTGG ACGAACTCTG TCTTTTCTGT GCATAGATTT   
  
  
+ GAGCTCCGAG CTCAATTTTT GGGATTTTTT TAGCTGACTT TTCTGCTTGG TGTTGTGAAA TTGTGATGGG   
  
  
+ TTTTGGCTTT GTTAAACTTG TTTATAACTT TTTCTGTCAA AACTTTATAG TAAATTGTTG CAATTTGAGA   
  
  
+ ATTATGCACT CAGTGGCTCT GTGATTTATC GTCCGCTGGC AAATTTTCTG ATGAAAGGAG ATGATTTATT   
  
  
+ AATTTTTCAG GCATGGTCAA ATTTTCTAGC CTTTCATGTA CATTGGTTGG TGATAGGCTG ATAGCTAAAT   
  
  
+ TATGTCTCAT TTCTCTAAAT TTATTGCCAG ATATGAAATT GGTGGAACAG TTTAAGGGTT TTGGTCGTCT   
  
  
+ TTTTTGCCCT TTTTTTTGGG TCCCCCCCCC GCCCGGGGGG GAACCCGGAG GGCAGAAAAA GTAACATAAC   
  
  
+ TTAAAATTAT CATTGACGTA GTCCTCCAAC CATAATGAAA GCAACTGAAA TAAGAGCATG CTAAGCTAGA   
  
  
+ GAGTAGGCTG CCTTGTCAGC CTCCCTTTTG ACATGAGAAA GATGGGAGAA GTTTAAAATA AAGTTCCTCG   
  
  
+ TCTTTGCATC CCGGACCACA ATTCGGAACT GGGTGAGGAC CTCCTTAGGC TTGTAAACAC AAGTTTCGAA   
  
  
+ CTTAGATTTG CGTACATGTC AATGCTTTAC TGGATAAGCT TAGCTAACAC CCCTCATCCT TCAACCTGTA   
  
  
+ CCTTCATATA AAAAAAAGGA CAATCCGGTG CACAAGCATC CCGCATTCAC GCAAGGTCTG GGGAAGGGCC   
  
  
+ GCACCCCAAA GGGTGAATTG TAGGCAGCCT AACCTGACTT TGTCAGTGGC TAATTCCACG ATTCGAACCC   
  
  
+ ATGATTTTTT GGTTACACGG GAACAACCTT ACCATTGCTC AAAGGCTCCC CTTCTTCAAC CTCTACCTTC   
  
  
+ ATATATGAAT GAAAATCCTT TGGCTAAGAA TTGAAAAGGA AATATGTGGT TATTGGGTTT GGGTTCTTAT   
  
  
+ TCCGTTGGTT GCCCCTTCAC ATTGAATGCA TCCTCTCGCT AAGAACTGAA AAGGACATAT GGGGATGTAT   
  
  
+ AGGTCAAATT TGACTCAATG ATAACTGAGG ATACAAGAAT TCTAGAAGTC TTTATCAGGG GAATTTTTTC   
  
  
+ TTTTTTCCAC TTGTGACTTT TTGATGCCTT GCTTTTTACT CTTCATTGAA TGAGTACCCT TCTTGACAGA   
  
  
+ TTCTTCCTCC CTTTTTTCTT AATTTCTTCT TGTAGTGGAA TATAAGTATG CTTCTAGGTA GTTGGATGCC   
  
  
+ ATGGAATCTC ATCCCTGTGT GTGCATTCTT CCCCTCTCCC GCCGCCCCCC CCCCCCCAAA AAGAAACCAA   
  
  
+ AAAAAAAAAA AAAATGGATG ATTCTTAGAA CTGACCATTT TTCCTTCCTC CTCCCATTTT ATTTTTTTCA   
  
  
+ GGCTGTTTTT CTTCTACCTG TTGCTTTAGC ATCACTCATA AAAACAAGGT GCAAGCGCAT GACATCGGGT   
  
  
+ ACTGAGCTTC TGAGTTTACA TGCATCGTCA TGAAATTCGG TAGACTTTGA AGTAAAGTTT CCCCATCGTA   
  
  
+ ACTGGTGGTG CATACCTGTC TTCACGCCCA TCAGTAGAGT GGTGAAGTGA AGCAATGTCG AATAGTTTGT   
  
  
+ ATTACGAGCC CAAGAGAGAA ACTGATGCAT ATTTTATGCC TCAATGTCAA TCCTTGAACC CCCAGCTCGA   
  
  
+ TTACAACACC ATTGACTATG GAGCTTTTAT GTGCTCAAAA GTTTTCCTGG GTCAGTACTG CACTCTGGAA   
  
  
+ TCATCCTCAG GAACTGGGAC TTGTCCGGTG TCTAGCTCCA CATCAACTCT CAGCTTCTCA TCCAATGGTA   
  
  
+ GCCCTGGATC ACAGCTTGAT TCCAACTCTT ATCCTTCTGA TCAAAATTGC TCTCCTGATA ATGCCAATTC   
  
  
+ CTCATCTTTA AGTCATTCCT GCGTCACAGA TGATGTGGAT GACCTGAAAT ACAGGCTGAG AGAGTTGGAA   
  
  
+ ACAGTGATGT TGGGACCTGA TCCCGATTTT GTCTACGGCA ATAGCAACCA CACATTTGGG GTTGGGATCC   
  
  
+ ATGTAGTGTC ATCAGAGTTT GACAGCTGGG GGAAAGTGAT GGAGATGATC GCTCGAAAGG ATTTGAAACT   
  
  
+ GGTGCTAATT GCCTGTGCAA GAGCAGTTGC TGAAAATGAT CAGTTGTTGG CCCAGTGGCT GATGGATGAA   
  
  
+ TTGAGACAGA TGGTTTCAGT TTCTGGTGAA CCAATTCAAA GATTGGGTGC TTATATGTTG GAAGGGCTCG   
  
  
+ TAGCGAGGCA GGCCTCCTCA GGTAGCTCCA TTTATAAAGC ATTGAGATGC AAGGAACCCG CGAGTGCTGA   
  
  
+ CCTTTTATCT TACATGCACA TACTCTTTGA GGTTTGCCCA TACATCAAAT TTGGCTACAT GTCAGCAAAT   
  
  
+ GGTGCCATTG CAGAAGCAAT GAAGGATGAA AAGAGAGTCC ACATTGTCGA TTTCCAAATT GGGCAGGGAA   
  
  
+ GCCAATGGGT GACCCTAATC CAGGCATTTG CAGCCCGGCC AGGGGGTCCA CCCCACATTC GCATTACCGG   
  
  
+ TATCGATGAC TCCTACTCTG CATATGCTCG TGGAGGTGGG TTGAACATTG TGGGCCATAG GCTCTTGAGG   
  
  
+ TTGGCTCAGT CATTTAAGGT CCCATTTGAG TTCAACGCAG TTGACGTCCC GGCTTGTCAA GTCCTGCTTA   
  
  
+ AAGATCTCGG CATTCAACAT GGTGAGGCCT TGGCTGTGAA CTTTGCCTTC ATTCTTCACC ACATGCCCGA   
  
  
+ TGAGAGCGTG AGCACAGAGA ATCACCGGGA TCGTCTTCTG AGGGTGGTAA AAGGGCTGAA GCCAAAAGTG   
  
  
+ GTAACGTTAG TGGAGCAAGA GTGTAACACA AACTCTGCTG CTTTCTTGCC TCGCTTTGTT GAGACATTGG   
  
  
+ AATACTACAC AGCGATGTTT GAGTCCATGG ATGTGACTTT GCCTAGGGAT CATAAAGACC GGATCAATGT   
  
  
+ TGAGCAGCAT TGTCTTGCTC GAGATGTTGT GAACTTGGTT GCGTGTGAAG GAGCTGAGAG GGTGGAGCGG   
  
  
+ CATGAGCTCC TTGGGAAATG GAGGTCGCGG TTCACAATGG CAGGGTTTAA GCCATACCCT TTAAGCACTC   
  
  
+ TGGTAAACAA CACAATAAGA ACTTTGTTGA AGAACTACTG TGATAGGTAT GGGCTAGAGG AGAGGAATGG   
  
  
+ AGGTCTTTAT CTTGGGTGGA TGAATAGAGA TTTGGTTGCT TCGAGTGCCT GGCAGTGTAG TAATTA  

- +Up\_Stream \_Len000GTAAAA TCATTATTTA AGGGTACACT CCTGGTGACG GATGACTAAG GCTTACCGTA   
  
  
- AAATCATTAT TTGAGGGTAC ACTCCTGGTG AACTACTCCT TAAGTCAAAA TATTAGGTGT CCACAGTTTC   
  
  
- GGATAAGGAT GAGAGTGGTG AGACGATAGA GAGGGGGGGG GGGGGGGGGC GGGGGGAGTT TTAGACGGAG   
  
  
- GAGGCAAGAG AGGTGTCGTC TAAGGAGTTA GTCCTTTCCA TACAGTCTCG AGTTTTGCTA AGTTGTAGTT   
  
  
- TTACCCTATT AAATAAGACG AAAGTCAAGA CCCAATAACA GTATACAAGT AAGTCAAGTC ACAAAGATCG   
  
  
- TACTAACGAA AAACTACGGA AGAAAAAACA AGACCCAACC TGCTTGAGAC AGAAAAGACA CGTATCTAAA   
  
  
- CTCGAGGCTC GAGTTAAAAA CCCTAAAAAA ATCGACTGAA AAGACGAACC ACAACACTTT AACACTACCC   
  
  
- AAAACCGAAA CAATTTGAAC AAATATTGAA AAAGACAGTT TTGAAATATC ATTTAACAAC GTTAAACTCT   
  
  
- TAATACGTGA GTCACCGAGA CACTAAATAG CAGGCGACCG TTTAAAAGAC TACTTTCCTC TACTAAATAA   
  
  
- TTAAAAAGTC CGTACCAGTT TAAAAGATCG GAAAGTACAT GTAACCAACC ACTATCCGAC TATCGATTTA   
  
  
- ATACAGAGTA AAGAGATTTA AATAACGGTC TATACTTTAA CCACCTTGTC AAATTCCCAA AACCAGCAGA   
  
  
- AAAAACGGGA AAAAAAACCC AGGGGGGGGG CGGGCCCCCC CTTGGGCCTC CCGTCTTTTT CATTGTATTG   
  
  
- AATTTTAATA GTAACTGCAT CAGGAGGTTG GTATTACTTT CGTTGACTTT ATTCTCGTAC GATTCGATCT   
  
  
- CTCATCCGAC GGAACAGTCG GAGGGAAAAC TGTACTCTTT CTACCCTCTT CAAATTTTAT TTCAAGGAGC   
  
  
- AGAAACGTAG GGCCTGGTGT TAAGCCTTGA CCCACTCCTG GAGGAATCCG AACATTTGTG TTCAAAGCTT   
  
  
- GAATCTAAAC GCATGTACAG TTACGAAATG ACCTATTCGA ATCGATTGTG GGGAGTAGGA AGTTGGACAT   
  
  
- GGAAGTATAT TTTTTTTCCT GTTAGGCCAC GTGTTCGTAG GGCGTAAGTG CGTTCCAGAC CCCTTCCCGG   
  
  
- CGTGGGGTTT CCCACTTAAC ATCCGTCGGA TTGGACTGAA ACAGTCACCG ATTAAGGTGC TAAGCTTGGG   
  
  
- TACTAAAAAA CCAATGTGCC CTTGTTGGAA TGGTAACGAG TTTCCGAGGG GAAGAAGTTG GAGATGGAAG   
  
  
- TATATACTTA CTTTTAGGAA ACCGATTCTT AACTTTTCCT TTATACACCA ATAACCCAAA CCCAAGAATA   
  
  
- AGGCAACCAA CGGGGAAGTG TAACTTACGT AGGAGAGCGA TTCTTGACTT TTCCTGTATA CCCCTACATA   
  
  
- TCCAGTTTAA ACTGAGTTAC TATTGACTCC TATGTTCTTA AGATCTTCAG AAATAGTCCC CTTAAAAAAG   
  
  
- AAAAAAGGTG AACACTGAAA AACTACGGAA CGAAAAATGA GAAGTAACTT ACTCATGGGA AGAACTGTCT   
  
  
- AAGAAGGAGG GAAAAAAGAA TTAAAGAAGA ACATCACCTT ATATTCATAC GAAGATCCAT CAACCTACGG   
  
  
- TACCTTAGAG TAGGGACACA CACGTAAGAA GGGGAGAGGG CGGCGGGGGG GGGGGGGTTT TTCTTTGGTT   
  
  
- TTTTTTTTTT TTTTACCTAC TAAGAATCTT GACTGGTAAA AAGGAAGGAG GAGGGTAAAA TAAAAAAAGT   
  
  
- CCGACAAAAA GAAGATGGAC AACGAAATCG TAGTGAGTAT TTTTGTTCCA CGTTCGCGTA CTGTAGCCCA   
  
  
- TGACTCGAAG ACTCAAATGT ACGTAGCAGT ACTTTAAGCC ATCTGAAACT TCATTTCAAA GGGGTAGCAT   
  
  
- TGACCACCAC GTATGGACAG AAGTGCGGGT AGTCATCTCA CCACTTCACT TCGTTACAGC TTATCAAACA   
  
  
- TAATGCTCGG GTTCTCTCTT TGACTACGTA TAAAATACGG AGTTACAGTT AGGAACTTGG GGGTCGAGCT   
  
  
- AATGTTGTGG TAACTGATAC CTCGAAAATA CACGAGTTTT CAAAAGGACC CAGTCATGAC GTGAGACCTT   
  
  
- AGTAGGAGTC CTTGACCCTG AACAGGCCAC AGATCGAGGT GTAGTTGAGA GTCGAAGAGT AGGTTACCAT   
  
  
- CGGGACCTAG TGTCGAACTA AGGTTGAGAA TAGGAAGACT AGTTTTAACG AGAGGACTAT TACGGTTAAG   
  
  
- GAGTAGAAAT TCAGTAAGGA CGCAGTGTCT ACTACACCTA CTGGACTTTA TGTCCGACTC TCTCAACCTT   
  
  
- TGTCACTACA ACCCTGGACT AGGGCTAAAA CAGATGCCGT TATCGTTGGT GTGTAAACCC CAACCCTAGG   
  
  
- TACATCACAG TAGTCTCAAA CTGTCGACCC CCTTTCACTA CCTCTACTAG CGAGCTTTCC TAAACTTTGA   
  
  
- CCACGATTAA CGGACACGTT CTCGTCAACG ACTTTTACTA GTCAACAACC GGGTCACCGA CTACCTACTT   
  
  
- AACTCTGTCT ACCAAAGTCA AAGACCACTT GGTTAAGTTT CTAACCCACG AATATACAAC CTTCCCGAGC   
  
  
- ATCGCTCCGT CCGGAGGAGT CCATCGAGGT AAATATTTCG TAACTCTACG TTCCTTGGGC GCTCACGACT   
  
  
- GGAAAATAGA ATGTACGTGT ATGAGAAACT CCAAACGGGT ATGTAGTTTA AACCGATGTA CAGTCGTTTA   
  
  
- CCACGGTAAC GTCTTCGTTA CTTCCTACTT TTCTCTCAGG TGTAACAGCT AAAGGTTTAA CCCGTCCCTT   
  
  
- CGGTTACCCA CTGGGATTAG GTCCGTAAAC GTCGGGCCGG TCCCCCAGGT GGGGTGTAAG CGTAATGGCC   
  
  
- ATAGCTACTG AGGATGAGAC GTATACGAGC ACCTCCACCC AACTTGTAAC ACCCGGTATC CGAGAACTCC   
  
  
- AACCGAGTCA GTAAATTCCA GGGTAAACTC AAGTTGCGTC AACTGCAGGG CCGAACAGTT CAGGACGAAT   
  
  
- TTCTAGAGCC GTAAGTTGTA CCACTCCGGA ACCGACACTT GAAACGGAAG TAAGAAGTGG TGTACGGGCT   
  
  
- ACTCTCGCAC TCGTGTCTCT TAGTGGCCCT AGCAGAAGAC TCCCACCATT TTCCCGACTT CGGTTTTCAC   
  
  
- CATTGCAATC ACCTCGTTCT CACATTGTGT TTGAGACGAC GAAAGAACGG AGCGAAACAA CTCTGTAACC   
  
  
- TTATGATGTG TCGCTACAAA CTCAGGTACC TACACTGAAA CGGATCCCTA GTATTTCTGG CCTAGTTACA   
  
  
- ACTCGTCGTA ACAGAACGAG CTCTACAACA CTTGAACCAA CGCACACTTC CTCGACTCTC CCACCTCGCC   
  
  
- GTACTCGAGG AACCCTTTAC CTCCAGCGCC AAGTGTTACC GTCCCAAATT CGGTATGGGA AATTCGTGAG   
  
  
- ACCATTTGTT GTGTTATTCT TGAAACAACT TCTTGATGAC ACTATCCATA CCCGATCTCC TCTCCTTACC   
  
  
- TCCAGAAATA GAACCCACCT ACTTATCTCT AAACCAACGA AGCTCACGGA CCGTCACATC ATTAAT

+     TCA-element

| Site Name | Organism | Position | Strand | Matrix score. | sequence | function |
| --- | --- | --- | --- | --- | --- | --- |
| TCA-element | Brassica oleracea | 3186 | - | 9 | TCAGAAGAGG | cis-acting element involved in salicylic acid responsiveness |
| TCA-element | Nicotiana tabacum | 950 | - | 9 | CCATCTTTTT | cis-acting element involved in salicylic acid responsiveness |
| TCA-element | Nicotiana tabacum | 372 | + | 9 | CCATCTTTTT | cis-acting element involved in salicylic acid responsiveness |
| TCA-element | Nicotiana tabacum | 1762 | - | 9 | CCATCTTTTT | cis-acting element involved in salicylic acid responsiveness |

>HU01G01391.1   
+ +Up\_Stream \_Len000CATTTT AGTAATAAAT TCCCATGTGA GGACCACTGC CTACTGATTC CGAATGGCAT   
  
  
+ TTTAGTAATA AACTCCCATG TGAGGACCAC TTGATGAGGA ATTCAGTTTT ATAATCCACA GGTGTCAAAG   
  
  
+ CCTATTCCTA CTCTCACCAC TCTGCTATCT CTCCCCCCCC CCCCCCCCCG CCCCCCTCAA AATCTGCCTC   
  
  
+ CTCCGTTCTC TCCACAGCAG ATTCCTCAAT CAGGAAAGGT ATGTCAGAGC TCAAAACGAT TCAACATCAA   
  
  
+ AATGGGATAA TTTATTCTGC TTTCAGTTCT GGGTTATTGT CATATGTTCA TTCAGTTCAG TGTTTCTAGC   
  
  
+ ATGATTGCTT TTTGATGCCT TCTTTTTTGT TCTGGGTTGG ACGAACTCTG TCTTTTCTGT GCATAGATTT   
  
  
+ GAGCTCCGAG CTCAATTTTT GGGATTTTTT TAGCTGACTT TTCTGCTTGG TGTTGTGAAA TTGTGATGGG   
  
  
+ TTTTGGCTTT GTTAAACTTG TTTATAACTT TTTCTGTCAA AACTTTATAG TAAATTGTTG CAATTTGAGA   
  
  
+ ATTATGCACT CAGTGGCTCT GTGATTTATC GTCCGCTGGC AAATTTTCTG ATGAAAGGAG ATGATTTATT   
  
  
+ AATTTTTCAG GCATGGTCAA ATTTTCTAGC CTTTCATGTA CATTGGTTGG TGATAGGCTG ATAGCTAAAT   
  
  
+ TATGTCTCAT TTCTCTAAAT TTATTGCCAG ATATGAAATT GGTGGAACAG TTTAAGGGTT TTGGTCGTCT   
  
  
+ TTTTTGCCCT TTTTTTTGGG TCCCCCCCCC GCCCGGGGGG GAACCCGGAG GGCAGAAAAA GTAACATAAC   
  
  
+ TTAAAATTAT CATTGACGTA GTCCTCCAAC CATAATGAAA GCAACTGAAA TAAGAGCATG CTAAGCTAGA   
  
  
+ GAGTAGGCTG CCTTGTCAGC CTCCCTTTTG ACATGAGAAA GATGGGAGAA GTTTAAAATA AAGTTCCTCG   
  
  
+ TCTTTGCATC CCGGACCACA ATTCGGAACT GGGTGAGGAC CTCCTTAGGC TTGTAAACAC AAGTTTCGAA   
  
  
+ CTTAGATTTG CGTACATGTC AATGCTTTAC TGGATAAGCT TAGCTAACAC CCCTCATCCT TCAACCTGTA   
  
  
+ CCTTCATATA AAAAAAAGGA CAATCCGGTG CACAAGCATC CCGCATTCAC GCAAGGTCTG GGGAAGGGCC   
  
  
+ GCACCCCAAA GGGTGAATTG TAGGCAGCCT AACCTGACTT TGTCAGTGGC TAATTCCACG ATTCGAACCC   
  
  
+ ATGATTTTTT GGTTACACGG GAACAACCTT ACCATTGCTC AAAGGCTCCC CTTCTTCAAC CTCTACCTTC   
  
  
+ ATATATGAAT GAAAATCCTT TGGCTAAGAA TTGAAAAGGA AATATGTGGT TATTGGGTTT GGGTTCTTAT   
  
  
+ TCCGTTGGTT GCCCCTTCAC ATTGAATGCA TCCTCTCGCT AAGAACTGAA AAGGACATAT GGGGATGTAT   
  
  
+ AGGTCAAATT TGACTCAATG ATAACTGAGG ATACAAGAAT TCTAGAAGTC TTTATCAGGG GAATTTTTTC   
  
  
+ TTTTTTCCAC TTGTGACTTT TTGATGCCTT GCTTTTTACT CTTCATTGAA TGAGTACCCT TCTTGACAGA   
  
  
+ TTCTTCCTCC CTTTTTTCTT AATTTCTTCT TGTAGTGGAA TATAAGTATG CTTCTAGGTA GTTGGATGCC   
  
  
+ ATGGAATCTC ATCCCTGTGT GTGCATTCTT CCCCTCTCCC GCCGCCCCCC CCCCCCCAAA AAGAAACCAA   
  
  
+ AAAAAAAAAA AAAATGGATG ATTCTTAGAA CTGACCATTT TTCCTTCCTC CTCCCATTTT ATTTTTTTCA   
  
  
+ GGCTGTTTTT CTTCTACCTG TTGCTTTAGC ATCACTCATA AAAACAAGGT GCAAGCGCAT GACATCGGGT   
  
  
+ ACTGAGCTTC TGAGTTTACA TGCATCGTCA TGAAATTCGG TAGACTTTGA AGTAAAGTTT CCCCATCGTA   
  
  
+ ACTGGTGGTG CATACCTGTC TTCACGCCCA TCAGTAGAGT GGTGAAGTGA AGCAATGTCG AATAGTTTGT   
  
  
+ ATTACGAGCC CAAGAGAGAA ACTGATGCAT ATTTTATGCC TCAATGTCAA TCCTTGAACC CCCAGCTCGA   
  
  
+ TTACAACACC ATTGACTATG GAGCTTTTAT GTGCTCAAAA GTTTTCCTGG GTCAGTACTG CACTCTGGAA   
  
  
+ TCATCCTCAG GAACTGGGAC TTGTCCGGTG TCTAGCTCCA CATCAACTCT CAGCTTCTCA TCCAATGGTA   
  
  
+ GCCCTGGATC ACAGCTTGAT TCCAACTCTT ATCCTTCTGA TCAAAATTGC TCTCCTGATA ATGCCAATTC   
  
  
+ CTCATCTTTA AGTCATTCCT GCGTCACAGA TGATGTGGAT GACCTGAAAT ACAGGCTGAG AGAGTTGGAA   
  
  
+ ACAGTGATGT TGGGACCTGA TCCCGATTTT GTCTACGGCA ATAGCAACCA CACATTTGGG GTTGGGATCC   
  
  
+ ATGTAGTGTC ATCAGAGTTT GACAGCTGGG GGAAAGTGAT GGAGATGATC GCTCGAAAGG ATTTGAAACT   
  
  
+ GGTGCTAATT GCCTGTGCAA GAGCAGTTGC TGAAAATGAT CAGTTGTTGG CCCAGTGGCT GATGGATGAA   
  
  
+ TTGAGACAGA TGGTTTCAGT TTCTGGTGAA CCAATTCAAA GATTGGGTGC TTATATGTTG GAAGGGCTCG   
  
  
+ TAGCGAGGCA GGCCTCCTCA GGTAGCTCCA TTTATAAAGC ATTGAGATGC AAGGAACCCG CGAGTGCTGA   
  
  
+ CCTTTTATCT TACATGCACA TACTCTTTGA GGTTTGCCCA TACATCAAAT TTGGCTACAT GTCAGCAAAT   
  
  
+ GGTGCCATTG CAGAAGCAAT GAAGGATGAA AAGAGAGTCC ACATTGTCGA TTTCCAAATT GGGCAGGGAA   
  
  
+ GCCAATGGGT GACCCTAATC CAGGCATTTG CAGCCCGGCC AGGGGGTCCA CCCCACATTC GCATTACCGG   
  
  
+ TATCGATGAC TCCTACTCTG CATATGCTCG TGGAGGTGGG TTGAACATTG TGGGCCATAG GCTCTTGAGG   
  
  
+ TTGGCTCAGT CATTTAAGGT CCCATTTGAG TTCAACGCAG TTGACGTCCC GGCTTGTCAA GTCCTGCTTA   
  
  
+ AAGATCTCGG CATTCAACAT GGTGAGGCCT TGGCTGTGAA CTTTGCCTTC ATTCTTCACC ACATGCCCGA   
  
  
+ TGAGAGCGTG AGCACAGAGA ATCACCGGGA TCGTCTTCTG AGGGTGGTAA AAGGGCTGAA GCCAAAAGTG   
  
  
+ GTAACGTTAG TGGAGCAAGA GTGTAACACA AACTCTGCTG CTTTCTTGCC TCGCTTTGTT GAGACATTGG   
  
  
+ AATACTACAC AGCGATGTTT GAGTCCATGG ATGTGACTTT GCCTAGGGAT CATAAAGACC GGATCAATGT   
  
  
+ TGAGCAGCAT TGTCTTGCTC GAGATGTTGT GAACTTGGTT GCGTGTGAAG GAGCTGAGAG GGTGGAGCGG   
  
  
+ CATGAGCTCC TTGGGAAATG GAGGTCGCGG TTCACAATGG CAGGGTTTAA GCCATACCCT TTAAGCACTC   
  
  
+ TGGTAAACAA CACAATAAGA ACTTTGTTGA AGAACTACTG TGATAGGTAT GGGCTAGAGG AGAGGAATGG   
  
  
+ AGGTCTTTAT CTTGGGTGGA TGAATAGAGA TTTGGTTGCT TCGAGTGCCT GGCAGTGTAG TAATTA  

- +Up\_Stream \_Len000GTAAAA TCATTATTTA AGGGTACACT CCTGGTGACG GATGACTAAG GCTTACCGTA   
  
  
- AAATCATTAT TTGAGGGTAC ACTCCTGGTG AACTACTCCT TAAGTCAAAA TATTAGGTGT CCACAGTTTC   
  
  
- GGATAAGGAT GAGAGTGGTG AGACGATAGA GAGGGGGGGG GGGGGGGGGC GGGGGGAGTT TTAGACGGAG   
  
  
- GAGGCAAGAG AGGTGTCGTC TAAGGAGTTA GTCCTTTCCA TACAGTCTCG AGTTTTGCTA AGTTGTAGTT   
  
  
- TTACCCTATT AAATAAGACG AAAGTCAAGA CCCAATAACA GTATACAAGT AAGTCAAGTC ACAAAGATCG   
  
  
- TACTAACGAA AAACTACGGA AGAAAAAACA AGACCCAACC TGCTTGAGAC AGAAAAGACA CGTATCTAAA   
  
  
- CTCGAGGCTC GAGTTAAAAA CCCTAAAAAA ATCGACTGAA AAGACGAACC ACAACACTTT AACACTACCC   
  
  
- AAAACCGAAA CAATTTGAAC AAATATTGAA AAAGACAGTT TTGAAATATC ATTTAACAAC GTTAAACTCT   
  
  
- TAATACGTGA GTCACCGAGA CACTAAATAG CAGGCGACCG TTTAAAAGAC TACTTTCCTC TACTAAATAA   
  
  
- TTAAAAAGTC CGTACCAGTT TAAAAGATCG GAAAGTACAT GTAACCAACC ACTATCCGAC TATCGATTTA   
  
  
- ATACAGAGTA AAGAGATTTA AATAACGGTC TATACTTTAA CCACCTTGTC AAATTCCCAA AACCAGCAGA   
  
  
- AAAAACGGGA AAAAAAACCC AGGGGGGGGG CGGGCCCCCC CTTGGGCCTC CCGTCTTTTT CATTGTATTG   
  
  
- AATTTTAATA GTAACTGCAT CAGGAGGTTG GTATTACTTT CGTTGACTTT ATTCTCGTAC GATTCGATCT   
  
  
- CTCATCCGAC GGAACAGTCG GAGGGAAAAC TGTACTCTTT CTACCCTCTT CAAATTTTAT TTCAAGGAGC   
  
  
- AGAAACGTAG GGCCTGGTGT TAAGCCTTGA CCCACTCCTG GAGGAATCCG AACATTTGTG TTCAAAGCTT   
  
  
- GAATCTAAAC GCATGTACAG TTACGAAATG ACCTATTCGA ATCGATTGTG GGGAGTAGGA AGTTGGACAT   
  
  
- GGAAGTATAT TTTTTTTCCT GTTAGGCCAC GTGTTCGTAG GGCGTAAGTG CGTTCCAGAC CCCTTCCCGG   
  
  
- CGTGGGGTTT CCCACTTAAC ATCCGTCGGA TTGGACTGAA ACAGTCACCG ATTAAGGTGC TAAGCTTGGG   
  
  
- TACTAAAAAA CCAATGTGCC CTTGTTGGAA TGGTAACGAG TTTCCGAGGG GAAGAAGTTG GAGATGGAAG   
  
  
- TATATACTTA CTTTTAGGAA ACCGATTCTT AACTTTTCCT TTATACACCA ATAACCCAAA CCCAAGAATA   
  
  
- AGGCAACCAA CGGGGAAGTG TAACTTACGT AGGAGAGCGA TTCTTGACTT TTCCTGTATA CCCCTACATA   
  
  
- TCCAGTTTAA ACTGAGTTAC TATTGACTCC TATGTTCTTA AGATCTTCAG AAATAGTCCC CTTAAAAAAG   
  
  
- AAAAAAGGTG AACACTGAAA AACTACGGAA CGAAAAATGA GAAGTAACTT ACTCATGGGA AGAACTGTCT   
  
  
- AAGAAGGAGG GAAAAAAGAA TTAAAGAAGA ACATCACCTT ATATTCATAC GAAGATCCAT CAACCTACGG   
  
  
- TACCTTAGAG TAGGGACACA CACGTAAGAA GGGGAGAGGG CGGCGGGGGG GGGGGGGTTT TTCTTTGGTT   
  
  
- TTTTTTTTTT TTTTACCTAC TAAGAATCTT GACTGGTAAA AAGGAAGGAG GAGGGTAAAA TAAAAAAAGT   
  
  
- CCGACAAAAA GAAGATGGAC AACGAAATCG TAGTGAGTAT TTTTGTTCCA CGTTCGCGTA CTGTAGCCCA   
  
  
- TGACTCGAAG ACTCAAATGT ACGTAGCAGT ACTTTAAGCC ATCTGAAACT TCATTTCAAA GGGGTAGCAT   
  
  
- TGACCACCAC GTATGGACAG AAGTGCGGGT AGTCATCTCA CCACTTCACT TCGTTACAGC TTATCAAACA   
  
  
- TAATGCTCGG GTTCTCTCTT TGACTACGTA TAAAATACGG AGTTACAGTT AGGAACTTGG GGGTCGAGCT   
  
  
- AATGTTGTGG TAACTGATAC CTCGAAAATA CACGAGTTTT CAAAAGGACC CAGTCATGAC GTGAGACCTT   
  
  
- AGTAGGAGTC CTTGACCCTG AACAGGCCAC AGATCGAGGT GTAGTTGAGA GTCGAAGAGT AGGTTACCAT   
  
  
- CGGGACCTAG TGTCGAACTA AGGTTGAGAA TAGGAAGACT AGTTTTAACG AGAGGACTAT TACGGTTAAG   
  
  
- GAGTAGAAAT TCAGTAAGGA CGCAGTGTCT ACTACACCTA CTGGACTTTA TGTCCGACTC TCTCAACCTT   
  
  
- TGTCACTACA ACCCTGGACT AGGGCTAAAA CAGATGCCGT TATCGTTGGT GTGTAAACCC CAACCCTAGG   
  
  
- TACATCACAG TAGTCTCAAA CTGTCGACCC CCTTTCACTA CCTCTACTAG CGAGCTTTCC TAAACTTTGA   
  
  
- CCACGATTAA CGGACACGTT CTCGTCAACG ACTTTTACTA GTCAACAACC GGGTCACCGA CTACCTACTT   
  
  
- AACTCTGTCT ACCAAAGTCA AAGACCACTT GGTTAAGTTT CTAACCCACG AATATACAAC CTTCCCGAGC   
  
  
- ATCGCTCCGT CCGGAGGAGT CCATCGAGGT AAATATTTCG TAACTCTACG TTCCTTGGGC GCTCACGACT   
  
  
- GGAAAATAGA ATGTACGTGT ATGAGAAACT CCAAACGGGT ATGTAGTTTA AACCGATGTA CAGTCGTTTA   
  
  
- CCACGGTAAC GTCTTCGTTA CTTCCTACTT TTCTCTCAGG TGTAACAGCT AAAGGTTTAA CCCGTCCCTT   
  
  
- CGGTTACCCA CTGGGATTAG GTCCGTAAAC GTCGGGCCGG TCCCCCAGGT GGGGTGTAAG CGTAATGGCC   
  
  
- ATAGCTACTG AGGATGAGAC GTATACGAGC ACCTCCACCC AACTTGTAAC ACCCGGTATC CGAGAACTCC   
  
  
- AACCGAGTCA GTAAATTCCA GGGTAAACTC AAGTTGCGTC AACTGCAGGG CCGAACAGTT CAGGACGAAT   
  
  
- TTCTAGAGCC GTAAGTTGTA CCACTCCGGA ACCGACACTT GAAACGGAAG TAAGAAGTGG TGTACGGGCT   
  
  
- ACTCTCGCAC TCGTGTCTCT TAGTGGCCCT AGCAGAAGAC TCCCACCATT TTCCCGACTT CGGTTTTCAC   
  
  
- CATTGCAATC ACCTCGTTCT CACATTGTGT TTGAGACGAC GAAAGAACGG AGCGAAACAA CTCTGTAACC   
  
  
- TTATGATGTG TCGCTACAAA CTCAGGTACC TACACTGAAA CGGATCCCTA GTATTTCTGG CCTAGTTACA   
  
  
- ACTCGTCGTA ACAGAACGAG CTCTACAACA CTTGAACCAA CGCACACTTC CTCGACTCTC CCACCTCGCC   
  
  
- GTACTCGAGG AACCCTTTAC CTCCAGCGCC AAGTGTTACC GTCCCAAATT CGGTATGGGA AATTCGTGAG   
  
  
- ACCATTTGTT GTGTTATTCT TGAAACAACT TCTTGATGAC ACTATCCATA CCCGATCTCC TCTCCTTACC   
  
  
- TCCAGAAATA GAACCCACCT ACTTATCTCT AAACCAACGA AGCTCACGGA CCGTCACATC ATTAAT

+     TCT-motif

| Site Name | Organism | Position | Strand | Matrix score. | sequence | function |
| --- | --- | --- | --- | --- | --- | --- |
| TCT-motif | Arabidopsis thaliana | 2742 | + | 6 | TCTTAC | part of a light responsive element |

>HU01G01391.1   
+ +Up\_Stream \_Len000CATTTT AGTAATAAAT TCCCATGTGA GGACCACTGC CTACTGATTC CGAATGGCAT   
  
  
+ TTTAGTAATA AACTCCCATG TGAGGACCAC TTGATGAGGA ATTCAGTTTT ATAATCCACA GGTGTCAAAG   
  
  
+ CCTATTCCTA CTCTCACCAC TCTGCTATCT CTCCCCCCCC CCCCCCCCCG CCCCCCTCAA AATCTGCCTC   
  
  
+ CTCCGTTCTC TCCACAGCAG ATTCCTCAAT CAGGAAAGGT ATGTCAGAGC TCAAAACGAT TCAACATCAA   
  
  
+ AATGGGATAA TTTATTCTGC TTTCAGTTCT GGGTTATTGT CATATGTTCA TTCAGTTCAG TGTTTCTAGC   
  
  
+ ATGATTGCTT TTTGATGCCT TCTTTTTTGT TCTGGGTTGG ACGAACTCTG TCTTTTCTGT GCATAGATTT   
  
  
+ GAGCTCCGAG CTCAATTTTT GGGATTTTTT TAGCTGACTT TTCTGCTTGG TGTTGTGAAA TTGTGATGGG   
  
  
+ TTTTGGCTTT GTTAAACTTG TTTATAACTT TTTCTGTCAA AACTTTATAG TAAATTGTTG CAATTTGAGA   
  
  
+ ATTATGCACT CAGTGGCTCT GTGATTTATC GTCCGCTGGC AAATTTTCTG ATGAAAGGAG ATGATTTATT   
  
  
+ AATTTTTCAG GCATGGTCAA ATTTTCTAGC CTTTCATGTA CATTGGTTGG TGATAGGCTG ATAGCTAAAT   
  
  
+ TATGTCTCAT TTCTCTAAAT TTATTGCCAG ATATGAAATT GGTGGAACAG TTTAAGGGTT TTGGTCGTCT   
  
  
+ TTTTTGCCCT TTTTTTTGGG TCCCCCCCCC GCCCGGGGGG GAACCCGGAG GGCAGAAAAA GTAACATAAC   
  
  
+ TTAAAATTAT CATTGACGTA GTCCTCCAAC CATAATGAAA GCAACTGAAA TAAGAGCATG CTAAGCTAGA   
  
  
+ GAGTAGGCTG CCTTGTCAGC CTCCCTTTTG ACATGAGAAA GATGGGAGAA GTTTAAAATA AAGTTCCTCG   
  
  
+ TCTTTGCATC CCGGACCACA ATTCGGAACT GGGTGAGGAC CTCCTTAGGC TTGTAAACAC AAGTTTCGAA   
  
  
+ CTTAGATTTG CGTACATGTC AATGCTTTAC TGGATAAGCT TAGCTAACAC CCCTCATCCT TCAACCTGTA   
  
  
+ CCTTCATATA AAAAAAAGGA CAATCCGGTG CACAAGCATC CCGCATTCAC GCAAGGTCTG GGGAAGGGCC   
  
  
+ GCACCCCAAA GGGTGAATTG TAGGCAGCCT AACCTGACTT TGTCAGTGGC TAATTCCACG ATTCGAACCC   
  
  
+ ATGATTTTTT GGTTACACGG GAACAACCTT ACCATTGCTC AAAGGCTCCC CTTCTTCAAC CTCTACCTTC   
  
  
+ ATATATGAAT GAAAATCCTT TGGCTAAGAA TTGAAAAGGA AATATGTGGT TATTGGGTTT GGGTTCTTAT   
  
  
+ TCCGTTGGTT GCCCCTTCAC ATTGAATGCA TCCTCTCGCT AAGAACTGAA AAGGACATAT GGGGATGTAT   
  
  
+ AGGTCAAATT TGACTCAATG ATAACTGAGG ATACAAGAAT TCTAGAAGTC TTTATCAGGG GAATTTTTTC   
  
  
+ TTTTTTCCAC TTGTGACTTT TTGATGCCTT GCTTTTTACT CTTCATTGAA TGAGTACCCT TCTTGACAGA   
  
  
+ TTCTTCCTCC CTTTTTTCTT AATTTCTTCT TGTAGTGGAA TATAAGTATG CTTCTAGGTA GTTGGATGCC   
  
  
+ ATGGAATCTC ATCCCTGTGT GTGCATTCTT CCCCTCTCCC GCCGCCCCCC CCCCCCCAAA AAGAAACCAA   
  
  
+ AAAAAAAAAA AAAATGGATG ATTCTTAGAA CTGACCATTT TTCCTTCCTC CTCCCATTTT ATTTTTTTCA   
  
  
+ GGCTGTTTTT CTTCTACCTG TTGCTTTAGC ATCACTCATA AAAACAAGGT GCAAGCGCAT GACATCGGGT   
  
  
+ ACTGAGCTTC TGAGTTTACA TGCATCGTCA TGAAATTCGG TAGACTTTGA AGTAAAGTTT CCCCATCGTA   
  
  
+ ACTGGTGGTG CATACCTGTC TTCACGCCCA TCAGTAGAGT GGTGAAGTGA AGCAATGTCG AATAGTTTGT   
  
  
+ ATTACGAGCC CAAGAGAGAA ACTGATGCAT ATTTTATGCC TCAATGTCAA TCCTTGAACC CCCAGCTCGA   
  
  
+ TTACAACACC ATTGACTATG GAGCTTTTAT GTGCTCAAAA GTTTTCCTGG GTCAGTACTG CACTCTGGAA   
  
  
+ TCATCCTCAG GAACTGGGAC TTGTCCGGTG TCTAGCTCCA CATCAACTCT CAGCTTCTCA TCCAATGGTA   
  
  
+ GCCCTGGATC ACAGCTTGAT TCCAACTCTT ATCCTTCTGA TCAAAATTGC TCTCCTGATA ATGCCAATTC   
  
  
+ CTCATCTTTA AGTCATTCCT GCGTCACAGA TGATGTGGAT GACCTGAAAT ACAGGCTGAG AGAGTTGGAA   
  
  
+ ACAGTGATGT TGGGACCTGA TCCCGATTTT GTCTACGGCA ATAGCAACCA CACATTTGGG GTTGGGATCC   
  
  
+ ATGTAGTGTC ATCAGAGTTT GACAGCTGGG GGAAAGTGAT GGAGATGATC GCTCGAAAGG ATTTGAAACT   
  
  
+ GGTGCTAATT GCCTGTGCAA GAGCAGTTGC TGAAAATGAT CAGTTGTTGG CCCAGTGGCT GATGGATGAA   
  
  
+ TTGAGACAGA TGGTTTCAGT TTCTGGTGAA CCAATTCAAA GATTGGGTGC TTATATGTTG GAAGGGCTCG   
  
  
+ TAGCGAGGCA GGCCTCCTCA GGTAGCTCCA TTTATAAAGC ATTGAGATGC AAGGAACCCG CGAGTGCTGA   
  
  
+ CCTTTTATCT TACATGCACA TACTCTTTGA GGTTTGCCCA TACATCAAAT TTGGCTACAT GTCAGCAAAT   
  
  
+ GGTGCCATTG CAGAAGCAAT GAAGGATGAA AAGAGAGTCC ACATTGTCGA TTTCCAAATT GGGCAGGGAA   
  
  
+ GCCAATGGGT GACCCTAATC CAGGCATTTG CAGCCCGGCC AGGGGGTCCA CCCCACATTC GCATTACCGG   
  
  
+ TATCGATGAC TCCTACTCTG CATATGCTCG TGGAGGTGGG TTGAACATTG TGGGCCATAG GCTCTTGAGG   
  
  
+ TTGGCTCAGT CATTTAAGGT CCCATTTGAG TTCAACGCAG TTGACGTCCC GGCTTGTCAA GTCCTGCTTA   
  
  
+ AAGATCTCGG CATTCAACAT GGTGAGGCCT TGGCTGTGAA CTTTGCCTTC ATTCTTCACC ACATGCCCGA   
  
  
+ TGAGAGCGTG AGCACAGAGA ATCACCGGGA TCGTCTTCTG AGGGTGGTAA AAGGGCTGAA GCCAAAAGTG   
  
  
+ GTAACGTTAG TGGAGCAAGA GTGTAACACA AACTCTGCTG CTTTCTTGCC TCGCTTTGTT GAGACATTGG   
  
  
+ AATACTACAC AGCGATGTTT GAGTCCATGG ATGTGACTTT GCCTAGGGAT CATAAAGACC GGATCAATGT   
  
  
+ TGAGCAGCAT TGTCTTGCTC GAGATGTTGT GAACTTGGTT GCGTGTGAAG GAGCTGAGAG GGTGGAGCGG   
  
  
+ CATGAGCTCC TTGGGAAATG GAGGTCGCGG TTCACAATGG CAGGGTTTAA GCCATACCCT TTAAGCACTC   
  
  
+ TGGTAAACAA CACAATAAGA ACTTTGTTGA AGAACTACTG TGATAGGTAT GGGCTAGAGG AGAGGAATGG   
  
  
+ AGGTCTTTAT CTTGGGTGGA TGAATAGAGA TTTGGTTGCT TCGAGTGCCT GGCAGTGTAG TAATTA  

- +Up\_Stream \_Len000GTAAAA TCATTATTTA AGGGTACACT CCTGGTGACG GATGACTAAG GCTTACCGTA   
  
  
- AAATCATTAT TTGAGGGTAC ACTCCTGGTG AACTACTCCT TAAGTCAAAA TATTAGGTGT CCACAGTTTC   
  
  
- GGATAAGGAT GAGAGTGGTG AGACGATAGA GAGGGGGGGG GGGGGGGGGC GGGGGGAGTT TTAGACGGAG   
  
  
- GAGGCAAGAG AGGTGTCGTC TAAGGAGTTA GTCCTTTCCA TACAGTCTCG AGTTTTGCTA AGTTGTAGTT   
  
  
- TTACCCTATT AAATAAGACG AAAGTCAAGA CCCAATAACA GTATACAAGT AAGTCAAGTC ACAAAGATCG   
  
  
- TACTAACGAA AAACTACGGA AGAAAAAACA AGACCCAACC TGCTTGAGAC AGAAAAGACA CGTATCTAAA   
  
  
- CTCGAGGCTC GAGTTAAAAA CCCTAAAAAA ATCGACTGAA AAGACGAACC ACAACACTTT AACACTACCC   
  
  
- AAAACCGAAA CAATTTGAAC AAATATTGAA AAAGACAGTT TTGAAATATC ATTTAACAAC GTTAAACTCT   
  
  
- TAATACGTGA GTCACCGAGA CACTAAATAG CAGGCGACCG TTTAAAAGAC TACTTTCCTC TACTAAATAA   
  
  
- TTAAAAAGTC CGTACCAGTT TAAAAGATCG GAAAGTACAT GTAACCAACC ACTATCCGAC TATCGATTTA   
  
  
- ATACAGAGTA AAGAGATTTA AATAACGGTC TATACTTTAA CCACCTTGTC AAATTCCCAA AACCAGCAGA   
  
  
- AAAAACGGGA AAAAAAACCC AGGGGGGGGG CGGGCCCCCC CTTGGGCCTC CCGTCTTTTT CATTGTATTG   
  
  
- AATTTTAATA GTAACTGCAT CAGGAGGTTG GTATTACTTT CGTTGACTTT ATTCTCGTAC GATTCGATCT   
  
  
- CTCATCCGAC GGAACAGTCG GAGGGAAAAC TGTACTCTTT CTACCCTCTT CAAATTTTAT TTCAAGGAGC   
  
  
- AGAAACGTAG GGCCTGGTGT TAAGCCTTGA CCCACTCCTG GAGGAATCCG AACATTTGTG TTCAAAGCTT   
  
  
- GAATCTAAAC GCATGTACAG TTACGAAATG ACCTATTCGA ATCGATTGTG GGGAGTAGGA AGTTGGACAT   
  
  
- GGAAGTATAT TTTTTTTCCT GTTAGGCCAC GTGTTCGTAG GGCGTAAGTG CGTTCCAGAC CCCTTCCCGG   
  
  
- CGTGGGGTTT CCCACTTAAC ATCCGTCGGA TTGGACTGAA ACAGTCACCG ATTAAGGTGC TAAGCTTGGG   
  
  
- TACTAAAAAA CCAATGTGCC CTTGTTGGAA TGGTAACGAG TTTCCGAGGG GAAGAAGTTG GAGATGGAAG   
  
  
- TATATACTTA CTTTTAGGAA ACCGATTCTT AACTTTTCCT TTATACACCA ATAACCCAAA CCCAAGAATA   
  
  
- AGGCAACCAA CGGGGAAGTG TAACTTACGT AGGAGAGCGA TTCTTGACTT TTCCTGTATA CCCCTACATA   
  
  
- TCCAGTTTAA ACTGAGTTAC TATTGACTCC TATGTTCTTA AGATCTTCAG AAATAGTCCC CTTAAAAAAG   
  
  
- AAAAAAGGTG AACACTGAAA AACTACGGAA CGAAAAATGA GAAGTAACTT ACTCATGGGA AGAACTGTCT   
  
  
- AAGAAGGAGG GAAAAAAGAA TTAAAGAAGA ACATCACCTT ATATTCATAC GAAGATCCAT CAACCTACGG   
  
  
- TACCTTAGAG TAGGGACACA CACGTAAGAA GGGGAGAGGG CGGCGGGGGG GGGGGGGTTT TTCTTTGGTT   
  
  
- TTTTTTTTTT TTTTACCTAC TAAGAATCTT GACTGGTAAA AAGGAAGGAG GAGGGTAAAA TAAAAAAAGT   
  
  
- CCGACAAAAA GAAGATGGAC AACGAAATCG TAGTGAGTAT TTTTGTTCCA CGTTCGCGTA CTGTAGCCCA   
  
  
- TGACTCGAAG ACTCAAATGT ACGTAGCAGT ACTTTAAGCC ATCTGAAACT TCATTTCAAA GGGGTAGCAT   
  
  
- TGACCACCAC GTATGGACAG AAGTGCGGGT AGTCATCTCA CCACTTCACT TCGTTACAGC TTATCAAACA   
  
  
- TAATGCTCGG GTTCTCTCTT TGACTACGTA TAAAATACGG AGTTACAGTT AGGAACTTGG GGGTCGAGCT   
  
  
- AATGTTGTGG TAACTGATAC CTCGAAAATA CACGAGTTTT CAAAAGGACC CAGTCATGAC GTGAGACCTT   
  
  
- AGTAGGAGTC CTTGACCCTG AACAGGCCAC AGATCGAGGT GTAGTTGAGA GTCGAAGAGT AGGTTACCAT   
  
  
- CGGGACCTAG TGTCGAACTA AGGTTGAGAA TAGGAAGACT AGTTTTAACG AGAGGACTAT TACGGTTAAG   
  
  
- GAGTAGAAAT TCAGTAAGGA CGCAGTGTCT ACTACACCTA CTGGACTTTA TGTCCGACTC TCTCAACCTT   
  
  
- TGTCACTACA ACCCTGGACT AGGGCTAAAA CAGATGCCGT TATCGTTGGT GTGTAAACCC CAACCCTAGG   
  
  
- TACATCACAG TAGTCTCAAA CTGTCGACCC CCTTTCACTA CCTCTACTAG CGAGCTTTCC TAAACTTTGA   
  
  
- CCACGATTAA CGGACACGTT CTCGTCAACG ACTTTTACTA GTCAACAACC GGGTCACCGA CTACCTACTT   
  
  
- AACTCTGTCT ACCAAAGTCA AAGACCACTT GGTTAAGTTT CTAACCCACG AATATACAAC CTTCCCGAGC   
  
  
- ATCGCTCCGT CCGGAGGAGT CCATCGAGGT AAATATTTCG TAACTCTACG TTCCTTGGGC GCTCACGACT   
  
  
- GGAAAATAGA ATGTACGTGT ATGAGAAACT CCAAACGGGT ATGTAGTTTA AACCGATGTA CAGTCGTTTA   
  
  
- CCACGGTAAC GTCTTCGTTA CTTCCTACTT TTCTCTCAGG TGTAACAGCT AAAGGTTTAA CCCGTCCCTT   
  
  
- CGGTTACCCA CTGGGATTAG GTCCGTAAAC GTCGGGCCGG TCCCCCAGGT GGGGTGTAAG CGTAATGGCC   
  
  
- ATAGCTACTG AGGATGAGAC GTATACGAGC ACCTCCACCC AACTTGTAAC ACCCGGTATC CGAGAACTCC   
  
  
- AACCGAGTCA GTAAATTCCA GGGTAAACTC AAGTTGCGTC AACTGCAGGG CCGAACAGTT CAGGACGAAT   
  
  
- TTCTAGAGCC GTAAGTTGTA CCACTCCGGA ACCGACACTT GAAACGGAAG TAAGAAGTGG TGTACGGGCT   
  
  
- ACTCTCGCAC TCGTGTCTCT TAGTGGCCCT AGCAGAAGAC TCCCACCATT TTCCCGACTT CGGTTTTCAC   
  
  
- CATTGCAATC ACCTCGTTCT CACATTGTGT TTGAGACGAC GAAAGAACGG AGCGAAACAA CTCTGTAACC   
  
  
- TTATGATGTG TCGCTACAAA CTCAGGTACC TACACTGAAA CGGATCCCTA GTATTTCTGG CCTAGTTACA   
  
  
- ACTCGTCGTA ACAGAACGAG CTCTACAACA CTTGAACCAA CGCACACTTC CTCGACTCTC CCACCTCGCC   
  
  
- GTACTCGAGG AACCCTTTAC CTCCAGCGCC AAGTGTTACC GTCCCAAATT CGGTATGGGA AATTCGTGAG   
  
  
- ACCATTTGTT GTGTTATTCT TGAAACAACT TCTTGATGAC ACTATCCATA CCCGATCTCC TCTCCTTACC   
  
  
- TCCAGAAATA GAACCCACCT ACTTATCTCT AAACCAACGA AGCTCACGGA CCGTCACATC ATTAAT

+     TGACG-motif

| Site Name | Organism | Position | Strand | Matrix score. | sequence | function |
| --- | --- | --- | --- | --- | --- | --- |
| TGACG-motif | Hordeum vulgare | 1920 | - | 5 | TGACG | cis-acting regulatory element involved in the MeJA-responsiveness |
| TGACG-motif | Hordeum vulgare | 858 | + | 5 | TGACG | cis-acting regulatory element involved in the MeJA-responsiveness |
| TGACG-motif | Hordeum vulgare | 3056 | + | 5 | TGACG | cis-acting regulatory element involved in the MeJA-responsiveness |
| TGACG-motif | Hordeum vulgare | 2336 | - | 5 | TGACG | cis-acting regulatory element involved in the MeJA-responsiveness |

>HU01G01391.1   
+ +Up\_Stream \_Len000CATTTT AGTAATAAAT TCCCATGTGA GGACCACTGC CTACTGATTC CGAATGGCAT   
  
  
+ TTTAGTAATA AACTCCCATG TGAGGACCAC TTGATGAGGA ATTCAGTTTT ATAATCCACA GGTGTCAAAG   
  
  
+ CCTATTCCTA CTCTCACCAC TCTGCTATCT CTCCCCCCCC CCCCCCCCCG CCCCCCTCAA AATCTGCCTC   
  
  
+ CTCCGTTCTC TCCACAGCAG ATTCCTCAAT CAGGAAAGGT ATGTCAGAGC TCAAAACGAT TCAACATCAA   
  
  
+ AATGGGATAA TTTATTCTGC TTTCAGTTCT GGGTTATTGT CATATGTTCA TTCAGTTCAG TGTTTCTAGC   
  
  
+ ATGATTGCTT TTTGATGCCT TCTTTTTTGT TCTGGGTTGG ACGAACTCTG TCTTTTCTGT GCATAGATTT   
  
  
+ GAGCTCCGAG CTCAATTTTT GGGATTTTTT TAGCTGACTT TTCTGCTTGG TGTTGTGAAA TTGTGATGGG   
  
  
+ TTTTGGCTTT GTTAAACTTG TTTATAACTT TTTCTGTCAA AACTTTATAG TAAATTGTTG CAATTTGAGA   
  
  
+ ATTATGCACT CAGTGGCTCT GTGATTTATC GTCCGCTGGC AAATTTTCTG ATGAAAGGAG ATGATTTATT   
  
  
+ AATTTTTCAG GCATGGTCAA ATTTTCTAGC CTTTCATGTA CATTGGTTGG TGATAGGCTG ATAGCTAAAT   
  
  
+ TATGTCTCAT TTCTCTAAAT TTATTGCCAG ATATGAAATT GGTGGAACAG TTTAAGGGTT TTGGTCGTCT   
  
  
+ TTTTTGCCCT TTTTTTTGGG TCCCCCCCCC GCCCGGGGGG GAACCCGGAG GGCAGAAAAA GTAACATAAC   
  
  
+ TTAAAATTAT CATTGACGTA GTCCTCCAAC CATAATGAAA GCAACTGAAA TAAGAGCATG CTAAGCTAGA   
  
  
+ GAGTAGGCTG CCTTGTCAGC CTCCCTTTTG ACATGAGAAA GATGGGAGAA GTTTAAAATA AAGTTCCTCG   
  
  
+ TCTTTGCATC CCGGACCACA ATTCGGAACT GGGTGAGGAC CTCCTTAGGC TTGTAAACAC AAGTTTCGAA   
  
  
+ CTTAGATTTG CGTACATGTC AATGCTTTAC TGGATAAGCT TAGCTAACAC CCCTCATCCT TCAACCTGTA   
  
  
+ CCTTCATATA AAAAAAAGGA CAATCCGGTG CACAAGCATC CCGCATTCAC GCAAGGTCTG GGGAAGGGCC   
  
  
+ GCACCCCAAA GGGTGAATTG TAGGCAGCCT AACCTGACTT TGTCAGTGGC TAATTCCACG ATTCGAACCC   
  
  
+ ATGATTTTTT GGTTACACGG GAACAACCTT ACCATTGCTC AAAGGCTCCC CTTCTTCAAC CTCTACCTTC   
  
  
+ ATATATGAAT GAAAATCCTT TGGCTAAGAA TTGAAAAGGA AATATGTGGT TATTGGGTTT GGGTTCTTAT   
  
  
+ TCCGTTGGTT GCCCCTTCAC ATTGAATGCA TCCTCTCGCT AAGAACTGAA AAGGACATAT GGGGATGTAT   
  
  
+ AGGTCAAATT TGACTCAATG ATAACTGAGG ATACAAGAAT TCTAGAAGTC TTTATCAGGG GAATTTTTTC   
  
  
+ TTTTTTCCAC TTGTGACTTT TTGATGCCTT GCTTTTTACT CTTCATTGAA TGAGTACCCT TCTTGACAGA   
  
  
+ TTCTTCCTCC CTTTTTTCTT AATTTCTTCT TGTAGTGGAA TATAAGTATG CTTCTAGGTA GTTGGATGCC   
  
  
+ ATGGAATCTC ATCCCTGTGT GTGCATTCTT CCCCTCTCCC GCCGCCCCCC CCCCCCCAAA AAGAAACCAA   
  
  
+ AAAAAAAAAA AAAATGGATG ATTCTTAGAA CTGACCATTT TTCCTTCCTC CTCCCATTTT ATTTTTTTCA   
  
  
+ GGCTGTTTTT CTTCTACCTG TTGCTTTAGC ATCACTCATA AAAACAAGGT GCAAGCGCAT GACATCGGGT   
  
  
+ ACTGAGCTTC TGAGTTTACA TGCATCGTCA TGAAATTCGG TAGACTTTGA AGTAAAGTTT CCCCATCGTA   
  
  
+ ACTGGTGGTG CATACCTGTC TTCACGCCCA TCAGTAGAGT GGTGAAGTGA AGCAATGTCG AATAGTTTGT   
  
  
+ ATTACGAGCC CAAGAGAGAA ACTGATGCAT ATTTTATGCC TCAATGTCAA TCCTTGAACC CCCAGCTCGA   
  
  
+ TTACAACACC ATTGACTATG GAGCTTTTAT GTGCTCAAAA GTTTTCCTGG GTCAGTACTG CACTCTGGAA   
  
  
+ TCATCCTCAG GAACTGGGAC TTGTCCGGTG TCTAGCTCCA CATCAACTCT CAGCTTCTCA TCCAATGGTA   
  
  
+ GCCCTGGATC ACAGCTTGAT TCCAACTCTT ATCCTTCTGA TCAAAATTGC TCTCCTGATA ATGCCAATTC   
  
  
+ CTCATCTTTA AGTCATTCCT GCGTCACAGA TGATGTGGAT GACCTGAAAT ACAGGCTGAG AGAGTTGGAA   
  
  
+ ACAGTGATGT TGGGACCTGA TCCCGATTTT GTCTACGGCA ATAGCAACCA CACATTTGGG GTTGGGATCC   
  
  
+ ATGTAGTGTC ATCAGAGTTT GACAGCTGGG GGAAAGTGAT GGAGATGATC GCTCGAAAGG ATTTGAAACT   
  
  
+ GGTGCTAATT GCCTGTGCAA GAGCAGTTGC TGAAAATGAT CAGTTGTTGG CCCAGTGGCT GATGGATGAA   
  
  
+ TTGAGACAGA TGGTTTCAGT TTCTGGTGAA CCAATTCAAA GATTGGGTGC TTATATGTTG GAAGGGCTCG   
  
  
+ TAGCGAGGCA GGCCTCCTCA GGTAGCTCCA TTTATAAAGC ATTGAGATGC AAGGAACCCG CGAGTGCTGA   
  
  
+ CCTTTTATCT TACATGCACA TACTCTTTGA GGTTTGCCCA TACATCAAAT TTGGCTACAT GTCAGCAAAT   
  
  
+ GGTGCCATTG CAGAAGCAAT GAAGGATGAA AAGAGAGTCC ACATTGTCGA TTTCCAAATT GGGCAGGGAA   
  
  
+ GCCAATGGGT GACCCTAATC CAGGCATTTG CAGCCCGGCC AGGGGGTCCA CCCCACATTC GCATTACCGG   
  
  
+ TATCGATGAC TCCTACTCTG CATATGCTCG TGGAGGTGGG TTGAACATTG TGGGCCATAG GCTCTTGAGG   
  
  
+ TTGGCTCAGT CATTTAAGGT CCCATTTGAG TTCAACGCAG TTGACGTCCC GGCTTGTCAA GTCCTGCTTA   
  
  
+ AAGATCTCGG CATTCAACAT GGTGAGGCCT TGGCTGTGAA CTTTGCCTTC ATTCTTCACC ACATGCCCGA   
  
  
+ TGAGAGCGTG AGCACAGAGA ATCACCGGGA TCGTCTTCTG AGGGTGGTAA AAGGGCTGAA GCCAAAAGTG   
  
  
+ GTAACGTTAG TGGAGCAAGA GTGTAACACA AACTCTGCTG CTTTCTTGCC TCGCTTTGTT GAGACATTGG   
  
  
+ AATACTACAC AGCGATGTTT GAGTCCATGG ATGTGACTTT GCCTAGGGAT CATAAAGACC GGATCAATGT   
  
  
+ TGAGCAGCAT TGTCTTGCTC GAGATGTTGT GAACTTGGTT GCGTGTGAAG GAGCTGAGAG GGTGGAGCGG   
  
  
+ CATGAGCTCC TTGGGAAATG GAGGTCGCGG TTCACAATGG CAGGGTTTAA GCCATACCCT TTAAGCACTC   
  
  
+ TGGTAAACAA CACAATAAGA ACTTTGTTGA AGAACTACTG TGATAGGTAT GGGCTAGAGG AGAGGAATGG   
  
  
+ AGGTCTTTAT CTTGGGTGGA TGAATAGAGA TTTGGTTGCT TCGAGTGCCT GGCAGTGTAG TAATTA  

- +Up\_Stream \_Len000GTAAAA TCATTATTTA AGGGTACACT CCTGGTGACG GATGACTAAG GCTTACCGTA   
  
  
- AAATCATTAT TTGAGGGTAC ACTCCTGGTG AACTACTCCT TAAGTCAAAA TATTAGGTGT CCACAGTTTC   
  
  
- GGATAAGGAT GAGAGTGGTG AGACGATAGA GAGGGGGGGG GGGGGGGGGC GGGGGGAGTT TTAGACGGAG   
  
  
- GAGGCAAGAG AGGTGTCGTC TAAGGAGTTA GTCCTTTCCA TACAGTCTCG AGTTTTGCTA AGTTGTAGTT   
  
  
- TTACCCTATT AAATAAGACG AAAGTCAAGA CCCAATAACA GTATACAAGT AAGTCAAGTC ACAAAGATCG   
  
  
- TACTAACGAA AAACTACGGA AGAAAAAACA AGACCCAACC TGCTTGAGAC AGAAAAGACA CGTATCTAAA   
  
  
- CTCGAGGCTC GAGTTAAAAA CCCTAAAAAA ATCGACTGAA AAGACGAACC ACAACACTTT AACACTACCC   
  
  
- AAAACCGAAA CAATTTGAAC AAATATTGAA AAAGACAGTT TTGAAATATC ATTTAACAAC GTTAAACTCT   
  
  
- TAATACGTGA GTCACCGAGA CACTAAATAG CAGGCGACCG TTTAAAAGAC TACTTTCCTC TACTAAATAA   
  
  
- TTAAAAAGTC CGTACCAGTT TAAAAGATCG GAAAGTACAT GTAACCAACC ACTATCCGAC TATCGATTTA   
  
  
- ATACAGAGTA AAGAGATTTA AATAACGGTC TATACTTTAA CCACCTTGTC AAATTCCCAA AACCAGCAGA   
  
  
- AAAAACGGGA AAAAAAACCC AGGGGGGGGG CGGGCCCCCC CTTGGGCCTC CCGTCTTTTT CATTGTATTG   
  
  
- AATTTTAATA GTAACTGCAT CAGGAGGTTG GTATTACTTT CGTTGACTTT ATTCTCGTAC GATTCGATCT   
  
  
- CTCATCCGAC GGAACAGTCG GAGGGAAAAC TGTACTCTTT CTACCCTCTT CAAATTTTAT TTCAAGGAGC   
  
  
- AGAAACGTAG GGCCTGGTGT TAAGCCTTGA CCCACTCCTG GAGGAATCCG AACATTTGTG TTCAAAGCTT   
  
  
- GAATCTAAAC GCATGTACAG TTACGAAATG ACCTATTCGA ATCGATTGTG GGGAGTAGGA AGTTGGACAT   
  
  
- GGAAGTATAT TTTTTTTCCT GTTAGGCCAC GTGTTCGTAG GGCGTAAGTG CGTTCCAGAC CCCTTCCCGG   
  
  
- CGTGGGGTTT CCCACTTAAC ATCCGTCGGA TTGGACTGAA ACAGTCACCG ATTAAGGTGC TAAGCTTGGG   
  
  
- TACTAAAAAA CCAATGTGCC CTTGTTGGAA TGGTAACGAG TTTCCGAGGG GAAGAAGTTG GAGATGGAAG   
  
  
- TATATACTTA CTTTTAGGAA ACCGATTCTT AACTTTTCCT TTATACACCA ATAACCCAAA CCCAAGAATA   
  
  
- AGGCAACCAA CGGGGAAGTG TAACTTACGT AGGAGAGCGA TTCTTGACTT TTCCTGTATA CCCCTACATA   
  
  
- TCCAGTTTAA ACTGAGTTAC TATTGACTCC TATGTTCTTA AGATCTTCAG AAATAGTCCC CTTAAAAAAG   
  
  
- AAAAAAGGTG AACACTGAAA AACTACGGAA CGAAAAATGA GAAGTAACTT ACTCATGGGA AGAACTGTCT   
  
  
- AAGAAGGAGG GAAAAAAGAA TTAAAGAAGA ACATCACCTT ATATTCATAC GAAGATCCAT CAACCTACGG   
  
  
- TACCTTAGAG TAGGGACACA CACGTAAGAA GGGGAGAGGG CGGCGGGGGG GGGGGGGTTT TTCTTTGGTT   
  
  
- TTTTTTTTTT TTTTACCTAC TAAGAATCTT GACTGGTAAA AAGGAAGGAG GAGGGTAAAA TAAAAAAAGT   
  
  
- CCGACAAAAA GAAGATGGAC AACGAAATCG TAGTGAGTAT TTTTGTTCCA CGTTCGCGTA CTGTAGCCCA   
  
  
- TGACTCGAAG ACTCAAATGT ACGTAGCAGT ACTTTAAGCC ATCTGAAACT TCATTTCAAA GGGGTAGCAT   
  
  
- TGACCACCAC GTATGGACAG AAGTGCGGGT AGTCATCTCA CCACTTCACT TCGTTACAGC TTATCAAACA   
  
  
- TAATGCTCGG GTTCTCTCTT TGACTACGTA TAAAATACGG AGTTACAGTT AGGAACTTGG GGGTCGAGCT   
  
  
- AATGTTGTGG TAACTGATAC CTCGAAAATA CACGAGTTTT CAAAAGGACC CAGTCATGAC GTGAGACCTT   
  
  
- AGTAGGAGTC CTTGACCCTG AACAGGCCAC AGATCGAGGT GTAGTTGAGA GTCGAAGAGT AGGTTACCAT   
  
  
- CGGGACCTAG TGTCGAACTA AGGTTGAGAA TAGGAAGACT AGTTTTAACG AGAGGACTAT TACGGTTAAG   
  
  
- GAGTAGAAAT TCAGTAAGGA CGCAGTGTCT ACTACACCTA CTGGACTTTA TGTCCGACTC TCTCAACCTT   
  
  
- TGTCACTACA ACCCTGGACT AGGGCTAAAA CAGATGCCGT TATCGTTGGT GTGTAAACCC CAACCCTAGG   
  
  
- TACATCACAG TAGTCTCAAA CTGTCGACCC CCTTTCACTA CCTCTACTAG CGAGCTTTCC TAAACTTTGA   
  
  
- CCACGATTAA CGGACACGTT CTCGTCAACG ACTTTTACTA GTCAACAACC GGGTCACCGA CTACCTACTT   
  
  
- AACTCTGTCT ACCAAAGTCA AAGACCACTT GGTTAAGTTT CTAACCCACG AATATACAAC CTTCCCGAGC   
  
  
- ATCGCTCCGT CCGGAGGAGT CCATCGAGGT AAATATTTCG TAACTCTACG TTCCTTGGGC GCTCACGACT   
  
  
- GGAAAATAGA ATGTACGTGT ATGAGAAACT CCAAACGGGT ATGTAGTTTA AACCGATGTA CAGTCGTTTA   
  
  
- CCACGGTAAC GTCTTCGTTA CTTCCTACTT TTCTCTCAGG TGTAACAGCT AAAGGTTTAA CCCGTCCCTT   
  
  
- CGGTTACCCA CTGGGATTAG GTCCGTAAAC GTCGGGCCGG TCCCCCAGGT GGGGTGTAAG CGTAATGGCC   
  
  
- ATAGCTACTG AGGATGAGAC GTATACGAGC ACCTCCACCC AACTTGTAAC ACCCGGTATC CGAGAACTCC   
  
  
- AACCGAGTCA GTAAATTCCA GGGTAAACTC AAGTTGCGTC AACTGCAGGG CCGAACAGTT CAGGACGAAT   
  
  
- TTCTAGAGCC GTAAGTTGTA CCACTCCGGA ACCGACACTT GAAACGGAAG TAAGAAGTGG TGTACGGGCT   
  
  
- ACTCTCGCAC TCGTGTCTCT TAGTGGCCCT AGCAGAAGAC TCCCACCATT TTCCCGACTT CGGTTTTCAC   
  
  
- CATTGCAATC ACCTCGTTCT CACATTGTGT TTGAGACGAC GAAAGAACGG AGCGAAACAA CTCTGTAACC   
  
  
- TTATGATGTG TCGCTACAAA CTCAGGTACC TACACTGAAA CGGATCCCTA GTATTTCTGG CCTAGTTACA   
  
  
- ACTCGTCGTA ACAGAACGAG CTCTACAACA CTTGAACCAA CGCACACTTC CTCGACTCTC CCACCTCGCC   
  
  
- GTACTCGAGG AACCCTTTAC CTCCAGCGCC AAGTGTTACC GTCCCAAATT CGGTATGGGA AATTCGTGAG   
  
  
- ACCATTTGTT GTGTTATTCT TGAAACAACT TCTTGATGAC ACTATCCATA CCCGATCTCC TCTCCTTACC   
  
  
- TCCAGAAATA GAACCCACCT ACTTATCTCT AAACCAACGA AGCTCACGGA CCGTCACATC ATTAAT

+     Unnamed\_\_1

| Site Name | Organism | Position | Strand | Matrix score. | sequence | function |
| --- | --- | --- | --- | --- | --- | --- |
| Unnamed\_\_1 | Zea mays | 1250 | - | 5 | CGTGG |  |
| Unnamed\_\_1 | Zea mays | 2973 | + | 5 | CGTGG |  |

>HU01G01391.1   
+ +Up\_Stream \_Len000CATTTT AGTAATAAAT TCCCATGTGA GGACCACTGC CTACTGATTC CGAATGGCAT   
  
  
+ TTTAGTAATA AACTCCCATG TGAGGACCAC TTGATGAGGA ATTCAGTTTT ATAATCCACA GGTGTCAAAG   
  
  
+ CCTATTCCTA CTCTCACCAC TCTGCTATCT CTCCCCCCCC CCCCCCCCCG CCCCCCTCAA AATCTGCCTC   
  
  
+ CTCCGTTCTC TCCACAGCAG ATTCCTCAAT CAGGAAAGGT ATGTCAGAGC TCAAAACGAT TCAACATCAA   
  
  
+ AATGGGATAA TTTATTCTGC TTTCAGTTCT GGGTTATTGT CATATGTTCA TTCAGTTCAG TGTTTCTAGC   
  
  
+ ATGATTGCTT TTTGATGCCT TCTTTTTTGT TCTGGGTTGG ACGAACTCTG TCTTTTCTGT GCATAGATTT   
  
  
+ GAGCTCCGAG CTCAATTTTT GGGATTTTTT TAGCTGACTT TTCTGCTTGG TGTTGTGAAA TTGTGATGGG   
  
  
+ TTTTGGCTTT GTTAAACTTG TTTATAACTT TTTCTGTCAA AACTTTATAG TAAATTGTTG CAATTTGAGA   
  
  
+ ATTATGCACT CAGTGGCTCT GTGATTTATC GTCCGCTGGC AAATTTTCTG ATGAAAGGAG ATGATTTATT   
  
  
+ AATTTTTCAG GCATGGTCAA ATTTTCTAGC CTTTCATGTA CATTGGTTGG TGATAGGCTG ATAGCTAAAT   
  
  
+ TATGTCTCAT TTCTCTAAAT TTATTGCCAG ATATGAAATT GGTGGAACAG TTTAAGGGTT TTGGTCGTCT   
  
  
+ TTTTTGCCCT TTTTTTTGGG TCCCCCCCCC GCCCGGGGGG GAACCCGGAG GGCAGAAAAA GTAACATAAC   
  
  
+ TTAAAATTAT CATTGACGTA GTCCTCCAAC CATAATGAAA GCAACTGAAA TAAGAGCATG CTAAGCTAGA   
  
  
+ GAGTAGGCTG CCTTGTCAGC CTCCCTTTTG ACATGAGAAA GATGGGAGAA GTTTAAAATA AAGTTCCTCG   
  
  
+ TCTTTGCATC CCGGACCACA ATTCGGAACT GGGTGAGGAC CTCCTTAGGC TTGTAAACAC AAGTTTCGAA   
  
  
+ CTTAGATTTG CGTACATGTC AATGCTTTAC TGGATAAGCT TAGCTAACAC CCCTCATCCT TCAACCTGTA   
  
  
+ CCTTCATATA AAAAAAAGGA CAATCCGGTG CACAAGCATC CCGCATTCAC GCAAGGTCTG GGGAAGGGCC   
  
  
+ GCACCCCAAA GGGTGAATTG TAGGCAGCCT AACCTGACTT TGTCAGTGGC TAATTCCACG ATTCGAACCC   
  
  
+ ATGATTTTTT GGTTACACGG GAACAACCTT ACCATTGCTC AAAGGCTCCC CTTCTTCAAC CTCTACCTTC   
  
  
+ ATATATGAAT GAAAATCCTT TGGCTAAGAA TTGAAAAGGA AATATGTGGT TATTGGGTTT GGGTTCTTAT   
  
  
+ TCCGTTGGTT GCCCCTTCAC ATTGAATGCA TCCTCTCGCT AAGAACTGAA AAGGACATAT GGGGATGTAT   
  
  
+ AGGTCAAATT TGACTCAATG ATAACTGAGG ATACAAGAAT TCTAGAAGTC TTTATCAGGG GAATTTTTTC   
  
  
+ TTTTTTCCAC TTGTGACTTT TTGATGCCTT GCTTTTTACT CTTCATTGAA TGAGTACCCT TCTTGACAGA   
  
  
+ TTCTTCCTCC CTTTTTTCTT AATTTCTTCT TGTAGTGGAA TATAAGTATG CTTCTAGGTA GTTGGATGCC   
  
  
+ ATGGAATCTC ATCCCTGTGT GTGCATTCTT CCCCTCTCCC GCCGCCCCCC CCCCCCCAAA AAGAAACCAA   
  
  
+ AAAAAAAAAA AAAATGGATG ATTCTTAGAA CTGACCATTT TTCCTTCCTC CTCCCATTTT ATTTTTTTCA   
  
  
+ GGCTGTTTTT CTTCTACCTG TTGCTTTAGC ATCACTCATA AAAACAAGGT GCAAGCGCAT GACATCGGGT   
  
  
+ ACTGAGCTTC TGAGTTTACA TGCATCGTCA TGAAATTCGG TAGACTTTGA AGTAAAGTTT CCCCATCGTA   
  
  
+ ACTGGTGGTG CATACCTGTC TTCACGCCCA TCAGTAGAGT GGTGAAGTGA AGCAATGTCG AATAGTTTGT   
  
  
+ ATTACGAGCC CAAGAGAGAA ACTGATGCAT ATTTTATGCC TCAATGTCAA TCCTTGAACC CCCAGCTCGA   
  
  
+ TTACAACACC ATTGACTATG GAGCTTTTAT GTGCTCAAAA GTTTTCCTGG GTCAGTACTG CACTCTGGAA   
  
  
+ TCATCCTCAG GAACTGGGAC TTGTCCGGTG TCTAGCTCCA CATCAACTCT CAGCTTCTCA TCCAATGGTA   
  
  
+ GCCCTGGATC ACAGCTTGAT TCCAACTCTT ATCCTTCTGA TCAAAATTGC TCTCCTGATA ATGCCAATTC   
  
  
+ CTCATCTTTA AGTCATTCCT GCGTCACAGA TGATGTGGAT GACCTGAAAT ACAGGCTGAG AGAGTTGGAA   
  
  
+ ACAGTGATGT TGGGACCTGA TCCCGATTTT GTCTACGGCA ATAGCAACCA CACATTTGGG GTTGGGATCC   
  
  
+ ATGTAGTGTC ATCAGAGTTT GACAGCTGGG GGAAAGTGAT GGAGATGATC GCTCGAAAGG ATTTGAAACT   
  
  
+ GGTGCTAATT GCCTGTGCAA GAGCAGTTGC TGAAAATGAT CAGTTGTTGG CCCAGTGGCT GATGGATGAA   
  
  
+ TTGAGACAGA TGGTTTCAGT TTCTGGTGAA CCAATTCAAA GATTGGGTGC TTATATGTTG GAAGGGCTCG   
  
  
+ TAGCGAGGCA GGCCTCCTCA GGTAGCTCCA TTTATAAAGC ATTGAGATGC AAGGAACCCG CGAGTGCTGA   
  
  
+ CCTTTTATCT TACATGCACA TACTCTTTGA GGTTTGCCCA TACATCAAAT TTGGCTACAT GTCAGCAAAT   
  
  
+ GGTGCCATTG CAGAAGCAAT GAAGGATGAA AAGAGAGTCC ACATTGTCGA TTTCCAAATT GGGCAGGGAA   
  
  
+ GCCAATGGGT GACCCTAATC CAGGCATTTG CAGCCCGGCC AGGGGGTCCA CCCCACATTC GCATTACCGG   
  
  
+ TATCGATGAC TCCTACTCTG CATATGCTCG TGGAGGTGGG TTGAACATTG TGGGCCATAG GCTCTTGAGG   
  
  
+ TTGGCTCAGT CATTTAAGGT CCCATTTGAG TTCAACGCAG TTGACGTCCC GGCTTGTCAA GTCCTGCTTA   
  
  
+ AAGATCTCGG CATTCAACAT GGTGAGGCCT TGGCTGTGAA CTTTGCCTTC ATTCTTCACC ACATGCCCGA   
  
  
+ TGAGAGCGTG AGCACAGAGA ATCACCGGGA TCGTCTTCTG AGGGTGGTAA AAGGGCTGAA GCCAAAAGTG   
  
  
+ GTAACGTTAG TGGAGCAAGA GTGTAACACA AACTCTGCTG CTTTCTTGCC TCGCTTTGTT GAGACATTGG   
  
  
+ AATACTACAC AGCGATGTTT GAGTCCATGG ATGTGACTTT GCCTAGGGAT CATAAAGACC GGATCAATGT   
  
  
+ TGAGCAGCAT TGTCTTGCTC GAGATGTTGT GAACTTGGTT GCGTGTGAAG GAGCTGAGAG GGTGGAGCGG   
  
  
+ CATGAGCTCC TTGGGAAATG GAGGTCGCGG TTCACAATGG CAGGGTTTAA GCCATACCCT TTAAGCACTC   
  
  
+ TGGTAAACAA CACAATAAGA ACTTTGTTGA AGAACTACTG TGATAGGTAT GGGCTAGAGG AGAGGAATGG   
  
  
+ AGGTCTTTAT CTTGGGTGGA TGAATAGAGA TTTGGTTGCT TCGAGTGCCT GGCAGTGTAG TAATTA  

- +Up\_Stream \_Len000GTAAAA TCATTATTTA AGGGTACACT CCTGGTGACG GATGACTAAG GCTTACCGTA   
  
  
- AAATCATTAT TTGAGGGTAC ACTCCTGGTG AACTACTCCT TAAGTCAAAA TATTAGGTGT CCACAGTTTC   
  
  
- GGATAAGGAT GAGAGTGGTG AGACGATAGA GAGGGGGGGG GGGGGGGGGC GGGGGGAGTT TTAGACGGAG   
  
  
- GAGGCAAGAG AGGTGTCGTC TAAGGAGTTA GTCCTTTCCA TACAGTCTCG AGTTTTGCTA AGTTGTAGTT   
  
  
- TTACCCTATT AAATAAGACG AAAGTCAAGA CCCAATAACA GTATACAAGT AAGTCAAGTC ACAAAGATCG   
  
  
- TACTAACGAA AAACTACGGA AGAAAAAACA AGACCCAACC TGCTTGAGAC AGAAAAGACA CGTATCTAAA   
  
  
- CTCGAGGCTC GAGTTAAAAA CCCTAAAAAA ATCGACTGAA AAGACGAACC ACAACACTTT AACACTACCC   
  
  
- AAAACCGAAA CAATTTGAAC AAATATTGAA AAAGACAGTT TTGAAATATC ATTTAACAAC GTTAAACTCT   
  
  
- TAATACGTGA GTCACCGAGA CACTAAATAG CAGGCGACCG TTTAAAAGAC TACTTTCCTC TACTAAATAA   
  
  
- TTAAAAAGTC CGTACCAGTT TAAAAGATCG GAAAGTACAT GTAACCAACC ACTATCCGAC TATCGATTTA   
  
  
- ATACAGAGTA AAGAGATTTA AATAACGGTC TATACTTTAA CCACCTTGTC AAATTCCCAA AACCAGCAGA   
  
  
- AAAAACGGGA AAAAAAACCC AGGGGGGGGG CGGGCCCCCC CTTGGGCCTC CCGTCTTTTT CATTGTATTG   
  
  
- AATTTTAATA GTAACTGCAT CAGGAGGTTG GTATTACTTT CGTTGACTTT ATTCTCGTAC GATTCGATCT   
  
  
- CTCATCCGAC GGAACAGTCG GAGGGAAAAC TGTACTCTTT CTACCCTCTT CAAATTTTAT TTCAAGGAGC   
  
  
- AGAAACGTAG GGCCTGGTGT TAAGCCTTGA CCCACTCCTG GAGGAATCCG AACATTTGTG TTCAAAGCTT   
  
  
- GAATCTAAAC GCATGTACAG TTACGAAATG ACCTATTCGA ATCGATTGTG GGGAGTAGGA AGTTGGACAT   
  
  
- GGAAGTATAT TTTTTTTCCT GTTAGGCCAC GTGTTCGTAG GGCGTAAGTG CGTTCCAGAC CCCTTCCCGG   
  
  
- CGTGGGGTTT CCCACTTAAC ATCCGTCGGA TTGGACTGAA ACAGTCACCG ATTAAGGTGC TAAGCTTGGG   
  
  
- TACTAAAAAA CCAATGTGCC CTTGTTGGAA TGGTAACGAG TTTCCGAGGG GAAGAAGTTG GAGATGGAAG   
  
  
- TATATACTTA CTTTTAGGAA ACCGATTCTT AACTTTTCCT TTATACACCA ATAACCCAAA CCCAAGAATA   
  
  
- AGGCAACCAA CGGGGAAGTG TAACTTACGT AGGAGAGCGA TTCTTGACTT TTCCTGTATA CCCCTACATA   
  
  
- TCCAGTTTAA ACTGAGTTAC TATTGACTCC TATGTTCTTA AGATCTTCAG AAATAGTCCC CTTAAAAAAG   
  
  
- AAAAAAGGTG AACACTGAAA AACTACGGAA CGAAAAATGA GAAGTAACTT ACTCATGGGA AGAACTGTCT   
  
  
- AAGAAGGAGG GAAAAAAGAA TTAAAGAAGA ACATCACCTT ATATTCATAC GAAGATCCAT CAACCTACGG   
  
  
- TACCTTAGAG TAGGGACACA CACGTAAGAA GGGGAGAGGG CGGCGGGGGG GGGGGGGTTT TTCTTTGGTT   
  
  
- TTTTTTTTTT TTTTACCTAC TAAGAATCTT GACTGGTAAA AAGGAAGGAG GAGGGTAAAA TAAAAAAAGT   
  
  
- CCGACAAAAA GAAGATGGAC AACGAAATCG TAGTGAGTAT TTTTGTTCCA CGTTCGCGTA CTGTAGCCCA   
  
  
- TGACTCGAAG ACTCAAATGT ACGTAGCAGT ACTTTAAGCC ATCTGAAACT TCATTTCAAA GGGGTAGCAT   
  
  
- TGACCACCAC GTATGGACAG AAGTGCGGGT AGTCATCTCA CCACTTCACT TCGTTACAGC TTATCAAACA   
  
  
- TAATGCTCGG GTTCTCTCTT TGACTACGTA TAAAATACGG AGTTACAGTT AGGAACTTGG GGGTCGAGCT   
  
  
- AATGTTGTGG TAACTGATAC CTCGAAAATA CACGAGTTTT CAAAAGGACC CAGTCATGAC GTGAGACCTT   
  
  
- AGTAGGAGTC CTTGACCCTG AACAGGCCAC AGATCGAGGT GTAGTTGAGA GTCGAAGAGT AGGTTACCAT   
  
  
- CGGGACCTAG TGTCGAACTA AGGTTGAGAA TAGGAAGACT AGTTTTAACG AGAGGACTAT TACGGTTAAG   
  
  
- GAGTAGAAAT TCAGTAAGGA CGCAGTGTCT ACTACACCTA CTGGACTTTA TGTCCGACTC TCTCAACCTT   
  
  
- TGTCACTACA ACCCTGGACT AGGGCTAAAA CAGATGCCGT TATCGTTGGT GTGTAAACCC CAACCCTAGG   
  
  
- TACATCACAG TAGTCTCAAA CTGTCGACCC CCTTTCACTA CCTCTACTAG CGAGCTTTCC TAAACTTTGA   
  
  
- CCACGATTAA CGGACACGTT CTCGTCAACG ACTTTTACTA GTCAACAACC GGGTCACCGA CTACCTACTT   
  
  
- AACTCTGTCT ACCAAAGTCA AAGACCACTT GGTTAAGTTT CTAACCCACG AATATACAAC CTTCCCGAGC   
  
  
- ATCGCTCCGT CCGGAGGAGT CCATCGAGGT AAATATTTCG TAACTCTACG TTCCTTGGGC GCTCACGACT   
  
  
- GGAAAATAGA ATGTACGTGT ATGAGAAACT CCAAACGGGT ATGTAGTTTA AACCGATGTA CAGTCGTTTA   
  
  
- CCACGGTAAC GTCTTCGTTA CTTCCTACTT TTCTCTCAGG TGTAACAGCT AAAGGTTTAA CCCGTCCCTT   
  
  
- CGGTTACCCA CTGGGATTAG GTCCGTAAAC GTCGGGCCGG TCCCCCAGGT GGGGTGTAAG CGTAATGGCC   
  
  
- ATAGCTACTG AGGATGAGAC GTATACGAGC ACCTCCACCC AACTTGTAAC ACCCGGTATC CGAGAACTCC   
  
  
- AACCGAGTCA GTAAATTCCA GGGTAAACTC AAGTTGCGTC AACTGCAGGG CCGAACAGTT CAGGACGAAT   
  
  
- TTCTAGAGCC GTAAGTTGTA CCACTCCGGA ACCGACACTT GAAACGGAAG TAAGAAGTGG TGTACGGGCT   
  
  
- ACTCTCGCAC TCGTGTCTCT TAGTGGCCCT AGCAGAAGAC TCCCACCATT TTCCCGACTT CGGTTTTCAC   
  
  
- CATTGCAATC ACCTCGTTCT CACATTGTGT TTGAGACGAC GAAAGAACGG AGCGAAACAA CTCTGTAACC   
  
  
- TTATGATGTG TCGCTACAAA CTCAGGTACC TACACTGAAA CGGATCCCTA GTATTTCTGG CCTAGTTACA   
  
  
- ACTCGTCGTA ACAGAACGAG CTCTACAACA CTTGAACCAA CGCACACTTC CTCGACTCTC CCACCTCGCC   
  
  
- GTACTCGAGG AACCCTTTAC CTCCAGCGCC AAGTGTTACC GTCCCAAATT CGGTATGGGA AATTCGTGAG   
  
  
- ACCATTTGTT GTGTTATTCT TGAAACAACT TCTTGATGAC ACTATCCATA CCCGATCTCC TCTCCTTACC   
  
  
- TCCAGAAATA GAACCCACCT ACTTATCTCT AAACCAACGA AGCTCACGGA CCGTCACATC ATTAAT

+     Unnamed\_\_2

| Site Name | Organism | Position | Strand | Matrix score. | sequence | function |
| --- | --- | --- | --- | --- | --- | --- |
| Unnamed\_\_2 | Zea mays | 807 | - | 6 | CCCCGG |  |
| Unnamed\_\_2 | Petroselinum hortense | 1220 | + | 9 | AACCTAACCT |  |

>HU01G01391.1   
+ +Up\_Stream \_Len000CATTTT AGTAATAAAT TCCCATGTGA GGACCACTGC CTACTGATTC CGAATGGCAT   
  
  
+ TTTAGTAATA AACTCCCATG TGAGGACCAC TTGATGAGGA ATTCAGTTTT ATAATCCACA GGTGTCAAAG   
  
  
+ CCTATTCCTA CTCTCACCAC TCTGCTATCT CTCCCCCCCC CCCCCCCCCG CCCCCCTCAA AATCTGCCTC   
  
  
+ CTCCGTTCTC TCCACAGCAG ATTCCTCAAT CAGGAAAGGT ATGTCAGAGC TCAAAACGAT TCAACATCAA   
  
  
+ AATGGGATAA TTTATTCTGC TTTCAGTTCT GGGTTATTGT CATATGTTCA TTCAGTTCAG TGTTTCTAGC   
  
  
+ ATGATTGCTT TTTGATGCCT TCTTTTTTGT TCTGGGTTGG ACGAACTCTG TCTTTTCTGT GCATAGATTT   
  
  
+ GAGCTCCGAG CTCAATTTTT GGGATTTTTT TAGCTGACTT TTCTGCTTGG TGTTGTGAAA TTGTGATGGG   
  
  
+ TTTTGGCTTT GTTAAACTTG TTTATAACTT TTTCTGTCAA AACTTTATAG TAAATTGTTG CAATTTGAGA   
  
  
+ ATTATGCACT CAGTGGCTCT GTGATTTATC GTCCGCTGGC AAATTTTCTG ATGAAAGGAG ATGATTTATT   
  
  
+ AATTTTTCAG GCATGGTCAA ATTTTCTAGC CTTTCATGTA CATTGGTTGG TGATAGGCTG ATAGCTAAAT   
  
  
+ TATGTCTCAT TTCTCTAAAT TTATTGCCAG ATATGAAATT GGTGGAACAG TTTAAGGGTT TTGGTCGTCT   
  
  
+ TTTTTGCCCT TTTTTTTGGG TCCCCCCCCC GCCCGGGGGG GAACCCGGAG GGCAGAAAAA GTAACATAAC   
  
  
+ TTAAAATTAT CATTGACGTA GTCCTCCAAC CATAATGAAA GCAACTGAAA TAAGAGCATG CTAAGCTAGA   
  
  
+ GAGTAGGCTG CCTTGTCAGC CTCCCTTTTG ACATGAGAAA GATGGGAGAA GTTTAAAATA AAGTTCCTCG   
  
  
+ TCTTTGCATC CCGGACCACA ATTCGGAACT GGGTGAGGAC CTCCTTAGGC TTGTAAACAC AAGTTTCGAA   
  
  
+ CTTAGATTTG CGTACATGTC AATGCTTTAC TGGATAAGCT TAGCTAACAC CCCTCATCCT TCAACCTGTA   
  
  
+ CCTTCATATA AAAAAAAGGA CAATCCGGTG CACAAGCATC CCGCATTCAC GCAAGGTCTG GGGAAGGGCC   
  
  
+ GCACCCCAAA GGGTGAATTG TAGGCAGCCT AACCTGACTT TGTCAGTGGC TAATTCCACG ATTCGAACCC   
  
  
+ ATGATTTTTT GGTTACACGG GAACAACCTT ACCATTGCTC AAAGGCTCCC CTTCTTCAAC CTCTACCTTC   
  
  
+ ATATATGAAT GAAAATCCTT TGGCTAAGAA TTGAAAAGGA AATATGTGGT TATTGGGTTT GGGTTCTTAT   
  
  
+ TCCGTTGGTT GCCCCTTCAC ATTGAATGCA TCCTCTCGCT AAGAACTGAA AAGGACATAT GGGGATGTAT   
  
  
+ AGGTCAAATT TGACTCAATG ATAACTGAGG ATACAAGAAT TCTAGAAGTC TTTATCAGGG GAATTTTTTC   
  
  
+ TTTTTTCCAC TTGTGACTTT TTGATGCCTT GCTTTTTACT CTTCATTGAA TGAGTACCCT TCTTGACAGA   
  
  
+ TTCTTCCTCC CTTTTTTCTT AATTTCTTCT TGTAGTGGAA TATAAGTATG CTTCTAGGTA GTTGGATGCC   
  
  
+ ATGGAATCTC ATCCCTGTGT GTGCATTCTT CCCCTCTCCC GCCGCCCCCC CCCCCCCAAA AAGAAACCAA   
  
  
+ AAAAAAAAAA AAAATGGATG ATTCTTAGAA CTGACCATTT TTCCTTCCTC CTCCCATTTT ATTTTTTTCA   
  
  
+ GGCTGTTTTT CTTCTACCTG TTGCTTTAGC ATCACTCATA AAAACAAGGT GCAAGCGCAT GACATCGGGT   
  
  
+ ACTGAGCTTC TGAGTTTACA TGCATCGTCA TGAAATTCGG TAGACTTTGA AGTAAAGTTT CCCCATCGTA   
  
  
+ ACTGGTGGTG CATACCTGTC TTCACGCCCA TCAGTAGAGT GGTGAAGTGA AGCAATGTCG AATAGTTTGT   
  
  
+ ATTACGAGCC CAAGAGAGAA ACTGATGCAT ATTTTATGCC TCAATGTCAA TCCTTGAACC CCCAGCTCGA   
  
  
+ TTACAACACC ATTGACTATG GAGCTTTTAT GTGCTCAAAA GTTTTCCTGG GTCAGTACTG CACTCTGGAA   
  
  
+ TCATCCTCAG GAACTGGGAC TTGTCCGGTG TCTAGCTCCA CATCAACTCT CAGCTTCTCA TCCAATGGTA   
  
  
+ GCCCTGGATC ACAGCTTGAT TCCAACTCTT ATCCTTCTGA TCAAAATTGC TCTCCTGATA ATGCCAATTC   
  
  
+ CTCATCTTTA AGTCATTCCT GCGTCACAGA TGATGTGGAT GACCTGAAAT ACAGGCTGAG AGAGTTGGAA   
  
  
+ ACAGTGATGT TGGGACCTGA TCCCGATTTT GTCTACGGCA ATAGCAACCA CACATTTGGG GTTGGGATCC   
  
  
+ ATGTAGTGTC ATCAGAGTTT GACAGCTGGG GGAAAGTGAT GGAGATGATC GCTCGAAAGG ATTTGAAACT   
  
  
+ GGTGCTAATT GCCTGTGCAA GAGCAGTTGC TGAAAATGAT CAGTTGTTGG CCCAGTGGCT GATGGATGAA   
  
  
+ TTGAGACAGA TGGTTTCAGT TTCTGGTGAA CCAATTCAAA GATTGGGTGC TTATATGTTG GAAGGGCTCG   
  
  
+ TAGCGAGGCA GGCCTCCTCA GGTAGCTCCA TTTATAAAGC ATTGAGATGC AAGGAACCCG CGAGTGCTGA   
  
  
+ CCTTTTATCT TACATGCACA TACTCTTTGA GGTTTGCCCA TACATCAAAT TTGGCTACAT GTCAGCAAAT   
  
  
+ GGTGCCATTG CAGAAGCAAT GAAGGATGAA AAGAGAGTCC ACATTGTCGA TTTCCAAATT GGGCAGGGAA   
  
  
+ GCCAATGGGT GACCCTAATC CAGGCATTTG CAGCCCGGCC AGGGGGTCCA CCCCACATTC GCATTACCGG   
  
  
+ TATCGATGAC TCCTACTCTG CATATGCTCG TGGAGGTGGG TTGAACATTG TGGGCCATAG GCTCTTGAGG   
  
  
+ TTGGCTCAGT CATTTAAGGT CCCATTTGAG TTCAACGCAG TTGACGTCCC GGCTTGTCAA GTCCTGCTTA   
  
  
+ AAGATCTCGG CATTCAACAT GGTGAGGCCT TGGCTGTGAA CTTTGCCTTC ATTCTTCACC ACATGCCCGA   
  
  
+ TGAGAGCGTG AGCACAGAGA ATCACCGGGA TCGTCTTCTG AGGGTGGTAA AAGGGCTGAA GCCAAAAGTG   
  
  
+ GTAACGTTAG TGGAGCAAGA GTGTAACACA AACTCTGCTG CTTTCTTGCC TCGCTTTGTT GAGACATTGG   
  
  
+ AATACTACAC AGCGATGTTT GAGTCCATGG ATGTGACTTT GCCTAGGGAT CATAAAGACC GGATCAATGT   
  
  
+ TGAGCAGCAT TGTCTTGCTC GAGATGTTGT GAACTTGGTT GCGTGTGAAG GAGCTGAGAG GGTGGAGCGG   
  
  
+ CATGAGCTCC TTGGGAAATG GAGGTCGCGG TTCACAATGG CAGGGTTTAA GCCATACCCT TTAAGCACTC   
  
  
+ TGGTAAACAA CACAATAAGA ACTTTGTTGA AGAACTACTG TGATAGGTAT GGGCTAGAGG AGAGGAATGG   
  
  
+ AGGTCTTTAT CTTGGGTGGA TGAATAGAGA TTTGGTTGCT TCGAGTGCCT GGCAGTGTAG TAATTA  

- +Up\_Stream \_Len000GTAAAA TCATTATTTA AGGGTACACT CCTGGTGACG GATGACTAAG GCTTACCGTA   
  
  
- AAATCATTAT TTGAGGGTAC ACTCCTGGTG AACTACTCCT TAAGTCAAAA TATTAGGTGT CCACAGTTTC   
  
  
- GGATAAGGAT GAGAGTGGTG AGACGATAGA GAGGGGGGGG GGGGGGGGGC GGGGGGAGTT TTAGACGGAG   
  
  
- GAGGCAAGAG AGGTGTCGTC TAAGGAGTTA GTCCTTTCCA TACAGTCTCG AGTTTTGCTA AGTTGTAGTT   
  
  
- TTACCCTATT AAATAAGACG AAAGTCAAGA CCCAATAACA GTATACAAGT AAGTCAAGTC ACAAAGATCG   
  
  
- TACTAACGAA AAACTACGGA AGAAAAAACA AGACCCAACC TGCTTGAGAC AGAAAAGACA CGTATCTAAA   
  
  
- CTCGAGGCTC GAGTTAAAAA CCCTAAAAAA ATCGACTGAA AAGACGAACC ACAACACTTT AACACTACCC   
  
  
- AAAACCGAAA CAATTTGAAC AAATATTGAA AAAGACAGTT TTGAAATATC ATTTAACAAC GTTAAACTCT   
  
  
- TAATACGTGA GTCACCGAGA CACTAAATAG CAGGCGACCG TTTAAAAGAC TACTTTCCTC TACTAAATAA   
  
  
- TTAAAAAGTC CGTACCAGTT TAAAAGATCG GAAAGTACAT GTAACCAACC ACTATCCGAC TATCGATTTA   
  
  
- ATACAGAGTA AAGAGATTTA AATAACGGTC TATACTTTAA CCACCTTGTC AAATTCCCAA AACCAGCAGA   
  
  
- AAAAACGGGA AAAAAAACCC AGGGGGGGGG CGGGCCCCCC CTTGGGCCTC CCGTCTTTTT CATTGTATTG   
  
  
- AATTTTAATA GTAACTGCAT CAGGAGGTTG GTATTACTTT CGTTGACTTT ATTCTCGTAC GATTCGATCT   
  
  
- CTCATCCGAC GGAACAGTCG GAGGGAAAAC TGTACTCTTT CTACCCTCTT CAAATTTTAT TTCAAGGAGC   
  
  
- AGAAACGTAG GGCCTGGTGT TAAGCCTTGA CCCACTCCTG GAGGAATCCG AACATTTGTG TTCAAAGCTT   
  
  
- GAATCTAAAC GCATGTACAG TTACGAAATG ACCTATTCGA ATCGATTGTG GGGAGTAGGA AGTTGGACAT   
  
  
- GGAAGTATAT TTTTTTTCCT GTTAGGCCAC GTGTTCGTAG GGCGTAAGTG CGTTCCAGAC CCCTTCCCGG   
  
  
- CGTGGGGTTT CCCACTTAAC ATCCGTCGGA TTGGACTGAA ACAGTCACCG ATTAAGGTGC TAAGCTTGGG   
  
  
- TACTAAAAAA CCAATGTGCC CTTGTTGGAA TGGTAACGAG TTTCCGAGGG GAAGAAGTTG GAGATGGAAG   
  
  
- TATATACTTA CTTTTAGGAA ACCGATTCTT AACTTTTCCT TTATACACCA ATAACCCAAA CCCAAGAATA   
  
  
- AGGCAACCAA CGGGGAAGTG TAACTTACGT AGGAGAGCGA TTCTTGACTT TTCCTGTATA CCCCTACATA   
  
  
- TCCAGTTTAA ACTGAGTTAC TATTGACTCC TATGTTCTTA AGATCTTCAG AAATAGTCCC CTTAAAAAAG   
  
  
- AAAAAAGGTG AACACTGAAA AACTACGGAA CGAAAAATGA GAAGTAACTT ACTCATGGGA AGAACTGTCT   
  
  
- AAGAAGGAGG GAAAAAAGAA TTAAAGAAGA ACATCACCTT ATATTCATAC GAAGATCCAT CAACCTACGG   
  
  
- TACCTTAGAG TAGGGACACA CACGTAAGAA GGGGAGAGGG CGGCGGGGGG GGGGGGGTTT TTCTTTGGTT   
  
  
- TTTTTTTTTT TTTTACCTAC TAAGAATCTT GACTGGTAAA AAGGAAGGAG GAGGGTAAAA TAAAAAAAGT   
  
  
- CCGACAAAAA GAAGATGGAC AACGAAATCG TAGTGAGTAT TTTTGTTCCA CGTTCGCGTA CTGTAGCCCA   
  
  
- TGACTCGAAG ACTCAAATGT ACGTAGCAGT ACTTTAAGCC ATCTGAAACT TCATTTCAAA GGGGTAGCAT   
  
  
- TGACCACCAC GTATGGACAG AAGTGCGGGT AGTCATCTCA CCACTTCACT TCGTTACAGC TTATCAAACA   
  
  
- TAATGCTCGG GTTCTCTCTT TGACTACGTA TAAAATACGG AGTTACAGTT AGGAACTTGG GGGTCGAGCT   
  
  
- AATGTTGTGG TAACTGATAC CTCGAAAATA CACGAGTTTT CAAAAGGACC CAGTCATGAC GTGAGACCTT   
  
  
- AGTAGGAGTC CTTGACCCTG AACAGGCCAC AGATCGAGGT GTAGTTGAGA GTCGAAGAGT AGGTTACCAT   
  
  
- CGGGACCTAG TGTCGAACTA AGGTTGAGAA TAGGAAGACT AGTTTTAACG AGAGGACTAT TACGGTTAAG   
  
  
- GAGTAGAAAT TCAGTAAGGA CGCAGTGTCT ACTACACCTA CTGGACTTTA TGTCCGACTC TCTCAACCTT   
  
  
- TGTCACTACA ACCCTGGACT AGGGCTAAAA CAGATGCCGT TATCGTTGGT GTGTAAACCC CAACCCTAGG   
  
  
- TACATCACAG TAGTCTCAAA CTGTCGACCC CCTTTCACTA CCTCTACTAG CGAGCTTTCC TAAACTTTGA   
  
  
- CCACGATTAA CGGACACGTT CTCGTCAACG ACTTTTACTA GTCAACAACC GGGTCACCGA CTACCTACTT   
  
  
- AACTCTGTCT ACCAAAGTCA AAGACCACTT GGTTAAGTTT CTAACCCACG AATATACAAC CTTCCCGAGC   
  
  
- ATCGCTCCGT CCGGAGGAGT CCATCGAGGT AAATATTTCG TAACTCTACG TTCCTTGGGC GCTCACGACT   
  
  
- GGAAAATAGA ATGTACGTGT ATGAGAAACT CCAAACGGGT ATGTAGTTTA AACCGATGTA CAGTCGTTTA   
  
  
- CCACGGTAAC GTCTTCGTTA CTTCCTACTT TTCTCTCAGG TGTAACAGCT AAAGGTTTAA CCCGTCCCTT   
  
  
- CGGTTACCCA CTGGGATTAG GTCCGTAAAC GTCGGGCCGG TCCCCCAGGT GGGGTGTAAG CGTAATGGCC   
  
  
- ATAGCTACTG AGGATGAGAC GTATACGAGC ACCTCCACCC AACTTGTAAC ACCCGGTATC CGAGAACTCC   
  
  
- AACCGAGTCA GTAAATTCCA GGGTAAACTC AAGTTGCGTC AACTGCAGGG CCGAACAGTT CAGGACGAAT   
  
  
- TTCTAGAGCC GTAAGTTGTA CCACTCCGGA ACCGACACTT GAAACGGAAG TAAGAAGTGG TGTACGGGCT   
  
  
- ACTCTCGCAC TCGTGTCTCT TAGTGGCCCT AGCAGAAGAC TCCCACCATT TTCCCGACTT CGGTTTTCAC   
  
  
- CATTGCAATC ACCTCGTTCT CACATTGTGT TTGAGACGAC GAAAGAACGG AGCGAAACAA CTCTGTAACC   
  
  
- TTATGATGTG TCGCTACAAA CTCAGGTACC TACACTGAAA CGGATCCCTA GTATTTCTGG CCTAGTTACA   
  
  
- ACTCGTCGTA ACAGAACGAG CTCTACAACA CTTGAACCAA CGCACACTTC CTCGACTCTC CCACCTCGCC   
  
  
- GTACTCGAGG AACCCTTTAC CTCCAGCGCC AAGTGTTACC GTCCCAAATT CGGTATGGGA AATTCGTGAG   
  
  
- ACCATTTGTT GTGTTATTCT TGAAACAACT TCTTGATGAC ACTATCCATA CCCGATCTCC TCTCCTTACC   
  
  
- TCCAGAAATA GAACCCACCT ACTTATCTCT AAACCAACGA AGCTCACGGA CCGTCACATC ATTAAT

+     Unnamed\_\_4

| Site Name | Organism | Position | Strand | Matrix score. | sequence | function |
| --- | --- | --- | --- | --- | --- | --- |
| Unnamed\_\_4 | Petroselinum hortense | 87 | + | 4 | CTCC |  |
| Unnamed\_\_4 | Petroselinum hortense | 3428 | - | 4 | CTCC |  |
| Unnamed\_\_4 | Petroselinum hortense | 2976 | - | 4 | CTCC |  |
| Unnamed\_\_4 | Petroselinum hortense | 3563 | - | 4 | CTCC |  |
| Unnamed\_\_4 | Petroselinum hortense | 2954 | + | 4 | CTCC |  |
| Unnamed\_\_4 | Petroselinum hortense | 3414 | - | 4 | CTCC |  |
| Unnamed\_\_4 | Petroselinum hortense | 3236 | - | 4 | CTCC |  |
| Unnamed\_\_4 | Petroselinum hortense | 3573 | - | 4 | CTCC |  |
| Unnamed\_\_4 | Petroselinum hortense | 175 | + | 4 | CTCC |  |
| Unnamed\_\_4 | Petroselinum hortense | 3441 | + | 4 | CTCC |  |
| Unnamed\_\_4 | Petroselinum hortense | 2690 | + | 4 | CTCC |  |
| Unnamed\_\_4 | Petroselinum hortense | 3454 | - | 4 | CTCC |  |
| Unnamed\_\_4 | Petroselinum hortense | 212 | + | 4 | CTCC |  |
| Unnamed\_\_4 | Petroselinum hortense | 215 | + | 4 | CTCC |  |
| Unnamed\_\_4 | Petroselinum hortense | 224 | + | 4 | CTCC |  |
| Unnamed\_\_4 | Petroselinum hortense | 428 | + | 4 | CTCC |  |
| Unnamed\_\_4 | Petroselinum hortense | 621 | - | 4 | CTCC |  |
| Unnamed\_\_4 | Petroselinum hortense | 821 | - | 4 | CTCC |  |
| Unnamed\_\_4 | Petroselinum hortense | 868 | + | 4 | CTCC |  |
| Unnamed\_\_4 | Petroselinum hortense | 935 | + | 4 | CTCC |  |
| Unnamed\_\_4 | Petroselinum hortense | 959 | - | 4 | CTCC |  |
| Unnamed\_\_4 | Petroselinum hortense | 1025 | + | 4 | CTCC |  |
| Unnamed\_\_4 | Petroselinum hortense | 1310 | + | 4 | CTCC |  |
| Unnamed\_\_4 | Petroselinum hortense | 1621 | + | 4 | CTCC |  |
| Unnamed\_\_4 | Petroselinum hortense | 1720 | + | 4 | CTCC |  |
| Unnamed\_\_4 | Petroselinum hortense | 1802 | + | 4 | CTCC |  |
| Unnamed\_\_4 | Petroselinum hortense | 1805 | + | 4 | CTCC |  |
| Unnamed\_\_4 | Petroselinum hortense | 2124 | - | 4 | CTCC |  |
| Unnamed\_\_4 | Petroselinum hortense | 2210 | + | 4 | CTCC |  |
| Unnamed\_\_4 | Petroselinum hortense | 2296 | + | 4 | CTCC |  |
| Unnamed\_\_4 | Petroselinum hortense | 2495 | - | 4 | CTCC |  |
| Unnamed\_\_4 | Petroselinum hortense | 2678 | + | 4 | CTCC |  |

>HU01G01391.1   
+ +Up\_Stream \_Len000CATTTT AGTAATAAAT TCCCATGTGA GGACCACTGC CTACTGATTC CGAATGGCAT   
  
  
+ TTTAGTAATA AACTCCCATG TGAGGACCAC TTGATGAGGA ATTCAGTTTT ATAATCCACA GGTGTCAAAG   
  
  
+ CCTATTCCTA CTCTCACCAC TCTGCTATCT CTCCCCCCCC CCCCCCCCCG CCCCCCTCAA AATCTGCCTC   
  
  
+ CTCCGTTCTC TCCACAGCAG ATTCCTCAAT CAGGAAAGGT ATGTCAGAGC TCAAAACGAT TCAACATCAA   
  
  
+ AATGGGATAA TTTATTCTGC TTTCAGTTCT GGGTTATTGT CATATGTTCA TTCAGTTCAG TGTTTCTAGC   
  
  
+ ATGATTGCTT TTTGATGCCT TCTTTTTTGT TCTGGGTTGG ACGAACTCTG TCTTTTCTGT GCATAGATTT   
  
  
+ GAGCTCCGAG CTCAATTTTT GGGATTTTTT TAGCTGACTT TTCTGCTTGG TGTTGTGAAA TTGTGATGGG   
  
  
+ TTTTGGCTTT GTTAAACTTG TTTATAACTT TTTCTGTCAA AACTTTATAG TAAATTGTTG CAATTTGAGA   
  
  
+ ATTATGCACT CAGTGGCTCT GTGATTTATC GTCCGCTGGC AAATTTTCTG ATGAAAGGAG ATGATTTATT   
  
  
+ AATTTTTCAG GCATGGTCAA ATTTTCTAGC CTTTCATGTA CATTGGTTGG TGATAGGCTG ATAGCTAAAT   
  
  
+ TATGTCTCAT TTCTCTAAAT TTATTGCCAG ATATGAAATT GGTGGAACAG TTTAAGGGTT TTGGTCGTCT   
  
  
+ TTTTTGCCCT TTTTTTTGGG TCCCCCCCCC GCCCGGGGGG GAACCCGGAG GGCAGAAAAA GTAACATAAC   
  
  
+ TTAAAATTAT CATTGACGTA GTCCTCCAAC CATAATGAAA GCAACTGAAA TAAGAGCATG CTAAGCTAGA   
  
  
+ GAGTAGGCTG CCTTGTCAGC CTCCCTTTTG ACATGAGAAA GATGGGAGAA GTTTAAAATA AAGTTCCTCG   
  
  
+ TCTTTGCATC CCGGACCACA ATTCGGAACT GGGTGAGGAC CTCCTTAGGC TTGTAAACAC AAGTTTCGAA   
  
  
+ CTTAGATTTG CGTACATGTC AATGCTTTAC TGGATAAGCT TAGCTAACAC CCCTCATCCT TCAACCTGTA   
  
  
+ CCTTCATATA AAAAAAAGGA CAATCCGGTG CACAAGCATC CCGCATTCAC GCAAGGTCTG GGGAAGGGCC   
  
  
+ GCACCCCAAA GGGTGAATTG TAGGCAGCCT AACCTGACTT TGTCAGTGGC TAATTCCACG ATTCGAACCC   
  
  
+ ATGATTTTTT GGTTACACGG GAACAACCTT ACCATTGCTC AAAGGCTCCC CTTCTTCAAC CTCTACCTTC   
  
  
+ ATATATGAAT GAAAATCCTT TGGCTAAGAA TTGAAAAGGA AATATGTGGT TATTGGGTTT GGGTTCTTAT   
  
  
+ TCCGTTGGTT GCCCCTTCAC ATTGAATGCA TCCTCTCGCT AAGAACTGAA AAGGACATAT GGGGATGTAT   
  
  
+ AGGTCAAATT TGACTCAATG ATAACTGAGG ATACAAGAAT TCTAGAAGTC TTTATCAGGG GAATTTTTTC   
  
  
+ TTTTTTCCAC TTGTGACTTT TTGATGCCTT GCTTTTTACT CTTCATTGAA TGAGTACCCT TCTTGACAGA   
  
  
+ TTCTTCCTCC CTTTTTTCTT AATTTCTTCT TGTAGTGGAA TATAAGTATG CTTCTAGGTA GTTGGATGCC   
  
  
+ ATGGAATCTC ATCCCTGTGT GTGCATTCTT CCCCTCTCCC GCCGCCCCCC CCCCCCCAAA AAGAAACCAA   
  
  
+ AAAAAAAAAA AAAATGGATG ATTCTTAGAA CTGACCATTT TTCCTTCCTC CTCCCATTTT ATTTTTTTCA   
  
  
+ GGCTGTTTTT CTTCTACCTG TTGCTTTAGC ATCACTCATA AAAACAAGGT GCAAGCGCAT GACATCGGGT   
  
  
+ ACTGAGCTTC TGAGTTTACA TGCATCGTCA TGAAATTCGG TAGACTTTGA AGTAAAGTTT CCCCATCGTA   
  
  
+ ACTGGTGGTG CATACCTGTC TTCACGCCCA TCAGTAGAGT GGTGAAGTGA AGCAATGTCG AATAGTTTGT   
  
  
+ ATTACGAGCC CAAGAGAGAA ACTGATGCAT ATTTTATGCC TCAATGTCAA TCCTTGAACC CCCAGCTCGA   
  
  
+ TTACAACACC ATTGACTATG GAGCTTTTAT GTGCTCAAAA GTTTTCCTGG GTCAGTACTG CACTCTGGAA   
  
  
+ TCATCCTCAG GAACTGGGAC TTGTCCGGTG TCTAGCTCCA CATCAACTCT CAGCTTCTCA TCCAATGGTA   
  
  
+ GCCCTGGATC ACAGCTTGAT TCCAACTCTT ATCCTTCTGA TCAAAATTGC TCTCCTGATA ATGCCAATTC   
  
  
+ CTCATCTTTA AGTCATTCCT GCGTCACAGA TGATGTGGAT GACCTGAAAT ACAGGCTGAG AGAGTTGGAA   
  
  
+ ACAGTGATGT TGGGACCTGA TCCCGATTTT GTCTACGGCA ATAGCAACCA CACATTTGGG GTTGGGATCC   
  
  
+ ATGTAGTGTC ATCAGAGTTT GACAGCTGGG GGAAAGTGAT GGAGATGATC GCTCGAAAGG ATTTGAAACT   
  
  
+ GGTGCTAATT GCCTGTGCAA GAGCAGTTGC TGAAAATGAT CAGTTGTTGG CCCAGTGGCT GATGGATGAA   
  
  
+ TTGAGACAGA TGGTTTCAGT TTCTGGTGAA CCAATTCAAA GATTGGGTGC TTATATGTTG GAAGGGCTCG   
  
  
+ TAGCGAGGCA GGCCTCCTCA GGTAGCTCCA TTTATAAAGC ATTGAGATGC AAGGAACCCG CGAGTGCTGA   
  
  
+ CCTTTTATCT TACATGCACA TACTCTTTGA GGTTTGCCCA TACATCAAAT TTGGCTACAT GTCAGCAAAT   
  
  
+ GGTGCCATTG CAGAAGCAAT GAAGGATGAA AAGAGAGTCC ACATTGTCGA TTTCCAAATT GGGCAGGGAA   
  
  
+ GCCAATGGGT GACCCTAATC CAGGCATTTG CAGCCCGGCC AGGGGGTCCA CCCCACATTC GCATTACCGG   
  
  
+ TATCGATGAC TCCTACTCTG CATATGCTCG TGGAGGTGGG TTGAACATTG TGGGCCATAG GCTCTTGAGG   
  
  
+ TTGGCTCAGT CATTTAAGGT CCCATTTGAG TTCAACGCAG TTGACGTCCC GGCTTGTCAA GTCCTGCTTA   
  
  
+ AAGATCTCGG CATTCAACAT GGTGAGGCCT TGGCTGTGAA CTTTGCCTTC ATTCTTCACC ACATGCCCGA   
  
  
+ TGAGAGCGTG AGCACAGAGA ATCACCGGGA TCGTCTTCTG AGGGTGGTAA AAGGGCTGAA GCCAAAAGTG   
  
  
+ GTAACGTTAG TGGAGCAAGA GTGTAACACA AACTCTGCTG CTTTCTTGCC TCGCTTTGTT GAGACATTGG   
  
  
+ AATACTACAC AGCGATGTTT GAGTCCATGG ATGTGACTTT GCCTAGGGAT CATAAAGACC GGATCAATGT   
  
  
+ TGAGCAGCAT TGTCTTGCTC GAGATGTTGT GAACTTGGTT GCGTGTGAAG GAGCTGAGAG GGTGGAGCGG   
  
  
+ CATGAGCTCC TTGGGAAATG GAGGTCGCGG TTCACAATGG CAGGGTTTAA GCCATACCCT TTAAGCACTC   
  
  
+ TGGTAAACAA CACAATAAGA ACTTTGTTGA AGAACTACTG TGATAGGTAT GGGCTAGAGG AGAGGAATGG   
  
  
+ AGGTCTTTAT CTTGGGTGGA TGAATAGAGA TTTGGTTGCT TCGAGTGCCT GGCAGTGTAG TAATTA  

- +Up\_Stream \_Len000GTAAAA TCATTATTTA AGGGTACACT CCTGGTGACG GATGACTAAG GCTTACCGTA   
  
  
- AAATCATTAT TTGAGGGTAC ACTCCTGGTG AACTACTCCT TAAGTCAAAA TATTAGGTGT CCACAGTTTC   
  
  
- GGATAAGGAT GAGAGTGGTG AGACGATAGA GAGGGGGGGG GGGGGGGGGC GGGGGGAGTT TTAGACGGAG   
  
  
- GAGGCAAGAG AGGTGTCGTC TAAGGAGTTA GTCCTTTCCA TACAGTCTCG AGTTTTGCTA AGTTGTAGTT   
  
  
- TTACCCTATT AAATAAGACG AAAGTCAAGA CCCAATAACA GTATACAAGT AAGTCAAGTC ACAAAGATCG   
  
  
- TACTAACGAA AAACTACGGA AGAAAAAACA AGACCCAACC TGCTTGAGAC AGAAAAGACA CGTATCTAAA   
  
  
- CTCGAGGCTC GAGTTAAAAA CCCTAAAAAA ATCGACTGAA AAGACGAACC ACAACACTTT AACACTACCC   
  
  
- AAAACCGAAA CAATTTGAAC AAATATTGAA AAAGACAGTT TTGAAATATC ATTTAACAAC GTTAAACTCT   
  
  
- TAATACGTGA GTCACCGAGA CACTAAATAG CAGGCGACCG TTTAAAAGAC TACTTTCCTC TACTAAATAA   
  
  
- TTAAAAAGTC CGTACCAGTT TAAAAGATCG GAAAGTACAT GTAACCAACC ACTATCCGAC TATCGATTTA   
  
  
- ATACAGAGTA AAGAGATTTA AATAACGGTC TATACTTTAA CCACCTTGTC AAATTCCCAA AACCAGCAGA   
  
  
- AAAAACGGGA AAAAAAACCC AGGGGGGGGG CGGGCCCCCC CTTGGGCCTC CCGTCTTTTT CATTGTATTG   
  
  
- AATTTTAATA GTAACTGCAT CAGGAGGTTG GTATTACTTT CGTTGACTTT ATTCTCGTAC GATTCGATCT   
  
  
- CTCATCCGAC GGAACAGTCG GAGGGAAAAC TGTACTCTTT CTACCCTCTT CAAATTTTAT TTCAAGGAGC   
  
  
- AGAAACGTAG GGCCTGGTGT TAAGCCTTGA CCCACTCCTG GAGGAATCCG AACATTTGTG TTCAAAGCTT   
  
  
- GAATCTAAAC GCATGTACAG TTACGAAATG ACCTATTCGA ATCGATTGTG GGGAGTAGGA AGTTGGACAT   
  
  
- GGAAGTATAT TTTTTTTCCT GTTAGGCCAC GTGTTCGTAG GGCGTAAGTG CGTTCCAGAC CCCTTCCCGG   
  
  
- CGTGGGGTTT CCCACTTAAC ATCCGTCGGA TTGGACTGAA ACAGTCACCG ATTAAGGTGC TAAGCTTGGG   
  
  
- TACTAAAAAA CCAATGTGCC CTTGTTGGAA TGGTAACGAG TTTCCGAGGG GAAGAAGTTG GAGATGGAAG   
  
  
- TATATACTTA CTTTTAGGAA ACCGATTCTT AACTTTTCCT TTATACACCA ATAACCCAAA CCCAAGAATA   
  
  
- AGGCAACCAA CGGGGAAGTG TAACTTACGT AGGAGAGCGA TTCTTGACTT TTCCTGTATA CCCCTACATA   
  
  
- TCCAGTTTAA ACTGAGTTAC TATTGACTCC TATGTTCTTA AGATCTTCAG AAATAGTCCC CTTAAAAAAG   
  
  
- AAAAAAGGTG AACACTGAAA AACTACGGAA CGAAAAATGA GAAGTAACTT ACTCATGGGA AGAACTGTCT   
  
  
- AAGAAGGAGG GAAAAAAGAA TTAAAGAAGA ACATCACCTT ATATTCATAC GAAGATCCAT CAACCTACGG   
  
  
- TACCTTAGAG TAGGGACACA CACGTAAGAA GGGGAGAGGG CGGCGGGGGG GGGGGGGTTT TTCTTTGGTT   
  
  
- TTTTTTTTTT TTTTACCTAC TAAGAATCTT GACTGGTAAA AAGGAAGGAG GAGGGTAAAA TAAAAAAAGT   
  
  
- CCGACAAAAA GAAGATGGAC AACGAAATCG TAGTGAGTAT TTTTGTTCCA CGTTCGCGTA CTGTAGCCCA   
  
  
- TGACTCGAAG ACTCAAATGT ACGTAGCAGT ACTTTAAGCC ATCTGAAACT TCATTTCAAA GGGGTAGCAT   
  
  
- TGACCACCAC GTATGGACAG AAGTGCGGGT AGTCATCTCA CCACTTCACT TCGTTACAGC TTATCAAACA   
  
  
- TAATGCTCGG GTTCTCTCTT TGACTACGTA TAAAATACGG AGTTACAGTT AGGAACTTGG GGGTCGAGCT   
  
  
- AATGTTGTGG TAACTGATAC CTCGAAAATA CACGAGTTTT CAAAAGGACC CAGTCATGAC GTGAGACCTT   
  
  
- AGTAGGAGTC CTTGACCCTG AACAGGCCAC AGATCGAGGT GTAGTTGAGA GTCGAAGAGT AGGTTACCAT   
  
  
- CGGGACCTAG TGTCGAACTA AGGTTGAGAA TAGGAAGACT AGTTTTAACG AGAGGACTAT TACGGTTAAG   
  
  
- GAGTAGAAAT TCAGTAAGGA CGCAGTGTCT ACTACACCTA CTGGACTTTA TGTCCGACTC TCTCAACCTT   
  
  
- TGTCACTACA ACCCTGGACT AGGGCTAAAA CAGATGCCGT TATCGTTGGT GTGTAAACCC CAACCCTAGG   
  
  
- TACATCACAG TAGTCTCAAA CTGTCGACCC CCTTTCACTA CCTCTACTAG CGAGCTTTCC TAAACTTTGA   
  
  
- CCACGATTAA CGGACACGTT CTCGTCAACG ACTTTTACTA GTCAACAACC GGGTCACCGA CTACCTACTT   
  
  
- AACTCTGTCT ACCAAAGTCA AAGACCACTT GGTTAAGTTT CTAACCCACG AATATACAAC CTTCCCGAGC   
  
  
- ATCGCTCCGT CCGGAGGAGT CCATCGAGGT AAATATTTCG TAACTCTACG TTCCTTGGGC GCTCACGACT   
  
  
- GGAAAATAGA ATGTACGTGT ATGAGAAACT CCAAACGGGT ATGTAGTTTA AACCGATGTA CAGTCGTTTA   
  
  
- CCACGGTAAC GTCTTCGTTA CTTCCTACTT TTCTCTCAGG TGTAACAGCT AAAGGTTTAA CCCGTCCCTT   
  
  
- CGGTTACCCA CTGGGATTAG GTCCGTAAAC GTCGGGCCGG TCCCCCAGGT GGGGTGTAAG CGTAATGGCC   
  
  
- ATAGCTACTG AGGATGAGAC GTATACGAGC ACCTCCACCC AACTTGTAAC ACCCGGTATC CGAGAACTCC   
  
  
- AACCGAGTCA GTAAATTCCA GGGTAAACTC AAGTTGCGTC AACTGCAGGG CCGAACAGTT CAGGACGAAT   
  
  
- TTCTAGAGCC GTAAGTTGTA CCACTCCGGA ACCGACACTT GAAACGGAAG TAAGAAGTGG TGTACGGGCT   
  
  
- ACTCTCGCAC TCGTGTCTCT TAGTGGCCCT AGCAGAAGAC TCCCACCATT TTCCCGACTT CGGTTTTCAC   
  
  
- CATTGCAATC ACCTCGTTCT CACATTGTGT TTGAGACGAC GAAAGAACGG AGCGAAACAA CTCTGTAACC   
  
  
- TTATGATGTG TCGCTACAAA CTCAGGTACC TACACTGAAA CGGATCCCTA GTATTTCTGG CCTAGTTACA   
  
  
- ACTCGTCGTA ACAGAACGAG CTCTACAACA CTTGAACCAA CGCACACTTC CTCGACTCTC CCACCTCGCC   
  
  
- GTACTCGAGG AACCCTTTAC CTCCAGCGCC AAGTGTTACC GTCCCAAATT CGGTATGGGA AATTCGTGAG   
  
  
- ACCATTTGTT GTGTTATTCT TGAAACAACT TCTTGATGAC ACTATCCATA CCCGATCTCC TCTCCTTACC   
  
  
- TCCAGAAATA GAACCCACCT ACTTATCTCT AAACCAACGA AGCTCACGGA CCGTCACATC ATTAAT

+     W box

| Site Name | Organism | Position | Strand | Matrix score. | sequence | function |
| --- | --- | --- | --- | --- | --- | --- |
| W box | Arabidopsis thaliana | 649 | - | 6 | TTGACC |  |
| W box | Arabidopsis thaliana | 1476 | - | 6 | TTGACC |  |

>HU01G01391.1   
+ +Up\_Stream \_Len000CATTTT AGTAATAAAT TCCCATGTGA GGACCACTGC CTACTGATTC CGAATGGCAT   
  
  
+ TTTAGTAATA AACTCCCATG TGAGGACCAC TTGATGAGGA ATTCAGTTTT ATAATCCACA GGTGTCAAAG   
  
  
+ CCTATTCCTA CTCTCACCAC TCTGCTATCT CTCCCCCCCC CCCCCCCCCG CCCCCCTCAA AATCTGCCTC   
  
  
+ CTCCGTTCTC TCCACAGCAG ATTCCTCAAT CAGGAAAGGT ATGTCAGAGC TCAAAACGAT TCAACATCAA   
  
  
+ AATGGGATAA TTTATTCTGC TTTCAGTTCT GGGTTATTGT CATATGTTCA TTCAGTTCAG TGTTTCTAGC   
  
  
+ ATGATTGCTT TTTGATGCCT TCTTTTTTGT TCTGGGTTGG ACGAACTCTG TCTTTTCTGT GCATAGATTT   
  
  
+ GAGCTCCGAG CTCAATTTTT GGGATTTTTT TAGCTGACTT TTCTGCTTGG TGTTGTGAAA TTGTGATGGG   
  
  
+ TTTTGGCTTT GTTAAACTTG TTTATAACTT TTTCTGTCAA AACTTTATAG TAAATTGTTG CAATTTGAGA   
  
  
+ ATTATGCACT CAGTGGCTCT GTGATTTATC GTCCGCTGGC AAATTTTCTG ATGAAAGGAG ATGATTTATT   
  
  
+ AATTTTTCAG GCATGGTCAA ATTTTCTAGC CTTTCATGTA CATTGGTTGG TGATAGGCTG ATAGCTAAAT   
  
  
+ TATGTCTCAT TTCTCTAAAT TTATTGCCAG ATATGAAATT GGTGGAACAG TTTAAGGGTT TTGGTCGTCT   
  
  
+ TTTTTGCCCT TTTTTTTGGG TCCCCCCCCC GCCCGGGGGG GAACCCGGAG GGCAGAAAAA GTAACATAAC   
  
  
+ TTAAAATTAT CATTGACGTA GTCCTCCAAC CATAATGAAA GCAACTGAAA TAAGAGCATG CTAAGCTAGA   
  
  
+ GAGTAGGCTG CCTTGTCAGC CTCCCTTTTG ACATGAGAAA GATGGGAGAA GTTTAAAATA AAGTTCCTCG   
  
  
+ TCTTTGCATC CCGGACCACA ATTCGGAACT GGGTGAGGAC CTCCTTAGGC TTGTAAACAC AAGTTTCGAA   
  
  
+ CTTAGATTTG CGTACATGTC AATGCTTTAC TGGATAAGCT TAGCTAACAC CCCTCATCCT TCAACCTGTA   
  
  
+ CCTTCATATA AAAAAAAGGA CAATCCGGTG CACAAGCATC CCGCATTCAC GCAAGGTCTG GGGAAGGGCC   
  
  
+ GCACCCCAAA GGGTGAATTG TAGGCAGCCT AACCTGACTT TGTCAGTGGC TAATTCCACG ATTCGAACCC   
  
  
+ ATGATTTTTT GGTTACACGG GAACAACCTT ACCATTGCTC AAAGGCTCCC CTTCTTCAAC CTCTACCTTC   
  
  
+ ATATATGAAT GAAAATCCTT TGGCTAAGAA TTGAAAAGGA AATATGTGGT TATTGGGTTT GGGTTCTTAT   
  
  
+ TCCGTTGGTT GCCCCTTCAC ATTGAATGCA TCCTCTCGCT AAGAACTGAA AAGGACATAT GGGGATGTAT   
  
  
+ AGGTCAAATT TGACTCAATG ATAACTGAGG ATACAAGAAT TCTAGAAGTC TTTATCAGGG GAATTTTTTC   
  
  
+ TTTTTTCCAC TTGTGACTTT TTGATGCCTT GCTTTTTACT CTTCATTGAA TGAGTACCCT TCTTGACAGA   
  
  
+ TTCTTCCTCC CTTTTTTCTT AATTTCTTCT TGTAGTGGAA TATAAGTATG CTTCTAGGTA GTTGGATGCC   
  
  
+ ATGGAATCTC ATCCCTGTGT GTGCATTCTT CCCCTCTCCC GCCGCCCCCC CCCCCCCAAA AAGAAACCAA   
  
  
+ AAAAAAAAAA AAAATGGATG ATTCTTAGAA CTGACCATTT TTCCTTCCTC CTCCCATTTT ATTTTTTTCA   
  
  
+ GGCTGTTTTT CTTCTACCTG TTGCTTTAGC ATCACTCATA AAAACAAGGT GCAAGCGCAT GACATCGGGT   
  
  
+ ACTGAGCTTC TGAGTTTACA TGCATCGTCA TGAAATTCGG TAGACTTTGA AGTAAAGTTT CCCCATCGTA   
  
  
+ ACTGGTGGTG CATACCTGTC TTCACGCCCA TCAGTAGAGT GGTGAAGTGA AGCAATGTCG AATAGTTTGT   
  
  
+ ATTACGAGCC CAAGAGAGAA ACTGATGCAT ATTTTATGCC TCAATGTCAA TCCTTGAACC CCCAGCTCGA   
  
  
+ TTACAACACC ATTGACTATG GAGCTTTTAT GTGCTCAAAA GTTTTCCTGG GTCAGTACTG CACTCTGGAA   
  
  
+ TCATCCTCAG GAACTGGGAC TTGTCCGGTG TCTAGCTCCA CATCAACTCT CAGCTTCTCA TCCAATGGTA   
  
  
+ GCCCTGGATC ACAGCTTGAT TCCAACTCTT ATCCTTCTGA TCAAAATTGC TCTCCTGATA ATGCCAATTC   
  
  
+ CTCATCTTTA AGTCATTCCT GCGTCACAGA TGATGTGGAT GACCTGAAAT ACAGGCTGAG AGAGTTGGAA   
  
  
+ ACAGTGATGT TGGGACCTGA TCCCGATTTT GTCTACGGCA ATAGCAACCA CACATTTGGG GTTGGGATCC   
  
  
+ ATGTAGTGTC ATCAGAGTTT GACAGCTGGG GGAAAGTGAT GGAGATGATC GCTCGAAAGG ATTTGAAACT   
  
  
+ GGTGCTAATT GCCTGTGCAA GAGCAGTTGC TGAAAATGAT CAGTTGTTGG CCCAGTGGCT GATGGATGAA   
  
  
+ TTGAGACAGA TGGTTTCAGT TTCTGGTGAA CCAATTCAAA GATTGGGTGC TTATATGTTG GAAGGGCTCG   
  
  
+ TAGCGAGGCA GGCCTCCTCA GGTAGCTCCA TTTATAAAGC ATTGAGATGC AAGGAACCCG CGAGTGCTGA   
  
  
+ CCTTTTATCT TACATGCACA TACTCTTTGA GGTTTGCCCA TACATCAAAT TTGGCTACAT GTCAGCAAAT   
  
  
+ GGTGCCATTG CAGAAGCAAT GAAGGATGAA AAGAGAGTCC ACATTGTCGA TTTCCAAATT GGGCAGGGAA   
  
  
+ GCCAATGGGT GACCCTAATC CAGGCATTTG CAGCCCGGCC AGGGGGTCCA CCCCACATTC GCATTACCGG   
  
  
+ TATCGATGAC TCCTACTCTG CATATGCTCG TGGAGGTGGG TTGAACATTG TGGGCCATAG GCTCTTGAGG   
  
  
+ TTGGCTCAGT CATTTAAGGT CCCATTTGAG TTCAACGCAG TTGACGTCCC GGCTTGTCAA GTCCTGCTTA   
  
  
+ AAGATCTCGG CATTCAACAT GGTGAGGCCT TGGCTGTGAA CTTTGCCTTC ATTCTTCACC ACATGCCCGA   
  
  
+ TGAGAGCGTG AGCACAGAGA ATCACCGGGA TCGTCTTCTG AGGGTGGTAA AAGGGCTGAA GCCAAAAGTG   
  
  
+ GTAACGTTAG TGGAGCAAGA GTGTAACACA AACTCTGCTG CTTTCTTGCC TCGCTTTGTT GAGACATTGG   
  
  
+ AATACTACAC AGCGATGTTT GAGTCCATGG ATGTGACTTT GCCTAGGGAT CATAAAGACC GGATCAATGT   
  
  
+ TGAGCAGCAT TGTCTTGCTC GAGATGTTGT GAACTTGGTT GCGTGTGAAG GAGCTGAGAG GGTGGAGCGG   
  
  
+ CATGAGCTCC TTGGGAAATG GAGGTCGCGG TTCACAATGG CAGGGTTTAA GCCATACCCT TTAAGCACTC   
  
  
+ TGGTAAACAA CACAATAAGA ACTTTGTTGA AGAACTACTG TGATAGGTAT GGGCTAGAGG AGAGGAATGG   
  
  
+ AGGTCTTTAT CTTGGGTGGA TGAATAGAGA TTTGGTTGCT TCGAGTGCCT GGCAGTGTAG TAATTA  

- +Up\_Stream \_Len000GTAAAA TCATTATTTA AGGGTACACT CCTGGTGACG GATGACTAAG GCTTACCGTA   
  
  
- AAATCATTAT TTGAGGGTAC ACTCCTGGTG AACTACTCCT TAAGTCAAAA TATTAGGTGT CCACAGTTTC   
  
  
- GGATAAGGAT GAGAGTGGTG AGACGATAGA GAGGGGGGGG GGGGGGGGGC GGGGGGAGTT TTAGACGGAG   
  
  
- GAGGCAAGAG AGGTGTCGTC TAAGGAGTTA GTCCTTTCCA TACAGTCTCG AGTTTTGCTA AGTTGTAGTT   
  
  
- TTACCCTATT AAATAAGACG AAAGTCAAGA CCCAATAACA GTATACAAGT AAGTCAAGTC ACAAAGATCG   
  
  
- TACTAACGAA AAACTACGGA AGAAAAAACA AGACCCAACC TGCTTGAGAC AGAAAAGACA CGTATCTAAA   
  
  
- CTCGAGGCTC GAGTTAAAAA CCCTAAAAAA ATCGACTGAA AAGACGAACC ACAACACTTT AACACTACCC   
  
  
- AAAACCGAAA CAATTTGAAC AAATATTGAA AAAGACAGTT TTGAAATATC ATTTAACAAC GTTAAACTCT   
  
  
- TAATACGTGA GTCACCGAGA CACTAAATAG CAGGCGACCG TTTAAAAGAC TACTTTCCTC TACTAAATAA   
  
  
- TTAAAAAGTC CGTACCAGTT TAAAAGATCG GAAAGTACAT GTAACCAACC ACTATCCGAC TATCGATTTA   
  
  
- ATACAGAGTA AAGAGATTTA AATAACGGTC TATACTTTAA CCACCTTGTC AAATTCCCAA AACCAGCAGA   
  
  
- AAAAACGGGA AAAAAAACCC AGGGGGGGGG CGGGCCCCCC CTTGGGCCTC CCGTCTTTTT CATTGTATTG   
  
  
- AATTTTAATA GTAACTGCAT CAGGAGGTTG GTATTACTTT CGTTGACTTT ATTCTCGTAC GATTCGATCT   
  
  
- CTCATCCGAC GGAACAGTCG GAGGGAAAAC TGTACTCTTT CTACCCTCTT CAAATTTTAT TTCAAGGAGC   
  
  
- AGAAACGTAG GGCCTGGTGT TAAGCCTTGA CCCACTCCTG GAGGAATCCG AACATTTGTG TTCAAAGCTT   
  
  
- GAATCTAAAC GCATGTACAG TTACGAAATG ACCTATTCGA ATCGATTGTG GGGAGTAGGA AGTTGGACAT   
  
  
- GGAAGTATAT TTTTTTTCCT GTTAGGCCAC GTGTTCGTAG GGCGTAAGTG CGTTCCAGAC CCCTTCCCGG   
  
  
- CGTGGGGTTT CCCACTTAAC ATCCGTCGGA TTGGACTGAA ACAGTCACCG ATTAAGGTGC TAAGCTTGGG   
  
  
- TACTAAAAAA CCAATGTGCC CTTGTTGGAA TGGTAACGAG TTTCCGAGGG GAAGAAGTTG GAGATGGAAG   
  
  
- TATATACTTA CTTTTAGGAA ACCGATTCTT AACTTTTCCT TTATACACCA ATAACCCAAA CCCAAGAATA   
  
  
- AGGCAACCAA CGGGGAAGTG TAACTTACGT AGGAGAGCGA TTCTTGACTT TTCCTGTATA CCCCTACATA   
  
  
- TCCAGTTTAA ACTGAGTTAC TATTGACTCC TATGTTCTTA AGATCTTCAG AAATAGTCCC CTTAAAAAAG   
  
  
- AAAAAAGGTG AACACTGAAA AACTACGGAA CGAAAAATGA GAAGTAACTT ACTCATGGGA AGAACTGTCT   
  
  
- AAGAAGGAGG GAAAAAAGAA TTAAAGAAGA ACATCACCTT ATATTCATAC GAAGATCCAT CAACCTACGG   
  
  
- TACCTTAGAG TAGGGACACA CACGTAAGAA GGGGAGAGGG CGGCGGGGGG GGGGGGGTTT TTCTTTGGTT   
  
  
- TTTTTTTTTT TTTTACCTAC TAAGAATCTT GACTGGTAAA AAGGAAGGAG GAGGGTAAAA TAAAAAAAGT   
  
  
- CCGACAAAAA GAAGATGGAC AACGAAATCG TAGTGAGTAT TTTTGTTCCA CGTTCGCGTA CTGTAGCCCA   
  
  
- TGACTCGAAG ACTCAAATGT ACGTAGCAGT ACTTTAAGCC ATCTGAAACT TCATTTCAAA GGGGTAGCAT   
  
  
- TGACCACCAC GTATGGACAG AAGTGCGGGT AGTCATCTCA CCACTTCACT TCGTTACAGC TTATCAAACA   
  
  
- TAATGCTCGG GTTCTCTCTT TGACTACGTA TAAAATACGG AGTTACAGTT AGGAACTTGG GGGTCGAGCT   
  
  
- AATGTTGTGG TAACTGATAC CTCGAAAATA CACGAGTTTT CAAAAGGACC CAGTCATGAC GTGAGACCTT   
  
  
- AGTAGGAGTC CTTGACCCTG AACAGGCCAC AGATCGAGGT GTAGTTGAGA GTCGAAGAGT AGGTTACCAT   
  
  
- CGGGACCTAG TGTCGAACTA AGGTTGAGAA TAGGAAGACT AGTTTTAACG AGAGGACTAT TACGGTTAAG   
  
  
- GAGTAGAAAT TCAGTAAGGA CGCAGTGTCT ACTACACCTA CTGGACTTTA TGTCCGACTC TCTCAACCTT   
  
  
- TGTCACTACA ACCCTGGACT AGGGCTAAAA CAGATGCCGT TATCGTTGGT GTGTAAACCC CAACCCTAGG   
  
  
- TACATCACAG TAGTCTCAAA CTGTCGACCC CCTTTCACTA CCTCTACTAG CGAGCTTTCC TAAACTTTGA   
  
  
- CCACGATTAA CGGACACGTT CTCGTCAACG ACTTTTACTA GTCAACAACC GGGTCACCGA CTACCTACTT   
  
  
- AACTCTGTCT ACCAAAGTCA AAGACCACTT GGTTAAGTTT CTAACCCACG AATATACAAC CTTCCCGAGC   
  
  
- ATCGCTCCGT CCGGAGGAGT CCATCGAGGT AAATATTTCG TAACTCTACG TTCCTTGGGC GCTCACGACT   
  
  
- GGAAAATAGA ATGTACGTGT ATGAGAAACT CCAAACGGGT ATGTAGTTTA AACCGATGTA CAGTCGTTTA   
  
  
- CCACGGTAAC GTCTTCGTTA CTTCCTACTT TTCTCTCAGG TGTAACAGCT AAAGGTTTAA CCCGTCCCTT   
  
  
- CGGTTACCCA CTGGGATTAG GTCCGTAAAC GTCGGGCCGG TCCCCCAGGT GGGGTGTAAG CGTAATGGCC   
  
  
- ATAGCTACTG AGGATGAGAC GTATACGAGC ACCTCCACCC AACTTGTAAC ACCCGGTATC CGAGAACTCC   
  
  
- AACCGAGTCA GTAAATTCCA GGGTAAACTC AAGTTGCGTC AACTGCAGGG CCGAACAGTT CAGGACGAAT   
  
  
- TTCTAGAGCC GTAAGTTGTA CCACTCCGGA ACCGACACTT GAAACGGAAG TAAGAAGTGG TGTACGGGCT   
  
  
- ACTCTCGCAC TCGTGTCTCT TAGTGGCCCT AGCAGAAGAC TCCCACCATT TTCCCGACTT CGGTTTTCAC   
  
  
- CATTGCAATC ACCTCGTTCT CACATTGTGT TTGAGACGAC GAAAGAACGG AGCGAAACAA CTCTGTAACC   
  
  
- TTATGATGTG TCGCTACAAA CTCAGGTACC TACACTGAAA CGGATCCCTA GTATTTCTGG CCTAGTTACA   
  
  
- ACTCGTCGTA ACAGAACGAG CTCTACAACA CTTGAACCAA CGCACACTTC CTCGACTCTC CCACCTCGCC   
  
  
- GTACTCGAGG AACCCTTTAC CTCCAGCGCC AAGTGTTACC GTCCCAAATT CGGTATGGGA AATTCGTGAG   
  
  
- ACCATTTGTT GTGTTATTCT TGAAACAACT TCTTGATGAC ACTATCCATA CCCGATCTCC TCTCCTTACC   
  
  
- TCCAGAAATA GAACCCACCT ACTTATCTCT AAACCAACGA AGCTCACGGA CCGTCACATC ATTAAT

+     WRE3

| Site Name | Organism | Position | Strand | Matrix score. | sequence | function |
| --- | --- | --- | --- | --- | --- | --- |
| WRE3 | Pisum sativum | 2978 | - | 6 | CCACCT |  |

>HU01G01391.1   
+ +Up\_Stream \_Len000CATTTT AGTAATAAAT TCCCATGTGA GGACCACTGC CTACTGATTC CGAATGGCAT   
  
  
+ TTTAGTAATA AACTCCCATG TGAGGACCAC TTGATGAGGA ATTCAGTTTT ATAATCCACA GGTGTCAAAG   
  
  
+ CCTATTCCTA CTCTCACCAC TCTGCTATCT CTCCCCCCCC CCCCCCCCCG CCCCCCTCAA AATCTGCCTC   
  
  
+ CTCCGTTCTC TCCACAGCAG ATTCCTCAAT CAGGAAAGGT ATGTCAGAGC TCAAAACGAT TCAACATCAA   
  
  
+ AATGGGATAA TTTATTCTGC TTTCAGTTCT GGGTTATTGT CATATGTTCA TTCAGTTCAG TGTTTCTAGC   
  
  
+ ATGATTGCTT TTTGATGCCT TCTTTTTTGT TCTGGGTTGG ACGAACTCTG TCTTTTCTGT GCATAGATTT   
  
  
+ GAGCTCCGAG CTCAATTTTT GGGATTTTTT TAGCTGACTT TTCTGCTTGG TGTTGTGAAA TTGTGATGGG   
  
  
+ TTTTGGCTTT GTTAAACTTG TTTATAACTT TTTCTGTCAA AACTTTATAG TAAATTGTTG CAATTTGAGA   
  
  
+ ATTATGCACT CAGTGGCTCT GTGATTTATC GTCCGCTGGC AAATTTTCTG ATGAAAGGAG ATGATTTATT   
  
  
+ AATTTTTCAG GCATGGTCAA ATTTTCTAGC CTTTCATGTA CATTGGTTGG TGATAGGCTG ATAGCTAAAT   
  
  
+ TATGTCTCAT TTCTCTAAAT TTATTGCCAG ATATGAAATT GGTGGAACAG TTTAAGGGTT TTGGTCGTCT   
  
  
+ TTTTTGCCCT TTTTTTTGGG TCCCCCCCCC GCCCGGGGGG GAACCCGGAG GGCAGAAAAA GTAACATAAC   
  
  
+ TTAAAATTAT CATTGACGTA GTCCTCCAAC CATAATGAAA GCAACTGAAA TAAGAGCATG CTAAGCTAGA   
  
  
+ GAGTAGGCTG CCTTGTCAGC CTCCCTTTTG ACATGAGAAA GATGGGAGAA GTTTAAAATA AAGTTCCTCG   
  
  
+ TCTTTGCATC CCGGACCACA ATTCGGAACT GGGTGAGGAC CTCCTTAGGC TTGTAAACAC AAGTTTCGAA   
  
  
+ CTTAGATTTG CGTACATGTC AATGCTTTAC TGGATAAGCT TAGCTAACAC CCCTCATCCT TCAACCTGTA   
  
  
+ CCTTCATATA AAAAAAAGGA CAATCCGGTG CACAAGCATC CCGCATTCAC GCAAGGTCTG GGGAAGGGCC   
  
  
+ GCACCCCAAA GGGTGAATTG TAGGCAGCCT AACCTGACTT TGTCAGTGGC TAATTCCACG ATTCGAACCC   
  
  
+ ATGATTTTTT GGTTACACGG GAACAACCTT ACCATTGCTC AAAGGCTCCC CTTCTTCAAC CTCTACCTTC   
  
  
+ ATATATGAAT GAAAATCCTT TGGCTAAGAA TTGAAAAGGA AATATGTGGT TATTGGGTTT GGGTTCTTAT   
  
  
+ TCCGTTGGTT GCCCCTTCAC ATTGAATGCA TCCTCTCGCT AAGAACTGAA AAGGACATAT GGGGATGTAT   
  
  
+ AGGTCAAATT TGACTCAATG ATAACTGAGG ATACAAGAAT TCTAGAAGTC TTTATCAGGG GAATTTTTTC   
  
  
+ TTTTTTCCAC TTGTGACTTT TTGATGCCTT GCTTTTTACT CTTCATTGAA TGAGTACCCT TCTTGACAGA   
  
  
+ TTCTTCCTCC CTTTTTTCTT AATTTCTTCT TGTAGTGGAA TATAAGTATG CTTCTAGGTA GTTGGATGCC   
  
  
+ ATGGAATCTC ATCCCTGTGT GTGCATTCTT CCCCTCTCCC GCCGCCCCCC CCCCCCCAAA AAGAAACCAA   
  
  
+ AAAAAAAAAA AAAATGGATG ATTCTTAGAA CTGACCATTT TTCCTTCCTC CTCCCATTTT ATTTTTTTCA   
  
  
+ GGCTGTTTTT CTTCTACCTG TTGCTTTAGC ATCACTCATA AAAACAAGGT GCAAGCGCAT GACATCGGGT   
  
  
+ ACTGAGCTTC TGAGTTTACA TGCATCGTCA TGAAATTCGG TAGACTTTGA AGTAAAGTTT CCCCATCGTA   
  
  
+ ACTGGTGGTG CATACCTGTC TTCACGCCCA TCAGTAGAGT GGTGAAGTGA AGCAATGTCG AATAGTTTGT   
  
  
+ ATTACGAGCC CAAGAGAGAA ACTGATGCAT ATTTTATGCC TCAATGTCAA TCCTTGAACC CCCAGCTCGA   
  
  
+ TTACAACACC ATTGACTATG GAGCTTTTAT GTGCTCAAAA GTTTTCCTGG GTCAGTACTG CACTCTGGAA   
  
  
+ TCATCCTCAG GAACTGGGAC TTGTCCGGTG TCTAGCTCCA CATCAACTCT CAGCTTCTCA TCCAATGGTA   
  
  
+ GCCCTGGATC ACAGCTTGAT TCCAACTCTT ATCCTTCTGA TCAAAATTGC TCTCCTGATA ATGCCAATTC   
  
  
+ CTCATCTTTA AGTCATTCCT GCGTCACAGA TGATGTGGAT GACCTGAAAT ACAGGCTGAG AGAGTTGGAA   
  
  
+ ACAGTGATGT TGGGACCTGA TCCCGATTTT GTCTACGGCA ATAGCAACCA CACATTTGGG GTTGGGATCC   
  
  
+ ATGTAGTGTC ATCAGAGTTT GACAGCTGGG GGAAAGTGAT GGAGATGATC GCTCGAAAGG ATTTGAAACT   
  
  
+ GGTGCTAATT GCCTGTGCAA GAGCAGTTGC TGAAAATGAT CAGTTGTTGG CCCAGTGGCT GATGGATGAA   
  
  
+ TTGAGACAGA TGGTTTCAGT TTCTGGTGAA CCAATTCAAA GATTGGGTGC TTATATGTTG GAAGGGCTCG   
  
  
+ TAGCGAGGCA GGCCTCCTCA GGTAGCTCCA TTTATAAAGC ATTGAGATGC AAGGAACCCG CGAGTGCTGA   
  
  
+ CCTTTTATCT TACATGCACA TACTCTTTGA GGTTTGCCCA TACATCAAAT TTGGCTACAT GTCAGCAAAT   
  
  
+ GGTGCCATTG CAGAAGCAAT GAAGGATGAA AAGAGAGTCC ACATTGTCGA TTTCCAAATT GGGCAGGGAA   
  
  
+ GCCAATGGGT GACCCTAATC CAGGCATTTG CAGCCCGGCC AGGGGGTCCA CCCCACATTC GCATTACCGG   
  
  
+ TATCGATGAC TCCTACTCTG CATATGCTCG TGGAGGTGGG TTGAACATTG TGGGCCATAG GCTCTTGAGG   
  
  
+ TTGGCTCAGT CATTTAAGGT CCCATTTGAG TTCAACGCAG TTGACGTCCC GGCTTGTCAA GTCCTGCTTA   
  
  
+ AAGATCTCGG CATTCAACAT GGTGAGGCCT TGGCTGTGAA CTTTGCCTTC ATTCTTCACC ACATGCCCGA   
  
  
+ TGAGAGCGTG AGCACAGAGA ATCACCGGGA TCGTCTTCTG AGGGTGGTAA AAGGGCTGAA GCCAAAAGTG   
  
  
+ GTAACGTTAG TGGAGCAAGA GTGTAACACA AACTCTGCTG CTTTCTTGCC TCGCTTTGTT GAGACATTGG   
  
  
+ AATACTACAC AGCGATGTTT GAGTCCATGG ATGTGACTTT GCCTAGGGAT CATAAAGACC GGATCAATGT   
  
  
+ TGAGCAGCAT TGTCTTGCTC GAGATGTTGT GAACTTGGTT GCGTGTGAAG GAGCTGAGAG GGTGGAGCGG   
  
  
+ CATGAGCTCC TTGGGAAATG GAGGTCGCGG TTCACAATGG CAGGGTTTAA GCCATACCCT TTAAGCACTC   
  
  
+ TGGTAAACAA CACAATAAGA ACTTTGTTGA AGAACTACTG TGATAGGTAT GGGCTAGAGG AGAGGAATGG   
  
  
+ AGGTCTTTAT CTTGGGTGGA TGAATAGAGA TTTGGTTGCT TCGAGTGCCT GGCAGTGTAG TAATTA  

- +Up\_Stream \_Len000GTAAAA TCATTATTTA AGGGTACACT CCTGGTGACG GATGACTAAG GCTTACCGTA   
  
  
- AAATCATTAT TTGAGGGTAC ACTCCTGGTG AACTACTCCT TAAGTCAAAA TATTAGGTGT CCACAGTTTC   
  
  
- GGATAAGGAT GAGAGTGGTG AGACGATAGA GAGGGGGGGG GGGGGGGGGC GGGGGGAGTT TTAGACGGAG   
  
  
- GAGGCAAGAG AGGTGTCGTC TAAGGAGTTA GTCCTTTCCA TACAGTCTCG AGTTTTGCTA AGTTGTAGTT   
  
  
- TTACCCTATT AAATAAGACG AAAGTCAAGA CCCAATAACA GTATACAAGT AAGTCAAGTC ACAAAGATCG   
  
  
- TACTAACGAA AAACTACGGA AGAAAAAACA AGACCCAACC TGCTTGAGAC AGAAAAGACA CGTATCTAAA   
  
  
- CTCGAGGCTC GAGTTAAAAA CCCTAAAAAA ATCGACTGAA AAGACGAACC ACAACACTTT AACACTACCC   
  
  
- AAAACCGAAA CAATTTGAAC AAATATTGAA AAAGACAGTT TTGAAATATC ATTTAACAAC GTTAAACTCT   
  
  
- TAATACGTGA GTCACCGAGA CACTAAATAG CAGGCGACCG TTTAAAAGAC TACTTTCCTC TACTAAATAA   
  
  
- TTAAAAAGTC CGTACCAGTT TAAAAGATCG GAAAGTACAT GTAACCAACC ACTATCCGAC TATCGATTTA   
  
  
- ATACAGAGTA AAGAGATTTA AATAACGGTC TATACTTTAA CCACCTTGTC AAATTCCCAA AACCAGCAGA   
  
  
- AAAAACGGGA AAAAAAACCC AGGGGGGGGG CGGGCCCCCC CTTGGGCCTC CCGTCTTTTT CATTGTATTG   
  
  
- AATTTTAATA GTAACTGCAT CAGGAGGTTG GTATTACTTT CGTTGACTTT ATTCTCGTAC GATTCGATCT   
  
  
- CTCATCCGAC GGAACAGTCG GAGGGAAAAC TGTACTCTTT CTACCCTCTT CAAATTTTAT TTCAAGGAGC   
  
  
- AGAAACGTAG GGCCTGGTGT TAAGCCTTGA CCCACTCCTG GAGGAATCCG AACATTTGTG TTCAAAGCTT   
  
  
- GAATCTAAAC GCATGTACAG TTACGAAATG ACCTATTCGA ATCGATTGTG GGGAGTAGGA AGTTGGACAT   
  
  
- GGAAGTATAT TTTTTTTCCT GTTAGGCCAC GTGTTCGTAG GGCGTAAGTG CGTTCCAGAC CCCTTCCCGG   
  
  
- CGTGGGGTTT CCCACTTAAC ATCCGTCGGA TTGGACTGAA ACAGTCACCG ATTAAGGTGC TAAGCTTGGG   
  
  
- TACTAAAAAA CCAATGTGCC CTTGTTGGAA TGGTAACGAG TTTCCGAGGG GAAGAAGTTG GAGATGGAAG   
  
  
- TATATACTTA CTTTTAGGAA ACCGATTCTT AACTTTTCCT TTATACACCA ATAACCCAAA CCCAAGAATA   
  
  
- AGGCAACCAA CGGGGAAGTG TAACTTACGT AGGAGAGCGA TTCTTGACTT TTCCTGTATA CCCCTACATA   
  
  
- TCCAGTTTAA ACTGAGTTAC TATTGACTCC TATGTTCTTA AGATCTTCAG AAATAGTCCC CTTAAAAAAG   
  
  
- AAAAAAGGTG AACACTGAAA AACTACGGAA CGAAAAATGA GAAGTAACTT ACTCATGGGA AGAACTGTCT   
  
  
- AAGAAGGAGG GAAAAAAGAA TTAAAGAAGA ACATCACCTT ATATTCATAC GAAGATCCAT CAACCTACGG   
  
  
- TACCTTAGAG TAGGGACACA CACGTAAGAA GGGGAGAGGG CGGCGGGGGG GGGGGGGTTT TTCTTTGGTT   
  
  
- TTTTTTTTTT TTTTACCTAC TAAGAATCTT GACTGGTAAA AAGGAAGGAG GAGGGTAAAA TAAAAAAAGT   
  
  
- CCGACAAAAA GAAGATGGAC AACGAAATCG TAGTGAGTAT TTTTGTTCCA CGTTCGCGTA CTGTAGCCCA   
  
  
- TGACTCGAAG ACTCAAATGT ACGTAGCAGT ACTTTAAGCC ATCTGAAACT TCATTTCAAA GGGGTAGCAT   
  
  
- TGACCACCAC GTATGGACAG AAGTGCGGGT AGTCATCTCA CCACTTCACT TCGTTACAGC TTATCAAACA   
  
  
- TAATGCTCGG GTTCTCTCTT TGACTACGTA TAAAATACGG AGTTACAGTT AGGAACTTGG GGGTCGAGCT   
  
  
- AATGTTGTGG TAACTGATAC CTCGAAAATA CACGAGTTTT CAAAAGGACC CAGTCATGAC GTGAGACCTT   
  
  
- AGTAGGAGTC CTTGACCCTG AACAGGCCAC AGATCGAGGT GTAGTTGAGA GTCGAAGAGT AGGTTACCAT   
  
  
- CGGGACCTAG TGTCGAACTA AGGTTGAGAA TAGGAAGACT AGTTTTAACG AGAGGACTAT TACGGTTAAG   
  
  
- GAGTAGAAAT TCAGTAAGGA CGCAGTGTCT ACTACACCTA CTGGACTTTA TGTCCGACTC TCTCAACCTT   
  
  
- TGTCACTACA ACCCTGGACT AGGGCTAAAA CAGATGCCGT TATCGTTGGT GTGTAAACCC CAACCCTAGG   
  
  
- TACATCACAG TAGTCTCAAA CTGTCGACCC CCTTTCACTA CCTCTACTAG CGAGCTTTCC TAAACTTTGA   
  
  
- CCACGATTAA CGGACACGTT CTCGTCAACG ACTTTTACTA GTCAACAACC GGGTCACCGA CTACCTACTT   
  
  
- AACTCTGTCT ACCAAAGTCA AAGACCACTT GGTTAAGTTT CTAACCCACG AATATACAAC CTTCCCGAGC   
  
  
- ATCGCTCCGT CCGGAGGAGT CCATCGAGGT AAATATTTCG TAACTCTACG TTCCTTGGGC GCTCACGACT   
  
  
- GGAAAATAGA ATGTACGTGT ATGAGAAACT CCAAACGGGT ATGTAGTTTA AACCGATGTA CAGTCGTTTA   
  
  
- CCACGGTAAC GTCTTCGTTA CTTCCTACTT TTCTCTCAGG TGTAACAGCT AAAGGTTTAA CCCGTCCCTT   
  
  
- CGGTTACCCA CTGGGATTAG GTCCGTAAAC GTCGGGCCGG TCCCCCAGGT GGGGTGTAAG CGTAATGGCC   
  
  
- ATAGCTACTG AGGATGAGAC GTATACGAGC ACCTCCACCC AACTTGTAAC ACCCGGTATC CGAGAACTCC   
  
  
- AACCGAGTCA GTAAATTCCA GGGTAAACTC AAGTTGCGTC AACTGCAGGG CCGAACAGTT CAGGACGAAT   
  
  
- TTCTAGAGCC GTAAGTTGTA CCACTCCGGA ACCGACACTT GAAACGGAAG TAAGAAGTGG TGTACGGGCT   
  
  
- ACTCTCGCAC TCGTGTCTCT TAGTGGCCCT AGCAGAAGAC TCCCACCATT TTCCCGACTT CGGTTTTCAC   
  
  
- CATTGCAATC ACCTCGTTCT CACATTGTGT TTGAGACGAC GAAAGAACGG AGCGAAACAA CTCTGTAACC   
  
  
- TTATGATGTG TCGCTACAAA CTCAGGTACC TACACTGAAA CGGATCCCTA GTATTTCTGG CCTAGTTACA   
  
  
- ACTCGTCGTA ACAGAACGAG CTCTACAACA CTTGAACCAA CGCACACTTC CTCGACTCTC CCACCTCGCC   
  
  
- GTACTCGAGG AACCCTTTAC CTCCAGCGCC AAGTGTTACC GTCCCAAATT CGGTATGGGA AATTCGTGAG   
  
  
- ACCATTTGTT GTGTTATTCT TGAAACAACT TCTTGATGAC ACTATCCATA CCCGATCTCC TCTCCTTACC   
  
  
- TCCAGAAATA GAACCCACCT ACTTATCTCT AAACCAACGA AGCTCACGGA CCGTCACATC ATTAAT

+     as-1

| Site Name | Organism | Position | Strand | Matrix score. | sequence | function |
| --- | --- | --- | --- | --- | --- | --- |
| as-1 | Arabidopsis thaliana | 3056 | + | 5 | TGACG |  |
| as-1 | Arabidopsis thaliana | 2336 | - | 5 | TGACG |  |
| as-1 | Arabidopsis thaliana | 1920 | - | 5 | TGACG |  |
| as-1 | Arabidopsis thaliana | 858 | + | 5 | TGACG |  |

>HU01G01391.1   
+ +Up\_Stream \_Len000CATTTT AGTAATAAAT TCCCATGTGA GGACCACTGC CTACTGATTC CGAATGGCAT   
  
  
+ TTTAGTAATA AACTCCCATG TGAGGACCAC TTGATGAGGA ATTCAGTTTT ATAATCCACA GGTGTCAAAG   
  
  
+ CCTATTCCTA CTCTCACCAC TCTGCTATCT CTCCCCCCCC CCCCCCCCCG CCCCCCTCAA AATCTGCCTC   
  
  
+ CTCCGTTCTC TCCACAGCAG ATTCCTCAAT CAGGAAAGGT ATGTCAGAGC TCAAAACGAT TCAACATCAA   
  
  
+ AATGGGATAA TTTATTCTGC TTTCAGTTCT GGGTTATTGT CATATGTTCA TTCAGTTCAG TGTTTCTAGC   
  
  
+ ATGATTGCTT TTTGATGCCT TCTTTTTTGT TCTGGGTTGG ACGAACTCTG TCTTTTCTGT GCATAGATTT   
  
  
+ GAGCTCCGAG CTCAATTTTT GGGATTTTTT TAGCTGACTT TTCTGCTTGG TGTTGTGAAA TTGTGATGGG   
  
  
+ TTTTGGCTTT GTTAAACTTG TTTATAACTT TTTCTGTCAA AACTTTATAG TAAATTGTTG CAATTTGAGA   
  
  
+ ATTATGCACT CAGTGGCTCT GTGATTTATC GTCCGCTGGC AAATTTTCTG ATGAAAGGAG ATGATTTATT   
  
  
+ AATTTTTCAG GCATGGTCAA ATTTTCTAGC CTTTCATGTA CATTGGTTGG TGATAGGCTG ATAGCTAAAT   
  
  
+ TATGTCTCAT TTCTCTAAAT TTATTGCCAG ATATGAAATT GGTGGAACAG TTTAAGGGTT TTGGTCGTCT   
  
  
+ TTTTTGCCCT TTTTTTTGGG TCCCCCCCCC GCCCGGGGGG GAACCCGGAG GGCAGAAAAA GTAACATAAC   
  
  
+ TTAAAATTAT CATTGACGTA GTCCTCCAAC CATAATGAAA GCAACTGAAA TAAGAGCATG CTAAGCTAGA   
  
  
+ GAGTAGGCTG CCTTGTCAGC CTCCCTTTTG ACATGAGAAA GATGGGAGAA GTTTAAAATA AAGTTCCTCG   
  
  
+ TCTTTGCATC CCGGACCACA ATTCGGAACT GGGTGAGGAC CTCCTTAGGC TTGTAAACAC AAGTTTCGAA   
  
  
+ CTTAGATTTG CGTACATGTC AATGCTTTAC TGGATAAGCT TAGCTAACAC CCCTCATCCT TCAACCTGTA   
  
  
+ CCTTCATATA AAAAAAAGGA CAATCCGGTG CACAAGCATC CCGCATTCAC GCAAGGTCTG GGGAAGGGCC   
  
  
+ GCACCCCAAA GGGTGAATTG TAGGCAGCCT AACCTGACTT TGTCAGTGGC TAATTCCACG ATTCGAACCC   
  
  
+ ATGATTTTTT GGTTACACGG GAACAACCTT ACCATTGCTC AAAGGCTCCC CTTCTTCAAC CTCTACCTTC   
  
  
+ ATATATGAAT GAAAATCCTT TGGCTAAGAA TTGAAAAGGA AATATGTGGT TATTGGGTTT GGGTTCTTAT   
  
  
+ TCCGTTGGTT GCCCCTTCAC ATTGAATGCA TCCTCTCGCT AAGAACTGAA AAGGACATAT GGGGATGTAT   
  
  
+ AGGTCAAATT TGACTCAATG ATAACTGAGG ATACAAGAAT TCTAGAAGTC TTTATCAGGG GAATTTTTTC   
  
  
+ TTTTTTCCAC TTGTGACTTT TTGATGCCTT GCTTTTTACT CTTCATTGAA TGAGTACCCT TCTTGACAGA   
  
  
+ TTCTTCCTCC CTTTTTTCTT AATTTCTTCT TGTAGTGGAA TATAAGTATG CTTCTAGGTA GTTGGATGCC   
  
  
+ ATGGAATCTC ATCCCTGTGT GTGCATTCTT CCCCTCTCCC GCCGCCCCCC CCCCCCCAAA AAGAAACCAA   
  
  
+ AAAAAAAAAA AAAATGGATG ATTCTTAGAA CTGACCATTT TTCCTTCCTC CTCCCATTTT ATTTTTTTCA   
  
  
+ GGCTGTTTTT CTTCTACCTG TTGCTTTAGC ATCACTCATA AAAACAAGGT GCAAGCGCAT GACATCGGGT   
  
  
+ ACTGAGCTTC TGAGTTTACA TGCATCGTCA TGAAATTCGG TAGACTTTGA AGTAAAGTTT CCCCATCGTA   
  
  
+ ACTGGTGGTG CATACCTGTC TTCACGCCCA TCAGTAGAGT GGTGAAGTGA AGCAATGTCG AATAGTTTGT   
  
  
+ ATTACGAGCC CAAGAGAGAA ACTGATGCAT ATTTTATGCC TCAATGTCAA TCCTTGAACC CCCAGCTCGA   
  
  
+ TTACAACACC ATTGACTATG GAGCTTTTAT GTGCTCAAAA GTTTTCCTGG GTCAGTACTG CACTCTGGAA   
  
  
+ TCATCCTCAG GAACTGGGAC TTGTCCGGTG TCTAGCTCCA CATCAACTCT CAGCTTCTCA TCCAATGGTA   
  
  
+ GCCCTGGATC ACAGCTTGAT TCCAACTCTT ATCCTTCTGA TCAAAATTGC TCTCCTGATA ATGCCAATTC   
  
  
+ CTCATCTTTA AGTCATTCCT GCGTCACAGA TGATGTGGAT GACCTGAAAT ACAGGCTGAG AGAGTTGGAA   
  
  
+ ACAGTGATGT TGGGACCTGA TCCCGATTTT GTCTACGGCA ATAGCAACCA CACATTTGGG GTTGGGATCC   
  
  
+ ATGTAGTGTC ATCAGAGTTT GACAGCTGGG GGAAAGTGAT GGAGATGATC GCTCGAAAGG ATTTGAAACT   
  
  
+ GGTGCTAATT GCCTGTGCAA GAGCAGTTGC TGAAAATGAT CAGTTGTTGG CCCAGTGGCT GATGGATGAA   
  
  
+ TTGAGACAGA TGGTTTCAGT TTCTGGTGAA CCAATTCAAA GATTGGGTGC TTATATGTTG GAAGGGCTCG   
  
  
+ TAGCGAGGCA GGCCTCCTCA GGTAGCTCCA TTTATAAAGC ATTGAGATGC AAGGAACCCG CGAGTGCTGA   
  
  
+ CCTTTTATCT TACATGCACA TACTCTTTGA GGTTTGCCCA TACATCAAAT TTGGCTACAT GTCAGCAAAT   
  
  
+ GGTGCCATTG CAGAAGCAAT GAAGGATGAA AAGAGAGTCC ACATTGTCGA TTTCCAAATT GGGCAGGGAA   
  
  
+ GCCAATGGGT GACCCTAATC CAGGCATTTG CAGCCCGGCC AGGGGGTCCA CCCCACATTC GCATTACCGG   
  
  
+ TATCGATGAC TCCTACTCTG CATATGCTCG TGGAGGTGGG TTGAACATTG TGGGCCATAG GCTCTTGAGG   
  
  
+ TTGGCTCAGT CATTTAAGGT CCCATTTGAG TTCAACGCAG TTGACGTCCC GGCTTGTCAA GTCCTGCTTA   
  
  
+ AAGATCTCGG CATTCAACAT GGTGAGGCCT TGGCTGTGAA CTTTGCCTTC ATTCTTCACC ACATGCCCGA   
  
  
+ TGAGAGCGTG AGCACAGAGA ATCACCGGGA TCGTCTTCTG AGGGTGGTAA AAGGGCTGAA GCCAAAAGTG   
  
  
+ GTAACGTTAG TGGAGCAAGA GTGTAACACA AACTCTGCTG CTTTCTTGCC TCGCTTTGTT GAGACATTGG   
  
  
+ AATACTACAC AGCGATGTTT GAGTCCATGG ATGTGACTTT GCCTAGGGAT CATAAAGACC GGATCAATGT   
  
  
+ TGAGCAGCAT TGTCTTGCTC GAGATGTTGT GAACTTGGTT GCGTGTGAAG GAGCTGAGAG GGTGGAGCGG   
  
  
+ CATGAGCTCC TTGGGAAATG GAGGTCGCGG TTCACAATGG CAGGGTTTAA GCCATACCCT TTAAGCACTC   
  
  
+ TGGTAAACAA CACAATAAGA ACTTTGTTGA AGAACTACTG TGATAGGTAT GGGCTAGAGG AGAGGAATGG   
  
  
+ AGGTCTTTAT CTTGGGTGGA TGAATAGAGA TTTGGTTGCT TCGAGTGCCT GGCAGTGTAG TAATTA  

- +Up\_Stream \_Len000GTAAAA TCATTATTTA AGGGTACACT CCTGGTGACG GATGACTAAG GCTTACCGTA   
  
  
- AAATCATTAT TTGAGGGTAC ACTCCTGGTG AACTACTCCT TAAGTCAAAA TATTAGGTGT CCACAGTTTC   
  
  
- GGATAAGGAT GAGAGTGGTG AGACGATAGA GAGGGGGGGG GGGGGGGGGC GGGGGGAGTT TTAGACGGAG   
  
  
- GAGGCAAGAG AGGTGTCGTC TAAGGAGTTA GTCCTTTCCA TACAGTCTCG AGTTTTGCTA AGTTGTAGTT   
  
  
- TTACCCTATT AAATAAGACG AAAGTCAAGA CCCAATAACA GTATACAAGT AAGTCAAGTC ACAAAGATCG   
  
  
- TACTAACGAA AAACTACGGA AGAAAAAACA AGACCCAACC TGCTTGAGAC AGAAAAGACA CGTATCTAAA   
  
  
- CTCGAGGCTC GAGTTAAAAA CCCTAAAAAA ATCGACTGAA AAGACGAACC ACAACACTTT AACACTACCC   
  
  
- AAAACCGAAA CAATTTGAAC AAATATTGAA AAAGACAGTT TTGAAATATC ATTTAACAAC GTTAAACTCT   
  
  
- TAATACGTGA GTCACCGAGA CACTAAATAG CAGGCGACCG TTTAAAAGAC TACTTTCCTC TACTAAATAA   
  
  
- TTAAAAAGTC CGTACCAGTT TAAAAGATCG GAAAGTACAT GTAACCAACC ACTATCCGAC TATCGATTTA   
  
  
- ATACAGAGTA AAGAGATTTA AATAACGGTC TATACTTTAA CCACCTTGTC AAATTCCCAA AACCAGCAGA   
  
  
- AAAAACGGGA AAAAAAACCC AGGGGGGGGG CGGGCCCCCC CTTGGGCCTC CCGTCTTTTT CATTGTATTG   
  
  
- AATTTTAATA GTAACTGCAT CAGGAGGTTG GTATTACTTT CGTTGACTTT ATTCTCGTAC GATTCGATCT   
  
  
- CTCATCCGAC GGAACAGTCG GAGGGAAAAC TGTACTCTTT CTACCCTCTT CAAATTTTAT TTCAAGGAGC   
  
  
- AGAAACGTAG GGCCTGGTGT TAAGCCTTGA CCCACTCCTG GAGGAATCCG AACATTTGTG TTCAAAGCTT   
  
  
- GAATCTAAAC GCATGTACAG TTACGAAATG ACCTATTCGA ATCGATTGTG GGGAGTAGGA AGTTGGACAT   
  
  
- GGAAGTATAT TTTTTTTCCT GTTAGGCCAC GTGTTCGTAG GGCGTAAGTG CGTTCCAGAC CCCTTCCCGG   
  
  
- CGTGGGGTTT CCCACTTAAC ATCCGTCGGA TTGGACTGAA ACAGTCACCG ATTAAGGTGC TAAGCTTGGG   
  
  
- TACTAAAAAA CCAATGTGCC CTTGTTGGAA TGGTAACGAG TTTCCGAGGG GAAGAAGTTG GAGATGGAAG   
  
  
- TATATACTTA CTTTTAGGAA ACCGATTCTT AACTTTTCCT TTATACACCA ATAACCCAAA CCCAAGAATA   
  
  
- AGGCAACCAA CGGGGAAGTG TAACTTACGT AGGAGAGCGA TTCTTGACTT TTCCTGTATA CCCCTACATA   
  
  
- TCCAGTTTAA ACTGAGTTAC TATTGACTCC TATGTTCTTA AGATCTTCAG AAATAGTCCC CTTAAAAAAG   
  
  
- AAAAAAGGTG AACACTGAAA AACTACGGAA CGAAAAATGA GAAGTAACTT ACTCATGGGA AGAACTGTCT   
  
  
- AAGAAGGAGG GAAAAAAGAA TTAAAGAAGA ACATCACCTT ATATTCATAC GAAGATCCAT CAACCTACGG   
  
  
- TACCTTAGAG TAGGGACACA CACGTAAGAA GGGGAGAGGG CGGCGGGGGG GGGGGGGTTT TTCTTTGGTT   
  
  
- TTTTTTTTTT TTTTACCTAC TAAGAATCTT GACTGGTAAA AAGGAAGGAG GAGGGTAAAA TAAAAAAAGT   
  
  
- CCGACAAAAA GAAGATGGAC AACGAAATCG TAGTGAGTAT TTTTGTTCCA CGTTCGCGTA CTGTAGCCCA   
  
  
- TGACTCGAAG ACTCAAATGT ACGTAGCAGT ACTTTAAGCC ATCTGAAACT TCATTTCAAA GGGGTAGCAT   
  
  
- TGACCACCAC GTATGGACAG AAGTGCGGGT AGTCATCTCA CCACTTCACT TCGTTACAGC TTATCAAACA   
  
  
- TAATGCTCGG GTTCTCTCTT TGACTACGTA TAAAATACGG AGTTACAGTT AGGAACTTGG GGGTCGAGCT   
  
  
- AATGTTGTGG TAACTGATAC CTCGAAAATA CACGAGTTTT CAAAAGGACC CAGTCATGAC GTGAGACCTT   
  
  
- AGTAGGAGTC CTTGACCCTG AACAGGCCAC AGATCGAGGT GTAGTTGAGA GTCGAAGAGT AGGTTACCAT   
  
  
- CGGGACCTAG TGTCGAACTA AGGTTGAGAA TAGGAAGACT AGTTTTAACG AGAGGACTAT TACGGTTAAG   
  
  
- GAGTAGAAAT TCAGTAAGGA CGCAGTGTCT ACTACACCTA CTGGACTTTA TGTCCGACTC TCTCAACCTT   
  
  
- TGTCACTACA ACCCTGGACT AGGGCTAAAA CAGATGCCGT TATCGTTGGT GTGTAAACCC CAACCCTAGG   
  
  
- TACATCACAG TAGTCTCAAA CTGTCGACCC CCTTTCACTA CCTCTACTAG CGAGCTTTCC TAAACTTTGA   
  
  
- CCACGATTAA CGGACACGTT CTCGTCAACG ACTTTTACTA GTCAACAACC GGGTCACCGA CTACCTACTT   
  
  
- AACTCTGTCT ACCAAAGTCA AAGACCACTT GGTTAAGTTT CTAACCCACG AATATACAAC CTTCCCGAGC   
  
  
- ATCGCTCCGT CCGGAGGAGT CCATCGAGGT AAATATTTCG TAACTCTACG TTCCTTGGGC GCTCACGACT   
  
  
- GGAAAATAGA ATGTACGTGT ATGAGAAACT CCAAACGGGT ATGTAGTTTA AACCGATGTA CAGTCGTTTA   
  
  
- CCACGGTAAC GTCTTCGTTA CTTCCTACTT TTCTCTCAGG TGTAACAGCT AAAGGTTTAA CCCGTCCCTT   
  
  
- CGGTTACCCA CTGGGATTAG GTCCGTAAAC GTCGGGCCGG TCCCCCAGGT GGGGTGTAAG CGTAATGGCC   
  
  
- ATAGCTACTG AGGATGAGAC GTATACGAGC ACCTCCACCC AACTTGTAAC ACCCGGTATC CGAGAACTCC   
  
  
- AACCGAGTCA GTAAATTCCA GGGTAAACTC AAGTTGCGTC AACTGCAGGG CCGAACAGTT CAGGACGAAT   
  
  
- TTCTAGAGCC GTAAGTTGTA CCACTCCGGA ACCGACACTT GAAACGGAAG TAAGAAGTGG TGTACGGGCT   
  
  
- ACTCTCGCAC TCGTGTCTCT TAGTGGCCCT AGCAGAAGAC TCCCACCATT TTCCCGACTT CGGTTTTCAC   
  
  
- CATTGCAATC ACCTCGTTCT CACATTGTGT TTGAGACGAC GAAAGAACGG AGCGAAACAA CTCTGTAACC   
  
  
- TTATGATGTG TCGCTACAAA CTCAGGTACC TACACTGAAA CGGATCCCTA GTATTTCTGG CCTAGTTACA   
  
  
- ACTCGTCGTA ACAGAACGAG CTCTACAACA CTTGAACCAA CGCACACTTC CTCGACTCTC CCACCTCGCC   
  
  
- GTACTCGAGG AACCCTTTAC CTCCAGCGCC AAGTGTTACC GTCCCAAATT CGGTATGGGA AATTCGTGAG   
  
  
- ACCATTTGTT GTGTTATTCT TGAAACAACT TCTTGATGAC ACTATCCATA CCCGATCTCC TCTCCTTACC   
  
  
- TCCAGAAATA GAACCCACCT ACTTATCTCT AAACCAACGA AGCTCACGGA CCGTCACATC ATTAAT
