# Supplementary material for: Genome-Wide Identification and Expression Pattern of the GRAS Gene Family in Pitaya (Selenicereus undatus L.)
Source: Biology (Basel). 2022 Dec 21;12(1):11. doi: 10.3390/biology12010011 (PMC9854919; doi:10.3390/biology12010011)
Supplement: Supplementary file 1 [file biology-12-00011-s001.zip › Supplementary file S5/HU01G01850.1_plantcare.html]

Content-Type: text/html; charset=ISO-8859-1


PlantCARE


Webmaster Firefox specific output  
To save the result:
click on the frame with the right mouse button and save the source code as a text file with extension .html  
REFERENCE:PlantCARE: a database of plant cis-acting regulatory elements and a portal to tools for in silico analysis of promoter sequences.  
Lescot, M., Déhais, P., Moreau, Y., De Moor, B., Rouzé ,P.,and Rombauts, S.  
Nucleic Acids Res., Database issue(2002), 30(1):325-327.   


---

>HU01G01850.1   
+ +Up\_Stream \_Len000TTTAAA TATTTTATTT TATTTTAAAT TAATATATTT TATAATTTAA AATTATTAAT   
  
  
+ AATTGATGAT GTTACCCAAT GTTTAAGTTT TTAAATATTT TTATTTTTTA ATTTAAAAAT ATCATAATAT   
  
  
+ ATAATATAAT TTTAAGTTAA AATAAGATAG TTAAATTATT TTATAGTAAA TATTTATTAT TTTTTTTAAG   
  
  
+ AAAATTGCCA TGTGATTGTC ATCAGTCAAA CACTGCGTTG ACAGAAGCTG TCCTTGCTGC TACTCTCACC   
  
  
+ TCTCAACATC GGACCCGCGC AGACGTTAAC ATCTCCGTAC AAATACAATT CCCTGGCCAA AATTCAAATC   
  
  
+ AACCCTACTC GGTAGTCGGT TCCTTCTTCT TCCTGCCCTC TATATATACA CCCACAGCTG CGCTGCATGT   
  
  
+ TTTTCCGAGA ATCCTCAAAA ATCTTATAAA CTTCCTCGTG TGGGTAGAAA AAAGAAAAAG AAAAGGGTTT   
  
  
+ TAAAATCTTG GGTCAAAACC CATCAACACC ACAATTTGAC TCTTTCTTTT TAATTCAACC ACCGCCAGTT   
  
  
+ CCTCGTTGAT TTTCAGGTAC CTCTTTTTCT TGCCCCTTTT TTTGCTCGGT TGTTGTTTAA TGGTAAAGAT   
  
  
+ TGAGTACAAA GAATGATATT TGATGAGTTG GTGTGTTTAT TTGATGAGCC CAGATGATTT AACTTGTAGA   
  
  
+ TTTTGTGGTT TTGGTTTCTG GGTATTCATT GGTTTGCTGA ATTTCTGGTT CATGATCTGG GGTTAGAAAA   
  
  
+ GTTGAAATGA CTTTCTGGAG GTTGGAATTT GACTAAAGCT AATTTCTTTT TGTCTTCAAT TCTTTTTTAG   
  
  
+ TTGTGAGCTG AATTTGTTGA CCACTTCCAG CCTGTTGGAA ATTAAACTTA CTTAGGAGAA GAGAATCTCA   
  
  
+ GATTCTGAGC AAAAGCTTTA TATGGTTGTT TGGGAAATGG TAGGAACTAA GTGAAAACAC TGAAATTTTA   
  
  
+ AGCAAGCAAT TTTGTTCATT TGATTGGTCG GATGGTCTGA TTTAGGTTCT TTTCTTGGTC TAAATTTCTG   
  
  
+ AGCATTGATT GTTTAATTGC GTGGGGAACT TGTATCCTTG TGAGTTCCTG GTTCATTTCT TGGTTGCTTA   
  
  
+ CTCTAGCTCC CTTTCACAGT TTCTATGGTT GGAACTTGGA ACTTGGAACT TGGAAGCCTA ATTTAGTTGA   
  
  
+ ATATCTTGGC TTTGAGTTAG CTTTTCTTAG GAAAAGATGG TTCTGCTCTC GAAAATCTAA TCTAATCGAT   
  
  
+ AGATGCGTTA AGGAAGCAAA ACTTTTGGGG AGAGGGAAGT TAAGCGAAGC AAGCTGCTGT TTCAAAGTTG   
  
  
+ GACTGTTTTA TTGGTAAGTA TGAAACAATA GTTTATAAGA GATCATTGGG TTCTTAAGGT GATAGATCAT   
  
  
+ GGGTTATGAA TGAGCTTAGT TGTTCTTTTG CTTTCAACTG TACGTAAGGA TGATAGTTAT CCCTCATCTT   
  
  
+ GCATCAAAAA CGGCAAACCG TGATGCCAAA ACTCAAAAAC TGATTCCTCT GACAAGGCTT CTACCTATGA   
  
  
+ AAACTATGCA GATCATATCC ATTTGGCCTT CCCTCTAGGA ACCAAGTAGA GTAAGTTGGT AAATAAATAG   
  
  
+ AGTTGAACGA AACACTACAG ATTTGAATTT GATTAAGGAG GGCTATTATA GACTAAGGTG TAGCTCAAAT   
  
  
+ CCTTCTGTAG GGATTGTCTT ATTGGCTTCT TTTGGTTGCT TCTGGCTCAA GTCCTCTTAT TAGTTAGTAT   
  
  
+ GCATTACCTT GAGACAAGAT AGTTGCTTAA GCTTGAGCTT CTGTACTTAT TTGCGTATTG TAGACAACTA   
  
  
+ GCTGTGGCAT ATGATTCTGA AATGTTCCGA TGTCTTTTTC TTTGGATGCT GGGTTGTGTC ATGAGCTTTA   
  
  
+ GGTTTGTTTA TTTGATGTCA GGAAGTATGT AAATAACATG TGGCATCTTG CAATCTATTA CAGGATGATA   
  
  
+ TTGGCCTTTG TGGGAAGAAA TTGAAGAAGT TGTTTCACTT ACTGGATAGG GAGCATGGAC TCACATCAGC   
  
  
+ TTTTGAGATT CGGTATTTCC AGATCATACT CATCCTATAA TTTCTCTCAG TCTAGTCCCC AATCAATTCC   
  
  
+ AAATAGGTTA TTTGAATCAC CGAACGTTCG TTCTAGAGAC TCTCCAATCT CGCCCTTCTC AACGCCCTTC   
  
  
+ AATTGCGACC CCACTGTTAT ATTGAGCGAC AGTCAGGAGC ACCACAGTTC AACAGGAAGT CTCTCGGCAC   
  
  
+ AAAGCTCATC TTCTAATTCT CCCCTTGAGA CTAGCAGTTA TTATAATCAG TTCAACTCAA GCCCTGTTGG   
  
  
+ AGAATCACCT CAGGGATCAT CACCCGAGAA TCTTTTTCAA CAAGCTGCAG TTAATAAAGT CAGCATCGAG   
  
  
+ CATGCATTGC AGGAGCTAGA AACTGCTCTA ATGAGCACAG ATTGTGAGGA GAATGAAGCA ACTGTCTCTA   
  
  
+ TCCCATCTAT GGGTGAACCT CATCAGCCCC AAGTCCCTAG CCAGAGATCA AGATTCTGGA ATCGAGATCC   
  
  
+ TCAGGGTTCA CGCCCGGCTG AAGTTCACTC ATCATTGAGA AGATTAGGAG ACGAGGCTCA GAGTGAGAAA   
  
  
+ CGCCTCAAGG CAGTGGAAGA ACCAATACGG CCCAGTGCAC CACCCGGCAA TTTGAAGCAG TTGCTCATAG   
  
  
+ AATGTGCTCG GGCTTTGTCA GAGAATCGAA TAGAGGATTT TGAGAAGTTA GTTGAACAGG CAAAGGGCAT   
  
  
+ GGTCTCCATT TCTGGAGACC CAATTCAACG ACTTGGTGCT TACTTGATCG AGGGGCTAGT GGCAAGGAAG   
  
  
+ GAGTCTTCAG GTACGAAAAT CTACCGAGCT CTTAGGTGCA AAGAGCCCCT TGGTCAAGAC TTGCTTTCGT   
  
  
+ ACATGCACAT CCTTTATGAA ATTTGCCCTT ACTTGAAATT CGGTTATATG GTTGCAAATG GGGCTATAGC   
  
  
+ TGAAGCTTGC AGAGATGAGG ATCACATACA TATCGTCGAC TACCAGATTG GTCAAGGAAC TCAATGGATG   
  
  
+ ACTCTGTTGC AAGCCCTAGC CACAAAACCT GGAAGACCCC CCACTGTGAG AATTACTGGC ATTGATGATC   
  
  
+ CCGTCTCTAG GCATGCTCGG GGAGCTTGCT TGGAGGCAGT GGGGAAACGT TTGGCAGTTC TGTCGGAGAA   
  
  
+ ATTTAACATG CCCATTGAGT TTCAGGCAGT GCCCGTTTAT GCTTCTGAGG TCACCCAAGA AATGCTTGAT   
  
  
+ GTGAGGCCAG GGTGGGCCTT GGCTGTGAAC TTCCCATTGC AGCTCCACCA CACTCCCGAT GAGAGTGTTG   
  
  
+ ATGTGAACAA CCCGAGGGAT GGACTCCTAA GAATGGTGAA ATCACTCGGT CCCAAGGTTG TAACTTTGGT   
  
  
+ AGAACAAGAG TCAAACACCA ACACTACCCC TTTGCTGACA AGGTTCATAG AAACGCTGGA CTTCTACTCA   
  
  
+ GCAATGTTTG AATCCATCGA TGTGACAATG CCAAGGGACC GAAAGGAAAG GATCAATGTC GAGCAGCATT   
  
  
+ GCCTAGCCAA GGACATTGTC AACATCATCG CGTGTGAGGG GAAGGACAGG GTGGAGCGAC ATGAGCTCTT   
  
  
+ TGGGAAATGG AAGTCAAGGT TCACCATGGC AGGATTCCAA CAATCCCCAT TGAGCTCGTA TGTTAACTCC   
  
  
+ GTGATAAAAG GATTACTTAG GTGTTATTCA GAGTACTATA CGTTGGTGGA GAAGGATGGC GCCTTGCTTC   
  
  
+ TGGGATGGAA GGACCGGATG CTGGTTTCGG CTTCAGCTTG GCATTG  

- +Up\_Stream \_Len000AAATTT ATAAAATAAA ATAAAATTTA ATTATATAAA ATATTAAATT TTAATAATTA   
  
  
- TTAACTACTA CAATGGGTTA CAAATTCAAA AATTTATAAA AATAAAAAAT TAAATTTTTA TAGTATTATA   
  
  
- TATTATATTA AAATTCAATT TTATTCTATC AATTTAATAA AATATCATTT ATAAATAATA AAAAAAATTC   
  
  
- TTTTAACGGT ACACTAACAG TAGTCAGTTT GTGACGCAAC TGTCTTCGAC AGGAACGACG ATGAGAGTGG   
  
  
- AGAGTTGTAG CCTGGGCGCG TCTGCAATTG TAGAGGCATG TTTATGTTAA GGGACCGGTT TTAAGTTTAG   
  
  
- TTGGGATGAG CCATCAGCCA AGGAAGAAGA AGGACGGGAG ATATATATGT GGGTGTCGAC GCGACGTACA   
  
  
- AAAAGGCTCT TAGGAGTTTT TAGAATATTT GAAGGAGCAC ACCCATCTTT TTTCTTTTTC TTTTCCCAAA   
  
  
- ATTTTAGAAC CCAGTTTTGG GTAGTTGTGG TGTTAAACTG AGAAAGAAAA ATTAAGTTGG TGGCGGTCAA   
  
  
- GGAGCAACTA AAAGTCCATG GAGAAAAAGA ACGGGGAAAA AAACGAGCCA ACAACAAATT ACCATTTCTA   
  
  
- ACTCATGTTT CTTACTATAA ACTACTCAAC CACACAAATA AACTACTCGG GTCTACTAAA TTGAACATCT   
  
  
- AAAACACCAA AACCAAAGAC CCATAAGTAA CCAAACGACT TAAAGACCAA GTACTAGACC CCAATCTTTT   
  
  
- CAACTTTACT GAAAGACCTC CAACCTTAAA CTGATTTCGA TTAAAGAAAA ACAGAAGTTA AGAAAAAATC   
  
  
- AACACTCGAC TTAAACAACT GGTGAAGGTC GGACAACCTT TAATTTGAAT GAATCCTCTT CTCTTAGAGT   
  
  
- CTAAGACTCG TTTTCGAAAT ATACCAACAA ACCCTTTACC ATCCTTGATT CACTTTTGTG ACTTTAAAAT   
  
  
- TCGTTCGTTA AAACAAGTAA ACTAACCAGC CTACCAGACT AAATCCAAGA AAAGAACCAG ATTTAAAGAC   
  
  
- TCGTAACTAA CAAATTAACG CACCCCTTGA ACATAGGAAC ACTCAAGGAC CAAGTAAAGA ACCAACGAAT   
  
  
- GAGATCGAGG GAAAGTGTCA AAGATACCAA CCTTGAACCT TGAACCTTGA ACCTTCGGAT TAAATCAACT   
  
  
- TATAGAACCG AAACTCAATC GAAAAGAATC CTTTTCTACC AAGACGAGAG CTTTTAGATT AGATTAGCTA   
  
  
- TCTACGCAAT TCCTTCGTTT TGAAAACCCC TCTCCCTTCA ATTCGCTTCG TTCGACGACA AAGTTTCAAC   
  
  
- CTGACAAAAT AACCATTCAT ACTTTGTTAT CAAATATTCT CTAGTAACCC AAGAATTCCA CTATCTAGTA   
  
  
- CCCAATACTT ACTCGAATCA ACAAGAAAAC GAAAGTTGAC ATGCATTCCT ACTATCAATA GGGAGTAGAA   
  
  
- CGTAGTTTTT GCCGTTTGGC ACTACGGTTT TGAGTTTTTG ACTAAGGAGA CTGTTCCGAA GATGGATACT   
  
  
- TTTGATACGT CTAGTATAGG TAAACCGGAA GGGAGATCCT TGGTTCATCT CATTCAACCA TTTATTTATC   
  
  
- TCAACTTGCT TTGTGATGTC TAAACTTAAA CTAATTCCTC CCGATAATAT CTGATTCCAC ATCGAGTTTA   
  
  
- GGAAGACATC CCTAACAGAA TAACCGAAGA AAACCAACGA AGACCGAGTT CAGGAGAATA ATCAATCATA   
  
  
- CGTAATGGAA CTCTGTTCTA TCAACGAATT CGAACTCGAA GACATGAATA AACGCATAAC ATCTGTTGAT   
  
  
- CGACACCGTA TACTAAGACT TTACAAGGCT ACAGAAAAAG AAACCTACGA CCCAACACAG TACTCGAAAT   
  
  
- CCAAACAAAT AAACTACAGT CCTTCATACA TTTATTGTAC ACCGTAGAAC GTTAGATAAT GTCCTACTAT   
  
  
- AACCGGAAAC ACCCTTCTTT AACTTCTTCA ACAAAGTGAA TGACCTATCC CTCGTACCTG AGTGTAGTCG   
  
  
- AAAACTCTAA GCCATAAAGG TCTAGTATGA GTAGGATATT AAAGAGAGTC AGATCAGGGG TTAGTTAAGG   
  
  
- TTTATCCAAT AAACTTAGTG GCTTGCAAGC AAGATCTCTG AGAGGTTAGA GCGGGAAGAG TTGCGGGAAG   
  
  
- TTAACGCTGG GGTGACAATA TAACTCGCTG TCAGTCCTCG TGGTGTCAAG TTGTCCTTCA GAGAGCCGTG   
  
  
- TTTCGAGTAG AAGATTAAGA GGGGAACTCT GATCGTCAAT AATATTAGTC AAGTTGAGTT CGGGACAACC   
  
  
- TCTTAGTGGA GTCCCTAGTA GTGGGCTCTT AGAAAAAGTT GTTCGACGTC AATTATTTCA GTCGTAGCTC   
  
  
- GTACGTAACG TCCTCGATCT TTGACGAGAT TACTCGTGTC TAACACTCCT CTTACTTCGT TGACAGAGAT   
  
  
- AGGGTAGATA CCCACTTGGA GTAGTCGGGG TTCAGGGATC GGTCTCTAGT TCTAAGACCT TAGCTCTAGG   
  
  
- AGTCCCAAGT GCGGGCCGAC TTCAAGTGAG TAGTAACTCT TCTAATCCTC TGCTCCGAGT CTCACTCTTT   
  
  
- GCGGAGTTCC GTCACCTTCT TGGTTATGCC GGGTCACGTG GTGGGCCGTT AAACTTCGTC AACGAGTATC   
  
  
- TTACACGAGC CCGAAACAGT CTCTTAGCTT ATCTCCTAAA ACTCTTCAAT CAACTTGTCC GTTTCCCGTA   
  
  
- CCAGAGGTAA AGACCTCTGG GTTAAGTTGC TGAACCACGA ATGAACTAGC TCCCCGATCA CCGTTCCTTC   
  
  
- CTCAGAAGTC CATGCTTTTA GATGGCTCGA GAATCCACGT TTCTCGGGGA ACCAGTTCTG AACGAAAGCA   
  
  
- TGTACGTGTA GGAAATACTT TAAACGGGAA TGAACTTTAA GCCAATATAC CAACGTTTAC CCCGATATCG   
  
  
- ACTTCGAACG TCTCTACTCC TAGTGTATGT ATAGCAGCTG ATGGTCTAAC CAGTTCCTTG AGTTACCTAC   
  
  
- TGAGACAACG TTCGGGATCG GTGTTTTGGA CCTTCTGGGG GGTGACACTC TTAATGACCG TAACTACTAG   
  
  
- GGCAGAGATC CGTACGAGCC CCTCGAACGA ACCTCCGTCA CCCCTTTGCA AACCGTCAAG ACAGCCTCTT   
  
  
- TAAATTGTAC GGGTAACTCA AAGTCCGTCA CGGGCAAATA CGAAGACTCC AGTGGGTTCT TTACGAACTA   
  
  
- CACTCCGGTC CCACCCGGAA CCGACACTTG AAGGGTAACG TCGAGGTGGT GTGAGGGCTA CTCTCACAAC   
  
  
- TACACTTGTT GGGCTCCCTA CCTGAGGATT CTTACCACTT TAGTGAGCCA GGGTTCCAAC ATTGAAACCA   
  
  
- TCTTGTTCTC AGTTTGTGGT TGTGATGGGG AAACGACTGT TCCAAGTATC TTTGCGACCT GAAGATGAGT   
  
  
- CGTTACAAAC TTAGGTAGCT ACACTGTTAC GGTTCCCTGG CTTTCCTTTC CTAGTTACAG CTCGTCGTAA   
  
  
- CGGATCGGTT CCTGTAACAG TTGTAGTAGC GCACACTCCC CTTCCTGTCC CACCTCGCTG TACTCGAGAA   
  
  
- ACCCTTTACC TTCAGTTCCA AGTGGTACCG TCCTAAGGTT GTTAGGGGTA ACTCGAGCAT ACAATTGAGG   
  
  
- CACTATTTTC CTAATGAATC CACAATAAGT CTCATGATAT GCAACCACCT CTTCCTACCG CGGAACGAAG   
  
  
- ACCCTACCTT CCTGGCCTAC GACCAAAGCC GAAGTCGAAC CGTAAC

  
  
Motifs Found  

+   

| Site Name | Organism | Position | Strand | Matrix score. | sequence | function |
| --- | --- | --- | --- | --- | --- | --- |
|  | organism | 2563 | - | 4 | motif\_sequence | short\_function |
|  | organism | 2953 | - | 4 | motif\_sequence | short\_function |
|  | organism | 3548 | - | 4 | motif\_sequence | short\_function |
|  | organism | 3515 | - | 4 | motif\_sequence | short\_function |
|  | organism | 2879 | - | 4 | motif\_sequence | short\_function |
|  | organism | 2873 | - | 4 | motif\_sequence | short\_function |
|  | organism | 2718 | - | 4 | motif\_sequence | short\_function |
|  | organism | 2823 | + | 4 | motif\_sequence | short\_function |
|  | organism | 2666 | + | 4 | motif\_sequence | short\_function |
|  | organism | 2419 | - | 4 | motif\_sequence | short\_function |
|  | organism | 1846 | + | 4 | motif\_sequence | short\_function |
|  | organism | 1795 | + | 4 | motif\_sequence | short\_function |
|  | organism | 1552 | - | 4 | motif\_sequence | short\_function |
|  | organism | 902 | - | 4 | motif\_sequence | short\_function |
|  | organism | 700 | - | 4 | motif\_sequence | short\_function |
|  | organism | 315 | + | 4 | motif\_sequence | short\_function |
|  | organism | 262 | + | 4 | motif\_sequence | short\_function |
|  | organism | 1293 | - | 4 | motif\_sequence | short\_function |
|  | organism | 1590 | - | 4 | motif\_sequence | short\_function |
|  | organism | 2252 | + | 4 | motif\_sequence | short\_function |
|  | organism | 1442 | + | 4 | motif\_sequence | short\_function |
|  | organism | 2144 | + | 4 | motif\_sequence | short\_function |

>HU01G01850.1   
+ +Up\_Stream \_Len000TTTAAA TATTTTATTT TATTTTAAAT TAATATATTT TATAATTTAA AATTATTAAT   
  
  
+ AATTGATGAT GTTACCCAAT GTTTAAGTTT TTAAATATTT TTATTTTTTA ATTTAAAAAT ATCATAATAT   
  
  
+ ATAATATAAT TTTAAGTTAA AATAAGATAG TTAAATTATT TTATAGTAAA TATTTATTAT TTTTTTTAAG   
  
  
+ AAAATTGCCA TGTGATTGTC ATCAGTCAAA CACTGCGTTG ACAGAAGCTG TCCTTGCTGC TACTCTCACC   
  
  
+ TCTCAACATC GGACCCGCGC AGACGTTAAC ATCTCCGTAC AAATACAATT CCCTGGCCAA AATTCAAATC   
  
  
+ AACCCTACTC GGTAGTCGGT TCCTTCTTCT TCCTGCCCTC TATATATACA CCCACAGCTG CGCTGCATGT   
  
  
+ TTTTCCGAGA ATCCTCAAAA ATCTTATAAA CTTCCTCGTG TGGGTAGAAA AAAGAAAAAG AAAAGGGTTT   
  
  
+ TAAAATCTTG GGTCAAAACC CATCAACACC ACAATTTGAC TCTTTCTTTT TAATTCAACC ACCGCCAGTT   
  
  
+ CCTCGTTGAT TTTCAGGTAC CTCTTTTTCT TGCCCCTTTT TTTGCTCGGT TGTTGTTTAA TGGTAAAGAT   
  
  
+ TGAGTACAAA GAATGATATT TGATGAGTTG GTGTGTTTAT TTGATGAGCC CAGATGATTT AACTTGTAGA   
  
  
+ TTTTGTGGTT TTGGTTTCTG GGTATTCATT GGTTTGCTGA ATTTCTGGTT CATGATCTGG GGTTAGAAAA   
  
  
+ GTTGAAATGA CTTTCTGGAG GTTGGAATTT GACTAAAGCT AATTTCTTTT TGTCTTCAAT TCTTTTTTAG   
  
  
+ TTGTGAGCTG AATTTGTTGA CCACTTCCAG CCTGTTGGAA ATTAAACTTA CTTAGGAGAA GAGAATCTCA   
  
  
+ GATTCTGAGC AAAAGCTTTA TATGGTTGTT TGGGAAATGG TAGGAACTAA GTGAAAACAC TGAAATTTTA   
  
  
+ AGCAAGCAAT TTTGTTCATT TGATTGGTCG GATGGTCTGA TTTAGGTTCT TTTCTTGGTC TAAATTTCTG   
  
  
+ AGCATTGATT GTTTAATTGC GTGGGGAACT TGTATCCTTG TGAGTTCCTG GTTCATTTCT TGGTTGCTTA   
  
  
+ CTCTAGCTCC CTTTCACAGT TTCTATGGTT GGAACTTGGA ACTTGGAACT TGGAAGCCTA ATTTAGTTGA   
  
  
+ ATATCTTGGC TTTGAGTTAG CTTTTCTTAG GAAAAGATGG TTCTGCTCTC GAAAATCTAA TCTAATCGAT   
  
  
+ AGATGCGTTA AGGAAGCAAA ACTTTTGGGG AGAGGGAAGT TAAGCGAAGC AAGCTGCTGT TTCAAAGTTG   
  
  
+ GACTGTTTTA TTGGTAAGTA TGAAACAATA GTTTATAAGA GATCATTGGG TTCTTAAGGT GATAGATCAT   
  
  
+ GGGTTATGAA TGAGCTTAGT TGTTCTTTTG CTTTCAACTG TACGTAAGGA TGATAGTTAT CCCTCATCTT   
  
  
+ GCATCAAAAA CGGCAAACCG TGATGCCAAA ACTCAAAAAC TGATTCCTCT GACAAGGCTT CTACCTATGA   
  
  
+ AAACTATGCA GATCATATCC ATTTGGCCTT CCCTCTAGGA ACCAAGTAGA GTAAGTTGGT AAATAAATAG   
  
  
+ AGTTGAACGA AACACTACAG ATTTGAATTT GATTAAGGAG GGCTATTATA GACTAAGGTG TAGCTCAAAT   
  
  
+ CCTTCTGTAG GGATTGTCTT ATTGGCTTCT TTTGGTTGCT TCTGGCTCAA GTCCTCTTAT TAGTTAGTAT   
  
  
+ GCATTACCTT GAGACAAGAT AGTTGCTTAA GCTTGAGCTT CTGTACTTAT TTGCGTATTG TAGACAACTA   
  
  
+ GCTGTGGCAT ATGATTCTGA AATGTTCCGA TGTCTTTTTC TTTGGATGCT GGGTTGTGTC ATGAGCTTTA   
  
  
+ GGTTTGTTTA TTTGATGTCA GGAAGTATGT AAATAACATG TGGCATCTTG CAATCTATTA CAGGATGATA   
  
  
+ TTGGCCTTTG TGGGAAGAAA TTGAAGAAGT TGTTTCACTT ACTGGATAGG GAGCATGGAC TCACATCAGC   
  
  
+ TTTTGAGATT CGGTATTTCC AGATCATACT CATCCTATAA TTTCTCTCAG TCTAGTCCCC AATCAATTCC   
  
  
+ AAATAGGTTA TTTGAATCAC CGAACGTTCG TTCTAGAGAC TCTCCAATCT CGCCCTTCTC AACGCCCTTC   
  
  
+ AATTGCGACC CCACTGTTAT ATTGAGCGAC AGTCAGGAGC ACCACAGTTC AACAGGAAGT CTCTCGGCAC   
  
  
+ AAAGCTCATC TTCTAATTCT CCCCTTGAGA CTAGCAGTTA TTATAATCAG TTCAACTCAA GCCCTGTTGG   
  
  
+ AGAATCACCT CAGGGATCAT CACCCGAGAA TCTTTTTCAA CAAGCTGCAG TTAATAAAGT CAGCATCGAG   
  
  
+ CATGCATTGC AGGAGCTAGA AACTGCTCTA ATGAGCACAG ATTGTGAGGA GAATGAAGCA ACTGTCTCTA   
  
  
+ TCCCATCTAT GGGTGAACCT CATCAGCCCC AAGTCCCTAG CCAGAGATCA AGATTCTGGA ATCGAGATCC   
  
  
+ TCAGGGTTCA CGCCCGGCTG AAGTTCACTC ATCATTGAGA AGATTAGGAG ACGAGGCTCA GAGTGAGAAA   
  
  
+ CGCCTCAAGG CAGTGGAAGA ACCAATACGG CCCAGTGCAC CACCCGGCAA TTTGAAGCAG TTGCTCATAG   
  
  
+ AATGTGCTCG GGCTTTGTCA GAGAATCGAA TAGAGGATTT TGAGAAGTTA GTTGAACAGG CAAAGGGCAT   
  
  
+ GGTCTCCATT TCTGGAGACC CAATTCAACG ACTTGGTGCT TACTTGATCG AGGGGCTAGT GGCAAGGAAG   
  
  
+ GAGTCTTCAG GTACGAAAAT CTACCGAGCT CTTAGGTGCA AAGAGCCCCT TGGTCAAGAC TTGCTTTCGT   
  
  
+ ACATGCACAT CCTTTATGAA ATTTGCCCTT ACTTGAAATT CGGTTATATG GTTGCAAATG GGGCTATAGC   
  
  
+ TGAAGCTTGC AGAGATGAGG ATCACATACA TATCGTCGAC TACCAGATTG GTCAAGGAAC TCAATGGATG   
  
  
+ ACTCTGTTGC AAGCCCTAGC CACAAAACCT GGAAGACCCC CCACTGTGAG AATTACTGGC ATTGATGATC   
  
  
+ CCGTCTCTAG GCATGCTCGG GGAGCTTGCT TGGAGGCAGT GGGGAAACGT TTGGCAGTTC TGTCGGAGAA   
  
  
+ ATTTAACATG CCCATTGAGT TTCAGGCAGT GCCCGTTTAT GCTTCTGAGG TCACCCAAGA AATGCTTGAT   
  
  
+ GTGAGGCCAG GGTGGGCCTT GGCTGTGAAC TTCCCATTGC AGCTCCACCA CACTCCCGAT GAGAGTGTTG   
  
  
+ ATGTGAACAA CCCGAGGGAT GGACTCCTAA GAATGGTGAA ATCACTCGGT CCCAAGGTTG TAACTTTGGT   
  
  
+ AGAACAAGAG TCAAACACCA ACACTACCCC TTTGCTGACA AGGTTCATAG AAACGCTGGA CTTCTACTCA   
  
  
+ GCAATGTTTG AATCCATCGA TGTGACAATG CCAAGGGACC GAAAGGAAAG GATCAATGTC GAGCAGCATT   
  
  
+ GCCTAGCCAA GGACATTGTC AACATCATCG CGTGTGAGGG GAAGGACAGG GTGGAGCGAC ATGAGCTCTT   
  
  
+ TGGGAAATGG AAGTCAAGGT TCACCATGGC AGGATTCCAA CAATCCCCAT TGAGCTCGTA TGTTAACTCC   
  
  
+ GTGATAAAAG GATTACTTAG GTGTTATTCA GAGTACTATA CGTTGGTGGA GAAGGATGGC GCCTTGCTTC   
  
  
+ TGGGATGGAA GGACCGGATG CTGGTTTCGG CTTCAGCTTG GCATTG  

- +Up\_Stream \_Len000AAATTT ATAAAATAAA ATAAAATTTA ATTATATAAA ATATTAAATT TTAATAATTA   
  
  
- TTAACTACTA CAATGGGTTA CAAATTCAAA AATTTATAAA AATAAAAAAT TAAATTTTTA TAGTATTATA   
  
  
- TATTATATTA AAATTCAATT TTATTCTATC AATTTAATAA AATATCATTT ATAAATAATA AAAAAAATTC   
  
  
- TTTTAACGGT ACACTAACAG TAGTCAGTTT GTGACGCAAC TGTCTTCGAC AGGAACGACG ATGAGAGTGG   
  
  
- AGAGTTGTAG CCTGGGCGCG TCTGCAATTG TAGAGGCATG TTTATGTTAA GGGACCGGTT TTAAGTTTAG   
  
  
- TTGGGATGAG CCATCAGCCA AGGAAGAAGA AGGACGGGAG ATATATATGT GGGTGTCGAC GCGACGTACA   
  
  
- AAAAGGCTCT TAGGAGTTTT TAGAATATTT GAAGGAGCAC ACCCATCTTT TTTCTTTTTC TTTTCCCAAA   
  
  
- ATTTTAGAAC CCAGTTTTGG GTAGTTGTGG TGTTAAACTG AGAAAGAAAA ATTAAGTTGG TGGCGGTCAA   
  
  
- GGAGCAACTA AAAGTCCATG GAGAAAAAGA ACGGGGAAAA AAACGAGCCA ACAACAAATT ACCATTTCTA   
  
  
- ACTCATGTTT CTTACTATAA ACTACTCAAC CACACAAATA AACTACTCGG GTCTACTAAA TTGAACATCT   
  
  
- AAAACACCAA AACCAAAGAC CCATAAGTAA CCAAACGACT TAAAGACCAA GTACTAGACC CCAATCTTTT   
  
  
- CAACTTTACT GAAAGACCTC CAACCTTAAA CTGATTTCGA TTAAAGAAAA ACAGAAGTTA AGAAAAAATC   
  
  
- AACACTCGAC TTAAACAACT GGTGAAGGTC GGACAACCTT TAATTTGAAT GAATCCTCTT CTCTTAGAGT   
  
  
- CTAAGACTCG TTTTCGAAAT ATACCAACAA ACCCTTTACC ATCCTTGATT CACTTTTGTG ACTTTAAAAT   
  
  
- TCGTTCGTTA AAACAAGTAA ACTAACCAGC CTACCAGACT AAATCCAAGA AAAGAACCAG ATTTAAAGAC   
  
  
- TCGTAACTAA CAAATTAACG CACCCCTTGA ACATAGGAAC ACTCAAGGAC CAAGTAAAGA ACCAACGAAT   
  
  
- GAGATCGAGG GAAAGTGTCA AAGATACCAA CCTTGAACCT TGAACCTTGA ACCTTCGGAT TAAATCAACT   
  
  
- TATAGAACCG AAACTCAATC GAAAAGAATC CTTTTCTACC AAGACGAGAG CTTTTAGATT AGATTAGCTA   
  
  
- TCTACGCAAT TCCTTCGTTT TGAAAACCCC TCTCCCTTCA ATTCGCTTCG TTCGACGACA AAGTTTCAAC   
  
  
- CTGACAAAAT AACCATTCAT ACTTTGTTAT CAAATATTCT CTAGTAACCC AAGAATTCCA CTATCTAGTA   
  
  
- CCCAATACTT ACTCGAATCA ACAAGAAAAC GAAAGTTGAC ATGCATTCCT ACTATCAATA GGGAGTAGAA   
  
  
- CGTAGTTTTT GCCGTTTGGC ACTACGGTTT TGAGTTTTTG ACTAAGGAGA CTGTTCCGAA GATGGATACT   
  
  
- TTTGATACGT CTAGTATAGG TAAACCGGAA GGGAGATCCT TGGTTCATCT CATTCAACCA TTTATTTATC   
  
  
- TCAACTTGCT TTGTGATGTC TAAACTTAAA CTAATTCCTC CCGATAATAT CTGATTCCAC ATCGAGTTTA   
  
  
- GGAAGACATC CCTAACAGAA TAACCGAAGA AAACCAACGA AGACCGAGTT CAGGAGAATA ATCAATCATA   
  
  
- CGTAATGGAA CTCTGTTCTA TCAACGAATT CGAACTCGAA GACATGAATA AACGCATAAC ATCTGTTGAT   
  
  
- CGACACCGTA TACTAAGACT TTACAAGGCT ACAGAAAAAG AAACCTACGA CCCAACACAG TACTCGAAAT   
  
  
- CCAAACAAAT AAACTACAGT CCTTCATACA TTTATTGTAC ACCGTAGAAC GTTAGATAAT GTCCTACTAT   
  
  
- AACCGGAAAC ACCCTTCTTT AACTTCTTCA ACAAAGTGAA TGACCTATCC CTCGTACCTG AGTGTAGTCG   
  
  
- AAAACTCTAA GCCATAAAGG TCTAGTATGA GTAGGATATT AAAGAGAGTC AGATCAGGGG TTAGTTAAGG   
  
  
- TTTATCCAAT AAACTTAGTG GCTTGCAAGC AAGATCTCTG AGAGGTTAGA GCGGGAAGAG TTGCGGGAAG   
  
  
- TTAACGCTGG GGTGACAATA TAACTCGCTG TCAGTCCTCG TGGTGTCAAG TTGTCCTTCA GAGAGCCGTG   
  
  
- TTTCGAGTAG AAGATTAAGA GGGGAACTCT GATCGTCAAT AATATTAGTC AAGTTGAGTT CGGGACAACC   
  
  
- TCTTAGTGGA GTCCCTAGTA GTGGGCTCTT AGAAAAAGTT GTTCGACGTC AATTATTTCA GTCGTAGCTC   
  
  
- GTACGTAACG TCCTCGATCT TTGACGAGAT TACTCGTGTC TAACACTCCT CTTACTTCGT TGACAGAGAT   
  
  
- AGGGTAGATA CCCACTTGGA GTAGTCGGGG TTCAGGGATC GGTCTCTAGT TCTAAGACCT TAGCTCTAGG   
  
  
- AGTCCCAAGT GCGGGCCGAC TTCAAGTGAG TAGTAACTCT TCTAATCCTC TGCTCCGAGT CTCACTCTTT   
  
  
- GCGGAGTTCC GTCACCTTCT TGGTTATGCC GGGTCACGTG GTGGGCCGTT AAACTTCGTC AACGAGTATC   
  
  
- TTACACGAGC CCGAAACAGT CTCTTAGCTT ATCTCCTAAA ACTCTTCAAT CAACTTGTCC GTTTCCCGTA   
  
  
- CCAGAGGTAA AGACCTCTGG GTTAAGTTGC TGAACCACGA ATGAACTAGC TCCCCGATCA CCGTTCCTTC   
  
  
- CTCAGAAGTC CATGCTTTTA GATGGCTCGA GAATCCACGT TTCTCGGGGA ACCAGTTCTG AACGAAAGCA   
  
  
- TGTACGTGTA GGAAATACTT TAAACGGGAA TGAACTTTAA GCCAATATAC CAACGTTTAC CCCGATATCG   
  
  
- ACTTCGAACG TCTCTACTCC TAGTGTATGT ATAGCAGCTG ATGGTCTAAC CAGTTCCTTG AGTTACCTAC   
  
  
- TGAGACAACG TTCGGGATCG GTGTTTTGGA CCTTCTGGGG GGTGACACTC TTAATGACCG TAACTACTAG   
  
  
- GGCAGAGATC CGTACGAGCC CCTCGAACGA ACCTCCGTCA CCCCTTTGCA AACCGTCAAG ACAGCCTCTT   
  
  
- TAAATTGTAC GGGTAACTCA AAGTCCGTCA CGGGCAAATA CGAAGACTCC AGTGGGTTCT TTACGAACTA   
  
  
- CACTCCGGTC CCACCCGGAA CCGACACTTG AAGGGTAACG TCGAGGTGGT GTGAGGGCTA CTCTCACAAC   
  
  
- TACACTTGTT GGGCTCCCTA CCTGAGGATT CTTACCACTT TAGTGAGCCA GGGTTCCAAC ATTGAAACCA   
  
  
- TCTTGTTCTC AGTTTGTGGT TGTGATGGGG AAACGACTGT TCCAAGTATC TTTGCGACCT GAAGATGAGT   
  
  
- CGTTACAAAC TTAGGTAGCT ACACTGTTAC GGTTCCCTGG CTTTCCTTTC CTAGTTACAG CTCGTCGTAA   
  
  
- CGGATCGGTT CCTGTAACAG TTGTAGTAGC GCACACTCCC CTTCCTGTCC CACCTCGCTG TACTCGAGAA   
  
  
- ACCCTTTACC TTCAGTTCCA AGTGGTACCG TCCTAAGGTT GTTAGGGGTA ACTCGAGCAT ACAATTGAGG   
  
  
- CACTATTTTC CTAATGAATC CACAATAAGT CTCATGATAT GCAACCACCT CTTCCTACCG CGGAACGAAG   
  
  
- ACCCTACCTT CCTGGCCTAC GACCAAAGCC GAAGTCGAAC CGTAAC

+     AAGAA-motif

| Site Name | Organism | Position | Strand | Matrix score. | sequence | function |
| --- | --- | --- | --- | --- | --- | --- |
| AAGAA-motif | Avena sativa | 1602 | + | 9 | gGTAAAGAAA |  |

>HU01G01850.1   
+ +Up\_Stream \_Len000TTTAAA TATTTTATTT TATTTTAAAT TAATATATTT TATAATTTAA AATTATTAAT   
  
  
+ AATTGATGAT GTTACCCAAT GTTTAAGTTT TTAAATATTT TTATTTTTTA ATTTAAAAAT ATCATAATAT   
  
  
+ ATAATATAAT TTTAAGTTAA AATAAGATAG TTAAATTATT TTATAGTAAA TATTTATTAT TTTTTTTAAG   
  
  
+ AAAATTGCCA TGTGATTGTC ATCAGTCAAA CACTGCGTTG ACAGAAGCTG TCCTTGCTGC TACTCTCACC   
  
  
+ TCTCAACATC GGACCCGCGC AGACGTTAAC ATCTCCGTAC AAATACAATT CCCTGGCCAA AATTCAAATC   
  
  
+ AACCCTACTC GGTAGTCGGT TCCTTCTTCT TCCTGCCCTC TATATATACA CCCACAGCTG CGCTGCATGT   
  
  
+ TTTTCCGAGA ATCCTCAAAA ATCTTATAAA CTTCCTCGTG TGGGTAGAAA AAAGAAAAAG AAAAGGGTTT   
  
  
+ TAAAATCTTG GGTCAAAACC CATCAACACC ACAATTTGAC TCTTTCTTTT TAATTCAACC ACCGCCAGTT   
  
  
+ CCTCGTTGAT TTTCAGGTAC CTCTTTTTCT TGCCCCTTTT TTTGCTCGGT TGTTGTTTAA TGGTAAAGAT   
  
  
+ TGAGTACAAA GAATGATATT TGATGAGTTG GTGTGTTTAT TTGATGAGCC CAGATGATTT AACTTGTAGA   
  
  
+ TTTTGTGGTT TTGGTTTCTG GGTATTCATT GGTTTGCTGA ATTTCTGGTT CATGATCTGG GGTTAGAAAA   
  
  
+ GTTGAAATGA CTTTCTGGAG GTTGGAATTT GACTAAAGCT AATTTCTTTT TGTCTTCAAT TCTTTTTTAG   
  
  
+ TTGTGAGCTG AATTTGTTGA CCACTTCCAG CCTGTTGGAA ATTAAACTTA CTTAGGAGAA GAGAATCTCA   
  
  
+ GATTCTGAGC AAAAGCTTTA TATGGTTGTT TGGGAAATGG TAGGAACTAA GTGAAAACAC TGAAATTTTA   
  
  
+ AGCAAGCAAT TTTGTTCATT TGATTGGTCG GATGGTCTGA TTTAGGTTCT TTTCTTGGTC TAAATTTCTG   
  
  
+ AGCATTGATT GTTTAATTGC GTGGGGAACT TGTATCCTTG TGAGTTCCTG GTTCATTTCT TGGTTGCTTA   
  
  
+ CTCTAGCTCC CTTTCACAGT TTCTATGGTT GGAACTTGGA ACTTGGAACT TGGAAGCCTA ATTTAGTTGA   
  
  
+ ATATCTTGGC TTTGAGTTAG CTTTTCTTAG GAAAAGATGG TTCTGCTCTC GAAAATCTAA TCTAATCGAT   
  
  
+ AGATGCGTTA AGGAAGCAAA ACTTTTGGGG AGAGGGAAGT TAAGCGAAGC AAGCTGCTGT TTCAAAGTTG   
  
  
+ GACTGTTTTA TTGGTAAGTA TGAAACAATA GTTTATAAGA GATCATTGGG TTCTTAAGGT GATAGATCAT   
  
  
+ GGGTTATGAA TGAGCTTAGT TGTTCTTTTG CTTTCAACTG TACGTAAGGA TGATAGTTAT CCCTCATCTT   
  
  
+ GCATCAAAAA CGGCAAACCG TGATGCCAAA ACTCAAAAAC TGATTCCTCT GACAAGGCTT CTACCTATGA   
  
  
+ AAACTATGCA GATCATATCC ATTTGGCCTT CCCTCTAGGA ACCAAGTAGA GTAAGTTGGT AAATAAATAG   
  
  
+ AGTTGAACGA AACACTACAG ATTTGAATTT GATTAAGGAG GGCTATTATA GACTAAGGTG TAGCTCAAAT   
  
  
+ CCTTCTGTAG GGATTGTCTT ATTGGCTTCT TTTGGTTGCT TCTGGCTCAA GTCCTCTTAT TAGTTAGTAT   
  
  
+ GCATTACCTT GAGACAAGAT AGTTGCTTAA GCTTGAGCTT CTGTACTTAT TTGCGTATTG TAGACAACTA   
  
  
+ GCTGTGGCAT ATGATTCTGA AATGTTCCGA TGTCTTTTTC TTTGGATGCT GGGTTGTGTC ATGAGCTTTA   
  
  
+ GGTTTGTTTA TTTGATGTCA GGAAGTATGT AAATAACATG TGGCATCTTG CAATCTATTA CAGGATGATA   
  
  
+ TTGGCCTTTG TGGGAAGAAA TTGAAGAAGT TGTTTCACTT ACTGGATAGG GAGCATGGAC TCACATCAGC   
  
  
+ TTTTGAGATT CGGTATTTCC AGATCATACT CATCCTATAA TTTCTCTCAG TCTAGTCCCC AATCAATTCC   
  
  
+ AAATAGGTTA TTTGAATCAC CGAACGTTCG TTCTAGAGAC TCTCCAATCT CGCCCTTCTC AACGCCCTTC   
  
  
+ AATTGCGACC CCACTGTTAT ATTGAGCGAC AGTCAGGAGC ACCACAGTTC AACAGGAAGT CTCTCGGCAC   
  
  
+ AAAGCTCATC TTCTAATTCT CCCCTTGAGA CTAGCAGTTA TTATAATCAG TTCAACTCAA GCCCTGTTGG   
  
  
+ AGAATCACCT CAGGGATCAT CACCCGAGAA TCTTTTTCAA CAAGCTGCAG TTAATAAAGT CAGCATCGAG   
  
  
+ CATGCATTGC AGGAGCTAGA AACTGCTCTA ATGAGCACAG ATTGTGAGGA GAATGAAGCA ACTGTCTCTA   
  
  
+ TCCCATCTAT GGGTGAACCT CATCAGCCCC AAGTCCCTAG CCAGAGATCA AGATTCTGGA ATCGAGATCC   
  
  
+ TCAGGGTTCA CGCCCGGCTG AAGTTCACTC ATCATTGAGA AGATTAGGAG ACGAGGCTCA GAGTGAGAAA   
  
  
+ CGCCTCAAGG CAGTGGAAGA ACCAATACGG CCCAGTGCAC CACCCGGCAA TTTGAAGCAG TTGCTCATAG   
  
  
+ AATGTGCTCG GGCTTTGTCA GAGAATCGAA TAGAGGATTT TGAGAAGTTA GTTGAACAGG CAAAGGGCAT   
  
  
+ GGTCTCCATT TCTGGAGACC CAATTCAACG ACTTGGTGCT TACTTGATCG AGGGGCTAGT GGCAAGGAAG   
  
  
+ GAGTCTTCAG GTACGAAAAT CTACCGAGCT CTTAGGTGCA AAGAGCCCCT TGGTCAAGAC TTGCTTTCGT   
  
  
+ ACATGCACAT CCTTTATGAA ATTTGCCCTT ACTTGAAATT CGGTTATATG GTTGCAAATG GGGCTATAGC   
  
  
+ TGAAGCTTGC AGAGATGAGG ATCACATACA TATCGTCGAC TACCAGATTG GTCAAGGAAC TCAATGGATG   
  
  
+ ACTCTGTTGC AAGCCCTAGC CACAAAACCT GGAAGACCCC CCACTGTGAG AATTACTGGC ATTGATGATC   
  
  
+ CCGTCTCTAG GCATGCTCGG GGAGCTTGCT TGGAGGCAGT GGGGAAACGT TTGGCAGTTC TGTCGGAGAA   
  
  
+ ATTTAACATG CCCATTGAGT TTCAGGCAGT GCCCGTTTAT GCTTCTGAGG TCACCCAAGA AATGCTTGAT   
  
  
+ GTGAGGCCAG GGTGGGCCTT GGCTGTGAAC TTCCCATTGC AGCTCCACCA CACTCCCGAT GAGAGTGTTG   
  
  
+ ATGTGAACAA CCCGAGGGAT GGACTCCTAA GAATGGTGAA ATCACTCGGT CCCAAGGTTG TAACTTTGGT   
  
  
+ AGAACAAGAG TCAAACACCA ACACTACCCC TTTGCTGACA AGGTTCATAG AAACGCTGGA CTTCTACTCA   
  
  
+ GCAATGTTTG AATCCATCGA TGTGACAATG CCAAGGGACC GAAAGGAAAG GATCAATGTC GAGCAGCATT   
  
  
+ GCCTAGCCAA GGACATTGTC AACATCATCG CGTGTGAGGG GAAGGACAGG GTGGAGCGAC ATGAGCTCTT   
  
  
+ TGGGAAATGG AAGTCAAGGT TCACCATGGC AGGATTCCAA CAATCCCCAT TGAGCTCGTA TGTTAACTCC   
  
  
+ GTGATAAAAG GATTACTTAG GTGTTATTCA GAGTACTATA CGTTGGTGGA GAAGGATGGC GCCTTGCTTC   
  
  
+ TGGGATGGAA GGACCGGATG CTGGTTTCGG CTTCAGCTTG GCATTG  

- +Up\_Stream \_Len000AAATTT ATAAAATAAA ATAAAATTTA ATTATATAAA ATATTAAATT TTAATAATTA   
  
  
- TTAACTACTA CAATGGGTTA CAAATTCAAA AATTTATAAA AATAAAAAAT TAAATTTTTA TAGTATTATA   
  
  
- TATTATATTA AAATTCAATT TTATTCTATC AATTTAATAA AATATCATTT ATAAATAATA AAAAAAATTC   
  
  
- TTTTAACGGT ACACTAACAG TAGTCAGTTT GTGACGCAAC TGTCTTCGAC AGGAACGACG ATGAGAGTGG   
  
  
- AGAGTTGTAG CCTGGGCGCG TCTGCAATTG TAGAGGCATG TTTATGTTAA GGGACCGGTT TTAAGTTTAG   
  
  
- TTGGGATGAG CCATCAGCCA AGGAAGAAGA AGGACGGGAG ATATATATGT GGGTGTCGAC GCGACGTACA   
  
  
- AAAAGGCTCT TAGGAGTTTT TAGAATATTT GAAGGAGCAC ACCCATCTTT TTTCTTTTTC TTTTCCCAAA   
  
  
- ATTTTAGAAC CCAGTTTTGG GTAGTTGTGG TGTTAAACTG AGAAAGAAAA ATTAAGTTGG TGGCGGTCAA   
  
  
- GGAGCAACTA AAAGTCCATG GAGAAAAAGA ACGGGGAAAA AAACGAGCCA ACAACAAATT ACCATTTCTA   
  
  
- ACTCATGTTT CTTACTATAA ACTACTCAAC CACACAAATA AACTACTCGG GTCTACTAAA TTGAACATCT   
  
  
- AAAACACCAA AACCAAAGAC CCATAAGTAA CCAAACGACT TAAAGACCAA GTACTAGACC CCAATCTTTT   
  
  
- CAACTTTACT GAAAGACCTC CAACCTTAAA CTGATTTCGA TTAAAGAAAA ACAGAAGTTA AGAAAAAATC   
  
  
- AACACTCGAC TTAAACAACT GGTGAAGGTC GGACAACCTT TAATTTGAAT GAATCCTCTT CTCTTAGAGT   
  
  
- CTAAGACTCG TTTTCGAAAT ATACCAACAA ACCCTTTACC ATCCTTGATT CACTTTTGTG ACTTTAAAAT   
  
  
- TCGTTCGTTA AAACAAGTAA ACTAACCAGC CTACCAGACT AAATCCAAGA AAAGAACCAG ATTTAAAGAC   
  
  
- TCGTAACTAA CAAATTAACG CACCCCTTGA ACATAGGAAC ACTCAAGGAC CAAGTAAAGA ACCAACGAAT   
  
  
- GAGATCGAGG GAAAGTGTCA AAGATACCAA CCTTGAACCT TGAACCTTGA ACCTTCGGAT TAAATCAACT   
  
  
- TATAGAACCG AAACTCAATC GAAAAGAATC CTTTTCTACC AAGACGAGAG CTTTTAGATT AGATTAGCTA   
  
  
- TCTACGCAAT TCCTTCGTTT TGAAAACCCC TCTCCCTTCA ATTCGCTTCG TTCGACGACA AAGTTTCAAC   
  
  
- CTGACAAAAT AACCATTCAT ACTTTGTTAT CAAATATTCT CTAGTAACCC AAGAATTCCA CTATCTAGTA   
  
  
- CCCAATACTT ACTCGAATCA ACAAGAAAAC GAAAGTTGAC ATGCATTCCT ACTATCAATA GGGAGTAGAA   
  
  
- CGTAGTTTTT GCCGTTTGGC ACTACGGTTT TGAGTTTTTG ACTAAGGAGA CTGTTCCGAA GATGGATACT   
  
  
- TTTGATACGT CTAGTATAGG TAAACCGGAA GGGAGATCCT TGGTTCATCT CATTCAACCA TTTATTTATC   
  
  
- TCAACTTGCT TTGTGATGTC TAAACTTAAA CTAATTCCTC CCGATAATAT CTGATTCCAC ATCGAGTTTA   
  
  
- GGAAGACATC CCTAACAGAA TAACCGAAGA AAACCAACGA AGACCGAGTT CAGGAGAATA ATCAATCATA   
  
  
- CGTAATGGAA CTCTGTTCTA TCAACGAATT CGAACTCGAA GACATGAATA AACGCATAAC ATCTGTTGAT   
  
  
- CGACACCGTA TACTAAGACT TTACAAGGCT ACAGAAAAAG AAACCTACGA CCCAACACAG TACTCGAAAT   
  
  
- CCAAACAAAT AAACTACAGT CCTTCATACA TTTATTGTAC ACCGTAGAAC GTTAGATAAT GTCCTACTAT   
  
  
- AACCGGAAAC ACCCTTCTTT AACTTCTTCA ACAAAGTGAA TGACCTATCC CTCGTACCTG AGTGTAGTCG   
  
  
- AAAACTCTAA GCCATAAAGG TCTAGTATGA GTAGGATATT AAAGAGAGTC AGATCAGGGG TTAGTTAAGG   
  
  
- TTTATCCAAT AAACTTAGTG GCTTGCAAGC AAGATCTCTG AGAGGTTAGA GCGGGAAGAG TTGCGGGAAG   
  
  
- TTAACGCTGG GGTGACAATA TAACTCGCTG TCAGTCCTCG TGGTGTCAAG TTGTCCTTCA GAGAGCCGTG   
  
  
- TTTCGAGTAG AAGATTAAGA GGGGAACTCT GATCGTCAAT AATATTAGTC AAGTTGAGTT CGGGACAACC   
  
  
- TCTTAGTGGA GTCCCTAGTA GTGGGCTCTT AGAAAAAGTT GTTCGACGTC AATTATTTCA GTCGTAGCTC   
  
  
- GTACGTAACG TCCTCGATCT TTGACGAGAT TACTCGTGTC TAACACTCCT CTTACTTCGT TGACAGAGAT   
  
  
- AGGGTAGATA CCCACTTGGA GTAGTCGGGG TTCAGGGATC GGTCTCTAGT TCTAAGACCT TAGCTCTAGG   
  
  
- AGTCCCAAGT GCGGGCCGAC TTCAAGTGAG TAGTAACTCT TCTAATCCTC TGCTCCGAGT CTCACTCTTT   
  
  
- GCGGAGTTCC GTCACCTTCT TGGTTATGCC GGGTCACGTG GTGGGCCGTT AAACTTCGTC AACGAGTATC   
  
  
- TTACACGAGC CCGAAACAGT CTCTTAGCTT ATCTCCTAAA ACTCTTCAAT CAACTTGTCC GTTTCCCGTA   
  
  
- CCAGAGGTAA AGACCTCTGG GTTAAGTTGC TGAACCACGA ATGAACTAGC TCCCCGATCA CCGTTCCTTC   
  
  
- CTCAGAAGTC CATGCTTTTA GATGGCTCGA GAATCCACGT TTCTCGGGGA ACCAGTTCTG AACGAAAGCA   
  
  
- TGTACGTGTA GGAAATACTT TAAACGGGAA TGAACTTTAA GCCAATATAC CAACGTTTAC CCCGATATCG   
  
  
- ACTTCGAACG TCTCTACTCC TAGTGTATGT ATAGCAGCTG ATGGTCTAAC CAGTTCCTTG AGTTACCTAC   
  
  
- TGAGACAACG TTCGGGATCG GTGTTTTGGA CCTTCTGGGG GGTGACACTC TTAATGACCG TAACTACTAG   
  
  
- GGCAGAGATC CGTACGAGCC CCTCGAACGA ACCTCCGTCA CCCCTTTGCA AACCGTCAAG ACAGCCTCTT   
  
  
- TAAATTGTAC GGGTAACTCA AAGTCCGTCA CGGGCAAATA CGAAGACTCC AGTGGGTTCT TTACGAACTA   
  
  
- CACTCCGGTC CCACCCGGAA CCGACACTTG AAGGGTAACG TCGAGGTGGT GTGAGGGCTA CTCTCACAAC   
  
  
- TACACTTGTT GGGCTCCCTA CCTGAGGATT CTTACCACTT TAGTGAGCCA GGGTTCCAAC ATTGAAACCA   
  
  
- TCTTGTTCTC AGTTTGTGGT TGTGATGGGG AAACGACTGT TCCAAGTATC TTTGCGACCT GAAGATGAGT   
  
  
- CGTTACAAAC TTAGGTAGCT ACACTGTTAC GGTTCCCTGG CTTTCCTTTC CTAGTTACAG CTCGTCGTAA   
  
  
- CGGATCGGTT CCTGTAACAG TTGTAGTAGC GCACACTCCC CTTCCTGTCC CACCTCGCTG TACTCGAGAA   
  
  
- ACCCTTTACC TTCAGTTCCA AGTGGTACCG TCCTAAGGTT GTTAGGGGTA ACTCGAGCAT ACAATTGAGG   
  
  
- CACTATTTTC CTAATGAATC CACAATAAGT CTCATGATAT GCAACCACCT CTTCCTACCG CGGAACGAAG   
  
  
- ACCCTACCTT CCTGGCCTAC GACCAAAGCC GAAGTCGAAC CGTAAC

+     ABRE

| Site Name | Organism | Position | Strand | Matrix score. | sequence | function |
| --- | --- | --- | --- | --- | --- | --- |
| ABRE | Hordeum vulgare | 2872 | + | 9 | CGTACGTGCA | cis-acting element involved in the abscisic acid responsiveness |

>HU01G01850.1   
+ +Up\_Stream \_Len000TTTAAA TATTTTATTT TATTTTAAAT TAATATATTT TATAATTTAA AATTATTAAT   
  
  
+ AATTGATGAT GTTACCCAAT GTTTAAGTTT TTAAATATTT TTATTTTTTA ATTTAAAAAT ATCATAATAT   
  
  
+ ATAATATAAT TTTAAGTTAA AATAAGATAG TTAAATTATT TTATAGTAAA TATTTATTAT TTTTTTTAAG   
  
  
+ AAAATTGCCA TGTGATTGTC ATCAGTCAAA CACTGCGTTG ACAGAAGCTG TCCTTGCTGC TACTCTCACC   
  
  
+ TCTCAACATC GGACCCGCGC AGACGTTAAC ATCTCCGTAC AAATACAATT CCCTGGCCAA AATTCAAATC   
  
  
+ AACCCTACTC GGTAGTCGGT TCCTTCTTCT TCCTGCCCTC TATATATACA CCCACAGCTG CGCTGCATGT   
  
  
+ TTTTCCGAGA ATCCTCAAAA ATCTTATAAA CTTCCTCGTG TGGGTAGAAA AAAGAAAAAG AAAAGGGTTT   
  
  
+ TAAAATCTTG GGTCAAAACC CATCAACACC ACAATTTGAC TCTTTCTTTT TAATTCAACC ACCGCCAGTT   
  
  
+ CCTCGTTGAT TTTCAGGTAC CTCTTTTTCT TGCCCCTTTT TTTGCTCGGT TGTTGTTTAA TGGTAAAGAT   
  
  
+ TGAGTACAAA GAATGATATT TGATGAGTTG GTGTGTTTAT TTGATGAGCC CAGATGATTT AACTTGTAGA   
  
  
+ TTTTGTGGTT TTGGTTTCTG GGTATTCATT GGTTTGCTGA ATTTCTGGTT CATGATCTGG GGTTAGAAAA   
  
  
+ GTTGAAATGA CTTTCTGGAG GTTGGAATTT GACTAAAGCT AATTTCTTTT TGTCTTCAAT TCTTTTTTAG   
  
  
+ TTGTGAGCTG AATTTGTTGA CCACTTCCAG CCTGTTGGAA ATTAAACTTA CTTAGGAGAA GAGAATCTCA   
  
  
+ GATTCTGAGC AAAAGCTTTA TATGGTTGTT TGGGAAATGG TAGGAACTAA GTGAAAACAC TGAAATTTTA   
  
  
+ AGCAAGCAAT TTTGTTCATT TGATTGGTCG GATGGTCTGA TTTAGGTTCT TTTCTTGGTC TAAATTTCTG   
  
  
+ AGCATTGATT GTTTAATTGC GTGGGGAACT TGTATCCTTG TGAGTTCCTG GTTCATTTCT TGGTTGCTTA   
  
  
+ CTCTAGCTCC CTTTCACAGT TTCTATGGTT GGAACTTGGA ACTTGGAACT TGGAAGCCTA ATTTAGTTGA   
  
  
+ ATATCTTGGC TTTGAGTTAG CTTTTCTTAG GAAAAGATGG TTCTGCTCTC GAAAATCTAA TCTAATCGAT   
  
  
+ AGATGCGTTA AGGAAGCAAA ACTTTTGGGG AGAGGGAAGT TAAGCGAAGC AAGCTGCTGT TTCAAAGTTG   
  
  
+ GACTGTTTTA TTGGTAAGTA TGAAACAATA GTTTATAAGA GATCATTGGG TTCTTAAGGT GATAGATCAT   
  
  
+ GGGTTATGAA TGAGCTTAGT TGTTCTTTTG CTTTCAACTG TACGTAAGGA TGATAGTTAT CCCTCATCTT   
  
  
+ GCATCAAAAA CGGCAAACCG TGATGCCAAA ACTCAAAAAC TGATTCCTCT GACAAGGCTT CTACCTATGA   
  
  
+ AAACTATGCA GATCATATCC ATTTGGCCTT CCCTCTAGGA ACCAAGTAGA GTAAGTTGGT AAATAAATAG   
  
  
+ AGTTGAACGA AACACTACAG ATTTGAATTT GATTAAGGAG GGCTATTATA GACTAAGGTG TAGCTCAAAT   
  
  
+ CCTTCTGTAG GGATTGTCTT ATTGGCTTCT TTTGGTTGCT TCTGGCTCAA GTCCTCTTAT TAGTTAGTAT   
  
  
+ GCATTACCTT GAGACAAGAT AGTTGCTTAA GCTTGAGCTT CTGTACTTAT TTGCGTATTG TAGACAACTA   
  
  
+ GCTGTGGCAT ATGATTCTGA AATGTTCCGA TGTCTTTTTC TTTGGATGCT GGGTTGTGTC ATGAGCTTTA   
  
  
+ GGTTTGTTTA TTTGATGTCA GGAAGTATGT AAATAACATG TGGCATCTTG CAATCTATTA CAGGATGATA   
  
  
+ TTGGCCTTTG TGGGAAGAAA TTGAAGAAGT TGTTTCACTT ACTGGATAGG GAGCATGGAC TCACATCAGC   
  
  
+ TTTTGAGATT CGGTATTTCC AGATCATACT CATCCTATAA TTTCTCTCAG TCTAGTCCCC AATCAATTCC   
  
  
+ AAATAGGTTA TTTGAATCAC CGAACGTTCG TTCTAGAGAC TCTCCAATCT CGCCCTTCTC AACGCCCTTC   
  
  
+ AATTGCGACC CCACTGTTAT ATTGAGCGAC AGTCAGGAGC ACCACAGTTC AACAGGAAGT CTCTCGGCAC   
  
  
+ AAAGCTCATC TTCTAATTCT CCCCTTGAGA CTAGCAGTTA TTATAATCAG TTCAACTCAA GCCCTGTTGG   
  
  
+ AGAATCACCT CAGGGATCAT CACCCGAGAA TCTTTTTCAA CAAGCTGCAG TTAATAAAGT CAGCATCGAG   
  
  
+ CATGCATTGC AGGAGCTAGA AACTGCTCTA ATGAGCACAG ATTGTGAGGA GAATGAAGCA ACTGTCTCTA   
  
  
+ TCCCATCTAT GGGTGAACCT CATCAGCCCC AAGTCCCTAG CCAGAGATCA AGATTCTGGA ATCGAGATCC   
  
  
+ TCAGGGTTCA CGCCCGGCTG AAGTTCACTC ATCATTGAGA AGATTAGGAG ACGAGGCTCA GAGTGAGAAA   
  
  
+ CGCCTCAAGG CAGTGGAAGA ACCAATACGG CCCAGTGCAC CACCCGGCAA TTTGAAGCAG TTGCTCATAG   
  
  
+ AATGTGCTCG GGCTTTGTCA GAGAATCGAA TAGAGGATTT TGAGAAGTTA GTTGAACAGG CAAAGGGCAT   
  
  
+ GGTCTCCATT TCTGGAGACC CAATTCAACG ACTTGGTGCT TACTTGATCG AGGGGCTAGT GGCAAGGAAG   
  
  
+ GAGTCTTCAG GTACGAAAAT CTACCGAGCT CTTAGGTGCA AAGAGCCCCT TGGTCAAGAC TTGCTTTCGT   
  
  
+ ACATGCACAT CCTTTATGAA ATTTGCCCTT ACTTGAAATT CGGTTATATG GTTGCAAATG GGGCTATAGC   
  
  
+ TGAAGCTTGC AGAGATGAGG ATCACATACA TATCGTCGAC TACCAGATTG GTCAAGGAAC TCAATGGATG   
  
  
+ ACTCTGTTGC AAGCCCTAGC CACAAAACCT GGAAGACCCC CCACTGTGAG AATTACTGGC ATTGATGATC   
  
  
+ CCGTCTCTAG GCATGCTCGG GGAGCTTGCT TGGAGGCAGT GGGGAAACGT TTGGCAGTTC TGTCGGAGAA   
  
  
+ ATTTAACATG CCCATTGAGT TTCAGGCAGT GCCCGTTTAT GCTTCTGAGG TCACCCAAGA AATGCTTGAT   
  
  
+ GTGAGGCCAG GGTGGGCCTT GGCTGTGAAC TTCCCATTGC AGCTCCACCA CACTCCCGAT GAGAGTGTTG   
  
  
+ ATGTGAACAA CCCGAGGGAT GGACTCCTAA GAATGGTGAA ATCACTCGGT CCCAAGGTTG TAACTTTGGT   
  
  
+ AGAACAAGAG TCAAACACCA ACACTACCCC TTTGCTGACA AGGTTCATAG AAACGCTGGA CTTCTACTCA   
  
  
+ GCAATGTTTG AATCCATCGA TGTGACAATG CCAAGGGACC GAAAGGAAAG GATCAATGTC GAGCAGCATT   
  
  
+ GCCTAGCCAA GGACATTGTC AACATCATCG CGTGTGAGGG GAAGGACAGG GTGGAGCGAC ATGAGCTCTT   
  
  
+ TGGGAAATGG AAGTCAAGGT TCACCATGGC AGGATTCCAA CAATCCCCAT TGAGCTCGTA TGTTAACTCC   
  
  
+ GTGATAAAAG GATTACTTAG GTGTTATTCA GAGTACTATA CGTTGGTGGA GAAGGATGGC GCCTTGCTTC   
  
  
+ TGGGATGGAA GGACCGGATG CTGGTTTCGG CTTCAGCTTG GCATTG  

- +Up\_Stream \_Len000AAATTT ATAAAATAAA ATAAAATTTA ATTATATAAA ATATTAAATT TTAATAATTA   
  
  
- TTAACTACTA CAATGGGTTA CAAATTCAAA AATTTATAAA AATAAAAAAT TAAATTTTTA TAGTATTATA   
  
  
- TATTATATTA AAATTCAATT TTATTCTATC AATTTAATAA AATATCATTT ATAAATAATA AAAAAAATTC   
  
  
- TTTTAACGGT ACACTAACAG TAGTCAGTTT GTGACGCAAC TGTCTTCGAC AGGAACGACG ATGAGAGTGG   
  
  
- AGAGTTGTAG CCTGGGCGCG TCTGCAATTG TAGAGGCATG TTTATGTTAA GGGACCGGTT TTAAGTTTAG   
  
  
- TTGGGATGAG CCATCAGCCA AGGAAGAAGA AGGACGGGAG ATATATATGT GGGTGTCGAC GCGACGTACA   
  
  
- AAAAGGCTCT TAGGAGTTTT TAGAATATTT GAAGGAGCAC ACCCATCTTT TTTCTTTTTC TTTTCCCAAA   
  
  
- ATTTTAGAAC CCAGTTTTGG GTAGTTGTGG TGTTAAACTG AGAAAGAAAA ATTAAGTTGG TGGCGGTCAA   
  
  
- GGAGCAACTA AAAGTCCATG GAGAAAAAGA ACGGGGAAAA AAACGAGCCA ACAACAAATT ACCATTTCTA   
  
  
- ACTCATGTTT CTTACTATAA ACTACTCAAC CACACAAATA AACTACTCGG GTCTACTAAA TTGAACATCT   
  
  
- AAAACACCAA AACCAAAGAC CCATAAGTAA CCAAACGACT TAAAGACCAA GTACTAGACC CCAATCTTTT   
  
  
- CAACTTTACT GAAAGACCTC CAACCTTAAA CTGATTTCGA TTAAAGAAAA ACAGAAGTTA AGAAAAAATC   
  
  
- AACACTCGAC TTAAACAACT GGTGAAGGTC GGACAACCTT TAATTTGAAT GAATCCTCTT CTCTTAGAGT   
  
  
- CTAAGACTCG TTTTCGAAAT ATACCAACAA ACCCTTTACC ATCCTTGATT CACTTTTGTG ACTTTAAAAT   
  
  
- TCGTTCGTTA AAACAAGTAA ACTAACCAGC CTACCAGACT AAATCCAAGA AAAGAACCAG ATTTAAAGAC   
  
  
- TCGTAACTAA CAAATTAACG CACCCCTTGA ACATAGGAAC ACTCAAGGAC CAAGTAAAGA ACCAACGAAT   
  
  
- GAGATCGAGG GAAAGTGTCA AAGATACCAA CCTTGAACCT TGAACCTTGA ACCTTCGGAT TAAATCAACT   
  
  
- TATAGAACCG AAACTCAATC GAAAAGAATC CTTTTCTACC AAGACGAGAG CTTTTAGATT AGATTAGCTA   
  
  
- TCTACGCAAT TCCTTCGTTT TGAAAACCCC TCTCCCTTCA ATTCGCTTCG TTCGACGACA AAGTTTCAAC   
  
  
- CTGACAAAAT AACCATTCAT ACTTTGTTAT CAAATATTCT CTAGTAACCC AAGAATTCCA CTATCTAGTA   
  
  
- CCCAATACTT ACTCGAATCA ACAAGAAAAC GAAAGTTGAC ATGCATTCCT ACTATCAATA GGGAGTAGAA   
  
  
- CGTAGTTTTT GCCGTTTGGC ACTACGGTTT TGAGTTTTTG ACTAAGGAGA CTGTTCCGAA GATGGATACT   
  
  
- TTTGATACGT CTAGTATAGG TAAACCGGAA GGGAGATCCT TGGTTCATCT CATTCAACCA TTTATTTATC   
  
  
- TCAACTTGCT TTGTGATGTC TAAACTTAAA CTAATTCCTC CCGATAATAT CTGATTCCAC ATCGAGTTTA   
  
  
- GGAAGACATC CCTAACAGAA TAACCGAAGA AAACCAACGA AGACCGAGTT CAGGAGAATA ATCAATCATA   
  
  
- CGTAATGGAA CTCTGTTCTA TCAACGAATT CGAACTCGAA GACATGAATA AACGCATAAC ATCTGTTGAT   
  
  
- CGACACCGTA TACTAAGACT TTACAAGGCT ACAGAAAAAG AAACCTACGA CCCAACACAG TACTCGAAAT   
  
  
- CCAAACAAAT AAACTACAGT CCTTCATACA TTTATTGTAC ACCGTAGAAC GTTAGATAAT GTCCTACTAT   
  
  
- AACCGGAAAC ACCCTTCTTT AACTTCTTCA ACAAAGTGAA TGACCTATCC CTCGTACCTG AGTGTAGTCG   
  
  
- AAAACTCTAA GCCATAAAGG TCTAGTATGA GTAGGATATT AAAGAGAGTC AGATCAGGGG TTAGTTAAGG   
  
  
- TTTATCCAAT AAACTTAGTG GCTTGCAAGC AAGATCTCTG AGAGGTTAGA GCGGGAAGAG TTGCGGGAAG   
  
  
- TTAACGCTGG GGTGACAATA TAACTCGCTG TCAGTCCTCG TGGTGTCAAG TTGTCCTTCA GAGAGCCGTG   
  
  
- TTTCGAGTAG AAGATTAAGA GGGGAACTCT GATCGTCAAT AATATTAGTC AAGTTGAGTT CGGGACAACC   
  
  
- TCTTAGTGGA GTCCCTAGTA GTGGGCTCTT AGAAAAAGTT GTTCGACGTC AATTATTTCA GTCGTAGCTC   
  
  
- GTACGTAACG TCCTCGATCT TTGACGAGAT TACTCGTGTC TAACACTCCT CTTACTTCGT TGACAGAGAT   
  
  
- AGGGTAGATA CCCACTTGGA GTAGTCGGGG TTCAGGGATC GGTCTCTAGT TCTAAGACCT TAGCTCTAGG   
  
  
- AGTCCCAAGT GCGGGCCGAC TTCAAGTGAG TAGTAACTCT TCTAATCCTC TGCTCCGAGT CTCACTCTTT   
  
  
- GCGGAGTTCC GTCACCTTCT TGGTTATGCC GGGTCACGTG GTGGGCCGTT AAACTTCGTC AACGAGTATC   
  
  
- TTACACGAGC CCGAAACAGT CTCTTAGCTT ATCTCCTAAA ACTCTTCAAT CAACTTGTCC GTTTCCCGTA   
  
  
- CCAGAGGTAA AGACCTCTGG GTTAAGTTGC TGAACCACGA ATGAACTAGC TCCCCGATCA CCGTTCCTTC   
  
  
- CTCAGAAGTC CATGCTTTTA GATGGCTCGA GAATCCACGT TTCTCGGGGA ACCAGTTCTG AACGAAAGCA   
  
  
- TGTACGTGTA GGAAATACTT TAAACGGGAA TGAACTTTAA GCCAATATAC CAACGTTTAC CCCGATATCG   
  
  
- ACTTCGAACG TCTCTACTCC TAGTGTATGT ATAGCAGCTG ATGGTCTAAC CAGTTCCTTG AGTTACCTAC   
  
  
- TGAGACAACG TTCGGGATCG GTGTTTTGGA CCTTCTGGGG GGTGACACTC TTAATGACCG TAACTACTAG   
  
  
- GGCAGAGATC CGTACGAGCC CCTCGAACGA ACCTCCGTCA CCCCTTTGCA AACCGTCAAG ACAGCCTCTT   
  
  
- TAAATTGTAC GGGTAACTCA AAGTCCGTCA CGGGCAAATA CGAAGACTCC AGTGGGTTCT TTACGAACTA   
  
  
- CACTCCGGTC CCACCCGGAA CCGACACTTG AAGGGTAACG TCGAGGTGGT GTGAGGGCTA CTCTCACAAC   
  
  
- TACACTTGTT GGGCTCCCTA CCTGAGGATT CTTACCACTT TAGTGAGCCA GGGTTCCAAC ATTGAAACCA   
  
  
- TCTTGTTCTC AGTTTGTGGT TGTGATGGGG AAACGACTGT TCCAAGTATC TTTGCGACCT GAAGATGAGT   
  
  
- CGTTACAAAC TTAGGTAGCT ACACTGTTAC GGTTCCCTGG CTTTCCTTTC CTAGTTACAG CTCGTCGTAA   
  
  
- CGGATCGGTT CCTGTAACAG TTGTAGTAGC GCACACTCCC CTTCCTGTCC CACCTCGCTG TACTCGAGAA   
  
  
- ACCCTTTACC TTCAGTTCCA AGTGGTACCG TCCTAAGGTT GTTAGGGGTA ACTCGAGCAT ACAATTGAGG   
  
  
- CACTATTTTC CTAATGAATC CACAATAAGT CTCATGATAT GCAACCACCT CTTCCTACCG CGGAACGAAG   
  
  
- ACCCTACCTT CCTGGCCTAC GACCAAAGCC GAAGTCGAAC CGTAAC

+     ACTCATCCT sequence

| Site Name | Organism | Position | Strand | Matrix score. | sequence | function |
| --- | --- | --- | --- | --- | --- | --- |
| ACTCATCCT sequence | Arabidopsis thaliana | 2062 | + | 9 | ACTCATCCT |  |

>HU01G01850.1   
+ +Up\_Stream \_Len000TTTAAA TATTTTATTT TATTTTAAAT TAATATATTT TATAATTTAA AATTATTAAT   
  
  
+ AATTGATGAT GTTACCCAAT GTTTAAGTTT TTAAATATTT TTATTTTTTA ATTTAAAAAT ATCATAATAT   
  
  
+ ATAATATAAT TTTAAGTTAA AATAAGATAG TTAAATTATT TTATAGTAAA TATTTATTAT TTTTTTTAAG   
  
  
+ AAAATTGCCA TGTGATTGTC ATCAGTCAAA CACTGCGTTG ACAGAAGCTG TCCTTGCTGC TACTCTCACC   
  
  
+ TCTCAACATC GGACCCGCGC AGACGTTAAC ATCTCCGTAC AAATACAATT CCCTGGCCAA AATTCAAATC   
  
  
+ AACCCTACTC GGTAGTCGGT TCCTTCTTCT TCCTGCCCTC TATATATACA CCCACAGCTG CGCTGCATGT   
  
  
+ TTTTCCGAGA ATCCTCAAAA ATCTTATAAA CTTCCTCGTG TGGGTAGAAA AAAGAAAAAG AAAAGGGTTT   
  
  
+ TAAAATCTTG GGTCAAAACC CATCAACACC ACAATTTGAC TCTTTCTTTT TAATTCAACC ACCGCCAGTT   
  
  
+ CCTCGTTGAT TTTCAGGTAC CTCTTTTTCT TGCCCCTTTT TTTGCTCGGT TGTTGTTTAA TGGTAAAGAT   
  
  
+ TGAGTACAAA GAATGATATT TGATGAGTTG GTGTGTTTAT TTGATGAGCC CAGATGATTT AACTTGTAGA   
  
  
+ TTTTGTGGTT TTGGTTTCTG GGTATTCATT GGTTTGCTGA ATTTCTGGTT CATGATCTGG GGTTAGAAAA   
  
  
+ GTTGAAATGA CTTTCTGGAG GTTGGAATTT GACTAAAGCT AATTTCTTTT TGTCTTCAAT TCTTTTTTAG   
  
  
+ TTGTGAGCTG AATTTGTTGA CCACTTCCAG CCTGTTGGAA ATTAAACTTA CTTAGGAGAA GAGAATCTCA   
  
  
+ GATTCTGAGC AAAAGCTTTA TATGGTTGTT TGGGAAATGG TAGGAACTAA GTGAAAACAC TGAAATTTTA   
  
  
+ AGCAAGCAAT TTTGTTCATT TGATTGGTCG GATGGTCTGA TTTAGGTTCT TTTCTTGGTC TAAATTTCTG   
  
  
+ AGCATTGATT GTTTAATTGC GTGGGGAACT TGTATCCTTG TGAGTTCCTG GTTCATTTCT TGGTTGCTTA   
  
  
+ CTCTAGCTCC CTTTCACAGT TTCTATGGTT GGAACTTGGA ACTTGGAACT TGGAAGCCTA ATTTAGTTGA   
  
  
+ ATATCTTGGC TTTGAGTTAG CTTTTCTTAG GAAAAGATGG TTCTGCTCTC GAAAATCTAA TCTAATCGAT   
  
  
+ AGATGCGTTA AGGAAGCAAA ACTTTTGGGG AGAGGGAAGT TAAGCGAAGC AAGCTGCTGT TTCAAAGTTG   
  
  
+ GACTGTTTTA TTGGTAAGTA TGAAACAATA GTTTATAAGA GATCATTGGG TTCTTAAGGT GATAGATCAT   
  
  
+ GGGTTATGAA TGAGCTTAGT TGTTCTTTTG CTTTCAACTG TACGTAAGGA TGATAGTTAT CCCTCATCTT   
  
  
+ GCATCAAAAA CGGCAAACCG TGATGCCAAA ACTCAAAAAC TGATTCCTCT GACAAGGCTT CTACCTATGA   
  
  
+ AAACTATGCA GATCATATCC ATTTGGCCTT CCCTCTAGGA ACCAAGTAGA GTAAGTTGGT AAATAAATAG   
  
  
+ AGTTGAACGA AACACTACAG ATTTGAATTT GATTAAGGAG GGCTATTATA GACTAAGGTG TAGCTCAAAT   
  
  
+ CCTTCTGTAG GGATTGTCTT ATTGGCTTCT TTTGGTTGCT TCTGGCTCAA GTCCTCTTAT TAGTTAGTAT   
  
  
+ GCATTACCTT GAGACAAGAT AGTTGCTTAA GCTTGAGCTT CTGTACTTAT TTGCGTATTG TAGACAACTA   
  
  
+ GCTGTGGCAT ATGATTCTGA AATGTTCCGA TGTCTTTTTC TTTGGATGCT GGGTTGTGTC ATGAGCTTTA   
  
  
+ GGTTTGTTTA TTTGATGTCA GGAAGTATGT AAATAACATG TGGCATCTTG CAATCTATTA CAGGATGATA   
  
  
+ TTGGCCTTTG TGGGAAGAAA TTGAAGAAGT TGTTTCACTT ACTGGATAGG GAGCATGGAC TCACATCAGC   
  
  
+ TTTTGAGATT CGGTATTTCC AGATCATACT CATCCTATAA TTTCTCTCAG TCTAGTCCCC AATCAATTCC   
  
  
+ AAATAGGTTA TTTGAATCAC CGAACGTTCG TTCTAGAGAC TCTCCAATCT CGCCCTTCTC AACGCCCTTC   
  
  
+ AATTGCGACC CCACTGTTAT ATTGAGCGAC AGTCAGGAGC ACCACAGTTC AACAGGAAGT CTCTCGGCAC   
  
  
+ AAAGCTCATC TTCTAATTCT CCCCTTGAGA CTAGCAGTTA TTATAATCAG TTCAACTCAA GCCCTGTTGG   
  
  
+ AGAATCACCT CAGGGATCAT CACCCGAGAA TCTTTTTCAA CAAGCTGCAG TTAATAAAGT CAGCATCGAG   
  
  
+ CATGCATTGC AGGAGCTAGA AACTGCTCTA ATGAGCACAG ATTGTGAGGA GAATGAAGCA ACTGTCTCTA   
  
  
+ TCCCATCTAT GGGTGAACCT CATCAGCCCC AAGTCCCTAG CCAGAGATCA AGATTCTGGA ATCGAGATCC   
  
  
+ TCAGGGTTCA CGCCCGGCTG AAGTTCACTC ATCATTGAGA AGATTAGGAG ACGAGGCTCA GAGTGAGAAA   
  
  
+ CGCCTCAAGG CAGTGGAAGA ACCAATACGG CCCAGTGCAC CACCCGGCAA TTTGAAGCAG TTGCTCATAG   
  
  
+ AATGTGCTCG GGCTTTGTCA GAGAATCGAA TAGAGGATTT TGAGAAGTTA GTTGAACAGG CAAAGGGCAT   
  
  
+ GGTCTCCATT TCTGGAGACC CAATTCAACG ACTTGGTGCT TACTTGATCG AGGGGCTAGT GGCAAGGAAG   
  
  
+ GAGTCTTCAG GTACGAAAAT CTACCGAGCT CTTAGGTGCA AAGAGCCCCT TGGTCAAGAC TTGCTTTCGT   
  
  
+ ACATGCACAT CCTTTATGAA ATTTGCCCTT ACTTGAAATT CGGTTATATG GTTGCAAATG GGGCTATAGC   
  
  
+ TGAAGCTTGC AGAGATGAGG ATCACATACA TATCGTCGAC TACCAGATTG GTCAAGGAAC TCAATGGATG   
  
  
+ ACTCTGTTGC AAGCCCTAGC CACAAAACCT GGAAGACCCC CCACTGTGAG AATTACTGGC ATTGATGATC   
  
  
+ CCGTCTCTAG GCATGCTCGG GGAGCTTGCT TGGAGGCAGT GGGGAAACGT TTGGCAGTTC TGTCGGAGAA   
  
  
+ ATTTAACATG CCCATTGAGT TTCAGGCAGT GCCCGTTTAT GCTTCTGAGG TCACCCAAGA AATGCTTGAT   
  
  
+ GTGAGGCCAG GGTGGGCCTT GGCTGTGAAC TTCCCATTGC AGCTCCACCA CACTCCCGAT GAGAGTGTTG   
  
  
+ ATGTGAACAA CCCGAGGGAT GGACTCCTAA GAATGGTGAA ATCACTCGGT CCCAAGGTTG TAACTTTGGT   
  
  
+ AGAACAAGAG TCAAACACCA ACACTACCCC TTTGCTGACA AGGTTCATAG AAACGCTGGA CTTCTACTCA   
  
  
+ GCAATGTTTG AATCCATCGA TGTGACAATG CCAAGGGACC GAAAGGAAAG GATCAATGTC GAGCAGCATT   
  
  
+ GCCTAGCCAA GGACATTGTC AACATCATCG CGTGTGAGGG GAAGGACAGG GTGGAGCGAC ATGAGCTCTT   
  
  
+ TGGGAAATGG AAGTCAAGGT TCACCATGGC AGGATTCCAA CAATCCCCAT TGAGCTCGTA TGTTAACTCC   
  
  
+ GTGATAAAAG GATTACTTAG GTGTTATTCA GAGTACTATA CGTTGGTGGA GAAGGATGGC GCCTTGCTTC   
  
  
+ TGGGATGGAA GGACCGGATG CTGGTTTCGG CTTCAGCTTG GCATTG  

- +Up\_Stream \_Len000AAATTT ATAAAATAAA ATAAAATTTA ATTATATAAA ATATTAAATT TTAATAATTA   
  
  
- TTAACTACTA CAATGGGTTA CAAATTCAAA AATTTATAAA AATAAAAAAT TAAATTTTTA TAGTATTATA   
  
  
- TATTATATTA AAATTCAATT TTATTCTATC AATTTAATAA AATATCATTT ATAAATAATA AAAAAAATTC   
  
  
- TTTTAACGGT ACACTAACAG TAGTCAGTTT GTGACGCAAC TGTCTTCGAC AGGAACGACG ATGAGAGTGG   
  
  
- AGAGTTGTAG CCTGGGCGCG TCTGCAATTG TAGAGGCATG TTTATGTTAA GGGACCGGTT TTAAGTTTAG   
  
  
- TTGGGATGAG CCATCAGCCA AGGAAGAAGA AGGACGGGAG ATATATATGT GGGTGTCGAC GCGACGTACA   
  
  
- AAAAGGCTCT TAGGAGTTTT TAGAATATTT GAAGGAGCAC ACCCATCTTT TTTCTTTTTC TTTTCCCAAA   
  
  
- ATTTTAGAAC CCAGTTTTGG GTAGTTGTGG TGTTAAACTG AGAAAGAAAA ATTAAGTTGG TGGCGGTCAA   
  
  
- GGAGCAACTA AAAGTCCATG GAGAAAAAGA ACGGGGAAAA AAACGAGCCA ACAACAAATT ACCATTTCTA   
  
  
- ACTCATGTTT CTTACTATAA ACTACTCAAC CACACAAATA AACTACTCGG GTCTACTAAA TTGAACATCT   
  
  
- AAAACACCAA AACCAAAGAC CCATAAGTAA CCAAACGACT TAAAGACCAA GTACTAGACC CCAATCTTTT   
  
  
- CAACTTTACT GAAAGACCTC CAACCTTAAA CTGATTTCGA TTAAAGAAAA ACAGAAGTTA AGAAAAAATC   
  
  
- AACACTCGAC TTAAACAACT GGTGAAGGTC GGACAACCTT TAATTTGAAT GAATCCTCTT CTCTTAGAGT   
  
  
- CTAAGACTCG TTTTCGAAAT ATACCAACAA ACCCTTTACC ATCCTTGATT CACTTTTGTG ACTTTAAAAT   
  
  
- TCGTTCGTTA AAACAAGTAA ACTAACCAGC CTACCAGACT AAATCCAAGA AAAGAACCAG ATTTAAAGAC   
  
  
- TCGTAACTAA CAAATTAACG CACCCCTTGA ACATAGGAAC ACTCAAGGAC CAAGTAAAGA ACCAACGAAT   
  
  
- GAGATCGAGG GAAAGTGTCA AAGATACCAA CCTTGAACCT TGAACCTTGA ACCTTCGGAT TAAATCAACT   
  
  
- TATAGAACCG AAACTCAATC GAAAAGAATC CTTTTCTACC AAGACGAGAG CTTTTAGATT AGATTAGCTA   
  
  
- TCTACGCAAT TCCTTCGTTT TGAAAACCCC TCTCCCTTCA ATTCGCTTCG TTCGACGACA AAGTTTCAAC   
  
  
- CTGACAAAAT AACCATTCAT ACTTTGTTAT CAAATATTCT CTAGTAACCC AAGAATTCCA CTATCTAGTA   
  
  
- CCCAATACTT ACTCGAATCA ACAAGAAAAC GAAAGTTGAC ATGCATTCCT ACTATCAATA GGGAGTAGAA   
  
  
- CGTAGTTTTT GCCGTTTGGC ACTACGGTTT TGAGTTTTTG ACTAAGGAGA CTGTTCCGAA GATGGATACT   
  
  
- TTTGATACGT CTAGTATAGG TAAACCGGAA GGGAGATCCT TGGTTCATCT CATTCAACCA TTTATTTATC   
  
  
- TCAACTTGCT TTGTGATGTC TAAACTTAAA CTAATTCCTC CCGATAATAT CTGATTCCAC ATCGAGTTTA   
  
  
- GGAAGACATC CCTAACAGAA TAACCGAAGA AAACCAACGA AGACCGAGTT CAGGAGAATA ATCAATCATA   
  
  
- CGTAATGGAA CTCTGTTCTA TCAACGAATT CGAACTCGAA GACATGAATA AACGCATAAC ATCTGTTGAT   
  
  
- CGACACCGTA TACTAAGACT TTACAAGGCT ACAGAAAAAG AAACCTACGA CCCAACACAG TACTCGAAAT   
  
  
- CCAAACAAAT AAACTACAGT CCTTCATACA TTTATTGTAC ACCGTAGAAC GTTAGATAAT GTCCTACTAT   
  
  
- AACCGGAAAC ACCCTTCTTT AACTTCTTCA ACAAAGTGAA TGACCTATCC CTCGTACCTG AGTGTAGTCG   
  
  
- AAAACTCTAA GCCATAAAGG TCTAGTATGA GTAGGATATT AAAGAGAGTC AGATCAGGGG TTAGTTAAGG   
  
  
- TTTATCCAAT AAACTTAGTG GCTTGCAAGC AAGATCTCTG AGAGGTTAGA GCGGGAAGAG TTGCGGGAAG   
  
  
- TTAACGCTGG GGTGACAATA TAACTCGCTG TCAGTCCTCG TGGTGTCAAG TTGTCCTTCA GAGAGCCGTG   
  
  
- TTTCGAGTAG AAGATTAAGA GGGGAACTCT GATCGTCAAT AATATTAGTC AAGTTGAGTT CGGGACAACC   
  
  
- TCTTAGTGGA GTCCCTAGTA GTGGGCTCTT AGAAAAAGTT GTTCGACGTC AATTATTTCA GTCGTAGCTC   
  
  
- GTACGTAACG TCCTCGATCT TTGACGAGAT TACTCGTGTC TAACACTCCT CTTACTTCGT TGACAGAGAT   
  
  
- AGGGTAGATA CCCACTTGGA GTAGTCGGGG TTCAGGGATC GGTCTCTAGT TCTAAGACCT TAGCTCTAGG   
  
  
- AGTCCCAAGT GCGGGCCGAC TTCAAGTGAG TAGTAACTCT TCTAATCCTC TGCTCCGAGT CTCACTCTTT   
  
  
- GCGGAGTTCC GTCACCTTCT TGGTTATGCC GGGTCACGTG GTGGGCCGTT AAACTTCGTC AACGAGTATC   
  
  
- TTACACGAGC CCGAAACAGT CTCTTAGCTT ATCTCCTAAA ACTCTTCAAT CAACTTGTCC GTTTCCCGTA   
  
  
- CCAGAGGTAA AGACCTCTGG GTTAAGTTGC TGAACCACGA ATGAACTAGC TCCCCGATCA CCGTTCCTTC   
  
  
- CTCAGAAGTC CATGCTTTTA GATGGCTCGA GAATCCACGT TTCTCGGGGA ACCAGTTCTG AACGAAAGCA   
  
  
- TGTACGTGTA GGAAATACTT TAAACGGGAA TGAACTTTAA GCCAATATAC CAACGTTTAC CCCGATATCG   
  
  
- ACTTCGAACG TCTCTACTCC TAGTGTATGT ATAGCAGCTG ATGGTCTAAC CAGTTCCTTG AGTTACCTAC   
  
  
- TGAGACAACG TTCGGGATCG GTGTTTTGGA CCTTCTGGGG GGTGACACTC TTAATGACCG TAACTACTAG   
  
  
- GGCAGAGATC CGTACGAGCC CCTCGAACGA ACCTCCGTCA CCCCTTTGCA AACCGTCAAG ACAGCCTCTT   
  
  
- TAAATTGTAC GGGTAACTCA AAGTCCGTCA CGGGCAAATA CGAAGACTCC AGTGGGTTCT TTACGAACTA   
  
  
- CACTCCGGTC CCACCCGGAA CCGACACTTG AAGGGTAACG TCGAGGTGGT GTGAGGGCTA CTCTCACAAC   
  
  
- TACACTTGTT GGGCTCCCTA CCTGAGGATT CTTACCACTT TAGTGAGCCA GGGTTCCAAC ATTGAAACCA   
  
  
- TCTTGTTCTC AGTTTGTGGT TGTGATGGGG AAACGACTGT TCCAAGTATC TTTGCGACCT GAAGATGAGT   
  
  
- CGTTACAAAC TTAGGTAGCT ACACTGTTAC GGTTCCCTGG CTTTCCTTTC CTAGTTACAG CTCGTCGTAA   
  
  
- CGGATCGGTT CCTGTAACAG TTGTAGTAGC GCACACTCCC CTTCCTGTCC CACCTCGCTG TACTCGAGAA   
  
  
- ACCCTTTACC TTCAGTTCCA AGTGGTACCG TCCTAAGGTT GTTAGGGGTA ACTCGAGCAT ACAATTGAGG   
  
  
- CACTATTTTC CTAATGAATC CACAATAAGT CTCATGATAT GCAACCACCT CTTCCTACCG CGGAACGAAG   
  
  
- ACCCTACCTT CCTGGCCTAC GACCAAAGCC GAAGTCGAAC CGTAAC

+     AP-1

| Site Name | Organism | Position | Strand | Matrix score. | sequence | function |
| --- | --- | --- | --- | --- | --- | --- |
| AP-1 | Arabidopsis thaliana | 1207 | + | 8 | TGAGTTAG |  |

>HU01G01850.1   
+ +Up\_Stream \_Len000TTTAAA TATTTTATTT TATTTTAAAT TAATATATTT TATAATTTAA AATTATTAAT   
  
  
+ AATTGATGAT GTTACCCAAT GTTTAAGTTT TTAAATATTT TTATTTTTTA ATTTAAAAAT ATCATAATAT   
  
  
+ ATAATATAAT TTTAAGTTAA AATAAGATAG TTAAATTATT TTATAGTAAA TATTTATTAT TTTTTTTAAG   
  
  
+ AAAATTGCCA TGTGATTGTC ATCAGTCAAA CACTGCGTTG ACAGAAGCTG TCCTTGCTGC TACTCTCACC   
  
  
+ TCTCAACATC GGACCCGCGC AGACGTTAAC ATCTCCGTAC AAATACAATT CCCTGGCCAA AATTCAAATC   
  
  
+ AACCCTACTC GGTAGTCGGT TCCTTCTTCT TCCTGCCCTC TATATATACA CCCACAGCTG CGCTGCATGT   
  
  
+ TTTTCCGAGA ATCCTCAAAA ATCTTATAAA CTTCCTCGTG TGGGTAGAAA AAAGAAAAAG AAAAGGGTTT   
  
  
+ TAAAATCTTG GGTCAAAACC CATCAACACC ACAATTTGAC TCTTTCTTTT TAATTCAACC ACCGCCAGTT   
  
  
+ CCTCGTTGAT TTTCAGGTAC CTCTTTTTCT TGCCCCTTTT TTTGCTCGGT TGTTGTTTAA TGGTAAAGAT   
  
  
+ TGAGTACAAA GAATGATATT TGATGAGTTG GTGTGTTTAT TTGATGAGCC CAGATGATTT AACTTGTAGA   
  
  
+ TTTTGTGGTT TTGGTTTCTG GGTATTCATT GGTTTGCTGA ATTTCTGGTT CATGATCTGG GGTTAGAAAA   
  
  
+ GTTGAAATGA CTTTCTGGAG GTTGGAATTT GACTAAAGCT AATTTCTTTT TGTCTTCAAT TCTTTTTTAG   
  
  
+ TTGTGAGCTG AATTTGTTGA CCACTTCCAG CCTGTTGGAA ATTAAACTTA CTTAGGAGAA GAGAATCTCA   
  
  
+ GATTCTGAGC AAAAGCTTTA TATGGTTGTT TGGGAAATGG TAGGAACTAA GTGAAAACAC TGAAATTTTA   
  
  
+ AGCAAGCAAT TTTGTTCATT TGATTGGTCG GATGGTCTGA TTTAGGTTCT TTTCTTGGTC TAAATTTCTG   
  
  
+ AGCATTGATT GTTTAATTGC GTGGGGAACT TGTATCCTTG TGAGTTCCTG GTTCATTTCT TGGTTGCTTA   
  
  
+ CTCTAGCTCC CTTTCACAGT TTCTATGGTT GGAACTTGGA ACTTGGAACT TGGAAGCCTA ATTTAGTTGA   
  
  
+ ATATCTTGGC TTTGAGTTAG CTTTTCTTAG GAAAAGATGG TTCTGCTCTC GAAAATCTAA TCTAATCGAT   
  
  
+ AGATGCGTTA AGGAAGCAAA ACTTTTGGGG AGAGGGAAGT TAAGCGAAGC AAGCTGCTGT TTCAAAGTTG   
  
  
+ GACTGTTTTA TTGGTAAGTA TGAAACAATA GTTTATAAGA GATCATTGGG TTCTTAAGGT GATAGATCAT   
  
  
+ GGGTTATGAA TGAGCTTAGT TGTTCTTTTG CTTTCAACTG TACGTAAGGA TGATAGTTAT CCCTCATCTT   
  
  
+ GCATCAAAAA CGGCAAACCG TGATGCCAAA ACTCAAAAAC TGATTCCTCT GACAAGGCTT CTACCTATGA   
  
  
+ AAACTATGCA GATCATATCC ATTTGGCCTT CCCTCTAGGA ACCAAGTAGA GTAAGTTGGT AAATAAATAG   
  
  
+ AGTTGAACGA AACACTACAG ATTTGAATTT GATTAAGGAG GGCTATTATA GACTAAGGTG TAGCTCAAAT   
  
  
+ CCTTCTGTAG GGATTGTCTT ATTGGCTTCT TTTGGTTGCT TCTGGCTCAA GTCCTCTTAT TAGTTAGTAT   
  
  
+ GCATTACCTT GAGACAAGAT AGTTGCTTAA GCTTGAGCTT CTGTACTTAT TTGCGTATTG TAGACAACTA   
  
  
+ GCTGTGGCAT ATGATTCTGA AATGTTCCGA TGTCTTTTTC TTTGGATGCT GGGTTGTGTC ATGAGCTTTA   
  
  
+ GGTTTGTTTA TTTGATGTCA GGAAGTATGT AAATAACATG TGGCATCTTG CAATCTATTA CAGGATGATA   
  
  
+ TTGGCCTTTG TGGGAAGAAA TTGAAGAAGT TGTTTCACTT ACTGGATAGG GAGCATGGAC TCACATCAGC   
  
  
+ TTTTGAGATT CGGTATTTCC AGATCATACT CATCCTATAA TTTCTCTCAG TCTAGTCCCC AATCAATTCC   
  
  
+ AAATAGGTTA TTTGAATCAC CGAACGTTCG TTCTAGAGAC TCTCCAATCT CGCCCTTCTC AACGCCCTTC   
  
  
+ AATTGCGACC CCACTGTTAT ATTGAGCGAC AGTCAGGAGC ACCACAGTTC AACAGGAAGT CTCTCGGCAC   
  
  
+ AAAGCTCATC TTCTAATTCT CCCCTTGAGA CTAGCAGTTA TTATAATCAG TTCAACTCAA GCCCTGTTGG   
  
  
+ AGAATCACCT CAGGGATCAT CACCCGAGAA TCTTTTTCAA CAAGCTGCAG TTAATAAAGT CAGCATCGAG   
  
  
+ CATGCATTGC AGGAGCTAGA AACTGCTCTA ATGAGCACAG ATTGTGAGGA GAATGAAGCA ACTGTCTCTA   
  
  
+ TCCCATCTAT GGGTGAACCT CATCAGCCCC AAGTCCCTAG CCAGAGATCA AGATTCTGGA ATCGAGATCC   
  
  
+ TCAGGGTTCA CGCCCGGCTG AAGTTCACTC ATCATTGAGA AGATTAGGAG ACGAGGCTCA GAGTGAGAAA   
  
  
+ CGCCTCAAGG CAGTGGAAGA ACCAATACGG CCCAGTGCAC CACCCGGCAA TTTGAAGCAG TTGCTCATAG   
  
  
+ AATGTGCTCG GGCTTTGTCA GAGAATCGAA TAGAGGATTT TGAGAAGTTA GTTGAACAGG CAAAGGGCAT   
  
  
+ GGTCTCCATT TCTGGAGACC CAATTCAACG ACTTGGTGCT TACTTGATCG AGGGGCTAGT GGCAAGGAAG   
  
  
+ GAGTCTTCAG GTACGAAAAT CTACCGAGCT CTTAGGTGCA AAGAGCCCCT TGGTCAAGAC TTGCTTTCGT   
  
  
+ ACATGCACAT CCTTTATGAA ATTTGCCCTT ACTTGAAATT CGGTTATATG GTTGCAAATG GGGCTATAGC   
  
  
+ TGAAGCTTGC AGAGATGAGG ATCACATACA TATCGTCGAC TACCAGATTG GTCAAGGAAC TCAATGGATG   
  
  
+ ACTCTGTTGC AAGCCCTAGC CACAAAACCT GGAAGACCCC CCACTGTGAG AATTACTGGC ATTGATGATC   
  
  
+ CCGTCTCTAG GCATGCTCGG GGAGCTTGCT TGGAGGCAGT GGGGAAACGT TTGGCAGTTC TGTCGGAGAA   
  
  
+ ATTTAACATG CCCATTGAGT TTCAGGCAGT GCCCGTTTAT GCTTCTGAGG TCACCCAAGA AATGCTTGAT   
  
  
+ GTGAGGCCAG GGTGGGCCTT GGCTGTGAAC TTCCCATTGC AGCTCCACCA CACTCCCGAT GAGAGTGTTG   
  
  
+ ATGTGAACAA CCCGAGGGAT GGACTCCTAA GAATGGTGAA ATCACTCGGT CCCAAGGTTG TAACTTTGGT   
  
  
+ AGAACAAGAG TCAAACACCA ACACTACCCC TTTGCTGACA AGGTTCATAG AAACGCTGGA CTTCTACTCA   
  
  
+ GCAATGTTTG AATCCATCGA TGTGACAATG CCAAGGGACC GAAAGGAAAG GATCAATGTC GAGCAGCATT   
  
  
+ GCCTAGCCAA GGACATTGTC AACATCATCG CGTGTGAGGG GAAGGACAGG GTGGAGCGAC ATGAGCTCTT   
  
  
+ TGGGAAATGG AAGTCAAGGT TCACCATGGC AGGATTCCAA CAATCCCCAT TGAGCTCGTA TGTTAACTCC   
  
  
+ GTGATAAAAG GATTACTTAG GTGTTATTCA GAGTACTATA CGTTGGTGGA GAAGGATGGC GCCTTGCTTC   
  
  
+ TGGGATGGAA GGACCGGATG CTGGTTTCGG CTTCAGCTTG GCATTG  

- +Up\_Stream \_Len000AAATTT ATAAAATAAA ATAAAATTTA ATTATATAAA ATATTAAATT TTAATAATTA   
  
  
- TTAACTACTA CAATGGGTTA CAAATTCAAA AATTTATAAA AATAAAAAAT TAAATTTTTA TAGTATTATA   
  
  
- TATTATATTA AAATTCAATT TTATTCTATC AATTTAATAA AATATCATTT ATAAATAATA AAAAAAATTC   
  
  
- TTTTAACGGT ACACTAACAG TAGTCAGTTT GTGACGCAAC TGTCTTCGAC AGGAACGACG ATGAGAGTGG   
  
  
- AGAGTTGTAG CCTGGGCGCG TCTGCAATTG TAGAGGCATG TTTATGTTAA GGGACCGGTT TTAAGTTTAG   
  
  
- TTGGGATGAG CCATCAGCCA AGGAAGAAGA AGGACGGGAG ATATATATGT GGGTGTCGAC GCGACGTACA   
  
  
- AAAAGGCTCT TAGGAGTTTT TAGAATATTT GAAGGAGCAC ACCCATCTTT TTTCTTTTTC TTTTCCCAAA   
  
  
- ATTTTAGAAC CCAGTTTTGG GTAGTTGTGG TGTTAAACTG AGAAAGAAAA ATTAAGTTGG TGGCGGTCAA   
  
  
- GGAGCAACTA AAAGTCCATG GAGAAAAAGA ACGGGGAAAA AAACGAGCCA ACAACAAATT ACCATTTCTA   
  
  
- ACTCATGTTT CTTACTATAA ACTACTCAAC CACACAAATA AACTACTCGG GTCTACTAAA TTGAACATCT   
  
  
- AAAACACCAA AACCAAAGAC CCATAAGTAA CCAAACGACT TAAAGACCAA GTACTAGACC CCAATCTTTT   
  
  
- CAACTTTACT GAAAGACCTC CAACCTTAAA CTGATTTCGA TTAAAGAAAA ACAGAAGTTA AGAAAAAATC   
  
  
- AACACTCGAC TTAAACAACT GGTGAAGGTC GGACAACCTT TAATTTGAAT GAATCCTCTT CTCTTAGAGT   
  
  
- CTAAGACTCG TTTTCGAAAT ATACCAACAA ACCCTTTACC ATCCTTGATT CACTTTTGTG ACTTTAAAAT   
  
  
- TCGTTCGTTA AAACAAGTAA ACTAACCAGC CTACCAGACT AAATCCAAGA AAAGAACCAG ATTTAAAGAC   
  
  
- TCGTAACTAA CAAATTAACG CACCCCTTGA ACATAGGAAC ACTCAAGGAC CAAGTAAAGA ACCAACGAAT   
  
  
- GAGATCGAGG GAAAGTGTCA AAGATACCAA CCTTGAACCT TGAACCTTGA ACCTTCGGAT TAAATCAACT   
  
  
- TATAGAACCG AAACTCAATC GAAAAGAATC CTTTTCTACC AAGACGAGAG CTTTTAGATT AGATTAGCTA   
  
  
- TCTACGCAAT TCCTTCGTTT TGAAAACCCC TCTCCCTTCA ATTCGCTTCG TTCGACGACA AAGTTTCAAC   
  
  
- CTGACAAAAT AACCATTCAT ACTTTGTTAT CAAATATTCT CTAGTAACCC AAGAATTCCA CTATCTAGTA   
  
  
- CCCAATACTT ACTCGAATCA ACAAGAAAAC GAAAGTTGAC ATGCATTCCT ACTATCAATA GGGAGTAGAA   
  
  
- CGTAGTTTTT GCCGTTTGGC ACTACGGTTT TGAGTTTTTG ACTAAGGAGA CTGTTCCGAA GATGGATACT   
  
  
- TTTGATACGT CTAGTATAGG TAAACCGGAA GGGAGATCCT TGGTTCATCT CATTCAACCA TTTATTTATC   
  
  
- TCAACTTGCT TTGTGATGTC TAAACTTAAA CTAATTCCTC CCGATAATAT CTGATTCCAC ATCGAGTTTA   
  
  
- GGAAGACATC CCTAACAGAA TAACCGAAGA AAACCAACGA AGACCGAGTT CAGGAGAATA ATCAATCATA   
  
  
- CGTAATGGAA CTCTGTTCTA TCAACGAATT CGAACTCGAA GACATGAATA AACGCATAAC ATCTGTTGAT   
  
  
- CGACACCGTA TACTAAGACT TTACAAGGCT ACAGAAAAAG AAACCTACGA CCCAACACAG TACTCGAAAT   
  
  
- CCAAACAAAT AAACTACAGT CCTTCATACA TTTATTGTAC ACCGTAGAAC GTTAGATAAT GTCCTACTAT   
  
  
- AACCGGAAAC ACCCTTCTTT AACTTCTTCA ACAAAGTGAA TGACCTATCC CTCGTACCTG AGTGTAGTCG   
  
  
- AAAACTCTAA GCCATAAAGG TCTAGTATGA GTAGGATATT AAAGAGAGTC AGATCAGGGG TTAGTTAAGG   
  
  
- TTTATCCAAT AAACTTAGTG GCTTGCAAGC AAGATCTCTG AGAGGTTAGA GCGGGAAGAG TTGCGGGAAG   
  
  
- TTAACGCTGG GGTGACAATA TAACTCGCTG TCAGTCCTCG TGGTGTCAAG TTGTCCTTCA GAGAGCCGTG   
  
  
- TTTCGAGTAG AAGATTAAGA GGGGAACTCT GATCGTCAAT AATATTAGTC AAGTTGAGTT CGGGACAACC   
  
  
- TCTTAGTGGA GTCCCTAGTA GTGGGCTCTT AGAAAAAGTT GTTCGACGTC AATTATTTCA GTCGTAGCTC   
  
  
- GTACGTAACG TCCTCGATCT TTGACGAGAT TACTCGTGTC TAACACTCCT CTTACTTCGT TGACAGAGAT   
  
  
- AGGGTAGATA CCCACTTGGA GTAGTCGGGG TTCAGGGATC GGTCTCTAGT TCTAAGACCT TAGCTCTAGG   
  
  
- AGTCCCAAGT GCGGGCCGAC TTCAAGTGAG TAGTAACTCT TCTAATCCTC TGCTCCGAGT CTCACTCTTT   
  
  
- GCGGAGTTCC GTCACCTTCT TGGTTATGCC GGGTCACGTG GTGGGCCGTT AAACTTCGTC AACGAGTATC   
  
  
- TTACACGAGC CCGAAACAGT CTCTTAGCTT ATCTCCTAAA ACTCTTCAAT CAACTTGTCC GTTTCCCGTA   
  
  
- CCAGAGGTAA AGACCTCTGG GTTAAGTTGC TGAACCACGA ATGAACTAGC TCCCCGATCA CCGTTCCTTC   
  
  
- CTCAGAAGTC CATGCTTTTA GATGGCTCGA GAATCCACGT TTCTCGGGGA ACCAGTTCTG AACGAAAGCA   
  
  
- TGTACGTGTA GGAAATACTT TAAACGGGAA TGAACTTTAA GCCAATATAC CAACGTTTAC CCCGATATCG   
  
  
- ACTTCGAACG TCTCTACTCC TAGTGTATGT ATAGCAGCTG ATGGTCTAAC CAGTTCCTTG AGTTACCTAC   
  
  
- TGAGACAACG TTCGGGATCG GTGTTTTGGA CCTTCTGGGG GGTGACACTC TTAATGACCG TAACTACTAG   
  
  
- GGCAGAGATC CGTACGAGCC CCTCGAACGA ACCTCCGTCA CCCCTTTGCA AACCGTCAAG ACAGCCTCTT   
  
  
- TAAATTGTAC GGGTAACTCA AAGTCCGTCA CGGGCAAATA CGAAGACTCC AGTGGGTTCT TTACGAACTA   
  
  
- CACTCCGGTC CCACCCGGAA CCGACACTTG AAGGGTAACG TCGAGGTGGT GTGAGGGCTA CTCTCACAAC   
  
  
- TACACTTGTT GGGCTCCCTA CCTGAGGATT CTTACCACTT TAGTGAGCCA GGGTTCCAAC ATTGAAACCA   
  
  
- TCTTGTTCTC AGTTTGTGGT TGTGATGGGG AAACGACTGT TCCAAGTATC TTTGCGACCT GAAGATGAGT   
  
  
- CGTTACAAAC TTAGGTAGCT ACACTGTTAC GGTTCCCTGG CTTTCCTTTC CTAGTTACAG CTCGTCGTAA   
  
  
- CGGATCGGTT CCTGTAACAG TTGTAGTAGC GCACACTCCC CTTCCTGTCC CACCTCGCTG TACTCGAGAA   
  
  
- ACCCTTTACC TTCAGTTCCA AGTGGTACCG TCCTAAGGTT GTTAGGGGTA ACTCGAGCAT ACAATTGAGG   
  
  
- CACTATTTTC CTAATGAATC CACAATAAGT CTCATGATAT GCAACCACCT CTTCCTACCG CGGAACGAAG   
  
  
- ACCCTACCTT CCTGGCCTAC GACCAAAGCC GAAGTCGAAC CGTAAC

+     ARE

| Site Name | Organism | Position | Strand | Matrix score. | sequence | function |
| --- | --- | --- | --- | --- | --- | --- |
| ARE | Zea mays | 716 | - | 6 | AAACCA | cis-acting regulatory element essential for the anaerobic induction |
| ARE | Zea mays | 734 | - | 6 | AAACCA | cis-acting regulatory element essential for the anaerobic induction |
| ARE | Zea mays | 710 | - | 6 | AAACCA | cis-acting regulatory element essential for the anaerobic induction |
| ARE | Zea mays | 3736 | - | 6 | AAACCA | cis-acting regulatory element essential for the anaerobic induction |

>HU01G01850.1   
+ +Up\_Stream \_Len000TTTAAA TATTTTATTT TATTTTAAAT TAATATATTT TATAATTTAA AATTATTAAT   
  
  
+ AATTGATGAT GTTACCCAAT GTTTAAGTTT TTAAATATTT TTATTTTTTA ATTTAAAAAT ATCATAATAT   
  
  
+ ATAATATAAT TTTAAGTTAA AATAAGATAG TTAAATTATT TTATAGTAAA TATTTATTAT TTTTTTTAAG   
  
  
+ AAAATTGCCA TGTGATTGTC ATCAGTCAAA CACTGCGTTG ACAGAAGCTG TCCTTGCTGC TACTCTCACC   
  
  
+ TCTCAACATC GGACCCGCGC AGACGTTAAC ATCTCCGTAC AAATACAATT CCCTGGCCAA AATTCAAATC   
  
  
+ AACCCTACTC GGTAGTCGGT TCCTTCTTCT TCCTGCCCTC TATATATACA CCCACAGCTG CGCTGCATGT   
  
  
+ TTTTCCGAGA ATCCTCAAAA ATCTTATAAA CTTCCTCGTG TGGGTAGAAA AAAGAAAAAG AAAAGGGTTT   
  
  
+ TAAAATCTTG GGTCAAAACC CATCAACACC ACAATTTGAC TCTTTCTTTT TAATTCAACC ACCGCCAGTT   
  
  
+ CCTCGTTGAT TTTCAGGTAC CTCTTTTTCT TGCCCCTTTT TTTGCTCGGT TGTTGTTTAA TGGTAAAGAT   
  
  
+ TGAGTACAAA GAATGATATT TGATGAGTTG GTGTGTTTAT TTGATGAGCC CAGATGATTT AACTTGTAGA   
  
  
+ TTTTGTGGTT TTGGTTTCTG GGTATTCATT GGTTTGCTGA ATTTCTGGTT CATGATCTGG GGTTAGAAAA   
  
  
+ GTTGAAATGA CTTTCTGGAG GTTGGAATTT GACTAAAGCT AATTTCTTTT TGTCTTCAAT TCTTTTTTAG   
  
  
+ TTGTGAGCTG AATTTGTTGA CCACTTCCAG CCTGTTGGAA ATTAAACTTA CTTAGGAGAA GAGAATCTCA   
  
  
+ GATTCTGAGC AAAAGCTTTA TATGGTTGTT TGGGAAATGG TAGGAACTAA GTGAAAACAC TGAAATTTTA   
  
  
+ AGCAAGCAAT TTTGTTCATT TGATTGGTCG GATGGTCTGA TTTAGGTTCT TTTCTTGGTC TAAATTTCTG   
  
  
+ AGCATTGATT GTTTAATTGC GTGGGGAACT TGTATCCTTG TGAGTTCCTG GTTCATTTCT TGGTTGCTTA   
  
  
+ CTCTAGCTCC CTTTCACAGT TTCTATGGTT GGAACTTGGA ACTTGGAACT TGGAAGCCTA ATTTAGTTGA   
  
  
+ ATATCTTGGC TTTGAGTTAG CTTTTCTTAG GAAAAGATGG TTCTGCTCTC GAAAATCTAA TCTAATCGAT   
  
  
+ AGATGCGTTA AGGAAGCAAA ACTTTTGGGG AGAGGGAAGT TAAGCGAAGC AAGCTGCTGT TTCAAAGTTG   
  
  
+ GACTGTTTTA TTGGTAAGTA TGAAACAATA GTTTATAAGA GATCATTGGG TTCTTAAGGT GATAGATCAT   
  
  
+ GGGTTATGAA TGAGCTTAGT TGTTCTTTTG CTTTCAACTG TACGTAAGGA TGATAGTTAT CCCTCATCTT   
  
  
+ GCATCAAAAA CGGCAAACCG TGATGCCAAA ACTCAAAAAC TGATTCCTCT GACAAGGCTT CTACCTATGA   
  
  
+ AAACTATGCA GATCATATCC ATTTGGCCTT CCCTCTAGGA ACCAAGTAGA GTAAGTTGGT AAATAAATAG   
  
  
+ AGTTGAACGA AACACTACAG ATTTGAATTT GATTAAGGAG GGCTATTATA GACTAAGGTG TAGCTCAAAT   
  
  
+ CCTTCTGTAG GGATTGTCTT ATTGGCTTCT TTTGGTTGCT TCTGGCTCAA GTCCTCTTAT TAGTTAGTAT   
  
  
+ GCATTACCTT GAGACAAGAT AGTTGCTTAA GCTTGAGCTT CTGTACTTAT TTGCGTATTG TAGACAACTA   
  
  
+ GCTGTGGCAT ATGATTCTGA AATGTTCCGA TGTCTTTTTC TTTGGATGCT GGGTTGTGTC ATGAGCTTTA   
  
  
+ GGTTTGTTTA TTTGATGTCA GGAAGTATGT AAATAACATG TGGCATCTTG CAATCTATTA CAGGATGATA   
  
  
+ TTGGCCTTTG TGGGAAGAAA TTGAAGAAGT TGTTTCACTT ACTGGATAGG GAGCATGGAC TCACATCAGC   
  
  
+ TTTTGAGATT CGGTATTTCC AGATCATACT CATCCTATAA TTTCTCTCAG TCTAGTCCCC AATCAATTCC   
  
  
+ AAATAGGTTA TTTGAATCAC CGAACGTTCG TTCTAGAGAC TCTCCAATCT CGCCCTTCTC AACGCCCTTC   
  
  
+ AATTGCGACC CCACTGTTAT ATTGAGCGAC AGTCAGGAGC ACCACAGTTC AACAGGAAGT CTCTCGGCAC   
  
  
+ AAAGCTCATC TTCTAATTCT CCCCTTGAGA CTAGCAGTTA TTATAATCAG TTCAACTCAA GCCCTGTTGG   
  
  
+ AGAATCACCT CAGGGATCAT CACCCGAGAA TCTTTTTCAA CAAGCTGCAG TTAATAAAGT CAGCATCGAG   
  
  
+ CATGCATTGC AGGAGCTAGA AACTGCTCTA ATGAGCACAG ATTGTGAGGA GAATGAAGCA ACTGTCTCTA   
  
  
+ TCCCATCTAT GGGTGAACCT CATCAGCCCC AAGTCCCTAG CCAGAGATCA AGATTCTGGA ATCGAGATCC   
  
  
+ TCAGGGTTCA CGCCCGGCTG AAGTTCACTC ATCATTGAGA AGATTAGGAG ACGAGGCTCA GAGTGAGAAA   
  
  
+ CGCCTCAAGG CAGTGGAAGA ACCAATACGG CCCAGTGCAC CACCCGGCAA TTTGAAGCAG TTGCTCATAG   
  
  
+ AATGTGCTCG GGCTTTGTCA GAGAATCGAA TAGAGGATTT TGAGAAGTTA GTTGAACAGG CAAAGGGCAT   
  
  
+ GGTCTCCATT TCTGGAGACC CAATTCAACG ACTTGGTGCT TACTTGATCG AGGGGCTAGT GGCAAGGAAG   
  
  
+ GAGTCTTCAG GTACGAAAAT CTACCGAGCT CTTAGGTGCA AAGAGCCCCT TGGTCAAGAC TTGCTTTCGT   
  
  
+ ACATGCACAT CCTTTATGAA ATTTGCCCTT ACTTGAAATT CGGTTATATG GTTGCAAATG GGGCTATAGC   
  
  
+ TGAAGCTTGC AGAGATGAGG ATCACATACA TATCGTCGAC TACCAGATTG GTCAAGGAAC TCAATGGATG   
  
  
+ ACTCTGTTGC AAGCCCTAGC CACAAAACCT GGAAGACCCC CCACTGTGAG AATTACTGGC ATTGATGATC   
  
  
+ CCGTCTCTAG GCATGCTCGG GGAGCTTGCT TGGAGGCAGT GGGGAAACGT TTGGCAGTTC TGTCGGAGAA   
  
  
+ ATTTAACATG CCCATTGAGT TTCAGGCAGT GCCCGTTTAT GCTTCTGAGG TCACCCAAGA AATGCTTGAT   
  
  
+ GTGAGGCCAG GGTGGGCCTT GGCTGTGAAC TTCCCATTGC AGCTCCACCA CACTCCCGAT GAGAGTGTTG   
  
  
+ ATGTGAACAA CCCGAGGGAT GGACTCCTAA GAATGGTGAA ATCACTCGGT CCCAAGGTTG TAACTTTGGT   
  
  
+ AGAACAAGAG TCAAACACCA ACACTACCCC TTTGCTGACA AGGTTCATAG AAACGCTGGA CTTCTACTCA   
  
  
+ GCAATGTTTG AATCCATCGA TGTGACAATG CCAAGGGACC GAAAGGAAAG GATCAATGTC GAGCAGCATT   
  
  
+ GCCTAGCCAA GGACATTGTC AACATCATCG CGTGTGAGGG GAAGGACAGG GTGGAGCGAC ATGAGCTCTT   
  
  
+ TGGGAAATGG AAGTCAAGGT TCACCATGGC AGGATTCCAA CAATCCCCAT TGAGCTCGTA TGTTAACTCC   
  
  
+ GTGATAAAAG GATTACTTAG GTGTTATTCA GAGTACTATA CGTTGGTGGA GAAGGATGGC GCCTTGCTTC   
  
  
+ TGGGATGGAA GGACCGGATG CTGGTTTCGG CTTCAGCTTG GCATTG  

- +Up\_Stream \_Len000AAATTT ATAAAATAAA ATAAAATTTA ATTATATAAA ATATTAAATT TTAATAATTA   
  
  
- TTAACTACTA CAATGGGTTA CAAATTCAAA AATTTATAAA AATAAAAAAT TAAATTTTTA TAGTATTATA   
  
  
- TATTATATTA AAATTCAATT TTATTCTATC AATTTAATAA AATATCATTT ATAAATAATA AAAAAAATTC   
  
  
- TTTTAACGGT ACACTAACAG TAGTCAGTTT GTGACGCAAC TGTCTTCGAC AGGAACGACG ATGAGAGTGG   
  
  
- AGAGTTGTAG CCTGGGCGCG TCTGCAATTG TAGAGGCATG TTTATGTTAA GGGACCGGTT TTAAGTTTAG   
  
  
- TTGGGATGAG CCATCAGCCA AGGAAGAAGA AGGACGGGAG ATATATATGT GGGTGTCGAC GCGACGTACA   
  
  
- AAAAGGCTCT TAGGAGTTTT TAGAATATTT GAAGGAGCAC ACCCATCTTT TTTCTTTTTC TTTTCCCAAA   
  
  
- ATTTTAGAAC CCAGTTTTGG GTAGTTGTGG TGTTAAACTG AGAAAGAAAA ATTAAGTTGG TGGCGGTCAA   
  
  
- GGAGCAACTA AAAGTCCATG GAGAAAAAGA ACGGGGAAAA AAACGAGCCA ACAACAAATT ACCATTTCTA   
  
  
- ACTCATGTTT CTTACTATAA ACTACTCAAC CACACAAATA AACTACTCGG GTCTACTAAA TTGAACATCT   
  
  
- AAAACACCAA AACCAAAGAC CCATAAGTAA CCAAACGACT TAAAGACCAA GTACTAGACC CCAATCTTTT   
  
  
- CAACTTTACT GAAAGACCTC CAACCTTAAA CTGATTTCGA TTAAAGAAAA ACAGAAGTTA AGAAAAAATC   
  
  
- AACACTCGAC TTAAACAACT GGTGAAGGTC GGACAACCTT TAATTTGAAT GAATCCTCTT CTCTTAGAGT   
  
  
- CTAAGACTCG TTTTCGAAAT ATACCAACAA ACCCTTTACC ATCCTTGATT CACTTTTGTG ACTTTAAAAT   
  
  
- TCGTTCGTTA AAACAAGTAA ACTAACCAGC CTACCAGACT AAATCCAAGA AAAGAACCAG ATTTAAAGAC   
  
  
- TCGTAACTAA CAAATTAACG CACCCCTTGA ACATAGGAAC ACTCAAGGAC CAAGTAAAGA ACCAACGAAT   
  
  
- GAGATCGAGG GAAAGTGTCA AAGATACCAA CCTTGAACCT TGAACCTTGA ACCTTCGGAT TAAATCAACT   
  
  
- TATAGAACCG AAACTCAATC GAAAAGAATC CTTTTCTACC AAGACGAGAG CTTTTAGATT AGATTAGCTA   
  
  
- TCTACGCAAT TCCTTCGTTT TGAAAACCCC TCTCCCTTCA ATTCGCTTCG TTCGACGACA AAGTTTCAAC   
  
  
- CTGACAAAAT AACCATTCAT ACTTTGTTAT CAAATATTCT CTAGTAACCC AAGAATTCCA CTATCTAGTA   
  
  
- CCCAATACTT ACTCGAATCA ACAAGAAAAC GAAAGTTGAC ATGCATTCCT ACTATCAATA GGGAGTAGAA   
  
  
- CGTAGTTTTT GCCGTTTGGC ACTACGGTTT TGAGTTTTTG ACTAAGGAGA CTGTTCCGAA GATGGATACT   
  
  
- TTTGATACGT CTAGTATAGG TAAACCGGAA GGGAGATCCT TGGTTCATCT CATTCAACCA TTTATTTATC   
  
  
- TCAACTTGCT TTGTGATGTC TAAACTTAAA CTAATTCCTC CCGATAATAT CTGATTCCAC ATCGAGTTTA   
  
  
- GGAAGACATC CCTAACAGAA TAACCGAAGA AAACCAACGA AGACCGAGTT CAGGAGAATA ATCAATCATA   
  
  
- CGTAATGGAA CTCTGTTCTA TCAACGAATT CGAACTCGAA GACATGAATA AACGCATAAC ATCTGTTGAT   
  
  
- CGACACCGTA TACTAAGACT TTACAAGGCT ACAGAAAAAG AAACCTACGA CCCAACACAG TACTCGAAAT   
  
  
- CCAAACAAAT AAACTACAGT CCTTCATACA TTTATTGTAC ACCGTAGAAC GTTAGATAAT GTCCTACTAT   
  
  
- AACCGGAAAC ACCCTTCTTT AACTTCTTCA ACAAAGTGAA TGACCTATCC CTCGTACCTG AGTGTAGTCG   
  
  
- AAAACTCTAA GCCATAAAGG TCTAGTATGA GTAGGATATT AAAGAGAGTC AGATCAGGGG TTAGTTAAGG   
  
  
- TTTATCCAAT AAACTTAGTG GCTTGCAAGC AAGATCTCTG AGAGGTTAGA GCGGGAAGAG TTGCGGGAAG   
  
  
- TTAACGCTGG GGTGACAATA TAACTCGCTG TCAGTCCTCG TGGTGTCAAG TTGTCCTTCA GAGAGCCGTG   
  
  
- TTTCGAGTAG AAGATTAAGA GGGGAACTCT GATCGTCAAT AATATTAGTC AAGTTGAGTT CGGGACAACC   
  
  
- TCTTAGTGGA GTCCCTAGTA GTGGGCTCTT AGAAAAAGTT GTTCGACGTC AATTATTTCA GTCGTAGCTC   
  
  
- GTACGTAACG TCCTCGATCT TTGACGAGAT TACTCGTGTC TAACACTCCT CTTACTTCGT TGACAGAGAT   
  
  
- AGGGTAGATA CCCACTTGGA GTAGTCGGGG TTCAGGGATC GGTCTCTAGT TCTAAGACCT TAGCTCTAGG   
  
  
- AGTCCCAAGT GCGGGCCGAC TTCAAGTGAG TAGTAACTCT TCTAATCCTC TGCTCCGAGT CTCACTCTTT   
  
  
- GCGGAGTTCC GTCACCTTCT TGGTTATGCC GGGTCACGTG GTGGGCCGTT AAACTTCGTC AACGAGTATC   
  
  
- TTACACGAGC CCGAAACAGT CTCTTAGCTT ATCTCCTAAA ACTCTTCAAT CAACTTGTCC GTTTCCCGTA   
  
  
- CCAGAGGTAA AGACCTCTGG GTTAAGTTGC TGAACCACGA ATGAACTAGC TCCCCGATCA CCGTTCCTTC   
  
  
- CTCAGAAGTC CATGCTTTTA GATGGCTCGA GAATCCACGT TTCTCGGGGA ACCAGTTCTG AACGAAAGCA   
  
  
- TGTACGTGTA GGAAATACTT TAAACGGGAA TGAACTTTAA GCCAATATAC CAACGTTTAC CCCGATATCG   
  
  
- ACTTCGAACG TCTCTACTCC TAGTGTATGT ATAGCAGCTG ATGGTCTAAC CAGTTCCTTG AGTTACCTAC   
  
  
- TGAGACAACG TTCGGGATCG GTGTTTTGGA CCTTCTGGGG GGTGACACTC TTAATGACCG TAACTACTAG   
  
  
- GGCAGAGATC CGTACGAGCC CCTCGAACGA ACCTCCGTCA CCCCTTTGCA AACCGTCAAG ACAGCCTCTT   
  
  
- TAAATTGTAC GGGTAACTCA AAGTCCGTCA CGGGCAAATA CGAAGACTCC AGTGGGTTCT TTACGAACTA   
  
  
- CACTCCGGTC CCACCCGGAA CCGACACTTG AAGGGTAACG TCGAGGTGGT GTGAGGGCTA CTCTCACAAC   
  
  
- TACACTTGTT GGGCTCCCTA CCTGAGGATT CTTACCACTT TAGTGAGCCA GGGTTCCAAC ATTGAAACCA   
  
  
- TCTTGTTCTC AGTTTGTGGT TGTGATGGGG AAACGACTGT TCCAAGTATC TTTGCGACCT GAAGATGAGT   
  
  
- CGTTACAAAC TTAGGTAGCT ACACTGTTAC GGTTCCCTGG CTTTCCTTTC CTAGTTACAG CTCGTCGTAA   
  
  
- CGGATCGGTT CCTGTAACAG TTGTAGTAGC GCACACTCCC CTTCCTGTCC CACCTCGCTG TACTCGAGAA   
  
  
- ACCCTTTACC TTCAGTTCCA AGTGGTACCG TCCTAAGGTT GTTAGGGGTA ACTCGAGCAT ACAATTGAGG   
  
  
- CACTATTTTC CTAATGAATC CACAATAAGT CTCATGATAT GCAACCACCT CTTCCTACCG CGGAACGAAG   
  
  
- ACCCTACCTT CCTGGCCTAC GACCAAAGCC GAAGTCGAAC CGTAAC

+     ATCT-motif

| Site Name | Organism | Position | Strand | Matrix score. | sequence | function |
| --- | --- | --- | --- | --- | --- | --- |
| ATCT-motif | Pisum sativum | 1253 | + | 9 | AATCTAATCC | part of a conserved DNA module involved in light responsiveness |
| ATCT-motif | Pisum sativum | 1248 | + | 9 | AATCTAATCC | part of a conserved DNA module involved in light responsiveness |

>HU01G01850.1   
+ +Up\_Stream \_Len000TTTAAA TATTTTATTT TATTTTAAAT TAATATATTT TATAATTTAA AATTATTAAT   
  
  
+ AATTGATGAT GTTACCCAAT GTTTAAGTTT TTAAATATTT TTATTTTTTA ATTTAAAAAT ATCATAATAT   
  
  
+ ATAATATAAT TTTAAGTTAA AATAAGATAG TTAAATTATT TTATAGTAAA TATTTATTAT TTTTTTTAAG   
  
  
+ AAAATTGCCA TGTGATTGTC ATCAGTCAAA CACTGCGTTG ACAGAAGCTG TCCTTGCTGC TACTCTCACC   
  
  
+ TCTCAACATC GGACCCGCGC AGACGTTAAC ATCTCCGTAC AAATACAATT CCCTGGCCAA AATTCAAATC   
  
  
+ AACCCTACTC GGTAGTCGGT TCCTTCTTCT TCCTGCCCTC TATATATACA CCCACAGCTG CGCTGCATGT   
  
  
+ TTTTCCGAGA ATCCTCAAAA ATCTTATAAA CTTCCTCGTG TGGGTAGAAA AAAGAAAAAG AAAAGGGTTT   
  
  
+ TAAAATCTTG GGTCAAAACC CATCAACACC ACAATTTGAC TCTTTCTTTT TAATTCAACC ACCGCCAGTT   
  
  
+ CCTCGTTGAT TTTCAGGTAC CTCTTTTTCT TGCCCCTTTT TTTGCTCGGT TGTTGTTTAA TGGTAAAGAT   
  
  
+ TGAGTACAAA GAATGATATT TGATGAGTTG GTGTGTTTAT TTGATGAGCC CAGATGATTT AACTTGTAGA   
  
  
+ TTTTGTGGTT TTGGTTTCTG GGTATTCATT GGTTTGCTGA ATTTCTGGTT CATGATCTGG GGTTAGAAAA   
  
  
+ GTTGAAATGA CTTTCTGGAG GTTGGAATTT GACTAAAGCT AATTTCTTTT TGTCTTCAAT TCTTTTTTAG   
  
  
+ TTGTGAGCTG AATTTGTTGA CCACTTCCAG CCTGTTGGAA ATTAAACTTA CTTAGGAGAA GAGAATCTCA   
  
  
+ GATTCTGAGC AAAAGCTTTA TATGGTTGTT TGGGAAATGG TAGGAACTAA GTGAAAACAC TGAAATTTTA   
  
  
+ AGCAAGCAAT TTTGTTCATT TGATTGGTCG GATGGTCTGA TTTAGGTTCT TTTCTTGGTC TAAATTTCTG   
  
  
+ AGCATTGATT GTTTAATTGC GTGGGGAACT TGTATCCTTG TGAGTTCCTG GTTCATTTCT TGGTTGCTTA   
  
  
+ CTCTAGCTCC CTTTCACAGT TTCTATGGTT GGAACTTGGA ACTTGGAACT TGGAAGCCTA ATTTAGTTGA   
  
  
+ ATATCTTGGC TTTGAGTTAG CTTTTCTTAG GAAAAGATGG TTCTGCTCTC GAAAATCTAA TCTAATCGAT   
  
  
+ AGATGCGTTA AGGAAGCAAA ACTTTTGGGG AGAGGGAAGT TAAGCGAAGC AAGCTGCTGT TTCAAAGTTG   
  
  
+ GACTGTTTTA TTGGTAAGTA TGAAACAATA GTTTATAAGA GATCATTGGG TTCTTAAGGT GATAGATCAT   
  
  
+ GGGTTATGAA TGAGCTTAGT TGTTCTTTTG CTTTCAACTG TACGTAAGGA TGATAGTTAT CCCTCATCTT   
  
  
+ GCATCAAAAA CGGCAAACCG TGATGCCAAA ACTCAAAAAC TGATTCCTCT GACAAGGCTT CTACCTATGA   
  
  
+ AAACTATGCA GATCATATCC ATTTGGCCTT CCCTCTAGGA ACCAAGTAGA GTAAGTTGGT AAATAAATAG   
  
  
+ AGTTGAACGA AACACTACAG ATTTGAATTT GATTAAGGAG GGCTATTATA GACTAAGGTG TAGCTCAAAT   
  
  
+ CCTTCTGTAG GGATTGTCTT ATTGGCTTCT TTTGGTTGCT TCTGGCTCAA GTCCTCTTAT TAGTTAGTAT   
  
  
+ GCATTACCTT GAGACAAGAT AGTTGCTTAA GCTTGAGCTT CTGTACTTAT TTGCGTATTG TAGACAACTA   
  
  
+ GCTGTGGCAT ATGATTCTGA AATGTTCCGA TGTCTTTTTC TTTGGATGCT GGGTTGTGTC ATGAGCTTTA   
  
  
+ GGTTTGTTTA TTTGATGTCA GGAAGTATGT AAATAACATG TGGCATCTTG CAATCTATTA CAGGATGATA   
  
  
+ TTGGCCTTTG TGGGAAGAAA TTGAAGAAGT TGTTTCACTT ACTGGATAGG GAGCATGGAC TCACATCAGC   
  
  
+ TTTTGAGATT CGGTATTTCC AGATCATACT CATCCTATAA TTTCTCTCAG TCTAGTCCCC AATCAATTCC   
  
  
+ AAATAGGTTA TTTGAATCAC CGAACGTTCG TTCTAGAGAC TCTCCAATCT CGCCCTTCTC AACGCCCTTC   
  
  
+ AATTGCGACC CCACTGTTAT ATTGAGCGAC AGTCAGGAGC ACCACAGTTC AACAGGAAGT CTCTCGGCAC   
  
  
+ AAAGCTCATC TTCTAATTCT CCCCTTGAGA CTAGCAGTTA TTATAATCAG TTCAACTCAA GCCCTGTTGG   
  
  
+ AGAATCACCT CAGGGATCAT CACCCGAGAA TCTTTTTCAA CAAGCTGCAG TTAATAAAGT CAGCATCGAG   
  
  
+ CATGCATTGC AGGAGCTAGA AACTGCTCTA ATGAGCACAG ATTGTGAGGA GAATGAAGCA ACTGTCTCTA   
  
  
+ TCCCATCTAT GGGTGAACCT CATCAGCCCC AAGTCCCTAG CCAGAGATCA AGATTCTGGA ATCGAGATCC   
  
  
+ TCAGGGTTCA CGCCCGGCTG AAGTTCACTC ATCATTGAGA AGATTAGGAG ACGAGGCTCA GAGTGAGAAA   
  
  
+ CGCCTCAAGG CAGTGGAAGA ACCAATACGG CCCAGTGCAC CACCCGGCAA TTTGAAGCAG TTGCTCATAG   
  
  
+ AATGTGCTCG GGCTTTGTCA GAGAATCGAA TAGAGGATTT TGAGAAGTTA GTTGAACAGG CAAAGGGCAT   
  
  
+ GGTCTCCATT TCTGGAGACC CAATTCAACG ACTTGGTGCT TACTTGATCG AGGGGCTAGT GGCAAGGAAG   
  
  
+ GAGTCTTCAG GTACGAAAAT CTACCGAGCT CTTAGGTGCA AAGAGCCCCT TGGTCAAGAC TTGCTTTCGT   
  
  
+ ACATGCACAT CCTTTATGAA ATTTGCCCTT ACTTGAAATT CGGTTATATG GTTGCAAATG GGGCTATAGC   
  
  
+ TGAAGCTTGC AGAGATGAGG ATCACATACA TATCGTCGAC TACCAGATTG GTCAAGGAAC TCAATGGATG   
  
  
+ ACTCTGTTGC AAGCCCTAGC CACAAAACCT GGAAGACCCC CCACTGTGAG AATTACTGGC ATTGATGATC   
  
  
+ CCGTCTCTAG GCATGCTCGG GGAGCTTGCT TGGAGGCAGT GGGGAAACGT TTGGCAGTTC TGTCGGAGAA   
  
  
+ ATTTAACATG CCCATTGAGT TTCAGGCAGT GCCCGTTTAT GCTTCTGAGG TCACCCAAGA AATGCTTGAT   
  
  
+ GTGAGGCCAG GGTGGGCCTT GGCTGTGAAC TTCCCATTGC AGCTCCACCA CACTCCCGAT GAGAGTGTTG   
  
  
+ ATGTGAACAA CCCGAGGGAT GGACTCCTAA GAATGGTGAA ATCACTCGGT CCCAAGGTTG TAACTTTGGT   
  
  
+ AGAACAAGAG TCAAACACCA ACACTACCCC TTTGCTGACA AGGTTCATAG AAACGCTGGA CTTCTACTCA   
  
  
+ GCAATGTTTG AATCCATCGA TGTGACAATG CCAAGGGACC GAAAGGAAAG GATCAATGTC GAGCAGCATT   
  
  
+ GCCTAGCCAA GGACATTGTC AACATCATCG CGTGTGAGGG GAAGGACAGG GTGGAGCGAC ATGAGCTCTT   
  
  
+ TGGGAAATGG AAGTCAAGGT TCACCATGGC AGGATTCCAA CAATCCCCAT TGAGCTCGTA TGTTAACTCC   
  
  
+ GTGATAAAAG GATTACTTAG GTGTTATTCA GAGTACTATA CGTTGGTGGA GAAGGATGGC GCCTTGCTTC   
  
  
+ TGGGATGGAA GGACCGGATG CTGGTTTCGG CTTCAGCTTG GCATTG  

- +Up\_Stream \_Len000AAATTT ATAAAATAAA ATAAAATTTA ATTATATAAA ATATTAAATT TTAATAATTA   
  
  
- TTAACTACTA CAATGGGTTA CAAATTCAAA AATTTATAAA AATAAAAAAT TAAATTTTTA TAGTATTATA   
  
  
- TATTATATTA AAATTCAATT TTATTCTATC AATTTAATAA AATATCATTT ATAAATAATA AAAAAAATTC   
  
  
- TTTTAACGGT ACACTAACAG TAGTCAGTTT GTGACGCAAC TGTCTTCGAC AGGAACGACG ATGAGAGTGG   
  
  
- AGAGTTGTAG CCTGGGCGCG TCTGCAATTG TAGAGGCATG TTTATGTTAA GGGACCGGTT TTAAGTTTAG   
  
  
- TTGGGATGAG CCATCAGCCA AGGAAGAAGA AGGACGGGAG ATATATATGT GGGTGTCGAC GCGACGTACA   
  
  
- AAAAGGCTCT TAGGAGTTTT TAGAATATTT GAAGGAGCAC ACCCATCTTT TTTCTTTTTC TTTTCCCAAA   
  
  
- ATTTTAGAAC CCAGTTTTGG GTAGTTGTGG TGTTAAACTG AGAAAGAAAA ATTAAGTTGG TGGCGGTCAA   
  
  
- GGAGCAACTA AAAGTCCATG GAGAAAAAGA ACGGGGAAAA AAACGAGCCA ACAACAAATT ACCATTTCTA   
  
  
- ACTCATGTTT CTTACTATAA ACTACTCAAC CACACAAATA AACTACTCGG GTCTACTAAA TTGAACATCT   
  
  
- AAAACACCAA AACCAAAGAC CCATAAGTAA CCAAACGACT TAAAGACCAA GTACTAGACC CCAATCTTTT   
  
  
- CAACTTTACT GAAAGACCTC CAACCTTAAA CTGATTTCGA TTAAAGAAAA ACAGAAGTTA AGAAAAAATC   
  
  
- AACACTCGAC TTAAACAACT GGTGAAGGTC GGACAACCTT TAATTTGAAT GAATCCTCTT CTCTTAGAGT   
  
  
- CTAAGACTCG TTTTCGAAAT ATACCAACAA ACCCTTTACC ATCCTTGATT CACTTTTGTG ACTTTAAAAT   
  
  
- TCGTTCGTTA AAACAAGTAA ACTAACCAGC CTACCAGACT AAATCCAAGA AAAGAACCAG ATTTAAAGAC   
  
  
- TCGTAACTAA CAAATTAACG CACCCCTTGA ACATAGGAAC ACTCAAGGAC CAAGTAAAGA ACCAACGAAT   
  
  
- GAGATCGAGG GAAAGTGTCA AAGATACCAA CCTTGAACCT TGAACCTTGA ACCTTCGGAT TAAATCAACT   
  
  
- TATAGAACCG AAACTCAATC GAAAAGAATC CTTTTCTACC AAGACGAGAG CTTTTAGATT AGATTAGCTA   
  
  
- TCTACGCAAT TCCTTCGTTT TGAAAACCCC TCTCCCTTCA ATTCGCTTCG TTCGACGACA AAGTTTCAAC   
  
  
- CTGACAAAAT AACCATTCAT ACTTTGTTAT CAAATATTCT CTAGTAACCC AAGAATTCCA CTATCTAGTA   
  
  
- CCCAATACTT ACTCGAATCA ACAAGAAAAC GAAAGTTGAC ATGCATTCCT ACTATCAATA GGGAGTAGAA   
  
  
- CGTAGTTTTT GCCGTTTGGC ACTACGGTTT TGAGTTTTTG ACTAAGGAGA CTGTTCCGAA GATGGATACT   
  
  
- TTTGATACGT CTAGTATAGG TAAACCGGAA GGGAGATCCT TGGTTCATCT CATTCAACCA TTTATTTATC   
  
  
- TCAACTTGCT TTGTGATGTC TAAACTTAAA CTAATTCCTC CCGATAATAT CTGATTCCAC ATCGAGTTTA   
  
  
- GGAAGACATC CCTAACAGAA TAACCGAAGA AAACCAACGA AGACCGAGTT CAGGAGAATA ATCAATCATA   
  
  
- CGTAATGGAA CTCTGTTCTA TCAACGAATT CGAACTCGAA GACATGAATA AACGCATAAC ATCTGTTGAT   
  
  
- CGACACCGTA TACTAAGACT TTACAAGGCT ACAGAAAAAG AAACCTACGA CCCAACACAG TACTCGAAAT   
  
  
- CCAAACAAAT AAACTACAGT CCTTCATACA TTTATTGTAC ACCGTAGAAC GTTAGATAAT GTCCTACTAT   
  
  
- AACCGGAAAC ACCCTTCTTT AACTTCTTCA ACAAAGTGAA TGACCTATCC CTCGTACCTG AGTGTAGTCG   
  
  
- AAAACTCTAA GCCATAAAGG TCTAGTATGA GTAGGATATT AAAGAGAGTC AGATCAGGGG TTAGTTAAGG   
  
  
- TTTATCCAAT AAACTTAGTG GCTTGCAAGC AAGATCTCTG AGAGGTTAGA GCGGGAAGAG TTGCGGGAAG   
  
  
- TTAACGCTGG GGTGACAATA TAACTCGCTG TCAGTCCTCG TGGTGTCAAG TTGTCCTTCA GAGAGCCGTG   
  
  
- TTTCGAGTAG AAGATTAAGA GGGGAACTCT GATCGTCAAT AATATTAGTC AAGTTGAGTT CGGGACAACC   
  
  
- TCTTAGTGGA GTCCCTAGTA GTGGGCTCTT AGAAAAAGTT GTTCGACGTC AATTATTTCA GTCGTAGCTC   
  
  
- GTACGTAACG TCCTCGATCT TTGACGAGAT TACTCGTGTC TAACACTCCT CTTACTTCGT TGACAGAGAT   
  
  
- AGGGTAGATA CCCACTTGGA GTAGTCGGGG TTCAGGGATC GGTCTCTAGT TCTAAGACCT TAGCTCTAGG   
  
  
- AGTCCCAAGT GCGGGCCGAC TTCAAGTGAG TAGTAACTCT TCTAATCCTC TGCTCCGAGT CTCACTCTTT   
  
  
- GCGGAGTTCC GTCACCTTCT TGGTTATGCC GGGTCACGTG GTGGGCCGTT AAACTTCGTC AACGAGTATC   
  
  
- TTACACGAGC CCGAAACAGT CTCTTAGCTT ATCTCCTAAA ACTCTTCAAT CAACTTGTCC GTTTCCCGTA   
  
  
- CCAGAGGTAA AGACCTCTGG GTTAAGTTGC TGAACCACGA ATGAACTAGC TCCCCGATCA CCGTTCCTTC   
  
  
- CTCAGAAGTC CATGCTTTTA GATGGCTCGA GAATCCACGT TTCTCGGGGA ACCAGTTCTG AACGAAAGCA   
  
  
- TGTACGTGTA GGAAATACTT TAAACGGGAA TGAACTTTAA GCCAATATAC CAACGTTTAC CCCGATATCG   
  
  
- ACTTCGAACG TCTCTACTCC TAGTGTATGT ATAGCAGCTG ATGGTCTAAC CAGTTCCTTG AGTTACCTAC   
  
  
- TGAGACAACG TTCGGGATCG GTGTTTTGGA CCTTCTGGGG GGTGACACTC TTAATGACCG TAACTACTAG   
  
  
- GGCAGAGATC CGTACGAGCC CCTCGAACGA ACCTCCGTCA CCCCTTTGCA AACCGTCAAG ACAGCCTCTT   
  
  
- TAAATTGTAC GGGTAACTCA AAGTCCGTCA CGGGCAAATA CGAAGACTCC AGTGGGTTCT TTACGAACTA   
  
  
- CACTCCGGTC CCACCCGGAA CCGACACTTG AAGGGTAACG TCGAGGTGGT GTGAGGGCTA CTCTCACAAC   
  
  
- TACACTTGTT GGGCTCCCTA CCTGAGGATT CTTACCACTT TAGTGAGCCA GGGTTCCAAC ATTGAAACCA   
  
  
- TCTTGTTCTC AGTTTGTGGT TGTGATGGGG AAACGACTGT TCCAAGTATC TTTGCGACCT GAAGATGAGT   
  
  
- CGTTACAAAC TTAGGTAGCT ACACTGTTAC GGTTCCCTGG CTTTCCTTTC CTAGTTACAG CTCGTCGTAA   
  
  
- CGGATCGGTT CCTGTAACAG TTGTAGTAGC GCACACTCCC CTTCCTGTCC CACCTCGCTG TACTCGAGAA   
  
  
- ACCCTTTACC TTCAGTTCCA AGTGGTACCG TCCTAAGGTT GTTAGGGGTA ACTCGAGCAT ACAATTGAGG   
  
  
- CACTATTTTC CTAATGAATC CACAATAAGT CTCATGATAT GCAACCACCT CTTCCTACCG CGGAACGAAG   
  
  
- ACCCTACCTT CCTGGCCTAC GACCAAAGCC GAAGTCGAAC CGTAAC

+     AT~TATA-box

| Site Name | Organism | Position | Strand | Matrix score. | sequence | function |
| --- | --- | --- | --- | --- | --- | --- |
| AT~TATA-box | Arabidopsis thaliana | 397 | + | 6 | TATATA |  |
| AT~TATA-box | Arabidopsis thaliana | 395 | + | 6 | TATATA |  |
| AT~TATA-box | Arabidopsis thaliana | 142 | + | 6 | TATATA |  |

>HU01G01850.1   
+ +Up\_Stream \_Len000TTTAAA TATTTTATTT TATTTTAAAT TAATATATTT TATAATTTAA AATTATTAAT   
  
  
+ AATTGATGAT GTTACCCAAT GTTTAAGTTT TTAAATATTT TTATTTTTTA ATTTAAAAAT ATCATAATAT   
  
  
+ ATAATATAAT TTTAAGTTAA AATAAGATAG TTAAATTATT TTATAGTAAA TATTTATTAT TTTTTTTAAG   
  
  
+ AAAATTGCCA TGTGATTGTC ATCAGTCAAA CACTGCGTTG ACAGAAGCTG TCCTTGCTGC TACTCTCACC   
  
  
+ TCTCAACATC GGACCCGCGC AGACGTTAAC ATCTCCGTAC AAATACAATT CCCTGGCCAA AATTCAAATC   
  
  
+ AACCCTACTC GGTAGTCGGT TCCTTCTTCT TCCTGCCCTC TATATATACA CCCACAGCTG CGCTGCATGT   
  
  
+ TTTTCCGAGA ATCCTCAAAA ATCTTATAAA CTTCCTCGTG TGGGTAGAAA AAAGAAAAAG AAAAGGGTTT   
  
  
+ TAAAATCTTG GGTCAAAACC CATCAACACC ACAATTTGAC TCTTTCTTTT TAATTCAACC ACCGCCAGTT   
  
  
+ CCTCGTTGAT TTTCAGGTAC CTCTTTTTCT TGCCCCTTTT TTTGCTCGGT TGTTGTTTAA TGGTAAAGAT   
  
  
+ TGAGTACAAA GAATGATATT TGATGAGTTG GTGTGTTTAT TTGATGAGCC CAGATGATTT AACTTGTAGA   
  
  
+ TTTTGTGGTT TTGGTTTCTG GGTATTCATT GGTTTGCTGA ATTTCTGGTT CATGATCTGG GGTTAGAAAA   
  
  
+ GTTGAAATGA CTTTCTGGAG GTTGGAATTT GACTAAAGCT AATTTCTTTT TGTCTTCAAT TCTTTTTTAG   
  
  
+ TTGTGAGCTG AATTTGTTGA CCACTTCCAG CCTGTTGGAA ATTAAACTTA CTTAGGAGAA GAGAATCTCA   
  
  
+ GATTCTGAGC AAAAGCTTTA TATGGTTGTT TGGGAAATGG TAGGAACTAA GTGAAAACAC TGAAATTTTA   
  
  
+ AGCAAGCAAT TTTGTTCATT TGATTGGTCG GATGGTCTGA TTTAGGTTCT TTTCTTGGTC TAAATTTCTG   
  
  
+ AGCATTGATT GTTTAATTGC GTGGGGAACT TGTATCCTTG TGAGTTCCTG GTTCATTTCT TGGTTGCTTA   
  
  
+ CTCTAGCTCC CTTTCACAGT TTCTATGGTT GGAACTTGGA ACTTGGAACT TGGAAGCCTA ATTTAGTTGA   
  
  
+ ATATCTTGGC TTTGAGTTAG CTTTTCTTAG GAAAAGATGG TTCTGCTCTC GAAAATCTAA TCTAATCGAT   
  
  
+ AGATGCGTTA AGGAAGCAAA ACTTTTGGGG AGAGGGAAGT TAAGCGAAGC AAGCTGCTGT TTCAAAGTTG   
  
  
+ GACTGTTTTA TTGGTAAGTA TGAAACAATA GTTTATAAGA GATCATTGGG TTCTTAAGGT GATAGATCAT   
  
  
+ GGGTTATGAA TGAGCTTAGT TGTTCTTTTG CTTTCAACTG TACGTAAGGA TGATAGTTAT CCCTCATCTT   
  
  
+ GCATCAAAAA CGGCAAACCG TGATGCCAAA ACTCAAAAAC TGATTCCTCT GACAAGGCTT CTACCTATGA   
  
  
+ AAACTATGCA GATCATATCC ATTTGGCCTT CCCTCTAGGA ACCAAGTAGA GTAAGTTGGT AAATAAATAG   
  
  
+ AGTTGAACGA AACACTACAG ATTTGAATTT GATTAAGGAG GGCTATTATA GACTAAGGTG TAGCTCAAAT   
  
  
+ CCTTCTGTAG GGATTGTCTT ATTGGCTTCT TTTGGTTGCT TCTGGCTCAA GTCCTCTTAT TAGTTAGTAT   
  
  
+ GCATTACCTT GAGACAAGAT AGTTGCTTAA GCTTGAGCTT CTGTACTTAT TTGCGTATTG TAGACAACTA   
  
  
+ GCTGTGGCAT ATGATTCTGA AATGTTCCGA TGTCTTTTTC TTTGGATGCT GGGTTGTGTC ATGAGCTTTA   
  
  
+ GGTTTGTTTA TTTGATGTCA GGAAGTATGT AAATAACATG TGGCATCTTG CAATCTATTA CAGGATGATA   
  
  
+ TTGGCCTTTG TGGGAAGAAA TTGAAGAAGT TGTTTCACTT ACTGGATAGG GAGCATGGAC TCACATCAGC   
  
  
+ TTTTGAGATT CGGTATTTCC AGATCATACT CATCCTATAA TTTCTCTCAG TCTAGTCCCC AATCAATTCC   
  
  
+ AAATAGGTTA TTTGAATCAC CGAACGTTCG TTCTAGAGAC TCTCCAATCT CGCCCTTCTC AACGCCCTTC   
  
  
+ AATTGCGACC CCACTGTTAT ATTGAGCGAC AGTCAGGAGC ACCACAGTTC AACAGGAAGT CTCTCGGCAC   
  
  
+ AAAGCTCATC TTCTAATTCT CCCCTTGAGA CTAGCAGTTA TTATAATCAG TTCAACTCAA GCCCTGTTGG   
  
  
+ AGAATCACCT CAGGGATCAT CACCCGAGAA TCTTTTTCAA CAAGCTGCAG TTAATAAAGT CAGCATCGAG   
  
  
+ CATGCATTGC AGGAGCTAGA AACTGCTCTA ATGAGCACAG ATTGTGAGGA GAATGAAGCA ACTGTCTCTA   
  
  
+ TCCCATCTAT GGGTGAACCT CATCAGCCCC AAGTCCCTAG CCAGAGATCA AGATTCTGGA ATCGAGATCC   
  
  
+ TCAGGGTTCA CGCCCGGCTG AAGTTCACTC ATCATTGAGA AGATTAGGAG ACGAGGCTCA GAGTGAGAAA   
  
  
+ CGCCTCAAGG CAGTGGAAGA ACCAATACGG CCCAGTGCAC CACCCGGCAA TTTGAAGCAG TTGCTCATAG   
  
  
+ AATGTGCTCG GGCTTTGTCA GAGAATCGAA TAGAGGATTT TGAGAAGTTA GTTGAACAGG CAAAGGGCAT   
  
  
+ GGTCTCCATT TCTGGAGACC CAATTCAACG ACTTGGTGCT TACTTGATCG AGGGGCTAGT GGCAAGGAAG   
  
  
+ GAGTCTTCAG GTACGAAAAT CTACCGAGCT CTTAGGTGCA AAGAGCCCCT TGGTCAAGAC TTGCTTTCGT   
  
  
+ ACATGCACAT CCTTTATGAA ATTTGCCCTT ACTTGAAATT CGGTTATATG GTTGCAAATG GGGCTATAGC   
  
  
+ TGAAGCTTGC AGAGATGAGG ATCACATACA TATCGTCGAC TACCAGATTG GTCAAGGAAC TCAATGGATG   
  
  
+ ACTCTGTTGC AAGCCCTAGC CACAAAACCT GGAAGACCCC CCACTGTGAG AATTACTGGC ATTGATGATC   
  
  
+ CCGTCTCTAG GCATGCTCGG GGAGCTTGCT TGGAGGCAGT GGGGAAACGT TTGGCAGTTC TGTCGGAGAA   
  
  
+ ATTTAACATG CCCATTGAGT TTCAGGCAGT GCCCGTTTAT GCTTCTGAGG TCACCCAAGA AATGCTTGAT   
  
  
+ GTGAGGCCAG GGTGGGCCTT GGCTGTGAAC TTCCCATTGC AGCTCCACCA CACTCCCGAT GAGAGTGTTG   
  
  
+ ATGTGAACAA CCCGAGGGAT GGACTCCTAA GAATGGTGAA ATCACTCGGT CCCAAGGTTG TAACTTTGGT   
  
  
+ AGAACAAGAG TCAAACACCA ACACTACCCC TTTGCTGACA AGGTTCATAG AAACGCTGGA CTTCTACTCA   
  
  
+ GCAATGTTTG AATCCATCGA TGTGACAATG CCAAGGGACC GAAAGGAAAG GATCAATGTC GAGCAGCATT   
  
  
+ GCCTAGCCAA GGACATTGTC AACATCATCG CGTGTGAGGG GAAGGACAGG GTGGAGCGAC ATGAGCTCTT   
  
  
+ TGGGAAATGG AAGTCAAGGT TCACCATGGC AGGATTCCAA CAATCCCCAT TGAGCTCGTA TGTTAACTCC   
  
  
+ GTGATAAAAG GATTACTTAG GTGTTATTCA GAGTACTATA CGTTGGTGGA GAAGGATGGC GCCTTGCTTC   
  
  
+ TGGGATGGAA GGACCGGATG CTGGTTTCGG CTTCAGCTTG GCATTG  

- +Up\_Stream \_Len000AAATTT ATAAAATAAA ATAAAATTTA ATTATATAAA ATATTAAATT TTAATAATTA   
  
  
- TTAACTACTA CAATGGGTTA CAAATTCAAA AATTTATAAA AATAAAAAAT TAAATTTTTA TAGTATTATA   
  
  
- TATTATATTA AAATTCAATT TTATTCTATC AATTTAATAA AATATCATTT ATAAATAATA AAAAAAATTC   
  
  
- TTTTAACGGT ACACTAACAG TAGTCAGTTT GTGACGCAAC TGTCTTCGAC AGGAACGACG ATGAGAGTGG   
  
  
- AGAGTTGTAG CCTGGGCGCG TCTGCAATTG TAGAGGCATG TTTATGTTAA GGGACCGGTT TTAAGTTTAG   
  
  
- TTGGGATGAG CCATCAGCCA AGGAAGAAGA AGGACGGGAG ATATATATGT GGGTGTCGAC GCGACGTACA   
  
  
- AAAAGGCTCT TAGGAGTTTT TAGAATATTT GAAGGAGCAC ACCCATCTTT TTTCTTTTTC TTTTCCCAAA   
  
  
- ATTTTAGAAC CCAGTTTTGG GTAGTTGTGG TGTTAAACTG AGAAAGAAAA ATTAAGTTGG TGGCGGTCAA   
  
  
- GGAGCAACTA AAAGTCCATG GAGAAAAAGA ACGGGGAAAA AAACGAGCCA ACAACAAATT ACCATTTCTA   
  
  
- ACTCATGTTT CTTACTATAA ACTACTCAAC CACACAAATA AACTACTCGG GTCTACTAAA TTGAACATCT   
  
  
- AAAACACCAA AACCAAAGAC CCATAAGTAA CCAAACGACT TAAAGACCAA GTACTAGACC CCAATCTTTT   
  
  
- CAACTTTACT GAAAGACCTC CAACCTTAAA CTGATTTCGA TTAAAGAAAA ACAGAAGTTA AGAAAAAATC   
  
  
- AACACTCGAC TTAAACAACT GGTGAAGGTC GGACAACCTT TAATTTGAAT GAATCCTCTT CTCTTAGAGT   
  
  
- CTAAGACTCG TTTTCGAAAT ATACCAACAA ACCCTTTACC ATCCTTGATT CACTTTTGTG ACTTTAAAAT   
  
  
- TCGTTCGTTA AAACAAGTAA ACTAACCAGC CTACCAGACT AAATCCAAGA AAAGAACCAG ATTTAAAGAC   
  
  
- TCGTAACTAA CAAATTAACG CACCCCTTGA ACATAGGAAC ACTCAAGGAC CAAGTAAAGA ACCAACGAAT   
  
  
- GAGATCGAGG GAAAGTGTCA AAGATACCAA CCTTGAACCT TGAACCTTGA ACCTTCGGAT TAAATCAACT   
  
  
- TATAGAACCG AAACTCAATC GAAAAGAATC CTTTTCTACC AAGACGAGAG CTTTTAGATT AGATTAGCTA   
  
  
- TCTACGCAAT TCCTTCGTTT TGAAAACCCC TCTCCCTTCA ATTCGCTTCG TTCGACGACA AAGTTTCAAC   
  
  
- CTGACAAAAT AACCATTCAT ACTTTGTTAT CAAATATTCT CTAGTAACCC AAGAATTCCA CTATCTAGTA   
  
  
- CCCAATACTT ACTCGAATCA ACAAGAAAAC GAAAGTTGAC ATGCATTCCT ACTATCAATA GGGAGTAGAA   
  
  
- CGTAGTTTTT GCCGTTTGGC ACTACGGTTT TGAGTTTTTG ACTAAGGAGA CTGTTCCGAA GATGGATACT   
  
  
- TTTGATACGT CTAGTATAGG TAAACCGGAA GGGAGATCCT TGGTTCATCT CATTCAACCA TTTATTTATC   
  
  
- TCAACTTGCT TTGTGATGTC TAAACTTAAA CTAATTCCTC CCGATAATAT CTGATTCCAC ATCGAGTTTA   
  
  
- GGAAGACATC CCTAACAGAA TAACCGAAGA AAACCAACGA AGACCGAGTT CAGGAGAATA ATCAATCATA   
  
  
- CGTAATGGAA CTCTGTTCTA TCAACGAATT CGAACTCGAA GACATGAATA AACGCATAAC ATCTGTTGAT   
  
  
- CGACACCGTA TACTAAGACT TTACAAGGCT ACAGAAAAAG AAACCTACGA CCCAACACAG TACTCGAAAT   
  
  
- CCAAACAAAT AAACTACAGT CCTTCATACA TTTATTGTAC ACCGTAGAAC GTTAGATAAT GTCCTACTAT   
  
  
- AACCGGAAAC ACCCTTCTTT AACTTCTTCA ACAAAGTGAA TGACCTATCC CTCGTACCTG AGTGTAGTCG   
  
  
- AAAACTCTAA GCCATAAAGG TCTAGTATGA GTAGGATATT AAAGAGAGTC AGATCAGGGG TTAGTTAAGG   
  
  
- TTTATCCAAT AAACTTAGTG GCTTGCAAGC AAGATCTCTG AGAGGTTAGA GCGGGAAGAG TTGCGGGAAG   
  
  
- TTAACGCTGG GGTGACAATA TAACTCGCTG TCAGTCCTCG TGGTGTCAAG TTGTCCTTCA GAGAGCCGTG   
  
  
- TTTCGAGTAG AAGATTAAGA GGGGAACTCT GATCGTCAAT AATATTAGTC AAGTTGAGTT CGGGACAACC   
  
  
- TCTTAGTGGA GTCCCTAGTA GTGGGCTCTT AGAAAAAGTT GTTCGACGTC AATTATTTCA GTCGTAGCTC   
  
  
- GTACGTAACG TCCTCGATCT TTGACGAGAT TACTCGTGTC TAACACTCCT CTTACTTCGT TGACAGAGAT   
  
  
- AGGGTAGATA CCCACTTGGA GTAGTCGGGG TTCAGGGATC GGTCTCTAGT TCTAAGACCT TAGCTCTAGG   
  
  
- AGTCCCAAGT GCGGGCCGAC TTCAAGTGAG TAGTAACTCT TCTAATCCTC TGCTCCGAGT CTCACTCTTT   
  
  
- GCGGAGTTCC GTCACCTTCT TGGTTATGCC GGGTCACGTG GTGGGCCGTT AAACTTCGTC AACGAGTATC   
  
  
- TTACACGAGC CCGAAACAGT CTCTTAGCTT ATCTCCTAAA ACTCTTCAAT CAACTTGTCC GTTTCCCGTA   
  
  
- CCAGAGGTAA AGACCTCTGG GTTAAGTTGC TGAACCACGA ATGAACTAGC TCCCCGATCA CCGTTCCTTC   
  
  
- CTCAGAAGTC CATGCTTTTA GATGGCTCGA GAATCCACGT TTCTCGGGGA ACCAGTTCTG AACGAAAGCA   
  
  
- TGTACGTGTA GGAAATACTT TAAACGGGAA TGAACTTTAA GCCAATATAC CAACGTTTAC CCCGATATCG   
  
  
- ACTTCGAACG TCTCTACTCC TAGTGTATGT ATAGCAGCTG ATGGTCTAAC CAGTTCCTTG AGTTACCTAC   
  
  
- TGAGACAACG TTCGGGATCG GTGTTTTGGA CCTTCTGGGG GGTGACACTC TTAATGACCG TAACTACTAG   
  
  
- GGCAGAGATC CGTACGAGCC CCTCGAACGA ACCTCCGTCA CCCCTTTGCA AACCGTCAAG ACAGCCTCTT   
  
  
- TAAATTGTAC GGGTAACTCA AAGTCCGTCA CGGGCAAATA CGAAGACTCC AGTGGGTTCT TTACGAACTA   
  
  
- CACTCCGGTC CCACCCGGAA CCGACACTTG AAGGGTAACG TCGAGGTGGT GTGAGGGCTA CTCTCACAAC   
  
  
- TACACTTGTT GGGCTCCCTA CCTGAGGATT CTTACCACTT TAGTGAGCCA GGGTTCCAAC ATTGAAACCA   
  
  
- TCTTGTTCTC AGTTTGTGGT TGTGATGGGG AAACGACTGT TCCAAGTATC TTTGCGACCT GAAGATGAGT   
  
  
- CGTTACAAAC TTAGGTAGCT ACACTGTTAC GGTTCCCTGG CTTTCCTTTC CTAGTTACAG CTCGTCGTAA   
  
  
- CGGATCGGTT CCTGTAACAG TTGTAGTAGC GCACACTCCC CTTCCTGTCC CACCTCGCTG TACTCGAGAA   
  
  
- ACCCTTTACC TTCAGTTCCA AGTGGTACCG TCCTAAGGTT GTTAGGGGTA ACTCGAGCAT ACAATTGAGG   
  
  
- CACTATTTTC CTAATGAATC CACAATAAGT CTCATGATAT GCAACCACCT CTTCCTACCG CGGAACGAAG   
  
  
- ACCCTACCTT CCTGGCCTAC GACCAAAGCC GAAGTCGAAC CGTAAC

+     Box 4

| Site Name | Organism | Position | Strand | Matrix score. | sequence | function |
| --- | --- | --- | --- | --- | --- | --- |
| Box 4 | Petroselinum crispum | 69 | + | 6 | ATTAAT | part of a conserved DNA module involved in light responsiveness |
| Box 4 | Petroselinum crispum | 43 | + | 6 | ATTAAT | part of a conserved DNA module involved in light responsiveness |

>HU01G01850.1   
+ +Up\_Stream \_Len000TTTAAA TATTTTATTT TATTTTAAAT TAATATATTT TATAATTTAA AATTATTAAT   
  
  
+ AATTGATGAT GTTACCCAAT GTTTAAGTTT TTAAATATTT TTATTTTTTA ATTTAAAAAT ATCATAATAT   
  
  
+ ATAATATAAT TTTAAGTTAA AATAAGATAG TTAAATTATT TTATAGTAAA TATTTATTAT TTTTTTTAAG   
  
  
+ AAAATTGCCA TGTGATTGTC ATCAGTCAAA CACTGCGTTG ACAGAAGCTG TCCTTGCTGC TACTCTCACC   
  
  
+ TCTCAACATC GGACCCGCGC AGACGTTAAC ATCTCCGTAC AAATACAATT CCCTGGCCAA AATTCAAATC   
  
  
+ AACCCTACTC GGTAGTCGGT TCCTTCTTCT TCCTGCCCTC TATATATACA CCCACAGCTG CGCTGCATGT   
  
  
+ TTTTCCGAGA ATCCTCAAAA ATCTTATAAA CTTCCTCGTG TGGGTAGAAA AAAGAAAAAG AAAAGGGTTT   
  
  
+ TAAAATCTTG GGTCAAAACC CATCAACACC ACAATTTGAC TCTTTCTTTT TAATTCAACC ACCGCCAGTT   
  
  
+ CCTCGTTGAT TTTCAGGTAC CTCTTTTTCT TGCCCCTTTT TTTGCTCGGT TGTTGTTTAA TGGTAAAGAT   
  
  
+ TGAGTACAAA GAATGATATT TGATGAGTTG GTGTGTTTAT TTGATGAGCC CAGATGATTT AACTTGTAGA   
  
  
+ TTTTGTGGTT TTGGTTTCTG GGTATTCATT GGTTTGCTGA ATTTCTGGTT CATGATCTGG GGTTAGAAAA   
  
  
+ GTTGAAATGA CTTTCTGGAG GTTGGAATTT GACTAAAGCT AATTTCTTTT TGTCTTCAAT TCTTTTTTAG   
  
  
+ TTGTGAGCTG AATTTGTTGA CCACTTCCAG CCTGTTGGAA ATTAAACTTA CTTAGGAGAA GAGAATCTCA   
  
  
+ GATTCTGAGC AAAAGCTTTA TATGGTTGTT TGGGAAATGG TAGGAACTAA GTGAAAACAC TGAAATTTTA   
  
  
+ AGCAAGCAAT TTTGTTCATT TGATTGGTCG GATGGTCTGA TTTAGGTTCT TTTCTTGGTC TAAATTTCTG   
  
  
+ AGCATTGATT GTTTAATTGC GTGGGGAACT TGTATCCTTG TGAGTTCCTG GTTCATTTCT TGGTTGCTTA   
  
  
+ CTCTAGCTCC CTTTCACAGT TTCTATGGTT GGAACTTGGA ACTTGGAACT TGGAAGCCTA ATTTAGTTGA   
  
  
+ ATATCTTGGC TTTGAGTTAG CTTTTCTTAG GAAAAGATGG TTCTGCTCTC GAAAATCTAA TCTAATCGAT   
  
  
+ AGATGCGTTA AGGAAGCAAA ACTTTTGGGG AGAGGGAAGT TAAGCGAAGC AAGCTGCTGT TTCAAAGTTG   
  
  
+ GACTGTTTTA TTGGTAAGTA TGAAACAATA GTTTATAAGA GATCATTGGG TTCTTAAGGT GATAGATCAT   
  
  
+ GGGTTATGAA TGAGCTTAGT TGTTCTTTTG CTTTCAACTG TACGTAAGGA TGATAGTTAT CCCTCATCTT   
  
  
+ GCATCAAAAA CGGCAAACCG TGATGCCAAA ACTCAAAAAC TGATTCCTCT GACAAGGCTT CTACCTATGA   
  
  
+ AAACTATGCA GATCATATCC ATTTGGCCTT CCCTCTAGGA ACCAAGTAGA GTAAGTTGGT AAATAAATAG   
  
  
+ AGTTGAACGA AACACTACAG ATTTGAATTT GATTAAGGAG GGCTATTATA GACTAAGGTG TAGCTCAAAT   
  
  
+ CCTTCTGTAG GGATTGTCTT ATTGGCTTCT TTTGGTTGCT TCTGGCTCAA GTCCTCTTAT TAGTTAGTAT   
  
  
+ GCATTACCTT GAGACAAGAT AGTTGCTTAA GCTTGAGCTT CTGTACTTAT TTGCGTATTG TAGACAACTA   
  
  
+ GCTGTGGCAT ATGATTCTGA AATGTTCCGA TGTCTTTTTC TTTGGATGCT GGGTTGTGTC ATGAGCTTTA   
  
  
+ GGTTTGTTTA TTTGATGTCA GGAAGTATGT AAATAACATG TGGCATCTTG CAATCTATTA CAGGATGATA   
  
  
+ TTGGCCTTTG TGGGAAGAAA TTGAAGAAGT TGTTTCACTT ACTGGATAGG GAGCATGGAC TCACATCAGC   
  
  
+ TTTTGAGATT CGGTATTTCC AGATCATACT CATCCTATAA TTTCTCTCAG TCTAGTCCCC AATCAATTCC   
  
  
+ AAATAGGTTA TTTGAATCAC CGAACGTTCG TTCTAGAGAC TCTCCAATCT CGCCCTTCTC AACGCCCTTC   
  
  
+ AATTGCGACC CCACTGTTAT ATTGAGCGAC AGTCAGGAGC ACCACAGTTC AACAGGAAGT CTCTCGGCAC   
  
  
+ AAAGCTCATC TTCTAATTCT CCCCTTGAGA CTAGCAGTTA TTATAATCAG TTCAACTCAA GCCCTGTTGG   
  
  
+ AGAATCACCT CAGGGATCAT CACCCGAGAA TCTTTTTCAA CAAGCTGCAG TTAATAAAGT CAGCATCGAG   
  
  
+ CATGCATTGC AGGAGCTAGA AACTGCTCTA ATGAGCACAG ATTGTGAGGA GAATGAAGCA ACTGTCTCTA   
  
  
+ TCCCATCTAT GGGTGAACCT CATCAGCCCC AAGTCCCTAG CCAGAGATCA AGATTCTGGA ATCGAGATCC   
  
  
+ TCAGGGTTCA CGCCCGGCTG AAGTTCACTC ATCATTGAGA AGATTAGGAG ACGAGGCTCA GAGTGAGAAA   
  
  
+ CGCCTCAAGG CAGTGGAAGA ACCAATACGG CCCAGTGCAC CACCCGGCAA TTTGAAGCAG TTGCTCATAG   
  
  
+ AATGTGCTCG GGCTTTGTCA GAGAATCGAA TAGAGGATTT TGAGAAGTTA GTTGAACAGG CAAAGGGCAT   
  
  
+ GGTCTCCATT TCTGGAGACC CAATTCAACG ACTTGGTGCT TACTTGATCG AGGGGCTAGT GGCAAGGAAG   
  
  
+ GAGTCTTCAG GTACGAAAAT CTACCGAGCT CTTAGGTGCA AAGAGCCCCT TGGTCAAGAC TTGCTTTCGT   
  
  
+ ACATGCACAT CCTTTATGAA ATTTGCCCTT ACTTGAAATT CGGTTATATG GTTGCAAATG GGGCTATAGC   
  
  
+ TGAAGCTTGC AGAGATGAGG ATCACATACA TATCGTCGAC TACCAGATTG GTCAAGGAAC TCAATGGATG   
  
  
+ ACTCTGTTGC AAGCCCTAGC CACAAAACCT GGAAGACCCC CCACTGTGAG AATTACTGGC ATTGATGATC   
  
  
+ CCGTCTCTAG GCATGCTCGG GGAGCTTGCT TGGAGGCAGT GGGGAAACGT TTGGCAGTTC TGTCGGAGAA   
  
  
+ ATTTAACATG CCCATTGAGT TTCAGGCAGT GCCCGTTTAT GCTTCTGAGG TCACCCAAGA AATGCTTGAT   
  
  
+ GTGAGGCCAG GGTGGGCCTT GGCTGTGAAC TTCCCATTGC AGCTCCACCA CACTCCCGAT GAGAGTGTTG   
  
  
+ ATGTGAACAA CCCGAGGGAT GGACTCCTAA GAATGGTGAA ATCACTCGGT CCCAAGGTTG TAACTTTGGT   
  
  
+ AGAACAAGAG TCAAACACCA ACACTACCCC TTTGCTGACA AGGTTCATAG AAACGCTGGA CTTCTACTCA   
  
  
+ GCAATGTTTG AATCCATCGA TGTGACAATG CCAAGGGACC GAAAGGAAAG GATCAATGTC GAGCAGCATT   
  
  
+ GCCTAGCCAA GGACATTGTC AACATCATCG CGTGTGAGGG GAAGGACAGG GTGGAGCGAC ATGAGCTCTT   
  
  
+ TGGGAAATGG AAGTCAAGGT TCACCATGGC AGGATTCCAA CAATCCCCAT TGAGCTCGTA TGTTAACTCC   
  
  
+ GTGATAAAAG GATTACTTAG GTGTTATTCA GAGTACTATA CGTTGGTGGA GAAGGATGGC GCCTTGCTTC   
  
  
+ TGGGATGGAA GGACCGGATG CTGGTTTCGG CTTCAGCTTG GCATTG  

- +Up\_Stream \_Len000AAATTT ATAAAATAAA ATAAAATTTA ATTATATAAA ATATTAAATT TTAATAATTA   
  
  
- TTAACTACTA CAATGGGTTA CAAATTCAAA AATTTATAAA AATAAAAAAT TAAATTTTTA TAGTATTATA   
  
  
- TATTATATTA AAATTCAATT TTATTCTATC AATTTAATAA AATATCATTT ATAAATAATA AAAAAAATTC   
  
  
- TTTTAACGGT ACACTAACAG TAGTCAGTTT GTGACGCAAC TGTCTTCGAC AGGAACGACG ATGAGAGTGG   
  
  
- AGAGTTGTAG CCTGGGCGCG TCTGCAATTG TAGAGGCATG TTTATGTTAA GGGACCGGTT TTAAGTTTAG   
  
  
- TTGGGATGAG CCATCAGCCA AGGAAGAAGA AGGACGGGAG ATATATATGT GGGTGTCGAC GCGACGTACA   
  
  
- AAAAGGCTCT TAGGAGTTTT TAGAATATTT GAAGGAGCAC ACCCATCTTT TTTCTTTTTC TTTTCCCAAA   
  
  
- ATTTTAGAAC CCAGTTTTGG GTAGTTGTGG TGTTAAACTG AGAAAGAAAA ATTAAGTTGG TGGCGGTCAA   
  
  
- GGAGCAACTA AAAGTCCATG GAGAAAAAGA ACGGGGAAAA AAACGAGCCA ACAACAAATT ACCATTTCTA   
  
  
- ACTCATGTTT CTTACTATAA ACTACTCAAC CACACAAATA AACTACTCGG GTCTACTAAA TTGAACATCT   
  
  
- AAAACACCAA AACCAAAGAC CCATAAGTAA CCAAACGACT TAAAGACCAA GTACTAGACC CCAATCTTTT   
  
  
- CAACTTTACT GAAAGACCTC CAACCTTAAA CTGATTTCGA TTAAAGAAAA ACAGAAGTTA AGAAAAAATC   
  
  
- AACACTCGAC TTAAACAACT GGTGAAGGTC GGACAACCTT TAATTTGAAT GAATCCTCTT CTCTTAGAGT   
  
  
- CTAAGACTCG TTTTCGAAAT ATACCAACAA ACCCTTTACC ATCCTTGATT CACTTTTGTG ACTTTAAAAT   
  
  
- TCGTTCGTTA AAACAAGTAA ACTAACCAGC CTACCAGACT AAATCCAAGA AAAGAACCAG ATTTAAAGAC   
  
  
- TCGTAACTAA CAAATTAACG CACCCCTTGA ACATAGGAAC ACTCAAGGAC CAAGTAAAGA ACCAACGAAT   
  
  
- GAGATCGAGG GAAAGTGTCA AAGATACCAA CCTTGAACCT TGAACCTTGA ACCTTCGGAT TAAATCAACT   
  
  
- TATAGAACCG AAACTCAATC GAAAAGAATC CTTTTCTACC AAGACGAGAG CTTTTAGATT AGATTAGCTA   
  
  
- TCTACGCAAT TCCTTCGTTT TGAAAACCCC TCTCCCTTCA ATTCGCTTCG TTCGACGACA AAGTTTCAAC   
  
  
- CTGACAAAAT AACCATTCAT ACTTTGTTAT CAAATATTCT CTAGTAACCC AAGAATTCCA CTATCTAGTA   
  
  
- CCCAATACTT ACTCGAATCA ACAAGAAAAC GAAAGTTGAC ATGCATTCCT ACTATCAATA GGGAGTAGAA   
  
  
- CGTAGTTTTT GCCGTTTGGC ACTACGGTTT TGAGTTTTTG ACTAAGGAGA CTGTTCCGAA GATGGATACT   
  
  
- TTTGATACGT CTAGTATAGG TAAACCGGAA GGGAGATCCT TGGTTCATCT CATTCAACCA TTTATTTATC   
  
  
- TCAACTTGCT TTGTGATGTC TAAACTTAAA CTAATTCCTC CCGATAATAT CTGATTCCAC ATCGAGTTTA   
  
  
- GGAAGACATC CCTAACAGAA TAACCGAAGA AAACCAACGA AGACCGAGTT CAGGAGAATA ATCAATCATA   
  
  
- CGTAATGGAA CTCTGTTCTA TCAACGAATT CGAACTCGAA GACATGAATA AACGCATAAC ATCTGTTGAT   
  
  
- CGACACCGTA TACTAAGACT TTACAAGGCT ACAGAAAAAG AAACCTACGA CCCAACACAG TACTCGAAAT   
  
  
- CCAAACAAAT AAACTACAGT CCTTCATACA TTTATTGTAC ACCGTAGAAC GTTAGATAAT GTCCTACTAT   
  
  
- AACCGGAAAC ACCCTTCTTT AACTTCTTCA ACAAAGTGAA TGACCTATCC CTCGTACCTG AGTGTAGTCG   
  
  
- AAAACTCTAA GCCATAAAGG TCTAGTATGA GTAGGATATT AAAGAGAGTC AGATCAGGGG TTAGTTAAGG   
  
  
- TTTATCCAAT AAACTTAGTG GCTTGCAAGC AAGATCTCTG AGAGGTTAGA GCGGGAAGAG TTGCGGGAAG   
  
  
- TTAACGCTGG GGTGACAATA TAACTCGCTG TCAGTCCTCG TGGTGTCAAG TTGTCCTTCA GAGAGCCGTG   
  
  
- TTTCGAGTAG AAGATTAAGA GGGGAACTCT GATCGTCAAT AATATTAGTC AAGTTGAGTT CGGGACAACC   
  
  
- TCTTAGTGGA GTCCCTAGTA GTGGGCTCTT AGAAAAAGTT GTTCGACGTC AATTATTTCA GTCGTAGCTC   
  
  
- GTACGTAACG TCCTCGATCT TTGACGAGAT TACTCGTGTC TAACACTCCT CTTACTTCGT TGACAGAGAT   
  
  
- AGGGTAGATA CCCACTTGGA GTAGTCGGGG TTCAGGGATC GGTCTCTAGT TCTAAGACCT TAGCTCTAGG   
  
  
- AGTCCCAAGT GCGGGCCGAC TTCAAGTGAG TAGTAACTCT TCTAATCCTC TGCTCCGAGT CTCACTCTTT   
  
  
- GCGGAGTTCC GTCACCTTCT TGGTTATGCC GGGTCACGTG GTGGGCCGTT AAACTTCGTC AACGAGTATC   
  
  
- TTACACGAGC CCGAAACAGT CTCTTAGCTT ATCTCCTAAA ACTCTTCAAT CAACTTGTCC GTTTCCCGTA   
  
  
- CCAGAGGTAA AGACCTCTGG GTTAAGTTGC TGAACCACGA ATGAACTAGC TCCCCGATCA CCGTTCCTTC   
  
  
- CTCAGAAGTC CATGCTTTTA GATGGCTCGA GAATCCACGT TTCTCGGGGA ACCAGTTCTG AACGAAAGCA   
  
  
- TGTACGTGTA GGAAATACTT TAAACGGGAA TGAACTTTAA GCCAATATAC CAACGTTTAC CCCGATATCG   
  
  
- ACTTCGAACG TCTCTACTCC TAGTGTATGT ATAGCAGCTG ATGGTCTAAC CAGTTCCTTG AGTTACCTAC   
  
  
- TGAGACAACG TTCGGGATCG GTGTTTTGGA CCTTCTGGGG GGTGACACTC TTAATGACCG TAACTACTAG   
  
  
- GGCAGAGATC CGTACGAGCC CCTCGAACGA ACCTCCGTCA CCCCTTTGCA AACCGTCAAG ACAGCCTCTT   
  
  
- TAAATTGTAC GGGTAACTCA AAGTCCGTCA CGGGCAAATA CGAAGACTCC AGTGGGTTCT TTACGAACTA   
  
  
- CACTCCGGTC CCACCCGGAA CCGACACTTG AAGGGTAACG TCGAGGTGGT GTGAGGGCTA CTCTCACAAC   
  
  
- TACACTTGTT GGGCTCCCTA CCTGAGGATT CTTACCACTT TAGTGAGCCA GGGTTCCAAC ATTGAAACCA   
  
  
- TCTTGTTCTC AGTTTGTGGT TGTGATGGGG AAACGACTGT TCCAAGTATC TTTGCGACCT GAAGATGAGT   
  
  
- CGTTACAAAC TTAGGTAGCT ACACTGTTAC GGTTCCCTGG CTTTCCTTTC CTAGTTACAG CTCGTCGTAA   
  
  
- CGGATCGGTT CCTGTAACAG TTGTAGTAGC GCACACTCCC CTTCCTGTCC CACCTCGCTG TACTCGAGAA   
  
  
- ACCCTTTACC TTCAGTTCCA AGTGGTACCG TCCTAAGGTT GTTAGGGGTA ACTCGAGCAT ACAATTGAGG   
  
  
- CACTATTTTC CTAATGAATC CACAATAAGT CTCATGATAT GCAACCACCT CTTCCTACCG CGGAACGAAG   
  
  
- ACCCTACCTT CCTGGCCTAC GACCAAAGCC GAAGTCGAAC CGTAAC

+     CAAT-box

| Site Name | Organism | Position | Strand | Matrix score. | sequence | function |
| --- | --- | --- | --- | --- | --- | --- |
| CAAT-box | Nicotiana glutinosa | 3623 | - | 4 | CAAT |  |
| CAAT-box | Nicotiana glutinosa | 3757 | - | 4 | CAAT |  |
| CAAT-box | Nicotiana glutinosa | 3519 | - | 4 | CAAT |  |
| CAAT-box | Nicotiana glutinosa | 3615 | + | 4 | CAAT |  |
| CAAT-box | Nicotiana glutinosa | 3502 | - | 4 | CAAT |  |
| CAAT-box | Nicotiana glutinosa | 3488 | + | 4 | CAAT |  |
| CAAT-box | Nicotiana glutinosa | 3436 | + | 4 | CAAT |  |
| CAAT-box | Nicotiana glutinosa | 3460 | + | 4 | CAAT |  |
| CAAT-box | Nicotiana glutinosa | 3168 | - | 4 | CAAT |  |
| CAAT-box | Nicotiana glutinosa | 3260 | - | 4 | CAAT |  |
| CAAT-box | Nicotiana glutinosa | 3006 | + | 4 | CAAT |  |
| CAAT-box | Nicotiana glutinosa | 3075 | - | 4 | CAAT |  |
| CAAT-box | Arabidopsis thaliana | 2991 | - | 5 | CCAAT | common cis-acting element in promoter and enhancer regions |
| CAAT-box | Nicotiana glutinosa | 2755 | + | 4 | CAAT |  |
| CAAT-box | Arabidopsis thaliana | 2754 | + | 5 | CCAAT | common cis-acting element in promoter and enhancer regions |
| CAAT-box | Nicotiana glutinosa | 2642 | + | 4 | CAAT |  |
| CAAT-box | Nicotiana glutinosa | 2617 | + | 4 | CAAT |  |
| CAAT-box | Arabidopsis thaliana | 2616 | + | 5 | CCAAT | common cis-acting element in promoter and enhancer regions |
| CAAT-box | Nicotiana glutinosa | 2558 | - | 4 | CAAT |  |
| CAAT-box | Nicotiana glutinosa | 2425 | - | 4 | CAAT |  |
| CAAT-box | Nicotiana glutinosa | 2390 | - | 4 | CAAT |  |
| CAAT-box | Nicotiana glutinosa | 2195 | - | 4 | CAAT |  |
| CAAT-box | Nicotiana glutinosa | 2176 | - | 4 | CAAT |  |
| CAAT-box | Nicotiana glutinosa | 2174 | + | 4 | CAAT |  |
| CAAT-box | Nicotiana glutinosa | 2149 | + | 4 | CAAT |  |
| CAAT-box | Arabidopsis thaliana | 2148 | + | 5 | CCAAT | common cis-acting element in promoter and enhancer regions |
| CAAT-box | Nicotiana glutinosa | 2098 | + | 4 | CAAT |  |
| CAAT-box | Nicotiana glutinosa | 2094 | + | 4 | CAAT |  |
| CAAT-box | Arabidopsis thaliana | 2093 | + | 5 | CCAAT | common cis-acting element in promoter and enhancer regions |
| CAAT-box | Nicotiana glutinosa | 1984 | - | 4 | CAAT |  |
| CAAT-box | Arabidopsis thaliana | 1964 | - | 5 | CCAAT | common cis-acting element in promoter and enhancer regions |
| CAAT-box | Nicotiana glutinosa | 1945 | + | 4 | CAAT |  |
| CAAT-box | Arabidopsis thaliana | 1705 | - | 5 | CCAAT | common cis-acting element in promoter and enhancer regions |
| CAAT-box | Nicotiana glutinosa | 1697 | - | 4 | CAAT |  |
| CAAT-box | Nicotiana glutinosa | 1360 | + | 4 | CAAT |  |
| CAAT-box | Arabidopsis thaliana | 1379 | - | 5 | CCAAT | common cis-acting element in promoter and enhancer regions |
| CAAT-box | Arabidopsis thaliana | 1344 | - | 5 | CCAAT | common cis-acting element in promoter and enhancer regions |
| CAAT-box | Nicotiana glutinosa | 1070 | - | 4 | CAAT |  |
| CAAT-box | Nicotiana glutinosa | 1058 | - | 4 | CAAT |  |
| CAAT-box | Nicotiana glutinosa | 1062 | - | 4 | CAAT |  |
| CAAT-box | Arabidopsis thaliana | 1007 | - | 5 | CCAAT | common cis-acting element in promoter and enhancer regions |
| CAAT-box | Nicotiana glutinosa | 991 | + | 4 | CAAT |  |
| CAAT-box | Pisum sativum | 1904 | - | 5 | CAAAT | common cis-acting element in promoter and enhancer regions |
| CAAT-box | Nicotiana glutinosa | 831 | + | 4 | CAAT |  |
| CAAT-box | Arabidopsis thaliana | 732 | - | 5 | CCAAT | common cis-acting element in promoter and enhancer regions |
| CAAT-box | Nicotiana glutinosa | 633 | - | 4 | CAAT |  |
| CAAT-box | Arabidopsis thaliana | 90 | + | 5 | CCAAT | common cis-acting element in promoter and enhancer regions |
| CAAT-box | Nicotiana glutinosa | 330 | + | 4 | CAAT |  |
| CAAT-box | Pisum sativum | 1565 | - | 5 | CAAAT | common cis-acting element in promoter and enhancer regions |
| CAAT-box | Pisum sativum | 801 | - | 5 | CAAAT | common cis-acting element in promoter and enhancer regions |
| CAAT-box | Pisum sativum | 1641 | - | 5 | CAAAT | common cis-acting element in promoter and enhancer regions |
| CAAT-box | Pisum sativum | 324 | + | 5 | CAAAT | common cis-acting element in promoter and enhancer regions |
| CAAT-box | Nicotiana glutinosa | 229 | - | 4 | CAAT |  |
| CAAT-box | Nicotiana glutinosa | 526 | + | 4 | CAAT |  |
| CAAT-box | Nicotiana glutinosa | 76 | - | 4 | CAAT |  |
| CAAT-box | Nicotiana glutinosa | 218 | - | 4 | CAAT |  |
| CAAT-box | Nicotiana glutinosa | 91 | + | 4 | CAAT |  |
| CAAT-box | Pisum sativum | 2929 | + | 5 | CAAAT | common cis-acting element in promoter and enhancer regions |
| CAAT-box | Pisum sativum | 1002 | - | 5 | CAAAT | common cis-acting element in promoter and enhancer regions |
| CAAT-box | Pisum sativum | 2114 | - | 5 | CAAAT | common cis-acting element in promoter and enhancer regions |
| CAAT-box | Pisum sativum | 856 | - | 5 | CAAAT | common cis-acting element in promoter and enhancer regions |
| CAAT-box | Pisum sativum | 2644 | - | 5 | CAAAT | common cis-acting element in promoter and enhancer regions |
| CAAT-box | Pisum sativum | 1803 | - | 5 | CAAAT | common cis-acting element in promoter and enhancer regions |
| CAAT-box | Nicotiana glutinosa | 1811 | - | 4 | CAAT |  |
| CAAT-box | Pisum sativum | 2104 | + | 5 | CAAAT | common cis-acting element in promoter and enhancer regions |
| CAAT-box | Pisum sativum | 349 | + | 5 | CAAAT | common cis-acting element in promoter and enhancer regions |
| CAAT-box | Pisum sativum | 2895 | - | 5 | CAAAT | common cis-acting element in promoter and enhancer regions |
| CAAT-box | Pisum sativum | 652 | - | 5 | CAAAT | common cis-acting element in promoter and enhancer regions |
| CAAT-box | Pisum sativum | 1635 | - | 5 | CAAAT | common cis-acting element in promoter and enhancer regions |
| CAAT-box | Pisum sativum | 673 | - | 5 | CAAAT | common cis-acting element in promoter and enhancer regions |
| CAAT-box | Pisum sativum | 1680 | + | 5 | CAAAT | common cis-acting element in promoter and enhancer regions |
| CAAT-box | Pisum sativum | 528 | - | 5 | CAAAT | common cis-acting element in promoter and enhancer regions |

>HU01G01850.1   
+ +Up\_Stream \_Len000TTTAAA TATTTTATTT TATTTTAAAT TAATATATTT TATAATTTAA AATTATTAAT   
  
  
+ AATTGATGAT GTTACCCAAT GTTTAAGTTT TTAAATATTT TTATTTTTTA ATTTAAAAAT ATCATAATAT   
  
  
+ ATAATATAAT TTTAAGTTAA AATAAGATAG TTAAATTATT TTATAGTAAA TATTTATTAT TTTTTTTAAG   
  
  
+ AAAATTGCCA TGTGATTGTC ATCAGTCAAA CACTGCGTTG ACAGAAGCTG TCCTTGCTGC TACTCTCACC   
  
  
+ TCTCAACATC GGACCCGCGC AGACGTTAAC ATCTCCGTAC AAATACAATT CCCTGGCCAA AATTCAAATC   
  
  
+ AACCCTACTC GGTAGTCGGT TCCTTCTTCT TCCTGCCCTC TATATATACA CCCACAGCTG CGCTGCATGT   
  
  
+ TTTTCCGAGA ATCCTCAAAA ATCTTATAAA CTTCCTCGTG TGGGTAGAAA AAAGAAAAAG AAAAGGGTTT   
  
  
+ TAAAATCTTG GGTCAAAACC CATCAACACC ACAATTTGAC TCTTTCTTTT TAATTCAACC ACCGCCAGTT   
  
  
+ CCTCGTTGAT TTTCAGGTAC CTCTTTTTCT TGCCCCTTTT TTTGCTCGGT TGTTGTTTAA TGGTAAAGAT   
  
  
+ TGAGTACAAA GAATGATATT TGATGAGTTG GTGTGTTTAT TTGATGAGCC CAGATGATTT AACTTGTAGA   
  
  
+ TTTTGTGGTT TTGGTTTCTG GGTATTCATT GGTTTGCTGA ATTTCTGGTT CATGATCTGG GGTTAGAAAA   
  
  
+ GTTGAAATGA CTTTCTGGAG GTTGGAATTT GACTAAAGCT AATTTCTTTT TGTCTTCAAT TCTTTTTTAG   
  
  
+ TTGTGAGCTG AATTTGTTGA CCACTTCCAG CCTGTTGGAA ATTAAACTTA CTTAGGAGAA GAGAATCTCA   
  
  
+ GATTCTGAGC AAAAGCTTTA TATGGTTGTT TGGGAAATGG TAGGAACTAA GTGAAAACAC TGAAATTTTA   
  
  
+ AGCAAGCAAT TTTGTTCATT TGATTGGTCG GATGGTCTGA TTTAGGTTCT TTTCTTGGTC TAAATTTCTG   
  
  
+ AGCATTGATT GTTTAATTGC GTGGGGAACT TGTATCCTTG TGAGTTCCTG GTTCATTTCT TGGTTGCTTA   
  
  
+ CTCTAGCTCC CTTTCACAGT TTCTATGGTT GGAACTTGGA ACTTGGAACT TGGAAGCCTA ATTTAGTTGA   
  
  
+ ATATCTTGGC TTTGAGTTAG CTTTTCTTAG GAAAAGATGG TTCTGCTCTC GAAAATCTAA TCTAATCGAT   
  
  
+ AGATGCGTTA AGGAAGCAAA ACTTTTGGGG AGAGGGAAGT TAAGCGAAGC AAGCTGCTGT TTCAAAGTTG   
  
  
+ GACTGTTTTA TTGGTAAGTA TGAAACAATA GTTTATAAGA GATCATTGGG TTCTTAAGGT GATAGATCAT   
  
  
+ GGGTTATGAA TGAGCTTAGT TGTTCTTTTG CTTTCAACTG TACGTAAGGA TGATAGTTAT CCCTCATCTT   
  
  
+ GCATCAAAAA CGGCAAACCG TGATGCCAAA ACTCAAAAAC TGATTCCTCT GACAAGGCTT CTACCTATGA   
  
  
+ AAACTATGCA GATCATATCC ATTTGGCCTT CCCTCTAGGA ACCAAGTAGA GTAAGTTGGT AAATAAATAG   
  
  
+ AGTTGAACGA AACACTACAG ATTTGAATTT GATTAAGGAG GGCTATTATA GACTAAGGTG TAGCTCAAAT   
  
  
+ CCTTCTGTAG GGATTGTCTT ATTGGCTTCT TTTGGTTGCT TCTGGCTCAA GTCCTCTTAT TAGTTAGTAT   
  
  
+ GCATTACCTT GAGACAAGAT AGTTGCTTAA GCTTGAGCTT CTGTACTTAT TTGCGTATTG TAGACAACTA   
  
  
+ GCTGTGGCAT ATGATTCTGA AATGTTCCGA TGTCTTTTTC TTTGGATGCT GGGTTGTGTC ATGAGCTTTA   
  
  
+ GGTTTGTTTA TTTGATGTCA GGAAGTATGT AAATAACATG TGGCATCTTG CAATCTATTA CAGGATGATA   
  
  
+ TTGGCCTTTG TGGGAAGAAA TTGAAGAAGT TGTTTCACTT ACTGGATAGG GAGCATGGAC TCACATCAGC   
  
  
+ TTTTGAGATT CGGTATTTCC AGATCATACT CATCCTATAA TTTCTCTCAG TCTAGTCCCC AATCAATTCC   
  
  
+ AAATAGGTTA TTTGAATCAC CGAACGTTCG TTCTAGAGAC TCTCCAATCT CGCCCTTCTC AACGCCCTTC   
  
  
+ AATTGCGACC CCACTGTTAT ATTGAGCGAC AGTCAGGAGC ACCACAGTTC AACAGGAAGT CTCTCGGCAC   
  
  
+ AAAGCTCATC TTCTAATTCT CCCCTTGAGA CTAGCAGTTA TTATAATCAG TTCAACTCAA GCCCTGTTGG   
  
  
+ AGAATCACCT CAGGGATCAT CACCCGAGAA TCTTTTTCAA CAAGCTGCAG TTAATAAAGT CAGCATCGAG   
  
  
+ CATGCATTGC AGGAGCTAGA AACTGCTCTA ATGAGCACAG ATTGTGAGGA GAATGAAGCA ACTGTCTCTA   
  
  
+ TCCCATCTAT GGGTGAACCT CATCAGCCCC AAGTCCCTAG CCAGAGATCA AGATTCTGGA ATCGAGATCC   
  
  
+ TCAGGGTTCA CGCCCGGCTG AAGTTCACTC ATCATTGAGA AGATTAGGAG ACGAGGCTCA GAGTGAGAAA   
  
  
+ CGCCTCAAGG CAGTGGAAGA ACCAATACGG CCCAGTGCAC CACCCGGCAA TTTGAAGCAG TTGCTCATAG   
  
  
+ AATGTGCTCG GGCTTTGTCA GAGAATCGAA TAGAGGATTT TGAGAAGTTA GTTGAACAGG CAAAGGGCAT   
  
  
+ GGTCTCCATT TCTGGAGACC CAATTCAACG ACTTGGTGCT TACTTGATCG AGGGGCTAGT GGCAAGGAAG   
  
  
+ GAGTCTTCAG GTACGAAAAT CTACCGAGCT CTTAGGTGCA AAGAGCCCCT TGGTCAAGAC TTGCTTTCGT   
  
  
+ ACATGCACAT CCTTTATGAA ATTTGCCCTT ACTTGAAATT CGGTTATATG GTTGCAAATG GGGCTATAGC   
  
  
+ TGAAGCTTGC AGAGATGAGG ATCACATACA TATCGTCGAC TACCAGATTG GTCAAGGAAC TCAATGGATG   
  
  
+ ACTCTGTTGC AAGCCCTAGC CACAAAACCT GGAAGACCCC CCACTGTGAG AATTACTGGC ATTGATGATC   
  
  
+ CCGTCTCTAG GCATGCTCGG GGAGCTTGCT TGGAGGCAGT GGGGAAACGT TTGGCAGTTC TGTCGGAGAA   
  
  
+ ATTTAACATG CCCATTGAGT TTCAGGCAGT GCCCGTTTAT GCTTCTGAGG TCACCCAAGA AATGCTTGAT   
  
  
+ GTGAGGCCAG GGTGGGCCTT GGCTGTGAAC TTCCCATTGC AGCTCCACCA CACTCCCGAT GAGAGTGTTG   
  
  
+ ATGTGAACAA CCCGAGGGAT GGACTCCTAA GAATGGTGAA ATCACTCGGT CCCAAGGTTG TAACTTTGGT   
  
  
+ AGAACAAGAG TCAAACACCA ACACTACCCC TTTGCTGACA AGGTTCATAG AAACGCTGGA CTTCTACTCA   
  
  
+ GCAATGTTTG AATCCATCGA TGTGACAATG CCAAGGGACC GAAAGGAAAG GATCAATGTC GAGCAGCATT   
  
  
+ GCCTAGCCAA GGACATTGTC AACATCATCG CGTGTGAGGG GAAGGACAGG GTGGAGCGAC ATGAGCTCTT   
  
  
+ TGGGAAATGG AAGTCAAGGT TCACCATGGC AGGATTCCAA CAATCCCCAT TGAGCTCGTA TGTTAACTCC   
  
  
+ GTGATAAAAG GATTACTTAG GTGTTATTCA GAGTACTATA CGTTGGTGGA GAAGGATGGC GCCTTGCTTC   
  
  
+ TGGGATGGAA GGACCGGATG CTGGTTTCGG CTTCAGCTTG GCATTG  

- +Up\_Stream \_Len000AAATTT ATAAAATAAA ATAAAATTTA ATTATATAAA ATATTAAATT TTAATAATTA   
  
  
- TTAACTACTA CAATGGGTTA CAAATTCAAA AATTTATAAA AATAAAAAAT TAAATTTTTA TAGTATTATA   
  
  
- TATTATATTA AAATTCAATT TTATTCTATC AATTTAATAA AATATCATTT ATAAATAATA AAAAAAATTC   
  
  
- TTTTAACGGT ACACTAACAG TAGTCAGTTT GTGACGCAAC TGTCTTCGAC AGGAACGACG ATGAGAGTGG   
  
  
- AGAGTTGTAG CCTGGGCGCG TCTGCAATTG TAGAGGCATG TTTATGTTAA GGGACCGGTT TTAAGTTTAG   
  
  
- TTGGGATGAG CCATCAGCCA AGGAAGAAGA AGGACGGGAG ATATATATGT GGGTGTCGAC GCGACGTACA   
  
  
- AAAAGGCTCT TAGGAGTTTT TAGAATATTT GAAGGAGCAC ACCCATCTTT TTTCTTTTTC TTTTCCCAAA   
  
  
- ATTTTAGAAC CCAGTTTTGG GTAGTTGTGG TGTTAAACTG AGAAAGAAAA ATTAAGTTGG TGGCGGTCAA   
  
  
- GGAGCAACTA AAAGTCCATG GAGAAAAAGA ACGGGGAAAA AAACGAGCCA ACAACAAATT ACCATTTCTA   
  
  
- ACTCATGTTT CTTACTATAA ACTACTCAAC CACACAAATA AACTACTCGG GTCTACTAAA TTGAACATCT   
  
  
- AAAACACCAA AACCAAAGAC CCATAAGTAA CCAAACGACT TAAAGACCAA GTACTAGACC CCAATCTTTT   
  
  
- CAACTTTACT GAAAGACCTC CAACCTTAAA CTGATTTCGA TTAAAGAAAA ACAGAAGTTA AGAAAAAATC   
  
  
- AACACTCGAC TTAAACAACT GGTGAAGGTC GGACAACCTT TAATTTGAAT GAATCCTCTT CTCTTAGAGT   
  
  
- CTAAGACTCG TTTTCGAAAT ATACCAACAA ACCCTTTACC ATCCTTGATT CACTTTTGTG ACTTTAAAAT   
  
  
- TCGTTCGTTA AAACAAGTAA ACTAACCAGC CTACCAGACT AAATCCAAGA AAAGAACCAG ATTTAAAGAC   
  
  
- TCGTAACTAA CAAATTAACG CACCCCTTGA ACATAGGAAC ACTCAAGGAC CAAGTAAAGA ACCAACGAAT   
  
  
- GAGATCGAGG GAAAGTGTCA AAGATACCAA CCTTGAACCT TGAACCTTGA ACCTTCGGAT TAAATCAACT   
  
  
- TATAGAACCG AAACTCAATC GAAAAGAATC CTTTTCTACC AAGACGAGAG CTTTTAGATT AGATTAGCTA   
  
  
- TCTACGCAAT TCCTTCGTTT TGAAAACCCC TCTCCCTTCA ATTCGCTTCG TTCGACGACA AAGTTTCAAC   
  
  
- CTGACAAAAT AACCATTCAT ACTTTGTTAT CAAATATTCT CTAGTAACCC AAGAATTCCA CTATCTAGTA   
  
  
- CCCAATACTT ACTCGAATCA ACAAGAAAAC GAAAGTTGAC ATGCATTCCT ACTATCAATA GGGAGTAGAA   
  
  
- CGTAGTTTTT GCCGTTTGGC ACTACGGTTT TGAGTTTTTG ACTAAGGAGA CTGTTCCGAA GATGGATACT   
  
  
- TTTGATACGT CTAGTATAGG TAAACCGGAA GGGAGATCCT TGGTTCATCT CATTCAACCA TTTATTTATC   
  
  
- TCAACTTGCT TTGTGATGTC TAAACTTAAA CTAATTCCTC CCGATAATAT CTGATTCCAC ATCGAGTTTA   
  
  
- GGAAGACATC CCTAACAGAA TAACCGAAGA AAACCAACGA AGACCGAGTT CAGGAGAATA ATCAATCATA   
  
  
- CGTAATGGAA CTCTGTTCTA TCAACGAATT CGAACTCGAA GACATGAATA AACGCATAAC ATCTGTTGAT   
  
  
- CGACACCGTA TACTAAGACT TTACAAGGCT ACAGAAAAAG AAACCTACGA CCCAACACAG TACTCGAAAT   
  
  
- CCAAACAAAT AAACTACAGT CCTTCATACA TTTATTGTAC ACCGTAGAAC GTTAGATAAT GTCCTACTAT   
  
  
- AACCGGAAAC ACCCTTCTTT AACTTCTTCA ACAAAGTGAA TGACCTATCC CTCGTACCTG AGTGTAGTCG   
  
  
- AAAACTCTAA GCCATAAAGG TCTAGTATGA GTAGGATATT AAAGAGAGTC AGATCAGGGG TTAGTTAAGG   
  
  
- TTTATCCAAT AAACTTAGTG GCTTGCAAGC AAGATCTCTG AGAGGTTAGA GCGGGAAGAG TTGCGGGAAG   
  
  
- TTAACGCTGG GGTGACAATA TAACTCGCTG TCAGTCCTCG TGGTGTCAAG TTGTCCTTCA GAGAGCCGTG   
  
  
- TTTCGAGTAG AAGATTAAGA GGGGAACTCT GATCGTCAAT AATATTAGTC AAGTTGAGTT CGGGACAACC   
  
  
- TCTTAGTGGA GTCCCTAGTA GTGGGCTCTT AGAAAAAGTT GTTCGACGTC AATTATTTCA GTCGTAGCTC   
  
  
- GTACGTAACG TCCTCGATCT TTGACGAGAT TACTCGTGTC TAACACTCCT CTTACTTCGT TGACAGAGAT   
  
  
- AGGGTAGATA CCCACTTGGA GTAGTCGGGG TTCAGGGATC GGTCTCTAGT TCTAAGACCT TAGCTCTAGG   
  
  
- AGTCCCAAGT GCGGGCCGAC TTCAAGTGAG TAGTAACTCT TCTAATCCTC TGCTCCGAGT CTCACTCTTT   
  
  
- GCGGAGTTCC GTCACCTTCT TGGTTATGCC GGGTCACGTG GTGGGCCGTT AAACTTCGTC AACGAGTATC   
  
  
- TTACACGAGC CCGAAACAGT CTCTTAGCTT ATCTCCTAAA ACTCTTCAAT CAACTTGTCC GTTTCCCGTA   
  
  
- CCAGAGGTAA AGACCTCTGG GTTAAGTTGC TGAACCACGA ATGAACTAGC TCCCCGATCA CCGTTCCTTC   
  
  
- CTCAGAAGTC CATGCTTTTA GATGGCTCGA GAATCCACGT TTCTCGGGGA ACCAGTTCTG AACGAAAGCA   
  
  
- TGTACGTGTA GGAAATACTT TAAACGGGAA TGAACTTTAA GCCAATATAC CAACGTTTAC CCCGATATCG   
  
  
- ACTTCGAACG TCTCTACTCC TAGTGTATGT ATAGCAGCTG ATGGTCTAAC CAGTTCCTTG AGTTACCTAC   
  
  
- TGAGACAACG TTCGGGATCG GTGTTTTGGA CCTTCTGGGG GGTGACACTC TTAATGACCG TAACTACTAG   
  
  
- GGCAGAGATC CGTACGAGCC CCTCGAACGA ACCTCCGTCA CCCCTTTGCA AACCGTCAAG ACAGCCTCTT   
  
  
- TAAATTGTAC GGGTAACTCA AAGTCCGTCA CGGGCAAATA CGAAGACTCC AGTGGGTTCT TTACGAACTA   
  
  
- CACTCCGGTC CCACCCGGAA CCGACACTTG AAGGGTAACG TCGAGGTGGT GTGAGGGCTA CTCTCACAAC   
  
  
- TACACTTGTT GGGCTCCCTA CCTGAGGATT CTTACCACTT TAGTGAGCCA GGGTTCCAAC ATTGAAACCA   
  
  
- TCTTGTTCTC AGTTTGTGGT TGTGATGGGG AAACGACTGT TCCAAGTATC TTTGCGACCT GAAGATGAGT   
  
  
- CGTTACAAAC TTAGGTAGCT ACACTGTTAC GGTTCCCTGG CTTTCCTTTC CTAGTTACAG CTCGTCGTAA   
  
  
- CGGATCGGTT CCTGTAACAG TTGTAGTAGC GCACACTCCC CTTCCTGTCC CACCTCGCTG TACTCGAGAA   
  
  
- ACCCTTTACC TTCAGTTCCA AGTGGTACCG TCCTAAGGTT GTTAGGGGTA ACTCGAGCAT ACAATTGAGG   
  
  
- CACTATTTTC CTAATGAATC CACAATAAGT CTCATGATAT GCAACCACCT CTTCCTACCG CGGAACGAAG   
  
  
- ACCCTACCTT CCTGGCCTAC GACCAAAGCC GAAGTCGAAC CGTAAC

+     CAT-box

| Site Name | Organism | Position | Strand | Matrix score. | sequence | function |
| --- | --- | --- | --- | --- | --- | --- |
| CAT-box | Arabidopsis thaliana | 2792 | - | 6 | GCCACT | cis-acting regulatory element related to meristem expression |

>HU01G01850.1   
+ +Up\_Stream \_Len000TTTAAA TATTTTATTT TATTTTAAAT TAATATATTT TATAATTTAA AATTATTAAT   
  
  
+ AATTGATGAT GTTACCCAAT GTTTAAGTTT TTAAATATTT TTATTTTTTA ATTTAAAAAT ATCATAATAT   
  
  
+ ATAATATAAT TTTAAGTTAA AATAAGATAG TTAAATTATT TTATAGTAAA TATTTATTAT TTTTTTTAAG   
  
  
+ AAAATTGCCA TGTGATTGTC ATCAGTCAAA CACTGCGTTG ACAGAAGCTG TCCTTGCTGC TACTCTCACC   
  
  
+ TCTCAACATC GGACCCGCGC AGACGTTAAC ATCTCCGTAC AAATACAATT CCCTGGCCAA AATTCAAATC   
  
  
+ AACCCTACTC GGTAGTCGGT TCCTTCTTCT TCCTGCCCTC TATATATACA CCCACAGCTG CGCTGCATGT   
  
  
+ TTTTCCGAGA ATCCTCAAAA ATCTTATAAA CTTCCTCGTG TGGGTAGAAA AAAGAAAAAG AAAAGGGTTT   
  
  
+ TAAAATCTTG GGTCAAAACC CATCAACACC ACAATTTGAC TCTTTCTTTT TAATTCAACC ACCGCCAGTT   
  
  
+ CCTCGTTGAT TTTCAGGTAC CTCTTTTTCT TGCCCCTTTT TTTGCTCGGT TGTTGTTTAA TGGTAAAGAT   
  
  
+ TGAGTACAAA GAATGATATT TGATGAGTTG GTGTGTTTAT TTGATGAGCC CAGATGATTT AACTTGTAGA   
  
  
+ TTTTGTGGTT TTGGTTTCTG GGTATTCATT GGTTTGCTGA ATTTCTGGTT CATGATCTGG GGTTAGAAAA   
  
  
+ GTTGAAATGA CTTTCTGGAG GTTGGAATTT GACTAAAGCT AATTTCTTTT TGTCTTCAAT TCTTTTTTAG   
  
  
+ TTGTGAGCTG AATTTGTTGA CCACTTCCAG CCTGTTGGAA ATTAAACTTA CTTAGGAGAA GAGAATCTCA   
  
  
+ GATTCTGAGC AAAAGCTTTA TATGGTTGTT TGGGAAATGG TAGGAACTAA GTGAAAACAC TGAAATTTTA   
  
  
+ AGCAAGCAAT TTTGTTCATT TGATTGGTCG GATGGTCTGA TTTAGGTTCT TTTCTTGGTC TAAATTTCTG   
  
  
+ AGCATTGATT GTTTAATTGC GTGGGGAACT TGTATCCTTG TGAGTTCCTG GTTCATTTCT TGGTTGCTTA   
  
  
+ CTCTAGCTCC CTTTCACAGT TTCTATGGTT GGAACTTGGA ACTTGGAACT TGGAAGCCTA ATTTAGTTGA   
  
  
+ ATATCTTGGC TTTGAGTTAG CTTTTCTTAG GAAAAGATGG TTCTGCTCTC GAAAATCTAA TCTAATCGAT   
  
  
+ AGATGCGTTA AGGAAGCAAA ACTTTTGGGG AGAGGGAAGT TAAGCGAAGC AAGCTGCTGT TTCAAAGTTG   
  
  
+ GACTGTTTTA TTGGTAAGTA TGAAACAATA GTTTATAAGA GATCATTGGG TTCTTAAGGT GATAGATCAT   
  
  
+ GGGTTATGAA TGAGCTTAGT TGTTCTTTTG CTTTCAACTG TACGTAAGGA TGATAGTTAT CCCTCATCTT   
  
  
+ GCATCAAAAA CGGCAAACCG TGATGCCAAA ACTCAAAAAC TGATTCCTCT GACAAGGCTT CTACCTATGA   
  
  
+ AAACTATGCA GATCATATCC ATTTGGCCTT CCCTCTAGGA ACCAAGTAGA GTAAGTTGGT AAATAAATAG   
  
  
+ AGTTGAACGA AACACTACAG ATTTGAATTT GATTAAGGAG GGCTATTATA GACTAAGGTG TAGCTCAAAT   
  
  
+ CCTTCTGTAG GGATTGTCTT ATTGGCTTCT TTTGGTTGCT TCTGGCTCAA GTCCTCTTAT TAGTTAGTAT   
  
  
+ GCATTACCTT GAGACAAGAT AGTTGCTTAA GCTTGAGCTT CTGTACTTAT TTGCGTATTG TAGACAACTA   
  
  
+ GCTGTGGCAT ATGATTCTGA AATGTTCCGA TGTCTTTTTC TTTGGATGCT GGGTTGTGTC ATGAGCTTTA   
  
  
+ GGTTTGTTTA TTTGATGTCA GGAAGTATGT AAATAACATG TGGCATCTTG CAATCTATTA CAGGATGATA   
  
  
+ TTGGCCTTTG TGGGAAGAAA TTGAAGAAGT TGTTTCACTT ACTGGATAGG GAGCATGGAC TCACATCAGC   
  
  
+ TTTTGAGATT CGGTATTTCC AGATCATACT CATCCTATAA TTTCTCTCAG TCTAGTCCCC AATCAATTCC   
  
  
+ AAATAGGTTA TTTGAATCAC CGAACGTTCG TTCTAGAGAC TCTCCAATCT CGCCCTTCTC AACGCCCTTC   
  
  
+ AATTGCGACC CCACTGTTAT ATTGAGCGAC AGTCAGGAGC ACCACAGTTC AACAGGAAGT CTCTCGGCAC   
  
  
+ AAAGCTCATC TTCTAATTCT CCCCTTGAGA CTAGCAGTTA TTATAATCAG TTCAACTCAA GCCCTGTTGG   
  
  
+ AGAATCACCT CAGGGATCAT CACCCGAGAA TCTTTTTCAA CAAGCTGCAG TTAATAAAGT CAGCATCGAG   
  
  
+ CATGCATTGC AGGAGCTAGA AACTGCTCTA ATGAGCACAG ATTGTGAGGA GAATGAAGCA ACTGTCTCTA   
  
  
+ TCCCATCTAT GGGTGAACCT CATCAGCCCC AAGTCCCTAG CCAGAGATCA AGATTCTGGA ATCGAGATCC   
  
  
+ TCAGGGTTCA CGCCCGGCTG AAGTTCACTC ATCATTGAGA AGATTAGGAG ACGAGGCTCA GAGTGAGAAA   
  
  
+ CGCCTCAAGG CAGTGGAAGA ACCAATACGG CCCAGTGCAC CACCCGGCAA TTTGAAGCAG TTGCTCATAG   
  
  
+ AATGTGCTCG GGCTTTGTCA GAGAATCGAA TAGAGGATTT TGAGAAGTTA GTTGAACAGG CAAAGGGCAT   
  
  
+ GGTCTCCATT TCTGGAGACC CAATTCAACG ACTTGGTGCT TACTTGATCG AGGGGCTAGT GGCAAGGAAG   
  
  
+ GAGTCTTCAG GTACGAAAAT CTACCGAGCT CTTAGGTGCA AAGAGCCCCT TGGTCAAGAC TTGCTTTCGT   
  
  
+ ACATGCACAT CCTTTATGAA ATTTGCCCTT ACTTGAAATT CGGTTATATG GTTGCAAATG GGGCTATAGC   
  
  
+ TGAAGCTTGC AGAGATGAGG ATCACATACA TATCGTCGAC TACCAGATTG GTCAAGGAAC TCAATGGATG   
  
  
+ ACTCTGTTGC AAGCCCTAGC CACAAAACCT GGAAGACCCC CCACTGTGAG AATTACTGGC ATTGATGATC   
  
  
+ CCGTCTCTAG GCATGCTCGG GGAGCTTGCT TGGAGGCAGT GGGGAAACGT TTGGCAGTTC TGTCGGAGAA   
  
  
+ ATTTAACATG CCCATTGAGT TTCAGGCAGT GCCCGTTTAT GCTTCTGAGG TCACCCAAGA AATGCTTGAT   
  
  
+ GTGAGGCCAG GGTGGGCCTT GGCTGTGAAC TTCCCATTGC AGCTCCACCA CACTCCCGAT GAGAGTGTTG   
  
  
+ ATGTGAACAA CCCGAGGGAT GGACTCCTAA GAATGGTGAA ATCACTCGGT CCCAAGGTTG TAACTTTGGT   
  
  
+ AGAACAAGAG TCAAACACCA ACACTACCCC TTTGCTGACA AGGTTCATAG AAACGCTGGA CTTCTACTCA   
  
  
+ GCAATGTTTG AATCCATCGA TGTGACAATG CCAAGGGACC GAAAGGAAAG GATCAATGTC GAGCAGCATT   
  
  
+ GCCTAGCCAA GGACATTGTC AACATCATCG CGTGTGAGGG GAAGGACAGG GTGGAGCGAC ATGAGCTCTT   
  
  
+ TGGGAAATGG AAGTCAAGGT TCACCATGGC AGGATTCCAA CAATCCCCAT TGAGCTCGTA TGTTAACTCC   
  
  
+ GTGATAAAAG GATTACTTAG GTGTTATTCA GAGTACTATA CGTTGGTGGA GAAGGATGGC GCCTTGCTTC   
  
  
+ TGGGATGGAA GGACCGGATG CTGGTTTCGG CTTCAGCTTG GCATTG  

- +Up\_Stream \_Len000AAATTT ATAAAATAAA ATAAAATTTA ATTATATAAA ATATTAAATT TTAATAATTA   
  
  
- TTAACTACTA CAATGGGTTA CAAATTCAAA AATTTATAAA AATAAAAAAT TAAATTTTTA TAGTATTATA   
  
  
- TATTATATTA AAATTCAATT TTATTCTATC AATTTAATAA AATATCATTT ATAAATAATA AAAAAAATTC   
  
  
- TTTTAACGGT ACACTAACAG TAGTCAGTTT GTGACGCAAC TGTCTTCGAC AGGAACGACG ATGAGAGTGG   
  
  
- AGAGTTGTAG CCTGGGCGCG TCTGCAATTG TAGAGGCATG TTTATGTTAA GGGACCGGTT TTAAGTTTAG   
  
  
- TTGGGATGAG CCATCAGCCA AGGAAGAAGA AGGACGGGAG ATATATATGT GGGTGTCGAC GCGACGTACA   
  
  
- AAAAGGCTCT TAGGAGTTTT TAGAATATTT GAAGGAGCAC ACCCATCTTT TTTCTTTTTC TTTTCCCAAA   
  
  
- ATTTTAGAAC CCAGTTTTGG GTAGTTGTGG TGTTAAACTG AGAAAGAAAA ATTAAGTTGG TGGCGGTCAA   
  
  
- GGAGCAACTA AAAGTCCATG GAGAAAAAGA ACGGGGAAAA AAACGAGCCA ACAACAAATT ACCATTTCTA   
  
  
- ACTCATGTTT CTTACTATAA ACTACTCAAC CACACAAATA AACTACTCGG GTCTACTAAA TTGAACATCT   
  
  
- AAAACACCAA AACCAAAGAC CCATAAGTAA CCAAACGACT TAAAGACCAA GTACTAGACC CCAATCTTTT   
  
  
- CAACTTTACT GAAAGACCTC CAACCTTAAA CTGATTTCGA TTAAAGAAAA ACAGAAGTTA AGAAAAAATC   
  
  
- AACACTCGAC TTAAACAACT GGTGAAGGTC GGACAACCTT TAATTTGAAT GAATCCTCTT CTCTTAGAGT   
  
  
- CTAAGACTCG TTTTCGAAAT ATACCAACAA ACCCTTTACC ATCCTTGATT CACTTTTGTG ACTTTAAAAT   
  
  
- TCGTTCGTTA AAACAAGTAA ACTAACCAGC CTACCAGACT AAATCCAAGA AAAGAACCAG ATTTAAAGAC   
  
  
- TCGTAACTAA CAAATTAACG CACCCCTTGA ACATAGGAAC ACTCAAGGAC CAAGTAAAGA ACCAACGAAT   
  
  
- GAGATCGAGG GAAAGTGTCA AAGATACCAA CCTTGAACCT TGAACCTTGA ACCTTCGGAT TAAATCAACT   
  
  
- TATAGAACCG AAACTCAATC GAAAAGAATC CTTTTCTACC AAGACGAGAG CTTTTAGATT AGATTAGCTA   
  
  
- TCTACGCAAT TCCTTCGTTT TGAAAACCCC TCTCCCTTCA ATTCGCTTCG TTCGACGACA AAGTTTCAAC   
  
  
- CTGACAAAAT AACCATTCAT ACTTTGTTAT CAAATATTCT CTAGTAACCC AAGAATTCCA CTATCTAGTA   
  
  
- CCCAATACTT ACTCGAATCA ACAAGAAAAC GAAAGTTGAC ATGCATTCCT ACTATCAATA GGGAGTAGAA   
  
  
- CGTAGTTTTT GCCGTTTGGC ACTACGGTTT TGAGTTTTTG ACTAAGGAGA CTGTTCCGAA GATGGATACT   
  
  
- TTTGATACGT CTAGTATAGG TAAACCGGAA GGGAGATCCT TGGTTCATCT CATTCAACCA TTTATTTATC   
  
  
- TCAACTTGCT TTGTGATGTC TAAACTTAAA CTAATTCCTC CCGATAATAT CTGATTCCAC ATCGAGTTTA   
  
  
- GGAAGACATC CCTAACAGAA TAACCGAAGA AAACCAACGA AGACCGAGTT CAGGAGAATA ATCAATCATA   
  
  
- CGTAATGGAA CTCTGTTCTA TCAACGAATT CGAACTCGAA GACATGAATA AACGCATAAC ATCTGTTGAT   
  
  
- CGACACCGTA TACTAAGACT TTACAAGGCT ACAGAAAAAG AAACCTACGA CCCAACACAG TACTCGAAAT   
  
  
- CCAAACAAAT AAACTACAGT CCTTCATACA TTTATTGTAC ACCGTAGAAC GTTAGATAAT GTCCTACTAT   
  
  
- AACCGGAAAC ACCCTTCTTT AACTTCTTCA ACAAAGTGAA TGACCTATCC CTCGTACCTG AGTGTAGTCG   
  
  
- AAAACTCTAA GCCATAAAGG TCTAGTATGA GTAGGATATT AAAGAGAGTC AGATCAGGGG TTAGTTAAGG   
  
  
- TTTATCCAAT AAACTTAGTG GCTTGCAAGC AAGATCTCTG AGAGGTTAGA GCGGGAAGAG TTGCGGGAAG   
  
  
- TTAACGCTGG GGTGACAATA TAACTCGCTG TCAGTCCTCG TGGTGTCAAG TTGTCCTTCA GAGAGCCGTG   
  
  
- TTTCGAGTAG AAGATTAAGA GGGGAACTCT GATCGTCAAT AATATTAGTC AAGTTGAGTT CGGGACAACC   
  
  
- TCTTAGTGGA GTCCCTAGTA GTGGGCTCTT AGAAAAAGTT GTTCGACGTC AATTATTTCA GTCGTAGCTC   
  
  
- GTACGTAACG TCCTCGATCT TTGACGAGAT TACTCGTGTC TAACACTCCT CTTACTTCGT TGACAGAGAT   
  
  
- AGGGTAGATA CCCACTTGGA GTAGTCGGGG TTCAGGGATC GGTCTCTAGT TCTAAGACCT TAGCTCTAGG   
  
  
- AGTCCCAAGT GCGGGCCGAC TTCAAGTGAG TAGTAACTCT TCTAATCCTC TGCTCCGAGT CTCACTCTTT   
  
  
- GCGGAGTTCC GTCACCTTCT TGGTTATGCC GGGTCACGTG GTGGGCCGTT AAACTTCGTC AACGAGTATC   
  
  
- TTACACGAGC CCGAAACAGT CTCTTAGCTT ATCTCCTAAA ACTCTTCAAT CAACTTGTCC GTTTCCCGTA   
  
  
- CCAGAGGTAA AGACCTCTGG GTTAAGTTGC TGAACCACGA ATGAACTAGC TCCCCGATCA CCGTTCCTTC   
  
  
- CTCAGAAGTC CATGCTTTTA GATGGCTCGA GAATCCACGT TTCTCGGGGA ACCAGTTCTG AACGAAAGCA   
  
  
- TGTACGTGTA GGAAATACTT TAAACGGGAA TGAACTTTAA GCCAATATAC CAACGTTTAC CCCGATATCG   
  
  
- ACTTCGAACG TCTCTACTCC TAGTGTATGT ATAGCAGCTG ATGGTCTAAC CAGTTCCTTG AGTTACCTAC   
  
  
- TGAGACAACG TTCGGGATCG GTGTTTTGGA CCTTCTGGGG GGTGACACTC TTAATGACCG TAACTACTAG   
  
  
- GGCAGAGATC CGTACGAGCC CCTCGAACGA ACCTCCGTCA CCCCTTTGCA AACCGTCAAG ACAGCCTCTT   
  
  
- TAAATTGTAC GGGTAACTCA AAGTCCGTCA CGGGCAAATA CGAAGACTCC AGTGGGTTCT TTACGAACTA   
  
  
- CACTCCGGTC CCACCCGGAA CCGACACTTG AAGGGTAACG TCGAGGTGGT GTGAGGGCTA CTCTCACAAC   
  
  
- TACACTTGTT GGGCTCCCTA CCTGAGGATT CTTACCACTT TAGTGAGCCA GGGTTCCAAC ATTGAAACCA   
  
  
- TCTTGTTCTC AGTTTGTGGT TGTGATGGGG AAACGACTGT TCCAAGTATC TTTGCGACCT GAAGATGAGT   
  
  
- CGTTACAAAC TTAGGTAGCT ACACTGTTAC GGTTCCCTGG CTTTCCTTTC CTAGTTACAG CTCGTCGTAA   
  
  
- CGGATCGGTT CCTGTAACAG TTGTAGTAGC GCACACTCCC CTTCCTGTCC CACCTCGCTG TACTCGAGAA   
  
  
- ACCCTTTACC TTCAGTTCCA AGTGGTACCG TCCTAAGGTT GTTAGGGGTA ACTCGAGCAT ACAATTGAGG   
  
  
- CACTATTTTC CTAATGAATC CACAATAAGT CTCATGATAT GCAACCACCT CTTCCTACCG CGGAACGAAG   
  
  
- ACCCTACCTT CCTGGCCTAC GACCAAAGCC GAAGTCGAAC CGTAAC

+     ERE

| Site Name | Organism | Position | Strand | Matrix score. | sequence | function |
| --- | --- | --- | --- | --- | --- | --- |
| ERE | Nicotiana glutinos | 2889 | - | 8 | ATTTCATA |  |
| ERE | Nicotiana glutinos | 493 | - | 8 | ATTTTAAA |  |
| ERE | Nicotiana glutinos | 60 | - | 8 | ATTTTAAA |  |
| ERE | Nicotiana glutinos | 36 | + | 8 | ATTTTAAA |  |

>HU01G01850.1   
+ +Up\_Stream \_Len000TTTAAA TATTTTATTT TATTTTAAAT TAATATATTT TATAATTTAA AATTATTAAT   
  
  
+ AATTGATGAT GTTACCCAAT GTTTAAGTTT TTAAATATTT TTATTTTTTA ATTTAAAAAT ATCATAATAT   
  
  
+ ATAATATAAT TTTAAGTTAA AATAAGATAG TTAAATTATT TTATAGTAAA TATTTATTAT TTTTTTTAAG   
  
  
+ AAAATTGCCA TGTGATTGTC ATCAGTCAAA CACTGCGTTG ACAGAAGCTG TCCTTGCTGC TACTCTCACC   
  
  
+ TCTCAACATC GGACCCGCGC AGACGTTAAC ATCTCCGTAC AAATACAATT CCCTGGCCAA AATTCAAATC   
  
  
+ AACCCTACTC GGTAGTCGGT TCCTTCTTCT TCCTGCCCTC TATATATACA CCCACAGCTG CGCTGCATGT   
  
  
+ TTTTCCGAGA ATCCTCAAAA ATCTTATAAA CTTCCTCGTG TGGGTAGAAA AAAGAAAAAG AAAAGGGTTT   
  
  
+ TAAAATCTTG GGTCAAAACC CATCAACACC ACAATTTGAC TCTTTCTTTT TAATTCAACC ACCGCCAGTT   
  
  
+ CCTCGTTGAT TTTCAGGTAC CTCTTTTTCT TGCCCCTTTT TTTGCTCGGT TGTTGTTTAA TGGTAAAGAT   
  
  
+ TGAGTACAAA GAATGATATT TGATGAGTTG GTGTGTTTAT TTGATGAGCC CAGATGATTT AACTTGTAGA   
  
  
+ TTTTGTGGTT TTGGTTTCTG GGTATTCATT GGTTTGCTGA ATTTCTGGTT CATGATCTGG GGTTAGAAAA   
  
  
+ GTTGAAATGA CTTTCTGGAG GTTGGAATTT GACTAAAGCT AATTTCTTTT TGTCTTCAAT TCTTTTTTAG   
  
  
+ TTGTGAGCTG AATTTGTTGA CCACTTCCAG CCTGTTGGAA ATTAAACTTA CTTAGGAGAA GAGAATCTCA   
  
  
+ GATTCTGAGC AAAAGCTTTA TATGGTTGTT TGGGAAATGG TAGGAACTAA GTGAAAACAC TGAAATTTTA   
  
  
+ AGCAAGCAAT TTTGTTCATT TGATTGGTCG GATGGTCTGA TTTAGGTTCT TTTCTTGGTC TAAATTTCTG   
  
  
+ AGCATTGATT GTTTAATTGC GTGGGGAACT TGTATCCTTG TGAGTTCCTG GTTCATTTCT TGGTTGCTTA   
  
  
+ CTCTAGCTCC CTTTCACAGT TTCTATGGTT GGAACTTGGA ACTTGGAACT TGGAAGCCTA ATTTAGTTGA   
  
  
+ ATATCTTGGC TTTGAGTTAG CTTTTCTTAG GAAAAGATGG TTCTGCTCTC GAAAATCTAA TCTAATCGAT   
  
  
+ AGATGCGTTA AGGAAGCAAA ACTTTTGGGG AGAGGGAAGT TAAGCGAAGC AAGCTGCTGT TTCAAAGTTG   
  
  
+ GACTGTTTTA TTGGTAAGTA TGAAACAATA GTTTATAAGA GATCATTGGG TTCTTAAGGT GATAGATCAT   
  
  
+ GGGTTATGAA TGAGCTTAGT TGTTCTTTTG CTTTCAACTG TACGTAAGGA TGATAGTTAT CCCTCATCTT   
  
  
+ GCATCAAAAA CGGCAAACCG TGATGCCAAA ACTCAAAAAC TGATTCCTCT GACAAGGCTT CTACCTATGA   
  
  
+ AAACTATGCA GATCATATCC ATTTGGCCTT CCCTCTAGGA ACCAAGTAGA GTAAGTTGGT AAATAAATAG   
  
  
+ AGTTGAACGA AACACTACAG ATTTGAATTT GATTAAGGAG GGCTATTATA GACTAAGGTG TAGCTCAAAT   
  
  
+ CCTTCTGTAG GGATTGTCTT ATTGGCTTCT TTTGGTTGCT TCTGGCTCAA GTCCTCTTAT TAGTTAGTAT   
  
  
+ GCATTACCTT GAGACAAGAT AGTTGCTTAA GCTTGAGCTT CTGTACTTAT TTGCGTATTG TAGACAACTA   
  
  
+ GCTGTGGCAT ATGATTCTGA AATGTTCCGA TGTCTTTTTC TTTGGATGCT GGGTTGTGTC ATGAGCTTTA   
  
  
+ GGTTTGTTTA TTTGATGTCA GGAAGTATGT AAATAACATG TGGCATCTTG CAATCTATTA CAGGATGATA   
  
  
+ TTGGCCTTTG TGGGAAGAAA TTGAAGAAGT TGTTTCACTT ACTGGATAGG GAGCATGGAC TCACATCAGC   
  
  
+ TTTTGAGATT CGGTATTTCC AGATCATACT CATCCTATAA TTTCTCTCAG TCTAGTCCCC AATCAATTCC   
  
  
+ AAATAGGTTA TTTGAATCAC CGAACGTTCG TTCTAGAGAC TCTCCAATCT CGCCCTTCTC AACGCCCTTC   
  
  
+ AATTGCGACC CCACTGTTAT ATTGAGCGAC AGTCAGGAGC ACCACAGTTC AACAGGAAGT CTCTCGGCAC   
  
  
+ AAAGCTCATC TTCTAATTCT CCCCTTGAGA CTAGCAGTTA TTATAATCAG TTCAACTCAA GCCCTGTTGG   
  
  
+ AGAATCACCT CAGGGATCAT CACCCGAGAA TCTTTTTCAA CAAGCTGCAG TTAATAAAGT CAGCATCGAG   
  
  
+ CATGCATTGC AGGAGCTAGA AACTGCTCTA ATGAGCACAG ATTGTGAGGA GAATGAAGCA ACTGTCTCTA   
  
  
+ TCCCATCTAT GGGTGAACCT CATCAGCCCC AAGTCCCTAG CCAGAGATCA AGATTCTGGA ATCGAGATCC   
  
  
+ TCAGGGTTCA CGCCCGGCTG AAGTTCACTC ATCATTGAGA AGATTAGGAG ACGAGGCTCA GAGTGAGAAA   
  
  
+ CGCCTCAAGG CAGTGGAAGA ACCAATACGG CCCAGTGCAC CACCCGGCAA TTTGAAGCAG TTGCTCATAG   
  
  
+ AATGTGCTCG GGCTTTGTCA GAGAATCGAA TAGAGGATTT TGAGAAGTTA GTTGAACAGG CAAAGGGCAT   
  
  
+ GGTCTCCATT TCTGGAGACC CAATTCAACG ACTTGGTGCT TACTTGATCG AGGGGCTAGT GGCAAGGAAG   
  
  
+ GAGTCTTCAG GTACGAAAAT CTACCGAGCT CTTAGGTGCA AAGAGCCCCT TGGTCAAGAC TTGCTTTCGT   
  
  
+ ACATGCACAT CCTTTATGAA ATTTGCCCTT ACTTGAAATT CGGTTATATG GTTGCAAATG GGGCTATAGC   
  
  
+ TGAAGCTTGC AGAGATGAGG ATCACATACA TATCGTCGAC TACCAGATTG GTCAAGGAAC TCAATGGATG   
  
  
+ ACTCTGTTGC AAGCCCTAGC CACAAAACCT GGAAGACCCC CCACTGTGAG AATTACTGGC ATTGATGATC   
  
  
+ CCGTCTCTAG GCATGCTCGG GGAGCTTGCT TGGAGGCAGT GGGGAAACGT TTGGCAGTTC TGTCGGAGAA   
  
  
+ ATTTAACATG CCCATTGAGT TTCAGGCAGT GCCCGTTTAT GCTTCTGAGG TCACCCAAGA AATGCTTGAT   
  
  
+ GTGAGGCCAG GGTGGGCCTT GGCTGTGAAC TTCCCATTGC AGCTCCACCA CACTCCCGAT GAGAGTGTTG   
  
  
+ ATGTGAACAA CCCGAGGGAT GGACTCCTAA GAATGGTGAA ATCACTCGGT CCCAAGGTTG TAACTTTGGT   
  
  
+ AGAACAAGAG TCAAACACCA ACACTACCCC TTTGCTGACA AGGTTCATAG AAACGCTGGA CTTCTACTCA   
  
  
+ GCAATGTTTG AATCCATCGA TGTGACAATG CCAAGGGACC GAAAGGAAAG GATCAATGTC GAGCAGCATT   
  
  
+ GCCTAGCCAA GGACATTGTC AACATCATCG CGTGTGAGGG GAAGGACAGG GTGGAGCGAC ATGAGCTCTT   
  
  
+ TGGGAAATGG AAGTCAAGGT TCACCATGGC AGGATTCCAA CAATCCCCAT TGAGCTCGTA TGTTAACTCC   
  
  
+ GTGATAAAAG GATTACTTAG GTGTTATTCA GAGTACTATA CGTTGGTGGA GAAGGATGGC GCCTTGCTTC   
  
  
+ TGGGATGGAA GGACCGGATG CTGGTTTCGG CTTCAGCTTG GCATTG  

- +Up\_Stream \_Len000AAATTT ATAAAATAAA ATAAAATTTA ATTATATAAA ATATTAAATT TTAATAATTA   
  
  
- TTAACTACTA CAATGGGTTA CAAATTCAAA AATTTATAAA AATAAAAAAT TAAATTTTTA TAGTATTATA   
  
  
- TATTATATTA AAATTCAATT TTATTCTATC AATTTAATAA AATATCATTT ATAAATAATA AAAAAAATTC   
  
  
- TTTTAACGGT ACACTAACAG TAGTCAGTTT GTGACGCAAC TGTCTTCGAC AGGAACGACG ATGAGAGTGG   
  
  
- AGAGTTGTAG CCTGGGCGCG TCTGCAATTG TAGAGGCATG TTTATGTTAA GGGACCGGTT TTAAGTTTAG   
  
  
- TTGGGATGAG CCATCAGCCA AGGAAGAAGA AGGACGGGAG ATATATATGT GGGTGTCGAC GCGACGTACA   
  
  
- AAAAGGCTCT TAGGAGTTTT TAGAATATTT GAAGGAGCAC ACCCATCTTT TTTCTTTTTC TTTTCCCAAA   
  
  
- ATTTTAGAAC CCAGTTTTGG GTAGTTGTGG TGTTAAACTG AGAAAGAAAA ATTAAGTTGG TGGCGGTCAA   
  
  
- GGAGCAACTA AAAGTCCATG GAGAAAAAGA ACGGGGAAAA AAACGAGCCA ACAACAAATT ACCATTTCTA   
  
  
- ACTCATGTTT CTTACTATAA ACTACTCAAC CACACAAATA AACTACTCGG GTCTACTAAA TTGAACATCT   
  
  
- AAAACACCAA AACCAAAGAC CCATAAGTAA CCAAACGACT TAAAGACCAA GTACTAGACC CCAATCTTTT   
  
  
- CAACTTTACT GAAAGACCTC CAACCTTAAA CTGATTTCGA TTAAAGAAAA ACAGAAGTTA AGAAAAAATC   
  
  
- AACACTCGAC TTAAACAACT GGTGAAGGTC GGACAACCTT TAATTTGAAT GAATCCTCTT CTCTTAGAGT   
  
  
- CTAAGACTCG TTTTCGAAAT ATACCAACAA ACCCTTTACC ATCCTTGATT CACTTTTGTG ACTTTAAAAT   
  
  
- TCGTTCGTTA AAACAAGTAA ACTAACCAGC CTACCAGACT AAATCCAAGA AAAGAACCAG ATTTAAAGAC   
  
  
- TCGTAACTAA CAAATTAACG CACCCCTTGA ACATAGGAAC ACTCAAGGAC CAAGTAAAGA ACCAACGAAT   
  
  
- GAGATCGAGG GAAAGTGTCA AAGATACCAA CCTTGAACCT TGAACCTTGA ACCTTCGGAT TAAATCAACT   
  
  
- TATAGAACCG AAACTCAATC GAAAAGAATC CTTTTCTACC AAGACGAGAG CTTTTAGATT AGATTAGCTA   
  
  
- TCTACGCAAT TCCTTCGTTT TGAAAACCCC TCTCCCTTCA ATTCGCTTCG TTCGACGACA AAGTTTCAAC   
  
  
- CTGACAAAAT AACCATTCAT ACTTTGTTAT CAAATATTCT CTAGTAACCC AAGAATTCCA CTATCTAGTA   
  
  
- CCCAATACTT ACTCGAATCA ACAAGAAAAC GAAAGTTGAC ATGCATTCCT ACTATCAATA GGGAGTAGAA   
  
  
- CGTAGTTTTT GCCGTTTGGC ACTACGGTTT TGAGTTTTTG ACTAAGGAGA CTGTTCCGAA GATGGATACT   
  
  
- TTTGATACGT CTAGTATAGG TAAACCGGAA GGGAGATCCT TGGTTCATCT CATTCAACCA TTTATTTATC   
  
  
- TCAACTTGCT TTGTGATGTC TAAACTTAAA CTAATTCCTC CCGATAATAT CTGATTCCAC ATCGAGTTTA   
  
  
- GGAAGACATC CCTAACAGAA TAACCGAAGA AAACCAACGA AGACCGAGTT CAGGAGAATA ATCAATCATA   
  
  
- CGTAATGGAA CTCTGTTCTA TCAACGAATT CGAACTCGAA GACATGAATA AACGCATAAC ATCTGTTGAT   
  
  
- CGACACCGTA TACTAAGACT TTACAAGGCT ACAGAAAAAG AAACCTACGA CCCAACACAG TACTCGAAAT   
  
  
- CCAAACAAAT AAACTACAGT CCTTCATACA TTTATTGTAC ACCGTAGAAC GTTAGATAAT GTCCTACTAT   
  
  
- AACCGGAAAC ACCCTTCTTT AACTTCTTCA ACAAAGTGAA TGACCTATCC CTCGTACCTG AGTGTAGTCG   
  
  
- AAAACTCTAA GCCATAAAGG TCTAGTATGA GTAGGATATT AAAGAGAGTC AGATCAGGGG TTAGTTAAGG   
  
  
- TTTATCCAAT AAACTTAGTG GCTTGCAAGC AAGATCTCTG AGAGGTTAGA GCGGGAAGAG TTGCGGGAAG   
  
  
- TTAACGCTGG GGTGACAATA TAACTCGCTG TCAGTCCTCG TGGTGTCAAG TTGTCCTTCA GAGAGCCGTG   
  
  
- TTTCGAGTAG AAGATTAAGA GGGGAACTCT GATCGTCAAT AATATTAGTC AAGTTGAGTT CGGGACAACC   
  
  
- TCTTAGTGGA GTCCCTAGTA GTGGGCTCTT AGAAAAAGTT GTTCGACGTC AATTATTTCA GTCGTAGCTC   
  
  
- GTACGTAACG TCCTCGATCT TTGACGAGAT TACTCGTGTC TAACACTCCT CTTACTTCGT TGACAGAGAT   
  
  
- AGGGTAGATA CCCACTTGGA GTAGTCGGGG TTCAGGGATC GGTCTCTAGT TCTAAGACCT TAGCTCTAGG   
  
  
- AGTCCCAAGT GCGGGCCGAC TTCAAGTGAG TAGTAACTCT TCTAATCCTC TGCTCCGAGT CTCACTCTTT   
  
  
- GCGGAGTTCC GTCACCTTCT TGGTTATGCC GGGTCACGTG GTGGGCCGTT AAACTTCGTC AACGAGTATC   
  
  
- TTACACGAGC CCGAAACAGT CTCTTAGCTT ATCTCCTAAA ACTCTTCAAT CAACTTGTCC GTTTCCCGTA   
  
  
- CCAGAGGTAA AGACCTCTGG GTTAAGTTGC TGAACCACGA ATGAACTAGC TCCCCGATCA CCGTTCCTTC   
  
  
- CTCAGAAGTC CATGCTTTTA GATGGCTCGA GAATCCACGT TTCTCGGGGA ACCAGTTCTG AACGAAAGCA   
  
  
- TGTACGTGTA GGAAATACTT TAAACGGGAA TGAACTTTAA GCCAATATAC CAACGTTTAC CCCGATATCG   
  
  
- ACTTCGAACG TCTCTACTCC TAGTGTATGT ATAGCAGCTG ATGGTCTAAC CAGTTCCTTG AGTTACCTAC   
  
  
- TGAGACAACG TTCGGGATCG GTGTTTTGGA CCTTCTGGGG GGTGACACTC TTAATGACCG TAACTACTAG   
  
  
- GGCAGAGATC CGTACGAGCC CCTCGAACGA ACCTCCGTCA CCCCTTTGCA AACCGTCAAG ACAGCCTCTT   
  
  
- TAAATTGTAC GGGTAACTCA AAGTCCGTCA CGGGCAAATA CGAAGACTCC AGTGGGTTCT TTACGAACTA   
  
  
- CACTCCGGTC CCACCCGGAA CCGACACTTG AAGGGTAACG TCGAGGTGGT GTGAGGGCTA CTCTCACAAC   
  
  
- TACACTTGTT GGGCTCCCTA CCTGAGGATT CTTACCACTT TAGTGAGCCA GGGTTCCAAC ATTGAAACCA   
  
  
- TCTTGTTCTC AGTTTGTGGT TGTGATGGGG AAACGACTGT TCCAAGTATC TTTGCGACCT GAAGATGAGT   
  
  
- CGTTACAAAC TTAGGTAGCT ACACTGTTAC GGTTCCCTGG CTTTCCTTTC CTAGTTACAG CTCGTCGTAA   
  
  
- CGGATCGGTT CCTGTAACAG TTGTAGTAGC GCACACTCCC CTTCCTGTCC CACCTCGCTG TACTCGAGAA   
  
  
- ACCCTTTACC TTCAGTTCCA AGTGGTACCG TCCTAAGGTT GTTAGGGGTA ACTCGAGCAT ACAATTGAGG   
  
  
- CACTATTTTC CTAATGAATC CACAATAAGT CTCATGATAT GCAACCACCT CTTCCTACCG CGGAACGAAG   
  
  
- ACCCTACCTT CCTGGCCTAC GACCAAAGCC GAAGTCGAAC CGTAAC

+     GARE-motif

| Site Name | Organism | Position | Strand | Matrix score. | sequence | function |
| --- | --- | --- | --- | --- | --- | --- |
| GARE-motif | Brassica oleracea | 3017 | + | 7 | TCTGTTG | gibberellin-responsive element |

>HU01G01850.1   
+ +Up\_Stream \_Len000TTTAAA TATTTTATTT TATTTTAAAT TAATATATTT TATAATTTAA AATTATTAAT   
  
  
+ AATTGATGAT GTTACCCAAT GTTTAAGTTT TTAAATATTT TTATTTTTTA ATTTAAAAAT ATCATAATAT   
  
  
+ ATAATATAAT TTTAAGTTAA AATAAGATAG TTAAATTATT TTATAGTAAA TATTTATTAT TTTTTTTAAG   
  
  
+ AAAATTGCCA TGTGATTGTC ATCAGTCAAA CACTGCGTTG ACAGAAGCTG TCCTTGCTGC TACTCTCACC   
  
  
+ TCTCAACATC GGACCCGCGC AGACGTTAAC ATCTCCGTAC AAATACAATT CCCTGGCCAA AATTCAAATC   
  
  
+ AACCCTACTC GGTAGTCGGT TCCTTCTTCT TCCTGCCCTC TATATATACA CCCACAGCTG CGCTGCATGT   
  
  
+ TTTTCCGAGA ATCCTCAAAA ATCTTATAAA CTTCCTCGTG TGGGTAGAAA AAAGAAAAAG AAAAGGGTTT   
  
  
+ TAAAATCTTG GGTCAAAACC CATCAACACC ACAATTTGAC TCTTTCTTTT TAATTCAACC ACCGCCAGTT   
  
  
+ CCTCGTTGAT TTTCAGGTAC CTCTTTTTCT TGCCCCTTTT TTTGCTCGGT TGTTGTTTAA TGGTAAAGAT   
  
  
+ TGAGTACAAA GAATGATATT TGATGAGTTG GTGTGTTTAT TTGATGAGCC CAGATGATTT AACTTGTAGA   
  
  
+ TTTTGTGGTT TTGGTTTCTG GGTATTCATT GGTTTGCTGA ATTTCTGGTT CATGATCTGG GGTTAGAAAA   
  
  
+ GTTGAAATGA CTTTCTGGAG GTTGGAATTT GACTAAAGCT AATTTCTTTT TGTCTTCAAT TCTTTTTTAG   
  
  
+ TTGTGAGCTG AATTTGTTGA CCACTTCCAG CCTGTTGGAA ATTAAACTTA CTTAGGAGAA GAGAATCTCA   
  
  
+ GATTCTGAGC AAAAGCTTTA TATGGTTGTT TGGGAAATGG TAGGAACTAA GTGAAAACAC TGAAATTTTA   
  
  
+ AGCAAGCAAT TTTGTTCATT TGATTGGTCG GATGGTCTGA TTTAGGTTCT TTTCTTGGTC TAAATTTCTG   
  
  
+ AGCATTGATT GTTTAATTGC GTGGGGAACT TGTATCCTTG TGAGTTCCTG GTTCATTTCT TGGTTGCTTA   
  
  
+ CTCTAGCTCC CTTTCACAGT TTCTATGGTT GGAACTTGGA ACTTGGAACT TGGAAGCCTA ATTTAGTTGA   
  
  
+ ATATCTTGGC TTTGAGTTAG CTTTTCTTAG GAAAAGATGG TTCTGCTCTC GAAAATCTAA TCTAATCGAT   
  
  
+ AGATGCGTTA AGGAAGCAAA ACTTTTGGGG AGAGGGAAGT TAAGCGAAGC AAGCTGCTGT TTCAAAGTTG   
  
  
+ GACTGTTTTA TTGGTAAGTA TGAAACAATA GTTTATAAGA GATCATTGGG TTCTTAAGGT GATAGATCAT   
  
  
+ GGGTTATGAA TGAGCTTAGT TGTTCTTTTG CTTTCAACTG TACGTAAGGA TGATAGTTAT CCCTCATCTT   
  
  
+ GCATCAAAAA CGGCAAACCG TGATGCCAAA ACTCAAAAAC TGATTCCTCT GACAAGGCTT CTACCTATGA   
  
  
+ AAACTATGCA GATCATATCC ATTTGGCCTT CCCTCTAGGA ACCAAGTAGA GTAAGTTGGT AAATAAATAG   
  
  
+ AGTTGAACGA AACACTACAG ATTTGAATTT GATTAAGGAG GGCTATTATA GACTAAGGTG TAGCTCAAAT   
  
  
+ CCTTCTGTAG GGATTGTCTT ATTGGCTTCT TTTGGTTGCT TCTGGCTCAA GTCCTCTTAT TAGTTAGTAT   
  
  
+ GCATTACCTT GAGACAAGAT AGTTGCTTAA GCTTGAGCTT CTGTACTTAT TTGCGTATTG TAGACAACTA   
  
  
+ GCTGTGGCAT ATGATTCTGA AATGTTCCGA TGTCTTTTTC TTTGGATGCT GGGTTGTGTC ATGAGCTTTA   
  
  
+ GGTTTGTTTA TTTGATGTCA GGAAGTATGT AAATAACATG TGGCATCTTG CAATCTATTA CAGGATGATA   
  
  
+ TTGGCCTTTG TGGGAAGAAA TTGAAGAAGT TGTTTCACTT ACTGGATAGG GAGCATGGAC TCACATCAGC   
  
  
+ TTTTGAGATT CGGTATTTCC AGATCATACT CATCCTATAA TTTCTCTCAG TCTAGTCCCC AATCAATTCC   
  
  
+ AAATAGGTTA TTTGAATCAC CGAACGTTCG TTCTAGAGAC TCTCCAATCT CGCCCTTCTC AACGCCCTTC   
  
  
+ AATTGCGACC CCACTGTTAT ATTGAGCGAC AGTCAGGAGC ACCACAGTTC AACAGGAAGT CTCTCGGCAC   
  
  
+ AAAGCTCATC TTCTAATTCT CCCCTTGAGA CTAGCAGTTA TTATAATCAG TTCAACTCAA GCCCTGTTGG   
  
  
+ AGAATCACCT CAGGGATCAT CACCCGAGAA TCTTTTTCAA CAAGCTGCAG TTAATAAAGT CAGCATCGAG   
  
  
+ CATGCATTGC AGGAGCTAGA AACTGCTCTA ATGAGCACAG ATTGTGAGGA GAATGAAGCA ACTGTCTCTA   
  
  
+ TCCCATCTAT GGGTGAACCT CATCAGCCCC AAGTCCCTAG CCAGAGATCA AGATTCTGGA ATCGAGATCC   
  
  
+ TCAGGGTTCA CGCCCGGCTG AAGTTCACTC ATCATTGAGA AGATTAGGAG ACGAGGCTCA GAGTGAGAAA   
  
  
+ CGCCTCAAGG CAGTGGAAGA ACCAATACGG CCCAGTGCAC CACCCGGCAA TTTGAAGCAG TTGCTCATAG   
  
  
+ AATGTGCTCG GGCTTTGTCA GAGAATCGAA TAGAGGATTT TGAGAAGTTA GTTGAACAGG CAAAGGGCAT   
  
  
+ GGTCTCCATT TCTGGAGACC CAATTCAACG ACTTGGTGCT TACTTGATCG AGGGGCTAGT GGCAAGGAAG   
  
  
+ GAGTCTTCAG GTACGAAAAT CTACCGAGCT CTTAGGTGCA AAGAGCCCCT TGGTCAAGAC TTGCTTTCGT   
  
  
+ ACATGCACAT CCTTTATGAA ATTTGCCCTT ACTTGAAATT CGGTTATATG GTTGCAAATG GGGCTATAGC   
  
  
+ TGAAGCTTGC AGAGATGAGG ATCACATACA TATCGTCGAC TACCAGATTG GTCAAGGAAC TCAATGGATG   
  
  
+ ACTCTGTTGC AAGCCCTAGC CACAAAACCT GGAAGACCCC CCACTGTGAG AATTACTGGC ATTGATGATC   
  
  
+ CCGTCTCTAG GCATGCTCGG GGAGCTTGCT TGGAGGCAGT GGGGAAACGT TTGGCAGTTC TGTCGGAGAA   
  
  
+ ATTTAACATG CCCATTGAGT TTCAGGCAGT GCCCGTTTAT GCTTCTGAGG TCACCCAAGA AATGCTTGAT   
  
  
+ GTGAGGCCAG GGTGGGCCTT GGCTGTGAAC TTCCCATTGC AGCTCCACCA CACTCCCGAT GAGAGTGTTG   
  
  
+ ATGTGAACAA CCCGAGGGAT GGACTCCTAA GAATGGTGAA ATCACTCGGT CCCAAGGTTG TAACTTTGGT   
  
  
+ AGAACAAGAG TCAAACACCA ACACTACCCC TTTGCTGACA AGGTTCATAG AAACGCTGGA CTTCTACTCA   
  
  
+ GCAATGTTTG AATCCATCGA TGTGACAATG CCAAGGGACC GAAAGGAAAG GATCAATGTC GAGCAGCATT   
  
  
+ GCCTAGCCAA GGACATTGTC AACATCATCG CGTGTGAGGG GAAGGACAGG GTGGAGCGAC ATGAGCTCTT   
  
  
+ TGGGAAATGG AAGTCAAGGT TCACCATGGC AGGATTCCAA CAATCCCCAT TGAGCTCGTA TGTTAACTCC   
  
  
+ GTGATAAAAG GATTACTTAG GTGTTATTCA GAGTACTATA CGTTGGTGGA GAAGGATGGC GCCTTGCTTC   
  
  
+ TGGGATGGAA GGACCGGATG CTGGTTTCGG CTTCAGCTTG GCATTG  

- +Up\_Stream \_Len000AAATTT ATAAAATAAA ATAAAATTTA ATTATATAAA ATATTAAATT TTAATAATTA   
  
  
- TTAACTACTA CAATGGGTTA CAAATTCAAA AATTTATAAA AATAAAAAAT TAAATTTTTA TAGTATTATA   
  
  
- TATTATATTA AAATTCAATT TTATTCTATC AATTTAATAA AATATCATTT ATAAATAATA AAAAAAATTC   
  
  
- TTTTAACGGT ACACTAACAG TAGTCAGTTT GTGACGCAAC TGTCTTCGAC AGGAACGACG ATGAGAGTGG   
  
  
- AGAGTTGTAG CCTGGGCGCG TCTGCAATTG TAGAGGCATG TTTATGTTAA GGGACCGGTT TTAAGTTTAG   
  
  
- TTGGGATGAG CCATCAGCCA AGGAAGAAGA AGGACGGGAG ATATATATGT GGGTGTCGAC GCGACGTACA   
  
  
- AAAAGGCTCT TAGGAGTTTT TAGAATATTT GAAGGAGCAC ACCCATCTTT TTTCTTTTTC TTTTCCCAAA   
  
  
- ATTTTAGAAC CCAGTTTTGG GTAGTTGTGG TGTTAAACTG AGAAAGAAAA ATTAAGTTGG TGGCGGTCAA   
  
  
- GGAGCAACTA AAAGTCCATG GAGAAAAAGA ACGGGGAAAA AAACGAGCCA ACAACAAATT ACCATTTCTA   
  
  
- ACTCATGTTT CTTACTATAA ACTACTCAAC CACACAAATA AACTACTCGG GTCTACTAAA TTGAACATCT   
  
  
- AAAACACCAA AACCAAAGAC CCATAAGTAA CCAAACGACT TAAAGACCAA GTACTAGACC CCAATCTTTT   
  
  
- CAACTTTACT GAAAGACCTC CAACCTTAAA CTGATTTCGA TTAAAGAAAA ACAGAAGTTA AGAAAAAATC   
  
  
- AACACTCGAC TTAAACAACT GGTGAAGGTC GGACAACCTT TAATTTGAAT GAATCCTCTT CTCTTAGAGT   
  
  
- CTAAGACTCG TTTTCGAAAT ATACCAACAA ACCCTTTACC ATCCTTGATT CACTTTTGTG ACTTTAAAAT   
  
  
- TCGTTCGTTA AAACAAGTAA ACTAACCAGC CTACCAGACT AAATCCAAGA AAAGAACCAG ATTTAAAGAC   
  
  
- TCGTAACTAA CAAATTAACG CACCCCTTGA ACATAGGAAC ACTCAAGGAC CAAGTAAAGA ACCAACGAAT   
  
  
- GAGATCGAGG GAAAGTGTCA AAGATACCAA CCTTGAACCT TGAACCTTGA ACCTTCGGAT TAAATCAACT   
  
  
- TATAGAACCG AAACTCAATC GAAAAGAATC CTTTTCTACC AAGACGAGAG CTTTTAGATT AGATTAGCTA   
  
  
- TCTACGCAAT TCCTTCGTTT TGAAAACCCC TCTCCCTTCA ATTCGCTTCG TTCGACGACA AAGTTTCAAC   
  
  
- CTGACAAAAT AACCATTCAT ACTTTGTTAT CAAATATTCT CTAGTAACCC AAGAATTCCA CTATCTAGTA   
  
  
- CCCAATACTT ACTCGAATCA ACAAGAAAAC GAAAGTTGAC ATGCATTCCT ACTATCAATA GGGAGTAGAA   
  
  
- CGTAGTTTTT GCCGTTTGGC ACTACGGTTT TGAGTTTTTG ACTAAGGAGA CTGTTCCGAA GATGGATACT   
  
  
- TTTGATACGT CTAGTATAGG TAAACCGGAA GGGAGATCCT TGGTTCATCT CATTCAACCA TTTATTTATC   
  
  
- TCAACTTGCT TTGTGATGTC TAAACTTAAA CTAATTCCTC CCGATAATAT CTGATTCCAC ATCGAGTTTA   
  
  
- GGAAGACATC CCTAACAGAA TAACCGAAGA AAACCAACGA AGACCGAGTT CAGGAGAATA ATCAATCATA   
  
  
- CGTAATGGAA CTCTGTTCTA TCAACGAATT CGAACTCGAA GACATGAATA AACGCATAAC ATCTGTTGAT   
  
  
- CGACACCGTA TACTAAGACT TTACAAGGCT ACAGAAAAAG AAACCTACGA CCCAACACAG TACTCGAAAT   
  
  
- CCAAACAAAT AAACTACAGT CCTTCATACA TTTATTGTAC ACCGTAGAAC GTTAGATAAT GTCCTACTAT   
  
  
- AACCGGAAAC ACCCTTCTTT AACTTCTTCA ACAAAGTGAA TGACCTATCC CTCGTACCTG AGTGTAGTCG   
  
  
- AAAACTCTAA GCCATAAAGG TCTAGTATGA GTAGGATATT AAAGAGAGTC AGATCAGGGG TTAGTTAAGG   
  
  
- TTTATCCAAT AAACTTAGTG GCTTGCAAGC AAGATCTCTG AGAGGTTAGA GCGGGAAGAG TTGCGGGAAG   
  
  
- TTAACGCTGG GGTGACAATA TAACTCGCTG TCAGTCCTCG TGGTGTCAAG TTGTCCTTCA GAGAGCCGTG   
  
  
- TTTCGAGTAG AAGATTAAGA GGGGAACTCT GATCGTCAAT AATATTAGTC AAGTTGAGTT CGGGACAACC   
  
  
- TCTTAGTGGA GTCCCTAGTA GTGGGCTCTT AGAAAAAGTT GTTCGACGTC AATTATTTCA GTCGTAGCTC   
  
  
- GTACGTAACG TCCTCGATCT TTGACGAGAT TACTCGTGTC TAACACTCCT CTTACTTCGT TGACAGAGAT   
  
  
- AGGGTAGATA CCCACTTGGA GTAGTCGGGG TTCAGGGATC GGTCTCTAGT TCTAAGACCT TAGCTCTAGG   
  
  
- AGTCCCAAGT GCGGGCCGAC TTCAAGTGAG TAGTAACTCT TCTAATCCTC TGCTCCGAGT CTCACTCTTT   
  
  
- GCGGAGTTCC GTCACCTTCT TGGTTATGCC GGGTCACGTG GTGGGCCGTT AAACTTCGTC AACGAGTATC   
  
  
- TTACACGAGC CCGAAACAGT CTCTTAGCTT ATCTCCTAAA ACTCTTCAAT CAACTTGTCC GTTTCCCGTA   
  
  
- CCAGAGGTAA AGACCTCTGG GTTAAGTTGC TGAACCACGA ATGAACTAGC TCCCCGATCA CCGTTCCTTC   
  
  
- CTCAGAAGTC CATGCTTTTA GATGGCTCGA GAATCCACGT TTCTCGGGGA ACCAGTTCTG AACGAAAGCA   
  
  
- TGTACGTGTA GGAAATACTT TAAACGGGAA TGAACTTTAA GCCAATATAC CAACGTTTAC CCCGATATCG   
  
  
- ACTTCGAACG TCTCTACTCC TAGTGTATGT ATAGCAGCTG ATGGTCTAAC CAGTTCCTTG AGTTACCTAC   
  
  
- TGAGACAACG TTCGGGATCG GTGTTTTGGA CCTTCTGGGG GGTGACACTC TTAATGACCG TAACTACTAG   
  
  
- GGCAGAGATC CGTACGAGCC CCTCGAACGA ACCTCCGTCA CCCCTTTGCA AACCGTCAAG ACAGCCTCTT   
  
  
- TAAATTGTAC GGGTAACTCA AAGTCCGTCA CGGGCAAATA CGAAGACTCC AGTGGGTTCT TTACGAACTA   
  
  
- CACTCCGGTC CCACCCGGAA CCGACACTTG AAGGGTAACG TCGAGGTGGT GTGAGGGCTA CTCTCACAAC   
  
  
- TACACTTGTT GGGCTCCCTA CCTGAGGATT CTTACCACTT TAGTGAGCCA GGGTTCCAAC ATTGAAACCA   
  
  
- TCTTGTTCTC AGTTTGTGGT TGTGATGGGG AAACGACTGT TCCAAGTATC TTTGCGACCT GAAGATGAGT   
  
  
- CGTTACAAAC TTAGGTAGCT ACACTGTTAC GGTTCCCTGG CTTTCCTTTC CTAGTTACAG CTCGTCGTAA   
  
  
- CGGATCGGTT CCTGTAACAG TTGTAGTAGC GCACACTCCC CTTCCTGTCC CACCTCGCTG TACTCGAGAA   
  
  
- ACCCTTTACC TTCAGTTCCA AGTGGTACCG TCCTAAGGTT GTTAGGGGTA ACTCGAGCAT ACAATTGAGG   
  
  
- CACTATTTTC CTAATGAATC CACAATAAGT CTCATGATAT GCAACCACCT CTTCCTACCG CGGAACGAAG   
  
  
- ACCCTACCTT CCTGGCCTAC GACCAAAGCC GAAGTCGAAC CGTAAC

+     GATA-motif

| Site Name | Organism | Position | Strand | Matrix score. | sequence | function |
| --- | --- | --- | --- | --- | --- | --- |
| GATA-motif | Pisum sativum | 2009 | + | 7 | GATAGGG | part of a light responsive element |

>HU01G01850.1   
+ +Up\_Stream \_Len000TTTAAA TATTTTATTT TATTTTAAAT TAATATATTT TATAATTTAA AATTATTAAT   
  
  
+ AATTGATGAT GTTACCCAAT GTTTAAGTTT TTAAATATTT TTATTTTTTA ATTTAAAAAT ATCATAATAT   
  
  
+ ATAATATAAT TTTAAGTTAA AATAAGATAG TTAAATTATT TTATAGTAAA TATTTATTAT TTTTTTTAAG   
  
  
+ AAAATTGCCA TGTGATTGTC ATCAGTCAAA CACTGCGTTG ACAGAAGCTG TCCTTGCTGC TACTCTCACC   
  
  
+ TCTCAACATC GGACCCGCGC AGACGTTAAC ATCTCCGTAC AAATACAATT CCCTGGCCAA AATTCAAATC   
  
  
+ AACCCTACTC GGTAGTCGGT TCCTTCTTCT TCCTGCCCTC TATATATACA CCCACAGCTG CGCTGCATGT   
  
  
+ TTTTCCGAGA ATCCTCAAAA ATCTTATAAA CTTCCTCGTG TGGGTAGAAA AAAGAAAAAG AAAAGGGTTT   
  
  
+ TAAAATCTTG GGTCAAAACC CATCAACACC ACAATTTGAC TCTTTCTTTT TAATTCAACC ACCGCCAGTT   
  
  
+ CCTCGTTGAT TTTCAGGTAC CTCTTTTTCT TGCCCCTTTT TTTGCTCGGT TGTTGTTTAA TGGTAAAGAT   
  
  
+ TGAGTACAAA GAATGATATT TGATGAGTTG GTGTGTTTAT TTGATGAGCC CAGATGATTT AACTTGTAGA   
  
  
+ TTTTGTGGTT TTGGTTTCTG GGTATTCATT GGTTTGCTGA ATTTCTGGTT CATGATCTGG GGTTAGAAAA   
  
  
+ GTTGAAATGA CTTTCTGGAG GTTGGAATTT GACTAAAGCT AATTTCTTTT TGTCTTCAAT TCTTTTTTAG   
  
  
+ TTGTGAGCTG AATTTGTTGA CCACTTCCAG CCTGTTGGAA ATTAAACTTA CTTAGGAGAA GAGAATCTCA   
  
  
+ GATTCTGAGC AAAAGCTTTA TATGGTTGTT TGGGAAATGG TAGGAACTAA GTGAAAACAC TGAAATTTTA   
  
  
+ AGCAAGCAAT TTTGTTCATT TGATTGGTCG GATGGTCTGA TTTAGGTTCT TTTCTTGGTC TAAATTTCTG   
  
  
+ AGCATTGATT GTTTAATTGC GTGGGGAACT TGTATCCTTG TGAGTTCCTG GTTCATTTCT TGGTTGCTTA   
  
  
+ CTCTAGCTCC CTTTCACAGT TTCTATGGTT GGAACTTGGA ACTTGGAACT TGGAAGCCTA ATTTAGTTGA   
  
  
+ ATATCTTGGC TTTGAGTTAG CTTTTCTTAG GAAAAGATGG TTCTGCTCTC GAAAATCTAA TCTAATCGAT   
  
  
+ AGATGCGTTA AGGAAGCAAA ACTTTTGGGG AGAGGGAAGT TAAGCGAAGC AAGCTGCTGT TTCAAAGTTG   
  
  
+ GACTGTTTTA TTGGTAAGTA TGAAACAATA GTTTATAAGA GATCATTGGG TTCTTAAGGT GATAGATCAT   
  
  
+ GGGTTATGAA TGAGCTTAGT TGTTCTTTTG CTTTCAACTG TACGTAAGGA TGATAGTTAT CCCTCATCTT   
  
  
+ GCATCAAAAA CGGCAAACCG TGATGCCAAA ACTCAAAAAC TGATTCCTCT GACAAGGCTT CTACCTATGA   
  
  
+ AAACTATGCA GATCATATCC ATTTGGCCTT CCCTCTAGGA ACCAAGTAGA GTAAGTTGGT AAATAAATAG   
  
  
+ AGTTGAACGA AACACTACAG ATTTGAATTT GATTAAGGAG GGCTATTATA GACTAAGGTG TAGCTCAAAT   
  
  
+ CCTTCTGTAG GGATTGTCTT ATTGGCTTCT TTTGGTTGCT TCTGGCTCAA GTCCTCTTAT TAGTTAGTAT   
  
  
+ GCATTACCTT GAGACAAGAT AGTTGCTTAA GCTTGAGCTT CTGTACTTAT TTGCGTATTG TAGACAACTA   
  
  
+ GCTGTGGCAT ATGATTCTGA AATGTTCCGA TGTCTTTTTC TTTGGATGCT GGGTTGTGTC ATGAGCTTTA   
  
  
+ GGTTTGTTTA TTTGATGTCA GGAAGTATGT AAATAACATG TGGCATCTTG CAATCTATTA CAGGATGATA   
  
  
+ TTGGCCTTTG TGGGAAGAAA TTGAAGAAGT TGTTTCACTT ACTGGATAGG GAGCATGGAC TCACATCAGC   
  
  
+ TTTTGAGATT CGGTATTTCC AGATCATACT CATCCTATAA TTTCTCTCAG TCTAGTCCCC AATCAATTCC   
  
  
+ AAATAGGTTA TTTGAATCAC CGAACGTTCG TTCTAGAGAC TCTCCAATCT CGCCCTTCTC AACGCCCTTC   
  
  
+ AATTGCGACC CCACTGTTAT ATTGAGCGAC AGTCAGGAGC ACCACAGTTC AACAGGAAGT CTCTCGGCAC   
  
  
+ AAAGCTCATC TTCTAATTCT CCCCTTGAGA CTAGCAGTTA TTATAATCAG TTCAACTCAA GCCCTGTTGG   
  
  
+ AGAATCACCT CAGGGATCAT CACCCGAGAA TCTTTTTCAA CAAGCTGCAG TTAATAAAGT CAGCATCGAG   
  
  
+ CATGCATTGC AGGAGCTAGA AACTGCTCTA ATGAGCACAG ATTGTGAGGA GAATGAAGCA ACTGTCTCTA   
  
  
+ TCCCATCTAT GGGTGAACCT CATCAGCCCC AAGTCCCTAG CCAGAGATCA AGATTCTGGA ATCGAGATCC   
  
  
+ TCAGGGTTCA CGCCCGGCTG AAGTTCACTC ATCATTGAGA AGATTAGGAG ACGAGGCTCA GAGTGAGAAA   
  
  
+ CGCCTCAAGG CAGTGGAAGA ACCAATACGG CCCAGTGCAC CACCCGGCAA TTTGAAGCAG TTGCTCATAG   
  
  
+ AATGTGCTCG GGCTTTGTCA GAGAATCGAA TAGAGGATTT TGAGAAGTTA GTTGAACAGG CAAAGGGCAT   
  
  
+ GGTCTCCATT TCTGGAGACC CAATTCAACG ACTTGGTGCT TACTTGATCG AGGGGCTAGT GGCAAGGAAG   
  
  
+ GAGTCTTCAG GTACGAAAAT CTACCGAGCT CTTAGGTGCA AAGAGCCCCT TGGTCAAGAC TTGCTTTCGT   
  
  
+ ACATGCACAT CCTTTATGAA ATTTGCCCTT ACTTGAAATT CGGTTATATG GTTGCAAATG GGGCTATAGC   
  
  
+ TGAAGCTTGC AGAGATGAGG ATCACATACA TATCGTCGAC TACCAGATTG GTCAAGGAAC TCAATGGATG   
  
  
+ ACTCTGTTGC AAGCCCTAGC CACAAAACCT GGAAGACCCC CCACTGTGAG AATTACTGGC ATTGATGATC   
  
  
+ CCGTCTCTAG GCATGCTCGG GGAGCTTGCT TGGAGGCAGT GGGGAAACGT TTGGCAGTTC TGTCGGAGAA   
  
  
+ ATTTAACATG CCCATTGAGT TTCAGGCAGT GCCCGTTTAT GCTTCTGAGG TCACCCAAGA AATGCTTGAT   
  
  
+ GTGAGGCCAG GGTGGGCCTT GGCTGTGAAC TTCCCATTGC AGCTCCACCA CACTCCCGAT GAGAGTGTTG   
  
  
+ ATGTGAACAA CCCGAGGGAT GGACTCCTAA GAATGGTGAA ATCACTCGGT CCCAAGGTTG TAACTTTGGT   
  
  
+ AGAACAAGAG TCAAACACCA ACACTACCCC TTTGCTGACA AGGTTCATAG AAACGCTGGA CTTCTACTCA   
  
  
+ GCAATGTTTG AATCCATCGA TGTGACAATG CCAAGGGACC GAAAGGAAAG GATCAATGTC GAGCAGCATT   
  
  
+ GCCTAGCCAA GGACATTGTC AACATCATCG CGTGTGAGGG GAAGGACAGG GTGGAGCGAC ATGAGCTCTT   
  
  
+ TGGGAAATGG AAGTCAAGGT TCACCATGGC AGGATTCCAA CAATCCCCAT TGAGCTCGTA TGTTAACTCC   
  
  
+ GTGATAAAAG GATTACTTAG GTGTTATTCA GAGTACTATA CGTTGGTGGA GAAGGATGGC GCCTTGCTTC   
  
  
+ TGGGATGGAA GGACCGGATG CTGGTTTCGG CTTCAGCTTG GCATTG  

- +Up\_Stream \_Len000AAATTT ATAAAATAAA ATAAAATTTA ATTATATAAA ATATTAAATT TTAATAATTA   
  
  
- TTAACTACTA CAATGGGTTA CAAATTCAAA AATTTATAAA AATAAAAAAT TAAATTTTTA TAGTATTATA   
  
  
- TATTATATTA AAATTCAATT TTATTCTATC AATTTAATAA AATATCATTT ATAAATAATA AAAAAAATTC   
  
  
- TTTTAACGGT ACACTAACAG TAGTCAGTTT GTGACGCAAC TGTCTTCGAC AGGAACGACG ATGAGAGTGG   
  
  
- AGAGTTGTAG CCTGGGCGCG TCTGCAATTG TAGAGGCATG TTTATGTTAA GGGACCGGTT TTAAGTTTAG   
  
  
- TTGGGATGAG CCATCAGCCA AGGAAGAAGA AGGACGGGAG ATATATATGT GGGTGTCGAC GCGACGTACA   
  
  
- AAAAGGCTCT TAGGAGTTTT TAGAATATTT GAAGGAGCAC ACCCATCTTT TTTCTTTTTC TTTTCCCAAA   
  
  
- ATTTTAGAAC CCAGTTTTGG GTAGTTGTGG TGTTAAACTG AGAAAGAAAA ATTAAGTTGG TGGCGGTCAA   
  
  
- GGAGCAACTA AAAGTCCATG GAGAAAAAGA ACGGGGAAAA AAACGAGCCA ACAACAAATT ACCATTTCTA   
  
  
- ACTCATGTTT CTTACTATAA ACTACTCAAC CACACAAATA AACTACTCGG GTCTACTAAA TTGAACATCT   
  
  
- AAAACACCAA AACCAAAGAC CCATAAGTAA CCAAACGACT TAAAGACCAA GTACTAGACC CCAATCTTTT   
  
  
- CAACTTTACT GAAAGACCTC CAACCTTAAA CTGATTTCGA TTAAAGAAAA ACAGAAGTTA AGAAAAAATC   
  
  
- AACACTCGAC TTAAACAACT GGTGAAGGTC GGACAACCTT TAATTTGAAT GAATCCTCTT CTCTTAGAGT   
  
  
- CTAAGACTCG TTTTCGAAAT ATACCAACAA ACCCTTTACC ATCCTTGATT CACTTTTGTG ACTTTAAAAT   
  
  
- TCGTTCGTTA AAACAAGTAA ACTAACCAGC CTACCAGACT AAATCCAAGA AAAGAACCAG ATTTAAAGAC   
  
  
- TCGTAACTAA CAAATTAACG CACCCCTTGA ACATAGGAAC ACTCAAGGAC CAAGTAAAGA ACCAACGAAT   
  
  
- GAGATCGAGG GAAAGTGTCA AAGATACCAA CCTTGAACCT TGAACCTTGA ACCTTCGGAT TAAATCAACT   
  
  
- TATAGAACCG AAACTCAATC GAAAAGAATC CTTTTCTACC AAGACGAGAG CTTTTAGATT AGATTAGCTA   
  
  
- TCTACGCAAT TCCTTCGTTT TGAAAACCCC TCTCCCTTCA ATTCGCTTCG TTCGACGACA AAGTTTCAAC   
  
  
- CTGACAAAAT AACCATTCAT ACTTTGTTAT CAAATATTCT CTAGTAACCC AAGAATTCCA CTATCTAGTA   
  
  
- CCCAATACTT ACTCGAATCA ACAAGAAAAC GAAAGTTGAC ATGCATTCCT ACTATCAATA GGGAGTAGAA   
  
  
- CGTAGTTTTT GCCGTTTGGC ACTACGGTTT TGAGTTTTTG ACTAAGGAGA CTGTTCCGAA GATGGATACT   
  
  
- TTTGATACGT CTAGTATAGG TAAACCGGAA GGGAGATCCT TGGTTCATCT CATTCAACCA TTTATTTATC   
  
  
- TCAACTTGCT TTGTGATGTC TAAACTTAAA CTAATTCCTC CCGATAATAT CTGATTCCAC ATCGAGTTTA   
  
  
- GGAAGACATC CCTAACAGAA TAACCGAAGA AAACCAACGA AGACCGAGTT CAGGAGAATA ATCAATCATA   
  
  
- CGTAATGGAA CTCTGTTCTA TCAACGAATT CGAACTCGAA GACATGAATA AACGCATAAC ATCTGTTGAT   
  
  
- CGACACCGTA TACTAAGACT TTACAAGGCT ACAGAAAAAG AAACCTACGA CCCAACACAG TACTCGAAAT   
  
  
- CCAAACAAAT AAACTACAGT CCTTCATACA TTTATTGTAC ACCGTAGAAC GTTAGATAAT GTCCTACTAT   
  
  
- AACCGGAAAC ACCCTTCTTT AACTTCTTCA ACAAAGTGAA TGACCTATCC CTCGTACCTG AGTGTAGTCG   
  
  
- AAAACTCTAA GCCATAAAGG TCTAGTATGA GTAGGATATT AAAGAGAGTC AGATCAGGGG TTAGTTAAGG   
  
  
- TTTATCCAAT AAACTTAGTG GCTTGCAAGC AAGATCTCTG AGAGGTTAGA GCGGGAAGAG TTGCGGGAAG   
  
  
- TTAACGCTGG GGTGACAATA TAACTCGCTG TCAGTCCTCG TGGTGTCAAG TTGTCCTTCA GAGAGCCGTG   
  
  
- TTTCGAGTAG AAGATTAAGA GGGGAACTCT GATCGTCAAT AATATTAGTC AAGTTGAGTT CGGGACAACC   
  
  
- TCTTAGTGGA GTCCCTAGTA GTGGGCTCTT AGAAAAAGTT GTTCGACGTC AATTATTTCA GTCGTAGCTC   
  
  
- GTACGTAACG TCCTCGATCT TTGACGAGAT TACTCGTGTC TAACACTCCT CTTACTTCGT TGACAGAGAT   
  
  
- AGGGTAGATA CCCACTTGGA GTAGTCGGGG TTCAGGGATC GGTCTCTAGT TCTAAGACCT TAGCTCTAGG   
  
  
- AGTCCCAAGT GCGGGCCGAC TTCAAGTGAG TAGTAACTCT TCTAATCCTC TGCTCCGAGT CTCACTCTTT   
  
  
- GCGGAGTTCC GTCACCTTCT TGGTTATGCC GGGTCACGTG GTGGGCCGTT AAACTTCGTC AACGAGTATC   
  
  
- TTACACGAGC CCGAAACAGT CTCTTAGCTT ATCTCCTAAA ACTCTTCAAT CAACTTGTCC GTTTCCCGTA   
  
  
- CCAGAGGTAA AGACCTCTGG GTTAAGTTGC TGAACCACGA ATGAACTAGC TCCCCGATCA CCGTTCCTTC   
  
  
- CTCAGAAGTC CATGCTTTTA GATGGCTCGA GAATCCACGT TTCTCGGGGA ACCAGTTCTG AACGAAAGCA   
  
  
- TGTACGTGTA GGAAATACTT TAAACGGGAA TGAACTTTAA GCCAATATAC CAACGTTTAC CCCGATATCG   
  
  
- ACTTCGAACG TCTCTACTCC TAGTGTATGT ATAGCAGCTG ATGGTCTAAC CAGTTCCTTG AGTTACCTAC   
  
  
- TGAGACAACG TTCGGGATCG GTGTTTTGGA CCTTCTGGGG GGTGACACTC TTAATGACCG TAACTACTAG   
  
  
- GGCAGAGATC CGTACGAGCC CCTCGAACGA ACCTCCGTCA CCCCTTTGCA AACCGTCAAG ACAGCCTCTT   
  
  
- TAAATTGTAC GGGTAACTCA AAGTCCGTCA CGGGCAAATA CGAAGACTCC AGTGGGTTCT TTACGAACTA   
  
  
- CACTCCGGTC CCACCCGGAA CCGACACTTG AAGGGTAACG TCGAGGTGGT GTGAGGGCTA CTCTCACAAC   
  
  
- TACACTTGTT GGGCTCCCTA CCTGAGGATT CTTACCACTT TAGTGAGCCA GGGTTCCAAC ATTGAAACCA   
  
  
- TCTTGTTCTC AGTTTGTGGT TGTGATGGGG AAACGACTGT TCCAAGTATC TTTGCGACCT GAAGATGAGT   
  
  
- CGTTACAAAC TTAGGTAGCT ACACTGTTAC GGTTCCCTGG CTTTCCTTTC CTAGTTACAG CTCGTCGTAA   
  
  
- CGGATCGGTT CCTGTAACAG TTGTAGTAGC GCACACTCCC CTTCCTGTCC CACCTCGCTG TACTCGAGAA   
  
  
- ACCCTTTACC TTCAGTTCCA AGTGGTACCG TCCTAAGGTT GTTAGGGGTA ACTCGAGCAT ACAATTGAGG   
  
  
- CACTATTTTC CTAATGAATC CACAATAAGT CTCATGATAT GCAACCACCT CTTCCTACCG CGGAACGAAG   
  
  
- ACCCTACCTT CCTGGCCTAC GACCAAAGCC GAAGTCGAAC CGTAAC

+     Gap-box

| Site Name | Organism | Position | Strand | Matrix score. | sequence | function |
| --- | --- | --- | --- | --- | --- | --- |
| Gap-box | Arabidopsis thaliana | 997 | - | 9 | CAAATGAA(A/G)A | part of a light responsive element |

>HU01G01850.1   
+ +Up\_Stream \_Len000TTTAAA TATTTTATTT TATTTTAAAT TAATATATTT TATAATTTAA AATTATTAAT   
  
  
+ AATTGATGAT GTTACCCAAT GTTTAAGTTT TTAAATATTT TTATTTTTTA ATTTAAAAAT ATCATAATAT   
  
  
+ ATAATATAAT TTTAAGTTAA AATAAGATAG TTAAATTATT TTATAGTAAA TATTTATTAT TTTTTTTAAG   
  
  
+ AAAATTGCCA TGTGATTGTC ATCAGTCAAA CACTGCGTTG ACAGAAGCTG TCCTTGCTGC TACTCTCACC   
  
  
+ TCTCAACATC GGACCCGCGC AGACGTTAAC ATCTCCGTAC AAATACAATT CCCTGGCCAA AATTCAAATC   
  
  
+ AACCCTACTC GGTAGTCGGT TCCTTCTTCT TCCTGCCCTC TATATATACA CCCACAGCTG CGCTGCATGT   
  
  
+ TTTTCCGAGA ATCCTCAAAA ATCTTATAAA CTTCCTCGTG TGGGTAGAAA AAAGAAAAAG AAAAGGGTTT   
  
  
+ TAAAATCTTG GGTCAAAACC CATCAACACC ACAATTTGAC TCTTTCTTTT TAATTCAACC ACCGCCAGTT   
  
  
+ CCTCGTTGAT TTTCAGGTAC CTCTTTTTCT TGCCCCTTTT TTTGCTCGGT TGTTGTTTAA TGGTAAAGAT   
  
  
+ TGAGTACAAA GAATGATATT TGATGAGTTG GTGTGTTTAT TTGATGAGCC CAGATGATTT AACTTGTAGA   
  
  
+ TTTTGTGGTT TTGGTTTCTG GGTATTCATT GGTTTGCTGA ATTTCTGGTT CATGATCTGG GGTTAGAAAA   
  
  
+ GTTGAAATGA CTTTCTGGAG GTTGGAATTT GACTAAAGCT AATTTCTTTT TGTCTTCAAT TCTTTTTTAG   
  
  
+ TTGTGAGCTG AATTTGTTGA CCACTTCCAG CCTGTTGGAA ATTAAACTTA CTTAGGAGAA GAGAATCTCA   
  
  
+ GATTCTGAGC AAAAGCTTTA TATGGTTGTT TGGGAAATGG TAGGAACTAA GTGAAAACAC TGAAATTTTA   
  
  
+ AGCAAGCAAT TTTGTTCATT TGATTGGTCG GATGGTCTGA TTTAGGTTCT TTTCTTGGTC TAAATTTCTG   
  
  
+ AGCATTGATT GTTTAATTGC GTGGGGAACT TGTATCCTTG TGAGTTCCTG GTTCATTTCT TGGTTGCTTA   
  
  
+ CTCTAGCTCC CTTTCACAGT TTCTATGGTT GGAACTTGGA ACTTGGAACT TGGAAGCCTA ATTTAGTTGA   
  
  
+ ATATCTTGGC TTTGAGTTAG CTTTTCTTAG GAAAAGATGG TTCTGCTCTC GAAAATCTAA TCTAATCGAT   
  
  
+ AGATGCGTTA AGGAAGCAAA ACTTTTGGGG AGAGGGAAGT TAAGCGAAGC AAGCTGCTGT TTCAAAGTTG   
  
  
+ GACTGTTTTA TTGGTAAGTA TGAAACAATA GTTTATAAGA GATCATTGGG TTCTTAAGGT GATAGATCAT   
  
  
+ GGGTTATGAA TGAGCTTAGT TGTTCTTTTG CTTTCAACTG TACGTAAGGA TGATAGTTAT CCCTCATCTT   
  
  
+ GCATCAAAAA CGGCAAACCG TGATGCCAAA ACTCAAAAAC TGATTCCTCT GACAAGGCTT CTACCTATGA   
  
  
+ AAACTATGCA GATCATATCC ATTTGGCCTT CCCTCTAGGA ACCAAGTAGA GTAAGTTGGT AAATAAATAG   
  
  
+ AGTTGAACGA AACACTACAG ATTTGAATTT GATTAAGGAG GGCTATTATA GACTAAGGTG TAGCTCAAAT   
  
  
+ CCTTCTGTAG GGATTGTCTT ATTGGCTTCT TTTGGTTGCT TCTGGCTCAA GTCCTCTTAT TAGTTAGTAT   
  
  
+ GCATTACCTT GAGACAAGAT AGTTGCTTAA GCTTGAGCTT CTGTACTTAT TTGCGTATTG TAGACAACTA   
  
  
+ GCTGTGGCAT ATGATTCTGA AATGTTCCGA TGTCTTTTTC TTTGGATGCT GGGTTGTGTC ATGAGCTTTA   
  
  
+ GGTTTGTTTA TTTGATGTCA GGAAGTATGT AAATAACATG TGGCATCTTG CAATCTATTA CAGGATGATA   
  
  
+ TTGGCCTTTG TGGGAAGAAA TTGAAGAAGT TGTTTCACTT ACTGGATAGG GAGCATGGAC TCACATCAGC   
  
  
+ TTTTGAGATT CGGTATTTCC AGATCATACT CATCCTATAA TTTCTCTCAG TCTAGTCCCC AATCAATTCC   
  
  
+ AAATAGGTTA TTTGAATCAC CGAACGTTCG TTCTAGAGAC TCTCCAATCT CGCCCTTCTC AACGCCCTTC   
  
  
+ AATTGCGACC CCACTGTTAT ATTGAGCGAC AGTCAGGAGC ACCACAGTTC AACAGGAAGT CTCTCGGCAC   
  
  
+ AAAGCTCATC TTCTAATTCT CCCCTTGAGA CTAGCAGTTA TTATAATCAG TTCAACTCAA GCCCTGTTGG   
  
  
+ AGAATCACCT CAGGGATCAT CACCCGAGAA TCTTTTTCAA CAAGCTGCAG TTAATAAAGT CAGCATCGAG   
  
  
+ CATGCATTGC AGGAGCTAGA AACTGCTCTA ATGAGCACAG ATTGTGAGGA GAATGAAGCA ACTGTCTCTA   
  
  
+ TCCCATCTAT GGGTGAACCT CATCAGCCCC AAGTCCCTAG CCAGAGATCA AGATTCTGGA ATCGAGATCC   
  
  
+ TCAGGGTTCA CGCCCGGCTG AAGTTCACTC ATCATTGAGA AGATTAGGAG ACGAGGCTCA GAGTGAGAAA   
  
  
+ CGCCTCAAGG CAGTGGAAGA ACCAATACGG CCCAGTGCAC CACCCGGCAA TTTGAAGCAG TTGCTCATAG   
  
  
+ AATGTGCTCG GGCTTTGTCA GAGAATCGAA TAGAGGATTT TGAGAAGTTA GTTGAACAGG CAAAGGGCAT   
  
  
+ GGTCTCCATT TCTGGAGACC CAATTCAACG ACTTGGTGCT TACTTGATCG AGGGGCTAGT GGCAAGGAAG   
  
  
+ GAGTCTTCAG GTACGAAAAT CTACCGAGCT CTTAGGTGCA AAGAGCCCCT TGGTCAAGAC TTGCTTTCGT   
  
  
+ ACATGCACAT CCTTTATGAA ATTTGCCCTT ACTTGAAATT CGGTTATATG GTTGCAAATG GGGCTATAGC   
  
  
+ TGAAGCTTGC AGAGATGAGG ATCACATACA TATCGTCGAC TACCAGATTG GTCAAGGAAC TCAATGGATG   
  
  
+ ACTCTGTTGC AAGCCCTAGC CACAAAACCT GGAAGACCCC CCACTGTGAG AATTACTGGC ATTGATGATC   
  
  
+ CCGTCTCTAG GCATGCTCGG GGAGCTTGCT TGGAGGCAGT GGGGAAACGT TTGGCAGTTC TGTCGGAGAA   
  
  
+ ATTTAACATG CCCATTGAGT TTCAGGCAGT GCCCGTTTAT GCTTCTGAGG TCACCCAAGA AATGCTTGAT   
  
  
+ GTGAGGCCAG GGTGGGCCTT GGCTGTGAAC TTCCCATTGC AGCTCCACCA CACTCCCGAT GAGAGTGTTG   
  
  
+ ATGTGAACAA CCCGAGGGAT GGACTCCTAA GAATGGTGAA ATCACTCGGT CCCAAGGTTG TAACTTTGGT   
  
  
+ AGAACAAGAG TCAAACACCA ACACTACCCC TTTGCTGACA AGGTTCATAG AAACGCTGGA CTTCTACTCA   
  
  
+ GCAATGTTTG AATCCATCGA TGTGACAATG CCAAGGGACC GAAAGGAAAG GATCAATGTC GAGCAGCATT   
  
  
+ GCCTAGCCAA GGACATTGTC AACATCATCG CGTGTGAGGG GAAGGACAGG GTGGAGCGAC ATGAGCTCTT   
  
  
+ TGGGAAATGG AAGTCAAGGT TCACCATGGC AGGATTCCAA CAATCCCCAT TGAGCTCGTA TGTTAACTCC   
  
  
+ GTGATAAAAG GATTACTTAG GTGTTATTCA GAGTACTATA CGTTGGTGGA GAAGGATGGC GCCTTGCTTC   
  
  
+ TGGGATGGAA GGACCGGATG CTGGTTTCGG CTTCAGCTTG GCATTG  

- +Up\_Stream \_Len000AAATTT ATAAAATAAA ATAAAATTTA ATTATATAAA ATATTAAATT TTAATAATTA   
  
  
- TTAACTACTA CAATGGGTTA CAAATTCAAA AATTTATAAA AATAAAAAAT TAAATTTTTA TAGTATTATA   
  
  
- TATTATATTA AAATTCAATT TTATTCTATC AATTTAATAA AATATCATTT ATAAATAATA AAAAAAATTC   
  
  
- TTTTAACGGT ACACTAACAG TAGTCAGTTT GTGACGCAAC TGTCTTCGAC AGGAACGACG ATGAGAGTGG   
  
  
- AGAGTTGTAG CCTGGGCGCG TCTGCAATTG TAGAGGCATG TTTATGTTAA GGGACCGGTT TTAAGTTTAG   
  
  
- TTGGGATGAG CCATCAGCCA AGGAAGAAGA AGGACGGGAG ATATATATGT GGGTGTCGAC GCGACGTACA   
  
  
- AAAAGGCTCT TAGGAGTTTT TAGAATATTT GAAGGAGCAC ACCCATCTTT TTTCTTTTTC TTTTCCCAAA   
  
  
- ATTTTAGAAC CCAGTTTTGG GTAGTTGTGG TGTTAAACTG AGAAAGAAAA ATTAAGTTGG TGGCGGTCAA   
  
  
- GGAGCAACTA AAAGTCCATG GAGAAAAAGA ACGGGGAAAA AAACGAGCCA ACAACAAATT ACCATTTCTA   
  
  
- ACTCATGTTT CTTACTATAA ACTACTCAAC CACACAAATA AACTACTCGG GTCTACTAAA TTGAACATCT   
  
  
- AAAACACCAA AACCAAAGAC CCATAAGTAA CCAAACGACT TAAAGACCAA GTACTAGACC CCAATCTTTT   
  
  
- CAACTTTACT GAAAGACCTC CAACCTTAAA CTGATTTCGA TTAAAGAAAA ACAGAAGTTA AGAAAAAATC   
  
  
- AACACTCGAC TTAAACAACT GGTGAAGGTC GGACAACCTT TAATTTGAAT GAATCCTCTT CTCTTAGAGT   
  
  
- CTAAGACTCG TTTTCGAAAT ATACCAACAA ACCCTTTACC ATCCTTGATT CACTTTTGTG ACTTTAAAAT   
  
  
- TCGTTCGTTA AAACAAGTAA ACTAACCAGC CTACCAGACT AAATCCAAGA AAAGAACCAG ATTTAAAGAC   
  
  
- TCGTAACTAA CAAATTAACG CACCCCTTGA ACATAGGAAC ACTCAAGGAC CAAGTAAAGA ACCAACGAAT   
  
  
- GAGATCGAGG GAAAGTGTCA AAGATACCAA CCTTGAACCT TGAACCTTGA ACCTTCGGAT TAAATCAACT   
  
  
- TATAGAACCG AAACTCAATC GAAAAGAATC CTTTTCTACC AAGACGAGAG CTTTTAGATT AGATTAGCTA   
  
  
- TCTACGCAAT TCCTTCGTTT TGAAAACCCC TCTCCCTTCA ATTCGCTTCG TTCGACGACA AAGTTTCAAC   
  
  
- CTGACAAAAT AACCATTCAT ACTTTGTTAT CAAATATTCT CTAGTAACCC AAGAATTCCA CTATCTAGTA   
  
  
- CCCAATACTT ACTCGAATCA ACAAGAAAAC GAAAGTTGAC ATGCATTCCT ACTATCAATA GGGAGTAGAA   
  
  
- CGTAGTTTTT GCCGTTTGGC ACTACGGTTT TGAGTTTTTG ACTAAGGAGA CTGTTCCGAA GATGGATACT   
  
  
- TTTGATACGT CTAGTATAGG TAAACCGGAA GGGAGATCCT TGGTTCATCT CATTCAACCA TTTATTTATC   
  
  
- TCAACTTGCT TTGTGATGTC TAAACTTAAA CTAATTCCTC CCGATAATAT CTGATTCCAC ATCGAGTTTA   
  
  
- GGAAGACATC CCTAACAGAA TAACCGAAGA AAACCAACGA AGACCGAGTT CAGGAGAATA ATCAATCATA   
  
  
- CGTAATGGAA CTCTGTTCTA TCAACGAATT CGAACTCGAA GACATGAATA AACGCATAAC ATCTGTTGAT   
  
  
- CGACACCGTA TACTAAGACT TTACAAGGCT ACAGAAAAAG AAACCTACGA CCCAACACAG TACTCGAAAT   
  
  
- CCAAACAAAT AAACTACAGT CCTTCATACA TTTATTGTAC ACCGTAGAAC GTTAGATAAT GTCCTACTAT   
  
  
- AACCGGAAAC ACCCTTCTTT AACTTCTTCA ACAAAGTGAA TGACCTATCC CTCGTACCTG AGTGTAGTCG   
  
  
- AAAACTCTAA GCCATAAAGG TCTAGTATGA GTAGGATATT AAAGAGAGTC AGATCAGGGG TTAGTTAAGG   
  
  
- TTTATCCAAT AAACTTAGTG GCTTGCAAGC AAGATCTCTG AGAGGTTAGA GCGGGAAGAG TTGCGGGAAG   
  
  
- TTAACGCTGG GGTGACAATA TAACTCGCTG TCAGTCCTCG TGGTGTCAAG TTGTCCTTCA GAGAGCCGTG   
  
  
- TTTCGAGTAG AAGATTAAGA GGGGAACTCT GATCGTCAAT AATATTAGTC AAGTTGAGTT CGGGACAACC   
  
  
- TCTTAGTGGA GTCCCTAGTA GTGGGCTCTT AGAAAAAGTT GTTCGACGTC AATTATTTCA GTCGTAGCTC   
  
  
- GTACGTAACG TCCTCGATCT TTGACGAGAT TACTCGTGTC TAACACTCCT CTTACTTCGT TGACAGAGAT   
  
  
- AGGGTAGATA CCCACTTGGA GTAGTCGGGG TTCAGGGATC GGTCTCTAGT TCTAAGACCT TAGCTCTAGG   
  
  
- AGTCCCAAGT GCGGGCCGAC TTCAAGTGAG TAGTAACTCT TCTAATCCTC TGCTCCGAGT CTCACTCTTT   
  
  
- GCGGAGTTCC GTCACCTTCT TGGTTATGCC GGGTCACGTG GTGGGCCGTT AAACTTCGTC AACGAGTATC   
  
  
- TTACACGAGC CCGAAACAGT CTCTTAGCTT ATCTCCTAAA ACTCTTCAAT CAACTTGTCC GTTTCCCGTA   
  
  
- CCAGAGGTAA AGACCTCTGG GTTAAGTTGC TGAACCACGA ATGAACTAGC TCCCCGATCA CCGTTCCTTC   
  
  
- CTCAGAAGTC CATGCTTTTA GATGGCTCGA GAATCCACGT TTCTCGGGGA ACCAGTTCTG AACGAAAGCA   
  
  
- TGTACGTGTA GGAAATACTT TAAACGGGAA TGAACTTTAA GCCAATATAC CAACGTTTAC CCCGATATCG   
  
  
- ACTTCGAACG TCTCTACTCC TAGTGTATGT ATAGCAGCTG ATGGTCTAAC CAGTTCCTTG AGTTACCTAC   
  
  
- TGAGACAACG TTCGGGATCG GTGTTTTGGA CCTTCTGGGG GGTGACACTC TTAATGACCG TAACTACTAG   
  
  
- GGCAGAGATC CGTACGAGCC CCTCGAACGA ACCTCCGTCA CCCCTTTGCA AACCGTCAAG ACAGCCTCTT   
  
  
- TAAATTGTAC GGGTAACTCA AAGTCCGTCA CGGGCAAATA CGAAGACTCC AGTGGGTTCT TTACGAACTA   
  
  
- CACTCCGGTC CCACCCGGAA CCGACACTTG AAGGGTAACG TCGAGGTGGT GTGAGGGCTA CTCTCACAAC   
  
  
- TACACTTGTT GGGCTCCCTA CCTGAGGATT CTTACCACTT TAGTGAGCCA GGGTTCCAAC ATTGAAACCA   
  
  
- TCTTGTTCTC AGTTTGTGGT TGTGATGGGG AAACGACTGT TCCAAGTATC TTTGCGACCT GAAGATGAGT   
  
  
- CGTTACAAAC TTAGGTAGCT ACACTGTTAC GGTTCCCTGG CTTTCCTTTC CTAGTTACAG CTCGTCGTAA   
  
  
- CGGATCGGTT CCTGTAACAG TTGTAGTAGC GCACACTCCC CTTCCTGTCC CACCTCGCTG TACTCGAGAA   
  
  
- ACCCTTTACC TTCAGTTCCA AGTGGTACCG TCCTAAGGTT GTTAGGGGTA ACTCGAGCAT ACAATTGAGG   
  
  
- CACTATTTTC CTAATGAATC CACAATAAGT CTCATGATAT GCAACCACCT CTTCCTACCG CGGAACGAAG   
  
  
- ACCCTACCTT CCTGGCCTAC GACCAAAGCC GAAGTCGAAC CGTAAC

+     LTR

| Site Name | Organism | Position | Strand | Matrix score. | sequence | function |
| --- | --- | --- | --- | --- | --- | --- |
| LTR | Hordeum vulgare | 3739 | - | 6 | CCGAAA | cis-acting element involved in low-temperature responsiveness |
| LTR | Hordeum vulgare | 3473 | + | 6 | CCGAAA | cis-acting element involved in low-temperature responsiveness |

>HU01G01850.1   
+ +Up\_Stream \_Len000TTTAAA TATTTTATTT TATTTTAAAT TAATATATTT TATAATTTAA AATTATTAAT   
  
  
+ AATTGATGAT GTTACCCAAT GTTTAAGTTT TTAAATATTT TTATTTTTTA ATTTAAAAAT ATCATAATAT   
  
  
+ ATAATATAAT TTTAAGTTAA AATAAGATAG TTAAATTATT TTATAGTAAA TATTTATTAT TTTTTTTAAG   
  
  
+ AAAATTGCCA TGTGATTGTC ATCAGTCAAA CACTGCGTTG ACAGAAGCTG TCCTTGCTGC TACTCTCACC   
  
  
+ TCTCAACATC GGACCCGCGC AGACGTTAAC ATCTCCGTAC AAATACAATT CCCTGGCCAA AATTCAAATC   
  
  
+ AACCCTACTC GGTAGTCGGT TCCTTCTTCT TCCTGCCCTC TATATATACA CCCACAGCTG CGCTGCATGT   
  
  
+ TTTTCCGAGA ATCCTCAAAA ATCTTATAAA CTTCCTCGTG TGGGTAGAAA AAAGAAAAAG AAAAGGGTTT   
  
  
+ TAAAATCTTG GGTCAAAACC CATCAACACC ACAATTTGAC TCTTTCTTTT TAATTCAACC ACCGCCAGTT   
  
  
+ CCTCGTTGAT TTTCAGGTAC CTCTTTTTCT TGCCCCTTTT TTTGCTCGGT TGTTGTTTAA TGGTAAAGAT   
  
  
+ TGAGTACAAA GAATGATATT TGATGAGTTG GTGTGTTTAT TTGATGAGCC CAGATGATTT AACTTGTAGA   
  
  
+ TTTTGTGGTT TTGGTTTCTG GGTATTCATT GGTTTGCTGA ATTTCTGGTT CATGATCTGG GGTTAGAAAA   
  
  
+ GTTGAAATGA CTTTCTGGAG GTTGGAATTT GACTAAAGCT AATTTCTTTT TGTCTTCAAT TCTTTTTTAG   
  
  
+ TTGTGAGCTG AATTTGTTGA CCACTTCCAG CCTGTTGGAA ATTAAACTTA CTTAGGAGAA GAGAATCTCA   
  
  
+ GATTCTGAGC AAAAGCTTTA TATGGTTGTT TGGGAAATGG TAGGAACTAA GTGAAAACAC TGAAATTTTA   
  
  
+ AGCAAGCAAT TTTGTTCATT TGATTGGTCG GATGGTCTGA TTTAGGTTCT TTTCTTGGTC TAAATTTCTG   
  
  
+ AGCATTGATT GTTTAATTGC GTGGGGAACT TGTATCCTTG TGAGTTCCTG GTTCATTTCT TGGTTGCTTA   
  
  
+ CTCTAGCTCC CTTTCACAGT TTCTATGGTT GGAACTTGGA ACTTGGAACT TGGAAGCCTA ATTTAGTTGA   
  
  
+ ATATCTTGGC TTTGAGTTAG CTTTTCTTAG GAAAAGATGG TTCTGCTCTC GAAAATCTAA TCTAATCGAT   
  
  
+ AGATGCGTTA AGGAAGCAAA ACTTTTGGGG AGAGGGAAGT TAAGCGAAGC AAGCTGCTGT TTCAAAGTTG   
  
  
+ GACTGTTTTA TTGGTAAGTA TGAAACAATA GTTTATAAGA GATCATTGGG TTCTTAAGGT GATAGATCAT   
  
  
+ GGGTTATGAA TGAGCTTAGT TGTTCTTTTG CTTTCAACTG TACGTAAGGA TGATAGTTAT CCCTCATCTT   
  
  
+ GCATCAAAAA CGGCAAACCG TGATGCCAAA ACTCAAAAAC TGATTCCTCT GACAAGGCTT CTACCTATGA   
  
  
+ AAACTATGCA GATCATATCC ATTTGGCCTT CCCTCTAGGA ACCAAGTAGA GTAAGTTGGT AAATAAATAG   
  
  
+ AGTTGAACGA AACACTACAG ATTTGAATTT GATTAAGGAG GGCTATTATA GACTAAGGTG TAGCTCAAAT   
  
  
+ CCTTCTGTAG GGATTGTCTT ATTGGCTTCT TTTGGTTGCT TCTGGCTCAA GTCCTCTTAT TAGTTAGTAT   
  
  
+ GCATTACCTT GAGACAAGAT AGTTGCTTAA GCTTGAGCTT CTGTACTTAT TTGCGTATTG TAGACAACTA   
  
  
+ GCTGTGGCAT ATGATTCTGA AATGTTCCGA TGTCTTTTTC TTTGGATGCT GGGTTGTGTC ATGAGCTTTA   
  
  
+ GGTTTGTTTA TTTGATGTCA GGAAGTATGT AAATAACATG TGGCATCTTG CAATCTATTA CAGGATGATA   
  
  
+ TTGGCCTTTG TGGGAAGAAA TTGAAGAAGT TGTTTCACTT ACTGGATAGG GAGCATGGAC TCACATCAGC   
  
  
+ TTTTGAGATT CGGTATTTCC AGATCATACT CATCCTATAA TTTCTCTCAG TCTAGTCCCC AATCAATTCC   
  
  
+ AAATAGGTTA TTTGAATCAC CGAACGTTCG TTCTAGAGAC TCTCCAATCT CGCCCTTCTC AACGCCCTTC   
  
  
+ AATTGCGACC CCACTGTTAT ATTGAGCGAC AGTCAGGAGC ACCACAGTTC AACAGGAAGT CTCTCGGCAC   
  
  
+ AAAGCTCATC TTCTAATTCT CCCCTTGAGA CTAGCAGTTA TTATAATCAG TTCAACTCAA GCCCTGTTGG   
  
  
+ AGAATCACCT CAGGGATCAT CACCCGAGAA TCTTTTTCAA CAAGCTGCAG TTAATAAAGT CAGCATCGAG   
  
  
+ CATGCATTGC AGGAGCTAGA AACTGCTCTA ATGAGCACAG ATTGTGAGGA GAATGAAGCA ACTGTCTCTA   
  
  
+ TCCCATCTAT GGGTGAACCT CATCAGCCCC AAGTCCCTAG CCAGAGATCA AGATTCTGGA ATCGAGATCC   
  
  
+ TCAGGGTTCA CGCCCGGCTG AAGTTCACTC ATCATTGAGA AGATTAGGAG ACGAGGCTCA GAGTGAGAAA   
  
  
+ CGCCTCAAGG CAGTGGAAGA ACCAATACGG CCCAGTGCAC CACCCGGCAA TTTGAAGCAG TTGCTCATAG   
  
  
+ AATGTGCTCG GGCTTTGTCA GAGAATCGAA TAGAGGATTT TGAGAAGTTA GTTGAACAGG CAAAGGGCAT   
  
  
+ GGTCTCCATT TCTGGAGACC CAATTCAACG ACTTGGTGCT TACTTGATCG AGGGGCTAGT GGCAAGGAAG   
  
  
+ GAGTCTTCAG GTACGAAAAT CTACCGAGCT CTTAGGTGCA AAGAGCCCCT TGGTCAAGAC TTGCTTTCGT   
  
  
+ ACATGCACAT CCTTTATGAA ATTTGCCCTT ACTTGAAATT CGGTTATATG GTTGCAAATG GGGCTATAGC   
  
  
+ TGAAGCTTGC AGAGATGAGG ATCACATACA TATCGTCGAC TACCAGATTG GTCAAGGAAC TCAATGGATG   
  
  
+ ACTCTGTTGC AAGCCCTAGC CACAAAACCT GGAAGACCCC CCACTGTGAG AATTACTGGC ATTGATGATC   
  
  
+ CCGTCTCTAG GCATGCTCGG GGAGCTTGCT TGGAGGCAGT GGGGAAACGT TTGGCAGTTC TGTCGGAGAA   
  
  
+ ATTTAACATG CCCATTGAGT TTCAGGCAGT GCCCGTTTAT GCTTCTGAGG TCACCCAAGA AATGCTTGAT   
  
  
+ GTGAGGCCAG GGTGGGCCTT GGCTGTGAAC TTCCCATTGC AGCTCCACCA CACTCCCGAT GAGAGTGTTG   
  
  
+ ATGTGAACAA CCCGAGGGAT GGACTCCTAA GAATGGTGAA ATCACTCGGT CCCAAGGTTG TAACTTTGGT   
  
  
+ AGAACAAGAG TCAAACACCA ACACTACCCC TTTGCTGACA AGGTTCATAG AAACGCTGGA CTTCTACTCA   
  
  
+ GCAATGTTTG AATCCATCGA TGTGACAATG CCAAGGGACC GAAAGGAAAG GATCAATGTC GAGCAGCATT   
  
  
+ GCCTAGCCAA GGACATTGTC AACATCATCG CGTGTGAGGG GAAGGACAGG GTGGAGCGAC ATGAGCTCTT   
  
  
+ TGGGAAATGG AAGTCAAGGT TCACCATGGC AGGATTCCAA CAATCCCCAT TGAGCTCGTA TGTTAACTCC   
  
  
+ GTGATAAAAG GATTACTTAG GTGTTATTCA GAGTACTATA CGTTGGTGGA GAAGGATGGC GCCTTGCTTC   
  
  
+ TGGGATGGAA GGACCGGATG CTGGTTTCGG CTTCAGCTTG GCATTG  

- +Up\_Stream \_Len000AAATTT ATAAAATAAA ATAAAATTTA ATTATATAAA ATATTAAATT TTAATAATTA   
  
  
- TTAACTACTA CAATGGGTTA CAAATTCAAA AATTTATAAA AATAAAAAAT TAAATTTTTA TAGTATTATA   
  
  
- TATTATATTA AAATTCAATT TTATTCTATC AATTTAATAA AATATCATTT ATAAATAATA AAAAAAATTC   
  
  
- TTTTAACGGT ACACTAACAG TAGTCAGTTT GTGACGCAAC TGTCTTCGAC AGGAACGACG ATGAGAGTGG   
  
  
- AGAGTTGTAG CCTGGGCGCG TCTGCAATTG TAGAGGCATG TTTATGTTAA GGGACCGGTT TTAAGTTTAG   
  
  
- TTGGGATGAG CCATCAGCCA AGGAAGAAGA AGGACGGGAG ATATATATGT GGGTGTCGAC GCGACGTACA   
  
  
- AAAAGGCTCT TAGGAGTTTT TAGAATATTT GAAGGAGCAC ACCCATCTTT TTTCTTTTTC TTTTCCCAAA   
  
  
- ATTTTAGAAC CCAGTTTTGG GTAGTTGTGG TGTTAAACTG AGAAAGAAAA ATTAAGTTGG TGGCGGTCAA   
  
  
- GGAGCAACTA AAAGTCCATG GAGAAAAAGA ACGGGGAAAA AAACGAGCCA ACAACAAATT ACCATTTCTA   
  
  
- ACTCATGTTT CTTACTATAA ACTACTCAAC CACACAAATA AACTACTCGG GTCTACTAAA TTGAACATCT   
  
  
- AAAACACCAA AACCAAAGAC CCATAAGTAA CCAAACGACT TAAAGACCAA GTACTAGACC CCAATCTTTT   
  
  
- CAACTTTACT GAAAGACCTC CAACCTTAAA CTGATTTCGA TTAAAGAAAA ACAGAAGTTA AGAAAAAATC   
  
  
- AACACTCGAC TTAAACAACT GGTGAAGGTC GGACAACCTT TAATTTGAAT GAATCCTCTT CTCTTAGAGT   
  
  
- CTAAGACTCG TTTTCGAAAT ATACCAACAA ACCCTTTACC ATCCTTGATT CACTTTTGTG ACTTTAAAAT   
  
  
- TCGTTCGTTA AAACAAGTAA ACTAACCAGC CTACCAGACT AAATCCAAGA AAAGAACCAG ATTTAAAGAC   
  
  
- TCGTAACTAA CAAATTAACG CACCCCTTGA ACATAGGAAC ACTCAAGGAC CAAGTAAAGA ACCAACGAAT   
  
  
- GAGATCGAGG GAAAGTGTCA AAGATACCAA CCTTGAACCT TGAACCTTGA ACCTTCGGAT TAAATCAACT   
  
  
- TATAGAACCG AAACTCAATC GAAAAGAATC CTTTTCTACC AAGACGAGAG CTTTTAGATT AGATTAGCTA   
  
  
- TCTACGCAAT TCCTTCGTTT TGAAAACCCC TCTCCCTTCA ATTCGCTTCG TTCGACGACA AAGTTTCAAC   
  
  
- CTGACAAAAT AACCATTCAT ACTTTGTTAT CAAATATTCT CTAGTAACCC AAGAATTCCA CTATCTAGTA   
  
  
- CCCAATACTT ACTCGAATCA ACAAGAAAAC GAAAGTTGAC ATGCATTCCT ACTATCAATA GGGAGTAGAA   
  
  
- CGTAGTTTTT GCCGTTTGGC ACTACGGTTT TGAGTTTTTG ACTAAGGAGA CTGTTCCGAA GATGGATACT   
  
  
- TTTGATACGT CTAGTATAGG TAAACCGGAA GGGAGATCCT TGGTTCATCT CATTCAACCA TTTATTTATC   
  
  
- TCAACTTGCT TTGTGATGTC TAAACTTAAA CTAATTCCTC CCGATAATAT CTGATTCCAC ATCGAGTTTA   
  
  
- GGAAGACATC CCTAACAGAA TAACCGAAGA AAACCAACGA AGACCGAGTT CAGGAGAATA ATCAATCATA   
  
  
- CGTAATGGAA CTCTGTTCTA TCAACGAATT CGAACTCGAA GACATGAATA AACGCATAAC ATCTGTTGAT   
  
  
- CGACACCGTA TACTAAGACT TTACAAGGCT ACAGAAAAAG AAACCTACGA CCCAACACAG TACTCGAAAT   
  
  
- CCAAACAAAT AAACTACAGT CCTTCATACA TTTATTGTAC ACCGTAGAAC GTTAGATAAT GTCCTACTAT   
  
  
- AACCGGAAAC ACCCTTCTTT AACTTCTTCA ACAAAGTGAA TGACCTATCC CTCGTACCTG AGTGTAGTCG   
  
  
- AAAACTCTAA GCCATAAAGG TCTAGTATGA GTAGGATATT AAAGAGAGTC AGATCAGGGG TTAGTTAAGG   
  
  
- TTTATCCAAT AAACTTAGTG GCTTGCAAGC AAGATCTCTG AGAGGTTAGA GCGGGAAGAG TTGCGGGAAG   
  
  
- TTAACGCTGG GGTGACAATA TAACTCGCTG TCAGTCCTCG TGGTGTCAAG TTGTCCTTCA GAGAGCCGTG   
  
  
- TTTCGAGTAG AAGATTAAGA GGGGAACTCT GATCGTCAAT AATATTAGTC AAGTTGAGTT CGGGACAACC   
  
  
- TCTTAGTGGA GTCCCTAGTA GTGGGCTCTT AGAAAAAGTT GTTCGACGTC AATTATTTCA GTCGTAGCTC   
  
  
- GTACGTAACG TCCTCGATCT TTGACGAGAT TACTCGTGTC TAACACTCCT CTTACTTCGT TGACAGAGAT   
  
  
- AGGGTAGATA CCCACTTGGA GTAGTCGGGG TTCAGGGATC GGTCTCTAGT TCTAAGACCT TAGCTCTAGG   
  
  
- AGTCCCAAGT GCGGGCCGAC TTCAAGTGAG TAGTAACTCT TCTAATCCTC TGCTCCGAGT CTCACTCTTT   
  
  
- GCGGAGTTCC GTCACCTTCT TGGTTATGCC GGGTCACGTG GTGGGCCGTT AAACTTCGTC AACGAGTATC   
  
  
- TTACACGAGC CCGAAACAGT CTCTTAGCTT ATCTCCTAAA ACTCTTCAAT CAACTTGTCC GTTTCCCGTA   
  
  
- CCAGAGGTAA AGACCTCTGG GTTAAGTTGC TGAACCACGA ATGAACTAGC TCCCCGATCA CCGTTCCTTC   
  
  
- CTCAGAAGTC CATGCTTTTA GATGGCTCGA GAATCCACGT TTCTCGGGGA ACCAGTTCTG AACGAAAGCA   
  
  
- TGTACGTGTA GGAAATACTT TAAACGGGAA TGAACTTTAA GCCAATATAC CAACGTTTAC CCCGATATCG   
  
  
- ACTTCGAACG TCTCTACTCC TAGTGTATGT ATAGCAGCTG ATGGTCTAAC CAGTTCCTTG AGTTACCTAC   
  
  
- TGAGACAACG TTCGGGATCG GTGTTTTGGA CCTTCTGGGG GGTGACACTC TTAATGACCG TAACTACTAG   
  
  
- GGCAGAGATC CGTACGAGCC CCTCGAACGA ACCTCCGTCA CCCCTTTGCA AACCGTCAAG ACAGCCTCTT   
  
  
- TAAATTGTAC GGGTAACTCA AAGTCCGTCA CGGGCAAATA CGAAGACTCC AGTGGGTTCT TTACGAACTA   
  
  
- CACTCCGGTC CCACCCGGAA CCGACACTTG AAGGGTAACG TCGAGGTGGT GTGAGGGCTA CTCTCACAAC   
  
  
- TACACTTGTT GGGCTCCCTA CCTGAGGATT CTTACCACTT TAGTGAGCCA GGGTTCCAAC ATTGAAACCA   
  
  
- TCTTGTTCTC AGTTTGTGGT TGTGATGGGG AAACGACTGT TCCAAGTATC TTTGCGACCT GAAGATGAGT   
  
  
- CGTTACAAAC TTAGGTAGCT ACACTGTTAC GGTTCCCTGG CTTTCCTTTC CTAGTTACAG CTCGTCGTAA   
  
  
- CGGATCGGTT CCTGTAACAG TTGTAGTAGC GCACACTCCC CTTCCTGTCC CACCTCGCTG TACTCGAGAA   
  
  
- ACCCTTTACC TTCAGTTCCA AGTGGTACCG TCCTAAGGTT GTTAGGGGTA ACTCGAGCAT ACAATTGAGG   
  
  
- CACTATTTTC CTAATGAATC CACAATAAGT CTCATGATAT GCAACCACCT CTTCCTACCG CGGAACGAAG   
  
  
- ACCCTACCTT CCTGGCCTAC GACCAAAGCC GAAGTCGAAC CGTAAC

+     MBS

| Site Name | Organism | Position | Strand | Matrix score. | sequence | function |
| --- | --- | --- | --- | --- | --- | --- |
| MBS | Arabidopsis thaliana | 2652 | - | 6 | CAACTG | MYB binding site involved in drought-inducibility |
| MBS | Arabidopsis thaliana | 2443 | + | 6 | CAACTG | MYB binding site involved in drought-inducibility |
| MBS | Arabidopsis thaliana | 1439 | + | 6 | CAACTG | MYB binding site involved in drought-inducibility |

>HU01G01850.1   
+ +Up\_Stream \_Len000TTTAAA TATTTTATTT TATTTTAAAT TAATATATTT TATAATTTAA AATTATTAAT   
  
  
+ AATTGATGAT GTTACCCAAT GTTTAAGTTT TTAAATATTT TTATTTTTTA ATTTAAAAAT ATCATAATAT   
  
  
+ ATAATATAAT TTTAAGTTAA AATAAGATAG TTAAATTATT TTATAGTAAA TATTTATTAT TTTTTTTAAG   
  
  
+ AAAATTGCCA TGTGATTGTC ATCAGTCAAA CACTGCGTTG ACAGAAGCTG TCCTTGCTGC TACTCTCACC   
  
  
+ TCTCAACATC GGACCCGCGC AGACGTTAAC ATCTCCGTAC AAATACAATT CCCTGGCCAA AATTCAAATC   
  
  
+ AACCCTACTC GGTAGTCGGT TCCTTCTTCT TCCTGCCCTC TATATATACA CCCACAGCTG CGCTGCATGT   
  
  
+ TTTTCCGAGA ATCCTCAAAA ATCTTATAAA CTTCCTCGTG TGGGTAGAAA AAAGAAAAAG AAAAGGGTTT   
  
  
+ TAAAATCTTG GGTCAAAACC CATCAACACC ACAATTTGAC TCTTTCTTTT TAATTCAACC ACCGCCAGTT   
  
  
+ CCTCGTTGAT TTTCAGGTAC CTCTTTTTCT TGCCCCTTTT TTTGCTCGGT TGTTGTTTAA TGGTAAAGAT   
  
  
+ TGAGTACAAA GAATGATATT TGATGAGTTG GTGTGTTTAT TTGATGAGCC CAGATGATTT AACTTGTAGA   
  
  
+ TTTTGTGGTT TTGGTTTCTG GGTATTCATT GGTTTGCTGA ATTTCTGGTT CATGATCTGG GGTTAGAAAA   
  
  
+ GTTGAAATGA CTTTCTGGAG GTTGGAATTT GACTAAAGCT AATTTCTTTT TGTCTTCAAT TCTTTTTTAG   
  
  
+ TTGTGAGCTG AATTTGTTGA CCACTTCCAG CCTGTTGGAA ATTAAACTTA CTTAGGAGAA GAGAATCTCA   
  
  
+ GATTCTGAGC AAAAGCTTTA TATGGTTGTT TGGGAAATGG TAGGAACTAA GTGAAAACAC TGAAATTTTA   
  
  
+ AGCAAGCAAT TTTGTTCATT TGATTGGTCG GATGGTCTGA TTTAGGTTCT TTTCTTGGTC TAAATTTCTG   
  
  
+ AGCATTGATT GTTTAATTGC GTGGGGAACT TGTATCCTTG TGAGTTCCTG GTTCATTTCT TGGTTGCTTA   
  
  
+ CTCTAGCTCC CTTTCACAGT TTCTATGGTT GGAACTTGGA ACTTGGAACT TGGAAGCCTA ATTTAGTTGA   
  
  
+ ATATCTTGGC TTTGAGTTAG CTTTTCTTAG GAAAAGATGG TTCTGCTCTC GAAAATCTAA TCTAATCGAT   
  
  
+ AGATGCGTTA AGGAAGCAAA ACTTTTGGGG AGAGGGAAGT TAAGCGAAGC AAGCTGCTGT TTCAAAGTTG   
  
  
+ GACTGTTTTA TTGGTAAGTA TGAAACAATA GTTTATAAGA GATCATTGGG TTCTTAAGGT GATAGATCAT   
  
  
+ GGGTTATGAA TGAGCTTAGT TGTTCTTTTG CTTTCAACTG TACGTAAGGA TGATAGTTAT CCCTCATCTT   
  
  
+ GCATCAAAAA CGGCAAACCG TGATGCCAAA ACTCAAAAAC TGATTCCTCT GACAAGGCTT CTACCTATGA   
  
  
+ AAACTATGCA GATCATATCC ATTTGGCCTT CCCTCTAGGA ACCAAGTAGA GTAAGTTGGT AAATAAATAG   
  
  
+ AGTTGAACGA AACACTACAG ATTTGAATTT GATTAAGGAG GGCTATTATA GACTAAGGTG TAGCTCAAAT   
  
  
+ CCTTCTGTAG GGATTGTCTT ATTGGCTTCT TTTGGTTGCT TCTGGCTCAA GTCCTCTTAT TAGTTAGTAT   
  
  
+ GCATTACCTT GAGACAAGAT AGTTGCTTAA GCTTGAGCTT CTGTACTTAT TTGCGTATTG TAGACAACTA   
  
  
+ GCTGTGGCAT ATGATTCTGA AATGTTCCGA TGTCTTTTTC TTTGGATGCT GGGTTGTGTC ATGAGCTTTA   
  
  
+ GGTTTGTTTA TTTGATGTCA GGAAGTATGT AAATAACATG TGGCATCTTG CAATCTATTA CAGGATGATA   
  
  
+ TTGGCCTTTG TGGGAAGAAA TTGAAGAAGT TGTTTCACTT ACTGGATAGG GAGCATGGAC TCACATCAGC   
  
  
+ TTTTGAGATT CGGTATTTCC AGATCATACT CATCCTATAA TTTCTCTCAG TCTAGTCCCC AATCAATTCC   
  
  
+ AAATAGGTTA TTTGAATCAC CGAACGTTCG TTCTAGAGAC TCTCCAATCT CGCCCTTCTC AACGCCCTTC   
  
  
+ AATTGCGACC CCACTGTTAT ATTGAGCGAC AGTCAGGAGC ACCACAGTTC AACAGGAAGT CTCTCGGCAC   
  
  
+ AAAGCTCATC TTCTAATTCT CCCCTTGAGA CTAGCAGTTA TTATAATCAG TTCAACTCAA GCCCTGTTGG   
  
  
+ AGAATCACCT CAGGGATCAT CACCCGAGAA TCTTTTTCAA CAAGCTGCAG TTAATAAAGT CAGCATCGAG   
  
  
+ CATGCATTGC AGGAGCTAGA AACTGCTCTA ATGAGCACAG ATTGTGAGGA GAATGAAGCA ACTGTCTCTA   
  
  
+ TCCCATCTAT GGGTGAACCT CATCAGCCCC AAGTCCCTAG CCAGAGATCA AGATTCTGGA ATCGAGATCC   
  
  
+ TCAGGGTTCA CGCCCGGCTG AAGTTCACTC ATCATTGAGA AGATTAGGAG ACGAGGCTCA GAGTGAGAAA   
  
  
+ CGCCTCAAGG CAGTGGAAGA ACCAATACGG CCCAGTGCAC CACCCGGCAA TTTGAAGCAG TTGCTCATAG   
  
  
+ AATGTGCTCG GGCTTTGTCA GAGAATCGAA TAGAGGATTT TGAGAAGTTA GTTGAACAGG CAAAGGGCAT   
  
  
+ GGTCTCCATT TCTGGAGACC CAATTCAACG ACTTGGTGCT TACTTGATCG AGGGGCTAGT GGCAAGGAAG   
  
  
+ GAGTCTTCAG GTACGAAAAT CTACCGAGCT CTTAGGTGCA AAGAGCCCCT TGGTCAAGAC TTGCTTTCGT   
  
  
+ ACATGCACAT CCTTTATGAA ATTTGCCCTT ACTTGAAATT CGGTTATATG GTTGCAAATG GGGCTATAGC   
  
  
+ TGAAGCTTGC AGAGATGAGG ATCACATACA TATCGTCGAC TACCAGATTG GTCAAGGAAC TCAATGGATG   
  
  
+ ACTCTGTTGC AAGCCCTAGC CACAAAACCT GGAAGACCCC CCACTGTGAG AATTACTGGC ATTGATGATC   
  
  
+ CCGTCTCTAG GCATGCTCGG GGAGCTTGCT TGGAGGCAGT GGGGAAACGT TTGGCAGTTC TGTCGGAGAA   
  
  
+ ATTTAACATG CCCATTGAGT TTCAGGCAGT GCCCGTTTAT GCTTCTGAGG TCACCCAAGA AATGCTTGAT   
  
  
+ GTGAGGCCAG GGTGGGCCTT GGCTGTGAAC TTCCCATTGC AGCTCCACCA CACTCCCGAT GAGAGTGTTG   
  
  
+ ATGTGAACAA CCCGAGGGAT GGACTCCTAA GAATGGTGAA ATCACTCGGT CCCAAGGTTG TAACTTTGGT   
  
  
+ AGAACAAGAG TCAAACACCA ACACTACCCC TTTGCTGACA AGGTTCATAG AAACGCTGGA CTTCTACTCA   
  
  
+ GCAATGTTTG AATCCATCGA TGTGACAATG CCAAGGGACC GAAAGGAAAG GATCAATGTC GAGCAGCATT   
  
  
+ GCCTAGCCAA GGACATTGTC AACATCATCG CGTGTGAGGG GAAGGACAGG GTGGAGCGAC ATGAGCTCTT   
  
  
+ TGGGAAATGG AAGTCAAGGT TCACCATGGC AGGATTCCAA CAATCCCCAT TGAGCTCGTA TGTTAACTCC   
  
  
+ GTGATAAAAG GATTACTTAG GTGTTATTCA GAGTACTATA CGTTGGTGGA GAAGGATGGC GCCTTGCTTC   
  
  
+ TGGGATGGAA GGACCGGATG CTGGTTTCGG CTTCAGCTTG GCATTG  

- +Up\_Stream \_Len000AAATTT ATAAAATAAA ATAAAATTTA ATTATATAAA ATATTAAATT TTAATAATTA   
  
  
- TTAACTACTA CAATGGGTTA CAAATTCAAA AATTTATAAA AATAAAAAAT TAAATTTTTA TAGTATTATA   
  
  
- TATTATATTA AAATTCAATT TTATTCTATC AATTTAATAA AATATCATTT ATAAATAATA AAAAAAATTC   
  
  
- TTTTAACGGT ACACTAACAG TAGTCAGTTT GTGACGCAAC TGTCTTCGAC AGGAACGACG ATGAGAGTGG   
  
  
- AGAGTTGTAG CCTGGGCGCG TCTGCAATTG TAGAGGCATG TTTATGTTAA GGGACCGGTT TTAAGTTTAG   
  
  
- TTGGGATGAG CCATCAGCCA AGGAAGAAGA AGGACGGGAG ATATATATGT GGGTGTCGAC GCGACGTACA   
  
  
- AAAAGGCTCT TAGGAGTTTT TAGAATATTT GAAGGAGCAC ACCCATCTTT TTTCTTTTTC TTTTCCCAAA   
  
  
- ATTTTAGAAC CCAGTTTTGG GTAGTTGTGG TGTTAAACTG AGAAAGAAAA ATTAAGTTGG TGGCGGTCAA   
  
  
- GGAGCAACTA AAAGTCCATG GAGAAAAAGA ACGGGGAAAA AAACGAGCCA ACAACAAATT ACCATTTCTA   
  
  
- ACTCATGTTT CTTACTATAA ACTACTCAAC CACACAAATA AACTACTCGG GTCTACTAAA TTGAACATCT   
  
  
- AAAACACCAA AACCAAAGAC CCATAAGTAA CCAAACGACT TAAAGACCAA GTACTAGACC CCAATCTTTT   
  
  
- CAACTTTACT GAAAGACCTC CAACCTTAAA CTGATTTCGA TTAAAGAAAA ACAGAAGTTA AGAAAAAATC   
  
  
- AACACTCGAC TTAAACAACT GGTGAAGGTC GGACAACCTT TAATTTGAAT GAATCCTCTT CTCTTAGAGT   
  
  
- CTAAGACTCG TTTTCGAAAT ATACCAACAA ACCCTTTACC ATCCTTGATT CACTTTTGTG ACTTTAAAAT   
  
  
- TCGTTCGTTA AAACAAGTAA ACTAACCAGC CTACCAGACT AAATCCAAGA AAAGAACCAG ATTTAAAGAC   
  
  
- TCGTAACTAA CAAATTAACG CACCCCTTGA ACATAGGAAC ACTCAAGGAC CAAGTAAAGA ACCAACGAAT   
  
  
- GAGATCGAGG GAAAGTGTCA AAGATACCAA CCTTGAACCT TGAACCTTGA ACCTTCGGAT TAAATCAACT   
  
  
- TATAGAACCG AAACTCAATC GAAAAGAATC CTTTTCTACC AAGACGAGAG CTTTTAGATT AGATTAGCTA   
  
  
- TCTACGCAAT TCCTTCGTTT TGAAAACCCC TCTCCCTTCA ATTCGCTTCG TTCGACGACA AAGTTTCAAC   
  
  
- CTGACAAAAT AACCATTCAT ACTTTGTTAT CAAATATTCT CTAGTAACCC AAGAATTCCA CTATCTAGTA   
  
  
- CCCAATACTT ACTCGAATCA ACAAGAAAAC GAAAGTTGAC ATGCATTCCT ACTATCAATA GGGAGTAGAA   
  
  
- CGTAGTTTTT GCCGTTTGGC ACTACGGTTT TGAGTTTTTG ACTAAGGAGA CTGTTCCGAA GATGGATACT   
  
  
- TTTGATACGT CTAGTATAGG TAAACCGGAA GGGAGATCCT TGGTTCATCT CATTCAACCA TTTATTTATC   
  
  
- TCAACTTGCT TTGTGATGTC TAAACTTAAA CTAATTCCTC CCGATAATAT CTGATTCCAC ATCGAGTTTA   
  
  
- GGAAGACATC CCTAACAGAA TAACCGAAGA AAACCAACGA AGACCGAGTT CAGGAGAATA ATCAATCATA   
  
  
- CGTAATGGAA CTCTGTTCTA TCAACGAATT CGAACTCGAA GACATGAATA AACGCATAAC ATCTGTTGAT   
  
  
- CGACACCGTA TACTAAGACT TTACAAGGCT ACAGAAAAAG AAACCTACGA CCCAACACAG TACTCGAAAT   
  
  
- CCAAACAAAT AAACTACAGT CCTTCATACA TTTATTGTAC ACCGTAGAAC GTTAGATAAT GTCCTACTAT   
  
  
- AACCGGAAAC ACCCTTCTTT AACTTCTTCA ACAAAGTGAA TGACCTATCC CTCGTACCTG AGTGTAGTCG   
  
  
- AAAACTCTAA GCCATAAAGG TCTAGTATGA GTAGGATATT AAAGAGAGTC AGATCAGGGG TTAGTTAAGG   
  
  
- TTTATCCAAT AAACTTAGTG GCTTGCAAGC AAGATCTCTG AGAGGTTAGA GCGGGAAGAG TTGCGGGAAG   
  
  
- TTAACGCTGG GGTGACAATA TAACTCGCTG TCAGTCCTCG TGGTGTCAAG TTGTCCTTCA GAGAGCCGTG   
  
  
- TTTCGAGTAG AAGATTAAGA GGGGAACTCT GATCGTCAAT AATATTAGTC AAGTTGAGTT CGGGACAACC   
  
  
- TCTTAGTGGA GTCCCTAGTA GTGGGCTCTT AGAAAAAGTT GTTCGACGTC AATTATTTCA GTCGTAGCTC   
  
  
- GTACGTAACG TCCTCGATCT TTGACGAGAT TACTCGTGTC TAACACTCCT CTTACTTCGT TGACAGAGAT   
  
  
- AGGGTAGATA CCCACTTGGA GTAGTCGGGG TTCAGGGATC GGTCTCTAGT TCTAAGACCT TAGCTCTAGG   
  
  
- AGTCCCAAGT GCGGGCCGAC TTCAAGTGAG TAGTAACTCT TCTAATCCTC TGCTCCGAGT CTCACTCTTT   
  
  
- GCGGAGTTCC GTCACCTTCT TGGTTATGCC GGGTCACGTG GTGGGCCGTT AAACTTCGTC AACGAGTATC   
  
  
- TTACACGAGC CCGAAACAGT CTCTTAGCTT ATCTCCTAAA ACTCTTCAAT CAACTTGTCC GTTTCCCGTA   
  
  
- CCAGAGGTAA AGACCTCTGG GTTAAGTTGC TGAACCACGA ATGAACTAGC TCCCCGATCA CCGTTCCTTC   
  
  
- CTCAGAAGTC CATGCTTTTA GATGGCTCGA GAATCCACGT TTCTCGGGGA ACCAGTTCTG AACGAAAGCA   
  
  
- TGTACGTGTA GGAAATACTT TAAACGGGAA TGAACTTTAA GCCAATATAC CAACGTTTAC CCCGATATCG   
  
  
- ACTTCGAACG TCTCTACTCC TAGTGTATGT ATAGCAGCTG ATGGTCTAAC CAGTTCCTTG AGTTACCTAC   
  
  
- TGAGACAACG TTCGGGATCG GTGTTTTGGA CCTTCTGGGG GGTGACACTC TTAATGACCG TAACTACTAG   
  
  
- GGCAGAGATC CGTACGAGCC CCTCGAACGA ACCTCCGTCA CCCCTTTGCA AACCGTCAAG ACAGCCTCTT   
  
  
- TAAATTGTAC GGGTAACTCA AAGTCCGTCA CGGGCAAATA CGAAGACTCC AGTGGGTTCT TTACGAACTA   
  
  
- CACTCCGGTC CCACCCGGAA CCGACACTTG AAGGGTAACG TCGAGGTGGT GTGAGGGCTA CTCTCACAAC   
  
  
- TACACTTGTT GGGCTCCCTA CCTGAGGATT CTTACCACTT TAGTGAGCCA GGGTTCCAAC ATTGAAACCA   
  
  
- TCTTGTTCTC AGTTTGTGGT TGTGATGGGG AAACGACTGT TCCAAGTATC TTTGCGACCT GAAGATGAGT   
  
  
- CGTTACAAAC TTAGGTAGCT ACACTGTTAC GGTTCCCTGG CTTTCCTTTC CTAGTTACAG CTCGTCGTAA   
  
  
- CGGATCGGTT CCTGTAACAG TTGTAGTAGC GCACACTCCC CTTCCTGTCC CACCTCGCTG TACTCGAGAA   
  
  
- ACCCTTTACC TTCAGTTCCA AGTGGTACCG TCCTAAGGTT GTTAGGGGTA ACTCGAGCAT ACAATTGAGG   
  
  
- CACTATTTTC CTAATGAATC CACAATAAGT CTCATGATAT GCAACCACCT CTTCCTACCG CGGAACGAAG   
  
  
- ACCCTACCTT CCTGGCCTAC GACCAAAGCC GAAGTCGAAC CGTAAC

+     MRE

| Site Name | Organism | Position | Strand | Matrix score. | sequence | function |
| --- | --- | --- | --- | --- | --- | --- |
| MRE | Petroselinum crispum | 1026 | - | 7 | AACCTAA | MYB binding site involved in light responsiveness |
| MRE | Petroselinum crispum | 1892 | - | 7 | AACCTAA | MYB binding site involved in light responsiveness |

>HU01G01850.1   
+ +Up\_Stream \_Len000TTTAAA TATTTTATTT TATTTTAAAT TAATATATTT TATAATTTAA AATTATTAAT   
  
  
+ AATTGATGAT GTTACCCAAT GTTTAAGTTT TTAAATATTT TTATTTTTTA ATTTAAAAAT ATCATAATAT   
  
  
+ ATAATATAAT TTTAAGTTAA AATAAGATAG TTAAATTATT TTATAGTAAA TATTTATTAT TTTTTTTAAG   
  
  
+ AAAATTGCCA TGTGATTGTC ATCAGTCAAA CACTGCGTTG ACAGAAGCTG TCCTTGCTGC TACTCTCACC   
  
  
+ TCTCAACATC GGACCCGCGC AGACGTTAAC ATCTCCGTAC AAATACAATT CCCTGGCCAA AATTCAAATC   
  
  
+ AACCCTACTC GGTAGTCGGT TCCTTCTTCT TCCTGCCCTC TATATATACA CCCACAGCTG CGCTGCATGT   
  
  
+ TTTTCCGAGA ATCCTCAAAA ATCTTATAAA CTTCCTCGTG TGGGTAGAAA AAAGAAAAAG AAAAGGGTTT   
  
  
+ TAAAATCTTG GGTCAAAACC CATCAACACC ACAATTTGAC TCTTTCTTTT TAATTCAACC ACCGCCAGTT   
  
  
+ CCTCGTTGAT TTTCAGGTAC CTCTTTTTCT TGCCCCTTTT TTTGCTCGGT TGTTGTTTAA TGGTAAAGAT   
  
  
+ TGAGTACAAA GAATGATATT TGATGAGTTG GTGTGTTTAT TTGATGAGCC CAGATGATTT AACTTGTAGA   
  
  
+ TTTTGTGGTT TTGGTTTCTG GGTATTCATT GGTTTGCTGA ATTTCTGGTT CATGATCTGG GGTTAGAAAA   
  
  
+ GTTGAAATGA CTTTCTGGAG GTTGGAATTT GACTAAAGCT AATTTCTTTT TGTCTTCAAT TCTTTTTTAG   
  
  
+ TTGTGAGCTG AATTTGTTGA CCACTTCCAG CCTGTTGGAA ATTAAACTTA CTTAGGAGAA GAGAATCTCA   
  
  
+ GATTCTGAGC AAAAGCTTTA TATGGTTGTT TGGGAAATGG TAGGAACTAA GTGAAAACAC TGAAATTTTA   
  
  
+ AGCAAGCAAT TTTGTTCATT TGATTGGTCG GATGGTCTGA TTTAGGTTCT TTTCTTGGTC TAAATTTCTG   
  
  
+ AGCATTGATT GTTTAATTGC GTGGGGAACT TGTATCCTTG TGAGTTCCTG GTTCATTTCT TGGTTGCTTA   
  
  
+ CTCTAGCTCC CTTTCACAGT TTCTATGGTT GGAACTTGGA ACTTGGAACT TGGAAGCCTA ATTTAGTTGA   
  
  
+ ATATCTTGGC TTTGAGTTAG CTTTTCTTAG GAAAAGATGG TTCTGCTCTC GAAAATCTAA TCTAATCGAT   
  
  
+ AGATGCGTTA AGGAAGCAAA ACTTTTGGGG AGAGGGAAGT TAAGCGAAGC AAGCTGCTGT TTCAAAGTTG   
  
  
+ GACTGTTTTA TTGGTAAGTA TGAAACAATA GTTTATAAGA GATCATTGGG TTCTTAAGGT GATAGATCAT   
  
  
+ GGGTTATGAA TGAGCTTAGT TGTTCTTTTG CTTTCAACTG TACGTAAGGA TGATAGTTAT CCCTCATCTT   
  
  
+ GCATCAAAAA CGGCAAACCG TGATGCCAAA ACTCAAAAAC TGATTCCTCT GACAAGGCTT CTACCTATGA   
  
  
+ AAACTATGCA GATCATATCC ATTTGGCCTT CCCTCTAGGA ACCAAGTAGA GTAAGTTGGT AAATAAATAG   
  
  
+ AGTTGAACGA AACACTACAG ATTTGAATTT GATTAAGGAG GGCTATTATA GACTAAGGTG TAGCTCAAAT   
  
  
+ CCTTCTGTAG GGATTGTCTT ATTGGCTTCT TTTGGTTGCT TCTGGCTCAA GTCCTCTTAT TAGTTAGTAT   
  
  
+ GCATTACCTT GAGACAAGAT AGTTGCTTAA GCTTGAGCTT CTGTACTTAT TTGCGTATTG TAGACAACTA   
  
  
+ GCTGTGGCAT ATGATTCTGA AATGTTCCGA TGTCTTTTTC TTTGGATGCT GGGTTGTGTC ATGAGCTTTA   
  
  
+ GGTTTGTTTA TTTGATGTCA GGAAGTATGT AAATAACATG TGGCATCTTG CAATCTATTA CAGGATGATA   
  
  
+ TTGGCCTTTG TGGGAAGAAA TTGAAGAAGT TGTTTCACTT ACTGGATAGG GAGCATGGAC TCACATCAGC   
  
  
+ TTTTGAGATT CGGTATTTCC AGATCATACT CATCCTATAA TTTCTCTCAG TCTAGTCCCC AATCAATTCC   
  
  
+ AAATAGGTTA TTTGAATCAC CGAACGTTCG TTCTAGAGAC TCTCCAATCT CGCCCTTCTC AACGCCCTTC   
  
  
+ AATTGCGACC CCACTGTTAT ATTGAGCGAC AGTCAGGAGC ACCACAGTTC AACAGGAAGT CTCTCGGCAC   
  
  
+ AAAGCTCATC TTCTAATTCT CCCCTTGAGA CTAGCAGTTA TTATAATCAG TTCAACTCAA GCCCTGTTGG   
  
  
+ AGAATCACCT CAGGGATCAT CACCCGAGAA TCTTTTTCAA CAAGCTGCAG TTAATAAAGT CAGCATCGAG   
  
  
+ CATGCATTGC AGGAGCTAGA AACTGCTCTA ATGAGCACAG ATTGTGAGGA GAATGAAGCA ACTGTCTCTA   
  
  
+ TCCCATCTAT GGGTGAACCT CATCAGCCCC AAGTCCCTAG CCAGAGATCA AGATTCTGGA ATCGAGATCC   
  
  
+ TCAGGGTTCA CGCCCGGCTG AAGTTCACTC ATCATTGAGA AGATTAGGAG ACGAGGCTCA GAGTGAGAAA   
  
  
+ CGCCTCAAGG CAGTGGAAGA ACCAATACGG CCCAGTGCAC CACCCGGCAA TTTGAAGCAG TTGCTCATAG   
  
  
+ AATGTGCTCG GGCTTTGTCA GAGAATCGAA TAGAGGATTT TGAGAAGTTA GTTGAACAGG CAAAGGGCAT   
  
  
+ GGTCTCCATT TCTGGAGACC CAATTCAACG ACTTGGTGCT TACTTGATCG AGGGGCTAGT GGCAAGGAAG   
  
  
+ GAGTCTTCAG GTACGAAAAT CTACCGAGCT CTTAGGTGCA AAGAGCCCCT TGGTCAAGAC TTGCTTTCGT   
  
  
+ ACATGCACAT CCTTTATGAA ATTTGCCCTT ACTTGAAATT CGGTTATATG GTTGCAAATG GGGCTATAGC   
  
  
+ TGAAGCTTGC AGAGATGAGG ATCACATACA TATCGTCGAC TACCAGATTG GTCAAGGAAC TCAATGGATG   
  
  
+ ACTCTGTTGC AAGCCCTAGC CACAAAACCT GGAAGACCCC CCACTGTGAG AATTACTGGC ATTGATGATC   
  
  
+ CCGTCTCTAG GCATGCTCGG GGAGCTTGCT TGGAGGCAGT GGGGAAACGT TTGGCAGTTC TGTCGGAGAA   
  
  
+ ATTTAACATG CCCATTGAGT TTCAGGCAGT GCCCGTTTAT GCTTCTGAGG TCACCCAAGA AATGCTTGAT   
  
  
+ GTGAGGCCAG GGTGGGCCTT GGCTGTGAAC TTCCCATTGC AGCTCCACCA CACTCCCGAT GAGAGTGTTG   
  
  
+ ATGTGAACAA CCCGAGGGAT GGACTCCTAA GAATGGTGAA ATCACTCGGT CCCAAGGTTG TAACTTTGGT   
  
  
+ AGAACAAGAG TCAAACACCA ACACTACCCC TTTGCTGACA AGGTTCATAG AAACGCTGGA CTTCTACTCA   
  
  
+ GCAATGTTTG AATCCATCGA TGTGACAATG CCAAGGGACC GAAAGGAAAG GATCAATGTC GAGCAGCATT   
  
  
+ GCCTAGCCAA GGACATTGTC AACATCATCG CGTGTGAGGG GAAGGACAGG GTGGAGCGAC ATGAGCTCTT   
  
  
+ TGGGAAATGG AAGTCAAGGT TCACCATGGC AGGATTCCAA CAATCCCCAT TGAGCTCGTA TGTTAACTCC   
  
  
+ GTGATAAAAG GATTACTTAG GTGTTATTCA GAGTACTATA CGTTGGTGGA GAAGGATGGC GCCTTGCTTC   
  
  
+ TGGGATGGAA GGACCGGATG CTGGTTTCGG CTTCAGCTTG GCATTG  

- +Up\_Stream \_Len000AAATTT ATAAAATAAA ATAAAATTTA ATTATATAAA ATATTAAATT TTAATAATTA   
  
  
- TTAACTACTA CAATGGGTTA CAAATTCAAA AATTTATAAA AATAAAAAAT TAAATTTTTA TAGTATTATA   
  
  
- TATTATATTA AAATTCAATT TTATTCTATC AATTTAATAA AATATCATTT ATAAATAATA AAAAAAATTC   
  
  
- TTTTAACGGT ACACTAACAG TAGTCAGTTT GTGACGCAAC TGTCTTCGAC AGGAACGACG ATGAGAGTGG   
  
  
- AGAGTTGTAG CCTGGGCGCG TCTGCAATTG TAGAGGCATG TTTATGTTAA GGGACCGGTT TTAAGTTTAG   
  
  
- TTGGGATGAG CCATCAGCCA AGGAAGAAGA AGGACGGGAG ATATATATGT GGGTGTCGAC GCGACGTACA   
  
  
- AAAAGGCTCT TAGGAGTTTT TAGAATATTT GAAGGAGCAC ACCCATCTTT TTTCTTTTTC TTTTCCCAAA   
  
  
- ATTTTAGAAC CCAGTTTTGG GTAGTTGTGG TGTTAAACTG AGAAAGAAAA ATTAAGTTGG TGGCGGTCAA   
  
  
- GGAGCAACTA AAAGTCCATG GAGAAAAAGA ACGGGGAAAA AAACGAGCCA ACAACAAATT ACCATTTCTA   
  
  
- ACTCATGTTT CTTACTATAA ACTACTCAAC CACACAAATA AACTACTCGG GTCTACTAAA TTGAACATCT   
  
  
- AAAACACCAA AACCAAAGAC CCATAAGTAA CCAAACGACT TAAAGACCAA GTACTAGACC CCAATCTTTT   
  
  
- CAACTTTACT GAAAGACCTC CAACCTTAAA CTGATTTCGA TTAAAGAAAA ACAGAAGTTA AGAAAAAATC   
  
  
- AACACTCGAC TTAAACAACT GGTGAAGGTC GGACAACCTT TAATTTGAAT GAATCCTCTT CTCTTAGAGT   
  
  
- CTAAGACTCG TTTTCGAAAT ATACCAACAA ACCCTTTACC ATCCTTGATT CACTTTTGTG ACTTTAAAAT   
  
  
- TCGTTCGTTA AAACAAGTAA ACTAACCAGC CTACCAGACT AAATCCAAGA AAAGAACCAG ATTTAAAGAC   
  
  
- TCGTAACTAA CAAATTAACG CACCCCTTGA ACATAGGAAC ACTCAAGGAC CAAGTAAAGA ACCAACGAAT   
  
  
- GAGATCGAGG GAAAGTGTCA AAGATACCAA CCTTGAACCT TGAACCTTGA ACCTTCGGAT TAAATCAACT   
  
  
- TATAGAACCG AAACTCAATC GAAAAGAATC CTTTTCTACC AAGACGAGAG CTTTTAGATT AGATTAGCTA   
  
  
- TCTACGCAAT TCCTTCGTTT TGAAAACCCC TCTCCCTTCA ATTCGCTTCG TTCGACGACA AAGTTTCAAC   
  
  
- CTGACAAAAT AACCATTCAT ACTTTGTTAT CAAATATTCT CTAGTAACCC AAGAATTCCA CTATCTAGTA   
  
  
- CCCAATACTT ACTCGAATCA ACAAGAAAAC GAAAGTTGAC ATGCATTCCT ACTATCAATA GGGAGTAGAA   
  
  
- CGTAGTTTTT GCCGTTTGGC ACTACGGTTT TGAGTTTTTG ACTAAGGAGA CTGTTCCGAA GATGGATACT   
  
  
- TTTGATACGT CTAGTATAGG TAAACCGGAA GGGAGATCCT TGGTTCATCT CATTCAACCA TTTATTTATC   
  
  
- TCAACTTGCT TTGTGATGTC TAAACTTAAA CTAATTCCTC CCGATAATAT CTGATTCCAC ATCGAGTTTA   
  
  
- GGAAGACATC CCTAACAGAA TAACCGAAGA AAACCAACGA AGACCGAGTT CAGGAGAATA ATCAATCATA   
  
  
- CGTAATGGAA CTCTGTTCTA TCAACGAATT CGAACTCGAA GACATGAATA AACGCATAAC ATCTGTTGAT   
  
  
- CGACACCGTA TACTAAGACT TTACAAGGCT ACAGAAAAAG AAACCTACGA CCCAACACAG TACTCGAAAT   
  
  
- CCAAACAAAT AAACTACAGT CCTTCATACA TTTATTGTAC ACCGTAGAAC GTTAGATAAT GTCCTACTAT   
  
  
- AACCGGAAAC ACCCTTCTTT AACTTCTTCA ACAAAGTGAA TGACCTATCC CTCGTACCTG AGTGTAGTCG   
  
  
- AAAACTCTAA GCCATAAAGG TCTAGTATGA GTAGGATATT AAAGAGAGTC AGATCAGGGG TTAGTTAAGG   
  
  
- TTTATCCAAT AAACTTAGTG GCTTGCAAGC AAGATCTCTG AGAGGTTAGA GCGGGAAGAG TTGCGGGAAG   
  
  
- TTAACGCTGG GGTGACAATA TAACTCGCTG TCAGTCCTCG TGGTGTCAAG TTGTCCTTCA GAGAGCCGTG   
  
  
- TTTCGAGTAG AAGATTAAGA GGGGAACTCT GATCGTCAAT AATATTAGTC AAGTTGAGTT CGGGACAACC   
  
  
- TCTTAGTGGA GTCCCTAGTA GTGGGCTCTT AGAAAAAGTT GTTCGACGTC AATTATTTCA GTCGTAGCTC   
  
  
- GTACGTAACG TCCTCGATCT TTGACGAGAT TACTCGTGTC TAACACTCCT CTTACTTCGT TGACAGAGAT   
  
  
- AGGGTAGATA CCCACTTGGA GTAGTCGGGG TTCAGGGATC GGTCTCTAGT TCTAAGACCT TAGCTCTAGG   
  
  
- AGTCCCAAGT GCGGGCCGAC TTCAAGTGAG TAGTAACTCT TCTAATCCTC TGCTCCGAGT CTCACTCTTT   
  
  
- GCGGAGTTCC GTCACCTTCT TGGTTATGCC GGGTCACGTG GTGGGCCGTT AAACTTCGTC AACGAGTATC   
  
  
- TTACACGAGC CCGAAACAGT CTCTTAGCTT ATCTCCTAAA ACTCTTCAAT CAACTTGTCC GTTTCCCGTA   
  
  
- CCAGAGGTAA AGACCTCTGG GTTAAGTTGC TGAACCACGA ATGAACTAGC TCCCCGATCA CCGTTCCTTC   
  
  
- CTCAGAAGTC CATGCTTTTA GATGGCTCGA GAATCCACGT TTCTCGGGGA ACCAGTTCTG AACGAAAGCA   
  
  
- TGTACGTGTA GGAAATACTT TAAACGGGAA TGAACTTTAA GCCAATATAC CAACGTTTAC CCCGATATCG   
  
  
- ACTTCGAACG TCTCTACTCC TAGTGTATGT ATAGCAGCTG ATGGTCTAAC CAGTTCCTTG AGTTACCTAC   
  
  
- TGAGACAACG TTCGGGATCG GTGTTTTGGA CCTTCTGGGG GGTGACACTC TTAATGACCG TAACTACTAG   
  
  
- GGCAGAGATC CGTACGAGCC CCTCGAACGA ACCTCCGTCA CCCCTTTGCA AACCGTCAAG ACAGCCTCTT   
  
  
- TAAATTGTAC GGGTAACTCA AAGTCCGTCA CGGGCAAATA CGAAGACTCC AGTGGGTTCT TTACGAACTA   
  
  
- CACTCCGGTC CCACCCGGAA CCGACACTTG AAGGGTAACG TCGAGGTGGT GTGAGGGCTA CTCTCACAAC   
  
  
- TACACTTGTT GGGCTCCCTA CCTGAGGATT CTTACCACTT TAGTGAGCCA GGGTTCCAAC ATTGAAACCA   
  
  
- TCTTGTTCTC AGTTTGTGGT TGTGATGGGG AAACGACTGT TCCAAGTATC TTTGCGACCT GAAGATGAGT   
  
  
- CGTTACAAAC TTAGGTAGCT ACACTGTTAC GGTTCCCTGG CTTTCCTTTC CTAGTTACAG CTCGTCGTAA   
  
  
- CGGATCGGTT CCTGTAACAG TTGTAGTAGC GCACACTCCC CTTCCTGTCC CACCTCGCTG TACTCGAGAA   
  
  
- ACCCTTTACC TTCAGTTCCA AGTGGTACCG TCCTAAGGTT GTTAGGGGTA ACTCGAGCAT ACAATTGAGG   
  
  
- CACTATTTTC CTAATGAATC CACAATAAGT CTCATGATAT GCAACCACCT CTTCCTACCG CGGAACGAAG   
  
  
- ACCCTACCTT CCTGGCCTAC GACCAAAGCC GAAGTCGAAC CGTAAC

+     MYB

| Site Name | Organism | Position | Strand | Matrix score. | sequence | function |
| --- | --- | --- | --- | --- | --- | --- |
| MYB | Arabidopsis thaliana | 3018 | - | 6 | CAACAG |  |
| MYB | Arabidopsis thaliana | 2923 | - | 6 | CAACCA |  |
| MYB | Arabidopsis thaliana | 2308 | - | 6 | CAACAG |  |
| MYB | Arabidopsis thaliana | 2224 | + | 6 | CAACAG |  |
| MYB | Arabidopsis thaliana | 1717 | - | 6 | CAACCA |  |
| MYB | Arabidopsis thaliana | 1150 | - | 6 | CAACCA |  |
| MYB | Arabidopsis thaliana | 1115 | - | 6 | CAACCA |  |
| MYB | Arabidopsis thaliana | 937 | - | 6 | CAACCA |  |
| MYB | Arabidopsis thaliana | 876 | - | 6 | CAACAG |  |
| MYB | Arabidopsis thaliana | 550 | + | 6 | CAACCA |  |

>HU01G01850.1   
+ +Up\_Stream \_Len000TTTAAA TATTTTATTT TATTTTAAAT TAATATATTT TATAATTTAA AATTATTAAT   
  
  
+ AATTGATGAT GTTACCCAAT GTTTAAGTTT TTAAATATTT TTATTTTTTA ATTTAAAAAT ATCATAATAT   
  
  
+ ATAATATAAT TTTAAGTTAA AATAAGATAG TTAAATTATT TTATAGTAAA TATTTATTAT TTTTTTTAAG   
  
  
+ AAAATTGCCA TGTGATTGTC ATCAGTCAAA CACTGCGTTG ACAGAAGCTG TCCTTGCTGC TACTCTCACC   
  
  
+ TCTCAACATC GGACCCGCGC AGACGTTAAC ATCTCCGTAC AAATACAATT CCCTGGCCAA AATTCAAATC   
  
  
+ AACCCTACTC GGTAGTCGGT TCCTTCTTCT TCCTGCCCTC TATATATACA CCCACAGCTG CGCTGCATGT   
  
  
+ TTTTCCGAGA ATCCTCAAAA ATCTTATAAA CTTCCTCGTG TGGGTAGAAA AAAGAAAAAG AAAAGGGTTT   
  
  
+ TAAAATCTTG GGTCAAAACC CATCAACACC ACAATTTGAC TCTTTCTTTT TAATTCAACC ACCGCCAGTT   
  
  
+ CCTCGTTGAT TTTCAGGTAC CTCTTTTTCT TGCCCCTTTT TTTGCTCGGT TGTTGTTTAA TGGTAAAGAT   
  
  
+ TGAGTACAAA GAATGATATT TGATGAGTTG GTGTGTTTAT TTGATGAGCC CAGATGATTT AACTTGTAGA   
  
  
+ TTTTGTGGTT TTGGTTTCTG GGTATTCATT GGTTTGCTGA ATTTCTGGTT CATGATCTGG GGTTAGAAAA   
  
  
+ GTTGAAATGA CTTTCTGGAG GTTGGAATTT GACTAAAGCT AATTTCTTTT TGTCTTCAAT TCTTTTTTAG   
  
  
+ TTGTGAGCTG AATTTGTTGA CCACTTCCAG CCTGTTGGAA ATTAAACTTA CTTAGGAGAA GAGAATCTCA   
  
  
+ GATTCTGAGC AAAAGCTTTA TATGGTTGTT TGGGAAATGG TAGGAACTAA GTGAAAACAC TGAAATTTTA   
  
  
+ AGCAAGCAAT TTTGTTCATT TGATTGGTCG GATGGTCTGA TTTAGGTTCT TTTCTTGGTC TAAATTTCTG   
  
  
+ AGCATTGATT GTTTAATTGC GTGGGGAACT TGTATCCTTG TGAGTTCCTG GTTCATTTCT TGGTTGCTTA   
  
  
+ CTCTAGCTCC CTTTCACAGT TTCTATGGTT GGAACTTGGA ACTTGGAACT TGGAAGCCTA ATTTAGTTGA   
  
  
+ ATATCTTGGC TTTGAGTTAG CTTTTCTTAG GAAAAGATGG TTCTGCTCTC GAAAATCTAA TCTAATCGAT   
  
  
+ AGATGCGTTA AGGAAGCAAA ACTTTTGGGG AGAGGGAAGT TAAGCGAAGC AAGCTGCTGT TTCAAAGTTG   
  
  
+ GACTGTTTTA TTGGTAAGTA TGAAACAATA GTTTATAAGA GATCATTGGG TTCTTAAGGT GATAGATCAT   
  
  
+ GGGTTATGAA TGAGCTTAGT TGTTCTTTTG CTTTCAACTG TACGTAAGGA TGATAGTTAT CCCTCATCTT   
  
  
+ GCATCAAAAA CGGCAAACCG TGATGCCAAA ACTCAAAAAC TGATTCCTCT GACAAGGCTT CTACCTATGA   
  
  
+ AAACTATGCA GATCATATCC ATTTGGCCTT CCCTCTAGGA ACCAAGTAGA GTAAGTTGGT AAATAAATAG   
  
  
+ AGTTGAACGA AACACTACAG ATTTGAATTT GATTAAGGAG GGCTATTATA GACTAAGGTG TAGCTCAAAT   
  
  
+ CCTTCTGTAG GGATTGTCTT ATTGGCTTCT TTTGGTTGCT TCTGGCTCAA GTCCTCTTAT TAGTTAGTAT   
  
  
+ GCATTACCTT GAGACAAGAT AGTTGCTTAA GCTTGAGCTT CTGTACTTAT TTGCGTATTG TAGACAACTA   
  
  
+ GCTGTGGCAT ATGATTCTGA AATGTTCCGA TGTCTTTTTC TTTGGATGCT GGGTTGTGTC ATGAGCTTTA   
  
  
+ GGTTTGTTTA TTTGATGTCA GGAAGTATGT AAATAACATG TGGCATCTTG CAATCTATTA CAGGATGATA   
  
  
+ TTGGCCTTTG TGGGAAGAAA TTGAAGAAGT TGTTTCACTT ACTGGATAGG GAGCATGGAC TCACATCAGC   
  
  
+ TTTTGAGATT CGGTATTTCC AGATCATACT CATCCTATAA TTTCTCTCAG TCTAGTCCCC AATCAATTCC   
  
  
+ AAATAGGTTA TTTGAATCAC CGAACGTTCG TTCTAGAGAC TCTCCAATCT CGCCCTTCTC AACGCCCTTC   
  
  
+ AATTGCGACC CCACTGTTAT ATTGAGCGAC AGTCAGGAGC ACCACAGTTC AACAGGAAGT CTCTCGGCAC   
  
  
+ AAAGCTCATC TTCTAATTCT CCCCTTGAGA CTAGCAGTTA TTATAATCAG TTCAACTCAA GCCCTGTTGG   
  
  
+ AGAATCACCT CAGGGATCAT CACCCGAGAA TCTTTTTCAA CAAGCTGCAG TTAATAAAGT CAGCATCGAG   
  
  
+ CATGCATTGC AGGAGCTAGA AACTGCTCTA ATGAGCACAG ATTGTGAGGA GAATGAAGCA ACTGTCTCTA   
  
  
+ TCCCATCTAT GGGTGAACCT CATCAGCCCC AAGTCCCTAG CCAGAGATCA AGATTCTGGA ATCGAGATCC   
  
  
+ TCAGGGTTCA CGCCCGGCTG AAGTTCACTC ATCATTGAGA AGATTAGGAG ACGAGGCTCA GAGTGAGAAA   
  
  
+ CGCCTCAAGG CAGTGGAAGA ACCAATACGG CCCAGTGCAC CACCCGGCAA TTTGAAGCAG TTGCTCATAG   
  
  
+ AATGTGCTCG GGCTTTGTCA GAGAATCGAA TAGAGGATTT TGAGAAGTTA GTTGAACAGG CAAAGGGCAT   
  
  
+ GGTCTCCATT TCTGGAGACC CAATTCAACG ACTTGGTGCT TACTTGATCG AGGGGCTAGT GGCAAGGAAG   
  
  
+ GAGTCTTCAG GTACGAAAAT CTACCGAGCT CTTAGGTGCA AAGAGCCCCT TGGTCAAGAC TTGCTTTCGT   
  
  
+ ACATGCACAT CCTTTATGAA ATTTGCCCTT ACTTGAAATT CGGTTATATG GTTGCAAATG GGGCTATAGC   
  
  
+ TGAAGCTTGC AGAGATGAGG ATCACATACA TATCGTCGAC TACCAGATTG GTCAAGGAAC TCAATGGATG   
  
  
+ ACTCTGTTGC AAGCCCTAGC CACAAAACCT GGAAGACCCC CCACTGTGAG AATTACTGGC ATTGATGATC   
  
  
+ CCGTCTCTAG GCATGCTCGG GGAGCTTGCT TGGAGGCAGT GGGGAAACGT TTGGCAGTTC TGTCGGAGAA   
  
  
+ ATTTAACATG CCCATTGAGT TTCAGGCAGT GCCCGTTTAT GCTTCTGAGG TCACCCAAGA AATGCTTGAT   
  
  
+ GTGAGGCCAG GGTGGGCCTT GGCTGTGAAC TTCCCATTGC AGCTCCACCA CACTCCCGAT GAGAGTGTTG   
  
  
+ ATGTGAACAA CCCGAGGGAT GGACTCCTAA GAATGGTGAA ATCACTCGGT CCCAAGGTTG TAACTTTGGT   
  
  
+ AGAACAAGAG TCAAACACCA ACACTACCCC TTTGCTGACA AGGTTCATAG AAACGCTGGA CTTCTACTCA   
  
  
+ GCAATGTTTG AATCCATCGA TGTGACAATG CCAAGGGACC GAAAGGAAAG GATCAATGTC GAGCAGCATT   
  
  
+ GCCTAGCCAA GGACATTGTC AACATCATCG CGTGTGAGGG GAAGGACAGG GTGGAGCGAC ATGAGCTCTT   
  
  
+ TGGGAAATGG AAGTCAAGGT TCACCATGGC AGGATTCCAA CAATCCCCAT TGAGCTCGTA TGTTAACTCC   
  
  
+ GTGATAAAAG GATTACTTAG GTGTTATTCA GAGTACTATA CGTTGGTGGA GAAGGATGGC GCCTTGCTTC   
  
  
+ TGGGATGGAA GGACCGGATG CTGGTTTCGG CTTCAGCTTG GCATTG  

- +Up\_Stream \_Len000AAATTT ATAAAATAAA ATAAAATTTA ATTATATAAA ATATTAAATT TTAATAATTA   
  
  
- TTAACTACTA CAATGGGTTA CAAATTCAAA AATTTATAAA AATAAAAAAT TAAATTTTTA TAGTATTATA   
  
  
- TATTATATTA AAATTCAATT TTATTCTATC AATTTAATAA AATATCATTT ATAAATAATA AAAAAAATTC   
  
  
- TTTTAACGGT ACACTAACAG TAGTCAGTTT GTGACGCAAC TGTCTTCGAC AGGAACGACG ATGAGAGTGG   
  
  
- AGAGTTGTAG CCTGGGCGCG TCTGCAATTG TAGAGGCATG TTTATGTTAA GGGACCGGTT TTAAGTTTAG   
  
  
- TTGGGATGAG CCATCAGCCA AGGAAGAAGA AGGACGGGAG ATATATATGT GGGTGTCGAC GCGACGTACA   
  
  
- AAAAGGCTCT TAGGAGTTTT TAGAATATTT GAAGGAGCAC ACCCATCTTT TTTCTTTTTC TTTTCCCAAA   
  
  
- ATTTTAGAAC CCAGTTTTGG GTAGTTGTGG TGTTAAACTG AGAAAGAAAA ATTAAGTTGG TGGCGGTCAA   
  
  
- GGAGCAACTA AAAGTCCATG GAGAAAAAGA ACGGGGAAAA AAACGAGCCA ACAACAAATT ACCATTTCTA   
  
  
- ACTCATGTTT CTTACTATAA ACTACTCAAC CACACAAATA AACTACTCGG GTCTACTAAA TTGAACATCT   
  
  
- AAAACACCAA AACCAAAGAC CCATAAGTAA CCAAACGACT TAAAGACCAA GTACTAGACC CCAATCTTTT   
  
  
- CAACTTTACT GAAAGACCTC CAACCTTAAA CTGATTTCGA TTAAAGAAAA ACAGAAGTTA AGAAAAAATC   
  
  
- AACACTCGAC TTAAACAACT GGTGAAGGTC GGACAACCTT TAATTTGAAT GAATCCTCTT CTCTTAGAGT   
  
  
- CTAAGACTCG TTTTCGAAAT ATACCAACAA ACCCTTTACC ATCCTTGATT CACTTTTGTG ACTTTAAAAT   
  
  
- TCGTTCGTTA AAACAAGTAA ACTAACCAGC CTACCAGACT AAATCCAAGA AAAGAACCAG ATTTAAAGAC   
  
  
- TCGTAACTAA CAAATTAACG CACCCCTTGA ACATAGGAAC ACTCAAGGAC CAAGTAAAGA ACCAACGAAT   
  
  
- GAGATCGAGG GAAAGTGTCA AAGATACCAA CCTTGAACCT TGAACCTTGA ACCTTCGGAT TAAATCAACT   
  
  
- TATAGAACCG AAACTCAATC GAAAAGAATC CTTTTCTACC AAGACGAGAG CTTTTAGATT AGATTAGCTA   
  
  
- TCTACGCAAT TCCTTCGTTT TGAAAACCCC TCTCCCTTCA ATTCGCTTCG TTCGACGACA AAGTTTCAAC   
  
  
- CTGACAAAAT AACCATTCAT ACTTTGTTAT CAAATATTCT CTAGTAACCC AAGAATTCCA CTATCTAGTA   
  
  
- CCCAATACTT ACTCGAATCA ACAAGAAAAC GAAAGTTGAC ATGCATTCCT ACTATCAATA GGGAGTAGAA   
  
  
- CGTAGTTTTT GCCGTTTGGC ACTACGGTTT TGAGTTTTTG ACTAAGGAGA CTGTTCCGAA GATGGATACT   
  
  
- TTTGATACGT CTAGTATAGG TAAACCGGAA GGGAGATCCT TGGTTCATCT CATTCAACCA TTTATTTATC   
  
  
- TCAACTTGCT TTGTGATGTC TAAACTTAAA CTAATTCCTC CCGATAATAT CTGATTCCAC ATCGAGTTTA   
  
  
- GGAAGACATC CCTAACAGAA TAACCGAAGA AAACCAACGA AGACCGAGTT CAGGAGAATA ATCAATCATA   
  
  
- CGTAATGGAA CTCTGTTCTA TCAACGAATT CGAACTCGAA GACATGAATA AACGCATAAC ATCTGTTGAT   
  
  
- CGACACCGTA TACTAAGACT TTACAAGGCT ACAGAAAAAG AAACCTACGA CCCAACACAG TACTCGAAAT   
  
  
- CCAAACAAAT AAACTACAGT CCTTCATACA TTTATTGTAC ACCGTAGAAC GTTAGATAAT GTCCTACTAT   
  
  
- AACCGGAAAC ACCCTTCTTT AACTTCTTCA ACAAAGTGAA TGACCTATCC CTCGTACCTG AGTGTAGTCG   
  
  
- AAAACTCTAA GCCATAAAGG TCTAGTATGA GTAGGATATT AAAGAGAGTC AGATCAGGGG TTAGTTAAGG   
  
  
- TTTATCCAAT AAACTTAGTG GCTTGCAAGC AAGATCTCTG AGAGGTTAGA GCGGGAAGAG TTGCGGGAAG   
  
  
- TTAACGCTGG GGTGACAATA TAACTCGCTG TCAGTCCTCG TGGTGTCAAG TTGTCCTTCA GAGAGCCGTG   
  
  
- TTTCGAGTAG AAGATTAAGA GGGGAACTCT GATCGTCAAT AATATTAGTC AAGTTGAGTT CGGGACAACC   
  
  
- TCTTAGTGGA GTCCCTAGTA GTGGGCTCTT AGAAAAAGTT GTTCGACGTC AATTATTTCA GTCGTAGCTC   
  
  
- GTACGTAACG TCCTCGATCT TTGACGAGAT TACTCGTGTC TAACACTCCT CTTACTTCGT TGACAGAGAT   
  
  
- AGGGTAGATA CCCACTTGGA GTAGTCGGGG TTCAGGGATC GGTCTCTAGT TCTAAGACCT TAGCTCTAGG   
  
  
- AGTCCCAAGT GCGGGCCGAC TTCAAGTGAG TAGTAACTCT TCTAATCCTC TGCTCCGAGT CTCACTCTTT   
  
  
- GCGGAGTTCC GTCACCTTCT TGGTTATGCC GGGTCACGTG GTGGGCCGTT AAACTTCGTC AACGAGTATC   
  
  
- TTACACGAGC CCGAAACAGT CTCTTAGCTT ATCTCCTAAA ACTCTTCAAT CAACTTGTCC GTTTCCCGTA   
  
  
- CCAGAGGTAA AGACCTCTGG GTTAAGTTGC TGAACCACGA ATGAACTAGC TCCCCGATCA CCGTTCCTTC   
  
  
- CTCAGAAGTC CATGCTTTTA GATGGCTCGA GAATCCACGT TTCTCGGGGA ACCAGTTCTG AACGAAAGCA   
  
  
- TGTACGTGTA GGAAATACTT TAAACGGGAA TGAACTTTAA GCCAATATAC CAACGTTTAC CCCGATATCG   
  
  
- ACTTCGAACG TCTCTACTCC TAGTGTATGT ATAGCAGCTG ATGGTCTAAC CAGTTCCTTG AGTTACCTAC   
  
  
- TGAGACAACG TTCGGGATCG GTGTTTTGGA CCTTCTGGGG GGTGACACTC TTAATGACCG TAACTACTAG   
  
  
- GGCAGAGATC CGTACGAGCC CCTCGAACGA ACCTCCGTCA CCCCTTTGCA AACCGTCAAG ACAGCCTCTT   
  
  
- TAAATTGTAC GGGTAACTCA AAGTCCGTCA CGGGCAAATA CGAAGACTCC AGTGGGTTCT TTACGAACTA   
  
  
- CACTCCGGTC CCACCCGGAA CCGACACTTG AAGGGTAACG TCGAGGTGGT GTGAGGGCTA CTCTCACAAC   
  
  
- TACACTTGTT GGGCTCCCTA CCTGAGGATT CTTACCACTT TAGTGAGCCA GGGTTCCAAC ATTGAAACCA   
  
  
- TCTTGTTCTC AGTTTGTGGT TGTGATGGGG AAACGACTGT TCCAAGTATC TTTGCGACCT GAAGATGAGT   
  
  
- CGTTACAAAC TTAGGTAGCT ACACTGTTAC GGTTCCCTGG CTTTCCTTTC CTAGTTACAG CTCGTCGTAA   
  
  
- CGGATCGGTT CCTGTAACAG TTGTAGTAGC GCACACTCCC CTTCCTGTCC CACCTCGCTG TACTCGAGAA   
  
  
- ACCCTTTACC TTCAGTTCCA AGTGGTACCG TCCTAAGGTT GTTAGGGGTA ACTCGAGCAT ACAATTGAGG   
  
  
- CACTATTTTC CTAATGAATC CACAATAAGT CTCATGATAT GCAACCACCT CTTCCTACCG CGGAACGAAG   
  
  
- ACCCTACCTT CCTGGCCTAC GACCAAAGCC GAAGTCGAAC CGTAAC

+     MYC

| Site Name | Organism | Position | Strand | Matrix score. | sequence | function |
| --- | --- | --- | --- | --- | --- | --- |
| MYC | Arabidopsis thaliana | 2929 | - | 6 | CATTTG |  |
| MYC | Arabidopsis thaliana | 2174 | - | 6 | CAATTG |  |
| MYC | Arabidopsis thaliana | 1931 | + | 6 | CATGTG |  |
| MYC | Arabidopsis thaliana | 1564 | + | 6 | CATTTG |  |
| MYC | Arabidopsis thaliana | 1001 | + | 6 | CATTTG |  |
| MYC | Arabidopsis thaliana | 223 | + | 6 | CATGTG |  |

>HU01G01850.1   
+ +Up\_Stream \_Len000TTTAAA TATTTTATTT TATTTTAAAT TAATATATTT TATAATTTAA AATTATTAAT   
  
  
+ AATTGATGAT GTTACCCAAT GTTTAAGTTT TTAAATATTT TTATTTTTTA ATTTAAAAAT ATCATAATAT   
  
  
+ ATAATATAAT TTTAAGTTAA AATAAGATAG TTAAATTATT TTATAGTAAA TATTTATTAT TTTTTTTAAG   
  
  
+ AAAATTGCCA TGTGATTGTC ATCAGTCAAA CACTGCGTTG ACAGAAGCTG TCCTTGCTGC TACTCTCACC   
  
  
+ TCTCAACATC GGACCCGCGC AGACGTTAAC ATCTCCGTAC AAATACAATT CCCTGGCCAA AATTCAAATC   
  
  
+ AACCCTACTC GGTAGTCGGT TCCTTCTTCT TCCTGCCCTC TATATATACA CCCACAGCTG CGCTGCATGT   
  
  
+ TTTTCCGAGA ATCCTCAAAA ATCTTATAAA CTTCCTCGTG TGGGTAGAAA AAAGAAAAAG AAAAGGGTTT   
  
  
+ TAAAATCTTG GGTCAAAACC CATCAACACC ACAATTTGAC TCTTTCTTTT TAATTCAACC ACCGCCAGTT   
  
  
+ CCTCGTTGAT TTTCAGGTAC CTCTTTTTCT TGCCCCTTTT TTTGCTCGGT TGTTGTTTAA TGGTAAAGAT   
  
  
+ TGAGTACAAA GAATGATATT TGATGAGTTG GTGTGTTTAT TTGATGAGCC CAGATGATTT AACTTGTAGA   
  
  
+ TTTTGTGGTT TTGGTTTCTG GGTATTCATT GGTTTGCTGA ATTTCTGGTT CATGATCTGG GGTTAGAAAA   
  
  
+ GTTGAAATGA CTTTCTGGAG GTTGGAATTT GACTAAAGCT AATTTCTTTT TGTCTTCAAT TCTTTTTTAG   
  
  
+ TTGTGAGCTG AATTTGTTGA CCACTTCCAG CCTGTTGGAA ATTAAACTTA CTTAGGAGAA GAGAATCTCA   
  
  
+ GATTCTGAGC AAAAGCTTTA TATGGTTGTT TGGGAAATGG TAGGAACTAA GTGAAAACAC TGAAATTTTA   
  
  
+ AGCAAGCAAT TTTGTTCATT TGATTGGTCG GATGGTCTGA TTTAGGTTCT TTTCTTGGTC TAAATTTCTG   
  
  
+ AGCATTGATT GTTTAATTGC GTGGGGAACT TGTATCCTTG TGAGTTCCTG GTTCATTTCT TGGTTGCTTA   
  
  
+ CTCTAGCTCC CTTTCACAGT TTCTATGGTT GGAACTTGGA ACTTGGAACT TGGAAGCCTA ATTTAGTTGA   
  
  
+ ATATCTTGGC TTTGAGTTAG CTTTTCTTAG GAAAAGATGG TTCTGCTCTC GAAAATCTAA TCTAATCGAT   
  
  
+ AGATGCGTTA AGGAAGCAAA ACTTTTGGGG AGAGGGAAGT TAAGCGAAGC AAGCTGCTGT TTCAAAGTTG   
  
  
+ GACTGTTTTA TTGGTAAGTA TGAAACAATA GTTTATAAGA GATCATTGGG TTCTTAAGGT GATAGATCAT   
  
  
+ GGGTTATGAA TGAGCTTAGT TGTTCTTTTG CTTTCAACTG TACGTAAGGA TGATAGTTAT CCCTCATCTT   
  
  
+ GCATCAAAAA CGGCAAACCG TGATGCCAAA ACTCAAAAAC TGATTCCTCT GACAAGGCTT CTACCTATGA   
  
  
+ AAACTATGCA GATCATATCC ATTTGGCCTT CCCTCTAGGA ACCAAGTAGA GTAAGTTGGT AAATAAATAG   
  
  
+ AGTTGAACGA AACACTACAG ATTTGAATTT GATTAAGGAG GGCTATTATA GACTAAGGTG TAGCTCAAAT   
  
  
+ CCTTCTGTAG GGATTGTCTT ATTGGCTTCT TTTGGTTGCT TCTGGCTCAA GTCCTCTTAT TAGTTAGTAT   
  
  
+ GCATTACCTT GAGACAAGAT AGTTGCTTAA GCTTGAGCTT CTGTACTTAT TTGCGTATTG TAGACAACTA   
  
  
+ GCTGTGGCAT ATGATTCTGA AATGTTCCGA TGTCTTTTTC TTTGGATGCT GGGTTGTGTC ATGAGCTTTA   
  
  
+ GGTTTGTTTA TTTGATGTCA GGAAGTATGT AAATAACATG TGGCATCTTG CAATCTATTA CAGGATGATA   
  
  
+ TTGGCCTTTG TGGGAAGAAA TTGAAGAAGT TGTTTCACTT ACTGGATAGG GAGCATGGAC TCACATCAGC   
  
  
+ TTTTGAGATT CGGTATTTCC AGATCATACT CATCCTATAA TTTCTCTCAG TCTAGTCCCC AATCAATTCC   
  
  
+ AAATAGGTTA TTTGAATCAC CGAACGTTCG TTCTAGAGAC TCTCCAATCT CGCCCTTCTC AACGCCCTTC   
  
  
+ AATTGCGACC CCACTGTTAT ATTGAGCGAC AGTCAGGAGC ACCACAGTTC AACAGGAAGT CTCTCGGCAC   
  
  
+ AAAGCTCATC TTCTAATTCT CCCCTTGAGA CTAGCAGTTA TTATAATCAG TTCAACTCAA GCCCTGTTGG   
  
  
+ AGAATCACCT CAGGGATCAT CACCCGAGAA TCTTTTTCAA CAAGCTGCAG TTAATAAAGT CAGCATCGAG   
  
  
+ CATGCATTGC AGGAGCTAGA AACTGCTCTA ATGAGCACAG ATTGTGAGGA GAATGAAGCA ACTGTCTCTA   
  
  
+ TCCCATCTAT GGGTGAACCT CATCAGCCCC AAGTCCCTAG CCAGAGATCA AGATTCTGGA ATCGAGATCC   
  
  
+ TCAGGGTTCA CGCCCGGCTG AAGTTCACTC ATCATTGAGA AGATTAGGAG ACGAGGCTCA GAGTGAGAAA   
  
  
+ CGCCTCAAGG CAGTGGAAGA ACCAATACGG CCCAGTGCAC CACCCGGCAA TTTGAAGCAG TTGCTCATAG   
  
  
+ AATGTGCTCG GGCTTTGTCA GAGAATCGAA TAGAGGATTT TGAGAAGTTA GTTGAACAGG CAAAGGGCAT   
  
  
+ GGTCTCCATT TCTGGAGACC CAATTCAACG ACTTGGTGCT TACTTGATCG AGGGGCTAGT GGCAAGGAAG   
  
  
+ GAGTCTTCAG GTACGAAAAT CTACCGAGCT CTTAGGTGCA AAGAGCCCCT TGGTCAAGAC TTGCTTTCGT   
  
  
+ ACATGCACAT CCTTTATGAA ATTTGCCCTT ACTTGAAATT CGGTTATATG GTTGCAAATG GGGCTATAGC   
  
  
+ TGAAGCTTGC AGAGATGAGG ATCACATACA TATCGTCGAC TACCAGATTG GTCAAGGAAC TCAATGGATG   
  
  
+ ACTCTGTTGC AAGCCCTAGC CACAAAACCT GGAAGACCCC CCACTGTGAG AATTACTGGC ATTGATGATC   
  
  
+ CCGTCTCTAG GCATGCTCGG GGAGCTTGCT TGGAGGCAGT GGGGAAACGT TTGGCAGTTC TGTCGGAGAA   
  
  
+ ATTTAACATG CCCATTGAGT TTCAGGCAGT GCCCGTTTAT GCTTCTGAGG TCACCCAAGA AATGCTTGAT   
  
  
+ GTGAGGCCAG GGTGGGCCTT GGCTGTGAAC TTCCCATTGC AGCTCCACCA CACTCCCGAT GAGAGTGTTG   
  
  
+ ATGTGAACAA CCCGAGGGAT GGACTCCTAA GAATGGTGAA ATCACTCGGT CCCAAGGTTG TAACTTTGGT   
  
  
+ AGAACAAGAG TCAAACACCA ACACTACCCC TTTGCTGACA AGGTTCATAG AAACGCTGGA CTTCTACTCA   
  
  
+ GCAATGTTTG AATCCATCGA TGTGACAATG CCAAGGGACC GAAAGGAAAG GATCAATGTC GAGCAGCATT   
  
  
+ GCCTAGCCAA GGACATTGTC AACATCATCG CGTGTGAGGG GAAGGACAGG GTGGAGCGAC ATGAGCTCTT   
  
  
+ TGGGAAATGG AAGTCAAGGT TCACCATGGC AGGATTCCAA CAATCCCCAT TGAGCTCGTA TGTTAACTCC   
  
  
+ GTGATAAAAG GATTACTTAG GTGTTATTCA GAGTACTATA CGTTGGTGGA GAAGGATGGC GCCTTGCTTC   
  
  
+ TGGGATGGAA GGACCGGATG CTGGTTTCGG CTTCAGCTTG GCATTG  

- +Up\_Stream \_Len000AAATTT ATAAAATAAA ATAAAATTTA ATTATATAAA ATATTAAATT TTAATAATTA   
  
  
- TTAACTACTA CAATGGGTTA CAAATTCAAA AATTTATAAA AATAAAAAAT TAAATTTTTA TAGTATTATA   
  
  
- TATTATATTA AAATTCAATT TTATTCTATC AATTTAATAA AATATCATTT ATAAATAATA AAAAAAATTC   
  
  
- TTTTAACGGT ACACTAACAG TAGTCAGTTT GTGACGCAAC TGTCTTCGAC AGGAACGACG ATGAGAGTGG   
  
  
- AGAGTTGTAG CCTGGGCGCG TCTGCAATTG TAGAGGCATG TTTATGTTAA GGGACCGGTT TTAAGTTTAG   
  
  
- TTGGGATGAG CCATCAGCCA AGGAAGAAGA AGGACGGGAG ATATATATGT GGGTGTCGAC GCGACGTACA   
  
  
- AAAAGGCTCT TAGGAGTTTT TAGAATATTT GAAGGAGCAC ACCCATCTTT TTTCTTTTTC TTTTCCCAAA   
  
  
- ATTTTAGAAC CCAGTTTTGG GTAGTTGTGG TGTTAAACTG AGAAAGAAAA ATTAAGTTGG TGGCGGTCAA   
  
  
- GGAGCAACTA AAAGTCCATG GAGAAAAAGA ACGGGGAAAA AAACGAGCCA ACAACAAATT ACCATTTCTA   
  
  
- ACTCATGTTT CTTACTATAA ACTACTCAAC CACACAAATA AACTACTCGG GTCTACTAAA TTGAACATCT   
  
  
- AAAACACCAA AACCAAAGAC CCATAAGTAA CCAAACGACT TAAAGACCAA GTACTAGACC CCAATCTTTT   
  
  
- CAACTTTACT GAAAGACCTC CAACCTTAAA CTGATTTCGA TTAAAGAAAA ACAGAAGTTA AGAAAAAATC   
  
  
- AACACTCGAC TTAAACAACT GGTGAAGGTC GGACAACCTT TAATTTGAAT GAATCCTCTT CTCTTAGAGT   
  
  
- CTAAGACTCG TTTTCGAAAT ATACCAACAA ACCCTTTACC ATCCTTGATT CACTTTTGTG ACTTTAAAAT   
  
  
- TCGTTCGTTA AAACAAGTAA ACTAACCAGC CTACCAGACT AAATCCAAGA AAAGAACCAG ATTTAAAGAC   
  
  
- TCGTAACTAA CAAATTAACG CACCCCTTGA ACATAGGAAC ACTCAAGGAC CAAGTAAAGA ACCAACGAAT   
  
  
- GAGATCGAGG GAAAGTGTCA AAGATACCAA CCTTGAACCT TGAACCTTGA ACCTTCGGAT TAAATCAACT   
  
  
- TATAGAACCG AAACTCAATC GAAAAGAATC CTTTTCTACC AAGACGAGAG CTTTTAGATT AGATTAGCTA   
  
  
- TCTACGCAAT TCCTTCGTTT TGAAAACCCC TCTCCCTTCA ATTCGCTTCG TTCGACGACA AAGTTTCAAC   
  
  
- CTGACAAAAT AACCATTCAT ACTTTGTTAT CAAATATTCT CTAGTAACCC AAGAATTCCA CTATCTAGTA   
  
  
- CCCAATACTT ACTCGAATCA ACAAGAAAAC GAAAGTTGAC ATGCATTCCT ACTATCAATA GGGAGTAGAA   
  
  
- CGTAGTTTTT GCCGTTTGGC ACTACGGTTT TGAGTTTTTG ACTAAGGAGA CTGTTCCGAA GATGGATACT   
  
  
- TTTGATACGT CTAGTATAGG TAAACCGGAA GGGAGATCCT TGGTTCATCT CATTCAACCA TTTATTTATC   
  
  
- TCAACTTGCT TTGTGATGTC TAAACTTAAA CTAATTCCTC CCGATAATAT CTGATTCCAC ATCGAGTTTA   
  
  
- GGAAGACATC CCTAACAGAA TAACCGAAGA AAACCAACGA AGACCGAGTT CAGGAGAATA ATCAATCATA   
  
  
- CGTAATGGAA CTCTGTTCTA TCAACGAATT CGAACTCGAA GACATGAATA AACGCATAAC ATCTGTTGAT   
  
  
- CGACACCGTA TACTAAGACT TTACAAGGCT ACAGAAAAAG AAACCTACGA CCCAACACAG TACTCGAAAT   
  
  
- CCAAACAAAT AAACTACAGT CCTTCATACA TTTATTGTAC ACCGTAGAAC GTTAGATAAT GTCCTACTAT   
  
  
- AACCGGAAAC ACCCTTCTTT AACTTCTTCA ACAAAGTGAA TGACCTATCC CTCGTACCTG AGTGTAGTCG   
  
  
- AAAACTCTAA GCCATAAAGG TCTAGTATGA GTAGGATATT AAAGAGAGTC AGATCAGGGG TTAGTTAAGG   
  
  
- TTTATCCAAT AAACTTAGTG GCTTGCAAGC AAGATCTCTG AGAGGTTAGA GCGGGAAGAG TTGCGGGAAG   
  
  
- TTAACGCTGG GGTGACAATA TAACTCGCTG TCAGTCCTCG TGGTGTCAAG TTGTCCTTCA GAGAGCCGTG   
  
  
- TTTCGAGTAG AAGATTAAGA GGGGAACTCT GATCGTCAAT AATATTAGTC AAGTTGAGTT CGGGACAACC   
  
  
- TCTTAGTGGA GTCCCTAGTA GTGGGCTCTT AGAAAAAGTT GTTCGACGTC AATTATTTCA GTCGTAGCTC   
  
  
- GTACGTAACG TCCTCGATCT TTGACGAGAT TACTCGTGTC TAACACTCCT CTTACTTCGT TGACAGAGAT   
  
  
- AGGGTAGATA CCCACTTGGA GTAGTCGGGG TTCAGGGATC GGTCTCTAGT TCTAAGACCT TAGCTCTAGG   
  
  
- AGTCCCAAGT GCGGGCCGAC TTCAAGTGAG TAGTAACTCT TCTAATCCTC TGCTCCGAGT CTCACTCTTT   
  
  
- GCGGAGTTCC GTCACCTTCT TGGTTATGCC GGGTCACGTG GTGGGCCGTT AAACTTCGTC AACGAGTATC   
  
  
- TTACACGAGC CCGAAACAGT CTCTTAGCTT ATCTCCTAAA ACTCTTCAAT CAACTTGTCC GTTTCCCGTA   
  
  
- CCAGAGGTAA AGACCTCTGG GTTAAGTTGC TGAACCACGA ATGAACTAGC TCCCCGATCA CCGTTCCTTC   
  
  
- CTCAGAAGTC CATGCTTTTA GATGGCTCGA GAATCCACGT TTCTCGGGGA ACCAGTTCTG AACGAAAGCA   
  
  
- TGTACGTGTA GGAAATACTT TAAACGGGAA TGAACTTTAA GCCAATATAC CAACGTTTAC CCCGATATCG   
  
  
- ACTTCGAACG TCTCTACTCC TAGTGTATGT ATAGCAGCTG ATGGTCTAAC CAGTTCCTTG AGTTACCTAC   
  
  
- TGAGACAACG TTCGGGATCG GTGTTTTGGA CCTTCTGGGG GGTGACACTC TTAATGACCG TAACTACTAG   
  
  
- GGCAGAGATC CGTACGAGCC CCTCGAACGA ACCTCCGTCA CCCCTTTGCA AACCGTCAAG ACAGCCTCTT   
  
  
- TAAATTGTAC GGGTAACTCA AAGTCCGTCA CGGGCAAATA CGAAGACTCC AGTGGGTTCT TTACGAACTA   
  
  
- CACTCCGGTC CCACCCGGAA CCGACACTTG AAGGGTAACG TCGAGGTGGT GTGAGGGCTA CTCTCACAAC   
  
  
- TACACTTGTT GGGCTCCCTA CCTGAGGATT CTTACCACTT TAGTGAGCCA GGGTTCCAAC ATTGAAACCA   
  
  
- TCTTGTTCTC AGTTTGTGGT TGTGATGGGG AAACGACTGT TCCAAGTATC TTTGCGACCT GAAGATGAGT   
  
  
- CGTTACAAAC TTAGGTAGCT ACACTGTTAC GGTTCCCTGG CTTTCCTTTC CTAGTTACAG CTCGTCGTAA   
  
  
- CGGATCGGTT CCTGTAACAG TTGTAGTAGC GCACACTCCC CTTCCTGTCC CACCTCGCTG TACTCGAGAA   
  
  
- ACCCTTTACC TTCAGTTCCA AGTGGTACCG TCCTAAGGTT GTTAGGGGTA ACTCGAGCAT ACAATTGAGG   
  
  
- CACTATTTTC CTAATGAATC CACAATAAGT CTCATGATAT GCAACCACCT CTTCCTACCG CGGAACGAAG   
  
  
- ACCCTACCTT CCTGGCCTAC GACCAAAGCC GAAGTCGAAC CGTAAC

+     Myb

| Site Name | Organism | Position | Strand | Matrix score. | sequence | function |
| --- | --- | --- | --- | --- | --- | --- |
| Myb | Arabidopsis thaliana | 2652 | - | 6 | CAACTG |  |
| Myb | Arabidopsis thaliana | 2362 | - | 6 | TAACTG |  |
| Myb | Arabidopsis thaliana | 2443 | + | 6 | CAACTG |  |
| Myb | Arabidopsis thaliana | 2279 | - | 6 | TAACTG |  |
| Myb | Arabidopsis thaliana | 1439 | + | 6 | CAACTG |  |

>HU01G01850.1   
+ +Up\_Stream \_Len000TTTAAA TATTTTATTT TATTTTAAAT TAATATATTT TATAATTTAA AATTATTAAT   
  
  
+ AATTGATGAT GTTACCCAAT GTTTAAGTTT TTAAATATTT TTATTTTTTA ATTTAAAAAT ATCATAATAT   
  
  
+ ATAATATAAT TTTAAGTTAA AATAAGATAG TTAAATTATT TTATAGTAAA TATTTATTAT TTTTTTTAAG   
  
  
+ AAAATTGCCA TGTGATTGTC ATCAGTCAAA CACTGCGTTG ACAGAAGCTG TCCTTGCTGC TACTCTCACC   
  
  
+ TCTCAACATC GGACCCGCGC AGACGTTAAC ATCTCCGTAC AAATACAATT CCCTGGCCAA AATTCAAATC   
  
  
+ AACCCTACTC GGTAGTCGGT TCCTTCTTCT TCCTGCCCTC TATATATACA CCCACAGCTG CGCTGCATGT   
  
  
+ TTTTCCGAGA ATCCTCAAAA ATCTTATAAA CTTCCTCGTG TGGGTAGAAA AAAGAAAAAG AAAAGGGTTT   
  
  
+ TAAAATCTTG GGTCAAAACC CATCAACACC ACAATTTGAC TCTTTCTTTT TAATTCAACC ACCGCCAGTT   
  
  
+ CCTCGTTGAT TTTCAGGTAC CTCTTTTTCT TGCCCCTTTT TTTGCTCGGT TGTTGTTTAA TGGTAAAGAT   
  
  
+ TGAGTACAAA GAATGATATT TGATGAGTTG GTGTGTTTAT TTGATGAGCC CAGATGATTT AACTTGTAGA   
  
  
+ TTTTGTGGTT TTGGTTTCTG GGTATTCATT GGTTTGCTGA ATTTCTGGTT CATGATCTGG GGTTAGAAAA   
  
  
+ GTTGAAATGA CTTTCTGGAG GTTGGAATTT GACTAAAGCT AATTTCTTTT TGTCTTCAAT TCTTTTTTAG   
  
  
+ TTGTGAGCTG AATTTGTTGA CCACTTCCAG CCTGTTGGAA ATTAAACTTA CTTAGGAGAA GAGAATCTCA   
  
  
+ GATTCTGAGC AAAAGCTTTA TATGGTTGTT TGGGAAATGG TAGGAACTAA GTGAAAACAC TGAAATTTTA   
  
  
+ AGCAAGCAAT TTTGTTCATT TGATTGGTCG GATGGTCTGA TTTAGGTTCT TTTCTTGGTC TAAATTTCTG   
  
  
+ AGCATTGATT GTTTAATTGC GTGGGGAACT TGTATCCTTG TGAGTTCCTG GTTCATTTCT TGGTTGCTTA   
  
  
+ CTCTAGCTCC CTTTCACAGT TTCTATGGTT GGAACTTGGA ACTTGGAACT TGGAAGCCTA ATTTAGTTGA   
  
  
+ ATATCTTGGC TTTGAGTTAG CTTTTCTTAG GAAAAGATGG TTCTGCTCTC GAAAATCTAA TCTAATCGAT   
  
  
+ AGATGCGTTA AGGAAGCAAA ACTTTTGGGG AGAGGGAAGT TAAGCGAAGC AAGCTGCTGT TTCAAAGTTG   
  
  
+ GACTGTTTTA TTGGTAAGTA TGAAACAATA GTTTATAAGA GATCATTGGG TTCTTAAGGT GATAGATCAT   
  
  
+ GGGTTATGAA TGAGCTTAGT TGTTCTTTTG CTTTCAACTG TACGTAAGGA TGATAGTTAT CCCTCATCTT   
  
  
+ GCATCAAAAA CGGCAAACCG TGATGCCAAA ACTCAAAAAC TGATTCCTCT GACAAGGCTT CTACCTATGA   
  
  
+ AAACTATGCA GATCATATCC ATTTGGCCTT CCCTCTAGGA ACCAAGTAGA GTAAGTTGGT AAATAAATAG   
  
  
+ AGTTGAACGA AACACTACAG ATTTGAATTT GATTAAGGAG GGCTATTATA GACTAAGGTG TAGCTCAAAT   
  
  
+ CCTTCTGTAG GGATTGTCTT ATTGGCTTCT TTTGGTTGCT TCTGGCTCAA GTCCTCTTAT TAGTTAGTAT   
  
  
+ GCATTACCTT GAGACAAGAT AGTTGCTTAA GCTTGAGCTT CTGTACTTAT TTGCGTATTG TAGACAACTA   
  
  
+ GCTGTGGCAT ATGATTCTGA AATGTTCCGA TGTCTTTTTC TTTGGATGCT GGGTTGTGTC ATGAGCTTTA   
  
  
+ GGTTTGTTTA TTTGATGTCA GGAAGTATGT AAATAACATG TGGCATCTTG CAATCTATTA CAGGATGATA   
  
  
+ TTGGCCTTTG TGGGAAGAAA TTGAAGAAGT TGTTTCACTT ACTGGATAGG GAGCATGGAC TCACATCAGC   
  
  
+ TTTTGAGATT CGGTATTTCC AGATCATACT CATCCTATAA TTTCTCTCAG TCTAGTCCCC AATCAATTCC   
  
  
+ AAATAGGTTA TTTGAATCAC CGAACGTTCG TTCTAGAGAC TCTCCAATCT CGCCCTTCTC AACGCCCTTC   
  
  
+ AATTGCGACC CCACTGTTAT ATTGAGCGAC AGTCAGGAGC ACCACAGTTC AACAGGAAGT CTCTCGGCAC   
  
  
+ AAAGCTCATC TTCTAATTCT CCCCTTGAGA CTAGCAGTTA TTATAATCAG TTCAACTCAA GCCCTGTTGG   
  
  
+ AGAATCACCT CAGGGATCAT CACCCGAGAA TCTTTTTCAA CAAGCTGCAG TTAATAAAGT CAGCATCGAG   
  
  
+ CATGCATTGC AGGAGCTAGA AACTGCTCTA ATGAGCACAG ATTGTGAGGA GAATGAAGCA ACTGTCTCTA   
  
  
+ TCCCATCTAT GGGTGAACCT CATCAGCCCC AAGTCCCTAG CCAGAGATCA AGATTCTGGA ATCGAGATCC   
  
  
+ TCAGGGTTCA CGCCCGGCTG AAGTTCACTC ATCATTGAGA AGATTAGGAG ACGAGGCTCA GAGTGAGAAA   
  
  
+ CGCCTCAAGG CAGTGGAAGA ACCAATACGG CCCAGTGCAC CACCCGGCAA TTTGAAGCAG TTGCTCATAG   
  
  
+ AATGTGCTCG GGCTTTGTCA GAGAATCGAA TAGAGGATTT TGAGAAGTTA GTTGAACAGG CAAAGGGCAT   
  
  
+ GGTCTCCATT TCTGGAGACC CAATTCAACG ACTTGGTGCT TACTTGATCG AGGGGCTAGT GGCAAGGAAG   
  
  
+ GAGTCTTCAG GTACGAAAAT CTACCGAGCT CTTAGGTGCA AAGAGCCCCT TGGTCAAGAC TTGCTTTCGT   
  
  
+ ACATGCACAT CCTTTATGAA ATTTGCCCTT ACTTGAAATT CGGTTATATG GTTGCAAATG GGGCTATAGC   
  
  
+ TGAAGCTTGC AGAGATGAGG ATCACATACA TATCGTCGAC TACCAGATTG GTCAAGGAAC TCAATGGATG   
  
  
+ ACTCTGTTGC AAGCCCTAGC CACAAAACCT GGAAGACCCC CCACTGTGAG AATTACTGGC ATTGATGATC   
  
  
+ CCGTCTCTAG GCATGCTCGG GGAGCTTGCT TGGAGGCAGT GGGGAAACGT TTGGCAGTTC TGTCGGAGAA   
  
  
+ ATTTAACATG CCCATTGAGT TTCAGGCAGT GCCCGTTTAT GCTTCTGAGG TCACCCAAGA AATGCTTGAT   
  
  
+ GTGAGGCCAG GGTGGGCCTT GGCTGTGAAC TTCCCATTGC AGCTCCACCA CACTCCCGAT GAGAGTGTTG   
  
  
+ ATGTGAACAA CCCGAGGGAT GGACTCCTAA GAATGGTGAA ATCACTCGGT CCCAAGGTTG TAACTTTGGT   
  
  
+ AGAACAAGAG TCAAACACCA ACACTACCCC TTTGCTGACA AGGTTCATAG AAACGCTGGA CTTCTACTCA   
  
  
+ GCAATGTTTG AATCCATCGA TGTGACAATG CCAAGGGACC GAAAGGAAAG GATCAATGTC GAGCAGCATT   
  
  
+ GCCTAGCCAA GGACATTGTC AACATCATCG CGTGTGAGGG GAAGGACAGG GTGGAGCGAC ATGAGCTCTT   
  
  
+ TGGGAAATGG AAGTCAAGGT TCACCATGGC AGGATTCCAA CAATCCCCAT TGAGCTCGTA TGTTAACTCC   
  
  
+ GTGATAAAAG GATTACTTAG GTGTTATTCA GAGTACTATA CGTTGGTGGA GAAGGATGGC GCCTTGCTTC   
  
  
+ TGGGATGGAA GGACCGGATG CTGGTTTCGG CTTCAGCTTG GCATTG  

- +Up\_Stream \_Len000AAATTT ATAAAATAAA ATAAAATTTA ATTATATAAA ATATTAAATT TTAATAATTA   
  
  
- TTAACTACTA CAATGGGTTA CAAATTCAAA AATTTATAAA AATAAAAAAT TAAATTTTTA TAGTATTATA   
  
  
- TATTATATTA AAATTCAATT TTATTCTATC AATTTAATAA AATATCATTT ATAAATAATA AAAAAAATTC   
  
  
- TTTTAACGGT ACACTAACAG TAGTCAGTTT GTGACGCAAC TGTCTTCGAC AGGAACGACG ATGAGAGTGG   
  
  
- AGAGTTGTAG CCTGGGCGCG TCTGCAATTG TAGAGGCATG TTTATGTTAA GGGACCGGTT TTAAGTTTAG   
  
  
- TTGGGATGAG CCATCAGCCA AGGAAGAAGA AGGACGGGAG ATATATATGT GGGTGTCGAC GCGACGTACA   
  
  
- AAAAGGCTCT TAGGAGTTTT TAGAATATTT GAAGGAGCAC ACCCATCTTT TTTCTTTTTC TTTTCCCAAA   
  
  
- ATTTTAGAAC CCAGTTTTGG GTAGTTGTGG TGTTAAACTG AGAAAGAAAA ATTAAGTTGG TGGCGGTCAA   
  
  
- GGAGCAACTA AAAGTCCATG GAGAAAAAGA ACGGGGAAAA AAACGAGCCA ACAACAAATT ACCATTTCTA   
  
  
- ACTCATGTTT CTTACTATAA ACTACTCAAC CACACAAATA AACTACTCGG GTCTACTAAA TTGAACATCT   
  
  
- AAAACACCAA AACCAAAGAC CCATAAGTAA CCAAACGACT TAAAGACCAA GTACTAGACC CCAATCTTTT   
  
  
- CAACTTTACT GAAAGACCTC CAACCTTAAA CTGATTTCGA TTAAAGAAAA ACAGAAGTTA AGAAAAAATC   
  
  
- AACACTCGAC TTAAACAACT GGTGAAGGTC GGACAACCTT TAATTTGAAT GAATCCTCTT CTCTTAGAGT   
  
  
- CTAAGACTCG TTTTCGAAAT ATACCAACAA ACCCTTTACC ATCCTTGATT CACTTTTGTG ACTTTAAAAT   
  
  
- TCGTTCGTTA AAACAAGTAA ACTAACCAGC CTACCAGACT AAATCCAAGA AAAGAACCAG ATTTAAAGAC   
  
  
- TCGTAACTAA CAAATTAACG CACCCCTTGA ACATAGGAAC ACTCAAGGAC CAAGTAAAGA ACCAACGAAT   
  
  
- GAGATCGAGG GAAAGTGTCA AAGATACCAA CCTTGAACCT TGAACCTTGA ACCTTCGGAT TAAATCAACT   
  
  
- TATAGAACCG AAACTCAATC GAAAAGAATC CTTTTCTACC AAGACGAGAG CTTTTAGATT AGATTAGCTA   
  
  
- TCTACGCAAT TCCTTCGTTT TGAAAACCCC TCTCCCTTCA ATTCGCTTCG TTCGACGACA AAGTTTCAAC   
  
  
- CTGACAAAAT AACCATTCAT ACTTTGTTAT CAAATATTCT CTAGTAACCC AAGAATTCCA CTATCTAGTA   
  
  
- CCCAATACTT ACTCGAATCA ACAAGAAAAC GAAAGTTGAC ATGCATTCCT ACTATCAATA GGGAGTAGAA   
  
  
- CGTAGTTTTT GCCGTTTGGC ACTACGGTTT TGAGTTTTTG ACTAAGGAGA CTGTTCCGAA GATGGATACT   
  
  
- TTTGATACGT CTAGTATAGG TAAACCGGAA GGGAGATCCT TGGTTCATCT CATTCAACCA TTTATTTATC   
  
  
- TCAACTTGCT TTGTGATGTC TAAACTTAAA CTAATTCCTC CCGATAATAT CTGATTCCAC ATCGAGTTTA   
  
  
- GGAAGACATC CCTAACAGAA TAACCGAAGA AAACCAACGA AGACCGAGTT CAGGAGAATA ATCAATCATA   
  
  
- CGTAATGGAA CTCTGTTCTA TCAACGAATT CGAACTCGAA GACATGAATA AACGCATAAC ATCTGTTGAT   
  
  
- CGACACCGTA TACTAAGACT TTACAAGGCT ACAGAAAAAG AAACCTACGA CCCAACACAG TACTCGAAAT   
  
  
- CCAAACAAAT AAACTACAGT CCTTCATACA TTTATTGTAC ACCGTAGAAC GTTAGATAAT GTCCTACTAT   
  
  
- AACCGGAAAC ACCCTTCTTT AACTTCTTCA ACAAAGTGAA TGACCTATCC CTCGTACCTG AGTGTAGTCG   
  
  
- AAAACTCTAA GCCATAAAGG TCTAGTATGA GTAGGATATT AAAGAGAGTC AGATCAGGGG TTAGTTAAGG   
  
  
- TTTATCCAAT AAACTTAGTG GCTTGCAAGC AAGATCTCTG AGAGGTTAGA GCGGGAAGAG TTGCGGGAAG   
  
  
- TTAACGCTGG GGTGACAATA TAACTCGCTG TCAGTCCTCG TGGTGTCAAG TTGTCCTTCA GAGAGCCGTG   
  
  
- TTTCGAGTAG AAGATTAAGA GGGGAACTCT GATCGTCAAT AATATTAGTC AAGTTGAGTT CGGGACAACC   
  
  
- TCTTAGTGGA GTCCCTAGTA GTGGGCTCTT AGAAAAAGTT GTTCGACGTC AATTATTTCA GTCGTAGCTC   
  
  
- GTACGTAACG TCCTCGATCT TTGACGAGAT TACTCGTGTC TAACACTCCT CTTACTTCGT TGACAGAGAT   
  
  
- AGGGTAGATA CCCACTTGGA GTAGTCGGGG TTCAGGGATC GGTCTCTAGT TCTAAGACCT TAGCTCTAGG   
  
  
- AGTCCCAAGT GCGGGCCGAC TTCAAGTGAG TAGTAACTCT TCTAATCCTC TGCTCCGAGT CTCACTCTTT   
  
  
- GCGGAGTTCC GTCACCTTCT TGGTTATGCC GGGTCACGTG GTGGGCCGTT AAACTTCGTC AACGAGTATC   
  
  
- TTACACGAGC CCGAAACAGT CTCTTAGCTT ATCTCCTAAA ACTCTTCAAT CAACTTGTCC GTTTCCCGTA   
  
  
- CCAGAGGTAA AGACCTCTGG GTTAAGTTGC TGAACCACGA ATGAACTAGC TCCCCGATCA CCGTTCCTTC   
  
  
- CTCAGAAGTC CATGCTTTTA GATGGCTCGA GAATCCACGT TTCTCGGGGA ACCAGTTCTG AACGAAAGCA   
  
  
- TGTACGTGTA GGAAATACTT TAAACGGGAA TGAACTTTAA GCCAATATAC CAACGTTTAC CCCGATATCG   
  
  
- ACTTCGAACG TCTCTACTCC TAGTGTATGT ATAGCAGCTG ATGGTCTAAC CAGTTCCTTG AGTTACCTAC   
  
  
- TGAGACAACG TTCGGGATCG GTGTTTTGGA CCTTCTGGGG GGTGACACTC TTAATGACCG TAACTACTAG   
  
  
- GGCAGAGATC CGTACGAGCC CCTCGAACGA ACCTCCGTCA CCCCTTTGCA AACCGTCAAG ACAGCCTCTT   
  
  
- TAAATTGTAC GGGTAACTCA AAGTCCGTCA CGGGCAAATA CGAAGACTCC AGTGGGTTCT TTACGAACTA   
  
  
- CACTCCGGTC CCACCCGGAA CCGACACTTG AAGGGTAACG TCGAGGTGGT GTGAGGGCTA CTCTCACAAC   
  
  
- TACACTTGTT GGGCTCCCTA CCTGAGGATT CTTACCACTT TAGTGAGCCA GGGTTCCAAC ATTGAAACCA   
  
  
- TCTTGTTCTC AGTTTGTGGT TGTGATGGGG AAACGACTGT TCCAAGTATC TTTGCGACCT GAAGATGAGT   
  
  
- CGTTACAAAC TTAGGTAGCT ACACTGTTAC GGTTCCCTGG CTTTCCTTTC CTAGTTACAG CTCGTCGTAA   
  
  
- CGGATCGGTT CCTGTAACAG TTGTAGTAGC GCACACTCCC CTTCCTGTCC CACCTCGCTG TACTCGAGAA   
  
  
- ACCCTTTACC TTCAGTTCCA AGTGGTACCG TCCTAAGGTT GTTAGGGGTA ACTCGAGCAT ACAATTGAGG   
  
  
- CACTATTTTC CTAATGAATC CACAATAAGT CTCATGATAT GCAACCACCT CTTCCTACCG CGGAACGAAG   
  
  
- ACCCTACCTT CCTGGCCTAC GACCAAAGCC GAAGTCGAAC CGTAAC

+     Myb-binding site

| Site Name | Organism | Position | Strand | Matrix score. | sequence | function |
| --- | --- | --- | --- | --- | --- | --- |
| Myb-binding site | Nicotiana tabacum | 3018 | - | 6 | CAACAG |  |
| Myb-binding site | Nicotiana tabacum | 2308 | - | 6 | CAACAG |  |
| Myb-binding site | Nicotiana tabacum | 2224 | + | 6 | CAACAG |  |
| Myb-binding site | Nicotiana tabacum | 876 | - | 6 | CAACAG |  |

>HU01G01850.1   
+ +Up\_Stream \_Len000TTTAAA TATTTTATTT TATTTTAAAT TAATATATTT TATAATTTAA AATTATTAAT   
  
  
+ AATTGATGAT GTTACCCAAT GTTTAAGTTT TTAAATATTT TTATTTTTTA ATTTAAAAAT ATCATAATAT   
  
  
+ ATAATATAAT TTTAAGTTAA AATAAGATAG TTAAATTATT TTATAGTAAA TATTTATTAT TTTTTTTAAG   
  
  
+ AAAATTGCCA TGTGATTGTC ATCAGTCAAA CACTGCGTTG ACAGAAGCTG TCCTTGCTGC TACTCTCACC   
  
  
+ TCTCAACATC GGACCCGCGC AGACGTTAAC ATCTCCGTAC AAATACAATT CCCTGGCCAA AATTCAAATC   
  
  
+ AACCCTACTC GGTAGTCGGT TCCTTCTTCT TCCTGCCCTC TATATATACA CCCACAGCTG CGCTGCATGT   
  
  
+ TTTTCCGAGA ATCCTCAAAA ATCTTATAAA CTTCCTCGTG TGGGTAGAAA AAAGAAAAAG AAAAGGGTTT   
  
  
+ TAAAATCTTG GGTCAAAACC CATCAACACC ACAATTTGAC TCTTTCTTTT TAATTCAACC ACCGCCAGTT   
  
  
+ CCTCGTTGAT TTTCAGGTAC CTCTTTTTCT TGCCCCTTTT TTTGCTCGGT TGTTGTTTAA TGGTAAAGAT   
  
  
+ TGAGTACAAA GAATGATATT TGATGAGTTG GTGTGTTTAT TTGATGAGCC CAGATGATTT AACTTGTAGA   
  
  
+ TTTTGTGGTT TTGGTTTCTG GGTATTCATT GGTTTGCTGA ATTTCTGGTT CATGATCTGG GGTTAGAAAA   
  
  
+ GTTGAAATGA CTTTCTGGAG GTTGGAATTT GACTAAAGCT AATTTCTTTT TGTCTTCAAT TCTTTTTTAG   
  
  
+ TTGTGAGCTG AATTTGTTGA CCACTTCCAG CCTGTTGGAA ATTAAACTTA CTTAGGAGAA GAGAATCTCA   
  
  
+ GATTCTGAGC AAAAGCTTTA TATGGTTGTT TGGGAAATGG TAGGAACTAA GTGAAAACAC TGAAATTTTA   
  
  
+ AGCAAGCAAT TTTGTTCATT TGATTGGTCG GATGGTCTGA TTTAGGTTCT TTTCTTGGTC TAAATTTCTG   
  
  
+ AGCATTGATT GTTTAATTGC GTGGGGAACT TGTATCCTTG TGAGTTCCTG GTTCATTTCT TGGTTGCTTA   
  
  
+ CTCTAGCTCC CTTTCACAGT TTCTATGGTT GGAACTTGGA ACTTGGAACT TGGAAGCCTA ATTTAGTTGA   
  
  
+ ATATCTTGGC TTTGAGTTAG CTTTTCTTAG GAAAAGATGG TTCTGCTCTC GAAAATCTAA TCTAATCGAT   
  
  
+ AGATGCGTTA AGGAAGCAAA ACTTTTGGGG AGAGGGAAGT TAAGCGAAGC AAGCTGCTGT TTCAAAGTTG   
  
  
+ GACTGTTTTA TTGGTAAGTA TGAAACAATA GTTTATAAGA GATCATTGGG TTCTTAAGGT GATAGATCAT   
  
  
+ GGGTTATGAA TGAGCTTAGT TGTTCTTTTG CTTTCAACTG TACGTAAGGA TGATAGTTAT CCCTCATCTT   
  
  
+ GCATCAAAAA CGGCAAACCG TGATGCCAAA ACTCAAAAAC TGATTCCTCT GACAAGGCTT CTACCTATGA   
  
  
+ AAACTATGCA GATCATATCC ATTTGGCCTT CCCTCTAGGA ACCAAGTAGA GTAAGTTGGT AAATAAATAG   
  
  
+ AGTTGAACGA AACACTACAG ATTTGAATTT GATTAAGGAG GGCTATTATA GACTAAGGTG TAGCTCAAAT   
  
  
+ CCTTCTGTAG GGATTGTCTT ATTGGCTTCT TTTGGTTGCT TCTGGCTCAA GTCCTCTTAT TAGTTAGTAT   
  
  
+ GCATTACCTT GAGACAAGAT AGTTGCTTAA GCTTGAGCTT CTGTACTTAT TTGCGTATTG TAGACAACTA   
  
  
+ GCTGTGGCAT ATGATTCTGA AATGTTCCGA TGTCTTTTTC TTTGGATGCT GGGTTGTGTC ATGAGCTTTA   
  
  
+ GGTTTGTTTA TTTGATGTCA GGAAGTATGT AAATAACATG TGGCATCTTG CAATCTATTA CAGGATGATA   
  
  
+ TTGGCCTTTG TGGGAAGAAA TTGAAGAAGT TGTTTCACTT ACTGGATAGG GAGCATGGAC TCACATCAGC   
  
  
+ TTTTGAGATT CGGTATTTCC AGATCATACT CATCCTATAA TTTCTCTCAG TCTAGTCCCC AATCAATTCC   
  
  
+ AAATAGGTTA TTTGAATCAC CGAACGTTCG TTCTAGAGAC TCTCCAATCT CGCCCTTCTC AACGCCCTTC   
  
  
+ AATTGCGACC CCACTGTTAT ATTGAGCGAC AGTCAGGAGC ACCACAGTTC AACAGGAAGT CTCTCGGCAC   
  
  
+ AAAGCTCATC TTCTAATTCT CCCCTTGAGA CTAGCAGTTA TTATAATCAG TTCAACTCAA GCCCTGTTGG   
  
  
+ AGAATCACCT CAGGGATCAT CACCCGAGAA TCTTTTTCAA CAAGCTGCAG TTAATAAAGT CAGCATCGAG   
  
  
+ CATGCATTGC AGGAGCTAGA AACTGCTCTA ATGAGCACAG ATTGTGAGGA GAATGAAGCA ACTGTCTCTA   
  
  
+ TCCCATCTAT GGGTGAACCT CATCAGCCCC AAGTCCCTAG CCAGAGATCA AGATTCTGGA ATCGAGATCC   
  
  
+ TCAGGGTTCA CGCCCGGCTG AAGTTCACTC ATCATTGAGA AGATTAGGAG ACGAGGCTCA GAGTGAGAAA   
  
  
+ CGCCTCAAGG CAGTGGAAGA ACCAATACGG CCCAGTGCAC CACCCGGCAA TTTGAAGCAG TTGCTCATAG   
  
  
+ AATGTGCTCG GGCTTTGTCA GAGAATCGAA TAGAGGATTT TGAGAAGTTA GTTGAACAGG CAAAGGGCAT   
  
  
+ GGTCTCCATT TCTGGAGACC CAATTCAACG ACTTGGTGCT TACTTGATCG AGGGGCTAGT GGCAAGGAAG   
  
  
+ GAGTCTTCAG GTACGAAAAT CTACCGAGCT CTTAGGTGCA AAGAGCCCCT TGGTCAAGAC TTGCTTTCGT   
  
  
+ ACATGCACAT CCTTTATGAA ATTTGCCCTT ACTTGAAATT CGGTTATATG GTTGCAAATG GGGCTATAGC   
  
  
+ TGAAGCTTGC AGAGATGAGG ATCACATACA TATCGTCGAC TACCAGATTG GTCAAGGAAC TCAATGGATG   
  
  
+ ACTCTGTTGC AAGCCCTAGC CACAAAACCT GGAAGACCCC CCACTGTGAG AATTACTGGC ATTGATGATC   
  
  
+ CCGTCTCTAG GCATGCTCGG GGAGCTTGCT TGGAGGCAGT GGGGAAACGT TTGGCAGTTC TGTCGGAGAA   
  
  
+ ATTTAACATG CCCATTGAGT TTCAGGCAGT GCCCGTTTAT GCTTCTGAGG TCACCCAAGA AATGCTTGAT   
  
  
+ GTGAGGCCAG GGTGGGCCTT GGCTGTGAAC TTCCCATTGC AGCTCCACCA CACTCCCGAT GAGAGTGTTG   
  
  
+ ATGTGAACAA CCCGAGGGAT GGACTCCTAA GAATGGTGAA ATCACTCGGT CCCAAGGTTG TAACTTTGGT   
  
  
+ AGAACAAGAG TCAAACACCA ACACTACCCC TTTGCTGACA AGGTTCATAG AAACGCTGGA CTTCTACTCA   
  
  
+ GCAATGTTTG AATCCATCGA TGTGACAATG CCAAGGGACC GAAAGGAAAG GATCAATGTC GAGCAGCATT   
  
  
+ GCCTAGCCAA GGACATTGTC AACATCATCG CGTGTGAGGG GAAGGACAGG GTGGAGCGAC ATGAGCTCTT   
  
  
+ TGGGAAATGG AAGTCAAGGT TCACCATGGC AGGATTCCAA CAATCCCCAT TGAGCTCGTA TGTTAACTCC   
  
  
+ GTGATAAAAG GATTACTTAG GTGTTATTCA GAGTACTATA CGTTGGTGGA GAAGGATGGC GCCTTGCTTC   
  
  
+ TGGGATGGAA GGACCGGATG CTGGTTTCGG CTTCAGCTTG GCATTG  

- +Up\_Stream \_Len000AAATTT ATAAAATAAA ATAAAATTTA ATTATATAAA ATATTAAATT TTAATAATTA   
  
  
- TTAACTACTA CAATGGGTTA CAAATTCAAA AATTTATAAA AATAAAAAAT TAAATTTTTA TAGTATTATA   
  
  
- TATTATATTA AAATTCAATT TTATTCTATC AATTTAATAA AATATCATTT ATAAATAATA AAAAAAATTC   
  
  
- TTTTAACGGT ACACTAACAG TAGTCAGTTT GTGACGCAAC TGTCTTCGAC AGGAACGACG ATGAGAGTGG   
  
  
- AGAGTTGTAG CCTGGGCGCG TCTGCAATTG TAGAGGCATG TTTATGTTAA GGGACCGGTT TTAAGTTTAG   
  
  
- TTGGGATGAG CCATCAGCCA AGGAAGAAGA AGGACGGGAG ATATATATGT GGGTGTCGAC GCGACGTACA   
  
  
- AAAAGGCTCT TAGGAGTTTT TAGAATATTT GAAGGAGCAC ACCCATCTTT TTTCTTTTTC TTTTCCCAAA   
  
  
- ATTTTAGAAC CCAGTTTTGG GTAGTTGTGG TGTTAAACTG AGAAAGAAAA ATTAAGTTGG TGGCGGTCAA   
  
  
- GGAGCAACTA AAAGTCCATG GAGAAAAAGA ACGGGGAAAA AAACGAGCCA ACAACAAATT ACCATTTCTA   
  
  
- ACTCATGTTT CTTACTATAA ACTACTCAAC CACACAAATA AACTACTCGG GTCTACTAAA TTGAACATCT   
  
  
- AAAACACCAA AACCAAAGAC CCATAAGTAA CCAAACGACT TAAAGACCAA GTACTAGACC CCAATCTTTT   
  
  
- CAACTTTACT GAAAGACCTC CAACCTTAAA CTGATTTCGA TTAAAGAAAA ACAGAAGTTA AGAAAAAATC   
  
  
- AACACTCGAC TTAAACAACT GGTGAAGGTC GGACAACCTT TAATTTGAAT GAATCCTCTT CTCTTAGAGT   
  
  
- CTAAGACTCG TTTTCGAAAT ATACCAACAA ACCCTTTACC ATCCTTGATT CACTTTTGTG ACTTTAAAAT   
  
  
- TCGTTCGTTA AAACAAGTAA ACTAACCAGC CTACCAGACT AAATCCAAGA AAAGAACCAG ATTTAAAGAC   
  
  
- TCGTAACTAA CAAATTAACG CACCCCTTGA ACATAGGAAC ACTCAAGGAC CAAGTAAAGA ACCAACGAAT   
  
  
- GAGATCGAGG GAAAGTGTCA AAGATACCAA CCTTGAACCT TGAACCTTGA ACCTTCGGAT TAAATCAACT   
  
  
- TATAGAACCG AAACTCAATC GAAAAGAATC CTTTTCTACC AAGACGAGAG CTTTTAGATT AGATTAGCTA   
  
  
- TCTACGCAAT TCCTTCGTTT TGAAAACCCC TCTCCCTTCA ATTCGCTTCG TTCGACGACA AAGTTTCAAC   
  
  
- CTGACAAAAT AACCATTCAT ACTTTGTTAT CAAATATTCT CTAGTAACCC AAGAATTCCA CTATCTAGTA   
  
  
- CCCAATACTT ACTCGAATCA ACAAGAAAAC GAAAGTTGAC ATGCATTCCT ACTATCAATA GGGAGTAGAA   
  
  
- CGTAGTTTTT GCCGTTTGGC ACTACGGTTT TGAGTTTTTG ACTAAGGAGA CTGTTCCGAA GATGGATACT   
  
  
- TTTGATACGT CTAGTATAGG TAAACCGGAA GGGAGATCCT TGGTTCATCT CATTCAACCA TTTATTTATC   
  
  
- TCAACTTGCT TTGTGATGTC TAAACTTAAA CTAATTCCTC CCGATAATAT CTGATTCCAC ATCGAGTTTA   
  
  
- GGAAGACATC CCTAACAGAA TAACCGAAGA AAACCAACGA AGACCGAGTT CAGGAGAATA ATCAATCATA   
  
  
- CGTAATGGAA CTCTGTTCTA TCAACGAATT CGAACTCGAA GACATGAATA AACGCATAAC ATCTGTTGAT   
  
  
- CGACACCGTA TACTAAGACT TTACAAGGCT ACAGAAAAAG AAACCTACGA CCCAACACAG TACTCGAAAT   
  
  
- CCAAACAAAT AAACTACAGT CCTTCATACA TTTATTGTAC ACCGTAGAAC GTTAGATAAT GTCCTACTAT   
  
  
- AACCGGAAAC ACCCTTCTTT AACTTCTTCA ACAAAGTGAA TGACCTATCC CTCGTACCTG AGTGTAGTCG   
  
  
- AAAACTCTAA GCCATAAAGG TCTAGTATGA GTAGGATATT AAAGAGAGTC AGATCAGGGG TTAGTTAAGG   
  
  
- TTTATCCAAT AAACTTAGTG GCTTGCAAGC AAGATCTCTG AGAGGTTAGA GCGGGAAGAG TTGCGGGAAG   
  
  
- TTAACGCTGG GGTGACAATA TAACTCGCTG TCAGTCCTCG TGGTGTCAAG TTGTCCTTCA GAGAGCCGTG   
  
  
- TTTCGAGTAG AAGATTAAGA GGGGAACTCT GATCGTCAAT AATATTAGTC AAGTTGAGTT CGGGACAACC   
  
  
- TCTTAGTGGA GTCCCTAGTA GTGGGCTCTT AGAAAAAGTT GTTCGACGTC AATTATTTCA GTCGTAGCTC   
  
  
- GTACGTAACG TCCTCGATCT TTGACGAGAT TACTCGTGTC TAACACTCCT CTTACTTCGT TGACAGAGAT   
  
  
- AGGGTAGATA CCCACTTGGA GTAGTCGGGG TTCAGGGATC GGTCTCTAGT TCTAAGACCT TAGCTCTAGG   
  
  
- AGTCCCAAGT GCGGGCCGAC TTCAAGTGAG TAGTAACTCT TCTAATCCTC TGCTCCGAGT CTCACTCTTT   
  
  
- GCGGAGTTCC GTCACCTTCT TGGTTATGCC GGGTCACGTG GTGGGCCGTT AAACTTCGTC AACGAGTATC   
  
  
- TTACACGAGC CCGAAACAGT CTCTTAGCTT ATCTCCTAAA ACTCTTCAAT CAACTTGTCC GTTTCCCGTA   
  
  
- CCAGAGGTAA AGACCTCTGG GTTAAGTTGC TGAACCACGA ATGAACTAGC TCCCCGATCA CCGTTCCTTC   
  
  
- CTCAGAAGTC CATGCTTTTA GATGGCTCGA GAATCCACGT TTCTCGGGGA ACCAGTTCTG AACGAAAGCA   
  
  
- TGTACGTGTA GGAAATACTT TAAACGGGAA TGAACTTTAA GCCAATATAC CAACGTTTAC CCCGATATCG   
  
  
- ACTTCGAACG TCTCTACTCC TAGTGTATGT ATAGCAGCTG ATGGTCTAAC CAGTTCCTTG AGTTACCTAC   
  
  
- TGAGACAACG TTCGGGATCG GTGTTTTGGA CCTTCTGGGG GGTGACACTC TTAATGACCG TAACTACTAG   
  
  
- GGCAGAGATC CGTACGAGCC CCTCGAACGA ACCTCCGTCA CCCCTTTGCA AACCGTCAAG ACAGCCTCTT   
  
  
- TAAATTGTAC GGGTAACTCA AAGTCCGTCA CGGGCAAATA CGAAGACTCC AGTGGGTTCT TTACGAACTA   
  
  
- CACTCCGGTC CCACCCGGAA CCGACACTTG AAGGGTAACG TCGAGGTGGT GTGAGGGCTA CTCTCACAAC   
  
  
- TACACTTGTT GGGCTCCCTA CCTGAGGATT CTTACCACTT TAGTGAGCCA GGGTTCCAAC ATTGAAACCA   
  
  
- TCTTGTTCTC AGTTTGTGGT TGTGATGGGG AAACGACTGT TCCAAGTATC TTTGCGACCT GAAGATGAGT   
  
  
- CGTTACAAAC TTAGGTAGCT ACACTGTTAC GGTTCCCTGG CTTTCCTTTC CTAGTTACAG CTCGTCGTAA   
  
  
- CGGATCGGTT CCTGTAACAG TTGTAGTAGC GCACACTCCC CTTCCTGTCC CACCTCGCTG TACTCGAGAA   
  
  
- ACCCTTTACC TTCAGTTCCA AGTGGTACCG TCCTAAGGTT GTTAGGGGTA ACTCGAGCAT ACAATTGAGG   
  
  
- CACTATTTTC CTAATGAATC CACAATAAGT CTCATGATAT GCAACCACCT CTTCCTACCG CGGAACGAAG   
  
  
- ACCCTACCTT CCTGGCCTAC GACCAAAGCC GAAGTCGAAC CGTAAC

+     Myc

| Site Name | Organism | Position | Strand | Matrix score. | sequence | function |
| --- | --- | --- | --- | --- | --- | --- |
| Myc | Arabidopsis thaliana | 1370 | - | 7 | TCTCTTA |  |

>HU01G01850.1   
+ +Up\_Stream \_Len000TTTAAA TATTTTATTT TATTTTAAAT TAATATATTT TATAATTTAA AATTATTAAT   
  
  
+ AATTGATGAT GTTACCCAAT GTTTAAGTTT TTAAATATTT TTATTTTTTA ATTTAAAAAT ATCATAATAT   
  
  
+ ATAATATAAT TTTAAGTTAA AATAAGATAG TTAAATTATT TTATAGTAAA TATTTATTAT TTTTTTTAAG   
  
  
+ AAAATTGCCA TGTGATTGTC ATCAGTCAAA CACTGCGTTG ACAGAAGCTG TCCTTGCTGC TACTCTCACC   
  
  
+ TCTCAACATC GGACCCGCGC AGACGTTAAC ATCTCCGTAC AAATACAATT CCCTGGCCAA AATTCAAATC   
  
  
+ AACCCTACTC GGTAGTCGGT TCCTTCTTCT TCCTGCCCTC TATATATACA CCCACAGCTG CGCTGCATGT   
  
  
+ TTTTCCGAGA ATCCTCAAAA ATCTTATAAA CTTCCTCGTG TGGGTAGAAA AAAGAAAAAG AAAAGGGTTT   
  
  
+ TAAAATCTTG GGTCAAAACC CATCAACACC ACAATTTGAC TCTTTCTTTT TAATTCAACC ACCGCCAGTT   
  
  
+ CCTCGTTGAT TTTCAGGTAC CTCTTTTTCT TGCCCCTTTT TTTGCTCGGT TGTTGTTTAA TGGTAAAGAT   
  
  
+ TGAGTACAAA GAATGATATT TGATGAGTTG GTGTGTTTAT TTGATGAGCC CAGATGATTT AACTTGTAGA   
  
  
+ TTTTGTGGTT TTGGTTTCTG GGTATTCATT GGTTTGCTGA ATTTCTGGTT CATGATCTGG GGTTAGAAAA   
  
  
+ GTTGAAATGA CTTTCTGGAG GTTGGAATTT GACTAAAGCT AATTTCTTTT TGTCTTCAAT TCTTTTTTAG   
  
  
+ TTGTGAGCTG AATTTGTTGA CCACTTCCAG CCTGTTGGAA ATTAAACTTA CTTAGGAGAA GAGAATCTCA   
  
  
+ GATTCTGAGC AAAAGCTTTA TATGGTTGTT TGGGAAATGG TAGGAACTAA GTGAAAACAC TGAAATTTTA   
  
  
+ AGCAAGCAAT TTTGTTCATT TGATTGGTCG GATGGTCTGA TTTAGGTTCT TTTCTTGGTC TAAATTTCTG   
  
  
+ AGCATTGATT GTTTAATTGC GTGGGGAACT TGTATCCTTG TGAGTTCCTG GTTCATTTCT TGGTTGCTTA   
  
  
+ CTCTAGCTCC CTTTCACAGT TTCTATGGTT GGAACTTGGA ACTTGGAACT TGGAAGCCTA ATTTAGTTGA   
  
  
+ ATATCTTGGC TTTGAGTTAG CTTTTCTTAG GAAAAGATGG TTCTGCTCTC GAAAATCTAA TCTAATCGAT   
  
  
+ AGATGCGTTA AGGAAGCAAA ACTTTTGGGG AGAGGGAAGT TAAGCGAAGC AAGCTGCTGT TTCAAAGTTG   
  
  
+ GACTGTTTTA TTGGTAAGTA TGAAACAATA GTTTATAAGA GATCATTGGG TTCTTAAGGT GATAGATCAT   
  
  
+ GGGTTATGAA TGAGCTTAGT TGTTCTTTTG CTTTCAACTG TACGTAAGGA TGATAGTTAT CCCTCATCTT   
  
  
+ GCATCAAAAA CGGCAAACCG TGATGCCAAA ACTCAAAAAC TGATTCCTCT GACAAGGCTT CTACCTATGA   
  
  
+ AAACTATGCA GATCATATCC ATTTGGCCTT CCCTCTAGGA ACCAAGTAGA GTAAGTTGGT AAATAAATAG   
  
  
+ AGTTGAACGA AACACTACAG ATTTGAATTT GATTAAGGAG GGCTATTATA GACTAAGGTG TAGCTCAAAT   
  
  
+ CCTTCTGTAG GGATTGTCTT ATTGGCTTCT TTTGGTTGCT TCTGGCTCAA GTCCTCTTAT TAGTTAGTAT   
  
  
+ GCATTACCTT GAGACAAGAT AGTTGCTTAA GCTTGAGCTT CTGTACTTAT TTGCGTATTG TAGACAACTA   
  
  
+ GCTGTGGCAT ATGATTCTGA AATGTTCCGA TGTCTTTTTC TTTGGATGCT GGGTTGTGTC ATGAGCTTTA   
  
  
+ GGTTTGTTTA TTTGATGTCA GGAAGTATGT AAATAACATG TGGCATCTTG CAATCTATTA CAGGATGATA   
  
  
+ TTGGCCTTTG TGGGAAGAAA TTGAAGAAGT TGTTTCACTT ACTGGATAGG GAGCATGGAC TCACATCAGC   
  
  
+ TTTTGAGATT CGGTATTTCC AGATCATACT CATCCTATAA TTTCTCTCAG TCTAGTCCCC AATCAATTCC   
  
  
+ AAATAGGTTA TTTGAATCAC CGAACGTTCG TTCTAGAGAC TCTCCAATCT CGCCCTTCTC AACGCCCTTC   
  
  
+ AATTGCGACC CCACTGTTAT ATTGAGCGAC AGTCAGGAGC ACCACAGTTC AACAGGAAGT CTCTCGGCAC   
  
  
+ AAAGCTCATC TTCTAATTCT CCCCTTGAGA CTAGCAGTTA TTATAATCAG TTCAACTCAA GCCCTGTTGG   
  
  
+ AGAATCACCT CAGGGATCAT CACCCGAGAA TCTTTTTCAA CAAGCTGCAG TTAATAAAGT CAGCATCGAG   
  
  
+ CATGCATTGC AGGAGCTAGA AACTGCTCTA ATGAGCACAG ATTGTGAGGA GAATGAAGCA ACTGTCTCTA   
  
  
+ TCCCATCTAT GGGTGAACCT CATCAGCCCC AAGTCCCTAG CCAGAGATCA AGATTCTGGA ATCGAGATCC   
  
  
+ TCAGGGTTCA CGCCCGGCTG AAGTTCACTC ATCATTGAGA AGATTAGGAG ACGAGGCTCA GAGTGAGAAA   
  
  
+ CGCCTCAAGG CAGTGGAAGA ACCAATACGG CCCAGTGCAC CACCCGGCAA TTTGAAGCAG TTGCTCATAG   
  
  
+ AATGTGCTCG GGCTTTGTCA GAGAATCGAA TAGAGGATTT TGAGAAGTTA GTTGAACAGG CAAAGGGCAT   
  
  
+ GGTCTCCATT TCTGGAGACC CAATTCAACG ACTTGGTGCT TACTTGATCG AGGGGCTAGT GGCAAGGAAG   
  
  
+ GAGTCTTCAG GTACGAAAAT CTACCGAGCT CTTAGGTGCA AAGAGCCCCT TGGTCAAGAC TTGCTTTCGT   
  
  
+ ACATGCACAT CCTTTATGAA ATTTGCCCTT ACTTGAAATT CGGTTATATG GTTGCAAATG GGGCTATAGC   
  
  
+ TGAAGCTTGC AGAGATGAGG ATCACATACA TATCGTCGAC TACCAGATTG GTCAAGGAAC TCAATGGATG   
  
  
+ ACTCTGTTGC AAGCCCTAGC CACAAAACCT GGAAGACCCC CCACTGTGAG AATTACTGGC ATTGATGATC   
  
  
+ CCGTCTCTAG GCATGCTCGG GGAGCTTGCT TGGAGGCAGT GGGGAAACGT TTGGCAGTTC TGTCGGAGAA   
  
  
+ ATTTAACATG CCCATTGAGT TTCAGGCAGT GCCCGTTTAT GCTTCTGAGG TCACCCAAGA AATGCTTGAT   
  
  
+ GTGAGGCCAG GGTGGGCCTT GGCTGTGAAC TTCCCATTGC AGCTCCACCA CACTCCCGAT GAGAGTGTTG   
  
  
+ ATGTGAACAA CCCGAGGGAT GGACTCCTAA GAATGGTGAA ATCACTCGGT CCCAAGGTTG TAACTTTGGT   
  
  
+ AGAACAAGAG TCAAACACCA ACACTACCCC TTTGCTGACA AGGTTCATAG AAACGCTGGA CTTCTACTCA   
  
  
+ GCAATGTTTG AATCCATCGA TGTGACAATG CCAAGGGACC GAAAGGAAAG GATCAATGTC GAGCAGCATT   
  
  
+ GCCTAGCCAA GGACATTGTC AACATCATCG CGTGTGAGGG GAAGGACAGG GTGGAGCGAC ATGAGCTCTT   
  
  
+ TGGGAAATGG AAGTCAAGGT TCACCATGGC AGGATTCCAA CAATCCCCAT TGAGCTCGTA TGTTAACTCC   
  
  
+ GTGATAAAAG GATTACTTAG GTGTTATTCA GAGTACTATA CGTTGGTGGA GAAGGATGGC GCCTTGCTTC   
  
  
+ TGGGATGGAA GGACCGGATG CTGGTTTCGG CTTCAGCTTG GCATTG  

- +Up\_Stream \_Len000AAATTT ATAAAATAAA ATAAAATTTA ATTATATAAA ATATTAAATT TTAATAATTA   
  
  
- TTAACTACTA CAATGGGTTA CAAATTCAAA AATTTATAAA AATAAAAAAT TAAATTTTTA TAGTATTATA   
  
  
- TATTATATTA AAATTCAATT TTATTCTATC AATTTAATAA AATATCATTT ATAAATAATA AAAAAAATTC   
  
  
- TTTTAACGGT ACACTAACAG TAGTCAGTTT GTGACGCAAC TGTCTTCGAC AGGAACGACG ATGAGAGTGG   
  
  
- AGAGTTGTAG CCTGGGCGCG TCTGCAATTG TAGAGGCATG TTTATGTTAA GGGACCGGTT TTAAGTTTAG   
  
  
- TTGGGATGAG CCATCAGCCA AGGAAGAAGA AGGACGGGAG ATATATATGT GGGTGTCGAC GCGACGTACA   
  
  
- AAAAGGCTCT TAGGAGTTTT TAGAATATTT GAAGGAGCAC ACCCATCTTT TTTCTTTTTC TTTTCCCAAA   
  
  
- ATTTTAGAAC CCAGTTTTGG GTAGTTGTGG TGTTAAACTG AGAAAGAAAA ATTAAGTTGG TGGCGGTCAA   
  
  
- GGAGCAACTA AAAGTCCATG GAGAAAAAGA ACGGGGAAAA AAACGAGCCA ACAACAAATT ACCATTTCTA   
  
  
- ACTCATGTTT CTTACTATAA ACTACTCAAC CACACAAATA AACTACTCGG GTCTACTAAA TTGAACATCT   
  
  
- AAAACACCAA AACCAAAGAC CCATAAGTAA CCAAACGACT TAAAGACCAA GTACTAGACC CCAATCTTTT   
  
  
- CAACTTTACT GAAAGACCTC CAACCTTAAA CTGATTTCGA TTAAAGAAAA ACAGAAGTTA AGAAAAAATC   
  
  
- AACACTCGAC TTAAACAACT GGTGAAGGTC GGACAACCTT TAATTTGAAT GAATCCTCTT CTCTTAGAGT   
  
  
- CTAAGACTCG TTTTCGAAAT ATACCAACAA ACCCTTTACC ATCCTTGATT CACTTTTGTG ACTTTAAAAT   
  
  
- TCGTTCGTTA AAACAAGTAA ACTAACCAGC CTACCAGACT AAATCCAAGA AAAGAACCAG ATTTAAAGAC   
  
  
- TCGTAACTAA CAAATTAACG CACCCCTTGA ACATAGGAAC ACTCAAGGAC CAAGTAAAGA ACCAACGAAT   
  
  
- GAGATCGAGG GAAAGTGTCA AAGATACCAA CCTTGAACCT TGAACCTTGA ACCTTCGGAT TAAATCAACT   
  
  
- TATAGAACCG AAACTCAATC GAAAAGAATC CTTTTCTACC AAGACGAGAG CTTTTAGATT AGATTAGCTA   
  
  
- TCTACGCAAT TCCTTCGTTT TGAAAACCCC TCTCCCTTCA ATTCGCTTCG TTCGACGACA AAGTTTCAAC   
  
  
- CTGACAAAAT AACCATTCAT ACTTTGTTAT CAAATATTCT CTAGTAACCC AAGAATTCCA CTATCTAGTA   
  
  
- CCCAATACTT ACTCGAATCA ACAAGAAAAC GAAAGTTGAC ATGCATTCCT ACTATCAATA GGGAGTAGAA   
  
  
- CGTAGTTTTT GCCGTTTGGC ACTACGGTTT TGAGTTTTTG ACTAAGGAGA CTGTTCCGAA GATGGATACT   
  
  
- TTTGATACGT CTAGTATAGG TAAACCGGAA GGGAGATCCT TGGTTCATCT CATTCAACCA TTTATTTATC   
  
  
- TCAACTTGCT TTGTGATGTC TAAACTTAAA CTAATTCCTC CCGATAATAT CTGATTCCAC ATCGAGTTTA   
  
  
- GGAAGACATC CCTAACAGAA TAACCGAAGA AAACCAACGA AGACCGAGTT CAGGAGAATA ATCAATCATA   
  
  
- CGTAATGGAA CTCTGTTCTA TCAACGAATT CGAACTCGAA GACATGAATA AACGCATAAC ATCTGTTGAT   
  
  
- CGACACCGTA TACTAAGACT TTACAAGGCT ACAGAAAAAG AAACCTACGA CCCAACACAG TACTCGAAAT   
  
  
- CCAAACAAAT AAACTACAGT CCTTCATACA TTTATTGTAC ACCGTAGAAC GTTAGATAAT GTCCTACTAT   
  
  
- AACCGGAAAC ACCCTTCTTT AACTTCTTCA ACAAAGTGAA TGACCTATCC CTCGTACCTG AGTGTAGTCG   
  
  
- AAAACTCTAA GCCATAAAGG TCTAGTATGA GTAGGATATT AAAGAGAGTC AGATCAGGGG TTAGTTAAGG   
  
  
- TTTATCCAAT AAACTTAGTG GCTTGCAAGC AAGATCTCTG AGAGGTTAGA GCGGGAAGAG TTGCGGGAAG   
  
  
- TTAACGCTGG GGTGACAATA TAACTCGCTG TCAGTCCTCG TGGTGTCAAG TTGTCCTTCA GAGAGCCGTG   
  
  
- TTTCGAGTAG AAGATTAAGA GGGGAACTCT GATCGTCAAT AATATTAGTC AAGTTGAGTT CGGGACAACC   
  
  
- TCTTAGTGGA GTCCCTAGTA GTGGGCTCTT AGAAAAAGTT GTTCGACGTC AATTATTTCA GTCGTAGCTC   
  
  
- GTACGTAACG TCCTCGATCT TTGACGAGAT TACTCGTGTC TAACACTCCT CTTACTTCGT TGACAGAGAT   
  
  
- AGGGTAGATA CCCACTTGGA GTAGTCGGGG TTCAGGGATC GGTCTCTAGT TCTAAGACCT TAGCTCTAGG   
  
  
- AGTCCCAAGT GCGGGCCGAC TTCAAGTGAG TAGTAACTCT TCTAATCCTC TGCTCCGAGT CTCACTCTTT   
  
  
- GCGGAGTTCC GTCACCTTCT TGGTTATGCC GGGTCACGTG GTGGGCCGTT AAACTTCGTC AACGAGTATC   
  
  
- TTACACGAGC CCGAAACAGT CTCTTAGCTT ATCTCCTAAA ACTCTTCAAT CAACTTGTCC GTTTCCCGTA   
  
  
- CCAGAGGTAA AGACCTCTGG GTTAAGTTGC TGAACCACGA ATGAACTAGC TCCCCGATCA CCGTTCCTTC   
  
  
- CTCAGAAGTC CATGCTTTTA GATGGCTCGA GAATCCACGT TTCTCGGGGA ACCAGTTCTG AACGAAAGCA   
  
  
- TGTACGTGTA GGAAATACTT TAAACGGGAA TGAACTTTAA GCCAATATAC CAACGTTTAC CCCGATATCG   
  
  
- ACTTCGAACG TCTCTACTCC TAGTGTATGT ATAGCAGCTG ATGGTCTAAC CAGTTCCTTG AGTTACCTAC   
  
  
- TGAGACAACG TTCGGGATCG GTGTTTTGGA CCTTCTGGGG GGTGACACTC TTAATGACCG TAACTACTAG   
  
  
- GGCAGAGATC CGTACGAGCC CCTCGAACGA ACCTCCGTCA CCCCTTTGCA AACCGTCAAG ACAGCCTCTT   
  
  
- TAAATTGTAC GGGTAACTCA AAGTCCGTCA CGGGCAAATA CGAAGACTCC AGTGGGTTCT TTACGAACTA   
  
  
- CACTCCGGTC CCACCCGGAA CCGACACTTG AAGGGTAACG TCGAGGTGGT GTGAGGGCTA CTCTCACAAC   
  
  
- TACACTTGTT GGGCTCCCTA CCTGAGGATT CTTACCACTT TAGTGAGCCA GGGTTCCAAC ATTGAAACCA   
  
  
- TCTTGTTCTC AGTTTGTGGT TGTGATGGGG AAACGACTGT TCCAAGTATC TTTGCGACCT GAAGATGAGT   
  
  
- CGTTACAAAC TTAGGTAGCT ACACTGTTAC GGTTCCCTGG CTTTCCTTTC CTAGTTACAG CTCGTCGTAA   
  
  
- CGGATCGGTT CCTGTAACAG TTGTAGTAGC GCACACTCCC CTTCCTGTCC CACCTCGCTG TACTCGAGAA   
  
  
- ACCCTTTACC TTCAGTTCCA AGTGGTACCG TCCTAAGGTT GTTAGGGGTA ACTCGAGCAT ACAATTGAGG   
  
  
- CACTATTTTC CTAATGAATC CACAATAAGT CTCATGATAT GCAACCACCT CTTCCTACCG CGGAACGAAG   
  
  
- ACCCTACCTT CCTGGCCTAC GACCAAAGCC GAAGTCGAAC CGTAAC

+     O2-site

| Site Name | Organism | Position | Strand | Matrix score. | sequence | function |
| --- | --- | --- | --- | --- | --- | --- |
| O2-site | Zea mays | 3291 | + | 9 | GTTGACGTGA | cis-acting regulatory element involved in zein metabolism regulation |
| O2-site | Zea mays | 3524 | - | 9 | GATGATGTGG | cis-acting regulatory element involved in zein metabolism regulation |

>HU01G01850.1   
+ +Up\_Stream \_Len000TTTAAA TATTTTATTT TATTTTAAAT TAATATATTT TATAATTTAA AATTATTAAT   
  
  
+ AATTGATGAT GTTACCCAAT GTTTAAGTTT TTAAATATTT TTATTTTTTA ATTTAAAAAT ATCATAATAT   
  
  
+ ATAATATAAT TTTAAGTTAA AATAAGATAG TTAAATTATT TTATAGTAAA TATTTATTAT TTTTTTTAAG   
  
  
+ AAAATTGCCA TGTGATTGTC ATCAGTCAAA CACTGCGTTG ACAGAAGCTG TCCTTGCTGC TACTCTCACC   
  
  
+ TCTCAACATC GGACCCGCGC AGACGTTAAC ATCTCCGTAC AAATACAATT CCCTGGCCAA AATTCAAATC   
  
  
+ AACCCTACTC GGTAGTCGGT TCCTTCTTCT TCCTGCCCTC TATATATACA CCCACAGCTG CGCTGCATGT   
  
  
+ TTTTCCGAGA ATCCTCAAAA ATCTTATAAA CTTCCTCGTG TGGGTAGAAA AAAGAAAAAG AAAAGGGTTT   
  
  
+ TAAAATCTTG GGTCAAAACC CATCAACACC ACAATTTGAC TCTTTCTTTT TAATTCAACC ACCGCCAGTT   
  
  
+ CCTCGTTGAT TTTCAGGTAC CTCTTTTTCT TGCCCCTTTT TTTGCTCGGT TGTTGTTTAA TGGTAAAGAT   
  
  
+ TGAGTACAAA GAATGATATT TGATGAGTTG GTGTGTTTAT TTGATGAGCC CAGATGATTT AACTTGTAGA   
  
  
+ TTTTGTGGTT TTGGTTTCTG GGTATTCATT GGTTTGCTGA ATTTCTGGTT CATGATCTGG GGTTAGAAAA   
  
  
+ GTTGAAATGA CTTTCTGGAG GTTGGAATTT GACTAAAGCT AATTTCTTTT TGTCTTCAAT TCTTTTTTAG   
  
  
+ TTGTGAGCTG AATTTGTTGA CCACTTCCAG CCTGTTGGAA ATTAAACTTA CTTAGGAGAA GAGAATCTCA   
  
  
+ GATTCTGAGC AAAAGCTTTA TATGGTTGTT TGGGAAATGG TAGGAACTAA GTGAAAACAC TGAAATTTTA   
  
  
+ AGCAAGCAAT TTTGTTCATT TGATTGGTCG GATGGTCTGA TTTAGGTTCT TTTCTTGGTC TAAATTTCTG   
  
  
+ AGCATTGATT GTTTAATTGC GTGGGGAACT TGTATCCTTG TGAGTTCCTG GTTCATTTCT TGGTTGCTTA   
  
  
+ CTCTAGCTCC CTTTCACAGT TTCTATGGTT GGAACTTGGA ACTTGGAACT TGGAAGCCTA ATTTAGTTGA   
  
  
+ ATATCTTGGC TTTGAGTTAG CTTTTCTTAG GAAAAGATGG TTCTGCTCTC GAAAATCTAA TCTAATCGAT   
  
  
+ AGATGCGTTA AGGAAGCAAA ACTTTTGGGG AGAGGGAAGT TAAGCGAAGC AAGCTGCTGT TTCAAAGTTG   
  
  
+ GACTGTTTTA TTGGTAAGTA TGAAACAATA GTTTATAAGA GATCATTGGG TTCTTAAGGT GATAGATCAT   
  
  
+ GGGTTATGAA TGAGCTTAGT TGTTCTTTTG CTTTCAACTG TACGTAAGGA TGATAGTTAT CCCTCATCTT   
  
  
+ GCATCAAAAA CGGCAAACCG TGATGCCAAA ACTCAAAAAC TGATTCCTCT GACAAGGCTT CTACCTATGA   
  
  
+ AAACTATGCA GATCATATCC ATTTGGCCTT CCCTCTAGGA ACCAAGTAGA GTAAGTTGGT AAATAAATAG   
  
  
+ AGTTGAACGA AACACTACAG ATTTGAATTT GATTAAGGAG GGCTATTATA GACTAAGGTG TAGCTCAAAT   
  
  
+ CCTTCTGTAG GGATTGTCTT ATTGGCTTCT TTTGGTTGCT TCTGGCTCAA GTCCTCTTAT TAGTTAGTAT   
  
  
+ GCATTACCTT GAGACAAGAT AGTTGCTTAA GCTTGAGCTT CTGTACTTAT TTGCGTATTG TAGACAACTA   
  
  
+ GCTGTGGCAT ATGATTCTGA AATGTTCCGA TGTCTTTTTC TTTGGATGCT GGGTTGTGTC ATGAGCTTTA   
  
  
+ GGTTTGTTTA TTTGATGTCA GGAAGTATGT AAATAACATG TGGCATCTTG CAATCTATTA CAGGATGATA   
  
  
+ TTGGCCTTTG TGGGAAGAAA TTGAAGAAGT TGTTTCACTT ACTGGATAGG GAGCATGGAC TCACATCAGC   
  
  
+ TTTTGAGATT CGGTATTTCC AGATCATACT CATCCTATAA TTTCTCTCAG TCTAGTCCCC AATCAATTCC   
  
  
+ AAATAGGTTA TTTGAATCAC CGAACGTTCG TTCTAGAGAC TCTCCAATCT CGCCCTTCTC AACGCCCTTC   
  
  
+ AATTGCGACC CCACTGTTAT ATTGAGCGAC AGTCAGGAGC ACCACAGTTC AACAGGAAGT CTCTCGGCAC   
  
  
+ AAAGCTCATC TTCTAATTCT CCCCTTGAGA CTAGCAGTTA TTATAATCAG TTCAACTCAA GCCCTGTTGG   
  
  
+ AGAATCACCT CAGGGATCAT CACCCGAGAA TCTTTTTCAA CAAGCTGCAG TTAATAAAGT CAGCATCGAG   
  
  
+ CATGCATTGC AGGAGCTAGA AACTGCTCTA ATGAGCACAG ATTGTGAGGA GAATGAAGCA ACTGTCTCTA   
  
  
+ TCCCATCTAT GGGTGAACCT CATCAGCCCC AAGTCCCTAG CCAGAGATCA AGATTCTGGA ATCGAGATCC   
  
  
+ TCAGGGTTCA CGCCCGGCTG AAGTTCACTC ATCATTGAGA AGATTAGGAG ACGAGGCTCA GAGTGAGAAA   
  
  
+ CGCCTCAAGG CAGTGGAAGA ACCAATACGG CCCAGTGCAC CACCCGGCAA TTTGAAGCAG TTGCTCATAG   
  
  
+ AATGTGCTCG GGCTTTGTCA GAGAATCGAA TAGAGGATTT TGAGAAGTTA GTTGAACAGG CAAAGGGCAT   
  
  
+ GGTCTCCATT TCTGGAGACC CAATTCAACG ACTTGGTGCT TACTTGATCG AGGGGCTAGT GGCAAGGAAG   
  
  
+ GAGTCTTCAG GTACGAAAAT CTACCGAGCT CTTAGGTGCA AAGAGCCCCT TGGTCAAGAC TTGCTTTCGT   
  
  
+ ACATGCACAT CCTTTATGAA ATTTGCCCTT ACTTGAAATT CGGTTATATG GTTGCAAATG GGGCTATAGC   
  
  
+ TGAAGCTTGC AGAGATGAGG ATCACATACA TATCGTCGAC TACCAGATTG GTCAAGGAAC TCAATGGATG   
  
  
+ ACTCTGTTGC AAGCCCTAGC CACAAAACCT GGAAGACCCC CCACTGTGAG AATTACTGGC ATTGATGATC   
  
  
+ CCGTCTCTAG GCATGCTCGG GGAGCTTGCT TGGAGGCAGT GGGGAAACGT TTGGCAGTTC TGTCGGAGAA   
  
  
+ ATTTAACATG CCCATTGAGT TTCAGGCAGT GCCCGTTTAT GCTTCTGAGG TCACCCAAGA AATGCTTGAT   
  
  
+ GTGAGGCCAG GGTGGGCCTT GGCTGTGAAC TTCCCATTGC AGCTCCACCA CACTCCCGAT GAGAGTGTTG   
  
  
+ ATGTGAACAA CCCGAGGGAT GGACTCCTAA GAATGGTGAA ATCACTCGGT CCCAAGGTTG TAACTTTGGT   
  
  
+ AGAACAAGAG TCAAACACCA ACACTACCCC TTTGCTGACA AGGTTCATAG AAACGCTGGA CTTCTACTCA   
  
  
+ GCAATGTTTG AATCCATCGA TGTGACAATG CCAAGGGACC GAAAGGAAAG GATCAATGTC GAGCAGCATT   
  
  
+ GCCTAGCCAA GGACATTGTC AACATCATCG CGTGTGAGGG GAAGGACAGG GTGGAGCGAC ATGAGCTCTT   
  
  
+ TGGGAAATGG AAGTCAAGGT TCACCATGGC AGGATTCCAA CAATCCCCAT TGAGCTCGTA TGTTAACTCC   
  
  
+ GTGATAAAAG GATTACTTAG GTGTTATTCA GAGTACTATA CGTTGGTGGA GAAGGATGGC GCCTTGCTTC   
  
  
+ TGGGATGGAA GGACCGGATG CTGGTTTCGG CTTCAGCTTG GCATTG  

- +Up\_Stream \_Len000AAATTT ATAAAATAAA ATAAAATTTA ATTATATAAA ATATTAAATT TTAATAATTA   
  
  
- TTAACTACTA CAATGGGTTA CAAATTCAAA AATTTATAAA AATAAAAAAT TAAATTTTTA TAGTATTATA   
  
  
- TATTATATTA AAATTCAATT TTATTCTATC AATTTAATAA AATATCATTT ATAAATAATA AAAAAAATTC   
  
  
- TTTTAACGGT ACACTAACAG TAGTCAGTTT GTGACGCAAC TGTCTTCGAC AGGAACGACG ATGAGAGTGG   
  
  
- AGAGTTGTAG CCTGGGCGCG TCTGCAATTG TAGAGGCATG TTTATGTTAA GGGACCGGTT TTAAGTTTAG   
  
  
- TTGGGATGAG CCATCAGCCA AGGAAGAAGA AGGACGGGAG ATATATATGT GGGTGTCGAC GCGACGTACA   
  
  
- AAAAGGCTCT TAGGAGTTTT TAGAATATTT GAAGGAGCAC ACCCATCTTT TTTCTTTTTC TTTTCCCAAA   
  
  
- ATTTTAGAAC CCAGTTTTGG GTAGTTGTGG TGTTAAACTG AGAAAGAAAA ATTAAGTTGG TGGCGGTCAA   
  
  
- GGAGCAACTA AAAGTCCATG GAGAAAAAGA ACGGGGAAAA AAACGAGCCA ACAACAAATT ACCATTTCTA   
  
  
- ACTCATGTTT CTTACTATAA ACTACTCAAC CACACAAATA AACTACTCGG GTCTACTAAA TTGAACATCT   
  
  
- AAAACACCAA AACCAAAGAC CCATAAGTAA CCAAACGACT TAAAGACCAA GTACTAGACC CCAATCTTTT   
  
  
- CAACTTTACT GAAAGACCTC CAACCTTAAA CTGATTTCGA TTAAAGAAAA ACAGAAGTTA AGAAAAAATC   
  
  
- AACACTCGAC TTAAACAACT GGTGAAGGTC GGACAACCTT TAATTTGAAT GAATCCTCTT CTCTTAGAGT   
  
  
- CTAAGACTCG TTTTCGAAAT ATACCAACAA ACCCTTTACC ATCCTTGATT CACTTTTGTG ACTTTAAAAT   
  
  
- TCGTTCGTTA AAACAAGTAA ACTAACCAGC CTACCAGACT AAATCCAAGA AAAGAACCAG ATTTAAAGAC   
  
  
- TCGTAACTAA CAAATTAACG CACCCCTTGA ACATAGGAAC ACTCAAGGAC CAAGTAAAGA ACCAACGAAT   
  
  
- GAGATCGAGG GAAAGTGTCA AAGATACCAA CCTTGAACCT TGAACCTTGA ACCTTCGGAT TAAATCAACT   
  
  
- TATAGAACCG AAACTCAATC GAAAAGAATC CTTTTCTACC AAGACGAGAG CTTTTAGATT AGATTAGCTA   
  
  
- TCTACGCAAT TCCTTCGTTT TGAAAACCCC TCTCCCTTCA ATTCGCTTCG TTCGACGACA AAGTTTCAAC   
  
  
- CTGACAAAAT AACCATTCAT ACTTTGTTAT CAAATATTCT CTAGTAACCC AAGAATTCCA CTATCTAGTA   
  
  
- CCCAATACTT ACTCGAATCA ACAAGAAAAC GAAAGTTGAC ATGCATTCCT ACTATCAATA GGGAGTAGAA   
  
  
- CGTAGTTTTT GCCGTTTGGC ACTACGGTTT TGAGTTTTTG ACTAAGGAGA CTGTTCCGAA GATGGATACT   
  
  
- TTTGATACGT CTAGTATAGG TAAACCGGAA GGGAGATCCT TGGTTCATCT CATTCAACCA TTTATTTATC   
  
  
- TCAACTTGCT TTGTGATGTC TAAACTTAAA CTAATTCCTC CCGATAATAT CTGATTCCAC ATCGAGTTTA   
  
  
- GGAAGACATC CCTAACAGAA TAACCGAAGA AAACCAACGA AGACCGAGTT CAGGAGAATA ATCAATCATA   
  
  
- CGTAATGGAA CTCTGTTCTA TCAACGAATT CGAACTCGAA GACATGAATA AACGCATAAC ATCTGTTGAT   
  
  
- CGACACCGTA TACTAAGACT TTACAAGGCT ACAGAAAAAG AAACCTACGA CCCAACACAG TACTCGAAAT   
  
  
- CCAAACAAAT AAACTACAGT CCTTCATACA TTTATTGTAC ACCGTAGAAC GTTAGATAAT GTCCTACTAT   
  
  
- AACCGGAAAC ACCCTTCTTT AACTTCTTCA ACAAAGTGAA TGACCTATCC CTCGTACCTG AGTGTAGTCG   
  
  
- AAAACTCTAA GCCATAAAGG TCTAGTATGA GTAGGATATT AAAGAGAGTC AGATCAGGGG TTAGTTAAGG   
  
  
- TTTATCCAAT AAACTTAGTG GCTTGCAAGC AAGATCTCTG AGAGGTTAGA GCGGGAAGAG TTGCGGGAAG   
  
  
- TTAACGCTGG GGTGACAATA TAACTCGCTG TCAGTCCTCG TGGTGTCAAG TTGTCCTTCA GAGAGCCGTG   
  
  
- TTTCGAGTAG AAGATTAAGA GGGGAACTCT GATCGTCAAT AATATTAGTC AAGTTGAGTT CGGGACAACC   
  
  
- TCTTAGTGGA GTCCCTAGTA GTGGGCTCTT AGAAAAAGTT GTTCGACGTC AATTATTTCA GTCGTAGCTC   
  
  
- GTACGTAACG TCCTCGATCT TTGACGAGAT TACTCGTGTC TAACACTCCT CTTACTTCGT TGACAGAGAT   
  
  
- AGGGTAGATA CCCACTTGGA GTAGTCGGGG TTCAGGGATC GGTCTCTAGT TCTAAGACCT TAGCTCTAGG   
  
  
- AGTCCCAAGT GCGGGCCGAC TTCAAGTGAG TAGTAACTCT TCTAATCCTC TGCTCCGAGT CTCACTCTTT   
  
  
- GCGGAGTTCC GTCACCTTCT TGGTTATGCC GGGTCACGTG GTGGGCCGTT AAACTTCGTC AACGAGTATC   
  
  
- TTACACGAGC CCGAAACAGT CTCTTAGCTT ATCTCCTAAA ACTCTTCAAT CAACTTGTCC GTTTCCCGTA   
  
  
- CCAGAGGTAA AGACCTCTGG GTTAAGTTGC TGAACCACGA ATGAACTAGC TCCCCGATCA CCGTTCCTTC   
  
  
- CTCAGAAGTC CATGCTTTTA GATGGCTCGA GAATCCACGT TTCTCGGGGA ACCAGTTCTG AACGAAAGCA   
  
  
- TGTACGTGTA GGAAATACTT TAAACGGGAA TGAACTTTAA GCCAATATAC CAACGTTTAC CCCGATATCG   
  
  
- ACTTCGAACG TCTCTACTCC TAGTGTATGT ATAGCAGCTG ATGGTCTAAC CAGTTCCTTG AGTTACCTAC   
  
  
- TGAGACAACG TTCGGGATCG GTGTTTTGGA CCTTCTGGGG GGTGACACTC TTAATGACCG TAACTACTAG   
  
  
- GGCAGAGATC CGTACGAGCC CCTCGAACGA ACCTCCGTCA CCCCTTTGCA AACCGTCAAG ACAGCCTCTT   
  
  
- TAAATTGTAC GGGTAACTCA AAGTCCGTCA CGGGCAAATA CGAAGACTCC AGTGGGTTCT TTACGAACTA   
  
  
- CACTCCGGTC CCACCCGGAA CCGACACTTG AAGGGTAACG TCGAGGTGGT GTGAGGGCTA CTCTCACAAC   
  
  
- TACACTTGTT GGGCTCCCTA CCTGAGGATT CTTACCACTT TAGTGAGCCA GGGTTCCAAC ATTGAAACCA   
  
  
- TCTTGTTCTC AGTTTGTGGT TGTGATGGGG AAACGACTGT TCCAAGTATC TTTGCGACCT GAAGATGAGT   
  
  
- CGTTACAAAC TTAGGTAGCT ACACTGTTAC GGTTCCCTGG CTTTCCTTTC CTAGTTACAG CTCGTCGTAA   
  
  
- CGGATCGGTT CCTGTAACAG TTGTAGTAGC GCACACTCCC CTTCCTGTCC CACCTCGCTG TACTCGAGAA   
  
  
- ACCCTTTACC TTCAGTTCCA AGTGGTACCG TCCTAAGGTT GTTAGGGGTA ACTCGAGCAT ACAATTGAGG   
  
  
- CACTATTTTC CTAATGAATC CACAATAAGT CTCATGATAT GCAACCACCT CTTCCTACCG CGGAACGAAG   
  
  
- ACCCTACCTT CCTGGCCTAC GACCAAAGCC GAAGTCGAAC CGTAAC

+     P-box

| Site Name | Organism | Position | Strand | Matrix score. | sequence | function |
| --- | --- | --- | --- | --- | --- | --- |
| P-box | Petroselinum crispum | 3609 | + | 13 | TTCCAACAAACCCC | gibberellin-responsive element and part of a light responsive element |

>HU01G01850.1   
+ +Up\_Stream \_Len000TTTAAA TATTTTATTT TATTTTAAAT TAATATATTT TATAATTTAA AATTATTAAT   
  
  
+ AATTGATGAT GTTACCCAAT GTTTAAGTTT TTAAATATTT TTATTTTTTA ATTTAAAAAT ATCATAATAT   
  
  
+ ATAATATAAT TTTAAGTTAA AATAAGATAG TTAAATTATT TTATAGTAAA TATTTATTAT TTTTTTTAAG   
  
  
+ AAAATTGCCA TGTGATTGTC ATCAGTCAAA CACTGCGTTG ACAGAAGCTG TCCTTGCTGC TACTCTCACC   
  
  
+ TCTCAACATC GGACCCGCGC AGACGTTAAC ATCTCCGTAC AAATACAATT CCCTGGCCAA AATTCAAATC   
  
  
+ AACCCTACTC GGTAGTCGGT TCCTTCTTCT TCCTGCCCTC TATATATACA CCCACAGCTG CGCTGCATGT   
  
  
+ TTTTCCGAGA ATCCTCAAAA ATCTTATAAA CTTCCTCGTG TGGGTAGAAA AAAGAAAAAG AAAAGGGTTT   
  
  
+ TAAAATCTTG GGTCAAAACC CATCAACACC ACAATTTGAC TCTTTCTTTT TAATTCAACC ACCGCCAGTT   
  
  
+ CCTCGTTGAT TTTCAGGTAC CTCTTTTTCT TGCCCCTTTT TTTGCTCGGT TGTTGTTTAA TGGTAAAGAT   
  
  
+ TGAGTACAAA GAATGATATT TGATGAGTTG GTGTGTTTAT TTGATGAGCC CAGATGATTT AACTTGTAGA   
  
  
+ TTTTGTGGTT TTGGTTTCTG GGTATTCATT GGTTTGCTGA ATTTCTGGTT CATGATCTGG GGTTAGAAAA   
  
  
+ GTTGAAATGA CTTTCTGGAG GTTGGAATTT GACTAAAGCT AATTTCTTTT TGTCTTCAAT TCTTTTTTAG   
  
  
+ TTGTGAGCTG AATTTGTTGA CCACTTCCAG CCTGTTGGAA ATTAAACTTA CTTAGGAGAA GAGAATCTCA   
  
  
+ GATTCTGAGC AAAAGCTTTA TATGGTTGTT TGGGAAATGG TAGGAACTAA GTGAAAACAC TGAAATTTTA   
  
  
+ AGCAAGCAAT TTTGTTCATT TGATTGGTCG GATGGTCTGA TTTAGGTTCT TTTCTTGGTC TAAATTTCTG   
  
  
+ AGCATTGATT GTTTAATTGC GTGGGGAACT TGTATCCTTG TGAGTTCCTG GTTCATTTCT TGGTTGCTTA   
  
  
+ CTCTAGCTCC CTTTCACAGT TTCTATGGTT GGAACTTGGA ACTTGGAACT TGGAAGCCTA ATTTAGTTGA   
  
  
+ ATATCTTGGC TTTGAGTTAG CTTTTCTTAG GAAAAGATGG TTCTGCTCTC GAAAATCTAA TCTAATCGAT   
  
  
+ AGATGCGTTA AGGAAGCAAA ACTTTTGGGG AGAGGGAAGT TAAGCGAAGC AAGCTGCTGT TTCAAAGTTG   
  
  
+ GACTGTTTTA TTGGTAAGTA TGAAACAATA GTTTATAAGA GATCATTGGG TTCTTAAGGT GATAGATCAT   
  
  
+ GGGTTATGAA TGAGCTTAGT TGTTCTTTTG CTTTCAACTG TACGTAAGGA TGATAGTTAT CCCTCATCTT   
  
  
+ GCATCAAAAA CGGCAAACCG TGATGCCAAA ACTCAAAAAC TGATTCCTCT GACAAGGCTT CTACCTATGA   
  
  
+ AAACTATGCA GATCATATCC ATTTGGCCTT CCCTCTAGGA ACCAAGTAGA GTAAGTTGGT AAATAAATAG   
  
  
+ AGTTGAACGA AACACTACAG ATTTGAATTT GATTAAGGAG GGCTATTATA GACTAAGGTG TAGCTCAAAT   
  
  
+ CCTTCTGTAG GGATTGTCTT ATTGGCTTCT TTTGGTTGCT TCTGGCTCAA GTCCTCTTAT TAGTTAGTAT   
  
  
+ GCATTACCTT GAGACAAGAT AGTTGCTTAA GCTTGAGCTT CTGTACTTAT TTGCGTATTG TAGACAACTA   
  
  
+ GCTGTGGCAT ATGATTCTGA AATGTTCCGA TGTCTTTTTC TTTGGATGCT GGGTTGTGTC ATGAGCTTTA   
  
  
+ GGTTTGTTTA TTTGATGTCA GGAAGTATGT AAATAACATG TGGCATCTTG CAATCTATTA CAGGATGATA   
  
  
+ TTGGCCTTTG TGGGAAGAAA TTGAAGAAGT TGTTTCACTT ACTGGATAGG GAGCATGGAC TCACATCAGC   
  
  
+ TTTTGAGATT CGGTATTTCC AGATCATACT CATCCTATAA TTTCTCTCAG TCTAGTCCCC AATCAATTCC   
  
  
+ AAATAGGTTA TTTGAATCAC CGAACGTTCG TTCTAGAGAC TCTCCAATCT CGCCCTTCTC AACGCCCTTC   
  
  
+ AATTGCGACC CCACTGTTAT ATTGAGCGAC AGTCAGGAGC ACCACAGTTC AACAGGAAGT CTCTCGGCAC   
  
  
+ AAAGCTCATC TTCTAATTCT CCCCTTGAGA CTAGCAGTTA TTATAATCAG TTCAACTCAA GCCCTGTTGG   
  
  
+ AGAATCACCT CAGGGATCAT CACCCGAGAA TCTTTTTCAA CAAGCTGCAG TTAATAAAGT CAGCATCGAG   
  
  
+ CATGCATTGC AGGAGCTAGA AACTGCTCTA ATGAGCACAG ATTGTGAGGA GAATGAAGCA ACTGTCTCTA   
  
  
+ TCCCATCTAT GGGTGAACCT CATCAGCCCC AAGTCCCTAG CCAGAGATCA AGATTCTGGA ATCGAGATCC   
  
  
+ TCAGGGTTCA CGCCCGGCTG AAGTTCACTC ATCATTGAGA AGATTAGGAG ACGAGGCTCA GAGTGAGAAA   
  
  
+ CGCCTCAAGG CAGTGGAAGA ACCAATACGG CCCAGTGCAC CACCCGGCAA TTTGAAGCAG TTGCTCATAG   
  
  
+ AATGTGCTCG GGCTTTGTCA GAGAATCGAA TAGAGGATTT TGAGAAGTTA GTTGAACAGG CAAAGGGCAT   
  
  
+ GGTCTCCATT TCTGGAGACC CAATTCAACG ACTTGGTGCT TACTTGATCG AGGGGCTAGT GGCAAGGAAG   
  
  
+ GAGTCTTCAG GTACGAAAAT CTACCGAGCT CTTAGGTGCA AAGAGCCCCT TGGTCAAGAC TTGCTTTCGT   
  
  
+ ACATGCACAT CCTTTATGAA ATTTGCCCTT ACTTGAAATT CGGTTATATG GTTGCAAATG GGGCTATAGC   
  
  
+ TGAAGCTTGC AGAGATGAGG ATCACATACA TATCGTCGAC TACCAGATTG GTCAAGGAAC TCAATGGATG   
  
  
+ ACTCTGTTGC AAGCCCTAGC CACAAAACCT GGAAGACCCC CCACTGTGAG AATTACTGGC ATTGATGATC   
  
  
+ CCGTCTCTAG GCATGCTCGG GGAGCTTGCT TGGAGGCAGT GGGGAAACGT TTGGCAGTTC TGTCGGAGAA   
  
  
+ ATTTAACATG CCCATTGAGT TTCAGGCAGT GCCCGTTTAT GCTTCTGAGG TCACCCAAGA AATGCTTGAT   
  
  
+ GTGAGGCCAG GGTGGGCCTT GGCTGTGAAC TTCCCATTGC AGCTCCACCA CACTCCCGAT GAGAGTGTTG   
  
  
+ ATGTGAACAA CCCGAGGGAT GGACTCCTAA GAATGGTGAA ATCACTCGGT CCCAAGGTTG TAACTTTGGT   
  
  
+ AGAACAAGAG TCAAACACCA ACACTACCCC TTTGCTGACA AGGTTCATAG AAACGCTGGA CTTCTACTCA   
  
  
+ GCAATGTTTG AATCCATCGA TGTGACAATG CCAAGGGACC GAAAGGAAAG GATCAATGTC GAGCAGCATT   
  
  
+ GCCTAGCCAA GGACATTGTC AACATCATCG CGTGTGAGGG GAAGGACAGG GTGGAGCGAC ATGAGCTCTT   
  
  
+ TGGGAAATGG AAGTCAAGGT TCACCATGGC AGGATTCCAA CAATCCCCAT TGAGCTCGTA TGTTAACTCC   
  
  
+ GTGATAAAAG GATTACTTAG GTGTTATTCA GAGTACTATA CGTTGGTGGA GAAGGATGGC GCCTTGCTTC   
  
  
+ TGGGATGGAA GGACCGGATG CTGGTTTCGG CTTCAGCTTG GCATTG  

- +Up\_Stream \_Len000AAATTT ATAAAATAAA ATAAAATTTA ATTATATAAA ATATTAAATT TTAATAATTA   
  
  
- TTAACTACTA CAATGGGTTA CAAATTCAAA AATTTATAAA AATAAAAAAT TAAATTTTTA TAGTATTATA   
  
  
- TATTATATTA AAATTCAATT TTATTCTATC AATTTAATAA AATATCATTT ATAAATAATA AAAAAAATTC   
  
  
- TTTTAACGGT ACACTAACAG TAGTCAGTTT GTGACGCAAC TGTCTTCGAC AGGAACGACG ATGAGAGTGG   
  
  
- AGAGTTGTAG CCTGGGCGCG TCTGCAATTG TAGAGGCATG TTTATGTTAA GGGACCGGTT TTAAGTTTAG   
  
  
- TTGGGATGAG CCATCAGCCA AGGAAGAAGA AGGACGGGAG ATATATATGT GGGTGTCGAC GCGACGTACA   
  
  
- AAAAGGCTCT TAGGAGTTTT TAGAATATTT GAAGGAGCAC ACCCATCTTT TTTCTTTTTC TTTTCCCAAA   
  
  
- ATTTTAGAAC CCAGTTTTGG GTAGTTGTGG TGTTAAACTG AGAAAGAAAA ATTAAGTTGG TGGCGGTCAA   
  
  
- GGAGCAACTA AAAGTCCATG GAGAAAAAGA ACGGGGAAAA AAACGAGCCA ACAACAAATT ACCATTTCTA   
  
  
- ACTCATGTTT CTTACTATAA ACTACTCAAC CACACAAATA AACTACTCGG GTCTACTAAA TTGAACATCT   
  
  
- AAAACACCAA AACCAAAGAC CCATAAGTAA CCAAACGACT TAAAGACCAA GTACTAGACC CCAATCTTTT   
  
  
- CAACTTTACT GAAAGACCTC CAACCTTAAA CTGATTTCGA TTAAAGAAAA ACAGAAGTTA AGAAAAAATC   
  
  
- AACACTCGAC TTAAACAACT GGTGAAGGTC GGACAACCTT TAATTTGAAT GAATCCTCTT CTCTTAGAGT   
  
  
- CTAAGACTCG TTTTCGAAAT ATACCAACAA ACCCTTTACC ATCCTTGATT CACTTTTGTG ACTTTAAAAT   
  
  
- TCGTTCGTTA AAACAAGTAA ACTAACCAGC CTACCAGACT AAATCCAAGA AAAGAACCAG ATTTAAAGAC   
  
  
- TCGTAACTAA CAAATTAACG CACCCCTTGA ACATAGGAAC ACTCAAGGAC CAAGTAAAGA ACCAACGAAT   
  
  
- GAGATCGAGG GAAAGTGTCA AAGATACCAA CCTTGAACCT TGAACCTTGA ACCTTCGGAT TAAATCAACT   
  
  
- TATAGAACCG AAACTCAATC GAAAAGAATC CTTTTCTACC AAGACGAGAG CTTTTAGATT AGATTAGCTA   
  
  
- TCTACGCAAT TCCTTCGTTT TGAAAACCCC TCTCCCTTCA ATTCGCTTCG TTCGACGACA AAGTTTCAAC   
  
  
- CTGACAAAAT AACCATTCAT ACTTTGTTAT CAAATATTCT CTAGTAACCC AAGAATTCCA CTATCTAGTA   
  
  
- CCCAATACTT ACTCGAATCA ACAAGAAAAC GAAAGTTGAC ATGCATTCCT ACTATCAATA GGGAGTAGAA   
  
  
- CGTAGTTTTT GCCGTTTGGC ACTACGGTTT TGAGTTTTTG ACTAAGGAGA CTGTTCCGAA GATGGATACT   
  
  
- TTTGATACGT CTAGTATAGG TAAACCGGAA GGGAGATCCT TGGTTCATCT CATTCAACCA TTTATTTATC   
  
  
- TCAACTTGCT TTGTGATGTC TAAACTTAAA CTAATTCCTC CCGATAATAT CTGATTCCAC ATCGAGTTTA   
  
  
- GGAAGACATC CCTAACAGAA TAACCGAAGA AAACCAACGA AGACCGAGTT CAGGAGAATA ATCAATCATA   
  
  
- CGTAATGGAA CTCTGTTCTA TCAACGAATT CGAACTCGAA GACATGAATA AACGCATAAC ATCTGTTGAT   
  
  
- CGACACCGTA TACTAAGACT TTACAAGGCT ACAGAAAAAG AAACCTACGA CCCAACACAG TACTCGAAAT   
  
  
- CCAAACAAAT AAACTACAGT CCTTCATACA TTTATTGTAC ACCGTAGAAC GTTAGATAAT GTCCTACTAT   
  
  
- AACCGGAAAC ACCCTTCTTT AACTTCTTCA ACAAAGTGAA TGACCTATCC CTCGTACCTG AGTGTAGTCG   
  
  
- AAAACTCTAA GCCATAAAGG TCTAGTATGA GTAGGATATT AAAGAGAGTC AGATCAGGGG TTAGTTAAGG   
  
  
- TTTATCCAAT AAACTTAGTG GCTTGCAAGC AAGATCTCTG AGAGGTTAGA GCGGGAAGAG TTGCGGGAAG   
  
  
- TTAACGCTGG GGTGACAATA TAACTCGCTG TCAGTCCTCG TGGTGTCAAG TTGTCCTTCA GAGAGCCGTG   
  
  
- TTTCGAGTAG AAGATTAAGA GGGGAACTCT GATCGTCAAT AATATTAGTC AAGTTGAGTT CGGGACAACC   
  
  
- TCTTAGTGGA GTCCCTAGTA GTGGGCTCTT AGAAAAAGTT GTTCGACGTC AATTATTTCA GTCGTAGCTC   
  
  
- GTACGTAACG TCCTCGATCT TTGACGAGAT TACTCGTGTC TAACACTCCT CTTACTTCGT TGACAGAGAT   
  
  
- AGGGTAGATA CCCACTTGGA GTAGTCGGGG TTCAGGGATC GGTCTCTAGT TCTAAGACCT TAGCTCTAGG   
  
  
- AGTCCCAAGT GCGGGCCGAC TTCAAGTGAG TAGTAACTCT TCTAATCCTC TGCTCCGAGT CTCACTCTTT   
  
  
- GCGGAGTTCC GTCACCTTCT TGGTTATGCC GGGTCACGTG GTGGGCCGTT AAACTTCGTC AACGAGTATC   
  
  
- TTACACGAGC CCGAAACAGT CTCTTAGCTT ATCTCCTAAA ACTCTTCAAT CAACTTGTCC GTTTCCCGTA   
  
  
- CCAGAGGTAA AGACCTCTGG GTTAAGTTGC TGAACCACGA ATGAACTAGC TCCCCGATCA CCGTTCCTTC   
  
  
- CTCAGAAGTC CATGCTTTTA GATGGCTCGA GAATCCACGT TTCTCGGGGA ACCAGTTCTG AACGAAAGCA   
  
  
- TGTACGTGTA GGAAATACTT TAAACGGGAA TGAACTTTAA GCCAATATAC CAACGTTTAC CCCGATATCG   
  
  
- ACTTCGAACG TCTCTACTCC TAGTGTATGT ATAGCAGCTG ATGGTCTAAC CAGTTCCTTG AGTTACCTAC   
  
  
- TGAGACAACG TTCGGGATCG GTGTTTTGGA CCTTCTGGGG GGTGACACTC TTAATGACCG TAACTACTAG   
  
  
- GGCAGAGATC CGTACGAGCC CCTCGAACGA ACCTCCGTCA CCCCTTTGCA AACCGTCAAG ACAGCCTCTT   
  
  
- TAAATTGTAC GGGTAACTCA AAGTCCGTCA CGGGCAAATA CGAAGACTCC AGTGGGTTCT TTACGAACTA   
  
  
- CACTCCGGTC CCACCCGGAA CCGACACTTG AAGGGTAACG TCGAGGTGGT GTGAGGGCTA CTCTCACAAC   
  
  
- TACACTTGTT GGGCTCCCTA CCTGAGGATT CTTACCACTT TAGTGAGCCA GGGTTCCAAC ATTGAAACCA   
  
  
- TCTTGTTCTC AGTTTGTGGT TGTGATGGGG AAACGACTGT TCCAAGTATC TTTGCGACCT GAAGATGAGT   
  
  
- CGTTACAAAC TTAGGTAGCT ACACTGTTAC GGTTCCCTGG CTTTCCTTTC CTAGTTACAG CTCGTCGTAA   
  
  
- CGGATCGGTT CCTGTAACAG TTGTAGTAGC GCACACTCCC CTTCCTGTCC CACCTCGCTG TACTCGAGAA   
  
  
- ACCCTTTACC TTCAGTTCCA AGTGGTACCG TCCTAAGGTT GTTAGGGGTA ACTCGAGCAT ACAATTGAGG   
  
  
- CACTATTTTC CTAATGAATC CACAATAAGT CTCATGATAT GCAACCACCT CTTCCTACCG CGGAACGAAG   
  
  
- ACCCTACCTT CCTGGCCTAC GACCAAAGCC GAAGTCGAAC CGTAAC

+     STRE

| Site Name | Organism | Position | Strand | Matrix score. | sequence | function |
| --- | --- | --- | --- | --- | --- | --- |
| STRE | Arabidopsis thaliana | 3541 | + | 5 | AGGGG |  |
| STRE | Arabidopsis thaliana | 3391 | - | 5 | AGGGG |  |
| STRE | Arabidopsis thaliana | 2850 | - | 5 | AGGGG |  |
| STRE | Arabidopsis thaliana | 2785 | + | 5 | AGGGG |  |
| STRE | Arabidopsis thaliana | 2265 | - | 5 | AGGGG |  |
| STRE | Arabidopsis thaliana | 597 | - | 5 | AGGGG |  |

>HU01G01850.1   
+ +Up\_Stream \_Len000TTTAAA TATTTTATTT TATTTTAAAT TAATATATTT TATAATTTAA AATTATTAAT   
  
  
+ AATTGATGAT GTTACCCAAT GTTTAAGTTT TTAAATATTT TTATTTTTTA ATTTAAAAAT ATCATAATAT   
  
  
+ ATAATATAAT TTTAAGTTAA AATAAGATAG TTAAATTATT TTATAGTAAA TATTTATTAT TTTTTTTAAG   
  
  
+ AAAATTGCCA TGTGATTGTC ATCAGTCAAA CACTGCGTTG ACAGAAGCTG TCCTTGCTGC TACTCTCACC   
  
  
+ TCTCAACATC GGACCCGCGC AGACGTTAAC ATCTCCGTAC AAATACAATT CCCTGGCCAA AATTCAAATC   
  
  
+ AACCCTACTC GGTAGTCGGT TCCTTCTTCT TCCTGCCCTC TATATATACA CCCACAGCTG CGCTGCATGT   
  
  
+ TTTTCCGAGA ATCCTCAAAA ATCTTATAAA CTTCCTCGTG TGGGTAGAAA AAAGAAAAAG AAAAGGGTTT   
  
  
+ TAAAATCTTG GGTCAAAACC CATCAACACC ACAATTTGAC TCTTTCTTTT TAATTCAACC ACCGCCAGTT   
  
  
+ CCTCGTTGAT TTTCAGGTAC CTCTTTTTCT TGCCCCTTTT TTTGCTCGGT TGTTGTTTAA TGGTAAAGAT   
  
  
+ TGAGTACAAA GAATGATATT TGATGAGTTG GTGTGTTTAT TTGATGAGCC CAGATGATTT AACTTGTAGA   
  
  
+ TTTTGTGGTT TTGGTTTCTG GGTATTCATT GGTTTGCTGA ATTTCTGGTT CATGATCTGG GGTTAGAAAA   
  
  
+ GTTGAAATGA CTTTCTGGAG GTTGGAATTT GACTAAAGCT AATTTCTTTT TGTCTTCAAT TCTTTTTTAG   
  
  
+ TTGTGAGCTG AATTTGTTGA CCACTTCCAG CCTGTTGGAA ATTAAACTTA CTTAGGAGAA GAGAATCTCA   
  
  
+ GATTCTGAGC AAAAGCTTTA TATGGTTGTT TGGGAAATGG TAGGAACTAA GTGAAAACAC TGAAATTTTA   
  
  
+ AGCAAGCAAT TTTGTTCATT TGATTGGTCG GATGGTCTGA TTTAGGTTCT TTTCTTGGTC TAAATTTCTG   
  
  
+ AGCATTGATT GTTTAATTGC GTGGGGAACT TGTATCCTTG TGAGTTCCTG GTTCATTTCT TGGTTGCTTA   
  
  
+ CTCTAGCTCC CTTTCACAGT TTCTATGGTT GGAACTTGGA ACTTGGAACT TGGAAGCCTA ATTTAGTTGA   
  
  
+ ATATCTTGGC TTTGAGTTAG CTTTTCTTAG GAAAAGATGG TTCTGCTCTC GAAAATCTAA TCTAATCGAT   
  
  
+ AGATGCGTTA AGGAAGCAAA ACTTTTGGGG AGAGGGAAGT TAAGCGAAGC AAGCTGCTGT TTCAAAGTTG   
  
  
+ GACTGTTTTA TTGGTAAGTA TGAAACAATA GTTTATAAGA GATCATTGGG TTCTTAAGGT GATAGATCAT   
  
  
+ GGGTTATGAA TGAGCTTAGT TGTTCTTTTG CTTTCAACTG TACGTAAGGA TGATAGTTAT CCCTCATCTT   
  
  
+ GCATCAAAAA CGGCAAACCG TGATGCCAAA ACTCAAAAAC TGATTCCTCT GACAAGGCTT CTACCTATGA   
  
  
+ AAACTATGCA GATCATATCC ATTTGGCCTT CCCTCTAGGA ACCAAGTAGA GTAAGTTGGT AAATAAATAG   
  
  
+ AGTTGAACGA AACACTACAG ATTTGAATTT GATTAAGGAG GGCTATTATA GACTAAGGTG TAGCTCAAAT   
  
  
+ CCTTCTGTAG GGATTGTCTT ATTGGCTTCT TTTGGTTGCT TCTGGCTCAA GTCCTCTTAT TAGTTAGTAT   
  
  
+ GCATTACCTT GAGACAAGAT AGTTGCTTAA GCTTGAGCTT CTGTACTTAT TTGCGTATTG TAGACAACTA   
  
  
+ GCTGTGGCAT ATGATTCTGA AATGTTCCGA TGTCTTTTTC TTTGGATGCT GGGTTGTGTC ATGAGCTTTA   
  
  
+ GGTTTGTTTA TTTGATGTCA GGAAGTATGT AAATAACATG TGGCATCTTG CAATCTATTA CAGGATGATA   
  
  
+ TTGGCCTTTG TGGGAAGAAA TTGAAGAAGT TGTTTCACTT ACTGGATAGG GAGCATGGAC TCACATCAGC   
  
  
+ TTTTGAGATT CGGTATTTCC AGATCATACT CATCCTATAA TTTCTCTCAG TCTAGTCCCC AATCAATTCC   
  
  
+ AAATAGGTTA TTTGAATCAC CGAACGTTCG TTCTAGAGAC TCTCCAATCT CGCCCTTCTC AACGCCCTTC   
  
  
+ AATTGCGACC CCACTGTTAT ATTGAGCGAC AGTCAGGAGC ACCACAGTTC AACAGGAAGT CTCTCGGCAC   
  
  
+ AAAGCTCATC TTCTAATTCT CCCCTTGAGA CTAGCAGTTA TTATAATCAG TTCAACTCAA GCCCTGTTGG   
  
  
+ AGAATCACCT CAGGGATCAT CACCCGAGAA TCTTTTTCAA CAAGCTGCAG TTAATAAAGT CAGCATCGAG   
  
  
+ CATGCATTGC AGGAGCTAGA AACTGCTCTA ATGAGCACAG ATTGTGAGGA GAATGAAGCA ACTGTCTCTA   
  
  
+ TCCCATCTAT GGGTGAACCT CATCAGCCCC AAGTCCCTAG CCAGAGATCA AGATTCTGGA ATCGAGATCC   
  
  
+ TCAGGGTTCA CGCCCGGCTG AAGTTCACTC ATCATTGAGA AGATTAGGAG ACGAGGCTCA GAGTGAGAAA   
  
  
+ CGCCTCAAGG CAGTGGAAGA ACCAATACGG CCCAGTGCAC CACCCGGCAA TTTGAAGCAG TTGCTCATAG   
  
  
+ AATGTGCTCG GGCTTTGTCA GAGAATCGAA TAGAGGATTT TGAGAAGTTA GTTGAACAGG CAAAGGGCAT   
  
  
+ GGTCTCCATT TCTGGAGACC CAATTCAACG ACTTGGTGCT TACTTGATCG AGGGGCTAGT GGCAAGGAAG   
  
  
+ GAGTCTTCAG GTACGAAAAT CTACCGAGCT CTTAGGTGCA AAGAGCCCCT TGGTCAAGAC TTGCTTTCGT   
  
  
+ ACATGCACAT CCTTTATGAA ATTTGCCCTT ACTTGAAATT CGGTTATATG GTTGCAAATG GGGCTATAGC   
  
  
+ TGAAGCTTGC AGAGATGAGG ATCACATACA TATCGTCGAC TACCAGATTG GTCAAGGAAC TCAATGGATG   
  
  
+ ACTCTGTTGC AAGCCCTAGC CACAAAACCT GGAAGACCCC CCACTGTGAG AATTACTGGC ATTGATGATC   
  
  
+ CCGTCTCTAG GCATGCTCGG GGAGCTTGCT TGGAGGCAGT GGGGAAACGT TTGGCAGTTC TGTCGGAGAA   
  
  
+ ATTTAACATG CCCATTGAGT TTCAGGCAGT GCCCGTTTAT GCTTCTGAGG TCACCCAAGA AATGCTTGAT   
  
  
+ GTGAGGCCAG GGTGGGCCTT GGCTGTGAAC TTCCCATTGC AGCTCCACCA CACTCCCGAT GAGAGTGTTG   
  
  
+ ATGTGAACAA CCCGAGGGAT GGACTCCTAA GAATGGTGAA ATCACTCGGT CCCAAGGTTG TAACTTTGGT   
  
  
+ AGAACAAGAG TCAAACACCA ACACTACCCC TTTGCTGACA AGGTTCATAG AAACGCTGGA CTTCTACTCA   
  
  
+ GCAATGTTTG AATCCATCGA TGTGACAATG CCAAGGGACC GAAAGGAAAG GATCAATGTC GAGCAGCATT   
  
  
+ GCCTAGCCAA GGACATTGTC AACATCATCG CGTGTGAGGG GAAGGACAGG GTGGAGCGAC ATGAGCTCTT   
  
  
+ TGGGAAATGG AAGTCAAGGT TCACCATGGC AGGATTCCAA CAATCCCCAT TGAGCTCGTA TGTTAACTCC   
  
  
+ GTGATAAAAG GATTACTTAG GTGTTATTCA GAGTACTATA CGTTGGTGGA GAAGGATGGC GCCTTGCTTC   
  
  
+ TGGGATGGAA GGACCGGATG CTGGTTTCGG CTTCAGCTTG GCATTG  

- +Up\_Stream \_Len000AAATTT ATAAAATAAA ATAAAATTTA ATTATATAAA ATATTAAATT TTAATAATTA   
  
  
- TTAACTACTA CAATGGGTTA CAAATTCAAA AATTTATAAA AATAAAAAAT TAAATTTTTA TAGTATTATA   
  
  
- TATTATATTA AAATTCAATT TTATTCTATC AATTTAATAA AATATCATTT ATAAATAATA AAAAAAATTC   
  
  
- TTTTAACGGT ACACTAACAG TAGTCAGTTT GTGACGCAAC TGTCTTCGAC AGGAACGACG ATGAGAGTGG   
  
  
- AGAGTTGTAG CCTGGGCGCG TCTGCAATTG TAGAGGCATG TTTATGTTAA GGGACCGGTT TTAAGTTTAG   
  
  
- TTGGGATGAG CCATCAGCCA AGGAAGAAGA AGGACGGGAG ATATATATGT GGGTGTCGAC GCGACGTACA   
  
  
- AAAAGGCTCT TAGGAGTTTT TAGAATATTT GAAGGAGCAC ACCCATCTTT TTTCTTTTTC TTTTCCCAAA   
  
  
- ATTTTAGAAC CCAGTTTTGG GTAGTTGTGG TGTTAAACTG AGAAAGAAAA ATTAAGTTGG TGGCGGTCAA   
  
  
- GGAGCAACTA AAAGTCCATG GAGAAAAAGA ACGGGGAAAA AAACGAGCCA ACAACAAATT ACCATTTCTA   
  
  
- ACTCATGTTT CTTACTATAA ACTACTCAAC CACACAAATA AACTACTCGG GTCTACTAAA TTGAACATCT   
  
  
- AAAACACCAA AACCAAAGAC CCATAAGTAA CCAAACGACT TAAAGACCAA GTACTAGACC CCAATCTTTT   
  
  
- CAACTTTACT GAAAGACCTC CAACCTTAAA CTGATTTCGA TTAAAGAAAA ACAGAAGTTA AGAAAAAATC   
  
  
- AACACTCGAC TTAAACAACT GGTGAAGGTC GGACAACCTT TAATTTGAAT GAATCCTCTT CTCTTAGAGT   
  
  
- CTAAGACTCG TTTTCGAAAT ATACCAACAA ACCCTTTACC ATCCTTGATT CACTTTTGTG ACTTTAAAAT   
  
  
- TCGTTCGTTA AAACAAGTAA ACTAACCAGC CTACCAGACT AAATCCAAGA AAAGAACCAG ATTTAAAGAC   
  
  
- TCGTAACTAA CAAATTAACG CACCCCTTGA ACATAGGAAC ACTCAAGGAC CAAGTAAAGA ACCAACGAAT   
  
  
- GAGATCGAGG GAAAGTGTCA AAGATACCAA CCTTGAACCT TGAACCTTGA ACCTTCGGAT TAAATCAACT   
  
  
- TATAGAACCG AAACTCAATC GAAAAGAATC CTTTTCTACC AAGACGAGAG CTTTTAGATT AGATTAGCTA   
  
  
- TCTACGCAAT TCCTTCGTTT TGAAAACCCC TCTCCCTTCA ATTCGCTTCG TTCGACGACA AAGTTTCAAC   
  
  
- CTGACAAAAT AACCATTCAT ACTTTGTTAT CAAATATTCT CTAGTAACCC AAGAATTCCA CTATCTAGTA   
  
  
- CCCAATACTT ACTCGAATCA ACAAGAAAAC GAAAGTTGAC ATGCATTCCT ACTATCAATA GGGAGTAGAA   
  
  
- CGTAGTTTTT GCCGTTTGGC ACTACGGTTT TGAGTTTTTG ACTAAGGAGA CTGTTCCGAA GATGGATACT   
  
  
- TTTGATACGT CTAGTATAGG TAAACCGGAA GGGAGATCCT TGGTTCATCT CATTCAACCA TTTATTTATC   
  
  
- TCAACTTGCT TTGTGATGTC TAAACTTAAA CTAATTCCTC CCGATAATAT CTGATTCCAC ATCGAGTTTA   
  
  
- GGAAGACATC CCTAACAGAA TAACCGAAGA AAACCAACGA AGACCGAGTT CAGGAGAATA ATCAATCATA   
  
  
- CGTAATGGAA CTCTGTTCTA TCAACGAATT CGAACTCGAA GACATGAATA AACGCATAAC ATCTGTTGAT   
  
  
- CGACACCGTA TACTAAGACT TTACAAGGCT ACAGAAAAAG AAACCTACGA CCCAACACAG TACTCGAAAT   
  
  
- CCAAACAAAT AAACTACAGT CCTTCATACA TTTATTGTAC ACCGTAGAAC GTTAGATAAT GTCCTACTAT   
  
  
- AACCGGAAAC ACCCTTCTTT AACTTCTTCA ACAAAGTGAA TGACCTATCC CTCGTACCTG AGTGTAGTCG   
  
  
- AAAACTCTAA GCCATAAAGG TCTAGTATGA GTAGGATATT AAAGAGAGTC AGATCAGGGG TTAGTTAAGG   
  
  
- TTTATCCAAT AAACTTAGTG GCTTGCAAGC AAGATCTCTG AGAGGTTAGA GCGGGAAGAG TTGCGGGAAG   
  
  
- TTAACGCTGG GGTGACAATA TAACTCGCTG TCAGTCCTCG TGGTGTCAAG TTGTCCTTCA GAGAGCCGTG   
  
  
- TTTCGAGTAG AAGATTAAGA GGGGAACTCT GATCGTCAAT AATATTAGTC AAGTTGAGTT CGGGACAACC   
  
  
- TCTTAGTGGA GTCCCTAGTA GTGGGCTCTT AGAAAAAGTT GTTCGACGTC AATTATTTCA GTCGTAGCTC   
  
  
- GTACGTAACG TCCTCGATCT TTGACGAGAT TACTCGTGTC TAACACTCCT CTTACTTCGT TGACAGAGAT   
  
  
- AGGGTAGATA CCCACTTGGA GTAGTCGGGG TTCAGGGATC GGTCTCTAGT TCTAAGACCT TAGCTCTAGG   
  
  
- AGTCCCAAGT GCGGGCCGAC TTCAAGTGAG TAGTAACTCT TCTAATCCTC TGCTCCGAGT CTCACTCTTT   
  
  
- GCGGAGTTCC GTCACCTTCT TGGTTATGCC GGGTCACGTG GTGGGCCGTT AAACTTCGTC AACGAGTATC   
  
  
- TTACACGAGC CCGAAACAGT CTCTTAGCTT ATCTCCTAAA ACTCTTCAAT CAACTTGTCC GTTTCCCGTA   
  
  
- CCAGAGGTAA AGACCTCTGG GTTAAGTTGC TGAACCACGA ATGAACTAGC TCCCCGATCA CCGTTCCTTC   
  
  
- CTCAGAAGTC CATGCTTTTA GATGGCTCGA GAATCCACGT TTCTCGGGGA ACCAGTTCTG AACGAAAGCA   
  
  
- TGTACGTGTA GGAAATACTT TAAACGGGAA TGAACTTTAA GCCAATATAC CAACGTTTAC CCCGATATCG   
  
  
- ACTTCGAACG TCTCTACTCC TAGTGTATGT ATAGCAGCTG ATGGTCTAAC CAGTTCCTTG AGTTACCTAC   
  
  
- TGAGACAACG TTCGGGATCG GTGTTTTGGA CCTTCTGGGG GGTGACACTC TTAATGACCG TAACTACTAG   
  
  
- GGCAGAGATC CGTACGAGCC CCTCGAACGA ACCTCCGTCA CCCCTTTGCA AACCGTCAAG ACAGCCTCTT   
  
  
- TAAATTGTAC GGGTAACTCA AAGTCCGTCA CGGGCAAATA CGAAGACTCC AGTGGGTTCT TTACGAACTA   
  
  
- CACTCCGGTC CCACCCGGAA CCGACACTTG AAGGGTAACG TCGAGGTGGT GTGAGGGCTA CTCTCACAAC   
  
  
- TACACTTGTT GGGCTCCCTA CCTGAGGATT CTTACCACTT TAGTGAGCCA GGGTTCCAAC ATTGAAACCA   
  
  
- TCTTGTTCTC AGTTTGTGGT TGTGATGGGG AAACGACTGT TCCAAGTATC TTTGCGACCT GAAGATGAGT   
  
  
- CGTTACAAAC TTAGGTAGCT ACACTGTTAC GGTTCCCTGG CTTTCCTTTC CTAGTTACAG CTCGTCGTAA   
  
  
- CGGATCGGTT CCTGTAACAG TTGTAGTAGC GCACACTCCC CTTCCTGTCC CACCTCGCTG TACTCGAGAA   
  
  
- ACCCTTTACC TTCAGTTCCA AGTGGTACCG TCCTAAGGTT GTTAGGGGTA ACTCGAGCAT ACAATTGAGG   
  
  
- CACTATTTTC CTAATGAATC CACAATAAGT CTCATGATAT GCAACCACCT CTTCCTACCG CGGAACGAAG   
  
  
- ACCCTACCTT CCTGGCCTAC GACCAAAGCC GAAGTCGAAC CGTAAC

+     TATA

| Site Name | Organism | Position | Strand | Matrix score. | sequence | function |
| --- | --- | --- | --- | --- | --- | --- |
| TATA | Arabidopsis thaliana | 182 | - | 8 | TATAAAAT |  |
| TATA | Arabidopsis thaliana | 51 | - | 8 | TATAAAAT |  |

>HU01G01850.1   
+ +Up\_Stream \_Len000TTTAAA TATTTTATTT TATTTTAAAT TAATATATTT TATAATTTAA AATTATTAAT   
  
  
+ AATTGATGAT GTTACCCAAT GTTTAAGTTT TTAAATATTT TTATTTTTTA ATTTAAAAAT ATCATAATAT   
  
  
+ ATAATATAAT TTTAAGTTAA AATAAGATAG TTAAATTATT TTATAGTAAA TATTTATTAT TTTTTTTAAG   
  
  
+ AAAATTGCCA TGTGATTGTC ATCAGTCAAA CACTGCGTTG ACAGAAGCTG TCCTTGCTGC TACTCTCACC   
  
  
+ TCTCAACATC GGACCCGCGC AGACGTTAAC ATCTCCGTAC AAATACAATT CCCTGGCCAA AATTCAAATC   
  
  
+ AACCCTACTC GGTAGTCGGT TCCTTCTTCT TCCTGCCCTC TATATATACA CCCACAGCTG CGCTGCATGT   
  
  
+ TTTTCCGAGA ATCCTCAAAA ATCTTATAAA CTTCCTCGTG TGGGTAGAAA AAAGAAAAAG AAAAGGGTTT   
  
  
+ TAAAATCTTG GGTCAAAACC CATCAACACC ACAATTTGAC TCTTTCTTTT TAATTCAACC ACCGCCAGTT   
  
  
+ CCTCGTTGAT TTTCAGGTAC CTCTTTTTCT TGCCCCTTTT TTTGCTCGGT TGTTGTTTAA TGGTAAAGAT   
  
  
+ TGAGTACAAA GAATGATATT TGATGAGTTG GTGTGTTTAT TTGATGAGCC CAGATGATTT AACTTGTAGA   
  
  
+ TTTTGTGGTT TTGGTTTCTG GGTATTCATT GGTTTGCTGA ATTTCTGGTT CATGATCTGG GGTTAGAAAA   
  
  
+ GTTGAAATGA CTTTCTGGAG GTTGGAATTT GACTAAAGCT AATTTCTTTT TGTCTTCAAT TCTTTTTTAG   
  
  
+ TTGTGAGCTG AATTTGTTGA CCACTTCCAG CCTGTTGGAA ATTAAACTTA CTTAGGAGAA GAGAATCTCA   
  
  
+ GATTCTGAGC AAAAGCTTTA TATGGTTGTT TGGGAAATGG TAGGAACTAA GTGAAAACAC TGAAATTTTA   
  
  
+ AGCAAGCAAT TTTGTTCATT TGATTGGTCG GATGGTCTGA TTTAGGTTCT TTTCTTGGTC TAAATTTCTG   
  
  
+ AGCATTGATT GTTTAATTGC GTGGGGAACT TGTATCCTTG TGAGTTCCTG GTTCATTTCT TGGTTGCTTA   
  
  
+ CTCTAGCTCC CTTTCACAGT TTCTATGGTT GGAACTTGGA ACTTGGAACT TGGAAGCCTA ATTTAGTTGA   
  
  
+ ATATCTTGGC TTTGAGTTAG CTTTTCTTAG GAAAAGATGG TTCTGCTCTC GAAAATCTAA TCTAATCGAT   
  
  
+ AGATGCGTTA AGGAAGCAAA ACTTTTGGGG AGAGGGAAGT TAAGCGAAGC AAGCTGCTGT TTCAAAGTTG   
  
  
+ GACTGTTTTA TTGGTAAGTA TGAAACAATA GTTTATAAGA GATCATTGGG TTCTTAAGGT GATAGATCAT   
  
  
+ GGGTTATGAA TGAGCTTAGT TGTTCTTTTG CTTTCAACTG TACGTAAGGA TGATAGTTAT CCCTCATCTT   
  
  
+ GCATCAAAAA CGGCAAACCG TGATGCCAAA ACTCAAAAAC TGATTCCTCT GACAAGGCTT CTACCTATGA   
  
  
+ AAACTATGCA GATCATATCC ATTTGGCCTT CCCTCTAGGA ACCAAGTAGA GTAAGTTGGT AAATAAATAG   
  
  
+ AGTTGAACGA AACACTACAG ATTTGAATTT GATTAAGGAG GGCTATTATA GACTAAGGTG TAGCTCAAAT   
  
  
+ CCTTCTGTAG GGATTGTCTT ATTGGCTTCT TTTGGTTGCT TCTGGCTCAA GTCCTCTTAT TAGTTAGTAT   
  
  
+ GCATTACCTT GAGACAAGAT AGTTGCTTAA GCTTGAGCTT CTGTACTTAT TTGCGTATTG TAGACAACTA   
  
  
+ GCTGTGGCAT ATGATTCTGA AATGTTCCGA TGTCTTTTTC TTTGGATGCT GGGTTGTGTC ATGAGCTTTA   
  
  
+ GGTTTGTTTA TTTGATGTCA GGAAGTATGT AAATAACATG TGGCATCTTG CAATCTATTA CAGGATGATA   
  
  
+ TTGGCCTTTG TGGGAAGAAA TTGAAGAAGT TGTTTCACTT ACTGGATAGG GAGCATGGAC TCACATCAGC   
  
  
+ TTTTGAGATT CGGTATTTCC AGATCATACT CATCCTATAA TTTCTCTCAG TCTAGTCCCC AATCAATTCC   
  
  
+ AAATAGGTTA TTTGAATCAC CGAACGTTCG TTCTAGAGAC TCTCCAATCT CGCCCTTCTC AACGCCCTTC   
  
  
+ AATTGCGACC CCACTGTTAT ATTGAGCGAC AGTCAGGAGC ACCACAGTTC AACAGGAAGT CTCTCGGCAC   
  
  
+ AAAGCTCATC TTCTAATTCT CCCCTTGAGA CTAGCAGTTA TTATAATCAG TTCAACTCAA GCCCTGTTGG   
  
  
+ AGAATCACCT CAGGGATCAT CACCCGAGAA TCTTTTTCAA CAAGCTGCAG TTAATAAAGT CAGCATCGAG   
  
  
+ CATGCATTGC AGGAGCTAGA AACTGCTCTA ATGAGCACAG ATTGTGAGGA GAATGAAGCA ACTGTCTCTA   
  
  
+ TCCCATCTAT GGGTGAACCT CATCAGCCCC AAGTCCCTAG CCAGAGATCA AGATTCTGGA ATCGAGATCC   
  
  
+ TCAGGGTTCA CGCCCGGCTG AAGTTCACTC ATCATTGAGA AGATTAGGAG ACGAGGCTCA GAGTGAGAAA   
  
  
+ CGCCTCAAGG CAGTGGAAGA ACCAATACGG CCCAGTGCAC CACCCGGCAA TTTGAAGCAG TTGCTCATAG   
  
  
+ AATGTGCTCG GGCTTTGTCA GAGAATCGAA TAGAGGATTT TGAGAAGTTA GTTGAACAGG CAAAGGGCAT   
  
  
+ GGTCTCCATT TCTGGAGACC CAATTCAACG ACTTGGTGCT TACTTGATCG AGGGGCTAGT GGCAAGGAAG   
  
  
+ GAGTCTTCAG GTACGAAAAT CTACCGAGCT CTTAGGTGCA AAGAGCCCCT TGGTCAAGAC TTGCTTTCGT   
  
  
+ ACATGCACAT CCTTTATGAA ATTTGCCCTT ACTTGAAATT CGGTTATATG GTTGCAAATG GGGCTATAGC   
  
  
+ TGAAGCTTGC AGAGATGAGG ATCACATACA TATCGTCGAC TACCAGATTG GTCAAGGAAC TCAATGGATG   
  
  
+ ACTCTGTTGC AAGCCCTAGC CACAAAACCT GGAAGACCCC CCACTGTGAG AATTACTGGC ATTGATGATC   
  
  
+ CCGTCTCTAG GCATGCTCGG GGAGCTTGCT TGGAGGCAGT GGGGAAACGT TTGGCAGTTC TGTCGGAGAA   
  
  
+ ATTTAACATG CCCATTGAGT TTCAGGCAGT GCCCGTTTAT GCTTCTGAGG TCACCCAAGA AATGCTTGAT   
  
  
+ GTGAGGCCAG GGTGGGCCTT GGCTGTGAAC TTCCCATTGC AGCTCCACCA CACTCCCGAT GAGAGTGTTG   
  
  
+ ATGTGAACAA CCCGAGGGAT GGACTCCTAA GAATGGTGAA ATCACTCGGT CCCAAGGTTG TAACTTTGGT   
  
  
+ AGAACAAGAG TCAAACACCA ACACTACCCC TTTGCTGACA AGGTTCATAG AAACGCTGGA CTTCTACTCA   
  
  
+ GCAATGTTTG AATCCATCGA TGTGACAATG CCAAGGGACC GAAAGGAAAG GATCAATGTC GAGCAGCATT   
  
  
+ GCCTAGCCAA GGACATTGTC AACATCATCG CGTGTGAGGG GAAGGACAGG GTGGAGCGAC ATGAGCTCTT   
  
  
+ TGGGAAATGG AAGTCAAGGT TCACCATGGC AGGATTCCAA CAATCCCCAT TGAGCTCGTA TGTTAACTCC   
  
  
+ GTGATAAAAG GATTACTTAG GTGTTATTCA GAGTACTATA CGTTGGTGGA GAAGGATGGC GCCTTGCTTC   
  
  
+ TGGGATGGAA GGACCGGATG CTGGTTTCGG CTTCAGCTTG GCATTG  

- +Up\_Stream \_Len000AAATTT ATAAAATAAA ATAAAATTTA ATTATATAAA ATATTAAATT TTAATAATTA   
  
  
- TTAACTACTA CAATGGGTTA CAAATTCAAA AATTTATAAA AATAAAAAAT TAAATTTTTA TAGTATTATA   
  
  
- TATTATATTA AAATTCAATT TTATTCTATC AATTTAATAA AATATCATTT ATAAATAATA AAAAAAATTC   
  
  
- TTTTAACGGT ACACTAACAG TAGTCAGTTT GTGACGCAAC TGTCTTCGAC AGGAACGACG ATGAGAGTGG   
  
  
- AGAGTTGTAG CCTGGGCGCG TCTGCAATTG TAGAGGCATG TTTATGTTAA GGGACCGGTT TTAAGTTTAG   
  
  
- TTGGGATGAG CCATCAGCCA AGGAAGAAGA AGGACGGGAG ATATATATGT GGGTGTCGAC GCGACGTACA   
  
  
- AAAAGGCTCT TAGGAGTTTT TAGAATATTT GAAGGAGCAC ACCCATCTTT TTTCTTTTTC TTTTCCCAAA   
  
  
- ATTTTAGAAC CCAGTTTTGG GTAGTTGTGG TGTTAAACTG AGAAAGAAAA ATTAAGTTGG TGGCGGTCAA   
  
  
- GGAGCAACTA AAAGTCCATG GAGAAAAAGA ACGGGGAAAA AAACGAGCCA ACAACAAATT ACCATTTCTA   
  
  
- ACTCATGTTT CTTACTATAA ACTACTCAAC CACACAAATA AACTACTCGG GTCTACTAAA TTGAACATCT   
  
  
- AAAACACCAA AACCAAAGAC CCATAAGTAA CCAAACGACT TAAAGACCAA GTACTAGACC CCAATCTTTT   
  
  
- CAACTTTACT GAAAGACCTC CAACCTTAAA CTGATTTCGA TTAAAGAAAA ACAGAAGTTA AGAAAAAATC   
  
  
- AACACTCGAC TTAAACAACT GGTGAAGGTC GGACAACCTT TAATTTGAAT GAATCCTCTT CTCTTAGAGT   
  
  
- CTAAGACTCG TTTTCGAAAT ATACCAACAA ACCCTTTACC ATCCTTGATT CACTTTTGTG ACTTTAAAAT   
  
  
- TCGTTCGTTA AAACAAGTAA ACTAACCAGC CTACCAGACT AAATCCAAGA AAAGAACCAG ATTTAAAGAC   
  
  
- TCGTAACTAA CAAATTAACG CACCCCTTGA ACATAGGAAC ACTCAAGGAC CAAGTAAAGA ACCAACGAAT   
  
  
- GAGATCGAGG GAAAGTGTCA AAGATACCAA CCTTGAACCT TGAACCTTGA ACCTTCGGAT TAAATCAACT   
  
  
- TATAGAACCG AAACTCAATC GAAAAGAATC CTTTTCTACC AAGACGAGAG CTTTTAGATT AGATTAGCTA   
  
  
- TCTACGCAAT TCCTTCGTTT TGAAAACCCC TCTCCCTTCA ATTCGCTTCG TTCGACGACA AAGTTTCAAC   
  
  
- CTGACAAAAT AACCATTCAT ACTTTGTTAT CAAATATTCT CTAGTAACCC AAGAATTCCA CTATCTAGTA   
  
  
- CCCAATACTT ACTCGAATCA ACAAGAAAAC GAAAGTTGAC ATGCATTCCT ACTATCAATA GGGAGTAGAA   
  
  
- CGTAGTTTTT GCCGTTTGGC ACTACGGTTT TGAGTTTTTG ACTAAGGAGA CTGTTCCGAA GATGGATACT   
  
  
- TTTGATACGT CTAGTATAGG TAAACCGGAA GGGAGATCCT TGGTTCATCT CATTCAACCA TTTATTTATC   
  
  
- TCAACTTGCT TTGTGATGTC TAAACTTAAA CTAATTCCTC CCGATAATAT CTGATTCCAC ATCGAGTTTA   
  
  
- GGAAGACATC CCTAACAGAA TAACCGAAGA AAACCAACGA AGACCGAGTT CAGGAGAATA ATCAATCATA   
  
  
- CGTAATGGAA CTCTGTTCTA TCAACGAATT CGAACTCGAA GACATGAATA AACGCATAAC ATCTGTTGAT   
  
  
- CGACACCGTA TACTAAGACT TTACAAGGCT ACAGAAAAAG AAACCTACGA CCCAACACAG TACTCGAAAT   
  
  
- CCAAACAAAT AAACTACAGT CCTTCATACA TTTATTGTAC ACCGTAGAAC GTTAGATAAT GTCCTACTAT   
  
  
- AACCGGAAAC ACCCTTCTTT AACTTCTTCA ACAAAGTGAA TGACCTATCC CTCGTACCTG AGTGTAGTCG   
  
  
- AAAACTCTAA GCCATAAAGG TCTAGTATGA GTAGGATATT AAAGAGAGTC AGATCAGGGG TTAGTTAAGG   
  
  
- TTTATCCAAT AAACTTAGTG GCTTGCAAGC AAGATCTCTG AGAGGTTAGA GCGGGAAGAG TTGCGGGAAG   
  
  
- TTAACGCTGG GGTGACAATA TAACTCGCTG TCAGTCCTCG TGGTGTCAAG TTGTCCTTCA GAGAGCCGTG   
  
  
- TTTCGAGTAG AAGATTAAGA GGGGAACTCT GATCGTCAAT AATATTAGTC AAGTTGAGTT CGGGACAACC   
  
  
- TCTTAGTGGA GTCCCTAGTA GTGGGCTCTT AGAAAAAGTT GTTCGACGTC AATTATTTCA GTCGTAGCTC   
  
  
- GTACGTAACG TCCTCGATCT TTGACGAGAT TACTCGTGTC TAACACTCCT CTTACTTCGT TGACAGAGAT   
  
  
- AGGGTAGATA CCCACTTGGA GTAGTCGGGG TTCAGGGATC GGTCTCTAGT TCTAAGACCT TAGCTCTAGG   
  
  
- AGTCCCAAGT GCGGGCCGAC TTCAAGTGAG TAGTAACTCT TCTAATCCTC TGCTCCGAGT CTCACTCTTT   
  
  
- GCGGAGTTCC GTCACCTTCT TGGTTATGCC GGGTCACGTG GTGGGCCGTT AAACTTCGTC AACGAGTATC   
  
  
- TTACACGAGC CCGAAACAGT CTCTTAGCTT ATCTCCTAAA ACTCTTCAAT CAACTTGTCC GTTTCCCGTA   
  
  
- CCAGAGGTAA AGACCTCTGG GTTAAGTTGC TGAACCACGA ATGAACTAGC TCCCCGATCA CCGTTCCTTC   
  
  
- CTCAGAAGTC CATGCTTTTA GATGGCTCGA GAATCCACGT TTCTCGGGGA ACCAGTTCTG AACGAAAGCA   
  
  
- TGTACGTGTA GGAAATACTT TAAACGGGAA TGAACTTTAA GCCAATATAC CAACGTTTAC CCCGATATCG   
  
  
- ACTTCGAACG TCTCTACTCC TAGTGTATGT ATAGCAGCTG ATGGTCTAAC CAGTTCCTTG AGTTACCTAC   
  
  
- TGAGACAACG TTCGGGATCG GTGTTTTGGA CCTTCTGGGG GGTGACACTC TTAATGACCG TAACTACTAG   
  
  
- GGCAGAGATC CGTACGAGCC CCTCGAACGA ACCTCCGTCA CCCCTTTGCA AACCGTCAAG ACAGCCTCTT   
  
  
- TAAATTGTAC GGGTAACTCA AAGTCCGTCA CGGGCAAATA CGAAGACTCC AGTGGGTTCT TTACGAACTA   
  
  
- CACTCCGGTC CCACCCGGAA CCGACACTTG AAGGGTAACG TCGAGGTGGT GTGAGGGCTA CTCTCACAAC   
  
  
- TACACTTGTT GGGCTCCCTA CCTGAGGATT CTTACCACTT TAGTGAGCCA GGGTTCCAAC ATTGAAACCA   
  
  
- TCTTGTTCTC AGTTTGTGGT TGTGATGGGG AAACGACTGT TCCAAGTATC TTTGCGACCT GAAGATGAGT   
  
  
- CGTTACAAAC TTAGGTAGCT ACACTGTTAC GGTTCCCTGG CTTTCCTTTC CTAGTTACAG CTCGTCGTAA   
  
  
- CGGATCGGTT CCTGTAACAG TTGTAGTAGC GCACACTCCC CTTCCTGTCC CACCTCGCTG TACTCGAGAA   
  
  
- ACCCTTTACC TTCAGTTCCA AGTGGTACCG TCCTAAGGTT GTTAGGGGTA ACTCGAGCAT ACAATTGAGG   
  
  
- CACTATTTTC CTAATGAATC CACAATAAGT CTCATGATAT GCAACCACCT CTTCCTACCG CGGAACGAAG   
  
  
- ACCCTACCTT CCTGGCCTAC GACCAAAGCC GAAGTCGAAC CGTAAC

+     TATA-box

| Site Name | Organism | Position | Strand | Matrix score. | sequence | function |
| --- | --- | --- | --- | --- | --- | --- |
| TATA-box | Arabidopsis thaliana | 3681 | - | 4 | TATA | core promoter element around -30 of transcription start |
| TATA-box | Arabidopsis thaliana | 2939 | - | 4 | TATA | core promoter element around -30 of transcription start |
| TATA-box | Arabidopsis thaliana | 2919 | - | 4 | TATA | core promoter element around -30 of transcription start |
| TATA-box | Arabidopsis thaliana | 2918 | - | 5 | TATAA | core promoter element around -30 of transcription start |
| TATA-box | Arabidopsis thaliana | 2286 | - | 4 | TATA | core promoter element around -30 of transcription start |
| TATA-box | Arabidopsis thaliana | 2285 | - | 5 | TATAA | core promoter element around -30 of transcription start |
| TATA-box | Brassica napus | 2284 | + | 6 | ATTATA | core promoter element around -30 of transcription start |
| TATA-box | Arabidopsis thaliana | 2192 | - | 4 | TATA | core promoter element around -30 of transcription start |
| TATA-box | Arabidopsis thaliana | 2191 | - | 5 | TATAA | core promoter element around -30 of transcription start |
| TATA-box | Arabidopsis thaliana | 2070 | - | 4 | TATA | core promoter element around -30 of transcription start |
| TATA-box | Arabidopsis thaliana | 1661 | + | 4 | TATA | core promoter element around -30 of transcription start |
| TATA-box | Arabidopsis thaliana | 1660 | - | 5 | TATAA | core promoter element around -30 of transcription start |
| TATA-box | Brassica napus | 1659 | + | 6 | ATTATA | core promoter element around -30 of transcription start |
| TATA-box | Arabidopsis thaliana | 1367 | - | 5 | TATAA | core promoter element around -30 of transcription start |
| TATA-box | Arabidopsis thaliana | 1538 | + | 9 | ccTATAAAaa | core promoter element around -30 of transcription start |
| TATA-box | Arabidopsis thaliana | 1368 | + | 4 | TATA | core promoter element around -30 of transcription start |
| TATA-box | Helianthus annuus | 1366 | - | 6 | TATAAA | core promoter element around -30 of transcription start |
| TATA-box | Arabidopsis thaliana | 933 | + | 4 | TATA | core promoter element around -30 of transcription start |
| TATA-box | Arabidopsis thaliana | 48 | + | 4 | TATA | core promoter element around -30 of transcription start |
| TATA-box | Arabidopsis thaliana | 932 | - | 5 | TATAA | core promoter element around -30 of transcription start |
| TATA-box | Helianthus annuus | 931 | - | 6 | TATAAA | core promoter element around -30 of transcription start |
| TATA-box | Arabidopsis thaliana | 628 | + | 8 | TAAAGATT | core promoter element around -30 of transcription start |
| TATA-box | Arabidopsis thaliana | 449 | + | 4 | TATA | core promoter element around -30 of transcription start |
| TATA-box | Arabidopsis thaliana | 448 | - | 5 | TATAA | core promoter element around -30 of transcription start |
| TATA-box | Arabidopsis thaliana | 399 | + | 4 | TATA | core promoter element around -30 of transcription start |
| TATA-box | Arabidopsis thaliana | 397 | + | 6 | TATATA | core promoter element around -30 of transcription start |
| TATA-box | Brassica napus | 396 | + | 6 | ATATAT | core promoter element around -30 of transcription start |
| TATA-box | Arabidopsis thaliana | 395 | + | 6 | TATATA | core promoter element around -30 of transcription start |
| TATA-box | Arabidopsis thaliana | 186 | + | 4 | TATA | core promoter element around -30 of transcription start |
| TATA-box | Brassica napus | 47 | + | 6 | ATATAT | core promoter element around -30 of transcription start |
| TATA-box | Arabidopsis thaliana | 185 | - | 5 | TATAA | core promoter element around -30 of transcription start |
| TATA-box | Pisum sativum | 52 | - | 7 | TATAAAA | core promoter element around -30 of transcription start |
| TATA-box | Arabidopsis thaliana | 19 | - | 8 | TATTTAAA | core promoter element around -30 of transcription start |
| TATA-box | Helianthus annuus | 184 | - | 6 | TATAAA | core promoter element around -30 of transcription start |
| TATA-box | Pisum sativum | 183 | - | 7 | TATAAAA | core promoter element around -30 of transcription start |
| TATA-box | Arabidopsis thaliana | 149 | + | 4 | TATA | core promoter element around -30 of transcription start |
| TATA-box | Brassica oleracea | 148 | + | 6 | ATATAA | core promoter element around -30 of transcription start |
| TATA-box | Arabidopsis thaliana | 144 | + | 4 | TATA | core promoter element around -30 of transcription start |
| TATA-box | Brassica oleracea | 143 | + | 6 | ATATAA | core promoter element around -30 of transcription start |
| TATA-box | Arabidopsis thaliana | 142 | + | 6 | TATATA | core promoter element around -30 of transcription start |
| TATA-box | Brassica napus | 141 | + | 6 | ATATAT | core promoter element around -30 of transcription start |
| TATA-box | Arabidopsis thaliana | 104 | - | 8 | TATTTAAA | core promoter element around -30 of transcription start |
| TATA-box | Arabidopsis thaliana | 55 | + | 4 | TATA | core promoter element around -30 of transcription start |
| TATA-box | Arabidopsis thaliana | 54 | - | 5 | TATAA | core promoter element around -30 of transcription start |
| TATA-box | Helianthus annuus | 53 | - | 6 | TATAAA | core promoter element around -30 of transcription start |

>HU01G01850.1   
+ +Up\_Stream \_Len000TTTAAA TATTTTATTT TATTTTAAAT TAATATATTT TATAATTTAA AATTATTAAT   
  
  
+ AATTGATGAT GTTACCCAAT GTTTAAGTTT TTAAATATTT TTATTTTTTA ATTTAAAAAT ATCATAATAT   
  
  
+ ATAATATAAT TTTAAGTTAA AATAAGATAG TTAAATTATT TTATAGTAAA TATTTATTAT TTTTTTTAAG   
  
  
+ AAAATTGCCA TGTGATTGTC ATCAGTCAAA CACTGCGTTG ACAGAAGCTG TCCTTGCTGC TACTCTCACC   
  
  
+ TCTCAACATC GGACCCGCGC AGACGTTAAC ATCTCCGTAC AAATACAATT CCCTGGCCAA AATTCAAATC   
  
  
+ AACCCTACTC GGTAGTCGGT TCCTTCTTCT TCCTGCCCTC TATATATACA CCCACAGCTG CGCTGCATGT   
  
  
+ TTTTCCGAGA ATCCTCAAAA ATCTTATAAA CTTCCTCGTG TGGGTAGAAA AAAGAAAAAG AAAAGGGTTT   
  
  
+ TAAAATCTTG GGTCAAAACC CATCAACACC ACAATTTGAC TCTTTCTTTT TAATTCAACC ACCGCCAGTT   
  
  
+ CCTCGTTGAT TTTCAGGTAC CTCTTTTTCT TGCCCCTTTT TTTGCTCGGT TGTTGTTTAA TGGTAAAGAT   
  
  
+ TGAGTACAAA GAATGATATT TGATGAGTTG GTGTGTTTAT TTGATGAGCC CAGATGATTT AACTTGTAGA   
  
  
+ TTTTGTGGTT TTGGTTTCTG GGTATTCATT GGTTTGCTGA ATTTCTGGTT CATGATCTGG GGTTAGAAAA   
  
  
+ GTTGAAATGA CTTTCTGGAG GTTGGAATTT GACTAAAGCT AATTTCTTTT TGTCTTCAAT TCTTTTTTAG   
  
  
+ TTGTGAGCTG AATTTGTTGA CCACTTCCAG CCTGTTGGAA ATTAAACTTA CTTAGGAGAA GAGAATCTCA   
  
  
+ GATTCTGAGC AAAAGCTTTA TATGGTTGTT TGGGAAATGG TAGGAACTAA GTGAAAACAC TGAAATTTTA   
  
  
+ AGCAAGCAAT TTTGTTCATT TGATTGGTCG GATGGTCTGA TTTAGGTTCT TTTCTTGGTC TAAATTTCTG   
  
  
+ AGCATTGATT GTTTAATTGC GTGGGGAACT TGTATCCTTG TGAGTTCCTG GTTCATTTCT TGGTTGCTTA   
  
  
+ CTCTAGCTCC CTTTCACAGT TTCTATGGTT GGAACTTGGA ACTTGGAACT TGGAAGCCTA ATTTAGTTGA   
  
  
+ ATATCTTGGC TTTGAGTTAG CTTTTCTTAG GAAAAGATGG TTCTGCTCTC GAAAATCTAA TCTAATCGAT   
  
  
+ AGATGCGTTA AGGAAGCAAA ACTTTTGGGG AGAGGGAAGT TAAGCGAAGC AAGCTGCTGT TTCAAAGTTG   
  
  
+ GACTGTTTTA TTGGTAAGTA TGAAACAATA GTTTATAAGA GATCATTGGG TTCTTAAGGT GATAGATCAT   
  
  
+ GGGTTATGAA TGAGCTTAGT TGTTCTTTTG CTTTCAACTG TACGTAAGGA TGATAGTTAT CCCTCATCTT   
  
  
+ GCATCAAAAA CGGCAAACCG TGATGCCAAA ACTCAAAAAC TGATTCCTCT GACAAGGCTT CTACCTATGA   
  
  
+ AAACTATGCA GATCATATCC ATTTGGCCTT CCCTCTAGGA ACCAAGTAGA GTAAGTTGGT AAATAAATAG   
  
  
+ AGTTGAACGA AACACTACAG ATTTGAATTT GATTAAGGAG GGCTATTATA GACTAAGGTG TAGCTCAAAT   
  
  
+ CCTTCTGTAG GGATTGTCTT ATTGGCTTCT TTTGGTTGCT TCTGGCTCAA GTCCTCTTAT TAGTTAGTAT   
  
  
+ GCATTACCTT GAGACAAGAT AGTTGCTTAA GCTTGAGCTT CTGTACTTAT TTGCGTATTG TAGACAACTA   
  
  
+ GCTGTGGCAT ATGATTCTGA AATGTTCCGA TGTCTTTTTC TTTGGATGCT GGGTTGTGTC ATGAGCTTTA   
  
  
+ GGTTTGTTTA TTTGATGTCA GGAAGTATGT AAATAACATG TGGCATCTTG CAATCTATTA CAGGATGATA   
  
  
+ TTGGCCTTTG TGGGAAGAAA TTGAAGAAGT TGTTTCACTT ACTGGATAGG GAGCATGGAC TCACATCAGC   
  
  
+ TTTTGAGATT CGGTATTTCC AGATCATACT CATCCTATAA TTTCTCTCAG TCTAGTCCCC AATCAATTCC   
  
  
+ AAATAGGTTA TTTGAATCAC CGAACGTTCG TTCTAGAGAC TCTCCAATCT CGCCCTTCTC AACGCCCTTC   
  
  
+ AATTGCGACC CCACTGTTAT ATTGAGCGAC AGTCAGGAGC ACCACAGTTC AACAGGAAGT CTCTCGGCAC   
  
  
+ AAAGCTCATC TTCTAATTCT CCCCTTGAGA CTAGCAGTTA TTATAATCAG TTCAACTCAA GCCCTGTTGG   
  
  
+ AGAATCACCT CAGGGATCAT CACCCGAGAA TCTTTTTCAA CAAGCTGCAG TTAATAAAGT CAGCATCGAG   
  
  
+ CATGCATTGC AGGAGCTAGA AACTGCTCTA ATGAGCACAG ATTGTGAGGA GAATGAAGCA ACTGTCTCTA   
  
  
+ TCCCATCTAT GGGTGAACCT CATCAGCCCC AAGTCCCTAG CCAGAGATCA AGATTCTGGA ATCGAGATCC   
  
  
+ TCAGGGTTCA CGCCCGGCTG AAGTTCACTC ATCATTGAGA AGATTAGGAG ACGAGGCTCA GAGTGAGAAA   
  
  
+ CGCCTCAAGG CAGTGGAAGA ACCAATACGG CCCAGTGCAC CACCCGGCAA TTTGAAGCAG TTGCTCATAG   
  
  
+ AATGTGCTCG GGCTTTGTCA GAGAATCGAA TAGAGGATTT TGAGAAGTTA GTTGAACAGG CAAAGGGCAT   
  
  
+ GGTCTCCATT TCTGGAGACC CAATTCAACG ACTTGGTGCT TACTTGATCG AGGGGCTAGT GGCAAGGAAG   
  
  
+ GAGTCTTCAG GTACGAAAAT CTACCGAGCT CTTAGGTGCA AAGAGCCCCT TGGTCAAGAC TTGCTTTCGT   
  
  
+ ACATGCACAT CCTTTATGAA ATTTGCCCTT ACTTGAAATT CGGTTATATG GTTGCAAATG GGGCTATAGC   
  
  
+ TGAAGCTTGC AGAGATGAGG ATCACATACA TATCGTCGAC TACCAGATTG GTCAAGGAAC TCAATGGATG   
  
  
+ ACTCTGTTGC AAGCCCTAGC CACAAAACCT GGAAGACCCC CCACTGTGAG AATTACTGGC ATTGATGATC   
  
  
+ CCGTCTCTAG GCATGCTCGG GGAGCTTGCT TGGAGGCAGT GGGGAAACGT TTGGCAGTTC TGTCGGAGAA   
  
  
+ ATTTAACATG CCCATTGAGT TTCAGGCAGT GCCCGTTTAT GCTTCTGAGG TCACCCAAGA AATGCTTGAT   
  
  
+ GTGAGGCCAG GGTGGGCCTT GGCTGTGAAC TTCCCATTGC AGCTCCACCA CACTCCCGAT GAGAGTGTTG   
  
  
+ ATGTGAACAA CCCGAGGGAT GGACTCCTAA GAATGGTGAA ATCACTCGGT CCCAAGGTTG TAACTTTGGT   
  
  
+ AGAACAAGAG TCAAACACCA ACACTACCCC TTTGCTGACA AGGTTCATAG AAACGCTGGA CTTCTACTCA   
  
  
+ GCAATGTTTG AATCCATCGA TGTGACAATG CCAAGGGACC GAAAGGAAAG GATCAATGTC GAGCAGCATT   
  
  
+ GCCTAGCCAA GGACATTGTC AACATCATCG CGTGTGAGGG GAAGGACAGG GTGGAGCGAC ATGAGCTCTT   
  
  
+ TGGGAAATGG AAGTCAAGGT TCACCATGGC AGGATTCCAA CAATCCCCAT TGAGCTCGTA TGTTAACTCC   
  
  
+ GTGATAAAAG GATTACTTAG GTGTTATTCA GAGTACTATA CGTTGGTGGA GAAGGATGGC GCCTTGCTTC   
  
  
+ TGGGATGGAA GGACCGGATG CTGGTTTCGG CTTCAGCTTG GCATTG  

- +Up\_Stream \_Len000AAATTT ATAAAATAAA ATAAAATTTA ATTATATAAA ATATTAAATT TTAATAATTA   
  
  
- TTAACTACTA CAATGGGTTA CAAATTCAAA AATTTATAAA AATAAAAAAT TAAATTTTTA TAGTATTATA   
  
  
- TATTATATTA AAATTCAATT TTATTCTATC AATTTAATAA AATATCATTT ATAAATAATA AAAAAAATTC   
  
  
- TTTTAACGGT ACACTAACAG TAGTCAGTTT GTGACGCAAC TGTCTTCGAC AGGAACGACG ATGAGAGTGG   
  
  
- AGAGTTGTAG CCTGGGCGCG TCTGCAATTG TAGAGGCATG TTTATGTTAA GGGACCGGTT TTAAGTTTAG   
  
  
- TTGGGATGAG CCATCAGCCA AGGAAGAAGA AGGACGGGAG ATATATATGT GGGTGTCGAC GCGACGTACA   
  
  
- AAAAGGCTCT TAGGAGTTTT TAGAATATTT GAAGGAGCAC ACCCATCTTT TTTCTTTTTC TTTTCCCAAA   
  
  
- ATTTTAGAAC CCAGTTTTGG GTAGTTGTGG TGTTAAACTG AGAAAGAAAA ATTAAGTTGG TGGCGGTCAA   
  
  
- GGAGCAACTA AAAGTCCATG GAGAAAAAGA ACGGGGAAAA AAACGAGCCA ACAACAAATT ACCATTTCTA   
  
  
- ACTCATGTTT CTTACTATAA ACTACTCAAC CACACAAATA AACTACTCGG GTCTACTAAA TTGAACATCT   
  
  
- AAAACACCAA AACCAAAGAC CCATAAGTAA CCAAACGACT TAAAGACCAA GTACTAGACC CCAATCTTTT   
  
  
- CAACTTTACT GAAAGACCTC CAACCTTAAA CTGATTTCGA TTAAAGAAAA ACAGAAGTTA AGAAAAAATC   
  
  
- AACACTCGAC TTAAACAACT GGTGAAGGTC GGACAACCTT TAATTTGAAT GAATCCTCTT CTCTTAGAGT   
  
  
- CTAAGACTCG TTTTCGAAAT ATACCAACAA ACCCTTTACC ATCCTTGATT CACTTTTGTG ACTTTAAAAT   
  
  
- TCGTTCGTTA AAACAAGTAA ACTAACCAGC CTACCAGACT AAATCCAAGA AAAGAACCAG ATTTAAAGAC   
  
  
- TCGTAACTAA CAAATTAACG CACCCCTTGA ACATAGGAAC ACTCAAGGAC CAAGTAAAGA ACCAACGAAT   
  
  
- GAGATCGAGG GAAAGTGTCA AAGATACCAA CCTTGAACCT TGAACCTTGA ACCTTCGGAT TAAATCAACT   
  
  
- TATAGAACCG AAACTCAATC GAAAAGAATC CTTTTCTACC AAGACGAGAG CTTTTAGATT AGATTAGCTA   
  
  
- TCTACGCAAT TCCTTCGTTT TGAAAACCCC TCTCCCTTCA ATTCGCTTCG TTCGACGACA AAGTTTCAAC   
  
  
- CTGACAAAAT AACCATTCAT ACTTTGTTAT CAAATATTCT CTAGTAACCC AAGAATTCCA CTATCTAGTA   
  
  
- CCCAATACTT ACTCGAATCA ACAAGAAAAC GAAAGTTGAC ATGCATTCCT ACTATCAATA GGGAGTAGAA   
  
  
- CGTAGTTTTT GCCGTTTGGC ACTACGGTTT TGAGTTTTTG ACTAAGGAGA CTGTTCCGAA GATGGATACT   
  
  
- TTTGATACGT CTAGTATAGG TAAACCGGAA GGGAGATCCT TGGTTCATCT CATTCAACCA TTTATTTATC   
  
  
- TCAACTTGCT TTGTGATGTC TAAACTTAAA CTAATTCCTC CCGATAATAT CTGATTCCAC ATCGAGTTTA   
  
  
- GGAAGACATC CCTAACAGAA TAACCGAAGA AAACCAACGA AGACCGAGTT CAGGAGAATA ATCAATCATA   
  
  
- CGTAATGGAA CTCTGTTCTA TCAACGAATT CGAACTCGAA GACATGAATA AACGCATAAC ATCTGTTGAT   
  
  
- CGACACCGTA TACTAAGACT TTACAAGGCT ACAGAAAAAG AAACCTACGA CCCAACACAG TACTCGAAAT   
  
  
- CCAAACAAAT AAACTACAGT CCTTCATACA TTTATTGTAC ACCGTAGAAC GTTAGATAAT GTCCTACTAT   
  
  
- AACCGGAAAC ACCCTTCTTT AACTTCTTCA ACAAAGTGAA TGACCTATCC CTCGTACCTG AGTGTAGTCG   
  
  
- AAAACTCTAA GCCATAAAGG TCTAGTATGA GTAGGATATT AAAGAGAGTC AGATCAGGGG TTAGTTAAGG   
  
  
- TTTATCCAAT AAACTTAGTG GCTTGCAAGC AAGATCTCTG AGAGGTTAGA GCGGGAAGAG TTGCGGGAAG   
  
  
- TTAACGCTGG GGTGACAATA TAACTCGCTG TCAGTCCTCG TGGTGTCAAG TTGTCCTTCA GAGAGCCGTG   
  
  
- TTTCGAGTAG AAGATTAAGA GGGGAACTCT GATCGTCAAT AATATTAGTC AAGTTGAGTT CGGGACAACC   
  
  
- TCTTAGTGGA GTCCCTAGTA GTGGGCTCTT AGAAAAAGTT GTTCGACGTC AATTATTTCA GTCGTAGCTC   
  
  
- GTACGTAACG TCCTCGATCT TTGACGAGAT TACTCGTGTC TAACACTCCT CTTACTTCGT TGACAGAGAT   
  
  
- AGGGTAGATA CCCACTTGGA GTAGTCGGGG TTCAGGGATC GGTCTCTAGT TCTAAGACCT TAGCTCTAGG   
  
  
- AGTCCCAAGT GCGGGCCGAC TTCAAGTGAG TAGTAACTCT TCTAATCCTC TGCTCCGAGT CTCACTCTTT   
  
  
- GCGGAGTTCC GTCACCTTCT TGGTTATGCC GGGTCACGTG GTGGGCCGTT AAACTTCGTC AACGAGTATC   
  
  
- TTACACGAGC CCGAAACAGT CTCTTAGCTT ATCTCCTAAA ACTCTTCAAT CAACTTGTCC GTTTCCCGTA   
  
  
- CCAGAGGTAA AGACCTCTGG GTTAAGTTGC TGAACCACGA ATGAACTAGC TCCCCGATCA CCGTTCCTTC   
  
  
- CTCAGAAGTC CATGCTTTTA GATGGCTCGA GAATCCACGT TTCTCGGGGA ACCAGTTCTG AACGAAAGCA   
  
  
- TGTACGTGTA GGAAATACTT TAAACGGGAA TGAACTTTAA GCCAATATAC CAACGTTTAC CCCGATATCG   
  
  
- ACTTCGAACG TCTCTACTCC TAGTGTATGT ATAGCAGCTG ATGGTCTAAC CAGTTCCTTG AGTTACCTAC   
  
  
- TGAGACAACG TTCGGGATCG GTGTTTTGGA CCTTCTGGGG GGTGACACTC TTAATGACCG TAACTACTAG   
  
  
- GGCAGAGATC CGTACGAGCC CCTCGAACGA ACCTCCGTCA CCCCTTTGCA AACCGTCAAG ACAGCCTCTT   
  
  
- TAAATTGTAC GGGTAACTCA AAGTCCGTCA CGGGCAAATA CGAAGACTCC AGTGGGTTCT TTACGAACTA   
  
  
- CACTCCGGTC CCACCCGGAA CCGACACTTG AAGGGTAACG TCGAGGTGGT GTGAGGGCTA CTCTCACAAC   
  
  
- TACACTTGTT GGGCTCCCTA CCTGAGGATT CTTACCACTT TAGTGAGCCA GGGTTCCAAC ATTGAAACCA   
  
  
- TCTTGTTCTC AGTTTGTGGT TGTGATGGGG AAACGACTGT TCCAAGTATC TTTGCGACCT GAAGATGAGT   
  
  
- CGTTACAAAC TTAGGTAGCT ACACTGTTAC GGTTCCCTGG CTTTCCTTTC CTAGTTACAG CTCGTCGTAA   
  
  
- CGGATCGGTT CCTGTAACAG TTGTAGTAGC GCACACTCCC CTTCCTGTCC CACCTCGCTG TACTCGAGAA   
  
  
- ACCCTTTACC TTCAGTTCCA AGTGGTACCG TCCTAAGGTT GTTAGGGGTA ACTCGAGCAT ACAATTGAGG   
  
  
- CACTATTTTC CTAATGAATC CACAATAAGT CTCATGATAT GCAACCACCT CTTCCTACCG CGGAACGAAG   
  
  
- ACCCTACCTT CCTGGCCTAC GACCAAAGCC GAAGTCGAAC CGTAAC

+     TATC-box

| Site Name | Organism | Position | Strand | Matrix score. | sequence | function |
| --- | --- | --- | --- | --- | --- | --- |
| TATC-box | Oryza sativa | 2453 | + | 7 | TATCCCA | cis-acting element involved in gibberellin-responsiveness |

>HU01G01850.1   
+ +Up\_Stream \_Len000TTTAAA TATTTTATTT TATTTTAAAT TAATATATTT TATAATTTAA AATTATTAAT   
  
  
+ AATTGATGAT GTTACCCAAT GTTTAAGTTT TTAAATATTT TTATTTTTTA ATTTAAAAAT ATCATAATAT   
  
  
+ ATAATATAAT TTTAAGTTAA AATAAGATAG TTAAATTATT TTATAGTAAA TATTTATTAT TTTTTTTAAG   
  
  
+ AAAATTGCCA TGTGATTGTC ATCAGTCAAA CACTGCGTTG ACAGAAGCTG TCCTTGCTGC TACTCTCACC   
  
  
+ TCTCAACATC GGACCCGCGC AGACGTTAAC ATCTCCGTAC AAATACAATT CCCTGGCCAA AATTCAAATC   
  
  
+ AACCCTACTC GGTAGTCGGT TCCTTCTTCT TCCTGCCCTC TATATATACA CCCACAGCTG CGCTGCATGT   
  
  
+ TTTTCCGAGA ATCCTCAAAA ATCTTATAAA CTTCCTCGTG TGGGTAGAAA AAAGAAAAAG AAAAGGGTTT   
  
  
+ TAAAATCTTG GGTCAAAACC CATCAACACC ACAATTTGAC TCTTTCTTTT TAATTCAACC ACCGCCAGTT   
  
  
+ CCTCGTTGAT TTTCAGGTAC CTCTTTTTCT TGCCCCTTTT TTTGCTCGGT TGTTGTTTAA TGGTAAAGAT   
  
  
+ TGAGTACAAA GAATGATATT TGATGAGTTG GTGTGTTTAT TTGATGAGCC CAGATGATTT AACTTGTAGA   
  
  
+ TTTTGTGGTT TTGGTTTCTG GGTATTCATT GGTTTGCTGA ATTTCTGGTT CATGATCTGG GGTTAGAAAA   
  
  
+ GTTGAAATGA CTTTCTGGAG GTTGGAATTT GACTAAAGCT AATTTCTTTT TGTCTTCAAT TCTTTTTTAG   
  
  
+ TTGTGAGCTG AATTTGTTGA CCACTTCCAG CCTGTTGGAA ATTAAACTTA CTTAGGAGAA GAGAATCTCA   
  
  
+ GATTCTGAGC AAAAGCTTTA TATGGTTGTT TGGGAAATGG TAGGAACTAA GTGAAAACAC TGAAATTTTA   
  
  
+ AGCAAGCAAT TTTGTTCATT TGATTGGTCG GATGGTCTGA TTTAGGTTCT TTTCTTGGTC TAAATTTCTG   
  
  
+ AGCATTGATT GTTTAATTGC GTGGGGAACT TGTATCCTTG TGAGTTCCTG GTTCATTTCT TGGTTGCTTA   
  
  
+ CTCTAGCTCC CTTTCACAGT TTCTATGGTT GGAACTTGGA ACTTGGAACT TGGAAGCCTA ATTTAGTTGA   
  
  
+ ATATCTTGGC TTTGAGTTAG CTTTTCTTAG GAAAAGATGG TTCTGCTCTC GAAAATCTAA TCTAATCGAT   
  
  
+ AGATGCGTTA AGGAAGCAAA ACTTTTGGGG AGAGGGAAGT TAAGCGAAGC AAGCTGCTGT TTCAAAGTTG   
  
  
+ GACTGTTTTA TTGGTAAGTA TGAAACAATA GTTTATAAGA GATCATTGGG TTCTTAAGGT GATAGATCAT   
  
  
+ GGGTTATGAA TGAGCTTAGT TGTTCTTTTG CTTTCAACTG TACGTAAGGA TGATAGTTAT CCCTCATCTT   
  
  
+ GCATCAAAAA CGGCAAACCG TGATGCCAAA ACTCAAAAAC TGATTCCTCT GACAAGGCTT CTACCTATGA   
  
  
+ AAACTATGCA GATCATATCC ATTTGGCCTT CCCTCTAGGA ACCAAGTAGA GTAAGTTGGT AAATAAATAG   
  
  
+ AGTTGAACGA AACACTACAG ATTTGAATTT GATTAAGGAG GGCTATTATA GACTAAGGTG TAGCTCAAAT   
  
  
+ CCTTCTGTAG GGATTGTCTT ATTGGCTTCT TTTGGTTGCT TCTGGCTCAA GTCCTCTTAT TAGTTAGTAT   
  
  
+ GCATTACCTT GAGACAAGAT AGTTGCTTAA GCTTGAGCTT CTGTACTTAT TTGCGTATTG TAGACAACTA   
  
  
+ GCTGTGGCAT ATGATTCTGA AATGTTCCGA TGTCTTTTTC TTTGGATGCT GGGTTGTGTC ATGAGCTTTA   
  
  
+ GGTTTGTTTA TTTGATGTCA GGAAGTATGT AAATAACATG TGGCATCTTG CAATCTATTA CAGGATGATA   
  
  
+ TTGGCCTTTG TGGGAAGAAA TTGAAGAAGT TGTTTCACTT ACTGGATAGG GAGCATGGAC TCACATCAGC   
  
  
+ TTTTGAGATT CGGTATTTCC AGATCATACT CATCCTATAA TTTCTCTCAG TCTAGTCCCC AATCAATTCC   
  
  
+ AAATAGGTTA TTTGAATCAC CGAACGTTCG TTCTAGAGAC TCTCCAATCT CGCCCTTCTC AACGCCCTTC   
  
  
+ AATTGCGACC CCACTGTTAT ATTGAGCGAC AGTCAGGAGC ACCACAGTTC AACAGGAAGT CTCTCGGCAC   
  
  
+ AAAGCTCATC TTCTAATTCT CCCCTTGAGA CTAGCAGTTA TTATAATCAG TTCAACTCAA GCCCTGTTGG   
  
  
+ AGAATCACCT CAGGGATCAT CACCCGAGAA TCTTTTTCAA CAAGCTGCAG TTAATAAAGT CAGCATCGAG   
  
  
+ CATGCATTGC AGGAGCTAGA AACTGCTCTA ATGAGCACAG ATTGTGAGGA GAATGAAGCA ACTGTCTCTA   
  
  
+ TCCCATCTAT GGGTGAACCT CATCAGCCCC AAGTCCCTAG CCAGAGATCA AGATTCTGGA ATCGAGATCC   
  
  
+ TCAGGGTTCA CGCCCGGCTG AAGTTCACTC ATCATTGAGA AGATTAGGAG ACGAGGCTCA GAGTGAGAAA   
  
  
+ CGCCTCAAGG CAGTGGAAGA ACCAATACGG CCCAGTGCAC CACCCGGCAA TTTGAAGCAG TTGCTCATAG   
  
  
+ AATGTGCTCG GGCTTTGTCA GAGAATCGAA TAGAGGATTT TGAGAAGTTA GTTGAACAGG CAAAGGGCAT   
  
  
+ GGTCTCCATT TCTGGAGACC CAATTCAACG ACTTGGTGCT TACTTGATCG AGGGGCTAGT GGCAAGGAAG   
  
  
+ GAGTCTTCAG GTACGAAAAT CTACCGAGCT CTTAGGTGCA AAGAGCCCCT TGGTCAAGAC TTGCTTTCGT   
  
  
+ ACATGCACAT CCTTTATGAA ATTTGCCCTT ACTTGAAATT CGGTTATATG GTTGCAAATG GGGCTATAGC   
  
  
+ TGAAGCTTGC AGAGATGAGG ATCACATACA TATCGTCGAC TACCAGATTG GTCAAGGAAC TCAATGGATG   
  
  
+ ACTCTGTTGC AAGCCCTAGC CACAAAACCT GGAAGACCCC CCACTGTGAG AATTACTGGC ATTGATGATC   
  
  
+ CCGTCTCTAG GCATGCTCGG GGAGCTTGCT TGGAGGCAGT GGGGAAACGT TTGGCAGTTC TGTCGGAGAA   
  
  
+ ATTTAACATG CCCATTGAGT TTCAGGCAGT GCCCGTTTAT GCTTCTGAGG TCACCCAAGA AATGCTTGAT   
  
  
+ GTGAGGCCAG GGTGGGCCTT GGCTGTGAAC TTCCCATTGC AGCTCCACCA CACTCCCGAT GAGAGTGTTG   
  
  
+ ATGTGAACAA CCCGAGGGAT GGACTCCTAA GAATGGTGAA ATCACTCGGT CCCAAGGTTG TAACTTTGGT   
  
  
+ AGAACAAGAG TCAAACACCA ACACTACCCC TTTGCTGACA AGGTTCATAG AAACGCTGGA CTTCTACTCA   
  
  
+ GCAATGTTTG AATCCATCGA TGTGACAATG CCAAGGGACC GAAAGGAAAG GATCAATGTC GAGCAGCATT   
  
  
+ GCCTAGCCAA GGACATTGTC AACATCATCG CGTGTGAGGG GAAGGACAGG GTGGAGCGAC ATGAGCTCTT   
  
  
+ TGGGAAATGG AAGTCAAGGT TCACCATGGC AGGATTCCAA CAATCCCCAT TGAGCTCGTA TGTTAACTCC   
  
  
+ GTGATAAAAG GATTACTTAG GTGTTATTCA GAGTACTATA CGTTGGTGGA GAAGGATGGC GCCTTGCTTC   
  
  
+ TGGGATGGAA GGACCGGATG CTGGTTTCGG CTTCAGCTTG GCATTG  

- +Up\_Stream \_Len000AAATTT ATAAAATAAA ATAAAATTTA ATTATATAAA ATATTAAATT TTAATAATTA   
  
  
- TTAACTACTA CAATGGGTTA CAAATTCAAA AATTTATAAA AATAAAAAAT TAAATTTTTA TAGTATTATA   
  
  
- TATTATATTA AAATTCAATT TTATTCTATC AATTTAATAA AATATCATTT ATAAATAATA AAAAAAATTC   
  
  
- TTTTAACGGT ACACTAACAG TAGTCAGTTT GTGACGCAAC TGTCTTCGAC AGGAACGACG ATGAGAGTGG   
  
  
- AGAGTTGTAG CCTGGGCGCG TCTGCAATTG TAGAGGCATG TTTATGTTAA GGGACCGGTT TTAAGTTTAG   
  
  
- TTGGGATGAG CCATCAGCCA AGGAAGAAGA AGGACGGGAG ATATATATGT GGGTGTCGAC GCGACGTACA   
  
  
- AAAAGGCTCT TAGGAGTTTT TAGAATATTT GAAGGAGCAC ACCCATCTTT TTTCTTTTTC TTTTCCCAAA   
  
  
- ATTTTAGAAC CCAGTTTTGG GTAGTTGTGG TGTTAAACTG AGAAAGAAAA ATTAAGTTGG TGGCGGTCAA   
  
  
- GGAGCAACTA AAAGTCCATG GAGAAAAAGA ACGGGGAAAA AAACGAGCCA ACAACAAATT ACCATTTCTA   
  
  
- ACTCATGTTT CTTACTATAA ACTACTCAAC CACACAAATA AACTACTCGG GTCTACTAAA TTGAACATCT   
  
  
- AAAACACCAA AACCAAAGAC CCATAAGTAA CCAAACGACT TAAAGACCAA GTACTAGACC CCAATCTTTT   
  
  
- CAACTTTACT GAAAGACCTC CAACCTTAAA CTGATTTCGA TTAAAGAAAA ACAGAAGTTA AGAAAAAATC   
  
  
- AACACTCGAC TTAAACAACT GGTGAAGGTC GGACAACCTT TAATTTGAAT GAATCCTCTT CTCTTAGAGT   
  
  
- CTAAGACTCG TTTTCGAAAT ATACCAACAA ACCCTTTACC ATCCTTGATT CACTTTTGTG ACTTTAAAAT   
  
  
- TCGTTCGTTA AAACAAGTAA ACTAACCAGC CTACCAGACT AAATCCAAGA AAAGAACCAG ATTTAAAGAC   
  
  
- TCGTAACTAA CAAATTAACG CACCCCTTGA ACATAGGAAC ACTCAAGGAC CAAGTAAAGA ACCAACGAAT   
  
  
- GAGATCGAGG GAAAGTGTCA AAGATACCAA CCTTGAACCT TGAACCTTGA ACCTTCGGAT TAAATCAACT   
  
  
- TATAGAACCG AAACTCAATC GAAAAGAATC CTTTTCTACC AAGACGAGAG CTTTTAGATT AGATTAGCTA   
  
  
- TCTACGCAAT TCCTTCGTTT TGAAAACCCC TCTCCCTTCA ATTCGCTTCG TTCGACGACA AAGTTTCAAC   
  
  
- CTGACAAAAT AACCATTCAT ACTTTGTTAT CAAATATTCT CTAGTAACCC AAGAATTCCA CTATCTAGTA   
  
  
- CCCAATACTT ACTCGAATCA ACAAGAAAAC GAAAGTTGAC ATGCATTCCT ACTATCAATA GGGAGTAGAA   
  
  
- CGTAGTTTTT GCCGTTTGGC ACTACGGTTT TGAGTTTTTG ACTAAGGAGA CTGTTCCGAA GATGGATACT   
  
  
- TTTGATACGT CTAGTATAGG TAAACCGGAA GGGAGATCCT TGGTTCATCT CATTCAACCA TTTATTTATC   
  
  
- TCAACTTGCT TTGTGATGTC TAAACTTAAA CTAATTCCTC CCGATAATAT CTGATTCCAC ATCGAGTTTA   
  
  
- GGAAGACATC CCTAACAGAA TAACCGAAGA AAACCAACGA AGACCGAGTT CAGGAGAATA ATCAATCATA   
  
  
- CGTAATGGAA CTCTGTTCTA TCAACGAATT CGAACTCGAA GACATGAATA AACGCATAAC ATCTGTTGAT   
  
  
- CGACACCGTA TACTAAGACT TTACAAGGCT ACAGAAAAAG AAACCTACGA CCCAACACAG TACTCGAAAT   
  
  
- CCAAACAAAT AAACTACAGT CCTTCATACA TTTATTGTAC ACCGTAGAAC GTTAGATAAT GTCCTACTAT   
  
  
- AACCGGAAAC ACCCTTCTTT AACTTCTTCA ACAAAGTGAA TGACCTATCC CTCGTACCTG AGTGTAGTCG   
  
  
- AAAACTCTAA GCCATAAAGG TCTAGTATGA GTAGGATATT AAAGAGAGTC AGATCAGGGG TTAGTTAAGG   
  
  
- TTTATCCAAT AAACTTAGTG GCTTGCAAGC AAGATCTCTG AGAGGTTAGA GCGGGAAGAG TTGCGGGAAG   
  
  
- TTAACGCTGG GGTGACAATA TAACTCGCTG TCAGTCCTCG TGGTGTCAAG TTGTCCTTCA GAGAGCCGTG   
  
  
- TTTCGAGTAG AAGATTAAGA GGGGAACTCT GATCGTCAAT AATATTAGTC AAGTTGAGTT CGGGACAACC   
  
  
- TCTTAGTGGA GTCCCTAGTA GTGGGCTCTT AGAAAAAGTT GTTCGACGTC AATTATTTCA GTCGTAGCTC   
  
  
- GTACGTAACG TCCTCGATCT TTGACGAGAT TACTCGTGTC TAACACTCCT CTTACTTCGT TGACAGAGAT   
  
  
- AGGGTAGATA CCCACTTGGA GTAGTCGGGG TTCAGGGATC GGTCTCTAGT TCTAAGACCT TAGCTCTAGG   
  
  
- AGTCCCAAGT GCGGGCCGAC TTCAAGTGAG TAGTAACTCT TCTAATCCTC TGCTCCGAGT CTCACTCTTT   
  
  
- GCGGAGTTCC GTCACCTTCT TGGTTATGCC GGGTCACGTG GTGGGCCGTT AAACTTCGTC AACGAGTATC   
  
  
- TTACACGAGC CCGAAACAGT CTCTTAGCTT ATCTCCTAAA ACTCTTCAAT CAACTTGTCC GTTTCCCGTA   
  
  
- CCAGAGGTAA AGACCTCTGG GTTAAGTTGC TGAACCACGA ATGAACTAGC TCCCCGATCA CCGTTCCTTC   
  
  
- CTCAGAAGTC CATGCTTTTA GATGGCTCGA GAATCCACGT TTCTCGGGGA ACCAGTTCTG AACGAAAGCA   
  
  
- TGTACGTGTA GGAAATACTT TAAACGGGAA TGAACTTTAA GCCAATATAC CAACGTTTAC CCCGATATCG   
  
  
- ACTTCGAACG TCTCTACTCC TAGTGTATGT ATAGCAGCTG ATGGTCTAAC CAGTTCCTTG AGTTACCTAC   
  
  
- TGAGACAACG TTCGGGATCG GTGTTTTGGA CCTTCTGGGG GGTGACACTC TTAATGACCG TAACTACTAG   
  
  
- GGCAGAGATC CGTACGAGCC CCTCGAACGA ACCTCCGTCA CCCCTTTGCA AACCGTCAAG ACAGCCTCTT   
  
  
- TAAATTGTAC GGGTAACTCA AAGTCCGTCA CGGGCAAATA CGAAGACTCC AGTGGGTTCT TTACGAACTA   
  
  
- CACTCCGGTC CCACCCGGAA CCGACACTTG AAGGGTAACG TCGAGGTGGT GTGAGGGCTA CTCTCACAAC   
  
  
- TACACTTGTT GGGCTCCCTA CCTGAGGATT CTTACCACTT TAGTGAGCCA GGGTTCCAAC ATTGAAACCA   
  
  
- TCTTGTTCTC AGTTTGTGGT TGTGATGGGG AAACGACTGT TCCAAGTATC TTTGCGACCT GAAGATGAGT   
  
  
- CGTTACAAAC TTAGGTAGCT ACACTGTTAC GGTTCCCTGG CTTTCCTTTC CTAGTTACAG CTCGTCGTAA   
  
  
- CGGATCGGTT CCTGTAACAG TTGTAGTAGC GCACACTCCC CTTCCTGTCC CACCTCGCTG TACTCGAGAA   
  
  
- ACCCTTTACC TTCAGTTCCA AGTGGTACCG TCCTAAGGTT GTTAGGGGTA ACTCGAGCAT ACAATTGAGG   
  
  
- CACTATTTTC CTAATGAATC CACAATAAGT CTCATGATAT GCAACCACCT CTTCCTACCG CGGAACGAAG   
  
  
- ACCCTACCTT CCTGGCCTAC GACCAAAGCC GAAGTCGAAC CGTAAC

+     TC-rich repeats

| Site Name | Organism | Position | Strand | Matrix score. | sequence | function |
| --- | --- | --- | --- | --- | --- | --- |
| TC-rich repeats | Nicotiana tabacum | 2681 | - | 9 | ATTCTCTAAC | cis-acting element involved in defense and stress responsiveness |
| TC-rich repeats | Nicotiana tabacum | 2310 | - | 9 | ATTCTCTAAC | cis-acting element involved in defense and stress responsiveness |

>HU01G01850.1   
+ +Up\_Stream \_Len000TTTAAA TATTTTATTT TATTTTAAAT TAATATATTT TATAATTTAA AATTATTAAT   
  
  
+ AATTGATGAT GTTACCCAAT GTTTAAGTTT TTAAATATTT TTATTTTTTA ATTTAAAAAT ATCATAATAT   
  
  
+ ATAATATAAT TTTAAGTTAA AATAAGATAG TTAAATTATT TTATAGTAAA TATTTATTAT TTTTTTTAAG   
  
  
+ AAAATTGCCA TGTGATTGTC ATCAGTCAAA CACTGCGTTG ACAGAAGCTG TCCTTGCTGC TACTCTCACC   
  
  
+ TCTCAACATC GGACCCGCGC AGACGTTAAC ATCTCCGTAC AAATACAATT CCCTGGCCAA AATTCAAATC   
  
  
+ AACCCTACTC GGTAGTCGGT TCCTTCTTCT TCCTGCCCTC TATATATACA CCCACAGCTG CGCTGCATGT   
  
  
+ TTTTCCGAGA ATCCTCAAAA ATCTTATAAA CTTCCTCGTG TGGGTAGAAA AAAGAAAAAG AAAAGGGTTT   
  
  
+ TAAAATCTTG GGTCAAAACC CATCAACACC ACAATTTGAC TCTTTCTTTT TAATTCAACC ACCGCCAGTT   
  
  
+ CCTCGTTGAT TTTCAGGTAC CTCTTTTTCT TGCCCCTTTT TTTGCTCGGT TGTTGTTTAA TGGTAAAGAT   
  
  
+ TGAGTACAAA GAATGATATT TGATGAGTTG GTGTGTTTAT TTGATGAGCC CAGATGATTT AACTTGTAGA   
  
  
+ TTTTGTGGTT TTGGTTTCTG GGTATTCATT GGTTTGCTGA ATTTCTGGTT CATGATCTGG GGTTAGAAAA   
  
  
+ GTTGAAATGA CTTTCTGGAG GTTGGAATTT GACTAAAGCT AATTTCTTTT TGTCTTCAAT TCTTTTTTAG   
  
  
+ TTGTGAGCTG AATTTGTTGA CCACTTCCAG CCTGTTGGAA ATTAAACTTA CTTAGGAGAA GAGAATCTCA   
  
  
+ GATTCTGAGC AAAAGCTTTA TATGGTTGTT TGGGAAATGG TAGGAACTAA GTGAAAACAC TGAAATTTTA   
  
  
+ AGCAAGCAAT TTTGTTCATT TGATTGGTCG GATGGTCTGA TTTAGGTTCT TTTCTTGGTC TAAATTTCTG   
  
  
+ AGCATTGATT GTTTAATTGC GTGGGGAACT TGTATCCTTG TGAGTTCCTG GTTCATTTCT TGGTTGCTTA   
  
  
+ CTCTAGCTCC CTTTCACAGT TTCTATGGTT GGAACTTGGA ACTTGGAACT TGGAAGCCTA ATTTAGTTGA   
  
  
+ ATATCTTGGC TTTGAGTTAG CTTTTCTTAG GAAAAGATGG TTCTGCTCTC GAAAATCTAA TCTAATCGAT   
  
  
+ AGATGCGTTA AGGAAGCAAA ACTTTTGGGG AGAGGGAAGT TAAGCGAAGC AAGCTGCTGT TTCAAAGTTG   
  
  
+ GACTGTTTTA TTGGTAAGTA TGAAACAATA GTTTATAAGA GATCATTGGG TTCTTAAGGT GATAGATCAT   
  
  
+ GGGTTATGAA TGAGCTTAGT TGTTCTTTTG CTTTCAACTG TACGTAAGGA TGATAGTTAT CCCTCATCTT   
  
  
+ GCATCAAAAA CGGCAAACCG TGATGCCAAA ACTCAAAAAC TGATTCCTCT GACAAGGCTT CTACCTATGA   
  
  
+ AAACTATGCA GATCATATCC ATTTGGCCTT CCCTCTAGGA ACCAAGTAGA GTAAGTTGGT AAATAAATAG   
  
  
+ AGTTGAACGA AACACTACAG ATTTGAATTT GATTAAGGAG GGCTATTATA GACTAAGGTG TAGCTCAAAT   
  
  
+ CCTTCTGTAG GGATTGTCTT ATTGGCTTCT TTTGGTTGCT TCTGGCTCAA GTCCTCTTAT TAGTTAGTAT   
  
  
+ GCATTACCTT GAGACAAGAT AGTTGCTTAA GCTTGAGCTT CTGTACTTAT TTGCGTATTG TAGACAACTA   
  
  
+ GCTGTGGCAT ATGATTCTGA AATGTTCCGA TGTCTTTTTC TTTGGATGCT GGGTTGTGTC ATGAGCTTTA   
  
  
+ GGTTTGTTTA TTTGATGTCA GGAAGTATGT AAATAACATG TGGCATCTTG CAATCTATTA CAGGATGATA   
  
  
+ TTGGCCTTTG TGGGAAGAAA TTGAAGAAGT TGTTTCACTT ACTGGATAGG GAGCATGGAC TCACATCAGC   
  
  
+ TTTTGAGATT CGGTATTTCC AGATCATACT CATCCTATAA TTTCTCTCAG TCTAGTCCCC AATCAATTCC   
  
  
+ AAATAGGTTA TTTGAATCAC CGAACGTTCG TTCTAGAGAC TCTCCAATCT CGCCCTTCTC AACGCCCTTC   
  
  
+ AATTGCGACC CCACTGTTAT ATTGAGCGAC AGTCAGGAGC ACCACAGTTC AACAGGAAGT CTCTCGGCAC   
  
  
+ AAAGCTCATC TTCTAATTCT CCCCTTGAGA CTAGCAGTTA TTATAATCAG TTCAACTCAA GCCCTGTTGG   
  
  
+ AGAATCACCT CAGGGATCAT CACCCGAGAA TCTTTTTCAA CAAGCTGCAG TTAATAAAGT CAGCATCGAG   
  
  
+ CATGCATTGC AGGAGCTAGA AACTGCTCTA ATGAGCACAG ATTGTGAGGA GAATGAAGCA ACTGTCTCTA   
  
  
+ TCCCATCTAT GGGTGAACCT CATCAGCCCC AAGTCCCTAG CCAGAGATCA AGATTCTGGA ATCGAGATCC   
  
  
+ TCAGGGTTCA CGCCCGGCTG AAGTTCACTC ATCATTGAGA AGATTAGGAG ACGAGGCTCA GAGTGAGAAA   
  
  
+ CGCCTCAAGG CAGTGGAAGA ACCAATACGG CCCAGTGCAC CACCCGGCAA TTTGAAGCAG TTGCTCATAG   
  
  
+ AATGTGCTCG GGCTTTGTCA GAGAATCGAA TAGAGGATTT TGAGAAGTTA GTTGAACAGG CAAAGGGCAT   
  
  
+ GGTCTCCATT TCTGGAGACC CAATTCAACG ACTTGGTGCT TACTTGATCG AGGGGCTAGT GGCAAGGAAG   
  
  
+ GAGTCTTCAG GTACGAAAAT CTACCGAGCT CTTAGGTGCA AAGAGCCCCT TGGTCAAGAC TTGCTTTCGT   
  
  
+ ACATGCACAT CCTTTATGAA ATTTGCCCTT ACTTGAAATT CGGTTATATG GTTGCAAATG GGGCTATAGC   
  
  
+ TGAAGCTTGC AGAGATGAGG ATCACATACA TATCGTCGAC TACCAGATTG GTCAAGGAAC TCAATGGATG   
  
  
+ ACTCTGTTGC AAGCCCTAGC CACAAAACCT GGAAGACCCC CCACTGTGAG AATTACTGGC ATTGATGATC   
  
  
+ CCGTCTCTAG GCATGCTCGG GGAGCTTGCT TGGAGGCAGT GGGGAAACGT TTGGCAGTTC TGTCGGAGAA   
  
  
+ ATTTAACATG CCCATTGAGT TTCAGGCAGT GCCCGTTTAT GCTTCTGAGG TCACCCAAGA AATGCTTGAT   
  
  
+ GTGAGGCCAG GGTGGGCCTT GGCTGTGAAC TTCCCATTGC AGCTCCACCA CACTCCCGAT GAGAGTGTTG   
  
  
+ ATGTGAACAA CCCGAGGGAT GGACTCCTAA GAATGGTGAA ATCACTCGGT CCCAAGGTTG TAACTTTGGT   
  
  
+ AGAACAAGAG TCAAACACCA ACACTACCCC TTTGCTGACA AGGTTCATAG AAACGCTGGA CTTCTACTCA   
  
  
+ GCAATGTTTG AATCCATCGA TGTGACAATG CCAAGGGACC GAAAGGAAAG GATCAATGTC GAGCAGCATT   
  
  
+ GCCTAGCCAA GGACATTGTC AACATCATCG CGTGTGAGGG GAAGGACAGG GTGGAGCGAC ATGAGCTCTT   
  
  
+ TGGGAAATGG AAGTCAAGGT TCACCATGGC AGGATTCCAA CAATCCCCAT TGAGCTCGTA TGTTAACTCC   
  
  
+ GTGATAAAAG GATTACTTAG GTGTTATTCA GAGTACTATA CGTTGGTGGA GAAGGATGGC GCCTTGCTTC   
  
  
+ TGGGATGGAA GGACCGGATG CTGGTTTCGG CTTCAGCTTG GCATTG  

- +Up\_Stream \_Len000AAATTT ATAAAATAAA ATAAAATTTA ATTATATAAA ATATTAAATT TTAATAATTA   
  
  
- TTAACTACTA CAATGGGTTA CAAATTCAAA AATTTATAAA AATAAAAAAT TAAATTTTTA TAGTATTATA   
  
  
- TATTATATTA AAATTCAATT TTATTCTATC AATTTAATAA AATATCATTT ATAAATAATA AAAAAAATTC   
  
  
- TTTTAACGGT ACACTAACAG TAGTCAGTTT GTGACGCAAC TGTCTTCGAC AGGAACGACG ATGAGAGTGG   
  
  
- AGAGTTGTAG CCTGGGCGCG TCTGCAATTG TAGAGGCATG TTTATGTTAA GGGACCGGTT TTAAGTTTAG   
  
  
- TTGGGATGAG CCATCAGCCA AGGAAGAAGA AGGACGGGAG ATATATATGT GGGTGTCGAC GCGACGTACA   
  
  
- AAAAGGCTCT TAGGAGTTTT TAGAATATTT GAAGGAGCAC ACCCATCTTT TTTCTTTTTC TTTTCCCAAA   
  
  
- ATTTTAGAAC CCAGTTTTGG GTAGTTGTGG TGTTAAACTG AGAAAGAAAA ATTAAGTTGG TGGCGGTCAA   
  
  
- GGAGCAACTA AAAGTCCATG GAGAAAAAGA ACGGGGAAAA AAACGAGCCA ACAACAAATT ACCATTTCTA   
  
  
- ACTCATGTTT CTTACTATAA ACTACTCAAC CACACAAATA AACTACTCGG GTCTACTAAA TTGAACATCT   
  
  
- AAAACACCAA AACCAAAGAC CCATAAGTAA CCAAACGACT TAAAGACCAA GTACTAGACC CCAATCTTTT   
  
  
- CAACTTTACT GAAAGACCTC CAACCTTAAA CTGATTTCGA TTAAAGAAAA ACAGAAGTTA AGAAAAAATC   
  
  
- AACACTCGAC TTAAACAACT GGTGAAGGTC GGACAACCTT TAATTTGAAT GAATCCTCTT CTCTTAGAGT   
  
  
- CTAAGACTCG TTTTCGAAAT ATACCAACAA ACCCTTTACC ATCCTTGATT CACTTTTGTG ACTTTAAAAT   
  
  
- TCGTTCGTTA AAACAAGTAA ACTAACCAGC CTACCAGACT AAATCCAAGA AAAGAACCAG ATTTAAAGAC   
  
  
- TCGTAACTAA CAAATTAACG CACCCCTTGA ACATAGGAAC ACTCAAGGAC CAAGTAAAGA ACCAACGAAT   
  
  
- GAGATCGAGG GAAAGTGTCA AAGATACCAA CCTTGAACCT TGAACCTTGA ACCTTCGGAT TAAATCAACT   
  
  
- TATAGAACCG AAACTCAATC GAAAAGAATC CTTTTCTACC AAGACGAGAG CTTTTAGATT AGATTAGCTA   
  
  
- TCTACGCAAT TCCTTCGTTT TGAAAACCCC TCTCCCTTCA ATTCGCTTCG TTCGACGACA AAGTTTCAAC   
  
  
- CTGACAAAAT AACCATTCAT ACTTTGTTAT CAAATATTCT CTAGTAACCC AAGAATTCCA CTATCTAGTA   
  
  
- CCCAATACTT ACTCGAATCA ACAAGAAAAC GAAAGTTGAC ATGCATTCCT ACTATCAATA GGGAGTAGAA   
  
  
- CGTAGTTTTT GCCGTTTGGC ACTACGGTTT TGAGTTTTTG ACTAAGGAGA CTGTTCCGAA GATGGATACT   
  
  
- TTTGATACGT CTAGTATAGG TAAACCGGAA GGGAGATCCT TGGTTCATCT CATTCAACCA TTTATTTATC   
  
  
- TCAACTTGCT TTGTGATGTC TAAACTTAAA CTAATTCCTC CCGATAATAT CTGATTCCAC ATCGAGTTTA   
  
  
- GGAAGACATC CCTAACAGAA TAACCGAAGA AAACCAACGA AGACCGAGTT CAGGAGAATA ATCAATCATA   
  
  
- CGTAATGGAA CTCTGTTCTA TCAACGAATT CGAACTCGAA GACATGAATA AACGCATAAC ATCTGTTGAT   
  
  
- CGACACCGTA TACTAAGACT TTACAAGGCT ACAGAAAAAG AAACCTACGA CCCAACACAG TACTCGAAAT   
  
  
- CCAAACAAAT AAACTACAGT CCTTCATACA TTTATTGTAC ACCGTAGAAC GTTAGATAAT GTCCTACTAT   
  
  
- AACCGGAAAC ACCCTTCTTT AACTTCTTCA ACAAAGTGAA TGACCTATCC CTCGTACCTG AGTGTAGTCG   
  
  
- AAAACTCTAA GCCATAAAGG TCTAGTATGA GTAGGATATT AAAGAGAGTC AGATCAGGGG TTAGTTAAGG   
  
  
- TTTATCCAAT AAACTTAGTG GCTTGCAAGC AAGATCTCTG AGAGGTTAGA GCGGGAAGAG TTGCGGGAAG   
  
  
- TTAACGCTGG GGTGACAATA TAACTCGCTG TCAGTCCTCG TGGTGTCAAG TTGTCCTTCA GAGAGCCGTG   
  
  
- TTTCGAGTAG AAGATTAAGA GGGGAACTCT GATCGTCAAT AATATTAGTC AAGTTGAGTT CGGGACAACC   
  
  
- TCTTAGTGGA GTCCCTAGTA GTGGGCTCTT AGAAAAAGTT GTTCGACGTC AATTATTTCA GTCGTAGCTC   
  
  
- GTACGTAACG TCCTCGATCT TTGACGAGAT TACTCGTGTC TAACACTCCT CTTACTTCGT TGACAGAGAT   
  
  
- AGGGTAGATA CCCACTTGGA GTAGTCGGGG TTCAGGGATC GGTCTCTAGT TCTAAGACCT TAGCTCTAGG   
  
  
- AGTCCCAAGT GCGGGCCGAC TTCAAGTGAG TAGTAACTCT TCTAATCCTC TGCTCCGAGT CTCACTCTTT   
  
  
- GCGGAGTTCC GTCACCTTCT TGGTTATGCC GGGTCACGTG GTGGGCCGTT AAACTTCGTC AACGAGTATC   
  
  
- TTACACGAGC CCGAAACAGT CTCTTAGCTT ATCTCCTAAA ACTCTTCAAT CAACTTGTCC GTTTCCCGTA   
  
  
- CCAGAGGTAA AGACCTCTGG GTTAAGTTGC TGAACCACGA ATGAACTAGC TCCCCGATCA CCGTTCCTTC   
  
  
- CTCAGAAGTC CATGCTTTTA GATGGCTCGA GAATCCACGT TTCTCGGGGA ACCAGTTCTG AACGAAAGCA   
  
  
- TGTACGTGTA GGAAATACTT TAAACGGGAA TGAACTTTAA GCCAATATAC CAACGTTTAC CCCGATATCG   
  
  
- ACTTCGAACG TCTCTACTCC TAGTGTATGT ATAGCAGCTG ATGGTCTAAC CAGTTCCTTG AGTTACCTAC   
  
  
- TGAGACAACG TTCGGGATCG GTGTTTTGGA CCTTCTGGGG GGTGACACTC TTAATGACCG TAACTACTAG   
  
  
- GGCAGAGATC CGTACGAGCC CCTCGAACGA ACCTCCGTCA CCCCTTTGCA AACCGTCAAG ACAGCCTCTT   
  
  
- TAAATTGTAC GGGTAACTCA AAGTCCGTCA CGGGCAAATA CGAAGACTCC AGTGGGTTCT TTACGAACTA   
  
  
- CACTCCGGTC CCACCCGGAA CCGACACTTG AAGGGTAACG TCGAGGTGGT GTGAGGGCTA CTCTCACAAC   
  
  
- TACACTTGTT GGGCTCCCTA CCTGAGGATT CTTACCACTT TAGTGAGCCA GGGTTCCAAC ATTGAAACCA   
  
  
- TCTTGTTCTC AGTTTGTGGT TGTGATGGGG AAACGACTGT TCCAAGTATC TTTGCGACCT GAAGATGAGT   
  
  
- CGTTACAAAC TTAGGTAGCT ACACTGTTAC GGTTCCCTGG CTTTCCTTTC CTAGTTACAG CTCGTCGTAA   
  
  
- CGGATCGGTT CCTGTAACAG TTGTAGTAGC GCACACTCCC CTTCCTGTCC CACCTCGCTG TACTCGAGAA   
  
  
- ACCCTTTACC TTCAGTTCCA AGTGGTACCG TCCTAAGGTT GTTAGGGGTA ACTCGAGCAT ACAATTGAGG   
  
  
- CACTATTTTC CTAATGAATC CACAATAAGT CTCATGATAT GCAACCACCT CTTCCTACCG CGGAACGAAG   
  
  
- ACCCTACCTT CCTGGCCTAC GACCAAAGCC GAAGTCGAAC CGTAAC

+     TCA-element

| Site Name | Organism | Position | Strand | Matrix score. | sequence | function |
| --- | --- | --- | --- | --- | --- | --- |
| TCA-element | Nicotiana tabacum | 1225 | - | 9 | CCATCTTTTT | cis-acting element involved in salicylic acid responsiveness |

>HU01G01850.1   
+ +Up\_Stream \_Len000TTTAAA TATTTTATTT TATTTTAAAT TAATATATTT TATAATTTAA AATTATTAAT   
  
  
+ AATTGATGAT GTTACCCAAT GTTTAAGTTT TTAAATATTT TTATTTTTTA ATTTAAAAAT ATCATAATAT   
  
  
+ ATAATATAAT TTTAAGTTAA AATAAGATAG TTAAATTATT TTATAGTAAA TATTTATTAT TTTTTTTAAG   
  
  
+ AAAATTGCCA TGTGATTGTC ATCAGTCAAA CACTGCGTTG ACAGAAGCTG TCCTTGCTGC TACTCTCACC   
  
  
+ TCTCAACATC GGACCCGCGC AGACGTTAAC ATCTCCGTAC AAATACAATT CCCTGGCCAA AATTCAAATC   
  
  
+ AACCCTACTC GGTAGTCGGT TCCTTCTTCT TCCTGCCCTC TATATATACA CCCACAGCTG CGCTGCATGT   
  
  
+ TTTTCCGAGA ATCCTCAAAA ATCTTATAAA CTTCCTCGTG TGGGTAGAAA AAAGAAAAAG AAAAGGGTTT   
  
  
+ TAAAATCTTG GGTCAAAACC CATCAACACC ACAATTTGAC TCTTTCTTTT TAATTCAACC ACCGCCAGTT   
  
  
+ CCTCGTTGAT TTTCAGGTAC CTCTTTTTCT TGCCCCTTTT TTTGCTCGGT TGTTGTTTAA TGGTAAAGAT   
  
  
+ TGAGTACAAA GAATGATATT TGATGAGTTG GTGTGTTTAT TTGATGAGCC CAGATGATTT AACTTGTAGA   
  
  
+ TTTTGTGGTT TTGGTTTCTG GGTATTCATT GGTTTGCTGA ATTTCTGGTT CATGATCTGG GGTTAGAAAA   
  
  
+ GTTGAAATGA CTTTCTGGAG GTTGGAATTT GACTAAAGCT AATTTCTTTT TGTCTTCAAT TCTTTTTTAG   
  
  
+ TTGTGAGCTG AATTTGTTGA CCACTTCCAG CCTGTTGGAA ATTAAACTTA CTTAGGAGAA GAGAATCTCA   
  
  
+ GATTCTGAGC AAAAGCTTTA TATGGTTGTT TGGGAAATGG TAGGAACTAA GTGAAAACAC TGAAATTTTA   
  
  
+ AGCAAGCAAT TTTGTTCATT TGATTGGTCG GATGGTCTGA TTTAGGTTCT TTTCTTGGTC TAAATTTCTG   
  
  
+ AGCATTGATT GTTTAATTGC GTGGGGAACT TGTATCCTTG TGAGTTCCTG GTTCATTTCT TGGTTGCTTA   
  
  
+ CTCTAGCTCC CTTTCACAGT TTCTATGGTT GGAACTTGGA ACTTGGAACT TGGAAGCCTA ATTTAGTTGA   
  
  
+ ATATCTTGGC TTTGAGTTAG CTTTTCTTAG GAAAAGATGG TTCTGCTCTC GAAAATCTAA TCTAATCGAT   
  
  
+ AGATGCGTTA AGGAAGCAAA ACTTTTGGGG AGAGGGAAGT TAAGCGAAGC AAGCTGCTGT TTCAAAGTTG   
  
  
+ GACTGTTTTA TTGGTAAGTA TGAAACAATA GTTTATAAGA GATCATTGGG TTCTTAAGGT GATAGATCAT   
  
  
+ GGGTTATGAA TGAGCTTAGT TGTTCTTTTG CTTTCAACTG TACGTAAGGA TGATAGTTAT CCCTCATCTT   
  
  
+ GCATCAAAAA CGGCAAACCG TGATGCCAAA ACTCAAAAAC TGATTCCTCT GACAAGGCTT CTACCTATGA   
  
  
+ AAACTATGCA GATCATATCC ATTTGGCCTT CCCTCTAGGA ACCAAGTAGA GTAAGTTGGT AAATAAATAG   
  
  
+ AGTTGAACGA AACACTACAG ATTTGAATTT GATTAAGGAG GGCTATTATA GACTAAGGTG TAGCTCAAAT   
  
  
+ CCTTCTGTAG GGATTGTCTT ATTGGCTTCT TTTGGTTGCT TCTGGCTCAA GTCCTCTTAT TAGTTAGTAT   
  
  
+ GCATTACCTT GAGACAAGAT AGTTGCTTAA GCTTGAGCTT CTGTACTTAT TTGCGTATTG TAGACAACTA   
  
  
+ GCTGTGGCAT ATGATTCTGA AATGTTCCGA TGTCTTTTTC TTTGGATGCT GGGTTGTGTC ATGAGCTTTA   
  
  
+ GGTTTGTTTA TTTGATGTCA GGAAGTATGT AAATAACATG TGGCATCTTG CAATCTATTA CAGGATGATA   
  
  
+ TTGGCCTTTG TGGGAAGAAA TTGAAGAAGT TGTTTCACTT ACTGGATAGG GAGCATGGAC TCACATCAGC   
  
  
+ TTTTGAGATT CGGTATTTCC AGATCATACT CATCCTATAA TTTCTCTCAG TCTAGTCCCC AATCAATTCC   
  
  
+ AAATAGGTTA TTTGAATCAC CGAACGTTCG TTCTAGAGAC TCTCCAATCT CGCCCTTCTC AACGCCCTTC   
  
  
+ AATTGCGACC CCACTGTTAT ATTGAGCGAC AGTCAGGAGC ACCACAGTTC AACAGGAAGT CTCTCGGCAC   
  
  
+ AAAGCTCATC TTCTAATTCT CCCCTTGAGA CTAGCAGTTA TTATAATCAG TTCAACTCAA GCCCTGTTGG   
  
  
+ AGAATCACCT CAGGGATCAT CACCCGAGAA TCTTTTTCAA CAAGCTGCAG TTAATAAAGT CAGCATCGAG   
  
  
+ CATGCATTGC AGGAGCTAGA AACTGCTCTA ATGAGCACAG ATTGTGAGGA GAATGAAGCA ACTGTCTCTA   
  
  
+ TCCCATCTAT GGGTGAACCT CATCAGCCCC AAGTCCCTAG CCAGAGATCA AGATTCTGGA ATCGAGATCC   
  
  
+ TCAGGGTTCA CGCCCGGCTG AAGTTCACTC ATCATTGAGA AGATTAGGAG ACGAGGCTCA GAGTGAGAAA   
  
  
+ CGCCTCAAGG CAGTGGAAGA ACCAATACGG CCCAGTGCAC CACCCGGCAA TTTGAAGCAG TTGCTCATAG   
  
  
+ AATGTGCTCG GGCTTTGTCA GAGAATCGAA TAGAGGATTT TGAGAAGTTA GTTGAACAGG CAAAGGGCAT   
  
  
+ GGTCTCCATT TCTGGAGACC CAATTCAACG ACTTGGTGCT TACTTGATCG AGGGGCTAGT GGCAAGGAAG   
  
  
+ GAGTCTTCAG GTACGAAAAT CTACCGAGCT CTTAGGTGCA AAGAGCCCCT TGGTCAAGAC TTGCTTTCGT   
  
  
+ ACATGCACAT CCTTTATGAA ATTTGCCCTT ACTTGAAATT CGGTTATATG GTTGCAAATG GGGCTATAGC   
  
  
+ TGAAGCTTGC AGAGATGAGG ATCACATACA TATCGTCGAC TACCAGATTG GTCAAGGAAC TCAATGGATG   
  
  
+ ACTCTGTTGC AAGCCCTAGC CACAAAACCT GGAAGACCCC CCACTGTGAG AATTACTGGC ATTGATGATC   
  
  
+ CCGTCTCTAG GCATGCTCGG GGAGCTTGCT TGGAGGCAGT GGGGAAACGT TTGGCAGTTC TGTCGGAGAA   
  
  
+ ATTTAACATG CCCATTGAGT TTCAGGCAGT GCCCGTTTAT GCTTCTGAGG TCACCCAAGA AATGCTTGAT   
  
  
+ GTGAGGCCAG GGTGGGCCTT GGCTGTGAAC TTCCCATTGC AGCTCCACCA CACTCCCGAT GAGAGTGTTG   
  
  
+ ATGTGAACAA CCCGAGGGAT GGACTCCTAA GAATGGTGAA ATCACTCGGT CCCAAGGTTG TAACTTTGGT   
  
  
+ AGAACAAGAG TCAAACACCA ACACTACCCC TTTGCTGACA AGGTTCATAG AAACGCTGGA CTTCTACTCA   
  
  
+ GCAATGTTTG AATCCATCGA TGTGACAATG CCAAGGGACC GAAAGGAAAG GATCAATGTC GAGCAGCATT   
  
  
+ GCCTAGCCAA GGACATTGTC AACATCATCG CGTGTGAGGG GAAGGACAGG GTGGAGCGAC ATGAGCTCTT   
  
  
+ TGGGAAATGG AAGTCAAGGT TCACCATGGC AGGATTCCAA CAATCCCCAT TGAGCTCGTA TGTTAACTCC   
  
  
+ GTGATAAAAG GATTACTTAG GTGTTATTCA GAGTACTATA CGTTGGTGGA GAAGGATGGC GCCTTGCTTC   
  
  
+ TGGGATGGAA GGACCGGATG CTGGTTTCGG CTTCAGCTTG GCATTG  

- +Up\_Stream \_Len000AAATTT ATAAAATAAA ATAAAATTTA ATTATATAAA ATATTAAATT TTAATAATTA   
  
  
- TTAACTACTA CAATGGGTTA CAAATTCAAA AATTTATAAA AATAAAAAAT TAAATTTTTA TAGTATTATA   
  
  
- TATTATATTA AAATTCAATT TTATTCTATC AATTTAATAA AATATCATTT ATAAATAATA AAAAAAATTC   
  
  
- TTTTAACGGT ACACTAACAG TAGTCAGTTT GTGACGCAAC TGTCTTCGAC AGGAACGACG ATGAGAGTGG   
  
  
- AGAGTTGTAG CCTGGGCGCG TCTGCAATTG TAGAGGCATG TTTATGTTAA GGGACCGGTT TTAAGTTTAG   
  
  
- TTGGGATGAG CCATCAGCCA AGGAAGAAGA AGGACGGGAG ATATATATGT GGGTGTCGAC GCGACGTACA   
  
  
- AAAAGGCTCT TAGGAGTTTT TAGAATATTT GAAGGAGCAC ACCCATCTTT TTTCTTTTTC TTTTCCCAAA   
  
  
- ATTTTAGAAC CCAGTTTTGG GTAGTTGTGG TGTTAAACTG AGAAAGAAAA ATTAAGTTGG TGGCGGTCAA   
  
  
- GGAGCAACTA AAAGTCCATG GAGAAAAAGA ACGGGGAAAA AAACGAGCCA ACAACAAATT ACCATTTCTA   
  
  
- ACTCATGTTT CTTACTATAA ACTACTCAAC CACACAAATA AACTACTCGG GTCTACTAAA TTGAACATCT   
  
  
- AAAACACCAA AACCAAAGAC CCATAAGTAA CCAAACGACT TAAAGACCAA GTACTAGACC CCAATCTTTT   
  
  
- CAACTTTACT GAAAGACCTC CAACCTTAAA CTGATTTCGA TTAAAGAAAA ACAGAAGTTA AGAAAAAATC   
  
  
- AACACTCGAC TTAAACAACT GGTGAAGGTC GGACAACCTT TAATTTGAAT GAATCCTCTT CTCTTAGAGT   
  
  
- CTAAGACTCG TTTTCGAAAT ATACCAACAA ACCCTTTACC ATCCTTGATT CACTTTTGTG ACTTTAAAAT   
  
  
- TCGTTCGTTA AAACAAGTAA ACTAACCAGC CTACCAGACT AAATCCAAGA AAAGAACCAG ATTTAAAGAC   
  
  
- TCGTAACTAA CAAATTAACG CACCCCTTGA ACATAGGAAC ACTCAAGGAC CAAGTAAAGA ACCAACGAAT   
  
  
- GAGATCGAGG GAAAGTGTCA AAGATACCAA CCTTGAACCT TGAACCTTGA ACCTTCGGAT TAAATCAACT   
  
  
- TATAGAACCG AAACTCAATC GAAAAGAATC CTTTTCTACC AAGACGAGAG CTTTTAGATT AGATTAGCTA   
  
  
- TCTACGCAAT TCCTTCGTTT TGAAAACCCC TCTCCCTTCA ATTCGCTTCG TTCGACGACA AAGTTTCAAC   
  
  
- CTGACAAAAT AACCATTCAT ACTTTGTTAT CAAATATTCT CTAGTAACCC AAGAATTCCA CTATCTAGTA   
  
  
- CCCAATACTT ACTCGAATCA ACAAGAAAAC GAAAGTTGAC ATGCATTCCT ACTATCAATA GGGAGTAGAA   
  
  
- CGTAGTTTTT GCCGTTTGGC ACTACGGTTT TGAGTTTTTG ACTAAGGAGA CTGTTCCGAA GATGGATACT   
  
  
- TTTGATACGT CTAGTATAGG TAAACCGGAA GGGAGATCCT TGGTTCATCT CATTCAACCA TTTATTTATC   
  
  
- TCAACTTGCT TTGTGATGTC TAAACTTAAA CTAATTCCTC CCGATAATAT CTGATTCCAC ATCGAGTTTA   
  
  
- GGAAGACATC CCTAACAGAA TAACCGAAGA AAACCAACGA AGACCGAGTT CAGGAGAATA ATCAATCATA   
  
  
- CGTAATGGAA CTCTGTTCTA TCAACGAATT CGAACTCGAA GACATGAATA AACGCATAAC ATCTGTTGAT   
  
  
- CGACACCGTA TACTAAGACT TTACAAGGCT ACAGAAAAAG AAACCTACGA CCCAACACAG TACTCGAAAT   
  
  
- CCAAACAAAT AAACTACAGT CCTTCATACA TTTATTGTAC ACCGTAGAAC GTTAGATAAT GTCCTACTAT   
  
  
- AACCGGAAAC ACCCTTCTTT AACTTCTTCA ACAAAGTGAA TGACCTATCC CTCGTACCTG AGTGTAGTCG   
  
  
- AAAACTCTAA GCCATAAAGG TCTAGTATGA GTAGGATATT AAAGAGAGTC AGATCAGGGG TTAGTTAAGG   
  
  
- TTTATCCAAT AAACTTAGTG GCTTGCAAGC AAGATCTCTG AGAGGTTAGA GCGGGAAGAG TTGCGGGAAG   
  
  
- TTAACGCTGG GGTGACAATA TAACTCGCTG TCAGTCCTCG TGGTGTCAAG TTGTCCTTCA GAGAGCCGTG   
  
  
- TTTCGAGTAG AAGATTAAGA GGGGAACTCT GATCGTCAAT AATATTAGTC AAGTTGAGTT CGGGACAACC   
  
  
- TCTTAGTGGA GTCCCTAGTA GTGGGCTCTT AGAAAAAGTT GTTCGACGTC AATTATTTCA GTCGTAGCTC   
  
  
- GTACGTAACG TCCTCGATCT TTGACGAGAT TACTCGTGTC TAACACTCCT CTTACTTCGT TGACAGAGAT   
  
  
- AGGGTAGATA CCCACTTGGA GTAGTCGGGG TTCAGGGATC GGTCTCTAGT TCTAAGACCT TAGCTCTAGG   
  
  
- AGTCCCAAGT GCGGGCCGAC TTCAAGTGAG TAGTAACTCT TCTAATCCTC TGCTCCGAGT CTCACTCTTT   
  
  
- GCGGAGTTCC GTCACCTTCT TGGTTATGCC GGGTCACGTG GTGGGCCGTT AAACTTCGTC AACGAGTATC   
  
  
- TTACACGAGC CCGAAACAGT CTCTTAGCTT ATCTCCTAAA ACTCTTCAAT CAACTTGTCC GTTTCCCGTA   
  
  
- CCAGAGGTAA AGACCTCTGG GTTAAGTTGC TGAACCACGA ATGAACTAGC TCCCCGATCA CCGTTCCTTC   
  
  
- CTCAGAAGTC CATGCTTTTA GATGGCTCGA GAATCCACGT TTCTCGGGGA ACCAGTTCTG AACGAAAGCA   
  
  
- TGTACGTGTA GGAAATACTT TAAACGGGAA TGAACTTTAA GCCAATATAC CAACGTTTAC CCCGATATCG   
  
  
- ACTTCGAACG TCTCTACTCC TAGTGTATGT ATAGCAGCTG ATGGTCTAAC CAGTTCCTTG AGTTACCTAC   
  
  
- TGAGACAACG TTCGGGATCG GTGTTTTGGA CCTTCTGGGG GGTGACACTC TTAATGACCG TAACTACTAG   
  
  
- GGCAGAGATC CGTACGAGCC CCTCGAACGA ACCTCCGTCA CCCCTTTGCA AACCGTCAAG ACAGCCTCTT   
  
  
- TAAATTGTAC GGGTAACTCA AAGTCCGTCA CGGGCAAATA CGAAGACTCC AGTGGGTTCT TTACGAACTA   
  
  
- CACTCCGGTC CCACCCGGAA CCGACACTTG AAGGGTAACG TCGAGGTGGT GTGAGGGCTA CTCTCACAAC   
  
  
- TACACTTGTT GGGCTCCCTA CCTGAGGATT CTTACCACTT TAGTGAGCCA GGGTTCCAAC ATTGAAACCA   
  
  
- TCTTGTTCTC AGTTTGTGGT TGTGATGGGG AAACGACTGT TCCAAGTATC TTTGCGACCT GAAGATGAGT   
  
  
- CGTTACAAAC TTAGGTAGCT ACACTGTTAC GGTTCCCTGG CTTTCCTTTC CTAGTTACAG CTCGTCGTAA   
  
  
- CGGATCGGTT CCTGTAACAG TTGTAGTAGC GCACACTCCC CTTCCTGTCC CACCTCGCTG TACTCGAGAA   
  
  
- ACCCTTTACC TTCAGTTCCA AGTGGTACCG TCCTAAGGTT GTTAGGGGTA ACTCGAGCAT ACAATTGAGG   
  
  
- CACTATTTTC CTAATGAATC CACAATAAGT CTCATGATAT GCAACCACCT CTTCCTACCG CGGAACGAAG   
  
  
- ACCCTACCTT CCTGGCCTAC GACCAAAGCC GAAGTCGAAC CGTAAC

+     TGA-element

| Site Name | Organism | Position | Strand | Matrix score. | sequence | function |
| --- | --- | --- | --- | --- | --- | --- |
| TGA-element | Brassica oleracea | 2761 | + | 6 | AACGAC | auxin-responsive element |

>HU01G01850.1   
+ +Up\_Stream \_Len000TTTAAA TATTTTATTT TATTTTAAAT TAATATATTT TATAATTTAA AATTATTAAT   
  
  
+ AATTGATGAT GTTACCCAAT GTTTAAGTTT TTAAATATTT TTATTTTTTA ATTTAAAAAT ATCATAATAT   
  
  
+ ATAATATAAT TTTAAGTTAA AATAAGATAG TTAAATTATT TTATAGTAAA TATTTATTAT TTTTTTTAAG   
  
  
+ AAAATTGCCA TGTGATTGTC ATCAGTCAAA CACTGCGTTG ACAGAAGCTG TCCTTGCTGC TACTCTCACC   
  
  
+ TCTCAACATC GGACCCGCGC AGACGTTAAC ATCTCCGTAC AAATACAATT CCCTGGCCAA AATTCAAATC   
  
  
+ AACCCTACTC GGTAGTCGGT TCCTTCTTCT TCCTGCCCTC TATATATACA CCCACAGCTG CGCTGCATGT   
  
  
+ TTTTCCGAGA ATCCTCAAAA ATCTTATAAA CTTCCTCGTG TGGGTAGAAA AAAGAAAAAG AAAAGGGTTT   
  
  
+ TAAAATCTTG GGTCAAAACC CATCAACACC ACAATTTGAC TCTTTCTTTT TAATTCAACC ACCGCCAGTT   
  
  
+ CCTCGTTGAT TTTCAGGTAC CTCTTTTTCT TGCCCCTTTT TTTGCTCGGT TGTTGTTTAA TGGTAAAGAT   
  
  
+ TGAGTACAAA GAATGATATT TGATGAGTTG GTGTGTTTAT TTGATGAGCC CAGATGATTT AACTTGTAGA   
  
  
+ TTTTGTGGTT TTGGTTTCTG GGTATTCATT GGTTTGCTGA ATTTCTGGTT CATGATCTGG GGTTAGAAAA   
  
  
+ GTTGAAATGA CTTTCTGGAG GTTGGAATTT GACTAAAGCT AATTTCTTTT TGTCTTCAAT TCTTTTTTAG   
  
  
+ TTGTGAGCTG AATTTGTTGA CCACTTCCAG CCTGTTGGAA ATTAAACTTA CTTAGGAGAA GAGAATCTCA   
  
  
+ GATTCTGAGC AAAAGCTTTA TATGGTTGTT TGGGAAATGG TAGGAACTAA GTGAAAACAC TGAAATTTTA   
  
  
+ AGCAAGCAAT TTTGTTCATT TGATTGGTCG GATGGTCTGA TTTAGGTTCT TTTCTTGGTC TAAATTTCTG   
  
  
+ AGCATTGATT GTTTAATTGC GTGGGGAACT TGTATCCTTG TGAGTTCCTG GTTCATTTCT TGGTTGCTTA   
  
  
+ CTCTAGCTCC CTTTCACAGT TTCTATGGTT GGAACTTGGA ACTTGGAACT TGGAAGCCTA ATTTAGTTGA   
  
  
+ ATATCTTGGC TTTGAGTTAG CTTTTCTTAG GAAAAGATGG TTCTGCTCTC GAAAATCTAA TCTAATCGAT   
  
  
+ AGATGCGTTA AGGAAGCAAA ACTTTTGGGG AGAGGGAAGT TAAGCGAAGC AAGCTGCTGT TTCAAAGTTG   
  
  
+ GACTGTTTTA TTGGTAAGTA TGAAACAATA GTTTATAAGA GATCATTGGG TTCTTAAGGT GATAGATCAT   
  
  
+ GGGTTATGAA TGAGCTTAGT TGTTCTTTTG CTTTCAACTG TACGTAAGGA TGATAGTTAT CCCTCATCTT   
  
  
+ GCATCAAAAA CGGCAAACCG TGATGCCAAA ACTCAAAAAC TGATTCCTCT GACAAGGCTT CTACCTATGA   
  
  
+ AAACTATGCA GATCATATCC ATTTGGCCTT CCCTCTAGGA ACCAAGTAGA GTAAGTTGGT AAATAAATAG   
  
  
+ AGTTGAACGA AACACTACAG ATTTGAATTT GATTAAGGAG GGCTATTATA GACTAAGGTG TAGCTCAAAT   
  
  
+ CCTTCTGTAG GGATTGTCTT ATTGGCTTCT TTTGGTTGCT TCTGGCTCAA GTCCTCTTAT TAGTTAGTAT   
  
  
+ GCATTACCTT GAGACAAGAT AGTTGCTTAA GCTTGAGCTT CTGTACTTAT TTGCGTATTG TAGACAACTA   
  
  
+ GCTGTGGCAT ATGATTCTGA AATGTTCCGA TGTCTTTTTC TTTGGATGCT GGGTTGTGTC ATGAGCTTTA   
  
  
+ GGTTTGTTTA TTTGATGTCA GGAAGTATGT AAATAACATG TGGCATCTTG CAATCTATTA CAGGATGATA   
  
  
+ TTGGCCTTTG TGGGAAGAAA TTGAAGAAGT TGTTTCACTT ACTGGATAGG GAGCATGGAC TCACATCAGC   
  
  
+ TTTTGAGATT CGGTATTTCC AGATCATACT CATCCTATAA TTTCTCTCAG TCTAGTCCCC AATCAATTCC   
  
  
+ AAATAGGTTA TTTGAATCAC CGAACGTTCG TTCTAGAGAC TCTCCAATCT CGCCCTTCTC AACGCCCTTC   
  
  
+ AATTGCGACC CCACTGTTAT ATTGAGCGAC AGTCAGGAGC ACCACAGTTC AACAGGAAGT CTCTCGGCAC   
  
  
+ AAAGCTCATC TTCTAATTCT CCCCTTGAGA CTAGCAGTTA TTATAATCAG TTCAACTCAA GCCCTGTTGG   
  
  
+ AGAATCACCT CAGGGATCAT CACCCGAGAA TCTTTTTCAA CAAGCTGCAG TTAATAAAGT CAGCATCGAG   
  
  
+ CATGCATTGC AGGAGCTAGA AACTGCTCTA ATGAGCACAG ATTGTGAGGA GAATGAAGCA ACTGTCTCTA   
  
  
+ TCCCATCTAT GGGTGAACCT CATCAGCCCC AAGTCCCTAG CCAGAGATCA AGATTCTGGA ATCGAGATCC   
  
  
+ TCAGGGTTCA CGCCCGGCTG AAGTTCACTC ATCATTGAGA AGATTAGGAG ACGAGGCTCA GAGTGAGAAA   
  
  
+ CGCCTCAAGG CAGTGGAAGA ACCAATACGG CCCAGTGCAC CACCCGGCAA TTTGAAGCAG TTGCTCATAG   
  
  
+ AATGTGCTCG GGCTTTGTCA GAGAATCGAA TAGAGGATTT TGAGAAGTTA GTTGAACAGG CAAAGGGCAT   
  
  
+ GGTCTCCATT TCTGGAGACC CAATTCAACG ACTTGGTGCT TACTTGATCG AGGGGCTAGT GGCAAGGAAG   
  
  
+ GAGTCTTCAG GTACGAAAAT CTACCGAGCT CTTAGGTGCA AAGAGCCCCT TGGTCAAGAC TTGCTTTCGT   
  
  
+ ACATGCACAT CCTTTATGAA ATTTGCCCTT ACTTGAAATT CGGTTATATG GTTGCAAATG GGGCTATAGC   
  
  
+ TGAAGCTTGC AGAGATGAGG ATCACATACA TATCGTCGAC TACCAGATTG GTCAAGGAAC TCAATGGATG   
  
  
+ ACTCTGTTGC AAGCCCTAGC CACAAAACCT GGAAGACCCC CCACTGTGAG AATTACTGGC ATTGATGATC   
  
  
+ CCGTCTCTAG GCATGCTCGG GGAGCTTGCT TGGAGGCAGT GGGGAAACGT TTGGCAGTTC TGTCGGAGAA   
  
  
+ ATTTAACATG CCCATTGAGT TTCAGGCAGT GCCCGTTTAT GCTTCTGAGG TCACCCAAGA AATGCTTGAT   
  
  
+ GTGAGGCCAG GGTGGGCCTT GGCTGTGAAC TTCCCATTGC AGCTCCACCA CACTCCCGAT GAGAGTGTTG   
  
  
+ ATGTGAACAA CCCGAGGGAT GGACTCCTAA GAATGGTGAA ATCACTCGGT CCCAAGGTTG TAACTTTGGT   
  
  
+ AGAACAAGAG TCAAACACCA ACACTACCCC TTTGCTGACA AGGTTCATAG AAACGCTGGA CTTCTACTCA   
  
  
+ GCAATGTTTG AATCCATCGA TGTGACAATG CCAAGGGACC GAAAGGAAAG GATCAATGTC GAGCAGCATT   
  
  
+ GCCTAGCCAA GGACATTGTC AACATCATCG CGTGTGAGGG GAAGGACAGG GTGGAGCGAC ATGAGCTCTT   
  
  
+ TGGGAAATGG AAGTCAAGGT TCACCATGGC AGGATTCCAA CAATCCCCAT TGAGCTCGTA TGTTAACTCC   
  
  
+ GTGATAAAAG GATTACTTAG GTGTTATTCA GAGTACTATA CGTTGGTGGA GAAGGATGGC GCCTTGCTTC   
  
  
+ TGGGATGGAA GGACCGGATG CTGGTTTCGG CTTCAGCTTG GCATTG  

- +Up\_Stream \_Len000AAATTT ATAAAATAAA ATAAAATTTA ATTATATAAA ATATTAAATT TTAATAATTA   
  
  
- TTAACTACTA CAATGGGTTA CAAATTCAAA AATTTATAAA AATAAAAAAT TAAATTTTTA TAGTATTATA   
  
  
- TATTATATTA AAATTCAATT TTATTCTATC AATTTAATAA AATATCATTT ATAAATAATA AAAAAAATTC   
  
  
- TTTTAACGGT ACACTAACAG TAGTCAGTTT GTGACGCAAC TGTCTTCGAC AGGAACGACG ATGAGAGTGG   
  
  
- AGAGTTGTAG CCTGGGCGCG TCTGCAATTG TAGAGGCATG TTTATGTTAA GGGACCGGTT TTAAGTTTAG   
  
  
- TTGGGATGAG CCATCAGCCA AGGAAGAAGA AGGACGGGAG ATATATATGT GGGTGTCGAC GCGACGTACA   
  
  
- AAAAGGCTCT TAGGAGTTTT TAGAATATTT GAAGGAGCAC ACCCATCTTT TTTCTTTTTC TTTTCCCAAA   
  
  
- ATTTTAGAAC CCAGTTTTGG GTAGTTGTGG TGTTAAACTG AGAAAGAAAA ATTAAGTTGG TGGCGGTCAA   
  
  
- GGAGCAACTA AAAGTCCATG GAGAAAAAGA ACGGGGAAAA AAACGAGCCA ACAACAAATT ACCATTTCTA   
  
  
- ACTCATGTTT CTTACTATAA ACTACTCAAC CACACAAATA AACTACTCGG GTCTACTAAA TTGAACATCT   
  
  
- AAAACACCAA AACCAAAGAC CCATAAGTAA CCAAACGACT TAAAGACCAA GTACTAGACC CCAATCTTTT   
  
  
- CAACTTTACT GAAAGACCTC CAACCTTAAA CTGATTTCGA TTAAAGAAAA ACAGAAGTTA AGAAAAAATC   
  
  
- AACACTCGAC TTAAACAACT GGTGAAGGTC GGACAACCTT TAATTTGAAT GAATCCTCTT CTCTTAGAGT   
  
  
- CTAAGACTCG TTTTCGAAAT ATACCAACAA ACCCTTTACC ATCCTTGATT CACTTTTGTG ACTTTAAAAT   
  
  
- TCGTTCGTTA AAACAAGTAA ACTAACCAGC CTACCAGACT AAATCCAAGA AAAGAACCAG ATTTAAAGAC   
  
  
- TCGTAACTAA CAAATTAACG CACCCCTTGA ACATAGGAAC ACTCAAGGAC CAAGTAAAGA ACCAACGAAT   
  
  
- GAGATCGAGG GAAAGTGTCA AAGATACCAA CCTTGAACCT TGAACCTTGA ACCTTCGGAT TAAATCAACT   
  
  
- TATAGAACCG AAACTCAATC GAAAAGAATC CTTTTCTACC AAGACGAGAG CTTTTAGATT AGATTAGCTA   
  
  
- TCTACGCAAT TCCTTCGTTT TGAAAACCCC TCTCCCTTCA ATTCGCTTCG TTCGACGACA AAGTTTCAAC   
  
  
- CTGACAAAAT AACCATTCAT ACTTTGTTAT CAAATATTCT CTAGTAACCC AAGAATTCCA CTATCTAGTA   
  
  
- CCCAATACTT ACTCGAATCA ACAAGAAAAC GAAAGTTGAC ATGCATTCCT ACTATCAATA GGGAGTAGAA   
  
  
- CGTAGTTTTT GCCGTTTGGC ACTACGGTTT TGAGTTTTTG ACTAAGGAGA CTGTTCCGAA GATGGATACT   
  
  
- TTTGATACGT CTAGTATAGG TAAACCGGAA GGGAGATCCT TGGTTCATCT CATTCAACCA TTTATTTATC   
  
  
- TCAACTTGCT TTGTGATGTC TAAACTTAAA CTAATTCCTC CCGATAATAT CTGATTCCAC ATCGAGTTTA   
  
  
- GGAAGACATC CCTAACAGAA TAACCGAAGA AAACCAACGA AGACCGAGTT CAGGAGAATA ATCAATCATA   
  
  
- CGTAATGGAA CTCTGTTCTA TCAACGAATT CGAACTCGAA GACATGAATA AACGCATAAC ATCTGTTGAT   
  
  
- CGACACCGTA TACTAAGACT TTACAAGGCT ACAGAAAAAG AAACCTACGA CCCAACACAG TACTCGAAAT   
  
  
- CCAAACAAAT AAACTACAGT CCTTCATACA TTTATTGTAC ACCGTAGAAC GTTAGATAAT GTCCTACTAT   
  
  
- AACCGGAAAC ACCCTTCTTT AACTTCTTCA ACAAAGTGAA TGACCTATCC CTCGTACCTG AGTGTAGTCG   
  
  
- AAAACTCTAA GCCATAAAGG TCTAGTATGA GTAGGATATT AAAGAGAGTC AGATCAGGGG TTAGTTAAGG   
  
  
- TTTATCCAAT AAACTTAGTG GCTTGCAAGC AAGATCTCTG AGAGGTTAGA GCGGGAAGAG TTGCGGGAAG   
  
  
- TTAACGCTGG GGTGACAATA TAACTCGCTG TCAGTCCTCG TGGTGTCAAG TTGTCCTTCA GAGAGCCGTG   
  
  
- TTTCGAGTAG AAGATTAAGA GGGGAACTCT GATCGTCAAT AATATTAGTC AAGTTGAGTT CGGGACAACC   
  
  
- TCTTAGTGGA GTCCCTAGTA GTGGGCTCTT AGAAAAAGTT GTTCGACGTC AATTATTTCA GTCGTAGCTC   
  
  
- GTACGTAACG TCCTCGATCT TTGACGAGAT TACTCGTGTC TAACACTCCT CTTACTTCGT TGACAGAGAT   
  
  
- AGGGTAGATA CCCACTTGGA GTAGTCGGGG TTCAGGGATC GGTCTCTAGT TCTAAGACCT TAGCTCTAGG   
  
  
- AGTCCCAAGT GCGGGCCGAC TTCAAGTGAG TAGTAACTCT TCTAATCCTC TGCTCCGAGT CTCACTCTTT   
  
  
- GCGGAGTTCC GTCACCTTCT TGGTTATGCC GGGTCACGTG GTGGGCCGTT AAACTTCGTC AACGAGTATC   
  
  
- TTACACGAGC CCGAAACAGT CTCTTAGCTT ATCTCCTAAA ACTCTTCAAT CAACTTGTCC GTTTCCCGTA   
  
  
- CCAGAGGTAA AGACCTCTGG GTTAAGTTGC TGAACCACGA ATGAACTAGC TCCCCGATCA CCGTTCCTTC   
  
  
- CTCAGAAGTC CATGCTTTTA GATGGCTCGA GAATCCACGT TTCTCGGGGA ACCAGTTCTG AACGAAAGCA   
  
  
- TGTACGTGTA GGAAATACTT TAAACGGGAA TGAACTTTAA GCCAATATAC CAACGTTTAC CCCGATATCG   
  
  
- ACTTCGAACG TCTCTACTCC TAGTGTATGT ATAGCAGCTG ATGGTCTAAC CAGTTCCTTG AGTTACCTAC   
  
  
- TGAGACAACG TTCGGGATCG GTGTTTTGGA CCTTCTGGGG GGTGACACTC TTAATGACCG TAACTACTAG   
  
  
- GGCAGAGATC CGTACGAGCC CCTCGAACGA ACCTCCGTCA CCCCTTTGCA AACCGTCAAG ACAGCCTCTT   
  
  
- TAAATTGTAC GGGTAACTCA AAGTCCGTCA CGGGCAAATA CGAAGACTCC AGTGGGTTCT TTACGAACTA   
  
  
- CACTCCGGTC CCACCCGGAA CCGACACTTG AAGGGTAACG TCGAGGTGGT GTGAGGGCTA CTCTCACAAC   
  
  
- TACACTTGTT GGGCTCCCTA CCTGAGGATT CTTACCACTT TAGTGAGCCA GGGTTCCAAC ATTGAAACCA   
  
  
- TCTTGTTCTC AGTTTGTGGT TGTGATGGGG AAACGACTGT TCCAAGTATC TTTGCGACCT GAAGATGAGT   
  
  
- CGTTACAAAC TTAGGTAGCT ACACTGTTAC GGTTCCCTGG CTTTCCTTTC CTAGTTACAG CTCGTCGTAA   
  
  
- CGGATCGGTT CCTGTAACAG TTGTAGTAGC GCACACTCCC CTTCCTGTCC CACCTCGCTG TACTCGAGAA   
  
  
- ACCCTTTACC TTCAGTTCCA AGTGGTACCG TCCTAAGGTT GTTAGGGGTA ACTCGAGCAT ACAATTGAGG   
  
  
- CACTATTTTC CTAATGAATC CACAATAAGT CTCATGATAT GCAACCACCT CTTCCTACCG CGGAACGAAG   
  
  
- ACCCTACCTT CCTGGCCTAC GACCAAAGCC GAAGTCGAAC CGTAAC

+     Unnamed\_\_1

| Site Name | Organism | Position | Strand | Matrix score. | sequence | function |
| --- | --- | --- | --- | --- | --- | --- |
| Unnamed\_\_1 | Zea mays | 1074 | + | 5 | CGTGG |  |
| Unnamed\_\_1 | Glycine max | 1639 | + | 11 | GAATTTAATTAA | 60K protein binding site |

>HU01G01850.1   
+ +Up\_Stream \_Len000TTTAAA TATTTTATTT TATTTTAAAT TAATATATTT TATAATTTAA AATTATTAAT   
  
  
+ AATTGATGAT GTTACCCAAT GTTTAAGTTT TTAAATATTT TTATTTTTTA ATTTAAAAAT ATCATAATAT   
  
  
+ ATAATATAAT TTTAAGTTAA AATAAGATAG TTAAATTATT TTATAGTAAA TATTTATTAT TTTTTTTAAG   
  
  
+ AAAATTGCCA TGTGATTGTC ATCAGTCAAA CACTGCGTTG ACAGAAGCTG TCCTTGCTGC TACTCTCACC   
  
  
+ TCTCAACATC GGACCCGCGC AGACGTTAAC ATCTCCGTAC AAATACAATT CCCTGGCCAA AATTCAAATC   
  
  
+ AACCCTACTC GGTAGTCGGT TCCTTCTTCT TCCTGCCCTC TATATATACA CCCACAGCTG CGCTGCATGT   
  
  
+ TTTTCCGAGA ATCCTCAAAA ATCTTATAAA CTTCCTCGTG TGGGTAGAAA AAAGAAAAAG AAAAGGGTTT   
  
  
+ TAAAATCTTG GGTCAAAACC CATCAACACC ACAATTTGAC TCTTTCTTTT TAATTCAACC ACCGCCAGTT   
  
  
+ CCTCGTTGAT TTTCAGGTAC CTCTTTTTCT TGCCCCTTTT TTTGCTCGGT TGTTGTTTAA TGGTAAAGAT   
  
  
+ TGAGTACAAA GAATGATATT TGATGAGTTG GTGTGTTTAT TTGATGAGCC CAGATGATTT AACTTGTAGA   
  
  
+ TTTTGTGGTT TTGGTTTCTG GGTATTCATT GGTTTGCTGA ATTTCTGGTT CATGATCTGG GGTTAGAAAA   
  
  
+ GTTGAAATGA CTTTCTGGAG GTTGGAATTT GACTAAAGCT AATTTCTTTT TGTCTTCAAT TCTTTTTTAG   
  
  
+ TTGTGAGCTG AATTTGTTGA CCACTTCCAG CCTGTTGGAA ATTAAACTTA CTTAGGAGAA GAGAATCTCA   
  
  
+ GATTCTGAGC AAAAGCTTTA TATGGTTGTT TGGGAAATGG TAGGAACTAA GTGAAAACAC TGAAATTTTA   
  
  
+ AGCAAGCAAT TTTGTTCATT TGATTGGTCG GATGGTCTGA TTTAGGTTCT TTTCTTGGTC TAAATTTCTG   
  
  
+ AGCATTGATT GTTTAATTGC GTGGGGAACT TGTATCCTTG TGAGTTCCTG GTTCATTTCT TGGTTGCTTA   
  
  
+ CTCTAGCTCC CTTTCACAGT TTCTATGGTT GGAACTTGGA ACTTGGAACT TGGAAGCCTA ATTTAGTTGA   
  
  
+ ATATCTTGGC TTTGAGTTAG CTTTTCTTAG GAAAAGATGG TTCTGCTCTC GAAAATCTAA TCTAATCGAT   
  
  
+ AGATGCGTTA AGGAAGCAAA ACTTTTGGGG AGAGGGAAGT TAAGCGAAGC AAGCTGCTGT TTCAAAGTTG   
  
  
+ GACTGTTTTA TTGGTAAGTA TGAAACAATA GTTTATAAGA GATCATTGGG TTCTTAAGGT GATAGATCAT   
  
  
+ GGGTTATGAA TGAGCTTAGT TGTTCTTTTG CTTTCAACTG TACGTAAGGA TGATAGTTAT CCCTCATCTT   
  
  
+ GCATCAAAAA CGGCAAACCG TGATGCCAAA ACTCAAAAAC TGATTCCTCT GACAAGGCTT CTACCTATGA   
  
  
+ AAACTATGCA GATCATATCC ATTTGGCCTT CCCTCTAGGA ACCAAGTAGA GTAAGTTGGT AAATAAATAG   
  
  
+ AGTTGAACGA AACACTACAG ATTTGAATTT GATTAAGGAG GGCTATTATA GACTAAGGTG TAGCTCAAAT   
  
  
+ CCTTCTGTAG GGATTGTCTT ATTGGCTTCT TTTGGTTGCT TCTGGCTCAA GTCCTCTTAT TAGTTAGTAT   
  
  
+ GCATTACCTT GAGACAAGAT AGTTGCTTAA GCTTGAGCTT CTGTACTTAT TTGCGTATTG TAGACAACTA   
  
  
+ GCTGTGGCAT ATGATTCTGA AATGTTCCGA TGTCTTTTTC TTTGGATGCT GGGTTGTGTC ATGAGCTTTA   
  
  
+ GGTTTGTTTA TTTGATGTCA GGAAGTATGT AAATAACATG TGGCATCTTG CAATCTATTA CAGGATGATA   
  
  
+ TTGGCCTTTG TGGGAAGAAA TTGAAGAAGT TGTTTCACTT ACTGGATAGG GAGCATGGAC TCACATCAGC   
  
  
+ TTTTGAGATT CGGTATTTCC AGATCATACT CATCCTATAA TTTCTCTCAG TCTAGTCCCC AATCAATTCC   
  
  
+ AAATAGGTTA TTTGAATCAC CGAACGTTCG TTCTAGAGAC TCTCCAATCT CGCCCTTCTC AACGCCCTTC   
  
  
+ AATTGCGACC CCACTGTTAT ATTGAGCGAC AGTCAGGAGC ACCACAGTTC AACAGGAAGT CTCTCGGCAC   
  
  
+ AAAGCTCATC TTCTAATTCT CCCCTTGAGA CTAGCAGTTA TTATAATCAG TTCAACTCAA GCCCTGTTGG   
  
  
+ AGAATCACCT CAGGGATCAT CACCCGAGAA TCTTTTTCAA CAAGCTGCAG TTAATAAAGT CAGCATCGAG   
  
  
+ CATGCATTGC AGGAGCTAGA AACTGCTCTA ATGAGCACAG ATTGTGAGGA GAATGAAGCA ACTGTCTCTA   
  
  
+ TCCCATCTAT GGGTGAACCT CATCAGCCCC AAGTCCCTAG CCAGAGATCA AGATTCTGGA ATCGAGATCC   
  
  
+ TCAGGGTTCA CGCCCGGCTG AAGTTCACTC ATCATTGAGA AGATTAGGAG ACGAGGCTCA GAGTGAGAAA   
  
  
+ CGCCTCAAGG CAGTGGAAGA ACCAATACGG CCCAGTGCAC CACCCGGCAA TTTGAAGCAG TTGCTCATAG   
  
  
+ AATGTGCTCG GGCTTTGTCA GAGAATCGAA TAGAGGATTT TGAGAAGTTA GTTGAACAGG CAAAGGGCAT   
  
  
+ GGTCTCCATT TCTGGAGACC CAATTCAACG ACTTGGTGCT TACTTGATCG AGGGGCTAGT GGCAAGGAAG   
  
  
+ GAGTCTTCAG GTACGAAAAT CTACCGAGCT CTTAGGTGCA AAGAGCCCCT TGGTCAAGAC TTGCTTTCGT   
  
  
+ ACATGCACAT CCTTTATGAA ATTTGCCCTT ACTTGAAATT CGGTTATATG GTTGCAAATG GGGCTATAGC   
  
  
+ TGAAGCTTGC AGAGATGAGG ATCACATACA TATCGTCGAC TACCAGATTG GTCAAGGAAC TCAATGGATG   
  
  
+ ACTCTGTTGC AAGCCCTAGC CACAAAACCT GGAAGACCCC CCACTGTGAG AATTACTGGC ATTGATGATC   
  
  
+ CCGTCTCTAG GCATGCTCGG GGAGCTTGCT TGGAGGCAGT GGGGAAACGT TTGGCAGTTC TGTCGGAGAA   
  
  
+ ATTTAACATG CCCATTGAGT TTCAGGCAGT GCCCGTTTAT GCTTCTGAGG TCACCCAAGA AATGCTTGAT   
  
  
+ GTGAGGCCAG GGTGGGCCTT GGCTGTGAAC TTCCCATTGC AGCTCCACCA CACTCCCGAT GAGAGTGTTG   
  
  
+ ATGTGAACAA CCCGAGGGAT GGACTCCTAA GAATGGTGAA ATCACTCGGT CCCAAGGTTG TAACTTTGGT   
  
  
+ AGAACAAGAG TCAAACACCA ACACTACCCC TTTGCTGACA AGGTTCATAG AAACGCTGGA CTTCTACTCA   
  
  
+ GCAATGTTTG AATCCATCGA TGTGACAATG CCAAGGGACC GAAAGGAAAG GATCAATGTC GAGCAGCATT   
  
  
+ GCCTAGCCAA GGACATTGTC AACATCATCG CGTGTGAGGG GAAGGACAGG GTGGAGCGAC ATGAGCTCTT   
  
  
+ TGGGAAATGG AAGTCAAGGT TCACCATGGC AGGATTCCAA CAATCCCCAT TGAGCTCGTA TGTTAACTCC   
  
  
+ GTGATAAAAG GATTACTTAG GTGTTATTCA GAGTACTATA CGTTGGTGGA GAAGGATGGC GCCTTGCTTC   
  
  
+ TGGGATGGAA GGACCGGATG CTGGTTTCGG CTTCAGCTTG GCATTG  

- +Up\_Stream \_Len000AAATTT ATAAAATAAA ATAAAATTTA ATTATATAAA ATATTAAATT TTAATAATTA   
  
  
- TTAACTACTA CAATGGGTTA CAAATTCAAA AATTTATAAA AATAAAAAAT TAAATTTTTA TAGTATTATA   
  
  
- TATTATATTA AAATTCAATT TTATTCTATC AATTTAATAA AATATCATTT ATAAATAATA AAAAAAATTC   
  
  
- TTTTAACGGT ACACTAACAG TAGTCAGTTT GTGACGCAAC TGTCTTCGAC AGGAACGACG ATGAGAGTGG   
  
  
- AGAGTTGTAG CCTGGGCGCG TCTGCAATTG TAGAGGCATG TTTATGTTAA GGGACCGGTT TTAAGTTTAG   
  
  
- TTGGGATGAG CCATCAGCCA AGGAAGAAGA AGGACGGGAG ATATATATGT GGGTGTCGAC GCGACGTACA   
  
  
- AAAAGGCTCT TAGGAGTTTT TAGAATATTT GAAGGAGCAC ACCCATCTTT TTTCTTTTTC TTTTCCCAAA   
  
  
- ATTTTAGAAC CCAGTTTTGG GTAGTTGTGG TGTTAAACTG AGAAAGAAAA ATTAAGTTGG TGGCGGTCAA   
  
  
- GGAGCAACTA AAAGTCCATG GAGAAAAAGA ACGGGGAAAA AAACGAGCCA ACAACAAATT ACCATTTCTA   
  
  
- ACTCATGTTT CTTACTATAA ACTACTCAAC CACACAAATA AACTACTCGG GTCTACTAAA TTGAACATCT   
  
  
- AAAACACCAA AACCAAAGAC CCATAAGTAA CCAAACGACT TAAAGACCAA GTACTAGACC CCAATCTTTT   
  
  
- CAACTTTACT GAAAGACCTC CAACCTTAAA CTGATTTCGA TTAAAGAAAA ACAGAAGTTA AGAAAAAATC   
  
  
- AACACTCGAC TTAAACAACT GGTGAAGGTC GGACAACCTT TAATTTGAAT GAATCCTCTT CTCTTAGAGT   
  
  
- CTAAGACTCG TTTTCGAAAT ATACCAACAA ACCCTTTACC ATCCTTGATT CACTTTTGTG ACTTTAAAAT   
  
  
- TCGTTCGTTA AAACAAGTAA ACTAACCAGC CTACCAGACT AAATCCAAGA AAAGAACCAG ATTTAAAGAC   
  
  
- TCGTAACTAA CAAATTAACG CACCCCTTGA ACATAGGAAC ACTCAAGGAC CAAGTAAAGA ACCAACGAAT   
  
  
- GAGATCGAGG GAAAGTGTCA AAGATACCAA CCTTGAACCT TGAACCTTGA ACCTTCGGAT TAAATCAACT   
  
  
- TATAGAACCG AAACTCAATC GAAAAGAATC CTTTTCTACC AAGACGAGAG CTTTTAGATT AGATTAGCTA   
  
  
- TCTACGCAAT TCCTTCGTTT TGAAAACCCC TCTCCCTTCA ATTCGCTTCG TTCGACGACA AAGTTTCAAC   
  
  
- CTGACAAAAT AACCATTCAT ACTTTGTTAT CAAATATTCT CTAGTAACCC AAGAATTCCA CTATCTAGTA   
  
  
- CCCAATACTT ACTCGAATCA ACAAGAAAAC GAAAGTTGAC ATGCATTCCT ACTATCAATA GGGAGTAGAA   
  
  
- CGTAGTTTTT GCCGTTTGGC ACTACGGTTT TGAGTTTTTG ACTAAGGAGA CTGTTCCGAA GATGGATACT   
  
  
- TTTGATACGT CTAGTATAGG TAAACCGGAA GGGAGATCCT TGGTTCATCT CATTCAACCA TTTATTTATC   
  
  
- TCAACTTGCT TTGTGATGTC TAAACTTAAA CTAATTCCTC CCGATAATAT CTGATTCCAC ATCGAGTTTA   
  
  
- GGAAGACATC CCTAACAGAA TAACCGAAGA AAACCAACGA AGACCGAGTT CAGGAGAATA ATCAATCATA   
  
  
- CGTAATGGAA CTCTGTTCTA TCAACGAATT CGAACTCGAA GACATGAATA AACGCATAAC ATCTGTTGAT   
  
  
- CGACACCGTA TACTAAGACT TTACAAGGCT ACAGAAAAAG AAACCTACGA CCCAACACAG TACTCGAAAT   
  
  
- CCAAACAAAT AAACTACAGT CCTTCATACA TTTATTGTAC ACCGTAGAAC GTTAGATAAT GTCCTACTAT   
  
  
- AACCGGAAAC ACCCTTCTTT AACTTCTTCA ACAAAGTGAA TGACCTATCC CTCGTACCTG AGTGTAGTCG   
  
  
- AAAACTCTAA GCCATAAAGG TCTAGTATGA GTAGGATATT AAAGAGAGTC AGATCAGGGG TTAGTTAAGG   
  
  
- TTTATCCAAT AAACTTAGTG GCTTGCAAGC AAGATCTCTG AGAGGTTAGA GCGGGAAGAG TTGCGGGAAG   
  
  
- TTAACGCTGG GGTGACAATA TAACTCGCTG TCAGTCCTCG TGGTGTCAAG TTGTCCTTCA GAGAGCCGTG   
  
  
- TTTCGAGTAG AAGATTAAGA GGGGAACTCT GATCGTCAAT AATATTAGTC AAGTTGAGTT CGGGACAACC   
  
  
- TCTTAGTGGA GTCCCTAGTA GTGGGCTCTT AGAAAAAGTT GTTCGACGTC AATTATTTCA GTCGTAGCTC   
  
  
- GTACGTAACG TCCTCGATCT TTGACGAGAT TACTCGTGTC TAACACTCCT CTTACTTCGT TGACAGAGAT   
  
  
- AGGGTAGATA CCCACTTGGA GTAGTCGGGG TTCAGGGATC GGTCTCTAGT TCTAAGACCT TAGCTCTAGG   
  
  
- AGTCCCAAGT GCGGGCCGAC TTCAAGTGAG TAGTAACTCT TCTAATCCTC TGCTCCGAGT CTCACTCTTT   
  
  
- GCGGAGTTCC GTCACCTTCT TGGTTATGCC GGGTCACGTG GTGGGCCGTT AAACTTCGTC AACGAGTATC   
  
  
- TTACACGAGC CCGAAACAGT CTCTTAGCTT ATCTCCTAAA ACTCTTCAAT CAACTTGTCC GTTTCCCGTA   
  
  
- CCAGAGGTAA AGACCTCTGG GTTAAGTTGC TGAACCACGA ATGAACTAGC TCCCCGATCA CCGTTCCTTC   
  
  
- CTCAGAAGTC CATGCTTTTA GATGGCTCGA GAATCCACGT TTCTCGGGGA ACCAGTTCTG AACGAAAGCA   
  
  
- TGTACGTGTA GGAAATACTT TAAACGGGAA TGAACTTTAA GCCAATATAC CAACGTTTAC CCCGATATCG   
  
  
- ACTTCGAACG TCTCTACTCC TAGTGTATGT ATAGCAGCTG ATGGTCTAAC CAGTTCCTTG AGTTACCTAC   
  
  
- TGAGACAACG TTCGGGATCG GTGTTTTGGA CCTTCTGGGG GGTGACACTC TTAATGACCG TAACTACTAG   
  
  
- GGCAGAGATC CGTACGAGCC CCTCGAACGA ACCTCCGTCA CCCCTTTGCA AACCGTCAAG ACAGCCTCTT   
  
  
- TAAATTGTAC GGGTAACTCA AAGTCCGTCA CGGGCAAATA CGAAGACTCC AGTGGGTTCT TTACGAACTA   
  
  
- CACTCCGGTC CCACCCGGAA CCGACACTTG AAGGGTAACG TCGAGGTGGT GTGAGGGCTA CTCTCACAAC   
  
  
- TACACTTGTT GGGCTCCCTA CCTGAGGATT CTTACCACTT TAGTGAGCCA GGGTTCCAAC ATTGAAACCA   
  
  
- TCTTGTTCTC AGTTTGTGGT TGTGATGGGG AAACGACTGT TCCAAGTATC TTTGCGACCT GAAGATGAGT   
  
  
- CGTTACAAAC TTAGGTAGCT ACACTGTTAC GGTTCCCTGG CTTTCCTTTC CTAGTTACAG CTCGTCGTAA   
  
  
- CGGATCGGTT CCTGTAACAG TTGTAGTAGC GCACACTCCC CTTCCTGTCC CACCTCGCTG TACTCGAGAA   
  
  
- ACCCTTTACC TTCAGTTCCA AGTGGTACCG TCCTAAGGTT GTTAGGGGTA ACTCGAGCAT ACAATTGAGG   
  
  
- CACTATTTTC CTAATGAATC CACAATAAGT CTCATGATAT GCAACCACCT CTTCCTACCG CGGAACGAAG   
  
  
- ACCCTACCTT CCTGGCCTAC GACCAAAGCC GAAGTCGAAC CGTAAC

+     Unnamed\_\_4

| Site Name | Organism | Position | Strand | Matrix score. | sequence | function |
| --- | --- | --- | --- | --- | --- | --- |
| Unnamed\_\_4 | Petroselinum hortense | 317 | + | 4 | CTCC |  |
| Unnamed\_\_4 | Petroselinum hortense | 3641 | + | 4 | CTCC |  |
| Unnamed\_\_4 | Petroselinum hortense | 3692 | - | 4 | CTCC |  |
| Unnamed\_\_4 | Petroselinum hortense | 3557 | - | 4 | CTCC |  |
| Unnamed\_\_4 | Petroselinum hortense | 791 | - | 4 | CTCC |  |
| Unnamed\_\_4 | Petroselinum hortense | 3318 | + | 4 | CTCC |  |
| Unnamed\_\_4 | Petroselinum hortense | 899 | - | 4 | CTCC |  |
| Unnamed\_\_4 | Petroselinum hortense | 1131 | + | 4 | CTCC |  |
| Unnamed\_\_4 | Petroselinum hortense | 1293 | - | 4 | CTCC |  |
| Unnamed\_\_4 | Petroselinum hortense | 1651 | - | 4 | CTCC |  |
| Unnamed\_\_4 | Petroselinum hortense | 2014 | - | 4 | CTCC |  |
| Unnamed\_\_4 | Petroselinum hortense | 2146 | + | 4 | CTCC |  |
| Unnamed\_\_4 | Petroselinum hortense | 2210 | - | 4 | CTCC |  |
| Unnamed\_\_4 | Petroselinum hortense | 2263 | + | 4 | CTCC |  |
| Unnamed\_\_4 | Petroselinum hortense | 2313 | - | 4 | CTCC |  |
| Unnamed\_\_4 | Petroselinum hortense | 2396 | - | 4 | CTCC |  |
| Unnamed\_\_4 | Petroselinum hortense | 2432 | - | 4 | CTCC |  |
| Unnamed\_\_4 | Petroselinum hortense | 2571 | - | 4 | CTCC |  |
| Unnamed\_\_4 | Petroselinum hortense | 2738 | + | 4 | CTCC |  |
| Unnamed\_\_4 | Petroselinum hortense | 2748 | - | 4 | CTCC |  |
| Unnamed\_\_4 | Petroselinum hortense | 2804 | - | 4 | CTCC |  |
| Unnamed\_\_4 | Petroselinum hortense | 3105 | - | 4 | CTCC |  |
| Unnamed\_\_4 | Petroselinum hortense | 3116 | - | 4 | CTCC |  |
| Unnamed\_\_4 | Petroselinum hortense | 3149 | - | 4 | CTCC |  |
| Unnamed\_\_4 | Petroselinum hortense | 3267 | + | 4 | CTCC |  |
| Unnamed\_\_4 | Petroselinum hortense | 3277 | + | 4 | CTCC |  |

>HU01G01850.1   
+ +Up\_Stream \_Len000TTTAAA TATTTTATTT TATTTTAAAT TAATATATTT TATAATTTAA AATTATTAAT   
  
  
+ AATTGATGAT GTTACCCAAT GTTTAAGTTT TTAAATATTT TTATTTTTTA ATTTAAAAAT ATCATAATAT   
  
  
+ ATAATATAAT TTTAAGTTAA AATAAGATAG TTAAATTATT TTATAGTAAA TATTTATTAT TTTTTTTAAG   
  
  
+ AAAATTGCCA TGTGATTGTC ATCAGTCAAA CACTGCGTTG ACAGAAGCTG TCCTTGCTGC TACTCTCACC   
  
  
+ TCTCAACATC GGACCCGCGC AGACGTTAAC ATCTCCGTAC AAATACAATT CCCTGGCCAA AATTCAAATC   
  
  
+ AACCCTACTC GGTAGTCGGT TCCTTCTTCT TCCTGCCCTC TATATATACA CCCACAGCTG CGCTGCATGT   
  
  
+ TTTTCCGAGA ATCCTCAAAA ATCTTATAAA CTTCCTCGTG TGGGTAGAAA AAAGAAAAAG AAAAGGGTTT   
  
  
+ TAAAATCTTG GGTCAAAACC CATCAACACC ACAATTTGAC TCTTTCTTTT TAATTCAACC ACCGCCAGTT   
  
  
+ CCTCGTTGAT TTTCAGGTAC CTCTTTTTCT TGCCCCTTTT TTTGCTCGGT TGTTGTTTAA TGGTAAAGAT   
  
  
+ TGAGTACAAA GAATGATATT TGATGAGTTG GTGTGTTTAT TTGATGAGCC CAGATGATTT AACTTGTAGA   
  
  
+ TTTTGTGGTT TTGGTTTCTG GGTATTCATT GGTTTGCTGA ATTTCTGGTT CATGATCTGG GGTTAGAAAA   
  
  
+ GTTGAAATGA CTTTCTGGAG GTTGGAATTT GACTAAAGCT AATTTCTTTT TGTCTTCAAT TCTTTTTTAG   
  
  
+ TTGTGAGCTG AATTTGTTGA CCACTTCCAG CCTGTTGGAA ATTAAACTTA CTTAGGAGAA GAGAATCTCA   
  
  
+ GATTCTGAGC AAAAGCTTTA TATGGTTGTT TGGGAAATGG TAGGAACTAA GTGAAAACAC TGAAATTTTA   
  
  
+ AGCAAGCAAT TTTGTTCATT TGATTGGTCG GATGGTCTGA TTTAGGTTCT TTTCTTGGTC TAAATTTCTG   
  
  
+ AGCATTGATT GTTTAATTGC GTGGGGAACT TGTATCCTTG TGAGTTCCTG GTTCATTTCT TGGTTGCTTA   
  
  
+ CTCTAGCTCC CTTTCACAGT TTCTATGGTT GGAACTTGGA ACTTGGAACT TGGAAGCCTA ATTTAGTTGA   
  
  
+ ATATCTTGGC TTTGAGTTAG CTTTTCTTAG GAAAAGATGG TTCTGCTCTC GAAAATCTAA TCTAATCGAT   
  
  
+ AGATGCGTTA AGGAAGCAAA ACTTTTGGGG AGAGGGAAGT TAAGCGAAGC AAGCTGCTGT TTCAAAGTTG   
  
  
+ GACTGTTTTA TTGGTAAGTA TGAAACAATA GTTTATAAGA GATCATTGGG TTCTTAAGGT GATAGATCAT   
  
  
+ GGGTTATGAA TGAGCTTAGT TGTTCTTTTG CTTTCAACTG TACGTAAGGA TGATAGTTAT CCCTCATCTT   
  
  
+ GCATCAAAAA CGGCAAACCG TGATGCCAAA ACTCAAAAAC TGATTCCTCT GACAAGGCTT CTACCTATGA   
  
  
+ AAACTATGCA GATCATATCC ATTTGGCCTT CCCTCTAGGA ACCAAGTAGA GTAAGTTGGT AAATAAATAG   
  
  
+ AGTTGAACGA AACACTACAG ATTTGAATTT GATTAAGGAG GGCTATTATA GACTAAGGTG TAGCTCAAAT   
  
  
+ CCTTCTGTAG GGATTGTCTT ATTGGCTTCT TTTGGTTGCT TCTGGCTCAA GTCCTCTTAT TAGTTAGTAT   
  
  
+ GCATTACCTT GAGACAAGAT AGTTGCTTAA GCTTGAGCTT CTGTACTTAT TTGCGTATTG TAGACAACTA   
  
  
+ GCTGTGGCAT ATGATTCTGA AATGTTCCGA TGTCTTTTTC TTTGGATGCT GGGTTGTGTC ATGAGCTTTA   
  
  
+ GGTTTGTTTA TTTGATGTCA GGAAGTATGT AAATAACATG TGGCATCTTG CAATCTATTA CAGGATGATA   
  
  
+ TTGGCCTTTG TGGGAAGAAA TTGAAGAAGT TGTTTCACTT ACTGGATAGG GAGCATGGAC TCACATCAGC   
  
  
+ TTTTGAGATT CGGTATTTCC AGATCATACT CATCCTATAA TTTCTCTCAG TCTAGTCCCC AATCAATTCC   
  
  
+ AAATAGGTTA TTTGAATCAC CGAACGTTCG TTCTAGAGAC TCTCCAATCT CGCCCTTCTC AACGCCCTTC   
  
  
+ AATTGCGACC CCACTGTTAT ATTGAGCGAC AGTCAGGAGC ACCACAGTTC AACAGGAAGT CTCTCGGCAC   
  
  
+ AAAGCTCATC TTCTAATTCT CCCCTTGAGA CTAGCAGTTA TTATAATCAG TTCAACTCAA GCCCTGTTGG   
  
  
+ AGAATCACCT CAGGGATCAT CACCCGAGAA TCTTTTTCAA CAAGCTGCAG TTAATAAAGT CAGCATCGAG   
  
  
+ CATGCATTGC AGGAGCTAGA AACTGCTCTA ATGAGCACAG ATTGTGAGGA GAATGAAGCA ACTGTCTCTA   
  
  
+ TCCCATCTAT GGGTGAACCT CATCAGCCCC AAGTCCCTAG CCAGAGATCA AGATTCTGGA ATCGAGATCC   
  
  
+ TCAGGGTTCA CGCCCGGCTG AAGTTCACTC ATCATTGAGA AGATTAGGAG ACGAGGCTCA GAGTGAGAAA   
  
  
+ CGCCTCAAGG CAGTGGAAGA ACCAATACGG CCCAGTGCAC CACCCGGCAA TTTGAAGCAG TTGCTCATAG   
  
  
+ AATGTGCTCG GGCTTTGTCA GAGAATCGAA TAGAGGATTT TGAGAAGTTA GTTGAACAGG CAAAGGGCAT   
  
  
+ GGTCTCCATT TCTGGAGACC CAATTCAACG ACTTGGTGCT TACTTGATCG AGGGGCTAGT GGCAAGGAAG   
  
  
+ GAGTCTTCAG GTACGAAAAT CTACCGAGCT CTTAGGTGCA AAGAGCCCCT TGGTCAAGAC TTGCTTTCGT   
  
  
+ ACATGCACAT CCTTTATGAA ATTTGCCCTT ACTTGAAATT CGGTTATATG GTTGCAAATG GGGCTATAGC   
  
  
+ TGAAGCTTGC AGAGATGAGG ATCACATACA TATCGTCGAC TACCAGATTG GTCAAGGAAC TCAATGGATG   
  
  
+ ACTCTGTTGC AAGCCCTAGC CACAAAACCT GGAAGACCCC CCACTGTGAG AATTACTGGC ATTGATGATC   
  
  
+ CCGTCTCTAG GCATGCTCGG GGAGCTTGCT TGGAGGCAGT GGGGAAACGT TTGGCAGTTC TGTCGGAGAA   
  
  
+ ATTTAACATG CCCATTGAGT TTCAGGCAGT GCCCGTTTAT GCTTCTGAGG TCACCCAAGA AATGCTTGAT   
  
  
+ GTGAGGCCAG GGTGGGCCTT GGCTGTGAAC TTCCCATTGC AGCTCCACCA CACTCCCGAT GAGAGTGTTG   
  
  
+ ATGTGAACAA CCCGAGGGAT GGACTCCTAA GAATGGTGAA ATCACTCGGT CCCAAGGTTG TAACTTTGGT   
  
  
+ AGAACAAGAG TCAAACACCA ACACTACCCC TTTGCTGACA AGGTTCATAG AAACGCTGGA CTTCTACTCA   
  
  
+ GCAATGTTTG AATCCATCGA TGTGACAATG CCAAGGGACC GAAAGGAAAG GATCAATGTC GAGCAGCATT   
  
  
+ GCCTAGCCAA GGACATTGTC AACATCATCG CGTGTGAGGG GAAGGACAGG GTGGAGCGAC ATGAGCTCTT   
  
  
+ TGGGAAATGG AAGTCAAGGT TCACCATGGC AGGATTCCAA CAATCCCCAT TGAGCTCGTA TGTTAACTCC   
  
  
+ GTGATAAAAG GATTACTTAG GTGTTATTCA GAGTACTATA CGTTGGTGGA GAAGGATGGC GCCTTGCTTC   
  
  
+ TGGGATGGAA GGACCGGATG CTGGTTTCGG CTTCAGCTTG GCATTG  

- +Up\_Stream \_Len000AAATTT ATAAAATAAA ATAAAATTTA ATTATATAAA ATATTAAATT TTAATAATTA   
  
  
- TTAACTACTA CAATGGGTTA CAAATTCAAA AATTTATAAA AATAAAAAAT TAAATTTTTA TAGTATTATA   
  
  
- TATTATATTA AAATTCAATT TTATTCTATC AATTTAATAA AATATCATTT ATAAATAATA AAAAAAATTC   
  
  
- TTTTAACGGT ACACTAACAG TAGTCAGTTT GTGACGCAAC TGTCTTCGAC AGGAACGACG ATGAGAGTGG   
  
  
- AGAGTTGTAG CCTGGGCGCG TCTGCAATTG TAGAGGCATG TTTATGTTAA GGGACCGGTT TTAAGTTTAG   
  
  
- TTGGGATGAG CCATCAGCCA AGGAAGAAGA AGGACGGGAG ATATATATGT GGGTGTCGAC GCGACGTACA   
  
  
- AAAAGGCTCT TAGGAGTTTT TAGAATATTT GAAGGAGCAC ACCCATCTTT TTTCTTTTTC TTTTCCCAAA   
  
  
- ATTTTAGAAC CCAGTTTTGG GTAGTTGTGG TGTTAAACTG AGAAAGAAAA ATTAAGTTGG TGGCGGTCAA   
  
  
- GGAGCAACTA AAAGTCCATG GAGAAAAAGA ACGGGGAAAA AAACGAGCCA ACAACAAATT ACCATTTCTA   
  
  
- ACTCATGTTT CTTACTATAA ACTACTCAAC CACACAAATA AACTACTCGG GTCTACTAAA TTGAACATCT   
  
  
- AAAACACCAA AACCAAAGAC CCATAAGTAA CCAAACGACT TAAAGACCAA GTACTAGACC CCAATCTTTT   
  
  
- CAACTTTACT GAAAGACCTC CAACCTTAAA CTGATTTCGA TTAAAGAAAA ACAGAAGTTA AGAAAAAATC   
  
  
- AACACTCGAC TTAAACAACT GGTGAAGGTC GGACAACCTT TAATTTGAAT GAATCCTCTT CTCTTAGAGT   
  
  
- CTAAGACTCG TTTTCGAAAT ATACCAACAA ACCCTTTACC ATCCTTGATT CACTTTTGTG ACTTTAAAAT   
  
  
- TCGTTCGTTA AAACAAGTAA ACTAACCAGC CTACCAGACT AAATCCAAGA AAAGAACCAG ATTTAAAGAC   
  
  
- TCGTAACTAA CAAATTAACG CACCCCTTGA ACATAGGAAC ACTCAAGGAC CAAGTAAAGA ACCAACGAAT   
  
  
- GAGATCGAGG GAAAGTGTCA AAGATACCAA CCTTGAACCT TGAACCTTGA ACCTTCGGAT TAAATCAACT   
  
  
- TATAGAACCG AAACTCAATC GAAAAGAATC CTTTTCTACC AAGACGAGAG CTTTTAGATT AGATTAGCTA   
  
  
- TCTACGCAAT TCCTTCGTTT TGAAAACCCC TCTCCCTTCA ATTCGCTTCG TTCGACGACA AAGTTTCAAC   
  
  
- CTGACAAAAT AACCATTCAT ACTTTGTTAT CAAATATTCT CTAGTAACCC AAGAATTCCA CTATCTAGTA   
  
  
- CCCAATACTT ACTCGAATCA ACAAGAAAAC GAAAGTTGAC ATGCATTCCT ACTATCAATA GGGAGTAGAA   
  
  
- CGTAGTTTTT GCCGTTTGGC ACTACGGTTT TGAGTTTTTG ACTAAGGAGA CTGTTCCGAA GATGGATACT   
  
  
- TTTGATACGT CTAGTATAGG TAAACCGGAA GGGAGATCCT TGGTTCATCT CATTCAACCA TTTATTTATC   
  
  
- TCAACTTGCT TTGTGATGTC TAAACTTAAA CTAATTCCTC CCGATAATAT CTGATTCCAC ATCGAGTTTA   
  
  
- GGAAGACATC CCTAACAGAA TAACCGAAGA AAACCAACGA AGACCGAGTT CAGGAGAATA ATCAATCATA   
  
  
- CGTAATGGAA CTCTGTTCTA TCAACGAATT CGAACTCGAA GACATGAATA AACGCATAAC ATCTGTTGAT   
  
  
- CGACACCGTA TACTAAGACT TTACAAGGCT ACAGAAAAAG AAACCTACGA CCCAACACAG TACTCGAAAT   
  
  
- CCAAACAAAT AAACTACAGT CCTTCATACA TTTATTGTAC ACCGTAGAAC GTTAGATAAT GTCCTACTAT   
  
  
- AACCGGAAAC ACCCTTCTTT AACTTCTTCA ACAAAGTGAA TGACCTATCC CTCGTACCTG AGTGTAGTCG   
  
  
- AAAACTCTAA GCCATAAAGG TCTAGTATGA GTAGGATATT AAAGAGAGTC AGATCAGGGG TTAGTTAAGG   
  
  
- TTTATCCAAT AAACTTAGTG GCTTGCAAGC AAGATCTCTG AGAGGTTAGA GCGGGAAGAG TTGCGGGAAG   
  
  
- TTAACGCTGG GGTGACAATA TAACTCGCTG TCAGTCCTCG TGGTGTCAAG TTGTCCTTCA GAGAGCCGTG   
  
  
- TTTCGAGTAG AAGATTAAGA GGGGAACTCT GATCGTCAAT AATATTAGTC AAGTTGAGTT CGGGACAACC   
  
  
- TCTTAGTGGA GTCCCTAGTA GTGGGCTCTT AGAAAAAGTT GTTCGACGTC AATTATTTCA GTCGTAGCTC   
  
  
- GTACGTAACG TCCTCGATCT TTGACGAGAT TACTCGTGTC TAACACTCCT CTTACTTCGT TGACAGAGAT   
  
  
- AGGGTAGATA CCCACTTGGA GTAGTCGGGG TTCAGGGATC GGTCTCTAGT TCTAAGACCT TAGCTCTAGG   
  
  
- AGTCCCAAGT GCGGGCCGAC TTCAAGTGAG TAGTAACTCT TCTAATCCTC TGCTCCGAGT CTCACTCTTT   
  
  
- GCGGAGTTCC GTCACCTTCT TGGTTATGCC GGGTCACGTG GTGGGCCGTT AAACTTCGTC AACGAGTATC   
  
  
- TTACACGAGC CCGAAACAGT CTCTTAGCTT ATCTCCTAAA ACTCTTCAAT CAACTTGTCC GTTTCCCGTA   
  
  
- CCAGAGGTAA AGACCTCTGG GTTAAGTTGC TGAACCACGA ATGAACTAGC TCCCCGATCA CCGTTCCTTC   
  
  
- CTCAGAAGTC CATGCTTTTA GATGGCTCGA GAATCCACGT TTCTCGGGGA ACCAGTTCTG AACGAAAGCA   
  
  
- TGTACGTGTA GGAAATACTT TAAACGGGAA TGAACTTTAA GCCAATATAC CAACGTTTAC CCCGATATCG   
  
  
- ACTTCGAACG TCTCTACTCC TAGTGTATGT ATAGCAGCTG ATGGTCTAAC CAGTTCCTTG AGTTACCTAC   
  
  
- TGAGACAACG TTCGGGATCG GTGTTTTGGA CCTTCTGGGG GGTGACACTC TTAATGACCG TAACTACTAG   
  
  
- GGCAGAGATC CGTACGAGCC CCTCGAACGA ACCTCCGTCA CCCCTTTGCA AACCGTCAAG ACAGCCTCTT   
  
  
- TAAATTGTAC GGGTAACTCA AAGTCCGTCA CGGGCAAATA CGAAGACTCC AGTGGGTTCT TTACGAACTA   
  
  
- CACTCCGGTC CCACCCGGAA CCGACACTTG AAGGGTAACG TCGAGGTGGT GTGAGGGCTA CTCTCACAAC   
  
  
- TACACTTGTT GGGCTCCCTA CCTGAGGATT CTTACCACTT TAGTGAGCCA GGGTTCCAAC ATTGAAACCA   
  
  
- TCTTGTTCTC AGTTTGTGGT TGTGATGGGG AAACGACTGT TCCAAGTATC TTTGCGACCT GAAGATGAGT   
  
  
- CGTTACAAAC TTAGGTAGCT ACACTGTTAC GGTTCCCTGG CTTTCCTTTC CTAGTTACAG CTCGTCGTAA   
  
  
- CGGATCGGTT CCTGTAACAG TTGTAGTAGC GCACACTCCC CTTCCTGTCC CACCTCGCTG TACTCGAGAA   
  
  
- ACCCTTTACC TTCAGTTCCA AGTGGTACCG TCCTAAGGTT GTTAGGGGTA ACTCGAGCAT ACAATTGAGG   
  
  
- CACTATTTTC CTAATGAATC CACAATAAGT CTCATGATAT GCAACCACCT CTTCCTACCG CGGAACGAAG   
  
  
- ACCCTACCTT CCTGGCCTAC GACCAAAGCC GAAGTCGAAC CGTAAC

+     W box

| Site Name | Organism | Position | Strand | Matrix score. | sequence | function |
| --- | --- | --- | --- | --- | --- | --- |
| W box | Arabidopsis thaliana | 2994 | - | 6 | TTGACC |  |
| W box | Arabidopsis thaliana | 2856 | - | 6 | TTGACC |  |
| W box | Arabidopsis thaliana | 861 | + | 6 | TTGACC |  |
| W box | Arabidopsis thaliana | 505 | - | 6 | TTGACC |  |

>HU01G01850.1   
+ +Up\_Stream \_Len000TTTAAA TATTTTATTT TATTTTAAAT TAATATATTT TATAATTTAA AATTATTAAT   
  
  
+ AATTGATGAT GTTACCCAAT GTTTAAGTTT TTAAATATTT TTATTTTTTA ATTTAAAAAT ATCATAATAT   
  
  
+ ATAATATAAT TTTAAGTTAA AATAAGATAG TTAAATTATT TTATAGTAAA TATTTATTAT TTTTTTTAAG   
  
  
+ AAAATTGCCA TGTGATTGTC ATCAGTCAAA CACTGCGTTG ACAGAAGCTG TCCTTGCTGC TACTCTCACC   
  
  
+ TCTCAACATC GGACCCGCGC AGACGTTAAC ATCTCCGTAC AAATACAATT CCCTGGCCAA AATTCAAATC   
  
  
+ AACCCTACTC GGTAGTCGGT TCCTTCTTCT TCCTGCCCTC TATATATACA CCCACAGCTG CGCTGCATGT   
  
  
+ TTTTCCGAGA ATCCTCAAAA ATCTTATAAA CTTCCTCGTG TGGGTAGAAA AAAGAAAAAG AAAAGGGTTT   
  
  
+ TAAAATCTTG GGTCAAAACC CATCAACACC ACAATTTGAC TCTTTCTTTT TAATTCAACC ACCGCCAGTT   
  
  
+ CCTCGTTGAT TTTCAGGTAC CTCTTTTTCT TGCCCCTTTT TTTGCTCGGT TGTTGTTTAA TGGTAAAGAT   
  
  
+ TGAGTACAAA GAATGATATT TGATGAGTTG GTGTGTTTAT TTGATGAGCC CAGATGATTT AACTTGTAGA   
  
  
+ TTTTGTGGTT TTGGTTTCTG GGTATTCATT GGTTTGCTGA ATTTCTGGTT CATGATCTGG GGTTAGAAAA   
  
  
+ GTTGAAATGA CTTTCTGGAG GTTGGAATTT GACTAAAGCT AATTTCTTTT TGTCTTCAAT TCTTTTTTAG   
  
  
+ TTGTGAGCTG AATTTGTTGA CCACTTCCAG CCTGTTGGAA ATTAAACTTA CTTAGGAGAA GAGAATCTCA   
  
  
+ GATTCTGAGC AAAAGCTTTA TATGGTTGTT TGGGAAATGG TAGGAACTAA GTGAAAACAC TGAAATTTTA   
  
  
+ AGCAAGCAAT TTTGTTCATT TGATTGGTCG GATGGTCTGA TTTAGGTTCT TTTCTTGGTC TAAATTTCTG   
  
  
+ AGCATTGATT GTTTAATTGC GTGGGGAACT TGTATCCTTG TGAGTTCCTG GTTCATTTCT TGGTTGCTTA   
  
  
+ CTCTAGCTCC CTTTCACAGT TTCTATGGTT GGAACTTGGA ACTTGGAACT TGGAAGCCTA ATTTAGTTGA   
  
  
+ ATATCTTGGC TTTGAGTTAG CTTTTCTTAG GAAAAGATGG TTCTGCTCTC GAAAATCTAA TCTAATCGAT   
  
  
+ AGATGCGTTA AGGAAGCAAA ACTTTTGGGG AGAGGGAAGT TAAGCGAAGC AAGCTGCTGT TTCAAAGTTG   
  
  
+ GACTGTTTTA TTGGTAAGTA TGAAACAATA GTTTATAAGA GATCATTGGG TTCTTAAGGT GATAGATCAT   
  
  
+ GGGTTATGAA TGAGCTTAGT TGTTCTTTTG CTTTCAACTG TACGTAAGGA TGATAGTTAT CCCTCATCTT   
  
  
+ GCATCAAAAA CGGCAAACCG TGATGCCAAA ACTCAAAAAC TGATTCCTCT GACAAGGCTT CTACCTATGA   
  
  
+ AAACTATGCA GATCATATCC ATTTGGCCTT CCCTCTAGGA ACCAAGTAGA GTAAGTTGGT AAATAAATAG   
  
  
+ AGTTGAACGA AACACTACAG ATTTGAATTT GATTAAGGAG GGCTATTATA GACTAAGGTG TAGCTCAAAT   
  
  
+ CCTTCTGTAG GGATTGTCTT ATTGGCTTCT TTTGGTTGCT TCTGGCTCAA GTCCTCTTAT TAGTTAGTAT   
  
  
+ GCATTACCTT GAGACAAGAT AGTTGCTTAA GCTTGAGCTT CTGTACTTAT TTGCGTATTG TAGACAACTA   
  
  
+ GCTGTGGCAT ATGATTCTGA AATGTTCCGA TGTCTTTTTC TTTGGATGCT GGGTTGTGTC ATGAGCTTTA   
  
  
+ GGTTTGTTTA TTTGATGTCA GGAAGTATGT AAATAACATG TGGCATCTTG CAATCTATTA CAGGATGATA   
  
  
+ TTGGCCTTTG TGGGAAGAAA TTGAAGAAGT TGTTTCACTT ACTGGATAGG GAGCATGGAC TCACATCAGC   
  
  
+ TTTTGAGATT CGGTATTTCC AGATCATACT CATCCTATAA TTTCTCTCAG TCTAGTCCCC AATCAATTCC   
  
  
+ AAATAGGTTA TTTGAATCAC CGAACGTTCG TTCTAGAGAC TCTCCAATCT CGCCCTTCTC AACGCCCTTC   
  
  
+ AATTGCGACC CCACTGTTAT ATTGAGCGAC AGTCAGGAGC ACCACAGTTC AACAGGAAGT CTCTCGGCAC   
  
  
+ AAAGCTCATC TTCTAATTCT CCCCTTGAGA CTAGCAGTTA TTATAATCAG TTCAACTCAA GCCCTGTTGG   
  
  
+ AGAATCACCT CAGGGATCAT CACCCGAGAA TCTTTTTCAA CAAGCTGCAG TTAATAAAGT CAGCATCGAG   
  
  
+ CATGCATTGC AGGAGCTAGA AACTGCTCTA ATGAGCACAG ATTGTGAGGA GAATGAAGCA ACTGTCTCTA   
  
  
+ TCCCATCTAT GGGTGAACCT CATCAGCCCC AAGTCCCTAG CCAGAGATCA AGATTCTGGA ATCGAGATCC   
  
  
+ TCAGGGTTCA CGCCCGGCTG AAGTTCACTC ATCATTGAGA AGATTAGGAG ACGAGGCTCA GAGTGAGAAA   
  
  
+ CGCCTCAAGG CAGTGGAAGA ACCAATACGG CCCAGTGCAC CACCCGGCAA TTTGAAGCAG TTGCTCATAG   
  
  
+ AATGTGCTCG GGCTTTGTCA GAGAATCGAA TAGAGGATTT TGAGAAGTTA GTTGAACAGG CAAAGGGCAT   
  
  
+ GGTCTCCATT TCTGGAGACC CAATTCAACG ACTTGGTGCT TACTTGATCG AGGGGCTAGT GGCAAGGAAG   
  
  
+ GAGTCTTCAG GTACGAAAAT CTACCGAGCT CTTAGGTGCA AAGAGCCCCT TGGTCAAGAC TTGCTTTCGT   
  
  
+ ACATGCACAT CCTTTATGAA ATTTGCCCTT ACTTGAAATT CGGTTATATG GTTGCAAATG GGGCTATAGC   
  
  
+ TGAAGCTTGC AGAGATGAGG ATCACATACA TATCGTCGAC TACCAGATTG GTCAAGGAAC TCAATGGATG   
  
  
+ ACTCTGTTGC AAGCCCTAGC CACAAAACCT GGAAGACCCC CCACTGTGAG AATTACTGGC ATTGATGATC   
  
  
+ CCGTCTCTAG GCATGCTCGG GGAGCTTGCT TGGAGGCAGT GGGGAAACGT TTGGCAGTTC TGTCGGAGAA   
  
  
+ ATTTAACATG CCCATTGAGT TTCAGGCAGT GCCCGTTTAT GCTTCTGAGG TCACCCAAGA AATGCTTGAT   
  
  
+ GTGAGGCCAG GGTGGGCCTT GGCTGTGAAC TTCCCATTGC AGCTCCACCA CACTCCCGAT GAGAGTGTTG   
  
  
+ ATGTGAACAA CCCGAGGGAT GGACTCCTAA GAATGGTGAA ATCACTCGGT CCCAAGGTTG TAACTTTGGT   
  
  
+ AGAACAAGAG TCAAACACCA ACACTACCCC TTTGCTGACA AGGTTCATAG AAACGCTGGA CTTCTACTCA   
  
  
+ GCAATGTTTG AATCCATCGA TGTGACAATG CCAAGGGACC GAAAGGAAAG GATCAATGTC GAGCAGCATT   
  
  
+ GCCTAGCCAA GGACATTGTC AACATCATCG CGTGTGAGGG GAAGGACAGG GTGGAGCGAC ATGAGCTCTT   
  
  
+ TGGGAAATGG AAGTCAAGGT TCACCATGGC AGGATTCCAA CAATCCCCAT TGAGCTCGTA TGTTAACTCC   
  
  
+ GTGATAAAAG GATTACTTAG GTGTTATTCA GAGTACTATA CGTTGGTGGA GAAGGATGGC GCCTTGCTTC   
  
  
+ TGGGATGGAA GGACCGGATG CTGGTTTCGG CTTCAGCTTG GCATTG  

- +Up\_Stream \_Len000AAATTT ATAAAATAAA ATAAAATTTA ATTATATAAA ATATTAAATT TTAATAATTA   
  
  
- TTAACTACTA CAATGGGTTA CAAATTCAAA AATTTATAAA AATAAAAAAT TAAATTTTTA TAGTATTATA   
  
  
- TATTATATTA AAATTCAATT TTATTCTATC AATTTAATAA AATATCATTT ATAAATAATA AAAAAAATTC   
  
  
- TTTTAACGGT ACACTAACAG TAGTCAGTTT GTGACGCAAC TGTCTTCGAC AGGAACGACG ATGAGAGTGG   
  
  
- AGAGTTGTAG CCTGGGCGCG TCTGCAATTG TAGAGGCATG TTTATGTTAA GGGACCGGTT TTAAGTTTAG   
  
  
- TTGGGATGAG CCATCAGCCA AGGAAGAAGA AGGACGGGAG ATATATATGT GGGTGTCGAC GCGACGTACA   
  
  
- AAAAGGCTCT TAGGAGTTTT TAGAATATTT GAAGGAGCAC ACCCATCTTT TTTCTTTTTC TTTTCCCAAA   
  
  
- ATTTTAGAAC CCAGTTTTGG GTAGTTGTGG TGTTAAACTG AGAAAGAAAA ATTAAGTTGG TGGCGGTCAA   
  
  
- GGAGCAACTA AAAGTCCATG GAGAAAAAGA ACGGGGAAAA AAACGAGCCA ACAACAAATT ACCATTTCTA   
  
  
- ACTCATGTTT CTTACTATAA ACTACTCAAC CACACAAATA AACTACTCGG GTCTACTAAA TTGAACATCT   
  
  
- AAAACACCAA AACCAAAGAC CCATAAGTAA CCAAACGACT TAAAGACCAA GTACTAGACC CCAATCTTTT   
  
  
- CAACTTTACT GAAAGACCTC CAACCTTAAA CTGATTTCGA TTAAAGAAAA ACAGAAGTTA AGAAAAAATC   
  
  
- AACACTCGAC TTAAACAACT GGTGAAGGTC GGACAACCTT TAATTTGAAT GAATCCTCTT CTCTTAGAGT   
  
  
- CTAAGACTCG TTTTCGAAAT ATACCAACAA ACCCTTTACC ATCCTTGATT CACTTTTGTG ACTTTAAAAT   
  
  
- TCGTTCGTTA AAACAAGTAA ACTAACCAGC CTACCAGACT AAATCCAAGA AAAGAACCAG ATTTAAAGAC   
  
  
- TCGTAACTAA CAAATTAACG CACCCCTTGA ACATAGGAAC ACTCAAGGAC CAAGTAAAGA ACCAACGAAT   
  
  
- GAGATCGAGG GAAAGTGTCA AAGATACCAA CCTTGAACCT TGAACCTTGA ACCTTCGGAT TAAATCAACT   
  
  
- TATAGAACCG AAACTCAATC GAAAAGAATC CTTTTCTACC AAGACGAGAG CTTTTAGATT AGATTAGCTA   
  
  
- TCTACGCAAT TCCTTCGTTT TGAAAACCCC TCTCCCTTCA ATTCGCTTCG TTCGACGACA AAGTTTCAAC   
  
  
- CTGACAAAAT AACCATTCAT ACTTTGTTAT CAAATATTCT CTAGTAACCC AAGAATTCCA CTATCTAGTA   
  
  
- CCCAATACTT ACTCGAATCA ACAAGAAAAC GAAAGTTGAC ATGCATTCCT ACTATCAATA GGGAGTAGAA   
  
  
- CGTAGTTTTT GCCGTTTGGC ACTACGGTTT TGAGTTTTTG ACTAAGGAGA CTGTTCCGAA GATGGATACT   
  
  
- TTTGATACGT CTAGTATAGG TAAACCGGAA GGGAGATCCT TGGTTCATCT CATTCAACCA TTTATTTATC   
  
  
- TCAACTTGCT TTGTGATGTC TAAACTTAAA CTAATTCCTC CCGATAATAT CTGATTCCAC ATCGAGTTTA   
  
  
- GGAAGACATC CCTAACAGAA TAACCGAAGA AAACCAACGA AGACCGAGTT CAGGAGAATA ATCAATCATA   
  
  
- CGTAATGGAA CTCTGTTCTA TCAACGAATT CGAACTCGAA GACATGAATA AACGCATAAC ATCTGTTGAT   
  
  
- CGACACCGTA TACTAAGACT TTACAAGGCT ACAGAAAAAG AAACCTACGA CCCAACACAG TACTCGAAAT   
  
  
- CCAAACAAAT AAACTACAGT CCTTCATACA TTTATTGTAC ACCGTAGAAC GTTAGATAAT GTCCTACTAT   
  
  
- AACCGGAAAC ACCCTTCTTT AACTTCTTCA ACAAAGTGAA TGACCTATCC CTCGTACCTG AGTGTAGTCG   
  
  
- AAAACTCTAA GCCATAAAGG TCTAGTATGA GTAGGATATT AAAGAGAGTC AGATCAGGGG TTAGTTAAGG   
  
  
- TTTATCCAAT AAACTTAGTG GCTTGCAAGC AAGATCTCTG AGAGGTTAGA GCGGGAAGAG TTGCGGGAAG   
  
  
- TTAACGCTGG GGTGACAATA TAACTCGCTG TCAGTCCTCG TGGTGTCAAG TTGTCCTTCA GAGAGCCGTG   
  
  
- TTTCGAGTAG AAGATTAAGA GGGGAACTCT GATCGTCAAT AATATTAGTC AAGTTGAGTT CGGGACAACC   
  
  
- TCTTAGTGGA GTCCCTAGTA GTGGGCTCTT AGAAAAAGTT GTTCGACGTC AATTATTTCA GTCGTAGCTC   
  
  
- GTACGTAACG TCCTCGATCT TTGACGAGAT TACTCGTGTC TAACACTCCT CTTACTTCGT TGACAGAGAT   
  
  
- AGGGTAGATA CCCACTTGGA GTAGTCGGGG TTCAGGGATC GGTCTCTAGT TCTAAGACCT TAGCTCTAGG   
  
  
- AGTCCCAAGT GCGGGCCGAC TTCAAGTGAG TAGTAACTCT TCTAATCCTC TGCTCCGAGT CTCACTCTTT   
  
  
- GCGGAGTTCC GTCACCTTCT TGGTTATGCC GGGTCACGTG GTGGGCCGTT AAACTTCGTC AACGAGTATC   
  
  
- TTACACGAGC CCGAAACAGT CTCTTAGCTT ATCTCCTAAA ACTCTTCAAT CAACTTGTCC GTTTCCCGTA   
  
  
- CCAGAGGTAA AGACCTCTGG GTTAAGTTGC TGAACCACGA ATGAACTAGC TCCCCGATCA CCGTTCCTTC   
  
  
- CTCAGAAGTC CATGCTTTTA GATGGCTCGA GAATCCACGT TTCTCGGGGA ACCAGTTCTG AACGAAAGCA   
  
  
- TGTACGTGTA GGAAATACTT TAAACGGGAA TGAACTTTAA GCCAATATAC CAACGTTTAC CCCGATATCG   
  
  
- ACTTCGAACG TCTCTACTCC TAGTGTATGT ATAGCAGCTG ATGGTCTAAC CAGTTCCTTG AGTTACCTAC   
  
  
- TGAGACAACG TTCGGGATCG GTGTTTTGGA CCTTCTGGGG GGTGACACTC TTAATGACCG TAACTACTAG   
  
  
- GGCAGAGATC CGTACGAGCC CCTCGAACGA ACCTCCGTCA CCCCTTTGCA AACCGTCAAG ACAGCCTCTT   
  
  
- TAAATTGTAC GGGTAACTCA AAGTCCGTCA CGGGCAAATA CGAAGACTCC AGTGGGTTCT TTACGAACTA   
  
  
- CACTCCGGTC CCACCCGGAA CCGACACTTG AAGGGTAACG TCGAGGTGGT GTGAGGGCTA CTCTCACAAC   
  
  
- TACACTTGTT GGGCTCCCTA CCTGAGGATT CTTACCACTT TAGTGAGCCA GGGTTCCAAC ATTGAAACCA   
  
  
- TCTTGTTCTC AGTTTGTGGT TGTGATGGGG AAACGACTGT TCCAAGTATC TTTGCGACCT GAAGATGAGT   
  
  
- CGTTACAAAC TTAGGTAGCT ACACTGTTAC GGTTCCCTGG CTTTCCTTTC CTAGTTACAG CTCGTCGTAA   
  
  
- CGGATCGGTT CCTGTAACAG TTGTAGTAGC GCACACTCCC CTTCCTGTCC CACCTCGCTG TACTCGAGAA   
  
  
- ACCCTTTACC TTCAGTTCCA AGTGGTACCG TCCTAAGGTT GTTAGGGGTA ACTCGAGCAT ACAATTGAGG   
  
  
- CACTATTTTC CTAATGAATC CACAATAAGT CTCATGATAT GCAACCACCT CTTCCTACCG CGGAACGAAG   
  
  
- ACCCTACCTT CCTGGCCTAC GACCAAAGCC GAAGTCGAAC CGTAAC
