# Supplementary material for: Genome-Wide Identification and Expression Pattern of the GRAS Gene Family in Pitaya (Selenicereus undatus L.)
Source: Biology (Basel). 2022 Dec 21;12(1):11. doi: 10.3390/biology12010011 (PMC9854919; doi:10.3390/biology12010011)
Supplement: Supplementary file 1 [file biology-12-00011-s001.zip › Supplementary file S5/HU02G01569.1_plantcare.html]

Content-Type: text/html; charset=ISO-8859-1


PlantCARE


Webmaster Firefox specific output  
To save the result:
click on the frame with the right mouse button and save the source code as a text file with extension .html  
REFERENCE:PlantCARE: a database of plant cis-acting regulatory elements and a portal to tools for in silico analysis of promoter sequences.  
Lescot, M., Déhais, P., Moreau, Y., De Moor, B., Rouzé ,P.,and Rombauts, S.  
Nucleic Acids Res., Database issue(2002), 30(1):325-327.   


---

>HU02G01569.1   
+ +Up\_Stream \_Len000AGCGTA GGAAGTGACG CCGCTATTGG AGGCGAAGAC GCTAGTACCA AGGCCAAAAC   
  
  
+ AAATGAGGAG ATAGATCAGT TAAACCGTTT GGTTAAGAAA ATTAAGCGAA CATCTCTTGA ACCTCCATCT   
  
  
+ GAACTCGATG ACATTGAGGA TGTGGATGCA GAAAAGATGG ATATGGGCTC TCCATCGGTG CCAGCAAATC   
  
  
+ AACCTAATGT TGGGTGGGGT CTCATTCGGT CAATGGGGGA CTAGGGACGT CATATCGTCA CCAATTACAA   
  
  
+ CGGAACAACC CTAAACCGCA TTTTAAAATC ACATCTTGGT AGCAGACAAA GATGTACGAC CCAAAAATTC   
  
  
+ AACTCTGTAA AGCACTAGCA AAGCTCGTAG TACACAGAAC AATCGCACAC TCATAGTGAT AGAAGTGTAC   
  
  
+ TATCCATTAT GCAGTGAATA TGACAAGGGA GGGGGTCCTG AGATGCCCAA ATTTGGCAGC CGAGTTGCCT   
  
  
+ TGACTGCCCA TCAATGTCCT AAACCAGTTG AGCATGTTAT TAATTTGCAA GAAAGATCGA ATTCTTCCTA   
  
  
+ TCTAAAAACT GACACAATAA AACAATACCA TGTATGGGAC ATTGCTAGGA TTGTTAACAC AATTATTGTC   
  
  
+ TTGTACGTTG CACCTGAAAA AAAAGAAAAT TGTTGCCATA GACAACATAT AAGTTAGGCA TTCATGACTA   
  
  
+ ATATCTGAAT TAGGTGTGTC GATCTGTGAT TCAAATTGAT ATAATTAGAT CTTTAAGGTA TCTAATTGCT   
  
  
+ TCAAGTTTTC TCTATAATGA AGATGTATAT TAGCTCACAT AGGTCTTAAG GTTAACTAAT GTCATATCAG   
  
  
+ GGGGTGTGGG CCGCCATTTT TGATAAGAAT TGAATTGAAC TTGCATTGTT TTTTCTTTGG CGAGAATGCA   
  
  
+ AGATTTGTTA ATTTATTCAT ATGCGTCATT ATACACTATA AGAAAAATAA TTGTAGGAAT ATATGAAGTA   
  
  
+ TTTTTACTTG TGCTCATGTT AAATGTGGAA TACTTTTTTA ATGTCGTATA GTACCACAGT ATTCTACAAT   
  
  
+ TTAAGAGATT AATCGATCCA TTAAAGTCAT GCAAACCTGA TGCATATTCA AGACATTGCA TGAAATGATC   
  
  
+ TTATTTTAGT CAAAGACTCC ATCATTTATA AGCAATATTT ACTTCGATAC ACGTGTCATT AAAAAAGACT   
  
  
+ ATAGAAATGT TACTGTTATT TTGTTTGAGC TAAATAGAAA ATTTAAAAGT TAATTAAGAT AATAATTAAA   
  
  
+ ACTAAATATT CAATGATGGC TAATAAATAC ATTGTCGTAT TAACTGACTA ATATTTGTAG ATGGTCGTGT   
  
  
+ TAATTTCTTT TTATTTTAAA GTTCAAATGA GTTACAAAAG GTTATAAAAT ATAAAGTAAA ATGGGAGACA   
  
  
+ AAGCAGACAG GGTGGGGGAA GCTAAGCTTA AAGTAGGATT TAGAAAGGCA ATGAAGGTGA ACCCTCAAAT   
  
  
+ CGATCCGCTG CCAATCACAG AAACCCAAAG CTTTGCTCAC CGACAACTCC CGGTTAACTG CAGTCACTCA   
  
  
+ CGGCACTGGG TAATACAGTA ATCGTGTACC CAGTTCATCC CCCTTTTCCT TTGGGAATAC AGTACCTCGA   
  
  
+ AAGTTCCTTT GAAATTCTAC TGGCCAGGCA TACCCATAAA TTGATCCTCT CGATATCATA AGATATGATA   
  
  
+ TTTCTCTGTC ATATCTGTTA GTTTCGGTAT CATATTTCTT CTTTATCTCT GTAATTTGAG GTATGCTTCA   
  
  
+ TTCCTGGCCG CTTGAATTAT CTTTCTTTCT TTGATTAGTA TTGTTTTTTT GGTTACCTAT TTTGCTCCAG   
  
  
+ TCCTGTTCAT TTGGGTCTTT TTGGATCTGG GTTTTTGCTG GGTTTGTTGA TTCTTTGAGA AATTTGGGTG   
  
  
+ CTTGAATTTG CCCTGCAATA TTCATGGGTT AGCGTTTTCC TGAATTTTGC TCTGTTCCTT AAGTGATTAT   
  
  
+ TTTTGATTGA TTACACCTTG GTGGTCCTGG TGGAATTTCC GAGGAGAAAT TGTCATGGGT TCCCACAACT   
  
  
+ TTGGAGAATT CCCTGATGAG ACTCTAAATG AGTATCAATC TACTTTGGGA ACCATGTCCC CCGGTTATGA   
  
  
+ TGGGTCTTTG AATTATACAA CCTTGTTCAA TTACAAAGAC CCATCTCAGG ATCTCACGGC ACTGAACCTT   
  
  
+ CCTAGCCCAT TGCCTGACCC TATGCCATTC AACATTGGTT CATATTCGGG TTTGAGCCCC GGGGTTGAAT   
  
  
+ CTTCGGATGA TAGCGATTCA GATGATGTTG TTAAGTACAT TGGCCAAGTG CTTATGGAAG AGGATATGAA   
  
  
+ GGAGAAGCCT TGTATGTTCC ATGACCCTTT AGCACTCCAA GCTGCTGAGA AACCCTTTTA TGATGTGTTG   
  
  
+ GGAAAGAAGT ATCCTCCTTC CCCTAATCAA CACCCACTTA TTGATCATTC TGTGGATAGC CCAGACAATC   
  
  
+ AGTCCTGTGG AACGAGCACA ATTAGTGATC TTAGTGGCAG TAACTGCACT TCCAGTTCAA CCAATTATAT   
  
  
+ TGATGCTGTG GCAGTCGCTG ATTCGAGTGA GAATACTAAG ACCTCTTTTG TGCAAAGTTC TCTGATTGAA   
  
  
+ TCGTTTTCTC AGTCGTCCAC CCTTCCACAG TGGTCATTTG GATCATTGGG TGCCTTGGGT GGCACAGCTT   
  
  
+ CTCAGGGTTC GAATTCAGTT ATCTCCTCCC GTGGTTTCCC TATGGCCATG AACGTTTTTA GCGAGCAGTC   
  
  
+ CATGATACAG TTTCAGAAGG GGGTGGAGGA GGCGAGCAAG TTCCTTCCAA AGAACAATAA CCTTGCGATT   
  
  
+ GACCTTGAGA GCGTCACTTT CCCAAATGAA AAGGAGGCGG CCCCCATGGT GGTGGTTAAG AAAGAGAAGG   
  
  
+ ATGAACACTC ACCTGATAGC TCGAGAGGTA GCAAGATTCA CTACCGTGAT GATGAGGACT TTGAGGATGG   
  
  
+ TAGGAGGAGT AAGCAGTCAG CTGTTTCTGT GGAGGAGGCT GAGTTGTCTG AAATGTTTGA CCGGGTTTTG   
  
  
+ CTTTGCAATC CCATGAAACA TGAAGCTCAT TGTACAAGTG GTTTGAAGTC CGAGAAGGGA ACATCCCTGC   
  
  
+ AGGCTGGCCA AGTACAGGTA CAGGAGGGTC AGAAGGCTCG TGCAAAGAAA CAGGGTAATA ATGATAATAA   
  
  
+ GAAGAATAAG AATGTGGTGG ATTTAAGGAC TCTGCTTATC CTCTGTGCGC AATCTGCTGC ATCTGATGAT   
  
  
+ CGCAGGACAG CTGATGAACT GCTGAAGCAG ATTAGGGAGC ACTCTTCTGC AGCTGGGGAT GGATCTCAAA   
  
  
+ GGTTGGCTCA TTACTTTGCT AATGCCTTGG AGGCGCGTTT AGCTGGAACT GGCTCACAGA TCTATACAGC   
  
  
+ CCTGAGTTCG AAGAGGACAA CAGCAGCTGA TATGATAAAA GCTTATCAGT TTTTCTTTCG TGCTTGCCCA   
  
  
+ TTTTTAAAGA TCTCTATTAT CTTTGCGAAC CATATGATTA TACAGAGAGC TGAAAAAGCA TCAAAGCTTC   
  
  
+ ATGTTATAGA TTTTGGCATC CTGTATGGTT TTCAGTGGCC CCTCCTCATT CAACGCCTGT CGGAGCGACC   
  
  
+ TGGTGGACCT CCAAAACTGT GCATTACTGG GATTGATCTT CCCCAACCTG GATTTAGGCC AACAGAAAGA   
  
  
+ GTTGAGGCAA CAGGGCTCCG CTTGGCAAAG TATTGTGAGC GCTTCAATGT TCCATTTGAA TACCATGCCA   
  
  
+ TTGCACAGAA ATGGGAAACC ATCAAAGCTG AAGACCTGAA GATAGAAGAT GATGAGGTGG TTGCCGTGAA   
  
  
+ CTGTCTCTTC AGGTTTAAGA ACCTGCTTGA TGAGACGATA GTGGTGGATA GTCCGAGGAA TGCAGTTCTC   
  
  
+ GGCTTGATTA GAAGGATAAA ACCTGATATC TTTGTTCACG GAGTAGTGAA CGGGTCTTAC AATGCGCCCT   
  
  
+ TCTTTGTTAC ACGTTTCAGG GAGGCCCTCT TCCATTACTC TACTGTATTC GATATGTTTG ATGCCAATGC   
  
  
+ TTCCCGGGAG GATCCTGAGA GATTGATGTT TGAGAAGGAG TTTTATGGGC GAGAAATTAT GAATGTGGTG   
  
  
+ GCTTGTGAGG GTACAGAGAG AGTTGAAAGA CCTGAGACAT ACAAGCAATG GCAGGCGCGG AATAGTAGGG   
  
  
+ CAGGGTTCAG GCAGCTGCCA TTGGACCAAG AGCTCGTCAG TAAACTGAGG AGCAAGGTCC AGATGCACTA   
  
  
+ TCCTAGGGAC TTCGTTGTTG ATGTAGATGG ACATTGGACA TTGCAGGGAT GGAAGGGGAG GATCATCTGT   
  
  
+ GCTGTGTCTG CATGGGGTCC TGCTTA  

- +Up\_Stream \_Len000TCGCAT CCTTCACTGC GGCGATAACC TCCGCTTCTG CGATCATGGT TCCGGTTTTG   
  
  
- TTTACTCCTC TATCTAGTCA ATTTGGCAAA CCAATTCTTT TAATTCGCTT GTAGAGAACT TGGAGGTAGA   
  
  
- CTTGAGCTAC TGTAACTCCT ACACCTACGT CTTTTCTACC TATACCCGAG AGGTAGCCAC GGTCGTTTAG   
  
  
- TTGGATTACA ACCCACCCCA GAGTAAGCCA GTTACCCCCT GATCCCTGCA GTATAGCAGT GGTTAATGTT   
  
  
- GCCTTGTTGG GATTTGGCGT AAAATTTTAG TGTAGAACCA TCGTCTGTTT CTACATGCTG GGTTTTTAAG   
  
  
- TTGAGACATT TCGTGATCGT TTCGAGCATC ATGTGTCTTG TTAGCGTGTG AGTATCACTA TCTTCACATG   
  
  
- ATAGGTAATA CGTCACTTAT ACTGTTCCCT CCCCCAGGAC TCTACGGGTT TAAACCGTCG GCTCAACGGA   
  
  
- ACTGACGGGT AGTTACAGGA TTTGGTCAAC TCGTACAATA ATTAAACGTT CTTTCTAGCT TAAGAAGGAT   
  
  
- AGATTTTTGA CTGTGTTATT TTGTTATGGT ACATACCCTG TAACGATCCT AACAATTGTG TTAATAACAG   
  
  
- AACATGCAAC GTGGACTTTT TTTTCTTTTA ACAACGGTAT CTGTTGTATA TTCAATCCGT AAGTACTGAT   
  
  
- TATAGACTTA ATCCACACAG CTAGACACTA AGTTTAACTA TATTAATCTA GAAATTCCAT AGATTAACGA   
  
  
- AGTTCAAAAG AGATATTACT TCTACATATA ATCGAGTGTA TCCAGAATTC CAATTGATTA CAGTATAGTC   
  
  
- CCCCACACCC GGCGGTAAAA ACTATTCTTA ACTTAACTTG AACGTAACAA AAAAGAAACC GCTCTTACGT   
  
  
- TCTAAACAAT TAAATAAGTA TACGCAGTAA TATGTGATAT TCTTTTTATT AACATCCTTA TATACTTCAT   
  
  
- AAAAATGAAC ACGAGTACAA TTTACACCTT ATGAAAAAAT TACAGCATAT CATGGTGTCA TAAGATGTTA   
  
  
- AATTCTCTAA TTAGCTAGGT AATTTCAGTA CGTTTGGACT ACGTATAAGT TCTGTAACGT ACTTTACTAG   
  
  
- AATAAAATCA GTTTCTGAGG TAGTAAATAT TCGTTATAAA TGAAGCTATG TGCACAGTAA TTTTTTCTGA   
  
  
- TATCTTTACA ATGACAATAA AACAAACTCG ATTTATCTTT TAAATTTTCA ATTAATTCTA TTATTAATTT   
  
  
- TGATTTATAA GTTACTACCG ATTATTTATG TAACAGCATA ATTGACTGAT TATAAACATC TACCAGCACA   
  
  
- ATTAAAGAAA AATAAAATTT CAAGTTTACT CAATGTTTTC CAATATTTTA TATTTCATTT TACCCTCTGT   
  
  
- TTCGTCTGTC CCACCCCCTT CGATTCGAAT TTCATCCTAA ATCTTTCCGT TACTTCCACT TGGGAGTTTA   
  
  
- GCTAGGCGAC GGTTAGTGTC TTTGGGTTTC GAAACGAGTG GCTGTTGAGG GCCAATTGAC GTCAGTGAGT   
  
  
- GCCGTGACCC ATTATGTCAT TAGCACATGG GTCAAGTAGG GGGAAAAGGA AACCCTTATG TCATGGAGCT   
  
  
- TTCAAGGAAA CTTTAAGATG ACCGGTCCGT ATGGGTATTT AACTAGGAGA GCTATAGTAT TCTATACTAT   
  
  
- AAAGAGACAG TATAGACAAT CAAAGCCATA GTATAAAGAA GAAATAGAGA CATTAAACTC CATACGAAGT   
  
  
- AAGGACCGGC GAACTTAATA GAAAGAAAGA AACTAATCAT AACAAAAAAA CCAATGGATA AAACGAGGTC   
  
  
- AGGACAAGTA AACCCAGAAA AACCTAGACC CAAAAACGAC CCAAACAACT AAGAAACTCT TTAAACCCAC   
  
  
- GAACTTAAAC GGGACGTTAT AAGTACCCAA TCGCAAAAGG ACTTAAAACG AGACAAGGAA TTCACTAATA   
  
  
- AAAACTAACT AATGTGGAAC CACCAGGACC ACCTTAAAGG CTCCTCTTTA ACAGTACCCA AGGGTGTTGA   
  
  
- AACCTCTTAA GGGACTACTC TGAGATTTAC TCATAGTTAG ATGAAACCCT TGGTACAGGG GGCCAATACT   
  
  
- ACCCAGAAAC TTAATATGTT GGAACAAGTT AATGTTTCTG GGTAGAGTCC TAGAGTGCCG TGACTTGGAA   
  
  
- GGATCGGGTA ACGGACTGGG ATACGGTAAG TTGTAACCAA GTATAAGCCC AAACTCGGGG CCCCAACTTA   
  
  
- GAAGCCTACT ATCGCTAAGT CTACTACAAC AATTCATGTA ACCGGTTCAC GAATACCTTC TCCTATACTT   
  
  
- CCTCTTCGGA ACATACAAGG TACTGGGAAA TCGTGAGGTT CGACGACTCT TTGGGAAAAT ACTACACAAC   
  
  
- CCTTTCTTCA TAGGAGGAAG GGGATTAGTT GTGGGTGAAT AACTAGTAAG ACACCTATCG GGTCTGTTAG   
  
  
- TCAGGACACC TTGCTCGTGT TAATCACTAG AATCACCGTC ATTGACGTGA AGGTCAAGTT GGTTAATATA   
  
  
- ACTACGACAC CGTCAGCGAC TAAGCTCACT CTTATGATTC TGGAGAAAAC ACGTTTCAAG AGACTAACTT   
  
  
- AGCAAAAGAG TCAGCAGGTG GGAAGGTGTC ACCAGTAAAC CTAGTAACCC ACGGAACCCA CCGTGTCGAA   
  
  
- GAGTCCCAAG CTTAAGTCAA TAGAGGAGGG CACCAAAGGG ATACCGGTAC TTGCAAAAAT CGCTCGTCAG   
  
  
- GTACTATGTC AAAGTCTTCC CCCACCTCCT CCGCTCGTTC AAGGAAGGTT TCTTGTTATT GGAACGCTAA   
  
  
- CTGGAACTCT CGCAGTGAAA GGGTTTACTT TTCCTCCGCC GGGGGTACCA CCACCAATTC TTTCTCTTCC   
  
  
- TACTTGTGAG TGGACTATCG AGCTCTCCAT CGTTCTAAGT GATGGCACTA CTACTCCTGA AACTCCTACC   
  
  
- ATCCTCCTCA TTCGTCAGTC GACAAAGACA CCTCCTCCGA CTCAACAGAC TTTACAAACT GGCCCAAAAC   
  
  
- GAAACGTTAG GGTACTTTGT ACTTCGAGTA ACATGTTCAC CAAACTTCAG GCTCTTCCCT TGTAGGGACG   
  
  
- TCCGACCGGT TCATGTCCAT GTCCTCCCAG TCTTCCGAGC ACGTTTCTTT GTCCCATTAT TACTATTATT   
  
  
- CTTCTTATTC TTACACCACC TAAATTCCTG AGACGAATAG GAGACACGCG TTAGACGACG TAGACTACTA   
  
  
- GCGTCCTGTC GACTACTTGA CGACTTCGTC TAATCCCTCG TGAGAAGACG TCGACCCCTA CCTAGAGTTT   
  
  
- CCAACCGAGT AATGAAACGA TTACGGAACC TCCGCGCAAA TCGACCTTGA CCGAGTGTCT AGATATGTCG   
  
  
- GGACTCAAGC TTCTCCTGTT GTCGTCGACT ATACTATTTT CGAATAGTCA AAAAGAAAGC ACGAACGGGT   
  
  
- AAAAATTTCT AGAGATAATA GAAACGCTTG GTATACTAAT ATGTCTCTCG ACTTTTTCGT AGTTTCGAAG   
  
  
- TACAATATCT AAAACCGTAG GACATACCAA AAGTCACCGG GGAGGAGTAA GTTGCGGACA GCCTCGCTGG   
  
  
- ACCACCTGGA GGTTTTGACA CGTAATGACC CTAACTAGAA GGGGTTGGAC CTAAATCCGG TTGTCTTTCT   
  
  
- CAACTCCGTT GTCCCGAGGC GAACCGTTTC ATAACACTCG CGAAGTTACA AGGTAAACTT ATGGTACGGT   
  
  
- AACGTGTCTT TACCCTTTGG TAGTTTCGAC TTCTGGACTT CTATCTTCTA CTACTCCACC AACGGCACTT   
  
  
- GACAGAGAAG TCCAAATTCT TGGACGAACT ACTCTGCTAT CACCACCTAT CAGGCTCCTT ACGTCAAGAG   
  
  
- CCGAACTAAT CTTCCTATTT TGGACTATAG AAACAAGTGC CTCATCACTT GCCCAGAATG TTACGCGGGA   
  
  
- AGAAACAATG TGCAAAGTCC CTCCGGGAGA AGGTAATGAG ATGACATAAG CTATACAAAC TACGGTTACG   
  
  
- AAGGGCCCTC CTAGGACTCT CTAACTACAA ACTCTTCCTC AAAATACCCG CTCTTTAATA CTTACACCAC   
  
  
- CGAACACTCC CATGTCTCTC TCAACTTTCT GGACTCTGTA TGTTCGTTAC CGTCCGCGCC TTATCATCCC   
  
  
- GTCCCAAGTC CGTCGACGGT AACCTGGTTC TCGAGCAGTC ATTTGACTCC TCGTTCCAGG TCTACGTGAT   
  
  
- AGGATCCCTG AAGCAACAAC TACATCTACC TGTAACCTGT AACGTCCCTA CCTTCCCCTC CTAGTAGACA   
  
  
- CGACACAGAC GTACCCCAGG ACGAAT

  
  
Motifs Found  

+   

| Site Name | Organism | Position | Strand | Matrix score. | sequence | function |
| --- | --- | --- | --- | --- | --- | --- |
|  | organism | 3073 | - | 4 | motif\_sequence | short\_function |
|  | organism | 3206 | + | 4 | motif\_sequence | short\_function |
|  | organism | 3759 | - | 4 | motif\_sequence | short\_function |
|  | organism | 3096 | - | 4 | motif\_sequence | short\_function |
|  | organism | 3184 | + | 4 | motif\_sequence | short\_function |
|  | organism | 4240 | - | 4 | motif\_sequence | short\_function |
|  | organism | 3102 | - | 4 | motif\_sequence | short\_function |
|  | organism | 3610 | + | 4 | motif\_sequence | short\_function |
|  | organism | 2328 | + | 4 | motif\_sequence | short\_function |
|  | organism | 3374 | - | 4 | motif\_sequence | short\_function |
|  | organism | 2656 | - | 4 | motif\_sequence | short\_function |
|  | organism | 3962 | + | 4 | motif\_sequence | short\_function |
|  | organism | 1946 | + | 4 | motif\_sequence | short\_function |
|  | organism | 3691 | + | 4 | motif\_sequence | short\_function |
|  | organism | 336 | + | 4 | motif\_sequence | short\_function |
|  | organism | 3251 | - | 4 | motif\_sequence | short\_function |
|  | organism | 2685 | + | 4 | motif\_sequence | short\_function |
|  | organism | 4227 | - | 4 | motif\_sequence | short\_function |
|  | organism | 192 | + | 4 | motif\_sequence | short\_function |
|  | organism | 3591 | + | 4 | motif\_sequence | short\_function |
|  | organism | 601 | - | 4 | motif\_sequence | short\_function |
|  | organism | 3789 | + | 4 | motif\_sequence | short\_function |
|  | organism | 2072 | + | 4 | motif\_sequence | short\_function |
|  | organism | 3266 | + | 4 | motif\_sequence | short\_function |
|  | organism | 2279 | - | 4 | motif\_sequence | short\_function |
|  | organism | 4233 | - | 4 | motif\_sequence | short\_function |
|  | organism | 508 | + | 4 | motif\_sequence | short\_function |
|  | organism | 3717 | - | 4 | motif\_sequence | short\_function |
|  | organism | 2243 | + | 4 | motif\_sequence | short\_function |
|  | organism | 4075 | - | 4 | motif\_sequence | short\_function |
|  | organism | 2088 | + | 4 | motif\_sequence | short\_function |
|  | organism | 3197 | + | 4 | motif\_sequence | short\_function |
|  | organism | 2301 | - | 4 | motif\_sequence | short\_function |
|  | organism | 4271 | + | 4 | motif\_sequence | short\_function |
|  | organism | 81 | - | 4 | motif\_sequence | short\_function |
|  | organism | 3229 | - | 4 | motif\_sequence | short\_function |
|  | organism | 1321 | - | 4 | motif\_sequence | short\_function |
|  | organism | 3752 | - | 4 | motif\_sequence | short\_function |
|  | organism | 122 | - | 4 | motif\_sequence | short\_function |
|  | organism | 1827 | + | 4 | motif\_sequence | short\_function |
|  | organism | 3951 | + | 4 | motif\_sequence | short\_function |
|  | organism | 793 | - | 4 | motif\_sequence | short\_function |

>HU02G01569.1   
+ +Up\_Stream \_Len000AGCGTA GGAAGTGACG CCGCTATTGG AGGCGAAGAC GCTAGTACCA AGGCCAAAAC   
  
  
+ AAATGAGGAG ATAGATCAGT TAAACCGTTT GGTTAAGAAA ATTAAGCGAA CATCTCTTGA ACCTCCATCT   
  
  
+ GAACTCGATG ACATTGAGGA TGTGGATGCA GAAAAGATGG ATATGGGCTC TCCATCGGTG CCAGCAAATC   
  
  
+ AACCTAATGT TGGGTGGGGT CTCATTCGGT CAATGGGGGA CTAGGGACGT CATATCGTCA CCAATTACAA   
  
  
+ CGGAACAACC CTAAACCGCA TTTTAAAATC ACATCTTGGT AGCAGACAAA GATGTACGAC CCAAAAATTC   
  
  
+ AACTCTGTAA AGCACTAGCA AAGCTCGTAG TACACAGAAC AATCGCACAC TCATAGTGAT AGAAGTGTAC   
  
  
+ TATCCATTAT GCAGTGAATA TGACAAGGGA GGGGGTCCTG AGATGCCCAA ATTTGGCAGC CGAGTTGCCT   
  
  
+ TGACTGCCCA TCAATGTCCT AAACCAGTTG AGCATGTTAT TAATTTGCAA GAAAGATCGA ATTCTTCCTA   
  
  
+ TCTAAAAACT GACACAATAA AACAATACCA TGTATGGGAC ATTGCTAGGA TTGTTAACAC AATTATTGTC   
  
  
+ TTGTACGTTG CACCTGAAAA AAAAGAAAAT TGTTGCCATA GACAACATAT AAGTTAGGCA TTCATGACTA   
  
  
+ ATATCTGAAT TAGGTGTGTC GATCTGTGAT TCAAATTGAT ATAATTAGAT CTTTAAGGTA TCTAATTGCT   
  
  
+ TCAAGTTTTC TCTATAATGA AGATGTATAT TAGCTCACAT AGGTCTTAAG GTTAACTAAT GTCATATCAG   
  
  
+ GGGGTGTGGG CCGCCATTTT TGATAAGAAT TGAATTGAAC TTGCATTGTT TTTTCTTTGG CGAGAATGCA   
  
  
+ AGATTTGTTA ATTTATTCAT ATGCGTCATT ATACACTATA AGAAAAATAA TTGTAGGAAT ATATGAAGTA   
  
  
+ TTTTTACTTG TGCTCATGTT AAATGTGGAA TACTTTTTTA ATGTCGTATA GTACCACAGT ATTCTACAAT   
  
  
+ TTAAGAGATT AATCGATCCA TTAAAGTCAT GCAAACCTGA TGCATATTCA AGACATTGCA TGAAATGATC   
  
  
+ TTATTTTAGT CAAAGACTCC ATCATTTATA AGCAATATTT ACTTCGATAC ACGTGTCATT AAAAAAGACT   
  
  
+ ATAGAAATGT TACTGTTATT TTGTTTGAGC TAAATAGAAA ATTTAAAAGT TAATTAAGAT AATAATTAAA   
  
  
+ ACTAAATATT CAATGATGGC TAATAAATAC ATTGTCGTAT TAACTGACTA ATATTTGTAG ATGGTCGTGT   
  
  
+ TAATTTCTTT TTATTTTAAA GTTCAAATGA GTTACAAAAG GTTATAAAAT ATAAAGTAAA ATGGGAGACA   
  
  
+ AAGCAGACAG GGTGGGGGAA GCTAAGCTTA AAGTAGGATT TAGAAAGGCA ATGAAGGTGA ACCCTCAAAT   
  
  
+ CGATCCGCTG CCAATCACAG AAACCCAAAG CTTTGCTCAC CGACAACTCC CGGTTAACTG CAGTCACTCA   
  
  
+ CGGCACTGGG TAATACAGTA ATCGTGTACC CAGTTCATCC CCCTTTTCCT TTGGGAATAC AGTACCTCGA   
  
  
+ AAGTTCCTTT GAAATTCTAC TGGCCAGGCA TACCCATAAA TTGATCCTCT CGATATCATA AGATATGATA   
  
  
+ TTTCTCTGTC ATATCTGTTA GTTTCGGTAT CATATTTCTT CTTTATCTCT GTAATTTGAG GTATGCTTCA   
  
  
+ TTCCTGGCCG CTTGAATTAT CTTTCTTTCT TTGATTAGTA TTGTTTTTTT GGTTACCTAT TTTGCTCCAG   
  
  
+ TCCTGTTCAT TTGGGTCTTT TTGGATCTGG GTTTTTGCTG GGTTTGTTGA TTCTTTGAGA AATTTGGGTG   
  
  
+ CTTGAATTTG CCCTGCAATA TTCATGGGTT AGCGTTTTCC TGAATTTTGC TCTGTTCCTT AAGTGATTAT   
  
  
+ TTTTGATTGA TTACACCTTG GTGGTCCTGG TGGAATTTCC GAGGAGAAAT TGTCATGGGT TCCCACAACT   
  
  
+ TTGGAGAATT CCCTGATGAG ACTCTAAATG AGTATCAATC TACTTTGGGA ACCATGTCCC CCGGTTATGA   
  
  
+ TGGGTCTTTG AATTATACAA CCTTGTTCAA TTACAAAGAC CCATCTCAGG ATCTCACGGC ACTGAACCTT   
  
  
+ CCTAGCCCAT TGCCTGACCC TATGCCATTC AACATTGGTT CATATTCGGG TTTGAGCCCC GGGGTTGAAT   
  
  
+ CTTCGGATGA TAGCGATTCA GATGATGTTG TTAAGTACAT TGGCCAAGTG CTTATGGAAG AGGATATGAA   
  
  
+ GGAGAAGCCT TGTATGTTCC ATGACCCTTT AGCACTCCAA GCTGCTGAGA AACCCTTTTA TGATGTGTTG   
  
  
+ GGAAAGAAGT ATCCTCCTTC CCCTAATCAA CACCCACTTA TTGATCATTC TGTGGATAGC CCAGACAATC   
  
  
+ AGTCCTGTGG AACGAGCACA ATTAGTGATC TTAGTGGCAG TAACTGCACT TCCAGTTCAA CCAATTATAT   
  
  
+ TGATGCTGTG GCAGTCGCTG ATTCGAGTGA GAATACTAAG ACCTCTTTTG TGCAAAGTTC TCTGATTGAA   
  
  
+ TCGTTTTCTC AGTCGTCCAC CCTTCCACAG TGGTCATTTG GATCATTGGG TGCCTTGGGT GGCACAGCTT   
  
  
+ CTCAGGGTTC GAATTCAGTT ATCTCCTCCC GTGGTTTCCC TATGGCCATG AACGTTTTTA GCGAGCAGTC   
  
  
+ CATGATACAG TTTCAGAAGG GGGTGGAGGA GGCGAGCAAG TTCCTTCCAA AGAACAATAA CCTTGCGATT   
  
  
+ GACCTTGAGA GCGTCACTTT CCCAAATGAA AAGGAGGCGG CCCCCATGGT GGTGGTTAAG AAAGAGAAGG   
  
  
+ ATGAACACTC ACCTGATAGC TCGAGAGGTA GCAAGATTCA CTACCGTGAT GATGAGGACT TTGAGGATGG   
  
  
+ TAGGAGGAGT AAGCAGTCAG CTGTTTCTGT GGAGGAGGCT GAGTTGTCTG AAATGTTTGA CCGGGTTTTG   
  
  
+ CTTTGCAATC CCATGAAACA TGAAGCTCAT TGTACAAGTG GTTTGAAGTC CGAGAAGGGA ACATCCCTGC   
  
  
+ AGGCTGGCCA AGTACAGGTA CAGGAGGGTC AGAAGGCTCG TGCAAAGAAA CAGGGTAATA ATGATAATAA   
  
  
+ GAAGAATAAG AATGTGGTGG ATTTAAGGAC TCTGCTTATC CTCTGTGCGC AATCTGCTGC ATCTGATGAT   
  
  
+ CGCAGGACAG CTGATGAACT GCTGAAGCAG ATTAGGGAGC ACTCTTCTGC AGCTGGGGAT GGATCTCAAA   
  
  
+ GGTTGGCTCA TTACTTTGCT AATGCCTTGG AGGCGCGTTT AGCTGGAACT GGCTCACAGA TCTATACAGC   
  
  
+ CCTGAGTTCG AAGAGGACAA CAGCAGCTGA TATGATAAAA GCTTATCAGT TTTTCTTTCG TGCTTGCCCA   
  
  
+ TTTTTAAAGA TCTCTATTAT CTTTGCGAAC CATATGATTA TACAGAGAGC TGAAAAAGCA TCAAAGCTTC   
  
  
+ ATGTTATAGA TTTTGGCATC CTGTATGGTT TTCAGTGGCC CCTCCTCATT CAACGCCTGT CGGAGCGACC   
  
  
+ TGGTGGACCT CCAAAACTGT GCATTACTGG GATTGATCTT CCCCAACCTG GATTTAGGCC AACAGAAAGA   
  
  
+ GTTGAGGCAA CAGGGCTCCG CTTGGCAAAG TATTGTGAGC GCTTCAATGT TCCATTTGAA TACCATGCCA   
  
  
+ TTGCACAGAA ATGGGAAACC ATCAAAGCTG AAGACCTGAA GATAGAAGAT GATGAGGTGG TTGCCGTGAA   
  
  
+ CTGTCTCTTC AGGTTTAAGA ACCTGCTTGA TGAGACGATA GTGGTGGATA GTCCGAGGAA TGCAGTTCTC   
  
  
+ GGCTTGATTA GAAGGATAAA ACCTGATATC TTTGTTCACG GAGTAGTGAA CGGGTCTTAC AATGCGCCCT   
  
  
+ TCTTTGTTAC ACGTTTCAGG GAGGCCCTCT TCCATTACTC TACTGTATTC GATATGTTTG ATGCCAATGC   
  
  
+ TTCCCGGGAG GATCCTGAGA GATTGATGTT TGAGAAGGAG TTTTATGGGC GAGAAATTAT GAATGTGGTG   
  
  
+ GCTTGTGAGG GTACAGAGAG AGTTGAAAGA CCTGAGACAT ACAAGCAATG GCAGGCGCGG AATAGTAGGG   
  
  
+ CAGGGTTCAG GCAGCTGCCA TTGGACCAAG AGCTCGTCAG TAAACTGAGG AGCAAGGTCC AGATGCACTA   
  
  
+ TCCTAGGGAC TTCGTTGTTG ATGTAGATGG ACATTGGACA TTGCAGGGAT GGAAGGGGAG GATCATCTGT   
  
  
+ GCTGTGTCTG CATGGGGTCC TGCTTA  

- +Up\_Stream \_Len000TCGCAT CCTTCACTGC GGCGATAACC TCCGCTTCTG CGATCATGGT TCCGGTTTTG   
  
  
- TTTACTCCTC TATCTAGTCA ATTTGGCAAA CCAATTCTTT TAATTCGCTT GTAGAGAACT TGGAGGTAGA   
  
  
- CTTGAGCTAC TGTAACTCCT ACACCTACGT CTTTTCTACC TATACCCGAG AGGTAGCCAC GGTCGTTTAG   
  
  
- TTGGATTACA ACCCACCCCA GAGTAAGCCA GTTACCCCCT GATCCCTGCA GTATAGCAGT GGTTAATGTT   
  
  
- GCCTTGTTGG GATTTGGCGT AAAATTTTAG TGTAGAACCA TCGTCTGTTT CTACATGCTG GGTTTTTAAG   
  
  
- TTGAGACATT TCGTGATCGT TTCGAGCATC ATGTGTCTTG TTAGCGTGTG AGTATCACTA TCTTCACATG   
  
  
- ATAGGTAATA CGTCACTTAT ACTGTTCCCT CCCCCAGGAC TCTACGGGTT TAAACCGTCG GCTCAACGGA   
  
  
- ACTGACGGGT AGTTACAGGA TTTGGTCAAC TCGTACAATA ATTAAACGTT CTTTCTAGCT TAAGAAGGAT   
  
  
- AGATTTTTGA CTGTGTTATT TTGTTATGGT ACATACCCTG TAACGATCCT AACAATTGTG TTAATAACAG   
  
  
- AACATGCAAC GTGGACTTTT TTTTCTTTTA ACAACGGTAT CTGTTGTATA TTCAATCCGT AAGTACTGAT   
  
  
- TATAGACTTA ATCCACACAG CTAGACACTA AGTTTAACTA TATTAATCTA GAAATTCCAT AGATTAACGA   
  
  
- AGTTCAAAAG AGATATTACT TCTACATATA ATCGAGTGTA TCCAGAATTC CAATTGATTA CAGTATAGTC   
  
  
- CCCCACACCC GGCGGTAAAA ACTATTCTTA ACTTAACTTG AACGTAACAA AAAAGAAACC GCTCTTACGT   
  
  
- TCTAAACAAT TAAATAAGTA TACGCAGTAA TATGTGATAT TCTTTTTATT AACATCCTTA TATACTTCAT   
  
  
- AAAAATGAAC ACGAGTACAA TTTACACCTT ATGAAAAAAT TACAGCATAT CATGGTGTCA TAAGATGTTA   
  
  
- AATTCTCTAA TTAGCTAGGT AATTTCAGTA CGTTTGGACT ACGTATAAGT TCTGTAACGT ACTTTACTAG   
  
  
- AATAAAATCA GTTTCTGAGG TAGTAAATAT TCGTTATAAA TGAAGCTATG TGCACAGTAA TTTTTTCTGA   
  
  
- TATCTTTACA ATGACAATAA AACAAACTCG ATTTATCTTT TAAATTTTCA ATTAATTCTA TTATTAATTT   
  
  
- TGATTTATAA GTTACTACCG ATTATTTATG TAACAGCATA ATTGACTGAT TATAAACATC TACCAGCACA   
  
  
- ATTAAAGAAA AATAAAATTT CAAGTTTACT CAATGTTTTC CAATATTTTA TATTTCATTT TACCCTCTGT   
  
  
- TTCGTCTGTC CCACCCCCTT CGATTCGAAT TTCATCCTAA ATCTTTCCGT TACTTCCACT TGGGAGTTTA   
  
  
- GCTAGGCGAC GGTTAGTGTC TTTGGGTTTC GAAACGAGTG GCTGTTGAGG GCCAATTGAC GTCAGTGAGT   
  
  
- GCCGTGACCC ATTATGTCAT TAGCACATGG GTCAAGTAGG GGGAAAAGGA AACCCTTATG TCATGGAGCT   
  
  
- TTCAAGGAAA CTTTAAGATG ACCGGTCCGT ATGGGTATTT AACTAGGAGA GCTATAGTAT TCTATACTAT   
  
  
- AAAGAGACAG TATAGACAAT CAAAGCCATA GTATAAAGAA GAAATAGAGA CATTAAACTC CATACGAAGT   
  
  
- AAGGACCGGC GAACTTAATA GAAAGAAAGA AACTAATCAT AACAAAAAAA CCAATGGATA AAACGAGGTC   
  
  
- AGGACAAGTA AACCCAGAAA AACCTAGACC CAAAAACGAC CCAAACAACT AAGAAACTCT TTAAACCCAC   
  
  
- GAACTTAAAC GGGACGTTAT AAGTACCCAA TCGCAAAAGG ACTTAAAACG AGACAAGGAA TTCACTAATA   
  
  
- AAAACTAACT AATGTGGAAC CACCAGGACC ACCTTAAAGG CTCCTCTTTA ACAGTACCCA AGGGTGTTGA   
  
  
- AACCTCTTAA GGGACTACTC TGAGATTTAC TCATAGTTAG ATGAAACCCT TGGTACAGGG GGCCAATACT   
  
  
- ACCCAGAAAC TTAATATGTT GGAACAAGTT AATGTTTCTG GGTAGAGTCC TAGAGTGCCG TGACTTGGAA   
  
  
- GGATCGGGTA ACGGACTGGG ATACGGTAAG TTGTAACCAA GTATAAGCCC AAACTCGGGG CCCCAACTTA   
  
  
- GAAGCCTACT ATCGCTAAGT CTACTACAAC AATTCATGTA ACCGGTTCAC GAATACCTTC TCCTATACTT   
  
  
- CCTCTTCGGA ACATACAAGG TACTGGGAAA TCGTGAGGTT CGACGACTCT TTGGGAAAAT ACTACACAAC   
  
  
- CCTTTCTTCA TAGGAGGAAG GGGATTAGTT GTGGGTGAAT AACTAGTAAG ACACCTATCG GGTCTGTTAG   
  
  
- TCAGGACACC TTGCTCGTGT TAATCACTAG AATCACCGTC ATTGACGTGA AGGTCAAGTT GGTTAATATA   
  
  
- ACTACGACAC CGTCAGCGAC TAAGCTCACT CTTATGATTC TGGAGAAAAC ACGTTTCAAG AGACTAACTT   
  
  
- AGCAAAAGAG TCAGCAGGTG GGAAGGTGTC ACCAGTAAAC CTAGTAACCC ACGGAACCCA CCGTGTCGAA   
  
  
- GAGTCCCAAG CTTAAGTCAA TAGAGGAGGG CACCAAAGGG ATACCGGTAC TTGCAAAAAT CGCTCGTCAG   
  
  
- GTACTATGTC AAAGTCTTCC CCCACCTCCT CCGCTCGTTC AAGGAAGGTT TCTTGTTATT GGAACGCTAA   
  
  
- CTGGAACTCT CGCAGTGAAA GGGTTTACTT TTCCTCCGCC GGGGGTACCA CCACCAATTC TTTCTCTTCC   
  
  
- TACTTGTGAG TGGACTATCG AGCTCTCCAT CGTTCTAAGT GATGGCACTA CTACTCCTGA AACTCCTACC   
  
  
- ATCCTCCTCA TTCGTCAGTC GACAAAGACA CCTCCTCCGA CTCAACAGAC TTTACAAACT GGCCCAAAAC   
  
  
- GAAACGTTAG GGTACTTTGT ACTTCGAGTA ACATGTTCAC CAAACTTCAG GCTCTTCCCT TGTAGGGACG   
  
  
- TCCGACCGGT TCATGTCCAT GTCCTCCCAG TCTTCCGAGC ACGTTTCTTT GTCCCATTAT TACTATTATT   
  
  
- CTTCTTATTC TTACACCACC TAAATTCCTG AGACGAATAG GAGACACGCG TTAGACGACG TAGACTACTA   
  
  
- GCGTCCTGTC GACTACTTGA CGACTTCGTC TAATCCCTCG TGAGAAGACG TCGACCCCTA CCTAGAGTTT   
  
  
- CCAACCGAGT AATGAAACGA TTACGGAACC TCCGCGCAAA TCGACCTTGA CCGAGTGTCT AGATATGTCG   
  
  
- GGACTCAAGC TTCTCCTGTT GTCGTCGACT ATACTATTTT CGAATAGTCA AAAAGAAAGC ACGAACGGGT   
  
  
- AAAAATTTCT AGAGATAATA GAAACGCTTG GTATACTAAT ATGTCTCTCG ACTTTTTCGT AGTTTCGAAG   
  
  
- TACAATATCT AAAACCGTAG GACATACCAA AAGTCACCGG GGAGGAGTAA GTTGCGGACA GCCTCGCTGG   
  
  
- ACCACCTGGA GGTTTTGACA CGTAATGACC CTAACTAGAA GGGGTTGGAC CTAAATCCGG TTGTCTTTCT   
  
  
- CAACTCCGTT GTCCCGAGGC GAACCGTTTC ATAACACTCG CGAAGTTACA AGGTAAACTT ATGGTACGGT   
  
  
- AACGTGTCTT TACCCTTTGG TAGTTTCGAC TTCTGGACTT CTATCTTCTA CTACTCCACC AACGGCACTT   
  
  
- GACAGAGAAG TCCAAATTCT TGGACGAACT ACTCTGCTAT CACCACCTAT CAGGCTCCTT ACGTCAAGAG   
  
  
- CCGAACTAAT CTTCCTATTT TGGACTATAG AAACAAGTGC CTCATCACTT GCCCAGAATG TTACGCGGGA   
  
  
- AGAAACAATG TGCAAAGTCC CTCCGGGAGA AGGTAATGAG ATGACATAAG CTATACAAAC TACGGTTACG   
  
  
- AAGGGCCCTC CTAGGACTCT CTAACTACAA ACTCTTCCTC AAAATACCCG CTCTTTAATA CTTACACCAC   
  
  
- CGAACACTCC CATGTCTCTC TCAACTTTCT GGACTCTGTA TGTTCGTTAC CGTCCGCGCC TTATCATCCC   
  
  
- GTCCCAAGTC CGTCGACGGT AACCTGGTTC TCGAGCAGTC ATTTGACTCC TCGTTCCAGG TCTACGTGAT   
  
  
- AGGATCCCTG AAGCAACAAC TACATCTACC TGTAACCTGT AACGTCCCTA CCTTCCCCTC CTAGTAGACA   
  
  
- CGACACAGAC GTACCCCAGG ACGAAT

+     AAGAA-motif

| Site Name | Organism | Position | Strand | Matrix score. | sequence | function |
| --- | --- | --- | --- | --- | --- | --- |
| AAGAA-motif | Avena sativa | 3417 | - | 7 | GAAAGAA |  |
| AAGAA-motif | Avena sativa | 2858 | + | 9 | gGTAAAGAAA |  |
| AAGAA-motif | Avena sativa | 2386 | + | 7 | GAAAGAA |  |
| AAGAA-motif | Avena sativa | 1777 | - | 7 | GAAAGAA |  |
| AAGAA-motif | Avena sativa | 105 | + | 9 | gGTAAAGAAA |  |

>HU02G01569.1   
+ +Up\_Stream \_Len000AGCGTA GGAAGTGACG CCGCTATTGG AGGCGAAGAC GCTAGTACCA AGGCCAAAAC   
  
  
+ AAATGAGGAG ATAGATCAGT TAAACCGTTT GGTTAAGAAA ATTAAGCGAA CATCTCTTGA ACCTCCATCT   
  
  
+ GAACTCGATG ACATTGAGGA TGTGGATGCA GAAAAGATGG ATATGGGCTC TCCATCGGTG CCAGCAAATC   
  
  
+ AACCTAATGT TGGGTGGGGT CTCATTCGGT CAATGGGGGA CTAGGGACGT CATATCGTCA CCAATTACAA   
  
  
+ CGGAACAACC CTAAACCGCA TTTTAAAATC ACATCTTGGT AGCAGACAAA GATGTACGAC CCAAAAATTC   
  
  
+ AACTCTGTAA AGCACTAGCA AAGCTCGTAG TACACAGAAC AATCGCACAC TCATAGTGAT AGAAGTGTAC   
  
  
+ TATCCATTAT GCAGTGAATA TGACAAGGGA GGGGGTCCTG AGATGCCCAA ATTTGGCAGC CGAGTTGCCT   
  
  
+ TGACTGCCCA TCAATGTCCT AAACCAGTTG AGCATGTTAT TAATTTGCAA GAAAGATCGA ATTCTTCCTA   
  
  
+ TCTAAAAACT GACACAATAA AACAATACCA TGTATGGGAC ATTGCTAGGA TTGTTAACAC AATTATTGTC   
  
  
+ TTGTACGTTG CACCTGAAAA AAAAGAAAAT TGTTGCCATA GACAACATAT AAGTTAGGCA TTCATGACTA   
  
  
+ ATATCTGAAT TAGGTGTGTC GATCTGTGAT TCAAATTGAT ATAATTAGAT CTTTAAGGTA TCTAATTGCT   
  
  
+ TCAAGTTTTC TCTATAATGA AGATGTATAT TAGCTCACAT AGGTCTTAAG GTTAACTAAT GTCATATCAG   
  
  
+ GGGGTGTGGG CCGCCATTTT TGATAAGAAT TGAATTGAAC TTGCATTGTT TTTTCTTTGG CGAGAATGCA   
  
  
+ AGATTTGTTA ATTTATTCAT ATGCGTCATT ATACACTATA AGAAAAATAA TTGTAGGAAT ATATGAAGTA   
  
  
+ TTTTTACTTG TGCTCATGTT AAATGTGGAA TACTTTTTTA ATGTCGTATA GTACCACAGT ATTCTACAAT   
  
  
+ TTAAGAGATT AATCGATCCA TTAAAGTCAT GCAAACCTGA TGCATATTCA AGACATTGCA TGAAATGATC   
  
  
+ TTATTTTAGT CAAAGACTCC ATCATTTATA AGCAATATTT ACTTCGATAC ACGTGTCATT AAAAAAGACT   
  
  
+ ATAGAAATGT TACTGTTATT TTGTTTGAGC TAAATAGAAA ATTTAAAAGT TAATTAAGAT AATAATTAAA   
  
  
+ ACTAAATATT CAATGATGGC TAATAAATAC ATTGTCGTAT TAACTGACTA ATATTTGTAG ATGGTCGTGT   
  
  
+ TAATTTCTTT TTATTTTAAA GTTCAAATGA GTTACAAAAG GTTATAAAAT ATAAAGTAAA ATGGGAGACA   
  
  
+ AAGCAGACAG GGTGGGGGAA GCTAAGCTTA AAGTAGGATT TAGAAAGGCA ATGAAGGTGA ACCCTCAAAT   
  
  
+ CGATCCGCTG CCAATCACAG AAACCCAAAG CTTTGCTCAC CGACAACTCC CGGTTAACTG CAGTCACTCA   
  
  
+ CGGCACTGGG TAATACAGTA ATCGTGTACC CAGTTCATCC CCCTTTTCCT TTGGGAATAC AGTACCTCGA   
  
  
+ AAGTTCCTTT GAAATTCTAC TGGCCAGGCA TACCCATAAA TTGATCCTCT CGATATCATA AGATATGATA   
  
  
+ TTTCTCTGTC ATATCTGTTA GTTTCGGTAT CATATTTCTT CTTTATCTCT GTAATTTGAG GTATGCTTCA   
  
  
+ TTCCTGGCCG CTTGAATTAT CTTTCTTTCT TTGATTAGTA TTGTTTTTTT GGTTACCTAT TTTGCTCCAG   
  
  
+ TCCTGTTCAT TTGGGTCTTT TTGGATCTGG GTTTTTGCTG GGTTTGTTGA TTCTTTGAGA AATTTGGGTG   
  
  
+ CTTGAATTTG CCCTGCAATA TTCATGGGTT AGCGTTTTCC TGAATTTTGC TCTGTTCCTT AAGTGATTAT   
  
  
+ TTTTGATTGA TTACACCTTG GTGGTCCTGG TGGAATTTCC GAGGAGAAAT TGTCATGGGT TCCCACAACT   
  
  
+ TTGGAGAATT CCCTGATGAG ACTCTAAATG AGTATCAATC TACTTTGGGA ACCATGTCCC CCGGTTATGA   
  
  
+ TGGGTCTTTG AATTATACAA CCTTGTTCAA TTACAAAGAC CCATCTCAGG ATCTCACGGC ACTGAACCTT   
  
  
+ CCTAGCCCAT TGCCTGACCC TATGCCATTC AACATTGGTT CATATTCGGG TTTGAGCCCC GGGGTTGAAT   
  
  
+ CTTCGGATGA TAGCGATTCA GATGATGTTG TTAAGTACAT TGGCCAAGTG CTTATGGAAG AGGATATGAA   
  
  
+ GGAGAAGCCT TGTATGTTCC ATGACCCTTT AGCACTCCAA GCTGCTGAGA AACCCTTTTA TGATGTGTTG   
  
  
+ GGAAAGAAGT ATCCTCCTTC CCCTAATCAA CACCCACTTA TTGATCATTC TGTGGATAGC CCAGACAATC   
  
  
+ AGTCCTGTGG AACGAGCACA ATTAGTGATC TTAGTGGCAG TAACTGCACT TCCAGTTCAA CCAATTATAT   
  
  
+ TGATGCTGTG GCAGTCGCTG ATTCGAGTGA GAATACTAAG ACCTCTTTTG TGCAAAGTTC TCTGATTGAA   
  
  
+ TCGTTTTCTC AGTCGTCCAC CCTTCCACAG TGGTCATTTG GATCATTGGG TGCCTTGGGT GGCACAGCTT   
  
  
+ CTCAGGGTTC GAATTCAGTT ATCTCCTCCC GTGGTTTCCC TATGGCCATG AACGTTTTTA GCGAGCAGTC   
  
  
+ CATGATACAG TTTCAGAAGG GGGTGGAGGA GGCGAGCAAG TTCCTTCCAA AGAACAATAA CCTTGCGATT   
  
  
+ GACCTTGAGA GCGTCACTTT CCCAAATGAA AAGGAGGCGG CCCCCATGGT GGTGGTTAAG AAAGAGAAGG   
  
  
+ ATGAACACTC ACCTGATAGC TCGAGAGGTA GCAAGATTCA CTACCGTGAT GATGAGGACT TTGAGGATGG   
  
  
+ TAGGAGGAGT AAGCAGTCAG CTGTTTCTGT GGAGGAGGCT GAGTTGTCTG AAATGTTTGA CCGGGTTTTG   
  
  
+ CTTTGCAATC CCATGAAACA TGAAGCTCAT TGTACAAGTG GTTTGAAGTC CGAGAAGGGA ACATCCCTGC   
  
  
+ AGGCTGGCCA AGTACAGGTA CAGGAGGGTC AGAAGGCTCG TGCAAAGAAA CAGGGTAATA ATGATAATAA   
  
  
+ GAAGAATAAG AATGTGGTGG ATTTAAGGAC TCTGCTTATC CTCTGTGCGC AATCTGCTGC ATCTGATGAT   
  
  
+ CGCAGGACAG CTGATGAACT GCTGAAGCAG ATTAGGGAGC ACTCTTCTGC AGCTGGGGAT GGATCTCAAA   
  
  
+ GGTTGGCTCA TTACTTTGCT AATGCCTTGG AGGCGCGTTT AGCTGGAACT GGCTCACAGA TCTATACAGC   
  
  
+ CCTGAGTTCG AAGAGGACAA CAGCAGCTGA TATGATAAAA GCTTATCAGT TTTTCTTTCG TGCTTGCCCA   
  
  
+ TTTTTAAAGA TCTCTATTAT CTTTGCGAAC CATATGATTA TACAGAGAGC TGAAAAAGCA TCAAAGCTTC   
  
  
+ ATGTTATAGA TTTTGGCATC CTGTATGGTT TTCAGTGGCC CCTCCTCATT CAACGCCTGT CGGAGCGACC   
  
  
+ TGGTGGACCT CCAAAACTGT GCATTACTGG GATTGATCTT CCCCAACCTG GATTTAGGCC AACAGAAAGA   
  
  
+ GTTGAGGCAA CAGGGCTCCG CTTGGCAAAG TATTGTGAGC GCTTCAATGT TCCATTTGAA TACCATGCCA   
  
  
+ TTGCACAGAA ATGGGAAACC ATCAAAGCTG AAGACCTGAA GATAGAAGAT GATGAGGTGG TTGCCGTGAA   
  
  
+ CTGTCTCTTC AGGTTTAAGA ACCTGCTTGA TGAGACGATA GTGGTGGATA GTCCGAGGAA TGCAGTTCTC   
  
  
+ GGCTTGATTA GAAGGATAAA ACCTGATATC TTTGTTCACG GAGTAGTGAA CGGGTCTTAC AATGCGCCCT   
  
  
+ TCTTTGTTAC ACGTTTCAGG GAGGCCCTCT TCCATTACTC TACTGTATTC GATATGTTTG ATGCCAATGC   
  
  
+ TTCCCGGGAG GATCCTGAGA GATTGATGTT TGAGAAGGAG TTTTATGGGC GAGAAATTAT GAATGTGGTG   
  
  
+ GCTTGTGAGG GTACAGAGAG AGTTGAAAGA CCTGAGACAT ACAAGCAATG GCAGGCGCGG AATAGTAGGG   
  
  
+ CAGGGTTCAG GCAGCTGCCA TTGGACCAAG AGCTCGTCAG TAAACTGAGG AGCAAGGTCC AGATGCACTA   
  
  
+ TCCTAGGGAC TTCGTTGTTG ATGTAGATGG ACATTGGACA TTGCAGGGAT GGAAGGGGAG GATCATCTGT   
  
  
+ GCTGTGTCTG CATGGGGTCC TGCTTA  

- +Up\_Stream \_Len000TCGCAT CCTTCACTGC GGCGATAACC TCCGCTTCTG CGATCATGGT TCCGGTTTTG   
  
  
- TTTACTCCTC TATCTAGTCA ATTTGGCAAA CCAATTCTTT TAATTCGCTT GTAGAGAACT TGGAGGTAGA   
  
  
- CTTGAGCTAC TGTAACTCCT ACACCTACGT CTTTTCTACC TATACCCGAG AGGTAGCCAC GGTCGTTTAG   
  
  
- TTGGATTACA ACCCACCCCA GAGTAAGCCA GTTACCCCCT GATCCCTGCA GTATAGCAGT GGTTAATGTT   
  
  
- GCCTTGTTGG GATTTGGCGT AAAATTTTAG TGTAGAACCA TCGTCTGTTT CTACATGCTG GGTTTTTAAG   
  
  
- TTGAGACATT TCGTGATCGT TTCGAGCATC ATGTGTCTTG TTAGCGTGTG AGTATCACTA TCTTCACATG   
  
  
- ATAGGTAATA CGTCACTTAT ACTGTTCCCT CCCCCAGGAC TCTACGGGTT TAAACCGTCG GCTCAACGGA   
  
  
- ACTGACGGGT AGTTACAGGA TTTGGTCAAC TCGTACAATA ATTAAACGTT CTTTCTAGCT TAAGAAGGAT   
  
  
- AGATTTTTGA CTGTGTTATT TTGTTATGGT ACATACCCTG TAACGATCCT AACAATTGTG TTAATAACAG   
  
  
- AACATGCAAC GTGGACTTTT TTTTCTTTTA ACAACGGTAT CTGTTGTATA TTCAATCCGT AAGTACTGAT   
  
  
- TATAGACTTA ATCCACACAG CTAGACACTA AGTTTAACTA TATTAATCTA GAAATTCCAT AGATTAACGA   
  
  
- AGTTCAAAAG AGATATTACT TCTACATATA ATCGAGTGTA TCCAGAATTC CAATTGATTA CAGTATAGTC   
  
  
- CCCCACACCC GGCGGTAAAA ACTATTCTTA ACTTAACTTG AACGTAACAA AAAAGAAACC GCTCTTACGT   
  
  
- TCTAAACAAT TAAATAAGTA TACGCAGTAA TATGTGATAT TCTTTTTATT AACATCCTTA TATACTTCAT   
  
  
- AAAAATGAAC ACGAGTACAA TTTACACCTT ATGAAAAAAT TACAGCATAT CATGGTGTCA TAAGATGTTA   
  
  
- AATTCTCTAA TTAGCTAGGT AATTTCAGTA CGTTTGGACT ACGTATAAGT TCTGTAACGT ACTTTACTAG   
  
  
- AATAAAATCA GTTTCTGAGG TAGTAAATAT TCGTTATAAA TGAAGCTATG TGCACAGTAA TTTTTTCTGA   
  
  
- TATCTTTACA ATGACAATAA AACAAACTCG ATTTATCTTT TAAATTTTCA ATTAATTCTA TTATTAATTT   
  
  
- TGATTTATAA GTTACTACCG ATTATTTATG TAACAGCATA ATTGACTGAT TATAAACATC TACCAGCACA   
  
  
- ATTAAAGAAA AATAAAATTT CAAGTTTACT CAATGTTTTC CAATATTTTA TATTTCATTT TACCCTCTGT   
  
  
- TTCGTCTGTC CCACCCCCTT CGATTCGAAT TTCATCCTAA ATCTTTCCGT TACTTCCACT TGGGAGTTTA   
  
  
- GCTAGGCGAC GGTTAGTGTC TTTGGGTTTC GAAACGAGTG GCTGTTGAGG GCCAATTGAC GTCAGTGAGT   
  
  
- GCCGTGACCC ATTATGTCAT TAGCACATGG GTCAAGTAGG GGGAAAAGGA AACCCTTATG TCATGGAGCT   
  
  
- TTCAAGGAAA CTTTAAGATG ACCGGTCCGT ATGGGTATTT AACTAGGAGA GCTATAGTAT TCTATACTAT   
  
  
- AAAGAGACAG TATAGACAAT CAAAGCCATA GTATAAAGAA GAAATAGAGA CATTAAACTC CATACGAAGT   
  
  
- AAGGACCGGC GAACTTAATA GAAAGAAAGA AACTAATCAT AACAAAAAAA CCAATGGATA AAACGAGGTC   
  
  
- AGGACAAGTA AACCCAGAAA AACCTAGACC CAAAAACGAC CCAAACAACT AAGAAACTCT TTAAACCCAC   
  
  
- GAACTTAAAC GGGACGTTAT AAGTACCCAA TCGCAAAAGG ACTTAAAACG AGACAAGGAA TTCACTAATA   
  
  
- AAAACTAACT AATGTGGAAC CACCAGGACC ACCTTAAAGG CTCCTCTTTA ACAGTACCCA AGGGTGTTGA   
  
  
- AACCTCTTAA GGGACTACTC TGAGATTTAC TCATAGTTAG ATGAAACCCT TGGTACAGGG GGCCAATACT   
  
  
- ACCCAGAAAC TTAATATGTT GGAACAAGTT AATGTTTCTG GGTAGAGTCC TAGAGTGCCG TGACTTGGAA   
  
  
- GGATCGGGTA ACGGACTGGG ATACGGTAAG TTGTAACCAA GTATAAGCCC AAACTCGGGG CCCCAACTTA   
  
  
- GAAGCCTACT ATCGCTAAGT CTACTACAAC AATTCATGTA ACCGGTTCAC GAATACCTTC TCCTATACTT   
  
  
- CCTCTTCGGA ACATACAAGG TACTGGGAAA TCGTGAGGTT CGACGACTCT TTGGGAAAAT ACTACACAAC   
  
  
- CCTTTCTTCA TAGGAGGAAG GGGATTAGTT GTGGGTGAAT AACTAGTAAG ACACCTATCG GGTCTGTTAG   
  
  
- TCAGGACACC TTGCTCGTGT TAATCACTAG AATCACCGTC ATTGACGTGA AGGTCAAGTT GGTTAATATA   
  
  
- ACTACGACAC CGTCAGCGAC TAAGCTCACT CTTATGATTC TGGAGAAAAC ACGTTTCAAG AGACTAACTT   
  
  
- AGCAAAAGAG TCAGCAGGTG GGAAGGTGTC ACCAGTAAAC CTAGTAACCC ACGGAACCCA CCGTGTCGAA   
  
  
- GAGTCCCAAG CTTAAGTCAA TAGAGGAGGG CACCAAAGGG ATACCGGTAC TTGCAAAAAT CGCTCGTCAG   
  
  
- GTACTATGTC AAAGTCTTCC CCCACCTCCT CCGCTCGTTC AAGGAAGGTT TCTTGTTATT GGAACGCTAA   
  
  
- CTGGAACTCT CGCAGTGAAA GGGTTTACTT TTCCTCCGCC GGGGGTACCA CCACCAATTC TTTCTCTTCC   
  
  
- TACTTGTGAG TGGACTATCG AGCTCTCCAT CGTTCTAAGT GATGGCACTA CTACTCCTGA AACTCCTACC   
  
  
- ATCCTCCTCA TTCGTCAGTC GACAAAGACA CCTCCTCCGA CTCAACAGAC TTTACAAACT GGCCCAAAAC   
  
  
- GAAACGTTAG GGTACTTTGT ACTTCGAGTA ACATGTTCAC CAAACTTCAG GCTCTTCCCT TGTAGGGACG   
  
  
- TCCGACCGGT TCATGTCCAT GTCCTCCCAG TCTTCCGAGC ACGTTTCTTT GTCCCATTAT TACTATTATT   
  
  
- CTTCTTATTC TTACACCACC TAAATTCCTG AGACGAATAG GAGACACGCG TTAGACGACG TAGACTACTA   
  
  
- GCGTCCTGTC GACTACTTGA CGACTTCGTC TAATCCCTCG TGAGAAGACG TCGACCCCTA CCTAGAGTTT   
  
  
- CCAACCGAGT AATGAAACGA TTACGGAACC TCCGCGCAAA TCGACCTTGA CCGAGTGTCT AGATATGTCG   
  
  
- GGACTCAAGC TTCTCCTGTT GTCGTCGACT ATACTATTTT CGAATAGTCA AAAAGAAAGC ACGAACGGGT   
  
  
- AAAAATTTCT AGAGATAATA GAAACGCTTG GTATACTAAT ATGTCTCTCG ACTTTTTCGT AGTTTCGAAG   
  
  
- TACAATATCT AAAACCGTAG GACATACCAA AAGTCACCGG GGAGGAGTAA GTTGCGGACA GCCTCGCTGG   
  
  
- ACCACCTGGA GGTTTTGACA CGTAATGACC CTAACTAGAA GGGGTTGGAC CTAAATCCGG TTGTCTTTCT   
  
  
- CAACTCCGTT GTCCCGAGGC GAACCGTTTC ATAACACTCG CGAAGTTACA AGGTAAACTT ATGGTACGGT   
  
  
- AACGTGTCTT TACCCTTTGG TAGTTTCGAC TTCTGGACTT CTATCTTCTA CTACTCCACC AACGGCACTT   
  
  
- GACAGAGAAG TCCAAATTCT TGGACGAACT ACTCTGCTAT CACCACCTAT CAGGCTCCTT ACGTCAAGAG   
  
  
- CCGAACTAAT CTTCCTATTT TGGACTATAG AAACAAGTGC CTCATCACTT GCCCAGAATG TTACGCGGGA   
  
  
- AGAAACAATG TGCAAAGTCC CTCCGGGAGA AGGTAATGAG ATGACATAAG CTATACAAAC TACGGTTACG   
  
  
- AAGGGCCCTC CTAGGACTCT CTAACTACAA ACTCTTCCTC AAAATACCCG CTCTTTAATA CTTACACCAC   
  
  
- CGAACACTCC CATGTCTCTC TCAACTTTCT GGACTCTGTA TGTTCGTTAC CGTCCGCGCC TTATCATCCC   
  
  
- GTCCCAAGTC CGTCGACGGT AACCTGGTTC TCGAGCAGTC ATTTGACTCC TCGTTCCAGG TCTACGTGAT   
  
  
- AGGATCCCTG AAGCAACAAC TACATCTACC TGTAACCTGT AACGTCCCTA CCTTCCCCTC CTAGTAGACA   
  
  
- CGACACAGAC GTACCCCAGG ACGAAT

+     ABRE

| Site Name | Organism | Position | Strand | Matrix score. | sequence | function |
| --- | --- | --- | --- | --- | --- | --- |
| ABRE | Arabidopsis thaliana | 3934 | - | 5 | ACGTG | cis-acting element involved in the abscisic acid responsiveness |
| ABRE | Arabidopsis thaliana | 3005 | - | 7 | AACCCGG | cis-acting element involved in the abscisic acid responsiveness |
| ABRE | Arabidopsis thaliana | 1175 | + | 5 | ACGTG | cis-acting element involved in the abscisic acid responsiveness |
| ABRE | Arabidopsis thaliana | 1174 | + | 6 | CACGTG | cis-acting element involved in the abscisic acid responsiveness |

>HU02G01569.1   
+ +Up\_Stream \_Len000AGCGTA GGAAGTGACG CCGCTATTGG AGGCGAAGAC GCTAGTACCA AGGCCAAAAC   
  
  
+ AAATGAGGAG ATAGATCAGT TAAACCGTTT GGTTAAGAAA ATTAAGCGAA CATCTCTTGA ACCTCCATCT   
  
  
+ GAACTCGATG ACATTGAGGA TGTGGATGCA GAAAAGATGG ATATGGGCTC TCCATCGGTG CCAGCAAATC   
  
  
+ AACCTAATGT TGGGTGGGGT CTCATTCGGT CAATGGGGGA CTAGGGACGT CATATCGTCA CCAATTACAA   
  
  
+ CGGAACAACC CTAAACCGCA TTTTAAAATC ACATCTTGGT AGCAGACAAA GATGTACGAC CCAAAAATTC   
  
  
+ AACTCTGTAA AGCACTAGCA AAGCTCGTAG TACACAGAAC AATCGCACAC TCATAGTGAT AGAAGTGTAC   
  
  
+ TATCCATTAT GCAGTGAATA TGACAAGGGA GGGGGTCCTG AGATGCCCAA ATTTGGCAGC CGAGTTGCCT   
  
  
+ TGACTGCCCA TCAATGTCCT AAACCAGTTG AGCATGTTAT TAATTTGCAA GAAAGATCGA ATTCTTCCTA   
  
  
+ TCTAAAAACT GACACAATAA AACAATACCA TGTATGGGAC ATTGCTAGGA TTGTTAACAC AATTATTGTC   
  
  
+ TTGTACGTTG CACCTGAAAA AAAAGAAAAT TGTTGCCATA GACAACATAT AAGTTAGGCA TTCATGACTA   
  
  
+ ATATCTGAAT TAGGTGTGTC GATCTGTGAT TCAAATTGAT ATAATTAGAT CTTTAAGGTA TCTAATTGCT   
  
  
+ TCAAGTTTTC TCTATAATGA AGATGTATAT TAGCTCACAT AGGTCTTAAG GTTAACTAAT GTCATATCAG   
  
  
+ GGGGTGTGGG CCGCCATTTT TGATAAGAAT TGAATTGAAC TTGCATTGTT TTTTCTTTGG CGAGAATGCA   
  
  
+ AGATTTGTTA ATTTATTCAT ATGCGTCATT ATACACTATA AGAAAAATAA TTGTAGGAAT ATATGAAGTA   
  
  
+ TTTTTACTTG TGCTCATGTT AAATGTGGAA TACTTTTTTA ATGTCGTATA GTACCACAGT ATTCTACAAT   
  
  
+ TTAAGAGATT AATCGATCCA TTAAAGTCAT GCAAACCTGA TGCATATTCA AGACATTGCA TGAAATGATC   
  
  
+ TTATTTTAGT CAAAGACTCC ATCATTTATA AGCAATATTT ACTTCGATAC ACGTGTCATT AAAAAAGACT   
  
  
+ ATAGAAATGT TACTGTTATT TTGTTTGAGC TAAATAGAAA ATTTAAAAGT TAATTAAGAT AATAATTAAA   
  
  
+ ACTAAATATT CAATGATGGC TAATAAATAC ATTGTCGTAT TAACTGACTA ATATTTGTAG ATGGTCGTGT   
  
  
+ TAATTTCTTT TTATTTTAAA GTTCAAATGA GTTACAAAAG GTTATAAAAT ATAAAGTAAA ATGGGAGACA   
  
  
+ AAGCAGACAG GGTGGGGGAA GCTAAGCTTA AAGTAGGATT TAGAAAGGCA ATGAAGGTGA ACCCTCAAAT   
  
  
+ CGATCCGCTG CCAATCACAG AAACCCAAAG CTTTGCTCAC CGACAACTCC CGGTTAACTG CAGTCACTCA   
  
  
+ CGGCACTGGG TAATACAGTA ATCGTGTACC CAGTTCATCC CCCTTTTCCT TTGGGAATAC AGTACCTCGA   
  
  
+ AAGTTCCTTT GAAATTCTAC TGGCCAGGCA TACCCATAAA TTGATCCTCT CGATATCATA AGATATGATA   
  
  
+ TTTCTCTGTC ATATCTGTTA GTTTCGGTAT CATATTTCTT CTTTATCTCT GTAATTTGAG GTATGCTTCA   
  
  
+ TTCCTGGCCG CTTGAATTAT CTTTCTTTCT TTGATTAGTA TTGTTTTTTT GGTTACCTAT TTTGCTCCAG   
  
  
+ TCCTGTTCAT TTGGGTCTTT TTGGATCTGG GTTTTTGCTG GGTTTGTTGA TTCTTTGAGA AATTTGGGTG   
  
  
+ CTTGAATTTG CCCTGCAATA TTCATGGGTT AGCGTTTTCC TGAATTTTGC TCTGTTCCTT AAGTGATTAT   
  
  
+ TTTTGATTGA TTACACCTTG GTGGTCCTGG TGGAATTTCC GAGGAGAAAT TGTCATGGGT TCCCACAACT   
  
  
+ TTGGAGAATT CCCTGATGAG ACTCTAAATG AGTATCAATC TACTTTGGGA ACCATGTCCC CCGGTTATGA   
  
  
+ TGGGTCTTTG AATTATACAA CCTTGTTCAA TTACAAAGAC CCATCTCAGG ATCTCACGGC ACTGAACCTT   
  
  
+ CCTAGCCCAT TGCCTGACCC TATGCCATTC AACATTGGTT CATATTCGGG TTTGAGCCCC GGGGTTGAAT   
  
  
+ CTTCGGATGA TAGCGATTCA GATGATGTTG TTAAGTACAT TGGCCAAGTG CTTATGGAAG AGGATATGAA   
  
  
+ GGAGAAGCCT TGTATGTTCC ATGACCCTTT AGCACTCCAA GCTGCTGAGA AACCCTTTTA TGATGTGTTG   
  
  
+ GGAAAGAAGT ATCCTCCTTC CCCTAATCAA CACCCACTTA TTGATCATTC TGTGGATAGC CCAGACAATC   
  
  
+ AGTCCTGTGG AACGAGCACA ATTAGTGATC TTAGTGGCAG TAACTGCACT TCCAGTTCAA CCAATTATAT   
  
  
+ TGATGCTGTG GCAGTCGCTG ATTCGAGTGA GAATACTAAG ACCTCTTTTG TGCAAAGTTC TCTGATTGAA   
  
  
+ TCGTTTTCTC AGTCGTCCAC CCTTCCACAG TGGTCATTTG GATCATTGGG TGCCTTGGGT GGCACAGCTT   
  
  
+ CTCAGGGTTC GAATTCAGTT ATCTCCTCCC GTGGTTTCCC TATGGCCATG AACGTTTTTA GCGAGCAGTC   
  
  
+ CATGATACAG TTTCAGAAGG GGGTGGAGGA GGCGAGCAAG TTCCTTCCAA AGAACAATAA CCTTGCGATT   
  
  
+ GACCTTGAGA GCGTCACTTT CCCAAATGAA AAGGAGGCGG CCCCCATGGT GGTGGTTAAG AAAGAGAAGG   
  
  
+ ATGAACACTC ACCTGATAGC TCGAGAGGTA GCAAGATTCA CTACCGTGAT GATGAGGACT TTGAGGATGG   
  
  
+ TAGGAGGAGT AAGCAGTCAG CTGTTTCTGT GGAGGAGGCT GAGTTGTCTG AAATGTTTGA CCGGGTTTTG   
  
  
+ CTTTGCAATC CCATGAAACA TGAAGCTCAT TGTACAAGTG GTTTGAAGTC CGAGAAGGGA ACATCCCTGC   
  
  
+ AGGCTGGCCA AGTACAGGTA CAGGAGGGTC AGAAGGCTCG TGCAAAGAAA CAGGGTAATA ATGATAATAA   
  
  
+ GAAGAATAAG AATGTGGTGG ATTTAAGGAC TCTGCTTATC CTCTGTGCGC AATCTGCTGC ATCTGATGAT   
  
  
+ CGCAGGACAG CTGATGAACT GCTGAAGCAG ATTAGGGAGC ACTCTTCTGC AGCTGGGGAT GGATCTCAAA   
  
  
+ GGTTGGCTCA TTACTTTGCT AATGCCTTGG AGGCGCGTTT AGCTGGAACT GGCTCACAGA TCTATACAGC   
  
  
+ CCTGAGTTCG AAGAGGACAA CAGCAGCTGA TATGATAAAA GCTTATCAGT TTTTCTTTCG TGCTTGCCCA   
  
  
+ TTTTTAAAGA TCTCTATTAT CTTTGCGAAC CATATGATTA TACAGAGAGC TGAAAAAGCA TCAAAGCTTC   
  
  
+ ATGTTATAGA TTTTGGCATC CTGTATGGTT TTCAGTGGCC CCTCCTCATT CAACGCCTGT CGGAGCGACC   
  
  
+ TGGTGGACCT CCAAAACTGT GCATTACTGG GATTGATCTT CCCCAACCTG GATTTAGGCC AACAGAAAGA   
  
  
+ GTTGAGGCAA CAGGGCTCCG CTTGGCAAAG TATTGTGAGC GCTTCAATGT TCCATTTGAA TACCATGCCA   
  
  
+ TTGCACAGAA ATGGGAAACC ATCAAAGCTG AAGACCTGAA GATAGAAGAT GATGAGGTGG TTGCCGTGAA   
  
  
+ CTGTCTCTTC AGGTTTAAGA ACCTGCTTGA TGAGACGATA GTGGTGGATA GTCCGAGGAA TGCAGTTCTC   
  
  
+ GGCTTGATTA GAAGGATAAA ACCTGATATC TTTGTTCACG GAGTAGTGAA CGGGTCTTAC AATGCGCCCT   
  
  
+ TCTTTGTTAC ACGTTTCAGG GAGGCCCTCT TCCATTACTC TACTGTATTC GATATGTTTG ATGCCAATGC   
  
  
+ TTCCCGGGAG GATCCTGAGA GATTGATGTT TGAGAAGGAG TTTTATGGGC GAGAAATTAT GAATGTGGTG   
  
  
+ GCTTGTGAGG GTACAGAGAG AGTTGAAAGA CCTGAGACAT ACAAGCAATG GCAGGCGCGG AATAGTAGGG   
  
  
+ CAGGGTTCAG GCAGCTGCCA TTGGACCAAG AGCTCGTCAG TAAACTGAGG AGCAAGGTCC AGATGCACTA   
  
  
+ TCCTAGGGAC TTCGTTGTTG ATGTAGATGG ACATTGGACA TTGCAGGGAT GGAAGGGGAG GATCATCTGT   
  
  
+ GCTGTGTCTG CATGGGGTCC TGCTTA  

- +Up\_Stream \_Len000TCGCAT CCTTCACTGC GGCGATAACC TCCGCTTCTG CGATCATGGT TCCGGTTTTG   
  
  
- TTTACTCCTC TATCTAGTCA ATTTGGCAAA CCAATTCTTT TAATTCGCTT GTAGAGAACT TGGAGGTAGA   
  
  
- CTTGAGCTAC TGTAACTCCT ACACCTACGT CTTTTCTACC TATACCCGAG AGGTAGCCAC GGTCGTTTAG   
  
  
- TTGGATTACA ACCCACCCCA GAGTAAGCCA GTTACCCCCT GATCCCTGCA GTATAGCAGT GGTTAATGTT   
  
  
- GCCTTGTTGG GATTTGGCGT AAAATTTTAG TGTAGAACCA TCGTCTGTTT CTACATGCTG GGTTTTTAAG   
  
  
- TTGAGACATT TCGTGATCGT TTCGAGCATC ATGTGTCTTG TTAGCGTGTG AGTATCACTA TCTTCACATG   
  
  
- ATAGGTAATA CGTCACTTAT ACTGTTCCCT CCCCCAGGAC TCTACGGGTT TAAACCGTCG GCTCAACGGA   
  
  
- ACTGACGGGT AGTTACAGGA TTTGGTCAAC TCGTACAATA ATTAAACGTT CTTTCTAGCT TAAGAAGGAT   
  
  
- AGATTTTTGA CTGTGTTATT TTGTTATGGT ACATACCCTG TAACGATCCT AACAATTGTG TTAATAACAG   
  
  
- AACATGCAAC GTGGACTTTT TTTTCTTTTA ACAACGGTAT CTGTTGTATA TTCAATCCGT AAGTACTGAT   
  
  
- TATAGACTTA ATCCACACAG CTAGACACTA AGTTTAACTA TATTAATCTA GAAATTCCAT AGATTAACGA   
  
  
- AGTTCAAAAG AGATATTACT TCTACATATA ATCGAGTGTA TCCAGAATTC CAATTGATTA CAGTATAGTC   
  
  
- CCCCACACCC GGCGGTAAAA ACTATTCTTA ACTTAACTTG AACGTAACAA AAAAGAAACC GCTCTTACGT   
  
  
- TCTAAACAAT TAAATAAGTA TACGCAGTAA TATGTGATAT TCTTTTTATT AACATCCTTA TATACTTCAT   
  
  
- AAAAATGAAC ACGAGTACAA TTTACACCTT ATGAAAAAAT TACAGCATAT CATGGTGTCA TAAGATGTTA   
  
  
- AATTCTCTAA TTAGCTAGGT AATTTCAGTA CGTTTGGACT ACGTATAAGT TCTGTAACGT ACTTTACTAG   
  
  
- AATAAAATCA GTTTCTGAGG TAGTAAATAT TCGTTATAAA TGAAGCTATG TGCACAGTAA TTTTTTCTGA   
  
  
- TATCTTTACA ATGACAATAA AACAAACTCG ATTTATCTTT TAAATTTTCA ATTAATTCTA TTATTAATTT   
  
  
- TGATTTATAA GTTACTACCG ATTATTTATG TAACAGCATA ATTGACTGAT TATAAACATC TACCAGCACA   
  
  
- ATTAAAGAAA AATAAAATTT CAAGTTTACT CAATGTTTTC CAATATTTTA TATTTCATTT TACCCTCTGT   
  
  
- TTCGTCTGTC CCACCCCCTT CGATTCGAAT TTCATCCTAA ATCTTTCCGT TACTTCCACT TGGGAGTTTA   
  
  
- GCTAGGCGAC GGTTAGTGTC TTTGGGTTTC GAAACGAGTG GCTGTTGAGG GCCAATTGAC GTCAGTGAGT   
  
  
- GCCGTGACCC ATTATGTCAT TAGCACATGG GTCAAGTAGG GGGAAAAGGA AACCCTTATG TCATGGAGCT   
  
  
- TTCAAGGAAA CTTTAAGATG ACCGGTCCGT ATGGGTATTT AACTAGGAGA GCTATAGTAT TCTATACTAT   
  
  
- AAAGAGACAG TATAGACAAT CAAAGCCATA GTATAAAGAA GAAATAGAGA CATTAAACTC CATACGAAGT   
  
  
- AAGGACCGGC GAACTTAATA GAAAGAAAGA AACTAATCAT AACAAAAAAA CCAATGGATA AAACGAGGTC   
  
  
- AGGACAAGTA AACCCAGAAA AACCTAGACC CAAAAACGAC CCAAACAACT AAGAAACTCT TTAAACCCAC   
  
  
- GAACTTAAAC GGGACGTTAT AAGTACCCAA TCGCAAAAGG ACTTAAAACG AGACAAGGAA TTCACTAATA   
  
  
- AAAACTAACT AATGTGGAAC CACCAGGACC ACCTTAAAGG CTCCTCTTTA ACAGTACCCA AGGGTGTTGA   
  
  
- AACCTCTTAA GGGACTACTC TGAGATTTAC TCATAGTTAG ATGAAACCCT TGGTACAGGG GGCCAATACT   
  
  
- ACCCAGAAAC TTAATATGTT GGAACAAGTT AATGTTTCTG GGTAGAGTCC TAGAGTGCCG TGACTTGGAA   
  
  
- GGATCGGGTA ACGGACTGGG ATACGGTAAG TTGTAACCAA GTATAAGCCC AAACTCGGGG CCCCAACTTA   
  
  
- GAAGCCTACT ATCGCTAAGT CTACTACAAC AATTCATGTA ACCGGTTCAC GAATACCTTC TCCTATACTT   
  
  
- CCTCTTCGGA ACATACAAGG TACTGGGAAA TCGTGAGGTT CGACGACTCT TTGGGAAAAT ACTACACAAC   
  
  
- CCTTTCTTCA TAGGAGGAAG GGGATTAGTT GTGGGTGAAT AACTAGTAAG ACACCTATCG GGTCTGTTAG   
  
  
- TCAGGACACC TTGCTCGTGT TAATCACTAG AATCACCGTC ATTGACGTGA AGGTCAAGTT GGTTAATATA   
  
  
- ACTACGACAC CGTCAGCGAC TAAGCTCACT CTTATGATTC TGGAGAAAAC ACGTTTCAAG AGACTAACTT   
  
  
- AGCAAAAGAG TCAGCAGGTG GGAAGGTGTC ACCAGTAAAC CTAGTAACCC ACGGAACCCA CCGTGTCGAA   
  
  
- GAGTCCCAAG CTTAAGTCAA TAGAGGAGGG CACCAAAGGG ATACCGGTAC TTGCAAAAAT CGCTCGTCAG   
  
  
- GTACTATGTC AAAGTCTTCC CCCACCTCCT CCGCTCGTTC AAGGAAGGTT TCTTGTTATT GGAACGCTAA   
  
  
- CTGGAACTCT CGCAGTGAAA GGGTTTACTT TTCCTCCGCC GGGGGTACCA CCACCAATTC TTTCTCTTCC   
  
  
- TACTTGTGAG TGGACTATCG AGCTCTCCAT CGTTCTAAGT GATGGCACTA CTACTCCTGA AACTCCTACC   
  
  
- ATCCTCCTCA TTCGTCAGTC GACAAAGACA CCTCCTCCGA CTCAACAGAC TTTACAAACT GGCCCAAAAC   
  
  
- GAAACGTTAG GGTACTTTGT ACTTCGAGTA ACATGTTCAC CAAACTTCAG GCTCTTCCCT TGTAGGGACG   
  
  
- TCCGACCGGT TCATGTCCAT GTCCTCCCAG TCTTCCGAGC ACGTTTCTTT GTCCCATTAT TACTATTATT   
  
  
- CTTCTTATTC TTACACCACC TAAATTCCTG AGACGAATAG GAGACACGCG TTAGACGACG TAGACTACTA   
  
  
- GCGTCCTGTC GACTACTTGA CGACTTCGTC TAATCCCTCG TGAGAAGACG TCGACCCCTA CCTAGAGTTT   
  
  
- CCAACCGAGT AATGAAACGA TTACGGAACC TCCGCGCAAA TCGACCTTGA CCGAGTGTCT AGATATGTCG   
  
  
- GGACTCAAGC TTCTCCTGTT GTCGTCGACT ATACTATTTT CGAATAGTCA AAAAGAAAGC ACGAACGGGT   
  
  
- AAAAATTTCT AGAGATAATA GAAACGCTTG GTATACTAAT ATGTCTCTCG ACTTTTTCGT AGTTTCGAAG   
  
  
- TACAATATCT AAAACCGTAG GACATACCAA AAGTCACCGG GGAGGAGTAA GTTGCGGACA GCCTCGCTGG   
  
  
- ACCACCTGGA GGTTTTGACA CGTAATGACC CTAACTAGAA GGGGTTGGAC CTAAATCCGG TTGTCTTTCT   
  
  
- CAACTCCGTT GTCCCGAGGC GAACCGTTTC ATAACACTCG CGAAGTTACA AGGTAAACTT ATGGTACGGT   
  
  
- AACGTGTCTT TACCCTTTGG TAGTTTCGAC TTCTGGACTT CTATCTTCTA CTACTCCACC AACGGCACTT   
  
  
- GACAGAGAAG TCCAAATTCT TGGACGAACT ACTCTGCTAT CACCACCTAT CAGGCTCCTT ACGTCAAGAG   
  
  
- CCGAACTAAT CTTCCTATTT TGGACTATAG AAACAAGTGC CTCATCACTT GCCCAGAATG TTACGCGGGA   
  
  
- AGAAACAATG TGCAAAGTCC CTCCGGGAGA AGGTAATGAG ATGACATAAG CTATACAAAC TACGGTTACG   
  
  
- AAGGGCCCTC CTAGGACTCT CTAACTACAA ACTCTTCCTC AAAATACCCG CTCTTTAATA CTTACACCAC   
  
  
- CGAACACTCC CATGTCTCTC TCAACTTTCT GGACTCTGTA TGTTCGTTAC CGTCCGCGCC TTATCATCCC   
  
  
- GTCCCAAGTC CGTCGACGGT AACCTGGTTC TCGAGCAGTC ATTTGACTCC TCGTTCCAGG TCTACGTGAT   
  
  
- AGGATCCCTG AAGCAACAAC TACATCTACC TGTAACCTGT AACGTCCCTA CCTTCCCCTC CTAGTAGACA   
  
  
- CGACACAGAC GTACCCCAGG ACGAAT

+     ARE

| Site Name | Organism | Position | Strand | Matrix score. | sequence | function |
| --- | --- | --- | --- | --- | --- | --- |
| ARE | Zea mays | 515 | + | 6 | AAACCA | cis-acting regulatory element essential for the anaerobic induction |
| ARE | Zea mays | 3730 | + | 6 | AAACCA | cis-acting regulatory element essential for the anaerobic induction |
| ARE | Zea mays | 3053 | - | 6 | AAACCA | cis-acting regulatory element essential for the anaerobic induction |
| ARE | Zea mays | 2696 | - | 6 | AAACCA | cis-acting regulatory element essential for the anaerobic induction |
| ARE | Zea mays | 3530 | - | 6 | AAACCA | cis-acting regulatory element essential for the anaerobic induction |

>HU02G01569.1   
+ +Up\_Stream \_Len000AGCGTA GGAAGTGACG CCGCTATTGG AGGCGAAGAC GCTAGTACCA AGGCCAAAAC   
  
  
+ AAATGAGGAG ATAGATCAGT TAAACCGTTT GGTTAAGAAA ATTAAGCGAA CATCTCTTGA ACCTCCATCT   
  
  
+ GAACTCGATG ACATTGAGGA TGTGGATGCA GAAAAGATGG ATATGGGCTC TCCATCGGTG CCAGCAAATC   
  
  
+ AACCTAATGT TGGGTGGGGT CTCATTCGGT CAATGGGGGA CTAGGGACGT CATATCGTCA CCAATTACAA   
  
  
+ CGGAACAACC CTAAACCGCA TTTTAAAATC ACATCTTGGT AGCAGACAAA GATGTACGAC CCAAAAATTC   
  
  
+ AACTCTGTAA AGCACTAGCA AAGCTCGTAG TACACAGAAC AATCGCACAC TCATAGTGAT AGAAGTGTAC   
  
  
+ TATCCATTAT GCAGTGAATA TGACAAGGGA GGGGGTCCTG AGATGCCCAA ATTTGGCAGC CGAGTTGCCT   
  
  
+ TGACTGCCCA TCAATGTCCT AAACCAGTTG AGCATGTTAT TAATTTGCAA GAAAGATCGA ATTCTTCCTA   
  
  
+ TCTAAAAACT GACACAATAA AACAATACCA TGTATGGGAC ATTGCTAGGA TTGTTAACAC AATTATTGTC   
  
  
+ TTGTACGTTG CACCTGAAAA AAAAGAAAAT TGTTGCCATA GACAACATAT AAGTTAGGCA TTCATGACTA   
  
  
+ ATATCTGAAT TAGGTGTGTC GATCTGTGAT TCAAATTGAT ATAATTAGAT CTTTAAGGTA TCTAATTGCT   
  
  
+ TCAAGTTTTC TCTATAATGA AGATGTATAT TAGCTCACAT AGGTCTTAAG GTTAACTAAT GTCATATCAG   
  
  
+ GGGGTGTGGG CCGCCATTTT TGATAAGAAT TGAATTGAAC TTGCATTGTT TTTTCTTTGG CGAGAATGCA   
  
  
+ AGATTTGTTA ATTTATTCAT ATGCGTCATT ATACACTATA AGAAAAATAA TTGTAGGAAT ATATGAAGTA   
  
  
+ TTTTTACTTG TGCTCATGTT AAATGTGGAA TACTTTTTTA ATGTCGTATA GTACCACAGT ATTCTACAAT   
  
  
+ TTAAGAGATT AATCGATCCA TTAAAGTCAT GCAAACCTGA TGCATATTCA AGACATTGCA TGAAATGATC   
  
  
+ TTATTTTAGT CAAAGACTCC ATCATTTATA AGCAATATTT ACTTCGATAC ACGTGTCATT AAAAAAGACT   
  
  
+ ATAGAAATGT TACTGTTATT TTGTTTGAGC TAAATAGAAA ATTTAAAAGT TAATTAAGAT AATAATTAAA   
  
  
+ ACTAAATATT CAATGATGGC TAATAAATAC ATTGTCGTAT TAACTGACTA ATATTTGTAG ATGGTCGTGT   
  
  
+ TAATTTCTTT TTATTTTAAA GTTCAAATGA GTTACAAAAG GTTATAAAAT ATAAAGTAAA ATGGGAGACA   
  
  
+ AAGCAGACAG GGTGGGGGAA GCTAAGCTTA AAGTAGGATT TAGAAAGGCA ATGAAGGTGA ACCCTCAAAT   
  
  
+ CGATCCGCTG CCAATCACAG AAACCCAAAG CTTTGCTCAC CGACAACTCC CGGTTAACTG CAGTCACTCA   
  
  
+ CGGCACTGGG TAATACAGTA ATCGTGTACC CAGTTCATCC CCCTTTTCCT TTGGGAATAC AGTACCTCGA   
  
  
+ AAGTTCCTTT GAAATTCTAC TGGCCAGGCA TACCCATAAA TTGATCCTCT CGATATCATA AGATATGATA   
  
  
+ TTTCTCTGTC ATATCTGTTA GTTTCGGTAT CATATTTCTT CTTTATCTCT GTAATTTGAG GTATGCTTCA   
  
  
+ TTCCTGGCCG CTTGAATTAT CTTTCTTTCT TTGATTAGTA TTGTTTTTTT GGTTACCTAT TTTGCTCCAG   
  
  
+ TCCTGTTCAT TTGGGTCTTT TTGGATCTGG GTTTTTGCTG GGTTTGTTGA TTCTTTGAGA AATTTGGGTG   
  
  
+ CTTGAATTTG CCCTGCAATA TTCATGGGTT AGCGTTTTCC TGAATTTTGC TCTGTTCCTT AAGTGATTAT   
  
  
+ TTTTGATTGA TTACACCTTG GTGGTCCTGG TGGAATTTCC GAGGAGAAAT TGTCATGGGT TCCCACAACT   
  
  
+ TTGGAGAATT CCCTGATGAG ACTCTAAATG AGTATCAATC TACTTTGGGA ACCATGTCCC CCGGTTATGA   
  
  
+ TGGGTCTTTG AATTATACAA CCTTGTTCAA TTACAAAGAC CCATCTCAGG ATCTCACGGC ACTGAACCTT   
  
  
+ CCTAGCCCAT TGCCTGACCC TATGCCATTC AACATTGGTT CATATTCGGG TTTGAGCCCC GGGGTTGAAT   
  
  
+ CTTCGGATGA TAGCGATTCA GATGATGTTG TTAAGTACAT TGGCCAAGTG CTTATGGAAG AGGATATGAA   
  
  
+ GGAGAAGCCT TGTATGTTCC ATGACCCTTT AGCACTCCAA GCTGCTGAGA AACCCTTTTA TGATGTGTTG   
  
  
+ GGAAAGAAGT ATCCTCCTTC CCCTAATCAA CACCCACTTA TTGATCATTC TGTGGATAGC CCAGACAATC   
  
  
+ AGTCCTGTGG AACGAGCACA ATTAGTGATC TTAGTGGCAG TAACTGCACT TCCAGTTCAA CCAATTATAT   
  
  
+ TGATGCTGTG GCAGTCGCTG ATTCGAGTGA GAATACTAAG ACCTCTTTTG TGCAAAGTTC TCTGATTGAA   
  
  
+ TCGTTTTCTC AGTCGTCCAC CCTTCCACAG TGGTCATTTG GATCATTGGG TGCCTTGGGT GGCACAGCTT   
  
  
+ CTCAGGGTTC GAATTCAGTT ATCTCCTCCC GTGGTTTCCC TATGGCCATG AACGTTTTTA GCGAGCAGTC   
  
  
+ CATGATACAG TTTCAGAAGG GGGTGGAGGA GGCGAGCAAG TTCCTTCCAA AGAACAATAA CCTTGCGATT   
  
  
+ GACCTTGAGA GCGTCACTTT CCCAAATGAA AAGGAGGCGG CCCCCATGGT GGTGGTTAAG AAAGAGAAGG   
  
  
+ ATGAACACTC ACCTGATAGC TCGAGAGGTA GCAAGATTCA CTACCGTGAT GATGAGGACT TTGAGGATGG   
  
  
+ TAGGAGGAGT AAGCAGTCAG CTGTTTCTGT GGAGGAGGCT GAGTTGTCTG AAATGTTTGA CCGGGTTTTG   
  
  
+ CTTTGCAATC CCATGAAACA TGAAGCTCAT TGTACAAGTG GTTTGAAGTC CGAGAAGGGA ACATCCCTGC   
  
  
+ AGGCTGGCCA AGTACAGGTA CAGGAGGGTC AGAAGGCTCG TGCAAAGAAA CAGGGTAATA ATGATAATAA   
  
  
+ GAAGAATAAG AATGTGGTGG ATTTAAGGAC TCTGCTTATC CTCTGTGCGC AATCTGCTGC ATCTGATGAT   
  
  
+ CGCAGGACAG CTGATGAACT GCTGAAGCAG ATTAGGGAGC ACTCTTCTGC AGCTGGGGAT GGATCTCAAA   
  
  
+ GGTTGGCTCA TTACTTTGCT AATGCCTTGG AGGCGCGTTT AGCTGGAACT GGCTCACAGA TCTATACAGC   
  
  
+ CCTGAGTTCG AAGAGGACAA CAGCAGCTGA TATGATAAAA GCTTATCAGT TTTTCTTTCG TGCTTGCCCA   
  
  
+ TTTTTAAAGA TCTCTATTAT CTTTGCGAAC CATATGATTA TACAGAGAGC TGAAAAAGCA TCAAAGCTTC   
  
  
+ ATGTTATAGA TTTTGGCATC CTGTATGGTT TTCAGTGGCC CCTCCTCATT CAACGCCTGT CGGAGCGACC   
  
  
+ TGGTGGACCT CCAAAACTGT GCATTACTGG GATTGATCTT CCCCAACCTG GATTTAGGCC AACAGAAAGA   
  
  
+ GTTGAGGCAA CAGGGCTCCG CTTGGCAAAG TATTGTGAGC GCTTCAATGT TCCATTTGAA TACCATGCCA   
  
  
+ TTGCACAGAA ATGGGAAACC ATCAAAGCTG AAGACCTGAA GATAGAAGAT GATGAGGTGG TTGCCGTGAA   
  
  
+ CTGTCTCTTC AGGTTTAAGA ACCTGCTTGA TGAGACGATA GTGGTGGATA GTCCGAGGAA TGCAGTTCTC   
  
  
+ GGCTTGATTA GAAGGATAAA ACCTGATATC TTTGTTCACG GAGTAGTGAA CGGGTCTTAC AATGCGCCCT   
  
  
+ TCTTTGTTAC ACGTTTCAGG GAGGCCCTCT TCCATTACTC TACTGTATTC GATATGTTTG ATGCCAATGC   
  
  
+ TTCCCGGGAG GATCCTGAGA GATTGATGTT TGAGAAGGAG TTTTATGGGC GAGAAATTAT GAATGTGGTG   
  
  
+ GCTTGTGAGG GTACAGAGAG AGTTGAAAGA CCTGAGACAT ACAAGCAATG GCAGGCGCGG AATAGTAGGG   
  
  
+ CAGGGTTCAG GCAGCTGCCA TTGGACCAAG AGCTCGTCAG TAAACTGAGG AGCAAGGTCC AGATGCACTA   
  
  
+ TCCTAGGGAC TTCGTTGTTG ATGTAGATGG ACATTGGACA TTGCAGGGAT GGAAGGGGAG GATCATCTGT   
  
  
+ GCTGTGTCTG CATGGGGTCC TGCTTA  

- +Up\_Stream \_Len000TCGCAT CCTTCACTGC GGCGATAACC TCCGCTTCTG CGATCATGGT TCCGGTTTTG   
  
  
- TTTACTCCTC TATCTAGTCA ATTTGGCAAA CCAATTCTTT TAATTCGCTT GTAGAGAACT TGGAGGTAGA   
  
  
- CTTGAGCTAC TGTAACTCCT ACACCTACGT CTTTTCTACC TATACCCGAG AGGTAGCCAC GGTCGTTTAG   
  
  
- TTGGATTACA ACCCACCCCA GAGTAAGCCA GTTACCCCCT GATCCCTGCA GTATAGCAGT GGTTAATGTT   
  
  
- GCCTTGTTGG GATTTGGCGT AAAATTTTAG TGTAGAACCA TCGTCTGTTT CTACATGCTG GGTTTTTAAG   
  
  
- TTGAGACATT TCGTGATCGT TTCGAGCATC ATGTGTCTTG TTAGCGTGTG AGTATCACTA TCTTCACATG   
  
  
- ATAGGTAATA CGTCACTTAT ACTGTTCCCT CCCCCAGGAC TCTACGGGTT TAAACCGTCG GCTCAACGGA   
  
  
- ACTGACGGGT AGTTACAGGA TTTGGTCAAC TCGTACAATA ATTAAACGTT CTTTCTAGCT TAAGAAGGAT   
  
  
- AGATTTTTGA CTGTGTTATT TTGTTATGGT ACATACCCTG TAACGATCCT AACAATTGTG TTAATAACAG   
  
  
- AACATGCAAC GTGGACTTTT TTTTCTTTTA ACAACGGTAT CTGTTGTATA TTCAATCCGT AAGTACTGAT   
  
  
- TATAGACTTA ATCCACACAG CTAGACACTA AGTTTAACTA TATTAATCTA GAAATTCCAT AGATTAACGA   
  
  
- AGTTCAAAAG AGATATTACT TCTACATATA ATCGAGTGTA TCCAGAATTC CAATTGATTA CAGTATAGTC   
  
  
- CCCCACACCC GGCGGTAAAA ACTATTCTTA ACTTAACTTG AACGTAACAA AAAAGAAACC GCTCTTACGT   
  
  
- TCTAAACAAT TAAATAAGTA TACGCAGTAA TATGTGATAT TCTTTTTATT AACATCCTTA TATACTTCAT   
  
  
- AAAAATGAAC ACGAGTACAA TTTACACCTT ATGAAAAAAT TACAGCATAT CATGGTGTCA TAAGATGTTA   
  
  
- AATTCTCTAA TTAGCTAGGT AATTTCAGTA CGTTTGGACT ACGTATAAGT TCTGTAACGT ACTTTACTAG   
  
  
- AATAAAATCA GTTTCTGAGG TAGTAAATAT TCGTTATAAA TGAAGCTATG TGCACAGTAA TTTTTTCTGA   
  
  
- TATCTTTACA ATGACAATAA AACAAACTCG ATTTATCTTT TAAATTTTCA ATTAATTCTA TTATTAATTT   
  
  
- TGATTTATAA GTTACTACCG ATTATTTATG TAACAGCATA ATTGACTGAT TATAAACATC TACCAGCACA   
  
  
- ATTAAAGAAA AATAAAATTT CAAGTTTACT CAATGTTTTC CAATATTTTA TATTTCATTT TACCCTCTGT   
  
  
- TTCGTCTGTC CCACCCCCTT CGATTCGAAT TTCATCCTAA ATCTTTCCGT TACTTCCACT TGGGAGTTTA   
  
  
- GCTAGGCGAC GGTTAGTGTC TTTGGGTTTC GAAACGAGTG GCTGTTGAGG GCCAATTGAC GTCAGTGAGT   
  
  
- GCCGTGACCC ATTATGTCAT TAGCACATGG GTCAAGTAGG GGGAAAAGGA AACCCTTATG TCATGGAGCT   
  
  
- TTCAAGGAAA CTTTAAGATG ACCGGTCCGT ATGGGTATTT AACTAGGAGA GCTATAGTAT TCTATACTAT   
  
  
- AAAGAGACAG TATAGACAAT CAAAGCCATA GTATAAAGAA GAAATAGAGA CATTAAACTC CATACGAAGT   
  
  
- AAGGACCGGC GAACTTAATA GAAAGAAAGA AACTAATCAT AACAAAAAAA CCAATGGATA AAACGAGGTC   
  
  
- AGGACAAGTA AACCCAGAAA AACCTAGACC CAAAAACGAC CCAAACAACT AAGAAACTCT TTAAACCCAC   
  
  
- GAACTTAAAC GGGACGTTAT AAGTACCCAA TCGCAAAAGG ACTTAAAACG AGACAAGGAA TTCACTAATA   
  
  
- AAAACTAACT AATGTGGAAC CACCAGGACC ACCTTAAAGG CTCCTCTTTA ACAGTACCCA AGGGTGTTGA   
  
  
- AACCTCTTAA GGGACTACTC TGAGATTTAC TCATAGTTAG ATGAAACCCT TGGTACAGGG GGCCAATACT   
  
  
- ACCCAGAAAC TTAATATGTT GGAACAAGTT AATGTTTCTG GGTAGAGTCC TAGAGTGCCG TGACTTGGAA   
  
  
- GGATCGGGTA ACGGACTGGG ATACGGTAAG TTGTAACCAA GTATAAGCCC AAACTCGGGG CCCCAACTTA   
  
  
- GAAGCCTACT ATCGCTAAGT CTACTACAAC AATTCATGTA ACCGGTTCAC GAATACCTTC TCCTATACTT   
  
  
- CCTCTTCGGA ACATACAAGG TACTGGGAAA TCGTGAGGTT CGACGACTCT TTGGGAAAAT ACTACACAAC   
  
  
- CCTTTCTTCA TAGGAGGAAG GGGATTAGTT GTGGGTGAAT AACTAGTAAG ACACCTATCG GGTCTGTTAG   
  
  
- TCAGGACACC TTGCTCGTGT TAATCACTAG AATCACCGTC ATTGACGTGA AGGTCAAGTT GGTTAATATA   
  
  
- ACTACGACAC CGTCAGCGAC TAAGCTCACT CTTATGATTC TGGAGAAAAC ACGTTTCAAG AGACTAACTT   
  
  
- AGCAAAAGAG TCAGCAGGTG GGAAGGTGTC ACCAGTAAAC CTAGTAACCC ACGGAACCCA CCGTGTCGAA   
  
  
- GAGTCCCAAG CTTAAGTCAA TAGAGGAGGG CACCAAAGGG ATACCGGTAC TTGCAAAAAT CGCTCGTCAG   
  
  
- GTACTATGTC AAAGTCTTCC CCCACCTCCT CCGCTCGTTC AAGGAAGGTT TCTTGTTATT GGAACGCTAA   
  
  
- CTGGAACTCT CGCAGTGAAA GGGTTTACTT TTCCTCCGCC GGGGGTACCA CCACCAATTC TTTCTCTTCC   
  
  
- TACTTGTGAG TGGACTATCG AGCTCTCCAT CGTTCTAAGT GATGGCACTA CTACTCCTGA AACTCCTACC   
  
  
- ATCCTCCTCA TTCGTCAGTC GACAAAGACA CCTCCTCCGA CTCAACAGAC TTTACAAACT GGCCCAAAAC   
  
  
- GAAACGTTAG GGTACTTTGT ACTTCGAGTA ACATGTTCAC CAAACTTCAG GCTCTTCCCT TGTAGGGACG   
  
  
- TCCGACCGGT TCATGTCCAT GTCCTCCCAG TCTTCCGAGC ACGTTTCTTT GTCCCATTAT TACTATTATT   
  
  
- CTTCTTATTC TTACACCACC TAAATTCCTG AGACGAATAG GAGACACGCG TTAGACGACG TAGACTACTA   
  
  
- GCGTCCTGTC GACTACTTGA CGACTTCGTC TAATCCCTCG TGAGAAGACG TCGACCCCTA CCTAGAGTTT   
  
  
- CCAACCGAGT AATGAAACGA TTACGGAACC TCCGCGCAAA TCGACCTTGA CCGAGTGTCT AGATATGTCG   
  
  
- GGACTCAAGC TTCTCCTGTT GTCGTCGACT ATACTATTTT CGAATAGTCA AAAAGAAAGC ACGAACGGGT   
  
  
- AAAAATTTCT AGAGATAATA GAAACGCTTG GTATACTAAT ATGTCTCTCG ACTTTTTCGT AGTTTCGAAG   
  
  
- TACAATATCT AAAACCGTAG GACATACCAA AAGTCACCGG GGAGGAGTAA GTTGCGGACA GCCTCGCTGG   
  
  
- ACCACCTGGA GGTTTTGACA CGTAATGACC CTAACTAGAA GGGGTTGGAC CTAAATCCGG TTGTCTTTCT   
  
  
- CAACTCCGTT GTCCCGAGGC GAACCGTTTC ATAACACTCG CGAAGTTACA AGGTAAACTT ATGGTACGGT   
  
  
- AACGTGTCTT TACCCTTTGG TAGTTTCGAC TTCTGGACTT CTATCTTCTA CTACTCCACC AACGGCACTT   
  
  
- GACAGAGAAG TCCAAATTCT TGGACGAACT ACTCTGCTAT CACCACCTAT CAGGCTCCTT ACGTCAAGAG   
  
  
- CCGAACTAAT CTTCCTATTT TGGACTATAG AAACAAGTGC CTCATCACTT GCCCAGAATG TTACGCGGGA   
  
  
- AGAAACAATG TGCAAAGTCC CTCCGGGAGA AGGTAATGAG ATGACATAAG CTATACAAAC TACGGTTACG   
  
  
- AAGGGCCCTC CTAGGACTCT CTAACTACAA ACTCTTCCTC AAAATACCCG CTCTTTAATA CTTACACCAC   
  
  
- CGAACACTCC CATGTCTCTC TCAACTTTCT GGACTCTGTA TGTTCGTTAC CGTCCGCGCC TTATCATCCC   
  
  
- GTCCCAAGTC CGTCGACGGT AACCTGGTTC TCGAGCAGTC ATTTGACTCC TCGTTCCAGG TCTACGTGAT   
  
  
- AGGATCCCTG AAGCAACAAC TACATCTACC TGTAACCTGT AACGTCCCTA CCTTCCCCTC CTAGTAGACA   
  
  
- CGACACAGAC GTACCCCAGG ACGAAT

+     Box 4

| Site Name | Organism | Position | Strand | Matrix score. | sequence | function |
| --- | --- | --- | --- | --- | --- | --- |
| Box 4 | Petroselinum crispum | 533 | + | 6 | ATTAAT | part of a conserved DNA module involved in light responsiveness |
| Box 4 | Petroselinum crispum | 1062 | + | 6 | ATTAAT | part of a conserved DNA module involved in light responsiveness |

>HU02G01569.1   
+ +Up\_Stream \_Len000AGCGTA GGAAGTGACG CCGCTATTGG AGGCGAAGAC GCTAGTACCA AGGCCAAAAC   
  
  
+ AAATGAGGAG ATAGATCAGT TAAACCGTTT GGTTAAGAAA ATTAAGCGAA CATCTCTTGA ACCTCCATCT   
  
  
+ GAACTCGATG ACATTGAGGA TGTGGATGCA GAAAAGATGG ATATGGGCTC TCCATCGGTG CCAGCAAATC   
  
  
+ AACCTAATGT TGGGTGGGGT CTCATTCGGT CAATGGGGGA CTAGGGACGT CATATCGTCA CCAATTACAA   
  
  
+ CGGAACAACC CTAAACCGCA TTTTAAAATC ACATCTTGGT AGCAGACAAA GATGTACGAC CCAAAAATTC   
  
  
+ AACTCTGTAA AGCACTAGCA AAGCTCGTAG TACACAGAAC AATCGCACAC TCATAGTGAT AGAAGTGTAC   
  
  
+ TATCCATTAT GCAGTGAATA TGACAAGGGA GGGGGTCCTG AGATGCCCAA ATTTGGCAGC CGAGTTGCCT   
  
  
+ TGACTGCCCA TCAATGTCCT AAACCAGTTG AGCATGTTAT TAATTTGCAA GAAAGATCGA ATTCTTCCTA   
  
  
+ TCTAAAAACT GACACAATAA AACAATACCA TGTATGGGAC ATTGCTAGGA TTGTTAACAC AATTATTGTC   
  
  
+ TTGTACGTTG CACCTGAAAA AAAAGAAAAT TGTTGCCATA GACAACATAT AAGTTAGGCA TTCATGACTA   
  
  
+ ATATCTGAAT TAGGTGTGTC GATCTGTGAT TCAAATTGAT ATAATTAGAT CTTTAAGGTA TCTAATTGCT   
  
  
+ TCAAGTTTTC TCTATAATGA AGATGTATAT TAGCTCACAT AGGTCTTAAG GTTAACTAAT GTCATATCAG   
  
  
+ GGGGTGTGGG CCGCCATTTT TGATAAGAAT TGAATTGAAC TTGCATTGTT TTTTCTTTGG CGAGAATGCA   
  
  
+ AGATTTGTTA ATTTATTCAT ATGCGTCATT ATACACTATA AGAAAAATAA TTGTAGGAAT ATATGAAGTA   
  
  
+ TTTTTACTTG TGCTCATGTT AAATGTGGAA TACTTTTTTA ATGTCGTATA GTACCACAGT ATTCTACAAT   
  
  
+ TTAAGAGATT AATCGATCCA TTAAAGTCAT GCAAACCTGA TGCATATTCA AGACATTGCA TGAAATGATC   
  
  
+ TTATTTTAGT CAAAGACTCC ATCATTTATA AGCAATATTT ACTTCGATAC ACGTGTCATT AAAAAAGACT   
  
  
+ ATAGAAATGT TACTGTTATT TTGTTTGAGC TAAATAGAAA ATTTAAAAGT TAATTAAGAT AATAATTAAA   
  
  
+ ACTAAATATT CAATGATGGC TAATAAATAC ATTGTCGTAT TAACTGACTA ATATTTGTAG ATGGTCGTGT   
  
  
+ TAATTTCTTT TTATTTTAAA GTTCAAATGA GTTACAAAAG GTTATAAAAT ATAAAGTAAA ATGGGAGACA   
  
  
+ AAGCAGACAG GGTGGGGGAA GCTAAGCTTA AAGTAGGATT TAGAAAGGCA ATGAAGGTGA ACCCTCAAAT   
  
  
+ CGATCCGCTG CCAATCACAG AAACCCAAAG CTTTGCTCAC CGACAACTCC CGGTTAACTG CAGTCACTCA   
  
  
+ CGGCACTGGG TAATACAGTA ATCGTGTACC CAGTTCATCC CCCTTTTCCT TTGGGAATAC AGTACCTCGA   
  
  
+ AAGTTCCTTT GAAATTCTAC TGGCCAGGCA TACCCATAAA TTGATCCTCT CGATATCATA AGATATGATA   
  
  
+ TTTCTCTGTC ATATCTGTTA GTTTCGGTAT CATATTTCTT CTTTATCTCT GTAATTTGAG GTATGCTTCA   
  
  
+ TTCCTGGCCG CTTGAATTAT CTTTCTTTCT TTGATTAGTA TTGTTTTTTT GGTTACCTAT TTTGCTCCAG   
  
  
+ TCCTGTTCAT TTGGGTCTTT TTGGATCTGG GTTTTTGCTG GGTTTGTTGA TTCTTTGAGA AATTTGGGTG   
  
  
+ CTTGAATTTG CCCTGCAATA TTCATGGGTT AGCGTTTTCC TGAATTTTGC TCTGTTCCTT AAGTGATTAT   
  
  
+ TTTTGATTGA TTACACCTTG GTGGTCCTGG TGGAATTTCC GAGGAGAAAT TGTCATGGGT TCCCACAACT   
  
  
+ TTGGAGAATT CCCTGATGAG ACTCTAAATG AGTATCAATC TACTTTGGGA ACCATGTCCC CCGGTTATGA   
  
  
+ TGGGTCTTTG AATTATACAA CCTTGTTCAA TTACAAAGAC CCATCTCAGG ATCTCACGGC ACTGAACCTT   
  
  
+ CCTAGCCCAT TGCCTGACCC TATGCCATTC AACATTGGTT CATATTCGGG TTTGAGCCCC GGGGTTGAAT   
  
  
+ CTTCGGATGA TAGCGATTCA GATGATGTTG TTAAGTACAT TGGCCAAGTG CTTATGGAAG AGGATATGAA   
  
  
+ GGAGAAGCCT TGTATGTTCC ATGACCCTTT AGCACTCCAA GCTGCTGAGA AACCCTTTTA TGATGTGTTG   
  
  
+ GGAAAGAAGT ATCCTCCTTC CCCTAATCAA CACCCACTTA TTGATCATTC TGTGGATAGC CCAGACAATC   
  
  
+ AGTCCTGTGG AACGAGCACA ATTAGTGATC TTAGTGGCAG TAACTGCACT TCCAGTTCAA CCAATTATAT   
  
  
+ TGATGCTGTG GCAGTCGCTG ATTCGAGTGA GAATACTAAG ACCTCTTTTG TGCAAAGTTC TCTGATTGAA   
  
  
+ TCGTTTTCTC AGTCGTCCAC CCTTCCACAG TGGTCATTTG GATCATTGGG TGCCTTGGGT GGCACAGCTT   
  
  
+ CTCAGGGTTC GAATTCAGTT ATCTCCTCCC GTGGTTTCCC TATGGCCATG AACGTTTTTA GCGAGCAGTC   
  
  
+ CATGATACAG TTTCAGAAGG GGGTGGAGGA GGCGAGCAAG TTCCTTCCAA AGAACAATAA CCTTGCGATT   
  
  
+ GACCTTGAGA GCGTCACTTT CCCAAATGAA AAGGAGGCGG CCCCCATGGT GGTGGTTAAG AAAGAGAAGG   
  
  
+ ATGAACACTC ACCTGATAGC TCGAGAGGTA GCAAGATTCA CTACCGTGAT GATGAGGACT TTGAGGATGG   
  
  
+ TAGGAGGAGT AAGCAGTCAG CTGTTTCTGT GGAGGAGGCT GAGTTGTCTG AAATGTTTGA CCGGGTTTTG   
  
  
+ CTTTGCAATC CCATGAAACA TGAAGCTCAT TGTACAAGTG GTTTGAAGTC CGAGAAGGGA ACATCCCTGC   
  
  
+ AGGCTGGCCA AGTACAGGTA CAGGAGGGTC AGAAGGCTCG TGCAAAGAAA CAGGGTAATA ATGATAATAA   
  
  
+ GAAGAATAAG AATGTGGTGG ATTTAAGGAC TCTGCTTATC CTCTGTGCGC AATCTGCTGC ATCTGATGAT   
  
  
+ CGCAGGACAG CTGATGAACT GCTGAAGCAG ATTAGGGAGC ACTCTTCTGC AGCTGGGGAT GGATCTCAAA   
  
  
+ GGTTGGCTCA TTACTTTGCT AATGCCTTGG AGGCGCGTTT AGCTGGAACT GGCTCACAGA TCTATACAGC   
  
  
+ CCTGAGTTCG AAGAGGACAA CAGCAGCTGA TATGATAAAA GCTTATCAGT TTTTCTTTCG TGCTTGCCCA   
  
  
+ TTTTTAAAGA TCTCTATTAT CTTTGCGAAC CATATGATTA TACAGAGAGC TGAAAAAGCA TCAAAGCTTC   
  
  
+ ATGTTATAGA TTTTGGCATC CTGTATGGTT TTCAGTGGCC CCTCCTCATT CAACGCCTGT CGGAGCGACC   
  
  
+ TGGTGGACCT CCAAAACTGT GCATTACTGG GATTGATCTT CCCCAACCTG GATTTAGGCC AACAGAAAGA   
  
  
+ GTTGAGGCAA CAGGGCTCCG CTTGGCAAAG TATTGTGAGC GCTTCAATGT TCCATTTGAA TACCATGCCA   
  
  
+ TTGCACAGAA ATGGGAAACC ATCAAAGCTG AAGACCTGAA GATAGAAGAT GATGAGGTGG TTGCCGTGAA   
  
  
+ CTGTCTCTTC AGGTTTAAGA ACCTGCTTGA TGAGACGATA GTGGTGGATA GTCCGAGGAA TGCAGTTCTC   
  
  
+ GGCTTGATTA GAAGGATAAA ACCTGATATC TTTGTTCACG GAGTAGTGAA CGGGTCTTAC AATGCGCCCT   
  
  
+ TCTTTGTTAC ACGTTTCAGG GAGGCCCTCT TCCATTACTC TACTGTATTC GATATGTTTG ATGCCAATGC   
  
  
+ TTCCCGGGAG GATCCTGAGA GATTGATGTT TGAGAAGGAG TTTTATGGGC GAGAAATTAT GAATGTGGTG   
  
  
+ GCTTGTGAGG GTACAGAGAG AGTTGAAAGA CCTGAGACAT ACAAGCAATG GCAGGCGCGG AATAGTAGGG   
  
  
+ CAGGGTTCAG GCAGCTGCCA TTGGACCAAG AGCTCGTCAG TAAACTGAGG AGCAAGGTCC AGATGCACTA   
  
  
+ TCCTAGGGAC TTCGTTGTTG ATGTAGATGG ACATTGGACA TTGCAGGGAT GGAAGGGGAG GATCATCTGT   
  
  
+ GCTGTGTCTG CATGGGGTCC TGCTTA  

- +Up\_Stream \_Len000TCGCAT CCTTCACTGC GGCGATAACC TCCGCTTCTG CGATCATGGT TCCGGTTTTG   
  
  
- TTTACTCCTC TATCTAGTCA ATTTGGCAAA CCAATTCTTT TAATTCGCTT GTAGAGAACT TGGAGGTAGA   
  
  
- CTTGAGCTAC TGTAACTCCT ACACCTACGT CTTTTCTACC TATACCCGAG AGGTAGCCAC GGTCGTTTAG   
  
  
- TTGGATTACA ACCCACCCCA GAGTAAGCCA GTTACCCCCT GATCCCTGCA GTATAGCAGT GGTTAATGTT   
  
  
- GCCTTGTTGG GATTTGGCGT AAAATTTTAG TGTAGAACCA TCGTCTGTTT CTACATGCTG GGTTTTTAAG   
  
  
- TTGAGACATT TCGTGATCGT TTCGAGCATC ATGTGTCTTG TTAGCGTGTG AGTATCACTA TCTTCACATG   
  
  
- ATAGGTAATA CGTCACTTAT ACTGTTCCCT CCCCCAGGAC TCTACGGGTT TAAACCGTCG GCTCAACGGA   
  
  
- ACTGACGGGT AGTTACAGGA TTTGGTCAAC TCGTACAATA ATTAAACGTT CTTTCTAGCT TAAGAAGGAT   
  
  
- AGATTTTTGA CTGTGTTATT TTGTTATGGT ACATACCCTG TAACGATCCT AACAATTGTG TTAATAACAG   
  
  
- AACATGCAAC GTGGACTTTT TTTTCTTTTA ACAACGGTAT CTGTTGTATA TTCAATCCGT AAGTACTGAT   
  
  
- TATAGACTTA ATCCACACAG CTAGACACTA AGTTTAACTA TATTAATCTA GAAATTCCAT AGATTAACGA   
  
  
- AGTTCAAAAG AGATATTACT TCTACATATA ATCGAGTGTA TCCAGAATTC CAATTGATTA CAGTATAGTC   
  
  
- CCCCACACCC GGCGGTAAAA ACTATTCTTA ACTTAACTTG AACGTAACAA AAAAGAAACC GCTCTTACGT   
  
  
- TCTAAACAAT TAAATAAGTA TACGCAGTAA TATGTGATAT TCTTTTTATT AACATCCTTA TATACTTCAT   
  
  
- AAAAATGAAC ACGAGTACAA TTTACACCTT ATGAAAAAAT TACAGCATAT CATGGTGTCA TAAGATGTTA   
  
  
- AATTCTCTAA TTAGCTAGGT AATTTCAGTA CGTTTGGACT ACGTATAAGT TCTGTAACGT ACTTTACTAG   
  
  
- AATAAAATCA GTTTCTGAGG TAGTAAATAT TCGTTATAAA TGAAGCTATG TGCACAGTAA TTTTTTCTGA   
  
  
- TATCTTTACA ATGACAATAA AACAAACTCG ATTTATCTTT TAAATTTTCA ATTAATTCTA TTATTAATTT   
  
  
- TGATTTATAA GTTACTACCG ATTATTTATG TAACAGCATA ATTGACTGAT TATAAACATC TACCAGCACA   
  
  
- ATTAAAGAAA AATAAAATTT CAAGTTTACT CAATGTTTTC CAATATTTTA TATTTCATTT TACCCTCTGT   
  
  
- TTCGTCTGTC CCACCCCCTT CGATTCGAAT TTCATCCTAA ATCTTTCCGT TACTTCCACT TGGGAGTTTA   
  
  
- GCTAGGCGAC GGTTAGTGTC TTTGGGTTTC GAAACGAGTG GCTGTTGAGG GCCAATTGAC GTCAGTGAGT   
  
  
- GCCGTGACCC ATTATGTCAT TAGCACATGG GTCAAGTAGG GGGAAAAGGA AACCCTTATG TCATGGAGCT   
  
  
- TTCAAGGAAA CTTTAAGATG ACCGGTCCGT ATGGGTATTT AACTAGGAGA GCTATAGTAT TCTATACTAT   
  
  
- AAAGAGACAG TATAGACAAT CAAAGCCATA GTATAAAGAA GAAATAGAGA CATTAAACTC CATACGAAGT   
  
  
- AAGGACCGGC GAACTTAATA GAAAGAAAGA AACTAATCAT AACAAAAAAA CCAATGGATA AAACGAGGTC   
  
  
- AGGACAAGTA AACCCAGAAA AACCTAGACC CAAAAACGAC CCAAACAACT AAGAAACTCT TTAAACCCAC   
  
  
- GAACTTAAAC GGGACGTTAT AAGTACCCAA TCGCAAAAGG ACTTAAAACG AGACAAGGAA TTCACTAATA   
  
  
- AAAACTAACT AATGTGGAAC CACCAGGACC ACCTTAAAGG CTCCTCTTTA ACAGTACCCA AGGGTGTTGA   
  
  
- AACCTCTTAA GGGACTACTC TGAGATTTAC TCATAGTTAG ATGAAACCCT TGGTACAGGG GGCCAATACT   
  
  
- ACCCAGAAAC TTAATATGTT GGAACAAGTT AATGTTTCTG GGTAGAGTCC TAGAGTGCCG TGACTTGGAA   
  
  
- GGATCGGGTA ACGGACTGGG ATACGGTAAG TTGTAACCAA GTATAAGCCC AAACTCGGGG CCCCAACTTA   
  
  
- GAAGCCTACT ATCGCTAAGT CTACTACAAC AATTCATGTA ACCGGTTCAC GAATACCTTC TCCTATACTT   
  
  
- CCTCTTCGGA ACATACAAGG TACTGGGAAA TCGTGAGGTT CGACGACTCT TTGGGAAAAT ACTACACAAC   
  
  
- CCTTTCTTCA TAGGAGGAAG GGGATTAGTT GTGGGTGAAT AACTAGTAAG ACACCTATCG GGTCTGTTAG   
  
  
- TCAGGACACC TTGCTCGTGT TAATCACTAG AATCACCGTC ATTGACGTGA AGGTCAAGTT GGTTAATATA   
  
  
- ACTACGACAC CGTCAGCGAC TAAGCTCACT CTTATGATTC TGGAGAAAAC ACGTTTCAAG AGACTAACTT   
  
  
- AGCAAAAGAG TCAGCAGGTG GGAAGGTGTC ACCAGTAAAC CTAGTAACCC ACGGAACCCA CCGTGTCGAA   
  
  
- GAGTCCCAAG CTTAAGTCAA TAGAGGAGGG CACCAAAGGG ATACCGGTAC TTGCAAAAAT CGCTCGTCAG   
  
  
- GTACTATGTC AAAGTCTTCC CCCACCTCCT CCGCTCGTTC AAGGAAGGTT TCTTGTTATT GGAACGCTAA   
  
  
- CTGGAACTCT CGCAGTGAAA GGGTTTACTT TTCCTCCGCC GGGGGTACCA CCACCAATTC TTTCTCTTCC   
  
  
- TACTTGTGAG TGGACTATCG AGCTCTCCAT CGTTCTAAGT GATGGCACTA CTACTCCTGA AACTCCTACC   
  
  
- ATCCTCCTCA TTCGTCAGTC GACAAAGACA CCTCCTCCGA CTCAACAGAC TTTACAAACT GGCCCAAAAC   
  
  
- GAAACGTTAG GGTACTTTGT ACTTCGAGTA ACATGTTCAC CAAACTTCAG GCTCTTCCCT TGTAGGGACG   
  
  
- TCCGACCGGT TCATGTCCAT GTCCTCCCAG TCTTCCGAGC ACGTTTCTTT GTCCCATTAT TACTATTATT   
  
  
- CTTCTTATTC TTACACCACC TAAATTCCTG AGACGAATAG GAGACACGCG TTAGACGACG TAGACTACTA   
  
  
- GCGTCCTGTC GACTACTTGA CGACTTCGTC TAATCCCTCG TGAGAAGACG TCGACCCCTA CCTAGAGTTT   
  
  
- CCAACCGAGT AATGAAACGA TTACGGAACC TCCGCGCAAA TCGACCTTGA CCGAGTGTCT AGATATGTCG   
  
  
- GGACTCAAGC TTCTCCTGTT GTCGTCGACT ATACTATTTT CGAATAGTCA AAAAGAAAGC ACGAACGGGT   
  
  
- AAAAATTTCT AGAGATAATA GAAACGCTTG GTATACTAAT ATGTCTCTCG ACTTTTTCGT AGTTTCGAAG   
  
  
- TACAATATCT AAAACCGTAG GACATACCAA AAGTCACCGG GGAGGAGTAA GTTGCGGACA GCCTCGCTGG   
  
  
- ACCACCTGGA GGTTTTGACA CGTAATGACC CTAACTAGAA GGGGTTGGAC CTAAATCCGG TTGTCTTTCT   
  
  
- CAACTCCGTT GTCCCGAGGC GAACCGTTTC ATAACACTCG CGAAGTTACA AGGTAAACTT ATGGTACGGT   
  
  
- AACGTGTCTT TACCCTTTGG TAGTTTCGAC TTCTGGACTT CTATCTTCTA CTACTCCACC AACGGCACTT   
  
  
- GACAGAGAAG TCCAAATTCT TGGACGAACT ACTCTGCTAT CACCACCTAT CAGGCTCCTT ACGTCAAGAG   
  
  
- CCGAACTAAT CTTCCTATTT TGGACTATAG AAACAAGTGC CTCATCACTT GCCCAGAATG TTACGCGGGA   
  
  
- AGAAACAATG TGCAAAGTCC CTCCGGGAGA AGGTAATGAG ATGACATAAG CTATACAAAC TACGGTTACG   
  
  
- AAGGGCCCTC CTAGGACTCT CTAACTACAA ACTCTTCCTC AAAATACCCG CTCTTTAATA CTTACACCAC   
  
  
- CGAACACTCC CATGTCTCTC TCAACTTTCT GGACTCTGTA TGTTCGTTAC CGTCCGCGCC TTATCATCCC   
  
  
- GTCCCAAGTC CGTCGACGGT AACCTGGTTC TCGAGCAGTC ATTTGACTCC TCGTTCCAGG TCTACGTGAT   
  
  
- AGGATCCCTG AAGCAACAAC TACATCTACC TGTAACCTGT AACGTCCCTA CCTTCCCCTC CTAGTAGACA   
  
  
- CGACACAGAC GTACCCCAGG ACGAAT

+     CAAT-box

| Site Name | Organism | Position | Strand | Matrix score. | sequence | function |
| --- | --- | --- | --- | --- | --- | --- |
| CAAT-box | Arabidopsis thaliana | 2515 | + | 5 | CCAAT | common cis-acting element in promoter and enhancer regions |
| CAAT-box | Arabidopsis thaliana | 2283 | - | 5 | CCAAT | common cis-acting element in promoter and enhancer regions |
| CAAT-box | Arabidopsis thaliana | 40 | - | 5 | CCAAT | common cis-acting element in promoter and enhancer regions |
| CAAT-box | Arabidopsis thaliana | 2208 | - | 5 | CCAAT | common cis-acting element in promoter and enhancer regions |
| CAAT-box | Arabidopsis thaliana | 275 | + | 5 | CCAAT | common cis-acting element in promoter and enhancer regions |
| CAAT-box | Arabidopsis thaliana | 4237 | - | 5 | CCAAT | common cis-acting element in promoter and enhancer regions |
| CAAT-box | Arabidopsis thaliana | 4154 | - | 5 | CCAAT | common cis-acting element in promoter and enhancer regions |
| CAAT-box | Nicotiana glutinosa | 769 | - | 4 | CAAT |  |
| CAAT-box | Nicotiana glutinosa | 663 | - | 4 | CAAT |  |
| CAAT-box | Nicotiana glutinosa | 2070 | + | 4 | CAAT |  |
| CAAT-box | Nicotiana glutinosa | 1295 | - | 4 | CAAT |  |
| CAAT-box | Nicotiana glutinosa | 2523 | - | 4 | CAAT |  |
| CAAT-box | Arabidopsis thaliana | 1485 | + | 5 | CCAAT | common cis-acting element in promoter and enhancer regions |
| CAAT-box | Nicotiana glutinosa | 3020 | + | 4 | CAAT |  |
| CAAT-box | Arabidopsis thaliana | 2639 | - | 5 | CCAAT | common cis-acting element in promoter and enhancer regions |
| CAAT-box | Nicotiana glutinosa | 2183 | - | 4 | CAAT |  |
| CAAT-box | Nicotiana glutinosa | 1794 | - | 4 | CAAT |  |
| CAAT-box | Nicotiana glutinosa | 4110 | + | 4 | CAAT |  |
| CAAT-box | Arabidopsis thaliana | 3988 | + | 5 | CCAAT | common cis-acting element in promoter and enhancer regions |
| CAAT-box | Nicotiana glutinosa | 276 | + | 4 | CAAT |  |
| CAAT-box | Nicotiana glutinosa | 2473 | + | 4 | CAAT |  |
| CAAT-box | Nicotiana glutinosa | 2424 | - | 4 | CAAT |  |
| CAAT-box | Pisum sativum | 736 | + | 5 | CAAAT | common cis-acting element in promoter and enhancer regions |
| CAAT-box | Nicotiana glutinosa | 964 | - | 4 | CAAT |  |
| CAAT-box | Nicotiana glutinosa | 3606 | - | 4 | CAAT |  |
| CAAT-box | Nicotiana glutinosa | 157 | - | 4 | CAAT |  |
| CAAT-box | Nicotiana glutinosa | 2516 | + | 4 | CAAT |  |
| CAAT-box | Nicotiana glutinosa | 889 | - | 4 | CAAT |  |
| CAAT-box | Nicotiana glutinosa | 605 | - | 4 | CAAT |  |
| CAAT-box | Nicotiana glutinosa | 2802 | - | 4 | CAAT |  |
| CAAT-box | Nicotiana glutinosa | 1970 | - | 4 | CAAT |  |
| CAAT-box | Pisum sativum | 1470 | + | 5 | CAAAT | common cis-acting element in promoter and enhancer regions |
| CAAT-box | Nicotiana glutinosa | 3989 | + | 4 | CAAT |  |
| CAAT-box | Pisum sativum | 472 | + | 5 | CAAAT | common cis-acting element in promoter and enhancer regions |
| CAAT-box | Nicotiana glutinosa | 1910 | + | 4 | CAAT |  |
| CAAT-box | Nicotiana glutinosa | 3689 | + | 4 | CAAT |  |
| CAAT-box | Nicotiana glutinosa | 2132 | + | 4 | CAAT |  |
| CAAT-box | Nicotiana glutinosa | 1109 | - | 4 | CAAT |  |
| CAAT-box | Nicotiana glutinosa | 614 | - | 4 | CAAT |  |
| CAAT-box | Nicotiana glutinosa | 4244 | - | 4 | CAAT |  |
| CAAT-box | Pisum sativum | 1738 | - | 5 | CAAAT | common cis-acting element in promoter and enhancer regions |
| CAAT-box | Nicotiana glutinosa | 2450 | + | 4 | CAAT |  |
| CAAT-box | Nicotiana glutinosa | 245 | + | 4 | CAAT |  |
| CAAT-box | Nicotiana glutinosa | 2789 | + | 4 | CAAT |  |
| CAAT-box | Pisum sativum | 1886 | - | 5 | CAAAT | common cis-acting element in promoter and enhancer regions |
| CAAT-box | Nicotiana glutinosa | 878 | - | 4 | CAAT |  |
| CAAT-box | Nicotiana glutinosa | 506 | + | 4 | CAAT |  |
| CAAT-box | Pisum sativum | 3698 | - | 5 | CAAAT | common cis-acting element in promoter and enhancer regions |
| CAAT-box | Pisum sativum | 1317 | - | 5 | CAAAT | common cis-acting element in promoter and enhancer regions |
| CAAT-box | Nicotiana glutinosa | 2589 | - | 4 | CAAT |  |
| CAAT-box | Nicotiana glutinosa | 3204 | + | 4 | CAAT |  |
| CAAT-box | Pisum sativum | 74 | + | 5 | CAAAT | common cis-acting element in promoter and enhancer regions |
| CAAT-box | Nicotiana glutinosa | 1051 | + | 4 | CAAT |  |
| CAAT-box | Nicotiana glutinosa | 2013 | - | 4 | CAAT |  |
| CAAT-box | Nicotiana glutinosa | 624 | + | 4 | CAAT |  |
| CAAT-box | Nicotiana glutinosa | 4016 | - | 4 | CAAT |  |
| CAAT-box | Nicotiana glutinosa | 1486 | + | 4 | CAAT |  |
| CAAT-box | Pisum sativum | 2630 | - | 5 | CAAAT | common cis-acting element in promoter and enhancer regions |
| CAAT-box | Pisum sativum | 209 | + | 5 | CAAAT | common cis-acting element in promoter and enhancer regions |
| CAAT-box | Nicotiana glutinosa | 3043 | - | 4 | CAAT |  |
| CAAT-box | Pisum sativum | 1833 | - | 5 | CAAAT | common cis-acting element in promoter and enhancer regions |
| CAAT-box | Pisum sativum | 917 | - | 5 | CAAAT | common cis-acting element in promoter and enhancer regions |
| CAAT-box | Pisum sativum | 537 | - | 5 | CAAAT | common cis-acting element in promoter and enhancer regions |
| CAAT-box | Nicotiana glutinosa | 3714 | - | 4 | CAAT |  |
| CAAT-box | Nicotiana glutinosa | 1453 | + | 4 | CAAT |  |
| CAAT-box | Pisum sativum | 1900 | - | 5 | CAAAT | common cis-acting element in promoter and enhancer regions |
| CAAT-box | Nicotiana glutinosa | 394 | + | 4 | CAAT |  |
| CAAT-box | Nicotiana glutinosa | 3676 | - | 4 | CAAT |  |
| CAAT-box | Nicotiana glutinosa | 3914 | + | 4 | CAAT |  |
| CAAT-box | Nicotiana glutinosa | 629 | - | 4 | CAAT |  |
| CAAT-box | Pisum sativum | 2827 | + | 5 | CAAAT | common cis-acting element in promoter and enhancer regions |
| CAAT-box | Nicotiana glutinosa | 1157 | + | 4 | CAAT |  |
| CAAT-box | Pisum sativum | 475 | - | 5 | CAAAT | common cis-acting element in promoter and enhancer regions |
| CAAT-box | Nicotiana glutinosa | 1654 | - | 4 | CAAT |  |
| CAAT-box | Nicotiana glutinosa | 579 | + | 4 | CAAT |  |
| CAAT-box | Nicotiana glutinosa | 739 | - | 4 | CAAT |  |
| CAAT-box | Nicotiana glutinosa | 587 | + | 4 | CAAT |  |
| CAAT-box | Nicotiana glutinosa | 1275 | + | 4 | CAAT |  |
| CAAT-box | Nicotiana glutinosa | 873 | - | 4 | CAAT |  |
| CAAT-box | Pisum sativum | 1358 | + | 5 | CAAAT | common cis-acting element in promoter and enhancer regions |

>HU02G01569.1   
+ +Up\_Stream \_Len000AGCGTA GGAAGTGACG CCGCTATTGG AGGCGAAGAC GCTAGTACCA AGGCCAAAAC   
  
  
+ AAATGAGGAG ATAGATCAGT TAAACCGTTT GGTTAAGAAA ATTAAGCGAA CATCTCTTGA ACCTCCATCT   
  
  
+ GAACTCGATG ACATTGAGGA TGTGGATGCA GAAAAGATGG ATATGGGCTC TCCATCGGTG CCAGCAAATC   
  
  
+ AACCTAATGT TGGGTGGGGT CTCATTCGGT CAATGGGGGA CTAGGGACGT CATATCGTCA CCAATTACAA   
  
  
+ CGGAACAACC CTAAACCGCA TTTTAAAATC ACATCTTGGT AGCAGACAAA GATGTACGAC CCAAAAATTC   
  
  
+ AACTCTGTAA AGCACTAGCA AAGCTCGTAG TACACAGAAC AATCGCACAC TCATAGTGAT AGAAGTGTAC   
  
  
+ TATCCATTAT GCAGTGAATA TGACAAGGGA GGGGGTCCTG AGATGCCCAA ATTTGGCAGC CGAGTTGCCT   
  
  
+ TGACTGCCCA TCAATGTCCT AAACCAGTTG AGCATGTTAT TAATTTGCAA GAAAGATCGA ATTCTTCCTA   
  
  
+ TCTAAAAACT GACACAATAA AACAATACCA TGTATGGGAC ATTGCTAGGA TTGTTAACAC AATTATTGTC   
  
  
+ TTGTACGTTG CACCTGAAAA AAAAGAAAAT TGTTGCCATA GACAACATAT AAGTTAGGCA TTCATGACTA   
  
  
+ ATATCTGAAT TAGGTGTGTC GATCTGTGAT TCAAATTGAT ATAATTAGAT CTTTAAGGTA TCTAATTGCT   
  
  
+ TCAAGTTTTC TCTATAATGA AGATGTATAT TAGCTCACAT AGGTCTTAAG GTTAACTAAT GTCATATCAG   
  
  
+ GGGGTGTGGG CCGCCATTTT TGATAAGAAT TGAATTGAAC TTGCATTGTT TTTTCTTTGG CGAGAATGCA   
  
  
+ AGATTTGTTA ATTTATTCAT ATGCGTCATT ATACACTATA AGAAAAATAA TTGTAGGAAT ATATGAAGTA   
  
  
+ TTTTTACTTG TGCTCATGTT AAATGTGGAA TACTTTTTTA ATGTCGTATA GTACCACAGT ATTCTACAAT   
  
  
+ TTAAGAGATT AATCGATCCA TTAAAGTCAT GCAAACCTGA TGCATATTCA AGACATTGCA TGAAATGATC   
  
  
+ TTATTTTAGT CAAAGACTCC ATCATTTATA AGCAATATTT ACTTCGATAC ACGTGTCATT AAAAAAGACT   
  
  
+ ATAGAAATGT TACTGTTATT TTGTTTGAGC TAAATAGAAA ATTTAAAAGT TAATTAAGAT AATAATTAAA   
  
  
+ ACTAAATATT CAATGATGGC TAATAAATAC ATTGTCGTAT TAACTGACTA ATATTTGTAG ATGGTCGTGT   
  
  
+ TAATTTCTTT TTATTTTAAA GTTCAAATGA GTTACAAAAG GTTATAAAAT ATAAAGTAAA ATGGGAGACA   
  
  
+ AAGCAGACAG GGTGGGGGAA GCTAAGCTTA AAGTAGGATT TAGAAAGGCA ATGAAGGTGA ACCCTCAAAT   
  
  
+ CGATCCGCTG CCAATCACAG AAACCCAAAG CTTTGCTCAC CGACAACTCC CGGTTAACTG CAGTCACTCA   
  
  
+ CGGCACTGGG TAATACAGTA ATCGTGTACC CAGTTCATCC CCCTTTTCCT TTGGGAATAC AGTACCTCGA   
  
  
+ AAGTTCCTTT GAAATTCTAC TGGCCAGGCA TACCCATAAA TTGATCCTCT CGATATCATA AGATATGATA   
  
  
+ TTTCTCTGTC ATATCTGTTA GTTTCGGTAT CATATTTCTT CTTTATCTCT GTAATTTGAG GTATGCTTCA   
  
  
+ TTCCTGGCCG CTTGAATTAT CTTTCTTTCT TTGATTAGTA TTGTTTTTTT GGTTACCTAT TTTGCTCCAG   
  
  
+ TCCTGTTCAT TTGGGTCTTT TTGGATCTGG GTTTTTGCTG GGTTTGTTGA TTCTTTGAGA AATTTGGGTG   
  
  
+ CTTGAATTTG CCCTGCAATA TTCATGGGTT AGCGTTTTCC TGAATTTTGC TCTGTTCCTT AAGTGATTAT   
  
  
+ TTTTGATTGA TTACACCTTG GTGGTCCTGG TGGAATTTCC GAGGAGAAAT TGTCATGGGT TCCCACAACT   
  
  
+ TTGGAGAATT CCCTGATGAG ACTCTAAATG AGTATCAATC TACTTTGGGA ACCATGTCCC CCGGTTATGA   
  
  
+ TGGGTCTTTG AATTATACAA CCTTGTTCAA TTACAAAGAC CCATCTCAGG ATCTCACGGC ACTGAACCTT   
  
  
+ CCTAGCCCAT TGCCTGACCC TATGCCATTC AACATTGGTT CATATTCGGG TTTGAGCCCC GGGGTTGAAT   
  
  
+ CTTCGGATGA TAGCGATTCA GATGATGTTG TTAAGTACAT TGGCCAAGTG CTTATGGAAG AGGATATGAA   
  
  
+ GGAGAAGCCT TGTATGTTCC ATGACCCTTT AGCACTCCAA GCTGCTGAGA AACCCTTTTA TGATGTGTTG   
  
  
+ GGAAAGAAGT ATCCTCCTTC CCCTAATCAA CACCCACTTA TTGATCATTC TGTGGATAGC CCAGACAATC   
  
  
+ AGTCCTGTGG AACGAGCACA ATTAGTGATC TTAGTGGCAG TAACTGCACT TCCAGTTCAA CCAATTATAT   
  
  
+ TGATGCTGTG GCAGTCGCTG ATTCGAGTGA GAATACTAAG ACCTCTTTTG TGCAAAGTTC TCTGATTGAA   
  
  
+ TCGTTTTCTC AGTCGTCCAC CCTTCCACAG TGGTCATTTG GATCATTGGG TGCCTTGGGT GGCACAGCTT   
  
  
+ CTCAGGGTTC GAATTCAGTT ATCTCCTCCC GTGGTTTCCC TATGGCCATG AACGTTTTTA GCGAGCAGTC   
  
  
+ CATGATACAG TTTCAGAAGG GGGTGGAGGA GGCGAGCAAG TTCCTTCCAA AGAACAATAA CCTTGCGATT   
  
  
+ GACCTTGAGA GCGTCACTTT CCCAAATGAA AAGGAGGCGG CCCCCATGGT GGTGGTTAAG AAAGAGAAGG   
  
  
+ ATGAACACTC ACCTGATAGC TCGAGAGGTA GCAAGATTCA CTACCGTGAT GATGAGGACT TTGAGGATGG   
  
  
+ TAGGAGGAGT AAGCAGTCAG CTGTTTCTGT GGAGGAGGCT GAGTTGTCTG AAATGTTTGA CCGGGTTTTG   
  
  
+ CTTTGCAATC CCATGAAACA TGAAGCTCAT TGTACAAGTG GTTTGAAGTC CGAGAAGGGA ACATCCCTGC   
  
  
+ AGGCTGGCCA AGTACAGGTA CAGGAGGGTC AGAAGGCTCG TGCAAAGAAA CAGGGTAATA ATGATAATAA   
  
  
+ GAAGAATAAG AATGTGGTGG ATTTAAGGAC TCTGCTTATC CTCTGTGCGC AATCTGCTGC ATCTGATGAT   
  
  
+ CGCAGGACAG CTGATGAACT GCTGAAGCAG ATTAGGGAGC ACTCTTCTGC AGCTGGGGAT GGATCTCAAA   
  
  
+ GGTTGGCTCA TTACTTTGCT AATGCCTTGG AGGCGCGTTT AGCTGGAACT GGCTCACAGA TCTATACAGC   
  
  
+ CCTGAGTTCG AAGAGGACAA CAGCAGCTGA TATGATAAAA GCTTATCAGT TTTTCTTTCG TGCTTGCCCA   
  
  
+ TTTTTAAAGA TCTCTATTAT CTTTGCGAAC CATATGATTA TACAGAGAGC TGAAAAAGCA TCAAAGCTTC   
  
  
+ ATGTTATAGA TTTTGGCATC CTGTATGGTT TTCAGTGGCC CCTCCTCATT CAACGCCTGT CGGAGCGACC   
  
  
+ TGGTGGACCT CCAAAACTGT GCATTACTGG GATTGATCTT CCCCAACCTG GATTTAGGCC AACAGAAAGA   
  
  
+ GTTGAGGCAA CAGGGCTCCG CTTGGCAAAG TATTGTGAGC GCTTCAATGT TCCATTTGAA TACCATGCCA   
  
  
+ TTGCACAGAA ATGGGAAACC ATCAAAGCTG AAGACCTGAA GATAGAAGAT GATGAGGTGG TTGCCGTGAA   
  
  
+ CTGTCTCTTC AGGTTTAAGA ACCTGCTTGA TGAGACGATA GTGGTGGATA GTCCGAGGAA TGCAGTTCTC   
  
  
+ GGCTTGATTA GAAGGATAAA ACCTGATATC TTTGTTCACG GAGTAGTGAA CGGGTCTTAC AATGCGCCCT   
  
  
+ TCTTTGTTAC ACGTTTCAGG GAGGCCCTCT TCCATTACTC TACTGTATTC GATATGTTTG ATGCCAATGC   
  
  
+ TTCCCGGGAG GATCCTGAGA GATTGATGTT TGAGAAGGAG TTTTATGGGC GAGAAATTAT GAATGTGGTG   
  
  
+ GCTTGTGAGG GTACAGAGAG AGTTGAAAGA CCTGAGACAT ACAAGCAATG GCAGGCGCGG AATAGTAGGG   
  
  
+ CAGGGTTCAG GCAGCTGCCA TTGGACCAAG AGCTCGTCAG TAAACTGAGG AGCAAGGTCC AGATGCACTA   
  
  
+ TCCTAGGGAC TTCGTTGTTG ATGTAGATGG ACATTGGACA TTGCAGGGAT GGAAGGGGAG GATCATCTGT   
  
  
+ GCTGTGTCTG CATGGGGTCC TGCTTA  

- +Up\_Stream \_Len000TCGCAT CCTTCACTGC GGCGATAACC TCCGCTTCTG CGATCATGGT TCCGGTTTTG   
  
  
- TTTACTCCTC TATCTAGTCA ATTTGGCAAA CCAATTCTTT TAATTCGCTT GTAGAGAACT TGGAGGTAGA   
  
  
- CTTGAGCTAC TGTAACTCCT ACACCTACGT CTTTTCTACC TATACCCGAG AGGTAGCCAC GGTCGTTTAG   
  
  
- TTGGATTACA ACCCACCCCA GAGTAAGCCA GTTACCCCCT GATCCCTGCA GTATAGCAGT GGTTAATGTT   
  
  
- GCCTTGTTGG GATTTGGCGT AAAATTTTAG TGTAGAACCA TCGTCTGTTT CTACATGCTG GGTTTTTAAG   
  
  
- TTGAGACATT TCGTGATCGT TTCGAGCATC ATGTGTCTTG TTAGCGTGTG AGTATCACTA TCTTCACATG   
  
  
- ATAGGTAATA CGTCACTTAT ACTGTTCCCT CCCCCAGGAC TCTACGGGTT TAAACCGTCG GCTCAACGGA   
  
  
- ACTGACGGGT AGTTACAGGA TTTGGTCAAC TCGTACAATA ATTAAACGTT CTTTCTAGCT TAAGAAGGAT   
  
  
- AGATTTTTGA CTGTGTTATT TTGTTATGGT ACATACCCTG TAACGATCCT AACAATTGTG TTAATAACAG   
  
  
- AACATGCAAC GTGGACTTTT TTTTCTTTTA ACAACGGTAT CTGTTGTATA TTCAATCCGT AAGTACTGAT   
  
  
- TATAGACTTA ATCCACACAG CTAGACACTA AGTTTAACTA TATTAATCTA GAAATTCCAT AGATTAACGA   
  
  
- AGTTCAAAAG AGATATTACT TCTACATATA ATCGAGTGTA TCCAGAATTC CAATTGATTA CAGTATAGTC   
  
  
- CCCCACACCC GGCGGTAAAA ACTATTCTTA ACTTAACTTG AACGTAACAA AAAAGAAACC GCTCTTACGT   
  
  
- TCTAAACAAT TAAATAAGTA TACGCAGTAA TATGTGATAT TCTTTTTATT AACATCCTTA TATACTTCAT   
  
  
- AAAAATGAAC ACGAGTACAA TTTACACCTT ATGAAAAAAT TACAGCATAT CATGGTGTCA TAAGATGTTA   
  
  
- AATTCTCTAA TTAGCTAGGT AATTTCAGTA CGTTTGGACT ACGTATAAGT TCTGTAACGT ACTTTACTAG   
  
  
- AATAAAATCA GTTTCTGAGG TAGTAAATAT TCGTTATAAA TGAAGCTATG TGCACAGTAA TTTTTTCTGA   
  
  
- TATCTTTACA ATGACAATAA AACAAACTCG ATTTATCTTT TAAATTTTCA ATTAATTCTA TTATTAATTT   
  
  
- TGATTTATAA GTTACTACCG ATTATTTATG TAACAGCATA ATTGACTGAT TATAAACATC TACCAGCACA   
  
  
- ATTAAAGAAA AATAAAATTT CAAGTTTACT CAATGTTTTC CAATATTTTA TATTTCATTT TACCCTCTGT   
  
  
- TTCGTCTGTC CCACCCCCTT CGATTCGAAT TTCATCCTAA ATCTTTCCGT TACTTCCACT TGGGAGTTTA   
  
  
- GCTAGGCGAC GGTTAGTGTC TTTGGGTTTC GAAACGAGTG GCTGTTGAGG GCCAATTGAC GTCAGTGAGT   
  
  
- GCCGTGACCC ATTATGTCAT TAGCACATGG GTCAAGTAGG GGGAAAAGGA AACCCTTATG TCATGGAGCT   
  
  
- TTCAAGGAAA CTTTAAGATG ACCGGTCCGT ATGGGTATTT AACTAGGAGA GCTATAGTAT TCTATACTAT   
  
  
- AAAGAGACAG TATAGACAAT CAAAGCCATA GTATAAAGAA GAAATAGAGA CATTAAACTC CATACGAAGT   
  
  
- AAGGACCGGC GAACTTAATA GAAAGAAAGA AACTAATCAT AACAAAAAAA CCAATGGATA AAACGAGGTC   
  
  
- AGGACAAGTA AACCCAGAAA AACCTAGACC CAAAAACGAC CCAAACAACT AAGAAACTCT TTAAACCCAC   
  
  
- GAACTTAAAC GGGACGTTAT AAGTACCCAA TCGCAAAAGG ACTTAAAACG AGACAAGGAA TTCACTAATA   
  
  
- AAAACTAACT AATGTGGAAC CACCAGGACC ACCTTAAAGG CTCCTCTTTA ACAGTACCCA AGGGTGTTGA   
  
  
- AACCTCTTAA GGGACTACTC TGAGATTTAC TCATAGTTAG ATGAAACCCT TGGTACAGGG GGCCAATACT   
  
  
- ACCCAGAAAC TTAATATGTT GGAACAAGTT AATGTTTCTG GGTAGAGTCC TAGAGTGCCG TGACTTGGAA   
  
  
- GGATCGGGTA ACGGACTGGG ATACGGTAAG TTGTAACCAA GTATAAGCCC AAACTCGGGG CCCCAACTTA   
  
  
- GAAGCCTACT ATCGCTAAGT CTACTACAAC AATTCATGTA ACCGGTTCAC GAATACCTTC TCCTATACTT   
  
  
- CCTCTTCGGA ACATACAAGG TACTGGGAAA TCGTGAGGTT CGACGACTCT TTGGGAAAAT ACTACACAAC   
  
  
- CCTTTCTTCA TAGGAGGAAG GGGATTAGTT GTGGGTGAAT AACTAGTAAG ACACCTATCG GGTCTGTTAG   
  
  
- TCAGGACACC TTGCTCGTGT TAATCACTAG AATCACCGTC ATTGACGTGA AGGTCAAGTT GGTTAATATA   
  
  
- ACTACGACAC CGTCAGCGAC TAAGCTCACT CTTATGATTC TGGAGAAAAC ACGTTTCAAG AGACTAACTT   
  
  
- AGCAAAAGAG TCAGCAGGTG GGAAGGTGTC ACCAGTAAAC CTAGTAACCC ACGGAACCCA CCGTGTCGAA   
  
  
- GAGTCCCAAG CTTAAGTCAA TAGAGGAGGG CACCAAAGGG ATACCGGTAC TTGCAAAAAT CGCTCGTCAG   
  
  
- GTACTATGTC AAAGTCTTCC CCCACCTCCT CCGCTCGTTC AAGGAAGGTT TCTTGTTATT GGAACGCTAA   
  
  
- CTGGAACTCT CGCAGTGAAA GGGTTTACTT TTCCTCCGCC GGGGGTACCA CCACCAATTC TTTCTCTTCC   
  
  
- TACTTGTGAG TGGACTATCG AGCTCTCCAT CGTTCTAAGT GATGGCACTA CTACTCCTGA AACTCCTACC   
  
  
- ATCCTCCTCA TTCGTCAGTC GACAAAGACA CCTCCTCCGA CTCAACAGAC TTTACAAACT GGCCCAAAAC   
  
  
- GAAACGTTAG GGTACTTTGT ACTTCGAGTA ACATGTTCAC CAAACTTCAG GCTCTTCCCT TGTAGGGACG   
  
  
- TCCGACCGGT TCATGTCCAT GTCCTCCCAG TCTTCCGAGC ACGTTTCTTT GTCCCATTAT TACTATTATT   
  
  
- CTTCTTATTC TTACACCACC TAAATTCCTG AGACGAATAG GAGACACGCG TTAGACGACG TAGACTACTA   
  
  
- GCGTCCTGTC GACTACTTGA CGACTTCGTC TAATCCCTCG TGAGAAGACG TCGACCCCTA CCTAGAGTTT   
  
  
- CCAACCGAGT AATGAAACGA TTACGGAACC TCCGCGCAAA TCGACCTTGA CCGAGTGTCT AGATATGTCG   
  
  
- GGACTCAAGC TTCTCCTGTT GTCGTCGACT ATACTATTTT CGAATAGTCA AAAAGAAAGC ACGAACGGGT   
  
  
- AAAAATTTCT AGAGATAATA GAAACGCTTG GTATACTAAT ATGTCTCTCG ACTTTTTCGT AGTTTCGAAG   
  
  
- TACAATATCT AAAACCGTAG GACATACCAA AAGTCACCGG GGAGGAGTAA GTTGCGGACA GCCTCGCTGG   
  
  
- ACCACCTGGA GGTTTTGACA CGTAATGACC CTAACTAGAA GGGGTTGGAC CTAAATCCGG TTGTCTTTCT   
  
  
- CAACTCCGTT GTCCCGAGGC GAACCGTTTC ATAACACTCG CGAAGTTACA AGGTAAACTT ATGGTACGGT   
  
  
- AACGTGTCTT TACCCTTTGG TAGTTTCGAC TTCTGGACTT CTATCTTCTA CTACTCCACC AACGGCACTT   
  
  
- GACAGAGAAG TCCAAATTCT TGGACGAACT ACTCTGCTAT CACCACCTAT CAGGCTCCTT ACGTCAAGAG   
  
  
- CCGAACTAAT CTTCCTATTT TGGACTATAG AAACAAGTGC CTCATCACTT GCCCAGAATG TTACGCGGGA   
  
  
- AGAAACAATG TGCAAAGTCC CTCCGGGAGA AGGTAATGAG ATGACATAAG CTATACAAAC TACGGTTACG   
  
  
- AAGGGCCCTC CTAGGACTCT CTAACTACAA ACTCTTCCTC AAAATACCCG CTCTTTAATA CTTACACCAC   
  
  
- CGAACACTCC CATGTCTCTC TCAACTTTCT GGACTCTGTA TGTTCGTTAC CGTCCGCGCC TTATCATCCC   
  
  
- GTCCCAAGTC CGTCGACGGT AACCTGGTTC TCGAGCAGTC ATTTGACTCC TCGTTCCAGG TCTACGTGAT   
  
  
- AGGATCCCTG AAGCAACAAC TACATCTACC TGTAACCTGT AACGTCCCTA CCTTCCCCTC CTAGTAGACA   
  
  
- CGACACAGAC GTACCCCAGG ACGAAT

+     CARE

| Site Name | Organism | Position | Strand | Matrix score. | sequence | function |
| --- | --- | --- | --- | --- | --- | --- |
| CARE | Oryza sativa | 1518 | + | 8 | CAACTCCC |  |

>HU02G01569.1   
+ +Up\_Stream \_Len000AGCGTA GGAAGTGACG CCGCTATTGG AGGCGAAGAC GCTAGTACCA AGGCCAAAAC   
  
  
+ AAATGAGGAG ATAGATCAGT TAAACCGTTT GGTTAAGAAA ATTAAGCGAA CATCTCTTGA ACCTCCATCT   
  
  
+ GAACTCGATG ACATTGAGGA TGTGGATGCA GAAAAGATGG ATATGGGCTC TCCATCGGTG CCAGCAAATC   
  
  
+ AACCTAATGT TGGGTGGGGT CTCATTCGGT CAATGGGGGA CTAGGGACGT CATATCGTCA CCAATTACAA   
  
  
+ CGGAACAACC CTAAACCGCA TTTTAAAATC ACATCTTGGT AGCAGACAAA GATGTACGAC CCAAAAATTC   
  
  
+ AACTCTGTAA AGCACTAGCA AAGCTCGTAG TACACAGAAC AATCGCACAC TCATAGTGAT AGAAGTGTAC   
  
  
+ TATCCATTAT GCAGTGAATA TGACAAGGGA GGGGGTCCTG AGATGCCCAA ATTTGGCAGC CGAGTTGCCT   
  
  
+ TGACTGCCCA TCAATGTCCT AAACCAGTTG AGCATGTTAT TAATTTGCAA GAAAGATCGA ATTCTTCCTA   
  
  
+ TCTAAAAACT GACACAATAA AACAATACCA TGTATGGGAC ATTGCTAGGA TTGTTAACAC AATTATTGTC   
  
  
+ TTGTACGTTG CACCTGAAAA AAAAGAAAAT TGTTGCCATA GACAACATAT AAGTTAGGCA TTCATGACTA   
  
  
+ ATATCTGAAT TAGGTGTGTC GATCTGTGAT TCAAATTGAT ATAATTAGAT CTTTAAGGTA TCTAATTGCT   
  
  
+ TCAAGTTTTC TCTATAATGA AGATGTATAT TAGCTCACAT AGGTCTTAAG GTTAACTAAT GTCATATCAG   
  
  
+ GGGGTGTGGG CCGCCATTTT TGATAAGAAT TGAATTGAAC TTGCATTGTT TTTTCTTTGG CGAGAATGCA   
  
  
+ AGATTTGTTA ATTTATTCAT ATGCGTCATT ATACACTATA AGAAAAATAA TTGTAGGAAT ATATGAAGTA   
  
  
+ TTTTTACTTG TGCTCATGTT AAATGTGGAA TACTTTTTTA ATGTCGTATA GTACCACAGT ATTCTACAAT   
  
  
+ TTAAGAGATT AATCGATCCA TTAAAGTCAT GCAAACCTGA TGCATATTCA AGACATTGCA TGAAATGATC   
  
  
+ TTATTTTAGT CAAAGACTCC ATCATTTATA AGCAATATTT ACTTCGATAC ACGTGTCATT AAAAAAGACT   
  
  
+ ATAGAAATGT TACTGTTATT TTGTTTGAGC TAAATAGAAA ATTTAAAAGT TAATTAAGAT AATAATTAAA   
  
  
+ ACTAAATATT CAATGATGGC TAATAAATAC ATTGTCGTAT TAACTGACTA ATATTTGTAG ATGGTCGTGT   
  
  
+ TAATTTCTTT TTATTTTAAA GTTCAAATGA GTTACAAAAG GTTATAAAAT ATAAAGTAAA ATGGGAGACA   
  
  
+ AAGCAGACAG GGTGGGGGAA GCTAAGCTTA AAGTAGGATT TAGAAAGGCA ATGAAGGTGA ACCCTCAAAT   
  
  
+ CGATCCGCTG CCAATCACAG AAACCCAAAG CTTTGCTCAC CGACAACTCC CGGTTAACTG CAGTCACTCA   
  
  
+ CGGCACTGGG TAATACAGTA ATCGTGTACC CAGTTCATCC CCCTTTTCCT TTGGGAATAC AGTACCTCGA   
  
  
+ AAGTTCCTTT GAAATTCTAC TGGCCAGGCA TACCCATAAA TTGATCCTCT CGATATCATA AGATATGATA   
  
  
+ TTTCTCTGTC ATATCTGTTA GTTTCGGTAT CATATTTCTT CTTTATCTCT GTAATTTGAG GTATGCTTCA   
  
  
+ TTCCTGGCCG CTTGAATTAT CTTTCTTTCT TTGATTAGTA TTGTTTTTTT GGTTACCTAT TTTGCTCCAG   
  
  
+ TCCTGTTCAT TTGGGTCTTT TTGGATCTGG GTTTTTGCTG GGTTTGTTGA TTCTTTGAGA AATTTGGGTG   
  
  
+ CTTGAATTTG CCCTGCAATA TTCATGGGTT AGCGTTTTCC TGAATTTTGC TCTGTTCCTT AAGTGATTAT   
  
  
+ TTTTGATTGA TTACACCTTG GTGGTCCTGG TGGAATTTCC GAGGAGAAAT TGTCATGGGT TCCCACAACT   
  
  
+ TTGGAGAATT CCCTGATGAG ACTCTAAATG AGTATCAATC TACTTTGGGA ACCATGTCCC CCGGTTATGA   
  
  
+ TGGGTCTTTG AATTATACAA CCTTGTTCAA TTACAAAGAC CCATCTCAGG ATCTCACGGC ACTGAACCTT   
  
  
+ CCTAGCCCAT TGCCTGACCC TATGCCATTC AACATTGGTT CATATTCGGG TTTGAGCCCC GGGGTTGAAT   
  
  
+ CTTCGGATGA TAGCGATTCA GATGATGTTG TTAAGTACAT TGGCCAAGTG CTTATGGAAG AGGATATGAA   
  
  
+ GGAGAAGCCT TGTATGTTCC ATGACCCTTT AGCACTCCAA GCTGCTGAGA AACCCTTTTA TGATGTGTTG   
  
  
+ GGAAAGAAGT ATCCTCCTTC CCCTAATCAA CACCCACTTA TTGATCATTC TGTGGATAGC CCAGACAATC   
  
  
+ AGTCCTGTGG AACGAGCACA ATTAGTGATC TTAGTGGCAG TAACTGCACT TCCAGTTCAA CCAATTATAT   
  
  
+ TGATGCTGTG GCAGTCGCTG ATTCGAGTGA GAATACTAAG ACCTCTTTTG TGCAAAGTTC TCTGATTGAA   
  
  
+ TCGTTTTCTC AGTCGTCCAC CCTTCCACAG TGGTCATTTG GATCATTGGG TGCCTTGGGT GGCACAGCTT   
  
  
+ CTCAGGGTTC GAATTCAGTT ATCTCCTCCC GTGGTTTCCC TATGGCCATG AACGTTTTTA GCGAGCAGTC   
  
  
+ CATGATACAG TTTCAGAAGG GGGTGGAGGA GGCGAGCAAG TTCCTTCCAA AGAACAATAA CCTTGCGATT   
  
  
+ GACCTTGAGA GCGTCACTTT CCCAAATGAA AAGGAGGCGG CCCCCATGGT GGTGGTTAAG AAAGAGAAGG   
  
  
+ ATGAACACTC ACCTGATAGC TCGAGAGGTA GCAAGATTCA CTACCGTGAT GATGAGGACT TTGAGGATGG   
  
  
+ TAGGAGGAGT AAGCAGTCAG CTGTTTCTGT GGAGGAGGCT GAGTTGTCTG AAATGTTTGA CCGGGTTTTG   
  
  
+ CTTTGCAATC CCATGAAACA TGAAGCTCAT TGTACAAGTG GTTTGAAGTC CGAGAAGGGA ACATCCCTGC   
  
  
+ AGGCTGGCCA AGTACAGGTA CAGGAGGGTC AGAAGGCTCG TGCAAAGAAA CAGGGTAATA ATGATAATAA   
  
  
+ GAAGAATAAG AATGTGGTGG ATTTAAGGAC TCTGCTTATC CTCTGTGCGC AATCTGCTGC ATCTGATGAT   
  
  
+ CGCAGGACAG CTGATGAACT GCTGAAGCAG ATTAGGGAGC ACTCTTCTGC AGCTGGGGAT GGATCTCAAA   
  
  
+ GGTTGGCTCA TTACTTTGCT AATGCCTTGG AGGCGCGTTT AGCTGGAACT GGCTCACAGA TCTATACAGC   
  
  
+ CCTGAGTTCG AAGAGGACAA CAGCAGCTGA TATGATAAAA GCTTATCAGT TTTTCTTTCG TGCTTGCCCA   
  
  
+ TTTTTAAAGA TCTCTATTAT CTTTGCGAAC CATATGATTA TACAGAGAGC TGAAAAAGCA TCAAAGCTTC   
  
  
+ ATGTTATAGA TTTTGGCATC CTGTATGGTT TTCAGTGGCC CCTCCTCATT CAACGCCTGT CGGAGCGACC   
  
  
+ TGGTGGACCT CCAAAACTGT GCATTACTGG GATTGATCTT CCCCAACCTG GATTTAGGCC AACAGAAAGA   
  
  
+ GTTGAGGCAA CAGGGCTCCG CTTGGCAAAG TATTGTGAGC GCTTCAATGT TCCATTTGAA TACCATGCCA   
  
  
+ TTGCACAGAA ATGGGAAACC ATCAAAGCTG AAGACCTGAA GATAGAAGAT GATGAGGTGG TTGCCGTGAA   
  
  
+ CTGTCTCTTC AGGTTTAAGA ACCTGCTTGA TGAGACGATA GTGGTGGATA GTCCGAGGAA TGCAGTTCTC   
  
  
+ GGCTTGATTA GAAGGATAAA ACCTGATATC TTTGTTCACG GAGTAGTGAA CGGGTCTTAC AATGCGCCCT   
  
  
+ TCTTTGTTAC ACGTTTCAGG GAGGCCCTCT TCCATTACTC TACTGTATTC GATATGTTTG ATGCCAATGC   
  
  
+ TTCCCGGGAG GATCCTGAGA GATTGATGTT TGAGAAGGAG TTTTATGGGC GAGAAATTAT GAATGTGGTG   
  
  
+ GCTTGTGAGG GTACAGAGAG AGTTGAAAGA CCTGAGACAT ACAAGCAATG GCAGGCGCGG AATAGTAGGG   
  
  
+ CAGGGTTCAG GCAGCTGCCA TTGGACCAAG AGCTCGTCAG TAAACTGAGG AGCAAGGTCC AGATGCACTA   
  
  
+ TCCTAGGGAC TTCGTTGTTG ATGTAGATGG ACATTGGACA TTGCAGGGAT GGAAGGGGAG GATCATCTGT   
  
  
+ GCTGTGTCTG CATGGGGTCC TGCTTA  

- +Up\_Stream \_Len000TCGCAT CCTTCACTGC GGCGATAACC TCCGCTTCTG CGATCATGGT TCCGGTTTTG   
  
  
- TTTACTCCTC TATCTAGTCA ATTTGGCAAA CCAATTCTTT TAATTCGCTT GTAGAGAACT TGGAGGTAGA   
  
  
- CTTGAGCTAC TGTAACTCCT ACACCTACGT CTTTTCTACC TATACCCGAG AGGTAGCCAC GGTCGTTTAG   
  
  
- TTGGATTACA ACCCACCCCA GAGTAAGCCA GTTACCCCCT GATCCCTGCA GTATAGCAGT GGTTAATGTT   
  
  
- GCCTTGTTGG GATTTGGCGT AAAATTTTAG TGTAGAACCA TCGTCTGTTT CTACATGCTG GGTTTTTAAG   
  
  
- TTGAGACATT TCGTGATCGT TTCGAGCATC ATGTGTCTTG TTAGCGTGTG AGTATCACTA TCTTCACATG   
  
  
- ATAGGTAATA CGTCACTTAT ACTGTTCCCT CCCCCAGGAC TCTACGGGTT TAAACCGTCG GCTCAACGGA   
  
  
- ACTGACGGGT AGTTACAGGA TTTGGTCAAC TCGTACAATA ATTAAACGTT CTTTCTAGCT TAAGAAGGAT   
  
  
- AGATTTTTGA CTGTGTTATT TTGTTATGGT ACATACCCTG TAACGATCCT AACAATTGTG TTAATAACAG   
  
  
- AACATGCAAC GTGGACTTTT TTTTCTTTTA ACAACGGTAT CTGTTGTATA TTCAATCCGT AAGTACTGAT   
  
  
- TATAGACTTA ATCCACACAG CTAGACACTA AGTTTAACTA TATTAATCTA GAAATTCCAT AGATTAACGA   
  
  
- AGTTCAAAAG AGATATTACT TCTACATATA ATCGAGTGTA TCCAGAATTC CAATTGATTA CAGTATAGTC   
  
  
- CCCCACACCC GGCGGTAAAA ACTATTCTTA ACTTAACTTG AACGTAACAA AAAAGAAACC GCTCTTACGT   
  
  
- TCTAAACAAT TAAATAAGTA TACGCAGTAA TATGTGATAT TCTTTTTATT AACATCCTTA TATACTTCAT   
  
  
- AAAAATGAAC ACGAGTACAA TTTACACCTT ATGAAAAAAT TACAGCATAT CATGGTGTCA TAAGATGTTA   
  
  
- AATTCTCTAA TTAGCTAGGT AATTTCAGTA CGTTTGGACT ACGTATAAGT TCTGTAACGT ACTTTACTAG   
  
  
- AATAAAATCA GTTTCTGAGG TAGTAAATAT TCGTTATAAA TGAAGCTATG TGCACAGTAA TTTTTTCTGA   
  
  
- TATCTTTACA ATGACAATAA AACAAACTCG ATTTATCTTT TAAATTTTCA ATTAATTCTA TTATTAATTT   
  
  
- TGATTTATAA GTTACTACCG ATTATTTATG TAACAGCATA ATTGACTGAT TATAAACATC TACCAGCACA   
  
  
- ATTAAAGAAA AATAAAATTT CAAGTTTACT CAATGTTTTC CAATATTTTA TATTTCATTT TACCCTCTGT   
  
  
- TTCGTCTGTC CCACCCCCTT CGATTCGAAT TTCATCCTAA ATCTTTCCGT TACTTCCACT TGGGAGTTTA   
  
  
- GCTAGGCGAC GGTTAGTGTC TTTGGGTTTC GAAACGAGTG GCTGTTGAGG GCCAATTGAC GTCAGTGAGT   
  
  
- GCCGTGACCC ATTATGTCAT TAGCACATGG GTCAAGTAGG GGGAAAAGGA AACCCTTATG TCATGGAGCT   
  
  
- TTCAAGGAAA CTTTAAGATG ACCGGTCCGT ATGGGTATTT AACTAGGAGA GCTATAGTAT TCTATACTAT   
  
  
- AAAGAGACAG TATAGACAAT CAAAGCCATA GTATAAAGAA GAAATAGAGA CATTAAACTC CATACGAAGT   
  
  
- AAGGACCGGC GAACTTAATA GAAAGAAAGA AACTAATCAT AACAAAAAAA CCAATGGATA AAACGAGGTC   
  
  
- AGGACAAGTA AACCCAGAAA AACCTAGACC CAAAAACGAC CCAAACAACT AAGAAACTCT TTAAACCCAC   
  
  
- GAACTTAAAC GGGACGTTAT AAGTACCCAA TCGCAAAAGG ACTTAAAACG AGACAAGGAA TTCACTAATA   
  
  
- AAAACTAACT AATGTGGAAC CACCAGGACC ACCTTAAAGG CTCCTCTTTA ACAGTACCCA AGGGTGTTGA   
  
  
- AACCTCTTAA GGGACTACTC TGAGATTTAC TCATAGTTAG ATGAAACCCT TGGTACAGGG GGCCAATACT   
  
  
- ACCCAGAAAC TTAATATGTT GGAACAAGTT AATGTTTCTG GGTAGAGTCC TAGAGTGCCG TGACTTGGAA   
  
  
- GGATCGGGTA ACGGACTGGG ATACGGTAAG TTGTAACCAA GTATAAGCCC AAACTCGGGG CCCCAACTTA   
  
  
- GAAGCCTACT ATCGCTAAGT CTACTACAAC AATTCATGTA ACCGGTTCAC GAATACCTTC TCCTATACTT   
  
  
- CCTCTTCGGA ACATACAAGG TACTGGGAAA TCGTGAGGTT CGACGACTCT TTGGGAAAAT ACTACACAAC   
  
  
- CCTTTCTTCA TAGGAGGAAG GGGATTAGTT GTGGGTGAAT AACTAGTAAG ACACCTATCG GGTCTGTTAG   
  
  
- TCAGGACACC TTGCTCGTGT TAATCACTAG AATCACCGTC ATTGACGTGA AGGTCAAGTT GGTTAATATA   
  
  
- ACTACGACAC CGTCAGCGAC TAAGCTCACT CTTATGATTC TGGAGAAAAC ACGTTTCAAG AGACTAACTT   
  
  
- AGCAAAAGAG TCAGCAGGTG GGAAGGTGTC ACCAGTAAAC CTAGTAACCC ACGGAACCCA CCGTGTCGAA   
  
  
- GAGTCCCAAG CTTAAGTCAA TAGAGGAGGG CACCAAAGGG ATACCGGTAC TTGCAAAAAT CGCTCGTCAG   
  
  
- GTACTATGTC AAAGTCTTCC CCCACCTCCT CCGCTCGTTC AAGGAAGGTT TCTTGTTATT GGAACGCTAA   
  
  
- CTGGAACTCT CGCAGTGAAA GGGTTTACTT TTCCTCCGCC GGGGGTACCA CCACCAATTC TTTCTCTTCC   
  
  
- TACTTGTGAG TGGACTATCG AGCTCTCCAT CGTTCTAAGT GATGGCACTA CTACTCCTGA AACTCCTACC   
  
  
- ATCCTCCTCA TTCGTCAGTC GACAAAGACA CCTCCTCCGA CTCAACAGAC TTTACAAACT GGCCCAAAAC   
  
  
- GAAACGTTAG GGTACTTTGT ACTTCGAGTA ACATGTTCAC CAAACTTCAG GCTCTTCCCT TGTAGGGACG   
  
  
- TCCGACCGGT TCATGTCCAT GTCCTCCCAG TCTTCCGAGC ACGTTTCTTT GTCCCATTAT TACTATTATT   
  
  
- CTTCTTATTC TTACACCACC TAAATTCCTG AGACGAATAG GAGACACGCG TTAGACGACG TAGACTACTA   
  
  
- GCGTCCTGTC GACTACTTGA CGACTTCGTC TAATCCCTCG TGAGAAGACG TCGACCCCTA CCTAGAGTTT   
  
  
- CCAACCGAGT AATGAAACGA TTACGGAACC TCCGCGCAAA TCGACCTTGA CCGAGTGTCT AGATATGTCG   
  
  
- GGACTCAAGC TTCTCCTGTT GTCGTCGACT ATACTATTTT CGAATAGTCA AAAAGAAAGC ACGAACGGGT   
  
  
- AAAAATTTCT AGAGATAATA GAAACGCTTG GTATACTAAT ATGTCTCTCG ACTTTTTCGT AGTTTCGAAG   
  
  
- TACAATATCT AAAACCGTAG GACATACCAA AAGTCACCGG GGAGGAGTAA GTTGCGGACA GCCTCGCTGG   
  
  
- ACCACCTGGA GGTTTTGACA CGTAATGACC CTAACTAGAA GGGGTTGGAC CTAAATCCGG TTGTCTTTCT   
  
  
- CAACTCCGTT GTCCCGAGGC GAACCGTTTC ATAACACTCG CGAAGTTACA AGGTAAACTT ATGGTACGGT   
  
  
- AACGTGTCTT TACCCTTTGG TAGTTTCGAC TTCTGGACTT CTATCTTCTA CTACTCCACC AACGGCACTT   
  
  
- GACAGAGAAG TCCAAATTCT TGGACGAACT ACTCTGCTAT CACCACCTAT CAGGCTCCTT ACGTCAAGAG   
  
  
- CCGAACTAAT CTTCCTATTT TGGACTATAG AAACAAGTGC CTCATCACTT GCCCAGAATG TTACGCGGGA   
  
  
- AGAAACAATG TGCAAAGTCC CTCCGGGAGA AGGTAATGAG ATGACATAAG CTATACAAAC TACGGTTACG   
  
  
- AAGGGCCCTC CTAGGACTCT CTAACTACAA ACTCTTCCTC AAAATACCCG CTCTTTAATA CTTACACCAC   
  
  
- CGAACACTCC CATGTCTCTC TCAACTTTCT GGACTCTGTA TGTTCGTTAC CGTCCGCGCC TTATCATCCC   
  
  
- GTCCCAAGTC CGTCGACGGT AACCTGGTTC TCGAGCAGTC ATTTGACTCC TCGTTCCAGG TCTACGTGAT   
  
  
- AGGATCCCTG AAGCAACAAC TACATCTACC TGTAACCTGT AACGTCCCTA CCTTCCCCTC CTAGTAGACA   
  
  
- CGACACAGAC GTACCCCAGG ACGAAT

+     CAT-box

| Site Name | Organism | Position | Strand | Matrix score. | sequence | function |
| --- | --- | --- | --- | --- | --- | --- |
| CAT-box | Arabidopsis thaliana | 2487 | - | 6 | GCCACT | cis-acting regulatory element related to meristem expression |
| CAT-box | Arabidopsis thaliana | 3538 | - | 6 | GCCACT | cis-acting regulatory element related to meristem expression |

>HU02G01569.1   
+ +Up\_Stream \_Len000AGCGTA GGAAGTGACG CCGCTATTGG AGGCGAAGAC GCTAGTACCA AGGCCAAAAC   
  
  
+ AAATGAGGAG ATAGATCAGT TAAACCGTTT GGTTAAGAAA ATTAAGCGAA CATCTCTTGA ACCTCCATCT   
  
  
+ GAACTCGATG ACATTGAGGA TGTGGATGCA GAAAAGATGG ATATGGGCTC TCCATCGGTG CCAGCAAATC   
  
  
+ AACCTAATGT TGGGTGGGGT CTCATTCGGT CAATGGGGGA CTAGGGACGT CATATCGTCA CCAATTACAA   
  
  
+ CGGAACAACC CTAAACCGCA TTTTAAAATC ACATCTTGGT AGCAGACAAA GATGTACGAC CCAAAAATTC   
  
  
+ AACTCTGTAA AGCACTAGCA AAGCTCGTAG TACACAGAAC AATCGCACAC TCATAGTGAT AGAAGTGTAC   
  
  
+ TATCCATTAT GCAGTGAATA TGACAAGGGA GGGGGTCCTG AGATGCCCAA ATTTGGCAGC CGAGTTGCCT   
  
  
+ TGACTGCCCA TCAATGTCCT AAACCAGTTG AGCATGTTAT TAATTTGCAA GAAAGATCGA ATTCTTCCTA   
  
  
+ TCTAAAAACT GACACAATAA AACAATACCA TGTATGGGAC ATTGCTAGGA TTGTTAACAC AATTATTGTC   
  
  
+ TTGTACGTTG CACCTGAAAA AAAAGAAAAT TGTTGCCATA GACAACATAT AAGTTAGGCA TTCATGACTA   
  
  
+ ATATCTGAAT TAGGTGTGTC GATCTGTGAT TCAAATTGAT ATAATTAGAT CTTTAAGGTA TCTAATTGCT   
  
  
+ TCAAGTTTTC TCTATAATGA AGATGTATAT TAGCTCACAT AGGTCTTAAG GTTAACTAAT GTCATATCAG   
  
  
+ GGGGTGTGGG CCGCCATTTT TGATAAGAAT TGAATTGAAC TTGCATTGTT TTTTCTTTGG CGAGAATGCA   
  
  
+ AGATTTGTTA ATTTATTCAT ATGCGTCATT ATACACTATA AGAAAAATAA TTGTAGGAAT ATATGAAGTA   
  
  
+ TTTTTACTTG TGCTCATGTT AAATGTGGAA TACTTTTTTA ATGTCGTATA GTACCACAGT ATTCTACAAT   
  
  
+ TTAAGAGATT AATCGATCCA TTAAAGTCAT GCAAACCTGA TGCATATTCA AGACATTGCA TGAAATGATC   
  
  
+ TTATTTTAGT CAAAGACTCC ATCATTTATA AGCAATATTT ACTTCGATAC ACGTGTCATT AAAAAAGACT   
  
  
+ ATAGAAATGT TACTGTTATT TTGTTTGAGC TAAATAGAAA ATTTAAAAGT TAATTAAGAT AATAATTAAA   
  
  
+ ACTAAATATT CAATGATGGC TAATAAATAC ATTGTCGTAT TAACTGACTA ATATTTGTAG ATGGTCGTGT   
  
  
+ TAATTTCTTT TTATTTTAAA GTTCAAATGA GTTACAAAAG GTTATAAAAT ATAAAGTAAA ATGGGAGACA   
  
  
+ AAGCAGACAG GGTGGGGGAA GCTAAGCTTA AAGTAGGATT TAGAAAGGCA ATGAAGGTGA ACCCTCAAAT   
  
  
+ CGATCCGCTG CCAATCACAG AAACCCAAAG CTTTGCTCAC CGACAACTCC CGGTTAACTG CAGTCACTCA   
  
  
+ CGGCACTGGG TAATACAGTA ATCGTGTACC CAGTTCATCC CCCTTTTCCT TTGGGAATAC AGTACCTCGA   
  
  
+ AAGTTCCTTT GAAATTCTAC TGGCCAGGCA TACCCATAAA TTGATCCTCT CGATATCATA AGATATGATA   
  
  
+ TTTCTCTGTC ATATCTGTTA GTTTCGGTAT CATATTTCTT CTTTATCTCT GTAATTTGAG GTATGCTTCA   
  
  
+ TTCCTGGCCG CTTGAATTAT CTTTCTTTCT TTGATTAGTA TTGTTTTTTT GGTTACCTAT TTTGCTCCAG   
  
  
+ TCCTGTTCAT TTGGGTCTTT TTGGATCTGG GTTTTTGCTG GGTTTGTTGA TTCTTTGAGA AATTTGGGTG   
  
  
+ CTTGAATTTG CCCTGCAATA TTCATGGGTT AGCGTTTTCC TGAATTTTGC TCTGTTCCTT AAGTGATTAT   
  
  
+ TTTTGATTGA TTACACCTTG GTGGTCCTGG TGGAATTTCC GAGGAGAAAT TGTCATGGGT TCCCACAACT   
  
  
+ TTGGAGAATT CCCTGATGAG ACTCTAAATG AGTATCAATC TACTTTGGGA ACCATGTCCC CCGGTTATGA   
  
  
+ TGGGTCTTTG AATTATACAA CCTTGTTCAA TTACAAAGAC CCATCTCAGG ATCTCACGGC ACTGAACCTT   
  
  
+ CCTAGCCCAT TGCCTGACCC TATGCCATTC AACATTGGTT CATATTCGGG TTTGAGCCCC GGGGTTGAAT   
  
  
+ CTTCGGATGA TAGCGATTCA GATGATGTTG TTAAGTACAT TGGCCAAGTG CTTATGGAAG AGGATATGAA   
  
  
+ GGAGAAGCCT TGTATGTTCC ATGACCCTTT AGCACTCCAA GCTGCTGAGA AACCCTTTTA TGATGTGTTG   
  
  
+ GGAAAGAAGT ATCCTCCTTC CCCTAATCAA CACCCACTTA TTGATCATTC TGTGGATAGC CCAGACAATC   
  
  
+ AGTCCTGTGG AACGAGCACA ATTAGTGATC TTAGTGGCAG TAACTGCACT TCCAGTTCAA CCAATTATAT   
  
  
+ TGATGCTGTG GCAGTCGCTG ATTCGAGTGA GAATACTAAG ACCTCTTTTG TGCAAAGTTC TCTGATTGAA   
  
  
+ TCGTTTTCTC AGTCGTCCAC CCTTCCACAG TGGTCATTTG GATCATTGGG TGCCTTGGGT GGCACAGCTT   
  
  
+ CTCAGGGTTC GAATTCAGTT ATCTCCTCCC GTGGTTTCCC TATGGCCATG AACGTTTTTA GCGAGCAGTC   
  
  
+ CATGATACAG TTTCAGAAGG GGGTGGAGGA GGCGAGCAAG TTCCTTCCAA AGAACAATAA CCTTGCGATT   
  
  
+ GACCTTGAGA GCGTCACTTT CCCAAATGAA AAGGAGGCGG CCCCCATGGT GGTGGTTAAG AAAGAGAAGG   
  
  
+ ATGAACACTC ACCTGATAGC TCGAGAGGTA GCAAGATTCA CTACCGTGAT GATGAGGACT TTGAGGATGG   
  
  
+ TAGGAGGAGT AAGCAGTCAG CTGTTTCTGT GGAGGAGGCT GAGTTGTCTG AAATGTTTGA CCGGGTTTTG   
  
  
+ CTTTGCAATC CCATGAAACA TGAAGCTCAT TGTACAAGTG GTTTGAAGTC CGAGAAGGGA ACATCCCTGC   
  
  
+ AGGCTGGCCA AGTACAGGTA CAGGAGGGTC AGAAGGCTCG TGCAAAGAAA CAGGGTAATA ATGATAATAA   
  
  
+ GAAGAATAAG AATGTGGTGG ATTTAAGGAC TCTGCTTATC CTCTGTGCGC AATCTGCTGC ATCTGATGAT   
  
  
+ CGCAGGACAG CTGATGAACT GCTGAAGCAG ATTAGGGAGC ACTCTTCTGC AGCTGGGGAT GGATCTCAAA   
  
  
+ GGTTGGCTCA TTACTTTGCT AATGCCTTGG AGGCGCGTTT AGCTGGAACT GGCTCACAGA TCTATACAGC   
  
  
+ CCTGAGTTCG AAGAGGACAA CAGCAGCTGA TATGATAAAA GCTTATCAGT TTTTCTTTCG TGCTTGCCCA   
  
  
+ TTTTTAAAGA TCTCTATTAT CTTTGCGAAC CATATGATTA TACAGAGAGC TGAAAAAGCA TCAAAGCTTC   
  
  
+ ATGTTATAGA TTTTGGCATC CTGTATGGTT TTCAGTGGCC CCTCCTCATT CAACGCCTGT CGGAGCGACC   
  
  
+ TGGTGGACCT CCAAAACTGT GCATTACTGG GATTGATCTT CCCCAACCTG GATTTAGGCC AACAGAAAGA   
  
  
+ GTTGAGGCAA CAGGGCTCCG CTTGGCAAAG TATTGTGAGC GCTTCAATGT TCCATTTGAA TACCATGCCA   
  
  
+ TTGCACAGAA ATGGGAAACC ATCAAAGCTG AAGACCTGAA GATAGAAGAT GATGAGGTGG TTGCCGTGAA   
  
  
+ CTGTCTCTTC AGGTTTAAGA ACCTGCTTGA TGAGACGATA GTGGTGGATA GTCCGAGGAA TGCAGTTCTC   
  
  
+ GGCTTGATTA GAAGGATAAA ACCTGATATC TTTGTTCACG GAGTAGTGAA CGGGTCTTAC AATGCGCCCT   
  
  
+ TCTTTGTTAC ACGTTTCAGG GAGGCCCTCT TCCATTACTC TACTGTATTC GATATGTTTG ATGCCAATGC   
  
  
+ TTCCCGGGAG GATCCTGAGA GATTGATGTT TGAGAAGGAG TTTTATGGGC GAGAAATTAT GAATGTGGTG   
  
  
+ GCTTGTGAGG GTACAGAGAG AGTTGAAAGA CCTGAGACAT ACAAGCAATG GCAGGCGCGG AATAGTAGGG   
  
  
+ CAGGGTTCAG GCAGCTGCCA TTGGACCAAG AGCTCGTCAG TAAACTGAGG AGCAAGGTCC AGATGCACTA   
  
  
+ TCCTAGGGAC TTCGTTGTTG ATGTAGATGG ACATTGGACA TTGCAGGGAT GGAAGGGGAG GATCATCTGT   
  
  
+ GCTGTGTCTG CATGGGGTCC TGCTTA  

- +Up\_Stream \_Len000TCGCAT CCTTCACTGC GGCGATAACC TCCGCTTCTG CGATCATGGT TCCGGTTTTG   
  
  
- TTTACTCCTC TATCTAGTCA ATTTGGCAAA CCAATTCTTT TAATTCGCTT GTAGAGAACT TGGAGGTAGA   
  
  
- CTTGAGCTAC TGTAACTCCT ACACCTACGT CTTTTCTACC TATACCCGAG AGGTAGCCAC GGTCGTTTAG   
  
  
- TTGGATTACA ACCCACCCCA GAGTAAGCCA GTTACCCCCT GATCCCTGCA GTATAGCAGT GGTTAATGTT   
  
  
- GCCTTGTTGG GATTTGGCGT AAAATTTTAG TGTAGAACCA TCGTCTGTTT CTACATGCTG GGTTTTTAAG   
  
  
- TTGAGACATT TCGTGATCGT TTCGAGCATC ATGTGTCTTG TTAGCGTGTG AGTATCACTA TCTTCACATG   
  
  
- ATAGGTAATA CGTCACTTAT ACTGTTCCCT CCCCCAGGAC TCTACGGGTT TAAACCGTCG GCTCAACGGA   
  
  
- ACTGACGGGT AGTTACAGGA TTTGGTCAAC TCGTACAATA ATTAAACGTT CTTTCTAGCT TAAGAAGGAT   
  
  
- AGATTTTTGA CTGTGTTATT TTGTTATGGT ACATACCCTG TAACGATCCT AACAATTGTG TTAATAACAG   
  
  
- AACATGCAAC GTGGACTTTT TTTTCTTTTA ACAACGGTAT CTGTTGTATA TTCAATCCGT AAGTACTGAT   
  
  
- TATAGACTTA ATCCACACAG CTAGACACTA AGTTTAACTA TATTAATCTA GAAATTCCAT AGATTAACGA   
  
  
- AGTTCAAAAG AGATATTACT TCTACATATA ATCGAGTGTA TCCAGAATTC CAATTGATTA CAGTATAGTC   
  
  
- CCCCACACCC GGCGGTAAAA ACTATTCTTA ACTTAACTTG AACGTAACAA AAAAGAAACC GCTCTTACGT   
  
  
- TCTAAACAAT TAAATAAGTA TACGCAGTAA TATGTGATAT TCTTTTTATT AACATCCTTA TATACTTCAT   
  
  
- AAAAATGAAC ACGAGTACAA TTTACACCTT ATGAAAAAAT TACAGCATAT CATGGTGTCA TAAGATGTTA   
  
  
- AATTCTCTAA TTAGCTAGGT AATTTCAGTA CGTTTGGACT ACGTATAAGT TCTGTAACGT ACTTTACTAG   
  
  
- AATAAAATCA GTTTCTGAGG TAGTAAATAT TCGTTATAAA TGAAGCTATG TGCACAGTAA TTTTTTCTGA   
  
  
- TATCTTTACA ATGACAATAA AACAAACTCG ATTTATCTTT TAAATTTTCA ATTAATTCTA TTATTAATTT   
  
  
- TGATTTATAA GTTACTACCG ATTATTTATG TAACAGCATA ATTGACTGAT TATAAACATC TACCAGCACA   
  
  
- ATTAAAGAAA AATAAAATTT CAAGTTTACT CAATGTTTTC CAATATTTTA TATTTCATTT TACCCTCTGT   
  
  
- TTCGTCTGTC CCACCCCCTT CGATTCGAAT TTCATCCTAA ATCTTTCCGT TACTTCCACT TGGGAGTTTA   
  
  
- GCTAGGCGAC GGTTAGTGTC TTTGGGTTTC GAAACGAGTG GCTGTTGAGG GCCAATTGAC GTCAGTGAGT   
  
  
- GCCGTGACCC ATTATGTCAT TAGCACATGG GTCAAGTAGG GGGAAAAGGA AACCCTTATG TCATGGAGCT   
  
  
- TTCAAGGAAA CTTTAAGATG ACCGGTCCGT ATGGGTATTT AACTAGGAGA GCTATAGTAT TCTATACTAT   
  
  
- AAAGAGACAG TATAGACAAT CAAAGCCATA GTATAAAGAA GAAATAGAGA CATTAAACTC CATACGAAGT   
  
  
- AAGGACCGGC GAACTTAATA GAAAGAAAGA AACTAATCAT AACAAAAAAA CCAATGGATA AAACGAGGTC   
  
  
- AGGACAAGTA AACCCAGAAA AACCTAGACC CAAAAACGAC CCAAACAACT AAGAAACTCT TTAAACCCAC   
  
  
- GAACTTAAAC GGGACGTTAT AAGTACCCAA TCGCAAAAGG ACTTAAAACG AGACAAGGAA TTCACTAATA   
  
  
- AAAACTAACT AATGTGGAAC CACCAGGACC ACCTTAAAGG CTCCTCTTTA ACAGTACCCA AGGGTGTTGA   
  
  
- AACCTCTTAA GGGACTACTC TGAGATTTAC TCATAGTTAG ATGAAACCCT TGGTACAGGG GGCCAATACT   
  
  
- ACCCAGAAAC TTAATATGTT GGAACAAGTT AATGTTTCTG GGTAGAGTCC TAGAGTGCCG TGACTTGGAA   
  
  
- GGATCGGGTA ACGGACTGGG ATACGGTAAG TTGTAACCAA GTATAAGCCC AAACTCGGGG CCCCAACTTA   
  
  
- GAAGCCTACT ATCGCTAAGT CTACTACAAC AATTCATGTA ACCGGTTCAC GAATACCTTC TCCTATACTT   
  
  
- CCTCTTCGGA ACATACAAGG TACTGGGAAA TCGTGAGGTT CGACGACTCT TTGGGAAAAT ACTACACAAC   
  
  
- CCTTTCTTCA TAGGAGGAAG GGGATTAGTT GTGGGTGAAT AACTAGTAAG ACACCTATCG GGTCTGTTAG   
  
  
- TCAGGACACC TTGCTCGTGT TAATCACTAG AATCACCGTC ATTGACGTGA AGGTCAAGTT GGTTAATATA   
  
  
- ACTACGACAC CGTCAGCGAC TAAGCTCACT CTTATGATTC TGGAGAAAAC ACGTTTCAAG AGACTAACTT   
  
  
- AGCAAAAGAG TCAGCAGGTG GGAAGGTGTC ACCAGTAAAC CTAGTAACCC ACGGAACCCA CCGTGTCGAA   
  
  
- GAGTCCCAAG CTTAAGTCAA TAGAGGAGGG CACCAAAGGG ATACCGGTAC TTGCAAAAAT CGCTCGTCAG   
  
  
- GTACTATGTC AAAGTCTTCC CCCACCTCCT CCGCTCGTTC AAGGAAGGTT TCTTGTTATT GGAACGCTAA   
  
  
- CTGGAACTCT CGCAGTGAAA GGGTTTACTT TTCCTCCGCC GGGGGTACCA CCACCAATTC TTTCTCTTCC   
  
  
- TACTTGTGAG TGGACTATCG AGCTCTCCAT CGTTCTAAGT GATGGCACTA CTACTCCTGA AACTCCTACC   
  
  
- ATCCTCCTCA TTCGTCAGTC GACAAAGACA CCTCCTCCGA CTCAACAGAC TTTACAAACT GGCCCAAAAC   
  
  
- GAAACGTTAG GGTACTTTGT ACTTCGAGTA ACATGTTCAC CAAACTTCAG GCTCTTCCCT TGTAGGGACG   
  
  
- TCCGACCGGT TCATGTCCAT GTCCTCCCAG TCTTCCGAGC ACGTTTCTTT GTCCCATTAT TACTATTATT   
  
  
- CTTCTTATTC TTACACCACC TAAATTCCTG AGACGAATAG GAGACACGCG TTAGACGACG TAGACTACTA   
  
  
- GCGTCCTGTC GACTACTTGA CGACTTCGTC TAATCCCTCG TGAGAAGACG TCGACCCCTA CCTAGAGTTT   
  
  
- CCAACCGAGT AATGAAACGA TTACGGAACC TCCGCGCAAA TCGACCTTGA CCGAGTGTCT AGATATGTCG   
  
  
- GGACTCAAGC TTCTCCTGTT GTCGTCGACT ATACTATTTT CGAATAGTCA AAAAGAAAGC ACGAACGGGT   
  
  
- AAAAATTTCT AGAGATAATA GAAACGCTTG GTATACTAAT ATGTCTCTCG ACTTTTTCGT AGTTTCGAAG   
  
  
- TACAATATCT AAAACCGTAG GACATACCAA AAGTCACCGG GGAGGAGTAA GTTGCGGACA GCCTCGCTGG   
  
  
- ACCACCTGGA GGTTTTGACA CGTAATGACC CTAACTAGAA GGGGTTGGAC CTAAATCCGG TTGTCTTTCT   
  
  
- CAACTCCGTT GTCCCGAGGC GAACCGTTTC ATAACACTCG CGAAGTTACA AGGTAAACTT ATGGTACGGT   
  
  
- AACGTGTCTT TACCCTTTGG TAGTTTCGAC TTCTGGACTT CTATCTTCTA CTACTCCACC AACGGCACTT   
  
  
- GACAGAGAAG TCCAAATTCT TGGACGAACT ACTCTGCTAT CACCACCTAT CAGGCTCCTT ACGTCAAGAG   
  
  
- CCGAACTAAT CTTCCTATTT TGGACTATAG AAACAAGTGC CTCATCACTT GCCCAGAATG TTACGCGGGA   
  
  
- AGAAACAATG TGCAAAGTCC CTCCGGGAGA AGGTAATGAG ATGACATAAG CTATACAAAC TACGGTTACG   
  
  
- AAGGGCCCTC CTAGGACTCT CTAACTACAA ACTCTTCCTC AAAATACCCG CTCTTTAATA CTTACACCAC   
  
  
- CGAACACTCC CATGTCTCTC TCAACTTTCT GGACTCTGTA TGTTCGTTAC CGTCCGCGCC TTATCATCCC   
  
  
- GTCCCAAGTC CGTCGACGGT AACCTGGTTC TCGAGCAGTC ATTTGACTCC TCGTTCCAGG TCTACGTGAT   
  
  
- AGGATCCCTG AAGCAACAAC TACATCTACC TGTAACCTGT AACGTCCCTA CCTTCCCCTC CTAGTAGACA   
  
  
- CGACACAGAC GTACCCCAGG ACGAAT

+     CCAAT-box

| Site Name | Organism | Position | Strand | Matrix score. | sequence | function |
| --- | --- | --- | --- | --- | --- | --- |
| CCAAT-box | Hordeum vulgare | 282 | + | 6 | CAACGG | MYBHv1 binding site |

>HU02G01569.1   
+ +Up\_Stream \_Len000AGCGTA GGAAGTGACG CCGCTATTGG AGGCGAAGAC GCTAGTACCA AGGCCAAAAC   
  
  
+ AAATGAGGAG ATAGATCAGT TAAACCGTTT GGTTAAGAAA ATTAAGCGAA CATCTCTTGA ACCTCCATCT   
  
  
+ GAACTCGATG ACATTGAGGA TGTGGATGCA GAAAAGATGG ATATGGGCTC TCCATCGGTG CCAGCAAATC   
  
  
+ AACCTAATGT TGGGTGGGGT CTCATTCGGT CAATGGGGGA CTAGGGACGT CATATCGTCA CCAATTACAA   
  
  
+ CGGAACAACC CTAAACCGCA TTTTAAAATC ACATCTTGGT AGCAGACAAA GATGTACGAC CCAAAAATTC   
  
  
+ AACTCTGTAA AGCACTAGCA AAGCTCGTAG TACACAGAAC AATCGCACAC TCATAGTGAT AGAAGTGTAC   
  
  
+ TATCCATTAT GCAGTGAATA TGACAAGGGA GGGGGTCCTG AGATGCCCAA ATTTGGCAGC CGAGTTGCCT   
  
  
+ TGACTGCCCA TCAATGTCCT AAACCAGTTG AGCATGTTAT TAATTTGCAA GAAAGATCGA ATTCTTCCTA   
  
  
+ TCTAAAAACT GACACAATAA AACAATACCA TGTATGGGAC ATTGCTAGGA TTGTTAACAC AATTATTGTC   
  
  
+ TTGTACGTTG CACCTGAAAA AAAAGAAAAT TGTTGCCATA GACAACATAT AAGTTAGGCA TTCATGACTA   
  
  
+ ATATCTGAAT TAGGTGTGTC GATCTGTGAT TCAAATTGAT ATAATTAGAT CTTTAAGGTA TCTAATTGCT   
  
  
+ TCAAGTTTTC TCTATAATGA AGATGTATAT TAGCTCACAT AGGTCTTAAG GTTAACTAAT GTCATATCAG   
  
  
+ GGGGTGTGGG CCGCCATTTT TGATAAGAAT TGAATTGAAC TTGCATTGTT TTTTCTTTGG CGAGAATGCA   
  
  
+ AGATTTGTTA ATTTATTCAT ATGCGTCATT ATACACTATA AGAAAAATAA TTGTAGGAAT ATATGAAGTA   
  
  
+ TTTTTACTTG TGCTCATGTT AAATGTGGAA TACTTTTTTA ATGTCGTATA GTACCACAGT ATTCTACAAT   
  
  
+ TTAAGAGATT AATCGATCCA TTAAAGTCAT GCAAACCTGA TGCATATTCA AGACATTGCA TGAAATGATC   
  
  
+ TTATTTTAGT CAAAGACTCC ATCATTTATA AGCAATATTT ACTTCGATAC ACGTGTCATT AAAAAAGACT   
  
  
+ ATAGAAATGT TACTGTTATT TTGTTTGAGC TAAATAGAAA ATTTAAAAGT TAATTAAGAT AATAATTAAA   
  
  
+ ACTAAATATT CAATGATGGC TAATAAATAC ATTGTCGTAT TAACTGACTA ATATTTGTAG ATGGTCGTGT   
  
  
+ TAATTTCTTT TTATTTTAAA GTTCAAATGA GTTACAAAAG GTTATAAAAT ATAAAGTAAA ATGGGAGACA   
  
  
+ AAGCAGACAG GGTGGGGGAA GCTAAGCTTA AAGTAGGATT TAGAAAGGCA ATGAAGGTGA ACCCTCAAAT   
  
  
+ CGATCCGCTG CCAATCACAG AAACCCAAAG CTTTGCTCAC CGACAACTCC CGGTTAACTG CAGTCACTCA   
  
  
+ CGGCACTGGG TAATACAGTA ATCGTGTACC CAGTTCATCC CCCTTTTCCT TTGGGAATAC AGTACCTCGA   
  
  
+ AAGTTCCTTT GAAATTCTAC TGGCCAGGCA TACCCATAAA TTGATCCTCT CGATATCATA AGATATGATA   
  
  
+ TTTCTCTGTC ATATCTGTTA GTTTCGGTAT CATATTTCTT CTTTATCTCT GTAATTTGAG GTATGCTTCA   
  
  
+ TTCCTGGCCG CTTGAATTAT CTTTCTTTCT TTGATTAGTA TTGTTTTTTT GGTTACCTAT TTTGCTCCAG   
  
  
+ TCCTGTTCAT TTGGGTCTTT TTGGATCTGG GTTTTTGCTG GGTTTGTTGA TTCTTTGAGA AATTTGGGTG   
  
  
+ CTTGAATTTG CCCTGCAATA TTCATGGGTT AGCGTTTTCC TGAATTTTGC TCTGTTCCTT AAGTGATTAT   
  
  
+ TTTTGATTGA TTACACCTTG GTGGTCCTGG TGGAATTTCC GAGGAGAAAT TGTCATGGGT TCCCACAACT   
  
  
+ TTGGAGAATT CCCTGATGAG ACTCTAAATG AGTATCAATC TACTTTGGGA ACCATGTCCC CCGGTTATGA   
  
  
+ TGGGTCTTTG AATTATACAA CCTTGTTCAA TTACAAAGAC CCATCTCAGG ATCTCACGGC ACTGAACCTT   
  
  
+ CCTAGCCCAT TGCCTGACCC TATGCCATTC AACATTGGTT CATATTCGGG TTTGAGCCCC GGGGTTGAAT   
  
  
+ CTTCGGATGA TAGCGATTCA GATGATGTTG TTAAGTACAT TGGCCAAGTG CTTATGGAAG AGGATATGAA   
  
  
+ GGAGAAGCCT TGTATGTTCC ATGACCCTTT AGCACTCCAA GCTGCTGAGA AACCCTTTTA TGATGTGTTG   
  
  
+ GGAAAGAAGT ATCCTCCTTC CCCTAATCAA CACCCACTTA TTGATCATTC TGTGGATAGC CCAGACAATC   
  
  
+ AGTCCTGTGG AACGAGCACA ATTAGTGATC TTAGTGGCAG TAACTGCACT TCCAGTTCAA CCAATTATAT   
  
  
+ TGATGCTGTG GCAGTCGCTG ATTCGAGTGA GAATACTAAG ACCTCTTTTG TGCAAAGTTC TCTGATTGAA   
  
  
+ TCGTTTTCTC AGTCGTCCAC CCTTCCACAG TGGTCATTTG GATCATTGGG TGCCTTGGGT GGCACAGCTT   
  
  
+ CTCAGGGTTC GAATTCAGTT ATCTCCTCCC GTGGTTTCCC TATGGCCATG AACGTTTTTA GCGAGCAGTC   
  
  
+ CATGATACAG TTTCAGAAGG GGGTGGAGGA GGCGAGCAAG TTCCTTCCAA AGAACAATAA CCTTGCGATT   
  
  
+ GACCTTGAGA GCGTCACTTT CCCAAATGAA AAGGAGGCGG CCCCCATGGT GGTGGTTAAG AAAGAGAAGG   
  
  
+ ATGAACACTC ACCTGATAGC TCGAGAGGTA GCAAGATTCA CTACCGTGAT GATGAGGACT TTGAGGATGG   
  
  
+ TAGGAGGAGT AAGCAGTCAG CTGTTTCTGT GGAGGAGGCT GAGTTGTCTG AAATGTTTGA CCGGGTTTTG   
  
  
+ CTTTGCAATC CCATGAAACA TGAAGCTCAT TGTACAAGTG GTTTGAAGTC CGAGAAGGGA ACATCCCTGC   
  
  
+ AGGCTGGCCA AGTACAGGTA CAGGAGGGTC AGAAGGCTCG TGCAAAGAAA CAGGGTAATA ATGATAATAA   
  
  
+ GAAGAATAAG AATGTGGTGG ATTTAAGGAC TCTGCTTATC CTCTGTGCGC AATCTGCTGC ATCTGATGAT   
  
  
+ CGCAGGACAG CTGATGAACT GCTGAAGCAG ATTAGGGAGC ACTCTTCTGC AGCTGGGGAT GGATCTCAAA   
  
  
+ GGTTGGCTCA TTACTTTGCT AATGCCTTGG AGGCGCGTTT AGCTGGAACT GGCTCACAGA TCTATACAGC   
  
  
+ CCTGAGTTCG AAGAGGACAA CAGCAGCTGA TATGATAAAA GCTTATCAGT TTTTCTTTCG TGCTTGCCCA   
  
  
+ TTTTTAAAGA TCTCTATTAT CTTTGCGAAC CATATGATTA TACAGAGAGC TGAAAAAGCA TCAAAGCTTC   
  
  
+ ATGTTATAGA TTTTGGCATC CTGTATGGTT TTCAGTGGCC CCTCCTCATT CAACGCCTGT CGGAGCGACC   
  
  
+ TGGTGGACCT CCAAAACTGT GCATTACTGG GATTGATCTT CCCCAACCTG GATTTAGGCC AACAGAAAGA   
  
  
+ GTTGAGGCAA CAGGGCTCCG CTTGGCAAAG TATTGTGAGC GCTTCAATGT TCCATTTGAA TACCATGCCA   
  
  
+ TTGCACAGAA ATGGGAAACC ATCAAAGCTG AAGACCTGAA GATAGAAGAT GATGAGGTGG TTGCCGTGAA   
  
  
+ CTGTCTCTTC AGGTTTAAGA ACCTGCTTGA TGAGACGATA GTGGTGGATA GTCCGAGGAA TGCAGTTCTC   
  
  
+ GGCTTGATTA GAAGGATAAA ACCTGATATC TTTGTTCACG GAGTAGTGAA CGGGTCTTAC AATGCGCCCT   
  
  
+ TCTTTGTTAC ACGTTTCAGG GAGGCCCTCT TCCATTACTC TACTGTATTC GATATGTTTG ATGCCAATGC   
  
  
+ TTCCCGGGAG GATCCTGAGA GATTGATGTT TGAGAAGGAG TTTTATGGGC GAGAAATTAT GAATGTGGTG   
  
  
+ GCTTGTGAGG GTACAGAGAG AGTTGAAAGA CCTGAGACAT ACAAGCAATG GCAGGCGCGG AATAGTAGGG   
  
  
+ CAGGGTTCAG GCAGCTGCCA TTGGACCAAG AGCTCGTCAG TAAACTGAGG AGCAAGGTCC AGATGCACTA   
  
  
+ TCCTAGGGAC TTCGTTGTTG ATGTAGATGG ACATTGGACA TTGCAGGGAT GGAAGGGGAG GATCATCTGT   
  
  
+ GCTGTGTCTG CATGGGGTCC TGCTTA  

- +Up\_Stream \_Len000TCGCAT CCTTCACTGC GGCGATAACC TCCGCTTCTG CGATCATGGT TCCGGTTTTG   
  
  
- TTTACTCCTC TATCTAGTCA ATTTGGCAAA CCAATTCTTT TAATTCGCTT GTAGAGAACT TGGAGGTAGA   
  
  
- CTTGAGCTAC TGTAACTCCT ACACCTACGT CTTTTCTACC TATACCCGAG AGGTAGCCAC GGTCGTTTAG   
  
  
- TTGGATTACA ACCCACCCCA GAGTAAGCCA GTTACCCCCT GATCCCTGCA GTATAGCAGT GGTTAATGTT   
  
  
- GCCTTGTTGG GATTTGGCGT AAAATTTTAG TGTAGAACCA TCGTCTGTTT CTACATGCTG GGTTTTTAAG   
  
  
- TTGAGACATT TCGTGATCGT TTCGAGCATC ATGTGTCTTG TTAGCGTGTG AGTATCACTA TCTTCACATG   
  
  
- ATAGGTAATA CGTCACTTAT ACTGTTCCCT CCCCCAGGAC TCTACGGGTT TAAACCGTCG GCTCAACGGA   
  
  
- ACTGACGGGT AGTTACAGGA TTTGGTCAAC TCGTACAATA ATTAAACGTT CTTTCTAGCT TAAGAAGGAT   
  
  
- AGATTTTTGA CTGTGTTATT TTGTTATGGT ACATACCCTG TAACGATCCT AACAATTGTG TTAATAACAG   
  
  
- AACATGCAAC GTGGACTTTT TTTTCTTTTA ACAACGGTAT CTGTTGTATA TTCAATCCGT AAGTACTGAT   
  
  
- TATAGACTTA ATCCACACAG CTAGACACTA AGTTTAACTA TATTAATCTA GAAATTCCAT AGATTAACGA   
  
  
- AGTTCAAAAG AGATATTACT TCTACATATA ATCGAGTGTA TCCAGAATTC CAATTGATTA CAGTATAGTC   
  
  
- CCCCACACCC GGCGGTAAAA ACTATTCTTA ACTTAACTTG AACGTAACAA AAAAGAAACC GCTCTTACGT   
  
  
- TCTAAACAAT TAAATAAGTA TACGCAGTAA TATGTGATAT TCTTTTTATT AACATCCTTA TATACTTCAT   
  
  
- AAAAATGAAC ACGAGTACAA TTTACACCTT ATGAAAAAAT TACAGCATAT CATGGTGTCA TAAGATGTTA   
  
  
- AATTCTCTAA TTAGCTAGGT AATTTCAGTA CGTTTGGACT ACGTATAAGT TCTGTAACGT ACTTTACTAG   
  
  
- AATAAAATCA GTTTCTGAGG TAGTAAATAT TCGTTATAAA TGAAGCTATG TGCACAGTAA TTTTTTCTGA   
  
  
- TATCTTTACA ATGACAATAA AACAAACTCG ATTTATCTTT TAAATTTTCA ATTAATTCTA TTATTAATTT   
  
  
- TGATTTATAA GTTACTACCG ATTATTTATG TAACAGCATA ATTGACTGAT TATAAACATC TACCAGCACA   
  
  
- ATTAAAGAAA AATAAAATTT CAAGTTTACT CAATGTTTTC CAATATTTTA TATTTCATTT TACCCTCTGT   
  
  
- TTCGTCTGTC CCACCCCCTT CGATTCGAAT TTCATCCTAA ATCTTTCCGT TACTTCCACT TGGGAGTTTA   
  
  
- GCTAGGCGAC GGTTAGTGTC TTTGGGTTTC GAAACGAGTG GCTGTTGAGG GCCAATTGAC GTCAGTGAGT   
  
  
- GCCGTGACCC ATTATGTCAT TAGCACATGG GTCAAGTAGG GGGAAAAGGA AACCCTTATG TCATGGAGCT   
  
  
- TTCAAGGAAA CTTTAAGATG ACCGGTCCGT ATGGGTATTT AACTAGGAGA GCTATAGTAT TCTATACTAT   
  
  
- AAAGAGACAG TATAGACAAT CAAAGCCATA GTATAAAGAA GAAATAGAGA CATTAAACTC CATACGAAGT   
  
  
- AAGGACCGGC GAACTTAATA GAAAGAAAGA AACTAATCAT AACAAAAAAA CCAATGGATA AAACGAGGTC   
  
  
- AGGACAAGTA AACCCAGAAA AACCTAGACC CAAAAACGAC CCAAACAACT AAGAAACTCT TTAAACCCAC   
  
  
- GAACTTAAAC GGGACGTTAT AAGTACCCAA TCGCAAAAGG ACTTAAAACG AGACAAGGAA TTCACTAATA   
  
  
- AAAACTAACT AATGTGGAAC CACCAGGACC ACCTTAAAGG CTCCTCTTTA ACAGTACCCA AGGGTGTTGA   
  
  
- AACCTCTTAA GGGACTACTC TGAGATTTAC TCATAGTTAG ATGAAACCCT TGGTACAGGG GGCCAATACT   
  
  
- ACCCAGAAAC TTAATATGTT GGAACAAGTT AATGTTTCTG GGTAGAGTCC TAGAGTGCCG TGACTTGGAA   
  
  
- GGATCGGGTA ACGGACTGGG ATACGGTAAG TTGTAACCAA GTATAAGCCC AAACTCGGGG CCCCAACTTA   
  
  
- GAAGCCTACT ATCGCTAAGT CTACTACAAC AATTCATGTA ACCGGTTCAC GAATACCTTC TCCTATACTT   
  
  
- CCTCTTCGGA ACATACAAGG TACTGGGAAA TCGTGAGGTT CGACGACTCT TTGGGAAAAT ACTACACAAC   
  
  
- CCTTTCTTCA TAGGAGGAAG GGGATTAGTT GTGGGTGAAT AACTAGTAAG ACACCTATCG GGTCTGTTAG   
  
  
- TCAGGACACC TTGCTCGTGT TAATCACTAG AATCACCGTC ATTGACGTGA AGGTCAAGTT GGTTAATATA   
  
  
- ACTACGACAC CGTCAGCGAC TAAGCTCACT CTTATGATTC TGGAGAAAAC ACGTTTCAAG AGACTAACTT   
  
  
- AGCAAAAGAG TCAGCAGGTG GGAAGGTGTC ACCAGTAAAC CTAGTAACCC ACGGAACCCA CCGTGTCGAA   
  
  
- GAGTCCCAAG CTTAAGTCAA TAGAGGAGGG CACCAAAGGG ATACCGGTAC TTGCAAAAAT CGCTCGTCAG   
  
  
- GTACTATGTC AAAGTCTTCC CCCACCTCCT CCGCTCGTTC AAGGAAGGTT TCTTGTTATT GGAACGCTAA   
  
  
- CTGGAACTCT CGCAGTGAAA GGGTTTACTT TTCCTCCGCC GGGGGTACCA CCACCAATTC TTTCTCTTCC   
  
  
- TACTTGTGAG TGGACTATCG AGCTCTCCAT CGTTCTAAGT GATGGCACTA CTACTCCTGA AACTCCTACC   
  
  
- ATCCTCCTCA TTCGTCAGTC GACAAAGACA CCTCCTCCGA CTCAACAGAC TTTACAAACT GGCCCAAAAC   
  
  
- GAAACGTTAG GGTACTTTGT ACTTCGAGTA ACATGTTCAC CAAACTTCAG GCTCTTCCCT TGTAGGGACG   
  
  
- TCCGACCGGT TCATGTCCAT GTCCTCCCAG TCTTCCGAGC ACGTTTCTTT GTCCCATTAT TACTATTATT   
  
  
- CTTCTTATTC TTACACCACC TAAATTCCTG AGACGAATAG GAGACACGCG TTAGACGACG TAGACTACTA   
  
  
- GCGTCCTGTC GACTACTTGA CGACTTCGTC TAATCCCTCG TGAGAAGACG TCGACCCCTA CCTAGAGTTT   
  
  
- CCAACCGAGT AATGAAACGA TTACGGAACC TCCGCGCAAA TCGACCTTGA CCGAGTGTCT AGATATGTCG   
  
  
- GGACTCAAGC TTCTCCTGTT GTCGTCGACT ATACTATTTT CGAATAGTCA AAAAGAAAGC ACGAACGGGT   
  
  
- AAAAATTTCT AGAGATAATA GAAACGCTTG GTATACTAAT ATGTCTCTCG ACTTTTTCGT AGTTTCGAAG   
  
  
- TACAATATCT AAAACCGTAG GACATACCAA AAGTCACCGG GGAGGAGTAA GTTGCGGACA GCCTCGCTGG   
  
  
- ACCACCTGGA GGTTTTGACA CGTAATGACC CTAACTAGAA GGGGTTGGAC CTAAATCCGG TTGTCTTTCT   
  
  
- CAACTCCGTT GTCCCGAGGC GAACCGTTTC ATAACACTCG CGAAGTTACA AGGTAAACTT ATGGTACGGT   
  
  
- AACGTGTCTT TACCCTTTGG TAGTTTCGAC TTCTGGACTT CTATCTTCTA CTACTCCACC AACGGCACTT   
  
  
- GACAGAGAAG TCCAAATTCT TGGACGAACT ACTCTGCTAT CACCACCTAT CAGGCTCCTT ACGTCAAGAG   
  
  
- CCGAACTAAT CTTCCTATTT TGGACTATAG AAACAAGTGC CTCATCACTT GCCCAGAATG TTACGCGGGA   
  
  
- AGAAACAATG TGCAAAGTCC CTCCGGGAGA AGGTAATGAG ATGACATAAG CTATACAAAC TACGGTTACG   
  
  
- AAGGGCCCTC CTAGGACTCT CTAACTACAA ACTCTTCCTC AAAATACCCG CTCTTTAATA CTTACACCAC   
  
  
- CGAACACTCC CATGTCTCTC TCAACTTTCT GGACTCTGTA TGTTCGTTAC CGTCCGCGCC TTATCATCCC   
  
  
- GTCCCAAGTC CGTCGACGGT AACCTGGTTC TCGAGCAGTC ATTTGACTCC TCGTTCCAGG TCTACGTGAT   
  
  
- AGGATCCCTG AAGCAACAAC TACATCTACC TGTAACCTGT AACGTCCCTA CCTTCCCCTC CTAGTAGACA   
  
  
- CGACACAGAC GTACCCCAGG ACGAAT

+     CGTCA-motif

| Site Name | Organism | Position | Strand | Matrix score. | sequence | function |
| --- | --- | --- | --- | --- | --- | --- |
| CGTCA-motif | Hordeum vulgare | 270 | + | 5 | CGTCA | cis-acting regulatory element involved in the MeJA-responsiveness |
| CGTCA-motif | Hordeum vulgare | 938 | + | 5 | CGTCA | cis-acting regulatory element involved in the MeJA-responsiveness |
| CGTCA-motif | Hordeum vulgare | 262 | + | 5 | CGTCA | cis-acting regulatory element involved in the MeJA-responsiveness |
| CGTCA-motif | Hordeum vulgare | 30 | - | 5 | CGTCA | cis-acting regulatory element involved in the MeJA-responsiveness |
| CGTCA-motif | Hordeum vulgare | 4169 | + | 5 | CGTCA | cis-acting regulatory element involved in the MeJA-responsiveness |
| CGTCA-motif | Hordeum vulgare | 2816 | + | 5 | CGTCA | cis-acting regulatory element involved in the MeJA-responsiveness |

>HU02G01569.1   
+ +Up\_Stream \_Len000AGCGTA GGAAGTGACG CCGCTATTGG AGGCGAAGAC GCTAGTACCA AGGCCAAAAC   
  
  
+ AAATGAGGAG ATAGATCAGT TAAACCGTTT GGTTAAGAAA ATTAAGCGAA CATCTCTTGA ACCTCCATCT   
  
  
+ GAACTCGATG ACATTGAGGA TGTGGATGCA GAAAAGATGG ATATGGGCTC TCCATCGGTG CCAGCAAATC   
  
  
+ AACCTAATGT TGGGTGGGGT CTCATTCGGT CAATGGGGGA CTAGGGACGT CATATCGTCA CCAATTACAA   
  
  
+ CGGAACAACC CTAAACCGCA TTTTAAAATC ACATCTTGGT AGCAGACAAA GATGTACGAC CCAAAAATTC   
  
  
+ AACTCTGTAA AGCACTAGCA AAGCTCGTAG TACACAGAAC AATCGCACAC TCATAGTGAT AGAAGTGTAC   
  
  
+ TATCCATTAT GCAGTGAATA TGACAAGGGA GGGGGTCCTG AGATGCCCAA ATTTGGCAGC CGAGTTGCCT   
  
  
+ TGACTGCCCA TCAATGTCCT AAACCAGTTG AGCATGTTAT TAATTTGCAA GAAAGATCGA ATTCTTCCTA   
  
  
+ TCTAAAAACT GACACAATAA AACAATACCA TGTATGGGAC ATTGCTAGGA TTGTTAACAC AATTATTGTC   
  
  
+ TTGTACGTTG CACCTGAAAA AAAAGAAAAT TGTTGCCATA GACAACATAT AAGTTAGGCA TTCATGACTA   
  
  
+ ATATCTGAAT TAGGTGTGTC GATCTGTGAT TCAAATTGAT ATAATTAGAT CTTTAAGGTA TCTAATTGCT   
  
  
+ TCAAGTTTTC TCTATAATGA AGATGTATAT TAGCTCACAT AGGTCTTAAG GTTAACTAAT GTCATATCAG   
  
  
+ GGGGTGTGGG CCGCCATTTT TGATAAGAAT TGAATTGAAC TTGCATTGTT TTTTCTTTGG CGAGAATGCA   
  
  
+ AGATTTGTTA ATTTATTCAT ATGCGTCATT ATACACTATA AGAAAAATAA TTGTAGGAAT ATATGAAGTA   
  
  
+ TTTTTACTTG TGCTCATGTT AAATGTGGAA TACTTTTTTA ATGTCGTATA GTACCACAGT ATTCTACAAT   
  
  
+ TTAAGAGATT AATCGATCCA TTAAAGTCAT GCAAACCTGA TGCATATTCA AGACATTGCA TGAAATGATC   
  
  
+ TTATTTTAGT CAAAGACTCC ATCATTTATA AGCAATATTT ACTTCGATAC ACGTGTCATT AAAAAAGACT   
  
  
+ ATAGAAATGT TACTGTTATT TTGTTTGAGC TAAATAGAAA ATTTAAAAGT TAATTAAGAT AATAATTAAA   
  
  
+ ACTAAATATT CAATGATGGC TAATAAATAC ATTGTCGTAT TAACTGACTA ATATTTGTAG ATGGTCGTGT   
  
  
+ TAATTTCTTT TTATTTTAAA GTTCAAATGA GTTACAAAAG GTTATAAAAT ATAAAGTAAA ATGGGAGACA   
  
  
+ AAGCAGACAG GGTGGGGGAA GCTAAGCTTA AAGTAGGATT TAGAAAGGCA ATGAAGGTGA ACCCTCAAAT   
  
  
+ CGATCCGCTG CCAATCACAG AAACCCAAAG CTTTGCTCAC CGACAACTCC CGGTTAACTG CAGTCACTCA   
  
  
+ CGGCACTGGG TAATACAGTA ATCGTGTACC CAGTTCATCC CCCTTTTCCT TTGGGAATAC AGTACCTCGA   
  
  
+ AAGTTCCTTT GAAATTCTAC TGGCCAGGCA TACCCATAAA TTGATCCTCT CGATATCATA AGATATGATA   
  
  
+ TTTCTCTGTC ATATCTGTTA GTTTCGGTAT CATATTTCTT CTTTATCTCT GTAATTTGAG GTATGCTTCA   
  
  
+ TTCCTGGCCG CTTGAATTAT CTTTCTTTCT TTGATTAGTA TTGTTTTTTT GGTTACCTAT TTTGCTCCAG   
  
  
+ TCCTGTTCAT TTGGGTCTTT TTGGATCTGG GTTTTTGCTG GGTTTGTTGA TTCTTTGAGA AATTTGGGTG   
  
  
+ CTTGAATTTG CCCTGCAATA TTCATGGGTT AGCGTTTTCC TGAATTTTGC TCTGTTCCTT AAGTGATTAT   
  
  
+ TTTTGATTGA TTACACCTTG GTGGTCCTGG TGGAATTTCC GAGGAGAAAT TGTCATGGGT TCCCACAACT   
  
  
+ TTGGAGAATT CCCTGATGAG ACTCTAAATG AGTATCAATC TACTTTGGGA ACCATGTCCC CCGGTTATGA   
  
  
+ TGGGTCTTTG AATTATACAA CCTTGTTCAA TTACAAAGAC CCATCTCAGG ATCTCACGGC ACTGAACCTT   
  
  
+ CCTAGCCCAT TGCCTGACCC TATGCCATTC AACATTGGTT CATATTCGGG TTTGAGCCCC GGGGTTGAAT   
  
  
+ CTTCGGATGA TAGCGATTCA GATGATGTTG TTAAGTACAT TGGCCAAGTG CTTATGGAAG AGGATATGAA   
  
  
+ GGAGAAGCCT TGTATGTTCC ATGACCCTTT AGCACTCCAA GCTGCTGAGA AACCCTTTTA TGATGTGTTG   
  
  
+ GGAAAGAAGT ATCCTCCTTC CCCTAATCAA CACCCACTTA TTGATCATTC TGTGGATAGC CCAGACAATC   
  
  
+ AGTCCTGTGG AACGAGCACA ATTAGTGATC TTAGTGGCAG TAACTGCACT TCCAGTTCAA CCAATTATAT   
  
  
+ TGATGCTGTG GCAGTCGCTG ATTCGAGTGA GAATACTAAG ACCTCTTTTG TGCAAAGTTC TCTGATTGAA   
  
  
+ TCGTTTTCTC AGTCGTCCAC CCTTCCACAG TGGTCATTTG GATCATTGGG TGCCTTGGGT GGCACAGCTT   
  
  
+ CTCAGGGTTC GAATTCAGTT ATCTCCTCCC GTGGTTTCCC TATGGCCATG AACGTTTTTA GCGAGCAGTC   
  
  
+ CATGATACAG TTTCAGAAGG GGGTGGAGGA GGCGAGCAAG TTCCTTCCAA AGAACAATAA CCTTGCGATT   
  
  
+ GACCTTGAGA GCGTCACTTT CCCAAATGAA AAGGAGGCGG CCCCCATGGT GGTGGTTAAG AAAGAGAAGG   
  
  
+ ATGAACACTC ACCTGATAGC TCGAGAGGTA GCAAGATTCA CTACCGTGAT GATGAGGACT TTGAGGATGG   
  
  
+ TAGGAGGAGT AAGCAGTCAG CTGTTTCTGT GGAGGAGGCT GAGTTGTCTG AAATGTTTGA CCGGGTTTTG   
  
  
+ CTTTGCAATC CCATGAAACA TGAAGCTCAT TGTACAAGTG GTTTGAAGTC CGAGAAGGGA ACATCCCTGC   
  
  
+ AGGCTGGCCA AGTACAGGTA CAGGAGGGTC AGAAGGCTCG TGCAAAGAAA CAGGGTAATA ATGATAATAA   
  
  
+ GAAGAATAAG AATGTGGTGG ATTTAAGGAC TCTGCTTATC CTCTGTGCGC AATCTGCTGC ATCTGATGAT   
  
  
+ CGCAGGACAG CTGATGAACT GCTGAAGCAG ATTAGGGAGC ACTCTTCTGC AGCTGGGGAT GGATCTCAAA   
  
  
+ GGTTGGCTCA TTACTTTGCT AATGCCTTGG AGGCGCGTTT AGCTGGAACT GGCTCACAGA TCTATACAGC   
  
  
+ CCTGAGTTCG AAGAGGACAA CAGCAGCTGA TATGATAAAA GCTTATCAGT TTTTCTTTCG TGCTTGCCCA   
  
  
+ TTTTTAAAGA TCTCTATTAT CTTTGCGAAC CATATGATTA TACAGAGAGC TGAAAAAGCA TCAAAGCTTC   
  
  
+ ATGTTATAGA TTTTGGCATC CTGTATGGTT TTCAGTGGCC CCTCCTCATT CAACGCCTGT CGGAGCGACC   
  
  
+ TGGTGGACCT CCAAAACTGT GCATTACTGG GATTGATCTT CCCCAACCTG GATTTAGGCC AACAGAAAGA   
  
  
+ GTTGAGGCAA CAGGGCTCCG CTTGGCAAAG TATTGTGAGC GCTTCAATGT TCCATTTGAA TACCATGCCA   
  
  
+ TTGCACAGAA ATGGGAAACC ATCAAAGCTG AAGACCTGAA GATAGAAGAT GATGAGGTGG TTGCCGTGAA   
  
  
+ CTGTCTCTTC AGGTTTAAGA ACCTGCTTGA TGAGACGATA GTGGTGGATA GTCCGAGGAA TGCAGTTCTC   
  
  
+ GGCTTGATTA GAAGGATAAA ACCTGATATC TTTGTTCACG GAGTAGTGAA CGGGTCTTAC AATGCGCCCT   
  
  
+ TCTTTGTTAC ACGTTTCAGG GAGGCCCTCT TCCATTACTC TACTGTATTC GATATGTTTG ATGCCAATGC   
  
  
+ TTCCCGGGAG GATCCTGAGA GATTGATGTT TGAGAAGGAG TTTTATGGGC GAGAAATTAT GAATGTGGTG   
  
  
+ GCTTGTGAGG GTACAGAGAG AGTTGAAAGA CCTGAGACAT ACAAGCAATG GCAGGCGCGG AATAGTAGGG   
  
  
+ CAGGGTTCAG GCAGCTGCCA TTGGACCAAG AGCTCGTCAG TAAACTGAGG AGCAAGGTCC AGATGCACTA   
  
  
+ TCCTAGGGAC TTCGTTGTTG ATGTAGATGG ACATTGGACA TTGCAGGGAT GGAAGGGGAG GATCATCTGT   
  
  
+ GCTGTGTCTG CATGGGGTCC TGCTTA  

- +Up\_Stream \_Len000TCGCAT CCTTCACTGC GGCGATAACC TCCGCTTCTG CGATCATGGT TCCGGTTTTG   
  
  
- TTTACTCCTC TATCTAGTCA ATTTGGCAAA CCAATTCTTT TAATTCGCTT GTAGAGAACT TGGAGGTAGA   
  
  
- CTTGAGCTAC TGTAACTCCT ACACCTACGT CTTTTCTACC TATACCCGAG AGGTAGCCAC GGTCGTTTAG   
  
  
- TTGGATTACA ACCCACCCCA GAGTAAGCCA GTTACCCCCT GATCCCTGCA GTATAGCAGT GGTTAATGTT   
  
  
- GCCTTGTTGG GATTTGGCGT AAAATTTTAG TGTAGAACCA TCGTCTGTTT CTACATGCTG GGTTTTTAAG   
  
  
- TTGAGACATT TCGTGATCGT TTCGAGCATC ATGTGTCTTG TTAGCGTGTG AGTATCACTA TCTTCACATG   
  
  
- ATAGGTAATA CGTCACTTAT ACTGTTCCCT CCCCCAGGAC TCTACGGGTT TAAACCGTCG GCTCAACGGA   
  
  
- ACTGACGGGT AGTTACAGGA TTTGGTCAAC TCGTACAATA ATTAAACGTT CTTTCTAGCT TAAGAAGGAT   
  
  
- AGATTTTTGA CTGTGTTATT TTGTTATGGT ACATACCCTG TAACGATCCT AACAATTGTG TTAATAACAG   
  
  
- AACATGCAAC GTGGACTTTT TTTTCTTTTA ACAACGGTAT CTGTTGTATA TTCAATCCGT AAGTACTGAT   
  
  
- TATAGACTTA ATCCACACAG CTAGACACTA AGTTTAACTA TATTAATCTA GAAATTCCAT AGATTAACGA   
  
  
- AGTTCAAAAG AGATATTACT TCTACATATA ATCGAGTGTA TCCAGAATTC CAATTGATTA CAGTATAGTC   
  
  
- CCCCACACCC GGCGGTAAAA ACTATTCTTA ACTTAACTTG AACGTAACAA AAAAGAAACC GCTCTTACGT   
  
  
- TCTAAACAAT TAAATAAGTA TACGCAGTAA TATGTGATAT TCTTTTTATT AACATCCTTA TATACTTCAT   
  
  
- AAAAATGAAC ACGAGTACAA TTTACACCTT ATGAAAAAAT TACAGCATAT CATGGTGTCA TAAGATGTTA   
  
  
- AATTCTCTAA TTAGCTAGGT AATTTCAGTA CGTTTGGACT ACGTATAAGT TCTGTAACGT ACTTTACTAG   
  
  
- AATAAAATCA GTTTCTGAGG TAGTAAATAT TCGTTATAAA TGAAGCTATG TGCACAGTAA TTTTTTCTGA   
  
  
- TATCTTTACA ATGACAATAA AACAAACTCG ATTTATCTTT TAAATTTTCA ATTAATTCTA TTATTAATTT   
  
  
- TGATTTATAA GTTACTACCG ATTATTTATG TAACAGCATA ATTGACTGAT TATAAACATC TACCAGCACA   
  
  
- ATTAAAGAAA AATAAAATTT CAAGTTTACT CAATGTTTTC CAATATTTTA TATTTCATTT TACCCTCTGT   
  
  
- TTCGTCTGTC CCACCCCCTT CGATTCGAAT TTCATCCTAA ATCTTTCCGT TACTTCCACT TGGGAGTTTA   
  
  
- GCTAGGCGAC GGTTAGTGTC TTTGGGTTTC GAAACGAGTG GCTGTTGAGG GCCAATTGAC GTCAGTGAGT   
  
  
- GCCGTGACCC ATTATGTCAT TAGCACATGG GTCAAGTAGG GGGAAAAGGA AACCCTTATG TCATGGAGCT   
  
  
- TTCAAGGAAA CTTTAAGATG ACCGGTCCGT ATGGGTATTT AACTAGGAGA GCTATAGTAT TCTATACTAT   
  
  
- AAAGAGACAG TATAGACAAT CAAAGCCATA GTATAAAGAA GAAATAGAGA CATTAAACTC CATACGAAGT   
  
  
- AAGGACCGGC GAACTTAATA GAAAGAAAGA AACTAATCAT AACAAAAAAA CCAATGGATA AAACGAGGTC   
  
  
- AGGACAAGTA AACCCAGAAA AACCTAGACC CAAAAACGAC CCAAACAACT AAGAAACTCT TTAAACCCAC   
  
  
- GAACTTAAAC GGGACGTTAT AAGTACCCAA TCGCAAAAGG ACTTAAAACG AGACAAGGAA TTCACTAATA   
  
  
- AAAACTAACT AATGTGGAAC CACCAGGACC ACCTTAAAGG CTCCTCTTTA ACAGTACCCA AGGGTGTTGA   
  
  
- AACCTCTTAA GGGACTACTC TGAGATTTAC TCATAGTTAG ATGAAACCCT TGGTACAGGG GGCCAATACT   
  
  
- ACCCAGAAAC TTAATATGTT GGAACAAGTT AATGTTTCTG GGTAGAGTCC TAGAGTGCCG TGACTTGGAA   
  
  
- GGATCGGGTA ACGGACTGGG ATACGGTAAG TTGTAACCAA GTATAAGCCC AAACTCGGGG CCCCAACTTA   
  
  
- GAAGCCTACT ATCGCTAAGT CTACTACAAC AATTCATGTA ACCGGTTCAC GAATACCTTC TCCTATACTT   
  
  
- CCTCTTCGGA ACATACAAGG TACTGGGAAA TCGTGAGGTT CGACGACTCT TTGGGAAAAT ACTACACAAC   
  
  
- CCTTTCTTCA TAGGAGGAAG GGGATTAGTT GTGGGTGAAT AACTAGTAAG ACACCTATCG GGTCTGTTAG   
  
  
- TCAGGACACC TTGCTCGTGT TAATCACTAG AATCACCGTC ATTGACGTGA AGGTCAAGTT GGTTAATATA   
  
  
- ACTACGACAC CGTCAGCGAC TAAGCTCACT CTTATGATTC TGGAGAAAAC ACGTTTCAAG AGACTAACTT   
  
  
- AGCAAAAGAG TCAGCAGGTG GGAAGGTGTC ACCAGTAAAC CTAGTAACCC ACGGAACCCA CCGTGTCGAA   
  
  
- GAGTCCCAAG CTTAAGTCAA TAGAGGAGGG CACCAAAGGG ATACCGGTAC TTGCAAAAAT CGCTCGTCAG   
  
  
- GTACTATGTC AAAGTCTTCC CCCACCTCCT CCGCTCGTTC AAGGAAGGTT TCTTGTTATT GGAACGCTAA   
  
  
- CTGGAACTCT CGCAGTGAAA GGGTTTACTT TTCCTCCGCC GGGGGTACCA CCACCAATTC TTTCTCTTCC   
  
  
- TACTTGTGAG TGGACTATCG AGCTCTCCAT CGTTCTAAGT GATGGCACTA CTACTCCTGA AACTCCTACC   
  
  
- ATCCTCCTCA TTCGTCAGTC GACAAAGACA CCTCCTCCGA CTCAACAGAC TTTACAAACT GGCCCAAAAC   
  
  
- GAAACGTTAG GGTACTTTGT ACTTCGAGTA ACATGTTCAC CAAACTTCAG GCTCTTCCCT TGTAGGGACG   
  
  
- TCCGACCGGT TCATGTCCAT GTCCTCCCAG TCTTCCGAGC ACGTTTCTTT GTCCCATTAT TACTATTATT   
  
  
- CTTCTTATTC TTACACCACC TAAATTCCTG AGACGAATAG GAGACACGCG TTAGACGACG TAGACTACTA   
  
  
- GCGTCCTGTC GACTACTTGA CGACTTCGTC TAATCCCTCG TGAGAAGACG TCGACCCCTA CCTAGAGTTT   
  
  
- CCAACCGAGT AATGAAACGA TTACGGAACC TCCGCGCAAA TCGACCTTGA CCGAGTGTCT AGATATGTCG   
  
  
- GGACTCAAGC TTCTCCTGTT GTCGTCGACT ATACTATTTT CGAATAGTCA AAAAGAAAGC ACGAACGGGT   
  
  
- AAAAATTTCT AGAGATAATA GAAACGCTTG GTATACTAAT ATGTCTCTCG ACTTTTTCGT AGTTTCGAAG   
  
  
- TACAATATCT AAAACCGTAG GACATACCAA AAGTCACCGG GGAGGAGTAA GTTGCGGACA GCCTCGCTGG   
  
  
- ACCACCTGGA GGTTTTGACA CGTAATGACC CTAACTAGAA GGGGTTGGAC CTAAATCCGG TTGTCTTTCT   
  
  
- CAACTCCGTT GTCCCGAGGC GAACCGTTTC ATAACACTCG CGAAGTTACA AGGTAAACTT ATGGTACGGT   
  
  
- AACGTGTCTT TACCCTTTGG TAGTTTCGAC TTCTGGACTT CTATCTTCTA CTACTCCACC AACGGCACTT   
  
  
- GACAGAGAAG TCCAAATTCT TGGACGAACT ACTCTGCTAT CACCACCTAT CAGGCTCCTT ACGTCAAGAG   
  
  
- CCGAACTAAT CTTCCTATTT TGGACTATAG AAACAAGTGC CTCATCACTT GCCCAGAATG TTACGCGGGA   
  
  
- AGAAACAATG TGCAAAGTCC CTCCGGGAGA AGGTAATGAG ATGACATAAG CTATACAAAC TACGGTTACG   
  
  
- AAGGGCCCTC CTAGGACTCT CTAACTACAA ACTCTTCCTC AAAATACCCG CTCTTTAATA CTTACACCAC   
  
  
- CGAACACTCC CATGTCTCTC TCAACTTTCT GGACTCTGTA TGTTCGTTAC CGTCCGCGCC TTATCATCCC   
  
  
- GTCCCAAGTC CGTCGACGGT AACCTGGTTC TCGAGCAGTC ATTTGACTCC TCGTTCCAGG TCTACGTGAT   
  
  
- AGGATCCCTG AAGCAACAAC TACATCTACC TGTAACCTGT AACGTCCCTA CCTTCCCCTC CTAGTAGACA   
  
  
- CGACACAGAC GTACCCCAGG ACGAAT

+     ERE

| Site Name | Organism | Position | Strand | Matrix score. | sequence | function |
| --- | --- | --- | --- | --- | --- | --- |
| ERE | Nicotiana glutinos | 1347 | + | 8 | ATTTTAAA |  |
| ERE | Nicotiana glutinos | 306 | - | 8 | ATTTTAAA |  |
| ERE | Nicotiana glutinos | 304 | + | 8 | ATTTTAAA |  |

>HU02G01569.1   
+ +Up\_Stream \_Len000AGCGTA GGAAGTGACG CCGCTATTGG AGGCGAAGAC GCTAGTACCA AGGCCAAAAC   
  
  
+ AAATGAGGAG ATAGATCAGT TAAACCGTTT GGTTAAGAAA ATTAAGCGAA CATCTCTTGA ACCTCCATCT   
  
  
+ GAACTCGATG ACATTGAGGA TGTGGATGCA GAAAAGATGG ATATGGGCTC TCCATCGGTG CCAGCAAATC   
  
  
+ AACCTAATGT TGGGTGGGGT CTCATTCGGT CAATGGGGGA CTAGGGACGT CATATCGTCA CCAATTACAA   
  
  
+ CGGAACAACC CTAAACCGCA TTTTAAAATC ACATCTTGGT AGCAGACAAA GATGTACGAC CCAAAAATTC   
  
  
+ AACTCTGTAA AGCACTAGCA AAGCTCGTAG TACACAGAAC AATCGCACAC TCATAGTGAT AGAAGTGTAC   
  
  
+ TATCCATTAT GCAGTGAATA TGACAAGGGA GGGGGTCCTG AGATGCCCAA ATTTGGCAGC CGAGTTGCCT   
  
  
+ TGACTGCCCA TCAATGTCCT AAACCAGTTG AGCATGTTAT TAATTTGCAA GAAAGATCGA ATTCTTCCTA   
  
  
+ TCTAAAAACT GACACAATAA AACAATACCA TGTATGGGAC ATTGCTAGGA TTGTTAACAC AATTATTGTC   
  
  
+ TTGTACGTTG CACCTGAAAA AAAAGAAAAT TGTTGCCATA GACAACATAT AAGTTAGGCA TTCATGACTA   
  
  
+ ATATCTGAAT TAGGTGTGTC GATCTGTGAT TCAAATTGAT ATAATTAGAT CTTTAAGGTA TCTAATTGCT   
  
  
+ TCAAGTTTTC TCTATAATGA AGATGTATAT TAGCTCACAT AGGTCTTAAG GTTAACTAAT GTCATATCAG   
  
  
+ GGGGTGTGGG CCGCCATTTT TGATAAGAAT TGAATTGAAC TTGCATTGTT TTTTCTTTGG CGAGAATGCA   
  
  
+ AGATTTGTTA ATTTATTCAT ATGCGTCATT ATACACTATA AGAAAAATAA TTGTAGGAAT ATATGAAGTA   
  
  
+ TTTTTACTTG TGCTCATGTT AAATGTGGAA TACTTTTTTA ATGTCGTATA GTACCACAGT ATTCTACAAT   
  
  
+ TTAAGAGATT AATCGATCCA TTAAAGTCAT GCAAACCTGA TGCATATTCA AGACATTGCA TGAAATGATC   
  
  
+ TTATTTTAGT CAAAGACTCC ATCATTTATA AGCAATATTT ACTTCGATAC ACGTGTCATT AAAAAAGACT   
  
  
+ ATAGAAATGT TACTGTTATT TTGTTTGAGC TAAATAGAAA ATTTAAAAGT TAATTAAGAT AATAATTAAA   
  
  
+ ACTAAATATT CAATGATGGC TAATAAATAC ATTGTCGTAT TAACTGACTA ATATTTGTAG ATGGTCGTGT   
  
  
+ TAATTTCTTT TTATTTTAAA GTTCAAATGA GTTACAAAAG GTTATAAAAT ATAAAGTAAA ATGGGAGACA   
  
  
+ AAGCAGACAG GGTGGGGGAA GCTAAGCTTA AAGTAGGATT TAGAAAGGCA ATGAAGGTGA ACCCTCAAAT   
  
  
+ CGATCCGCTG CCAATCACAG AAACCCAAAG CTTTGCTCAC CGACAACTCC CGGTTAACTG CAGTCACTCA   
  
  
+ CGGCACTGGG TAATACAGTA ATCGTGTACC CAGTTCATCC CCCTTTTCCT TTGGGAATAC AGTACCTCGA   
  
  
+ AAGTTCCTTT GAAATTCTAC TGGCCAGGCA TACCCATAAA TTGATCCTCT CGATATCATA AGATATGATA   
  
  
+ TTTCTCTGTC ATATCTGTTA GTTTCGGTAT CATATTTCTT CTTTATCTCT GTAATTTGAG GTATGCTTCA   
  
  
+ TTCCTGGCCG CTTGAATTAT CTTTCTTTCT TTGATTAGTA TTGTTTTTTT GGTTACCTAT TTTGCTCCAG   
  
  
+ TCCTGTTCAT TTGGGTCTTT TTGGATCTGG GTTTTTGCTG GGTTTGTTGA TTCTTTGAGA AATTTGGGTG   
  
  
+ CTTGAATTTG CCCTGCAATA TTCATGGGTT AGCGTTTTCC TGAATTTTGC TCTGTTCCTT AAGTGATTAT   
  
  
+ TTTTGATTGA TTACACCTTG GTGGTCCTGG TGGAATTTCC GAGGAGAAAT TGTCATGGGT TCCCACAACT   
  
  
+ TTGGAGAATT CCCTGATGAG ACTCTAAATG AGTATCAATC TACTTTGGGA ACCATGTCCC CCGGTTATGA   
  
  
+ TGGGTCTTTG AATTATACAA CCTTGTTCAA TTACAAAGAC CCATCTCAGG ATCTCACGGC ACTGAACCTT   
  
  
+ CCTAGCCCAT TGCCTGACCC TATGCCATTC AACATTGGTT CATATTCGGG TTTGAGCCCC GGGGTTGAAT   
  
  
+ CTTCGGATGA TAGCGATTCA GATGATGTTG TTAAGTACAT TGGCCAAGTG CTTATGGAAG AGGATATGAA   
  
  
+ GGAGAAGCCT TGTATGTTCC ATGACCCTTT AGCACTCCAA GCTGCTGAGA AACCCTTTTA TGATGTGTTG   
  
  
+ GGAAAGAAGT ATCCTCCTTC CCCTAATCAA CACCCACTTA TTGATCATTC TGTGGATAGC CCAGACAATC   
  
  
+ AGTCCTGTGG AACGAGCACA ATTAGTGATC TTAGTGGCAG TAACTGCACT TCCAGTTCAA CCAATTATAT   
  
  
+ TGATGCTGTG GCAGTCGCTG ATTCGAGTGA GAATACTAAG ACCTCTTTTG TGCAAAGTTC TCTGATTGAA   
  
  
+ TCGTTTTCTC AGTCGTCCAC CCTTCCACAG TGGTCATTTG GATCATTGGG TGCCTTGGGT GGCACAGCTT   
  
  
+ CTCAGGGTTC GAATTCAGTT ATCTCCTCCC GTGGTTTCCC TATGGCCATG AACGTTTTTA GCGAGCAGTC   
  
  
+ CATGATACAG TTTCAGAAGG GGGTGGAGGA GGCGAGCAAG TTCCTTCCAA AGAACAATAA CCTTGCGATT   
  
  
+ GACCTTGAGA GCGTCACTTT CCCAAATGAA AAGGAGGCGG CCCCCATGGT GGTGGTTAAG AAAGAGAAGG   
  
  
+ ATGAACACTC ACCTGATAGC TCGAGAGGTA GCAAGATTCA CTACCGTGAT GATGAGGACT TTGAGGATGG   
  
  
+ TAGGAGGAGT AAGCAGTCAG CTGTTTCTGT GGAGGAGGCT GAGTTGTCTG AAATGTTTGA CCGGGTTTTG   
  
  
+ CTTTGCAATC CCATGAAACA TGAAGCTCAT TGTACAAGTG GTTTGAAGTC CGAGAAGGGA ACATCCCTGC   
  
  
+ AGGCTGGCCA AGTACAGGTA CAGGAGGGTC AGAAGGCTCG TGCAAAGAAA CAGGGTAATA ATGATAATAA   
  
  
+ GAAGAATAAG AATGTGGTGG ATTTAAGGAC TCTGCTTATC CTCTGTGCGC AATCTGCTGC ATCTGATGAT   
  
  
+ CGCAGGACAG CTGATGAACT GCTGAAGCAG ATTAGGGAGC ACTCTTCTGC AGCTGGGGAT GGATCTCAAA   
  
  
+ GGTTGGCTCA TTACTTTGCT AATGCCTTGG AGGCGCGTTT AGCTGGAACT GGCTCACAGA TCTATACAGC   
  
  
+ CCTGAGTTCG AAGAGGACAA CAGCAGCTGA TATGATAAAA GCTTATCAGT TTTTCTTTCG TGCTTGCCCA   
  
  
+ TTTTTAAAGA TCTCTATTAT CTTTGCGAAC CATATGATTA TACAGAGAGC TGAAAAAGCA TCAAAGCTTC   
  
  
+ ATGTTATAGA TTTTGGCATC CTGTATGGTT TTCAGTGGCC CCTCCTCATT CAACGCCTGT CGGAGCGACC   
  
  
+ TGGTGGACCT CCAAAACTGT GCATTACTGG GATTGATCTT CCCCAACCTG GATTTAGGCC AACAGAAAGA   
  
  
+ GTTGAGGCAA CAGGGCTCCG CTTGGCAAAG TATTGTGAGC GCTTCAATGT TCCATTTGAA TACCATGCCA   
  
  
+ TTGCACAGAA ATGGGAAACC ATCAAAGCTG AAGACCTGAA GATAGAAGAT GATGAGGTGG TTGCCGTGAA   
  
  
+ CTGTCTCTTC AGGTTTAAGA ACCTGCTTGA TGAGACGATA GTGGTGGATA GTCCGAGGAA TGCAGTTCTC   
  
  
+ GGCTTGATTA GAAGGATAAA ACCTGATATC TTTGTTCACG GAGTAGTGAA CGGGTCTTAC AATGCGCCCT   
  
  
+ TCTTTGTTAC ACGTTTCAGG GAGGCCCTCT TCCATTACTC TACTGTATTC GATATGTTTG ATGCCAATGC   
  
  
+ TTCCCGGGAG GATCCTGAGA GATTGATGTT TGAGAAGGAG TTTTATGGGC GAGAAATTAT GAATGTGGTG   
  
  
+ GCTTGTGAGG GTACAGAGAG AGTTGAAAGA CCTGAGACAT ACAAGCAATG GCAGGCGCGG AATAGTAGGG   
  
  
+ CAGGGTTCAG GCAGCTGCCA TTGGACCAAG AGCTCGTCAG TAAACTGAGG AGCAAGGTCC AGATGCACTA   
  
  
+ TCCTAGGGAC TTCGTTGTTG ATGTAGATGG ACATTGGACA TTGCAGGGAT GGAAGGGGAG GATCATCTGT   
  
  
+ GCTGTGTCTG CATGGGGTCC TGCTTA  

- +Up\_Stream \_Len000TCGCAT CCTTCACTGC GGCGATAACC TCCGCTTCTG CGATCATGGT TCCGGTTTTG   
  
  
- TTTACTCCTC TATCTAGTCA ATTTGGCAAA CCAATTCTTT TAATTCGCTT GTAGAGAACT TGGAGGTAGA   
  
  
- CTTGAGCTAC TGTAACTCCT ACACCTACGT CTTTTCTACC TATACCCGAG AGGTAGCCAC GGTCGTTTAG   
  
  
- TTGGATTACA ACCCACCCCA GAGTAAGCCA GTTACCCCCT GATCCCTGCA GTATAGCAGT GGTTAATGTT   
  
  
- GCCTTGTTGG GATTTGGCGT AAAATTTTAG TGTAGAACCA TCGTCTGTTT CTACATGCTG GGTTTTTAAG   
  
  
- TTGAGACATT TCGTGATCGT TTCGAGCATC ATGTGTCTTG TTAGCGTGTG AGTATCACTA TCTTCACATG   
  
  
- ATAGGTAATA CGTCACTTAT ACTGTTCCCT CCCCCAGGAC TCTACGGGTT TAAACCGTCG GCTCAACGGA   
  
  
- ACTGACGGGT AGTTACAGGA TTTGGTCAAC TCGTACAATA ATTAAACGTT CTTTCTAGCT TAAGAAGGAT   
  
  
- AGATTTTTGA CTGTGTTATT TTGTTATGGT ACATACCCTG TAACGATCCT AACAATTGTG TTAATAACAG   
  
  
- AACATGCAAC GTGGACTTTT TTTTCTTTTA ACAACGGTAT CTGTTGTATA TTCAATCCGT AAGTACTGAT   
  
  
- TATAGACTTA ATCCACACAG CTAGACACTA AGTTTAACTA TATTAATCTA GAAATTCCAT AGATTAACGA   
  
  
- AGTTCAAAAG AGATATTACT TCTACATATA ATCGAGTGTA TCCAGAATTC CAATTGATTA CAGTATAGTC   
  
  
- CCCCACACCC GGCGGTAAAA ACTATTCTTA ACTTAACTTG AACGTAACAA AAAAGAAACC GCTCTTACGT   
  
  
- TCTAAACAAT TAAATAAGTA TACGCAGTAA TATGTGATAT TCTTTTTATT AACATCCTTA TATACTTCAT   
  
  
- AAAAATGAAC ACGAGTACAA TTTACACCTT ATGAAAAAAT TACAGCATAT CATGGTGTCA TAAGATGTTA   
  
  
- AATTCTCTAA TTAGCTAGGT AATTTCAGTA CGTTTGGACT ACGTATAAGT TCTGTAACGT ACTTTACTAG   
  
  
- AATAAAATCA GTTTCTGAGG TAGTAAATAT TCGTTATAAA TGAAGCTATG TGCACAGTAA TTTTTTCTGA   
  
  
- TATCTTTACA ATGACAATAA AACAAACTCG ATTTATCTTT TAAATTTTCA ATTAATTCTA TTATTAATTT   
  
  
- TGATTTATAA GTTACTACCG ATTATTTATG TAACAGCATA ATTGACTGAT TATAAACATC TACCAGCACA   
  
  
- ATTAAAGAAA AATAAAATTT CAAGTTTACT CAATGTTTTC CAATATTTTA TATTTCATTT TACCCTCTGT   
  
  
- TTCGTCTGTC CCACCCCCTT CGATTCGAAT TTCATCCTAA ATCTTTCCGT TACTTCCACT TGGGAGTTTA   
  
  
- GCTAGGCGAC GGTTAGTGTC TTTGGGTTTC GAAACGAGTG GCTGTTGAGG GCCAATTGAC GTCAGTGAGT   
  
  
- GCCGTGACCC ATTATGTCAT TAGCACATGG GTCAAGTAGG GGGAAAAGGA AACCCTTATG TCATGGAGCT   
  
  
- TTCAAGGAAA CTTTAAGATG ACCGGTCCGT ATGGGTATTT AACTAGGAGA GCTATAGTAT TCTATACTAT   
  
  
- AAAGAGACAG TATAGACAAT CAAAGCCATA GTATAAAGAA GAAATAGAGA CATTAAACTC CATACGAAGT   
  
  
- AAGGACCGGC GAACTTAATA GAAAGAAAGA AACTAATCAT AACAAAAAAA CCAATGGATA AAACGAGGTC   
  
  
- AGGACAAGTA AACCCAGAAA AACCTAGACC CAAAAACGAC CCAAACAACT AAGAAACTCT TTAAACCCAC   
  
  
- GAACTTAAAC GGGACGTTAT AAGTACCCAA TCGCAAAAGG ACTTAAAACG AGACAAGGAA TTCACTAATA   
  
  
- AAAACTAACT AATGTGGAAC CACCAGGACC ACCTTAAAGG CTCCTCTTTA ACAGTACCCA AGGGTGTTGA   
  
  
- AACCTCTTAA GGGACTACTC TGAGATTTAC TCATAGTTAG ATGAAACCCT TGGTACAGGG GGCCAATACT   
  
  
- ACCCAGAAAC TTAATATGTT GGAACAAGTT AATGTTTCTG GGTAGAGTCC TAGAGTGCCG TGACTTGGAA   
  
  
- GGATCGGGTA ACGGACTGGG ATACGGTAAG TTGTAACCAA GTATAAGCCC AAACTCGGGG CCCCAACTTA   
  
  
- GAAGCCTACT ATCGCTAAGT CTACTACAAC AATTCATGTA ACCGGTTCAC GAATACCTTC TCCTATACTT   
  
  
- CCTCTTCGGA ACATACAAGG TACTGGGAAA TCGTGAGGTT CGACGACTCT TTGGGAAAAT ACTACACAAC   
  
  
- CCTTTCTTCA TAGGAGGAAG GGGATTAGTT GTGGGTGAAT AACTAGTAAG ACACCTATCG GGTCTGTTAG   
  
  
- TCAGGACACC TTGCTCGTGT TAATCACTAG AATCACCGTC ATTGACGTGA AGGTCAAGTT GGTTAATATA   
  
  
- ACTACGACAC CGTCAGCGAC TAAGCTCACT CTTATGATTC TGGAGAAAAC ACGTTTCAAG AGACTAACTT   
  
  
- AGCAAAAGAG TCAGCAGGTG GGAAGGTGTC ACCAGTAAAC CTAGTAACCC ACGGAACCCA CCGTGTCGAA   
  
  
- GAGTCCCAAG CTTAAGTCAA TAGAGGAGGG CACCAAAGGG ATACCGGTAC TTGCAAAAAT CGCTCGTCAG   
  
  
- GTACTATGTC AAAGTCTTCC CCCACCTCCT CCGCTCGTTC AAGGAAGGTT TCTTGTTATT GGAACGCTAA   
  
  
- CTGGAACTCT CGCAGTGAAA GGGTTTACTT TTCCTCCGCC GGGGGTACCA CCACCAATTC TTTCTCTTCC   
  
  
- TACTTGTGAG TGGACTATCG AGCTCTCCAT CGTTCTAAGT GATGGCACTA CTACTCCTGA AACTCCTACC   
  
  
- ATCCTCCTCA TTCGTCAGTC GACAAAGACA CCTCCTCCGA CTCAACAGAC TTTACAAACT GGCCCAAAAC   
  
  
- GAAACGTTAG GGTACTTTGT ACTTCGAGTA ACATGTTCAC CAAACTTCAG GCTCTTCCCT TGTAGGGACG   
  
  
- TCCGACCGGT TCATGTCCAT GTCCTCCCAG TCTTCCGAGC ACGTTTCTTT GTCCCATTAT TACTATTATT   
  
  
- CTTCTTATTC TTACACCACC TAAATTCCTG AGACGAATAG GAGACACGCG TTAGACGACG TAGACTACTA   
  
  
- GCGTCCTGTC GACTACTTGA CGACTTCGTC TAATCCCTCG TGAGAAGACG TCGACCCCTA CCTAGAGTTT   
  
  
- CCAACCGAGT AATGAAACGA TTACGGAACC TCCGCGCAAA TCGACCTTGA CCGAGTGTCT AGATATGTCG   
  
  
- GGACTCAAGC TTCTCCTGTT GTCGTCGACT ATACTATTTT CGAATAGTCA AAAAGAAAGC ACGAACGGGT   
  
  
- AAAAATTTCT AGAGATAATA GAAACGCTTG GTATACTAAT ATGTCTCTCG ACTTTTTCGT AGTTTCGAAG   
  
  
- TACAATATCT AAAACCGTAG GACATACCAA AAGTCACCGG GGAGGAGTAA GTTGCGGACA GCCTCGCTGG   
  
  
- ACCACCTGGA GGTTTTGACA CGTAATGACC CTAACTAGAA GGGGTTGGAC CTAAATCCGG TTGTCTTTCT   
  
  
- CAACTCCGTT GTCCCGAGGC GAACCGTTTC ATAACACTCG CGAAGTTACA AGGTAAACTT ATGGTACGGT   
  
  
- AACGTGTCTT TACCCTTTGG TAGTTTCGAC TTCTGGACTT CTATCTTCTA CTACTCCACC AACGGCACTT   
  
  
- GACAGAGAAG TCCAAATTCT TGGACGAACT ACTCTGCTAT CACCACCTAT CAGGCTCCTT ACGTCAAGAG   
  
  
- CCGAACTAAT CTTCCTATTT TGGACTATAG AAACAAGTGC CTCATCACTT GCCCAGAATG TTACGCGGGA   
  
  
- AGAAACAATG TGCAAAGTCC CTCCGGGAGA AGGTAATGAG ATGACATAAG CTATACAAAC TACGGTTACG   
  
  
- AAGGGCCCTC CTAGGACTCT CTAACTACAA ACTCTTCCTC AAAATACCCG CTCTTTAATA CTTACACCAC   
  
  
- CGAACACTCC CATGTCTCTC TCAACTTTCT GGACTCTGTA TGTTCGTTAC CGTCCGCGCC TTATCATCCC   
  
  
- GTCCCAAGTC CGTCGACGGT AACCTGGTTC TCGAGCAGTC ATTTGACTCC TCGTTCCAGG TCTACGTGAT   
  
  
- AGGATCCCTG AAGCAACAAC TACATCTACC TGTAACCTGT AACGTCCCTA CCTTCCCCTC CTAGTAGACA   
  
  
- CGACACAGAC GTACCCCAGG ACGAAT

+     G-Box

| Site Name | Organism | Position | Strand | Matrix score. | sequence | function |
| --- | --- | --- | --- | --- | --- | --- |
| G-Box | Pisum sativum | 1174 | + | 6 | CACGTG | cis-acting regulatory element involved in light responsiveness |
| G-Box | Pisum sativum | 3934 | + | 6 | CACGTT | cis-acting regulatory element involved in light responsiveness |

>HU02G01569.1   
+ +Up\_Stream \_Len000AGCGTA GGAAGTGACG CCGCTATTGG AGGCGAAGAC GCTAGTACCA AGGCCAAAAC   
  
  
+ AAATGAGGAG ATAGATCAGT TAAACCGTTT GGTTAAGAAA ATTAAGCGAA CATCTCTTGA ACCTCCATCT   
  
  
+ GAACTCGATG ACATTGAGGA TGTGGATGCA GAAAAGATGG ATATGGGCTC TCCATCGGTG CCAGCAAATC   
  
  
+ AACCTAATGT TGGGTGGGGT CTCATTCGGT CAATGGGGGA CTAGGGACGT CATATCGTCA CCAATTACAA   
  
  
+ CGGAACAACC CTAAACCGCA TTTTAAAATC ACATCTTGGT AGCAGACAAA GATGTACGAC CCAAAAATTC   
  
  
+ AACTCTGTAA AGCACTAGCA AAGCTCGTAG TACACAGAAC AATCGCACAC TCATAGTGAT AGAAGTGTAC   
  
  
+ TATCCATTAT GCAGTGAATA TGACAAGGGA GGGGGTCCTG AGATGCCCAA ATTTGGCAGC CGAGTTGCCT   
  
  
+ TGACTGCCCA TCAATGTCCT AAACCAGTTG AGCATGTTAT TAATTTGCAA GAAAGATCGA ATTCTTCCTA   
  
  
+ TCTAAAAACT GACACAATAA AACAATACCA TGTATGGGAC ATTGCTAGGA TTGTTAACAC AATTATTGTC   
  
  
+ TTGTACGTTG CACCTGAAAA AAAAGAAAAT TGTTGCCATA GACAACATAT AAGTTAGGCA TTCATGACTA   
  
  
+ ATATCTGAAT TAGGTGTGTC GATCTGTGAT TCAAATTGAT ATAATTAGAT CTTTAAGGTA TCTAATTGCT   
  
  
+ TCAAGTTTTC TCTATAATGA AGATGTATAT TAGCTCACAT AGGTCTTAAG GTTAACTAAT GTCATATCAG   
  
  
+ GGGGTGTGGG CCGCCATTTT TGATAAGAAT TGAATTGAAC TTGCATTGTT TTTTCTTTGG CGAGAATGCA   
  
  
+ AGATTTGTTA ATTTATTCAT ATGCGTCATT ATACACTATA AGAAAAATAA TTGTAGGAAT ATATGAAGTA   
  
  
+ TTTTTACTTG TGCTCATGTT AAATGTGGAA TACTTTTTTA ATGTCGTATA GTACCACAGT ATTCTACAAT   
  
  
+ TTAAGAGATT AATCGATCCA TTAAAGTCAT GCAAACCTGA TGCATATTCA AGACATTGCA TGAAATGATC   
  
  
+ TTATTTTAGT CAAAGACTCC ATCATTTATA AGCAATATTT ACTTCGATAC ACGTGTCATT AAAAAAGACT   
  
  
+ ATAGAAATGT TACTGTTATT TTGTTTGAGC TAAATAGAAA ATTTAAAAGT TAATTAAGAT AATAATTAAA   
  
  
+ ACTAAATATT CAATGATGGC TAATAAATAC ATTGTCGTAT TAACTGACTA ATATTTGTAG ATGGTCGTGT   
  
  
+ TAATTTCTTT TTATTTTAAA GTTCAAATGA GTTACAAAAG GTTATAAAAT ATAAAGTAAA ATGGGAGACA   
  
  
+ AAGCAGACAG GGTGGGGGAA GCTAAGCTTA AAGTAGGATT TAGAAAGGCA ATGAAGGTGA ACCCTCAAAT   
  
  
+ CGATCCGCTG CCAATCACAG AAACCCAAAG CTTTGCTCAC CGACAACTCC CGGTTAACTG CAGTCACTCA   
  
  
+ CGGCACTGGG TAATACAGTA ATCGTGTACC CAGTTCATCC CCCTTTTCCT TTGGGAATAC AGTACCTCGA   
  
  
+ AAGTTCCTTT GAAATTCTAC TGGCCAGGCA TACCCATAAA TTGATCCTCT CGATATCATA AGATATGATA   
  
  
+ TTTCTCTGTC ATATCTGTTA GTTTCGGTAT CATATTTCTT CTTTATCTCT GTAATTTGAG GTATGCTTCA   
  
  
+ TTCCTGGCCG CTTGAATTAT CTTTCTTTCT TTGATTAGTA TTGTTTTTTT GGTTACCTAT TTTGCTCCAG   
  
  
+ TCCTGTTCAT TTGGGTCTTT TTGGATCTGG GTTTTTGCTG GGTTTGTTGA TTCTTTGAGA AATTTGGGTG   
  
  
+ CTTGAATTTG CCCTGCAATA TTCATGGGTT AGCGTTTTCC TGAATTTTGC TCTGTTCCTT AAGTGATTAT   
  
  
+ TTTTGATTGA TTACACCTTG GTGGTCCTGG TGGAATTTCC GAGGAGAAAT TGTCATGGGT TCCCACAACT   
  
  
+ TTGGAGAATT CCCTGATGAG ACTCTAAATG AGTATCAATC TACTTTGGGA ACCATGTCCC CCGGTTATGA   
  
  
+ TGGGTCTTTG AATTATACAA CCTTGTTCAA TTACAAAGAC CCATCTCAGG ATCTCACGGC ACTGAACCTT   
  
  
+ CCTAGCCCAT TGCCTGACCC TATGCCATTC AACATTGGTT CATATTCGGG TTTGAGCCCC GGGGTTGAAT   
  
  
+ CTTCGGATGA TAGCGATTCA GATGATGTTG TTAAGTACAT TGGCCAAGTG CTTATGGAAG AGGATATGAA   
  
  
+ GGAGAAGCCT TGTATGTTCC ATGACCCTTT AGCACTCCAA GCTGCTGAGA AACCCTTTTA TGATGTGTTG   
  
  
+ GGAAAGAAGT ATCCTCCTTC CCCTAATCAA CACCCACTTA TTGATCATTC TGTGGATAGC CCAGACAATC   
  
  
+ AGTCCTGTGG AACGAGCACA ATTAGTGATC TTAGTGGCAG TAACTGCACT TCCAGTTCAA CCAATTATAT   
  
  
+ TGATGCTGTG GCAGTCGCTG ATTCGAGTGA GAATACTAAG ACCTCTTTTG TGCAAAGTTC TCTGATTGAA   
  
  
+ TCGTTTTCTC AGTCGTCCAC CCTTCCACAG TGGTCATTTG GATCATTGGG TGCCTTGGGT GGCACAGCTT   
  
  
+ CTCAGGGTTC GAATTCAGTT ATCTCCTCCC GTGGTTTCCC TATGGCCATG AACGTTTTTA GCGAGCAGTC   
  
  
+ CATGATACAG TTTCAGAAGG GGGTGGAGGA GGCGAGCAAG TTCCTTCCAA AGAACAATAA CCTTGCGATT   
  
  
+ GACCTTGAGA GCGTCACTTT CCCAAATGAA AAGGAGGCGG CCCCCATGGT GGTGGTTAAG AAAGAGAAGG   
  
  
+ ATGAACACTC ACCTGATAGC TCGAGAGGTA GCAAGATTCA CTACCGTGAT GATGAGGACT TTGAGGATGG   
  
  
+ TAGGAGGAGT AAGCAGTCAG CTGTTTCTGT GGAGGAGGCT GAGTTGTCTG AAATGTTTGA CCGGGTTTTG   
  
  
+ CTTTGCAATC CCATGAAACA TGAAGCTCAT TGTACAAGTG GTTTGAAGTC CGAGAAGGGA ACATCCCTGC   
  
  
+ AGGCTGGCCA AGTACAGGTA CAGGAGGGTC AGAAGGCTCG TGCAAAGAAA CAGGGTAATA ATGATAATAA   
  
  
+ GAAGAATAAG AATGTGGTGG ATTTAAGGAC TCTGCTTATC CTCTGTGCGC AATCTGCTGC ATCTGATGAT   
  
  
+ CGCAGGACAG CTGATGAACT GCTGAAGCAG ATTAGGGAGC ACTCTTCTGC AGCTGGGGAT GGATCTCAAA   
  
  
+ GGTTGGCTCA TTACTTTGCT AATGCCTTGG AGGCGCGTTT AGCTGGAACT GGCTCACAGA TCTATACAGC   
  
  
+ CCTGAGTTCG AAGAGGACAA CAGCAGCTGA TATGATAAAA GCTTATCAGT TTTTCTTTCG TGCTTGCCCA   
  
  
+ TTTTTAAAGA TCTCTATTAT CTTTGCGAAC CATATGATTA TACAGAGAGC TGAAAAAGCA TCAAAGCTTC   
  
  
+ ATGTTATAGA TTTTGGCATC CTGTATGGTT TTCAGTGGCC CCTCCTCATT CAACGCCTGT CGGAGCGACC   
  
  
+ TGGTGGACCT CCAAAACTGT GCATTACTGG GATTGATCTT CCCCAACCTG GATTTAGGCC AACAGAAAGA   
  
  
+ GTTGAGGCAA CAGGGCTCCG CTTGGCAAAG TATTGTGAGC GCTTCAATGT TCCATTTGAA TACCATGCCA   
  
  
+ TTGCACAGAA ATGGGAAACC ATCAAAGCTG AAGACCTGAA GATAGAAGAT GATGAGGTGG TTGCCGTGAA   
  
  
+ CTGTCTCTTC AGGTTTAAGA ACCTGCTTGA TGAGACGATA GTGGTGGATA GTCCGAGGAA TGCAGTTCTC   
  
  
+ GGCTTGATTA GAAGGATAAA ACCTGATATC TTTGTTCACG GAGTAGTGAA CGGGTCTTAC AATGCGCCCT   
  
  
+ TCTTTGTTAC ACGTTTCAGG GAGGCCCTCT TCCATTACTC TACTGTATTC GATATGTTTG ATGCCAATGC   
  
  
+ TTCCCGGGAG GATCCTGAGA GATTGATGTT TGAGAAGGAG TTTTATGGGC GAGAAATTAT GAATGTGGTG   
  
  
+ GCTTGTGAGG GTACAGAGAG AGTTGAAAGA CCTGAGACAT ACAAGCAATG GCAGGCGCGG AATAGTAGGG   
  
  
+ CAGGGTTCAG GCAGCTGCCA TTGGACCAAG AGCTCGTCAG TAAACTGAGG AGCAAGGTCC AGATGCACTA   
  
  
+ TCCTAGGGAC TTCGTTGTTG ATGTAGATGG ACATTGGACA TTGCAGGGAT GGAAGGGGAG GATCATCTGT   
  
  
+ GCTGTGTCTG CATGGGGTCC TGCTTA  

- +Up\_Stream \_Len000TCGCAT CCTTCACTGC GGCGATAACC TCCGCTTCTG CGATCATGGT TCCGGTTTTG   
  
  
- TTTACTCCTC TATCTAGTCA ATTTGGCAAA CCAATTCTTT TAATTCGCTT GTAGAGAACT TGGAGGTAGA   
  
  
- CTTGAGCTAC TGTAACTCCT ACACCTACGT CTTTTCTACC TATACCCGAG AGGTAGCCAC GGTCGTTTAG   
  
  
- TTGGATTACA ACCCACCCCA GAGTAAGCCA GTTACCCCCT GATCCCTGCA GTATAGCAGT GGTTAATGTT   
  
  
- GCCTTGTTGG GATTTGGCGT AAAATTTTAG TGTAGAACCA TCGTCTGTTT CTACATGCTG GGTTTTTAAG   
  
  
- TTGAGACATT TCGTGATCGT TTCGAGCATC ATGTGTCTTG TTAGCGTGTG AGTATCACTA TCTTCACATG   
  
  
- ATAGGTAATA CGTCACTTAT ACTGTTCCCT CCCCCAGGAC TCTACGGGTT TAAACCGTCG GCTCAACGGA   
  
  
- ACTGACGGGT AGTTACAGGA TTTGGTCAAC TCGTACAATA ATTAAACGTT CTTTCTAGCT TAAGAAGGAT   
  
  
- AGATTTTTGA CTGTGTTATT TTGTTATGGT ACATACCCTG TAACGATCCT AACAATTGTG TTAATAACAG   
  
  
- AACATGCAAC GTGGACTTTT TTTTCTTTTA ACAACGGTAT CTGTTGTATA TTCAATCCGT AAGTACTGAT   
  
  
- TATAGACTTA ATCCACACAG CTAGACACTA AGTTTAACTA TATTAATCTA GAAATTCCAT AGATTAACGA   
  
  
- AGTTCAAAAG AGATATTACT TCTACATATA ATCGAGTGTA TCCAGAATTC CAATTGATTA CAGTATAGTC   
  
  
- CCCCACACCC GGCGGTAAAA ACTATTCTTA ACTTAACTTG AACGTAACAA AAAAGAAACC GCTCTTACGT   
  
  
- TCTAAACAAT TAAATAAGTA TACGCAGTAA TATGTGATAT TCTTTTTATT AACATCCTTA TATACTTCAT   
  
  
- AAAAATGAAC ACGAGTACAA TTTACACCTT ATGAAAAAAT TACAGCATAT CATGGTGTCA TAAGATGTTA   
  
  
- AATTCTCTAA TTAGCTAGGT AATTTCAGTA CGTTTGGACT ACGTATAAGT TCTGTAACGT ACTTTACTAG   
  
  
- AATAAAATCA GTTTCTGAGG TAGTAAATAT TCGTTATAAA TGAAGCTATG TGCACAGTAA TTTTTTCTGA   
  
  
- TATCTTTACA ATGACAATAA AACAAACTCG ATTTATCTTT TAAATTTTCA ATTAATTCTA TTATTAATTT   
  
  
- TGATTTATAA GTTACTACCG ATTATTTATG TAACAGCATA ATTGACTGAT TATAAACATC TACCAGCACA   
  
  
- ATTAAAGAAA AATAAAATTT CAAGTTTACT CAATGTTTTC CAATATTTTA TATTTCATTT TACCCTCTGT   
  
  
- TTCGTCTGTC CCACCCCCTT CGATTCGAAT TTCATCCTAA ATCTTTCCGT TACTTCCACT TGGGAGTTTA   
  
  
- GCTAGGCGAC GGTTAGTGTC TTTGGGTTTC GAAACGAGTG GCTGTTGAGG GCCAATTGAC GTCAGTGAGT   
  
  
- GCCGTGACCC ATTATGTCAT TAGCACATGG GTCAAGTAGG GGGAAAAGGA AACCCTTATG TCATGGAGCT   
  
  
- TTCAAGGAAA CTTTAAGATG ACCGGTCCGT ATGGGTATTT AACTAGGAGA GCTATAGTAT TCTATACTAT   
  
  
- AAAGAGACAG TATAGACAAT CAAAGCCATA GTATAAAGAA GAAATAGAGA CATTAAACTC CATACGAAGT   
  
  
- AAGGACCGGC GAACTTAATA GAAAGAAAGA AACTAATCAT AACAAAAAAA CCAATGGATA AAACGAGGTC   
  
  
- AGGACAAGTA AACCCAGAAA AACCTAGACC CAAAAACGAC CCAAACAACT AAGAAACTCT TTAAACCCAC   
  
  
- GAACTTAAAC GGGACGTTAT AAGTACCCAA TCGCAAAAGG ACTTAAAACG AGACAAGGAA TTCACTAATA   
  
  
- AAAACTAACT AATGTGGAAC CACCAGGACC ACCTTAAAGG CTCCTCTTTA ACAGTACCCA AGGGTGTTGA   
  
  
- AACCTCTTAA GGGACTACTC TGAGATTTAC TCATAGTTAG ATGAAACCCT TGGTACAGGG GGCCAATACT   
  
  
- ACCCAGAAAC TTAATATGTT GGAACAAGTT AATGTTTCTG GGTAGAGTCC TAGAGTGCCG TGACTTGGAA   
  
  
- GGATCGGGTA ACGGACTGGG ATACGGTAAG TTGTAACCAA GTATAAGCCC AAACTCGGGG CCCCAACTTA   
  
  
- GAAGCCTACT ATCGCTAAGT CTACTACAAC AATTCATGTA ACCGGTTCAC GAATACCTTC TCCTATACTT   
  
  
- CCTCTTCGGA ACATACAAGG TACTGGGAAA TCGTGAGGTT CGACGACTCT TTGGGAAAAT ACTACACAAC   
  
  
- CCTTTCTTCA TAGGAGGAAG GGGATTAGTT GTGGGTGAAT AACTAGTAAG ACACCTATCG GGTCTGTTAG   
  
  
- TCAGGACACC TTGCTCGTGT TAATCACTAG AATCACCGTC ATTGACGTGA AGGTCAAGTT GGTTAATATA   
  
  
- ACTACGACAC CGTCAGCGAC TAAGCTCACT CTTATGATTC TGGAGAAAAC ACGTTTCAAG AGACTAACTT   
  
  
- AGCAAAAGAG TCAGCAGGTG GGAAGGTGTC ACCAGTAAAC CTAGTAACCC ACGGAACCCA CCGTGTCGAA   
  
  
- GAGTCCCAAG CTTAAGTCAA TAGAGGAGGG CACCAAAGGG ATACCGGTAC TTGCAAAAAT CGCTCGTCAG   
  
  
- GTACTATGTC AAAGTCTTCC CCCACCTCCT CCGCTCGTTC AAGGAAGGTT TCTTGTTATT GGAACGCTAA   
  
  
- CTGGAACTCT CGCAGTGAAA GGGTTTACTT TTCCTCCGCC GGGGGTACCA CCACCAATTC TTTCTCTTCC   
  
  
- TACTTGTGAG TGGACTATCG AGCTCTCCAT CGTTCTAAGT GATGGCACTA CTACTCCTGA AACTCCTACC   
  
  
- ATCCTCCTCA TTCGTCAGTC GACAAAGACA CCTCCTCCGA CTCAACAGAC TTTACAAACT GGCCCAAAAC   
  
  
- GAAACGTTAG GGTACTTTGT ACTTCGAGTA ACATGTTCAC CAAACTTCAG GCTCTTCCCT TGTAGGGACG   
  
  
- TCCGACCGGT TCATGTCCAT GTCCTCCCAG TCTTCCGAGC ACGTTTCTTT GTCCCATTAT TACTATTATT   
  
  
- CTTCTTATTC TTACACCACC TAAATTCCTG AGACGAATAG GAGACACGCG TTAGACGACG TAGACTACTA   
  
  
- GCGTCCTGTC GACTACTTGA CGACTTCGTC TAATCCCTCG TGAGAAGACG TCGACCCCTA CCTAGAGTTT   
  
  
- CCAACCGAGT AATGAAACGA TTACGGAACC TCCGCGCAAA TCGACCTTGA CCGAGTGTCT AGATATGTCG   
  
  
- GGACTCAAGC TTCTCCTGTT GTCGTCGACT ATACTATTTT CGAATAGTCA AAAAGAAAGC ACGAACGGGT   
  
  
- AAAAATTTCT AGAGATAATA GAAACGCTTG GTATACTAAT ATGTCTCTCG ACTTTTTCGT AGTTTCGAAG   
  
  
- TACAATATCT AAAACCGTAG GACATACCAA AAGTCACCGG GGAGGAGTAA GTTGCGGACA GCCTCGCTGG   
  
  
- ACCACCTGGA GGTTTTGACA CGTAATGACC CTAACTAGAA GGGGTTGGAC CTAAATCCGG TTGTCTTTCT   
  
  
- CAACTCCGTT GTCCCGAGGC GAACCGTTTC ATAACACTCG CGAAGTTACA AGGTAAACTT ATGGTACGGT   
  
  
- AACGTGTCTT TACCCTTTGG TAGTTTCGAC TTCTGGACTT CTATCTTCTA CTACTCCACC AACGGCACTT   
  
  
- GACAGAGAAG TCCAAATTCT TGGACGAACT ACTCTGCTAT CACCACCTAT CAGGCTCCTT ACGTCAAGAG   
  
  
- CCGAACTAAT CTTCCTATTT TGGACTATAG AAACAAGTGC CTCATCACTT GCCCAGAATG TTACGCGGGA   
  
  
- AGAAACAATG TGCAAAGTCC CTCCGGGAGA AGGTAATGAG ATGACATAAG CTATACAAAC TACGGTTACG   
  
  
- AAGGGCCCTC CTAGGACTCT CTAACTACAA ACTCTTCCTC AAAATACCCG CTCTTTAATA CTTACACCAC   
  
  
- CGAACACTCC CATGTCTCTC TCAACTTTCT GGACTCTGTA TGTTCGTTAC CGTCCGCGCC TTATCATCCC   
  
  
- GTCCCAAGTC CGTCGACGGT AACCTGGTTC TCGAGCAGTC ATTTGACTCC TCGTTCCAGG TCTACGTGAT   
  
  
- AGGATCCCTG AAGCAACAAC TACATCTACC TGTAACCTGT AACGTCCCTA CCTTCCCCTC CTAGTAGACA   
  
  
- CGACACAGAC GTACCCCAGG ACGAAT

+     G-box

| Site Name | Organism | Position | Strand | Matrix score. | sequence | function |
| --- | --- | --- | --- | --- | --- | --- |
| G-box | Zea mays | 1328 | - | 6 | CACGAC | cis-acting regulatory element involved in light responsiveness |
| G-box | Arabidopsis thaliana | 1174 | + | 6 | CACGTG | cis-acting regulatory element involved in light responsiveness |
| G-box | Brassica napus | 1173 | + | 8 | ACACGTGT | cis-acting regulatory element involved in light responsiveness |

>HU02G01569.1   
+ +Up\_Stream \_Len000AGCGTA GGAAGTGACG CCGCTATTGG AGGCGAAGAC GCTAGTACCA AGGCCAAAAC   
  
  
+ AAATGAGGAG ATAGATCAGT TAAACCGTTT GGTTAAGAAA ATTAAGCGAA CATCTCTTGA ACCTCCATCT   
  
  
+ GAACTCGATG ACATTGAGGA TGTGGATGCA GAAAAGATGG ATATGGGCTC TCCATCGGTG CCAGCAAATC   
  
  
+ AACCTAATGT TGGGTGGGGT CTCATTCGGT CAATGGGGGA CTAGGGACGT CATATCGTCA CCAATTACAA   
  
  
+ CGGAACAACC CTAAACCGCA TTTTAAAATC ACATCTTGGT AGCAGACAAA GATGTACGAC CCAAAAATTC   
  
  
+ AACTCTGTAA AGCACTAGCA AAGCTCGTAG TACACAGAAC AATCGCACAC TCATAGTGAT AGAAGTGTAC   
  
  
+ TATCCATTAT GCAGTGAATA TGACAAGGGA GGGGGTCCTG AGATGCCCAA ATTTGGCAGC CGAGTTGCCT   
  
  
+ TGACTGCCCA TCAATGTCCT AAACCAGTTG AGCATGTTAT TAATTTGCAA GAAAGATCGA ATTCTTCCTA   
  
  
+ TCTAAAAACT GACACAATAA AACAATACCA TGTATGGGAC ATTGCTAGGA TTGTTAACAC AATTATTGTC   
  
  
+ TTGTACGTTG CACCTGAAAA AAAAGAAAAT TGTTGCCATA GACAACATAT AAGTTAGGCA TTCATGACTA   
  
  
+ ATATCTGAAT TAGGTGTGTC GATCTGTGAT TCAAATTGAT ATAATTAGAT CTTTAAGGTA TCTAATTGCT   
  
  
+ TCAAGTTTTC TCTATAATGA AGATGTATAT TAGCTCACAT AGGTCTTAAG GTTAACTAAT GTCATATCAG   
  
  
+ GGGGTGTGGG CCGCCATTTT TGATAAGAAT TGAATTGAAC TTGCATTGTT TTTTCTTTGG CGAGAATGCA   
  
  
+ AGATTTGTTA ATTTATTCAT ATGCGTCATT ATACACTATA AGAAAAATAA TTGTAGGAAT ATATGAAGTA   
  
  
+ TTTTTACTTG TGCTCATGTT AAATGTGGAA TACTTTTTTA ATGTCGTATA GTACCACAGT ATTCTACAAT   
  
  
+ TTAAGAGATT AATCGATCCA TTAAAGTCAT GCAAACCTGA TGCATATTCA AGACATTGCA TGAAATGATC   
  
  
+ TTATTTTAGT CAAAGACTCC ATCATTTATA AGCAATATTT ACTTCGATAC ACGTGTCATT AAAAAAGACT   
  
  
+ ATAGAAATGT TACTGTTATT TTGTTTGAGC TAAATAGAAA ATTTAAAAGT TAATTAAGAT AATAATTAAA   
  
  
+ ACTAAATATT CAATGATGGC TAATAAATAC ATTGTCGTAT TAACTGACTA ATATTTGTAG ATGGTCGTGT   
  
  
+ TAATTTCTTT TTATTTTAAA GTTCAAATGA GTTACAAAAG GTTATAAAAT ATAAAGTAAA ATGGGAGACA   
  
  
+ AAGCAGACAG GGTGGGGGAA GCTAAGCTTA AAGTAGGATT TAGAAAGGCA ATGAAGGTGA ACCCTCAAAT   
  
  
+ CGATCCGCTG CCAATCACAG AAACCCAAAG CTTTGCTCAC CGACAACTCC CGGTTAACTG CAGTCACTCA   
  
  
+ CGGCACTGGG TAATACAGTA ATCGTGTACC CAGTTCATCC CCCTTTTCCT TTGGGAATAC AGTACCTCGA   
  
  
+ AAGTTCCTTT GAAATTCTAC TGGCCAGGCA TACCCATAAA TTGATCCTCT CGATATCATA AGATATGATA   
  
  
+ TTTCTCTGTC ATATCTGTTA GTTTCGGTAT CATATTTCTT CTTTATCTCT GTAATTTGAG GTATGCTTCA   
  
  
+ TTCCTGGCCG CTTGAATTAT CTTTCTTTCT TTGATTAGTA TTGTTTTTTT GGTTACCTAT TTTGCTCCAG   
  
  
+ TCCTGTTCAT TTGGGTCTTT TTGGATCTGG GTTTTTGCTG GGTTTGTTGA TTCTTTGAGA AATTTGGGTG   
  
  
+ CTTGAATTTG CCCTGCAATA TTCATGGGTT AGCGTTTTCC TGAATTTTGC TCTGTTCCTT AAGTGATTAT   
  
  
+ TTTTGATTGA TTACACCTTG GTGGTCCTGG TGGAATTTCC GAGGAGAAAT TGTCATGGGT TCCCACAACT   
  
  
+ TTGGAGAATT CCCTGATGAG ACTCTAAATG AGTATCAATC TACTTTGGGA ACCATGTCCC CCGGTTATGA   
  
  
+ TGGGTCTTTG AATTATACAA CCTTGTTCAA TTACAAAGAC CCATCTCAGG ATCTCACGGC ACTGAACCTT   
  
  
+ CCTAGCCCAT TGCCTGACCC TATGCCATTC AACATTGGTT CATATTCGGG TTTGAGCCCC GGGGTTGAAT   
  
  
+ CTTCGGATGA TAGCGATTCA GATGATGTTG TTAAGTACAT TGGCCAAGTG CTTATGGAAG AGGATATGAA   
  
  
+ GGAGAAGCCT TGTATGTTCC ATGACCCTTT AGCACTCCAA GCTGCTGAGA AACCCTTTTA TGATGTGTTG   
  
  
+ GGAAAGAAGT ATCCTCCTTC CCCTAATCAA CACCCACTTA TTGATCATTC TGTGGATAGC CCAGACAATC   
  
  
+ AGTCCTGTGG AACGAGCACA ATTAGTGATC TTAGTGGCAG TAACTGCACT TCCAGTTCAA CCAATTATAT   
  
  
+ TGATGCTGTG GCAGTCGCTG ATTCGAGTGA GAATACTAAG ACCTCTTTTG TGCAAAGTTC TCTGATTGAA   
  
  
+ TCGTTTTCTC AGTCGTCCAC CCTTCCACAG TGGTCATTTG GATCATTGGG TGCCTTGGGT GGCACAGCTT   
  
  
+ CTCAGGGTTC GAATTCAGTT ATCTCCTCCC GTGGTTTCCC TATGGCCATG AACGTTTTTA GCGAGCAGTC   
  
  
+ CATGATACAG TTTCAGAAGG GGGTGGAGGA GGCGAGCAAG TTCCTTCCAA AGAACAATAA CCTTGCGATT   
  
  
+ GACCTTGAGA GCGTCACTTT CCCAAATGAA AAGGAGGCGG CCCCCATGGT GGTGGTTAAG AAAGAGAAGG   
  
  
+ ATGAACACTC ACCTGATAGC TCGAGAGGTA GCAAGATTCA CTACCGTGAT GATGAGGACT TTGAGGATGG   
  
  
+ TAGGAGGAGT AAGCAGTCAG CTGTTTCTGT GGAGGAGGCT GAGTTGTCTG AAATGTTTGA CCGGGTTTTG   
  
  
+ CTTTGCAATC CCATGAAACA TGAAGCTCAT TGTACAAGTG GTTTGAAGTC CGAGAAGGGA ACATCCCTGC   
  
  
+ AGGCTGGCCA AGTACAGGTA CAGGAGGGTC AGAAGGCTCG TGCAAAGAAA CAGGGTAATA ATGATAATAA   
  
  
+ GAAGAATAAG AATGTGGTGG ATTTAAGGAC TCTGCTTATC CTCTGTGCGC AATCTGCTGC ATCTGATGAT   
  
  
+ CGCAGGACAG CTGATGAACT GCTGAAGCAG ATTAGGGAGC ACTCTTCTGC AGCTGGGGAT GGATCTCAAA   
  
  
+ GGTTGGCTCA TTACTTTGCT AATGCCTTGG AGGCGCGTTT AGCTGGAACT GGCTCACAGA TCTATACAGC   
  
  
+ CCTGAGTTCG AAGAGGACAA CAGCAGCTGA TATGATAAAA GCTTATCAGT TTTTCTTTCG TGCTTGCCCA   
  
  
+ TTTTTAAAGA TCTCTATTAT CTTTGCGAAC CATATGATTA TACAGAGAGC TGAAAAAGCA TCAAAGCTTC   
  
  
+ ATGTTATAGA TTTTGGCATC CTGTATGGTT TTCAGTGGCC CCTCCTCATT CAACGCCTGT CGGAGCGACC   
  
  
+ TGGTGGACCT CCAAAACTGT GCATTACTGG GATTGATCTT CCCCAACCTG GATTTAGGCC AACAGAAAGA   
  
  
+ GTTGAGGCAA CAGGGCTCCG CTTGGCAAAG TATTGTGAGC GCTTCAATGT TCCATTTGAA TACCATGCCA   
  
  
+ TTGCACAGAA ATGGGAAACC ATCAAAGCTG AAGACCTGAA GATAGAAGAT GATGAGGTGG TTGCCGTGAA   
  
  
+ CTGTCTCTTC AGGTTTAAGA ACCTGCTTGA TGAGACGATA GTGGTGGATA GTCCGAGGAA TGCAGTTCTC   
  
  
+ GGCTTGATTA GAAGGATAAA ACCTGATATC TTTGTTCACG GAGTAGTGAA CGGGTCTTAC AATGCGCCCT   
  
  
+ TCTTTGTTAC ACGTTTCAGG GAGGCCCTCT TCCATTACTC TACTGTATTC GATATGTTTG ATGCCAATGC   
  
  
+ TTCCCGGGAG GATCCTGAGA GATTGATGTT TGAGAAGGAG TTTTATGGGC GAGAAATTAT GAATGTGGTG   
  
  
+ GCTTGTGAGG GTACAGAGAG AGTTGAAAGA CCTGAGACAT ACAAGCAATG GCAGGCGCGG AATAGTAGGG   
  
  
+ CAGGGTTCAG GCAGCTGCCA TTGGACCAAG AGCTCGTCAG TAAACTGAGG AGCAAGGTCC AGATGCACTA   
  
  
+ TCCTAGGGAC TTCGTTGTTG ATGTAGATGG ACATTGGACA TTGCAGGGAT GGAAGGGGAG GATCATCTGT   
  
  
+ GCTGTGTCTG CATGGGGTCC TGCTTA  

- +Up\_Stream \_Len000TCGCAT CCTTCACTGC GGCGATAACC TCCGCTTCTG CGATCATGGT TCCGGTTTTG   
  
  
- TTTACTCCTC TATCTAGTCA ATTTGGCAAA CCAATTCTTT TAATTCGCTT GTAGAGAACT TGGAGGTAGA   
  
  
- CTTGAGCTAC TGTAACTCCT ACACCTACGT CTTTTCTACC TATACCCGAG AGGTAGCCAC GGTCGTTTAG   
  
  
- TTGGATTACA ACCCACCCCA GAGTAAGCCA GTTACCCCCT GATCCCTGCA GTATAGCAGT GGTTAATGTT   
  
  
- GCCTTGTTGG GATTTGGCGT AAAATTTTAG TGTAGAACCA TCGTCTGTTT CTACATGCTG GGTTTTTAAG   
  
  
- TTGAGACATT TCGTGATCGT TTCGAGCATC ATGTGTCTTG TTAGCGTGTG AGTATCACTA TCTTCACATG   
  
  
- ATAGGTAATA CGTCACTTAT ACTGTTCCCT CCCCCAGGAC TCTACGGGTT TAAACCGTCG GCTCAACGGA   
  
  
- ACTGACGGGT AGTTACAGGA TTTGGTCAAC TCGTACAATA ATTAAACGTT CTTTCTAGCT TAAGAAGGAT   
  
  
- AGATTTTTGA CTGTGTTATT TTGTTATGGT ACATACCCTG TAACGATCCT AACAATTGTG TTAATAACAG   
  
  
- AACATGCAAC GTGGACTTTT TTTTCTTTTA ACAACGGTAT CTGTTGTATA TTCAATCCGT AAGTACTGAT   
  
  
- TATAGACTTA ATCCACACAG CTAGACACTA AGTTTAACTA TATTAATCTA GAAATTCCAT AGATTAACGA   
  
  
- AGTTCAAAAG AGATATTACT TCTACATATA ATCGAGTGTA TCCAGAATTC CAATTGATTA CAGTATAGTC   
  
  
- CCCCACACCC GGCGGTAAAA ACTATTCTTA ACTTAACTTG AACGTAACAA AAAAGAAACC GCTCTTACGT   
  
  
- TCTAAACAAT TAAATAAGTA TACGCAGTAA TATGTGATAT TCTTTTTATT AACATCCTTA TATACTTCAT   
  
  
- AAAAATGAAC ACGAGTACAA TTTACACCTT ATGAAAAAAT TACAGCATAT CATGGTGTCA TAAGATGTTA   
  
  
- AATTCTCTAA TTAGCTAGGT AATTTCAGTA CGTTTGGACT ACGTATAAGT TCTGTAACGT ACTTTACTAG   
  
  
- AATAAAATCA GTTTCTGAGG TAGTAAATAT TCGTTATAAA TGAAGCTATG TGCACAGTAA TTTTTTCTGA   
  
  
- TATCTTTACA ATGACAATAA AACAAACTCG ATTTATCTTT TAAATTTTCA ATTAATTCTA TTATTAATTT   
  
  
- TGATTTATAA GTTACTACCG ATTATTTATG TAACAGCATA ATTGACTGAT TATAAACATC TACCAGCACA   
  
  
- ATTAAAGAAA AATAAAATTT CAAGTTTACT CAATGTTTTC CAATATTTTA TATTTCATTT TACCCTCTGT   
  
  
- TTCGTCTGTC CCACCCCCTT CGATTCGAAT TTCATCCTAA ATCTTTCCGT TACTTCCACT TGGGAGTTTA   
  
  
- GCTAGGCGAC GGTTAGTGTC TTTGGGTTTC GAAACGAGTG GCTGTTGAGG GCCAATTGAC GTCAGTGAGT   
  
  
- GCCGTGACCC ATTATGTCAT TAGCACATGG GTCAAGTAGG GGGAAAAGGA AACCCTTATG TCATGGAGCT   
  
  
- TTCAAGGAAA CTTTAAGATG ACCGGTCCGT ATGGGTATTT AACTAGGAGA GCTATAGTAT TCTATACTAT   
  
  
- AAAGAGACAG TATAGACAAT CAAAGCCATA GTATAAAGAA GAAATAGAGA CATTAAACTC CATACGAAGT   
  
  
- AAGGACCGGC GAACTTAATA GAAAGAAAGA AACTAATCAT AACAAAAAAA CCAATGGATA AAACGAGGTC   
  
  
- AGGACAAGTA AACCCAGAAA AACCTAGACC CAAAAACGAC CCAAACAACT AAGAAACTCT TTAAACCCAC   
  
  
- GAACTTAAAC GGGACGTTAT AAGTACCCAA TCGCAAAAGG ACTTAAAACG AGACAAGGAA TTCACTAATA   
  
  
- AAAACTAACT AATGTGGAAC CACCAGGACC ACCTTAAAGG CTCCTCTTTA ACAGTACCCA AGGGTGTTGA   
  
  
- AACCTCTTAA GGGACTACTC TGAGATTTAC TCATAGTTAG ATGAAACCCT TGGTACAGGG GGCCAATACT   
  
  
- ACCCAGAAAC TTAATATGTT GGAACAAGTT AATGTTTCTG GGTAGAGTCC TAGAGTGCCG TGACTTGGAA   
  
  
- GGATCGGGTA ACGGACTGGG ATACGGTAAG TTGTAACCAA GTATAAGCCC AAACTCGGGG CCCCAACTTA   
  
  
- GAAGCCTACT ATCGCTAAGT CTACTACAAC AATTCATGTA ACCGGTTCAC GAATACCTTC TCCTATACTT   
  
  
- CCTCTTCGGA ACATACAAGG TACTGGGAAA TCGTGAGGTT CGACGACTCT TTGGGAAAAT ACTACACAAC   
  
  
- CCTTTCTTCA TAGGAGGAAG GGGATTAGTT GTGGGTGAAT AACTAGTAAG ACACCTATCG GGTCTGTTAG   
  
  
- TCAGGACACC TTGCTCGTGT TAATCACTAG AATCACCGTC ATTGACGTGA AGGTCAAGTT GGTTAATATA   
  
  
- ACTACGACAC CGTCAGCGAC TAAGCTCACT CTTATGATTC TGGAGAAAAC ACGTTTCAAG AGACTAACTT   
  
  
- AGCAAAAGAG TCAGCAGGTG GGAAGGTGTC ACCAGTAAAC CTAGTAACCC ACGGAACCCA CCGTGTCGAA   
  
  
- GAGTCCCAAG CTTAAGTCAA TAGAGGAGGG CACCAAAGGG ATACCGGTAC TTGCAAAAAT CGCTCGTCAG   
  
  
- GTACTATGTC AAAGTCTTCC CCCACCTCCT CCGCTCGTTC AAGGAAGGTT TCTTGTTATT GGAACGCTAA   
  
  
- CTGGAACTCT CGCAGTGAAA GGGTTTACTT TTCCTCCGCC GGGGGTACCA CCACCAATTC TTTCTCTTCC   
  
  
- TACTTGTGAG TGGACTATCG AGCTCTCCAT CGTTCTAAGT GATGGCACTA CTACTCCTGA AACTCCTACC   
  
  
- ATCCTCCTCA TTCGTCAGTC GACAAAGACA CCTCCTCCGA CTCAACAGAC TTTACAAACT GGCCCAAAAC   
  
  
- GAAACGTTAG GGTACTTTGT ACTTCGAGTA ACATGTTCAC CAAACTTCAG GCTCTTCCCT TGTAGGGACG   
  
  
- TCCGACCGGT TCATGTCCAT GTCCTCCCAG TCTTCCGAGC ACGTTTCTTT GTCCCATTAT TACTATTATT   
  
  
- CTTCTTATTC TTACACCACC TAAATTCCTG AGACGAATAG GAGACACGCG TTAGACGACG TAGACTACTA   
  
  
- GCGTCCTGTC GACTACTTGA CGACTTCGTC TAATCCCTCG TGAGAAGACG TCGACCCCTA CCTAGAGTTT   
  
  
- CCAACCGAGT AATGAAACGA TTACGGAACC TCCGCGCAAA TCGACCTTGA CCGAGTGTCT AGATATGTCG   
  
  
- GGACTCAAGC TTCTCCTGTT GTCGTCGACT ATACTATTTT CGAATAGTCA AAAAGAAAGC ACGAACGGGT   
  
  
- AAAAATTTCT AGAGATAATA GAAACGCTTG GTATACTAAT ATGTCTCTCG ACTTTTTCGT AGTTTCGAAG   
  
  
- TACAATATCT AAAACCGTAG GACATACCAA AAGTCACCGG GGAGGAGTAA GTTGCGGACA GCCTCGCTGG   
  
  
- ACCACCTGGA GGTTTTGACA CGTAATGACC CTAACTAGAA GGGGTTGGAC CTAAATCCGG TTGTCTTTCT   
  
  
- CAACTCCGTT GTCCCGAGGC GAACCGTTTC ATAACACTCG CGAAGTTACA AGGTAAACTT ATGGTACGGT   
  
  
- AACGTGTCTT TACCCTTTGG TAGTTTCGAC TTCTGGACTT CTATCTTCTA CTACTCCACC AACGGCACTT   
  
  
- GACAGAGAAG TCCAAATTCT TGGACGAACT ACTCTGCTAT CACCACCTAT CAGGCTCCTT ACGTCAAGAG   
  
  
- CCGAACTAAT CTTCCTATTT TGGACTATAG AAACAAGTGC CTCATCACTT GCCCAGAATG TTACGCGGGA   
  
  
- AGAAACAATG TGCAAAGTCC CTCCGGGAGA AGGTAATGAG ATGACATAAG CTATACAAAC TACGGTTACG   
  
  
- AAGGGCCCTC CTAGGACTCT CTAACTACAA ACTCTTCCTC AAAATACCCG CTCTTTAATA CTTACACCAC   
  
  
- CGAACACTCC CATGTCTCTC TCAACTTTCT GGACTCTGTA TGTTCGTTAC CGTCCGCGCC TTATCATCCC   
  
  
- GTCCCAAGTC CGTCGACGGT AACCTGGTTC TCGAGCAGTC ATTTGACTCC TCGTTCCAGG TCTACGTGAT   
  
  
- AGGATCCCTG AAGCAACAAC TACATCTACC TGTAACCTGT AACGTCCCTA CCTTCCCCTC CTAGTAGACA   
  
  
- CGACACAGAC GTACCCCAGG ACGAAT

+     GARE-motif

| Site Name | Organism | Position | Strand | Matrix score. | sequence | function |
| --- | --- | --- | --- | --- | --- | --- |
| GARE-motif | Brassica oleracea | 3634 | - | 7 | TCTGTTG | gibberellin-responsive element |

>HU02G01569.1   
+ +Up\_Stream \_Len000AGCGTA GGAAGTGACG CCGCTATTGG AGGCGAAGAC GCTAGTACCA AGGCCAAAAC   
  
  
+ AAATGAGGAG ATAGATCAGT TAAACCGTTT GGTTAAGAAA ATTAAGCGAA CATCTCTTGA ACCTCCATCT   
  
  
+ GAACTCGATG ACATTGAGGA TGTGGATGCA GAAAAGATGG ATATGGGCTC TCCATCGGTG CCAGCAAATC   
  
  
+ AACCTAATGT TGGGTGGGGT CTCATTCGGT CAATGGGGGA CTAGGGACGT CATATCGTCA CCAATTACAA   
  
  
+ CGGAACAACC CTAAACCGCA TTTTAAAATC ACATCTTGGT AGCAGACAAA GATGTACGAC CCAAAAATTC   
  
  
+ AACTCTGTAA AGCACTAGCA AAGCTCGTAG TACACAGAAC AATCGCACAC TCATAGTGAT AGAAGTGTAC   
  
  
+ TATCCATTAT GCAGTGAATA TGACAAGGGA GGGGGTCCTG AGATGCCCAA ATTTGGCAGC CGAGTTGCCT   
  
  
+ TGACTGCCCA TCAATGTCCT AAACCAGTTG AGCATGTTAT TAATTTGCAA GAAAGATCGA ATTCTTCCTA   
  
  
+ TCTAAAAACT GACACAATAA AACAATACCA TGTATGGGAC ATTGCTAGGA TTGTTAACAC AATTATTGTC   
  
  
+ TTGTACGTTG CACCTGAAAA AAAAGAAAAT TGTTGCCATA GACAACATAT AAGTTAGGCA TTCATGACTA   
  
  
+ ATATCTGAAT TAGGTGTGTC GATCTGTGAT TCAAATTGAT ATAATTAGAT CTTTAAGGTA TCTAATTGCT   
  
  
+ TCAAGTTTTC TCTATAATGA AGATGTATAT TAGCTCACAT AGGTCTTAAG GTTAACTAAT GTCATATCAG   
  
  
+ GGGGTGTGGG CCGCCATTTT TGATAAGAAT TGAATTGAAC TTGCATTGTT TTTTCTTTGG CGAGAATGCA   
  
  
+ AGATTTGTTA ATTTATTCAT ATGCGTCATT ATACACTATA AGAAAAATAA TTGTAGGAAT ATATGAAGTA   
  
  
+ TTTTTACTTG TGCTCATGTT AAATGTGGAA TACTTTTTTA ATGTCGTATA GTACCACAGT ATTCTACAAT   
  
  
+ TTAAGAGATT AATCGATCCA TTAAAGTCAT GCAAACCTGA TGCATATTCA AGACATTGCA TGAAATGATC   
  
  
+ TTATTTTAGT CAAAGACTCC ATCATTTATA AGCAATATTT ACTTCGATAC ACGTGTCATT AAAAAAGACT   
  
  
+ ATAGAAATGT TACTGTTATT TTGTTTGAGC TAAATAGAAA ATTTAAAAGT TAATTAAGAT AATAATTAAA   
  
  
+ ACTAAATATT CAATGATGGC TAATAAATAC ATTGTCGTAT TAACTGACTA ATATTTGTAG ATGGTCGTGT   
  
  
+ TAATTTCTTT TTATTTTAAA GTTCAAATGA GTTACAAAAG GTTATAAAAT ATAAAGTAAA ATGGGAGACA   
  
  
+ AAGCAGACAG GGTGGGGGAA GCTAAGCTTA AAGTAGGATT TAGAAAGGCA ATGAAGGTGA ACCCTCAAAT   
  
  
+ CGATCCGCTG CCAATCACAG AAACCCAAAG CTTTGCTCAC CGACAACTCC CGGTTAACTG CAGTCACTCA   
  
  
+ CGGCACTGGG TAATACAGTA ATCGTGTACC CAGTTCATCC CCCTTTTCCT TTGGGAATAC AGTACCTCGA   
  
  
+ AAGTTCCTTT GAAATTCTAC TGGCCAGGCA TACCCATAAA TTGATCCTCT CGATATCATA AGATATGATA   
  
  
+ TTTCTCTGTC ATATCTGTTA GTTTCGGTAT CATATTTCTT CTTTATCTCT GTAATTTGAG GTATGCTTCA   
  
  
+ TTCCTGGCCG CTTGAATTAT CTTTCTTTCT TTGATTAGTA TTGTTTTTTT GGTTACCTAT TTTGCTCCAG   
  
  
+ TCCTGTTCAT TTGGGTCTTT TTGGATCTGG GTTTTTGCTG GGTTTGTTGA TTCTTTGAGA AATTTGGGTG   
  
  
+ CTTGAATTTG CCCTGCAATA TTCATGGGTT AGCGTTTTCC TGAATTTTGC TCTGTTCCTT AAGTGATTAT   
  
  
+ TTTTGATTGA TTACACCTTG GTGGTCCTGG TGGAATTTCC GAGGAGAAAT TGTCATGGGT TCCCACAACT   
  
  
+ TTGGAGAATT CCCTGATGAG ACTCTAAATG AGTATCAATC TACTTTGGGA ACCATGTCCC CCGGTTATGA   
  
  
+ TGGGTCTTTG AATTATACAA CCTTGTTCAA TTACAAAGAC CCATCTCAGG ATCTCACGGC ACTGAACCTT   
  
  
+ CCTAGCCCAT TGCCTGACCC TATGCCATTC AACATTGGTT CATATTCGGG TTTGAGCCCC GGGGTTGAAT   
  
  
+ CTTCGGATGA TAGCGATTCA GATGATGTTG TTAAGTACAT TGGCCAAGTG CTTATGGAAG AGGATATGAA   
  
  
+ GGAGAAGCCT TGTATGTTCC ATGACCCTTT AGCACTCCAA GCTGCTGAGA AACCCTTTTA TGATGTGTTG   
  
  
+ GGAAAGAAGT ATCCTCCTTC CCCTAATCAA CACCCACTTA TTGATCATTC TGTGGATAGC CCAGACAATC   
  
  
+ AGTCCTGTGG AACGAGCACA ATTAGTGATC TTAGTGGCAG TAACTGCACT TCCAGTTCAA CCAATTATAT   
  
  
+ TGATGCTGTG GCAGTCGCTG ATTCGAGTGA GAATACTAAG ACCTCTTTTG TGCAAAGTTC TCTGATTGAA   
  
  
+ TCGTTTTCTC AGTCGTCCAC CCTTCCACAG TGGTCATTTG GATCATTGGG TGCCTTGGGT GGCACAGCTT   
  
  
+ CTCAGGGTTC GAATTCAGTT ATCTCCTCCC GTGGTTTCCC TATGGCCATG AACGTTTTTA GCGAGCAGTC   
  
  
+ CATGATACAG TTTCAGAAGG GGGTGGAGGA GGCGAGCAAG TTCCTTCCAA AGAACAATAA CCTTGCGATT   
  
  
+ GACCTTGAGA GCGTCACTTT CCCAAATGAA AAGGAGGCGG CCCCCATGGT GGTGGTTAAG AAAGAGAAGG   
  
  
+ ATGAACACTC ACCTGATAGC TCGAGAGGTA GCAAGATTCA CTACCGTGAT GATGAGGACT TTGAGGATGG   
  
  
+ TAGGAGGAGT AAGCAGTCAG CTGTTTCTGT GGAGGAGGCT GAGTTGTCTG AAATGTTTGA CCGGGTTTTG   
  
  
+ CTTTGCAATC CCATGAAACA TGAAGCTCAT TGTACAAGTG GTTTGAAGTC CGAGAAGGGA ACATCCCTGC   
  
  
+ AGGCTGGCCA AGTACAGGTA CAGGAGGGTC AGAAGGCTCG TGCAAAGAAA CAGGGTAATA ATGATAATAA   
  
  
+ GAAGAATAAG AATGTGGTGG ATTTAAGGAC TCTGCTTATC CTCTGTGCGC AATCTGCTGC ATCTGATGAT   
  
  
+ CGCAGGACAG CTGATGAACT GCTGAAGCAG ATTAGGGAGC ACTCTTCTGC AGCTGGGGAT GGATCTCAAA   
  
  
+ GGTTGGCTCA TTACTTTGCT AATGCCTTGG AGGCGCGTTT AGCTGGAACT GGCTCACAGA TCTATACAGC   
  
  
+ CCTGAGTTCG AAGAGGACAA CAGCAGCTGA TATGATAAAA GCTTATCAGT TTTTCTTTCG TGCTTGCCCA   
  
  
+ TTTTTAAAGA TCTCTATTAT CTTTGCGAAC CATATGATTA TACAGAGAGC TGAAAAAGCA TCAAAGCTTC   
  
  
+ ATGTTATAGA TTTTGGCATC CTGTATGGTT TTCAGTGGCC CCTCCTCATT CAACGCCTGT CGGAGCGACC   
  
  
+ TGGTGGACCT CCAAAACTGT GCATTACTGG GATTGATCTT CCCCAACCTG GATTTAGGCC AACAGAAAGA   
  
  
+ GTTGAGGCAA CAGGGCTCCG CTTGGCAAAG TATTGTGAGC GCTTCAATGT TCCATTTGAA TACCATGCCA   
  
  
+ TTGCACAGAA ATGGGAAACC ATCAAAGCTG AAGACCTGAA GATAGAAGAT GATGAGGTGG TTGCCGTGAA   
  
  
+ CTGTCTCTTC AGGTTTAAGA ACCTGCTTGA TGAGACGATA GTGGTGGATA GTCCGAGGAA TGCAGTTCTC   
  
  
+ GGCTTGATTA GAAGGATAAA ACCTGATATC TTTGTTCACG GAGTAGTGAA CGGGTCTTAC AATGCGCCCT   
  
  
+ TCTTTGTTAC ACGTTTCAGG GAGGCCCTCT TCCATTACTC TACTGTATTC GATATGTTTG ATGCCAATGC   
  
  
+ TTCCCGGGAG GATCCTGAGA GATTGATGTT TGAGAAGGAG TTTTATGGGC GAGAAATTAT GAATGTGGTG   
  
  
+ GCTTGTGAGG GTACAGAGAG AGTTGAAAGA CCTGAGACAT ACAAGCAATG GCAGGCGCGG AATAGTAGGG   
  
  
+ CAGGGTTCAG GCAGCTGCCA TTGGACCAAG AGCTCGTCAG TAAACTGAGG AGCAAGGTCC AGATGCACTA   
  
  
+ TCCTAGGGAC TTCGTTGTTG ATGTAGATGG ACATTGGACA TTGCAGGGAT GGAAGGGGAG GATCATCTGT   
  
  
+ GCTGTGTCTG CATGGGGTCC TGCTTA  

- +Up\_Stream \_Len000TCGCAT CCTTCACTGC GGCGATAACC TCCGCTTCTG CGATCATGGT TCCGGTTTTG   
  
  
- TTTACTCCTC TATCTAGTCA ATTTGGCAAA CCAATTCTTT TAATTCGCTT GTAGAGAACT TGGAGGTAGA   
  
  
- CTTGAGCTAC TGTAACTCCT ACACCTACGT CTTTTCTACC TATACCCGAG AGGTAGCCAC GGTCGTTTAG   
  
  
- TTGGATTACA ACCCACCCCA GAGTAAGCCA GTTACCCCCT GATCCCTGCA GTATAGCAGT GGTTAATGTT   
  
  
- GCCTTGTTGG GATTTGGCGT AAAATTTTAG TGTAGAACCA TCGTCTGTTT CTACATGCTG GGTTTTTAAG   
  
  
- TTGAGACATT TCGTGATCGT TTCGAGCATC ATGTGTCTTG TTAGCGTGTG AGTATCACTA TCTTCACATG   
  
  
- ATAGGTAATA CGTCACTTAT ACTGTTCCCT CCCCCAGGAC TCTACGGGTT TAAACCGTCG GCTCAACGGA   
  
  
- ACTGACGGGT AGTTACAGGA TTTGGTCAAC TCGTACAATA ATTAAACGTT CTTTCTAGCT TAAGAAGGAT   
  
  
- AGATTTTTGA CTGTGTTATT TTGTTATGGT ACATACCCTG TAACGATCCT AACAATTGTG TTAATAACAG   
  
  
- AACATGCAAC GTGGACTTTT TTTTCTTTTA ACAACGGTAT CTGTTGTATA TTCAATCCGT AAGTACTGAT   
  
  
- TATAGACTTA ATCCACACAG CTAGACACTA AGTTTAACTA TATTAATCTA GAAATTCCAT AGATTAACGA   
  
  
- AGTTCAAAAG AGATATTACT TCTACATATA ATCGAGTGTA TCCAGAATTC CAATTGATTA CAGTATAGTC   
  
  
- CCCCACACCC GGCGGTAAAA ACTATTCTTA ACTTAACTTG AACGTAACAA AAAAGAAACC GCTCTTACGT   
  
  
- TCTAAACAAT TAAATAAGTA TACGCAGTAA TATGTGATAT TCTTTTTATT AACATCCTTA TATACTTCAT   
  
  
- AAAAATGAAC ACGAGTACAA TTTACACCTT ATGAAAAAAT TACAGCATAT CATGGTGTCA TAAGATGTTA   
  
  
- AATTCTCTAA TTAGCTAGGT AATTTCAGTA CGTTTGGACT ACGTATAAGT TCTGTAACGT ACTTTACTAG   
  
  
- AATAAAATCA GTTTCTGAGG TAGTAAATAT TCGTTATAAA TGAAGCTATG TGCACAGTAA TTTTTTCTGA   
  
  
- TATCTTTACA ATGACAATAA AACAAACTCG ATTTATCTTT TAAATTTTCA ATTAATTCTA TTATTAATTT   
  
  
- TGATTTATAA GTTACTACCG ATTATTTATG TAACAGCATA ATTGACTGAT TATAAACATC TACCAGCACA   
  
  
- ATTAAAGAAA AATAAAATTT CAAGTTTACT CAATGTTTTC CAATATTTTA TATTTCATTT TACCCTCTGT   
  
  
- TTCGTCTGTC CCACCCCCTT CGATTCGAAT TTCATCCTAA ATCTTTCCGT TACTTCCACT TGGGAGTTTA   
  
  
- GCTAGGCGAC GGTTAGTGTC TTTGGGTTTC GAAACGAGTG GCTGTTGAGG GCCAATTGAC GTCAGTGAGT   
  
  
- GCCGTGACCC ATTATGTCAT TAGCACATGG GTCAAGTAGG GGGAAAAGGA AACCCTTATG TCATGGAGCT   
  
  
- TTCAAGGAAA CTTTAAGATG ACCGGTCCGT ATGGGTATTT AACTAGGAGA GCTATAGTAT TCTATACTAT   
  
  
- AAAGAGACAG TATAGACAAT CAAAGCCATA GTATAAAGAA GAAATAGAGA CATTAAACTC CATACGAAGT   
  
  
- AAGGACCGGC GAACTTAATA GAAAGAAAGA AACTAATCAT AACAAAAAAA CCAATGGATA AAACGAGGTC   
  
  
- AGGACAAGTA AACCCAGAAA AACCTAGACC CAAAAACGAC CCAAACAACT AAGAAACTCT TTAAACCCAC   
  
  
- GAACTTAAAC GGGACGTTAT AAGTACCCAA TCGCAAAAGG ACTTAAAACG AGACAAGGAA TTCACTAATA   
  
  
- AAAACTAACT AATGTGGAAC CACCAGGACC ACCTTAAAGG CTCCTCTTTA ACAGTACCCA AGGGTGTTGA   
  
  
- AACCTCTTAA GGGACTACTC TGAGATTTAC TCATAGTTAG ATGAAACCCT TGGTACAGGG GGCCAATACT   
  
  
- ACCCAGAAAC TTAATATGTT GGAACAAGTT AATGTTTCTG GGTAGAGTCC TAGAGTGCCG TGACTTGGAA   
  
  
- GGATCGGGTA ACGGACTGGG ATACGGTAAG TTGTAACCAA GTATAAGCCC AAACTCGGGG CCCCAACTTA   
  
  
- GAAGCCTACT ATCGCTAAGT CTACTACAAC AATTCATGTA ACCGGTTCAC GAATACCTTC TCCTATACTT   
  
  
- CCTCTTCGGA ACATACAAGG TACTGGGAAA TCGTGAGGTT CGACGACTCT TTGGGAAAAT ACTACACAAC   
  
  
- CCTTTCTTCA TAGGAGGAAG GGGATTAGTT GTGGGTGAAT AACTAGTAAG ACACCTATCG GGTCTGTTAG   
  
  
- TCAGGACACC TTGCTCGTGT TAATCACTAG AATCACCGTC ATTGACGTGA AGGTCAAGTT GGTTAATATA   
  
  
- ACTACGACAC CGTCAGCGAC TAAGCTCACT CTTATGATTC TGGAGAAAAC ACGTTTCAAG AGACTAACTT   
  
  
- AGCAAAAGAG TCAGCAGGTG GGAAGGTGTC ACCAGTAAAC CTAGTAACCC ACGGAACCCA CCGTGTCGAA   
  
  
- GAGTCCCAAG CTTAAGTCAA TAGAGGAGGG CACCAAAGGG ATACCGGTAC TTGCAAAAAT CGCTCGTCAG   
  
  
- GTACTATGTC AAAGTCTTCC CCCACCTCCT CCGCTCGTTC AAGGAAGGTT TCTTGTTATT GGAACGCTAA   
  
  
- CTGGAACTCT CGCAGTGAAA GGGTTTACTT TTCCTCCGCC GGGGGTACCA CCACCAATTC TTTCTCTTCC   
  
  
- TACTTGTGAG TGGACTATCG AGCTCTCCAT CGTTCTAAGT GATGGCACTA CTACTCCTGA AACTCCTACC   
  
  
- ATCCTCCTCA TTCGTCAGTC GACAAAGACA CCTCCTCCGA CTCAACAGAC TTTACAAACT GGCCCAAAAC   
  
  
- GAAACGTTAG GGTACTTTGT ACTTCGAGTA ACATGTTCAC CAAACTTCAG GCTCTTCCCT TGTAGGGACG   
  
  
- TCCGACCGGT TCATGTCCAT GTCCTCCCAG TCTTCCGAGC ACGTTTCTTT GTCCCATTAT TACTATTATT   
  
  
- CTTCTTATTC TTACACCACC TAAATTCCTG AGACGAATAG GAGACACGCG TTAGACGACG TAGACTACTA   
  
  
- GCGTCCTGTC GACTACTTGA CGACTTCGTC TAATCCCTCG TGAGAAGACG TCGACCCCTA CCTAGAGTTT   
  
  
- CCAACCGAGT AATGAAACGA TTACGGAACC TCCGCGCAAA TCGACCTTGA CCGAGTGTCT AGATATGTCG   
  
  
- GGACTCAAGC TTCTCCTGTT GTCGTCGACT ATACTATTTT CGAATAGTCA AAAAGAAAGC ACGAACGGGT   
  
  
- AAAAATTTCT AGAGATAATA GAAACGCTTG GTATACTAAT ATGTCTCTCG ACTTTTTCGT AGTTTCGAAG   
  
  
- TACAATATCT AAAACCGTAG GACATACCAA AAGTCACCGG GGAGGAGTAA GTTGCGGACA GCCTCGCTGG   
  
  
- ACCACCTGGA GGTTTTGACA CGTAATGACC CTAACTAGAA GGGGTTGGAC CTAAATCCGG TTGTCTTTCT   
  
  
- CAACTCCGTT GTCCCGAGGC GAACCGTTTC ATAACACTCG CGAAGTTACA AGGTAAACTT ATGGTACGGT   
  
  
- AACGTGTCTT TACCCTTTGG TAGTTTCGAC TTCTGGACTT CTATCTTCTA CTACTCCACC AACGGCACTT   
  
  
- GACAGAGAAG TCCAAATTCT TGGACGAACT ACTCTGCTAT CACCACCTAT CAGGCTCCTT ACGTCAAGAG   
  
  
- CCGAACTAAT CTTCCTATTT TGGACTATAG AAACAAGTGC CTCATCACTT GCCCAGAATG TTACGCGGGA   
  
  
- AGAAACAATG TGCAAAGTCC CTCCGGGAGA AGGTAATGAG ATGACATAAG CTATACAAAC TACGGTTACG   
  
  
- AAGGGCCCTC CTAGGACTCT CTAACTACAA ACTCTTCCTC AAAATACCCG CTCTTTAATA CTTACACCAC   
  
  
- CGAACACTCC CATGTCTCTC TCAACTTTCT GGACTCTGTA TGTTCGTTAC CGTCCGCGCC TTATCATCCC   
  
  
- GTCCCAAGTC CGTCGACGGT AACCTGGTTC TCGAGCAGTC ATTTGACTCC TCGTTCCAGG TCTACGTGAT   
  
  
- AGGATCCCTG AAGCAACAAC TACATCTACC TGTAACCTGT AACGTCCCTA CCTTCCCCTC CTAGTAGACA   
  
  
- CGACACAGAC GTACCCCAGG ACGAAT

+     GATA-motif

| Site Name | Organism | Position | Strand | Matrix score. | sequence | function |
| --- | --- | --- | --- | --- | --- | --- |
| GATA-motif | Arabidopsis thaliana | 560 | - | 7 | GATAGGA | part of a light responsive element |
| GATA-motif | Solanum tuberosum | 1586 | - | 9 | AAGGATAAGG | part of a light responsive element |

>HU02G01569.1   
+ +Up\_Stream \_Len000AGCGTA GGAAGTGACG CCGCTATTGG AGGCGAAGAC GCTAGTACCA AGGCCAAAAC   
  
  
+ AAATGAGGAG ATAGATCAGT TAAACCGTTT GGTTAAGAAA ATTAAGCGAA CATCTCTTGA ACCTCCATCT   
  
  
+ GAACTCGATG ACATTGAGGA TGTGGATGCA GAAAAGATGG ATATGGGCTC TCCATCGGTG CCAGCAAATC   
  
  
+ AACCTAATGT TGGGTGGGGT CTCATTCGGT CAATGGGGGA CTAGGGACGT CATATCGTCA CCAATTACAA   
  
  
+ CGGAACAACC CTAAACCGCA TTTTAAAATC ACATCTTGGT AGCAGACAAA GATGTACGAC CCAAAAATTC   
  
  
+ AACTCTGTAA AGCACTAGCA AAGCTCGTAG TACACAGAAC AATCGCACAC TCATAGTGAT AGAAGTGTAC   
  
  
+ TATCCATTAT GCAGTGAATA TGACAAGGGA GGGGGTCCTG AGATGCCCAA ATTTGGCAGC CGAGTTGCCT   
  
  
+ TGACTGCCCA TCAATGTCCT AAACCAGTTG AGCATGTTAT TAATTTGCAA GAAAGATCGA ATTCTTCCTA   
  
  
+ TCTAAAAACT GACACAATAA AACAATACCA TGTATGGGAC ATTGCTAGGA TTGTTAACAC AATTATTGTC   
  
  
+ TTGTACGTTG CACCTGAAAA AAAAGAAAAT TGTTGCCATA GACAACATAT AAGTTAGGCA TTCATGACTA   
  
  
+ ATATCTGAAT TAGGTGTGTC GATCTGTGAT TCAAATTGAT ATAATTAGAT CTTTAAGGTA TCTAATTGCT   
  
  
+ TCAAGTTTTC TCTATAATGA AGATGTATAT TAGCTCACAT AGGTCTTAAG GTTAACTAAT GTCATATCAG   
  
  
+ GGGGTGTGGG CCGCCATTTT TGATAAGAAT TGAATTGAAC TTGCATTGTT TTTTCTTTGG CGAGAATGCA   
  
  
+ AGATTTGTTA ATTTATTCAT ATGCGTCATT ATACACTATA AGAAAAATAA TTGTAGGAAT ATATGAAGTA   
  
  
+ TTTTTACTTG TGCTCATGTT AAATGTGGAA TACTTTTTTA ATGTCGTATA GTACCACAGT ATTCTACAAT   
  
  
+ TTAAGAGATT AATCGATCCA TTAAAGTCAT GCAAACCTGA TGCATATTCA AGACATTGCA TGAAATGATC   
  
  
+ TTATTTTAGT CAAAGACTCC ATCATTTATA AGCAATATTT ACTTCGATAC ACGTGTCATT AAAAAAGACT   
  
  
+ ATAGAAATGT TACTGTTATT TTGTTTGAGC TAAATAGAAA ATTTAAAAGT TAATTAAGAT AATAATTAAA   
  
  
+ ACTAAATATT CAATGATGGC TAATAAATAC ATTGTCGTAT TAACTGACTA ATATTTGTAG ATGGTCGTGT   
  
  
+ TAATTTCTTT TTATTTTAAA GTTCAAATGA GTTACAAAAG GTTATAAAAT ATAAAGTAAA ATGGGAGACA   
  
  
+ AAGCAGACAG GGTGGGGGAA GCTAAGCTTA AAGTAGGATT TAGAAAGGCA ATGAAGGTGA ACCCTCAAAT   
  
  
+ CGATCCGCTG CCAATCACAG AAACCCAAAG CTTTGCTCAC CGACAACTCC CGGTTAACTG CAGTCACTCA   
  
  
+ CGGCACTGGG TAATACAGTA ATCGTGTACC CAGTTCATCC CCCTTTTCCT TTGGGAATAC AGTACCTCGA   
  
  
+ AAGTTCCTTT GAAATTCTAC TGGCCAGGCA TACCCATAAA TTGATCCTCT CGATATCATA AGATATGATA   
  
  
+ TTTCTCTGTC ATATCTGTTA GTTTCGGTAT CATATTTCTT CTTTATCTCT GTAATTTGAG GTATGCTTCA   
  
  
+ TTCCTGGCCG CTTGAATTAT CTTTCTTTCT TTGATTAGTA TTGTTTTTTT GGTTACCTAT TTTGCTCCAG   
  
  
+ TCCTGTTCAT TTGGGTCTTT TTGGATCTGG GTTTTTGCTG GGTTTGTTGA TTCTTTGAGA AATTTGGGTG   
  
  
+ CTTGAATTTG CCCTGCAATA TTCATGGGTT AGCGTTTTCC TGAATTTTGC TCTGTTCCTT AAGTGATTAT   
  
  
+ TTTTGATTGA TTACACCTTG GTGGTCCTGG TGGAATTTCC GAGGAGAAAT TGTCATGGGT TCCCACAACT   
  
  
+ TTGGAGAATT CCCTGATGAG ACTCTAAATG AGTATCAATC TACTTTGGGA ACCATGTCCC CCGGTTATGA   
  
  
+ TGGGTCTTTG AATTATACAA CCTTGTTCAA TTACAAAGAC CCATCTCAGG ATCTCACGGC ACTGAACCTT   
  
  
+ CCTAGCCCAT TGCCTGACCC TATGCCATTC AACATTGGTT CATATTCGGG TTTGAGCCCC GGGGTTGAAT   
  
  
+ CTTCGGATGA TAGCGATTCA GATGATGTTG TTAAGTACAT TGGCCAAGTG CTTATGGAAG AGGATATGAA   
  
  
+ GGAGAAGCCT TGTATGTTCC ATGACCCTTT AGCACTCCAA GCTGCTGAGA AACCCTTTTA TGATGTGTTG   
  
  
+ GGAAAGAAGT ATCCTCCTTC CCCTAATCAA CACCCACTTA TTGATCATTC TGTGGATAGC CCAGACAATC   
  
  
+ AGTCCTGTGG AACGAGCACA ATTAGTGATC TTAGTGGCAG TAACTGCACT TCCAGTTCAA CCAATTATAT   
  
  
+ TGATGCTGTG GCAGTCGCTG ATTCGAGTGA GAATACTAAG ACCTCTTTTG TGCAAAGTTC TCTGATTGAA   
  
  
+ TCGTTTTCTC AGTCGTCCAC CCTTCCACAG TGGTCATTTG GATCATTGGG TGCCTTGGGT GGCACAGCTT   
  
  
+ CTCAGGGTTC GAATTCAGTT ATCTCCTCCC GTGGTTTCCC TATGGCCATG AACGTTTTTA GCGAGCAGTC   
  
  
+ CATGATACAG TTTCAGAAGG GGGTGGAGGA GGCGAGCAAG TTCCTTCCAA AGAACAATAA CCTTGCGATT   
  
  
+ GACCTTGAGA GCGTCACTTT CCCAAATGAA AAGGAGGCGG CCCCCATGGT GGTGGTTAAG AAAGAGAAGG   
  
  
+ ATGAACACTC ACCTGATAGC TCGAGAGGTA GCAAGATTCA CTACCGTGAT GATGAGGACT TTGAGGATGG   
  
  
+ TAGGAGGAGT AAGCAGTCAG CTGTTTCTGT GGAGGAGGCT GAGTTGTCTG AAATGTTTGA CCGGGTTTTG   
  
  
+ CTTTGCAATC CCATGAAACA TGAAGCTCAT TGTACAAGTG GTTTGAAGTC CGAGAAGGGA ACATCCCTGC   
  
  
+ AGGCTGGCCA AGTACAGGTA CAGGAGGGTC AGAAGGCTCG TGCAAAGAAA CAGGGTAATA ATGATAATAA   
  
  
+ GAAGAATAAG AATGTGGTGG ATTTAAGGAC TCTGCTTATC CTCTGTGCGC AATCTGCTGC ATCTGATGAT   
  
  
+ CGCAGGACAG CTGATGAACT GCTGAAGCAG ATTAGGGAGC ACTCTTCTGC AGCTGGGGAT GGATCTCAAA   
  
  
+ GGTTGGCTCA TTACTTTGCT AATGCCTTGG AGGCGCGTTT AGCTGGAACT GGCTCACAGA TCTATACAGC   
  
  
+ CCTGAGTTCG AAGAGGACAA CAGCAGCTGA TATGATAAAA GCTTATCAGT TTTTCTTTCG TGCTTGCCCA   
  
  
+ TTTTTAAAGA TCTCTATTAT CTTTGCGAAC CATATGATTA TACAGAGAGC TGAAAAAGCA TCAAAGCTTC   
  
  
+ ATGTTATAGA TTTTGGCATC CTGTATGGTT TTCAGTGGCC CCTCCTCATT CAACGCCTGT CGGAGCGACC   
  
  
+ TGGTGGACCT CCAAAACTGT GCATTACTGG GATTGATCTT CCCCAACCTG GATTTAGGCC AACAGAAAGA   
  
  
+ GTTGAGGCAA CAGGGCTCCG CTTGGCAAAG TATTGTGAGC GCTTCAATGT TCCATTTGAA TACCATGCCA   
  
  
+ TTGCACAGAA ATGGGAAACC ATCAAAGCTG AAGACCTGAA GATAGAAGAT GATGAGGTGG TTGCCGTGAA   
  
  
+ CTGTCTCTTC AGGTTTAAGA ACCTGCTTGA TGAGACGATA GTGGTGGATA GTCCGAGGAA TGCAGTTCTC   
  
  
+ GGCTTGATTA GAAGGATAAA ACCTGATATC TTTGTTCACG GAGTAGTGAA CGGGTCTTAC AATGCGCCCT   
  
  
+ TCTTTGTTAC ACGTTTCAGG GAGGCCCTCT TCCATTACTC TACTGTATTC GATATGTTTG ATGCCAATGC   
  
  
+ TTCCCGGGAG GATCCTGAGA GATTGATGTT TGAGAAGGAG TTTTATGGGC GAGAAATTAT GAATGTGGTG   
  
  
+ GCTTGTGAGG GTACAGAGAG AGTTGAAAGA CCTGAGACAT ACAAGCAATG GCAGGCGCGG AATAGTAGGG   
  
  
+ CAGGGTTCAG GCAGCTGCCA TTGGACCAAG AGCTCGTCAG TAAACTGAGG AGCAAGGTCC AGATGCACTA   
  
  
+ TCCTAGGGAC TTCGTTGTTG ATGTAGATGG ACATTGGACA TTGCAGGGAT GGAAGGGGAG GATCATCTGT   
  
  
+ GCTGTGTCTG CATGGGGTCC TGCTTA  

- +Up\_Stream \_Len000TCGCAT CCTTCACTGC GGCGATAACC TCCGCTTCTG CGATCATGGT TCCGGTTTTG   
  
  
- TTTACTCCTC TATCTAGTCA ATTTGGCAAA CCAATTCTTT TAATTCGCTT GTAGAGAACT TGGAGGTAGA   
  
  
- CTTGAGCTAC TGTAACTCCT ACACCTACGT CTTTTCTACC TATACCCGAG AGGTAGCCAC GGTCGTTTAG   
  
  
- TTGGATTACA ACCCACCCCA GAGTAAGCCA GTTACCCCCT GATCCCTGCA GTATAGCAGT GGTTAATGTT   
  
  
- GCCTTGTTGG GATTTGGCGT AAAATTTTAG TGTAGAACCA TCGTCTGTTT CTACATGCTG GGTTTTTAAG   
  
  
- TTGAGACATT TCGTGATCGT TTCGAGCATC ATGTGTCTTG TTAGCGTGTG AGTATCACTA TCTTCACATG   
  
  
- ATAGGTAATA CGTCACTTAT ACTGTTCCCT CCCCCAGGAC TCTACGGGTT TAAACCGTCG GCTCAACGGA   
  
  
- ACTGACGGGT AGTTACAGGA TTTGGTCAAC TCGTACAATA ATTAAACGTT CTTTCTAGCT TAAGAAGGAT   
  
  
- AGATTTTTGA CTGTGTTATT TTGTTATGGT ACATACCCTG TAACGATCCT AACAATTGTG TTAATAACAG   
  
  
- AACATGCAAC GTGGACTTTT TTTTCTTTTA ACAACGGTAT CTGTTGTATA TTCAATCCGT AAGTACTGAT   
  
  
- TATAGACTTA ATCCACACAG CTAGACACTA AGTTTAACTA TATTAATCTA GAAATTCCAT AGATTAACGA   
  
  
- AGTTCAAAAG AGATATTACT TCTACATATA ATCGAGTGTA TCCAGAATTC CAATTGATTA CAGTATAGTC   
  
  
- CCCCACACCC GGCGGTAAAA ACTATTCTTA ACTTAACTTG AACGTAACAA AAAAGAAACC GCTCTTACGT   
  
  
- TCTAAACAAT TAAATAAGTA TACGCAGTAA TATGTGATAT TCTTTTTATT AACATCCTTA TATACTTCAT   
  
  
- AAAAATGAAC ACGAGTACAA TTTACACCTT ATGAAAAAAT TACAGCATAT CATGGTGTCA TAAGATGTTA   
  
  
- AATTCTCTAA TTAGCTAGGT AATTTCAGTA CGTTTGGACT ACGTATAAGT TCTGTAACGT ACTTTACTAG   
  
  
- AATAAAATCA GTTTCTGAGG TAGTAAATAT TCGTTATAAA TGAAGCTATG TGCACAGTAA TTTTTTCTGA   
  
  
- TATCTTTACA ATGACAATAA AACAAACTCG ATTTATCTTT TAAATTTTCA ATTAATTCTA TTATTAATTT   
  
  
- TGATTTATAA GTTACTACCG ATTATTTATG TAACAGCATA ATTGACTGAT TATAAACATC TACCAGCACA   
  
  
- ATTAAAGAAA AATAAAATTT CAAGTTTACT CAATGTTTTC CAATATTTTA TATTTCATTT TACCCTCTGT   
  
  
- TTCGTCTGTC CCACCCCCTT CGATTCGAAT TTCATCCTAA ATCTTTCCGT TACTTCCACT TGGGAGTTTA   
  
  
- GCTAGGCGAC GGTTAGTGTC TTTGGGTTTC GAAACGAGTG GCTGTTGAGG GCCAATTGAC GTCAGTGAGT   
  
  
- GCCGTGACCC ATTATGTCAT TAGCACATGG GTCAAGTAGG GGGAAAAGGA AACCCTTATG TCATGGAGCT   
  
  
- TTCAAGGAAA CTTTAAGATG ACCGGTCCGT ATGGGTATTT AACTAGGAGA GCTATAGTAT TCTATACTAT   
  
  
- AAAGAGACAG TATAGACAAT CAAAGCCATA GTATAAAGAA GAAATAGAGA CATTAAACTC CATACGAAGT   
  
  
- AAGGACCGGC GAACTTAATA GAAAGAAAGA AACTAATCAT AACAAAAAAA CCAATGGATA AAACGAGGTC   
  
  
- AGGACAAGTA AACCCAGAAA AACCTAGACC CAAAAACGAC CCAAACAACT AAGAAACTCT TTAAACCCAC   
  
  
- GAACTTAAAC GGGACGTTAT AAGTACCCAA TCGCAAAAGG ACTTAAAACG AGACAAGGAA TTCACTAATA   
  
  
- AAAACTAACT AATGTGGAAC CACCAGGACC ACCTTAAAGG CTCCTCTTTA ACAGTACCCA AGGGTGTTGA   
  
  
- AACCTCTTAA GGGACTACTC TGAGATTTAC TCATAGTTAG ATGAAACCCT TGGTACAGGG GGCCAATACT   
  
  
- ACCCAGAAAC TTAATATGTT GGAACAAGTT AATGTTTCTG GGTAGAGTCC TAGAGTGCCG TGACTTGGAA   
  
  
- GGATCGGGTA ACGGACTGGG ATACGGTAAG TTGTAACCAA GTATAAGCCC AAACTCGGGG CCCCAACTTA   
  
  
- GAAGCCTACT ATCGCTAAGT CTACTACAAC AATTCATGTA ACCGGTTCAC GAATACCTTC TCCTATACTT   
  
  
- CCTCTTCGGA ACATACAAGG TACTGGGAAA TCGTGAGGTT CGACGACTCT TTGGGAAAAT ACTACACAAC   
  
  
- CCTTTCTTCA TAGGAGGAAG GGGATTAGTT GTGGGTGAAT AACTAGTAAG ACACCTATCG GGTCTGTTAG   
  
  
- TCAGGACACC TTGCTCGTGT TAATCACTAG AATCACCGTC ATTGACGTGA AGGTCAAGTT GGTTAATATA   
  
  
- ACTACGACAC CGTCAGCGAC TAAGCTCACT CTTATGATTC TGGAGAAAAC ACGTTTCAAG AGACTAACTT   
  
  
- AGCAAAAGAG TCAGCAGGTG GGAAGGTGTC ACCAGTAAAC CTAGTAACCC ACGGAACCCA CCGTGTCGAA   
  
  
- GAGTCCCAAG CTTAAGTCAA TAGAGGAGGG CACCAAAGGG ATACCGGTAC TTGCAAAAAT CGCTCGTCAG   
  
  
- GTACTATGTC AAAGTCTTCC CCCACCTCCT CCGCTCGTTC AAGGAAGGTT TCTTGTTATT GGAACGCTAA   
  
  
- CTGGAACTCT CGCAGTGAAA GGGTTTACTT TTCCTCCGCC GGGGGTACCA CCACCAATTC TTTCTCTTCC   
  
  
- TACTTGTGAG TGGACTATCG AGCTCTCCAT CGTTCTAAGT GATGGCACTA CTACTCCTGA AACTCCTACC   
  
  
- ATCCTCCTCA TTCGTCAGTC GACAAAGACA CCTCCTCCGA CTCAACAGAC TTTACAAACT GGCCCAAAAC   
  
  
- GAAACGTTAG GGTACTTTGT ACTTCGAGTA ACATGTTCAC CAAACTTCAG GCTCTTCCCT TGTAGGGACG   
  
  
- TCCGACCGGT TCATGTCCAT GTCCTCCCAG TCTTCCGAGC ACGTTTCTTT GTCCCATTAT TACTATTATT   
  
  
- CTTCTTATTC TTACACCACC TAAATTCCTG AGACGAATAG GAGACACGCG TTAGACGACG TAGACTACTA   
  
  
- GCGTCCTGTC GACTACTTGA CGACTTCGTC TAATCCCTCG TGAGAAGACG TCGACCCCTA CCTAGAGTTT   
  
  
- CCAACCGAGT AATGAAACGA TTACGGAACC TCCGCGCAAA TCGACCTTGA CCGAGTGTCT AGATATGTCG   
  
  
- GGACTCAAGC TTCTCCTGTT GTCGTCGACT ATACTATTTT CGAATAGTCA AAAAGAAAGC ACGAACGGGT   
  
  
- AAAAATTTCT AGAGATAATA GAAACGCTTG GTATACTAAT ATGTCTCTCG ACTTTTTCGT AGTTTCGAAG   
  
  
- TACAATATCT AAAACCGTAG GACATACCAA AAGTCACCGG GGAGGAGTAA GTTGCGGACA GCCTCGCTGG   
  
  
- ACCACCTGGA GGTTTTGACA CGTAATGACC CTAACTAGAA GGGGTTGGAC CTAAATCCGG TTGTCTTTCT   
  
  
- CAACTCCGTT GTCCCGAGGC GAACCGTTTC ATAACACTCG CGAAGTTACA AGGTAAACTT ATGGTACGGT   
  
  
- AACGTGTCTT TACCCTTTGG TAGTTTCGAC TTCTGGACTT CTATCTTCTA CTACTCCACC AACGGCACTT   
  
  
- GACAGAGAAG TCCAAATTCT TGGACGAACT ACTCTGCTAT CACCACCTAT CAGGCTCCTT ACGTCAAGAG   
  
  
- CCGAACTAAT CTTCCTATTT TGGACTATAG AAACAAGTGC CTCATCACTT GCCCAGAATG TTACGCGGGA   
  
  
- AGAAACAATG TGCAAAGTCC CTCCGGGAGA AGGTAATGAG ATGACATAAG CTATACAAAC TACGGTTACG   
  
  
- AAGGGCCCTC CTAGGACTCT CTAACTACAA ACTCTTCCTC AAAATACCCG CTCTTTAATA CTTACACCAC   
  
  
- CGAACACTCC CATGTCTCTC TCAACTTTCT GGACTCTGTA TGTTCGTTAC CGTCCGCGCC TTATCATCCC   
  
  
- GTCCCAAGTC CGTCGACGGT AACCTGGTTC TCGAGCAGTC ATTTGACTCC TCGTTCCAGG TCTACGTGAT   
  
  
- AGGATCCCTG AAGCAACAAC TACATCTACC TGTAACCTGT AACGTCCCTA CCTTCCCCTC CTAGTAGACA   
  
  
- CGACACAGAC GTACCCCAGG ACGAAT

+     GC-motif

| Site Name | Organism | Position | Strand | Matrix score. | sequence | function |
| --- | --- | --- | --- | --- | --- | --- |
| GC-motif | Zea mays | 2092 | + | 6 | CCCCCG | enhancer-like element involved in anoxic specific inducibility |

>HU02G01569.1   
+ +Up\_Stream \_Len000AGCGTA GGAAGTGACG CCGCTATTGG AGGCGAAGAC GCTAGTACCA AGGCCAAAAC   
  
  
+ AAATGAGGAG ATAGATCAGT TAAACCGTTT GGTTAAGAAA ATTAAGCGAA CATCTCTTGA ACCTCCATCT   
  
  
+ GAACTCGATG ACATTGAGGA TGTGGATGCA GAAAAGATGG ATATGGGCTC TCCATCGGTG CCAGCAAATC   
  
  
+ AACCTAATGT TGGGTGGGGT CTCATTCGGT CAATGGGGGA CTAGGGACGT CATATCGTCA CCAATTACAA   
  
  
+ CGGAACAACC CTAAACCGCA TTTTAAAATC ACATCTTGGT AGCAGACAAA GATGTACGAC CCAAAAATTC   
  
  
+ AACTCTGTAA AGCACTAGCA AAGCTCGTAG TACACAGAAC AATCGCACAC TCATAGTGAT AGAAGTGTAC   
  
  
+ TATCCATTAT GCAGTGAATA TGACAAGGGA GGGGGTCCTG AGATGCCCAA ATTTGGCAGC CGAGTTGCCT   
  
  
+ TGACTGCCCA TCAATGTCCT AAACCAGTTG AGCATGTTAT TAATTTGCAA GAAAGATCGA ATTCTTCCTA   
  
  
+ TCTAAAAACT GACACAATAA AACAATACCA TGTATGGGAC ATTGCTAGGA TTGTTAACAC AATTATTGTC   
  
  
+ TTGTACGTTG CACCTGAAAA AAAAGAAAAT TGTTGCCATA GACAACATAT AAGTTAGGCA TTCATGACTA   
  
  
+ ATATCTGAAT TAGGTGTGTC GATCTGTGAT TCAAATTGAT ATAATTAGAT CTTTAAGGTA TCTAATTGCT   
  
  
+ TCAAGTTTTC TCTATAATGA AGATGTATAT TAGCTCACAT AGGTCTTAAG GTTAACTAAT GTCATATCAG   
  
  
+ GGGGTGTGGG CCGCCATTTT TGATAAGAAT TGAATTGAAC TTGCATTGTT TTTTCTTTGG CGAGAATGCA   
  
  
+ AGATTTGTTA ATTTATTCAT ATGCGTCATT ATACACTATA AGAAAAATAA TTGTAGGAAT ATATGAAGTA   
  
  
+ TTTTTACTTG TGCTCATGTT AAATGTGGAA TACTTTTTTA ATGTCGTATA GTACCACAGT ATTCTACAAT   
  
  
+ TTAAGAGATT AATCGATCCA TTAAAGTCAT GCAAACCTGA TGCATATTCA AGACATTGCA TGAAATGATC   
  
  
+ TTATTTTAGT CAAAGACTCC ATCATTTATA AGCAATATTT ACTTCGATAC ACGTGTCATT AAAAAAGACT   
  
  
+ ATAGAAATGT TACTGTTATT TTGTTTGAGC TAAATAGAAA ATTTAAAAGT TAATTAAGAT AATAATTAAA   
  
  
+ ACTAAATATT CAATGATGGC TAATAAATAC ATTGTCGTAT TAACTGACTA ATATTTGTAG ATGGTCGTGT   
  
  
+ TAATTTCTTT TTATTTTAAA GTTCAAATGA GTTACAAAAG GTTATAAAAT ATAAAGTAAA ATGGGAGACA   
  
  
+ AAGCAGACAG GGTGGGGGAA GCTAAGCTTA AAGTAGGATT TAGAAAGGCA ATGAAGGTGA ACCCTCAAAT   
  
  
+ CGATCCGCTG CCAATCACAG AAACCCAAAG CTTTGCTCAC CGACAACTCC CGGTTAACTG CAGTCACTCA   
  
  
+ CGGCACTGGG TAATACAGTA ATCGTGTACC CAGTTCATCC CCCTTTTCCT TTGGGAATAC AGTACCTCGA   
  
  
+ AAGTTCCTTT GAAATTCTAC TGGCCAGGCA TACCCATAAA TTGATCCTCT CGATATCATA AGATATGATA   
  
  
+ TTTCTCTGTC ATATCTGTTA GTTTCGGTAT CATATTTCTT CTTTATCTCT GTAATTTGAG GTATGCTTCA   
  
  
+ TTCCTGGCCG CTTGAATTAT CTTTCTTTCT TTGATTAGTA TTGTTTTTTT GGTTACCTAT TTTGCTCCAG   
  
  
+ TCCTGTTCAT TTGGGTCTTT TTGGATCTGG GTTTTTGCTG GGTTTGTTGA TTCTTTGAGA AATTTGGGTG   
  
  
+ CTTGAATTTG CCCTGCAATA TTCATGGGTT AGCGTTTTCC TGAATTTTGC TCTGTTCCTT AAGTGATTAT   
  
  
+ TTTTGATTGA TTACACCTTG GTGGTCCTGG TGGAATTTCC GAGGAGAAAT TGTCATGGGT TCCCACAACT   
  
  
+ TTGGAGAATT CCCTGATGAG ACTCTAAATG AGTATCAATC TACTTTGGGA ACCATGTCCC CCGGTTATGA   
  
  
+ TGGGTCTTTG AATTATACAA CCTTGTTCAA TTACAAAGAC CCATCTCAGG ATCTCACGGC ACTGAACCTT   
  
  
+ CCTAGCCCAT TGCCTGACCC TATGCCATTC AACATTGGTT CATATTCGGG TTTGAGCCCC GGGGTTGAAT   
  
  
+ CTTCGGATGA TAGCGATTCA GATGATGTTG TTAAGTACAT TGGCCAAGTG CTTATGGAAG AGGATATGAA   
  
  
+ GGAGAAGCCT TGTATGTTCC ATGACCCTTT AGCACTCCAA GCTGCTGAGA AACCCTTTTA TGATGTGTTG   
  
  
+ GGAAAGAAGT ATCCTCCTTC CCCTAATCAA CACCCACTTA TTGATCATTC TGTGGATAGC CCAGACAATC   
  
  
+ AGTCCTGTGG AACGAGCACA ATTAGTGATC TTAGTGGCAG TAACTGCACT TCCAGTTCAA CCAATTATAT   
  
  
+ TGATGCTGTG GCAGTCGCTG ATTCGAGTGA GAATACTAAG ACCTCTTTTG TGCAAAGTTC TCTGATTGAA   
  
  
+ TCGTTTTCTC AGTCGTCCAC CCTTCCACAG TGGTCATTTG GATCATTGGG TGCCTTGGGT GGCACAGCTT   
  
  
+ CTCAGGGTTC GAATTCAGTT ATCTCCTCCC GTGGTTTCCC TATGGCCATG AACGTTTTTA GCGAGCAGTC   
  
  
+ CATGATACAG TTTCAGAAGG GGGTGGAGGA GGCGAGCAAG TTCCTTCCAA AGAACAATAA CCTTGCGATT   
  
  
+ GACCTTGAGA GCGTCACTTT CCCAAATGAA AAGGAGGCGG CCCCCATGGT GGTGGTTAAG AAAGAGAAGG   
  
  
+ ATGAACACTC ACCTGATAGC TCGAGAGGTA GCAAGATTCA CTACCGTGAT GATGAGGACT TTGAGGATGG   
  
  
+ TAGGAGGAGT AAGCAGTCAG CTGTTTCTGT GGAGGAGGCT GAGTTGTCTG AAATGTTTGA CCGGGTTTTG   
  
  
+ CTTTGCAATC CCATGAAACA TGAAGCTCAT TGTACAAGTG GTTTGAAGTC CGAGAAGGGA ACATCCCTGC   
  
  
+ AGGCTGGCCA AGTACAGGTA CAGGAGGGTC AGAAGGCTCG TGCAAAGAAA CAGGGTAATA ATGATAATAA   
  
  
+ GAAGAATAAG AATGTGGTGG ATTTAAGGAC TCTGCTTATC CTCTGTGCGC AATCTGCTGC ATCTGATGAT   
  
  
+ CGCAGGACAG CTGATGAACT GCTGAAGCAG ATTAGGGAGC ACTCTTCTGC AGCTGGGGAT GGATCTCAAA   
  
  
+ GGTTGGCTCA TTACTTTGCT AATGCCTTGG AGGCGCGTTT AGCTGGAACT GGCTCACAGA TCTATACAGC   
  
  
+ CCTGAGTTCG AAGAGGACAA CAGCAGCTGA TATGATAAAA GCTTATCAGT TTTTCTTTCG TGCTTGCCCA   
  
  
+ TTTTTAAAGA TCTCTATTAT CTTTGCGAAC CATATGATTA TACAGAGAGC TGAAAAAGCA TCAAAGCTTC   
  
  
+ ATGTTATAGA TTTTGGCATC CTGTATGGTT TTCAGTGGCC CCTCCTCATT CAACGCCTGT CGGAGCGACC   
  
  
+ TGGTGGACCT CCAAAACTGT GCATTACTGG GATTGATCTT CCCCAACCTG GATTTAGGCC AACAGAAAGA   
  
  
+ GTTGAGGCAA CAGGGCTCCG CTTGGCAAAG TATTGTGAGC GCTTCAATGT TCCATTTGAA TACCATGCCA   
  
  
+ TTGCACAGAA ATGGGAAACC ATCAAAGCTG AAGACCTGAA GATAGAAGAT GATGAGGTGG TTGCCGTGAA   
  
  
+ CTGTCTCTTC AGGTTTAAGA ACCTGCTTGA TGAGACGATA GTGGTGGATA GTCCGAGGAA TGCAGTTCTC   
  
  
+ GGCTTGATTA GAAGGATAAA ACCTGATATC TTTGTTCACG GAGTAGTGAA CGGGTCTTAC AATGCGCCCT   
  
  
+ TCTTTGTTAC ACGTTTCAGG GAGGCCCTCT TCCATTACTC TACTGTATTC GATATGTTTG ATGCCAATGC   
  
  
+ TTCCCGGGAG GATCCTGAGA GATTGATGTT TGAGAAGGAG TTTTATGGGC GAGAAATTAT GAATGTGGTG   
  
  
+ GCTTGTGAGG GTACAGAGAG AGTTGAAAGA CCTGAGACAT ACAAGCAATG GCAGGCGCGG AATAGTAGGG   
  
  
+ CAGGGTTCAG GCAGCTGCCA TTGGACCAAG AGCTCGTCAG TAAACTGAGG AGCAAGGTCC AGATGCACTA   
  
  
+ TCCTAGGGAC TTCGTTGTTG ATGTAGATGG ACATTGGACA TTGCAGGGAT GGAAGGGGAG GATCATCTGT   
  
  
+ GCTGTGTCTG CATGGGGTCC TGCTTA  

- +Up\_Stream \_Len000TCGCAT CCTTCACTGC GGCGATAACC TCCGCTTCTG CGATCATGGT TCCGGTTTTG   
  
  
- TTTACTCCTC TATCTAGTCA ATTTGGCAAA CCAATTCTTT TAATTCGCTT GTAGAGAACT TGGAGGTAGA   
  
  
- CTTGAGCTAC TGTAACTCCT ACACCTACGT CTTTTCTACC TATACCCGAG AGGTAGCCAC GGTCGTTTAG   
  
  
- TTGGATTACA ACCCACCCCA GAGTAAGCCA GTTACCCCCT GATCCCTGCA GTATAGCAGT GGTTAATGTT   
  
  
- GCCTTGTTGG GATTTGGCGT AAAATTTTAG TGTAGAACCA TCGTCTGTTT CTACATGCTG GGTTTTTAAG   
  
  
- TTGAGACATT TCGTGATCGT TTCGAGCATC ATGTGTCTTG TTAGCGTGTG AGTATCACTA TCTTCACATG   
  
  
- ATAGGTAATA CGTCACTTAT ACTGTTCCCT CCCCCAGGAC TCTACGGGTT TAAACCGTCG GCTCAACGGA   
  
  
- ACTGACGGGT AGTTACAGGA TTTGGTCAAC TCGTACAATA ATTAAACGTT CTTTCTAGCT TAAGAAGGAT   
  
  
- AGATTTTTGA CTGTGTTATT TTGTTATGGT ACATACCCTG TAACGATCCT AACAATTGTG TTAATAACAG   
  
  
- AACATGCAAC GTGGACTTTT TTTTCTTTTA ACAACGGTAT CTGTTGTATA TTCAATCCGT AAGTACTGAT   
  
  
- TATAGACTTA ATCCACACAG CTAGACACTA AGTTTAACTA TATTAATCTA GAAATTCCAT AGATTAACGA   
  
  
- AGTTCAAAAG AGATATTACT TCTACATATA ATCGAGTGTA TCCAGAATTC CAATTGATTA CAGTATAGTC   
  
  
- CCCCACACCC GGCGGTAAAA ACTATTCTTA ACTTAACTTG AACGTAACAA AAAAGAAACC GCTCTTACGT   
  
  
- TCTAAACAAT TAAATAAGTA TACGCAGTAA TATGTGATAT TCTTTTTATT AACATCCTTA TATACTTCAT   
  
  
- AAAAATGAAC ACGAGTACAA TTTACACCTT ATGAAAAAAT TACAGCATAT CATGGTGTCA TAAGATGTTA   
  
  
- AATTCTCTAA TTAGCTAGGT AATTTCAGTA CGTTTGGACT ACGTATAAGT TCTGTAACGT ACTTTACTAG   
  
  
- AATAAAATCA GTTTCTGAGG TAGTAAATAT TCGTTATAAA TGAAGCTATG TGCACAGTAA TTTTTTCTGA   
  
  
- TATCTTTACA ATGACAATAA AACAAACTCG ATTTATCTTT TAAATTTTCA ATTAATTCTA TTATTAATTT   
  
  
- TGATTTATAA GTTACTACCG ATTATTTATG TAACAGCATA ATTGACTGAT TATAAACATC TACCAGCACA   
  
  
- ATTAAAGAAA AATAAAATTT CAAGTTTACT CAATGTTTTC CAATATTTTA TATTTCATTT TACCCTCTGT   
  
  
- TTCGTCTGTC CCACCCCCTT CGATTCGAAT TTCATCCTAA ATCTTTCCGT TACTTCCACT TGGGAGTTTA   
  
  
- GCTAGGCGAC GGTTAGTGTC TTTGGGTTTC GAAACGAGTG GCTGTTGAGG GCCAATTGAC GTCAGTGAGT   
  
  
- GCCGTGACCC ATTATGTCAT TAGCACATGG GTCAAGTAGG GGGAAAAGGA AACCCTTATG TCATGGAGCT   
  
  
- TTCAAGGAAA CTTTAAGATG ACCGGTCCGT ATGGGTATTT AACTAGGAGA GCTATAGTAT TCTATACTAT   
  
  
- AAAGAGACAG TATAGACAAT CAAAGCCATA GTATAAAGAA GAAATAGAGA CATTAAACTC CATACGAAGT   
  
  
- AAGGACCGGC GAACTTAATA GAAAGAAAGA AACTAATCAT AACAAAAAAA CCAATGGATA AAACGAGGTC   
  
  
- AGGACAAGTA AACCCAGAAA AACCTAGACC CAAAAACGAC CCAAACAACT AAGAAACTCT TTAAACCCAC   
  
  
- GAACTTAAAC GGGACGTTAT AAGTACCCAA TCGCAAAAGG ACTTAAAACG AGACAAGGAA TTCACTAATA   
  
  
- AAAACTAACT AATGTGGAAC CACCAGGACC ACCTTAAAGG CTCCTCTTTA ACAGTACCCA AGGGTGTTGA   
  
  
- AACCTCTTAA GGGACTACTC TGAGATTTAC TCATAGTTAG ATGAAACCCT TGGTACAGGG GGCCAATACT   
  
  
- ACCCAGAAAC TTAATATGTT GGAACAAGTT AATGTTTCTG GGTAGAGTCC TAGAGTGCCG TGACTTGGAA   
  
  
- GGATCGGGTA ACGGACTGGG ATACGGTAAG TTGTAACCAA GTATAAGCCC AAACTCGGGG CCCCAACTTA   
  
  
- GAAGCCTACT ATCGCTAAGT CTACTACAAC AATTCATGTA ACCGGTTCAC GAATACCTTC TCCTATACTT   
  
  
- CCTCTTCGGA ACATACAAGG TACTGGGAAA TCGTGAGGTT CGACGACTCT TTGGGAAAAT ACTACACAAC   
  
  
- CCTTTCTTCA TAGGAGGAAG GGGATTAGTT GTGGGTGAAT AACTAGTAAG ACACCTATCG GGTCTGTTAG   
  
  
- TCAGGACACC TTGCTCGTGT TAATCACTAG AATCACCGTC ATTGACGTGA AGGTCAAGTT GGTTAATATA   
  
  
- ACTACGACAC CGTCAGCGAC TAAGCTCACT CTTATGATTC TGGAGAAAAC ACGTTTCAAG AGACTAACTT   
  
  
- AGCAAAAGAG TCAGCAGGTG GGAAGGTGTC ACCAGTAAAC CTAGTAACCC ACGGAACCCA CCGTGTCGAA   
  
  
- GAGTCCCAAG CTTAAGTCAA TAGAGGAGGG CACCAAAGGG ATACCGGTAC TTGCAAAAAT CGCTCGTCAG   
  
  
- GTACTATGTC AAAGTCTTCC CCCACCTCCT CCGCTCGTTC AAGGAAGGTT TCTTGTTATT GGAACGCTAA   
  
  
- CTGGAACTCT CGCAGTGAAA GGGTTTACTT TTCCTCCGCC GGGGGTACCA CCACCAATTC TTTCTCTTCC   
  
  
- TACTTGTGAG TGGACTATCG AGCTCTCCAT CGTTCTAAGT GATGGCACTA CTACTCCTGA AACTCCTACC   
  
  
- ATCCTCCTCA TTCGTCAGTC GACAAAGACA CCTCCTCCGA CTCAACAGAC TTTACAAACT GGCCCAAAAC   
  
  
- GAAACGTTAG GGTACTTTGT ACTTCGAGTA ACATGTTCAC CAAACTTCAG GCTCTTCCCT TGTAGGGACG   
  
  
- TCCGACCGGT TCATGTCCAT GTCCTCCCAG TCTTCCGAGC ACGTTTCTTT GTCCCATTAT TACTATTATT   
  
  
- CTTCTTATTC TTACACCACC TAAATTCCTG AGACGAATAG GAGACACGCG TTAGACGACG TAGACTACTA   
  
  
- GCGTCCTGTC GACTACTTGA CGACTTCGTC TAATCCCTCG TGAGAAGACG TCGACCCCTA CCTAGAGTTT   
  
  
- CCAACCGAGT AATGAAACGA TTACGGAACC TCCGCGCAAA TCGACCTTGA CCGAGTGTCT AGATATGTCG   
  
  
- GGACTCAAGC TTCTCCTGTT GTCGTCGACT ATACTATTTT CGAATAGTCA AAAAGAAAGC ACGAACGGGT   
  
  
- AAAAATTTCT AGAGATAATA GAAACGCTTG GTATACTAAT ATGTCTCTCG ACTTTTTCGT AGTTTCGAAG   
  
  
- TACAATATCT AAAACCGTAG GACATACCAA AAGTCACCGG GGAGGAGTAA GTTGCGGACA GCCTCGCTGG   
  
  
- ACCACCTGGA GGTTTTGACA CGTAATGACC CTAACTAGAA GGGGTTGGAC CTAAATCCGG TTGTCTTTCT   
  
  
- CAACTCCGTT GTCCCGAGGC GAACCGTTTC ATAACACTCG CGAAGTTACA AGGTAAACTT ATGGTACGGT   
  
  
- AACGTGTCTT TACCCTTTGG TAGTTTCGAC TTCTGGACTT CTATCTTCTA CTACTCCACC AACGGCACTT   
  
  
- GACAGAGAAG TCCAAATTCT TGGACGAACT ACTCTGCTAT CACCACCTAT CAGGCTCCTT ACGTCAAGAG   
  
  
- CCGAACTAAT CTTCCTATTT TGGACTATAG AAACAAGTGC CTCATCACTT GCCCAGAATG TTACGCGGGA   
  
  
- AGAAACAATG TGCAAAGTCC CTCCGGGAGA AGGTAATGAG ATGACATAAG CTATACAAAC TACGGTTACG   
  
  
- AAGGGCCCTC CTAGGACTCT CTAACTACAA ACTCTTCCTC AAAATACCCG CTCTTTAATA CTTACACCAC   
  
  
- CGAACACTCC CATGTCTCTC TCAACTTTCT GGACTCTGTA TGTTCGTTAC CGTCCGCGCC TTATCATCCC   
  
  
- GTCCCAAGTC CGTCGACGGT AACCTGGTTC TCGAGCAGTC ATTTGACTCC TCGTTCCAGG TCTACGTGAT   
  
  
- AGGATCCCTG AAGCAACAAC TACATCTACC TGTAACCTGT AACGTCCCTA CCTTCCCCTC CTAGTAGACA   
  
  
- CGACACAGAC GTACCCCAGG ACGAAT

+     GT1-motif

| Site Name | Organism | Position | Strand | Matrix score. | sequence | function |
| --- | --- | --- | --- | --- | --- | --- |
| GT1-motif | Arabidopsis thaliana | 2858 | + | 6 | GGTTAA | light responsive element |
| GT1-motif | Arabidopsis thaliana | 1526 | + | 6 | GGTTAA | light responsive element |
| GT1-motif | Arabidopsis thaliana | 824 | + | 6 | GGTTAA | light responsive element |
| GT1-motif | Arabidopsis thaliana | 105 | + | 6 | GGTTAA | light responsive element |

>HU02G01569.1   
+ +Up\_Stream \_Len000AGCGTA GGAAGTGACG CCGCTATTGG AGGCGAAGAC GCTAGTACCA AGGCCAAAAC   
  
  
+ AAATGAGGAG ATAGATCAGT TAAACCGTTT GGTTAAGAAA ATTAAGCGAA CATCTCTTGA ACCTCCATCT   
  
  
+ GAACTCGATG ACATTGAGGA TGTGGATGCA GAAAAGATGG ATATGGGCTC TCCATCGGTG CCAGCAAATC   
  
  
+ AACCTAATGT TGGGTGGGGT CTCATTCGGT CAATGGGGGA CTAGGGACGT CATATCGTCA CCAATTACAA   
  
  
+ CGGAACAACC CTAAACCGCA TTTTAAAATC ACATCTTGGT AGCAGACAAA GATGTACGAC CCAAAAATTC   
  
  
+ AACTCTGTAA AGCACTAGCA AAGCTCGTAG TACACAGAAC AATCGCACAC TCATAGTGAT AGAAGTGTAC   
  
  
+ TATCCATTAT GCAGTGAATA TGACAAGGGA GGGGGTCCTG AGATGCCCAA ATTTGGCAGC CGAGTTGCCT   
  
  
+ TGACTGCCCA TCAATGTCCT AAACCAGTTG AGCATGTTAT TAATTTGCAA GAAAGATCGA ATTCTTCCTA   
  
  
+ TCTAAAAACT GACACAATAA AACAATACCA TGTATGGGAC ATTGCTAGGA TTGTTAACAC AATTATTGTC   
  
  
+ TTGTACGTTG CACCTGAAAA AAAAGAAAAT TGTTGCCATA GACAACATAT AAGTTAGGCA TTCATGACTA   
  
  
+ ATATCTGAAT TAGGTGTGTC GATCTGTGAT TCAAATTGAT ATAATTAGAT CTTTAAGGTA TCTAATTGCT   
  
  
+ TCAAGTTTTC TCTATAATGA AGATGTATAT TAGCTCACAT AGGTCTTAAG GTTAACTAAT GTCATATCAG   
  
  
+ GGGGTGTGGG CCGCCATTTT TGATAAGAAT TGAATTGAAC TTGCATTGTT TTTTCTTTGG CGAGAATGCA   
  
  
+ AGATTTGTTA ATTTATTCAT ATGCGTCATT ATACACTATA AGAAAAATAA TTGTAGGAAT ATATGAAGTA   
  
  
+ TTTTTACTTG TGCTCATGTT AAATGTGGAA TACTTTTTTA ATGTCGTATA GTACCACAGT ATTCTACAAT   
  
  
+ TTAAGAGATT AATCGATCCA TTAAAGTCAT GCAAACCTGA TGCATATTCA AGACATTGCA TGAAATGATC   
  
  
+ TTATTTTAGT CAAAGACTCC ATCATTTATA AGCAATATTT ACTTCGATAC ACGTGTCATT AAAAAAGACT   
  
  
+ ATAGAAATGT TACTGTTATT TTGTTTGAGC TAAATAGAAA ATTTAAAAGT TAATTAAGAT AATAATTAAA   
  
  
+ ACTAAATATT CAATGATGGC TAATAAATAC ATTGTCGTAT TAACTGACTA ATATTTGTAG ATGGTCGTGT   
  
  
+ TAATTTCTTT TTATTTTAAA GTTCAAATGA GTTACAAAAG GTTATAAAAT ATAAAGTAAA ATGGGAGACA   
  
  
+ AAGCAGACAG GGTGGGGGAA GCTAAGCTTA AAGTAGGATT TAGAAAGGCA ATGAAGGTGA ACCCTCAAAT   
  
  
+ CGATCCGCTG CCAATCACAG AAACCCAAAG CTTTGCTCAC CGACAACTCC CGGTTAACTG CAGTCACTCA   
  
  
+ CGGCACTGGG TAATACAGTA ATCGTGTACC CAGTTCATCC CCCTTTTCCT TTGGGAATAC AGTACCTCGA   
  
  
+ AAGTTCCTTT GAAATTCTAC TGGCCAGGCA TACCCATAAA TTGATCCTCT CGATATCATA AGATATGATA   
  
  
+ TTTCTCTGTC ATATCTGTTA GTTTCGGTAT CATATTTCTT CTTTATCTCT GTAATTTGAG GTATGCTTCA   
  
  
+ TTCCTGGCCG CTTGAATTAT CTTTCTTTCT TTGATTAGTA TTGTTTTTTT GGTTACCTAT TTTGCTCCAG   
  
  
+ TCCTGTTCAT TTGGGTCTTT TTGGATCTGG GTTTTTGCTG GGTTTGTTGA TTCTTTGAGA AATTTGGGTG   
  
  
+ CTTGAATTTG CCCTGCAATA TTCATGGGTT AGCGTTTTCC TGAATTTTGC TCTGTTCCTT AAGTGATTAT   
  
  
+ TTTTGATTGA TTACACCTTG GTGGTCCTGG TGGAATTTCC GAGGAGAAAT TGTCATGGGT TCCCACAACT   
  
  
+ TTGGAGAATT CCCTGATGAG ACTCTAAATG AGTATCAATC TACTTTGGGA ACCATGTCCC CCGGTTATGA   
  
  
+ TGGGTCTTTG AATTATACAA CCTTGTTCAA TTACAAAGAC CCATCTCAGG ATCTCACGGC ACTGAACCTT   
  
  
+ CCTAGCCCAT TGCCTGACCC TATGCCATTC AACATTGGTT CATATTCGGG TTTGAGCCCC GGGGTTGAAT   
  
  
+ CTTCGGATGA TAGCGATTCA GATGATGTTG TTAAGTACAT TGGCCAAGTG CTTATGGAAG AGGATATGAA   
  
  
+ GGAGAAGCCT TGTATGTTCC ATGACCCTTT AGCACTCCAA GCTGCTGAGA AACCCTTTTA TGATGTGTTG   
  
  
+ GGAAAGAAGT ATCCTCCTTC CCCTAATCAA CACCCACTTA TTGATCATTC TGTGGATAGC CCAGACAATC   
  
  
+ AGTCCTGTGG AACGAGCACA ATTAGTGATC TTAGTGGCAG TAACTGCACT TCCAGTTCAA CCAATTATAT   
  
  
+ TGATGCTGTG GCAGTCGCTG ATTCGAGTGA GAATACTAAG ACCTCTTTTG TGCAAAGTTC TCTGATTGAA   
  
  
+ TCGTTTTCTC AGTCGTCCAC CCTTCCACAG TGGTCATTTG GATCATTGGG TGCCTTGGGT GGCACAGCTT   
  
  
+ CTCAGGGTTC GAATTCAGTT ATCTCCTCCC GTGGTTTCCC TATGGCCATG AACGTTTTTA GCGAGCAGTC   
  
  
+ CATGATACAG TTTCAGAAGG GGGTGGAGGA GGCGAGCAAG TTCCTTCCAA AGAACAATAA CCTTGCGATT   
  
  
+ GACCTTGAGA GCGTCACTTT CCCAAATGAA AAGGAGGCGG CCCCCATGGT GGTGGTTAAG AAAGAGAAGG   
  
  
+ ATGAACACTC ACCTGATAGC TCGAGAGGTA GCAAGATTCA CTACCGTGAT GATGAGGACT TTGAGGATGG   
  
  
+ TAGGAGGAGT AAGCAGTCAG CTGTTTCTGT GGAGGAGGCT GAGTTGTCTG AAATGTTTGA CCGGGTTTTG   
  
  
+ CTTTGCAATC CCATGAAACA TGAAGCTCAT TGTACAAGTG GTTTGAAGTC CGAGAAGGGA ACATCCCTGC   
  
  
+ AGGCTGGCCA AGTACAGGTA CAGGAGGGTC AGAAGGCTCG TGCAAAGAAA CAGGGTAATA ATGATAATAA   
  
  
+ GAAGAATAAG AATGTGGTGG ATTTAAGGAC TCTGCTTATC CTCTGTGCGC AATCTGCTGC ATCTGATGAT   
  
  
+ CGCAGGACAG CTGATGAACT GCTGAAGCAG ATTAGGGAGC ACTCTTCTGC AGCTGGGGAT GGATCTCAAA   
  
  
+ GGTTGGCTCA TTACTTTGCT AATGCCTTGG AGGCGCGTTT AGCTGGAACT GGCTCACAGA TCTATACAGC   
  
  
+ CCTGAGTTCG AAGAGGACAA CAGCAGCTGA TATGATAAAA GCTTATCAGT TTTTCTTTCG TGCTTGCCCA   
  
  
+ TTTTTAAAGA TCTCTATTAT CTTTGCGAAC CATATGATTA TACAGAGAGC TGAAAAAGCA TCAAAGCTTC   
  
  
+ ATGTTATAGA TTTTGGCATC CTGTATGGTT TTCAGTGGCC CCTCCTCATT CAACGCCTGT CGGAGCGACC   
  
  
+ TGGTGGACCT CCAAAACTGT GCATTACTGG GATTGATCTT CCCCAACCTG GATTTAGGCC AACAGAAAGA   
  
  
+ GTTGAGGCAA CAGGGCTCCG CTTGGCAAAG TATTGTGAGC GCTTCAATGT TCCATTTGAA TACCATGCCA   
  
  
+ TTGCACAGAA ATGGGAAACC ATCAAAGCTG AAGACCTGAA GATAGAAGAT GATGAGGTGG TTGCCGTGAA   
  
  
+ CTGTCTCTTC AGGTTTAAGA ACCTGCTTGA TGAGACGATA GTGGTGGATA GTCCGAGGAA TGCAGTTCTC   
  
  
+ GGCTTGATTA GAAGGATAAA ACCTGATATC TTTGTTCACG GAGTAGTGAA CGGGTCTTAC AATGCGCCCT   
  
  
+ TCTTTGTTAC ACGTTTCAGG GAGGCCCTCT TCCATTACTC TACTGTATTC GATATGTTTG ATGCCAATGC   
  
  
+ TTCCCGGGAG GATCCTGAGA GATTGATGTT TGAGAAGGAG TTTTATGGGC GAGAAATTAT GAATGTGGTG   
  
  
+ GCTTGTGAGG GTACAGAGAG AGTTGAAAGA CCTGAGACAT ACAAGCAATG GCAGGCGCGG AATAGTAGGG   
  
  
+ CAGGGTTCAG GCAGCTGCCA TTGGACCAAG AGCTCGTCAG TAAACTGAGG AGCAAGGTCC AGATGCACTA   
  
  
+ TCCTAGGGAC TTCGTTGTTG ATGTAGATGG ACATTGGACA TTGCAGGGAT GGAAGGGGAG GATCATCTGT   
  
  
+ GCTGTGTCTG CATGGGGTCC TGCTTA  

- +Up\_Stream \_Len000TCGCAT CCTTCACTGC GGCGATAACC TCCGCTTCTG CGATCATGGT TCCGGTTTTG   
  
  
- TTTACTCCTC TATCTAGTCA ATTTGGCAAA CCAATTCTTT TAATTCGCTT GTAGAGAACT TGGAGGTAGA   
  
  
- CTTGAGCTAC TGTAACTCCT ACACCTACGT CTTTTCTACC TATACCCGAG AGGTAGCCAC GGTCGTTTAG   
  
  
- TTGGATTACA ACCCACCCCA GAGTAAGCCA GTTACCCCCT GATCCCTGCA GTATAGCAGT GGTTAATGTT   
  
  
- GCCTTGTTGG GATTTGGCGT AAAATTTTAG TGTAGAACCA TCGTCTGTTT CTACATGCTG GGTTTTTAAG   
  
  
- TTGAGACATT TCGTGATCGT TTCGAGCATC ATGTGTCTTG TTAGCGTGTG AGTATCACTA TCTTCACATG   
  
  
- ATAGGTAATA CGTCACTTAT ACTGTTCCCT CCCCCAGGAC TCTACGGGTT TAAACCGTCG GCTCAACGGA   
  
  
- ACTGACGGGT AGTTACAGGA TTTGGTCAAC TCGTACAATA ATTAAACGTT CTTTCTAGCT TAAGAAGGAT   
  
  
- AGATTTTTGA CTGTGTTATT TTGTTATGGT ACATACCCTG TAACGATCCT AACAATTGTG TTAATAACAG   
  
  
- AACATGCAAC GTGGACTTTT TTTTCTTTTA ACAACGGTAT CTGTTGTATA TTCAATCCGT AAGTACTGAT   
  
  
- TATAGACTTA ATCCACACAG CTAGACACTA AGTTTAACTA TATTAATCTA GAAATTCCAT AGATTAACGA   
  
  
- AGTTCAAAAG AGATATTACT TCTACATATA ATCGAGTGTA TCCAGAATTC CAATTGATTA CAGTATAGTC   
  
  
- CCCCACACCC GGCGGTAAAA ACTATTCTTA ACTTAACTTG AACGTAACAA AAAAGAAACC GCTCTTACGT   
  
  
- TCTAAACAAT TAAATAAGTA TACGCAGTAA TATGTGATAT TCTTTTTATT AACATCCTTA TATACTTCAT   
  
  
- AAAAATGAAC ACGAGTACAA TTTACACCTT ATGAAAAAAT TACAGCATAT CATGGTGTCA TAAGATGTTA   
  
  
- AATTCTCTAA TTAGCTAGGT AATTTCAGTA CGTTTGGACT ACGTATAAGT TCTGTAACGT ACTTTACTAG   
  
  
- AATAAAATCA GTTTCTGAGG TAGTAAATAT TCGTTATAAA TGAAGCTATG TGCACAGTAA TTTTTTCTGA   
  
  
- TATCTTTACA ATGACAATAA AACAAACTCG ATTTATCTTT TAAATTTTCA ATTAATTCTA TTATTAATTT   
  
  
- TGATTTATAA GTTACTACCG ATTATTTATG TAACAGCATA ATTGACTGAT TATAAACATC TACCAGCACA   
  
  
- ATTAAAGAAA AATAAAATTT CAAGTTTACT CAATGTTTTC CAATATTTTA TATTTCATTT TACCCTCTGT   
  
  
- TTCGTCTGTC CCACCCCCTT CGATTCGAAT TTCATCCTAA ATCTTTCCGT TACTTCCACT TGGGAGTTTA   
  
  
- GCTAGGCGAC GGTTAGTGTC TTTGGGTTTC GAAACGAGTG GCTGTTGAGG GCCAATTGAC GTCAGTGAGT   
  
  
- GCCGTGACCC ATTATGTCAT TAGCACATGG GTCAAGTAGG GGGAAAAGGA AACCCTTATG TCATGGAGCT   
  
  
- TTCAAGGAAA CTTTAAGATG ACCGGTCCGT ATGGGTATTT AACTAGGAGA GCTATAGTAT TCTATACTAT   
  
  
- AAAGAGACAG TATAGACAAT CAAAGCCATA GTATAAAGAA GAAATAGAGA CATTAAACTC CATACGAAGT   
  
  
- AAGGACCGGC GAACTTAATA GAAAGAAAGA AACTAATCAT AACAAAAAAA CCAATGGATA AAACGAGGTC   
  
  
- AGGACAAGTA AACCCAGAAA AACCTAGACC CAAAAACGAC CCAAACAACT AAGAAACTCT TTAAACCCAC   
  
  
- GAACTTAAAC GGGACGTTAT AAGTACCCAA TCGCAAAAGG ACTTAAAACG AGACAAGGAA TTCACTAATA   
  
  
- AAAACTAACT AATGTGGAAC CACCAGGACC ACCTTAAAGG CTCCTCTTTA ACAGTACCCA AGGGTGTTGA   
  
  
- AACCTCTTAA GGGACTACTC TGAGATTTAC TCATAGTTAG ATGAAACCCT TGGTACAGGG GGCCAATACT   
  
  
- ACCCAGAAAC TTAATATGTT GGAACAAGTT AATGTTTCTG GGTAGAGTCC TAGAGTGCCG TGACTTGGAA   
  
  
- GGATCGGGTA ACGGACTGGG ATACGGTAAG TTGTAACCAA GTATAAGCCC AAACTCGGGG CCCCAACTTA   
  
  
- GAAGCCTACT ATCGCTAAGT CTACTACAAC AATTCATGTA ACCGGTTCAC GAATACCTTC TCCTATACTT   
  
  
- CCTCTTCGGA ACATACAAGG TACTGGGAAA TCGTGAGGTT CGACGACTCT TTGGGAAAAT ACTACACAAC   
  
  
- CCTTTCTTCA TAGGAGGAAG GGGATTAGTT GTGGGTGAAT AACTAGTAAG ACACCTATCG GGTCTGTTAG   
  
  
- TCAGGACACC TTGCTCGTGT TAATCACTAG AATCACCGTC ATTGACGTGA AGGTCAAGTT GGTTAATATA   
  
  
- ACTACGACAC CGTCAGCGAC TAAGCTCACT CTTATGATTC TGGAGAAAAC ACGTTTCAAG AGACTAACTT   
  
  
- AGCAAAAGAG TCAGCAGGTG GGAAGGTGTC ACCAGTAAAC CTAGTAACCC ACGGAACCCA CCGTGTCGAA   
  
  
- GAGTCCCAAG CTTAAGTCAA TAGAGGAGGG CACCAAAGGG ATACCGGTAC TTGCAAAAAT CGCTCGTCAG   
  
  
- GTACTATGTC AAAGTCTTCC CCCACCTCCT CCGCTCGTTC AAGGAAGGTT TCTTGTTATT GGAACGCTAA   
  
  
- CTGGAACTCT CGCAGTGAAA GGGTTTACTT TTCCTCCGCC GGGGGTACCA CCACCAATTC TTTCTCTTCC   
  
  
- TACTTGTGAG TGGACTATCG AGCTCTCCAT CGTTCTAAGT GATGGCACTA CTACTCCTGA AACTCCTACC   
  
  
- ATCCTCCTCA TTCGTCAGTC GACAAAGACA CCTCCTCCGA CTCAACAGAC TTTACAAACT GGCCCAAAAC   
  
  
- GAAACGTTAG GGTACTTTGT ACTTCGAGTA ACATGTTCAC CAAACTTCAG GCTCTTCCCT TGTAGGGACG   
  
  
- TCCGACCGGT TCATGTCCAT GTCCTCCCAG TCTTCCGAGC ACGTTTCTTT GTCCCATTAT TACTATTATT   
  
  
- CTTCTTATTC TTACACCACC TAAATTCCTG AGACGAATAG GAGACACGCG TTAGACGACG TAGACTACTA   
  
  
- GCGTCCTGTC GACTACTTGA CGACTTCGTC TAATCCCTCG TGAGAAGACG TCGACCCCTA CCTAGAGTTT   
  
  
- CCAACCGAGT AATGAAACGA TTACGGAACC TCCGCGCAAA TCGACCTTGA CCGAGTGTCT AGATATGTCG   
  
  
- GGACTCAAGC TTCTCCTGTT GTCGTCGACT ATACTATTTT CGAATAGTCA AAAAGAAAGC ACGAACGGGT   
  
  
- AAAAATTTCT AGAGATAATA GAAACGCTTG GTATACTAAT ATGTCTCTCG ACTTTTTCGT AGTTTCGAAG   
  
  
- TACAATATCT AAAACCGTAG GACATACCAA AAGTCACCGG GGAGGAGTAA GTTGCGGACA GCCTCGCTGG   
  
  
- ACCACCTGGA GGTTTTGACA CGTAATGACC CTAACTAGAA GGGGTTGGAC CTAAATCCGG TTGTCTTTCT   
  
  
- CAACTCCGTT GTCCCGAGGC GAACCGTTTC ATAACACTCG CGAAGTTACA AGGTAAACTT ATGGTACGGT   
  
  
- AACGTGTCTT TACCCTTTGG TAGTTTCGAC TTCTGGACTT CTATCTTCTA CTACTCCACC AACGGCACTT   
  
  
- GACAGAGAAG TCCAAATTCT TGGACGAACT ACTCTGCTAT CACCACCTAT CAGGCTCCTT ACGTCAAGAG   
  
  
- CCGAACTAAT CTTCCTATTT TGGACTATAG AAACAAGTGC CTCATCACTT GCCCAGAATG TTACGCGGGA   
  
  
- AGAAACAATG TGCAAAGTCC CTCCGGGAGA AGGTAATGAG ATGACATAAG CTATACAAAC TACGGTTACG   
  
  
- AAGGGCCCTC CTAGGACTCT CTAACTACAA ACTCTTCCTC AAAATACCCG CTCTTTAATA CTTACACCAC   
  
  
- CGAACACTCC CATGTCTCTC TCAACTTTCT GGACTCTGTA TGTTCGTTAC CGTCCGCGCC TTATCATCCC   
  
  
- GTCCCAAGTC CGTCGACGGT AACCTGGTTC TCGAGCAGTC ATTTGACTCC TCGTTCCAGG TCTACGTGAT   
  
  
- AGGATCCCTG AAGCAACAAC TACATCTACC TGTAACCTGT AACGTCCCTA CCTTCCCCTC CTAGTAGACA   
  
  
- CGACACAGAC GTACCCCAGG ACGAAT

+     GTGGC-motif

| Site Name | Organism | Position | Strand | Matrix score. | sequence | function |
| --- | --- | --- | --- | --- | --- | --- |
| GTGGC-motif | Spinacia oleracea | 2526 | + | 10 | GATTCTGTGGC | part of a light responsive element |

>HU02G01569.1   
+ +Up\_Stream \_Len000AGCGTA GGAAGTGACG CCGCTATTGG AGGCGAAGAC GCTAGTACCA AGGCCAAAAC   
  
  
+ AAATGAGGAG ATAGATCAGT TAAACCGTTT GGTTAAGAAA ATTAAGCGAA CATCTCTTGA ACCTCCATCT   
  
  
+ GAACTCGATG ACATTGAGGA TGTGGATGCA GAAAAGATGG ATATGGGCTC TCCATCGGTG CCAGCAAATC   
  
  
+ AACCTAATGT TGGGTGGGGT CTCATTCGGT CAATGGGGGA CTAGGGACGT CATATCGTCA CCAATTACAA   
  
  
+ CGGAACAACC CTAAACCGCA TTTTAAAATC ACATCTTGGT AGCAGACAAA GATGTACGAC CCAAAAATTC   
  
  
+ AACTCTGTAA AGCACTAGCA AAGCTCGTAG TACACAGAAC AATCGCACAC TCATAGTGAT AGAAGTGTAC   
  
  
+ TATCCATTAT GCAGTGAATA TGACAAGGGA GGGGGTCCTG AGATGCCCAA ATTTGGCAGC CGAGTTGCCT   
  
  
+ TGACTGCCCA TCAATGTCCT AAACCAGTTG AGCATGTTAT TAATTTGCAA GAAAGATCGA ATTCTTCCTA   
  
  
+ TCTAAAAACT GACACAATAA AACAATACCA TGTATGGGAC ATTGCTAGGA TTGTTAACAC AATTATTGTC   
  
  
+ TTGTACGTTG CACCTGAAAA AAAAGAAAAT TGTTGCCATA GACAACATAT AAGTTAGGCA TTCATGACTA   
  
  
+ ATATCTGAAT TAGGTGTGTC GATCTGTGAT TCAAATTGAT ATAATTAGAT CTTTAAGGTA TCTAATTGCT   
  
  
+ TCAAGTTTTC TCTATAATGA AGATGTATAT TAGCTCACAT AGGTCTTAAG GTTAACTAAT GTCATATCAG   
  
  
+ GGGGTGTGGG CCGCCATTTT TGATAAGAAT TGAATTGAAC TTGCATTGTT TTTTCTTTGG CGAGAATGCA   
  
  
+ AGATTTGTTA ATTTATTCAT ATGCGTCATT ATACACTATA AGAAAAATAA TTGTAGGAAT ATATGAAGTA   
  
  
+ TTTTTACTTG TGCTCATGTT AAATGTGGAA TACTTTTTTA ATGTCGTATA GTACCACAGT ATTCTACAAT   
  
  
+ TTAAGAGATT AATCGATCCA TTAAAGTCAT GCAAACCTGA TGCATATTCA AGACATTGCA TGAAATGATC   
  
  
+ TTATTTTAGT CAAAGACTCC ATCATTTATA AGCAATATTT ACTTCGATAC ACGTGTCATT AAAAAAGACT   
  
  
+ ATAGAAATGT TACTGTTATT TTGTTTGAGC TAAATAGAAA ATTTAAAAGT TAATTAAGAT AATAATTAAA   
  
  
+ ACTAAATATT CAATGATGGC TAATAAATAC ATTGTCGTAT TAACTGACTA ATATTTGTAG ATGGTCGTGT   
  
  
+ TAATTTCTTT TTATTTTAAA GTTCAAATGA GTTACAAAAG GTTATAAAAT ATAAAGTAAA ATGGGAGACA   
  
  
+ AAGCAGACAG GGTGGGGGAA GCTAAGCTTA AAGTAGGATT TAGAAAGGCA ATGAAGGTGA ACCCTCAAAT   
  
  
+ CGATCCGCTG CCAATCACAG AAACCCAAAG CTTTGCTCAC CGACAACTCC CGGTTAACTG CAGTCACTCA   
  
  
+ CGGCACTGGG TAATACAGTA ATCGTGTACC CAGTTCATCC CCCTTTTCCT TTGGGAATAC AGTACCTCGA   
  
  
+ AAGTTCCTTT GAAATTCTAC TGGCCAGGCA TACCCATAAA TTGATCCTCT CGATATCATA AGATATGATA   
  
  
+ TTTCTCTGTC ATATCTGTTA GTTTCGGTAT CATATTTCTT CTTTATCTCT GTAATTTGAG GTATGCTTCA   
  
  
+ TTCCTGGCCG CTTGAATTAT CTTTCTTTCT TTGATTAGTA TTGTTTTTTT GGTTACCTAT TTTGCTCCAG   
  
  
+ TCCTGTTCAT TTGGGTCTTT TTGGATCTGG GTTTTTGCTG GGTTTGTTGA TTCTTTGAGA AATTTGGGTG   
  
  
+ CTTGAATTTG CCCTGCAATA TTCATGGGTT AGCGTTTTCC TGAATTTTGC TCTGTTCCTT AAGTGATTAT   
  
  
+ TTTTGATTGA TTACACCTTG GTGGTCCTGG TGGAATTTCC GAGGAGAAAT TGTCATGGGT TCCCACAACT   
  
  
+ TTGGAGAATT CCCTGATGAG ACTCTAAATG AGTATCAATC TACTTTGGGA ACCATGTCCC CCGGTTATGA   
  
  
+ TGGGTCTTTG AATTATACAA CCTTGTTCAA TTACAAAGAC CCATCTCAGG ATCTCACGGC ACTGAACCTT   
  
  
+ CCTAGCCCAT TGCCTGACCC TATGCCATTC AACATTGGTT CATATTCGGG TTTGAGCCCC GGGGTTGAAT   
  
  
+ CTTCGGATGA TAGCGATTCA GATGATGTTG TTAAGTACAT TGGCCAAGTG CTTATGGAAG AGGATATGAA   
  
  
+ GGAGAAGCCT TGTATGTTCC ATGACCCTTT AGCACTCCAA GCTGCTGAGA AACCCTTTTA TGATGTGTTG   
  
  
+ GGAAAGAAGT ATCCTCCTTC CCCTAATCAA CACCCACTTA TTGATCATTC TGTGGATAGC CCAGACAATC   
  
  
+ AGTCCTGTGG AACGAGCACA ATTAGTGATC TTAGTGGCAG TAACTGCACT TCCAGTTCAA CCAATTATAT   
  
  
+ TGATGCTGTG GCAGTCGCTG ATTCGAGTGA GAATACTAAG ACCTCTTTTG TGCAAAGTTC TCTGATTGAA   
  
  
+ TCGTTTTCTC AGTCGTCCAC CCTTCCACAG TGGTCATTTG GATCATTGGG TGCCTTGGGT GGCACAGCTT   
  
  
+ CTCAGGGTTC GAATTCAGTT ATCTCCTCCC GTGGTTTCCC TATGGCCATG AACGTTTTTA GCGAGCAGTC   
  
  
+ CATGATACAG TTTCAGAAGG GGGTGGAGGA GGCGAGCAAG TTCCTTCCAA AGAACAATAA CCTTGCGATT   
  
  
+ GACCTTGAGA GCGTCACTTT CCCAAATGAA AAGGAGGCGG CCCCCATGGT GGTGGTTAAG AAAGAGAAGG   
  
  
+ ATGAACACTC ACCTGATAGC TCGAGAGGTA GCAAGATTCA CTACCGTGAT GATGAGGACT TTGAGGATGG   
  
  
+ TAGGAGGAGT AAGCAGTCAG CTGTTTCTGT GGAGGAGGCT GAGTTGTCTG AAATGTTTGA CCGGGTTTTG   
  
  
+ CTTTGCAATC CCATGAAACA TGAAGCTCAT TGTACAAGTG GTTTGAAGTC CGAGAAGGGA ACATCCCTGC   
  
  
+ AGGCTGGCCA AGTACAGGTA CAGGAGGGTC AGAAGGCTCG TGCAAAGAAA CAGGGTAATA ATGATAATAA   
  
  
+ GAAGAATAAG AATGTGGTGG ATTTAAGGAC TCTGCTTATC CTCTGTGCGC AATCTGCTGC ATCTGATGAT   
  
  
+ CGCAGGACAG CTGATGAACT GCTGAAGCAG ATTAGGGAGC ACTCTTCTGC AGCTGGGGAT GGATCTCAAA   
  
  
+ GGTTGGCTCA TTACTTTGCT AATGCCTTGG AGGCGCGTTT AGCTGGAACT GGCTCACAGA TCTATACAGC   
  
  
+ CCTGAGTTCG AAGAGGACAA CAGCAGCTGA TATGATAAAA GCTTATCAGT TTTTCTTTCG TGCTTGCCCA   
  
  
+ TTTTTAAAGA TCTCTATTAT CTTTGCGAAC CATATGATTA TACAGAGAGC TGAAAAAGCA TCAAAGCTTC   
  
  
+ ATGTTATAGA TTTTGGCATC CTGTATGGTT TTCAGTGGCC CCTCCTCATT CAACGCCTGT CGGAGCGACC   
  
  
+ TGGTGGACCT CCAAAACTGT GCATTACTGG GATTGATCTT CCCCAACCTG GATTTAGGCC AACAGAAAGA   
  
  
+ GTTGAGGCAA CAGGGCTCCG CTTGGCAAAG TATTGTGAGC GCTTCAATGT TCCATTTGAA TACCATGCCA   
  
  
+ TTGCACAGAA ATGGGAAACC ATCAAAGCTG AAGACCTGAA GATAGAAGAT GATGAGGTGG TTGCCGTGAA   
  
  
+ CTGTCTCTTC AGGTTTAAGA ACCTGCTTGA TGAGACGATA GTGGTGGATA GTCCGAGGAA TGCAGTTCTC   
  
  
+ GGCTTGATTA GAAGGATAAA ACCTGATATC TTTGTTCACG GAGTAGTGAA CGGGTCTTAC AATGCGCCCT   
  
  
+ TCTTTGTTAC ACGTTTCAGG GAGGCCCTCT TCCATTACTC TACTGTATTC GATATGTTTG ATGCCAATGC   
  
  
+ TTCCCGGGAG GATCCTGAGA GATTGATGTT TGAGAAGGAG TTTTATGGGC GAGAAATTAT GAATGTGGTG   
  
  
+ GCTTGTGAGG GTACAGAGAG AGTTGAAAGA CCTGAGACAT ACAAGCAATG GCAGGCGCGG AATAGTAGGG   
  
  
+ CAGGGTTCAG GCAGCTGCCA TTGGACCAAG AGCTCGTCAG TAAACTGAGG AGCAAGGTCC AGATGCACTA   
  
  
+ TCCTAGGGAC TTCGTTGTTG ATGTAGATGG ACATTGGACA TTGCAGGGAT GGAAGGGGAG GATCATCTGT   
  
  
+ GCTGTGTCTG CATGGGGTCC TGCTTA  

- +Up\_Stream \_Len000TCGCAT CCTTCACTGC GGCGATAACC TCCGCTTCTG CGATCATGGT TCCGGTTTTG   
  
  
- TTTACTCCTC TATCTAGTCA ATTTGGCAAA CCAATTCTTT TAATTCGCTT GTAGAGAACT TGGAGGTAGA   
  
  
- CTTGAGCTAC TGTAACTCCT ACACCTACGT CTTTTCTACC TATACCCGAG AGGTAGCCAC GGTCGTTTAG   
  
  
- TTGGATTACA ACCCACCCCA GAGTAAGCCA GTTACCCCCT GATCCCTGCA GTATAGCAGT GGTTAATGTT   
  
  
- GCCTTGTTGG GATTTGGCGT AAAATTTTAG TGTAGAACCA TCGTCTGTTT CTACATGCTG GGTTTTTAAG   
  
  
- TTGAGACATT TCGTGATCGT TTCGAGCATC ATGTGTCTTG TTAGCGTGTG AGTATCACTA TCTTCACATG   
  
  
- ATAGGTAATA CGTCACTTAT ACTGTTCCCT CCCCCAGGAC TCTACGGGTT TAAACCGTCG GCTCAACGGA   
  
  
- ACTGACGGGT AGTTACAGGA TTTGGTCAAC TCGTACAATA ATTAAACGTT CTTTCTAGCT TAAGAAGGAT   
  
  
- AGATTTTTGA CTGTGTTATT TTGTTATGGT ACATACCCTG TAACGATCCT AACAATTGTG TTAATAACAG   
  
  
- AACATGCAAC GTGGACTTTT TTTTCTTTTA ACAACGGTAT CTGTTGTATA TTCAATCCGT AAGTACTGAT   
  
  
- TATAGACTTA ATCCACACAG CTAGACACTA AGTTTAACTA TATTAATCTA GAAATTCCAT AGATTAACGA   
  
  
- AGTTCAAAAG AGATATTACT TCTACATATA ATCGAGTGTA TCCAGAATTC CAATTGATTA CAGTATAGTC   
  
  
- CCCCACACCC GGCGGTAAAA ACTATTCTTA ACTTAACTTG AACGTAACAA AAAAGAAACC GCTCTTACGT   
  
  
- TCTAAACAAT TAAATAAGTA TACGCAGTAA TATGTGATAT TCTTTTTATT AACATCCTTA TATACTTCAT   
  
  
- AAAAATGAAC ACGAGTACAA TTTACACCTT ATGAAAAAAT TACAGCATAT CATGGTGTCA TAAGATGTTA   
  
  
- AATTCTCTAA TTAGCTAGGT AATTTCAGTA CGTTTGGACT ACGTATAAGT TCTGTAACGT ACTTTACTAG   
  
  
- AATAAAATCA GTTTCTGAGG TAGTAAATAT TCGTTATAAA TGAAGCTATG TGCACAGTAA TTTTTTCTGA   
  
  
- TATCTTTACA ATGACAATAA AACAAACTCG ATTTATCTTT TAAATTTTCA ATTAATTCTA TTATTAATTT   
  
  
- TGATTTATAA GTTACTACCG ATTATTTATG TAACAGCATA ATTGACTGAT TATAAACATC TACCAGCACA   
  
  
- ATTAAAGAAA AATAAAATTT CAAGTTTACT CAATGTTTTC CAATATTTTA TATTTCATTT TACCCTCTGT   
  
  
- TTCGTCTGTC CCACCCCCTT CGATTCGAAT TTCATCCTAA ATCTTTCCGT TACTTCCACT TGGGAGTTTA   
  
  
- GCTAGGCGAC GGTTAGTGTC TTTGGGTTTC GAAACGAGTG GCTGTTGAGG GCCAATTGAC GTCAGTGAGT   
  
  
- GCCGTGACCC ATTATGTCAT TAGCACATGG GTCAAGTAGG GGGAAAAGGA AACCCTTATG TCATGGAGCT   
  
  
- TTCAAGGAAA CTTTAAGATG ACCGGTCCGT ATGGGTATTT AACTAGGAGA GCTATAGTAT TCTATACTAT   
  
  
- AAAGAGACAG TATAGACAAT CAAAGCCATA GTATAAAGAA GAAATAGAGA CATTAAACTC CATACGAAGT   
  
  
- AAGGACCGGC GAACTTAATA GAAAGAAAGA AACTAATCAT AACAAAAAAA CCAATGGATA AAACGAGGTC   
  
  
- AGGACAAGTA AACCCAGAAA AACCTAGACC CAAAAACGAC CCAAACAACT AAGAAACTCT TTAAACCCAC   
  
  
- GAACTTAAAC GGGACGTTAT AAGTACCCAA TCGCAAAAGG ACTTAAAACG AGACAAGGAA TTCACTAATA   
  
  
- AAAACTAACT AATGTGGAAC CACCAGGACC ACCTTAAAGG CTCCTCTTTA ACAGTACCCA AGGGTGTTGA   
  
  
- AACCTCTTAA GGGACTACTC TGAGATTTAC TCATAGTTAG ATGAAACCCT TGGTACAGGG GGCCAATACT   
  
  
- ACCCAGAAAC TTAATATGTT GGAACAAGTT AATGTTTCTG GGTAGAGTCC TAGAGTGCCG TGACTTGGAA   
  
  
- GGATCGGGTA ACGGACTGGG ATACGGTAAG TTGTAACCAA GTATAAGCCC AAACTCGGGG CCCCAACTTA   
  
  
- GAAGCCTACT ATCGCTAAGT CTACTACAAC AATTCATGTA ACCGGTTCAC GAATACCTTC TCCTATACTT   
  
  
- CCTCTTCGGA ACATACAAGG TACTGGGAAA TCGTGAGGTT CGACGACTCT TTGGGAAAAT ACTACACAAC   
  
  
- CCTTTCTTCA TAGGAGGAAG GGGATTAGTT GTGGGTGAAT AACTAGTAAG ACACCTATCG GGTCTGTTAG   
  
  
- TCAGGACACC TTGCTCGTGT TAATCACTAG AATCACCGTC ATTGACGTGA AGGTCAAGTT GGTTAATATA   
  
  
- ACTACGACAC CGTCAGCGAC TAAGCTCACT CTTATGATTC TGGAGAAAAC ACGTTTCAAG AGACTAACTT   
  
  
- AGCAAAAGAG TCAGCAGGTG GGAAGGTGTC ACCAGTAAAC CTAGTAACCC ACGGAACCCA CCGTGTCGAA   
  
  
- GAGTCCCAAG CTTAAGTCAA TAGAGGAGGG CACCAAAGGG ATACCGGTAC TTGCAAAAAT CGCTCGTCAG   
  
  
- GTACTATGTC AAAGTCTTCC CCCACCTCCT CCGCTCGTTC AAGGAAGGTT TCTTGTTATT GGAACGCTAA   
  
  
- CTGGAACTCT CGCAGTGAAA GGGTTTACTT TTCCTCCGCC GGGGGTACCA CCACCAATTC TTTCTCTTCC   
  
  
- TACTTGTGAG TGGACTATCG AGCTCTCCAT CGTTCTAAGT GATGGCACTA CTACTCCTGA AACTCCTACC   
  
  
- ATCCTCCTCA TTCGTCAGTC GACAAAGACA CCTCCTCCGA CTCAACAGAC TTTACAAACT GGCCCAAAAC   
  
  
- GAAACGTTAG GGTACTTTGT ACTTCGAGTA ACATGTTCAC CAAACTTCAG GCTCTTCCCT TGTAGGGACG   
  
  
- TCCGACCGGT TCATGTCCAT GTCCTCCCAG TCTTCCGAGC ACGTTTCTTT GTCCCATTAT TACTATTATT   
  
  
- CTTCTTATTC TTACACCACC TAAATTCCTG AGACGAATAG GAGACACGCG TTAGACGACG TAGACTACTA   
  
  
- GCGTCCTGTC GACTACTTGA CGACTTCGTC TAATCCCTCG TGAGAAGACG TCGACCCCTA CCTAGAGTTT   
  
  
- CCAACCGAGT AATGAAACGA TTACGGAACC TCCGCGCAAA TCGACCTTGA CCGAGTGTCT AGATATGTCG   
  
  
- GGACTCAAGC TTCTCCTGTT GTCGTCGACT ATACTATTTT CGAATAGTCA AAAAGAAAGC ACGAACGGGT   
  
  
- AAAAATTTCT AGAGATAATA GAAACGCTTG GTATACTAAT ATGTCTCTCG ACTTTTTCGT AGTTTCGAAG   
  
  
- TACAATATCT AAAACCGTAG GACATACCAA AAGTCACCGG GGAGGAGTAA GTTGCGGACA GCCTCGCTGG   
  
  
- ACCACCTGGA GGTTTTGACA CGTAATGACC CTAACTAGAA GGGGTTGGAC CTAAATCCGG TTGTCTTTCT   
  
  
- CAACTCCGTT GTCCCGAGGC GAACCGTTTC ATAACACTCG CGAAGTTACA AGGTAAACTT ATGGTACGGT   
  
  
- AACGTGTCTT TACCCTTTGG TAGTTTCGAC TTCTGGACTT CTATCTTCTA CTACTCCACC AACGGCACTT   
  
  
- GACAGAGAAG TCCAAATTCT TGGACGAACT ACTCTGCTAT CACCACCTAT CAGGCTCCTT ACGTCAAGAG   
  
  
- CCGAACTAAT CTTCCTATTT TGGACTATAG AAACAAGTGC CTCATCACTT GCCCAGAATG TTACGCGGGA   
  
  
- AGAAACAATG TGCAAAGTCC CTCCGGGAGA AGGTAATGAG ATGACATAAG CTATACAAAC TACGGTTACG   
  
  
- AAGGGCCCTC CTAGGACTCT CTAACTACAA ACTCTTCCTC AAAATACCCG CTCTTTAATA CTTACACCAC   
  
  
- CGAACACTCC CATGTCTCTC TCAACTTTCT GGACTCTGTA TGTTCGTTAC CGTCCGCGCC TTATCATCCC   
  
  
- GTCCCAAGTC CGTCGACGGT AACCTGGTTC TCGAGCAGTC ATTTGACTCC TCGTTCCAGG TCTACGTGAT   
  
  
- AGGATCCCTG AAGCAACAAC TACATCTACC TGTAACCTGT AACGTCCCTA CCTTCCCCTC CTAGTAGACA   
  
  
- CGACACAGAC GTACCCCAGG ACGAAT

+     Gap-box

| Site Name | Organism | Position | Strand | Matrix score. | sequence | function |
| --- | --- | --- | --- | --- | --- | --- |
| Gap-box | Arabidopsis thaliana | 1828 | - | 9 | CAAATGAA(A/G)A | part of a light responsive element |
| Gap-box | Arabidopsis thaliana | 2827 | + | 9.5 | CAAATGAA(A/G)A | part of a light responsive element |

>HU02G01569.1   
+ +Up\_Stream \_Len000AGCGTA GGAAGTGACG CCGCTATTGG AGGCGAAGAC GCTAGTACCA AGGCCAAAAC   
  
  
+ AAATGAGGAG ATAGATCAGT TAAACCGTTT GGTTAAGAAA ATTAAGCGAA CATCTCTTGA ACCTCCATCT   
  
  
+ GAACTCGATG ACATTGAGGA TGTGGATGCA GAAAAGATGG ATATGGGCTC TCCATCGGTG CCAGCAAATC   
  
  
+ AACCTAATGT TGGGTGGGGT CTCATTCGGT CAATGGGGGA CTAGGGACGT CATATCGTCA CCAATTACAA   
  
  
+ CGGAACAACC CTAAACCGCA TTTTAAAATC ACATCTTGGT AGCAGACAAA GATGTACGAC CCAAAAATTC   
  
  
+ AACTCTGTAA AGCACTAGCA AAGCTCGTAG TACACAGAAC AATCGCACAC TCATAGTGAT AGAAGTGTAC   
  
  
+ TATCCATTAT GCAGTGAATA TGACAAGGGA GGGGGTCCTG AGATGCCCAA ATTTGGCAGC CGAGTTGCCT   
  
  
+ TGACTGCCCA TCAATGTCCT AAACCAGTTG AGCATGTTAT TAATTTGCAA GAAAGATCGA ATTCTTCCTA   
  
  
+ TCTAAAAACT GACACAATAA AACAATACCA TGTATGGGAC ATTGCTAGGA TTGTTAACAC AATTATTGTC   
  
  
+ TTGTACGTTG CACCTGAAAA AAAAGAAAAT TGTTGCCATA GACAACATAT AAGTTAGGCA TTCATGACTA   
  
  
+ ATATCTGAAT TAGGTGTGTC GATCTGTGAT TCAAATTGAT ATAATTAGAT CTTTAAGGTA TCTAATTGCT   
  
  
+ TCAAGTTTTC TCTATAATGA AGATGTATAT TAGCTCACAT AGGTCTTAAG GTTAACTAAT GTCATATCAG   
  
  
+ GGGGTGTGGG CCGCCATTTT TGATAAGAAT TGAATTGAAC TTGCATTGTT TTTTCTTTGG CGAGAATGCA   
  
  
+ AGATTTGTTA ATTTATTCAT ATGCGTCATT ATACACTATA AGAAAAATAA TTGTAGGAAT ATATGAAGTA   
  
  
+ TTTTTACTTG TGCTCATGTT AAATGTGGAA TACTTTTTTA ATGTCGTATA GTACCACAGT ATTCTACAAT   
  
  
+ TTAAGAGATT AATCGATCCA TTAAAGTCAT GCAAACCTGA TGCATATTCA AGACATTGCA TGAAATGATC   
  
  
+ TTATTTTAGT CAAAGACTCC ATCATTTATA AGCAATATTT ACTTCGATAC ACGTGTCATT AAAAAAGACT   
  
  
+ ATAGAAATGT TACTGTTATT TTGTTTGAGC TAAATAGAAA ATTTAAAAGT TAATTAAGAT AATAATTAAA   
  
  
+ ACTAAATATT CAATGATGGC TAATAAATAC ATTGTCGTAT TAACTGACTA ATATTTGTAG ATGGTCGTGT   
  
  
+ TAATTTCTTT TTATTTTAAA GTTCAAATGA GTTACAAAAG GTTATAAAAT ATAAAGTAAA ATGGGAGACA   
  
  
+ AAGCAGACAG GGTGGGGGAA GCTAAGCTTA AAGTAGGATT TAGAAAGGCA ATGAAGGTGA ACCCTCAAAT   
  
  
+ CGATCCGCTG CCAATCACAG AAACCCAAAG CTTTGCTCAC CGACAACTCC CGGTTAACTG CAGTCACTCA   
  
  
+ CGGCACTGGG TAATACAGTA ATCGTGTACC CAGTTCATCC CCCTTTTCCT TTGGGAATAC AGTACCTCGA   
  
  
+ AAGTTCCTTT GAAATTCTAC TGGCCAGGCA TACCCATAAA TTGATCCTCT CGATATCATA AGATATGATA   
  
  
+ TTTCTCTGTC ATATCTGTTA GTTTCGGTAT CATATTTCTT CTTTATCTCT GTAATTTGAG GTATGCTTCA   
  
  
+ TTCCTGGCCG CTTGAATTAT CTTTCTTTCT TTGATTAGTA TTGTTTTTTT GGTTACCTAT TTTGCTCCAG   
  
  
+ TCCTGTTCAT TTGGGTCTTT TTGGATCTGG GTTTTTGCTG GGTTTGTTGA TTCTTTGAGA AATTTGGGTG   
  
  
+ CTTGAATTTG CCCTGCAATA TTCATGGGTT AGCGTTTTCC TGAATTTTGC TCTGTTCCTT AAGTGATTAT   
  
  
+ TTTTGATTGA TTACACCTTG GTGGTCCTGG TGGAATTTCC GAGGAGAAAT TGTCATGGGT TCCCACAACT   
  
  
+ TTGGAGAATT CCCTGATGAG ACTCTAAATG AGTATCAATC TACTTTGGGA ACCATGTCCC CCGGTTATGA   
  
  
+ TGGGTCTTTG AATTATACAA CCTTGTTCAA TTACAAAGAC CCATCTCAGG ATCTCACGGC ACTGAACCTT   
  
  
+ CCTAGCCCAT TGCCTGACCC TATGCCATTC AACATTGGTT CATATTCGGG TTTGAGCCCC GGGGTTGAAT   
  
  
+ CTTCGGATGA TAGCGATTCA GATGATGTTG TTAAGTACAT TGGCCAAGTG CTTATGGAAG AGGATATGAA   
  
  
+ GGAGAAGCCT TGTATGTTCC ATGACCCTTT AGCACTCCAA GCTGCTGAGA AACCCTTTTA TGATGTGTTG   
  
  
+ GGAAAGAAGT ATCCTCCTTC CCCTAATCAA CACCCACTTA TTGATCATTC TGTGGATAGC CCAGACAATC   
  
  
+ AGTCCTGTGG AACGAGCACA ATTAGTGATC TTAGTGGCAG TAACTGCACT TCCAGTTCAA CCAATTATAT   
  
  
+ TGATGCTGTG GCAGTCGCTG ATTCGAGTGA GAATACTAAG ACCTCTTTTG TGCAAAGTTC TCTGATTGAA   
  
  
+ TCGTTTTCTC AGTCGTCCAC CCTTCCACAG TGGTCATTTG GATCATTGGG TGCCTTGGGT GGCACAGCTT   
  
  
+ CTCAGGGTTC GAATTCAGTT ATCTCCTCCC GTGGTTTCCC TATGGCCATG AACGTTTTTA GCGAGCAGTC   
  
  
+ CATGATACAG TTTCAGAAGG GGGTGGAGGA GGCGAGCAAG TTCCTTCCAA AGAACAATAA CCTTGCGATT   
  
  
+ GACCTTGAGA GCGTCACTTT CCCAAATGAA AAGGAGGCGG CCCCCATGGT GGTGGTTAAG AAAGAGAAGG   
  
  
+ ATGAACACTC ACCTGATAGC TCGAGAGGTA GCAAGATTCA CTACCGTGAT GATGAGGACT TTGAGGATGG   
  
  
+ TAGGAGGAGT AAGCAGTCAG CTGTTTCTGT GGAGGAGGCT GAGTTGTCTG AAATGTTTGA CCGGGTTTTG   
  
  
+ CTTTGCAATC CCATGAAACA TGAAGCTCAT TGTACAAGTG GTTTGAAGTC CGAGAAGGGA ACATCCCTGC   
  
  
+ AGGCTGGCCA AGTACAGGTA CAGGAGGGTC AGAAGGCTCG TGCAAAGAAA CAGGGTAATA ATGATAATAA   
  
  
+ GAAGAATAAG AATGTGGTGG ATTTAAGGAC TCTGCTTATC CTCTGTGCGC AATCTGCTGC ATCTGATGAT   
  
  
+ CGCAGGACAG CTGATGAACT GCTGAAGCAG ATTAGGGAGC ACTCTTCTGC AGCTGGGGAT GGATCTCAAA   
  
  
+ GGTTGGCTCA TTACTTTGCT AATGCCTTGG AGGCGCGTTT AGCTGGAACT GGCTCACAGA TCTATACAGC   
  
  
+ CCTGAGTTCG AAGAGGACAA CAGCAGCTGA TATGATAAAA GCTTATCAGT TTTTCTTTCG TGCTTGCCCA   
  
  
+ TTTTTAAAGA TCTCTATTAT CTTTGCGAAC CATATGATTA TACAGAGAGC TGAAAAAGCA TCAAAGCTTC   
  
  
+ ATGTTATAGA TTTTGGCATC CTGTATGGTT TTCAGTGGCC CCTCCTCATT CAACGCCTGT CGGAGCGACC   
  
  
+ TGGTGGACCT CCAAAACTGT GCATTACTGG GATTGATCTT CCCCAACCTG GATTTAGGCC AACAGAAAGA   
  
  
+ GTTGAGGCAA CAGGGCTCCG CTTGGCAAAG TATTGTGAGC GCTTCAATGT TCCATTTGAA TACCATGCCA   
  
  
+ TTGCACAGAA ATGGGAAACC ATCAAAGCTG AAGACCTGAA GATAGAAGAT GATGAGGTGG TTGCCGTGAA   
  
  
+ CTGTCTCTTC AGGTTTAAGA ACCTGCTTGA TGAGACGATA GTGGTGGATA GTCCGAGGAA TGCAGTTCTC   
  
  
+ GGCTTGATTA GAAGGATAAA ACCTGATATC TTTGTTCACG GAGTAGTGAA CGGGTCTTAC AATGCGCCCT   
  
  
+ TCTTTGTTAC ACGTTTCAGG GAGGCCCTCT TCCATTACTC TACTGTATTC GATATGTTTG ATGCCAATGC   
  
  
+ TTCCCGGGAG GATCCTGAGA GATTGATGTT TGAGAAGGAG TTTTATGGGC GAGAAATTAT GAATGTGGTG   
  
  
+ GCTTGTGAGG GTACAGAGAG AGTTGAAAGA CCTGAGACAT ACAAGCAATG GCAGGCGCGG AATAGTAGGG   
  
  
+ CAGGGTTCAG GCAGCTGCCA TTGGACCAAG AGCTCGTCAG TAAACTGAGG AGCAAGGTCC AGATGCACTA   
  
  
+ TCCTAGGGAC TTCGTTGTTG ATGTAGATGG ACATTGGACA TTGCAGGGAT GGAAGGGGAG GATCATCTGT   
  
  
+ GCTGTGTCTG CATGGGGTCC TGCTTA  

- +Up\_Stream \_Len000TCGCAT CCTTCACTGC GGCGATAACC TCCGCTTCTG CGATCATGGT TCCGGTTTTG   
  
  
- TTTACTCCTC TATCTAGTCA ATTTGGCAAA CCAATTCTTT TAATTCGCTT GTAGAGAACT TGGAGGTAGA   
  
  
- CTTGAGCTAC TGTAACTCCT ACACCTACGT CTTTTCTACC TATACCCGAG AGGTAGCCAC GGTCGTTTAG   
  
  
- TTGGATTACA ACCCACCCCA GAGTAAGCCA GTTACCCCCT GATCCCTGCA GTATAGCAGT GGTTAATGTT   
  
  
- GCCTTGTTGG GATTTGGCGT AAAATTTTAG TGTAGAACCA TCGTCTGTTT CTACATGCTG GGTTTTTAAG   
  
  
- TTGAGACATT TCGTGATCGT TTCGAGCATC ATGTGTCTTG TTAGCGTGTG AGTATCACTA TCTTCACATG   
  
  
- ATAGGTAATA CGTCACTTAT ACTGTTCCCT CCCCCAGGAC TCTACGGGTT TAAACCGTCG GCTCAACGGA   
  
  
- ACTGACGGGT AGTTACAGGA TTTGGTCAAC TCGTACAATA ATTAAACGTT CTTTCTAGCT TAAGAAGGAT   
  
  
- AGATTTTTGA CTGTGTTATT TTGTTATGGT ACATACCCTG TAACGATCCT AACAATTGTG TTAATAACAG   
  
  
- AACATGCAAC GTGGACTTTT TTTTCTTTTA ACAACGGTAT CTGTTGTATA TTCAATCCGT AAGTACTGAT   
  
  
- TATAGACTTA ATCCACACAG CTAGACACTA AGTTTAACTA TATTAATCTA GAAATTCCAT AGATTAACGA   
  
  
- AGTTCAAAAG AGATATTACT TCTACATATA ATCGAGTGTA TCCAGAATTC CAATTGATTA CAGTATAGTC   
  
  
- CCCCACACCC GGCGGTAAAA ACTATTCTTA ACTTAACTTG AACGTAACAA AAAAGAAACC GCTCTTACGT   
  
  
- TCTAAACAAT TAAATAAGTA TACGCAGTAA TATGTGATAT TCTTTTTATT AACATCCTTA TATACTTCAT   
  
  
- AAAAATGAAC ACGAGTACAA TTTACACCTT ATGAAAAAAT TACAGCATAT CATGGTGTCA TAAGATGTTA   
  
  
- AATTCTCTAA TTAGCTAGGT AATTTCAGTA CGTTTGGACT ACGTATAAGT TCTGTAACGT ACTTTACTAG   
  
  
- AATAAAATCA GTTTCTGAGG TAGTAAATAT TCGTTATAAA TGAAGCTATG TGCACAGTAA TTTTTTCTGA   
  
  
- TATCTTTACA ATGACAATAA AACAAACTCG ATTTATCTTT TAAATTTTCA ATTAATTCTA TTATTAATTT   
  
  
- TGATTTATAA GTTACTACCG ATTATTTATG TAACAGCATA ATTGACTGAT TATAAACATC TACCAGCACA   
  
  
- ATTAAAGAAA AATAAAATTT CAAGTTTACT CAATGTTTTC CAATATTTTA TATTTCATTT TACCCTCTGT   
  
  
- TTCGTCTGTC CCACCCCCTT CGATTCGAAT TTCATCCTAA ATCTTTCCGT TACTTCCACT TGGGAGTTTA   
  
  
- GCTAGGCGAC GGTTAGTGTC TTTGGGTTTC GAAACGAGTG GCTGTTGAGG GCCAATTGAC GTCAGTGAGT   
  
  
- GCCGTGACCC ATTATGTCAT TAGCACATGG GTCAAGTAGG GGGAAAAGGA AACCCTTATG TCATGGAGCT   
  
  
- TTCAAGGAAA CTTTAAGATG ACCGGTCCGT ATGGGTATTT AACTAGGAGA GCTATAGTAT TCTATACTAT   
  
  
- AAAGAGACAG TATAGACAAT CAAAGCCATA GTATAAAGAA GAAATAGAGA CATTAAACTC CATACGAAGT   
  
  
- AAGGACCGGC GAACTTAATA GAAAGAAAGA AACTAATCAT AACAAAAAAA CCAATGGATA AAACGAGGTC   
  
  
- AGGACAAGTA AACCCAGAAA AACCTAGACC CAAAAACGAC CCAAACAACT AAGAAACTCT TTAAACCCAC   
  
  
- GAACTTAAAC GGGACGTTAT AAGTACCCAA TCGCAAAAGG ACTTAAAACG AGACAAGGAA TTCACTAATA   
  
  
- AAAACTAACT AATGTGGAAC CACCAGGACC ACCTTAAAGG CTCCTCTTTA ACAGTACCCA AGGGTGTTGA   
  
  
- AACCTCTTAA GGGACTACTC TGAGATTTAC TCATAGTTAG ATGAAACCCT TGGTACAGGG GGCCAATACT   
  
  
- ACCCAGAAAC TTAATATGTT GGAACAAGTT AATGTTTCTG GGTAGAGTCC TAGAGTGCCG TGACTTGGAA   
  
  
- GGATCGGGTA ACGGACTGGG ATACGGTAAG TTGTAACCAA GTATAAGCCC AAACTCGGGG CCCCAACTTA   
  
  
- GAAGCCTACT ATCGCTAAGT CTACTACAAC AATTCATGTA ACCGGTTCAC GAATACCTTC TCCTATACTT   
  
  
- CCTCTTCGGA ACATACAAGG TACTGGGAAA TCGTGAGGTT CGACGACTCT TTGGGAAAAT ACTACACAAC   
  
  
- CCTTTCTTCA TAGGAGGAAG GGGATTAGTT GTGGGTGAAT AACTAGTAAG ACACCTATCG GGTCTGTTAG   
  
  
- TCAGGACACC TTGCTCGTGT TAATCACTAG AATCACCGTC ATTGACGTGA AGGTCAAGTT GGTTAATATA   
  
  
- ACTACGACAC CGTCAGCGAC TAAGCTCACT CTTATGATTC TGGAGAAAAC ACGTTTCAAG AGACTAACTT   
  
  
- AGCAAAAGAG TCAGCAGGTG GGAAGGTGTC ACCAGTAAAC CTAGTAACCC ACGGAACCCA CCGTGTCGAA   
  
  
- GAGTCCCAAG CTTAAGTCAA TAGAGGAGGG CACCAAAGGG ATACCGGTAC TTGCAAAAAT CGCTCGTCAG   
  
  
- GTACTATGTC AAAGTCTTCC CCCACCTCCT CCGCTCGTTC AAGGAAGGTT TCTTGTTATT GGAACGCTAA   
  
  
- CTGGAACTCT CGCAGTGAAA GGGTTTACTT TTCCTCCGCC GGGGGTACCA CCACCAATTC TTTCTCTTCC   
  
  
- TACTTGTGAG TGGACTATCG AGCTCTCCAT CGTTCTAAGT GATGGCACTA CTACTCCTGA AACTCCTACC   
  
  
- ATCCTCCTCA TTCGTCAGTC GACAAAGACA CCTCCTCCGA CTCAACAGAC TTTACAAACT GGCCCAAAAC   
  
  
- GAAACGTTAG GGTACTTTGT ACTTCGAGTA ACATGTTCAC CAAACTTCAG GCTCTTCCCT TGTAGGGACG   
  
  
- TCCGACCGGT TCATGTCCAT GTCCTCCCAG TCTTCCGAGC ACGTTTCTTT GTCCCATTAT TACTATTATT   
  
  
- CTTCTTATTC TTACACCACC TAAATTCCTG AGACGAATAG GAGACACGCG TTAGACGACG TAGACTACTA   
  
  
- GCGTCCTGTC GACTACTTGA CGACTTCGTC TAATCCCTCG TGAGAAGACG TCGACCCCTA CCTAGAGTTT   
  
  
- CCAACCGAGT AATGAAACGA TTACGGAACC TCCGCGCAAA TCGACCTTGA CCGAGTGTCT AGATATGTCG   
  
  
- GGACTCAAGC TTCTCCTGTT GTCGTCGACT ATACTATTTT CGAATAGTCA AAAAGAAAGC ACGAACGGGT   
  
  
- AAAAATTTCT AGAGATAATA GAAACGCTTG GTATACTAAT ATGTCTCTCG ACTTTTTCGT AGTTTCGAAG   
  
  
- TACAATATCT AAAACCGTAG GACATACCAA AAGTCACCGG GGAGGAGTAA GTTGCGGACA GCCTCGCTGG   
  
  
- ACCACCTGGA GGTTTTGACA CGTAATGACC CTAACTAGAA GGGGTTGGAC CTAAATCCGG TTGTCTTTCT   
  
  
- CAACTCCGTT GTCCCGAGGC GAACCGTTTC ATAACACTCG CGAAGTTACA AGGTAAACTT ATGGTACGGT   
  
  
- AACGTGTCTT TACCCTTTGG TAGTTTCGAC TTCTGGACTT CTATCTTCTA CTACTCCACC AACGGCACTT   
  
  
- GACAGAGAAG TCCAAATTCT TGGACGAACT ACTCTGCTAT CACCACCTAT CAGGCTCCTT ACGTCAAGAG   
  
  
- CCGAACTAAT CTTCCTATTT TGGACTATAG AAACAAGTGC CTCATCACTT GCCCAGAATG TTACGCGGGA   
  
  
- AGAAACAATG TGCAAAGTCC CTCCGGGAGA AGGTAATGAG ATGACATAAG CTATACAAAC TACGGTTACG   
  
  
- AAGGGCCCTC CTAGGACTCT CTAACTACAA ACTCTTCCTC AAAATACCCG CTCTTTAATA CTTACACCAC   
  
  
- CGAACACTCC CATGTCTCTC TCAACTTTCT GGACTCTGTA TGTTCGTTAC CGTCCGCGCC TTATCATCCC   
  
  
- GTCCCAAGTC CGTCGACGGT AACCTGGTTC TCGAGCAGTC ATTTGACTCC TCGTTCCAGG TCTACGTGAT   
  
  
- AGGATCCCTG AAGCAACAAC TACATCTACC TGTAACCTGT AACGTCCCTA CCTTCCCCTC CTAGTAGACA   
  
  
- CGACACAGAC GTACCCCAGG ACGAAT

+     HD-Zip 1

| Site Name | Organism | Position | Strand | Matrix score. | sequence | function |
| --- | --- | --- | --- | --- | --- | --- |
| HD-Zip 1 | Arabidopsis thaliana | 624 | + | 8.5 | CAAT(A/T)ATTG | element involved in differentiation of the palisade mesophyll cells |

>HU02G01569.1   
+ +Up\_Stream \_Len000AGCGTA GGAAGTGACG CCGCTATTGG AGGCGAAGAC GCTAGTACCA AGGCCAAAAC   
  
  
+ AAATGAGGAG ATAGATCAGT TAAACCGTTT GGTTAAGAAA ATTAAGCGAA CATCTCTTGA ACCTCCATCT   
  
  
+ GAACTCGATG ACATTGAGGA TGTGGATGCA GAAAAGATGG ATATGGGCTC TCCATCGGTG CCAGCAAATC   
  
  
+ AACCTAATGT TGGGTGGGGT CTCATTCGGT CAATGGGGGA CTAGGGACGT CATATCGTCA CCAATTACAA   
  
  
+ CGGAACAACC CTAAACCGCA TTTTAAAATC ACATCTTGGT AGCAGACAAA GATGTACGAC CCAAAAATTC   
  
  
+ AACTCTGTAA AGCACTAGCA AAGCTCGTAG TACACAGAAC AATCGCACAC TCATAGTGAT AGAAGTGTAC   
  
  
+ TATCCATTAT GCAGTGAATA TGACAAGGGA GGGGGTCCTG AGATGCCCAA ATTTGGCAGC CGAGTTGCCT   
  
  
+ TGACTGCCCA TCAATGTCCT AAACCAGTTG AGCATGTTAT TAATTTGCAA GAAAGATCGA ATTCTTCCTA   
  
  
+ TCTAAAAACT GACACAATAA AACAATACCA TGTATGGGAC ATTGCTAGGA TTGTTAACAC AATTATTGTC   
  
  
+ TTGTACGTTG CACCTGAAAA AAAAGAAAAT TGTTGCCATA GACAACATAT AAGTTAGGCA TTCATGACTA   
  
  
+ ATATCTGAAT TAGGTGTGTC GATCTGTGAT TCAAATTGAT ATAATTAGAT CTTTAAGGTA TCTAATTGCT   
  
  
+ TCAAGTTTTC TCTATAATGA AGATGTATAT TAGCTCACAT AGGTCTTAAG GTTAACTAAT GTCATATCAG   
  
  
+ GGGGTGTGGG CCGCCATTTT TGATAAGAAT TGAATTGAAC TTGCATTGTT TTTTCTTTGG CGAGAATGCA   
  
  
+ AGATTTGTTA ATTTATTCAT ATGCGTCATT ATACACTATA AGAAAAATAA TTGTAGGAAT ATATGAAGTA   
  
  
+ TTTTTACTTG TGCTCATGTT AAATGTGGAA TACTTTTTTA ATGTCGTATA GTACCACAGT ATTCTACAAT   
  
  
+ TTAAGAGATT AATCGATCCA TTAAAGTCAT GCAAACCTGA TGCATATTCA AGACATTGCA TGAAATGATC   
  
  
+ TTATTTTAGT CAAAGACTCC ATCATTTATA AGCAATATTT ACTTCGATAC ACGTGTCATT AAAAAAGACT   
  
  
+ ATAGAAATGT TACTGTTATT TTGTTTGAGC TAAATAGAAA ATTTAAAAGT TAATTAAGAT AATAATTAAA   
  
  
+ ACTAAATATT CAATGATGGC TAATAAATAC ATTGTCGTAT TAACTGACTA ATATTTGTAG ATGGTCGTGT   
  
  
+ TAATTTCTTT TTATTTTAAA GTTCAAATGA GTTACAAAAG GTTATAAAAT ATAAAGTAAA ATGGGAGACA   
  
  
+ AAGCAGACAG GGTGGGGGAA GCTAAGCTTA AAGTAGGATT TAGAAAGGCA ATGAAGGTGA ACCCTCAAAT   
  
  
+ CGATCCGCTG CCAATCACAG AAACCCAAAG CTTTGCTCAC CGACAACTCC CGGTTAACTG CAGTCACTCA   
  
  
+ CGGCACTGGG TAATACAGTA ATCGTGTACC CAGTTCATCC CCCTTTTCCT TTGGGAATAC AGTACCTCGA   
  
  
+ AAGTTCCTTT GAAATTCTAC TGGCCAGGCA TACCCATAAA TTGATCCTCT CGATATCATA AGATATGATA   
  
  
+ TTTCTCTGTC ATATCTGTTA GTTTCGGTAT CATATTTCTT CTTTATCTCT GTAATTTGAG GTATGCTTCA   
  
  
+ TTCCTGGCCG CTTGAATTAT CTTTCTTTCT TTGATTAGTA TTGTTTTTTT GGTTACCTAT TTTGCTCCAG   
  
  
+ TCCTGTTCAT TTGGGTCTTT TTGGATCTGG GTTTTTGCTG GGTTTGTTGA TTCTTTGAGA AATTTGGGTG   
  
  
+ CTTGAATTTG CCCTGCAATA TTCATGGGTT AGCGTTTTCC TGAATTTTGC TCTGTTCCTT AAGTGATTAT   
  
  
+ TTTTGATTGA TTACACCTTG GTGGTCCTGG TGGAATTTCC GAGGAGAAAT TGTCATGGGT TCCCACAACT   
  
  
+ TTGGAGAATT CCCTGATGAG ACTCTAAATG AGTATCAATC TACTTTGGGA ACCATGTCCC CCGGTTATGA   
  
  
+ TGGGTCTTTG AATTATACAA CCTTGTTCAA TTACAAAGAC CCATCTCAGG ATCTCACGGC ACTGAACCTT   
  
  
+ CCTAGCCCAT TGCCTGACCC TATGCCATTC AACATTGGTT CATATTCGGG TTTGAGCCCC GGGGTTGAAT   
  
  
+ CTTCGGATGA TAGCGATTCA GATGATGTTG TTAAGTACAT TGGCCAAGTG CTTATGGAAG AGGATATGAA   
  
  
+ GGAGAAGCCT TGTATGTTCC ATGACCCTTT AGCACTCCAA GCTGCTGAGA AACCCTTTTA TGATGTGTTG   
  
  
+ GGAAAGAAGT ATCCTCCTTC CCCTAATCAA CACCCACTTA TTGATCATTC TGTGGATAGC CCAGACAATC   
  
  
+ AGTCCTGTGG AACGAGCACA ATTAGTGATC TTAGTGGCAG TAACTGCACT TCCAGTTCAA CCAATTATAT   
  
  
+ TGATGCTGTG GCAGTCGCTG ATTCGAGTGA GAATACTAAG ACCTCTTTTG TGCAAAGTTC TCTGATTGAA   
  
  
+ TCGTTTTCTC AGTCGTCCAC CCTTCCACAG TGGTCATTTG GATCATTGGG TGCCTTGGGT GGCACAGCTT   
  
  
+ CTCAGGGTTC GAATTCAGTT ATCTCCTCCC GTGGTTTCCC TATGGCCATG AACGTTTTTA GCGAGCAGTC   
  
  
+ CATGATACAG TTTCAGAAGG GGGTGGAGGA GGCGAGCAAG TTCCTTCCAA AGAACAATAA CCTTGCGATT   
  
  
+ GACCTTGAGA GCGTCACTTT CCCAAATGAA AAGGAGGCGG CCCCCATGGT GGTGGTTAAG AAAGAGAAGG   
  
  
+ ATGAACACTC ACCTGATAGC TCGAGAGGTA GCAAGATTCA CTACCGTGAT GATGAGGACT TTGAGGATGG   
  
  
+ TAGGAGGAGT AAGCAGTCAG CTGTTTCTGT GGAGGAGGCT GAGTTGTCTG AAATGTTTGA CCGGGTTTTG   
  
  
+ CTTTGCAATC CCATGAAACA TGAAGCTCAT TGTACAAGTG GTTTGAAGTC CGAGAAGGGA ACATCCCTGC   
  
  
+ AGGCTGGCCA AGTACAGGTA CAGGAGGGTC AGAAGGCTCG TGCAAAGAAA CAGGGTAATA ATGATAATAA   
  
  
+ GAAGAATAAG AATGTGGTGG ATTTAAGGAC TCTGCTTATC CTCTGTGCGC AATCTGCTGC ATCTGATGAT   
  
  
+ CGCAGGACAG CTGATGAACT GCTGAAGCAG ATTAGGGAGC ACTCTTCTGC AGCTGGGGAT GGATCTCAAA   
  
  
+ GGTTGGCTCA TTACTTTGCT AATGCCTTGG AGGCGCGTTT AGCTGGAACT GGCTCACAGA TCTATACAGC   
  
  
+ CCTGAGTTCG AAGAGGACAA CAGCAGCTGA TATGATAAAA GCTTATCAGT TTTTCTTTCG TGCTTGCCCA   
  
  
+ TTTTTAAAGA TCTCTATTAT CTTTGCGAAC CATATGATTA TACAGAGAGC TGAAAAAGCA TCAAAGCTTC   
  
  
+ ATGTTATAGA TTTTGGCATC CTGTATGGTT TTCAGTGGCC CCTCCTCATT CAACGCCTGT CGGAGCGACC   
  
  
+ TGGTGGACCT CCAAAACTGT GCATTACTGG GATTGATCTT CCCCAACCTG GATTTAGGCC AACAGAAAGA   
  
  
+ GTTGAGGCAA CAGGGCTCCG CTTGGCAAAG TATTGTGAGC GCTTCAATGT TCCATTTGAA TACCATGCCA   
  
  
+ TTGCACAGAA ATGGGAAACC ATCAAAGCTG AAGACCTGAA GATAGAAGAT GATGAGGTGG TTGCCGTGAA   
  
  
+ CTGTCTCTTC AGGTTTAAGA ACCTGCTTGA TGAGACGATA GTGGTGGATA GTCCGAGGAA TGCAGTTCTC   
  
  
+ GGCTTGATTA GAAGGATAAA ACCTGATATC TTTGTTCACG GAGTAGTGAA CGGGTCTTAC AATGCGCCCT   
  
  
+ TCTTTGTTAC ACGTTTCAGG GAGGCCCTCT TCCATTACTC TACTGTATTC GATATGTTTG ATGCCAATGC   
  
  
+ TTCCCGGGAG GATCCTGAGA GATTGATGTT TGAGAAGGAG TTTTATGGGC GAGAAATTAT GAATGTGGTG   
  
  
+ GCTTGTGAGG GTACAGAGAG AGTTGAAAGA CCTGAGACAT ACAAGCAATG GCAGGCGCGG AATAGTAGGG   
  
  
+ CAGGGTTCAG GCAGCTGCCA TTGGACCAAG AGCTCGTCAG TAAACTGAGG AGCAAGGTCC AGATGCACTA   
  
  
+ TCCTAGGGAC TTCGTTGTTG ATGTAGATGG ACATTGGACA TTGCAGGGAT GGAAGGGGAG GATCATCTGT   
  
  
+ GCTGTGTCTG CATGGGGTCC TGCTTA  

- +Up\_Stream \_Len000TCGCAT CCTTCACTGC GGCGATAACC TCCGCTTCTG CGATCATGGT TCCGGTTTTG   
  
  
- TTTACTCCTC TATCTAGTCA ATTTGGCAAA CCAATTCTTT TAATTCGCTT GTAGAGAACT TGGAGGTAGA   
  
  
- CTTGAGCTAC TGTAACTCCT ACACCTACGT CTTTTCTACC TATACCCGAG AGGTAGCCAC GGTCGTTTAG   
  
  
- TTGGATTACA ACCCACCCCA GAGTAAGCCA GTTACCCCCT GATCCCTGCA GTATAGCAGT GGTTAATGTT   
  
  
- GCCTTGTTGG GATTTGGCGT AAAATTTTAG TGTAGAACCA TCGTCTGTTT CTACATGCTG GGTTTTTAAG   
  
  
- TTGAGACATT TCGTGATCGT TTCGAGCATC ATGTGTCTTG TTAGCGTGTG AGTATCACTA TCTTCACATG   
  
  
- ATAGGTAATA CGTCACTTAT ACTGTTCCCT CCCCCAGGAC TCTACGGGTT TAAACCGTCG GCTCAACGGA   
  
  
- ACTGACGGGT AGTTACAGGA TTTGGTCAAC TCGTACAATA ATTAAACGTT CTTTCTAGCT TAAGAAGGAT   
  
  
- AGATTTTTGA CTGTGTTATT TTGTTATGGT ACATACCCTG TAACGATCCT AACAATTGTG TTAATAACAG   
  
  
- AACATGCAAC GTGGACTTTT TTTTCTTTTA ACAACGGTAT CTGTTGTATA TTCAATCCGT AAGTACTGAT   
  
  
- TATAGACTTA ATCCACACAG CTAGACACTA AGTTTAACTA TATTAATCTA GAAATTCCAT AGATTAACGA   
  
  
- AGTTCAAAAG AGATATTACT TCTACATATA ATCGAGTGTA TCCAGAATTC CAATTGATTA CAGTATAGTC   
  
  
- CCCCACACCC GGCGGTAAAA ACTATTCTTA ACTTAACTTG AACGTAACAA AAAAGAAACC GCTCTTACGT   
  
  
- TCTAAACAAT TAAATAAGTA TACGCAGTAA TATGTGATAT TCTTTTTATT AACATCCTTA TATACTTCAT   
  
  
- AAAAATGAAC ACGAGTACAA TTTACACCTT ATGAAAAAAT TACAGCATAT CATGGTGTCA TAAGATGTTA   
  
  
- AATTCTCTAA TTAGCTAGGT AATTTCAGTA CGTTTGGACT ACGTATAAGT TCTGTAACGT ACTTTACTAG   
  
  
- AATAAAATCA GTTTCTGAGG TAGTAAATAT TCGTTATAAA TGAAGCTATG TGCACAGTAA TTTTTTCTGA   
  
  
- TATCTTTACA ATGACAATAA AACAAACTCG ATTTATCTTT TAAATTTTCA ATTAATTCTA TTATTAATTT   
  
  
- TGATTTATAA GTTACTACCG ATTATTTATG TAACAGCATA ATTGACTGAT TATAAACATC TACCAGCACA   
  
  
- ATTAAAGAAA AATAAAATTT CAAGTTTACT CAATGTTTTC CAATATTTTA TATTTCATTT TACCCTCTGT   
  
  
- TTCGTCTGTC CCACCCCCTT CGATTCGAAT TTCATCCTAA ATCTTTCCGT TACTTCCACT TGGGAGTTTA   
  
  
- GCTAGGCGAC GGTTAGTGTC TTTGGGTTTC GAAACGAGTG GCTGTTGAGG GCCAATTGAC GTCAGTGAGT   
  
  
- GCCGTGACCC ATTATGTCAT TAGCACATGG GTCAAGTAGG GGGAAAAGGA AACCCTTATG TCATGGAGCT   
  
  
- TTCAAGGAAA CTTTAAGATG ACCGGTCCGT ATGGGTATTT AACTAGGAGA GCTATAGTAT TCTATACTAT   
  
  
- AAAGAGACAG TATAGACAAT CAAAGCCATA GTATAAAGAA GAAATAGAGA CATTAAACTC CATACGAAGT   
  
  
- AAGGACCGGC GAACTTAATA GAAAGAAAGA AACTAATCAT AACAAAAAAA CCAATGGATA AAACGAGGTC   
  
  
- AGGACAAGTA AACCCAGAAA AACCTAGACC CAAAAACGAC CCAAACAACT AAGAAACTCT TTAAACCCAC   
  
  
- GAACTTAAAC GGGACGTTAT AAGTACCCAA TCGCAAAAGG ACTTAAAACG AGACAAGGAA TTCACTAATA   
  
  
- AAAACTAACT AATGTGGAAC CACCAGGACC ACCTTAAAGG CTCCTCTTTA ACAGTACCCA AGGGTGTTGA   
  
  
- AACCTCTTAA GGGACTACTC TGAGATTTAC TCATAGTTAG ATGAAACCCT TGGTACAGGG GGCCAATACT   
  
  
- ACCCAGAAAC TTAATATGTT GGAACAAGTT AATGTTTCTG GGTAGAGTCC TAGAGTGCCG TGACTTGGAA   
  
  
- GGATCGGGTA ACGGACTGGG ATACGGTAAG TTGTAACCAA GTATAAGCCC AAACTCGGGG CCCCAACTTA   
  
  
- GAAGCCTACT ATCGCTAAGT CTACTACAAC AATTCATGTA ACCGGTTCAC GAATACCTTC TCCTATACTT   
  
  
- CCTCTTCGGA ACATACAAGG TACTGGGAAA TCGTGAGGTT CGACGACTCT TTGGGAAAAT ACTACACAAC   
  
  
- CCTTTCTTCA TAGGAGGAAG GGGATTAGTT GTGGGTGAAT AACTAGTAAG ACACCTATCG GGTCTGTTAG   
  
  
- TCAGGACACC TTGCTCGTGT TAATCACTAG AATCACCGTC ATTGACGTGA AGGTCAAGTT GGTTAATATA   
  
  
- ACTACGACAC CGTCAGCGAC TAAGCTCACT CTTATGATTC TGGAGAAAAC ACGTTTCAAG AGACTAACTT   
  
  
- AGCAAAAGAG TCAGCAGGTG GGAAGGTGTC ACCAGTAAAC CTAGTAACCC ACGGAACCCA CCGTGTCGAA   
  
  
- GAGTCCCAAG CTTAAGTCAA TAGAGGAGGG CACCAAAGGG ATACCGGTAC TTGCAAAAAT CGCTCGTCAG   
  
  
- GTACTATGTC AAAGTCTTCC CCCACCTCCT CCGCTCGTTC AAGGAAGGTT TCTTGTTATT GGAACGCTAA   
  
  
- CTGGAACTCT CGCAGTGAAA GGGTTTACTT TTCCTCCGCC GGGGGTACCA CCACCAATTC TTTCTCTTCC   
  
  
- TACTTGTGAG TGGACTATCG AGCTCTCCAT CGTTCTAAGT GATGGCACTA CTACTCCTGA AACTCCTACC   
  
  
- ATCCTCCTCA TTCGTCAGTC GACAAAGACA CCTCCTCCGA CTCAACAGAC TTTACAAACT GGCCCAAAAC   
  
  
- GAAACGTTAG GGTACTTTGT ACTTCGAGTA ACATGTTCAC CAAACTTCAG GCTCTTCCCT TGTAGGGACG   
  
  
- TCCGACCGGT TCATGTCCAT GTCCTCCCAG TCTTCCGAGC ACGTTTCTTT GTCCCATTAT TACTATTATT   
  
  
- CTTCTTATTC TTACACCACC TAAATTCCTG AGACGAATAG GAGACACGCG TTAGACGACG TAGACTACTA   
  
  
- GCGTCCTGTC GACTACTTGA CGACTTCGTC TAATCCCTCG TGAGAAGACG TCGACCCCTA CCTAGAGTTT   
  
  
- CCAACCGAGT AATGAAACGA TTACGGAACC TCCGCGCAAA TCGACCTTGA CCGAGTGTCT AGATATGTCG   
  
  
- GGACTCAAGC TTCTCCTGTT GTCGTCGACT ATACTATTTT CGAATAGTCA AAAAGAAAGC ACGAACGGGT   
  
  
- AAAAATTTCT AGAGATAATA GAAACGCTTG GTATACTAAT ATGTCTCTCG ACTTTTTCGT AGTTTCGAAG   
  
  
- TACAATATCT AAAACCGTAG GACATACCAA AAGTCACCGG GGAGGAGTAA GTTGCGGACA GCCTCGCTGG   
  
  
- ACCACCTGGA GGTTTTGACA CGTAATGACC CTAACTAGAA GGGGTTGGAC CTAAATCCGG TTGTCTTTCT   
  
  
- CAACTCCGTT GTCCCGAGGC GAACCGTTTC ATAACACTCG CGAAGTTACA AGGTAAACTT ATGGTACGGT   
  
  
- AACGTGTCTT TACCCTTTGG TAGTTTCGAC TTCTGGACTT CTATCTTCTA CTACTCCACC AACGGCACTT   
  
  
- GACAGAGAAG TCCAAATTCT TGGACGAACT ACTCTGCTAT CACCACCTAT CAGGCTCCTT ACGTCAAGAG   
  
  
- CCGAACTAAT CTTCCTATTT TGGACTATAG AAACAAGTGC CTCATCACTT GCCCAGAATG TTACGCGGGA   
  
  
- AGAAACAATG TGCAAAGTCC CTCCGGGAGA AGGTAATGAG ATGACATAAG CTATACAAAC TACGGTTACG   
  
  
- AAGGGCCCTC CTAGGACTCT CTAACTACAA ACTCTTCCTC AAAATACCCG CTCTTTAATA CTTACACCAC   
  
  
- CGAACACTCC CATGTCTCTC TCAACTTTCT GGACTCTGTA TGTTCGTTAC CGTCCGCGCC TTATCATCCC   
  
  
- GTCCCAAGTC CGTCGACGGT AACCTGGTTC TCGAGCAGTC ATTTGACTCC TCGTTCCAGG TCTACGTGAT   
  
  
- AGGATCCCTG AAGCAACAAC TACATCTACC TGTAACCTGT AACGTCCCTA CCTTCCCCTC CTAGTAGACA   
  
  
- CGACACAGAC GTACCCCAGG ACGAAT

+     LTR

| Site Name | Organism | Position | Strand | Matrix score. | sequence | function |
| --- | --- | --- | --- | --- | --- | --- |
| LTR | Hordeum vulgare | 1706 | - | 6 | CCGAAA | cis-acting element involved in low-temperature responsiveness |

>HU02G01569.1   
+ +Up\_Stream \_Len000AGCGTA GGAAGTGACG CCGCTATTGG AGGCGAAGAC GCTAGTACCA AGGCCAAAAC   
  
  
+ AAATGAGGAG ATAGATCAGT TAAACCGTTT GGTTAAGAAA ATTAAGCGAA CATCTCTTGA ACCTCCATCT   
  
  
+ GAACTCGATG ACATTGAGGA TGTGGATGCA GAAAAGATGG ATATGGGCTC TCCATCGGTG CCAGCAAATC   
  
  
+ AACCTAATGT TGGGTGGGGT CTCATTCGGT CAATGGGGGA CTAGGGACGT CATATCGTCA CCAATTACAA   
  
  
+ CGGAACAACC CTAAACCGCA TTTTAAAATC ACATCTTGGT AGCAGACAAA GATGTACGAC CCAAAAATTC   
  
  
+ AACTCTGTAA AGCACTAGCA AAGCTCGTAG TACACAGAAC AATCGCACAC TCATAGTGAT AGAAGTGTAC   
  
  
+ TATCCATTAT GCAGTGAATA TGACAAGGGA GGGGGTCCTG AGATGCCCAA ATTTGGCAGC CGAGTTGCCT   
  
  
+ TGACTGCCCA TCAATGTCCT AAACCAGTTG AGCATGTTAT TAATTTGCAA GAAAGATCGA ATTCTTCCTA   
  
  
+ TCTAAAAACT GACACAATAA AACAATACCA TGTATGGGAC ATTGCTAGGA TTGTTAACAC AATTATTGTC   
  
  
+ TTGTACGTTG CACCTGAAAA AAAAGAAAAT TGTTGCCATA GACAACATAT AAGTTAGGCA TTCATGACTA   
  
  
+ ATATCTGAAT TAGGTGTGTC GATCTGTGAT TCAAATTGAT ATAATTAGAT CTTTAAGGTA TCTAATTGCT   
  
  
+ TCAAGTTTTC TCTATAATGA AGATGTATAT TAGCTCACAT AGGTCTTAAG GTTAACTAAT GTCATATCAG   
  
  
+ GGGGTGTGGG CCGCCATTTT TGATAAGAAT TGAATTGAAC TTGCATTGTT TTTTCTTTGG CGAGAATGCA   
  
  
+ AGATTTGTTA ATTTATTCAT ATGCGTCATT ATACACTATA AGAAAAATAA TTGTAGGAAT ATATGAAGTA   
  
  
+ TTTTTACTTG TGCTCATGTT AAATGTGGAA TACTTTTTTA ATGTCGTATA GTACCACAGT ATTCTACAAT   
  
  
+ TTAAGAGATT AATCGATCCA TTAAAGTCAT GCAAACCTGA TGCATATTCA AGACATTGCA TGAAATGATC   
  
  
+ TTATTTTAGT CAAAGACTCC ATCATTTATA AGCAATATTT ACTTCGATAC ACGTGTCATT AAAAAAGACT   
  
  
+ ATAGAAATGT TACTGTTATT TTGTTTGAGC TAAATAGAAA ATTTAAAAGT TAATTAAGAT AATAATTAAA   
  
  
+ ACTAAATATT CAATGATGGC TAATAAATAC ATTGTCGTAT TAACTGACTA ATATTTGTAG ATGGTCGTGT   
  
  
+ TAATTTCTTT TTATTTTAAA GTTCAAATGA GTTACAAAAG GTTATAAAAT ATAAAGTAAA ATGGGAGACA   
  
  
+ AAGCAGACAG GGTGGGGGAA GCTAAGCTTA AAGTAGGATT TAGAAAGGCA ATGAAGGTGA ACCCTCAAAT   
  
  
+ CGATCCGCTG CCAATCACAG AAACCCAAAG CTTTGCTCAC CGACAACTCC CGGTTAACTG CAGTCACTCA   
  
  
+ CGGCACTGGG TAATACAGTA ATCGTGTACC CAGTTCATCC CCCTTTTCCT TTGGGAATAC AGTACCTCGA   
  
  
+ AAGTTCCTTT GAAATTCTAC TGGCCAGGCA TACCCATAAA TTGATCCTCT CGATATCATA AGATATGATA   
  
  
+ TTTCTCTGTC ATATCTGTTA GTTTCGGTAT CATATTTCTT CTTTATCTCT GTAATTTGAG GTATGCTTCA   
  
  
+ TTCCTGGCCG CTTGAATTAT CTTTCTTTCT TTGATTAGTA TTGTTTTTTT GGTTACCTAT TTTGCTCCAG   
  
  
+ TCCTGTTCAT TTGGGTCTTT TTGGATCTGG GTTTTTGCTG GGTTTGTTGA TTCTTTGAGA AATTTGGGTG   
  
  
+ CTTGAATTTG CCCTGCAATA TTCATGGGTT AGCGTTTTCC TGAATTTTGC TCTGTTCCTT AAGTGATTAT   
  
  
+ TTTTGATTGA TTACACCTTG GTGGTCCTGG TGGAATTTCC GAGGAGAAAT TGTCATGGGT TCCCACAACT   
  
  
+ TTGGAGAATT CCCTGATGAG ACTCTAAATG AGTATCAATC TACTTTGGGA ACCATGTCCC CCGGTTATGA   
  
  
+ TGGGTCTTTG AATTATACAA CCTTGTTCAA TTACAAAGAC CCATCTCAGG ATCTCACGGC ACTGAACCTT   
  
  
+ CCTAGCCCAT TGCCTGACCC TATGCCATTC AACATTGGTT CATATTCGGG TTTGAGCCCC GGGGTTGAAT   
  
  
+ CTTCGGATGA TAGCGATTCA GATGATGTTG TTAAGTACAT TGGCCAAGTG CTTATGGAAG AGGATATGAA   
  
  
+ GGAGAAGCCT TGTATGTTCC ATGACCCTTT AGCACTCCAA GCTGCTGAGA AACCCTTTTA TGATGTGTTG   
  
  
+ GGAAAGAAGT ATCCTCCTTC CCCTAATCAA CACCCACTTA TTGATCATTC TGTGGATAGC CCAGACAATC   
  
  
+ AGTCCTGTGG AACGAGCACA ATTAGTGATC TTAGTGGCAG TAACTGCACT TCCAGTTCAA CCAATTATAT   
  
  
+ TGATGCTGTG GCAGTCGCTG ATTCGAGTGA GAATACTAAG ACCTCTTTTG TGCAAAGTTC TCTGATTGAA   
  
  
+ TCGTTTTCTC AGTCGTCCAC CCTTCCACAG TGGTCATTTG GATCATTGGG TGCCTTGGGT GGCACAGCTT   
  
  
+ CTCAGGGTTC GAATTCAGTT ATCTCCTCCC GTGGTTTCCC TATGGCCATG AACGTTTTTA GCGAGCAGTC   
  
  
+ CATGATACAG TTTCAGAAGG GGGTGGAGGA GGCGAGCAAG TTCCTTCCAA AGAACAATAA CCTTGCGATT   
  
  
+ GACCTTGAGA GCGTCACTTT CCCAAATGAA AAGGAGGCGG CCCCCATGGT GGTGGTTAAG AAAGAGAAGG   
  
  
+ ATGAACACTC ACCTGATAGC TCGAGAGGTA GCAAGATTCA CTACCGTGAT GATGAGGACT TTGAGGATGG   
  
  
+ TAGGAGGAGT AAGCAGTCAG CTGTTTCTGT GGAGGAGGCT GAGTTGTCTG AAATGTTTGA CCGGGTTTTG   
  
  
+ CTTTGCAATC CCATGAAACA TGAAGCTCAT TGTACAAGTG GTTTGAAGTC CGAGAAGGGA ACATCCCTGC   
  
  
+ AGGCTGGCCA AGTACAGGTA CAGGAGGGTC AGAAGGCTCG TGCAAAGAAA CAGGGTAATA ATGATAATAA   
  
  
+ GAAGAATAAG AATGTGGTGG ATTTAAGGAC TCTGCTTATC CTCTGTGCGC AATCTGCTGC ATCTGATGAT   
  
  
+ CGCAGGACAG CTGATGAACT GCTGAAGCAG ATTAGGGAGC ACTCTTCTGC AGCTGGGGAT GGATCTCAAA   
  
  
+ GGTTGGCTCA TTACTTTGCT AATGCCTTGG AGGCGCGTTT AGCTGGAACT GGCTCACAGA TCTATACAGC   
  
  
+ CCTGAGTTCG AAGAGGACAA CAGCAGCTGA TATGATAAAA GCTTATCAGT TTTTCTTTCG TGCTTGCCCA   
  
  
+ TTTTTAAAGA TCTCTATTAT CTTTGCGAAC CATATGATTA TACAGAGAGC TGAAAAAGCA TCAAAGCTTC   
  
  
+ ATGTTATAGA TTTTGGCATC CTGTATGGTT TTCAGTGGCC CCTCCTCATT CAACGCCTGT CGGAGCGACC   
  
  
+ TGGTGGACCT CCAAAACTGT GCATTACTGG GATTGATCTT CCCCAACCTG GATTTAGGCC AACAGAAAGA   
  
  
+ GTTGAGGCAA CAGGGCTCCG CTTGGCAAAG TATTGTGAGC GCTTCAATGT TCCATTTGAA TACCATGCCA   
  
  
+ TTGCACAGAA ATGGGAAACC ATCAAAGCTG AAGACCTGAA GATAGAAGAT GATGAGGTGG TTGCCGTGAA   
  
  
+ CTGTCTCTTC AGGTTTAAGA ACCTGCTTGA TGAGACGATA GTGGTGGATA GTCCGAGGAA TGCAGTTCTC   
  
  
+ GGCTTGATTA GAAGGATAAA ACCTGATATC TTTGTTCACG GAGTAGTGAA CGGGTCTTAC AATGCGCCCT   
  
  
+ TCTTTGTTAC ACGTTTCAGG GAGGCCCTCT TCCATTACTC TACTGTATTC GATATGTTTG ATGCCAATGC   
  
  
+ TTCCCGGGAG GATCCTGAGA GATTGATGTT TGAGAAGGAG TTTTATGGGC GAGAAATTAT GAATGTGGTG   
  
  
+ GCTTGTGAGG GTACAGAGAG AGTTGAAAGA CCTGAGACAT ACAAGCAATG GCAGGCGCGG AATAGTAGGG   
  
  
+ CAGGGTTCAG GCAGCTGCCA TTGGACCAAG AGCTCGTCAG TAAACTGAGG AGCAAGGTCC AGATGCACTA   
  
  
+ TCCTAGGGAC TTCGTTGTTG ATGTAGATGG ACATTGGACA TTGCAGGGAT GGAAGGGGAG GATCATCTGT   
  
  
+ GCTGTGTCTG CATGGGGTCC TGCTTA  

- +Up\_Stream \_Len000TCGCAT CCTTCACTGC GGCGATAACC TCCGCTTCTG CGATCATGGT TCCGGTTTTG   
  
  
- TTTACTCCTC TATCTAGTCA ATTTGGCAAA CCAATTCTTT TAATTCGCTT GTAGAGAACT TGGAGGTAGA   
  
  
- CTTGAGCTAC TGTAACTCCT ACACCTACGT CTTTTCTACC TATACCCGAG AGGTAGCCAC GGTCGTTTAG   
  
  
- TTGGATTACA ACCCACCCCA GAGTAAGCCA GTTACCCCCT GATCCCTGCA GTATAGCAGT GGTTAATGTT   
  
  
- GCCTTGTTGG GATTTGGCGT AAAATTTTAG TGTAGAACCA TCGTCTGTTT CTACATGCTG GGTTTTTAAG   
  
  
- TTGAGACATT TCGTGATCGT TTCGAGCATC ATGTGTCTTG TTAGCGTGTG AGTATCACTA TCTTCACATG   
  
  
- ATAGGTAATA CGTCACTTAT ACTGTTCCCT CCCCCAGGAC TCTACGGGTT TAAACCGTCG GCTCAACGGA   
  
  
- ACTGACGGGT AGTTACAGGA TTTGGTCAAC TCGTACAATA ATTAAACGTT CTTTCTAGCT TAAGAAGGAT   
  
  
- AGATTTTTGA CTGTGTTATT TTGTTATGGT ACATACCCTG TAACGATCCT AACAATTGTG TTAATAACAG   
  
  
- AACATGCAAC GTGGACTTTT TTTTCTTTTA ACAACGGTAT CTGTTGTATA TTCAATCCGT AAGTACTGAT   
  
  
- TATAGACTTA ATCCACACAG CTAGACACTA AGTTTAACTA TATTAATCTA GAAATTCCAT AGATTAACGA   
  
  
- AGTTCAAAAG AGATATTACT TCTACATATA ATCGAGTGTA TCCAGAATTC CAATTGATTA CAGTATAGTC   
  
  
- CCCCACACCC GGCGGTAAAA ACTATTCTTA ACTTAACTTG AACGTAACAA AAAAGAAACC GCTCTTACGT   
  
  
- TCTAAACAAT TAAATAAGTA TACGCAGTAA TATGTGATAT TCTTTTTATT AACATCCTTA TATACTTCAT   
  
  
- AAAAATGAAC ACGAGTACAA TTTACACCTT ATGAAAAAAT TACAGCATAT CATGGTGTCA TAAGATGTTA   
  
  
- AATTCTCTAA TTAGCTAGGT AATTTCAGTA CGTTTGGACT ACGTATAAGT TCTGTAACGT ACTTTACTAG   
  
  
- AATAAAATCA GTTTCTGAGG TAGTAAATAT TCGTTATAAA TGAAGCTATG TGCACAGTAA TTTTTTCTGA   
  
  
- TATCTTTACA ATGACAATAA AACAAACTCG ATTTATCTTT TAAATTTTCA ATTAATTCTA TTATTAATTT   
  
  
- TGATTTATAA GTTACTACCG ATTATTTATG TAACAGCATA ATTGACTGAT TATAAACATC TACCAGCACA   
  
  
- ATTAAAGAAA AATAAAATTT CAAGTTTACT CAATGTTTTC CAATATTTTA TATTTCATTT TACCCTCTGT   
  
  
- TTCGTCTGTC CCACCCCCTT CGATTCGAAT TTCATCCTAA ATCTTTCCGT TACTTCCACT TGGGAGTTTA   
  
  
- GCTAGGCGAC GGTTAGTGTC TTTGGGTTTC GAAACGAGTG GCTGTTGAGG GCCAATTGAC GTCAGTGAGT   
  
  
- GCCGTGACCC ATTATGTCAT TAGCACATGG GTCAAGTAGG GGGAAAAGGA AACCCTTATG TCATGGAGCT   
  
  
- TTCAAGGAAA CTTTAAGATG ACCGGTCCGT ATGGGTATTT AACTAGGAGA GCTATAGTAT TCTATACTAT   
  
  
- AAAGAGACAG TATAGACAAT CAAAGCCATA GTATAAAGAA GAAATAGAGA CATTAAACTC CATACGAAGT   
  
  
- AAGGACCGGC GAACTTAATA GAAAGAAAGA AACTAATCAT AACAAAAAAA CCAATGGATA AAACGAGGTC   
  
  
- AGGACAAGTA AACCCAGAAA AACCTAGACC CAAAAACGAC CCAAACAACT AAGAAACTCT TTAAACCCAC   
  
  
- GAACTTAAAC GGGACGTTAT AAGTACCCAA TCGCAAAAGG ACTTAAAACG AGACAAGGAA TTCACTAATA   
  
  
- AAAACTAACT AATGTGGAAC CACCAGGACC ACCTTAAAGG CTCCTCTTTA ACAGTACCCA AGGGTGTTGA   
  
  
- AACCTCTTAA GGGACTACTC TGAGATTTAC TCATAGTTAG ATGAAACCCT TGGTACAGGG GGCCAATACT   
  
  
- ACCCAGAAAC TTAATATGTT GGAACAAGTT AATGTTTCTG GGTAGAGTCC TAGAGTGCCG TGACTTGGAA   
  
  
- GGATCGGGTA ACGGACTGGG ATACGGTAAG TTGTAACCAA GTATAAGCCC AAACTCGGGG CCCCAACTTA   
  
  
- GAAGCCTACT ATCGCTAAGT CTACTACAAC AATTCATGTA ACCGGTTCAC GAATACCTTC TCCTATACTT   
  
  
- CCTCTTCGGA ACATACAAGG TACTGGGAAA TCGTGAGGTT CGACGACTCT TTGGGAAAAT ACTACACAAC   
  
  
- CCTTTCTTCA TAGGAGGAAG GGGATTAGTT GTGGGTGAAT AACTAGTAAG ACACCTATCG GGTCTGTTAG   
  
  
- TCAGGACACC TTGCTCGTGT TAATCACTAG AATCACCGTC ATTGACGTGA AGGTCAAGTT GGTTAATATA   
  
  
- ACTACGACAC CGTCAGCGAC TAAGCTCACT CTTATGATTC TGGAGAAAAC ACGTTTCAAG AGACTAACTT   
  
  
- AGCAAAAGAG TCAGCAGGTG GGAAGGTGTC ACCAGTAAAC CTAGTAACCC ACGGAACCCA CCGTGTCGAA   
  
  
- GAGTCCCAAG CTTAAGTCAA TAGAGGAGGG CACCAAAGGG ATACCGGTAC TTGCAAAAAT CGCTCGTCAG   
  
  
- GTACTATGTC AAAGTCTTCC CCCACCTCCT CCGCTCGTTC AAGGAAGGTT TCTTGTTATT GGAACGCTAA   
  
  
- CTGGAACTCT CGCAGTGAAA GGGTTTACTT TTCCTCCGCC GGGGGTACCA CCACCAATTC TTTCTCTTCC   
  
  
- TACTTGTGAG TGGACTATCG AGCTCTCCAT CGTTCTAAGT GATGGCACTA CTACTCCTGA AACTCCTACC   
  
  
- ATCCTCCTCA TTCGTCAGTC GACAAAGACA CCTCCTCCGA CTCAACAGAC TTTACAAACT GGCCCAAAAC   
  
  
- GAAACGTTAG GGTACTTTGT ACTTCGAGTA ACATGTTCAC CAAACTTCAG GCTCTTCCCT TGTAGGGACG   
  
  
- TCCGACCGGT TCATGTCCAT GTCCTCCCAG TCTTCCGAGC ACGTTTCTTT GTCCCATTAT TACTATTATT   
  
  
- CTTCTTATTC TTACACCACC TAAATTCCTG AGACGAATAG GAGACACGCG TTAGACGACG TAGACTACTA   
  
  
- GCGTCCTGTC GACTACTTGA CGACTTCGTC TAATCCCTCG TGAGAAGACG TCGACCCCTA CCTAGAGTTT   
  
  
- CCAACCGAGT AATGAAACGA TTACGGAACC TCCGCGCAAA TCGACCTTGA CCGAGTGTCT AGATATGTCG   
  
  
- GGACTCAAGC TTCTCCTGTT GTCGTCGACT ATACTATTTT CGAATAGTCA AAAAGAAAGC ACGAACGGGT   
  
  
- AAAAATTTCT AGAGATAATA GAAACGCTTG GTATACTAAT ATGTCTCTCG ACTTTTTCGT AGTTTCGAAG   
  
  
- TACAATATCT AAAACCGTAG GACATACCAA AAGTCACCGG GGAGGAGTAA GTTGCGGACA GCCTCGCTGG   
  
  
- ACCACCTGGA GGTTTTGACA CGTAATGACC CTAACTAGAA GGGGTTGGAC CTAAATCCGG TTGTCTTTCT   
  
  
- CAACTCCGTT GTCCCGAGGC GAACCGTTTC ATAACACTCG CGAAGTTACA AGGTAAACTT ATGGTACGGT   
  
  
- AACGTGTCTT TACCCTTTGG TAGTTTCGAC TTCTGGACTT CTATCTTCTA CTACTCCACC AACGGCACTT   
  
  
- GACAGAGAAG TCCAAATTCT TGGACGAACT ACTCTGCTAT CACCACCTAT CAGGCTCCTT ACGTCAAGAG   
  
  
- CCGAACTAAT CTTCCTATTT TGGACTATAG AAACAAGTGC CTCATCACTT GCCCAGAATG TTACGCGGGA   
  
  
- AGAAACAATG TGCAAAGTCC CTCCGGGAGA AGGTAATGAG ATGACATAAG CTATACAAAC TACGGTTACG   
  
  
- AAGGGCCCTC CTAGGACTCT CTAACTACAA ACTCTTCCTC AAAATACCCG CTCTTTAATA CTTACACCAC   
  
  
- CGAACACTCC CATGTCTCTC TCAACTTTCT GGACTCTGTA TGTTCGTTAC CGTCCGCGCC TTATCATCCC   
  
  
- GTCCCAAGTC CGTCGACGGT AACCTGGTTC TCGAGCAGTC ATTTGACTCC TCGTTCCAGG TCTACGTGAT   
  
  
- AGGATCCCTG AAGCAACAAC TACATCTACC TGTAACCTGT AACGTCCCTA CCTTCCCCTC CTAGTAGACA   
  
  
- CGACACAGAC GTACCCCAGG ACGAAT

+     MBS

| Site Name | Organism | Position | Strand | Matrix score. | sequence | function |
| --- | --- | --- | --- | --- | --- | --- |
| MBS | Arabidopsis thaliana | 519 | - | 6 | CAACTG | MYB binding site involved in drought-inducibility |

>HU02G01569.1   
+ +Up\_Stream \_Len000AGCGTA GGAAGTGACG CCGCTATTGG AGGCGAAGAC GCTAGTACCA AGGCCAAAAC   
  
  
+ AAATGAGGAG ATAGATCAGT TAAACCGTTT GGTTAAGAAA ATTAAGCGAA CATCTCTTGA ACCTCCATCT   
  
  
+ GAACTCGATG ACATTGAGGA TGTGGATGCA GAAAAGATGG ATATGGGCTC TCCATCGGTG CCAGCAAATC   
  
  
+ AACCTAATGT TGGGTGGGGT CTCATTCGGT CAATGGGGGA CTAGGGACGT CATATCGTCA CCAATTACAA   
  
  
+ CGGAACAACC CTAAACCGCA TTTTAAAATC ACATCTTGGT AGCAGACAAA GATGTACGAC CCAAAAATTC   
  
  
+ AACTCTGTAA AGCACTAGCA AAGCTCGTAG TACACAGAAC AATCGCACAC TCATAGTGAT AGAAGTGTAC   
  
  
+ TATCCATTAT GCAGTGAATA TGACAAGGGA GGGGGTCCTG AGATGCCCAA ATTTGGCAGC CGAGTTGCCT   
  
  
+ TGACTGCCCA TCAATGTCCT AAACCAGTTG AGCATGTTAT TAATTTGCAA GAAAGATCGA ATTCTTCCTA   
  
  
+ TCTAAAAACT GACACAATAA AACAATACCA TGTATGGGAC ATTGCTAGGA TTGTTAACAC AATTATTGTC   
  
  
+ TTGTACGTTG CACCTGAAAA AAAAGAAAAT TGTTGCCATA GACAACATAT AAGTTAGGCA TTCATGACTA   
  
  
+ ATATCTGAAT TAGGTGTGTC GATCTGTGAT TCAAATTGAT ATAATTAGAT CTTTAAGGTA TCTAATTGCT   
  
  
+ TCAAGTTTTC TCTATAATGA AGATGTATAT TAGCTCACAT AGGTCTTAAG GTTAACTAAT GTCATATCAG   
  
  
+ GGGGTGTGGG CCGCCATTTT TGATAAGAAT TGAATTGAAC TTGCATTGTT TTTTCTTTGG CGAGAATGCA   
  
  
+ AGATTTGTTA ATTTATTCAT ATGCGTCATT ATACACTATA AGAAAAATAA TTGTAGGAAT ATATGAAGTA   
  
  
+ TTTTTACTTG TGCTCATGTT AAATGTGGAA TACTTTTTTA ATGTCGTATA GTACCACAGT ATTCTACAAT   
  
  
+ TTAAGAGATT AATCGATCCA TTAAAGTCAT GCAAACCTGA TGCATATTCA AGACATTGCA TGAAATGATC   
  
  
+ TTATTTTAGT CAAAGACTCC ATCATTTATA AGCAATATTT ACTTCGATAC ACGTGTCATT AAAAAAGACT   
  
  
+ ATAGAAATGT TACTGTTATT TTGTTTGAGC TAAATAGAAA ATTTAAAAGT TAATTAAGAT AATAATTAAA   
  
  
+ ACTAAATATT CAATGATGGC TAATAAATAC ATTGTCGTAT TAACTGACTA ATATTTGTAG ATGGTCGTGT   
  
  
+ TAATTTCTTT TTATTTTAAA GTTCAAATGA GTTACAAAAG GTTATAAAAT ATAAAGTAAA ATGGGAGACA   
  
  
+ AAGCAGACAG GGTGGGGGAA GCTAAGCTTA AAGTAGGATT TAGAAAGGCA ATGAAGGTGA ACCCTCAAAT   
  
  
+ CGATCCGCTG CCAATCACAG AAACCCAAAG CTTTGCTCAC CGACAACTCC CGGTTAACTG CAGTCACTCA   
  
  
+ CGGCACTGGG TAATACAGTA ATCGTGTACC CAGTTCATCC CCCTTTTCCT TTGGGAATAC AGTACCTCGA   
  
  
+ AAGTTCCTTT GAAATTCTAC TGGCCAGGCA TACCCATAAA TTGATCCTCT CGATATCATA AGATATGATA   
  
  
+ TTTCTCTGTC ATATCTGTTA GTTTCGGTAT CATATTTCTT CTTTATCTCT GTAATTTGAG GTATGCTTCA   
  
  
+ TTCCTGGCCG CTTGAATTAT CTTTCTTTCT TTGATTAGTA TTGTTTTTTT GGTTACCTAT TTTGCTCCAG   
  
  
+ TCCTGTTCAT TTGGGTCTTT TTGGATCTGG GTTTTTGCTG GGTTTGTTGA TTCTTTGAGA AATTTGGGTG   
  
  
+ CTTGAATTTG CCCTGCAATA TTCATGGGTT AGCGTTTTCC TGAATTTTGC TCTGTTCCTT AAGTGATTAT   
  
  
+ TTTTGATTGA TTACACCTTG GTGGTCCTGG TGGAATTTCC GAGGAGAAAT TGTCATGGGT TCCCACAACT   
  
  
+ TTGGAGAATT CCCTGATGAG ACTCTAAATG AGTATCAATC TACTTTGGGA ACCATGTCCC CCGGTTATGA   
  
  
+ TGGGTCTTTG AATTATACAA CCTTGTTCAA TTACAAAGAC CCATCTCAGG ATCTCACGGC ACTGAACCTT   
  
  
+ CCTAGCCCAT TGCCTGACCC TATGCCATTC AACATTGGTT CATATTCGGG TTTGAGCCCC GGGGTTGAAT   
  
  
+ CTTCGGATGA TAGCGATTCA GATGATGTTG TTAAGTACAT TGGCCAAGTG CTTATGGAAG AGGATATGAA   
  
  
+ GGAGAAGCCT TGTATGTTCC ATGACCCTTT AGCACTCCAA GCTGCTGAGA AACCCTTTTA TGATGTGTTG   
  
  
+ GGAAAGAAGT ATCCTCCTTC CCCTAATCAA CACCCACTTA TTGATCATTC TGTGGATAGC CCAGACAATC   
  
  
+ AGTCCTGTGG AACGAGCACA ATTAGTGATC TTAGTGGCAG TAACTGCACT TCCAGTTCAA CCAATTATAT   
  
  
+ TGATGCTGTG GCAGTCGCTG ATTCGAGTGA GAATACTAAG ACCTCTTTTG TGCAAAGTTC TCTGATTGAA   
  
  
+ TCGTTTTCTC AGTCGTCCAC CCTTCCACAG TGGTCATTTG GATCATTGGG TGCCTTGGGT GGCACAGCTT   
  
  
+ CTCAGGGTTC GAATTCAGTT ATCTCCTCCC GTGGTTTCCC TATGGCCATG AACGTTTTTA GCGAGCAGTC   
  
  
+ CATGATACAG TTTCAGAAGG GGGTGGAGGA GGCGAGCAAG TTCCTTCCAA AGAACAATAA CCTTGCGATT   
  
  
+ GACCTTGAGA GCGTCACTTT CCCAAATGAA AAGGAGGCGG CCCCCATGGT GGTGGTTAAG AAAGAGAAGG   
  
  
+ ATGAACACTC ACCTGATAGC TCGAGAGGTA GCAAGATTCA CTACCGTGAT GATGAGGACT TTGAGGATGG   
  
  
+ TAGGAGGAGT AAGCAGTCAG CTGTTTCTGT GGAGGAGGCT GAGTTGTCTG AAATGTTTGA CCGGGTTTTG   
  
  
+ CTTTGCAATC CCATGAAACA TGAAGCTCAT TGTACAAGTG GTTTGAAGTC CGAGAAGGGA ACATCCCTGC   
  
  
+ AGGCTGGCCA AGTACAGGTA CAGGAGGGTC AGAAGGCTCG TGCAAAGAAA CAGGGTAATA ATGATAATAA   
  
  
+ GAAGAATAAG AATGTGGTGG ATTTAAGGAC TCTGCTTATC CTCTGTGCGC AATCTGCTGC ATCTGATGAT   
  
  
+ CGCAGGACAG CTGATGAACT GCTGAAGCAG ATTAGGGAGC ACTCTTCTGC AGCTGGGGAT GGATCTCAAA   
  
  
+ GGTTGGCTCA TTACTTTGCT AATGCCTTGG AGGCGCGTTT AGCTGGAACT GGCTCACAGA TCTATACAGC   
  
  
+ CCTGAGTTCG AAGAGGACAA CAGCAGCTGA TATGATAAAA GCTTATCAGT TTTTCTTTCG TGCTTGCCCA   
  
  
+ TTTTTAAAGA TCTCTATTAT CTTTGCGAAC CATATGATTA TACAGAGAGC TGAAAAAGCA TCAAAGCTTC   
  
  
+ ATGTTATAGA TTTTGGCATC CTGTATGGTT TTCAGTGGCC CCTCCTCATT CAACGCCTGT CGGAGCGACC   
  
  
+ TGGTGGACCT CCAAAACTGT GCATTACTGG GATTGATCTT CCCCAACCTG GATTTAGGCC AACAGAAAGA   
  
  
+ GTTGAGGCAA CAGGGCTCCG CTTGGCAAAG TATTGTGAGC GCTTCAATGT TCCATTTGAA TACCATGCCA   
  
  
+ TTGCACAGAA ATGGGAAACC ATCAAAGCTG AAGACCTGAA GATAGAAGAT GATGAGGTGG TTGCCGTGAA   
  
  
+ CTGTCTCTTC AGGTTTAAGA ACCTGCTTGA TGAGACGATA GTGGTGGATA GTCCGAGGAA TGCAGTTCTC   
  
  
+ GGCTTGATTA GAAGGATAAA ACCTGATATC TTTGTTCACG GAGTAGTGAA CGGGTCTTAC AATGCGCCCT   
  
  
+ TCTTTGTTAC ACGTTTCAGG GAGGCCCTCT TCCATTACTC TACTGTATTC GATATGTTTG ATGCCAATGC   
  
  
+ TTCCCGGGAG GATCCTGAGA GATTGATGTT TGAGAAGGAG TTTTATGGGC GAGAAATTAT GAATGTGGTG   
  
  
+ GCTTGTGAGG GTACAGAGAG AGTTGAAAGA CCTGAGACAT ACAAGCAATG GCAGGCGCGG AATAGTAGGG   
  
  
+ CAGGGTTCAG GCAGCTGCCA TTGGACCAAG AGCTCGTCAG TAAACTGAGG AGCAAGGTCC AGATGCACTA   
  
  
+ TCCTAGGGAC TTCGTTGTTG ATGTAGATGG ACATTGGACA TTGCAGGGAT GGAAGGGGAG GATCATCTGT   
  
  
+ GCTGTGTCTG CATGGGGTCC TGCTTA  

- +Up\_Stream \_Len000TCGCAT CCTTCACTGC GGCGATAACC TCCGCTTCTG CGATCATGGT TCCGGTTTTG   
  
  
- TTTACTCCTC TATCTAGTCA ATTTGGCAAA CCAATTCTTT TAATTCGCTT GTAGAGAACT TGGAGGTAGA   
  
  
- CTTGAGCTAC TGTAACTCCT ACACCTACGT CTTTTCTACC TATACCCGAG AGGTAGCCAC GGTCGTTTAG   
  
  
- TTGGATTACA ACCCACCCCA GAGTAAGCCA GTTACCCCCT GATCCCTGCA GTATAGCAGT GGTTAATGTT   
  
  
- GCCTTGTTGG GATTTGGCGT AAAATTTTAG TGTAGAACCA TCGTCTGTTT CTACATGCTG GGTTTTTAAG   
  
  
- TTGAGACATT TCGTGATCGT TTCGAGCATC ATGTGTCTTG TTAGCGTGTG AGTATCACTA TCTTCACATG   
  
  
- ATAGGTAATA CGTCACTTAT ACTGTTCCCT CCCCCAGGAC TCTACGGGTT TAAACCGTCG GCTCAACGGA   
  
  
- ACTGACGGGT AGTTACAGGA TTTGGTCAAC TCGTACAATA ATTAAACGTT CTTTCTAGCT TAAGAAGGAT   
  
  
- AGATTTTTGA CTGTGTTATT TTGTTATGGT ACATACCCTG TAACGATCCT AACAATTGTG TTAATAACAG   
  
  
- AACATGCAAC GTGGACTTTT TTTTCTTTTA ACAACGGTAT CTGTTGTATA TTCAATCCGT AAGTACTGAT   
  
  
- TATAGACTTA ATCCACACAG CTAGACACTA AGTTTAACTA TATTAATCTA GAAATTCCAT AGATTAACGA   
  
  
- AGTTCAAAAG AGATATTACT TCTACATATA ATCGAGTGTA TCCAGAATTC CAATTGATTA CAGTATAGTC   
  
  
- CCCCACACCC GGCGGTAAAA ACTATTCTTA ACTTAACTTG AACGTAACAA AAAAGAAACC GCTCTTACGT   
  
  
- TCTAAACAAT TAAATAAGTA TACGCAGTAA TATGTGATAT TCTTTTTATT AACATCCTTA TATACTTCAT   
  
  
- AAAAATGAAC ACGAGTACAA TTTACACCTT ATGAAAAAAT TACAGCATAT CATGGTGTCA TAAGATGTTA   
  
  
- AATTCTCTAA TTAGCTAGGT AATTTCAGTA CGTTTGGACT ACGTATAAGT TCTGTAACGT ACTTTACTAG   
  
  
- AATAAAATCA GTTTCTGAGG TAGTAAATAT TCGTTATAAA TGAAGCTATG TGCACAGTAA TTTTTTCTGA   
  
  
- TATCTTTACA ATGACAATAA AACAAACTCG ATTTATCTTT TAAATTTTCA ATTAATTCTA TTATTAATTT   
  
  
- TGATTTATAA GTTACTACCG ATTATTTATG TAACAGCATA ATTGACTGAT TATAAACATC TACCAGCACA   
  
  
- ATTAAAGAAA AATAAAATTT CAAGTTTACT CAATGTTTTC CAATATTTTA TATTTCATTT TACCCTCTGT   
  
  
- TTCGTCTGTC CCACCCCCTT CGATTCGAAT TTCATCCTAA ATCTTTCCGT TACTTCCACT TGGGAGTTTA   
  
  
- GCTAGGCGAC GGTTAGTGTC TTTGGGTTTC GAAACGAGTG GCTGTTGAGG GCCAATTGAC GTCAGTGAGT   
  
  
- GCCGTGACCC ATTATGTCAT TAGCACATGG GTCAAGTAGG GGGAAAAGGA AACCCTTATG TCATGGAGCT   
  
  
- TTCAAGGAAA CTTTAAGATG ACCGGTCCGT ATGGGTATTT AACTAGGAGA GCTATAGTAT TCTATACTAT   
  
  
- AAAGAGACAG TATAGACAAT CAAAGCCATA GTATAAAGAA GAAATAGAGA CATTAAACTC CATACGAAGT   
  
  
- AAGGACCGGC GAACTTAATA GAAAGAAAGA AACTAATCAT AACAAAAAAA CCAATGGATA AAACGAGGTC   
  
  
- AGGACAAGTA AACCCAGAAA AACCTAGACC CAAAAACGAC CCAAACAACT AAGAAACTCT TTAAACCCAC   
  
  
- GAACTTAAAC GGGACGTTAT AAGTACCCAA TCGCAAAAGG ACTTAAAACG AGACAAGGAA TTCACTAATA   
  
  
- AAAACTAACT AATGTGGAAC CACCAGGACC ACCTTAAAGG CTCCTCTTTA ACAGTACCCA AGGGTGTTGA   
  
  
- AACCTCTTAA GGGACTACTC TGAGATTTAC TCATAGTTAG ATGAAACCCT TGGTACAGGG GGCCAATACT   
  
  
- ACCCAGAAAC TTAATATGTT GGAACAAGTT AATGTTTCTG GGTAGAGTCC TAGAGTGCCG TGACTTGGAA   
  
  
- GGATCGGGTA ACGGACTGGG ATACGGTAAG TTGTAACCAA GTATAAGCCC AAACTCGGGG CCCCAACTTA   
  
  
- GAAGCCTACT ATCGCTAAGT CTACTACAAC AATTCATGTA ACCGGTTCAC GAATACCTTC TCCTATACTT   
  
  
- CCTCTTCGGA ACATACAAGG TACTGGGAAA TCGTGAGGTT CGACGACTCT TTGGGAAAAT ACTACACAAC   
  
  
- CCTTTCTTCA TAGGAGGAAG GGGATTAGTT GTGGGTGAAT AACTAGTAAG ACACCTATCG GGTCTGTTAG   
  
  
- TCAGGACACC TTGCTCGTGT TAATCACTAG AATCACCGTC ATTGACGTGA AGGTCAAGTT GGTTAATATA   
  
  
- ACTACGACAC CGTCAGCGAC TAAGCTCACT CTTATGATTC TGGAGAAAAC ACGTTTCAAG AGACTAACTT   
  
  
- AGCAAAAGAG TCAGCAGGTG GGAAGGTGTC ACCAGTAAAC CTAGTAACCC ACGGAACCCA CCGTGTCGAA   
  
  
- GAGTCCCAAG CTTAAGTCAA TAGAGGAGGG CACCAAAGGG ATACCGGTAC TTGCAAAAAT CGCTCGTCAG   
  
  
- GTACTATGTC AAAGTCTTCC CCCACCTCCT CCGCTCGTTC AAGGAAGGTT TCTTGTTATT GGAACGCTAA   
  
  
- CTGGAACTCT CGCAGTGAAA GGGTTTACTT TTCCTCCGCC GGGGGTACCA CCACCAATTC TTTCTCTTCC   
  
  
- TACTTGTGAG TGGACTATCG AGCTCTCCAT CGTTCTAAGT GATGGCACTA CTACTCCTGA AACTCCTACC   
  
  
- ATCCTCCTCA TTCGTCAGTC GACAAAGACA CCTCCTCCGA CTCAACAGAC TTTACAAACT GGCCCAAAAC   
  
  
- GAAACGTTAG GGTACTTTGT ACTTCGAGTA ACATGTTCAC CAAACTTCAG GCTCTTCCCT TGTAGGGACG   
  
  
- TCCGACCGGT TCATGTCCAT GTCCTCCCAG TCTTCCGAGC ACGTTTCTTT GTCCCATTAT TACTATTATT   
  
  
- CTTCTTATTC TTACACCACC TAAATTCCTG AGACGAATAG GAGACACGCG TTAGACGACG TAGACTACTA   
  
  
- GCGTCCTGTC GACTACTTGA CGACTTCGTC TAATCCCTCG TGAGAAGACG TCGACCCCTA CCTAGAGTTT   
  
  
- CCAACCGAGT AATGAAACGA TTACGGAACC TCCGCGCAAA TCGACCTTGA CCGAGTGTCT AGATATGTCG   
  
  
- GGACTCAAGC TTCTCCTGTT GTCGTCGACT ATACTATTTT CGAATAGTCA AAAAGAAAGC ACGAACGGGT   
  
  
- AAAAATTTCT AGAGATAATA GAAACGCTTG GTATACTAAT ATGTCTCTCG ACTTTTTCGT AGTTTCGAAG   
  
  
- TACAATATCT AAAACCGTAG GACATACCAA AAGTCACCGG GGAGGAGTAA GTTGCGGACA GCCTCGCTGG   
  
  
- ACCACCTGGA GGTTTTGACA CGTAATGACC CTAACTAGAA GGGGTTGGAC CTAAATCCGG TTGTCTTTCT   
  
  
- CAACTCCGTT GTCCCGAGGC GAACCGTTTC ATAACACTCG CGAAGTTACA AGGTAAACTT ATGGTACGGT   
  
  
- AACGTGTCTT TACCCTTTGG TAGTTTCGAC TTCTGGACTT CTATCTTCTA CTACTCCACC AACGGCACTT   
  
  
- GACAGAGAAG TCCAAATTCT TGGACGAACT ACTCTGCTAT CACCACCTAT CAGGCTCCTT ACGTCAAGAG   
  
  
- CCGAACTAAT CTTCCTATTT TGGACTATAG AAACAAGTGC CTCATCACTT GCCCAGAATG TTACGCGGGA   
  
  
- AGAAACAATG TGCAAAGTCC CTCCGGGAGA AGGTAATGAG ATGACATAAG CTATACAAAC TACGGTTACG   
  
  
- AAGGGCCCTC CTAGGACTCT CTAACTACAA ACTCTTCCTC AAAATACCCG CTCTTTAATA CTTACACCAC   
  
  
- CGAACACTCC CATGTCTCTC TCAACTTTCT GGACTCTGTA TGTTCGTTAC CGTCCGCGCC TTATCATCCC   
  
  
- GTCCCAAGTC CGTCGACGGT AACCTGGTTC TCGAGCAGTC ATTTGACTCC TCGTTCCAGG TCTACGTGAT   
  
  
- AGGATCCCTG AAGCAACAAC TACATCTACC TGTAACCTGT AACGTCCCTA CCTTCCCCTC CTAGTAGACA   
  
  
- CGACACAGAC GTACCCCAGG ACGAAT

+     MRE

| Site Name | Organism | Position | Strand | Matrix score. | sequence | function |
| --- | --- | --- | --- | --- | --- | --- |
| MRE | Petroselinum crispum | 215 | + | 7 | AACCTAA | MYB binding site involved in light responsiveness |

>HU02G01569.1   
+ +Up\_Stream \_Len000AGCGTA GGAAGTGACG CCGCTATTGG AGGCGAAGAC GCTAGTACCA AGGCCAAAAC   
  
  
+ AAATGAGGAG ATAGATCAGT TAAACCGTTT GGTTAAGAAA ATTAAGCGAA CATCTCTTGA ACCTCCATCT   
  
  
+ GAACTCGATG ACATTGAGGA TGTGGATGCA GAAAAGATGG ATATGGGCTC TCCATCGGTG CCAGCAAATC   
  
  
+ AACCTAATGT TGGGTGGGGT CTCATTCGGT CAATGGGGGA CTAGGGACGT CATATCGTCA CCAATTACAA   
  
  
+ CGGAACAACC CTAAACCGCA TTTTAAAATC ACATCTTGGT AGCAGACAAA GATGTACGAC CCAAAAATTC   
  
  
+ AACTCTGTAA AGCACTAGCA AAGCTCGTAG TACACAGAAC AATCGCACAC TCATAGTGAT AGAAGTGTAC   
  
  
+ TATCCATTAT GCAGTGAATA TGACAAGGGA GGGGGTCCTG AGATGCCCAA ATTTGGCAGC CGAGTTGCCT   
  
  
+ TGACTGCCCA TCAATGTCCT AAACCAGTTG AGCATGTTAT TAATTTGCAA GAAAGATCGA ATTCTTCCTA   
  
  
+ TCTAAAAACT GACACAATAA AACAATACCA TGTATGGGAC ATTGCTAGGA TTGTTAACAC AATTATTGTC   
  
  
+ TTGTACGTTG CACCTGAAAA AAAAGAAAAT TGTTGCCATA GACAACATAT AAGTTAGGCA TTCATGACTA   
  
  
+ ATATCTGAAT TAGGTGTGTC GATCTGTGAT TCAAATTGAT ATAATTAGAT CTTTAAGGTA TCTAATTGCT   
  
  
+ TCAAGTTTTC TCTATAATGA AGATGTATAT TAGCTCACAT AGGTCTTAAG GTTAACTAAT GTCATATCAG   
  
  
+ GGGGTGTGGG CCGCCATTTT TGATAAGAAT TGAATTGAAC TTGCATTGTT TTTTCTTTGG CGAGAATGCA   
  
  
+ AGATTTGTTA ATTTATTCAT ATGCGTCATT ATACACTATA AGAAAAATAA TTGTAGGAAT ATATGAAGTA   
  
  
+ TTTTTACTTG TGCTCATGTT AAATGTGGAA TACTTTTTTA ATGTCGTATA GTACCACAGT ATTCTACAAT   
  
  
+ TTAAGAGATT AATCGATCCA TTAAAGTCAT GCAAACCTGA TGCATATTCA AGACATTGCA TGAAATGATC   
  
  
+ TTATTTTAGT CAAAGACTCC ATCATTTATA AGCAATATTT ACTTCGATAC ACGTGTCATT AAAAAAGACT   
  
  
+ ATAGAAATGT TACTGTTATT TTGTTTGAGC TAAATAGAAA ATTTAAAAGT TAATTAAGAT AATAATTAAA   
  
  
+ ACTAAATATT CAATGATGGC TAATAAATAC ATTGTCGTAT TAACTGACTA ATATTTGTAG ATGGTCGTGT   
  
  
+ TAATTTCTTT TTATTTTAAA GTTCAAATGA GTTACAAAAG GTTATAAAAT ATAAAGTAAA ATGGGAGACA   
  
  
+ AAGCAGACAG GGTGGGGGAA GCTAAGCTTA AAGTAGGATT TAGAAAGGCA ATGAAGGTGA ACCCTCAAAT   
  
  
+ CGATCCGCTG CCAATCACAG AAACCCAAAG CTTTGCTCAC CGACAACTCC CGGTTAACTG CAGTCACTCA   
  
  
+ CGGCACTGGG TAATACAGTA ATCGTGTACC CAGTTCATCC CCCTTTTCCT TTGGGAATAC AGTACCTCGA   
  
  
+ AAGTTCCTTT GAAATTCTAC TGGCCAGGCA TACCCATAAA TTGATCCTCT CGATATCATA AGATATGATA   
  
  
+ TTTCTCTGTC ATATCTGTTA GTTTCGGTAT CATATTTCTT CTTTATCTCT GTAATTTGAG GTATGCTTCA   
  
  
+ TTCCTGGCCG CTTGAATTAT CTTTCTTTCT TTGATTAGTA TTGTTTTTTT GGTTACCTAT TTTGCTCCAG   
  
  
+ TCCTGTTCAT TTGGGTCTTT TTGGATCTGG GTTTTTGCTG GGTTTGTTGA TTCTTTGAGA AATTTGGGTG   
  
  
+ CTTGAATTTG CCCTGCAATA TTCATGGGTT AGCGTTTTCC TGAATTTTGC TCTGTTCCTT AAGTGATTAT   
  
  
+ TTTTGATTGA TTACACCTTG GTGGTCCTGG TGGAATTTCC GAGGAGAAAT TGTCATGGGT TCCCACAACT   
  
  
+ TTGGAGAATT CCCTGATGAG ACTCTAAATG AGTATCAATC TACTTTGGGA ACCATGTCCC CCGGTTATGA   
  
  
+ TGGGTCTTTG AATTATACAA CCTTGTTCAA TTACAAAGAC CCATCTCAGG ATCTCACGGC ACTGAACCTT   
  
  
+ CCTAGCCCAT TGCCTGACCC TATGCCATTC AACATTGGTT CATATTCGGG TTTGAGCCCC GGGGTTGAAT   
  
  
+ CTTCGGATGA TAGCGATTCA GATGATGTTG TTAAGTACAT TGGCCAAGTG CTTATGGAAG AGGATATGAA   
  
  
+ GGAGAAGCCT TGTATGTTCC ATGACCCTTT AGCACTCCAA GCTGCTGAGA AACCCTTTTA TGATGTGTTG   
  
  
+ GGAAAGAAGT ATCCTCCTTC CCCTAATCAA CACCCACTTA TTGATCATTC TGTGGATAGC CCAGACAATC   
  
  
+ AGTCCTGTGG AACGAGCACA ATTAGTGATC TTAGTGGCAG TAACTGCACT TCCAGTTCAA CCAATTATAT   
  
  
+ TGATGCTGTG GCAGTCGCTG ATTCGAGTGA GAATACTAAG ACCTCTTTTG TGCAAAGTTC TCTGATTGAA   
  
  
+ TCGTTTTCTC AGTCGTCCAC CCTTCCACAG TGGTCATTTG GATCATTGGG TGCCTTGGGT GGCACAGCTT   
  
  
+ CTCAGGGTTC GAATTCAGTT ATCTCCTCCC GTGGTTTCCC TATGGCCATG AACGTTTTTA GCGAGCAGTC   
  
  
+ CATGATACAG TTTCAGAAGG GGGTGGAGGA GGCGAGCAAG TTCCTTCCAA AGAACAATAA CCTTGCGATT   
  
  
+ GACCTTGAGA GCGTCACTTT CCCAAATGAA AAGGAGGCGG CCCCCATGGT GGTGGTTAAG AAAGAGAAGG   
  
  
+ ATGAACACTC ACCTGATAGC TCGAGAGGTA GCAAGATTCA CTACCGTGAT GATGAGGACT TTGAGGATGG   
  
  
+ TAGGAGGAGT AAGCAGTCAG CTGTTTCTGT GGAGGAGGCT GAGTTGTCTG AAATGTTTGA CCGGGTTTTG   
  
  
+ CTTTGCAATC CCATGAAACA TGAAGCTCAT TGTACAAGTG GTTTGAAGTC CGAGAAGGGA ACATCCCTGC   
  
  
+ AGGCTGGCCA AGTACAGGTA CAGGAGGGTC AGAAGGCTCG TGCAAAGAAA CAGGGTAATA ATGATAATAA   
  
  
+ GAAGAATAAG AATGTGGTGG ATTTAAGGAC TCTGCTTATC CTCTGTGCGC AATCTGCTGC ATCTGATGAT   
  
  
+ CGCAGGACAG CTGATGAACT GCTGAAGCAG ATTAGGGAGC ACTCTTCTGC AGCTGGGGAT GGATCTCAAA   
  
  
+ GGTTGGCTCA TTACTTTGCT AATGCCTTGG AGGCGCGTTT AGCTGGAACT GGCTCACAGA TCTATACAGC   
  
  
+ CCTGAGTTCG AAGAGGACAA CAGCAGCTGA TATGATAAAA GCTTATCAGT TTTTCTTTCG TGCTTGCCCA   
  
  
+ TTTTTAAAGA TCTCTATTAT CTTTGCGAAC CATATGATTA TACAGAGAGC TGAAAAAGCA TCAAAGCTTC   
  
  
+ ATGTTATAGA TTTTGGCATC CTGTATGGTT TTCAGTGGCC CCTCCTCATT CAACGCCTGT CGGAGCGACC   
  
  
+ TGGTGGACCT CCAAAACTGT GCATTACTGG GATTGATCTT CCCCAACCTG GATTTAGGCC AACAGAAAGA   
  
  
+ GTTGAGGCAA CAGGGCTCCG CTTGGCAAAG TATTGTGAGC GCTTCAATGT TCCATTTGAA TACCATGCCA   
  
  
+ TTGCACAGAA ATGGGAAACC ATCAAAGCTG AAGACCTGAA GATAGAAGAT GATGAGGTGG TTGCCGTGAA   
  
  
+ CTGTCTCTTC AGGTTTAAGA ACCTGCTTGA TGAGACGATA GTGGTGGATA GTCCGAGGAA TGCAGTTCTC   
  
  
+ GGCTTGATTA GAAGGATAAA ACCTGATATC TTTGTTCACG GAGTAGTGAA CGGGTCTTAC AATGCGCCCT   
  
  
+ TCTTTGTTAC ACGTTTCAGG GAGGCCCTCT TCCATTACTC TACTGTATTC GATATGTTTG ATGCCAATGC   
  
  
+ TTCCCGGGAG GATCCTGAGA GATTGATGTT TGAGAAGGAG TTTTATGGGC GAGAAATTAT GAATGTGGTG   
  
  
+ GCTTGTGAGG GTACAGAGAG AGTTGAAAGA CCTGAGACAT ACAAGCAATG GCAGGCGCGG AATAGTAGGG   
  
  
+ CAGGGTTCAG GCAGCTGCCA TTGGACCAAG AGCTCGTCAG TAAACTGAGG AGCAAGGTCC AGATGCACTA   
  
  
+ TCCTAGGGAC TTCGTTGTTG ATGTAGATGG ACATTGGACA TTGCAGGGAT GGAAGGGGAG GATCATCTGT   
  
  
+ GCTGTGTCTG CATGGGGTCC TGCTTA  

- +Up\_Stream \_Len000TCGCAT CCTTCACTGC GGCGATAACC TCCGCTTCTG CGATCATGGT TCCGGTTTTG   
  
  
- TTTACTCCTC TATCTAGTCA ATTTGGCAAA CCAATTCTTT TAATTCGCTT GTAGAGAACT TGGAGGTAGA   
  
  
- CTTGAGCTAC TGTAACTCCT ACACCTACGT CTTTTCTACC TATACCCGAG AGGTAGCCAC GGTCGTTTAG   
  
  
- TTGGATTACA ACCCACCCCA GAGTAAGCCA GTTACCCCCT GATCCCTGCA GTATAGCAGT GGTTAATGTT   
  
  
- GCCTTGTTGG GATTTGGCGT AAAATTTTAG TGTAGAACCA TCGTCTGTTT CTACATGCTG GGTTTTTAAG   
  
  
- TTGAGACATT TCGTGATCGT TTCGAGCATC ATGTGTCTTG TTAGCGTGTG AGTATCACTA TCTTCACATG   
  
  
- ATAGGTAATA CGTCACTTAT ACTGTTCCCT CCCCCAGGAC TCTACGGGTT TAAACCGTCG GCTCAACGGA   
  
  
- ACTGACGGGT AGTTACAGGA TTTGGTCAAC TCGTACAATA ATTAAACGTT CTTTCTAGCT TAAGAAGGAT   
  
  
- AGATTTTTGA CTGTGTTATT TTGTTATGGT ACATACCCTG TAACGATCCT AACAATTGTG TTAATAACAG   
  
  
- AACATGCAAC GTGGACTTTT TTTTCTTTTA ACAACGGTAT CTGTTGTATA TTCAATCCGT AAGTACTGAT   
  
  
- TATAGACTTA ATCCACACAG CTAGACACTA AGTTTAACTA TATTAATCTA GAAATTCCAT AGATTAACGA   
  
  
- AGTTCAAAAG AGATATTACT TCTACATATA ATCGAGTGTA TCCAGAATTC CAATTGATTA CAGTATAGTC   
  
  
- CCCCACACCC GGCGGTAAAA ACTATTCTTA ACTTAACTTG AACGTAACAA AAAAGAAACC GCTCTTACGT   
  
  
- TCTAAACAAT TAAATAAGTA TACGCAGTAA TATGTGATAT TCTTTTTATT AACATCCTTA TATACTTCAT   
  
  
- AAAAATGAAC ACGAGTACAA TTTACACCTT ATGAAAAAAT TACAGCATAT CATGGTGTCA TAAGATGTTA   
  
  
- AATTCTCTAA TTAGCTAGGT AATTTCAGTA CGTTTGGACT ACGTATAAGT TCTGTAACGT ACTTTACTAG   
  
  
- AATAAAATCA GTTTCTGAGG TAGTAAATAT TCGTTATAAA TGAAGCTATG TGCACAGTAA TTTTTTCTGA   
  
  
- TATCTTTACA ATGACAATAA AACAAACTCG ATTTATCTTT TAAATTTTCA ATTAATTCTA TTATTAATTT   
  
  
- TGATTTATAA GTTACTACCG ATTATTTATG TAACAGCATA ATTGACTGAT TATAAACATC TACCAGCACA   
  
  
- ATTAAAGAAA AATAAAATTT CAAGTTTACT CAATGTTTTC CAATATTTTA TATTTCATTT TACCCTCTGT   
  
  
- TTCGTCTGTC CCACCCCCTT CGATTCGAAT TTCATCCTAA ATCTTTCCGT TACTTCCACT TGGGAGTTTA   
  
  
- GCTAGGCGAC GGTTAGTGTC TTTGGGTTTC GAAACGAGTG GCTGTTGAGG GCCAATTGAC GTCAGTGAGT   
  
  
- GCCGTGACCC ATTATGTCAT TAGCACATGG GTCAAGTAGG GGGAAAAGGA AACCCTTATG TCATGGAGCT   
  
  
- TTCAAGGAAA CTTTAAGATG ACCGGTCCGT ATGGGTATTT AACTAGGAGA GCTATAGTAT TCTATACTAT   
  
  
- AAAGAGACAG TATAGACAAT CAAAGCCATA GTATAAAGAA GAAATAGAGA CATTAAACTC CATACGAAGT   
  
  
- AAGGACCGGC GAACTTAATA GAAAGAAAGA AACTAATCAT AACAAAAAAA CCAATGGATA AAACGAGGTC   
  
  
- AGGACAAGTA AACCCAGAAA AACCTAGACC CAAAAACGAC CCAAACAACT AAGAAACTCT TTAAACCCAC   
  
  
- GAACTTAAAC GGGACGTTAT AAGTACCCAA TCGCAAAAGG ACTTAAAACG AGACAAGGAA TTCACTAATA   
  
  
- AAAACTAACT AATGTGGAAC CACCAGGACC ACCTTAAAGG CTCCTCTTTA ACAGTACCCA AGGGTGTTGA   
  
  
- AACCTCTTAA GGGACTACTC TGAGATTTAC TCATAGTTAG ATGAAACCCT TGGTACAGGG GGCCAATACT   
  
  
- ACCCAGAAAC TTAATATGTT GGAACAAGTT AATGTTTCTG GGTAGAGTCC TAGAGTGCCG TGACTTGGAA   
  
  
- GGATCGGGTA ACGGACTGGG ATACGGTAAG TTGTAACCAA GTATAAGCCC AAACTCGGGG CCCCAACTTA   
  
  
- GAAGCCTACT ATCGCTAAGT CTACTACAAC AATTCATGTA ACCGGTTCAC GAATACCTTC TCCTATACTT   
  
  
- CCTCTTCGGA ACATACAAGG TACTGGGAAA TCGTGAGGTT CGACGACTCT TTGGGAAAAT ACTACACAAC   
  
  
- CCTTTCTTCA TAGGAGGAAG GGGATTAGTT GTGGGTGAAT AACTAGTAAG ACACCTATCG GGTCTGTTAG   
  
  
- TCAGGACACC TTGCTCGTGT TAATCACTAG AATCACCGTC ATTGACGTGA AGGTCAAGTT GGTTAATATA   
  
  
- ACTACGACAC CGTCAGCGAC TAAGCTCACT CTTATGATTC TGGAGAAAAC ACGTTTCAAG AGACTAACTT   
  
  
- AGCAAAAGAG TCAGCAGGTG GGAAGGTGTC ACCAGTAAAC CTAGTAACCC ACGGAACCCA CCGTGTCGAA   
  
  
- GAGTCCCAAG CTTAAGTCAA TAGAGGAGGG CACCAAAGGG ATACCGGTAC TTGCAAAAAT CGCTCGTCAG   
  
  
- GTACTATGTC AAAGTCTTCC CCCACCTCCT CCGCTCGTTC AAGGAAGGTT TCTTGTTATT GGAACGCTAA   
  
  
- CTGGAACTCT CGCAGTGAAA GGGTTTACTT TTCCTCCGCC GGGGGTACCA CCACCAATTC TTTCTCTTCC   
  
  
- TACTTGTGAG TGGACTATCG AGCTCTCCAT CGTTCTAAGT GATGGCACTA CTACTCCTGA AACTCCTACC   
  
  
- ATCCTCCTCA TTCGTCAGTC GACAAAGACA CCTCCTCCGA CTCAACAGAC TTTACAAACT GGCCCAAAAC   
  
  
- GAAACGTTAG GGTACTTTGT ACTTCGAGTA ACATGTTCAC CAAACTTCAG GCTCTTCCCT TGTAGGGACG   
  
  
- TCCGACCGGT TCATGTCCAT GTCCTCCCAG TCTTCCGAGC ACGTTTCTTT GTCCCATTAT TACTATTATT   
  
  
- CTTCTTATTC TTACACCACC TAAATTCCTG AGACGAATAG GAGACACGCG TTAGACGACG TAGACTACTA   
  
  
- GCGTCCTGTC GACTACTTGA CGACTTCGTC TAATCCCTCG TGAGAAGACG TCGACCCCTA CCTAGAGTTT   
  
  
- CCAACCGAGT AATGAAACGA TTACGGAACC TCCGCGCAAA TCGACCTTGA CCGAGTGTCT AGATATGTCG   
  
  
- GGACTCAAGC TTCTCCTGTT GTCGTCGACT ATACTATTTT CGAATAGTCA AAAAGAAAGC ACGAACGGGT   
  
  
- AAAAATTTCT AGAGATAATA GAAACGCTTG GTATACTAAT ATGTCTCTCG ACTTTTTCGT AGTTTCGAAG   
  
  
- TACAATATCT AAAACCGTAG GACATACCAA AAGTCACCGG GGAGGAGTAA GTTGCGGACA GCCTCGCTGG   
  
  
- ACCACCTGGA GGTTTTGACA CGTAATGACC CTAACTAGAA GGGGTTGGAC CTAAATCCGG TTGTCTTTCT   
  
  
- CAACTCCGTT GTCCCGAGGC GAACCGTTTC ATAACACTCG CGAAGTTACA AGGTAAACTT ATGGTACGGT   
  
  
- AACGTGTCTT TACCCTTTGG TAGTTTCGAC TTCTGGACTT CTATCTTCTA CTACTCCACC AACGGCACTT   
  
  
- GACAGAGAAG TCCAAATTCT TGGACGAACT ACTCTGCTAT CACCACCTAT CAGGCTCCTT ACGTCAAGAG   
  
  
- CCGAACTAAT CTTCCTATTT TGGACTATAG AAACAAGTGC CTCATCACTT GCCCAGAATG TTACGCGGGA   
  
  
- AGAAACAATG TGCAAAGTCC CTCCGGGAGA AGGTAATGAG ATGACATAAG CTATACAAAC TACGGTTACG   
  
  
- AAGGGCCCTC CTAGGACTCT CTAACTACAA ACTCTTCCTC AAAATACCCG CTCTTTAATA CTTACACCAC   
  
  
- CGAACACTCC CATGTCTCTC TCAACTTTCT GGACTCTGTA TGTTCGTTAC CGTCCGCGCC TTATCATCCC   
  
  
- GTCCCAAGTC CGTCGACGGT AACCTGGTTC TCGAGCAGTC ATTTGACTCC TCGTTCCAGG TCTACGTGAT   
  
  
- AGGATCCCTG AAGCAACAAC TACATCTACC TGTAACCTGT AACGTCCCTA CCTTCCCCTC CTAGTAGACA   
  
  
- CGACACAGAC GTACCCCAGG ACGAAT

+     MYB

| Site Name | Organism | Position | Strand | Matrix score. | sequence | function |
| --- | --- | --- | --- | --- | --- | --- |
| MYB | Arabidopsis thaliana | 3772 | - | 6 | CAACCA |  |
| MYB | Arabidopsis thaliana | 3652 | + | 6 | CAACAG |  |
| MYB | Arabidopsis thaliana | 3634 | + | 6 | CAACAG |  |
| MYB | Arabidopsis thaliana | 3382 | + | 6 | CAACAG |  |
| MYB | Arabidopsis thaliana | 2857 | - | 6 | TAACCA |  |
| MYB | Arabidopsis thaliana | 2512 | + | 6 | CAACCA |  |
| MYB | Arabidopsis thaliana | 1804 | - | 6 | TAACCA |  |
| MYB | Arabidopsis thaliana | 104 | - | 6 | TAACCA |  |

>HU02G01569.1   
+ +Up\_Stream \_Len000AGCGTA GGAAGTGACG CCGCTATTGG AGGCGAAGAC GCTAGTACCA AGGCCAAAAC   
  
  
+ AAATGAGGAG ATAGATCAGT TAAACCGTTT GGTTAAGAAA ATTAAGCGAA CATCTCTTGA ACCTCCATCT   
  
  
+ GAACTCGATG ACATTGAGGA TGTGGATGCA GAAAAGATGG ATATGGGCTC TCCATCGGTG CCAGCAAATC   
  
  
+ AACCTAATGT TGGGTGGGGT CTCATTCGGT CAATGGGGGA CTAGGGACGT CATATCGTCA CCAATTACAA   
  
  
+ CGGAACAACC CTAAACCGCA TTTTAAAATC ACATCTTGGT AGCAGACAAA GATGTACGAC CCAAAAATTC   
  
  
+ AACTCTGTAA AGCACTAGCA AAGCTCGTAG TACACAGAAC AATCGCACAC TCATAGTGAT AGAAGTGTAC   
  
  
+ TATCCATTAT GCAGTGAATA TGACAAGGGA GGGGGTCCTG AGATGCCCAA ATTTGGCAGC CGAGTTGCCT   
  
  
+ TGACTGCCCA TCAATGTCCT AAACCAGTTG AGCATGTTAT TAATTTGCAA GAAAGATCGA ATTCTTCCTA   
  
  
+ TCTAAAAACT GACACAATAA AACAATACCA TGTATGGGAC ATTGCTAGGA TTGTTAACAC AATTATTGTC   
  
  
+ TTGTACGTTG CACCTGAAAA AAAAGAAAAT TGTTGCCATA GACAACATAT AAGTTAGGCA TTCATGACTA   
  
  
+ ATATCTGAAT TAGGTGTGTC GATCTGTGAT TCAAATTGAT ATAATTAGAT CTTTAAGGTA TCTAATTGCT   
  
  
+ TCAAGTTTTC TCTATAATGA AGATGTATAT TAGCTCACAT AGGTCTTAAG GTTAACTAAT GTCATATCAG   
  
  
+ GGGGTGTGGG CCGCCATTTT TGATAAGAAT TGAATTGAAC TTGCATTGTT TTTTCTTTGG CGAGAATGCA   
  
  
+ AGATTTGTTA ATTTATTCAT ATGCGTCATT ATACACTATA AGAAAAATAA TTGTAGGAAT ATATGAAGTA   
  
  
+ TTTTTACTTG TGCTCATGTT AAATGTGGAA TACTTTTTTA ATGTCGTATA GTACCACAGT ATTCTACAAT   
  
  
+ TTAAGAGATT AATCGATCCA TTAAAGTCAT GCAAACCTGA TGCATATTCA AGACATTGCA TGAAATGATC   
  
  
+ TTATTTTAGT CAAAGACTCC ATCATTTATA AGCAATATTT ACTTCGATAC ACGTGTCATT AAAAAAGACT   
  
  
+ ATAGAAATGT TACTGTTATT TTGTTTGAGC TAAATAGAAA ATTTAAAAGT TAATTAAGAT AATAATTAAA   
  
  
+ ACTAAATATT CAATGATGGC TAATAAATAC ATTGTCGTAT TAACTGACTA ATATTTGTAG ATGGTCGTGT   
  
  
+ TAATTTCTTT TTATTTTAAA GTTCAAATGA GTTACAAAAG GTTATAAAAT ATAAAGTAAA ATGGGAGACA   
  
  
+ AAGCAGACAG GGTGGGGGAA GCTAAGCTTA AAGTAGGATT TAGAAAGGCA ATGAAGGTGA ACCCTCAAAT   
  
  
+ CGATCCGCTG CCAATCACAG AAACCCAAAG CTTTGCTCAC CGACAACTCC CGGTTAACTG CAGTCACTCA   
  
  
+ CGGCACTGGG TAATACAGTA ATCGTGTACC CAGTTCATCC CCCTTTTCCT TTGGGAATAC AGTACCTCGA   
  
  
+ AAGTTCCTTT GAAATTCTAC TGGCCAGGCA TACCCATAAA TTGATCCTCT CGATATCATA AGATATGATA   
  
  
+ TTTCTCTGTC ATATCTGTTA GTTTCGGTAT CATATTTCTT CTTTATCTCT GTAATTTGAG GTATGCTTCA   
  
  
+ TTCCTGGCCG CTTGAATTAT CTTTCTTTCT TTGATTAGTA TTGTTTTTTT GGTTACCTAT TTTGCTCCAG   
  
  
+ TCCTGTTCAT TTGGGTCTTT TTGGATCTGG GTTTTTGCTG GGTTTGTTGA TTCTTTGAGA AATTTGGGTG   
  
  
+ CTTGAATTTG CCCTGCAATA TTCATGGGTT AGCGTTTTCC TGAATTTTGC TCTGTTCCTT AAGTGATTAT   
  
  
+ TTTTGATTGA TTACACCTTG GTGGTCCTGG TGGAATTTCC GAGGAGAAAT TGTCATGGGT TCCCACAACT   
  
  
+ TTGGAGAATT CCCTGATGAG ACTCTAAATG AGTATCAATC TACTTTGGGA ACCATGTCCC CCGGTTATGA   
  
  
+ TGGGTCTTTG AATTATACAA CCTTGTTCAA TTACAAAGAC CCATCTCAGG ATCTCACGGC ACTGAACCTT   
  
  
+ CCTAGCCCAT TGCCTGACCC TATGCCATTC AACATTGGTT CATATTCGGG TTTGAGCCCC GGGGTTGAAT   
  
  
+ CTTCGGATGA TAGCGATTCA GATGATGTTG TTAAGTACAT TGGCCAAGTG CTTATGGAAG AGGATATGAA   
  
  
+ GGAGAAGCCT TGTATGTTCC ATGACCCTTT AGCACTCCAA GCTGCTGAGA AACCCTTTTA TGATGTGTTG   
  
  
+ GGAAAGAAGT ATCCTCCTTC CCCTAATCAA CACCCACTTA TTGATCATTC TGTGGATAGC CCAGACAATC   
  
  
+ AGTCCTGTGG AACGAGCACA ATTAGTGATC TTAGTGGCAG TAACTGCACT TCCAGTTCAA CCAATTATAT   
  
  
+ TGATGCTGTG GCAGTCGCTG ATTCGAGTGA GAATACTAAG ACCTCTTTTG TGCAAAGTTC TCTGATTGAA   
  
  
+ TCGTTTTCTC AGTCGTCCAC CCTTCCACAG TGGTCATTTG GATCATTGGG TGCCTTGGGT GGCACAGCTT   
  
  
+ CTCAGGGTTC GAATTCAGTT ATCTCCTCCC GTGGTTTCCC TATGGCCATG AACGTTTTTA GCGAGCAGTC   
  
  
+ CATGATACAG TTTCAGAAGG GGGTGGAGGA GGCGAGCAAG TTCCTTCCAA AGAACAATAA CCTTGCGATT   
  
  
+ GACCTTGAGA GCGTCACTTT CCCAAATGAA AAGGAGGCGG CCCCCATGGT GGTGGTTAAG AAAGAGAAGG   
  
  
+ ATGAACACTC ACCTGATAGC TCGAGAGGTA GCAAGATTCA CTACCGTGAT GATGAGGACT TTGAGGATGG   
  
  
+ TAGGAGGAGT AAGCAGTCAG CTGTTTCTGT GGAGGAGGCT GAGTTGTCTG AAATGTTTGA CCGGGTTTTG   
  
  
+ CTTTGCAATC CCATGAAACA TGAAGCTCAT TGTACAAGTG GTTTGAAGTC CGAGAAGGGA ACATCCCTGC   
  
  
+ AGGCTGGCCA AGTACAGGTA CAGGAGGGTC AGAAGGCTCG TGCAAAGAAA CAGGGTAATA ATGATAATAA   
  
  
+ GAAGAATAAG AATGTGGTGG ATTTAAGGAC TCTGCTTATC CTCTGTGCGC AATCTGCTGC ATCTGATGAT   
  
  
+ CGCAGGACAG CTGATGAACT GCTGAAGCAG ATTAGGGAGC ACTCTTCTGC AGCTGGGGAT GGATCTCAAA   
  
  
+ GGTTGGCTCA TTACTTTGCT AATGCCTTGG AGGCGCGTTT AGCTGGAACT GGCTCACAGA TCTATACAGC   
  
  
+ CCTGAGTTCG AAGAGGACAA CAGCAGCTGA TATGATAAAA GCTTATCAGT TTTTCTTTCG TGCTTGCCCA   
  
  
+ TTTTTAAAGA TCTCTATTAT CTTTGCGAAC CATATGATTA TACAGAGAGC TGAAAAAGCA TCAAAGCTTC   
  
  
+ ATGTTATAGA TTTTGGCATC CTGTATGGTT TTCAGTGGCC CCTCCTCATT CAACGCCTGT CGGAGCGACC   
  
  
+ TGGTGGACCT CCAAAACTGT GCATTACTGG GATTGATCTT CCCCAACCTG GATTTAGGCC AACAGAAAGA   
  
  
+ GTTGAGGCAA CAGGGCTCCG CTTGGCAAAG TATTGTGAGC GCTTCAATGT TCCATTTGAA TACCATGCCA   
  
  
+ TTGCACAGAA ATGGGAAACC ATCAAAGCTG AAGACCTGAA GATAGAAGAT GATGAGGTGG TTGCCGTGAA   
  
  
+ CTGTCTCTTC AGGTTTAAGA ACCTGCTTGA TGAGACGATA GTGGTGGATA GTCCGAGGAA TGCAGTTCTC   
  
  
+ GGCTTGATTA GAAGGATAAA ACCTGATATC TTTGTTCACG GAGTAGTGAA CGGGTCTTAC AATGCGCCCT   
  
  
+ TCTTTGTTAC ACGTTTCAGG GAGGCCCTCT TCCATTACTC TACTGTATTC GATATGTTTG ATGCCAATGC   
  
  
+ TTCCCGGGAG GATCCTGAGA GATTGATGTT TGAGAAGGAG TTTTATGGGC GAGAAATTAT GAATGTGGTG   
  
  
+ GCTTGTGAGG GTACAGAGAG AGTTGAAAGA CCTGAGACAT ACAAGCAATG GCAGGCGCGG AATAGTAGGG   
  
  
+ CAGGGTTCAG GCAGCTGCCA TTGGACCAAG AGCTCGTCAG TAAACTGAGG AGCAAGGTCC AGATGCACTA   
  
  
+ TCCTAGGGAC TTCGTTGTTG ATGTAGATGG ACATTGGACA TTGCAGGGAT GGAAGGGGAG GATCATCTGT   
  
  
+ GCTGTGTCTG CATGGGGTCC TGCTTA  

- +Up\_Stream \_Len000TCGCAT CCTTCACTGC GGCGATAACC TCCGCTTCTG CGATCATGGT TCCGGTTTTG   
  
  
- TTTACTCCTC TATCTAGTCA ATTTGGCAAA CCAATTCTTT TAATTCGCTT GTAGAGAACT TGGAGGTAGA   
  
  
- CTTGAGCTAC TGTAACTCCT ACACCTACGT CTTTTCTACC TATACCCGAG AGGTAGCCAC GGTCGTTTAG   
  
  
- TTGGATTACA ACCCACCCCA GAGTAAGCCA GTTACCCCCT GATCCCTGCA GTATAGCAGT GGTTAATGTT   
  
  
- GCCTTGTTGG GATTTGGCGT AAAATTTTAG TGTAGAACCA TCGTCTGTTT CTACATGCTG GGTTTTTAAG   
  
  
- TTGAGACATT TCGTGATCGT TTCGAGCATC ATGTGTCTTG TTAGCGTGTG AGTATCACTA TCTTCACATG   
  
  
- ATAGGTAATA CGTCACTTAT ACTGTTCCCT CCCCCAGGAC TCTACGGGTT TAAACCGTCG GCTCAACGGA   
  
  
- ACTGACGGGT AGTTACAGGA TTTGGTCAAC TCGTACAATA ATTAAACGTT CTTTCTAGCT TAAGAAGGAT   
  
  
- AGATTTTTGA CTGTGTTATT TTGTTATGGT ACATACCCTG TAACGATCCT AACAATTGTG TTAATAACAG   
  
  
- AACATGCAAC GTGGACTTTT TTTTCTTTTA ACAACGGTAT CTGTTGTATA TTCAATCCGT AAGTACTGAT   
  
  
- TATAGACTTA ATCCACACAG CTAGACACTA AGTTTAACTA TATTAATCTA GAAATTCCAT AGATTAACGA   
  
  
- AGTTCAAAAG AGATATTACT TCTACATATA ATCGAGTGTA TCCAGAATTC CAATTGATTA CAGTATAGTC   
  
  
- CCCCACACCC GGCGGTAAAA ACTATTCTTA ACTTAACTTG AACGTAACAA AAAAGAAACC GCTCTTACGT   
  
  
- TCTAAACAAT TAAATAAGTA TACGCAGTAA TATGTGATAT TCTTTTTATT AACATCCTTA TATACTTCAT   
  
  
- AAAAATGAAC ACGAGTACAA TTTACACCTT ATGAAAAAAT TACAGCATAT CATGGTGTCA TAAGATGTTA   
  
  
- AATTCTCTAA TTAGCTAGGT AATTTCAGTA CGTTTGGACT ACGTATAAGT TCTGTAACGT ACTTTACTAG   
  
  
- AATAAAATCA GTTTCTGAGG TAGTAAATAT TCGTTATAAA TGAAGCTATG TGCACAGTAA TTTTTTCTGA   
  
  
- TATCTTTACA ATGACAATAA AACAAACTCG ATTTATCTTT TAAATTTTCA ATTAATTCTA TTATTAATTT   
  
  
- TGATTTATAA GTTACTACCG ATTATTTATG TAACAGCATA ATTGACTGAT TATAAACATC TACCAGCACA   
  
  
- ATTAAAGAAA AATAAAATTT CAAGTTTACT CAATGTTTTC CAATATTTTA TATTTCATTT TACCCTCTGT   
  
  
- TTCGTCTGTC CCACCCCCTT CGATTCGAAT TTCATCCTAA ATCTTTCCGT TACTTCCACT TGGGAGTTTA   
  
  
- GCTAGGCGAC GGTTAGTGTC TTTGGGTTTC GAAACGAGTG GCTGTTGAGG GCCAATTGAC GTCAGTGAGT   
  
  
- GCCGTGACCC ATTATGTCAT TAGCACATGG GTCAAGTAGG GGGAAAAGGA AACCCTTATG TCATGGAGCT   
  
  
- TTCAAGGAAA CTTTAAGATG ACCGGTCCGT ATGGGTATTT AACTAGGAGA GCTATAGTAT TCTATACTAT   
  
  
- AAAGAGACAG TATAGACAAT CAAAGCCATA GTATAAAGAA GAAATAGAGA CATTAAACTC CATACGAAGT   
  
  
- AAGGACCGGC GAACTTAATA GAAAGAAAGA AACTAATCAT AACAAAAAAA CCAATGGATA AAACGAGGTC   
  
  
- AGGACAAGTA AACCCAGAAA AACCTAGACC CAAAAACGAC CCAAACAACT AAGAAACTCT TTAAACCCAC   
  
  
- GAACTTAAAC GGGACGTTAT AAGTACCCAA TCGCAAAAGG ACTTAAAACG AGACAAGGAA TTCACTAATA   
  
  
- AAAACTAACT AATGTGGAAC CACCAGGACC ACCTTAAAGG CTCCTCTTTA ACAGTACCCA AGGGTGTTGA   
  
  
- AACCTCTTAA GGGACTACTC TGAGATTTAC TCATAGTTAG ATGAAACCCT TGGTACAGGG GGCCAATACT   
  
  
- ACCCAGAAAC TTAATATGTT GGAACAAGTT AATGTTTCTG GGTAGAGTCC TAGAGTGCCG TGACTTGGAA   
  
  
- GGATCGGGTA ACGGACTGGG ATACGGTAAG TTGTAACCAA GTATAAGCCC AAACTCGGGG CCCCAACTTA   
  
  
- GAAGCCTACT ATCGCTAAGT CTACTACAAC AATTCATGTA ACCGGTTCAC GAATACCTTC TCCTATACTT   
  
  
- CCTCTTCGGA ACATACAAGG TACTGGGAAA TCGTGAGGTT CGACGACTCT TTGGGAAAAT ACTACACAAC   
  
  
- CCTTTCTTCA TAGGAGGAAG GGGATTAGTT GTGGGTGAAT AACTAGTAAG ACACCTATCG GGTCTGTTAG   
  
  
- TCAGGACACC TTGCTCGTGT TAATCACTAG AATCACCGTC ATTGACGTGA AGGTCAAGTT GGTTAATATA   
  
  
- ACTACGACAC CGTCAGCGAC TAAGCTCACT CTTATGATTC TGGAGAAAAC ACGTTTCAAG AGACTAACTT   
  
  
- AGCAAAAGAG TCAGCAGGTG GGAAGGTGTC ACCAGTAAAC CTAGTAACCC ACGGAACCCA CCGTGTCGAA   
  
  
- GAGTCCCAAG CTTAAGTCAA TAGAGGAGGG CACCAAAGGG ATACCGGTAC TTGCAAAAAT CGCTCGTCAG   
  
  
- GTACTATGTC AAAGTCTTCC CCCACCTCCT CCGCTCGTTC AAGGAAGGTT TCTTGTTATT GGAACGCTAA   
  
  
- CTGGAACTCT CGCAGTGAAA GGGTTTACTT TTCCTCCGCC GGGGGTACCA CCACCAATTC TTTCTCTTCC   
  
  
- TACTTGTGAG TGGACTATCG AGCTCTCCAT CGTTCTAAGT GATGGCACTA CTACTCCTGA AACTCCTACC   
  
  
- ATCCTCCTCA TTCGTCAGTC GACAAAGACA CCTCCTCCGA CTCAACAGAC TTTACAAACT GGCCCAAAAC   
  
  
- GAAACGTTAG GGTACTTTGT ACTTCGAGTA ACATGTTCAC CAAACTTCAG GCTCTTCCCT TGTAGGGACG   
  
  
- TCCGACCGGT TCATGTCCAT GTCCTCCCAG TCTTCCGAGC ACGTTTCTTT GTCCCATTAT TACTATTATT   
  
  
- CTTCTTATTC TTACACCACC TAAATTCCTG AGACGAATAG GAGACACGCG TTAGACGACG TAGACTACTA   
  
  
- GCGTCCTGTC GACTACTTGA CGACTTCGTC TAATCCCTCG TGAGAAGACG TCGACCCCTA CCTAGAGTTT   
  
  
- CCAACCGAGT AATGAAACGA TTACGGAACC TCCGCGCAAA TCGACCTTGA CCGAGTGTCT AGATATGTCG   
  
  
- GGACTCAAGC TTCTCCTGTT GTCGTCGACT ATACTATTTT CGAATAGTCA AAAAGAAAGC ACGAACGGGT   
  
  
- AAAAATTTCT AGAGATAATA GAAACGCTTG GTATACTAAT ATGTCTCTCG ACTTTTTCGT AGTTTCGAAG   
  
  
- TACAATATCT AAAACCGTAG GACATACCAA AAGTCACCGG GGAGGAGTAA GTTGCGGACA GCCTCGCTGG   
  
  
- ACCACCTGGA GGTTTTGACA CGTAATGACC CTAACTAGAA GGGGTTGGAC CTAAATCCGG TTGTCTTTCT   
  
  
- CAACTCCGTT GTCCCGAGGC GAACCGTTTC ATAACACTCG CGAAGTTACA AGGTAAACTT ATGGTACGGT   
  
  
- AACGTGTCTT TACCCTTTGG TAGTTTCGAC TTCTGGACTT CTATCTTCTA CTACTCCACC AACGGCACTT   
  
  
- GACAGAGAAG TCCAAATTCT TGGACGAACT ACTCTGCTAT CACCACCTAT CAGGCTCCTT ACGTCAAGAG   
  
  
- CCGAACTAAT CTTCCTATTT TGGACTATAG AAACAAGTGC CTCATCACTT GCCCAGAATG TTACGCGGGA   
  
  
- AGAAACAATG TGCAAAGTCC CTCCGGGAGA AGGTAATGAG ATGACATAAG CTATACAAAC TACGGTTACG   
  
  
- AAGGGCCCTC CTAGGACTCT CTAACTACAA ACTCTTCCTC AAAATACCCG CTCTTTAATA CTTACACCAC   
  
  
- CGAACACTCC CATGTCTCTC TCAACTTTCT GGACTCTGTA TGTTCGTTAC CGTCCGCGCC TTATCATCCC   
  
  
- GTCCCAAGTC CGTCGACGGT AACCTGGTTC TCGAGCAGTC ATTTGACTCC TCGTTCCAGG TCTACGTGAT   
  
  
- AGGATCCCTG AAGCAACAAC TACATCTACC TGTAACCTGT AACGTCCCTA CCTTCCCCTC CTAGTAGACA   
  
  
- CGACACAGAC GTACCCCAGG ACGAAT

+     MYB recognition site

| Site Name | Organism | Position | Strand | Matrix score. | sequence | function |
| --- | --- | --- | --- | --- | --- | --- |
| MYB recognition site | Arabidopsis thaliana | 282 | - | 6 | CCGTTG |  |

>HU02G01569.1   
+ +Up\_Stream \_Len000AGCGTA GGAAGTGACG CCGCTATTGG AGGCGAAGAC GCTAGTACCA AGGCCAAAAC   
  
  
+ AAATGAGGAG ATAGATCAGT TAAACCGTTT GGTTAAGAAA ATTAAGCGAA CATCTCTTGA ACCTCCATCT   
  
  
+ GAACTCGATG ACATTGAGGA TGTGGATGCA GAAAAGATGG ATATGGGCTC TCCATCGGTG CCAGCAAATC   
  
  
+ AACCTAATGT TGGGTGGGGT CTCATTCGGT CAATGGGGGA CTAGGGACGT CATATCGTCA CCAATTACAA   
  
  
+ CGGAACAACC CTAAACCGCA TTTTAAAATC ACATCTTGGT AGCAGACAAA GATGTACGAC CCAAAAATTC   
  
  
+ AACTCTGTAA AGCACTAGCA AAGCTCGTAG TACACAGAAC AATCGCACAC TCATAGTGAT AGAAGTGTAC   
  
  
+ TATCCATTAT GCAGTGAATA TGACAAGGGA GGGGGTCCTG AGATGCCCAA ATTTGGCAGC CGAGTTGCCT   
  
  
+ TGACTGCCCA TCAATGTCCT AAACCAGTTG AGCATGTTAT TAATTTGCAA GAAAGATCGA ATTCTTCCTA   
  
  
+ TCTAAAAACT GACACAATAA AACAATACCA TGTATGGGAC ATTGCTAGGA TTGTTAACAC AATTATTGTC   
  
  
+ TTGTACGTTG CACCTGAAAA AAAAGAAAAT TGTTGCCATA GACAACATAT AAGTTAGGCA TTCATGACTA   
  
  
+ ATATCTGAAT TAGGTGTGTC GATCTGTGAT TCAAATTGAT ATAATTAGAT CTTTAAGGTA TCTAATTGCT   
  
  
+ TCAAGTTTTC TCTATAATGA AGATGTATAT TAGCTCACAT AGGTCTTAAG GTTAACTAAT GTCATATCAG   
  
  
+ GGGGTGTGGG CCGCCATTTT TGATAAGAAT TGAATTGAAC TTGCATTGTT TTTTCTTTGG CGAGAATGCA   
  
  
+ AGATTTGTTA ATTTATTCAT ATGCGTCATT ATACACTATA AGAAAAATAA TTGTAGGAAT ATATGAAGTA   
  
  
+ TTTTTACTTG TGCTCATGTT AAATGTGGAA TACTTTTTTA ATGTCGTATA GTACCACAGT ATTCTACAAT   
  
  
+ TTAAGAGATT AATCGATCCA TTAAAGTCAT GCAAACCTGA TGCATATTCA AGACATTGCA TGAAATGATC   
  
  
+ TTATTTTAGT CAAAGACTCC ATCATTTATA AGCAATATTT ACTTCGATAC ACGTGTCATT AAAAAAGACT   
  
  
+ ATAGAAATGT TACTGTTATT TTGTTTGAGC TAAATAGAAA ATTTAAAAGT TAATTAAGAT AATAATTAAA   
  
  
+ ACTAAATATT CAATGATGGC TAATAAATAC ATTGTCGTAT TAACTGACTA ATATTTGTAG ATGGTCGTGT   
  
  
+ TAATTTCTTT TTATTTTAAA GTTCAAATGA GTTACAAAAG GTTATAAAAT ATAAAGTAAA ATGGGAGACA   
  
  
+ AAGCAGACAG GGTGGGGGAA GCTAAGCTTA AAGTAGGATT TAGAAAGGCA ATGAAGGTGA ACCCTCAAAT   
  
  
+ CGATCCGCTG CCAATCACAG AAACCCAAAG CTTTGCTCAC CGACAACTCC CGGTTAACTG CAGTCACTCA   
  
  
+ CGGCACTGGG TAATACAGTA ATCGTGTACC CAGTTCATCC CCCTTTTCCT TTGGGAATAC AGTACCTCGA   
  
  
+ AAGTTCCTTT GAAATTCTAC TGGCCAGGCA TACCCATAAA TTGATCCTCT CGATATCATA AGATATGATA   
  
  
+ TTTCTCTGTC ATATCTGTTA GTTTCGGTAT CATATTTCTT CTTTATCTCT GTAATTTGAG GTATGCTTCA   
  
  
+ TTCCTGGCCG CTTGAATTAT CTTTCTTTCT TTGATTAGTA TTGTTTTTTT GGTTACCTAT TTTGCTCCAG   
  
  
+ TCCTGTTCAT TTGGGTCTTT TTGGATCTGG GTTTTTGCTG GGTTTGTTGA TTCTTTGAGA AATTTGGGTG   
  
  
+ CTTGAATTTG CCCTGCAATA TTCATGGGTT AGCGTTTTCC TGAATTTTGC TCTGTTCCTT AAGTGATTAT   
  
  
+ TTTTGATTGA TTACACCTTG GTGGTCCTGG TGGAATTTCC GAGGAGAAAT TGTCATGGGT TCCCACAACT   
  
  
+ TTGGAGAATT CCCTGATGAG ACTCTAAATG AGTATCAATC TACTTTGGGA ACCATGTCCC CCGGTTATGA   
  
  
+ TGGGTCTTTG AATTATACAA CCTTGTTCAA TTACAAAGAC CCATCTCAGG ATCTCACGGC ACTGAACCTT   
  
  
+ CCTAGCCCAT TGCCTGACCC TATGCCATTC AACATTGGTT CATATTCGGG TTTGAGCCCC GGGGTTGAAT   
  
  
+ CTTCGGATGA TAGCGATTCA GATGATGTTG TTAAGTACAT TGGCCAAGTG CTTATGGAAG AGGATATGAA   
  
  
+ GGAGAAGCCT TGTATGTTCC ATGACCCTTT AGCACTCCAA GCTGCTGAGA AACCCTTTTA TGATGTGTTG   
  
  
+ GGAAAGAAGT ATCCTCCTTC CCCTAATCAA CACCCACTTA TTGATCATTC TGTGGATAGC CCAGACAATC   
  
  
+ AGTCCTGTGG AACGAGCACA ATTAGTGATC TTAGTGGCAG TAACTGCACT TCCAGTTCAA CCAATTATAT   
  
  
+ TGATGCTGTG GCAGTCGCTG ATTCGAGTGA GAATACTAAG ACCTCTTTTG TGCAAAGTTC TCTGATTGAA   
  
  
+ TCGTTTTCTC AGTCGTCCAC CCTTCCACAG TGGTCATTTG GATCATTGGG TGCCTTGGGT GGCACAGCTT   
  
  
+ CTCAGGGTTC GAATTCAGTT ATCTCCTCCC GTGGTTTCCC TATGGCCATG AACGTTTTTA GCGAGCAGTC   
  
  
+ CATGATACAG TTTCAGAAGG GGGTGGAGGA GGCGAGCAAG TTCCTTCCAA AGAACAATAA CCTTGCGATT   
  
  
+ GACCTTGAGA GCGTCACTTT CCCAAATGAA AAGGAGGCGG CCCCCATGGT GGTGGTTAAG AAAGAGAAGG   
  
  
+ ATGAACACTC ACCTGATAGC TCGAGAGGTA GCAAGATTCA CTACCGTGAT GATGAGGACT TTGAGGATGG   
  
  
+ TAGGAGGAGT AAGCAGTCAG CTGTTTCTGT GGAGGAGGCT GAGTTGTCTG AAATGTTTGA CCGGGTTTTG   
  
  
+ CTTTGCAATC CCATGAAACA TGAAGCTCAT TGTACAAGTG GTTTGAAGTC CGAGAAGGGA ACATCCCTGC   
  
  
+ AGGCTGGCCA AGTACAGGTA CAGGAGGGTC AGAAGGCTCG TGCAAAGAAA CAGGGTAATA ATGATAATAA   
  
  
+ GAAGAATAAG AATGTGGTGG ATTTAAGGAC TCTGCTTATC CTCTGTGCGC AATCTGCTGC ATCTGATGAT   
  
  
+ CGCAGGACAG CTGATGAACT GCTGAAGCAG ATTAGGGAGC ACTCTTCTGC AGCTGGGGAT GGATCTCAAA   
  
  
+ GGTTGGCTCA TTACTTTGCT AATGCCTTGG AGGCGCGTTT AGCTGGAACT GGCTCACAGA TCTATACAGC   
  
  
+ CCTGAGTTCG AAGAGGACAA CAGCAGCTGA TATGATAAAA GCTTATCAGT TTTTCTTTCG TGCTTGCCCA   
  
  
+ TTTTTAAAGA TCTCTATTAT CTTTGCGAAC CATATGATTA TACAGAGAGC TGAAAAAGCA TCAAAGCTTC   
  
  
+ ATGTTATAGA TTTTGGCATC CTGTATGGTT TTCAGTGGCC CCTCCTCATT CAACGCCTGT CGGAGCGACC   
  
  
+ TGGTGGACCT CCAAAACTGT GCATTACTGG GATTGATCTT CCCCAACCTG GATTTAGGCC AACAGAAAGA   
  
  
+ GTTGAGGCAA CAGGGCTCCG CTTGGCAAAG TATTGTGAGC GCTTCAATGT TCCATTTGAA TACCATGCCA   
  
  
+ TTGCACAGAA ATGGGAAACC ATCAAAGCTG AAGACCTGAA GATAGAAGAT GATGAGGTGG TTGCCGTGAA   
  
  
+ CTGTCTCTTC AGGTTTAAGA ACCTGCTTGA TGAGACGATA GTGGTGGATA GTCCGAGGAA TGCAGTTCTC   
  
  
+ GGCTTGATTA GAAGGATAAA ACCTGATATC TTTGTTCACG GAGTAGTGAA CGGGTCTTAC AATGCGCCCT   
  
  
+ TCTTTGTTAC ACGTTTCAGG GAGGCCCTCT TCCATTACTC TACTGTATTC GATATGTTTG ATGCCAATGC   
  
  
+ TTCCCGGGAG GATCCTGAGA GATTGATGTT TGAGAAGGAG TTTTATGGGC GAGAAATTAT GAATGTGGTG   
  
  
+ GCTTGTGAGG GTACAGAGAG AGTTGAAAGA CCTGAGACAT ACAAGCAATG GCAGGCGCGG AATAGTAGGG   
  
  
+ CAGGGTTCAG GCAGCTGCCA TTGGACCAAG AGCTCGTCAG TAAACTGAGG AGCAAGGTCC AGATGCACTA   
  
  
+ TCCTAGGGAC TTCGTTGTTG ATGTAGATGG ACATTGGACA TTGCAGGGAT GGAAGGGGAG GATCATCTGT   
  
  
+ GCTGTGTCTG CATGGGGTCC TGCTTA  

- +Up\_Stream \_Len000TCGCAT CCTTCACTGC GGCGATAACC TCCGCTTCTG CGATCATGGT TCCGGTTTTG   
  
  
- TTTACTCCTC TATCTAGTCA ATTTGGCAAA CCAATTCTTT TAATTCGCTT GTAGAGAACT TGGAGGTAGA   
  
  
- CTTGAGCTAC TGTAACTCCT ACACCTACGT CTTTTCTACC TATACCCGAG AGGTAGCCAC GGTCGTTTAG   
  
  
- TTGGATTACA ACCCACCCCA GAGTAAGCCA GTTACCCCCT GATCCCTGCA GTATAGCAGT GGTTAATGTT   
  
  
- GCCTTGTTGG GATTTGGCGT AAAATTTTAG TGTAGAACCA TCGTCTGTTT CTACATGCTG GGTTTTTAAG   
  
  
- TTGAGACATT TCGTGATCGT TTCGAGCATC ATGTGTCTTG TTAGCGTGTG AGTATCACTA TCTTCACATG   
  
  
- ATAGGTAATA CGTCACTTAT ACTGTTCCCT CCCCCAGGAC TCTACGGGTT TAAACCGTCG GCTCAACGGA   
  
  
- ACTGACGGGT AGTTACAGGA TTTGGTCAAC TCGTACAATA ATTAAACGTT CTTTCTAGCT TAAGAAGGAT   
  
  
- AGATTTTTGA CTGTGTTATT TTGTTATGGT ACATACCCTG TAACGATCCT AACAATTGTG TTAATAACAG   
  
  
- AACATGCAAC GTGGACTTTT TTTTCTTTTA ACAACGGTAT CTGTTGTATA TTCAATCCGT AAGTACTGAT   
  
  
- TATAGACTTA ATCCACACAG CTAGACACTA AGTTTAACTA TATTAATCTA GAAATTCCAT AGATTAACGA   
  
  
- AGTTCAAAAG AGATATTACT TCTACATATA ATCGAGTGTA TCCAGAATTC CAATTGATTA CAGTATAGTC   
  
  
- CCCCACACCC GGCGGTAAAA ACTATTCTTA ACTTAACTTG AACGTAACAA AAAAGAAACC GCTCTTACGT   
  
  
- TCTAAACAAT TAAATAAGTA TACGCAGTAA TATGTGATAT TCTTTTTATT AACATCCTTA TATACTTCAT   
  
  
- AAAAATGAAC ACGAGTACAA TTTACACCTT ATGAAAAAAT TACAGCATAT CATGGTGTCA TAAGATGTTA   
  
  
- AATTCTCTAA TTAGCTAGGT AATTTCAGTA CGTTTGGACT ACGTATAAGT TCTGTAACGT ACTTTACTAG   
  
  
- AATAAAATCA GTTTCTGAGG TAGTAAATAT TCGTTATAAA TGAAGCTATG TGCACAGTAA TTTTTTCTGA   
  
  
- TATCTTTACA ATGACAATAA AACAAACTCG ATTTATCTTT TAAATTTTCA ATTAATTCTA TTATTAATTT   
  
  
- TGATTTATAA GTTACTACCG ATTATTTATG TAACAGCATA ATTGACTGAT TATAAACATC TACCAGCACA   
  
  
- ATTAAAGAAA AATAAAATTT CAAGTTTACT CAATGTTTTC CAATATTTTA TATTTCATTT TACCCTCTGT   
  
  
- TTCGTCTGTC CCACCCCCTT CGATTCGAAT TTCATCCTAA ATCTTTCCGT TACTTCCACT TGGGAGTTTA   
  
  
- GCTAGGCGAC GGTTAGTGTC TTTGGGTTTC GAAACGAGTG GCTGTTGAGG GCCAATTGAC GTCAGTGAGT   
  
  
- GCCGTGACCC ATTATGTCAT TAGCACATGG GTCAAGTAGG GGGAAAAGGA AACCCTTATG TCATGGAGCT   
  
  
- TTCAAGGAAA CTTTAAGATG ACCGGTCCGT ATGGGTATTT AACTAGGAGA GCTATAGTAT TCTATACTAT   
  
  
- AAAGAGACAG TATAGACAAT CAAAGCCATA GTATAAAGAA GAAATAGAGA CATTAAACTC CATACGAAGT   
  
  
- AAGGACCGGC GAACTTAATA GAAAGAAAGA AACTAATCAT AACAAAAAAA CCAATGGATA AAACGAGGTC   
  
  
- AGGACAAGTA AACCCAGAAA AACCTAGACC CAAAAACGAC CCAAACAACT AAGAAACTCT TTAAACCCAC   
  
  
- GAACTTAAAC GGGACGTTAT AAGTACCCAA TCGCAAAAGG ACTTAAAACG AGACAAGGAA TTCACTAATA   
  
  
- AAAACTAACT AATGTGGAAC CACCAGGACC ACCTTAAAGG CTCCTCTTTA ACAGTACCCA AGGGTGTTGA   
  
  
- AACCTCTTAA GGGACTACTC TGAGATTTAC TCATAGTTAG ATGAAACCCT TGGTACAGGG GGCCAATACT   
  
  
- ACCCAGAAAC TTAATATGTT GGAACAAGTT AATGTTTCTG GGTAGAGTCC TAGAGTGCCG TGACTTGGAA   
  
  
- GGATCGGGTA ACGGACTGGG ATACGGTAAG TTGTAACCAA GTATAAGCCC AAACTCGGGG CCCCAACTTA   
  
  
- GAAGCCTACT ATCGCTAAGT CTACTACAAC AATTCATGTA ACCGGTTCAC GAATACCTTC TCCTATACTT   
  
  
- CCTCTTCGGA ACATACAAGG TACTGGGAAA TCGTGAGGTT CGACGACTCT TTGGGAAAAT ACTACACAAC   
  
  
- CCTTTCTTCA TAGGAGGAAG GGGATTAGTT GTGGGTGAAT AACTAGTAAG ACACCTATCG GGTCTGTTAG   
  
  
- TCAGGACACC TTGCTCGTGT TAATCACTAG AATCACCGTC ATTGACGTGA AGGTCAAGTT GGTTAATATA   
  
  
- ACTACGACAC CGTCAGCGAC TAAGCTCACT CTTATGATTC TGGAGAAAAC ACGTTTCAAG AGACTAACTT   
  
  
- AGCAAAAGAG TCAGCAGGTG GGAAGGTGTC ACCAGTAAAC CTAGTAACCC ACGGAACCCA CCGTGTCGAA   
  
  
- GAGTCCCAAG CTTAAGTCAA TAGAGGAGGG CACCAAAGGG ATACCGGTAC TTGCAAAAAT CGCTCGTCAG   
  
  
- GTACTATGTC AAAGTCTTCC CCCACCTCCT CCGCTCGTTC AAGGAAGGTT TCTTGTTATT GGAACGCTAA   
  
  
- CTGGAACTCT CGCAGTGAAA GGGTTTACTT TTCCTCCGCC GGGGGTACCA CCACCAATTC TTTCTCTTCC   
  
  
- TACTTGTGAG TGGACTATCG AGCTCTCCAT CGTTCTAAGT GATGGCACTA CTACTCCTGA AACTCCTACC   
  
  
- ATCCTCCTCA TTCGTCAGTC GACAAAGACA CCTCCTCCGA CTCAACAGAC TTTACAAACT GGCCCAAAAC   
  
  
- GAAACGTTAG GGTACTTTGT ACTTCGAGTA ACATGTTCAC CAAACTTCAG GCTCTTCCCT TGTAGGGACG   
  
  
- TCCGACCGGT TCATGTCCAT GTCCTCCCAG TCTTCCGAGC ACGTTTCTTT GTCCCATTAT TACTATTATT   
  
  
- CTTCTTATTC TTACACCACC TAAATTCCTG AGACGAATAG GAGACACGCG TTAGACGACG TAGACTACTA   
  
  
- GCGTCCTGTC GACTACTTGA CGACTTCGTC TAATCCCTCG TGAGAAGACG TCGACCCCTA CCTAGAGTTT   
  
  
- CCAACCGAGT AATGAAACGA TTACGGAACC TCCGCGCAAA TCGACCTTGA CCGAGTGTCT AGATATGTCG   
  
  
- GGACTCAAGC TTCTCCTGTT GTCGTCGACT ATACTATTTT CGAATAGTCA AAAAGAAAGC ACGAACGGGT   
  
  
- AAAAATTTCT AGAGATAATA GAAACGCTTG GTATACTAAT ATGTCTCTCG ACTTTTTCGT AGTTTCGAAG   
  
  
- TACAATATCT AAAACCGTAG GACATACCAA AAGTCACCGG GGAGGAGTAA GTTGCGGACA GCCTCGCTGG   
  
  
- ACCACCTGGA GGTTTTGACA CGTAATGACC CTAACTAGAA GGGGTTGGAC CTAAATCCGG TTGTCTTTCT   
  
  
- CAACTCCGTT GTCCCGAGGC GAACCGTTTC ATAACACTCG CGAAGTTACA AGGTAAACTT ATGGTACGGT   
  
  
- AACGTGTCTT TACCCTTTGG TAGTTTCGAC TTCTGGACTT CTATCTTCTA CTACTCCACC AACGGCACTT   
  
  
- GACAGAGAAG TCCAAATTCT TGGACGAACT ACTCTGCTAT CACCACCTAT CAGGCTCCTT ACGTCAAGAG   
  
  
- CCGAACTAAT CTTCCTATTT TGGACTATAG AAACAAGTGC CTCATCACTT GCCCAGAATG TTACGCGGGA   
  
  
- AGAAACAATG TGCAAAGTCC CTCCGGGAGA AGGTAATGAG ATGACATAAG CTATACAAAC TACGGTTACG   
  
  
- AAGGGCCCTC CTAGGACTCT CTAACTACAA ACTCTTCCTC AAAATACCCG CTCTTTAATA CTTACACCAC   
  
  
- CGAACACTCC CATGTCTCTC TCAACTTTCT GGACTCTGTA TGTTCGTTAC CGTCCGCGCC TTATCATCCC   
  
  
- GTCCCAAGTC CGTCGACGGT AACCTGGTTC TCGAGCAGTC ATTTGACTCC TCGTTCCAGG TCTACGTGAT   
  
  
- AGGATCCCTG AAGCAACAAC TACATCTACC TGTAACCTGT AACGTCCCTA CCTTCCCCTC CTAGTAGACA   
  
  
- CGACACAGAC GTACCCCAGG ACGAAT

+     MYB-like sequence

| Site Name | Organism | Position | Strand | Matrix score. | sequence | function |
| --- | --- | --- | --- | --- | --- | --- |
| MYB-like sequence | Arabidopsis thaliana | 104 | - | 6 | TAACCA |  |
| MYB-like sequence | Arabidopsis thaliana | 2857 | - | 6 | TAACCA |  |
| MYB-like sequence | Arabidopsis thaliana | 1804 | - | 6 | TAACCA |  |

>HU02G01569.1   
+ +Up\_Stream \_Len000AGCGTA GGAAGTGACG CCGCTATTGG AGGCGAAGAC GCTAGTACCA AGGCCAAAAC   
  
  
+ AAATGAGGAG ATAGATCAGT TAAACCGTTT GGTTAAGAAA ATTAAGCGAA CATCTCTTGA ACCTCCATCT   
  
  
+ GAACTCGATG ACATTGAGGA TGTGGATGCA GAAAAGATGG ATATGGGCTC TCCATCGGTG CCAGCAAATC   
  
  
+ AACCTAATGT TGGGTGGGGT CTCATTCGGT CAATGGGGGA CTAGGGACGT CATATCGTCA CCAATTACAA   
  
  
+ CGGAACAACC CTAAACCGCA TTTTAAAATC ACATCTTGGT AGCAGACAAA GATGTACGAC CCAAAAATTC   
  
  
+ AACTCTGTAA AGCACTAGCA AAGCTCGTAG TACACAGAAC AATCGCACAC TCATAGTGAT AGAAGTGTAC   
  
  
+ TATCCATTAT GCAGTGAATA TGACAAGGGA GGGGGTCCTG AGATGCCCAA ATTTGGCAGC CGAGTTGCCT   
  
  
+ TGACTGCCCA TCAATGTCCT AAACCAGTTG AGCATGTTAT TAATTTGCAA GAAAGATCGA ATTCTTCCTA   
  
  
+ TCTAAAAACT GACACAATAA AACAATACCA TGTATGGGAC ATTGCTAGGA TTGTTAACAC AATTATTGTC   
  
  
+ TTGTACGTTG CACCTGAAAA AAAAGAAAAT TGTTGCCATA GACAACATAT AAGTTAGGCA TTCATGACTA   
  
  
+ ATATCTGAAT TAGGTGTGTC GATCTGTGAT TCAAATTGAT ATAATTAGAT CTTTAAGGTA TCTAATTGCT   
  
  
+ TCAAGTTTTC TCTATAATGA AGATGTATAT TAGCTCACAT AGGTCTTAAG GTTAACTAAT GTCATATCAG   
  
  
+ GGGGTGTGGG CCGCCATTTT TGATAAGAAT TGAATTGAAC TTGCATTGTT TTTTCTTTGG CGAGAATGCA   
  
  
+ AGATTTGTTA ATTTATTCAT ATGCGTCATT ATACACTATA AGAAAAATAA TTGTAGGAAT ATATGAAGTA   
  
  
+ TTTTTACTTG TGCTCATGTT AAATGTGGAA TACTTTTTTA ATGTCGTATA GTACCACAGT ATTCTACAAT   
  
  
+ TTAAGAGATT AATCGATCCA TTAAAGTCAT GCAAACCTGA TGCATATTCA AGACATTGCA TGAAATGATC   
  
  
+ TTATTTTAGT CAAAGACTCC ATCATTTATA AGCAATATTT ACTTCGATAC ACGTGTCATT AAAAAAGACT   
  
  
+ ATAGAAATGT TACTGTTATT TTGTTTGAGC TAAATAGAAA ATTTAAAAGT TAATTAAGAT AATAATTAAA   
  
  
+ ACTAAATATT CAATGATGGC TAATAAATAC ATTGTCGTAT TAACTGACTA ATATTTGTAG ATGGTCGTGT   
  
  
+ TAATTTCTTT TTATTTTAAA GTTCAAATGA GTTACAAAAG GTTATAAAAT ATAAAGTAAA ATGGGAGACA   
  
  
+ AAGCAGACAG GGTGGGGGAA GCTAAGCTTA AAGTAGGATT TAGAAAGGCA ATGAAGGTGA ACCCTCAAAT   
  
  
+ CGATCCGCTG CCAATCACAG AAACCCAAAG CTTTGCTCAC CGACAACTCC CGGTTAACTG CAGTCACTCA   
  
  
+ CGGCACTGGG TAATACAGTA ATCGTGTACC CAGTTCATCC CCCTTTTCCT TTGGGAATAC AGTACCTCGA   
  
  
+ AAGTTCCTTT GAAATTCTAC TGGCCAGGCA TACCCATAAA TTGATCCTCT CGATATCATA AGATATGATA   
  
  
+ TTTCTCTGTC ATATCTGTTA GTTTCGGTAT CATATTTCTT CTTTATCTCT GTAATTTGAG GTATGCTTCA   
  
  
+ TTCCTGGCCG CTTGAATTAT CTTTCTTTCT TTGATTAGTA TTGTTTTTTT GGTTACCTAT TTTGCTCCAG   
  
  
+ TCCTGTTCAT TTGGGTCTTT TTGGATCTGG GTTTTTGCTG GGTTTGTTGA TTCTTTGAGA AATTTGGGTG   
  
  
+ CTTGAATTTG CCCTGCAATA TTCATGGGTT AGCGTTTTCC TGAATTTTGC TCTGTTCCTT AAGTGATTAT   
  
  
+ TTTTGATTGA TTACACCTTG GTGGTCCTGG TGGAATTTCC GAGGAGAAAT TGTCATGGGT TCCCACAACT   
  
  
+ TTGGAGAATT CCCTGATGAG ACTCTAAATG AGTATCAATC TACTTTGGGA ACCATGTCCC CCGGTTATGA   
  
  
+ TGGGTCTTTG AATTATACAA CCTTGTTCAA TTACAAAGAC CCATCTCAGG ATCTCACGGC ACTGAACCTT   
  
  
+ CCTAGCCCAT TGCCTGACCC TATGCCATTC AACATTGGTT CATATTCGGG TTTGAGCCCC GGGGTTGAAT   
  
  
+ CTTCGGATGA TAGCGATTCA GATGATGTTG TTAAGTACAT TGGCCAAGTG CTTATGGAAG AGGATATGAA   
  
  
+ GGAGAAGCCT TGTATGTTCC ATGACCCTTT AGCACTCCAA GCTGCTGAGA AACCCTTTTA TGATGTGTTG   
  
  
+ GGAAAGAAGT ATCCTCCTTC CCCTAATCAA CACCCACTTA TTGATCATTC TGTGGATAGC CCAGACAATC   
  
  
+ AGTCCTGTGG AACGAGCACA ATTAGTGATC TTAGTGGCAG TAACTGCACT TCCAGTTCAA CCAATTATAT   
  
  
+ TGATGCTGTG GCAGTCGCTG ATTCGAGTGA GAATACTAAG ACCTCTTTTG TGCAAAGTTC TCTGATTGAA   
  
  
+ TCGTTTTCTC AGTCGTCCAC CCTTCCACAG TGGTCATTTG GATCATTGGG TGCCTTGGGT GGCACAGCTT   
  
  
+ CTCAGGGTTC GAATTCAGTT ATCTCCTCCC GTGGTTTCCC TATGGCCATG AACGTTTTTA GCGAGCAGTC   
  
  
+ CATGATACAG TTTCAGAAGG GGGTGGAGGA GGCGAGCAAG TTCCTTCCAA AGAACAATAA CCTTGCGATT   
  
  
+ GACCTTGAGA GCGTCACTTT CCCAAATGAA AAGGAGGCGG CCCCCATGGT GGTGGTTAAG AAAGAGAAGG   
  
  
+ ATGAACACTC ACCTGATAGC TCGAGAGGTA GCAAGATTCA CTACCGTGAT GATGAGGACT TTGAGGATGG   
  
  
+ TAGGAGGAGT AAGCAGTCAG CTGTTTCTGT GGAGGAGGCT GAGTTGTCTG AAATGTTTGA CCGGGTTTTG   
  
  
+ CTTTGCAATC CCATGAAACA TGAAGCTCAT TGTACAAGTG GTTTGAAGTC CGAGAAGGGA ACATCCCTGC   
  
  
+ AGGCTGGCCA AGTACAGGTA CAGGAGGGTC AGAAGGCTCG TGCAAAGAAA CAGGGTAATA ATGATAATAA   
  
  
+ GAAGAATAAG AATGTGGTGG ATTTAAGGAC TCTGCTTATC CTCTGTGCGC AATCTGCTGC ATCTGATGAT   
  
  
+ CGCAGGACAG CTGATGAACT GCTGAAGCAG ATTAGGGAGC ACTCTTCTGC AGCTGGGGAT GGATCTCAAA   
  
  
+ GGTTGGCTCA TTACTTTGCT AATGCCTTGG AGGCGCGTTT AGCTGGAACT GGCTCACAGA TCTATACAGC   
  
  
+ CCTGAGTTCG AAGAGGACAA CAGCAGCTGA TATGATAAAA GCTTATCAGT TTTTCTTTCG TGCTTGCCCA   
  
  
+ TTTTTAAAGA TCTCTATTAT CTTTGCGAAC CATATGATTA TACAGAGAGC TGAAAAAGCA TCAAAGCTTC   
  
  
+ ATGTTATAGA TTTTGGCATC CTGTATGGTT TTCAGTGGCC CCTCCTCATT CAACGCCTGT CGGAGCGACC   
  
  
+ TGGTGGACCT CCAAAACTGT GCATTACTGG GATTGATCTT CCCCAACCTG GATTTAGGCC AACAGAAAGA   
  
  
+ GTTGAGGCAA CAGGGCTCCG CTTGGCAAAG TATTGTGAGC GCTTCAATGT TCCATTTGAA TACCATGCCA   
  
  
+ TTGCACAGAA ATGGGAAACC ATCAAAGCTG AAGACCTGAA GATAGAAGAT GATGAGGTGG TTGCCGTGAA   
  
  
+ CTGTCTCTTC AGGTTTAAGA ACCTGCTTGA TGAGACGATA GTGGTGGATA GTCCGAGGAA TGCAGTTCTC   
  
  
+ GGCTTGATTA GAAGGATAAA ACCTGATATC TTTGTTCACG GAGTAGTGAA CGGGTCTTAC AATGCGCCCT   
  
  
+ TCTTTGTTAC ACGTTTCAGG GAGGCCCTCT TCCATTACTC TACTGTATTC GATATGTTTG ATGCCAATGC   
  
  
+ TTCCCGGGAG GATCCTGAGA GATTGATGTT TGAGAAGGAG TTTTATGGGC GAGAAATTAT GAATGTGGTG   
  
  
+ GCTTGTGAGG GTACAGAGAG AGTTGAAAGA CCTGAGACAT ACAAGCAATG GCAGGCGCGG AATAGTAGGG   
  
  
+ CAGGGTTCAG GCAGCTGCCA TTGGACCAAG AGCTCGTCAG TAAACTGAGG AGCAAGGTCC AGATGCACTA   
  
  
+ TCCTAGGGAC TTCGTTGTTG ATGTAGATGG ACATTGGACA TTGCAGGGAT GGAAGGGGAG GATCATCTGT   
  
  
+ GCTGTGTCTG CATGGGGTCC TGCTTA  

- +Up\_Stream \_Len000TCGCAT CCTTCACTGC GGCGATAACC TCCGCTTCTG CGATCATGGT TCCGGTTTTG   
  
  
- TTTACTCCTC TATCTAGTCA ATTTGGCAAA CCAATTCTTT TAATTCGCTT GTAGAGAACT TGGAGGTAGA   
  
  
- CTTGAGCTAC TGTAACTCCT ACACCTACGT CTTTTCTACC TATACCCGAG AGGTAGCCAC GGTCGTTTAG   
  
  
- TTGGATTACA ACCCACCCCA GAGTAAGCCA GTTACCCCCT GATCCCTGCA GTATAGCAGT GGTTAATGTT   
  
  
- GCCTTGTTGG GATTTGGCGT AAAATTTTAG TGTAGAACCA TCGTCTGTTT CTACATGCTG GGTTTTTAAG   
  
  
- TTGAGACATT TCGTGATCGT TTCGAGCATC ATGTGTCTTG TTAGCGTGTG AGTATCACTA TCTTCACATG   
  
  
- ATAGGTAATA CGTCACTTAT ACTGTTCCCT CCCCCAGGAC TCTACGGGTT TAAACCGTCG GCTCAACGGA   
  
  
- ACTGACGGGT AGTTACAGGA TTTGGTCAAC TCGTACAATA ATTAAACGTT CTTTCTAGCT TAAGAAGGAT   
  
  
- AGATTTTTGA CTGTGTTATT TTGTTATGGT ACATACCCTG TAACGATCCT AACAATTGTG TTAATAACAG   
  
  
- AACATGCAAC GTGGACTTTT TTTTCTTTTA ACAACGGTAT CTGTTGTATA TTCAATCCGT AAGTACTGAT   
  
  
- TATAGACTTA ATCCACACAG CTAGACACTA AGTTTAACTA TATTAATCTA GAAATTCCAT AGATTAACGA   
  
  
- AGTTCAAAAG AGATATTACT TCTACATATA ATCGAGTGTA TCCAGAATTC CAATTGATTA CAGTATAGTC   
  
  
- CCCCACACCC GGCGGTAAAA ACTATTCTTA ACTTAACTTG AACGTAACAA AAAAGAAACC GCTCTTACGT   
  
  
- TCTAAACAAT TAAATAAGTA TACGCAGTAA TATGTGATAT TCTTTTTATT AACATCCTTA TATACTTCAT   
  
  
- AAAAATGAAC ACGAGTACAA TTTACACCTT ATGAAAAAAT TACAGCATAT CATGGTGTCA TAAGATGTTA   
  
  
- AATTCTCTAA TTAGCTAGGT AATTTCAGTA CGTTTGGACT ACGTATAAGT TCTGTAACGT ACTTTACTAG   
  
  
- AATAAAATCA GTTTCTGAGG TAGTAAATAT TCGTTATAAA TGAAGCTATG TGCACAGTAA TTTTTTCTGA   
  
  
- TATCTTTACA ATGACAATAA AACAAACTCG ATTTATCTTT TAAATTTTCA ATTAATTCTA TTATTAATTT   
  
  
- TGATTTATAA GTTACTACCG ATTATTTATG TAACAGCATA ATTGACTGAT TATAAACATC TACCAGCACA   
  
  
- ATTAAAGAAA AATAAAATTT CAAGTTTACT CAATGTTTTC CAATATTTTA TATTTCATTT TACCCTCTGT   
  
  
- TTCGTCTGTC CCACCCCCTT CGATTCGAAT TTCATCCTAA ATCTTTCCGT TACTTCCACT TGGGAGTTTA   
  
  
- GCTAGGCGAC GGTTAGTGTC TTTGGGTTTC GAAACGAGTG GCTGTTGAGG GCCAATTGAC GTCAGTGAGT   
  
  
- GCCGTGACCC ATTATGTCAT TAGCACATGG GTCAAGTAGG GGGAAAAGGA AACCCTTATG TCATGGAGCT   
  
  
- TTCAAGGAAA CTTTAAGATG ACCGGTCCGT ATGGGTATTT AACTAGGAGA GCTATAGTAT TCTATACTAT   
  
  
- AAAGAGACAG TATAGACAAT CAAAGCCATA GTATAAAGAA GAAATAGAGA CATTAAACTC CATACGAAGT   
  
  
- AAGGACCGGC GAACTTAATA GAAAGAAAGA AACTAATCAT AACAAAAAAA CCAATGGATA AAACGAGGTC   
  
  
- AGGACAAGTA AACCCAGAAA AACCTAGACC CAAAAACGAC CCAAACAACT AAGAAACTCT TTAAACCCAC   
  
  
- GAACTTAAAC GGGACGTTAT AAGTACCCAA TCGCAAAAGG ACTTAAAACG AGACAAGGAA TTCACTAATA   
  
  
- AAAACTAACT AATGTGGAAC CACCAGGACC ACCTTAAAGG CTCCTCTTTA ACAGTACCCA AGGGTGTTGA   
  
  
- AACCTCTTAA GGGACTACTC TGAGATTTAC TCATAGTTAG ATGAAACCCT TGGTACAGGG GGCCAATACT   
  
  
- ACCCAGAAAC TTAATATGTT GGAACAAGTT AATGTTTCTG GGTAGAGTCC TAGAGTGCCG TGACTTGGAA   
  
  
- GGATCGGGTA ACGGACTGGG ATACGGTAAG TTGTAACCAA GTATAAGCCC AAACTCGGGG CCCCAACTTA   
  
  
- GAAGCCTACT ATCGCTAAGT CTACTACAAC AATTCATGTA ACCGGTTCAC GAATACCTTC TCCTATACTT   
  
  
- CCTCTTCGGA ACATACAAGG TACTGGGAAA TCGTGAGGTT CGACGACTCT TTGGGAAAAT ACTACACAAC   
  
  
- CCTTTCTTCA TAGGAGGAAG GGGATTAGTT GTGGGTGAAT AACTAGTAAG ACACCTATCG GGTCTGTTAG   
  
  
- TCAGGACACC TTGCTCGTGT TAATCACTAG AATCACCGTC ATTGACGTGA AGGTCAAGTT GGTTAATATA   
  
  
- ACTACGACAC CGTCAGCGAC TAAGCTCACT CTTATGATTC TGGAGAAAAC ACGTTTCAAG AGACTAACTT   
  
  
- AGCAAAAGAG TCAGCAGGTG GGAAGGTGTC ACCAGTAAAC CTAGTAACCC ACGGAACCCA CCGTGTCGAA   
  
  
- GAGTCCCAAG CTTAAGTCAA TAGAGGAGGG CACCAAAGGG ATACCGGTAC TTGCAAAAAT CGCTCGTCAG   
  
  
- GTACTATGTC AAAGTCTTCC CCCACCTCCT CCGCTCGTTC AAGGAAGGTT TCTTGTTATT GGAACGCTAA   
  
  
- CTGGAACTCT CGCAGTGAAA GGGTTTACTT TTCCTCCGCC GGGGGTACCA CCACCAATTC TTTCTCTTCC   
  
  
- TACTTGTGAG TGGACTATCG AGCTCTCCAT CGTTCTAAGT GATGGCACTA CTACTCCTGA AACTCCTACC   
  
  
- ATCCTCCTCA TTCGTCAGTC GACAAAGACA CCTCCTCCGA CTCAACAGAC TTTACAAACT GGCCCAAAAC   
  
  
- GAAACGTTAG GGTACTTTGT ACTTCGAGTA ACATGTTCAC CAAACTTCAG GCTCTTCCCT TGTAGGGACG   
  
  
- TCCGACCGGT TCATGTCCAT GTCCTCCCAG TCTTCCGAGC ACGTTTCTTT GTCCCATTAT TACTATTATT   
  
  
- CTTCTTATTC TTACACCACC TAAATTCCTG AGACGAATAG GAGACACGCG TTAGACGACG TAGACTACTA   
  
  
- GCGTCCTGTC GACTACTTGA CGACTTCGTC TAATCCCTCG TGAGAAGACG TCGACCCCTA CCTAGAGTTT   
  
  
- CCAACCGAGT AATGAAACGA TTACGGAACC TCCGCGCAAA TCGACCTTGA CCGAGTGTCT AGATATGTCG   
  
  
- GGACTCAAGC TTCTCCTGTT GTCGTCGACT ATACTATTTT CGAATAGTCA AAAAGAAAGC ACGAACGGGT   
  
  
- AAAAATTTCT AGAGATAATA GAAACGCTTG GTATACTAAT ATGTCTCTCG ACTTTTTCGT AGTTTCGAAG   
  
  
- TACAATATCT AAAACCGTAG GACATACCAA AAGTCACCGG GGAGGAGTAA GTTGCGGACA GCCTCGCTGG   
  
  
- ACCACCTGGA GGTTTTGACA CGTAATGACC CTAACTAGAA GGGGTTGGAC CTAAATCCGG TTGTCTTTCT   
  
  
- CAACTCCGTT GTCCCGAGGC GAACCGTTTC ATAACACTCG CGAAGTTACA AGGTAAACTT ATGGTACGGT   
  
  
- AACGTGTCTT TACCCTTTGG TAGTTTCGAC TTCTGGACTT CTATCTTCTA CTACTCCACC AACGGCACTT   
  
  
- GACAGAGAAG TCCAAATTCT TGGACGAACT ACTCTGCTAT CACCACCTAT CAGGCTCCTT ACGTCAAGAG   
  
  
- CCGAACTAAT CTTCCTATTT TGGACTATAG AAACAAGTGC CTCATCACTT GCCCAGAATG TTACGCGGGA   
  
  
- AGAAACAATG TGCAAAGTCC CTCCGGGAGA AGGTAATGAG ATGACATAAG CTATACAAAC TACGGTTACG   
  
  
- AAGGGCCCTC CTAGGACTCT CTAACTACAA ACTCTTCCTC AAAATACCCG CTCTTTAATA CTTACACCAC   
  
  
- CGAACACTCC CATGTCTCTC TCAACTTTCT GGACTCTGTA TGTTCGTTAC CGTCCGCGCC TTATCATCCC   
  
  
- GTCCCAAGTC CGTCGACGGT AACCTGGTTC TCGAGCAGTC ATTTGACTCC TCGTTCCAGG TCTACGTGAT   
  
  
- AGGATCCCTG AAGCAACAAC TACATCTACC TGTAACCTGT AACGTCCCTA CCTTCCCCTC CTAGTAGACA   
  
  
- CGACACAGAC GTACCCCAGG ACGAAT

+     MYC

| Site Name | Organism | Position | Strand | Matrix score. | sequence | function |
| --- | --- | --- | --- | --- | --- | --- |
| MYC | Arabidopsis thaliana | 2629 | + | 6 | CATTTG |  |
| MYC | Arabidopsis thaliana | 2827 | - | 6 | CATTTG |  |
| MYC | Arabidopsis thaliana | 74 | - | 6 | CATTTG |  |
| MYC | Arabidopsis thaliana | 3697 | + | 6 | CATTTG |  |
| MYC | Arabidopsis thaliana | 1358 | - | 6 | CATTTG |  |
| MYC | Arabidopsis thaliana | 1832 | + | 6 | CATTTG |  |

>HU02G01569.1   
+ +Up\_Stream \_Len000AGCGTA GGAAGTGACG CCGCTATTGG AGGCGAAGAC GCTAGTACCA AGGCCAAAAC   
  
  
+ AAATGAGGAG ATAGATCAGT TAAACCGTTT GGTTAAGAAA ATTAAGCGAA CATCTCTTGA ACCTCCATCT   
  
  
+ GAACTCGATG ACATTGAGGA TGTGGATGCA GAAAAGATGG ATATGGGCTC TCCATCGGTG CCAGCAAATC   
  
  
+ AACCTAATGT TGGGTGGGGT CTCATTCGGT CAATGGGGGA CTAGGGACGT CATATCGTCA CCAATTACAA   
  
  
+ CGGAACAACC CTAAACCGCA TTTTAAAATC ACATCTTGGT AGCAGACAAA GATGTACGAC CCAAAAATTC   
  
  
+ AACTCTGTAA AGCACTAGCA AAGCTCGTAG TACACAGAAC AATCGCACAC TCATAGTGAT AGAAGTGTAC   
  
  
+ TATCCATTAT GCAGTGAATA TGACAAGGGA GGGGGTCCTG AGATGCCCAA ATTTGGCAGC CGAGTTGCCT   
  
  
+ TGACTGCCCA TCAATGTCCT AAACCAGTTG AGCATGTTAT TAATTTGCAA GAAAGATCGA ATTCTTCCTA   
  
  
+ TCTAAAAACT GACACAATAA AACAATACCA TGTATGGGAC ATTGCTAGGA TTGTTAACAC AATTATTGTC   
  
  
+ TTGTACGTTG CACCTGAAAA AAAAGAAAAT TGTTGCCATA GACAACATAT AAGTTAGGCA TTCATGACTA   
  
  
+ ATATCTGAAT TAGGTGTGTC GATCTGTGAT TCAAATTGAT ATAATTAGAT CTTTAAGGTA TCTAATTGCT   
  
  
+ TCAAGTTTTC TCTATAATGA AGATGTATAT TAGCTCACAT AGGTCTTAAG GTTAACTAAT GTCATATCAG   
  
  
+ GGGGTGTGGG CCGCCATTTT TGATAAGAAT TGAATTGAAC TTGCATTGTT TTTTCTTTGG CGAGAATGCA   
  
  
+ AGATTTGTTA ATTTATTCAT ATGCGTCATT ATACACTATA AGAAAAATAA TTGTAGGAAT ATATGAAGTA   
  
  
+ TTTTTACTTG TGCTCATGTT AAATGTGGAA TACTTTTTTA ATGTCGTATA GTACCACAGT ATTCTACAAT   
  
  
+ TTAAGAGATT AATCGATCCA TTAAAGTCAT GCAAACCTGA TGCATATTCA AGACATTGCA TGAAATGATC   
  
  
+ TTATTTTAGT CAAAGACTCC ATCATTTATA AGCAATATTT ACTTCGATAC ACGTGTCATT AAAAAAGACT   
  
  
+ ATAGAAATGT TACTGTTATT TTGTTTGAGC TAAATAGAAA ATTTAAAAGT TAATTAAGAT AATAATTAAA   
  
  
+ ACTAAATATT CAATGATGGC TAATAAATAC ATTGTCGTAT TAACTGACTA ATATTTGTAG ATGGTCGTGT   
  
  
+ TAATTTCTTT TTATTTTAAA GTTCAAATGA GTTACAAAAG GTTATAAAAT ATAAAGTAAA ATGGGAGACA   
  
  
+ AAGCAGACAG GGTGGGGGAA GCTAAGCTTA AAGTAGGATT TAGAAAGGCA ATGAAGGTGA ACCCTCAAAT   
  
  
+ CGATCCGCTG CCAATCACAG AAACCCAAAG CTTTGCTCAC CGACAACTCC CGGTTAACTG CAGTCACTCA   
  
  
+ CGGCACTGGG TAATACAGTA ATCGTGTACC CAGTTCATCC CCCTTTTCCT TTGGGAATAC AGTACCTCGA   
  
  
+ AAGTTCCTTT GAAATTCTAC TGGCCAGGCA TACCCATAAA TTGATCCTCT CGATATCATA AGATATGATA   
  
  
+ TTTCTCTGTC ATATCTGTTA GTTTCGGTAT CATATTTCTT CTTTATCTCT GTAATTTGAG GTATGCTTCA   
  
  
+ TTCCTGGCCG CTTGAATTAT CTTTCTTTCT TTGATTAGTA TTGTTTTTTT GGTTACCTAT TTTGCTCCAG   
  
  
+ TCCTGTTCAT TTGGGTCTTT TTGGATCTGG GTTTTTGCTG GGTTTGTTGA TTCTTTGAGA AATTTGGGTG   
  
  
+ CTTGAATTTG CCCTGCAATA TTCATGGGTT AGCGTTTTCC TGAATTTTGC TCTGTTCCTT AAGTGATTAT   
  
  
+ TTTTGATTGA TTACACCTTG GTGGTCCTGG TGGAATTTCC GAGGAGAAAT TGTCATGGGT TCCCACAACT   
  
  
+ TTGGAGAATT CCCTGATGAG ACTCTAAATG AGTATCAATC TACTTTGGGA ACCATGTCCC CCGGTTATGA   
  
  
+ TGGGTCTTTG AATTATACAA CCTTGTTCAA TTACAAAGAC CCATCTCAGG ATCTCACGGC ACTGAACCTT   
  
  
+ CCTAGCCCAT TGCCTGACCC TATGCCATTC AACATTGGTT CATATTCGGG TTTGAGCCCC GGGGTTGAAT   
  
  
+ CTTCGGATGA TAGCGATTCA GATGATGTTG TTAAGTACAT TGGCCAAGTG CTTATGGAAG AGGATATGAA   
  
  
+ GGAGAAGCCT TGTATGTTCC ATGACCCTTT AGCACTCCAA GCTGCTGAGA AACCCTTTTA TGATGTGTTG   
  
  
+ GGAAAGAAGT ATCCTCCTTC CCCTAATCAA CACCCACTTA TTGATCATTC TGTGGATAGC CCAGACAATC   
  
  
+ AGTCCTGTGG AACGAGCACA ATTAGTGATC TTAGTGGCAG TAACTGCACT TCCAGTTCAA CCAATTATAT   
  
  
+ TGATGCTGTG GCAGTCGCTG ATTCGAGTGA GAATACTAAG ACCTCTTTTG TGCAAAGTTC TCTGATTGAA   
  
  
+ TCGTTTTCTC AGTCGTCCAC CCTTCCACAG TGGTCATTTG GATCATTGGG TGCCTTGGGT GGCACAGCTT   
  
  
+ CTCAGGGTTC GAATTCAGTT ATCTCCTCCC GTGGTTTCCC TATGGCCATG AACGTTTTTA GCGAGCAGTC   
  
  
+ CATGATACAG TTTCAGAAGG GGGTGGAGGA GGCGAGCAAG TTCCTTCCAA AGAACAATAA CCTTGCGATT   
  
  
+ GACCTTGAGA GCGTCACTTT CCCAAATGAA AAGGAGGCGG CCCCCATGGT GGTGGTTAAG AAAGAGAAGG   
  
  
+ ATGAACACTC ACCTGATAGC TCGAGAGGTA GCAAGATTCA CTACCGTGAT GATGAGGACT TTGAGGATGG   
  
  
+ TAGGAGGAGT AAGCAGTCAG CTGTTTCTGT GGAGGAGGCT GAGTTGTCTG AAATGTTTGA CCGGGTTTTG   
  
  
+ CTTTGCAATC CCATGAAACA TGAAGCTCAT TGTACAAGTG GTTTGAAGTC CGAGAAGGGA ACATCCCTGC   
  
  
+ AGGCTGGCCA AGTACAGGTA CAGGAGGGTC AGAAGGCTCG TGCAAAGAAA CAGGGTAATA ATGATAATAA   
  
  
+ GAAGAATAAG AATGTGGTGG ATTTAAGGAC TCTGCTTATC CTCTGTGCGC AATCTGCTGC ATCTGATGAT   
  
  
+ CGCAGGACAG CTGATGAACT GCTGAAGCAG ATTAGGGAGC ACTCTTCTGC AGCTGGGGAT GGATCTCAAA   
  
  
+ GGTTGGCTCA TTACTTTGCT AATGCCTTGG AGGCGCGTTT AGCTGGAACT GGCTCACAGA TCTATACAGC   
  
  
+ CCTGAGTTCG AAGAGGACAA CAGCAGCTGA TATGATAAAA GCTTATCAGT TTTTCTTTCG TGCTTGCCCA   
  
  
+ TTTTTAAAGA TCTCTATTAT CTTTGCGAAC CATATGATTA TACAGAGAGC TGAAAAAGCA TCAAAGCTTC   
  
  
+ ATGTTATAGA TTTTGGCATC CTGTATGGTT TTCAGTGGCC CCTCCTCATT CAACGCCTGT CGGAGCGACC   
  
  
+ TGGTGGACCT CCAAAACTGT GCATTACTGG GATTGATCTT CCCCAACCTG GATTTAGGCC AACAGAAAGA   
  
  
+ GTTGAGGCAA CAGGGCTCCG CTTGGCAAAG TATTGTGAGC GCTTCAATGT TCCATTTGAA TACCATGCCA   
  
  
+ TTGCACAGAA ATGGGAAACC ATCAAAGCTG AAGACCTGAA GATAGAAGAT GATGAGGTGG TTGCCGTGAA   
  
  
+ CTGTCTCTTC AGGTTTAAGA ACCTGCTTGA TGAGACGATA GTGGTGGATA GTCCGAGGAA TGCAGTTCTC   
  
  
+ GGCTTGATTA GAAGGATAAA ACCTGATATC TTTGTTCACG GAGTAGTGAA CGGGTCTTAC AATGCGCCCT   
  
  
+ TCTTTGTTAC ACGTTTCAGG GAGGCCCTCT TCCATTACTC TACTGTATTC GATATGTTTG ATGCCAATGC   
  
  
+ TTCCCGGGAG GATCCTGAGA GATTGATGTT TGAGAAGGAG TTTTATGGGC GAGAAATTAT GAATGTGGTG   
  
  
+ GCTTGTGAGG GTACAGAGAG AGTTGAAAGA CCTGAGACAT ACAAGCAATG GCAGGCGCGG AATAGTAGGG   
  
  
+ CAGGGTTCAG GCAGCTGCCA TTGGACCAAG AGCTCGTCAG TAAACTGAGG AGCAAGGTCC AGATGCACTA   
  
  
+ TCCTAGGGAC TTCGTTGTTG ATGTAGATGG ACATTGGACA TTGCAGGGAT GGAAGGGGAG GATCATCTGT   
  
  
+ GCTGTGTCTG CATGGGGTCC TGCTTA  

- +Up\_Stream \_Len000TCGCAT CCTTCACTGC GGCGATAACC TCCGCTTCTG CGATCATGGT TCCGGTTTTG   
  
  
- TTTACTCCTC TATCTAGTCA ATTTGGCAAA CCAATTCTTT TAATTCGCTT GTAGAGAACT TGGAGGTAGA   
  
  
- CTTGAGCTAC TGTAACTCCT ACACCTACGT CTTTTCTACC TATACCCGAG AGGTAGCCAC GGTCGTTTAG   
  
  
- TTGGATTACA ACCCACCCCA GAGTAAGCCA GTTACCCCCT GATCCCTGCA GTATAGCAGT GGTTAATGTT   
  
  
- GCCTTGTTGG GATTTGGCGT AAAATTTTAG TGTAGAACCA TCGTCTGTTT CTACATGCTG GGTTTTTAAG   
  
  
- TTGAGACATT TCGTGATCGT TTCGAGCATC ATGTGTCTTG TTAGCGTGTG AGTATCACTA TCTTCACATG   
  
  
- ATAGGTAATA CGTCACTTAT ACTGTTCCCT CCCCCAGGAC TCTACGGGTT TAAACCGTCG GCTCAACGGA   
  
  
- ACTGACGGGT AGTTACAGGA TTTGGTCAAC TCGTACAATA ATTAAACGTT CTTTCTAGCT TAAGAAGGAT   
  
  
- AGATTTTTGA CTGTGTTATT TTGTTATGGT ACATACCCTG TAACGATCCT AACAATTGTG TTAATAACAG   
  
  
- AACATGCAAC GTGGACTTTT TTTTCTTTTA ACAACGGTAT CTGTTGTATA TTCAATCCGT AAGTACTGAT   
  
  
- TATAGACTTA ATCCACACAG CTAGACACTA AGTTTAACTA TATTAATCTA GAAATTCCAT AGATTAACGA   
  
  
- AGTTCAAAAG AGATATTACT TCTACATATA ATCGAGTGTA TCCAGAATTC CAATTGATTA CAGTATAGTC   
  
  
- CCCCACACCC GGCGGTAAAA ACTATTCTTA ACTTAACTTG AACGTAACAA AAAAGAAACC GCTCTTACGT   
  
  
- TCTAAACAAT TAAATAAGTA TACGCAGTAA TATGTGATAT TCTTTTTATT AACATCCTTA TATACTTCAT   
  
  
- AAAAATGAAC ACGAGTACAA TTTACACCTT ATGAAAAAAT TACAGCATAT CATGGTGTCA TAAGATGTTA   
  
  
- AATTCTCTAA TTAGCTAGGT AATTTCAGTA CGTTTGGACT ACGTATAAGT TCTGTAACGT ACTTTACTAG   
  
  
- AATAAAATCA GTTTCTGAGG TAGTAAATAT TCGTTATAAA TGAAGCTATG TGCACAGTAA TTTTTTCTGA   
  
  
- TATCTTTACA ATGACAATAA AACAAACTCG ATTTATCTTT TAAATTTTCA ATTAATTCTA TTATTAATTT   
  
  
- TGATTTATAA GTTACTACCG ATTATTTATG TAACAGCATA ATTGACTGAT TATAAACATC TACCAGCACA   
  
  
- ATTAAAGAAA AATAAAATTT CAAGTTTACT CAATGTTTTC CAATATTTTA TATTTCATTT TACCCTCTGT   
  
  
- TTCGTCTGTC CCACCCCCTT CGATTCGAAT TTCATCCTAA ATCTTTCCGT TACTTCCACT TGGGAGTTTA   
  
  
- GCTAGGCGAC GGTTAGTGTC TTTGGGTTTC GAAACGAGTG GCTGTTGAGG GCCAATTGAC GTCAGTGAGT   
  
  
- GCCGTGACCC ATTATGTCAT TAGCACATGG GTCAAGTAGG GGGAAAAGGA AACCCTTATG TCATGGAGCT   
  
  
- TTCAAGGAAA CTTTAAGATG ACCGGTCCGT ATGGGTATTT AACTAGGAGA GCTATAGTAT TCTATACTAT   
  
  
- AAAGAGACAG TATAGACAAT CAAAGCCATA GTATAAAGAA GAAATAGAGA CATTAAACTC CATACGAAGT   
  
  
- AAGGACCGGC GAACTTAATA GAAAGAAAGA AACTAATCAT AACAAAAAAA CCAATGGATA AAACGAGGTC   
  
  
- AGGACAAGTA AACCCAGAAA AACCTAGACC CAAAAACGAC CCAAACAACT AAGAAACTCT TTAAACCCAC   
  
  
- GAACTTAAAC GGGACGTTAT AAGTACCCAA TCGCAAAAGG ACTTAAAACG AGACAAGGAA TTCACTAATA   
  
  
- AAAACTAACT AATGTGGAAC CACCAGGACC ACCTTAAAGG CTCCTCTTTA ACAGTACCCA AGGGTGTTGA   
  
  
- AACCTCTTAA GGGACTACTC TGAGATTTAC TCATAGTTAG ATGAAACCCT TGGTACAGGG GGCCAATACT   
  
  
- ACCCAGAAAC TTAATATGTT GGAACAAGTT AATGTTTCTG GGTAGAGTCC TAGAGTGCCG TGACTTGGAA   
  
  
- GGATCGGGTA ACGGACTGGG ATACGGTAAG TTGTAACCAA GTATAAGCCC AAACTCGGGG CCCCAACTTA   
  
  
- GAAGCCTACT ATCGCTAAGT CTACTACAAC AATTCATGTA ACCGGTTCAC GAATACCTTC TCCTATACTT   
  
  
- CCTCTTCGGA ACATACAAGG TACTGGGAAA TCGTGAGGTT CGACGACTCT TTGGGAAAAT ACTACACAAC   
  
  
- CCTTTCTTCA TAGGAGGAAG GGGATTAGTT GTGGGTGAAT AACTAGTAAG ACACCTATCG GGTCTGTTAG   
  
  
- TCAGGACACC TTGCTCGTGT TAATCACTAG AATCACCGTC ATTGACGTGA AGGTCAAGTT GGTTAATATA   
  
  
- ACTACGACAC CGTCAGCGAC TAAGCTCACT CTTATGATTC TGGAGAAAAC ACGTTTCAAG AGACTAACTT   
  
  
- AGCAAAAGAG TCAGCAGGTG GGAAGGTGTC ACCAGTAAAC CTAGTAACCC ACGGAACCCA CCGTGTCGAA   
  
  
- GAGTCCCAAG CTTAAGTCAA TAGAGGAGGG CACCAAAGGG ATACCGGTAC TTGCAAAAAT CGCTCGTCAG   
  
  
- GTACTATGTC AAAGTCTTCC CCCACCTCCT CCGCTCGTTC AAGGAAGGTT TCTTGTTATT GGAACGCTAA   
  
  
- CTGGAACTCT CGCAGTGAAA GGGTTTACTT TTCCTCCGCC GGGGGTACCA CCACCAATTC TTTCTCTTCC   
  
  
- TACTTGTGAG TGGACTATCG AGCTCTCCAT CGTTCTAAGT GATGGCACTA CTACTCCTGA AACTCCTACC   
  
  
- ATCCTCCTCA TTCGTCAGTC GACAAAGACA CCTCCTCCGA CTCAACAGAC TTTACAAACT GGCCCAAAAC   
  
  
- GAAACGTTAG GGTACTTTGT ACTTCGAGTA ACATGTTCAC CAAACTTCAG GCTCTTCCCT TGTAGGGACG   
  
  
- TCCGACCGGT TCATGTCCAT GTCCTCCCAG TCTTCCGAGC ACGTTTCTTT GTCCCATTAT TACTATTATT   
  
  
- CTTCTTATTC TTACACCACC TAAATTCCTG AGACGAATAG GAGACACGCG TTAGACGACG TAGACTACTA   
  
  
- GCGTCCTGTC GACTACTTGA CGACTTCGTC TAATCCCTCG TGAGAAGACG TCGACCCCTA CCTAGAGTTT   
  
  
- CCAACCGAGT AATGAAACGA TTACGGAACC TCCGCGCAAA TCGACCTTGA CCGAGTGTCT AGATATGTCG   
  
  
- GGACTCAAGC TTCTCCTGTT GTCGTCGACT ATACTATTTT CGAATAGTCA AAAAGAAAGC ACGAACGGGT   
  
  
- AAAAATTTCT AGAGATAATA GAAACGCTTG GTATACTAAT ATGTCTCTCG ACTTTTTCGT AGTTTCGAAG   
  
  
- TACAATATCT AAAACCGTAG GACATACCAA AAGTCACCGG GGAGGAGTAA GTTGCGGACA GCCTCGCTGG   
  
  
- ACCACCTGGA GGTTTTGACA CGTAATGACC CTAACTAGAA GGGGTTGGAC CTAAATCCGG TTGTCTTTCT   
  
  
- CAACTCCGTT GTCCCGAGGC GAACCGTTTC ATAACACTCG CGAAGTTACA AGGTAAACTT ATGGTACGGT   
  
  
- AACGTGTCTT TACCCTTTGG TAGTTTCGAC TTCTGGACTT CTATCTTCTA CTACTCCACC AACGGCACTT   
  
  
- GACAGAGAAG TCCAAATTCT TGGACGAACT ACTCTGCTAT CACCACCTAT CAGGCTCCTT ACGTCAAGAG   
  
  
- CCGAACTAAT CTTCCTATTT TGGACTATAG AAACAAGTGC CTCATCACTT GCCCAGAATG TTACGCGGGA   
  
  
- AGAAACAATG TGCAAAGTCC CTCCGGGAGA AGGTAATGAG ATGACATAAG CTATACAAAC TACGGTTACG   
  
  
- AAGGGCCCTC CTAGGACTCT CTAACTACAA ACTCTTCCTC AAAATACCCG CTCTTTAATA CTTACACCAC   
  
  
- CGAACACTCC CATGTCTCTC TCAACTTTCT GGACTCTGTA TGTTCGTTAC CGTCCGCGCC TTATCATCCC   
  
  
- GTCCCAAGTC CGTCGACGGT AACCTGGTTC TCGAGCAGTC ATTTGACTCC TCGTTCCAGG TCTACGTGAT   
  
  
- AGGATCCCTG AAGCAACAAC TACATCTACC TGTAACCTGT AACGTCCCTA CCTTCCCCTC CTAGTAGACA   
  
  
- CGACACAGAC GTACCCCAGG ACGAAT

+     Myb

| Site Name | Organism | Position | Strand | Matrix score. | sequence | function |
| --- | --- | --- | --- | --- | --- | --- |
| Myb | Arabidopsis thaliana | 2680 | - | 6 | TAACTG |  |
| Myb | Arabidopsis thaliana | 1529 | + | 6 | TAACTG |  |
| Myb | Arabidopsis thaliana | 1305 | + | 6 | TAACTG |  |
| Myb | Arabidopsis thaliana | 519 | - | 6 | CAACTG |  |
| Myb | Arabidopsis thaliana | 2495 | + | 6 | TAACTG |  |
| Myb | Arabidopsis thaliana | 91 | - | 6 | TAACTG |  |

>HU02G01569.1   
+ +Up\_Stream \_Len000AGCGTA GGAAGTGACG CCGCTATTGG AGGCGAAGAC GCTAGTACCA AGGCCAAAAC   
  
  
+ AAATGAGGAG ATAGATCAGT TAAACCGTTT GGTTAAGAAA ATTAAGCGAA CATCTCTTGA ACCTCCATCT   
  
  
+ GAACTCGATG ACATTGAGGA TGTGGATGCA GAAAAGATGG ATATGGGCTC TCCATCGGTG CCAGCAAATC   
  
  
+ AACCTAATGT TGGGTGGGGT CTCATTCGGT CAATGGGGGA CTAGGGACGT CATATCGTCA CCAATTACAA   
  
  
+ CGGAACAACC CTAAACCGCA TTTTAAAATC ACATCTTGGT AGCAGACAAA GATGTACGAC CCAAAAATTC   
  
  
+ AACTCTGTAA AGCACTAGCA AAGCTCGTAG TACACAGAAC AATCGCACAC TCATAGTGAT AGAAGTGTAC   
  
  
+ TATCCATTAT GCAGTGAATA TGACAAGGGA GGGGGTCCTG AGATGCCCAA ATTTGGCAGC CGAGTTGCCT   
  
  
+ TGACTGCCCA TCAATGTCCT AAACCAGTTG AGCATGTTAT TAATTTGCAA GAAAGATCGA ATTCTTCCTA   
  
  
+ TCTAAAAACT GACACAATAA AACAATACCA TGTATGGGAC ATTGCTAGGA TTGTTAACAC AATTATTGTC   
  
  
+ TTGTACGTTG CACCTGAAAA AAAAGAAAAT TGTTGCCATA GACAACATAT AAGTTAGGCA TTCATGACTA   
  
  
+ ATATCTGAAT TAGGTGTGTC GATCTGTGAT TCAAATTGAT ATAATTAGAT CTTTAAGGTA TCTAATTGCT   
  
  
+ TCAAGTTTTC TCTATAATGA AGATGTATAT TAGCTCACAT AGGTCTTAAG GTTAACTAAT GTCATATCAG   
  
  
+ GGGGTGTGGG CCGCCATTTT TGATAAGAAT TGAATTGAAC TTGCATTGTT TTTTCTTTGG CGAGAATGCA   
  
  
+ AGATTTGTTA ATTTATTCAT ATGCGTCATT ATACACTATA AGAAAAATAA TTGTAGGAAT ATATGAAGTA   
  
  
+ TTTTTACTTG TGCTCATGTT AAATGTGGAA TACTTTTTTA ATGTCGTATA GTACCACAGT ATTCTACAAT   
  
  
+ TTAAGAGATT AATCGATCCA TTAAAGTCAT GCAAACCTGA TGCATATTCA AGACATTGCA TGAAATGATC   
  
  
+ TTATTTTAGT CAAAGACTCC ATCATTTATA AGCAATATTT ACTTCGATAC ACGTGTCATT AAAAAAGACT   
  
  
+ ATAGAAATGT TACTGTTATT TTGTTTGAGC TAAATAGAAA ATTTAAAAGT TAATTAAGAT AATAATTAAA   
  
  
+ ACTAAATATT CAATGATGGC TAATAAATAC ATTGTCGTAT TAACTGACTA ATATTTGTAG ATGGTCGTGT   
  
  
+ TAATTTCTTT TTATTTTAAA GTTCAAATGA GTTACAAAAG GTTATAAAAT ATAAAGTAAA ATGGGAGACA   
  
  
+ AAGCAGACAG GGTGGGGGAA GCTAAGCTTA AAGTAGGATT TAGAAAGGCA ATGAAGGTGA ACCCTCAAAT   
  
  
+ CGATCCGCTG CCAATCACAG AAACCCAAAG CTTTGCTCAC CGACAACTCC CGGTTAACTG CAGTCACTCA   
  
  
+ CGGCACTGGG TAATACAGTA ATCGTGTACC CAGTTCATCC CCCTTTTCCT TTGGGAATAC AGTACCTCGA   
  
  
+ AAGTTCCTTT GAAATTCTAC TGGCCAGGCA TACCCATAAA TTGATCCTCT CGATATCATA AGATATGATA   
  
  
+ TTTCTCTGTC ATATCTGTTA GTTTCGGTAT CATATTTCTT CTTTATCTCT GTAATTTGAG GTATGCTTCA   
  
  
+ TTCCTGGCCG CTTGAATTAT CTTTCTTTCT TTGATTAGTA TTGTTTTTTT GGTTACCTAT TTTGCTCCAG   
  
  
+ TCCTGTTCAT TTGGGTCTTT TTGGATCTGG GTTTTTGCTG GGTTTGTTGA TTCTTTGAGA AATTTGGGTG   
  
  
+ CTTGAATTTG CCCTGCAATA TTCATGGGTT AGCGTTTTCC TGAATTTTGC TCTGTTCCTT AAGTGATTAT   
  
  
+ TTTTGATTGA TTACACCTTG GTGGTCCTGG TGGAATTTCC GAGGAGAAAT TGTCATGGGT TCCCACAACT   
  
  
+ TTGGAGAATT CCCTGATGAG ACTCTAAATG AGTATCAATC TACTTTGGGA ACCATGTCCC CCGGTTATGA   
  
  
+ TGGGTCTTTG AATTATACAA CCTTGTTCAA TTACAAAGAC CCATCTCAGG ATCTCACGGC ACTGAACCTT   
  
  
+ CCTAGCCCAT TGCCTGACCC TATGCCATTC AACATTGGTT CATATTCGGG TTTGAGCCCC GGGGTTGAAT   
  
  
+ CTTCGGATGA TAGCGATTCA GATGATGTTG TTAAGTACAT TGGCCAAGTG CTTATGGAAG AGGATATGAA   
  
  
+ GGAGAAGCCT TGTATGTTCC ATGACCCTTT AGCACTCCAA GCTGCTGAGA AACCCTTTTA TGATGTGTTG   
  
  
+ GGAAAGAAGT ATCCTCCTTC CCCTAATCAA CACCCACTTA TTGATCATTC TGTGGATAGC CCAGACAATC   
  
  
+ AGTCCTGTGG AACGAGCACA ATTAGTGATC TTAGTGGCAG TAACTGCACT TCCAGTTCAA CCAATTATAT   
  
  
+ TGATGCTGTG GCAGTCGCTG ATTCGAGTGA GAATACTAAG ACCTCTTTTG TGCAAAGTTC TCTGATTGAA   
  
  
+ TCGTTTTCTC AGTCGTCCAC CCTTCCACAG TGGTCATTTG GATCATTGGG TGCCTTGGGT GGCACAGCTT   
  
  
+ CTCAGGGTTC GAATTCAGTT ATCTCCTCCC GTGGTTTCCC TATGGCCATG AACGTTTTTA GCGAGCAGTC   
  
  
+ CATGATACAG TTTCAGAAGG GGGTGGAGGA GGCGAGCAAG TTCCTTCCAA AGAACAATAA CCTTGCGATT   
  
  
+ GACCTTGAGA GCGTCACTTT CCCAAATGAA AAGGAGGCGG CCCCCATGGT GGTGGTTAAG AAAGAGAAGG   
  
  
+ ATGAACACTC ACCTGATAGC TCGAGAGGTA GCAAGATTCA CTACCGTGAT GATGAGGACT TTGAGGATGG   
  
  
+ TAGGAGGAGT AAGCAGTCAG CTGTTTCTGT GGAGGAGGCT GAGTTGTCTG AAATGTTTGA CCGGGTTTTG   
  
  
+ CTTTGCAATC CCATGAAACA TGAAGCTCAT TGTACAAGTG GTTTGAAGTC CGAGAAGGGA ACATCCCTGC   
  
  
+ AGGCTGGCCA AGTACAGGTA CAGGAGGGTC AGAAGGCTCG TGCAAAGAAA CAGGGTAATA ATGATAATAA   
  
  
+ GAAGAATAAG AATGTGGTGG ATTTAAGGAC TCTGCTTATC CTCTGTGCGC AATCTGCTGC ATCTGATGAT   
  
  
+ CGCAGGACAG CTGATGAACT GCTGAAGCAG ATTAGGGAGC ACTCTTCTGC AGCTGGGGAT GGATCTCAAA   
  
  
+ GGTTGGCTCA TTACTTTGCT AATGCCTTGG AGGCGCGTTT AGCTGGAACT GGCTCACAGA TCTATACAGC   
  
  
+ CCTGAGTTCG AAGAGGACAA CAGCAGCTGA TATGATAAAA GCTTATCAGT TTTTCTTTCG TGCTTGCCCA   
  
  
+ TTTTTAAAGA TCTCTATTAT CTTTGCGAAC CATATGATTA TACAGAGAGC TGAAAAAGCA TCAAAGCTTC   
  
  
+ ATGTTATAGA TTTTGGCATC CTGTATGGTT TTCAGTGGCC CCTCCTCATT CAACGCCTGT CGGAGCGACC   
  
  
+ TGGTGGACCT CCAAAACTGT GCATTACTGG GATTGATCTT CCCCAACCTG GATTTAGGCC AACAGAAAGA   
  
  
+ GTTGAGGCAA CAGGGCTCCG CTTGGCAAAG TATTGTGAGC GCTTCAATGT TCCATTTGAA TACCATGCCA   
  
  
+ TTGCACAGAA ATGGGAAACC ATCAAAGCTG AAGACCTGAA GATAGAAGAT GATGAGGTGG TTGCCGTGAA   
  
  
+ CTGTCTCTTC AGGTTTAAGA ACCTGCTTGA TGAGACGATA GTGGTGGATA GTCCGAGGAA TGCAGTTCTC   
  
  
+ GGCTTGATTA GAAGGATAAA ACCTGATATC TTTGTTCACG GAGTAGTGAA CGGGTCTTAC AATGCGCCCT   
  
  
+ TCTTTGTTAC ACGTTTCAGG GAGGCCCTCT TCCATTACTC TACTGTATTC GATATGTTTG ATGCCAATGC   
  
  
+ TTCCCGGGAG GATCCTGAGA GATTGATGTT TGAGAAGGAG TTTTATGGGC GAGAAATTAT GAATGTGGTG   
  
  
+ GCTTGTGAGG GTACAGAGAG AGTTGAAAGA CCTGAGACAT ACAAGCAATG GCAGGCGCGG AATAGTAGGG   
  
  
+ CAGGGTTCAG GCAGCTGCCA TTGGACCAAG AGCTCGTCAG TAAACTGAGG AGCAAGGTCC AGATGCACTA   
  
  
+ TCCTAGGGAC TTCGTTGTTG ATGTAGATGG ACATTGGACA TTGCAGGGAT GGAAGGGGAG GATCATCTGT   
  
  
+ GCTGTGTCTG CATGGGGTCC TGCTTA  

- +Up\_Stream \_Len000TCGCAT CCTTCACTGC GGCGATAACC TCCGCTTCTG CGATCATGGT TCCGGTTTTG   
  
  
- TTTACTCCTC TATCTAGTCA ATTTGGCAAA CCAATTCTTT TAATTCGCTT GTAGAGAACT TGGAGGTAGA   
  
  
- CTTGAGCTAC TGTAACTCCT ACACCTACGT CTTTTCTACC TATACCCGAG AGGTAGCCAC GGTCGTTTAG   
  
  
- TTGGATTACA ACCCACCCCA GAGTAAGCCA GTTACCCCCT GATCCCTGCA GTATAGCAGT GGTTAATGTT   
  
  
- GCCTTGTTGG GATTTGGCGT AAAATTTTAG TGTAGAACCA TCGTCTGTTT CTACATGCTG GGTTTTTAAG   
  
  
- TTGAGACATT TCGTGATCGT TTCGAGCATC ATGTGTCTTG TTAGCGTGTG AGTATCACTA TCTTCACATG   
  
  
- ATAGGTAATA CGTCACTTAT ACTGTTCCCT CCCCCAGGAC TCTACGGGTT TAAACCGTCG GCTCAACGGA   
  
  
- ACTGACGGGT AGTTACAGGA TTTGGTCAAC TCGTACAATA ATTAAACGTT CTTTCTAGCT TAAGAAGGAT   
  
  
- AGATTTTTGA CTGTGTTATT TTGTTATGGT ACATACCCTG TAACGATCCT AACAATTGTG TTAATAACAG   
  
  
- AACATGCAAC GTGGACTTTT TTTTCTTTTA ACAACGGTAT CTGTTGTATA TTCAATCCGT AAGTACTGAT   
  
  
- TATAGACTTA ATCCACACAG CTAGACACTA AGTTTAACTA TATTAATCTA GAAATTCCAT AGATTAACGA   
  
  
- AGTTCAAAAG AGATATTACT TCTACATATA ATCGAGTGTA TCCAGAATTC CAATTGATTA CAGTATAGTC   
  
  
- CCCCACACCC GGCGGTAAAA ACTATTCTTA ACTTAACTTG AACGTAACAA AAAAGAAACC GCTCTTACGT   
  
  
- TCTAAACAAT TAAATAAGTA TACGCAGTAA TATGTGATAT TCTTTTTATT AACATCCTTA TATACTTCAT   
  
  
- AAAAATGAAC ACGAGTACAA TTTACACCTT ATGAAAAAAT TACAGCATAT CATGGTGTCA TAAGATGTTA   
  
  
- AATTCTCTAA TTAGCTAGGT AATTTCAGTA CGTTTGGACT ACGTATAAGT TCTGTAACGT ACTTTACTAG   
  
  
- AATAAAATCA GTTTCTGAGG TAGTAAATAT TCGTTATAAA TGAAGCTATG TGCACAGTAA TTTTTTCTGA   
  
  
- TATCTTTACA ATGACAATAA AACAAACTCG ATTTATCTTT TAAATTTTCA ATTAATTCTA TTATTAATTT   
  
  
- TGATTTATAA GTTACTACCG ATTATTTATG TAACAGCATA ATTGACTGAT TATAAACATC TACCAGCACA   
  
  
- ATTAAAGAAA AATAAAATTT CAAGTTTACT CAATGTTTTC CAATATTTTA TATTTCATTT TACCCTCTGT   
  
  
- TTCGTCTGTC CCACCCCCTT CGATTCGAAT TTCATCCTAA ATCTTTCCGT TACTTCCACT TGGGAGTTTA   
  
  
- GCTAGGCGAC GGTTAGTGTC TTTGGGTTTC GAAACGAGTG GCTGTTGAGG GCCAATTGAC GTCAGTGAGT   
  
  
- GCCGTGACCC ATTATGTCAT TAGCACATGG GTCAAGTAGG GGGAAAAGGA AACCCTTATG TCATGGAGCT   
  
  
- TTCAAGGAAA CTTTAAGATG ACCGGTCCGT ATGGGTATTT AACTAGGAGA GCTATAGTAT TCTATACTAT   
  
  
- AAAGAGACAG TATAGACAAT CAAAGCCATA GTATAAAGAA GAAATAGAGA CATTAAACTC CATACGAAGT   
  
  
- AAGGACCGGC GAACTTAATA GAAAGAAAGA AACTAATCAT AACAAAAAAA CCAATGGATA AAACGAGGTC   
  
  
- AGGACAAGTA AACCCAGAAA AACCTAGACC CAAAAACGAC CCAAACAACT AAGAAACTCT TTAAACCCAC   
  
  
- GAACTTAAAC GGGACGTTAT AAGTACCCAA TCGCAAAAGG ACTTAAAACG AGACAAGGAA TTCACTAATA   
  
  
- AAAACTAACT AATGTGGAAC CACCAGGACC ACCTTAAAGG CTCCTCTTTA ACAGTACCCA AGGGTGTTGA   
  
  
- AACCTCTTAA GGGACTACTC TGAGATTTAC TCATAGTTAG ATGAAACCCT TGGTACAGGG GGCCAATACT   
  
  
- ACCCAGAAAC TTAATATGTT GGAACAAGTT AATGTTTCTG GGTAGAGTCC TAGAGTGCCG TGACTTGGAA   
  
  
- GGATCGGGTA ACGGACTGGG ATACGGTAAG TTGTAACCAA GTATAAGCCC AAACTCGGGG CCCCAACTTA   
  
  
- GAAGCCTACT ATCGCTAAGT CTACTACAAC AATTCATGTA ACCGGTTCAC GAATACCTTC TCCTATACTT   
  
  
- CCTCTTCGGA ACATACAAGG TACTGGGAAA TCGTGAGGTT CGACGACTCT TTGGGAAAAT ACTACACAAC   
  
  
- CCTTTCTTCA TAGGAGGAAG GGGATTAGTT GTGGGTGAAT AACTAGTAAG ACACCTATCG GGTCTGTTAG   
  
  
- TCAGGACACC TTGCTCGTGT TAATCACTAG AATCACCGTC ATTGACGTGA AGGTCAAGTT GGTTAATATA   
  
  
- ACTACGACAC CGTCAGCGAC TAAGCTCACT CTTATGATTC TGGAGAAAAC ACGTTTCAAG AGACTAACTT   
  
  
- AGCAAAAGAG TCAGCAGGTG GGAAGGTGTC ACCAGTAAAC CTAGTAACCC ACGGAACCCA CCGTGTCGAA   
  
  
- GAGTCCCAAG CTTAAGTCAA TAGAGGAGGG CACCAAAGGG ATACCGGTAC TTGCAAAAAT CGCTCGTCAG   
  
  
- GTACTATGTC AAAGTCTTCC CCCACCTCCT CCGCTCGTTC AAGGAAGGTT TCTTGTTATT GGAACGCTAA   
  
  
- CTGGAACTCT CGCAGTGAAA GGGTTTACTT TTCCTCCGCC GGGGGTACCA CCACCAATTC TTTCTCTTCC   
  
  
- TACTTGTGAG TGGACTATCG AGCTCTCCAT CGTTCTAAGT GATGGCACTA CTACTCCTGA AACTCCTACC   
  
  
- ATCCTCCTCA TTCGTCAGTC GACAAAGACA CCTCCTCCGA CTCAACAGAC TTTACAAACT GGCCCAAAAC   
  
  
- GAAACGTTAG GGTACTTTGT ACTTCGAGTA ACATGTTCAC CAAACTTCAG GCTCTTCCCT TGTAGGGACG   
  
  
- TCCGACCGGT TCATGTCCAT GTCCTCCCAG TCTTCCGAGC ACGTTTCTTT GTCCCATTAT TACTATTATT   
  
  
- CTTCTTATTC TTACACCACC TAAATTCCTG AGACGAATAG GAGACACGCG TTAGACGACG TAGACTACTA   
  
  
- GCGTCCTGTC GACTACTTGA CGACTTCGTC TAATCCCTCG TGAGAAGACG TCGACCCCTA CCTAGAGTTT   
  
  
- CCAACCGAGT AATGAAACGA TTACGGAACC TCCGCGCAAA TCGACCTTGA CCGAGTGTCT AGATATGTCG   
  
  
- GGACTCAAGC TTCTCCTGTT GTCGTCGACT ATACTATTTT CGAATAGTCA AAAAGAAAGC ACGAACGGGT   
  
  
- AAAAATTTCT AGAGATAATA GAAACGCTTG GTATACTAAT ATGTCTCTCG ACTTTTTCGT AGTTTCGAAG   
  
  
- TACAATATCT AAAACCGTAG GACATACCAA AAGTCACCGG GGAGGAGTAA GTTGCGGACA GCCTCGCTGG   
  
  
- ACCACCTGGA GGTTTTGACA CGTAATGACC CTAACTAGAA GGGGTTGGAC CTAAATCCGG TTGTCTTTCT   
  
  
- CAACTCCGTT GTCCCGAGGC GAACCGTTTC ATAACACTCG CGAAGTTACA AGGTAAACTT ATGGTACGGT   
  
  
- AACGTGTCTT TACCCTTTGG TAGTTTCGAC TTCTGGACTT CTATCTTCTA CTACTCCACC AACGGCACTT   
  
  
- GACAGAGAAG TCCAAATTCT TGGACGAACT ACTCTGCTAT CACCACCTAT CAGGCTCCTT ACGTCAAGAG   
  
  
- CCGAACTAAT CTTCCTATTT TGGACTATAG AAACAAGTGC CTCATCACTT GCCCAGAATG TTACGCGGGA   
  
  
- AGAAACAATG TGCAAAGTCC CTCCGGGAGA AGGTAATGAG ATGACATAAG CTATACAAAC TACGGTTACG   
  
  
- AAGGGCCCTC CTAGGACTCT CTAACTACAA ACTCTTCCTC AAAATACCCG CTCTTTAATA CTTACACCAC   
  
  
- CGAACACTCC CATGTCTCTC TCAACTTTCT GGACTCTGTA TGTTCGTTAC CGTCCGCGCC TTATCATCCC   
  
  
- GTCCCAAGTC CGTCGACGGT AACCTGGTTC TCGAGCAGTC ATTTGACTCC TCGTTCCAGG TCTACGTGAT   
  
  
- AGGATCCCTG AAGCAACAAC TACATCTACC TGTAACCTGT AACGTCCCTA CCTTCCCCTC CTAGTAGACA   
  
  
- CGACACAGAC GTACCCCAGG ACGAAT

+     Myb-binding site

| Site Name | Organism | Position | Strand | Matrix score. | sequence | function |
| --- | --- | --- | --- | --- | --- | --- |
| Myb-binding site | Nicotiana tabacum | 3652 | + | 6 | CAACAG |  |
| Myb-binding site | Nicotiana tabacum | 3634 | + | 6 | CAACAG |  |
| Myb-binding site | Nicotiana tabacum | 3382 | + | 6 | CAACAG |  |

>HU02G01569.1   
+ +Up\_Stream \_Len000AGCGTA GGAAGTGACG CCGCTATTGG AGGCGAAGAC GCTAGTACCA AGGCCAAAAC   
  
  
+ AAATGAGGAG ATAGATCAGT TAAACCGTTT GGTTAAGAAA ATTAAGCGAA CATCTCTTGA ACCTCCATCT   
  
  
+ GAACTCGATG ACATTGAGGA TGTGGATGCA GAAAAGATGG ATATGGGCTC TCCATCGGTG CCAGCAAATC   
  
  
+ AACCTAATGT TGGGTGGGGT CTCATTCGGT CAATGGGGGA CTAGGGACGT CATATCGTCA CCAATTACAA   
  
  
+ CGGAACAACC CTAAACCGCA TTTTAAAATC ACATCTTGGT AGCAGACAAA GATGTACGAC CCAAAAATTC   
  
  
+ AACTCTGTAA AGCACTAGCA AAGCTCGTAG TACACAGAAC AATCGCACAC TCATAGTGAT AGAAGTGTAC   
  
  
+ TATCCATTAT GCAGTGAATA TGACAAGGGA GGGGGTCCTG AGATGCCCAA ATTTGGCAGC CGAGTTGCCT   
  
  
+ TGACTGCCCA TCAATGTCCT AAACCAGTTG AGCATGTTAT TAATTTGCAA GAAAGATCGA ATTCTTCCTA   
  
  
+ TCTAAAAACT GACACAATAA AACAATACCA TGTATGGGAC ATTGCTAGGA TTGTTAACAC AATTATTGTC   
  
  
+ TTGTACGTTG CACCTGAAAA AAAAGAAAAT TGTTGCCATA GACAACATAT AAGTTAGGCA TTCATGACTA   
  
  
+ ATATCTGAAT TAGGTGTGTC GATCTGTGAT TCAAATTGAT ATAATTAGAT CTTTAAGGTA TCTAATTGCT   
  
  
+ TCAAGTTTTC TCTATAATGA AGATGTATAT TAGCTCACAT AGGTCTTAAG GTTAACTAAT GTCATATCAG   
  
  
+ GGGGTGTGGG CCGCCATTTT TGATAAGAAT TGAATTGAAC TTGCATTGTT TTTTCTTTGG CGAGAATGCA   
  
  
+ AGATTTGTTA ATTTATTCAT ATGCGTCATT ATACACTATA AGAAAAATAA TTGTAGGAAT ATATGAAGTA   
  
  
+ TTTTTACTTG TGCTCATGTT AAATGTGGAA TACTTTTTTA ATGTCGTATA GTACCACAGT ATTCTACAAT   
  
  
+ TTAAGAGATT AATCGATCCA TTAAAGTCAT GCAAACCTGA TGCATATTCA AGACATTGCA TGAAATGATC   
  
  
+ TTATTTTAGT CAAAGACTCC ATCATTTATA AGCAATATTT ACTTCGATAC ACGTGTCATT AAAAAAGACT   
  
  
+ ATAGAAATGT TACTGTTATT TTGTTTGAGC TAAATAGAAA ATTTAAAAGT TAATTAAGAT AATAATTAAA   
  
  
+ ACTAAATATT CAATGATGGC TAATAAATAC ATTGTCGTAT TAACTGACTA ATATTTGTAG ATGGTCGTGT   
  
  
+ TAATTTCTTT TTATTTTAAA GTTCAAATGA GTTACAAAAG GTTATAAAAT ATAAAGTAAA ATGGGAGACA   
  
  
+ AAGCAGACAG GGTGGGGGAA GCTAAGCTTA AAGTAGGATT TAGAAAGGCA ATGAAGGTGA ACCCTCAAAT   
  
  
+ CGATCCGCTG CCAATCACAG AAACCCAAAG CTTTGCTCAC CGACAACTCC CGGTTAACTG CAGTCACTCA   
  
  
+ CGGCACTGGG TAATACAGTA ATCGTGTACC CAGTTCATCC CCCTTTTCCT TTGGGAATAC AGTACCTCGA   
  
  
+ AAGTTCCTTT GAAATTCTAC TGGCCAGGCA TACCCATAAA TTGATCCTCT CGATATCATA AGATATGATA   
  
  
+ TTTCTCTGTC ATATCTGTTA GTTTCGGTAT CATATTTCTT CTTTATCTCT GTAATTTGAG GTATGCTTCA   
  
  
+ TTCCTGGCCG CTTGAATTAT CTTTCTTTCT TTGATTAGTA TTGTTTTTTT GGTTACCTAT TTTGCTCCAG   
  
  
+ TCCTGTTCAT TTGGGTCTTT TTGGATCTGG GTTTTTGCTG GGTTTGTTGA TTCTTTGAGA AATTTGGGTG   
  
  
+ CTTGAATTTG CCCTGCAATA TTCATGGGTT AGCGTTTTCC TGAATTTTGC TCTGTTCCTT AAGTGATTAT   
  
  
+ TTTTGATTGA TTACACCTTG GTGGTCCTGG TGGAATTTCC GAGGAGAAAT TGTCATGGGT TCCCACAACT   
  
  
+ TTGGAGAATT CCCTGATGAG ACTCTAAATG AGTATCAATC TACTTTGGGA ACCATGTCCC CCGGTTATGA   
  
  
+ TGGGTCTTTG AATTATACAA CCTTGTTCAA TTACAAAGAC CCATCTCAGG ATCTCACGGC ACTGAACCTT   
  
  
+ CCTAGCCCAT TGCCTGACCC TATGCCATTC AACATTGGTT CATATTCGGG TTTGAGCCCC GGGGTTGAAT   
  
  
+ CTTCGGATGA TAGCGATTCA GATGATGTTG TTAAGTACAT TGGCCAAGTG CTTATGGAAG AGGATATGAA   
  
  
+ GGAGAAGCCT TGTATGTTCC ATGACCCTTT AGCACTCCAA GCTGCTGAGA AACCCTTTTA TGATGTGTTG   
  
  
+ GGAAAGAAGT ATCCTCCTTC CCCTAATCAA CACCCACTTA TTGATCATTC TGTGGATAGC CCAGACAATC   
  
  
+ AGTCCTGTGG AACGAGCACA ATTAGTGATC TTAGTGGCAG TAACTGCACT TCCAGTTCAA CCAATTATAT   
  
  
+ TGATGCTGTG GCAGTCGCTG ATTCGAGTGA GAATACTAAG ACCTCTTTTG TGCAAAGTTC TCTGATTGAA   
  
  
+ TCGTTTTCTC AGTCGTCCAC CCTTCCACAG TGGTCATTTG GATCATTGGG TGCCTTGGGT GGCACAGCTT   
  
  
+ CTCAGGGTTC GAATTCAGTT ATCTCCTCCC GTGGTTTCCC TATGGCCATG AACGTTTTTA GCGAGCAGTC   
  
  
+ CATGATACAG TTTCAGAAGG GGGTGGAGGA GGCGAGCAAG TTCCTTCCAA AGAACAATAA CCTTGCGATT   
  
  
+ GACCTTGAGA GCGTCACTTT CCCAAATGAA AAGGAGGCGG CCCCCATGGT GGTGGTTAAG AAAGAGAAGG   
  
  
+ ATGAACACTC ACCTGATAGC TCGAGAGGTA GCAAGATTCA CTACCGTGAT GATGAGGACT TTGAGGATGG   
  
  
+ TAGGAGGAGT AAGCAGTCAG CTGTTTCTGT GGAGGAGGCT GAGTTGTCTG AAATGTTTGA CCGGGTTTTG   
  
  
+ CTTTGCAATC CCATGAAACA TGAAGCTCAT TGTACAAGTG GTTTGAAGTC CGAGAAGGGA ACATCCCTGC   
  
  
+ AGGCTGGCCA AGTACAGGTA CAGGAGGGTC AGAAGGCTCG TGCAAAGAAA CAGGGTAATA ATGATAATAA   
  
  
+ GAAGAATAAG AATGTGGTGG ATTTAAGGAC TCTGCTTATC CTCTGTGCGC AATCTGCTGC ATCTGATGAT   
  
  
+ CGCAGGACAG CTGATGAACT GCTGAAGCAG ATTAGGGAGC ACTCTTCTGC AGCTGGGGAT GGATCTCAAA   
  
  
+ GGTTGGCTCA TTACTTTGCT AATGCCTTGG AGGCGCGTTT AGCTGGAACT GGCTCACAGA TCTATACAGC   
  
  
+ CCTGAGTTCG AAGAGGACAA CAGCAGCTGA TATGATAAAA GCTTATCAGT TTTTCTTTCG TGCTTGCCCA   
  
  
+ TTTTTAAAGA TCTCTATTAT CTTTGCGAAC CATATGATTA TACAGAGAGC TGAAAAAGCA TCAAAGCTTC   
  
  
+ ATGTTATAGA TTTTGGCATC CTGTATGGTT TTCAGTGGCC CCTCCTCATT CAACGCCTGT CGGAGCGACC   
  
  
+ TGGTGGACCT CCAAAACTGT GCATTACTGG GATTGATCTT CCCCAACCTG GATTTAGGCC AACAGAAAGA   
  
  
+ GTTGAGGCAA CAGGGCTCCG CTTGGCAAAG TATTGTGAGC GCTTCAATGT TCCATTTGAA TACCATGCCA   
  
  
+ TTGCACAGAA ATGGGAAACC ATCAAAGCTG AAGACCTGAA GATAGAAGAT GATGAGGTGG TTGCCGTGAA   
  
  
+ CTGTCTCTTC AGGTTTAAGA ACCTGCTTGA TGAGACGATA GTGGTGGATA GTCCGAGGAA TGCAGTTCTC   
  
  
+ GGCTTGATTA GAAGGATAAA ACCTGATATC TTTGTTCACG GAGTAGTGAA CGGGTCTTAC AATGCGCCCT   
  
  
+ TCTTTGTTAC ACGTTTCAGG GAGGCCCTCT TCCATTACTC TACTGTATTC GATATGTTTG ATGCCAATGC   
  
  
+ TTCCCGGGAG GATCCTGAGA GATTGATGTT TGAGAAGGAG TTTTATGGGC GAGAAATTAT GAATGTGGTG   
  
  
+ GCTTGTGAGG GTACAGAGAG AGTTGAAAGA CCTGAGACAT ACAAGCAATG GCAGGCGCGG AATAGTAGGG   
  
  
+ CAGGGTTCAG GCAGCTGCCA TTGGACCAAG AGCTCGTCAG TAAACTGAGG AGCAAGGTCC AGATGCACTA   
  
  
+ TCCTAGGGAC TTCGTTGTTG ATGTAGATGG ACATTGGACA TTGCAGGGAT GGAAGGGGAG GATCATCTGT   
  
  
+ GCTGTGTCTG CATGGGGTCC TGCTTA  

- +Up\_Stream \_Len000TCGCAT CCTTCACTGC GGCGATAACC TCCGCTTCTG CGATCATGGT TCCGGTTTTG   
  
  
- TTTACTCCTC TATCTAGTCA ATTTGGCAAA CCAATTCTTT TAATTCGCTT GTAGAGAACT TGGAGGTAGA   
  
  
- CTTGAGCTAC TGTAACTCCT ACACCTACGT CTTTTCTACC TATACCCGAG AGGTAGCCAC GGTCGTTTAG   
  
  
- TTGGATTACA ACCCACCCCA GAGTAAGCCA GTTACCCCCT GATCCCTGCA GTATAGCAGT GGTTAATGTT   
  
  
- GCCTTGTTGG GATTTGGCGT AAAATTTTAG TGTAGAACCA TCGTCTGTTT CTACATGCTG GGTTTTTAAG   
  
  
- TTGAGACATT TCGTGATCGT TTCGAGCATC ATGTGTCTTG TTAGCGTGTG AGTATCACTA TCTTCACATG   
  
  
- ATAGGTAATA CGTCACTTAT ACTGTTCCCT CCCCCAGGAC TCTACGGGTT TAAACCGTCG GCTCAACGGA   
  
  
- ACTGACGGGT AGTTACAGGA TTTGGTCAAC TCGTACAATA ATTAAACGTT CTTTCTAGCT TAAGAAGGAT   
  
  
- AGATTTTTGA CTGTGTTATT TTGTTATGGT ACATACCCTG TAACGATCCT AACAATTGTG TTAATAACAG   
  
  
- AACATGCAAC GTGGACTTTT TTTTCTTTTA ACAACGGTAT CTGTTGTATA TTCAATCCGT AAGTACTGAT   
  
  
- TATAGACTTA ATCCACACAG CTAGACACTA AGTTTAACTA TATTAATCTA GAAATTCCAT AGATTAACGA   
  
  
- AGTTCAAAAG AGATATTACT TCTACATATA ATCGAGTGTA TCCAGAATTC CAATTGATTA CAGTATAGTC   
  
  
- CCCCACACCC GGCGGTAAAA ACTATTCTTA ACTTAACTTG AACGTAACAA AAAAGAAACC GCTCTTACGT   
  
  
- TCTAAACAAT TAAATAAGTA TACGCAGTAA TATGTGATAT TCTTTTTATT AACATCCTTA TATACTTCAT   
  
  
- AAAAATGAAC ACGAGTACAA TTTACACCTT ATGAAAAAAT TACAGCATAT CATGGTGTCA TAAGATGTTA   
  
  
- AATTCTCTAA TTAGCTAGGT AATTTCAGTA CGTTTGGACT ACGTATAAGT TCTGTAACGT ACTTTACTAG   
  
  
- AATAAAATCA GTTTCTGAGG TAGTAAATAT TCGTTATAAA TGAAGCTATG TGCACAGTAA TTTTTTCTGA   
  
  
- TATCTTTACA ATGACAATAA AACAAACTCG ATTTATCTTT TAAATTTTCA ATTAATTCTA TTATTAATTT   
  
  
- TGATTTATAA GTTACTACCG ATTATTTATG TAACAGCATA ATTGACTGAT TATAAACATC TACCAGCACA   
  
  
- ATTAAAGAAA AATAAAATTT CAAGTTTACT CAATGTTTTC CAATATTTTA TATTTCATTT TACCCTCTGT   
  
  
- TTCGTCTGTC CCACCCCCTT CGATTCGAAT TTCATCCTAA ATCTTTCCGT TACTTCCACT TGGGAGTTTA   
  
  
- GCTAGGCGAC GGTTAGTGTC TTTGGGTTTC GAAACGAGTG GCTGTTGAGG GCCAATTGAC GTCAGTGAGT   
  
  
- GCCGTGACCC ATTATGTCAT TAGCACATGG GTCAAGTAGG GGGAAAAGGA AACCCTTATG TCATGGAGCT   
  
  
- TTCAAGGAAA CTTTAAGATG ACCGGTCCGT ATGGGTATTT AACTAGGAGA GCTATAGTAT TCTATACTAT   
  
  
- AAAGAGACAG TATAGACAAT CAAAGCCATA GTATAAAGAA GAAATAGAGA CATTAAACTC CATACGAAGT   
  
  
- AAGGACCGGC GAACTTAATA GAAAGAAAGA AACTAATCAT AACAAAAAAA CCAATGGATA AAACGAGGTC   
  
  
- AGGACAAGTA AACCCAGAAA AACCTAGACC CAAAAACGAC CCAAACAACT AAGAAACTCT TTAAACCCAC   
  
  
- GAACTTAAAC GGGACGTTAT AAGTACCCAA TCGCAAAAGG ACTTAAAACG AGACAAGGAA TTCACTAATA   
  
  
- AAAACTAACT AATGTGGAAC CACCAGGACC ACCTTAAAGG CTCCTCTTTA ACAGTACCCA AGGGTGTTGA   
  
  
- AACCTCTTAA GGGACTACTC TGAGATTTAC TCATAGTTAG ATGAAACCCT TGGTACAGGG GGCCAATACT   
  
  
- ACCCAGAAAC TTAATATGTT GGAACAAGTT AATGTTTCTG GGTAGAGTCC TAGAGTGCCG TGACTTGGAA   
  
  
- GGATCGGGTA ACGGACTGGG ATACGGTAAG TTGTAACCAA GTATAAGCCC AAACTCGGGG CCCCAACTTA   
  
  
- GAAGCCTACT ATCGCTAAGT CTACTACAAC AATTCATGTA ACCGGTTCAC GAATACCTTC TCCTATACTT   
  
  
- CCTCTTCGGA ACATACAAGG TACTGGGAAA TCGTGAGGTT CGACGACTCT TTGGGAAAAT ACTACACAAC   
  
  
- CCTTTCTTCA TAGGAGGAAG GGGATTAGTT GTGGGTGAAT AACTAGTAAG ACACCTATCG GGTCTGTTAG   
  
  
- TCAGGACACC TTGCTCGTGT TAATCACTAG AATCACCGTC ATTGACGTGA AGGTCAAGTT GGTTAATATA   
  
  
- ACTACGACAC CGTCAGCGAC TAAGCTCACT CTTATGATTC TGGAGAAAAC ACGTTTCAAG AGACTAACTT   
  
  
- AGCAAAAGAG TCAGCAGGTG GGAAGGTGTC ACCAGTAAAC CTAGTAACCC ACGGAACCCA CCGTGTCGAA   
  
  
- GAGTCCCAAG CTTAAGTCAA TAGAGGAGGG CACCAAAGGG ATACCGGTAC TTGCAAAAAT CGCTCGTCAG   
  
  
- GTACTATGTC AAAGTCTTCC CCCACCTCCT CCGCTCGTTC AAGGAAGGTT TCTTGTTATT GGAACGCTAA   
  
  
- CTGGAACTCT CGCAGTGAAA GGGTTTACTT TTCCTCCGCC GGGGGTACCA CCACCAATTC TTTCTCTTCC   
  
  
- TACTTGTGAG TGGACTATCG AGCTCTCCAT CGTTCTAAGT GATGGCACTA CTACTCCTGA AACTCCTACC   
  
  
- ATCCTCCTCA TTCGTCAGTC GACAAAGACA CCTCCTCCGA CTCAACAGAC TTTACAAACT GGCCCAAAAC   
  
  
- GAAACGTTAG GGTACTTTGT ACTTCGAGTA ACATGTTCAC CAAACTTCAG GCTCTTCCCT TGTAGGGACG   
  
  
- TCCGACCGGT TCATGTCCAT GTCCTCCCAG TCTTCCGAGC ACGTTTCTTT GTCCCATTAT TACTATTATT   
  
  
- CTTCTTATTC TTACACCACC TAAATTCCTG AGACGAATAG GAGACACGCG TTAGACGACG TAGACTACTA   
  
  
- GCGTCCTGTC GACTACTTGA CGACTTCGTC TAATCCCTCG TGAGAAGACG TCGACCCCTA CCTAGAGTTT   
  
  
- CCAACCGAGT AATGAAACGA TTACGGAACC TCCGCGCAAA TCGACCTTGA CCGAGTGTCT AGATATGTCG   
  
  
- GGACTCAAGC TTCTCCTGTT GTCGTCGACT ATACTATTTT CGAATAGTCA AAAAGAAAGC ACGAACGGGT   
  
  
- AAAAATTTCT AGAGATAATA GAAACGCTTG GTATACTAAT ATGTCTCTCG ACTTTTTCGT AGTTTCGAAG   
  
  
- TACAATATCT AAAACCGTAG GACATACCAA AAGTCACCGG GGAGGAGTAA GTTGCGGACA GCCTCGCTGG   
  
  
- ACCACCTGGA GGTTTTGACA CGTAATGACC CTAACTAGAA GGGGTTGGAC CTAAATCCGG TTGTCTTTCT   
  
  
- CAACTCCGTT GTCCCGAGGC GAACCGTTTC ATAACACTCG CGAAGTTACA AGGTAAACTT ATGGTACGGT   
  
  
- AACGTGTCTT TACCCTTTGG TAGTTTCGAC TTCTGGACTT CTATCTTCTA CTACTCCACC AACGGCACTT   
  
  
- GACAGAGAAG TCCAAATTCT TGGACGAACT ACTCTGCTAT CACCACCTAT CAGGCTCCTT ACGTCAAGAG   
  
  
- CCGAACTAAT CTTCCTATTT TGGACTATAG AAACAAGTGC CTCATCACTT GCCCAGAATG TTACGCGGGA   
  
  
- AGAAACAATG TGCAAAGTCC CTCCGGGAGA AGGTAATGAG ATGACATAAG CTATACAAAC TACGGTTACG   
  
  
- AAGGGCCCTC CTAGGACTCT CTAACTACAA ACTCTTCCTC AAAATACCCG CTCTTTAATA CTTACACCAC   
  
  
- CGAACACTCC CATGTCTCTC TCAACTTTCT GGACTCTGTA TGTTCGTTAC CGTCCGCGCC TTATCATCCC   
  
  
- GTCCCAAGTC CGTCGACGGT AACCTGGTTC TCGAGCAGTC ATTTGACTCC TCGTTCCAGG TCTACGTGAT   
  
  
- AGGATCCCTG AAGCAACAAC TACATCTACC TGTAACCTGT AACGTCCCTA CCTTCCCCTC CTAGTAGACA   
  
  
- CGACACAGAC GTACCCCAGG ACGAAT

+     Myc

| Site Name | Organism | Position | Strand | Matrix score. | sequence | function |
| --- | --- | --- | --- | --- | --- | --- |
| Myc | Arabidopsis thaliana | 1056 | - | 7 | TCTCTTA |  |

>HU02G01569.1   
+ +Up\_Stream \_Len000AGCGTA GGAAGTGACG CCGCTATTGG AGGCGAAGAC GCTAGTACCA AGGCCAAAAC   
  
  
+ AAATGAGGAG ATAGATCAGT TAAACCGTTT GGTTAAGAAA ATTAAGCGAA CATCTCTTGA ACCTCCATCT   
  
  
+ GAACTCGATG ACATTGAGGA TGTGGATGCA GAAAAGATGG ATATGGGCTC TCCATCGGTG CCAGCAAATC   
  
  
+ AACCTAATGT TGGGTGGGGT CTCATTCGGT CAATGGGGGA CTAGGGACGT CATATCGTCA CCAATTACAA   
  
  
+ CGGAACAACC CTAAACCGCA TTTTAAAATC ACATCTTGGT AGCAGACAAA GATGTACGAC CCAAAAATTC   
  
  
+ AACTCTGTAA AGCACTAGCA AAGCTCGTAG TACACAGAAC AATCGCACAC TCATAGTGAT AGAAGTGTAC   
  
  
+ TATCCATTAT GCAGTGAATA TGACAAGGGA GGGGGTCCTG AGATGCCCAA ATTTGGCAGC CGAGTTGCCT   
  
  
+ TGACTGCCCA TCAATGTCCT AAACCAGTTG AGCATGTTAT TAATTTGCAA GAAAGATCGA ATTCTTCCTA   
  
  
+ TCTAAAAACT GACACAATAA AACAATACCA TGTATGGGAC ATTGCTAGGA TTGTTAACAC AATTATTGTC   
  
  
+ TTGTACGTTG CACCTGAAAA AAAAGAAAAT TGTTGCCATA GACAACATAT AAGTTAGGCA TTCATGACTA   
  
  
+ ATATCTGAAT TAGGTGTGTC GATCTGTGAT TCAAATTGAT ATAATTAGAT CTTTAAGGTA TCTAATTGCT   
  
  
+ TCAAGTTTTC TCTATAATGA AGATGTATAT TAGCTCACAT AGGTCTTAAG GTTAACTAAT GTCATATCAG   
  
  
+ GGGGTGTGGG CCGCCATTTT TGATAAGAAT TGAATTGAAC TTGCATTGTT TTTTCTTTGG CGAGAATGCA   
  
  
+ AGATTTGTTA ATTTATTCAT ATGCGTCATT ATACACTATA AGAAAAATAA TTGTAGGAAT ATATGAAGTA   
  
  
+ TTTTTACTTG TGCTCATGTT AAATGTGGAA TACTTTTTTA ATGTCGTATA GTACCACAGT ATTCTACAAT   
  
  
+ TTAAGAGATT AATCGATCCA TTAAAGTCAT GCAAACCTGA TGCATATTCA AGACATTGCA TGAAATGATC   
  
  
+ TTATTTTAGT CAAAGACTCC ATCATTTATA AGCAATATTT ACTTCGATAC ACGTGTCATT AAAAAAGACT   
  
  
+ ATAGAAATGT TACTGTTATT TTGTTTGAGC TAAATAGAAA ATTTAAAAGT TAATTAAGAT AATAATTAAA   
  
  
+ ACTAAATATT CAATGATGGC TAATAAATAC ATTGTCGTAT TAACTGACTA ATATTTGTAG ATGGTCGTGT   
  
  
+ TAATTTCTTT TTATTTTAAA GTTCAAATGA GTTACAAAAG GTTATAAAAT ATAAAGTAAA ATGGGAGACA   
  
  
+ AAGCAGACAG GGTGGGGGAA GCTAAGCTTA AAGTAGGATT TAGAAAGGCA ATGAAGGTGA ACCCTCAAAT   
  
  
+ CGATCCGCTG CCAATCACAG AAACCCAAAG CTTTGCTCAC CGACAACTCC CGGTTAACTG CAGTCACTCA   
  
  
+ CGGCACTGGG TAATACAGTA ATCGTGTACC CAGTTCATCC CCCTTTTCCT TTGGGAATAC AGTACCTCGA   
  
  
+ AAGTTCCTTT GAAATTCTAC TGGCCAGGCA TACCCATAAA TTGATCCTCT CGATATCATA AGATATGATA   
  
  
+ TTTCTCTGTC ATATCTGTTA GTTTCGGTAT CATATTTCTT CTTTATCTCT GTAATTTGAG GTATGCTTCA   
  
  
+ TTCCTGGCCG CTTGAATTAT CTTTCTTTCT TTGATTAGTA TTGTTTTTTT GGTTACCTAT TTTGCTCCAG   
  
  
+ TCCTGTTCAT TTGGGTCTTT TTGGATCTGG GTTTTTGCTG GGTTTGTTGA TTCTTTGAGA AATTTGGGTG   
  
  
+ CTTGAATTTG CCCTGCAATA TTCATGGGTT AGCGTTTTCC TGAATTTTGC TCTGTTCCTT AAGTGATTAT   
  
  
+ TTTTGATTGA TTACACCTTG GTGGTCCTGG TGGAATTTCC GAGGAGAAAT TGTCATGGGT TCCCACAACT   
  
  
+ TTGGAGAATT CCCTGATGAG ACTCTAAATG AGTATCAATC TACTTTGGGA ACCATGTCCC CCGGTTATGA   
  
  
+ TGGGTCTTTG AATTATACAA CCTTGTTCAA TTACAAAGAC CCATCTCAGG ATCTCACGGC ACTGAACCTT   
  
  
+ CCTAGCCCAT TGCCTGACCC TATGCCATTC AACATTGGTT CATATTCGGG TTTGAGCCCC GGGGTTGAAT   
  
  
+ CTTCGGATGA TAGCGATTCA GATGATGTTG TTAAGTACAT TGGCCAAGTG CTTATGGAAG AGGATATGAA   
  
  
+ GGAGAAGCCT TGTATGTTCC ATGACCCTTT AGCACTCCAA GCTGCTGAGA AACCCTTTTA TGATGTGTTG   
  
  
+ GGAAAGAAGT ATCCTCCTTC CCCTAATCAA CACCCACTTA TTGATCATTC TGTGGATAGC CCAGACAATC   
  
  
+ AGTCCTGTGG AACGAGCACA ATTAGTGATC TTAGTGGCAG TAACTGCACT TCCAGTTCAA CCAATTATAT   
  
  
+ TGATGCTGTG GCAGTCGCTG ATTCGAGTGA GAATACTAAG ACCTCTTTTG TGCAAAGTTC TCTGATTGAA   
  
  
+ TCGTTTTCTC AGTCGTCCAC CCTTCCACAG TGGTCATTTG GATCATTGGG TGCCTTGGGT GGCACAGCTT   
  
  
+ CTCAGGGTTC GAATTCAGTT ATCTCCTCCC GTGGTTTCCC TATGGCCATG AACGTTTTTA GCGAGCAGTC   
  
  
+ CATGATACAG TTTCAGAAGG GGGTGGAGGA GGCGAGCAAG TTCCTTCCAA AGAACAATAA CCTTGCGATT   
  
  
+ GACCTTGAGA GCGTCACTTT CCCAAATGAA AAGGAGGCGG CCCCCATGGT GGTGGTTAAG AAAGAGAAGG   
  
  
+ ATGAACACTC ACCTGATAGC TCGAGAGGTA GCAAGATTCA CTACCGTGAT GATGAGGACT TTGAGGATGG   
  
  
+ TAGGAGGAGT AAGCAGTCAG CTGTTTCTGT GGAGGAGGCT GAGTTGTCTG AAATGTTTGA CCGGGTTTTG   
  
  
+ CTTTGCAATC CCATGAAACA TGAAGCTCAT TGTACAAGTG GTTTGAAGTC CGAGAAGGGA ACATCCCTGC   
  
  
+ AGGCTGGCCA AGTACAGGTA CAGGAGGGTC AGAAGGCTCG TGCAAAGAAA CAGGGTAATA ATGATAATAA   
  
  
+ GAAGAATAAG AATGTGGTGG ATTTAAGGAC TCTGCTTATC CTCTGTGCGC AATCTGCTGC ATCTGATGAT   
  
  
+ CGCAGGACAG CTGATGAACT GCTGAAGCAG ATTAGGGAGC ACTCTTCTGC AGCTGGGGAT GGATCTCAAA   
  
  
+ GGTTGGCTCA TTACTTTGCT AATGCCTTGG AGGCGCGTTT AGCTGGAACT GGCTCACAGA TCTATACAGC   
  
  
+ CCTGAGTTCG AAGAGGACAA CAGCAGCTGA TATGATAAAA GCTTATCAGT TTTTCTTTCG TGCTTGCCCA   
  
  
+ TTTTTAAAGA TCTCTATTAT CTTTGCGAAC CATATGATTA TACAGAGAGC TGAAAAAGCA TCAAAGCTTC   
  
  
+ ATGTTATAGA TTTTGGCATC CTGTATGGTT TTCAGTGGCC CCTCCTCATT CAACGCCTGT CGGAGCGACC   
  
  
+ TGGTGGACCT CCAAAACTGT GCATTACTGG GATTGATCTT CCCCAACCTG GATTTAGGCC AACAGAAAGA   
  
  
+ GTTGAGGCAA CAGGGCTCCG CTTGGCAAAG TATTGTGAGC GCTTCAATGT TCCATTTGAA TACCATGCCA   
  
  
+ TTGCACAGAA ATGGGAAACC ATCAAAGCTG AAGACCTGAA GATAGAAGAT GATGAGGTGG TTGCCGTGAA   
  
  
+ CTGTCTCTTC AGGTTTAAGA ACCTGCTTGA TGAGACGATA GTGGTGGATA GTCCGAGGAA TGCAGTTCTC   
  
  
+ GGCTTGATTA GAAGGATAAA ACCTGATATC TTTGTTCACG GAGTAGTGAA CGGGTCTTAC AATGCGCCCT   
  
  
+ TCTTTGTTAC ACGTTTCAGG GAGGCCCTCT TCCATTACTC TACTGTATTC GATATGTTTG ATGCCAATGC   
  
  
+ TTCCCGGGAG GATCCTGAGA GATTGATGTT TGAGAAGGAG TTTTATGGGC GAGAAATTAT GAATGTGGTG   
  
  
+ GCTTGTGAGG GTACAGAGAG AGTTGAAAGA CCTGAGACAT ACAAGCAATG GCAGGCGCGG AATAGTAGGG   
  
  
+ CAGGGTTCAG GCAGCTGCCA TTGGACCAAG AGCTCGTCAG TAAACTGAGG AGCAAGGTCC AGATGCACTA   
  
  
+ TCCTAGGGAC TTCGTTGTTG ATGTAGATGG ACATTGGACA TTGCAGGGAT GGAAGGGGAG GATCATCTGT   
  
  
+ GCTGTGTCTG CATGGGGTCC TGCTTA  

- +Up\_Stream \_Len000TCGCAT CCTTCACTGC GGCGATAACC TCCGCTTCTG CGATCATGGT TCCGGTTTTG   
  
  
- TTTACTCCTC TATCTAGTCA ATTTGGCAAA CCAATTCTTT TAATTCGCTT GTAGAGAACT TGGAGGTAGA   
  
  
- CTTGAGCTAC TGTAACTCCT ACACCTACGT CTTTTCTACC TATACCCGAG AGGTAGCCAC GGTCGTTTAG   
  
  
- TTGGATTACA ACCCACCCCA GAGTAAGCCA GTTACCCCCT GATCCCTGCA GTATAGCAGT GGTTAATGTT   
  
  
- GCCTTGTTGG GATTTGGCGT AAAATTTTAG TGTAGAACCA TCGTCTGTTT CTACATGCTG GGTTTTTAAG   
  
  
- TTGAGACATT TCGTGATCGT TTCGAGCATC ATGTGTCTTG TTAGCGTGTG AGTATCACTA TCTTCACATG   
  
  
- ATAGGTAATA CGTCACTTAT ACTGTTCCCT CCCCCAGGAC TCTACGGGTT TAAACCGTCG GCTCAACGGA   
  
  
- ACTGACGGGT AGTTACAGGA TTTGGTCAAC TCGTACAATA ATTAAACGTT CTTTCTAGCT TAAGAAGGAT   
  
  
- AGATTTTTGA CTGTGTTATT TTGTTATGGT ACATACCCTG TAACGATCCT AACAATTGTG TTAATAACAG   
  
  
- AACATGCAAC GTGGACTTTT TTTTCTTTTA ACAACGGTAT CTGTTGTATA TTCAATCCGT AAGTACTGAT   
  
  
- TATAGACTTA ATCCACACAG CTAGACACTA AGTTTAACTA TATTAATCTA GAAATTCCAT AGATTAACGA   
  
  
- AGTTCAAAAG AGATATTACT TCTACATATA ATCGAGTGTA TCCAGAATTC CAATTGATTA CAGTATAGTC   
  
  
- CCCCACACCC GGCGGTAAAA ACTATTCTTA ACTTAACTTG AACGTAACAA AAAAGAAACC GCTCTTACGT   
  
  
- TCTAAACAAT TAAATAAGTA TACGCAGTAA TATGTGATAT TCTTTTTATT AACATCCTTA TATACTTCAT   
  
  
- AAAAATGAAC ACGAGTACAA TTTACACCTT ATGAAAAAAT TACAGCATAT CATGGTGTCA TAAGATGTTA   
  
  
- AATTCTCTAA TTAGCTAGGT AATTTCAGTA CGTTTGGACT ACGTATAAGT TCTGTAACGT ACTTTACTAG   
  
  
- AATAAAATCA GTTTCTGAGG TAGTAAATAT TCGTTATAAA TGAAGCTATG TGCACAGTAA TTTTTTCTGA   
  
  
- TATCTTTACA ATGACAATAA AACAAACTCG ATTTATCTTT TAAATTTTCA ATTAATTCTA TTATTAATTT   
  
  
- TGATTTATAA GTTACTACCG ATTATTTATG TAACAGCATA ATTGACTGAT TATAAACATC TACCAGCACA   
  
  
- ATTAAAGAAA AATAAAATTT CAAGTTTACT CAATGTTTTC CAATATTTTA TATTTCATTT TACCCTCTGT   
  
  
- TTCGTCTGTC CCACCCCCTT CGATTCGAAT TTCATCCTAA ATCTTTCCGT TACTTCCACT TGGGAGTTTA   
  
  
- GCTAGGCGAC GGTTAGTGTC TTTGGGTTTC GAAACGAGTG GCTGTTGAGG GCCAATTGAC GTCAGTGAGT   
  
  
- GCCGTGACCC ATTATGTCAT TAGCACATGG GTCAAGTAGG GGGAAAAGGA AACCCTTATG TCATGGAGCT   
  
  
- TTCAAGGAAA CTTTAAGATG ACCGGTCCGT ATGGGTATTT AACTAGGAGA GCTATAGTAT TCTATACTAT   
  
  
- AAAGAGACAG TATAGACAAT CAAAGCCATA GTATAAAGAA GAAATAGAGA CATTAAACTC CATACGAAGT   
  
  
- AAGGACCGGC GAACTTAATA GAAAGAAAGA AACTAATCAT AACAAAAAAA CCAATGGATA AAACGAGGTC   
  
  
- AGGACAAGTA AACCCAGAAA AACCTAGACC CAAAAACGAC CCAAACAACT AAGAAACTCT TTAAACCCAC   
  
  
- GAACTTAAAC GGGACGTTAT AAGTACCCAA TCGCAAAAGG ACTTAAAACG AGACAAGGAA TTCACTAATA   
  
  
- AAAACTAACT AATGTGGAAC CACCAGGACC ACCTTAAAGG CTCCTCTTTA ACAGTACCCA AGGGTGTTGA   
  
  
- AACCTCTTAA GGGACTACTC TGAGATTTAC TCATAGTTAG ATGAAACCCT TGGTACAGGG GGCCAATACT   
  
  
- ACCCAGAAAC TTAATATGTT GGAACAAGTT AATGTTTCTG GGTAGAGTCC TAGAGTGCCG TGACTTGGAA   
  
  
- GGATCGGGTA ACGGACTGGG ATACGGTAAG TTGTAACCAA GTATAAGCCC AAACTCGGGG CCCCAACTTA   
  
  
- GAAGCCTACT ATCGCTAAGT CTACTACAAC AATTCATGTA ACCGGTTCAC GAATACCTTC TCCTATACTT   
  
  
- CCTCTTCGGA ACATACAAGG TACTGGGAAA TCGTGAGGTT CGACGACTCT TTGGGAAAAT ACTACACAAC   
  
  
- CCTTTCTTCA TAGGAGGAAG GGGATTAGTT GTGGGTGAAT AACTAGTAAG ACACCTATCG GGTCTGTTAG   
  
  
- TCAGGACACC TTGCTCGTGT TAATCACTAG AATCACCGTC ATTGACGTGA AGGTCAAGTT GGTTAATATA   
  
  
- ACTACGACAC CGTCAGCGAC TAAGCTCACT CTTATGATTC TGGAGAAAAC ACGTTTCAAG AGACTAACTT   
  
  
- AGCAAAAGAG TCAGCAGGTG GGAAGGTGTC ACCAGTAAAC CTAGTAACCC ACGGAACCCA CCGTGTCGAA   
  
  
- GAGTCCCAAG CTTAAGTCAA TAGAGGAGGG CACCAAAGGG ATACCGGTAC TTGCAAAAAT CGCTCGTCAG   
  
  
- GTACTATGTC AAAGTCTTCC CCCACCTCCT CCGCTCGTTC AAGGAAGGTT TCTTGTTATT GGAACGCTAA   
  
  
- CTGGAACTCT CGCAGTGAAA GGGTTTACTT TTCCTCCGCC GGGGGTACCA CCACCAATTC TTTCTCTTCC   
  
  
- TACTTGTGAG TGGACTATCG AGCTCTCCAT CGTTCTAAGT GATGGCACTA CTACTCCTGA AACTCCTACC   
  
  
- ATCCTCCTCA TTCGTCAGTC GACAAAGACA CCTCCTCCGA CTCAACAGAC TTTACAAACT GGCCCAAAAC   
  
  
- GAAACGTTAG GGTACTTTGT ACTTCGAGTA ACATGTTCAC CAAACTTCAG GCTCTTCCCT TGTAGGGACG   
  
  
- TCCGACCGGT TCATGTCCAT GTCCTCCCAG TCTTCCGAGC ACGTTTCTTT GTCCCATTAT TACTATTATT   
  
  
- CTTCTTATTC TTACACCACC TAAATTCCTG AGACGAATAG GAGACACGCG TTAGACGACG TAGACTACTA   
  
  
- GCGTCCTGTC GACTACTTGA CGACTTCGTC TAATCCCTCG TGAGAAGACG TCGACCCCTA CCTAGAGTTT   
  
  
- CCAACCGAGT AATGAAACGA TTACGGAACC TCCGCGCAAA TCGACCTTGA CCGAGTGTCT AGATATGTCG   
  
  
- GGACTCAAGC TTCTCCTGTT GTCGTCGACT ATACTATTTT CGAATAGTCA AAAAGAAAGC ACGAACGGGT   
  
  
- AAAAATTTCT AGAGATAATA GAAACGCTTG GTATACTAAT ATGTCTCTCG ACTTTTTCGT AGTTTCGAAG   
  
  
- TACAATATCT AAAACCGTAG GACATACCAA AAGTCACCGG GGAGGAGTAA GTTGCGGACA GCCTCGCTGG   
  
  
- ACCACCTGGA GGTTTTGACA CGTAATGACC CTAACTAGAA GGGGTTGGAC CTAAATCCGG TTGTCTTTCT   
  
  
- CAACTCCGTT GTCCCGAGGC GAACCGTTTC ATAACACTCG CGAAGTTACA AGGTAAACTT ATGGTACGGT   
  
  
- AACGTGTCTT TACCCTTTGG TAGTTTCGAC TTCTGGACTT CTATCTTCTA CTACTCCACC AACGGCACTT   
  
  
- GACAGAGAAG TCCAAATTCT TGGACGAACT ACTCTGCTAT CACCACCTAT CAGGCTCCTT ACGTCAAGAG   
  
  
- CCGAACTAAT CTTCCTATTT TGGACTATAG AAACAAGTGC CTCATCACTT GCCCAGAATG TTACGCGGGA   
  
  
- AGAAACAATG TGCAAAGTCC CTCCGGGAGA AGGTAATGAG ATGACATAAG CTATACAAAC TACGGTTACG   
  
  
- AAGGGCCCTC CTAGGACTCT CTAACTACAA ACTCTTCCTC AAAATACCCG CTCTTTAATA CTTACACCAC   
  
  
- CGAACACTCC CATGTCTCTC TCAACTTTCT GGACTCTGTA TGTTCGTTAC CGTCCGCGCC TTATCATCCC   
  
  
- GTCCCAAGTC CGTCGACGGT AACCTGGTTC TCGAGCAGTC ATTTGACTCC TCGTTCCAGG TCTACGTGAT   
  
  
- AGGATCCCTG AAGCAACAAC TACATCTACC TGTAACCTGT AACGTCCCTA CCTTCCCCTC CTAGTAGACA   
  
  
- CGACACAGAC GTACCCCAGG ACGAAT

+     O2-site

| Site Name | Organism | Position | Strand | Matrix score. | sequence | function |
| --- | --- | --- | --- | --- | --- | --- |
| O2-site | Zea mays | 2922 | + | 9 | GATGATGTGG | cis-acting regulatory element involved in zein metabolism regulation |
| O2-site | Zea mays | 3762 | + | 9 | GATGATGTGG | cis-acting regulatory element involved in zein metabolism regulation |
| O2-site | Zea mays | 160 | + | 9 | GATGATGTGG | cis-acting regulatory element involved in zein metabolism regulation |
| O2-site | Zea mays | 3765 | + | 9 | GATGATGTGG | cis-acting regulatory element involved in zein metabolism regulation |
| O2-site | Zea mays | 3774 | + | 9 | GTTGACGTGA | cis-acting regulatory element involved in zein metabolism regulation |
| O2-site | Zea mays | 151 | + | 9 | GATGACATGG | cis-acting regulatory element involved in zein metabolism regulation |
| O2-site | Zea mays | 2265 | + | 9 | GATGATGTGG | cis-acting regulatory element involved in zein metabolism regulation |
| O2-site | Zea mays | 2526 | + | 9 | GATGATGTGG | cis-acting regulatory element involved in zein metabolism regulation |

>HU02G01569.1   
+ +Up\_Stream \_Len000AGCGTA GGAAGTGACG CCGCTATTGG AGGCGAAGAC GCTAGTACCA AGGCCAAAAC   
  
  
+ AAATGAGGAG ATAGATCAGT TAAACCGTTT GGTTAAGAAA ATTAAGCGAA CATCTCTTGA ACCTCCATCT   
  
  
+ GAACTCGATG ACATTGAGGA TGTGGATGCA GAAAAGATGG ATATGGGCTC TCCATCGGTG CCAGCAAATC   
  
  
+ AACCTAATGT TGGGTGGGGT CTCATTCGGT CAATGGGGGA CTAGGGACGT CATATCGTCA CCAATTACAA   
  
  
+ CGGAACAACC CTAAACCGCA TTTTAAAATC ACATCTTGGT AGCAGACAAA GATGTACGAC CCAAAAATTC   
  
  
+ AACTCTGTAA AGCACTAGCA AAGCTCGTAG TACACAGAAC AATCGCACAC TCATAGTGAT AGAAGTGTAC   
  
  
+ TATCCATTAT GCAGTGAATA TGACAAGGGA GGGGGTCCTG AGATGCCCAA ATTTGGCAGC CGAGTTGCCT   
  
  
+ TGACTGCCCA TCAATGTCCT AAACCAGTTG AGCATGTTAT TAATTTGCAA GAAAGATCGA ATTCTTCCTA   
  
  
+ TCTAAAAACT GACACAATAA AACAATACCA TGTATGGGAC ATTGCTAGGA TTGTTAACAC AATTATTGTC   
  
  
+ TTGTACGTTG CACCTGAAAA AAAAGAAAAT TGTTGCCATA GACAACATAT AAGTTAGGCA TTCATGACTA   
  
  
+ ATATCTGAAT TAGGTGTGTC GATCTGTGAT TCAAATTGAT ATAATTAGAT CTTTAAGGTA TCTAATTGCT   
  
  
+ TCAAGTTTTC TCTATAATGA AGATGTATAT TAGCTCACAT AGGTCTTAAG GTTAACTAAT GTCATATCAG   
  
  
+ GGGGTGTGGG CCGCCATTTT TGATAAGAAT TGAATTGAAC TTGCATTGTT TTTTCTTTGG CGAGAATGCA   
  
  
+ AGATTTGTTA ATTTATTCAT ATGCGTCATT ATACACTATA AGAAAAATAA TTGTAGGAAT ATATGAAGTA   
  
  
+ TTTTTACTTG TGCTCATGTT AAATGTGGAA TACTTTTTTA ATGTCGTATA GTACCACAGT ATTCTACAAT   
  
  
+ TTAAGAGATT AATCGATCCA TTAAAGTCAT GCAAACCTGA TGCATATTCA AGACATTGCA TGAAATGATC   
  
  
+ TTATTTTAGT CAAAGACTCC ATCATTTATA AGCAATATTT ACTTCGATAC ACGTGTCATT AAAAAAGACT   
  
  
+ ATAGAAATGT TACTGTTATT TTGTTTGAGC TAAATAGAAA ATTTAAAAGT TAATTAAGAT AATAATTAAA   
  
  
+ ACTAAATATT CAATGATGGC TAATAAATAC ATTGTCGTAT TAACTGACTA ATATTTGTAG ATGGTCGTGT   
  
  
+ TAATTTCTTT TTATTTTAAA GTTCAAATGA GTTACAAAAG GTTATAAAAT ATAAAGTAAA ATGGGAGACA   
  
  
+ AAGCAGACAG GGTGGGGGAA GCTAAGCTTA AAGTAGGATT TAGAAAGGCA ATGAAGGTGA ACCCTCAAAT   
  
  
+ CGATCCGCTG CCAATCACAG AAACCCAAAG CTTTGCTCAC CGACAACTCC CGGTTAACTG CAGTCACTCA   
  
  
+ CGGCACTGGG TAATACAGTA ATCGTGTACC CAGTTCATCC CCCTTTTCCT TTGGGAATAC AGTACCTCGA   
  
  
+ AAGTTCCTTT GAAATTCTAC TGGCCAGGCA TACCCATAAA TTGATCCTCT CGATATCATA AGATATGATA   
  
  
+ TTTCTCTGTC ATATCTGTTA GTTTCGGTAT CATATTTCTT CTTTATCTCT GTAATTTGAG GTATGCTTCA   
  
  
+ TTCCTGGCCG CTTGAATTAT CTTTCTTTCT TTGATTAGTA TTGTTTTTTT GGTTACCTAT TTTGCTCCAG   
  
  
+ TCCTGTTCAT TTGGGTCTTT TTGGATCTGG GTTTTTGCTG GGTTTGTTGA TTCTTTGAGA AATTTGGGTG   
  
  
+ CTTGAATTTG CCCTGCAATA TTCATGGGTT AGCGTTTTCC TGAATTTTGC TCTGTTCCTT AAGTGATTAT   
  
  
+ TTTTGATTGA TTACACCTTG GTGGTCCTGG TGGAATTTCC GAGGAGAAAT TGTCATGGGT TCCCACAACT   
  
  
+ TTGGAGAATT CCCTGATGAG ACTCTAAATG AGTATCAATC TACTTTGGGA ACCATGTCCC CCGGTTATGA   
  
  
+ TGGGTCTTTG AATTATACAA CCTTGTTCAA TTACAAAGAC CCATCTCAGG ATCTCACGGC ACTGAACCTT   
  
  
+ CCTAGCCCAT TGCCTGACCC TATGCCATTC AACATTGGTT CATATTCGGG TTTGAGCCCC GGGGTTGAAT   
  
  
+ CTTCGGATGA TAGCGATTCA GATGATGTTG TTAAGTACAT TGGCCAAGTG CTTATGGAAG AGGATATGAA   
  
  
+ GGAGAAGCCT TGTATGTTCC ATGACCCTTT AGCACTCCAA GCTGCTGAGA AACCCTTTTA TGATGTGTTG   
  
  
+ GGAAAGAAGT ATCCTCCTTC CCCTAATCAA CACCCACTTA TTGATCATTC TGTGGATAGC CCAGACAATC   
  
  
+ AGTCCTGTGG AACGAGCACA ATTAGTGATC TTAGTGGCAG TAACTGCACT TCCAGTTCAA CCAATTATAT   
  
  
+ TGATGCTGTG GCAGTCGCTG ATTCGAGTGA GAATACTAAG ACCTCTTTTG TGCAAAGTTC TCTGATTGAA   
  
  
+ TCGTTTTCTC AGTCGTCCAC CCTTCCACAG TGGTCATTTG GATCATTGGG TGCCTTGGGT GGCACAGCTT   
  
  
+ CTCAGGGTTC GAATTCAGTT ATCTCCTCCC GTGGTTTCCC TATGGCCATG AACGTTTTTA GCGAGCAGTC   
  
  
+ CATGATACAG TTTCAGAAGG GGGTGGAGGA GGCGAGCAAG TTCCTTCCAA AGAACAATAA CCTTGCGATT   
  
  
+ GACCTTGAGA GCGTCACTTT CCCAAATGAA AAGGAGGCGG CCCCCATGGT GGTGGTTAAG AAAGAGAAGG   
  
  
+ ATGAACACTC ACCTGATAGC TCGAGAGGTA GCAAGATTCA CTACCGTGAT GATGAGGACT TTGAGGATGG   
  
  
+ TAGGAGGAGT AAGCAGTCAG CTGTTTCTGT GGAGGAGGCT GAGTTGTCTG AAATGTTTGA CCGGGTTTTG   
  
  
+ CTTTGCAATC CCATGAAACA TGAAGCTCAT TGTACAAGTG GTTTGAAGTC CGAGAAGGGA ACATCCCTGC   
  
  
+ AGGCTGGCCA AGTACAGGTA CAGGAGGGTC AGAAGGCTCG TGCAAAGAAA CAGGGTAATA ATGATAATAA   
  
  
+ GAAGAATAAG AATGTGGTGG ATTTAAGGAC TCTGCTTATC CTCTGTGCGC AATCTGCTGC ATCTGATGAT   
  
  
+ CGCAGGACAG CTGATGAACT GCTGAAGCAG ATTAGGGAGC ACTCTTCTGC AGCTGGGGAT GGATCTCAAA   
  
  
+ GGTTGGCTCA TTACTTTGCT AATGCCTTGG AGGCGCGTTT AGCTGGAACT GGCTCACAGA TCTATACAGC   
  
  
+ CCTGAGTTCG AAGAGGACAA CAGCAGCTGA TATGATAAAA GCTTATCAGT TTTTCTTTCG TGCTTGCCCA   
  
  
+ TTTTTAAAGA TCTCTATTAT CTTTGCGAAC CATATGATTA TACAGAGAGC TGAAAAAGCA TCAAAGCTTC   
  
  
+ ATGTTATAGA TTTTGGCATC CTGTATGGTT TTCAGTGGCC CCTCCTCATT CAACGCCTGT CGGAGCGACC   
  
  
+ TGGTGGACCT CCAAAACTGT GCATTACTGG GATTGATCTT CCCCAACCTG GATTTAGGCC AACAGAAAGA   
  
  
+ GTTGAGGCAA CAGGGCTCCG CTTGGCAAAG TATTGTGAGC GCTTCAATGT TCCATTTGAA TACCATGCCA   
  
  
+ TTGCACAGAA ATGGGAAACC ATCAAAGCTG AAGACCTGAA GATAGAAGAT GATGAGGTGG TTGCCGTGAA   
  
  
+ CTGTCTCTTC AGGTTTAAGA ACCTGCTTGA TGAGACGATA GTGGTGGATA GTCCGAGGAA TGCAGTTCTC   
  
  
+ GGCTTGATTA GAAGGATAAA ACCTGATATC TTTGTTCACG GAGTAGTGAA CGGGTCTTAC AATGCGCCCT   
  
  
+ TCTTTGTTAC ACGTTTCAGG GAGGCCCTCT TCCATTACTC TACTGTATTC GATATGTTTG ATGCCAATGC   
  
  
+ TTCCCGGGAG GATCCTGAGA GATTGATGTT TGAGAAGGAG TTTTATGGGC GAGAAATTAT GAATGTGGTG   
  
  
+ GCTTGTGAGG GTACAGAGAG AGTTGAAAGA CCTGAGACAT ACAAGCAATG GCAGGCGCGG AATAGTAGGG   
  
  
+ CAGGGTTCAG GCAGCTGCCA TTGGACCAAG AGCTCGTCAG TAAACTGAGG AGCAAGGTCC AGATGCACTA   
  
  
+ TCCTAGGGAC TTCGTTGTTG ATGTAGATGG ACATTGGACA TTGCAGGGAT GGAAGGGGAG GATCATCTGT   
  
  
+ GCTGTGTCTG CATGGGGTCC TGCTTA  

- +Up\_Stream \_Len000TCGCAT CCTTCACTGC GGCGATAACC TCCGCTTCTG CGATCATGGT TCCGGTTTTG   
  
  
- TTTACTCCTC TATCTAGTCA ATTTGGCAAA CCAATTCTTT TAATTCGCTT GTAGAGAACT TGGAGGTAGA   
  
  
- CTTGAGCTAC TGTAACTCCT ACACCTACGT CTTTTCTACC TATACCCGAG AGGTAGCCAC GGTCGTTTAG   
  
  
- TTGGATTACA ACCCACCCCA GAGTAAGCCA GTTACCCCCT GATCCCTGCA GTATAGCAGT GGTTAATGTT   
  
  
- GCCTTGTTGG GATTTGGCGT AAAATTTTAG TGTAGAACCA TCGTCTGTTT CTACATGCTG GGTTTTTAAG   
  
  
- TTGAGACATT TCGTGATCGT TTCGAGCATC ATGTGTCTTG TTAGCGTGTG AGTATCACTA TCTTCACATG   
  
  
- ATAGGTAATA CGTCACTTAT ACTGTTCCCT CCCCCAGGAC TCTACGGGTT TAAACCGTCG GCTCAACGGA   
  
  
- ACTGACGGGT AGTTACAGGA TTTGGTCAAC TCGTACAATA ATTAAACGTT CTTTCTAGCT TAAGAAGGAT   
  
  
- AGATTTTTGA CTGTGTTATT TTGTTATGGT ACATACCCTG TAACGATCCT AACAATTGTG TTAATAACAG   
  
  
- AACATGCAAC GTGGACTTTT TTTTCTTTTA ACAACGGTAT CTGTTGTATA TTCAATCCGT AAGTACTGAT   
  
  
- TATAGACTTA ATCCACACAG CTAGACACTA AGTTTAACTA TATTAATCTA GAAATTCCAT AGATTAACGA   
  
  
- AGTTCAAAAG AGATATTACT TCTACATATA ATCGAGTGTA TCCAGAATTC CAATTGATTA CAGTATAGTC   
  
  
- CCCCACACCC GGCGGTAAAA ACTATTCTTA ACTTAACTTG AACGTAACAA AAAAGAAACC GCTCTTACGT   
  
  
- TCTAAACAAT TAAATAAGTA TACGCAGTAA TATGTGATAT TCTTTTTATT AACATCCTTA TATACTTCAT   
  
  
- AAAAATGAAC ACGAGTACAA TTTACACCTT ATGAAAAAAT TACAGCATAT CATGGTGTCA TAAGATGTTA   
  
  
- AATTCTCTAA TTAGCTAGGT AATTTCAGTA CGTTTGGACT ACGTATAAGT TCTGTAACGT ACTTTACTAG   
  
  
- AATAAAATCA GTTTCTGAGG TAGTAAATAT TCGTTATAAA TGAAGCTATG TGCACAGTAA TTTTTTCTGA   
  
  
- TATCTTTACA ATGACAATAA AACAAACTCG ATTTATCTTT TAAATTTTCA ATTAATTCTA TTATTAATTT   
  
  
- TGATTTATAA GTTACTACCG ATTATTTATG TAACAGCATA ATTGACTGAT TATAAACATC TACCAGCACA   
  
  
- ATTAAAGAAA AATAAAATTT CAAGTTTACT CAATGTTTTC CAATATTTTA TATTTCATTT TACCCTCTGT   
  
  
- TTCGTCTGTC CCACCCCCTT CGATTCGAAT TTCATCCTAA ATCTTTCCGT TACTTCCACT TGGGAGTTTA   
  
  
- GCTAGGCGAC GGTTAGTGTC TTTGGGTTTC GAAACGAGTG GCTGTTGAGG GCCAATTGAC GTCAGTGAGT   
  
  
- GCCGTGACCC ATTATGTCAT TAGCACATGG GTCAAGTAGG GGGAAAAGGA AACCCTTATG TCATGGAGCT   
  
  
- TTCAAGGAAA CTTTAAGATG ACCGGTCCGT ATGGGTATTT AACTAGGAGA GCTATAGTAT TCTATACTAT   
  
  
- AAAGAGACAG TATAGACAAT CAAAGCCATA GTATAAAGAA GAAATAGAGA CATTAAACTC CATACGAAGT   
  
  
- AAGGACCGGC GAACTTAATA GAAAGAAAGA AACTAATCAT AACAAAAAAA CCAATGGATA AAACGAGGTC   
  
  
- AGGACAAGTA AACCCAGAAA AACCTAGACC CAAAAACGAC CCAAACAACT AAGAAACTCT TTAAACCCAC   
  
  
- GAACTTAAAC GGGACGTTAT AAGTACCCAA TCGCAAAAGG ACTTAAAACG AGACAAGGAA TTCACTAATA   
  
  
- AAAACTAACT AATGTGGAAC CACCAGGACC ACCTTAAAGG CTCCTCTTTA ACAGTACCCA AGGGTGTTGA   
  
  
- AACCTCTTAA GGGACTACTC TGAGATTTAC TCATAGTTAG ATGAAACCCT TGGTACAGGG GGCCAATACT   
  
  
- ACCCAGAAAC TTAATATGTT GGAACAAGTT AATGTTTCTG GGTAGAGTCC TAGAGTGCCG TGACTTGGAA   
  
  
- GGATCGGGTA ACGGACTGGG ATACGGTAAG TTGTAACCAA GTATAAGCCC AAACTCGGGG CCCCAACTTA   
  
  
- GAAGCCTACT ATCGCTAAGT CTACTACAAC AATTCATGTA ACCGGTTCAC GAATACCTTC TCCTATACTT   
  
  
- CCTCTTCGGA ACATACAAGG TACTGGGAAA TCGTGAGGTT CGACGACTCT TTGGGAAAAT ACTACACAAC   
  
  
- CCTTTCTTCA TAGGAGGAAG GGGATTAGTT GTGGGTGAAT AACTAGTAAG ACACCTATCG GGTCTGTTAG   
  
  
- TCAGGACACC TTGCTCGTGT TAATCACTAG AATCACCGTC ATTGACGTGA AGGTCAAGTT GGTTAATATA   
  
  
- ACTACGACAC CGTCAGCGAC TAAGCTCACT CTTATGATTC TGGAGAAAAC ACGTTTCAAG AGACTAACTT   
  
  
- AGCAAAAGAG TCAGCAGGTG GGAAGGTGTC ACCAGTAAAC CTAGTAACCC ACGGAACCCA CCGTGTCGAA   
  
  
- GAGTCCCAAG CTTAAGTCAA TAGAGGAGGG CACCAAAGGG ATACCGGTAC TTGCAAAAAT CGCTCGTCAG   
  
  
- GTACTATGTC AAAGTCTTCC CCCACCTCCT CCGCTCGTTC AAGGAAGGTT TCTTGTTATT GGAACGCTAA   
  
  
- CTGGAACTCT CGCAGTGAAA GGGTTTACTT TTCCTCCGCC GGGGGTACCA CCACCAATTC TTTCTCTTCC   
  
  
- TACTTGTGAG TGGACTATCG AGCTCTCCAT CGTTCTAAGT GATGGCACTA CTACTCCTGA AACTCCTACC   
  
  
- ATCCTCCTCA TTCGTCAGTC GACAAAGACA CCTCCTCCGA CTCAACAGAC TTTACAAACT GGCCCAAAAC   
  
  
- GAAACGTTAG GGTACTTTGT ACTTCGAGTA ACATGTTCAC CAAACTTCAG GCTCTTCCCT TGTAGGGACG   
  
  
- TCCGACCGGT TCATGTCCAT GTCCTCCCAG TCTTCCGAGC ACGTTTCTTT GTCCCATTAT TACTATTATT   
  
  
- CTTCTTATTC TTACACCACC TAAATTCCTG AGACGAATAG GAGACACGCG TTAGACGACG TAGACTACTA   
  
  
- GCGTCCTGTC GACTACTTGA CGACTTCGTC TAATCCCTCG TGAGAAGACG TCGACCCCTA CCTAGAGTTT   
  
  
- CCAACCGAGT AATGAAACGA TTACGGAACC TCCGCGCAAA TCGACCTTGA CCGAGTGTCT AGATATGTCG   
  
  
- GGACTCAAGC TTCTCCTGTT GTCGTCGACT ATACTATTTT CGAATAGTCA AAAAGAAAGC ACGAACGGGT   
  
  
- AAAAATTTCT AGAGATAATA GAAACGCTTG GTATACTAAT ATGTCTCTCG ACTTTTTCGT AGTTTCGAAG   
  
  
- TACAATATCT AAAACCGTAG GACATACCAA AAGTCACCGG GGAGGAGTAA GTTGCGGACA GCCTCGCTGG   
  
  
- ACCACCTGGA GGTTTTGACA CGTAATGACC CTAACTAGAA GGGGTTGGAC CTAAATCCGG TTGTCTTTCT   
  
  
- CAACTCCGTT GTCCCGAGGC GAACCGTTTC ATAACACTCG CGAAGTTACA AGGTAAACTT ATGGTACGGT   
  
  
- AACGTGTCTT TACCCTTTGG TAGTTTCGAC TTCTGGACTT CTATCTTCTA CTACTCCACC AACGGCACTT   
  
  
- GACAGAGAAG TCCAAATTCT TGGACGAACT ACTCTGCTAT CACCACCTAT CAGGCTCCTT ACGTCAAGAG   
  
  
- CCGAACTAAT CTTCCTATTT TGGACTATAG AAACAAGTGC CTCATCACTT GCCCAGAATG TTACGCGGGA   
  
  
- AGAAACAATG TGCAAAGTCC CTCCGGGAGA AGGTAATGAG ATGACATAAG CTATACAAAC TACGGTTACG   
  
  
- AAGGGCCCTC CTAGGACTCT CTAACTACAA ACTCTTCCTC AAAATACCCG CTCTTTAATA CTTACACCAC   
  
  
- CGAACACTCC CATGTCTCTC TCAACTTTCT GGACTCTGTA TGTTCGTTAC CGTCCGCGCC TTATCATCCC   
  
  
- GTCCCAAGTC CGTCGACGGT AACCTGGTTC TCGAGCAGTC ATTTGACTCC TCGTTCCAGG TCTACGTGAT   
  
  
- AGGATCCCTG AAGCAACAAC TACATCTACC TGTAACCTGT AACGTCCCTA CCTTCCCCTC CTAGTAGACA   
  
  
- CGACACAGAC GTACCCCAGG ACGAAT

+     P-box

| Site Name | Organism | Position | Strand | Matrix score. | sequence | function |
| --- | --- | --- | --- | --- | --- | --- |
| P-box | Oryza sativa | 1369 | - | 7 | CCTTTTG | gibberellin-responsive element |

>HU02G01569.1   
+ +Up\_Stream \_Len000AGCGTA GGAAGTGACG CCGCTATTGG AGGCGAAGAC GCTAGTACCA AGGCCAAAAC   
  
  
+ AAATGAGGAG ATAGATCAGT TAAACCGTTT GGTTAAGAAA ATTAAGCGAA CATCTCTTGA ACCTCCATCT   
  
  
+ GAACTCGATG ACATTGAGGA TGTGGATGCA GAAAAGATGG ATATGGGCTC TCCATCGGTG CCAGCAAATC   
  
  
+ AACCTAATGT TGGGTGGGGT CTCATTCGGT CAATGGGGGA CTAGGGACGT CATATCGTCA CCAATTACAA   
  
  
+ CGGAACAACC CTAAACCGCA TTTTAAAATC ACATCTTGGT AGCAGACAAA GATGTACGAC CCAAAAATTC   
  
  
+ AACTCTGTAA AGCACTAGCA AAGCTCGTAG TACACAGAAC AATCGCACAC TCATAGTGAT AGAAGTGTAC   
  
  
+ TATCCATTAT GCAGTGAATA TGACAAGGGA GGGGGTCCTG AGATGCCCAA ATTTGGCAGC CGAGTTGCCT   
  
  
+ TGACTGCCCA TCAATGTCCT AAACCAGTTG AGCATGTTAT TAATTTGCAA GAAAGATCGA ATTCTTCCTA   
  
  
+ TCTAAAAACT GACACAATAA AACAATACCA TGTATGGGAC ATTGCTAGGA TTGTTAACAC AATTATTGTC   
  
  
+ TTGTACGTTG CACCTGAAAA AAAAGAAAAT TGTTGCCATA GACAACATAT AAGTTAGGCA TTCATGACTA   
  
  
+ ATATCTGAAT TAGGTGTGTC GATCTGTGAT TCAAATTGAT ATAATTAGAT CTTTAAGGTA TCTAATTGCT   
  
  
+ TCAAGTTTTC TCTATAATGA AGATGTATAT TAGCTCACAT AGGTCTTAAG GTTAACTAAT GTCATATCAG   
  
  
+ GGGGTGTGGG CCGCCATTTT TGATAAGAAT TGAATTGAAC TTGCATTGTT TTTTCTTTGG CGAGAATGCA   
  
  
+ AGATTTGTTA ATTTATTCAT ATGCGTCATT ATACACTATA AGAAAAATAA TTGTAGGAAT ATATGAAGTA   
  
  
+ TTTTTACTTG TGCTCATGTT AAATGTGGAA TACTTTTTTA ATGTCGTATA GTACCACAGT ATTCTACAAT   
  
  
+ TTAAGAGATT AATCGATCCA TTAAAGTCAT GCAAACCTGA TGCATATTCA AGACATTGCA TGAAATGATC   
  
  
+ TTATTTTAGT CAAAGACTCC ATCATTTATA AGCAATATTT ACTTCGATAC ACGTGTCATT AAAAAAGACT   
  
  
+ ATAGAAATGT TACTGTTATT TTGTTTGAGC TAAATAGAAA ATTTAAAAGT TAATTAAGAT AATAATTAAA   
  
  
+ ACTAAATATT CAATGATGGC TAATAAATAC ATTGTCGTAT TAACTGACTA ATATTTGTAG ATGGTCGTGT   
  
  
+ TAATTTCTTT TTATTTTAAA GTTCAAATGA GTTACAAAAG GTTATAAAAT ATAAAGTAAA ATGGGAGACA   
  
  
+ AAGCAGACAG GGTGGGGGAA GCTAAGCTTA AAGTAGGATT TAGAAAGGCA ATGAAGGTGA ACCCTCAAAT   
  
  
+ CGATCCGCTG CCAATCACAG AAACCCAAAG CTTTGCTCAC CGACAACTCC CGGTTAACTG CAGTCACTCA   
  
  
+ CGGCACTGGG TAATACAGTA ATCGTGTACC CAGTTCATCC CCCTTTTCCT TTGGGAATAC AGTACCTCGA   
  
  
+ AAGTTCCTTT GAAATTCTAC TGGCCAGGCA TACCCATAAA TTGATCCTCT CGATATCATA AGATATGATA   
  
  
+ TTTCTCTGTC ATATCTGTTA GTTTCGGTAT CATATTTCTT CTTTATCTCT GTAATTTGAG GTATGCTTCA   
  
  
+ TTCCTGGCCG CTTGAATTAT CTTTCTTTCT TTGATTAGTA TTGTTTTTTT GGTTACCTAT TTTGCTCCAG   
  
  
+ TCCTGTTCAT TTGGGTCTTT TTGGATCTGG GTTTTTGCTG GGTTTGTTGA TTCTTTGAGA AATTTGGGTG   
  
  
+ CTTGAATTTG CCCTGCAATA TTCATGGGTT AGCGTTTTCC TGAATTTTGC TCTGTTCCTT AAGTGATTAT   
  
  
+ TTTTGATTGA TTACACCTTG GTGGTCCTGG TGGAATTTCC GAGGAGAAAT TGTCATGGGT TCCCACAACT   
  
  
+ TTGGAGAATT CCCTGATGAG ACTCTAAATG AGTATCAATC TACTTTGGGA ACCATGTCCC CCGGTTATGA   
  
  
+ TGGGTCTTTG AATTATACAA CCTTGTTCAA TTACAAAGAC CCATCTCAGG ATCTCACGGC ACTGAACCTT   
  
  
+ CCTAGCCCAT TGCCTGACCC TATGCCATTC AACATTGGTT CATATTCGGG TTTGAGCCCC GGGGTTGAAT   
  
  
+ CTTCGGATGA TAGCGATTCA GATGATGTTG TTAAGTACAT TGGCCAAGTG CTTATGGAAG AGGATATGAA   
  
  
+ GGAGAAGCCT TGTATGTTCC ATGACCCTTT AGCACTCCAA GCTGCTGAGA AACCCTTTTA TGATGTGTTG   
  
  
+ GGAAAGAAGT ATCCTCCTTC CCCTAATCAA CACCCACTTA TTGATCATTC TGTGGATAGC CCAGACAATC   
  
  
+ AGTCCTGTGG AACGAGCACA ATTAGTGATC TTAGTGGCAG TAACTGCACT TCCAGTTCAA CCAATTATAT   
  
  
+ TGATGCTGTG GCAGTCGCTG ATTCGAGTGA GAATACTAAG ACCTCTTTTG TGCAAAGTTC TCTGATTGAA   
  
  
+ TCGTTTTCTC AGTCGTCCAC CCTTCCACAG TGGTCATTTG GATCATTGGG TGCCTTGGGT GGCACAGCTT   
  
  
+ CTCAGGGTTC GAATTCAGTT ATCTCCTCCC GTGGTTTCCC TATGGCCATG AACGTTTTTA GCGAGCAGTC   
  
  
+ CATGATACAG TTTCAGAAGG GGGTGGAGGA GGCGAGCAAG TTCCTTCCAA AGAACAATAA CCTTGCGATT   
  
  
+ GACCTTGAGA GCGTCACTTT CCCAAATGAA AAGGAGGCGG CCCCCATGGT GGTGGTTAAG AAAGAGAAGG   
  
  
+ ATGAACACTC ACCTGATAGC TCGAGAGGTA GCAAGATTCA CTACCGTGAT GATGAGGACT TTGAGGATGG   
  
  
+ TAGGAGGAGT AAGCAGTCAG CTGTTTCTGT GGAGGAGGCT GAGTTGTCTG AAATGTTTGA CCGGGTTTTG   
  
  
+ CTTTGCAATC CCATGAAACA TGAAGCTCAT TGTACAAGTG GTTTGAAGTC CGAGAAGGGA ACATCCCTGC   
  
  
+ AGGCTGGCCA AGTACAGGTA CAGGAGGGTC AGAAGGCTCG TGCAAAGAAA CAGGGTAATA ATGATAATAA   
  
  
+ GAAGAATAAG AATGTGGTGG ATTTAAGGAC TCTGCTTATC CTCTGTGCGC AATCTGCTGC ATCTGATGAT   
  
  
+ CGCAGGACAG CTGATGAACT GCTGAAGCAG ATTAGGGAGC ACTCTTCTGC AGCTGGGGAT GGATCTCAAA   
  
  
+ GGTTGGCTCA TTACTTTGCT AATGCCTTGG AGGCGCGTTT AGCTGGAACT GGCTCACAGA TCTATACAGC   
  
  
+ CCTGAGTTCG AAGAGGACAA CAGCAGCTGA TATGATAAAA GCTTATCAGT TTTTCTTTCG TGCTTGCCCA   
  
  
+ TTTTTAAAGA TCTCTATTAT CTTTGCGAAC CATATGATTA TACAGAGAGC TGAAAAAGCA TCAAAGCTTC   
  
  
+ ATGTTATAGA TTTTGGCATC CTGTATGGTT TTCAGTGGCC CCTCCTCATT CAACGCCTGT CGGAGCGACC   
  
  
+ TGGTGGACCT CCAAAACTGT GCATTACTGG GATTGATCTT CCCCAACCTG GATTTAGGCC AACAGAAAGA   
  
  
+ GTTGAGGCAA CAGGGCTCCG CTTGGCAAAG TATTGTGAGC GCTTCAATGT TCCATTTGAA TACCATGCCA   
  
  
+ TTGCACAGAA ATGGGAAACC ATCAAAGCTG AAGACCTGAA GATAGAAGAT GATGAGGTGG TTGCCGTGAA   
  
  
+ CTGTCTCTTC AGGTTTAAGA ACCTGCTTGA TGAGACGATA GTGGTGGATA GTCCGAGGAA TGCAGTTCTC   
  
  
+ GGCTTGATTA GAAGGATAAA ACCTGATATC TTTGTTCACG GAGTAGTGAA CGGGTCTTAC AATGCGCCCT   
  
  
+ TCTTTGTTAC ACGTTTCAGG GAGGCCCTCT TCCATTACTC TACTGTATTC GATATGTTTG ATGCCAATGC   
  
  
+ TTCCCGGGAG GATCCTGAGA GATTGATGTT TGAGAAGGAG TTTTATGGGC GAGAAATTAT GAATGTGGTG   
  
  
+ GCTTGTGAGG GTACAGAGAG AGTTGAAAGA CCTGAGACAT ACAAGCAATG GCAGGCGCGG AATAGTAGGG   
  
  
+ CAGGGTTCAG GCAGCTGCCA TTGGACCAAG AGCTCGTCAG TAAACTGAGG AGCAAGGTCC AGATGCACTA   
  
  
+ TCCTAGGGAC TTCGTTGTTG ATGTAGATGG ACATTGGACA TTGCAGGGAT GGAAGGGGAG GATCATCTGT   
  
  
+ GCTGTGTCTG CATGGGGTCC TGCTTA  

- +Up\_Stream \_Len000TCGCAT CCTTCACTGC GGCGATAACC TCCGCTTCTG CGATCATGGT TCCGGTTTTG   
  
  
- TTTACTCCTC TATCTAGTCA ATTTGGCAAA CCAATTCTTT TAATTCGCTT GTAGAGAACT TGGAGGTAGA   
  
  
- CTTGAGCTAC TGTAACTCCT ACACCTACGT CTTTTCTACC TATACCCGAG AGGTAGCCAC GGTCGTTTAG   
  
  
- TTGGATTACA ACCCACCCCA GAGTAAGCCA GTTACCCCCT GATCCCTGCA GTATAGCAGT GGTTAATGTT   
  
  
- GCCTTGTTGG GATTTGGCGT AAAATTTTAG TGTAGAACCA TCGTCTGTTT CTACATGCTG GGTTTTTAAG   
  
  
- TTGAGACATT TCGTGATCGT TTCGAGCATC ATGTGTCTTG TTAGCGTGTG AGTATCACTA TCTTCACATG   
  
  
- ATAGGTAATA CGTCACTTAT ACTGTTCCCT CCCCCAGGAC TCTACGGGTT TAAACCGTCG GCTCAACGGA   
  
  
- ACTGACGGGT AGTTACAGGA TTTGGTCAAC TCGTACAATA ATTAAACGTT CTTTCTAGCT TAAGAAGGAT   
  
  
- AGATTTTTGA CTGTGTTATT TTGTTATGGT ACATACCCTG TAACGATCCT AACAATTGTG TTAATAACAG   
  
  
- AACATGCAAC GTGGACTTTT TTTTCTTTTA ACAACGGTAT CTGTTGTATA TTCAATCCGT AAGTACTGAT   
  
  
- TATAGACTTA ATCCACACAG CTAGACACTA AGTTTAACTA TATTAATCTA GAAATTCCAT AGATTAACGA   
  
  
- AGTTCAAAAG AGATATTACT TCTACATATA ATCGAGTGTA TCCAGAATTC CAATTGATTA CAGTATAGTC   
  
  
- CCCCACACCC GGCGGTAAAA ACTATTCTTA ACTTAACTTG AACGTAACAA AAAAGAAACC GCTCTTACGT   
  
  
- TCTAAACAAT TAAATAAGTA TACGCAGTAA TATGTGATAT TCTTTTTATT AACATCCTTA TATACTTCAT   
  
  
- AAAAATGAAC ACGAGTACAA TTTACACCTT ATGAAAAAAT TACAGCATAT CATGGTGTCA TAAGATGTTA   
  
  
- AATTCTCTAA TTAGCTAGGT AATTTCAGTA CGTTTGGACT ACGTATAAGT TCTGTAACGT ACTTTACTAG   
  
  
- AATAAAATCA GTTTCTGAGG TAGTAAATAT TCGTTATAAA TGAAGCTATG TGCACAGTAA TTTTTTCTGA   
  
  
- TATCTTTACA ATGACAATAA AACAAACTCG ATTTATCTTT TAAATTTTCA ATTAATTCTA TTATTAATTT   
  
  
- TGATTTATAA GTTACTACCG ATTATTTATG TAACAGCATA ATTGACTGAT TATAAACATC TACCAGCACA   
  
  
- ATTAAAGAAA AATAAAATTT CAAGTTTACT CAATGTTTTC CAATATTTTA TATTTCATTT TACCCTCTGT   
  
  
- TTCGTCTGTC CCACCCCCTT CGATTCGAAT TTCATCCTAA ATCTTTCCGT TACTTCCACT TGGGAGTTTA   
  
  
- GCTAGGCGAC GGTTAGTGTC TTTGGGTTTC GAAACGAGTG GCTGTTGAGG GCCAATTGAC GTCAGTGAGT   
  
  
- GCCGTGACCC ATTATGTCAT TAGCACATGG GTCAAGTAGG GGGAAAAGGA AACCCTTATG TCATGGAGCT   
  
  
- TTCAAGGAAA CTTTAAGATG ACCGGTCCGT ATGGGTATTT AACTAGGAGA GCTATAGTAT TCTATACTAT   
  
  
- AAAGAGACAG TATAGACAAT CAAAGCCATA GTATAAAGAA GAAATAGAGA CATTAAACTC CATACGAAGT   
  
  
- AAGGACCGGC GAACTTAATA GAAAGAAAGA AACTAATCAT AACAAAAAAA CCAATGGATA AAACGAGGTC   
  
  
- AGGACAAGTA AACCCAGAAA AACCTAGACC CAAAAACGAC CCAAACAACT AAGAAACTCT TTAAACCCAC   
  
  
- GAACTTAAAC GGGACGTTAT AAGTACCCAA TCGCAAAAGG ACTTAAAACG AGACAAGGAA TTCACTAATA   
  
  
- AAAACTAACT AATGTGGAAC CACCAGGACC ACCTTAAAGG CTCCTCTTTA ACAGTACCCA AGGGTGTTGA   
  
  
- AACCTCTTAA GGGACTACTC TGAGATTTAC TCATAGTTAG ATGAAACCCT TGGTACAGGG GGCCAATACT   
  
  
- ACCCAGAAAC TTAATATGTT GGAACAAGTT AATGTTTCTG GGTAGAGTCC TAGAGTGCCG TGACTTGGAA   
  
  
- GGATCGGGTA ACGGACTGGG ATACGGTAAG TTGTAACCAA GTATAAGCCC AAACTCGGGG CCCCAACTTA   
  
  
- GAAGCCTACT ATCGCTAAGT CTACTACAAC AATTCATGTA ACCGGTTCAC GAATACCTTC TCCTATACTT   
  
  
- CCTCTTCGGA ACATACAAGG TACTGGGAAA TCGTGAGGTT CGACGACTCT TTGGGAAAAT ACTACACAAC   
  
  
- CCTTTCTTCA TAGGAGGAAG GGGATTAGTT GTGGGTGAAT AACTAGTAAG ACACCTATCG GGTCTGTTAG   
  
  
- TCAGGACACC TTGCTCGTGT TAATCACTAG AATCACCGTC ATTGACGTGA AGGTCAAGTT GGTTAATATA   
  
  
- ACTACGACAC CGTCAGCGAC TAAGCTCACT CTTATGATTC TGGAGAAAAC ACGTTTCAAG AGACTAACTT   
  
  
- AGCAAAAGAG TCAGCAGGTG GGAAGGTGTC ACCAGTAAAC CTAGTAACCC ACGGAACCCA CCGTGTCGAA   
  
  
- GAGTCCCAAG CTTAAGTCAA TAGAGGAGGG CACCAAAGGG ATACCGGTAC TTGCAAAAAT CGCTCGTCAG   
  
  
- GTACTATGTC AAAGTCTTCC CCCACCTCCT CCGCTCGTTC AAGGAAGGTT TCTTGTTATT GGAACGCTAA   
  
  
- CTGGAACTCT CGCAGTGAAA GGGTTTACTT TTCCTCCGCC GGGGGTACCA CCACCAATTC TTTCTCTTCC   
  
  
- TACTTGTGAG TGGACTATCG AGCTCTCCAT CGTTCTAAGT GATGGCACTA CTACTCCTGA AACTCCTACC   
  
  
- ATCCTCCTCA TTCGTCAGTC GACAAAGACA CCTCCTCCGA CTCAACAGAC TTTACAAACT GGCCCAAAAC   
  
  
- GAAACGTTAG GGTACTTTGT ACTTCGAGTA ACATGTTCAC CAAACTTCAG GCTCTTCCCT TGTAGGGACG   
  
  
- TCCGACCGGT TCATGTCCAT GTCCTCCCAG TCTTCCGAGC ACGTTTCTTT GTCCCATTAT TACTATTATT   
  
  
- CTTCTTATTC TTACACCACC TAAATTCCTG AGACGAATAG GAGACACGCG TTAGACGACG TAGACTACTA   
  
  
- GCGTCCTGTC GACTACTTGA CGACTTCGTC TAATCCCTCG TGAGAAGACG TCGACCCCTA CCTAGAGTTT   
  
  
- CCAACCGAGT AATGAAACGA TTACGGAACC TCCGCGCAAA TCGACCTTGA CCGAGTGTCT AGATATGTCG   
  
  
- GGACTCAAGC TTCTCCTGTT GTCGTCGACT ATACTATTTT CGAATAGTCA AAAAGAAAGC ACGAACGGGT   
  
  
- AAAAATTTCT AGAGATAATA GAAACGCTTG GTATACTAAT ATGTCTCTCG ACTTTTTCGT AGTTTCGAAG   
  
  
- TACAATATCT AAAACCGTAG GACATACCAA AAGTCACCGG GGAGGAGTAA GTTGCGGACA GCCTCGCTGG   
  
  
- ACCACCTGGA GGTTTTGACA CGTAATGACC CTAACTAGAA GGGGTTGGAC CTAAATCCGG TTGTCTTTCT   
  
  
- CAACTCCGTT GTCCCGAGGC GAACCGTTTC ATAACACTCG CGAAGTTACA AGGTAAACTT ATGGTACGGT   
  
  
- AACGTGTCTT TACCCTTTGG TAGTTTCGAC TTCTGGACTT CTATCTTCTA CTACTCCACC AACGGCACTT   
  
  
- GACAGAGAAG TCCAAATTCT TGGACGAACT ACTCTGCTAT CACCACCTAT CAGGCTCCTT ACGTCAAGAG   
  
  
- CCGAACTAAT CTTCCTATTT TGGACTATAG AAACAAGTGC CTCATCACTT GCCCAGAATG TTACGCGGGA   
  
  
- AGAAACAATG TGCAAAGTCC CTCCGGGAGA AGGTAATGAG ATGACATAAG CTATACAAAC TACGGTTACG   
  
  
- AAGGGCCCTC CTAGGACTCT CTAACTACAA ACTCTTCCTC AAAATACCCG CTCTTTAATA CTTACACCAC   
  
  
- CGAACACTCC CATGTCTCTC TCAACTTTCT GGACTCTGTA TGTTCGTTAC CGTCCGCGCC TTATCATCCC   
  
  
- GTCCCAAGTC CGTCGACGGT AACCTGGTTC TCGAGCAGTC ATTTGACTCC TCGTTCCAGG TCTACGTGAT   
  
  
- AGGATCCCTG AAGCAACAAC TACATCTACC TGTAACCTGT AACGTCCCTA CCTTCCCCTC CTAGTAGACA   
  
  
- CGACACAGAC GTACCCCAGG ACGAAT

+     Pc-CMA2a

| Site Name | Organism | Position | Strand | Matrix score. | sequence | function |
| --- | --- | --- | --- | --- | --- | --- |
| Pc-CMA2a | Pisum sativum | 1482 | + | 12 | CAGCCAATCACAG | part of a light responsive element |

>HU02G01569.1   
+ +Up\_Stream \_Len000AGCGTA GGAAGTGACG CCGCTATTGG AGGCGAAGAC GCTAGTACCA AGGCCAAAAC   
  
  
+ AAATGAGGAG ATAGATCAGT TAAACCGTTT GGTTAAGAAA ATTAAGCGAA CATCTCTTGA ACCTCCATCT   
  
  
+ GAACTCGATG ACATTGAGGA TGTGGATGCA GAAAAGATGG ATATGGGCTC TCCATCGGTG CCAGCAAATC   
  
  
+ AACCTAATGT TGGGTGGGGT CTCATTCGGT CAATGGGGGA CTAGGGACGT CATATCGTCA CCAATTACAA   
  
  
+ CGGAACAACC CTAAACCGCA TTTTAAAATC ACATCTTGGT AGCAGACAAA GATGTACGAC CCAAAAATTC   
  
  
+ AACTCTGTAA AGCACTAGCA AAGCTCGTAG TACACAGAAC AATCGCACAC TCATAGTGAT AGAAGTGTAC   
  
  
+ TATCCATTAT GCAGTGAATA TGACAAGGGA GGGGGTCCTG AGATGCCCAA ATTTGGCAGC CGAGTTGCCT   
  
  
+ TGACTGCCCA TCAATGTCCT AAACCAGTTG AGCATGTTAT TAATTTGCAA GAAAGATCGA ATTCTTCCTA   
  
  
+ TCTAAAAACT GACACAATAA AACAATACCA TGTATGGGAC ATTGCTAGGA TTGTTAACAC AATTATTGTC   
  
  
+ TTGTACGTTG CACCTGAAAA AAAAGAAAAT TGTTGCCATA GACAACATAT AAGTTAGGCA TTCATGACTA   
  
  
+ ATATCTGAAT TAGGTGTGTC GATCTGTGAT TCAAATTGAT ATAATTAGAT CTTTAAGGTA TCTAATTGCT   
  
  
+ TCAAGTTTTC TCTATAATGA AGATGTATAT TAGCTCACAT AGGTCTTAAG GTTAACTAAT GTCATATCAG   
  
  
+ GGGGTGTGGG CCGCCATTTT TGATAAGAAT TGAATTGAAC TTGCATTGTT TTTTCTTTGG CGAGAATGCA   
  
  
+ AGATTTGTTA ATTTATTCAT ATGCGTCATT ATACACTATA AGAAAAATAA TTGTAGGAAT ATATGAAGTA   
  
  
+ TTTTTACTTG TGCTCATGTT AAATGTGGAA TACTTTTTTA ATGTCGTATA GTACCACAGT ATTCTACAAT   
  
  
+ TTAAGAGATT AATCGATCCA TTAAAGTCAT GCAAACCTGA TGCATATTCA AGACATTGCA TGAAATGATC   
  
  
+ TTATTTTAGT CAAAGACTCC ATCATTTATA AGCAATATTT ACTTCGATAC ACGTGTCATT AAAAAAGACT   
  
  
+ ATAGAAATGT TACTGTTATT TTGTTTGAGC TAAATAGAAA ATTTAAAAGT TAATTAAGAT AATAATTAAA   
  
  
+ ACTAAATATT CAATGATGGC TAATAAATAC ATTGTCGTAT TAACTGACTA ATATTTGTAG ATGGTCGTGT   
  
  
+ TAATTTCTTT TTATTTTAAA GTTCAAATGA GTTACAAAAG GTTATAAAAT ATAAAGTAAA ATGGGAGACA   
  
  
+ AAGCAGACAG GGTGGGGGAA GCTAAGCTTA AAGTAGGATT TAGAAAGGCA ATGAAGGTGA ACCCTCAAAT   
  
  
+ CGATCCGCTG CCAATCACAG AAACCCAAAG CTTTGCTCAC CGACAACTCC CGGTTAACTG CAGTCACTCA   
  
  
+ CGGCACTGGG TAATACAGTA ATCGTGTACC CAGTTCATCC CCCTTTTCCT TTGGGAATAC AGTACCTCGA   
  
  
+ AAGTTCCTTT GAAATTCTAC TGGCCAGGCA TACCCATAAA TTGATCCTCT CGATATCATA AGATATGATA   
  
  
+ TTTCTCTGTC ATATCTGTTA GTTTCGGTAT CATATTTCTT CTTTATCTCT GTAATTTGAG GTATGCTTCA   
  
  
+ TTCCTGGCCG CTTGAATTAT CTTTCTTTCT TTGATTAGTA TTGTTTTTTT GGTTACCTAT TTTGCTCCAG   
  
  
+ TCCTGTTCAT TTGGGTCTTT TTGGATCTGG GTTTTTGCTG GGTTTGTTGA TTCTTTGAGA AATTTGGGTG   
  
  
+ CTTGAATTTG CCCTGCAATA TTCATGGGTT AGCGTTTTCC TGAATTTTGC TCTGTTCCTT AAGTGATTAT   
  
  
+ TTTTGATTGA TTACACCTTG GTGGTCCTGG TGGAATTTCC GAGGAGAAAT TGTCATGGGT TCCCACAACT   
  
  
+ TTGGAGAATT CCCTGATGAG ACTCTAAATG AGTATCAATC TACTTTGGGA ACCATGTCCC CCGGTTATGA   
  
  
+ TGGGTCTTTG AATTATACAA CCTTGTTCAA TTACAAAGAC CCATCTCAGG ATCTCACGGC ACTGAACCTT   
  
  
+ CCTAGCCCAT TGCCTGACCC TATGCCATTC AACATTGGTT CATATTCGGG TTTGAGCCCC GGGGTTGAAT   
  
  
+ CTTCGGATGA TAGCGATTCA GATGATGTTG TTAAGTACAT TGGCCAAGTG CTTATGGAAG AGGATATGAA   
  
  
+ GGAGAAGCCT TGTATGTTCC ATGACCCTTT AGCACTCCAA GCTGCTGAGA AACCCTTTTA TGATGTGTTG   
  
  
+ GGAAAGAAGT ATCCTCCTTC CCCTAATCAA CACCCACTTA TTGATCATTC TGTGGATAGC CCAGACAATC   
  
  
+ AGTCCTGTGG AACGAGCACA ATTAGTGATC TTAGTGGCAG TAACTGCACT TCCAGTTCAA CCAATTATAT   
  
  
+ TGATGCTGTG GCAGTCGCTG ATTCGAGTGA GAATACTAAG ACCTCTTTTG TGCAAAGTTC TCTGATTGAA   
  
  
+ TCGTTTTCTC AGTCGTCCAC CCTTCCACAG TGGTCATTTG GATCATTGGG TGCCTTGGGT GGCACAGCTT   
  
  
+ CTCAGGGTTC GAATTCAGTT ATCTCCTCCC GTGGTTTCCC TATGGCCATG AACGTTTTTA GCGAGCAGTC   
  
  
+ CATGATACAG TTTCAGAAGG GGGTGGAGGA GGCGAGCAAG TTCCTTCCAA AGAACAATAA CCTTGCGATT   
  
  
+ GACCTTGAGA GCGTCACTTT CCCAAATGAA AAGGAGGCGG CCCCCATGGT GGTGGTTAAG AAAGAGAAGG   
  
  
+ ATGAACACTC ACCTGATAGC TCGAGAGGTA GCAAGATTCA CTACCGTGAT GATGAGGACT TTGAGGATGG   
  
  
+ TAGGAGGAGT AAGCAGTCAG CTGTTTCTGT GGAGGAGGCT GAGTTGTCTG AAATGTTTGA CCGGGTTTTG   
  
  
+ CTTTGCAATC CCATGAAACA TGAAGCTCAT TGTACAAGTG GTTTGAAGTC CGAGAAGGGA ACATCCCTGC   
  
  
+ AGGCTGGCCA AGTACAGGTA CAGGAGGGTC AGAAGGCTCG TGCAAAGAAA CAGGGTAATA ATGATAATAA   
  
  
+ GAAGAATAAG AATGTGGTGG ATTTAAGGAC TCTGCTTATC CTCTGTGCGC AATCTGCTGC ATCTGATGAT   
  
  
+ CGCAGGACAG CTGATGAACT GCTGAAGCAG ATTAGGGAGC ACTCTTCTGC AGCTGGGGAT GGATCTCAAA   
  
  
+ GGTTGGCTCA TTACTTTGCT AATGCCTTGG AGGCGCGTTT AGCTGGAACT GGCTCACAGA TCTATACAGC   
  
  
+ CCTGAGTTCG AAGAGGACAA CAGCAGCTGA TATGATAAAA GCTTATCAGT TTTTCTTTCG TGCTTGCCCA   
  
  
+ TTTTTAAAGA TCTCTATTAT CTTTGCGAAC CATATGATTA TACAGAGAGC TGAAAAAGCA TCAAAGCTTC   
  
  
+ ATGTTATAGA TTTTGGCATC CTGTATGGTT TTCAGTGGCC CCTCCTCATT CAACGCCTGT CGGAGCGACC   
  
  
+ TGGTGGACCT CCAAAACTGT GCATTACTGG GATTGATCTT CCCCAACCTG GATTTAGGCC AACAGAAAGA   
  
  
+ GTTGAGGCAA CAGGGCTCCG CTTGGCAAAG TATTGTGAGC GCTTCAATGT TCCATTTGAA TACCATGCCA   
  
  
+ TTGCACAGAA ATGGGAAACC ATCAAAGCTG AAGACCTGAA GATAGAAGAT GATGAGGTGG TTGCCGTGAA   
  
  
+ CTGTCTCTTC AGGTTTAAGA ACCTGCTTGA TGAGACGATA GTGGTGGATA GTCCGAGGAA TGCAGTTCTC   
  
  
+ GGCTTGATTA GAAGGATAAA ACCTGATATC TTTGTTCACG GAGTAGTGAA CGGGTCTTAC AATGCGCCCT   
  
  
+ TCTTTGTTAC ACGTTTCAGG GAGGCCCTCT TCCATTACTC TACTGTATTC GATATGTTTG ATGCCAATGC   
  
  
+ TTCCCGGGAG GATCCTGAGA GATTGATGTT TGAGAAGGAG TTTTATGGGC GAGAAATTAT GAATGTGGTG   
  
  
+ GCTTGTGAGG GTACAGAGAG AGTTGAAAGA CCTGAGACAT ACAAGCAATG GCAGGCGCGG AATAGTAGGG   
  
  
+ CAGGGTTCAG GCAGCTGCCA TTGGACCAAG AGCTCGTCAG TAAACTGAGG AGCAAGGTCC AGATGCACTA   
  
  
+ TCCTAGGGAC TTCGTTGTTG ATGTAGATGG ACATTGGACA TTGCAGGGAT GGAAGGGGAG GATCATCTGT   
  
  
+ GCTGTGTCTG CATGGGGTCC TGCTTA  

- +Up\_Stream \_Len000TCGCAT CCTTCACTGC GGCGATAACC TCCGCTTCTG CGATCATGGT TCCGGTTTTG   
  
  
- TTTACTCCTC TATCTAGTCA ATTTGGCAAA CCAATTCTTT TAATTCGCTT GTAGAGAACT TGGAGGTAGA   
  
  
- CTTGAGCTAC TGTAACTCCT ACACCTACGT CTTTTCTACC TATACCCGAG AGGTAGCCAC GGTCGTTTAG   
  
  
- TTGGATTACA ACCCACCCCA GAGTAAGCCA GTTACCCCCT GATCCCTGCA GTATAGCAGT GGTTAATGTT   
  
  
- GCCTTGTTGG GATTTGGCGT AAAATTTTAG TGTAGAACCA TCGTCTGTTT CTACATGCTG GGTTTTTAAG   
  
  
- TTGAGACATT TCGTGATCGT TTCGAGCATC ATGTGTCTTG TTAGCGTGTG AGTATCACTA TCTTCACATG   
  
  
- ATAGGTAATA CGTCACTTAT ACTGTTCCCT CCCCCAGGAC TCTACGGGTT TAAACCGTCG GCTCAACGGA   
  
  
- ACTGACGGGT AGTTACAGGA TTTGGTCAAC TCGTACAATA ATTAAACGTT CTTTCTAGCT TAAGAAGGAT   
  
  
- AGATTTTTGA CTGTGTTATT TTGTTATGGT ACATACCCTG TAACGATCCT AACAATTGTG TTAATAACAG   
  
  
- AACATGCAAC GTGGACTTTT TTTTCTTTTA ACAACGGTAT CTGTTGTATA TTCAATCCGT AAGTACTGAT   
  
  
- TATAGACTTA ATCCACACAG CTAGACACTA AGTTTAACTA TATTAATCTA GAAATTCCAT AGATTAACGA   
  
  
- AGTTCAAAAG AGATATTACT TCTACATATA ATCGAGTGTA TCCAGAATTC CAATTGATTA CAGTATAGTC   
  
  
- CCCCACACCC GGCGGTAAAA ACTATTCTTA ACTTAACTTG AACGTAACAA AAAAGAAACC GCTCTTACGT   
  
  
- TCTAAACAAT TAAATAAGTA TACGCAGTAA TATGTGATAT TCTTTTTATT AACATCCTTA TATACTTCAT   
  
  
- AAAAATGAAC ACGAGTACAA TTTACACCTT ATGAAAAAAT TACAGCATAT CATGGTGTCA TAAGATGTTA   
  
  
- AATTCTCTAA TTAGCTAGGT AATTTCAGTA CGTTTGGACT ACGTATAAGT TCTGTAACGT ACTTTACTAG   
  
  
- AATAAAATCA GTTTCTGAGG TAGTAAATAT TCGTTATAAA TGAAGCTATG TGCACAGTAA TTTTTTCTGA   
  
  
- TATCTTTACA ATGACAATAA AACAAACTCG ATTTATCTTT TAAATTTTCA ATTAATTCTA TTATTAATTT   
  
  
- TGATTTATAA GTTACTACCG ATTATTTATG TAACAGCATA ATTGACTGAT TATAAACATC TACCAGCACA   
  
  
- ATTAAAGAAA AATAAAATTT CAAGTTTACT CAATGTTTTC CAATATTTTA TATTTCATTT TACCCTCTGT   
  
  
- TTCGTCTGTC CCACCCCCTT CGATTCGAAT TTCATCCTAA ATCTTTCCGT TACTTCCACT TGGGAGTTTA   
  
  
- GCTAGGCGAC GGTTAGTGTC TTTGGGTTTC GAAACGAGTG GCTGTTGAGG GCCAATTGAC GTCAGTGAGT   
  
  
- GCCGTGACCC ATTATGTCAT TAGCACATGG GTCAAGTAGG GGGAAAAGGA AACCCTTATG TCATGGAGCT   
  
  
- TTCAAGGAAA CTTTAAGATG ACCGGTCCGT ATGGGTATTT AACTAGGAGA GCTATAGTAT TCTATACTAT   
  
  
- AAAGAGACAG TATAGACAAT CAAAGCCATA GTATAAAGAA GAAATAGAGA CATTAAACTC CATACGAAGT   
  
  
- AAGGACCGGC GAACTTAATA GAAAGAAAGA AACTAATCAT AACAAAAAAA CCAATGGATA AAACGAGGTC   
  
  
- AGGACAAGTA AACCCAGAAA AACCTAGACC CAAAAACGAC CCAAACAACT AAGAAACTCT TTAAACCCAC   
  
  
- GAACTTAAAC GGGACGTTAT AAGTACCCAA TCGCAAAAGG ACTTAAAACG AGACAAGGAA TTCACTAATA   
  
  
- AAAACTAACT AATGTGGAAC CACCAGGACC ACCTTAAAGG CTCCTCTTTA ACAGTACCCA AGGGTGTTGA   
  
  
- AACCTCTTAA GGGACTACTC TGAGATTTAC TCATAGTTAG ATGAAACCCT TGGTACAGGG GGCCAATACT   
  
  
- ACCCAGAAAC TTAATATGTT GGAACAAGTT AATGTTTCTG GGTAGAGTCC TAGAGTGCCG TGACTTGGAA   
  
  
- GGATCGGGTA ACGGACTGGG ATACGGTAAG TTGTAACCAA GTATAAGCCC AAACTCGGGG CCCCAACTTA   
  
  
- GAAGCCTACT ATCGCTAAGT CTACTACAAC AATTCATGTA ACCGGTTCAC GAATACCTTC TCCTATACTT   
  
  
- CCTCTTCGGA ACATACAAGG TACTGGGAAA TCGTGAGGTT CGACGACTCT TTGGGAAAAT ACTACACAAC   
  
  
- CCTTTCTTCA TAGGAGGAAG GGGATTAGTT GTGGGTGAAT AACTAGTAAG ACACCTATCG GGTCTGTTAG   
  
  
- TCAGGACACC TTGCTCGTGT TAATCACTAG AATCACCGTC ATTGACGTGA AGGTCAAGTT GGTTAATATA   
  
  
- ACTACGACAC CGTCAGCGAC TAAGCTCACT CTTATGATTC TGGAGAAAAC ACGTTTCAAG AGACTAACTT   
  
  
- AGCAAAAGAG TCAGCAGGTG GGAAGGTGTC ACCAGTAAAC CTAGTAACCC ACGGAACCCA CCGTGTCGAA   
  
  
- GAGTCCCAAG CTTAAGTCAA TAGAGGAGGG CACCAAAGGG ATACCGGTAC TTGCAAAAAT CGCTCGTCAG   
  
  
- GTACTATGTC AAAGTCTTCC CCCACCTCCT CCGCTCGTTC AAGGAAGGTT TCTTGTTATT GGAACGCTAA   
  
  
- CTGGAACTCT CGCAGTGAAA GGGTTTACTT TTCCTCCGCC GGGGGTACCA CCACCAATTC TTTCTCTTCC   
  
  
- TACTTGTGAG TGGACTATCG AGCTCTCCAT CGTTCTAAGT GATGGCACTA CTACTCCTGA AACTCCTACC   
  
  
- ATCCTCCTCA TTCGTCAGTC GACAAAGACA CCTCCTCCGA CTCAACAGAC TTTACAAACT GGCCCAAAAC   
  
  
- GAAACGTTAG GGTACTTTGT ACTTCGAGTA ACATGTTCAC CAAACTTCAG GCTCTTCCCT TGTAGGGACG   
  
  
- TCCGACCGGT TCATGTCCAT GTCCTCCCAG TCTTCCGAGC ACGTTTCTTT GTCCCATTAT TACTATTATT   
  
  
- CTTCTTATTC TTACACCACC TAAATTCCTG AGACGAATAG GAGACACGCG TTAGACGACG TAGACTACTA   
  
  
- GCGTCCTGTC GACTACTTGA CGACTTCGTC TAATCCCTCG TGAGAAGACG TCGACCCCTA CCTAGAGTTT   
  
  
- CCAACCGAGT AATGAAACGA TTACGGAACC TCCGCGCAAA TCGACCTTGA CCGAGTGTCT AGATATGTCG   
  
  
- GGACTCAAGC TTCTCCTGTT GTCGTCGACT ATACTATTTT CGAATAGTCA AAAAGAAAGC ACGAACGGGT   
  
  
- AAAAATTTCT AGAGATAATA GAAACGCTTG GTATACTAAT ATGTCTCTCG ACTTTTTCGT AGTTTCGAAG   
  
  
- TACAATATCT AAAACCGTAG GACATACCAA AAGTCACCGG GGAGGAGTAA GTTGCGGACA GCCTCGCTGG   
  
  
- ACCACCTGGA GGTTTTGACA CGTAATGACC CTAACTAGAA GGGGTTGGAC CTAAATCCGG TTGTCTTTCT   
  
  
- CAACTCCGTT GTCCCGAGGC GAACCGTTTC ATAACACTCG CGAAGTTACA AGGTAAACTT ATGGTACGGT   
  
  
- AACGTGTCTT TACCCTTTGG TAGTTTCGAC TTCTGGACTT CTATCTTCTA CTACTCCACC AACGGCACTT   
  
  
- GACAGAGAAG TCCAAATTCT TGGACGAACT ACTCTGCTAT CACCACCTAT CAGGCTCCTT ACGTCAAGAG   
  
  
- CCGAACTAAT CTTCCTATTT TGGACTATAG AAACAAGTGC CTCATCACTT GCCCAGAATG TTACGCGGGA   
  
  
- AGAAACAATG TGCAAAGTCC CTCCGGGAGA AGGTAATGAG ATGACATAAG CTATACAAAC TACGGTTACG   
  
  
- AAGGGCCCTC CTAGGACTCT CTAACTACAA ACTCTTCCTC AAAATACCCG CTCTTTAATA CTTACACCAC   
  
  
- CGAACACTCC CATGTCTCTC TCAACTTTCT GGACTCTGTA TGTTCGTTAC CGTCCGCGCC TTATCATCCC   
  
  
- GTCCCAAGTC CGTCGACGGT AACCTGGTTC TCGAGCAGTC ATTTGACTCC TCGTTCCAGG TCTACGTGAT   
  
  
- AGGATCCCTG AAGCAACAAC TACATCTACC TGTAACCTGT AACGTCCCTA CCTTCCCCTC CTAGTAGACA   
  
  
- CGACACAGAC GTACCCCAGG ACGAAT

+     STRE

| Site Name | Organism | Position | Strand | Matrix score. | sequence | function |
| --- | --- | --- | --- | --- | --- | --- |
| STRE | Arabidopsis thaliana | 4258 | + | 5 | AGGGG |  |
| STRE | Arabidopsis thaliana | 3543 | - | 5 | AGGGG |  |
| STRE | Arabidopsis thaliana | 2404 | - | 5 | AGGGG |  |
| STRE | Arabidopsis thaliana | 1584 | - | 5 | AGGGG |  |
| STRE | Arabidopsis thaliana | 843 | + | 5 | AGGGG |  |
| STRE | Arabidopsis thaliana | 454 | + | 5 | AGGGG |  |
| STRE | Arabidopsis thaliana | 2752 | + | 5 | AGGGG |  |

>HU02G01569.1   
+ +Up\_Stream \_Len000AGCGTA GGAAGTGACG CCGCTATTGG AGGCGAAGAC GCTAGTACCA AGGCCAAAAC   
  
  
+ AAATGAGGAG ATAGATCAGT TAAACCGTTT GGTTAAGAAA ATTAAGCGAA CATCTCTTGA ACCTCCATCT   
  
  
+ GAACTCGATG ACATTGAGGA TGTGGATGCA GAAAAGATGG ATATGGGCTC TCCATCGGTG CCAGCAAATC   
  
  
+ AACCTAATGT TGGGTGGGGT CTCATTCGGT CAATGGGGGA CTAGGGACGT CATATCGTCA CCAATTACAA   
  
  
+ CGGAACAACC CTAAACCGCA TTTTAAAATC ACATCTTGGT AGCAGACAAA GATGTACGAC CCAAAAATTC   
  
  
+ AACTCTGTAA AGCACTAGCA AAGCTCGTAG TACACAGAAC AATCGCACAC TCATAGTGAT AGAAGTGTAC   
  
  
+ TATCCATTAT GCAGTGAATA TGACAAGGGA GGGGGTCCTG AGATGCCCAA ATTTGGCAGC CGAGTTGCCT   
  
  
+ TGACTGCCCA TCAATGTCCT AAACCAGTTG AGCATGTTAT TAATTTGCAA GAAAGATCGA ATTCTTCCTA   
  
  
+ TCTAAAAACT GACACAATAA AACAATACCA TGTATGGGAC ATTGCTAGGA TTGTTAACAC AATTATTGTC   
  
  
+ TTGTACGTTG CACCTGAAAA AAAAGAAAAT TGTTGCCATA GACAACATAT AAGTTAGGCA TTCATGACTA   
  
  
+ ATATCTGAAT TAGGTGTGTC GATCTGTGAT TCAAATTGAT ATAATTAGAT CTTTAAGGTA TCTAATTGCT   
  
  
+ TCAAGTTTTC TCTATAATGA AGATGTATAT TAGCTCACAT AGGTCTTAAG GTTAACTAAT GTCATATCAG   
  
  
+ GGGGTGTGGG CCGCCATTTT TGATAAGAAT TGAATTGAAC TTGCATTGTT TTTTCTTTGG CGAGAATGCA   
  
  
+ AGATTTGTTA ATTTATTCAT ATGCGTCATT ATACACTATA AGAAAAATAA TTGTAGGAAT ATATGAAGTA   
  
  
+ TTTTTACTTG TGCTCATGTT AAATGTGGAA TACTTTTTTA ATGTCGTATA GTACCACAGT ATTCTACAAT   
  
  
+ TTAAGAGATT AATCGATCCA TTAAAGTCAT GCAAACCTGA TGCATATTCA AGACATTGCA TGAAATGATC   
  
  
+ TTATTTTAGT CAAAGACTCC ATCATTTATA AGCAATATTT ACTTCGATAC ACGTGTCATT AAAAAAGACT   
  
  
+ ATAGAAATGT TACTGTTATT TTGTTTGAGC TAAATAGAAA ATTTAAAAGT TAATTAAGAT AATAATTAAA   
  
  
+ ACTAAATATT CAATGATGGC TAATAAATAC ATTGTCGTAT TAACTGACTA ATATTTGTAG ATGGTCGTGT   
  
  
+ TAATTTCTTT TTATTTTAAA GTTCAAATGA GTTACAAAAG GTTATAAAAT ATAAAGTAAA ATGGGAGACA   
  
  
+ AAGCAGACAG GGTGGGGGAA GCTAAGCTTA AAGTAGGATT TAGAAAGGCA ATGAAGGTGA ACCCTCAAAT   
  
  
+ CGATCCGCTG CCAATCACAG AAACCCAAAG CTTTGCTCAC CGACAACTCC CGGTTAACTG CAGTCACTCA   
  
  
+ CGGCACTGGG TAATACAGTA ATCGTGTACC CAGTTCATCC CCCTTTTCCT TTGGGAATAC AGTACCTCGA   
  
  
+ AAGTTCCTTT GAAATTCTAC TGGCCAGGCA TACCCATAAA TTGATCCTCT CGATATCATA AGATATGATA   
  
  
+ TTTCTCTGTC ATATCTGTTA GTTTCGGTAT CATATTTCTT CTTTATCTCT GTAATTTGAG GTATGCTTCA   
  
  
+ TTCCTGGCCG CTTGAATTAT CTTTCTTTCT TTGATTAGTA TTGTTTTTTT GGTTACCTAT TTTGCTCCAG   
  
  
+ TCCTGTTCAT TTGGGTCTTT TTGGATCTGG GTTTTTGCTG GGTTTGTTGA TTCTTTGAGA AATTTGGGTG   
  
  
+ CTTGAATTTG CCCTGCAATA TTCATGGGTT AGCGTTTTCC TGAATTTTGC TCTGTTCCTT AAGTGATTAT   
  
  
+ TTTTGATTGA TTACACCTTG GTGGTCCTGG TGGAATTTCC GAGGAGAAAT TGTCATGGGT TCCCACAACT   
  
  
+ TTGGAGAATT CCCTGATGAG ACTCTAAATG AGTATCAATC TACTTTGGGA ACCATGTCCC CCGGTTATGA   
  
  
+ TGGGTCTTTG AATTATACAA CCTTGTTCAA TTACAAAGAC CCATCTCAGG ATCTCACGGC ACTGAACCTT   
  
  
+ CCTAGCCCAT TGCCTGACCC TATGCCATTC AACATTGGTT CATATTCGGG TTTGAGCCCC GGGGTTGAAT   
  
  
+ CTTCGGATGA TAGCGATTCA GATGATGTTG TTAAGTACAT TGGCCAAGTG CTTATGGAAG AGGATATGAA   
  
  
+ GGAGAAGCCT TGTATGTTCC ATGACCCTTT AGCACTCCAA GCTGCTGAGA AACCCTTTTA TGATGTGTTG   
  
  
+ GGAAAGAAGT ATCCTCCTTC CCCTAATCAA CACCCACTTA TTGATCATTC TGTGGATAGC CCAGACAATC   
  
  
+ AGTCCTGTGG AACGAGCACA ATTAGTGATC TTAGTGGCAG TAACTGCACT TCCAGTTCAA CCAATTATAT   
  
  
+ TGATGCTGTG GCAGTCGCTG ATTCGAGTGA GAATACTAAG ACCTCTTTTG TGCAAAGTTC TCTGATTGAA   
  
  
+ TCGTTTTCTC AGTCGTCCAC CCTTCCACAG TGGTCATTTG GATCATTGGG TGCCTTGGGT GGCACAGCTT   
  
  
+ CTCAGGGTTC GAATTCAGTT ATCTCCTCCC GTGGTTTCCC TATGGCCATG AACGTTTTTA GCGAGCAGTC   
  
  
+ CATGATACAG TTTCAGAAGG GGGTGGAGGA GGCGAGCAAG TTCCTTCCAA AGAACAATAA CCTTGCGATT   
  
  
+ GACCTTGAGA GCGTCACTTT CCCAAATGAA AAGGAGGCGG CCCCCATGGT GGTGGTTAAG AAAGAGAAGG   
  
  
+ ATGAACACTC ACCTGATAGC TCGAGAGGTA GCAAGATTCA CTACCGTGAT GATGAGGACT TTGAGGATGG   
  
  
+ TAGGAGGAGT AAGCAGTCAG CTGTTTCTGT GGAGGAGGCT GAGTTGTCTG AAATGTTTGA CCGGGTTTTG   
  
  
+ CTTTGCAATC CCATGAAACA TGAAGCTCAT TGTACAAGTG GTTTGAAGTC CGAGAAGGGA ACATCCCTGC   
  
  
+ AGGCTGGCCA AGTACAGGTA CAGGAGGGTC AGAAGGCTCG TGCAAAGAAA CAGGGTAATA ATGATAATAA   
  
  
+ GAAGAATAAG AATGTGGTGG ATTTAAGGAC TCTGCTTATC CTCTGTGCGC AATCTGCTGC ATCTGATGAT   
  
  
+ CGCAGGACAG CTGATGAACT GCTGAAGCAG ATTAGGGAGC ACTCTTCTGC AGCTGGGGAT GGATCTCAAA   
  
  
+ GGTTGGCTCA TTACTTTGCT AATGCCTTGG AGGCGCGTTT AGCTGGAACT GGCTCACAGA TCTATACAGC   
  
  
+ CCTGAGTTCG AAGAGGACAA CAGCAGCTGA TATGATAAAA GCTTATCAGT TTTTCTTTCG TGCTTGCCCA   
  
  
+ TTTTTAAAGA TCTCTATTAT CTTTGCGAAC CATATGATTA TACAGAGAGC TGAAAAAGCA TCAAAGCTTC   
  
  
+ ATGTTATAGA TTTTGGCATC CTGTATGGTT TTCAGTGGCC CCTCCTCATT CAACGCCTGT CGGAGCGACC   
  
  
+ TGGTGGACCT CCAAAACTGT GCATTACTGG GATTGATCTT CCCCAACCTG GATTTAGGCC AACAGAAAGA   
  
  
+ GTTGAGGCAA CAGGGCTCCG CTTGGCAAAG TATTGTGAGC GCTTCAATGT TCCATTTGAA TACCATGCCA   
  
  
+ TTGCACAGAA ATGGGAAACC ATCAAAGCTG AAGACCTGAA GATAGAAGAT GATGAGGTGG TTGCCGTGAA   
  
  
+ CTGTCTCTTC AGGTTTAAGA ACCTGCTTGA TGAGACGATA GTGGTGGATA GTCCGAGGAA TGCAGTTCTC   
  
  
+ GGCTTGATTA GAAGGATAAA ACCTGATATC TTTGTTCACG GAGTAGTGAA CGGGTCTTAC AATGCGCCCT   
  
  
+ TCTTTGTTAC ACGTTTCAGG GAGGCCCTCT TCCATTACTC TACTGTATTC GATATGTTTG ATGCCAATGC   
  
  
+ TTCCCGGGAG GATCCTGAGA GATTGATGTT TGAGAAGGAG TTTTATGGGC GAGAAATTAT GAATGTGGTG   
  
  
+ GCTTGTGAGG GTACAGAGAG AGTTGAAAGA CCTGAGACAT ACAAGCAATG GCAGGCGCGG AATAGTAGGG   
  
  
+ CAGGGTTCAG GCAGCTGCCA TTGGACCAAG AGCTCGTCAG TAAACTGAGG AGCAAGGTCC AGATGCACTA   
  
  
+ TCCTAGGGAC TTCGTTGTTG ATGTAGATGG ACATTGGACA TTGCAGGGAT GGAAGGGGAG GATCATCTGT   
  
  
+ GCTGTGTCTG CATGGGGTCC TGCTTA  

- +Up\_Stream \_Len000TCGCAT CCTTCACTGC GGCGATAACC TCCGCTTCTG CGATCATGGT TCCGGTTTTG   
  
  
- TTTACTCCTC TATCTAGTCA ATTTGGCAAA CCAATTCTTT TAATTCGCTT GTAGAGAACT TGGAGGTAGA   
  
  
- CTTGAGCTAC TGTAACTCCT ACACCTACGT CTTTTCTACC TATACCCGAG AGGTAGCCAC GGTCGTTTAG   
  
  
- TTGGATTACA ACCCACCCCA GAGTAAGCCA GTTACCCCCT GATCCCTGCA GTATAGCAGT GGTTAATGTT   
  
  
- GCCTTGTTGG GATTTGGCGT AAAATTTTAG TGTAGAACCA TCGTCTGTTT CTACATGCTG GGTTTTTAAG   
  
  
- TTGAGACATT TCGTGATCGT TTCGAGCATC ATGTGTCTTG TTAGCGTGTG AGTATCACTA TCTTCACATG   
  
  
- ATAGGTAATA CGTCACTTAT ACTGTTCCCT CCCCCAGGAC TCTACGGGTT TAAACCGTCG GCTCAACGGA   
  
  
- ACTGACGGGT AGTTACAGGA TTTGGTCAAC TCGTACAATA ATTAAACGTT CTTTCTAGCT TAAGAAGGAT   
  
  
- AGATTTTTGA CTGTGTTATT TTGTTATGGT ACATACCCTG TAACGATCCT AACAATTGTG TTAATAACAG   
  
  
- AACATGCAAC GTGGACTTTT TTTTCTTTTA ACAACGGTAT CTGTTGTATA TTCAATCCGT AAGTACTGAT   
  
  
- TATAGACTTA ATCCACACAG CTAGACACTA AGTTTAACTA TATTAATCTA GAAATTCCAT AGATTAACGA   
  
  
- AGTTCAAAAG AGATATTACT TCTACATATA ATCGAGTGTA TCCAGAATTC CAATTGATTA CAGTATAGTC   
  
  
- CCCCACACCC GGCGGTAAAA ACTATTCTTA ACTTAACTTG AACGTAACAA AAAAGAAACC GCTCTTACGT   
  
  
- TCTAAACAAT TAAATAAGTA TACGCAGTAA TATGTGATAT TCTTTTTATT AACATCCTTA TATACTTCAT   
  
  
- AAAAATGAAC ACGAGTACAA TTTACACCTT ATGAAAAAAT TACAGCATAT CATGGTGTCA TAAGATGTTA   
  
  
- AATTCTCTAA TTAGCTAGGT AATTTCAGTA CGTTTGGACT ACGTATAAGT TCTGTAACGT ACTTTACTAG   
  
  
- AATAAAATCA GTTTCTGAGG TAGTAAATAT TCGTTATAAA TGAAGCTATG TGCACAGTAA TTTTTTCTGA   
  
  
- TATCTTTACA ATGACAATAA AACAAACTCG ATTTATCTTT TAAATTTTCA ATTAATTCTA TTATTAATTT   
  
  
- TGATTTATAA GTTACTACCG ATTATTTATG TAACAGCATA ATTGACTGAT TATAAACATC TACCAGCACA   
  
  
- ATTAAAGAAA AATAAAATTT CAAGTTTACT CAATGTTTTC CAATATTTTA TATTTCATTT TACCCTCTGT   
  
  
- TTCGTCTGTC CCACCCCCTT CGATTCGAAT TTCATCCTAA ATCTTTCCGT TACTTCCACT TGGGAGTTTA   
  
  
- GCTAGGCGAC GGTTAGTGTC TTTGGGTTTC GAAACGAGTG GCTGTTGAGG GCCAATTGAC GTCAGTGAGT   
  
  
- GCCGTGACCC ATTATGTCAT TAGCACATGG GTCAAGTAGG GGGAAAAGGA AACCCTTATG TCATGGAGCT   
  
  
- TTCAAGGAAA CTTTAAGATG ACCGGTCCGT ATGGGTATTT AACTAGGAGA GCTATAGTAT TCTATACTAT   
  
  
- AAAGAGACAG TATAGACAAT CAAAGCCATA GTATAAAGAA GAAATAGAGA CATTAAACTC CATACGAAGT   
  
  
- AAGGACCGGC GAACTTAATA GAAAGAAAGA AACTAATCAT AACAAAAAAA CCAATGGATA AAACGAGGTC   
  
  
- AGGACAAGTA AACCCAGAAA AACCTAGACC CAAAAACGAC CCAAACAACT AAGAAACTCT TTAAACCCAC   
  
  
- GAACTTAAAC GGGACGTTAT AAGTACCCAA TCGCAAAAGG ACTTAAAACG AGACAAGGAA TTCACTAATA   
  
  
- AAAACTAACT AATGTGGAAC CACCAGGACC ACCTTAAAGG CTCCTCTTTA ACAGTACCCA AGGGTGTTGA   
  
  
- AACCTCTTAA GGGACTACTC TGAGATTTAC TCATAGTTAG ATGAAACCCT TGGTACAGGG GGCCAATACT   
  
  
- ACCCAGAAAC TTAATATGTT GGAACAAGTT AATGTTTCTG GGTAGAGTCC TAGAGTGCCG TGACTTGGAA   
  
  
- GGATCGGGTA ACGGACTGGG ATACGGTAAG TTGTAACCAA GTATAAGCCC AAACTCGGGG CCCCAACTTA   
  
  
- GAAGCCTACT ATCGCTAAGT CTACTACAAC AATTCATGTA ACCGGTTCAC GAATACCTTC TCCTATACTT   
  
  
- CCTCTTCGGA ACATACAAGG TACTGGGAAA TCGTGAGGTT CGACGACTCT TTGGGAAAAT ACTACACAAC   
  
  
- CCTTTCTTCA TAGGAGGAAG GGGATTAGTT GTGGGTGAAT AACTAGTAAG ACACCTATCG GGTCTGTTAG   
  
  
- TCAGGACACC TTGCTCGTGT TAATCACTAG AATCACCGTC ATTGACGTGA AGGTCAAGTT GGTTAATATA   
  
  
- ACTACGACAC CGTCAGCGAC TAAGCTCACT CTTATGATTC TGGAGAAAAC ACGTTTCAAG AGACTAACTT   
  
  
- AGCAAAAGAG TCAGCAGGTG GGAAGGTGTC ACCAGTAAAC CTAGTAACCC ACGGAACCCA CCGTGTCGAA   
  
  
- GAGTCCCAAG CTTAAGTCAA TAGAGGAGGG CACCAAAGGG ATACCGGTAC TTGCAAAAAT CGCTCGTCAG   
  
  
- GTACTATGTC AAAGTCTTCC CCCACCTCCT CCGCTCGTTC AAGGAAGGTT TCTTGTTATT GGAACGCTAA   
  
  
- CTGGAACTCT CGCAGTGAAA GGGTTTACTT TTCCTCCGCC GGGGGTACCA CCACCAATTC TTTCTCTTCC
[truncated: 164,298 more chars]
