# Supplementary material for: Genome-Wide Identification and Expression Pattern of the GRAS Gene Family in Pitaya (Selenicereus undatus L.)
Source: Biology (Basel). 2022 Dec 21;12(1):11. doi: 10.3390/biology12010011 (PMC9854919; doi:10.3390/biology12010011)
Supplement: Supplementary file 1 [file biology-12-00011-s001.zip › Supplementary file S5/HU02G01570.1_plantcare.html]

Content-Type: text/html; charset=ISO-8859-1


PlantCARE


Webmaster Firefox specific output  
To save the result:
click on the frame with the right mouse button and save the source code as a text file with extension .html  
REFERENCE:PlantCARE: a database of plant cis-acting regulatory elements and a portal to tools for in silico analysis of promoter sequences.  
Lescot, M., Déhais, P., Moreau, Y., De Moor, B., Rouzé ,P.,and Rombauts, S.  
Nucleic Acids Res., Database issue(2002), 30(1):325-327.   


---

>HU02G01570.1   
+ +Up\_Stream \_Len000ATTATT ATTCTAATAT ATAGATAATA ATATAATAAT AATAATAATA ATAATAATAA   
  
  
+ TTATTATTAT TATTATTATT TATTAAATAT AATAGTTATT ATAATAATAA TAATTATAAT AATAATAAGA   
  
  
+ TTATTATCAT TATATTATTA TTATTATTAT TGTAATTTAT TATTATTATA GTAATTATTA TTGTTATATA   
  
  
+ GTAAAATTAT TATTAGTATT GTTATTATAA ATATTCATAC TTGCATATAA TATTTCTAAA ATAAAAACAG   
  
  
+ AGTTAATTCC ATTCCAAAAC TACTCACCAA ACAGCAAGGA GTGGGACCTG ATTCCTTTCC AATTTTCACC   
  
  
+ AAACAACTGG GTTCAGAATT CAAGTTCCAT ATCCAACCCC CCAGGATTCA AGTCCTGATT CCATTCCATT   
  
  
+ CCAGTTTCCC TCACCAAACT GGGCCTTAGA TTGATGGTTG TTCAGTTGGA TGAGGGTTGT TCCATGCCAA   
  
  
+ GTTAGATCAT TGTTGTCAAT TTTGAGAACG ACTATCTTAA TAAGCCTAAC GATGTTGATT TGATTTAGTT   
  
  
+ CAAACCATTT CAAATAATAC AAATCAAATT CTATTTAGAC CGACACTAAA ATAAATAGAT TGAGAAAAAT   
  
  
+ TATAGATTTT AAAGTTCAGA TTAAAATCCC TAAAGATCAA CAGATTGAAA TGCATATTTC ATAGTGCACA   
  
  
+ TGGACTTGCT TTGAATCTCG TCTAGAGAAC CTAAGAAAAA GCAAGAGTGG TTCAGAAGGT TAGAATTGTT   
  
  
+ CCAGCACATG ACAAGTACAA TCCCAACTTT TTTCAAAAGA TTTGCTACTT TTAATTGGAT TCCCATGTTG   
  
  
+ AATGAAGTTC TGGGTTTAAG GCTACAACTT TCTTGGGAGG ATAAGGTTCT TAAGTTAAGG TGGTCCTCTT   
  
  
+ TTCTTTTGAT CTTTCTTATG GGTACAACCT CATGGGTCTC ATCAACACAT GAACTCTTTT TATCTTTTAT   
  
  
+ CTTTCCCCTT TCCTTTATGC AGGATTGACA GGTGCATGGA AAACAAGAAG CAATTAAATA CTAGTGGCTT   
  
  
+ AGCCCCATCC CTAATATTTT GGCTATTCTG TTTCACCATC CTCCCCTGTC TCCATTTTCT TTGTTTCCTT   
  
  
+ TTTTTCTAGG AACTTGTGAT GGGCAAGGGA AGATCAGTAC AATGTGATGG TTGTTAGTAG CGAGAAGATG   
  
  
+ AAGTGTCACC GCAAAAATTT TGTGCCATAA TTTGTCGTAA GGGAAACAAG TATCAAACAA CAATGTCATT   
  
  
+ TATGATAAAC AAGTAACAGG AGATATGTAC TCTGAAAAAA TATAAGGTAC CTGAGAAGTT GTTAACTTAG   
  
  
+ CTTGAAAAAC CCTCCTAACT TGATAAACCC TCTTCGCTAT GTTATCCCGC ACCCTGCCAT AGAGTCACCG   
  
  
+ ATTCAGTATA TATGTAACAT ACCCAATCTT GTCTGGATAT ATAGTTAACC CTTTTGGAAT ATCTGAAAAT   
  
  
+ CCACACGAGA GTCATCCTTC TAGACTAGGA AACATGCGCC ACATTTTGGA TTCTAACCAA GTATAATAAG   
  
  
+ CATATCTTAA AGCCAAAAAT GCAAGGATAG ATACAAATAC CCATCACATG CATGTAAATG TCCCTTGTCA   
  
  
+ TGGCTCTGAT TCTCAAAATA TTAATTTTCA ATAAATTAAA TGAAATTGCA TTATTTTAAA GATTTCTGAA   
  
  
+ TGCATCCATT ATTGCAATTT TTTAAAGACT GCACAATGAT GACAATGTCA ATGGTTTCCT AAAGTTGCGC   
  
  
+ CTGATGGGCT ATTGACAGTG AAGCAACACT CCCTTTGTTC CCATTTATGT GTCTCCACTT GCCCCTTTGA   
  
  
+ AACTCTCTAC ATTTGTATGT CATCTGAATT TGATTCTCAA ATCTCAGATG ATTTGAATTC CCTTTATGCC   
  
  
+ ATCTTTACTA TCTATTGAAT GATTCTTTCT TTTCCACTTT CTTATCTTCC ACTACCTTCT TCTTGGATGC   
  
  
+ CCTTTTGCTG CTTATAAAGG GCGACACCTG AACAAGTTGC TGTATTCTCA AACCATGAAT CTGTACAGGT   
  
  
+ TTAGCCCCGA TATGTGTCCT AGCTTTGTGG GTCACGAAGA AATTGGTCCT AATTTAATAG GACACCAAGA   
  
  
+ CCCTTTTCAA AATGCATACA ATTGCACTGA TTTTGGTGAT GCTTACTTGT TGCAATTCCC AGTTGAAGAT   
  
  
+ TTATGTCAAT ACCGTGTTCC TGATCTAATG GGGCTACCTG TTGAAGATGG GGATCCTACA GAGGTCCCTG   
  
  
+ ACATTCCTGA TACATGCTTC AACTACATAA GCAATCTCCT AATGGAAGAG GGCTCGGATG ACCGCCCTGG   
  
  
+ CGCCACCCTG CAGGACTATA TGGCTGCTCA AGTCTCCTAC AGCAAGTCCT CCAATGCCGC CTTTCCCGGT   
  
  
+ GAGAGTTGCT CATTCTTTGG TAATAATCAA TGCCCTCCAC CTGCTTATCA AAGCAACCCT TATTGCCTTG   
  
  
+ ATGATTACTT CAACTTGCTA TCCAATACTA CTGGCAATAG TGTTAACAAT GCTGGTGTTC ATGACAGCAA   
  
  
+ CAACTTTGTT CAGAACAGTT TGATTGGGAA TAGGGGTACT AGTTCTAGTT CTAGGGGCAG AAGTGATTTT   
  
  
+ GGATCTACCC TTATTAGGAA TTTGCCATTA GGGACCGACA CACTTGATGT GGTCAATCCA GCACCTACTC   
  
  
+ CTCGAGCCCC TCTGGTTACG ACTCAGTGTC AGGTGTTTGA AACACACCAG TATGAGCAGG CCTTCGAACT   
  
  
+ GATCACTAAT GGAAGCTGGG AATTCAGTCA TTCTGTTGCT AATACATCAA ATGATGGAAT TCAAAATCAA   
  
  
+ CCAATTCAAG CAGTTTCAAC CCAAGAACGA TGGAATATGA AAACATTTTC CGATGACCAA CTTGAGCCTG   
  
  
+ CTCTTTTGCG AAGTCTTGTG ATGTCCTCAA ACTGTACTGA TATCTCTGAT CAGGGTGTAC ACAGGATAGT   
  
  
+ GAACCCTGAG CCACCCAACA ACAGAAATAC GGAGATTGTG TGCAATGACT CATCCAGTAA GAGTAAGACA   
  
  
+ AGACGAAGAG AGCATGGTGC AGGAGAAAAC CACGATGAAG GAGAAAACCA CGATGAGACA GGAAGGAGTA   
  
  
+ ACAAGCAATC AGCACCATCC AATGAAGTGT TCGATGAAAT TGAGCAGTAT GGTGATGTGC TGCTCTGCCC   
  
  
+ TGAAGGAAGA AGTAAACCAG CAGGGTTATG CAGCAGCAGC AACGCTAACA GAACTAGCAA CTCACAGAAC   
  
  
+ TCGGAAGACT CAAAGTCACA GCAAAATGGG AGTTCCTCCA AGGTGTCAAA AAAGCGAGGC AATAAAAAGC   
  
  
+ AAGTGGTTGA TTTGAGGACT CTCCTAACTC GATGTGCACA ATGTGTTGCT CGTGTGGATT TAACTGGTTC   
  
  
+ TTACCAGTTG CTTCAGGAGA TTCGGCAGCA TTCTTCTCCC TATGGTGATT ATCTCCAAAG AATGGCTCAT   
  
  
+ TACCTTGCTA ATGGCCTTGA GGCGCGCTTA GAAGGCAAAG GATATGAACT AACTCGTGCC AATGAGCATA   
  
  
+ TCCCACCTAC TGAGATCCTC AAGGCTAACC GGGTATATGT GGCAGCGGTC CCTTTCAAGA TAATGTCTTA   
  
  
+ TTACACAACA AACAAGACAA TTGCAAGCCT AGTTGAGGAG GCACCAAGTA TACACATAAT TGATTTTGGT   
  
  
+ ATTTTCTATG GACTACAATG GCCCTGCATC ATTCAGAACC TCTCCAAGAG GCCCAATGGG CCTCCCAGAA   
  
  
+ TCCGCATCAC AGGCATCGAT TTCCCCCAAC CTGGGTTCCG GCCAGCAGAG AGGGTTGAAG AGACAGGGCG   
  
  
+ GTGTTTAGCC AAGTATTGTG ACAGGTACAA TGTGCCCTTT GAATACCACC CCATAGCCAA GAAGTGGGAG   
  
  
+ ACCATCCAAC TCGAGGAGCT CAAAATTGAA AGGAATGAGC CCCTTGTAGT GAACTGTCTG TATAGATCGC   
  
  
+ ACAACCTATT TGATGAAAGC GTAGAGGAAA ACAGCCCAAG AGACGCGTTC CTGAGCTTGG TTAGGAAAAT   
  
  
+ AAATCCCGAT ATATTCATGC ATGGGGTGGT GAATACCGCA TCAAGTGTAC CTTTCTTCCT GAACAGGTTC   
  
  
+ AAGGAGGCCA TGTTTCACTA CACGGCCTTG TTTGACTTGT TCGAGGCCAC AATGAGCCGC GAGGATCAAG   
  
  
+ AGAGGGTGTT ACTAGAGAGC AAGTTGCATG GGAATCAGGC GTTGAATGTG ATCGCCTGCG AAGGAGCGGA   
  
  
+ GAGAGTGGAG AGGCCTGAGT CATACAAACA ATGGCAGGTT AGGACTCAAA GGGCCGGGTT TGTTCAAGTG   
  
  
+ TCGATGGATC GCGAGCTTTT GAGAAGGGCA AGGGCCATGG TCAGGAGGAA TTTTAGGGAA GAGTTCAGTT   
  
  
+ ATGATGAAGA TGGGCATTGG GTGGTTCAAG GATGGAAAGG CAGGATAATG TATGCCATTT CTTGTTGGAA   
  
  
+ ACCTTCATA  

- +Up\_Stream \_Len000TAATAA TAAGATTATA TATCTATTAT TATATTATTA TTATTATTAT TATTATTATT   
  
  
- AATAATAATA ATAATAATAA ATAATTTATA TTATCAATAA TATTATTATT ATTAATATTA TTATTATTCT   
  
  
- AATAATAGTA ATATAATAAT AATAATAATA ACATTAAATA ATAATAATAT CATTAATAAT AACAATATAT   
  
  
- CATTTTAATA ATAATCATAA CAATAATATT TATAAGTATG AACGTATATT ATAAAGATTT TATTTTTGTC   
  
  
- TCAATTAAGG TAAGGTTTTG ATGAGTGGTT TGTCGTTCCT CACCCTGGAC TAAGGAAAGG TTAAAAGTGG   
  
  
- TTTGTTGACC CAAGTCTTAA GTTCAAGGTA TAGGTTGGGG GGTCCTAAGT TCAGGACTAA GGTAAGGTAA   
  
  
- GGTCAAAGGG AGTGGTTTGA CCCGGAATCT AACTACCAAC AAGTCAACCT ACTCCCAACA AGGTACGGTT   
  
  
- CAATCTAGTA ACAACAGTTA AAACTCTTGC TGATAGAATT ATTCGGATTG CTACAACTAA ACTAAATCAA   
  
  
- GTTTGGTAAA GTTTATTATG TTTAGTTTAA GATAAATCTG GCTGTGATTT TATTTATCTA ACTCTTTTTA   
  
  
- ATATCTAAAA TTTCAAGTCT AATTTTAGGG ATTTCTAGTT GTCTAACTTT ACGTATAAAG TATCACGTGT   
  
  
- ACCTGAACGA AACTTAGAGC AGATCTCTTG GATTCTTTTT CGTTCTCACC AAGTCTTCCA ATCTTAACAA   
  
  
- GGTCGTGTAC TGTTCATGTT AGGGTTGAAA AAAGTTTTCT AAACGATGAA AATTAACCTA AGGGTACAAC   
  
  
- TTACTTCAAG ACCCAAATTC CGATGTTGAA AGAACCCTCC TATTCCAAGA ATTCAATTCC ACCAGGAGAA   
  
  
- AAGAAAACTA GAAAGAATAC CCATGTTGGA GTACCCAGAG TAGTTGTGTA CTTGAGAAAA ATAGAAAATA   
  
  
- GAAAGGGGAA AGGAAATACG TCCTAACTGT CCACGTACCT TTTGTTCTTC GTTAATTTAT GATCACCGAA   
  
  
- TCGGGGTAGG GATTATAAAA CCGATAAGAC AAAGTGGTAG GAGGGGACAG AGGTAAAAGA AACAAAGGAA   
  
  
- AAAAAGATCC TTGAACACTA CCCGTTCCCT TCTAGTCATG TTACACTACC AACAATCATC GCTCTTCTAC   
  
  
- TTCACAGTGG CGTTTTTAAA ACACGGTATT AAACAGCATT CCCTTTGTTC ATAGTTTGTT GTTACAGTAA   
  
  
- ATACTATTTG TTCATTGTCC TCTATACATG AGACTTTTTT ATATTCCATG GACTCTTCAA CAATTGAATC   
  
  
- GAACTTTTTG GGAGGATTGA ACTATTTGGG AGAAGCGATA CAATAGGGCG TGGGACGGTA TCTCAGTGGC   
  
  
- TAAGTCATAT ATACATTGTA TGGGTTAGAA CAGACCTATA TATCAATTGG GAAAACCTTA TAGACTTTTA   
  
  
- GGTGTGCTCT CAGTAGGAAG ATCTGATCCT TTGTACGCGG TGTAAAACCT AAGATTGGTT CATATTATTC   
  
  
- GTATAGAATT TCGGTTTTTA CGTTCCTATC TATGTTTATG GGTAGTGTAC GTACATTTAC AGGGAACAGT   
  
  
- ACCGAGACTA AGAGTTTTAT AATTAAAAGT TATTTAATTT ACTTTAACGT AATAAAATTT CTAAAGACTT   
  
  
- ACGTAGGTAA TAACGTTAAA AAATTTCTGA CGTGTTACTA CTGTTACAGT TACCAAAGGA TTTCAACGCG   
  
  
- GACTACCCGA TAACTGTCAC TTCGTTGTGA GGGAAACAAG GGTAAATACA CAGAGGTGAA CGGGGAAACT   
  
  
- TTGAGAGATG TAAACATACA GTAGACTTAA ACTAAGAGTT TAGAGTCTAC TAAACTTAAG GGAAATACGG   
  
  
- TAGAAATGAT AGATAACTTA CTAAGAAAGA AAAGGTGAAA GAATAGAAGG TGATGGAAGA AGAACCTACG   
  
  
- GGAAAACGAC GAATATTTCC CGCTGTGGAC TTGTTCAACG ACATAAGAGT TTGGTACTTA GACATGTCCA   
  
  
- AATCGGGGCT ATACACAGGA TCGAAACACC CAGTGCTTCT TTAACCAGGA TTAAATTATC CTGTGGTTCT   
  
  
- GGGAAAAGTT TTACGTATGT TAACGTGACT AAAACCACTA CGAATGAACA ACGTTAAGGG TCAACTTCTA   
  
  
- AATACAGTTA TGGCACAAGG ACTAGATTAC CCCGATGGAC AACTTCTACC CCTAGGATGT CTCCAGGGAC   
  
  
- TGTAAGGACT ATGTACGAAG TTGATGTATT CGTTAGAGGA TTACCTTCTC CCGAGCCTAC TGGCGGGACC   
  
  
- GCGGTGGGAC GTCCTGATAT ACCGACGAGT TCAGAGGATG TCGTTCAGGA GGTTACGGCG GAAAGGGCCA   
  
  
- CTCTCAACGA GTAAGAAACC ATTATTAGTT ACGGGAGGTG GACGAATAGT TTCGTTGGGA ATAACGGAAC   
  
  
- TACTAATGAA GTTGAACGAT AGGTTATGAT GACCGTTATC ACAATTGTTA CGACCACAAG TACTGTCGTT   
  
  
- GTTGAAACAA GTCTTGTCAA ACTAACCCTT ATCCCCATGA TCAAGATCAA GATCCCCGTC TTCACTAAAA   
  
  
- CCTAGATGGG AATAATCCTT AAACGGTAAT CCCTGGCTGT GTGAACTACA CCAGTTAGGT CGTGGATGAG   
  
  
- GAGCTCGGGG AGACCAATGC TGAGTCACAG TCCACAAACT TTGTGTGGTC ATACTCGTCC GGAAGCTTGA   
  
  
- CTAGTGATTA CCTTCGACCC TTAAGTCAGT AAGACAACGA TTATGTAGTT TACTACCTTA AGTTTTAGTT   
  
  
- GGTTAAGTTC GTCAAAGTTG GGTTCTTGCT ACCTTATACT TTTGTAAAAG GCTACTGGTT GAACTCGGAC   
  
  
- GAGAAAACGC TTCAGAACAC TACAGGAGTT TGACATGACT ATAGAGACTA GTCCCACATG TGTCCTATCA   
  
  
- CTTGGGACTC GGTGGGTTGT TGTCTTTATG CCTCTAACAC ACGTTACTGA GTAGGTCATT CTCATTCTGT   
  
  
- TCTGCTTCTC TCGTACCACG TCCTCTTTTG GTGCTACTTC CTCTTTTGGT GCTACTCTGT CCTTCCTCAT   
  
  
- TGTTCGTTAG TCGTGGTAGG TTACTTCACA AGCTACTTTA ACTCGTCATA CCACTACACG ACGAGACGGG   
  
  
- ACTTCCTTCT TCATTTGGTC GTCCCAATAC GTCGTCGTCG TTGCGATTGT CTTGATCGTT GAGTGTCTTG   
  
  
- AGCCTTCTGA GTTTCAGTGT CGTTTTACCC TCAAGGAGGT TCCACAGTTT TTTCGCTCCG TTATTTTTCG   
  
  
- TTCACCAACT AAACTCCTGA GAGGATTGAG CTACACGTGT TACACAACGA GCACACCTAA ATTGACCAAG   
  
  
- AATGGTCAAC GAAGTCCTCT AAGCCGTCGT AAGAAGAGGG ATACCACTAA TAGAGGTTTC TTACCGAGTA   
  
  
- ATGGAACGAT TACCGGAACT CCGCGCGAAT CTTCCGTTTC CTATACTTGA TTGAGCACGG TTACTCGTAT   
  
  
- AGGGTGGATG ACTCTAGGAG TTCCGATTGG CCCATATACA CCGTCGCCAG GGAAAGTTCT ATTACAGAAT   
  
  
- AATGTGTTGT TTGTTCTGTT AACGTTCGGA TCAACTCCTC CGTGGTTCAT ATGTGTATTA ACTAAAACCA   
  
  
- TAAAAGATAC CTGATGTTAC CGGGACGTAG TAAGTCTTGG AGAGGTTCTC CGGGTTACCC GGAGGGTCTT   
  
  
- AGGCGTAGTG TCCGTAGCTA AAGGGGGTTG GACCCAAGGC CGGTCGTCTC TCCCAACTTC TCTGTCCCGC   
  
  
- CACAAATCGG TTCATAACAC TGTCCATGTT ACACGGGAAA CTTATGGTGG GGTATCGGTT CTTCACCCTC   
  
  
- TGGTAGGTTG AGCTCCTCGA GTTTTAACTT TCCTTACTCG GGGAACATCA CTTGACAGAC ATATCTAGCG   
  
  
- TGTTGGATAA ACTACTTTCG CATCTCCTTT TGTCGGGTTC TCTGCGCAAG GACTCGAACC AATCCTTTTA   
  
  
- TTTAGGGCTA TATAAGTACG TACCCCACCA CTTATGGCGT AGTTCACATG GAAAGAAGGA CTTGTCCAAG   
  
  
- TTCCTCCGGT ACAAAGTGAT GTGCCGGAAC AAACTGAACA AGCTCCGGTG TTACTCGGCG CTCCTAGTTC   
  
  
- TCTCCCACAA TGATCTCTCG TTCAACGTAC CCTTAGTCCG CAACTTACAC TAGCGGACGC TTCCTCGCCT   
  
  
- CTCTCACCTC TCCGGACTCA GTATGTTTGT TACCGTCCAA TCCTGAGTTT CCCGGCCCAA ACAAGTTCAC   
  
  
- AGCTACCTAG CGCTCGAAAA CTCTTCCCGT TCCCGGTACC AGTCCTCCTT AAAATCCCTT CTCAAGTCAA   
  
  
- TACTACTTCT ACCCGTAACC CACCAAGTTC CTACCTTTCC GTCCTATTAC ATACGGTAAA GAACAACCTT   
  
  
- TGGAAGTAT

  
  
Motifs Found  

+   

| Site Name | Organism | Position | Strand | Matrix score. | sequence | function |
| --- | --- | --- | --- | --- | --- | --- |
|  | organism | 4350 | - | 4 | motif\_sequence | short\_function |
|  | organism | 4332 | - | 4 | motif\_sequence | short\_function |
|  | organism | 4211 | - | 4 | motif\_sequence | short\_function |
|  | organism | 4202 | - | 4 | motif\_sequence | short\_function |
|  | organism | 3945 | - | 4 | motif\_sequence | short\_function |
|  | organism | 4055 | - | 4 | motif\_sequence | short\_function |
|  | organism | 3814 | + | 4 | motif\_sequence | short\_function |
|  | organism | 3771 | - | 4 | motif\_sequence | short\_function |
|  | organism | 3759 | - | 4 | motif\_sequence | short\_function |
|  | organism | 3684 | + | 4 | motif\_sequence | short\_function |
|  | organism | 3380 | - | 4 | motif\_sequence | short\_function |
|  | organism | 3415 | + | 4 | motif\_sequence | short\_function |
|  | organism | 3313 | + | 4 | motif\_sequence | short\_function |
|  | organism | 3326 | + | 4 | motif\_sequence | short\_function |
|  | organism | 3139 | + | 4 | motif\_sequence | short\_function |
|  | organism | 3147 | + | 4 | motif\_sequence | short\_function |
|  | organism | 3019 | - | 4 | motif\_sequence | short\_function |
|  | organism | 2975 | - | 4 | motif\_sequence | short\_function |
|  | organism | 2906 | + | 4 | motif\_sequence | short\_function |
|  | organism | 1602 | + | 4 | motif\_sequence | short\_function |
|  | organism | 1153 | - | 4 | motif\_sequence | short\_function |
|  | organism | 1289 | + | 4 | motif\_sequence | short\_function |
|  | organism | 2895 | + | 4 | motif\_sequence | short\_function |
|  | organism | 1364 | + | 4 | motif\_sequence | short\_function |
|  | organism | 2278 | + | 4 | motif\_sequence | short\_function |
|  | organism | 1938 | + | 4 | motif\_sequence | short\_function |
|  | organism | 2289 | - | 4 | motif\_sequence | short\_function |
|  | organism | 1829 | + | 4 | motif\_sequence | short\_function |
|  | organism | 2537 | - | 4 | motif\_sequence | short\_function |
|  | organism | 1283 | - | 4 | motif\_sequence | short\_function |
|  | organism | 778 | - | 4 | motif\_sequence | short\_function |
|  | organism | 2597 | + | 4 | motif\_sequence | short\_function |
|  | organism | 2169 | - | 4 | motif\_sequence | short\_function |
|  | organism | 2217 | - | 4 | motif\_sequence | short\_function |
|  | organism | 2025 | + | 4 | motif\_sequence | short\_function |
|  | organism | 2027 | - | 4 | motif\_sequence | short\_function |
|  | organism | 700 | - | 4 | motif\_sequence | short\_function |
|  | organism | 1188 | - | 4 | motif\_sequence | short\_function |

>HU02G01570.1   
+ +Up\_Stream \_Len000ATTATT ATTCTAATAT ATAGATAATA ATATAATAAT AATAATAATA ATAATAATAA   
  
  
+ TTATTATTAT TATTATTATT TATTAAATAT AATAGTTATT ATAATAATAA TAATTATAAT AATAATAAGA   
  
  
+ TTATTATCAT TATATTATTA TTATTATTAT TGTAATTTAT TATTATTATA GTAATTATTA TTGTTATATA   
  
  
+ GTAAAATTAT TATTAGTATT GTTATTATAA ATATTCATAC TTGCATATAA TATTTCTAAA ATAAAAACAG   
  
  
+ AGTTAATTCC ATTCCAAAAC TACTCACCAA ACAGCAAGGA GTGGGACCTG ATTCCTTTCC AATTTTCACC   
  
  
+ AAACAACTGG GTTCAGAATT CAAGTTCCAT ATCCAACCCC CCAGGATTCA AGTCCTGATT CCATTCCATT   
  
  
+ CCAGTTTCCC TCACCAAACT GGGCCTTAGA TTGATGGTTG TTCAGTTGGA TGAGGGTTGT TCCATGCCAA   
  
  
+ GTTAGATCAT TGTTGTCAAT TTTGAGAACG ACTATCTTAA TAAGCCTAAC GATGTTGATT TGATTTAGTT   
  
  
+ CAAACCATTT CAAATAATAC AAATCAAATT CTATTTAGAC CGACACTAAA ATAAATAGAT TGAGAAAAAT   
  
  
+ TATAGATTTT AAAGTTCAGA TTAAAATCCC TAAAGATCAA CAGATTGAAA TGCATATTTC ATAGTGCACA   
  
  
+ TGGACTTGCT TTGAATCTCG TCTAGAGAAC CTAAGAAAAA GCAAGAGTGG TTCAGAAGGT TAGAATTGTT   
  
  
+ CCAGCACATG ACAAGTACAA TCCCAACTTT TTTCAAAAGA TTTGCTACTT TTAATTGGAT TCCCATGTTG   
  
  
+ AATGAAGTTC TGGGTTTAAG GCTACAACTT TCTTGGGAGG ATAAGGTTCT TAAGTTAAGG TGGTCCTCTT   
  
  
+ TTCTTTTGAT CTTTCTTATG GGTACAACCT CATGGGTCTC ATCAACACAT GAACTCTTTT TATCTTTTAT   
  
  
+ CTTTCCCCTT TCCTTTATGC AGGATTGACA GGTGCATGGA AAACAAGAAG CAATTAAATA CTAGTGGCTT   
  
  
+ AGCCCCATCC CTAATATTTT GGCTATTCTG TTTCACCATC CTCCCCTGTC TCCATTTTCT TTGTTTCCTT   
  
  
+ TTTTTCTAGG AACTTGTGAT GGGCAAGGGA AGATCAGTAC AATGTGATGG TTGTTAGTAG CGAGAAGATG   
  
  
+ AAGTGTCACC GCAAAAATTT TGTGCCATAA TTTGTCGTAA GGGAAACAAG TATCAAACAA CAATGTCATT   
  
  
+ TATGATAAAC AAGTAACAGG AGATATGTAC TCTGAAAAAA TATAAGGTAC CTGAGAAGTT GTTAACTTAG   
  
  
+ CTTGAAAAAC CCTCCTAACT TGATAAACCC TCTTCGCTAT GTTATCCCGC ACCCTGCCAT AGAGTCACCG   
  
  
+ ATTCAGTATA TATGTAACAT ACCCAATCTT GTCTGGATAT ATAGTTAACC CTTTTGGAAT ATCTGAAAAT   
  
  
+ CCACACGAGA GTCATCCTTC TAGACTAGGA AACATGCGCC ACATTTTGGA TTCTAACCAA GTATAATAAG   
  
  
+ CATATCTTAA AGCCAAAAAT GCAAGGATAG ATACAAATAC CCATCACATG CATGTAAATG TCCCTTGTCA   
  
  
+ TGGCTCTGAT TCTCAAAATA TTAATTTTCA ATAAATTAAA TGAAATTGCA TTATTTTAAA GATTTCTGAA   
  
  
+ TGCATCCATT ATTGCAATTT TTTAAAGACT GCACAATGAT GACAATGTCA ATGGTTTCCT AAAGTTGCGC   
  
  
+ CTGATGGGCT ATTGACAGTG AAGCAACACT CCCTTTGTTC CCATTTATGT GTCTCCACTT GCCCCTTTGA   
  
  
+ AACTCTCTAC ATTTGTATGT CATCTGAATT TGATTCTCAA ATCTCAGATG ATTTGAATTC CCTTTATGCC   
  
  
+ ATCTTTACTA TCTATTGAAT GATTCTTTCT TTTCCACTTT CTTATCTTCC ACTACCTTCT TCTTGGATGC   
  
  
+ CCTTTTGCTG CTTATAAAGG GCGACACCTG AACAAGTTGC TGTATTCTCA AACCATGAAT CTGTACAGGT   
  
  
+ TTAGCCCCGA TATGTGTCCT AGCTTTGTGG GTCACGAAGA AATTGGTCCT AATTTAATAG GACACCAAGA   
  
  
+ CCCTTTTCAA AATGCATACA ATTGCACTGA TTTTGGTGAT GCTTACTTGT TGCAATTCCC AGTTGAAGAT   
  
  
+ TTATGTCAAT ACCGTGTTCC TGATCTAATG GGGCTACCTG TTGAAGATGG GGATCCTACA GAGGTCCCTG   
  
  
+ ACATTCCTGA TACATGCTTC AACTACATAA GCAATCTCCT AATGGAAGAG GGCTCGGATG ACCGCCCTGG   
  
  
+ CGCCACCCTG CAGGACTATA TGGCTGCTCA AGTCTCCTAC AGCAAGTCCT CCAATGCCGC CTTTCCCGGT   
  
  
+ GAGAGTTGCT CATTCTTTGG TAATAATCAA TGCCCTCCAC CTGCTTATCA AAGCAACCCT TATTGCCTTG   
  
  
+ ATGATTACTT CAACTTGCTA TCCAATACTA CTGGCAATAG TGTTAACAAT GCTGGTGTTC ATGACAGCAA   
  
  
+ CAACTTTGTT CAGAACAGTT TGATTGGGAA TAGGGGTACT AGTTCTAGTT CTAGGGGCAG AAGTGATTTT   
  
  
+ GGATCTACCC TTATTAGGAA TTTGCCATTA GGGACCGACA CACTTGATGT GGTCAATCCA GCACCTACTC   
  
  
+ CTCGAGCCCC TCTGGTTACG ACTCAGTGTC AGGTGTTTGA AACACACCAG TATGAGCAGG CCTTCGAACT   
  
  
+ GATCACTAAT GGAAGCTGGG AATTCAGTCA TTCTGTTGCT AATACATCAA ATGATGGAAT TCAAAATCAA   
  
  
+ CCAATTCAAG CAGTTTCAAC CCAAGAACGA TGGAATATGA AAACATTTTC CGATGACCAA CTTGAGCCTG   
  
  
+ CTCTTTTGCG AAGTCTTGTG ATGTCCTCAA ACTGTACTGA TATCTCTGAT CAGGGTGTAC ACAGGATAGT   
  
  
+ GAACCCTGAG CCACCCAACA ACAGAAATAC GGAGATTGTG TGCAATGACT CATCCAGTAA GAGTAAGACA   
  
  
+ AGACGAAGAG AGCATGGTGC AGGAGAAAAC CACGATGAAG GAGAAAACCA CGATGAGACA GGAAGGAGTA   
  
  
+ ACAAGCAATC AGCACCATCC AATGAAGTGT TCGATGAAAT TGAGCAGTAT GGTGATGTGC TGCTCTGCCC   
  
  
+ TGAAGGAAGA AGTAAACCAG CAGGGTTATG CAGCAGCAGC AACGCTAACA GAACTAGCAA CTCACAGAAC   
  
  
+ TCGGAAGACT CAAAGTCACA GCAAAATGGG AGTTCCTCCA AGGTGTCAAA AAAGCGAGGC AATAAAAAGC   
  
  
+ AAGTGGTTGA TTTGAGGACT CTCCTAACTC GATGTGCACA ATGTGTTGCT CGTGTGGATT TAACTGGTTC   
  
  
+ TTACCAGTTG CTTCAGGAGA TTCGGCAGCA TTCTTCTCCC TATGGTGATT ATCTCCAAAG AATGGCTCAT   
  
  
+ TACCTTGCTA ATGGCCTTGA GGCGCGCTTA GAAGGCAAAG GATATGAACT AACTCGTGCC AATGAGCATA   
  
  
+ TCCCACCTAC TGAGATCCTC AAGGCTAACC GGGTATATGT GGCAGCGGTC CCTTTCAAGA TAATGTCTTA   
  
  
+ TTACACAACA AACAAGACAA TTGCAAGCCT AGTTGAGGAG GCACCAAGTA TACACATAAT TGATTTTGGT   
  
  
+ ATTTTCTATG GACTACAATG GCCCTGCATC ATTCAGAACC TCTCCAAGAG GCCCAATGGG CCTCCCAGAA   
  
  
+ TCCGCATCAC AGGCATCGAT TTCCCCCAAC CTGGGTTCCG GCCAGCAGAG AGGGTTGAAG AGACAGGGCG   
  
  
+ GTGTTTAGCC AAGTATTGTG ACAGGTACAA TGTGCCCTTT GAATACCACC CCATAGCCAA GAAGTGGGAG   
  
  
+ ACCATCCAAC TCGAGGAGCT CAAAATTGAA AGGAATGAGC CCCTTGTAGT GAACTGTCTG TATAGATCGC   
  
  
+ ACAACCTATT TGATGAAAGC GTAGAGGAAA ACAGCCCAAG AGACGCGTTC CTGAGCTTGG TTAGGAAAAT   
  
  
+ AAATCCCGAT ATATTCATGC ATGGGGTGGT GAATACCGCA TCAAGTGTAC CTTTCTTCCT GAACAGGTTC   
  
  
+ AAGGAGGCCA TGTTTCACTA CACGGCCTTG TTTGACTTGT TCGAGGCCAC AATGAGCCGC GAGGATCAAG   
  
  
+ AGAGGGTGTT ACTAGAGAGC AAGTTGCATG GGAATCAGGC GTTGAATGTG ATCGCCTGCG AAGGAGCGGA   
  
  
+ GAGAGTGGAG AGGCCTGAGT CATACAAACA ATGGCAGGTT AGGACTCAAA GGGCCGGGTT TGTTCAAGTG   
  
  
+ TCGATGGATC GCGAGCTTTT GAGAAGGGCA AGGGCCATGG TCAGGAGGAA TTTTAGGGAA GAGTTCAGTT   
  
  
+ ATGATGAAGA TGGGCATTGG GTGGTTCAAG GATGGAAAGG CAGGATAATG TATGCCATTT CTTGTTGGAA   
  
  
+ ACCTTCATA  

- +Up\_Stream \_Len000TAATAA TAAGATTATA TATCTATTAT TATATTATTA TTATTATTAT TATTATTATT   
  
  
- AATAATAATA ATAATAATAA ATAATTTATA TTATCAATAA TATTATTATT ATTAATATTA TTATTATTCT   
  
  
- AATAATAGTA ATATAATAAT AATAATAATA ACATTAAATA ATAATAATAT CATTAATAAT AACAATATAT   
  
  
- CATTTTAATA ATAATCATAA CAATAATATT TATAAGTATG AACGTATATT ATAAAGATTT TATTTTTGTC   
  
  
- TCAATTAAGG TAAGGTTTTG ATGAGTGGTT TGTCGTTCCT CACCCTGGAC TAAGGAAAGG TTAAAAGTGG   
  
  
- TTTGTTGACC CAAGTCTTAA GTTCAAGGTA TAGGTTGGGG GGTCCTAAGT TCAGGACTAA GGTAAGGTAA   
  
  
- GGTCAAAGGG AGTGGTTTGA CCCGGAATCT AACTACCAAC AAGTCAACCT ACTCCCAACA AGGTACGGTT   
  
  
- CAATCTAGTA ACAACAGTTA AAACTCTTGC TGATAGAATT ATTCGGATTG CTACAACTAA ACTAAATCAA   
  
  
- GTTTGGTAAA GTTTATTATG TTTAGTTTAA GATAAATCTG GCTGTGATTT TATTTATCTA ACTCTTTTTA   
  
  
- ATATCTAAAA TTTCAAGTCT AATTTTAGGG ATTTCTAGTT GTCTAACTTT ACGTATAAAG TATCACGTGT   
  
  
- ACCTGAACGA AACTTAGAGC AGATCTCTTG GATTCTTTTT CGTTCTCACC AAGTCTTCCA ATCTTAACAA   
  
  
- GGTCGTGTAC TGTTCATGTT AGGGTTGAAA AAAGTTTTCT AAACGATGAA AATTAACCTA AGGGTACAAC   
  
  
- TTACTTCAAG ACCCAAATTC CGATGTTGAA AGAACCCTCC TATTCCAAGA ATTCAATTCC ACCAGGAGAA   
  
  
- AAGAAAACTA GAAAGAATAC CCATGTTGGA GTACCCAGAG TAGTTGTGTA CTTGAGAAAA ATAGAAAATA   
  
  
- GAAAGGGGAA AGGAAATACG TCCTAACTGT CCACGTACCT TTTGTTCTTC GTTAATTTAT GATCACCGAA   
  
  
- TCGGGGTAGG GATTATAAAA CCGATAAGAC AAAGTGGTAG GAGGGGACAG AGGTAAAAGA AACAAAGGAA   
  
  
- AAAAAGATCC TTGAACACTA CCCGTTCCCT TCTAGTCATG TTACACTACC AACAATCATC GCTCTTCTAC   
  
  
- TTCACAGTGG CGTTTTTAAA ACACGGTATT AAACAGCATT CCCTTTGTTC ATAGTTTGTT GTTACAGTAA   
  
  
- ATACTATTTG TTCATTGTCC TCTATACATG AGACTTTTTT ATATTCCATG GACTCTTCAA CAATTGAATC   
  
  
- GAACTTTTTG GGAGGATTGA ACTATTTGGG AGAAGCGATA CAATAGGGCG TGGGACGGTA TCTCAGTGGC   
  
  
- TAAGTCATAT ATACATTGTA TGGGTTAGAA CAGACCTATA TATCAATTGG GAAAACCTTA TAGACTTTTA   
  
  
- GGTGTGCTCT CAGTAGGAAG ATCTGATCCT TTGTACGCGG TGTAAAACCT AAGATTGGTT CATATTATTC   
  
  
- GTATAGAATT TCGGTTTTTA CGTTCCTATC TATGTTTATG GGTAGTGTAC GTACATTTAC AGGGAACAGT   
  
  
- ACCGAGACTA AGAGTTTTAT AATTAAAAGT TATTTAATTT ACTTTAACGT AATAAAATTT CTAAAGACTT   
  
  
- ACGTAGGTAA TAACGTTAAA AAATTTCTGA CGTGTTACTA CTGTTACAGT TACCAAAGGA TTTCAACGCG   
  
  
- GACTACCCGA TAACTGTCAC TTCGTTGTGA GGGAAACAAG GGTAAATACA CAGAGGTGAA CGGGGAAACT   
  
  
- TTGAGAGATG TAAACATACA GTAGACTTAA ACTAAGAGTT TAGAGTCTAC TAAACTTAAG GGAAATACGG   
  
  
- TAGAAATGAT AGATAACTTA CTAAGAAAGA AAAGGTGAAA GAATAGAAGG TGATGGAAGA AGAACCTACG   
  
  
- GGAAAACGAC GAATATTTCC CGCTGTGGAC TTGTTCAACG ACATAAGAGT TTGGTACTTA GACATGTCCA   
  
  
- AATCGGGGCT ATACACAGGA TCGAAACACC CAGTGCTTCT TTAACCAGGA TTAAATTATC CTGTGGTTCT   
  
  
- GGGAAAAGTT TTACGTATGT TAACGTGACT AAAACCACTA CGAATGAACA ACGTTAAGGG TCAACTTCTA   
  
  
- AATACAGTTA TGGCACAAGG ACTAGATTAC CCCGATGGAC AACTTCTACC CCTAGGATGT CTCCAGGGAC   
  
  
- TGTAAGGACT ATGTACGAAG TTGATGTATT CGTTAGAGGA TTACCTTCTC CCGAGCCTAC TGGCGGGACC   
  
  
- GCGGTGGGAC GTCCTGATAT ACCGACGAGT TCAGAGGATG TCGTTCAGGA GGTTACGGCG GAAAGGGCCA   
  
  
- CTCTCAACGA GTAAGAAACC ATTATTAGTT ACGGGAGGTG GACGAATAGT TTCGTTGGGA ATAACGGAAC   
  
  
- TACTAATGAA GTTGAACGAT AGGTTATGAT GACCGTTATC ACAATTGTTA CGACCACAAG TACTGTCGTT   
  
  
- GTTGAAACAA GTCTTGTCAA ACTAACCCTT ATCCCCATGA TCAAGATCAA GATCCCCGTC TTCACTAAAA   
  
  
- CCTAGATGGG AATAATCCTT AAACGGTAAT CCCTGGCTGT GTGAACTACA CCAGTTAGGT CGTGGATGAG   
  
  
- GAGCTCGGGG AGACCAATGC TGAGTCACAG TCCACAAACT TTGTGTGGTC ATACTCGTCC GGAAGCTTGA   
  
  
- CTAGTGATTA CCTTCGACCC TTAAGTCAGT AAGACAACGA TTATGTAGTT TACTACCTTA AGTTTTAGTT   
  
  
- GGTTAAGTTC GTCAAAGTTG GGTTCTTGCT ACCTTATACT TTTGTAAAAG GCTACTGGTT GAACTCGGAC   
  
  
- GAGAAAACGC TTCAGAACAC TACAGGAGTT TGACATGACT ATAGAGACTA GTCCCACATG TGTCCTATCA   
  
  
- CTTGGGACTC GGTGGGTTGT TGTCTTTATG CCTCTAACAC ACGTTACTGA GTAGGTCATT CTCATTCTGT   
  
  
- TCTGCTTCTC TCGTACCACG TCCTCTTTTG GTGCTACTTC CTCTTTTGGT GCTACTCTGT CCTTCCTCAT   
  
  
- TGTTCGTTAG TCGTGGTAGG TTACTTCACA AGCTACTTTA ACTCGTCATA CCACTACACG ACGAGACGGG   
  
  
- ACTTCCTTCT TCATTTGGTC GTCCCAATAC GTCGTCGTCG TTGCGATTGT CTTGATCGTT GAGTGTCTTG   
  
  
- AGCCTTCTGA GTTTCAGTGT CGTTTTACCC TCAAGGAGGT TCCACAGTTT TTTCGCTCCG TTATTTTTCG   
  
  
- TTCACCAACT AAACTCCTGA GAGGATTGAG CTACACGTGT TACACAACGA GCACACCTAA ATTGACCAAG   
  
  
- AATGGTCAAC GAAGTCCTCT AAGCCGTCGT AAGAAGAGGG ATACCACTAA TAGAGGTTTC TTACCGAGTA   
  
  
- ATGGAACGAT TACCGGAACT CCGCGCGAAT CTTCCGTTTC CTATACTTGA TTGAGCACGG TTACTCGTAT   
  
  
- AGGGTGGATG ACTCTAGGAG TTCCGATTGG CCCATATACA CCGTCGCCAG GGAAAGTTCT ATTACAGAAT   
  
  
- AATGTGTTGT TTGTTCTGTT AACGTTCGGA TCAACTCCTC CGTGGTTCAT ATGTGTATTA ACTAAAACCA   
  
  
- TAAAAGATAC CTGATGTTAC CGGGACGTAG TAAGTCTTGG AGAGGTTCTC CGGGTTACCC GGAGGGTCTT   
  
  
- AGGCGTAGTG TCCGTAGCTA AAGGGGGTTG GACCCAAGGC CGGTCGTCTC TCCCAACTTC TCTGTCCCGC   
  
  
- CACAAATCGG TTCATAACAC TGTCCATGTT ACACGGGAAA CTTATGGTGG GGTATCGGTT CTTCACCCTC   
  
  
- TGGTAGGTTG AGCTCCTCGA GTTTTAACTT TCCTTACTCG GGGAACATCA CTTGACAGAC ATATCTAGCG   
  
  
- TGTTGGATAA ACTACTTTCG CATCTCCTTT TGTCGGGTTC TCTGCGCAAG GACTCGAACC AATCCTTTTA   
  
  
- TTTAGGGCTA TATAAGTACG TACCCCACCA CTTATGGCGT AGTTCACATG GAAAGAAGGA CTTGTCCAAG   
  
  
- TTCCTCCGGT ACAAAGTGAT GTGCCGGAAC AAACTGAACA AGCTCCGGTG TTACTCGGCG CTCCTAGTTC   
  
  
- TCTCCCACAA TGATCTCTCG TTCAACGTAC CCTTAGTCCG CAACTTACAC TAGCGGACGC TTCCTCGCCT   
  
  
- CTCTCACCTC TCCGGACTCA GTATGTTTGT TACCGTCCAA TCCTGAGTTT CCCGGCCCAA ACAAGTTCAC   
  
  
- AGCTACCTAG CGCTCGAAAA CTCTTCCCGT TCCCGGTACC AGTCCTCCTT AAAATCCCTT CTCAAGTCAA   
  
  
- TACTACTTCT ACCCGTAACC CACCAAGTTC CTACCTTTCC GTCCTATTAC ATACGGTAAA GAACAACCTT   
  
  
- TGGAAGTAT

+     AAGAA-motif

| Site Name | Organism | Position | Strand | Matrix score. | sequence | function |
| --- | --- | --- | --- | --- | --- | --- |
| AAGAA-motif | Avena sativa | 1920 | - | 9 | gGTAAAGAAA |  |
| AAGAA-motif | Avena sativa | 1917 | - | 7 | GAAAGAA |  |

>HU02G01570.1   
+ +Up\_Stream \_Len000ATTATT ATTCTAATAT ATAGATAATA ATATAATAAT AATAATAATA ATAATAATAA   
  
  
+ TTATTATTAT TATTATTATT TATTAAATAT AATAGTTATT ATAATAATAA TAATTATAAT AATAATAAGA   
  
  
+ TTATTATCAT TATATTATTA TTATTATTAT TGTAATTTAT TATTATTATA GTAATTATTA TTGTTATATA   
  
  
+ GTAAAATTAT TATTAGTATT GTTATTATAA ATATTCATAC TTGCATATAA TATTTCTAAA ATAAAAACAG   
  
  
+ AGTTAATTCC ATTCCAAAAC TACTCACCAA ACAGCAAGGA GTGGGACCTG ATTCCTTTCC AATTTTCACC   
  
  
+ AAACAACTGG GTTCAGAATT CAAGTTCCAT ATCCAACCCC CCAGGATTCA AGTCCTGATT CCATTCCATT   
  
  
+ CCAGTTTCCC TCACCAAACT GGGCCTTAGA TTGATGGTTG TTCAGTTGGA TGAGGGTTGT TCCATGCCAA   
  
  
+ GTTAGATCAT TGTTGTCAAT TTTGAGAACG ACTATCTTAA TAAGCCTAAC GATGTTGATT TGATTTAGTT   
  
  
+ CAAACCATTT CAAATAATAC AAATCAAATT CTATTTAGAC CGACACTAAA ATAAATAGAT TGAGAAAAAT   
  
  
+ TATAGATTTT AAAGTTCAGA TTAAAATCCC TAAAGATCAA CAGATTGAAA TGCATATTTC ATAGTGCACA   
  
  
+ TGGACTTGCT TTGAATCTCG TCTAGAGAAC CTAAGAAAAA GCAAGAGTGG TTCAGAAGGT TAGAATTGTT   
  
  
+ CCAGCACATG ACAAGTACAA TCCCAACTTT TTTCAAAAGA TTTGCTACTT TTAATTGGAT TCCCATGTTG   
  
  
+ AATGAAGTTC TGGGTTTAAG GCTACAACTT TCTTGGGAGG ATAAGGTTCT TAAGTTAAGG TGGTCCTCTT   
  
  
+ TTCTTTTGAT CTTTCTTATG GGTACAACCT CATGGGTCTC ATCAACACAT GAACTCTTTT TATCTTTTAT   
  
  
+ CTTTCCCCTT TCCTTTATGC AGGATTGACA GGTGCATGGA AAACAAGAAG CAATTAAATA CTAGTGGCTT   
  
  
+ AGCCCCATCC CTAATATTTT GGCTATTCTG TTTCACCATC CTCCCCTGTC TCCATTTTCT TTGTTTCCTT   
  
  
+ TTTTTCTAGG AACTTGTGAT GGGCAAGGGA AGATCAGTAC AATGTGATGG TTGTTAGTAG CGAGAAGATG   
  
  
+ AAGTGTCACC GCAAAAATTT TGTGCCATAA TTTGTCGTAA GGGAAACAAG TATCAAACAA CAATGTCATT   
  
  
+ TATGATAAAC AAGTAACAGG AGATATGTAC TCTGAAAAAA TATAAGGTAC CTGAGAAGTT GTTAACTTAG   
  
  
+ CTTGAAAAAC CCTCCTAACT TGATAAACCC TCTTCGCTAT GTTATCCCGC ACCCTGCCAT AGAGTCACCG   
  
  
+ ATTCAGTATA TATGTAACAT ACCCAATCTT GTCTGGATAT ATAGTTAACC CTTTTGGAAT ATCTGAAAAT   
  
  
+ CCACACGAGA GTCATCCTTC TAGACTAGGA AACATGCGCC ACATTTTGGA TTCTAACCAA GTATAATAAG   
  
  
+ CATATCTTAA AGCCAAAAAT GCAAGGATAG ATACAAATAC CCATCACATG CATGTAAATG TCCCTTGTCA   
  
  
+ TGGCTCTGAT TCTCAAAATA TTAATTTTCA ATAAATTAAA TGAAATTGCA TTATTTTAAA GATTTCTGAA   
  
  
+ TGCATCCATT ATTGCAATTT TTTAAAGACT GCACAATGAT GACAATGTCA ATGGTTTCCT AAAGTTGCGC   
  
  
+ CTGATGGGCT ATTGACAGTG AAGCAACACT CCCTTTGTTC CCATTTATGT GTCTCCACTT GCCCCTTTGA   
  
  
+ AACTCTCTAC ATTTGTATGT CATCTGAATT TGATTCTCAA ATCTCAGATG ATTTGAATTC CCTTTATGCC   
  
  
+ ATCTTTACTA TCTATTGAAT GATTCTTTCT TTTCCACTTT CTTATCTTCC ACTACCTTCT TCTTGGATGC   
  
  
+ CCTTTTGCTG CTTATAAAGG GCGACACCTG AACAAGTTGC TGTATTCTCA AACCATGAAT CTGTACAGGT   
  
  
+ TTAGCCCCGA TATGTGTCCT AGCTTTGTGG GTCACGAAGA AATTGGTCCT AATTTAATAG GACACCAAGA   
  
  
+ CCCTTTTCAA AATGCATACA ATTGCACTGA TTTTGGTGAT GCTTACTTGT TGCAATTCCC AGTTGAAGAT   
  
  
+ TTATGTCAAT ACCGTGTTCC TGATCTAATG GGGCTACCTG TTGAAGATGG GGATCCTACA GAGGTCCCTG   
  
  
+ ACATTCCTGA TACATGCTTC AACTACATAA GCAATCTCCT AATGGAAGAG GGCTCGGATG ACCGCCCTGG   
  
  
+ CGCCACCCTG CAGGACTATA TGGCTGCTCA AGTCTCCTAC AGCAAGTCCT CCAATGCCGC CTTTCCCGGT   
  
  
+ GAGAGTTGCT CATTCTTTGG TAATAATCAA TGCCCTCCAC CTGCTTATCA AAGCAACCCT TATTGCCTTG   
  
  
+ ATGATTACTT CAACTTGCTA TCCAATACTA CTGGCAATAG TGTTAACAAT GCTGGTGTTC ATGACAGCAA   
  
  
+ CAACTTTGTT CAGAACAGTT TGATTGGGAA TAGGGGTACT AGTTCTAGTT CTAGGGGCAG AAGTGATTTT   
  
  
+ GGATCTACCC TTATTAGGAA TTTGCCATTA GGGACCGACA CACTTGATGT GGTCAATCCA GCACCTACTC   
  
  
+ CTCGAGCCCC TCTGGTTACG ACTCAGTGTC AGGTGTTTGA AACACACCAG TATGAGCAGG CCTTCGAACT   
  
  
+ GATCACTAAT GGAAGCTGGG AATTCAGTCA TTCTGTTGCT AATACATCAA ATGATGGAAT TCAAAATCAA   
  
  
+ CCAATTCAAG CAGTTTCAAC CCAAGAACGA TGGAATATGA AAACATTTTC CGATGACCAA CTTGAGCCTG   
  
  
+ CTCTTTTGCG AAGTCTTGTG ATGTCCTCAA ACTGTACTGA TATCTCTGAT CAGGGTGTAC ACAGGATAGT   
  
  
+ GAACCCTGAG CCACCCAACA ACAGAAATAC GGAGATTGTG TGCAATGACT CATCCAGTAA GAGTAAGACA   
  
  
+ AGACGAAGAG AGCATGGTGC AGGAGAAAAC CACGATGAAG GAGAAAACCA CGATGAGACA GGAAGGAGTA   
  
  
+ ACAAGCAATC AGCACCATCC AATGAAGTGT TCGATGAAAT TGAGCAGTAT GGTGATGTGC TGCTCTGCCC   
  
  
+ TGAAGGAAGA AGTAAACCAG CAGGGTTATG CAGCAGCAGC AACGCTAACA GAACTAGCAA CTCACAGAAC   
  
  
+ TCGGAAGACT CAAAGTCACA GCAAAATGGG AGTTCCTCCA AGGTGTCAAA AAAGCGAGGC AATAAAAAGC   
  
  
+ AAGTGGTTGA TTTGAGGACT CTCCTAACTC GATGTGCACA ATGTGTTGCT CGTGTGGATT TAACTGGTTC   
  
  
+ TTACCAGTTG CTTCAGGAGA TTCGGCAGCA TTCTTCTCCC TATGGTGATT ATCTCCAAAG AATGGCTCAT   
  
  
+ TACCTTGCTA ATGGCCTTGA GGCGCGCTTA GAAGGCAAAG GATATGAACT AACTCGTGCC AATGAGCATA   
  
  
+ TCCCACCTAC TGAGATCCTC AAGGCTAACC GGGTATATGT GGCAGCGGTC CCTTTCAAGA TAATGTCTTA   
  
  
+ TTACACAACA AACAAGACAA TTGCAAGCCT AGTTGAGGAG GCACCAAGTA TACACATAAT TGATTTTGGT   
  
  
+ ATTTTCTATG GACTACAATG GCCCTGCATC ATTCAGAACC TCTCCAAGAG GCCCAATGGG CCTCCCAGAA   
  
  
+ TCCGCATCAC AGGCATCGAT TTCCCCCAAC CTGGGTTCCG GCCAGCAGAG AGGGTTGAAG AGACAGGGCG   
  
  
+ GTGTTTAGCC AAGTATTGTG ACAGGTACAA TGTGCCCTTT GAATACCACC CCATAGCCAA GAAGTGGGAG   
  
  
+ ACCATCCAAC TCGAGGAGCT CAAAATTGAA AGGAATGAGC CCCTTGTAGT GAACTGTCTG TATAGATCGC   
  
  
+ ACAACCTATT TGATGAAAGC GTAGAGGAAA ACAGCCCAAG AGACGCGTTC CTGAGCTTGG TTAGGAAAAT   
  
  
+ AAATCCCGAT ATATTCATGC ATGGGGTGGT GAATACCGCA TCAAGTGTAC CTTTCTTCCT GAACAGGTTC   
  
  
+ AAGGAGGCCA TGTTTCACTA CACGGCCTTG TTTGACTTGT TCGAGGCCAC AATGAGCCGC GAGGATCAAG   
  
  
+ AGAGGGTGTT ACTAGAGAGC AAGTTGCATG GGAATCAGGC GTTGAATGTG ATCGCCTGCG AAGGAGCGGA   
  
  
+ GAGAGTGGAG AGGCCTGAGT CATACAAACA ATGGCAGGTT AGGACTCAAA GGGCCGGGTT TGTTCAAGTG   
  
  
+ TCGATGGATC GCGAGCTTTT GAGAAGGGCA AGGGCCATGG TCAGGAGGAA TTTTAGGGAA GAGTTCAGTT   
  
  
+ ATGATGAAGA TGGGCATTGG GTGGTTCAAG GATGGAAAGG CAGGATAATG TATGCCATTT CTTGTTGGAA   
  
  
+ ACCTTCATA  

- +Up\_Stream \_Len000TAATAA TAAGATTATA TATCTATTAT TATATTATTA TTATTATTAT TATTATTATT   
  
  
- AATAATAATA ATAATAATAA ATAATTTATA TTATCAATAA TATTATTATT ATTAATATTA TTATTATTCT   
  
  
- AATAATAGTA ATATAATAAT AATAATAATA ACATTAAATA ATAATAATAT CATTAATAAT AACAATATAT   
  
  
- CATTTTAATA ATAATCATAA CAATAATATT TATAAGTATG AACGTATATT ATAAAGATTT TATTTTTGTC   
  
  
- TCAATTAAGG TAAGGTTTTG ATGAGTGGTT TGTCGTTCCT CACCCTGGAC TAAGGAAAGG TTAAAAGTGG   
  
  
- TTTGTTGACC CAAGTCTTAA GTTCAAGGTA TAGGTTGGGG GGTCCTAAGT TCAGGACTAA GGTAAGGTAA   
  
  
- GGTCAAAGGG AGTGGTTTGA CCCGGAATCT AACTACCAAC AAGTCAACCT ACTCCCAACA AGGTACGGTT   
  
  
- CAATCTAGTA ACAACAGTTA AAACTCTTGC TGATAGAATT ATTCGGATTG CTACAACTAA ACTAAATCAA   
  
  
- GTTTGGTAAA GTTTATTATG TTTAGTTTAA GATAAATCTG GCTGTGATTT TATTTATCTA ACTCTTTTTA   
  
  
- ATATCTAAAA TTTCAAGTCT AATTTTAGGG ATTTCTAGTT GTCTAACTTT ACGTATAAAG TATCACGTGT   
  
  
- ACCTGAACGA AACTTAGAGC AGATCTCTTG GATTCTTTTT CGTTCTCACC AAGTCTTCCA ATCTTAACAA   
  
  
- GGTCGTGTAC TGTTCATGTT AGGGTTGAAA AAAGTTTTCT AAACGATGAA AATTAACCTA AGGGTACAAC   
  
  
- TTACTTCAAG ACCCAAATTC CGATGTTGAA AGAACCCTCC TATTCCAAGA ATTCAATTCC ACCAGGAGAA   
  
  
- AAGAAAACTA GAAAGAATAC CCATGTTGGA GTACCCAGAG TAGTTGTGTA CTTGAGAAAA ATAGAAAATA   
  
  
- GAAAGGGGAA AGGAAATACG TCCTAACTGT CCACGTACCT TTTGTTCTTC GTTAATTTAT GATCACCGAA   
  
  
- TCGGGGTAGG GATTATAAAA CCGATAAGAC AAAGTGGTAG GAGGGGACAG AGGTAAAAGA AACAAAGGAA   
  
  
- AAAAAGATCC TTGAACACTA CCCGTTCCCT TCTAGTCATG TTACACTACC AACAATCATC GCTCTTCTAC   
  
  
- TTCACAGTGG CGTTTTTAAA ACACGGTATT AAACAGCATT CCCTTTGTTC ATAGTTTGTT GTTACAGTAA   
  
  
- ATACTATTTG TTCATTGTCC TCTATACATG AGACTTTTTT ATATTCCATG GACTCTTCAA CAATTGAATC   
  
  
- GAACTTTTTG GGAGGATTGA ACTATTTGGG AGAAGCGATA CAATAGGGCG TGGGACGGTA TCTCAGTGGC   
  
  
- TAAGTCATAT ATACATTGTA TGGGTTAGAA CAGACCTATA TATCAATTGG GAAAACCTTA TAGACTTTTA   
  
  
- GGTGTGCTCT CAGTAGGAAG ATCTGATCCT TTGTACGCGG TGTAAAACCT AAGATTGGTT CATATTATTC   
  
  
- GTATAGAATT TCGGTTTTTA CGTTCCTATC TATGTTTATG GGTAGTGTAC GTACATTTAC AGGGAACAGT   
  
  
- ACCGAGACTA AGAGTTTTAT AATTAAAAGT TATTTAATTT ACTTTAACGT AATAAAATTT CTAAAGACTT   
  
  
- ACGTAGGTAA TAACGTTAAA AAATTTCTGA CGTGTTACTA CTGTTACAGT TACCAAAGGA TTTCAACGCG   
  
  
- GACTACCCGA TAACTGTCAC TTCGTTGTGA GGGAAACAAG GGTAAATACA CAGAGGTGAA CGGGGAAACT   
  
  
- TTGAGAGATG TAAACATACA GTAGACTTAA ACTAAGAGTT TAGAGTCTAC TAAACTTAAG GGAAATACGG   
  
  
- TAGAAATGAT AGATAACTTA CTAAGAAAGA AAAGGTGAAA GAATAGAAGG TGATGGAAGA AGAACCTACG   
  
  
- GGAAAACGAC GAATATTTCC CGCTGTGGAC TTGTTCAACG ACATAAGAGT TTGGTACTTA GACATGTCCA   
  
  
- AATCGGGGCT ATACACAGGA TCGAAACACC CAGTGCTTCT TTAACCAGGA TTAAATTATC CTGTGGTTCT   
  
  
- GGGAAAAGTT TTACGTATGT TAACGTGACT AAAACCACTA CGAATGAACA ACGTTAAGGG TCAACTTCTA   
  
  
- AATACAGTTA TGGCACAAGG ACTAGATTAC CCCGATGGAC AACTTCTACC CCTAGGATGT CTCCAGGGAC   
  
  
- TGTAAGGACT ATGTACGAAG TTGATGTATT CGTTAGAGGA TTACCTTCTC CCGAGCCTAC TGGCGGGACC   
  
  
- GCGGTGGGAC GTCCTGATAT ACCGACGAGT TCAGAGGATG TCGTTCAGGA GGTTACGGCG GAAAGGGCCA   
  
  
- CTCTCAACGA GTAAGAAACC ATTATTAGTT ACGGGAGGTG GACGAATAGT TTCGTTGGGA ATAACGGAAC   
  
  
- TACTAATGAA GTTGAACGAT AGGTTATGAT GACCGTTATC ACAATTGTTA CGACCACAAG TACTGTCGTT   
  
  
- GTTGAAACAA GTCTTGTCAA ACTAACCCTT ATCCCCATGA TCAAGATCAA GATCCCCGTC TTCACTAAAA   
  
  
- CCTAGATGGG AATAATCCTT AAACGGTAAT CCCTGGCTGT GTGAACTACA CCAGTTAGGT CGTGGATGAG   
  
  
- GAGCTCGGGG AGACCAATGC TGAGTCACAG TCCACAAACT TTGTGTGGTC ATACTCGTCC GGAAGCTTGA   
  
  
- CTAGTGATTA CCTTCGACCC TTAAGTCAGT AAGACAACGA TTATGTAGTT TACTACCTTA AGTTTTAGTT   
  
  
- GGTTAAGTTC GTCAAAGTTG GGTTCTTGCT ACCTTATACT TTTGTAAAAG GCTACTGGTT GAACTCGGAC   
  
  
- GAGAAAACGC TTCAGAACAC TACAGGAGTT TGACATGACT ATAGAGACTA GTCCCACATG TGTCCTATCA   
  
  
- CTTGGGACTC GGTGGGTTGT TGTCTTTATG CCTCTAACAC ACGTTACTGA GTAGGTCATT CTCATTCTGT   
  
  
- TCTGCTTCTC TCGTACCACG TCCTCTTTTG GTGCTACTTC CTCTTTTGGT GCTACTCTGT CCTTCCTCAT   
  
  
- TGTTCGTTAG TCGTGGTAGG TTACTTCACA AGCTACTTTA ACTCGTCATA CCACTACACG ACGAGACGGG   
  
  
- ACTTCCTTCT TCATTTGGTC GTCCCAATAC GTCGTCGTCG TTGCGATTGT CTTGATCGTT GAGTGTCTTG   
  
  
- AGCCTTCTGA GTTTCAGTGT CGTTTTACCC TCAAGGAGGT TCCACAGTTT TTTCGCTCCG TTATTTTTCG   
  
  
- TTCACCAACT AAACTCCTGA GAGGATTGAG CTACACGTGT TACACAACGA GCACACCTAA ATTGACCAAG   
  
  
- AATGGTCAAC GAAGTCCTCT AAGCCGTCGT AAGAAGAGGG ATACCACTAA TAGAGGTTTC TTACCGAGTA   
  
  
- ATGGAACGAT TACCGGAACT CCGCGCGAAT CTTCCGTTTC CTATACTTGA TTGAGCACGG TTACTCGTAT   
  
  
- AGGGTGGATG ACTCTAGGAG TTCCGATTGG CCCATATACA CCGTCGCCAG GGAAAGTTCT ATTACAGAAT   
  
  
- AATGTGTTGT TTGTTCTGTT AACGTTCGGA TCAACTCCTC CGTGGTTCAT ATGTGTATTA ACTAAAACCA   
  
  
- TAAAAGATAC CTGATGTTAC CGGGACGTAG TAAGTCTTGG AGAGGTTCTC CGGGTTACCC GGAGGGTCTT   
  
  
- AGGCGTAGTG TCCGTAGCTA AAGGGGGTTG GACCCAAGGC CGGTCGTCTC TCCCAACTTC TCTGTCCCGC   
  
  
- CACAAATCGG TTCATAACAC TGTCCATGTT ACACGGGAAA CTTATGGTGG GGTATCGGTT CTTCACCCTC   
  
  
- TGGTAGGTTG AGCTCCTCGA GTTTTAACTT TCCTTACTCG GGGAACATCA CTTGACAGAC ATATCTAGCG   
  
  
- TGTTGGATAA ACTACTTTCG CATCTCCTTT TGTCGGGTTC TCTGCGCAAG GACTCGAACC AATCCTTTTA   
  
  
- TTTAGGGCTA TATAAGTACG TACCCCACCA CTTATGGCGT AGTTCACATG GAAAGAAGGA CTTGTCCAAG   
  
  
- TTCCTCCGGT ACAAAGTGAT GTGCCGGAAC AAACTGAACA AGCTCCGGTG TTACTCGGCG CTCCTAGTTC   
  
  
- TCTCCCACAA TGATCTCTCG TTCAACGTAC CCTTAGTCCG CAACTTACAC TAGCGGACGC TTCCTCGCCT   
  
  
- CTCTCACCTC TCCGGACTCA GTATGTTTGT TACCGTCCAA TCCTGAGTTT CCCGGCCCAA ACAAGTTCAC   
  
  
- AGCTACCTAG CGCTCGAAAA CTCTTCCCGT TCCCGGTACC AGTCCTCCTT AAAATCCCTT CTCAAGTCAA   
  
  
- TACTACTTCT ACCCGTAACC CACCAAGTTC CTACCTTTCC GTCCTATTAC ATACGGTAAA GAACAACCTT   
  
  
- TGGAAGTAT

+     ABRE

| Site Name | Organism | Position | Strand | Matrix score. | sequence | function |
| --- | --- | --- | --- | --- | --- | --- |
| ABRE | Arabidopsis thaliana | 4258 | - | 7 | AACCCGG | cis-acting element involved in the abscisic acid responsiveness |

>HU02G01570.1   
+ +Up\_Stream \_Len000ATTATT ATTCTAATAT ATAGATAATA ATATAATAAT AATAATAATA ATAATAATAA   
  
  
+ TTATTATTAT TATTATTATT TATTAAATAT AATAGTTATT ATAATAATAA TAATTATAAT AATAATAAGA   
  
  
+ TTATTATCAT TATATTATTA TTATTATTAT TGTAATTTAT TATTATTATA GTAATTATTA TTGTTATATA   
  
  
+ GTAAAATTAT TATTAGTATT GTTATTATAA ATATTCATAC TTGCATATAA TATTTCTAAA ATAAAAACAG   
  
  
+ AGTTAATTCC ATTCCAAAAC TACTCACCAA ACAGCAAGGA GTGGGACCTG ATTCCTTTCC AATTTTCACC   
  
  
+ AAACAACTGG GTTCAGAATT CAAGTTCCAT ATCCAACCCC CCAGGATTCA AGTCCTGATT CCATTCCATT   
  
  
+ CCAGTTTCCC TCACCAAACT GGGCCTTAGA TTGATGGTTG TTCAGTTGGA TGAGGGTTGT TCCATGCCAA   
  
  
+ GTTAGATCAT TGTTGTCAAT TTTGAGAACG ACTATCTTAA TAAGCCTAAC GATGTTGATT TGATTTAGTT   
  
  
+ CAAACCATTT CAAATAATAC AAATCAAATT CTATTTAGAC CGACACTAAA ATAAATAGAT TGAGAAAAAT   
  
  
+ TATAGATTTT AAAGTTCAGA TTAAAATCCC TAAAGATCAA CAGATTGAAA TGCATATTTC ATAGTGCACA   
  
  
+ TGGACTTGCT TTGAATCTCG TCTAGAGAAC CTAAGAAAAA GCAAGAGTGG TTCAGAAGGT TAGAATTGTT   
  
  
+ CCAGCACATG ACAAGTACAA TCCCAACTTT TTTCAAAAGA TTTGCTACTT TTAATTGGAT TCCCATGTTG   
  
  
+ AATGAAGTTC TGGGTTTAAG GCTACAACTT TCTTGGGAGG ATAAGGTTCT TAAGTTAAGG TGGTCCTCTT   
  
  
+ TTCTTTTGAT CTTTCTTATG GGTACAACCT CATGGGTCTC ATCAACACAT GAACTCTTTT TATCTTTTAT   
  
  
+ CTTTCCCCTT TCCTTTATGC AGGATTGACA GGTGCATGGA AAACAAGAAG CAATTAAATA CTAGTGGCTT   
  
  
+ AGCCCCATCC CTAATATTTT GGCTATTCTG TTTCACCATC CTCCCCTGTC TCCATTTTCT TTGTTTCCTT   
  
  
+ TTTTTCTAGG AACTTGTGAT GGGCAAGGGA AGATCAGTAC AATGTGATGG TTGTTAGTAG CGAGAAGATG   
  
  
+ AAGTGTCACC GCAAAAATTT TGTGCCATAA TTTGTCGTAA GGGAAACAAG TATCAAACAA CAATGTCATT   
  
  
+ TATGATAAAC AAGTAACAGG AGATATGTAC TCTGAAAAAA TATAAGGTAC CTGAGAAGTT GTTAACTTAG   
  
  
+ CTTGAAAAAC CCTCCTAACT TGATAAACCC TCTTCGCTAT GTTATCCCGC ACCCTGCCAT AGAGTCACCG   
  
  
+ ATTCAGTATA TATGTAACAT ACCCAATCTT GTCTGGATAT ATAGTTAACC CTTTTGGAAT ATCTGAAAAT   
  
  
+ CCACACGAGA GTCATCCTTC TAGACTAGGA AACATGCGCC ACATTTTGGA TTCTAACCAA GTATAATAAG   
  
  
+ CATATCTTAA AGCCAAAAAT GCAAGGATAG ATACAAATAC CCATCACATG CATGTAAATG TCCCTTGTCA   
  
  
+ TGGCTCTGAT TCTCAAAATA TTAATTTTCA ATAAATTAAA TGAAATTGCA TTATTTTAAA GATTTCTGAA   
  
  
+ TGCATCCATT ATTGCAATTT TTTAAAGACT GCACAATGAT GACAATGTCA ATGGTTTCCT AAAGTTGCGC   
  
  
+ CTGATGGGCT ATTGACAGTG AAGCAACACT CCCTTTGTTC CCATTTATGT GTCTCCACTT GCCCCTTTGA   
  
  
+ AACTCTCTAC ATTTGTATGT CATCTGAATT TGATTCTCAA ATCTCAGATG ATTTGAATTC CCTTTATGCC   
  
  
+ ATCTTTACTA TCTATTGAAT GATTCTTTCT TTTCCACTTT CTTATCTTCC ACTACCTTCT TCTTGGATGC   
  
  
+ CCTTTTGCTG CTTATAAAGG GCGACACCTG AACAAGTTGC TGTATTCTCA AACCATGAAT CTGTACAGGT   
  
  
+ TTAGCCCCGA TATGTGTCCT AGCTTTGTGG GTCACGAAGA AATTGGTCCT AATTTAATAG GACACCAAGA   
  
  
+ CCCTTTTCAA AATGCATACA ATTGCACTGA TTTTGGTGAT GCTTACTTGT TGCAATTCCC AGTTGAAGAT   
  
  
+ TTATGTCAAT ACCGTGTTCC TGATCTAATG GGGCTACCTG TTGAAGATGG GGATCCTACA GAGGTCCCTG   
  
  
+ ACATTCCTGA TACATGCTTC AACTACATAA GCAATCTCCT AATGGAAGAG GGCTCGGATG ACCGCCCTGG   
  
  
+ CGCCACCCTG CAGGACTATA TGGCTGCTCA AGTCTCCTAC AGCAAGTCCT CCAATGCCGC CTTTCCCGGT   
  
  
+ GAGAGTTGCT CATTCTTTGG TAATAATCAA TGCCCTCCAC CTGCTTATCA AAGCAACCCT TATTGCCTTG   
  
  
+ ATGATTACTT CAACTTGCTA TCCAATACTA CTGGCAATAG TGTTAACAAT GCTGGTGTTC ATGACAGCAA   
  
  
+ CAACTTTGTT CAGAACAGTT TGATTGGGAA TAGGGGTACT AGTTCTAGTT CTAGGGGCAG AAGTGATTTT   
  
  
+ GGATCTACCC TTATTAGGAA TTTGCCATTA GGGACCGACA CACTTGATGT GGTCAATCCA GCACCTACTC   
  
  
+ CTCGAGCCCC TCTGGTTACG ACTCAGTGTC AGGTGTTTGA AACACACCAG TATGAGCAGG CCTTCGAACT   
  
  
+ GATCACTAAT GGAAGCTGGG AATTCAGTCA TTCTGTTGCT AATACATCAA ATGATGGAAT TCAAAATCAA   
  
  
+ CCAATTCAAG CAGTTTCAAC CCAAGAACGA TGGAATATGA AAACATTTTC CGATGACCAA CTTGAGCCTG   
  
  
+ CTCTTTTGCG AAGTCTTGTG ATGTCCTCAA ACTGTACTGA TATCTCTGAT CAGGGTGTAC ACAGGATAGT   
  
  
+ GAACCCTGAG CCACCCAACA ACAGAAATAC GGAGATTGTG TGCAATGACT CATCCAGTAA GAGTAAGACA   
  
  
+ AGACGAAGAG AGCATGGTGC AGGAGAAAAC CACGATGAAG GAGAAAACCA CGATGAGACA GGAAGGAGTA   
  
  
+ ACAAGCAATC AGCACCATCC AATGAAGTGT TCGATGAAAT TGAGCAGTAT GGTGATGTGC TGCTCTGCCC   
  
  
+ TGAAGGAAGA AGTAAACCAG CAGGGTTATG CAGCAGCAGC AACGCTAACA GAACTAGCAA CTCACAGAAC   
  
  
+ TCGGAAGACT CAAAGTCACA GCAAAATGGG AGTTCCTCCA AGGTGTCAAA AAAGCGAGGC AATAAAAAGC   
  
  
+ AAGTGGTTGA TTTGAGGACT CTCCTAACTC GATGTGCACA ATGTGTTGCT CGTGTGGATT TAACTGGTTC   
  
  
+ TTACCAGTTG CTTCAGGAGA TTCGGCAGCA TTCTTCTCCC TATGGTGATT ATCTCCAAAG AATGGCTCAT   
  
  
+ TACCTTGCTA ATGGCCTTGA GGCGCGCTTA GAAGGCAAAG GATATGAACT AACTCGTGCC AATGAGCATA   
  
  
+ TCCCACCTAC TGAGATCCTC AAGGCTAACC GGGTATATGT GGCAGCGGTC CCTTTCAAGA TAATGTCTTA   
  
  
+ TTACACAACA AACAAGACAA TTGCAAGCCT AGTTGAGGAG GCACCAAGTA TACACATAAT TGATTTTGGT   
  
  
+ ATTTTCTATG GACTACAATG GCCCTGCATC ATTCAGAACC TCTCCAAGAG GCCCAATGGG CCTCCCAGAA   
  
  
+ TCCGCATCAC AGGCATCGAT TTCCCCCAAC CTGGGTTCCG GCCAGCAGAG AGGGTTGAAG AGACAGGGCG   
  
  
+ GTGTTTAGCC AAGTATTGTG ACAGGTACAA TGTGCCCTTT GAATACCACC CCATAGCCAA GAAGTGGGAG   
  
  
+ ACCATCCAAC TCGAGGAGCT CAAAATTGAA AGGAATGAGC CCCTTGTAGT GAACTGTCTG TATAGATCGC   
  
  
+ ACAACCTATT TGATGAAAGC GTAGAGGAAA ACAGCCCAAG AGACGCGTTC CTGAGCTTGG TTAGGAAAAT   
  
  
+ AAATCCCGAT ATATTCATGC ATGGGGTGGT GAATACCGCA TCAAGTGTAC CTTTCTTCCT GAACAGGTTC   
  
  
+ AAGGAGGCCA TGTTTCACTA CACGGCCTTG TTTGACTTGT TCGAGGCCAC AATGAGCCGC GAGGATCAAG   
  
  
+ AGAGGGTGTT ACTAGAGAGC AAGTTGCATG GGAATCAGGC GTTGAATGTG ATCGCCTGCG AAGGAGCGGA   
  
  
+ GAGAGTGGAG AGGCCTGAGT CATACAAACA ATGGCAGGTT AGGACTCAAA GGGCCGGGTT TGTTCAAGTG   
  
  
+ TCGATGGATC GCGAGCTTTT GAGAAGGGCA AGGGCCATGG TCAGGAGGAA TTTTAGGGAA GAGTTCAGTT   
  
  
+ ATGATGAAGA TGGGCATTGG GTGGTTCAAG GATGGAAAGG CAGGATAATG TATGCCATTT CTTGTTGGAA   
  
  
+ ACCTTCATA  

- +Up\_Stream \_Len000TAATAA TAAGATTATA TATCTATTAT TATATTATTA TTATTATTAT TATTATTATT   
  
  
- AATAATAATA ATAATAATAA ATAATTTATA TTATCAATAA TATTATTATT ATTAATATTA TTATTATTCT   
  
  
- AATAATAGTA ATATAATAAT AATAATAATA ACATTAAATA ATAATAATAT CATTAATAAT AACAATATAT   
  
  
- CATTTTAATA ATAATCATAA CAATAATATT TATAAGTATG AACGTATATT ATAAAGATTT TATTTTTGTC   
  
  
- TCAATTAAGG TAAGGTTTTG ATGAGTGGTT TGTCGTTCCT CACCCTGGAC TAAGGAAAGG TTAAAAGTGG   
  
  
- TTTGTTGACC CAAGTCTTAA GTTCAAGGTA TAGGTTGGGG GGTCCTAAGT TCAGGACTAA GGTAAGGTAA   
  
  
- GGTCAAAGGG AGTGGTTTGA CCCGGAATCT AACTACCAAC AAGTCAACCT ACTCCCAACA AGGTACGGTT   
  
  
- CAATCTAGTA ACAACAGTTA AAACTCTTGC TGATAGAATT ATTCGGATTG CTACAACTAA ACTAAATCAA   
  
  
- GTTTGGTAAA GTTTATTATG TTTAGTTTAA GATAAATCTG GCTGTGATTT TATTTATCTA ACTCTTTTTA   
  
  
- ATATCTAAAA TTTCAAGTCT AATTTTAGGG ATTTCTAGTT GTCTAACTTT ACGTATAAAG TATCACGTGT   
  
  
- ACCTGAACGA AACTTAGAGC AGATCTCTTG GATTCTTTTT CGTTCTCACC AAGTCTTCCA ATCTTAACAA   
  
  
- GGTCGTGTAC TGTTCATGTT AGGGTTGAAA AAAGTTTTCT AAACGATGAA AATTAACCTA AGGGTACAAC   
  
  
- TTACTTCAAG ACCCAAATTC CGATGTTGAA AGAACCCTCC TATTCCAAGA ATTCAATTCC ACCAGGAGAA   
  
  
- AAGAAAACTA GAAAGAATAC CCATGTTGGA GTACCCAGAG TAGTTGTGTA CTTGAGAAAA ATAGAAAATA   
  
  
- GAAAGGGGAA AGGAAATACG TCCTAACTGT CCACGTACCT TTTGTTCTTC GTTAATTTAT GATCACCGAA   
  
  
- TCGGGGTAGG GATTATAAAA CCGATAAGAC AAAGTGGTAG GAGGGGACAG AGGTAAAAGA AACAAAGGAA   
  
  
- AAAAAGATCC TTGAACACTA CCCGTTCCCT TCTAGTCATG TTACACTACC AACAATCATC GCTCTTCTAC   
  
  
- TTCACAGTGG CGTTTTTAAA ACACGGTATT AAACAGCATT CCCTTTGTTC ATAGTTTGTT GTTACAGTAA   
  
  
- ATACTATTTG TTCATTGTCC TCTATACATG AGACTTTTTT ATATTCCATG GACTCTTCAA CAATTGAATC   
  
  
- GAACTTTTTG GGAGGATTGA ACTATTTGGG AGAAGCGATA CAATAGGGCG TGGGACGGTA TCTCAGTGGC   
  
  
- TAAGTCATAT ATACATTGTA TGGGTTAGAA CAGACCTATA TATCAATTGG GAAAACCTTA TAGACTTTTA   
  
  
- GGTGTGCTCT CAGTAGGAAG ATCTGATCCT TTGTACGCGG TGTAAAACCT AAGATTGGTT CATATTATTC   
  
  
- GTATAGAATT TCGGTTTTTA CGTTCCTATC TATGTTTATG GGTAGTGTAC GTACATTTAC AGGGAACAGT   
  
  
- ACCGAGACTA AGAGTTTTAT AATTAAAAGT TATTTAATTT ACTTTAACGT AATAAAATTT CTAAAGACTT   
  
  
- ACGTAGGTAA TAACGTTAAA AAATTTCTGA CGTGTTACTA CTGTTACAGT TACCAAAGGA TTTCAACGCG   
  
  
- GACTACCCGA TAACTGTCAC TTCGTTGTGA GGGAAACAAG GGTAAATACA CAGAGGTGAA CGGGGAAACT   
  
  
- TTGAGAGATG TAAACATACA GTAGACTTAA ACTAAGAGTT TAGAGTCTAC TAAACTTAAG GGAAATACGG   
  
  
- TAGAAATGAT AGATAACTTA CTAAGAAAGA AAAGGTGAAA GAATAGAAGG TGATGGAAGA AGAACCTACG   
  
  
- GGAAAACGAC GAATATTTCC CGCTGTGGAC TTGTTCAACG ACATAAGAGT TTGGTACTTA GACATGTCCA   
  
  
- AATCGGGGCT ATACACAGGA TCGAAACACC CAGTGCTTCT TTAACCAGGA TTAAATTATC CTGTGGTTCT   
  
  
- GGGAAAAGTT TTACGTATGT TAACGTGACT AAAACCACTA CGAATGAACA ACGTTAAGGG TCAACTTCTA   
  
  
- AATACAGTTA TGGCACAAGG ACTAGATTAC CCCGATGGAC AACTTCTACC CCTAGGATGT CTCCAGGGAC   
  
  
- TGTAAGGACT ATGTACGAAG TTGATGTATT CGTTAGAGGA TTACCTTCTC CCGAGCCTAC TGGCGGGACC   
  
  
- GCGGTGGGAC GTCCTGATAT ACCGACGAGT TCAGAGGATG TCGTTCAGGA GGTTACGGCG GAAAGGGCCA   
  
  
- CTCTCAACGA GTAAGAAACC ATTATTAGTT ACGGGAGGTG GACGAATAGT TTCGTTGGGA ATAACGGAAC   
  
  
- TACTAATGAA GTTGAACGAT AGGTTATGAT GACCGTTATC ACAATTGTTA CGACCACAAG TACTGTCGTT   
  
  
- GTTGAAACAA GTCTTGTCAA ACTAACCCTT ATCCCCATGA TCAAGATCAA GATCCCCGTC TTCACTAAAA   
  
  
- CCTAGATGGG AATAATCCTT AAACGGTAAT CCCTGGCTGT GTGAACTACA CCAGTTAGGT CGTGGATGAG   
  
  
- GAGCTCGGGG AGACCAATGC TGAGTCACAG TCCACAAACT TTGTGTGGTC ATACTCGTCC GGAAGCTTGA   
  
  
- CTAGTGATTA CCTTCGACCC TTAAGTCAGT AAGACAACGA TTATGTAGTT TACTACCTTA AGTTTTAGTT   
  
  
- GGTTAAGTTC GTCAAAGTTG GGTTCTTGCT ACCTTATACT TTTGTAAAAG GCTACTGGTT GAACTCGGAC   
  
  
- GAGAAAACGC TTCAGAACAC TACAGGAGTT TGACATGACT ATAGAGACTA GTCCCACATG TGTCCTATCA   
  
  
- CTTGGGACTC GGTGGGTTGT TGTCTTTATG CCTCTAACAC ACGTTACTGA GTAGGTCATT CTCATTCTGT   
  
  
- TCTGCTTCTC TCGTACCACG TCCTCTTTTG GTGCTACTTC CTCTTTTGGT GCTACTCTGT CCTTCCTCAT   
  
  
- TGTTCGTTAG TCGTGGTAGG TTACTTCACA AGCTACTTTA ACTCGTCATA CCACTACACG ACGAGACGGG   
  
  
- ACTTCCTTCT TCATTTGGTC GTCCCAATAC GTCGTCGTCG TTGCGATTGT CTTGATCGTT GAGTGTCTTG   
  
  
- AGCCTTCTGA GTTTCAGTGT CGTTTTACCC TCAAGGAGGT TCCACAGTTT TTTCGCTCCG TTATTTTTCG   
  
  
- TTCACCAACT AAACTCCTGA GAGGATTGAG CTACACGTGT TACACAACGA GCACACCTAA ATTGACCAAG   
  
  
- AATGGTCAAC GAAGTCCTCT AAGCCGTCGT AAGAAGAGGG ATACCACTAA TAGAGGTTTC TTACCGAGTA   
  
  
- ATGGAACGAT TACCGGAACT CCGCGCGAAT CTTCCGTTTC CTATACTTGA TTGAGCACGG TTACTCGTAT   
  
  
- AGGGTGGATG ACTCTAGGAG TTCCGATTGG CCCATATACA CCGTCGCCAG GGAAAGTTCT ATTACAGAAT   
  
  
- AATGTGTTGT TTGTTCTGTT AACGTTCGGA TCAACTCCTC CGTGGTTCAT ATGTGTATTA ACTAAAACCA   
  
  
- TAAAAGATAC CTGATGTTAC CGGGACGTAG TAAGTCTTGG AGAGGTTCTC CGGGTTACCC GGAGGGTCTT   
  
  
- AGGCGTAGTG TCCGTAGCTA AAGGGGGTTG GACCCAAGGC CGGTCGTCTC TCCCAACTTC TCTGTCCCGC   
  
  
- CACAAATCGG TTCATAACAC TGTCCATGTT ACACGGGAAA CTTATGGTGG GGTATCGGTT CTTCACCCTC   
  
  
- TGGTAGGTTG AGCTCCTCGA GTTTTAACTT TCCTTACTCG GGGAACATCA CTTGACAGAC ATATCTAGCG   
  
  
- TGTTGGATAA ACTACTTTCG CATCTCCTTT TGTCGGGTTC TCTGCGCAAG GACTCGAACC AATCCTTTTA   
  
  
- TTTAGGGCTA TATAAGTACG TACCCCACCA CTTATGGCGT AGTTCACATG GAAAGAAGGA CTTGTCCAAG   
  
  
- TTCCTCCGGT ACAAAGTGAT GTGCCGGAAC AAACTGAACA AGCTCCGGTG TTACTCGGCG CTCCTAGTTC   
  
  
- TCTCCCACAA TGATCTCTCG TTCAACGTAC CCTTAGTCCG CAACTTACAC TAGCGGACGC TTCCTCGCCT   
  
  
- CTCTCACCTC TCCGGACTCA GTATGTTTGT TACCGTCCAA TCCTGAGTTT CCCGGCCCAA ACAAGTTCAC   
  
  
- AGCTACCTAG CGCTCGAAAA CTCTTCCCGT TCCCGGTACC AGTCCTCCTT AAAATCCCTT CTCAAGTCAA   
  
  
- TACTACTTCT ACCCGTAACC CACCAAGTTC CTACCTTTCC GTCCTATTAC ATACGGTAAA GAACAACCTT   
  
  
- TGGAAGTAT

+     ARE

| Site Name | Organism | Position | Strand | Matrix score. | sequence | function |
| --- | --- | --- | --- | --- | --- | --- |
| ARE | Zea mays | 566 | + | 6 | AAACCA | cis-acting regulatory element essential for the anaerobic induction |
| ARE | Zea mays | 3059 | + | 6 | AAACCA | cis-acting regulatory element essential for the anaerobic induction |
| ARE | Zea mays | 2014 | + | 6 | AAACCA | cis-acting regulatory element essential for the anaerobic induction |
| ARE | Zea mays | 3168 | + | 6 | AAACCA | cis-acting regulatory element essential for the anaerobic induction |
| ARE | Zea mays | 1736 | - | 6 | AAACCA | cis-acting regulatory element essential for the anaerobic induction |
| ARE | Zea mays | 3041 | + | 6 | AAACCA | cis-acting regulatory element essential for the anaerobic induction |

>HU02G01570.1   
+ +Up\_Stream \_Len000ATTATT ATTCTAATAT ATAGATAATA ATATAATAAT AATAATAATA ATAATAATAA   
  
  
+ TTATTATTAT TATTATTATT TATTAAATAT AATAGTTATT ATAATAATAA TAATTATAAT AATAATAAGA   
  
  
+ TTATTATCAT TATATTATTA TTATTATTAT TGTAATTTAT TATTATTATA GTAATTATTA TTGTTATATA   
  
  
+ GTAAAATTAT TATTAGTATT GTTATTATAA ATATTCATAC TTGCATATAA TATTTCTAAA ATAAAAACAG   
  
  
+ AGTTAATTCC ATTCCAAAAC TACTCACCAA ACAGCAAGGA GTGGGACCTG ATTCCTTTCC AATTTTCACC   
  
  
+ AAACAACTGG GTTCAGAATT CAAGTTCCAT ATCCAACCCC CCAGGATTCA AGTCCTGATT CCATTCCATT   
  
  
+ CCAGTTTCCC TCACCAAACT GGGCCTTAGA TTGATGGTTG TTCAGTTGGA TGAGGGTTGT TCCATGCCAA   
  
  
+ GTTAGATCAT TGTTGTCAAT TTTGAGAACG ACTATCTTAA TAAGCCTAAC GATGTTGATT TGATTTAGTT   
  
  
+ CAAACCATTT CAAATAATAC AAATCAAATT CTATTTAGAC CGACACTAAA ATAAATAGAT TGAGAAAAAT   
  
  
+ TATAGATTTT AAAGTTCAGA TTAAAATCCC TAAAGATCAA CAGATTGAAA TGCATATTTC ATAGTGCACA   
  
  
+ TGGACTTGCT TTGAATCTCG TCTAGAGAAC CTAAGAAAAA GCAAGAGTGG TTCAGAAGGT TAGAATTGTT   
  
  
+ CCAGCACATG ACAAGTACAA TCCCAACTTT TTTCAAAAGA TTTGCTACTT TTAATTGGAT TCCCATGTTG   
  
  
+ AATGAAGTTC TGGGTTTAAG GCTACAACTT TCTTGGGAGG ATAAGGTTCT TAAGTTAAGG TGGTCCTCTT   
  
  
+ TTCTTTTGAT CTTTCTTATG GGTACAACCT CATGGGTCTC ATCAACACAT GAACTCTTTT TATCTTTTAT   
  
  
+ CTTTCCCCTT TCCTTTATGC AGGATTGACA GGTGCATGGA AAACAAGAAG CAATTAAATA CTAGTGGCTT   
  
  
+ AGCCCCATCC CTAATATTTT GGCTATTCTG TTTCACCATC CTCCCCTGTC TCCATTTTCT TTGTTTCCTT   
  
  
+ TTTTTCTAGG AACTTGTGAT GGGCAAGGGA AGATCAGTAC AATGTGATGG TTGTTAGTAG CGAGAAGATG   
  
  
+ AAGTGTCACC GCAAAAATTT TGTGCCATAA TTTGTCGTAA GGGAAACAAG TATCAAACAA CAATGTCATT   
  
  
+ TATGATAAAC AAGTAACAGG AGATATGTAC TCTGAAAAAA TATAAGGTAC CTGAGAAGTT GTTAACTTAG   
  
  
+ CTTGAAAAAC CCTCCTAACT TGATAAACCC TCTTCGCTAT GTTATCCCGC ACCCTGCCAT AGAGTCACCG   
  
  
+ ATTCAGTATA TATGTAACAT ACCCAATCTT GTCTGGATAT ATAGTTAACC CTTTTGGAAT ATCTGAAAAT   
  
  
+ CCACACGAGA GTCATCCTTC TAGACTAGGA AACATGCGCC ACATTTTGGA TTCTAACCAA GTATAATAAG   
  
  
+ CATATCTTAA AGCCAAAAAT GCAAGGATAG ATACAAATAC CCATCACATG CATGTAAATG TCCCTTGTCA   
  
  
+ TGGCTCTGAT TCTCAAAATA TTAATTTTCA ATAAATTAAA TGAAATTGCA TTATTTTAAA GATTTCTGAA   
  
  
+ TGCATCCATT ATTGCAATTT TTTAAAGACT GCACAATGAT GACAATGTCA ATGGTTTCCT AAAGTTGCGC   
  
  
+ CTGATGGGCT ATTGACAGTG AAGCAACACT CCCTTTGTTC CCATTTATGT GTCTCCACTT GCCCCTTTGA   
  
  
+ AACTCTCTAC ATTTGTATGT CATCTGAATT TGATTCTCAA ATCTCAGATG ATTTGAATTC CCTTTATGCC   
  
  
+ ATCTTTACTA TCTATTGAAT GATTCTTTCT TTTCCACTTT CTTATCTTCC ACTACCTTCT TCTTGGATGC   
  
  
+ CCTTTTGCTG CTTATAAAGG GCGACACCTG AACAAGTTGC TGTATTCTCA AACCATGAAT CTGTACAGGT   
  
  
+ TTAGCCCCGA TATGTGTCCT AGCTTTGTGG GTCACGAAGA AATTGGTCCT AATTTAATAG GACACCAAGA   
  
  
+ CCCTTTTCAA AATGCATACA ATTGCACTGA TTTTGGTGAT GCTTACTTGT TGCAATTCCC AGTTGAAGAT   
  
  
+ TTATGTCAAT ACCGTGTTCC TGATCTAATG GGGCTACCTG TTGAAGATGG GGATCCTACA GAGGTCCCTG   
  
  
+ ACATTCCTGA TACATGCTTC AACTACATAA GCAATCTCCT AATGGAAGAG GGCTCGGATG ACCGCCCTGG   
  
  
+ CGCCACCCTG CAGGACTATA TGGCTGCTCA AGTCTCCTAC AGCAAGTCCT CCAATGCCGC CTTTCCCGGT   
  
  
+ GAGAGTTGCT CATTCTTTGG TAATAATCAA TGCCCTCCAC CTGCTTATCA AAGCAACCCT TATTGCCTTG   
  
  
+ ATGATTACTT CAACTTGCTA TCCAATACTA CTGGCAATAG TGTTAACAAT GCTGGTGTTC ATGACAGCAA   
  
  
+ CAACTTTGTT CAGAACAGTT TGATTGGGAA TAGGGGTACT AGTTCTAGTT CTAGGGGCAG AAGTGATTTT   
  
  
+ GGATCTACCC TTATTAGGAA TTTGCCATTA GGGACCGACA CACTTGATGT GGTCAATCCA GCACCTACTC   
  
  
+ CTCGAGCCCC TCTGGTTACG ACTCAGTGTC AGGTGTTTGA AACACACCAG TATGAGCAGG CCTTCGAACT   
  
  
+ GATCACTAAT GGAAGCTGGG AATTCAGTCA TTCTGTTGCT AATACATCAA ATGATGGAAT TCAAAATCAA   
  
  
+ CCAATTCAAG CAGTTTCAAC CCAAGAACGA TGGAATATGA AAACATTTTC CGATGACCAA CTTGAGCCTG   
  
  
+ CTCTTTTGCG AAGTCTTGTG ATGTCCTCAA ACTGTACTGA TATCTCTGAT CAGGGTGTAC ACAGGATAGT   
  
  
+ GAACCCTGAG CCACCCAACA ACAGAAATAC GGAGATTGTG TGCAATGACT CATCCAGTAA GAGTAAGACA   
  
  
+ AGACGAAGAG AGCATGGTGC AGGAGAAAAC CACGATGAAG GAGAAAACCA CGATGAGACA GGAAGGAGTA   
  
  
+ ACAAGCAATC AGCACCATCC AATGAAGTGT TCGATGAAAT TGAGCAGTAT GGTGATGTGC TGCTCTGCCC   
  
  
+ TGAAGGAAGA AGTAAACCAG CAGGGTTATG CAGCAGCAGC AACGCTAACA GAACTAGCAA CTCACAGAAC   
  
  
+ TCGGAAGACT CAAAGTCACA GCAAAATGGG AGTTCCTCCA AGGTGTCAAA AAAGCGAGGC AATAAAAAGC   
  
  
+ AAGTGGTTGA TTTGAGGACT CTCCTAACTC GATGTGCACA ATGTGTTGCT CGTGTGGATT TAACTGGTTC   
  
  
+ TTACCAGTTG CTTCAGGAGA TTCGGCAGCA TTCTTCTCCC TATGGTGATT ATCTCCAAAG AATGGCTCAT   
  
  
+ TACCTTGCTA ATGGCCTTGA GGCGCGCTTA GAAGGCAAAG GATATGAACT AACTCGTGCC AATGAGCATA   
  
  
+ TCCCACCTAC TGAGATCCTC AAGGCTAACC GGGTATATGT GGCAGCGGTC CCTTTCAAGA TAATGTCTTA   
  
  
+ TTACACAACA AACAAGACAA TTGCAAGCCT AGTTGAGGAG GCACCAAGTA TACACATAAT TGATTTTGGT   
  
  
+ ATTTTCTATG GACTACAATG GCCCTGCATC ATTCAGAACC TCTCCAAGAG GCCCAATGGG CCTCCCAGAA   
  
  
+ TCCGCATCAC AGGCATCGAT TTCCCCCAAC CTGGGTTCCG GCCAGCAGAG AGGGTTGAAG AGACAGGGCG   
  
  
+ GTGTTTAGCC AAGTATTGTG ACAGGTACAA TGTGCCCTTT GAATACCACC CCATAGCCAA GAAGTGGGAG   
  
  
+ ACCATCCAAC TCGAGGAGCT CAAAATTGAA AGGAATGAGC CCCTTGTAGT GAACTGTCTG TATAGATCGC   
  
  
+ ACAACCTATT TGATGAAAGC GTAGAGGAAA ACAGCCCAAG AGACGCGTTC CTGAGCTTGG TTAGGAAAAT   
  
  
+ AAATCCCGAT ATATTCATGC ATGGGGTGGT GAATACCGCA TCAAGTGTAC CTTTCTTCCT GAACAGGTTC   
  
  
+ AAGGAGGCCA TGTTTCACTA CACGGCCTTG TTTGACTTGT TCGAGGCCAC AATGAGCCGC GAGGATCAAG   
  
  
+ AGAGGGTGTT ACTAGAGAGC AAGTTGCATG GGAATCAGGC GTTGAATGTG ATCGCCTGCG AAGGAGCGGA   
  
  
+ GAGAGTGGAG AGGCCTGAGT CATACAAACA ATGGCAGGTT AGGACTCAAA GGGCCGGGTT TGTTCAAGTG   
  
  
+ TCGATGGATC GCGAGCTTTT GAGAAGGGCA AGGGCCATGG TCAGGAGGAA TTTTAGGGAA GAGTTCAGTT   
  
  
+ ATGATGAAGA TGGGCATTGG GTGGTTCAAG GATGGAAAGG CAGGATAATG TATGCCATTT CTTGTTGGAA   
  
  
+ ACCTTCATA  

- +Up\_Stream \_Len000TAATAA TAAGATTATA TATCTATTAT TATATTATTA TTATTATTAT TATTATTATT   
  
  
- AATAATAATA ATAATAATAA ATAATTTATA TTATCAATAA TATTATTATT ATTAATATTA TTATTATTCT   
  
  
- AATAATAGTA ATATAATAAT AATAATAATA ACATTAAATA ATAATAATAT CATTAATAAT AACAATATAT   
  
  
- CATTTTAATA ATAATCATAA CAATAATATT TATAAGTATG AACGTATATT ATAAAGATTT TATTTTTGTC   
  
  
- TCAATTAAGG TAAGGTTTTG ATGAGTGGTT TGTCGTTCCT CACCCTGGAC TAAGGAAAGG TTAAAAGTGG   
  
  
- TTTGTTGACC CAAGTCTTAA GTTCAAGGTA TAGGTTGGGG GGTCCTAAGT TCAGGACTAA GGTAAGGTAA   
  
  
- GGTCAAAGGG AGTGGTTTGA CCCGGAATCT AACTACCAAC AAGTCAACCT ACTCCCAACA AGGTACGGTT   
  
  
- CAATCTAGTA ACAACAGTTA AAACTCTTGC TGATAGAATT ATTCGGATTG CTACAACTAA ACTAAATCAA   
  
  
- GTTTGGTAAA GTTTATTATG TTTAGTTTAA GATAAATCTG GCTGTGATTT TATTTATCTA ACTCTTTTTA   
  
  
- ATATCTAAAA TTTCAAGTCT AATTTTAGGG ATTTCTAGTT GTCTAACTTT ACGTATAAAG TATCACGTGT   
  
  
- ACCTGAACGA AACTTAGAGC AGATCTCTTG GATTCTTTTT CGTTCTCACC AAGTCTTCCA ATCTTAACAA   
  
  
- GGTCGTGTAC TGTTCATGTT AGGGTTGAAA AAAGTTTTCT AAACGATGAA AATTAACCTA AGGGTACAAC   
  
  
- TTACTTCAAG ACCCAAATTC CGATGTTGAA AGAACCCTCC TATTCCAAGA ATTCAATTCC ACCAGGAGAA   
  
  
- AAGAAAACTA GAAAGAATAC CCATGTTGGA GTACCCAGAG TAGTTGTGTA CTTGAGAAAA ATAGAAAATA   
  
  
- GAAAGGGGAA AGGAAATACG TCCTAACTGT CCACGTACCT TTTGTTCTTC GTTAATTTAT GATCACCGAA   
  
  
- TCGGGGTAGG GATTATAAAA CCGATAAGAC AAAGTGGTAG GAGGGGACAG AGGTAAAAGA AACAAAGGAA   
  
  
- AAAAAGATCC TTGAACACTA CCCGTTCCCT TCTAGTCATG TTACACTACC AACAATCATC GCTCTTCTAC   
  
  
- TTCACAGTGG CGTTTTTAAA ACACGGTATT AAACAGCATT CCCTTTGTTC ATAGTTTGTT GTTACAGTAA   
  
  
- ATACTATTTG TTCATTGTCC TCTATACATG AGACTTTTTT ATATTCCATG GACTCTTCAA CAATTGAATC   
  
  
- GAACTTTTTG GGAGGATTGA ACTATTTGGG AGAAGCGATA CAATAGGGCG TGGGACGGTA TCTCAGTGGC   
  
  
- TAAGTCATAT ATACATTGTA TGGGTTAGAA CAGACCTATA TATCAATTGG GAAAACCTTA TAGACTTTTA   
  
  
- GGTGTGCTCT CAGTAGGAAG ATCTGATCCT TTGTACGCGG TGTAAAACCT AAGATTGGTT CATATTATTC   
  
  
- GTATAGAATT TCGGTTTTTA CGTTCCTATC TATGTTTATG GGTAGTGTAC GTACATTTAC AGGGAACAGT   
  
  
- ACCGAGACTA AGAGTTTTAT AATTAAAAGT TATTTAATTT ACTTTAACGT AATAAAATTT CTAAAGACTT   
  
  
- ACGTAGGTAA TAACGTTAAA AAATTTCTGA CGTGTTACTA CTGTTACAGT TACCAAAGGA TTTCAACGCG   
  
  
- GACTACCCGA TAACTGTCAC TTCGTTGTGA GGGAAACAAG GGTAAATACA CAGAGGTGAA CGGGGAAACT   
  
  
- TTGAGAGATG TAAACATACA GTAGACTTAA ACTAAGAGTT TAGAGTCTAC TAAACTTAAG GGAAATACGG   
  
  
- TAGAAATGAT AGATAACTTA CTAAGAAAGA AAAGGTGAAA GAATAGAAGG TGATGGAAGA AGAACCTACG   
  
  
- GGAAAACGAC GAATATTTCC CGCTGTGGAC TTGTTCAACG ACATAAGAGT TTGGTACTTA GACATGTCCA   
  
  
- AATCGGGGCT ATACACAGGA TCGAAACACC CAGTGCTTCT TTAACCAGGA TTAAATTATC CTGTGGTTCT   
  
  
- GGGAAAAGTT TTACGTATGT TAACGTGACT AAAACCACTA CGAATGAACA ACGTTAAGGG TCAACTTCTA   
  
  
- AATACAGTTA TGGCACAAGG ACTAGATTAC CCCGATGGAC AACTTCTACC CCTAGGATGT CTCCAGGGAC   
  
  
- TGTAAGGACT ATGTACGAAG TTGATGTATT CGTTAGAGGA TTACCTTCTC CCGAGCCTAC TGGCGGGACC   
  
  
- GCGGTGGGAC GTCCTGATAT ACCGACGAGT TCAGAGGATG TCGTTCAGGA GGTTACGGCG GAAAGGGCCA   
  
  
- CTCTCAACGA GTAAGAAACC ATTATTAGTT ACGGGAGGTG GACGAATAGT TTCGTTGGGA ATAACGGAAC   
  
  
- TACTAATGAA GTTGAACGAT AGGTTATGAT GACCGTTATC ACAATTGTTA CGACCACAAG TACTGTCGTT   
  
  
- GTTGAAACAA GTCTTGTCAA ACTAACCCTT ATCCCCATGA TCAAGATCAA GATCCCCGTC TTCACTAAAA   
  
  
- CCTAGATGGG AATAATCCTT AAACGGTAAT CCCTGGCTGT GTGAACTACA CCAGTTAGGT CGTGGATGAG   
  
  
- GAGCTCGGGG AGACCAATGC TGAGTCACAG TCCACAAACT TTGTGTGGTC ATACTCGTCC GGAAGCTTGA   
  
  
- CTAGTGATTA CCTTCGACCC TTAAGTCAGT AAGACAACGA TTATGTAGTT TACTACCTTA AGTTTTAGTT   
  
  
- GGTTAAGTTC GTCAAAGTTG GGTTCTTGCT ACCTTATACT TTTGTAAAAG GCTACTGGTT GAACTCGGAC   
  
  
- GAGAAAACGC TTCAGAACAC TACAGGAGTT TGACATGACT ATAGAGACTA GTCCCACATG TGTCCTATCA   
  
  
- CTTGGGACTC GGTGGGTTGT TGTCTTTATG CCTCTAACAC ACGTTACTGA GTAGGTCATT CTCATTCTGT   
  
  
- TCTGCTTCTC TCGTACCACG TCCTCTTTTG GTGCTACTTC CTCTTTTGGT GCTACTCTGT CCTTCCTCAT   
  
  
- TGTTCGTTAG TCGTGGTAGG TTACTTCACA AGCTACTTTA ACTCGTCATA CCACTACACG ACGAGACGGG   
  
  
- ACTTCCTTCT TCATTTGGTC GTCCCAATAC GTCGTCGTCG TTGCGATTGT CTTGATCGTT GAGTGTCTTG   
  
  
- AGCCTTCTGA GTTTCAGTGT CGTTTTACCC TCAAGGAGGT TCCACAGTTT TTTCGCTCCG TTATTTTTCG   
  
  
- TTCACCAACT AAACTCCTGA GAGGATTGAG CTACACGTGT TACACAACGA GCACACCTAA ATTGACCAAG   
  
  
- AATGGTCAAC GAAGTCCTCT AAGCCGTCGT AAGAAGAGGG ATACCACTAA TAGAGGTTTC TTACCGAGTA   
  
  
- ATGGAACGAT TACCGGAACT CCGCGCGAAT CTTCCGTTTC CTATACTTGA TTGAGCACGG TTACTCGTAT   
  
  
- AGGGTGGATG ACTCTAGGAG TTCCGATTGG CCCATATACA CCGTCGCCAG GGAAAGTTCT ATTACAGAAT   
  
  
- AATGTGTTGT TTGTTCTGTT AACGTTCGGA TCAACTCCTC CGTGGTTCAT ATGTGTATTA ACTAAAACCA   
  
  
- TAAAAGATAC CTGATGTTAC CGGGACGTAG TAAGTCTTGG AGAGGTTCTC CGGGTTACCC GGAGGGTCTT   
  
  
- AGGCGTAGTG TCCGTAGCTA AAGGGGGTTG GACCCAAGGC CGGTCGTCTC TCCCAACTTC TCTGTCCCGC   
  
  
- CACAAATCGG TTCATAACAC TGTCCATGTT ACACGGGAAA CTTATGGTGG GGTATCGGTT CTTCACCCTC   
  
  
- TGGTAGGTTG AGCTCCTCGA GTTTTAACTT TCCTTACTCG GGGAACATCA CTTGACAGAC ATATCTAGCG   
  
  
- TGTTGGATAA ACTACTTTCG CATCTCCTTT TGTCGGGTTC TCTGCGCAAG GACTCGAACC AATCCTTTTA   
  
  
- TTTAGGGCTA TATAAGTACG TACCCCACCA CTTATGGCGT AGTTCACATG GAAAGAAGGA CTTGTCCAAG   
  
  
- TTCCTCCGGT ACAAAGTGAT GTGCCGGAAC AAACTGAACA AGCTCCGGTG TTACTCGGCG CTCCTAGTTC   
  
  
- TCTCCCACAA TGATCTCTCG TTCAACGTAC CCTTAGTCCG CAACTTACAC TAGCGGACGC TTCCTCGCCT   
  
  
- CTCTCACCTC TCCGGACTCA GTATGTTTGT TACCGTCCAA TCCTGAGTTT CCCGGCCCAA ACAAGTTCAC   
  
  
- AGCTACCTAG CGCTCGAAAA CTCTTCCCGT TCCCGGTACC AGTCCTCCTT AAAATCCCTT CTCAAGTCAA   
  
  
- TACTACTTCT ACCCGTAACC CACCAAGTTC CTACCTTTCC GTCCTATTAC ATACGGTAAA GAACAACCTT   
  
  
- TGGAAGTAT

+     AT-rich element

| Site Name | Organism | Position | Strand | Matrix score. | sequence | function |
| --- | --- | --- | --- | --- | --- | --- |
| AT-rich element | Glycine max | 581 | + | 10 | ATAGAAATCAA | binding site of AT-rich DNA binding protein (ATBP-1) |

>HU02G01570.1   
+ +Up\_Stream \_Len000ATTATT ATTCTAATAT ATAGATAATA ATATAATAAT AATAATAATA ATAATAATAA   
  
  
+ TTATTATTAT TATTATTATT TATTAAATAT AATAGTTATT ATAATAATAA TAATTATAAT AATAATAAGA   
  
  
+ TTATTATCAT TATATTATTA TTATTATTAT TGTAATTTAT TATTATTATA GTAATTATTA TTGTTATATA   
  
  
+ GTAAAATTAT TATTAGTATT GTTATTATAA ATATTCATAC TTGCATATAA TATTTCTAAA ATAAAAACAG   
  
  
+ AGTTAATTCC ATTCCAAAAC TACTCACCAA ACAGCAAGGA GTGGGACCTG ATTCCTTTCC AATTTTCACC   
  
  
+ AAACAACTGG GTTCAGAATT CAAGTTCCAT ATCCAACCCC CCAGGATTCA AGTCCTGATT CCATTCCATT   
  
  
+ CCAGTTTCCC TCACCAAACT GGGCCTTAGA TTGATGGTTG TTCAGTTGGA TGAGGGTTGT TCCATGCCAA   
  
  
+ GTTAGATCAT TGTTGTCAAT TTTGAGAACG ACTATCTTAA TAAGCCTAAC GATGTTGATT TGATTTAGTT   
  
  
+ CAAACCATTT CAAATAATAC AAATCAAATT CTATTTAGAC CGACACTAAA ATAAATAGAT TGAGAAAAAT   
  
  
+ TATAGATTTT AAAGTTCAGA TTAAAATCCC TAAAGATCAA CAGATTGAAA TGCATATTTC ATAGTGCACA   
  
  
+ TGGACTTGCT TTGAATCTCG TCTAGAGAAC CTAAGAAAAA GCAAGAGTGG TTCAGAAGGT TAGAATTGTT   
  
  
+ CCAGCACATG ACAAGTACAA TCCCAACTTT TTTCAAAAGA TTTGCTACTT TTAATTGGAT TCCCATGTTG   
  
  
+ AATGAAGTTC TGGGTTTAAG GCTACAACTT TCTTGGGAGG ATAAGGTTCT TAAGTTAAGG TGGTCCTCTT   
  
  
+ TTCTTTTGAT CTTTCTTATG GGTACAACCT CATGGGTCTC ATCAACACAT GAACTCTTTT TATCTTTTAT   
  
  
+ CTTTCCCCTT TCCTTTATGC AGGATTGACA GGTGCATGGA AAACAAGAAG CAATTAAATA CTAGTGGCTT   
  
  
+ AGCCCCATCC CTAATATTTT GGCTATTCTG TTTCACCATC CTCCCCTGTC TCCATTTTCT TTGTTTCCTT   
  
  
+ TTTTTCTAGG AACTTGTGAT GGGCAAGGGA AGATCAGTAC AATGTGATGG TTGTTAGTAG CGAGAAGATG   
  
  
+ AAGTGTCACC GCAAAAATTT TGTGCCATAA TTTGTCGTAA GGGAAACAAG TATCAAACAA CAATGTCATT   
  
  
+ TATGATAAAC AAGTAACAGG AGATATGTAC TCTGAAAAAA TATAAGGTAC CTGAGAAGTT GTTAACTTAG   
  
  
+ CTTGAAAAAC CCTCCTAACT TGATAAACCC TCTTCGCTAT GTTATCCCGC ACCCTGCCAT AGAGTCACCG   
  
  
+ ATTCAGTATA TATGTAACAT ACCCAATCTT GTCTGGATAT ATAGTTAACC CTTTTGGAAT ATCTGAAAAT   
  
  
+ CCACACGAGA GTCATCCTTC TAGACTAGGA AACATGCGCC ACATTTTGGA TTCTAACCAA GTATAATAAG   
  
  
+ CATATCTTAA AGCCAAAAAT GCAAGGATAG ATACAAATAC CCATCACATG CATGTAAATG TCCCTTGTCA   
  
  
+ TGGCTCTGAT TCTCAAAATA TTAATTTTCA ATAAATTAAA TGAAATTGCA TTATTTTAAA GATTTCTGAA   
  
  
+ TGCATCCATT ATTGCAATTT TTTAAAGACT GCACAATGAT GACAATGTCA ATGGTTTCCT AAAGTTGCGC   
  
  
+ CTGATGGGCT ATTGACAGTG AAGCAACACT CCCTTTGTTC CCATTTATGT GTCTCCACTT GCCCCTTTGA   
  
  
+ AACTCTCTAC ATTTGTATGT CATCTGAATT TGATTCTCAA ATCTCAGATG ATTTGAATTC CCTTTATGCC   
  
  
+ ATCTTTACTA TCTATTGAAT GATTCTTTCT TTTCCACTTT CTTATCTTCC ACTACCTTCT TCTTGGATGC   
  
  
+ CCTTTTGCTG CTTATAAAGG GCGACACCTG AACAAGTTGC TGTATTCTCA AACCATGAAT CTGTACAGGT   
  
  
+ TTAGCCCCGA TATGTGTCCT AGCTTTGTGG GTCACGAAGA AATTGGTCCT AATTTAATAG GACACCAAGA   
  
  
+ CCCTTTTCAA AATGCATACA ATTGCACTGA TTTTGGTGAT GCTTACTTGT TGCAATTCCC AGTTGAAGAT   
  
  
+ TTATGTCAAT ACCGTGTTCC TGATCTAATG GGGCTACCTG TTGAAGATGG GGATCCTACA GAGGTCCCTG   
  
  
+ ACATTCCTGA TACATGCTTC AACTACATAA GCAATCTCCT AATGGAAGAG GGCTCGGATG ACCGCCCTGG   
  
  
+ CGCCACCCTG CAGGACTATA TGGCTGCTCA AGTCTCCTAC AGCAAGTCCT CCAATGCCGC CTTTCCCGGT   
  
  
+ GAGAGTTGCT CATTCTTTGG TAATAATCAA TGCCCTCCAC CTGCTTATCA AAGCAACCCT TATTGCCTTG   
  
  
+ ATGATTACTT CAACTTGCTA TCCAATACTA CTGGCAATAG TGTTAACAAT GCTGGTGTTC ATGACAGCAA   
  
  
+ CAACTTTGTT CAGAACAGTT TGATTGGGAA TAGGGGTACT AGTTCTAGTT CTAGGGGCAG AAGTGATTTT   
  
  
+ GGATCTACCC TTATTAGGAA TTTGCCATTA GGGACCGACA CACTTGATGT GGTCAATCCA GCACCTACTC   
  
  
+ CTCGAGCCCC TCTGGTTACG ACTCAGTGTC AGGTGTTTGA AACACACCAG TATGAGCAGG CCTTCGAACT   
  
  
+ GATCACTAAT GGAAGCTGGG AATTCAGTCA TTCTGTTGCT AATACATCAA ATGATGGAAT TCAAAATCAA   
  
  
+ CCAATTCAAG CAGTTTCAAC CCAAGAACGA TGGAATATGA AAACATTTTC CGATGACCAA CTTGAGCCTG   
  
  
+ CTCTTTTGCG AAGTCTTGTG ATGTCCTCAA ACTGTACTGA TATCTCTGAT CAGGGTGTAC ACAGGATAGT   
  
  
+ GAACCCTGAG CCACCCAACA ACAGAAATAC GGAGATTGTG TGCAATGACT CATCCAGTAA GAGTAAGACA   
  
  
+ AGACGAAGAG AGCATGGTGC AGGAGAAAAC CACGATGAAG GAGAAAACCA CGATGAGACA GGAAGGAGTA   
  
  
+ ACAAGCAATC AGCACCATCC AATGAAGTGT TCGATGAAAT TGAGCAGTAT GGTGATGTGC TGCTCTGCCC   
  
  
+ TGAAGGAAGA AGTAAACCAG CAGGGTTATG CAGCAGCAGC AACGCTAACA GAACTAGCAA CTCACAGAAC   
  
  
+ TCGGAAGACT CAAAGTCACA GCAAAATGGG AGTTCCTCCA AGGTGTCAAA AAAGCGAGGC AATAAAAAGC   
  
  
+ AAGTGGTTGA TTTGAGGACT CTCCTAACTC GATGTGCACA ATGTGTTGCT CGTGTGGATT TAACTGGTTC   
  
  
+ TTACCAGTTG CTTCAGGAGA TTCGGCAGCA TTCTTCTCCC TATGGTGATT ATCTCCAAAG AATGGCTCAT   
  
  
+ TACCTTGCTA ATGGCCTTGA GGCGCGCTTA GAAGGCAAAG GATATGAACT AACTCGTGCC AATGAGCATA   
  
  
+ TCCCACCTAC TGAGATCCTC AAGGCTAACC GGGTATATGT GGCAGCGGTC CCTTTCAAGA TAATGTCTTA   
  
  
+ TTACACAACA AACAAGACAA TTGCAAGCCT AGTTGAGGAG GCACCAAGTA TACACATAAT TGATTTTGGT   
  
  
+ ATTTTCTATG GACTACAATG GCCCTGCATC ATTCAGAACC TCTCCAAGAG GCCCAATGGG CCTCCCAGAA   
  
  
+ TCCGCATCAC AGGCATCGAT TTCCCCCAAC CTGGGTTCCG GCCAGCAGAG AGGGTTGAAG AGACAGGGCG   
  
  
+ GTGTTTAGCC AAGTATTGTG ACAGGTACAA TGTGCCCTTT GAATACCACC CCATAGCCAA GAAGTGGGAG   
  
  
+ ACCATCCAAC TCGAGGAGCT CAAAATTGAA AGGAATGAGC CCCTTGTAGT GAACTGTCTG TATAGATCGC   
  
  
+ ACAACCTATT TGATGAAAGC GTAGAGGAAA ACAGCCCAAG AGACGCGTTC CTGAGCTTGG TTAGGAAAAT   
  
  
+ AAATCCCGAT ATATTCATGC ATGGGGTGGT GAATACCGCA TCAAGTGTAC CTTTCTTCCT GAACAGGTTC   
  
  
+ AAGGAGGCCA TGTTTCACTA CACGGCCTTG TTTGACTTGT TCGAGGCCAC AATGAGCCGC GAGGATCAAG   
  
  
+ AGAGGGTGTT ACTAGAGAGC AAGTTGCATG GGAATCAGGC GTTGAATGTG ATCGCCTGCG AAGGAGCGGA   
  
  
+ GAGAGTGGAG AGGCCTGAGT CATACAAACA ATGGCAGGTT AGGACTCAAA GGGCCGGGTT TGTTCAAGTG   
  
  
+ TCGATGGATC GCGAGCTTTT GAGAAGGGCA AGGGCCATGG TCAGGAGGAA TTTTAGGGAA GAGTTCAGTT   
  
  
+ ATGATGAAGA TGGGCATTGG GTGGTTCAAG GATGGAAAGG CAGGATAATG TATGCCATTT CTTGTTGGAA   
  
  
+ ACCTTCATA  

- +Up\_Stream \_Len000TAATAA TAAGATTATA TATCTATTAT TATATTATTA TTATTATTAT TATTATTATT   
  
  
- AATAATAATA ATAATAATAA ATAATTTATA TTATCAATAA TATTATTATT ATTAATATTA TTATTATTCT   
  
  
- AATAATAGTA ATATAATAAT AATAATAATA ACATTAAATA ATAATAATAT CATTAATAAT AACAATATAT   
  
  
- CATTTTAATA ATAATCATAA CAATAATATT TATAAGTATG AACGTATATT ATAAAGATTT TATTTTTGTC   
  
  
- TCAATTAAGG TAAGGTTTTG ATGAGTGGTT TGTCGTTCCT CACCCTGGAC TAAGGAAAGG TTAAAAGTGG   
  
  
- TTTGTTGACC CAAGTCTTAA GTTCAAGGTA TAGGTTGGGG GGTCCTAAGT TCAGGACTAA GGTAAGGTAA   
  
  
- GGTCAAAGGG AGTGGTTTGA CCCGGAATCT AACTACCAAC AAGTCAACCT ACTCCCAACA AGGTACGGTT   
  
  
- CAATCTAGTA ACAACAGTTA AAACTCTTGC TGATAGAATT ATTCGGATTG CTACAACTAA ACTAAATCAA   
  
  
- GTTTGGTAAA GTTTATTATG TTTAGTTTAA GATAAATCTG GCTGTGATTT TATTTATCTA ACTCTTTTTA   
  
  
- ATATCTAAAA TTTCAAGTCT AATTTTAGGG ATTTCTAGTT GTCTAACTTT ACGTATAAAG TATCACGTGT   
  
  
- ACCTGAACGA AACTTAGAGC AGATCTCTTG GATTCTTTTT CGTTCTCACC AAGTCTTCCA ATCTTAACAA   
  
  
- GGTCGTGTAC TGTTCATGTT AGGGTTGAAA AAAGTTTTCT AAACGATGAA AATTAACCTA AGGGTACAAC   
  
  
- TTACTTCAAG ACCCAAATTC CGATGTTGAA AGAACCCTCC TATTCCAAGA ATTCAATTCC ACCAGGAGAA   
  
  
- AAGAAAACTA GAAAGAATAC CCATGTTGGA GTACCCAGAG TAGTTGTGTA CTTGAGAAAA ATAGAAAATA   
  
  
- GAAAGGGGAA AGGAAATACG TCCTAACTGT CCACGTACCT TTTGTTCTTC GTTAATTTAT GATCACCGAA   
  
  
- TCGGGGTAGG GATTATAAAA CCGATAAGAC AAAGTGGTAG GAGGGGACAG AGGTAAAAGA AACAAAGGAA   
  
  
- AAAAAGATCC TTGAACACTA CCCGTTCCCT TCTAGTCATG TTACACTACC AACAATCATC GCTCTTCTAC   
  
  
- TTCACAGTGG CGTTTTTAAA ACACGGTATT AAACAGCATT CCCTTTGTTC ATAGTTTGTT GTTACAGTAA   
  
  
- ATACTATTTG TTCATTGTCC TCTATACATG AGACTTTTTT ATATTCCATG GACTCTTCAA CAATTGAATC   
  
  
- GAACTTTTTG GGAGGATTGA ACTATTTGGG AGAAGCGATA CAATAGGGCG TGGGACGGTA TCTCAGTGGC   
  
  
- TAAGTCATAT ATACATTGTA TGGGTTAGAA CAGACCTATA TATCAATTGG GAAAACCTTA TAGACTTTTA   
  
  
- GGTGTGCTCT CAGTAGGAAG ATCTGATCCT TTGTACGCGG TGTAAAACCT AAGATTGGTT CATATTATTC   
  
  
- GTATAGAATT TCGGTTTTTA CGTTCCTATC TATGTTTATG GGTAGTGTAC GTACATTTAC AGGGAACAGT   
  
  
- ACCGAGACTA AGAGTTTTAT AATTAAAAGT TATTTAATTT ACTTTAACGT AATAAAATTT CTAAAGACTT   
  
  
- ACGTAGGTAA TAACGTTAAA AAATTTCTGA CGTGTTACTA CTGTTACAGT TACCAAAGGA TTTCAACGCG   
  
  
- GACTACCCGA TAACTGTCAC TTCGTTGTGA GGGAAACAAG GGTAAATACA CAGAGGTGAA CGGGGAAACT   
  
  
- TTGAGAGATG TAAACATACA GTAGACTTAA ACTAAGAGTT TAGAGTCTAC TAAACTTAAG GGAAATACGG   
  
  
- TAGAAATGAT AGATAACTTA CTAAGAAAGA AAAGGTGAAA GAATAGAAGG TGATGGAAGA AGAACCTACG   
  
  
- GGAAAACGAC GAATATTTCC CGCTGTGGAC TTGTTCAACG ACATAAGAGT TTGGTACTTA GACATGTCCA   
  
  
- AATCGGGGCT ATACACAGGA TCGAAACACC CAGTGCTTCT TTAACCAGGA TTAAATTATC CTGTGGTTCT   
  
  
- GGGAAAAGTT TTACGTATGT TAACGTGACT AAAACCACTA CGAATGAACA ACGTTAAGGG TCAACTTCTA   
  
  
- AATACAGTTA TGGCACAAGG ACTAGATTAC CCCGATGGAC AACTTCTACC CCTAGGATGT CTCCAGGGAC   
  
  
- TGTAAGGACT ATGTACGAAG TTGATGTATT CGTTAGAGGA TTACCTTCTC CCGAGCCTAC TGGCGGGACC   
  
  
- GCGGTGGGAC GTCCTGATAT ACCGACGAGT TCAGAGGATG TCGTTCAGGA GGTTACGGCG GAAAGGGCCA   
  
  
- CTCTCAACGA GTAAGAAACC ATTATTAGTT ACGGGAGGTG GACGAATAGT TTCGTTGGGA ATAACGGAAC   
  
  
- TACTAATGAA GTTGAACGAT AGGTTATGAT GACCGTTATC ACAATTGTTA CGACCACAAG TACTGTCGTT   
  
  
- GTTGAAACAA GTCTTGTCAA ACTAACCCTT ATCCCCATGA TCAAGATCAA GATCCCCGTC TTCACTAAAA   
  
  
- CCTAGATGGG AATAATCCTT AAACGGTAAT CCCTGGCTGT GTGAACTACA CCAGTTAGGT CGTGGATGAG   
  
  
- GAGCTCGGGG AGACCAATGC TGAGTCACAG TCCACAAACT TTGTGTGGTC ATACTCGTCC GGAAGCTTGA   
  
  
- CTAGTGATTA CCTTCGACCC TTAAGTCAGT AAGACAACGA TTATGTAGTT TACTACCTTA AGTTTTAGTT   
  
  
- GGTTAAGTTC GTCAAAGTTG GGTTCTTGCT ACCTTATACT TTTGTAAAAG GCTACTGGTT GAACTCGGAC   
  
  
- GAGAAAACGC TTCAGAACAC TACAGGAGTT TGACATGACT ATAGAGACTA GTCCCACATG TGTCCTATCA   
  
  
- CTTGGGACTC GGTGGGTTGT TGTCTTTATG CCTCTAACAC ACGTTACTGA GTAGGTCATT CTCATTCTGT   
  
  
- TCTGCTTCTC TCGTACCACG TCCTCTTTTG GTGCTACTTC CTCTTTTGGT GCTACTCTGT CCTTCCTCAT   
  
  
- TGTTCGTTAG TCGTGGTAGG TTACTTCACA AGCTACTTTA ACTCGTCATA CCACTACACG ACGAGACGGG   
  
  
- ACTTCCTTCT TCATTTGGTC GTCCCAATAC GTCGTCGTCG TTGCGATTGT CTTGATCGTT GAGTGTCTTG   
  
  
- AGCCTTCTGA GTTTCAGTGT CGTTTTACCC TCAAGGAGGT TCCACAGTTT TTTCGCTCCG TTATTTTTCG   
  
  
- TTCACCAACT AAACTCCTGA GAGGATTGAG CTACACGTGT TACACAACGA GCACACCTAA ATTGACCAAG   
  
  
- AATGGTCAAC GAAGTCCTCT AAGCCGTCGT AAGAAGAGGG ATACCACTAA TAGAGGTTTC TTACCGAGTA   
  
  
- ATGGAACGAT TACCGGAACT CCGCGCGAAT CTTCCGTTTC CTATACTTGA TTGAGCACGG TTACTCGTAT   
  
  
- AGGGTGGATG ACTCTAGGAG TTCCGATTGG CCCATATACA CCGTCGCCAG GGAAAGTTCT ATTACAGAAT   
  
  
- AATGTGTTGT TTGTTCTGTT AACGTTCGGA TCAACTCCTC CGTGGTTCAT ATGTGTATTA ACTAAAACCA   
  
  
- TAAAAGATAC CTGATGTTAC CGGGACGTAG TAAGTCTTGG AGAGGTTCTC CGGGTTACCC GGAGGGTCTT   
  
  
- AGGCGTAGTG TCCGTAGCTA AAGGGGGTTG GACCCAAGGC CGGTCGTCTC TCCCAACTTC TCTGTCCCGC   
  
  
- CACAAATCGG TTCATAACAC TGTCCATGTT ACACGGGAAA CTTATGGTGG GGTATCGGTT CTTCACCCTC   
  
  
- TGGTAGGTTG AGCTCCTCGA GTTTTAACTT TCCTTACTCG GGGAACATCA CTTGACAGAC ATATCTAGCG   
  
  
- TGTTGGATAA ACTACTTTCG CATCTCCTTT TGTCGGGTTC TCTGCGCAAG GACTCGAACC AATCCTTTTA   
  
  
- TTTAGGGCTA TATAAGTACG TACCCCACCA CTTATGGCGT AGTTCACATG GAAAGAAGGA CTTGTCCAAG   
  
  
- TTCCTCCGGT ACAAAGTGAT GTGCCGGAAC AAACTGAACA AGCTCCGGTG TTACTCGGCG CTCCTAGTTC   
  
  
- TCTCCCACAA TGATCTCTCG TTCAACGTAC CCTTAGTCCG CAACTTACAC TAGCGGACGC TTCCTCGCCT   
  
  
- CTCTCACCTC TCCGGACTCA GTATGTTTGT TACCGTCCAA TCCTGAGTTT CCCGGCCCAA ACAAGTTCAC   
  
  
- AGCTACCTAG CGCTCGAAAA CTCTTCCCGT TCCCGGTACC AGTCCTCCTT AAAATCCCTT CTCAAGTCAA   
  
  
- TACTACTTCT ACCCGTAACC CACCAAGTTC CTACCTTTCC GTCCTATTAC ATACGGTAAA GAACAACCTT   
  
  
- TGGAAGTAT

+     ATC-motif

| Site Name | Organism | Position | Strand | Matrix score. | sequence | function |
| --- | --- | --- | --- | --- | --- | --- |
| ATC-motif | Pisum sativum | 2470 | + | 9 | TGCTATCCA | part of a conserved DNA module involved in light responsiveness |

>HU02G01570.1   
+ +Up\_Stream \_Len000ATTATT ATTCTAATAT ATAGATAATA ATATAATAAT AATAATAATA ATAATAATAA   
  
  
+ TTATTATTAT TATTATTATT TATTAAATAT AATAGTTATT ATAATAATAA TAATTATAAT AATAATAAGA   
  
  
+ TTATTATCAT TATATTATTA TTATTATTAT TGTAATTTAT TATTATTATA GTAATTATTA TTGTTATATA   
  
  
+ GTAAAATTAT TATTAGTATT GTTATTATAA ATATTCATAC TTGCATATAA TATTTCTAAA ATAAAAACAG   
  
  
+ AGTTAATTCC ATTCCAAAAC TACTCACCAA ACAGCAAGGA GTGGGACCTG ATTCCTTTCC AATTTTCACC   
  
  
+ AAACAACTGG GTTCAGAATT CAAGTTCCAT ATCCAACCCC CCAGGATTCA AGTCCTGATT CCATTCCATT   
  
  
+ CCAGTTTCCC TCACCAAACT GGGCCTTAGA TTGATGGTTG TTCAGTTGGA TGAGGGTTGT TCCATGCCAA   
  
  
+ GTTAGATCAT TGTTGTCAAT TTTGAGAACG ACTATCTTAA TAAGCCTAAC GATGTTGATT TGATTTAGTT   
  
  
+ CAAACCATTT CAAATAATAC AAATCAAATT CTATTTAGAC CGACACTAAA ATAAATAGAT TGAGAAAAAT   
  
  
+ TATAGATTTT AAAGTTCAGA TTAAAATCCC TAAAGATCAA CAGATTGAAA TGCATATTTC ATAGTGCACA   
  
  
+ TGGACTTGCT TTGAATCTCG TCTAGAGAAC CTAAGAAAAA GCAAGAGTGG TTCAGAAGGT TAGAATTGTT   
  
  
+ CCAGCACATG ACAAGTACAA TCCCAACTTT TTTCAAAAGA TTTGCTACTT TTAATTGGAT TCCCATGTTG   
  
  
+ AATGAAGTTC TGGGTTTAAG GCTACAACTT TCTTGGGAGG ATAAGGTTCT TAAGTTAAGG TGGTCCTCTT   
  
  
+ TTCTTTTGAT CTTTCTTATG GGTACAACCT CATGGGTCTC ATCAACACAT GAACTCTTTT TATCTTTTAT   
  
  
+ CTTTCCCCTT TCCTTTATGC AGGATTGACA GGTGCATGGA AAACAAGAAG CAATTAAATA CTAGTGGCTT   
  
  
+ AGCCCCATCC CTAATATTTT GGCTATTCTG TTTCACCATC CTCCCCTGTC TCCATTTTCT TTGTTTCCTT   
  
  
+ TTTTTCTAGG AACTTGTGAT GGGCAAGGGA AGATCAGTAC AATGTGATGG TTGTTAGTAG CGAGAAGATG   
  
  
+ AAGTGTCACC GCAAAAATTT TGTGCCATAA TTTGTCGTAA GGGAAACAAG TATCAAACAA CAATGTCATT   
  
  
+ TATGATAAAC AAGTAACAGG AGATATGTAC TCTGAAAAAA TATAAGGTAC CTGAGAAGTT GTTAACTTAG   
  
  
+ CTTGAAAAAC CCTCCTAACT TGATAAACCC TCTTCGCTAT GTTATCCCGC ACCCTGCCAT AGAGTCACCG   
  
  
+ ATTCAGTATA TATGTAACAT ACCCAATCTT GTCTGGATAT ATAGTTAACC CTTTTGGAAT ATCTGAAAAT   
  
  
+ CCACACGAGA GTCATCCTTC TAGACTAGGA AACATGCGCC ACATTTTGGA TTCTAACCAA GTATAATAAG   
  
  
+ CATATCTTAA AGCCAAAAAT GCAAGGATAG ATACAAATAC CCATCACATG CATGTAAATG TCCCTTGTCA   
  
  
+ TGGCTCTGAT TCTCAAAATA TTAATTTTCA ATAAATTAAA TGAAATTGCA TTATTTTAAA GATTTCTGAA   
  
  
+ TGCATCCATT ATTGCAATTT TTTAAAGACT GCACAATGAT GACAATGTCA ATGGTTTCCT AAAGTTGCGC   
  
  
+ CTGATGGGCT ATTGACAGTG AAGCAACACT CCCTTTGTTC CCATTTATGT GTCTCCACTT GCCCCTTTGA   
  
  
+ AACTCTCTAC ATTTGTATGT CATCTGAATT TGATTCTCAA ATCTCAGATG ATTTGAATTC CCTTTATGCC   
  
  
+ ATCTTTACTA TCTATTGAAT GATTCTTTCT TTTCCACTTT CTTATCTTCC ACTACCTTCT TCTTGGATGC   
  
  
+ CCTTTTGCTG CTTATAAAGG GCGACACCTG AACAAGTTGC TGTATTCTCA AACCATGAAT CTGTACAGGT   
  
  
+ TTAGCCCCGA TATGTGTCCT AGCTTTGTGG GTCACGAAGA AATTGGTCCT AATTTAATAG GACACCAAGA   
  
  
+ CCCTTTTCAA AATGCATACA ATTGCACTGA TTTTGGTGAT GCTTACTTGT TGCAATTCCC AGTTGAAGAT   
  
  
+ TTATGTCAAT ACCGTGTTCC TGATCTAATG GGGCTACCTG TTGAAGATGG GGATCCTACA GAGGTCCCTG   
  
  
+ ACATTCCTGA TACATGCTTC AACTACATAA GCAATCTCCT AATGGAAGAG GGCTCGGATG ACCGCCCTGG   
  
  
+ CGCCACCCTG CAGGACTATA TGGCTGCTCA AGTCTCCTAC AGCAAGTCCT CCAATGCCGC CTTTCCCGGT   
  
  
+ GAGAGTTGCT CATTCTTTGG TAATAATCAA TGCCCTCCAC CTGCTTATCA AAGCAACCCT TATTGCCTTG   
  
  
+ ATGATTACTT CAACTTGCTA TCCAATACTA CTGGCAATAG TGTTAACAAT GCTGGTGTTC ATGACAGCAA   
  
  
+ CAACTTTGTT CAGAACAGTT TGATTGGGAA TAGGGGTACT AGTTCTAGTT CTAGGGGCAG AAGTGATTTT   
  
  
+ GGATCTACCC TTATTAGGAA TTTGCCATTA GGGACCGACA CACTTGATGT GGTCAATCCA GCACCTACTC   
  
  
+ CTCGAGCCCC TCTGGTTACG ACTCAGTGTC AGGTGTTTGA AACACACCAG TATGAGCAGG CCTTCGAACT   
  
  
+ GATCACTAAT GGAAGCTGGG AATTCAGTCA TTCTGTTGCT AATACATCAA ATGATGGAAT TCAAAATCAA   
  
  
+ CCAATTCAAG CAGTTTCAAC CCAAGAACGA TGGAATATGA AAACATTTTC CGATGACCAA CTTGAGCCTG   
  
  
+ CTCTTTTGCG AAGTCTTGTG ATGTCCTCAA ACTGTACTGA TATCTCTGAT CAGGGTGTAC ACAGGATAGT   
  
  
+ GAACCCTGAG CCACCCAACA ACAGAAATAC GGAGATTGTG TGCAATGACT CATCCAGTAA GAGTAAGACA   
  
  
+ AGACGAAGAG AGCATGGTGC AGGAGAAAAC CACGATGAAG GAGAAAACCA CGATGAGACA GGAAGGAGTA   
  
  
+ ACAAGCAATC AGCACCATCC AATGAAGTGT TCGATGAAAT TGAGCAGTAT GGTGATGTGC TGCTCTGCCC   
  
  
+ TGAAGGAAGA AGTAAACCAG CAGGGTTATG CAGCAGCAGC AACGCTAACA GAACTAGCAA CTCACAGAAC   
  
  
+ TCGGAAGACT CAAAGTCACA GCAAAATGGG AGTTCCTCCA AGGTGTCAAA AAAGCGAGGC AATAAAAAGC   
  
  
+ AAGTGGTTGA TTTGAGGACT CTCCTAACTC GATGTGCACA ATGTGTTGCT CGTGTGGATT TAACTGGTTC   
  
  
+ TTACCAGTTG CTTCAGGAGA TTCGGCAGCA TTCTTCTCCC TATGGTGATT ATCTCCAAAG AATGGCTCAT   
  
  
+ TACCTTGCTA ATGGCCTTGA GGCGCGCTTA GAAGGCAAAG GATATGAACT AACTCGTGCC AATGAGCATA   
  
  
+ TCCCACCTAC TGAGATCCTC AAGGCTAACC GGGTATATGT GGCAGCGGTC CCTTTCAAGA TAATGTCTTA   
  
  
+ TTACACAACA AACAAGACAA TTGCAAGCCT AGTTGAGGAG GCACCAAGTA TACACATAAT TGATTTTGGT   
  
  
+ ATTTTCTATG GACTACAATG GCCCTGCATC ATTCAGAACC TCTCCAAGAG GCCCAATGGG CCTCCCAGAA   
  
  
+ TCCGCATCAC AGGCATCGAT TTCCCCCAAC CTGGGTTCCG GCCAGCAGAG AGGGTTGAAG AGACAGGGCG   
  
  
+ GTGTTTAGCC AAGTATTGTG ACAGGTACAA TGTGCCCTTT GAATACCACC CCATAGCCAA GAAGTGGGAG   
  
  
+ ACCATCCAAC TCGAGGAGCT CAAAATTGAA AGGAATGAGC CCCTTGTAGT GAACTGTCTG TATAGATCGC   
  
  
+ ACAACCTATT TGATGAAAGC GTAGAGGAAA ACAGCCCAAG AGACGCGTTC CTGAGCTTGG TTAGGAAAAT   
  
  
+ AAATCCCGAT ATATTCATGC ATGGGGTGGT GAATACCGCA TCAAGTGTAC CTTTCTTCCT GAACAGGTTC   
  
  
+ AAGGAGGCCA TGTTTCACTA CACGGCCTTG TTTGACTTGT TCGAGGCCAC AATGAGCCGC GAGGATCAAG   
  
  
+ AGAGGGTGTT ACTAGAGAGC AAGTTGCATG GGAATCAGGC GTTGAATGTG ATCGCCTGCG AAGGAGCGGA   
  
  
+ GAGAGTGGAG AGGCCTGAGT CATACAAACA ATGGCAGGTT AGGACTCAAA GGGCCGGGTT TGTTCAAGTG   
  
  
+ TCGATGGATC GCGAGCTTTT GAGAAGGGCA AGGGCCATGG TCAGGAGGAA TTTTAGGGAA GAGTTCAGTT   
  
  
+ ATGATGAAGA TGGGCATTGG GTGGTTCAAG GATGGAAAGG CAGGATAATG TATGCCATTT CTTGTTGGAA   
  
  
+ ACCTTCATA  

- +Up\_Stream \_Len000TAATAA TAAGATTATA TATCTATTAT TATATTATTA TTATTATTAT TATTATTATT   
  
  
- AATAATAATA ATAATAATAA ATAATTTATA TTATCAATAA TATTATTATT ATTAATATTA TTATTATTCT   
  
  
- AATAATAGTA ATATAATAAT AATAATAATA ACATTAAATA ATAATAATAT CATTAATAAT AACAATATAT   
  
  
- CATTTTAATA ATAATCATAA CAATAATATT TATAAGTATG AACGTATATT ATAAAGATTT TATTTTTGTC   
  
  
- TCAATTAAGG TAAGGTTTTG ATGAGTGGTT TGTCGTTCCT CACCCTGGAC TAAGGAAAGG TTAAAAGTGG   
  
  
- TTTGTTGACC CAAGTCTTAA GTTCAAGGTA TAGGTTGGGG GGTCCTAAGT TCAGGACTAA GGTAAGGTAA   
  
  
- GGTCAAAGGG AGTGGTTTGA CCCGGAATCT AACTACCAAC AAGTCAACCT ACTCCCAACA AGGTACGGTT   
  
  
- CAATCTAGTA ACAACAGTTA AAACTCTTGC TGATAGAATT ATTCGGATTG CTACAACTAA ACTAAATCAA   
  
  
- GTTTGGTAAA GTTTATTATG TTTAGTTTAA GATAAATCTG GCTGTGATTT TATTTATCTA ACTCTTTTTA   
  
  
- ATATCTAAAA TTTCAAGTCT AATTTTAGGG ATTTCTAGTT GTCTAACTTT ACGTATAAAG TATCACGTGT   
  
  
- ACCTGAACGA AACTTAGAGC AGATCTCTTG GATTCTTTTT CGTTCTCACC AAGTCTTCCA ATCTTAACAA   
  
  
- GGTCGTGTAC TGTTCATGTT AGGGTTGAAA AAAGTTTTCT AAACGATGAA AATTAACCTA AGGGTACAAC   
  
  
- TTACTTCAAG ACCCAAATTC CGATGTTGAA AGAACCCTCC TATTCCAAGA ATTCAATTCC ACCAGGAGAA   
  
  
- AAGAAAACTA GAAAGAATAC CCATGTTGGA GTACCCAGAG TAGTTGTGTA CTTGAGAAAA ATAGAAAATA   
  
  
- GAAAGGGGAA AGGAAATACG TCCTAACTGT CCACGTACCT TTTGTTCTTC GTTAATTTAT GATCACCGAA   
  
  
- TCGGGGTAGG GATTATAAAA CCGATAAGAC AAAGTGGTAG GAGGGGACAG AGGTAAAAGA AACAAAGGAA   
  
  
- AAAAAGATCC TTGAACACTA CCCGTTCCCT TCTAGTCATG TTACACTACC AACAATCATC GCTCTTCTAC   
  
  
- TTCACAGTGG CGTTTTTAAA ACACGGTATT AAACAGCATT CCCTTTGTTC ATAGTTTGTT GTTACAGTAA   
  
  
- ATACTATTTG TTCATTGTCC TCTATACATG AGACTTTTTT ATATTCCATG GACTCTTCAA CAATTGAATC   
  
  
- GAACTTTTTG GGAGGATTGA ACTATTTGGG AGAAGCGATA CAATAGGGCG TGGGACGGTA TCTCAGTGGC   
  
  
- TAAGTCATAT ATACATTGTA TGGGTTAGAA CAGACCTATA TATCAATTGG GAAAACCTTA TAGACTTTTA   
  
  
- GGTGTGCTCT CAGTAGGAAG ATCTGATCCT TTGTACGCGG TGTAAAACCT AAGATTGGTT CATATTATTC   
  
  
- GTATAGAATT TCGGTTTTTA CGTTCCTATC TATGTTTATG GGTAGTGTAC GTACATTTAC AGGGAACAGT   
  
  
- ACCGAGACTA AGAGTTTTAT AATTAAAAGT TATTTAATTT ACTTTAACGT AATAAAATTT CTAAAGACTT   
  
  
- ACGTAGGTAA TAACGTTAAA AAATTTCTGA CGTGTTACTA CTGTTACAGT TACCAAAGGA TTTCAACGCG   
  
  
- GACTACCCGA TAACTGTCAC TTCGTTGTGA GGGAAACAAG GGTAAATACA CAGAGGTGAA CGGGGAAACT   
  
  
- TTGAGAGATG TAAACATACA GTAGACTTAA ACTAAGAGTT TAGAGTCTAC TAAACTTAAG GGAAATACGG   
  
  
- TAGAAATGAT AGATAACTTA CTAAGAAAGA AAAGGTGAAA GAATAGAAGG TGATGGAAGA AGAACCTACG   
  
  
- GGAAAACGAC GAATATTTCC CGCTGTGGAC TTGTTCAACG ACATAAGAGT TTGGTACTTA GACATGTCCA   
  
  
- AATCGGGGCT ATACACAGGA TCGAAACACC CAGTGCTTCT TTAACCAGGA TTAAATTATC CTGTGGTTCT   
  
  
- GGGAAAAGTT TTACGTATGT TAACGTGACT AAAACCACTA CGAATGAACA ACGTTAAGGG TCAACTTCTA   
  
  
- AATACAGTTA TGGCACAAGG ACTAGATTAC CCCGATGGAC AACTTCTACC CCTAGGATGT CTCCAGGGAC   
  
  
- TGTAAGGACT ATGTACGAAG TTGATGTATT CGTTAGAGGA TTACCTTCTC CCGAGCCTAC TGGCGGGACC   
  
  
- GCGGTGGGAC GTCCTGATAT ACCGACGAGT TCAGAGGATG TCGTTCAGGA GGTTACGGCG GAAAGGGCCA   
  
  
- CTCTCAACGA GTAAGAAACC ATTATTAGTT ACGGGAGGTG GACGAATAGT TTCGTTGGGA ATAACGGAAC   
  
  
- TACTAATGAA GTTGAACGAT AGGTTATGAT GACCGTTATC ACAATTGTTA CGACCACAAG TACTGTCGTT   
  
  
- GTTGAAACAA GTCTTGTCAA ACTAACCCTT ATCCCCATGA TCAAGATCAA GATCCCCGTC TTCACTAAAA   
  
  
- CCTAGATGGG AATAATCCTT AAACGGTAAT CCCTGGCTGT GTGAACTACA CCAGTTAGGT CGTGGATGAG   
  
  
- GAGCTCGGGG AGACCAATGC TGAGTCACAG TCCACAAACT TTGTGTGGTC ATACTCGTCC GGAAGCTTGA   
  
  
- CTAGTGATTA CCTTCGACCC TTAAGTCAGT AAGACAACGA TTATGTAGTT TACTACCTTA AGTTTTAGTT   
  
  
- GGTTAAGTTC GTCAAAGTTG GGTTCTTGCT ACCTTATACT TTTGTAAAAG GCTACTGGTT GAACTCGGAC   
  
  
- GAGAAAACGC TTCAGAACAC TACAGGAGTT TGACATGACT ATAGAGACTA GTCCCACATG TGTCCTATCA   
  
  
- CTTGGGACTC GGTGGGTTGT TGTCTTTATG CCTCTAACAC ACGTTACTGA GTAGGTCATT CTCATTCTGT   
  
  
- TCTGCTTCTC TCGTACCACG TCCTCTTTTG GTGCTACTTC CTCTTTTGGT GCTACTCTGT CCTTCCTCAT   
  
  
- TGTTCGTTAG TCGTGGTAGG TTACTTCACA AGCTACTTTA ACTCGTCATA CCACTACACG ACGAGACGGG   
  
  
- ACTTCCTTCT TCATTTGGTC GTCCCAATAC GTCGTCGTCG TTGCGATTGT CTTGATCGTT GAGTGTCTTG   
  
  
- AGCCTTCTGA GTTTCAGTGT CGTTTTACCC TCAAGGAGGT TCCACAGTTT TTTCGCTCCG TTATTTTTCG   
  
  
- TTCACCAACT AAACTCCTGA GAGGATTGAG CTACACGTGT TACACAACGA GCACACCTAA ATTGACCAAG   
  
  
- AATGGTCAAC GAAGTCCTCT AAGCCGTCGT AAGAAGAGGG ATACCACTAA TAGAGGTTTC TTACCGAGTA   
  
  
- ATGGAACGAT TACCGGAACT CCGCGCGAAT CTTCCGTTTC CTATACTTGA TTGAGCACGG TTACTCGTAT   
  
  
- AGGGTGGATG ACTCTAGGAG TTCCGATTGG CCCATATACA CCGTCGCCAG GGAAAGTTCT ATTACAGAAT   
  
  
- AATGTGTTGT TTGTTCTGTT AACGTTCGGA TCAACTCCTC CGTGGTTCAT ATGTGTATTA ACTAAAACCA   
  
  
- TAAAAGATAC CTGATGTTAC CGGGACGTAG TAAGTCTTGG AGAGGTTCTC CGGGTTACCC GGAGGGTCTT   
  
  
- AGGCGTAGTG TCCGTAGCTA AAGGGGGTTG GACCCAAGGC CGGTCGTCTC TCCCAACTTC TCTGTCCCGC   
  
  
- CACAAATCGG TTCATAACAC TGTCCATGTT ACACGGGAAA CTTATGGTGG GGTATCGGTT CTTCACCCTC   
  
  
- TGGTAGGTTG AGCTCCTCGA GTTTTAACTT TCCTTACTCG GGGAACATCA CTTGACAGAC ATATCTAGCG   
  
  
- TGTTGGATAA ACTACTTTCG CATCTCCTTT TGTCGGGTTC TCTGCGCAAG GACTCGAACC AATCCTTTTA   
  
  
- TTTAGGGCTA TATAAGTACG TACCCCACCA CTTATGGCGT AGTTCACATG GAAAGAAGGA CTTGTCCAAG   
  
  
- TTCCTCCGGT ACAAAGTGAT GTGCCGGAAC AAACTGAACA AGCTCCGGTG TTACTCGGCG CTCCTAGTTC   
  
  
- TCTCCCACAA TGATCTCTCG TTCAACGTAC CCTTAGTCCG CAACTTACAC TAGCGGACGC TTCCTCGCCT   
  
  
- CTCTCACCTC TCCGGACTCA GTATGTTTGT TACCGTCCAA TCCTGAGTTT CCCGGCCCAA ACAAGTTCAC   
  
  
- AGCTACCTAG CGCTCGAAAA CTCTTCCCGT TCCCGGTACC AGTCCTCCTT AAAATCCCTT CTCAAGTCAA   
  
  
- TACTACTTCT ACCCGTAACC CACCAAGTTC CTACCTTTCC GTCCTATTAC ATACGGTAAA GAACAACCTT   
  
  
- TGGAAGTAT

+     AT~TATA-box

| Site Name | Organism | Position | Strand | Matrix score. | sequence | function |
| --- | --- | --- | --- | --- | --- | --- |
| AT~TATA-box | Arabidopsis thaliana | 1442 | + | 6 | TATATA |  |
| AT~TATA-box | Arabidopsis thaliana | 1411 | + | 6 | TATATA |  |
| AT~TATA-box | Arabidopsis thaliana | 209 | + | 6 | TATATA |  |
| AT~TATA-box | Arabidopsis thaliana | 32 | + | 6 | TATATA |  |

>HU02G01570.1   
+ +Up\_Stream \_Len000ATTATT ATTCTAATAT ATAGATAATA ATATAATAAT AATAATAATA ATAATAATAA   
  
  
+ TTATTATTAT TATTATTATT TATTAAATAT AATAGTTATT ATAATAATAA TAATTATAAT AATAATAAGA   
  
  
+ TTATTATCAT TATATTATTA TTATTATTAT TGTAATTTAT TATTATTATA GTAATTATTA TTGTTATATA   
  
  
+ GTAAAATTAT TATTAGTATT GTTATTATAA ATATTCATAC TTGCATATAA TATTTCTAAA ATAAAAACAG   
  
  
+ AGTTAATTCC ATTCCAAAAC TACTCACCAA ACAGCAAGGA GTGGGACCTG ATTCCTTTCC AATTTTCACC   
  
  
+ AAACAACTGG GTTCAGAATT CAAGTTCCAT ATCCAACCCC CCAGGATTCA AGTCCTGATT CCATTCCATT   
  
  
+ CCAGTTTCCC TCACCAAACT GGGCCTTAGA TTGATGGTTG TTCAGTTGGA TGAGGGTTGT TCCATGCCAA   
  
  
+ GTTAGATCAT TGTTGTCAAT TTTGAGAACG ACTATCTTAA TAAGCCTAAC GATGTTGATT TGATTTAGTT   
  
  
+ CAAACCATTT CAAATAATAC AAATCAAATT CTATTTAGAC CGACACTAAA ATAAATAGAT TGAGAAAAAT   
  
  
+ TATAGATTTT AAAGTTCAGA TTAAAATCCC TAAAGATCAA CAGATTGAAA TGCATATTTC ATAGTGCACA   
  
  
+ TGGACTTGCT TTGAATCTCG TCTAGAGAAC CTAAGAAAAA GCAAGAGTGG TTCAGAAGGT TAGAATTGTT   
  
  
+ CCAGCACATG ACAAGTACAA TCCCAACTTT TTTCAAAAGA TTTGCTACTT TTAATTGGAT TCCCATGTTG   
  
  
+ AATGAAGTTC TGGGTTTAAG GCTACAACTT TCTTGGGAGG ATAAGGTTCT TAAGTTAAGG TGGTCCTCTT   
  
  
+ TTCTTTTGAT CTTTCTTATG GGTACAACCT CATGGGTCTC ATCAACACAT GAACTCTTTT TATCTTTTAT   
  
  
+ CTTTCCCCTT TCCTTTATGC AGGATTGACA GGTGCATGGA AAACAAGAAG CAATTAAATA CTAGTGGCTT   
  
  
+ AGCCCCATCC CTAATATTTT GGCTATTCTG TTTCACCATC CTCCCCTGTC TCCATTTTCT TTGTTTCCTT   
  
  
+ TTTTTCTAGG AACTTGTGAT GGGCAAGGGA AGATCAGTAC AATGTGATGG TTGTTAGTAG CGAGAAGATG   
  
  
+ AAGTGTCACC GCAAAAATTT TGTGCCATAA TTTGTCGTAA GGGAAACAAG TATCAAACAA CAATGTCATT   
  
  
+ TATGATAAAC AAGTAACAGG AGATATGTAC TCTGAAAAAA TATAAGGTAC CTGAGAAGTT GTTAACTTAG   
  
  
+ CTTGAAAAAC CCTCCTAACT TGATAAACCC TCTTCGCTAT GTTATCCCGC ACCCTGCCAT AGAGTCACCG   
  
  
+ ATTCAGTATA TATGTAACAT ACCCAATCTT GTCTGGATAT ATAGTTAACC CTTTTGGAAT ATCTGAAAAT   
  
  
+ CCACACGAGA GTCATCCTTC TAGACTAGGA AACATGCGCC ACATTTTGGA TTCTAACCAA GTATAATAAG   
  
  
+ CATATCTTAA AGCCAAAAAT GCAAGGATAG ATACAAATAC CCATCACATG CATGTAAATG TCCCTTGTCA   
  
  
+ TGGCTCTGAT TCTCAAAATA TTAATTTTCA ATAAATTAAA TGAAATTGCA TTATTTTAAA GATTTCTGAA   
  
  
+ TGCATCCATT ATTGCAATTT TTTAAAGACT GCACAATGAT GACAATGTCA ATGGTTTCCT AAAGTTGCGC   
  
  
+ CTGATGGGCT ATTGACAGTG AAGCAACACT CCCTTTGTTC CCATTTATGT GTCTCCACTT GCCCCTTTGA   
  
  
+ AACTCTCTAC ATTTGTATGT CATCTGAATT TGATTCTCAA ATCTCAGATG ATTTGAATTC CCTTTATGCC   
  
  
+ ATCTTTACTA TCTATTGAAT GATTCTTTCT TTTCCACTTT CTTATCTTCC ACTACCTTCT TCTTGGATGC   
  
  
+ CCTTTTGCTG CTTATAAAGG GCGACACCTG AACAAGTTGC TGTATTCTCA AACCATGAAT CTGTACAGGT   
  
  
+ TTAGCCCCGA TATGTGTCCT AGCTTTGTGG GTCACGAAGA AATTGGTCCT AATTTAATAG GACACCAAGA   
  
  
+ CCCTTTTCAA AATGCATACA ATTGCACTGA TTTTGGTGAT GCTTACTTGT TGCAATTCCC AGTTGAAGAT   
  
  
+ TTATGTCAAT ACCGTGTTCC TGATCTAATG GGGCTACCTG TTGAAGATGG GGATCCTACA GAGGTCCCTG   
  
  
+ ACATTCCTGA TACATGCTTC AACTACATAA GCAATCTCCT AATGGAAGAG GGCTCGGATG ACCGCCCTGG   
  
  
+ CGCCACCCTG CAGGACTATA TGGCTGCTCA AGTCTCCTAC AGCAAGTCCT CCAATGCCGC CTTTCCCGGT   
  
  
+ GAGAGTTGCT CATTCTTTGG TAATAATCAA TGCCCTCCAC CTGCTTATCA AAGCAACCCT TATTGCCTTG   
  
  
+ ATGATTACTT CAACTTGCTA TCCAATACTA CTGGCAATAG TGTTAACAAT GCTGGTGTTC ATGACAGCAA   
  
  
+ CAACTTTGTT CAGAACAGTT TGATTGGGAA TAGGGGTACT AGTTCTAGTT CTAGGGGCAG AAGTGATTTT   
  
  
+ GGATCTACCC TTATTAGGAA TTTGCCATTA GGGACCGACA CACTTGATGT GGTCAATCCA GCACCTACTC   
  
  
+ CTCGAGCCCC TCTGGTTACG ACTCAGTGTC AGGTGTTTGA AACACACCAG TATGAGCAGG CCTTCGAACT   
  
  
+ GATCACTAAT GGAAGCTGGG AATTCAGTCA TTCTGTTGCT AATACATCAA ATGATGGAAT TCAAAATCAA   
  
  
+ CCAATTCAAG CAGTTTCAAC CCAAGAACGA TGGAATATGA AAACATTTTC CGATGACCAA CTTGAGCCTG   
  
  
+ CTCTTTTGCG AAGTCTTGTG ATGTCCTCAA ACTGTACTGA TATCTCTGAT CAGGGTGTAC ACAGGATAGT   
  
  
+ GAACCCTGAG CCACCCAACA ACAGAAATAC GGAGATTGTG TGCAATGACT CATCCAGTAA GAGTAAGACA   
  
  
+ AGACGAAGAG AGCATGGTGC AGGAGAAAAC CACGATGAAG GAGAAAACCA CGATGAGACA GGAAGGAGTA   
  
  
+ ACAAGCAATC AGCACCATCC AATGAAGTGT TCGATGAAAT TGAGCAGTAT GGTGATGTGC TGCTCTGCCC   
  
  
+ TGAAGGAAGA AGTAAACCAG CAGGGTTATG CAGCAGCAGC AACGCTAACA GAACTAGCAA CTCACAGAAC   
  
  
+ TCGGAAGACT CAAAGTCACA GCAAAATGGG AGTTCCTCCA AGGTGTCAAA AAAGCGAGGC AATAAAAAGC   
  
  
+ AAGTGGTTGA TTTGAGGACT CTCCTAACTC GATGTGCACA ATGTGTTGCT CGTGTGGATT TAACTGGTTC   
  
  
+ TTACCAGTTG CTTCAGGAGA TTCGGCAGCA TTCTTCTCCC TATGGTGATT ATCTCCAAAG AATGGCTCAT   
  
  
+ TACCTTGCTA ATGGCCTTGA GGCGCGCTTA GAAGGCAAAG GATATGAACT AACTCGTGCC AATGAGCATA   
  
  
+ TCCCACCTAC TGAGATCCTC AAGGCTAACC GGGTATATGT GGCAGCGGTC CCTTTCAAGA TAATGTCTTA   
  
  
+ TTACACAACA AACAAGACAA TTGCAAGCCT AGTTGAGGAG GCACCAAGTA TACACATAAT TGATTTTGGT   
  
  
+ ATTTTCTATG GACTACAATG GCCCTGCATC ATTCAGAACC TCTCCAAGAG GCCCAATGGG CCTCCCAGAA   
  
  
+ TCCGCATCAC AGGCATCGAT TTCCCCCAAC CTGGGTTCCG GCCAGCAGAG AGGGTTGAAG AGACAGGGCG   
  
  
+ GTGTTTAGCC AAGTATTGTG ACAGGTACAA TGTGCCCTTT GAATACCACC CCATAGCCAA GAAGTGGGAG   
  
  
+ ACCATCCAAC TCGAGGAGCT CAAAATTGAA AGGAATGAGC CCCTTGTAGT GAACTGTCTG TATAGATCGC   
  
  
+ ACAACCTATT TGATGAAAGC GTAGAGGAAA ACAGCCCAAG AGACGCGTTC CTGAGCTTGG TTAGGAAAAT   
  
  
+ AAATCCCGAT ATATTCATGC ATGGGGTGGT GAATACCGCA TCAAGTGTAC CTTTCTTCCT GAACAGGTTC   
  
  
+ AAGGAGGCCA TGTTTCACTA CACGGCCTTG TTTGACTTGT TCGAGGCCAC AATGAGCCGC GAGGATCAAG   
  
  
+ AGAGGGTGTT ACTAGAGAGC AAGTTGCATG GGAATCAGGC GTTGAATGTG ATCGCCTGCG AAGGAGCGGA   
  
  
+ GAGAGTGGAG AGGCCTGAGT CATACAAACA ATGGCAGGTT AGGACTCAAA GGGCCGGGTT TGTTCAAGTG   
  
  
+ TCGATGGATC GCGAGCTTTT GAGAAGGGCA AGGGCCATGG TCAGGAGGAA TTTTAGGGAA GAGTTCAGTT   
  
  
+ ATGATGAAGA TGGGCATTGG GTGGTTCAAG GATGGAAAGG CAGGATAATG TATGCCATTT CTTGTTGGAA   
  
  
+ ACCTTCATA  

- +Up\_Stream \_Len000TAATAA TAAGATTATA TATCTATTAT TATATTATTA TTATTATTAT TATTATTATT   
  
  
- AATAATAATA ATAATAATAA ATAATTTATA TTATCAATAA TATTATTATT ATTAATATTA TTATTATTCT   
  
  
- AATAATAGTA ATATAATAAT AATAATAATA ACATTAAATA ATAATAATAT CATTAATAAT AACAATATAT   
  
  
- CATTTTAATA ATAATCATAA CAATAATATT TATAAGTATG AACGTATATT ATAAAGATTT TATTTTTGTC   
  
  
- TCAATTAAGG TAAGGTTTTG ATGAGTGGTT TGTCGTTCCT CACCCTGGAC TAAGGAAAGG TTAAAAGTGG   
  
  
- TTTGTTGACC CAAGTCTTAA GTTCAAGGTA TAGGTTGGGG GGTCCTAAGT TCAGGACTAA GGTAAGGTAA   
  
  
- GGTCAAAGGG AGTGGTTTGA CCCGGAATCT AACTACCAAC AAGTCAACCT ACTCCCAACA AGGTACGGTT   
  
  
- CAATCTAGTA ACAACAGTTA AAACTCTTGC TGATAGAATT ATTCGGATTG CTACAACTAA ACTAAATCAA   
  
  
- GTTTGGTAAA GTTTATTATG TTTAGTTTAA GATAAATCTG GCTGTGATTT TATTTATCTA ACTCTTTTTA   
  
  
- ATATCTAAAA TTTCAAGTCT AATTTTAGGG ATTTCTAGTT GTCTAACTTT ACGTATAAAG TATCACGTGT   
  
  
- ACCTGAACGA AACTTAGAGC AGATCTCTTG GATTCTTTTT CGTTCTCACC AAGTCTTCCA ATCTTAACAA   
  
  
- GGTCGTGTAC TGTTCATGTT AGGGTTGAAA AAAGTTTTCT AAACGATGAA AATTAACCTA AGGGTACAAC   
  
  
- TTACTTCAAG ACCCAAATTC CGATGTTGAA AGAACCCTCC TATTCCAAGA ATTCAATTCC ACCAGGAGAA   
  
  
- AAGAAAACTA GAAAGAATAC CCATGTTGGA GTACCCAGAG TAGTTGTGTA CTTGAGAAAA ATAGAAAATA   
  
  
- GAAAGGGGAA AGGAAATACG TCCTAACTGT CCACGTACCT TTTGTTCTTC GTTAATTTAT GATCACCGAA   
  
  
- TCGGGGTAGG GATTATAAAA CCGATAAGAC AAAGTGGTAG GAGGGGACAG AGGTAAAAGA AACAAAGGAA   
  
  
- AAAAAGATCC TTGAACACTA CCCGTTCCCT TCTAGTCATG TTACACTACC AACAATCATC GCTCTTCTAC   
  
  
- TTCACAGTGG CGTTTTTAAA ACACGGTATT AAACAGCATT CCCTTTGTTC ATAGTTTGTT GTTACAGTAA   
  
  
- ATACTATTTG TTCATTGTCC TCTATACATG AGACTTTTTT ATATTCCATG GACTCTTCAA CAATTGAATC   
  
  
- GAACTTTTTG GGAGGATTGA ACTATTTGGG AGAAGCGATA CAATAGGGCG TGGGACGGTA TCTCAGTGGC   
  
  
- TAAGTCATAT ATACATTGTA TGGGTTAGAA CAGACCTATA TATCAATTGG GAAAACCTTA TAGACTTTTA   
  
  
- GGTGTGCTCT CAGTAGGAAG ATCTGATCCT TTGTACGCGG TGTAAAACCT AAGATTGGTT CATATTATTC   
  
  
- GTATAGAATT TCGGTTTTTA CGTTCCTATC TATGTTTATG GGTAGTGTAC GTACATTTAC AGGGAACAGT   
  
  
- ACCGAGACTA AGAGTTTTAT AATTAAAAGT TATTTAATTT ACTTTAACGT AATAAAATTT CTAAAGACTT   
  
  
- ACGTAGGTAA TAACGTTAAA AAATTTCTGA CGTGTTACTA CTGTTACAGT TACCAAAGGA TTTCAACGCG   
  
  
- GACTACCCGA TAACTGTCAC TTCGTTGTGA GGGAAACAAG GGTAAATACA CAGAGGTGAA CGGGGAAACT   
  
  
- TTGAGAGATG TAAACATACA GTAGACTTAA ACTAAGAGTT TAGAGTCTAC TAAACTTAAG GGAAATACGG   
  
  
- TAGAAATGAT AGATAACTTA CTAAGAAAGA AAAGGTGAAA GAATAGAAGG TGATGGAAGA AGAACCTACG   
  
  
- GGAAAACGAC GAATATTTCC CGCTGTGGAC TTGTTCAACG ACATAAGAGT TTGGTACTTA GACATGTCCA   
  
  
- AATCGGGGCT ATACACAGGA TCGAAACACC CAGTGCTTCT TTAACCAGGA TTAAATTATC CTGTGGTTCT   
  
  
- GGGAAAAGTT TTACGTATGT TAACGTGACT AAAACCACTA CGAATGAACA ACGTTAAGGG TCAACTTCTA   
  
  
- AATACAGTTA TGGCACAAGG ACTAGATTAC CCCGATGGAC AACTTCTACC CCTAGGATGT CTCCAGGGAC   
  
  
- TGTAAGGACT ATGTACGAAG TTGATGTATT CGTTAGAGGA TTACCTTCTC CCGAGCCTAC TGGCGGGACC   
  
  
- GCGGTGGGAC GTCCTGATAT ACCGACGAGT TCAGAGGATG TCGTTCAGGA GGTTACGGCG GAAAGGGCCA   
  
  
- CTCTCAACGA GTAAGAAACC ATTATTAGTT ACGGGAGGTG GACGAATAGT TTCGTTGGGA ATAACGGAAC   
  
  
- TACTAATGAA GTTGAACGAT AGGTTATGAT GACCGTTATC ACAATTGTTA CGACCACAAG TACTGTCGTT   
  
  
- GTTGAAACAA GTCTTGTCAA ACTAACCCTT ATCCCCATGA TCAAGATCAA GATCCCCGTC TTCACTAAAA   
  
  
- CCTAGATGGG AATAATCCTT AAACGGTAAT CCCTGGCTGT GTGAACTACA CCAGTTAGGT CGTGGATGAG   
  
  
- GAGCTCGGGG AGACCAATGC TGAGTCACAG TCCACAAACT TTGTGTGGTC ATACTCGTCC GGAAGCTTGA   
  
  
- CTAGTGATTA CCTTCGACCC TTAAGTCAGT AAGACAACGA TTATGTAGTT TACTACCTTA AGTTTTAGTT   
  
  
- GGTTAAGTTC GTCAAAGTTG GGTTCTTGCT ACCTTATACT TTTGTAAAAG GCTACTGGTT GAACTCGGAC   
  
  
- GAGAAAACGC TTCAGAACAC TACAGGAGTT TGACATGACT ATAGAGACTA GTCCCACATG TGTCCTATCA   
  
  
- CTTGGGACTC GGTGGGTTGT TGTCTTTATG CCTCTAACAC ACGTTACTGA GTAGGTCATT CTCATTCTGT   
  
  
- TCTGCTTCTC TCGTACCACG TCCTCTTTTG GTGCTACTTC CTCTTTTGGT GCTACTCTGT CCTTCCTCAT   
  
  
- TGTTCGTTAG TCGTGGTAGG TTACTTCACA AGCTACTTTA ACTCGTCATA CCACTACACG ACGAGACGGG   
  
  
- ACTTCCTTCT TCATTTGGTC GTCCCAATAC GTCGTCGTCG TTGCGATTGT CTTGATCGTT GAGTGTCTTG   
  
  
- AGCCTTCTGA GTTTCAGTGT CGTTTTACCC TCAAGGAGGT TCCACAGTTT TTTCGCTCCG TTATTTTTCG   
  
  
- TTCACCAACT AAACTCCTGA GAGGATTGAG CTACACGTGT TACACAACGA GCACACCTAA ATTGACCAAG   
  
  
- AATGGTCAAC GAAGTCCTCT AAGCCGTCGT AAGAAGAGGG ATACCACTAA TAGAGGTTTC TTACCGAGTA   
  
  
- ATGGAACGAT TACCGGAACT CCGCGCGAAT CTTCCGTTTC CTATACTTGA TTGAGCACGG TTACTCGTAT   
  
  
- AGGGTGGATG ACTCTAGGAG TTCCGATTGG CCCATATACA CCGTCGCCAG GGAAAGTTCT ATTACAGAAT   
  
  
- AATGTGTTGT TTGTTCTGTT AACGTTCGGA TCAACTCCTC CGTGGTTCAT ATGTGTATTA ACTAAAACCA   
  
  
- TAAAAGATAC CTGATGTTAC CGGGACGTAG TAAGTCTTGG AGAGGTTCTC CGGGTTACCC GGAGGGTCTT   
  
  
- AGGCGTAGTG TCCGTAGCTA AAGGGGGTTG GACCCAAGGC CGGTCGTCTC TCCCAACTTC TCTGTCCCGC   
  
  
- CACAAATCGG TTCATAACAC TGTCCATGTT ACACGGGAAA CTTATGGTGG GGTATCGGTT CTTCACCCTC   
  
  
- TGGTAGGTTG AGCTCCTCGA GTTTTAACTT TCCTTACTCG GGGAACATCA CTTGACAGAC ATATCTAGCG   
  
  
- TGTTGGATAA ACTACTTTCG CATCTCCTTT TGTCGGGTTC TCTGCGCAAG GACTCGAACC AATCCTTTTA   
  
  
- TTTAGGGCTA TATAAGTACG TACCCCACCA CTTATGGCGT AGTTCACATG GAAAGAAGGA CTTGTCCAAG   
  
  
- TTCCTCCGGT ACAAAGTGAT GTGCCGGAAC AAACTGAACA AGCTCCGGTG TTACTCGGCG CTCCTAGTTC   
  
  
- TCTCCCACAA TGATCTCTCG TTCAACGTAC CCTTAGTCCG CAACTTACAC TAGCGGACGC TTCCTCGCCT   
  
  
- CTCTCACCTC TCCGGACTCA GTATGTTTGT TACCGTCCAA TCCTGAGTTT CCCGGCCCAA ACAAGTTCAC   
  
  
- AGCTACCTAG CGCTCGAAAA CTCTTCCCGT TCCCGGTACC AGTCCTCCTT AAAATCCCTT CTCAAGTCAA   
  
  
- TACTACTTCT ACCCGTAACC CACCAAGTTC CTACCTTTCC GTCCTATTAC ATACGGTAAA GAACAACCTT   
  
  
- TGGAAGTAT

+     Box 4

| Site Name | Organism | Position | Strand | Matrix score. | sequence | function |
| --- | --- | --- | --- | --- | --- | --- |
| Box 4 | Petroselinum crispum | 1634 | + | 6 | ATTAAT | part of a conserved DNA module involved in light responsiveness |

>HU02G01570.1   
+ +Up\_Stream \_Len000ATTATT ATTCTAATAT ATAGATAATA ATATAATAAT AATAATAATA ATAATAATAA   
  
  
+ TTATTATTAT TATTATTATT TATTAAATAT AATAGTTATT ATAATAATAA TAATTATAAT AATAATAAGA   
  
  
+ TTATTATCAT TATATTATTA TTATTATTAT TGTAATTTAT TATTATTATA GTAATTATTA TTGTTATATA   
  
  
+ GTAAAATTAT TATTAGTATT GTTATTATAA ATATTCATAC TTGCATATAA TATTTCTAAA ATAAAAACAG   
  
  
+ AGTTAATTCC ATTCCAAAAC TACTCACCAA ACAGCAAGGA GTGGGACCTG ATTCCTTTCC AATTTTCACC   
  
  
+ AAACAACTGG GTTCAGAATT CAAGTTCCAT ATCCAACCCC CCAGGATTCA AGTCCTGATT CCATTCCATT   
  
  
+ CCAGTTTCCC TCACCAAACT GGGCCTTAGA TTGATGGTTG TTCAGTTGGA TGAGGGTTGT TCCATGCCAA   
  
  
+ GTTAGATCAT TGTTGTCAAT TTTGAGAACG ACTATCTTAA TAAGCCTAAC GATGTTGATT TGATTTAGTT   
  
  
+ CAAACCATTT CAAATAATAC AAATCAAATT CTATTTAGAC CGACACTAAA ATAAATAGAT TGAGAAAAAT   
  
  
+ TATAGATTTT AAAGTTCAGA TTAAAATCCC TAAAGATCAA CAGATTGAAA TGCATATTTC ATAGTGCACA   
  
  
+ TGGACTTGCT TTGAATCTCG TCTAGAGAAC CTAAGAAAAA GCAAGAGTGG TTCAGAAGGT TAGAATTGTT   
  
  
+ CCAGCACATG ACAAGTACAA TCCCAACTTT TTTCAAAAGA TTTGCTACTT TTAATTGGAT TCCCATGTTG   
  
  
+ AATGAAGTTC TGGGTTTAAG GCTACAACTT TCTTGGGAGG ATAAGGTTCT TAAGTTAAGG TGGTCCTCTT   
  
  
+ TTCTTTTGAT CTTTCTTATG GGTACAACCT CATGGGTCTC ATCAACACAT GAACTCTTTT TATCTTTTAT   
  
  
+ CTTTCCCCTT TCCTTTATGC AGGATTGACA GGTGCATGGA AAACAAGAAG CAATTAAATA CTAGTGGCTT   
  
  
+ AGCCCCATCC CTAATATTTT GGCTATTCTG TTTCACCATC CTCCCCTGTC TCCATTTTCT TTGTTTCCTT   
  
  
+ TTTTTCTAGG AACTTGTGAT GGGCAAGGGA AGATCAGTAC AATGTGATGG TTGTTAGTAG CGAGAAGATG   
  
  
+ AAGTGTCACC GCAAAAATTT TGTGCCATAA TTTGTCGTAA GGGAAACAAG TATCAAACAA CAATGTCATT   
  
  
+ TATGATAAAC AAGTAACAGG AGATATGTAC TCTGAAAAAA TATAAGGTAC CTGAGAAGTT GTTAACTTAG   
  
  
+ CTTGAAAAAC CCTCCTAACT TGATAAACCC TCTTCGCTAT GTTATCCCGC ACCCTGCCAT AGAGTCACCG   
  
  
+ ATTCAGTATA TATGTAACAT ACCCAATCTT GTCTGGATAT ATAGTTAACC CTTTTGGAAT ATCTGAAAAT   
  
  
+ CCACACGAGA GTCATCCTTC TAGACTAGGA AACATGCGCC ACATTTTGGA TTCTAACCAA GTATAATAAG   
  
  
+ CATATCTTAA AGCCAAAAAT GCAAGGATAG ATACAAATAC CCATCACATG CATGTAAATG TCCCTTGTCA   
  
  
+ TGGCTCTGAT TCTCAAAATA TTAATTTTCA ATAAATTAAA TGAAATTGCA TTATTTTAAA GATTTCTGAA   
  
  
+ TGCATCCATT ATTGCAATTT TTTAAAGACT GCACAATGAT GACAATGTCA ATGGTTTCCT AAAGTTGCGC   
  
  
+ CTGATGGGCT ATTGACAGTG AAGCAACACT CCCTTTGTTC CCATTTATGT GTCTCCACTT GCCCCTTTGA   
  
  
+ AACTCTCTAC ATTTGTATGT CATCTGAATT TGATTCTCAA ATCTCAGATG ATTTGAATTC CCTTTATGCC   
  
  
+ ATCTTTACTA TCTATTGAAT GATTCTTTCT TTTCCACTTT CTTATCTTCC ACTACCTTCT TCTTGGATGC   
  
  
+ CCTTTTGCTG CTTATAAAGG GCGACACCTG AACAAGTTGC TGTATTCTCA AACCATGAAT CTGTACAGGT   
  
  
+ TTAGCCCCGA TATGTGTCCT AGCTTTGTGG GTCACGAAGA AATTGGTCCT AATTTAATAG GACACCAAGA   
  
  
+ CCCTTTTCAA AATGCATACA ATTGCACTGA TTTTGGTGAT GCTTACTTGT TGCAATTCCC AGTTGAAGAT   
  
  
+ TTATGTCAAT ACCGTGTTCC TGATCTAATG GGGCTACCTG TTGAAGATGG GGATCCTACA GAGGTCCCTG   
  
  
+ ACATTCCTGA TACATGCTTC AACTACATAA GCAATCTCCT AATGGAAGAG GGCTCGGATG ACCGCCCTGG   
  
  
+ CGCCACCCTG CAGGACTATA TGGCTGCTCA AGTCTCCTAC AGCAAGTCCT CCAATGCCGC CTTTCCCGGT   
  
  
+ GAGAGTTGCT CATTCTTTGG TAATAATCAA TGCCCTCCAC CTGCTTATCA AAGCAACCCT TATTGCCTTG   
  
  
+ ATGATTACTT CAACTTGCTA TCCAATACTA CTGGCAATAG TGTTAACAAT GCTGGTGTTC ATGACAGCAA   
  
  
+ CAACTTTGTT CAGAACAGTT TGATTGGGAA TAGGGGTACT AGTTCTAGTT CTAGGGGCAG AAGTGATTTT   
  
  
+ GGATCTACCC TTATTAGGAA TTTGCCATTA GGGACCGACA CACTTGATGT GGTCAATCCA GCACCTACTC   
  
  
+ CTCGAGCCCC TCTGGTTACG ACTCAGTGTC AGGTGTTTGA AACACACCAG TATGAGCAGG CCTTCGAACT   
  
  
+ GATCACTAAT GGAAGCTGGG AATTCAGTCA TTCTGTTGCT AATACATCAA ATGATGGAAT TCAAAATCAA   
  
  
+ CCAATTCAAG CAGTTTCAAC CCAAGAACGA TGGAATATGA AAACATTTTC CGATGACCAA CTTGAGCCTG   
  
  
+ CTCTTTTGCG AAGTCTTGTG ATGTCCTCAA ACTGTACTGA TATCTCTGAT CAGGGTGTAC ACAGGATAGT   
  
  
+ GAACCCTGAG CCACCCAACA ACAGAAATAC GGAGATTGTG TGCAATGACT CATCCAGTAA GAGTAAGACA   
  
  
+ AGACGAAGAG AGCATGGTGC AGGAGAAAAC CACGATGAAG GAGAAAACCA CGATGAGACA GGAAGGAGTA   
  
  
+ ACAAGCAATC AGCACCATCC AATGAAGTGT TCGATGAAAT TGAGCAGTAT GGTGATGTGC TGCTCTGCCC   
  
  
+ TGAAGGAAGA AGTAAACCAG CAGGGTTATG CAGCAGCAGC AACGCTAACA GAACTAGCAA CTCACAGAAC   
  
  
+ TCGGAAGACT CAAAGTCACA GCAAAATGGG AGTTCCTCCA AGGTGTCAAA AAAGCGAGGC AATAAAAAGC   
  
  
+ AAGTGGTTGA TTTGAGGACT CTCCTAACTC GATGTGCACA ATGTGTTGCT CGTGTGGATT TAACTGGTTC   
  
  
+ TTACCAGTTG CTTCAGGAGA TTCGGCAGCA TTCTTCTCCC TATGGTGATT ATCTCCAAAG AATGGCTCAT   
  
  
+ TACCTTGCTA ATGGCCTTGA GGCGCGCTTA GAAGGCAAAG GATATGAACT AACTCGTGCC AATGAGCATA   
  
  
+ TCCCACCTAC TGAGATCCTC AAGGCTAACC GGGTATATGT GGCAGCGGTC CCTTTCAAGA TAATGTCTTA   
  
  
+ TTACACAACA AACAAGACAA TTGCAAGCCT AGTTGAGGAG GCACCAAGTA TACACATAAT TGATTTTGGT   
  
  
+ ATTTTCTATG GACTACAATG GCCCTGCATC ATTCAGAACC TCTCCAAGAG GCCCAATGGG CCTCCCAGAA   
  
  
+ TCCGCATCAC AGGCATCGAT TTCCCCCAAC CTGGGTTCCG GCCAGCAGAG AGGGTTGAAG AGACAGGGCG   
  
  
+ GTGTTTAGCC AAGTATTGTG ACAGGTACAA TGTGCCCTTT GAATACCACC CCATAGCCAA GAAGTGGGAG   
  
  
+ ACCATCCAAC TCGAGGAGCT CAAAATTGAA AGGAATGAGC CCCTTGTAGT GAACTGTCTG TATAGATCGC   
  
  
+ ACAACCTATT TGATGAAAGC GTAGAGGAAA ACAGCCCAAG AGACGCGTTC CTGAGCTTGG TTAGGAAAAT   
  
  
+ AAATCCCGAT ATATTCATGC ATGGGGTGGT GAATACCGCA TCAAGTGTAC CTTTCTTCCT GAACAGGTTC   
  
  
+ AAGGAGGCCA TGTTTCACTA CACGGCCTTG TTTGACTTGT TCGAGGCCAC AATGAGCCGC GAGGATCAAG   
  
  
+ AGAGGGTGTT ACTAGAGAGC AAGTTGCATG GGAATCAGGC GTTGAATGTG ATCGCCTGCG AAGGAGCGGA   
  
  
+ GAGAGTGGAG AGGCCTGAGT CATACAAACA ATGGCAGGTT AGGACTCAAA GGGCCGGGTT TGTTCAAGTG   
  
  
+ TCGATGGATC GCGAGCTTTT GAGAAGGGCA AGGGCCATGG TCAGGAGGAA TTTTAGGGAA GAGTTCAGTT   
  
  
+ ATGATGAAGA TGGGCATTGG GTGGTTCAAG GATGGAAAGG CAGGATAATG TATGCCATTT CTTGTTGGAA   
  
  
+ ACCTTCATA  

- +Up\_Stream \_Len000TAATAA TAAGATTATA TATCTATTAT TATATTATTA TTATTATTAT TATTATTATT   
  
  
- AATAATAATA ATAATAATAA ATAATTTATA TTATCAATAA TATTATTATT ATTAATATTA TTATTATTCT   
  
  
- AATAATAGTA ATATAATAAT AATAATAATA ACATTAAATA ATAATAATAT CATTAATAAT AACAATATAT   
  
  
- CATTTTAATA ATAATCATAA CAATAATATT TATAAGTATG AACGTATATT ATAAAGATTT TATTTTTGTC   
  
  
- TCAATTAAGG TAAGGTTTTG ATGAGTGGTT TGTCGTTCCT CACCCTGGAC TAAGGAAAGG TTAAAAGTGG   
  
  
- TTTGTTGACC CAAGTCTTAA GTTCAAGGTA TAGGTTGGGG GGTCCTAAGT TCAGGACTAA GGTAAGGTAA   
  
  
- GGTCAAAGGG AGTGGTTTGA CCCGGAATCT AACTACCAAC AAGTCAACCT ACTCCCAACA AGGTACGGTT   
  
  
- CAATCTAGTA ACAACAGTTA AAACTCTTGC TGATAGAATT ATTCGGATTG CTACAACTAA ACTAAATCAA   
  
  
- GTTTGGTAAA GTTTATTATG TTTAGTTTAA GATAAATCTG GCTGTGATTT TATTTATCTA ACTCTTTTTA   
  
  
- ATATCTAAAA TTTCAAGTCT AATTTTAGGG ATTTCTAGTT GTCTAACTTT ACGTATAAAG TATCACGTGT   
  
  
- ACCTGAACGA AACTTAGAGC AGATCTCTTG GATTCTTTTT CGTTCTCACC AAGTCTTCCA ATCTTAACAA   
  
  
- GGTCGTGTAC TGTTCATGTT AGGGTTGAAA AAAGTTTTCT AAACGATGAA AATTAACCTA AGGGTACAAC   
  
  
- TTACTTCAAG ACCCAAATTC CGATGTTGAA AGAACCCTCC TATTCCAAGA ATTCAATTCC ACCAGGAGAA   
  
  
- AAGAAAACTA GAAAGAATAC CCATGTTGGA GTACCCAGAG TAGTTGTGTA CTTGAGAAAA ATAGAAAATA   
  
  
- GAAAGGGGAA AGGAAATACG TCCTAACTGT CCACGTACCT TTTGTTCTTC GTTAATTTAT GATCACCGAA   
  
  
- TCGGGGTAGG GATTATAAAA CCGATAAGAC AAAGTGGTAG GAGGGGACAG AGGTAAAAGA AACAAAGGAA   
  
  
- AAAAAGATCC TTGAACACTA CCCGTTCCCT TCTAGTCATG TTACACTACC AACAATCATC GCTCTTCTAC   
  
  
- TTCACAGTGG CGTTTTTAAA ACACGGTATT AAACAGCATT CCCTTTGTTC ATAGTTTGTT GTTACAGTAA   
  
  
- ATACTATTTG TTCATTGTCC TCTATACATG AGACTTTTTT ATATTCCATG GACTCTTCAA CAATTGAATC   
  
  
- GAACTTTTTG GGAGGATTGA ACTATTTGGG AGAAGCGATA CAATAGGGCG TGGGACGGTA TCTCAGTGGC   
  
  
- TAAGTCATAT ATACATTGTA TGGGTTAGAA CAGACCTATA TATCAATTGG GAAAACCTTA TAGACTTTTA   
  
  
- GGTGTGCTCT CAGTAGGAAG ATCTGATCCT TTGTACGCGG TGTAAAACCT AAGATTGGTT CATATTATTC   
  
  
- GTATAGAATT TCGGTTTTTA CGTTCCTATC TATGTTTATG GGTAGTGTAC GTACATTTAC AGGGAACAGT   
  
  
- ACCGAGACTA AGAGTTTTAT AATTAAAAGT TATTTAATTT ACTTTAACGT AATAAAATTT CTAAAGACTT   
  
  
- ACGTAGGTAA TAACGTTAAA AAATTTCTGA CGTGTTACTA CTGTTACAGT TACCAAAGGA TTTCAACGCG   
  
  
- GACTACCCGA TAACTGTCAC TTCGTTGTGA GGGAAACAAG GGTAAATACA CAGAGGTGAA CGGGGAAACT   
  
  
- TTGAGAGATG TAAACATACA GTAGACTTAA ACTAAGAGTT TAGAGTCTAC TAAACTTAAG GGAAATACGG   
  
  
- TAGAAATGAT AGATAACTTA CTAAGAAAGA AAAGGTGAAA GAATAGAAGG TGATGGAAGA AGAACCTACG   
  
  
- GGAAAACGAC GAATATTTCC CGCTGTGGAC TTGTTCAACG ACATAAGAGT TTGGTACTTA GACATGTCCA   
  
  
- AATCGGGGCT ATACACAGGA TCGAAACACC CAGTGCTTCT TTAACCAGGA TTAAATTATC CTGTGGTTCT   
  
  
- GGGAAAAGTT TTACGTATGT TAACGTGACT AAAACCACTA CGAATGAACA ACGTTAAGGG TCAACTTCTA   
  
  
- AATACAGTTA TGGCACAAGG ACTAGATTAC CCCGATGGAC AACTTCTACC CCTAGGATGT CTCCAGGGAC   
  
  
- TGTAAGGACT ATGTACGAAG TTGATGTATT CGTTAGAGGA TTACCTTCTC CCGAGCCTAC TGGCGGGACC   
  
  
- GCGGTGGGAC GTCCTGATAT ACCGACGAGT TCAGAGGATG TCGTTCAGGA GGTTACGGCG GAAAGGGCCA   
  
  
- CTCTCAACGA GTAAGAAACC ATTATTAGTT ACGGGAGGTG GACGAATAGT TTCGTTGGGA ATAACGGAAC   
  
  
- TACTAATGAA GTTGAACGAT AGGTTATGAT GACCGTTATC ACAATTGTTA CGACCACAAG TACTGTCGTT   
  
  
- GTTGAAACAA GTCTTGTCAA ACTAACCCTT ATCCCCATGA TCAAGATCAA GATCCCCGTC TTCACTAAAA   
  
  
- CCTAGATGGG AATAATCCTT AAACGGTAAT CCCTGGCTGT GTGAACTACA CCAGTTAGGT CGTGGATGAG   
  
  
- GAGCTCGGGG AGACCAATGC TGAGTCACAG TCCACAAACT TTGTGTGGTC ATACTCGTCC GGAAGCTTGA   
  
  
- CTAGTGATTA CCTTCGACCC TTAAGTCAGT AAGACAACGA TTATGTAGTT TACTACCTTA AGTTTTAGTT   
  
  
- GGTTAAGTTC GTCAAAGTTG GGTTCTTGCT ACCTTATACT TTTGTAAAAG GCTACTGGTT GAACTCGGAC   
  
  
- GAGAAAACGC TTCAGAACAC TACAGGAGTT TGACATGACT ATAGAGACTA GTCCCACATG TGTCCTATCA   
  
  
- CTTGGGACTC GGTGGGTTGT TGTCTTTATG CCTCTAACAC ACGTTACTGA GTAGGTCATT CTCATTCTGT   
  
  
- TCTGCTTCTC TCGTACCACG TCCTCTTTTG GTGCTACTTC CTCTTTTGGT GCTACTCTGT CCTTCCTCAT   
  
  
- TGTTCGTTAG TCGTGGTAGG TTACTTCACA AGCTACTTTA ACTCGTCATA CCACTACACG ACGAGACGGG   
  
  
- ACTTCCTTCT TCATTTGGTC GTCCCAATAC GTCGTCGTCG TTGCGATTGT CTTGATCGTT GAGTGTCTTG   
  
  
- AGCCTTCTGA GTTTCAGTGT CGTTTTACCC TCAAGGAGGT TCCACAGTTT TTTCGCTCCG TTATTTTTCG   
  
  
- TTCACCAACT AAACTCCTGA GAGGATTGAG CTACACGTGT TACACAACGA GCACACCTAA ATTGACCAAG   
  
  
- AATGGTCAAC GAAGTCCTCT AAGCCGTCGT AAGAAGAGGG ATACCACTAA TAGAGGTTTC TTACCGAGTA   
  
  
- ATGGAACGAT TACCGGAACT CCGCGCGAAT CTTCCGTTTC CTATACTTGA TTGAGCACGG TTACTCGTAT   
  
  
- AGGGTGGATG ACTCTAGGAG TTCCGATTGG CCCATATACA CCGTCGCCAG GGAAAGTTCT ATTACAGAAT   
  
  
- AATGTGTTGT TTGTTCTGTT AACGTTCGGA TCAACTCCTC CGTGGTTCAT ATGTGTATTA ACTAAAACCA   
  
  
- TAAAAGATAC CTGATGTTAC CGGGACGTAG TAAGTCTTGG AGAGGTTCTC CGGGTTACCC GGAGGGTCTT   
  
  
- AGGCGTAGTG TCCGTAGCTA AAGGGGGTTG GACCCAAGGC CGGTCGTCTC TCCCAACTTC TCTGTCCCGC   
  
  
- CACAAATCGG TTCATAACAC TGTCCATGTT ACACGGGAAA CTTATGGTGG GGTATCGGTT CTTCACCCTC   
  
  
- TGGTAGGTTG AGCTCCTCGA GTTTTAACTT TCCTTACTCG GGGAACATCA CTTGACAGAC ATATCTAGCG   
  
  
- TGTTGGATAA ACTACTTTCG CATCTCCTTT TGTCGGGTTC TCTGCGCAAG GACTCGAACC AATCCTTTTA   
  
  
- TTTAGGGCTA TATAAGTACG TACCCCACCA CTTATGGCGT AGTTCACATG GAAAGAAGGA CTTGTCCAAG   
  
  
- TTCCTCCGGT ACAAAGTGAT GTGCCGGAAC AAACTGAACA AGCTCCGGTG TTACTCGGCG CTCCTAGTTC   
  
  
- TCTCCCACAA TGATCTCTCG TTCAACGTAC CCTTAGTCCG CAACTTACAC TAGCGGACGC TTCCTCGCCT   
  
  
- CTCTCACCTC TCCGGACTCA GTATGTTTGT TACCGTCCAA TCCTGAGTTT CCCGGCCCAA ACAAGTTCAC   
  
  
- AGCTACCTAG CGCTCGAAAA CTCTTCCCGT TCCCGGTACC AGTCCTCCTT AAAATCCCTT CTCAAGTCAA   
  
  
- TACTACTTCT ACCCGTAACC CACCAAGTTC CTACCTTTCC GTCCTATTAC ATACGGTAAA GAACAACCTT   
  
  
- TGGAAGTAT

+     Box II

| Site Name | Organism | Position | Strand | Matrix score. | sequence | function |
| --- | --- | --- | --- | --- | --- | --- |
| Box II | Solanum tuberosum | 2402 | + | 9 | TGGTAATAA | part of a light responsive element |

>HU02G01570.1   
+ +Up\_Stream \_Len000ATTATT ATTCTAATAT ATAGATAATA ATATAATAAT AATAATAATA ATAATAATAA   
  
  
+ TTATTATTAT TATTATTATT TATTAAATAT AATAGTTATT ATAATAATAA TAATTATAAT AATAATAAGA   
  
  
+ TTATTATCAT TATATTATTA TTATTATTAT TGTAATTTAT TATTATTATA GTAATTATTA TTGTTATATA   
  
  
+ GTAAAATTAT TATTAGTATT GTTATTATAA ATATTCATAC TTGCATATAA TATTTCTAAA ATAAAAACAG   
  
  
+ AGTTAATTCC ATTCCAAAAC TACTCACCAA ACAGCAAGGA GTGGGACCTG ATTCCTTTCC AATTTTCACC   
  
  
+ AAACAACTGG GTTCAGAATT CAAGTTCCAT ATCCAACCCC CCAGGATTCA AGTCCTGATT CCATTCCATT   
  
  
+ CCAGTTTCCC TCACCAAACT GGGCCTTAGA TTGATGGTTG TTCAGTTGGA TGAGGGTTGT TCCATGCCAA   
  
  
+ GTTAGATCAT TGTTGTCAAT TTTGAGAACG ACTATCTTAA TAAGCCTAAC GATGTTGATT TGATTTAGTT   
  
  
+ CAAACCATTT CAAATAATAC AAATCAAATT CTATTTAGAC CGACACTAAA ATAAATAGAT TGAGAAAAAT   
  
  
+ TATAGATTTT AAAGTTCAGA TTAAAATCCC TAAAGATCAA CAGATTGAAA TGCATATTTC ATAGTGCACA   
  
  
+ TGGACTTGCT TTGAATCTCG TCTAGAGAAC CTAAGAAAAA GCAAGAGTGG TTCAGAAGGT TAGAATTGTT   
  
  
+ CCAGCACATG ACAAGTACAA TCCCAACTTT TTTCAAAAGA TTTGCTACTT TTAATTGGAT TCCCATGTTG   
  
  
+ AATGAAGTTC TGGGTTTAAG GCTACAACTT TCTTGGGAGG ATAAGGTTCT TAAGTTAAGG TGGTCCTCTT   
  
  
+ TTCTTTTGAT CTTTCTTATG GGTACAACCT CATGGGTCTC ATCAACACAT GAACTCTTTT TATCTTTTAT   
  
  
+ CTTTCCCCTT TCCTTTATGC AGGATTGACA GGTGCATGGA AAACAAGAAG CAATTAAATA CTAGTGGCTT   
  
  
+ AGCCCCATCC CTAATATTTT GGCTATTCTG TTTCACCATC CTCCCCTGTC TCCATTTTCT TTGTTTCCTT   
  
  
+ TTTTTCTAGG AACTTGTGAT GGGCAAGGGA AGATCAGTAC AATGTGATGG TTGTTAGTAG CGAGAAGATG   
  
  
+ AAGTGTCACC GCAAAAATTT TGTGCCATAA TTTGTCGTAA GGGAAACAAG TATCAAACAA CAATGTCATT   
  
  
+ TATGATAAAC AAGTAACAGG AGATATGTAC TCTGAAAAAA TATAAGGTAC CTGAGAAGTT GTTAACTTAG   
  
  
+ CTTGAAAAAC CCTCCTAACT TGATAAACCC TCTTCGCTAT GTTATCCCGC ACCCTGCCAT AGAGTCACCG   
  
  
+ ATTCAGTATA TATGTAACAT ACCCAATCTT GTCTGGATAT ATAGTTAACC CTTTTGGAAT ATCTGAAAAT   
  
  
+ CCACACGAGA GTCATCCTTC TAGACTAGGA AACATGCGCC ACATTTTGGA TTCTAACCAA GTATAATAAG   
  
  
+ CATATCTTAA AGCCAAAAAT GCAAGGATAG ATACAAATAC CCATCACATG CATGTAAATG TCCCTTGTCA   
  
  
+ TGGCTCTGAT TCTCAAAATA TTAATTTTCA ATAAATTAAA TGAAATTGCA TTATTTTAAA GATTTCTGAA   
  
  
+ TGCATCCATT ATTGCAATTT TTTAAAGACT GCACAATGAT GACAATGTCA ATGGTTTCCT AAAGTTGCGC   
  
  
+ CTGATGGGCT ATTGACAGTG AAGCAACACT CCCTTTGTTC CCATTTATGT GTCTCCACTT GCCCCTTTGA   
  
  
+ AACTCTCTAC ATTTGTATGT CATCTGAATT TGATTCTCAA ATCTCAGATG ATTTGAATTC CCTTTATGCC   
  
  
+ ATCTTTACTA TCTATTGAAT GATTCTTTCT TTTCCACTTT CTTATCTTCC ACTACCTTCT TCTTGGATGC   
  
  
+ CCTTTTGCTG CTTATAAAGG GCGACACCTG AACAAGTTGC TGTATTCTCA AACCATGAAT CTGTACAGGT   
  
  
+ TTAGCCCCGA TATGTGTCCT AGCTTTGTGG GTCACGAAGA AATTGGTCCT AATTTAATAG GACACCAAGA   
  
  
+ CCCTTTTCAA AATGCATACA ATTGCACTGA TTTTGGTGAT GCTTACTTGT TGCAATTCCC AGTTGAAGAT   
  
  
+ TTATGTCAAT ACCGTGTTCC TGATCTAATG GGGCTACCTG TTGAAGATGG GGATCCTACA GAGGTCCCTG   
  
  
+ ACATTCCTGA TACATGCTTC AACTACATAA GCAATCTCCT AATGGAAGAG GGCTCGGATG ACCGCCCTGG   
  
  
+ CGCCACCCTG CAGGACTATA TGGCTGCTCA AGTCTCCTAC AGCAAGTCCT CCAATGCCGC CTTTCCCGGT   
  
  
+ GAGAGTTGCT CATTCTTTGG TAATAATCAA TGCCCTCCAC CTGCTTATCA AAGCAACCCT TATTGCCTTG   
  
  
+ ATGATTACTT CAACTTGCTA TCCAATACTA CTGGCAATAG TGTTAACAAT GCTGGTGTTC ATGACAGCAA   
  
  
+ CAACTTTGTT CAGAACAGTT TGATTGGGAA TAGGGGTACT AGTTCTAGTT CTAGGGGCAG AAGTGATTTT   
  
  
+ GGATCTACCC TTATTAGGAA TTTGCCATTA GGGACCGACA CACTTGATGT GGTCAATCCA GCACCTACTC   
  
  
+ CTCGAGCCCC TCTGGTTACG ACTCAGTGTC AGGTGTTTGA AACACACCAG TATGAGCAGG CCTTCGAACT   
  
  
+ GATCACTAAT GGAAGCTGGG AATTCAGTCA TTCTGTTGCT AATACATCAA ATGATGGAAT TCAAAATCAA   
  
  
+ CCAATTCAAG CAGTTTCAAC CCAAGAACGA TGGAATATGA AAACATTTTC CGATGACCAA CTTGAGCCTG   
  
  
+ CTCTTTTGCG AAGTCTTGTG ATGTCCTCAA ACTGTACTGA TATCTCTGAT CAGGGTGTAC ACAGGATAGT   
  
  
+ GAACCCTGAG CCACCCAACA ACAGAAATAC GGAGATTGTG TGCAATGACT CATCCAGTAA GAGTAAGACA   
  
  
+ AGACGAAGAG AGCATGGTGC AGGAGAAAAC CACGATGAAG GAGAAAACCA CGATGAGACA GGAAGGAGTA   
  
  
+ ACAAGCAATC AGCACCATCC AATGAAGTGT TCGATGAAAT TGAGCAGTAT GGTGATGTGC TGCTCTGCCC   
  
  
+ TGAAGGAAGA AGTAAACCAG CAGGGTTATG CAGCAGCAGC AACGCTAACA GAACTAGCAA CTCACAGAAC   
  
  
+ TCGGAAGACT CAAAGTCACA GCAAAATGGG AGTTCCTCCA AGGTGTCAAA AAAGCGAGGC AATAAAAAGC   
  
  
+ AAGTGGTTGA TTTGAGGACT CTCCTAACTC GATGTGCACA ATGTGTTGCT CGTGTGGATT TAACTGGTTC   
  
  
+ TTACCAGTTG CTTCAGGAGA TTCGGCAGCA TTCTTCTCCC TATGGTGATT ATCTCCAAAG AATGGCTCAT   
  
  
+ TACCTTGCTA ATGGCCTTGA GGCGCGCTTA GAAGGCAAAG GATATGAACT AACTCGTGCC AATGAGCATA   
  
  
+ TCCCACCTAC TGAGATCCTC AAGGCTAACC GGGTATATGT GGCAGCGGTC CCTTTCAAGA TAATGTCTTA   
  
  
+ TTACACAACA AACAAGACAA TTGCAAGCCT AGTTGAGGAG GCACCAAGTA TACACATAAT TGATTTTGGT   
  
  
+ ATTTTCTATG GACTACAATG GCCCTGCATC ATTCAGAACC TCTCCAAGAG GCCCAATGGG CCTCCCAGAA   
  
  
+ TCCGCATCAC AGGCATCGAT TTCCCCCAAC CTGGGTTCCG GCCAGCAGAG AGGGTTGAAG AGACAGGGCG   
  
  
+ GTGTTTAGCC AAGTATTGTG ACAGGTACAA TGTGCCCTTT GAATACCACC CCATAGCCAA GAAGTGGGAG   
  
  
+ ACCATCCAAC TCGAGGAGCT CAAAATTGAA AGGAATGAGC CCCTTGTAGT GAACTGTCTG TATAGATCGC   
  
  
+ ACAACCTATT TGATGAAAGC GTAGAGGAAA ACAGCCCAAG AGACGCGTTC CTGAGCTTGG TTAGGAAAAT   
  
  
+ AAATCCCGAT ATATTCATGC ATGGGGTGGT GAATACCGCA TCAAGTGTAC CTTTCTTCCT GAACAGGTTC   
  
  
+ AAGGAGGCCA TGTTTCACTA CACGGCCTTG TTTGACTTGT TCGAGGCCAC AATGAGCCGC GAGGATCAAG   
  
  
+ AGAGGGTGTT ACTAGAGAGC AAGTTGCATG GGAATCAGGC GTTGAATGTG ATCGCCTGCG AAGGAGCGGA   
  
  
+ GAGAGTGGAG AGGCCTGAGT CATACAAACA ATGGCAGGTT AGGACTCAAA GGGCCGGGTT TGTTCAAGTG   
  
  
+ TCGATGGATC GCGAGCTTTT GAGAAGGGCA AGGGCCATGG TCAGGAGGAA TTTTAGGGAA GAGTTCAGTT   
  
  
+ ATGATGAAGA TGGGCATTGG GTGGTTCAAG GATGGAAAGG CAGGATAATG TATGCCATTT CTTGTTGGAA   
  
  
+ ACCTTCATA  

- +Up\_Stream \_Len000TAATAA TAAGATTATA TATCTATTAT TATATTATTA TTATTATTAT TATTATTATT   
  
  
- AATAATAATA ATAATAATAA ATAATTTATA TTATCAATAA TATTATTATT ATTAATATTA TTATTATTCT   
  
  
- AATAATAGTA ATATAATAAT AATAATAATA ACATTAAATA ATAATAATAT CATTAATAAT AACAATATAT   
  
  
- CATTTTAATA ATAATCATAA CAATAATATT TATAAGTATG AACGTATATT ATAAAGATTT TATTTTTGTC   
  
  
- TCAATTAAGG TAAGGTTTTG ATGAGTGGTT TGTCGTTCCT CACCCTGGAC TAAGGAAAGG TTAAAAGTGG   
  
  
- TTTGTTGACC CAAGTCTTAA GTTCAAGGTA TAGGTTGGGG GGTCCTAAGT TCAGGACTAA GGTAAGGTAA   
  
  
- GGTCAAAGGG AGTGGTTTGA CCCGGAATCT AACTACCAAC AAGTCAACCT ACTCCCAACA AGGTACGGTT   
  
  
- CAATCTAGTA ACAACAGTTA AAACTCTTGC TGATAGAATT ATTCGGATTG CTACAACTAA ACTAAATCAA   
  
  
- GTTTGGTAAA GTTTATTATG TTTAGTTTAA GATAAATCTG GCTGTGATTT TATTTATCTA ACTCTTTTTA   
  
  
- ATATCTAAAA TTTCAAGTCT AATTTTAGGG ATTTCTAGTT GTCTAACTTT ACGTATAAAG TATCACGTGT   
  
  
- ACCTGAACGA AACTTAGAGC AGATCTCTTG GATTCTTTTT CGTTCTCACC AAGTCTTCCA ATCTTAACAA   
  
  
- GGTCGTGTAC TGTTCATGTT AGGGTTGAAA AAAGTTTTCT AAACGATGAA AATTAACCTA AGGGTACAAC   
  
  
- TTACTTCAAG ACCCAAATTC CGATGTTGAA AGAACCCTCC TATTCCAAGA ATTCAATTCC ACCAGGAGAA   
  
  
- AAGAAAACTA GAAAGAATAC CCATGTTGGA GTACCCAGAG TAGTTGTGTA CTTGAGAAAA ATAGAAAATA   
  
  
- GAAAGGGGAA AGGAAATACG TCCTAACTGT CCACGTACCT TTTGTTCTTC GTTAATTTAT GATCACCGAA   
  
  
- TCGGGGTAGG GATTATAAAA CCGATAAGAC AAAGTGGTAG GAGGGGACAG AGGTAAAAGA AACAAAGGAA   
  
  
- AAAAAGATCC TTGAACACTA CCCGTTCCCT TCTAGTCATG TTACACTACC AACAATCATC GCTCTTCTAC   
  
  
- TTCACAGTGG CGTTTTTAAA ACACGGTATT AAACAGCATT CCCTTTGTTC ATAGTTTGTT GTTACAGTAA   
  
  
- ATACTATTTG TTCATTGTCC TCTATACATG AGACTTTTTT ATATTCCATG GACTCTTCAA CAATTGAATC   
  
  
- GAACTTTTTG GGAGGATTGA ACTATTTGGG AGAAGCGATA CAATAGGGCG TGGGACGGTA TCTCAGTGGC   
  
  
- TAAGTCATAT ATACATTGTA TGGGTTAGAA CAGACCTATA TATCAATTGG GAAAACCTTA TAGACTTTTA   
  
  
- GGTGTGCTCT CAGTAGGAAG ATCTGATCCT TTGTACGCGG TGTAAAACCT AAGATTGGTT CATATTATTC   
  
  
- GTATAGAATT TCGGTTTTTA CGTTCCTATC TATGTTTATG GGTAGTGTAC GTACATTTAC AGGGAACAGT   
  
  
- ACCGAGACTA AGAGTTTTAT AATTAAAAGT TATTTAATTT ACTTTAACGT AATAAAATTT CTAAAGACTT   
  
  
- ACGTAGGTAA TAACGTTAAA AAATTTCTGA CGTGTTACTA CTGTTACAGT TACCAAAGGA TTTCAACGCG   
  
  
- GACTACCCGA TAACTGTCAC TTCGTTGTGA GGGAAACAAG GGTAAATACA CAGAGGTGAA CGGGGAAACT   
  
  
- TTGAGAGATG TAAACATACA GTAGACTTAA ACTAAGAGTT TAGAGTCTAC TAAACTTAAG GGAAATACGG   
  
  
- TAGAAATGAT AGATAACTTA CTAAGAAAGA AAAGGTGAAA GAATAGAAGG TGATGGAAGA AGAACCTACG   
  
  
- GGAAAACGAC GAATATTTCC CGCTGTGGAC TTGTTCAACG ACATAAGAGT TTGGTACTTA GACATGTCCA   
  
  
- AATCGGGGCT ATACACAGGA TCGAAACACC CAGTGCTTCT TTAACCAGGA TTAAATTATC CTGTGGTTCT   
  
  
- GGGAAAAGTT TTACGTATGT TAACGTGACT AAAACCACTA CGAATGAACA ACGTTAAGGG TCAACTTCTA   
  
  
- AATACAGTTA TGGCACAAGG ACTAGATTAC CCCGATGGAC AACTTCTACC CCTAGGATGT CTCCAGGGAC   
  
  
- TGTAAGGACT ATGTACGAAG TTGATGTATT CGTTAGAGGA TTACCTTCTC CCGAGCCTAC TGGCGGGACC   
  
  
- GCGGTGGGAC GTCCTGATAT ACCGACGAGT TCAGAGGATG TCGTTCAGGA GGTTACGGCG GAAAGGGCCA   
  
  
- CTCTCAACGA GTAAGAAACC ATTATTAGTT ACGGGAGGTG GACGAATAGT TTCGTTGGGA ATAACGGAAC   
  
  
- TACTAATGAA GTTGAACGAT AGGTTATGAT GACCGTTATC ACAATTGTTA CGACCACAAG TACTGTCGTT   
  
  
- GTTGAAACAA GTCTTGTCAA ACTAACCCTT ATCCCCATGA TCAAGATCAA GATCCCCGTC TTCACTAAAA   
  
  
- CCTAGATGGG AATAATCCTT AAACGGTAAT CCCTGGCTGT GTGAACTACA CCAGTTAGGT CGTGGATGAG   
  
  
- GAGCTCGGGG AGACCAATGC TGAGTCACAG TCCACAAACT TTGTGTGGTC ATACTCGTCC GGAAGCTTGA   
  
  
- CTAGTGATTA CCTTCGACCC TTAAGTCAGT AAGACAACGA TTATGTAGTT TACTACCTTA AGTTTTAGTT   
  
  
- GGTTAAGTTC GTCAAAGTTG GGTTCTTGCT ACCTTATACT TTTGTAAAAG GCTACTGGTT GAACTCGGAC   
  
  
- GAGAAAACGC TTCAGAACAC TACAGGAGTT TGACATGACT ATAGAGACTA GTCCCACATG TGTCCTATCA   
  
  
- CTTGGGACTC GGTGGGTTGT TGTCTTTATG CCTCTAACAC ACGTTACTGA GTAGGTCATT CTCATTCTGT   
  
  
- TCTGCTTCTC TCGTACCACG TCCTCTTTTG GTGCTACTTC CTCTTTTGGT GCTACTCTGT CCTTCCTCAT   
  
  
- TGTTCGTTAG TCGTGGTAGG TTACTTCACA AGCTACTTTA ACTCGTCATA CCACTACACG ACGAGACGGG   
  
  
- ACTTCCTTCT TCATTTGGTC GTCCCAATAC GTCGTCGTCG TTGCGATTGT CTTGATCGTT GAGTGTCTTG   
  
  
- AGCCTTCTGA GTTTCAGTGT CGTTTTACCC TCAAGGAGGT TCCACAGTTT TTTCGCTCCG TTATTTTTCG   
  
  
- TTCACCAACT AAACTCCTGA GAGGATTGAG CTACACGTGT TACACAACGA GCACACCTAA ATTGACCAAG   
  
  
- AATGGTCAAC GAAGTCCTCT AAGCCGTCGT AAGAAGAGGG ATACCACTAA TAGAGGTTTC TTACCGAGTA   
  
  
- ATGGAACGAT TACCGGAACT CCGCGCGAAT CTTCCGTTTC CTATACTTGA TTGAGCACGG TTACTCGTAT   
  
  
- AGGGTGGATG ACTCTAGGAG TTCCGATTGG CCCATATACA CCGTCGCCAG GGAAAGTTCT ATTACAGAAT   
  
  
- AATGTGTTGT TTGTTCTGTT AACGTTCGGA TCAACTCCTC CGTGGTTCAT ATGTGTATTA ACTAAAACCA   
  
  
- TAAAAGATAC CTGATGTTAC CGGGACGTAG TAAGTCTTGG AGAGGTTCTC CGGGTTACCC GGAGGGTCTT   
  
  
- AGGCGTAGTG TCCGTAGCTA AAGGGGGTTG GACCCAAGGC CGGTCGTCTC TCCCAACTTC TCTGTCCCGC   
  
  
- CACAAATCGG TTCATAACAC TGTCCATGTT ACACGGGAAA CTTATGGTGG GGTATCGGTT CTTCACCCTC   
  
  
- TGGTAGGTTG AGCTCCTCGA GTTTTAACTT TCCTTACTCG GGGAACATCA CTTGACAGAC ATATCTAGCG   
  
  
- TGTTGGATAA ACTACTTTCG CATCTCCTTT TGTCGGGTTC TCTGCGCAAG GACTCGAACC AATCCTTTTA   
  
  
- TTTAGGGCTA TATAAGTACG TACCCCACCA CTTATGGCGT AGTTCACATG GAAAGAAGGA CTTGTCCAAG   
  
  
- TTCCTCCGGT ACAAAGTGAT GTGCCGGAAC AAACTGAACA AGCTCCGGTG TTACTCGGCG CTCCTAGTTC   
  
  
- TCTCCCACAA TGATCTCTCG TTCAACGTAC CCTTAGTCCG CAACTTACAC TAGCGGACGC TTCCTCGCCT   
  
  
- CTCTCACCTC TCCGGACTCA GTATGTTTGT TACCGTCCAA TCCTGAGTTT CCCGGCCCAA ACAAGTTCAC   
  
  
- AGCTACCTAG CGCTCGAAAA CTCTTCCCGT TCCCGGTACC AGTCCTCCTT AAAATCCCTT CTCAAGTCAA   
  
  
- TACTACTTCT ACCCGTAACC CACCAAGTTC CTACCTTTCC GTCCTATTAC ATACGGTAAA GAACAACCTT   
  
  
- TGGAAGTAT

+     CAAT-box

| Site Name | Organism | Position | Strand | Matrix score. | sequence | function |
| --- | --- | --- | --- | --- | --- | --- |
| CAAT-box | Arabidopsis thaliana | 4360 | - | 5 | CCAAT | common cis-acting element in promoter and enhancer regions |
| CAAT-box | Nicotiana glutinosa | 4233 | + | 4 | CAAT |  |
| CAAT-box | Nicotiana glutinosa | 4114 | + | 4 | CAAT |  |
| CAAT-box | Nicotiana glutinosa | 3812 | + | 4 | CAAT |  |
| CAAT-box | Nicotiana glutinosa | 3879 | - | 4 | CAAT |  |
| CAAT-box | Nicotiana glutinosa | 3799 | - | 4 | CAAT |  |
| CAAT-box | Nicotiana glutinosa | 3698 | + | 4 | CAAT |  |
| CAAT-box | Arabidopsis thaliana | 3697 | + | 5 | CCAAT | common cis-acting element in promoter and enhancer regions |
| CAAT-box | Nicotiana glutinosa | 3660 | + | 4 | CAAT |  |
| CAAT-box | Nicotiana glutinosa | 3633 | - | 4 | CAAT |  |
| CAAT-box | Pisum sativum | 3932 | - | 5 | CAAAT | common cis-acting element in promoter and enhancer regions |
| CAAT-box | Nicotiana glutinosa | 3594 | - | 4 | CAAT |  |
| CAAT-box | Nicotiana glutinosa | 3592 | + | 4 | CAAT |  |
| CAAT-box | Nicotiana glutinosa | 3494 | + | 4 | CAAT |  |
| CAAT-box | Nicotiana glutinosa | 3333 | + | 4 | CAAT |  |
| CAAT-box | Arabidopsis thaliana | 3493 | + | 5 | CCAAT | common cis-acting element in promoter and enhancer regions |
| CAAT-box | Nicotiana glutinosa | 3284 | + | 4 | CAAT |  |
| CAAT-box | Nicotiana glutinosa | 3123 | - | 4 | CAAT |  |
| CAAT-box | Daucus carota | 3119 | - | 11 | AGCTCAATTTCA | common cis-acting element in promoter and enhancer regions |
| CAAT-box | Nicotiana glutinosa | 3090 | + | 4 | CAAT |  |
| CAAT-box | Nicotiana glutinosa | 2987 | + | 4 | CAAT |  |
| CAAT-box | Nicotiana glutinosa | 3104 | + | 4 | CAAT |  |
| CAAT-box | Arabidopsis thaliana | 3103 | + | 5 | CCAAT | common cis-acting element in promoter and enhancer regions |
| CAAT-box | Nicotiana glutinosa | 2979 | - | 4 | CAAT |  |
| CAAT-box | Nicotiana glutinosa | 2806 | + | 4 | CAAT |  |
| CAAT-box | Arabidopsis thaliana | 2805 | + | 5 | CCAAT | common cis-acting element in promoter and enhancer regions |
| CAAT-box | Nicotiana glutinosa | 2501 | + | 4 | CAAT |  |
| CAAT-box | Nicotiana glutinosa | 2648 | + | 4 | CAAT |  |
| CAAT-box | Arabidopsis thaliana | 2547 | - | 5 | CCAAT | common cis-acting element in promoter and enhancer regions |
| CAAT-box | Nicotiana glutinosa | 2489 | + | 4 | CAAT |  |
| CAAT-box | Nicotiana glutinosa | 2477 | + | 4 | CAAT |  |
| CAAT-box | Nicotiana glutinosa | 2446 | - | 4 | CAAT |  |
| CAAT-box | Arabidopsis thaliana | 2476 | + | 5 | CCAAT | common cis-acting element in promoter and enhancer regions |
| CAAT-box | Nicotiana glutinosa | 2412 | + | 4 | CAAT |  |
| CAAT-box | Nicotiana glutinosa | 2366 | + | 4 | CAAT |  |
| CAAT-box | Arabidopsis thaliana | 2365 | + | 5 | CCAAT | common cis-acting element in promoter and enhancer regions |
| CAAT-box | Nicotiana glutinosa | 2276 | + | 4 | CAAT |  |
| CAAT-box | Nicotiana glutinosa | 2157 | + | 4 | CAAT |  |
| CAAT-box | Nicotiana glutinosa | 2181 | + | 4 | CAAT |  |
| CAAT-box | Nicotiana glutinosa | 2123 | + | 4 | CAAT |  |
| CAAT-box | Nicotiana glutinosa | 2125 | - | 4 | CAAT |  |
| CAAT-box | Arabidopsis thaliana | 2076 | - | 5 | CCAAT | common cis-acting element in promoter and enhancer regions |
| CAAT-box | Nicotiana glutinosa | 1908 | - | 4 | CAAT |  |
| CAAT-box | Nicotiana glutinosa | 1733 | + | 4 | CAAT |  |
| CAAT-box | Nicotiana glutinosa | 1765 | - | 4 | CAAT |  |
| CAAT-box | Nicotiana glutinosa | 1727 | + | 4 | CAAT |  |
| CAAT-box | Nicotiana glutinosa | 1718 | + | 4 | CAAT |  |
| CAAT-box | Nicotiana glutinosa | 1695 | - | 4 | CAAT |  |
| CAAT-box | Nicotiana glutinosa | 1699 | + | 4 | CAAT |  |
| CAAT-box | Nicotiana glutinosa | 1643 | + | 4 | CAAT |  |
| CAAT-box | Nicotiana glutinosa | 1428 | + | 4 | CAAT |  |
| CAAT-box | Nicotiana glutinosa | 1659 | - | 4 | CAAT |  |
| CAAT-box | Arabidopsis thaliana | 1427 | + | 5 | CCAAT | common cis-acting element in promoter and enhancer regions |
| CAAT-box | Nicotiana glutinosa | 1255 | + | 4 | CAAT |  |
| CAAT-box | Nicotiana glutinosa | 1164 | + | 4 | CAAT |  |
| CAAT-box | Nicotiana glutinosa | 1035 | + | 4 | CAAT |  |
| CAAT-box | Nicotiana glutinosa | 1008 | - | 4 | CAAT |  |
| CAAT-box | Arabidopsis thaliana | 828 | - | 5 | CCAAT | common cis-acting element in promoter and enhancer regions |
| CAAT-box | Nicotiana glutinosa | 792 | + | 4 | CAAT |  |
| CAAT-box | Nicotiana glutinosa | 678 | - | 4 | CAAT |  |
| CAAT-box | Nicotiana glutinosa | 344 | + | 4 | CAAT |  |
| CAAT-box | Nicotiana glutinosa | 454 | - | 4 | CAAT |  |
| CAAT-box | Arabidopsis thaliana | 343 | + | 5 | CCAAT | common cis-acting element in promoter and enhancer regions |
| CAAT-box | Nicotiana glutinosa | 232 | - | 4 | CAAT |  |
| CAAT-box | Pisum sativum | 1862 | + | 5 | CAAAT | common cis-acting element in promoter and enhancer regions |
| CAAT-box | Pisum sativum | 1578 | + | 5 | CAAAT | common cis-acting element in promoter and enhancer regions |
| CAAT-box | Pisum sativum | 1224 | - | 5 | CAAAT | common cis-acting element in promoter and enhancer regions |
| CAAT-box | Pisum sativum | 552 | - | 5 | CAAAT | common cis-acting element in promoter and enhancer regions |
| CAAT-box | Nicotiana glutinosa | 173 | - | 4 | CAAT |  |
| CAAT-box | Pisum sativum | 1835 | - | 5 | CAAAT | common cis-acting element in promoter and enhancer regions |
| CAAT-box | Pisum sativum | 814 | - | 5 | CAAAT | common cis-acting element in promoter and enhancer regions |
| CAAT-box | Nicotiana glutinosa | 623 | - | 4 | CAAT |  |
| CAAT-box | Nicotiana glutinosa | 769 | - | 4 | CAAT |  |
| CAAT-box | Pisum sativum | 3304 | - | 5 | CAAAT | common cis-acting element in promoter and enhancer regions |
| CAAT-box | Pisum sativum | 1875 | - | 5 | CAAAT | common cis-acting element in promoter and enhancer regions |
| CAAT-box | Pisum sativum | 2614 | - | 5 | CAAAT | common cis-acting element in promoter and enhancer regions |
| CAAT-box | Pisum sativum | 584 | + | 5 | CAAAT | common cis-acting element in promoter and enhancer regions |
| CAAT-box | Nicotiana glutinosa | 204 | - | 4 | CAAT |  |
| CAAT-box | Pisum sativum | 2782 | + | 5 | CAAAT | common cis-acting element in promoter and enhancer regions |
| CAAT-box | Pisum sativum | 1852 | - | 5 | CAAAT | common cis-acting element in promoter and enhancer regions |
| CAAT-box | Pisum sativum | 575 | + | 5 | CAAAT | common cis-acting element in promoter and enhancer regions |
| CAAT-box | Pisum sativum | 589 | + | 5 | CAAAT | common cis-acting element in promoter and enhancer regions |
| CAAT-box | Nicotiana glutinosa | 511 | + | 4 | CAAT |  |
| CAAT-box | Nicotiana glutinosa | 503 | - | 4 | CAAT |  |

>HU02G01570.1   
+ +Up\_Stream \_Len000ATTATT ATTCTAATAT ATAGATAATA ATATAATAAT AATAATAATA ATAATAATAA   
  
  
+ TTATTATTAT TATTATTATT TATTAAATAT AATAGTTATT ATAATAATAA TAATTATAAT AATAATAAGA   
  
  
+ TTATTATCAT TATATTATTA TTATTATTAT TGTAATTTAT TATTATTATA GTAATTATTA TTGTTATATA   
  
  
+ GTAAAATTAT TATTAGTATT GTTATTATAA ATATTCATAC TTGCATATAA TATTTCTAAA ATAAAAACAG   
  
  
+ AGTTAATTCC ATTCCAAAAC TACTCACCAA ACAGCAAGGA GTGGGACCTG ATTCCTTTCC AATTTTCACC   
  
  
+ AAACAACTGG GTTCAGAATT CAAGTTCCAT ATCCAACCCC CCAGGATTCA AGTCCTGATT CCATTCCATT   
  
  
+ CCAGTTTCCC TCACCAAACT GGGCCTTAGA TTGATGGTTG TTCAGTTGGA TGAGGGTTGT TCCATGCCAA   
  
  
+ GTTAGATCAT TGTTGTCAAT TTTGAGAACG ACTATCTTAA TAAGCCTAAC GATGTTGATT TGATTTAGTT   
  
  
+ CAAACCATTT CAAATAATAC AAATCAAATT CTATTTAGAC CGACACTAAA ATAAATAGAT TGAGAAAAAT   
  
  
+ TATAGATTTT AAAGTTCAGA TTAAAATCCC TAAAGATCAA CAGATTGAAA TGCATATTTC ATAGTGCACA   
  
  
+ TGGACTTGCT TTGAATCTCG TCTAGAGAAC CTAAGAAAAA GCAAGAGTGG TTCAGAAGGT TAGAATTGTT   
  
  
+ CCAGCACATG ACAAGTACAA TCCCAACTTT TTTCAAAAGA TTTGCTACTT TTAATTGGAT TCCCATGTTG   
  
  
+ AATGAAGTTC TGGGTTTAAG GCTACAACTT TCTTGGGAGG ATAAGGTTCT TAAGTTAAGG TGGTCCTCTT   
  
  
+ TTCTTTTGAT CTTTCTTATG GGTACAACCT CATGGGTCTC ATCAACACAT GAACTCTTTT TATCTTTTAT   
  
  
+ CTTTCCCCTT TCCTTTATGC AGGATTGACA GGTGCATGGA AAACAAGAAG CAATTAAATA CTAGTGGCTT   
  
  
+ AGCCCCATCC CTAATATTTT GGCTATTCTG TTTCACCATC CTCCCCTGTC TCCATTTTCT TTGTTTCCTT   
  
  
+ TTTTTCTAGG AACTTGTGAT GGGCAAGGGA AGATCAGTAC AATGTGATGG TTGTTAGTAG CGAGAAGATG   
  
  
+ AAGTGTCACC GCAAAAATTT TGTGCCATAA TTTGTCGTAA GGGAAACAAG TATCAAACAA CAATGTCATT   
  
  
+ TATGATAAAC AAGTAACAGG AGATATGTAC TCTGAAAAAA TATAAGGTAC CTGAGAAGTT GTTAACTTAG   
  
  
+ CTTGAAAAAC CCTCCTAACT TGATAAACCC TCTTCGCTAT GTTATCCCGC ACCCTGCCAT AGAGTCACCG   
  
  
+ ATTCAGTATA TATGTAACAT ACCCAATCTT GTCTGGATAT ATAGTTAACC CTTTTGGAAT ATCTGAAAAT   
  
  
+ CCACACGAGA GTCATCCTTC TAGACTAGGA AACATGCGCC ACATTTTGGA TTCTAACCAA GTATAATAAG   
  
  
+ CATATCTTAA AGCCAAAAAT GCAAGGATAG ATACAAATAC CCATCACATG CATGTAAATG TCCCTTGTCA   
  
  
+ TGGCTCTGAT TCTCAAAATA TTAATTTTCA ATAAATTAAA TGAAATTGCA TTATTTTAAA GATTTCTGAA   
  
  
+ TGCATCCATT ATTGCAATTT TTTAAAGACT GCACAATGAT GACAATGTCA ATGGTTTCCT AAAGTTGCGC   
  
  
+ CTGATGGGCT ATTGACAGTG AAGCAACACT CCCTTTGTTC CCATTTATGT GTCTCCACTT GCCCCTTTGA   
  
  
+ AACTCTCTAC ATTTGTATGT CATCTGAATT TGATTCTCAA ATCTCAGATG ATTTGAATTC CCTTTATGCC   
  
  
+ ATCTTTACTA TCTATTGAAT GATTCTTTCT TTTCCACTTT CTTATCTTCC ACTACCTTCT TCTTGGATGC   
  
  
+ CCTTTTGCTG CTTATAAAGG GCGACACCTG AACAAGTTGC TGTATTCTCA AACCATGAAT CTGTACAGGT   
  
  
+ TTAGCCCCGA TATGTGTCCT AGCTTTGTGG GTCACGAAGA AATTGGTCCT AATTTAATAG GACACCAAGA   
  
  
+ CCCTTTTCAA AATGCATACA ATTGCACTGA TTTTGGTGAT GCTTACTTGT TGCAATTCCC AGTTGAAGAT   
  
  
+ TTATGTCAAT ACCGTGTTCC TGATCTAATG GGGCTACCTG TTGAAGATGG GGATCCTACA GAGGTCCCTG   
  
  
+ ACATTCCTGA TACATGCTTC AACTACATAA GCAATCTCCT AATGGAAGAG GGCTCGGATG ACCGCCCTGG   
  
  
+ CGCCACCCTG CAGGACTATA TGGCTGCTCA AGTCTCCTAC AGCAAGTCCT CCAATGCCGC CTTTCCCGGT   
  
  
+ GAGAGTTGCT CATTCTTTGG TAATAATCAA TGCCCTCCAC CTGCTTATCA AAGCAACCCT TATTGCCTTG   
  
  
+ ATGATTACTT CAACTTGCTA TCCAATACTA CTGGCAATAG TGTTAACAAT GCTGGTGTTC ATGACAGCAA   
  
  
+ CAACTTTGTT CAGAACAGTT TGATTGGGAA TAGGGGTACT AGTTCTAGTT CTAGGGGCAG AAGTGATTTT   
  
  
+ GGATCTACCC TTATTAGGAA TTTGCCATTA GGGACCGACA CACTTGATGT GGTCAATCCA GCACCTACTC   
  
  
+ CTCGAGCCCC TCTGGTTACG ACTCAGTGTC AGGTGTTTGA AACACACCAG TATGAGCAGG CCTTCGAACT   
  
  
+ GATCACTAAT GGAAGCTGGG AATTCAGTCA TTCTGTTGCT AATACATCAA ATGATGGAAT TCAAAATCAA   
  
  
+ CCAATTCAAG CAGTTTCAAC CCAAGAACGA TGGAATATGA AAACATTTTC CGATGACCAA CTTGAGCCTG   
  
  
+ CTCTTTTGCG AAGTCTTGTG ATGTCCTCAA ACTGTACTGA TATCTCTGAT CAGGGTGTAC ACAGGATAGT   
  
  
+ GAACCCTGAG CCACCCAACA ACAGAAATAC GGAGATTGTG TGCAATGACT CATCCAGTAA GAGTAAGACA   
  
  
+ AGACGAAGAG AGCATGGTGC AGGAGAAAAC CACGATGAAG GAGAAAACCA CGATGAGACA GGAAGGAGTA   
  
  
+ ACAAGCAATC AGCACCATCC AATGAAGTGT TCGATGAAAT TGAGCAGTAT GGTGATGTGC TGCTCTGCCC   
  
  
+ TGAAGGAAGA AGTAAACCAG CAGGGTTATG CAGCAGCAGC AACGCTAACA GAACTAGCAA CTCACAGAAC   
  
  
+ TCGGAAGACT CAAAGTCACA GCAAAATGGG AGTTCCTCCA AGGTGTCAAA AAAGCGAGGC AATAAAAAGC   
  
  
+ AAGTGGTTGA TTTGAGGACT CTCCTAACTC GATGTGCACA ATGTGTTGCT CGTGTGGATT TAACTGGTTC   
  
  
+ TTACCAGTTG CTTCAGGAGA TTCGGCAGCA TTCTTCTCCC TATGGTGATT ATCTCCAAAG AATGGCTCAT   
  
  
+ TACCTTGCTA ATGGCCTTGA GGCGCGCTTA GAAGGCAAAG GATATGAACT AACTCGTGCC AATGAGCATA   
  
  
+ TCCCACCTAC TGAGATCCTC AAGGCTAACC GGGTATATGT GGCAGCGGTC CCTTTCAAGA TAATGTCTTA   
  
  
+ TTACACAACA AACAAGACAA TTGCAAGCCT AGTTGAGGAG GCACCAAGTA TACACATAAT TGATTTTGGT   
  
  
+ ATTTTCTATG GACTACAATG GCCCTGCATC ATTCAGAACC TCTCCAAGAG GCCCAATGGG CCTCCCAGAA   
  
  
+ TCCGCATCAC AGGCATCGAT TTCCCCCAAC CTGGGTTCCG GCCAGCAGAG AGGGTTGAAG AGACAGGGCG   
  
  
+ GTGTTTAGCC AAGTATTGTG ACAGGTACAA TGTGCCCTTT GAATACCACC CCATAGCCAA GAAGTGGGAG   
  
  
+ ACCATCCAAC TCGAGGAGCT CAAAATTGAA AGGAATGAGC CCCTTGTAGT GAACTGTCTG TATAGATCGC   
  
  
+ ACAACCTATT TGATGAAAGC GTAGAGGAAA ACAGCCCAAG AGACGCGTTC CTGAGCTTGG TTAGGAAAAT   
  
  
+ AAATCCCGAT ATATTCATGC ATGGGGTGGT GAATACCGCA TCAAGTGTAC CTTTCTTCCT GAACAGGTTC   
  
  
+ AAGGAGGCCA TGTTTCACTA CACGGCCTTG TTTGACTTGT TCGAGGCCAC AATGAGCCGC GAGGATCAAG   
  
  
+ AGAGGGTGTT ACTAGAGAGC AAGTTGCATG GGAATCAGGC GTTGAATGTG ATCGCCTGCG AAGGAGCGGA   
  
  
+ GAGAGTGGAG AGGCCTGAGT CATACAAACA ATGGCAGGTT AGGACTCAAA GGGCCGGGTT TGTTCAAGTG   
  
  
+ TCGATGGATC GCGAGCTTTT GAGAAGGGCA AGGGCCATGG TCAGGAGGAA TTTTAGGGAA GAGTTCAGTT   
  
  
+ ATGATGAAGA TGGGCATTGG GTGGTTCAAG GATGGAAAGG CAGGATAATG TATGCCATTT CTTGTTGGAA   
  
  
+ ACCTTCATA  

- +Up\_Stream \_Len000TAATAA TAAGATTATA TATCTATTAT TATATTATTA TTATTATTAT TATTATTATT   
  
  
- AATAATAATA ATAATAATAA ATAATTTATA TTATCAATAA TATTATTATT ATTAATATTA TTATTATTCT   
  
  
- AATAATAGTA ATATAATAAT AATAATAATA ACATTAAATA ATAATAATAT CATTAATAAT AACAATATAT   
  
  
- CATTTTAATA ATAATCATAA CAATAATATT TATAAGTATG AACGTATATT ATAAAGATTT TATTTTTGTC   
  
  
- TCAATTAAGG TAAGGTTTTG ATGAGTGGTT TGTCGTTCCT CACCCTGGAC TAAGGAAAGG TTAAAAGTGG   
  
  
- TTTGTTGACC CAAGTCTTAA GTTCAAGGTA TAGGTTGGGG GGTCCTAAGT TCAGGACTAA GGTAAGGTAA   
  
  
- GGTCAAAGGG AGTGGTTTGA CCCGGAATCT AACTACCAAC AAGTCAACCT ACTCCCAACA AGGTACGGTT   
  
  
- CAATCTAGTA ACAACAGTTA AAACTCTTGC TGATAGAATT ATTCGGATTG CTACAACTAA ACTAAATCAA   
  
  
- GTTTGGTAAA GTTTATTATG TTTAGTTTAA GATAAATCTG GCTGTGATTT TATTTATCTA ACTCTTTTTA   
  
  
- ATATCTAAAA TTTCAAGTCT AATTTTAGGG ATTTCTAGTT GTCTAACTTT ACGTATAAAG TATCACGTGT   
  
  
- ACCTGAACGA AACTTAGAGC AGATCTCTTG GATTCTTTTT CGTTCTCACC AAGTCTTCCA ATCTTAACAA   
  
  
- GGTCGTGTAC TGTTCATGTT AGGGTTGAAA AAAGTTTTCT AAACGATGAA AATTAACCTA AGGGTACAAC   
  
  
- TTACTTCAAG ACCCAAATTC CGATGTTGAA AGAACCCTCC TATTCCAAGA ATTCAATTCC ACCAGGAGAA   
  
  
- AAGAAAACTA GAAAGAATAC CCATGTTGGA GTACCCAGAG TAGTTGTGTA CTTGAGAAAA ATAGAAAATA   
  
  
- GAAAGGGGAA AGGAAATACG TCCTAACTGT CCACGTACCT TTTGTTCTTC GTTAATTTAT GATCACCGAA   
  
  
- TCGGGGTAGG GATTATAAAA CCGATAAGAC AAAGTGGTAG GAGGGGACAG AGGTAAAAGA AACAAAGGAA   
  
  
- AAAAAGATCC TTGAACACTA CCCGTTCCCT TCTAGTCATG TTACACTACC AACAATCATC GCTCTTCTAC   
  
  
- TTCACAGTGG CGTTTTTAAA ACACGGTATT AAACAGCATT CCCTTTGTTC ATAGTTTGTT GTTACAGTAA   
  
  
- ATACTATTTG TTCATTGTCC TCTATACATG AGACTTTTTT ATATTCCATG GACTCTTCAA CAATTGAATC   
  
  
- GAACTTTTTG GGAGGATTGA ACTATTTGGG AGAAGCGATA CAATAGGGCG TGGGACGGTA TCTCAGTGGC   
  
  
- TAAGTCATAT ATACATTGTA TGGGTTAGAA CAGACCTATA TATCAATTGG GAAAACCTTA TAGACTTTTA   
  
  
- GGTGTGCTCT CAGTAGGAAG ATCTGATCCT TTGTACGCGG TGTAAAACCT AAGATTGGTT CATATTATTC   
  
  
- GTATAGAATT TCGGTTTTTA CGTTCCTATC TATGTTTATG GGTAGTGTAC GTACATTTAC AGGGAACAGT   
  
  
- ACCGAGACTA AGAGTTTTAT AATTAAAAGT TATTTAATTT ACTTTAACGT AATAAAATTT CTAAAGACTT   
  
  
- ACGTAGGTAA TAACGTTAAA AAATTTCTGA CGTGTTACTA CTGTTACAGT TACCAAAGGA TTTCAACGCG   
  
  
- GACTACCCGA TAACTGTCAC TTCGTTGTGA GGGAAACAAG GGTAAATACA CAGAGGTGAA CGGGGAAACT   
  
  
- TTGAGAGATG TAAACATACA GTAGACTTAA ACTAAGAGTT TAGAGTCTAC TAAACTTAAG GGAAATACGG   
  
  
- TAGAAATGAT AGATAACTTA CTAAGAAAGA AAAGGTGAAA GAATAGAAGG TGATGGAAGA AGAACCTACG   
  
  
- GGAAAACGAC GAATATTTCC CGCTGTGGAC TTGTTCAACG ACATAAGAGT TTGGTACTTA GACATGTCCA   
  
  
- AATCGGGGCT ATACACAGGA TCGAAACACC CAGTGCTTCT TTAACCAGGA TTAAATTATC CTGTGGTTCT   
  
  
- GGGAAAAGTT TTACGTATGT TAACGTGACT AAAACCACTA CGAATGAACA ACGTTAAGGG TCAACTTCTA   
  
  
- AATACAGTTA TGGCACAAGG ACTAGATTAC CCCGATGGAC AACTTCTACC CCTAGGATGT CTCCAGGGAC   
  
  
- TGTAAGGACT ATGTACGAAG TTGATGTATT CGTTAGAGGA TTACCTTCTC CCGAGCCTAC TGGCGGGACC   
  
  
- GCGGTGGGAC GTCCTGATAT ACCGACGAGT TCAGAGGATG TCGTTCAGGA GGTTACGGCG GAAAGGGCCA   
  
  
- CTCTCAACGA GTAAGAAACC ATTATTAGTT ACGGGAGGTG GACGAATAGT TTCGTTGGGA ATAACGGAAC   
  
  
- TACTAATGAA GTTGAACGAT AGGTTATGAT GACCGTTATC ACAATTGTTA CGACCACAAG TACTGTCGTT   
  
  
- GTTGAAACAA GTCTTGTCAA ACTAACCCTT ATCCCCATGA TCAAGATCAA GATCCCCGTC TTCACTAAAA   
  
  
- CCTAGATGGG AATAATCCTT AAACGGTAAT CCCTGGCTGT GTGAACTACA CCAGTTAGGT CGTGGATGAG   
  
  
- GAGCTCGGGG AGACCAATGC TGAGTCACAG TCCACAAACT TTGTGTGGTC ATACTCGTCC GGAAGCTTGA   
  
  
- CTAGTGATTA CCTTCGACCC TTAAGTCAGT AAGACAACGA TTATGTAGTT TACTACCTTA AGTTTTAGTT   
  
  
- GGTTAAGTTC GTCAAAGTTG GGTTCTTGCT ACCTTATACT TTTGTAAAAG GCTACTGGTT GAACTCGGAC   
  
  
- GAGAAAACGC TTCAGAACAC TACAGGAGTT TGACATGACT ATAGAGACTA GTCCCACATG TGTCCTATCA   
  
  
- CTTGGGACTC GGTGGGTTGT TGTCTTTATG CCTCTAACAC ACGTTACTGA GTAGGTCATT CTCATTCTGT   
  
  
- TCTGCTTCTC TCGTACCACG TCCTCTTTTG GTGCTACTTC CTCTTTTGGT GCTACTCTGT CCTTCCTCAT   
  
  
- TGTTCGTTAG TCGTGGTAGG TTACTTCACA AGCTACTTTA ACTCGTCATA CCACTACACG ACGAGACGGG   
  
  
- ACTTCCTTCT TCATTTGGTC GTCCCAATAC GTCGTCGTCG TTGCGATTGT CTTGATCGTT GAGTGTCTTG   
  
  
- AGCCTTCTGA GTTTCAGTGT CGTTTTACCC TCAAGGAGGT TCCACAGTTT TTTCGCTCCG TTATTTTTCG   
  
  
- TTCACCAACT AAACTCCTGA GAGGATTGAG CTACACGTGT TACACAACGA GCACACCTAA ATTGACCAAG   
  
  
- AATGGTCAAC GAAGTCCTCT AAGCCGTCGT AAGAAGAGGG ATACCACTAA TAGAGGTTTC TTACCGAGTA   
  
  
- ATGGAACGAT TACCGGAACT CCGCGCGAAT CTTCCGTTTC CTATACTTGA TTGAGCACGG TTACTCGTAT   
  
  
- AGGGTGGATG ACTCTAGGAG TTCCGATTGG CCCATATACA CCGTCGCCAG GGAAAGTTCT ATTACAGAAT   
  
  
- AATGTGTTGT TTGTTCTGTT AACGTTCGGA TCAACTCCTC CGTGGTTCAT ATGTGTATTA ACTAAAACCA   
  
  
- TAAAAGATAC CTGATGTTAC CGGGACGTAG TAAGTCTTGG AGAGGTTCTC CGGGTTACCC GGAGGGTCTT   
  
  
- AGGCGTAGTG TCCGTAGCTA AAGGGGGTTG GACCCAAGGC CGGTCGTCTC TCCCAACTTC TCTGTCCCGC   
  
  
- CACAAATCGG TTCATAACAC TGTCCATGTT ACACGGGAAA CTTATGGTGG GGTATCGGTT CTTCACCCTC   
  
  
- TGGTAGGTTG AGCTCCTCGA GTTTTAACTT TCCTTACTCG GGGAACATCA CTTGACAGAC ATATCTAGCG   
  
  
- TGTTGGATAA ACTACTTTCG CATCTCCTTT TGTCGGGTTC TCTGCGCAAG GACTCGAACC AATCCTTTTA   
  
  
- TTTAGGGCTA TATAAGTACG TACCCCACCA CTTATGGCGT AGTTCACATG GAAAGAAGGA CTTGTCCAAG   
  
  
- TTCCTCCGGT ACAAAGTGAT GTGCCGGAAC AAACTGAACA AGCTCCGGTG TTACTCGGCG CTCCTAGTTC   
  
  
- TCTCCCACAA TGATCTCTCG TTCAACGTAC CCTTAGTCCG CAACTTACAC TAGCGGACGC TTCCTCGCCT   
  
  
- CTCTCACCTC TCCGGACTCA GTATGTTTGT TACCGTCCAA TCCTGAGTTT CCCGGCCCAA ACAAGTTCAC   
  
  
- AGCTACCTAG CGCTCGAAAA CTCTTCCCGT TCCCGGTACC AGTCCTCCTT AAAATCCCTT CTCAAGTCAA   
  
  
- TACTACTTCT ACCCGTAACC CACCAAGTTC CTACCTTTCC GTCCTATTAC ATACGGTAAA GAACAACCTT   
  
  
- TGGAAGTAT

+     CARE

| Site Name | Organism | Position | Strand | Matrix score. | sequence | function |
| --- | --- | --- | --- | --- | --- | --- |
| CARE | Oryza sativa | 3212 | + | 8 | CAACTCAC |  |

>HU02G01570.1   
+ +Up\_Stream \_Len000ATTATT ATTCTAATAT ATAGATAATA ATATAATAAT AATAATAATA ATAATAATAA   
  
  
+ TTATTATTAT TATTATTATT TATTAAATAT AATAGTTATT ATAATAATAA TAATTATAAT AATAATAAGA   
  
  
+ TTATTATCAT TATATTATTA TTATTATTAT TGTAATTTAT TATTATTATA GTAATTATTA TTGTTATATA   
  
  
+ GTAAAATTAT TATTAGTATT GTTATTATAA ATATTCATAC TTGCATATAA TATTTCTAAA ATAAAAACAG   
  
  
+ AGTTAATTCC ATTCCAAAAC TACTCACCAA ACAGCAAGGA GTGGGACCTG ATTCCTTTCC AATTTTCACC   
  
  
+ AAACAACTGG GTTCAGAATT CAAGTTCCAT ATCCAACCCC CCAGGATTCA AGTCCTGATT CCATTCCATT   
  
  
+ CCAGTTTCCC TCACCAAACT GGGCCTTAGA TTGATGGTTG TTCAGTTGGA TGAGGGTTGT TCCATGCCAA   
  
  
+ GTTAGATCAT TGTTGTCAAT TTTGAGAACG ACTATCTTAA TAAGCCTAAC GATGTTGATT TGATTTAGTT   
  
  
+ CAAACCATTT CAAATAATAC AAATCAAATT CTATTTAGAC CGACACTAAA ATAAATAGAT TGAGAAAAAT   
  
  
+ TATAGATTTT AAAGTTCAGA TTAAAATCCC TAAAGATCAA CAGATTGAAA TGCATATTTC ATAGTGCACA   
  
  
+ TGGACTTGCT TTGAATCTCG TCTAGAGAAC CTAAGAAAAA GCAAGAGTGG TTCAGAAGGT TAGAATTGTT   
  
  
+ CCAGCACATG ACAAGTACAA TCCCAACTTT TTTCAAAAGA TTTGCTACTT TTAATTGGAT TCCCATGTTG   
  
  
+ AATGAAGTTC TGGGTTTAAG GCTACAACTT TCTTGGGAGG ATAAGGTTCT TAAGTTAAGG TGGTCCTCTT   
  
  
+ TTCTTTTGAT CTTTCTTATG GGTACAACCT CATGGGTCTC ATCAACACAT GAACTCTTTT TATCTTTTAT   
  
  
+ CTTTCCCCTT TCCTTTATGC AGGATTGACA GGTGCATGGA AAACAAGAAG CAATTAAATA CTAGTGGCTT   
  
  
+ AGCCCCATCC CTAATATTTT GGCTATTCTG TTTCACCATC CTCCCCTGTC TCCATTTTCT TTGTTTCCTT   
  
  
+ TTTTTCTAGG AACTTGTGAT GGGCAAGGGA AGATCAGTAC AATGTGATGG TTGTTAGTAG CGAGAAGATG   
  
  
+ AAGTGTCACC GCAAAAATTT TGTGCCATAA TTTGTCGTAA GGGAAACAAG TATCAAACAA CAATGTCATT   
  
  
+ TATGATAAAC AAGTAACAGG AGATATGTAC TCTGAAAAAA TATAAGGTAC CTGAGAAGTT GTTAACTTAG   
  
  
+ CTTGAAAAAC CCTCCTAACT TGATAAACCC TCTTCGCTAT GTTATCCCGC ACCCTGCCAT AGAGTCACCG   
  
  
+ ATTCAGTATA TATGTAACAT ACCCAATCTT GTCTGGATAT ATAGTTAACC CTTTTGGAAT ATCTGAAAAT   
  
  
+ CCACACGAGA GTCATCCTTC TAGACTAGGA AACATGCGCC ACATTTTGGA TTCTAACCAA GTATAATAAG   
  
  
+ CATATCTTAA AGCCAAAAAT GCAAGGATAG ATACAAATAC CCATCACATG CATGTAAATG TCCCTTGTCA   
  
  
+ TGGCTCTGAT TCTCAAAATA TTAATTTTCA ATAAATTAAA TGAAATTGCA TTATTTTAAA GATTTCTGAA   
  
  
+ TGCATCCATT ATTGCAATTT TTTAAAGACT GCACAATGAT GACAATGTCA ATGGTTTCCT AAAGTTGCGC   
  
  
+ CTGATGGGCT ATTGACAGTG AAGCAACACT CCCTTTGTTC CCATTTATGT GTCTCCACTT GCCCCTTTGA   
  
  
+ AACTCTCTAC ATTTGTATGT CATCTGAATT TGATTCTCAA ATCTCAGATG ATTTGAATTC CCTTTATGCC   
  
  
+ ATCTTTACTA TCTATTGAAT GATTCTTTCT TTTCCACTTT CTTATCTTCC ACTACCTTCT TCTTGGATGC   
  
  
+ CCTTTTGCTG CTTATAAAGG GCGACACCTG AACAAGTTGC TGTATTCTCA AACCATGAAT CTGTACAGGT   
  
  
+ TTAGCCCCGA TATGTGTCCT AGCTTTGTGG GTCACGAAGA AATTGGTCCT AATTTAATAG GACACCAAGA   
  
  
+ CCCTTTTCAA AATGCATACA ATTGCACTGA TTTTGGTGAT GCTTACTTGT TGCAATTCCC AGTTGAAGAT   
  
  
+ TTATGTCAAT ACCGTGTTCC TGATCTAATG GGGCTACCTG TTGAAGATGG GGATCCTACA GAGGTCCCTG   
  
  
+ ACATTCCTGA TACATGCTTC AACTACATAA GCAATCTCCT AATGGAAGAG GGCTCGGATG ACCGCCCTGG   
  
  
+ CGCCACCCTG CAGGACTATA TGGCTGCTCA AGTCTCCTAC AGCAAGTCCT CCAATGCCGC CTTTCCCGGT   
  
  
+ GAGAGTTGCT CATTCTTTGG TAATAATCAA TGCCCTCCAC CTGCTTATCA AAGCAACCCT TATTGCCTTG   
  
  
+ ATGATTACTT CAACTTGCTA TCCAATACTA CTGGCAATAG TGTTAACAAT GCTGGTGTTC ATGACAGCAA   
  
  
+ CAACTTTGTT CAGAACAGTT TGATTGGGAA TAGGGGTACT AGTTCTAGTT CTAGGGGCAG AAGTGATTTT   
  
  
+ GGATCTACCC TTATTAGGAA TTTGCCATTA GGGACCGACA CACTTGATGT GGTCAATCCA GCACCTACTC   
  
  
+ CTCGAGCCCC TCTGGTTACG ACTCAGTGTC AGGTGTTTGA AACACACCAG TATGAGCAGG CCTTCGAACT   
  
  
+ GATCACTAAT GGAAGCTGGG AATTCAGTCA TTCTGTTGCT AATACATCAA ATGATGGAAT TCAAAATCAA   
  
  
+ CCAATTCAAG CAGTTTCAAC CCAAGAACGA TGGAATATGA AAACATTTTC CGATGACCAA CTTGAGCCTG   
  
  
+ CTCTTTTGCG AAGTCTTGTG ATGTCCTCAA ACTGTACTGA TATCTCTGAT CAGGGTGTAC ACAGGATAGT   
  
  
+ GAACCCTGAG CCACCCAACA ACAGAAATAC GGAGATTGTG TGCAATGACT CATCCAGTAA GAGTAAGACA   
  
  
+ AGACGAAGAG AGCATGGTGC AGGAGAAAAC CACGATGAAG GAGAAAACCA CGATGAGACA GGAAGGAGTA   
  
  
+ ACAAGCAATC AGCACCATCC AATGAAGTGT TCGATGAAAT TGAGCAGTAT GGTGATGTGC TGCTCTGCCC   
  
  
+ TGAAGGAAGA AGTAAACCAG CAGGGTTATG CAGCAGCAGC AACGCTAACA GAACTAGCAA CTCACAGAAC   
  
  
+ TCGGAAGACT CAAAGTCACA GCAAAATGGG AGTTCCTCCA AGGTGTCAAA AAAGCGAGGC AATAAAAAGC   
  
  
+ AAGTGGTTGA TTTGAGGACT CTCCTAACTC GATGTGCACA ATGTGTTGCT CGTGTGGATT TAACTGGTTC   
  
  
+ TTACCAGTTG CTTCAGGAGA TTCGGCAGCA TTCTTCTCCC TATGGTGATT ATCTCCAAAG AATGGCTCAT   
  
  
+ TACCTTGCTA ATGGCCTTGA GGCGCGCTTA GAAGGCAAAG GATATGAACT AACTCGTGCC AATGAGCATA   
  
  
+ TCCCACCTAC TGAGATCCTC AAGGCTAACC GGGTATATGT GGCAGCGGTC CCTTTCAAGA TAATGTCTTA   
  
  
+ TTACACAACA AACAAGACAA TTGCAAGCCT AGTTGAGGAG GCACCAAGTA TACACATAAT TGATTTTGGT   
  
  
+ ATTTTCTATG GACTACAATG GCCCTGCATC ATTCAGAACC TCTCCAAGAG GCCCAATGGG CCTCCCAGAA   
  
  
+ TCCGCATCAC AGGCATCGAT TTCCCCCAAC CTGGGTTCCG GCCAGCAGAG AGGGTTGAAG AGACAGGGCG   
  
  
+ GTGTTTAGCC AAGTATTGTG ACAGGTACAA TGTGCCCTTT GAATACCACC CCATAGCCAA GAAGTGGGAG   
  
  
+ ACCATCCAAC TCGAGGAGCT CAAAATTGAA AGGAATGAGC CCCTTGTAGT GAACTGTCTG TATAGATCGC   
  
  
+ ACAACCTATT TGATGAAAGC GTAGAGGAAA ACAGCCCAAG AGACGCGTTC CTGAGCTTGG TTAGGAAAAT   
  
  
+ AAATCCCGAT ATATTCATGC ATGGGGTGGT GAATACCGCA TCAAGTGTAC CTTTCTTCCT GAACAGGTTC   
  
  
+ AAGGAGGCCA TGTTTCACTA CACGGCCTTG TTTGACTTGT TCGAGGCCAC AATGAGCCGC GAGGATCAAG   
  
  
+ AGAGGGTGTT ACTAGAGAGC AAGTTGCATG GGAATCAGGC GTTGAATGTG ATCGCCTGCG AAGGAGCGGA   
  
  
+ GAGAGTGGAG AGGCCTGAGT CATACAAACA ATGGCAGGTT AGGACTCAAA GGGCCGGGTT TGTTCAAGTG   
  
  
+ TCGATGGATC GCGAGCTTTT GAGAAGGGCA AGGGCCATGG TCAGGAGGAA TTTTAGGGAA GAGTTCAGTT   
  
  
+ ATGATGAAGA TGGGCATTGG GTGGTTCAAG GATGGAAAGG CAGGATAATG TATGCCATTT CTTGTTGGAA   
  
  
+ ACCTTCATA  

- +Up\_Stream \_Len000TAATAA TAAGATTATA TATCTATTAT TATATTATTA TTATTATTAT TATTATTATT   
  
  
- AATAATAATA ATAATAATAA ATAATTTATA TTATCAATAA TATTATTATT ATTAATATTA TTATTATTCT   
  
  
- AATAATAGTA ATATAATAAT AATAATAATA ACATTAAATA ATAATAATAT CATTAATAAT AACAATATAT   
  
  
- CATTTTAATA ATAATCATAA CAATAATATT TATAAGTATG AACGTATATT ATAAAGATTT TATTTTTGTC   
  
  
- TCAATTAAGG TAAGGTTTTG ATGAGTGGTT TGTCGTTCCT CACCCTGGAC TAAGGAAAGG TTAAAAGTGG   
  
  
- TTTGTTGACC CAAGTCTTAA GTTCAAGGTA TAGGTTGGGG GGTCCTAAGT TCAGGACTAA GGTAAGGTAA   
  
  
- GGTCAAAGGG AGTGGTTTGA CCCGGAATCT AACTACCAAC AAGTCAACCT ACTCCCAACA AGGTACGGTT   
  
  
- CAATCTAGTA ACAACAGTTA AAACTCTTGC TGATAGAATT ATTCGGATTG CTACAACTAA ACTAAATCAA   
  
  
- GTTTGGTAAA GTTTATTATG TTTAGTTTAA GATAAATCTG GCTGTGATTT TATTTATCTA ACTCTTTTTA   
  
  
- ATATCTAAAA TTTCAAGTCT AATTTTAGGG ATTTCTAGTT GTCTAACTTT ACGTATAAAG TATCACGTGT   
  
  
- ACCTGAACGA AACTTAGAGC AGATCTCTTG GATTCTTTTT CGTTCTCACC AAGTCTTCCA ATCTTAACAA   
  
  
- GGTCGTGTAC TGTTCATGTT AGGGTTGAAA AAAGTTTTCT AAACGATGAA AATTAACCTA AGGGTACAAC   
  
  
- TTACTTCAAG ACCCAAATTC CGATGTTGAA AGAACCCTCC TATTCCAAGA ATTCAATTCC ACCAGGAGAA   
  
  
- AAGAAAACTA GAAAGAATAC CCATGTTGGA GTACCCAGAG TAGTTGTGTA CTTGAGAAAA ATAGAAAATA   
  
  
- GAAAGGGGAA AGGAAATACG TCCTAACTGT CCACGTACCT TTTGTTCTTC GTTAATTTAT GATCACCGAA   
  
  
- TCGGGGTAGG GATTATAAAA CCGATAAGAC AAAGTGGTAG GAGGGGACAG AGGTAAAAGA AACAAAGGAA   
  
  
- AAAAAGATCC TTGAACACTA CCCGTTCCCT TCTAGTCATG TTACACTACC AACAATCATC GCTCTTCTAC   
  
  
- TTCACAGTGG CGTTTTTAAA ACACGGTATT AAACAGCATT CCCTTTGTTC ATAGTTTGTT GTTACAGTAA   
  
  
- ATACTATTTG TTCATTGTCC TCTATACATG AGACTTTTTT ATATTCCATG GACTCTTCAA CAATTGAATC   
  
  
- GAACTTTTTG GGAGGATTGA ACTATTTGGG AGAAGCGATA CAATAGGGCG TGGGACGGTA TCTCAGTGGC   
  
  
- TAAGTCATAT ATACATTGTA TGGGTTAGAA CAGACCTATA TATCAATTGG GAAAACCTTA TAGACTTTTA   
  
  
- GGTGTGCTCT CAGTAGGAAG ATCTGATCCT TTGTACGCGG TGTAAAACCT AAGATTGGTT CATATTATTC   
  
  
- GTATAGAATT TCGGTTTTTA CGTTCCTATC TATGTTTATG GGTAGTGTAC GTACATTTAC AGGGAACAGT   
  
  
- ACCGAGACTA AGAGTTTTAT AATTAAAAGT TATTTAATTT ACTTTAACGT AATAAAATTT CTAAAGACTT   
  
  
- ACGTAGGTAA TAACGTTAAA AAATTTCTGA CGTGTTACTA CTGTTACAGT TACCAAAGGA TTTCAACGCG   
  
  
- GACTACCCGA TAACTGTCAC TTCGTTGTGA GGGAAACAAG GGTAAATACA CAGAGGTGAA CGGGGAAACT   
  
  
- TTGAGAGATG TAAACATACA GTAGACTTAA ACTAAGAGTT TAGAGTCTAC TAAACTTAAG GGAAATACGG   
  
  
- TAGAAATGAT AGATAACTTA CTAAGAAAGA AAAGGTGAAA GAATAGAAGG TGATGGAAGA AGAACCTACG   
  
  
- GGAAAACGAC GAATATTTCC CGCTGTGGAC TTGTTCAACG ACATAAGAGT TTGGTACTTA GACATGTCCA   
  
  
- AATCGGGGCT ATACACAGGA TCGAAACACC CAGTGCTTCT TTAACCAGGA TTAAATTATC CTGTGGTTCT   
  
  
- GGGAAAAGTT TTACGTATGT TAACGTGACT AAAACCACTA CGAATGAACA ACGTTAAGGG TCAACTTCTA   
  
  
- AATACAGTTA TGGCACAAGG ACTAGATTAC CCCGATGGAC AACTTCTACC CCTAGGATGT CTCCAGGGAC   
  
  
- TGTAAGGACT ATGTACGAAG TTGATGTATT CGTTAGAGGA TTACCTTCTC CCGAGCCTAC TGGCGGGACC   
  
  
- GCGGTGGGAC GTCCTGATAT ACCGACGAGT TCAGAGGATG TCGTTCAGGA GGTTACGGCG GAAAGGGCCA   
  
  
- CTCTCAACGA GTAAGAAACC ATTATTAGTT ACGGGAGGTG GACGAATAGT TTCGTTGGGA ATAACGGAAC   
  
  
- TACTAATGAA GTTGAACGAT AGGTTATGAT GACCGTTATC ACAATTGTTA CGACCACAAG TACTGTCGTT   
  
  
- GTTGAAACAA GTCTTGTCAA ACTAACCCTT ATCCCCATGA TCAAGATCAA GATCCCCGTC TTCACTAAAA   
  
  
- CCTAGATGGG AATAATCCTT AAACGGTAAT CCCTGGCTGT GTGAACTACA CCAGTTAGGT CGTGGATGAG   
  
  
- GAGCTCGGGG AGACCAATGC TGAGTCACAG TCCACAAACT TTGTGTGGTC ATACTCGTCC GGAAGCTTGA   
  
  
- CTAGTGATTA CCTTCGACCC TTAAGTCAGT AAGACAACGA TTATGTAGTT TACTACCTTA AGTTTTAGTT   
  
  
- GGTTAAGTTC GTCAAAGTTG GGTTCTTGCT ACCTTATACT TTTGTAAAAG GCTACTGGTT GAACTCGGAC   
  
  
- GAGAAAACGC TTCAGAACAC TACAGGAGTT TGACATGACT ATAGAGACTA GTCCCACATG TGTCCTATCA   
  
  
- CTTGGGACTC GGTGGGTTGT TGTCTTTATG CCTCTAACAC ACGTTACTGA GTAGGTCATT CTCATTCTGT   
  
  
- TCTGCTTCTC TCGTACCACG TCCTCTTTTG GTGCTACTTC CTCTTTTGGT GCTACTCTGT CCTTCCTCAT   
  
  
- TGTTCGTTAG TCGTGGTAGG TTACTTCACA AGCTACTTTA ACTCGTCATA CCACTACACG ACGAGACGGG   
  
  
- ACTTCCTTCT TCATTTGGTC GTCCCAATAC GTCGTCGTCG TTGCGATTGT CTTGATCGTT GAGTGTCTTG   
  
  
- AGCCTTCTGA GTTTCAGTGT CGTTTTACCC TCAAGGAGGT TCCACAGTTT TTTCGCTCCG TTATTTTTCG   
  
  
- TTCACCAACT AAACTCCTGA GAGGATTGAG CTACACGTGT TACACAACGA GCACACCTAA ATTGACCAAG   
  
  
- AATGGTCAAC GAAGTCCTCT AAGCCGTCGT AAGAAGAGGG ATACCACTAA TAGAGGTTTC TTACCGAGTA   
  
  
- ATGGAACGAT TACCGGAACT CCGCGCGAAT CTTCCGTTTC CTATACTTGA TTGAGCACGG TTACTCGTAT   
  
  
- AGGGTGGATG ACTCTAGGAG TTCCGATTGG CCCATATACA CCGTCGCCAG GGAAAGTTCT ATTACAGAAT   
  
  
- AATGTGTTGT TTGTTCTGTT AACGTTCGGA TCAACTCCTC CGTGGTTCAT ATGTGTATTA ACTAAAACCA   
  
  
- TAAAAGATAC CTGATGTTAC CGGGACGTAG TAAGTCTTGG AGAGGTTCTC CGGGTTACCC GGAGGGTCTT   
  
  
- AGGCGTAGTG TCCGTAGCTA AAGGGGGTTG GACCCAAGGC CGGTCGTCTC TCCCAACTTC TCTGTCCCGC   
  
  
- CACAAATCGG TTCATAACAC TGTCCATGTT ACACGGGAAA CTTATGGTGG GGTATCGGTT CTTCACCCTC   
  
  
- TGGTAGGTTG AGCTCCTCGA GTTTTAACTT TCCTTACTCG GGGAACATCA CTTGACAGAC ATATCTAGCG   
  
  
- TGTTGGATAA ACTACTTTCG CATCTCCTTT TGTCGGGTTC TCTGCGCAAG GACTCGAACC AATCCTTTTA   
  
  
- TTTAGGGCTA TATAAGTACG TACCCCACCA CTTATGGCGT AGTTCACATG GAAAGAAGGA CTTGTCCAAG   
  
  
- TTCCTCCGGT ACAAAGTGAT GTGCCGGAAC AAACTGAACA AGCTCCGGTG TTACTCGGCG CTCCTAGTTC   
  
  
- TCTCCCACAA TGATCTCTCG TTCAACGTAC CCTTAGTCCG CAACTTACAC TAGCGGACGC TTCCTCGCCT   
  
  
- CTCTCACCTC TCCGGACTCA GTATGTTTGT TACCGTCCAA TCCTGAGTTT CCCGGCCCAA ACAAGTTCAC   
  
  
- AGCTACCTAG CGCTCGAAAA CTCTTCCCGT TCCCGGTACC AGTCCTCCTT AAAATCCCTT CTCAAGTCAA   
  
  
- TACTACTTCT ACCCGTAACC CACCAAGTTC CTACCTTTCC GTCCTATTAC ATACGGTAAA GAACAACCTT   
  
  
- TGGAAGTAT

+     CAT-box

| Site Name | Organism | Position | Strand | Matrix score. | sequence | function |
| --- | --- | --- | --- | --- | --- | --- |
| CAT-box | Arabidopsis thaliana | 1047 | - | 6 | GCCACT | cis-acting regulatory element related to meristem expression |

>HU02G01570.1   
+ +Up\_Stream \_Len000ATTATT ATTCTAATAT ATAGATAATA ATATAATAAT AATAATAATA ATAATAATAA   
  
  
+ TTATTATTAT TATTATTATT TATTAAATAT AATAGTTATT ATAATAATAA TAATTATAAT AATAATAAGA   
  
  
+ TTATTATCAT TATATTATTA TTATTATTAT TGTAATTTAT TATTATTATA GTAATTATTA TTGTTATATA   
  
  
+ GTAAAATTAT TATTAGTATT GTTATTATAA ATATTCATAC TTGCATATAA TATTTCTAAA ATAAAAACAG   
  
  
+ AGTTAATTCC ATTCCAAAAC TACTCACCAA ACAGCAAGGA GTGGGACCTG ATTCCTTTCC AATTTTCACC   
  
  
+ AAACAACTGG GTTCAGAATT CAAGTTCCAT ATCCAACCCC CCAGGATTCA AGTCCTGATT CCATTCCATT   
  
  
+ CCAGTTTCCC TCACCAAACT GGGCCTTAGA TTGATGGTTG TTCAGTTGGA TGAGGGTTGT TCCATGCCAA   
  
  
+ GTTAGATCAT TGTTGTCAAT TTTGAGAACG ACTATCTTAA TAAGCCTAAC GATGTTGATT TGATTTAGTT   
  
  
+ CAAACCATTT CAAATAATAC AAATCAAATT CTATTTAGAC CGACACTAAA ATAAATAGAT TGAGAAAAAT   
  
  
+ TATAGATTTT AAAGTTCAGA TTAAAATCCC TAAAGATCAA CAGATTGAAA TGCATATTTC ATAGTGCACA   
  
  
+ TGGACTTGCT TTGAATCTCG TCTAGAGAAC CTAAGAAAAA GCAAGAGTGG TTCAGAAGGT TAGAATTGTT   
  
  
+ CCAGCACATG ACAAGTACAA TCCCAACTTT TTTCAAAAGA TTTGCTACTT TTAATTGGAT TCCCATGTTG   
  
  
+ AATGAAGTTC TGGGTTTAAG GCTACAACTT TCTTGGGAGG ATAAGGTTCT TAAGTTAAGG TGGTCCTCTT   
  
  
+ TTCTTTTGAT CTTTCTTATG GGTACAACCT CATGGGTCTC ATCAACACAT GAACTCTTTT TATCTTTTAT   
  
  
+ CTTTCCCCTT TCCTTTATGC AGGATTGACA GGTGCATGGA AAACAAGAAG CAATTAAATA CTAGTGGCTT   
  
  
+ AGCCCCATCC CTAATATTTT GGCTATTCTG TTTCACCATC CTCCCCTGTC TCCATTTTCT TTGTTTCCTT   
  
  
+ TTTTTCTAGG AACTTGTGAT GGGCAAGGGA AGATCAGTAC AATGTGATGG TTGTTAGTAG CGAGAAGATG   
  
  
+ AAGTGTCACC GCAAAAATTT TGTGCCATAA TTTGTCGTAA GGGAAACAAG TATCAAACAA CAATGTCATT   
  
  
+ TATGATAAAC AAGTAACAGG AGATATGTAC TCTGAAAAAA TATAAGGTAC CTGAGAAGTT GTTAACTTAG   
  
  
+ CTTGAAAAAC CCTCCTAACT TGATAAACCC TCTTCGCTAT GTTATCCCGC ACCCTGCCAT AGAGTCACCG   
  
  
+ ATTCAGTATA TATGTAACAT ACCCAATCTT GTCTGGATAT ATAGTTAACC CTTTTGGAAT ATCTGAAAAT   
  
  
+ CCACACGAGA GTCATCCTTC TAGACTAGGA AACATGCGCC ACATTTTGGA TTCTAACCAA GTATAATAAG   
  
  
+ CATATCTTAA AGCCAAAAAT GCAAGGATAG ATACAAATAC CCATCACATG CATGTAAATG TCCCTTGTCA   
  
  
+ TGGCTCTGAT TCTCAAAATA TTAATTTTCA ATAAATTAAA TGAAATTGCA TTATTTTAAA GATTTCTGAA   
  
  
+ TGCATCCATT ATTGCAATTT TTTAAAGACT GCACAATGAT GACAATGTCA ATGGTTTCCT AAAGTTGCGC   
  
  
+ CTGATGGGCT ATTGACAGTG AAGCAACACT CCCTTTGTTC CCATTTATGT GTCTCCACTT GCCCCTTTGA   
  
  
+ AACTCTCTAC ATTTGTATGT CATCTGAATT TGATTCTCAA ATCTCAGATG ATTTGAATTC CCTTTATGCC   
  
  
+ ATCTTTACTA TCTATTGAAT GATTCTTTCT TTTCCACTTT CTTATCTTCC ACTACCTTCT TCTTGGATGC   
  
  
+ CCTTTTGCTG CTTATAAAGG GCGACACCTG AACAAGTTGC TGTATTCTCA AACCATGAAT CTGTACAGGT   
  
  
+ TTAGCCCCGA TATGTGTCCT AGCTTTGTGG GTCACGAAGA AATTGGTCCT AATTTAATAG GACACCAAGA   
  
  
+ CCCTTTTCAA AATGCATACA ATTGCACTGA TTTTGGTGAT GCTTACTTGT TGCAATTCCC AGTTGAAGAT   
  
  
+ TTATGTCAAT ACCGTGTTCC TGATCTAATG GGGCTACCTG TTGAAGATGG GGATCCTACA GAGGTCCCTG   
  
  
+ ACATTCCTGA TACATGCTTC AACTACATAA GCAATCTCCT AATGGAAGAG GGCTCGGATG ACCGCCCTGG   
  
  
+ CGCCACCCTG CAGGACTATA TGGCTGCTCA AGTCTCCTAC AGCAAGTCCT CCAATGCCGC CTTTCCCGGT   
  
  
+ GAGAGTTGCT CATTCTTTGG TAATAATCAA TGCCCTCCAC CTGCTTATCA AAGCAACCCT TATTGCCTTG   
  
  
+ ATGATTACTT CAACTTGCTA TCCAATACTA CTGGCAATAG TGTTAACAAT GCTGGTGTTC ATGACAGCAA   
  
  
+ CAACTTTGTT CAGAACAGTT TGATTGGGAA TAGGGGTACT AGTTCTAGTT CTAGGGGCAG AAGTGATTTT   
  
  
+ GGATCTACCC TTATTAGGAA TTTGCCATTA GGGACCGACA CACTTGATGT GGTCAATCCA GCACCTACTC   
  
  
+ CTCGAGCCCC TCTGGTTACG ACTCAGTGTC AGGTGTTTGA AACACACCAG TATGAGCAGG CCTTCGAACT   
  
  
+ GATCACTAAT GGAAGCTGGG AATTCAGTCA TTCTGTTGCT AATACATCAA ATGATGGAAT TCAAAATCAA   
  
  
+ CCAATTCAAG CAGTTTCAAC CCAAGAACGA TGGAATATGA AAACATTTTC CGATGACCAA CTTGAGCCTG   
  
  
+ CTCTTTTGCG AAGTCTTGTG ATGTCCTCAA ACTGTACTGA TATCTCTGAT CAGGGTGTAC ACAGGATAGT   
  
  
+ GAACCCTGAG CCACCCAACA ACAGAAATAC GGAGATTGTG TGCAATGACT CATCCAGTAA GAGTAAGACA   
  
  
+ AGACGAAGAG AGCATGGTGC AGGAGAAAAC CACGATGAAG GAGAAAACCA CGATGAGACA GGAAGGAGTA   
  
  
+ ACAAGCAATC AGCACCATCC AATGAAGTGT TCGATGAAAT TGAGCAGTAT GGTGATGTGC TGCTCTGCCC   
  
  
+ TGAAGGAAGA AGTAAACCAG CAGGGTTATG CAGCAGCAGC AACGCTAACA GAACTAGCAA CTCACAGAAC   
  
  
+ TCGGAAGACT CAAAGTCACA GCAAAATGGG AGTTCCTCCA AGGTGTCAAA AAAGCGAGGC AATAAAAAGC   
  
  
+ AAGTGGTTGA TTTGAGGACT CTCCTAACTC GATGTGCACA ATGTGTTGCT CGTGTGGATT TAACTGGTTC   
  
  
+ TTACCAGTTG CTTCAGGAGA TTCGGCAGCA TTCTTCTCCC TATGGTGATT ATCTCCAAAG AATGGCTCAT   
  
  
+ TACCTTGCTA ATGGCCTTGA GGCGCGCTTA GAAGGCAAAG GATATGAACT AACTCGTGCC AATGAGCATA   
  
  
+ TCCCACCTAC TGAGATCCTC AAGGCTAACC GGGTATATGT GGCAGCGGTC CCTTTCAAGA TAATGTCTTA   
  
  
+ TTACACAACA AACAAGACAA TTGCAAGCCT AGTTGAGGAG GCACCAAGTA TACACATAAT TGATTTTGGT   
  
  
+ ATTTTCTATG GACTACAATG GCCCTGCATC ATTCAGAACC TCTCCAAGAG GCCCAATGGG CCTCCCAGAA   
  
  
+ TCCGCATCAC AGGCATCGAT TTCCCCCAAC CTGGGTTCCG GCCAGCAGAG AGGGTTGAAG AGACAGGGCG   
  
  
+ GTGTTTAGCC AAGTATTGTG ACAGGTACAA TGTGCCCTTT GAATACCACC CCATAGCCAA GAAGTGGGAG   
  
  
+ ACCATCCAAC TCGAGGAGCT CAAAATTGAA AGGAATGAGC CCCTTGTAGT GAACTGTCTG TATAGATCGC   
  
  
+ ACAACCTATT TGATGAAAGC GTAGAGGAAA ACAGCCCAAG AGACGCGTTC CTGAGCTTGG TTAGGAAAAT   
  
  
+ AAATCCCGAT ATATTCATGC ATGGGGTGGT GAATACCGCA TCAAGTGTAC CTTTCTTCCT GAACAGGTTC   
  
  
+ AAGGAGGCCA TGTTTCACTA CACGGCCTTG TTTGACTTGT TCGAGGCCAC AATGAGCCGC GAGGATCAAG   
  
  
+ AGAGGGTGTT ACTAGAGAGC AAGTTGCATG GGAATCAGGC GTTGAATGTG ATCGCCTGCG AAGGAGCGGA   
  
  
+ GAGAGTGGAG AGGCCTGAGT CATACAAACA ATGGCAGGTT AGGACTCAAA GGGCCGGGTT TGTTCAAGTG   
  
  
+ TCGATGGATC GCGAGCTTTT GAGAAGGGCA AGGGCCATGG TCAGGAGGAA TTTTAGGGAA GAGTTCAGTT   
  
  
+ ATGATGAAGA TGGGCATTGG GTGGTTCAAG GATGGAAAGG CAGGATAATG TATGCCATTT CTTGTTGGAA   
  
  
+ ACCTTCATA  

- +Up\_Stream \_Len000TAATAA TAAGATTATA TATCTATTAT TATATTATTA TTATTATTAT TATTATTATT   
  
  
- AATAATAATA ATAATAATAA ATAATTTATA TTATCAATAA TATTATTATT ATTAATATTA TTATTATTCT   
  
  
- AATAATAGTA ATATAATAAT AATAATAATA ACATTAAATA ATAATAATAT CATTAATAAT AACAATATAT   
  
  
- CATTTTAATA ATAATCATAA CAATAATATT TATAAGTATG AACGTATATT ATAAAGATTT TATTTTTGTC   
  
  
- TCAATTAAGG TAAGGTTTTG ATGAGTGGTT TGTCGTTCCT CACCCTGGAC TAAGGAAAGG TTAAAAGTGG   
  
  
- TTTGTTGACC CAAGTCTTAA GTTCAAGGTA TAGGTTGGGG GGTCCTAAGT TCAGGACTAA GGTAAGGTAA   
  
  
- GGTCAAAGGG AGTGGTTTGA CCCGGAATCT AACTACCAAC AAGTCAACCT ACTCCCAACA AGGTACGGTT   
  
  
- CAATCTAGTA ACAACAGTTA AAACTCTTGC TGATAGAATT ATTCGGATTG CTACAACTAA ACTAAATCAA   
  
  
- GTTTGGTAAA GTTTATTATG TTTAGTTTAA GATAAATCTG GCTGTGATTT TATTTATCTA ACTCTTTTTA   
  
  
- ATATCTAAAA TTTCAAGTCT AATTTTAGGG ATTTCTAGTT GTCTAACTTT ACGTATAAAG TATCACGTGT   
  
  
- ACCTGAACGA AACTTAGAGC AGATCTCTTG GATTCTTTTT CGTTCTCACC AAGTCTTCCA ATCTTAACAA   
  
  
- GGTCGTGTAC TGTTCATGTT AGGGTTGAAA AAAGTTTTCT AAACGATGAA AATTAACCTA AGGGTACAAC   
  
  
- TTACTTCAAG ACCCAAATTC CGATGTTGAA AGAACCCTCC TATTCCAAGA ATTCAATTCC ACCAGGAGAA   
  
  
- AAGAAAACTA GAAAGAATAC CCATGTTGGA GTACCCAGAG TAGTTGTGTA CTTGAGAAAA ATAGAAAATA   
  
  
- GAAAGGGGAA AGGAAATACG TCCTAACTGT CCACGTACCT TTTGTTCTTC GTTAATTTAT GATCACCGAA   
  
  
- TCGGGGTAGG GATTATAAAA CCGATAAGAC AAAGTGGTAG GAGGGGACAG AGGTAAAAGA AACAAAGGAA   
  
  
- AAAAAGATCC TTGAACACTA CCCGTTCCCT TCTAGTCATG TTACACTACC AACAATCATC GCTCTTCTAC   
  
  
- TTCACAGTGG CGTTTTTAAA ACACGGTATT AAACAGCATT CCCTTTGTTC ATAGTTTGTT GTTACAGTAA   
  
  
- ATACTATTTG TTCATTGTCC TCTATACATG AGACTTTTTT ATATTCCATG GACTCTTCAA CAATTGAATC   
  
  
- GAACTTTTTG GGAGGATTGA ACTATTTGGG AGAAGCGATA CAATAGGGCG TGGGACGGTA TCTCAGTGGC   
  
  
- TAAGTCATAT ATACATTGTA TGGGTTAGAA CAGACCTATA TATCAATTGG GAAAACCTTA TAGACTTTTA   
  
  
- GGTGTGCTCT CAGTAGGAAG ATCTGATCCT TTGTACGCGG TGTAAAACCT AAGATTGGTT CATATTATTC   
  
  
- GTATAGAATT TCGGTTTTTA CGTTCCTATC TATGTTTATG GGTAGTGTAC GTACATTTAC AGGGAACAGT   
  
  
- ACCGAGACTA AGAGTTTTAT AATTAAAAGT TATTTAATTT ACTTTAACGT AATAAAATTT CTAAAGACTT   
  
  
- ACGTAGGTAA TAACGTTAAA AAATTTCTGA CGTGTTACTA CTGTTACAGT TACCAAAGGA TTTCAACGCG   
  
  
- GACTACCCGA TAACTGTCAC TTCGTTGTGA GGGAAACAAG GGTAAATACA CAGAGGTGAA CGGGGAAACT   
  
  
- TTGAGAGATG TAAACATACA GTAGACTTAA ACTAAGAGTT TAGAGTCTAC TAAACTTAAG GGAAATACGG   
  
  
- TAGAAATGAT AGATAACTTA CTAAGAAAGA AAAGGTGAAA GAATAGAAGG TGATGGAAGA AGAACCTACG   
  
  
- GGAAAACGAC GAATATTTCC CGCTGTGGAC TTGTTCAACG ACATAAGAGT TTGGTACTTA GACATGTCCA   
  
  
- AATCGGGGCT ATACACAGGA TCGAAACACC CAGTGCTTCT TTAACCAGGA TTAAATTATC CTGTGGTTCT   
  
  
- GGGAAAAGTT TTACGTATGT TAACGTGACT AAAACCACTA CGAATGAACA ACGTTAAGGG TCAACTTCTA   
  
  
- AATACAGTTA TGGCACAAGG ACTAGATTAC CCCGATGGAC AACTTCTACC CCTAGGATGT CTCCAGGGAC   
  
  
- TGTAAGGACT ATGTACGAAG TTGATGTATT CGTTAGAGGA TTACCTTCTC CCGAGCCTAC TGGCGGGACC   
  
  
- GCGGTGGGAC GTCCTGATAT ACCGACGAGT TCAGAGGATG TCGTTCAGGA GGTTACGGCG GAAAGGGCCA   
  
  
- CTCTCAACGA GTAAGAAACC ATTATTAGTT ACGGGAGGTG GACGAATAGT TTCGTTGGGA ATAACGGAAC   
  
  
- TACTAATGAA GTTGAACGAT AGGTTATGAT GACCGTTATC ACAATTGTTA CGACCACAAG TACTGTCGTT   
  
  
- GTTGAAACAA GTCTTGTCAA ACTAACCCTT ATCCCCATGA TCAAGATCAA GATCCCCGTC TTCACTAAAA   
  
  
- CCTAGATGGG AATAATCCTT AAACGGTAAT CCCTGGCTGT GTGAACTACA CCAGTTAGGT CGTGGATGAG   
  
  
- GAGCTCGGGG AGACCAATGC TGAGTCACAG TCCACAAACT TTGTGTGGTC ATACTCGTCC GGAAGCTTGA   
  
  
- CTAGTGATTA CCTTCGACCC TTAAGTCAGT AAGACAACGA TTATGTAGTT TACTACCTTA AGTTTTAGTT   
  
  
- GGTTAAGTTC GTCAAAGTTG GGTTCTTGCT ACCTTATACT TTTGTAAAAG GCTACTGGTT GAACTCGGAC   
  
  
- GAGAAAACGC TTCAGAACAC TACAGGAGTT TGACATGACT ATAGAGACTA GTCCCACATG TGTCCTATCA   
  
  
- CTTGGGACTC GGTGGGTTGT TGTCTTTATG CCTCTAACAC ACGTTACTGA GTAGGTCATT CTCATTCTGT   
  
  
- TCTGCTTCTC TCGTACCACG TCCTCTTTTG GTGCTACTTC CTCTTTTGGT GCTACTCTGT CCTTCCTCAT   
  
  
- TGTTCGTTAG TCGTGGTAGG TTACTTCACA AGCTACTTTA ACTCGTCATA CCACTACACG ACGAGACGGG   
  
  
- ACTTCCTTCT TCATTTGGTC GTCCCAATAC GTCGTCGTCG TTGCGATTGT CTTGATCGTT GAGTGTCTTG   
  
  
- AGCCTTCTGA GTTTCAGTGT CGTTTTACCC TCAAGGAGGT TCCACAGTTT TTTCGCTCCG TTATTTTTCG   
  
  
- TTCACCAACT AAACTCCTGA GAGGATTGAG CTACACGTGT TACACAACGA GCACACCTAA ATTGACCAAG   
  
  
- AATGGTCAAC GAAGTCCTCT AAGCCGTCGT AAGAAGAGGG ATACCACTAA TAGAGGTTTC TTACCGAGTA   
  
  
- ATGGAACGAT TACCGGAACT CCGCGCGAAT CTTCCGTTTC CTATACTTGA TTGAGCACGG TTACTCGTAT   
  
  
- AGGGTGGATG ACTCTAGGAG TTCCGATTGG CCCATATACA CCGTCGCCAG GGAAAGTTCT ATTACAGAAT   
  
  
- AATGTGTTGT TTGTTCTGTT AACGTTCGGA TCAACTCCTC CGTGGTTCAT ATGTGTATTA ACTAAAACCA   
  
  
- TAAAAGATAC CTGATGTTAC CGGGACGTAG TAAGTCTTGG AGAGGTTCTC CGGGTTACCC GGAGGGTCTT   
  
  
- AGGCGTAGTG TCCGTAGCTA AAGGGGGTTG GACCCAAGGC CGGTCGTCTC TCCCAACTTC TCTGTCCCGC   
  
  
- CACAAATCGG TTCATAACAC TGTCCATGTT ACACGGGAAA CTTATGGTGG GGTATCGGTT CTTCACCCTC   
  
  
- TGGTAGGTTG AGCTCCTCGA GTTTTAACTT TCCTTACTCG GGGAACATCA CTTGACAGAC ATATCTAGCG   
  
  
- TGTTGGATAA ACTACTTTCG CATCTCCTTT TGTCGGGTTC TCTGCGCAAG GACTCGAACC AATCCTTTTA   
  
  
- TTTAGGGCTA TATAAGTACG TACCCCACCA CTTATGGCGT AGTTCACATG GAAAGAAGGA CTTGTCCAAG   
  
  
- TTCCTCCGGT ACAAAGTGAT GTGCCGGAAC AAACTGAACA AGCTCCGGTG TTACTCGGCG CTCCTAGTTC   
  
  
- TCTCCCACAA TGATCTCTCG TTCAACGTAC CCTTAGTCCG CAACTTACAC TAGCGGACGC TTCCTCGCCT   
  
  
- CTCTCACCTC TCCGGACTCA GTATGTTTGT TACCGTCCAA TCCTGAGTTT CCCGGCCCAA ACAAGTTCAC   
  
  
- AGCTACCTAG CGCTCGAAAA CTCTTCCCGT TCCCGGTACC AGTCCTCCTT AAAATCCCTT CTCAAGTCAA   
  
  
- TACTACTTCT ACCCGTAACC CACCAAGTTC CTACCTTTCC GTCCTATTAC ATACGGTAAA GAACAACCTT   
  
  
- TGGAAGTAT

+     ERE

| Site Name | Organism | Position | Strand | Matrix score. | sequence | function |
| --- | --- | --- | --- | --- | --- | --- |
| ERE | Nicotiana glutinos | 1667 | + | 8 | ATTTTAAA |  |
| ERE | Nicotiana glutinos | 690 | + | 8 | ATTTCATA |  |
| ERE | Nicotiana glutinos | 640 | + | 8 | ATTTTAAA |  |

>HU02G01570.1   
+ +Up\_Stream \_Len000ATTATT ATTCTAATAT ATAGATAATA ATATAATAAT AATAATAATA ATAATAATAA   
  
  
+ TTATTATTAT TATTATTATT TATTAAATAT AATAGTTATT ATAATAATAA TAATTATAAT AATAATAAGA   
  
  
+ TTATTATCAT TATATTATTA TTATTATTAT TGTAATTTAT TATTATTATA GTAATTATTA TTGTTATATA   
  
  
+ GTAAAATTAT TATTAGTATT GTTATTATAA ATATTCATAC TTGCATATAA TATTTCTAAA ATAAAAACAG   
  
  
+ AGTTAATTCC ATTCCAAAAC TACTCACCAA ACAGCAAGGA GTGGGACCTG ATTCCTTTCC AATTTTCACC   
  
  
+ AAACAACTGG GTTCAGAATT CAAGTTCCAT ATCCAACCCC CCAGGATTCA AGTCCTGATT CCATTCCATT   
  
  
+ CCAGTTTCCC TCACCAAACT GGGCCTTAGA TTGATGGTTG TTCAGTTGGA TGAGGGTTGT TCCATGCCAA   
  
  
+ GTTAGATCAT TGTTGTCAAT TTTGAGAACG ACTATCTTAA TAAGCCTAAC GATGTTGATT TGATTTAGTT   
  
  
+ CAAACCATTT CAAATAATAC AAATCAAATT CTATTTAGAC CGACACTAAA ATAAATAGAT TGAGAAAAAT   
  
  
+ TATAGATTTT AAAGTTCAGA TTAAAATCCC TAAAGATCAA CAGATTGAAA TGCATATTTC ATAGTGCACA   
  
  
+ TGGACTTGCT TTGAATCTCG TCTAGAGAAC CTAAGAAAAA GCAAGAGTGG TTCAGAAGGT TAGAATTGTT   
  
  
+ CCAGCACATG ACAAGTACAA TCCCAACTTT TTTCAAAAGA TTTGCTACTT TTAATTGGAT TCCCATGTTG   
  
  
+ AATGAAGTTC TGGGTTTAAG GCTACAACTT TCTTGGGAGG ATAAGGTTCT TAAGTTAAGG TGGTCCTCTT   
  
  
+ TTCTTTTGAT CTTTCTTATG GGTACAACCT CATGGGTCTC ATCAACACAT GAACTCTTTT TATCTTTTAT   
  
  
+ CTTTCCCCTT TCCTTTATGC AGGATTGACA GGTGCATGGA AAACAAGAAG CAATTAAATA CTAGTGGCTT   
  
  
+ AGCCCCATCC CTAATATTTT GGCTATTCTG TTTCACCATC CTCCCCTGTC TCCATTTTCT TTGTTTCCTT   
  
  
+ TTTTTCTAGG AACTTGTGAT GGGCAAGGGA AGATCAGTAC AATGTGATGG TTGTTAGTAG CGAGAAGATG   
  
  
+ AAGTGTCACC GCAAAAATTT TGTGCCATAA TTTGTCGTAA GGGAAACAAG TATCAAACAA CAATGTCATT   
  
  
+ TATGATAAAC AAGTAACAGG AGATATGTAC TCTGAAAAAA TATAAGGTAC CTGAGAAGTT GTTAACTTAG   
  
  
+ CTTGAAAAAC CCTCCTAACT TGATAAACCC TCTTCGCTAT GTTATCCCGC ACCCTGCCAT AGAGTCACCG   
  
  
+ ATTCAGTATA TATGTAACAT ACCCAATCTT GTCTGGATAT ATAGTTAACC CTTTTGGAAT ATCTGAAAAT   
  
  
+ CCACACGAGA GTCATCCTTC TAGACTAGGA AACATGCGCC ACATTTTGGA TTCTAACCAA GTATAATAAG   
  
  
+ CATATCTTAA AGCCAAAAAT GCAAGGATAG ATACAAATAC CCATCACATG CATGTAAATG TCCCTTGTCA   
  
  
+ TGGCTCTGAT TCTCAAAATA TTAATTTTCA ATAAATTAAA TGAAATTGCA TTATTTTAAA GATTTCTGAA   
  
  
+ TGCATCCATT ATTGCAATTT TTTAAAGACT GCACAATGAT GACAATGTCA ATGGTTTCCT AAAGTTGCGC   
  
  
+ CTGATGGGCT ATTGACAGTG AAGCAACACT CCCTTTGTTC CCATTTATGT GTCTCCACTT GCCCCTTTGA   
  
  
+ AACTCTCTAC ATTTGTATGT CATCTGAATT TGATTCTCAA ATCTCAGATG ATTTGAATTC CCTTTATGCC   
  
  
+ ATCTTTACTA TCTATTGAAT GATTCTTTCT TTTCCACTTT CTTATCTTCC ACTACCTTCT TCTTGGATGC   
  
  
+ CCTTTTGCTG CTTATAAAGG GCGACACCTG AACAAGTTGC TGTATTCTCA AACCATGAAT CTGTACAGGT   
  
  
+ TTAGCCCCGA TATGTGTCCT AGCTTTGTGG GTCACGAAGA AATTGGTCCT AATTTAATAG GACACCAAGA   
  
  
+ CCCTTTTCAA AATGCATACA ATTGCACTGA TTTTGGTGAT GCTTACTTGT TGCAATTCCC AGTTGAAGAT   
  
  
+ TTATGTCAAT ACCGTGTTCC TGATCTAATG GGGCTACCTG TTGAAGATGG GGATCCTACA GAGGTCCCTG   
  
  
+ ACATTCCTGA TACATGCTTC AACTACATAA GCAATCTCCT AATGGAAGAG GGCTCGGATG ACCGCCCTGG   
  
  
+ CGCCACCCTG CAGGACTATA TGGCTGCTCA AGTCTCCTAC AGCAAGTCCT CCAATGCCGC CTTTCCCGGT   
  
  
+ GAGAGTTGCT CATTCTTTGG TAATAATCAA TGCCCTCCAC CTGCTTATCA AAGCAACCCT TATTGCCTTG   
  
  
+ ATGATTACTT CAACTTGCTA TCCAATACTA CTGGCAATAG TGTTAACAAT GCTGGTGTTC ATGACAGCAA   
  
  
+ CAACTTTGTT CAGAACAGTT TGATTGGGAA TAGGGGTACT AGTTCTAGTT CTAGGGGCAG AAGTGATTTT   
  
  
+ GGATCTACCC TTATTAGGAA TTTGCCATTA GGGACCGACA CACTTGATGT GGTCAATCCA GCACCTACTC   
  
  
+ CTCGAGCCCC TCTGGTTACG ACTCAGTGTC AGGTGTTTGA AACACACCAG TATGAGCAGG CCTTCGAACT   
  
  
+ GATCACTAAT GGAAGCTGGG AATTCAGTCA TTCTGTTGCT AATACATCAA ATGATGGAAT TCAAAATCAA   
  
  
+ CCAATTCAAG CAGTTTCAAC CCAAGAACGA TGGAATATGA AAACATTTTC CGATGACCAA CTTGAGCCTG   
  
  
+ CTCTTTTGCG AAGTCTTGTG ATGTCCTCAA ACTGTACTGA TATCTCTGAT CAGGGTGTAC ACAGGATAGT   
  
  
+ GAACCCTGAG CCACCCAACA ACAGAAATAC GGAGATTGTG TGCAATGACT CATCCAGTAA GAGTAAGACA   
  
  
+ AGACGAAGAG AGCATGGTGC AGGAGAAAAC CACGATGAAG GAGAAAACCA CGATGAGACA GGAAGGAGTA   
  
  
+ ACAAGCAATC AGCACCATCC AATGAAGTGT TCGATGAAAT TGAGCAGTAT GGTGATGTGC TGCTCTGCCC   
  
  
+ TGAAGGAAGA AGTAAACCAG CAGGGTTATG CAGCAGCAGC AACGCTAACA GAACTAGCAA CTCACAGAAC   
  
  
+ TCGGAAGACT CAAAGTCACA GCAAAATGGG AGTTCCTCCA AGGTGTCAAA AAAGCGAGGC AATAAAAAGC   
  
  
+ AAGTGGTTGA TTTGAGGACT CTCCTAACTC GATGTGCACA ATGTGTTGCT CGTGTGGATT TAACTGGTTC   
  
  
+ TTACCAGTTG CTTCAGGAGA TTCGGCAGCA TTCTTCTCCC TATGGTGATT ATCTCCAAAG AATGGCTCAT   
  
  
+ TACCTTGCTA ATGGCCTTGA GGCGCGCTTA GAAGGCAAAG GATATGAACT AACTCGTGCC AATGAGCATA   
  
  
+ TCCCACCTAC TGAGATCCTC AAGGCTAACC GGGTATATGT GGCAGCGGTC CCTTTCAAGA TAATGTCTTA   
  
  
+ TTACACAACA AACAAGACAA TTGCAAGCCT AGTTGAGGAG GCACCAAGTA TACACATAAT TGATTTTGGT   
  
  
+ ATTTTCTATG GACTACAATG GCCCTGCATC ATTCAGAACC TCTCCAAGAG GCCCAATGGG CCTCCCAGAA   
  
  
+ TCCGCATCAC AGGCATCGAT TTCCCCCAAC CTGGGTTCCG GCCAGCAGAG AGGGTTGAAG AGACAGGGCG   
  
  
+ GTGTTTAGCC AAGTATTGTG ACAGGTACAA TGTGCCCTTT GAATACCACC CCATAGCCAA GAAGTGGGAG   
  
  
+ ACCATCCAAC TCGAGGAGCT CAAAATTGAA AGGAATGAGC CCCTTGTAGT GAACTGTCTG TATAGATCGC   
  
  
+ ACAACCTATT TGATGAAAGC GTAGAGGAAA ACAGCCCAAG AGACGCGTTC CTGAGCTTGG TTAGGAAAAT   
  
  
+ AAATCCCGAT ATATTCATGC ATGGGGTGGT GAATACCGCA TCAAGTGTAC CTTTCTTCCT GAACAGGTTC   
  
  
+ AAGGAGGCCA TGTTTCACTA CACGGCCTTG TTTGACTTGT TCGAGGCCAC AATGAGCCGC GAGGATCAAG   
  
  
+ AGAGGGTGTT ACTAGAGAGC AAGTTGCATG GGAATCAGGC GTTGAATGTG ATCGCCTGCG AAGGAGCGGA   
  
  
+ GAGAGTGGAG AGGCCTGAGT CATACAAACA ATGGCAGGTT AGGACTCAAA GGGCCGGGTT TGTTCAAGTG   
  
  
+ TCGATGGATC GCGAGCTTTT GAGAAGGGCA AGGGCCATGG TCAGGAGGAA TTTTAGGGAA GAGTTCAGTT   
  
  
+ ATGATGAAGA TGGGCATTGG GTGGTTCAAG GATGGAAAGG CAGGATAATG TATGCCATTT CTTGTTGGAA   
  
  
+ ACCTTCATA  

- +Up\_Stream \_Len000TAATAA TAAGATTATA TATCTATTAT TATATTATTA TTATTATTAT TATTATTATT   
  
  
- AATAATAATA ATAATAATAA ATAATTTATA TTATCAATAA TATTATTATT ATTAATATTA TTATTATTCT   
  
  
- AATAATAGTA ATATAATAAT AATAATAATA ACATTAAATA ATAATAATAT CATTAATAAT AACAATATAT   
  
  
- CATTTTAATA ATAATCATAA CAATAATATT TATAAGTATG AACGTATATT ATAAAGATTT TATTTTTGTC   
  
  
- TCAATTAAGG TAAGGTTTTG ATGAGTGGTT TGTCGTTCCT CACCCTGGAC TAAGGAAAGG TTAAAAGTGG   
  
  
- TTTGTTGACC CAAGTCTTAA GTTCAAGGTA TAGGTTGGGG GGTCCTAAGT TCAGGACTAA GGTAAGGTAA   
  
  
- GGTCAAAGGG AGTGGTTTGA CCCGGAATCT AACTACCAAC AAGTCAACCT ACTCCCAACA AGGTACGGTT   
  
  
- CAATCTAGTA ACAACAGTTA AAACTCTTGC TGATAGAATT ATTCGGATTG CTACAACTAA ACTAAATCAA   
  
  
- GTTTGGTAAA GTTTATTATG TTTAGTTTAA GATAAATCTG GCTGTGATTT TATTTATCTA ACTCTTTTTA   
  
  
- ATATCTAAAA TTTCAAGTCT AATTTTAGGG ATTTCTAGTT GTCTAACTTT ACGTATAAAG TATCACGTGT   
  
  
- ACCTGAACGA AACTTAGAGC AGATCTCTTG GATTCTTTTT CGTTCTCACC AAGTCTTCCA ATCTTAACAA   
  
  
- GGTCGTGTAC TGTTCATGTT AGGGTTGAAA AAAGTTTTCT AAACGATGAA AATTAACCTA AGGGTACAAC   
  
  
- TTACTTCAAG ACCCAAATTC CGATGTTGAA AGAACCCTCC TATTCCAAGA ATTCAATTCC ACCAGGAGAA   
  
  
- AAGAAAACTA GAAAGAATAC CCATGTTGGA GTACCCAGAG TAGTTGTGTA CTTGAGAAAA ATAGAAAATA   
  
  
- GAAAGGGGAA AGGAAATACG TCCTAACTGT CCACGTACCT TTTGTTCTTC GTTAATTTAT GATCACCGAA   
  
  
- TCGGGGTAGG GATTATAAAA CCGATAAGAC AAAGTGGTAG GAGGGGACAG AGGTAAAAGA AACAAAGGAA   
  
  
- AAAAAGATCC TTGAACACTA CCCGTTCCCT TCTAGTCATG TTACACTACC AACAATCATC GCTCTTCTAC   
  
  
- TTCACAGTGG CGTTTTTAAA ACACGGTATT AAACAGCATT CCCTTTGTTC ATAGTTTGTT GTTACAGTAA   
  
  
- ATACTATTTG TTCATTGTCC TCTATACATG AGACTTTTTT ATATTCCATG GACTCTTCAA CAATTGAATC   
  
  
- GAACTTTTTG GGAGGATTGA ACTATTTGGG AGAAGCGATA CAATAGGGCG TGGGACGGTA TCTCAGTGGC   
  
  
- TAAGTCATAT ATACATTGTA TGGGTTAGAA CAGACCTATA TATCAATTGG GAAAACCTTA TAGACTTTTA   
  
  
- GGTGTGCTCT CAGTAGGAAG ATCTGATCCT TTGTACGCGG TGTAAAACCT AAGATTGGTT CATATTATTC   
  
  
- GTATAGAATT TCGGTTTTTA CGTTCCTATC TATGTTTATG GGTAGTGTAC GTACATTTAC AGGGAACAGT   
  
  
- ACCGAGACTA AGAGTTTTAT AATTAAAAGT TATTTAATTT ACTTTAACGT AATAAAATTT CTAAAGACTT   
  
  
- ACGTAGGTAA TAACGTTAAA AAATTTCTGA CGTGTTACTA CTGTTACAGT TACCAAAGGA TTTCAACGCG   
  
  
- GACTACCCGA TAACTGTCAC TTCGTTGTGA GGGAAACAAG GGTAAATACA CAGAGGTGAA CGGGGAAACT   
  
  
- TTGAGAGATG TAAACATACA GTAGACTTAA ACTAAGAGTT TAGAGTCTAC TAAACTTAAG GGAAATACGG   
  
  
- TAGAAATGAT AGATAACTTA CTAAGAAAGA AAAGGTGAAA GAATAGAAGG TGATGGAAGA AGAACCTACG   
  
  
- GGAAAACGAC GAATATTTCC CGCTGTGGAC TTGTTCAACG ACATAAGAGT TTGGTACTTA GACATGTCCA   
  
  
- AATCGGGGCT ATACACAGGA TCGAAACACC CAGTGCTTCT TTAACCAGGA TTAAATTATC CTGTGGTTCT   
  
  
- GGGAAAAGTT TTACGTATGT TAACGTGACT AAAACCACTA CGAATGAACA ACGTTAAGGG TCAACTTCTA   
  
  
- AATACAGTTA TGGCACAAGG ACTAGATTAC CCCGATGGAC AACTTCTACC CCTAGGATGT CTCCAGGGAC   
  
  
- TGTAAGGACT ATGTACGAAG TTGATGTATT CGTTAGAGGA TTACCTTCTC CCGAGCCTAC TGGCGGGACC   
  
  
- GCGGTGGGAC GTCCTGATAT ACCGACGAGT TCAGAGGATG TCGTTCAGGA GGTTACGGCG GAAAGGGCCA   
  
  
- CTCTCAACGA GTAAGAAACC ATTATTAGTT ACGGGAGGTG GACGAATAGT TTCGTTGGGA ATAACGGAAC   
  
  
- TACTAATGAA GTTGAACGAT AGGTTATGAT GACCGTTATC ACAATTGTTA CGACCACAAG TACTGTCGTT   
  
  
- GTTGAAACAA GTCTTGTCAA ACTAACCCTT ATCCCCATGA TCAAGATCAA GATCCCCGTC TTCACTAAAA   
  
  
- CCTAGATGGG AATAATCCTT AAACGGTAAT CCCTGGCTGT GTGAACTACA CCAGTTAGGT CGTGGATGAG   
  
  
- GAGCTCGGGG AGACCAATGC TGAGTCACAG TCCACAAACT TTGTGTGGTC ATACTCGTCC GGAAGCTTGA   
  
  
- CTAGTGATTA CCTTCGACCC TTAAGTCAGT AAGACAACGA TTATGTAGTT TACTACCTTA AGTTTTAGTT   
  
  
- GGTTAAGTTC GTCAAAGTTG GGTTCTTGCT ACCTTATACT TTTGTAAAAG GCTACTGGTT GAACTCGGAC   
  
  
- GAGAAAACGC TTCAGAACAC TACAGGAGTT TGACATGACT ATAGAGACTA GTCCCACATG TGTCCTATCA   
  
  
- CTTGGGACTC GGTGGGTTGT TGTCTTTATG CCTCTAACAC ACGTTACTGA GTAGGTCATT CTCATTCTGT   
  
  
- TCTGCTTCTC TCGTACCACG TCCTCTTTTG GTGCTACTTC CTCTTTTGGT GCTACTCTGT CCTTCCTCAT   
  
  
- TGTTCGTTAG TCGTGGTAGG TTACTTCACA AGCTACTTTA ACTCGTCATA CCACTACACG ACGAGACGGG   
  
  
- ACTTCCTTCT TCATTTGGTC GTCCCAATAC GTCGTCGTCG TTGCGATTGT CTTGATCGTT GAGTGTCTTG   
  
  
- AGCCTTCTGA GTTTCAGTGT CGTTTTACCC TCAAGGAGGT TCCACAGTTT TTTCGCTCCG TTATTTTTCG   
  
  
- TTCACCAACT AAACTCCTGA GAGGATTGAG CTACACGTGT TACACAACGA GCACACCTAA ATTGACCAAG   
  
  
- AATGGTCAAC GAAGTCCTCT AAGCCGTCGT AAGAAGAGGG ATACCACTAA TAGAGGTTTC TTACCGAGTA   
  
  
- ATGGAACGAT TACCGGAACT CCGCGCGAAT CTTCCGTTTC CTATACTTGA TTGAGCACGG TTACTCGTAT   
  
  
- AGGGTGGATG ACTCTAGGAG TTCCGATTGG CCCATATACA CCGTCGCCAG GGAAAGTTCT ATTACAGAAT   
  
  
- AATGTGTTGT TTGTTCTGTT AACGTTCGGA TCAACTCCTC CGTGGTTCAT ATGTGTATTA ACTAAAACCA   
  
  
- TAAAAGATAC CTGATGTTAC CGGGACGTAG TAAGTCTTGG AGAGGTTCTC CGGGTTACCC GGAGGGTCTT   
  
  
- AGGCGTAGTG TCCGTAGCTA AAGGGGGTTG GACCCAAGGC CGGTCGTCTC TCCCAACTTC TCTGTCCCGC   
  
  
- CACAAATCGG TTCATAACAC TGTCCATGTT ACACGGGAAA CTTATGGTGG GGTATCGGTT CTTCACCCTC   
  
  
- TGGTAGGTTG AGCTCCTCGA GTTTTAACTT TCCTTACTCG GGGAACATCA CTTGACAGAC ATATCTAGCG   
  
  
- TGTTGGATAA ACTACTTTCG CATCTCCTTT TGTCGGGTTC TCTGCGCAAG GACTCGAACC AATCCTTTTA   
  
  
- TTTAGGGCTA TATAAGTACG TACCCCACCA CTTATGGCGT AGTTCACATG GAAAGAAGGA CTTGTCCAAG   
  
  
- TTCCTCCGGT ACAAAGTGAT GTGCCGGAAC AAACTGAACA AGCTCCGGTG TTACTCGGCG CTCCTAGTTC   
  
  
- TCTCCCACAA TGATCTCTCG TTCAACGTAC CCTTAGTCCG CAACTTACAC TAGCGGACGC TTCCTCGCCT   
  
  
- CTCTCACCTC TCCGGACTCA GTATGTTTGT TACCGTCCAA TCCTGAGTTT CCCGGCCCAA ACAAGTTCAC   
  
  
- AGCTACCTAG CGCTCGAAAA CTCTTCCCGT TCCCGGTACC AGTCCTCCTT AAAATCCCTT CTCAAGTCAA   
  
  
- TACTACTTCT ACCCGTAACC CACCAAGTTC CTACCTTTCC GTCCTATTAC ATACGGTAAA GAACAACCTT   
  
  
- TGGAAGTAT

+     GA-motif

| Site Name | Organism | Position | Strand | Matrix score. | sequence | function |
| --- | --- | --- | --- | --- | --- | --- |
| GA-motif | Arabidopsis thaliana | 35 | + | 8 | ATAGATAA | part of a light responsive element |

>HU02G01570.1   
+ +Up\_Stream \_Len000ATTATT ATTCTAATAT ATAGATAATA ATATAATAAT AATAATAATA ATAATAATAA   
  
  
+ TTATTATTAT TATTATTATT TATTAAATAT AATAGTTATT ATAATAATAA TAATTATAAT AATAATAAGA   
  
  
+ TTATTATCAT TATATTATTA TTATTATTAT TGTAATTTAT TATTATTATA GTAATTATTA TTGTTATATA   
  
  
+ GTAAAATTAT TATTAGTATT GTTATTATAA ATATTCATAC TTGCATATAA TATTTCTAAA ATAAAAACAG   
  
  
+ AGTTAATTCC ATTCCAAAAC TACTCACCAA ACAGCAAGGA GTGGGACCTG ATTCCTTTCC AATTTTCACC   
  
  
+ AAACAACTGG GTTCAGAATT CAAGTTCCAT ATCCAACCCC CCAGGATTCA AGTCCTGATT CCATTCCATT   
  
  
+ CCAGTTTCCC TCACCAAACT GGGCCTTAGA TTGATGGTTG TTCAGTTGGA TGAGGGTTGT TCCATGCCAA   
  
  
+ GTTAGATCAT TGTTGTCAAT TTTGAGAACG ACTATCTTAA TAAGCCTAAC GATGTTGATT TGATTTAGTT   
  
  
+ CAAACCATTT CAAATAATAC AAATCAAATT CTATTTAGAC CGACACTAAA ATAAATAGAT TGAGAAAAAT   
  
  
+ TATAGATTTT AAAGTTCAGA TTAAAATCCC TAAAGATCAA CAGATTGAAA TGCATATTTC ATAGTGCACA   
  
  
+ TGGACTTGCT TTGAATCTCG TCTAGAGAAC CTAAGAAAAA GCAAGAGTGG TTCAGAAGGT TAGAATTGTT   
  
  
+ CCAGCACATG ACAAGTACAA TCCCAACTTT TTTCAAAAGA TTTGCTACTT TTAATTGGAT TCCCATGTTG   
  
  
+ AATGAAGTTC TGGGTTTAAG GCTACAACTT TCTTGGGAGG ATAAGGTTCT TAAGTTAAGG TGGTCCTCTT   
  
  
+ TTCTTTTGAT CTTTCTTATG GGTACAACCT CATGGGTCTC ATCAACACAT GAACTCTTTT TATCTTTTAT   
  
  
+ CTTTCCCCTT TCCTTTATGC AGGATTGACA GGTGCATGGA AAACAAGAAG CAATTAAATA CTAGTGGCTT   
  
  
+ AGCCCCATCC CTAATATTTT GGCTATTCTG TTTCACCATC CTCCCCTGTC TCCATTTTCT TTGTTTCCTT   
  
  
+ TTTTTCTAGG AACTTGTGAT GGGCAAGGGA AGATCAGTAC AATGTGATGG TTGTTAGTAG CGAGAAGATG   
  
  
+ AAGTGTCACC GCAAAAATTT TGTGCCATAA TTTGTCGTAA GGGAAACAAG TATCAAACAA CAATGTCATT   
  
  
+ TATGATAAAC AAGTAACAGG AGATATGTAC TCTGAAAAAA TATAAGGTAC CTGAGAAGTT GTTAACTTAG   
  
  
+ CTTGAAAAAC CCTCCTAACT TGATAAACCC TCTTCGCTAT GTTATCCCGC ACCCTGCCAT AGAGTCACCG   
  
  
+ ATTCAGTATA TATGTAACAT ACCCAATCTT GTCTGGATAT ATAGTTAACC CTTTTGGAAT ATCTGAAAAT   
  
  
+ CCACACGAGA GTCATCCTTC TAGACTAGGA AACATGCGCC ACATTTTGGA TTCTAACCAA GTATAATAAG   
  
  
+ CATATCTTAA AGCCAAAAAT GCAAGGATAG ATACAAATAC CCATCACATG CATGTAAATG TCCCTTGTCA   
  
  
+ TGGCTCTGAT TCTCAAAATA TTAATTTTCA ATAAATTAAA TGAAATTGCA TTATTTTAAA GATTTCTGAA   
  
  
+ TGCATCCATT ATTGCAATTT TTTAAAGACT GCACAATGAT GACAATGTCA ATGGTTTCCT AAAGTTGCGC   
  
  
+ CTGATGGGCT ATTGACAGTG AAGCAACACT CCCTTTGTTC CCATTTATGT GTCTCCACTT GCCCCTTTGA   
  
  
+ AACTCTCTAC ATTTGTATGT CATCTGAATT TGATTCTCAA ATCTCAGATG ATTTGAATTC CCTTTATGCC   
  
  
+ ATCTTTACTA TCTATTGAAT GATTCTTTCT TTTCCACTTT CTTATCTTCC ACTACCTTCT TCTTGGATGC   
  
  
+ CCTTTTGCTG CTTATAAAGG GCGACACCTG AACAAGTTGC TGTATTCTCA AACCATGAAT CTGTACAGGT   
  
  
+ TTAGCCCCGA TATGTGTCCT AGCTTTGTGG GTCACGAAGA AATTGGTCCT AATTTAATAG GACACCAAGA   
  
  
+ CCCTTTTCAA AATGCATACA ATTGCACTGA TTTTGGTGAT GCTTACTTGT TGCAATTCCC AGTTGAAGAT   
  
  
+ TTATGTCAAT ACCGTGTTCC TGATCTAATG GGGCTACCTG TTGAAGATGG GGATCCTACA GAGGTCCCTG   
  
  
+ ACATTCCTGA TACATGCTTC AACTACATAA GCAATCTCCT AATGGAAGAG GGCTCGGATG ACCGCCCTGG   
  
  
+ CGCCACCCTG CAGGACTATA TGGCTGCTCA AGTCTCCTAC AGCAAGTCCT CCAATGCCGC CTTTCCCGGT   
  
  
+ GAGAGTTGCT CATTCTTTGG TAATAATCAA TGCCCTCCAC CTGCTTATCA AAGCAACCCT TATTGCCTTG   
  
  
+ ATGATTACTT CAACTTGCTA TCCAATACTA CTGGCAATAG TGTTAACAAT GCTGGTGTTC ATGACAGCAA   
  
  
+ CAACTTTGTT CAGAACAGTT TGATTGGGAA TAGGGGTACT AGTTCTAGTT CTAGGGGCAG AAGTGATTTT   
  
  
+ GGATCTACCC TTATTAGGAA TTTGCCATTA GGGACCGACA CACTTGATGT GGTCAATCCA GCACCTACTC   
  
  
+ CTCGAGCCCC TCTGGTTACG ACTCAGTGTC AGGTGTTTGA AACACACCAG TATGAGCAGG CCTTCGAACT   
  
  
+ GATCACTAAT GGAAGCTGGG AATTCAGTCA TTCTGTTGCT AATACATCAA ATGATGGAAT TCAAAATCAA   
  
  
+ CCAATTCAAG CAGTTTCAAC CCAAGAACGA TGGAATATGA AAACATTTTC CGATGACCAA CTTGAGCCTG   
  
  
+ CTCTTTTGCG AAGTCTTGTG ATGTCCTCAA ACTGTACTGA TATCTCTGAT CAGGGTGTAC ACAGGATAGT   
  
  
+ GAACCCTGAG CCACCCAACA ACAGAAATAC GGAGATTGTG TGCAATGACT CATCCAGTAA GAGTAAGACA   
  
  
+ AGACGAAGAG AGCATGGTGC AGGAGAAAAC CACGATGAAG GAGAAAACCA CGATGAGACA GGAAGGAGTA   
  
  
+ ACAAGCAATC AGCACCATCC AATGAAGTGT TCGATGAAAT TGAGCAGTAT GGTGATGTGC TGCTCTGCCC   
  
  
+ TGAAGGAAGA AGTAAACCAG CAGGGTTATG CAGCAGCAGC AACGCTAACA GAACTAGCAA CTCACAGAAC   
  
  
+ TCGGAAGACT CAAAGTCACA GCAAAATGGG AGTTCCTCCA AGGTGTCAAA AAAGCGAGGC AATAAAAAGC   
  
  
+ AAGTGGTTGA TTTGAGGACT CTCCTAACTC GATGTGCACA ATGTGTTGCT CGTGTGGATT TAACTGGTTC   
  
  
+ TTACCAGTTG CTTCAGGAGA TTCGGCAGCA TTCTTCTCCC TATGGTGATT ATCTCCAAAG AATGGCTCAT   
  
  
+ TACCTTGCTA ATGGCCTTGA GGCGCGCTTA GAAGGCAAAG GATATGAACT AACTCGTGCC AATGAGCATA   
  
  
+ TCCCACCTAC TGAGATCCTC AAGGCTAACC GGGTATATGT GGCAGCGGTC CCTTTCAAGA TAATGTCTTA   
  
  
+ TTACACAACA AACAAGACAA TTGCAAGCCT AGTTGAGGAG GCACCAAGTA TACACATAAT TGATTTTGGT   
  
  
+ ATTTTCTATG GACTACAATG GCCCTGCATC ATTCAGAACC TCTCCAAGAG GCCCAATGGG CCTCCCAGAA   
  
  
+ TCCGCATCAC AGGCATCGAT TTCCCCCAAC CTGGGTTCCG GCCAGCAGAG AGGGTTGAAG AGACAGGGCG   
  
  
+ GTGTTTAGCC AAGTATTGTG ACAGGTACAA TGTGCCCTTT GAATACCACC CCATAGCCAA GAAGTGGGAG   
  
  
+ ACCATCCAAC TCGAGGAGCT CAAAATTGAA AGGAATGAGC CCCTTGTAGT GAACTGTCTG TATAGATCGC   
  
  
+ ACAACCTATT TGATGAAAGC GTAGAGGAAA ACAGCCCAAG AGACGCGTTC CTGAGCTTGG TTAGGAAAAT   
  
  
+ AAATCCCGAT ATATTCATGC ATGGGGTGGT GAATACCGCA TCAAGTGTAC CTTTCTTCCT GAACAGGTTC   
  
  
+ AAGGAGGCCA TGTTTCACTA CACGGCCTTG TTTGACTTGT TCGAGGCCAC AATGAGCCGC GAGGATCAAG   
  
  
+ AGAGGGTGTT ACTAGAGAGC AAGTTGCATG GGAATCAGGC GTTGAATGTG ATCGCCTGCG AAGGAGCGGA   
  
  
+ GAGAGTGGAG AGGCCTGAGT CATACAAACA ATGGCAGGTT AGGACTCAAA GGGCCGGGTT TGTTCAAGTG   
  
  
+ TCGATGGATC GCGAGCTTTT GAGAAGGGCA AGGGCCATGG TCAGGAGGAA TTTTAGGGAA GAGTTCAGTT   
  
  
+ ATGATGAAGA TGGGCATTGG GTGGTTCAAG GATGGAAAGG CAGGATAATG TATGCCATTT CTTGTTGGAA   
  
  
+ ACCTTCATA  

- +Up\_Stream \_Len000TAATAA TAAGATTATA TATCTATTAT TATATTATTA TTATTATTAT TATTATTATT   
  
  
- AATAATAATA ATAATAATAA ATAATTTATA TTATCAATAA TATTATTATT ATTAATATTA TTATTATTCT   
  
  
- AATAATAGTA ATATAATAAT AATAATAATA ACATTAAATA ATAATAATAT CATTAATAAT AACAATATAT   
  
  
- CATTTTAATA ATAATCATAA CAATAATATT TATAAGTATG AACGTATATT ATAAAGATTT TATTTTTGTC   
  
  
- TCAATTAAGG TAAGGTTTTG ATGAGTGGTT TGTCGTTCCT CACCCTGGAC TAAGGAAAGG TTAAAAGTGG   
  
  
- TTTGTTGACC CAAGTCTTAA GTTCAAGGTA TAGGTTGGGG GGTCCTAAGT TCAGGACTAA GGTAAGGTAA   
  
  
- GGTCAAAGGG AGTGGTTTGA CCCGGAATCT AACTACCAAC AAGTCAACCT ACTCCCAACA AGGTACGGTT   
  
  
- CAATCTAGTA ACAACAGTTA AAACTCTTGC TGATAGAATT ATTCGGATTG CTACAACTAA ACTAAATCAA   
  
  
- GTTTGGTAAA GTTTATTATG TTTAGTTTAA GATAAATCTG GCTGTGATTT TATTTATCTA ACTCTTTTTA   
  
  
- ATATCTAAAA TTTCAAGTCT AATTTTAGGG ATTTCTAGTT GTCTAACTTT ACGTATAAAG TATCACGTGT   
  
  
- ACCTGAACGA AACTTAGAGC AGATCTCTTG GATTCTTTTT CGTTCTCACC AAGTCTTCCA ATCTTAACAA   
  
  
- GGTCGTGTAC TGTTCATGTT AGGGTTGAAA AAAGTTTTCT AAACGATGAA AATTAACCTA AGGGTACAAC   
  
  
- TTACTTCAAG ACCCAAATTC CGATGTTGAA AGAACCCTCC TATTCCAAGA ATTCAATTCC ACCAGGAGAA   
  
  
- AAGAAAACTA GAAAGAATAC CCATGTTGGA GTACCCAGAG TAGTTGTGTA CTTGAGAAAA ATAGAAAATA   
  
  
- GAAAGGGGAA AGGAAATACG TCCTAACTGT CCACGTACCT TTTGTTCTTC GTTAATTTAT GATCACCGAA   
  
  
- TCGGGGTAGG GATTATAAAA CCGATAAGAC AAAGTGGTAG GAGGGGACAG AGGTAAAAGA AACAAAGGAA   
  
  
- AAAAAGATCC TTGAACACTA CCCGTTCCCT TCTAGTCATG TTACACTACC AACAATCATC GCTCTTCTAC   
  
  
- TTCACAGTGG CGTTTTTAAA ACACGGTATT AAACAGCATT CCCTTTGTTC ATAGTTTGTT GTTACAGTAA   
  
  
- ATACTATTTG TTCATTGTCC TCTATACATG AGACTTTTTT ATATTCCATG GACTCTTCAA CAATTGAATC   
  
  
- GAACTTTTTG GGAGGATTGA ACTATTTGGG AGAAGCGATA CAATAGGGCG TGGGACGGTA TCTCAGTGGC   
  
  
- TAAGTCATAT ATACATTGTA TGGGTTAGAA CAGACCTATA TATCAATTGG GAAAACCTTA TAGACTTTTA   
  
  
- GGTGTGCTCT CAGTAGGAAG ATCTGATCCT TTGTACGCGG TGTAAAACCT AAGATTGGTT CATATTATTC   
  
  
- GTATAGAATT TCGGTTTTTA CGTTCCTATC TATGTTTATG GGTAGTGTAC GTACATTTAC AGGGAACAGT   
  
  
- ACCGAGACTA AGAGTTTTAT AATTAAAAGT TATTTAATTT ACTTTAACGT AATAAAATTT CTAAAGACTT   
  
  
- ACGTAGGTAA TAACGTTAAA AAATTTCTGA CGTGTTACTA CTGTTACAGT TACCAAAGGA TTTCAACGCG   
  
  
- GACTACCCGA TAACTGTCAC TTCGTTGTGA GGGAAACAAG GGTAAATACA CAGAGGTGAA CGGGGAAACT   
  
  
- TTGAGAGATG TAAACATACA GTAGACTTAA ACTAAGAGTT TAGAGTCTAC TAAACTTAAG GGAAATACGG   
  
  
- TAGAAATGAT AGATAACTTA CTAAGAAAGA AAAGGTGAAA GAATAGAAGG TGATGGAAGA AGAACCTACG   
  
  
- GGAAAACGAC GAATATTTCC CGCTGTGGAC TTGTTCAACG ACATAAGAGT TTGGTACTTA GACATGTCCA   
  
  
- AATCGGGGCT ATACACAGGA TCGAAACACC CAGTGCTTCT TTAACCAGGA TTAAATTATC CTGTGGTTCT   
  
  
- GGGAAAAGTT TTACGTATGT TAACGTGACT AAAACCACTA CGAATGAACA ACGTTAAGGG TCAACTTCTA   
  
  
- AATACAGTTA TGGCACAAGG ACTAGATTAC CCCGATGGAC AACTTCTACC CCTAGGATGT CTCCAGGGAC   
  
  
- TGTAAGGACT ATGTACGAAG TTGATGTATT CGTTAGAGGA TTACCTTCTC CCGAGCCTAC TGGCGGGACC   
  
  
- GCGGTGGGAC GTCCTGATAT ACCGACGAGT TCAGAGGATG TCGTTCAGGA GGTTACGGCG GAAAGGGCCA   
  
  
- CTCTCAACGA GTAAGAAACC ATTATTAGTT ACGGGAGGTG GACGAATAGT TTCGTTGGGA ATAACGGAAC   
  
  
- TACTAATGAA GTTGAACGAT AGGTTATGAT GACCGTTATC ACAATTGTTA CGACCACAAG TACTGTCGTT   
  
  
- GTTGAAACAA GTCTTGTCAA ACTAACCCTT ATCCCCATGA TCAAGATCAA GATCCCCGTC TTCACTAAAA   
  
  
- CCTAGATGGG AATAATCCTT AAACGGTAAT CCCTGGCTGT GTGAACTACA CCAGTTAGGT CGTGGATGAG   
  
  
- GAGCTCGGGG AGACCAATGC TGAGTCACAG TCCACAAACT TTGTGTGGTC ATACTCGTCC GGAAGCTTGA   
  
  
- CTAGTGATTA CCTTCGACCC TTAAGTCAGT AAGACAACGA TTATGTAGTT TACTACCTTA AGTTTTAGTT   
  
  
- GGTTAAGTTC GTCAAAGTTG GGTTCTTGCT ACCTTATACT TTTGTAAAAG GCTACTGGTT GAACTCGGAC   
  
  
- GAGAAAACGC TTCAGAACAC TACAGGAGTT TGACATGACT ATAGAGACTA GTCCCACATG TGTCCTATCA   
  
  
- CTTGGGACTC GGTGGGTTGT TGTCTTTATG CCTCTAACAC ACGTTACTGA GTAGGTCATT CTCATTCTGT   
  
  
- TCTGCTTCTC TCGTACCACG TCCTCTTTTG GTGCTACTTC CTCTTTTGGT GCTACTCTGT CCTTCCTCAT   
  
  
- TGTTCGTTAG TCGTGGTAGG TTACTTCACA AGCTACTTTA ACTCGTCATA CCACTACACG ACGAGACGGG   
  
  
- ACTTCCTTCT TCATTTGGTC GTCCCAATAC GTCGTCGTCG TTGCGATTGT CTTGATCGTT GAGTGTCTTG   
  
  
- AGCCTTCTGA GTTTCAGTGT CGTTTTACCC TCAAGGAGGT TCCACAGTTT TTTCGCTCCG TTATTTTTCG   
  
  
- TTCACCAACT AAACTCCTGA GAGGATTGAG CTACACGTGT TACACAACGA GCACACCTAA ATTGACCAAG   
  
  
- AATGGTCAAC GAAGTCCTCT AAGCCGTCGT AAGAAGAGGG ATACCACTAA TAGAGGTTTC TTACCGAGTA   
  
  
- ATGGAACGAT TACCGGAACT CCGCGCGAAT CTTCCGTTTC CTATACTTGA TTGAGCACGG TTACTCGTAT   
  
  
- AGGGTGGATG ACTCTAGGAG TTCCGATTGG CCCATATACA CCGTCGCCAG GGAAAGTTCT ATTACAGAAT   
  
  
- AATGTGTTGT TTGTTCTGTT AACGTTCGGA TCAACTCCTC CGTGGTTCAT ATGTGTATTA ACTAAAACCA   
  
  
- TAAAAGATAC CTGATGTTAC CGGGACGTAG TAAGTCTTGG AGAGGTTCTC CGGGTTACCC GGAGGGTCTT   
  
  
- AGGCGTAGTG TCCGTAGCTA AAGGGGGTTG GACCCAAGGC CGGTCGTCTC TCCCAACTTC TCTGTCCCGC   
  
  
- CACAAATCGG TTCATAACAC TGTCCATGTT ACACGGGAAA CTTATGGTGG GGTATCGGTT CTTCACCCTC   
  
  
- TGGTAGGTTG AGCTCCTCGA GTTTTAACTT TCCTTACTCG GGGAACATCA CTTGACAGAC ATATCTAGCG   
  
  
- TGTTGGATAA ACTACTTTCG CATCTCCTTT TGTCGGGTTC TCTGCGCAAG GACTCGAACC AATCCTTTTA   
  
  
- TTTAGGGCTA TATAAGTACG TACCCCACCA CTTATGGCGT AGTTCACATG GAAAGAAGGA CTTGTCCAAG   
  
  
- TTCCTCCGGT ACAAAGTGAT GTGCCGGAAC AAACTGAACA AGCTCCGGTG TTACTCGGCG CTCCTAGTTC   
  
  
- TCTCCCACAA TGATCTCTCG TTCAACGTAC CCTTAGTCCG CAACTTACAC TAGCGGACGC TTCCTCGCCT   
  
  
- CTCTCACCTC TCCGGACTCA GTATGTTTGT TACCGTCCAA TCCTGAGTTT CCCGGCCCAA ACAAGTTCAC   
  
  
- AGCTACCTAG CGCTCGAAAA CTCTTCCCGT TCCCGGTACC AGTCCTCCTT AAAATCCCTT CTCAAGTCAA   
  
  
- TACTACTTCT ACCCGTAACC CACCAAGTTC CTACCTTTCC GTCCTATTAC ATACGGTAAA GAACAACCTT   
  
  
- TGGAAGTAT

+     GARE-motif

| Site Name | Organism | Position | Strand | Matrix score. | sequence | function |
| --- | --- | --- | --- | --- | --- | --- |
| GARE-motif | Brassica oleracea | 2963 | - | 7 | TCTGTTG | gibberellin-responsive element |
| GARE-motif | Brassica oleracea | 2766 | + | 7 | TCTGTTG | gibberellin-responsive element |
| GARE-motif | Brassica oleracea | 672 | - | 7 | TCTGTTG | gibberellin-responsive element |

>HU02G01570.1   
+ +Up\_Stream \_Len000ATTATT ATTCTAATAT ATAGATAATA ATATAATAAT AATAATAATA ATAATAATAA   
  
  
+ TTATTATTAT TATTATTATT TATTAAATAT AATAGTTATT ATAATAATAA TAATTATAAT AATAATAAGA   
  
  
+ TTATTATCAT TATATTATTA TTATTATTAT TGTAATTTAT TATTATTATA GTAATTATTA TTGTTATATA   
  
  
+ GTAAAATTAT TATTAGTATT GTTATTATAA ATATTCATAC TTGCATATAA TATTTCTAAA ATAAAAACAG   
  
  
+ AGTTAATTCC ATTCCAAAAC TACTCACCAA ACAGCAAGGA GTGGGACCTG ATTCCTTTCC AATTTTCACC   
  
  
+ AAACAACTGG GTTCAGAATT CAAGTTCCAT ATCCAACCCC CCAGGATTCA AGTCCTGATT CCATTCCATT   
  
  
+ CCAGTTTCCC TCACCAAACT GGGCCTTAGA TTGATGGTTG TTCAGTTGGA TGAGGGTTGT TCCATGCCAA   
  
  
+ GTTAGATCAT TGTTGTCAAT TTTGAGAACG ACTATCTTAA TAAGCCTAAC GATGTTGATT TGATTTAGTT   
  
  
+ CAAACCATTT CAAATAATAC AAATCAAATT CTATTTAGAC CGACACTAAA ATAAATAGAT TGAGAAAAAT   
  
  
+ TATAGATTTT AAAGTTCAGA TTAAAATCCC TAAAGATCAA CAGATTGAAA TGCATATTTC ATAGTGCACA   
  
  
+ TGGACTTGCT TTGAATCTCG TCTAGAGAAC CTAAGAAAAA GCAAGAGTGG TTCAGAAGGT TAGAATTGTT   
  
  
+ CCAGCACATG ACAAGTACAA TCCCAACTTT TTTCAAAAGA TTTGCTACTT TTAATTGGAT TCCCATGTTG   
  
  
+ AATGAAGTTC TGGGTTTAAG GCTACAACTT TCTTGGGAGG ATAAGGTTCT TAAGTTAAGG TGGTCCTCTT   
  
  
+ TTCTTTTGAT CTTTCTTATG GGTACAACCT CATGGGTCTC ATCAACACAT GAACTCTTTT TATCTTTTAT   
  
  
+ CTTTCCCCTT TCCTTTATGC AGGATTGACA GGTGCATGGA AAACAAGAAG CAATTAAATA CTAGTGGCTT   
  
  
+ AGCCCCATCC CTAATATTTT GGCTATTCTG TTTCACCATC CTCCCCTGTC TCCATTTTCT TTGTTTCCTT   
  
  
+ TTTTTCTAGG AACTTGTGAT GGGCAAGGGA AGATCAGTAC AATGTGATGG TTGTTAGTAG CGAGAAGATG   
  
  
+ AAGTGTCACC GCAAAAATTT TGTGCCATAA TTTGTCGTAA GGGAAACAAG TATCAAACAA CAATGTCATT   
  
  
+ TATGATAAAC AAGTAACAGG AGATATGTAC TCTGAAAAAA TATAAGGTAC CTGAGAAGTT GTTAACTTAG   
  
  
+ CTTGAAAAAC CCTCCTAACT TGATAAACCC TCTTCGCTAT GTTATCCCGC ACCCTGCCAT AGAGTCACCG   
  
  
+ ATTCAGTATA TATGTAACAT ACCCAATCTT GTCTGGATAT ATAGTTAACC CTTTTGGAAT ATCTGAAAAT   
  
  
+ CCACACGAGA GTCATCCTTC TAGACTAGGA AACATGCGCC ACATTTTGGA TTCTAACCAA GTATAATAAG   
  
  
+ CATATCTTAA AGCCAAAAAT GCAAGGATAG ATACAAATAC CCATCACATG CATGTAAATG TCCCTTGTCA   
  
  
+ TGGCTCTGAT TCTCAAAATA TTAATTTTCA ATAAATTAAA TGAAATTGCA TTATTTTAAA GATTTCTGAA   
  
  
+ TGCATCCATT ATTGCAATTT TTTAAAGACT GCACAATGAT GACAATGTCA ATGGTTTCCT AAAGTTGCGC   
  
  
+ CTGATGGGCT ATTGACAGTG AAGCAACACT CCCTTTGTTC CCATTTATGT GTCTCCACTT GCCCCTTTGA   
  
  
+ AACTCTCTAC ATTTGTATGT CATCTGAATT TGATTCTCAA ATCTCAGATG ATTTGAATTC CCTTTATGCC   
  
  
+ ATCTTTACTA TCTATTGAAT GATTCTTTCT TTTCCACTTT CTTATCTTCC ACTACCTTCT TCTTGGATGC   
  
  
+ CCTTTTGCTG CTTATAAAGG GCGACACCTG AACAAGTTGC TGTATTCTCA AACCATGAAT CTGTACAGGT   
  
  
+ TTAGCCCCGA TATGTGTCCT AGCTTTGTGG GTCACGAAGA AATTGGTCCT AATTTAATAG GACACCAAGA   
  
  
+ CCCTTTTCAA AATGCATACA ATTGCACTGA TTTTGGTGAT GCTTACTTGT TGCAATTCCC AGTTGAAGAT   
  
  
+ TTATGTCAAT ACCGTGTTCC TGATCTAATG GGGCTACCTG TTGAAGATGG GGATCCTACA GAGGTCCCTG   
  
  
+ ACATTCCTGA TACATGCTTC AACTACATAA GCAATCTCCT AATGGAAGAG GGCTCGGATG ACCGCCCTGG   
  
  
+ CGCCACCCTG CAGGACTATA TGGCTGCTCA AGTCTCCTAC AGCAAGTCCT CCAATGCCGC CTTTCCCGGT   
  
  
+ GAGAGTTGCT CATTCTTTGG TAATAATCAA TGCCCTCCAC CTGCTTATCA AAGCAACCCT TATTGCCTTG   
  
  
+ ATGATTACTT CAACTTGCTA TCCAATACTA CTGGCAATAG TGTTAACAAT GCTGGTGTTC ATGACAGCAA   
  
  
+ CAACTTTGTT CAGAACAGTT TGATTGGGAA TAGGGGTACT AGTTCTAGTT CTAGGGGCAG AAGTGATTTT   
  
  
+ GGATCTACCC TTATTAGGAA TTTGCCATTA GGGACCGACA CACTTGATGT GGTCAATCCA GCACCTACTC   
  
  
+ CTCGAGCCCC TCTGGTTACG ACTCAGTGTC AGGTGTTTGA AACACACCAG TATGAGCAGG CCTTCGAACT   
  
  
+ GATCACTAAT GGAAGCTGGG AATTCAGTCA TTCTGTTGCT AATACATCAA ATGATGGAAT TCAAAATCAA   
  
  
+ CCAATTCAAG CAGTTTCAAC CCAAGAACGA TGGAATATGA AAACATTTTC CGATGACCAA CTTGAGCCTG   
  
  
+ CTCTTTTGCG AAGTCTTGTG ATGTCCTCAA ACTGTACTGA TATCTCTGAT CAGGGTGTAC ACAGGATAGT   
  
  
+ GAACCCTGAG CCACCCAACA ACAGAAATAC GGAGATTGTG TGCAATGACT CATCCAGTAA GAGTAAGACA   
  
  
+ AGACGAAGAG AGCATGGTGC AGGAGAAAAC CACGATGAAG GAGAAAACCA CGATGAGACA GGAAGGAGTA   
  
  
+ ACAAGCAATC AGCACCATCC AATGAAGTGT TCGATGAAAT TGAGCAGTAT GGTGATGTGC TGCTCTGCCC   
  
  
+ TGAAGGAAGA AGTAAACCAG CAGGGTTATG CAGCAGCAGC AACGCTAACA GAACTAGCAA CTCACAGAAC   
  
  
+ TCGGAAGACT CAAAGTCACA GCAAAATGGG AGTTCCTCCA AGGTGTCAAA AAAGCGAGGC AATAAAAAGC   
  
  
+ AAGTGGTTGA TTTGAGGACT CTCCTAACTC GATGTGCACA ATGTGTTGCT CGTGTGGATT TAACTGGTTC   
  
  
+ TTACCAGTTG CTTCAGGAGA TTCGGCAGCA TTCTTCTCCC TATGGTGATT ATCTCCAAAG AATGGCTCAT   
  
  
+ TACCTTGCTA ATGGCCTTGA GGCGCGCTTA GAAGGCAAAG GATATGAACT AACTCGTGCC AATGAGCATA   
  
  
+ TCCCACCTAC TGAGATCCTC AAGGCTAACC GGGTATATGT GGCAGCGGTC CCTTTCAAGA TAATGTCTTA   
  
  
+ TTACACAACA AACAAGACAA TTGCAAGCCT AGTTGAGGAG GCACCAAGTA TACACATAAT TGATTTTGGT   
  
  
+ ATTTTCTATG GACTACAATG GCCCTGCATC ATTCAGAACC TCTCCAAGAG GCCCAATGGG CCTCCCAGAA   
  
  
+ TCCGCATCAC AGGCATCGAT TTCCCCCAAC CTGGGTTCCG GCCAGCAGAG AGGGTTGAAG AGACAGGGCG   
  
  
+ GTGTTTAGCC AAGTATTGTG ACAGGTACAA TGTGCCCTTT GAATACCACC CCATAGCCAA GAAGTGGGAG   
  
  
+ ACCATCCAAC TCGAGGAGCT CAAAATTGAA AGGAATGAGC CCCTTGTAGT GAACTGTCTG TATAGATCGC   
  
  
+ ACAACCTATT TGATGAAAGC GTAGAGGAAA ACAGCCCAAG AGACGCGTTC CTGAGCTTGG TTAGGAAAAT   
  
  
+ AAATCCCGAT ATATTCATGC ATGGGGTGGT GAATACCGCA TCAAGTGTAC CTTTCTTCCT GAACAGGTTC   
  
  
+ AAGGAGGCCA TGTTTCACTA CACGGCCTTG TTTGACTTGT TCGAGGCCAC AATGAGCCGC GAGGATCAAG   
  
  
+ AGAGGGTGTT ACTAGAGAGC AAGTTGCATG GGAATCAGGC GTTGAATGTG ATCGCCTGCG AAGGAGCGGA   
  
  
+ GAGAGTGGAG AGGCCTGAGT CATACAAACA ATGGCAGGTT AGGACTCAAA GGGCCGGGTT TGTTCAAGTG   
  
  
+ TCGATGGATC GCGAGCTTTT GAGAAGGGCA AGGGCCATGG TCAGGAGGAA TTTTAGGGAA GAGTTCAGTT   
  
  
+ ATGATGAAGA TGGGCATTGG GTGGTTCAAG GATGGAAAGG CAGGATAATG TATGCCATTT CTTGTTGGAA   
  
  
+ ACCTTCATA  

- +Up\_Stream \_Len000TAATAA TAAGATTATA TATCTATTAT TATATTATTA TTATTATTAT TATTATTATT   
  
  
- AATAATAATA ATAATAATAA ATAATTTATA TTATCAATAA TATTATTATT ATTAATATTA TTATTATTCT   
  
  
- AATAATAGTA ATATAATAAT AATAATAATA ACATTAAATA ATAATAATAT CATTAATAAT AACAATATAT   
  
  
- CATTTTAATA ATAATCATAA CAATAATATT TATAAGTATG AACGTATATT ATAAAGATTT TATTTTTGTC   
  
  
- TCAATTAAGG TAAGGTTTTG ATGAGTGGTT TGTCGTTCCT CACCCTGGAC TAAGGAAAGG TTAAAAGTGG   
  
  
- TTTGTTGACC CAAGTCTTAA GTTCAAGGTA TAGGTTGGGG GGTCCTAAGT TCAGGACTAA GGTAAGGTAA   
  
  
- GGTCAAAGGG AGTGGTTTGA CCCGGAATCT AACTACCAAC AAGTCAACCT ACTCCCAACA AGGTACGGTT   
  
  
- CAATCTAGTA ACAACAGTTA AAACTCTTGC TGATAGAATT ATTCGGATTG CTACAACTAA ACTAAATCAA   
  
  
- GTTTGGTAAA GTTTATTATG TTTAGTTTAA GATAAATCTG GCTGTGATTT TATTTATCTA ACTCTTTTTA   
  
  
- ATATCTAAAA TTTCAAGTCT AATTTTAGGG ATTTCTAGTT GTCTAACTTT ACGTATAAAG TATCACGTGT   
  
  
- ACCTGAACGA AACTTAGAGC AGATCTCTTG GATTCTTTTT CGTTCTCACC AAGTCTTCCA ATCTTAACAA   
  
  
- GGTCGTGTAC TGTTCATGTT AGGGTTGAAA AAAGTTTTCT AAACGATGAA AATTAACCTA AGGGTACAAC   
  
  
- TTACTTCAAG ACCCAAATTC CGATGTTGAA AGAACCCTCC TATTCCAAGA ATTCAATTCC ACCAGGAGAA   
  
  
- AAGAAAACTA GAAAGAATAC CCATGTTGGA GTACCCAGAG TAGTTGTGTA CTTGAGAAAA ATAGAAAATA   
  
  
- GAAAGGGGAA AGGAAATACG TCCTAACTGT CCACGTACCT TTTGTTCTTC GTTAATTTAT GATCACCGAA   
  
  
- TCGGGGTAGG GATTATAAAA CCGATAAGAC AAAGTGGTAG GAGGGGACAG AGGTAAAAGA AACAAAGGAA   
  
  
- AAAAAGATCC TTGAACACTA CCCGTTCCCT TCTAGTCATG TTACACTACC AACAATCATC GCTCTTCTAC   
  
  
- TTCACAGTGG CGTTTTTAAA ACACGGTATT AAACAGCATT CCCTTTGTTC ATAGTTTGTT GTTACAGTAA   
  
  
- ATACTATTTG TTCATTGTCC TCTATACATG AGACTTTTTT ATATTCCATG GACTCTTCAA CAATTGAATC   
  
  
- GAACTTTTTG GGAGGATTGA ACTATTTGGG AGAAGCGATA CAATAGGGCG TGGGACGGTA TCTCAGTGGC   
  
  
- TAAGTCATAT ATACATTGTA TGGGTTAGAA CAGACCTATA TATCAATTGG GAAAACCTTA TAGACTTTTA   
  
  
- GGTGTGCTCT CAGTAGGAAG ATCTGATCCT TTGTACGCGG TGTAAAACCT AAGATTGGTT CATATTATTC   
  
  
- GTATAGAATT TCGGTTTTTA CGTTCCTATC TATGTTTATG GGTAGTGTAC GTACATTTAC AGGGAACAGT   
  
  
- ACCGAGACTA AGAGTTTTAT AATTAAAAGT TATTTAATTT ACTTTAACGT AATAAAATTT CTAAAGACTT   
  
  
- ACGTAGGTAA TAACGTTAAA AAATTTCTGA CGTGTTACTA CTGTTACAGT TACCAAAGGA TTTCAACGCG   
  
  
- GACTACCCGA TAACTGTCAC TTCGTTGTGA GGGAAACAAG GGTAAATACA CAGAGGTGAA CGGGGAAACT   
  
  
- TTGAGAGATG TAAACATACA GTAGACTTAA ACTAAGAGTT TAGAGTCTAC TAAACTTAAG GGAAATACGG   
  
  
- TAGAAATGAT AGATAACTTA CTAAGAAAGA AAAGGTGAAA GAATAGAAGG TGATGGAAGA AGAACCTACG   
  
  
- GGAAAACGAC GAATATTTCC CGCTGTGGAC TTGTTCAACG ACATAAGAGT TTGGTACTTA GACATGTCCA   
  
  
- AATCGGGGCT ATACACAGGA TCGAAACACC CAGTGCTTCT TTAACCAGGA TTAAATTATC CTGTGGTTCT   
  
  
- GGGAAAAGTT TTACGTATGT TAACGTGACT AAAACCACTA CGAATGAACA ACGTTAAGGG TCAACTTCTA   
  
  
- AATACAGTTA TGGCACAAGG ACTAGATTAC CCCGATGGAC AACTTCTACC CCTAGGATGT CTCCAGGGAC   
  
  
- TGTAAGGACT ATGTACGAAG TTGATGTATT CGTTAGAGGA TTACCTTCTC CCGAGCCTAC TGGCGGGACC   
  
  
- GCGGTGGGAC GTCCTGATAT ACCGACGAGT TCAGAGGATG TCGTTCAGGA GGTTACGGCG GAAAGGGCCA   
  
  
- CTCTCAACGA GTAAGAAACC ATTATTAGTT ACGGGAGGTG GACGAATAGT TTCGTTGGGA ATAACGGAAC   
  
  
- TACTAATGAA GTTGAACGAT AGGTTATGAT GACCGTTATC ACAATTGTTA CGACCACAAG TACTGTCGTT   
  
  
- GTTGAAACAA GTCTTGTCAA ACTAACCCTT ATCCCCATGA TCAAGATCAA GATCCCCGTC TTCACTAAAA   
  
  
- CCTAGATGGG AATAATCCTT AAACGGTAAT CCCTGGCTGT GTGAACTACA CCAGTTAGGT CGTGGATGAG   
  
  
- GAGCTCGGGG AGACCAATGC TGAGTCACAG TCCACAAACT TTGTGTGGTC ATACTCGTCC GGAAGCTTGA   
  
  
- CTAGTGATTA CCTTCGACCC TTAAGTCAGT AAGACAACGA TTATGTAGTT TACTACCTTA AGTTTTAGTT   
  
  
- GGTTAAGTTC GTCAAAGTTG GGTTCTTGCT ACCTTATACT TTTGTAAAAG GCTACTGGTT GAACTCGGAC   
  
  
- GAGAAAACGC TTCAGAACAC TACAGGAGTT TGACATGACT ATAGAGACTA GTCCCACATG TGTCCTATCA   
  
  
- CTTGGGACTC GGTGGGTTGT TGTCTTTATG CCTCTAACAC ACGTTACTGA GTAGGTCATT CTCATTCTGT   
  
  
- TCTGCTTCTC TCGTACCACG TCCTCTTTTG GTGCTACTTC CTCTTTTGGT GCTACTCTGT CCTTCCTCAT   
  
  
- TGTTCGTTAG TCGTGGTAGG TTACTTCACA AGCTACTTTA ACTCGTCATA CCACTACACG ACGAGACGGG   
  
  
- ACTTCCTTCT TCATTTGGTC GTCCCAATAC GTCGTCGTCG TTGCGATTGT CTTGATCGTT GAGTGTCTTG   
  
  
- AGCCTTCTGA GTTTCAGTGT CGTTTTACCC TCAAGGAGGT TCCACAGTTT TTTCGCTCCG TTATTTTTCG   
  
  
- TTCACCAACT AAACTCCTGA GAGGATTGAG CTACACGTGT TACACAACGA GCACACCTAA ATTGACCAAG   
  
  
- AATGGTCAAC GAAGTCCTCT AAGCCGTCGT AAGAAGAGGG ATACCACTAA TAGAGGTTTC TTACCGAGTA   
  
  
- ATGGAACGAT TACCGGAACT CCGCGCGAAT CTTCCGTTTC CTATACTTGA TTGAGCACGG TTACTCGTAT   
  
  
- AGGGTGGATG ACTCTAGGAG TTCCGATTGG CCCATATACA CCGTCGCCAG GGAAAGTTCT ATTACAGAAT   
  
  
- AATGTGTTGT TTGTTCTGTT AACGTTCGGA TCAACTCCTC CGTGGTTCAT ATGTGTATTA ACTAAAACCA   
  
  
- TAAAAGATAC CTGATGTTAC CGGGACGTAG TAAGTCTTGG AGAGGTTCTC CGGGTTACCC GGAGGGTCTT   
  
  
- AGGCGTAGTG TCCGTAGCTA AAGGGGGTTG GACCCAAGGC CGGTCGTCTC TCCCAACTTC TCTGTCCCGC   
  
  
- CACAAATCGG TTCATAACAC TGTCCATGTT ACACGGGAAA CTTATGGTGG GGTATCGGTT CTTCACCCTC   
  
  
- TGGTAGGTTG AGCTCCTCGA GTTTTAACTT TCCTTACTCG GGGAACATCA CTTGACAGAC ATATCTAGCG   
  
  
- TGTTGGATAA ACTACTTTCG CATCTCCTTT TGTCGGGTTC TCTGCGCAAG GACTCGAACC AATCCTTTTA   
  
  
- TTTAGGGCTA TATAAGTACG TACCCCACCA CTTATGGCGT AGTTCACATG GAAAGAAGGA CTTGTCCAAG   
  
  
- TTCCTCCGGT ACAAAGTGAT GTGCCGGAAC AAACTGAACA AGCTCCGGTG TTACTCGGCG CTCCTAGTTC   
  
  
- TCTCCCACAA TGATCTCTCG TTCAACGTAC CCTTAGTCCG CAACTTACAC TAGCGGACGC TTCCTCGCCT   
  
  
- CTCTCACCTC TCCGGACTCA GTATGTTTGT TACCGTCCAA TCCTGAGTTT CCCGGCCCAA ACAAGTTCAC   
  
  
- AGCTACCTAG CGCTCGAAAA CTCTTCCCGT TCCCGGTACC AGTCCTCCTT AAAATCCCTT CTCAAGTCAA   
  
  
- TACTACTTCT ACCCGTAACC CACCAAGTTC CTACCTTTCC GTCCTATTAC ATACGGTAAA GAACAACCTT   
  
  
- TGGAAGTAT

+     GATA-motif

| Site Name | Organism | Position | Strand | Matrix score. | sequence | function |
| --- | --- | --- | --- | --- | --- | --- |
| GATA-motif | Solanum tuberosum | 881 | + | 9 | AAGGATAAGG | part of a light responsive element |

>HU02G01570.1   
+ +Up\_Stream \_Len000ATTATT ATTCTAATAT ATAGATAATA ATATAATAAT AATAATAATA ATAATAATAA   
  
  
+ TTATTATTAT TATTATTATT TATTAAATAT AATAGTTATT ATAATAATAA TAATTATAAT AATAATAAGA   
  
  
+ TTATTATCAT TATATTATTA TTATTATTAT TGTAATTTAT TATTATTATA GTAATTATTA TTGTTATATA   
  
  
+ GTAAAATTAT TATTAGTATT GTTATTATAA ATATTCATAC TTGCATATAA TATTTCTAAA ATAAAAACAG   
  
  
+ AGTTAATTCC ATTCCAAAAC TACTCACCAA ACAGCAAGGA GTGGGACCTG ATTCCTTTCC AATTTTCACC   
  
  
+ AAACAACTGG GTTCAGAATT CAAGTTCCAT ATCCAACCCC CCAGGATTCA AGTCCTGATT CCATTCCATT   
  
  
+ CCAGTTTCCC TCACCAAACT GGGCCTTAGA TTGATGGTTG TTCAGTTGGA TGAGGGTTGT TCCATGCCAA   
  
  
+ GTTAGATCAT TGTTGTCAAT TTTGAGAACG ACTATCTTAA TAAGCCTAAC GATGTTGATT TGATTTAGTT   
  
  
+ CAAACCATTT CAAATAATAC AAATCAAATT CTATTTAGAC CGACACTAAA ATAAATAGAT TGAGAAAAAT   
  
  
+ TATAGATTTT AAAGTTCAGA TTAAAATCCC TAAAGATCAA CAGATTGAAA TGCATATTTC ATAGTGCACA   
  
  
+ TGGACTTGCT TTGAATCTCG TCTAGAGAAC CTAAGAAAAA GCAAGAGTGG TTCAGAAGGT TAGAATTGTT   
  
  
+ CCAGCACATG ACAAGTACAA TCCCAACTTT TTTCAAAAGA TTTGCTACTT TTAATTGGAT TCCCATGTTG   
  
  
+ AATGAAGTTC TGGGTTTAAG GCTACAACTT TCTTGGGAGG ATAAGGTTCT TAAGTTAAGG TGGTCCTCTT   
  
  
+ TTCTTTTGAT CTTTCTTATG GGTACAACCT CATGGGTCTC ATCAACACAT GAACTCTTTT TATCTTTTAT   
  
  
+ CTTTCCCCTT TCCTTTATGC AGGATTGACA GGTGCATGGA AAACAAGAAG CAATTAAATA CTAGTGGCTT   
  
  
+ AGCCCCATCC CTAATATTTT GGCTATTCTG TTTCACCATC CTCCCCTGTC TCCATTTTCT TTGTTTCCTT   
  
  
+ TTTTTCTAGG AACTTGTGAT GGGCAAGGGA AGATCAGTAC AATGTGATGG TTGTTAGTAG CGAGAAGATG   
  
  
+ AAGTGTCACC GCAAAAATTT TGTGCCATAA TTTGTCGTAA GGGAAACAAG TATCAAACAA CAATGTCATT   
  
  
+ TATGATAAAC AAGTAACAGG AGATATGTAC TCTGAAAAAA TATAAGGTAC CTGAGAAGTT GTTAACTTAG   
  
  
+ CTTGAAAAAC CCTCCTAACT TGATAAACCC TCTTCGCTAT GTTATCCCGC ACCCTGCCAT AGAGTCACCG   
  
  
+ ATTCAGTATA TATGTAACAT ACCCAATCTT GTCTGGATAT ATAGTTAACC CTTTTGGAAT ATCTGAAAAT   
  
  
+ CCACACGAGA GTCATCCTTC TAGACTAGGA AACATGCGCC ACATTTTGGA TTCTAACCAA GTATAATAAG   
  
  
+ CATATCTTAA AGCCAAAAAT GCAAGGATAG ATACAAATAC CCATCACATG CATGTAAATG TCCCTTGTCA   
  
  
+ TGGCTCTGAT TCTCAAAATA TTAATTTTCA ATAAATTAAA TGAAATTGCA TTATTTTAAA GATTTCTGAA   
  
  
+ TGCATCCATT ATTGCAATTT TTTAAAGACT GCACAATGAT GACAATGTCA ATGGTTTCCT AAAGTTGCGC   
  
  
+ CTGATGGGCT ATTGACAGTG AAGCAACACT CCCTTTGTTC CCATTTATGT GTCTCCACTT GCCCCTTTGA   
  
  
+ AACTCTCTAC ATTTGTATGT CATCTGAATT TGATTCTCAA ATCTCAGATG ATTTGAATTC CCTTTATGCC   
  
  
+ ATCTTTACTA TCTATTGAAT GATTCTTTCT TTTCCACTTT CTTATCTTCC ACTACCTTCT TCTTGGATGC   
  
  
+ CCTTTTGCTG CTTATAAAGG GCGACACCTG AACAAGTTGC TGTATTCTCA AACCATGAAT CTGTACAGGT   
  
  
+ TTAGCCCCGA TATGTGTCCT AGCTTTGTGG GTCACGAAGA AATTGGTCCT AATTTAATAG GACACCAAGA   
  
  
+ CCCTTTTCAA AATGCATACA ATTGCACTGA TTTTGGTGAT GCTTACTTGT TGCAATTCCC AGTTGAAGAT   
  
  
+ TTATGTCAAT ACCGTGTTCC TGATCTAATG GGGCTACCTG TTGAAGATGG GGATCCTACA GAGGTCCCTG   
  
  
+ ACATTCCTGA TACATGCTTC AACTACATAA GCAATCTCCT AATGGAAGAG GGCTCGGATG ACCGCCCTGG   
  
  
+ CGCCACCCTG CAGGACTATA TGGCTGCTCA AGTCTCCTAC AGCAAGTCCT CCAATGCCGC CTTTCCCGGT   
  
  
+ GAGAGTTGCT CATTCTTTGG TAATAATCAA TGCCCTCCAC CTGCTTATCA AAGCAACCCT TATTGCCTTG   
  
  
+ ATGATTACTT CAACTTGCTA TCCAATACTA CTGGCAATAG TGTTAACAAT GCTGGTGTTC ATGACAGCAA   
  
  
+ CAACTTTGTT CAGAACAGTT TGATTGGGAA TAGGGGTACT AGTTCTAGTT CTAGGGGCAG AAGTGATTTT   
  
  
+ GGATCTACCC TTATTAGGAA TTTGCCATTA GGGACCGACA CACTTGATGT GGTCAATCCA GCACCTACTC   
  
  
+ CTCGAGCCCC TCTGGTTACG ACTCAGTGTC AGGTGTTTGA AACACACCAG TATGAGCAGG CCTTCGAACT   
  
  
+ GATCACTAAT GGAAGCTGGG AATTCAGTCA TTCTGTTGCT AATACATCAA ATGATGGAAT TCAAAATCAA   
  
  
+ CCAATTCAAG CAGTTTCAAC CCAAGAACGA TGGAATATGA AAACATTTTC CGATGACCAA CTTGAGCCTG   
  
  
+ CTCTTTTGCG AAGTCTTGTG ATGTCCTCAA ACTGTACTGA TATCTCTGAT CAGGGTGTAC ACAGGATAGT   
  
  
+ GAACCCTGAG CCACCCAACA ACAGAAATAC GGAGATTGTG TGCAATGACT CATCCAGTAA GAGTAAGACA   
  
  
+ AGACGAAGAG AGCATGGTGC AGGAGAAAAC CACGATGAAG GAGAAAACCA CGATGAGACA GGAAGGAGTA   
  
  
+ ACAAGCAATC AGCACCATCC AATGAAGTGT TCGATGAAAT TGAGCAGTAT GGTGATGTGC TGCTCTGCCC   
  
  
+ TGAAGGAAGA AGTAAACCAG CAGGGTTATG CAGCAGCAGC AACGCTAACA GAACTAGCAA CTCACAGAAC   
  
  
+ TCGGAAGACT CAAAGTCACA GCAAAATGGG AGTTCCTCCA AGGTGTCAAA AAAGCGAGGC AATAAAAAGC   
  
  
+ AAGTGGTTGA TTTGAGGACT CTCCTAACTC GATGTGCACA ATGTGTTGCT CGTGTGGATT TAACTGGTTC   
  
  
+ TTACCAGTTG CTTCAGGAGA TTCGGCAGCA TTCTTCTCCC TATGGTGATT ATCTCCAAAG AATGGCTCAT   
  
  
+ TACCTTGCTA ATGGCCTTGA GGCGCGCTTA GAAGGCAAAG GATATGAACT AACTCGTGCC AATGAGCATA   
  
  
+ TCCCACCTAC TGAGATCCTC AAGGCTAACC GGGTATATGT GGCAGCGGTC CCTTTCAAGA TAATGTCTTA   
  
  
+ TTACACAACA AACAAGACAA TTGCAAGCCT AGTTGAGGAG GCACCAAGTA TACACATAAT TGATTTTGGT   
  
  
+ ATTTTCTATG GACTACAATG GCCCTGCATC ATTCAGAACC TCTCCAAGAG GCCCAATGGG CCTCCCAGAA   
  
  
+ TCCGCATCAC AGGCATCGAT TTCCCCCAAC CTGGGTTCCG GCCAGCAGAG AGGGTTGAAG AGACAGGGCG   
  
  
+ GTGTTTAGCC AAGTATTGTG ACAGGTACAA TGTGCCCTTT GAATACCACC CCATAGCCAA GAAGTGGGAG   
  
  
+ ACCATCCAAC TCGAGGAGCT CAAAATTGAA AGGAATGAGC CCCTTGTAGT GAACTGTCTG TATAGATCGC   
  
  
+ ACAACCTATT TGATGAAAGC GTAGAGGAAA ACAGCCCAAG AGACGCGTTC CTGAGCTTGG TTAGGAAAAT   
  
  
+ AAATCCCGAT ATATTCATGC ATGGGGTGGT GAATACCGCA TCAAGTGTAC CTTTCTTCCT GAACAGGTTC   
  
  
+ AAGGAGGCCA TGTTTCACTA CACGGCCTTG TTTGACTTGT TCGAGGCCAC AATGAGCCGC GAGGATCAAG   
  
  
+ AGAGGGTGTT ACTAGAGAGC AAGTTGCATG GGAATCAGGC GTTGAATGTG ATCGCCTGCG AAGGAGCGGA   
  
  
+ GAGAGTGGAG AGGCCTGAGT CATACAAACA ATGGCAGGTT AGGACTCAAA GGGCCGGGTT TGTTCAAGTG   
  
  
+ TCGATGGATC GCGAGCTTTT GAGAAGGGCA AGGGCCATGG TCAGGAGGAA TTTTAGGGAA GAGTTCAGTT   
  
  
+ ATGATGAAGA TGGGCATTGG GTGGTTCAAG GATGGAAAGG CAGGATAATG TATGCCATTT CTTGTTGGAA   
  
  
+ ACCTTCATA  

- +Up\_Stream \_Len000TAATAA TAAGATTATA TATCTATTAT TATATTATTA TTATTATTAT TATTATTATT   
  
  
- AATAATAATA ATAATAATAA ATAATTTATA TTATCAATAA TATTATTATT ATTAATATTA TTATTATTCT   
  
  
- AATAATAGTA ATATAATAAT AATAATAATA ACATTAAATA ATAATAATAT CATTAATAAT AACAATATAT   
  
  
- CATTTTAATA ATAATCATAA CAATAATATT TATAAGTATG AACGTATATT ATAAAGATTT TATTTTTGTC   
  
  
- TCAATTAAGG TAAGGTTTTG ATGAGTGGTT TGTCGTTCCT CACCCTGGAC TAAGGAAAGG TTAAAAGTGG   
  
  
- TTTGTTGACC CAAGTCTTAA GTTCAAGGTA TAGGTTGGGG GGTCCTAAGT TCAGGACTAA GGTAAGGTAA   
  
  
- GGTCAAAGGG AGTGGTTTGA CCCGGAATCT AACTACCAAC AAGTCAACCT ACTCCCAACA AGGTACGGTT   
  
  
- CAATCTAGTA ACAACAGTTA AAACTCTTGC TGATAGAATT ATTCGGATTG CTACAACTAA ACTAAATCAA   
  
  
- GTTTGGTAAA GTTTATTATG TTTAGTTTAA GATAAATCTG GCTGTGATTT TATTTATCTA ACTCTTTTTA   
  
  
- ATATCTAAAA TTTCAAGTCT AATTTTAGGG ATTTCTAGTT GTCTAACTTT ACGTATAAAG TATCACGTGT   
  
  
- ACCTGAACGA AACTTAGAGC AGATCTCTTG GATTCTTTTT CGTTCTCACC AAGTCTTCCA ATCTTAACAA   
  
  
- GGTCGTGTAC TGTTCATGTT AGGGTTGAAA AAAGTTTTCT AAACGATGAA AATTAACCTA AGGGTACAAC   
  
  
- TTACTTCAAG ACCCAAATTC CGATGTTGAA AGAACCCTCC TATTCCAAGA ATTCAATTCC ACCAGGAGAA   
  
  
- AAGAAAACTA GAAAGAATAC CCATGTTGGA GTACCCAGAG TAGTTGTGTA CTTGAGAAAA ATAGAAAATA   
  
  
- GAAAGGGGAA AGGAAATACG TCCTAACTGT CCACGTACCT TTTGTTCTTC GTTAATTTAT GATCACCGAA   
  
  
- TCGGGGTAGG GATTATAAAA CCGATAAGAC AAAGTGGTAG GAGGGGACAG AGGTAAAAGA AACAAAGGAA   
  
  
- AAAAAGATCC TTGAACACTA CCCGTTCCCT TCTAGTCATG TTACACTACC AACAATCATC GCTCTTCTAC   
  
  
- TTCACAGTGG CGTTTTTAAA ACACGGTATT AAACAGCATT CCCTTTGTTC ATAGTTTGTT GTTACAGTAA   
  
  
- ATACTATTTG TTCATTGTCC TCTATACATG AGACTTTTTT ATATTCCATG GACTCTTCAA CAATTGAATC   
  
  
- GAACTTTTTG GGAGGATTGA ACTATTTGGG AGAAGCGATA CAATAGGGCG TGGGACGGTA TCTCAGTGGC   
  
  
- TAAGTCATAT ATACATTGTA TGGGTTAGAA CAGACCTATA TATCAATTGG GAAAACCTTA TAGACTTTTA   
  
  
- GGTGTGCTCT CAGTAGGAAG ATCTGATCCT TTGTACGCGG TGTAAAACCT AAGATTGGTT CATATTATTC   
  
  
- GTATAGAATT TCGGTTTTTA CGTTCCTATC TATGTTTATG GGTAGTGTAC GTACATTTAC AGGGAACAGT   
  
  
- ACCGAGACTA AGAGTTTTAT AATTAAAAGT TATTTAATTT ACTTTAACGT AATAAAATTT CTAAAGACTT   
  
  
- ACGTAGGTAA TAACGTTAAA AAATTTCTGA CGTGTTACTA CTGTTACAGT TACCAAAGGA TTTCAACGCG   
  
  
- GACTACCCGA TAACTGTCAC TTCGTTGTGA GGGAAACAAG GGTAAATACA CAGAGGTGAA CGGGGAAACT   
  
  
- TTGAGAGATG TAAACATACA GTAGACTTAA ACTAAGAGTT TAGAGTCTAC TAAACTTAAG GGAAATACGG   
  
  
- TAGAAATGAT AGATAACTTA CTAAGAAAGA AAAGGTGAAA GAATAGAAGG TGATGGAAGA AGAACCTACG   
  
  
- GGAAAACGAC GAATATTTCC CGCTGTGGAC TTGTTCAACG ACATAAGAGT TTGGTACTTA GACATGTCCA   
  
  
- AATCGGGGCT ATACACAGGA TCGAAACACC CAGTGCTTCT TTAACCAGGA TTAAATTATC CTGTGGTTCT   
  
  
- GGGAAAAGTT TTACGTATGT TAACGTGACT AAAACCACTA CGAATGAACA ACGTTAAGGG TCAACTTCTA   
  
  
- AATACAGTTA TGGCACAAGG ACTAGATTAC CCCGATGGAC AACTTCTACC CCTAGGATGT CTCCAGGGAC   
  
  
- TGTAAGGACT ATGTACGAAG TTGATGTATT CGTTAGAGGA TTACCTTCTC CCGAGCCTAC TGGCGGGACC   
  
  
- GCGGTGGGAC GTCCTGATAT ACCGACGAGT TCAGAGGATG TCGTTCAGGA GGTTACGGCG GAAAGGGCCA   
  
  
- CTCTCAACGA GTAAGAAACC ATTATTAGTT ACGGGAGGTG GACGAATAGT TTCGTTGGGA ATAACGGAAC   
  
  
- TACTAATGAA GTTGAACGAT AGGTTATGAT GACCGTTATC ACAATTGTTA CGACCACAAG TACTGTCGTT   
  
  
- GTTGAAACAA GTCTTGTCAA ACTAACCCTT ATCCCCATGA TCAAGATCAA GATCCCCGTC TTCACTAAAA   
  
  
- CCTAGATGGG AATAATCCTT AAACGGTAAT CCCTGGCTGT GTGAACTACA CCAGTTAGGT CGTGGATGAG   
  
  
- GAGCTCGGGG AGACCAATGC TGAGTCACAG TCCACAAACT TTGTGTGGTC ATACTCGTCC GGAAGCTTGA   
  
  
- CTAGTGATTA CCTTCGACCC TTAAGTCAGT AAGACAACGA TTATGTAGTT TACTACCTTA AGTTTTAGTT   
  
  
- GGTTAAGTTC GTCAAAGTTG GGTTCTTGCT ACCTTATACT TTTGTAAAAG GCTACTGGTT GAACTCGGAC   
  
  
- GAGAAAACGC TTCAGAACAC TACAGGAGTT TGACATGACT ATAGAGACTA GTCCCACATG TGTCCTATCA   
  
  
- CTTGGGACTC GGTGGGTTGT TGTCTTTATG CCTCTAACAC ACGTTACTGA GTAGGTCATT CTCATTCTGT   
  
  
- TCTGCTTCTC TCGTACCACG TCCTCTTTTG GTGCTACTTC CTCTTTTGGT GCTACTCTGT CCTTCCTCAT   
  
  
- TGTTCGTTAG TCGTGGTAGG TTACTTCACA AGCTACTTTA ACTCGTCATA CCACTACACG ACGAGACGGG   
  
  
- ACTTCCTTCT TCATTTGGTC GTCCCAATAC GTCGTCGTCG TTGCGATTGT CTTGATCGTT GAGTGTCTTG   
  
  
- AGCCTTCTGA GTTTCAGTGT CGTTTTACCC TCAAGGAGGT TCCACAGTTT TTTCGCTCCG TTATTTTTCG   
  
  
- TTCACCAACT AAACTCCTGA GAGGATTGAG CTACACGTGT TACACAACGA GCACACCTAA ATTGACCAAG   
  
  
- AATGGTCAAC GAAGTCCTCT AAGCCGTCGT AAGAAGAGGG ATACCACTAA TAGAGGTTTC TTACCGAGTA   
  
  
- ATGGAACGAT TACCGGAACT CCGCGCGAAT CTTCCGTTTC CTATACTTGA TTGAGCACGG TTACTCGTAT   
  
  
- AGGGTGGATG ACTCTAGGAG TTCCGATTGG CCCATATACA CCGTCGCCAG GGAAAGTTCT ATTACAGAAT   
  
  
- AATGTGTTGT TTGTTCTGTT AACGTTCGGA TCAACTCCTC CGTGGTTCAT ATGTGTATTA ACTAAAACCA   
  
  
- TAAAAGATAC CTGATGTTAC CGGGACGTAG TAAGTCTTGG AGAGGTTCTC CGGGTTACCC GGAGGGTCTT   
  
  
- AGGCGTAGTG TCCGTAGCTA AAGGGGGTTG GACCCAAGGC CGGTCGTCTC TCCCAACTTC TCTGTCCCGC   
  
  
- CACAAATCGG TTCATAACAC TGTCCATGTT ACACGGGAAA CTTATGGTGG GGTATCGGTT CTTCACCCTC   
  
  
- TGGTAGGTTG AGCTCCTCGA GTTTTAACTT TCCTTACTCG GGGAACATCA CTTGACAGAC ATATCTAGCG   
  
  
- TGTTGGATAA ACTACTTTCG CATCTCCTTT TGTCGGGTTC TCTGCGCAAG GACTCGAACC AATCCTTTTA   
  
  
- TTTAGGGCTA TATAAGTACG TACCCCACCA CTTATGGCGT AGTTCACATG GAAAGAAGGA CTTGTCCAAG   
  
  
- TTCCTCCGGT ACAAAGTGAT GTGCCGGAAC AAACTGAACA AGCTCCGGTG TTACTCGGCG CTCCTAGTTC   
  
  
- TCTCCCACAA TGATCTCTCG TTCAACGTAC CCTTAGTCCG CAACTTACAC TAGCGGACGC TTCCTCGCCT   
  
  
- CTCTCACCTC TCCGGACTCA GTATGTTTGT TACCGTCCAA TCCTGAGTTT CCCGGCCCAA ACAAGTTCAC   
  
  
- AGCTACCTAG CGCTCGAAAA CTCTTCCCGT TCCCGGTACC AGTCCTCCTT AAAATCCCTT CTCAAGTCAA   
  
  
- TACTACTTCT ACCCGTAACC CACCAAGTTC CTACCTTTCC GTCCTATTAC ATACGGTAAA GAACAACCTT   
  
  
- TGGAAGTAT

+     GCN4\_motif

| Site Name | Organism | Position | Strand | Matrix score. | sequence | function |
| --- | --- | --- | --- | --- | --- | --- |
| GCN4\_motif | Oryza sativa | 4220 | + | 7 | TGAGTCA | cis-regulatory element involved in endosperm expression |
| GCN4\_motif | Oryza sativa | 2990 | - | 7 | TGAGTCA | cis-regulatory element involved in endosperm expression |

>HU02G01570.1   
+ +Up\_Stream \_Len000ATTATT ATTCTAATAT ATAGATAATA ATATAATAAT AATAATAATA ATAATAATAA   
  
  
+ TTATTATTAT TATTATTATT TATTAAATAT AATAGTTATT ATAATAATAA TAATTATAAT AATAATAAGA   
  
  
+ TTATTATCAT TATATTATTA TTATTATTAT TGTAATTTAT TATTATTATA GTAATTATTA TTGTTATATA   
  
  
+ GTAAAATTAT TATTAGTATT GTTATTATAA ATATTCATAC TTGCATATAA TATTTCTAAA ATAAAAACAG   
  
  
+ AGTTAATTCC ATTCCAAAAC TACTCACCAA ACAGCAAGGA GTGGGACCTG ATTCCTTTCC AATTTTCACC   
  
  
+ AAACAACTGG GTTCAGAATT CAAGTTCCAT ATCCAACCCC CCAGGATTCA AGTCCTGATT CCATTCCATT   
  
  
+ CCAGTTTCCC TCACCAAACT GGGCCTTAGA TTGATGGTTG TTCAGTTGGA TGAGGGTTGT TCCATGCCAA   
  
  
+ GTTAGATCAT TGTTGTCAAT TTTGAGAACG ACTATCTTAA TAAGCCTAAC GATGTTGATT TGATTTAGTT   
  
  
+ CAAACCATTT CAAATAATAC AAATCAAATT CTATTTAGAC CGACACTAAA ATAAATAGAT TGAGAAAAAT   
  
  
+ TATAGATTTT AAAGTTCAGA TTAAAATCCC TAAAGATCAA CAGATTGAAA TGCATATTTC ATAGTGCACA   
  
  
+ TGGACTTGCT TTGAATCTCG TCTAGAGAAC CTAAGAAAAA GCAAGAGTGG TTCAGAAGGT TAGAATTGTT   
  
  
+ CCAGCACATG ACAAGTACAA TCCCAACTTT TTTCAAAAGA TTTGCTACTT TTAATTGGAT TCCCATGTTG   
  
  
+ AATGAAGTTC TGGGTTTAAG GCTACAACTT TCTTGGGAGG ATAAGGTTCT TAAGTTAAGG TGGTCCTCTT   
  
  
+ TTCTTTTGAT CTTTCTTATG GGTACAACCT CATGGGTCTC ATCAACACAT GAACTCTTTT TATCTTTTAT   
  
  
+ CTTTCCCCTT TCCTTTATGC AGGATTGACA GGTGCATGGA AAACAAGAAG CAATTAAATA CTAGTGGCTT   
  
  
+ AGCCCCATCC CTAATATTTT GGCTATTCTG TTTCACCATC CTCCCCTGTC TCCATTTTCT TTGTTTCCTT   
  
  
+ TTTTTCTAGG AACTTGTGAT GGGCAAGGGA AGATCAGTAC AATGTGATGG TTGTTAGTAG CGAGAAGATG   
  
  
+ AAGTGTCACC GCAAAAATTT TGTGCCATAA TTTGTCGTAA GGGAAACAAG TATCAAACAA CAATGTCATT   
  
  
+ TATGATAAAC AAGTAACAGG AGATATGTAC TCTGAAAAAA TATAAGGTAC CTGAGAAGTT GTTAACTTAG   
  
  
+ CTTGAAAAAC CCTCCTAACT TGATAAACCC TCTTCGCTAT GTTATCCCGC ACCCTGCCAT AGAGTCACCG   
  
  
+ ATTCAGTATA TATGTAACAT ACCCAATCTT GTCTGGATAT ATAGTTAACC CTTTTGGAAT ATCTGAAAAT   
  
  
+ CCACACGAGA GTCATCCTTC TAGACTAGGA AACATGCGCC ACATTTTGGA TTCTAACCAA GTATAATAAG   
  
  
+ CATATCTTAA AGCCAAAAAT GCAAGGATAG ATACAAATAC CCATCACATG CATGTAAATG TCCCTTGTCA   
  
  
+ TGGCTCTGAT TCTCAAAATA TTAATTTTCA ATAAATTAAA TGAAATTGCA TTATTTTAAA GATTTCTGAA   
  
  
+ TGCATCCATT ATTGCAATTT TTTAAAGACT GCACAATGAT GACAATGTCA ATGGTTTCCT AAAGTTGCGC   
  
  
+ CTGATGGGCT ATTGACAGTG AAGCAACACT CCCTTTGTTC CCATTTATGT GTCTCCACTT GCCCCTTTGA   
  
  
+ AACTCTCTAC ATTTGTATGT CATCTGAATT TGATTCTCAA ATCTCAGATG ATTTGAATTC CCTTTATGCC   
  
  
+ ATCTTTACTA TCTATTGAAT GATTCTTTCT TTTCCACTTT CTTATCTTCC ACTACCTTCT TCTTGGATGC   
  
  
+ CCTTTTGCTG CTTATAAAGG GCGACACCTG AACAAGTTGC TGTATTCTCA AACCATGAAT CTGTACAGGT   
  
  
+ TTAGCCCCGA TATGTGTCCT AGCTTTGTGG GTCACGAAGA AATTGGTCCT AATTTAATAG GACACCAAGA   
  
  
+ CCCTTTTCAA AATGCATACA ATTGCACTGA TTTTGGTGAT GCTTACTTGT TGCAATTCCC AGTTGAAGAT   
  
  
+ TTATGTCAAT ACCGTGTTCC TGATCTAATG GGGCTACCTG TTGAAGATGG GGATCCTACA GAGGTCCCTG   
  
  
+ ACATTCCTGA TACATGCTTC AACTACATAA GCAATCTCCT AATGGAAGAG GGCTCGGATG ACCGCCCTGG   
  
  
+ CGCCACCCTG CAGGACTATA TGGCTGCTCA AGTCTCCTAC AGCAAGTCCT CCAATGCCGC CTTTCCCGGT   
  
  
+ GAGAGTTGCT CATTCTTTGG TAATAATCAA TGCCCTCCAC CTGCTTATCA AAGCAACCCT TATTGCCTTG   
  
  
+ ATGATTACTT CAACTTGCTA TCCAATACTA CTGGCAATAG TGTTAACAAT GCTGGTGTTC ATGACAGCAA   
  
  
+ CAACTTTGTT CAGAACAGTT TGATTGGGAA TAGGGGTACT AGTTCTAGTT CTAGGGGCAG AAGTGATTTT   
  
  
+ GGATCTACCC TTATTAGGAA TTTGCCATTA GGGACCGACA CACTTGATGT GGTCAATCCA GCACCTACTC   
  
  
+ CTCGAGCCCC TCTGGTTACG ACTCAGTGTC AGGTGTTTGA AACACACCAG TATGAGCAGG CCTTCGAACT   
  
  
+ GATCACTAAT GGAAGCTGGG AATTCAGTCA TTCTGTTGCT AATACATCAA ATGATGGAAT TCAAAATCAA   
  
  
+ CCAATTCAAG CAGTTTCAAC CCAAGAACGA TGGAATATGA AAACATTTTC CGATGACCAA CTTGAGCCTG   
  
  
+ CTCTTTTGCG AAGTCTTGTG ATGTCCTCAA ACTGTACTGA TATCTCTGAT CAGGGTGTAC ACAGGATAGT   
  
  
+ GAACCCTGAG CCACCCAACA ACAGAAATAC GGAGATTGTG TGCAATGACT CATCCAGTAA GAGTAAGACA   
  
  
+ AGACGAAGAG AGCATGGTGC AGGAGAAAAC CACGATGAAG GAGAAAACCA CGATGAGACA GGAAGGAGTA   
  
  
+ ACAAGCAATC AGCACCATCC AATGAAGTGT TCGATGAAAT TGAGCAGTAT GGTGATGTGC TGCTCTGCCC   
  
  
+ TGAAGGAAGA AGTAAACCAG CAGGGTTATG CAGCAGCAGC AACGCTAACA GAACTAGCAA CTCACAGAAC   
  
  
+ TCGGAAGACT CAAAGTCACA GCAAAATGGG AGTTCCTCCA AGGTGTCAAA AAAGCGAGGC AATAAAAAGC   
  
  
+ AAGTGGTTGA TTTGAGGACT CTCCTAACTC GATGTGCACA ATGTGTTGCT CGTGTGGATT TAACTGGTTC   
  
  
+ TTACCAGTTG CTTCAGGAGA TTCGGCAGCA TTCTTCTCCC TATGGTGATT ATCTCCAAAG AATGGCTCAT   
  
  
+ TACCTTGCTA ATGGCCTTGA GGCGCGCTTA GAAGGCAAAG GATATGAACT AACTCGTGCC AATGAGCATA   
  
  
+ TCCCACCTAC TGAGATCCTC AAGGCTAACC GGGTATATGT GGCAGCGGTC CCTTTCAAGA TAATGTCTTA   
  
  
+ TTACACAACA AACAAGACAA TTGCAAGCCT AGTTGAGGAG GCACCAAGTA TACACATAAT TGATTTTGGT   
  
  
+ ATTTTCTATG GACTACAATG GCCCTGCATC ATTCAGAACC TCTCCAAGAG GCCCAATGGG CCTCCCAGAA   
  
  
+ TCCGCATCAC AGGCATCGAT TTCCCCCAAC CTGGGTTCCG GCCAGCAGAG AGGGTTGAAG AGACAGGGCG   
  
  
+ GTGTTTAGCC AAGTATTGTG ACAGGTACAA TGTGCCCTTT GAATACCACC CCATAGCCAA GAAGTGGGAG   
  
  
+ ACCATCCAAC TCGAGGAGCT CAAAATTGAA AGGAATGAGC CCCTTGTAGT GAACTGTCTG TATAGATCGC   
  
  
+ ACAACCTATT TGATGAAAGC GTAGAGGAAA ACAGCCCAAG AGACGCGTTC CTGAGCTTGG TTAGGAAAAT   
  
  
+ AAATCCCGAT ATATTCATGC ATGGGGTGGT GAATACCGCA TCAAGTGTAC CTTTCTTCCT GAACAGGTTC   
  
  
+ AAGGAGGCCA TGTTTCACTA CACGGCCTTG TTTGACTTGT TCGAGGCCAC AATGAGCCGC GAGGATCAAG   
  
  
+ AGAGGGTGTT ACTAGAGAGC AAGTTGCATG GGAATCAGGC GTTGAATGTG ATCGCCTGCG AAGGAGCGGA   
  
  
+ GAGAGTGGAG AGGCCTGAGT CATACAAACA ATGGCAGGTT AGGACTCAAA GGGCCGGGTT TGTTCAAGTG   
  
  
+ TCGATGGATC GCGAGCTTTT GAGAAGGGCA AGGGCCATGG TCAGGAGGAA TTTTAGGGAA GAGTTCAGTT   
  
  
+ ATGATGAAGA TGGGCATTGG GTGGTTCAAG GATGGAAAGG CAGGATAATG TATGCCATTT CTTGTTGGAA   
  
  
+ ACCTTCATA  

- +Up\_Stream \_Len000TAATAA TAAGATTATA TATCTATTAT TATATTATTA TTATTATTAT TATTATTATT   
  
  
- AATAATAATA ATAATAATAA ATAATTTATA TTATCAATAA TATTATTATT ATTAATATTA TTATTATTCT   
  
  
- AATAATAGTA ATATAATAAT AATAATAATA ACATTAAATA ATAATAATAT CATTAATAAT AACAATATAT   
  
  
- CATTTTAATA ATAATCATAA CAATAATATT TATAAGTATG AACGTATATT ATAAAGATTT TATTTTTGTC   
  
  
- TCAATTAAGG TAAGGTTTTG ATGAGTGGTT TGTCGTTCCT CACCCTGGAC TAAGGAAAGG TTAAAAGTGG   
  
  
- TTTGTTGACC CAAGTCTTAA GTTCAAGGTA TAGGTTGGGG GGTCCTAAGT TCAGGACTAA GGTAAGGTAA   
  
  
- GGTCAAAGGG AGTGGTTTGA CCCGGAATCT AACTACCAAC AAGTCAACCT ACTCCCAACA AGGTACGGTT   
  
  
- CAATCTAGTA ACAACAGTTA AAACTCTTGC TGATAGAATT ATTCGGATTG CTACAACTAA ACTAAATCAA   
  
  
- GTTTGGTAAA GTTTATTATG TTTAGTTTAA GATAAATCTG GCTGTGATTT TATTTATCTA ACTCTTTTTA   
  
  
- ATATCTAAAA TTTCAAGTCT AATTTTAGGG ATTTCTAGTT GTCTAACTTT ACGTATAAAG TATCACGTGT   
  
  
- ACCTGAACGA AACTTAGAGC AGATCTCTTG GATTCTTTTT CGTTCTCACC AAGTCTTCCA ATCTTAACAA   
  
  
- GGTCGTGTAC TGTTCATGTT AGGGTTGAAA AAAGTTTTCT AAACGATGAA AATTAACCTA AGGGTACAAC   
  
  
- TTACTTCAAG ACCCAAATTC CGATGTTGAA AGAACCCTCC TATTCCAAGA ATTCAATTCC ACCAGGAGAA   
  
  
- AAGAAAACTA GAAAGAATAC CCATGTTGGA GTACCCAGAG TAGTTGTGTA CTTGAGAAAA ATAGAAAATA   
  
  
- GAAAGGGGAA AGGAAATACG TCCTAACTGT CCACGTACCT TTTGTTCTTC GTTAATTTAT GATCACCGAA   
  
  
- TCGGGGTAGG GATTATAAAA CCGATAAGAC AAAGTGGTAG GAGGGGACAG AGGTAAAAGA AACAAAGGAA   
  
  
- AAAAAGATCC TTGAACACTA CCCGTTCCCT TCTAGTCATG TTACACTACC AACAATCATC GCTCTTCTAC   
  
  
- TTCACAGTGG CGTTTTTAAA ACACGGTATT AAACAGCATT CCCTTTGTTC ATAGTTTGTT GTTACAGTAA   
  
  
- ATACTATTTG TTCATTGTCC TCTATACATG AGACTTTTTT ATATTCCATG GACTCTTCAA CAATTGAATC   
  
  
- GAACTTTTTG GGAGGATTGA ACTATTTGGG AGAAGCGATA CAATAGGGCG TGGGACGGTA TCTCAGTGGC   
  
  
- TAAGTCATAT ATACATTGTA TGGGTTAGAA CAGACCTATA TATCAATTGG GAAAACCTTA TAGACTTTTA   
  
  
- GGTGTGCTCT CAGTAGGAAG ATCTGATCCT TTGTACGCGG TGTAAAACCT AAGATTGGTT CATATTATTC   
  
  
- GTATAGAATT TCGGTTTTTA CGTTCCTATC TATGTTTATG GGTAGTGTAC GTACATTTAC AGGGAACAGT   
  
  
- ACCGAGACTA AGAGTTTTAT AATTAAAAGT TATTTAATTT ACTTTAACGT AATAAAATTT CTAAAGACTT   
  
  
- ACGTAGGTAA TAACGTTAAA AAATTTCTGA CGTGTTACTA CTGTTACAGT TACCAAAGGA TTTCAACGCG   
  
  
- GACTACCCGA TAACTGTCAC TTCGTTGTGA GGGAAACAAG GGTAAATACA CAGAGGTGAA CGGGGAAACT   
  
  
- TTGAGAGATG TAAACATACA GTAGACTTAA ACTAAGAGTT TAGAGTCTAC TAAACTTAAG GGAAATACGG   
  
  
- TAGAAATGAT AGATAACTTA CTAAGAAAGA AAAGGTGAAA GAATAGAAGG TGATGGAAGA AGAACCTACG   
  
  
- GGAAAACGAC GAATATTTCC CGCTGTGGAC TTGTTCAACG ACATAAGAGT TTGGTACTTA GACATGTCCA   
  
  
- AATCGGGGCT ATACACAGGA TCGAAACACC CAGTGCTTCT TTAACCAGGA TTAAATTATC CTGTGGTTCT   
  
  
- GGGAAAAGTT TTACGTATGT TAACGTGACT AAAACCACTA CGAATGAACA ACGTTAAGGG TCAACTTCTA   
  
  
- AATACAGTTA TGGCACAAGG ACTAGATTAC CCCGATGGAC AACTTCTACC CCTAGGATGT CTCCAGGGAC   
  
  
- TGTAAGGACT ATGTACGAAG TTGATGTATT CGTTAGAGGA TTACCTTCTC CCGAGCCTAC TGGCGGGACC   
  
  
- GCGGTGGGAC GTCCTGATAT ACCGACGAGT TCAGAGGATG TCGTTCAGGA GGTTACGGCG GAAAGGGCCA   
  
  
- CTCTCAACGA GTAAGAAACC ATTATTAGTT ACGGGAGGTG GACGAATAGT TTCGTTGGGA ATAACGGAAC   
  
  
- TACTAATGAA GTTGAACGAT AGGTTATGAT GACCGTTATC ACAATTGTTA CGACCACAAG TACTGTCGTT   
  
  
- GTTGAAACAA GTCTTGTCAA ACTAACCCTT ATCCCCATGA TCAAGATCAA GATCCCCGTC TTCACTAAAA   
  
  
- CCTAGATGGG AATAATCCTT AAACGGTAAT CCCTGGCTGT GTGAACTACA CCAGTTAGGT CGTGGATGAG   
  
  
- GAGCTCGGGG AGACCAATGC TGAGTCACAG TCCACAAACT TTGTGTGGTC ATACTCGTCC GGAAGCTTGA   
  
  
- CTAGTGATTA CCTTCGACCC TTAAGTCAGT AAGACAACGA TTATGTAGTT TACTACCTTA AGTTTTAGTT   
  
  
- GGTTAAGTTC GTCAAAGTTG GGTTCTTGCT ACCTTATACT TTTGTAAAAG GCTACTGGTT GAACTCGGAC   
  
  
- GAGAAAACGC TTCAGAACAC TACAGGAGTT TGACATGACT ATAGAGACTA GTCCCACATG TGTCCTATCA   
  
  
- CTTGGGACTC GGTGGGTTGT TGTCTTTATG CCTCTAACAC ACGTTACTGA GTAGGTCATT CTCATTCTGT   
  
  
- TCTGCTTCTC TCGTACCACG TCCTCTTTTG GTGCTACTTC CTCTTTTGGT GCTACTCTGT CCTTCCTCAT   
  
  
- TGTTCGTTAG TCGTGGTAGG TTACTTCACA AGCTACTTTA ACTCGTCATA CCACTACACG ACGAGACGGG   
  
  
- ACTTCCTTCT TCATTTGGTC GTCCCAATAC GTCGTCGTCG TTGCGATTGT CTTGATCGTT GAGTGTCTTG   
  
  
- AGCCTTCTGA GTTTCAGTGT CGTTTTACCC TCAAGGAGGT TCCACAGTTT TTTCGCTCCG TTATTTTTCG   
  
  
- TTCACCAACT AAACTCCTGA GAGGATTGAG CTACACGTGT TACACAACGA GCACACCTAA ATTGACCAAG   
  
  
- AATGGTCAAC GAAGTCCTCT AAGCCGTCGT AAGAAGAGGG ATACCACTAA TAGAGGTTTC TTACCGAGTA   
  
  
- ATGGAACGAT TACCGGAACT CCGCGCGAAT CTTCCGTTTC CTATACTTGA TTGAGCACGG TTACTCGTAT   
  
  
- AGGGTGGATG ACTCTAGGAG TTCCGATTGG CCCATATACA CCGTCGCCAG GGAAAGTTCT ATTACAGAAT   
  
  
- AATGTGTTGT TTGTTCTGTT AACGTTCGGA TCAACTCCTC CGTGGTTCAT ATGTGTATTA ACTAAAACCA   
  
  
- TAAAAGATAC CTGATGTTAC CGGGACGTAG TAAGTCTTGG AGAGGTTCTC CGGGTTACCC GGAGGGTCTT   
  
  
- AGGCGTAGTG TCCGTAGCTA AAGGGGGTTG GACCCAAGGC CGGTCGTCTC TCCCAACTTC TCTGTCCCGC   
  
  
- CACAAATCGG TTCATAACAC TGTCCATGTT ACACGGGAAA CTTATGGTGG GGTATCGGTT CTTCACCCTC   
  
  
- TGGTAGGTTG AGCTCCTCGA GTTTTAACTT TCCTTACTCG GGGAACATCA CTTGACAGAC ATATCTAGCG   
  
  
- TGTTGGATAA ACTACTTTCG CATCTCCTTT TGTCGGGTTC TCTGCGCAAG GACTCGAACC AATCCTTTTA   
  
  
- TTTAGGGCTA TATAAGTACG TACCCCACCA CTTATGGCGT AGTTCACATG GAAAGAAGGA CTTGTCCAAG   
  
  
- TTCCTCCGGT ACAAAGTGAT GTGCCGGAAC AAACTGAACA AGCTCCGGTG TTACTCGGCG CTCCTAGTTC   
  
  
- TCTCCCACAA TGATCTCTCG TTCAACGTAC CCTTAGTCCG CAACTTACAC TAGCGGACGC TTCCTCGCCT   
  
  
- CTCTCACCTC TCCGGACTCA GTATGTTTGT TACCGTCCAA TCCTGAGTTT CCCGGCCCAA ACAAGTTCAC   
  
  
- AGCTACCTAG CGCTCGAAAA CTCTTCCCGT TCCCGGTACC AGTCCTCCTT AAAATCCCTT CTCAAGTCAA   
  
  
- TACTACTTCT ACCCGTAACC CACCAAGTTC CTACCTTTCC GTCCTATTAC ATACGGTAAA GAACAACCTT   
  
  
- TGGAAGTAT

+     GT1-motif

| Site Name | Organism | Position | Strand | Matrix score. | sequence | function |
| --- | --- | --- | --- | --- | --- | --- |
| GT1-motif | Arabidopsis thaliana | 1449 | - | 6 | GGTTAA | light responsive element |

>HU02G01570.1   
+ +Up\_Stream \_Len000ATTATT ATTCTAATAT ATAGATAATA ATATAATAAT AATAATAATA ATAATAATAA   
  
  
+ TTATTATTAT TATTATTATT TATTAAATAT AATAGTTATT ATAATAATAA TAATTATAAT AATAATAAGA   
  
  
+ TTATTATCAT TATATTATTA TTATTATTAT TGTAATTTAT TATTATTATA GTAATTATTA TTGTTATATA   
  
  
+ GTAAAATTAT TATTAGTATT GTTATTATAA ATATTCATAC TTGCATATAA TATTTCTAAA ATAAAAACAG   
  
  
+ AGTTAATTCC ATTCCAAAAC TACTCACCAA ACAGCAAGGA GTGGGACCTG ATTCCTTTCC AATTTTCACC   
  
  
+ AAACAACTGG GTTCAGAATT CAAGTTCCAT ATCCAACCCC CCAGGATTCA AGTCCTGATT CCATTCCATT   
  
  
+ CCAGTTTCCC TCACCAAACT GGGCCTTAGA TTGATGGTTG TTCAGTTGGA TGAGGGTTGT TCCATGCCAA   
  
  
+ GTTAGATCAT TGTTGTCAAT TTTGAGAACG ACTATCTTAA TAAGCCTAAC GATGTTGATT TGATTTAGTT   
  
  
+ CAAACCATTT CAAATAATAC AAATCAAATT CTATTTAGAC CGACACTAAA ATAAATAGAT TGAGAAAAAT   
  
  
+ TATAGATTTT AAAGTTCAGA TTAAAATCCC TAAAGATCAA CAGATTGAAA TGCATATTTC ATAGTGCACA   
  
  
+ TGGACTTGCT TTGAATCTCG TCTAGAGAAC CTAAGAAAAA GCAAGAGTGG TTCAGAAGGT TAGAATTGTT   
  
  
+ CCAGCACATG ACAAGTACAA TCCCAACTTT TTTCAAAAGA TTTGCTACTT TTAATTGGAT TCCCATGTTG   
  
  
+ AATGAAGTTC TGGGTTTAAG GCTACAACTT TCTTGGGAGG ATAAGGTTCT TAAGTTAAGG TGGTCCTCTT   
  
  
+ TTCTTTTGAT CTTTCTTATG GGTACAACCT CATGGGTCTC ATCAACACAT GAACTCTTTT TATCTTTTAT   
  
  
+ CTTTCCCCTT TCCTTTATGC AGGATTGACA GGTGCATGGA AAACAAGAAG CAATTAAATA CTAGTGGCTT   
  
  
+ AGCCCCATCC CTAATATTTT GGCTATTCTG TTTCACCATC CTCCCCTGTC TCCATTTTCT TTGTTTCCTT   
  
  
+ TTTTTCTAGG AACTTGTGAT GGGCAAGGGA AGATCAGTAC AATGTGATGG TTGTTAGTAG CGAGAAGATG   
  
  
+ AAGTGTCACC GCAAAAATTT TGTGCCATAA TTTGTCGTAA GGGAAACAAG TATCAAACAA CAATGTCATT   
  
  
+ TATGATAAAC AAGTAACAGG AGATATGTAC TCTGAAAAAA TATAAGGTAC CTGAGAAGTT GTTAACTTAG   
  
  
+ CTTGAAAAAC CCTCCTAACT TGATAAACCC TCTTCGCTAT GTTATCCCGC ACCCTGCCAT AGAGTCACCG   
  
  
+ ATTCAGTATA TATGTAACAT ACCCAATCTT GTCTGGATAT ATAGTTAACC CTTTTGGAAT ATCTGAAAAT   
  
  
+ CCACACGAGA GTCATCCTTC TAGACTAGGA AACATGCGCC ACATTTTGGA TTCTAACCAA GTATAATAAG   
  
  
+ CATATCTTAA AGCCAAAAAT GCAAGGATAG ATACAAATAC CCATCACATG CATGTAAATG TCCCTTGTCA   
  
  
+ TGGCTCTGAT TCTCAAAATA TTAATTTTCA ATAAATTAAA TGAAATTGCA TTATTTTAAA GATTTCTGAA   
  
  
+ TGCATCCATT ATTGCAATTT TTTAAAGACT GCACAATGAT GACAATGTCA ATGGTTTCCT AAAGTTGCGC   
  
  
+ CTGATGGGCT ATTGACAGTG AAGCAACACT CCCTTTGTTC CCATTTATGT GTCTCCACTT GCCCCTTTGA   
  
  
+ AACTCTCTAC ATTTGTATGT CATCTGAATT TGATTCTCAA ATCTCAGATG ATTTGAATTC CCTTTATGCC   
  
  
+ ATCTTTACTA TCTATTGAAT GATTCTTTCT TTTCCACTTT CTTATCTTCC ACTACCTTCT TCTTGGATGC   
  
  
+ CCTTTTGCTG CTTATAAAGG GCGACACCTG AACAAGTTGC TGTATTCTCA AACCATGAAT CTGTACAGGT   
  
  
+ TTAGCCCCGA TATGTGTCCT AGCTTTGTGG GTCACGAAGA AATTGGTCCT AATTTAATAG GACACCAAGA   
  
  
+ CCCTTTTCAA AATGCATACA ATTGCACTGA TTTTGGTGAT GCTTACTTGT TGCAATTCCC AGTTGAAGAT   
  
  
+ TTATGTCAAT ACCGTGTTCC TGATCTAATG GGGCTACCTG TTGAAGATGG GGATCCTACA GAGGTCCCTG   
  
  
+ ACATTCCTGA TACATGCTTC AACTACATAA GCAATCTCCT AATGGAAGAG GGCTCGGATG ACCGCCCTGG   
  
  
+ CGCCACCCTG CAGGACTATA TGGCTGCTCA AGTCTCCTAC AGCAAGTCCT CCAATGCCGC CTTTCCCGGT   
  
  
+ GAGAGTTGCT CATTCTTTGG TAATAATCAA TGCCCTCCAC CTGCTTATCA AAGCAACCCT TATTGCCTTG   
  
  
+ ATGATTACTT CAACTTGCTA TCCAATACTA CTGGCAATAG TGTTAACAAT GCTGGTGTTC ATGACAGCAA   
  
  
+ CAACTTTGTT CAGAACAGTT TGATTGGGAA TAGGGGTACT AGTTCTAGTT CTAGGGGCAG AAGTGATTTT   
  
  
+ GGATCTACCC TTATTAGGAA TTTGCCATTA GGGACCGACA CACTTGATGT GGTCAATCCA GCACCTACTC   
  
  
+ CTCGAGCCCC TCTGGTTACG ACTCAGTGTC AGGTGTTTGA AACACACCAG TATGAGCAGG CCTTCGAACT   
  
  
+ GATCACTAAT GGAAGCTGGG AATTCAGTCA TTCTGTTGCT AATACATCAA ATGATGGAAT TCAAAATCAA   
  
  
+ CCAATTCAAG CAGTTTCAAC CCAAGAACGA TGGAATATGA AAACATTTTC CGATGACCAA CTTGAGCCTG   
  
  
+ CTCTTTTGCG AAGTCTTGTG ATGTCCTCAA ACTGTACTGA TATCTCTGAT CAGGGTGTAC ACAGGATAGT   
  
  
+ GAACCCTGAG CCACCCAACA ACAGAAATAC GGAGATTGTG TGCAATGACT CATCCAGTAA GAGTAAGACA   
  
  
+ AGACGAAGAG AGCATGGTGC AGGAGAAAAC CACGATGAAG GAGAAAACCA CGATGAGACA GGAAGGAGTA   
  
  
+ ACAAGCAATC AGCACCATCC AATGAAGTGT TCGATGAAAT TGAGCAGTAT GGTGATGTGC TGCTCTGCCC   
  
  
+ TGAAGGAAGA AGTAAACCAG CAGGGTTATG CAGCAGCAGC AACGCTAACA GAACTAGCAA CTCACAGAAC   
  
  
+ TCGGAAGACT CAAAGTCACA GCAAAATGGG AGTTCCTCCA AGGTGTCAAA AAAGCGAGGC AATAAAAAGC   
  
  
+ AAGTGGTTGA TTTGAGGACT CTCCTAACTC GATGTGCACA ATGTGTTGCT CGTGTGGATT TAACTGGTTC   
  
  
+ TTACCAGTTG CTTCAGGAGA TTCGGCAGCA TTCTTCTCCC TATGGTGATT ATCTCCAAAG AATGGCTCAT   
  
  
+ TACCTTGCTA ATGGCCTTGA GGCGCGCTTA GAAGGCAAAG GATATGAACT AACTCGTGCC AATGAGCATA   
  
  
+ TCCCACCTAC TGAGATCCTC AAGGCTAACC GGGTATATGT GGCAGCGGTC CCTTTCAAGA TAATGTCTTA   
  
  
+ TTACACAACA AACAAGACAA TTGCAAGCCT AGTTGAGGAG GCACCAAGTA TACACATAAT TGATTTTGGT   
  
  
+ ATTTTCTATG GACTACAATG GCCCTGCATC ATTCAGAACC TCTCCAAGAG GCCCAATGGG CCTCCCAGAA   
  
  
+ TCCGCATCAC AGGCATCGAT TTCCCCCAAC CTGGGTTCCG GCCAGCAGAG AGGGTTGAAG AGACAGGGCG   
  
  
+ GTGTTTAGCC AAGTATTGTG ACAGGTACAA TGTGCCCTTT GAATACCACC CCATAGCCAA GAAGTGGGAG   
  
  
+ ACCATCCAAC TCGAGGAGCT CAAAATTGAA AGGAATGAGC CCCTTGTAGT GAACTGTCTG TATAGATCGC   
  
  
+ ACAACCTATT TGATGAAAGC GTAGAGGAAA ACAGCCCAAG AGACGCGTTC CTGAGCTTGG TTAGGAAAAT   
  
  
+ AAATCCCGAT ATATTCATGC ATGGGGTGGT GAATACCGCA TCAAGTGTAC CTTTCTTCCT GAACAGGTTC   
  
  
+ AAGGAGGCCA TGTTTCACTA CACGGCCTTG TTTGACTTGT TCGAGGCCAC AATGAGCCGC GAGGATCAAG   
  
  
+ AGAGGGTGTT ACTAGAGAGC AAGTTGCATG GGAATCAGGC GTTGAATGTG ATCGCCTGCG AAGGAGCGGA   
  
  
+ GAGAGTGGAG AGGCCTGAGT CATACAAACA ATGGCAGGTT AGGACTCAAA GGGCCGGGTT TGTTCAAGTG   
  
  
+ TCGATGGATC GCGAGCTTTT GAGAAGGGCA AGGGCCATGG TCAGGAGGAA TTTTAGGGAA GAGTTCAGTT   
  
  
+ ATGATGAAGA TGGGCATTGG GTGGTTCAAG GATGGAAAGG CAGGATAATG TATGCCATTT CTTGTTGGAA   
  
  
+ ACCTTCATA  

- +Up\_Stream \_Len000TAATAA TAAGATTATA TATCTATTAT TATATTATTA TTATTATTAT TATTATTATT   
  
  
- AATAATAATA ATAATAATAA ATAATTTATA TTATCAATAA TATTATTATT ATTAATATTA TTATTATTCT   
  
  
- AATAATAGTA ATATAATAAT AATAATAATA ACATTAAATA ATAATAATAT CATTAATAAT AACAATATAT   
  
  
- CATTTTAATA ATAATCATAA CAATAATATT TATAAGTATG AACGTATATT ATAAAGATTT TATTTTTGTC   
  
  
- TCAATTAAGG TAAGGTTTTG ATGAGTGGTT TGTCGTTCCT CACCCTGGAC TAAGGAAAGG TTAAAAGTGG   
  
  
- TTTGTTGACC CAAGTCTTAA GTTCAAGGTA TAGGTTGGGG GGTCCTAAGT TCAGGACTAA GGTAAGGTAA   
  
  
- GGTCAAAGGG AGTGGTTTGA CCCGGAATCT AACTACCAAC AAGTCAACCT ACTCCCAACA AGGTACGGTT   
  
  
- CAATCTAGTA ACAACAGTTA AAACTCTTGC TGATAGAATT ATTCGGATTG CTACAACTAA ACTAAATCAA   
  
  
- GTTTGGTAAA GTTTATTATG TTTAGTTTAA GATAAATCTG GCTGTGATTT TATTTATCTA ACTCTTTTTA   
  
  
- ATATCTAAAA TTTCAAGTCT AATTTTAGGG ATTTCTAGTT GTCTAACTTT ACGTATAAAG TATCACGTGT   
  
  
- ACCTGAACGA AACTTAGAGC AGATCTCTTG GATTCTTTTT CGTTCTCACC AAGTCTTCCA ATCTTAACAA   
  
  
- GGTCGTGTAC TGTTCATGTT AGGGTTGAAA AAAGTTTTCT AAACGATGAA AATTAACCTA AGGGTACAAC   
  
  
- TTACTTCAAG ACCCAAATTC CGATGTTGAA AGAACCCTCC TATTCCAAGA ATTCAATTCC ACCAGGAGAA   
  
  
- AAGAAAACTA GAAAGAATAC CCATGTTGGA GTACCCAGAG TAGTTGTGTA CTTGAGAAAA ATAGAAAATA   
  
  
- GAAAGGGGAA AGGAAATACG TCCTAACTGT CCACGTACCT TTTGTTCTTC GTTAATTTAT GATCACCGAA   
  
  
- TCGGGGTAGG GATTATAAAA CCGATAAGAC AAAGTGGTAG GAGGGGACAG AGGTAAAAGA AACAAAGGAA   
  
  
- AAAAAGATCC TTGAACACTA CCCGTTCCCT TCTAGTCATG TTACACTACC AACAATCATC GCTCTTCTAC   
  
  
- TTCACAGTGG CGTTTTTAAA ACACGGTATT AAACAGCATT CCCTTTGTTC ATAGTTTGTT GTTACAGTAA   
  
  
- ATACTATTTG TTCATTGTCC TCTATACATG AGACTTTTTT ATATTCCATG GACTCTTCAA CAATTGAATC   
  
  
- GAACTTTTTG GGAGGATTGA ACTATTTGGG AGAAGCGATA CAATAGGGCG TGGGACGGTA TCTCAGTGGC   
  
  
- TAAGTCATAT ATACATTGTA TGGGTTAGAA CAGACCTATA TATCAATTGG GAAAACCTTA TAGACTTTTA   
  
  
- GGTGTGCTCT CAGTAGGAAG ATCTGATCCT TTGTACGCGG TGTAAAACCT AAGATTGGTT CATATTATTC   
  
  
- GTATAGAATT TCGGTTTTTA CGTTCCTATC TATGTTTATG GGTAGTGTAC GTACATTTAC AGGGAACAGT   
  
  
- ACCGAGACTA AGAGTTTTAT AATTAAAAGT TATTTAATTT ACTTTAACGT AATAAAATTT CTAAAGACTT   
  
  
- ACGTAGGTAA TAACGTTAAA AAATTTCTGA CGTGTTACTA CTGTTACAGT TACCAAAGGA TTTCAACGCG   
  
  
- GACTACCCGA TAACTGTCAC TTCGTTGTGA GGGAAACAAG GGTAAATACA CAGAGGTGAA CGGGGAAACT   
  
  
- TTGAGAGATG TAAACATACA GTAGACTTAA ACTAAGAGTT TAGAGTCTAC TAAACTTAAG GGAAATACGG   
  
  
- TAGAAATGAT AGATAACTTA CTAAGAAAGA AAAGGTGAAA GAATAGAAGG TGATGGAAGA AGAACCTACG   
  
  
- GGAAAACGAC GAATATTTCC CGCTGTGGAC TTGTTCAACG ACATAAGAGT TTGGTACTTA GACATGTCCA   
  
  
- AATCGGGGCT ATACACAGGA TCGAAACACC CAGTGCTTCT TTAACCAGGA TTAAATTATC CTGTGGTTCT   
  
  
- GGGAAAAGTT TTACGTATGT TAACGTGACT AAAACCACTA CGAATGAACA ACGTTAAGGG TCAACTTCTA   
  
  
- AATACAGTTA TGGCACAAGG ACTAGATTAC CCCGATGGAC AACTTCTACC CCTAGGATGT CTCCAGGGAC   
  
  
- TGTAAGGACT ATGTACGAAG TTGATGTATT CGTTAGAGGA TTACCTTCTC CCGAGCCTAC TGGCGGGACC   
  
  
- GCGGTGGGAC GTCCTGATAT ACCGACGAGT TCAGAGGATG TCGTTCAGGA GGTTACGGCG GAAAGGGCCA   
  
  
- CTCTCAACGA GTAAGAAACC ATTATTAGTT ACGGGAGGTG GACGAATAGT TTCGTTGGGA ATAACGGAAC   
  
  
- TACTAATGAA GTTGAACGAT AGGTTATGAT GACCGTTATC ACAATTGTTA CGACCACAAG TACTGTCGTT   
  
  
- GTTGAAACAA GTCTTGTCAA ACTAACCCTT ATCCCCATGA TCAAGATCAA GATCCCCGTC TTCACTAAAA   
  
  
- CCTAGATGGG AATAATCCTT AAACGGTAAT CCCTGGCTGT GTGAACTACA CCAGTTAGGT CGTGGATGAG   
  
  
- GAGCTCGGGG AGACCAATGC TGAGTCACAG TCCACAAACT TTGTGTGGTC ATACTCGTCC GGAAGCTTGA   
  
  
- CTAGTGATTA CCTTCGACCC TTAAGTCAGT AAGACAACGA TTATGTAGTT TACTACCTTA AGTTTTAGTT   
  
  
- GGTTAAGTTC GTCAAAGTTG GGTTCTTGCT ACCTTATACT TTTGTAAAAG GCTACTGGTT GAACTCGGAC   
  
  
- GAGAAAACGC TTCAGAACAC TACAGGAGTT TGACATGACT ATAGAGACTA GTCCCACATG TGTCCTATCA   
  
  
- CTTGGGACTC GGTGGGTTGT TGTCTTTATG CCTCTAACAC ACGTTACTGA GTAGGTCATT CTCATTCTGT   
  
  
- TCTGCTTCTC TCGTACCACG TCCTCTTTTG GTGCTACTTC CTCTTTTGGT GCTACTCTGT CCTTCCTCAT   
  
  
- TGTTCGTTAG TCGTGGTAGG TTACTTCACA AGCTACTTTA ACTCGTCATA CCACTACACG ACGAGACGGG   
  
  
- ACTTCCTTCT TCATTTGGTC GTCCCAATAC GTCGTCGTCG TTGCGATTGT CTTGATCGTT GAGTGTCTTG   
  
  
- AGCCTTCTGA GTTTCAGTGT CGTTTTACCC TCAAGGAGGT TCCACAGTTT TTTCGCTCCG TTATTTTTCG   
  
  
- TTCACCAACT AAACTCCTGA GAGGATTGAG CTACACGTGT TACACAACGA GCACACCTAA ATTGACCAAG   
  
  
- AATGGTCAAC GAAGTCCTCT AAGCCGTCGT AAGAAGAGGG ATACCACTAA TAGAGGTTTC TTACCGAGTA   
  
  
- ATGGAACGAT TACCGGAACT CCGCGCGAAT CTTCCGTTTC CTATACTTGA TTGAGCACGG TTACTCGTAT   
  
  
- AGGGTGGATG ACTCTAGGAG TTCCGATTGG CCCATATACA CCGTCGCCAG GGAAAGTTCT ATTACAGAAT   
  
  
- AATGTGTTGT TTGTTCTGTT AACGTTCGGA TCAACTCCTC CGTGGTTCAT ATGTGTATTA ACTAAAACCA   
  
  
- TAAAAGATAC CTGATGTTAC CGGGACGTAG TAAGTCTTGG AGAGGTTCTC CGGGTTACCC GGAGGGTCTT   
  
  
- AGGCGTAGTG TCCGTAGCTA AAGGGGGTTG GACCCAAGGC CGGTCGTCTC TCCCAACTTC TCTGTCCCGC   
  
  
- CACAAATCGG TTCATAACAC TGTCCATGTT ACACGGGAAA CTTATGGTGG GGTATCGGTT CTTCACCCTC   
  
  
- TGGTAGGTTG AGCTCCTCGA GTTTTAACTT TCCTTACTCG GGGAACATCA CTTGACAGAC ATATCTAGCG   
  
  
- TGTTGGATAA ACTACTTTCG CATCTCCTTT TGTCGGGTTC TCTGCGCAAG GACTCGAACC AATCCTTTTA   
  
  
- TTTAGGGCTA TATAAGTACG TACCCCACCA CTTATGGCGT AGTTCACATG GAAAGAAGGA CTTGTCCAAG   
  
  
- TTCCTCCGGT ACAAAGTGAT GTGCCGGAAC AAACTGAACA AGCTCCGGTG TTACTCGGCG CTCCTAGTTC   
  
  
- TCTCCCACAA TGATCTCTCG TTCAACGTAC CCTTAGTCCG CAACTTACAC TAGCGGACGC TTCCTCGCCT   
  
  
- CTCTCACCTC TCCGGACTCA GTATGTTTGT TACCGTCCAA TCCTGAGTTT CCCGGCCCAA ACAAGTTCAC   
  
  
- AGCTACCTAG CGCTCGAAAA CTCTTCCCGT TCCCGGTACC AGTCCTCCTT AAAATCCCTT CTCAAGTCAA   
  
  
- TACTACTTCT ACCCGTAACC CACCAAGTTC CTACCTTTCC GTCCTATTAC ATACGGTAAA GAACAACCTT   
  
  
- TGGAAGTAT

+     I-box

| Site Name | Organism | Position | Strand | Matrix score. | sequence | function |
| --- | --- | --- | --- | --- | --- | --- |
| I-box | Arabidopsis thaliana | 882 | - | 9 | ccttatcct | part of a light responsive element |
| I-box | Zea mays | 883 | + | 9 | gGATAAGGTG | part of a light responsive element |
| I-box | Flaveria trinervia | 381 | + | 10 | cCATATCCAAT | part of a light responsive element |

>HU02G01570.1   
+ +Up\_Stream \_Len000ATTATT ATTCTAATAT ATAGATAATA ATATAATAAT AATAATAATA ATAATAATAA   
  
  
+ TTATTATTAT TATTATTATT TATTAAATAT AATAGTTATT ATAATAATAA TAATTATAAT AATAATAAGA   
  
  
+ TTATTATCAT TATATTATTA TTATTATTAT TGTAATTTAT TATTATTATA GTAATTATTA TTGTTATATA   
  
  
+ GTAAAATTAT TATTAGTATT GTTATTATAA ATATTCATAC TTGCATATAA TATTTCTAAA ATAAAAACAG   
  
  
+ AGTTAATTCC ATTCCAAAAC TACTCACCAA ACAGCAAGGA GTGGGACCTG ATTCCTTTCC AATTTTCACC   
  
  
+ AAACAACTGG GTTCAGAATT CAAGTTCCAT ATCCAACCCC CCAGGATTCA AGTCCTGATT CCATTCCATT   
  
  
+ CCAGTTTCCC TCACCAAACT GGGCCTTAGA TTGATGGTTG TTCAGTTGGA TGAGGGTTGT TCCATGCCAA   
  
  
+ GTTAGATCAT TGTTGTCAAT TTTGAGAACG ACTATCTTAA TAAGCCTAAC GATGTTGATT TGATTTAGTT   
  
  
+ CAAACCATTT CAAATAATAC AAATCAAATT CTATTTAGAC CGACACTAAA ATAAATAGAT TGAGAAAAAT   
  
  
+ TATAGATTTT AAAGTTCAGA TTAAAATCCC TAAAGATCAA CAGATTGAAA TGCATATTTC ATAGTGCACA   
  
  
+ TGGACTTGCT TTGAATCTCG TCTAGAGAAC CTAAGAAAAA GCAAGAGTGG TTCAGAAGGT TAGAATTGTT   
  
  
+ CCAGCACATG ACAAGTACAA TCCCAACTTT TTTCAAAAGA TTTGCTACTT TTAATTGGAT TCCCATGTTG   
  
  
+ AATGAAGTTC TGGGTTTAAG GCTACAACTT TCTTGGGAGG ATAAGGTTCT TAAGTTAAGG TGGTCCTCTT   
  
  
+ TTCTTTTGAT CTTTCTTATG GGTACAACCT CATGGGTCTC ATCAACACAT GAACTCTTTT TATCTTTTAT   
  
  
+ CTTTCCCCTT TCCTTTATGC AGGATTGACA GGTGCATGGA AAACAAGAAG CAATTAAATA CTAGTGGCTT   
  
  
+ AGCCCCATCC CTAATATTTT GGCTATTCTG TTTCACCATC CTCCCCTGTC TCCATTTTCT TTGTTTCCTT   
  
  
+ TTTTTCTAGG AACTTGTGAT GGGCAAGGGA AGATCAGTAC AATGTGATGG TTGTTAGTAG CGAGAAGATG   
  
  
+ AAGTGTCACC GCAAAAATTT TGTGCCATAA TTTGTCGTAA GGGAAACAAG TATCAAACAA CAATGTCATT   
  
  
+ TATGATAAAC AAGTAACAGG AGATATGTAC TCTGAAAAAA TATAAGGTAC CTGAGAAGTT GTTAACTTAG   
  
  
+ CTTGAAAAAC CCTCCTAACT TGATAAACCC TCTTCGCTAT GTTATCCCGC ACCCTGCCAT AGAGTCACCG   
  
  
+ ATTCAGTATA TATGTAACAT ACCCAATCTT GTCTGGATAT ATAGTTAACC CTTTTGGAAT ATCTGAAAAT   
  
  
+ CCACACGAGA GTCATCCTTC TAGACTAGGA AACATGCGCC ACATTTTGGA TTCTAACCAA GTATAATAAG   
  
  
+ CATATCTTAA AGCCAAAAAT GCAAGGATAG ATACAAATAC CCATCACATG CATGTAAATG TCCCTTGTCA   
  
  
+ TGGCTCTGAT TCTCAAAATA TTAATTTTCA ATAAATTAAA TGAAATTGCA TTATTTTAAA GATTTCTGAA   
  
  
+ TGCATCCATT ATTGCAATTT TTTAAAGACT GCACAATGAT GACAATGTCA ATGGTTTCCT AAAGTTGCGC   
  
  
+ CTGATGGGCT ATTGACAGTG AAGCAACACT CCCTTTGTTC CCATTTATGT GTCTCCACTT GCCCCTTTGA   
  
  
+ AACTCTCTAC ATTTGTATGT CATCTGAATT TGATTCTCAA ATCTCAGATG ATTTGAATTC CCTTTATGCC   
  
  
+ ATCTTTACTA TCTATTGAAT GATTCTTTCT TTTCCACTTT CTTATCTTCC ACTACCTTCT TCTTGGATGC   
  
  
+ CCTTTTGCTG CTTATAAAGG GCGACACCTG AACAAGTTGC TGTATTCTCA AACCATGAAT CTGTACAGGT   
  
  
+ TTAGCCCCGA TATGTGTCCT AGCTTTGTGG GTCACGAAGA AATTGGTCCT AATTTAATAG GACACCAAGA   
  
  
+ CCCTTTTCAA AATGCATACA ATTGCACTGA TTTTGGTGAT GCTTACTTGT TGCAATTCCC AGTTGAAGAT   
  
  
+ TTATGTCAAT ACCGTGTTCC TGATCTAATG GGGCTACCTG TTGAAGATGG GGATCCTACA GAGGTCCCTG   
  
  
+ ACATTCCTGA TACATGCTTC AACTACATAA GCAATCTCCT AATGGAAGAG GGCTCGGATG ACCGCCCTGG   
  
  
+ CGCCACCCTG CAGGACTATA TGGCTGCTCA AGTCTCCTAC AGCAAGTCCT CCAATGCCGC CTTTCCCGGT   
  
  
+ GAGAGTTGCT CATTCTTTGG TAATAATCAA TGCCCTCCAC CTGCTTATCA AAGCAACCCT TATTGCCTTG   
  
  
+ ATGATTACTT CAACTTGCTA TCCAATACTA CTGGCAATAG TGTTAACAAT GCTGGTGTTC ATGACAGCAA   
  
  
+ CAACTTTGTT CAGAACAGTT TGATTGGGAA TAGGGGTACT AGTTCTAGTT CTAGGGGCAG AAGTGATTTT   
  
  
+ GGATCTACCC TTATTAGGAA TTTGCCATTA GGGACCGACA CACTTGATGT GGTCAATCCA GCACCTACTC   
  
  
+ CTCGAGCCCC TCTGGTTACG ACTCAGTGTC AGGTGTTTGA AACACACCAG TATGAGCAGG CCTTCGAACT   
  
  
+ GATCACTAAT GGAAGCTGGG AATTCAGTCA TTCTGTTGCT AATACATCAA ATGATGGAAT TCAAAATCAA   
  
  
+ CCAATTCAAG CAGTTTCAAC CCAAGAACGA TGGAATATGA AAACATTTTC CGATGACCAA CTTGAGCCTG   
  
  
+ CTCTTTTGCG AAGTCTTGTG ATGTCCTCAA ACTGTACTGA TATCTCTGAT CAGGGTGTAC ACAGGATAGT   
  
  
+ GAACCCTGAG CCACCCAACA ACAGAAATAC GGAGATTGTG TGCAATGACT CATCCAGTAA GAGTAAGACA   
  
  
+ AGACGAAGAG AGCATGGTGC AGGAGAAAAC CACGATGAAG GAGAAAACCA CGATGAGACA GGAAGGAGTA   
  
  
+ ACAAGCAATC AGCACCATCC AATGAAGTGT TCGATGAAAT TGAGCAGTAT GGTGATGTGC TGCTCTGCCC   
  
  
+ TGAAGGAAGA AGTAAACCAG CAGGGTTATG CAGCAGCAGC AACGCTAACA GAACTAGCAA CTCACAGAAC   
  
  
+ TCGGAAGACT CAAAGTCACA GCAAAATGGG AGTTCCTCCA AGGTGTCAAA AAAGCGAGGC AATAAAAAGC   
  
  
+ AAGTGGTTGA TTTGAGGACT CTCCTAACTC GATGTGCACA ATGTGTTGCT CGTGTGGATT TAACTGGTTC   
  
  
+ TTACCAGTTG CTTCAGGAGA TTCGGCAGCA TTCTTCTCCC TATGGTGATT ATCTCCAAAG AATGGCTCAT   
  
  
+ TACCTTGCTA ATGGCCTTGA GGCGCGCTTA GAAGGCAAAG GATATGAACT AACTCGTGCC AATGAGCATA   
  
  
+ TCCCACCTAC TGAGATCCTC AAGGCTAACC GGGTATATGT GGCAGCGGTC CCTTTCAAGA TAATGTCTTA   
  
  
+ TTACACAACA AACAAGACAA TTGCAAGCCT AGTTGAGGAG GCACCAAGTA TACACATAAT TGATTTTGGT   
  
  
+ ATTTTCTATG GACTACAATG GCCCTGCATC ATTCAGAACC TCTCCAAGAG GCCCAATGGG CCTCCCAGAA   
  
  
+ TCCGCATCAC AGGCATCGAT TTCCCCCAAC CTGGGTTCCG GCCAGCAGAG AGGGTTGAAG AGACAGGGCG   
  
  
+ GTGTTTAGCC AAGTATTGTG ACAGGTACAA TGTGCCCTTT GAATACCACC CCATAGCCAA GAAGTGGGAG   
  
  
+ ACCATCCAAC TCGAGGAGCT CAAAATTGAA AGGAATGAGC CCCTTGTAGT GAACTGTCTG TATAGATCGC   
  
  
+ ACAACCTATT TGATGAAAGC GTAGAGGAAA ACAGCCCAAG AGACGCGTTC CTGAGCTTGG TTAGGAAAAT   
  
  
+ AAATCCCGAT ATATTCATGC ATGGGGTGGT GAATACCGCA TCAAGTGTAC CTTTCTTCCT GAACAGGTTC   
  
  
+ AAGGAGGCCA TGTTTCACTA CACGGCCTTG TTTGACTTGT TCGAGGCCAC AATGAGCCGC GAGGATCAAG   
  
  
+ AGAGGGTGTT ACTAGAGAGC AAGTTGCATG GGAATCAGGC GTTGAATGTG ATCGCCTGCG AAGGAGCGGA   
  
  
+ GAGAGTGGAG AGGCCTGAGT CATACAAACA ATGGCAGGTT AGGACTCAAA GGGCCGGGTT TGTTCAAGTG   
  
  
+ TCGATGGATC GCGAGCTTTT GAGAAGGGCA AGGGCCATGG TCAGGAGGAA TTTTAGGGAA GAGTTCAGTT   
  
  
+ ATGATGAAGA TGGGCATTGG GTGGTTCAAG GATGGAAAGG CAGGATAATG TATGCCATTT CTTGTTGGAA   
  
  
+ ACCTTCATA  

- +Up\_Stream \_Len000TAATAA TAAGATTATA TATCTATTAT TATATTATTA TTATTATTAT TATTATTATT   
  
  
- AATAATAATA ATAATAATAA ATAATTTATA TTATCAATAA TATTATTATT ATTAATATTA TTATTATTCT   
  
  
- AATAATAGTA ATATAATAAT AATAATAATA ACATTAAATA ATAATAATAT CATTAATAAT AACAATATAT   
  
  
- CATTTTAATA ATAATCATAA CAATAATATT TATAAGTATG AACGTATATT ATAAAGATTT TATTTTTGTC   
  
  
- TCAATTAAGG TAAGGTTTTG ATGAGTGGTT TGTCGTTCCT CACCCTGGAC TAAGGAAAGG TTAAAAGTGG   
  
  
- TTTGTTGACC CAAGTCTTAA GTTCAAGGTA TAGGTTGGGG GGTCCTAAGT TCAGGACTAA GGTAAGGTAA   
  
  
- GGTCAAAGGG AGTGGTTTGA CCCGGAATCT AACTACCAAC AAGTCAACCT ACTCCCAACA AGGTACGGTT   
  
  
- CAATCTAGTA ACAACAGTTA AAACTCTTGC TGATAGAATT ATTCGGATTG CTACAACTAA ACTAAATCAA   
  
  
- GTTTGGTAAA GTTTATTATG TTTAGTTTAA GATAAATCTG GCTGTGATTT TATTTATCTA ACTCTTTTTA   
  
  
- ATATCTAAAA TTTCAAGTCT AATTTTAGGG ATTTCTAGTT GTCTAACTTT ACGTATAAAG TATCACGTGT   
  
  
- ACCTGAACGA AACTTAGAGC AGATCTCTTG GATTCTTTTT CGTTCTCACC AAGTCTTCCA ATCTTAACAA   
  
  
- GGTCGTGTAC TGTTCATGTT AGGGTTGAAA AAAGTTTTCT AAACGATGAA AATTAACCTA AGGGTACAAC   
  
  
- TTACTTCAAG ACCCAAATTC CGATGTTGAA AGAACCCTCC TATTCCAAGA ATTCAATTCC ACCAGGAGAA   
  
  
- AAGAAAACTA GAAAGAATAC CCATGTTGGA GTACCCAGAG TAGTTGTGTA CTTGAGAAAA ATAGAAAATA   
  
  
- GAAAGGGGAA AGGAAATACG TCCTAACTGT CCACGTACCT TTTGTTCTTC GTTAATTTAT GATCACCGAA   
  
  
- TCGGGGTAGG GATTATAAAA CCGATAAGAC AAAGTGGTAG GAGGGGACAG AGGTAAAAGA AACAAAGGAA   
  
  
- AAAAAGATCC TTGAACACTA CCCGTTCCCT TCTAGTCATG TTACACTACC AACAATCATC GCTCTTCTAC   
  
  
- TTCACAGTGG CGTTTTTAAA ACACGGTATT AAACAGCATT CCCTTTGTTC ATAGTTTGTT GTTACAGTAA   
  
  
- ATACTATTTG TTCATTGTCC TCTATACATG AGACTTTTTT ATATTCCATG GACTCTTCAA CAATTGAATC   
  
  
- GAACTTTTTG GGAGGATTGA ACTATTTGGG AGAAGCGATA CAATAGGGCG TGGGACGGTA TCTCAGTGGC   
  
  
- TAAGTCATAT ATACATTGTA TGGGTTAGAA CAGACCTATA TATCAATTGG GAAAACCTTA TAGACTTTTA   
  
  
- GGTGTGCTCT CAGTAGGAAG ATCTGATCCT TTGTACGCGG TGTAAAACCT AAGATTGGTT CATATTATTC   
  
  
- GTATAGAATT TCGGTTTTTA CGTTCCTATC TATGTTTATG GGTAGTGTAC GTACATTTAC AGGGAACAGT   
  
  
- ACCGAGACTA AGAGTTTTAT AATTAAAAGT TATTTAATTT ACTTTAACGT AATAAAATTT CTAAAGACTT   
  
  
- ACGTAGGTAA TAACGTTAAA AAATTTCTGA CGTGTTACTA CTGTTACAGT TACCAAAGGA TTTCAACGCG   
  
  
- GACTACCCGA TAACTGTCAC TTCGTTGTGA GGGAAACAAG GGTAAATACA CAGAGGTGAA CGGGGAAACT   
  
  
- TTGAGAGATG TAAACATACA GTAGACTTAA ACTAAGAGTT TAGAGTCTAC TAAACTTAAG GGAAATACGG   
  
  
- TAGAAATGAT AGATAACTTA CTAAGAAAGA AAAGGTGAAA GAATAGAAGG TGATGGAAGA AGAACCTACG   
  
  
- GGAAAACGAC GAATATTTCC CGCTGTGGAC TTGTTCAACG ACATAAGAGT TTGGTACTTA GACATGTCCA   
  
  
- AATCGGGGCT ATACACAGGA TCGAAACACC CAGTGCTTCT TTAACCAGGA TTAAATTATC CTGTGGTTCT   
  
  
- GGGAAAAGTT TTACGTATGT TAACGTGACT AAAACCACTA CGAATGAACA ACGTTAAGGG TCAACTTCTA   
  
  
- AATACAGTTA TGGCACAAGG ACTAGATTAC CCCGATGGAC AACTTCTACC CCTAGGATGT CTCCAGGGAC   
  
  
- TGTAAGGACT ATGTACGAAG TTGATGTATT CGTTAGAGGA TTACCTTCTC CCGAGCCTAC TGGCGGGACC   
  
  
- GCGGTGGGAC GTCCTGATAT ACCGACGAGT TCAGAGGATG TCGTTCAGGA GGTTACGGCG GAAAGGGCCA   
  
  
- CTCTCAACGA GTAAGAAACC ATTATTAGTT ACGGGAGGTG GACGAATAGT TTCGTTGGGA ATAACGGAAC   
  
  
- TACTAATGAA GTTGAACGAT AGGTTATGAT GACCGTTATC ACAATTGTTA CGACCACAAG TACTGTCGTT   
  
  
- GTTGAAACAA GTCTTGTCAA ACTAACCCTT ATCCCCATGA TCAAGATCAA GATCCCCGTC TTCACTAAAA   
  
  
- CCTAGATGGG AATAATCCTT AAACGGTAAT CCCTGGCTGT GTGAACTACA CCAGTTAGGT CGTGGATGAG   
  
  
- GAGCTCGGGG AGACCAATGC TGAGTCACAG TCCACAAACT TTGTGTGGTC ATACTCGTCC GGAAGCTTGA   
  
  
- CTAGTGATTA CCTTCGACCC TTAAGTCAGT AAGACAACGA TTATGTAGTT TACTACCTTA AGTTTTAGTT   
  
  
- GGTTAAGTTC GTCAAAGTTG GGTTCTTGCT ACCTTATACT TTTGTAAAAG GCTACTGGTT GAACTCGGAC   
  
  
- GAGAAAACGC TTCAGAACAC TACAGGAGTT TGACATGACT ATAGAGACTA GTCCCACATG TGTCCTATCA   
  
  
- CTTGGGACTC GGTGGGTTGT TGTCTTTATG CCTCTAACAC ACGTTACTGA GTAGGTCATT CTCATTCTGT   
  
  
- TCTGCTTCTC TCGTACCACG TCCTCTTTTG GTGCTACTTC CTCTTTTGGT GCTACTCTGT CCTTCCTCAT   
  
  
- TGTTCGTTAG TCGTGGTAGG TTACTTCACA AGCTACTTTA ACTCGTCATA CCACTACACG ACGAGACGGG   
  
  
- ACTTCCTTCT TCATTTGGTC GTCCCAATAC GTCGTCGTCG TTGCGATTGT CTTGATCGTT GAGTGTCTTG   
  
  
- AGCCTTCTGA GTTTCAGTGT CGTTTTACCC TCAAGGAGGT TCCACAGTTT TTTCGCTCCG TTATTTTTCG   
  
  
- TTCACCAACT AAACTCCTGA GAGGATTGAG CTACACGTGT TACACAACGA GCACACCTAA ATTGACCAAG   
  
  
- AATGGTCAAC GAAGTCCTCT AAGCCGTCGT AAGAAGAGGG ATACCACTAA TAGAGGTTTC TTACCGAGTA   
  
  
- ATGGAACGAT TACCGGAACT CCGCGCGAAT CTTCCGTTTC CTATACTTGA TTGAGCACGG TTACTCGTAT   
  
  
- AGGGTGGATG ACTCTAGGAG TTCCGATTGG CCCATATACA CCGTCGCCAG GGAAAGTTCT ATTACAGAAT   
  
  
- AATGTGTTGT TTGTTCTGTT AACGTTCGGA TCAACTCCTC CGTGGTTCAT ATGTGTATTA ACTAAAACCA   
  
  
- TAAAAGATAC CTGATGTTAC CGGGACGTAG TAAGTCTTGG AGAGGTTCTC CGGGTTACCC GGAGGGTCTT   
  
  
- AGGCGTAGTG TCCGTAGCTA AAGGGGGTTG GACCCAAGGC CGGTCGTCTC TCCCAACTTC TCTGTCCCGC   
  
  
- CACAAATCGG TTCATAACAC TGTCCATGTT ACACGGGAAA CTTATGGTGG GGTATCGGTT CTTCACCCTC   
  
  
- TGGTAGGTTG AGCTCCTCGA GTTTTAACTT TCCTTACTCG GGGAACATCA CTTGACAGAC ATATCTAGCG   
  
  
- TGTTGGATAA ACTACTTTCG CATCTCCTTT TGTCGGGTTC TCTGCGCAAG GACTCGAACC AATCCTTTTA   
  
  
- TTTAGGGCTA TATAAGTACG TACCCCACCA CTTATGGCGT AGTTCACATG GAAAGAAGGA CTTGTCCAAG   
  
  
- TTCCTCCGGT ACAAAGTGAT GTGCCGGAAC AAACTGAACA AGCTCCGGTG TTACTCGGCG CTCCTAGTTC   
  
  
- TCTCCCACAA TGATCTCTCG TTCAACGTAC CCTTAGTCCG CAACTTACAC TAGCGGACGC TTCCTCGCCT   
  
  
- CTCTCACCTC TCCGGACTCA GTATGTTTGT TACCGTCCAA TCCTGAGTTT CCCGGCCCAA ACAAGTTCAC   
  
  
- AGCTACCTAG CGCTCGAAAA CTCTTCCCGT TCCCGGTACC AGTCCTCCTT AAAATCCCTT CTCAAGTCAA   
  
  
- TACTACTTCT ACCCGTAACC CACCAAGTTC CTACCTTTCC GTCCTATTAC ATACGGTAAA GAACAACCTT   
  
  
- TGGAAGTAT

+     L-box

| Site Name | Organism | Position | Strand | Matrix score. | sequence | function |
| --- | --- | --- | --- | --- | --- | --- |
| L-box | Petroselinum crispum | 3504 | + | 11 | ATCCCACCTAC | part of a light responsive element |

>HU02G01570.1   
+ +Up\_Stream \_Len000ATTATT ATTCTAATAT ATAGATAATA ATATAATAAT AATAATAATA ATAATAATAA   
  
  
+ TTATTATTAT TATTATTATT TATTAAATAT AATAGTTATT ATAATAATAA TAATTATAAT AATAATAAGA   
  
  
+ TTATTATCAT TATATTATTA TTATTATTAT TGTAATTTAT TATTATTATA GTAATTATTA TTGTTATATA   
  
  
+ GTAAAATTAT TATTAGTATT GTTATTATAA ATATTCATAC TTGCATATAA TATTTCTAAA ATAAAAACAG   
  
  
+ AGTTAATTCC ATTCCAAAAC TACTCACCAA ACAGCAAGGA GTGGGACCTG ATTCCTTTCC AATTTTCACC   
  
  
+ AAACAACTGG GTTCAGAATT CAAGTTCCAT ATCCAACCCC CCAGGATTCA AGTCCTGATT CCATTCCATT   
  
  
+ CCAGTTTCCC TCACCAAACT GGGCCTTAGA TTGATGGTTG TTCAGTTGGA TGAGGGTTGT TCCATGCCAA   
  
  
+ GTTAGATCAT TGTTGTCAAT TTTGAGAACG ACTATCTTAA TAAGCCTAAC GATGTTGATT TGATTTAGTT   
  
  
+ CAAACCATTT CAAATAATAC AAATCAAATT CTATTTAGAC CGACACTAAA ATAAATAGAT TGAGAAAAAT   
  
  
+ TATAGATTTT AAAGTTCAGA TTAAAATCCC TAAAGATCAA CAGATTGAAA TGCATATTTC ATAGTGCACA   
  
  
+ TGGACTTGCT TTGAATCTCG TCTAGAGAAC CTAAGAAAAA GCAAGAGTGG TTCAGAAGGT TAGAATTGTT   
  
  
+ CCAGCACATG ACAAGTACAA TCCCAACTTT TTTCAAAAGA TTTGCTACTT TTAATTGGAT TCCCATGTTG   
  
  
+ AATGAAGTTC TGGGTTTAAG GCTACAACTT TCTTGGGAGG ATAAGGTTCT TAAGTTAAGG TGGTCCTCTT   
  
  
+ TTCTTTTGAT CTTTCTTATG GGTACAACCT CATGGGTCTC ATCAACACAT GAACTCTTTT TATCTTTTAT   
  
  
+ CTTTCCCCTT TCCTTTATGC AGGATTGACA GGTGCATGGA AAACAAGAAG CAATTAAATA CTAGTGGCTT   
  
  
+ AGCCCCATCC CTAATATTTT GGCTATTCTG TTTCACCATC CTCCCCTGTC TCCATTTTCT TTGTTTCCTT   
  
  
+ TTTTTCTAGG AACTTGTGAT GGGCAAGGGA AGATCAGTAC AATGTGATGG TTGTTAGTAG CGAGAAGATG   
  
  
+ AAGTGTCACC GCAAAAATTT TGTGCCATAA TTTGTCGTAA GGGAAACAAG TATCAAACAA CAATGTCATT   
  
  
+ TATGATAAAC AAGTAACAGG AGATATGTAC TCTGAAAAAA TATAAGGTAC CTGAGAAGTT GTTAACTTAG   
  
  
+ CTTGAAAAAC CCTCCTAACT TGATAAACCC TCTTCGCTAT GTTATCCCGC ACCCTGCCAT AGAGTCACCG   
  
  
+ ATTCAGTATA TATGTAACAT ACCCAATCTT GTCTGGATAT ATAGTTAACC CTTTTGGAAT ATCTGAAAAT   
  
  
+ CCACACGAGA GTCATCCTTC TAGACTAGGA AACATGCGCC ACATTTTGGA TTCTAACCAA GTATAATAAG   
  
  
+ CATATCTTAA AGCCAAAAAT GCAAGGATAG ATACAAATAC CCATCACATG CATGTAAATG TCCCTTGTCA   
  
  
+ TGGCTCTGAT TCTCAAAATA TTAATTTTCA ATAAATTAAA TGAAATTGCA TTATTTTAAA GATTTCTGAA   
  
  
+ TGCATCCATT ATTGCAATTT TTTAAAGACT GCACAATGAT GACAATGTCA ATGGTTTCCT AAAGTTGCGC   
  
  
+ CTGATGGGCT ATTGACAGTG AAGCAACACT CCCTTTGTTC CCATTTATGT GTCTCCACTT GCCCCTTTGA   
  
  
+ AACTCTCTAC ATTTGTATGT CATCTGAATT TGATTCTCAA ATCTCAGATG ATTTGAATTC CCTTTATGCC   
  
  
+ ATCTTTACTA TCTATTGAAT GATTCTTTCT TTTCCACTTT CTTATCTTCC ACTACCTTCT TCTTGGATGC   
  
  
+ CCTTTTGCTG CTTATAAAGG GCGACACCTG AACAAGTTGC TGTATTCTCA AACCATGAAT CTGTACAGGT   
  
  
+ TTAGCCCCGA TATGTGTCCT AGCTTTGTGG GTCACGAAGA AATTGGTCCT AATTTAATAG GACACCAAGA   
  
  
+ CCCTTTTCAA AATGCATACA ATTGCACTGA TTTTGGTGAT GCTTACTTGT TGCAATTCCC AGTTGAAGAT   
  
  
+ TTATGTCAAT ACCGTGTTCC TGATCTAATG GGGCTACCTG TTGAAGATGG GGATCCTACA GAGGTCCCTG   
  
  
+ ACATTCCTGA TACATGCTTC AACTACATAA GCAATCTCCT AATGGAAGAG GGCTCGGATG ACCGCCCTGG   
  
  
+ CGCCACCCTG CAGGACTATA TGGCTGCTCA AGTCTCCTAC AGCAAGTCCT CCAATGCCGC CTTTCCCGGT   
  
  
+ GAGAGTTGCT CATTCTTTGG TAATAATCAA TGCCCTCCAC CTGCTTATCA AAGCAACCCT TATTGCCTTG   
  
  
+ ATGATTACTT CAACTTGCTA TCCAATACTA CTGGCAATAG TGTTAACAAT GCTGGTGTTC ATGACAGCAA   
  
  
+ CAACTTTGTT CAGAACAGTT TGATTGGGAA TAGGGGTACT AGTTCTAGTT CTAGGGGCAG AAGTGATTTT   
  
  
+ GGATCTACCC TTATTAGGAA TTTGCCATTA GGGACCGACA CACTTGATGT GGTCAATCCA GCACCTACTC   
  
  
+ CTCGAGCCCC TCTGGTTACG ACTCAGTGTC AGGTGTTTGA AACACACCAG TATGAGCAGG CCTTCGAACT   
  
  
+ GATCACTAAT GGAAGCTGGG AATTCAGTCA TTCTGTTGCT AATACATCAA ATGATGGAAT TCAAAATCAA   
  
  
+ CCAATTCAAG CAGTTTCAAC CCAAGAACGA TGGAATATGA AAACATTTTC CGATGACCAA CTTGAGCCTG   
  
  
+ CTCTTTTGCG AAGTCTTGTG ATGTCCTCAA ACTGTACTGA TATCTCTGAT CAGGGTGTAC ACAGGATAGT   
  
  
+ GAACCCTGAG CCACCCAACA ACAGAAATAC GGAGATTGTG TGCAATGACT CATCCAGTAA GAGTAAGACA   
  
  
+ AGACGAAGAG AGCATGGTGC AGGAGAAAAC CACGATGAAG GAGAAAACCA CGATGAGACA GGAAGGAGTA   
  
  
+ ACAAGCAATC AGCACCATCC AATGAAGTGT TCGATGAAAT TGAGCAGTAT GGTGATGTGC TGCTCTGCCC   
  
  
+ TGAAGGAAGA AGTAAACCAG CAGGGTTATG CAGCAGCAGC AACGCTAACA GAACTAGCAA CTCACAGAAC   
  
  
+ TCGGAAGACT CAAAGTCACA GCAAAATGGG AGTTCCTCCA AGGTGTCAAA AAAGCGAGGC AATAAAAAGC   
  
  
+ AAGTGGTTGA TTTGAGGACT CTCCTAACTC GATGTGCACA ATGTGTTGCT CGTGTGGATT TAACTGGTTC   
  
  
+ TTACCAGTTG CTTCAGGAGA TTCGGCAGCA TTCTTCTCCC TATGGTGATT ATCTCCAAAG AATGGCTCAT   
  
  
+ TACCTTGCTA ATGGCCTTGA GGCGCGCTTA GAAGGCAAAG GATATGAACT AACTCGTGCC AATGAGCATA   
  
  
+ TCCCACCTAC TGAGATCCTC AAGGCTAACC GGGTATATGT GGCAGCGGTC CCTTTCAAGA TAATGTCTTA   
  
  
+ TTACACAACA AACAAGACAA TTGCAAGCCT AGTTGAGGAG GCACCAAGTA TACACATAAT TGATTTTGGT   
  
  
+ ATTTTCTATG GACTACAATG GCCCTGCATC ATTCAGAACC TCTCCAAGAG GCCCAATGGG CCTCCCAGAA   
  
  
+ TCCGCATCAC AGGCATCGAT TTCCCCCAAC CTGGGTTCCG GCCAGCAGAG AGGGTTGAAG AGACAGGGCG   
  
  
+ GTGTTTAGCC AAGTATTGTG ACAGGTACAA TGTGCCCTTT GAATACCACC CCATAGCCAA GAAGTGGGAG   
  
  
+ ACCATCCAAC TCGAGGAGCT CAAAATTGAA AGGAATGAGC CCCTTGTAGT GAACTGTCTG TATAGATCGC   
  
  
+ ACAACCTATT TGATGAAAGC GTAGAGGAAA ACAGCCCAAG AGACGCGTTC CTGAGCTTGG TTAGGAAAAT   
  
  
+ AAATCCCGAT ATATTCATGC ATGGGGTGGT GAATACCGCA TCAAGTGTAC CTTTCTTCCT GAACAGGTTC   
  
  
+ AAGGAGGCCA TGTTTCACTA CACGGCCTTG TTTGACTTGT TCGAGGCCAC AATGAGCCGC GAGGATCAAG   
  
  
+ AGAGGGTGTT ACTAGAGAGC AAGTTGCATG GGAATCAGGC GTTGAATGTG ATCGCCTGCG AAGGAGCGGA   
  
  
+ GAGAGTGGAG AGGCCTGAGT CATACAAACA ATGGCAGGTT AGGACTCAAA GGGCCGGGTT TGTTCAAGTG   
  
  
+ TCGATGGATC GCGAGCTTTT GAGAAGGGCA AGGGCCATGG TCAGGAGGAA TTTTAGGGAA GAGTTCAGTT   
  
  
+ ATGATGAAGA TGGGCATTGG GTGGTTCAAG GATGGAAAGG CAGGATAATG TATGCCATTT CTTGTTGGAA   
  
  
+ ACCTTCATA  

- +Up\_Stream \_Len000TAATAA TAAGATTATA TATCTATTAT TATATTATTA TTATTATTAT TATTATTATT   
  
  
- AATAATAATA ATAATAATAA ATAATTTATA TTATCAATAA TATTATTATT ATTAATATTA TTATTATTCT   
  
  
- AATAATAGTA ATATAATAAT AATAATAATA ACATTAAATA ATAATAATAT CATTAATAAT AACAATATAT   
  
  
- CATTTTAATA ATAATCATAA CAATAATATT TATAAGTATG AACGTATATT ATAAAGATTT TATTTTTGTC   
  
  
- TCAATTAAGG TAAGGTTTTG ATGAGTGGTT TGTCGTTCCT CACCCTGGAC TAAGGAAAGG TTAAAAGTGG   
  
  
- TTTGTTGACC CAAGTCTTAA GTTCAAGGTA TAGGTTGGGG GGTCCTAAGT TCAGGACTAA GGTAAGGTAA   
  
  
- GGTCAAAGGG AGTGGTTTGA CCCGGAATCT AACTACCAAC AAGTCAACCT ACTCCCAACA AGGTACGGTT   
  
  
- CAATCTAGTA ACAACAGTTA AAACTCTTGC TGATAGAATT ATTCGGATTG CTACAACTAA ACTAAATCAA   
  
  
- GTTTGGTAAA GTTTATTATG TTTAGTTTAA GATAAATCTG GCTGTGATTT TATTTATCTA ACTCTTTTTA   
  
  
- ATATCTAAAA TTTCAAGTCT AATTTTAGGG ATTTCTAGTT GTCTAACTTT ACGTATAAAG TATCACGTGT   
  
  
- ACCTGAACGA AACTTAGAGC AGATCTCTTG GATTCTTTTT CGTTCTCACC AAGTCTTCCA ATCTTAACAA   
  
  
- GGTCGTGTAC TGTTCATGTT AGGGTTGAAA AAAGTTTTCT AAACGATGAA AATTAACCTA AGGGTACAAC   
  
  
- TTACTTCAAG ACCCAAATTC CGATGTTGAA AGAACCCTCC TATTCCAAGA ATTCAATTCC ACCAGGAGAA   
  
  
- AAGAAAACTA GAAAGAATAC CCATGTTGGA GTACCCAGAG TAGTTGTGTA CTTGAGAAAA ATAGAAAATA   
  
  
- GAAAGGGGAA AGGAAATACG TCCTAACTGT CCACGTACCT TTTGTTCTTC GTTAATTTAT GATCACCGAA   
  
  
- TCGGGGTAGG GATTATAAAA CCGATAAGAC AAAGTGGTAG GAGGGGACAG AGGTAAAAGA AACAAAGGAA   
  
  
- AAAAAGATCC TTGAACACTA CCCGTTCCCT TCTAGTCATG TTACACTACC AACAATCATC GCTCTTCTAC   
  
  
- TTCACAGTGG CGTTTTTAAA ACACGGTATT AAACAGCATT CCCTTTGTTC ATAGTTTGTT GTTACAGTAA   
  
  
- ATACTATTTG TTCATTGTCC TCTATACATG AGACTTTTTT ATATTCCATG GACTCTTCAA CAATTGAATC   
  
  
- GAACTTTTTG GGAGGATTGA ACTATTTGGG AGAAGCGATA CAATAGGGCG TGGGACGGTA TCTCAGTGGC   
  
  
- TAAGTCATAT ATACATTGTA TGGGTTAGAA CAGACCTATA TATCAATTGG GAAAACCTTA TAGACTTTTA   
  
  
- GGTGTGCTCT CAGTAGGAAG ATCTGATCCT TTGTACGCGG TGTAAAACCT AAGATTGGTT CATATTATTC   
  
  
- GTATAGAATT TCGGTTTTTA CGTTCCTATC TATGTTTATG GGTAGTGTAC GTACATTTAC AGGGAACAGT   
  
  
- ACCGAGACTA AGAGTTTTAT AATTAAAAGT TATTTAATTT ACTTTAACGT AATAAAATTT CTAAAGACTT   
  
  
- ACGTAGGTAA TAACGTTAAA AAATTTCTGA CGTGTTACTA CTGTTACAGT TACCAAAGGA TTTCAACGCG   
  
  
- GACTACCCGA TAACTGTCAC TTCGTTGTGA GGGAAACAAG GGTAAATACA CAGAGGTGAA CGGGGAAACT   
  
  
- TTGAGAGATG TAAACATACA GTAGACTTAA ACTAAGAGTT TAGAGTCTAC TAAACTTAAG GGAAATACGG   
  
  
- TAGAAATGAT AGATAACTTA CTAAGAAAGA AAAGGTGAAA GAATAGAAGG TGATGGAAGA AGAACCTACG   
  
  
- GGAAAACGAC GAATATTTCC CGCTGTGGAC TTGTTCAACG ACATAAGAGT TTGGTACTTA GACATGTCCA   
  
  
- AATCGGGGCT ATACACAGGA TCGAAACACC CAGTGCTTCT TTAACCAGGA TTAAATTATC CTGTGGTTCT   
  
  
- GGGAAAAGTT TTACGTATGT TAACGTGACT AAAACCACTA CGAATGAACA ACGTTAAGGG TCAACTTCTA   
  
  
- AATACAGTTA TGGCACAAGG ACTAGATTAC CCCGATGGAC AACTTCTACC CCTAGGATGT CTCCAGGGAC   
  
  
- TGTAAGGACT ATGTACGAAG TTGATGTATT CGTTAGAGGA TTACCTTCTC CCGAGCCTAC TGGCGGGACC   
  
  
- GCGGTGGGAC GTCCTGATAT ACCGACGAGT TCAGAGGATG TCGTTCAGGA GGTTACGGCG GAAAGGGCCA   
  
  
- CTCTCAACGA GTAAGAAACC ATTATTAGTT ACGGGAGGTG GACGAATAGT TTCGTTGGGA ATAACGGAAC   
  
  
- TACTAATGAA GTTGAACGAT AGGTTATGAT GACCGTTATC ACAATTGTTA CGACCACAAG TACTGTCGTT   
  
  
- GTTGAAACAA GTCTTGTCAA ACTAACCCTT ATCCCCATGA TCAAGATCAA GATCCCCGTC TTCACTAAAA   
  
  
- CCTAGATGGG AATAATCCTT AAACGGTAAT CCCTGGCTGT GTGAACTACA CCAGTTAGGT CGTGGATGAG   
  
  
- GAGCTCGGGG AGACCAATGC TGAGTCACAG TCCACAAACT TTGTGTGGTC ATACTCGTCC GGAAGCTTGA   
  
  
- CTAGTGATTA CCTTCGACCC TTAAGTCAGT AAGACAACGA TTATGTAGTT TACTACCTTA AGTTTTAGTT   
  
  
- GGTTAAGTTC GTCAAAGTTG GGTTCTTGCT ACCTTATACT TTTGTAAAAG GCTACTGGTT GAACTCGGAC   
  
  
- GAGAAAACGC TTCAGAACAC TACAGGAGTT TGACATGACT ATAGAGACTA GTCCCACATG TGTCCTATCA   
  
  
- CTTGGGACTC GGTGGGTTGT TGTCTTTATG CCTCTAACAC ACGTTACTGA GTAGGTCATT CTCATTCTGT   
  
  
- TCTGCTTCTC TCGTACCACG TCCTCTTTTG GTGCTACTTC CTCTTTTGGT GCTACTCTGT CCTTCCTCAT   
  
  
- TGTTCGTTAG TCGTGGTAGG TTACTTCACA AGCTACTTTA ACTCGTCATA CCACTACACG ACGAGACGGG   
  
  
- ACTTCCTTCT TCATTTGGTC GTCCCAATAC GTCGTCGTCG TTGCGATTGT CTTGATCGTT GAGTGTCTTG   
  
  
- AGCCTTCTGA GTTTCAGTGT CGTTTTACCC TCAAGGAGGT TCCACAGTTT TTTCGCTCCG TTATTTTTCG   
  
  
- TTCACCAACT AAACTCCTGA GAGGATTGAG CTACACGTGT TACACAACGA GCACACCTAA ATTGACCAAG   
  
  
- AATGGTCAAC GAAGTCCTCT AAGCCGTCGT AAGAAGAGGG ATACCACTAA TAGAGGTTTC TTACCGAGTA   
  
  
- ATGGAACGAT TACCGGAACT CCGCGCGAAT CTTCCGTTTC CTATACTTGA TTGAGCACGG TTACTCGTAT   
  
  
- AGGGTGGATG ACTCTAGGAG TTCCGATTGG CCCATATACA CCGTCGCCAG GGAAAGTTCT ATTACAGAAT   
  
  
- AATGTGTTGT TTGTTCTGTT AACGTTCGGA TCAACTCCTC CGTGGTTCAT ATGTGTATTA ACTAAAACCA   
  
  
- TAAAAGATAC CTGATGTTAC CGGGACGTAG TAAGTCTTGG AGAGGTTCTC CGGGTTACCC GGAGGGTCTT   
  
  
- AGGCGTAGTG TCCGTAGCTA AAGGGGGTTG GACCCAAGGC CGGTCGTCTC TCCCAACTTC TCTGTCCCGC   
  
  
- CACAAATCGG TTCATAACAC TGTCCATGTT ACACGGGAAA CTTATGGTGG GGTATCGGTT CTTCACCCTC   
  
  
- TGGTAGGTTG AGCTCCTCGA GTTTTAACTT TCCTTACTCG GGGAACATCA CTTGACAGAC ATATCTAGCG   
  
  
- TGTTGGATAA ACTACTTTCG CATCTCCTTT TGTCGGGTTC TCTGCGCAAG GACTCGAACC AATCCTTTTA   
  
  
- TTTAGGGCTA TATAAGTACG TACCCCACCA CTTATGGCGT AGTTCACATG GAAAGAAGGA CTTGTCCAAG   
  
  
- TTCCTCCGGT ACAAAGTGAT GTGCCGGAAC AAACTGAACA AGCTCCGGTG TTACTCGGCG CTCCTAGTTC   
  
  
- TCTCCCACAA TGATCTCTCG TTCAACGTAC CCTTAGTCCG CAACTTACAC TAGCGGACGC TTCCTCGCCT   
  
  
- CTCTCACCTC TCCGGACTCA GTATGTTTGT TACCGTCCAA TCCTGAGTTT CCCGGCCCAA ACAAGTTCAC   
  
  
- AGCTACCTAG CGCTCGAAAA CTCTTCCCGT TCCCGGTACC AGTCCTCCTT AAAATCCCTT CTCAAGTCAA   
  
  
- TACTACTTCT ACCCGTAACC CACCAAGTTC CTACCTTTCC GTCCTATTAC ATACGGTAAA GAACAACCTT   
  
  
- TGGAAGTAT

+     MBS

| Site Name | Organism | Position | Strand | Matrix score. | sequence | function |
| --- | --- | --- | --- | --- | --- | --- |
| MBS | Arabidopsis thaliana | 3369 | - | 6 | CAACTG | MYB binding site involved in drought-inducibility |
| MBS | Arabidopsis thaliana | 2164 | - | 6 | CAACTG | MYB binding site involved in drought-inducibility |
| MBS | Arabidopsis thaliana | 467 | - | 6 | CAACTG | MYB binding site involved in drought-inducibility |
| MBS | Arabidopsis thaliana | 358 | + | 6 | CAACTG | MYB binding site involved in drought-inducibility |

>HU02G01570.1   
+ +Up\_Stream \_Len000ATTATT ATTCTAATAT ATAGATAATA ATATAATAAT AATAATAATA ATAATAATAA   
  
  
+ TTATTATTAT TATTATTATT TATTAAATAT AATAGTTATT ATAATAATAA TAATTATAAT AATAATAAGA   
  
  
+ TTATTATCAT TATATTATTA TTATTATTAT TGTAATTTAT TATTATTATA GTAATTATTA TTGTTATATA   
  
  
+ GTAAAATTAT TATTAGTATT GTTATTATAA ATATTCATAC TTGCATATAA TATTTCTAAA ATAAAAACAG   
  
  
+ AGTTAATTCC ATTCCAAAAC TACTCACCAA ACAGCAAGGA GTGGGACCTG ATTCCTTTCC AATTTTCACC   
  
  
+ AAACAACTGG GTTCAGAATT CAAGTTCCAT ATCCAACCCC CCAGGATTCA AGTCCTGATT CCATTCCATT   
  
  
+ CCAGTTTCCC TCACCAAACT GGGCCTTAGA TTGATGGTTG TTCAGTTGGA TGAGGGTTGT TCCATGCCAA   
  
  
+ GTTAGATCAT TGTTGTCAAT TTTGAGAACG ACTATCTTAA TAAGCCTAAC GATGTTGATT TGATTTAGTT   
  
  
+ CAAACCATTT CAAATAATAC AAATCAAATT CTATTTAGAC CGACACTAAA ATAAATAGAT TGAGAAAAAT   
  
  
+ TATAGATTTT AAAGTTCAGA TTAAAATCCC TAAAGATCAA CAGATTGAAA TGCATATTTC ATAGTGCACA   
  
  
+ TGGACTTGCT TTGAATCTCG TCTAGAGAAC CTAAGAAAAA GCAAGAGTGG TTCAGAAGGT TAGAATTGTT   
  
  
+ CCAGCACATG ACAAGTACAA TCCCAACTTT TTTCAAAAGA TTTGCTACTT TTAATTGGAT TCCCATGTTG   
  
  
+ AATGAAGTTC TGGGTTTAAG GCTACAACTT TCTTGGGAGG ATAAGGTTCT TAAGTTAAGG TGGTCCTCTT   
  
  
+ TTCTTTTGAT CTTTCTTATG GGTACAACCT CATGGGTCTC ATCAACACAT GAACTCTTTT TATCTTTTAT   
  
  
+ CTTTCCCCTT TCCTTTATGC AGGATTGACA GGTGCATGGA AAACAAGAAG CAATTAAATA CTAGTGGCTT   
  
  
+ AGCCCCATCC CTAATATTTT GGCTATTCTG TTTCACCATC CTCCCCTGTC TCCATTTTCT TTGTTTCCTT   
  
  
+ TTTTTCTAGG AACTTGTGAT GGGCAAGGGA AGATCAGTAC AATGTGATGG TTGTTAGTAG CGAGAAGATG   
  
  
+ AAGTGTCACC GCAAAAATTT TGTGCCATAA TTTGTCGTAA GGGAAACAAG TATCAAACAA CAATGTCATT   
  
  
+ TATGATAAAC AAGTAACAGG AGATATGTAC TCTGAAAAAA TATAAGGTAC CTGAGAAGTT GTTAACTTAG   
  
  
+ CTTGAAAAAC CCTCCTAACT TGATAAACCC TCTTCGCTAT GTTATCCCGC ACCCTGCCAT AGAGTCACCG   
  
  
+ ATTCAGTATA TATGTAACAT ACCCAATCTT GTCTGGATAT ATAGTTAACC CTTTTGGAAT ATCTGAAAAT   
  
  
+ CCACACGAGA GTCATCCTTC TAGACTAGGA AACATGCGCC ACATTTTGGA TTCTAACCAA GTATAATAAG   
  
  
+ CATATCTTAA AGCCAAAAAT GCAAGGATAG ATACAAATAC CCATCACATG CATGTAAATG TCCCTTGTCA   
  
  
+ TGGCTCTGAT TCTCAAAATA TTAATTTTCA ATAAATTAAA TGAAATTGCA TTATTTTAAA GATTTCTGAA   
  
  
+ TGCATCCATT ATTGCAATTT TTTAAAGACT GCACAATGAT GACAATGTCA ATGGTTTCCT AAAGTTGCGC   
  
  
+ CTGATGGGCT ATTGACAGTG AAGCAACACT CCCTTTGTTC CCATTTATGT GTCTCCACTT GCCCCTTTGA   
  
  
+ AACTCTCTAC ATTTGTATGT CATCTGAATT TGATTCTCAA ATCTCAGATG ATTTGAATTC CCTTTATGCC   
  
  
+ ATCTTTACTA TCTATTGAAT GATTCTTTCT TTTCCACTTT CTTATCTTCC ACTACCTTCT TCTTGGATGC   
  
  
+ CCTTTTGCTG CTTATAAAGG GCGACACCTG AACAAGTTGC TGTATTCTCA AACCATGAAT CTGTACAGGT   
  
  
+ TTAGCCCCGA TATGTGTCCT AGCTTTGTGG GTCACGAAGA AATTGGTCCT AATTTAATAG GACACCAAGA   
  
  
+ CCCTTTTCAA AATGCATACA ATTGCACTGA TTTTGGTGAT GCTTACTTGT TGCAATTCCC AGTTGAAGAT   
  
  
+ TTATGTCAAT ACCGTGTTCC TGATCTAATG GGGCTACCTG TTGAAGATGG GGATCCTACA GAGGTCCCTG   
  
  
+ ACATTCCTGA TACATGCTTC AACTACATAA GCAATCTCCT AATGGAAGAG GGCTCGGATG ACCGCCCTGG   
  
  
+ CGCCACCCTG CAGGACTATA TGGCTGCTCA AGTCTCCTAC AGCAAGTCCT CCAATGCCGC CTTTCCCGGT   
  
  
+ GAGAGTTGCT CATTCTTTGG TAATAATCAA TGCCCTCCAC CTGCTTATCA AAGCAACCCT TATTGCCTTG   
  
  
+ ATGATTACTT CAACTTGCTA TCCAATACTA CTGGCAATAG TGTTAACAAT GCTGGTGTTC ATGACAGCAA   
  
  
+ CAACTTTGTT CAGAACAGTT TGATTGGGAA TAGGGGTACT AGTTCTAGTT CTAGGGGCAG AAGTGATTTT   
  
  
+ GGATCTACCC TTATTAGGAA TTTGCCATTA GGGACCGACA CACTTGATGT GGTCAATCCA GCACCTACTC   
  
  
+ CTCGAGCCCC TCTGGTTACG ACTCAGTGTC AGGTGTTTGA AACACACCAG TATGAGCAGG CCTTCGAACT   
  
  
+ GATCACTAAT GGAAGCTGGG AATTCAGTCA TTCTGTTGCT AATACATCAA ATGATGGAAT TCAAAATCAA   
  
  
+ CCAATTCAAG CAGTTTCAAC CCAAGAACGA TGGAATATGA AAACATTTTC CGATGACCAA CTTGAGCCTG   
  
  
+ CTCTTTTGCG AAGTCTTGTG ATGTCCTCAA ACTGTACTGA TATCTCTGAT CAGGGTGTAC ACAGGATAGT   
  
  
+ GAACCCTGAG CCACCCAACA ACAGAAATAC GGAGATTGTG TGCAATGACT CATCCAGTAA GAGTAAGACA   
  
  
+ AGACGAAGAG AGCATGGTGC AGGAGAAAAC CACGATGAAG GAGAAAACCA CGATGAGACA GGAAGGAGTA   
  
  
+ ACAAGCAATC AGCACCATCC AATGAAGTGT TCGATGAAAT TGAGCAGTAT GGTGATGTGC TGCTCTGCCC   
  
  
+ TGAAGGAAGA AGTAAACCAG CAGGGTTATG CAGCAGCAGC AACGCTAACA GAACTAGCAA CTCACAGAAC   
  
  
+ TCGGAAGACT CAAAGTCACA GCAAAATGGG AGTTCCTCCA AGGTGTCAAA AAAGCGAGGC AATAAAAAGC   
  
  
+ AAGTGGTTGA TTTGAGGACT CTCCTAACTC GATGTGCACA ATGTGTTGCT CGTGTGGATT TAACTGGTTC   
  
  
+ TTACCAGTTG CTTCAGGAGA TTCGGCAGCA TTCTTCTCCC TATGGTGATT ATCTCCAAAG AATGGCTCAT   
  
  
+ TACCTTGCTA ATGGCCTTGA GGCGCGCTTA GAAGGCAAAG GATATGAACT AACTCGTGCC AATGAGCATA   
  
  
+ TCCCACCTAC TGAGATCCTC AAGGCTAACC GGGTATATGT GGCAGCGGTC CCTTTCAAGA TAATGTCTTA   
  
  
+ TTACACAACA AACAAGACAA TTGCAAGCCT AGTTGAGGAG GCACCAAGTA TACACATAAT TGATTTTGGT   
  
  
+ ATTTTCTATG GACTACAATG GCCCTGCATC ATTCAGAACC TCTCCAAGAG GCCCAATGGG CCTCCCAGAA   
  
  
+ TCCGCATCAC AGGCATCGAT TTCCCCCAAC CTGGGTTCCG GCCAGCAGAG AGGGTTGAAG AGACAGGGCG   
  
  
+ GTGTTTAGCC AAGTATTGTG ACAGGTACAA TGTGCCCTTT GAATACCACC CCATAGCCAA GAAGTGGGAG   
  
  
+ ACCATCCAAC TCGAGGAGCT CAAAATTGAA AGGAATGAGC CCCTTGTAGT GAACTGTCTG TATAGATCGC   
  
  
+ ACAACCTATT TGATGAAAGC GTAGAGGAAA ACAGCCCAAG AGACGCGTTC CTGAGCTTGG TTAGGAAAAT   
  
  
+ AAATCCCGAT ATATTCATGC ATGGGGTGGT GAATACCGCA TCAAGTGTAC CTTTCTTCCT GAACAGGTTC   
  
  
+ AAGGAGGCCA TGTTTCACTA CACGGCCTTG TTTGACTTGT TCGAGGCCAC AATGAGCCGC GAGGATCAAG   
  
  
+ AGAGGGTGTT ACTAGAGAGC AAGTTGCATG GGAATCAGGC GTTGAATGTG ATCGCCTGCG AAGGAGCGGA   
  
  
+ GAGAGTGGAG AGGCCTGAGT CATACAAACA ATGGCAGGTT AGGACTCAAA GGGCCGGGTT TGTTCAAGTG   
  
  
+ TCGATGGATC GCGAGCTTTT GAGAAGGGCA AGGGCCATGG TCAGGAGGAA TTTTAGGGAA GAGTTCAGTT   
  
  
+ ATGATGAAGA TGGGCATTGG GTGGTTCAAG GATGGAAAGG CAGGATAATG TATGCCATTT CTTGTTGGAA   
  
  
+ ACCTTCATA  

- +Up\_Stream \_Len000TAATAA TAAGATTATA TATCTATTAT TATATTATTA TTATTATTAT TATTATTATT   
  
  
- AATAATAATA ATAATAATAA ATAATTTATA TTATCAATAA TATTATTATT ATTAATATTA TTATTATTCT   
  
  
- AATAATAGTA ATATAATAAT AATAATAATA ACATTAAATA ATAATAATAT CATTAATAAT AACAATATAT   
  
  
- CATTTTAATA ATAATCATAA CAATAATATT TATAAGTATG AACGTATATT ATAAAGATTT TATTTTTGTC   
  
  
- TCAATTAAGG TAAGGTTTTG ATGAGTGGTT TGTCGTTCCT CACCCTGGAC TAAGGAAAGG TTAAAAGTGG   
  
  
- TTTGTTGACC CAAGTCTTAA GTTCAAGGTA TAGGTTGGGG GGTCCTAAGT TCAGGACTAA GGTAAGGTAA   
  
  
- GGTCAAAGGG AGTGGTTTGA CCCGGAATCT AACTACCAAC AAGTCAACCT ACTCCCAACA AGGTACGGTT   
  
  
- CAATCTAGTA ACAACAGTTA AAACTCTTGC TGATAGAATT ATTCGGATTG CTACAACTAA ACTAAATCAA   
  
  
- GTTTGGTAAA GTTTATTATG TTTAGTTTAA GATAAATCTG GCTGTGATTT TATTTATCTA ACTCTTTTTA   
  
  
- ATATCTAAAA TTTCAAGTCT AATTTTAGGG ATTTCTAGTT GTCTAACTTT ACGTATAAAG TATCACGTGT   
  
  
- ACCTGAACGA AACTTAGAGC AGATCTCTTG GATTCTTTTT CGTTCTCACC AAGTCTTCCA ATCTTAACAA   
  
  
- GGTCGTGTAC TGTTCATGTT AGGGTTGAAA AAAGTTTTCT AAACGATGAA AATTAACCTA AGGGTACAAC   
  
  
- TTACTTCAAG ACCCAAATTC CGATGTTGAA AGAACCCTCC TATTCCAAGA ATTCAATTCC ACCAGGAGAA   
  
  
- AAGAAAACTA GAAAGAATAC CCATGTTGGA GTACCCAGAG TAGTTGTGTA CTTGAGAAAA ATAGAAAATA   
  
  
- GAAAGGGGAA AGGAAATACG TCCTAACTGT CCACGTACCT TTTGTTCTTC GTTAATTTAT GATCACCGAA   
  
  
- TCGGGGTAGG GATTATAAAA CCGATAAGAC AAAGTGGTAG GAGGGGACAG AGGTAAAAGA AACAAAGGAA   
  
  
- AAAAAGATCC TTGAACACTA CCCGTTCCCT TCTAGTCATG TTACACTACC AACAATCATC GCTCTTCTAC   
  
  
- TTCACAGTGG CGTTTTTAAA ACACGGTATT AAACAGCATT CCCTTTGTTC ATAGTTTGTT GTTACAGTAA   
  
  
- ATACTATTTG TTCATTGTCC TCTATACATG AGACTTTTTT ATATTCCATG GACTCTTCAA CAATTGAATC   
  
  
- GAACTTTTTG GGAGGATTGA ACTATTTGGG AGAAGCGATA CAATAGGGCG TGGGACGGTA TCTCAGTGGC   
  
  
- TAAGTCATAT ATACATTGTA TGGGTTAGAA CAGACCTATA TATCAATTGG GAAAACCTTA TAGACTTTTA   
  
  
- GGTGTGCTCT CAGTAGGAAG ATCTGATCCT TTGTACGCGG TGTAAAACCT AAGATTGGTT CATATTATTC   
  
  
- GTATAGAATT TCGGTTTTTA CGTTCCTATC TATGTTTATG GGTAGTGTAC GTACATTTAC AGGGAACAGT   
  
  
- ACCGAGACTA AGAGTTTTAT AATTAAAAGT TATTTAATTT ACTTTAACGT AATAAAATTT CTAAAGACTT   
  
  
- ACGTAGGTAA TAACGTTAAA AAATTTCTGA CGTGTTACTA CTGTTACAGT TACCAAAGGA TTTCAACGCG   
  
  
- GACTACCCGA TAACTGTCAC TTCGTTGTGA GGGAAACAAG GGTAAATACA CAGAGGTGAA CGGGGAAACT   
  
  
- TTGAGAGATG TAAACATACA GTAGACTTAA ACTAAGAGTT TAGAGTCTAC TAAACTTAAG GGAAATACGG   
  
  
- TAGAAATGAT AGATAACTTA CTAAGAAAGA AAAGGTGAAA GAATAGAAGG TGATGGAAGA AGAACCTACG   
  
  
- GGAAAACGAC GAATATTTCC CGCTGTGGAC TTGTTCAACG ACATAAGAGT TTGGTACTTA GACATGTCCA   
  
  
- AATCGGGGCT ATACACAGGA TCGAAACACC CAGTGCTTCT TTAACCAGGA TTAAATTATC CTGTGGTTCT   
  
  
- GGGAAAAGTT TTACGTATGT TAACGTGACT AAAACCACTA CGAATGAACA ACGTTAAGGG TCAACTTCTA   
  
  
- AATACAGTTA TGGCACAAGG ACTAGATTAC CCCGATGGAC AACTTCTACC CCTAGGATGT CTCCAGGGAC   
  
  
- TGTAAGGACT ATGTACGAAG TTGATGTATT CGTTAGAGGA TTACCTTCTC CCGAGCCTAC TGGCGGGACC   
  
  
- GCGGTGGGAC GTCCTGATAT ACCGACGAGT TCAGAGGATG TCGTTCAGGA GGTTACGGCG GAAAGGGCCA   
  
  
- CTCTCAACGA GTAAGAAACC ATTATTAGTT ACGGGAGGTG GACGAATAGT TTCGTTGGGA ATAACGGAAC   
  
  
- TACTAATGAA GTTGAACGAT AGGTTATGAT GACCGTTATC ACAATTGTTA CGACCACAAG TACTGTCGTT   
  
  
- GTTGAAACAA GTCTTGTCAA ACTAACCCTT ATCCCCATGA TCAAGATCAA GATCCCCGTC TTCACTAAAA   
  
  
- CCTAGATGGG AATAATCCTT AAACGGTAAT CCCTGGCTGT GTGAACTACA CCAGTTAGGT CGTGGATGAG   
  
  
- GAGCTCGGGG AGACCAATGC TGAGTCACAG TCCACAAACT TTGTGTGGTC ATACTCGTCC GGAAGCTTGA   
  
  
- CTAGTGATTA CCTTCGACCC TTAAGTCAGT AAGACAACGA TTATGTAGTT TACTACCTTA AGTTTTAGTT   
  
  
- GGTTAAGTTC GTCAAAGTTG GGTTCTTGCT ACCTTATACT TTTGTAAAAG GCTACTGGTT GAACTCGGAC   
  
  
- GAGAAAACGC TTCAGAACAC TACAGGAGTT TGACATGACT ATAGAGACTA GTCCCACATG TGTCCTATCA   
  
  
- CTTGGGACTC GGTGGGTTGT TGTCTTTATG CCTCTAACAC ACGTTACTGA GTAGGTCATT CTCATTCTGT   
  
  
- TCTGCTTCTC TCGTACCACG TCCTCTTTTG GTGCTACTTC CTCTTTTGGT GCTACTCTGT CCTTCCTCAT   
  
  
- TGTTCGTTAG TCGTGGTAGG TTACTTCACA AGCTACTTTA ACTCGTCATA CCACTACACG ACGAGACGGG   
  
  
- ACTTCCTTCT TCATTTGGTC GTCCCAATAC GTCGTCGTCG TTGCGATTGT CTTGATCGTT GAGTGTCTTG   
  
  
- AGCCTTCTGA GTTTCAGTGT CGTTTTACCC TCAAGGAGGT TCCACAGTTT TTTCGCTCCG TTATTTTTCG   
  
  
- TTCACCAACT AAACTCCTGA GAGGATTGAG CTACACGTGT TACACAACGA GCACACCTAA ATTGACCAAG   
  
  
- AATGGTCAAC GAAGTCCTCT AAGCCGTCGT AAGAAGAGGG ATACCACTAA TAGAGGTTTC TTACCGAGTA   
  
  
- ATGGAACGAT TACCGGAACT CCGCGCGAAT CTTCCGTTTC CTATACTTGA TTGAGCACGG TTACTCGTAT   
  
  
- AGGGTGGATG ACTCTAGGAG TTCCGATTGG CCCATATACA CCGTCGCCAG GGAAAGTTCT ATTACAGAAT   
  
  
- AATGTGTTGT TTGTTCTGTT AACGTTCGGA TCAACTCCTC CGTGGTTCAT ATGTGTATTA ACTAAAACCA   
  
  
- TAAAAGATAC CTGATGTTAC CGGGACGTAG TAAGTCTTGG AGAGGTTCTC CGGGTTACCC GGAGGGTCTT   
  
  
- AGGCGTAGTG TCCGTAGCTA AAGGGGGTTG GACCCAAGGC CGGTCGTCTC TCCCAACTTC TCTGTCCCGC   
  
  
- CACAAATCGG TTCATAACAC TGTCCATGTT ACACGGGAAA CTTATGGTGG GGTATCGGTT CTTCACCCTC   
  
  
- TGGTAGGTTG AGCTCCTCGA GTTTTAACTT TCCTTACTCG GGGAACATCA CTTGACAGAC ATATCTAGCG   
  
  
- TGTTGGATAA ACTACTTTCG CATCTCCTTT TGTCGGGTTC TCTGCGCAAG GACTCGAACC AATCCTTTTA   
  
  
- TTTAGGGCTA TATAAGTACG TACCCCACCA CTTATGGCGT AGTTCACATG GAAAGAAGGA CTTGTCCAAG   
  
  
- TTCCTCCGGT ACAAAGTGAT GTGCCGGAAC AAACTGAACA AGCTCCGGTG TTACTCGGCG CTCCTAGTTC   
  
  
- TCTCCCACAA TGATCTCTCG TTCAACGTAC CCTTAGTCCG CAACTTACAC TAGCGGACGC TTCCTCGCCT   
  
  
- CTCTCACCTC TCCGGACTCA GTATGTTTGT TACCGTCCAA TCCTGAGTTT CCCGGCCCAA ACAAGTTCAC   
  
  
- AGCTACCTAG CGCTCGAAAA CTCTTCCCGT TCCCGGTACC AGTCCTCCTT AAAATCCCTT CTCAAGTCAA   
  
  
- TACTACTTCT ACCCGTAACC CACCAAGTTC CTACCTTTCC GTCCTATTAC ATACGGTAAA GAACAACCTT   
  
  
- TGGAAGTAT

+     MRE

| Site Name | Organism | Position | Strand | Matrix score. | sequence | function |
| --- | --- | --- | --- | --- | --- | --- |
| MRE | Petroselinum crispum | 732 | + | 7 | AACCTAA | MYB binding site involved in light responsiveness |

>HU02G01570.1   
+ +Up\_Stream \_Len000ATTATT ATTCTAATAT ATAGATAATA ATATAATAAT AATAATAATA ATAATAATAA   
  
  
+ TTATTATTAT TATTATTATT TATTAAATAT AATAGTTATT ATAATAATAA TAATTATAAT AATAATAAGA   
  
  
+ TTATTATCAT TATATTATTA TTATTATTAT TGTAATTTAT TATTATTATA GTAATTATTA TTGTTATATA   
  
  
+ GTAAAATTAT TATTAGTATT GTTATTATAA ATATTCATAC TTGCATATAA TATTTCTAAA ATAAAAACAG   
  
  
+ AGTTAATTCC ATTCCAAAAC TACTCACCAA ACAGCAAGGA GTGGGACCTG ATTCCTTTCC AATTTTCACC   
  
  
+ AAACAACTGG GTTCAGAATT CAAGTTCCAT ATCCAACCCC CCAGGATTCA AGTCCTGATT CCATTCCATT   
  
  
+ CCAGTTTCCC TCACCAAACT GGGCCTTAGA TTGATGGTTG TTCAGTTGGA TGAGGGTTGT TCCATGCCAA   
  
  
+ GTTAGATCAT TGTTGTCAAT TTTGAGAACG ACTATCTTAA TAAGCCTAAC GATGTTGATT TGATTTAGTT   
  
  
+ CAAACCATTT CAAATAATAC AAATCAAATT CTATTTAGAC CGACACTAAA ATAAATAGAT TGAGAAAAAT   
  
  
+ TATAGATTTT AAAGTTCAGA TTAAAATCCC TAAAGATCAA CAGATTGAAA TGCATATTTC ATAGTGCACA   
  
  
+ TGGACTTGCT TTGAATCTCG TCTAGAGAAC CTAAGAAAAA GCAAGAGTGG TTCAGAAGGT TAGAATTGTT   
  
  
+ CCAGCACATG ACAAGTACAA TCCCAACTTT TTTCAAAAGA TTTGCTACTT TTAATTGGAT TCCCATGTTG   
  
  
+ AATGAAGTTC TGGGTTTAAG GCTACAACTT TCTTGGGAGG ATAAGGTTCT TAAGTTAAGG TGGTCCTCTT   
  
  
+ TTCTTTTGAT CTTTCTTATG GGTACAACCT CATGGGTCTC ATCAACACAT GAACTCTTTT TATCTTTTAT   
  
  
+ CTTTCCCCTT TCCTTTATGC AGGATTGACA GGTGCATGGA AAACAAGAAG CAATTAAATA CTAGTGGCTT   
  
  
+ AGCCCCATCC CTAATATTTT GGCTATTCTG TTTCACCATC CTCCCCTGTC TCCATTTTCT TTGTTTCCTT   
  
  
+ TTTTTCTAGG AACTTGTGAT GGGCAAGGGA AGATCAGTAC AATGTGATGG TTGTTAGTAG CGAGAAGATG   
  
  
+ AAGTGTCACC GCAAAAATTT TGTGCCATAA TTTGTCGTAA GGGAAACAAG TATCAAACAA CAATGTCATT   
  
  
+ TATGATAAAC AAGTAACAGG AGATATGTAC TCTGAAAAAA TATAAGGTAC CTGAGAAGTT GTTAACTTAG   
  
  
+ CTTGAAAAAC CCTCCTAACT TGATAAACCC TCTTCGCTAT GTTATCCCGC ACCCTGCCAT AGAGTCACCG   
  
  
+ ATTCAGTATA TATGTAACAT ACCCAATCTT GTCTGGATAT ATAGTTAACC CTTTTGGAAT ATCTGAAAAT   
  
  
+ CCACACGAGA GTCATCCTTC TAGACTAGGA AACATGCGCC ACATTTTGGA TTCTAACCAA GTATAATAAG   
  
  
+ CATATCTTAA AGCCAAAAAT GCAAGGATAG ATACAAATAC CCATCACATG CATGTAAATG TCCCTTGTCA   
  
  
+ TGGCTCTGAT TCTCAAAATA TTAATTTTCA ATAAATTAAA TGAAATTGCA TTATTTTAAA GATTTCTGAA   
  
  
+ TGCATCCATT ATTGCAATTT TTTAAAGACT GCACAATGAT GACAATGTCA ATGGTTTCCT AAAGTTGCGC   
  
  
+ CTGATGGGCT ATTGACAGTG AAGCAACACT CCCTTTGTTC CCATTTATGT GTCTCCACTT GCCCCTTTGA   
  
  
+ AACTCTCTAC ATTTGTATGT CATCTGAATT TGATTCTCAA ATCTCAGATG ATTTGAATTC CCTTTATGCC   
  
  
+ ATCTTTACTA TCTATTGAAT GATTCTTTCT TTTCCACTTT CTTATCTTCC ACTACCTTCT TCTTGGATGC   
  
  
+ CCTTTTGCTG CTTATAAAGG GCGACACCTG AACAAGTTGC TGTATTCTCA AACCATGAAT CTGTACAGGT   
  
  
+ TTAGCCCCGA TATGTGTCCT AGCTTTGTGG GTCACGAAGA AATTGGTCCT AATTTAATAG GACACCAAGA   
  
  
+ CCCTTTTCAA AATGCATACA ATTGCACTGA TTTTGGTGAT GCTTACTTGT TGCAATTCCC AGTTGAAGAT   
  
  
+ TTATGTCAAT ACCGTGTTCC TGATCTAATG GGGCTACCTG TTGAAGATGG GGATCCTACA GAGGTCCCTG   
  
  
+ ACATTCCTGA TACATGCTTC AACTACATAA GCAATCTCCT AATGGAAGAG GGCTCGGATG ACCGCCCTGG   
  
  
+ CGCCACCCTG CAGGACTATA TGGCTGCTCA AGTCTCCTAC AGCAAGTCCT CCAATGCCGC CTTTCCCGGT   
  
  
+ GAGAGTTGCT CATTCTTTGG TAATAATCAA TGCCCTCCAC CTGCTTATCA AAGCAACCCT TATTGCCTTG   
  
  
+ ATGATTACTT CAACTTGCTA TCCAATACTA CTGGCAATAG TGTTAACAAT GCTGGTGTTC ATGACAGCAA   
  
  
+ CAACTTTGTT CAGAACAGTT TGATTGGGAA TAGGGGTACT AGTTCTAGTT CTAGGGGCAG AAGTGATTTT   
  
  
+ GGATCTACCC TTATTAGGAA TTTGCCATTA GGGACCGACA CACTTGATGT GGTCAATCCA GCACCTACTC   
  
  
+ CTCGAGCCCC TCTGGTTACG ACTCAGTGTC AGGTGTTTGA AACACACCAG TATGAGCAGG CCTTCGAACT   
  
  
+ GATCACTAAT GGAAGCTGGG AATTCAGTCA TTCTGTTGCT AATACATCAA ATGATGGAAT TCAAAATCAA   
  
  
+ CCAATTCAAG CAGTTTCAAC CCAAGAACGA TGGAATATGA AAACATTTTC CGATGACCAA CTTGAGCCTG   
  
  
+ CTCTTTTGCG AAGTCTTGTG ATGTCCTCAA ACTGTACTGA TATCTCTGAT CAGGGTGTAC ACAGGATAGT   
  
  
+ GAACCCTGAG CCACCCAACA ACAGAAATAC GGAGATTGTG TGCAATGACT CATCCAGTAA GAGTAAGACA   
  
  
+ AGACGAAGAG AGCATGGTGC AGGAGAAAAC CACGATGAAG GAGAAAACCA CGATGAGACA GGAAGGAGTA   
  
  
+ ACAAGCAATC AGCACCATCC AATGAAGTGT TCGATGAAAT TGAGCAGTAT GGTGATGTGC TGCTCTGCCC   
  
  
+ TGAAGGAAGA AGTAAACCAG CAGGGTTATG CAGCAGCAGC AACGCTAACA GAACTAGCAA CTCACAGAAC   
  
  
+ TCGGAAGACT CAAAGTCACA GCAAAATGGG AGTTCCTCCA AGGTGTCAAA AAAGCGAGGC AATAAAAAGC   
  
  
+ AAGTGGTTGA TTTGAGGACT CTCCTAACTC GATGTGCACA ATGTGTTGCT CGTGTGGATT TAACTGGTTC   
  
  
+ TTACCAGTTG CTTCAGGAGA TTCGGCAGCA TTCTTCTCCC TATGGTGATT ATCTCCAAAG AATGGCTCAT   
  
  
+ TACCTTGCTA ATGGCCTTGA GGCGCGCTTA GAAGGCAAAG GATATGAACT AACTCGTGCC AATGAGCATA   
  
  
+ TCCCACCTAC TGAGATCCTC AAGGCTAACC GGGTATATGT GGCAGCGGTC CCTTTCAAGA TAATGTCTTA   
  
  
+ TTACACAACA AACAAGACAA TTGCAAGCCT AGTTGAGGAG GCACCAAGTA TACACATAAT TGATTTTGGT   
  
  
+ ATTTTCTATG GACTACAATG GCCCTGCATC ATTCAGAACC TCTCCAAGAG GCCCAATGGG CCTCCCAGAA   
  
  
+ TCCGCATCAC AGGCATCGAT TTCCCCCAAC CTGGGTTCCG GCCAGCAGAG AGGGTTGAAG AGACAGGGCG   
  
  
+ GTGTTTAGCC AAGTATTGTG ACAGGTACAA TGTGCCCTTT GAATACCACC CCATAGCCAA GAAGTGGGAG   
  
  
+ ACCATCCAAC TCGAGGAGCT CAAAATTGAA AGGAATGAGC CCCTTGTAGT GAACTGTCTG TATAGATCGC   
  
  
+ ACAACCTATT TGATGAAAGC GTAGAGGAAA ACAGCCCAAG AGACGCGTTC CTGAGCTTGG TTAGGAAAAT   
  
  
+ AAATCCCGAT ATATTCATGC ATGGGGTGGT GAATACCGCA TCAAGTGTAC CTTTCTTCCT GAACAGGTTC   
  
  
+ AAGGAGGCCA TGTTTCACTA CACGGCCTTG TTTGACTTGT TCGAGGCCAC AATGAGCCGC GAGGATCAAG   
  
  
+ AGAGGGTGTT ACTAGAGAGC AAGTTGCATG GGAATCAGGC GTTGAATGTG ATCGCCTGCG AAGGAGCGGA   
  
  
+ GAGAGTGGAG AGGCCTGAGT CATACAAACA ATGGCAGGTT AGGACTCAAA GGGCCGGGTT TGTTCAAGTG   
  
  
+ TCGATGGATC GCGAGCTTTT GAGAAGGGCA AGGGCCATGG TCAGGAGGAA TTTTAGGGAA GAGTTCAGTT   
  
  
+ ATGATGAAGA TGGGCATTGG GTGGTTCAAG GATGGAAAGG CAGGATAATG TATGCCATTT CTTGTTGGAA   
  
  
+ ACCTTCATA  

- +Up\_Stream \_Len000TAATAA TAAGATTATA TATCTATTAT TATATTATTA TTATTATTAT TATTATTATT   
  
  
- AATAATAATA ATAATAATAA ATAATTTATA TTATCAATAA TATTATTATT ATTAATATTA TTATTATTCT   
  
  
- AATAATAGTA ATATAATAAT AATAATAATA ACATTAAATA ATAATAATAT CATTAATAAT AACAATATAT   
  
  
- CATTTTAATA ATAATCATAA CAATAATATT TATAAGTATG AACGTATATT ATAAAGATTT TATTTTTGTC   
  
  
- TCAATTAAGG TAAGGTTTTG ATGAGTGGTT TGTCGTTCCT CACCCTGGAC TAAGGAAAGG TTAAAAGTGG   
  
  
- TTTGTTGACC CAAGTCTTAA GTTCAAGGTA TAGGTTGGGG GGTCCTAAGT TCAGGACTAA GGTAAGGTAA   
  
  
- GGTCAAAGGG AGTGGTTTGA CCCGGAATCT AACTACCAAC AAGTCAACCT ACTCCCAACA AGGTACGGTT   
  
  
- CAATCTAGTA ACAACAGTTA AAACTCTTGC TGATAGAATT ATTCGGATTG CTACAACTAA ACTAAATCAA   
  
  
- GTTTGGTAAA GTTTATTATG TTTAGTTTAA GATAAATCTG GCTGTGATTT TATTTATCTA ACTCTTTTTA   
  
  
- ATATCTAAAA TTTCAAGTCT AATTTTAGGG ATTTCTAGTT GTCTAACTTT ACGTATAAAG TATCACGTGT   
  
  
- ACCTGAACGA AACTTAGAGC AGATCTCTTG GATTCTTTTT CGTTCTCACC AAGTCTTCCA ATCTTAACAA   
  
  
- GGTCGTGTAC TGTTCATGTT AGGGTTGAAA AAAGTTTTCT AAACGATGAA AATTAACCTA AGGGTACAAC   
  
  
- TTACTTCAAG ACCCAAATTC CGATGTTGAA AGAACCCTCC TATTCCAAGA ATTCAATTCC ACCAGGAGAA   
  
  
- AAGAAAACTA GAAAGAATAC CCATGTTGGA GTACCCAGAG TAGTTGTGTA CTTGAGAAAA ATAGAAAATA   
  
  
- GAAAGGGGAA AGGAAATACG TCCTAACTGT CCACGTACCT TTTGTTCTTC GTTAATTTAT GATCACCGAA   
  
  
- TCGGGGTAGG GATTATAAAA CCGATAAGAC AAAGTGGTAG GAGGGGACAG AGGTAAAAGA AACAAAGGAA   
  
  
- AAAAAGATCC TTGAACACTA CCCGTTCCCT TCTAGTCATG TTACACTACC AACAATCATC GCTCTTCTAC   
  
  
- TTCACAGTGG CGTTTTTAAA ACACGGTATT AAACAGCATT CCCTTTGTTC ATAGTTTGTT GTTACAGTAA   
  
  
- ATACTATTTG TTCATTGTCC TCTATACATG AGACTTTTTT ATATTCCATG GACTCTTCAA CAATTGAATC   
  
  
- GAACTTTTTG GGAGGATTGA ACTATTTGGG AGAAGCGATA CAATAGGGCG TGGGACGGTA TCTCAGTGGC   
  
  
- TAAGTCATAT ATACATTGTA TGGGTTAGAA CAGACCTATA TATCAATTGG GAAAACCTTA TAGACTTTTA   
  
  
- GGTGTGCTCT CAGTAGGAAG ATCTGATCCT TTGTACGCGG TGTAAAACCT AAGATTGGTT CATATTATTC   
  
  
- GTATAGAATT TCGGTTTTTA CGTTCCTATC TATGTTTATG GGTAGTGTAC GTACATTTAC AGGGAACAGT   
  
  
- ACCGAGACTA AGAGTTTTAT AATTAAAAGT TATTTAATTT ACTTTAACGT AATAAAATTT CTAAAGACTT   
  
  
- ACGTAGGTAA TAACGTTAAA AAATTTCTGA CGTGTTACTA CTGTTACAGT TACCAAAGGA TTTCAACGCG   
  
  
- GACTACCCGA TAACTGTCAC TTCGTTGTGA GGGAAACAAG GGTAAATACA CAGAGGTGAA CGGGGAAACT   
  
  
- TTGAGAGATG TAAACATACA GTAGACTTAA ACTAAGAGTT TAGAGTCTAC TAAACTTAAG GGAAATACGG   
  
  
- TAGAAATGAT AGATAACTTA CTAAGAAAGA AAAGGTGAAA GAATAGAAGG TGATGGAAGA AGAACCTACG   
  
  
- GGAAAACGAC GAATATTTCC CGCTGTGGAC TTGTTCAACG ACATAAGAGT TTGGTACTTA GACATGTCCA   
  
  
- AATCGGGGCT ATACACAGGA TCGAAACACC CAGTGCTTCT TTAACCAGGA TTAAATTATC CTGTGGTTCT   
  
  
- GGGAAAAGTT TTACGTATGT TAACGTGACT AAAACCACTA CGAATGAACA ACGTTAAGGG TCAACTTCTA   
  
  
- AATACAGTTA TGGCACAAGG ACTAGATTAC CCCGATGGAC AACTTCTACC CCTAGGATGT CTCCAGGGAC   
  
  
- TGTAAGGACT ATGTACGAAG TTGATGTATT CGTTAGAGGA TTACCTTCTC CCGAGCCTAC TGGCGGGACC   
  
  
- GCGGTGGGAC GTCCTGATAT ACCGACGAGT TCAGAGGATG TCGTTCAGGA GGTTACGGCG GAAAGGGCCA   
  
  
- CTCTCAACGA GTAAGAAACC ATTATTAGTT ACGGGAGGTG GACGAATAGT TTCGTTGGGA ATAACGGAAC   
  
  
- TACTAATGAA GTTGAACGAT AGGTTATGAT GACCGTTATC ACAATTGTTA CGACCACAAG TACTGTCGTT   
  
  
- GTTGAAACAA GTCTTGTCAA ACTAACCCTT ATCCCCATGA TCAAGATCAA GATCCCCGTC TTCACTAAAA   
  
  
- CCTAGATGGG AATAATCCTT AAACGGTAAT CCCTGGCTGT GTGAACTACA CCAGTTAGGT CGTGGATGAG   
  
  
- GAGCTCGGGG AGACCAATGC TGAGTCACAG TCCACAAACT TTGTGTGGTC ATACTCGTCC GGAAGCTTGA   
  
  
- CTAGTGATTA CCTTCGACCC TTAAGTCAGT AAGACAACGA TTATGTAGTT TACTACCTTA AGTTTTAGTT   
  
  
- GGTTAAGTTC GTCAAAGTTG GGTTCTTGCT ACCTTATACT TTTGTAAAAG GCTACTGGTT GAACTCGGAC   
  
  
- GAGAAAACGC TTCAGAACAC TACAGGAGTT TGACATGACT ATAGAGACTA GTCCCACATG TGTCCTATCA   
  
  
- CTTGGGACTC GGTGGGTTGT TGTCTTTATG CCTCTAACAC ACGTTACTGA GTAGGTCATT CTCATTCTGT   
  
  
- TCTGCTTCTC TCGTACCACG TCCTCTTTTG GTGCTACTTC CTCTTTTGGT GCTACTCTGT CCTTCCTCAT   
  
  
- TGTTCGTTAG TCGTGGTAGG TTACTTCACA AGCTACTTTA ACTCGTCATA CCACTACACG ACGAGACGGG   
  
  
- ACTTCCTTCT TCATTTGGTC GTCCCAATAC GTCGTCGTCG TTGCGATTGT CTTGATCGTT GAGTGTCTTG   
  
  
- AGCCTTCTGA GTTTCAGTGT CGTTTTACCC TCAAGGAGGT TCCACAGTTT TTTCGCTCCG TTATTTTTCG   
  
  
- TTCACCAACT AAACTCCTGA GAGGATTGAG CTACACGTGT TACACAACGA GCACACCTAA ATTGACCAAG   
  
  
- AATGGTCAAC GAAGTCCTCT AAGCCGTCGT AAGAAGAGGG ATACCACTAA TAGAGGTTTC TTACCGAGTA   
  
  
- ATGGAACGAT TACCGGAACT CCGCGCGAAT CTTCCGTTTC CTATACTTGA TTGAGCACGG TTACTCGTAT   
  
  
- AGGGTGGATG ACTCTAGGAG TTCCGATTGG CCCATATACA CCGTCGCCAG GGAAAGTTCT ATTACAGAAT   
  
  
- AATGTGTTGT TTGTTCTGTT AACGTTCGGA TCAACTCCTC CGTGGTTCAT ATGTGTATTA ACTAAAACCA   
  
  
- TAAAAGATAC CTGATGTTAC CGGGACGTAG TAAGTCTTGG AGAGGTTCTC CGGGTTACCC GGAGGGTCTT   
  
  
- AGGCGTAGTG TCCGTAGCTA AAGGGGGTTG GACCCAAGGC CGGTCGTCTC TCCCAACTTC TCTGTCCCGC   
  
  
- CACAAATCGG TTCATAACAC TGTCCATGTT ACACGGGAAA CTTATGGTGG GGTATCGGTT CTTCACCCTC   
  
  
- TGGTAGGTTG AGCTCCTCGA GTTTTAACTT TCCTTACTCG GGGAACATCA CTTGACAGAC ATATCTAGCG   
  
  
- TGTTGGATAA ACTACTTTCG CATCTCCTTT TGTCGGGTTC TCTGCGCAAG GACTCGAACC AATCCTTTTA   
  
  
- TTTAGGGCTA TATAAGTACG TACCCCACCA CTTATGGCGT AGTTCACATG GAAAGAAGGA CTTGTCCAAG   
  
  
- TTCCTCCGGT ACAAAGTGAT GTGCCGGAAC AAACTGAACA AGCTCCGGTG TTACTCGGCG CTCCTAGTTC   
  
  
- TCTCCCACAA TGATCTCTCG TTCAACGTAC CCTTAGTCCG CAACTTACAC TAGCGGACGC TTCCTCGCCT   
  
  
- CTCTCACCTC TCCGGACTCA GTATGTTTGT TACCGTCCAA TCCTGAGTTT CCCGGCCCAA ACAAGTTCAC   
  
  
- AGCTACCTAG CGCTCGAAAA CTCTTCCCGT TCCCGGTACC AGTCCTCCTT AAAATCCCTT CTCAAGTCAA   
  
  
- TACTACTTCT ACCCGTAACC CACCAAGTTC CTACCTTTCC GTCCTATTAC ATACGGTAAA GAACAACCTT   
  
  
- TGGAAGTAT

+     MYB

| Site Name | Organism | Position | Strand | Matrix score. | sequence | function |
| --- | --- | --- | --- | --- | --- | --- |
| MYB | Arabidopsis thaliana | 3982 | - | 6 | TAACCA |  |
| MYB | Arabidopsis thaliana | 3298 | - | 6 | CAACCA |  |
| MYB | Arabidopsis thaliana | 2963 | + | 6 | CAACAG |  |
| MYB | Arabidopsis thaliana | 2802 | + | 6 | CAACCA |  |
| MYB | Arabidopsis thaliana | 2767 | - | 6 | CAACAG |  |
| MYB | Arabidopsis thaliana | 2677 | - | 6 | TAACCA |  |
| MYB | Arabidopsis thaliana | 2212 | - | 6 | CAACAG |  |
| MYB | Arabidopsis thaliana | 1528 | + | 6 | TAACCA |  |
| MYB | Arabidopsis thaliana | 1172 | - | 6 | CAACCA |  |
| MYB | Arabidopsis thaliana | 672 | + | 6 | CAACAG |  |
| MYB | Arabidopsis thaliana | 459 | - | 6 | CAACCA |  |

>HU02G01570.1   
+ +Up\_Stream \_Len000ATTATT ATTCTAATAT ATAGATAATA ATATAATAAT AATAATAATA ATAATAATAA   
  
  
+ TTATTATTAT TATTATTATT TATTAAATAT AATAGTTATT ATAATAATAA TAATTATAAT AATAATAAGA   
  
  
+ TTATTATCAT TATATTATTA TTATTATTAT TGTAATTTAT TATTATTATA GTAATTATTA TTGTTATATA   
  
  
+ GTAAAATTAT TATTAGTATT GTTATTATAA ATATTCATAC TTGCATATAA TATTTCTAAA ATAAAAACAG   
  
  
+ AGTTAATTCC ATTCCAAAAC TACTCACCAA ACAGCAAGGA GTGGGACCTG ATTCCTTTCC AATTTTCACC   
  
  
+ AAACAACTGG GTTCAGAATT CAAGTTCCAT ATCCAACCCC CCAGGATTCA AGTCCTGATT CCATTCCATT   
  
  
+ CCAGTTTCCC TCACCAAACT GGGCCTTAGA TTGATGGTTG TTCAGTTGGA TGAGGGTTGT TCCATGCCAA   
  
  
+ GTTAGATCAT TGTTGTCAAT TTTGAGAACG ACTATCTTAA TAAGCCTAAC GATGTTGATT TGATTTAGTT   
  
  
+ CAAACCATTT CAAATAATAC AAATCAAATT CTATTTAGAC CGACACTAAA ATAAATAGAT TGAGAAAAAT   
  
  
+ TATAGATTTT AAAGTTCAGA TTAAAATCCC TAAAGATCAA CAGATTGAAA TGCATATTTC ATAGTGCACA   
  
  
+ TGGACTTGCT TTGAATCTCG TCTAGAGAAC CTAAGAAAAA GCAAGAGTGG TTCAGAAGGT TAGAATTGTT   
  
  
+ CCAGCACATG ACAAGTACAA TCCCAACTTT TTTCAAAAGA TTTGCTACTT TTAATTGGAT TCCCATGTTG   
  
  
+ AATGAAGTTC TGGGTTTAAG GCTACAACTT TCTTGGGAGG ATAAGGTTCT TAAGTTAAGG TGGTCCTCTT   
  
  
+ TTCTTTTGAT CTTTCTTATG GGTACAACCT CATGGGTCTC ATCAACACAT GAACTCTTTT TATCTTTTAT   
  
  
+ CTTTCCCCTT TCCTTTATGC AGGATTGACA GGTGCATGGA AAACAAGAAG CAATTAAATA CTAGTGGCTT   
  
  
+ AGCCCCATCC CTAATATTTT GGCTATTCTG TTTCACCATC CTCCCCTGTC TCCATTTTCT TTGTTTCCTT   
  
  
+ TTTTTCTAGG AACTTGTGAT GGGCAAGGGA AGATCAGTAC AATGTGATGG TTGTTAGTAG CGAGAAGATG   
  
  
+ AAGTGTCACC GCAAAAATTT TGTGCCATAA TTTGTCGTAA GGGAAACAAG TATCAAACAA CAATGTCATT   
  
  
+ TATGATAAAC AAGTAACAGG AGATATGTAC TCTGAAAAAA TATAAGGTAC CTGAGAAGTT GTTAACTTAG   
  
  
+ CTTGAAAAAC CCTCCTAACT TGATAAACCC TCTTCGCTAT GTTATCCCGC ACCCTGCCAT AGAGTCACCG   
  
  
+ ATTCAGTATA TATGTAACAT ACCCAATCTT GTCTGGATAT ATAGTTAACC CTTTTGGAAT ATCTGAAAAT   
  
  
+ CCACACGAGA GTCATCCTTC TAGACTAGGA AACATGCGCC ACATTTTGGA TTCTAACCAA GTATAATAAG   
  
  
+ CATATCTTAA AGCCAAAAAT GCAAGGATAG ATACAAATAC CCATCACATG CATGTAAATG TCCCTTGTCA   
  
  
+ TGGCTCTGAT TCTCAAAATA TTAATTTTCA ATAAATTAAA TGAAATTGCA TTATTTTAAA GATTTCTGAA   
  
  
+ TGCATCCATT ATTGCAATTT TTTAAAGACT GCACAATGAT GACAATGTCA ATGGTTTCCT AAAGTTGCGC   
  
  
+ CTGATGGGCT ATTGACAGTG AAGCAACACT CCCTTTGTTC CCATTTATGT GTCTCCACTT GCCCCTTTGA   
  
  
+ AACTCTCTAC ATTTGTATGT CATCTGAATT TGATTCTCAA ATCTCAGATG ATTTGAATTC CCTTTATGCC   
  
  
+ ATCTTTACTA TCTATTGAAT GATTCTTTCT TTTCCACTTT CTTATCTTCC ACTACCTTCT TCTTGGATGC   
  
  
+ CCTTTTGCTG CTTATAAAGG GCGACACCTG AACAAGTTGC TGTATTCTCA AACCATGAAT CTGTACAGGT   
  
  
+ TTAGCCCCGA TATGTGTCCT AGCTTTGTGG GTCACGAAGA AATTGGTCCT AATTTAATAG GACACCAAGA   
  
  
+ CCCTTTTCAA AATGCATACA ATTGCACTGA TTTTGGTGAT GCTTACTTGT TGCAATTCCC AGTTGAAGAT   
  
  
+ TTATGTCAAT ACCGTGTTCC TGATCTAATG GGGCTACCTG TTGAAGATGG GGATCCTACA GAGGTCCCTG   
  
  
+ ACATTCCTGA TACATGCTTC AACTACATAA GCAATCTCCT AATGGAAGAG GGCTCGGATG ACCGCCCTGG   
  
  
+ CGCCACCCTG CAGGACTATA TGGCTGCTCA AGTCTCCTAC AGCAAGTCCT CCAATGCCGC CTTTCCCGGT   
  
  
+ GAGAGTTGCT CATTCTTTGG TAATAATCAA TGCCCTCCAC CTGCTTATCA AAGCAACCCT TATTGCCTTG   
  
  
+ ATGATTACTT CAACTTGCTA TCCAATACTA CTGGCAATAG TGTTAACAAT GCTGGTGTTC ATGACAGCAA   
  
  
+ CAACTTTGTT CAGAACAGTT TGATTGGGAA TAGGGGTACT AGTTCTAGTT CTAGGGGCAG AAGTGATTTT   
  
  
+ GGATCTACCC TTATTAGGAA TTTGCCATTA GGGACCGACA CACTTGATGT GGTCAATCCA GCACCTACTC   
  
  
+ CTCGAGCCCC TCTGGTTACG ACTCAGTGTC AGGTGTTTGA AACACACCAG TATGAGCAGG CCTTCGAACT   
  
  
+ GATCACTAAT GGAAGCTGGG AATTCAGTCA TTCTGTTGCT AATACATCAA ATGATGGAAT TCAAAATCAA   
  
  
+ CCAATTCAAG CAGTTTCAAC CCAAGAACGA TGGAATATGA AAACATTTTC CGATGACCAA CTTGAGCCTG   
  
  
+ CTCTTTTGCG AAGTCTTGTG ATGTCCTCAA ACTGTACTGA TATCTCTGAT CAGGGTGTAC ACAGGATAGT   
  
  
+ GAACCCTGAG CCACCCAACA ACAGAAATAC GGAGATTGTG TGCAATGACT CATCCAGTAA GAGTAAGACA   
  
  
+ AGACGAAGAG AGCATGGTGC AGGAGAAAAC CACGATGAAG GAGAAAACCA CGATGAGACA GGAAGGAGTA   
  
  
+ ACAAGCAATC AGCACCATCC AATGAAGTGT TCGATGAAAT TGAGCAGTAT GGTGATGTGC TGCTCTGCCC   
  
  
+ TGAAGGAAGA AGTAAACCAG CAGGGTTATG CAGCAGCAGC AACGCTAACA GAACTAGCAA CTCACAGAAC   
  
  
+ TCGGAAGACT CAAAGTCACA GCAAAATGGG AGTTCCTCCA AGGTGTCAAA AAAGCGAGGC AATAAAAAGC   
  
  
+ AAGTGGTTGA TTTGAGGACT CTCCTAACTC GATGTGCACA ATGTGTTGCT CGTGTGGATT TAACTGGTTC   
  
  
+ TTACCAGTTG CTTCAGGAGA TTCGGCAGCA TTCTTCTCCC TATGGTGATT ATCTCCAAAG AATGGCTCAT   
  
  
+ TACCTTGCTA ATGGCCTTGA GGCGCGCTTA GAAGGCAAAG GATATGAACT AACTCGTGCC AATGAGCATA   
  
  
+ TCCCACCTAC TGAGATCCTC AAGGCTAACC GGGTATATGT GGCAGCGGTC CCTTTCAAGA TAATGTCTTA   
  
  
+ TTACACAACA AACAAGACAA TTGCAAGCCT AGTTGAGGAG GCACCAAGTA TACACATAAT TGATTTTGGT   
  
  
+ ATTTTCTATG GACTACAATG GCCCTGCATC ATTCAGAACC TCTCCAAGAG GCCCAATGGG CCTCCCAGAA   
  
  
+ TCCGCATCAC AGGCATCGAT TTCCCCCAAC CTGGGTTCCG GCCAGCAGAG AGGGTTGAAG AGACAGGGCG   
  
  
+ GTGTTTAGCC AAGTATTGTG ACAGGTACAA TGTGCCCTTT GAATACCACC CCATAGCCAA GAAGTGGGAG   
  
  
+ ACCATCCAAC TCGAGGAGCT CAAAATTGAA AGGAATGAGC CCCTTGTAGT GAACTGTCTG TATAGATCGC   
  
  
+ ACAACCTATT TGATGAAAGC GTAGAGGAAA ACAGCCCAAG AGACGCGTTC CTGAGCTTGG TTAGGAAAAT   
  
  
+ AAATCCCGAT ATATTCATGC ATGGGGTGGT GAATACCGCA TCAAGTGTAC CTTTCTTCCT GAACAGGTTC   
  
  
+ AAGGAGGCCA TGTTTCACTA CACGGCCTTG TTTGACTTGT TCGAGGCCAC AATGAGCCGC GAGGATCAAG   
  
  
+ AGAGGGTGTT ACTAGAGAGC AAGTTGCATG GGAATCAGGC GTTGAATGTG ATCGCCTGCG AAGGAGCGGA   
  
  
+ GAGAGTGGAG AGGCCTGAGT CATACAAACA ATGGCAGGTT AGGACTCAAA GGGCCGGGTT TGTTCAAGTG   
  
  
+ TCGATGGATC GCGAGCTTTT GAGAAGGGCA AGGGCCATGG TCAGGAGGAA TTTTAGGGAA GAGTTCAGTT   
  
  
+ ATGATGAAGA TGGGCATTGG GTGGTTCAAG GATGGAAAGG CAGGATAATG TATGCCATTT CTTGTTGGAA   
  
  
+ ACCTTCATA  

- +Up\_Stream \_Len000TAATAA TAAGATTATA TATCTATTAT TATATTATTA TTATTATTAT TATTATTATT   
  
  
- AATAATAATA ATAATAATAA ATAATTTATA TTATCAATAA TATTATTATT ATTAATATTA TTATTATTCT   
  
  
- AATAATAGTA ATATAATAAT AATAATAATA ACATTAAATA ATAATAATAT CATTAATAAT AACAATATAT   
  
  
- CATTTTAATA ATAATCATAA CAATAATATT TATAAGTATG AACGTATATT ATAAAGATTT TATTTTTGTC   
  
  
- TCAATTAAGG TAAGGTTTTG ATGAGTGGTT TGTCGTTCCT CACCCTGGAC TAAGGAAAGG TTAAAAGTGG   
  
  
- TTTGTTGACC CAAGTCTTAA GTTCAAGGTA TAGGTTGGGG GGTCCTAAGT TCAGGACTAA GGTAAGGTAA   
  
  
- GGTCAAAGGG AGTGGTTTGA CCCGGAATCT AACTACCAAC AAGTCAACCT ACTCCCAACA AGGTACGGTT   
  
  
- CAATCTAGTA ACAACAGTTA AAACTCTTGC TGATAGAATT ATTCGGATTG CTACAACTAA ACTAAATCAA   
  
  
- GTTTGGTAAA GTTTATTATG TTTAGTTTAA GATAAATCTG GCTGTGATTT TATTTATCTA ACTCTTTTTA   
  
  
- ATATCTAAAA TTTCAAGTCT AATTTTAGGG ATTTCTAGTT GTCTAACTTT ACGTATAAAG TATCACGTGT   
  
  
- ACCTGAACGA AACTTAGAGC AGATCTCTTG GATTCTTTTT CGTTCTCACC AAGTCTTCCA ATCTTAACAA   
  
  
- GGTCGTGTAC TGTTCATGTT AGGGTTGAAA AAAGTTTTCT AAACGATGAA AATTAACCTA AGGGTACAAC   
  
  
- TTACTTCAAG ACCCAAATTC CGATGTTGAA AGAACCCTCC TATTCCAAGA ATTCAATTCC ACCAGGAGAA   
  
  
- AAGAAAACTA GAAAGAATAC CCATGTTGGA GTACCCAGAG TAGTTGTGTA CTTGAGAAAA ATAGAAAATA   
  
  
- GAAAGGGGAA AGGAAATACG TCCTAACTGT CCACGTACCT TTTGTTCTTC GTTAATTTAT GATCACCGAA   
  
  
- TCGGGGTAGG GATTATAAAA CCGATAAGAC AAAGTGGTAG GAGGGGACAG AGGTAAAAGA AACAAAGGAA   
  
  
- AAAAAGATCC TTGAACACTA CCCGTTCCCT TCTAGTCATG TTACACTACC AACAATCATC GCTCTTCTAC   
  
  
- TTCACAGTGG CGTTTTTAAA ACACGGTATT AAACAGCATT CCCTTTGTTC ATAGTTTGTT GTTACAGTAA   
  
  
- ATACTATTTG TTCATTGTCC TCTATACATG AGACTTTTTT ATATTCCATG GACTCTTCAA CAATTGAATC   
  
  
- GAACTTTTTG GGAGGATTGA ACTATTTGGG AGAAGCGATA CAATAGGGCG TGGGACGGTA TCTCAGTGGC   
  
  
- TAAGTCATAT ATACATTGTA TGGGTTAGAA CAGACCTATA TATCAATTGG GAAAACCTTA TAGACTTTTA   
  
  
- GGTGTGCTCT CAGTAGGAAG ATCTGATCCT TTGTACGCGG TGTAAAACCT AAGATTGGTT CATATTATTC   
  
  
- GTATAGAATT TCGGTTTTTA CGTTCCTATC TATGTTTATG GGTAGTGTAC GTACATTTAC AGGGAACAGT   
  
  
- ACCGAGACTA AGAGTTTTAT AATTAAAAGT TATTTAATTT ACTTTAACGT AATAAAATTT CTAAAGACTT   
  
  
- ACGTAGGTAA TAACGTTAAA AAATTTCTGA CGTGTTACTA CTGTTACAGT TACCAAAGGA TTTCAACGCG   
  
  
- GACTACCCGA TAACTGTCAC TTCGTTGTGA GGGAAACAAG GGTAAATACA CAGAGGTGAA CGGGGAAACT   
  
  
- TTGAGAGATG TAAACATACA GTAGACTTAA ACTAAGAGTT TAGAGTCTAC TAAACTTAAG GGAAATACGG   
  
  
- TAGAAATGAT AGATAACTTA CTAAGAAAGA AAAGGTGAAA GAATAGAAGG TGATGGAAGA AGAACCTACG   
  
  
- GGAAAACGAC GAATATTTCC CGCTGTGGAC TTGTTCAACG ACATAAGAGT TTGGTACTTA GACATGTCCA   
  
  
- AATCGGGGCT ATACACAGGA TCGAAACACC CAGTGCTTCT TTAACCAGGA TTAAATTATC CTGTGGTTCT   
  
  
- GGGAAAAGTT TTACGTATGT TAACGTGACT AAAACCACTA CGAATGAACA ACGTTAAGGG TCAACTTCTA   
  
  
- AATACAGTTA TGGCACAAGG ACTAGATTAC CCCGATGGAC AACTTCTACC CCTAGGATGT CTCCAGGGAC   
  
  
- TGTAAGGACT ATGTACGAAG TTGATGTATT CGTTAGAGGA TTACCTTCTC CCGAGCCTAC TGGCGGGACC   
  
  
- GCGGTGGGAC GTCCTGATAT ACCGACGAGT TCAGAGGATG TCGTTCAGGA GGTTACGGCG GAAAGGGCCA   
  
  
- CTCTCAACGA GTAAGAAACC ATTATTAGTT ACGGGAGGTG GACGAATAGT TTCGTTGGGA ATAACGGAAC   
  
  
- TACTAATGAA GTTGAACGAT AGGTTATGAT GACCGTTATC ACAATTGTTA CGACCACAAG TACTGTCGTT   
  
  
- GTTGAAACAA GTCTTGTCAA ACTAACCCTT ATCCCCATGA TCAAGATCAA GATCCCCGTC TTCACTAAAA   
  
  
- CCTAGATGGG AATAATCCTT AAACGGTAAT CCCTGGCTGT GTGAACTACA CCAGTTAGGT CGTGGATGAG   
  
  
- GAGCTCGGGG AGACCAATGC TGAGTCACAG TCCACAAACT TTGTGTGGTC ATACTCGTCC GGAAGCTTGA   
  
  
- CTAGTGATTA CCTTCGACCC TTAAGTCAGT AAGACAACGA TTATGTAGTT TACTACCTTA AGTTTTAGTT   
  
  
- GGTTAAGTTC GTCAAAGTTG GGTTCTTGCT ACCTTATACT TTTGTAAAAG GCTACTGGTT GAACTCGGAC   
  
  
- GAGAAAACGC TTCAGAACAC TACAGGAGTT TGACATGACT ATAGAGACTA GTCCCACATG TGTCCTATCA   
  
  
- CTTGGGACTC GGTGGGTTGT TGTCTTTATG CCTCTAACAC ACGTTACTGA GTAGGTCATT CTCATTCTGT   
  
  
- TCTGCTTCTC TCGTACCACG TCCTCTTTTG GTGCTACTTC CTCTTTTGGT GCTACTCTGT CCTTCCTCAT   
  
  
- TGTTCGTTAG TCGTGGTAGG TTACTTCACA AGCTACTTTA ACTCGTCATA CCACTACACG ACGAGACGGG   
  
  
- ACTTCCTTCT TCATTTGGTC GTCCCAATAC GTCGTCGTCG TTGCGATTGT CTTGATCGTT GAGTGTCTTG   
  
  
- AGCCTTCTGA GTTTCAGTGT CGTTTTACCC TCAAGGAGGT TCCACAGTTT TTTCGCTCCG TTATTTTTCG   
  
  
- TTCACCAACT AAACTCCTGA GAGGATTGAG CTACACGTGT TACACAACGA GCACACCTAA ATTGACCAAG   
  
  
- AATGGTCAAC GAAGTCCTCT AAGCCGTCGT AAGAAGAGGG ATACCACTAA TAGAGGTTTC TTACCGAGTA   
  
  
- ATGGAACGAT TACCGGAACT CCGCGCGAAT CTTCCGTTTC CTATACTTGA TTGAGCACGG TTACTCGTAT   
  
  
- AGGGTGGATG ACTCTAGGAG TTCCGATTGG CCCATATACA CCGTCGCCAG GGAAAGTTCT ATTACAGAAT   
  
  
- AATGTGTTGT TTGTTCTGTT AACGTTCGGA TCAACTCCTC CGTGGTTCAT ATGTGTATTA ACTAAAACCA   
  
  
- TAAAAGATAC CTGATGTTAC CGGGACGTAG TAAGTCTTGG AGAGGTTCTC CGGGTTACCC GGAGGGTCTT   
  
  
- AGGCGTAGTG TCCGTAGCTA AAGGGGGTTG GACCCAAGGC CGGTCGTCTC TCCCAACTTC TCTGTCCCGC   
  
  
- CACAAATCGG TTCATAACAC TGTCCATGTT ACACGGGAAA CTTATGGTGG GGTATCGGTT CTTCACCCTC   
  
  
- TGGTAGGTTG AGCTCCTCGA GTTTTAACTT TCCTTACTCG GGGAACATCA CTTGACAGAC ATATCTAGCG   
  
  
- TGTTGGATAA ACTACTTTCG CATCTCCTTT TGTCGGGTTC TCTGCGCAAG GACTCGAACC AATCCTTTTA   
  
  
- TTTAGGGCTA TATAAGTACG TACCCCACCA CTTATGGCGT AGTTCACATG GAAAGAAGGA CTTGTCCAAG   
  
  
- TTCCTCCGGT ACAAAGTGAT GTGCCGGAAC AAACTGAACA AGCTCCGGTG TTACTCGGCG CTCCTAGTTC   
  
  
- TCTCCCACAA TGATCTCTCG TTCAACGTAC CCTTAGTCCG CAACTTACAC TAGCGGACGC TTCCTCGCCT   
  
  
- CTCTCACCTC TCCGGACTCA GTATGTTTGT TACCGTCCAA TCCTGAGTTT CCCGGCCCAA ACAAGTTCAC   
  
  
- AGCTACCTAG CGCTCGAAAA CTCTTCCCGT TCCCGGTACC AGTCCTCCTT AAAATCCCTT CTCAAGTCAA   
  
  
- TACTACTTCT ACCCGTAACC CACCAAGTTC CTACCTTTCC GTCCTATTAC ATACGGTAAA GAACAACCTT   
  
  
- TGGAAGTAT

+     MYB-like sequence

| Site Name | Organism | Position | Strand | Matrix score. | sequence | function |
| --- | --- | --- | --- | --- | --- | --- |
| MYB-like sequence | Arabidopsis thaliana | 2677 | - | 6 | TAACCA |  |
| MYB-like sequence | Arabidopsis thaliana | 1528 | + | 6 | TAACCA |  |
| MYB-like sequence | Arabidopsis thaliana | 3982 | - | 6 | TAACCA |  |

>HU02G01570.1   
+ +Up\_Stream \_Len000ATTATT ATTCTAATAT ATAGATAATA ATATAATAAT AATAATAATA ATAATAATAA   
  
  
+ TTATTATTAT TATTATTATT TATTAAATAT AATAGTTATT ATAATAATAA TAATTATAAT AATAATAAGA   
  
  
+ TTATTATCAT TATATTATTA TTATTATTAT TGTAATTTAT TATTATTATA GTAATTATTA TTGTTATATA   
  
  
+ GTAAAATTAT TATTAGTATT GTTATTATAA ATATTCATAC TTGCATATAA TATTTCTAAA ATAAAAACAG   
  
  
+ AGTTAATTCC ATTCCAAAAC TACTCACCAA ACAGCAAGGA GTGGGACCTG ATTCCTTTCC AATTTTCACC   
  
  
+ AAACAACTGG GTTCAGAATT CAAGTTCCAT ATCCAACCCC CCAGGATTCA AGTCCTGATT CCATTCCATT   
  
  
+ CCAGTTTCCC TCACCAAACT GGGCCTTAGA TTGATGGTTG TTCAGTTGGA TGAGGGTTGT TCCATGCCAA   
  
  
+ GTTAGATCAT TGTTGTCAAT TTTGAGAACG ACTATCTTAA TAAGCCTAAC GATGTTGATT TGATTTAGTT   
  
  
+ CAAACCATTT CAAATAATAC AAATCAAATT CTATTTAGAC CGACACTAAA ATAAATAGAT TGAGAAAAAT   
  
  
+ TATAGATTTT AAAGTTCAGA TTAAAATCCC TAAAGATCAA CAGATTGAAA TGCATATTTC ATAGTGCACA   
  
  
+ TGGACTTGCT TTGAATCTCG TCTAGAGAAC CTAAGAAAAA GCAAGAGTGG TTCAGAAGGT TAGAATTGTT   
  
  
+ CCAGCACATG ACAAGTACAA TCCCAACTTT TTTCAAAAGA TTTGCTACTT TTAATTGGAT TCCCATGTTG   
  
  
+ AATGAAGTTC TGGGTTTAAG GCTACAACTT TCTTGGGAGG ATAAGGTTCT TAAGTTAAGG TGGTCCTCTT   
  
  
+ TTCTTTTGAT CTTTCTTATG GGTACAACCT CATGGGTCTC ATCAACACAT GAACTCTTTT TATCTTTTAT   
  
  
+ CTTTCCCCTT TCCTTTATGC AGGATTGACA GGTGCATGGA AAACAAGAAG CAATTAAATA CTAGTGGCTT   
  
  
+ AGCCCCATCC CTAATATTTT GGCTATTCTG TTTCACCATC CTCCCCTGTC TCCATTTTCT TTGTTTCCTT   
  
  
+ TTTTTCTAGG AACTTGTGAT GGGCAAGGGA AGATCAGTAC AATGTGATGG TTGTTAGTAG CGAGAAGATG   
  
  
+ AAGTGTCACC GCAAAAATTT TGTGCCATAA TTTGTCGTAA GGGAAACAAG TATCAAACAA CAATGTCATT   
  
  
+ TATGATAAAC AAGTAACAGG AGATATGTAC TCTGAAAAAA TATAAGGTAC CTGAGAAGTT GTTAACTTAG   
  
  
+ CTTGAAAAAC CCTCCTAACT TGATAAACCC TCTTCGCTAT GTTATCCCGC ACCCTGCCAT AGAGTCACCG   
  
  
+ ATTCAGTATA TATGTAACAT ACCCAATCTT GTCTGGATAT ATAGTTAACC CTTTTGGAAT ATCTGAAAAT   
  
  
+ CCACACGAGA GTCATCCTTC TAGACTAGGA AACATGCGCC ACATTTTGGA TTCTAACCAA GTATAATAAG   
  
  
+ CATATCTTAA AGCCAAAAAT GCAAGGATAG ATACAAATAC CCATCACATG CATGTAAATG TCCCTTGTCA   
  
  
+ TGGCTCTGAT TCTCAAAATA TTAATTTTCA ATAAATTAAA TGAAATTGCA TTATTTTAAA GATTTCTGAA   
  
  
+ TGCATCCATT ATTGCAATTT TTTAAAGACT GCACAATGAT GACAATGTCA ATGGTTTCCT AAAGTTGCGC   
  
  
+ CTGATGGGCT ATTGACAGTG AAGCAACACT CCCTTTGTTC CCATTTATGT GTCTCCACTT GCCCCTTTGA   
  
  
+ AACTCTCTAC ATTTGTATGT CATCTGAATT TGATTCTCAA ATCTCAGATG ATTTGAATTC CCTTTATGCC   
  
  
+ ATCTTTACTA TCTATTGAAT GATTCTTTCT TTTCCACTTT CTTATCTTCC ACTACCTTCT TCTTGGATGC   
  
  
+ CCTTTTGCTG CTTATAAAGG GCGACACCTG AACAAGTTGC TGTATTCTCA AACCATGAAT CTGTACAGGT   
  
  
+ TTAGCCCCGA TATGTGTCCT AGCTTTGTGG GTCACGAAGA AATTGGTCCT AATTTAATAG GACACCAAGA   
  
  
+ CCCTTTTCAA AATGCATACA ATTGCACTGA TTTTGGTGAT GCTTACTTGT TGCAATTCCC AGTTGAAGAT   
  
  
+ TTATGTCAAT ACCGTGTTCC TGATCTAATG GGGCTACCTG TTGAAGATGG GGATCCTACA GAGGTCCCTG   
  
  
+ ACATTCCTGA TACATGCTTC AACTACATAA GCAATCTCCT AATGGAAGAG GGCTCGGATG ACCGCCCTGG   
  
  
+ CGCCACCCTG CAGGACTATA TGGCTGCTCA AGTCTCCTAC AGCAAGTCCT CCAATGCCGC CTTTCCCGGT   
  
  
+ GAGAGTTGCT CATTCTTTGG TAATAATCAA TGCCCTCCAC CTGCTTATCA AAGCAACCCT TATTGCCTTG   
  
  
+ ATGATTACTT CAACTTGCTA TCCAATACTA CTGGCAATAG TGTTAACAAT GCTGGTGTTC ATGACAGCAA   
  
  
+ CAACTTTGTT CAGAACAGTT TGATTGGGAA TAGGGGTACT AGTTCTAGTT CTAGGGGCAG AAGTGATTTT   
  
  
+ GGATCTACCC TTATTAGGAA TTTGCCATTA GGGACCGACA CACTTGATGT GGTCAATCCA GCACCTACTC   
  
  
+ CTCGAGCCCC TCTGGTTACG ACTCAGTGTC AGGTGTTTGA AACACACCAG TATGAGCAGG CCTTCGAACT   
  
  
+ GATCACTAAT GGAAGCTGGG AATTCAGTCA TTCTGTTGCT AATACATCAA ATGATGGAAT TCAAAATCAA   
  
  
+ CCAATTCAAG CAGTTTCAAC CCAAGAACGA TGGAATATGA AAACATTTTC CGATGACCAA CTTGAGCCTG   
  
  
+ CTCTTTTGCG AAGTCTTGTG ATGTCCTCAA ACTGTACTGA TATCTCTGAT CAGGGTGTAC ACAGGATAGT   
  
  
+ GAACCCTGAG CCACCCAACA ACAGAAATAC GGAGATTGTG TGCAATGACT CATCCAGTAA GAGTAAGACA   
  
  
+ AGACGAAGAG AGCATGGTGC AGGAGAAAAC CACGATGAAG GAGAAAACCA CGATGAGACA GGAAGGAGTA   
  
  
+ ACAAGCAATC AGCACCATCC AATGAAGTGT TCGATGAAAT TGAGCAGTAT GGTGATGTGC TGCTCTGCCC   
  
  
+ TGAAGGAAGA AGTAAACCAG CAGGGTTATG CAGCAGCAGC AACGCTAACA GAACTAGCAA CTCACAGAAC   
  
  
+ TCGGAAGACT CAAAGTCACA GCAAAATGGG AGTTCCTCCA AGGTGTCAAA AAAGCGAGGC AATAAAAAGC   
  
  
+ AAGTGGTTGA TTTGAGGACT CTCCTAACTC GATGTGCACA ATGTGTTGCT CGTGTGGATT TAACTGGTTC   
  
  
+ TTACCAGTTG CTTCAGGAGA TTCGGCAGCA TTCTTCTCCC TATGGTGATT ATCTCCAAAG AATGGCTCAT   
  
  
+ TACCTTGCTA ATGGCCTTGA GGCGCGCTTA GAAGGCAAAG GATATGAACT AACTCGTGCC AATGAGCATA   
  
  
+ TCCCACCTAC TGAGATCCTC AAGGCTAACC GGGTATATGT GGCAGCGGTC CCTTTCAAGA TAATGTCTTA   
  
  
+ TTACACAACA AACAAGACAA TTGCAAGCCT AGTTGAGGAG GCACCAAGTA TACACATAAT TGATTTTGGT   
  
  
+ ATTTTCTATG GACTACAATG GCCCTGCATC ATTCAGAACC TCTCCAAGAG GCCCAATGGG CCTCCCAGAA   
  
  
+ TCCGCATCAC AGGCATCGAT TTCCCCCAAC CTGGGTTCCG GCCAGCAGAG AGGGTTGAAG AGACAGGGCG   
  
  
+ GTGTTTAGCC AAGTATTGTG ACAGGTACAA TGTGCCCTTT GAATACCACC CCATAGCCAA GAAGTGGGAG   
  
  
+ ACCATCCAAC TCGAGGAGCT CAAAATTGAA AGGAATGAGC CCCTTGTAGT GAACTGTCTG TATAGATCGC   
  
  
+ ACAACCTATT TGATGAAAGC GTAGAGGAAA ACAGCCCAAG AGACGCGTTC CTGAGCTTGG TTAGGAAAAT   
  
  
+ AAATCCCGAT ATATTCATGC ATGGGGTGGT GAATACCGCA TCAAGTGTAC CTTTCTTCCT GAACAGGTTC   
  
  
+ AAGGAGGCCA TGTTTCACTA CACGGCCTTG TTTGACTTGT TCGAGGCCAC AATGAGCCGC GAGGATCAAG   
  
  
+ AGAGGGTGTT ACTAGAGAGC AAGTTGCATG GGAATCAGGC GTTGAATGTG ATCGCCTGCG AAGGAGCGGA   
  
  
+ GAGAGTGGAG AGGCCTGAGT CATACAAACA ATGGCAGGTT AGGACTCAAA GGGCCGGGTT TGTTCAAGTG   
  
  
+ TCGATGGATC GCGAGCTTTT GAGAAGGGCA AGGGCCATGG TCAGGAGGAA TTTTAGGGAA GAGTTCAGTT   
  
  
+ ATGATGAAGA TGGGCATTGG GTGGTTCAAG GATGGAAAGG CAGGATAATG TATGCCATTT CTTGTTGGAA   
  
  
+ ACCTTCATA  

- +Up\_Stream \_Len000TAATAA TAAGATTATA TATCTATTAT TATATTATTA TTATTATTAT TATTATTATT   
  
  
- AATAATAATA ATAATAATAA ATAATTTATA TTATCAATAA TATTATTATT ATTAATATTA TTATTATTCT   
  
  
- AATAATAGTA ATATAATAAT AATAATAATA ACATTAAATA ATAATAATAT CATTAATAAT AACAATATAT   
  
  
- CATTTTAATA ATAATCATAA CAATAATATT TATAAGTATG AACGTATATT ATAAAGATTT TATTTTTGTC   
  
  
- TCAATTAAGG TAAGGTTTTG ATGAGTGGTT TGTCGTTCCT CACCCTGGAC TAAGGAAAGG TTAAAAGTGG   
  
  
- TTTGTTGACC CAAGTCTTAA GTTCAAGGTA TAGGTTGGGG GGTCCTAAGT TCAGGACTAA GGTAAGGTAA   
  
  
- GGTCAAAGGG AGTGGTTTGA CCCGGAATCT AACTACCAAC AAGTCAACCT ACTCCCAACA AGGTACGGTT   
  
  
- CAATCTAGTA ACAACAGTTA AAACTCTTGC TGATAGAATT ATTCGGATTG CTACAACTAA ACTAAATCAA   
  
  
- GTTTGGTAAA GTTTATTATG TTTAGTTTAA GATAAATCTG GCTGTGATTT TATTTATCTA ACTCTTTTTA   
  
  
- ATATCTAAAA TTTCAAGTCT AATTTTAGGG ATTTCTAGTT GTCTAACTTT ACGTATAAAG TATCACGTGT   
  
  
- ACCTGAACGA AACTTAGAGC AGATCTCTTG GATTCTTTTT CGTTCTCACC AAGTCTTCCA ATCTTAACAA   
  
  
- GGTCGTGTAC TGTTCATGTT AGGGTTGAAA AAAGTTTTCT AAACGATGAA AATTAACCTA AGGGTACAAC   
  
  
- TTACTTCAAG ACCCAAATTC CGATGTTGAA AGAACCCTCC TATTCCAAGA ATTCAATTCC ACCAGGAGAA   
  
  
- AAGAAAACTA GAAAGAATAC CCATGTTGGA GTACCCAGAG TAGTTGTGTA CTTGAGAAAA ATAGAAAATA   
  
  
- GAAAGGGGAA AGGAAATACG TCCTAACTGT CCACGTACCT TTTGTTCTTC GTTAATTTAT GATCACCGAA   
  
  
- TCGGGGTAGG GATTATAAAA CCGATAAGAC AAAGTGGTAG GAGGGGACAG AGGTAAAAGA AACAAAGGAA   
  
  
- AAAAAGATCC TTGAACACTA CCCGTTCCCT TCTAGTCATG TTACACTACC AACAATCATC GCTCTTCTAC   
  
  
- TTCACAGTGG CGTTTTTAAA ACACGGTATT AAACAGCATT CCCTTTGTTC ATAGTTTGTT GTTACAGTAA   
  
  
- ATACTATTTG TTCATTGTCC TCTATACATG AGACTTTTTT ATATTCCATG GACTCTTCAA CAATTGAATC   
  
  
- GAACTTTTTG GGAGGATTGA ACTATTTGGG AGAAGCGATA CAATAGGGCG TGGGACGGTA TCTCAGTGGC   
  
  
- TAAGTCATAT ATACATTGTA TGGGTTAGAA CAGACCTATA TATCAATTGG GAAAACCTTA TAGACTTTTA   
  
  
- GGTGTGCTCT CAGTAGGAAG ATCTGATCCT TTGTACGCGG TGTAAAACCT AAGATTGGTT CATATTATTC   
  
  
- GTATAGAATT TCGGTTTTTA CGTTCCTATC TATGTTTATG GGTAGTGTAC GTACATTTAC AGGGAACAGT   
  
  
- ACCGAGACTA AGAGTTTTAT AATTAAAAGT TATTTAATTT ACTTTAACGT AATAAAATTT CTAAAGACTT   
  
  
- ACGTAGGTAA TAACGTTAAA AAATTTCTGA CGTGTTACTA CTGTTACAGT TACCAAAGGA TTTCAACGCG   
  
  
- GACTACCCGA TAACTGTCAC TTCGTTGTGA GGGAAACAAG GGTAAATACA CAGAGGTGAA CGGGGAAACT   
  
  
- TTGAGAGATG TAAACATACA GTAGACTTAA ACTAAGAGTT TAGAGTCTAC TAAACTTAAG GGAAATACGG   
  
  
- TAGAAATGAT AGATAACTTA CTAAGAAAGA AAAGGTGAAA GAATAGAAGG TGATGGAAGA AGAACCTACG   
  
  
- GGAAAACGAC GAATATTTCC CGCTGTGGAC TTGTTCAACG ACATAAGAGT TTGGTACTTA GACATGTCCA   
  
  
- AATCGGGGCT ATACACAGGA TCGAAACACC CAGTGCTTCT TTAACCAGGA TTAAATTATC CTGTGGTTCT   
  
  
- GGGAAAAGTT TTACGTATGT TAACGTGACT AAAACCACTA CGAATGAACA ACGTTAAGGG TCAACTTCTA   
  
  
- AATACAGTTA TGGCACAAGG ACTAGATTAC CCCGATGGAC AACTTCTACC CCTAGGATGT CTCCAGGGAC   
  
  
- TGTAAGGACT ATGTACGAAG TTGATGTATT CGTTAGAGGA TTACCTTCTC CCGAGCCTAC TGGCGGGACC   
  
  
- GCGGTGGGAC GTCCTGATAT ACCGACGAGT TCAGAGGATG TCGTTCAGGA GGTTACGGCG GAAAGGGCCA   
  
  
- CTCTCAACGA GTAAGAAACC ATTATTAGTT ACGGGAGGTG GACGAATAGT TTCGTTGGGA ATAACGGAAC   
  
  
- TACTAATGAA GTTGAACGAT AGGTTATGAT GACCGTTATC ACAATTGTTA CGACCACAAG TACTGTCGTT   
  
  
- GTTGAAACAA GTCTTGTCAA ACTAACCCTT ATCCCCATGA TCAAGATCAA GATCCCCGTC TTCACTAAAA   
  
  
- CCTAGATGGG AATAATCCTT AAACGGTAAT CCCTGGCTGT GTGAACTACA CCAGTTAGGT CGTGGATGAG   
  
  
- GAGCTCGGGG AGACCAATGC TGAGTCACAG TCCACAAACT TTGTGTGGTC ATACTCGTCC GGAAGCTTGA   
  
  
- CTAGTGATTA CCTTCGACCC TTAAGTCAGT AAGACAACGA TTATGTAGTT TACTACCTTA AGTTTTAGTT   
  
  
- GGTTAAGTTC GTCAAAGTTG GGTTCTTGCT ACCTTATACT TTTGTAAAAG GCTACTGGTT GAACTCGGAC   
  
  
- GAGAAAACGC TTCAGAACAC TACAGGAGTT TGACATGACT ATAGAGACTA GTCCCACATG TGTCCTATCA   
  
  
- CTTGGGACTC GGTGGGTTGT TGTCTTTATG CCTCTAACAC ACGTTACTGA GTAGGTCATT CTCATTCTGT   
  
  
- TCTGCTTCTC TCGTACCACG TCCTCTTTTG GTGCTACTTC CTCTTTTGGT GCTACTCTGT CCTTCCTCAT   
  
  
- TGTTCGTTAG TCGTGGTAGG TTACTTCACA AGCTACTTTA ACTCGTCATA CCACTACACG ACGAGACGGG   
  
  
- ACTTCCTTCT TCATTTGGTC GTCCCAATAC GTCGTCGTCG TTGCGATTGT CTTGATCGTT GAGTGTCTTG   
  
  
- AGCCTTCTGA GTTTCAGTGT CGTTTTACCC TCAAGGAGGT TCCACAGTTT TTTCGCTCCG TTATTTTTCG   
  
  
- TTCACCAACT AAACTCCTGA GAGGATTGAG CTACACGTGT TACACAACGA GCACACCTAA ATTGACCAAG   
  
  
- AATGGTCAAC GAAGTCCTCT AAGCCGTCGT AAGAAGAGGG ATACCACTAA TAGAGGTTTC TTACCGAGTA   
  
  
- ATGGAACGAT TACCGGAACT CCGCGCGAAT CTTCCGTTTC CTATACTTGA TTGAGCACGG TTACTCGTAT   
  
  
- AGGGTGGATG ACTCTAGGAG TTCCGATTGG CCCATATACA CCGTCGCCAG GGAAAGTTCT ATTACAGAAT   
  
  
- AATGTGTTGT TTGTTCTGTT AACGTTCGGA TCAACTCCTC CGTGGTTCAT ATGTGTATTA ACTAAAACCA   
  
  
- TAAAAGATAC CTGATGTTAC CGGGACGTAG TAAGTCTTGG AGAGGTTCTC CGGGTTACCC GGAGGGTCTT   
  
  
- AGGCGTAGTG TCCGTAGCTA AAGGGGGTTG GACCCAAGGC CGGTCGTCTC TCCCAACTTC TCTGTCCCGC   
  
  
- CACAAATCGG TTCATAACAC TGTCCATGTT ACACGGGAAA CTTATGGTGG GGTATCGGTT CTTCACCCTC   
  
  
- TGGTAGGTTG AGCTCCTCGA GTTTTAACTT TCCTTACTCG GGGAACATCA CTTGACAGAC ATATCTAGCG   
  
  
- TGTTGGATAA ACTACTTTCG CATCTCCTTT TGTCGGGTTC TCTGCGCAAG GACTCGAACC AATCCTTTTA   
  
  
- TTTAGGGCTA TATAAGTACG TACCCCACCA CTTATGGCGT AGTTCACATG GAAAGAAGGA CTTGTCCAAG   
  
  
- TTCCTCCGGT ACAAAGTGAT GTGCCGGAAC AAACTGAACA AGCTCCGGTG TTACTCGGCG CTCCTAGTTC   
  
  
- TCTCCCACAA TGATCTCTCG TTCAACGTAC CCTTAGTCCG CAACTTACAC TAGCGGACGC TTCCTCGCCT   
  
  
- CTCTCACCTC TCCGGACTCA GTATGTTTGT TACCGTCCAA TCCTGAGTTT CCCGGCCCAA ACAAGTTCAC   
  
  
- AGCTACCTAG CGCTCGAAAA CTCTTCCCGT TCCCGGTACC AGTCCTCCTT AAAATCCCTT CTCAAGTCAA   
  
  
- TACTACTTCT ACCCGTAACC CACCAAGTTC CTACCTTTCC GTCCTATTAC ATACGGTAAA GAACAACCTT   
  
  
- TGGAAGTAT

+     MYC

| Site Name | Organism | Position | Strand | Matrix score. | sequence | function |
| --- | --- | --- | --- | --- | --- | --- |
| MYC | Arabidopsis thaliana | 2782 | - | 6 | CATTTG |  |
| MYC | Arabidopsis thaliana | 3592 | - | 6 | CAATTG |  |
| MYC | Arabidopsis thaliana | 2123 | + | 6 | CAATTG |  |
| MYC | Arabidopsis thaliana | 1834 | + | 6 | CATTTG |  |
| MYC | Arabidopsis thaliana | 1589 | - | 6 | CATGTG |  |
| MYC | Arabidopsis thaliana | 960 | - | 6 | CATGTG |  |
| MYC | Arabidopsis thaliana | 779 | - | 6 | CATGTG |  |
| MYC | Arabidopsis thaliana | 701 | - | 6 | CATGTG |  |

>HU02G01570.1   
+ +Up\_Stream \_Len000ATTATT ATTCTAATAT ATAGATAATA ATATAATAAT AATAATAATA ATAATAATAA   
  
  
+ TTATTATTAT TATTATTATT TATTAAATAT AATAGTTATT ATAATAATAA TAATTATAAT AATAATAAGA   
  
  
+ TTATTATCAT TATATTATTA TTATTATTAT TGTAATTTAT TATTATTATA GTAATTATTA TTGTTATATA   
  
  
+ GTAAAATTAT TATTAGTATT GTTATTATAA ATATTCATAC TTGCATATAA TATTTCTAAA ATAAAAACAG   
  
  
+ AGTTAATTCC ATTCCAAAAC TACTCACCAA ACAGCAAGGA GTGGGACCTG ATTCCTTTCC AATTTTCACC   
  
  
+ AAACAACTGG GTTCAGAATT CAAGTTCCAT ATCCAACCCC CCAGGATTCA AGTCCTGATT CCATTCCATT   
  
  
+ CCAGTTTCCC TCACCAAACT GGGCCTTAGA TTGATGGTTG TTCAGTTGGA TGAGGGTTGT TCCATGCCAA   
  
  
+ GTTAGATCAT TGTTGTCAAT TTTGAGAACG ACTATCTTAA TAAGCCTAAC GATGTTGATT TGATTTAGTT   
  
  
+ CAAACCATTT CAAATAATAC AAATCAAATT CTATTTAGAC CGACACTAAA ATAAATAGAT TGAGAAAAAT   
  
  
+ TATAGATTTT AAAGTTCAGA TTAAAATCCC TAAAGATCAA CAGATTGAAA TGCATATTTC ATAGTGCACA   
  
  
+ TGGACTTGCT TTGAATCTCG TCTAGAGAAC CTAAGAAAAA GCAAGAGTGG TTCAGAAGGT TAGAATTGTT   
  
  
+ CCAGCACATG ACAAGTACAA TCCCAACTTT TTTCAAAAGA TTTGCTACTT TTAATTGGAT TCCCATGTTG   
  
  
+ AATGAAGTTC TGGGTTTAAG GCTACAACTT TCTTGGGAGG ATAAGGTTCT TAAGTTAAGG TGGTCCTCTT   
  
  
+ TTCTTTTGAT CTTTCTTATG GGTACAACCT CATGGGTCTC ATCAACACAT GAACTCTTTT TATCTTTTAT   
  
  
+ CTTTCCCCTT TCCTTTATGC AGGATTGACA GGTGCATGGA AAACAAGAAG CAATTAAATA CTAGTGGCTT   
  
  
+ AGCCCCATCC CTAATATTTT GGCTATTCTG TTTCACCATC CTCCCCTGTC TCCATTTTCT TTGTTTCCTT   
  
  
+ TTTTTCTAGG AACTTGTGAT GGGCAAGGGA AGATCAGTAC AATGTGATGG TTGTTAGTAG CGAGAAGATG   
  
  
+ AAGTGTCACC GCAAAAATTT TGTGCCATAA TTTGTCGTAA GGGAAACAAG TATCAAACAA CAATGTCATT   
  
  
+ TATGATAAAC AAGTAACAGG AGATATGTAC TCTGAAAAAA TATAAGGTAC CTGAGAAGTT GTTAACTTAG   
  
  
+ CTTGAAAAAC CCTCCTAACT TGATAAACCC TCTTCGCTAT GTTATCCCGC ACCCTGCCAT AGAGTCACCG   
  
  
+ ATTCAGTATA TATGTAACAT ACCCAATCTT GTCTGGATAT ATAGTTAACC CTTTTGGAAT ATCTGAAAAT   
  
  
+ CCACACGAGA GTCATCCTTC TAGACTAGGA AACATGCGCC ACATTTTGGA TTCTAACCAA GTATAATAAG   
  
  
+ CATATCTTAA AGCCAAAAAT GCAAGGATAG ATACAAATAC CCATCACATG CATGTAAATG TCCCTTGTCA   
  
  
+ TGGCTCTGAT TCTCAAAATA TTAATTTTCA ATAAATTAAA TGAAATTGCA TTATTTTAAA GATTTCTGAA   
  
  
+ TGCATCCATT ATTGCAATTT TTTAAAGACT GCACAATGAT GACAATGTCA ATGGTTTCCT AAAGTTGCGC   
  
  
+ CTGATGGGCT ATTGACAGTG AAGCAACACT CCCTTTGTTC CCATTTATGT GTCTCCACTT GCCCCTTTGA   
  
  
+ AACTCTCTAC ATTTGTATGT CATCTGAATT TGATTCTCAA ATCTCAGATG ATTTGAATTC CCTTTATGCC   
  
  
+ ATCTTTACTA TCTATTGAAT GATTCTTTCT TTTCCACTTT CTTATCTTCC ACTACCTTCT TCTTGGATGC   
  
  
+ CCTTTTGCTG CTTATAAAGG GCGACACCTG AACAAGTTGC TGTATTCTCA AACCATGAAT CTGTACAGGT   
  
  
+ TTAGCCCCGA TATGTGTCCT AGCTTTGTGG GTCACGAAGA AATTGGTCCT AATTTAATAG GACACCAAGA   
  
  
+ CCCTTTTCAA AATGCATACA ATTGCACTGA TTTTGGTGAT GCTTACTTGT TGCAATTCCC AGTTGAAGAT   
  
  
+ TTATGTCAAT ACCGTGTTCC TGATCTAATG GGGCTACCTG TTGAAGATGG GGATCCTACA GAGGTCCCTG   
  
  
+ ACATTCCTGA TACATGCTTC AACTACATAA GCAATCTCCT AATGGAAGAG GGCTCGGATG ACCGCCCTGG   
  
  
+ CGCCACCCTG CAGGACTATA TGGCTGCTCA AGTCTCCTAC AGCAAGTCCT CCAATGCCGC CTTTCCCGGT   
  
  
+ GAGAGTTGCT CATTCTTTGG TAATAATCAA TGCCCTCCAC CTGCTTATCA AAGCAACCCT TATTGCCTTG   
  
  
+ ATGATTACTT CAACTTGCTA TCCAATACTA CTGGCAATAG TGTTAACAAT GCTGGTGTTC ATGACAGCAA   
  
  
+ CAACTTTGTT CAGAACAGTT TGATTGGGAA TAGGGGTACT AGTTCTAGTT CTAGGGGCAG AAGTGATTTT   
  
  
+ GGATCTACCC TTATTAGGAA TTTGCCATTA GGGACCGACA CACTTGATGT GGTCAATCCA GCACCTACTC   
  
  
+ CTCGAGCCCC TCTGGTTACG ACTCAGTGTC AGGTGTTTGA AACACACCAG TATGAGCAGG CCTTCGAACT   
  
  
+ GATCACTAAT GGAAGCTGGG AATTCAGTCA TTCTGTTGCT AATACATCAA ATGATGGAAT TCAAAATCAA   
  
  
+ CCAATTCAAG CAGTTTCAAC CCAAGAACGA TGGAATATGA AAACATTTTC CGATGACCAA CTTGAGCCTG   
  
  
+ CTCTTTTGCG AAGTCTTGTG ATGTCCTCAA ACTGTACTGA TATCTCTGAT CAGGGTGTAC ACAGGATAGT   
  
  
+ GAACCCTGAG CCACCCAACA ACAGAAATAC GGAGATTGTG TGCAATGACT CATCCAGTAA GAGTAAGACA   
  
  
+ AGACGAAGAG AGCATGGTGC AGGAGAAAAC CACGATGAAG GAGAAAACCA CGATGAGACA GGAAGGAGTA   
  
  
+ ACAAGCAATC AGCACCATCC AATGAAGTGT TCGATGAAAT TGAGCAGTAT GGTGATGTGC TGCTCTGCCC   
  
  
+ TGAAGGAAGA AGTAAACCAG CAGGGTTATG CAGCAGCAGC AACGCTAACA GAACTAGCAA CTCACAGAAC   
  
  
+ TCGGAAGACT CAAAGTCACA GCAAAATGGG AGTTCCTCCA AGGTGTCAAA AAAGCGAGGC AATAAAAAGC   
  
  
+ AAGTGGTTGA TTTGAGGACT CTCCTAACTC GATGTGCACA ATGTGTTGCT CGTGTGGATT TAACTGGTTC   
  
  
+ TTACCAGTTG CTTCAGGAGA TTCGGCAGCA TTCTTCTCCC TATGGTGATT ATCTCCAAAG AATGGCTCAT   
  
  
+ TACCTTGCTA ATGGCCTTGA GGCGCGCTTA GAAGGCAAAG GATATGAACT AACTCGTGCC AATGAGCATA   
  
  
+ TCCCACCTAC TGAGATCCTC AAGGCTAACC GGGTATATGT GGCAGCGGTC CCTTTCAAGA TAATGTCTTA   
  
  
+ TTACACAACA AACAAGACAA TTGCAAGCCT AGTTGAGGAG GCACCAAGTA TACACATAAT TGATTTTGGT   
  
  
+ ATTTTCTATG GACTACAATG GCCCTGCATC ATTCAGAACC TCTCCAAGAG GCCCAATGGG CCTCCCAGAA   
  
  
+ TCCGCATCAC AGGCATCGAT TTCCCCCAAC CTGGGTTCCG GCCAGCAGAG AGGGTTGAAG AGACAGGGCG   
  
  
+ GTGTTTAGCC AAGTATTGTG ACAGGTACAA TGTGCCCTTT GAATACCACC CCATAGCCAA GAAGTGGGAG   
  
  
+ ACCATCCAAC TCGAGGAGCT CAAAATTGAA AGGAATGAGC CCCTTGTAGT GAACTGTCTG TATAGATCGC   
  
  
+ ACAACCTATT TGATGAAAGC GTAGAGGAAA ACAGCCCAAG AGACGCGTTC CTGAGCTTGG TTAGGAAAAT   
  
  
+ AAATCCCGAT ATATTCATGC ATGGGGTGGT GAATACCGCA TCAAGTGTAC CTTTCTTCCT GAACAGGTTC   
  
  
+ AAGGAGGCCA TGTTTCACTA CACGGCCTTG TTTGACTTGT TCGAGGCCAC AATGAGCCGC GAGGATCAAG   
  
  
+ AGAGGGTGTT ACTAGAGAGC AAGTTGCATG GGAATCAGGC GTTGAATGTG ATCGCCTGCG AAGGAGCGGA   
  
  
+ GAGAGTGGAG AGGCCTGAGT CATACAAACA ATGGCAGGTT AGGACTCAAA GGGCCGGGTT TGTTCAAGTG   
  
  
+ TCGATGGATC GCGAGCTTTT GAGAAGGGCA AGGGCCATGG TCAGGAGGAA TTTTAGGGAA GAGTTCAGTT   
  
  
+ ATGATGAAGA TGGGCATTGG GTGGTTCAAG GATGGAAAGG CAGGATAATG TATGCCATTT CTTGTTGGAA   
  
  
+ ACCTTCATA  

- +Up\_Stream \_Len000TAATAA TAAGATTATA TATCTATTAT TATATTATTA TTATTATTAT TATTATTATT   
  
  
- AATAATAATA ATAATAATAA ATAATTTATA TTATCAATAA TATTATTATT ATTAATATTA TTATTATTCT   
  
  
- AATAATAGTA ATATAATAAT AATAATAATA ACATTAAATA ATAATAATAT CATTAATAAT AACAATATAT   
  
  
- CATTTTAATA ATAATCATAA CAATAATATT TATAAGTATG AACGTATATT ATAAAGATTT TATTTTTGTC   
  
  
- TCAATTAAGG TAAGGTTTTG ATGAGTGGTT TGTCGTTCCT CACCCTGGAC TAAGGAAAGG TTAAAAGTGG   
  
  
- TTTGTTGACC CAAGTCTTAA GTTCAAGGTA TAGGTTGGGG GGTCCTAAGT TCAGGACTAA GGTAAGGTAA   
  
  
- GGTCAAAGGG AGTGGTTTGA CCCGGAATCT AACTACCAAC AAGTCAACCT ACTCCCAACA AGGTACGGTT   
  
  
- CAATCTAGTA ACAACAGTTA AAACTCTTGC TGATAGAATT ATTCGGATTG CTACAACTAA ACTAAATCAA   
  
  
- GTTTGGTAAA GTTTATTATG TTTAGTTTAA GATAAATCTG GCTGTGATTT TATTTATCTA ACTCTTTTTA   
  
  
- ATATCTAAAA TTTCAAGTCT AATTTTAGGG ATTTCTAGTT GTCTAACTTT ACGTATAAAG TATCACGTGT   
  
  
- ACCTGAACGA AACTTAGAGC AGATCTCTTG GATTCTTTTT CGTTCTCACC AAGTCTTCCA ATCTTAACAA   
  
  
- GGTCGTGTAC TGTTCATGTT AGGGTTGAAA AAAGTTTTCT AAACGATGAA AATTAACCTA AGGGTACAAC   
  
  
- TTACTTCAAG ACCCAAATTC CGATGTTGAA AGAACCCTCC TATTCCAAGA ATTCAATTCC ACCAGGAGAA   
  
  
- AAGAAAACTA GAAAGAATAC CCATGTTGGA GTACCCAGAG TAGTTGTGTA CTTGAGAAAA ATAGAAAATA   
  
  
- GAAAGGGGAA AGGAAATACG TCCTAACTGT CCACGTACCT TTTGTTCTTC GTTAATTTAT GATCACCGAA   
  
  
- TCGGGGTAGG GATTATAAAA CCGATAAGAC AAAGTGGTAG GAGGGGACAG AGGTAAAAGA AACAAAGGAA   
  
  
- AAAAAGATCC TTGAACACTA CCCGTTCCCT TCTAGTCATG TTACACTACC AACAATCATC GCTCTTCTAC   
  
  
- TTCACAGTGG CGTTTTTAAA ACACGGTATT AAACAGCATT CCCTTTGTTC ATAGTTTGTT GTTACAGTAA   
  
  
- ATACTATTTG TTCATTGTCC TCTATACATG AGACTTTTTT ATATTCCATG GACTCTTCAA CAATTGAATC   
  
  
- GAACTTTTTG GGAGGATTGA ACTATTTGGG AGAAGCGATA CAATAGGGCG TGGGACGGTA TCTCAGTGGC   
  
  
- TAAGTCATAT ATACATTGTA TGGGTTAGAA CAGACCTATA TATCAATTGG GAAAACCTTA TAGACTTTTA   
  
  
- GGTGTGCTCT CAGTAGGAAG ATCTGATCCT TTGTACGCGG TGTAAAACCT AAGATTGGTT CATATTATTC   
  
  
- GTATAGAATT TCGGTTTTTA CGTTCCTATC TATGTTTATG GGTAGTGTAC GTACATTTAC AGGGAACAGT   
  
  
- ACCGAGACTA AGAGTTTTAT AATTAAAAGT TATTTAATTT ACTTTAACGT AATAAAATTT CTAAAGACTT   
  
  
- ACGTAGGTAA TAACGTTAAA AAATTTCTGA CGTGTTACTA CTGTTACAGT TACCAAAGGA TTTCAACGCG   
  
  
- GACTACCCGA TAACTGTCAC TTCGTTGTGA GGGAAACAAG GGTAAATACA CAGAGGTGAA CGGGGAAACT   
  
  
- TTGAGAGATG TAAACATACA GTAGACTTAA ACTAAGAGTT TAGAGTCTAC TAAACTTAAG GGAAATACGG   
  
  
- TAGAAATGAT AGATAACTTA CTAAGAAAGA AAAGGTGAAA GAATAGAAGG TGATGGAAGA AGAACCTACG   
  
  
- GGAAAACGAC GAATATTTCC CGCTGTGGAC TTGTTCAACG ACATAAGAGT TTGGTACTTA GACATGTCCA   
  
  
- AATCGGGGCT ATACACAGGA TCGAAACACC CAGTGCTTCT TTAACCAGGA TTAAATTATC CTGTGGTTCT   
  
  
- GGGAAAAGTT TTACGTATGT TAACGTGACT AAAACCACTA CGAATGAACA ACGTTAAGGG TCAACTTCTA   
  
  
- AATACAGTTA TGGCACAAGG ACTAGATTAC CCCGATGGAC AACTTCTACC CCTAGGATGT CTCCAGGGAC   
  
  
- TGTAAGGACT ATGTACGAAG TTGATGTATT CGTTAGAGGA TTACCTTCTC CCGAGCCTAC TGGCGGGACC   
  
  
- GCGGTGGGAC GTCCTGATAT ACCGACGAGT TCAGAGGATG TCGTTCAGGA GGTTACGGCG GAAAGGGCCA   
  
  
- CTCTCAACGA GTAAGAAACC ATTATTAGTT ACGGGAGGTG GACGAATAGT TTCGTTGGGA ATAACGGAAC   
  
  
- TACTAATGAA GTTGAACGAT AGGTTATGAT GACCGTTATC ACAATTGTTA CGACCACAAG TACTGTCGTT   
  
  
- GTTGAAACAA GTCTTGTCAA ACTAACCCTT ATCCCCATGA TCAAGATCAA GATCCCCGTC TTCACTAAAA   
  
  
- CCTAGATGGG AATAATCCTT AAACGGTAAT CCCTGGCTGT GTGAACTACA CCAGTTAGGT CGTGGATGAG   
  
  
- GAGCTCGGGG AGACCAATGC TGAGTCACAG TCCACAAACT TTGTGTGGTC ATACTCGTCC GGAAGCTTGA   
  
  
- CTAGTGATTA CCTTCGACCC TTAAGTCAGT AAGACAACGA TTATGTAGTT TACTACCTTA AGTTTTAGTT   
  
  
- GGTTAAGTTC GTCAAAGTTG GGTTCTTGCT ACCTTATACT TTTGTAAAAG GCTACTGGTT GAACTCGGAC   
  
  
- GAGAAAACGC TTCAGAACAC TACAGGAGTT TGACATGACT ATAGAGACTA GTCCCACATG TGTCCTATCA   
  
  
- CTTGGGACTC GGTGGGTTGT TGTCTTTATG CCTCTAACAC ACGTTACTGA GTAGGTCATT CTCATTCTGT   
  
  
- TCTGCTTCTC TCGTACCACG TCCTCTTTTG GTGCTACTTC CTCTTTTGGT GCTACTCTGT CCTTCCTCAT   
  
  
- TGTTCGTTAG TCGTGGTAGG TTACTTCACA AGCTACTTTA ACTCGTCATA CCACTACACG ACGAGACGGG   
  
  
- ACTTCCTTCT TCATTTGGTC GTCCCAATAC GTCGTCGTCG TTGCGATTGT CTTGATCGTT GAGTGTCTTG   
  
  
- AGCCTTCTGA GTTTCAGTGT CGTTTTACCC TCAAGGAGGT TCCACAGTTT TTTCGCTCCG TTATTTTTCG   
  
  
- TTCACCAACT AAACTCCTGA GAGGATTGAG CTACACGTGT TACACAACGA GCACACCTAA ATTGACCAAG   
  
  
- AATGGTCAAC GAAGTCCTCT AAGCCGTCGT AAGAAGAGGG ATACCACTAA TAGAGGTTTC TTACCGAGTA   
  
  
- ATGGAACGAT TACCGGAACT CCGCGCGAAT CTTCCGTTTC CTATACTTGA TTGAGCACGG TTACTCGTAT   
  
  
- AGGGTGGATG ACTCTAGGAG TTCCGATTGG CCCATATACA CCGTCGCCAG GGAAAGTTCT ATTACAGAAT   
  
  
- AATGTGTTGT TTGTTCTGTT AACGTTCGGA TCAACTCCTC CGTGGTTCAT ATGTGTATTA ACTAAAACCA   
  
  
- TAAAAGATAC CTGATGTTAC CGGGACGTAG TAAGTCTTGG AGAGGTTCTC CGGGTTACCC GGAGGGTCTT   
  
  
- AGGCGTAGTG TCCGTAGCTA AAGGGGGTTG GACCCAAGGC CGGTCGTCTC TCCCAACTTC TCTGTCCCGC   
  
  
- CACAAATCGG TTCATAACAC TGTCCATGTT ACACGGGAAA CTTATGGTGG GGTATCGGTT CTTCACCCTC   
  
  
- TGGTAGGTTG AGCTCCTCGA GTTTTAACTT TCCTTACTCG GGGAACATCA CTTGACAGAC ATATCTAGCG   
  
  
- TGTTGGATAA ACTACTTTCG CATCTCCTTT TGTCGGGTTC TCTGCGCAAG GACTCGAACC AATCCTTTTA   
  
  
- TTTAGGGCTA TATAAGTACG TACCCCACCA CTTATGGCGT AGTTCACATG GAAAGAAGGA CTTGTCCAAG   
  
  
- TTCCTCCGGT ACAAAGTGAT GTGCCGGAAC AAACTGAACA AGCTCCGGTG TTACTCGGCG CTCCTAGTTC   
  
  
- TCTCCCACAA TGATCTCTCG TTCAACGTAC CCTTAGTCCG CAACTTACAC TAGCGGACGC TTCCTCGCCT   
  
  
- CTCTCACCTC TCCGGACTCA GTATGTTTGT TACCGTCCAA TCCTGAGTTT CCCGGCCCAA ACAAGTTCAC   
  
  
- AGCTACCTAG CGCTCGAAAA CTCTTCCCGT TCCCGGTACC AGTCCTCCTT AAAATCCCTT CTCAAGTCAA   
  
  
- TACTACTTCT ACCCGTAACC CACCAAGTTC CTACCTTTCC GTCCTATTAC ATACGGTAAA GAACAACCTT   
  
  
- TGGAAGTAT

+     Myb

| Site Name | Organism | Position | Strand | Matrix score. | sequence | function |
| --- | --- | --- | --- | --- | --- | --- |
| Myb | Arabidopsis thaliana | 4340 | - | 6 | TAACTG |  |
| Myb | Arabidopsis thaliana | 3369 | - | 6 | CAACTG |  |
| Myb | Arabidopsis thaliana | 3355 | + | 6 | TAACTG |  |
| Myb | Arabidopsis thaliana | 2164 | - | 6 | CAACTG |  |
| Myb | Arabidopsis thaliana | 467 | - | 6 | CAACTG |  |
| Myb | Arabidopsis thaliana | 358 | + | 6 | CAACTG |  |

>HU02G01570.1   
+ +Up\_Stream \_Len000ATTATT ATTCTAATAT ATAGATAATA ATATAATAAT AATAATAATA ATAATAATAA   
  
  
+ TTATTATTAT TATTATTATT TATTAAATAT AATAGTTATT ATAATAATAA TAATTATAAT AATAATAAGA   
  
  
+ TTATTATCAT TATATTATTA TTATTATTAT TGTAATTTAT TATTATTATA GTAATTATTA TTGTTATATA   
  
  
+ GTAAAATTAT TATTAGTATT GTTATTATAA ATATTCATAC TTGCATATAA TATTTCTAAA ATAAAAACAG   
  
  
+ AGTTAATTCC ATTCCAAAAC TACTCACCAA ACAGCAAGGA GTGGGACCTG ATTCCTTTCC AATTTTCACC   
  
  
+ AAACAACTGG GTTCAGAATT CAAGTTCCAT ATCCAACCCC CCAGGATTCA AGTCCTGATT CCATTCCATT   
  
  
+ CCAGTTTCCC TCACCAAACT GGGCCTTAGA TTGATGGTTG TTCAGTTGGA TGAGGGTTGT TCCATGCCAA   
  
  
+ GTTAGATCAT TGTTGTCAAT TTTGAGAACG ACTATCTTAA TAAGCCTAAC GATGTTGATT TGATTTAGTT   
  
  
+ CAAACCATTT CAAATAATAC AAATCAAATT CTATTTAGAC CGACACTAAA ATAAATAGAT TGAGAAAAAT   
  
  
+ TATAGATTTT AAAGTTCAGA TTAAAATCCC TAAAGATCAA CAGATTGAAA TGCATATTTC ATAGTGCACA   
  
  
+ TGGACTTGCT TTGAATCTCG TCTAGAGAAC CTAAGAAAAA GCAAGAGTGG TTCAGAAGGT TAGAATTGTT   
  
  
+ CCAGCACATG ACAAGTACAA TCCCAACTTT TTTCAAAAGA TTTGCTACTT TTAATTGGAT TCCCATGTTG   
  
  
+ AATGAAGTTC TGGGTTTAAG GCTACAACTT TCTTGGGAGG ATAAGGTTCT TAAGTTAAGG TGGTCCTCTT   
  
  
+ TTCTTTTGAT CTTTCTTATG GGTACAACCT CATGGGTCTC ATCAACACAT GAACTCTTTT TATCTTTTAT   
  
  
+ CTTTCCCCTT TCCTTTATGC AGGATTGACA GGTGCATGGA AAACAAGAAG CAATTAAATA CTAGTGGCTT   
  
  
+ AGCCCCATCC CTAATATTTT GGCTATTCTG TTTCACCATC CTCCCCTGTC TCCATTTTCT TTGTTTCCTT   
  
  
+ TTTTTCTAGG AACTTGTGAT GGGCAAGGGA AGATCAGTAC AATGTGATGG TTGTTAGTAG CGAGAAGATG   
  
  
+ AAGTGTCACC GCAAAAATTT TGTGCCATAA TTTGTCGTAA GGGAAACAAG TATCAAACAA CAATGTCATT   
  
  
+ TATGATAAAC AAGTAACAGG AGATATGTAC TCTGAAAAAA TATAAGGTAC CTGAGAAGTT GTTAACTTAG   
  
  
+ CTTGAAAAAC CCTCCTAACT TGATAAACCC TCTTCGCTAT GTTATCCCGC ACCCTGCCAT AGAGTCACCG   
  
  
+ ATTCAGTATA TATGTAACAT ACCCAATCTT GTCTGGATAT ATAGTTAACC CTTTTGGAAT ATCTGAAAAT   
  
  
+ CCACACGAGA GTCATCCTTC TAGACTAGGA AACATGCGCC ACATTTTGGA TTCTAACCAA GTATAATAAG   
  
  
+ CATATCTTAA AGCCAAAAAT GCAAGGATAG ATACAAATAC CCATCACATG CATGTAAATG TCCCTTGTCA   
  
  
+ TGGCTCTGAT TCTCAAAATA TTAATTTTCA ATAAATTAAA TGAAATTGCA TTATTTTAAA GATTTCTGAA   
  
  
+ TGCATCCATT ATTGCAATTT TTTAAAGACT GCACAATGAT GACAATGTCA ATGGTTTCCT AAAGTTGCGC   
  
  
+ CTGATGGGCT ATTGACAGTG AAGCAACACT CCCTTTGTTC CCATTTATGT GTCTCCACTT GCCCCTTTGA   
  
  
+ AACTCTCTAC ATTTGTATGT CATCTGAATT TGATTCTCAA ATCTCAGATG ATTTGAATTC CCTTTATGCC   
  
  
+ ATCTTTACTA TCTATTGAAT GATTCTTTCT TTTCCACTTT CTTATCTTCC ACTACCTTCT TCTTGGATGC   
  
  
+ CCTTTTGCTG CTTATAAAGG GCGACACCTG AACAAGTTGC TGTATTCTCA AACCATGAAT CTGTACAGGT   
  
  
+ TTAGCCCCGA TATGTGTCCT AGCTTTGTGG GTCACGAAGA AATTGGTCCT AATTTAATAG GACACCAAGA   
  
  
+ CCCTTTTCAA AATGCATACA ATTGCACTGA TTTTGGTGAT GCTTACTTGT TGCAATTCCC AGTTGAAGAT   
  
  
+ TTATGTCAAT ACCGTGTTCC TGATCTAATG GGGCTACCTG TTGAAGATGG GGATCCTACA GAGGTCCCTG   
  
  
+ ACATTCCTGA TACATGCTTC AACTACATAA GCAATCTCCT AATGGAAGAG GGCTCGGATG ACCGCCCTGG   
  
  
+ CGCCACCCTG CAGGACTATA TGGCTGCTCA AGTCTCCTAC AGCAAGTCCT CCAATGCCGC CTTTCCCGGT   
  
  
+ GAGAGTTGCT CATTCTTTGG TAATAATCAA TGCCCTCCAC CTGCTTATCA AAGCAACCCT TATTGCCTTG   
  
  
+ ATGATTACTT CAACTTGCTA TCCAATACTA CTGGCAATAG TGTTAACAAT GCTGGTGTTC ATGACAGCAA   
  
  
+ CAACTTTGTT CAGAACAGTT TGATTGGGAA TAGGGGTACT AGTTCTAGTT CTAGGGGCAG AAGTGATTTT   
  
  
+ GGATCTACCC TTATTAGGAA TTTGCCATTA GGGACCGACA CACTTGATGT GGTCAATCCA GCACCTACTC   
  
  
+ CTCGAGCCCC TCTGGTTACG ACTCAGTGTC AGGTGTTTGA AACACACCAG TATGAGCAGG CCTTCGAACT   
  
  
+ GATCACTAAT GGAAGCTGGG AATTCAGTCA TTCTGTTGCT AATACATCAA ATGATGGAAT TCAAAATCAA   
  
  
+ CCAATTCAAG CAGTTTCAAC CCAAGAACGA TGGAATATGA AAACATTTTC CGATGACCAA CTTGAGCCTG   
  
  
+ CTCTTTTGCG AAGTCTTGTG ATGTCCTCAA ACTGTACTGA TATCTCTGAT CAGGGTGTAC ACAGGATAGT   
  
  
+ GAACCCTGAG CCACCCAACA ACAGAAATAC GGAGATTGTG TGCAATGACT CATCCAGTAA GAGTAAGACA   
  
  
+ AGACGAAGAG AGCATGGTGC AGGAGAAAAC CACGATGAAG GAGAAAACCA CGATGAGACA GGAAGGAGTA   
  
  
+ ACAAGCAATC AGCACCATCC AATGAAGTGT TCGATGAAAT TGAGCAGTAT GGTGATGTGC TGCTCTGCCC   
  
  
+ TGAAGGAAGA AGTAAACCAG CAGGGTTATG CAGCAGCAGC AACGCTAACA GAACTAGCAA CTCACAGAAC   
  
  
+ TCGGAAGACT CAAAGTCACA GCAAAATGGG AGTTCCTCCA AGGTGTCAAA AAAGCGAGGC AATAAAAAGC   
  
  
+ AAGTGGTTGA TTTGAGGACT CTCCTAACTC GATGTGCACA ATGTGTTGCT CGTGTGGATT TAACTGGTTC   
  
  
+ TTACCAGTTG CTTCAGGAGA TTCGGCAGCA TTCTTCTCCC TATGGTGATT ATCTCCAAAG AATGGCTCAT   
  
  
+ TACCTTGCTA ATGGCCTTGA GGCGCGCTTA GAAGGCAAAG GATATGAACT AACTCGTGCC AATGAGCATA   
  
  
+ TCCCACCTAC TGAGATCCTC AAGGCTAACC GGGTATATGT GGCAGCGGTC CCTTTCAAGA TAATGTCTTA   
  
  
+ TTACACAACA AACAAGACAA TTGCAAGCCT AGTTGAGGAG GCACCAAGTA TACACATAAT TGATTTTGGT   
  
  
+ ATTTTCTATG GACTACAATG GCCCTGCATC ATTCAGAACC TCTCCAAGAG GCCCAATGGG CCTCCCAGAA   
  
  
+ TCCGCATCAC AGGCATCGAT TTCCCCCAAC CTGGGTTCCG GCCAGCAGAG AGGGTTGAAG AGACAGGGCG   
  
  
+ GTGTTTAGCC AAGTATTGTG ACAGGTACAA TGTGCCCTTT GAATACCACC CCATAGCCAA GAAGTGGGAG   
  
  
+ ACCATCCAAC TCGAGGAGCT CAAAATTGAA AGGAATGAGC CCCTTGTAGT GAACTGTCTG TATAGATCGC   
  
  
+ ACAACCTATT TGATGAAAGC GTAGAGGAAA ACAGCCCAAG AGACGCGTTC CTGAGCTTGG TTAGGAAAAT   
  
  
+ AAATCCCGAT ATATTCATGC ATGGGGTGGT GAATACCGCA TCAAGTGTAC CTTTCTTCCT GAACAGGTTC   
  
  
+ AAGGAGGCCA TGTTTCACTA CACGGCCTTG TTTGACTTGT TCGAGGCCAC AATGAGCCGC GAGGATCAAG   
  
  
+ AGAGGGTGTT ACTAGAGAGC AAGTTGCATG GGAATCAGGC GTTGAATGTG ATCGCCTGCG AAGGAGCGGA   
  
  
+ GAGAGTGGAG AGGCCTGAGT CATACAAACA ATGGCAGGTT AGGACTCAAA GGGCCGGGTT TGTTCAAGTG   
  
  
+ TCGATGGATC GCGAGCTTTT GAGAAGGGCA AGGGCCATGG TCAGGAGGAA TTTTAGGGAA GAGTTCAGTT   
  
  
+ ATGATGAAGA TGGGCATTGG GTGGTTCAAG GATGGAAAGG CAGGATAATG TATGCCATTT CTTGTTGGAA   
  
  
+ ACCTTCATA  

- +Up\_Stream \_Len000TAATAA TAAGATTATA TATCTATTAT TATATTATTA TTATTATTAT TATTATTATT   
  
  
- AATAATAATA ATAATAATAA ATAATTTATA TTATCAATAA TATTATTATT ATTAATATTA TTATTATTCT   
  
  
- AATAATAGTA ATATAATAAT AATAATAATA ACATTAAATA ATAATAATAT CATTAATAAT AACAATATAT   
  
  
- CATTTTAATA ATAATCATAA CAATAATATT TATAAGTATG AACGTATATT ATAAAGATTT TATTTTTGTC   
  
  
- TCAATTAAGG TAAGGTTTTG ATGAGTGGTT TGTCGTTCCT CACCCTGGAC TAAGGAAAGG TTAAAAGTGG   
  
  
- TTTGTTGACC CAAGTCTTAA GTTCAAGGTA TAGGTTGGGG GGTCCTAAGT TCAGGACTAA GGTAAGGTAA   
  
  
- GGTCAAAGGG AGTGGTTTGA CCCGGAATCT AACTACCAAC AAGTCAACCT ACTCCCAACA AGGTACGGTT   
  
  
- CAATCTAGTA ACAACAGTTA AAACTCTTGC TGATAGAATT ATTCGGATTG CTACAACTAA ACTAAATCAA   
  
  
- GTTTGGTAAA GTTTATTATG TTTAGTTTAA GATAAATCTG GCTGTGATTT TATTTATCTA ACTCTTTTTA   
  
  
- ATATCTAAAA TTTCAAGTCT AATTTTAGGG ATTTCTAGTT GTCTAACTTT ACGTATAAAG TATCACGTGT   
  
  
- ACCTGAACGA AACTTAGAGC AGATCTCTTG GATTCTTTTT CGTTCTCACC AAGTCTTCCA ATCTTAACAA   
  
  
- GGTCGTGTAC TGTTCATGTT AGGGTTGAAA AAAGTTTTCT AAACGATGAA AATTAACCTA AGGGTACAAC   
  
  
- TTACTTCAAG ACCCAAATTC CGATGTTGAA AGAACCCTCC TATTCCAAGA ATTCAATTCC ACCAGGAGAA   
  
  
- AAGAAAACTA GAAAGAATAC CCATGTTGGA GTACCCAGAG TAGTTGTGTA CTTGAGAAAA ATAGAAAATA   
  
  
- GAAAGGGGAA AGGAAATACG TCCTAACTGT CCACGTACCT TTTGTTCTTC GTTAATTTAT GATCACCGAA   
  
  
- TCGGGGTAGG GATTATAAAA CCGATAAGAC AAAGTGGTAG GAGGGGACAG AGGTAAAAGA AACAAAGGAA   
  
  
- AAAAAGATCC TTGAACACTA CCCGTTCCCT TCTAGTCATG TTACACTACC AACAATCATC GCTCTTCTAC   
  
  
- TTCACAGTGG CGTTTTTAAA ACACGGTATT AAACAGCATT CCCTTTGTTC ATAGTTTGTT GTTACAGTAA   
  
  
- ATACTATTTG TTCATTGTCC TCTATACATG AGACTTTTTT ATATTCCATG GACTCTTCAA CAATTGAATC   
  
  
- GAACTTTTTG GGAGGATTGA ACTATTTGGG AGAAGCGATA CAATAGGGCG TGGGACGGTA TCTCAGTGGC   
  
  
- TAAGTCATAT ATACATTGTA TGGGTTAGAA CAGACCTATA TATCAATTGG GAAAACCTTA TAGACTTTTA   
  
  
- GGTGTGCTCT CAGTAGGAAG ATCTGATCCT TTGTACGCGG TGTAAAACCT AAGATTGGTT CATATTATTC   
  
  
- GTATAGAATT TCGGTTTTTA CGTTCCTATC TATGTTTATG GGTAGTGTAC GTACATTTAC AGGGAACAGT   
  
  
- ACCGAGACTA AGAGTTTTAT AATTAAAAGT TATTTAATTT ACTTTAACGT AATAAAATTT CTAAAGACTT   
  
  
- ACGTAGGTAA TAACGTTAAA AAATTTCTGA CGTGTTACTA CTGTTACAGT TACCAAAGGA TTTCAACGCG   
  
  
- GACTACCCGA TAACTGTCAC TTCGTTGTGA GGGAAACAAG GGTAAATACA CAGAGGTGAA CGGGGAAACT   
  
  
- TTGAGAGATG TAAACATACA GTAGACTTAA ACTAAGAGTT TAGAGTCTAC TAAACTTAAG GGAAATACGG   
  
  
- TAGAAATGAT AGATAACTTA CTAAGAAAGA AAAGGTGAAA GAATAGAAGG TGATGGAAGA AGAACCTACG   
  
  
- GGAAAACGAC GAATATTTCC CGCTGTGGAC TTGTTCAACG ACATAAGAGT TTGGTACTTA GACATGTCCA   
  
  
- AATCGGGGCT ATACACAGGA TCGAAACACC CAGTGCTTCT TTAACCAGGA TTAAATTATC CTGTGGTTCT   
  
  
- GGGAAAAGTT TTACGTATGT TAACGTGACT AAAACCACTA CGAATGAACA ACGTTAAGGG TCAACTTCTA   
  
  
- AATACAGTTA TGGCACAAGG ACTAGATTAC CCCGATGGAC AACTTCTACC CCTAGGATGT CTCCAGGGAC   
  
  
- TGTAAGGACT ATGTACGAAG TTGATGTATT CGTTAGAGGA TTACCTTCTC CCGAGCCTAC TGGCGGGACC   
  
  
- GCGGTGGGAC GTCCTGATAT ACCGACGAGT TCAGAGGATG TCGTTCAGGA GGTTACGGCG GAAAGGGCCA   
  
  
- CTCTCAACGA GTAAGAAACC ATTATTAGTT ACGGGAGGTG GACGAATAGT TTCGTTGGGA ATAACGGAAC   
  
  
- TACTAATGAA GTTGAACGAT AGGTTATGAT GACCGTTATC ACAATTGTTA CGACCACAAG TACTGTCGTT   
  
  
- GTTGAAACAA GTCTTGTCAA ACTAACCCTT ATCCCCATGA TCAAGATCAA GATCCCCGTC TTCACTAAAA   
  
  
- CCTAGATGGG AATAATCCTT AAACGGTAAT CCCTGGCTGT GTGAACTACA CCAGTTAGGT CGTGGATGAG   
  
  
- GAGCTCGGGG AGACCAATGC TGAGTCACAG TCCACAAACT TTGTGTGGTC ATACTCGTCC GGAAGCTTGA   
  
  
- CTAGTGATTA CCTTCGACCC TTAAGTCAGT AAGACAACGA TTATGTAGTT TACTACCTTA AGTTTTAGTT   
  
  
- GGTTAAGTTC GTCAAAGTTG GGTTCTTGCT ACCTTATACT TTTGTAAAAG GCTACTGGTT GAACTCGGAC   
  
  
- GAGAAAACGC TTCAGAACAC TACAGGAGTT TGACATGACT ATAGAGACTA GTCCCACATG TGTCCTATCA   
  
  
- CTTGGGACTC GGTGGGTTGT TGTCTTTATG CCTCTAACAC ACGTTACTGA GTAGGTCATT CTCATTCTGT   
  
  
- TCTGCTTCTC TCGTACCACG TCCTCTTTTG GTGCTACTTC CTCTTTTGGT GCTACTCTGT CCTTCCTCAT   
  
  
- TGTTCGTTAG TCGTGGTAGG TTACTTCACA AGCTACTTTA ACTCGTCATA CCACTACACG ACGAGACGGG   
  
  
- ACTTCCTTCT TCATTTGGTC GTCCCAATAC GTCGTCGTCG TTGCGATTGT CTTGATCGTT GAGTGTCTTG   
  
  
- AGCCTTCTGA GTTTCAGTGT CGTTTTACCC TCAAGGAGGT TCCACAGTTT TTTCGCTCCG TTATTTTTCG   
  
  
- TTCACCAACT AAACTCCTGA GAGGATTGAG CTACACGTGT TACACAACGA GCACACCTAA ATTGACCAAG   
  
  
- AATGGTCAAC GAAGTCCTCT AAGCCGTCGT AAGAAGAGGG ATACCACTAA TAGAGGTTTC TTACCGAGTA   
  
  
- ATGGAACGAT TACCGGAACT CCGCGCGAAT CTTCCGTTTC CTATACTTGA TTGAGCACGG TTACTCGTAT   
  
  
- AGGGTGGATG ACTCTAGGAG TTCCGATTGG CCCATATACA CCGTCGCCAG GGAAAGTTCT ATTACAGAAT   
  
  
- AATGTGTTGT TTGTTCTGTT AACGTTCGGA TCAACTCCTC CGTGGTTCAT ATGTGTATTA ACTAAAACCA   
  
  
- TAAAAGATAC CTGATGTTAC CGGGACGTAG TAAGTCTTGG AGAGGTTCTC CGGGTTACCC GGAGGGTCTT   
  
  
- AGGCGTAGTG TCCGTAGCTA AAGGGGGTTG GACCCAAGGC CGGTCGTCTC TCCCAACTTC TCTGTCCCGC   
  
  
- CACAAATCGG TTCATAACAC TGTCCATGTT ACACGGGAAA CTTATGGTGG GGTATCGGTT CTTCACCCTC   
  
  
- TGGTAGGTTG AGCTCCTCGA GTTTTAACTT TCCTTACTCG GGGAACATCA CTTGACAGAC ATATCTAGCG   
  
  
- TGTTGGATAA ACTACTTTCG CATCTCCTTT TGTCGGGTTC TCTGCGCAAG GACTCGAACC AATCCTTTTA   
  
  
- TTTAGGGCTA TATAAGTACG TACCCCACCA CTTATGGCGT AGTTCACATG GAAAGAAGGA CTTGTCCAAG   
  
  
- TTCCTCCGGT ACAAAGTGAT GTGCCGGAAC AAACTGAACA AGCTCCGGTG TTACTCGGCG CTCCTAGTTC   
  
  
- TCTCCCACAA TGATCTCTCG TTCAACGTAC CCTTAGTCCG CAACTTACAC TAGCGGACGC TTCCTCGCCT   
  
  
- CTCTCACCTC TCCGGACTCA GTATGTTTGT TACCGTCCAA TCCTGAGTTT CCCGGCCCAA ACAAGTTCAC   
  
  
- AGCTACCTAG CGCTCGAAAA CTCTTCCCGT TCCCGGTACC AGTCCTCCTT AAAATCCCTT CTCAAGTCAA   
  
  
- TACTACTTCT ACCCGTAACC CACCAAGTTC CTACCTTTCC GTCCTATTAC ATACGGTAAA GAACAACCTT   
  
  
- TGGAAGTAT

+     Myb-binding site

| Site Name | Organism | Position | Strand | Matrix score. | sequence | function |
| --- | --- | --- | --- | --- | --- | --- |
| Myb-binding site | Nicotiana tabacum | 2767 | - | 6 | CAACAG |  |
| Myb-binding site | Nicotiana tabacum | 2963 | + | 6 | CAACAG |  |
| Myb-binding site | Nicotiana tabacum | 2212 | - | 6 | CAACAG |  |
| Myb-binding site | Nicotiana tabacum | 672 | + | 6 | CAACAG |  |

>HU02G01570.1   
+ +Up\_Stream \_Len000ATTATT ATTCTAATAT ATAGATAATA ATATAATAAT AATAATAATA ATAATAATAA   
  
  
+ TTATTATTAT TATTATTATT TATTAAATAT AATAGTTATT ATAATAATAA TAATTATAAT AATAATAAGA   
  
  
+ TTATTATCAT TATATTATTA TTATTATTAT TGTAATTTAT TATTATTATA GTAATTATTA TTGTTATATA   
  
  
+ GTAAAATTAT TATTAGTATT GTTATTATAA ATATTCATAC TTGCATATAA TATTTCTAAA ATAAAAACAG   
  
  
+ AGTTAATTCC ATTCCAAAAC TACTCACCAA ACAGCAAGGA GTGGGACCTG ATTCCTTTCC AATTTTCACC   
  
  
+ AAACAACTGG GTTCAGAATT CAAGTTCCAT ATCCAACCCC CCAGGATTCA AGTCCTGATT CCATTCCATT   
  
  
+ CCAGTTTCCC TCACCAAACT GGGCCTTAGA TTGATGGTTG TTCAGTTGGA TGAGGGTTGT TCCATGCCAA   
  
  
+ GTTAGATCAT TGTTGTCAAT TTTGAGAACG ACTATCTTAA TAAGCCTAAC GATGTTGATT TGATTTAGTT   
  
  
+ CAAACCATTT CAAATAATAC AAATCAAATT CTATTTAGAC CGACACTAAA ATAAATAGAT TGAGAAAAAT   
  
  
+ TATAGATTTT AAAGTTCAGA TTAAAATCCC TAAAGATCAA CAGATTGAAA TGCATATTTC ATAGTGCACA   
  
  
+ TGGACTTGCT TTGAATCTCG TCTAGAGAAC CTAAGAAAAA GCAAGAGTGG TTCAGAAGGT TAGAATTGTT   
  
  
+ CCAGCACATG ACAAGTACAA TCCCAACTTT TTTCAAAAGA TTTGCTACTT TTAATTGGAT TCCCATGTTG   
  
  
+ AATGAAGTTC TGGGTTTAAG GCTACAACTT TCTTGGGAGG ATAAGGTTCT TAAGTTAAGG TGGTCCTCTT   
  
  
+ TTCTTTTGAT CTTTCTTATG GGTACAACCT CATGGGTCTC ATCAACACAT GAACTCTTTT TATCTTTTAT   
  
  
+ CTTTCCCCTT TCCTTTATGC AGGATTGACA GGTGCATGGA AAACAAGAAG CAATTAAATA CTAGTGGCTT   
  
  
+ AGCCCCATCC CTAATATTTT GGCTATTCTG TTTCACCATC CTCCCCTGTC TCCATTTTCT TTGTTTCCTT   
  
  
+ TTTTTCTAGG AACTTGTGAT GGGCAAGGGA AGATCAGTAC AATGTGATGG TTGTTAGTAG CGAGAAGATG   
  
  
+ AAGTGTCACC GCAAAAATTT TGTGCCATAA TTTGTCGTAA GGGAAACAAG TATCAAACAA CAATGTCATT   
  
  
+ TATGATAAAC AAGTAACAGG AGATATGTAC TCTGAAAAAA TATAAGGTAC CTGAGAAGTT GTTAACTTAG   
  
  
+ CTTGAAAAAC CCTCCTAACT TGATAAACCC TCTTCGCTAT GTTATCCCGC ACCCTGCCAT AGAGTCACCG   
  
  
+ ATTCAGTATA TATGTAACAT ACCCAATCTT GTCTGGATAT ATAGTTAACC CTTTTGGAAT ATCTGAAAAT   
  
  
+ CCACACGAGA GTCATCCTTC TAGACTAGGA AACATGCGCC ACATTTTGGA TTCTAACCAA GTATAATAAG   
  
  
+ CATATCTTAA AGCCAAAAAT GCAAGGATAG ATACAAATAC CCATCACATG CATGTAAATG TCCCTTGTCA   
  
  
+ TGGCTCTGAT TCTCAAAATA TTAATTTTCA ATAAATTAAA TGAAATTGCA TTATTTTAAA GATTTCTGAA   
  
  
+ TGCATCCATT ATTGCAATTT TTTAAAGACT GCACAATGAT GACAATGTCA ATGGTTTCCT AAAGTTGCGC   
  
  
+ CTGATGGGCT ATTGACAGTG AAGCAACACT CCCTTTGTTC CCATTTATGT GTCTCCACTT GCCCCTTTGA   
  
  
+ AACTCTCTAC ATTTGTATGT CATCTGAATT TGATTCTCAA ATCTCAGATG ATTTGAATTC CCTTTATGCC   
  
  
+ ATCTTTACTA TCTATTGAAT GATTCTTTCT TTTCCACTTT CTTATCTTCC ACTACCTTCT TCTTGGATGC   
  
  
+ CCTTTTGCTG CTTATAAAGG GCGACACCTG AACAAGTTGC TGTATTCTCA AACCATGAAT CTGTACAGGT   
  
  
+ TTAGCCCCGA TATGTGTCCT AGCTTTGTGG GTCACGAAGA AATTGGTCCT AATTTAATAG GACACCAAGA   
  
  
+ CCCTTTTCAA AATGCATACA ATTGCACTGA TTTTGGTGAT GCTTACTTGT TGCAATTCCC AGTTGAAGAT   
  
  
+ TTATGTCAAT ACCGTGTTCC TGATCTAATG GGGCTACCTG TTGAAGATGG GGATCCTACA GAGGTCCCTG   
  
  
+ ACATTCCTGA TACATGCTTC AACTACATAA GCAATCTCCT AATGGAAGAG GGCTCGGATG ACCGCCCTGG   
  
  
+ CGCCACCCTG CAGGACTATA TGGCTGCTCA AGTCTCCTAC AGCAAGTCCT CCAATGCCGC CTTTCCCGGT   
  
  
+ GAGAGTTGCT CATTCTTTGG TAATAATCAA TGCCCTCCAC CTGCTTATCA AAGCAACCCT TATTGCCTTG   
  
  
+ ATGATTACTT CAACTTGCTA TCCAATACTA CTGGCAATAG TGTTAACAAT GCTGGTGTTC ATGACAGCAA   
  
  
+ CAACTTTGTT CAGAACAGTT TGATTGGGAA TAGGGGTACT AGTTCTAGTT CTAGGGGCAG AAGTGATTTT   
  
  
+ GGATCTACCC TTATTAGGAA TTTGCCATTA GGGACCGACA CACTTGATGT GGTCAATCCA GCACCTACTC   
  
  
+ CTCGAGCCCC TCTGGTTACG ACTCAGTGTC AGGTGTTTGA AACACACCAG TATGAGCAGG CCTTCGAACT   
  
  
+ GATCACTAAT GGAAGCTGGG AATTCAGTCA TTCTGTTGCT AATACATCAA ATGATGGAAT TCAAAATCAA   
  
  
+ CCAATTCAAG CAGTTTCAAC CCAAGAACGA TGGAATATGA AAACATTTTC CGATGACCAA CTTGAGCCTG   
  
  
+ CTCTTTTGCG AAGTCTTGTG ATGTCCTCAA ACTGTACTGA TATCTCTGAT CAGGGTGTAC ACAGGATAGT   
  
  
+ GAACCCTGAG CCACCCAACA ACAGAAATAC GGAGATTGTG TGCAATGACT CATCCAGTAA GAGTAAGACA   
  
  
+ AGACGAAGAG AGCATGGTGC AGGAGAAAAC CACGATGAAG GAGAAAACCA CGATGAGACA GGAAGGAGTA   
  
  
+ ACAAGCAATC AGCACCATCC AATGAAGTGT TCGATGAAAT TGAGCAGTAT GGTGATGTGC TGCTCTGCCC   
  
  
+ TGAAGGAAGA AGTAAACCAG CAGGGTTATG CAGCAGCAGC AACGCTAACA GAACTAGCAA CTCACAGAAC   
  
  
+ TCGGAAGACT CAAAGTCACA GCAAAATGGG AGTTCCTCCA AGGTGTCAAA AAAGCGAGGC AATAAAAAGC   
  
  
+ AAGTGGTTGA TTTGAGGACT CTCCTAACTC GATGTGCACA ATGTGTTGCT CGTGTGGATT TAACTGGTTC   
  
  
+ TTACCAGTTG CTTCAGGAGA TTCGGCAGCA TTCTTCTCCC TATGGTGATT ATCTCCAAAG AATGGCTCAT   
  
  
+ TACCTTGCTA ATGGCCTTGA GGCGCGCTTA GAAGGCAAAG GATATGAACT AACTCGTGCC AATGAGCATA   
  
  
+ TCCCACCTAC TGAGATCCTC AAGGCTAACC GGGTATATGT GGCAGCGGTC CCTTTCAAGA TAATGTCTTA   
  
  
+ TTACACAACA AACAAGACAA TTGCAAGCCT AGTTGAGGAG GCACCAAGTA TACACATAAT TGATTTTGGT   
  
  
+ ATTTTCTATG GACTACAATG GCCCTGCATC ATTCAGAACC TCTCCAAGAG GCCCAATGGG CCTCCCAGAA   
  
  
+ TCCGCATCAC AGGCATCGAT TTCCCCCAAC CTGGGTTCCG GCCAGCAGAG AGGGTTGAAG AGACAGGGCG   
  
  
+ GTGTTTAGCC AAGTATTGTG ACAGGTACAA TGTGCCCTTT GAATACCACC CCATAGCCAA GAAGTGGGAG   
  
  
+ ACCATCCAAC TCGAGGAGCT CAAAATTGAA AGGAATGAGC CCCTTGTAGT GAACTGTCTG TATAGATCGC   
  
  
+ ACAACCTATT TGATGAAAGC GTAGAGGAAA ACAGCCCAAG AGACGCGTTC CTGAGCTTGG TTAGGAAAAT   
  
  
+ AAATCCCGAT ATATTCATGC ATGGGGTGGT GAATACCGCA TCAAGTGTAC CTTTCTTCCT GAACAGGTTC   
  
  
+ AAGGAGGCCA TGTTTCACTA CACGGCCTTG TTTGACTTGT TCGAGGCCAC AATGAGCCGC GAGGATCAAG   
  
  
+ AGAGGGTGTT ACTAGAGAGC AAGTTGCATG GGAATCAGGC GTTGAATGTG ATCGCCTGCG AAGGAGCGGA   
  
  
+ GAGAGTGGAG AGGCCTGAGT CATACAAACA ATGGCAGGTT AGGACTCAAA GGGCCGGGTT TGTTCAAGTG   
  
  
+ TCGATGGATC GCGAGCTTTT GAGAAGGGCA AGGGCCATGG TCAGGAGGAA TTTTAGGGAA GAGTTCAGTT   
  
  
+ ATGATGAAGA TGGGCATTGG GTGGTTCAAG GATGGAAAGG CAGGATAATG TATGCCATTT CTTGTTGGAA   
  
  
+ ACCTTCATA  

- +Up\_Stream \_Len000TAATAA TAAGATTATA TATCTATTAT TATATTATTA TTATTATTAT TATTATTATT   
  
  
- AATAATAATA ATAATAATAA ATAATTTATA TTATCAATAA TATTATTATT ATTAATATTA TTATTATTCT   
  
  
- AATAATAGTA ATATAATAAT AATAATAATA ACATTAAATA ATAATAATAT CATTAATAAT AACAATATAT   
  
  
- CATTTTAATA ATAATCATAA CAATAATATT TATAAGTATG AACGTATATT ATAAAGATTT TATTTTTGTC   
  
  
- TCAATTAAGG TAAGGTTTTG ATGAGTGGTT TGTCGTTCCT CACCCTGGAC TAAGGAAAGG TTAAAAGTGG   
  
  
- TTTGTTGACC CAAGTCTTAA GTTCAAGGTA TAGGTTGGGG GGTCCTAAGT TCAGGACTAA GGTAAGGTAA   
  
  
- GGTCAAAGGG AGTGGTTTGA CCCGGAATCT AACTACCAAC AAGTCAACCT ACTCCCAACA AGGTACGGTT   
  
  
- CAATCTAGTA ACAACAGTTA AAACTCTTGC TGATAGAATT ATTCGGATTG CTACAACTAA ACTAAATCAA   
  
  
- GTTTGGTAAA GTTTATTATG TTTAGTTTAA GATAAATCTG GCTGTGATTT TATTTATCTA ACTCTTTTTA   
  
  
- ATATCTAAAA TTTCAAGTCT AATTTTAGGG ATTTCTAGTT GTCTAACTTT ACGTATAAAG TATCACGTGT   
  
  
- ACCTGAACGA AACTTAGAGC AGATCTCTTG GATTCTTTTT CGTTCTCACC AAGTCTTCCA ATCTTAACAA   
  
  
- GGTCGTGTAC TGTTCATGTT AGGGTTGAAA AAAGTTTTCT AAACGATGAA AATTAACCTA AGGGTACAAC   
  
  
- TTACTTCAAG ACCCAAATTC CGATGTTGAA AGAACCCTCC TATTCCAAGA ATTCAATTCC ACCAGGAGAA   
  
  
- AAGAAAACTA GAAAGAATAC CCATGTTGGA GTACCCAGAG TAGTTGTGTA CTTGAGAAAA ATAGAAAATA   
  
  
- GAAAGGGGAA AGGAAATACG TCCTAACTGT CCACGTACCT TTTGTTCTTC GTTAATTTAT GATCACCGAA   
  
  
- TCGGGGTAGG GATTATAAAA CCGATAAGAC AAAGTGGTAG GAGGGGACAG AGGTAAAAGA AACAAAGGAA   
  
  
- AAAAAGATCC TTGAACACTA CCCGTTCCCT TCTAGTCATG TTACACTACC AACAATCATC GCTCTTCTAC   
  
  
- TTCACAGTGG CGTTTTTAAA ACACGGTATT AAACAGCATT CCCTTTGTTC ATAGTTTGTT GTTACAGTAA   
  
  
- ATACTATTTG TTCATTGTCC TCTATACATG AGACTTTTTT ATATTCCATG GACTCTTCAA CAATTGAATC   
  
  
- GAACTTTTTG GGAGGATTGA ACTATTTGGG AGAAGCGATA CAATAGGGCG TGGGACGGTA TCTCAGTGGC   
  
  
- TAAGTCATAT ATACATTGTA TGGGTTAGAA CAGACCTATA TATCAATTGG GAAAACCTTA TAGACTTTTA   
  
  
- GGTGTGCTCT CAGTAGGAAG ATCTGATCCT TTGTACGCGG TGTAAAACCT AAGATTGGTT CATATTATTC   
  
  
- GTATAGAATT TCGGTTTTTA CGTTCCTATC TATGTTTATG GGTAGTGTAC GTACATTTAC AGGGAACAGT   
  
  
- ACCGAGACTA AGAGTTTTAT AATTAAAAGT TATTTAATTT ACTTTAACGT AATAAAATTT CTAAAGACTT   
  
  
- ACGTAGGTAA TAACGTTAAA AAATTTCTGA CGTGTTACTA CTGTTACAGT TACCAAAGGA TTTCAACGCG   
  
  
- GACTACCCGA TAACTGTCAC TTCGTTGTGA GGGAAACAAG GGTAAATACA CAGAGGTGAA CGGGGAAACT   
  
  
- TTGAGAGATG TAAACATACA GTAGACTTAA ACTAAGAGTT TAGAGTCTAC TAAACTTAAG GGAAATACGG   
  
  
- TAGAAATGAT AGATAACTTA CTAAGAAAGA AAAGGTGAAA GAATAGAAGG TGATGGAAGA AGAACCTACG   
  
  
- GGAAAACGAC GAATATTTCC CGCTGTGGAC TTGTTCAACG ACATAAGAGT TTGGTACTTA GACATGTCCA   
  
  
- AATCGGGGCT ATACACAGGA TCGAAACACC CAGTGCTTCT TTAACCAGGA TTAAATTATC CTGTGGTTCT   
  
  
- GGGAAAAGTT TTACGTATGT TAACGTGACT AAAACCACTA CGAATGAACA ACGTTAAGGG TCAACTTCTA   
  
  
- AATACAGTTA TGGCACAAGG ACTAGATTAC CCCGATGGAC AACTTCTACC CCTAGGATGT CTCCAGGGAC   
  
  
- TGTAAGGACT ATGTACGAAG TTGATGTATT CGTTAGAGGA TTACCTTCTC CCGAGCCTAC TGGCGGGACC   
  
  
- GCGGTGGGAC GTCCTGATAT ACCGACGAGT TCAGAGGATG TCGTTCAGGA GGTTACGGCG GAAAGGGCCA   
  
  
- CTCTCAACGA GTAAGAAACC ATTATTAGTT ACGGGAGGTG GACGAATAGT TTCGTTGGGA ATAACGGAAC   
  
  
- TACTAATGAA GTTGAACGAT AGGTTATGAT GACCGTTATC ACAATTGTTA CGACCACAAG TACTGTCGTT   
  
  
- GTTGAAACAA GTCTTGTCAA ACTAACCCTT ATCCCCATGA TCAAGATCAA GATCCCCGTC TTCACTAAAA   
  
  
- CCTAGATGGG AATAATCCTT AAACGGTAAT CCCTGGCTGT GTGAACTACA CCAGTTAGGT CGTGGATGAG   
  
  
- GAGCTCGGGG AGACCAATGC TGAGTCACAG TCCACAAACT TTGTGTGGTC ATACTCGTCC GGAAGCTTGA   
  
  
- CTAGTGATTA CCTTCGACCC TTAAGTCAGT AAGACAACGA TTATGTAGTT TACTACCTTA AGTTTTAGTT   
  
  
- GGTTAAGTTC GTCAAAGTTG GGTTCTTGCT ACCTTATACT TTTGTAAAAG GCTACTGGTT GAACTCGGAC   
  
  
- GAGAAAACGC TTCAGAACAC TACAGGAGTT TGACATGACT ATAGAGACTA GTCCCACATG TGTCCTATCA   
  
  
- CTTGGGACTC GGTGGGTTGT TGTCTTTATG CCTCTAACAC ACGTTACTGA GTAGGTCATT CTCATTCTGT   
  
  
- TCTGCTTCTC TCGTACCACG TCCTCTTTTG GTGCTACTTC CTCTTTTGGT GCTACTCTGT CCTTCCTCAT   
  
  
- TGTTCGTTAG TCGTGGTAGG TTACTTCACA AGCTACTTTA ACTCGTCATA CCACTACACG ACGAGACGGG   
  
  
- ACTTCCTTCT TCATTTGGTC GTCCCAATAC GTCGTCGTCG TTGCGATTGT CTTGATCGTT GAGTGTCTTG   
  
  
- AGCCTTCTGA GTTTCAGTGT CGTTTTACCC TCAAGGAGGT TCCACAGTTT TTTCGCTCCG TTATTTTTCG   
  
  
- TTCACCAACT AAACTCCTGA GAGGATTGAG CTACACGTGT TACACAACGA GCACACCTAA ATTGACCAAG   
  
  
- AATGGTCAAC GAAGTCCTCT AAGCCGTCGT AAGAAGAGGG ATACCACTAA TAGAGGTTTC TTACCGAGTA   
  
  
- ATGGAACGAT TACCGGAACT CCGCGCGAAT CTTCCGTTTC CTATACTTGA TTGAGCACGG TTACTCGTAT   
  
  
- AGGGTGGATG ACTCTAGGAG TTCCGATTGG CCCATATACA CCGTCGCCAG GGAAAGTTCT ATTACAGAAT   
  
  
- AATGTGTTGT TTGTTCTGTT AACGTTCGGA TCAACTCCTC CGTGGTTCAT ATGTGTATTA ACTAAAACCA   
  
  
- TAAAAGATAC CTGATGTTAC CGGGACGTAG TAAGTCTTGG AGAGGTTCTC CGGGTTACCC GGAGGGTCTT   
  
  
- AGGCGTAGTG TCCGTAGCTA AAGGGGGTTG GACCCAAGGC CGGTCGTCTC TCCCAACTTC TCTGTCCCGC   
  
  
- CACAAATCGG TTCATAACAC TGTCCATGTT ACACGGGAAA CTTATGGTGG GGTATCGGTT CTTCACCCTC   
  
  
- TGGTAGGTTG AGCTCCTCGA GTTTTAACTT TCCTTACTCG GGGAACATCA CTTGACAGAC ATATCTAGCG   
  
  
- TGTTGGATAA ACTACTTTCG CATCTCCTTT TGTCGGGTTC TCTGCGCAAG GACTCGAACC AATCCTTTTA   
  
  
- TTTAGGGCTA TATAAGTACG TACCCCACCA CTTATGGCGT AGTTCACATG GAAAGAAGGA CTTGTCCAAG   
  
  
- TTCCTCCGGT ACAAAGTGAT GTGCCGGAAC AAACTGAACA AGCTCCGGTG TTACTCGGCG CTCCTAGTTC   
  
  
- TCTCCCACAA TGATCTCTCG TTCAACGTAC CCTTAGTCCG CAACTTACAC TAGCGGACGC TTCCTCGCCT   
  
  
- CTCTCACCTC TCCGGACTCA GTATGTTTGT TACCGTCCAA TCCTGAGTTT CCCGGCCCAA ACAAGTTCAC   
  
  
- AGCTACCTAG CGCTCGAAAA CTCTTCCCGT TCCCGGTACC AGTCCTCCTT AAAATCCCTT CTCAAGTCAA   
  
  
- TACTACTTCT ACCCGTAACC CACCAAGTTC CTACCTTTCC GTCCTATTAC ATACGGTAAA GAACAACCTT   
  
  
- TGGAAGTAT

+     O2-site

| Site Name | Organism | Position | Strand | Matrix score. | sequence | function |
| --- | --- | --- | --- | --- | --- | --- |
| O2-site | Zea mays | 1871 | + | 8 | GATGA(C/T)(A/G)TG(A/G) | cis-acting regulatory element involved in zein metabolism regulation |

>HU02G01570.1   
+ +Up\_Stream \_Len000ATTATT ATTCTAATAT ATAGATAATA ATATAATAAT AATAATAATA ATAATAATAA   
  
  
+ TTATTATTAT TATTATTATT TATTAAATAT AATAGTTATT ATAATAATAA TAATTATAAT AATAATAAGA   
  
  
+ TTATTATCAT TATATTATTA TTATTATTAT TGTAATTTAT TATTATTATA GTAATTATTA TTGTTATATA   
  
  
+ GTAAAATTAT TATTAGTATT GTTATTATAA ATATTCATAC TTGCATATAA TATTTCTAAA ATAAAAACAG   
  
  
+ AGTTAATTCC ATTCCAAAAC TACTCACCAA ACAGCAAGGA GTGGGACCTG ATTCCTTTCC AATTTTCACC   
  
  
+ AAACAACTGG GTTCAGAATT CAAGTTCCAT ATCCAACCCC CCAGGATTCA AGTCCTGATT CCATTCCATT   
  
  
+ CCAGTTTCCC TCACCAAACT GGGCCTTAGA TTGATGGTTG TTCAGTTGGA TGAGGGTTGT TCCATGCCAA   
  
  
+ GTTAGATCAT TGTTGTCAAT TTTGAGAACG ACTATCTTAA TAAGCCTAAC GATGTTGATT TGATTTAGTT   
  
  
+ CAAACCATTT CAAATAATAC AAATCAAATT CTATTTAGAC CGACACTAAA ATAAATAGAT TGAGAAAAAT   
  
  
+ TATAGATTTT AAAGTTCAGA TTAAAATCCC TAAAGATCAA CAGATTGAAA TGCATATTTC ATAGTGCACA   
  
  
+ TGGACTTGCT TTGAATCTCG TCTAGAGAAC CTAAGAAAAA GCAAGAGTGG TTCAGAAGGT TAGAATTGTT   
  
  
+ CCAGCACATG ACAAGTACAA TCCCAACTTT TTTCAAAAGA TTTGCTACTT TTAATTGGAT TCCCATGTTG   
  
  
+ AATGAAGTTC TGGGTTTAAG GCTACAACTT TCTTGGGAGG ATAAGGTTCT TAAGTTAAGG TGGTCCTCTT   
  
  
+ TTCTTTTGAT CTTTCTTATG GGTACAACCT CATGGGTCTC ATCAACACAT GAACTCTTTT TATCTTTTAT   
  
  
+ CTTTCCCCTT TCCTTTATGC AGGATTGACA GGTGCATGGA AAACAAGAAG CAATTAAATA CTAGTGGCTT   
  
  
+ AGCCCCATCC CTAATATTTT GGCTATTCTG TTTCACCATC CTCCCCTGTC TCCATTTTCT TTGTTTCCTT   
  
  
+ TTTTTCTAGG AACTTGTGAT GGGCAAGGGA AGATCAGTAC AATGTGATGG TTGTTAGTAG CGAGAAGATG   
  
  
+ AAGTGTCACC GCAAAAATTT TGTGCCATAA TTTGTCGTAA GGGAAACAAG TATCAAACAA CAATGTCATT   
  
  
+ TATGATAAAC AAGTAACAGG AGATATGTAC TCTGAAAAAA TATAAGGTAC CTGAGAAGTT GTTAACTTAG   
  
  
+ CTTGAAAAAC CCTCCTAACT TGATAAACCC TCTTCGCTAT GTTATCCCGC ACCCTGCCAT AGAGTCACCG   
  
  
+ ATTCAGTATA TATGTAACAT ACCCAATCTT GTCTGGATAT ATAGTTAACC CTTTTGGAAT ATCTGAAAAT   
  
  
+ CCACACGAGA GTCATCCTTC TAGACTAGGA AACATGCGCC ACATTTTGGA TTCTAACCAA GTATAATAAG   
  
  
+ CATATCTTAA AGCCAAAAAT GCAAGGATAG ATACAAATAC CCATCACATG CATGTAAATG TCCCTTGTCA   
  
  
+ TGGCTCTGAT TCTCAAAATA TTAATTTTCA ATAAATTAAA TGAAATTGCA TTATTTTAAA GATTTCTGAA   
  
  
+ TGCATCCATT ATTGCAATTT TTTAAAGACT GCACAATGAT GACAATGTCA ATGGTTTCCT AAAGTTGCGC   
  
  
+ CTGATGGGCT ATTGACAGTG AAGCAACACT CCCTTTGTTC CCATTTATGT GTCTCCACTT GCCCCTTTGA   
  
  
+ AACTCTCTAC ATTTGTATGT CATCTGAATT TGATTCTCAA ATCTCAGATG ATTTGAATTC CCTTTATGCC   
  
  
+ ATCTTTACTA TCTATTGAAT GATTCTTTCT TTTCCACTTT CTTATCTTCC ACTACCTTCT TCTTGGATGC   
  
  
+ CCTTTTGCTG CTTATAAAGG GCGACACCTG AACAAGTTGC TGTATTCTCA AACCATGAAT CTGTACAGGT   
  
  
+ TTAGCCCCGA TATGTGTCCT AGCTTTGTGG GTCACGAAGA AATTGGTCCT AATTTAATAG GACACCAAGA   
  
  
+ CCCTTTTCAA AATGCATACA ATTGCACTGA TTTTGGTGAT GCTTACTTGT TGCAATTCCC AGTTGAAGAT   
  
  
+ TTATGTCAAT ACCGTGTTCC TGATCTAATG GGGCTACCTG TTGAAGATGG GGATCCTACA GAGGTCCCTG   
  
  
+ ACATTCCTGA TACATGCTTC AACTACATAA GCAATCTCCT AATGGAAGAG GGCTCGGATG ACCGCCCTGG   
  
  
+ CGCCACCCTG CAGGACTATA TGGCTGCTCA AGTCTCCTAC AGCAAGTCCT CCAATGCCGC CTTTCCCGGT   
  
  
+ GAGAGTTGCT CATTCTTTGG TAATAATCAA TGCCCTCCAC CTGCTTATCA AAGCAACCCT TATTGCCTTG   
  
  
+ ATGATTACTT CAACTTGCTA TCCAATACTA CTGGCAATAG TGTTAACAAT GCTGGTGTTC ATGACAGCAA   
  
  
+ CAACTTTGTT CAGAACAGTT TGATTGGGAA TAGGGGTACT AGTTCTAGTT CTAGGGGCAG AAGTGATTTT   
  
  
+ GGATCTACCC TTATTAGGAA TTTGCCATTA GGGACCGACA CACTTGATGT GGTCAATCCA GCACCTACTC   
  
  
+ CTCGAGCCCC TCTGGTTACG ACTCAGTGTC AGGTGTTTGA AACACACCAG TATGAGCAGG CCTTCGAACT   
  
  
+ GATCACTAAT GGAAGCTGGG AATTCAGTCA TTCTGTTGCT AATACATCAA ATGATGGAAT TCAAAATCAA   
  
  
+ CCAATTCAAG CAGTTTCAAC CCAAGAACGA TGGAATATGA AAACATTTTC CGATGACCAA CTTGAGCCTG   
  
  
+ CTCTTTTGCG AAGTCTTGTG ATGTCCTCAA ACTGTACTGA TATCTCTGAT CAGGGTGTAC ACAGGATAGT   
  
  
+ GAACCCTGAG CCACCCAACA ACAGAAATAC GGAGATTGTG TGCAATGACT CATCCAGTAA GAGTAAGACA   
  
  
+ AGACGAAGAG AGCATGGTGC AGGAGAAAAC CACGATGAAG GAGAAAACCA CGATGAGACA GGAAGGAGTA   
  
  
+ ACAAGCAATC AGCACCATCC AATGAAGTGT TCGATGAAAT TGAGCAGTAT GGTGATGTGC TGCTCTGCCC   
  
  
+ TGAAGGAAGA AGTAAACCAG CAGGGTTATG CAGCAGCAGC AACGCTAACA GAACTAGCAA CTCACAGAAC   
  
  
+ TCGGAAGACT CAAAGTCACA GCAAAATGGG AGTTCCTCCA AGGTGTCAAA AAAGCGAGGC AATAAAAAGC   
  
  
+ AAGTGGTTGA TTTGAGGACT CTCCTAACTC GATGTGCACA ATGTGTTGCT CGTGTGGATT TAACTGGTTC   
  
  
+ TTACCAGTTG CTTCAGGAGA TTCGGCAGCA TTCTTCTCCC TATGGTGATT ATCTCCAAAG AATGGCTCAT   
  
  
+ TACCTTGCTA ATGGCCTTGA GGCGCGCTTA GAAGGCAAAG GATATGAACT AACTCGTGCC AATGAGCATA   
  
  
+ TCCCACCTAC TGAGATCCTC AAGGCTAACC GGGTATATGT GGCAGCGGTC CCTTTCAAGA TAATGTCTTA   
  
  
+ TTACACAACA AACAAGACAA TTGCAAGCCT AGTTGAGGAG GCACCAAGTA TACACATAAT TGATTTTGGT   
  
  
+ ATTTTCTATG GACTACAATG GCCCTGCATC ATTCAGAACC TCTCCAAGAG GCCCAATGGG CCTCCCAGAA   
  
  
+ TCCGCATCAC AGGCATCGAT TTCCCCCAAC CTGGGTTCCG GCCAGCAGAG AGGGTTGAAG AGACAGGGCG   
  
  
+ GTGTTTAGCC AAGTATTGTG ACAGGTACAA TGTGCCCTTT GAATACCACC CCATAGCCAA GAAGTGGGAG   
  
  
+ ACCATCCAAC TCGAGGAGCT CAAAATTGAA AGGAATGAGC CCCTTGTAGT GAACTGTCTG TATAGATCGC   
  
  
+ ACAACCTATT TGATGAAAGC GTAGAGGAAA ACAGCCCAAG AGACGCGTTC CTGAGCTTGG TTAGGAAAAT   
  
  
+ AAATCCCGAT ATATTCATGC ATGGGGTGGT GAATACCGCA TCAAGTGTAC CTTTCTTCCT GAACAGGTTC   
  
  
+ AAGGAGGCCA TGTTTCACTA CACGGCCTTG TTTGACTTGT TCGAGGCCAC AATGAGCCGC GAGGATCAAG   
  
  
+ AGAGGGTGTT ACTAGAGAGC AAGTTGCATG GGAATCAGGC GTTGAATGTG ATCGCCTGCG AAGGAGCGGA   
  
  
+ GAGAGTGGAG AGGCCTGAGT CATACAAACA ATGGCAGGTT AGGACTCAAA GGGCCGGGTT TGTTCAAGTG   
  
  
+ TCGATGGATC GCGAGCTTTT GAGAAGGGCA AGGGCCATGG TCAGGAGGAA TTTTAGGGAA GAGTTCAGTT   
  
  
+ ATGATGAAGA TGGGCATTGG GTGGTTCAAG GATGGAAAGG CAGGATAATG TATGCCATTT CTTGTTGGAA   
  
  
+ ACCTTCATA  

- +Up\_Stream \_Len000TAATAA TAAGATTATA TATCTATTAT TATATTATTA TTATTATTAT TATTATTATT   
  
  
- AATAATAATA ATAATAATAA ATAATTTATA TTATCAATAA TATTATTATT ATTAATATTA TTATTATTCT   
  
  
- AATAATAGTA ATATAATAAT AATAATAATA ACATTAAATA ATAATAATAT CATTAATAAT AACAATATAT   
  
  
- CATTTTAATA ATAATCATAA CAATAATATT TATAAGTATG AACGTATATT ATAAAGATTT TATTTTTGTC   
  
  
- TCAATTAAGG TAAGGTTTTG ATGAGTGGTT TGTCGTTCCT CACCCTGGAC TAAGGAAAGG TTAAAAGTGG   
  
  
- TTTGTTGACC CAAGTCTTAA GTTCAAGGTA TAGGTTGGGG GGTCCTAAGT TCAGGACTAA GGTAAGGTAA   
  
  
- GGTCAAAGGG AGTGGTTTGA CCCGGAATCT AACTACCAAC AAGTCAACCT ACTCCCAACA AGGTACGGTT   
  
  
- CAATCTAGTA ACAACAGTTA AAACTCTTGC TGATAGAATT ATTCGGATTG CTACAACTAA ACTAAATCAA   
  
  
- GTTTGGTAAA GTTTATTATG TTTAGTTTAA GATAAATCTG GCTGTGATTT TATTTATCTA ACTCTTTTTA   
  
  
- ATATCTAAAA TTTCAAGTCT AATTTTAGGG ATTTCTAGTT GTCTAACTTT ACGTATAAAG TATCACGTGT   
  
  
- ACCTGAACGA AACTTAGAGC AGATCTCTTG GATTCTTTTT CGTTCTCACC AAGTCTTCCA ATCTTAACAA   
  
  
- GGTCGTGTAC TGTTCATGTT AGGGTTGAAA AAAGTTTTCT AAACGATGAA AATTAACCTA AGGGTACAAC   
  
  
- TTACTTCAAG ACCCAAATTC CGATGTTGAA AGAACCCTCC TATTCCAAGA ATTCAATTCC ACCAGGAGAA   
  
  
- AAGAAAACTA GAAAGAATAC CCATGTTGGA GTACCCAGAG TAGTTGTGTA CTTGAGAAAA ATAGAAAATA   
  
  
- GAAAGGGGAA AGGAAATACG TCCTAACTGT CCACGTACCT TTTGTTCTTC GTTAATTTAT GATCACCGAA   
  
  
- TCGGGGTAGG GATTATAAAA CCGATAAGAC AAAGTGGTAG GAGGGGACAG AGGTAAAAGA AACAAAGGAA   
  
  
- AAAAAGATCC TTGAACACTA CCCGTTCCCT TCTAGTCATG TTACACTACC AACAATCATC GCTCTTCTAC   
  
  
- TTCACAGTGG CGTTTTTAAA ACACGGTATT AAACAGCATT CCCTTTGTTC ATAGTTTGTT GTTACAGTAA   
  
  
- ATACTATTTG TTCATTGTCC TCTATACATG AGACTTTTTT ATATTCCATG GACTCTTCAA CAATTGAATC   
  
  
- GAACTTTTTG GGAGGATTGA ACTATTTGGG AGAAGCGATA CAATAGGGCG TGGGACGGTA TCTCAGTGGC   
  
  
- TAAGTCATAT ATACATTGTA TGGGTTAGAA CAGACCTATA TATCAATTGG GAAAACCTTA TAGACTTTTA   
  
  
- GGTGTGCTCT CAGTAGGAAG ATCTGATCCT TTGTACGCGG TGTAAAACCT AAGATTGGTT CATATTATTC   
  
  
- GTATAGAATT TCGGTTTTTA CGTTCCTATC TATGTTTATG GGTAGTGTAC GTACATTTAC AGGGAACAGT   
  
  
- ACCGAGACTA AGAGTTTTAT AATTAAAAGT TATTTAATTT ACTTTAACGT AATAAAATTT CTAAAGACTT   
  
  
- ACGTAGGTAA TAACGTTAAA AAATTTCTGA CGTGTTACTA CTGTTACAGT TACCAAAGGA TTTCAACGCG   
  
  
- GACTACCCGA TAACTGTCAC TTCGTTGTGA GGGAAACAAG GGTAAATACA CAGAGGTGAA CGGGGAAACT   
  
  
- TTGAGAGATG TAAACATACA GTAGACTTAA ACTAAGAGTT TAGAGTCTAC TAAACTTAAG GGAAATACGG   
  
  
- TAGAAATGAT AGATAACTTA CTAAGAAAGA AAAGGTGAAA GAATAGAAGG TGATGGAAGA AGAACCTACG   
  
  
- GGAAAACGAC GAATATTTCC CGCTGTGGAC TTGTTCAACG ACATAAGAGT TTGGTACTTA GACATGTCCA   
  
  
- AATCGGGGCT ATACACAGGA TCGAAACACC CAGTGCTTCT TTAACCAGGA TTAAATTATC CTGTGGTTCT   
  
  
- GGGAAAAGTT TTACGTATGT TAACGTGACT AAAACCACTA CGAATGAACA ACGTTAAGGG TCAACTTCTA   
  
  
- AATACAGTTA TGGCACAAGG ACTAGATTAC CCCGATGGAC AACTTCTACC CCTAGGATGT CTCCAGGGAC   
  
  
- TGTAAGGACT ATGTACGAAG TTGATGTATT CGTTAGAGGA TTACCTTCTC CCGAGCCTAC TGGCGGGACC   
  
  
- GCGGTGGGAC GTCCTGATAT ACCGACGAGT TCAGAGGATG TCGTTCAGGA GGTTACGGCG GAAAGGGCCA   
  
  
- CTCTCAACGA GTAAGAAACC ATTATTAGTT ACGGGAGGTG GACGAATAGT TTCGTTGGGA ATAACGGAAC   
  
  
- TACTAATGAA GTTGAACGAT AGGTTATGAT GACCGTTATC ACAATTGTTA CGACCACAAG TACTGTCGTT   
  
  
- GTTGAAACAA GTCTTGTCAA ACTAACCCTT ATCCCCATGA TCAAGATCAA GATCCCCGTC TTCACTAAAA   
  
  
- CCTAGATGGG AATAATCCTT AAACGGTAAT CCCTGGCTGT GTGAACTACA CCAGTTAGGT CGTGGATGAG   
  
  
- GAGCTCGGGG AGACCAATGC TGAGTCACAG TCCACAAACT TTGTGTGGTC ATACTCGTCC GGAAGCTTGA   
  
  
- CTAGTGATTA CCTTCGACCC TTAAGTCAGT AAGACAACGA TTATGTAGTT TACTACCTTA AGTTTTAGTT   
  
  
- GGTTAAGTTC GTCAAAGTTG GGTTCTTGCT ACCTTATACT TTTGTAAAAG GCTACTGGTT GAACTCGGAC   
  
  
- GAGAAAACGC TTCAGAACAC TACAGGAGTT TGACATGACT ATAGAGACTA GTCCCACATG TGTCCTATCA   
  
  
- CTTGGGACTC GGTGGGTTGT TGTCTTTATG CCTCTAACAC ACGTTACTGA GTAGGTCATT CTCATTCTGT   
  
  
- TCTGCTTCTC TCGTACCACG TCCTCTTTTG GTGCTACTTC CTCTTTTGGT GCTACTCTGT CCTTCCTCAT   
  
  
- TGTTCGTTAG TCGTGGTAGG TTACTTCACA AGCTACTTTA ACTCGTCATA CCACTACACG ACGAGACGGG   
  
  
- ACTTCCTTCT TCATTTGGTC GTCCCAATAC GTCGTCGTCG TTGCGATTGT CTTGATCGTT GAGTGTCTTG   
  
  
- AGCCTTCTGA GTTTCAGTGT CGTTTTACCC TCAAGGAGGT TCCACAGTTT TTTCGCTCCG TTATTTTTCG   
  
  
- TTCACCAACT AAACTCCTGA GAGGATTGAG CTACACGTGT TACACAACGA GCACACCTAA ATTGACCAAG   
  
  
- AATGGTCAAC GAAGTCCTCT AAGCCGTCGT AAGAAGAGGG ATACCACTAA TAGAGGTTTC TTACCGAGTA   
  
  
- ATGGAACGAT TACCGGAACT CCGCGCGAAT CTTCCGTTTC CTATACTTGA TTGAGCACGG TTACTCGTAT   
  
  
- AGGGTGGATG ACTCTAGGAG TTCCGATTGG CCCATATACA CCGTCGCCAG GGAAAGTTCT ATTACAGAAT   
  
  
- AATGTGTTGT TTGTTCTGTT AACGTTCGGA TCAACTCCTC CGTGGTTCAT ATGTGTATTA ACTAAAACCA   
  
  
- TAAAAGATAC CTGATGTTAC CGGGACGTAG TAAGTCTTGG AGAGGTTCTC CGGGTTACCC GGAGGGTCTT   
  
  
- AGGCGTAGTG TCCGTAGCTA AAGGGGGTTG GACCCAAGGC CGGTCGTCTC TCCCAACTTC TCTGTCCCGC   
  
  
- CACAAATCGG TTCATAACAC TGTCCATGTT ACACGGGAAA CTTATGGTGG GGTATCGGTT CTTCACCCTC   
  
  
- TGGTAGGTTG AGCTCCTCGA GTTTTAACTT TCCTTACTCG GGGAACATCA CTTGACAGAC ATATCTAGCG   
  
  
- TGTTGGATAA ACTACTTTCG CATCTCCTTT TGTCGGGTTC TCTGCGCAAG GACTCGAACC AATCCTTTTA   
  
  
- TTTAGGGCTA TATAAGTACG TACCCCACCA CTTATGGCGT AGTTCACATG GAAAGAAGGA CTTGTCCAAG   
  
  
- TTCCTCCGGT ACAAAGTGAT GTGCCGGAAC AAACTGAACA AGCTCCGGTG TTACTCGGCG CTCCTAGTTC   
  
  
- TCTCCCACAA TGATCTCTCG TTCAACGTAC CCTTAGTCCG CAACTTACAC TAGCGGACGC TTCCTCGCCT   
  
  
- CTCTCACCTC TCCGGACTCA GTATGTTTGT TACCGTCCAA TCCTGAGTTT CCCGGCCCAA ACAAGTTCAC   
  
  
- AGCTACCTAG CGCTCGAAAA CTCTTCCCGT TCCCGGTACC AGTCCTCCTT AAAATCCCTT CTCAAGTCAA   
  
  
- TACTACTTCT ACCCGTAACC CACCAAGTTC CTACCTTTCC GTCCTATTAC ATACGGTAAA GAACAACCTT   
  
  
- TGGAAGTAT

+     P-box

| Site Name | Organism | Position | Strand | Matrix score. | sequence | function |
| --- | --- | --- | --- | --- | --- | --- |
| P-box | Oryza sativa | 1965 | + | 7 | CCTTTTG | gibberellin-responsive element |
| P-box | Oryza sativa | 1454 | + | 7 | CCTTTTG | gibberellin-responsive element |

>HU02G01570.1   
+ +Up\_Stream \_Len000ATTATT ATTCTAATAT ATAGATAATA ATATAATAAT AATAATAATA ATAATAATAA   
  
  
+ TTATTATTAT TATTATTATT TATTAAATAT AATAGTTATT ATAATAATAA TAATTATAAT AATAATAAGA   
  
  
+ TTATTATCAT TATATTATTA TTATTATTAT TGTAATTTAT TATTATTATA GTAATTATTA TTGTTATATA   
  
  
+ GTAAAATTAT TATTAGTATT GTTATTATAA ATATTCATAC TTGCATATAA TATTTCTAAA ATAAAAACAG   
  
  
+ AGTTAATTCC ATTCCAAAAC TACTCACCAA ACAGCAAGGA GTGGGACCTG ATTCCTTTCC AATTTTCACC   
  
  
+ AAACAACTGG GTTCAGAATT CAAGTTCCAT ATCCAACCCC CCAGGATTCA AGTCCTGATT CCATTCCATT   
  
  
+ CCAGTTTCCC TCACCAAACT GGGCCTTAGA TTGATGGTTG TTCAGTTGGA TGAGGGTTGT TCCATGCCAA   
  
  
+ GTTAGATCAT TGTTGTCAAT TTTGAGAACG ACTATCTTAA TAAGCCTAAC GATGTTGATT TGATTTAGTT   
  
  
+ CAAACCATTT CAAATAATAC AAATCAAATT CTATTTAGAC CGACACTAAA ATAAATAGAT TGAGAAAAAT   
  
  
+ TATAGATTTT AAAGTTCAGA TTAAAATCCC TAAAGATCAA CAGATTGAAA TGCATATTTC ATAGTGCACA   
  
  
+ TGGACTTGCT TTGAATCTCG TCTAGAGAAC CTAAGAAAAA GCAAGAGTGG TTCAGAAGGT TAGAATTGTT   
  
  
+ CCAGCACATG ACAAGTACAA TCCCAACTTT TTTCAAAAGA TTTGCTACTT TTAATTGGAT TCCCATGTTG   
  
  
+ AATGAAGTTC TGGGTTTAAG GCTACAACTT TCTTGGGAGG ATAAGGTTCT TAAGTTAAGG TGGTCCTCTT   
  
  
+ TTCTTTTGAT CTTTCTTATG GGTACAACCT CATGGGTCTC ATCAACACAT GAACTCTTTT TATCTTTTAT   
  
  
+ CTTTCCCCTT TCCTTTATGC AGGATTGACA GGTGCATGGA AAACAAGAAG CAATTAAATA CTAGTGGCTT   
  
  
+ AGCCCCATCC CTAATATTTT GGCTATTCTG TTTCACCATC CTCCCCTGTC TCCATTTTCT TTGTTTCCTT   
  
  
+ TTTTTCTAGG AACTTGTGAT GGGCAAGGGA AGATCAGTAC AATGTGATGG TTGTTAGTAG CGAGAAGATG   
  
  
+ AAGTGTCACC GCAAAAATTT TGTGCCATAA TTTGTCGTAA GGGAAACAAG TATCAAACAA CAATGTCATT   
  
  
+ TATGATAAAC AAGTAACAGG AGATATGTAC TCTGAAAAAA TATAAGGTAC CTGAGAAGTT GTTAACTTAG   
  
  
+ CTTGAAAAAC CCTCCTAACT TGATAAACCC TCTTCGCTAT GTTATCCCGC ACCCTGCCAT AGAGTCACCG   
  
  
+ ATTCAGTATA TATGTAACAT ACCCAATCTT GTCTGGATAT ATAGTTAACC CTTTTGGAAT ATCTGAAAAT   
  
  
+ CCACACGAGA GTCATCCTTC TAGACTAGGA AACATGCGCC ACATTTTGGA TTCTAACCAA GTATAATAAG   
  
  
+ CATATCTTAA AGCCAAAAAT GCAAGGATAG ATACAAATAC CCATCACATG CATGTAAATG TCCCTTGTCA   
  
  
+ TGGCTCTGAT TCTCAAAATA TTAATTTTCA ATAAATTAAA TGAAATTGCA TTATTTTAAA GATTTCTGAA   
  
  
+ TGCATCCATT ATTGCAATTT TTTAAAGACT GCACAATGAT GACAATGTCA ATGGTTTCCT AAAGTTGCGC   
  
  
+ CTGATGGGCT ATTGACAGTG AAGCAACACT CCCTTTGTTC CCATTTATGT GTCTCCACTT GCCCCTTTGA   
  
  
+ AACTCTCTAC ATTTGTATGT CATCTGAATT TGATTCTCAA ATCTCAGATG ATTTGAATTC CCTTTATGCC   
  
  
+ ATCTTTACTA TCTATTGAAT GATTCTTTCT TTTCCACTTT CTTATCTTCC ACTACCTTCT TCTTGGATGC   
  
  
+ CCTTTTGCTG CTTATAAAGG GCGACACCTG AACAAGTTGC TGTATTCTCA AACCATGAAT CTGTACAGGT   
  
  
+ TTAGCCCCGA TATGTGTCCT AGCTTTGTGG GTCACGAAGA AATTGGTCCT AATTTAATAG GACACCAAGA   
  
  
+ CCCTTTTCAA AATGCATACA ATTGCACTGA TTTTGGTGAT GCTTACTTGT TGCAATTCCC AGTTGAAGAT   
  
  
+ TTATGTCAAT ACCGTGTTCC TGATCTAATG GGGCTACCTG TTGAAGATGG GGATCCTACA GAGGTCCCTG   
  
  
+ ACATTCCTGA TACATGCTTC AACTACATAA GCAATCTCCT AATGGAAGAG GGCTCGGATG ACCGCCCTGG   
  
  
+ CGCCACCCTG CAGGACTATA TGGCTGCTCA AGTCTCCTAC AGCAAGTCCT CCAATGCCGC CTTTCCCGGT   
  
  
+ GAGAGTTGCT CATTCTTTGG TAATAATCAA TGCCCTCCAC CTGCTTATCA AAGCAACCCT TATTGCCTTG   
  
  
+ ATGATTACTT CAACTTGCTA TCCAATACTA CTGGCAATAG TGTTAACAAT GCTGGTGTTC ATGACAGCAA   
  
  
+ CAACTTTGTT CAGAACAGTT TGATTGGGAA TAGGGGTACT AGTTCTAGTT CTAGGGGCAG AAGTGATTTT   
  
  
+ GGATCTACCC TTATTAGGAA TTTGCCATTA GGGACCGACA CACTTGATGT GGTCAATCCA GCACCTACTC   
  
  
+ CTCGAGCCCC TCTGGTTACG ACTCAGTGTC AGGTGTTTGA AACACACCAG TATGAGCAGG CCTTCGAACT   
  
  
+ GATCACTAAT GGAAGCTGGG AATTCAGTCA TTCTGTTGCT AATACATCAA ATGATGGAAT TCAAAATCAA   
  
  
+ CCAATTCAAG CAGTTTCAAC CCAAGAACGA TGGAATATGA AAACATTTTC CGATGACCAA CTTGAGCCTG   
  
  
+ CTCTTTTGCG AAGTCTTGTG ATGTCCTCAA ACTGTACTGA TATCTCTGAT CAGGGTGTAC ACAGGATAGT   
  
  
+ GAACCCTGAG CCACCCAACA ACAGAAATAC GGAGATTGTG TGCAATGACT CATCCAGTAA GAGTAAGACA   
  
  
+ AGACGAAGAG AGCATGGTGC AGGAGAAAAC CACGATGAAG GAGAAAACCA CGATGAGACA GGAAGGAGTA   
  
  
+ ACAAGCAATC AGCACCATCC AATGAAGTGT TCGATGAAAT TGAGCAGTAT GGTGATGTGC TGCTCTGCCC   
  
  
+ TGAAGGAAGA AGTAAACCAG CAGGGTTATG CAGCAGCAGC AACGCTAACA GAACTAGCAA CTCACAGAAC   
  
  
+ TCGGAAGACT CAAAGTCACA GCAAAATGGG AGTTCCTCCA AGGTGTCAAA AAAGCGAGGC AATAAAAAGC   
  
  
+ AAGTGGTTGA TTTGAGGACT CTCCTAACTC GATGTGCACA ATGTGTTGCT CGTGTGGATT TAACTGGTTC   
  
  
+ TTACCAGTTG CTTCAGGAGA TTCGGCAGCA TTCTTCTCCC TATGGTGATT ATCTCCAAAG AATGGCTCAT   
  
  
+ TACCTTGCTA ATGGCCTTGA GGCGCGCTTA GAAGGCAAAG GATATGAACT AACTCGTGCC AATGAGCATA   
  
  
+ TCCCACCTAC TGAGATCCTC AAGGCTAACC GGGTATATGT GGCAGCGGTC CCTTTCAAGA TAATGTCTTA   
  
  
+ TTACACAACA AACAAGACAA TTGCAAGCCT AGTTGAGGAG GCACCAAGTA TACACATAAT TGATTTTGGT   
  
  
+ ATTTTCTATG GACTACAATG GCCCTGCATC ATTCAGAACC TCTCCAAGAG GCCCAATGGG CCTCCCAGAA   
  
  
+ TCCGCATCAC AGGCATCGAT TTCCCCCAAC CTGGGTTCCG GCCAGCAGAG AGGGTTGAAG AGACAGGGCG   
  
  
+ GTGTTTAGCC AAGTATTGTG ACAGGTACAA TGTGCCCTTT GAATACCACC CCATAGCCAA GAAGTGGGAG   
  
  
+ ACCATCCAAC TCGAGGAGCT CAAAATTGAA AGGAATGAGC CCCTTGTAGT GAACTGTCTG TATAGATCGC   
  
  
+ ACAACCTATT TGATGAAAGC GTAGAGGAAA ACAGCCCAAG AGACGCGTTC CTGAGCTTGG TTAGGAAAAT   
  
  
+ AAATCCCGAT ATATTCATGC ATGGGGTGGT GAATACCGCA TCAAGTGTAC CTTTCTTCCT GAACAGGTTC   
  
  
+ AAGGAGGCCA TGTTTCACTA CACGGCCTTG TTTGACTTGT TCGAGGCCAC AATGAGCCGC GAGGATCAAG   
  
  
+ AGAGGGTGTT ACTAGAGAGC AAGTTGCATG GGAATCAGGC GTTGAATGTG ATCGCCTGCG AAGGAGCGGA   
  
  
+ GAGAGTGGAG AGGCCTGAGT CATACAAACA ATGGCAGGTT AGGACTCAAA GGGCCGGGTT TGTTCAAGTG   
  
  
+ TCGATGGATC GCGAGCTTTT GAGAAGGGCA AGGGCCATGG TCAGGAGGAA TTTTAGGGAA GAGTTCAGTT   
  
  
+ ATGATGAAGA TGGGCATTGG GTGGTTCAAG GATGGAAAGG CAGGATAATG TATGCCATTT CTTGTTGGAA   
  
  
+ ACCTTCATA  

- +Up\_Stream \_Len000TAATAA TAAGATTATA TATCTATTAT TATATTATTA TTATTATTAT TATTATTATT   
  
  
- AATAATAATA ATAATAATAA ATAATTTATA TTATCAATAA TATTATTATT ATTAATATTA TTATTATTCT   
  
  
- AATAATAGTA ATATAATAAT AATAATAATA ACATTAAATA ATAATAATAT CATTAATAAT AACAATATAT   
  
  
- CATTTTAATA ATAATCATAA CAATAATATT TATAAGTATG AACGTATATT ATAAAGATTT TATTTTTGTC   
  
  
- TCAATTAAGG TAAGGTTTTG ATGAGTGGTT TGTCGTTCCT CACCCTGGAC TAAGGAAAGG TTAAAAGTGG   
  
  
- TTTGTTGACC CAAGTCTTAA GTTCAAGGTA TAGGTTGGGG GGTCCTAAGT TCAGGACTAA GGTAAGGTAA   
  
  
- GGTCAAAGGG AGTGGTTTGA CCCGGAATCT AACTACCAAC AAGTCAACCT ACTCCCAACA AGGTACGGTT   
  
  
- CAATCTAGTA ACAACAGTTA AAACTCTTGC TGATAGAATT ATTCGGATTG CTACAACTAA ACTAAATCAA   
  
  
- GTTTGGTAAA GTTTATTATG TTTAGTTTAA GATAAATCTG GCTGTGATTT TATTTATCTA ACTCTTTTTA   
  
  
- ATATCTAAAA TTTCAAGTCT AATTTTAGGG ATTTCTAGTT GTCTAACTTT ACGTATAAAG TATCACGTGT   
  
  
- ACCTGAACGA AACTTAGAGC AGATCTCTTG GATTCTTTTT CGTTCTCACC AAGTCTTCCA ATCTTAACAA   
  
  
- GGTCGTGTAC TGTTCATGTT AGGGTTGAAA AAAGTTTTCT AAACGATGAA AATTAACCTA AGGGTACAAC   
  
  
- TTACTTCAAG ACCCAAATTC CGATGTTGAA AGAACCCTCC TATTCCAAGA ATTCAATTCC ACCAGGAGAA   
  
  
- AAGAAAACTA GAAAGAATAC CCATGTTGGA GTACCCAGAG TAGTTGTGTA CTTGAGAAAA ATAGAAAATA   
  
  
- GAAAGGGGAA AGGAAATACG TCCTAACTGT CCACGTACCT TTTGTTCTTC GTTAATTTAT GATCACCGAA   
  
  
- TCGGGGTAGG GATTATAAAA CCGATAAGAC AAAGTGGTAG GAGGGGACAG AGGTAAAAGA AACAAAGGAA   
  
  
- AAAAAGATCC TTGAACACTA CCCGTTCCCT TCTAGTCATG TTACACTACC AACAATCATC GCTCTTCTAC   
  
  
- TTCACAGTGG CGTTTTTAAA ACACGGTATT AAACAGCATT CCCTTTGTTC ATAGTTTGTT GTTACAGTAA   
  
  
- ATACTATTTG TTCATTGTCC TCTATACATG AGACTTTTTT ATATTCCATG GACTCTTCAA CAATTGAATC   
  
  
- GAACTTTTTG GGAGGATTGA ACTATTTGGG AGAAGCGATA CAATAGGGCG TGGGACGGTA TCTCAGTGGC   
  
  
- TAAGTCATAT ATACATTGTA TGGGTTAGAA CAGACCTATA TATCAATTGG GAAAACCTTA TAGACTTTTA   
  
  
- GGTGTGCTCT CAGTAGGAAG ATCTGATCCT TTGTACGCGG TGTAAAACCT AAGATTGGTT CATATTATTC   
  
  
- GTATAGAATT TCGGTTTTTA CGTTCCTATC TATGTTTATG GGTAGTGTAC GTACATTTAC AGGGAACAGT   
  
  
- ACCGAGACTA AGAGTTTTAT AATTAAAAGT TATTTAATTT ACTTTAACGT AATAAAATTT CTAAAGACTT   
  
  
- ACGTAGGTAA TAACGTTAAA AAATTTCTGA CGTGTTACTA CTGTTACAGT TACCAAAGGA TTTCAACGCG   
  
  
- GACTACCCGA TAACTGTCAC TTCGTTGTGA GGGAAACAAG GGTAAATACA CAGAGGTGAA CGGGGAAACT   
  
  
- TTGAGAGATG TAAACATACA GTAGACTTAA ACTAAGAGTT TAGAGTCTAC TAAACTTAAG GGAAATACGG   
  
  
- TAGAAATGAT AGATAACTTA CTAAGAAAGA AAAGGTGAAA GAATAGAAGG TGATGGAAGA AGAACCTACG   
  
  
- GGAAAACGAC GAATATTTCC CGCTGTGGAC TTGTTCAACG ACATAAGAGT TTGGTACTTA GACATGTCCA   
  
  
- AATCGGGGCT ATACACAGGA TCGAAACACC CAGTGCTTCT TTAACCAGGA TTAAATTATC CTGTGGTTCT   
  
  
- GGGAAAAGTT TTACGTATGT TAACGTGACT AAAACCACTA CGAATGAACA ACGTTAAGGG TCAACTTCTA   
  
  
- AATACAGTTA TGGCACAAGG ACTAGATTAC CCCGATGGAC AACTTCTACC CCTAGGATGT CTCCAGGGAC   
  
  
- TGTAAGGACT ATGTACGAAG TTGATGTATT CGTTAGAGGA TTACCTTCTC CCGAGCCTAC TGGCGGGACC   
  
  
- GCGGTGGGAC GTCCTGATAT ACCGACGAGT TCAGAGGATG TCGTTCAGGA GGTTACGGCG GAAAGGGCCA   
  
  
- CTCTCAACGA GTAAGAAACC ATTATTAGTT ACGGGAGGTG GACGAATAGT TTCGTTGGGA ATAACGGAAC   
  
  
- TACTAATGAA GTTGAACGAT AGGTTATGAT GACCGTTATC ACAATTGTTA CGACCACAAG TACTGTCGTT   
  
  
- GTTGAAACAA GTCTTGTCAA ACTAACCCTT ATCCCCATGA TCAAGATCAA GATCCCCGTC TTCACTAAAA   
  
  
- CCTAGATGGG AATAATCCTT AAACGGTAAT CCCTGGCTGT GTGAACTACA CCAGTTAGGT CGTGGATGAG   
  
  
- GAGCTCGGGG AGACCAATGC TGAGTCACAG TCCACAAACT TTGTGTGGTC ATACTCGTCC GGAAGCTTGA   
  
  
- CTAGTGATTA CCTTCGACCC TTAAGTCAGT AAGACAACGA TTATGTAGTT TACTACCTTA AGTTTTAGTT   
  
  
- GGTTAAGTTC GTCAAAGTTG GGTTCTTGCT ACCTTATACT TTTGTAAAAG GCTACTGGTT GAACTCGGAC   
  
  
- GAGAAAACGC TTCAGAACAC TACAGGAGTT TGACATGACT ATAGAGACTA GTCCCACATG TGTCCTATCA   
  
  
- CTTGGGACTC GGTGGGTTGT TGTCTTTATG CCTCTAACAC ACGTTACTGA GTAGGTCATT CTCATTCTGT   
  
  
- TCTGCTTCTC TCGTACCACG TCCTCTTTTG GTGCTACTTC CTCTTTTGGT GCTACTCTGT CCTTCCTCAT   
  
  
- TGTTCGTTAG TCGTGGTAGG TTACTTCACA AGCTACTTTA ACTCGTCATA CCACTACACG ACGAGACGGG   
  
  
- ACTTCCTTCT TCATTTGGTC GTCCCAATAC GTCGTCGTCG TTGCGATTGT CTTGATCGTT GAGTGTCTTG   
  
  
- AGCCTTCTGA GTTTCAGTGT CGTTTTACCC TCAAGGAGGT TCCACAGTTT TTTCGCTCCG TTATTTTTCG   
  
  
- TTCACCAACT AAACTCCTGA GAGGATTGAG CTACACGTGT TACACAACGA GCACACCTAA ATTGACCAAG   
  
  
- AATGGTCAAC GAAGTCCTCT AAGCCGTCGT AAGAAGAGGG ATACCACTAA TAGAGGTTTC TTACCGAGTA   
  
  
- ATGGAACGAT TACCGGAACT CCGCGCGAAT CTTCCGTTTC CTATACTTGA TTGAGCACGG TTACTCGTAT   
  
  
- AGGGTGGATG ACTCTAGGAG TTCCGATTGG CCCATATACA CCGTCGCCAG GGAAAGTTCT ATTACAGAAT   
  
  
- AATGTGTTGT TTGTTCTGTT AACGTTCGGA TCAACTCCTC CGTGGTTCAT ATGTGTATTA ACTAAAACCA   
  
  
- TAAAAGATAC CTGATGTTAC CGGGACGTAG TAAGTCTTGG AGAGGTTCTC CGGGTTACCC GGAGGGTCTT   
  
  
- AGGCGTAGTG TCCGTAGCTA AAGGGGGTTG GACCCAAGGC CGGTCGTCTC TCCCAACTTC TCTGTCCCGC   
  
  
- CACAAATCGG TTCATAACAC TGTCCATGTT ACACGGGAAA CTTATGGTGG GGTATCGGTT CTTCACCCTC   
  
  
- TGGTAGGTTG AGCTCCTCGA GTTTTAACTT TCCTTACTCG GGGAACATCA CTTGACAGAC ATATCTAGCG   
  
  
- TGTTGGATAA ACTACTTTCG CATCTCCTTT TGTCGGGTTC TCTGCGCAAG GACTCGAACC AATCCTTTTA   
  
  
- TTTAGGGCTA TATAAGTACG TACCCCACCA CTTATGGCGT AGTTCACATG GAAAGAAGGA CTTGTCCAAG   
  
  
- TTCCTCCGGT ACAAAGTGAT GTGCCGGAAC AAACTGAACA AGCTCCGGTG TTACTCGGCG CTCCTAGTTC   
  
  
- TCTCCCACAA TGATCTCTCG TTCAACGTAC CCTTAGTCCG CAACTTACAC TAGCGGACGC TTCCTCGCCT   
  
  
- CTCTCACCTC TCCGGACTCA GTATGTTTGT TACCGTCCAA TCCTGAGTTT CCCGGCCCAA ACAAGTTCAC   
  
  
- AGCTACCTAG CGCTCGAAAA CTCTTCCCGT TCCCGGTACC AGTCCTCCTT AAAATCCCTT CTCAAGTCAA   
  
  
- TACTACTTCT ACCCGTAACC CACCAAGTTC CTACCTTTCC GTCCTATTAC ATACGGTAAA GAACAACCTT   
  
  
- TGGAAGTAT

+     RY-element

| Site Name | Organism | Position | Strand | Matrix score. | sequence | function |
| --- | --- | --- | --- | --- | --- | --- |
| RY-element | Helianthus annuus | 4010 | - | 8 | CATGCATG | cis-acting regulatory element involved in seed-specific regulation |
| RY-element | Helianthus annuus | 1591 | + | 8 | CATGCATG | cis-acting regulatory element involved in seed-specific regulation |

>HU02G01570.1   
+ +Up\_Stream \_Len000ATTATT ATTCTAATAT ATAGATAATA ATATAATAAT AATAATAATA ATAATAATAA   
  
  
+ TTATTATTAT TATTATTATT TATTAAATAT AATAGTTATT ATAATAATAA TAATTATAAT AATAATAAGA   
  
  
+ TTATTATCAT TATATTATTA TTATTATTAT TGTAATTTAT TATTATTATA GTAATTATTA TTGTTATATA   
  
  
+ GTAAAATTAT TATTAGTATT GTTATTATAA ATATTCATAC TTGCATATAA TATTTCTAAA ATAAAAACAG   
  
  
+ AGTTAATTCC ATTCCAAAAC TACTCACCAA ACAGCAAGGA GTGGGACCTG ATTCCTTTCC AATTTTCACC   
  
  
+ AAACAACTGG GTTCAGAATT CAAGTTCCAT ATCCAACCCC CCAGGATTCA AGTCCTGATT CCATTCCATT   
  
  
+ CCAGTTTCCC TCACCAAACT GGGCCTTAGA TTGATGGTTG TTCAGTTGGA TGAGGGTTGT TCCATGCCAA   
  
  
+ GTTAGATCAT TGTTGTCAAT TTTGAGAACG ACTATCTTAA TAAGCCTAAC GATGTTGATT TGATTTAGTT   
  
  
+ CAAACCATTT CAAATAATAC AAATCAAATT CTATTTAGAC CGACACTAAA ATAAATAGAT TGAGAAAAAT   
  
  
+ TATAGATTTT AAAGTTCAGA TTAAAATCCC TAAAGATCAA CAGATTGAAA TGCATATTTC ATAGTGCACA   
  
  
+ TGGACTTGCT TTGAATCTCG TCTAGAGAAC CTAAGAAAAA GCAAGAGTGG TTCAGAAGGT TAGAATTGTT   
  
  
+ CCAGCACATG ACAAGTACAA TCCCAACTTT TTTCAAAAGA TTTGCTACTT TTAATTGGAT TCCCATGTTG   
  
  
+ AATGAAGTTC TGGGTTTAAG GCTACAACTT TCTTGGGAGG ATAAGGTTCT TAAGTTAAGG TGGTCCTCTT   
  
  
+ TTCTTTTGAT CTTTCTTATG GGTACAACCT CATGGGTCTC ATCAACACAT GAACTCTTTT TATCTTTTAT   
  
  
+ CTTTCCCCTT TCCTTTATGC AGGATTGACA GGTGCATGGA AAACAAGAAG CAATTAAATA CTAGTGGCTT   
  
  
+ AGCCCCATCC CTAATATTTT GGCTATTCTG TTTCACCATC CTCCCCTGTC TCCATTTTCT TTGTTTCCTT   
  
  
+ TTTTTCTAGG AACTTGTGAT GGGCAAGGGA AGATCAGTAC AATGTGATGG TTGTTAGTAG CGAGAAGATG   
  
  
+ AAGTGTCACC GCAAAAATTT TGTGCCATAA TTTGTCGTAA GGGAAACAAG TATCAAACAA CAATGTCATT   
  
  
+ TATGATAAAC AAGTAACAGG AGATATGTAC TCTGAAAAAA TATAAGGTAC CTGAGAAGTT GTTAACTTAG   
  
  
+ CTTGAAAAAC CCTCCTAACT TGATAAACCC TCTTCGCTAT GTTATCCCGC ACCCTGCCAT AGAGTCACCG   
  
  
+ ATTCAGTATA TATGTAACAT ACCCAATCTT GTCTGGATAT ATAGTTAACC CTTTTGGAAT ATCTGAAAAT   
  
  
+ CCACACGAGA GTCATCCTTC TAGACTAGGA AACATGCGCC ACATTTTGGA TTCTAACCAA GTATAATAAG   
  
  
+ CATATCTTAA AGCCAAAAAT GCAAGGATAG ATACAAATAC CCATCACATG CATGTAAATG TCCCTTGTCA   
  
  
+ TGGCTCTGAT TCTCAAAATA TTAATTTTCA ATAAATTAAA TGAAATTGCA TTATTTTAAA GATTTCTGAA   
  
  
+ TGCATCCATT ATTGCAATTT TTTAAAGACT GCACAATGAT GACAATGTCA ATGGTTTCCT AAAGTTGCGC   
  
  
+ CTGATGGGCT ATTGACAGTG AAGCAACACT CCCTTTGTTC CCATTTATGT GTCTCCACTT GCCCCTTTGA   
  
  
+ AACTCTCTAC ATTTGTATGT CATCTGAATT TGATTCTCAA ATCTCAGATG ATTTGAATTC CCTTTATGCC   
  
  
+ ATCTTTACTA TCTATTGAAT GATTCTTTCT TTTCCACTTT CTTATCTTCC ACTACCTTCT TCTTGGATGC   
  
  
+ CCTTTTGCTG CTTATAAAGG GCGACACCTG AACAAGTTGC TGTATTCTCA AACCATGAAT CTGTACAGGT   
  
  
+ TTAGCCCCGA TATGTGTCCT AGCTTTGTGG GTCACGAAGA AATTGGTCCT AATTTAATAG GACACCAAGA   
  
  
+ CCCTTTTCAA AATGCATACA ATTGCACTGA TTTTGGTGAT GCTTACTTGT TGCAATTCCC AGTTGAAGAT   
  
  
+ TTATGTCAAT ACCGTGTTCC TGATCTAATG GGGCTACCTG TTGAAGATGG GGATCCTACA GAGGTCCCTG   
  
  
+ ACATTCCTGA TACATGCTTC AACTACATAA GCAATCTCCT AATGGAAGAG GGCTCGGATG ACCGCCCTGG   
  
  
+ CGCCACCCTG CAGGACTATA TGGCTGCTCA AGTCTCCTAC AGCAAGTCCT CCAATGCCGC CTTTCCCGGT   
  
  
+ GAGAGTTGCT CATTCTTTGG TAATAATCAA TGCCCTCCAC CTGCTTATCA AAGCAACCCT TATTGCCTTG   
  
  
+ ATGATTACTT CAACTTGCTA TCCAATACTA CTGGCAATAG TGTTAACAAT GCTGGTGTTC ATGACAGCAA   
  
  
+ CAACTTTGTT CAGAACAGTT TGATTGGGAA TAGGGGTACT AGTTCTAGTT CTAGGGGCAG AAGTGATTTT   
  
  
+ GGATCTACCC TTATTAGGAA TTTGCCATTA GGGACCGACA CACTTGATGT GGTCAATCCA GCACCTACTC   
  
  
+ CTCGAGCCCC TCTGGTTACG ACTCAGTGTC AGGTGTTTGA AACACACCAG TATGAGCAGG CCTTCGAACT   
  
  
+ GATCACTAAT GGAAGCTGGG AATTCAGTCA TTCTGTTGCT AATACATCAA ATGATGGAAT TCAAAATCAA   
  
  
+ CCAATTCAAG CAGTTTCAAC CCAAGAACGA TGGAATATGA AAACATTTTC CGATGACCAA CTTGAGCCTG   
  
  
+ CTCTTTTGCG AAGTCTTGTG ATGTCCTCAA ACTGTACTGA TATCTCTGAT CAGGGTGTAC ACAGGATAGT   
  
  
+ GAACCCTGAG CCACCCAACA ACAGAAATAC GGAGATTGTG TGCAATGACT CATCCAGTAA GAGTAAGACA   
  
  
+ AGACGAAGAG AGCATGGTGC AGGAGAAAAC CACGATGAAG GAGAAAACCA CGATGAGACA GGAAGGAGTA   
  
  
+ ACAAGCAATC AGCACCATCC AATGAAGTGT TCGATGAAAT TGAGCAGTAT GGTGATGTGC TGCTCTGCCC   
  
  
+ TGAAGGAAGA AGTAAACCAG CAGGGTTATG CAGCAGCAGC AACGCTAACA GAACTAGCAA CTCACAGAAC   
  
  
+ TCGGAAGACT CAAAGTCACA GCAAAATGGG AGTTCCTCCA AGGTGTCAAA AAAGCGAGGC AATAAAAAGC   
  
  
+ AAGTGGTTGA TTTGAGGACT CTCCTAACTC GATGTGCACA ATGTGTTGCT CGTGTGGATT TAACTGGTTC   
  
  
+ TTACCAGTTG CTTCAGGAGA TTCGGCAGCA TTCTTCTCCC TATGGTGATT ATCTCCAAAG AATGGCTCAT   
  
  
+ TACCTTGCTA ATGGCCTTGA GGCGCGCTTA GAAGGCAAAG GATATGAACT AACTCGTGCC AATGAGCATA   
  
  
+ TCCCACCTAC TGAGATCCTC AAGGCTAACC GGGTATATGT GGCAGCGGTC CCTTTCAAGA TAATGTCTTA   
  
  
+ TTACACAACA AACAAGACAA TTGCAAGCCT AGTTGAGGAG GCACCAAGTA TACACATAAT TGATTTTGGT   
  
  
+ ATTTTCTATG GACTACAATG GCCCTGCATC ATTCAGAACC TCTCCAAGAG GCCCAATGGG CCTCCCAGAA   
  
  
+ TCCGCATCAC AGGCATCGAT TTCCCCCAAC CTGGGTTCCG GCCAGCAGAG AGGGTTGAAG AGACAGGGCG   
  
  
+ GTGTTTAGCC AAGTATTGTG ACAGGTACAA TGTGCCCTTT GAATACCACC CCATAGCCAA GAAGTGGGAG   
  
  
+ ACCATCCAAC TCGAGGAGCT CAAAATTGAA AGGAATGAGC CCCTTGTAGT GAACTGTCTG TATAGATCGC   
  
  
+ ACAACCTATT TGATGAAAGC GTAGAGGAAA ACAGCCCAAG AGACGCGTTC CTGAGCTTGG TTAGGAAAAT   
  
  
+ AAATCCCGAT ATATTCATGC ATGGGGTGGT GAATACCGCA TCAAGTGTAC CTTTCTTCCT GAACAGGTTC   
  
  
+ AAGGAGGCCA TGTTTCACTA CACGGCCTTG TTTGACTTGT TCGAGGCCAC AATGAGCCGC GAGGATCAAG   
  
  
+ AGAGGGTGTT ACTAGAGAGC AAGTTGCATG GGAATCAGGC GTTGAATGTG ATCGCCTGCG AAGGAGCGGA   
  
  
+ GAGAGTGGAG AGGCCTGAGT CATACAAACA ATGGCAGGTT AGGACTCAAA GGGCCGGGTT TGTTCAAGTG   
  
  
+ TCGATGGATC GCGAGCTTTT GAGAAGGGCA AGGGCCATGG TCAGGAGGAA TTTTAGGGAA GAGTTCAGTT   
  
  
+ ATGATGAAGA TGGGCATTGG GTGGTTCAAG GATGGAAAGG CAGGATAATG TATGCCATTT CTTGTTGGAA   
  
  
+ ACCTTCATA  

- +Up\_Stream \_Len000TAATAA TAAGATTATA TATCTATTAT TATATTATTA TTATTATTAT TATTATTATT   
  
  
- AATAATAATA ATAATAATAA ATAATTTATA TTATCAATAA TATTATTATT ATTAATATTA TTATTATTCT   
  
  
- AATAATAGTA ATATAATAAT AATAATAATA ACATTAAATA ATAATAATAT CATTAATAAT AACAATATAT   
  
  
- CATTTTAATA ATAATCATAA CAATAATATT TATAAGTATG AACGTATATT ATAAAGATTT TATTTTTGTC   
  
  
- TCAATTAAGG TAAGGTTTTG ATGAGTGGTT TGTCGTTCCT CACCCTGGAC TAAGGAAAGG TTAAAAGTGG   
  
  
- TTTGTTGACC CAAGTCTTAA GTTCAAGGTA TAGGTTGGGG GGTCCTAAGT TCAGGACTAA GGTAAGGTAA   
  
  
- GGTCAAAGGG AGTGGTTTGA CCCGGAATCT AACTACCAAC AAGTCAACCT ACTCCCAACA AGGTACGGTT   
  
  
- CAATCTAGTA ACAACAGTTA AAACTCTTGC TGATAGAATT ATTCGGATTG CTACAACTAA ACTAAATCAA   
  
  
- GTTTGGTAAA GTTTATTATG TTTAGTTTAA GATAAATCTG GCTGTGATTT TATTTATCTA ACTCTTTTTA   
  
  
- ATATCTAAAA TTTCAAGTCT AATTTTAGGG ATTTCTAGTT GTCTAACTTT ACGTATAAAG TATCACGTGT   
  
  
- ACCTGAACGA AACTTAGAGC AGATCTCTTG GATTCTTTTT CGTTCTCACC AAGTCTTCCA ATCTTAACAA   
  
  
- GGTCGTGTAC TGTTCATGTT AGGGTTGAAA AAAGTTTTCT AAACGATGAA AATTAACCTA AGGGTACAAC   
  
  
- TTACTTCAAG ACCCAAATTC CGATGTTGAA AGAACCCTCC TATTCCAAGA ATTCAATTCC ACCAGGAGAA   
  
  
- AAGAAAACTA GAAAGAATAC CCATGTTGGA GTACCCAGAG TAGTTGTGTA CTTGAGAAAA ATAGAAAATA   
  
  
- GAAAGGGGAA AGGAAATACG TCCTAACTGT CCACGTACCT TTTGTTCTTC GTTAATTTAT GATCACCGAA   
  
  
- TCGGGGTAGG GATTATAAAA CCGATAAGAC AAAGTGGTAG GAGGGGACAG AGGTAAAAGA AACAAAGGAA   
  
  
- AAAAAGATCC TTGAACACTA CCCGTTCCCT TCTAGTCATG TTACACTACC AACAATCATC GCTCTTCTAC   
  
  
- TTCACAGTGG CGTTTTTAAA ACACGGTATT AAACAGCATT CCCTTTGTTC ATAGTTTGTT GTTACAGTAA   
  
  
- ATACTATTTG TTCATTGTCC TCTATACATG AGACTTTTTT ATATTCCATG GACTCTTCAA CAATTGAATC   
  
  
- GAACTTTTTG GGAGGATTGA ACTATTTGGG AGAAGCGATA CAATAGGGCG TGGGACGGTA TCTCAGTGGC   
  
  
- TAAGTCATAT ATACATTGTA TGGGTTAGAA CAGACCTATA TATCAATTGG GAAAACCTTA TAGACTTTTA   
  
  
- GGTGTGCTCT CAGTAGGAAG ATCTGATCCT TTGTACGCGG TGTAAAACCT AAGATTGGTT CATATTATTC   
  
  
- GTATAGAATT TCGGTTTTTA CGTTCCTATC TATGTTTATG GGTAGTGTAC GTACATTTAC AGGGAACAGT   
  
  
- ACCGAGACTA AGAGTTTTAT AATTAAAAGT TATTTAATTT ACTTTAACGT AATAAAATTT CTAAAGACTT   
  
  
- ACGTAGGTAA TAACGTTAAA AAATTTCTGA CGTGTTACTA CTGTTACAGT TACCAAAGGA TTTCAACGCG   
  
  
- GACTACCCGA TAACTGTCAC TTCGTTGTGA GGGAAACAAG GGTAAATACA CAGAGGTGAA CGGGGAAACT   
  
  
- TTGAGAGATG TAAACATACA GTAGACTTAA ACTAAGAGTT TAGAGTCTAC TAAACTTAAG GGAAATACGG   
  
  
- TAGAAATGAT AGATAACTTA CTAAGAAAGA AAAGGTGAAA GAATAGAAGG TGATGGAAGA AGAACCTACG   
  
  
- GGAAAACGAC GAATATTTCC CGCTGTGGAC TTGTTCAACG ACATAAGAGT TTGGTACTTA GACATGTCCA   
  
  
- AATCGGGGCT ATACACAGGA TCGAAACACC CAGTGCTTCT TTAACCAGGA TTAAATTATC CTGTGGTTCT   
  
  
- GGGAAAAGTT TTACGTATGT TAACGTGACT AAAACCACTA CGAATGAACA ACGTTAAGGG TCAACTTCTA   
  
  
- AATACAGTTA TGGCACAAGG ACTAGATTAC CCCGATGGAC AACTTCTACC CCTAGGATGT CTCCAGGGAC   
  
  
- TGTAAGGACT ATGTACGAAG TTGATGTATT CGTTAGAGGA TTACCTTCTC CCGAGCCTAC TGGCGGGACC   
  
  
- GCGGTGGGAC GTCCTGATAT ACCGACGAGT TCAGAGGATG TCGTTCAGGA GGTTACGGCG GAAAGGGCCA   
  
  
- CTCTCAACGA GTAAGAAACC ATTATTAGTT ACGGGAGGTG GACGAATAGT TTCGTTGGGA ATAACGGAAC   
  
  
- TACTAATGAA GTTGAACGAT AGGTTATGAT GACCGTTATC ACAATTGTTA CGACCACAAG TACTGTCGTT   
  
  
- GTTGAAACAA GTCTTGTCAA ACTAACCCTT ATCCCCATGA TCAAGATCAA GATCCCCGTC TTCACTAAAA   
  
  
- CCTAGATGGG AATAATCCTT AAACGGTAAT CCCTGGCTGT GTGAACTACA CCAGTTAGGT CGTGGATGAG   
  
  
- GAGCTCGGGG AGACCAATGC TGAGTCACAG TCCACAAACT TTGTGTGGTC ATACTCGTCC GGAAGCTTGA   
  
  
- CTAGTGATTA CCTTCGACCC TTAAGTCAGT AAGACAACGA TTATGTAGTT TACTACCTTA AGTTTTAGTT   
  
  
- GGTTAAGTTC GTCAAAGTTG GGTTCTTGCT ACCTTATACT TTTGTAAAAG GCTACTGGTT GAACTCGGAC   
  
  
- GAGAAAACGC TTCAGAACAC TACAGGAGTT TGACATGACT ATAGAGACTA GTCCCACATG TGTCCTATCA   
  
  
- CTTGGGACTC GGTGGGTTGT TGTCTTTATG CCTCTAACAC ACGTTACTGA GTAGGTCATT CTCATTCTGT   
  
  
- TCTGCTTCTC TCGTACCACG TCCTCTTTTG GTGCTACTTC CTCTTTTGGT GCTACTCTGT CCTTCCTCAT   
  
  
- TGTTCGTTAG TCGTGGTAGG TTACTTCACA AGCTACTTTA ACTCGTCATA CCACTACACG ACGAGACGGG   
  
  
- ACTTCCTTCT TCATTTGGTC GTCCCAATAC GTCGTCGTCG TTGCGATTGT CTTGATCGTT GAGTGTCTTG   
  
  
- AGCCTTCTGA GTTTCAGTGT CGTTTTACCC TCAAGGAGGT TCCACAGTTT TTTCGCTCCG TTATTTTTCG   
  
  
- TTCACCAACT AAACTCCTGA GAGGATTGAG CTACACGTGT TACACAACGA GCACACCTAA ATTGACCAAG   
  
  
- AATGGTCAAC GAAGTCCTCT AAGCCGTCGT AAGAAGAGGG ATACCACTAA TAGAGGTTTC TTACCGAGTA   
  
  
- ATGGAACGAT TACCGGAACT CCGCGCGAAT CTTCCGTTTC CTATACTTGA TTGAGCACGG TTACTCGTAT   
  
  
- AGGGTGGATG ACTCTAGGAG TTCCGATTGG CCCATATACA CCGTCGCCAG GGAAAGTTCT ATTACAGAAT   
  
  
- AATGTGTTGT TTGTTCTGTT AACGTTCGGA TCAACTCCTC CGTGGTTCAT ATGTGTATTA ACTAAAACCA   
  
  
- TAAAAGATAC CTGATGTTAC CGGGACGTAG TAAGTCTTGG AGAGGTTCTC CGGGTTACCC GGAGGGTCTT   
  
  
- AGGCGTAGTG TCCGTAGCTA AAGGGGGTTG GACCCAAGGC CGGTCGTCTC TCCCAACTTC TCTGTCCCGC   
  
  
- CACAAATCGG TTCATAACAC TGTCCATGTT ACACGGGAAA CTTATGGTGG GGTATCGGTT CTTCACCCTC   
  
  
- TGGTAGGTTG AGCTCCTCGA GTTTTAACTT TCCTTACTCG GGGAACATCA CTTGACAGAC ATATCTAGCG   
  
  
- TGTTGGATAA ACTACTTTCG CATCTCCTTT TGTCGGGTTC TCTGCGCAAG GACTCGAACC AATCCTTTTA   
  
  
- TTTAGGGCTA TATAAGTACG TACCCCACCA CTTATGGCGT AGTTCACATG GAAAGAAGGA CTTGTCCAAG   
  
  
- TTCCTCCGGT ACAAAGTGAT GTGCCGGAAC AAACTGAACA AGCTCCGGTG TTACTCGGCG CTCCTAGTTC   
  
  
- TCTCCCACAA TGATCTCTCG TTCAACGTAC CCTTAGTCCG CAACTTACAC TAGCGGACGC TTCCTCGCCT   
  
  
- CTCTCACCTC TCCGGACTCA GTATGTTTGT TACCGTCCAA TCCTGAGTTT CCCGGCCCAA ACAAGTTCAC   
  
  
- AGCTACCTAG CGCTCGAAAA CTCTTCCCGT TCCCGGTACC AGTCCTCCTT AAAATCCCTT CTCAAGTCAA   
  
  
- TACTACTTCT ACCCGTAACC CACCAAGTTC CTACCTTTCC GTCCTATTAC ATACGGTAAA GAACAACCTT   
  
  
- TGGAAGTAT

+     STRE

| Site Name | Organism | Position | Strand | Matrix score. | sequence | function |
| --- | --- | --- | --- | --- | --- | --- |
| STRE | Arabidopsis thaliana | 2556 | + | 5 | AGGGG |  |
| STRE | Arabidopsis thaliana | 1816 | - | 5 | AGGGG |  |
| STRE | Arabidopsis thaliana | 1097 | - | 5 | AGGGG |  |
| STRE | Arabidopsis thaliana | 989 | - | 5 | AGGGG |  |
| STRE | Arabidopsis thaliana | 3894 | - | 5 | AGGGG |  |
| STRE | Arabidopsis thaliana | 2577 | + | 5 | AGGGG |  |
| STRE | Arabidopsis thaliana | 2671 | - | 5 | AGGGG |  |

>HU02G01570.1   
+ +Up\_Stream \_Len000ATTATT ATTCTAATAT ATAGATAATA ATATAATAAT AATAATAATA ATAATAATAA   
  
  
+ TTATTATTAT TATTATTATT TATTAAATAT AATAGTTATT ATAATAATAA TAATTATAAT AATAATAAGA   
  
  
+ TTATTATCAT TATATTATTA TTATTATTAT TGTAATTTAT TATTATTATA GTAATTATTA TTGTTATATA   
  
  
+ GTAAAATTAT TATTAGTATT GTTATTATAA ATATTCATAC TTGCATATAA TATTTCTAAA ATAAAAACAG   
  
  
+ AGTTAATTCC ATTCCAAAAC TACTCACCAA ACAGCAAGGA GTGGGACCTG ATTCCTTTCC AATTTTCACC   
  
  
+ AAACAACTGG GTTCAGAATT CAAGTTCCAT ATCCAACCCC CCAGGATTCA AGTCCTGATT CCATTCCATT   
  
  
+ CCAGTTTCCC TCACCAAACT GGGCCTTAGA TTGATGGTTG TTCAGTTGGA TGAGGGTTGT TCCATGCCAA   
  
  
+ GTTAGATCAT TGTTGTCAAT TTTGAGAACG ACTATCTTAA TAAGCCTAAC GATGTTGATT TGATTTAGTT   
  
  
+ CAAACCATTT CAAATAATAC AAATCAAATT CTATTTAGAC CGACACTAAA ATAAATAGAT TGAGAAAAAT   
  
  
+ TATAGATTTT AAAGTTCAGA TTAAAATCCC TAAAGATCAA CAGATTGAAA TGCATATTTC ATAGTGCACA   
  
  
+ TGGACTTGCT TTGAATCTCG TCTAGAGAAC CTAAGAAAAA GCAAGAGTGG TTCAGAAGGT TAGAATTGTT   
  
  
+ CCAGCACATG ACAAGTACAA TCCCAACTTT TTTCAAAAGA TTTGCTACTT TTAATTGGAT TCCCATGTTG   
  
  
+ AATGAAGTTC TGGGTTTAAG GCTACAACTT TCTTGGGAGG ATAAGGTTCT TAAGTTAAGG TGGTCCTCTT   
  
  
+ TTCTTTTGAT CTTTCTTATG GGTACAACCT CATGGGTCTC ATCAACACAT GAACTCTTTT TATCTTTTAT   
  
  
+ CTTTCCCCTT TCCTTTATGC AGGATTGACA GGTGCATGGA AAACAAGAAG CAATTAAATA CTAGTGGCTT   
  
  
+ AGCCCCATCC CTAATATTTT GGCTATTCTG TTTCACCATC CTCCCCTGTC TCCATTTTCT TTGTTTCCTT   
  
  
+ TTTTTCTAGG AACTTGTGAT GGGCAAGGGA AGATCAGTAC AATGTGATGG TTGTTAGTAG CGAGAAGATG   
  
  
+ AAGTGTCACC GCAAAAATTT TGTGCCATAA TTTGTCGTAA GGGAAACAAG TATCAAACAA CAATGTCATT   
  
  
+ TATGATAAAC AAGTAACAGG AGATATGTAC TCTGAAAAAA TATAAGGTAC CTGAGAAGTT GTTAACTTAG   
  
  
+ CTTGAAAAAC CCTCCTAACT TGATAAACCC TCTTCGCTAT GTTATCCCGC ACCCTGCCAT AGAGTCACCG   
  
  
+ ATTCAGTATA TATGTAACAT ACCCAATCTT GTCTGGATAT ATAGTTAACC CTTTTGGAAT ATCTGAAAAT   
  
  
+ CCACACGAGA GTCATCCTTC TAGACTAGGA AACATGCGCC ACATTTTGGA TTCTAACCAA GTATAATAAG   
  
  
+ CATATCTTAA AGCCAAAAAT GCAAGGATAG ATACAAATAC CCATCACATG CATGTAAATG TCCCTTGTCA   
  
  
+ TGGCTCTGAT TCTCAAAATA TTAATTTTCA ATAAATTAAA TGAAATTGCA TTATTTTAAA GATTTCTGAA   
  
  
+ TGCATCCATT ATTGCAATTT TTTAAAGACT GCACAATGAT GACAATGTCA ATGGTTTCCT AAAGTTGCGC   
  
  
+ CTGATGGGCT ATTGACAGTG AAGCAACACT CCCTTTGTTC CCATTTATGT GTCTCCACTT GCCCCTTTGA   
  
  
+ AACTCTCTAC ATTTGTATGT CATCTGAATT TGATTCTCAA ATCTCAGATG ATTTGAATTC CCTTTATGCC   
  
  
+ ATCTTTACTA TCTATTGAAT GATTCTTTCT TTTCCACTTT CTTATCTTCC ACTACCTTCT TCTTGGATGC   
  
  
+ CCTTTTGCTG CTTATAAAGG GCGACACCTG AACAAGTTGC TGTATTCTCA AACCATGAAT CTGTACAGGT   
  
  
+ TTAGCCCCGA TATGTGTCCT AGCTTTGTGG GTCACGAAGA AATTGGTCCT AATTTAATAG GACACCAAGA   
  
  
+ CCCTTTTCAA AATGCATACA ATTGCACTGA TTTTGGTGAT GCTTACTTGT TGCAATTCCC AGTTGAAGAT   
  
  
+ TTATGTCAAT ACCGTGTTCC TGATCTAATG GGGCTACCTG TTGAAGATGG GGATCCTACA GAGGTCCCTG   
  
  
+ ACATTCCTGA TACATGCTTC AACTACATAA GCAATCTCCT AATGGAAGAG GGCTCGGATG ACCGCCCTGG   
  
  
+ CGCCACCCTG CAGGACTATA TGGCTGCTCA AGTCTCCTAC AGCAAGTCCT CCAATGCCGC CTTTCCCGGT   
  
  
+ GAGAGTTGCT CATTCTTTGG TAATAATCAA TGCCCTCCAC CTGCTTATCA AAGCAACCCT TATTGCCTTG   
  
  
+ ATGATTACTT CAACTTGCTA TCCAATACTA CTGGCAATAG TGTTAACAAT GCTGGTGTTC ATGACAGCAA   
  
  
+ CAACTTTGTT CAGAACAGTT TGATTGGGAA TAGGGGTACT AGTTCTAGTT CTAGGGGCAG AAGTGATTTT   
  
  
+ GGATCTACCC TTATTAGGAA TTTGCCATTA GGGACCGACA CACTTGATGT GGTCAATCCA GCACCTACTC   
  
  
+ CTCGAGCCCC TCTGGTTACG ACTCAGTGTC AGGTGTTTGA AACACACCAG TATGAGCAGG CCTTCGAACT   
  
  
+ GATCACTAAT GGAAGCTGGG AATTCAGTCA TTCTGTTGCT AATACATCAA ATGATGGAAT TCAAAATCAA   
  
  
+ CCAATTCAAG CAGTTTCAAC CCAAGAACGA TGGAATATGA AAACATTTTC CGATGACCAA CTTGAGCCTG   
  
  
+ CTCTTTTGCG AAGTCTTGTG ATGTCCTCAA ACTGTACTGA TATCTCTGAT CAGGGTGTAC ACAGGATAGT   
  
  
+ GAACCCTGAG CCACCCAACA ACAGAAATAC GGAGATTGTG TGCAATGACT CATCCAGTAA GAGTAAGACA   
  
  
+ AGACGAAGAG AGCATGGTGC AGGAGAAAAC CACGATGAAG GAGAAAACCA CGATGAGACA GGAAGGAGTA   
  
  
+ ACAAGCAATC AGCACCATCC AATGAAGTGT TCGATGAAAT TGAGCAGTAT GGTGATGTGC TGCTCTGCCC   
  
  
+ TGAAGGAAGA AGTAAACCAG CAGGGTTATG CAGCAGCAGC AACGCTAACA GAACTAGCAA CTCACAGAAC   
  
  
+ TCGGAAGACT CAAAGTCACA GCAAAATGGG AGTTCCTCCA AGGTGTCAAA AAAGCGAGGC AATAAAAAGC   
  
  
+ AAGTGGTTGA TTTGAGGACT CTCCTAACTC GATGTGCACA ATGTGTTGCT CGTGTGGATT TAACTGGTTC   
  
  
+ TTACCAGTTG CTTCAGGAGA TTCGGCAGCA TTCTTCTCCC TATGGTGATT ATCTCCAAAG AATGGCTCAT   
  
  
+ TACCTTGCTA ATGGCCTTGA GGCGCGCTTA GAAGGCAAAG GATATGAACT AACTCGTGCC AATGAGCATA   
  
  
+ TCCCACCTAC TGAGATCCTC AAGGCTAACC GGGTATATGT GGCAGCGGTC CCTTTCAAGA TAATGTCTTA   
  
  
+ TTACACAACA AACAAGACAA TTGCAAGCCT AGTTGAGGAG GCACCAAGTA TACACATAAT TGATTTTGGT   
  
  
+ ATTTTCTATG GACTACAATG GCCCTGCATC ATTCAGAACC TCTCCAAGAG GCCCAATGGG CCTCCCAGAA   
  
  
+ TCCGCATCAC AGGCATCGAT TTCCCCCAAC CTGGGTTCCG GCCAGCAGAG AGGGTTGAAG AGACAGGGCG   
  
  
+ GTGTTTAGCC AAGTATTGTG ACAGGTACAA TGTGCCCTTT GAATACCACC CCATAGCCAA GAAGTGGGAG   
  
  
+ ACCATCCAAC TCGAGGAGCT CAAAATTGAA AGGAATGAGC CCCTTGTAGT GAACTGTCTG TATAGATCGC   
  
  
+ ACAACCTATT TGATGAAAGC GTAGAGGAAA ACAGCCCAAG AGACGCGTTC CTGAGCTTGG TTAGGAAAAT   
  
  
+ AAATCCCGAT ATATTCATGC ATGGGGTGGT GAATACCGCA TCAAGTGTAC CTTTCTTCCT GAACAGGTTC   
  
  
+ AAGGAGGCCA TGTTTCACTA CACGGCCTTG TTTGACTTGT TCGAGGCCAC AATGAGCCGC GAGGATCAAG   
  
  
+ AGAGGGTGTT ACTAGAGAGC AAGTTGCATG GGAATCAGGC GTTGAATGTG ATCGCCTGCG AAGGAGCGGA   
  
  
+ GAGAGTGGAG AGGCCTGAGT CATACAAACA ATGGCAGGTT AGGACTCAAA GGGCCGGGTT TGTTCAAGTG   
  
  
+ TCGATGGATC GCGAGCTTTT GAGAAGGGCA AGGGCCATGG TCAGGAGGAA TTTTAGGGAA GAGTTCAGTT   
  
  
+ ATGATGAAGA TGGGCATTGG GTGGTTCAAG GATGGAAAGG CAGGATAATG TATGCCATTT CTTGTTGGAA   
  
  
+ ACCTTCATA  

- +Up\_Stream \_Len000TAATAA TAAGATTATA TATCTATTAT TATATTATTA TTATTATTAT TATTATTATT   
  
  
- AATAATAATA ATAATAATAA ATAATTTATA TTATCAATAA TATTATTATT ATTAATATTA TTATTATTCT   
  
  
- AATAATAGTA ATATAATAAT AATAATAATA ACATTAAATA ATAATAATAT CATTAATAAT AACAATATAT   
  
  
- CATTTTAATA ATAATCATAA CAATAATATT TATAAGTATG AACGTATATT ATAAAGATTT TATTTTTGTC   
  
  
- TCAATTAAGG TAAGGTTTTG ATGAGTGGTT TGTCGTTCCT CACCCTGGAC TAAGGAAAGG TTAAAAGTGG   
  
  
- TTTGTTGACC CAAGTCTTAA GTTCAAGGTA TAGGTTGGGG GGTCCTAAGT TCAGGACTAA GGTAAGGTAA   
  
  
- GGTCAAAGGG AGTGGTTTGA CCCGGAATCT AACTACCAAC AAGTCAACCT ACTCCCAACA AGGTACGGTT   
  
  
- CAATCTAGTA ACAACAGTTA AAACTCTTGC TGATAGAATT ATTCGGATTG CTACAACTAA ACTAAATCAA   
  
  
- GTTTGGTAAA GTTTATTATG TTTAGTTTAA GATAAATCTG GCTGTGATTT TATTTATCTA ACTCTTTTTA   
  
  
- ATATCTAAAA TTTCAAGTCT AATTTTAGGG ATTTCTAGTT GTCTAACTTT ACGTATAAAG TATCACGTGT   
  
  
- ACCTGAACGA AACTTAGAGC AGATCTCTTG GATTCTTTTT CGTTCTCACC AAGTCTTCCA ATCTTAACAA   
  
  
- GGTCGTGTAC TGTTCATGTT AGGGTTGAAA AAAGTTTTCT AAACGATGAA AATTAACCTA AGGGTACAAC   
  
  
- TTACTTCAAG ACCCAAATTC CGATGTTGAA AGAACCCTCC TATTCCAAGA ATTCAATTCC ACCAGGAGAA   
  
  
- AAGAAAACTA GAAAGAATAC CCATGTTGGA GTACCCAGAG TAGTTGTGTA CTTGAGAAAA ATAGAAAATA   
  
  
- GAAAGGGGAA AGGAAATACG TCCTAACTGT CCACGTACCT TTTGTTCTTC GTTAATTTAT GATCACCGAA   
  
  
- TCGGGGTAGG GATTATAAAA CCGATAAGAC AAAGTGGTAG GAGGGGACAG AGGTAAAAGA AACAAAGGAA   
  
  
- AAAAAGATCC TTGAACACTA CCCGTTCCCT TCTAGTCATG TTACACTACC AACAATCATC GCTCTTCTAC   
  
  
- TTCACAGTGG CGTTTTTAAA ACACGGTATT AAACAGCATT CCCTTTGTTC ATAGTTTGTT GTTACAGTAA   
  
  
- ATACTATTTG TTCATTGTCC TCTATACATG AGACTTTTTT ATATTCCATG GACTCTTCAA CAATTGAATC   
  
  
- GAACTTTTTG GGAGGATTGA ACTATTTGGG AGAAGCGATA CAATAGGGCG TGGGACGGTA TCTCAGTGGC   
  
  
- TAAGTCATAT ATACATTGTA TGGGTTAGAA CAGACCTATA TATCAATTGG GAAAACCTTA TAGACTTTTA   
  
  
- GGTGTGCTCT CAGTAGGAAG ATCTGATCCT TTGTACGCGG TGTAAAACCT AAGATTGGTT CATATTATTC   
  
  
- GTATAGAATT TCGGTTTTTA CGTTCCTATC TATGTTTATG GGTAGTGTAC GTACATTTAC AGGGAACAGT   
  
  
- ACCGAGACTA AGAGTTTTAT AATTAAAAGT TATTTAATTT ACTTTAACGT AATAAAATTT CTAAAGACTT   
  
  
- ACGTAGGTAA TAACGTTAAA AAATTTCTGA CGTGTTACTA CTGTTACAGT TACCAAAGGA TTTCAACGCG   
  
  
- GACTACCCGA TAACTGTCAC TTCGTTGTGA GGGAAACAAG GGTAAATACA CAGAGGTGAA CGGGGAAACT   
  
  
- TTGAGAGATG TAAACATACA GTAGACTTAA ACTAAGAGTT TAGAGTCTAC TAAACTTAAG GGAAATACGG   
  
  
- TAGAAATGAT AGATAACTTA CTAAGAAAGA AAAGGTGAAA GAATAGAAGG TGATGGAAGA AGAACCTACG   
  
  
- GGAAAACGAC GAATATTTCC CGCTGTGGAC TTGTTCAACG ACATAAGAGT TTGGTACTTA GACATGTCCA   
  
  
- AATCGGGGCT ATACACAGGA TCGAAACACC CAGTGCTTCT TTAACCAGGA TTAAATTATC CTGTGGTTCT   
  
  
- GGGAAAAGTT TTACGTATGT TAACGTGACT AAAACCACTA CGAATGAACA ACGTTAAGGG TCAACTTCTA   
  
  
- AATACAGTTA TGGCACAAGG ACTAGATTAC CCCGATGGAC AACTTCTACC CCTAGGATGT CTCCAGGGAC   
  
  
- TGTAAGGACT ATGTACGAAG TTGATGTATT CGTTAGAGGA TTACCTTCTC CCGAGCCTAC TGGCGGGACC   
  
  
- GCGGTGGGAC GTCCTGATAT ACCGACGAGT TCAGAGGATG TCGTTCAGGA GGTTACGGCG GAAAGGGCCA   
  
  
- CTCTCAACGA GTAAGAAACC ATTATTAGTT ACGGGAGGTG GACGAATAGT TTCGTTGGGA ATAACGGAAC   
  
  
- TACTAATGAA GTTGAACGAT AGGTTATGAT GACCGTTATC ACAATTGTTA CGACCACAAG TACTGTCGTT   
  
  
- GTTGAAACAA GTCTTGTCAA ACTAACCCTT ATCCCCATGA TCAAGATCAA GATCCCCGTC TTCACTAAAA   
  
  
- CCTAGATGGG AATAATCCTT AAACGGTAAT CCCTGGCTGT GTGAACTACA CCAGTTAGGT CGTGGATGAG   
  
  
- GAGCTCGGGG AGACCAATGC TGAGTCACAG TCCACAAACT TTGTGTGGTC ATACTCGTCC GGAAGCTTGA   
  
  
- CTAGTGATTA CCTTCGACCC TTAAGTCAGT AAGACAACGA TTATGTAGTT TACTACCTTA AGTTTTAGTT   
  
  
- GGTTAAGTTC GTCAAAGTTG GGTTCTTGCT ACCTTATACT TTTGTAAAAG GCTACTGGTT GAACTCGGAC   
  
  
- GAGAAAACGC TTCAGAACAC TACAGGAGTT TGACATGACT ATAGAGACTA GTCCCACATG TGTCCTATCA   
  
  
- CTTGGGACTC GGTGGGTTGT TGTCTTTATG CCTCTAACAC ACGTTACTGA GTAGGTCATT CTCATTCTGT   
  
  
- TCTGCTTCTC TCGTACCACG TCCTCTTTTG GTGCTACTTC CTCTTTTGGT GCTACTCTGT CCTTCCTCAT   
  
  
- TGTTCGTTAG TCGTGGTAGG TTACTTCACA AGCTACTTTA ACTCGTCATA CCACTACACG ACGAGACGGG   
  
  
- ACTTCCTTCT TCATTTGGTC GTCCCAATAC GTCGTCGTCG TTGCGATTGT CTTGATCGTT GAGTGTCTTG   
  
  
- AGCCTTCTGA GTTTCAGTGT CGTTTTACCC TCAAGGAGGT TCCACAGTTT TTTCGCTCCG TTATTTTTCG   
  
  
- TTCACCAACT AAACTCCTGA GAGGATTGAG CTACACGTGT TACACAACGA GCACACCTAA ATTGACCAAG   
  
  
- AATGGTCAAC GAAGTCCTCT AAGCCGTCGT AAGAAGAGGG ATACCACTAA TAGAGGTTTC TTACCGAGTA   
  
  
- ATGGAACGAT TACCGGAACT CCGCGCGAAT CTTCCGTTTC CTATACTTGA TTGAGCACGG TTACTCGTAT   
  
  
- AGGGTGGATG ACTCTAGGAG TTCCGATTGG CCCATATACA CCGTCGCCAG GGAAAGTTCT ATTACAGAAT   
  
  
- AATGTGTTGT TTGTTCTGTT AACGTTCGGA TCAACTCCTC CGTGGTTCAT ATGTGTATTA ACTAAAACCA   
  
  
- TAAAAGATAC CTGATGTTAC CGGGACGTAG TAAGTCTTGG AGAGGTTCTC CGGGTTACCC GGAGGGTCTT   
  
  
- AGGCGTAGTG TCCGTAGCTA AAGGGGGTTG GACCCAAGGC CGGTCGTCTC TCCCAACTTC TCTGTCCCGC   
  
  
- CACAAATCGG TTCATAACAC TGTCCATGTT ACACGGGAAA CTTATGGTGG GGTATCGGTT CTTCACCCTC   
  
  
- TGGTAGGTTG AGCTCCTCGA GTTTTAACTT TCCTTACTCG GGGAACATCA CTTGACAGAC ATATCTAGCG   
  
  
- TGTTGGATAA ACTACTTTCG CATCTCCTTT TGTCGGGTTC TCTGCGCAAG GACTCGAACC AATCCTTTTA   
  
  
- TTTAGGGCTA TATAAGTACG TACCCCACCA CTTATGGCGT AGTTCACATG GAAAGAAGGA CTTGTCCAAG   
  
  
- TTCCTCCGGT ACAAAGTGAT GTGCCGGAAC AAACTGAACA AGCTCCGGTG TTACTCGGCG CTCCTAGTTC   
  
  
- TCTCCCACAA TGATCTCTCG TTCAACGTAC CCTTAGTCCG CAACTTACAC TAGCGGACGC TTCCTCGCCT   
  
  
- CTCTCACCTC TCCGGACTCA GTATGTTTGT TACCGTCCAA TCCTGAGTTT CCCGGCCCAA ACAAGTTCAC   
  
  
- AGCTACCTAG CGCTCGAAAA CTCTTCCCGT TCCCGGTACC AGTCCTCCTT AAAATCCCTT CTCAAGTCAA   
  
  
- TACTACTTCT ACCCGTAACC CACCAAGTTC CTACCTTTCC GTCCTATTAC ATACGGTAAA GAACAACCTT   
  
  
- TGGAAGTAT

+     Sp1

| Site Name | Organism | Position | Strand | Matrix score. | sequence | function |
| --- | --- | --- | --- | --- | --- | --- |
| Sp1 | Oryza sativa | 2306 | - | 6 | GGGCGG | light responsive element |
| Sp1 | Oryza sativa | 3780 | + | 6 | GGGCGG | light responsive element |

>HU02G01570.1   
+ +Up\_Stream \_Len000ATTATT ATTCTAATAT ATAGATAATA ATATAATAAT AATAATAATA ATAATAATAA   
  
  
+ TTATTATTAT TATTATTATT TATTAAATAT AATAGTTATT ATAATAATAA TAATTATAAT AATAATAAGA   
  
  
+ TTATTATCAT TATATTATTA TTATTATTAT TGTAATTTAT TATTATTATA GTAATTATTA TTGTTATATA   
  
  
+ GTAAAATTAT TATTAGTATT GTTATTATAA ATATTCATAC TTGCATATAA TATTTCTAAA ATAAAAACAG   
  
  
+ AGTTAATTCC ATTCCAAAAC TACTCACCAA ACAGCAAGGA GTGGGACCTG ATTCCTTTCC AATTTTCACC   
  
  
+ AAACAACTGG GTTCAGAATT CAAGTTCCAT ATCCAACCCC CCAGGATTCA AGTCCTGATT CCATTCCATT   
  
  
+ CCAGTTTCCC TCACCAAACT GGGCCTTAGA TTGATGGTTG TTCAGTTGGA TGAGGGTTGT TCCATGCCAA   
  
  
+ GTTAGATCAT TGTTGTCAAT TTTGAGAACG ACTATCTTAA TAAGCCTAAC GATGTTGATT TGATTTAGTT   
  
  
+ CAAACCATTT CAAATAATAC AAATCAAATT CTATTTAGAC CGACACTAAA ATAAATAGAT TGAGAAAAAT   
  
  
+ TATAGATTTT AAAGTTCAGA TTAAAATCCC TAAAGATCAA CAGATTGAAA TGCATATTTC ATAGTGCACA   
  
  
+ TGGACTTGCT TTGAATCTCG TCTAGAGAAC CTAAGAAAAA GCAAGAGTGG TTCAGAAGGT TAGAATTGTT   
  
  
+ CCAGCACATG ACAAGTACAA TCCCAACTTT TTTCAAAAGA TTTGCTACTT TTAATTGGAT TCCCATGTTG   
  
  
+ AATGAAGTTC TGGGTTTAAG GCTACAACTT TCTTGGGAGG ATAAGGTTCT TAAGTTAAGG TGGTCCTCTT   
  
  
+ TTCTTTTGAT CTTTCTTATG GGTACAACCT CATGGGTCTC ATCAACACAT GAACTCTTTT TATCTTTTAT   
  
  
+ CTTTCCCCTT TCCTTTATGC AGGATTGACA GGTGCATGGA AAACAAGAAG CAATTAAATA CTAGTGGCTT   
  
  
+ AGCCCCATCC CTAATATTTT GGCTATTCTG TTTCACCATC CTCCCCTGTC TCCATTTTCT TTGTTTCCTT   
  
  
+ TTTTTCTAGG AACTTGTGAT GGGCAAGGGA AGATCAGTAC AATGTGATGG TTGTTAGTAG CGAGAAGATG   
  
  
+ AAGTGTCACC GCAAAAATTT TGTGCCATAA TTTGTCGTAA GGGAAACAAG TATCAAACAA CAATGTCATT   
  
  
+ TATGATAAAC AAGTAACAGG AGATATGTAC TCTGAAAAAA TATAAGGTAC CTGAGAAGTT GTTAACTTAG   
  
  
+ CTTGAAAAAC CCTCCTAACT TGATAAACCC TCTTCGCTAT GTTATCCCGC ACCCTGCCAT AGAGTCACCG   
  
  
+ ATTCAGTATA TATGTAACAT ACCCAATCTT GTCTGGATAT ATAGTTAACC CTTTTGGAAT ATCTGAAAAT   
  
  
+ CCACACGAGA GTCATCCTTC TAGACTAGGA AACATGCGCC ACATTTTGGA TTCTAACCAA GTATAATAAG   
  
  
+ CATATCTTAA AGCCAAAAAT GCAAGGATAG ATACAAATAC CCATCACATG CATGTAAATG TCCCTTGTCA   
  
  
+ TGGCTCTGAT TCTCAAAATA TTAATTTTCA ATAAATTAAA TGAAATTGCA TTATTTTAAA GATTTCTGAA   
  
  
+ TGCATCCATT ATTGCAATTT TTTAAAGACT GCACAATGAT GACAATGTCA ATGGTTTCCT AAAGTTGCGC   
  
  
+ CTGATGGGCT ATTGACAGTG AAGCAACACT CCCTTTGTTC CCATTTATGT GTCTCCACTT GCCCCTTTGA   
  
  
+ AACTCTCTAC ATTTGTATGT CATCTGAATT TGATTCTCAA ATCTCAGATG ATTTGAATTC CCTTTATGCC   
  
  
+ ATCTTTACTA TCTATTGAAT GATTCTTTCT TTTCCACTTT CTTATCTTCC ACTACCTTCT TCTTGGATGC   
  
  
+ CCTTTTGCTG CTTATAAAGG GCGACACCTG AACAAGTTGC TGTATTCTCA AACCATGAAT CTGTACAGGT   
  
  
+ TTAGCCCCGA TATGTGTCCT AGCTTTGTGG GTCACGAAGA AATTGGTCCT AATTTAATAG GACACCAAGA   
  
  
+ CCCTTTTCAA AATGCATACA ATTGCACTGA TTTTGGTGAT GCTTACTTGT TGCAATTCCC AGTTGAAGAT   
  
  
+ TTATGTCAAT ACCGTGTTCC TGATCTAATG GGGCTACCTG TTGAAGATGG GGATCCTACA GAGGTCCCTG   
  
  
+ ACATTCCTGA TACATGCTTC AACTACATAA GCAATCTCCT AATGGAAGAG GGCTCGGATG ACCGCCCTGG   
  
  
+ CGCCACCCTG CAGGACTATA TGGCTGCTCA AGTCTCCTAC AGCAAGTCCT CCAATGCCGC CTTTCCCGGT   
  
  
+ GAGAGTTGCT CATTCTTTGG TAATAATCAA TGCCCTCCAC CTGCTTATCA AAGCAACCCT TATTGCCTTG   
  
  
+ ATGATTACTT CAACTTGCTA TCCAATACTA CTGGCAATAG TGTTAACAAT GCTGGTGTTC ATGACAGCAA   
  
  
+ CAACTTTGTT CAGAACAGTT TGATTGGGAA TAGGGGTACT AGTTCTAGTT CTAGGGGCAG AAGTGATTTT   
  
  
+ GGATCTACCC TTATTAGGAA TTTGCCATTA GGGACCGACA CACTTGATGT GGTCAATCCA GCACCTACTC   
  
  
+ CTCGAGCCCC TCTGGTTACG ACTCAGTGTC AGGTGTTTGA AACACACCAG TATGAGCAGG CCTTCGAACT   
  
  
+ GATCACTAAT GGAAGCTGGG AATTCAGTCA TTCTGTTGCT AATACATCAA ATGATGGAAT TCAAAATCAA   
  
  
+ CCAATTCAAG CAGTTTCAAC CCAAGAACGA TGGAATATGA AAACATTTTC CGATGACCAA CTTGAGCCTG   
  
  
+ CTCTTTTGCG AAGTCTTGTG ATGTCCTCAA ACTGTACTGA TATCTCTGAT CAGGGTGTAC ACAGGATAGT   
  
  
+ GAACCCTGAG CCACCCAACA ACAGAAATAC GGAGATTGTG TGCAATGACT CATCCAGTAA GAGTAAGACA   
  
  
+ AGACGAAGAG AGCATGGTGC AGGAGAAAAC CACGATGAAG GAGAAAACCA CGATGAGACA GGAAGGAGTA   
  
  
+ ACAAGCAATC AGCACCATCC AATGAAGTGT TCGATGAAAT TGAGCAGTAT GGTGATGTGC TGCTCTGCCC   
  
  
+ TGAAGGAAGA AGTAAACCAG CAGGGTTATG CAGCAGCAGC AACGCTAACA GAACTAGCAA CTCACAGAAC   
  
  
+ TCGGAAGACT CAAAGTCACA GCAAAATGGG AGTTCCTCCA AGGTGTCAAA AAAGCGAGGC AATAAAAAGC   
  
  
+ AAGTGGTTGA TTTGAGGACT CTCCTAACTC GATGTGCACA ATGTGTTGCT CGTGTGGATT TAACTGGTTC   
  
  
+ TTACCAGTTG CTTCAGGAGA TTCGGCAGCA TTCTTCTCCC TATGGTGATT ATCTCCAAAG AATGGCTCAT   
  
  
+ TACCTTGCTA ATGGCCTTGA GGCGCGCTTA GAAGGCAAAG GATATGAACT AACTCGTGCC AATGAGCATA   
  
  
+ TCCCACCTAC TGAGATCCTC AAGGCTAACC GGGTATATGT GGCAGCGGTC CCTTTCAAGA TAATGTCTTA   
  
  
+ TTACACAACA AACAAGACAA TTGCAAGCCT AGTTGAGGAG GCACCAAGTA TACACATAAT TGATTTTGGT   
  
  
+ ATTTTCTATG GACTACAATG GCCCTGCATC ATTCAGAACC TCTCCAAGAG GCCCAATGGG CCTCCCAGAA   
  
  
+ TCCGCATCAC AGGCATCGAT TTCCCCCAAC CTGGGTTCCG GCCAGCAGAG AGGGTTGAAG AGACAGGGCG   
  
  
+ GTGTTTAGCC AAGTATTGTG ACAGGTACAA TGTGCCCTTT GAATACCACC CCATAGCCAA GAAGTGGGAG   
  
  
+ ACCATCCAAC TCGAGGAGCT CAAAATTGAA AGGAATGAGC CCCTTGTAGT GAACTGTCTG TATAGATCGC   
  
  
+ ACAACCTATT TGATGAAAGC GTAGAGGAAA ACAGCCCAAG AGACGCGTTC CTGAGCTTGG TTAGGAAAAT   
  
  
+ AAATCCCGAT ATATTCATGC ATGGGGTGGT GAATACCGCA TCAAGTGTAC CTTTCTTCCT GAACAGGTTC   
  
  
+ AAGGAGGCCA TGTTTCACTA CACGGCCTTG TTTGACTTGT TCGAGGCCAC AATGAGCCGC GAGGATCAAG   
  
  
+ AGAGGGTGTT ACTAGAGAGC AAGTTGCATG GGAATCAGGC GTTGAATGTG ATCGCCTGCG AAGGAGCGGA   
  
  
+ GAGAGTGGAG AGGCCTGAGT CATACAAACA ATGGCAGGTT AGGACTCAAA GGGCCGGGTT TGTTCAAGTG   
  
  
+ TCGATGGATC GCGAGCTTTT GAGAAGGGCA AGGGCCATGG TCAGGAGGAA TTTTAGGGAA GAGTTCAGTT   
  
  
+ ATGATGAAGA TGGGCATTGG GTGGTTCAAG GATGGAAAGG CAGGATAATG TATGCCATTT CTTGTTGGAA   
  
  
+ ACCTTCATA  

- +Up\_Stream \_Len000TAATAA TAAGATTATA TATCTATTAT TATATTATTA TTATTATTAT TATTATTATT   
  
  
- AATAATAATA ATAATAATAA ATAATTTATA TTATCAATAA TATTATTATT ATTAATATTA TTATTATTCT   
  
  
- AATAATAGTA ATATAATAAT AATAATAATA ACATTAAATA ATAATAATAT CATTAATAAT AACAATATAT   
  
  
- CATTTTAATA ATAATCATAA CAATAATATT TATAAGTATG AACGTATATT ATAAAGATTT TATTTTTGTC   
  
  
- TCAATTAAGG TAAGGTTTTG ATGAGTGGTT TGTCGTTCCT CACCCTGGAC TAAGGAAAGG TTAAAAGTGG   
  
  
- TTTGTTGACC CAAGTCTTAA GTTCAAGGTA TAGGTTGGGG GGTCCTAAGT TCAGGACTAA GGTAAGGTAA   
  
  
- GGTCAAAGGG AGTGGTTTGA CCCGGAATCT AACTACCAAC AAGTCAACCT ACTCCCAACA AGGTACGGTT   
  
  
- CAATCTAGTA ACAACAGTTA AAACTCTTGC TGATAGAATT ATTCGGATTG CTACAACTAA ACTAAATCAA   
  
  
- GTTTGGTAAA GTTTATTATG TTTAGTTTAA GATAAATCTG GCTGTGATTT TATTTATCTA ACTCTTTTTA   
  
  
- ATATCTAAAA TTTCAAGTCT AATTTTAGGG ATTTCTAGTT GTCTAACTTT ACGTATAAAG TATCACGTGT   
  
  
- ACCTGAACGA AACTTAGAGC AGATCTCTTG GATTCTTTTT CGTTCTCACC AAGTCTTCCA ATCTTAACAA   
  
  
- GGTCGTGTAC TGTTCATGTT AGGGTTGAAA AAAGTTTTCT AAACGATGAA AATTAACCTA AGGGTACAAC   
  
  
- TTACTTCAAG ACCCAAATTC CGATGTTGAA AGAACCCTCC TATTCCAAGA ATTCAATTCC ACCAGGAGAA   
  
  
- AAGAAAACTA GAAAGAATAC CCATGTTGGA GTACCCAGAG TAGTTGTGTA CTTGAGAAAA ATAGAAAATA   
  
  
- GAAAGGGGAA AGGAAATACG TCCTAACTGT CCACGTACCT TTTGTTCTTC GTTAATTTAT GATCACCGAA   
  
  
- TCGGGGTAGG GATTATAAAA CCGATAAGAC AAAGTGGTAG GAGGGGACAG AGGTAAAAGA AACAAAGGAA   
  
  
- AAAAAGATCC TTGAACACTA CCCGTTCCCT TCTAGTCATG TTACACTACC AACAATCATC GCTCTTCTAC   
  
  
- TTCACAGTGG CGTTTTTAAA ACACGGTATT AAACAGCATT CCCTTTGTTC ATAGTTTGTT GTTACAGTAA   
  
  
- ATACTATTTG TTCATTGTCC TCTATACATG AGACTTTTTT ATATTCCATG GACTCTTCAA CAATTGAATC   
  
  
- GAACTTTTTG GGAGGATTGA ACTATTTGGG AGAAGCGATA CAATAGGGCG TGGGACGGTA TCTCAGTGGC   
  
  
- TAAGTCATAT ATACATTGTA TGGGTTAGAA CAGACCTATA TATCAATTGG GAAAACCTTA TAGACTTTTA   
  
  
- GGTGTGCTCT CAGTAGGAAG ATCTGATCCT TTGTACGCGG TGTAAAACCT AAGATTGGTT CATATTATTC   
  
  
- GTATAGAATT TCGGTTTTTA CGTTCCTATC TATGTTTATG GGTAGTGTAC GTACATTTAC AGGGAACAGT   
  
  
- ACCGAGACTA AGAGTTTTAT AATTAAAAGT TATTTAATTT ACTTTAACGT AATAAAATTT CTAAAGACTT   
  
  
- ACGTAGGTAA TAACGTTAAA AAATTTCTGA CGTGTTACTA CTGTTACAGT TACCAAAGGA TTTCAACGCG   
  
  
- GACTACCCGA TAACTGTCAC TTCGTTGTGA GGGAAACAAG GGTAAATACA CAGAGGTGAA CGGGGAAACT   
  
  
- TTGAGAGATG TAAACATACA GTAGACTTAA ACTAAGAGTT TAGAGTCTAC TAAACTTAAG GGAAATACGG   
  
  
- TAGAAATGAT AGATAACTTA CTAAGAAAGA AAAGGTGAAA GAATAGAAGG TGATGGAAGA AGAACCTACG   
  
  
- GGAAAACGAC GAATATTTCC CGCTGTGGAC TTGTTCAACG ACATAAGAGT TTGGTACTTA GACATGTCCA   
  
  
- AATCGGGGCT ATACACAGGA TCGAAACACC CAGTGCTTCT TTAACCAGGA TTAAATTATC CTGTGGTTCT   
  
  
- GGGAAAAGTT TTACGTATGT TAACGTGACT AAAACCACTA CGAATGAACA ACGTTAAGGG TCAACTTCTA   
  
  
- AATACAGTTA TGGCACAAGG ACTAGATTAC CCCGATGGAC AACTTCTACC CCTAGGATGT CTCCAGGGAC   
  
  
- TGTAAGGACT ATGTACGAAG TTGATGTATT CGTTAGAGGA TTACCTTCTC CCGAGCCTAC TGGCGGGACC   
  
  
- GCGGTGGGAC GTCCTGATAT ACCGACGAGT TCAGAGGATG TCGTTCAGGA GGTTACGGCG GAAAGGGCCA   
  
  
- CTCTCAACGA GTAAGAAACC ATTATTAGTT ACGGGAGGTG GACGAATAGT TTCGTTGGGA ATAACGGAAC   
  
  
- TACTAATGAA GTTGAACGAT AGGTTATGAT GACCGTTATC ACAATTGTTA CGACCACAAG TACTGTCGTT   
  
  
- GTTGAAACAA GTCTTGTCAA ACTAACCCTT ATCCCCATGA TCAAGATCAA GATCCCCGTC TTCACTAAAA   
  
  
- CCTAGATGGG AATAATCCTT AAACGGTAAT CCCTGGCTGT GTGAACTACA CCAGTTAGGT CGTGGATGAG   
  
  
- GAGCTCGGGG AGACCAATGC TGAGTCACAG TCCACAAACT TTGTGTGGTC ATACTCGTCC GGAAGCTTGA   
  
  
- CTAGTGATTA CCTTCGACCC TTAAGTCAGT AAGACAACGA TTATGTAGTT TACTACCTTA AGTTTTAGTT   
  
  
- GGTTAAGTTC GTCAAAGTTG GGTTCTTGCT ACCTTATACT TTTGTAAAAG GCTACTGGTT GAACTCGGAC   
  
  
- GAGAAAACGC TTCAGAACAC TACAGGAGTT TGACATGACT ATAGAGACTA GTCCCACATG TGTCCTATCA   
  
  
- CTTGGGACTC GGTGGGTTGT TGTCTTTATG CCTCTAACAC ACGTTACTGA GTAGGTCATT CTCATTCTGT   
  
  
- TCTGCTTCTC TCGTACCACG TCCTCTTTTG GTGCTACTTC CTCTTTTGGT GCTACTCTGT CCTTCCTCAT   
  
  
- TGTTCGTTAG TCGTGGTAGG TTACTTCACA AGCTACTTTA ACTCGTCATA CCACTACACG ACGAGACGGG   
  
  
- ACTTCCTTCT TCATTTGGTC GTCCCAATAC GTCGTCGTCG TTGCGATTGT CTTGATCGTT GAGTGTCTTG   
  
  
- AGCCTTCTGA GTTTCAGTGT CGTTTTACCC TCAAGGAGGT TCCACAGTTT TTTCGCTCCG TTATTTTTCG   
  
  
- TTCACCAACT AAACTCCTGA GAGGATTGAG CTACACGTGT TACACAACGA GCACACCTAA ATTGACCAAG   
  
  
- AATGGTCAAC GAAGTCCTCT AAGCCGTCGT AAGAAGAGGG ATACCACTAA TAGAGGTTTC TTACCGAGTA   
  
  
- ATGGAACGAT TACCGGAACT CCGCGCGAAT CTTCCGTTTC CTATACTTGA TTGAGCACGG TTACTCGTAT   
  
  
- AGGGTGGATG ACTCTAGGAG TTCCGATTGG CCCATATACA CCGTCGCCAG GGAAAGTTCT ATTACAGAAT   
  
  
- AATGTGTTGT TTGTTCTGTT AACGTTCGGA TCAACTCCTC CGTGGTTCAT ATGTGTATTA ACTAAAACCA   
  
  
- TAAAAGATAC CTGATGTTAC CGGGACGTAG TAAGTCTTGG AGAGGTTCTC CGGGTTACCC GGAGGGTCTT   
  
  
- AGGCGTAGTG TCCGTAGCTA AAGGGGGTTG GACCCAAGGC CGGTCGTCTC TCCCAACTTC TCTGTCCCGC   
  
  
- CACAAATCGG TTCATAACAC TGTCCATGTT ACACGGGAAA CTTATGGTGG GGTATCGGTT CTTCACCCTC   
  
  
- TGGTAGGTTG AGCTCCTCGA GTTTTAACTT TCCTTACTCG GGGAACATCA CTTGACAGAC ATATCTAGCG   
  
  
- TGTTGGATAA ACTACTTTCG CATCTCCTTT TGTCGGGTTC TCTGCGCAAG GACTCGAACC AATCCTTTTA   
  
  
- TTTAGGGCTA TATAAGTACG TACCCCACCA CTTATGGCGT AGTTCACATG GAAAGAAGGA CTTGTCCAAG   
  
  
- TTCCTCCGGT ACAAAGTGAT GTGCCGGAAC AAACTGAACA AGCTCCGGTG TTACTCGGCG CTCCTAGTTC   
  
  
- TCTCCCACAA TGATCTCTCG TTCAACGTAC CCTTAGTCCG CAACTTACAC TAGCGGACGC TTCCTCGCCT   
  
  
- CTCTCACCTC TCCGGACTCA GTATGTTTGT TACCGTCCAA TCCTGAGTTT CCCGGCCCAA ACAAGTTCAC   
  
  
- AGCTACCTAG CGCTCGAAAA CTCTTCCCGT TCCCGGTACC AGTCCTCCTT AAAATCCCTT CTCAAGTCAA   
  
  
- TACTACTTCT ACCCGTAACC CACCAAGTTC CTACCTTTCC GTCCTATTAC ATACGGTAAA GAACAACCTT   
  
  
- TGGAAGTAT

+     TATA-box

| Site Name | Organism | Position | Strand | Matrix score. | sequence | function |
| --- | --- | --- | --- | --- | --- | --- |
| TATA-box | Arabidopsis thaliana | 3623 | - | 4 | TATA | core promoter element around -30 of transcription start |
| TATA-box | Arabidopsis thaliana | 1442 | + | 6 | TATATA | core promoter element around -30 of transcription start |
| TATA-box | Arabidopsis thaliana | 3538 | - | 4 | TATA | core promoter element around -30 of transcription start |
| TATA-box | Arabidopsis thaliana | 2331 | - | 4 | TATA | core promoter element around -30 of transcription start |
| TATA-box | Arabidopsis thaliana | 1977 | + | 4 | TATA | core promoter element around -30 of transcription start |
| TATA-box | Arabidopsis thaliana | 1976 | - | 5 | TATAA | core promoter element around -30 of transcription start |
| TATA-box | Arabidopsis thaliana | 4004 | - | 4 | TATA | core promoter element around -30 of transcription start |
| TATA-box | Brassica napus | 4003 | - | 6 | ATATAT | core promoter element around -30 of transcription start |
| TATA-box | Arabidopsis thaliana | 3915 | - | 4 | TATA | core promoter element around -30 of transcription start |
| TATA-box | Helianthus annuus | 3913 | - | 6 | TATACA | core promoter element around -30 of transcription start |
| TATA-box | Arabidopsis thaliana | 1671 | + | 8 | TAAAGATT | core promoter element around -30 of transcription start |
| TATA-box | Arabidopsis thaliana | 1536 | + | 4 | TATA | core promoter element around -30 of transcription start |
| TATA-box | Arabidopsis thaliana | 1444 | + | 4 | TATA | core promoter element around -30 of transcription start |
| TATA-box | Brassica napus | 1441 | + | 6 | ATATAT | core promoter element around -30 of transcription start |
| TATA-box | Arabidopsis thaliana | 1413 | + | 4 | TATA | core promoter element around -30 of transcription start |
| TATA-box | Brassica napus | 1412 | + | 6 | ATATAT | core promoter element around -30 of transcription start |
| TATA-box | Brassica napus | 112 | + | 6 | ATTATA | core promoter element around -30 of transcription start |
| TATA-box | Brassica oleracea | 101 | + | 6 | ATATAA | core promoter element around -30 of transcription start |
| TATA-box | Arabidopsis thaliana | 102 | + | 4 | TATA | core promoter element around -30 of transcription start |
| TATA-box | Arabidopsis thaliana | 46 | + | 4 | TATA | core promoter element around -30 of transcription start |
| TATA-box | Brassica oleracea | 45 | + | 6 | ATATAA | core promoter element around -30 of transcription start |
| TATA-box | Arabidopsis thaliana | 34 | + | 4 | TATA | core promoter element around -30 of transcription start |
| TATA-box | Arabidopsis thaliana | 32 | + | 6 | TATATA | core promoter element around -30 of transcription start |
| TATA-box | Brassica napus | 31 | + | 6 | ATATAT | core promoter element around -30 of transcription start |
| TATA-box | Arabidopsis thaliana | 128 | - | 5 | TATAA | core promoter element around -30 of transcription start |
| TATA-box | Brassica napus | 127 | + | 6 | ATTATA | core promoter element around -30 of transcription start |
| TATA-box | Arabidopsis thaliana | 114 | + | 4 | TATA | core promoter element around -30 of transcription start |
| TATA-box | Arabidopsis thaliana | 113 | - | 5 | TATAA | core promoter element around -30 of transcription start |
| TATA-box | Arabidopsis thaliana | 1411 | + | 6 | TATATA | core promoter element around -30 of transcription start |
| TATA-box | Arabidopsis thaliana | 1305 | + | 4 | TATA | core promoter element around -30 of transcription start |
| TATA-box | Brassica oleracea | 1304 | + | 6 | ATATAA | core promoter element around -30 of transcription start |
| TATA-box | Arabidopsis thaliana | 1125 | - | 9 | ccTATAAAaa | core promoter element around -30 of transcription start |
| TATA-box | Arabidopsis thaliana | 635 | + | 4 | TATA | core promoter element around -30 of transcription start |
| TATA-box | Arabidopsis thaliana | 634 | - | 5 | TATAA | core promoter element around -30 of transcription start |
| TATA-box | Brassica napus | 633 | + | 6 | ATTATA | core promoter element around -30 of transcription start |
| TATA-box | Arabidopsis thaliana | 260 | + | 4 | TATA | core promoter element around -30 of transcription start |
| TATA-box | Brassica oleracea | 259 | + | 6 | ATATAA | core promoter element around -30 of transcription start |
| TATA-box | Arabidopsis thaliana | 240 | + | 4 | TATA | core promoter element around -30 of transcription start |
| TATA-box | Arabidopsis thaliana | 239 | - | 5 | TATAA | core promoter element around -30 of transcription start |
| TATA-box | Brassica napus | 238 | + | 6 | ATTATA | core promoter element around -30 of transcription start |
| TATA-box | Arabidopsis thaliana | 211 | + | 4 | TATA | core promoter element around -30 of transcription start |
| TATA-box | Arabidopsis thaliana | 209 | + | 6 | TATATA | core promoter element around -30 of transcription start |
| TATA-box | Arabidopsis thaliana | 208 | - | 7 | TATATAA | core promoter element around -30 of transcription start |
| TATA-box | Arabidopsis thaliana | 191 | + | 4 | TATA | core promoter element around -30 of transcription start |
| TATA-box | Arabidopsis thaliana | 190 | - | 5 | TATAA | core promoter element around -30 of transcription start |
| TATA-box | Brassica napus | 189 | + | 6 | ATTATA | core promoter element around -30 of transcription start |
| TATA-box | Arabidopsis thaliana | 155 | + | 4 | TATA | core promoter element around -30 of transcription start |
| TATA-box | Arabidopsis thaliana | 154 | - | 5 | TATAA | core promoter element around -30 of transcription start |
| TATA-box | Brassica napus | 153 | + | 6 | ATTATA | core promoter element around -30 of transcription start |
| TATA-box | Arabidopsis thaliana | 129 | + | 4 | TATA | core promoter element around -30 of transcription start |

>HU02G01570.1   
+ +Up\_Stream \_Len000ATTATT ATTCTAATAT ATAGATAATA ATATAATAAT AATAATAATA ATAATAATAA   
  
  
+ TTATTATTAT TATTATTATT TATTAAATAT AATAGTTATT ATAATAATAA TAATTATAAT AATAATAAGA   
  
  
+ TTATTATCAT TATATTATTA TTATTATTAT TGTAATTTAT TATTATTATA GTAATTATTA TTGTTATATA   
  
  
+ GTAAAATTAT TATTAGTATT GTTATTATAA ATATTCATAC TTGCATATAA TATTTCTAAA ATAAAAACAG   
  
  
+ AGTTAATTCC ATTCCAAAAC TACTCACCAA ACAGCAAGGA GTGGGACCTG ATTCCTTTCC AATTTTCACC   
  
  
+ AAACAACTGG GTTCAGAATT CAAGTTCCAT ATCCAACCCC CCAGGATTCA AGTCCTGATT CCATTCCATT   
  
  
+ CCAGTTTCCC TCACCAAACT GGGCCTTAGA TTGATGGTTG TTCAGTTGGA TGAGGGTTGT TCCATGCCAA   
  
  
+ GTTAGATCAT TGTTGTCAAT TTTGAGAACG ACTATCTTAA TAAGCCTAAC GATGTTGATT TGATTTAGTT   
  
  
+ CAAACCATTT CAAATAATAC AAATCAAATT CTATTTAGAC CGACACTAAA ATAAATAGAT TGAGAAAAAT   
  
  
+ TATAGATTTT AAAGTTCAGA TTAAAATCCC TAAAGATCAA CAGATTGAAA TGCATATTTC ATAGTGCACA   
  
  
+ TGGACTTGCT TTGAATCTCG TCTAGAGAAC CTAAGAAAAA GCAAGAGTGG TTCAGAAGGT TAGAATTGTT   
  
  
+ CCAGCACATG ACAAGTACAA TCCCAACTTT TTTCAAAAGA TTTGCTACTT TTAATTGGAT TCCCATGTTG   
  
  
+ AATGAAGTTC TGGGTTTAAG GCTACAACTT TCTTGGGAGG ATAAGGTTCT TAAGTTAAGG TGGTCCTCTT   
  
  
+ TTCTTTTGAT CTTTCTTATG GGTACAACCT CATGGGTCTC ATCAACACAT GAACTCTTTT TATCTTTTAT   
  
  
+ CTTTCCCCTT TCCTTTATGC AGGATTGACA GGTGCATGGA AAACAAGAAG CAATTAAATA CTAGTGGCTT   
  
  
+ AGCCCCATCC CTAATATTTT GGCTATTCTG TTTCACCATC CTCCCCTGTC TCCATTTTCT TTGTTTCCTT   
  
  
+ TTTTTCTAGG AACTTGTGAT GGGCAAGGGA AGATCAGTAC AATGTGATGG TTGTTAGTAG CGAGAAGATG   
  
  
+ AAGTGTCACC GCAAAAATTT TGTGCCATAA TTTGTCGTAA GGGAAACAAG TATCAAACAA CAATGTCATT   
  
  
+ TATGATAAAC AAGTAACAGG AGATATGTAC TCTGAAAAAA TATAAGGTAC CTGAGAAGTT GTTAACTTAG   
  
  
+ CTTGAAAAAC CCTCCTAACT TGATAAACCC TCTTCGCTAT GTTATCCCGC ACCCTGCCAT AGAGTCACCG   
  
  
+ ATTCAGTATA TATGTAACAT ACCCAATCTT GTCTGGATAT ATAGTTAACC CTTTTGGAAT ATCTGAAAAT   
  
  
+ CCACACGAGA GTCATCCTTC TAGACTAGGA AACATGCGCC ACATTTTGGA TTCTAACCAA GTATAATAAG   
  
  
+ CATATCTTAA AGCCAAAAAT GCAAGGATAG ATACAAATAC CCATCACATG CATGTAAATG TCCCTTGTCA   
  
  
+ TGGCTCTGAT TCTCAAAATA TTAATTTTCA ATAAATTAAA TGAAATTGCA TTATTTTAAA GATTTCTGAA   
  
  
+ TGCATCCATT ATTGCAATTT TTTAAAGACT GCACAATGAT GACAATGTCA ATGGTTTCCT AAAGTTGCGC   
  
  
+ CTGATGGGCT ATTGACAGTG AAGCAACACT CCCTTTGTTC CCATTTATGT GTCTCCACTT GCCCCTTTGA   
  
  
+ AACTCTCTAC ATTTGTATGT CATCTGAATT TGATTCTCAA ATCTCAGATG ATTTGAATTC CCTTTATGCC   
  
  
+ ATCTTTACTA TCTATTGAAT GATTCTTTCT TTTCCACTTT CTTATCTTCC ACTACCTTCT TCTTGGATGC   
  
  
+ CCTTTTGCTG CTTATAAAGG GCGACACCTG AACAAGTTGC TGTATTCTCA AACCATGAAT CTGTACAGGT   
  
  
+ TTAGCCCCGA TATGTGTCCT AGCTTTGTGG GTCACGAAGA AATTGGTCCT AATTTAATAG GACACCAAGA   
  
  
+ CCCTTTTCAA AATGCATACA ATTGCACTGA TTTTGGTGAT GCTTACTTGT TGCAATTCCC AGTTGAAGAT   
  
  
+ TTATGTCAAT ACCGTGTTCC TGATCTAATG GGGCTACCTG TTGAAGATGG GGATCCTACA GAGGTCCCTG   
  
  
+ ACATTCCTGA TACATGCTTC AACTACATAA GCAATCTCCT AATGGAAGAG GGCTCGGATG ACCGCCCTGG   
  
  
+ CGCCACCCTG CAGGACTATA TGGCTGCTCA AGTCTCCTAC AGCAAGTCCT CCAATGCCGC CTTTCCCGGT   
  
  
+ GAGAGTTGCT CATTCTTTGG TAATAATCAA TGCCCTCCAC CTGCTTATCA AAGCAACCCT TATTGCCTTG   
  
  
+ ATGATTACTT CAACTTGCTA TCCAATACTA CTGGCAATAG TGTTAACAAT GCTGGTGTTC ATGACAGCAA   
  
  
+ CAACTTTGTT CAGAACAGTT TGATTGGGAA TAGGGGTACT AGTTCTAGTT CTAGGGGCAG AAGTGATTTT   
  
  
+ GGATCTACCC TTATTAGGAA TTTGCCATTA GGGACCGACA CACTTGATGT GGTCAATCCA GCACCTACTC   
  
  
+ CTCGAGCCCC TCTGGTTACG ACTCAGTGTC AGGTGTTTGA AACACACCAG TATGAGCAGG CCTTCGAACT   
  
  
+ GATCACTAAT GGAAGCTGGG AATTCAGTCA TTCTGTTGCT AATACATCAA ATGATGGAAT TCAAAATCAA   
  
  
+ CCAATTCAAG CAGTTTCAAC CCAAGAACGA TGGAATATGA AAACATTTTC CGATGACCAA CTTGAGCCTG   
  
  
+ CTCTTTTGCG AAGTCTTGTG ATGTCCTCAA ACTGTACTGA TATCTCTGAT CAGGGTGTAC ACAGGATAGT   
  
  
+ GAACCCTGAG CCACCCAACA ACAGAAATAC GGAGATTGTG TGCAATGACT CATCCAGTAA GAGTAAGACA   
  
  
+ AGACGAAGAG AGCATGGTGC AGGAGAAAAC CACGATGAAG GAGAAAACCA CGATGAGACA GGAAGGAGTA   
  
  
+ ACAAGCAATC AGCACCATCC AATGAAGTGT TCGATGAAAT TGAGCAGTAT GGTGATGTGC TGCTCTGCCC   
  
  
+ TGAAGGAAGA AGTAAACCAG CAGGGTTATG CAGCAGCAGC AACGCTAACA GAACTAGCAA CTCACAGAAC   
  
  
+ TCGGAAGACT CAAAGTCACA GCAAAATGGG AGTTCCTCCA AGGTGTCAAA AAAGCGAGGC AATAAAAAGC   
  
  
+ AAGTGGTTGA TTTGAGGACT CTCCTAACTC GATGTGCACA ATGTGTTGCT CGTGTGGATT TAACTGGTTC   
  
  
+ TTACCAGTTG CTTCAGGAGA TTCGGCAGCA TTCTTCTCCC TATGGTGATT ATCTCCAAAG AATGGCTCAT   
  
  
+ TACCTTGCTA ATGGCCTTGA GGCGCGCTTA GAAGGCAAAG GATATGAACT AACTCGTGCC AATGAGCATA   
  
  
+ TCCCACCTAC TGAGATCCTC AAGGCTAACC GGGTATATGT GGCAGCGGTC CCTTTCAAGA TAATGTCTTA   
  
  
+ TTACACAACA AACAAGACAA TTGCAAGCCT AGTTGAGGAG GCACCAAGTA TACACATAAT TGATTTTGGT   
  
  
+ ATTTTCTATG GACTACAATG GCCCTGCATC ATTCAGAACC TCTCCAAGAG GCCCAATGGG CCTCCCAGAA   
  
  
+ TCCGCATCAC AGGCATCGAT TTCCCCCAAC CTGGGTTCCG GCCAGCAGAG AGGGTTGAAG AGACAGGGCG   
  
  
+ GTGTTTAGCC AAGTATTGTG ACAGGTACAA TGTGCCCTTT GAATACCACC CCATAGCCAA GAAGTGGGAG   
  
  
+ ACCATCCAAC TCGAGGAGCT CAAAATTGAA AGGAATGAGC CCCTTGTAGT GAACTGTCTG TATAGATCGC   
  
  
+ ACAACCTATT TGATGAAAGC GTAGAGGAAA ACAGCCCAAG AGACGCGTTC CTGAGCTTGG TTAGGAAAAT   
  
  
+ AAATCCCGAT ATATTCATGC ATGGGGTGGT GAATACCGCA TCAAGTGTAC CTTTCTTCCT GAACAGGTTC   
  
  
+ AAGGAGGCCA TGTTTCACTA CACGGCCTTG TTTGACTTGT TCGAGGCCAC AATGAGCCGC GAGGATCAAG   
  
  
+ AGAGGGTGTT ACTAGAGAGC AAGTTGCATG GGAATCAGGC GTTGAATGTG ATCGCCTGCG AAGGAGCGGA   
  
  
+ GAGAGTGGAG AGGCCTGAGT CATACAAACA ATGGCAGGTT AGGACTCAAA GGGCCGGGTT TGTTCAAGTG
[truncated: 167,442 more chars]
