# Supplementary material for: Genome-Wide Identification and Expression Pattern of the GRAS Gene Family in Pitaya (Selenicereus undatus L.)
Source: Biology (Basel). 2022 Dec 21;12(1):11. doi: 10.3390/biology12010011 (PMC9854919; doi:10.3390/biology12010011)
Supplement: Supplementary file 1 [file biology-12-00011-s001.zip › Supplementary file S5/HU02G01571.1_plantcare.html]

Content-Type: text/html; charset=ISO-8859-1


PlantCARE


Webmaster Firefox specific output  
To save the result:
click on the frame with the right mouse button and save the source code as a text file with extension .html  
REFERENCE:PlantCARE: a database of plant cis-acting regulatory elements and a portal to tools for in silico analysis of promoter sequences.  
Lescot, M., Déhais, P., Moreau, Y., De Moor, B., Rouzé ,P.,and Rombauts, S.  
Nucleic Acids Res., Database issue(2002), 30(1):325-327.   


---

>HU02G01571.1   
+ +Up\_Stream \_Len000CTAGGG GTTGAGAGAA TCCGATAGCT ATGCAGTCCA AACATTATCT CGTCAAGCAT   
  
  
+ TACTATTTAA ATAATTAATA TTGAACTTGA TAGTTACACT TTTTGAATAC TAATTCCACA CCTCATTGCC   
  
  
+ TTTATTTCCA AACCTCGGGT TGCATTTGGA CCACATACCA GGAAGCTTGG ACGACAAAAA TTTCTTTTCT   
  
  
+ TGAGTATTAT TTTGTTTTAT TATTATTATT TTTTACTTTA TTTGGACCAC TTCACTTGGG TAACAAGATT   
  
  
+ GAATATTTGA CTATTATATT ATATGTCACG TATGGATATG GTAGTTGCTT AGAGCTTTGA CCGTTTGCAG   
  
  
+ GTTTAAGTAT GATTCCCTTG GAAAGGAGCC CCTCTTTATC TTAATTCAGA AGTAACTTTT GTTTTTTTAG   
  
  
+ ATAAATTGAA AAGAAAAATA ATAAATGTCT TAGTTGAGTT TGGTTATTCA ATCCGAAATT TGATCCTGCT   
  
  
+ TAATGTATAG TATATAATAC TAATACGCAT GTAACACAAC ACCTCAGATG GTCATTGTTT TACAATTTTT   
  
  
+ TTGAGTTTAG TATAAAAATA AAATCAATTA TTTTTAGAAG TGATGTTTAA TTTGCATTTT TTTTCCACCT   
  
  
+ AAAATCACGC ACTCGAACAT TAGGGGTTTG GCTAACTCAA AGACTCAAGG TAGTGACTTA TCCAAACTTA   
  
  
+ TTACATCCTA TAGATCTATA AAAACATAAA TCTCTTACAA TCAATCCAAA TTGATTCGGT CTAATTTGAT   
  
  
+ GACCCTGATG AGGGTCCTAC AATTACACAA GTGGGCCTTA GCCCCATGGC CGTTTTTTAA TCTTCATGGG   
  
  
+ TCCAGGCGGA GTTACCATCT CCAGGTCCAT TGAGGGTGTA TCACGTCTCA AATTACGAGT TTGCCTCTTT   
  
  
+ CTACGGGGAT TATAAATATC TACCTGCATA CCTCGAAAAG GAGGAGTTGA GATCAGCGAA ACTTGGCCAT   
  
  
+ CCCAGAGCAT TGAAATGCTC AGAACCCTTT TTCTAGAAAA ATATCGTTTT TCCTGAAAAT ATCCGACCTT   
  
  
+ ATGTCATTTA TTGTCGCTTT CTTGACTCTA ACCCGGGACT CCACACTAAC TTGACTATCG GAAAGGCGTT   
  
  
+ CCCTGGATCA CCATCCAAGA TAACCTCTTT TGCAGGGTTG ACGCTCGTTG GAAGAATCGT CGAATGTAGA   
  
  
+ GCCCCTATCA TCACATAAGC CCGCTCTTAC TTCATGCCAT CCTTGAATAG CAGTTTATCC CGAAGCAAAA   
  
  
+ ATAATTCTAA CTTTCTTATA TTGTAAACCC ACATCTAAAT TTAAATTCAT ACTCCAAAAT TCCGCCACGC   
  
  
+ AAATAAGGCT TTTTAATAGG CTTACATATA ATGGCATGCT ATAGAGACAC TGTTTTGAAG AAACTATTTA   
  
  
+ AACATCAGAC CTCATATTGA GTAGCAAATC AAGACTTCTT TCCTCCAGGC CATATTGAGT AGCAAATTAA   
  
  
+ GAATAACCAG AAGCTTGTGA AAAGAGTTAG AAAATAAAAG GTATAATAGA GACTTATGTT CTTAAGATAT   
  
  
+ CTCCTAGATA TGAATGTGAC CTCCTAGTCG ATATTTGTGC TGGATTAGCG AGTTGTGTGA CTGAGTTTGT   
  
  
+ ATGTGTCTCT TAGCATATGA GTTATTCTAG CTGCTTCTTT AAGCTATGTA GTGACTGTCT TCCTTCTTAT   
  
  
+ TTATAAGTGC TATGTCCAAC TAATAGCCGA AAAAAATGGT TCGCCTTTTC TGGTTTTCTT CTTTTTGAAG   
  
  
+ GTTGATTGGC TAATTTATAT AATGGTTTCA ATACTTATCC TTTATTGTTC CACAAATCCA CCTAACCACG   
  
  
+ TACCATTGAC AATAATTACC CACTAATTTC GGTTGGACTT GATTTCCTTT TGTTTGGTAC CTCAGGCATC   
  
  
+ TTCTGTGGAC CTTCATTCAC AGTTTAATTT TCACATTTTT ATAAGGCTGC TCTTGTGTTG CAAATTTTCC   
  
  
+ TATCCATTCC ACTCTTTGCC CATTCCCAGG CTAATTAGTG TTCTCTGTTC AACCATGGAT TCTGTGCTGG   
  
  
+ TTGATCCTGA GTTCATGAAA AATCTCTACA AATTCAAACC TGAATTGCTC TCAAACTTTT CAATGAATCC   
  
  
+ AAATGACGAC ATCTTTCAAG CCCTTCATTC AGAAAATGAT CCTTTACAGT TCCTCTCATT TGATGAAGGA   
  
  
+ ACCTGTCTTA ACAGCTGCAC TAGTCAACAA GTGCCAGATT TCCCTGATGC TTGTCTCAAG TTCATCAGTG   
  
  
+ ATATTCTTCT CGAAGAGGGT TTAGATGCAA ATCCTGCATC TGCACAGGCT CTCGAAGCCA CCGAGAAGTC   
  
  
+ CTTGTATGAT GCTCTCGGGC TTGGAGAGCC ATACCCCCTT TCATGTGATC ACTTTGCGCC ATCTATCTCT   
  
  
+ ACAAGTATTG AGAGCCCAGA TGACAGTTCT TCCAATAAAA GTTATAGCAG CAATCCCGAG ATAGATGGTT   
  
  
+ CTTATGCTAT CGCTGAGCCC AGTTTCGAGT CCAACCCCAA CTGTGTGCTT GATCAACCCC AGTTGAACTC   
  
  
+ CTTTCCAGCT CTACATGAGA TTTCTCGGTC CTTGGTGGAA CTGGGTTCTC AAGCCTCTGA GTTGAGCTTC   
  
  
+ GATGATGCAG GGAGTGCCCG TGTAGAGAAA AAGGGCAAAT CGATAAAGGG CTCGAGGAGG AAGAAGAGTC   
  
  
+ GTCAAAGAGA GGGTGAAGCG TGTTATGGAG GAAGGAGCCA TAAGGTTCAA GCTTCCTTCA ATGATGATTA   
  
  
+ CTATGAGATG GAACAGTATG ATGATGTAGT ATTGCTCTGT AATAATGAAC TAACGGGCAA TAGCCGTTTC   
  
  
+ AACACCGGGA AATCTTCACC TGAGGAGGGA TGGAGGAGAT TGCAGAGAAG CCGAGGAAAG AAGCAGAACA   
  
  
+ GTTTAGCAGT TGAAGTTGAT CTGATGACCC TGCTGACTCA GTGTGCACAA GCTGTATCGA GCTTTGATCT   
  
  
+ TCGAGGTGCA AACGAGCTAC TTAGGCAAAT CAGGCAGAAT GCTTCGCCCT ATGGTGGCAG CATCCAGAGG   
  
  
+ CTCGCCCATC ATGTAGCCAA TGCTCTCGAG GCACGTATAG CTGGCACAGG CTCTACAGTC TCTACTAACC   
  
  
+ TTGTTGATGC AAAGTTCTCA GCTTCTGACT TCCTAAAGGC TTACAGGTTA TATGTCTCAG CTGTTCCTTA   
  
  
+ CAAAAGGATG TCTTTCTTTC TTGCTAACTG CTCGATTGCA AAGTTGGCAG AGAAAGCAAC AAAGATCCAT   
  
  
+ ATCATTGATT TTGGTGTTTT CCTAGGTTTA CAATGGCCTT GTTTCATACA ACACCTATCA AAAAGGCCAA   
  
  
+ ATGGACCCCC AAAACTCCGA ATCACAGGAA TCGACTACCC CCAGCAGGGT TTCAGACCTG CACAAAGGGT   
  
  
+ TGAAGCTACA GGACACCGAT TATCTGGGTA CTGTGAGCGA TTTGGGGTGC CTTTTTCTTA TCAGGGCATT   
  
  
+ GCTCAGAAGT GGGAAACTAT TCAGCCGGAG GATCTCAAGA TCGAACAAGA CGAGCTGGTG ATTGTCAACT   
  
  
+ GTTTGTTCAG GTCAGGAACA CTGCTCGATG AGACAGTCGA AGCAAACAGT CCAAGAGATG CTTTCTTAGC   
  
  
+ TTTGGTTAGA AAGCTGAATC CCAGCCTATT CATTCACGGG GTTGTCAATG GCACATTCAA CGCTCCATTC   
  
  
+ TTCGTGACTC GATTCAGAGA GGCATTGTTT CATTATTCAT CAGTGTTTGA TGTGTCTGAA GAGACAATTC   
  
  
+ CACGAGATGC CCATGAGAGG TTCTTGATTG AGAGCGAGAT TTGTGGGAAA GAACTGTTCA ATGTGGTTGC   
  
  
+ TTGTGAGGGT GCAGAGAGGG TTCAAAGGCC TGAGACATAC AAGCAGTGGC AAGTGAGGAC AACGCGGGCC   
  
  
+ GGGTTAAGGC AGGTTGCCTT GGACCAGGAG CTTATGAAGG AAGCAACGGC AATGGTGAAG GCAAATTATC   
  
  
+ ATAAGGATTT TATGGTGGAT ATAAATAGGC ATTGGATGCT TCAAGGTTGG AAGGGTAGAA CCTTGTGTGC   
  
  
+ TCTCTCATTT TGGCAACCTG CCTG  

- +Up\_Stream \_Len000GATCCC CAACTCTCTT AGGCTATCGA TACGTCAGGT TTGTAATAGA GCAGTTCGTA   
  
  
- ATGATAAATT TATTAATTAT AACTTGAACT ATCAATGTGA AAAACTTATG ATTAAGGTGT GGAGTAACGG   
  
  
- AAATAAAGGT TTGGAGCCCA ACGTAAACCT GGTGTATGGT CCTTCGAACC TGCTGTTTTT AAAGAAAAGA   
  
  
- ACTCATAATA AAACAAAATA ATAATAATAA AAAATGAAAT AAACCTGGTG AAGTGAACCC ATTGTTCTAA   
  
  
- CTTATAAACT GATAATATAA TATACAGTGC ATACCTATAC CATCAACGAA TCTCGAAACT GGCAAACGTC   
  
  
- CAAATTCATA CTAAGGGAAC CTTTCCTCGG GGAGAAATAG AATTAAGTCT TCATTGAAAA CAAAAAAATC   
  
  
- TATTTAACTT TTCTTTTTAT TATTTACAGA ATCAACTCAA ACCAATAAGT TAGGCTTTAA ACTAGGACGA   
  
  
- ATTACATATC ATATATTATG ATTATGCGTA CATTGTGTTG TGGAGTCTAC CAGTAACAAA ATGTTAAAAA   
  
  
- AACTCAAATC ATATTTTTAT TTTAGTTAAT AAAAATCTTC ACTACAAATT AAACGTAAAA AAAAGGTGGA   
  
  
- TTTTAGTGCG TGAGCTTGTA ATCCCCAAAC CGATTGAGTT TCTGAGTTCC ATCACTGAAT AGGTTTGAAT   
  
  
- AATGTAGGAT ATCTAGATAT TTTTGTATTT AGAGAATGTT AGTTAGGTTT AACTAAGCCA GATTAAACTA   
  
  
- CTGGGACTAC TCCCAGGATG TTAATGTGTT CACCCGGAAT CGGGGTACCG GCAAAAAATT AGAAGTACCC   
  
  
- AGGTCCGCCT CAATGGTAGA GGTCCAGGTA ACTCCCACAT AGTGCAGAGT TTAATGCTCA AACGGAGAAA   
  
  
- GATGCCCCTA ATATTTATAG ATGGACGTAT GGAGCTTTTC CTCCTCAACT CTAGTCGCTT TGAACCGGTA   
  
  
- GGGTCTCGTA ACTTTACGAG TCTTGGGAAA AAGATCTTTT TATAGCAAAA AGGACTTTTA TAGGCTGGAA   
  
  
- TACAGTAAAT AACAGCGAAA GAACTGAGAT TGGGCCCTGA GGTGTGATTG AACTGATAGC CTTTCCGCAA   
  
  
- GGGACCTAGT GGTAGGTTCT ATTGGAGAAA ACGTCCCAAC TGCGAGCAAC CTTCTTAGCA GCTTACATCT   
  
  
- CGGGGATAGT AGTGTATTCG GGCGAGAATG AAGTACGGTA GGAACTTATC GTCAAATAGG GCTTCGTTTT   
  
  
- TATTAAGATT GAAAGAATAT AACATTTGGG TGTAGATTTA AATTTAAGTA TGAGGTTTTA AGGCGGTGCG   
  
  
- TTTATTCCGA AAAATTATCC GAATGTATAT TACCGTACGA TATCTCTGTG ACAAAACTTC TTTGATAAAT   
  
  
- TTGTAGTCTG GAGTATAACT CATCGTTTAG TTCTGAAGAA AGGAGGTCCG GTATAACTCA TCGTTTAATT   
  
  
- CTTATTGGTC TTCGAACACT TTTCTCAATC TTTTATTTTC CATATTATCT CTGAATACAA GAATTCTATA   
  
  
- GAGGATCTAT ACTTACACTG GAGGATCAGC TATAAACACG ACCTAATCGC TCAACACACT GACTCAAACA   
  
  
- TACACAGAGA ATCGTATACT CAATAAGATC GACGAAGAAA TTCGATACAT CACTGACAGA AGGAAGAATA   
  
  
- AATATTCACG ATACAGGTTG ATTATCGGCT TTTTTTACCA AGCGGAAAAG ACCAAAAGAA GAAAAACTTC   
  
  
- CAACTAACCG ATTAAATATA TTACCAAAGT TATGAATAGG AAATAACAAG GTGTTTAGGT GGATTGGTGC   
  
  
- ATGGTAACTG TTATTAATGG GTGATTAAAG CCAACCTGAA CTAAAGGAAA ACAAACCATG GAGTCCGTAG   
  
  
- AAGACACCTG GAAGTAAGTG TCAAATTAAA AGTGTAAAAA TATTCCGACG AGAACACAAC GTTTAAAAGG   
  
  
- ATAGGTAAGG TGAGAAACGG GTAAGGGTCC GATTAATCAC AAGAGACAAG TTGGTACCTA AGACACGACC   
  
  
- AACTAGGACT CAAGTACTTT TTAGAGATGT TTAAGTTTGG ACTTAACGAG AGTTTGAAAA GTTACTTAGG   
  
  
- TTTACTGCTG TAGAAAGTTC GGGAAGTAAG TCTTTTACTA GGAAATGTCA AGGAGAGTAA ACTACTTCCT   
  
  
- TGGACAGAAT TGTCGACGTG ATCAGTTGTT CACGGTCTAA AGGGACTACG AACAGAGTTC AAGTAGTCAC   
  
  
- TATAAGAAGA GCTTCTCCCA AATCTACGTT TAGGACGTAG ACGTGTCCGA GAGCTTCGGT GGCTCTTCAG   
  
  
- GAACATACTA CGAGAGCCCG AACCTCTCGG TATGGGGGAA AGTACACTAG TGAAACGCGG TAGATAGAGA   
  
  
- TGTTCATAAC TCTCGGGTCT ACTGTCAAGA AGGTTATTTT CAATATCGTC GTTAGGGCTC TATCTACCAA   
  
  
- GAATACGATA GCGACTCGGG TCAAAGCTCA GGTTGGGGTT GACACACGAA CTAGTTGGGG TCAACTTGAG   
  
  
- GAAAGGTCGA GATGTACTCT AAAGAGCCAG GAACCACCTT GACCCAAGAG TTCGGAGACT CAACTCGAAG   
  
  
- CTACTACGTC CCTCACGGGC ACATCTCTTT TTCCCGTTTA GCTATTTCCC GAGCTCCTCC TTCTTCTCAG   
  
  
- CAGTTTCTCT CCCACTTCGC ACAATACCTC CTTCCTCGGT ATTCCAAGTT CGAAGGAAGT TACTACTAAT   
  
  
- GATACTCTAC CTTGTCATAC TACTACATCA TAACGAGACA TTATTACTTG ATTGCCCGTT ATCGGCAAAG   
  
  
- TTGTGGCCCT TTAGAAGTGG ACTCCTCCCT ACCTCCTCTA ACGTCTCTTC GGCTCCTTTC TTCGTCTTGT   
  
  
- CAAATCGTCA ACTTCAACTA GACTACTGGG ACGACTGAGT CACACGTGTT CGACATAGCT CGAAACTAGA   
  
  
- AGCTCCACGT TTGCTCGATG AATCCGTTTA GTCCGTCTTA CGAAGCGGGA TACCACCGTC GTAGGTCTCC   
  
  
- GAGCGGGTAG TACATCGGTT ACGAGAGCTC CGTGCATATC GACCGTGTCC GAGATGTCAG AGATGATTGG   
  
  
- AACAACTACG TTTCAAGAGT CGAAGACTGA AGGATTTCCG AATGTCCAAT ATACAGAGTC GACAAGGAAT   
  
  
- GTTTTCCTAC AGAAAGAAAG AACGATTGAC GAGCTAACGT TTCAACCGTC TCTTTCGTTG TTTCTAGGTA   
  
  
- TAGTAACTAA AACCACAAAA GGATCCAAAT GTTACCGGAA CAAAGTATGT TGTGGATAGT TTTTCCGGTT   
  
  
- TACCTGGGGG TTTTGAGGCT TAGTGTCCTT AGCTGATGGG GGTCGTCCCA AAGTCTGGAC GTGTTTCCCA   
  
  
- ACTTCGATGT CCTGTGGCTA ATAGACCCAT GACACTCGCT AAACCCCACG GAAAAAGAAT AGTCCCGTAA   
  
  
- CGAGTCTTCA CCCTTTGATA AGTCGGCCTC CTAGAGTTCT AGCTTGTTCT GCTCGACCAC TAACAGTTGA   
  
  
- CAAACAAGTC CAGTCCTTGT GACGAGCTAC TCTGTCAGCT TCGTTTGTCA GGTTCTCTAC GAAAGAATCG   
  
  
- AAACCAATCT TTCGACTTAG GGTCGGATAA GTAAGTGCCC CAACAGTTAC CGTGTAAGTT GCGAGGTAAG   
  
  
- AAGCACTGAG CTAAGTCTCT CCGTAACAAA GTAATAAGTA GTCACAAACT ACACAGACTT CTCTGTTAAG   
  
  
- GTGCTCTACG GGTACTCTCC AAGAACTAAC TCTCGCTCTA AACACCCTTT CTTGACAAGT TACACCAACG   
  
  
- AACACTCCCA CGTCTCTCCC AAGTTTCCGG ACTCTGTATG TTCGTCACCG TTCACTCCTG TTGCGCCCGG   
  
  
- CCCAATTCCG TCCAACGGAA CCTGGTCCTC GAATACTTCC TTCGTTGCCG TTACCACTTC CGTTTAATAG   
  
  
- TATTCCTAAA ATACCACCTA TATTTATCCG TAACCTACGA AGTTCCAACC TTCCCATCTT GGAACACACG   
  
  
- AGAGAGTAAA ACCGTTGGAC GGAC

  
  
Motifs Found  

+   

| Site Name | Organism | Position | Strand | Matrix score. | sequence | function |
| --- | --- | --- | --- | --- | --- | --- |
|  | organism | 2282 | + | 4 | motif\_sequence | short\_function |
|  | organism | 2286 | - | 4 | motif\_sequence | short\_function |
|  | organism | 1543 | + | 4 | motif\_sequence | short\_function |
|  | organism | 3074 | + | 4 | motif\_sequence | short\_function |
|  | organism | 2381 | + | 4 | motif\_sequence | short\_function |
|  | organism | 2256 | - | 4 | motif\_sequence | short\_function |
|  | organism | 1892 | + | 4 | motif\_sequence | short\_function |
|  | organism | 3145 | + | 4 | motif\_sequence | short\_function |
|  | organism | 3795 | - | 4 | motif\_sequence | short\_function |
|  | organism | 3201 | - | 4 | motif\_sequence | short\_function |
|  | organism | 3065 | + | 4 | motif\_sequence | short\_function |
|  | organism | 3625 | - | 4 | motif\_sequence | short\_function |
|  | organism | 3702 | - | 4 | motif\_sequence | short\_function |
|  | organism | 3768 | + | 4 | motif\_sequence | short\_function |
|  | organism | 2839 | - | 4 | motif\_sequence | short\_function |
|  | organism | 2846 | - | 4 | motif\_sequence | short\_function |
|  | organism | 834 | + | 4 | motif\_sequence | short\_function |
|  | organism | 1530 | + | 4 | motif\_sequence | short\_function |
|  | organism | 861 | + | 4 | motif\_sequence | short\_function |
|  | organism | 3058 | - | 4 | motif\_sequence | short\_function |
|  | organism | 2870 | - | 4 | motif\_sequence | short\_function |
|  | organism | 1190 | - | 4 | motif\_sequence | short\_function |
|  | organism | 2941 | + | 4 | motif\_sequence | short\_function |
|  | organism | 2058 | + | 4 | motif\_sequence | short\_function |
|  | organism | 2026 | + | 4 | motif\_sequence | short\_function |
|  | organism | 1696 | + | 4 | motif\_sequence | short\_function |
|  | organism | 2616 | - | 4 | motif\_sequence | short\_function |
|  | organism | 649 | - | 4 | motif\_sequence | short\_function |
|  | organism | 2657 | - | 4 | motif\_sequence | short\_function |
|  | organism | 2816 | + | 4 | motif\_sequence | short\_function |
|  | organism | 2337 | - | 4 | motif\_sequence | short\_function |
|  | organism | 2009 | + | 4 | motif\_sequence | short\_function |
|  | organism | 2745 | - | 4 | motif\_sequence | short\_function |
|  | organism | 2533 | + | 4 | motif\_sequence | short\_function |
|  | organism | 932 | + | 4 | motif\_sequence | short\_function |

>HU02G01571.1   
+ +Up\_Stream \_Len000CTAGGG GTTGAGAGAA TCCGATAGCT ATGCAGTCCA AACATTATCT CGTCAAGCAT   
  
  
+ TACTATTTAA ATAATTAATA TTGAACTTGA TAGTTACACT TTTTGAATAC TAATTCCACA CCTCATTGCC   
  
  
+ TTTATTTCCA AACCTCGGGT TGCATTTGGA CCACATACCA GGAAGCTTGG ACGACAAAAA TTTCTTTTCT   
  
  
+ TGAGTATTAT TTTGTTTTAT TATTATTATT TTTTACTTTA TTTGGACCAC TTCACTTGGG TAACAAGATT   
  
  
+ GAATATTTGA CTATTATATT ATATGTCACG TATGGATATG GTAGTTGCTT AGAGCTTTGA CCGTTTGCAG   
  
  
+ GTTTAAGTAT GATTCCCTTG GAAAGGAGCC CCTCTTTATC TTAATTCAGA AGTAACTTTT GTTTTTTTAG   
  
  
+ ATAAATTGAA AAGAAAAATA ATAAATGTCT TAGTTGAGTT TGGTTATTCA ATCCGAAATT TGATCCTGCT   
  
  
+ TAATGTATAG TATATAATAC TAATACGCAT GTAACACAAC ACCTCAGATG GTCATTGTTT TACAATTTTT   
  
  
+ TTGAGTTTAG TATAAAAATA AAATCAATTA TTTTTAGAAG TGATGTTTAA TTTGCATTTT TTTTCCACCT   
  
  
+ AAAATCACGC ACTCGAACAT TAGGGGTTTG GCTAACTCAA AGACTCAAGG TAGTGACTTA TCCAAACTTA   
  
  
+ TTACATCCTA TAGATCTATA AAAACATAAA TCTCTTACAA TCAATCCAAA TTGATTCGGT CTAATTTGAT   
  
  
+ GACCCTGATG AGGGTCCTAC AATTACACAA GTGGGCCTTA GCCCCATGGC CGTTTTTTAA TCTTCATGGG   
  
  
+ TCCAGGCGGA GTTACCATCT CCAGGTCCAT TGAGGGTGTA TCACGTCTCA AATTACGAGT TTGCCTCTTT   
  
  
+ CTACGGGGAT TATAAATATC TACCTGCATA CCTCGAAAAG GAGGAGTTGA GATCAGCGAA ACTTGGCCAT   
  
  
+ CCCAGAGCAT TGAAATGCTC AGAACCCTTT TTCTAGAAAA ATATCGTTTT TCCTGAAAAT ATCCGACCTT   
  
  
+ ATGTCATTTA TTGTCGCTTT CTTGACTCTA ACCCGGGACT CCACACTAAC TTGACTATCG GAAAGGCGTT   
  
  
+ CCCTGGATCA CCATCCAAGA TAACCTCTTT TGCAGGGTTG ACGCTCGTTG GAAGAATCGT CGAATGTAGA   
  
  
+ GCCCCTATCA TCACATAAGC CCGCTCTTAC TTCATGCCAT CCTTGAATAG CAGTTTATCC CGAAGCAAAA   
  
  
+ ATAATTCTAA CTTTCTTATA TTGTAAACCC ACATCTAAAT TTAAATTCAT ACTCCAAAAT TCCGCCACGC   
  
  
+ AAATAAGGCT TTTTAATAGG CTTACATATA ATGGCATGCT ATAGAGACAC TGTTTTGAAG AAACTATTTA   
  
  
+ AACATCAGAC CTCATATTGA GTAGCAAATC AAGACTTCTT TCCTCCAGGC CATATTGAGT AGCAAATTAA   
  
  
+ GAATAACCAG AAGCTTGTGA AAAGAGTTAG AAAATAAAAG GTATAATAGA GACTTATGTT CTTAAGATAT   
  
  
+ CTCCTAGATA TGAATGTGAC CTCCTAGTCG ATATTTGTGC TGGATTAGCG AGTTGTGTGA CTGAGTTTGT   
  
  
+ ATGTGTCTCT TAGCATATGA GTTATTCTAG CTGCTTCTTT AAGCTATGTA GTGACTGTCT TCCTTCTTAT   
  
  
+ TTATAAGTGC TATGTCCAAC TAATAGCCGA AAAAAATGGT TCGCCTTTTC TGGTTTTCTT CTTTTTGAAG   
  
  
+ GTTGATTGGC TAATTTATAT AATGGTTTCA ATACTTATCC TTTATTGTTC CACAAATCCA CCTAACCACG   
  
  
+ TACCATTGAC AATAATTACC CACTAATTTC GGTTGGACTT GATTTCCTTT TGTTTGGTAC CTCAGGCATC   
  
  
+ TTCTGTGGAC CTTCATTCAC AGTTTAATTT TCACATTTTT ATAAGGCTGC TCTTGTGTTG CAAATTTTCC   
  
  
+ TATCCATTCC ACTCTTTGCC CATTCCCAGG CTAATTAGTG TTCTCTGTTC AACCATGGAT TCTGTGCTGG   
  
  
+ TTGATCCTGA GTTCATGAAA AATCTCTACA AATTCAAACC TGAATTGCTC TCAAACTTTT CAATGAATCC   
  
  
+ AAATGACGAC ATCTTTCAAG CCCTTCATTC AGAAAATGAT CCTTTACAGT TCCTCTCATT TGATGAAGGA   
  
  
+ ACCTGTCTTA ACAGCTGCAC TAGTCAACAA GTGCCAGATT TCCCTGATGC TTGTCTCAAG TTCATCAGTG   
  
  
+ ATATTCTTCT CGAAGAGGGT TTAGATGCAA ATCCTGCATC TGCACAGGCT CTCGAAGCCA CCGAGAAGTC   
  
  
+ CTTGTATGAT GCTCTCGGGC TTGGAGAGCC ATACCCCCTT TCATGTGATC ACTTTGCGCC ATCTATCTCT   
  
  
+ ACAAGTATTG AGAGCCCAGA TGACAGTTCT TCCAATAAAA GTTATAGCAG CAATCCCGAG ATAGATGGTT   
  
  
+ CTTATGCTAT CGCTGAGCCC AGTTTCGAGT CCAACCCCAA CTGTGTGCTT GATCAACCCC AGTTGAACTC   
  
  
+ CTTTCCAGCT CTACATGAGA TTTCTCGGTC CTTGGTGGAA CTGGGTTCTC AAGCCTCTGA GTTGAGCTTC   
  
  
+ GATGATGCAG GGAGTGCCCG TGTAGAGAAA AAGGGCAAAT CGATAAAGGG CTCGAGGAGG AAGAAGAGTC   
  
  
+ GTCAAAGAGA GGGTGAAGCG TGTTATGGAG GAAGGAGCCA TAAGGTTCAA GCTTCCTTCA ATGATGATTA   
  
  
+ CTATGAGATG GAACAGTATG ATGATGTAGT ATTGCTCTGT AATAATGAAC TAACGGGCAA TAGCCGTTTC   
  
  
+ AACACCGGGA AATCTTCACC TGAGGAGGGA TGGAGGAGAT TGCAGAGAAG CCGAGGAAAG AAGCAGAACA   
  
  
+ GTTTAGCAGT TGAAGTTGAT CTGATGACCC TGCTGACTCA GTGTGCACAA GCTGTATCGA GCTTTGATCT   
  
  
+ TCGAGGTGCA AACGAGCTAC TTAGGCAAAT CAGGCAGAAT GCTTCGCCCT ATGGTGGCAG CATCCAGAGG   
  
  
+ CTCGCCCATC ATGTAGCCAA TGCTCTCGAG GCACGTATAG CTGGCACAGG CTCTACAGTC TCTACTAACC   
  
  
+ TTGTTGATGC AAAGTTCTCA GCTTCTGACT TCCTAAAGGC TTACAGGTTA TATGTCTCAG CTGTTCCTTA   
  
  
+ CAAAAGGATG TCTTTCTTTC TTGCTAACTG CTCGATTGCA AAGTTGGCAG AGAAAGCAAC AAAGATCCAT   
  
  
+ ATCATTGATT TTGGTGTTTT CCTAGGTTTA CAATGGCCTT GTTTCATACA ACACCTATCA AAAAGGCCAA   
  
  
+ ATGGACCCCC AAAACTCCGA ATCACAGGAA TCGACTACCC CCAGCAGGGT TTCAGACCTG CACAAAGGGT   
  
  
+ TGAAGCTACA GGACACCGAT TATCTGGGTA CTGTGAGCGA TTTGGGGTGC CTTTTTCTTA TCAGGGCATT   
  
  
+ GCTCAGAAGT GGGAAACTAT TCAGCCGGAG GATCTCAAGA TCGAACAAGA CGAGCTGGTG ATTGTCAACT   
  
  
+ GTTTGTTCAG GTCAGGAACA CTGCTCGATG AGACAGTCGA AGCAAACAGT CCAAGAGATG CTTTCTTAGC   
  
  
+ TTTGGTTAGA AAGCTGAATC CCAGCCTATT CATTCACGGG GTTGTCAATG GCACATTCAA CGCTCCATTC   
  
  
+ TTCGTGACTC GATTCAGAGA GGCATTGTTT CATTATTCAT CAGTGTTTGA TGTGTCTGAA GAGACAATTC   
  
  
+ CACGAGATGC CCATGAGAGG TTCTTGATTG AGAGCGAGAT TTGTGGGAAA GAACTGTTCA ATGTGGTTGC   
  
  
+ TTGTGAGGGT GCAGAGAGGG TTCAAAGGCC TGAGACATAC AAGCAGTGGC AAGTGAGGAC AACGCGGGCC   
  
  
+ GGGTTAAGGC AGGTTGCCTT GGACCAGGAG CTTATGAAGG AAGCAACGGC AATGGTGAAG GCAAATTATC   
  
  
+ ATAAGGATTT TATGGTGGAT ATAAATAGGC ATTGGATGCT TCAAGGTTGG AAGGGTAGAA CCTTGTGTGC   
  
  
+ TCTCTCATTT TGGCAACCTG CCTG  

- +Up\_Stream \_Len000GATCCC CAACTCTCTT AGGCTATCGA TACGTCAGGT TTGTAATAGA GCAGTTCGTA   
  
  
- ATGATAAATT TATTAATTAT AACTTGAACT ATCAATGTGA AAAACTTATG ATTAAGGTGT GGAGTAACGG   
  
  
- AAATAAAGGT TTGGAGCCCA ACGTAAACCT GGTGTATGGT CCTTCGAACC TGCTGTTTTT AAAGAAAAGA   
  
  
- ACTCATAATA AAACAAAATA ATAATAATAA AAAATGAAAT AAACCTGGTG AAGTGAACCC ATTGTTCTAA   
  
  
- CTTATAAACT GATAATATAA TATACAGTGC ATACCTATAC CATCAACGAA TCTCGAAACT GGCAAACGTC   
  
  
- CAAATTCATA CTAAGGGAAC CTTTCCTCGG GGAGAAATAG AATTAAGTCT TCATTGAAAA CAAAAAAATC   
  
  
- TATTTAACTT TTCTTTTTAT TATTTACAGA ATCAACTCAA ACCAATAAGT TAGGCTTTAA ACTAGGACGA   
  
  
- ATTACATATC ATATATTATG ATTATGCGTA CATTGTGTTG TGGAGTCTAC CAGTAACAAA ATGTTAAAAA   
  
  
- AACTCAAATC ATATTTTTAT TTTAGTTAAT AAAAATCTTC ACTACAAATT AAACGTAAAA AAAAGGTGGA   
  
  
- TTTTAGTGCG TGAGCTTGTA ATCCCCAAAC CGATTGAGTT TCTGAGTTCC ATCACTGAAT AGGTTTGAAT   
  
  
- AATGTAGGAT ATCTAGATAT TTTTGTATTT AGAGAATGTT AGTTAGGTTT AACTAAGCCA GATTAAACTA   
  
  
- CTGGGACTAC TCCCAGGATG TTAATGTGTT CACCCGGAAT CGGGGTACCG GCAAAAAATT AGAAGTACCC   
  
  
- AGGTCCGCCT CAATGGTAGA GGTCCAGGTA ACTCCCACAT AGTGCAGAGT TTAATGCTCA AACGGAGAAA   
  
  
- GATGCCCCTA ATATTTATAG ATGGACGTAT GGAGCTTTTC CTCCTCAACT CTAGTCGCTT TGAACCGGTA   
  
  
- GGGTCTCGTA ACTTTACGAG TCTTGGGAAA AAGATCTTTT TATAGCAAAA AGGACTTTTA TAGGCTGGAA   
  
  
- TACAGTAAAT AACAGCGAAA GAACTGAGAT TGGGCCCTGA GGTGTGATTG AACTGATAGC CTTTCCGCAA   
  
  
- GGGACCTAGT GGTAGGTTCT ATTGGAGAAA ACGTCCCAAC TGCGAGCAAC CTTCTTAGCA GCTTACATCT   
  
  
- CGGGGATAGT AGTGTATTCG GGCGAGAATG AAGTACGGTA GGAACTTATC GTCAAATAGG GCTTCGTTTT   
  
  
- TATTAAGATT GAAAGAATAT AACATTTGGG TGTAGATTTA AATTTAAGTA TGAGGTTTTA AGGCGGTGCG   
  
  
- TTTATTCCGA AAAATTATCC GAATGTATAT TACCGTACGA TATCTCTGTG ACAAAACTTC TTTGATAAAT   
  
  
- TTGTAGTCTG GAGTATAACT CATCGTTTAG TTCTGAAGAA AGGAGGTCCG GTATAACTCA TCGTTTAATT   
  
  
- CTTATTGGTC TTCGAACACT TTTCTCAATC TTTTATTTTC CATATTATCT CTGAATACAA GAATTCTATA   
  
  
- GAGGATCTAT ACTTACACTG GAGGATCAGC TATAAACACG ACCTAATCGC TCAACACACT GACTCAAACA   
  
  
- TACACAGAGA ATCGTATACT CAATAAGATC GACGAAGAAA TTCGATACAT CACTGACAGA AGGAAGAATA   
  
  
- AATATTCACG ATACAGGTTG ATTATCGGCT TTTTTTACCA AGCGGAAAAG ACCAAAAGAA GAAAAACTTC   
  
  
- CAACTAACCG ATTAAATATA TTACCAAAGT TATGAATAGG AAATAACAAG GTGTTTAGGT GGATTGGTGC   
  
  
- ATGGTAACTG TTATTAATGG GTGATTAAAG CCAACCTGAA CTAAAGGAAA ACAAACCATG GAGTCCGTAG   
  
  
- AAGACACCTG GAAGTAAGTG TCAAATTAAA AGTGTAAAAA TATTCCGACG AGAACACAAC GTTTAAAAGG   
  
  
- ATAGGTAAGG TGAGAAACGG GTAAGGGTCC GATTAATCAC AAGAGACAAG TTGGTACCTA AGACACGACC   
  
  
- AACTAGGACT CAAGTACTTT TTAGAGATGT TTAAGTTTGG ACTTAACGAG AGTTTGAAAA GTTACTTAGG   
  
  
- TTTACTGCTG TAGAAAGTTC GGGAAGTAAG TCTTTTACTA GGAAATGTCA AGGAGAGTAA ACTACTTCCT   
  
  
- TGGACAGAAT TGTCGACGTG ATCAGTTGTT CACGGTCTAA AGGGACTACG AACAGAGTTC AAGTAGTCAC   
  
  
- TATAAGAAGA GCTTCTCCCA AATCTACGTT TAGGACGTAG ACGTGTCCGA GAGCTTCGGT GGCTCTTCAG   
  
  
- GAACATACTA CGAGAGCCCG AACCTCTCGG TATGGGGGAA AGTACACTAG TGAAACGCGG TAGATAGAGA   
  
  
- TGTTCATAAC TCTCGGGTCT ACTGTCAAGA AGGTTATTTT CAATATCGTC GTTAGGGCTC TATCTACCAA   
  
  
- GAATACGATA GCGACTCGGG TCAAAGCTCA GGTTGGGGTT GACACACGAA CTAGTTGGGG TCAACTTGAG   
  
  
- GAAAGGTCGA GATGTACTCT AAAGAGCCAG GAACCACCTT GACCCAAGAG TTCGGAGACT CAACTCGAAG   
  
  
- CTACTACGTC CCTCACGGGC ACATCTCTTT TTCCCGTTTA GCTATTTCCC GAGCTCCTCC TTCTTCTCAG   
  
  
- CAGTTTCTCT CCCACTTCGC ACAATACCTC CTTCCTCGGT ATTCCAAGTT CGAAGGAAGT TACTACTAAT   
  
  
- GATACTCTAC CTTGTCATAC TACTACATCA TAACGAGACA TTATTACTTG ATTGCCCGTT ATCGGCAAAG   
  
  
- TTGTGGCCCT TTAGAAGTGG ACTCCTCCCT ACCTCCTCTA ACGTCTCTTC GGCTCCTTTC TTCGTCTTGT   
  
  
- CAAATCGTCA ACTTCAACTA GACTACTGGG ACGACTGAGT CACACGTGTT CGACATAGCT CGAAACTAGA   
  
  
- AGCTCCACGT TTGCTCGATG AATCCGTTTA GTCCGTCTTA CGAAGCGGGA TACCACCGTC GTAGGTCTCC   
  
  
- GAGCGGGTAG TACATCGGTT ACGAGAGCTC CGTGCATATC GACCGTGTCC GAGATGTCAG AGATGATTGG   
  
  
- AACAACTACG TTTCAAGAGT CGAAGACTGA AGGATTTCCG AATGTCCAAT ATACAGAGTC GACAAGGAAT   
  
  
- GTTTTCCTAC AGAAAGAAAG AACGATTGAC GAGCTAACGT TTCAACCGTC TCTTTCGTTG TTTCTAGGTA   
  
  
- TAGTAACTAA AACCACAAAA GGATCCAAAT GTTACCGGAA CAAAGTATGT TGTGGATAGT TTTTCCGGTT   
  
  
- TACCTGGGGG TTTTGAGGCT TAGTGTCCTT AGCTGATGGG GGTCGTCCCA AAGTCTGGAC GTGTTTCCCA   
  
  
- ACTTCGATGT CCTGTGGCTA ATAGACCCAT GACACTCGCT AAACCCCACG GAAAAAGAAT AGTCCCGTAA   
  
  
- CGAGTCTTCA CCCTTTGATA AGTCGGCCTC CTAGAGTTCT AGCTTGTTCT GCTCGACCAC TAACAGTTGA   
  
  
- CAAACAAGTC CAGTCCTTGT GACGAGCTAC TCTGTCAGCT TCGTTTGTCA GGTTCTCTAC GAAAGAATCG   
  
  
- AAACCAATCT TTCGACTTAG GGTCGGATAA GTAAGTGCCC CAACAGTTAC CGTGTAAGTT GCGAGGTAAG   
  
  
- AAGCACTGAG CTAAGTCTCT CCGTAACAAA GTAATAAGTA GTCACAAACT ACACAGACTT CTCTGTTAAG   
  
  
- GTGCTCTACG GGTACTCTCC AAGAACTAAC TCTCGCTCTA AACACCCTTT CTTGACAAGT TACACCAACG   
  
  
- AACACTCCCA CGTCTCTCCC AAGTTTCCGG ACTCTGTATG TTCGTCACCG TTCACTCCTG TTGCGCCCGG   
  
  
- CCCAATTCCG TCCAACGGAA CCTGGTCCTC GAATACTTCC TTCGTTGCCG TTACCACTTC CGTTTAATAG   
  
  
- TATTCCTAAA ATACCACCTA TATTTATCCG TAACCTACGA AGTTCCAACC TTCCCATCTT GGAACACACG   
  
  
- AGAGAGTAAA ACCGTTGGAC GGAC

+     AAGAA-motif

| Site Name | Organism | Position | Strand | Matrix score. | sequence | function |
| --- | --- | --- | --- | --- | --- | --- |
| AAGAA-motif | Avena sativa | 2860 | + | 7 | GAAAGAA |  |
| AAGAA-motif | Avena sativa | 1440 | - | 7 | GAAAGAA |  |
| AAGAA-motif | Avena sativa | 3168 | - | 7 | GAAAGAA |  |
| AAGAA-motif | Avena sativa | 3761 | + | 7 | GAAAGAA |  |

>HU02G01571.1   
+ +Up\_Stream \_Len000CTAGGG GTTGAGAGAA TCCGATAGCT ATGCAGTCCA AACATTATCT CGTCAAGCAT   
  
  
+ TACTATTTAA ATAATTAATA TTGAACTTGA TAGTTACACT TTTTGAATAC TAATTCCACA CCTCATTGCC   
  
  
+ TTTATTTCCA AACCTCGGGT TGCATTTGGA CCACATACCA GGAAGCTTGG ACGACAAAAA TTTCTTTTCT   
  
  
+ TGAGTATTAT TTTGTTTTAT TATTATTATT TTTTACTTTA TTTGGACCAC TTCACTTGGG TAACAAGATT   
  
  
+ GAATATTTGA CTATTATATT ATATGTCACG TATGGATATG GTAGTTGCTT AGAGCTTTGA CCGTTTGCAG   
  
  
+ GTTTAAGTAT GATTCCCTTG GAAAGGAGCC CCTCTTTATC TTAATTCAGA AGTAACTTTT GTTTTTTTAG   
  
  
+ ATAAATTGAA AAGAAAAATA ATAAATGTCT TAGTTGAGTT TGGTTATTCA ATCCGAAATT TGATCCTGCT   
  
  
+ TAATGTATAG TATATAATAC TAATACGCAT GTAACACAAC ACCTCAGATG GTCATTGTTT TACAATTTTT   
  
  
+ TTGAGTTTAG TATAAAAATA AAATCAATTA TTTTTAGAAG TGATGTTTAA TTTGCATTTT TTTTCCACCT   
  
  
+ AAAATCACGC ACTCGAACAT TAGGGGTTTG GCTAACTCAA AGACTCAAGG TAGTGACTTA TCCAAACTTA   
  
  
+ TTACATCCTA TAGATCTATA AAAACATAAA TCTCTTACAA TCAATCCAAA TTGATTCGGT CTAATTTGAT   
  
  
+ GACCCTGATG AGGGTCCTAC AATTACACAA GTGGGCCTTA GCCCCATGGC CGTTTTTTAA TCTTCATGGG   
  
  
+ TCCAGGCGGA GTTACCATCT CCAGGTCCAT TGAGGGTGTA TCACGTCTCA AATTACGAGT TTGCCTCTTT   
  
  
+ CTACGGGGAT TATAAATATC TACCTGCATA CCTCGAAAAG GAGGAGTTGA GATCAGCGAA ACTTGGCCAT   
  
  
+ CCCAGAGCAT TGAAATGCTC AGAACCCTTT TTCTAGAAAA ATATCGTTTT TCCTGAAAAT ATCCGACCTT   
  
  
+ ATGTCATTTA TTGTCGCTTT CTTGACTCTA ACCCGGGACT CCACACTAAC TTGACTATCG GAAAGGCGTT   
  
  
+ CCCTGGATCA CCATCCAAGA TAACCTCTTT TGCAGGGTTG ACGCTCGTTG GAAGAATCGT CGAATGTAGA   
  
  
+ GCCCCTATCA TCACATAAGC CCGCTCTTAC TTCATGCCAT CCTTGAATAG CAGTTTATCC CGAAGCAAAA   
  
  
+ ATAATTCTAA CTTTCTTATA TTGTAAACCC ACATCTAAAT TTAAATTCAT ACTCCAAAAT TCCGCCACGC   
  
  
+ AAATAAGGCT TTTTAATAGG CTTACATATA ATGGCATGCT ATAGAGACAC TGTTTTGAAG AAACTATTTA   
  
  
+ AACATCAGAC CTCATATTGA GTAGCAAATC AAGACTTCTT TCCTCCAGGC CATATTGAGT AGCAAATTAA   
  
  
+ GAATAACCAG AAGCTTGTGA AAAGAGTTAG AAAATAAAAG GTATAATAGA GACTTATGTT CTTAAGATAT   
  
  
+ CTCCTAGATA TGAATGTGAC CTCCTAGTCG ATATTTGTGC TGGATTAGCG AGTTGTGTGA CTGAGTTTGT   
  
  
+ ATGTGTCTCT TAGCATATGA GTTATTCTAG CTGCTTCTTT AAGCTATGTA GTGACTGTCT TCCTTCTTAT   
  
  
+ TTATAAGTGC TATGTCCAAC TAATAGCCGA AAAAAATGGT TCGCCTTTTC TGGTTTTCTT CTTTTTGAAG   
  
  
+ GTTGATTGGC TAATTTATAT AATGGTTTCA ATACTTATCC TTTATTGTTC CACAAATCCA CCTAACCACG   
  
  
+ TACCATTGAC AATAATTACC CACTAATTTC GGTTGGACTT GATTTCCTTT TGTTTGGTAC CTCAGGCATC   
  
  
+ TTCTGTGGAC CTTCATTCAC AGTTTAATTT TCACATTTTT ATAAGGCTGC TCTTGTGTTG CAAATTTTCC   
  
  
+ TATCCATTCC ACTCTTTGCC CATTCCCAGG CTAATTAGTG TTCTCTGTTC AACCATGGAT TCTGTGCTGG   
  
  
+ TTGATCCTGA GTTCATGAAA AATCTCTACA AATTCAAACC TGAATTGCTC TCAAACTTTT CAATGAATCC   
  
  
+ AAATGACGAC ATCTTTCAAG CCCTTCATTC AGAAAATGAT CCTTTACAGT TCCTCTCATT TGATGAAGGA   
  
  
+ ACCTGTCTTA ACAGCTGCAC TAGTCAACAA GTGCCAGATT TCCCTGATGC TTGTCTCAAG TTCATCAGTG   
  
  
+ ATATTCTTCT CGAAGAGGGT TTAGATGCAA ATCCTGCATC TGCACAGGCT CTCGAAGCCA CCGAGAAGTC   
  
  
+ CTTGTATGAT GCTCTCGGGC TTGGAGAGCC ATACCCCCTT TCATGTGATC ACTTTGCGCC ATCTATCTCT   
  
  
+ ACAAGTATTG AGAGCCCAGA TGACAGTTCT TCCAATAAAA GTTATAGCAG CAATCCCGAG ATAGATGGTT   
  
  
+ CTTATGCTAT CGCTGAGCCC AGTTTCGAGT CCAACCCCAA CTGTGTGCTT GATCAACCCC AGTTGAACTC   
  
  
+ CTTTCCAGCT CTACATGAGA TTTCTCGGTC CTTGGTGGAA CTGGGTTCTC AAGCCTCTGA GTTGAGCTTC   
  
  
+ GATGATGCAG GGAGTGCCCG TGTAGAGAAA AAGGGCAAAT CGATAAAGGG CTCGAGGAGG AAGAAGAGTC   
  
  
+ GTCAAAGAGA GGGTGAAGCG TGTTATGGAG GAAGGAGCCA TAAGGTTCAA GCTTCCTTCA ATGATGATTA   
  
  
+ CTATGAGATG GAACAGTATG ATGATGTAGT ATTGCTCTGT AATAATGAAC TAACGGGCAA TAGCCGTTTC   
  
  
+ AACACCGGGA AATCTTCACC TGAGGAGGGA TGGAGGAGAT TGCAGAGAAG CCGAGGAAAG AAGCAGAACA   
  
  
+ GTTTAGCAGT TGAAGTTGAT CTGATGACCC TGCTGACTCA GTGTGCACAA GCTGTATCGA GCTTTGATCT   
  
  
+ TCGAGGTGCA AACGAGCTAC TTAGGCAAAT CAGGCAGAAT GCTTCGCCCT ATGGTGGCAG CATCCAGAGG   
  
  
+ CTCGCCCATC ATGTAGCCAA TGCTCTCGAG GCACGTATAG CTGGCACAGG CTCTACAGTC TCTACTAACC   
  
  
+ TTGTTGATGC AAAGTTCTCA GCTTCTGACT TCCTAAAGGC TTACAGGTTA TATGTCTCAG CTGTTCCTTA   
  
  
+ CAAAAGGATG TCTTTCTTTC TTGCTAACTG CTCGATTGCA AAGTTGGCAG AGAAAGCAAC AAAGATCCAT   
  
  
+ ATCATTGATT TTGGTGTTTT CCTAGGTTTA CAATGGCCTT GTTTCATACA ACACCTATCA AAAAGGCCAA   
  
  
+ ATGGACCCCC AAAACTCCGA ATCACAGGAA TCGACTACCC CCAGCAGGGT TTCAGACCTG CACAAAGGGT   
  
  
+ TGAAGCTACA GGACACCGAT TATCTGGGTA CTGTGAGCGA TTTGGGGTGC CTTTTTCTTA TCAGGGCATT   
  
  
+ GCTCAGAAGT GGGAAACTAT TCAGCCGGAG GATCTCAAGA TCGAACAAGA CGAGCTGGTG ATTGTCAACT   
  
  
+ GTTTGTTCAG GTCAGGAACA CTGCTCGATG AGACAGTCGA AGCAAACAGT CCAAGAGATG CTTTCTTAGC   
  
  
+ TTTGGTTAGA AAGCTGAATC CCAGCCTATT CATTCACGGG GTTGTCAATG GCACATTCAA CGCTCCATTC   
  
  
+ TTCGTGACTC GATTCAGAGA GGCATTGTTT CATTATTCAT CAGTGTTTGA TGTGTCTGAA GAGACAATTC   
  
  
+ CACGAGATGC CCATGAGAGG TTCTTGATTG AGAGCGAGAT TTGTGGGAAA GAACTGTTCA ATGTGGTTGC   
  
  
+ TTGTGAGGGT GCAGAGAGGG TTCAAAGGCC TGAGACATAC AAGCAGTGGC AAGTGAGGAC AACGCGGGCC   
  
  
+ GGGTTAAGGC AGGTTGCCTT GGACCAGGAG CTTATGAAGG AAGCAACGGC AATGGTGAAG GCAAATTATC   
  
  
+ ATAAGGATTT TATGGTGGAT ATAAATAGGC ATTGGATGCT TCAAGGTTGG AAGGGTAGAA CCTTGTGTGC   
  
  
+ TCTCTCATTT TGGCAACCTG CCTG  

- +Up\_Stream \_Len000GATCCC CAACTCTCTT AGGCTATCGA TACGTCAGGT TTGTAATAGA GCAGTTCGTA   
  
  
- ATGATAAATT TATTAATTAT AACTTGAACT ATCAATGTGA AAAACTTATG ATTAAGGTGT GGAGTAACGG   
  
  
- AAATAAAGGT TTGGAGCCCA ACGTAAACCT GGTGTATGGT CCTTCGAACC TGCTGTTTTT AAAGAAAAGA   
  
  
- ACTCATAATA AAACAAAATA ATAATAATAA AAAATGAAAT AAACCTGGTG AAGTGAACCC ATTGTTCTAA   
  
  
- CTTATAAACT GATAATATAA TATACAGTGC ATACCTATAC CATCAACGAA TCTCGAAACT GGCAAACGTC   
  
  
- CAAATTCATA CTAAGGGAAC CTTTCCTCGG GGAGAAATAG AATTAAGTCT TCATTGAAAA CAAAAAAATC   
  
  
- TATTTAACTT TTCTTTTTAT TATTTACAGA ATCAACTCAA ACCAATAAGT TAGGCTTTAA ACTAGGACGA   
  
  
- ATTACATATC ATATATTATG ATTATGCGTA CATTGTGTTG TGGAGTCTAC CAGTAACAAA ATGTTAAAAA   
  
  
- AACTCAAATC ATATTTTTAT TTTAGTTAAT AAAAATCTTC ACTACAAATT AAACGTAAAA AAAAGGTGGA   
  
  
- TTTTAGTGCG TGAGCTTGTA ATCCCCAAAC CGATTGAGTT TCTGAGTTCC ATCACTGAAT AGGTTTGAAT   
  
  
- AATGTAGGAT ATCTAGATAT TTTTGTATTT AGAGAATGTT AGTTAGGTTT AACTAAGCCA GATTAAACTA   
  
  
- CTGGGACTAC TCCCAGGATG TTAATGTGTT CACCCGGAAT CGGGGTACCG GCAAAAAATT AGAAGTACCC   
  
  
- AGGTCCGCCT CAATGGTAGA GGTCCAGGTA ACTCCCACAT AGTGCAGAGT TTAATGCTCA AACGGAGAAA   
  
  
- GATGCCCCTA ATATTTATAG ATGGACGTAT GGAGCTTTTC CTCCTCAACT CTAGTCGCTT TGAACCGGTA   
  
  
- GGGTCTCGTA ACTTTACGAG TCTTGGGAAA AAGATCTTTT TATAGCAAAA AGGACTTTTA TAGGCTGGAA   
  
  
- TACAGTAAAT AACAGCGAAA GAACTGAGAT TGGGCCCTGA GGTGTGATTG AACTGATAGC CTTTCCGCAA   
  
  
- GGGACCTAGT GGTAGGTTCT ATTGGAGAAA ACGTCCCAAC TGCGAGCAAC CTTCTTAGCA GCTTACATCT   
  
  
- CGGGGATAGT AGTGTATTCG GGCGAGAATG AAGTACGGTA GGAACTTATC GTCAAATAGG GCTTCGTTTT   
  
  
- TATTAAGATT GAAAGAATAT AACATTTGGG TGTAGATTTA AATTTAAGTA TGAGGTTTTA AGGCGGTGCG   
  
  
- TTTATTCCGA AAAATTATCC GAATGTATAT TACCGTACGA TATCTCTGTG ACAAAACTTC TTTGATAAAT   
  
  
- TTGTAGTCTG GAGTATAACT CATCGTTTAG TTCTGAAGAA AGGAGGTCCG GTATAACTCA TCGTTTAATT   
  
  
- CTTATTGGTC TTCGAACACT TTTCTCAATC TTTTATTTTC CATATTATCT CTGAATACAA GAATTCTATA   
  
  
- GAGGATCTAT ACTTACACTG GAGGATCAGC TATAAACACG ACCTAATCGC TCAACACACT GACTCAAACA   
  
  
- TACACAGAGA ATCGTATACT CAATAAGATC GACGAAGAAA TTCGATACAT CACTGACAGA AGGAAGAATA   
  
  
- AATATTCACG ATACAGGTTG ATTATCGGCT TTTTTTACCA AGCGGAAAAG ACCAAAAGAA GAAAAACTTC   
  
  
- CAACTAACCG ATTAAATATA TTACCAAAGT TATGAATAGG AAATAACAAG GTGTTTAGGT GGATTGGTGC   
  
  
- ATGGTAACTG TTATTAATGG GTGATTAAAG CCAACCTGAA CTAAAGGAAA ACAAACCATG GAGTCCGTAG   
  
  
- AAGACACCTG GAAGTAAGTG TCAAATTAAA AGTGTAAAAA TATTCCGACG AGAACACAAC GTTTAAAAGG   
  
  
- ATAGGTAAGG TGAGAAACGG GTAAGGGTCC GATTAATCAC AAGAGACAAG TTGGTACCTA AGACACGACC   
  
  
- AACTAGGACT CAAGTACTTT TTAGAGATGT TTAAGTTTGG ACTTAACGAG AGTTTGAAAA GTTACTTAGG   
  
  
- TTTACTGCTG TAGAAAGTTC GGGAAGTAAG TCTTTTACTA GGAAATGTCA AGGAGAGTAA ACTACTTCCT   
  
  
- TGGACAGAAT TGTCGACGTG ATCAGTTGTT CACGGTCTAA AGGGACTACG AACAGAGTTC AAGTAGTCAC   
  
  
- TATAAGAAGA GCTTCTCCCA AATCTACGTT TAGGACGTAG ACGTGTCCGA GAGCTTCGGT GGCTCTTCAG   
  
  
- GAACATACTA CGAGAGCCCG AACCTCTCGG TATGGGGGAA AGTACACTAG TGAAACGCGG TAGATAGAGA   
  
  
- TGTTCATAAC TCTCGGGTCT ACTGTCAAGA AGGTTATTTT CAATATCGTC GTTAGGGCTC TATCTACCAA   
  
  
- GAATACGATA GCGACTCGGG TCAAAGCTCA GGTTGGGGTT GACACACGAA CTAGTTGGGG TCAACTTGAG   
  
  
- GAAAGGTCGA GATGTACTCT AAAGAGCCAG GAACCACCTT GACCCAAGAG TTCGGAGACT CAACTCGAAG   
  
  
- CTACTACGTC CCTCACGGGC ACATCTCTTT TTCCCGTTTA GCTATTTCCC GAGCTCCTCC TTCTTCTCAG   
  
  
- CAGTTTCTCT CCCACTTCGC ACAATACCTC CTTCCTCGGT ATTCCAAGTT CGAAGGAAGT TACTACTAAT   
  
  
- GATACTCTAC CTTGTCATAC TACTACATCA TAACGAGACA TTATTACTTG ATTGCCCGTT ATCGGCAAAG   
  
  
- TTGTGGCCCT TTAGAAGTGG ACTCCTCCCT ACCTCCTCTA ACGTCTCTTC GGCTCCTTTC TTCGTCTTGT   
  
  
- CAAATCGTCA ACTTCAACTA GACTACTGGG ACGACTGAGT CACACGTGTT CGACATAGCT CGAAACTAGA   
  
  
- AGCTCCACGT TTGCTCGATG AATCCGTTTA GTCCGTCTTA CGAAGCGGGA TACCACCGTC GTAGGTCTCC   
  
  
- GAGCGGGTAG TACATCGGTT ACGAGAGCTC CGTGCATATC GACCGTGTCC GAGATGTCAG AGATGATTGG   
  
  
- AACAACTACG TTTCAAGAGT CGAAGACTGA AGGATTTCCG AATGTCCAAT ATACAGAGTC GACAAGGAAT   
  
  
- GTTTTCCTAC AGAAAGAAAG AACGATTGAC GAGCTAACGT TTCAACCGTC TCTTTCGTTG TTTCTAGGTA   
  
  
- TAGTAACTAA AACCACAAAA GGATCCAAAT GTTACCGGAA CAAAGTATGT TGTGGATAGT TTTTCCGGTT   
  
  
- TACCTGGGGG TTTTGAGGCT TAGTGTCCTT AGCTGATGGG GGTCGTCCCA AAGTCTGGAC GTGTTTCCCA   
  
  
- ACTTCGATGT CCTGTGGCTA ATAGACCCAT GACACTCGCT AAACCCCACG GAAAAAGAAT AGTCCCGTAA   
  
  
- CGAGTCTTCA CCCTTTGATA AGTCGGCCTC CTAGAGTTCT AGCTTGTTCT GCTCGACCAC TAACAGTTGA   
  
  
- CAAACAAGTC CAGTCCTTGT GACGAGCTAC TCTGTCAGCT TCGTTTGTCA GGTTCTCTAC GAAAGAATCG   
  
  
- AAACCAATCT TTCGACTTAG GGTCGGATAA GTAAGTGCCC CAACAGTTAC CGTGTAAGTT GCGAGGTAAG   
  
  
- AAGCACTGAG CTAAGTCTCT CCGTAACAAA GTAATAAGTA GTCACAAACT ACACAGACTT CTCTGTTAAG   
  
  
- GTGCTCTACG GGTACTCTCC AAGAACTAAC TCTCGCTCTA AACACCCTTT CTTGACAAGT TACACCAACG   
  
  
- AACACTCCCA CGTCTCTCCC AAGTTTCCGG ACTCTGTATG TTCGTCACCG TTCACTCCTG TTGCGCCCGG   
  
  
- CCCAATTCCG TCCAACGGAA CCTGGTCCTC GAATACTTCC TTCGTTGCCG TTACCACTTC CGTTTAATAG   
  
  
- TATTCCTAAA ATACCACCTA TATTTATCCG TAACCTACGA AGTTCCAACC TTCCCATCTT GGAACACACG   
  
  
- AGAGAGTAAA ACCGTTGGAC GGAC

+     ABRE

| Site Name | Organism | Position | Strand | Matrix score. | sequence | function |
| --- | --- | --- | --- | --- | --- | --- |
| ABRE | Arabidopsis thaliana | 3046 | - | 5 | ACGTG | cis-acting element involved in the abscisic acid responsiveness |
| ABRE | Arabidopsis thaliana | 3853 | - | 7 | AACCCGG | cis-acting element involved in the abscisic acid responsiveness |
| ABRE | Arabidopsis thaliana | 886 | - | 5 | ACGTG | cis-acting element involved in the abscisic acid responsiveness |
| ABRE | Arabidopsis thaliana | 311 | - | 5 | ACGTG | cis-acting element involved in the abscisic acid responsiveness |
| ABRE | Arabidopsis thaliana | 1821 | - | 5 | ACGTG | cis-acting element involved in the abscisic acid responsiveness |
| ABRE | Arabidopsis thaliana | 1084 | + | 7 | AACCCGG | cis-acting element involved in the abscisic acid responsiveness |

>HU02G01571.1   
+ +Up\_Stream \_Len000CTAGGG GTTGAGAGAA TCCGATAGCT ATGCAGTCCA AACATTATCT CGTCAAGCAT   
  
  
+ TACTATTTAA ATAATTAATA TTGAACTTGA TAGTTACACT TTTTGAATAC TAATTCCACA CCTCATTGCC   
  
  
+ TTTATTTCCA AACCTCGGGT TGCATTTGGA CCACATACCA GGAAGCTTGG ACGACAAAAA TTTCTTTTCT   
  
  
+ TGAGTATTAT TTTGTTTTAT TATTATTATT TTTTACTTTA TTTGGACCAC TTCACTTGGG TAACAAGATT   
  
  
+ GAATATTTGA CTATTATATT ATATGTCACG TATGGATATG GTAGTTGCTT AGAGCTTTGA CCGTTTGCAG   
  
  
+ GTTTAAGTAT GATTCCCTTG GAAAGGAGCC CCTCTTTATC TTAATTCAGA AGTAACTTTT GTTTTTTTAG   
  
  
+ ATAAATTGAA AAGAAAAATA ATAAATGTCT TAGTTGAGTT TGGTTATTCA ATCCGAAATT TGATCCTGCT   
  
  
+ TAATGTATAG TATATAATAC TAATACGCAT GTAACACAAC ACCTCAGATG GTCATTGTTT TACAATTTTT   
  
  
+ TTGAGTTTAG TATAAAAATA AAATCAATTA TTTTTAGAAG TGATGTTTAA TTTGCATTTT TTTTCCACCT   
  
  
+ AAAATCACGC ACTCGAACAT TAGGGGTTTG GCTAACTCAA AGACTCAAGG TAGTGACTTA TCCAAACTTA   
  
  
+ TTACATCCTA TAGATCTATA AAAACATAAA TCTCTTACAA TCAATCCAAA TTGATTCGGT CTAATTTGAT   
  
  
+ GACCCTGATG AGGGTCCTAC AATTACACAA GTGGGCCTTA GCCCCATGGC CGTTTTTTAA TCTTCATGGG   
  
  
+ TCCAGGCGGA GTTACCATCT CCAGGTCCAT TGAGGGTGTA TCACGTCTCA AATTACGAGT TTGCCTCTTT   
  
  
+ CTACGGGGAT TATAAATATC TACCTGCATA CCTCGAAAAG GAGGAGTTGA GATCAGCGAA ACTTGGCCAT   
  
  
+ CCCAGAGCAT TGAAATGCTC AGAACCCTTT TTCTAGAAAA ATATCGTTTT TCCTGAAAAT ATCCGACCTT   
  
  
+ ATGTCATTTA TTGTCGCTTT CTTGACTCTA ACCCGGGACT CCACACTAAC TTGACTATCG GAAAGGCGTT   
  
  
+ CCCTGGATCA CCATCCAAGA TAACCTCTTT TGCAGGGTTG ACGCTCGTTG GAAGAATCGT CGAATGTAGA   
  
  
+ GCCCCTATCA TCACATAAGC CCGCTCTTAC TTCATGCCAT CCTTGAATAG CAGTTTATCC CGAAGCAAAA   
  
  
+ ATAATTCTAA CTTTCTTATA TTGTAAACCC ACATCTAAAT TTAAATTCAT ACTCCAAAAT TCCGCCACGC   
  
  
+ AAATAAGGCT TTTTAATAGG CTTACATATA ATGGCATGCT ATAGAGACAC TGTTTTGAAG AAACTATTTA   
  
  
+ AACATCAGAC CTCATATTGA GTAGCAAATC AAGACTTCTT TCCTCCAGGC CATATTGAGT AGCAAATTAA   
  
  
+ GAATAACCAG AAGCTTGTGA AAAGAGTTAG AAAATAAAAG GTATAATAGA GACTTATGTT CTTAAGATAT   
  
  
+ CTCCTAGATA TGAATGTGAC CTCCTAGTCG ATATTTGTGC TGGATTAGCG AGTTGTGTGA CTGAGTTTGT   
  
  
+ ATGTGTCTCT TAGCATATGA GTTATTCTAG CTGCTTCTTT AAGCTATGTA GTGACTGTCT TCCTTCTTAT   
  
  
+ TTATAAGTGC TATGTCCAAC TAATAGCCGA AAAAAATGGT TCGCCTTTTC TGGTTTTCTT CTTTTTGAAG   
  
  
+ GTTGATTGGC TAATTTATAT AATGGTTTCA ATACTTATCC TTTATTGTTC CACAAATCCA CCTAACCACG   
  
  
+ TACCATTGAC AATAATTACC CACTAATTTC GGTTGGACTT GATTTCCTTT TGTTTGGTAC CTCAGGCATC   
  
  
+ TTCTGTGGAC CTTCATTCAC AGTTTAATTT TCACATTTTT ATAAGGCTGC TCTTGTGTTG CAAATTTTCC   
  
  
+ TATCCATTCC ACTCTTTGCC CATTCCCAGG CTAATTAGTG TTCTCTGTTC AACCATGGAT TCTGTGCTGG   
  
  
+ TTGATCCTGA GTTCATGAAA AATCTCTACA AATTCAAACC TGAATTGCTC TCAAACTTTT CAATGAATCC   
  
  
+ AAATGACGAC ATCTTTCAAG CCCTTCATTC AGAAAATGAT CCTTTACAGT TCCTCTCATT TGATGAAGGA   
  
  
+ ACCTGTCTTA ACAGCTGCAC TAGTCAACAA GTGCCAGATT TCCCTGATGC TTGTCTCAAG TTCATCAGTG   
  
  
+ ATATTCTTCT CGAAGAGGGT TTAGATGCAA ATCCTGCATC TGCACAGGCT CTCGAAGCCA CCGAGAAGTC   
  
  
+ CTTGTATGAT GCTCTCGGGC TTGGAGAGCC ATACCCCCTT TCATGTGATC ACTTTGCGCC ATCTATCTCT   
  
  
+ ACAAGTATTG AGAGCCCAGA TGACAGTTCT TCCAATAAAA GTTATAGCAG CAATCCCGAG ATAGATGGTT   
  
  
+ CTTATGCTAT CGCTGAGCCC AGTTTCGAGT CCAACCCCAA CTGTGTGCTT GATCAACCCC AGTTGAACTC   
  
  
+ CTTTCCAGCT CTACATGAGA TTTCTCGGTC CTTGGTGGAA CTGGGTTCTC AAGCCTCTGA GTTGAGCTTC   
  
  
+ GATGATGCAG GGAGTGCCCG TGTAGAGAAA AAGGGCAAAT CGATAAAGGG CTCGAGGAGG AAGAAGAGTC   
  
  
+ GTCAAAGAGA GGGTGAAGCG TGTTATGGAG GAAGGAGCCA TAAGGTTCAA GCTTCCTTCA ATGATGATTA   
  
  
+ CTATGAGATG GAACAGTATG ATGATGTAGT ATTGCTCTGT AATAATGAAC TAACGGGCAA TAGCCGTTTC   
  
  
+ AACACCGGGA AATCTTCACC TGAGGAGGGA TGGAGGAGAT TGCAGAGAAG CCGAGGAAAG AAGCAGAACA   
  
  
+ GTTTAGCAGT TGAAGTTGAT CTGATGACCC TGCTGACTCA GTGTGCACAA GCTGTATCGA GCTTTGATCT   
  
  
+ TCGAGGTGCA AACGAGCTAC TTAGGCAAAT CAGGCAGAAT GCTTCGCCCT ATGGTGGCAG CATCCAGAGG   
  
  
+ CTCGCCCATC ATGTAGCCAA TGCTCTCGAG GCACGTATAG CTGGCACAGG CTCTACAGTC TCTACTAACC   
  
  
+ TTGTTGATGC AAAGTTCTCA GCTTCTGACT TCCTAAAGGC TTACAGGTTA TATGTCTCAG CTGTTCCTTA   
  
  
+ CAAAAGGATG TCTTTCTTTC TTGCTAACTG CTCGATTGCA AAGTTGGCAG AGAAAGCAAC AAAGATCCAT   
  
  
+ ATCATTGATT TTGGTGTTTT CCTAGGTTTA CAATGGCCTT GTTTCATACA ACACCTATCA AAAAGGCCAA   
  
  
+ ATGGACCCCC AAAACTCCGA ATCACAGGAA TCGACTACCC CCAGCAGGGT TTCAGACCTG CACAAAGGGT   
  
  
+ TGAAGCTACA GGACACCGAT TATCTGGGTA CTGTGAGCGA TTTGGGGTGC CTTTTTCTTA TCAGGGCATT   
  
  
+ GCTCAGAAGT GGGAAACTAT TCAGCCGGAG GATCTCAAGA TCGAACAAGA CGAGCTGGTG ATTGTCAACT   
  
  
+ GTTTGTTCAG GTCAGGAACA CTGCTCGATG AGACAGTCGA AGCAAACAGT CCAAGAGATG CTTTCTTAGC   
  
  
+ TTTGGTTAGA AAGCTGAATC CCAGCCTATT CATTCACGGG GTTGTCAATG GCACATTCAA CGCTCCATTC   
  
  
+ TTCGTGACTC GATTCAGAGA GGCATTGTTT CATTATTCAT CAGTGTTTGA TGTGTCTGAA GAGACAATTC   
  
  
+ CACGAGATGC CCATGAGAGG TTCTTGATTG AGAGCGAGAT TTGTGGGAAA GAACTGTTCA ATGTGGTTGC   
  
  
+ TTGTGAGGGT GCAGAGAGGG TTCAAAGGCC TGAGACATAC AAGCAGTGGC AAGTGAGGAC AACGCGGGCC   
  
  
+ GGGTTAAGGC AGGTTGCCTT GGACCAGGAG CTTATGAAGG AAGCAACGGC AATGGTGAAG GCAAATTATC   
  
  
+ ATAAGGATTT TATGGTGGAT ATAAATAGGC ATTGGATGCT TCAAGGTTGG AAGGGTAGAA CCTTGTGTGC   
  
  
+ TCTCTCATTT TGGCAACCTG CCTG  

- +Up\_Stream \_Len000GATCCC CAACTCTCTT AGGCTATCGA TACGTCAGGT TTGTAATAGA GCAGTTCGTA   
  
  
- ATGATAAATT TATTAATTAT AACTTGAACT ATCAATGTGA AAAACTTATG ATTAAGGTGT GGAGTAACGG   
  
  
- AAATAAAGGT TTGGAGCCCA ACGTAAACCT GGTGTATGGT CCTTCGAACC TGCTGTTTTT AAAGAAAAGA   
  
  
- ACTCATAATA AAACAAAATA ATAATAATAA AAAATGAAAT AAACCTGGTG AAGTGAACCC ATTGTTCTAA   
  
  
- CTTATAAACT GATAATATAA TATACAGTGC ATACCTATAC CATCAACGAA TCTCGAAACT GGCAAACGTC   
  
  
- CAAATTCATA CTAAGGGAAC CTTTCCTCGG GGAGAAATAG AATTAAGTCT TCATTGAAAA CAAAAAAATC   
  
  
- TATTTAACTT TTCTTTTTAT TATTTACAGA ATCAACTCAA ACCAATAAGT TAGGCTTTAA ACTAGGACGA   
  
  
- ATTACATATC ATATATTATG ATTATGCGTA CATTGTGTTG TGGAGTCTAC CAGTAACAAA ATGTTAAAAA   
  
  
- AACTCAAATC ATATTTTTAT TTTAGTTAAT AAAAATCTTC ACTACAAATT AAACGTAAAA AAAAGGTGGA   
  
  
- TTTTAGTGCG TGAGCTTGTA ATCCCCAAAC CGATTGAGTT TCTGAGTTCC ATCACTGAAT AGGTTTGAAT   
  
  
- AATGTAGGAT ATCTAGATAT TTTTGTATTT AGAGAATGTT AGTTAGGTTT AACTAAGCCA GATTAAACTA   
  
  
- CTGGGACTAC TCCCAGGATG TTAATGTGTT CACCCGGAAT CGGGGTACCG GCAAAAAATT AGAAGTACCC   
  
  
- AGGTCCGCCT CAATGGTAGA GGTCCAGGTA ACTCCCACAT AGTGCAGAGT TTAATGCTCA AACGGAGAAA   
  
  
- GATGCCCCTA ATATTTATAG ATGGACGTAT GGAGCTTTTC CTCCTCAACT CTAGTCGCTT TGAACCGGTA   
  
  
- GGGTCTCGTA ACTTTACGAG TCTTGGGAAA AAGATCTTTT TATAGCAAAA AGGACTTTTA TAGGCTGGAA   
  
  
- TACAGTAAAT AACAGCGAAA GAACTGAGAT TGGGCCCTGA GGTGTGATTG AACTGATAGC CTTTCCGCAA   
  
  
- GGGACCTAGT GGTAGGTTCT ATTGGAGAAA ACGTCCCAAC TGCGAGCAAC CTTCTTAGCA GCTTACATCT   
  
  
- CGGGGATAGT AGTGTATTCG GGCGAGAATG AAGTACGGTA GGAACTTATC GTCAAATAGG GCTTCGTTTT   
  
  
- TATTAAGATT GAAAGAATAT AACATTTGGG TGTAGATTTA AATTTAAGTA TGAGGTTTTA AGGCGGTGCG   
  
  
- TTTATTCCGA AAAATTATCC GAATGTATAT TACCGTACGA TATCTCTGTG ACAAAACTTC TTTGATAAAT   
  
  
- TTGTAGTCTG GAGTATAACT CATCGTTTAG TTCTGAAGAA AGGAGGTCCG GTATAACTCA TCGTTTAATT   
  
  
- CTTATTGGTC TTCGAACACT TTTCTCAATC TTTTATTTTC CATATTATCT CTGAATACAA GAATTCTATA   
  
  
- GAGGATCTAT ACTTACACTG GAGGATCAGC TATAAACACG ACCTAATCGC TCAACACACT GACTCAAACA   
  
  
- TACACAGAGA ATCGTATACT CAATAAGATC GACGAAGAAA TTCGATACAT CACTGACAGA AGGAAGAATA   
  
  
- AATATTCACG ATACAGGTTG ATTATCGGCT TTTTTTACCA AGCGGAAAAG ACCAAAAGAA GAAAAACTTC   
  
  
- CAACTAACCG ATTAAATATA TTACCAAAGT TATGAATAGG AAATAACAAG GTGTTTAGGT GGATTGGTGC   
  
  
- ATGGTAACTG TTATTAATGG GTGATTAAAG CCAACCTGAA CTAAAGGAAA ACAAACCATG GAGTCCGTAG   
  
  
- AAGACACCTG GAAGTAAGTG TCAAATTAAA AGTGTAAAAA TATTCCGACG AGAACACAAC GTTTAAAAGG   
  
  
- ATAGGTAAGG TGAGAAACGG GTAAGGGTCC GATTAATCAC AAGAGACAAG TTGGTACCTA AGACACGACC   
  
  
- AACTAGGACT CAAGTACTTT TTAGAGATGT TTAAGTTTGG ACTTAACGAG AGTTTGAAAA GTTACTTAGG   
  
  
- TTTACTGCTG TAGAAAGTTC GGGAAGTAAG TCTTTTACTA GGAAATGTCA AGGAGAGTAA ACTACTTCCT   
  
  
- TGGACAGAAT TGTCGACGTG ATCAGTTGTT CACGGTCTAA AGGGACTACG AACAGAGTTC AAGTAGTCAC   
  
  
- TATAAGAAGA GCTTCTCCCA AATCTACGTT TAGGACGTAG ACGTGTCCGA GAGCTTCGGT GGCTCTTCAG   
  
  
- GAACATACTA CGAGAGCCCG AACCTCTCGG TATGGGGGAA AGTACACTAG TGAAACGCGG TAGATAGAGA   
  
  
- TGTTCATAAC TCTCGGGTCT ACTGTCAAGA AGGTTATTTT CAATATCGTC GTTAGGGCTC TATCTACCAA   
  
  
- GAATACGATA GCGACTCGGG TCAAAGCTCA GGTTGGGGTT GACACACGAA CTAGTTGGGG TCAACTTGAG   
  
  
- GAAAGGTCGA GATGTACTCT AAAGAGCCAG GAACCACCTT GACCCAAGAG TTCGGAGACT CAACTCGAAG   
  
  
- CTACTACGTC CCTCACGGGC ACATCTCTTT TTCCCGTTTA GCTATTTCCC GAGCTCCTCC TTCTTCTCAG   
  
  
- CAGTTTCTCT CCCACTTCGC ACAATACCTC CTTCCTCGGT ATTCCAAGTT CGAAGGAAGT TACTACTAAT   
  
  
- GATACTCTAC CTTGTCATAC TACTACATCA TAACGAGACA TTATTACTTG ATTGCCCGTT ATCGGCAAAG   
  
  
- TTGTGGCCCT TTAGAAGTGG ACTCCTCCCT ACCTCCTCTA ACGTCTCTTC GGCTCCTTTC TTCGTCTTGT   
  
  
- CAAATCGTCA ACTTCAACTA GACTACTGGG ACGACTGAGT CACACGTGTT CGACATAGCT CGAAACTAGA   
  
  
- AGCTCCACGT TTGCTCGATG AATCCGTTTA GTCCGTCTTA CGAAGCGGGA TACCACCGTC GTAGGTCTCC   
  
  
- GAGCGGGTAG TACATCGGTT ACGAGAGCTC CGTGCATATC GACCGTGTCC GAGATGTCAG AGATGATTGG   
  
  
- AACAACTACG TTTCAAGAGT CGAAGACTGA AGGATTTCCG AATGTCCAAT ATACAGAGTC GACAAGGAAT   
  
  
- GTTTTCCTAC AGAAAGAAAG AACGATTGAC GAGCTAACGT TTCAACCGTC TCTTTCGTTG TTTCTAGGTA   
  
  
- TAGTAACTAA AACCACAAAA GGATCCAAAT GTTACCGGAA CAAAGTATGT TGTGGATAGT TTTTCCGGTT   
  
  
- TACCTGGGGG TTTTGAGGCT TAGTGTCCTT AGCTGATGGG GGTCGTCCCA AAGTCTGGAC GTGTTTCCCA   
  
  
- ACTTCGATGT CCTGTGGCTA ATAGACCCAT GACACTCGCT AAACCCCACG GAAAAAGAAT AGTCCCGTAA   
  
  
- CGAGTCTTCA CCCTTTGATA AGTCGGCCTC CTAGAGTTCT AGCTTGTTCT GCTCGACCAC TAACAGTTGA   
  
  
- CAAACAAGTC CAGTCCTTGT GACGAGCTAC TCTGTCAGCT TCGTTTGTCA GGTTCTCTAC GAAAGAATCG   
  
  
- AAACCAATCT TTCGACTTAG GGTCGGATAA GTAAGTGCCC CAACAGTTAC CGTGTAAGTT GCGAGGTAAG   
  
  
- AAGCACTGAG CTAAGTCTCT CCGTAACAAA GTAATAAGTA GTCACAAACT ACACAGACTT CTCTGTTAAG   
  
  
- GTGCTCTACG GGTACTCTCC AAGAACTAAC TCTCGCTCTA AACACCCTTT CTTGACAAGT TACACCAACG   
  
  
- AACACTCCCA CGTCTCTCCC AAGTTTCCGG ACTCTGTATG TTCGTCACCG TTCACTCCTG TTGCGCCCGG   
  
  
- CCCAATTCCG TCCAACGGAA CCTGGTCCTC GAATACTTCC TTCGTTGCCG TTACCACTTC CGTTTAATAG   
  
  
- TATTCCTAAA ATACCACCTA TATTTATCCG TAACCTACGA AGTTCCAACC TTCCCATCTT GGAACACACG   
  
  
- AGAGAGTAAA ACCGTTGGAC GGAC

+     ABRE3a

| Site Name | Organism | Position | Strand | Matrix score. | sequence | function |
| --- | --- | --- | --- | --- | --- | --- |
| ABRE3a | Zea mays | 3046 | - | 6 | TACGTG |  |
| ABRE3a | Zea mays | 1821 | - | 6 | TACGTG |  |
| ABRE3a | Zea mays | 311 | - | 6 | TACGTG |  |

>HU02G01571.1   
+ +Up\_Stream \_Len000CTAGGG GTTGAGAGAA TCCGATAGCT ATGCAGTCCA AACATTATCT CGTCAAGCAT   
  
  
+ TACTATTTAA ATAATTAATA TTGAACTTGA TAGTTACACT TTTTGAATAC TAATTCCACA CCTCATTGCC   
  
  
+ TTTATTTCCA AACCTCGGGT TGCATTTGGA CCACATACCA GGAAGCTTGG ACGACAAAAA TTTCTTTTCT   
  
  
+ TGAGTATTAT TTTGTTTTAT TATTATTATT TTTTACTTTA TTTGGACCAC TTCACTTGGG TAACAAGATT   
  
  
+ GAATATTTGA CTATTATATT ATATGTCACG TATGGATATG GTAGTTGCTT AGAGCTTTGA CCGTTTGCAG   
  
  
+ GTTTAAGTAT GATTCCCTTG GAAAGGAGCC CCTCTTTATC TTAATTCAGA AGTAACTTTT GTTTTTTTAG   
  
  
+ ATAAATTGAA AAGAAAAATA ATAAATGTCT TAGTTGAGTT TGGTTATTCA ATCCGAAATT TGATCCTGCT   
  
  
+ TAATGTATAG TATATAATAC TAATACGCAT GTAACACAAC ACCTCAGATG GTCATTGTTT TACAATTTTT   
  
  
+ TTGAGTTTAG TATAAAAATA AAATCAATTA TTTTTAGAAG TGATGTTTAA TTTGCATTTT TTTTCCACCT   
  
  
+ AAAATCACGC ACTCGAACAT TAGGGGTTTG GCTAACTCAA AGACTCAAGG TAGTGACTTA TCCAAACTTA   
  
  
+ TTACATCCTA TAGATCTATA AAAACATAAA TCTCTTACAA TCAATCCAAA TTGATTCGGT CTAATTTGAT   
  
  
+ GACCCTGATG AGGGTCCTAC AATTACACAA GTGGGCCTTA GCCCCATGGC CGTTTTTTAA TCTTCATGGG   
  
  
+ TCCAGGCGGA GTTACCATCT CCAGGTCCAT TGAGGGTGTA TCACGTCTCA AATTACGAGT TTGCCTCTTT   
  
  
+ CTACGGGGAT TATAAATATC TACCTGCATA CCTCGAAAAG GAGGAGTTGA GATCAGCGAA ACTTGGCCAT   
  
  
+ CCCAGAGCAT TGAAATGCTC AGAACCCTTT TTCTAGAAAA ATATCGTTTT TCCTGAAAAT ATCCGACCTT   
  
  
+ ATGTCATTTA TTGTCGCTTT CTTGACTCTA ACCCGGGACT CCACACTAAC TTGACTATCG GAAAGGCGTT   
  
  
+ CCCTGGATCA CCATCCAAGA TAACCTCTTT TGCAGGGTTG ACGCTCGTTG GAAGAATCGT CGAATGTAGA   
  
  
+ GCCCCTATCA TCACATAAGC CCGCTCTTAC TTCATGCCAT CCTTGAATAG CAGTTTATCC CGAAGCAAAA   
  
  
+ ATAATTCTAA CTTTCTTATA TTGTAAACCC ACATCTAAAT TTAAATTCAT ACTCCAAAAT TCCGCCACGC   
  
  
+ AAATAAGGCT TTTTAATAGG CTTACATATA ATGGCATGCT ATAGAGACAC TGTTTTGAAG AAACTATTTA   
  
  
+ AACATCAGAC CTCATATTGA GTAGCAAATC AAGACTTCTT TCCTCCAGGC CATATTGAGT AGCAAATTAA   
  
  
+ GAATAACCAG AAGCTTGTGA AAAGAGTTAG AAAATAAAAG GTATAATAGA GACTTATGTT CTTAAGATAT   
  
  
+ CTCCTAGATA TGAATGTGAC CTCCTAGTCG ATATTTGTGC TGGATTAGCG AGTTGTGTGA CTGAGTTTGT   
  
  
+ ATGTGTCTCT TAGCATATGA GTTATTCTAG CTGCTTCTTT AAGCTATGTA GTGACTGTCT TCCTTCTTAT   
  
  
+ TTATAAGTGC TATGTCCAAC TAATAGCCGA AAAAAATGGT TCGCCTTTTC TGGTTTTCTT CTTTTTGAAG   
  
  
+ GTTGATTGGC TAATTTATAT AATGGTTTCA ATACTTATCC TTTATTGTTC CACAAATCCA CCTAACCACG   
  
  
+ TACCATTGAC AATAATTACC CACTAATTTC GGTTGGACTT GATTTCCTTT TGTTTGGTAC CTCAGGCATC   
  
  
+ TTCTGTGGAC CTTCATTCAC AGTTTAATTT TCACATTTTT ATAAGGCTGC TCTTGTGTTG CAAATTTTCC   
  
  
+ TATCCATTCC ACTCTTTGCC CATTCCCAGG CTAATTAGTG TTCTCTGTTC AACCATGGAT TCTGTGCTGG   
  
  
+ TTGATCCTGA GTTCATGAAA AATCTCTACA AATTCAAACC TGAATTGCTC TCAAACTTTT CAATGAATCC   
  
  
+ AAATGACGAC ATCTTTCAAG CCCTTCATTC AGAAAATGAT CCTTTACAGT TCCTCTCATT TGATGAAGGA   
  
  
+ ACCTGTCTTA ACAGCTGCAC TAGTCAACAA GTGCCAGATT TCCCTGATGC TTGTCTCAAG TTCATCAGTG   
  
  
+ ATATTCTTCT CGAAGAGGGT TTAGATGCAA ATCCTGCATC TGCACAGGCT CTCGAAGCCA CCGAGAAGTC   
  
  
+ CTTGTATGAT GCTCTCGGGC TTGGAGAGCC ATACCCCCTT TCATGTGATC ACTTTGCGCC ATCTATCTCT   
  
  
+ ACAAGTATTG AGAGCCCAGA TGACAGTTCT TCCAATAAAA GTTATAGCAG CAATCCCGAG ATAGATGGTT   
  
  
+ CTTATGCTAT CGCTGAGCCC AGTTTCGAGT CCAACCCCAA CTGTGTGCTT GATCAACCCC AGTTGAACTC   
  
  
+ CTTTCCAGCT CTACATGAGA TTTCTCGGTC CTTGGTGGAA CTGGGTTCTC AAGCCTCTGA GTTGAGCTTC   
  
  
+ GATGATGCAG GGAGTGCCCG TGTAGAGAAA AAGGGCAAAT CGATAAAGGG CTCGAGGAGG AAGAAGAGTC   
  
  
+ GTCAAAGAGA GGGTGAAGCG TGTTATGGAG GAAGGAGCCA TAAGGTTCAA GCTTCCTTCA ATGATGATTA   
  
  
+ CTATGAGATG GAACAGTATG ATGATGTAGT ATTGCTCTGT AATAATGAAC TAACGGGCAA TAGCCGTTTC   
  
  
+ AACACCGGGA AATCTTCACC TGAGGAGGGA TGGAGGAGAT TGCAGAGAAG CCGAGGAAAG AAGCAGAACA   
  
  
+ GTTTAGCAGT TGAAGTTGAT CTGATGACCC TGCTGACTCA GTGTGCACAA GCTGTATCGA GCTTTGATCT   
  
  
+ TCGAGGTGCA AACGAGCTAC TTAGGCAAAT CAGGCAGAAT GCTTCGCCCT ATGGTGGCAG CATCCAGAGG   
  
  
+ CTCGCCCATC ATGTAGCCAA TGCTCTCGAG GCACGTATAG CTGGCACAGG CTCTACAGTC TCTACTAACC   
  
  
+ TTGTTGATGC AAAGTTCTCA GCTTCTGACT TCCTAAAGGC TTACAGGTTA TATGTCTCAG CTGTTCCTTA   
  
  
+ CAAAAGGATG TCTTTCTTTC TTGCTAACTG CTCGATTGCA AAGTTGGCAG AGAAAGCAAC AAAGATCCAT   
  
  
+ ATCATTGATT TTGGTGTTTT CCTAGGTTTA CAATGGCCTT GTTTCATACA ACACCTATCA AAAAGGCCAA   
  
  
+ ATGGACCCCC AAAACTCCGA ATCACAGGAA TCGACTACCC CCAGCAGGGT TTCAGACCTG CACAAAGGGT   
  
  
+ TGAAGCTACA GGACACCGAT TATCTGGGTA CTGTGAGCGA TTTGGGGTGC CTTTTTCTTA TCAGGGCATT   
  
  
+ GCTCAGAAGT GGGAAACTAT TCAGCCGGAG GATCTCAAGA TCGAACAAGA CGAGCTGGTG ATTGTCAACT   
  
  
+ GTTTGTTCAG GTCAGGAACA CTGCTCGATG AGACAGTCGA AGCAAACAGT CCAAGAGATG CTTTCTTAGC   
  
  
+ TTTGGTTAGA AAGCTGAATC CCAGCCTATT CATTCACGGG GTTGTCAATG GCACATTCAA CGCTCCATTC   
  
  
+ TTCGTGACTC GATTCAGAGA GGCATTGTTT CATTATTCAT CAGTGTTTGA TGTGTCTGAA GAGACAATTC   
  
  
+ CACGAGATGC CCATGAGAGG TTCTTGATTG AGAGCGAGAT TTGTGGGAAA GAACTGTTCA ATGTGGTTGC   
  
  
+ TTGTGAGGGT GCAGAGAGGG TTCAAAGGCC TGAGACATAC AAGCAGTGGC AAGTGAGGAC AACGCGGGCC   
  
  
+ GGGTTAAGGC AGGTTGCCTT GGACCAGGAG CTTATGAAGG AAGCAACGGC AATGGTGAAG GCAAATTATC   
  
  
+ ATAAGGATTT TATGGTGGAT ATAAATAGGC ATTGGATGCT TCAAGGTTGG AAGGGTAGAA CCTTGTGTGC   
  
  
+ TCTCTCATTT TGGCAACCTG CCTG  

- +Up\_Stream \_Len000GATCCC CAACTCTCTT AGGCTATCGA TACGTCAGGT TTGTAATAGA GCAGTTCGTA   
  
  
- ATGATAAATT TATTAATTAT AACTTGAACT ATCAATGTGA AAAACTTATG ATTAAGGTGT GGAGTAACGG   
  
  
- AAATAAAGGT TTGGAGCCCA ACGTAAACCT GGTGTATGGT CCTTCGAACC TGCTGTTTTT AAAGAAAAGA   
  
  
- ACTCATAATA AAACAAAATA ATAATAATAA AAAATGAAAT AAACCTGGTG AAGTGAACCC ATTGTTCTAA   
  
  
- CTTATAAACT GATAATATAA TATACAGTGC ATACCTATAC CATCAACGAA TCTCGAAACT GGCAAACGTC   
  
  
- CAAATTCATA CTAAGGGAAC CTTTCCTCGG GGAGAAATAG AATTAAGTCT TCATTGAAAA CAAAAAAATC   
  
  
- TATTTAACTT TTCTTTTTAT TATTTACAGA ATCAACTCAA ACCAATAAGT TAGGCTTTAA ACTAGGACGA   
  
  
- ATTACATATC ATATATTATG ATTATGCGTA CATTGTGTTG TGGAGTCTAC CAGTAACAAA ATGTTAAAAA   
  
  
- AACTCAAATC ATATTTTTAT TTTAGTTAAT AAAAATCTTC ACTACAAATT AAACGTAAAA AAAAGGTGGA   
  
  
- TTTTAGTGCG TGAGCTTGTA ATCCCCAAAC CGATTGAGTT TCTGAGTTCC ATCACTGAAT AGGTTTGAAT   
  
  
- AATGTAGGAT ATCTAGATAT TTTTGTATTT AGAGAATGTT AGTTAGGTTT AACTAAGCCA GATTAAACTA   
  
  
- CTGGGACTAC TCCCAGGATG TTAATGTGTT CACCCGGAAT CGGGGTACCG GCAAAAAATT AGAAGTACCC   
  
  
- AGGTCCGCCT CAATGGTAGA GGTCCAGGTA ACTCCCACAT AGTGCAGAGT TTAATGCTCA AACGGAGAAA   
  
  
- GATGCCCCTA ATATTTATAG ATGGACGTAT GGAGCTTTTC CTCCTCAACT CTAGTCGCTT TGAACCGGTA   
  
  
- GGGTCTCGTA ACTTTACGAG TCTTGGGAAA AAGATCTTTT TATAGCAAAA AGGACTTTTA TAGGCTGGAA   
  
  
- TACAGTAAAT AACAGCGAAA GAACTGAGAT TGGGCCCTGA GGTGTGATTG AACTGATAGC CTTTCCGCAA   
  
  
- GGGACCTAGT GGTAGGTTCT ATTGGAGAAA ACGTCCCAAC TGCGAGCAAC CTTCTTAGCA GCTTACATCT   
  
  
- CGGGGATAGT AGTGTATTCG GGCGAGAATG AAGTACGGTA GGAACTTATC GTCAAATAGG GCTTCGTTTT   
  
  
- TATTAAGATT GAAAGAATAT AACATTTGGG TGTAGATTTA AATTTAAGTA TGAGGTTTTA AGGCGGTGCG   
  
  
- TTTATTCCGA AAAATTATCC GAATGTATAT TACCGTACGA TATCTCTGTG ACAAAACTTC TTTGATAAAT   
  
  
- TTGTAGTCTG GAGTATAACT CATCGTTTAG TTCTGAAGAA AGGAGGTCCG GTATAACTCA TCGTTTAATT   
  
  
- CTTATTGGTC TTCGAACACT TTTCTCAATC TTTTATTTTC CATATTATCT CTGAATACAA GAATTCTATA   
  
  
- GAGGATCTAT ACTTACACTG GAGGATCAGC TATAAACACG ACCTAATCGC TCAACACACT GACTCAAACA   
  
  
- TACACAGAGA ATCGTATACT CAATAAGATC GACGAAGAAA TTCGATACAT CACTGACAGA AGGAAGAATA   
  
  
- AATATTCACG ATACAGGTTG ATTATCGGCT TTTTTTACCA AGCGGAAAAG ACCAAAAGAA GAAAAACTTC   
  
  
- CAACTAACCG ATTAAATATA TTACCAAAGT TATGAATAGG AAATAACAAG GTGTTTAGGT GGATTGGTGC   
  
  
- ATGGTAACTG TTATTAATGG GTGATTAAAG CCAACCTGAA CTAAAGGAAA ACAAACCATG GAGTCCGTAG   
  
  
- AAGACACCTG GAAGTAAGTG TCAAATTAAA AGTGTAAAAA TATTCCGACG AGAACACAAC GTTTAAAAGG   
  
  
- ATAGGTAAGG TGAGAAACGG GTAAGGGTCC GATTAATCAC AAGAGACAAG TTGGTACCTA AGACACGACC   
  
  
- AACTAGGACT CAAGTACTTT TTAGAGATGT TTAAGTTTGG ACTTAACGAG AGTTTGAAAA GTTACTTAGG   
  
  
- TTTACTGCTG TAGAAAGTTC GGGAAGTAAG TCTTTTACTA GGAAATGTCA AGGAGAGTAA ACTACTTCCT   
  
  
- TGGACAGAAT TGTCGACGTG ATCAGTTGTT CACGGTCTAA AGGGACTACG AACAGAGTTC AAGTAGTCAC   
  
  
- TATAAGAAGA GCTTCTCCCA AATCTACGTT TAGGACGTAG ACGTGTCCGA GAGCTTCGGT GGCTCTTCAG   
  
  
- GAACATACTA CGAGAGCCCG AACCTCTCGG TATGGGGGAA AGTACACTAG TGAAACGCGG TAGATAGAGA   
  
  
- TGTTCATAAC TCTCGGGTCT ACTGTCAAGA AGGTTATTTT CAATATCGTC GTTAGGGCTC TATCTACCAA   
  
  
- GAATACGATA GCGACTCGGG TCAAAGCTCA GGTTGGGGTT GACACACGAA CTAGTTGGGG TCAACTTGAG   
  
  
- GAAAGGTCGA GATGTACTCT AAAGAGCCAG GAACCACCTT GACCCAAGAG TTCGGAGACT CAACTCGAAG   
  
  
- CTACTACGTC CCTCACGGGC ACATCTCTTT TTCCCGTTTA GCTATTTCCC GAGCTCCTCC TTCTTCTCAG   
  
  
- CAGTTTCTCT CCCACTTCGC ACAATACCTC CTTCCTCGGT ATTCCAAGTT CGAAGGAAGT TACTACTAAT   
  
  
- GATACTCTAC CTTGTCATAC TACTACATCA TAACGAGACA TTATTACTTG ATTGCCCGTT ATCGGCAAAG   
  
  
- TTGTGGCCCT TTAGAAGTGG ACTCCTCCCT ACCTCCTCTA ACGTCTCTTC GGCTCCTTTC TTCGTCTTGT   
  
  
- CAAATCGTCA ACTTCAACTA GACTACTGGG ACGACTGAGT CACACGTGTT CGACATAGCT CGAAACTAGA   
  
  
- AGCTCCACGT TTGCTCGATG AATCCGTTTA GTCCGTCTTA CGAAGCGGGA TACCACCGTC GTAGGTCTCC   
  
  
- GAGCGGGTAG TACATCGGTT ACGAGAGCTC CGTGCATATC GACCGTGTCC GAGATGTCAG AGATGATTGG   
  
  
- AACAACTACG TTTCAAGAGT CGAAGACTGA AGGATTTCCG AATGTCCAAT ATACAGAGTC GACAAGGAAT   
  
  
- GTTTTCCTAC AGAAAGAAAG AACGATTGAC GAGCTAACGT TTCAACCGTC TCTTTCGTTG TTTCTAGGTA   
  
  
- TAGTAACTAA AACCACAAAA GGATCCAAAT GTTACCGGAA CAAAGTATGT TGTGGATAGT TTTTCCGGTT   
  
  
- TACCTGGGGG TTTTGAGGCT TAGTGTCCTT AGCTGATGGG GGTCGTCCCA AAGTCTGGAC GTGTTTCCCA   
  
  
- ACTTCGATGT CCTGTGGCTA ATAGACCCAT GACACTCGCT AAACCCCACG GAAAAAGAAT AGTCCCGTAA   
  
  
- CGAGTCTTCA CCCTTTGATA AGTCGGCCTC CTAGAGTTCT AGCTTGTTCT GCTCGACCAC TAACAGTTGA   
  
  
- CAAACAAGTC CAGTCCTTGT GACGAGCTAC TCTGTCAGCT TCGTTTGTCA GGTTCTCTAC GAAAGAATCG   
  
  
- AAACCAATCT TTCGACTTAG GGTCGGATAA GTAAGTGCCC CAACAGTTAC CGTGTAAGTT GCGAGGTAAG   
  
  
- AAGCACTGAG CTAAGTCTCT CCGTAACAAA GTAATAAGTA GTCACAAACT ACACAGACTT CTCTGTTAAG   
  
  
- GTGCTCTACG GGTACTCTCC AAGAACTAAC TCTCGCTCTA AACACCCTTT CTTGACAAGT TACACCAACG   
  
  
- AACACTCCCA CGTCTCTCCC AAGTTTCCGG ACTCTGTATG TTCGTCACCG TTCACTCCTG TTGCGCCCGG   
  
  
- CCCAATTCCG TCCAACGGAA CCTGGTCCTC GAATACTTCC TTCGTTGCCG TTACCACTTC CGTTTAATAG   
  
  
- TATTCCTAAA ATACCACCTA TATTTATCCG TAACCTACGA AGTTCCAACC TTCCCATCTT GGAACACACG   
  
  
- AGAGAGTAAA ACCGTTGGAC GGAC

+     ABRE4

| Site Name | Organism | Position | Strand | Matrix score. | sequence | function |
| --- | --- | --- | --- | --- | --- | --- |
| ABRE4 | Zea mays | 3046 | + | 6 | CACGTA |  |
| ABRE4 | Zea mays | 1821 | + | 6 | CACGTA |  |
| ABRE4 | Zea mays | 311 | + | 6 | CACGTA |  |

>HU02G01571.1   
+ +Up\_Stream \_Len000CTAGGG GTTGAGAGAA TCCGATAGCT ATGCAGTCCA AACATTATCT CGTCAAGCAT   
  
  
+ TACTATTTAA ATAATTAATA TTGAACTTGA TAGTTACACT TTTTGAATAC TAATTCCACA CCTCATTGCC   
  
  
+ TTTATTTCCA AACCTCGGGT TGCATTTGGA CCACATACCA GGAAGCTTGG ACGACAAAAA TTTCTTTTCT   
  
  
+ TGAGTATTAT TTTGTTTTAT TATTATTATT TTTTACTTTA TTTGGACCAC TTCACTTGGG TAACAAGATT   
  
  
+ GAATATTTGA CTATTATATT ATATGTCACG TATGGATATG GTAGTTGCTT AGAGCTTTGA CCGTTTGCAG   
  
  
+ GTTTAAGTAT GATTCCCTTG GAAAGGAGCC CCTCTTTATC TTAATTCAGA AGTAACTTTT GTTTTTTTAG   
  
  
+ ATAAATTGAA AAGAAAAATA ATAAATGTCT TAGTTGAGTT TGGTTATTCA ATCCGAAATT TGATCCTGCT   
  
  
+ TAATGTATAG TATATAATAC TAATACGCAT GTAACACAAC ACCTCAGATG GTCATTGTTT TACAATTTTT   
  
  
+ TTGAGTTTAG TATAAAAATA AAATCAATTA TTTTTAGAAG TGATGTTTAA TTTGCATTTT TTTTCCACCT   
  
  
+ AAAATCACGC ACTCGAACAT TAGGGGTTTG GCTAACTCAA AGACTCAAGG TAGTGACTTA TCCAAACTTA   
  
  
+ TTACATCCTA TAGATCTATA AAAACATAAA TCTCTTACAA TCAATCCAAA TTGATTCGGT CTAATTTGAT   
  
  
+ GACCCTGATG AGGGTCCTAC AATTACACAA GTGGGCCTTA GCCCCATGGC CGTTTTTTAA TCTTCATGGG   
  
  
+ TCCAGGCGGA GTTACCATCT CCAGGTCCAT TGAGGGTGTA TCACGTCTCA AATTACGAGT TTGCCTCTTT   
  
  
+ CTACGGGGAT TATAAATATC TACCTGCATA CCTCGAAAAG GAGGAGTTGA GATCAGCGAA ACTTGGCCAT   
  
  
+ CCCAGAGCAT TGAAATGCTC AGAACCCTTT TTCTAGAAAA ATATCGTTTT TCCTGAAAAT ATCCGACCTT   
  
  
+ ATGTCATTTA TTGTCGCTTT CTTGACTCTA ACCCGGGACT CCACACTAAC TTGACTATCG GAAAGGCGTT   
  
  
+ CCCTGGATCA CCATCCAAGA TAACCTCTTT TGCAGGGTTG ACGCTCGTTG GAAGAATCGT CGAATGTAGA   
  
  
+ GCCCCTATCA TCACATAAGC CCGCTCTTAC TTCATGCCAT CCTTGAATAG CAGTTTATCC CGAAGCAAAA   
  
  
+ ATAATTCTAA CTTTCTTATA TTGTAAACCC ACATCTAAAT TTAAATTCAT ACTCCAAAAT TCCGCCACGC   
  
  
+ AAATAAGGCT TTTTAATAGG CTTACATATA ATGGCATGCT ATAGAGACAC TGTTTTGAAG AAACTATTTA   
  
  
+ AACATCAGAC CTCATATTGA GTAGCAAATC AAGACTTCTT TCCTCCAGGC CATATTGAGT AGCAAATTAA   
  
  
+ GAATAACCAG AAGCTTGTGA AAAGAGTTAG AAAATAAAAG GTATAATAGA GACTTATGTT CTTAAGATAT   
  
  
+ CTCCTAGATA TGAATGTGAC CTCCTAGTCG ATATTTGTGC TGGATTAGCG AGTTGTGTGA CTGAGTTTGT   
  
  
+ ATGTGTCTCT TAGCATATGA GTTATTCTAG CTGCTTCTTT AAGCTATGTA GTGACTGTCT TCCTTCTTAT   
  
  
+ TTATAAGTGC TATGTCCAAC TAATAGCCGA AAAAAATGGT TCGCCTTTTC TGGTTTTCTT CTTTTTGAAG   
  
  
+ GTTGATTGGC TAATTTATAT AATGGTTTCA ATACTTATCC TTTATTGTTC CACAAATCCA CCTAACCACG   
  
  
+ TACCATTGAC AATAATTACC CACTAATTTC GGTTGGACTT GATTTCCTTT TGTTTGGTAC CTCAGGCATC   
  
  
+ TTCTGTGGAC CTTCATTCAC AGTTTAATTT TCACATTTTT ATAAGGCTGC TCTTGTGTTG CAAATTTTCC   
  
  
+ TATCCATTCC ACTCTTTGCC CATTCCCAGG CTAATTAGTG TTCTCTGTTC AACCATGGAT TCTGTGCTGG   
  
  
+ TTGATCCTGA GTTCATGAAA AATCTCTACA AATTCAAACC TGAATTGCTC TCAAACTTTT CAATGAATCC   
  
  
+ AAATGACGAC ATCTTTCAAG CCCTTCATTC AGAAAATGAT CCTTTACAGT TCCTCTCATT TGATGAAGGA   
  
  
+ ACCTGTCTTA ACAGCTGCAC TAGTCAACAA GTGCCAGATT TCCCTGATGC TTGTCTCAAG TTCATCAGTG   
  
  
+ ATATTCTTCT CGAAGAGGGT TTAGATGCAA ATCCTGCATC TGCACAGGCT CTCGAAGCCA CCGAGAAGTC   
  
  
+ CTTGTATGAT GCTCTCGGGC TTGGAGAGCC ATACCCCCTT TCATGTGATC ACTTTGCGCC ATCTATCTCT   
  
  
+ ACAAGTATTG AGAGCCCAGA TGACAGTTCT TCCAATAAAA GTTATAGCAG CAATCCCGAG ATAGATGGTT   
  
  
+ CTTATGCTAT CGCTGAGCCC AGTTTCGAGT CCAACCCCAA CTGTGTGCTT GATCAACCCC AGTTGAACTC   
  
  
+ CTTTCCAGCT CTACATGAGA TTTCTCGGTC CTTGGTGGAA CTGGGTTCTC AAGCCTCTGA GTTGAGCTTC   
  
  
+ GATGATGCAG GGAGTGCCCG TGTAGAGAAA AAGGGCAAAT CGATAAAGGG CTCGAGGAGG AAGAAGAGTC   
  
  
+ GTCAAAGAGA GGGTGAAGCG TGTTATGGAG GAAGGAGCCA TAAGGTTCAA GCTTCCTTCA ATGATGATTA   
  
  
+ CTATGAGATG GAACAGTATG ATGATGTAGT ATTGCTCTGT AATAATGAAC TAACGGGCAA TAGCCGTTTC   
  
  
+ AACACCGGGA AATCTTCACC TGAGGAGGGA TGGAGGAGAT TGCAGAGAAG CCGAGGAAAG AAGCAGAACA   
  
  
+ GTTTAGCAGT TGAAGTTGAT CTGATGACCC TGCTGACTCA GTGTGCACAA GCTGTATCGA GCTTTGATCT   
  
  
+ TCGAGGTGCA AACGAGCTAC TTAGGCAAAT CAGGCAGAAT GCTTCGCCCT ATGGTGGCAG CATCCAGAGG   
  
  
+ CTCGCCCATC ATGTAGCCAA TGCTCTCGAG GCACGTATAG CTGGCACAGG CTCTACAGTC TCTACTAACC   
  
  
+ TTGTTGATGC AAAGTTCTCA GCTTCTGACT TCCTAAAGGC TTACAGGTTA TATGTCTCAG CTGTTCCTTA   
  
  
+ CAAAAGGATG TCTTTCTTTC TTGCTAACTG CTCGATTGCA AAGTTGGCAG AGAAAGCAAC AAAGATCCAT   
  
  
+ ATCATTGATT TTGGTGTTTT CCTAGGTTTA CAATGGCCTT GTTTCATACA ACACCTATCA AAAAGGCCAA   
  
  
+ ATGGACCCCC AAAACTCCGA ATCACAGGAA TCGACTACCC CCAGCAGGGT TTCAGACCTG CACAAAGGGT   
  
  
+ TGAAGCTACA GGACACCGAT TATCTGGGTA CTGTGAGCGA TTTGGGGTGC CTTTTTCTTA TCAGGGCATT   
  
  
+ GCTCAGAAGT GGGAAACTAT TCAGCCGGAG GATCTCAAGA TCGAACAAGA CGAGCTGGTG ATTGTCAACT   
  
  
+ GTTTGTTCAG GTCAGGAACA CTGCTCGATG AGACAGTCGA AGCAAACAGT CCAAGAGATG CTTTCTTAGC   
  
  
+ TTTGGTTAGA AAGCTGAATC CCAGCCTATT CATTCACGGG GTTGTCAATG GCACATTCAA CGCTCCATTC   
  
  
+ TTCGTGACTC GATTCAGAGA GGCATTGTTT CATTATTCAT CAGTGTTTGA TGTGTCTGAA GAGACAATTC   
  
  
+ CACGAGATGC CCATGAGAGG TTCTTGATTG AGAGCGAGAT TTGTGGGAAA GAACTGTTCA ATGTGGTTGC   
  
  
+ TTGTGAGGGT GCAGAGAGGG TTCAAAGGCC TGAGACATAC AAGCAGTGGC AAGTGAGGAC AACGCGGGCC   
  
  
+ GGGTTAAGGC AGGTTGCCTT GGACCAGGAG CTTATGAAGG AAGCAACGGC AATGGTGAAG GCAAATTATC   
  
  
+ ATAAGGATTT TATGGTGGAT ATAAATAGGC ATTGGATGCT TCAAGGTTGG AAGGGTAGAA CCTTGTGTGC   
  
  
+ TCTCTCATTT TGGCAACCTG CCTG  

- +Up\_Stream \_Len000GATCCC CAACTCTCTT AGGCTATCGA TACGTCAGGT TTGTAATAGA GCAGTTCGTA   
  
  
- ATGATAAATT TATTAATTAT AACTTGAACT ATCAATGTGA AAAACTTATG ATTAAGGTGT GGAGTAACGG   
  
  
- AAATAAAGGT TTGGAGCCCA ACGTAAACCT GGTGTATGGT CCTTCGAACC TGCTGTTTTT AAAGAAAAGA   
  
  
- ACTCATAATA AAACAAAATA ATAATAATAA AAAATGAAAT AAACCTGGTG AAGTGAACCC ATTGTTCTAA   
  
  
- CTTATAAACT GATAATATAA TATACAGTGC ATACCTATAC CATCAACGAA TCTCGAAACT GGCAAACGTC   
  
  
- CAAATTCATA CTAAGGGAAC CTTTCCTCGG GGAGAAATAG AATTAAGTCT TCATTGAAAA CAAAAAAATC   
  
  
- TATTTAACTT TTCTTTTTAT TATTTACAGA ATCAACTCAA ACCAATAAGT TAGGCTTTAA ACTAGGACGA   
  
  
- ATTACATATC ATATATTATG ATTATGCGTA CATTGTGTTG TGGAGTCTAC CAGTAACAAA ATGTTAAAAA   
  
  
- AACTCAAATC ATATTTTTAT TTTAGTTAAT AAAAATCTTC ACTACAAATT AAACGTAAAA AAAAGGTGGA   
  
  
- TTTTAGTGCG TGAGCTTGTA ATCCCCAAAC CGATTGAGTT TCTGAGTTCC ATCACTGAAT AGGTTTGAAT   
  
  
- AATGTAGGAT ATCTAGATAT TTTTGTATTT AGAGAATGTT AGTTAGGTTT AACTAAGCCA GATTAAACTA   
  
  
- CTGGGACTAC TCCCAGGATG TTAATGTGTT CACCCGGAAT CGGGGTACCG GCAAAAAATT AGAAGTACCC   
  
  
- AGGTCCGCCT CAATGGTAGA GGTCCAGGTA ACTCCCACAT AGTGCAGAGT TTAATGCTCA AACGGAGAAA   
  
  
- GATGCCCCTA ATATTTATAG ATGGACGTAT GGAGCTTTTC CTCCTCAACT CTAGTCGCTT TGAACCGGTA   
  
  
- GGGTCTCGTA ACTTTACGAG TCTTGGGAAA AAGATCTTTT TATAGCAAAA AGGACTTTTA TAGGCTGGAA   
  
  
- TACAGTAAAT AACAGCGAAA GAACTGAGAT TGGGCCCTGA GGTGTGATTG AACTGATAGC CTTTCCGCAA   
  
  
- GGGACCTAGT GGTAGGTTCT ATTGGAGAAA ACGTCCCAAC TGCGAGCAAC CTTCTTAGCA GCTTACATCT   
  
  
- CGGGGATAGT AGTGTATTCG GGCGAGAATG AAGTACGGTA GGAACTTATC GTCAAATAGG GCTTCGTTTT   
  
  
- TATTAAGATT GAAAGAATAT AACATTTGGG TGTAGATTTA AATTTAAGTA TGAGGTTTTA AGGCGGTGCG   
  
  
- TTTATTCCGA AAAATTATCC GAATGTATAT TACCGTACGA TATCTCTGTG ACAAAACTTC TTTGATAAAT   
  
  
- TTGTAGTCTG GAGTATAACT CATCGTTTAG TTCTGAAGAA AGGAGGTCCG GTATAACTCA TCGTTTAATT   
  
  
- CTTATTGGTC TTCGAACACT TTTCTCAATC TTTTATTTTC CATATTATCT CTGAATACAA GAATTCTATA   
  
  
- GAGGATCTAT ACTTACACTG GAGGATCAGC TATAAACACG ACCTAATCGC TCAACACACT GACTCAAACA   
  
  
- TACACAGAGA ATCGTATACT CAATAAGATC GACGAAGAAA TTCGATACAT CACTGACAGA AGGAAGAATA   
  
  
- AATATTCACG ATACAGGTTG ATTATCGGCT TTTTTTACCA AGCGGAAAAG ACCAAAAGAA GAAAAACTTC   
  
  
- CAACTAACCG ATTAAATATA TTACCAAAGT TATGAATAGG AAATAACAAG GTGTTTAGGT GGATTGGTGC   
  
  
- ATGGTAACTG TTATTAATGG GTGATTAAAG CCAACCTGAA CTAAAGGAAA ACAAACCATG GAGTCCGTAG   
  
  
- AAGACACCTG GAAGTAAGTG TCAAATTAAA AGTGTAAAAA TATTCCGACG AGAACACAAC GTTTAAAAGG   
  
  
- ATAGGTAAGG TGAGAAACGG GTAAGGGTCC GATTAATCAC AAGAGACAAG TTGGTACCTA AGACACGACC   
  
  
- AACTAGGACT CAAGTACTTT TTAGAGATGT TTAAGTTTGG ACTTAACGAG AGTTTGAAAA GTTACTTAGG   
  
  
- TTTACTGCTG TAGAAAGTTC GGGAAGTAAG TCTTTTACTA GGAAATGTCA AGGAGAGTAA ACTACTTCCT   
  
  
- TGGACAGAAT TGTCGACGTG ATCAGTTGTT CACGGTCTAA AGGGACTACG AACAGAGTTC AAGTAGTCAC   
  
  
- TATAAGAAGA GCTTCTCCCA AATCTACGTT TAGGACGTAG ACGTGTCCGA GAGCTTCGGT GGCTCTTCAG   
  
  
- GAACATACTA CGAGAGCCCG AACCTCTCGG TATGGGGGAA AGTACACTAG TGAAACGCGG TAGATAGAGA   
  
  
- TGTTCATAAC TCTCGGGTCT ACTGTCAAGA AGGTTATTTT CAATATCGTC GTTAGGGCTC TATCTACCAA   
  
  
- GAATACGATA GCGACTCGGG TCAAAGCTCA GGTTGGGGTT GACACACGAA CTAGTTGGGG TCAACTTGAG   
  
  
- GAAAGGTCGA GATGTACTCT AAAGAGCCAG GAACCACCTT GACCCAAGAG TTCGGAGACT CAACTCGAAG   
  
  
- CTACTACGTC CCTCACGGGC ACATCTCTTT TTCCCGTTTA GCTATTTCCC GAGCTCCTCC TTCTTCTCAG   
  
  
- CAGTTTCTCT CCCACTTCGC ACAATACCTC CTTCCTCGGT ATTCCAAGTT CGAAGGAAGT TACTACTAAT   
  
  
- GATACTCTAC CTTGTCATAC TACTACATCA TAACGAGACA TTATTACTTG ATTGCCCGTT ATCGGCAAAG   
  
  
- TTGTGGCCCT TTAGAAGTGG ACTCCTCCCT ACCTCCTCTA ACGTCTCTTC GGCTCCTTTC TTCGTCTTGT   
  
  
- CAAATCGTCA ACTTCAACTA GACTACTGGG ACGACTGAGT CACACGTGTT CGACATAGCT CGAAACTAGA   
  
  
- AGCTCCACGT TTGCTCGATG AATCCGTTTA GTCCGTCTTA CGAAGCGGGA TACCACCGTC GTAGGTCTCC   
  
  
- GAGCGGGTAG TACATCGGTT ACGAGAGCTC CGTGCATATC GACCGTGTCC GAGATGTCAG AGATGATTGG   
  
  
- AACAACTACG TTTCAAGAGT CGAAGACTGA AGGATTTCCG AATGTCCAAT ATACAGAGTC GACAAGGAAT   
  
  
- GTTTTCCTAC AGAAAGAAAG AACGATTGAC GAGCTAACGT TTCAACCGTC TCTTTCGTTG TTTCTAGGTA   
  
  
- TAGTAACTAA AACCACAAAA GGATCCAAAT GTTACCGGAA CAAAGTATGT TGTGGATAGT TTTTCCGGTT   
  
  
- TACCTGGGGG TTTTGAGGCT TAGTGTCCTT AGCTGATGGG GGTCGTCCCA AAGTCTGGAC GTGTTTCCCA   
  
  
- ACTTCGATGT CCTGTGGCTA ATAGACCCAT GACACTCGCT AAACCCCACG GAAAAAGAAT AGTCCCGTAA   
  
  
- CGAGTCTTCA CCCTTTGATA AGTCGGCCTC CTAGAGTTCT AGCTTGTTCT GCTCGACCAC TAACAGTTGA   
  
  
- CAAACAAGTC CAGTCCTTGT GACGAGCTAC TCTGTCAGCT TCGTTTGTCA GGTTCTCTAC GAAAGAATCG   
  
  
- AAACCAATCT TTCGACTTAG GGTCGGATAA GTAAGTGCCC CAACAGTTAC CGTGTAAGTT GCGAGGTAAG   
  
  
- AAGCACTGAG CTAAGTCTCT CCGTAACAAA GTAATAAGTA GTCACAAACT ACACAGACTT CTCTGTTAAG   
  
  
- GTGCTCTACG GGTACTCTCC AAGAACTAAC TCTCGCTCTA AACACCCTTT CTTGACAAGT TACACCAACG   
  
  
- AACACTCCCA CGTCTCTCCC AAGTTTCCGG ACTCTGTATG TTCGTCACCG TTCACTCCTG TTGCGCCCGG   
  
  
- CCCAATTCCG TCCAACGGAA CCTGGTCCTC GAATACTTCC TTCGTTGCCG TTACCACTTC CGTTTAATAG   
  
  
- TATTCCTAAA ATACCACCTA TATTTATCCG TAACCTACGA AGTTCCAACC TTCCCATCTT GGAACACACG   
  
  
- AGAGAGTAAA ACCGTTGGAC GGAC

+     ACE

| Site Name | Organism | Position | Strand | Matrix score. | sequence | function |
| --- | --- | --- | --- | --- | --- | --- |
| ACE | Petroselinum crispum | 309 | + | 9 | GACACGTATG | cis-acting element involved in light responsiveness |

>HU02G01571.1   
+ +Up\_Stream \_Len000CTAGGG GTTGAGAGAA TCCGATAGCT ATGCAGTCCA AACATTATCT CGTCAAGCAT   
  
  
+ TACTATTTAA ATAATTAATA TTGAACTTGA TAGTTACACT TTTTGAATAC TAATTCCACA CCTCATTGCC   
  
  
+ TTTATTTCCA AACCTCGGGT TGCATTTGGA CCACATACCA GGAAGCTTGG ACGACAAAAA TTTCTTTTCT   
  
  
+ TGAGTATTAT TTTGTTTTAT TATTATTATT TTTTACTTTA TTTGGACCAC TTCACTTGGG TAACAAGATT   
  
  
+ GAATATTTGA CTATTATATT ATATGTCACG TATGGATATG GTAGTTGCTT AGAGCTTTGA CCGTTTGCAG   
  
  
+ GTTTAAGTAT GATTCCCTTG GAAAGGAGCC CCTCTTTATC TTAATTCAGA AGTAACTTTT GTTTTTTTAG   
  
  
+ ATAAATTGAA AAGAAAAATA ATAAATGTCT TAGTTGAGTT TGGTTATTCA ATCCGAAATT TGATCCTGCT   
  
  
+ TAATGTATAG TATATAATAC TAATACGCAT GTAACACAAC ACCTCAGATG GTCATTGTTT TACAATTTTT   
  
  
+ TTGAGTTTAG TATAAAAATA AAATCAATTA TTTTTAGAAG TGATGTTTAA TTTGCATTTT TTTTCCACCT   
  
  
+ AAAATCACGC ACTCGAACAT TAGGGGTTTG GCTAACTCAA AGACTCAAGG TAGTGACTTA TCCAAACTTA   
  
  
+ TTACATCCTA TAGATCTATA AAAACATAAA TCTCTTACAA TCAATCCAAA TTGATTCGGT CTAATTTGAT   
  
  
+ GACCCTGATG AGGGTCCTAC AATTACACAA GTGGGCCTTA GCCCCATGGC CGTTTTTTAA TCTTCATGGG   
  
  
+ TCCAGGCGGA GTTACCATCT CCAGGTCCAT TGAGGGTGTA TCACGTCTCA AATTACGAGT TTGCCTCTTT   
  
  
+ CTACGGGGAT TATAAATATC TACCTGCATA CCTCGAAAAG GAGGAGTTGA GATCAGCGAA ACTTGGCCAT   
  
  
+ CCCAGAGCAT TGAAATGCTC AGAACCCTTT TTCTAGAAAA ATATCGTTTT TCCTGAAAAT ATCCGACCTT   
  
  
+ ATGTCATTTA TTGTCGCTTT CTTGACTCTA ACCCGGGACT CCACACTAAC TTGACTATCG GAAAGGCGTT   
  
  
+ CCCTGGATCA CCATCCAAGA TAACCTCTTT TGCAGGGTTG ACGCTCGTTG GAAGAATCGT CGAATGTAGA   
  
  
+ GCCCCTATCA TCACATAAGC CCGCTCTTAC TTCATGCCAT CCTTGAATAG CAGTTTATCC CGAAGCAAAA   
  
  
+ ATAATTCTAA CTTTCTTATA TTGTAAACCC ACATCTAAAT TTAAATTCAT ACTCCAAAAT TCCGCCACGC   
  
  
+ AAATAAGGCT TTTTAATAGG CTTACATATA ATGGCATGCT ATAGAGACAC TGTTTTGAAG AAACTATTTA   
  
  
+ AACATCAGAC CTCATATTGA GTAGCAAATC AAGACTTCTT TCCTCCAGGC CATATTGAGT AGCAAATTAA   
  
  
+ GAATAACCAG AAGCTTGTGA AAAGAGTTAG AAAATAAAAG GTATAATAGA GACTTATGTT CTTAAGATAT   
  
  
+ CTCCTAGATA TGAATGTGAC CTCCTAGTCG ATATTTGTGC TGGATTAGCG AGTTGTGTGA CTGAGTTTGT   
  
  
+ ATGTGTCTCT TAGCATATGA GTTATTCTAG CTGCTTCTTT AAGCTATGTA GTGACTGTCT TCCTTCTTAT   
  
  
+ TTATAAGTGC TATGTCCAAC TAATAGCCGA AAAAAATGGT TCGCCTTTTC TGGTTTTCTT CTTTTTGAAG   
  
  
+ GTTGATTGGC TAATTTATAT AATGGTTTCA ATACTTATCC TTTATTGTTC CACAAATCCA CCTAACCACG   
  
  
+ TACCATTGAC AATAATTACC CACTAATTTC GGTTGGACTT GATTTCCTTT TGTTTGGTAC CTCAGGCATC   
  
  
+ TTCTGTGGAC CTTCATTCAC AGTTTAATTT TCACATTTTT ATAAGGCTGC TCTTGTGTTG CAAATTTTCC   
  
  
+ TATCCATTCC ACTCTTTGCC CATTCCCAGG CTAATTAGTG TTCTCTGTTC AACCATGGAT TCTGTGCTGG   
  
  
+ TTGATCCTGA GTTCATGAAA AATCTCTACA AATTCAAACC TGAATTGCTC TCAAACTTTT CAATGAATCC   
  
  
+ AAATGACGAC ATCTTTCAAG CCCTTCATTC AGAAAATGAT CCTTTACAGT TCCTCTCATT TGATGAAGGA   
  
  
+ ACCTGTCTTA ACAGCTGCAC TAGTCAACAA GTGCCAGATT TCCCTGATGC TTGTCTCAAG TTCATCAGTG   
  
  
+ ATATTCTTCT CGAAGAGGGT TTAGATGCAA ATCCTGCATC TGCACAGGCT CTCGAAGCCA CCGAGAAGTC   
  
  
+ CTTGTATGAT GCTCTCGGGC TTGGAGAGCC ATACCCCCTT TCATGTGATC ACTTTGCGCC ATCTATCTCT   
  
  
+ ACAAGTATTG AGAGCCCAGA TGACAGTTCT TCCAATAAAA GTTATAGCAG CAATCCCGAG ATAGATGGTT   
  
  
+ CTTATGCTAT CGCTGAGCCC AGTTTCGAGT CCAACCCCAA CTGTGTGCTT GATCAACCCC AGTTGAACTC   
  
  
+ CTTTCCAGCT CTACATGAGA TTTCTCGGTC CTTGGTGGAA CTGGGTTCTC AAGCCTCTGA GTTGAGCTTC   
  
  
+ GATGATGCAG GGAGTGCCCG TGTAGAGAAA AAGGGCAAAT CGATAAAGGG CTCGAGGAGG AAGAAGAGTC   
  
  
+ GTCAAAGAGA GGGTGAAGCG TGTTATGGAG GAAGGAGCCA TAAGGTTCAA GCTTCCTTCA ATGATGATTA   
  
  
+ CTATGAGATG GAACAGTATG ATGATGTAGT ATTGCTCTGT AATAATGAAC TAACGGGCAA TAGCCGTTTC   
  
  
+ AACACCGGGA AATCTTCACC TGAGGAGGGA TGGAGGAGAT TGCAGAGAAG CCGAGGAAAG AAGCAGAACA   
  
  
+ GTTTAGCAGT TGAAGTTGAT CTGATGACCC TGCTGACTCA GTGTGCACAA GCTGTATCGA GCTTTGATCT   
  
  
+ TCGAGGTGCA AACGAGCTAC TTAGGCAAAT CAGGCAGAAT GCTTCGCCCT ATGGTGGCAG CATCCAGAGG   
  
  
+ CTCGCCCATC ATGTAGCCAA TGCTCTCGAG GCACGTATAG CTGGCACAGG CTCTACAGTC TCTACTAACC   
  
  
+ TTGTTGATGC AAAGTTCTCA GCTTCTGACT TCCTAAAGGC TTACAGGTTA TATGTCTCAG CTGTTCCTTA   
  
  
+ CAAAAGGATG TCTTTCTTTC TTGCTAACTG CTCGATTGCA AAGTTGGCAG AGAAAGCAAC AAAGATCCAT   
  
  
+ ATCATTGATT TTGGTGTTTT CCTAGGTTTA CAATGGCCTT GTTTCATACA ACACCTATCA AAAAGGCCAA   
  
  
+ ATGGACCCCC AAAACTCCGA ATCACAGGAA TCGACTACCC CCAGCAGGGT TTCAGACCTG CACAAAGGGT   
  
  
+ TGAAGCTACA GGACACCGAT TATCTGGGTA CTGTGAGCGA TTTGGGGTGC CTTTTTCTTA TCAGGGCATT   
  
  
+ GCTCAGAAGT GGGAAACTAT TCAGCCGGAG GATCTCAAGA TCGAACAAGA CGAGCTGGTG ATTGTCAACT   
  
  
+ GTTTGTTCAG GTCAGGAACA CTGCTCGATG AGACAGTCGA AGCAAACAGT CCAAGAGATG CTTTCTTAGC   
  
  
+ TTTGGTTAGA AAGCTGAATC CCAGCCTATT CATTCACGGG GTTGTCAATG GCACATTCAA CGCTCCATTC   
  
  
+ TTCGTGACTC GATTCAGAGA GGCATTGTTT CATTATTCAT CAGTGTTTGA TGTGTCTGAA GAGACAATTC   
  
  
+ CACGAGATGC CCATGAGAGG TTCTTGATTG AGAGCGAGAT TTGTGGGAAA GAACTGTTCA ATGTGGTTGC   
  
  
+ TTGTGAGGGT GCAGAGAGGG TTCAAAGGCC TGAGACATAC AAGCAGTGGC AAGTGAGGAC AACGCGGGCC   
  
  
+ GGGTTAAGGC AGGTTGCCTT GGACCAGGAG CTTATGAAGG AAGCAACGGC AATGGTGAAG GCAAATTATC   
  
  
+ ATAAGGATTT TATGGTGGAT ATAAATAGGC ATTGGATGCT TCAAGGTTGG AAGGGTAGAA CCTTGTGTGC   
  
  
+ TCTCTCATTT TGGCAACCTG CCTG  

- +Up\_Stream \_Len000GATCCC CAACTCTCTT AGGCTATCGA TACGTCAGGT TTGTAATAGA GCAGTTCGTA   
  
  
- ATGATAAATT TATTAATTAT AACTTGAACT ATCAATGTGA AAAACTTATG ATTAAGGTGT GGAGTAACGG   
  
  
- AAATAAAGGT TTGGAGCCCA ACGTAAACCT GGTGTATGGT CCTTCGAACC TGCTGTTTTT AAAGAAAAGA   
  
  
- ACTCATAATA AAACAAAATA ATAATAATAA AAAATGAAAT AAACCTGGTG AAGTGAACCC ATTGTTCTAA   
  
  
- CTTATAAACT GATAATATAA TATACAGTGC ATACCTATAC CATCAACGAA TCTCGAAACT GGCAAACGTC   
  
  
- CAAATTCATA CTAAGGGAAC CTTTCCTCGG GGAGAAATAG AATTAAGTCT TCATTGAAAA CAAAAAAATC   
  
  
- TATTTAACTT TTCTTTTTAT TATTTACAGA ATCAACTCAA ACCAATAAGT TAGGCTTTAA ACTAGGACGA   
  
  
- ATTACATATC ATATATTATG ATTATGCGTA CATTGTGTTG TGGAGTCTAC CAGTAACAAA ATGTTAAAAA   
  
  
- AACTCAAATC ATATTTTTAT TTTAGTTAAT AAAAATCTTC ACTACAAATT AAACGTAAAA AAAAGGTGGA   
  
  
- TTTTAGTGCG TGAGCTTGTA ATCCCCAAAC CGATTGAGTT TCTGAGTTCC ATCACTGAAT AGGTTTGAAT   
  
  
- AATGTAGGAT ATCTAGATAT TTTTGTATTT AGAGAATGTT AGTTAGGTTT AACTAAGCCA GATTAAACTA   
  
  
- CTGGGACTAC TCCCAGGATG TTAATGTGTT CACCCGGAAT CGGGGTACCG GCAAAAAATT AGAAGTACCC   
  
  
- AGGTCCGCCT CAATGGTAGA GGTCCAGGTA ACTCCCACAT AGTGCAGAGT TTAATGCTCA AACGGAGAAA   
  
  
- GATGCCCCTA ATATTTATAG ATGGACGTAT GGAGCTTTTC CTCCTCAACT CTAGTCGCTT TGAACCGGTA   
  
  
- GGGTCTCGTA ACTTTACGAG TCTTGGGAAA AAGATCTTTT TATAGCAAAA AGGACTTTTA TAGGCTGGAA   
  
  
- TACAGTAAAT AACAGCGAAA GAACTGAGAT TGGGCCCTGA GGTGTGATTG AACTGATAGC CTTTCCGCAA   
  
  
- GGGACCTAGT GGTAGGTTCT ATTGGAGAAA ACGTCCCAAC TGCGAGCAAC CTTCTTAGCA GCTTACATCT   
  
  
- CGGGGATAGT AGTGTATTCG GGCGAGAATG AAGTACGGTA GGAACTTATC GTCAAATAGG GCTTCGTTTT   
  
  
- TATTAAGATT GAAAGAATAT AACATTTGGG TGTAGATTTA AATTTAAGTA TGAGGTTTTA AGGCGGTGCG   
  
  
- TTTATTCCGA AAAATTATCC GAATGTATAT TACCGTACGA TATCTCTGTG ACAAAACTTC TTTGATAAAT   
  
  
- TTGTAGTCTG GAGTATAACT CATCGTTTAG TTCTGAAGAA AGGAGGTCCG GTATAACTCA TCGTTTAATT   
  
  
- CTTATTGGTC TTCGAACACT TTTCTCAATC TTTTATTTTC CATATTATCT CTGAATACAA GAATTCTATA   
  
  
- GAGGATCTAT ACTTACACTG GAGGATCAGC TATAAACACG ACCTAATCGC TCAACACACT GACTCAAACA   
  
  
- TACACAGAGA ATCGTATACT CAATAAGATC GACGAAGAAA TTCGATACAT CACTGACAGA AGGAAGAATA   
  
  
- AATATTCACG ATACAGGTTG ATTATCGGCT TTTTTTACCA AGCGGAAAAG ACCAAAAGAA GAAAAACTTC   
  
  
- CAACTAACCG ATTAAATATA TTACCAAAGT TATGAATAGG AAATAACAAG GTGTTTAGGT GGATTGGTGC   
  
  
- ATGGTAACTG TTATTAATGG GTGATTAAAG CCAACCTGAA CTAAAGGAAA ACAAACCATG GAGTCCGTAG   
  
  
- AAGACACCTG GAAGTAAGTG TCAAATTAAA AGTGTAAAAA TATTCCGACG AGAACACAAC GTTTAAAAGG   
  
  
- ATAGGTAAGG TGAGAAACGG GTAAGGGTCC GATTAATCAC AAGAGACAAG TTGGTACCTA AGACACGACC   
  
  
- AACTAGGACT CAAGTACTTT TTAGAGATGT TTAAGTTTGG ACTTAACGAG AGTTTGAAAA GTTACTTAGG   
  
  
- TTTACTGCTG TAGAAAGTTC GGGAAGTAAG TCTTTTACTA GGAAATGTCA AGGAGAGTAA ACTACTTCCT   
  
  
- TGGACAGAAT TGTCGACGTG ATCAGTTGTT CACGGTCTAA AGGGACTACG AACAGAGTTC AAGTAGTCAC   
  
  
- TATAAGAAGA GCTTCTCCCA AATCTACGTT TAGGACGTAG ACGTGTCCGA GAGCTTCGGT GGCTCTTCAG   
  
  
- GAACATACTA CGAGAGCCCG AACCTCTCGG TATGGGGGAA AGTACACTAG TGAAACGCGG TAGATAGAGA   
  
  
- TGTTCATAAC TCTCGGGTCT ACTGTCAAGA AGGTTATTTT CAATATCGTC GTTAGGGCTC TATCTACCAA   
  
  
- GAATACGATA GCGACTCGGG TCAAAGCTCA GGTTGGGGTT GACACACGAA CTAGTTGGGG TCAACTTGAG   
  
  
- GAAAGGTCGA GATGTACTCT AAAGAGCCAG GAACCACCTT GACCCAAGAG TTCGGAGACT CAACTCGAAG   
  
  
- CTACTACGTC CCTCACGGGC ACATCTCTTT TTCCCGTTTA GCTATTTCCC GAGCTCCTCC TTCTTCTCAG   
  
  
- CAGTTTCTCT CCCACTTCGC ACAATACCTC CTTCCTCGGT ATTCCAAGTT CGAAGGAAGT TACTACTAAT   
  
  
- GATACTCTAC CTTGTCATAC TACTACATCA TAACGAGACA TTATTACTTG ATTGCCCGTT ATCGGCAAAG   
  
  
- TTGTGGCCCT TTAGAAGTGG ACTCCTCCCT ACCTCCTCTA ACGTCTCTTC GGCTCCTTTC TTCGTCTTGT   
  
  
- CAAATCGTCA ACTTCAACTA GACTACTGGG ACGACTGAGT CACACGTGTT CGACATAGCT CGAAACTAGA   
  
  
- AGCTCCACGT TTGCTCGATG AATCCGTTTA GTCCGTCTTA CGAAGCGGGA TACCACCGTC GTAGGTCTCC   
  
  
- GAGCGGGTAG TACATCGGTT ACGAGAGCTC CGTGCATATC GACCGTGTCC GAGATGTCAG AGATGATTGG   
  
  
- AACAACTACG TTTCAAGAGT CGAAGACTGA AGGATTTCCG AATGTCCAAT ATACAGAGTC GACAAGGAAT   
  
  
- GTTTTCCTAC AGAAAGAAAG AACGATTGAC GAGCTAACGT TTCAACCGTC TCTTTCGTTG TTTCTAGGTA   
  
  
- TAGTAACTAA AACCACAAAA GGATCCAAAT GTTACCGGAA CAAAGTATGT TGTGGATAGT TTTTCCGGTT   
  
  
- TACCTGGGGG TTTTGAGGCT TAGTGTCCTT AGCTGATGGG GGTCGTCCCA AAGTCTGGAC GTGTTTCCCA   
  
  
- ACTTCGATGT CCTGTGGCTA ATAGACCCAT GACACTCGCT AAACCCCACG GAAAAAGAAT AGTCCCGTAA   
  
  
- CGAGTCTTCA CCCTTTGATA AGTCGGCCTC CTAGAGTTCT AGCTTGTTCT GCTCGACCAC TAACAGTTGA   
  
  
- CAAACAAGTC CAGTCCTTGT GACGAGCTAC TCTGTCAGCT TCGTTTGTCA GGTTCTCTAC GAAAGAATCG   
  
  
- AAACCAATCT TTCGACTTAG GGTCGGATAA GTAAGTGCCC CAACAGTTAC CGTGTAAGTT GCGAGGTAAG   
  
  
- AAGCACTGAG CTAAGTCTCT CCGTAACAAA GTAATAAGTA GTCACAAACT ACACAGACTT CTCTGTTAAG   
  
  
- GTGCTCTACG GGTACTCTCC AAGAACTAAC TCTCGCTCTA AACACCCTTT CTTGACAAGT TACACCAACG   
  
  
- AACACTCCCA CGTCTCTCCC AAGTTTCCGG ACTCTGTATG TTCGTCACCG TTCACTCCTG TTGCGCCCGG   
  
  
- CCCAATTCCG TCCAACGGAA CCTGGTCCTC GAATACTTCC TTCGTTGCCG TTACCACTTC CGTTTAATAG   
  
  
- TATTCCTAAA ATACCACCTA TATTTATCCG TAACCTACGA AGTTCCAACC TTCCCATCTT GGAACACACG   
  
  
- AGAGAGTAAA ACCGTTGGAC GGAC

+     AP-1

| Site Name | Organism | Position | Strand | Matrix score. | sequence | function |
| --- | --- | --- | --- | --- | --- | --- |
| AP-1 | Arabidopsis thaliana | 666 | - | 8 | TGAGTTAG |  |

>HU02G01571.1   
+ +Up\_Stream \_Len000CTAGGG GTTGAGAGAA TCCGATAGCT ATGCAGTCCA AACATTATCT CGTCAAGCAT   
  
  
+ TACTATTTAA ATAATTAATA TTGAACTTGA TAGTTACACT TTTTGAATAC TAATTCCACA CCTCATTGCC   
  
  
+ TTTATTTCCA AACCTCGGGT TGCATTTGGA CCACATACCA GGAAGCTTGG ACGACAAAAA TTTCTTTTCT   
  
  
+ TGAGTATTAT TTTGTTTTAT TATTATTATT TTTTACTTTA TTTGGACCAC TTCACTTGGG TAACAAGATT   
  
  
+ GAATATTTGA CTATTATATT ATATGTCACG TATGGATATG GTAGTTGCTT AGAGCTTTGA CCGTTTGCAG   
  
  
+ GTTTAAGTAT GATTCCCTTG GAAAGGAGCC CCTCTTTATC TTAATTCAGA AGTAACTTTT GTTTTTTTAG   
  
  
+ ATAAATTGAA AAGAAAAATA ATAAATGTCT TAGTTGAGTT TGGTTATTCA ATCCGAAATT TGATCCTGCT   
  
  
+ TAATGTATAG TATATAATAC TAATACGCAT GTAACACAAC ACCTCAGATG GTCATTGTTT TACAATTTTT   
  
  
+ TTGAGTTTAG TATAAAAATA AAATCAATTA TTTTTAGAAG TGATGTTTAA TTTGCATTTT TTTTCCACCT   
  
  
+ AAAATCACGC ACTCGAACAT TAGGGGTTTG GCTAACTCAA AGACTCAAGG TAGTGACTTA TCCAAACTTA   
  
  
+ TTACATCCTA TAGATCTATA AAAACATAAA TCTCTTACAA TCAATCCAAA TTGATTCGGT CTAATTTGAT   
  
  
+ GACCCTGATG AGGGTCCTAC AATTACACAA GTGGGCCTTA GCCCCATGGC CGTTTTTTAA TCTTCATGGG   
  
  
+ TCCAGGCGGA GTTACCATCT CCAGGTCCAT TGAGGGTGTA TCACGTCTCA AATTACGAGT TTGCCTCTTT   
  
  
+ CTACGGGGAT TATAAATATC TACCTGCATA CCTCGAAAAG GAGGAGTTGA GATCAGCGAA ACTTGGCCAT   
  
  
+ CCCAGAGCAT TGAAATGCTC AGAACCCTTT TTCTAGAAAA ATATCGTTTT TCCTGAAAAT ATCCGACCTT   
  
  
+ ATGTCATTTA TTGTCGCTTT CTTGACTCTA ACCCGGGACT CCACACTAAC TTGACTATCG GAAAGGCGTT   
  
  
+ CCCTGGATCA CCATCCAAGA TAACCTCTTT TGCAGGGTTG ACGCTCGTTG GAAGAATCGT CGAATGTAGA   
  
  
+ GCCCCTATCA TCACATAAGC CCGCTCTTAC TTCATGCCAT CCTTGAATAG CAGTTTATCC CGAAGCAAAA   
  
  
+ ATAATTCTAA CTTTCTTATA TTGTAAACCC ACATCTAAAT TTAAATTCAT ACTCCAAAAT TCCGCCACGC   
  
  
+ AAATAAGGCT TTTTAATAGG CTTACATATA ATGGCATGCT ATAGAGACAC TGTTTTGAAG AAACTATTTA   
  
  
+ AACATCAGAC CTCATATTGA GTAGCAAATC AAGACTTCTT TCCTCCAGGC CATATTGAGT AGCAAATTAA   
  
  
+ GAATAACCAG AAGCTTGTGA AAAGAGTTAG AAAATAAAAG GTATAATAGA GACTTATGTT CTTAAGATAT   
  
  
+ CTCCTAGATA TGAATGTGAC CTCCTAGTCG ATATTTGTGC TGGATTAGCG AGTTGTGTGA CTGAGTTTGT   
  
  
+ ATGTGTCTCT TAGCATATGA GTTATTCTAG CTGCTTCTTT AAGCTATGTA GTGACTGTCT TCCTTCTTAT   
  
  
+ TTATAAGTGC TATGTCCAAC TAATAGCCGA AAAAAATGGT TCGCCTTTTC TGGTTTTCTT CTTTTTGAAG   
  
  
+ GTTGATTGGC TAATTTATAT AATGGTTTCA ATACTTATCC TTTATTGTTC CACAAATCCA CCTAACCACG   
  
  
+ TACCATTGAC AATAATTACC CACTAATTTC GGTTGGACTT GATTTCCTTT TGTTTGGTAC CTCAGGCATC   
  
  
+ TTCTGTGGAC CTTCATTCAC AGTTTAATTT TCACATTTTT ATAAGGCTGC TCTTGTGTTG CAAATTTTCC   
  
  
+ TATCCATTCC ACTCTTTGCC CATTCCCAGG CTAATTAGTG TTCTCTGTTC AACCATGGAT TCTGTGCTGG   
  
  
+ TTGATCCTGA GTTCATGAAA AATCTCTACA AATTCAAACC TGAATTGCTC TCAAACTTTT CAATGAATCC   
  
  
+ AAATGACGAC ATCTTTCAAG CCCTTCATTC AGAAAATGAT CCTTTACAGT TCCTCTCATT TGATGAAGGA   
  
  
+ ACCTGTCTTA ACAGCTGCAC TAGTCAACAA GTGCCAGATT TCCCTGATGC TTGTCTCAAG TTCATCAGTG   
  
  
+ ATATTCTTCT CGAAGAGGGT TTAGATGCAA ATCCTGCATC TGCACAGGCT CTCGAAGCCA CCGAGAAGTC   
  
  
+ CTTGTATGAT GCTCTCGGGC TTGGAGAGCC ATACCCCCTT TCATGTGATC ACTTTGCGCC ATCTATCTCT   
  
  
+ ACAAGTATTG AGAGCCCAGA TGACAGTTCT TCCAATAAAA GTTATAGCAG CAATCCCGAG ATAGATGGTT   
  
  
+ CTTATGCTAT CGCTGAGCCC AGTTTCGAGT CCAACCCCAA CTGTGTGCTT GATCAACCCC AGTTGAACTC   
  
  
+ CTTTCCAGCT CTACATGAGA TTTCTCGGTC CTTGGTGGAA CTGGGTTCTC AAGCCTCTGA GTTGAGCTTC   
  
  
+ GATGATGCAG GGAGTGCCCG TGTAGAGAAA AAGGGCAAAT CGATAAAGGG CTCGAGGAGG AAGAAGAGTC   
  
  
+ GTCAAAGAGA GGGTGAAGCG TGTTATGGAG GAAGGAGCCA TAAGGTTCAA GCTTCCTTCA ATGATGATTA   
  
  
+ CTATGAGATG GAACAGTATG ATGATGTAGT ATTGCTCTGT AATAATGAAC TAACGGGCAA TAGCCGTTTC   
  
  
+ AACACCGGGA AATCTTCACC TGAGGAGGGA TGGAGGAGAT TGCAGAGAAG CCGAGGAAAG AAGCAGAACA   
  
  
+ GTTTAGCAGT TGAAGTTGAT CTGATGACCC TGCTGACTCA GTGTGCACAA GCTGTATCGA GCTTTGATCT   
  
  
+ TCGAGGTGCA AACGAGCTAC TTAGGCAAAT CAGGCAGAAT GCTTCGCCCT ATGGTGGCAG CATCCAGAGG   
  
  
+ CTCGCCCATC ATGTAGCCAA TGCTCTCGAG GCACGTATAG CTGGCACAGG CTCTACAGTC TCTACTAACC   
  
  
+ TTGTTGATGC AAAGTTCTCA GCTTCTGACT TCCTAAAGGC TTACAGGTTA TATGTCTCAG CTGTTCCTTA   
  
  
+ CAAAAGGATG TCTTTCTTTC TTGCTAACTG CTCGATTGCA AAGTTGGCAG AGAAAGCAAC AAAGATCCAT   
  
  
+ ATCATTGATT TTGGTGTTTT CCTAGGTTTA CAATGGCCTT GTTTCATACA ACACCTATCA AAAAGGCCAA   
  
  
+ ATGGACCCCC AAAACTCCGA ATCACAGGAA TCGACTACCC CCAGCAGGGT TTCAGACCTG CACAAAGGGT   
  
  
+ TGAAGCTACA GGACACCGAT TATCTGGGTA CTGTGAGCGA TTTGGGGTGC CTTTTTCTTA TCAGGGCATT   
  
  
+ GCTCAGAAGT GGGAAACTAT TCAGCCGGAG GATCTCAAGA TCGAACAAGA CGAGCTGGTG ATTGTCAACT   
  
  
+ GTTTGTTCAG GTCAGGAACA CTGCTCGATG AGACAGTCGA AGCAAACAGT CCAAGAGATG CTTTCTTAGC   
  
  
+ TTTGGTTAGA AAGCTGAATC CCAGCCTATT CATTCACGGG GTTGTCAATG GCACATTCAA CGCTCCATTC   
  
  
+ TTCGTGACTC GATTCAGAGA GGCATTGTTT CATTATTCAT CAGTGTTTGA TGTGTCTGAA GAGACAATTC   
  
  
+ CACGAGATGC CCATGAGAGG TTCTTGATTG AGAGCGAGAT TTGTGGGAAA GAACTGTTCA ATGTGGTTGC   
  
  
+ TTGTGAGGGT GCAGAGAGGG TTCAAAGGCC TGAGACATAC AAGCAGTGGC AAGTGAGGAC AACGCGGGCC   
  
  
+ GGGTTAAGGC AGGTTGCCTT GGACCAGGAG CTTATGAAGG AAGCAACGGC AATGGTGAAG GCAAATTATC   
  
  
+ ATAAGGATTT TATGGTGGAT ATAAATAGGC ATTGGATGCT TCAAGGTTGG AAGGGTAGAA CCTTGTGTGC   
  
  
+ TCTCTCATTT TGGCAACCTG CCTG  

- +Up\_Stream \_Len000GATCCC CAACTCTCTT AGGCTATCGA TACGTCAGGT TTGTAATAGA GCAGTTCGTA   
  
  
- ATGATAAATT TATTAATTAT AACTTGAACT ATCAATGTGA AAAACTTATG ATTAAGGTGT GGAGTAACGG   
  
  
- AAATAAAGGT TTGGAGCCCA ACGTAAACCT GGTGTATGGT CCTTCGAACC TGCTGTTTTT AAAGAAAAGA   
  
  
- ACTCATAATA AAACAAAATA ATAATAATAA AAAATGAAAT AAACCTGGTG AAGTGAACCC ATTGTTCTAA   
  
  
- CTTATAAACT GATAATATAA TATACAGTGC ATACCTATAC CATCAACGAA TCTCGAAACT GGCAAACGTC   
  
  
- CAAATTCATA CTAAGGGAAC CTTTCCTCGG GGAGAAATAG AATTAAGTCT TCATTGAAAA CAAAAAAATC   
  
  
- TATTTAACTT TTCTTTTTAT TATTTACAGA ATCAACTCAA ACCAATAAGT TAGGCTTTAA ACTAGGACGA   
  
  
- ATTACATATC ATATATTATG ATTATGCGTA CATTGTGTTG TGGAGTCTAC CAGTAACAAA ATGTTAAAAA   
  
  
- AACTCAAATC ATATTTTTAT TTTAGTTAAT AAAAATCTTC ACTACAAATT AAACGTAAAA AAAAGGTGGA   
  
  
- TTTTAGTGCG TGAGCTTGTA ATCCCCAAAC CGATTGAGTT TCTGAGTTCC ATCACTGAAT AGGTTTGAAT   
  
  
- AATGTAGGAT ATCTAGATAT TTTTGTATTT AGAGAATGTT AGTTAGGTTT AACTAAGCCA GATTAAACTA   
  
  
- CTGGGACTAC TCCCAGGATG TTAATGTGTT CACCCGGAAT CGGGGTACCG GCAAAAAATT AGAAGTACCC   
  
  
- AGGTCCGCCT CAATGGTAGA GGTCCAGGTA ACTCCCACAT AGTGCAGAGT TTAATGCTCA AACGGAGAAA   
  
  
- GATGCCCCTA ATATTTATAG ATGGACGTAT GGAGCTTTTC CTCCTCAACT CTAGTCGCTT TGAACCGGTA   
  
  
- GGGTCTCGTA ACTTTACGAG TCTTGGGAAA AAGATCTTTT TATAGCAAAA AGGACTTTTA TAGGCTGGAA   
  
  
- TACAGTAAAT AACAGCGAAA GAACTGAGAT TGGGCCCTGA GGTGTGATTG AACTGATAGC CTTTCCGCAA   
  
  
- GGGACCTAGT GGTAGGTTCT ATTGGAGAAA ACGTCCCAAC TGCGAGCAAC CTTCTTAGCA GCTTACATCT   
  
  
- CGGGGATAGT AGTGTATTCG GGCGAGAATG AAGTACGGTA GGAACTTATC GTCAAATAGG GCTTCGTTTT   
  
  
- TATTAAGATT GAAAGAATAT AACATTTGGG TGTAGATTTA AATTTAAGTA TGAGGTTTTA AGGCGGTGCG   
  
  
- TTTATTCCGA AAAATTATCC GAATGTATAT TACCGTACGA TATCTCTGTG ACAAAACTTC TTTGATAAAT   
  
  
- TTGTAGTCTG GAGTATAACT CATCGTTTAG TTCTGAAGAA AGGAGGTCCG GTATAACTCA TCGTTTAATT   
  
  
- CTTATTGGTC TTCGAACACT TTTCTCAATC TTTTATTTTC CATATTATCT CTGAATACAA GAATTCTATA   
  
  
- GAGGATCTAT ACTTACACTG GAGGATCAGC TATAAACACG ACCTAATCGC TCAACACACT GACTCAAACA   
  
  
- TACACAGAGA ATCGTATACT CAATAAGATC GACGAAGAAA TTCGATACAT CACTGACAGA AGGAAGAATA   
  
  
- AATATTCACG ATACAGGTTG ATTATCGGCT TTTTTTACCA AGCGGAAAAG ACCAAAAGAA GAAAAACTTC   
  
  
- CAACTAACCG ATTAAATATA TTACCAAAGT TATGAATAGG AAATAACAAG GTGTTTAGGT GGATTGGTGC   
  
  
- ATGGTAACTG TTATTAATGG GTGATTAAAG CCAACCTGAA CTAAAGGAAA ACAAACCATG GAGTCCGTAG   
  
  
- AAGACACCTG GAAGTAAGTG TCAAATTAAA AGTGTAAAAA TATTCCGACG AGAACACAAC GTTTAAAAGG   
  
  
- ATAGGTAAGG TGAGAAACGG GTAAGGGTCC GATTAATCAC AAGAGACAAG TTGGTACCTA AGACACGACC   
  
  
- AACTAGGACT CAAGTACTTT TTAGAGATGT TTAAGTTTGG ACTTAACGAG AGTTTGAAAA GTTACTTAGG   
  
  
- TTTACTGCTG TAGAAAGTTC GGGAAGTAAG TCTTTTACTA GGAAATGTCA AGGAGAGTAA ACTACTTCCT   
  
  
- TGGACAGAAT TGTCGACGTG ATCAGTTGTT CACGGTCTAA AGGGACTACG AACAGAGTTC AAGTAGTCAC   
  
  
- TATAAGAAGA GCTTCTCCCA AATCTACGTT TAGGACGTAG ACGTGTCCGA GAGCTTCGGT GGCTCTTCAG   
  
  
- GAACATACTA CGAGAGCCCG AACCTCTCGG TATGGGGGAA AGTACACTAG TGAAACGCGG TAGATAGAGA   
  
  
- TGTTCATAAC TCTCGGGTCT ACTGTCAAGA AGGTTATTTT CAATATCGTC GTTAGGGCTC TATCTACCAA   
  
  
- GAATACGATA GCGACTCGGG TCAAAGCTCA GGTTGGGGTT GACACACGAA CTAGTTGGGG TCAACTTGAG   
  
  
- GAAAGGTCGA GATGTACTCT AAAGAGCCAG GAACCACCTT GACCCAAGAG TTCGGAGACT CAACTCGAAG   
  
  
- CTACTACGTC CCTCACGGGC ACATCTCTTT TTCCCGTTTA GCTATTTCCC GAGCTCCTCC TTCTTCTCAG   
  
  
- CAGTTTCTCT CCCACTTCGC ACAATACCTC CTTCCTCGGT ATTCCAAGTT CGAAGGAAGT TACTACTAAT   
  
  
- GATACTCTAC CTTGTCATAC TACTACATCA TAACGAGACA TTATTACTTG ATTGCCCGTT ATCGGCAAAG   
  
  
- TTGTGGCCCT TTAGAAGTGG ACTCCTCCCT ACCTCCTCTA ACGTCTCTTC GGCTCCTTTC TTCGTCTTGT   
  
  
- CAAATCGTCA ACTTCAACTA GACTACTGGG ACGACTGAGT CACACGTGTT CGACATAGCT CGAAACTAGA   
  
  
- AGCTCCACGT TTGCTCGATG AATCCGTTTA GTCCGTCTTA CGAAGCGGGA TACCACCGTC GTAGGTCTCC   
  
  
- GAGCGGGTAG TACATCGGTT ACGAGAGCTC CGTGCATATC GACCGTGTCC GAGATGTCAG AGATGATTGG   
  
  
- AACAACTACG TTTCAAGAGT CGAAGACTGA AGGATTTCCG AATGTCCAAT ATACAGAGTC GACAAGGAAT   
  
  
- GTTTTCCTAC AGAAAGAAAG AACGATTGAC GAGCTAACGT TTCAACCGTC TCTTTCGTTG TTTCTAGGTA   
  
  
- TAGTAACTAA AACCACAAAA GGATCCAAAT GTTACCGGAA CAAAGTATGT TGTGGATAGT TTTTCCGGTT   
  
  
- TACCTGGGGG TTTTGAGGCT TAGTGTCCTT AGCTGATGGG GGTCGTCCCA AAGTCTGGAC GTGTTTCCCA   
  
  
- ACTTCGATGT CCTGTGGCTA ATAGACCCAT GACACTCGCT AAACCCCACG GAAAAAGAAT AGTCCCGTAA   
  
  
- CGAGTCTTCA CCCTTTGATA AGTCGGCCTC CTAGAGTTCT AGCTTGTTCT GCTCGACCAC TAACAGTTGA   
  
  
- CAAACAAGTC CAGTCCTTGT GACGAGCTAC TCTGTCAGCT TCGTTTGTCA GGTTCTCTAC GAAAGAATCG   
  
  
- AAACCAATCT TTCGACTTAG GGTCGGATAA GTAAGTGCCC CAACAGTTAC CGTGTAAGTT GCGAGGTAAG   
  
  
- AAGCACTGAG CTAAGTCTCT CCGTAACAAA GTAATAAGTA GTCACAAACT ACACAGACTT CTCTGTTAAG   
  
  
- GTGCTCTACG GGTACTCTCC AAGAACTAAC TCTCGCTCTA AACACCCTTT CTTGACAAGT TACACCAACG   
  
  
- AACACTCCCA CGTCTCTCCC AAGTTTCCGG ACTCTGTATG TTCGTCACCG TTCACTCCTG TTGCGCCCGG   
  
  
- CCCAATTCCG TCCAACGGAA CCTGGTCCTC GAATACTTCC TTCGTTGCCG TTACCACTTC CGTTTAATAG   
  
  
- TATTCCTAAA ATACCACCTA TATTTATCCG TAACCTACGA AGTTCCAACC TTCCCATCTT GGAACACACG   
  
  
- AGAGAGTAAA ACCGTTGGAC GGAC

+     ARE

| Site Name | Organism | Position | Strand | Matrix score. | sequence | function |
| --- | --- | --- | --- | --- | --- | --- |
| ARE | Zea mays | 1777 | - | 6 | AAACCA | cis-acting regulatory element essential for the anaerobic induction |
| ARE | Zea mays | 1735 | - | 6 | AAACCA | cis-acting regulatory element essential for the anaerobic induction |

>HU02G01571.1   
+ +Up\_Stream \_Len000CTAGGG GTTGAGAGAA TCCGATAGCT ATGCAGTCCA AACATTATCT CGTCAAGCAT   
  
  
+ TACTATTTAA ATAATTAATA TTGAACTTGA TAGTTACACT TTTTGAATAC TAATTCCACA CCTCATTGCC   
  
  
+ TTTATTTCCA AACCTCGGGT TGCATTTGGA CCACATACCA GGAAGCTTGG ACGACAAAAA TTTCTTTTCT   
  
  
+ TGAGTATTAT TTTGTTTTAT TATTATTATT TTTTACTTTA TTTGGACCAC TTCACTTGGG TAACAAGATT   
  
  
+ GAATATTTGA CTATTATATT ATATGTCACG TATGGATATG GTAGTTGCTT AGAGCTTTGA CCGTTTGCAG   
  
  
+ GTTTAAGTAT GATTCCCTTG GAAAGGAGCC CCTCTTTATC TTAATTCAGA AGTAACTTTT GTTTTTTTAG   
  
  
+ ATAAATTGAA AAGAAAAATA ATAAATGTCT TAGTTGAGTT TGGTTATTCA ATCCGAAATT TGATCCTGCT   
  
  
+ TAATGTATAG TATATAATAC TAATACGCAT GTAACACAAC ACCTCAGATG GTCATTGTTT TACAATTTTT   
  
  
+ TTGAGTTTAG TATAAAAATA AAATCAATTA TTTTTAGAAG TGATGTTTAA TTTGCATTTT TTTTCCACCT   
  
  
+ AAAATCACGC ACTCGAACAT TAGGGGTTTG GCTAACTCAA AGACTCAAGG TAGTGACTTA TCCAAACTTA   
  
  
+ TTACATCCTA TAGATCTATA AAAACATAAA TCTCTTACAA TCAATCCAAA TTGATTCGGT CTAATTTGAT   
  
  
+ GACCCTGATG AGGGTCCTAC AATTACACAA GTGGGCCTTA GCCCCATGGC CGTTTTTTAA TCTTCATGGG   
  
  
+ TCCAGGCGGA GTTACCATCT CCAGGTCCAT TGAGGGTGTA TCACGTCTCA AATTACGAGT TTGCCTCTTT   
  
  
+ CTACGGGGAT TATAAATATC TACCTGCATA CCTCGAAAAG GAGGAGTTGA GATCAGCGAA ACTTGGCCAT   
  
  
+ CCCAGAGCAT TGAAATGCTC AGAACCCTTT TTCTAGAAAA ATATCGTTTT TCCTGAAAAT ATCCGACCTT   
  
  
+ ATGTCATTTA TTGTCGCTTT CTTGACTCTA ACCCGGGACT CCACACTAAC TTGACTATCG GAAAGGCGTT   
  
  
+ CCCTGGATCA CCATCCAAGA TAACCTCTTT TGCAGGGTTG ACGCTCGTTG GAAGAATCGT CGAATGTAGA   
  
  
+ GCCCCTATCA TCACATAAGC CCGCTCTTAC TTCATGCCAT CCTTGAATAG CAGTTTATCC CGAAGCAAAA   
  
  
+ ATAATTCTAA CTTTCTTATA TTGTAAACCC ACATCTAAAT TTAAATTCAT ACTCCAAAAT TCCGCCACGC   
  
  
+ AAATAAGGCT TTTTAATAGG CTTACATATA ATGGCATGCT ATAGAGACAC TGTTTTGAAG AAACTATTTA   
  
  
+ AACATCAGAC CTCATATTGA GTAGCAAATC AAGACTTCTT TCCTCCAGGC CATATTGAGT AGCAAATTAA   
  
  
+ GAATAACCAG AAGCTTGTGA AAAGAGTTAG AAAATAAAAG GTATAATAGA GACTTATGTT CTTAAGATAT   
  
  
+ CTCCTAGATA TGAATGTGAC CTCCTAGTCG ATATTTGTGC TGGATTAGCG AGTTGTGTGA CTGAGTTTGT   
  
  
+ ATGTGTCTCT TAGCATATGA GTTATTCTAG CTGCTTCTTT AAGCTATGTA GTGACTGTCT TCCTTCTTAT   
  
  
+ TTATAAGTGC TATGTCCAAC TAATAGCCGA AAAAAATGGT TCGCCTTTTC TGGTTTTCTT CTTTTTGAAG   
  
  
+ GTTGATTGGC TAATTTATAT AATGGTTTCA ATACTTATCC TTTATTGTTC CACAAATCCA CCTAACCACG   
  
  
+ TACCATTGAC AATAATTACC CACTAATTTC GGTTGGACTT GATTTCCTTT TGTTTGGTAC CTCAGGCATC   
  
  
+ TTCTGTGGAC CTTCATTCAC AGTTTAATTT TCACATTTTT ATAAGGCTGC TCTTGTGTTG CAAATTTTCC   
  
  
+ TATCCATTCC ACTCTTTGCC CATTCCCAGG CTAATTAGTG TTCTCTGTTC AACCATGGAT TCTGTGCTGG   
  
  
+ TTGATCCTGA GTTCATGAAA AATCTCTACA AATTCAAACC TGAATTGCTC TCAAACTTTT CAATGAATCC   
  
  
+ AAATGACGAC ATCTTTCAAG CCCTTCATTC AGAAAATGAT CCTTTACAGT TCCTCTCATT TGATGAAGGA   
  
  
+ ACCTGTCTTA ACAGCTGCAC TAGTCAACAA GTGCCAGATT TCCCTGATGC TTGTCTCAAG TTCATCAGTG   
  
  
+ ATATTCTTCT CGAAGAGGGT TTAGATGCAA ATCCTGCATC TGCACAGGCT CTCGAAGCCA CCGAGAAGTC   
  
  
+ CTTGTATGAT GCTCTCGGGC TTGGAGAGCC ATACCCCCTT TCATGTGATC ACTTTGCGCC ATCTATCTCT   
  
  
+ ACAAGTATTG AGAGCCCAGA TGACAGTTCT TCCAATAAAA GTTATAGCAG CAATCCCGAG ATAGATGGTT   
  
  
+ CTTATGCTAT CGCTGAGCCC AGTTTCGAGT CCAACCCCAA CTGTGTGCTT GATCAACCCC AGTTGAACTC   
  
  
+ CTTTCCAGCT CTACATGAGA TTTCTCGGTC CTTGGTGGAA CTGGGTTCTC AAGCCTCTGA GTTGAGCTTC   
  
  
+ GATGATGCAG GGAGTGCCCG TGTAGAGAAA AAGGGCAAAT CGATAAAGGG CTCGAGGAGG AAGAAGAGTC   
  
  
+ GTCAAAGAGA GGGTGAAGCG TGTTATGGAG GAAGGAGCCA TAAGGTTCAA GCTTCCTTCA ATGATGATTA   
  
  
+ CTATGAGATG GAACAGTATG ATGATGTAGT ATTGCTCTGT AATAATGAAC TAACGGGCAA TAGCCGTTTC   
  
  
+ AACACCGGGA AATCTTCACC TGAGGAGGGA TGGAGGAGAT TGCAGAGAAG CCGAGGAAAG AAGCAGAACA   
  
  
+ GTTTAGCAGT TGAAGTTGAT CTGATGACCC TGCTGACTCA GTGTGCACAA GCTGTATCGA GCTTTGATCT   
  
  
+ TCGAGGTGCA AACGAGCTAC TTAGGCAAAT CAGGCAGAAT GCTTCGCCCT ATGGTGGCAG CATCCAGAGG   
  
  
+ CTCGCCCATC ATGTAGCCAA TGCTCTCGAG GCACGTATAG CTGGCACAGG CTCTACAGTC TCTACTAACC   
  
  
+ TTGTTGATGC AAAGTTCTCA GCTTCTGACT TCCTAAAGGC TTACAGGTTA TATGTCTCAG CTGTTCCTTA   
  
  
+ CAAAAGGATG TCTTTCTTTC TTGCTAACTG CTCGATTGCA AAGTTGGCAG AGAAAGCAAC AAAGATCCAT   
  
  
+ ATCATTGATT TTGGTGTTTT CCTAGGTTTA CAATGGCCTT GTTTCATACA ACACCTATCA AAAAGGCCAA   
  
  
+ ATGGACCCCC AAAACTCCGA ATCACAGGAA TCGACTACCC CCAGCAGGGT TTCAGACCTG CACAAAGGGT   
  
  
+ TGAAGCTACA GGACACCGAT TATCTGGGTA CTGTGAGCGA TTTGGGGTGC CTTTTTCTTA TCAGGGCATT   
  
  
+ GCTCAGAAGT GGGAAACTAT TCAGCCGGAG GATCTCAAGA TCGAACAAGA CGAGCTGGTG ATTGTCAACT   
  
  
+ GTTTGTTCAG GTCAGGAACA CTGCTCGATG AGACAGTCGA AGCAAACAGT CCAAGAGATG CTTTCTTAGC   
  
  
+ TTTGGTTAGA AAGCTGAATC CCAGCCTATT CATTCACGGG GTTGTCAATG GCACATTCAA CGCTCCATTC   
  
  
+ TTCGTGACTC GATTCAGAGA GGCATTGTTT CATTATTCAT CAGTGTTTGA TGTGTCTGAA GAGACAATTC   
  
  
+ CACGAGATGC CCATGAGAGG TTCTTGATTG AGAGCGAGAT TTGTGGGAAA GAACTGTTCA ATGTGGTTGC   
  
  
+ TTGTGAGGGT GCAGAGAGGG TTCAAAGGCC TGAGACATAC AAGCAGTGGC AAGTGAGGAC AACGCGGGCC   
  
  
+ GGGTTAAGGC AGGTTGCCTT GGACCAGGAG CTTATGAAGG AAGCAACGGC AATGGTGAAG GCAAATTATC   
  
  
+ ATAAGGATTT TATGGTGGAT ATAAATAGGC ATTGGATGCT TCAAGGTTGG AAGGGTAGAA CCTTGTGTGC   
  
  
+ TCTCTCATTT TGGCAACCTG CCTG  

- +Up\_Stream \_Len000GATCCC CAACTCTCTT AGGCTATCGA TACGTCAGGT TTGTAATAGA GCAGTTCGTA   
  
  
- ATGATAAATT TATTAATTAT AACTTGAACT ATCAATGTGA AAAACTTATG ATTAAGGTGT GGAGTAACGG   
  
  
- AAATAAAGGT TTGGAGCCCA ACGTAAACCT GGTGTATGGT CCTTCGAACC TGCTGTTTTT AAAGAAAAGA   
  
  
- ACTCATAATA AAACAAAATA ATAATAATAA AAAATGAAAT AAACCTGGTG AAGTGAACCC ATTGTTCTAA   
  
  
- CTTATAAACT GATAATATAA TATACAGTGC ATACCTATAC CATCAACGAA TCTCGAAACT GGCAAACGTC   
  
  
- CAAATTCATA CTAAGGGAAC CTTTCCTCGG GGAGAAATAG AATTAAGTCT TCATTGAAAA CAAAAAAATC   
  
  
- TATTTAACTT TTCTTTTTAT TATTTACAGA ATCAACTCAA ACCAATAAGT TAGGCTTTAA ACTAGGACGA   
  
  
- ATTACATATC ATATATTATG ATTATGCGTA CATTGTGTTG TGGAGTCTAC CAGTAACAAA ATGTTAAAAA   
  
  
- AACTCAAATC ATATTTTTAT TTTAGTTAAT AAAAATCTTC ACTACAAATT AAACGTAAAA AAAAGGTGGA   
  
  
- TTTTAGTGCG TGAGCTTGTA ATCCCCAAAC CGATTGAGTT TCTGAGTTCC ATCACTGAAT AGGTTTGAAT   
  
  
- AATGTAGGAT ATCTAGATAT TTTTGTATTT AGAGAATGTT AGTTAGGTTT AACTAAGCCA GATTAAACTA   
  
  
- CTGGGACTAC TCCCAGGATG TTAATGTGTT CACCCGGAAT CGGGGTACCG GCAAAAAATT AGAAGTACCC   
  
  
- AGGTCCGCCT CAATGGTAGA GGTCCAGGTA ACTCCCACAT AGTGCAGAGT TTAATGCTCA AACGGAGAAA   
  
  
- GATGCCCCTA ATATTTATAG ATGGACGTAT GGAGCTTTTC CTCCTCAACT CTAGTCGCTT TGAACCGGTA   
  
  
- GGGTCTCGTA ACTTTACGAG TCTTGGGAAA AAGATCTTTT TATAGCAAAA AGGACTTTTA TAGGCTGGAA   
  
  
- TACAGTAAAT AACAGCGAAA GAACTGAGAT TGGGCCCTGA GGTGTGATTG AACTGATAGC CTTTCCGCAA   
  
  
- GGGACCTAGT GGTAGGTTCT ATTGGAGAAA ACGTCCCAAC TGCGAGCAAC CTTCTTAGCA GCTTACATCT   
  
  
- CGGGGATAGT AGTGTATTCG GGCGAGAATG AAGTACGGTA GGAACTTATC GTCAAATAGG GCTTCGTTTT   
  
  
- TATTAAGATT GAAAGAATAT AACATTTGGG TGTAGATTTA AATTTAAGTA TGAGGTTTTA AGGCGGTGCG   
  
  
- TTTATTCCGA AAAATTATCC GAATGTATAT TACCGTACGA TATCTCTGTG ACAAAACTTC TTTGATAAAT   
  
  
- TTGTAGTCTG GAGTATAACT CATCGTTTAG TTCTGAAGAA AGGAGGTCCG GTATAACTCA TCGTTTAATT   
  
  
- CTTATTGGTC TTCGAACACT TTTCTCAATC TTTTATTTTC CATATTATCT CTGAATACAA GAATTCTATA   
  
  
- GAGGATCTAT ACTTACACTG GAGGATCAGC TATAAACACG ACCTAATCGC TCAACACACT GACTCAAACA   
  
  
- TACACAGAGA ATCGTATACT CAATAAGATC GACGAAGAAA TTCGATACAT CACTGACAGA AGGAAGAATA   
  
  
- AATATTCACG ATACAGGTTG ATTATCGGCT TTTTTTACCA AGCGGAAAAG ACCAAAAGAA GAAAAACTTC   
  
  
- CAACTAACCG ATTAAATATA TTACCAAAGT TATGAATAGG AAATAACAAG GTGTTTAGGT GGATTGGTGC   
  
  
- ATGGTAACTG TTATTAATGG GTGATTAAAG CCAACCTGAA CTAAAGGAAA ACAAACCATG GAGTCCGTAG   
  
  
- AAGACACCTG GAAGTAAGTG TCAAATTAAA AGTGTAAAAA TATTCCGACG AGAACACAAC GTTTAAAAGG   
  
  
- ATAGGTAAGG TGAGAAACGG GTAAGGGTCC GATTAATCAC AAGAGACAAG TTGGTACCTA AGACACGACC   
  
  
- AACTAGGACT CAAGTACTTT TTAGAGATGT TTAAGTTTGG ACTTAACGAG AGTTTGAAAA GTTACTTAGG   
  
  
- TTTACTGCTG TAGAAAGTTC GGGAAGTAAG TCTTTTACTA GGAAATGTCA AGGAGAGTAA ACTACTTCCT   
  
  
- TGGACAGAAT TGTCGACGTG ATCAGTTGTT CACGGTCTAA AGGGACTACG AACAGAGTTC AAGTAGTCAC   
  
  
- TATAAGAAGA GCTTCTCCCA AATCTACGTT TAGGACGTAG ACGTGTCCGA GAGCTTCGGT GGCTCTTCAG   
  
  
- GAACATACTA CGAGAGCCCG AACCTCTCGG TATGGGGGAA AGTACACTAG TGAAACGCGG TAGATAGAGA   
  
  
- TGTTCATAAC TCTCGGGTCT ACTGTCAAGA AGGTTATTTT CAATATCGTC GTTAGGGCTC TATCTACCAA   
  
  
- GAATACGATA GCGACTCGGG TCAAAGCTCA GGTTGGGGTT GACACACGAA CTAGTTGGGG TCAACTTGAG   
  
  
- GAAAGGTCGA GATGTACTCT AAAGAGCCAG GAACCACCTT GACCCAAGAG TTCGGAGACT CAACTCGAAG   
  
  
- CTACTACGTC CCTCACGGGC ACATCTCTTT TTCCCGTTTA GCTATTTCCC GAGCTCCTCC TTCTTCTCAG   
  
  
- CAGTTTCTCT CCCACTTCGC ACAATACCTC CTTCCTCGGT ATTCCAAGTT CGAAGGAAGT TACTACTAAT   
  
  
- GATACTCTAC CTTGTCATAC TACTACATCA TAACGAGACA TTATTACTTG ATTGCCCGTT ATCGGCAAAG   
  
  
- TTGTGGCCCT TTAGAAGTGG ACTCCTCCCT ACCTCCTCTA ACGTCTCTTC GGCTCCTTTC TTCGTCTTGT   
  
  
- CAAATCGTCA ACTTCAACTA GACTACTGGG ACGACTGAGT CACACGTGTT CGACATAGCT CGAAACTAGA   
  
  
- AGCTCCACGT TTGCTCGATG AATCCGTTTA GTCCGTCTTA CGAAGCGGGA TACCACCGTC GTAGGTCTCC   
  
  
- GAGCGGGTAG TACATCGGTT ACGAGAGCTC CGTGCATATC GACCGTGTCC GAGATGTCAG AGATGATTGG   
  
  
- AACAACTACG TTTCAAGAGT CGAAGACTGA AGGATTTCCG AATGTCCAAT ATACAGAGTC GACAAGGAAT   
  
  
- GTTTTCCTAC AGAAAGAAAG AACGATTGAC GAGCTAACGT TTCAACCGTC TCTTTCGTTG TTTCTAGGTA   
  
  
- TAGTAACTAA AACCACAAAA GGATCCAAAT GTTACCGGAA CAAAGTATGT TGTGGATAGT TTTTCCGGTT   
  
  
- TACCTGGGGG TTTTGAGGCT TAGTGTCCTT AGCTGATGGG GGTCGTCCCA AAGTCTGGAC GTGTTTCCCA   
  
  
- ACTTCGATGT CCTGTGGCTA ATAGACCCAT GACACTCGCT AAACCCCACG GAAAAAGAAT AGTCCCGTAA   
  
  
- CGAGTCTTCA CCCTTTGATA AGTCGGCCTC CTAGAGTTCT AGCTTGTTCT GCTCGACCAC TAACAGTTGA   
  
  
- CAAACAAGTC CAGTCCTTGT GACGAGCTAC TCTGTCAGCT TCGTTTGTCA GGTTCTCTAC GAAAGAATCG   
  
  
- AAACCAATCT TTCGACTTAG GGTCGGATAA GTAAGTGCCC CAACAGTTAC CGTGTAAGTT GCGAGGTAAG   
  
  
- AAGCACTGAG CTAAGTCTCT CCGTAACAAA GTAATAAGTA GTCACAAACT ACACAGACTT CTCTGTTAAG   
  
  
- GTGCTCTACG GGTACTCTCC AAGAACTAAC TCTCGCTCTA AACACCCTTT CTTGACAAGT TACACCAACG   
  
  
- AACACTCCCA CGTCTCTCCC AAGTTTCCGG ACTCTGTATG TTCGTCACCG TTCACTCCTG TTGCGCCCGG   
  
  
- CCCAATTCCG TCCAACGGAA CCTGGTCCTC GAATACTTCC TTCGTTGCCG TTACCACTTC CGTTTAATAG   
  
  
- TATTCCTAAA ATACCACCTA TATTTATCCG TAACCTACGA AGTTCCAACC TTCCCATCTT GGAACACACG   
  
  
- AGAGAGTAAA ACCGTTGGAC GGAC

+     AT~TATA-box

| Site Name | Organism | Position | Strand | Matrix score. | sequence | function |
| --- | --- | --- | --- | --- | --- | --- |
| AT~TATA-box | Arabidopsis thaliana | 1770 | + | 6 | TATATA |  |
| AT~TATA-box | Arabidopsis thaliana | 1768 | - | 8 | TATATAAA |  |
| AT~TATA-box | Arabidopsis thaliana | 505 | + | 6 | TATATA |  |

>HU02G01571.1   
+ +Up\_Stream \_Len000CTAGGG GTTGAGAGAA TCCGATAGCT ATGCAGTCCA AACATTATCT CGTCAAGCAT   
  
  
+ TACTATTTAA ATAATTAATA TTGAACTTGA TAGTTACACT TTTTGAATAC TAATTCCACA CCTCATTGCC   
  
  
+ TTTATTTCCA AACCTCGGGT TGCATTTGGA CCACATACCA GGAAGCTTGG ACGACAAAAA TTTCTTTTCT   
  
  
+ TGAGTATTAT TTTGTTTTAT TATTATTATT TTTTACTTTA TTTGGACCAC TTCACTTGGG TAACAAGATT   
  
  
+ GAATATTTGA CTATTATATT ATATGTCACG TATGGATATG GTAGTTGCTT AGAGCTTTGA CCGTTTGCAG   
  
  
+ GTTTAAGTAT GATTCCCTTG GAAAGGAGCC CCTCTTTATC TTAATTCAGA AGTAACTTTT GTTTTTTTAG   
  
  
+ ATAAATTGAA AAGAAAAATA ATAAATGTCT TAGTTGAGTT TGGTTATTCA ATCCGAAATT TGATCCTGCT   
  
  
+ TAATGTATAG TATATAATAC TAATACGCAT GTAACACAAC ACCTCAGATG GTCATTGTTT TACAATTTTT   
  
  
+ TTGAGTTTAG TATAAAAATA AAATCAATTA TTTTTAGAAG TGATGTTTAA TTTGCATTTT TTTTCCACCT   
  
  
+ AAAATCACGC ACTCGAACAT TAGGGGTTTG GCTAACTCAA AGACTCAAGG TAGTGACTTA TCCAAACTTA   
  
  
+ TTACATCCTA TAGATCTATA AAAACATAAA TCTCTTACAA TCAATCCAAA TTGATTCGGT CTAATTTGAT   
  
  
+ GACCCTGATG AGGGTCCTAC AATTACACAA GTGGGCCTTA GCCCCATGGC CGTTTTTTAA TCTTCATGGG   
  
  
+ TCCAGGCGGA GTTACCATCT CCAGGTCCAT TGAGGGTGTA TCACGTCTCA AATTACGAGT TTGCCTCTTT   
  
  
+ CTACGGGGAT TATAAATATC TACCTGCATA CCTCGAAAAG GAGGAGTTGA GATCAGCGAA ACTTGGCCAT   
  
  
+ CCCAGAGCAT TGAAATGCTC AGAACCCTTT TTCTAGAAAA ATATCGTTTT TCCTGAAAAT ATCCGACCTT   
  
  
+ ATGTCATTTA TTGTCGCTTT CTTGACTCTA ACCCGGGACT CCACACTAAC TTGACTATCG GAAAGGCGTT   
  
  
+ CCCTGGATCA CCATCCAAGA TAACCTCTTT TGCAGGGTTG ACGCTCGTTG GAAGAATCGT CGAATGTAGA   
  
  
+ GCCCCTATCA TCACATAAGC CCGCTCTTAC TTCATGCCAT CCTTGAATAG CAGTTTATCC CGAAGCAAAA   
  
  
+ ATAATTCTAA CTTTCTTATA TTGTAAACCC ACATCTAAAT TTAAATTCAT ACTCCAAAAT TCCGCCACGC   
  
  
+ AAATAAGGCT TTTTAATAGG CTTACATATA ATGGCATGCT ATAGAGACAC TGTTTTGAAG AAACTATTTA   
  
  
+ AACATCAGAC CTCATATTGA GTAGCAAATC AAGACTTCTT TCCTCCAGGC CATATTGAGT AGCAAATTAA   
  
  
+ GAATAACCAG AAGCTTGTGA AAAGAGTTAG AAAATAAAAG GTATAATAGA GACTTATGTT CTTAAGATAT   
  
  
+ CTCCTAGATA TGAATGTGAC CTCCTAGTCG ATATTTGTGC TGGATTAGCG AGTTGTGTGA CTGAGTTTGT   
  
  
+ ATGTGTCTCT TAGCATATGA GTTATTCTAG CTGCTTCTTT AAGCTATGTA GTGACTGTCT TCCTTCTTAT   
  
  
+ TTATAAGTGC TATGTCCAAC TAATAGCCGA AAAAAATGGT TCGCCTTTTC TGGTTTTCTT CTTTTTGAAG   
  
  
+ GTTGATTGGC TAATTTATAT AATGGTTTCA ATACTTATCC TTTATTGTTC CACAAATCCA CCTAACCACG   
  
  
+ TACCATTGAC AATAATTACC CACTAATTTC GGTTGGACTT GATTTCCTTT TGTTTGGTAC CTCAGGCATC   
  
  
+ TTCTGTGGAC CTTCATTCAC AGTTTAATTT TCACATTTTT ATAAGGCTGC TCTTGTGTTG CAAATTTTCC   
  
  
+ TATCCATTCC ACTCTTTGCC CATTCCCAGG CTAATTAGTG TTCTCTGTTC AACCATGGAT TCTGTGCTGG   
  
  
+ TTGATCCTGA GTTCATGAAA AATCTCTACA AATTCAAACC TGAATTGCTC TCAAACTTTT CAATGAATCC   
  
  
+ AAATGACGAC ATCTTTCAAG CCCTTCATTC AGAAAATGAT CCTTTACAGT TCCTCTCATT TGATGAAGGA   
  
  
+ ACCTGTCTTA ACAGCTGCAC TAGTCAACAA GTGCCAGATT TCCCTGATGC TTGTCTCAAG TTCATCAGTG   
  
  
+ ATATTCTTCT CGAAGAGGGT TTAGATGCAA ATCCTGCATC TGCACAGGCT CTCGAAGCCA CCGAGAAGTC   
  
  
+ CTTGTATGAT GCTCTCGGGC TTGGAGAGCC ATACCCCCTT TCATGTGATC ACTTTGCGCC ATCTATCTCT   
  
  
+ ACAAGTATTG AGAGCCCAGA TGACAGTTCT TCCAATAAAA GTTATAGCAG CAATCCCGAG ATAGATGGTT   
  
  
+ CTTATGCTAT CGCTGAGCCC AGTTTCGAGT CCAACCCCAA CTGTGTGCTT GATCAACCCC AGTTGAACTC   
  
  
+ CTTTCCAGCT CTACATGAGA TTTCTCGGTC CTTGGTGGAA CTGGGTTCTC AAGCCTCTGA GTTGAGCTTC   
  
  
+ GATGATGCAG GGAGTGCCCG TGTAGAGAAA AAGGGCAAAT CGATAAAGGG CTCGAGGAGG AAGAAGAGTC   
  
  
+ GTCAAAGAGA GGGTGAAGCG TGTTATGGAG GAAGGAGCCA TAAGGTTCAA GCTTCCTTCA ATGATGATTA   
  
  
+ CTATGAGATG GAACAGTATG ATGATGTAGT ATTGCTCTGT AATAATGAAC TAACGGGCAA TAGCCGTTTC   
  
  
+ AACACCGGGA AATCTTCACC TGAGGAGGGA TGGAGGAGAT TGCAGAGAAG CCGAGGAAAG AAGCAGAACA   
  
  
+ GTTTAGCAGT TGAAGTTGAT CTGATGACCC TGCTGACTCA GTGTGCACAA GCTGTATCGA GCTTTGATCT   
  
  
+ TCGAGGTGCA AACGAGCTAC TTAGGCAAAT CAGGCAGAAT GCTTCGCCCT ATGGTGGCAG CATCCAGAGG   
  
  
+ CTCGCCCATC ATGTAGCCAA TGCTCTCGAG GCACGTATAG CTGGCACAGG CTCTACAGTC TCTACTAACC   
  
  
+ TTGTTGATGC AAAGTTCTCA GCTTCTGACT TCCTAAAGGC TTACAGGTTA TATGTCTCAG CTGTTCCTTA   
  
  
+ CAAAAGGATG TCTTTCTTTC TTGCTAACTG CTCGATTGCA AAGTTGGCAG AGAAAGCAAC AAAGATCCAT   
  
  
+ ATCATTGATT TTGGTGTTTT CCTAGGTTTA CAATGGCCTT GTTTCATACA ACACCTATCA AAAAGGCCAA   
  
  
+ ATGGACCCCC AAAACTCCGA ATCACAGGAA TCGACTACCC CCAGCAGGGT TTCAGACCTG CACAAAGGGT   
  
  
+ TGAAGCTACA GGACACCGAT TATCTGGGTA CTGTGAGCGA TTTGGGGTGC CTTTTTCTTA TCAGGGCATT   
  
  
+ GCTCAGAAGT GGGAAACTAT TCAGCCGGAG GATCTCAAGA TCGAACAAGA CGAGCTGGTG ATTGTCAACT   
  
  
+ GTTTGTTCAG GTCAGGAACA CTGCTCGATG AGACAGTCGA AGCAAACAGT CCAAGAGATG CTTTCTTAGC   
  
  
+ TTTGGTTAGA AAGCTGAATC CCAGCCTATT CATTCACGGG GTTGTCAATG GCACATTCAA CGCTCCATTC   
  
  
+ TTCGTGACTC GATTCAGAGA GGCATTGTTT CATTATTCAT CAGTGTTTGA TGTGTCTGAA GAGACAATTC   
  
  
+ CACGAGATGC CCATGAGAGG TTCTTGATTG AGAGCGAGAT TTGTGGGAAA GAACTGTTCA ATGTGGTTGC   
  
  
+ TTGTGAGGGT GCAGAGAGGG TTCAAAGGCC TGAGACATAC AAGCAGTGGC AAGTGAGGAC AACGCGGGCC   
  
  
+ GGGTTAAGGC AGGTTGCCTT GGACCAGGAG CTTATGAAGG AAGCAACGGC AATGGTGAAG GCAAATTATC   
  
  
+ ATAAGGATTT TATGGTGGAT ATAAATAGGC ATTGGATGCT TCAAGGTTGG AAGGGTAGAA CCTTGTGTGC   
  
  
+ TCTCTCATTT TGGCAACCTG CCTG  

- +Up\_Stream \_Len000GATCCC CAACTCTCTT AGGCTATCGA TACGTCAGGT TTGTAATAGA GCAGTTCGTA   
  
  
- ATGATAAATT TATTAATTAT AACTTGAACT ATCAATGTGA AAAACTTATG ATTAAGGTGT GGAGTAACGG   
  
  
- AAATAAAGGT TTGGAGCCCA ACGTAAACCT GGTGTATGGT CCTTCGAACC TGCTGTTTTT AAAGAAAAGA   
  
  
- ACTCATAATA AAACAAAATA ATAATAATAA AAAATGAAAT AAACCTGGTG AAGTGAACCC ATTGTTCTAA   
  
  
- CTTATAAACT GATAATATAA TATACAGTGC ATACCTATAC CATCAACGAA TCTCGAAACT GGCAAACGTC   
  
  
- CAAATTCATA CTAAGGGAAC CTTTCCTCGG GGAGAAATAG AATTAAGTCT TCATTGAAAA CAAAAAAATC   
  
  
- TATTTAACTT TTCTTTTTAT TATTTACAGA ATCAACTCAA ACCAATAAGT TAGGCTTTAA ACTAGGACGA   
  
  
- ATTACATATC ATATATTATG ATTATGCGTA CATTGTGTTG TGGAGTCTAC CAGTAACAAA ATGTTAAAAA   
  
  
- AACTCAAATC ATATTTTTAT TTTAGTTAAT AAAAATCTTC ACTACAAATT AAACGTAAAA AAAAGGTGGA   
  
  
- TTTTAGTGCG TGAGCTTGTA ATCCCCAAAC CGATTGAGTT TCTGAGTTCC ATCACTGAAT AGGTTTGAAT   
  
  
- AATGTAGGAT ATCTAGATAT TTTTGTATTT AGAGAATGTT AGTTAGGTTT AACTAAGCCA GATTAAACTA   
  
  
- CTGGGACTAC TCCCAGGATG TTAATGTGTT CACCCGGAAT CGGGGTACCG GCAAAAAATT AGAAGTACCC   
  
  
- AGGTCCGCCT CAATGGTAGA GGTCCAGGTA ACTCCCACAT AGTGCAGAGT TTAATGCTCA AACGGAGAAA   
  
  
- GATGCCCCTA ATATTTATAG ATGGACGTAT GGAGCTTTTC CTCCTCAACT CTAGTCGCTT TGAACCGGTA   
  
  
- GGGTCTCGTA ACTTTACGAG TCTTGGGAAA AAGATCTTTT TATAGCAAAA AGGACTTTTA TAGGCTGGAA   
  
  
- TACAGTAAAT AACAGCGAAA GAACTGAGAT TGGGCCCTGA GGTGTGATTG AACTGATAGC CTTTCCGCAA   
  
  
- GGGACCTAGT GGTAGGTTCT ATTGGAGAAA ACGTCCCAAC TGCGAGCAAC CTTCTTAGCA GCTTACATCT   
  
  
- CGGGGATAGT AGTGTATTCG GGCGAGAATG AAGTACGGTA GGAACTTATC GTCAAATAGG GCTTCGTTTT   
  
  
- TATTAAGATT GAAAGAATAT AACATTTGGG TGTAGATTTA AATTTAAGTA TGAGGTTTTA AGGCGGTGCG   
  
  
- TTTATTCCGA AAAATTATCC GAATGTATAT TACCGTACGA TATCTCTGTG ACAAAACTTC TTTGATAAAT   
  
  
- TTGTAGTCTG GAGTATAACT CATCGTTTAG TTCTGAAGAA AGGAGGTCCG GTATAACTCA TCGTTTAATT   
  
  
- CTTATTGGTC TTCGAACACT TTTCTCAATC TTTTATTTTC CATATTATCT CTGAATACAA GAATTCTATA   
  
  
- GAGGATCTAT ACTTACACTG GAGGATCAGC TATAAACACG ACCTAATCGC TCAACACACT GACTCAAACA   
  
  
- TACACAGAGA ATCGTATACT CAATAAGATC GACGAAGAAA TTCGATACAT CACTGACAGA AGGAAGAATA   
  
  
- AATATTCACG ATACAGGTTG ATTATCGGCT TTTTTTACCA AGCGGAAAAG ACCAAAAGAA GAAAAACTTC   
  
  
- CAACTAACCG ATTAAATATA TTACCAAAGT TATGAATAGG AAATAACAAG GTGTTTAGGT GGATTGGTGC   
  
  
- ATGGTAACTG TTATTAATGG GTGATTAAAG CCAACCTGAA CTAAAGGAAA ACAAACCATG GAGTCCGTAG   
  
  
- AAGACACCTG GAAGTAAGTG TCAAATTAAA AGTGTAAAAA TATTCCGACG AGAACACAAC GTTTAAAAGG   
  
  
- ATAGGTAAGG TGAGAAACGG GTAAGGGTCC GATTAATCAC AAGAGACAAG TTGGTACCTA AGACACGACC   
  
  
- AACTAGGACT CAAGTACTTT TTAGAGATGT TTAAGTTTGG ACTTAACGAG AGTTTGAAAA GTTACTTAGG   
  
  
- TTTACTGCTG TAGAAAGTTC GGGAAGTAAG TCTTTTACTA GGAAATGTCA AGGAGAGTAA ACTACTTCCT   
  
  
- TGGACAGAAT TGTCGACGTG ATCAGTTGTT CACGGTCTAA AGGGACTACG AACAGAGTTC AAGTAGTCAC   
  
  
- TATAAGAAGA GCTTCTCCCA AATCTACGTT TAGGACGTAG ACGTGTCCGA GAGCTTCGGT GGCTCTTCAG   
  
  
- GAACATACTA CGAGAGCCCG AACCTCTCGG TATGGGGGAA AGTACACTAG TGAAACGCGG TAGATAGAGA   
  
  
- TGTTCATAAC TCTCGGGTCT ACTGTCAAGA AGGTTATTTT CAATATCGTC GTTAGGGCTC TATCTACCAA   
  
  
- GAATACGATA GCGACTCGGG TCAAAGCTCA GGTTGGGGTT GACACACGAA CTAGTTGGGG TCAACTTGAG   
  
  
- GAAAGGTCGA GATGTACTCT AAAGAGCCAG GAACCACCTT GACCCAAGAG TTCGGAGACT CAACTCGAAG   
  
  
- CTACTACGTC CCTCACGGGC ACATCTCTTT TTCCCGTTTA GCTATTTCCC GAGCTCCTCC TTCTTCTCAG   
  
  
- CAGTTTCTCT CCCACTTCGC ACAATACCTC CTTCCTCGGT ATTCCAAGTT CGAAGGAAGT TACTACTAAT   
  
  
- GATACTCTAC CTTGTCATAC TACTACATCA TAACGAGACA TTATTACTTG ATTGCCCGTT ATCGGCAAAG   
  
  
- TTGTGGCCCT TTAGAAGTGG ACTCCTCCCT ACCTCCTCTA ACGTCTCTTC GGCTCCTTTC TTCGTCTTGT   
  
  
- CAAATCGTCA ACTTCAACTA GACTACTGGG ACGACTGAGT CACACGTGTT CGACATAGCT CGAAACTAGA   
  
  
- AGCTCCACGT TTGCTCGATG AATCCGTTTA GTCCGTCTTA CGAAGCGGGA TACCACCGTC GTAGGTCTCC   
  
  
- GAGCGGGTAG TACATCGGTT ACGAGAGCTC CGTGCATATC GACCGTGTCC GAGATGTCAG AGATGATTGG   
  
  
- AACAACTACG TTTCAAGAGT CGAAGACTGA AGGATTTCCG AATGTCCAAT ATACAGAGTC GACAAGGAAT   
  
  
- GTTTTCCTAC AGAAAGAAAG AACGATTGAC GAGCTAACGT TTCAACCGTC TCTTTCGTTG TTTCTAGGTA   
  
  
- TAGTAACTAA AACCACAAAA GGATCCAAAT GTTACCGGAA CAAAGTATGT TGTGGATAGT TTTTCCGGTT   
  
  
- TACCTGGGGG TTTTGAGGCT TAGTGTCCTT AGCTGATGGG GGTCGTCCCA AAGTCTGGAC GTGTTTCCCA   
  
  
- ACTTCGATGT CCTGTGGCTA ATAGACCCAT GACACTCGCT AAACCCCACG GAAAAAGAAT AGTCCCGTAA   
  
  
- CGAGTCTTCA CCCTTTGATA AGTCGGCCTC CTAGAGTTCT AGCTTGTTCT GCTCGACCAC TAACAGTTGA   
  
  
- CAAACAAGTC CAGTCCTTGT GACGAGCTAC TCTGTCAGCT TCGTTTGTCA GGTTCTCTAC GAAAGAATCG   
  
  
- AAACCAATCT TTCGACTTAG GGTCGGATAA GTAAGTGCCC CAACAGTTAC CGTGTAAGTT GCGAGGTAAG   
  
  
- AAGCACTGAG CTAAGTCTCT CCGTAACAAA GTAATAAGTA GTCACAAACT ACACAGACTT CTCTGTTAAG   
  
  
- GTGCTCTACG GGTACTCTCC AAGAACTAAC TCTCGCTCTA AACACCCTTT CTTGACAAGT TACACCAACG   
  
  
- AACACTCCCA CGTCTCTCCC AAGTTTCCGG ACTCTGTATG TTCGTCACCG TTCACTCCTG TTGCGCCCGG   
  
  
- CCCAATTCCG TCCAACGGAA CCTGGTCCTC GAATACTTCC TTCGTTGCCG TTACCACTTC CGTTTAATAG   
  
  
- TATTCCTAAA ATACCACCTA TATTTATCCG TAACCTACGA AGTTCCAACC TTCCCATCTT GGAACACACG   
  
  
- AGAGAGTAAA ACCGTTGGAC GGAC

+     AuxRR-core

| Site Name | Organism | Position | Strand | Matrix score. | sequence | function |
| --- | --- | --- | --- | --- | --- | --- |
| AuxRR-core | Nicotiana tabacum | 868 | + | 7 | GGTCCAT | cis-acting regulatory element involved in auxin responsiveness |
| AuxRR-core | Nicotiana tabacum | 3295 | - | 7 | GGTCCAT | cis-acting regulatory element involved in auxin responsiveness |

>HU02G01571.1   
+ +Up\_Stream \_Len000CTAGGG GTTGAGAGAA TCCGATAGCT ATGCAGTCCA AACATTATCT CGTCAAGCAT   
  
  
+ TACTATTTAA ATAATTAATA TTGAACTTGA TAGTTACACT TTTTGAATAC TAATTCCACA CCTCATTGCC   
  
  
+ TTTATTTCCA AACCTCGGGT TGCATTTGGA CCACATACCA GGAAGCTTGG ACGACAAAAA TTTCTTTTCT   
  
  
+ TGAGTATTAT TTTGTTTTAT TATTATTATT TTTTACTTTA TTTGGACCAC TTCACTTGGG TAACAAGATT   
  
  
+ GAATATTTGA CTATTATATT ATATGTCACG TATGGATATG GTAGTTGCTT AGAGCTTTGA CCGTTTGCAG   
  
  
+ GTTTAAGTAT GATTCCCTTG GAAAGGAGCC CCTCTTTATC TTAATTCAGA AGTAACTTTT GTTTTTTTAG   
  
  
+ ATAAATTGAA AAGAAAAATA ATAAATGTCT TAGTTGAGTT TGGTTATTCA ATCCGAAATT TGATCCTGCT   
  
  
+ TAATGTATAG TATATAATAC TAATACGCAT GTAACACAAC ACCTCAGATG GTCATTGTTT TACAATTTTT   
  
  
+ TTGAGTTTAG TATAAAAATA AAATCAATTA TTTTTAGAAG TGATGTTTAA TTTGCATTTT TTTTCCACCT   
  
  
+ AAAATCACGC ACTCGAACAT TAGGGGTTTG GCTAACTCAA AGACTCAAGG TAGTGACTTA TCCAAACTTA   
  
  
+ TTACATCCTA TAGATCTATA AAAACATAAA TCTCTTACAA TCAATCCAAA TTGATTCGGT CTAATTTGAT   
  
  
+ GACCCTGATG AGGGTCCTAC AATTACACAA GTGGGCCTTA GCCCCATGGC CGTTTTTTAA TCTTCATGGG   
  
  
+ TCCAGGCGGA GTTACCATCT CCAGGTCCAT TGAGGGTGTA TCACGTCTCA AATTACGAGT TTGCCTCTTT   
  
  
+ CTACGGGGAT TATAAATATC TACCTGCATA CCTCGAAAAG GAGGAGTTGA GATCAGCGAA ACTTGGCCAT   
  
  
+ CCCAGAGCAT TGAAATGCTC AGAACCCTTT TTCTAGAAAA ATATCGTTTT TCCTGAAAAT ATCCGACCTT   
  
  
+ ATGTCATTTA TTGTCGCTTT CTTGACTCTA ACCCGGGACT CCACACTAAC TTGACTATCG GAAAGGCGTT   
  
  
+ CCCTGGATCA CCATCCAAGA TAACCTCTTT TGCAGGGTTG ACGCTCGTTG GAAGAATCGT CGAATGTAGA   
  
  
+ GCCCCTATCA TCACATAAGC CCGCTCTTAC TTCATGCCAT CCTTGAATAG CAGTTTATCC CGAAGCAAAA   
  
  
+ ATAATTCTAA CTTTCTTATA TTGTAAACCC ACATCTAAAT TTAAATTCAT ACTCCAAAAT TCCGCCACGC   
  
  
+ AAATAAGGCT TTTTAATAGG CTTACATATA ATGGCATGCT ATAGAGACAC TGTTTTGAAG AAACTATTTA   
  
  
+ AACATCAGAC CTCATATTGA GTAGCAAATC AAGACTTCTT TCCTCCAGGC CATATTGAGT AGCAAATTAA   
  
  
+ GAATAACCAG AAGCTTGTGA AAAGAGTTAG AAAATAAAAG GTATAATAGA GACTTATGTT CTTAAGATAT   
  
  
+ CTCCTAGATA TGAATGTGAC CTCCTAGTCG ATATTTGTGC TGGATTAGCG AGTTGTGTGA CTGAGTTTGT   
  
  
+ ATGTGTCTCT TAGCATATGA GTTATTCTAG CTGCTTCTTT AAGCTATGTA GTGACTGTCT TCCTTCTTAT   
  
  
+ TTATAAGTGC TATGTCCAAC TAATAGCCGA AAAAAATGGT TCGCCTTTTC TGGTTTTCTT CTTTTTGAAG   
  
  
+ GTTGATTGGC TAATTTATAT AATGGTTTCA ATACTTATCC TTTATTGTTC CACAAATCCA CCTAACCACG   
  
  
+ TACCATTGAC AATAATTACC CACTAATTTC GGTTGGACTT GATTTCCTTT TGTTTGGTAC CTCAGGCATC   
  
  
+ TTCTGTGGAC CTTCATTCAC AGTTTAATTT TCACATTTTT ATAAGGCTGC TCTTGTGTTG CAAATTTTCC   
  
  
+ TATCCATTCC ACTCTTTGCC CATTCCCAGG CTAATTAGTG TTCTCTGTTC AACCATGGAT TCTGTGCTGG   
  
  
+ TTGATCCTGA GTTCATGAAA AATCTCTACA AATTCAAACC TGAATTGCTC TCAAACTTTT CAATGAATCC   
  
  
+ AAATGACGAC ATCTTTCAAG CCCTTCATTC AGAAAATGAT CCTTTACAGT TCCTCTCATT TGATGAAGGA   
  
  
+ ACCTGTCTTA ACAGCTGCAC TAGTCAACAA GTGCCAGATT TCCCTGATGC TTGTCTCAAG TTCATCAGTG   
  
  
+ ATATTCTTCT CGAAGAGGGT TTAGATGCAA ATCCTGCATC TGCACAGGCT CTCGAAGCCA CCGAGAAGTC   
  
  
+ CTTGTATGAT GCTCTCGGGC TTGGAGAGCC ATACCCCCTT TCATGTGATC ACTTTGCGCC ATCTATCTCT   
  
  
+ ACAAGTATTG AGAGCCCAGA TGACAGTTCT TCCAATAAAA GTTATAGCAG CAATCCCGAG ATAGATGGTT   
  
  
+ CTTATGCTAT CGCTGAGCCC AGTTTCGAGT CCAACCCCAA CTGTGTGCTT GATCAACCCC AGTTGAACTC   
  
  
+ CTTTCCAGCT CTACATGAGA TTTCTCGGTC CTTGGTGGAA CTGGGTTCTC AAGCCTCTGA GTTGAGCTTC   
  
  
+ GATGATGCAG GGAGTGCCCG TGTAGAGAAA AAGGGCAAAT CGATAAAGGG CTCGAGGAGG AAGAAGAGTC   
  
  
+ GTCAAAGAGA GGGTGAAGCG TGTTATGGAG GAAGGAGCCA TAAGGTTCAA GCTTCCTTCA ATGATGATTA   
  
  
+ CTATGAGATG GAACAGTATG ATGATGTAGT ATTGCTCTGT AATAATGAAC TAACGGGCAA TAGCCGTTTC   
  
  
+ AACACCGGGA AATCTTCACC TGAGGAGGGA TGGAGGAGAT TGCAGAGAAG CCGAGGAAAG AAGCAGAACA   
  
  
+ GTTTAGCAGT TGAAGTTGAT CTGATGACCC TGCTGACTCA GTGTGCACAA GCTGTATCGA GCTTTGATCT   
  
  
+ TCGAGGTGCA AACGAGCTAC TTAGGCAAAT CAGGCAGAAT GCTTCGCCCT ATGGTGGCAG CATCCAGAGG   
  
  
+ CTCGCCCATC ATGTAGCCAA TGCTCTCGAG GCACGTATAG CTGGCACAGG CTCTACAGTC TCTACTAACC   
  
  
+ TTGTTGATGC AAAGTTCTCA GCTTCTGACT TCCTAAAGGC TTACAGGTTA TATGTCTCAG CTGTTCCTTA   
  
  
+ CAAAAGGATG TCTTTCTTTC TTGCTAACTG CTCGATTGCA AAGTTGGCAG AGAAAGCAAC AAAGATCCAT   
  
  
+ ATCATTGATT TTGGTGTTTT CCTAGGTTTA CAATGGCCTT GTTTCATACA ACACCTATCA AAAAGGCCAA   
  
  
+ ATGGACCCCC AAAACTCCGA ATCACAGGAA TCGACTACCC CCAGCAGGGT TTCAGACCTG CACAAAGGGT   
  
  
+ TGAAGCTACA GGACACCGAT TATCTGGGTA CTGTGAGCGA TTTGGGGTGC CTTTTTCTTA TCAGGGCATT   
  
  
+ GCTCAGAAGT GGGAAACTAT TCAGCCGGAG GATCTCAAGA TCGAACAAGA CGAGCTGGTG ATTGTCAACT   
  
  
+ GTTTGTTCAG GTCAGGAACA CTGCTCGATG AGACAGTCGA AGCAAACAGT CCAAGAGATG CTTTCTTAGC   
  
  
+ TTTGGTTAGA AAGCTGAATC CCAGCCTATT CATTCACGGG GTTGTCAATG GCACATTCAA CGCTCCATTC   
  
  
+ TTCGTGACTC GATTCAGAGA GGCATTGTTT CATTATTCAT CAGTGTTTGA TGTGTCTGAA GAGACAATTC   
  
  
+ CACGAGATGC CCATGAGAGG TTCTTGATTG AGAGCGAGAT TTGTGGGAAA GAACTGTTCA ATGTGGTTGC   
  
  
+ TTGTGAGGGT GCAGAGAGGG TTCAAAGGCC TGAGACATAC AAGCAGTGGC AAGTGAGGAC AACGCGGGCC   
  
  
+ GGGTTAAGGC AGGTTGCCTT GGACCAGGAG CTTATGAAGG AAGCAACGGC AATGGTGAAG GCAAATTATC   
  
  
+ ATAAGGATTT TATGGTGGAT ATAAATAGGC ATTGGATGCT TCAAGGTTGG AAGGGTAGAA CCTTGTGTGC   
  
  
+ TCTCTCATTT TGGCAACCTG CCTG  

- +Up\_Stream \_Len000GATCCC CAACTCTCTT AGGCTATCGA TACGTCAGGT TTGTAATAGA GCAGTTCGTA   
  
  
- ATGATAAATT TATTAATTAT AACTTGAACT ATCAATGTGA AAAACTTATG ATTAAGGTGT GGAGTAACGG   
  
  
- AAATAAAGGT TTGGAGCCCA ACGTAAACCT GGTGTATGGT CCTTCGAACC TGCTGTTTTT AAAGAAAAGA   
  
  
- ACTCATAATA AAACAAAATA ATAATAATAA AAAATGAAAT AAACCTGGTG AAGTGAACCC ATTGTTCTAA   
  
  
- CTTATAAACT GATAATATAA TATACAGTGC ATACCTATAC CATCAACGAA TCTCGAAACT GGCAAACGTC   
  
  
- CAAATTCATA CTAAGGGAAC CTTTCCTCGG GGAGAAATAG AATTAAGTCT TCATTGAAAA CAAAAAAATC   
  
  
- TATTTAACTT TTCTTTTTAT TATTTACAGA ATCAACTCAA ACCAATAAGT TAGGCTTTAA ACTAGGACGA   
  
  
- ATTACATATC ATATATTATG ATTATGCGTA CATTGTGTTG TGGAGTCTAC CAGTAACAAA ATGTTAAAAA   
  
  
- AACTCAAATC ATATTTTTAT TTTAGTTAAT AAAAATCTTC ACTACAAATT AAACGTAAAA AAAAGGTGGA   
  
  
- TTTTAGTGCG TGAGCTTGTA ATCCCCAAAC CGATTGAGTT TCTGAGTTCC ATCACTGAAT AGGTTTGAAT   
  
  
- AATGTAGGAT ATCTAGATAT TTTTGTATTT AGAGAATGTT AGTTAGGTTT AACTAAGCCA GATTAAACTA   
  
  
- CTGGGACTAC TCCCAGGATG TTAATGTGTT CACCCGGAAT CGGGGTACCG GCAAAAAATT AGAAGTACCC   
  
  
- AGGTCCGCCT CAATGGTAGA GGTCCAGGTA ACTCCCACAT AGTGCAGAGT TTAATGCTCA AACGGAGAAA   
  
  
- GATGCCCCTA ATATTTATAG ATGGACGTAT GGAGCTTTTC CTCCTCAACT CTAGTCGCTT TGAACCGGTA   
  
  
- GGGTCTCGTA ACTTTACGAG TCTTGGGAAA AAGATCTTTT TATAGCAAAA AGGACTTTTA TAGGCTGGAA   
  
  
- TACAGTAAAT AACAGCGAAA GAACTGAGAT TGGGCCCTGA GGTGTGATTG AACTGATAGC CTTTCCGCAA   
  
  
- GGGACCTAGT GGTAGGTTCT ATTGGAGAAA ACGTCCCAAC TGCGAGCAAC CTTCTTAGCA GCTTACATCT   
  
  
- CGGGGATAGT AGTGTATTCG GGCGAGAATG AAGTACGGTA GGAACTTATC GTCAAATAGG GCTTCGTTTT   
  
  
- TATTAAGATT GAAAGAATAT AACATTTGGG TGTAGATTTA AATTTAAGTA TGAGGTTTTA AGGCGGTGCG   
  
  
- TTTATTCCGA AAAATTATCC GAATGTATAT TACCGTACGA TATCTCTGTG ACAAAACTTC TTTGATAAAT   
  
  
- TTGTAGTCTG GAGTATAACT CATCGTTTAG TTCTGAAGAA AGGAGGTCCG GTATAACTCA TCGTTTAATT   
  
  
- CTTATTGGTC TTCGAACACT TTTCTCAATC TTTTATTTTC CATATTATCT CTGAATACAA GAATTCTATA   
  
  
- GAGGATCTAT ACTTACACTG GAGGATCAGC TATAAACACG ACCTAATCGC TCAACACACT GACTCAAACA   
  
  
- TACACAGAGA ATCGTATACT CAATAAGATC GACGAAGAAA TTCGATACAT CACTGACAGA AGGAAGAATA   
  
  
- AATATTCACG ATACAGGTTG ATTATCGGCT TTTTTTACCA AGCGGAAAAG ACCAAAAGAA GAAAAACTTC   
  
  
- CAACTAACCG ATTAAATATA TTACCAAAGT TATGAATAGG AAATAACAAG GTGTTTAGGT GGATTGGTGC   
  
  
- ATGGTAACTG TTATTAATGG GTGATTAAAG CCAACCTGAA CTAAAGGAAA ACAAACCATG GAGTCCGTAG   
  
  
- AAGACACCTG GAAGTAAGTG TCAAATTAAA AGTGTAAAAA TATTCCGACG AGAACACAAC GTTTAAAAGG   
  
  
- ATAGGTAAGG TGAGAAACGG GTAAGGGTCC GATTAATCAC AAGAGACAAG TTGGTACCTA AGACACGACC   
  
  
- AACTAGGACT CAAGTACTTT TTAGAGATGT TTAAGTTTGG ACTTAACGAG AGTTTGAAAA GTTACTTAGG   
  
  
- TTTACTGCTG TAGAAAGTTC GGGAAGTAAG TCTTTTACTA GGAAATGTCA AGGAGAGTAA ACTACTTCCT   
  
  
- TGGACAGAAT TGTCGACGTG ATCAGTTGTT CACGGTCTAA AGGGACTACG AACAGAGTTC AAGTAGTCAC   
  
  
- TATAAGAAGA GCTTCTCCCA AATCTACGTT TAGGACGTAG ACGTGTCCGA GAGCTTCGGT GGCTCTTCAG   
  
  
- GAACATACTA CGAGAGCCCG AACCTCTCGG TATGGGGGAA AGTACACTAG TGAAACGCGG TAGATAGAGA   
  
  
- TGTTCATAAC TCTCGGGTCT ACTGTCAAGA AGGTTATTTT CAATATCGTC GTTAGGGCTC TATCTACCAA   
  
  
- GAATACGATA GCGACTCGGG TCAAAGCTCA GGTTGGGGTT GACACACGAA CTAGTTGGGG TCAACTTGAG   
  
  
- GAAAGGTCGA GATGTACTCT AAAGAGCCAG GAACCACCTT GACCCAAGAG TTCGGAGACT CAACTCGAAG   
  
  
- CTACTACGTC CCTCACGGGC ACATCTCTTT TTCCCGTTTA GCTATTTCCC GAGCTCCTCC TTCTTCTCAG   
  
  
- CAGTTTCTCT CCCACTTCGC ACAATACCTC CTTCCTCGGT ATTCCAAGTT CGAAGGAAGT TACTACTAAT   
  
  
- GATACTCTAC CTTGTCATAC TACTACATCA TAACGAGACA TTATTACTTG ATTGCCCGTT ATCGGCAAAG   
  
  
- TTGTGGCCCT TTAGAAGTGG ACTCCTCCCT ACCTCCTCTA ACGTCTCTTC GGCTCCTTTC TTCGTCTTGT   
  
  
- CAAATCGTCA ACTTCAACTA GACTACTGGG ACGACTGAGT CACACGTGTT CGACATAGCT CGAAACTAGA   
  
  
- AGCTCCACGT TTGCTCGATG AATCCGTTTA GTCCGTCTTA CGAAGCGGGA TACCACCGTC GTAGGTCTCC   
  
  
- GAGCGGGTAG TACATCGGTT ACGAGAGCTC CGTGCATATC GACCGTGTCC GAGATGTCAG AGATGATTGG   
  
  
- AACAACTACG TTTCAAGAGT CGAAGACTGA AGGATTTCCG AATGTCCAAT ATACAGAGTC GACAAGGAAT   
  
  
- GTTTTCCTAC AGAAAGAAAG AACGATTGAC GAGCTAACGT TTCAACCGTC TCTTTCGTTG TTTCTAGGTA   
  
  
- TAGTAACTAA AACCACAAAA GGATCCAAAT GTTACCGGAA CAAAGTATGT TGTGGATAGT TTTTCCGGTT   
  
  
- TACCTGGGGG TTTTGAGGCT TAGTGTCCTT AGCTGATGGG GGTCGTCCCA AAGTCTGGAC GTGTTTCCCA   
  
  
- ACTTCGATGT CCTGTGGCTA ATAGACCCAT GACACTCGCT AAACCCCACG GAAAAAGAAT AGTCCCGTAA   
  
  
- CGAGTCTTCA CCCTTTGATA AGTCGGCCTC CTAGAGTTCT AGCTTGTTCT GCTCGACCAC TAACAGTTGA   
  
  
- CAAACAAGTC CAGTCCTTGT GACGAGCTAC TCTGTCAGCT TCGTTTGTCA GGTTCTCTAC GAAAGAATCG   
  
  
- AAACCAATCT TTCGACTTAG GGTCGGATAA GTAAGTGCCC CAACAGTTAC CGTGTAAGTT GCGAGGTAAG   
  
  
- AAGCACTGAG CTAAGTCTCT CCGTAACAAA GTAATAAGTA GTCACAAACT ACACAGACTT CTCTGTTAAG   
  
  
- GTGCTCTACG GGTACTCTCC AAGAACTAAC TCTCGCTCTA AACACCCTTT CTTGACAAGT TACACCAACG   
  
  
- AACACTCCCA CGTCTCTCCC AAGTTTCCGG ACTCTGTATG TTCGTCACCG TTCACTCCTG TTGCGCCCGG   
  
  
- CCCAATTCCG TCCAACGGAA CCTGGTCCTC GAATACTTCC TTCGTTGCCG TTACCACTTC CGTTTAATAG   
  
  
- TATTCCTAAA ATACCACCTA TATTTATCCG TAACCTACGA AGTTCCAACC TTCCCATCTT GGAACACACG   
  
  
- AGAGAGTAAA ACCGTTGGAC GGAC

+     Box 4

| Site Name | Organism | Position | Strand | Matrix score. | sequence | function |
| --- | --- | --- | --- | --- | --- | --- |
| Box 4 | Petroselinum crispum | 88 | + | 6 | ATTAAT | part of a conserved DNA module involved in light responsiveness |

>HU02G01571.1   
+ +Up\_Stream \_Len000CTAGGG GTTGAGAGAA TCCGATAGCT ATGCAGTCCA AACATTATCT CGTCAAGCAT   
  
  
+ TACTATTTAA ATAATTAATA TTGAACTTGA TAGTTACACT TTTTGAATAC TAATTCCACA CCTCATTGCC   
  
  
+ TTTATTTCCA AACCTCGGGT TGCATTTGGA CCACATACCA GGAAGCTTGG ACGACAAAAA TTTCTTTTCT   
  
  
+ TGAGTATTAT TTTGTTTTAT TATTATTATT TTTTACTTTA TTTGGACCAC TTCACTTGGG TAACAAGATT   
  
  
+ GAATATTTGA CTATTATATT ATATGTCACG TATGGATATG GTAGTTGCTT AGAGCTTTGA CCGTTTGCAG   
  
  
+ GTTTAAGTAT GATTCCCTTG GAAAGGAGCC CCTCTTTATC TTAATTCAGA AGTAACTTTT GTTTTTTTAG   
  
  
+ ATAAATTGAA AAGAAAAATA ATAAATGTCT TAGTTGAGTT TGGTTATTCA ATCCGAAATT TGATCCTGCT   
  
  
+ TAATGTATAG TATATAATAC TAATACGCAT GTAACACAAC ACCTCAGATG GTCATTGTTT TACAATTTTT   
  
  
+ TTGAGTTTAG TATAAAAATA AAATCAATTA TTTTTAGAAG TGATGTTTAA TTTGCATTTT TTTTCCACCT   
  
  
+ AAAATCACGC ACTCGAACAT TAGGGGTTTG GCTAACTCAA AGACTCAAGG TAGTGACTTA TCCAAACTTA   
  
  
+ TTACATCCTA TAGATCTATA AAAACATAAA TCTCTTACAA TCAATCCAAA TTGATTCGGT CTAATTTGAT   
  
  
+ GACCCTGATG AGGGTCCTAC AATTACACAA GTGGGCCTTA GCCCCATGGC CGTTTTTTAA TCTTCATGGG   
  
  
+ TCCAGGCGGA GTTACCATCT CCAGGTCCAT TGAGGGTGTA TCACGTCTCA AATTACGAGT TTGCCTCTTT   
  
  
+ CTACGGGGAT TATAAATATC TACCTGCATA CCTCGAAAAG GAGGAGTTGA GATCAGCGAA ACTTGGCCAT   
  
  
+ CCCAGAGCAT TGAAATGCTC AGAACCCTTT TTCTAGAAAA ATATCGTTTT TCCTGAAAAT ATCCGACCTT   
  
  
+ ATGTCATTTA TTGTCGCTTT CTTGACTCTA ACCCGGGACT CCACACTAAC TTGACTATCG GAAAGGCGTT   
  
  
+ CCCTGGATCA CCATCCAAGA TAACCTCTTT TGCAGGGTTG ACGCTCGTTG GAAGAATCGT CGAATGTAGA   
  
  
+ GCCCCTATCA TCACATAAGC CCGCTCTTAC TTCATGCCAT CCTTGAATAG CAGTTTATCC CGAAGCAAAA   
  
  
+ ATAATTCTAA CTTTCTTATA TTGTAAACCC ACATCTAAAT TTAAATTCAT ACTCCAAAAT TCCGCCACGC   
  
  
+ AAATAAGGCT TTTTAATAGG CTTACATATA ATGGCATGCT ATAGAGACAC TGTTTTGAAG AAACTATTTA   
  
  
+ AACATCAGAC CTCATATTGA GTAGCAAATC AAGACTTCTT TCCTCCAGGC CATATTGAGT AGCAAATTAA   
  
  
+ GAATAACCAG AAGCTTGTGA AAAGAGTTAG AAAATAAAAG GTATAATAGA GACTTATGTT CTTAAGATAT   
  
  
+ CTCCTAGATA TGAATGTGAC CTCCTAGTCG ATATTTGTGC TGGATTAGCG AGTTGTGTGA CTGAGTTTGT   
  
  
+ ATGTGTCTCT TAGCATATGA GTTATTCTAG CTGCTTCTTT AAGCTATGTA GTGACTGTCT TCCTTCTTAT   
  
  
+ TTATAAGTGC TATGTCCAAC TAATAGCCGA AAAAAATGGT TCGCCTTTTC TGGTTTTCTT CTTTTTGAAG   
  
  
+ GTTGATTGGC TAATTTATAT AATGGTTTCA ATACTTATCC TTTATTGTTC CACAAATCCA CCTAACCACG   
  
  
+ TACCATTGAC AATAATTACC CACTAATTTC GGTTGGACTT GATTTCCTTT TGTTTGGTAC CTCAGGCATC   
  
  
+ TTCTGTGGAC CTTCATTCAC AGTTTAATTT TCACATTTTT ATAAGGCTGC TCTTGTGTTG CAAATTTTCC   
  
  
+ TATCCATTCC ACTCTTTGCC CATTCCCAGG CTAATTAGTG TTCTCTGTTC AACCATGGAT TCTGTGCTGG   
  
  
+ TTGATCCTGA GTTCATGAAA AATCTCTACA AATTCAAACC TGAATTGCTC TCAAACTTTT CAATGAATCC   
  
  
+ AAATGACGAC ATCTTTCAAG CCCTTCATTC AGAAAATGAT CCTTTACAGT TCCTCTCATT TGATGAAGGA   
  
  
+ ACCTGTCTTA ACAGCTGCAC TAGTCAACAA GTGCCAGATT TCCCTGATGC TTGTCTCAAG TTCATCAGTG   
  
  
+ ATATTCTTCT CGAAGAGGGT TTAGATGCAA ATCCTGCATC TGCACAGGCT CTCGAAGCCA CCGAGAAGTC   
  
  
+ CTTGTATGAT GCTCTCGGGC TTGGAGAGCC ATACCCCCTT TCATGTGATC ACTTTGCGCC ATCTATCTCT   
  
  
+ ACAAGTATTG AGAGCCCAGA TGACAGTTCT TCCAATAAAA GTTATAGCAG CAATCCCGAG ATAGATGGTT   
  
  
+ CTTATGCTAT CGCTGAGCCC AGTTTCGAGT CCAACCCCAA CTGTGTGCTT GATCAACCCC AGTTGAACTC   
  
  
+ CTTTCCAGCT CTACATGAGA TTTCTCGGTC CTTGGTGGAA CTGGGTTCTC AAGCCTCTGA GTTGAGCTTC   
  
  
+ GATGATGCAG GGAGTGCCCG TGTAGAGAAA AAGGGCAAAT CGATAAAGGG CTCGAGGAGG AAGAAGAGTC   
  
  
+ GTCAAAGAGA GGGTGAAGCG TGTTATGGAG GAAGGAGCCA TAAGGTTCAA GCTTCCTTCA ATGATGATTA   
  
  
+ CTATGAGATG GAACAGTATG ATGATGTAGT ATTGCTCTGT AATAATGAAC TAACGGGCAA TAGCCGTTTC   
  
  
+ AACACCGGGA AATCTTCACC TGAGGAGGGA TGGAGGAGAT TGCAGAGAAG CCGAGGAAAG AAGCAGAACA   
  
  
+ GTTTAGCAGT TGAAGTTGAT CTGATGACCC TGCTGACTCA GTGTGCACAA GCTGTATCGA GCTTTGATCT   
  
  
+ TCGAGGTGCA AACGAGCTAC TTAGGCAAAT CAGGCAGAAT GCTTCGCCCT ATGGTGGCAG CATCCAGAGG   
  
  
+ CTCGCCCATC ATGTAGCCAA TGCTCTCGAG GCACGTATAG CTGGCACAGG CTCTACAGTC TCTACTAACC   
  
  
+ TTGTTGATGC AAAGTTCTCA GCTTCTGACT TCCTAAAGGC TTACAGGTTA TATGTCTCAG CTGTTCCTTA   
  
  
+ CAAAAGGATG TCTTTCTTTC TTGCTAACTG CTCGATTGCA AAGTTGGCAG AGAAAGCAAC AAAGATCCAT   
  
  
+ ATCATTGATT TTGGTGTTTT CCTAGGTTTA CAATGGCCTT GTTTCATACA ACACCTATCA AAAAGGCCAA   
  
  
+ ATGGACCCCC AAAACTCCGA ATCACAGGAA TCGACTACCC CCAGCAGGGT TTCAGACCTG CACAAAGGGT   
  
  
+ TGAAGCTACA GGACACCGAT TATCTGGGTA CTGTGAGCGA TTTGGGGTGC CTTTTTCTTA TCAGGGCATT   
  
  
+ GCTCAGAAGT GGGAAACTAT TCAGCCGGAG GATCTCAAGA TCGAACAAGA CGAGCTGGTG ATTGTCAACT   
  
  
+ GTTTGTTCAG GTCAGGAACA CTGCTCGATG AGACAGTCGA AGCAAACAGT CCAAGAGATG CTTTCTTAGC   
  
  
+ TTTGGTTAGA AAGCTGAATC CCAGCCTATT CATTCACGGG GTTGTCAATG GCACATTCAA CGCTCCATTC   
  
  
+ TTCGTGACTC GATTCAGAGA GGCATTGTTT CATTATTCAT CAGTGTTTGA TGTGTCTGAA GAGACAATTC   
  
  
+ CACGAGATGC CCATGAGAGG TTCTTGATTG AGAGCGAGAT TTGTGGGAAA GAACTGTTCA ATGTGGTTGC   
  
  
+ TTGTGAGGGT GCAGAGAGGG TTCAAAGGCC TGAGACATAC AAGCAGTGGC AAGTGAGGAC AACGCGGGCC   
  
  
+ GGGTTAAGGC AGGTTGCCTT GGACCAGGAG CTTATGAAGG AAGCAACGGC AATGGTGAAG GCAAATTATC   
  
  
+ ATAAGGATTT TATGGTGGAT ATAAATAGGC ATTGGATGCT TCAAGGTTGG AAGGGTAGAA CCTTGTGTGC   
  
  
+ TCTCTCATTT TGGCAACCTG CCTG  

- +Up\_Stream \_Len000GATCCC CAACTCTCTT AGGCTATCGA TACGTCAGGT TTGTAATAGA GCAGTTCGTA   
  
  
- ATGATAAATT TATTAATTAT AACTTGAACT ATCAATGTGA AAAACTTATG ATTAAGGTGT GGAGTAACGG   
  
  
- AAATAAAGGT TTGGAGCCCA ACGTAAACCT GGTGTATGGT CCTTCGAACC TGCTGTTTTT AAAGAAAAGA   
  
  
- ACTCATAATA AAACAAAATA ATAATAATAA AAAATGAAAT AAACCTGGTG AAGTGAACCC ATTGTTCTAA   
  
  
- CTTATAAACT GATAATATAA TATACAGTGC ATACCTATAC CATCAACGAA TCTCGAAACT GGCAAACGTC   
  
  
- CAAATTCATA CTAAGGGAAC CTTTCCTCGG GGAGAAATAG AATTAAGTCT TCATTGAAAA CAAAAAAATC   
  
  
- TATTTAACTT TTCTTTTTAT TATTTACAGA ATCAACTCAA ACCAATAAGT TAGGCTTTAA ACTAGGACGA   
  
  
- ATTACATATC ATATATTATG ATTATGCGTA CATTGTGTTG TGGAGTCTAC CAGTAACAAA ATGTTAAAAA   
  
  
- AACTCAAATC ATATTTTTAT TTTAGTTAAT AAAAATCTTC ACTACAAATT AAACGTAAAA AAAAGGTGGA   
  
  
- TTTTAGTGCG TGAGCTTGTA ATCCCCAAAC CGATTGAGTT TCTGAGTTCC ATCACTGAAT AGGTTTGAAT   
  
  
- AATGTAGGAT ATCTAGATAT TTTTGTATTT AGAGAATGTT AGTTAGGTTT AACTAAGCCA GATTAAACTA   
  
  
- CTGGGACTAC TCCCAGGATG TTAATGTGTT CACCCGGAAT CGGGGTACCG GCAAAAAATT AGAAGTACCC   
  
  
- AGGTCCGCCT CAATGGTAGA GGTCCAGGTA ACTCCCACAT AGTGCAGAGT TTAATGCTCA AACGGAGAAA   
  
  
- GATGCCCCTA ATATTTATAG ATGGACGTAT GGAGCTTTTC CTCCTCAACT CTAGTCGCTT TGAACCGGTA   
  
  
- GGGTCTCGTA ACTTTACGAG TCTTGGGAAA AAGATCTTTT TATAGCAAAA AGGACTTTTA TAGGCTGGAA   
  
  
- TACAGTAAAT AACAGCGAAA GAACTGAGAT TGGGCCCTGA GGTGTGATTG AACTGATAGC CTTTCCGCAA   
  
  
- GGGACCTAGT GGTAGGTTCT ATTGGAGAAA ACGTCCCAAC TGCGAGCAAC CTTCTTAGCA GCTTACATCT   
  
  
- CGGGGATAGT AGTGTATTCG GGCGAGAATG AAGTACGGTA GGAACTTATC GTCAAATAGG GCTTCGTTTT   
  
  
- TATTAAGATT GAAAGAATAT AACATTTGGG TGTAGATTTA AATTTAAGTA TGAGGTTTTA AGGCGGTGCG   
  
  
- TTTATTCCGA AAAATTATCC GAATGTATAT TACCGTACGA TATCTCTGTG ACAAAACTTC TTTGATAAAT   
  
  
- TTGTAGTCTG GAGTATAACT CATCGTTTAG TTCTGAAGAA AGGAGGTCCG GTATAACTCA TCGTTTAATT   
  
  
- CTTATTGGTC TTCGAACACT TTTCTCAATC TTTTATTTTC CATATTATCT CTGAATACAA GAATTCTATA   
  
  
- GAGGATCTAT ACTTACACTG GAGGATCAGC TATAAACACG ACCTAATCGC TCAACACACT GACTCAAACA   
  
  
- TACACAGAGA ATCGTATACT CAATAAGATC GACGAAGAAA TTCGATACAT CACTGACAGA AGGAAGAATA   
  
  
- AATATTCACG ATACAGGTTG ATTATCGGCT TTTTTTACCA AGCGGAAAAG ACCAAAAGAA GAAAAACTTC   
  
  
- CAACTAACCG ATTAAATATA TTACCAAAGT TATGAATAGG AAATAACAAG GTGTTTAGGT GGATTGGTGC   
  
  
- ATGGTAACTG TTATTAATGG GTGATTAAAG CCAACCTGAA CTAAAGGAAA ACAAACCATG GAGTCCGTAG   
  
  
- AAGACACCTG GAAGTAAGTG TCAAATTAAA AGTGTAAAAA TATTCCGACG AGAACACAAC GTTTAAAAGG   
  
  
- ATAGGTAAGG TGAGAAACGG GTAAGGGTCC GATTAATCAC AAGAGACAAG TTGGTACCTA AGACACGACC   
  
  
- AACTAGGACT CAAGTACTTT TTAGAGATGT TTAAGTTTGG ACTTAACGAG AGTTTGAAAA GTTACTTAGG   
  
  
- TTTACTGCTG TAGAAAGTTC GGGAAGTAAG TCTTTTACTA GGAAATGTCA AGGAGAGTAA ACTACTTCCT   
  
  
- TGGACAGAAT TGTCGACGTG ATCAGTTGTT CACGGTCTAA AGGGACTACG AACAGAGTTC AAGTAGTCAC   
  
  
- TATAAGAAGA GCTTCTCCCA AATCTACGTT TAGGACGTAG ACGTGTCCGA GAGCTTCGGT GGCTCTTCAG   
  
  
- GAACATACTA CGAGAGCCCG AACCTCTCGG TATGGGGGAA AGTACACTAG TGAAACGCGG TAGATAGAGA   
  
  
- TGTTCATAAC TCTCGGGTCT ACTGTCAAGA AGGTTATTTT CAATATCGTC GTTAGGGCTC TATCTACCAA   
  
  
- GAATACGATA GCGACTCGGG TCAAAGCTCA GGTTGGGGTT GACACACGAA CTAGTTGGGG TCAACTTGAG   
  
  
- GAAAGGTCGA GATGTACTCT AAAGAGCCAG GAACCACCTT GACCCAAGAG TTCGGAGACT CAACTCGAAG   
  
  
- CTACTACGTC CCTCACGGGC ACATCTCTTT TTCCCGTTTA GCTATTTCCC GAGCTCCTCC TTCTTCTCAG   
  
  
- CAGTTTCTCT CCCACTTCGC ACAATACCTC CTTCCTCGGT ATTCCAAGTT CGAAGGAAGT TACTACTAAT   
  
  
- GATACTCTAC CTTGTCATAC TACTACATCA TAACGAGACA TTATTACTTG ATTGCCCGTT ATCGGCAAAG   
  
  
- TTGTGGCCCT TTAGAAGTGG ACTCCTCCCT ACCTCCTCTA ACGTCTCTTC GGCTCCTTTC TTCGTCTTGT   
  
  
- CAAATCGTCA ACTTCAACTA GACTACTGGG ACGACTGAGT CACACGTGTT CGACATAGCT CGAAACTAGA   
  
  
- AGCTCCACGT TTGCTCGATG AATCCGTTTA GTCCGTCTTA CGAAGCGGGA TACCACCGTC GTAGGTCTCC   
  
  
- GAGCGGGTAG TACATCGGTT ACGAGAGCTC CGTGCATATC GACCGTGTCC GAGATGTCAG AGATGATTGG   
  
  
- AACAACTACG TTTCAAGAGT CGAAGACTGA AGGATTTCCG AATGTCCAAT ATACAGAGTC GACAAGGAAT   
  
  
- GTTTTCCTAC AGAAAGAAAG AACGATTGAC GAGCTAACGT TTCAACCGTC TCTTTCGTTG TTTCTAGGTA   
  
  
- TAGTAACTAA AACCACAAAA GGATCCAAAT GTTACCGGAA CAAAGTATGT TGTGGATAGT TTTTCCGGTT   
  
  
- TACCTGGGGG TTTTGAGGCT TAGTGTCCTT AGCTGATGGG GGTCGTCCCA AAGTCTGGAC GTGTTTCCCA   
  
  
- ACTTCGATGT CCTGTGGCTA ATAGACCCAT GACACTCGCT AAACCCCACG GAAAAAGAAT AGTCCCGTAA   
  
  
- CGAGTCTTCA CCCTTTGATA AGTCGGCCTC CTAGAGTTCT AGCTTGTTCT GCTCGACCAC TAACAGTTGA   
  
  
- CAAACAAGTC CAGTCCTTGT GACGAGCTAC TCTGTCAGCT TCGTTTGTCA GGTTCTCTAC GAAAGAATCG   
  
  
- AAACCAATCT TTCGACTTAG GGTCGGATAA GTAAGTGCCC CAACAGTTAC CGTGTAAGTT GCGAGGTAAG   
  
  
- AAGCACTGAG CTAAGTCTCT CCGTAACAAA GTAATAAGTA GTCACAAACT ACACAGACTT CTCTGTTAAG   
  
  
- GTGCTCTACG GGTACTCTCC AAGAACTAAC TCTCGCTCTA AACACCCTTT CTTGACAAGT TACACCAACG   
  
  
- AACACTCCCA CGTCTCTCCC AAGTTTCCGG ACTCTGTATG TTCGTCACCG TTCACTCCTG TTGCGCCCGG   
  
  
- CCCAATTCCG TCCAACGGAA CCTGGTCCTC GAATACTTCC TTCGTTGCCG TTACCACTTC CGTTTAATAG   
  
  
- TATTCCTAAA ATACCACCTA TATTTATCCG TAACCTACGA AGTTCCAACC TTCCCATCTT GGAACACACG   
  
  
- AGAGAGTAAA ACCGTTGGAC GGAC

+     CAAT-box

| Site Name | Organism | Position | Strand | Matrix score. | sequence | function |
| --- | --- | --- | --- | --- | --- | --- |
| CAAT-box | Nicotiana glutinosa | 3620 | + | 4 | CAAT |  |
| CAAT-box | Nicotiana glutinosa | 3495 | - | 4 | CAAT |  |
| CAAT-box | Pisum sativum | 3404 | - | 5 | CAAAT | common cis-acting element in promoter and enhancer regions |
| CAAT-box | Pisum sativum | 1429 | + | 5 | CAAAT | common cis-acting element in promoter and enhancer regions |
| CAAT-box | Nicotiana glutinosa | 1458 | - | 4 | CAAT |  |
| CAAT-box | Pisum sativum | 2970 | + | 5 | CAAAT | common cis-acting element in promoter and enhancer regions |
| CAAT-box | Nicotiana glutinosa | 1829 | - | 4 | CAAT |  |
| CAAT-box | Pisum sativum | 1467 | + | 5 | CAAAT | common cis-acting element in promoter and enhancer regions |
| CAAT-box | Pisum sativum | 751 | + | 5 | CAAAT | common cis-acting element in promoter and enhancer regions |
| CAAT-box | Nicotiana glutinosa | 3432 | - | 4 | CAAT |  |
| CAAT-box | Pisum sativum | 3292 | + | 5 | CAAAT | common cis-acting element in promoter and enhancer regions |
| CAAT-box | Nicotiana glutinosa | 3255 | + | 4 | CAAT |  |
| CAAT-box | Nicotiana glutinosa | 3228 | - | 4 | CAAT |  |
| CAAT-box | Arabidopsis thaliana | 1759 | - | 5 | CCAAT | common cis-acting element in promoter and enhancer regions |
| CAAT-box | Petunia hybrida | 3197 | - | 7 | TGCCAAC | common cis-acting element in promoter and enhancer regions |
| CAAT-box | Nicotiana glutinosa | 1420 | - | 4 | CAAT |  |
| CAAT-box | Pisum sativum | 1577 | - | 5 | CAAAT | common cis-acting element in promoter and enhancer regions |
| CAAT-box | Pisum sativum | 1334 | + | 5 | CAAAT | common cis-acting element in promoter and enhancer regions |
| CAAT-box | Pisum sativum | 768 | - | 5 | CAAAT | common cis-acting element in promoter and enhancer regions |
| CAAT-box | Nicotiana glutinosa | 1064 | - | 4 | CAAT |  |
| CAAT-box | Pisum sativum | 893 | + | 5 | CAAAT | common cis-acting element in promoter and enhancer regions |
| CAAT-box | Nicotiana glutinosa | 873 | - | 4 | CAAT |  |
| CAAT-box | Pisum sativum | 254 | - | 5 | CAAAT | common cis-acting element in promoter and enhancer regions |
| CAAT-box | Nicotiana glutinosa | 282 | - | 4 | CAAT |  |
| CAAT-box | Nicotiana glutinosa | 548 | - | 4 | CAAT |  |
| CAAT-box | Pisum sativum | 168 | - | 5 | CAAAT | common cis-acting element in promoter and enhancer regions |
| CAAT-box | Nicotiana glutinosa | 742 | + | 4 | CAAT |  |
| CAAT-box | Nicotiana glutinosa | 473 | + | 4 | CAAT |  |
| CAAT-box | Pisum sativum | 289 | - | 5 | CAAAT | common cis-acting element in promoter and enhancer regions |
| CAAT-box | Nicotiana glutinosa | 429 | - | 4 | CAAT |  |
| CAAT-box | Pisum sativum | 614 | - | 5 | CAAAT | common cis-acting element in promoter and enhancer regions |
| CAAT-box | Nicotiana glutinosa | 94 | - | 4 | CAAT |  |
| CAAT-box | Pisum sativum | 482 | - | 5 | CAAAT | common cis-acting element in promoter and enhancer regions |
| CAAT-box | Nicotiana glutinosa | 746 | + | 4 | CAAT |  |
| CAAT-box | Nicotiana glutinosa | 557 | + | 4 | CAAT |  |
| CAAT-box | Arabidopsis thaliana | 3031 | + | 5 | CCAAT | common cis-acting element in promoter and enhancer regions |
| CAAT-box | Nicotiana glutinosa | 3189 | - | 4 | CAAT |  |
| CAAT-box | Nicotiana glutinosa | 3032 | + | 4 | CAAT |  |
| CAAT-box | Pisum sativum | 3753 | - | 5 | CAAAT | common cis-acting element in promoter and enhancer regions |
| CAAT-box | Nicotiana glutinosa | 139 | - | 4 | CAAT |  |
| CAAT-box | Nicotiana glutinosa | 1798 | - | 4 | CAAT |  |
| CAAT-box | Pisum sativum | 1807 | + | 5 | CAAAT | common cis-acting element in promoter and enhancer regions |
| CAAT-box | Nicotiana glutinosa | 3741 | - | 4 | CAAT |  |
| CAAT-box | Nicotiana glutinosa | 754 | - | 4 | CAAT |  |
| CAAT-box | Nicotiana glutinosa | 3709 | + | 4 | CAAT |  |
| CAAT-box | Nicotiana glutinosa | 2792 | + | 4 | CAAT |  |
| CAAT-box | Nicotiana glutinosa | 3668 | - | 4 | CAAT |  |
| CAAT-box | Arabidopsis thaliana | 3955 | - | 5 | CCAAT | common cis-acting element in promoter and enhancer regions |
| CAAT-box | Pisum sativum | 3916 | + | 5 | CAAAT | common cis-acting element in promoter and enhancer regions |
| CAAT-box | Arabidopsis thaliana | 2416 | + | 5 | CCAAT | common cis-acting element in promoter and enhancer regions |
| CAAT-box | Nicotiana glutinosa | 589 | + | 4 | CAAT |  |
| CAAT-box | Nicotiana glutinosa | 1284 | - | 4 | CAAT |  |
| CAAT-box | Nicotiana glutinosa | 3904 | + | 4 | CAAT |  |
| CAAT-box | Nicotiana glutinosa | 3773 | + | 4 | CAAT |  |
| CAAT-box | Pisum sativum | 2162 | - | 5 | CAAAT | common cis-acting element in promoter and enhancer regions |
| CAAT-box | Pisum sativum | 2104 | + | 5 | CAAAT | common cis-acting element in promoter and enhancer regions |
| CAAT-box | Pisum sativum | 2063 | + | 5 | CAAAT | common cis-acting element in promoter and enhancer regions |
| CAAT-box | Pisum sativum | 1955 | + | 5 | CAAAT | common cis-acting element in promoter and enhancer regions |
| CAAT-box | Nicotiana glutinosa | 1783 | + | 4 | CAAT |  |
| CAAT-box | Nicotiana glutinosa | 794 | + | 4 | CAAT |  |
| CAAT-box | Nicotiana glutinosa | 993 | - | 4 | CAAT |  |
| CAAT-box | Nicotiana glutinosa | 2843 | - | 4 | CAAT |  |
| CAAT-box | Nicotiana glutinosa | 1834 | + | 4 | CAAT |  |
| CAAT-box | Nicotiana glutinosa | 2078 | - | 4 | CAAT |  |
| CAAT-box | Nicotiana glutinosa | 2391 | - | 4 | CAAT |  |
| CAAT-box | Pisum sativum | 2272 | + | 5 | CAAAT | common cis-acting element in promoter and enhancer regions |
| CAAT-box | Nicotiana glutinosa | 2417 | + | 4 | CAAT |  |
| CAAT-box | Nicotiana glutinosa | 2435 | + | 4 | CAAT |  |
| CAAT-box | Pisum sativum | 2630 | + | 5 | CAAAT | common cis-acting element in promoter and enhancer regions |
| CAAT-box | Nicotiana glutinosa | 2095 | + | 4 | CAAT |  |
| CAAT-box | Nicotiana glutinosa | 2723 | + | 4 | CAAT |  |
| CAAT-box | Nicotiana glutinosa | 2765 | - | 4 | CAAT |  |

>HU02G01571.1   
+ +Up\_Stream \_Len000CTAGGG GTTGAGAGAA TCCGATAGCT ATGCAGTCCA AACATTATCT CGTCAAGCAT   
  
  
+ TACTATTTAA ATAATTAATA TTGAACTTGA TAGTTACACT TTTTGAATAC TAATTCCACA CCTCATTGCC   
  
  
+ TTTATTTCCA AACCTCGGGT TGCATTTGGA CCACATACCA GGAAGCTTGG ACGACAAAAA TTTCTTTTCT   
  
  
+ TGAGTATTAT TTTGTTTTAT TATTATTATT TTTTACTTTA TTTGGACCAC TTCACTTGGG TAACAAGATT   
  
  
+ GAATATTTGA CTATTATATT ATATGTCACG TATGGATATG GTAGTTGCTT AGAGCTTTGA CCGTTTGCAG   
  
  
+ GTTTAAGTAT GATTCCCTTG GAAAGGAGCC CCTCTTTATC TTAATTCAGA AGTAACTTTT GTTTTTTTAG   
  
  
+ ATAAATTGAA AAGAAAAATA ATAAATGTCT TAGTTGAGTT TGGTTATTCA ATCCGAAATT TGATCCTGCT   
  
  
+ TAATGTATAG TATATAATAC TAATACGCAT GTAACACAAC ACCTCAGATG GTCATTGTTT TACAATTTTT   
  
  
+ TTGAGTTTAG TATAAAAATA AAATCAATTA TTTTTAGAAG TGATGTTTAA TTTGCATTTT TTTTCCACCT   
  
  
+ AAAATCACGC ACTCGAACAT TAGGGGTTTG GCTAACTCAA AGACTCAAGG TAGTGACTTA TCCAAACTTA   
  
  
+ TTACATCCTA TAGATCTATA AAAACATAAA TCTCTTACAA TCAATCCAAA TTGATTCGGT CTAATTTGAT   
  
  
+ GACCCTGATG AGGGTCCTAC AATTACACAA GTGGGCCTTA GCCCCATGGC CGTTTTTTAA TCTTCATGGG   
  
  
+ TCCAGGCGGA GTTACCATCT CCAGGTCCAT TGAGGGTGTA TCACGTCTCA AATTACGAGT TTGCCTCTTT   
  
  
+ CTACGGGGAT TATAAATATC TACCTGCATA CCTCGAAAAG GAGGAGTTGA GATCAGCGAA ACTTGGCCAT   
  
  
+ CCCAGAGCAT TGAAATGCTC AGAACCCTTT TTCTAGAAAA ATATCGTTTT TCCTGAAAAT ATCCGACCTT   
  
  
+ ATGTCATTTA TTGTCGCTTT CTTGACTCTA ACCCGGGACT CCACACTAAC TTGACTATCG GAAAGGCGTT   
  
  
+ CCCTGGATCA CCATCCAAGA TAACCTCTTT TGCAGGGTTG ACGCTCGTTG GAAGAATCGT CGAATGTAGA   
  
  
+ GCCCCTATCA TCACATAAGC CCGCTCTTAC TTCATGCCAT CCTTGAATAG CAGTTTATCC CGAAGCAAAA   
  
  
+ ATAATTCTAA CTTTCTTATA TTGTAAACCC ACATCTAAAT TTAAATTCAT ACTCCAAAAT TCCGCCACGC   
  
  
+ AAATAAGGCT TTTTAATAGG CTTACATATA ATGGCATGCT ATAGAGACAC TGTTTTGAAG AAACTATTTA   
  
  
+ AACATCAGAC CTCATATTGA GTAGCAAATC AAGACTTCTT TCCTCCAGGC CATATTGAGT AGCAAATTAA   
  
  
+ GAATAACCAG AAGCTTGTGA AAAGAGTTAG AAAATAAAAG GTATAATAGA GACTTATGTT CTTAAGATAT   
  
  
+ CTCCTAGATA TGAATGTGAC CTCCTAGTCG ATATTTGTGC TGGATTAGCG AGTTGTGTGA CTGAGTTTGT   
  
  
+ ATGTGTCTCT TAGCATATGA GTTATTCTAG CTGCTTCTTT AAGCTATGTA GTGACTGTCT TCCTTCTTAT   
  
  
+ TTATAAGTGC TATGTCCAAC TAATAGCCGA AAAAAATGGT TCGCCTTTTC TGGTTTTCTT CTTTTTGAAG   
  
  
+ GTTGATTGGC TAATTTATAT AATGGTTTCA ATACTTATCC TTTATTGTTC CACAAATCCA CCTAACCACG   
  
  
+ TACCATTGAC AATAATTACC CACTAATTTC GGTTGGACTT GATTTCCTTT TGTTTGGTAC CTCAGGCATC   
  
  
+ TTCTGTGGAC CTTCATTCAC AGTTTAATTT TCACATTTTT ATAAGGCTGC TCTTGTGTTG CAAATTTTCC   
  
  
+ TATCCATTCC ACTCTTTGCC CATTCCCAGG CTAATTAGTG TTCTCTGTTC AACCATGGAT TCTGTGCTGG   
  
  
+ TTGATCCTGA GTTCATGAAA AATCTCTACA AATTCAAACC TGAATTGCTC TCAAACTTTT CAATGAATCC   
  
  
+ AAATGACGAC ATCTTTCAAG CCCTTCATTC AGAAAATGAT CCTTTACAGT TCCTCTCATT TGATGAAGGA   
  
  
+ ACCTGTCTTA ACAGCTGCAC TAGTCAACAA GTGCCAGATT TCCCTGATGC TTGTCTCAAG TTCATCAGTG   
  
  
+ ATATTCTTCT CGAAGAGGGT TTAGATGCAA ATCCTGCATC TGCACAGGCT CTCGAAGCCA CCGAGAAGTC   
  
  
+ CTTGTATGAT GCTCTCGGGC TTGGAGAGCC ATACCCCCTT TCATGTGATC ACTTTGCGCC ATCTATCTCT   
  
  
+ ACAAGTATTG AGAGCCCAGA TGACAGTTCT TCCAATAAAA GTTATAGCAG CAATCCCGAG ATAGATGGTT   
  
  
+ CTTATGCTAT CGCTGAGCCC AGTTTCGAGT CCAACCCCAA CTGTGTGCTT GATCAACCCC AGTTGAACTC   
  
  
+ CTTTCCAGCT CTACATGAGA TTTCTCGGTC CTTGGTGGAA CTGGGTTCTC AAGCCTCTGA GTTGAGCTTC   
  
  
+ GATGATGCAG GGAGTGCCCG TGTAGAGAAA AAGGGCAAAT CGATAAAGGG CTCGAGGAGG AAGAAGAGTC   
  
  
+ GTCAAAGAGA GGGTGAAGCG TGTTATGGAG GAAGGAGCCA TAAGGTTCAA GCTTCCTTCA ATGATGATTA   
  
  
+ CTATGAGATG GAACAGTATG ATGATGTAGT ATTGCTCTGT AATAATGAAC TAACGGGCAA TAGCCGTTTC   
  
  
+ AACACCGGGA AATCTTCACC TGAGGAGGGA TGGAGGAGAT TGCAGAGAAG CCGAGGAAAG AAGCAGAACA   
  
  
+ GTTTAGCAGT TGAAGTTGAT CTGATGACCC TGCTGACTCA GTGTGCACAA GCTGTATCGA GCTTTGATCT   
  
  
+ TCGAGGTGCA AACGAGCTAC TTAGGCAAAT CAGGCAGAAT GCTTCGCCCT ATGGTGGCAG CATCCAGAGG   
  
  
+ CTCGCCCATC ATGTAGCCAA TGCTCTCGAG GCACGTATAG CTGGCACAGG CTCTACAGTC TCTACTAACC   
  
  
+ TTGTTGATGC AAAGTTCTCA GCTTCTGACT TCCTAAAGGC TTACAGGTTA TATGTCTCAG CTGTTCCTTA   
  
  
+ CAAAAGGATG TCTTTCTTTC TTGCTAACTG CTCGATTGCA AAGTTGGCAG AGAAAGCAAC AAAGATCCAT   
  
  
+ ATCATTGATT TTGGTGTTTT CCTAGGTTTA CAATGGCCTT GTTTCATACA ACACCTATCA AAAAGGCCAA   
  
  
+ ATGGACCCCC AAAACTCCGA ATCACAGGAA TCGACTACCC CCAGCAGGGT TTCAGACCTG CACAAAGGGT   
  
  
+ TGAAGCTACA GGACACCGAT TATCTGGGTA CTGTGAGCGA TTTGGGGTGC CTTTTTCTTA TCAGGGCATT   
  
  
+ GCTCAGAAGT GGGAAACTAT TCAGCCGGAG GATCTCAAGA TCGAACAAGA CGAGCTGGTG ATTGTCAACT   
  
  
+ GTTTGTTCAG GTCAGGAACA CTGCTCGATG AGACAGTCGA AGCAAACAGT CCAAGAGATG CTTTCTTAGC   
  
  
+ TTTGGTTAGA AAGCTGAATC CCAGCCTATT CATTCACGGG GTTGTCAATG GCACATTCAA CGCTCCATTC   
  
  
+ TTCGTGACTC GATTCAGAGA GGCATTGTTT CATTATTCAT CAGTGTTTGA TGTGTCTGAA GAGACAATTC   
  
  
+ CACGAGATGC CCATGAGAGG TTCTTGATTG AGAGCGAGAT TTGTGGGAAA GAACTGTTCA ATGTGGTTGC   
  
  
+ TTGTGAGGGT GCAGAGAGGG TTCAAAGGCC TGAGACATAC AAGCAGTGGC AAGTGAGGAC AACGCGGGCC   
  
  
+ GGGTTAAGGC AGGTTGCCTT GGACCAGGAG CTTATGAAGG AAGCAACGGC AATGGTGAAG GCAAATTATC   
  
  
+ ATAAGGATTT TATGGTGGAT ATAAATAGGC ATTGGATGCT TCAAGGTTGG AAGGGTAGAA CCTTGTGTGC   
  
  
+ TCTCTCATTT TGGCAACCTG CCTG  

- +Up\_Stream \_Len000GATCCC CAACTCTCTT AGGCTATCGA TACGTCAGGT TTGTAATAGA GCAGTTCGTA   
  
  
- ATGATAAATT TATTAATTAT AACTTGAACT ATCAATGTGA AAAACTTATG ATTAAGGTGT GGAGTAACGG   
  
  
- AAATAAAGGT TTGGAGCCCA ACGTAAACCT GGTGTATGGT CCTTCGAACC TGCTGTTTTT AAAGAAAAGA   
  
  
- ACTCATAATA AAACAAAATA ATAATAATAA AAAATGAAAT AAACCTGGTG AAGTGAACCC ATTGTTCTAA   
  
  
- CTTATAAACT GATAATATAA TATACAGTGC ATACCTATAC CATCAACGAA TCTCGAAACT GGCAAACGTC   
  
  
- CAAATTCATA CTAAGGGAAC CTTTCCTCGG GGAGAAATAG AATTAAGTCT TCATTGAAAA CAAAAAAATC   
  
  
- TATTTAACTT TTCTTTTTAT TATTTACAGA ATCAACTCAA ACCAATAAGT TAGGCTTTAA ACTAGGACGA   
  
  
- ATTACATATC ATATATTATG ATTATGCGTA CATTGTGTTG TGGAGTCTAC CAGTAACAAA ATGTTAAAAA   
  
  
- AACTCAAATC ATATTTTTAT TTTAGTTAAT AAAAATCTTC ACTACAAATT AAACGTAAAA AAAAGGTGGA   
  
  
- TTTTAGTGCG TGAGCTTGTA ATCCCCAAAC CGATTGAGTT TCTGAGTTCC ATCACTGAAT AGGTTTGAAT   
  
  
- AATGTAGGAT ATCTAGATAT TTTTGTATTT AGAGAATGTT AGTTAGGTTT AACTAAGCCA GATTAAACTA   
  
  
- CTGGGACTAC TCCCAGGATG TTAATGTGTT CACCCGGAAT CGGGGTACCG GCAAAAAATT AGAAGTACCC   
  
  
- AGGTCCGCCT CAATGGTAGA GGTCCAGGTA ACTCCCACAT AGTGCAGAGT TTAATGCTCA AACGGAGAAA   
  
  
- GATGCCCCTA ATATTTATAG ATGGACGTAT GGAGCTTTTC CTCCTCAACT CTAGTCGCTT TGAACCGGTA   
  
  
- GGGTCTCGTA ACTTTACGAG TCTTGGGAAA AAGATCTTTT TATAGCAAAA AGGACTTTTA TAGGCTGGAA   
  
  
- TACAGTAAAT AACAGCGAAA GAACTGAGAT TGGGCCCTGA GGTGTGATTG AACTGATAGC CTTTCCGCAA   
  
  
- GGGACCTAGT GGTAGGTTCT ATTGGAGAAA ACGTCCCAAC TGCGAGCAAC CTTCTTAGCA GCTTACATCT   
  
  
- CGGGGATAGT AGTGTATTCG GGCGAGAATG AAGTACGGTA GGAACTTATC GTCAAATAGG GCTTCGTTTT   
  
  
- TATTAAGATT GAAAGAATAT AACATTTGGG TGTAGATTTA AATTTAAGTA TGAGGTTTTA AGGCGGTGCG   
  
  
- TTTATTCCGA AAAATTATCC GAATGTATAT TACCGTACGA TATCTCTGTG ACAAAACTTC TTTGATAAAT   
  
  
- TTGTAGTCTG GAGTATAACT CATCGTTTAG TTCTGAAGAA AGGAGGTCCG GTATAACTCA TCGTTTAATT   
  
  
- CTTATTGGTC TTCGAACACT TTTCTCAATC TTTTATTTTC CATATTATCT CTGAATACAA GAATTCTATA   
  
  
- GAGGATCTAT ACTTACACTG GAGGATCAGC TATAAACACG ACCTAATCGC TCAACACACT GACTCAAACA   
  
  
- TACACAGAGA ATCGTATACT CAATAAGATC GACGAAGAAA TTCGATACAT CACTGACAGA AGGAAGAATA   
  
  
- AATATTCACG ATACAGGTTG ATTATCGGCT TTTTTTACCA AGCGGAAAAG ACCAAAAGAA GAAAAACTTC   
  
  
- CAACTAACCG ATTAAATATA TTACCAAAGT TATGAATAGG AAATAACAAG GTGTTTAGGT GGATTGGTGC   
  
  
- ATGGTAACTG TTATTAATGG GTGATTAAAG CCAACCTGAA CTAAAGGAAA ACAAACCATG GAGTCCGTAG   
  
  
- AAGACACCTG GAAGTAAGTG TCAAATTAAA AGTGTAAAAA TATTCCGACG AGAACACAAC GTTTAAAAGG   
  
  
- ATAGGTAAGG TGAGAAACGG GTAAGGGTCC GATTAATCAC AAGAGACAAG TTGGTACCTA AGACACGACC   
  
  
- AACTAGGACT CAAGTACTTT TTAGAGATGT TTAAGTTTGG ACTTAACGAG AGTTTGAAAA GTTACTTAGG   
  
  
- TTTACTGCTG TAGAAAGTTC GGGAAGTAAG TCTTTTACTA GGAAATGTCA AGGAGAGTAA ACTACTTCCT   
  
  
- TGGACAGAAT TGTCGACGTG ATCAGTTGTT CACGGTCTAA AGGGACTACG AACAGAGTTC AAGTAGTCAC   
  
  
- TATAAGAAGA GCTTCTCCCA AATCTACGTT TAGGACGTAG ACGTGTCCGA GAGCTTCGGT GGCTCTTCAG   
  
  
- GAACATACTA CGAGAGCCCG AACCTCTCGG TATGGGGGAA AGTACACTAG TGAAACGCGG TAGATAGAGA   
  
  
- TGTTCATAAC TCTCGGGTCT ACTGTCAAGA AGGTTATTTT CAATATCGTC GTTAGGGCTC TATCTACCAA   
  
  
- GAATACGATA GCGACTCGGG TCAAAGCTCA GGTTGGGGTT GACACACGAA CTAGTTGGGG TCAACTTGAG   
  
  
- GAAAGGTCGA GATGTACTCT AAAGAGCCAG GAACCACCTT GACCCAAGAG TTCGGAGACT CAACTCGAAG   
  
  
- CTACTACGTC CCTCACGGGC ACATCTCTTT TTCCCGTTTA GCTATTTCCC GAGCTCCTCC TTCTTCTCAG   
  
  
- CAGTTTCTCT CCCACTTCGC ACAATACCTC CTTCCTCGGT ATTCCAAGTT CGAAGGAAGT TACTACTAAT   
  
  
- GATACTCTAC CTTGTCATAC TACTACATCA TAACGAGACA TTATTACTTG ATTGCCCGTT ATCGGCAAAG   
  
  
- TTGTGGCCCT TTAGAAGTGG ACTCCTCCCT ACCTCCTCTA ACGTCTCTTC GGCTCCTTTC TTCGTCTTGT   
  
  
- CAAATCGTCA ACTTCAACTA GACTACTGGG ACGACTGAGT CACACGTGTT CGACATAGCT CGAAACTAGA   
  
  
- AGCTCCACGT TTGCTCGATG AATCCGTTTA GTCCGTCTTA CGAAGCGGGA TACCACCGTC GTAGGTCTCC   
  
  
- GAGCGGGTAG TACATCGGTT ACGAGAGCTC CGTGCATATC GACCGTGTCC GAGATGTCAG AGATGATTGG   
  
  
- AACAACTACG TTTCAAGAGT CGAAGACTGA AGGATTTCCG AATGTCCAAT ATACAGAGTC GACAAGGAAT   
  
  
- GTTTTCCTAC AGAAAGAAAG AACGATTGAC GAGCTAACGT TTCAACCGTC TCTTTCGTTG TTTCTAGGTA   
  
  
- TAGTAACTAA AACCACAAAA GGATCCAAAT GTTACCGGAA CAAAGTATGT TGTGGATAGT TTTTCCGGTT   
  
  
- TACCTGGGGG TTTTGAGGCT TAGTGTCCTT AGCTGATGGG GGTCGTCCCA AAGTCTGGAC GTGTTTCCCA   
  
  
- ACTTCGATGT CCTGTGGCTA ATAGACCCAT GACACTCGCT AAACCCCACG GAAAAAGAAT AGTCCCGTAA   
  
  
- CGAGTCTTCA CCCTTTGATA AGTCGGCCTC CTAGAGTTCT AGCTTGTTCT GCTCGACCAC TAACAGTTGA   
  
  
- CAAACAAGTC CAGTCCTTGT GACGAGCTAC TCTGTCAGCT TCGTTTGTCA GGTTCTCTAC GAAAGAATCG   
  
  
- AAACCAATCT TTCGACTTAG GGTCGGATAA GTAAGTGCCC CAACAGTTAC CGTGTAAGTT GCGAGGTAAG   
  
  
- AAGCACTGAG CTAAGTCTCT CCGTAACAAA GTAATAAGTA GTCACAAACT ACACAGACTT CTCTGTTAAG   
  
  
- GTGCTCTACG GGTACTCTCC AAGAACTAAC TCTCGCTCTA AACACCCTTT CTTGACAAGT TACACCAACG   
  
  
- AACACTCCCA CGTCTCTCCC AAGTTTCCGG ACTCTGTATG TTCGTCACCG TTCACTCCTG TTGCGCCCGG   
  
  
- CCCAATTCCG TCCAACGGAA CCTGGTCCTC GAATACTTCC TTCGTTGCCG TTACCACTTC CGTTTAATAG   
  
  
- TATTCCTAAA ATACCACCTA TATTTATCCG TAACCTACGA AGTTCCAACC TTCCCATCTT GGAACACACG   
  
  
- AGAGAGTAAA ACCGTTGGAC GGAC

+     CAT-box

| Site Name | Organism | Position | Strand | Matrix score. | sequence | function |
| --- | --- | --- | --- | --- | --- | --- |
| CAT-box | Arabidopsis thaliana | 3829 | - | 6 | GCCACT | cis-acting regulatory element related to meristem expression |

>HU02G01571.1   
+ +Up\_Stream \_Len000CTAGGG GTTGAGAGAA TCCGATAGCT ATGCAGTCCA AACATTATCT CGTCAAGCAT   
  
  
+ TACTATTTAA ATAATTAATA TTGAACTTGA TAGTTACACT TTTTGAATAC TAATTCCACA CCTCATTGCC   
  
  
+ TTTATTTCCA AACCTCGGGT TGCATTTGGA CCACATACCA GGAAGCTTGG ACGACAAAAA TTTCTTTTCT   
  
  
+ TGAGTATTAT TTTGTTTTAT TATTATTATT TTTTACTTTA TTTGGACCAC TTCACTTGGG TAACAAGATT   
  
  
+ GAATATTTGA CTATTATATT ATATGTCACG TATGGATATG GTAGTTGCTT AGAGCTTTGA CCGTTTGCAG   
  
  
+ GTTTAAGTAT GATTCCCTTG GAAAGGAGCC CCTCTTTATC TTAATTCAGA AGTAACTTTT GTTTTTTTAG   
  
  
+ ATAAATTGAA AAGAAAAATA ATAAATGTCT TAGTTGAGTT TGGTTATTCA ATCCGAAATT TGATCCTGCT   
  
  
+ TAATGTATAG TATATAATAC TAATACGCAT GTAACACAAC ACCTCAGATG GTCATTGTTT TACAATTTTT   
  
  
+ TTGAGTTTAG TATAAAAATA AAATCAATTA TTTTTAGAAG TGATGTTTAA TTTGCATTTT TTTTCCACCT   
  
  
+ AAAATCACGC ACTCGAACAT TAGGGGTTTG GCTAACTCAA AGACTCAAGG TAGTGACTTA TCCAAACTTA   
  
  
+ TTACATCCTA TAGATCTATA AAAACATAAA TCTCTTACAA TCAATCCAAA TTGATTCGGT CTAATTTGAT   
  
  
+ GACCCTGATG AGGGTCCTAC AATTACACAA GTGGGCCTTA GCCCCATGGC CGTTTTTTAA TCTTCATGGG   
  
  
+ TCCAGGCGGA GTTACCATCT CCAGGTCCAT TGAGGGTGTA TCACGTCTCA AATTACGAGT TTGCCTCTTT   
  
  
+ CTACGGGGAT TATAAATATC TACCTGCATA CCTCGAAAAG GAGGAGTTGA GATCAGCGAA ACTTGGCCAT   
  
  
+ CCCAGAGCAT TGAAATGCTC AGAACCCTTT TTCTAGAAAA ATATCGTTTT TCCTGAAAAT ATCCGACCTT   
  
  
+ ATGTCATTTA TTGTCGCTTT CTTGACTCTA ACCCGGGACT CCACACTAAC TTGACTATCG GAAAGGCGTT   
  
  
+ CCCTGGATCA CCATCCAAGA TAACCTCTTT TGCAGGGTTG ACGCTCGTTG GAAGAATCGT CGAATGTAGA   
  
  
+ GCCCCTATCA TCACATAAGC CCGCTCTTAC TTCATGCCAT CCTTGAATAG CAGTTTATCC CGAAGCAAAA   
  
  
+ ATAATTCTAA CTTTCTTATA TTGTAAACCC ACATCTAAAT TTAAATTCAT ACTCCAAAAT TCCGCCACGC   
  
  
+ AAATAAGGCT TTTTAATAGG CTTACATATA ATGGCATGCT ATAGAGACAC TGTTTTGAAG AAACTATTTA   
  
  
+ AACATCAGAC CTCATATTGA GTAGCAAATC AAGACTTCTT TCCTCCAGGC CATATTGAGT AGCAAATTAA   
  
  
+ GAATAACCAG AAGCTTGTGA AAAGAGTTAG AAAATAAAAG GTATAATAGA GACTTATGTT CTTAAGATAT   
  
  
+ CTCCTAGATA TGAATGTGAC CTCCTAGTCG ATATTTGTGC TGGATTAGCG AGTTGTGTGA CTGAGTTTGT   
  
  
+ ATGTGTCTCT TAGCATATGA GTTATTCTAG CTGCTTCTTT AAGCTATGTA GTGACTGTCT TCCTTCTTAT   
  
  
+ TTATAAGTGC TATGTCCAAC TAATAGCCGA AAAAAATGGT TCGCCTTTTC TGGTTTTCTT CTTTTTGAAG   
  
  
+ GTTGATTGGC TAATTTATAT AATGGTTTCA ATACTTATCC TTTATTGTTC CACAAATCCA CCTAACCACG   
  
  
+ TACCATTGAC AATAATTACC CACTAATTTC GGTTGGACTT GATTTCCTTT TGTTTGGTAC CTCAGGCATC   
  
  
+ TTCTGTGGAC CTTCATTCAC AGTTTAATTT TCACATTTTT ATAAGGCTGC TCTTGTGTTG CAAATTTTCC   
  
  
+ TATCCATTCC ACTCTTTGCC CATTCCCAGG CTAATTAGTG TTCTCTGTTC AACCATGGAT TCTGTGCTGG   
  
  
+ TTGATCCTGA GTTCATGAAA AATCTCTACA AATTCAAACC TGAATTGCTC TCAAACTTTT CAATGAATCC   
  
  
+ AAATGACGAC ATCTTTCAAG CCCTTCATTC AGAAAATGAT CCTTTACAGT TCCTCTCATT TGATGAAGGA   
  
  
+ ACCTGTCTTA ACAGCTGCAC TAGTCAACAA GTGCCAGATT TCCCTGATGC TTGTCTCAAG TTCATCAGTG   
  
  
+ ATATTCTTCT CGAAGAGGGT TTAGATGCAA ATCCTGCATC TGCACAGGCT CTCGAAGCCA CCGAGAAGTC   
  
  
+ CTTGTATGAT GCTCTCGGGC TTGGAGAGCC ATACCCCCTT TCATGTGATC ACTTTGCGCC ATCTATCTCT   
  
  
+ ACAAGTATTG AGAGCCCAGA TGACAGTTCT TCCAATAAAA GTTATAGCAG CAATCCCGAG ATAGATGGTT   
  
  
+ CTTATGCTAT CGCTGAGCCC AGTTTCGAGT CCAACCCCAA CTGTGTGCTT GATCAACCCC AGTTGAACTC   
  
  
+ CTTTCCAGCT CTACATGAGA TTTCTCGGTC CTTGGTGGAA CTGGGTTCTC AAGCCTCTGA GTTGAGCTTC   
  
  
+ GATGATGCAG GGAGTGCCCG TGTAGAGAAA AAGGGCAAAT CGATAAAGGG CTCGAGGAGG AAGAAGAGTC   
  
  
+ GTCAAAGAGA GGGTGAAGCG TGTTATGGAG GAAGGAGCCA TAAGGTTCAA GCTTCCTTCA ATGATGATTA   
  
  
+ CTATGAGATG GAACAGTATG ATGATGTAGT ATTGCTCTGT AATAATGAAC TAACGGGCAA TAGCCGTTTC   
  
  
+ AACACCGGGA AATCTTCACC TGAGGAGGGA TGGAGGAGAT TGCAGAGAAG CCGAGGAAAG AAGCAGAACA   
  
  
+ GTTTAGCAGT TGAAGTTGAT CTGATGACCC TGCTGACTCA GTGTGCACAA GCTGTATCGA GCTTTGATCT   
  
  
+ TCGAGGTGCA AACGAGCTAC TTAGGCAAAT CAGGCAGAAT GCTTCGCCCT ATGGTGGCAG CATCCAGAGG   
  
  
+ CTCGCCCATC ATGTAGCCAA TGCTCTCGAG GCACGTATAG CTGGCACAGG CTCTACAGTC TCTACTAACC   
  
  
+ TTGTTGATGC AAAGTTCTCA GCTTCTGACT TCCTAAAGGC TTACAGGTTA TATGTCTCAG CTGTTCCTTA   
  
  
+ CAAAAGGATG TCTTTCTTTC TTGCTAACTG CTCGATTGCA AAGTTGGCAG AGAAAGCAAC AAAGATCCAT   
  
  
+ ATCATTGATT TTGGTGTTTT CCTAGGTTTA CAATGGCCTT GTTTCATACA ACACCTATCA AAAAGGCCAA   
  
  
+ ATGGACCCCC AAAACTCCGA ATCACAGGAA TCGACTACCC CCAGCAGGGT TTCAGACCTG CACAAAGGGT   
  
  
+ TGAAGCTACA GGACACCGAT TATCTGGGTA CTGTGAGCGA TTTGGGGTGC CTTTTTCTTA TCAGGGCATT   
  
  
+ GCTCAGAAGT GGGAAACTAT TCAGCCGGAG GATCTCAAGA TCGAACAAGA CGAGCTGGTG ATTGTCAACT   
  
  
+ GTTTGTTCAG GTCAGGAACA CTGCTCGATG AGACAGTCGA AGCAAACAGT CCAAGAGATG CTTTCTTAGC   
  
  
+ TTTGGTTAGA AAGCTGAATC CCAGCCTATT CATTCACGGG GTTGTCAATG GCACATTCAA CGCTCCATTC   
  
  
+ TTCGTGACTC GATTCAGAGA GGCATTGTTT CATTATTCAT CAGTGTTTGA TGTGTCTGAA GAGACAATTC   
  
  
+ CACGAGATGC CCATGAGAGG TTCTTGATTG AGAGCGAGAT TTGTGGGAAA GAACTGTTCA ATGTGGTTGC   
  
  
+ TTGTGAGGGT GCAGAGAGGG TTCAAAGGCC TGAGACATAC AAGCAGTGGC AAGTGAGGAC AACGCGGGCC   
  
  
+ GGGTTAAGGC AGGTTGCCTT GGACCAGGAG CTTATGAAGG AAGCAACGGC AATGGTGAAG GCAAATTATC   
  
  
+ ATAAGGATTT TATGGTGGAT ATAAATAGGC ATTGGATGCT TCAAGGTTGG AAGGGTAGAA CCTTGTGTGC   
  
  
+ TCTCTCATTT TGGCAACCTG CCTG  

- +Up\_Stream \_Len000GATCCC CAACTCTCTT AGGCTATCGA TACGTCAGGT TTGTAATAGA GCAGTTCGTA   
  
  
- ATGATAAATT TATTAATTAT AACTTGAACT ATCAATGTGA AAAACTTATG ATTAAGGTGT GGAGTAACGG   
  
  
- AAATAAAGGT TTGGAGCCCA ACGTAAACCT GGTGTATGGT CCTTCGAACC TGCTGTTTTT AAAGAAAAGA   
  
  
- ACTCATAATA AAACAAAATA ATAATAATAA AAAATGAAAT AAACCTGGTG AAGTGAACCC ATTGTTCTAA   
  
  
- CTTATAAACT GATAATATAA TATACAGTGC ATACCTATAC CATCAACGAA TCTCGAAACT GGCAAACGTC   
  
  
- CAAATTCATA CTAAGGGAAC CTTTCCTCGG GGAGAAATAG AATTAAGTCT TCATTGAAAA CAAAAAAATC   
  
  
- TATTTAACTT TTCTTTTTAT TATTTACAGA ATCAACTCAA ACCAATAAGT TAGGCTTTAA ACTAGGACGA   
  
  
- ATTACATATC ATATATTATG ATTATGCGTA CATTGTGTTG TGGAGTCTAC CAGTAACAAA ATGTTAAAAA   
  
  
- AACTCAAATC ATATTTTTAT TTTAGTTAAT AAAAATCTTC ACTACAAATT AAACGTAAAA AAAAGGTGGA   
  
  
- TTTTAGTGCG TGAGCTTGTA ATCCCCAAAC CGATTGAGTT TCTGAGTTCC ATCACTGAAT AGGTTTGAAT   
  
  
- AATGTAGGAT ATCTAGATAT TTTTGTATTT AGAGAATGTT AGTTAGGTTT AACTAAGCCA GATTAAACTA   
  
  
- CTGGGACTAC TCCCAGGATG TTAATGTGTT CACCCGGAAT CGGGGTACCG GCAAAAAATT AGAAGTACCC   
  
  
- AGGTCCGCCT CAATGGTAGA GGTCCAGGTA ACTCCCACAT AGTGCAGAGT TTAATGCTCA AACGGAGAAA   
  
  
- GATGCCCCTA ATATTTATAG ATGGACGTAT GGAGCTTTTC CTCCTCAACT CTAGTCGCTT TGAACCGGTA   
  
  
- GGGTCTCGTA ACTTTACGAG TCTTGGGAAA AAGATCTTTT TATAGCAAAA AGGACTTTTA TAGGCTGGAA   
  
  
- TACAGTAAAT AACAGCGAAA GAACTGAGAT TGGGCCCTGA GGTGTGATTG AACTGATAGC CTTTCCGCAA   
  
  
- GGGACCTAGT GGTAGGTTCT ATTGGAGAAA ACGTCCCAAC TGCGAGCAAC CTTCTTAGCA GCTTACATCT   
  
  
- CGGGGATAGT AGTGTATTCG GGCGAGAATG AAGTACGGTA GGAACTTATC GTCAAATAGG GCTTCGTTTT   
  
  
- TATTAAGATT GAAAGAATAT AACATTTGGG TGTAGATTTA AATTTAAGTA TGAGGTTTTA AGGCGGTGCG   
  
  
- TTTATTCCGA AAAATTATCC GAATGTATAT TACCGTACGA TATCTCTGTG ACAAAACTTC TTTGATAAAT   
  
  
- TTGTAGTCTG GAGTATAACT CATCGTTTAG TTCTGAAGAA AGGAGGTCCG GTATAACTCA TCGTTTAATT   
  
  
- CTTATTGGTC TTCGAACACT TTTCTCAATC TTTTATTTTC CATATTATCT CTGAATACAA GAATTCTATA   
  
  
- GAGGATCTAT ACTTACACTG GAGGATCAGC TATAAACACG ACCTAATCGC TCAACACACT GACTCAAACA   
  
  
- TACACAGAGA ATCGTATACT CAATAAGATC GACGAAGAAA TTCGATACAT CACTGACAGA AGGAAGAATA   
  
  
- AATATTCACG ATACAGGTTG ATTATCGGCT TTTTTTACCA AGCGGAAAAG ACCAAAAGAA GAAAAACTTC   
  
  
- CAACTAACCG ATTAAATATA TTACCAAAGT TATGAATAGG AAATAACAAG GTGTTTAGGT GGATTGGTGC   
  
  
- ATGGTAACTG TTATTAATGG GTGATTAAAG CCAACCTGAA CTAAAGGAAA ACAAACCATG GAGTCCGTAG   
  
  
- AAGACACCTG GAAGTAAGTG TCAAATTAAA AGTGTAAAAA TATTCCGACG AGAACACAAC GTTTAAAAGG   
  
  
- ATAGGTAAGG TGAGAAACGG GTAAGGGTCC GATTAATCAC AAGAGACAAG TTGGTACCTA AGACACGACC   
  
  
- AACTAGGACT CAAGTACTTT TTAGAGATGT TTAAGTTTGG ACTTAACGAG AGTTTGAAAA GTTACTTAGG   
  
  
- TTTACTGCTG TAGAAAGTTC GGGAAGTAAG TCTTTTACTA GGAAATGTCA AGGAGAGTAA ACTACTTCCT   
  
  
- TGGACAGAAT TGTCGACGTG ATCAGTTGTT CACGGTCTAA AGGGACTACG AACAGAGTTC AAGTAGTCAC   
  
  
- TATAAGAAGA GCTTCTCCCA AATCTACGTT TAGGACGTAG ACGTGTCCGA GAGCTTCGGT GGCTCTTCAG   
  
  
- GAACATACTA CGAGAGCCCG AACCTCTCGG TATGGGGGAA AGTACACTAG TGAAACGCGG TAGATAGAGA   
  
  
- TGTTCATAAC TCTCGGGTCT ACTGTCAAGA AGGTTATTTT CAATATCGTC GTTAGGGCTC TATCTACCAA   
  
  
- GAATACGATA GCGACTCGGG TCAAAGCTCA GGTTGGGGTT GACACACGAA CTAGTTGGGG TCAACTTGAG   
  
  
- GAAAGGTCGA GATGTACTCT AAAGAGCCAG GAACCACCTT GACCCAAGAG TTCGGAGACT CAACTCGAAG   
  
  
- CTACTACGTC CCTCACGGGC ACATCTCTTT TTCCCGTTTA GCTATTTCCC GAGCTCCTCC TTCTTCTCAG   
  
  
- CAGTTTCTCT CCCACTTCGC ACAATACCTC CTTCCTCGGT ATTCCAAGTT CGAAGGAAGT TACTACTAAT   
  
  
- GATACTCTAC CTTGTCATAC TACTACATCA TAACGAGACA TTATTACTTG ATTGCCCGTT ATCGGCAAAG   
  
  
- TTGTGGCCCT TTAGAAGTGG ACTCCTCCCT ACCTCCTCTA ACGTCTCTTC GGCTCCTTTC TTCGTCTTGT   
  
  
- CAAATCGTCA ACTTCAACTA GACTACTGGG ACGACTGAGT CACACGTGTT CGACATAGCT CGAAACTAGA   
  
  
- AGCTCCACGT TTGCTCGATG AATCCGTTTA GTCCGTCTTA CGAAGCGGGA TACCACCGTC GTAGGTCTCC   
  
  
- GAGCGGGTAG TACATCGGTT ACGAGAGCTC CGTGCATATC GACCGTGTCC GAGATGTCAG AGATGATTGG   
  
  
- AACAACTACG TTTCAAGAGT CGAAGACTGA AGGATTTCCG AATGTCCAAT ATACAGAGTC GACAAGGAAT   
  
  
- GTTTTCCTAC AGAAAGAAAG AACGATTGAC GAGCTAACGT TTCAACCGTC TCTTTCGTTG TTTCTAGGTA   
  
  
- TAGTAACTAA AACCACAAAA GGATCCAAAT GTTACCGGAA CAAAGTATGT TGTGGATAGT TTTTCCGGTT   
  
  
- TACCTGGGGG TTTTGAGGCT TAGTGTCCTT AGCTGATGGG GGTCGTCCCA AAGTCTGGAC GTGTTTCCCA   
  
  
- ACTTCGATGT CCTGTGGCTA ATAGACCCAT GACACTCGCT AAACCCCACG GAAAAAGAAT AGTCCCGTAA   
  
  
- CGAGTCTTCA CCCTTTGATA AGTCGGCCTC CTAGAGTTCT AGCTTGTTCT GCTCGACCAC TAACAGTTGA   
  
  
- CAAACAAGTC CAGTCCTTGT GACGAGCTAC TCTGTCAGCT TCGTTTGTCA GGTTCTCTAC GAAAGAATCG   
  
  
- AAACCAATCT TTCGACTTAG GGTCGGATAA GTAAGTGCCC CAACAGTTAC CGTGTAAGTT GCGAGGTAAG   
  
  
- AAGCACTGAG CTAAGTCTCT CCGTAACAAA GTAATAAGTA GTCACAAACT ACACAGACTT CTCTGTTAAG   
  
  
- GTGCTCTACG GGTACTCTCC AAGAACTAAC TCTCGCTCTA AACACCCTTT CTTGACAAGT TACACCAACG   
  
  
- AACACTCCCA CGTCTCTCCC AAGTTTCCGG ACTCTGTATG TTCGTCACCG TTCACTCCTG TTGCGCCCGG   
  
  
- CCCAATTCCG TCCAACGGAA CCTGGTCCTC GAATACTTCC TTCGTTGCCG TTACCACTTC CGTTTAATAG   
  
  
- TATTCCTAAA ATACCACCTA TATTTATCCG TAACCTACGA AGTTCCAACC TTCCCATCTT GGAACACACG   
  
  
- AGAGAGTAAA ACCGTTGGAC GGAC

+     CCAAT-box

| Site Name | Organism | Position | Strand | Matrix score. | sequence | function |
| --- | --- | --- | --- | --- | --- | --- |
| CCAAT-box | Hordeum vulgare | 3898 | + | 6 | CAACGG | MYBHv1 binding site |

>HU02G01571.1   
+ +Up\_Stream \_Len000CTAGGG GTTGAGAGAA TCCGATAGCT ATGCAGTCCA AACATTATCT CGTCAAGCAT   
  
  
+ TACTATTTAA ATAATTAATA TTGAACTTGA TAGTTACACT TTTTGAATAC TAATTCCACA CCTCATTGCC   
  
  
+ TTTATTTCCA AACCTCGGGT TGCATTTGGA CCACATACCA GGAAGCTTGG ACGACAAAAA TTTCTTTTCT   
  
  
+ TGAGTATTAT TTTGTTTTAT TATTATTATT TTTTACTTTA TTTGGACCAC TTCACTTGGG TAACAAGATT   
  
  
+ GAATATTTGA CTATTATATT ATATGTCACG TATGGATATG GTAGTTGCTT AGAGCTTTGA CCGTTTGCAG   
  
  
+ GTTTAAGTAT GATTCCCTTG GAAAGGAGCC CCTCTTTATC TTAATTCAGA AGTAACTTTT GTTTTTTTAG   
  
  
+ ATAAATTGAA AAGAAAAATA ATAAATGTCT TAGTTGAGTT TGGTTATTCA ATCCGAAATT TGATCCTGCT   
  
  
+ TAATGTATAG TATATAATAC TAATACGCAT GTAACACAAC ACCTCAGATG GTCATTGTTT TACAATTTTT   
  
  
+ TTGAGTTTAG TATAAAAATA AAATCAATTA TTTTTAGAAG TGATGTTTAA TTTGCATTTT TTTTCCACCT   
  
  
+ AAAATCACGC ACTCGAACAT TAGGGGTTTG GCTAACTCAA AGACTCAAGG TAGTGACTTA TCCAAACTTA   
  
  
+ TTACATCCTA TAGATCTATA AAAACATAAA TCTCTTACAA TCAATCCAAA TTGATTCGGT CTAATTTGAT   
  
  
+ GACCCTGATG AGGGTCCTAC AATTACACAA GTGGGCCTTA GCCCCATGGC CGTTTTTTAA TCTTCATGGG   
  
  
+ TCCAGGCGGA GTTACCATCT CCAGGTCCAT TGAGGGTGTA TCACGTCTCA AATTACGAGT TTGCCTCTTT   
  
  
+ CTACGGGGAT TATAAATATC TACCTGCATA CCTCGAAAAG GAGGAGTTGA GATCAGCGAA ACTTGGCCAT   
  
  
+ CCCAGAGCAT TGAAATGCTC AGAACCCTTT TTCTAGAAAA ATATCGTTTT TCCTGAAAAT ATCCGACCTT   
  
  
+ ATGTCATTTA TTGTCGCTTT CTTGACTCTA ACCCGGGACT CCACACTAAC TTGACTATCG GAAAGGCGTT   
  
  
+ CCCTGGATCA CCATCCAAGA TAACCTCTTT TGCAGGGTTG ACGCTCGTTG GAAGAATCGT CGAATGTAGA   
  
  
+ GCCCCTATCA TCACATAAGC CCGCTCTTAC TTCATGCCAT CCTTGAATAG CAGTTTATCC CGAAGCAAAA   
  
  
+ ATAATTCTAA CTTTCTTATA TTGTAAACCC ACATCTAAAT TTAAATTCAT ACTCCAAAAT TCCGCCACGC   
  
  
+ AAATAAGGCT TTTTAATAGG CTTACATATA ATGGCATGCT ATAGAGACAC TGTTTTGAAG AAACTATTTA   
  
  
+ AACATCAGAC CTCATATTGA GTAGCAAATC AAGACTTCTT TCCTCCAGGC CATATTGAGT AGCAAATTAA   
  
  
+ GAATAACCAG AAGCTTGTGA AAAGAGTTAG AAAATAAAAG GTATAATAGA GACTTATGTT CTTAAGATAT   
  
  
+ CTCCTAGATA TGAATGTGAC CTCCTAGTCG ATATTTGTGC TGGATTAGCG AGTTGTGTGA CTGAGTTTGT   
  
  
+ ATGTGTCTCT TAGCATATGA GTTATTCTAG CTGCTTCTTT AAGCTATGTA GTGACTGTCT TCCTTCTTAT   
  
  
+ TTATAAGTGC TATGTCCAAC TAATAGCCGA AAAAAATGGT TCGCCTTTTC TGGTTTTCTT CTTTTTGAAG   
  
  
+ GTTGATTGGC TAATTTATAT AATGGTTTCA ATACTTATCC TTTATTGTTC CACAAATCCA CCTAACCACG   
  
  
+ TACCATTGAC AATAATTACC CACTAATTTC GGTTGGACTT GATTTCCTTT TGTTTGGTAC CTCAGGCATC   
  
  
+ TTCTGTGGAC CTTCATTCAC AGTTTAATTT TCACATTTTT ATAAGGCTGC TCTTGTGTTG CAAATTTTCC   
  
  
+ TATCCATTCC ACTCTTTGCC CATTCCCAGG CTAATTAGTG TTCTCTGTTC AACCATGGAT TCTGTGCTGG   
  
  
+ TTGATCCTGA GTTCATGAAA AATCTCTACA AATTCAAACC TGAATTGCTC TCAAACTTTT CAATGAATCC   
  
  
+ AAATGACGAC ATCTTTCAAG CCCTTCATTC AGAAAATGAT CCTTTACAGT TCCTCTCATT TGATGAAGGA   
  
  
+ ACCTGTCTTA ACAGCTGCAC TAGTCAACAA GTGCCAGATT TCCCTGATGC TTGTCTCAAG TTCATCAGTG   
  
  
+ ATATTCTTCT CGAAGAGGGT TTAGATGCAA ATCCTGCATC TGCACAGGCT CTCGAAGCCA CCGAGAAGTC   
  
  
+ CTTGTATGAT GCTCTCGGGC TTGGAGAGCC ATACCCCCTT TCATGTGATC ACTTTGCGCC ATCTATCTCT   
  
  
+ ACAAGTATTG AGAGCCCAGA TGACAGTTCT TCCAATAAAA GTTATAGCAG CAATCCCGAG ATAGATGGTT   
  
  
+ CTTATGCTAT CGCTGAGCCC AGTTTCGAGT CCAACCCCAA CTGTGTGCTT GATCAACCCC AGTTGAACTC   
  
  
+ CTTTCCAGCT CTACATGAGA TTTCTCGGTC CTTGGTGGAA CTGGGTTCTC AAGCCTCTGA GTTGAGCTTC   
  
  
+ GATGATGCAG GGAGTGCCCG TGTAGAGAAA AAGGGCAAAT CGATAAAGGG CTCGAGGAGG AAGAAGAGTC   
  
  
+ GTCAAAGAGA GGGTGAAGCG TGTTATGGAG GAAGGAGCCA TAAGGTTCAA GCTTCCTTCA ATGATGATTA   
  
  
+ CTATGAGATG GAACAGTATG ATGATGTAGT ATTGCTCTGT AATAATGAAC TAACGGGCAA TAGCCGTTTC   
  
  
+ AACACCGGGA AATCTTCACC TGAGGAGGGA TGGAGGAGAT TGCAGAGAAG CCGAGGAAAG AAGCAGAACA   
  
  
+ GTTTAGCAGT TGAAGTTGAT CTGATGACCC TGCTGACTCA GTGTGCACAA GCTGTATCGA GCTTTGATCT   
  
  
+ TCGAGGTGCA AACGAGCTAC TTAGGCAAAT CAGGCAGAAT GCTTCGCCCT ATGGTGGCAG CATCCAGAGG   
  
  
+ CTCGCCCATC ATGTAGCCAA TGCTCTCGAG GCACGTATAG CTGGCACAGG CTCTACAGTC TCTACTAACC   
  
  
+ TTGTTGATGC AAAGTTCTCA GCTTCTGACT TCCTAAAGGC TTACAGGTTA TATGTCTCAG CTGTTCCTTA   
  
  
+ CAAAAGGATG TCTTTCTTTC TTGCTAACTG CTCGATTGCA AAGTTGGCAG AGAAAGCAAC AAAGATCCAT   
  
  
+ ATCATTGATT TTGGTGTTTT CCTAGGTTTA CAATGGCCTT GTTTCATACA ACACCTATCA AAAAGGCCAA   
  
  
+ ATGGACCCCC AAAACTCCGA ATCACAGGAA TCGACTACCC CCAGCAGGGT TTCAGACCTG CACAAAGGGT   
  
  
+ TGAAGCTACA GGACACCGAT TATCTGGGTA CTGTGAGCGA TTTGGGGTGC CTTTTTCTTA TCAGGGCATT   
  
  
+ GCTCAGAAGT GGGAAACTAT TCAGCCGGAG GATCTCAAGA TCGAACAAGA CGAGCTGGTG ATTGTCAACT   
  
  
+ GTTTGTTCAG GTCAGGAACA CTGCTCGATG AGACAGTCGA AGCAAACAGT CCAAGAGATG CTTTCTTAGC   
  
  
+ TTTGGTTAGA AAGCTGAATC CCAGCCTATT CATTCACGGG GTTGTCAATG GCACATTCAA CGCTCCATTC   
  
  
+ TTCGTGACTC GATTCAGAGA GGCATTGTTT CATTATTCAT CAGTGTTTGA TGTGTCTGAA GAGACAATTC   
  
  
+ CACGAGATGC CCATGAGAGG TTCTTGATTG AGAGCGAGAT TTGTGGGAAA GAACTGTTCA ATGTGGTTGC   
  
  
+ TTGTGAGGGT GCAGAGAGGG TTCAAAGGCC TGAGACATAC AAGCAGTGGC AAGTGAGGAC AACGCGGGCC   
  
  
+ GGGTTAAGGC AGGTTGCCTT GGACCAGGAG CTTATGAAGG AAGCAACGGC AATGGTGAAG GCAAATTATC   
  
  
+ ATAAGGATTT TATGGTGGAT ATAAATAGGC ATTGGATGCT TCAAGGTTGG AAGGGTAGAA CCTTGTGTGC   
  
  
+ TCTCTCATTT TGGCAACCTG CCTG  

- +Up\_Stream \_Len000GATCCC CAACTCTCTT AGGCTATCGA TACGTCAGGT TTGTAATAGA GCAGTTCGTA   
  
  
- ATGATAAATT TATTAATTAT AACTTGAACT ATCAATGTGA AAAACTTATG ATTAAGGTGT GGAGTAACGG   
  
  
- AAATAAAGGT TTGGAGCCCA ACGTAAACCT GGTGTATGGT CCTTCGAACC TGCTGTTTTT AAAGAAAAGA   
  
  
- ACTCATAATA AAACAAAATA ATAATAATAA AAAATGAAAT AAACCTGGTG AAGTGAACCC ATTGTTCTAA   
  
  
- CTTATAAACT GATAATATAA TATACAGTGC ATACCTATAC CATCAACGAA TCTCGAAACT GGCAAACGTC   
  
  
- CAAATTCATA CTAAGGGAAC CTTTCCTCGG GGAGAAATAG AATTAAGTCT TCATTGAAAA CAAAAAAATC   
  
  
- TATTTAACTT TTCTTTTTAT TATTTACAGA ATCAACTCAA ACCAATAAGT TAGGCTTTAA ACTAGGACGA   
  
  
- ATTACATATC ATATATTATG ATTATGCGTA CATTGTGTTG TGGAGTCTAC CAGTAACAAA ATGTTAAAAA   
  
  
- AACTCAAATC ATATTTTTAT TTTAGTTAAT AAAAATCTTC ACTACAAATT AAACGTAAAA AAAAGGTGGA   
  
  
- TTTTAGTGCG TGAGCTTGTA ATCCCCAAAC CGATTGAGTT TCTGAGTTCC ATCACTGAAT AGGTTTGAAT   
  
  
- AATGTAGGAT ATCTAGATAT TTTTGTATTT AGAGAATGTT AGTTAGGTTT AACTAAGCCA GATTAAACTA   
  
  
- CTGGGACTAC TCCCAGGATG TTAATGTGTT CACCCGGAAT CGGGGTACCG GCAAAAAATT AGAAGTACCC   
  
  
- AGGTCCGCCT CAATGGTAGA GGTCCAGGTA ACTCCCACAT AGTGCAGAGT TTAATGCTCA AACGGAGAAA   
  
  
- GATGCCCCTA ATATTTATAG ATGGACGTAT GGAGCTTTTC CTCCTCAACT CTAGTCGCTT TGAACCGGTA   
  
  
- GGGTCTCGTA ACTTTACGAG TCTTGGGAAA AAGATCTTTT TATAGCAAAA AGGACTTTTA TAGGCTGGAA   
  
  
- TACAGTAAAT AACAGCGAAA GAACTGAGAT TGGGCCCTGA GGTGTGATTG AACTGATAGC CTTTCCGCAA   
  
  
- GGGACCTAGT GGTAGGTTCT ATTGGAGAAA ACGTCCCAAC TGCGAGCAAC CTTCTTAGCA GCTTACATCT   
  
  
- CGGGGATAGT AGTGTATTCG GGCGAGAATG AAGTACGGTA GGAACTTATC GTCAAATAGG GCTTCGTTTT   
  
  
- TATTAAGATT GAAAGAATAT AACATTTGGG TGTAGATTTA AATTTAAGTA TGAGGTTTTA AGGCGGTGCG   
  
  
- TTTATTCCGA AAAATTATCC GAATGTATAT TACCGTACGA TATCTCTGTG ACAAAACTTC TTTGATAAAT   
  
  
- TTGTAGTCTG GAGTATAACT CATCGTTTAG TTCTGAAGAA AGGAGGTCCG GTATAACTCA TCGTTTAATT   
  
  
- CTTATTGGTC TTCGAACACT TTTCTCAATC TTTTATTTTC CATATTATCT CTGAATACAA GAATTCTATA   
  
  
- GAGGATCTAT ACTTACACTG GAGGATCAGC TATAAACACG ACCTAATCGC TCAACACACT GACTCAAACA   
  
  
- TACACAGAGA ATCGTATACT CAATAAGATC GACGAAGAAA TTCGATACAT CACTGACAGA AGGAAGAATA   
  
  
- AATATTCACG ATACAGGTTG ATTATCGGCT TTTTTTACCA AGCGGAAAAG ACCAAAAGAA GAAAAACTTC   
  
  
- CAACTAACCG ATTAAATATA TTACCAAAGT TATGAATAGG AAATAACAAG GTGTTTAGGT GGATTGGTGC   
  
  
- ATGGTAACTG TTATTAATGG GTGATTAAAG CCAACCTGAA CTAAAGGAAA ACAAACCATG GAGTCCGTAG   
  
  
- AAGACACCTG GAAGTAAGTG TCAAATTAAA AGTGTAAAAA TATTCCGACG AGAACACAAC GTTTAAAAGG   
  
  
- ATAGGTAAGG TGAGAAACGG GTAAGGGTCC GATTAATCAC AAGAGACAAG TTGGTACCTA AGACACGACC   
  
  
- AACTAGGACT CAAGTACTTT TTAGAGATGT TTAAGTTTGG ACTTAACGAG AGTTTGAAAA GTTACTTAGG   
  
  
- TTTACTGCTG TAGAAAGTTC GGGAAGTAAG TCTTTTACTA GGAAATGTCA AGGAGAGTAA ACTACTTCCT   
  
  
- TGGACAGAAT TGTCGACGTG ATCAGTTGTT CACGGTCTAA AGGGACTACG AACAGAGTTC AAGTAGTCAC   
  
  
- TATAAGAAGA GCTTCTCCCA AATCTACGTT TAGGACGTAG ACGTGTCCGA GAGCTTCGGT GGCTCTTCAG   
  
  
- GAACATACTA CGAGAGCCCG AACCTCTCGG TATGGGGGAA AGTACACTAG TGAAACGCGG TAGATAGAGA   
  
  
- TGTTCATAAC TCTCGGGTCT ACTGTCAAGA AGGTTATTTT CAATATCGTC GTTAGGGCTC TATCTACCAA   
  
  
- GAATACGATA GCGACTCGGG TCAAAGCTCA GGTTGGGGTT GACACACGAA CTAGTTGGGG TCAACTTGAG   
  
  
- GAAAGGTCGA GATGTACTCT AAAGAGCCAG GAACCACCTT GACCCAAGAG TTCGGAGACT CAACTCGAAG   
  
  
- CTACTACGTC CCTCACGGGC ACATCTCTTT TTCCCGTTTA GCTATTTCCC GAGCTCCTCC TTCTTCTCAG   
  
  
- CAGTTTCTCT CCCACTTCGC ACAATACCTC CTTCCTCGGT ATTCCAAGTT CGAAGGAAGT TACTACTAAT   
  
  
- GATACTCTAC CTTGTCATAC TACTACATCA TAACGAGACA TTATTACTTG ATTGCCCGTT ATCGGCAAAG   
  
  
- TTGTGGCCCT TTAGAAGTGG ACTCCTCCCT ACCTCCTCTA ACGTCTCTTC GGCTCCTTTC TTCGTCTTGT   
  
  
- CAAATCGTCA ACTTCAACTA GACTACTGGG ACGACTGAGT CACACGTGTT CGACATAGCT CGAAACTAGA   
  
  
- AGCTCCACGT TTGCTCGATG AATCCGTTTA GTCCGTCTTA CGAAGCGGGA TACCACCGTC GTAGGTCTCC   
  
  
- GAGCGGGTAG TACATCGGTT ACGAGAGCTC CGTGCATATC GACCGTGTCC GAGATGTCAG AGATGATTGG   
  
  
- AACAACTACG TTTCAAGAGT CGAAGACTGA AGGATTTCCG AATGTCCAAT ATACAGAGTC GACAAGGAAT   
  
  
- GTTTTCCTAC AGAAAGAAAG AACGATTGAC GAGCTAACGT TTCAACCGTC TCTTTCGTTG TTTCTAGGTA   
  
  
- TAGTAACTAA AACCACAAAA GGATCCAAAT GTTACCGGAA CAAAGTATGT TGTGGATAGT TTTTCCGGTT   
  
  
- TACCTGGGGG TTTTGAGGCT TAGTGTCCTT AGCTGATGGG GGTCGTCCCA AAGTCTGGAC GTGTTTCCCA   
  
  
- ACTTCGATGT CCTGTGGCTA ATAGACCCAT GACACTCGCT AAACCCCACG GAAAAAGAAT AGTCCCGTAA   
  
  
- CGAGTCTTCA CCCTTTGATA AGTCGGCCTC CTAGAGTTCT AGCTTGTTCT GCTCGACCAC TAACAGTTGA   
  
  
- CAAACAAGTC CAGTCCTTGT GACGAGCTAC TCTGTCAGCT TCGTTTGTCA GGTTCTCTAC GAAAGAATCG   
  
  
- AAACCAATCT TTCGACTTAG GGTCGGATAA GTAAGTGCCC CAACAGTTAC CGTGTAAGTT GCGAGGTAAG   
  
  
- AAGCACTGAG CTAAGTCTCT CCGTAACAAA GTAATAAGTA GTCACAAACT ACACAGACTT CTCTGTTAAG   
  
  
- GTGCTCTACG GGTACTCTCC AAGAACTAAC TCTCGCTCTA AACACCCTTT CTTGACAAGT TACACCAACG   
  
  
- AACACTCCCA CGTCTCTCCC AAGTTTCCGG ACTCTGTATG TTCGTCACCG TTCACTCCTG TTGCGCCCGG   
  
  
- CCCAATTCCG TCCAACGGAA CCTGGTCCTC GAATACTTCC TTCGTTGCCG TTACCACTTC CGTTTAATAG   
  
  
- TATTCCTAAA ATACCACCTA TATTTATCCG TAACCTACGA AGTTCCAACC TTCCCATCTT GGAACACACG   
  
  
- AGAGAGTAAA ACCGTTGGAC GGAC

+     CGTCA-motif

| Site Name | Organism | Position | Strand | Matrix score. | sequence | function |
| --- | --- | --- | --- | --- | --- | --- |
| CGTCA-motif | Hordeum vulgare | 2664 | + | 5 | CGTCA | cis-acting regulatory element involved in the MeJA-responsiveness |
| CGTCA-motif | Hordeum vulgare | 2108 | - | 5 | CGTCA | cis-acting regulatory element involved in the MeJA-responsiveness |
| CGTCA-motif | Hordeum vulgare | 65 | + | 5 | CGTCA | cis-acting regulatory element involved in the MeJA-responsiveness |
| CGTCA-motif | Hordeum vulgare | 1163 | - | 5 | CGTCA | cis-acting regulatory element involved in the MeJA-responsiveness |

>HU02G01571.1   
+ +Up\_Stream \_Len000CTAGGG GTTGAGAGAA TCCGATAGCT ATGCAGTCCA AACATTATCT CGTCAAGCAT   
  
  
+ TACTATTTAA ATAATTAATA TTGAACTTGA TAGTTACACT TTTTGAATAC TAATTCCACA CCTCATTGCC   
  
  
+ TTTATTTCCA AACCTCGGGT TGCATTTGGA CCACATACCA GGAAGCTTGG ACGACAAAAA TTTCTTTTCT   
  
  
+ TGAGTATTAT TTTGTTTTAT TATTATTATT TTTTACTTTA TTTGGACCAC TTCACTTGGG TAACAAGATT   
  
  
+ GAATATTTGA CTATTATATT ATATGTCACG TATGGATATG GTAGTTGCTT AGAGCTTTGA CCGTTTGCAG   
  
  
+ GTTTAAGTAT GATTCCCTTG GAAAGGAGCC CCTCTTTATC TTAATTCAGA AGTAACTTTT GTTTTTTTAG   
  
  
+ ATAAATTGAA AAGAAAAATA ATAAATGTCT TAGTTGAGTT TGGTTATTCA ATCCGAAATT TGATCCTGCT   
  
  
+ TAATGTATAG TATATAATAC TAATACGCAT GTAACACAAC ACCTCAGATG GTCATTGTTT TACAATTTTT   
  
  
+ TTGAGTTTAG TATAAAAATA AAATCAATTA TTTTTAGAAG TGATGTTTAA TTTGCATTTT TTTTCCACCT   
  
  
+ AAAATCACGC ACTCGAACAT TAGGGGTTTG GCTAACTCAA AGACTCAAGG TAGTGACTTA TCCAAACTTA   
  
  
+ TTACATCCTA TAGATCTATA AAAACATAAA TCTCTTACAA TCAATCCAAA TTGATTCGGT CTAATTTGAT   
  
  
+ GACCCTGATG AGGGTCCTAC AATTACACAA GTGGGCCTTA GCCCCATGGC CGTTTTTTAA TCTTCATGGG   
  
  
+ TCCAGGCGGA GTTACCATCT CCAGGTCCAT TGAGGGTGTA TCACGTCTCA AATTACGAGT TTGCCTCTTT   
  
  
+ CTACGGGGAT TATAAATATC TACCTGCATA CCTCGAAAAG GAGGAGTTGA GATCAGCGAA ACTTGGCCAT   
  
  
+ CCCAGAGCAT TGAAATGCTC AGAACCCTTT TTCTAGAAAA ATATCGTTTT TCCTGAAAAT ATCCGACCTT   
  
  
+ ATGTCATTTA TTGTCGCTTT CTTGACTCTA ACCCGGGACT CCACACTAAC TTGACTATCG GAAAGGCGTT   
  
  
+ CCCTGGATCA CCATCCAAGA TAACCTCTTT TGCAGGGTTG ACGCTCGTTG GAAGAATCGT CGAATGTAGA   
  
  
+ GCCCCTATCA TCACATAAGC CCGCTCTTAC TTCATGCCAT CCTTGAATAG CAGTTTATCC CGAAGCAAAA   
  
  
+ ATAATTCTAA CTTTCTTATA TTGTAAACCC ACATCTAAAT TTAAATTCAT ACTCCAAAAT TCCGCCACGC   
  
  
+ AAATAAGGCT TTTTAATAGG CTTACATATA ATGGCATGCT ATAGAGACAC TGTTTTGAAG AAACTATTTA   
  
  
+ AACATCAGAC CTCATATTGA GTAGCAAATC AAGACTTCTT TCCTCCAGGC CATATTGAGT AGCAAATTAA   
  
  
+ GAATAACCAG AAGCTTGTGA AAAGAGTTAG AAAATAAAAG GTATAATAGA GACTTATGTT CTTAAGATAT   
  
  
+ CTCCTAGATA TGAATGTGAC CTCCTAGTCG ATATTTGTGC TGGATTAGCG AGTTGTGTGA CTGAGTTTGT   
  
  
+ ATGTGTCTCT TAGCATATGA GTTATTCTAG CTGCTTCTTT AAGCTATGTA GTGACTGTCT TCCTTCTTAT   
  
  
+ TTATAAGTGC TATGTCCAAC TAATAGCCGA AAAAAATGGT TCGCCTTTTC TGGTTTTCTT CTTTTTGAAG   
  
  
+ GTTGATTGGC TAATTTATAT AATGGTTTCA ATACTTATCC TTTATTGTTC CACAAATCCA CCTAACCACG   
  
  
+ TACCATTGAC AATAATTACC CACTAATTTC GGTTGGACTT GATTTCCTTT TGTTTGGTAC CTCAGGCATC   
  
  
+ TTCTGTGGAC CTTCATTCAC AGTTTAATTT TCACATTTTT ATAAGGCTGC TCTTGTGTTG CAAATTTTCC   
  
  
+ TATCCATTCC ACTCTTTGCC CATTCCCAGG CTAATTAGTG TTCTCTGTTC AACCATGGAT TCTGTGCTGG   
  
  
+ TTGATCCTGA GTTCATGAAA AATCTCTACA AATTCAAACC TGAATTGCTC TCAAACTTTT CAATGAATCC   
  
  
+ AAATGACGAC ATCTTTCAAG CCCTTCATTC AGAAAATGAT CCTTTACAGT TCCTCTCATT TGATGAAGGA   
  
  
+ ACCTGTCTTA ACAGCTGCAC TAGTCAACAA GTGCCAGATT TCCCTGATGC TTGTCTCAAG TTCATCAGTG   
  
  
+ ATATTCTTCT CGAAGAGGGT TTAGATGCAA ATCCTGCATC TGCACAGGCT CTCGAAGCCA CCGAGAAGTC   
  
  
+ CTTGTATGAT GCTCTCGGGC TTGGAGAGCC ATACCCCCTT TCATGTGATC ACTTTGCGCC ATCTATCTCT   
  
  
+ ACAAGTATTG AGAGCCCAGA TGACAGTTCT TCCAATAAAA GTTATAGCAG CAATCCCGAG ATAGATGGTT   
  
  
+ CTTATGCTAT CGCTGAGCCC AGTTTCGAGT CCAACCCCAA CTGTGTGCTT GATCAACCCC AGTTGAACTC   
  
  
+ CTTTCCAGCT CTACATGAGA TTTCTCGGTC CTTGGTGGAA CTGGGTTCTC AAGCCTCTGA GTTGAGCTTC   
  
  
+ GATGATGCAG GGAGTGCCCG TGTAGAGAAA AAGGGCAAAT CGATAAAGGG CTCGAGGAGG AAGAAGAGTC   
  
  
+ GTCAAAGAGA GGGTGAAGCG TGTTATGGAG GAAGGAGCCA TAAGGTTCAA GCTTCCTTCA ATGATGATTA   
  
  
+ CTATGAGATG GAACAGTATG ATGATGTAGT ATTGCTCTGT AATAATGAAC TAACGGGCAA TAGCCGTTTC   
  
  
+ AACACCGGGA AATCTTCACC TGAGGAGGGA TGGAGGAGAT TGCAGAGAAG CCGAGGAAAG AAGCAGAACA   
  
  
+ GTTTAGCAGT TGAAGTTGAT CTGATGACCC TGCTGACTCA GTGTGCACAA GCTGTATCGA GCTTTGATCT   
  
  
+ TCGAGGTGCA AACGAGCTAC TTAGGCAAAT CAGGCAGAAT GCTTCGCCCT ATGGTGGCAG CATCCAGAGG   
  
  
+ CTCGCCCATC ATGTAGCCAA TGCTCTCGAG GCACGTATAG CTGGCACAGG CTCTACAGTC TCTACTAACC   
  
  
+ TTGTTGATGC AAAGTTCTCA GCTTCTGACT TCCTAAAGGC TTACAGGTTA TATGTCTCAG CTGTTCCTTA   
  
  
+ CAAAAGGATG TCTTTCTTTC TTGCTAACTG CTCGATTGCA AAGTTGGCAG AGAAAGCAAC AAAGATCCAT   
  
  
+ ATCATTGATT TTGGTGTTTT CCTAGGTTTA CAATGGCCTT GTTTCATACA ACACCTATCA AAAAGGCCAA   
  
  
+ ATGGACCCCC AAAACTCCGA ATCACAGGAA TCGACTACCC CCAGCAGGGT TTCAGACCTG CACAAAGGGT   
  
  
+ TGAAGCTACA GGACACCGAT TATCTGGGTA CTGTGAGCGA TTTGGGGTGC CTTTTTCTTA TCAGGGCATT   
  
  
+ GCTCAGAAGT GGGAAACTAT TCAGCCGGAG GATCTCAAGA TCGAACAAGA CGAGCTGGTG ATTGTCAACT   
  
  
+ GTTTGTTCAG GTCAGGAACA CTGCTCGATG AGACAGTCGA AGCAAACAGT CCAAGAGATG CTTTCTTAGC   
  
  
+ TTTGGTTAGA AAGCTGAATC CCAGCCTATT CATTCACGGG GTTGTCAATG GCACATTCAA CGCTCCATTC   
  
  
+ TTCGTGACTC GATTCAGAGA GGCATTGTTT CATTATTCAT CAGTGTTTGA TGTGTCTGAA GAGACAATTC   
  
  
+ CACGAGATGC CCATGAGAGG TTCTTGATTG AGAGCGAGAT TTGTGGGAAA GAACTGTTCA ATGTGGTTGC   
  
  
+ TTGTGAGGGT GCAGAGAGGG TTCAAAGGCC TGAGACATAC AAGCAGTGGC AAGTGAGGAC AACGCGGGCC   
  
  
+ GGGTTAAGGC AGGTTGCCTT GGACCAGGAG CTTATGAAGG AAGCAACGGC AATGGTGAAG GCAAATTATC   
  
  
+ ATAAGGATTT TATGGTGGAT ATAAATAGGC ATTGGATGCT TCAAGGTTGG AAGGGTAGAA CCTTGTGTGC   
  
  
+ TCTCTCATTT TGGCAACCTG CCTG  

- +Up\_Stream \_Len000GATCCC CAACTCTCTT AGGCTATCGA TACGTCAGGT TTGTAATAGA GCAGTTCGTA   
  
  
- ATGATAAATT TATTAATTAT AACTTGAACT ATCAATGTGA AAAACTTATG ATTAAGGTGT GGAGTAACGG   
  
  
- AAATAAAGGT TTGGAGCCCA ACGTAAACCT GGTGTATGGT CCTTCGAACC TGCTGTTTTT AAAGAAAAGA   
  
  
- ACTCATAATA AAACAAAATA ATAATAATAA AAAATGAAAT AAACCTGGTG AAGTGAACCC ATTGTTCTAA   
  
  
- CTTATAAACT GATAATATAA TATACAGTGC ATACCTATAC CATCAACGAA TCTCGAAACT GGCAAACGTC   
  
  
- CAAATTCATA CTAAGGGAAC CTTTCCTCGG GGAGAAATAG AATTAAGTCT TCATTGAAAA CAAAAAAATC   
  
  
- TATTTAACTT TTCTTTTTAT TATTTACAGA ATCAACTCAA ACCAATAAGT TAGGCTTTAA ACTAGGACGA   
  
  
- ATTACATATC ATATATTATG ATTATGCGTA CATTGTGTTG TGGAGTCTAC CAGTAACAAA ATGTTAAAAA   
  
  
- AACTCAAATC ATATTTTTAT TTTAGTTAAT AAAAATCTTC ACTACAAATT AAACGTAAAA AAAAGGTGGA   
  
  
- TTTTAGTGCG TGAGCTTGTA ATCCCCAAAC CGATTGAGTT TCTGAGTTCC ATCACTGAAT AGGTTTGAAT   
  
  
- AATGTAGGAT ATCTAGATAT TTTTGTATTT AGAGAATGTT AGTTAGGTTT AACTAAGCCA GATTAAACTA   
  
  
- CTGGGACTAC TCCCAGGATG TTAATGTGTT CACCCGGAAT CGGGGTACCG GCAAAAAATT AGAAGTACCC   
  
  
- AGGTCCGCCT CAATGGTAGA GGTCCAGGTA ACTCCCACAT AGTGCAGAGT TTAATGCTCA AACGGAGAAA   
  
  
- GATGCCCCTA ATATTTATAG ATGGACGTAT GGAGCTTTTC CTCCTCAACT CTAGTCGCTT TGAACCGGTA   
  
  
- GGGTCTCGTA ACTTTACGAG TCTTGGGAAA AAGATCTTTT TATAGCAAAA AGGACTTTTA TAGGCTGGAA   
  
  
- TACAGTAAAT AACAGCGAAA GAACTGAGAT TGGGCCCTGA GGTGTGATTG AACTGATAGC CTTTCCGCAA   
  
  
- GGGACCTAGT GGTAGGTTCT ATTGGAGAAA ACGTCCCAAC TGCGAGCAAC CTTCTTAGCA GCTTACATCT   
  
  
- CGGGGATAGT AGTGTATTCG GGCGAGAATG AAGTACGGTA GGAACTTATC GTCAAATAGG GCTTCGTTTT   
  
  
- TATTAAGATT GAAAGAATAT AACATTTGGG TGTAGATTTA AATTTAAGTA TGAGGTTTTA AGGCGGTGCG   
  
  
- TTTATTCCGA AAAATTATCC GAATGTATAT TACCGTACGA TATCTCTGTG ACAAAACTTC TTTGATAAAT   
  
  
- TTGTAGTCTG GAGTATAACT CATCGTTTAG TTCTGAAGAA AGGAGGTCCG GTATAACTCA TCGTTTAATT   
  
  
- CTTATTGGTC TTCGAACACT TTTCTCAATC TTTTATTTTC CATATTATCT CTGAATACAA GAATTCTATA   
  
  
- GAGGATCTAT ACTTACACTG GAGGATCAGC TATAAACACG ACCTAATCGC TCAACACACT GACTCAAACA   
  
  
- TACACAGAGA ATCGTATACT CAATAAGATC GACGAAGAAA TTCGATACAT CACTGACAGA AGGAAGAATA   
  
  
- AATATTCACG ATACAGGTTG ATTATCGGCT TTTTTTACCA AGCGGAAAAG ACCAAAAGAA GAAAAACTTC   
  
  
- CAACTAACCG ATTAAATATA TTACCAAAGT TATGAATAGG AAATAACAAG GTGTTTAGGT GGATTGGTGC   
  
  
- ATGGTAACTG TTATTAATGG GTGATTAAAG CCAACCTGAA CTAAAGGAAA ACAAACCATG GAGTCCGTAG   
  
  
- AAGACACCTG GAAGTAAGTG TCAAATTAAA AGTGTAAAAA TATTCCGACG AGAACACAAC GTTTAAAAGG   
  
  
- ATAGGTAAGG TGAGAAACGG GTAAGGGTCC GATTAATCAC AAGAGACAAG TTGGTACCTA AGACACGACC   
  
  
- AACTAGGACT CAAGTACTTT TTAGAGATGT TTAAGTTTGG ACTTAACGAG AGTTTGAAAA GTTACTTAGG   
  
  
- TTTACTGCTG TAGAAAGTTC GGGAAGTAAG TCTTTTACTA GGAAATGTCA AGGAGAGTAA ACTACTTCCT   
  
  
- TGGACAGAAT TGTCGACGTG ATCAGTTGTT CACGGTCTAA AGGGACTACG AACAGAGTTC AAGTAGTCAC   
  
  
- TATAAGAAGA GCTTCTCCCA AATCTACGTT TAGGACGTAG ACGTGTCCGA GAGCTTCGGT GGCTCTTCAG   
  
  
- GAACATACTA CGAGAGCCCG AACCTCTCGG TATGGGGGAA AGTACACTAG TGAAACGCGG TAGATAGAGA   
  
  
- TGTTCATAAC TCTCGGGTCT ACTGTCAAGA AGGTTATTTT CAATATCGTC GTTAGGGCTC TATCTACCAA   
  
  
- GAATACGATA GCGACTCGGG TCAAAGCTCA GGTTGGGGTT GACACACGAA CTAGTTGGGG TCAACTTGAG   
  
  
- GAAAGGTCGA GATGTACTCT AAAGAGCCAG GAACCACCTT GACCCAAGAG TTCGGAGACT CAACTCGAAG   
  
  
- CTACTACGTC CCTCACGGGC ACATCTCTTT TTCCCGTTTA GCTATTTCCC GAGCTCCTCC TTCTTCTCAG   
  
  
- CAGTTTCTCT CCCACTTCGC ACAATACCTC CTTCCTCGGT ATTCCAAGTT CGAAGGAAGT TACTACTAAT   
  
  
- GATACTCTAC CTTGTCATAC TACTACATCA TAACGAGACA TTATTACTTG ATTGCCCGTT ATCGGCAAAG   
  
  
- TTGTGGCCCT TTAGAAGTGG ACTCCTCCCT ACCTCCTCTA ACGTCTCTTC GGCTCCTTTC TTCGTCTTGT   
  
  
- CAAATCGTCA ACTTCAACTA GACTACTGGG ACGACTGAGT CACACGTGTT CGACATAGCT CGAAACTAGA   
  
  
- AGCTCCACGT TTGCTCGATG AATCCGTTTA GTCCGTCTTA CGAAGCGGGA TACCACCGTC GTAGGTCTCC   
  
  
- GAGCGGGTAG TACATCGGTT ACGAGAGCTC CGTGCATATC GACCGTGTCC GAGATGTCAG AGATGATTGG   
  
  
- AACAACTACG TTTCAAGAGT CGAAGACTGA AGGATTTCCG AATGTCCAAT ATACAGAGTC GACAAGGAAT   
  
  
- GTTTTCCTAC AGAAAGAAAG AACGATTGAC GAGCTAACGT TTCAACCGTC TCTTTCGTTG TTTCTAGGTA   
  
  
- TAGTAACTAA AACCACAAAA GGATCCAAAT GTTACCGGAA CAAAGTATGT TGTGGATAGT TTTTCCGGTT   
  
  
- TACCTGGGGG TTTTGAGGCT TAGTGTCCTT AGCTGATGGG GGTCGTCCCA AAGTCTGGAC GTGTTTCCCA   
  
  
- ACTTCGATGT CCTGTGGCTA ATAGACCCAT GACACTCGCT AAACCCCACG GAAAAAGAAT AGTCCCGTAA   
  
  
- CGAGTCTTCA CCCTTTGATA AGTCGGCCTC CTAGAGTTCT AGCTTGTTCT GCTCGACCAC TAACAGTTGA   
  
  
- CAAACAAGTC CAGTCCTTGT GACGAGCTAC TCTGTCAGCT TCGTTTGTCA GGTTCTCTAC GAAAGAATCG   
  
  
- AAACCAATCT TTCGACTTAG GGTCGGATAA GTAAGTGCCC CAACAGTTAC CGTGTAAGTT GCGAGGTAAG   
  
  
- AAGCACTGAG CTAAGTCTCT CCGTAACAAA GTAATAAGTA GTCACAAACT ACACAGACTT CTCTGTTAAG   
  
  
- GTGCTCTACG GGTACTCTCC AAGAACTAAC TCTCGCTCTA AACACCCTTT CTTGACAAGT TACACCAACG   
  
  
- AACACTCCCA CGTCTCTCCC AAGTTTCCGG ACTCTGTATG TTCGTCACCG TTCACTCCTG TTGCGCCCGG   
  
  
- CCCAATTCCG TCCAACGGAA CCTGGTCCTC GAATACTTCC TTCGTTGCCG TTACCACTTC CGTTTAATAG   
  
  
- TATTCCTAAA ATACCACCTA TATTTATCCG TAACCTACGA AGTTCCAACC TTCCCATCTT GGAACACACG   
  
  
- AGAGAGTAAA ACCGTTGGAC GGAC

+     DRE1

| Site Name | Organism | Position | Strand | Matrix score. | sequence | function |
| --- | --- | --- | --- | --- | --- | --- |
| DRE1 | Zea mays | 2547 | - | 7 | ACCGAGA |  |
| DRE1 | Zea mays | 2304 | + | 7 | ACCGAGA |  |

>HU02G01571.1   
+ +Up\_Stream \_Len000CTAGGG GTTGAGAGAA TCCGATAGCT ATGCAGTCCA AACATTATCT CGTCAAGCAT   
  
  
+ TACTATTTAA ATAATTAATA TTGAACTTGA TAGTTACACT TTTTGAATAC TAATTCCACA CCTCATTGCC   
  
  
+ TTTATTTCCA AACCTCGGGT TGCATTTGGA CCACATACCA GGAAGCTTGG ACGACAAAAA TTTCTTTTCT   
  
  
+ TGAGTATTAT TTTGTTTTAT TATTATTATT TTTTACTTTA TTTGGACCAC TTCACTTGGG TAACAAGATT   
  
  
+ GAATATTTGA CTATTATATT ATATGTCACG TATGGATATG GTAGTTGCTT AGAGCTTTGA CCGTTTGCAG   
  
  
+ GTTTAAGTAT GATTCCCTTG GAAAGGAGCC CCTCTTTATC TTAATTCAGA AGTAACTTTT GTTTTTTTAG   
  
  
+ ATAAATTGAA AAGAAAAATA ATAAATGTCT TAGTTGAGTT TGGTTATTCA ATCCGAAATT TGATCCTGCT   
  
  
+ TAATGTATAG TATATAATAC TAATACGCAT GTAACACAAC ACCTCAGATG GTCATTGTTT TACAATTTTT   
  
  
+ TTGAGTTTAG TATAAAAATA AAATCAATTA TTTTTAGAAG TGATGTTTAA TTTGCATTTT TTTTCCACCT   
  
  
+ AAAATCACGC ACTCGAACAT TAGGGGTTTG GCTAACTCAA AGACTCAAGG TAGTGACTTA TCCAAACTTA   
  
  
+ TTACATCCTA TAGATCTATA AAAACATAAA TCTCTTACAA TCAATCCAAA TTGATTCGGT CTAATTTGAT   
  
  
+ GACCCTGATG AGGGTCCTAC AATTACACAA GTGGGCCTTA GCCCCATGGC CGTTTTTTAA TCTTCATGGG   
  
  
+ TCCAGGCGGA GTTACCATCT CCAGGTCCAT TGAGGGTGTA TCACGTCTCA AATTACGAGT TTGCCTCTTT   
  
  
+ CTACGGGGAT TATAAATATC TACCTGCATA CCTCGAAAAG GAGGAGTTGA GATCAGCGAA ACTTGGCCAT   
  
  
+ CCCAGAGCAT TGAAATGCTC AGAACCCTTT TTCTAGAAAA ATATCGTTTT TCCTGAAAAT ATCCGACCTT   
  
  
+ ATGTCATTTA TTGTCGCTTT CTTGACTCTA ACCCGGGACT CCACACTAAC TTGACTATCG GAAAGGCGTT   
  
  
+ CCCTGGATCA CCATCCAAGA TAACCTCTTT TGCAGGGTTG ACGCTCGTTG GAAGAATCGT CGAATGTAGA   
  
  
+ GCCCCTATCA TCACATAAGC CCGCTCTTAC TTCATGCCAT CCTTGAATAG CAGTTTATCC CGAAGCAAAA   
  
  
+ ATAATTCTAA CTTTCTTATA TTGTAAACCC ACATCTAAAT TTAAATTCAT ACTCCAAAAT TCCGCCACGC   
  
  
+ AAATAAGGCT TTTTAATAGG CTTACATATA ATGGCATGCT ATAGAGACAC TGTTTTGAAG AAACTATTTA   
  
  
+ AACATCAGAC CTCATATTGA GTAGCAAATC AAGACTTCTT TCCTCCAGGC CATATTGAGT AGCAAATTAA   
  
  
+ GAATAACCAG AAGCTTGTGA AAAGAGTTAG AAAATAAAAG GTATAATAGA GACTTATGTT CTTAAGATAT   
  
  
+ CTCCTAGATA TGAATGTGAC CTCCTAGTCG ATATTTGTGC TGGATTAGCG AGTTGTGTGA CTGAGTTTGT   
  
  
+ ATGTGTCTCT TAGCATATGA GTTATTCTAG CTGCTTCTTT AAGCTATGTA GTGACTGTCT TCCTTCTTAT   
  
  
+ TTATAAGTGC TATGTCCAAC TAATAGCCGA AAAAAATGGT TCGCCTTTTC TGGTTTTCTT CTTTTTGAAG   
  
  
+ GTTGATTGGC TAATTTATAT AATGGTTTCA ATACTTATCC TTTATTGTTC CACAAATCCA CCTAACCACG   
  
  
+ TACCATTGAC AATAATTACC CACTAATTTC GGTTGGACTT GATTTCCTTT TGTTTGGTAC CTCAGGCATC   
  
  
+ TTCTGTGGAC CTTCATTCAC AGTTTAATTT TCACATTTTT ATAAGGCTGC TCTTGTGTTG CAAATTTTCC   
  
  
+ TATCCATTCC ACTCTTTGCC CATTCCCAGG CTAATTAGTG TTCTCTGTTC AACCATGGAT TCTGTGCTGG   
  
  
+ TTGATCCTGA GTTCATGAAA AATCTCTACA AATTCAAACC TGAATTGCTC TCAAACTTTT CAATGAATCC   
  
  
+ AAATGACGAC ATCTTTCAAG CCCTTCATTC AGAAAATGAT CCTTTACAGT TCCTCTCATT TGATGAAGGA   
  
  
+ ACCTGTCTTA ACAGCTGCAC TAGTCAACAA GTGCCAGATT TCCCTGATGC TTGTCTCAAG TTCATCAGTG   
  
  
+ ATATTCTTCT CGAAGAGGGT TTAGATGCAA ATCCTGCATC TGCACAGGCT CTCGAAGCCA CCGAGAAGTC   
  
  
+ CTTGTATGAT GCTCTCGGGC TTGGAGAGCC ATACCCCCTT TCATGTGATC ACTTTGCGCC ATCTATCTCT   
  
  
+ ACAAGTATTG AGAGCCCAGA TGACAGTTCT TCCAATAAAA GTTATAGCAG CAATCCCGAG ATAGATGGTT   
  
  
+ CTTATGCTAT CGCTGAGCCC AGTTTCGAGT CCAACCCCAA CTGTGTGCTT GATCAACCCC AGTTGAACTC   
  
  
+ CTTTCCAGCT CTACATGAGA TTTCTCGGTC CTTGGTGGAA CTGGGTTCTC AAGCCTCTGA GTTGAGCTTC   
  
  
+ GATGATGCAG GGAGTGCCCG TGTAGAGAAA AAGGGCAAAT CGATAAAGGG CTCGAGGAGG AAGAAGAGTC   
  
  
+ GTCAAAGAGA GGGTGAAGCG TGTTATGGAG GAAGGAGCCA TAAGGTTCAA GCTTCCTTCA ATGATGATTA   
  
  
+ CTATGAGATG GAACAGTATG ATGATGTAGT ATTGCTCTGT AATAATGAAC TAACGGGCAA TAGCCGTTTC   
  
  
+ AACACCGGGA AATCTTCACC TGAGGAGGGA TGGAGGAGAT TGCAGAGAAG CCGAGGAAAG AAGCAGAACA   
  
  
+ GTTTAGCAGT TGAAGTTGAT CTGATGACCC TGCTGACTCA GTGTGCACAA GCTGTATCGA GCTTTGATCT   
  
  
+ TCGAGGTGCA AACGAGCTAC TTAGGCAAAT CAGGCAGAAT GCTTCGCCCT ATGGTGGCAG CATCCAGAGG   
  
  
+ CTCGCCCATC ATGTAGCCAA TGCTCTCGAG GCACGTATAG CTGGCACAGG CTCTACAGTC TCTACTAACC   
  
  
+ TTGTTGATGC AAAGTTCTCA GCTTCTGACT TCCTAAAGGC TTACAGGTTA TATGTCTCAG CTGTTCCTTA   
  
  
+ CAAAAGGATG TCTTTCTTTC TTGCTAACTG CTCGATTGCA AAGTTGGCAG AGAAAGCAAC AAAGATCCAT   
  
  
+ ATCATTGATT TTGGTGTTTT CCTAGGTTTA CAATGGCCTT GTTTCATACA ACACCTATCA AAAAGGCCAA   
  
  
+ ATGGACCCCC AAAACTCCGA ATCACAGGAA TCGACTACCC CCAGCAGGGT TTCAGACCTG CACAAAGGGT   
  
  
+ TGAAGCTACA GGACACCGAT TATCTGGGTA CTGTGAGCGA TTTGGGGTGC CTTTTTCTTA TCAGGGCATT   
  
  
+ GCTCAGAAGT GGGAAACTAT TCAGCCGGAG GATCTCAAGA TCGAACAAGA CGAGCTGGTG ATTGTCAACT   
  
  
+ GTTTGTTCAG GTCAGGAACA CTGCTCGATG AGACAGTCGA AGCAAACAGT CCAAGAGATG CTTTCTTAGC   
  
  
+ TTTGGTTAGA AAGCTGAATC CCAGCCTATT CATTCACGGG GTTGTCAATG GCACATTCAA CGCTCCATTC   
  
  
+ TTCGTGACTC GATTCAGAGA GGCATTGTTT CATTATTCAT CAGTGTTTGA TGTGTCTGAA GAGACAATTC   
  
  
+ CACGAGATGC CCATGAGAGG TTCTTGATTG AGAGCGAGAT TTGTGGGAAA GAACTGTTCA ATGTGGTTGC   
  
  
+ TTGTGAGGGT GCAGAGAGGG TTCAAAGGCC TGAGACATAC AAGCAGTGGC AAGTGAGGAC AACGCGGGCC   
  
  
+ GGGTTAAGGC AGGTTGCCTT GGACCAGGAG CTTATGAAGG AAGCAACGGC AATGGTGAAG GCAAATTATC   
  
  
+ ATAAGGATTT TATGGTGGAT ATAAATAGGC ATTGGATGCT TCAAGGTTGG AAGGGTAGAA CCTTGTGTGC   
  
  
+ TCTCTCATTT TGGCAACCTG CCTG  

- +Up\_Stream \_Len000GATCCC CAACTCTCTT AGGCTATCGA TACGTCAGGT TTGTAATAGA GCAGTTCGTA   
  
  
- ATGATAAATT TATTAATTAT AACTTGAACT ATCAATGTGA AAAACTTATG ATTAAGGTGT GGAGTAACGG   
  
  
- AAATAAAGGT TTGGAGCCCA ACGTAAACCT GGTGTATGGT CCTTCGAACC TGCTGTTTTT AAAGAAAAGA   
  
  
- ACTCATAATA AAACAAAATA ATAATAATAA AAAATGAAAT AAACCTGGTG AAGTGAACCC ATTGTTCTAA   
  
  
- CTTATAAACT GATAATATAA TATACAGTGC ATACCTATAC CATCAACGAA TCTCGAAACT GGCAAACGTC   
  
  
- CAAATTCATA CTAAGGGAAC CTTTCCTCGG GGAGAAATAG AATTAAGTCT TCATTGAAAA CAAAAAAATC   
  
  
- TATTTAACTT TTCTTTTTAT TATTTACAGA ATCAACTCAA ACCAATAAGT TAGGCTTTAA ACTAGGACGA   
  
  
- ATTACATATC ATATATTATG ATTATGCGTA CATTGTGTTG TGGAGTCTAC CAGTAACAAA ATGTTAAAAA   
  
  
- AACTCAAATC ATATTTTTAT TTTAGTTAAT AAAAATCTTC ACTACAAATT AAACGTAAAA AAAAGGTGGA   
  
  
- TTTTAGTGCG TGAGCTTGTA ATCCCCAAAC CGATTGAGTT TCTGAGTTCC ATCACTGAAT AGGTTTGAAT   
  
  
- AATGTAGGAT ATCTAGATAT TTTTGTATTT AGAGAATGTT AGTTAGGTTT AACTAAGCCA GATTAAACTA   
  
  
- CTGGGACTAC TCCCAGGATG TTAATGTGTT CACCCGGAAT CGGGGTACCG GCAAAAAATT AGAAGTACCC   
  
  
- AGGTCCGCCT CAATGGTAGA GGTCCAGGTA ACTCCCACAT AGTGCAGAGT TTAATGCTCA AACGGAGAAA   
  
  
- GATGCCCCTA ATATTTATAG ATGGACGTAT GGAGCTTTTC CTCCTCAACT CTAGTCGCTT TGAACCGGTA   
  
  
- GGGTCTCGTA ACTTTACGAG TCTTGGGAAA AAGATCTTTT TATAGCAAAA AGGACTTTTA TAGGCTGGAA   
  
  
- TACAGTAAAT AACAGCGAAA GAACTGAGAT TGGGCCCTGA GGTGTGATTG AACTGATAGC CTTTCCGCAA   
  
  
- GGGACCTAGT GGTAGGTTCT ATTGGAGAAA ACGTCCCAAC TGCGAGCAAC CTTCTTAGCA GCTTACATCT   
  
  
- CGGGGATAGT AGTGTATTCG GGCGAGAATG AAGTACGGTA GGAACTTATC GTCAAATAGG GCTTCGTTTT   
  
  
- TATTAAGATT GAAAGAATAT AACATTTGGG TGTAGATTTA AATTTAAGTA TGAGGTTTTA AGGCGGTGCG   
  
  
- TTTATTCCGA AAAATTATCC GAATGTATAT TACCGTACGA TATCTCTGTG ACAAAACTTC TTTGATAAAT   
  
  
- TTGTAGTCTG GAGTATAACT CATCGTTTAG TTCTGAAGAA AGGAGGTCCG GTATAACTCA TCGTTTAATT   
  
  
- CTTATTGGTC TTCGAACACT TTTCTCAATC TTTTATTTTC CATATTATCT CTGAATACAA GAATTCTATA   
  
  
- GAGGATCTAT ACTTACACTG GAGGATCAGC TATAAACACG ACCTAATCGC TCAACACACT GACTCAAACA   
  
  
- TACACAGAGA ATCGTATACT CAATAAGATC GACGAAGAAA TTCGATACAT CACTGACAGA AGGAAGAATA   
  
  
- AATATTCACG ATACAGGTTG ATTATCGGCT TTTTTTACCA AGCGGAAAAG ACCAAAAGAA GAAAAACTTC   
  
  
- CAACTAACCG ATTAAATATA TTACCAAAGT TATGAATAGG AAATAACAAG GTGTTTAGGT GGATTGGTGC   
  
  
- ATGGTAACTG TTATTAATGG GTGATTAAAG CCAACCTGAA CTAAAGGAAA ACAAACCATG GAGTCCGTAG   
  
  
- AAGACACCTG GAAGTAAGTG TCAAATTAAA AGTGTAAAAA TATTCCGACG AGAACACAAC GTTTAAAAGG   
  
  
- ATAGGTAAGG TGAGAAACGG GTAAGGGTCC GATTAATCAC AAGAGACAAG TTGGTACCTA AGACACGACC   
  
  
- AACTAGGACT CAAGTACTTT TTAGAGATGT TTAAGTTTGG ACTTAACGAG AGTTTGAAAA GTTACTTAGG   
  
  
- TTTACTGCTG TAGAAAGTTC GGGAAGTAAG TCTTTTACTA GGAAATGTCA AGGAGAGTAA ACTACTTCCT   
  
  
- TGGACAGAAT TGTCGACGTG ATCAGTTGTT CACGGTCTAA AGGGACTACG AACAGAGTTC AAGTAGTCAC   
  
  
- TATAAGAAGA GCTTCTCCCA AATCTACGTT TAGGACGTAG ACGTGTCCGA GAGCTTCGGT GGCTCTTCAG   
  
  
- GAACATACTA CGAGAGCCCG AACCTCTCGG TATGGGGGAA AGTACACTAG TGAAACGCGG TAGATAGAGA   
  
  
- TGTTCATAAC TCTCGGGTCT ACTGTCAAGA AGGTTATTTT CAATATCGTC GTTAGGGCTC TATCTACCAA   
  
  
- GAATACGATA GCGACTCGGG TCAAAGCTCA GGTTGGGGTT GACACACGAA CTAGTTGGGG TCAACTTGAG   
  
  
- GAAAGGTCGA GATGTACTCT AAAGAGCCAG GAACCACCTT GACCCAAGAG TTCGGAGACT CAACTCGAAG   
  
  
- CTACTACGTC CCTCACGGGC ACATCTCTTT TTCCCGTTTA GCTATTTCCC GAGCTCCTCC TTCTTCTCAG   
  
  
- CAGTTTCTCT CCCACTTCGC ACAATACCTC CTTCCTCGGT ATTCCAAGTT CGAAGGAAGT TACTACTAAT   
  
  
- GATACTCTAC CTTGTCATAC TACTACATCA TAACGAGACA TTATTACTTG ATTGCCCGTT ATCGGCAAAG   
  
  
- TTGTGGCCCT TTAGAAGTGG ACTCCTCCCT ACCTCCTCTA ACGTCTCTTC GGCTCCTTTC TTCGTCTTGT   
  
  
- CAAATCGTCA ACTTCAACTA GACTACTGGG ACGACTGAGT CACACGTGTT CGACATAGCT CGAAACTAGA   
  
  
- AGCTCCACGT TTGCTCGATG AATCCGTTTA GTCCGTCTTA CGAAGCGGGA TACCACCGTC GTAGGTCTCC   
  
  
- GAGCGGGTAG TACATCGGTT ACGAGAGCTC CGTGCATATC GACCGTGTCC GAGATGTCAG AGATGATTGG   
  
  
- AACAACTACG TTTCAAGAGT CGAAGACTGA AGGATTTCCG AATGTCCAAT ATACAGAGTC GACAAGGAAT   
  
  
- GTTTTCCTAC AGAAAGAAAG AACGATTGAC GAGCTAACGT TTCAACCGTC TCTTTCGTTG TTTCTAGGTA   
  
  
- TAGTAACTAA AACCACAAAA GGATCCAAAT GTTACCGGAA CAAAGTATGT TGTGGATAGT TTTTCCGGTT   
  
  
- TACCTGGGGG TTTTGAGGCT TAGTGTCCTT AGCTGATGGG GGTCGTCCCA AAGTCTGGAC GTGTTTCCCA   
  
  
- ACTTCGATGT CCTGTGGCTA ATAGACCCAT GACACTCGCT AAACCCCACG GAAAAAGAAT AGTCCCGTAA   
  
  
- CGAGTCTTCA CCCTTTGATA AGTCGGCCTC CTAGAGTTCT AGCTTGTTCT GCTCGACCAC TAACAGTTGA   
  
  
- CAAACAAGTC CAGTCCTTGT GACGAGCTAC TCTGTCAGCT TCGTTTGTCA GGTTCTCTAC GAAAGAATCG   
  
  
- AAACCAATCT TTCGACTTAG GGTCGGATAA GTAAGTGCCC CAACAGTTAC CGTGTAAGTT GCGAGGTAAG   
  
  
- AAGCACTGAG CTAAGTCTCT CCGTAACAAA GTAATAAGTA GTCACAAACT ACACAGACTT CTCTGTTAAG   
  
  
- GTGCTCTACG GGTACTCTCC AAGAACTAAC TCTCGCTCTA AACACCCTTT CTTGACAAGT TACACCAACG   
  
  
- AACACTCCCA CGTCTCTCCC AAGTTTCCGG ACTCTGTATG TTCGTCACCG TTCACTCCTG TTGCGCCCGG   
  
  
- CCCAATTCCG TCCAACGGAA CCTGGTCCTC GAATACTTCC TTCGTTGCCG TTACCACTTC CGTTTAATAG   
  
  
- TATTCCTAAA ATACCACCTA TATTTATCCG TAACCTACGA AGTTCCAACC TTCCCATCTT GGAACACACG   
  
  
- AGAGAGTAAA ACCGTTGGAC GGAC

+     G-box

| Site Name | Organism | Position | Strand | Matrix score. | sequence | function |
| --- | --- | --- | --- | --- | --- | --- |
| G-box | Zea mays | 886 | + | 6 | CACGTC | cis-acting regulatory element involved in light responsiveness |
| G-box | Arabidopsis thaliana | 3046 | - | 6 | TACGTG | cis-acting regulatory element involved in light responsiveness |
| G-box | Arabidopsis thaliana | 1821 | - | 6 | TACGTG | cis-acting regulatory element involved in light responsiveness |
| G-box | Arabidopsis thaliana | 311 | - | 6 | TACGTG | cis-acting regulatory element involved in light responsiveness |

>HU02G01571.1   
+ +Up\_Stream \_Len000CTAGGG GTTGAGAGAA TCCGATAGCT ATGCAGTCCA AACATTATCT CGTCAAGCAT   
  
  
+ TACTATTTAA ATAATTAATA TTGAACTTGA TAGTTACACT TTTTGAATAC TAATTCCACA CCTCATTGCC   
  
  
+ TTTATTTCCA AACCTCGGGT TGCATTTGGA CCACATACCA GGAAGCTTGG ACGACAAAAA TTTCTTTTCT   
  
  
+ TGAGTATTAT TTTGTTTTAT TATTATTATT TTTTACTTTA TTTGGACCAC TTCACTTGGG TAACAAGATT   
  
  
+ GAATATTTGA CTATTATATT ATATGTCACG TATGGATATG GTAGTTGCTT AGAGCTTTGA CCGTTTGCAG   
  
  
+ GTTTAAGTAT GATTCCCTTG GAAAGGAGCC CCTCTTTATC TTAATTCAGA AGTAACTTTT GTTTTTTTAG   
  
  
+ ATAAATTGAA AAGAAAAATA ATAAATGTCT TAGTTGAGTT TGGTTATTCA ATCCGAAATT TGATCCTGCT   
  
  
+ TAATGTATAG TATATAATAC TAATACGCAT GTAACACAAC ACCTCAGATG GTCATTGTTT TACAATTTTT   
  
  
+ TTGAGTTTAG TATAAAAATA AAATCAATTA TTTTTAGAAG TGATGTTTAA TTTGCATTTT TTTTCCACCT   
  
  
+ AAAATCACGC ACTCGAACAT TAGGGGTTTG GCTAACTCAA AGACTCAAGG TAGTGACTTA TCCAAACTTA   
  
  
+ TTACATCCTA TAGATCTATA AAAACATAAA TCTCTTACAA TCAATCCAAA TTGATTCGGT CTAATTTGAT   
  
  
+ GACCCTGATG AGGGTCCTAC AATTACACAA GTGGGCCTTA GCCCCATGGC CGTTTTTTAA TCTTCATGGG   
  
  
+ TCCAGGCGGA GTTACCATCT CCAGGTCCAT TGAGGGTGTA TCACGTCTCA AATTACGAGT TTGCCTCTTT   
  
  
+ CTACGGGGAT TATAAATATC TACCTGCATA CCTCGAAAAG GAGGAGTTGA GATCAGCGAA ACTTGGCCAT   
  
  
+ CCCAGAGCAT TGAAATGCTC AGAACCCTTT TTCTAGAAAA ATATCGTTTT TCCTGAAAAT ATCCGACCTT   
  
  
+ ATGTCATTTA TTGTCGCTTT CTTGACTCTA ACCCGGGACT CCACACTAAC TTGACTATCG GAAAGGCGTT   
  
  
+ CCCTGGATCA CCATCCAAGA TAACCTCTTT TGCAGGGTTG ACGCTCGTTG GAAGAATCGT CGAATGTAGA   
  
  
+ GCCCCTATCA TCACATAAGC CCGCTCTTAC TTCATGCCAT CCTTGAATAG CAGTTTATCC CGAAGCAAAA   
  
  
+ ATAATTCTAA CTTTCTTATA TTGTAAACCC ACATCTAAAT TTAAATTCAT ACTCCAAAAT TCCGCCACGC   
  
  
+ AAATAAGGCT TTTTAATAGG CTTACATATA ATGGCATGCT ATAGAGACAC TGTTTTGAAG AAACTATTTA   
  
  
+ AACATCAGAC CTCATATTGA GTAGCAAATC AAGACTTCTT TCCTCCAGGC CATATTGAGT AGCAAATTAA   
  
  
+ GAATAACCAG AAGCTTGTGA AAAGAGTTAG AAAATAAAAG GTATAATAGA GACTTATGTT CTTAAGATAT   
  
  
+ CTCCTAGATA TGAATGTGAC CTCCTAGTCG ATATTTGTGC TGGATTAGCG AGTTGTGTGA CTGAGTTTGT   
  
  
+ ATGTGTCTCT TAGCATATGA GTTATTCTAG CTGCTTCTTT AAGCTATGTA GTGACTGTCT TCCTTCTTAT   
  
  
+ TTATAAGTGC TATGTCCAAC TAATAGCCGA AAAAAATGGT TCGCCTTTTC TGGTTTTCTT CTTTTTGAAG   
  
  
+ GTTGATTGGC TAATTTATAT AATGGTTTCA ATACTTATCC TTTATTGTTC CACAAATCCA CCTAACCACG   
  
  
+ TACCATTGAC AATAATTACC CACTAATTTC GGTTGGACTT GATTTCCTTT TGTTTGGTAC CTCAGGCATC   
  
  
+ TTCTGTGGAC CTTCATTCAC AGTTTAATTT TCACATTTTT ATAAGGCTGC TCTTGTGTTG CAAATTTTCC   
  
  
+ TATCCATTCC ACTCTTTGCC CATTCCCAGG CTAATTAGTG TTCTCTGTTC AACCATGGAT TCTGTGCTGG   
  
  
+ TTGATCCTGA GTTCATGAAA AATCTCTACA AATTCAAACC TGAATTGCTC TCAAACTTTT CAATGAATCC   
  
  
+ AAATGACGAC ATCTTTCAAG CCCTTCATTC AGAAAATGAT CCTTTACAGT TCCTCTCATT TGATGAAGGA   
  
  
+ ACCTGTCTTA ACAGCTGCAC TAGTCAACAA GTGCCAGATT TCCCTGATGC TTGTCTCAAG TTCATCAGTG   
  
  
+ ATATTCTTCT CGAAGAGGGT TTAGATGCAA ATCCTGCATC TGCACAGGCT CTCGAAGCCA CCGAGAAGTC   
  
  
+ CTTGTATGAT GCTCTCGGGC TTGGAGAGCC ATACCCCCTT TCATGTGATC ACTTTGCGCC ATCTATCTCT   
  
  
+ ACAAGTATTG AGAGCCCAGA TGACAGTTCT TCCAATAAAA GTTATAGCAG CAATCCCGAG ATAGATGGTT   
  
  
+ CTTATGCTAT CGCTGAGCCC AGTTTCGAGT CCAACCCCAA CTGTGTGCTT GATCAACCCC AGTTGAACTC   
  
  
+ CTTTCCAGCT CTACATGAGA TTTCTCGGTC CTTGGTGGAA CTGGGTTCTC AAGCCTCTGA GTTGAGCTTC   
  
  
+ GATGATGCAG GGAGTGCCCG TGTAGAGAAA AAGGGCAAAT CGATAAAGGG CTCGAGGAGG AAGAAGAGTC   
  
  
+ GTCAAAGAGA GGGTGAAGCG TGTTATGGAG GAAGGAGCCA TAAGGTTCAA GCTTCCTTCA ATGATGATTA   
  
  
+ CTATGAGATG GAACAGTATG ATGATGTAGT ATTGCTCTGT AATAATGAAC TAACGGGCAA TAGCCGTTTC   
  
  
+ AACACCGGGA AATCTTCACC TGAGGAGGGA TGGAGGAGAT TGCAGAGAAG CCGAGGAAAG AAGCAGAACA   
  
  
+ GTTTAGCAGT TGAAGTTGAT CTGATGACCC TGCTGACTCA GTGTGCACAA GCTGTATCGA GCTTTGATCT   
  
  
+ TCGAGGTGCA AACGAGCTAC TTAGGCAAAT CAGGCAGAAT GCTTCGCCCT ATGGTGGCAG CATCCAGAGG   
  
  
+ CTCGCCCATC ATGTAGCCAA TGCTCTCGAG GCACGTATAG CTGGCACAGG CTCTACAGTC TCTACTAACC   
  
  
+ TTGTTGATGC AAAGTTCTCA GCTTCTGACT TCCTAAAGGC TTACAGGTTA TATGTCTCAG CTGTTCCTTA   
  
  
+ CAAAAGGATG TCTTTCTTTC TTGCTAACTG CTCGATTGCA AAGTTGGCAG AGAAAGCAAC AAAGATCCAT   
  
  
+ ATCATTGATT TTGGTGTTTT CCTAGGTTTA CAATGGCCTT GTTTCATACA ACACCTATCA AAAAGGCCAA   
  
  
+ ATGGACCCCC AAAACTCCGA ATCACAGGAA TCGACTACCC CCAGCAGGGT TTCAGACCTG CACAAAGGGT   
  
  
+ TGAAGCTACA GGACACCGAT TATCTGGGTA CTGTGAGCGA TTTGGGGTGC CTTTTTCTTA TCAGGGCATT   
  
  
+ GCTCAGAAGT GGGAAACTAT TCAGCCGGAG GATCTCAAGA TCGAACAAGA CGAGCTGGTG ATTGTCAACT   
  
  
+ GTTTGTTCAG GTCAGGAACA CTGCTCGATG AGACAGTCGA AGCAAACAGT CCAAGAGATG CTTTCTTAGC   
  
  
+ TTTGGTTAGA AAGCTGAATC CCAGCCTATT CATTCACGGG GTTGTCAATG GCACATTCAA CGCTCCATTC   
  
  
+ TTCGTGACTC GATTCAGAGA GGCATTGTTT CATTATTCAT CAGTGTTTGA TGTGTCTGAA GAGACAATTC   
  
  
+ CACGAGATGC CCATGAGAGG TTCTTGATTG AGAGCGAGAT TTGTGGGAAA GAACTGTTCA ATGTGGTTGC   
  
  
+ TTGTGAGGGT GCAGAGAGGG TTCAAAGGCC TGAGACATAC AAGCAGTGGC AAGTGAGGAC AACGCGGGCC   
  
  
+ GGGTTAAGGC AGGTTGCCTT GGACCAGGAG CTTATGAAGG AAGCAACGGC AATGGTGAAG GCAAATTATC   
  
  
+ ATAAGGATTT TATGGTGGAT ATAAATAGGC ATTGGATGCT TCAAGGTTGG AAGGGTAGAA CCTTGTGTGC   
  
  
+ TCTCTCATTT TGGCAACCTG CCTG  

- +Up\_Stream \_Len000GATCCC CAACTCTCTT AGGCTATCGA TACGTCAGGT TTGTAATAGA GCAGTTCGTA   
  
  
- ATGATAAATT TATTAATTAT AACTTGAACT ATCAATGTGA AAAACTTATG ATTAAGGTGT GGAGTAACGG   
  
  
- AAATAAAGGT TTGGAGCCCA ACGTAAACCT GGTGTATGGT CCTTCGAACC TGCTGTTTTT AAAGAAAAGA   
  
  
- ACTCATAATA AAACAAAATA ATAATAATAA AAAATGAAAT AAACCTGGTG AAGTGAACCC ATTGTTCTAA   
  
  
- CTTATAAACT GATAATATAA TATACAGTGC ATACCTATAC CATCAACGAA TCTCGAAACT GGCAAACGTC   
  
  
- CAAATTCATA CTAAGGGAAC CTTTCCTCGG GGAGAAATAG AATTAAGTCT TCATTGAAAA CAAAAAAATC   
  
  
- TATTTAACTT TTCTTTTTAT TATTTACAGA ATCAACTCAA ACCAATAAGT TAGGCTTTAA ACTAGGACGA   
  
  
- ATTACATATC ATATATTATG ATTATGCGTA CATTGTGTTG TGGAGTCTAC CAGTAACAAA ATGTTAAAAA   
  
  
- AACTCAAATC ATATTTTTAT TTTAGTTAAT AAAAATCTTC ACTACAAATT AAACGTAAAA AAAAGGTGGA   
  
  
- TTTTAGTGCG TGAGCTTGTA ATCCCCAAAC CGATTGAGTT TCTGAGTTCC ATCACTGAAT AGGTTTGAAT   
  
  
- AATGTAGGAT ATCTAGATAT TTTTGTATTT AGAGAATGTT AGTTAGGTTT AACTAAGCCA GATTAAACTA   
  
  
- CTGGGACTAC TCCCAGGATG TTAATGTGTT CACCCGGAAT CGGGGTACCG GCAAAAAATT AGAAGTACCC   
  
  
- AGGTCCGCCT CAATGGTAGA GGTCCAGGTA ACTCCCACAT AGTGCAGAGT TTAATGCTCA AACGGAGAAA   
  
  
- GATGCCCCTA ATATTTATAG ATGGACGTAT GGAGCTTTTC CTCCTCAACT CTAGTCGCTT TGAACCGGTA   
  
  
- GGGTCTCGTA ACTTTACGAG TCTTGGGAAA AAGATCTTTT TATAGCAAAA AGGACTTTTA TAGGCTGGAA   
  
  
- TACAGTAAAT AACAGCGAAA GAACTGAGAT TGGGCCCTGA GGTGTGATTG AACTGATAGC CTTTCCGCAA   
  
  
- GGGACCTAGT GGTAGGTTCT ATTGGAGAAA ACGTCCCAAC TGCGAGCAAC CTTCTTAGCA GCTTACATCT   
  
  
- CGGGGATAGT AGTGTATTCG GGCGAGAATG AAGTACGGTA GGAACTTATC GTCAAATAGG GCTTCGTTTT   
  
  
- TATTAAGATT GAAAGAATAT AACATTTGGG TGTAGATTTA AATTTAAGTA TGAGGTTTTA AGGCGGTGCG   
  
  
- TTTATTCCGA AAAATTATCC GAATGTATAT TACCGTACGA TATCTCTGTG ACAAAACTTC TTTGATAAAT   
  
  
- TTGTAGTCTG GAGTATAACT CATCGTTTAG TTCTGAAGAA AGGAGGTCCG GTATAACTCA TCGTTTAATT   
  
  
- CTTATTGGTC TTCGAACACT TTTCTCAATC TTTTATTTTC CATATTATCT CTGAATACAA GAATTCTATA   
  
  
- GAGGATCTAT ACTTACACTG GAGGATCAGC TATAAACACG ACCTAATCGC TCAACACACT GACTCAAACA   
  
  
- TACACAGAGA ATCGTATACT CAATAAGATC GACGAAGAAA TTCGATACAT CACTGACAGA AGGAAGAATA   
  
  
- AATATTCACG ATACAGGTTG ATTATCGGCT TTTTTTACCA AGCGGAAAAG ACCAAAAGAA GAAAAACTTC   
  
  
- CAACTAACCG ATTAAATATA TTACCAAAGT TATGAATAGG AAATAACAAG GTGTTTAGGT GGATTGGTGC   
  
  
- ATGGTAACTG TTATTAATGG GTGATTAAAG CCAACCTGAA CTAAAGGAAA ACAAACCATG GAGTCCGTAG   
  
  
- AAGACACCTG GAAGTAAGTG TCAAATTAAA AGTGTAAAAA TATTCCGACG AGAACACAAC GTTTAAAAGG   
  
  
- ATAGGTAAGG TGAGAAACGG GTAAGGGTCC GATTAATCAC AAGAGACAAG TTGGTACCTA AGACACGACC   
  
  
- AACTAGGACT CAAGTACTTT TTAGAGATGT TTAAGTTTGG ACTTAACGAG AGTTTGAAAA GTTACTTAGG   
  
  
- TTTACTGCTG TAGAAAGTTC GGGAAGTAAG TCTTTTACTA GGAAATGTCA AGGAGAGTAA ACTACTTCCT   
  
  
- TGGACAGAAT TGTCGACGTG ATCAGTTGTT CACGGTCTAA AGGGACTACG AACAGAGTTC AAGTAGTCAC   
  
  
- TATAAGAAGA GCTTCTCCCA AATCTACGTT TAGGACGTAG ACGTGTCCGA GAGCTTCGGT GGCTCTTCAG   
  
  
- GAACATACTA CGAGAGCCCG AACCTCTCGG TATGGGGGAA AGTACACTAG TGAAACGCGG TAGATAGAGA   
  
  
- TGTTCATAAC TCTCGGGTCT ACTGTCAAGA AGGTTATTTT CAATATCGTC GTTAGGGCTC TATCTACCAA   
  
  
- GAATACGATA GCGACTCGGG TCAAAGCTCA GGTTGGGGTT GACACACGAA CTAGTTGGGG TCAACTTGAG   
  
  
- GAAAGGTCGA GATGTACTCT AAAGAGCCAG GAACCACCTT GACCCAAGAG TTCGGAGACT CAACTCGAAG   
  
  
- CTACTACGTC CCTCACGGGC ACATCTCTTT TTCCCGTTTA GCTATTTCCC GAGCTCCTCC TTCTTCTCAG   
  
  
- CAGTTTCTCT CCCACTTCGC ACAATACCTC CTTCCTCGGT ATTCCAAGTT CGAAGGAAGT TACTACTAAT   
  
  
- GATACTCTAC CTTGTCATAC TACTACATCA TAACGAGACA TTATTACTTG ATTGCCCGTT ATCGGCAAAG   
  
  
- TTGTGGCCCT TTAGAAGTGG ACTCCTCCCT ACCTCCTCTA ACGTCTCTTC GGCTCCTTTC TTCGTCTTGT   
  
  
- CAAATCGTCA ACTTCAACTA GACTACTGGG ACGACTGAGT CACACGTGTT CGACATAGCT CGAAACTAGA   
  
  
- AGCTCCACGT TTGCTCGATG AATCCGTTTA GTCCGTCTTA CGAAGCGGGA TACCACCGTC GTAGGTCTCC   
  
  
- GAGCGGGTAG TACATCGGTT ACGAGAGCTC CGTGCATATC GACCGTGTCC GAGATGTCAG AGATGATTGG   
  
  
- AACAACTACG TTTCAAGAGT CGAAGACTGA AGGATTTCCG AATGTCCAAT ATACAGAGTC GACAAGGAAT   
  
  
- GTTTTCCTAC AGAAAGAAAG AACGATTGAC GAGCTAACGT TTCAACCGTC TCTTTCGTTG TTTCTAGGTA   
  
  
- TAGTAACTAA AACCACAAAA GGATCCAAAT GTTACCGGAA CAAAGTATGT TGTGGATAGT TTTTCCGGTT   
  
  
- TACCTGGGGG TTTTGAGGCT TAGTGTCCTT AGCTGATGGG GGTCGTCCCA AAGTCTGGAC GTGTTTCCCA   
  
  
- ACTTCGATGT CCTGTGGCTA ATAGACCCAT GACACTCGCT AAACCCCACG GAAAAAGAAT AGTCCCGTAA   
  
  
- CGAGTCTTCA CCCTTTGATA AGTCGGCCTC CTAGAGTTCT AGCTTGTTCT GCTCGACCAC TAACAGTTGA   
  
  
- CAAACAAGTC CAGTCCTTGT GACGAGCTAC TCTGTCAGCT TCGTTTGTCA GGTTCTCTAC GAAAGAATCG   
  
  
- AAACCAATCT TTCGACTTAG GGTCGGATAA GTAAGTGCCC CAACAGTTAC CGTGTAAGTT GCGAGGTAAG   
  
  
- AAGCACTGAG CTAAGTCTCT CCGTAACAAA GTAATAAGTA GTCACAAACT ACACAGACTT CTCTGTTAAG   
  
  
- GTGCTCTACG GGTACTCTCC AAGAACTAAC TCTCGCTCTA AACACCCTTT CTTGACAAGT TACACCAACG   
  
  
- AACACTCCCA CGTCTCTCCC AAGTTTCCGG ACTCTGTATG TTCGTCACCG TTCACTCCTG TTGCGCCCGG   
  
  
- CCCAATTCCG TCCAACGGAA CCTGGTCCTC GAATACTTCC TTCGTTGCCG TTACCACTTC CGTTTAATAG   
  
  
- TATTCCTAAA ATACCACCTA TATTTATCCG TAACCTACGA AGTTCCAACC TTCCCATCTT GGAACACACG   
  
  
- AGAGAGTAAA ACCGTTGGAC GGAC

+     GATA-motif

| Site Name | Organism | Position | Strand | Matrix score. | sequence | function |
| --- | --- | --- | --- | --- | --- | --- |
| GATA-motif | Solanum tuberosum | 1787 | - | 9 | AAGGATAAGG | part of a light responsive element |
| GATA-motif | Pisum sativum | 1197 | - | 7 | GATAGGG | part of a light responsive element |
| GATA-motif | Arabidopsis thaliana | 1962 | - | 7 | GATAGGA | part of a light responsive element |

>HU02G01571.1   
+ +Up\_Stream \_Len000CTAGGG GTTGAGAGAA TCCGATAGCT ATGCAGTCCA AACATTATCT CGTCAAGCAT   
  
  
+ TACTATTTAA ATAATTAATA TTGAACTTGA TAGTTACACT TTTTGAATAC TAATTCCACA CCTCATTGCC   
  
  
+ TTTATTTCCA AACCTCGGGT TGCATTTGGA CCACATACCA GGAAGCTTGG ACGACAAAAA TTTCTTTTCT   
  
  
+ TGAGTATTAT TTTGTTTTAT TATTATTATT TTTTACTTTA TTTGGACCAC TTCACTTGGG TAACAAGATT   
  
  
+ GAATATTTGA CTATTATATT ATATGTCACG TATGGATATG GTAGTTGCTT AGAGCTTTGA CCGTTTGCAG   
  
  
+ GTTTAAGTAT GATTCCCTTG GAAAGGAGCC CCTCTTTATC TTAATTCAGA AGTAACTTTT GTTTTTTTAG   
  
  
+ ATAAATTGAA AAGAAAAATA ATAAATGTCT TAGTTGAGTT TGGTTATTCA ATCCGAAATT TGATCCTGCT   
  
  
+ TAATGTATAG TATATAATAC TAATACGCAT GTAACACAAC ACCTCAGATG GTCATTGTTT TACAATTTTT   
  
  
+ TTGAGTTTAG TATAAAAATA AAATCAATTA TTTTTAGAAG TGATGTTTAA TTTGCATTTT TTTTCCACCT   
  
  
+ AAAATCACGC ACTCGAACAT TAGGGGTTTG GCTAACTCAA AGACTCAAGG TAGTGACTTA TCCAAACTTA   
  
  
+ TTACATCCTA TAGATCTATA AAAACATAAA TCTCTTACAA TCAATCCAAA TTGATTCGGT CTAATTTGAT   
  
  
+ GACCCTGATG AGGGTCCTAC AATTACACAA GTGGGCCTTA GCCCCATGGC CGTTTTTTAA TCTTCATGGG   
  
  
+ TCCAGGCGGA GTTACCATCT CCAGGTCCAT TGAGGGTGTA TCACGTCTCA AATTACGAGT TTGCCTCTTT   
  
  
+ CTACGGGGAT TATAAATATC TACCTGCATA CCTCGAAAAG GAGGAGTTGA GATCAGCGAA ACTTGGCCAT   
  
  
+ CCCAGAGCAT TGAAATGCTC AGAACCCTTT TTCTAGAAAA ATATCGTTTT TCCTGAAAAT ATCCGACCTT   
  
  
+ ATGTCATTTA TTGTCGCTTT CTTGACTCTA ACCCGGGACT CCACACTAAC TTGACTATCG GAAAGGCGTT   
  
  
+ CCCTGGATCA CCATCCAAGA TAACCTCTTT TGCAGGGTTG ACGCTCGTTG GAAGAATCGT CGAATGTAGA   
  
  
+ GCCCCTATCA TCACATAAGC CCGCTCTTAC TTCATGCCAT CCTTGAATAG CAGTTTATCC CGAAGCAAAA   
  
  
+ ATAATTCTAA CTTTCTTATA TTGTAAACCC ACATCTAAAT TTAAATTCAT ACTCCAAAAT TCCGCCACGC   
  
  
+ AAATAAGGCT TTTTAATAGG CTTACATATA ATGGCATGCT ATAGAGACAC TGTTTTGAAG AAACTATTTA   
  
  
+ AACATCAGAC CTCATATTGA GTAGCAAATC AAGACTTCTT TCCTCCAGGC CATATTGAGT AGCAAATTAA   
  
  
+ GAATAACCAG AAGCTTGTGA AAAGAGTTAG AAAATAAAAG GTATAATAGA GACTTATGTT CTTAAGATAT   
  
  
+ CTCCTAGATA TGAATGTGAC CTCCTAGTCG ATATTTGTGC TGGATTAGCG AGTTGTGTGA CTGAGTTTGT   
  
  
+ ATGTGTCTCT TAGCATATGA GTTATTCTAG CTGCTTCTTT AAGCTATGTA GTGACTGTCT TCCTTCTTAT   
  
  
+ TTATAAGTGC TATGTCCAAC TAATAGCCGA AAAAAATGGT TCGCCTTTTC TGGTTTTCTT CTTTTTGAAG   
  
  
+ GTTGATTGGC TAATTTATAT AATGGTTTCA ATACTTATCC TTTATTGTTC CACAAATCCA CCTAACCACG   
  
  
+ TACCATTGAC AATAATTACC CACTAATTTC GGTTGGACTT GATTTCCTTT TGTTTGGTAC CTCAGGCATC   
  
  
+ TTCTGTGGAC CTTCATTCAC AGTTTAATTT TCACATTTTT ATAAGGCTGC TCTTGTGTTG CAAATTTTCC   
  
  
+ TATCCATTCC ACTCTTTGCC CATTCCCAGG CTAATTAGTG TTCTCTGTTC AACCATGGAT TCTGTGCTGG   
  
  
+ TTGATCCTGA GTTCATGAAA AATCTCTACA AATTCAAACC TGAATTGCTC TCAAACTTTT CAATGAATCC   
  
  
+ AAATGACGAC ATCTTTCAAG CCCTTCATTC AGAAAATGAT CCTTTACAGT TCCTCTCATT TGATGAAGGA   
  
  
+ ACCTGTCTTA ACAGCTGCAC TAGTCAACAA GTGCCAGATT TCCCTGATGC TTGTCTCAAG TTCATCAGTG   
  
  
+ ATATTCTTCT CGAAGAGGGT TTAGATGCAA ATCCTGCATC TGCACAGGCT CTCGAAGCCA CCGAGAAGTC   
  
  
+ CTTGTATGAT GCTCTCGGGC TTGGAGAGCC ATACCCCCTT TCATGTGATC ACTTTGCGCC ATCTATCTCT   
  
  
+ ACAAGTATTG AGAGCCCAGA TGACAGTTCT TCCAATAAAA GTTATAGCAG CAATCCCGAG ATAGATGGTT   
  
  
+ CTTATGCTAT CGCTGAGCCC AGTTTCGAGT CCAACCCCAA CTGTGTGCTT GATCAACCCC AGTTGAACTC   
  
  
+ CTTTCCAGCT CTACATGAGA TTTCTCGGTC CTTGGTGGAA CTGGGTTCTC AAGCCTCTGA GTTGAGCTTC   
  
  
+ GATGATGCAG GGAGTGCCCG TGTAGAGAAA AAGGGCAAAT CGATAAAGGG CTCGAGGAGG AAGAAGAGTC   
  
  
+ GTCAAAGAGA GGGTGAAGCG TGTTATGGAG GAAGGAGCCA TAAGGTTCAA GCTTCCTTCA ATGATGATTA   
  
  
+ CTATGAGATG GAACAGTATG ATGATGTAGT ATTGCTCTGT AATAATGAAC TAACGGGCAA TAGCCGTTTC   
  
  
+ AACACCGGGA AATCTTCACC TGAGGAGGGA TGGAGGAGAT TGCAGAGAAG CCGAGGAAAG AAGCAGAACA   
  
  
+ GTTTAGCAGT TGAAGTTGAT CTGATGACCC TGCTGACTCA GTGTGCACAA GCTGTATCGA GCTTTGATCT   
  
  
+ TCGAGGTGCA AACGAGCTAC TTAGGCAAAT CAGGCAGAAT GCTTCGCCCT ATGGTGGCAG CATCCAGAGG   
  
  
+ CTCGCCCATC ATGTAGCCAA TGCTCTCGAG GCACGTATAG CTGGCACAGG CTCTACAGTC TCTACTAACC   
  
  
+ TTGTTGATGC AAAGTTCTCA GCTTCTGACT TCCTAAAGGC TTACAGGTTA TATGTCTCAG CTGTTCCTTA   
  
  
+ CAAAAGGATG TCTTTCTTTC TTGCTAACTG CTCGATTGCA AAGTTGGCAG AGAAAGCAAC AAAGATCCAT   
  
  
+ ATCATTGATT TTGGTGTTTT CCTAGGTTTA CAATGGCCTT GTTTCATACA ACACCTATCA AAAAGGCCAA   
  
  
+ ATGGACCCCC AAAACTCCGA ATCACAGGAA TCGACTACCC CCAGCAGGGT TTCAGACCTG CACAAAGGGT   
  
  
+ TGAAGCTACA GGACACCGAT TATCTGGGTA CTGTGAGCGA TTTGGGGTGC CTTTTTCTTA TCAGGGCATT   
  
  
+ GCTCAGAAGT GGGAAACTAT TCAGCCGGAG GATCTCAAGA TCGAACAAGA CGAGCTGGTG ATTGTCAACT   
  
  
+ GTTTGTTCAG GTCAGGAACA CTGCTCGATG AGACAGTCGA AGCAAACAGT CCAAGAGATG CTTTCTTAGC   
  
  
+ TTTGGTTAGA AAGCTGAATC CCAGCCTATT CATTCACGGG GTTGTCAATG GCACATTCAA CGCTCCATTC   
  
  
+ TTCGTGACTC GATTCAGAGA GGCATTGTTT CATTATTCAT CAGTGTTTGA TGTGTCTGAA GAGACAATTC   
  
  
+ CACGAGATGC CCATGAGAGG TTCTTGATTG AGAGCGAGAT TTGTGGGAAA GAACTGTTCA ATGTGGTTGC   
  
  
+ TTGTGAGGGT GCAGAGAGGG TTCAAAGGCC TGAGACATAC AAGCAGTGGC AAGTGAGGAC AACGCGGGCC   
  
  
+ GGGTTAAGGC AGGTTGCCTT GGACCAGGAG CTTATGAAGG AAGCAACGGC AATGGTGAAG GCAAATTATC   
  
  
+ ATAAGGATTT TATGGTGGAT ATAAATAGGC ATTGGATGCT TCAAGGTTGG AAGGGTAGAA CCTTGTGTGC   
  
  
+ TCTCTCATTT TGGCAACCTG CCTG  

- +Up\_Stream \_Len000GATCCC CAACTCTCTT AGGCTATCGA TACGTCAGGT TTGTAATAGA GCAGTTCGTA   
  
  
- ATGATAAATT TATTAATTAT AACTTGAACT ATCAATGTGA AAAACTTATG ATTAAGGTGT GGAGTAACGG   
  
  
- AAATAAAGGT TTGGAGCCCA ACGTAAACCT GGTGTATGGT CCTTCGAACC TGCTGTTTTT AAAGAAAAGA   
  
  
- ACTCATAATA AAACAAAATA ATAATAATAA AAAATGAAAT AAACCTGGTG AAGTGAACCC ATTGTTCTAA   
  
  
- CTTATAAACT GATAATATAA TATACAGTGC ATACCTATAC CATCAACGAA TCTCGAAACT GGCAAACGTC   
  
  
- CAAATTCATA CTAAGGGAAC CTTTCCTCGG GGAGAAATAG AATTAAGTCT TCATTGAAAA CAAAAAAATC   
  
  
- TATTTAACTT TTCTTTTTAT TATTTACAGA ATCAACTCAA ACCAATAAGT TAGGCTTTAA ACTAGGACGA   
  
  
- ATTACATATC ATATATTATG ATTATGCGTA CATTGTGTTG TGGAGTCTAC CAGTAACAAA ATGTTAAAAA   
  
  
- AACTCAAATC ATATTTTTAT TTTAGTTAAT AAAAATCTTC ACTACAAATT AAACGTAAAA AAAAGGTGGA   
  
  
- TTTTAGTGCG TGAGCTTGTA ATCCCCAAAC CGATTGAGTT TCTGAGTTCC ATCACTGAAT AGGTTTGAAT   
  
  
- AATGTAGGAT ATCTAGATAT TTTTGTATTT AGAGAATGTT AGTTAGGTTT AACTAAGCCA GATTAAACTA   
  
  
- CTGGGACTAC TCCCAGGATG TTAATGTGTT CACCCGGAAT CGGGGTACCG GCAAAAAATT AGAAGTACCC   
  
  
- AGGTCCGCCT CAATGGTAGA GGTCCAGGTA ACTCCCACAT AGTGCAGAGT TTAATGCTCA AACGGAGAAA   
  
  
- GATGCCCCTA ATATTTATAG ATGGACGTAT GGAGCTTTTC CTCCTCAACT CTAGTCGCTT TGAACCGGTA   
  
  
- GGGTCTCGTA ACTTTACGAG TCTTGGGAAA AAGATCTTTT TATAGCAAAA AGGACTTTTA TAGGCTGGAA   
  
  
- TACAGTAAAT AACAGCGAAA GAACTGAGAT TGGGCCCTGA GGTGTGATTG AACTGATAGC CTTTCCGCAA   
  
  
- GGGACCTAGT GGTAGGTTCT ATTGGAGAAA ACGTCCCAAC TGCGAGCAAC CTTCTTAGCA GCTTACATCT   
  
  
- CGGGGATAGT AGTGTATTCG GGCGAGAATG AAGTACGGTA GGAACTTATC GTCAAATAGG GCTTCGTTTT   
  
  
- TATTAAGATT GAAAGAATAT AACATTTGGG TGTAGATTTA AATTTAAGTA TGAGGTTTTA AGGCGGTGCG   
  
  
- TTTATTCCGA AAAATTATCC GAATGTATAT TACCGTACGA TATCTCTGTG ACAAAACTTC TTTGATAAAT   
  
  
- TTGTAGTCTG GAGTATAACT CATCGTTTAG TTCTGAAGAA AGGAGGTCCG GTATAACTCA TCGTTTAATT   
  
  
- CTTATTGGTC TTCGAACACT TTTCTCAATC TTTTATTTTC CATATTATCT CTGAATACAA GAATTCTATA   
  
  
- GAGGATCTAT ACTTACACTG GAGGATCAGC TATAAACACG ACCTAATCGC TCAACACACT GACTCAAACA   
  
  
- TACACAGAGA ATCGTATACT CAATAAGATC GACGAAGAAA TTCGATACAT CACTGACAGA AGGAAGAATA   
  
  
- AATATTCACG ATACAGGTTG ATTATCGGCT TTTTTTACCA AGCGGAAAAG ACCAAAAGAA GAAAAACTTC   
  
  
- CAACTAACCG ATTAAATATA TTACCAAAGT TATGAATAGG AAATAACAAG GTGTTTAGGT GGATTGGTGC   
  
  
- ATGGTAACTG TTATTAATGG GTGATTAAAG CCAACCTGAA CTAAAGGAAA ACAAACCATG GAGTCCGTAG   
  
  
- AAGACACCTG GAAGTAAGTG TCAAATTAAA AGTGTAAAAA TATTCCGACG AGAACACAAC GTTTAAAAGG   
  
  
- ATAGGTAAGG TGAGAAACGG GTAAGGGTCC GATTAATCAC AAGAGACAAG TTGGTACCTA AGACACGACC   
  
  
- AACTAGGACT CAAGTACTTT TTAGAGATGT TTAAGTTTGG ACTTAACGAG AGTTTGAAAA GTTACTTAGG   
  
  
- TTTACTGCTG TAGAAAGTTC GGGAAGTAAG TCTTTTACTA GGAAATGTCA AGGAGAGTAA ACTACTTCCT   
  
  
- TGGACAGAAT TGTCGACGTG ATCAGTTGTT CACGGTCTAA AGGGACTACG AACAGAGTTC AAGTAGTCAC   
  
  
- TATAAGAAGA GCTTCTCCCA AATCTACGTT TAGGACGTAG ACGTGTCCGA GAGCTTCGGT GGCTCTTCAG   
  
  
- GAACATACTA CGAGAGCCCG AACCTCTCGG TATGGGGGAA AGTACACTAG TGAAACGCGG TAGATAGAGA   
  
  
- TGTTCATAAC TCTCGGGTCT ACTGTCAAGA AGGTTATTTT CAATATCGTC GTTAGGGCTC TATCTACCAA   
  
  
- GAATACGATA GCGACTCGGG TCAAAGCTCA GGTTGGGGTT GACACACGAA CTAGTTGGGG TCAACTTGAG   
  
  
- GAAAGGTCGA GATGTACTCT AAAGAGCCAG GAACCACCTT GACCCAAGAG TTCGGAGACT CAACTCGAAG   
  
  
- CTACTACGTC CCTCACGGGC ACATCTCTTT TTCCCGTTTA GCTATTTCCC GAGCTCCTCC TTCTTCTCAG   
  
  
- CAGTTTCTCT CCCACTTCGC ACAATACCTC CTTCCTCGGT ATTCCAAGTT CGAAGGAAGT TACTACTAAT   
  
  
- GATACTCTAC CTTGTCATAC TACTACATCA TAACGAGACA TTATTACTTG ATTGCCCGTT ATCGGCAAAG   
  
  
- TTGTGGCCCT TTAGAAGTGG ACTCCTCCCT ACCTCCTCTA ACGTCTCTTC GGCTCCTTTC TTCGTCTTGT   
  
  
- CAAATCGTCA ACTTCAACTA GACTACTGGG ACGACTGAGT CACACGTGTT CGACATAGCT CGAAACTAGA   
  
  
- AGCTCCACGT TTGCTCGATG AATCCGTTTA GTCCGTCTTA CGAAGCGGGA TACCACCGTC GTAGGTCTCC   
  
  
- GAGCGGGTAG TACATCGGTT ACGAGAGCTC CGTGCATATC GACCGTGTCC GAGATGTCAG AGATGATTGG   
  
  
- AACAACTACG TTTCAAGAGT CGAAGACTGA AGGATTTCCG AATGTCCAAT ATACAGAGTC GACAAGGAAT   
  
  
- GTTTTCCTAC AGAAAGAAAG AACGATTGAC GAGCTAACGT TTCAACCGTC TCTTTCGTTG TTTCTAGGTA   
  
  
- TAGTAACTAA AACCACAAAA GGATCCAAAT GTTACCGGAA CAAAGTATGT TGTGGATAGT TTTTCCGGTT   
  
  
- TACCTGGGGG TTTTGAGGCT TAGTGTCCTT AGCTGATGGG GGTCGTCCCA AAGTCTGGAC GTGTTTCCCA   
  
  
- ACTTCGATGT CCTGTGGCTA ATAGACCCAT GACACTCGCT AAACCCCACG GAAAAAGAAT AGTCCCGTAA   
  
  
- CGAGTCTTCA CCCTTTGATA AGTCGGCCTC CTAGAGTTCT AGCTTGTTCT GCTCGACCAC TAACAGTTGA   
  
  
- CAAACAAGTC CAGTCCTTGT GACGAGCTAC TCTGTCAGCT TCGTTTGTCA GGTTCTCTAC GAAAGAATCG   
  
  
- AAACCAATCT TTCGACTTAG GGTCGGATAA GTAAGTGCCC CAACAGTTAC CGTGTAAGTT GCGAGGTAAG   
  
  
- AAGCACTGAG CTAAGTCTCT CCGTAACAAA GTAATAAGTA GTCACAAACT ACACAGACTT CTCTGTTAAG   
  
  
- GTGCTCTACG GGTACTCTCC AAGAACTAAC TCTCGCTCTA AACACCCTTT CTTGACAAGT TACACCAACG   
  
  
- AACACTCCCA CGTCTCTCCC AAGTTTCCGG ACTCTGTATG TTCGTCACCG TTCACTCCTG TTGCGCCCGG   
  
  
- CCCAATTCCG TCCAACGGAA CCTGGTCCTC GAATACTTCC TTCGTTGCCG TTACCACTTC CGTTTAATAG   
  
  
- TATTCCTAAA ATACCACCTA TATTTATCCG TAACCTACGA AGTTCCAACC TTCCCATCTT GGAACACACG   
  
  
- AGAGAGTAAA ACCGTTGGAC GGAC

+     GCN4\_motif

| Site Name | Organism | Position | Strand | Matrix score. | sequence | function |
| --- | --- | --- | --- | --- | --- | --- |
| GCN4\_motif | Oryza sativa | 2908 | - | 7 | TGAGTCA | cis-regulatory element involved in endosperm expression |

>HU02G01571.1   
+ +Up\_Stream \_Len000CTAGGG GTTGAGAGAA TCCGATAGCT ATGCAGTCCA AACATTATCT CGTCAAGCAT   
  
  
+ TACTATTTAA ATAATTAATA TTGAACTTGA TAGTTACACT TTTTGAATAC TAATTCCACA CCTCATTGCC   
  
  
+ TTTATTTCCA AACCTCGGGT TGCATTTGGA CCACATACCA GGAAGCTTGG ACGACAAAAA TTTCTTTTCT   
  
  
+ TGAGTATTAT TTTGTTTTAT TATTATTATT TTTTACTTTA TTTGGACCAC TTCACTTGGG TAACAAGATT   
  
  
+ GAATATTTGA CTATTATATT ATATGTCACG TATGGATATG GTAGTTGCTT AGAGCTTTGA CCGTTTGCAG   
  
  
+ GTTTAAGTAT GATTCCCTTG GAAAGGAGCC CCTCTTTATC TTAATTCAGA AGTAACTTTT GTTTTTTTAG   
  
  
+ ATAAATTGAA AAGAAAAATA ATAAATGTCT TAGTTGAGTT TGGTTATTCA ATCCGAAATT TGATCCTGCT   
  
  
+ TAATGTATAG TATATAATAC TAATACGCAT GTAACACAAC ACCTCAGATG GTCATTGTTT TACAATTTTT   
  
  
+ TTGAGTTTAG TATAAAAATA AAATCAATTA TTTTTAGAAG TGATGTTTAA TTTGCATTTT TTTTCCACCT   
  
  
+ AAAATCACGC ACTCGAACAT TAGGGGTTTG GCTAACTCAA AGACTCAAGG TAGTGACTTA TCCAAACTTA   
  
  
+ TTACATCCTA TAGATCTATA AAAACATAAA TCTCTTACAA TCAATCCAAA TTGATTCGGT CTAATTTGAT   
  
  
+ GACCCTGATG AGGGTCCTAC AATTACACAA GTGGGCCTTA GCCCCATGGC CGTTTTTTAA TCTTCATGGG   
  
  
+ TCCAGGCGGA GTTACCATCT CCAGGTCCAT TGAGGGTGTA TCACGTCTCA AATTACGAGT TTGCCTCTTT   
  
  
+ CTACGGGGAT TATAAATATC TACCTGCATA CCTCGAAAAG GAGGAGTTGA GATCAGCGAA ACTTGGCCAT   
  
  
+ CCCAGAGCAT TGAAATGCTC AGAACCCTTT TTCTAGAAAA ATATCGTTTT TCCTGAAAAT ATCCGACCTT   
  
  
+ ATGTCATTTA TTGTCGCTTT CTTGACTCTA ACCCGGGACT CCACACTAAC TTGACTATCG GAAAGGCGTT   
  
  
+ CCCTGGATCA CCATCCAAGA TAACCTCTTT TGCAGGGTTG ACGCTCGTTG GAAGAATCGT CGAATGTAGA   
  
  
+ GCCCCTATCA TCACATAAGC CCGCTCTTAC TTCATGCCAT CCTTGAATAG CAGTTTATCC CGAAGCAAAA   
  
  
+ ATAATTCTAA CTTTCTTATA TTGTAAACCC ACATCTAAAT TTAAATTCAT ACTCCAAAAT TCCGCCACGC   
  
  
+ AAATAAGGCT TTTTAATAGG CTTACATATA ATGGCATGCT ATAGAGACAC TGTTTTGAAG AAACTATTTA   
  
  
+ AACATCAGAC CTCATATTGA GTAGCAAATC AAGACTTCTT TCCTCCAGGC CATATTGAGT AGCAAATTAA   
  
  
+ GAATAACCAG AAGCTTGTGA AAAGAGTTAG AAAATAAAAG GTATAATAGA GACTTATGTT CTTAAGATAT   
  
  
+ CTCCTAGATA TGAATGTGAC CTCCTAGTCG ATATTTGTGC TGGATTAGCG AGTTGTGTGA CTGAGTTTGT   
  
  
+ ATGTGTCTCT TAGCATATGA GTTATTCTAG CTGCTTCTTT AAGCTATGTA GTGACTGTCT TCCTTCTTAT   
  
  
+ TTATAAGTGC TATGTCCAAC TAATAGCCGA AAAAAATGGT TCGCCTTTTC TGGTTTTCTT CTTTTTGAAG   
  
  
+ GTTGATTGGC TAATTTATAT AATGGTTTCA ATACTTATCC TTTATTGTTC CACAAATCCA CCTAACCACG   
  
  
+ TACCATTGAC AATAATTACC CACTAATTTC GGTTGGACTT GATTTCCTTT TGTTTGGTAC CTCAGGCATC   
  
  
+ TTCTGTGGAC CTTCATTCAC AGTTTAATTT TCACATTTTT ATAAGGCTGC TCTTGTGTTG CAAATTTTCC   
  
  
+ TATCCATTCC ACTCTTTGCC CATTCCCAGG CTAATTAGTG TTCTCTGTTC AACCATGGAT TCTGTGCTGG   
  
  
+ TTGATCCTGA GTTCATGAAA AATCTCTACA AATTCAAACC TGAATTGCTC TCAAACTTTT CAATGAATCC   
  
  
+ AAATGACGAC ATCTTTCAAG CCCTTCATTC AGAAAATGAT CCTTTACAGT TCCTCTCATT TGATGAAGGA   
  
  
+ ACCTGTCTTA ACAGCTGCAC TAGTCAACAA GTGCCAGATT TCCCTGATGC TTGTCTCAAG TTCATCAGTG   
  
  
+ ATATTCTTCT CGAAGAGGGT TTAGATGCAA ATCCTGCATC TGCACAGGCT CTCGAAGCCA CCGAGAAGTC   
  
  
+ CTTGTATGAT GCTCTCGGGC TTGGAGAGCC ATACCCCCTT TCATGTGATC ACTTTGCGCC ATCTATCTCT   
  
  
+ ACAAGTATTG AGAGCCCAGA TGACAGTTCT TCCAATAAAA GTTATAGCAG CAATCCCGAG ATAGATGGTT   
  
  
+ CTTATGCTAT CGCTGAGCCC AGTTTCGAGT CCAACCCCAA CTGTGTGCTT GATCAACCCC AGTTGAACTC   
  
  
+ CTTTCCAGCT CTACATGAGA TTTCTCGGTC CTTGGTGGAA CTGGGTTCTC AAGCCTCTGA GTTGAGCTTC   
  
  
+ GATGATGCAG GGAGTGCCCG TGTAGAGAAA AAGGGCAAAT CGATAAAGGG CTCGAGGAGG AAGAAGAGTC   
  
  
+ GTCAAAGAGA GGGTGAAGCG TGTTATGGAG GAAGGAGCCA TAAGGTTCAA GCTTCCTTCA ATGATGATTA   
  
  
+ CTATGAGATG GAACAGTATG ATGATGTAGT ATTGCTCTGT AATAATGAAC TAACGGGCAA TAGCCGTTTC   
  
  
+ AACACCGGGA AATCTTCACC TGAGGAGGGA TGGAGGAGAT TGCAGAGAAG CCGAGGAAAG AAGCAGAACA   
  
  
+ GTTTAGCAGT TGAAGTTGAT CTGATGACCC TGCTGACTCA GTGTGCACAA GCTGTATCGA GCTTTGATCT   
  
  
+ TCGAGGTGCA AACGAGCTAC TTAGGCAAAT CAGGCAGAAT GCTTCGCCCT ATGGTGGCAG CATCCAGAGG   
  
  
+ CTCGCCCATC ATGTAGCCAA TGCTCTCGAG GCACGTATAG CTGGCACAGG CTCTACAGTC TCTACTAACC   
  
  
+ TTGTTGATGC AAAGTTCTCA GCTTCTGACT TCCTAAAGGC TTACAGGTTA TATGTCTCAG CTGTTCCTTA   
  
  
+ CAAAAGGATG TCTTTCTTTC TTGCTAACTG CTCGATTGCA AAGTTGGCAG AGAAAGCAAC AAAGATCCAT   
  
  
+ ATCATTGATT TTGGTGTTTT CCTAGGTTTA CAATGGCCTT GTTTCATACA ACACCTATCA AAAAGGCCAA   
  
  
+ ATGGACCCCC AAAACTCCGA ATCACAGGAA TCGACTACCC CCAGCAGGGT TTCAGACCTG CACAAAGGGT   
  
  
+ TGAAGCTACA GGACACCGAT TATCTGGGTA CTGTGAGCGA TTTGGGGTGC CTTTTTCTTA TCAGGGCATT   
  
  
+ GCTCAGAAGT GGGAAACTAT TCAGCCGGAG GATCTCAAGA TCGAACAAGA CGAGCTGGTG ATTGTCAACT   
  
  
+ GTTTGTTCAG GTCAGGAACA CTGCTCGATG AGACAGTCGA AGCAAACAGT CCAAGAGATG CTTTCTTAGC   
  
  
+ TTTGGTTAGA AAGCTGAATC CCAGCCTATT CATTCACGGG GTTGTCAATG GCACATTCAA CGCTCCATTC   
  
  
+ TTCGTGACTC GATTCAGAGA GGCATTGTTT CATTATTCAT CAGTGTTTGA TGTGTCTGAA GAGACAATTC   
  
  
+ CACGAGATGC CCATGAGAGG TTCTTGATTG AGAGCGAGAT TTGTGGGAAA GAACTGTTCA ATGTGGTTGC   
  
  
+ TTGTGAGGGT GCAGAGAGGG TTCAAAGGCC TGAGACATAC AAGCAGTGGC AAGTGAGGAC AACGCGGGCC   
  
  
+ GGGTTAAGGC AGGTTGCCTT GGACCAGGAG CTTATGAAGG AAGCAACGGC AATGGTGAAG GCAAATTATC   
  
  
+ ATAAGGATTT TATGGTGGAT ATAAATAGGC ATTGGATGCT TCAAGGTTGG AAGGGTAGAA CCTTGTGTGC   
  
  
+ TCTCTCATTT TGGCAACCTG CCTG  

- +Up\_Stream \_Len000GATCCC CAACTCTCTT AGGCTATCGA TACGTCAGGT TTGTAATAGA GCAGTTCGTA   
  
  
- ATGATAAATT TATTAATTAT AACTTGAACT ATCAATGTGA AAAACTTATG ATTAAGGTGT GGAGTAACGG   
  
  
- AAATAAAGGT TTGGAGCCCA ACGTAAACCT GGTGTATGGT CCTTCGAACC TGCTGTTTTT AAAGAAAAGA   
  
  
- ACTCATAATA AAACAAAATA ATAATAATAA AAAATGAAAT AAACCTGGTG AAGTGAACCC ATTGTTCTAA   
  
  
- CTTATAAACT GATAATATAA TATACAGTGC ATACCTATAC CATCAACGAA TCTCGAAACT GGCAAACGTC   
  
  
- CAAATTCATA CTAAGGGAAC CTTTCCTCGG GGAGAAATAG AATTAAGTCT TCATTGAAAA CAAAAAAATC   
  
  
- TATTTAACTT TTCTTTTTAT TATTTACAGA ATCAACTCAA ACCAATAAGT TAGGCTTTAA ACTAGGACGA   
  
  
- ATTACATATC ATATATTATG ATTATGCGTA CATTGTGTTG TGGAGTCTAC CAGTAACAAA ATGTTAAAAA   
  
  
- AACTCAAATC ATATTTTTAT TTTAGTTAAT AAAAATCTTC ACTACAAATT AAACGTAAAA AAAAGGTGGA   
  
  
- TTTTAGTGCG TGAGCTTGTA ATCCCCAAAC CGATTGAGTT TCTGAGTTCC ATCACTGAAT AGGTTTGAAT   
  
  
- AATGTAGGAT ATCTAGATAT TTTTGTATTT AGAGAATGTT AGTTAGGTTT AACTAAGCCA GATTAAACTA   
  
  
- CTGGGACTAC TCCCAGGATG TTAATGTGTT CACCCGGAAT CGGGGTACCG GCAAAAAATT AGAAGTACCC   
  
  
- AGGTCCGCCT CAATGGTAGA GGTCCAGGTA ACTCCCACAT AGTGCAGAGT TTAATGCTCA AACGGAGAAA   
  
  
- GATGCCCCTA ATATTTATAG ATGGACGTAT GGAGCTTTTC CTCCTCAACT CTAGTCGCTT TGAACCGGTA   
  
  
- GGGTCTCGTA ACTTTACGAG TCTTGGGAAA AAGATCTTTT TATAGCAAAA AGGACTTTTA TAGGCTGGAA   
  
  
- TACAGTAAAT AACAGCGAAA GAACTGAGAT TGGGCCCTGA GGTGTGATTG AACTGATAGC CTTTCCGCAA   
  
  
- GGGACCTAGT GGTAGGTTCT ATTGGAGAAA ACGTCCCAAC TGCGAGCAAC CTTCTTAGCA GCTTACATCT   
  
  
- CGGGGATAGT AGTGTATTCG GGCGAGAATG AAGTACGGTA GGAACTTATC GTCAAATAGG GCTTCGTTTT   
  
  
- TATTAAGATT GAAAGAATAT AACATTTGGG TGTAGATTTA AATTTAAGTA TGAGGTTTTA AGGCGGTGCG   
  
  
- TTTATTCCGA AAAATTATCC GAATGTATAT TACCGTACGA TATCTCTGTG ACAAAACTTC TTTGATAAAT   
  
  
- TTGTAGTCTG GAGTATAACT CATCGTTTAG TTCTGAAGAA AGGAGGTCCG GTATAACTCA TCGTTTAATT   
  
  
- CTTATTGGTC TTCGAACACT TTTCTCAATC TTTTATTTTC CATATTATCT CTGAATACAA GAATTCTATA   
  
  
- GAGGATCTAT ACTTACACTG GAGGATCAGC TATAAACACG ACCTAATCGC TCAACACACT GACTCAAACA   
  
  
- TACACAGAGA ATCGTATACT CAATAAGATC GACGAAGAAA TTCGATACAT CACTGACAGA AGGAAGAATA   
  
  
- AATATTCACG ATACAGGTTG ATTATCGGCT TTTTTTACCA AGCGGAAAAG ACCAAAAGAA GAAAAACTTC   
  
  
- CAACTAACCG ATTAAATATA TTACCAAAGT TATGAATAGG AAATAACAAG GTGTTTAGGT GGATTGGTGC   
  
  
- ATGGTAACTG TTATTAATGG GTGATTAAAG CCAACCTGAA CTAAAGGAAA ACAAACCATG GAGTCCGTAG   
  
  
- AAGACACCTG GAAGTAAGTG TCAAATTAAA AGTGTAAAAA TATTCCGACG AGAACACAAC GTTTAAAAGG   
  
  
- ATAGGTAAGG TGAGAAACGG GTAAGGGTCC GATTAATCAC AAGAGACAAG TTGGTACCTA AGACACGACC   
  
  
- AACTAGGACT CAAGTACTTT TTAGAGATGT TTAAGTTTGG ACTTAACGAG AGTTTGAAAA GTTACTTAGG   
  
  
- TTTACTGCTG TAGAAAGTTC GGGAAGTAAG TCTTTTACTA GGAAATGTCA AGGAGAGTAA ACTACTTCCT   
  
  
- TGGACAGAAT TGTCGACGTG ATCAGTTGTT CACGGTCTAA AGGGACTACG AACAGAGTTC AAGTAGTCAC   
  
  
- TATAAGAAGA GCTTCTCCCA AATCTACGTT TAGGACGTAG ACGTGTCCGA GAGCTTCGGT GGCTCTTCAG   
  
  
- GAACATACTA CGAGAGCCCG AACCTCTCGG TATGGGGGAA AGTACACTAG TGAAACGCGG TAGATAGAGA   
  
  
- TGTTCATAAC TCTCGGGTCT ACTGTCAAGA AGGTTATTTT CAATATCGTC GTTAGGGCTC TATCTACCAA   
  
  
- GAATACGATA GCGACTCGGG TCAAAGCTCA GGTTGGGGTT GACACACGAA CTAGTTGGGG TCAACTTGAG   
  
  
- GAAAGGTCGA GATGTACTCT AAAGAGCCAG GAACCACCTT GACCCAAGAG TTCGGAGACT CAACTCGAAG   
  
  
- CTACTACGTC CCTCACGGGC ACATCTCTTT TTCCCGTTTA GCTATTTCCC GAGCTCCTCC TTCTTCTCAG   
  
  
- CAGTTTCTCT CCCACTTCGC ACAATACCTC CTTCCTCGGT ATTCCAAGTT CGAAGGAAGT TACTACTAAT   
  
  
- GATACTCTAC CTTGTCATAC TACTACATCA TAACGAGACA TTATTACTTG ATTGCCCGTT ATCGGCAAAG   
  
  
- TTGTGGCCCT TTAGAAGTGG ACTCCTCCCT ACCTCCTCTA ACGTCTCTTC GGCTCCTTTC TTCGTCTTGT   
  
  
- CAAATCGTCA ACTTCAACTA GACTACTGGG ACGACTGAGT CACACGTGTT CGACATAGCT CGAAACTAGA   
  
  
- AGCTCCACGT TTGCTCGATG AATCCGTTTA GTCCGTCTTA CGAAGCGGGA TACCACCGTC GTAGGTCTCC   
  
  
- GAGCGGGTAG TACATCGGTT ACGAGAGCTC CGTGCATATC GACCGTGTCC GAGATGTCAG AGATGATTGG   
  
  
- AACAACTACG TTTCAAGAGT CGAAGACTGA AGGATTTCCG AATGTCCAAT ATACAGAGTC GACAAGGAAT   
  
  
- GTTTTCCTAC AGAAAGAAAG AACGATTGAC GAGCTAACGT TTCAACCGTC TCTTTCGTTG TTTCTAGGTA   
  
  
- TAGTAACTAA AACCACAAAA GGATCCAAAT GTTACCGGAA CAAAGTATGT TGTGGATAGT TTTTCCGGTT   
  
  
- TACCTGGGGG TTTTGAGGCT TAGTGTCCTT AGCTGATGGG GGTCGTCCCA AAGTCTGGAC GTGTTTCCCA   
  
  
- ACTTCGATGT CCTGTGGCTA ATAGACCCAT GACACTCGCT AAACCCCACG GAAAAAGAAT AGTCCCGTAA   
  
  
- CGAGTCTTCA CCCTTTGATA AGTCGGCCTC CTAGAGTTCT AGCTTGTTCT GCTCGACCAC TAACAGTTGA   
  
  
- CAAACAAGTC CAGTCCTTGT GACGAGCTAC TCTGTCAGCT TCGTTTGTCA GGTTCTCTAC GAAAGAATCG   
  
  
- AAACCAATCT TTCGACTTAG GGTCGGATAA GTAAGTGCCC CAACAGTTAC CGTGTAAGTT GCGAGGTAAG   
  
  
- AAGCACTGAG CTAAGTCTCT CCGTAACAAA GTAATAAGTA GTCACAAACT ACACAGACTT CTCTGTTAAG   
  
  
- GTGCTCTACG GGTACTCTCC AAGAACTAAC TCTCGCTCTA AACACCCTTT CTTGACAAGT TACACCAACG   
  
  
- AACACTCCCA CGTCTCTCCC AAGTTTCCGG ACTCTGTATG TTCGTCACCG TTCACTCCTG TTGCGCCCGG   
  
  
- CCCAATTCCG TCCAACGGAA CCTGGTCCTC GAATACTTCC TTCGTTGCCG TTACCACTTC CGTTTAATAG   
  
  
- TATTCCTAAA ATACCACCTA TATTTATCCG TAACCTACGA AGTTCCAACC TTCCCATCTT GGAACACACG   
  
  
- AGAGAGTAAA ACCGTTGGAC GGAC

+     GT1-motif

| Site Name | Organism | Position | Strand | Matrix score. | sequence | function |
| --- | --- | --- | --- | --- | --- | --- |
| GT1-motif | Arabidopsis thaliana | 3856 | + | 6 | GGTTAA | light responsive element |

>HU02G01571.1   
+ +Up\_Stream \_Len000CTAGGG GTTGAGAGAA TCCGATAGCT ATGCAGTCCA AACATTATCT CGTCAAGCAT   
  
  
+ TACTATTTAA ATAATTAATA TTGAACTTGA TAGTTACACT TTTTGAATAC TAATTCCACA CCTCATTGCC   
  
  
+ TTTATTTCCA AACCTCGGGT TGCATTTGGA CCACATACCA GGAAGCTTGG ACGACAAAAA TTTCTTTTCT   
  
  
+ TGAGTATTAT TTTGTTTTAT TATTATTATT TTTTACTTTA TTTGGACCAC TTCACTTGGG TAACAAGATT   
  
  
+ GAATATTTGA CTATTATATT ATATGTCACG TATGGATATG GTAGTTGCTT AGAGCTTTGA CCGTTTGCAG   
  
  
+ GTTTAAGTAT GATTCCCTTG GAAAGGAGCC CCTCTTTATC TTAATTCAGA AGTAACTTTT GTTTTTTTAG   
  
  
+ ATAAATTGAA AAGAAAAATA ATAAATGTCT TAGTTGAGTT TGGTTATTCA ATCCGAAATT TGATCCTGCT   
  
  
+ TAATGTATAG TATATAATAC TAATACGCAT GTAACACAAC ACCTCAGATG GTCATTGTTT TACAATTTTT   
  
  
+ TTGAGTTTAG TATAAAAATA AAATCAATTA TTTTTAGAAG TGATGTTTAA TTTGCATTTT TTTTCCACCT   
  
  
+ AAAATCACGC ACTCGAACAT TAGGGGTTTG GCTAACTCAA AGACTCAAGG TAGTGACTTA TCCAAACTTA   
  
  
+ TTACATCCTA TAGATCTATA AAAACATAAA TCTCTTACAA TCAATCCAAA TTGATTCGGT CTAATTTGAT   
  
  
+ GACCCTGATG AGGGTCCTAC AATTACACAA GTGGGCCTTA GCCCCATGGC CGTTTTTTAA TCTTCATGGG   
  
  
+ TCCAGGCGGA GTTACCATCT CCAGGTCCAT TGAGGGTGTA TCACGTCTCA AATTACGAGT TTGCCTCTTT   
  
  
+ CTACGGGGAT TATAAATATC TACCTGCATA CCTCGAAAAG GAGGAGTTGA GATCAGCGAA ACTTGGCCAT   
  
  
+ CCCAGAGCAT TGAAATGCTC AGAACCCTTT TTCTAGAAAA ATATCGTTTT TCCTGAAAAT ATCCGACCTT   
  
  
+ ATGTCATTTA TTGTCGCTTT CTTGACTCTA ACCCGGGACT CCACACTAAC TTGACTATCG GAAAGGCGTT   
  
  
+ CCCTGGATCA CCATCCAAGA TAACCTCTTT TGCAGGGTTG ACGCTCGTTG GAAGAATCGT CGAATGTAGA   
  
  
+ GCCCCTATCA TCACATAAGC CCGCTCTTAC TTCATGCCAT CCTTGAATAG CAGTTTATCC CGAAGCAAAA   
  
  
+ ATAATTCTAA CTTTCTTATA TTGTAAACCC ACATCTAAAT TTAAATTCAT ACTCCAAAAT TCCGCCACGC   
  
  
+ AAATAAGGCT TTTTAATAGG CTTACATATA ATGGCATGCT ATAGAGACAC TGTTTTGAAG AAACTATTTA   
  
  
+ AACATCAGAC CTCATATTGA GTAGCAAATC AAGACTTCTT TCCTCCAGGC CATATTGAGT AGCAAATTAA   
  
  
+ GAATAACCAG AAGCTTGTGA AAAGAGTTAG AAAATAAAAG GTATAATAGA GACTTATGTT CTTAAGATAT   
  
  
+ CTCCTAGATA TGAATGTGAC CTCCTAGTCG ATATTTGTGC TGGATTAGCG AGTTGTGTGA CTGAGTTTGT   
  
  
+ ATGTGTCTCT TAGCATATGA GTTATTCTAG CTGCTTCTTT AAGCTATGTA GTGACTGTCT TCCTTCTTAT   
  
  
+ TTATAAGTGC TATGTCCAAC TAATAGCCGA AAAAAATGGT TCGCCTTTTC TGGTTTTCTT CTTTTTGAAG   
  
  
+ GTTGATTGGC TAATTTATAT AATGGTTTCA ATACTTATCC TTTATTGTTC CACAAATCCA CCTAACCACG   
  
  
+ TACCATTGAC AATAATTACC CACTAATTTC GGTTGGACTT GATTTCCTTT TGTTTGGTAC CTCAGGCATC   
  
  
+ TTCTGTGGAC CTTCATTCAC AGTTTAATTT TCACATTTTT ATAAGGCTGC TCTTGTGTTG CAAATTTTCC   
  
  
+ TATCCATTCC ACTCTTTGCC CATTCCCAGG CTAATTAGTG TTCTCTGTTC AACCATGGAT TCTGTGCTGG   
  
  
+ TTGATCCTGA GTTCATGAAA AATCTCTACA AATTCAAACC TGAATTGCTC TCAAACTTTT CAATGAATCC   
  
  
+ AAATGACGAC ATCTTTCAAG CCCTTCATTC AGAAAATGAT CCTTTACAGT TCCTCTCATT TGATGAAGGA   
  
  
+ ACCTGTCTTA ACAGCTGCAC TAGTCAACAA GTGCCAGATT TCCCTGATGC TTGTCTCAAG TTCATCAGTG   
  
  
+ ATATTCTTCT CGAAGAGGGT TTAGATGCAA ATCCTGCATC TGCACAGGCT CTCGAAGCCA CCGAGAAGTC   
  
  
+ CTTGTATGAT GCTCTCGGGC TTGGAGAGCC ATACCCCCTT TCATGTGATC ACTTTGCGCC ATCTATCTCT   
  
  
+ ACAAGTATTG AGAGCCCAGA TGACAGTTCT TCCAATAAAA GTTATAGCAG CAATCCCGAG ATAGATGGTT   
  
  
+ CTTATGCTAT CGCTGAGCCC AGTTTCGAGT CCAACCCCAA CTGTGTGCTT GATCAACCCC AGTTGAACTC   
  
  
+ CTTTCCAGCT CTACATGAGA TTTCTCGGTC CTTGGTGGAA CTGGGTTCTC AAGCCTCTGA GTTGAGCTTC   
  
  
+ GATGATGCAG GGAGTGCCCG TGTAGAGAAA AAGGGCAAAT CGATAAAGGG CTCGAGGAGG AAGAAGAGTC   
  
  
+ GTCAAAGAGA GGGTGAAGCG TGTTATGGAG GAAGGAGCCA TAAGGTTCAA GCTTCCTTCA ATGATGATTA   
  
  
+ CTATGAGATG GAACAGTATG ATGATGTAGT ATTGCTCTGT AATAATGAAC TAACGGGCAA TAGCCGTTTC   
  
  
+ AACACCGGGA AATCTTCACC TGAGGAGGGA TGGAGGAGAT TGCAGAGAAG CCGAGGAAAG AAGCAGAACA   
  
  
+ GTTTAGCAGT TGAAGTTGAT CTGATGACCC TGCTGACTCA GTGTGCACAA GCTGTATCGA GCTTTGATCT   
  
  
+ TCGAGGTGCA AACGAGCTAC TTAGGCAAAT CAGGCAGAAT GCTTCGCCCT ATGGTGGCAG CATCCAGAGG   
  
  
+ CTCGCCCATC ATGTAGCCAA TGCTCTCGAG GCACGTATAG CTGGCACAGG CTCTACAGTC TCTACTAACC   
  
  
+ TTGTTGATGC AAAGTTCTCA GCTTCTGACT TCCTAAAGGC TTACAGGTTA TATGTCTCAG CTGTTCCTTA   
  
  
+ CAAAAGGATG TCTTTCTTTC TTGCTAACTG CTCGATTGCA AAGTTGGCAG AGAAAGCAAC AAAGATCCAT   
  
  
+ ATCATTGATT TTGGTGTTTT CCTAGGTTTA CAATGGCCTT GTTTCATACA ACACCTATCA AAAAGGCCAA   
  
  
+ ATGGACCCCC AAAACTCCGA ATCACAGGAA TCGACTACCC CCAGCAGGGT TTCAGACCTG CACAAAGGGT   
  
  
+ TGAAGCTACA GGACACCGAT TATCTGGGTA CTGTGAGCGA TTTGGGGTGC CTTTTTCTTA TCAGGGCATT   
  
  
+ GCTCAGAAGT GGGAAACTAT TCAGCCGGAG GATCTCAAGA TCGAACAAGA CGAGCTGGTG ATTGTCAACT   
  
  
+ GTTTGTTCAG GTCAGGAACA CTGCTCGATG AGACAGTCGA AGCAAACAGT CCAAGAGATG CTTTCTTAGC   
  
  
+ TTTGGTTAGA AAGCTGAATC CCAGCCTATT CATTCACGGG GTTGTCAATG GCACATTCAA CGCTCCATTC   
  
  
+ TTCGTGACTC GATTCAGAGA GGCATTGTTT CATTATTCAT CAGTGTTTGA TGTGTCTGAA GAGACAATTC   
  
  
+ CACGAGATGC CCATGAGAGG TTCTTGATTG AGAGCGAGAT TTGTGGGAAA GAACTGTTCA ATGTGGTTGC   
  
  
+ TTGTGAGGGT GCAGAGAGGG TTCAAAGGCC TGAGACATAC AAGCAGTGGC AAGTGAGGAC AACGCGGGCC   
  
  
+ GGGTTAAGGC AGGTTGCCTT GGACCAGGAG CTTATGAAGG AAGCAACGGC AATGGTGAAG GCAAATTATC   
  
  
+ ATAAGGATTT TATGGTGGAT ATAAATAGGC ATTGGATGCT TCAAGGTTGG AAGGGTAGAA CCTTGTGTGC   
  
  
+ TCTCTCATTT TGGCAACCTG CCTG  

- +Up\_Stream \_Len000GATCCC CAACTCTCTT AGGCTATCGA TACGTCAGGT TTGTAATAGA GCAGTTCGTA   
  
  
- ATGATAAATT TATTAATTAT AACTTGAACT ATCAATGTGA AAAACTTATG ATTAAGGTGT GGAGTAACGG   
  
  
- AAATAAAGGT TTGGAGCCCA ACGTAAACCT GGTGTATGGT CCTTCGAACC TGCTGTTTTT AAAGAAAAGA   
  
  
- ACTCATAATA AAACAAAATA ATAATAATAA AAAATGAAAT AAACCTGGTG AAGTGAACCC ATTGTTCTAA   
  
  
- CTTATAAACT GATAATATAA TATACAGTGC ATACCTATAC CATCAACGAA TCTCGAAACT GGCAAACGTC   
  
  
- CAAATTCATA CTAAGGGAAC CTTTCCTCGG GGAGAAATAG AATTAAGTCT TCATTGAAAA CAAAAAAATC   
  
  
- TATTTAACTT TTCTTTTTAT TATTTACAGA ATCAACTCAA ACCAATAAGT TAGGCTTTAA ACTAGGACGA   
  
  
- ATTACATATC ATATATTATG ATTATGCGTA CATTGTGTTG TGGAGTCTAC CAGTAACAAA ATGTTAAAAA   
  
  
- AACTCAAATC ATATTTTTAT TTTAGTTAAT AAAAATCTTC ACTACAAATT AAACGTAAAA AAAAGGTGGA   
  
  
- TTTTAGTGCG TGAGCTTGTA ATCCCCAAAC CGATTGAGTT TCTGAGTTCC ATCACTGAAT AGGTTTGAAT   
  
  
- AATGTAGGAT ATCTAGATAT TTTTGTATTT AGAGAATGTT AGTTAGGTTT AACTAAGCCA GATTAAACTA   
  
  
- CTGGGACTAC TCCCAGGATG TTAATGTGTT CACCCGGAAT CGGGGTACCG GCAAAAAATT AGAAGTACCC   
  
  
- AGGTCCGCCT CAATGGTAGA GGTCCAGGTA ACTCCCACAT AGTGCAGAGT TTAATGCTCA AACGGAGAAA   
  
  
- GATGCCCCTA ATATTTATAG ATGGACGTAT GGAGCTTTTC CTCCTCAACT CTAGTCGCTT TGAACCGGTA   
  
  
- GGGTCTCGTA ACTTTACGAG TCTTGGGAAA AAGATCTTTT TATAGCAAAA AGGACTTTTA TAGGCTGGAA   
  
  
- TACAGTAAAT AACAGCGAAA GAACTGAGAT TGGGCCCTGA GGTGTGATTG AACTGATAGC CTTTCCGCAA   
  
  
- GGGACCTAGT GGTAGGTTCT ATTGGAGAAA ACGTCCCAAC TGCGAGCAAC CTTCTTAGCA GCTTACATCT   
  
  
- CGGGGATAGT AGTGTATTCG GGCGAGAATG AAGTACGGTA GGAACTTATC GTCAAATAGG GCTTCGTTTT   
  
  
- TATTAAGATT GAAAGAATAT AACATTTGGG TGTAGATTTA AATTTAAGTA TGAGGTTTTA AGGCGGTGCG   
  
  
- TTTATTCCGA AAAATTATCC GAATGTATAT TACCGTACGA TATCTCTGTG ACAAAACTTC TTTGATAAAT   
  
  
- TTGTAGTCTG GAGTATAACT CATCGTTTAG TTCTGAAGAA AGGAGGTCCG GTATAACTCA TCGTTTAATT   
  
  
- CTTATTGGTC TTCGAACACT TTTCTCAATC TTTTATTTTC CATATTATCT CTGAATACAA GAATTCTATA   
  
  
- GAGGATCTAT ACTTACACTG GAGGATCAGC TATAAACACG ACCTAATCGC TCAACACACT GACTCAAACA   
  
  
- TACACAGAGA ATCGTATACT CAATAAGATC GACGAAGAAA TTCGATACAT CACTGACAGA AGGAAGAATA   
  
  
- AATATTCACG ATACAGGTTG ATTATCGGCT TTTTTTACCA AGCGGAAAAG ACCAAAAGAA GAAAAACTTC   
  
  
- CAACTAACCG ATTAAATATA TTACCAAAGT TATGAATAGG AAATAACAAG GTGTTTAGGT GGATTGGTGC   
  
  
- ATGGTAACTG TTATTAATGG GTGATTAAAG CCAACCTGAA CTAAAGGAAA ACAAACCATG GAGTCCGTAG   
  
  
- AAGACACCTG GAAGTAAGTG TCAAATTAAA AGTGTAAAAA TATTCCGACG AGAACACAAC GTTTAAAAGG   
  
  
- ATAGGTAAGG TGAGAAACGG GTAAGGGTCC GATTAATCAC AAGAGACAAG TTGGTACCTA AGACACGACC   
  
  
- AACTAGGACT CAAGTACTTT TTAGAGATGT TTAAGTTTGG ACTTAACGAG AGTTTGAAAA GTTACTTAGG   
  
  
- TTTACTGCTG TAGAAAGTTC GGGAAGTAAG TCTTTTACTA GGAAATGTCA AGGAGAGTAA ACTACTTCCT   
  
  
- TGGACAGAAT TGTCGACGTG ATCAGTTGTT CACGGTCTAA AGGGACTACG AACAGAGTTC AAGTAGTCAC   
  
  
- TATAAGAAGA GCTTCTCCCA AATCTACGTT TAGGACGTAG ACGTGTCCGA GAGCTTCGGT GGCTCTTCAG   
  
  
- GAACATACTA CGAGAGCCCG AACCTCTCGG TATGGGGGAA AGTACACTAG TGAAACGCGG TAGATAGAGA   
  
  
- TGTTCATAAC TCTCGGGTCT ACTGTCAAGA AGGTTATTTT CAATATCGTC GTTAGGGCTC TATCTACCAA   
  
  
- GAATACGATA GCGACTCGGG TCAAAGCTCA GGTTGGGGTT GACACACGAA CTAGTTGGGG TCAACTTGAG   
  
  
- GAAAGGTCGA GATGTACTCT AAAGAGCCAG GAACCACCTT GACCCAAGAG TTCGGAGACT CAACTCGAAG   
  
  
- CTACTACGTC CCTCACGGGC ACATCTCTTT TTCCCGTTTA GCTATTTCCC GAGCTCCTCC TTCTTCTCAG   
  
  
- CAGTTTCTCT CCCACTTCGC ACAATACCTC CTTCCTCGGT ATTCCAAGTT CGAAGGAAGT TACTACTAAT   
  
  
- GATACTCTAC CTTGTCATAC TACTACATCA TAACGAGACA TTATTACTTG ATTGCCCGTT ATCGGCAAAG   
  
  
- TTGTGGCCCT TTAGAAGTGG ACTCCTCCCT ACCTCCTCTA ACGTCTCTTC GGCTCCTTTC TTCGTCTTGT   
  
  
- CAAATCGTCA ACTTCAACTA GACTACTGGG ACGACTGAGT CACACGTGTT CGACATAGCT CGAAACTAGA   
  
  
- AGCTCCACGT TTGCTCGATG AATCCGTTTA GTCCGTCTTA CGAAGCGGGA TACCACCGTC GTAGGTCTCC   
  
  
- GAGCGGGTAG TACATCGGTT ACGAGAGCTC CGTGCATATC GACCGTGTCC GAGATGTCAG AGATGATTGG   
  
  
- AACAACTACG TTTCAAGAGT CGAAGACTGA AGGATTTCCG AATGTCCAAT ATACAGAGTC GACAAGGAAT   
  
  
- GTTTTCCTAC AGAAAGAAAG AACGATTGAC GAGCTAACGT TTCAACCGTC TCTTTCGTTG TTTCTAGGTA   
  
  
- TAGTAACTAA AACCACAAAA GGATCCAAAT GTTACCGGAA CAAAGTATGT TGTGGATAGT TTTTCCGGTT   
  
  
- TACCTGGGGG TTTTGAGGCT TAGTGTCCTT AGCTGATGGG GGTCGTCCCA AAGTCTGGAC GTGTTTCCCA   
  
  
- ACTTCGATGT CCTGTGGCTA ATAGACCCAT GACACTCGCT AAACCCCACG GAAAAAGAAT AGTCCCGTAA   
  
  
- CGAGTCTTCA CCCTTTGATA AGTCGGCCTC CTAGAGTTCT AGCTTGTTCT GCTCGACCAC TAACAGTTGA   
  
  
- CAAACAAGTC CAGTCCTTGT GACGAGCTAC TCTGTCAGCT TCGTTTGTCA GGTTCTCTAC GAAAGAATCG   
  
  
- AAACCAATCT TTCGACTTAG GGTCGGATAA GTAAGTGCCC CAACAGTTAC CGTGTAAGTT GCGAGGTAAG   
  
  
- AAGCACTGAG CTAAGTCTCT CCGTAACAAA GTAATAAGTA GTCACAAACT ACACAGACTT CTCTGTTAAG   
  
  
- GTGCTCTACG GGTACTCTCC AAGAACTAAC TCTCGCTCTA AACACCCTTT CTTGACAAGT TACACCAACG   
  
  
- AACACTCCCA CGTCTCTCCC AAGTTTCCGG ACTCTGTATG TTCGTCACCG TTCACTCCTG TTGCGCCCGG   
  
  
- CCCAATTCCG TCCAACGGAA CCTGGTCCTC GAATACTTCC TTCGTTGCCG TTACCACTTC CGTTTAATAG   
  
  
- TATTCCTAAA ATACCACCTA TATTTATCCG TAACCTACGA AGTTCCAACC TTCCCATCTT GGAACACACG   
  
  
- AGAGAGTAAA ACCGTTGGAC GGAC

+     LTR

| Site Name | Organism | Position | Strand | Matrix score. | sequence | function |
| --- | --- | --- | --- | --- | --- | --- |
| LTR | Hordeum vulgare | 1851 | - | 6 | CCGAAA | cis-acting element involved in low-temperature responsiveness |
| LTR | Hordeum vulgare | 1711 | + | 6 | CCGAAA | cis-acting element involved in low-temperature responsiveness |
| LTR | Hordeum vulgare | 477 | + | 6 | CCGAAA | cis-acting element involved in low-temperature responsiveness |

>HU02G01571.1   
+ +Up\_Stream \_Len000CTAGGG GTTGAGAGAA TCCGATAGCT ATGCAGTCCA AACATTATCT CGTCAAGCAT   
  
  
+ TACTATTTAA ATAATTAATA TTGAACTTGA TAGTTACACT TTTTGAATAC TAATTCCACA CCTCATTGCC   
  
  
+ TTTATTTCCA AACCTCGGGT TGCATTTGGA CCACATACCA GGAAGCTTGG ACGACAAAAA TTTCTTTTCT   
  
  
+ TGAGTATTAT TTTGTTTTAT TATTATTATT TTTTACTTTA TTTGGACCAC TTCACTTGGG TAACAAGATT   
  
  
+ GAATATTTGA CTATTATATT ATATGTCACG TATGGATATG GTAGTTGCTT AGAGCTTTGA CCGTTTGCAG   
  
  
+ GTTTAAGTAT GATTCCCTTG GAAAGGAGCC CCTCTTTATC TTAATTCAGA AGTAACTTTT GTTTTTTTAG   
  
  
+ ATAAATTGAA AAGAAAAATA ATAAATGTCT TAGTTGAGTT TGGTTATTCA ATCCGAAATT TGATCCTGCT   
  
  
+ TAATGTATAG TATATAATAC TAATACGCAT GTAACACAAC ACCTCAGATG GTCATTGTTT TACAATTTTT   
  
  
+ TTGAGTTTAG TATAAAAATA AAATCAATTA TTTTTAGAAG TGATGTTTAA TTTGCATTTT TTTTCCACCT   
  
  
+ AAAATCACGC ACTCGAACAT TAGGGGTTTG GCTAACTCAA AGACTCAAGG TAGTGACTTA TCCAAACTTA   
  
  
+ TTACATCCTA TAGATCTATA AAAACATAAA TCTCTTACAA TCAATCCAAA TTGATTCGGT CTAATTTGAT   
  
  
+ GACCCTGATG AGGGTCCTAC AATTACACAA GTGGGCCTTA GCCCCATGGC CGTTTTTTAA TCTTCATGGG   
  
  
+ TCCAGGCGGA GTTACCATCT CCAGGTCCAT TGAGGGTGTA TCACGTCTCA AATTACGAGT TTGCCTCTTT   
  
  
+ CTACGGGGAT TATAAATATC TACCTGCATA CCTCGAAAAG GAGGAGTTGA GATCAGCGAA ACTTGGCCAT   
  
  
+ CCCAGAGCAT TGAAATGCTC AGAACCCTTT TTCTAGAAAA ATATCGTTTT TCCTGAAAAT ATCCGACCTT   
  
  
+ ATGTCATTTA TTGTCGCTTT CTTGACTCTA ACCCGGGACT CCACACTAAC TTGACTATCG GAAAGGCGTT   
  
  
+ CCCTGGATCA CCATCCAAGA TAACCTCTTT TGCAGGGTTG ACGCTCGTTG GAAGAATCGT CGAATGTAGA   
  
  
+ GCCCCTATCA TCACATAAGC CCGCTCTTAC TTCATGCCAT CCTTGAATAG CAGTTTATCC CGAAGCAAAA   
  
  
+ ATAATTCTAA CTTTCTTATA TTGTAAACCC ACATCTAAAT TTAAATTCAT ACTCCAAAAT TCCGCCACGC   
  
  
+ AAATAAGGCT TTTTAATAGG CTTACATATA ATGGCATGCT ATAGAGACAC TGTTTTGAAG AAACTATTTA   
  
  
+ AACATCAGAC CTCATATTGA GTAGCAAATC AAGACTTCTT TCCTCCAGGC CATATTGAGT AGCAAATTAA   
  
  
+ GAATAACCAG AAGCTTGTGA AAAGAGTTAG AAAATAAAAG GTATAATAGA GACTTATGTT CTTAAGATAT   
  
  
+ CTCCTAGATA TGAATGTGAC CTCCTAGTCG ATATTTGTGC TGGATTAGCG AGTTGTGTGA CTGAGTTTGT   
  
  
+ ATGTGTCTCT TAGCATATGA GTTATTCTAG CTGCTTCTTT AAGCTATGTA GTGACTGTCT TCCTTCTTAT   
  
  
+ TTATAAGTGC TATGTCCAAC TAATAGCCGA AAAAAATGGT TCGCCTTTTC TGGTTTTCTT CTTTTTGAAG   
  
  
+ GTTGATTGGC TAATTTATAT AATGGTTTCA ATACTTATCC TTTATTGTTC CACAAATCCA CCTAACCACG   
  
  
+ TACCATTGAC AATAATTACC CACTAATTTC GGTTGGACTT GATTTCCTTT TGTTTGGTAC CTCAGGCATC   
  
  
+ TTCTGTGGAC CTTCATTCAC AGTTTAATTT TCACATTTTT ATAAGGCTGC TCTTGTGTTG CAAATTTTCC   
  
  
+ TATCCATTCC ACTCTTTGCC CATTCCCAGG CTAATTAGTG TTCTCTGTTC AACCATGGAT TCTGTGCTGG   
  
  
+ TTGATCCTGA GTTCATGAAA AATCTCTACA AATTCAAACC TGAATTGCTC TCAAACTTTT CAATGAATCC   
  
  
+ AAATGACGAC ATCTTTCAAG CCCTTCATTC AGAAAATGAT CCTTTACAGT TCCTCTCATT TGATGAAGGA   
  
  
+ ACCTGTCTTA ACAGCTGCAC TAGTCAACAA GTGCCAGATT TCCCTGATGC TTGTCTCAAG TTCATCAGTG   
  
  
+ ATATTCTTCT CGAAGAGGGT TTAGATGCAA ATCCTGCATC TGCACAGGCT CTCGAAGCCA CCGAGAAGTC   
  
  
+ CTTGTATGAT GCTCTCGGGC TTGGAGAGCC ATACCCCCTT TCATGTGATC ACTTTGCGCC ATCTATCTCT   
  
  
+ ACAAGTATTG AGAGCCCAGA TGACAGTTCT TCCAATAAAA GTTATAGCAG CAATCCCGAG ATAGATGGTT   
  
  
+ CTTATGCTAT CGCTGAGCCC AGTTTCGAGT CCAACCCCAA CTGTGTGCTT GATCAACCCC AGTTGAACTC   
  
  
+ CTTTCCAGCT CTACATGAGA TTTCTCGGTC CTTGGTGGAA CTGGGTTCTC AAGCCTCTGA GTTGAGCTTC   
  
  
+ GATGATGCAG GGAGTGCCCG TGTAGAGAAA AAGGGCAAAT CGATAAAGGG CTCGAGGAGG AAGAAGAGTC   
  
  
+ GTCAAAGAGA GGGTGAAGCG TGTTATGGAG GAAGGAGCCA TAAGGTTCAA GCTTCCTTCA ATGATGATTA   
  
  
+ CTATGAGATG GAACAGTATG ATGATGTAGT ATTGCTCTGT AATAATGAAC TAACGGGCAA TAGCCGTTTC   
  
  
+ AACACCGGGA AATCTTCACC TGAGGAGGGA TGGAGGAGAT TGCAGAGAAG CCGAGGAAAG AAGCAGAACA   
  
  
+ GTTTAGCAGT TGAAGTTGAT CTGATGACCC TGCTGACTCA GTGTGCACAA GCTGTATCGA GCTTTGATCT   
  
  
+ TCGAGGTGCA AACGAGCTAC TTAGGCAAAT CAGGCAGAAT GCTTCGCCCT ATGGTGGCAG CATCCAGAGG   
  
  
+ CTCGCCCATC ATGTAGCCAA TGCTCTCGAG GCACGTATAG CTGGCACAGG CTCTACAGTC TCTACTAACC   
  
  
+ TTGTTGATGC AAAGTTCTCA GCTTCTGACT TCCTAAAGGC TTACAGGTTA TATGTCTCAG CTGTTCCTTA   
  
  
+ CAAAAGGATG TCTTTCTTTC TTGCTAACTG CTCGATTGCA AAGTTGGCAG AGAAAGCAAC AAAGATCCAT   
  
  
+ ATCATTGATT TTGGTGTTTT CCTAGGTTTA CAATGGCCTT GTTTCATACA ACACCTATCA AAAAGGCCAA   
  
  
+ ATGGACCCCC AAAACTCCGA ATCACAGGAA TCGACTACCC CCAGCAGGGT TTCAGACCTG CACAAAGGGT   
  
  
+ TGAAGCTACA GGACACCGAT TATCTGGGTA CTGTGAGCGA TTTGGGGTGC CTTTTTCTTA TCAGGGCATT   
  
  
+ GCTCAGAAGT GGGAAACTAT TCAGCCGGAG GATCTCAAGA TCGAACAAGA CGAGCTGGTG ATTGTCAACT   
  
  
+ GTTTGTTCAG GTCAGGAACA CTGCTCGATG AGACAGTCGA AGCAAACAGT CCAAGAGATG CTTTCTTAGC   
  
  
+ TTTGGTTAGA AAGCTGAATC CCAGCCTATT CATTCACGGG GTTGTCAATG GCACATTCAA CGCTCCATTC   
  
  
+ TTCGTGACTC GATTCAGAGA GGCATTGTTT CATTATTCAT CAGTGTTTGA TGTGTCTGAA GAGACAATTC   
  
  
+ CACGAGATGC CCATGAGAGG TTCTTGATTG AGAGCGAGAT TTGTGGGAAA GAACTGTTCA ATGTGGTTGC   
  
  
+ TTGTGAGGGT GCAGAGAGGG TTCAAAGGCC TGAGACATAC AAGCAGTGGC AAGTGAGGAC AACGCGGGCC   
  
  
+ GGGTTAAGGC AGGTTGCCTT GGACCAGGAG CTTATGAAGG AAGCAACGGC AATGGTGAAG GCAAATTATC   
  
  
+ ATAAGGATTT TATGGTGGAT ATAAATAGGC ATTGGATGCT TCAAGGTTGG AAGGGTAGAA CCTTGTGTGC   
  
  
+ TCTCTCATTT TGGCAACCTG CCTG  

- +Up\_Stream \_Len000GATCCC CAACTCTCTT AGGCTATCGA TACGTCAGGT TTGTAATAGA GCAGTTCGTA   
  
  
- ATGATAAATT TATTAATTAT AACTTGAACT ATCAATGTGA AAAACTTATG ATTAAGGTGT GGAGTAACGG   
  
  
- AAATAAAGGT TTGGAGCCCA ACGTAAACCT GGTGTATGGT CCTTCGAACC TGCTGTTTTT AAAGAAAAGA   
  
  
- ACTCATAATA AAACAAAATA ATAATAATAA AAAATGAAAT AAACCTGGTG AAGTGAACCC ATTGTTCTAA   
  
  
- CTTATAAACT GATAATATAA TATACAGTGC ATACCTATAC CATCAACGAA TCTCGAAACT GGCAAACGTC   
  
  
- CAAATTCATA CTAAGGGAAC CTTTCCTCGG GGAGAAATAG AATTAAGTCT TCATTGAAAA CAAAAAAATC   
  
  
- TATTTAACTT TTCTTTTTAT TATTTACAGA ATCAACTCAA ACCAATAAGT TAGGCTTTAA ACTAGGACGA   
  
  
- ATTACATATC ATATATTATG ATTATGCGTA CATTGTGTTG TGGAGTCTAC CAGTAACAAA ATGTTAAAAA   
  
  
- AACTCAAATC ATATTTTTAT TTTAGTTAAT AAAAATCTTC ACTACAAATT AAACGTAAAA AAAAGGTGGA   
  
  
- TTTTAGTGCG TGAGCTTGTA ATCCCCAAAC CGATTGAGTT TCTGAGTTCC ATCACTGAAT AGGTTTGAAT   
  
  
- AATGTAGGAT ATCTAGATAT TTTTGTATTT AGAGAATGTT AGTTAGGTTT AACTAAGCCA GATTAAACTA   
  
  
- CTGGGACTAC TCCCAGGATG TTAATGTGTT CACCCGGAAT CGGGGTACCG GCAAAAAATT AGAAGTACCC   
  
  
- AGGTCCGCCT CAATGGTAGA GGTCCAGGTA ACTCCCACAT AGTGCAGAGT TTAATGCTCA AACGGAGAAA   
  
  
- GATGCCCCTA ATATTTATAG ATGGACGTAT GGAGCTTTTC CTCCTCAACT CTAGTCGCTT TGAACCGGTA   
  
  
- GGGTCTCGTA ACTTTACGAG TCTTGGGAAA AAGATCTTTT TATAGCAAAA AGGACTTTTA TAGGCTGGAA   
  
  
- TACAGTAAAT AACAGCGAAA GAACTGAGAT TGGGCCCTGA GGTGTGATTG AACTGATAGC CTTTCCGCAA   
  
  
- GGGACCTAGT GGTAGGTTCT ATTGGAGAAA ACGTCCCAAC TGCGAGCAAC CTTCTTAGCA GCTTACATCT   
  
  
- CGGGGATAGT AGTGTATTCG GGCGAGAATG AAGTACGGTA GGAACTTATC GTCAAATAGG GCTTCGTTTT   
  
  
- TATTAAGATT GAAAGAATAT AACATTTGGG TGTAGATTTA AATTTAAGTA TGAGGTTTTA AGGCGGTGCG   
  
  
- TTTATTCCGA AAAATTATCC GAATGTATAT TACCGTACGA TATCTCTGTG ACAAAACTTC TTTGATAAAT   
  
  
- TTGTAGTCTG GAGTATAACT CATCGTTTAG TTCTGAAGAA AGGAGGTCCG GTATAACTCA TCGTTTAATT   
  
  
- CTTATTGGTC TTCGAACACT TTTCTCAATC TTTTATTTTC CATATTATCT CTGAATACAA GAATTCTATA   
  
  
- GAGGATCTAT ACTTACACTG GAGGATCAGC TATAAACACG ACCTAATCGC TCAACACACT GACTCAAACA   
  
  
- TACACAGAGA ATCGTATACT CAATAAGATC GACGAAGAAA TTCGATACAT CACTGACAGA AGGAAGAATA   
  
  
- AATATTCACG ATACAGGTTG ATTATCGGCT TTTTTTACCA AGCGGAAAAG ACCAAAAGAA GAAAAACTTC   
  
  
- CAACTAACCG ATTAAATATA TTACCAAAGT TATGAATAGG AAATAACAAG GTGTTTAGGT GGATTGGTGC   
  
  
- ATGGTAACTG TTATTAATGG GTGATTAAAG CCAACCTGAA CTAAAGGAAA ACAAACCATG GAGTCCGTAG   
  
  
- AAGACACCTG GAAGTAAGTG TCAAATTAAA AGTGTAAAAA TATTCCGACG AGAACACAAC GTTTAAAAGG   
  
  
- ATAGGTAAGG TGAGAAACGG GTAAGGGTCC GATTAATCAC AAGAGACAAG TTGGTACCTA AGACACGACC   
  
  
- AACTAGGACT CAAGTACTTT TTAGAGATGT TTAAGTTTGG ACTTAACGAG AGTTTGAAAA GTTACTTAGG   
  
  
- TTTACTGCTG TAGAAAGTTC GGGAAGTAAG TCTTTTACTA GGAAATGTCA AGGAGAGTAA ACTACTTCCT   
  
  
- TGGACAGAAT TGTCGACGTG ATCAGTTGTT CACGGTCTAA AGGGACTACG AACAGAGTTC AAGTAGTCAC   
  
  
- TATAAGAAGA GCTTCTCCCA AATCTACGTT TAGGACGTAG ACGTGTCCGA GAGCTTCGGT GGCTCTTCAG   
  
  
- GAACATACTA CGAGAGCCCG AACCTCTCGG TATGGGGGAA AGTACACTAG TGAAACGCGG TAGATAGAGA   
  
  
- TGTTCATAAC TCTCGGGTCT ACTGTCAAGA AGGTTATTTT CAATATCGTC GTTAGGGCTC TATCTACCAA   
  
  
- GAATACGATA GCGACTCGGG TCAAAGCTCA GGTTGGGGTT GACACACGAA CTAGTTGGGG TCAACTTGAG   
  
  
- GAAAGGTCGA GATGTACTCT AAAGAGCCAG GAACCACCTT GACCCAAGAG TTCGGAGACT CAACTCGAAG   
  
  
- CTACTACGTC CCTCACGGGC ACATCTCTTT TTCCCGTTTA GCTATTTCCC GAGCTCCTCC TTCTTCTCAG   
  
  
- CAGTTTCTCT CCCACTTCGC ACAATACCTC CTTCCTCGGT ATTCCAAGTT CGAAGGAAGT TACTACTAAT   
  
  
- GATACTCTAC CTTGTCATAC TACTACATCA TAACGAGACA TTATTACTTG ATTGCCCGTT ATCGGCAAAG   
  
  
- TTGTGGCCCT TTAGAAGTGG ACTCCTCCCT ACCTCCTCTA ACGTCTCTTC GGCTCCTTTC TTCGTCTTGT   
  
  
- CAAATCGTCA ACTTCAACTA GACTACTGGG ACGACTGAGT CACACGTGTT CGACATAGCT CGAAACTAGA   
  
  
- AGCTCCACGT TTGCTCGATG AATCCGTTTA GTCCGTCTTA CGAAGCGGGA TACCACCGTC GTAGGTCTCC   
  
  
- GAGCGGGTAG TACATCGGTT ACGAGAGCTC CGTGCATATC GACCGTGTCC GAGATGTCAG AGATGATTGG   
  
  
- AACAACTACG TTTCAAGAGT CGAAGACTGA AGGATTTCCG AATGTCCAAT ATACAGAGTC GACAAGGAAT   
  
  
- GTTTTCCTAC AGAAAGAAAG AACGATTGAC GAGCTAACGT TTCAACCGTC TCTTTCGTTG TTTCTAGGTA   
  
  
- TAGTAACTAA AACCACAAAA GGATCCAAAT GTTACCGGAA CAAAGTATGT TGTGGATAGT TTTTCCGGTT   
  
  
- TACCTGGGGG TTTTGAGGCT TAGTGTCCTT AGCTGATGGG GGTCGTCCCA AAGTCTGGAC GTGTTTCCCA   
  
  
- ACTTCGATGT CCTGTGGCTA ATAGACCCAT GACACTCGCT AAACCCCACG GAAAAAGAAT AGTCCCGTAA   
  
  
- CGAGTCTTCA CCCTTTGATA AGTCGGCCTC CTAGAGTTCT AGCTTGTTCT GCTCGACCAC TAACAGTTGA   
  
  
- CAAACAAGTC CAGTCCTTGT GACGAGCTAC TCTGTCAGCT TCGTTTGTCA GGTTCTCTAC GAAAGAATCG   
  
  
- AAACCAATCT TTCGACTTAG GGTCGGATAA GTAAGTGCCC CAACAGTTAC CGTGTAAGTT GCGAGGTAAG   
  
  
- AAGCACTGAG CTAAGTCTCT CCGTAACAAA GTAATAAGTA GTCACAAACT ACACAGACTT CTCTGTTAAG   
  
  
- GTGCTCTACG GGTACTCTCC AAGAACTAAC TCTCGCTCTA AACACCCTTT CTTGACAAGT TACACCAACG   
  
  
- AACACTCCCA CGTCTCTCCC AAGTTTCCGG ACTCTGTATG TTCGTCACCG TTCACTCCTG TTGCGCCCGG   
  
  
- CCCAATTCCG TCCAACGGAA CCTGGTCCTC GAATACTTCC TTCGTTGCCG TTACCACTTC CGTTTAATAG   
  
  
- TATTCCTAAA ATACCACCTA TATTTATCCG TAACCTACGA AGTTCCAACC TTCCCATCTT GGAACACACG   
  
  
- AGAGAGTAAA ACCGTTGGAC GGAC

+     MBS

| Site Name | Organism | Position | Strand | Matrix score. | sequence | function |
| --- | --- | --- | --- | --- | --- | --- |
| MBS | Arabidopsis thaliana | 3500 | + | 6 | CAACTG | MYB binding site involved in drought-inducibility |
| MBS | Arabidopsis thaliana | 2881 | - | 6 | CAACTG | MYB binding site involved in drought-inducibility |
| MBS | Arabidopsis thaliana | 2492 | + | 6 | CAACTG | MYB binding site involved in drought-inducibility |
| MBS | Arabidopsis thaliana | 2514 | - | 6 | CAACTG | MYB binding site involved in drought-inducibility |

>HU02G01571.1   
+ +Up\_Stream \_Len000CTAGGG GTTGAGAGAA TCCGATAGCT ATGCAGTCCA AACATTATCT CGTCAAGCAT   
  
  
+ TACTATTTAA ATAATTAATA TTGAACTTGA TAGTTACACT TTTTGAATAC TAATTCCACA CCTCATTGCC   
  
  
+ TTTATTTCCA AACCTCGGGT TGCATTTGGA CCACATACCA GGAAGCTTGG ACGACAAAAA TTTCTTTTCT   
  
  
+ TGAGTATTAT TTTGTTTTAT TATTATTATT TTTTACTTTA TTTGGACCAC TTCACTTGGG TAACAAGATT   
  
  
+ GAATATTTGA CTATTATATT ATATGTCACG TATGGATATG GTAGTTGCTT AGAGCTTTGA CCGTTTGCAG   
  
  
+ GTTTAAGTAT GATTCCCTTG GAAAGGAGCC CCTCTTTATC TTAATTCAGA AGTAACTTTT GTTTTTTTAG   
  
  
+ ATAAATTGAA AAGAAAAATA ATAAATGTCT TAGTTGAGTT TGGTTATTCA ATCCGAAATT TGATCCTGCT   
  
  
+ TAATGTATAG TATATAATAC TAATACGCAT GTAACACAAC ACCTCAGATG GTCATTGTTT TACAATTTTT   
  
  
+ TTGAGTTTAG TATAAAAATA AAATCAATTA TTTTTAGAAG TGATGTTTAA TTTGCATTTT TTTTCCACCT   
  
  
+ AAAATCACGC ACTCGAACAT TAGGGGTTTG GCTAACTCAA AGACTCAAGG TAGTGACTTA TCCAAACTTA   
  
  
+ TTACATCCTA TAGATCTATA AAAACATAAA TCTCTTACAA TCAATCCAAA TTGATTCGGT CTAATTTGAT   
  
  
+ GACCCTGATG AGGGTCCTAC AATTACACAA GTGGGCCTTA GCCCCATGGC CGTTTTTTAA TCTTCATGGG   
  
  
+ TCCAGGCGGA GTTACCATCT CCAGGTCCAT TGAGGGTGTA TCACGTCTCA AATTACGAGT TTGCCTCTTT   
  
  
+ CTACGGGGAT TATAAATATC TACCTGCATA CCTCGAAAAG GAGGAGTTGA GATCAGCGAA ACTTGGCCAT   
  
  
+ CCCAGAGCAT TGAAATGCTC AGAACCCTTT TTCTAGAAAA ATATCGTTTT TCCTGAAAAT ATCCGACCTT   
  
  
+ ATGTCATTTA TTGTCGCTTT CTTGACTCTA ACCCGGGACT CCACACTAAC TTGACTATCG GAAAGGCGTT   
  
  
+ CCCTGGATCA CCATCCAAGA TAACCTCTTT TGCAGGGTTG ACGCTCGTTG GAAGAATCGT CGAATGTAGA   
  
  
+ GCCCCTATCA TCACATAAGC CCGCTCTTAC TTCATGCCAT CCTTGAATAG CAGTTTATCC CGAAGCAAAA   
  
  
+ ATAATTCTAA CTTTCTTATA TTGTAAACCC ACATCTAAAT TTAAATTCAT ACTCCAAAAT TCCGCCACGC   
  
  
+ AAATAAGGCT TTTTAATAGG CTTACATATA ATGGCATGCT ATAGAGACAC TGTTTTGAAG AAACTATTTA   
  
  
+ AACATCAGAC CTCATATTGA GTAGCAAATC AAGACTTCTT TCCTCCAGGC CATATTGAGT AGCAAATTAA   
  
  
+ GAATAACCAG AAGCTTGTGA AAAGAGTTAG AAAATAAAAG GTATAATAGA GACTTATGTT CTTAAGATAT   
  
  
+ CTCCTAGATA TGAATGTGAC CTCCTAGTCG ATATTTGTGC TGGATTAGCG AGTTGTGTGA CTGAGTTTGT   
  
  
+ ATGTGTCTCT TAGCATATGA GTTATTCTAG CTGCTTCTTT AAGCTATGTA GTGACTGTCT TCCTTCTTAT   
  
  
+ TTATAAGTGC TATGTCCAAC TAATAGCCGA AAAAAATGGT TCGCCTTTTC TGGTTTTCTT CTTTTTGAAG   
  
  
+ GTTGATTGGC TAATTTATAT AATGGTTTCA ATACTTATCC TTTATTGTTC CACAAATCCA CCTAACCACG   
  
  
+ TACCATTGAC AATAATTACC CACTAATTTC GGTTGGACTT GATTTCCTTT TGTTTGGTAC CTCAGGCATC   
  
  
+ TTCTGTGGAC CTTCATTCAC AGTTTAATTT TCACATTTTT ATAAGGCTGC TCTTGTGTTG CAAATTTTCC   
  
  
+ TATCCATTCC ACTCTTTGCC CATTCCCAGG CTAATTAGTG TTCTCTGTTC AACCATGGAT TCTGTGCTGG   
  
  
+ TTGATCCTGA GTTCATGAAA AATCTCTACA AATTCAAACC TGAATTGCTC TCAAACTTTT CAATGAATCC   
  
  
+ AAATGACGAC ATCTTTCAAG CCCTTCATTC AGAAAATGAT CCTTTACAGT TCCTCTCATT TGATGAAGGA   
  
  
+ ACCTGTCTTA ACAGCTGCAC TAGTCAACAA GTGCCAGATT TCCCTGATGC TTGTCTCAAG TTCATCAGTG   
  
  
+ ATATTCTTCT CGAAGAGGGT TTAGATGCAA ATCCTGCATC TGCACAGGCT CTCGAAGCCA CCGAGAAGTC   
  
  
+ CTTGTATGAT GCTCTCGGGC TTGGAGAGCC ATACCCCCTT TCATGTGATC ACTTTGCGCC ATCTATCTCT   
  
  
+ ACAAGTATTG AGAGCCCAGA TGACAGTTCT TCCAATAAAA GTTATAGCAG CAATCCCGAG ATAGATGGTT   
  
  
+ CTTATGCTAT CGCTGAGCCC AGTTTCGAGT CCAACCCCAA CTGTGTGCTT GATCAACCCC AGTTGAACTC   
  
  
+ CTTTCCAGCT CTACATGAGA TTTCTCGGTC CTTGGTGGAA CTGGGTTCTC AAGCCTCTGA GTTGAGCTTC   
  
  
+ GATGATGCAG GGAGTGCCCG TGTAGAGAAA AAGGGCAAAT CGATAAAGGG CTCGAGGAGG AAGAAGAGTC   
  
  
+ GTCAAAGAGA GGGTGAAGCG TGTTATGGAG GAAGGAGCCA TAAGGTTCAA GCTTCCTTCA ATGATGATTA   
  
  
+ CTATGAGATG GAACAGTATG ATGATGTAGT ATTGCTCTGT AATAATGAAC TAACGGGCAA TAGCCGTTTC   
  
  
+ AACACCGGGA AATCTTCACC TGAGGAGGGA TGGAGGAGAT TGCAGAGAAG CCGAGGAAAG AAGCAGAACA   
  
  
+ GTTTAGCAGT TGAAGTTGAT CTGATGACCC TGCTGACTCA GTGTGCACAA GCTGTATCGA GCTTTGATCT   
  
  
+ TCGAGGTGCA AACGAGCTAC TTAGGCAAAT CAGGCAGAAT GCTTCGCCCT ATGGTGGCAG CATCCAGAGG   
  
  
+ CTCGCCCATC ATGTAGCCAA TGCTCTCGAG GCACGTATAG CTGGCACAGG CTCTACAGTC TCTACTAACC   
  
  
+ TTGTTGATGC AAAGTTCTCA GCTTCTGACT TCCTAAAGGC TTACAGGTTA TATGTCTCAG CTGTTCCTTA   
  
  
+ CAAAAGGATG TCTTTCTTTC TTGCTAACTG CTCGATTGCA AAGTTGGCAG AGAAAGCAAC AAAGATCCAT   
  
  
+ ATCATTGATT TTGGTGTTTT CCTAGGTTTA CAATGGCCTT GTTTCATACA ACACCTATCA AAAAGGCCAA   
  
  
+ ATGGACCCCC AAAACTCCGA ATCACAGGAA TCGACTACCC CCAGCAGGGT TTCAGACCTG CACAAAGGGT   
  
  
+ TGAAGCTACA GGACACCGAT TATCTGGGTA CTGTGAGCGA TTTGGGGTGC CTTTTTCTTA TCAGGGCATT   
  
  
+ GCTCAGAAGT GGGAAACTAT TCAGCCGGAG GATCTCAAGA TCGAACAAGA CGAGCTGGTG ATTGTCAACT   
  
  
+ GTTTGTTCAG GTCAGGAACA CTGCTCGATG AGACAGTCGA AGCAAACAGT CCAAGAGATG CTTTCTTAGC   
  
  
+ TTTGGTTAGA AAGCTGAATC CCAGCCTATT CATTCACGGG GTTGTCAATG GCACATTCAA CGCTCCATTC   
  
  
+ TTCGTGACTC GATTCAGAGA GGCATTGTTT CATTATTCAT CAGTGTTTGA TGTGTCTGAA GAGACAATTC   
  
  
+ CACGAGATGC CCATGAGAGG TTCTTGATTG AGAGCGAGAT TTGTGGGAAA GAACTGTTCA ATGTGGTTGC   
  
  
+ TTGTGAGGGT GCAGAGAGGG TTCAAAGGCC TGAGACATAC AAGCAGTGGC AAGTGAGGAC AACGCGGGCC   
  
  
+ GGGTTAAGGC AGGTTGCCTT GGACCAGGAG CTTATGAAGG AAGCAACGGC AATGGTGAAG GCAAATTATC   
  
  
+ ATAAGGATTT TATGGTGGAT ATAAATAGGC ATTGGATGCT TCAAGGTTGG AAGGGTAGAA CCTTGTGTGC   
  
  
+ TCTCTCATTT TGGCAACCTG CCTG  

- +Up\_Stream \_Len000GATCCC CAACTCTCTT AGGCTATCGA TACGTCAGGT TTGTAATAGA GCAGTTCGTA   
  
  
- ATGATAAATT TATTAATTAT AACTTGAACT ATCAATGTGA AAAACTTATG ATTAAGGTGT GGAGTAACGG   
  
  
- AAATAAAGGT TTGGAGCCCA ACGTAAACCT GGTGTATGGT CCTTCGAACC TGCTGTTTTT AAAGAAAAGA   
  
  
- ACTCATAATA AAACAAAATA ATAATAATAA AAAATGAAAT AAACCTGGTG AAGTGAACCC ATTGTTCTAA   
  
  
- CTTATAAACT GATAATATAA TATACAGTGC ATACCTATAC CATCAACGAA TCTCGAAACT GGCAAACGTC   
  
  
- CAAATTCATA CTAAGGGAAC CTTTCCTCGG GGAGAAATAG AATTAAGTCT TCATTGAAAA CAAAAAAATC   
  
  
- TATTTAACTT TTCTTTTTAT TATTTACAGA ATCAACTCAA ACCAATAAGT TAGGCTTTAA ACTAGGACGA   
  
  
- ATTACATATC ATATATTATG ATTATGCGTA CATTGTGTTG TGGAGTCTAC CAGTAACAAA ATGTTAAAAA   
  
  
- AACTCAAATC ATATTTTTAT TTTAGTTAAT AAAAATCTTC ACTACAAATT AAACGTAAAA AAAAGGTGGA   
  
  
- TTTTAGTGCG TGAGCTTGTA ATCCCCAAAC CGATTGAGTT TCTGAGTTCC ATCACTGAAT AGGTTTGAAT   
  
  
- AATGTAGGAT ATCTAGATAT TTTTGTATTT AGAGAATGTT AGTTAGGTTT AACTAAGCCA GATTAAACTA   
  
  
- CTGGGACTAC TCCCAGGATG TTAATGTGTT CACCCGGAAT CGGGGTACCG GCAAAAAATT AGAAGTACCC   
  
  
- AGGTCCGCCT CAATGGTAGA GGTCCAGGTA ACTCCCACAT AGTGCAGAGT TTAATGCTCA AACGGAGAAA   
  
  
- GATGCCCCTA ATATTTATAG ATGGACGTAT GGAGCTTTTC CTCCTCAACT CTAGTCGCTT TGAACCGGTA   
  
  
- GGGTCTCGTA ACTTTACGAG TCTTGGGAAA AAGATCTTTT TATAGCAAAA AGGACTTTTA TAGGCTGGAA   
  
  
- TACAGTAAAT AACAGCGAAA GAACTGAGAT TGGGCCCTGA GGTGTGATTG AACTGATAGC CTTTCCGCAA   
  
  
- GGGACCTAGT GGTAGGTTCT ATTGGAGAAA ACGTCCCAAC TGCGAGCAAC CTTCTTAGCA GCTTACATCT   
  
  
- CGGGGATAGT AGTGTATTCG GGCGAGAATG AAGTACGGTA GGAACTTATC GTCAAATAGG GCTTCGTTTT   
  
  
- TATTAAGATT GAAAGAATAT AACATTTGGG TGTAGATTTA AATTTAAGTA TGAGGTTTTA AGGCGGTGCG   
  
  
- TTTATTCCGA AAAATTATCC GAATGTATAT TACCGTACGA TATCTCTGTG ACAAAACTTC TTTGATAAAT   
  
  
- TTGTAGTCTG GAGTATAACT CATCGTTTAG TTCTGAAGAA AGGAGGTCCG GTATAACTCA TCGTTTAATT   
  
  
- CTTATTGGTC TTCGAACACT TTTCTCAATC TTTTATTTTC CATATTATCT CTGAATACAA GAATTCTATA   
  
  
- GAGGATCTAT ACTTACACTG GAGGATCAGC TATAAACACG ACCTAATCGC TCAACACACT GACTCAAACA   
  
  
- TACACAGAGA ATCGTATACT CAATAAGATC GACGAAGAAA TTCGATACAT CACTGACAGA AGGAAGAATA   
  
  
- AATATTCACG ATACAGGTTG ATTATCGGCT TTTTTTACCA AGCGGAAAAG ACCAAAAGAA GAAAAACTTC   
  
  
- CAACTAACCG ATTAAATATA TTACCAAAGT TATGAATAGG AAATAACAAG GTGTTTAGGT GGATTGGTGC   
  
  
- ATGGTAACTG TTATTAATGG GTGATTAAAG CCAACCTGAA CTAAAGGAAA ACAAACCATG GAGTCCGTAG   
  
  
- AAGACACCTG GAAGTAAGTG TCAAATTAAA AGTGTAAAAA TATTCCGACG AGAACACAAC GTTTAAAAGG   
  
  
- ATAGGTAAGG TGAGAAACGG GTAAGGGTCC GATTAATCAC AAGAGACAAG TTGGTACCTA AGACACGACC   
  
  
- AACTAGGACT CAAGTACTTT TTAGAGATGT TTAAGTTTGG ACTTAACGAG AGTTTGAAAA GTTACTTAGG   
  
  
- TTTACTGCTG TAGAAAGTTC GGGAAGTAAG TCTTTTACTA GGAAATGTCA AGGAGAGTAA ACTACTTCCT   
  
  
- TGGACAGAAT TGTCGACGTG ATCAGTTGTT CACGGTCTAA AGGGACTACG AACAGAGTTC AAGTAGTCAC   
  
  
- TATAAGAAGA GCTTCTCCCA AATCTACGTT TAGGACGTAG ACGTGTCCGA GAGCTTCGGT GGCTCTTCAG   
  
  
- GAACATACTA CGAGAGCCCG AACCTCTCGG TATGGGGGAA AGTACACTAG TGAAACGCGG TAGATAGAGA   
  
  
- TGTTCATAAC TCTCGGGTCT ACTGTCAAGA AGGTTATTTT CAATATCGTC GTTAGGGCTC TATCTACCAA   
  
  
- GAATACGATA GCGACTCGGG TCAAAGCTCA GGTTGGGGTT GACACACGAA CTAGTTGGGG TCAACTTGAG   
  
  
- GAAAGGTCGA GATGTACTCT AAAGAGCCAG GAACCACCTT GACCCAAGAG TTCGGAGACT CAACTCGAAG   
  
  
- CTACTACGTC CCTCACGGGC ACATCTCTTT TTCCCGTTTA GCTATTTCCC GAGCTCCTCC TTCTTCTCAG   
  
  
- CAGTTTCTCT CCCACTTCGC ACAATACCTC CTTCCTCGGT ATTCCAAGTT CGAAGGAAGT TACTACTAAT   
  
  
- GATACTCTAC CTTGTCATAC TACTACATCA TAACGAGACA TTATTACTTG ATTGCCCGTT ATCGGCAAAG   
  
  
- TTGTGGCCCT TTAGAAGTGG ACTCCTCCCT ACCTCCTCTA ACGTCTCTTC GGCTCCTTTC TTCGTCTTGT   
  
  
- CAAATCGTCA ACTTCAACTA GACTACTGGG ACGACTGAGT CACACGTGTT CGACATAGCT CGAAACTAGA   
  
  
- AGCTCCACGT TTGCTCGATG AATCCGTTTA GTCCGTCTTA CGAAGCGGGA TACCACCGTC GTAGGTCTCC   
  
  
- GAGCGGGTAG TACATCGGTT ACGAGAGCTC CGTGCATATC GACCGTGTCC GAGATGTCAG AGATGATTGG   
  
  
- AACAACTACG TTTCAAGAGT CGAAGACTGA AGGATTTCCG AATGTCCAAT ATACAGAGTC GACAAGGAAT   
  
  
- GTTTTCCTAC AGAAAGAAAG AACGATTGAC GAGCTAACGT TTCAACCGTC TCTTTCGTTG TTTCTAGGTA   
  
  
- TAGTAACTAA AACCACAAAA GGATCCAAAT GTTACCGGAA CAAAGTATGT TGTGGATAGT TTTTCCGGTT   
  
  
- TACCTGGGGG TTTTGAGGCT TAGTGTCCTT AGCTGATGGG GGTCGTCCCA AAGTCTGGAC GTGTTTCCCA   
  
  
- ACTTCGATGT CCTGTGGCTA ATAGACCCAT GACACTCGCT AAACCCCACG GAAAAAGAAT AGTCCCGTAA   
  
  
- CGAGTCTTCA CCCTTTGATA AGTCGGCCTC CTAGAGTTCT AGCTTGTTCT GCTCGACCAC TAACAGTTGA   
  
  
- CAAACAAGTC CAGTCCTTGT GACGAGCTAC TCTGTCAGCT TCGTTTGTCA GGTTCTCTAC GAAAGAATCG   
  
  
- AAACCAATCT TTCGACTTAG GGTCGGATAA GTAAGTGCCC CAACAGTTAC CGTGTAAGTT GCGAGGTAAG   
  
  
- AAGCACTGAG CTAAGTCTCT CCGTAACAAA GTAATAAGTA GTCACAAACT ACACAGACTT CTCTGTTAAG   
  
  
- GTGCTCTACG GGTACTCTCC AAGAACTAAC TCTCGCTCTA AACACCCTTT CTTGACAAGT TACACCAACG   
  
  
- AACACTCCCA CGTCTCTCCC AAGTTTCCGG ACTCTGTATG TTCGTCACCG TTCACTCCTG TTGCGCCCGG   
  
  
- CCCAATTCCG TCCAACGGAA CCTGGTCCTC GAATACTTCC TTCGTTGCCG TTACCACTTC CGTTTAATAG   
  
  
- TATTCCTAAA ATACCACCTA TATTTATCCG TAACCTACGA AGTTCCAACC TTCCCATCTT GGAACACACG   
  
  
- AGAGAGTAAA ACCGTTGGAC GGAC

+     MYB

| Site Name | Organism | Position | Strand | Matrix score. | sequence | function |
| --- | --- | --- | --- | --- | --- | --- |
| MYB | Arabidopsis thaliana | 3778 | - | 6 | CAACCA |  |
| MYB | Arabidopsis thaliana | 3577 | - | 6 | TAACCA |  |
| MYB | Arabidopsis thaliana | 2032 | - | 6 | CAACCA |  |
| MYB | Arabidopsis thaliana | 465 | - | 6 | TAACCA |  |
| MYB | Arabidopsis thaliana | 1478 | + | 6 | TAACCA |  |
| MYB | Arabidopsis thaliana | 2014 | + | 6 | CAACCA |  |
| MYB | Arabidopsis thaliana | 1817 | + | 6 | TAACCA |  |

>HU02G01571.1   
+ +Up\_Stream \_Len000CTAGGG GTTGAGAGAA TCCGATAGCT ATGCAGTCCA AACATTATCT CGTCAAGCAT   
  
  
+ TACTATTTAA ATAATTAATA TTGAACTTGA TAGTTACACT TTTTGAATAC TAATTCCACA CCTCATTGCC   
  
  
+ TTTATTTCCA AACCTCGGGT TGCATTTGGA CCACATACCA GGAAGCTTGG ACGACAAAAA TTTCTTTTCT   
  
  
+ TGAGTATTAT TTTGTTTTAT TATTATTATT TTTTACTTTA TTTGGACCAC TTCACTTGGG TAACAAGATT   
  
  
+ GAATATTTGA CTATTATATT ATATGTCACG TATGGATATG GTAGTTGCTT AGAGCTTTGA CCGTTTGCAG   
  
  
+ GTTTAAGTAT GATTCCCTTG GAAAGGAGCC CCTCTTTATC TTAATTCAGA AGTAACTTTT GTTTTTTTAG   
  
  
+ ATAAATTGAA AAGAAAAATA ATAAATGTCT TAGTTGAGTT TGGTTATTCA ATCCGAAATT TGATCCTGCT   
  
  
+ TAATGTATAG TATATAATAC TAATACGCAT GTAACACAAC ACCTCAGATG GTCATTGTTT TACAATTTTT   
  
  
+ TTGAGTTTAG TATAAAAATA AAATCAATTA TTTTTAGAAG TGATGTTTAA TTTGCATTTT TTTTCCACCT   
  
  
+ AAAATCACGC ACTCGAACAT TAGGGGTTTG GCTAACTCAA AGACTCAAGG TAGTGACTTA TCCAAACTTA   
  
  
+ TTACATCCTA TAGATCTATA AAAACATAAA TCTCTTACAA TCAATCCAAA TTGATTCGGT CTAATTTGAT   
  
  
+ GACCCTGATG AGGGTCCTAC AATTACACAA GTGGGCCTTA GCCCCATGGC CGTTTTTTAA TCTTCATGGG   
  
  
+ TCCAGGCGGA GTTACCATCT CCAGGTCCAT TGAGGGTGTA TCACGTCTCA AATTACGAGT TTGCCTCTTT   
  
  
+ CTACGGGGAT TATAAATATC TACCTGCATA CCTCGAAAAG GAGGAGTTGA GATCAGCGAA ACTTGGCCAT   
  
  
+ CCCAGAGCAT TGAAATGCTC AGAACCCTTT TTCTAGAAAA ATATCGTTTT TCCTGAAAAT ATCCGACCTT   
  
  
+ ATGTCATTTA TTGTCGCTTT CTTGACTCTA ACCCGGGACT CCACACTAAC TTGACTATCG GAAAGGCGTT   
  
  
+ CCCTGGATCA CCATCCAAGA TAACCTCTTT TGCAGGGTTG ACGCTCGTTG GAAGAATCGT CGAATGTAGA   
  
  
+ GCCCCTATCA TCACATAAGC CCGCTCTTAC TTCATGCCAT CCTTGAATAG CAGTTTATCC CGAAGCAAAA   
  
  
+ ATAATTCTAA CTTTCTTATA TTGTAAACCC ACATCTAAAT TTAAATTCAT ACTCCAAAAT TCCGCCACGC   
  
  
+ AAATAAGGCT TTTTAATAGG CTTACATATA ATGGCATGCT ATAGAGACAC TGTTTTGAAG AAACTATTTA   
  
  
+ AACATCAGAC CTCATATTGA GTAGCAAATC AAGACTTCTT TCCTCCAGGC CATATTGAGT AGCAAATTAA   
  
  
+ GAATAACCAG AAGCTTGTGA AAAGAGTTAG AAAATAAAAG GTATAATAGA GACTTATGTT CTTAAGATAT   
  
  
+ CTCCTAGATA TGAATGTGAC CTCCTAGTCG ATATTTGTGC TGGATTAGCG AGTTGTGTGA CTGAGTTTGT   
  
  
+ ATGTGTCTCT TAGCATATGA GTTATTCTAG CTGCTTCTTT AAGCTATGTA GTGACTGTCT TCCTTCTTAT   
  
  
+ TTATAAGTGC TATGTCCAAC TAATAGCCGA AAAAAATGGT TCGCCTTTTC TGGTTTTCTT CTTTTTGAAG   
  
  
+ GTTGATTGGC TAATTTATAT AATGGTTTCA ATACTTATCC TTTATTGTTC CACAAATCCA CCTAACCACG   
  
  
+ TACCATTGAC AATAATTACC CACTAATTTC GGTTGGACTT GATTTCCTTT TGTTTGGTAC CTCAGGCATC   
  
  
+ TTCTGTGGAC CTTCATTCAC AGTTTAATTT TCACATTTTT ATAAGGCTGC TCTTGTGTTG CAAATTTTCC   
  
  
+ TATCCATTCC ACTCTTTGCC CATTCCCAGG CTAATTAGTG TTCTCTGTTC AACCATGGAT TCTGTGCTGG   
  
  
+ TTGATCCTGA GTTCATGAAA AATCTCTACA AATTCAAACC TGAATTGCTC TCAAACTTTT CAATGAATCC   
  
  
+ AAATGACGAC ATCTTTCAAG CCCTTCATTC AGAAAATGAT CCTTTACAGT TCCTCTCATT TGATGAAGGA   
  
  
+ ACCTGTCTTA ACAGCTGCAC TAGTCAACAA GTGCCAGATT TCCCTGATGC TTGTCTCAAG TTCATCAGTG   
  
  
+ ATATTCTTCT CGAAGAGGGT TTAGATGCAA ATCCTGCATC TGCACAGGCT CTCGAAGCCA CCGAGAAGTC   
  
  
+ CTTGTATGAT GCTCTCGGGC TTGGAGAGCC ATACCCCCTT TCATGTGATC ACTTTGCGCC ATCTATCTCT   
  
  
+ ACAAGTATTG AGAGCCCAGA TGACAGTTCT TCCAATAAAA GTTATAGCAG CAATCCCGAG ATAGATGGTT   
  
  
+ CTTATGCTAT CGCTGAGCCC AGTTTCGAGT CCAACCCCAA CTGTGTGCTT GATCAACCCC AGTTGAACTC   
  
  
+ CTTTCCAGCT CTACATGAGA TTTCTCGGTC CTTGGTGGAA CTGGGTTCTC AAGCCTCTGA GTTGAGCTTC   
  
  
+ GATGATGCAG GGAGTGCCCG TGTAGAGAAA AAGGGCAAAT CGATAAAGGG CTCGAGGAGG AAGAAGAGTC   
  
  
+ GTCAAAGAGA GGGTGAAGCG TGTTATGGAG GAAGGAGCCA TAAGGTTCAA GCTTCCTTCA ATGATGATTA   
  
  
+ CTATGAGATG GAACAGTATG ATGATGTAGT ATTGCTCTGT AATAATGAAC TAACGGGCAA TAGCCGTTTC   
  
  
+ AACACCGGGA AATCTTCACC TGAGGAGGGA TGGAGGAGAT TGCAGAGAAG CCGAGGAAAG AAGCAGAACA   
  
  
+ GTTTAGCAGT TGAAGTTGAT CTGATGACCC TGCTGACTCA GTGTGCACAA GCTGTATCGA GCTTTGATCT   
  
  
+ TCGAGGTGCA AACGAGCTAC TTAGGCAAAT CAGGCAGAAT GCTTCGCCCT ATGGTGGCAG CATCCAGAGG   
  
  
+ CTCGCCCATC ATGTAGCCAA TGCTCTCGAG GCACGTATAG CTGGCACAGG CTCTACAGTC TCTACTAACC   
  
  
+ TTGTTGATGC AAAGTTCTCA GCTTCTGACT TCCTAAAGGC TTACAGGTTA TATGTCTCAG CTGTTCCTTA   
  
  
+ CAAAAGGATG TCTTTCTTTC TTGCTAACTG CTCGATTGCA AAGTTGGCAG AGAAAGCAAC AAAGATCCAT   
  
  
+ ATCATTGATT TTGGTGTTTT CCTAGGTTTA CAATGGCCTT GTTTCATACA ACACCTATCA AAAAGGCCAA   
  
  
+ ATGGACCCCC AAAACTCCGA ATCACAGGAA TCGACTACCC CCAGCAGGGT TTCAGACCTG CACAAAGGGT   
  
  
+ TGAAGCTACA GGACACCGAT TATCTGGGTA CTGTGAGCGA TTTGGGGTGC CTTTTTCTTA TCAGGGCATT   
  
  
+ GCTCAGAAGT GGGAAACTAT TCAGCCGGAG GATCTCAAGA TCGAACAAGA CGAGCTGGTG ATTGTCAACT   
  
  
+ GTTTGTTCAG GTCAGGAACA CTGCTCGATG AGACAGTCGA AGCAAACAGT CCAAGAGATG CTTTCTTAGC   
  
  
+ TTTGGTTAGA AAGCTGAATC CCAGCCTATT CATTCACGGG GTTGTCAATG GCACATTCAA CGCTCCATTC   
  
  
+ TTCGTGACTC GATTCAGAGA GGCATTGTTT CATTATTCAT CAGTGTTTGA TGTGTCTGAA GAGACAATTC   
  
  
+ CACGAGATGC CCATGAGAGG TTCTTGATTG AGAGCGAGAT TTGTGGGAAA GAACTGTTCA ATGTGGTTGC   
  
  
+ TTGTGAGGGT GCAGAGAGGG TTCAAAGGCC TGAGACATAC AAGCAGTGGC AAGTGAGGAC AACGCGGGCC   
  
  
+ GGGTTAAGGC AGGTTGCCTT GGACCAGGAG CTTATGAAGG AAGCAACGGC AATGGTGAAG GCAAATTATC   
  
  
+ ATAAGGATTT TATGGTGGAT ATAAATAGGC ATTGGATGCT TCAAGGTTGG AAGGGTAGAA CCTTGTGTGC   
  
  
+ TCTCTCATTT TGGCAACCTG CCTG  

- +Up\_Stream \_Len000GATCCC CAACTCTCTT AGGCTATCGA TACGTCAGGT TTGTAATAGA GCAGTTCGTA   
  
  
- ATGATAAATT TATTAATTAT AACTTGAACT ATCAATGTGA AAAACTTATG ATTAAGGTGT GGAGTAACGG   
  
  
- AAATAAAGGT TTGGAGCCCA ACGTAAACCT GGTGTATGGT CCTTCGAACC TGCTGTTTTT AAAGAAAAGA   
  
  
- ACTCATAATA AAACAAAATA ATAATAATAA AAAATGAAAT AAACCTGGTG AAGTGAACCC ATTGTTCTAA   
  
  
- CTTATAAACT GATAATATAA TATACAGTGC ATACCTATAC CATCAACGAA TCTCGAAACT GGCAAACGTC   
  
  
- CAAATTCATA CTAAGGGAAC CTTTCCTCGG GGAGAAATAG AATTAAGTCT TCATTGAAAA CAAAAAAATC   
  
  
- TATTTAACTT TTCTTTTTAT TATTTACAGA ATCAACTCAA ACCAATAAGT TAGGCTTTAA ACTAGGACGA   
  
  
- ATTACATATC ATATATTATG ATTATGCGTA CATTGTGTTG TGGAGTCTAC CAGTAACAAA ATGTTAAAAA   
  
  
- AACTCAAATC ATATTTTTAT TTTAGTTAAT AAAAATCTTC ACTACAAATT AAACGTAAAA AAAAGGTGGA   
  
  
- TTTTAGTGCG TGAGCTTGTA ATCCCCAAAC CGATTGAGTT TCTGAGTTCC ATCACTGAAT AGGTTTGAAT   
  
  
- AATGTAGGAT ATCTAGATAT TTTTGTATTT AGAGAATGTT AGTTAGGTTT AACTAAGCCA GATTAAACTA   
  
  
- CTGGGACTAC TCCCAGGATG TTAATGTGTT CACCCGGAAT CGGGGTACCG GCAAAAAATT AGAAGTACCC   
  
  
- AGGTCCGCCT CAATGGTAGA GGTCCAGGTA ACTCCCACAT AGTGCAGAGT TTAATGCTCA AACGGAGAAA   
  
  
- GATGCCCCTA ATATTTATAG ATGGACGTAT GGAGCTTTTC CTCCTCAACT CTAGTCGCTT TGAACCGGTA   
  
  
- GGGTCTCGTA ACTTTACGAG TCTTGGGAAA AAGATCTTTT TATAGCAAAA AGGACTTTTA TAGGCTGGAA   
  
  
- TACAGTAAAT AACAGCGAAA GAACTGAGAT TGGGCCCTGA GGTGTGATTG AACTGATAGC CTTTCCGCAA   
  
  
- GGGACCTAGT GGTAGGTTCT ATTGGAGAAA ACGTCCCAAC TGCGAGCAAC CTTCTTAGCA GCTTACATCT   
  
  
- CGGGGATAGT AGTGTATTCG GGCGAGAATG AAGTACGGTA GGAACTTATC GTCAAATAGG GCTTCGTTTT   
  
  
- TATTAAGATT GAAAGAATAT AACATTTGGG TGTAGATTTA AATTTAAGTA TGAGGTTTTA AGGCGGTGCG   
  
  
- TTTATTCCGA AAAATTATCC GAATGTATAT TACCGTACGA TATCTCTGTG ACAAAACTTC TTTGATAAAT   
  
  
- TTGTAGTCTG GAGTATAACT CATCGTTTAG TTCTGAAGAA AGGAGGTCCG GTATAACTCA TCGTTTAATT   
  
  
- CTTATTGGTC TTCGAACACT TTTCTCAATC TTTTATTTTC CATATTATCT CTGAATACAA GAATTCTATA   
  
  
- GAGGATCTAT ACTTACACTG GAGGATCAGC TATAAACACG ACCTAATCGC TCAACACACT GACTCAAACA   
  
  
- TACACAGAGA ATCGTATACT CAATAAGATC GACGAAGAAA TTCGATACAT CACTGACAGA AGGAAGAATA   
  
  
- AATATTCACG ATACAGGTTG ATTATCGGCT TTTTTTACCA AGCGGAAAAG ACCAAAAGAA GAAAAACTTC   
  
  
- CAACTAACCG ATTAAATATA TTACCAAAGT TATGAATAGG AAATAACAAG GTGTTTAGGT GGATTGGTGC   
  
  
- ATGGTAACTG TTATTAATGG GTGATTAAAG CCAACCTGAA CTAAAGGAAA ACAAACCATG GAGTCCGTAG   
  
  
- AAGACACCTG GAAGTAAGTG TCAAATTAAA AGTGTAAAAA TATTCCGACG AGAACACAAC GTTTAAAAGG   
  
  
- ATAGGTAAGG TGAGAAACGG GTAAGGGTCC GATTAATCAC AAGAGACAAG TTGGTACCTA AGACACGACC   
  
  
- AACTAGGACT CAAGTACTTT TTAGAGATGT TTAAGTTTGG ACTTAACGAG AGTTTGAAAA GTTACTTAGG   
  
  
- TTTACTGCTG TAGAAAGTTC GGGAAGTAAG TCTTTTACTA GGAAATGTCA AGGAGAGTAA ACTACTTCCT   
  
  
- TGGACAGAAT TGTCGACGTG ATCAGTTGTT CACGGTCTAA AGGGACTACG AACAGAGTTC AAGTAGTCAC   
  
  
- TATAAGAAGA GCTTCTCCCA AATCTACGTT TAGGACGTAG ACGTGTCCGA GAGCTTCGGT GGCTCTTCAG   
  
  
- GAACATACTA CGAGAGCCCG AACCTCTCGG TATGGGGGAA AGTACACTAG TGAAACGCGG TAGATAGAGA   
  
  
- TGTTCATAAC TCTCGGGTCT ACTGTCAAGA AGGTTATTTT CAATATCGTC GTTAGGGCTC TATCTACCAA   
  
  
- GAATACGATA GCGACTCGGG TCAAAGCTCA GGTTGGGGTT GACACACGAA CTAGTTGGGG TCAACTTGAG   
  
  
- GAAAGGTCGA GATGTACTCT AAAGAGCCAG GAACCACCTT GACCCAAGAG TTCGGAGACT CAACTCGAAG   
  
  
- CTACTACGTC CCTCACGGGC ACATCTCTTT TTCCCGTTTA GCTATTTCCC GAGCTCCTCC TTCTTCTCAG   
  
  
- CAGTTTCTCT CCCACTTCGC ACAATACCTC CTTCCTCGGT ATTCCAAGTT CGAAGGAAGT TACTACTAAT   
  
  
- GATACTCTAC CTTGTCATAC TACTACATCA TAACGAGACA TTATTACTTG ATTGCCCGTT ATCGGCAAAG   
  
  
- TTGTGGCCCT TTAGAAGTGG ACTCCTCCCT ACCTCCTCTA ACGTCTCTTC GGCTCCTTTC TTCGTCTTGT   
  
  
- CAAATCGTCA ACTTCAACTA GACTACTGGG ACGACTGAGT CACACGTGTT CGACATAGCT CGAAACTAGA   
  
  
- AGCTCCACGT TTGCTCGATG AATCCGTTTA GTCCGTCTTA CGAAGCGGGA TACCACCGTC GTAGGTCTCC   
  
  
- GAGCGGGTAG TACATCGGTT ACGAGAGCTC CGTGCATATC GACCGTGTCC GAGATGTCAG AGATGATTGG   
  
  
- AACAACTACG TTTCAAGAGT CGAAGACTGA AGGATTTCCG AATGTCCAAT ATACAGAGTC GACAAGGAAT   
  
  
- GTTTTCCTAC AGAAAGAAAG AACGATTGAC GAGCTAACGT TTCAACCGTC TCTTTCGTTG TTTCTAGGTA   
  
  
- TAGTAACTAA AACCACAAAA GGATCCAAAT GTTACCGGAA CAAAGTATGT TGTGGATAGT TTTTCCGGTT   
  
  
- TACCTGGGGG TTTTGAGGCT TAGTGTCCTT AGCTGATGGG GGTCGTCCCA AAGTCTGGAC GTGTTTCCCA   
  
  
- ACTTCGATGT CCTGTGGCTA ATAGACCCAT GACACTCGCT AAACCCCACG GAAAAAGAAT AGTCCCGTAA   
  
  
- CGAGTCTTCA CCCTTTGATA AGTCGGCCTC CTAGAGTTCT AGCTTGTTCT GCTCGACCAC TAACAGTTGA   
  
  
- CAAACAAGTC CAGTCCTTGT GACGAGCTAC TCTGTCAGCT TCGTTTGTCA GGTTCTCTAC GAAAGAATCG   
  
  
- AAACCAATCT TTCGACTTAG GGTCGGATAA GTAAGTGCCC CAACAGTTAC CGTGTAAGTT GCGAGGTAAG   
  
  
- AAGCACTGAG CTAAGTCTCT CCGTAACAAA GTAATAAGTA GTCACAAACT ACACAGACTT CTCTGTTAAG   
  
  
- GTGCTCTACG GGTACTCTCC AAGAACTAAC TCTCGCTCTA AACACCCTTT CTTGACAAGT TACACCAACG   
  
  
- AACACTCCCA CGTCTCTCCC AAGTTTCCGG ACTCTGTATG TTCGTCACCG TTCACTCCTG TTGCGCCCGG   
  
  
- CCCAATTCCG TCCAACGGAA CCTGGTCCTC GAATACTTCC TTCGTTGCCG TTACCACTTC CGTTTAATAG   
  
  
- TATTCCTAAA ATACCACCTA TATTTATCCG TAACCTACGA AGTTCCAACC TTCCCATCTT GGAACACACG   
  
  
- AGAGAGTAAA ACCGTTGGAC GGAC

+     MYB recognition site

| Site Name | Organism | Position | Strand | Matrix score. | sequence | function |
| --- | --- | --- | --- | --- | --- | --- |
| MYB recognition site | Arabidopsis thaliana | 3898 | - | 6 | CCGTTG |  |

>HU02G01571.1   
+ +Up\_Stream \_Len000CTAGGG GTTGAGAGAA TCCGATAGCT ATGCAGTCCA AACATTATCT CGTCAAGCAT   
  
  
+ TACTATTTAA ATAATTAATA TTGAACTTGA TAGTTACACT TTTTGAATAC TAATTCCACA CCTCATTGCC   
  
  
+ TTTATTTCCA AACCTCGGGT TGCATTTGGA CCACATACCA GGAAGCTTGG ACGACAAAAA TTTCTTTTCT   
  
  
+ TGAGTATTAT TTTGTTTTAT TATTATTATT TTTTACTTTA TTTGGACCAC TTCACTTGGG TAACAAGATT   
  
  
+ GAATATTTGA CTATTATATT ATATGTCACG TATGGATATG GTAGTTGCTT AGAGCTTTGA CCGTTTGCAG   
  
  
+ GTTTAAGTAT GATTCCCTTG GAAAGGAGCC CCTCTTTATC TTAATTCAGA AGTAACTTTT GTTTTTTTAG   
  
  
+ ATAAATTGAA AAGAAAAATA ATAAATGTCT TAGTTGAGTT TGGTTATTCA ATCCGAAATT TGATCCTGCT   
  
  
+ TAATGTATAG TATATAATAC TAATACGCAT GTAACACAAC ACCTCAGATG GTCATTGTTT TACAATTTTT   
  
  
+ TTGAGTTTAG TATAAAAATA AAATCAATTA TTTTTAGAAG TGATGTTTAA TTTGCATTTT TTTTCCACCT   
  
  
+ AAAATCACGC ACTCGAACAT TAGGGGTTTG GCTAACTCAA AGACTCAAGG TAGTGACTTA TCCAAACTTA   
  
  
+ TTACATCCTA TAGATCTATA AAAACATAAA TCTCTTACAA TCAATCCAAA TTGATTCGGT CTAATTTGAT   
  
  
+ GACCCTGATG AGGGTCCTAC AATTACACAA GTGGGCCTTA GCCCCATGGC CGTTTTTTAA TCTTCATGGG   
  
  
+ TCCAGGCGGA GTTACCATCT CCAGGTCCAT TGAGGGTGTA TCACGTCTCA AATTACGAGT TTGCCTCTTT   
  
  
+ CTACGGGGAT TATAAATATC TACCTGCATA CCTCGAAAAG GAGGAGTTGA GATCAGCGAA ACTTGGCCAT   
  
  
+ CCCAGAGCAT TGAAATGCTC AGAACCCTTT TTCTAGAAAA ATATCGTTTT TCCTGAAAAT ATCCGACCTT   
  
  
+ ATGTCATTTA TTGTCGCTTT CTTGACTCTA ACCCGGGACT CCACACTAAC TTGACTATCG GAAAGGCGTT   
  
  
+ CCCTGGATCA CCATCCAAGA TAACCTCTTT TGCAGGGTTG ACGCTCGTTG GAAGAATCGT CGAATGTAGA   
  
  
+ GCCCCTATCA TCACATAAGC CCGCTCTTAC TTCATGCCAT CCTTGAATAG CAGTTTATCC CGAAGCAAAA   
  
  
+ ATAATTCTAA CTTTCTTATA TTGTAAACCC ACATCTAAAT TTAAATTCAT ACTCCAAAAT TCCGCCACGC   
  
  
+ AAATAAGGCT TTTTAATAGG CTTACATATA ATGGCATGCT ATAGAGACAC TGTTTTGAAG AAACTATTTA   
  
  
+ AACATCAGAC CTCATATTGA GTAGCAAATC AAGACTTCTT TCCTCCAGGC CATATTGAGT AGCAAATTAA   
  
  
+ GAATAACCAG AAGCTTGTGA AAAGAGTTAG AAAATAAAAG GTATAATAGA GACTTATGTT CTTAAGATAT   
  
  
+ CTCCTAGATA TGAATGTGAC CTCCTAGTCG ATATTTGTGC TGGATTAGCG AGTTGTGTGA CTGAGTTTGT   
  
  
+ ATGTGTCTCT TAGCATATGA GTTATTCTAG CTGCTTCTTT AAGCTATGTA GTGACTGTCT TCCTTCTTAT   
  
  
+ TTATAAGTGC TATGTCCAAC TAATAGCCGA AAAAAATGGT TCGCCTTTTC TGGTTTTCTT CTTTTTGAAG   
  
  
+ GTTGATTGGC TAATTTATAT AATGGTTTCA ATACTTATCC TTTATTGTTC CACAAATCCA CCTAACCACG   
  
  
+ TACCATTGAC AATAATTACC CACTAATTTC GGTTGGACTT GATTTCCTTT TGTTTGGTAC CTCAGGCATC   
  
  
+ TTCTGTGGAC CTTCATTCAC AGTTTAATTT TCACATTTTT ATAAGGCTGC TCTTGTGTTG CAAATTTTCC   
  
  
+ TATCCATTCC ACTCTTTGCC CATTCCCAGG CTAATTAGTG TTCTCTGTTC AACCATGGAT TCTGTGCTGG   
  
  
+ TTGATCCTGA GTTCATGAAA AATCTCTACA AATTCAAACC TGAATTGCTC TCAAACTTTT CAATGAATCC   
  
  
+ AAATGACGAC ATCTTTCAAG CCCTTCATTC AGAAAATGAT CCTTTACAGT TCCTCTCATT TGATGAAGGA   
  
  
+ ACCTGTCTTA ACAGCTGCAC TAGTCAACAA GTGCCAGATT TCCCTGATGC TTGTCTCAAG TTCATCAGTG   
  
  
+ ATATTCTTCT CGAAGAGGGT TTAGATGCAA ATCCTGCATC TGCACAGGCT CTCGAAGCCA CCGAGAAGTC   
  
  
+ CTTGTATGAT GCTCTCGGGC TTGGAGAGCC ATACCCCCTT TCATGTGATC ACTTTGCGCC ATCTATCTCT   
  
  
+ ACAAGTATTG AGAGCCCAGA TGACAGTTCT TCCAATAAAA GTTATAGCAG CAATCCCGAG ATAGATGGTT   
  
  
+ CTTATGCTAT CGCTGAGCCC AGTTTCGAGT CCAACCCCAA CTGTGTGCTT GATCAACCCC AGTTGAACTC   
  
  
+ CTTTCCAGCT CTACATGAGA TTTCTCGGTC CTTGGTGGAA CTGGGTTCTC AAGCCTCTGA GTTGAGCTTC   
  
  
+ GATGATGCAG GGAGTGCCCG TGTAGAGAAA AAGGGCAAAT CGATAAAGGG CTCGAGGAGG AAGAAGAGTC   
  
  
+ GTCAAAGAGA GGGTGAAGCG TGTTATGGAG GAAGGAGCCA TAAGGTTCAA GCTTCCTTCA ATGATGATTA   
  
  
+ CTATGAGATG GAACAGTATG ATGATGTAGT ATTGCTCTGT AATAATGAAC TAACGGGCAA TAGCCGTTTC   
  
  
+ AACACCGGGA AATCTTCACC TGAGGAGGGA TGGAGGAGAT TGCAGAGAAG CCGAGGAAAG AAGCAGAACA   
  
  
+ GTTTAGCAGT TGAAGTTGAT CTGATGACCC TGCTGACTCA GTGTGCACAA GCTGTATCGA GCTTTGATCT   
  
  
+ TCGAGGTGCA AACGAGCTAC TTAGGCAAAT CAGGCAGAAT GCTTCGCCCT ATGGTGGCAG CATCCAGAGG   
  
  
+ CTCGCCCATC ATGTAGCCAA TGCTCTCGAG GCACGTATAG CTGGCACAGG CTCTACAGTC TCTACTAACC   
  
  
+ TTGTTGATGC AAAGTTCTCA GCTTCTGACT TCCTAAAGGC TTACAGGTTA TATGTCTCAG CTGTTCCTTA   
  
  
+ CAAAAGGATG TCTTTCTTTC TTGCTAACTG CTCGATTGCA AAGTTGGCAG AGAAAGCAAC AAAGATCCAT   
  
  
+ ATCATTGATT TTGGTGTTTT CCTAGGTTTA CAATGGCCTT GTTTCATACA ACACCTATCA AAAAGGCCAA   
  
  
+ ATGGACCCCC AAAACTCCGA ATCACAGGAA TCGACTACCC CCAGCAGGGT TTCAGACCTG CACAAAGGGT   
  
  
+ TGAAGCTACA GGACACCGAT TATCTGGGTA CTGTGAGCGA TTTGGGGTGC CTTTTTCTTA TCAGGGCATT   
  
  
+ GCTCAGAAGT GGGAAACTAT TCAGCCGGAG GATCTCAAGA TCGAACAAGA CGAGCTGGTG ATTGTCAACT   
  
  
+ GTTTGTTCAG GTCAGGAACA CTGCTCGATG AGACAGTCGA AGCAAACAGT CCAAGAGATG CTTTCTTAGC   
  
  
+ TTTGGTTAGA AAGCTGAATC CCAGCCTATT CATTCACGGG GTTGTCAATG GCACATTCAA CGCTCCATTC   
  
  
+ TTCGTGACTC GATTCAGAGA GGCATTGTTT CATTATTCAT CAGTGTTTGA TGTGTCTGAA GAGACAATTC   
  
  
+ CACGAGATGC CCATGAGAGG TTCTTGATTG AGAGCGAGAT TTGTGGGAAA GAACTGTTCA ATGTGGTTGC   
  
  
+ TTGTGAGGGT GCAGAGAGGG TTCAAAGGCC TGAGACATAC AAGCAGTGGC AAGTGAGGAC AACGCGGGCC   
  
  
+ GGGTTAAGGC AGGTTGCCTT GGACCAGGAG CTTATGAAGG AAGCAACGGC AATGGTGAAG GCAAATTATC   
  
  
+ ATAAGGATTT TATGGTGGAT ATAAATAGGC ATTGGATGCT TCAAGGTTGG AAGGGTAGAA CCTTGTGTGC   
  
  
+ TCTCTCATTT TGGCAACCTG CCTG  

- +Up\_Stream \_Len000GATCCC CAACTCTCTT AGGCTATCGA TACGTCAGGT TTGTAATAGA GCAGTTCGTA   
  
  
- ATGATAAATT TATTAATTAT AACTTGAACT ATCAATGTGA AAAACTTATG ATTAAGGTGT GGAGTAACGG   
  
  
- AAATAAAGGT TTGGAGCCCA ACGTAAACCT GGTGTATGGT CCTTCGAACC TGCTGTTTTT AAAGAAAAGA   
  
  
- ACTCATAATA AAACAAAATA ATAATAATAA AAAATGAAAT AAACCTGGTG AAGTGAACCC ATTGTTCTAA   
  
  
- CTTATAAACT GATAATATAA TATACAGTGC ATACCTATAC CATCAACGAA TCTCGAAACT GGCAAACGTC   
  
  
- CAAATTCATA CTAAGGGAAC CTTTCCTCGG GGAGAAATAG AATTAAGTCT TCATTGAAAA CAAAAAAATC   
  
  
- TATTTAACTT TTCTTTTTAT TATTTACAGA ATCAACTCAA ACCAATAAGT TAGGCTTTAA ACTAGGACGA   
  
  
- ATTACATATC ATATATTATG ATTATGCGTA CATTGTGTTG TGGAGTCTAC CAGTAACAAA ATGTTAAAAA   
  
  
- AACTCAAATC ATATTTTTAT TTTAGTTAAT AAAAATCTTC ACTACAAATT AAACGTAAAA AAAAGGTGGA   
  
  
- TTTTAGTGCG TGAGCTTGTA ATCCCCAAAC CGATTGAGTT TCTGAGTTCC ATCACTGAAT AGGTTTGAAT   
  
  
- AATGTAGGAT ATCTAGATAT TTTTGTATTT AGAGAATGTT AGTTAGGTTT AACTAAGCCA GATTAAACTA   
  
  
- CTGGGACTAC TCCCAGGATG TTAATGTGTT CACCCGGAAT CGGGGTACCG GCAAAAAATT AGAAGTACCC   
  
  
- AGGTCCGCCT CAATGGTAGA GGTCCAGGTA ACTCCCACAT AGTGCAGAGT TTAATGCTCA AACGGAGAAA   
  
  
- GATGCCCCTA ATATTTATAG ATGGACGTAT GGAGCTTTTC CTCCTCAACT CTAGTCGCTT TGAACCGGTA   
  
  
- GGGTCTCGTA ACTTTACGAG TCTTGGGAAA AAGATCTTTT TATAGCAAAA AGGACTTTTA TAGGCTGGAA   
  
  
- TACAGTAAAT AACAGCGAAA GAACTGAGAT TGGGCCCTGA GGTGTGATTG AACTGATAGC CTTTCCGCAA   
  
  
- GGGACCTAGT GGTAGGTTCT ATTGGAGAAA ACGTCCCAAC TGCGAGCAAC CTTCTTAGCA GCTTACATCT   
  
  
- CGGGGATAGT AGTGTATTCG GGCGAGAATG AAGTACGGTA GGAACTTATC GTCAAATAGG GCTTCGTTTT   
  
  
- TATTAAGATT GAAAGAATAT AACATTTGGG TGTAGATTTA AATTTAAGTA TGAGGTTTTA AGGCGGTGCG   
  
  
- TTTATTCCGA AAAATTATCC GAATGTATAT TACCGTACGA TATCTCTGTG ACAAAACTTC TTTGATAAAT   
  
  
- TTGTAGTCTG GAGTATAACT CATCGTTTAG TTCTGAAGAA AGGAGGTCCG GTATAACTCA TCGTTTAATT   
  
  
- CTTATTGGTC TTCGAACACT TTTCTCAATC TTTTATTTTC CATATTATCT CTGAATACAA GAATTCTATA   
  
  
- GAGGATCTAT ACTTACACTG GAGGATCAGC TATAAACACG ACCTAATCGC TCAACACACT GACTCAAACA   
  
  
- TACACAGAGA ATCGTATACT CAATAAGATC GACGAAGAAA TTCGATACAT CACTGACAGA AGGAAGAATA   
  
  
- AATATTCACG ATACAGGTTG ATTATCGGCT TTTTTTACCA AGCGGAAAAG ACCAAAAGAA GAAAAACTTC   
  
  
- CAACTAACCG ATTAAATATA TTACCAAAGT TATGAATAGG AAATAACAAG GTGTTTAGGT GGATTGGTGC   
  
  
- ATGGTAACTG TTATTAATGG GTGATTAAAG CCAACCTGAA CTAAAGGAAA ACAAACCATG GAGTCCGTAG   
  
  
- AAGACACCTG GAAGTAAGTG TCAAATTAAA AGTGTAAAAA TATTCCGACG AGAACACAAC GTTTAAAAGG   
  
  
- ATAGGTAAGG TGAGAAACGG GTAAGGGTCC GATTAATCAC AAGAGACAAG TTGGTACCTA AGACACGACC   
  
  
- AACTAGGACT CAAGTACTTT TTAGAGATGT TTAAGTTTGG ACTTAACGAG AGTTTGAAAA GTTACTTAGG   
  
  
- TTTACTGCTG TAGAAAGTTC GGGAAGTAAG TCTTTTACTA GGAAATGTCA AGGAGAGTAA ACTACTTCCT   
  
  
- TGGACAGAAT TGTCGACGTG ATCAGTTGTT CACGGTCTAA AGGGACTACG AACAGAGTTC AAGTAGTCAC   
  
  
- TATAAGAAGA GCTTCTCCCA AATCTACGTT TAGGACGTAG ACGTGTCCGA GAGCTTCGGT GGCTCTTCAG   
  
  
- GAACATACTA CGAGAGCCCG AACCTCTCGG TATGGGGGAA AGTACACTAG TGAAACGCGG TAGATAGAGA   
  
  
- TGTTCATAAC TCTCGGGTCT ACTGTCAAGA AGGTTATTTT CAATATCGTC GTTAGGGCTC TATCTACCAA   
  
  
- GAATACGATA GCGACTCGGG TCAAAGCTCA GGTTGGGGTT GACACACGAA CTAGTTGGGG TCAACTTGAG   
  
  
- GAAAGGTCGA GATGTACTCT AAAGAGCCAG GAACCACCTT GACCCAAGAG TTCGGAGACT CAACTCGAAG   
  
  
- CTACTACGTC CCTCACGGGC ACATCTCTTT TTCCCGTTTA GCTATTTCCC GAGCTCCTCC TTCTTCTCAG   
  
  
- CAGTTTCTCT CCCACTTCGC ACAATACCTC CTTCCTCGGT ATTCCAAGTT CGAAGGAAGT TACTACTAAT   
  
  
- GATACTCTAC CTTGTCATAC TACTACATCA TAACGAGACA TTATTACTTG ATTGCCCGTT ATCGGCAAAG   
  
  
- TTGTGGCCCT TTAGAAGTGG ACTCCTCCCT ACCTCCTCTA ACGTCTCTTC GGCTCCTTTC TTCGTCTTGT   
  
  
- CAAATCGTCA ACTTCAACTA GACTACTGGG ACGACTGAGT CACACGTGTT CGACATAGCT CGAAACTAGA   
  
  
- AGCTCCACGT TTGCTCGATG AATCCGTTTA GTCCGTCTTA CGAAGCGGGA TACCACCGTC GTAGGTCTCC   
  
  
- GAGCGGGTAG TACATCGGTT ACGAGAGCTC CGTGCATATC GACCGTGTCC GAGATGTCAG AGATGATTGG   
  
  
- AACAACTACG TTTCAAGAGT CGAAGACTGA AGGATTTCCG AATGTCCAAT ATACAGAGTC GACAAGGAAT   
  
  
- GTTTTCCTAC AGAAAGAAAG AACGATTGAC GAGCTAACGT TTCAACCGTC TCTTTCGTTG TTTCTAGGTA   
  
  
- TAGTAACTAA AACCACAAAA GGATCCAAAT GTTACCGGAA CAAAGTATGT TGTGGATAGT TTTTCCGGTT   
  
  
- TACCTGGGGG TTTTGAGGCT TAGTGTCCTT AGCTGATGGG GGTCGTCCCA AAGTCTGGAC GTGTTTCCCA   
  
  
- ACTTCGATGT CCTGTGGCTA ATAGACCCAT GACACTCGCT AAACCCCACG GAAAAAGAAT AGTCCCGTAA   
  
  
- CGAGTCTTCA CCCTTTGATA AGTCGGCCTC CTAGAGTTCT AGCTTGTTCT GCTCGACCAC TAACAGTTGA   
  
  
- CAAACAAGTC CAGTCCTTGT GACGAGCTAC TCTGTCAGCT TCGTTTGTCA GGTTCTCTAC GAAAGAATCG   
  
  
- AAACCAATCT TTCGACTTAG GGTCGGATAA GTAAGTGCCC CAACAGTTAC CGTGTAAGTT GCGAGGTAAG   
  
  
- AAGCACTGAG CTAAGTCTCT CCGTAACAAA GTAATAAGTA GTCACAAACT ACACAGACTT CTCTGTTAAG   
  
  
- GTGCTCTACG GGTACTCTCC AAGAACTAAC TCTCGCTCTA AACACCCTTT CTTGACAAGT TACACCAACG   
  
  
- AACACTCCCA CGTCTCTCCC AAGTTTCCGG ACTCTGTATG TTCGTCACCG TTCACTCCTG TTGCGCCCGG   
  
  
- CCCAATTCCG TCCAACGGAA CCTGGTCCTC GAATACTTCC TTCGTTGCCG TTACCACTTC CGTTTAATAG   
  
  
- TATTCCTAAA ATACCACCTA TATTTATCCG TAACCTACGA AGTTCCAACC TTCCCATCTT GGAACACACG   
  
  
- AGAGAGTAAA ACCGTTGGAC GGAC

+     MYB-like sequence

| Site Name | Organism | Position | Strand | Matrix score. | sequence | function |
| --- | --- | --- | --- | --- | --- | --- |
| MYB-like sequence | Arabidopsis thaliana | 465 | - | 6 | TAACCA |  |
| MYB-like sequence | Arabidopsis thaliana | 3577 | - | 6 | TAACCA |  |
| MYB-like sequence | Arabidopsis thaliana | 1817 | + | 6 | TAACCA |  |
| MYB-like sequence | Arabidopsis thaliana | 1478 | + | 6 | TAACCA |  |

>HU02G01571.1   
+ +Up\_Stream \_Len000CTAGGG GTTGAGAGAA TCCGATAGCT ATGCAGTCCA AACATTATCT CGTCAAGCAT   
  
  
+ TACTATTTAA ATAATTAATA TTGAACTTGA TAGTTACACT TTTTGAATAC TAATTCCACA CCTCATTGCC   
  
  
+ TTTATTTCCA AACCTCGGGT TGCATTTGGA CCACATACCA GGAAGCTTGG ACGACAAAAA TTTCTTTTCT   
  
  
+ TGAGTATTAT TTTGTTTTAT TATTATTATT TTTTACTTTA TTTGGACCAC TTCACTTGGG TAACAAGATT   
  
  
+ GAATATTTGA CTATTATATT ATATGTCACG TATGGATATG GTAGTTGCTT AGAGCTTTGA CCGTTTGCAG   
  
  
+ GTTTAAGTAT GATTCCCTTG GAAAGGAGCC CCTCTTTATC TTAATTCAGA AGTAACTTTT GTTTTTTTAG   
  
  
+ ATAAATTGAA AAGAAAAATA ATAAATGTCT TAGTTGAGTT TGGTTATTCA ATCCGAAATT TGATCCTGCT   
  
  
+ TAATGTATAG TATATAATAC TAATACGCAT GTAACACAAC ACCTCAGATG GTCATTGTTT TACAATTTTT   
  
  
+ TTGAGTTTAG TATAAAAATA AAATCAATTA TTTTTAGAAG TGATGTTTAA TTTGCATTTT TTTTCCACCT   
  
  
+ AAAATCACGC ACTCGAACAT TAGGGGTTTG GCTAACTCAA AGACTCAAGG TAGTGACTTA TCCAAACTTA   
  
  
+ TTACATCCTA TAGATCTATA AAAACATAAA TCTCTTACAA TCAATCCAAA TTGATTCGGT CTAATTTGAT   
  
  
+ GACCCTGATG AGGGTCCTAC AATTACACAA GTGGGCCTTA GCCCCATGGC CGTTTTTTAA TCTTCATGGG   
  
  
+ TCCAGGCGGA GTTACCATCT CCAGGTCCAT TGAGGGTGTA TCACGTCTCA AATTACGAGT TTGCCTCTTT   
  
  
+ CTACGGGGAT TATAAATATC TACCTGCATA CCTCGAAAAG GAGGAGTTGA GATCAGCGAA ACTTGGCCAT   
  
  
+ CCCAGAGCAT TGAAATGCTC AGAACCCTTT TTCTAGAAAA ATATCGTTTT TCCTGAAAAT ATCCGACCTT   
  
  
+ ATGTCATTTA TTGTCGCTTT CTTGACTCTA ACCCGGGACT CCACACTAAC TTGACTATCG GAAAGGCGTT   
  
  
+ CCCTGGATCA CCATCCAAGA TAACCTCTTT TGCAGGGTTG ACGCTCGTTG GAAGAATCGT CGAATGTAGA   
  
  
+ GCCCCTATCA TCACATAAGC CCGCTCTTAC TTCATGCCAT CCTTGAATAG CAGTTTATCC CGAAGCAAAA   
  
  
+ ATAATTCTAA CTTTCTTATA TTGTAAACCC ACATCTAAAT TTAAATTCAT ACTCCAAAAT TCCGCCACGC   
  
  
+ AAATAAGGCT TTTTAATAGG CTTACATATA ATGGCATGCT ATAGAGACAC TGTTTTGAAG AAACTATTTA   
  
  
+ AACATCAGAC CTCATATTGA GTAGCAAATC AAGACTTCTT TCCTCCAGGC CATATTGAGT AGCAAATTAA   
  
  
+ GAATAACCAG AAGCTTGTGA AAAGAGTTAG AAAATAAAAG GTATAATAGA GACTTATGTT CTTAAGATAT   
  
  
+ CTCCTAGATA TGAATGTGAC CTCCTAGTCG ATATTTGTGC TGGATTAGCG AGTTGTGTGA CTGAGTTTGT   
  
  
+ ATGTGTCTCT TAGCATATGA GTTATTCTAG CTGCTTCTTT AAGCTATGTA GTGACTGTCT TCCTTCTTAT   
  
  
+ TTATAAGTGC TATGTCCAAC TAATAGCCGA AAAAAATGGT TCGCCTTTTC TGGTTTTCTT CTTTTTGAAG   
  
  
+ GTTGATTGGC TAATTTATAT AATGGTTTCA ATACTTATCC TTTATTGTTC CACAAATCCA CCTAACCACG   
  
  
+ TACCATTGAC AATAATTACC CACTAATTTC GGTTGGACTT GATTTCCTTT TGTTTGGTAC CTCAGGCATC   
  
  
+ TTCTGTGGAC CTTCATTCAC AGTTTAATTT TCACATTTTT ATAAGGCTGC TCTTGTGTTG CAAATTTTCC   
  
  
+ TATCCATTCC ACTCTTTGCC CATTCCCAGG CTAATTAGTG TTCTCTGTTC AACCATGGAT TCTGTGCTGG   
  
  
+ TTGATCCTGA GTTCATGAAA AATCTCTACA AATTCAAACC TGAATTGCTC TCAAACTTTT CAATGAATCC   
  
  
+ AAATGACGAC ATCTTTCAAG CCCTTCATTC AGAAAATGAT CCTTTACAGT TCCTCTCATT TGATGAAGGA   
  
  
+ ACCTGTCTTA ACAGCTGCAC TAGTCAACAA GTGCCAGATT TCCCTGATGC TTGTCTCAAG TTCATCAGTG   
  
  
+ ATATTCTTCT CGAAGAGGGT TTAGATGCAA ATCCTGCATC TGCACAGGCT CTCGAAGCCA CCGAGAAGTC   
  
  
+ CTTGTATGAT GCTCTCGGGC TTGGAGAGCC ATACCCCCTT TCATGTGATC ACTTTGCGCC ATCTATCTCT   
  
  
+ ACAAGTATTG AGAGCCCAGA TGACAGTTCT TCCAATAAAA GTTATAGCAG CAATCCCGAG ATAGATGGTT   
  
  
+ CTTATGCTAT CGCTGAGCCC AGTTTCGAGT CCAACCCCAA CTGTGTGCTT GATCAACCCC AGTTGAACTC   
  
  
+ CTTTCCAGCT CTACATGAGA TTTCTCGGTC CTTGGTGGAA CTGGGTTCTC AAGCCTCTGA GTTGAGCTTC   
  
  
+ GATGATGCAG GGAGTGCCCG TGTAGAGAAA AAGGGCAAAT CGATAAAGGG CTCGAGGAGG AAGAAGAGTC   
  
  
+ GTCAAAGAGA GGGTGAAGCG TGTTATGGAG GAAGGAGCCA TAAGGTTCAA GCTTCCTTCA ATGATGATTA   
  
  
+ CTATGAGATG GAACAGTATG ATGATGTAGT ATTGCTCTGT AATAATGAAC TAACGGGCAA TAGCCGTTTC   
  
  
+ AACACCGGGA AATCTTCACC TGAGGAGGGA TGGAGGAGAT TGCAGAGAAG CCGAGGAAAG AAGCAGAACA   
  
  
+ GTTTAGCAGT TGAAGTTGAT CTGATGACCC TGCTGACTCA GTGTGCACAA GCTGTATCGA GCTTTGATCT   
  
  
+ TCGAGGTGCA AACGAGCTAC TTAGGCAAAT CAGGCAGAAT GCTTCGCCCT ATGGTGGCAG CATCCAGAGG   
  
  
+ CTCGCCCATC ATGTAGCCAA TGCTCTCGAG GCACGTATAG CTGGCACAGG CTCTACAGTC TCTACTAACC   
  
  
+ TTGTTGATGC AAAGTTCTCA GCTTCTGACT TCCTAAAGGC TTACAGGTTA TATGTCTCAG CTGTTCCTTA   
  
  
+ CAAAAGGATG TCTTTCTTTC TTGCTAACTG CTCGATTGCA AAGTTGGCAG AGAAAGCAAC AAAGATCCAT   
  
  
+ ATCATTGATT TTGGTGTTTT CCTAGGTTTA CAATGGCCTT GTTTCATACA ACACCTATCA AAAAGGCCAA   
  
  
+ ATGGACCCCC AAAACTCCGA ATCACAGGAA TCGACTACCC CCAGCAGGGT TTCAGACCTG CACAAAGGGT   
  
  
+ TGAAGCTACA GGACACCGAT TATCTGGGTA CTGTGAGCGA TTTGGGGTGC CTTTTTCTTA TCAGGGCATT   
  
  
+ GCTCAGAAGT GGGAAACTAT TCAGCCGGAG GATCTCAAGA TCGAACAAGA CGAGCTGGTG ATTGTCAACT   
  
  
+ GTTTGTTCAG GTCAGGAACA CTGCTCGATG AGACAGTCGA AGCAAACAGT CCAAGAGATG CTTTCTTAGC   
  
  
+ TTTGGTTAGA AAGCTGAATC CCAGCCTATT CATTCACGGG GTTGTCAATG GCACATTCAA CGCTCCATTC   
  
  
+ TTCGTGACTC GATTCAGAGA GGCATTGTTT CATTATTCAT CAGTGTTTGA TGTGTCTGAA GAGACAATTC   
  
  
+ CACGAGATGC CCATGAGAGG TTCTTGATTG AGAGCGAGAT TTGTGGGAAA GAACTGTTCA ATGTGGTTGC   
  
  
+ TTGTGAGGGT GCAGAGAGGG TTCAAAGGCC TGAGACATAC AAGCAGTGGC AAGTGAGGAC AACGCGGGCC   
  
  
+ GGGTTAAGGC AGGTTGCCTT GGACCAGGAG CTTATGAAGG AAGCAACGGC AATGGTGAAG GCAAATTATC   
  
  
+ ATAAGGATTT TATGGTGGAT ATAAATAGGC ATTGGATGCT TCAAGGTTGG AAGGGTAGAA CCTTGTGTGC   
  
  
+ TCTCTCATTT TGGCAACCTG CCTG  

- +Up\_Stream \_Len000GATCCC CAACTCTCTT AGGCTATCGA TACGTCAGGT TTGTAATAGA GCAGTTCGTA   
  
  
- ATGATAAATT TATTAATTAT AACTTGAACT ATCAATGTGA AAAACTTATG ATTAAGGTGT GGAGTAACGG   
  
  
- AAATAAAGGT TTGGAGCCCA ACGTAAACCT GGTGTATGGT CCTTCGAACC TGCTGTTTTT AAAGAAAAGA   
  
  
- ACTCATAATA AAACAAAATA ATAATAATAA AAAATGAAAT AAACCTGGTG AAGTGAACCC ATTGTTCTAA   
  
  
- CTTATAAACT GATAATATAA TATACAGTGC ATACCTATAC CATCAACGAA TCTCGAAACT GGCAAACGTC   
  
  
- CAAATTCATA CTAAGGGAAC CTTTCCTCGG GGAGAAATAG AATTAAGTCT TCATTGAAAA CAAAAAAATC   
  
  
- TATTTAACTT TTCTTTTTAT TATTTACAGA ATCAACTCAA ACCAATAAGT TAGGCTTTAA ACTAGGACGA   
  
  
- ATTACATATC ATATATTATG ATTATGCGTA CATTGTGTTG TGGAGTCTAC CAGTAACAAA ATGTTAAAAA   
  
  
- AACTCAAATC ATATTTTTAT TTTAGTTAAT AAAAATCTTC ACTACAAATT AAACGTAAAA AAAAGGTGGA   
  
  
- TTTTAGTGCG TGAGCTTGTA ATCCCCAAAC CGATTGAGTT TCTGAGTTCC ATCACTGAAT AGGTTTGAAT   
  
  
- AATGTAGGAT ATCTAGATAT TTTTGTATTT AGAGAATGTT AGTTAGGTTT AACTAAGCCA GATTAAACTA   
  
  
- CTGGGACTAC TCCCAGGATG TTAATGTGTT CACCCGGAAT CGGGGTACCG GCAAAAAATT AGAAGTACCC   
  
  
- AGGTCCGCCT CAATGGTAGA GGTCCAGGTA ACTCCCACAT AGTGCAGAGT TTAATGCTCA AACGGAGAAA   
  
  
- GATGCCCCTA ATATTTATAG ATGGACGTAT GGAGCTTTTC CTCCTCAACT CTAGTCGCTT TGAACCGGTA   
  
  
- GGGTCTCGTA ACTTTACGAG TCTTGGGAAA AAGATCTTTT TATAGCAAAA AGGACTTTTA TAGGCTGGAA   
  
  
- TACAGTAAAT AACAGCGAAA GAACTGAGAT TGGGCCCTGA GGTGTGATTG AACTGATAGC CTTTCCGCAA   
  
  
- GGGACCTAGT GGTAGGTTCT ATTGGAGAAA ACGTCCCAAC TGCGAGCAAC CTTCTTAGCA GCTTACATCT   
  
  
- CGGGGATAGT AGTGTATTCG GGCGAGAATG AAGTACGGTA GGAACTTATC GTCAAATAGG GCTTCGTTTT   
  
  
- TATTAAGATT GAAAGAATAT AACATTTGGG TGTAGATTTA AATTTAAGTA TGAGGTTTTA AGGCGGTGCG   
  
  
- TTTATTCCGA AAAATTATCC GAATGTATAT TACCGTACGA TATCTCTGTG ACAAAACTTC TTTGATAAAT   
  
  
- TTGTAGTCTG GAGTATAACT CATCGTTTAG TTCTGAAGAA AGGAGGTCCG GTATAACTCA TCGTTTAATT   
  
  
- CTTATTGGTC TTCGAACACT TTTCTCAATC TTTTATTTTC CATATTATCT CTGAATACAA GAATTCTATA   
  
  
- GAGGATCTAT ACTTACACTG GAGGATCAGC TATAAACACG ACCTAATCGC TCAACACACT GACTCAAACA   
  
  
- TACACAGAGA ATCGTATACT CAATAAGATC GACGAAGAAA TTCGATACAT CACTGACAGA AGGAAGAATA   
  
  
- AATATTCACG ATACAGGTTG ATTATCGGCT TTTTTTACCA AGCGGAAAAG ACCAAAAGAA GAAAAACTTC   
  
  
- CAACTAACCG ATTAAATATA TTACCAAAGT TATGAATAGG AAATAACAAG GTGTTTAGGT GGATTGGTGC   
  
  
- ATGGTAACTG TTATTAATGG GTGATTAAAG CCAACCTGAA CTAAAGGAAA ACAAACCATG GAGTCCGTAG   
  
  
- AAGACACCTG GAAGTAAGTG TCAAATTAAA AGTGTAAAAA TATTCCGACG AGAACACAAC GTTTAAAAGG   
  
  
- ATAGGTAAGG TGAGAAACGG GTAAGGGTCC GATTAATCAC AAGAGACAAG TTGGTACCTA AGACACGACC   
  
  
- AACTAGGACT CAAGTACTTT TTAGAGATGT TTAAGTTTGG ACTTAACGAG AGTTTGAAAA GTTACTTAGG   
  
  
- TTTACTGCTG TAGAAAGTTC GGGAAGTAAG TCTTTTACTA GGAAATGTCA AGGAGAGTAA ACTACTTCCT   
  
  
- TGGACAGAAT TGTCGACGTG ATCAGTTGTT CACGGTCTAA AGGGACTACG AACAGAGTTC AAGTAGTCAC   
  
  
- TATAAGAAGA GCTTCTCCCA AATCTACGTT TAGGACGTAG ACGTGTCCGA GAGCTTCGGT GGCTCTTCAG   
  
  
- GAACATACTA CGAGAGCCCG AACCTCTCGG TATGGGGGAA AGTACACTAG TGAAACGCGG TAGATAGAGA   
  
  
- TGTTCATAAC TCTCGGGTCT ACTGTCAAGA AGGTTATTTT CAATATCGTC GTTAGGGCTC TATCTACCAA   
  
  
- GAATACGATA GCGACTCGGG TCAAAGCTCA GGTTGGGGTT GACACACGAA CTAGTTGGGG TCAACTTGAG   
  
  
- GAAAGGTCGA GATGTACTCT AAAGAGCCAG GAACCACCTT GACCCAAGAG TTCGGAGACT CAACTCGAAG   
  
  
- CTACTACGTC CCTCACGGGC ACATCTCTTT TTCCCGTTTA GCTATTTCCC GAGCTCCTCC TTCTTCTCAG   
  
  
- CAGTTTCTCT CCCACTTCGC ACAATACCTC CTTCCTCGGT ATTCCAAGTT CGAAGGAAGT TACTACTAAT   
  
  
- GATACTCTAC CTTGTCATAC TACTACATCA TAACGAGACA TTATTACTTG ATTGCCCGTT ATCGGCAAAG   
  
  
- TTGTGGCCCT TTAGAAGTGG ACTCCTCCCT ACCTCCTCTA ACGTCTCTTC GGCTCCTTTC TTCGTCTTGT   
  
  
- CAAATCGTCA ACTTCAACTA GACTACTGGG ACGACTGAGT CACACGTGTT CGACATAGCT CGAAACTAGA   
  
  
- AGCTCCACGT TTGCTCGATG AATCCGTTTA GTCCGTCTTA CGAAGCGGGA TACCACCGTC GTAGGTCTCC   
  
  
- GAGCGGGTAG TACATCGGTT ACGAGAGCTC CGTGCATATC GACCGTGTCC GAGATGTCAG AGATGATTGG   
  
  
- AACAACTACG TTTCAAGAGT CGAAGACTGA AGGATTTCCG AATGTCCAAT ATACAGAGTC GACAAGGAAT   
  
  
- GTTTTCCTAC AGAAAGAAAG AACGATTGAC GAGCTAACGT TTCAACCGTC TCTTTCGTTG TTTCTAGGTA   
  
  
- TAGTAACTAA AACCACAAAA GGATCCAAAT GTTACCGGAA CAAAGTATGT TGTGGATAGT TTTTCCGGTT   
  
  
- TACCTGGGGG TTTTGAGGCT TAGTGTCCTT AGCTGATGGG GGTCGTCCCA AAGTCTGGAC GTGTTTCCCA   
  
  
- ACTTCGATGT CCTGTGGCTA ATAGACCCAT GACACTCGCT AAACCCCACG GAAAAAGAAT AGTCCCGTAA   
  
  
- CGAGTCTTCA CCCTTTGATA AGTCGGCCTC CTAGAGTTCT AGCTTGTTCT GCTCGACCAC TAACAGTTGA   
  
  
- CAAACAAGTC CAGTCCTTGT GACGAGCTAC TCTGTCAGCT TCGTTTGTCA GGTTCTCTAC GAAAGAATCG   
  
  
- AAACCAATCT TTCGACTTAG GGTCGGATAA GTAAGTGCCC CAACAGTTAC CGTGTAAGTT GCGAGGTAAG   
  
  
- AAGCACTGAG CTAAGTCTCT CCGTAACAAA GTAATAAGTA GTCACAAACT ACACAGACTT CTCTGTTAAG   
  
  
- GTGCTCTACG GGTACTCTCC AAGAACTAAC TCTCGCTCTA AACACCCTTT CTTGACAAGT TACACCAACG   
  
  
- AACACTCCCA CGTCTCTCCC AAGTTTCCGG ACTCTGTATG TTCGTCACCG TTCACTCCTG TTGCGCCCGG   
  
  
- CCCAATTCCG TCCAACGGAA CCTGGTCCTC GAATACTTCC TTCGTTGCCG TTACCACTTC CGTTTAATAG   
  
  
- TATTCCTAAA ATACCACCTA TATTTATCCG TAACCTACGA AGTTCCAACC TTCCCATCTT GGAACACACG   
  
  
- AGAGAGTAAA ACCGTTGGAC GGAC

+     MYC

| Site Name | Organism | Position | Strand | Matrix score. | sequence | function |
| --- | --- | --- | --- | --- | --- | --- |
| MYC | Arabidopsis thaliana | 3292 | - | 6 | CATTTG |  |
| MYC | Arabidopsis thaliana | 2356 | + | 6 | CATGTG |  |
| MYC | Arabidopsis thaliana | 2104 | - | 6 | CATTTG |  |
| MYC | Arabidopsis thaliana | 167 | + | 6 | CATTTG |  |
| MYC | Arabidopsis thaliana | 2161 | + | 6 | CATTTG |  |

>HU02G01571.1   
+ +Up\_Stream \_Len000CTAGGG GTTGAGAGAA TCCGATAGCT ATGCAGTCCA AACATTATCT CGTCAAGCAT   
  
  
+ TACTATTTAA ATAATTAATA TTGAACTTGA TAGTTACACT TTTTGAATAC TAATTCCACA CCTCATTGCC   
  
  
+ TTTATTTCCA AACCTCGGGT TGCATTTGGA CCACATACCA GGAAGCTTGG ACGACAAAAA TTTCTTTTCT   
  
  
+ TGAGTATTAT TTTGTTTTAT TATTATTATT TTTTACTTTA TTTGGACCAC TTCACTTGGG TAACAAGATT   
  
  
+ GAATATTTGA CTATTATATT ATATGTCACG TATGGATATG GTAGTTGCTT AGAGCTTTGA CCGTTTGCAG   
  
  
+ GTTTAAGTAT GATTCCCTTG GAAAGGAGCC CCTCTTTATC TTAATTCAGA AGTAACTTTT GTTTTTTTAG   
  
  
+ ATAAATTGAA AAGAAAAATA ATAAATGTCT TAGTTGAGTT TGGTTATTCA ATCCGAAATT TGATCCTGCT   
  
  
+ TAATGTATAG TATATAATAC TAATACGCAT GTAACACAAC ACCTCAGATG GTCATTGTTT TACAATTTTT   
  
  
+ TTGAGTTTAG TATAAAAATA AAATCAATTA TTTTTAGAAG TGATGTTTAA TTTGCATTTT TTTTCCACCT   
  
  
+ AAAATCACGC ACTCGAACAT TAGGGGTTTG GCTAACTCAA AGACTCAAGG TAGTGACTTA TCCAAACTTA   
  
  
+ TTACATCCTA TAGATCTATA AAAACATAAA TCTCTTACAA TCAATCCAAA TTGATTCGGT CTAATTTGAT   
  
  
+ GACCCTGATG AGGGTCCTAC AATTACACAA GTGGGCCTTA GCCCCATGGC CGTTTTTTAA TCTTCATGGG   
  
  
+ TCCAGGCGGA GTTACCATCT CCAGGTCCAT TGAGGGTGTA TCACGTCTCA AATTACGAGT TTGCCTCTTT   
  
  
+ CTACGGGGAT TATAAATATC TACCTGCATA CCTCGAAAAG GAGGAGTTGA GATCAGCGAA ACTTGGCCAT   
  
  
+ CCCAGAGCAT TGAAATGCTC AGAACCCTTT TTCTAGAAAA ATATCGTTTT TCCTGAAAAT ATCCGACCTT   
  
  
+ ATGTCATTTA TTGTCGCTTT CTTGACTCTA ACCCGGGACT CCACACTAAC TTGACTATCG GAAAGGCGTT   
  
  
+ CCCTGGATCA CCATCCAAGA TAACCTCTTT TGCAGGGTTG ACGCTCGTTG GAAGAATCGT CGAATGTAGA   
  
  
+ GCCCCTATCA TCACATAAGC CCGCTCTTAC TTCATGCCAT CCTTGAATAG CAGTTTATCC CGAAGCAAAA   
  
  
+ ATAATTCTAA CTTTCTTATA TTGTAAACCC ACATCTAAAT TTAAATTCAT ACTCCAAAAT TCCGCCACGC   
  
  
+ AAATAAGGCT TTTTAATAGG CTTACATATA ATGGCATGCT ATAGAGACAC TGTTTTGAAG AAACTATTTA   
  
  
+ AACATCAGAC CTCATATTGA GTAGCAAATC AAGACTTCTT TCCTCCAGGC CATATTGAGT AGCAAATTAA   
  
  
+ GAATAACCAG AAGCTTGTGA AAAGAGTTAG AAAATAAAAG GTATAATAGA GACTTATGTT CTTAAGATAT   
  
  
+ CTCCTAGATA TGAATGTGAC CTCCTAGTCG ATATTTGTGC TGGATTAGCG AGTTGTGTGA CTGAGTTTGT   
  
  
+ ATGTGTCTCT TAGCATATGA GTTATTCTAG CTGCTTCTTT AAGCTATGTA GTGACTGTCT TCCTTCTTAT   
  
  
+ TTATAAGTGC TATGTCCAAC TAATAGCCGA AAAAAATGGT TCGCCTTTTC TGGTTTTCTT CTTTTTGAAG   
  
  
+ GTTGATTGGC TAATTTATAT AATGGTTTCA ATACTTATCC TTTATTGTTC CACAAATCCA CCTAACCACG   
  
  
+ TACCATTGAC AATAATTACC CACTAATTTC GGTTGGACTT GATTTCCTTT TGTTTGGTAC CTCAGGCATC   
  
  
+ TTCTGTGGAC CTTCATTCAC AGTTTAATTT TCACATTTTT ATAAGGCTGC TCTTGTGTTG CAAATTTTCC   
  
  
+ TATCCATTCC ACTCTTTGCC CATTCCCAGG CTAATTAGTG TTCTCTGTTC AACCATGGAT TCTGTGCTGG   
  
  
+ TTGATCCTGA GTTCATGAAA AATCTCTACA AATTCAAACC TGAATTGCTC TCAAACTTTT CAATGAATCC   
  
  
+ AAATGACGAC ATCTTTCAAG CCCTTCATTC AGAAAATGAT CCTTTACAGT TCCTCTCATT TGATGAAGGA   
  
  
+ ACCTGTCTTA ACAGCTGCAC TAGTCAACAA GTGCCAGATT TCCCTGATGC TTGTCTCAAG TTCATCAGTG   
  
  
+ ATATTCTTCT CGAAGAGGGT TTAGATGCAA ATCCTGCATC TGCACAGGCT CTCGAAGCCA CCGAGAAGTC   
  
  
+ CTTGTATGAT GCTCTCGGGC TTGGAGAGCC ATACCCCCTT TCATGTGATC ACTTTGCGCC ATCTATCTCT   
  
  
+ ACAAGTATTG AGAGCCCAGA TGACAGTTCT TCCAATAAAA GTTATAGCAG CAATCCCGAG ATAGATGGTT   
  
  
+ CTTATGCTAT CGCTGAGCCC AGTTTCGAGT CCAACCCCAA CTGTGTGCTT GATCAACCCC AGTTGAACTC   
  
  
+ CTTTCCAGCT CTACATGAGA TTTCTCGGTC CTTGGTGGAA CTGGGTTCTC AAGCCTCTGA GTTGAGCTTC   
  
  
+ GATGATGCAG GGAGTGCCCG TGTAGAGAAA AAGGGCAAAT CGATAAAGGG CTCGAGGAGG AAGAAGAGTC   
  
  
+ GTCAAAGAGA GGGTGAAGCG TGTTATGGAG GAAGGAGCCA TAAGGTTCAA GCTTCCTTCA ATGATGATTA   
  
  
+ CTATGAGATG GAACAGTATG ATGATGTAGT ATTGCTCTGT AATAATGAAC TAACGGGCAA TAGCCGTTTC   
  
  
+ AACACCGGGA AATCTTCACC TGAGGAGGGA TGGAGGAGAT TGCAGAGAAG CCGAGGAAAG AAGCAGAACA   
  
  
+ GTTTAGCAGT TGAAGTTGAT CTGATGACCC TGCTGACTCA GTGTGCACAA GCTGTATCGA GCTTTGATCT   
  
  
+ TCGAGGTGCA AACGAGCTAC TTAGGCAAAT CAGGCAGAAT GCTTCGCCCT ATGGTGGCAG CATCCAGAGG   
  
  
+ CTCGCCCATC ATGTAGCCAA TGCTCTCGAG GCACGTATAG CTGGCACAGG CTCTACAGTC TCTACTAACC   
  
  
+ TTGTTGATGC AAAGTTCTCA GCTTCTGACT TCCTAAAGGC TTACAGGTTA TATGTCTCAG CTGTTCCTTA   
  
  
+ CAAAAGGATG TCTTTCTTTC TTGCTAACTG CTCGATTGCA AAGTTGGCAG AGAAAGCAAC AAAGATCCAT   
  
  
+ ATCATTGATT TTGGTGTTTT CCTAGGTTTA CAATGGCCTT GTTTCATACA ACACCTATCA AAAAGGCCAA   
  
  
+ ATGGACCCCC AAAACTCCGA ATCACAGGAA TCGACTACCC CCAGCAGGGT TTCAGACCTG CACAAAGGGT   
  
  
+ TGAAGCTACA GGACACCGAT TATCTGGGTA CTGTGAGCGA TTTGGGGTGC CTTTTTCTTA TCAGGGCATT   
  
  
+ GCTCAGAAGT GGGAAACTAT TCAGCCGGAG GATCTCAAGA TCGAACAAGA CGAGCTGGTG ATTGTCAACT   
  
  
+ GTTTGTTCAG GTCAGGAACA CTGCTCGATG AGACAGTCGA AGCAAACAGT CCAAGAGATG CTTTCTTAGC   
  
  
+ TTTGGTTAGA AAGCTGAATC CCAGCCTATT CATTCACGGG GTTGTCAATG GCACATTCAA CGCTCCATTC   
  
  
+ TTCGTGACTC GATTCAGAGA GGCATTGTTT CATTATTCAT CAGTGTTTGA TGTGTCTGAA GAGACAATTC   
  
  
+ CACGAGATGC CCATGAGAGG TTCTTGATTG AGAGCGAGAT TTGTGGGAAA GAACTGTTCA ATGTGGTTGC   
  
  
+ TTGTGAGGGT GCAGAGAGGG TTCAAAGGCC TGAGACATAC AAGCAGTGGC AAGTGAGGAC AACGCGGGCC   
  
  
+ GGGTTAAGGC AGGTTGCCTT GGACCAGGAG CTTATGAAGG AAGCAACGGC AATGGTGAAG GCAAATTATC   
  
  
+ ATAAGGATTT TATGGTGGAT ATAAATAGGC ATTGGATGCT TCAAGGTTGG AAGGGTAGAA CCTTGTGTGC   
  
  
+ TCTCTCATTT TGGCAACCTG CCTG  

- +Up\_Stream \_Len000GATCCC CAACTCTCTT AGGCTATCGA TACGTCAGGT TTGTAATAGA GCAGTTCGTA   
  
  
- ATGATAAATT TATTAATTAT AACTTGAACT ATCAATGTGA AAAACTTATG ATTAAGGTGT GGAGTAACGG   
  
  
- AAATAAAGGT TTGGAGCCCA ACGTAAACCT GGTGTATGGT CCTTCGAACC TGCTGTTTTT AAAGAAAAGA   
  
  
- ACTCATAATA AAACAAAATA ATAATAATAA AAAATGAAAT AAACCTGGTG AAGTGAACCC ATTGTTCTAA   
  
  
- CTTATAAACT GATAATATAA TATACAGTGC ATACCTATAC CATCAACGAA TCTCGAAACT GGCAAACGTC   
  
  
- CAAATTCATA CTAAGGGAAC CTTTCCTCGG GGAGAAATAG AATTAAGTCT TCATTGAAAA CAAAAAAATC   
  
  
- TATTTAACTT TTCTTTTTAT TATTTACAGA ATCAACTCAA ACCAATAAGT TAGGCTTTAA ACTAGGACGA   
  
  
- ATTACATATC ATATATTATG ATTATGCGTA CATTGTGTTG TGGAGTCTAC CAGTAACAAA ATGTTAAAAA   
  
  
- AACTCAAATC ATATTTTTAT TTTAGTTAAT AAAAATCTTC ACTACAAATT AAACGTAAAA AAAAGGTGGA   
  
  
- TTTTAGTGCG TGAGCTTGTA ATCCCCAAAC CGATTGAGTT TCTGAGTTCC ATCACTGAAT AGGTTTGAAT   
  
  
- AATGTAGGAT ATCTAGATAT TTTTGTATTT AGAGAATGTT AGTTAGGTTT AACTAAGCCA GATTAAACTA   
  
  
- CTGGGACTAC TCCCAGGATG TTAATGTGTT CACCCGGAAT CGGGGTACCG GCAAAAAATT AGAAGTACCC   
  
  
- AGGTCCGCCT CAATGGTAGA GGTCCAGGTA ACTCCCACAT AGTGCAGAGT TTAATGCTCA AACGGAGAAA   
  
  
- GATGCCCCTA ATATTTATAG ATGGACGTAT GGAGCTTTTC CTCCTCAACT CTAGTCGCTT TGAACCGGTA   
  
  
- GGGTCTCGTA ACTTTACGAG TCTTGGGAAA AAGATCTTTT TATAGCAAAA AGGACTTTTA TAGGCTGGAA   
  
  
- TACAGTAAAT AACAGCGAAA GAACTGAGAT TGGGCCCTGA GGTGTGATTG AACTGATAGC CTTTCCGCAA   
  
  
- GGGACCTAGT GGTAGGTTCT ATTGGAGAAA ACGTCCCAAC TGCGAGCAAC CTTCTTAGCA GCTTACATCT   
  
  
- CGGGGATAGT AGTGTATTCG GGCGAGAATG AAGTACGGTA GGAACTTATC GTCAAATAGG GCTTCGTTTT   
  
  
- TATTAAGATT GAAAGAATAT AACATTTGGG TGTAGATTTA AATTTAAGTA TGAGGTTTTA AGGCGGTGCG   
  
  
- TTTATTCCGA AAAATTATCC GAATGTATAT TACCGTACGA TATCTCTGTG ACAAAACTTC TTTGATAAAT   
  
  
- TTGTAGTCTG GAGTATAACT CATCGTTTAG TTCTGAAGAA AGGAGGTCCG GTATAACTCA TCGTTTAATT   
  
  
- CTTATTGGTC TTCGAACACT TTTCTCAATC TTTTATTTTC CATATTATCT CTGAATACAA GAATTCTATA   
  
  
- GAGGATCTAT ACTTACACTG GAGGATCAGC TATAAACACG ACCTAATCGC TCAACACACT GACTCAAACA   
  
  
- TACACAGAGA ATCGTATACT CAATAAGATC GACGAAGAAA TTCGATACAT CACTGACAGA AGGAAGAATA   
  
  
- AATATTCACG ATACAGGTTG ATTATCGGCT TTTTTTACCA AGCGGAAAAG ACCAAAAGAA GAAAAACTTC   
  
  
- CAACTAACCG ATTAAATATA TTACCAAAGT TATGAATAGG AAATAACAAG GTGTTTAGGT GGATTGGTGC   
  
  
- ATGGTAACTG TTATTAATGG GTGATTAAAG CCAACCTGAA CTAAAGGAAA ACAAACCATG GAGTCCGTAG   
  
  
- AAGACACCTG GAAGTAAGTG TCAAATTAAA AGTGTAAAAA TATTCCGACG AGAACACAAC GTTTAAAAGG   
  
  
- ATAGGTAAGG TGAGAAACGG GTAAGGGTCC GATTAATCAC AAGAGACAAG TTGGTACCTA AGACACGACC   
  
  
- AACTAGGACT CAAGTACTTT TTAGAGATGT TTAAGTTTGG ACTTAACGAG AGTTTGAAAA GTTACTTAGG   
  
  
- TTTACTGCTG TAGAAAGTTC GGGAAGTAAG TCTTTTACTA GGAAATGTCA AGGAGAGTAA ACTACTTCCT   
  
  
- TGGACAGAAT TGTCGACGTG ATCAGTTGTT CACGGTCTAA AGGGACTACG AACAGAGTTC AAGTAGTCAC   
  
  
- TATAAGAAGA GCTTCTCCCA AATCTACGTT TAGGACGTAG ACGTGTCCGA GAGCTTCGGT GGCTCTTCAG   
  
  
- GAACATACTA CGAGAGCCCG AACCTCTCGG TATGGGGGAA AGTACACTAG TGAAACGCGG TAGATAGAGA   
  
  
- TGTTCATAAC TCTCGGGTCT ACTGTCAAGA AGGTTATTTT CAATATCGTC GTTAGGGCTC TATCTACCAA   
  
  
- GAATACGATA GCGACTCGGG TCAAAGCTCA GGTTGGGGTT GACACACGAA CTAGTTGGGG TCAACTTGAG   
  
  
- GAAAGGTCGA GATGTACTCT AAAGAGCCAG GAACCACCTT GACCCAAGAG TTCGGAGACT CAACTCGAAG   
  
  
- CTACTACGTC CCTCACGGGC ACATCTCTTT TTCCCGTTTA GCTATTTCCC GAGCTCCTCC TTCTTCTCAG   
  
  
- CAGTTTCTCT CCCACTTCGC ACAATACCTC CTTCCTCGGT ATTCCAAGTT CGAAGGAAGT TACTACTAAT   
  
  
- GATACTCTAC CTTGTCATAC TACTACATCA TAACGAGACA TTATTACTTG ATTGCCCGTT ATCGGCAAAG   
  
  
- TTGTGGCCCT TTAGAAGTGG ACTCCTCCCT ACCTCCTCTA ACGTCTCTTC GGCTCCTTTC TTCGTCTTGT   
  
  
- CAAATCGTCA ACTTCAACTA GACTACTGGG ACGACTGAGT CACACGTGTT CGACATAGCT CGAAACTAGA   
  
  
- AGCTCCACGT TTGCTCGATG AATCCGTTTA GTCCGTCTTA CGAAGCGGGA TACCACCGTC GTAGGTCTCC   
  
  
- GAGCGGGTAG TACATCGGTT ACGAGAGCTC CGTGCATATC GACCGTGTCC GAGATGTCAG AGATGATTGG   
  
  
- AACAACTACG TTTCAAGAGT CGAAGACTGA AGGATTTCCG AATGTCCAAT ATACAGAGTC GACAAGGAAT   
  
  
- GTTTTCCTAC AGAAAGAAAG AACGATTGAC GAGCTAACGT TTCAACCGTC TCTTTCGTTG TTTCTAGGTA   
  
  
- TAGTAACTAA AACCACAAAA GGATCCAAAT GTTACCGGAA CAAAGTATGT TGTGGATAGT TTTTCCGGTT   
  
  
- TACCTGGGGG TTTTGAGGCT TAGTGTCCTT AGCTGATGGG GGTCGTCCCA AAGTCTGGAC GTGTTTCCCA   
  
  
- ACTTCGATGT CCTGTGGCTA ATAGACCCAT GACACTCGCT AAACCCCACG GAAAAAGAAT AGTCCCGTAA   
  
  
- CGAGTCTTCA CCCTTTGATA AGTCGGCCTC CTAGAGTTCT AGCTTGTTCT GCTCGACCAC TAACAGTTGA   
  
  
- CAAACAAGTC CAGTCCTTGT GACGAGCTAC TCTGTCAGCT TCGTTTGTCA GGTTCTCTAC GAAAGAATCG   
  
  
- AAACCAATCT TTCGACTTAG GGTCGGATAA GTAAGTGCCC CAACAGTTAC CGTGTAAGTT GCGAGGTAAG   
  
  
- AAGCACTGAG CTAAGTCTCT CCGTAACAAA GTAATAAGTA GTCACAAACT ACACAGACTT CTCTGTTAAG   
  
  
- GTGCTCTACG GGTACTCTCC AAGAACTAAC TCTCGCTCTA AACACCCTTT CTTGACAAGT TACACCAACG   
  
  
- AACACTCCCA CGTCTCTCCC AAGTTTCCGG ACTCTGTATG TTCGTCACCG TTCACTCCTG TTGCGCCCGG   
  
  
- CCCAATTCCG TCCAACGGAA CCTGGTCCTC GAATACTTCC TTCGTTGCCG TTACCACTTC CGTTTAATAG   
  
  
- TATTCCTAAA ATACCACCTA TATTTATCCG TAACCTACGA AGTTCCAACC TTCCCATCTT GGAACACACG   
  
  
- AGAGAGTAAA ACCGTTGGAC GGAC

+     Myb

| Site Name | Organism | Position | Strand | Matrix score. | sequence | function |
| --- | --- | --- | --- | --- | --- | --- |
| Myb | Arabidopsis thaliana | 3500 | + | 6 | CAACTG |  |
| Myb | Arabidopsis thaliana | 3179 | + | 6 | TAACTG |  |
| Myb | Arabidopsis thaliana | 2514 | - | 6 | CAACTG |  |
| Myb | Arabidopsis thaliana | 2881 | - | 6 | CAACTG |  |
| Myb | Arabidopsis thaliana | 2492 | + | 6 | CAACTG |  |

>HU02G01571.1   
+ +Up\_Stream \_Len000CTAGGG GTTGAGAGAA TCCGATAGCT ATGCAGTCCA AACATTATCT CGTCAAGCAT   
  
  
+ TACTATTTAA ATAATTAATA TTGAACTTGA TAGTTACACT TTTTGAATAC TAATTCCACA CCTCATTGCC   
  
  
+ TTTATTTCCA AACCTCGGGT TGCATTTGGA CCACATACCA GGAAGCTTGG ACGACAAAAA TTTCTTTTCT   
  
  
+ TGAGTATTAT TTTGTTTTAT TATTATTATT TTTTACTTTA TTTGGACCAC TTCACTTGGG TAACAAGATT   
  
  
+ GAATATTTGA CTATTATATT ATATGTCACG TATGGATATG GTAGTTGCTT AGAGCTTTGA CCGTTTGCAG   
  
  
+ GTTTAAGTAT GATTCCCTTG GAAAGGAGCC CCTCTTTATC TTAATTCAGA AGTAACTTTT GTTTTTTTAG   
  
  
+ ATAAATTGAA AAGAAAAATA ATAAATGTCT TAGTTGAGTT TGGTTATTCA ATCCGAAATT TGATCCTGCT   
  
  
+ TAATGTATAG TATATAATAC TAATACGCAT GTAACACAAC ACCTCAGATG GTCATTGTTT TACAATTTTT   
  
  
+ TTGAGTTTAG TATAAAAATA AAATCAATTA TTTTTAGAAG TGATGTTTAA TTTGCATTTT TTTTCCACCT   
  
  
+ AAAATCACGC ACTCGAACAT TAGGGGTTTG GCTAACTCAA AGACTCAAGG TAGTGACTTA TCCAAACTTA   
  
  
+ TTACATCCTA TAGATCTATA AAAACATAAA TCTCTTACAA TCAATCCAAA TTGATTCGGT CTAATTTGAT   
  
  
+ GACCCTGATG AGGGTCCTAC AATTACACAA GTGGGCCTTA GCCCCATGGC CGTTTTTTAA TCTTCATGGG   
  
  
+ TCCAGGCGGA GTTACCATCT CCAGGTCCAT TGAGGGTGTA TCACGTCTCA AATTACGAGT TTGCCTCTTT   
  
  
+ CTACGGGGAT TATAAATATC TACCTGCATA CCTCGAAAAG GAGGAGTTGA GATCAGCGAA ACTTGGCCAT   
  
  
+ CCCAGAGCAT TGAAATGCTC AGAACCCTTT TTCTAGAAAA ATATCGTTTT TCCTGAAAAT ATCCGACCTT   
  
  
+ ATGTCATTTA TTGTCGCTTT CTTGACTCTA ACCCGGGACT CCACACTAAC TTGACTATCG GAAAGGCGTT   
  
  
+ CCCTGGATCA CCATCCAAGA TAACCTCTTT TGCAGGGTTG ACGCTCGTTG GAAGAATCGT CGAATGTAGA   
  
  
+ GCCCCTATCA TCACATAAGC CCGCTCTTAC TTCATGCCAT CCTTGAATAG CAGTTTATCC CGAAGCAAAA   
  
  
+ ATAATTCTAA CTTTCTTATA TTGTAAACCC ACATCTAAAT TTAAATTCAT ACTCCAAAAT TCCGCCACGC   
  
  
+ AAATAAGGCT TTTTAATAGG CTTACATATA ATGGCATGCT ATAGAGACAC TGTTTTGAAG AAACTATTTA   
  
  
+ AACATCAGAC CTCATATTGA GTAGCAAATC AAGACTTCTT TCCTCCAGGC CATATTGAGT AGCAAATTAA   
  
  
+ GAATAACCAG AAGCTTGTGA AAAGAGTTAG AAAATAAAAG GTATAATAGA GACTTATGTT CTTAAGATAT   
  
  
+ CTCCTAGATA TGAATGTGAC CTCCTAGTCG ATATTTGTGC TGGATTAGCG AGTTGTGTGA CTGAGTTTGT   
  
  
+ ATGTGTCTCT TAGCATATGA GTTATTCTAG CTGCTTCTTT AAGCTATGTA GTGACTGTCT TCCTTCTTAT   
  
  
+ TTATAAGTGC TATGTCCAAC TAATAGCCGA AAAAAATGGT TCGCCTTTTC TGGTTTTCTT CTTTTTGAAG   
  
  
+ GTTGATTGGC TAATTTATAT AATGGTTTCA ATACTTATCC TTTATTGTTC CACAAATCCA CCTAACCACG   
  
  
+ TACCATTGAC AATAATTACC CACTAATTTC GGTTGGACTT GATTTCCTTT TGTTTGGTAC CTCAGGCATC   
  
  
+ TTCTGTGGAC CTTCATTCAC AGTTTAATTT TCACATTTTT ATAAGGCTGC TCTTGTGTTG CAAATTTTCC   
  
  
+ TATCCATTCC ACTCTTTGCC CATTCCCAGG CTAATTAGTG TTCTCTGTTC AACCATGGAT TCTGTGCTGG   
  
  
+ TTGATCCTGA GTTCATGAAA AATCTCTACA AATTCAAACC TGAATTGCTC TCAAACTTTT CAATGAATCC   
  
  
+ AAATGACGAC ATCTTTCAAG CCCTTCATTC AGAAAATGAT CCTTTACAGT TCCTCTCATT TGATGAAGGA   
  
  
+ ACCTGTCTTA ACAGCTGCAC TAGTCAACAA GTGCCAGATT TCCCTGATGC TTGTCTCAAG TTCATCAGTG   
  
  
+ ATATTCTTCT CGAAGAGGGT TTAGATGCAA ATCCTGCATC TGCACAGGCT CTCGAAGCCA CCGAGAAGTC   
  
  
+ CTTGTATGAT GCTCTCGGGC TTGGAGAGCC ATACCCCCTT TCATGTGATC ACTTTGCGCC ATCTATCTCT   
  
  
+ ACAAGTATTG AGAGCCCAGA TGACAGTTCT TCCAATAAAA GTTATAGCAG CAATCCCGAG ATAGATGGTT   
  
  
+ CTTATGCTAT CGCTGAGCCC AGTTTCGAGT CCAACCCCAA CTGTGTGCTT GATCAACCCC AGTTGAACTC   
  
  
+ CTTTCCAGCT CTACATGAGA TTTCTCGGTC CTTGGTGGAA CTGGGTTCTC AAGCCTCTGA GTTGAGCTTC   
  
  
+ GATGATGCAG GGAGTGCCCG TGTAGAGAAA AAGGGCAAAT CGATAAAGGG CTCGAGGAGG AAGAAGAGTC   
  
  
+ GTCAAAGAGA GGGTGAAGCG TGTTATGGAG GAAGGAGCCA TAAGGTTCAA GCTTCCTTCA ATGATGATTA   
  
  
+ CTATGAGATG GAACAGTATG ATGATGTAGT ATTGCTCTGT AATAATGAAC TAACGGGCAA TAGCCGTTTC   
  
  
+ AACACCGGGA AATCTTCACC TGAGGAGGGA TGGAGGAGAT TGCAGAGAAG CCGAGGAAAG AAGCAGAACA   
  
  
+ GTTTAGCAGT TGAAGTTGAT CTGATGACCC TGCTGACTCA GTGTGCACAA GCTGTATCGA GCTTTGATCT   
  
  
+ TCGAGGTGCA AACGAGCTAC TTAGGCAAAT CAGGCAGAAT GCTTCGCCCT ATGGTGGCAG CATCCAGAGG   
  
  
+ CTCGCCCATC ATGTAGCCAA TGCTCTCGAG GCACGTATAG CTGGCACAGG CTCTACAGTC TCTACTAACC   
  
  
+ TTGTTGATGC AAAGTTCTCA GCTTCTGACT TCCTAAAGGC TTACAGGTTA TATGTCTCAG CTGTTCCTTA   
  
  
+ CAAAAGGATG TCTTTCTTTC TTGCTAACTG CTCGATTGCA AAGTTGGCAG AGAAAGCAAC AAAGATCCAT   
  
  
+ ATCATTGATT TTGGTGTTTT CCTAGGTTTA CAATGGCCTT GTTTCATACA ACACCTATCA AAAAGGCCAA   
  
  
+ ATGGACCCCC AAAACTCCGA ATCACAGGAA TCGACTACCC CCAGCAGGGT TTCAGACCTG CACAAAGGGT   
  
  
+ TGAAGCTACA GGACACCGAT TATCTGGGTA CTGTGAGCGA TTTGGGGTGC CTTTTTCTTA TCAGGGCATT   
  
  
+ GCTCAGAAGT GGGAAACTAT TCAGCCGGAG GATCTCAAGA TCGAACAAGA CGAGCTGGTG ATTGTCAACT   
  
  
+ GTTTGTTCAG GTCAGGAACA CTGCTCGATG AGACAGTCGA AGCAAACAGT CCAAGAGATG CTTTCTTAGC   
  
  
+ TTTGGTTAGA AAGCTGAATC CCAGCCTATT CATTCACGGG GTTGTCAATG GCACATTCAA CGCTCCATTC   
  
  
+ TTCGTGACTC GATTCAGAGA GGCATTGTTT CATTATTCAT CAGTGTTTGA TGTGTCTGAA GAGACAATTC   
  
  
+ CACGAGATGC CCATGAGAGG TTCTTGATTG AGAGCGAGAT TTGTGGGAAA GAACTGTTCA ATGTGGTTGC   
  
  
+ TTGTGAGGGT GCAGAGAGGG TTCAAAGGCC TGAGACATAC AAGCAGTGGC AAGTGAGGAC AACGCGGGCC   
  
  
+ GGGTTAAGGC AGGTTGCCTT GGACCAGGAG CTTATGAAGG AAGCAACGGC AATGGTGAAG GCAAATTATC   
  
  
+ ATAAGGATTT TATGGTGGAT ATAAATAGGC ATTGGATGCT TCAAGGTTGG AAGGGTAGAA CCTTGTGTGC   
  
  
+ TCTCTCATTT TGGCAACCTG CCTG  

- +Up\_Stream \_Len000GATCCC CAACTCTCTT AGGCTATCGA TACGTCAGGT TTGTAATAGA GCAGTTCGTA   
  
  
- ATGATAAATT TATTAATTAT AACTTGAACT ATCAATGTGA AAAACTTATG ATTAAGGTGT GGAGTAACGG   
  
  
- AAATAAAGGT TTGGAGCCCA ACGTAAACCT GGTGTATGGT CCTTCGAACC TGCTGTTTTT AAAGAAAAGA   
  
  
- ACTCATAATA AAACAAAATA ATAATAATAA AAAATGAAAT AAACCTGGTG AAGTGAACCC ATTGTTCTAA   
  
  
- CTTATAAACT GATAATATAA TATACAGTGC ATACCTATAC CATCAACGAA TCTCGAAACT GGCAAACGTC   
  
  
- CAAATTCATA CTAAGGGAAC CTTTCCTCGG GGAGAAATAG AATTAAGTCT TCATTGAAAA CAAAAAAATC   
  
  
- TATTTAACTT TTCTTTTTAT TATTTACAGA ATCAACTCAA ACCAATAAGT TAGGCTTTAA ACTAGGACGA   
  
  
- ATTACATATC ATATATTATG ATTATGCGTA CATTGTGTTG TGGAGTCTAC CAGTAACAAA ATGTTAAAAA   
  
  
- AACTCAAATC ATATTTTTAT TTTAGTTAAT AAAAATCTTC ACTACAAATT AAACGTAAAA AAAAGGTGGA   
  
  
- TTTTAGTGCG TGAGCTTGTA ATCCCCAAAC CGATTGAGTT TCTGAGTTCC ATCACTGAAT AGGTTTGAAT   
  
  
- AATGTAGGAT ATCTAGATAT TTTTGTATTT AGAGAATGTT AGTTAGGTTT AACTAAGCCA GATTAAACTA   
  
  
- CTGGGACTAC TCCCAGGATG TTAATGTGTT CACCCGGAAT CGGGGTACCG GCAAAAAATT AGAAGTACCC   
  
  
- AGGTCCGCCT CAATGGTAGA GGTCCAGGTA ACTCCCACAT AGTGCAGAGT TTAATGCTCA AACGGAGAAA   
  
  
- GATGCCCCTA ATATTTATAG ATGGACGTAT GGAGCTTTTC CTCCTCAACT CTAGTCGCTT TGAACCGGTA   
  
  
- GGGTCTCGTA ACTTTACGAG TCTTGGGAAA AAGATCTTTT TATAGCAAAA AGGACTTTTA TAGGCTGGAA   
  
  
- TACAGTAAAT AACAGCGAAA GAACTGAGAT TGGGCCCTGA GGTGTGATTG AACTGATAGC CTTTCCGCAA   
  
  
- GGGACCTAGT GGTAGGTTCT ATTGGAGAAA ACGTCCCAAC TGCGAGCAAC CTTCTTAGCA GCTTACATCT   
  
  
- CGGGGATAGT AGTGTATTCG GGCGAGAATG AAGTACGGTA GGAACTTATC GTCAAATAGG GCTTCGTTTT   
  
  
- TATTAAGATT GAAAGAATAT AACATTTGGG TGTAGATTTA AATTTAAGTA TGAGGTTTTA AGGCGGTGCG   
  
  
- TTTATTCCGA AAAATTATCC GAATGTATAT TACCGTACGA TATCTCTGTG ACAAAACTTC TTTGATAAAT   
  
  
- TTGTAGTCTG GAGTATAACT CATCGTTTAG TTCTGAAGAA AGGAGGTCCG GTATAACTCA TCGTTTAATT   
  
  
- CTTATTGGTC TTCGAACACT TTTCTCAATC TTTTATTTTC CATATTATCT CTGAATACAA GAATTCTATA   
  
  
- GAGGATCTAT ACTTACACTG GAGGATCAGC TATAAACACG ACCTAATCGC TCAACACACT GACTCAAACA   
  
  
- TACACAGAGA ATCGTATACT CAATAAGATC GACGAAGAAA TTCGATACAT CACTGACAGA AGGAAGAATA   
  
  
- AATATTCACG ATACAGGTTG ATTATCGGCT TTTTTTACCA AGCGGAAAAG ACCAAAAGAA GAAAAACTTC   
  
  
- CAACTAACCG ATTAAATATA TTACCAAAGT TATGAATAGG AAATAACAAG GTGTTTAGGT GGATTGGTGC   
  
  
- ATGGTAACTG TTATTAATGG GTGATTAAAG CCAACCTGAA CTAAAGGAAA ACAAACCATG GAGTCCGTAG   
  
  
- AAGACACCTG GAAGTAAGTG TCAAATTAAA AGTGTAAAAA TATTCCGACG AGAACACAAC GTTTAAAAGG   
  
  
- ATAGGTAAGG TGAGAAACGG GTAAGGGTCC GATTAATCAC AAGAGACAAG TTGGTACCTA AGACACGACC   
  
  
- AACTAGGACT CAAGTACTTT TTAGAGATGT TTAAGTTTGG ACTTAACGAG AGTTTGAAAA GTTACTTAGG   
  
  
- TTTACTGCTG TAGAAAGTTC GGGAAGTAAG TCTTTTACTA GGAAATGTCA AGGAGAGTAA ACTACTTCCT   
  
  
- TGGACAGAAT TGTCGACGTG ATCAGTTGTT CACGGTCTAA AGGGACTACG AACAGAGTTC AAGTAGTCAC   
  
  
- TATAAGAAGA GCTTCTCCCA AATCTACGTT TAGGACGTAG ACGTGTCCGA GAGCTTCGGT GGCTCTTCAG   
  
  
- GAACATACTA CGAGAGCCCG AACCTCTCGG TATGGGGGAA AGTACACTAG TGAAACGCGG TAGATAGAGA   
  
  
- TGTTCATAAC TCTCGGGTCT ACTGTCAAGA AGGTTATTTT CAATATCGTC GTTAGGGCTC TATCTACCAA   
  
  
- GAATACGATA GCGACTCGGG TCAAAGCTCA GGTTGGGGTT GACACACGAA CTAGTTGGGG TCAACTTGAG   
  
  
- GAAAGGTCGA GATGTACTCT AAAGAGCCAG GAACCACCTT GACCCAAGAG TTCGGAGACT CAACTCGAAG   
  
  
- CTACTACGTC CCTCACGGGC ACATCTCTTT TTCCCGTTTA GCTATTTCCC GAGCTCCTCC TTCTTCTCAG   
  
  
- CAGTTTCTCT CCCACTTCGC ACAATACCTC CTTCCTCGGT ATTCCAAGTT CGAAGGAAGT TACTACTAAT   
  
  
- GATACTCTAC CTTGTCATAC TACTACATCA TAACGAGACA TTATTACTTG ATTGCCCGTT ATCGGCAAAG   
  
  
- TTGTGGCCCT TTAGAAGTGG ACTCCTCCCT ACCTCCTCTA ACGTCTCTTC GGCTCCTTTC TTCGTCTTGT   
  
  
- CAAATCGTCA ACTTCAACTA GACTACTGGG ACGACTGAGT CACACGTGTT CGACATAGCT CGAAACTAGA   
  
  
- AGCTCCACGT TTGCTCGATG AATCCGTTTA GTCCGTCTTA CGAAGCGGGA TACCACCGTC GTAGGTCTCC   
  
  
- GAGCGGGTAG TACATCGGTT ACGAGAGCTC CGTGCATATC GACCGTGTCC GAGATGTCAG AGATGATTGG   
  
  
- AACAACTACG TTTCAAGAGT CGAAGACTGA AGGATTTCCG AATGTCCAAT ATACAGAGTC GACAAGGAAT   
  
  
- GTTTTCCTAC AGAAAGAAAG AACGATTGAC GAGCTAACGT TTCAACCGTC TCTTTCGTTG TTTCTAGGTA   
  
  
- TAGTAACTAA AACCACAAAA GGATCCAAAT GTTACCGGAA CAAAGTATGT TGTGGATAGT TTTTCCGGTT   
  
  
- TACCTGGGGG TTTTGAGGCT TAGTGTCCTT AGCTGATGGG GGTCGTCCCA AAGTCTGGAC GTGTTTCCCA   
  
  
- ACTTCGATGT CCTGTGGCTA ATAGACCCAT GACACTCGCT AAACCCCACG GAAAAAGAAT AGTCCCGTAA   
  
  
- CGAGTCTTCA CCCTTTGATA AGTCGGCCTC CTAGAGTTCT AGCTTGTTCT GCTCGACCAC TAACAGTTGA   
  
  
- CAAACAAGTC CAGTCCTTGT GACGAGCTAC TCTGTCAGCT TCGTTTGTCA GGTTCTCTAC GAAAGAATCG   
  
  
- AAACCAATCT TTCGACTTAG GGTCGGATAA GTAAGTGCCC CAACAGTTAC CGTGTAAGTT GCGAGGTAAG   
  
  
- AAGCACTGAG CTAAGTCTCT CCGTAACAAA GTAATAAGTA GTCACAAACT ACACAGACTT CTCTGTTAAG   
  
  
- GTGCTCTACG GGTACTCTCC AAGAACTAAC TCTCGCTCTA AACACCCTTT CTTGACAAGT TACACCAACG   
  
  
- AACACTCCCA CGTCTCTCCC AAGTTTCCGG ACTCTGTATG TTCGTCACCG TTCACTCCTG TTGCGCCCGG   
  
  
- CCCAATTCCG TCCAACGGAA CCTGGTCCTC GAATACTTCC TTCGTTGCCG TTACCACTTC CGTTTAATAG   
  
  
- TATTCCTAAA ATACCACCTA TATTTATCCG TAACCTACGA AGTTCCAACC TTCCCATCTT GGAACACACG   
  
  
- AGAGAGTAAA ACCGTTGGAC GGAC

+     Myc

| Site Name | Organism | Position | Strand | Matrix score. | sequence | function |
| --- | --- | --- | --- | --- | --- | --- |
| Myc | Arabidopsis thaliana | 735 | + | 7 | TCTCTTA |  |
| Myc | Arabidopsis thaliana | 1620 | + | 7 | TCTCTTA |  |

>HU02G01571.1   
+ +Up\_Stream \_Len000CTAGGG GTTGAGAGAA TCCGATAGCT ATGCAGTCCA AACATTATCT CGTCAAGCAT   
  
  
+ TACTATTTAA ATAATTAATA TTGAACTTGA TAGTTACACT TTTTGAATAC TAATTCCACA CCTCATTGCC   
  
  
+ TTTATTTCCA AACCTCGGGT TGCATTTGGA CCACATACCA GGAAGCTTGG ACGACAAAAA TTTCTTTTCT   
  
  
+ TGAGTATTAT TTTGTTTTAT TATTATTATT TTTTACTTTA TTTGGACCAC TTCACTTGGG TAACAAGATT   
  
  
+ GAATATTTGA CTATTATATT ATATGTCACG TATGGATATG GTAGTTGCTT AGAGCTTTGA CCGTTTGCAG   
  
  
+ GTTTAAGTAT GATTCCCTTG GAAAGGAGCC CCTCTTTATC TTAATTCAGA AGTAACTTTT GTTTTTTTAG   
  
  
+ ATAAATTGAA AAGAAAAATA ATAAATGTCT TAGTTGAGTT TGGTTATTCA ATCCGAAATT TGATCCTGCT   
  
  
+ TAATGTATAG TATATAATAC TAATACGCAT GTAACACAAC ACCTCAGATG GTCATTGTTT TACAATTTTT   
  
  
+ TTGAGTTTAG TATAAAAATA AAATCAATTA TTTTTAGAAG TGATGTTTAA TTTGCATTTT TTTTCCACCT   
  
  
+ AAAATCACGC ACTCGAACAT TAGGGGTTTG GCTAACTCAA AGACTCAAGG TAGTGACTTA TCCAAACTTA   
  
  
+ TTACATCCTA TAGATCTATA AAAACATAAA TCTCTTACAA TCAATCCAAA TTGATTCGGT CTAATTTGAT   
  
  
+ GACCCTGATG AGGGTCCTAC AATTACACAA GTGGGCCTTA GCCCCATGGC CGTTTTTTAA TCTTCATGGG   
  
  
+ TCCAGGCGGA GTTACCATCT CCAGGTCCAT TGAGGGTGTA TCACGTCTCA AATTACGAGT TTGCCTCTTT   
  
  
+ CTACGGGGAT TATAAATATC TACCTGCATA CCTCGAAAAG GAGGAGTTGA GATCAGCGAA ACTTGGCCAT   
  
  
+ CCCAGAGCAT TGAAATGCTC AGAACCCTTT TTCTAGAAAA ATATCGTTTT TCCTGAAAAT ATCCGACCTT   
  
  
+ ATGTCATTTA TTGTCGCTTT CTTGACTCTA ACCCGGGACT CCACACTAAC TTGACTATCG GAAAGGCGTT   
  
  
+ CCCTGGATCA CCATCCAAGA TAACCTCTTT TGCAGGGTTG ACGCTCGTTG GAAGAATCGT CGAATGTAGA   
  
  
+ GCCCCTATCA TCACATAAGC CCGCTCTTAC TTCATGCCAT CCTTGAATAG CAGTTTATCC CGAAGCAAAA   
  
  
+ ATAATTCTAA CTTTCTTATA TTGTAAACCC ACATCTAAAT TTAAATTCAT ACTCCAAAAT TCCGCCACGC   
  
  
+ AAATAAGGCT TTTTAATAGG CTTACATATA ATGGCATGCT ATAGAGACAC TGTTTTGAAG AAACTATTTA   
  
  
+ AACATCAGAC CTCATATTGA GTAGCAAATC AAGACTTCTT TCCTCCAGGC CATATTGAGT AGCAAATTAA   
  
  
+ GAATAACCAG AAGCTTGTGA AAAGAGTTAG AAAATAAAAG GTATAATAGA GACTTATGTT CTTAAGATAT   
  
  
+ CTCCTAGATA TGAATGTGAC CTCCTAGTCG ATATTTGTGC TGGATTAGCG AGTTGTGTGA CTGAGTTTGT   
  
  
+ ATGTGTCTCT TAGCATATGA GTTATTCTAG CTGCTTCTTT AAGCTATGTA GTGACTGTCT TCCTTCTTAT   
  
  
+ TTATAAGTGC TATGTCCAAC TAATAGCCGA AAAAAATGGT TCGCCTTTTC TGGTTTTCTT CTTTTTGAAG   
  
  
+ GTTGATTGGC TAATTTATAT AATGGTTTCA ATACTTATCC TTTATTGTTC CACAAATCCA CCTAACCACG   
  
  
+ TACCATTGAC AATAATTACC CACTAATTTC GGTTGGACTT GATTTCCTTT TGTTTGGTAC CTCAGGCATC   
  
  
+ TTCTGTGGAC CTTCATTCAC AGTTTAATTT TCACATTTTT ATAAGGCTGC TCTTGTGTTG CAAATTTTCC   
  
  
+ TATCCATTCC ACTCTTTGCC CATTCCCAGG CTAATTAGTG TTCTCTGTTC AACCATGGAT TCTGTGCTGG   
  
  
+ TTGATCCTGA GTTCATGAAA AATCTCTACA AATTCAAACC TGAATTGCTC TCAAACTTTT CAATGAATCC   
  
  
+ AAATGACGAC ATCTTTCAAG CCCTTCATTC AGAAAATGAT CCTTTACAGT TCCTCTCATT TGATGAAGGA   
  
  
+ ACCTGTCTTA ACAGCTGCAC TAGTCAACAA GTGCCAGATT TCCCTGATGC TTGTCTCAAG TTCATCAGTG   
  
  
+ ATATTCTTCT CGAAGAGGGT TTAGATGCAA ATCCTGCATC TGCACAGGCT CTCGAAGCCA CCGAGAAGTC   
  
  
+ CTTGTATGAT GCTCTCGGGC TTGGAGAGCC ATACCCCCTT TCATGTGATC ACTTTGCGCC ATCTATCTCT   
  
  
+ ACAAGTATTG AGAGCCCAGA TGACAGTTCT TCCAATAAAA GTTATAGCAG CAATCCCGAG ATAGATGGTT   
  
  
+ CTTATGCTAT CGCTGAGCCC AGTTTCGAGT CCAACCCCAA CTGTGTGCTT GATCAACCCC AGTTGAACTC   
  
  
+ CTTTCCAGCT CTACATGAGA TTTCTCGGTC CTTGGTGGAA CTGGGTTCTC AAGCCTCTGA GTTGAGCTTC   
  
  
+ GATGATGCAG GGAGTGCCCG TGTAGAGAAA AAGGGCAAAT CGATAAAGGG CTCGAGGAGG AAGAAGAGTC   
  
  
+ GTCAAAGAGA GGGTGAAGCG TGTTATGGAG GAAGGAGCCA TAAGGTTCAA GCTTCCTTCA ATGATGATTA   
  
  
+ CTATGAGATG GAACAGTATG ATGATGTAGT ATTGCTCTGT AATAATGAAC TAACGGGCAA TAGCCGTTTC   
  
  
+ AACACCGGGA AATCTTCACC TGAGGAGGGA TGGAGGAGAT TGCAGAGAAG CCGAGGAAAG AAGCAGAACA   
  
  
+ GTTTAGCAGT TGAAGTTGAT CTGATGACCC TGCTGACTCA GTGTGCACAA GCTGTATCGA GCTTTGATCT   
  
  
+ TCGAGGTGCA AACGAGCTAC TTAGGCAAAT CAGGCAGAAT GCTTCGCCCT ATGGTGGCAG CATCCAGAGG   
  
  
+ CTCGCCCATC ATGTAGCCAA TGCTCTCGAG GCACGTATAG CTGGCACAGG CTCTACAGTC TCTACTAACC   
  
  
+ TTGTTGATGC AAAGTTCTCA GCTTCTGACT TCCTAAAGGC TTACAGGTTA TATGTCTCAG CTGTTCCTTA   
  
  
+ CAAAAGGATG TCTTTCTTTC TTGCTAACTG CTCGATTGCA AAGTTGGCAG AGAAAGCAAC AAAGATCCAT   
  
  
+ ATCATTGATT TTGGTGTTTT CCTAGGTTTA CAATGGCCTT GTTTCATACA ACACCTATCA AAAAGGCCAA   
  
  
+ ATGGACCCCC AAAACTCCGA ATCACAGGAA TCGACTACCC CCAGCAGGGT TTCAGACCTG CACAAAGGGT   
  
  
+ TGAAGCTACA GGACACCGAT TATCTGGGTA CTGTGAGCGA TTTGGGGTGC CTTTTTCTTA TCAGGGCATT   
  
  
+ GCTCAGAAGT GGGAAACTAT TCAGCCGGAG GATCTCAAGA TCGAACAAGA CGAGCTGGTG ATTGTCAACT   
  
  
+ GTTTGTTCAG GTCAGGAACA CTGCTCGATG AGACAGTCGA AGCAAACAGT CCAAGAGATG CTTTCTTAGC   
  
  
+ TTTGGTTAGA AAGCTGAATC CCAGCCTATT CATTCACGGG GTTGTCAATG GCACATTCAA CGCTCCATTC   
  
  
+ TTCGTGACTC GATTCAGAGA GGCATTGTTT CATTATTCAT CAGTGTTTGA TGTGTCTGAA GAGACAATTC   
  
  
+ CACGAGATGC CCATGAGAGG TTCTTGATTG AGAGCGAGAT TTGTGGGAAA GAACTGTTCA ATGTGGTTGC   
  
  
+ TTGTGAGGGT GCAGAGAGGG TTCAAAGGCC TGAGACATAC AAGCAGTGGC AAGTGAGGAC AACGCGGGCC   
  
  
+ GGGTTAAGGC AGGTTGCCTT GGACCAGGAG CTTATGAAGG AAGCAACGGC AATGGTGAAG GCAAATTATC   
  
  
+ ATAAGGATTT TATGGTGGAT ATAAATAGGC ATTGGATGCT TCAAGGTTGG AAGGGTAGAA CCTTGTGTGC   
  
  
+ TCTCTCATTT TGGCAACCTG CCTG  

- +Up\_Stream \_Len000GATCCC CAACTCTCTT AGGCTATCGA TACGTCAGGT TTGTAATAGA GCAGTTCGTA   
  
  
- ATGATAAATT TATTAATTAT AACTTGAACT ATCAATGTGA AAAACTTATG ATTAAGGTGT GGAGTAACGG   
  
  
- AAATAAAGGT TTGGAGCCCA ACGTAAACCT GGTGTATGGT CCTTCGAACC TGCTGTTTTT AAAGAAAAGA   
  
  
- ACTCATAATA AAACAAAATA ATAATAATAA AAAATGAAAT AAACCTGGTG AAGTGAACCC ATTGTTCTAA   
  
  
- CTTATAAACT GATAATATAA TATACAGTGC ATACCTATAC CATCAACGAA TCTCGAAACT GGCAAACGTC   
  
  
- CAAATTCATA CTAAGGGAAC CTTTCCTCGG GGAGAAATAG AATTAAGTCT TCATTGAAAA CAAAAAAATC   
  
  
- TATTTAACTT TTCTTTTTAT TATTTACAGA ATCAACTCAA ACCAATAAGT TAGGCTTTAA ACTAGGACGA   
  
  
- ATTACATATC ATATATTATG ATTATGCGTA CATTGTGTTG TGGAGTCTAC CAGTAACAAA ATGTTAAAAA   
  
  
- AACTCAAATC ATATTTTTAT TTTAGTTAAT AAAAATCTTC ACTACAAATT AAACGTAAAA AAAAGGTGGA   
  
  
- TTTTAGTGCG TGAGCTTGTA ATCCCCAAAC CGATTGAGTT TCTGAGTTCC ATCACTGAAT AGGTTTGAAT   
  
  
- AATGTAGGAT ATCTAGATAT TTTTGTATTT AGAGAATGTT AGTTAGGTTT AACTAAGCCA GATTAAACTA   
  
  
- CTGGGACTAC TCCCAGGATG TTAATGTGTT CACCCGGAAT CGGGGTACCG GCAAAAAATT AGAAGTACCC   
  
  
- AGGTCCGCCT CAATGGTAGA GGTCCAGGTA ACTCCCACAT AGTGCAGAGT TTAATGCTCA AACGGAGAAA   
  
  
- GATGCCCCTA ATATTTATAG ATGGACGTAT GGAGCTTTTC CTCCTCAACT CTAGTCGCTT TGAACCGGTA   
  
  
- GGGTCTCGTA ACTTTACGAG TCTTGGGAAA AAGATCTTTT TATAGCAAAA AGGACTTTTA TAGGCTGGAA   
  
  
- TACAGTAAAT AACAGCGAAA GAACTGAGAT TGGGCCCTGA GGTGTGATTG AACTGATAGC CTTTCCGCAA   
  
  
- GGGACCTAGT GGTAGGTTCT ATTGGAGAAA ACGTCCCAAC TGCGAGCAAC CTTCTTAGCA GCTTACATCT   
  
  
- CGGGGATAGT AGTGTATTCG GGCGAGAATG AAGTACGGTA GGAACTTATC GTCAAATAGG GCTTCGTTTT   
  
  
- TATTAAGATT GAAAGAATAT AACATTTGGG TGTAGATTTA AATTTAAGTA TGAGGTTTTA AGGCGGTGCG   
  
  
- TTTATTCCGA AAAATTATCC GAATGTATAT TACCGTACGA TATCTCTGTG ACAAAACTTC TTTGATAAAT   
  
  
- TTGTAGTCTG GAGTATAACT CATCGTTTAG TTCTGAAGAA AGGAGGTCCG GTATAACTCA TCGTTTAATT   
  
  
- CTTATTGGTC TTCGAACACT TTTCTCAATC TTTTATTTTC CATATTATCT CTGAATACAA GAATTCTATA   
  
  
- GAGGATCTAT ACTTACACTG GAGGATCAGC TATAAACACG ACCTAATCGC TCAACACACT GACTCAAACA   
  
  
- TACACAGAGA ATCGTATACT CAATAAGATC GACGAAGAAA TTCGATACAT CACTGACAGA AGGAAGAATA   
  
  
- AATATTCACG ATACAGGTTG ATTATCGGCT TTTTTTACCA AGCGGAAAAG ACCAAAAGAA GAAAAACTTC   
  
  
- CAACTAACCG ATTAAATATA TTACCAAAGT TATGAATAGG AAATAACAAG GTGTTTAGGT GGATTGGTGC   
  
  
- ATGGTAACTG TTATTAATGG GTGATTAAAG CCAACCTGAA CTAAAGGAAA ACAAACCATG GAGTCCGTAG   
  
  
- AAGACACCTG GAAGTAAGTG TCAAATTAAA AGTGTAAAAA TATTCCGACG AGAACACAAC GTTTAAAAGG   
  
  
- ATAGGTAAGG TGAGAAACGG GTAAGGGTCC GATTAATCAC AAGAGACAAG TTGGTACCTA AGACACGACC   
  
  
- AACTAGGACT CAAGTACTTT TTAGAGATGT TTAAGTTTGG ACTTAACGAG AGTTTGAAAA GTTACTTAGG   
  
  
- TTTACTGCTG TAGAAAGTTC GGGAAGTAAG TCTTTTACTA GGAAATGTCA AGGAGAGTAA ACTACTTCCT   
  
  
- TGGACAGAAT TGTCGACGTG ATCAGTTGTT CACGGTCTAA AGGGACTACG AACAGAGTTC AAGTAGTCAC   
  
  
- TATAAGAAGA GCTTCTCCCA AATCTACGTT TAGGACGTAG ACGTGTCCGA GAGCTTCGGT GGCTCTTCAG   
  
  
- GAACATACTA CGAGAGCCCG AACCTCTCGG TATGGGGGAA AGTACACTAG TGAAACGCGG TAGATAGAGA   
  
  
- TGTTCATAAC TCTCGGGTCT ACTGTCAAGA AGGTTATTTT CAATATCGTC GTTAGGGCTC TATCTACCAA   
  
  
- GAATACGATA GCGACTCGGG TCAAAGCTCA GGTTGGGGTT GACACACGAA CTAGTTGGGG TCAACTTGAG   
  
  
- GAAAGGTCGA GATGTACTCT AAAGAGCCAG GAACCACCTT GACCCAAGAG TTCGGAGACT CAACTCGAAG   
  
  
- CTACTACGTC CCTCACGGGC ACATCTCTTT TTCCCGTTTA GCTATTTCCC GAGCTCCTCC TTCTTCTCAG   
  
  
- CAGTTTCTCT CCCACTTCGC ACAATACCTC CTTCCTCGGT ATTCCAAGTT CGAAGGAAGT TACTACTAAT   
  
  
- GATACTCTAC CTTGTCATAC TACTACATCA TAACGAGACA TTATTACTTG ATTGCCCGTT ATCGGCAAAG   
  
  
- TTGTGGCCCT TTAGAAGTGG ACTCCTCCCT ACCTCCTCTA ACGTCTCTTC GGCTCCTTTC TTCGTCTTGT   
  
  
- CAAATCGTCA ACTTCAACTA GACTACTGGG ACGACTGAGT CACACGTGTT CGACATAGCT CGAAACTAGA   
  
  
- AGCTCCACGT TTGCTCGATG AATCCGTTTA GTCCGTCTTA CGAAGCGGGA TACCACCGTC GTAGGTCTCC   
  
  
- GAGCGGGTAG TACATCGGTT ACGAGAGCTC CGTGCATATC GACCGTGTCC GAGATGTCAG AGATGATTGG   
  
  
- AACAACTACG TTTCAAGAGT CGAAGACTGA AGGATTTCCG AATGTCCAAT ATACAGAGTC GACAAGGAAT   
  
  
- GTTTTCCTAC AGAAAGAAAG AACGATTGAC GAGCTAACGT TTCAACCGTC TCTTTCGTTG TTTCTAGGTA   
  
  
- TAGTAACTAA AACCACAAAA GGATCCAAAT GTTACCGGAA CAAAGTATGT TGTGGATAGT TTTTCCGGTT   
  
  
- TACCTGGGGG TTTTGAGGCT TAGTGTCCTT AGCTGATGGG GGTCGTCCCA AAGTCTGGAC GTGTTTCCCA   
  
  
- ACTTCGATGT CCTGTGGCTA ATAGACCCAT GACACTCGCT AAACCCCACG GAAAAAGAAT AGTCCCGTAA   
  
  
- CGAGTCTTCA CCCTTTGATA AGTCGGCCTC CTAGAGTTCT AGCTTGTTCT GCTCGACCAC TAACAGTTGA   
  
  
- CAAACAAGTC CAGTCCTTGT GACGAGCTAC TCTGTCAGCT TCGTTTGTCA GGTTCTCTAC GAAAGAATCG   
  
  
- AAACCAATCT TTCGACTTAG GGTCGGATAA GTAAGTGCCC CAACAGTTAC CGTGTAAGTT GCGAGGTAAG   
  
  
- AAGCACTGAG CTAAGTCTCT CCGTAACAAA GTAATAAGTA GTCACAAACT ACACAGACTT CTCTGTTAAG   
  
  
- GTGCTCTACG GGTACTCTCC AAGAACTAAC TCTCGCTCTA AACACCCTTT CTTGACAAGT TACACCAACG   
  
  
- AACACTCCCA CGTCTCTCCC AAGTTTCCGG ACTCTGTATG TTCGTCACCG TTCACTCCTG TTGCGCCCGG   
  
  
- CCCAATTCCG TCCAACGGAA CCTGGTCCTC GAATACTTCC TTCGTTGCCG TTACCACTTC CGTTTAATAG   
  
  
- TATTCCTAAA ATACCACCTA TATTTATCCG TAACCTACGA AGTTCCAACC TTCCCATCTT GGAACACACG   
  
  
- AGAGAGTAAA ACCGTTGGAC GGAC

+     O2-site

| Site Name | Organism | Position | Strand | Matrix score. | sequence | function |
| --- | --- | --- | --- | --- | --- | --- |
| O2-site | Zea mays | 2754 | + | 9 | GATGATGTGG | cis-acting regulatory element involved in zein metabolism regulation |

>HU02G01571.1   
+ +Up\_Stream \_Len000CTAGGG GTTGAGAGAA TCCGATAGCT ATGCAGTCCA AACATTATCT CGTCAAGCAT   
  
  
+ TACTATTTAA ATAATTAATA TTGAACTTGA TAGTTACACT TTTTGAATAC TAATTCCACA CCTCATTGCC   
  
  
+ TTTATTTCCA AACCTCGGGT TGCATTTGGA CCACATACCA GGAAGCTTGG ACGACAAAAA TTTCTTTTCT   
  
  
+ TGAGTATTAT TTTGTTTTAT TATTATTATT TTTTACTTTA TTTGGACCAC TTCACTTGGG TAACAAGATT   
  
  
+ GAATATTTGA CTATTATATT ATATGTCACG TATGGATATG GTAGTTGCTT AGAGCTTTGA CCGTTTGCAG   
  
  
+ GTTTAAGTAT GATTCCCTTG GAAAGGAGCC CCTCTTTATC TTAATTCAGA AGTAACTTTT GTTTTTTTAG   
  
  
+ ATAAATTGAA AAGAAAAATA ATAAATGTCT TAGTTGAGTT TGGTTATTCA ATCCGAAATT TGATCCTGCT   
  
  
+ TAATGTATAG TATATAATAC TAATACGCAT GTAACACAAC ACCTCAGATG GTCATTGTTT TACAATTTTT   
  
  
+ TTGAGTTTAG TATAAAAATA AAATCAATTA TTTTTAGAAG TGATGTTTAA TTTGCATTTT TTTTCCACCT   
  
  
+ AAAATCACGC ACTCGAACAT TAGGGGTTTG GCTAACTCAA AGACTCAAGG TAGTGACTTA TCCAAACTTA   
  
  
+ TTACATCCTA TAGATCTATA AAAACATAAA TCTCTTACAA TCAATCCAAA TTGATTCGGT CTAATTTGAT   
  
  
+ GACCCTGATG AGGGTCCTAC AATTACACAA GTGGGCCTTA GCCCCATGGC CGTTTTTTAA TCTTCATGGG   
  
  
+ TCCAGGCGGA GTTACCATCT CCAGGTCCAT TGAGGGTGTA TCACGTCTCA AATTACGAGT TTGCCTCTTT   
  
  
+ CTACGGGGAT TATAAATATC TACCTGCATA CCTCGAAAAG GAGGAGTTGA GATCAGCGAA ACTTGGCCAT   
  
  
+ CCCAGAGCAT TGAAATGCTC AGAACCCTTT TTCTAGAAAA ATATCGTTTT TCCTGAAAAT ATCCGACCTT   
  
  
+ ATGTCATTTA TTGTCGCTTT CTTGACTCTA ACCCGGGACT CCACACTAAC TTGACTATCG GAAAGGCGTT   
  
  
+ CCCTGGATCA CCATCCAAGA TAACCTCTTT TGCAGGGTTG ACGCTCGTTG GAAGAATCGT CGAATGTAGA   
  
  
+ GCCCCTATCA TCACATAAGC CCGCTCTTAC TTCATGCCAT CCTTGAATAG CAGTTTATCC CGAAGCAAAA   
  
  
+ ATAATTCTAA CTTTCTTATA TTGTAAACCC ACATCTAAAT TTAAATTCAT ACTCCAAAAT TCCGCCACGC   
  
  
+ AAATAAGGCT TTTTAATAGG CTTACATATA ATGGCATGCT ATAGAGACAC TGTTTTGAAG AAACTATTTA   
  
  
+ AACATCAGAC CTCATATTGA GTAGCAAATC AAGACTTCTT TCCTCCAGGC CATATTGAGT AGCAAATTAA   
  
  
+ GAATAACCAG AAGCTTGTGA AAAGAGTTAG AAAATAAAAG GTATAATAGA GACTTATGTT CTTAAGATAT   
  
  
+ CTCCTAGATA TGAATGTGAC CTCCTAGTCG ATATTTGTGC TGGATTAGCG AGTTGTGTGA CTGAGTTTGT   
  
  
+ ATGTGTCTCT TAGCATATGA GTTATTCTAG CTGCTTCTTT AAGCTATGTA GTGACTGTCT TCCTTCTTAT   
  
  
+ TTATAAGTGC TATGTCCAAC TAATAGCCGA AAAAAATGGT TCGCCTTTTC TGGTTTTCTT CTTTTTGAAG   
  
  
+ GTTGATTGGC TAATTTATAT AATGGTTTCA ATACTTATCC TTTATTGTTC CACAAATCCA CCTAACCACG   
  
  
+ TACCATTGAC AATAATTACC CACTAATTTC GGTTGGACTT GATTTCCTTT TGTTTGGTAC CTCAGGCATC   
  
  
+ TTCTGTGGAC CTTCATTCAC AGTTTAATTT TCACATTTTT ATAAGGCTGC TCTTGTGTTG CAAATTTTCC   
  
  
+ TATCCATTCC ACTCTTTGCC CATTCCCAGG CTAATTAGTG TTCTCTGTTC AACCATGGAT TCTGTGCTGG   
  
  
+ TTGATCCTGA GTTCATGAAA AATCTCTACA AATTCAAACC TGAATTGCTC TCAAACTTTT CAATGAATCC   
  
  
+ AAATGACGAC ATCTTTCAAG CCCTTCATTC AGAAAATGAT CCTTTACAGT TCCTCTCATT TGATGAAGGA   
  
  
+ ACCTGTCTTA ACAGCTGCAC TAGTCAACAA GTGCCAGATT TCCCTGATGC TTGTCTCAAG TTCATCAGTG   
  
  
+ ATATTCTTCT CGAAGAGGGT TTAGATGCAA ATCCTGCATC TGCACAGGCT CTCGAAGCCA CCGAGAAGTC   
  
  
+ CTTGTATGAT GCTCTCGGGC TTGGAGAGCC ATACCCCCTT TCATGTGATC ACTTTGCGCC ATCTATCTCT   
  
  
+ ACAAGTATTG AGAGCCCAGA TGACAGTTCT TCCAATAAAA GTTATAGCAG CAATCCCGAG ATAGATGGTT   
  
  
+ CTTATGCTAT CGCTGAGCCC AGTTTCGAGT CCAACCCCAA CTGTGTGCTT GATCAACCCC AGTTGAACTC   
  
  
+ CTTTCCAGCT CTACATGAGA TTTCTCGGTC CTTGGTGGAA CTGGGTTCTC AAGCCTCTGA GTTGAGCTTC   
  
  
+ GATGATGCAG GGAGTGCCCG TGTAGAGAAA AAGGGCAAAT CGATAAAGGG CTCGAGGAGG AAGAAGAGTC   
  
  
+ GTCAAAGAGA GGGTGAAGCG TGTTATGGAG GAAGGAGCCA TAAGGTTCAA GCTTCCTTCA ATGATGATTA   
  
  
+ CTATGAGATG GAACAGTATG ATGATGTAGT ATTGCTCTGT AATAATGAAC TAACGGGCAA TAGCCGTTTC   
  
  
+ AACACCGGGA AATCTTCACC TGAGGAGGGA TGGAGGAGAT TGCAGAGAAG CCGAGGAAAG AAGCAGAACA   
  
  
+ GTTTAGCAGT TGAAGTTGAT CTGATGACCC TGCTGACTCA GTGTGCACAA GCTGTATCGA GCTTTGATCT   
  
  
+ TCGAGGTGCA AACGAGCTAC TTAGGCAAAT CAGGCAGAAT GCTTCGCCCT ATGGTGGCAG CATCCAGAGG   
  
  
+ CTCGCCCATC ATGTAGCCAA TGCTCTCGAG GCACGTATAG CTGGCACAGG CTCTACAGTC TCTACTAACC   
  
  
+ TTGTTGATGC AAAGTTCTCA GCTTCTGACT TCCTAAAGGC TTACAGGTTA TATGTCTCAG CTGTTCCTTA   
  
  
+ CAAAAGGATG TCTTTCTTTC TTGCTAACTG CTCGATTGCA AAGTTGGCAG AGAAAGCAAC AAAGATCCAT   
  
  
+ ATCATTGATT TTGGTGTTTT CCTAGGTTTA CAATGGCCTT GTTTCATACA ACACCTATCA AAAAGGCCAA   
  
  
+ ATGGACCCCC AAAACTCCGA ATCACAGGAA TCGACTACCC CCAGCAGGGT TTCAGACCTG CACAAAGGGT   
  
  
+ TGAAGCTACA GGACACCGAT TATCTGGGTA CTGTGAGCGA TTTGGGGTGC CTTTTTCTTA TCAGGGCATT   
  
  
+ GCTCAGAAGT GGGAAACTAT TCAGCCGGAG GATCTCAAGA TCGAACAAGA CGAGCTGGTG ATTGTCAACT   
  
  
+ GTTTGTTCAG GTCAGGAACA CTGCTCGATG AGACAGTCGA AGCAAACAGT CCAAGAGATG CTTTCTTAGC   
  
  
+ TTTGGTTAGA AAGCTGAATC CCAGCCTATT CATTCACGGG GTTGTCAATG GCACATTCAA CGCTCCATTC   
  
  
+ TTCGTGACTC GATTCAGAGA GGCATTGTTT CATTATTCAT CAGTGTTTGA TGTGTCTGAA GAGACAATTC   
  
  
+ CACGAGATGC CCATGAGAGG TTCTTGATTG AGAGCGAGAT TTGTGGGAAA GAACTGTTCA ATGTGGTTGC   
  
  
+ TTGTGAGGGT GCAGAGAGGG TTCAAAGGCC TGAGACATAC AAGCAGTGGC AAGTGAGGAC AACGCGGGCC   
  
  
+ GGGTTAAGGC AGGTTGCCTT GGACCAGGAG CTTATGAAGG AAGCAACGGC AATGGTGAAG GCAAATTATC   
  
  
+ ATAAGGATTT TATGGTGGAT ATAAATAGGC ATTGGATGCT TCAAGGTTGG AAGGGTAGAA CCTTGTGTGC   
  
  
+ TCTCTCATTT TGGCAACCTG CCTG  

- +Up\_Stream \_Len000GATCCC CAACTCTCTT AGGCTATCGA TACGTCAGGT TTGTAATAGA GCAGTTCGTA   
  
  
- ATGATAAATT TATTAATTAT AACTTGAACT ATCAATGTGA AAAACTTATG ATTAAGGTGT GGAGTAACGG   
  
  
- AAATAAAGGT TTGGAGCCCA ACGTAAACCT GGTGTATGGT CCTTCGAACC TGCTGTTTTT AAAGAAAAGA   
  
  
- ACTCATAATA AAACAAAATA ATAATAATAA AAAATGAAAT AAACCTGGTG AAGTGAACCC ATTGTTCTAA   
  
  
- CTTATAAACT GATAATATAA TATACAGTGC ATACCTATAC CATCAACGAA TCTCGAAACT GGCAAACGTC   
  
  
- CAAATTCATA CTAAGGGAAC CTTTCCTCGG GGAGAAATAG AATTAAGTCT TCATTGAAAA CAAAAAAATC   
  
  
- TATTTAACTT TTCTTTTTAT TATTTACAGA ATCAACTCAA ACCAATAAGT TAGGCTTTAA ACTAGGACGA   
  
  
- ATTACATATC ATATATTATG ATTATGCGTA CATTGTGTTG TGGAGTCTAC CAGTAACAAA ATGTTAAAAA   
  
  
- AACTCAAATC ATATTTTTAT TTTAGTTAAT AAAAATCTTC ACTACAAATT AAACGTAAAA AAAAGGTGGA   
  
  
- TTTTAGTGCG TGAGCTTGTA ATCCCCAAAC CGATTGAGTT TCTGAGTTCC ATCACTGAAT AGGTTTGAAT   
  
  
- AATGTAGGAT ATCTAGATAT TTTTGTATTT AGAGAATGTT AGTTAGGTTT AACTAAGCCA GATTAAACTA   
  
  
- CTGGGACTAC TCCCAGGATG TTAATGTGTT CACCCGGAAT CGGGGTACCG GCAAAAAATT AGAAGTACCC   
  
  
- AGGTCCGCCT CAATGGTAGA GGTCCAGGTA ACTCCCACAT AGTGCAGAGT TTAATGCTCA AACGGAGAAA   
  
  
- GATGCCCCTA ATATTTATAG ATGGACGTAT GGAGCTTTTC CTCCTCAACT CTAGTCGCTT TGAACCGGTA   
  
  
- GGGTCTCGTA ACTTTACGAG TCTTGGGAAA AAGATCTTTT TATAGCAAAA AGGACTTTTA TAGGCTGGAA   
  
  
- TACAGTAAAT AACAGCGAAA GAACTGAGAT TGGGCCCTGA GGTGTGATTG AACTGATAGC CTTTCCGCAA   
  
  
- GGGACCTAGT GGTAGGTTCT ATTGGAGAAA ACGTCCCAAC TGCGAGCAAC CTTCTTAGCA GCTTACATCT   
  
  
- CGGGGATAGT AGTGTATTCG GGCGAGAATG AAGTACGGTA GGAACTTATC GTCAAATAGG GCTTCGTTTT   
  
  
- TATTAAGATT GAAAGAATAT AACATTTGGG TGTAGATTTA AATTTAAGTA TGAGGTTTTA AGGCGGTGCG   
  
  
- TTTATTCCGA AAAATTATCC GAATGTATAT TACCGTACGA TATCTCTGTG ACAAAACTTC TTTGATAAAT   
  
  
- TTGTAGTCTG GAGTATAACT CATCGTTTAG TTCTGAAGAA AGGAGGTCCG GTATAACTCA TCGTTTAATT   
  
  
- CTTATTGGTC TTCGAACACT TTTCTCAATC TTTTATTTTC CATATTATCT CTGAATACAA GAATTCTATA   
  
  
- GAGGATCTAT ACTTACACTG GAGGATCAGC TATAAACACG ACCTAATCGC TCAACACACT GACTCAAACA   
  
  
- TACACAGAGA ATCGTATACT CAATAAGATC GACGAAGAAA TTCGATACAT CACTGACAGA AGGAAGAATA   
  
  
- AATATTCACG ATACAGGTTG ATTATCGGCT TTTTTTACCA AGCGGAAAAG ACCAAAAGAA GAAAAACTTC   
  
  
- CAACTAACCG ATTAAATATA TTACCAAAGT TATGAATAGG AAATAACAAG GTGTTTAGGT GGATTGGTGC   
  
  
- ATGGTAACTG TTATTAATGG GTGATTAAAG CCAACCTGAA CTAAAGGAAA ACAAACCATG GAGTCCGTAG   
  
  
- AAGACACCTG GAAGTAAGTG TCAAATTAAA AGTGTAAAAA TATTCCGACG AGAACACAAC GTTTAAAAGG   
  
  
- ATAGGTAAGG TGAGAAACGG GTAAGGGTCC GATTAATCAC AAGAGACAAG TTGGTACCTA AGACACGACC   
  
  
- AACTAGGACT CAAGTACTTT TTAGAGATGT TTAAGTTTGG ACTTAACGAG AGTTTGAAAA GTTACTTAGG   
  
  
- TTTACTGCTG TAGAAAGTTC GGGAAGTAAG TCTTTTACTA GGAAATGTCA AGGAGAGTAA ACTACTTCCT   
  
  
- TGGACAGAAT TGTCGACGTG ATCAGTTGTT CACGGTCTAA AGGGACTACG AACAGAGTTC AAGTAGTCAC   
  
  
- TATAAGAAGA GCTTCTCCCA AATCTACGTT TAGGACGTAG ACGTGTCCGA GAGCTTCGGT GGCTCTTCAG   
  
  
- GAACATACTA CGAGAGCCCG AACCTCTCGG TATGGGGGAA AGTACACTAG TGAAACGCGG TAGATAGAGA   
  
  
- TGTTCATAAC TCTCGGGTCT ACTGTCAAGA AGGTTATTTT CAATATCGTC GTTAGGGCTC TATCTACCAA   
  
  
- GAATACGATA GCGACTCGGG TCAAAGCTCA GGTTGGGGTT GACACACGAA CTAGTTGGGG TCAACTTGAG   
  
  
- GAAAGGTCGA GATGTACTCT AAAGAGCCAG GAACCACCTT GACCCAAGAG TTCGGAGACT CAACTCGAAG   
  
  
- CTACTACGTC CCTCACGGGC ACATCTCTTT TTCCCGTTTA GCTATTTCCC GAGCTCCTCC TTCTTCTCAG   
  
  
- CAGTTTCTCT CCCACTTCGC ACAATACCTC CTTCCTCGGT ATTCCAAGTT CGAAGGAAGT TACTACTAAT   
  
  
- GATACTCTAC CTTGTCATAC TACTACATCA TAACGAGACA TTATTACTTG ATTGCCCGTT ATCGGCAAAG   
  
  
- TTGTGGCCCT TTAGAAGTGG ACTCCTCCCT ACCTCCTCTA ACGTCTCTTC GGCTCCTTTC TTCGTCTTGT   
  
  
- CAAATCGTCA ACTTCAACTA GACTACTGGG ACGACTGAGT CACACGTGTT CGACATAGCT CGAAACTAGA   
  
  
- AGCTCCACGT TTGCTCGATG AATCCGTTTA GTCCGTCTTA CGAAGCGGGA TACCACCGTC GTAGGTCTCC   
  
  
- GAGCGGGTAG TACATCGGTT ACGAGAGCTC CGTGCATATC GACCGTGTCC GAGATGTCAG AGATGATTGG   
  
  
- AACAACTACG TTTCAAGAGT CGAAGACTGA AGGATTTCCG AATGTCCAAT ATACAGAGTC GACAAGGAAT   
  
  
- GTTTTCCTAC AGAAAGAAAG AACGATTGAC GAGCTAACGT TTCAACCGTC TCTTTCGTTG TTTCTAGGTA   
  
  
- TAGTAACTAA AACCACAAAA GGATCCAAAT GTTACCGGAA CAAAGTATGT TGTGGATAGT TTTTCCGGTT   
  
  
- TACCTGGGGG TTTTGAGGCT TAGTGTCCTT AGCTGATGGG GGTCGTCCCA AAGTCTGGAC GTGTTTCCCA   
  
  
- ACTTCGATGT CCTGTGGCTA ATAGACCCAT GACACTCGCT AAACCCCACG GAAAAAGAAT AGTCCCGTAA   
  
  
- CGAGTCTTCA CCCTTTGATA AGTCGGCCTC CTAGAGTTCT AGCTTGTTCT GCTCGACCAC TAACAGTTGA   
  
  
- CAAACAAGTC CAGTCCTTGT GACGAGCTAC TCTGTCAGCT TCGTTTGTCA GGTTCTCTAC GAAAGAATCG   
  
  
- AAACCAATCT TTCGACTTAG GGTCGGATAA GTAAGTGCCC CAACAGTTAC CGTGTAAGTT GCGAGGTAAG   
  
  
- AAGCACTGAG CTAAGTCTCT CCGTAACAAA GTAATAAGTA GTCACAAACT ACACAGACTT CTCTGTTAAG   
  
  
- GTGCTCTACG GGTACTCTCC AAGAACTAAC TCTCGCTCTA AACACCCTTT CTTGACAAGT TACACCAACG   
  
  
- AACACTCCCA CGTCTCTCCC AAGTTTCCGG ACTCTGTATG TTCGTCACCG TTCACTCCTG TTGCGCCCGG   
  
  
- CCCAATTCCG TCCAACGGAA CCTGGTCCTC GAATACTTCC TTCGTTGCCG TTACCACTTC CGTTTAATAG   
  
  
- TATTCCTAAA ATACCACCTA TATTTATCCG TAACCTACGA AGTTCCAACC TTCCCATCTT GGAACACACG   
  
  
- AGAGAGTAAA ACCGTTGGAC GGAC

+     P-box

| Site Name | Organism | Position | Strand | Matrix score. | sequence | function |
| --- | --- | --- | --- | --- | --- | --- |
| P-box | Oryza sativa | 1870 | + | 7 | CCTTTTG | gibberellin-responsive element |
| P-box | Oryza sativa | 3155 | - | 7 | CCTTTTG | gibberellin-responsive element |

>HU02G01571.1   
+ +Up\_Stream \_Len000CTAGGG GTTGAGAGAA TCCGATAGCT ATGCAGTCCA AACATTATCT CGTCAAGCAT   
  
  
+ TACTATTTAA ATAATTAATA TTGAACTTGA TAGTTACACT TTTTGAATAC TAATTCCACA CCTCATTGCC   
  
  
+ TTTATTTCCA AACCTCGGGT TGCATTTGGA CCACATACCA GGAAGCTTGG ACGACAAAAA TTTCTTTTCT   
  
  
+ TGAGTATTAT TTTGTTTTAT TATTATTATT TTTTACTTTA TTTGGACCAC TTCACTTGGG TAACAAGATT   
  
  
+ GAATATTTGA CTATTATATT ATATGTCACG TATGGATATG GTAGTTGCTT AGAGCTTTGA CCGTTTGCAG   
  
  
+ GTTTAAGTAT GATTCCCTTG GAAAGGAGCC CCTCTTTATC TTAATTCAGA AGTAACTTTT GTTTTTTTAG   
  
  
+ ATAAATTGAA AAGAAAAATA ATAAATGTCT TAGTTGAGTT TGGTTATTCA ATCCGAAATT TGATCCTGCT   
  
  
+ TAATGTATAG TATATAATAC TAATACGCAT GTAACACAAC ACCTCAGATG GTCATTGTTT TACAATTTTT   
  
  
+ TTGAGTTTAG TATAAAAATA AAATCAATTA TTTTTAGAAG TGATGTTTAA TTTGCATTTT TTTTCCACCT   
  
  
+ AAAATCACGC ACTCGAACAT TAGGGGTTTG GCTAACTCAA AGACTCAAGG TAGTGACTTA TCCAAACTTA   
  
  
+ TTACATCCTA TAGATCTATA AAAACATAAA TCTCTTACAA TCAATCCAAA TTGATTCGGT CTAATTTGAT   
  
  
+ GACCCTGATG AGGGTCCTAC AATTACACAA GTGGGCCTTA GCCCCATGGC CGTTTTTTAA TCTTCATGGG   
  
  
+ TCCAGGCGGA GTTACCATCT CCAGGTCCAT TGAGGGTGTA TCACGTCTCA AATTACGAGT TTGCCTCTTT   
  
  
+ CTACGGGGAT TATAAATATC TACCTGCATA CCTCGAAAAG GAGGAGTTGA GATCAGCGAA ACTTGGCCAT   
  
  
+ CCCAGAGCAT TGAAATGCTC AGAACCCTTT TTCTAGAAAA ATATCGTTTT TCCTGAAAAT ATCCGACCTT   
  
  
+ ATGTCATTTA TTGTCGCTTT CTTGACTCTA ACCCGGGACT CCACACTAAC TTGACTATCG GAAAGGCGTT   
  
  
+ CCCTGGATCA CCATCCAAGA TAACCTCTTT TGCAGGGTTG ACGCTCGTTG GAAGAATCGT CGAATGTAGA   
  
  
+ GCCCCTATCA TCACATAAGC CCGCTCTTAC TTCATGCCAT CCTTGAATAG CAGTTTATCC CGAAGCAAAA   
  
  
+ ATAATTCTAA CTTTCTTATA TTGTAAACCC ACATCTAAAT TTAAATTCAT ACTCCAAAAT TCCGCCACGC   
  
  
+ AAATAAGGCT TTTTAATAGG CTTACATATA ATGGCATGCT ATAGAGACAC TGTTTTGAAG AAACTATTTA   
  
  
+ AACATCAGAC CTCATATTGA GTAGCAAATC AAGACTTCTT TCCTCCAGGC CATATTGAGT AGCAAATTAA   
  
  
+ GAATAACCAG AAGCTTGTGA AAAGAGTTAG AAAATAAAAG GTATAATAGA GACTTATGTT CTTAAGATAT   
  
  
+ CTCCTAGATA TGAATGTGAC CTCCTAGTCG ATATTTGTGC TGGATTAGCG AGTTGTGTGA CTGAGTTTGT   
  
  
+ ATGTGTCTCT TAGCATATGA GTTATTCTAG CTGCTTCTTT AAGCTATGTA GTGACTGTCT TCCTTCTTAT   
  
  
+ TTATAAGTGC TATGTCCAAC TAATAGCCGA AAAAAATGGT TCGCCTTTTC TGGTTTTCTT CTTTTTGAAG   
  
  
+ GTTGATTGGC TAATTTATAT AATGGTTTCA ATACTTATCC TTTATTGTTC CACAAATCCA CCTAACCACG   
  
  
+ TACCATTGAC AATAATTACC CACTAATTTC GGTTGGACTT GATTTCCTTT TGTTTGGTAC CTCAGGCATC   
  
  
+ TTCTGTGGAC CTTCATTCAC AGTTTAATTT TCACATTTTT ATAAGGCTGC TCTTGTGTTG CAAATTTTCC   
  
  
+ TATCCATTCC ACTCTTTGCC CATTCCCAGG CTAATTAGTG TTCTCTGTTC AACCATGGAT TCTGTGCTGG   
  
  
+ TTGATCCTGA GTTCATGAAA AATCTCTACA AATTCAAACC TGAATTGCTC TCAAACTTTT CAATGAATCC   
  
  
+ AAATGACGAC ATCTTTCAAG CCCTTCATTC AGAAAATGAT CCTTTACAGT TCCTCTCATT TGATGAAGGA   
  
  
+ ACCTGTCTTA ACAGCTGCAC TAGTCAACAA GTGCCAGATT TCCCTGATGC TTGTCTCAAG TTCATCAGTG   
  
  
+ ATATTCTTCT CGAAGAGGGT TTAGATGCAA ATCCTGCATC TGCACAGGCT CTCGAAGCCA CCGAGAAGTC   
  
  
+ CTTGTATGAT GCTCTCGGGC TTGGAGAGCC ATACCCCCTT TCATGTGATC ACTTTGCGCC ATCTATCTCT   
  
  
+ ACAAGTATTG AGAGCCCAGA TGACAGTTCT TCCAATAAAA GTTATAGCAG CAATCCCGAG ATAGATGGTT   
  
  
+ CTTATGCTAT CGCTGAGCCC AGTTTCGAGT CCAACCCCAA CTGTGTGCTT GATCAACCCC AGTTGAACTC   
  
  
+ CTTTCCAGCT CTACATGAGA TTTCTCGGTC CTTGGTGGAA CTGGGTTCTC AAGCCTCTGA GTTGAGCTTC   
  
  
+ GATGATGCAG GGAGTGCCCG TGTAGAGAAA AAGGGCAAAT CGATAAAGGG CTCGAGGAGG AAGAAGAGTC   
  
  
+ GTCAAAGAGA GGGTGAAGCG TGTTATGGAG GAAGGAGCCA TAAGGTTCAA GCTTCCTTCA ATGATGATTA   
  
  
+ CTATGAGATG GAACAGTATG ATGATGTAGT ATTGCTCTGT AATAATGAAC TAACGGGCAA TAGCCGTTTC   
  
  
+ AACACCGGGA AATCTTCACC TGAGGAGGGA TGGAGGAGAT TGCAGAGAAG CCGAGGAAAG AAGCAGAACA   
  
  
+ GTTTAGCAGT TGAAGTTGAT CTGATGACCC TGCTGACTCA GTGTGCACAA GCTGTATCGA GCTTTGATCT   
  
  
+ TCGAGGTGCA AACGAGCTAC TTAGGCAAAT CAGGCAGAAT GCTTCGCCCT ATGGTGGCAG CATCCAGAGG   
  
  
+ CTCGCCCATC ATGTAGCCAA TGCTCTCGAG GCACGTATAG CTGGCACAGG CTCTACAGTC TCTACTAACC   
  
  
+ TTGTTGATGC AAAGTTCTCA GCTTCTGACT TCCTAAAGGC TTACAGGTTA TATGTCTCAG CTGTTCCTTA   
  
  
+ CAAAAGGATG TCTTTCTTTC TTGCTAACTG CTCGATTGCA AAGTTGGCAG AGAAAGCAAC AAAGATCCAT   
  
  
+ ATCATTGATT TTGGTGTTTT CCTAGGTTTA CAATGGCCTT GTTTCATACA ACACCTATCA AAAAGGCCAA   
  
  
+ ATGGACCCCC AAAACTCCGA ATCACAGGAA TCGACTACCC CCAGCAGGGT TTCAGACCTG CACAAAGGGT   
  
  
+ TGAAGCTACA GGACACCGAT TATCTGGGTA CTGTGAGCGA TTTGGGGTGC CTTTTTCTTA TCAGGGCATT   
  
  
+ GCTCAGAAGT GGGAAACTAT TCAGCCGGAG GATCTCAAGA TCGAACAAGA CGAGCTGGTG ATTGTCAACT   
  
  
+ GTTTGTTCAG GTCAGGAACA CTGCTCGATG AGACAGTCGA AGCAAACAGT CCAAGAGATG CTTTCTTAGC   
  
  
+ TTTGGTTAGA AAGCTGAATC CCAGCCTATT CATTCACGGG GTTGTCAATG GCACATTCAA CGCTCCATTC   
  
  
+ TTCGTGACTC GATTCAGAGA GGCATTGTTT CATTATTCAT CAGTGTTTGA TGTGTCTGAA GAGACAATTC   
  
  
+ CACGAGATGC CCATGAGAGG TTCTTGATTG AGAGCGAGAT TTGTGGGAAA GAACTGTTCA ATGTGGTTGC   
  
  
+ TTGTGAGGGT GCAGAGAGGG TTCAAAGGCC TGAGACATAC AAGCAGTGGC AAGTGAGGAC AACGCGGGCC   
  
  
+ GGGTTAAGGC AGGTTGCCTT GGACCAGGAG CTTATGAAGG AAGCAACGGC AATGGTGAAG GCAAATTATC   
  
  
+ ATAAGGATTT TATGGTGGAT ATAAATAGGC ATTGGATGCT TCAAGGTTGG AAGGGTAGAA CCTTGTGTGC   
  
  
+ TCTCTCATTT TGGCAACCTG CCTG  

- +Up\_Stream \_Len000GATCCC CAACTCTCTT AGGCTATCGA TACGTCAGGT TTGTAATAGA GCAGTTCGTA   
  
  
- ATGATAAATT TATTAATTAT AACTTGAACT ATCAATGTGA AAAACTTATG ATTAAGGTGT GGAGTAACGG   
  
  
- AAATAAAGGT TTGGAGCCCA ACGTAAACCT GGTGTATGGT CCTTCGAACC TGCTGTTTTT AAAGAAAAGA   
  
  
- ACTCATAATA AAACAAAATA ATAATAATAA AAAATGAAAT AAACCTGGTG AAGTGAACCC ATTGTTCTAA   
  
  
- CTTATAAACT GATAATATAA TATACAGTGC ATACCTATAC CATCAACGAA TCTCGAAACT GGCAAACGTC   
  
  
- CAAATTCATA CTAAGGGAAC CTTTCCTCGG GGAGAAATAG AATTAAGTCT TCATTGAAAA CAAAAAAATC   
  
  
- TATTTAACTT TTCTTTTTAT TATTTACAGA ATCAACTCAA ACCAATAAGT TAGGCTTTAA ACTAGGACGA   
  
  
- ATTACATATC ATATATTATG ATTATGCGTA CATTGTGTTG TGGAGTCTAC CAGTAACAAA ATGTTAAAAA   
  
  
- AACTCAAATC ATATTTTTAT TTTAGTTAAT AAAAATCTTC ACTACAAATT AAACGTAAAA AAAAGGTGGA   
  
  
- TTTTAGTGCG TGAGCTTGTA ATCCCCAAAC CGATTGAGTT TCTGAGTTCC ATCACTGAAT AGGTTTGAAT   
  
  
- AATGTAGGAT ATCTAGATAT TTTTGTATTT AGAGAATGTT AGTTAGGTTT AACTAAGCCA GATTAAACTA   
  
  
- CTGGGACTAC TCCCAGGATG TTAATGTGTT CACCCGGAAT CGGGGTACCG GCAAAAAATT AGAAGTACCC   
  
  
- AGGTCCGCCT CAATGGTAGA GGTCCAGGTA ACTCCCACAT AGTGCAGAGT TTAATGCTCA AACGGAGAAA   
  
  
- GATGCCCCTA ATATTTATAG ATGGACGTAT GGAGCTTTTC CTCCTCAACT CTAGTCGCTT TGAACCGGTA   
  
  
- GGGTCTCGTA ACTTTACGAG TCTTGGGAAA AAGATCTTTT TATAGCAAAA AGGACTTTTA TAGGCTGGAA   
  
  
- TACAGTAAAT AACAGCGAAA GAACTGAGAT TGGGCCCTGA GGTGTGATTG AACTGATAGC CTTTCCGCAA   
  
  
- GGGACCTAGT GGTAGGTTCT ATTGGAGAAA ACGTCCCAAC TGCGAGCAAC CTTCTTAGCA GCTTACATCT   
  
  
- CGGGGATAGT AGTGTATTCG GGCGAGAATG AAGTACGGTA GGAACTTATC GTCAAATAGG GCTTCGTTTT   
  
  
- TATTAAGATT GAAAGAATAT AACATTTGGG TGTAGATTTA AATTTAAGTA TGAGGTTTTA AGGCGGTGCG   
  
  
- TTTATTCCGA AAAATTATCC GAATGTATAT TACCGTACGA TATCTCTGTG ACAAAACTTC TTTGATAAAT   
  
  
- TTGTAGTCTG GAGTATAACT CATCGTTTAG TTCTGAAGAA AGGAGGTCCG GTATAACTCA TCGTTTAATT   
  
  
- CTTATTGGTC TTCGAACACT TTTCTCAATC TTTTATTTTC CATATTATCT CTGAATACAA GAATTCTATA   
  
  
- GAGGATCTAT ACTTACACTG GAGGATCAGC TATAAACACG ACCTAATCGC TCAACACACT GACTCAAACA   
  
  
- TACACAGAGA ATCGTATACT CAATAAGATC GACGAAGAAA TTCGATACAT CACTGACAGA AGGAAGAATA   
  
  
- AATATTCACG ATACAGGTTG ATTATCGGCT TTTTTTACCA AGCGGAAAAG ACCAAAAGAA GAAAAACTTC   
  
  
- CAACTAACCG ATTAAATATA TTACCAAAGT TATGAATAGG AAATAACAAG GTGTTTAGGT GGATTGGTGC   
  
  
- ATGGTAACTG TTATTAATGG GTGATTAAAG CCAACCTGAA CTAAAGGAAA ACAAACCATG GAGTCCGTAG   
  
  
- AAGACACCTG GAAGTAAGTG TCAAATTAAA AGTGTAAAAA TATTCCGACG AGAACACAAC GTTTAAAAGG   
  
  
- ATAGGTAAGG TGAGAAACGG GTAAGGGTCC GATTAATCAC AAGAGACAAG TTGGTACCTA AGACACGACC   
  
  
- AACTAGGACT CAAGTACTTT TTAGAGATGT TTAAGTTTGG ACTTAACGAG AGTTTGAAAA GTTACTTAGG   
  
  
- TTTACTGCTG TAGAAAGTTC GGGAAGTAAG TCTTTTACTA GGAAATGTCA AGGAGAGTAA ACTACTTCCT   
  
  
- TGGACAGAAT TGTCGACGTG ATCAGTTGTT CACGGTCTAA AGGGACTACG AACAGAGTTC AAGTAGTCAC   
  
  
- TATAAGAAGA GCTTCTCCCA AATCTACGTT TAGGACGTAG ACGTGTCCGA GAGCTTCGGT GGCTCTTCAG   
  
  
- GAACATACTA CGAGAGCCCG AACCTCTCGG TATGGGGGAA AGTACACTAG TGAAACGCGG TAGATAGAGA   
  
  
- TGTTCATAAC TCTCGGGTCT ACTGTCAAGA AGGTTATTTT CAATATCGTC GTTAGGGCTC TATCTACCAA   
  
  
- GAATACGATA GCGACTCGGG TCAAAGCTCA GGTTGGGGTT GACACACGAA CTAGTTGGGG TCAACTTGAG   
  
  
- GAAAGGTCGA GATGTACTCT AAAGAGCCAG GAACCACCTT GACCCAAGAG TTCGGAGACT CAACTCGAAG   
  
  
- CTACTACGTC CCTCACGGGC ACATCTCTTT TTCCCGTTTA GCTATTTCCC GAGCTCCTCC TTCTTCTCAG   
  
  
- CAGTTTCTCT CCCACTTCGC ACAATACCTC CTTCCTCGGT ATTCCAAGTT CGAAGGAAGT TACTACTAAT   
  
  
- GATACTCTAC CTTGTCATAC TACTACATCA TAACGAGACA TTATTACTTG ATTGCCCGTT ATCGGCAAAG   
  
  
- TTGTGGCCCT TTAGAAGTGG ACTCCTCCCT ACCTCCTCTA ACGTCTCTTC GGCTCCTTTC TTCGTCTTGT   
  
  
- CAAATCGTCA ACTTCAACTA GACTACTGGG ACGACTGAGT CACACGTGTT CGACATAGCT CGAAACTAGA   
  
  
- AGCTCCACGT TTGCTCGATG AATCCGTTTA GTCCGTCTTA CGAAGCGGGA TACCACCGTC GTAGGTCTCC   
  
  
- GAGCGGGTAG TACATCGGTT ACGAGAGCTC CGTGCATATC GACCGTGTCC GAGATGTCAG AGATGATTGG   
  
  
- AACAACTACG TTTCAAGAGT CGAAGACTGA AGGATTTCCG AATGTCCAAT ATACAGAGTC GACAAGGAAT   
  
  
- GTTTTCCTAC AGAAAGAAAG AACGATTGAC GAGCTAACGT TTCAACCGTC TCTTTCGTTG TTTCTAGGTA   
  
  
- TAGTAACTAA AACCACAAAA GGATCCAAAT GTTACCGGAA CAAAGTATGT TGTGGATAGT TTTTCCGGTT   
  
  
- TACCTGGGGG TTTTGAGGCT TAGTGTCCTT AGCTGATGGG GGTCGTCCCA AAGTCTGGAC GTGTTTCCCA   
  
  
- ACTTCGATGT CCTGTGGCTA ATAGACCCAT GACACTCGCT AAACCCCACG GAAAAAGAAT AGTCCCGTAA   
  
  
- CGAGTCTTCA CCCTTTGATA AGTCGGCCTC CTAGAGTTCT AGCTTGTTCT GCTCGACCAC TAACAGTTGA   
  
  
- CAAACAAGTC CAGTCCTTGT GACGAGCTAC TCTGTCAGCT TCGTTTGTCA GGTTCTCTAC GAAAGAATCG   
  
  
- AAACCAATCT TTCGACTTAG GGTCGGATAA GTAAGTGCCC CAACAGTTAC CGTGTAAGTT GCGAGGTAAG   
  
  
- AAGCACTGAG CTAAGTCTCT CCGTAACAAA GTAATAAGTA GTCACAAACT ACACAGACTT CTCTGTTAAG   
  
  
- GTGCTCTACG GGTACTCTCC AAGAACTAAC TCTCGCTCTA AACACCCTTT CTTGACAAGT TACACCAACG   
  
  
- AACACTCCCA CGTCTCTCCC AAGTTTCCGG ACTCTGTATG TTCGTCACCG TTCACTCCTG TTGCGCCCGG   
  
  
- CCCAATTCCG TCCAACGGAA CCTGGTCCTC GAATACTTCC TTCGTTGCCG TTACCACTTC CGTTTAATAG   
  
  
- TATTCCTAAA ATACCACCTA TATTTATCCG TAACCTACGA AGTTCCAACC TTCCCATCTT GGAACACACG   
  
  
- AGAGAGTAAA ACCGTTGGAC GGAC

+     STRE

| Site Name | Organism | Position | Strand | Matrix score. | sequence | function |
| --- | --- | --- | --- | --- | --- | --- |
| STRE | Arabidopsis thaliana | 1196 | - | 5 | AGGGG |  |
| STRE | Arabidopsis thaliana | 656 | + | 5 | AGGGG |  |
| STRE | Arabidopsis thaliana | 383 | - | 5 | AGGGG |  |
| STRE | Arabidopsis thaliana | 21 | + | 5 | AGGGG |  |
| STRE | Arabidopsis thaliana | 2349 | - | 5 | AGGGG |  |

>HU02G01571.1   
+ +Up\_Stream \_Len000CTAGGG GTTGAGAGAA TCCGATAGCT ATGCAGTCCA AACATTATCT CGTCAAGCAT   
  
  
+ TACTATTTAA ATAATTAATA TTGAACTTGA TAGTTACACT TTTTGAATAC TAATTCCACA CCTCATTGCC   
  
  
+ TTTATTTCCA AACCTCGGGT TGCATTTGGA CCACATACCA GGAAGCTTGG ACGACAAAAA TTTCTTTTCT   
  
  
+ TGAGTATTAT TTTGTTTTAT TATTATTATT TTTTACTTTA TTTGGACCAC TTCACTTGGG TAACAAGATT   
  
  
+ GAATATTTGA CTATTATATT ATATGTCACG TATGGATATG GTAGTTGCTT AGAGCTTTGA CCGTTTGCAG   
  
  
+ GTTTAAGTAT GATTCCCTTG GAAAGGAGCC CCTCTTTATC TTAATTCAGA AGTAACTTTT GTTTTTTTAG   
  
  
+ ATAAATTGAA AAGAAAAATA ATAAATGTCT TAGTTGAGTT TGGTTATTCA ATCCGAAATT TGATCCTGCT   
  
  
+ TAATGTATAG TATATAATAC TAATACGCAT GTAACACAAC ACCTCAGATG GTCATTGTTT TACAATTTTT   
  
  
+ TTGAGTTTAG TATAAAAATA AAATCAATTA TTTTTAGAAG TGATGTTTAA TTTGCATTTT TTTTCCACCT   
  
  
+ AAAATCACGC ACTCGAACAT TAGGGGTTTG GCTAACTCAA AGACTCAAGG TAGTGACTTA TCCAAACTTA   
  
  
+ TTACATCCTA TAGATCTATA AAAACATAAA TCTCTTACAA TCAATCCAAA TTGATTCGGT CTAATTTGAT   
  
  
+ GACCCTGATG AGGGTCCTAC AATTACACAA GTGGGCCTTA GCCCCATGGC CGTTTTTTAA TCTTCATGGG   
  
  
+ TCCAGGCGGA GTTACCATCT CCAGGTCCAT TGAGGGTGTA TCACGTCTCA AATTACGAGT TTGCCTCTTT   
  
  
+ CTACGGGGAT TATAAATATC TACCTGCATA CCTCGAAAAG GAGGAGTTGA GATCAGCGAA ACTTGGCCAT   
  
  
+ CCCAGAGCAT TGAAATGCTC AGAACCCTTT TTCTAGAAAA ATATCGTTTT TCCTGAAAAT ATCCGACCTT   
  
  
+ ATGTCATTTA TTGTCGCTTT CTTGACTCTA ACCCGGGACT CCACACTAAC TTGACTATCG GAAAGGCGTT   
  
  
+ CCCTGGATCA CCATCCAAGA TAACCTCTTT TGCAGGGTTG ACGCTCGTTG GAAGAATCGT CGAATGTAGA   
  
  
+ GCCCCTATCA TCACATAAGC CCGCTCTTAC TTCATGCCAT CCTTGAATAG CAGTTTATCC CGAAGCAAAA   
  
  
+ ATAATTCTAA CTTTCTTATA TTGTAAACCC ACATCTAAAT TTAAATTCAT ACTCCAAAAT TCCGCCACGC   
  
  
+ AAATAAGGCT TTTTAATAGG CTTACATATA ATGGCATGCT ATAGAGACAC TGTTTTGAAG AAACTATTTA   
  
  
+ AACATCAGAC CTCATATTGA GTAGCAAATC AAGACTTCTT TCCTCCAGGC CATATTGAGT AGCAAATTAA   
  
  
+ GAATAACCAG AAGCTTGTGA AAAGAGTTAG AAAATAAAAG GTATAATAGA GACTTATGTT CTTAAGATAT   
  
  
+ CTCCTAGATA TGAATGTGAC CTCCTAGTCG ATATTTGTGC TGGATTAGCG AGTTGTGTGA CTGAGTTTGT   
  
  
+ ATGTGTCTCT TAGCATATGA GTTATTCTAG CTGCTTCTTT AAGCTATGTA GTGACTGTCT TCCTTCTTAT   
  
  
+ TTATAAGTGC TATGTCCAAC TAATAGCCGA AAAAAATGGT TCGCCTTTTC TGGTTTTCTT CTTTTTGAAG   
  
  
+ GTTGATTGGC TAATTTATAT AATGGTTTCA ATACTTATCC TTTATTGTTC CACAAATCCA CCTAACCACG   
  
  
+ TACCATTGAC AATAATTACC CACTAATTTC GGTTGGACTT GATTTCCTTT TGTTTGGTAC CTCAGGCATC   
  
  
+ TTCTGTGGAC CTTCATTCAC AGTTTAATTT TCACATTTTT ATAAGGCTGC TCTTGTGTTG CAAATTTTCC   
  
  
+ TATCCATTCC ACTCTTTGCC CATTCCCAGG CTAATTAGTG TTCTCTGTTC AACCATGGAT TCTGTGCTGG   
  
  
+ TTGATCCTGA GTTCATGAAA AATCTCTACA AATTCAAACC TGAATTGCTC TCAAACTTTT CAATGAATCC   
  
  
+ AAATGACGAC ATCTTTCAAG CCCTTCATTC AGAAAATGAT CCTTTACAGT TCCTCTCATT TGATGAAGGA   
  
  
+ ACCTGTCTTA ACAGCTGCAC TAGTCAACAA GTGCCAGATT TCCCTGATGC TTGTCTCAAG TTCATCAGTG   
  
  
+ ATATTCTTCT CGAAGAGGGT TTAGATGCAA ATCCTGCATC TGCACAGGCT CTCGAAGCCA CCGAGAAGTC   
  
  
+ CTTGTATGAT GCTCTCGGGC TTGGAGAGCC ATACCCCCTT TCATGTGATC ACTTTGCGCC ATCTATCTCT   
  
  
+ ACAAGTATTG AGAGCCCAGA TGACAGTTCT TCCAATAAAA GTTATAGCAG CAATCCCGAG ATAGATGGTT   
  
  
+ CTTATGCTAT CGCTGAGCCC AGTTTCGAGT CCAACCCCAA CTGTGTGCTT GATCAACCCC AGTTGAACTC   
  
  
+ CTTTCCAGCT CTACATGAGA TTTCTCGGTC CTTGGTGGAA CTGGGTTCTC AAGCCTCTGA GTTGAGCTTC   
  
  
+ GATGATGCAG GGAGTGCCCG TGTAGAGAAA AAGGGCAAAT CGATAAAGGG CTCGAGGAGG AAGAAGAGTC   
  
  
+ GTCAAAGAGA GGGTGAAGCG TGTTATGGAG GAAGGAGCCA TAAGGTTCAA GCTTCCTTCA ATGATGATTA   
  
  
+ CTATGAGATG GAACAGTATG ATGATGTAGT ATTGCTCTGT AATAATGAAC TAACGGGCAA TAGCCGTTTC   
  
  
+ AACACCGGGA AATCTTCACC TGAGGAGGGA TGGAGGAGAT TGCAGAGAAG CCGAGGAAAG AAGCAGAACA   
  
  
+ GTTTAGCAGT TGAAGTTGAT CTGATGACCC TGCTGACTCA GTGTGCACAA GCTGTATCGA GCTTTGATCT   
  
  
+ TCGAGGTGCA AACGAGCTAC TTAGGCAAAT CAGGCAGAAT GCTTCGCCCT ATGGTGGCAG CATCCAGAGG   
  
  
+ CTCGCCCATC ATGTAGCCAA TGCTCTCGAG GCACGTATAG CTGGCACAGG CTCTACAGTC TCTACTAACC   
  
  
+ TTGTTGATGC AAAGTTCTCA GCTTCTGACT TCCTAAAGGC TTACAGGTTA TATGTCTCAG CTGTTCCTTA   
  
  
+ CAAAAGGATG TCTTTCTTTC TTGCTAACTG CTCGATTGCA AAGTTGGCAG AGAAAGCAAC AAAGATCCAT   
  
  
+ ATCATTGATT TTGGTGTTTT CCTAGGTTTA CAATGGCCTT GTTTCATACA ACACCTATCA AAAAGGCCAA   
  
  
+ ATGGACCCCC AAAACTCCGA ATCACAGGAA TCGACTACCC CCAGCAGGGT TTCAGACCTG CACAAAGGGT   
  
  
+ TGAAGCTACA GGACACCGAT TATCTGGGTA CTGTGAGCGA TTTGGGGTGC CTTTTTCTTA TCAGGGCATT   
  
  
+ GCTCAGAAGT GGGAAACTAT TCAGCCGGAG GATCTCAAGA TCGAACAAGA CGAGCTGGTG ATTGTCAACT   
  
  
+ GTTTGTTCAG GTCAGGAACA CTGCTCGATG AGACAGTCGA AGCAAACAGT CCAAGAGATG CTTTCTTAGC   
  
  
+ TTTGGTTAGA AAGCTGAATC CCAGCCTATT CATTCACGGG GTTGTCAATG GCACATTCAA CGCTCCATTC   
  
  
+ TTCGTGACTC GATTCAGAGA GGCATTGTTT CATTATTCAT CAGTGTTTGA TGTGTCTGAA GAGACAATTC   
  
  
+ CACGAGATGC CCATGAGAGG TTCTTGATTG AGAGCGAGAT TTGTGGGAAA GAACTGTTCA ATGTGGTTGC   
  
  
+ TTGTGAGGGT GCAGAGAGGG TTCAAAGGCC TGAGACATAC AAGCAGTGGC AAGTGAGGAC AACGCGGGCC   
  
  
+ GGGTTAAGGC AGGTTGCCTT GGACCAGGAG CTTATGAAGG AAGCAACGGC AATGGTGAAG GCAAATTATC   
  
  
+ ATAAGGATTT TATGGTGGAT ATAAATAGGC ATTGGATGCT TCAAGGTTGG AAGGGTAGAA CCTTGTGTGC   
  
  
+ TCTCTCATTT TGGCAACCTG CCTG  

- +Up\_Stream \_Len000GATCCC CAACTCTCTT AGGCTATCGA TACGTCAGGT TTGTAATAGA GCAGTTCGTA   
  
  
- ATGATAAATT TATTAATTAT AACTTGAACT ATCAATGTGA AAAACTTATG ATTAAGGTGT GGAGTAACGG   
  
  
- AAATAAAGGT TTGGAGCCCA ACGTAAACCT GGTGTATGGT CCTTCGAACC TGCTGTTTTT AAAGAAAAGA   
  
  
- ACTCATAATA AAACAAAATA ATAATAATAA AAAATGAAAT AAACCTGGTG AAGTGAACCC ATTGTTCTAA   
  
  
- CTTATAAACT GATAATATAA TATACAGTGC ATACCTATAC CATCAACGAA TCTCGAAACT GGCAAACGTC   
  
  
- CAAATTCATA CTAAGGGAAC CTTTCCTCGG GGAGAAATAG AATTAAGTCT TCATTGAAAA CAAAAAAATC   
  
  
- TATTTAACTT TTCTTTTTAT TATTTACAGA ATCAACTCAA ACCAATAAGT TAGGCTTTAA ACTAGGACGA   
  
  
- ATTACATATC ATATATTATG ATTATGCGTA CATTGTGTTG TGGAGTCTAC CAGTAACAAA ATGTTAAAAA   
  
  
- AACTCAAATC ATATTTTTAT TTTAGTTAAT AAAAATCTTC ACTACAAATT AAACGTAAAA AAAAGGTGGA   
  
  
- TTTTAGTGCG TGAGCTTGTA ATCCCCAAAC CGATTGAGTT TCTGAGTTCC ATCACTGAAT AGGTTTGAAT   
  
  
- AATGTAGGAT ATCTAGATAT TTTTGTATTT AGAGAATGTT AGTTAGGTTT AACTAAGCCA GATTAAACTA   
  
  
- CTGGGACTAC TCCCAGGATG TTAATGTGTT CACCCGGAAT CGGGGTACCG GCAAAAAATT AGAAGTACCC   
  
  
- AGGTCCGCCT CAATGGTAGA GGTCCAGGTA ACTCCCACAT AGTGCAGAGT TTAATGCTCA AACGGAGAAA   
  
  
- GATGCCCCTA ATATTTATAG ATGGACGTAT GGAGCTTTTC CTCCTCAACT CTAGTCGCTT TGAACCGGTA   
  
  
- GGGTCTCGTA ACTTTACGAG TCTTGGGAAA AAGATCTTTT TATAGCAAAA AGGACTTTTA TAGGCTGGAA   
  
  
- TACAGTAAAT AACAGCGAAA GAACTGAGAT TGGGCCCTGA GGTGTGATTG AACTGATAGC CTTTCCGCAA   
  
  
- GGGACCTAGT GGTAGGTTCT ATTGGAGAAA ACGTCCCAAC TGCGAGCAAC CTTCTTAGCA GCTTACATCT   
  
  
- CGGGGATAGT AGTGTATTCG GGCGAGAATG AAGTACGGTA GGAACTTATC GTCAAATAGG GCTTCGTTTT   
  
  
- TATTAAGATT GAAAGAATAT AACATTTGGG TGTAGATTTA AATTTAAGTA TGAGGTTTTA AGGCGGTGCG   
  
  
- TTTATTCCGA AAAATTATCC GAATGTATAT TACCGTACGA TATCTCTGTG ACAAAACTTC TTTGATAAAT   
  
  
- TTGTAGTCTG GAGTATAACT CATCGTTTAG TTCTGAAGAA AGGAGGTCCG GTATAACTCA TCGTTTAATT   
  
  
- CTTATTGGTC TTCGAACACT TTTCTCAATC TTTTATTTTC CATATTATCT CTGAATACAA GAATTCTATA   
  
  
- GAGGATCTAT ACTTACACTG GAGGATCAGC TATAAACACG ACCTAATCGC TCAACACACT GACTCAAACA   
  
  
- TACACAGAGA ATCGTATACT CAATAAGATC GACGAAGAAA TTCGATACAT CACTGACAGA AGGAAGAATA   
  
  
- AATATTCACG ATACAGGTTG ATTATCGGCT TTTTTTACCA AGCGGAAAAG ACCAAAAGAA GAAAAACTTC   
  
  
- CAACTAACCG ATTAAATATA TTACCAAAGT TATGAATAGG AAATAACAAG GTGTTTAGGT GGATTGGTGC   
  
  
- ATGGTAACTG TTATTAATGG GTGATTAAAG CCAACCTGAA CTAAAGGAAA ACAAACCATG GAGTCCGTAG   
  
  
- AAGACACCTG GAAGTAAGTG TCAAATTAAA AGTGTAAAAA TATTCCGACG AGAACACAAC GTTTAAAAGG   
  
  
- ATAGGTAAGG TGAGAAACGG GTAAGGGTCC GATTAATCAC AAGAGACAAG TTGGTACCTA AGACACGACC   
  
  
- AACTAGGACT CAAGTACTTT TTAGAGATGT TTAAGTTTGG ACTTAACGAG AGTTTGAAAA GTTACTTAGG   
  
  
- TTTACTGCTG TAGAAAGTTC GGGAAGTAAG TCTTTTACTA GGAAATGTCA AGGAGAGTAA ACTACTTCCT   
  
  
- TGGACAGAAT TGTCGACGTG ATCAGTTGTT CACGGTCTAA AGGGACTACG AACAGAGTTC AAGTAGTCAC   
  
  
- TATAAGAAGA GCTTCTCCCA AATCTACGTT TAGGACGTAG ACGTGTCCGA GAGCTTCGGT GGCTCTTCAG   
  
  
- GAACATACTA CGAGAGCCCG AACCTCTCGG TATGGGGGAA AGTACACTAG TGAAACGCGG TAGATAGAGA   
  
  
- TGTTCATAAC TCTCGGGTCT ACTGTCAAGA AGGTTATTTT CAATATCGTC GTTAGGGCTC TATCTACCAA   
  
  
- GAATACGATA GCGACTCGGG TCAAAGCTCA GGTTGGGGTT GACACACGAA CTAGTTGGGG TCAACTTGAG   
  
  
- GAAAGGTCGA GATGTACTCT AAAGAGCCAG GAACCACCTT GACCCAAGAG TTCGGAGACT CAACTCGAAG   
  
  
- CTACTACGTC CCTCACGGGC ACATCTCTTT TTCCCGTTTA GCTATTTCCC GAGCTCCTCC TTCTTCTCAG   
  
  
- CAGTTTCTCT CCCACTTCGC ACAATACCTC CTTCCTCGGT ATTCCAAGTT CGAAGGAAGT TACTACTAAT   
  
  
- GATACTCTAC CTTGTCATAC TACTACATCA TAACGAGACA TTATTACTTG ATTGCCCGTT ATCGGCAAAG   
  
  
- TTGTGGCCCT TTAGAAGTGG ACTCCTCCCT ACCTCCTCTA ACGTCTCTTC GGCTCCTTTC TTCGTCTTGT   
  
  
- CAAATCGTCA ACTTCAACTA GACTACTGGG ACGACTGAGT CACACGTGTT CGACATAGCT CGAAACTAGA   
  
  
- AGCTCCACGT TTGCTCGATG AATCCGTTTA GTCCGTCTTA CGAAGCGGGA TACCACCGTC GTAGGTCTCC   
  
  
- GAGCGGGTAG TACATCGGTT ACGAGAGCTC CGTGCATATC GACCGTGTCC GAGATGTCAG AGATGATTGG   
  
  
- AACAACTACG TTTCAAGAGT CGAAGACTGA AGGATTTCCG AATGTCCAAT ATACAGAGTC GACAAGGAAT   
  
  
- GTTTTCCTAC AGAAAGAAAG AACGATTGAC GAGCTAACGT TTCAACCGTC TCTTTCGTTG TTTCTAGGTA   
  
  
- TAGTAACTAA AACCACAAAA GGATCCAAAT GTTACCGGAA CAAAGTATGT TGTGGATAGT TTTTCCGGTT   
  
  
- TACCTGGGGG TTTTGAGGCT TAGTGTCCTT AGCTGATGGG GGTCGTCCCA AAGTCTGGAC GTGTTTCCCA   
  
  
- ACTTCGATGT CCTGTGGCTA ATAGACCCAT GACACTCGCT AAACCCCACG GAAAAAGAAT AGTCCCGTAA   
  
  
- CGAGTCTTCA CCCTTTGATA AGTCGGCCTC CTAGAGTTCT AGCTTGTTCT GCTCGACCAC TAACAGTTGA   
  
  
- CAAACAAGTC CAGTCCTTGT GACGAGCTAC TCTGTCAGCT TCGTTTGTCA GGTTCTCTAC GAAAGAATCG   
  
  
- AAACCAATCT TTCGACTTAG GGTCGGATAA GTAAGTGCCC CAACAGTTAC CGTGTAAGTT GCGAGGTAAG   
  
  
- AAGCACTGAG CTAAGTCTCT CCGTAACAAA GTAATAAGTA GTCACAAACT ACACAGACTT CTCTGTTAAG   
  
  
- GTGCTCTACG GGTACTCTCC AAGAACTAAC TCTCGCTCTA AACACCCTTT CTTGACAAGT TACACCAACG   
  
  
- AACACTCCCA CGTCTCTCCC AAGTTTCCGG ACTCTGTATG TTCGTCACCG TTCACTCCTG TTGCGCCCGG   
  
  
- CCCAATTCCG TCCAACGGAA CCTGGTCCTC GAATACTTCC TTCGTTGCCG TTACCACTTC CGTTTAATAG   
  
  
- TATTCCTAAA ATACCACCTA TATTTATCCG TAACCTACGA AGTTCCAACC TTCCCATCTT GGAACACACG   
  
  
- AGAGAGTAAA ACCGTTGGAC GGAC

+     TATA-box

| Site Name | Organism | Position | Strand | Matrix score. | sequence | function |
| --- | --- | --- | --- | --- | --- | --- |
| TATA-box | Arabidopsis thaliana | 3944 | - | 4 | TATA | core promoter element around -30 of transcription start |
| TATA-box | Arabidopsis thaliana | 3278 | + | 9 | ccTATAAAaa | core promoter element around -30 of transcription start |
| TATA-box | Arabidopsis thaliana | 575 | + | 4 | TATA | core promoter element around -30 of transcription start |
| TATA-box | Helianthus annuus | 1768 | - | 6 | TATAAA | core promoter element around -30 of transcription start |
| TATA-box | Oryza sativa | 3153 | + | 7 | TACAAAA | core promoter element around -30 of transcription start |
| TATA-box | Arabidopsis thaliana | 3133 | - | 4 | TATA | core promoter element around -30 of transcription start |
| TATA-box | Arabidopsis thaliana | 3050 | - | 4 | TATA | core promoter element around -30 of transcription start |
| TATA-box | Brassica juncea | 1767 | - | 7 | TATAAAT | core promoter element around -30 of transcription start |
| TATA-box | Brassica oleracea | 3943 | + | 6 | ATATAA | core promoter element around -30 of transcription start |
| TATA-box | Arabidopsis thaliana | 2427 | - | 4 | TATA | core promoter element around -30 of transcription start |
| TATA-box | Helianthus annuus | 1932 | - | 6 | TATAAA | core promoter element around -30 of transcription start |
| TATA-box | Arabidopsis thaliana | 2426 | - | 5 | TATAA | core promoter element around -30 of transcription start |
| TATA-box | Arabidopsis thaliana | 1766 | - | 9 | taTATAAAtc | core promoter element around -30 of transcription start |
| TATA-box | Pisum sativum | 1931 | - | 7 | TATAAAA | core promoter element around -30 of transcription start |
| TATA-box | Helianthus annuus | 1684 | - | 6 | TATAAA | core promoter element around -30 of transcription start |
| TATA-box | Arabidopsis thaliana | 507 | + | 4 | TATA | core promoter element around -30 of transcription start |
| TATA-box | Brassica juncea | 1683 | - | 7 | TATAAAT | core promoter element around -30 of transcription start |
| TATA-box | Arabidopsis thaliana | 3132 | - | 5 | TATAA | core promoter element around -30 of transcription start |
| TATA-box | Arabidopsis thaliana | 1934 | + | 4 | TATA | core promoter element around -30 of transcription start |
| TATA-box | Arabidopsis thaliana | 1933 | - | 5 | TATAA | core promoter element around -30 of transcription start |
| TATA-box | Arabidopsis thaliana | 304 | + | 4 | TATA | core promoter element around -30 of transcription start |
| TATA-box | Arabidopsis thaliana | 1772 | + | 4 | TATA | core promoter element around -30 of transcription start |
| TATA-box | Brassica napus | 302 | + | 6 | ATTATA | core promoter element around -30 of transcription start |
| TATA-box | Arabidopsis thaliana | 299 | + | 4 | TATA | core promoter element around -30 of transcription start |
| TATA-box | Arabidopsis thaliana | 500 | + | 4 | TATA | core promoter element around -30 of transcription start |
| TATA-box | Arabidopsis thaliana | 1685 | - | 5 | TATAA | core promoter element around -30 of transcription start |
| TATA-box | Arabidopsis thaliana | 298 | - | 5 | TATAA | core promoter element around -30 of transcription start |
| TATA-box | Arabidopsis thaliana | 303 | - | 5 | TATAA | core promoter element around -30 of transcription start |
| TATA-box | Arabidopsis thaliana | 1399 | + | 8 | TATTTAAA | core promoter element around -30 of transcription start |
| TATA-box | Brassica oleracea | 1771 | + | 6 | ATATAA | core promoter element around -30 of transcription start |
| TATA-box | Arabidopsis thaliana | 1374 | + | 4 | TATA | core promoter element around -30 of transcription start |
| TATA-box | Arabidopsis thaliana | 1361 | + | 4 | TATA | core promoter element around -30 of transcription start |
| TATA-box | Arabidopsis thaliana | 925 | + | 4 | TATA | core promoter element around -30 of transcription start |
| TATA-box | Brassica oleracea | 1360 | + | 6 | ATATAA | core promoter element around -30 of transcription start |
| TATA-box | Arabidopsis thaliana | 721 | + | 4 | TATA | core promoter element around -30 of transcription start |
| TATA-box | Arabidopsis thaliana | 1345 | - | 9 | ccTATAAAaa | core promoter element around -30 of transcription start |
| TATA-box | Arabidopsis thaliana | 1281 | + | 4 | TATA | core promoter element around -30 of transcription start |
| TATA-box | Arabidopsis thaliana | 713 | + | 4 | TATA | core promoter element around -30 of transcription start |
| TATA-box | Arabidopsis thaliana | 719 | + | 9 | ccTATAAAaa | core promoter element around -30 of transcription start |
| TATA-box | Arabidopsis thaliana | 1280 | - | 5 | TATAA | core promoter element around -30 of transcription start |
| TATA-box | Zea mays | 1277 | - | 8 | TATAAGAA | core promoter element around -30 of transcription start |
| TATA-box | Brassica oleracea | 506 | + | 6 | ATATAA | core promoter element around -30 of transcription start |
| TATA-box | Arabidopsis thaliana | 80 | - | 8 | TATTTAAA | core promoter element around -30 of transcription start |
| TATA-box | Arabidopsis thaliana | 78 | + | 8 | TATTTAAA | core promoter element around -30 of transcription start |
| TATA-box | Helianthus annuus | 498 | - | 6 | TATACA | core promoter element around -30 of transcription start |
| TATA-box | Brassica napus | 923 | + | 6 | ATTATA | core promoter element around -30 of transcription start |
| TATA-box | Arabidopsis thaliana | 924 | - | 5 | TATAA | core promoter element around -30 of transcription start |
| TATA-box | Brassica napus | 297 | + | 6 | ATTATA | core promoter element around -30 of transcription start |
| TATA-box | Daucus carota | 1682 | - | 8 | TATAAATA | core promoter element around -30 of transcription start |
| TATA-box | Arabidopsis thaliana | 505 | + | 6 | TATATA | core promoter element around -30 of transcription start |
| TATA-box | Arabidopsis thaliana | 1930 | - | 9 | ccTATAAAaa | core promoter element around -30 of transcription start |
| TATA-box | Arabidopsis thaliana | 1770 | + | 6 | TATATA | core promoter element around -30 of transcription start |
| TATA-box | Arabidopsis thaliana | 1686 | + | 4 | TATA | core promoter element around -30 of transcription start |
| TATA-box | Arabidopsis thaliana | 1769 | - | 7 | TATATAA | core promoter element around -30 of transcription start |
| TATA-box | Arabidopsis thaliana | 1516 | + | 4 | TATA | core promoter element around -30 of transcription start |

>HU02G01571.1   
+ +Up\_Stream \_Len000CTAGGG GTTGAGAGAA TCCGATAGCT ATGCAGTCCA AACATTATCT CGTCAAGCAT   
  
  
+ TACTATTTAA ATAATTAATA TTGAACTTGA TAGTTACACT TTTTGAATAC TAATTCCACA CCTCATTGCC   
  
  
+ TTTATTTCCA AACCTCGGGT TGCATTTGGA CCACATACCA GGAAGCTTGG ACGACAAAAA TTTCTTTTCT   
  
  
+ TGAGTATTAT TTTGTTTTAT TATTATTATT TTTTACTTTA TTTGGACCAC TTCACTTGGG TAACAAGATT   
  
  
+ GAATATTTGA CTATTATATT ATATGTCACG TATGGATATG GTAGTTGCTT AGAGCTTTGA CCGTTTGCAG   
  
  
+ GTTTAAGTAT GATTCCCTTG GAAAGGAGCC CCTCTTTATC TTAATTCAGA AGTAACTTTT GTTTTTTTAG   
  
  
+ ATAAATTGAA AAGAAAAATA ATAAATGTCT TAGTTGAGTT TGGTTATTCA ATCCGAAATT TGATCCTGCT   
  
  
+ TAATGTATAG TATATAATAC TAATACGCAT GTAACACAAC ACCTCAGATG GTCATTGTTT TACAATTTTT   
  
  
+ TTGAGTTTAG TATAAAAATA AAATCAATTA TTTTTAGAAG TGATGTTTAA TTTGCATTTT TTTTCCACCT   
  
  
+ AAAATCACGC ACTCGAACAT TAGGGGTTTG GCTAACTCAA AGACTCAAGG TAGTGACTTA TCCAAACTTA   
  
  
+ TTACATCCTA TAGATCTATA AAAACATAAA TCTCTTACAA TCAATCCAAA TTGATTCGGT CTAATTTGAT   
  
  
+ GACCCTGATG AGGGTCCTAC AATTACACAA GTGGGCCTTA GCCCCATGGC CGTTTTTTAA TCTTCATGGG   
  
  
+ TCCAGGCGGA GTTACCATCT CCAGGTCCAT TGAGGGTGTA TCACGTCTCA AATTACGAGT TTGCCTCTTT   
  
  
+ CTACGGGGAT TATAAATATC TACCTGCATA CCTCGAAAAG GAGGAGTTGA GATCAGCGAA ACTTGGCCAT   
  
  
+ CCCAGAGCAT TGAAATGCTC AGAACCCTTT TTCTAGAAAA ATATCGTTTT TCCTGAAAAT ATCCGACCTT   
  
  
+ ATGTCATTTA TTGTCGCTTT CTTGACTCTA ACCCGGGACT CCACACTAAC TTGACTATCG GAAAGGCGTT   
  
  
+ CCCTGGATCA CCATCCAAGA TAACCTCTTT TGCAGGGTTG ACGCTCGTTG GAAGAATCGT CGAATGTAGA   
  
  
+ GCCCCTATCA TCACATAAGC CCGCTCTTAC TTCATGCCAT CCTTGAATAG CAGTTTATCC CGAAGCAAAA   
  
  
+ ATAATTCTAA CTTTCTTATA TTGTAAACCC ACATCTAAAT TTAAATTCAT ACTCCAAAAT TCCGCCACGC   
  
  
+ AAATAAGGCT TTTTAATAGG CTTACATATA ATGGCATGCT ATAGAGACAC TGTTTTGAAG AAACTATTTA   
  
  
+ AACATCAGAC CTCATATTGA GTAGCAAATC AAGACTTCTT TCCTCCAGGC CATATTGAGT AGCAAATTAA   
  
  
+ GAATAACCAG AAGCTTGTGA AAAGAGTTAG AAAATAAAAG GTATAATAGA GACTTATGTT CTTAAGATAT   
  
  
+ CTCCTAGATA TGAATGTGAC CTCCTAGTCG ATATTTGTGC TGGATTAGCG AGTTGTGTGA CTGAGTTTGT   
  
  
+ ATGTGTCTCT TAGCATATGA GTTATTCTAG CTGCTTCTTT AAGCTATGTA GTGACTGTCT TCCTTCTTAT   
  
  
+ TTATAAGTGC TATGTCCAAC TAATAGCCGA AAAAAATGGT TCGCCTTTTC TGGTTTTCTT CTTTTTGAAG   
  
  
+ GTTGATTGGC TAATTTATAT AATGGTTTCA ATACTTATCC TTTATTGTTC CACAAATCCA CCTAACCACG   
  
  
+ TACCATTGAC AATAATTACC CACTAATTTC GGTTGGACTT GATTTCCTTT TGTTTGGTAC CTCAGGCATC   
  
  
+ TTCTGTGGAC CTTCATTCAC AGTTTAATTT TCACATTTTT ATAAGGCTGC TCTTGTGTTG CAAATTTTCC   
  
  
+ TATCCATTCC ACTCTTTGCC CATTCCCAGG CTAATTAGTG TTCTCTGTTC AACCATGGAT TCTGTGCTGG   
  
  
+ TTGATCCTGA GTTCATGAAA AATCTCTACA AATTCAAACC TGAATTGCTC TCAAACTTTT CAATGAATCC   
  
  
+ AAATGACGAC ATCTTTCAAG CCCTTCATTC AGAAAATGAT CCTTTACAGT TCCTCTCATT TGATGAAGGA   
  
  
+ ACCTGTCTTA ACAGCTGCAC TAGTCAACAA GTGCCAGATT TCCCTGATGC TTGTCTCAAG TTCATCAGTG   
  
  
+ ATATTCTTCT CGAAGAGGGT TTAGATGCAA ATCCTGCATC TGCACAGGCT CTCGAAGCCA CCGAGAAGTC   
  
  
+ CTTGTATGAT GCTCTCGGGC TTGGAGAGCC ATACCCCCTT TCATGTGATC ACTTTGCGCC ATCTATCTCT   
  
  
+ ACAAGTATTG AGAGCCCAGA TGACAGTTCT TCCAATAAAA GTTATAGCAG CAATCCCGAG ATAGATGGTT   
  
  
+ CTTATGCTAT CGCTGAGCCC AGTTTCGAGT CCAACCCCAA CTGTGTGCTT GATCAACCCC AGTTGAACTC   
  
  
+ CTTTCCAGCT CTACATGAGA TTTCTCGGTC CTTGGTGGAA CTGGGTTCTC AAGCCTCTGA GTTGAGCTTC   
  
  
+ GATGATGCAG GGAGTGCCCG TGTAGAGAAA AAGGGCAAAT CGATAAAGGG CTCGAGGAGG AAGAAGAGTC   
  
  
+ GTCAAAGAGA GGGTGAAGCG TGTTATGGAG GAAGGAGCCA TAAGGTTCAA GCTTCCTTCA ATGATGATTA   
  
  
+ CTATGAGATG GAACAGTATG ATGATGTAGT ATTGCTCTGT AATAATGAAC TAACGGGCAA TAGCCGTTTC   
  
  
+ AACACCGGGA AATCTTCACC TGAGGAGGGA TGGAGGAGAT TGCAGAGAAG CCGAGGAAAG AAGCAGAACA   
  
  
+ GTTTAGCAGT TGAAGTTGAT CTGATGACCC TGCTGACTCA GTGTGCACAA GCTGTATCGA GCTTTGATCT   
  
  
+ TCGAGGTGCA AACGAGCTAC TTAGGCAAAT CAGGCAGAAT GCTTCGCCCT ATGGTGGCAG CATCCAGAGG   
  
  
+ CTCGCCCATC ATGTAGCCAA TGCTCTCGAG GCACGTATAG CTGGCACAGG CTCTACAGTC TCTACTAACC   
  
  
+ TTGTTGATGC AAAGTTCTCA GCTTCTGACT TCCTAAAGGC TTACAGGTTA TATGTCTCAG CTGTTCCTTA   
  
  
+ CAAAAGGATG TCTTTCTTTC TTGCTAACTG CTCGATTGCA AAGTTGGCAG AGAAAGCAAC AAAGATCCAT   
  
  
+ ATCATTGATT TTGGTGTTTT CCTAGGTTTA CAATGGCCTT GTTTCATACA ACACCTATCA AAAAGGCCAA   
  
  
+ ATGGACCCCC AAAACTCCGA ATCACAGGAA TCGACTACCC CCAGCAGGGT TTCAGACCTG CACAAAGGGT   
  
  
+ TGAAGCTACA GGACACCGAT TATCTGGGTA CTGTGAGCGA TTTGGGGTGC CTTTTTCTTA TCAGGGCATT   
  
  
+ GCTCAGAAGT GGGAAACTAT TCAGCCGGAG GATCTCAAGA TCGAACAAGA CGAGCTGGTG ATTGTCAACT   
  
  
+ GTTTGTTCAG GTCAGGAACA CTGCTCGATG AGACAGTCGA AGCAAACAGT CCAAGAGATG CTTTCTTAGC   
  
  
+ TTTGGTTAGA AAGCTGAATC CCAGCCTATT CATTCACGGG GTTGTCAATG GCACATTCAA CGCTCCATTC   
  
  
+ TTCGTGACTC GATTCAGAGA GGCATTGTTT CATTATTCAT CAGTGTTTGA TGTGTCTGAA GAGACAATTC   
  
  
+ CACGAGATGC CCATGAGAGG TTCTTGATTG AGAGCGAGAT TTGTGGGAAA GAACTGTTCA ATGTGGTTGC   
  
  
+ TTGTGAGGGT GCAGAGAGGG TTCAAAGGCC TGAGACATAC AAGCAGTGGC AAGTGAGGAC AACGCGGGCC   
  
  
+ GGGTTAAGGC AGGTTGCCTT GGACCAGGAG CTTATGAAGG AAGCAACGGC AATGGTGAAG GCAAATTATC   
  
  
+ ATAAGGATTT TATGGTGGAT ATAAATAGGC ATTGGATGCT TCAAGGTTGG AAGGGTAGAA CCTTGTGTGC   
  
  
+ TCTCTCATTT TGGCAACCTG CCTG  

- +Up\_Stream \_Len000GATCCC CAACTCTCTT AGGCTATCGA TACGTCAGGT TTGTAATAGA GCAGTTCGTA   
  
  
- ATGATAAATT TATTAATTAT AACTTGAACT ATCAATGTGA AAAACTTATG ATTAAGGTGT GGAGTAACGG   
  
  
- AAATAAAGGT TTGGAGCCCA ACGTAAACCT GGTGTATGGT CCTTCGAACC TGCTGTTTTT AAAGAAAAGA   
  
  
- ACTCATAATA AAACAAAATA ATAATAATAA AAAATGAAAT AAACCTGGTG AAGTGAACCC ATTGTTCTAA   
  
  
- CTTATAAACT GATAATATAA TATACAGTGC ATACCTATAC CATCAACGAA TCTCGAAACT GGCAAACGTC   
  
  
- CAAATTCATA CTAAGGGAAC CTTTCCTCGG GGAGAAATAG AATTAAGTCT TCATTGAAAA CAAAAAAATC   
  
  
- TATTTAACTT TTCTTTTTAT TATTTACAGA ATCAACTCAA ACCAATAAGT TAGGCTTTAA ACTAGGACGA   
  
  
- ATTACATATC ATATATTATG ATTATGCGTA CATTGTGTTG TGGAGTCTAC CAGTAACAAA ATGTTAAAAA   
  
  
- AACTCAAATC ATATTTTTAT TTTAGTTAAT AAAAATCTTC ACTACAAATT AAACGTAAAA AAAAGGTGGA   
  
  
- TTTTAGTGCG TGAGCTTGTA ATCCCCAAAC CGATTGAGTT TCTGAGTTCC ATCACTGAAT AGGTTTGAAT   
  
  
- AATGTAGGAT ATCTAGATAT TTTTGTATTT AGAGAATGTT AGTTAGGTTT AACTAAGCCA GATTAAACTA   
  
  
- CTGGGACTAC TCCCAGGATG TTAATGTGTT CACCCGGAAT CGGGGTACCG GCAAAAAATT AGAAGTACCC   
  
  
- AGGTCCGCCT CAATGGTAGA GGTCCAGGTA ACTCCCACAT AGTGCAGAGT TTAATGCTCA AACGGAGAAA   
  
  
- GATGCCCCTA ATATTTATAG ATGGACGTAT GGAGCTTTTC CTCCTCAACT CTAGTCGCTT TGAACCGGTA   
  
  
- GGGTCTCGTA ACTTTACGAG TCTTGGGAAA AAGATCTTTT TATAGCAAAA AGGACTTTTA TAGGCTGGAA   
  
  
- TACAGTAAAT AACAGCGAAA GAACTGAGAT TGGGCCCTGA GGTGTGATTG AACTGATAGC CTTTCCGCAA   
  
  
- GGGACCTAGT GGTAGGTTCT ATTGGAGAAA ACGTCCCAAC TGCGAGCAAC CTTCTTAGCA GCTTACATCT   
  
  
- CGGGGATAGT AGTGTATTCG GGCGAGAATG AAGTACGGTA GGAACTTATC GTCAAATAGG GCTTCGTTTT   
  
  
- TATTAAGATT GAAAGAATAT AACATTTGGG TGTAGATTTA AATTTAAGTA TGAGGTTTTA AGGCGGTGCG   
  
  
- TTTATTCCGA AAAATTATCC GAATGTATAT TACCGTACGA TATCTCTGTG ACAAAACTTC TTTGATAAAT   
  
  
- TTGTAGTCTG GAGTATAACT CATCGTTTAG TTCTGAAGAA AGGAGGTCCG GTATAACTCA TCGTTTAATT   
  
  
- CTTATTGGTC TTCGAACACT TTTCTCAATC TTTTATTTTC CATATTATCT CTGAATACAA GAATTCTATA   
  
  
- GAGGATCTAT ACTTACACTG GAGGATCAGC TATAAACACG ACCTAATCGC TCAACACACT GACTCAAACA   
  
  
- TACACAGAGA ATCGTATACT CAATAAGATC GACGAAGAAA TTCGATACAT CACTGACAGA AGGAAGAATA   
  
  
- AATATTCACG ATACAGGTTG ATTATCGGCT TTTTTTACCA AGCGGAAAAG ACCAAAAGAA GAAAAACTTC   
  
  
- CAACTAACCG ATTAAATATA TTACCAAAGT TATGAATAGG AAATAACAAG GTGTTTAGGT GGATTGGTGC   
  
  
- ATGGTAACTG TTATTAATGG GTGATTAAAG CCAACCTGAA CTAAAGGAAA ACAAACCATG GAGTCCGTAG   
  
  
- AAGACACCTG GAAGTAAGTG TCAAATTAAA AGTGTAAAAA TATTCCGACG AGAACACAAC GTTTAAAAGG   
  
  
- ATAGGTAAGG TGAGAAACGG GTAAGGGTCC GATTAATCAC AAGAGACAAG TTGGTACCTA AGACACGACC   
  
  
- AACTAGGACT CAAGTACTTT TTAGAGATGT TTAAGTTTGG ACTTAACGAG AGTTTGAAAA GTTACTTAGG   
  
  
- TTTACTGCTG TAGAAAGTTC GGGAAGTAAG TCTTTTACTA GGAAATGTCA AGGAGAGTAA ACTACTTCCT   
  
  
- TGGACAGAAT TGTCGACGTG ATCAGTTGTT CACGGTCTAA AGGGACTACG AACAGAGTTC AAGTAGTCAC   
  
  
- TATAAGAAGA GCTTCTCCCA AATCTACGTT TAGGACGTAG ACGTGTCCGA GAGCTTCGGT GGCTCTTCAG   
  
  
- GAACATACTA CGAGAGCCCG AACCTCTCGG TATGGGGGAA AGTACACTAG TGAAACGCGG TAGATAGAGA   
  
  
- TGTTCATAAC TCTCGGGTCT ACTGTCAAGA AGGTTATTTT CAATATCGTC GTTAGGGCTC TATCTACCAA   
  
  
- GAATACGATA GCGACTCGGG TCAAAGCTCA GGTTGGGGTT GACACACGAA CTAGTTGGGG TCAACTTGAG   
  
  
- GAAAGGTCGA GATGTACTCT AAAGAGCCAG GAACCACCTT GACCCAAGAG TTCGGAGACT CAACTCGAAG   
  
  
- CTACTACGTC CCTCACGGGC ACATCTCTTT TTCCCGTTTA GCTATTTCCC GAGCTCCTCC TTCTTCTCAG   
  
  
- CAGTTTCTCT CCCACTTCGC ACAATACCTC CTTCCTCGGT ATTCCAAGTT CGAAGGAAGT TACTACTAAT   
  
  
- GATACTCTAC CTTGTCATAC TACTACATCA TAACGAGACA TTATTACTTG ATTGCCCGTT ATCGGCAAAG   
  
  
- TTGTGGCCCT TTAGAAGTGG ACTCCTCCCT ACCTCCTCTA ACGTCTCTTC GGCTCCTTTC TTCGTCTTGT   
  
  
- CAAATCGTCA ACTTCAACTA GACTACTGGG ACGACTGAGT CACACGTGTT CGACATAGCT CGAAACTAGA   
  
  
- AGCTCCACGT TTGCTCGATG AATCCGTTTA GTCCGTCTTA CGAAGCGGGA TACCACCGTC GTAGGTCTCC   
  
  
- GAGCGGGTAG TACATCGGTT ACGAGAGCTC CGTGCATATC GACCGTGTCC GAGATGTCAG AGATGATTGG   
  
  
- AACAACTACG TTTCAAGAGT CGAAGACTGA AGGATTTCCG AATGTCCAAT ATACAGAGTC GACAAGGAAT   
  
  
- GTTTTCCTAC AGAAAGAAAG AACGATTGAC GAGCTAACGT TTCAACCGTC TCTTTCGTTG TTTCTAGGTA   
  
  
- TAGTAACTAA AACCACAAAA GGATCCAAAT GTTACCGGAA CAAAGTATGT TGTGGATAGT TTTTCCGGTT   
  
  
- TACCTGGGGG TTTTGAGGCT TAGTGTCCTT AGCTGATGGG GGTCGTCCCA AAGTCTGGAC GTGTTTCCCA   
  
  
- ACTTCGATGT CCTGTGGCTA ATAGACCCAT GACACTCGCT AAACCCCACG GAAAAAGAAT AGTCCCGTAA   
  
  
- CGAGTCTTCA CCCTTTGATA AGTCGGCCTC CTAGAGTTCT AGCTTGTTCT GCTCGACCAC TAACAGTTGA   
  
  
- CAAACAAGTC CAGTCCTTGT GACGAGCTAC TCTGTCAGCT TCGTTTGTCA GGTTCTCTAC GAAAGAATCG   
  
  
- AAACCAATCT TTCGACTTAG GGTCGGATAA GTAAGTGCCC CAACAGTTAC CGTGTAAGTT GCGAGGTAAG   
  
  
- AAGCACTGAG CTAAGTCTCT CCGTAACAAA GTAATAAGTA GTCACAAACT ACACAGACTT CTCTGTTAAG   
  
  
- GTGCTCTACG GGTACTCTCC AAGAACTAAC TCTCGCTCTA AACACCCTTT CTTGACAAGT TACACCAACG   
  
  
- AACACTCCCA CGTCTCTCCC AAGTTTCCGG ACTCTGTATG TTCGTCACCG TTCACTCCTG TTGCGCCCGG   
  
  
- CCCAATTCCG TCCAACGGAA CCTGGTCCTC GAATACTTCC TTCGTTGCCG TTACCACTTC CGTTTAATAG   
  
  
- TATTCCTAAA ATACCACCTA TATTTATCCG TAACCTACGA AGTTCCAACC TTCCCATCTT GGAACACACG   
  
  
- AGAGAGTAAA ACCGTTGGAC GGAC

+     TC-rich repeats

| Site Name | Organism | Position | Strand | Matrix score. | sequence | function |
| --- | --- | --- | --- | --- | --- | --- |
| TC-rich repeats | Nicotiana tabacum | 1500 | - | 9 | ATTCTCTAAC | cis-acting element involved in defense and stress responsiveness |

>HU02G01571.1   
+ +Up\_Stream \_Len000CTAGGG GTTGAGAGAA TCCGATAGCT ATGCAGTCCA AACATTATCT CGTCAAGCAT   
  
  
+ TACTATTTAA ATAATTAATA TTGAACTTGA TAGTTACACT TTTTGAATAC TAATTCCACA CCTCATTGCC   
  
  
+ TTTATTTCCA AACCTCGGGT TGCATTTGGA CCACATACCA GGAAGCTTGG ACGACAAAAA TTTCTTTTCT   
  
  
+ TGAGTATTAT TTTGTTTTAT TATTATTATT TTTTACTTTA TTTGGACCAC TTCACTTGGG TAACAAGATT   
  
  
+ GAATATTTGA CTATTATATT ATATGTCACG TATGGATATG GTAGTTGCTT AGAGCTTTGA CCGTTTGCAG   
  
  
+ GTTTAAGTAT GATTCCCTTG GAAAGGAGCC CCTCTTTATC TTAATTCAGA AGTAACTTTT GTTTTTTTAG   
  
  
+ ATAAATTGAA AAGAAAAATA ATAAATGTCT TAGTTGAGTT TGGTTATTCA ATCCGAAATT TGATCCTGCT   
  
  
+ TAATGTATAG TATATAATAC TAATACGCAT GTAACACAAC ACCTCAGATG GTCATTGTTT TACAATTTTT   
  
  
+ TTGAGTTTAG TATAAAAATA AAATCAATTA TTTTTAGAAG TGATGTTTAA TTTGCATTTT TTTTCCACCT   
  
  
+ AAAATCACGC ACTCGAACAT TAGGGGTTTG GCTAACTCAA AGACTCAAGG TAGTGACTTA TCCAAACTTA   
  
  
+ TTACATCCTA TAGATCTATA AAAACATAAA TCTCTTACAA TCAATCCAAA TTGATTCGGT CTAATTTGAT   
  
  
+ GACCCTGATG AGGGTCCTAC AATTACACAA GTGGGCCTTA GCCCCATGGC CGTTTTTTAA TCTTCATGGG   
  
  
+ TCCAGGCGGA GTTACCATCT CCAGGTCCAT TGAGGGTGTA TCACGTCTCA AATTACGAGT TTGCCTCTTT   
  
  
+ CTACGGGGAT TATAAATATC TACCTGCATA CCTCGAAAAG GAGGAGTTGA GATCAGCGAA ACTTGGCCAT   
  
  
+ CCCAGAGCAT TGAAATGCTC AGAACCCTTT TTCTAGAAAA ATATCGTTTT TCCTGAAAAT ATCCGACCTT   
  
  
+ ATGTCATTTA TTGTCGCTTT CTTGACTCTA ACCCGGGACT CCACACTAAC TTGACTATCG GAAAGGCGTT   
  
  
+ CCCTGGATCA CCATCCAAGA TAACCTCTTT TGCAGGGTTG ACGCTCGTTG GAAGAATCGT CGAATGTAGA   
  
  
+ GCCCCTATCA TCACATAAGC CCGCTCTTAC TTCATGCCAT CCTTGAATAG CAGTTTATCC CGAAGCAAAA   
  
  
+ ATAATTCTAA CTTTCTTATA TTGTAAACCC ACATCTAAAT TTAAATTCAT ACTCCAAAAT TCCGCCACGC   
  
  
+ AAATAAGGCT TTTTAATAGG CTTACATATA ATGGCATGCT ATAGAGACAC TGTTTTGAAG AAACTATTTA   
  
  
+ AACATCAGAC CTCATATTGA GTAGCAAATC AAGACTTCTT TCCTCCAGGC CATATTGAGT AGCAAATTAA   
  
  
+ GAATAACCAG AAGCTTGTGA AAAGAGTTAG AAAATAAAAG GTATAATAGA GACTTATGTT CTTAAGATAT   
  
  
+ CTCCTAGATA TGAATGTGAC CTCCTAGTCG ATATTTGTGC TGGATTAGCG AGTTGTGTGA CTGAGTTTGT   
  
  
+ ATGTGTCTCT TAGCATATGA GTTATTCTAG CTGCTTCTTT AAGCTATGTA GTGACTGTCT TCCTTCTTAT   
  
  
+ TTATAAGTGC TATGTCCAAC TAATAGCCGA AAAAAATGGT TCGCCTTTTC TGGTTTTCTT CTTTTTGAAG   
  
  
+ GTTGATTGGC TAATTTATAT AATGGTTTCA ATACTTATCC TTTATTGTTC CACAAATCCA CCTAACCACG   
  
  
+ TACCATTGAC AATAATTACC CACTAATTTC GGTTGGACTT GATTTCCTTT TGTTTGGTAC CTCAGGCATC   
  
  
+ TTCTGTGGAC CTTCATTCAC AGTTTAATTT TCACATTTTT ATAAGGCTGC TCTTGTGTTG CAAATTTTCC   
  
  
+ TATCCATTCC ACTCTTTGCC CATTCCCAGG CTAATTAGTG TTCTCTGTTC AACCATGGAT TCTGTGCTGG   
  
  
+ TTGATCCTGA GTTCATGAAA AATCTCTACA AATTCAAACC TGAATTGCTC TCAAACTTTT CAATGAATCC   
  
  
+ AAATGACGAC ATCTTTCAAG CCCTTCATTC AGAAAATGAT CCTTTACAGT TCCTCTCATT TGATGAAGGA   
  
  
+ ACCTGTCTTA ACAGCTGCAC TAGTCAACAA GTGCCAGATT TCCCTGATGC TTGTCTCAAG TTCATCAGTG   
  
  
+ ATATTCTTCT CGAAGAGGGT TTAGATGCAA ATCCTGCATC TGCACAGGCT CTCGAAGCCA CCGAGAAGTC   
  
  
+ CTTGTATGAT GCTCTCGGGC TTGGAGAGCC ATACCCCCTT TCATGTGATC ACTTTGCGCC ATCTATCTCT   
  
  
+ ACAAGTATTG AGAGCCCAGA TGACAGTTCT TCCAATAAAA GTTATAGCAG CAATCCCGAG ATAGATGGTT   
  
  
+ CTTATGCTAT CGCTGAGCCC AGTTTCGAGT CCAACCCCAA CTGTGTGCTT GATCAACCCC AGTTGAACTC   
  
  
+ CTTTCCAGCT CTACATGAGA TTTCTCGGTC CTTGGTGGAA CTGGGTTCTC AAGCCTCTGA GTTGAGCTTC   
  
  
+ GATGATGCAG GGAGTGCCCG TGTAGAGAAA AAGGGCAAAT CGATAAAGGG CTCGAGGAGG AAGAAGAGTC   
  
  
+ GTCAAAGAGA GGGTGAAGCG TGTTATGGAG GAAGGAGCCA TAAGGTTCAA GCTTCCTTCA ATGATGATTA   
  
  
+ CTATGAGATG GAACAGTATG ATGATGTAGT ATTGCTCTGT AATAATGAAC TAACGGGCAA TAGCCGTTTC   
  
  
+ AACACCGGGA AATCTTCACC TGAGGAGGGA TGGAGGAGAT TGCAGAGAAG CCGAGGAAAG AAGCAGAACA   
  
  
+ GTTTAGCAGT TGAAGTTGAT CTGATGACCC TGCTGACTCA GTGTGCACAA GCTGTATCGA GCTTTGATCT   
  
  
+ TCGAGGTGCA AACGAGCTAC TTAGGCAAAT CAGGCAGAAT GCTTCGCCCT ATGGTGGCAG CATCCAGAGG   
  
  
+ CTCGCCCATC ATGTAGCCAA TGCTCTCGAG GCACGTATAG CTGGCACAGG CTCTACAGTC TCTACTAACC   
  
  
+ TTGTTGATGC AAAGTTCTCA GCTTCTGACT TCCTAAAGGC TTACAGGTTA TATGTCTCAG CTGTTCCTTA   
  
  
+ CAAAAGGATG TCTTTCTTTC TTGCTAACTG CTCGATTGCA AAGTTGGCAG AGAAAGCAAC AAAGATCCAT   
  
  
+ ATCATTGATT TTGGTGTTTT CCTAGGTTTA CAATGGCCTT GTTTCATACA ACACCTATCA AAAAGGCCAA   
  
  
+ ATGGACCCCC AAAACTCCGA ATCACAGGAA TCGACTACCC CCAGCAGGGT TTCAGACCTG CACAAAGGGT   
  
  
+ TGAAGCTACA GGACACCGAT TATCTGGGTA CTGTGAGCGA TTTGGGGTGC CTTTTTCTTA TCAGGGCATT   
  
  
+ GCTCAGAAGT GGGAAACTAT TCAGCCGGAG GATCTCAAGA TCGAACAAGA CGAGCTGGTG ATTGTCAACT   
  
  
+ GTTTGTTCAG GTCAGGAACA CTGCTCGATG AGACAGTCGA AGCAAACAGT CCAAGAGATG CTTTCTTAGC   
  
  
+ TTTGGTTAGA AAGCTGAATC CCAGCCTATT CATTCACGGG GTTGTCAATG GCACATTCAA CGCTCCATTC   
  
  
+ TTCGTGACTC GATTCAGAGA GGCATTGTTT CATTATTCAT CAGTGTTTGA TGTGTCTGAA GAGACAATTC   
  
  
+ CACGAGATGC CCATGAGAGG TTCTTGATTG AGAGCGAGAT TTGTGGGAAA GAACTGTTCA ATGTGGTTGC   
  
  
+ TTGTGAGGGT GCAGAGAGGG TTCAAAGGCC TGAGACATAC AAGCAGTGGC AAGTGAGGAC AACGCGGGCC   
  
  
+ GGGTTAAGGC AGGTTGCCTT GGACCAGGAG CTTATGAAGG AAGCAACGGC AATGGTGAAG GCAAATTATC   
  
  
+ ATAAGGATTT TATGGTGGAT ATAAATAGGC ATTGGATGCT TCAAGGTTGG AAGGGTAGAA CCTTGTGTGC   
  
  
+ TCTCTCATTT TGGCAACCTG CCTG  

- +Up\_Stream \_Len000GATCCC CAACTCTCTT AGGCTATCGA TACGTCAGGT TTGTAATAGA GCAGTTCGTA   
  
  
- ATGATAAATT TATTAATTAT AACTTGAACT ATCAATGTGA AAAACTTATG ATTAAGGTGT GGAGTAACGG   
  
  
- AAATAAAGGT TTGGAGCCCA ACGTAAACCT GGTGTATGGT CCTTCGAACC TGCTGTTTTT AAAGAAAAGA   
  
  
- ACTCATAATA AAACAAAATA ATAATAATAA AAAATGAAAT AAACCTGGTG AAGTGAACCC ATTGTTCTAA   
  
  
- CTTATAAACT GATAATATAA TATACAGTGC ATACCTATAC CATCAACGAA TCTCGAAACT GGCAAACGTC   
  
  
- CAAATTCATA CTAAGGGAAC CTTTCCTCGG GGAGAAATAG AATTAAGTCT TCATTGAAAA CAAAAAAATC   
  
  
- TATTTAACTT TTCTTTTTAT TATTTACAGA ATCAACTCAA ACCAATAAGT TAGGCTTTAA ACTAGGACGA   
  
  
- ATTACATATC ATATATTATG ATTATGCGTA CATTGTGTTG TGGAGTCTAC CAGTAACAAA ATGTTAAAAA   
  
  
- AACTCAAATC ATATTTTTAT TTTAGTTAAT AAAAATCTTC ACTACAAATT AAACGTAAAA AAAAGGTGGA   
  
  
- TTTTAGTGCG TGAGCTTGTA ATCCCCAAAC CGATTGAGTT TCTGAGTTCC ATCACTGAAT AGGTTTGAAT   
  
  
- AATGTAGGAT ATCTAGATAT TTTTGTATTT AGAGAATGTT AGTTAGGTTT AACTAAGCCA GATTAAACTA   
  
  
- CTGGGACTAC TCCCAGGATG TTAATGTGTT CACCCGGAAT CGGGGTACCG GCAAAAAATT AGAAGTACCC   
  
  
- AGGTCCGCCT CAATGGTAGA GGTCCAGGTA ACTCCCACAT AGTGCAGAGT TTAATGCTCA AACGGAGAAA   
  
  
- GATGCCCCTA ATATTTATAG ATGGACGTAT GGAGCTTTTC CTCCTCAACT CTAGTCGCTT TGAACCGGTA   
  
  
- GGGTCTCGTA ACTTTACGAG TCTTGGGAAA AAGATCTTTT TATAGCAAAA AGGACTTTTA TAGGCTGGAA   
  
  
- TACAGTAAAT AACAGCGAAA GAACTGAGAT TGGGCCCTGA GGTGTGATTG AACTGATAGC CTTTCCGCAA   
  
  
- GGGACCTAGT GGTAGGTTCT ATTGGAGAAA ACGTCCCAAC TGCGAGCAAC CTTCTTAGCA GCTTACATCT   
  
  
- CGGGGATAGT AGTGTATTCG GGCGAGAATG AAGTACGGTA GGAACTTATC GTCAAATAGG GCTTCGTTTT   
  
  
- TATTAAGATT GAAAGAATAT AACATTTGGG TGTAGATTTA AATTTAAGTA TGAGGTTTTA AGGCGGTGCG   
  
  
- TTTATTCCGA AAAATTATCC GAATGTATAT TACCGTACGA TATCTCTGTG ACAAAACTTC TTTGATAAAT   
  
  
- TTGTAGTCTG GAGTATAACT CATCGTTTAG TTCTGAAGAA AGGAGGTCCG GTATAACTCA TCGTTTAATT   
  
  
- CTTATTGGTC TTCGAACACT TTTCTCAATC TTTTATTTTC CATATTATCT CTGAATACAA GAATTCTATA   
  
  
- GAGGATCTAT ACTTACACTG GAGGATCAGC TATAAACACG ACCTAATCGC TCAACACACT GACTCAAACA   
  
  
- TACACAGAGA ATCGTATACT CAATAAGATC GACGAAGAAA TTCGATACAT CACTGACAGA AGGAAGAATA   
  
  
- AATATTCACG ATACAGGTTG ATTATCGGCT TTTTTTACCA AGCGGAAAAG ACCAAAAGAA GAAAAACTTC   
  
  
- CAACTAACCG ATTAAATATA TTACCAAAGT TATGAATAGG AAATAACAAG GTGTTTAGGT GGATTGGTGC   
  
  
- ATGGTAACTG TTATTAATGG GTGATTAAAG CCAACCTGAA CTAAAGGAAA ACAAACCATG GAGTCCGTAG   
  
  
- AAGACACCTG GAAGTAAGTG TCAAATTAAA AGTGTAAAAA TATTCCGACG AGAACACAAC GTTTAAAAGG   
  
  
- ATAGGTAAGG TGAGAAACGG GTAAGGGTCC GATTAATCAC AAGAGACAAG TTGGTACCTA AGACACGACC   
  
  
- AACTAGGACT CAAGTACTTT TTAGAGATGT TTAAGTTTGG ACTTAACGAG AGTTTGAAAA GTTACTTAGG   
  
  
- TTTACTGCTG TAGAAAGTTC GGGAAGTAAG TCTTTTACTA GGAAATGTCA AGGAGAGTAA ACTACTTCCT   
  
  
- TGGACAGAAT TGTCGACGTG ATCAGTTGTT CACGGTCTAA AGGGACTACG AACAGAGTTC AAGTAGTCAC   
  
  
- TATAAGAAGA GCTTCTCCCA AATCTACGTT TAGGACGTAG ACGTGTCCGA GAGCTTCGGT GGCTCTTCAG   
  
  
- GAACATACTA CGAGAGCCCG AACCTCTCGG TATGGGGGAA AGTACACTAG TGAAACGCGG TAGATAGAGA   
  
  
- TGTTCATAAC TCTCGGGTCT ACTGTCAAGA AGGTTATTTT CAATATCGTC GTTAGGGCTC TATCTACCAA   
  
  
- GAATACGATA GCGACTCGGG TCAAAGCTCA GGTTGGGGTT GACACACGAA CTAGTTGGGG TCAACTTGAG   
  
  
- GAAAGGTCGA GATGTACTCT AAAGAGCCAG GAACCACCTT GACCCAAGAG TTCGGAGACT CAACTCGAAG   
  
  
- CTACTACGTC CCTCACGGGC ACATCTCTTT TTCCCGTTTA GCTATTTCCC GAGCTCCTCC TTCTTCTCAG   
  
  
- CAGTTTCTCT CCCACTTCGC ACAATACCTC CTTCCTCGGT ATTCCAAGTT CGAAGGAAGT TACTACTAAT   
  
  
- GATACTCTAC CTTGTCATAC TACTACATCA TAACGAGACA TTATTACTTG ATTGCCCGTT ATCGGCAAAG   
  
  
- TTGTGGCCCT TTAGAAGTGG ACTCCTCCCT ACCTCCTCTA ACGTCTCTTC GGCTCCTTTC TTCGTCTTGT   
  
  
- CAAATCGTCA ACTTCAACTA GACTACTGGG ACGACTGAGT CACACGTGTT CGACATAGCT CGAAACTAGA   
  
  
- AGCTCCACGT TTGCTCGATG AATCCGTTTA GTCCGTCTTA CGAAGCGGGA TACCACCGTC GTAGGTCTCC   
  
  
- GAGCGGGTAG TACATCGGTT ACGAGAGCTC CGTGCATATC GACCGTGTCC GAGATGTCAG AGATGATTGG   
  
  
- AACAACTACG TTTCAAGAGT CGAAGACTGA AGGATTTCCG AATGTCCAAT ATACAGAGTC GACAAGGAAT   
  
  
- GTTTTCCTAC AGAAAGAAAG AACGATTGAC GAGCTAACGT TTCAACCGTC TCTTTCGTTG TTTCTAGGTA   
  
  
- TAGTAACTAA AACCACAAAA GGATCCAAAT GTTACCGGAA CAAAGTATGT TGTGGATAGT TTTTCCGGTT   
  
  
- TACCTGGGGG TTTTGAGGCT TAGTGTCCTT AGCTGATGGG GGTCGTCCCA AAGTCTGGAC GTGTTTCCCA   
  
  
- ACTTCGATGT CCTGTGGCTA ATAGACCCAT GACACTCGCT AAACCCCACG GAAAAAGAAT AGTCCCGTAA   
  
  
- CGAGTCTTCA CCCTTTGATA AGTCGGCCTC CTAGAGTTCT AGCTTGTTCT GCTCGACCAC TAACAGTTGA   
  
  
- CAAACAAGTC CAGTCCTTGT GACGAGCTAC TCTGTCAGCT TCGTTTGTCA GGTTCTCTAC GAAAGAATCG   
  
  
- AAACCAATCT TTCGACTTAG GGTCGGATAA GTAAGTGCCC CAACAGTTAC CGTGTAAGTT GCGAGGTAAG   
  
  
- AAGCACTGAG CTAAGTCTCT CCGTAACAAA GTAATAAGTA GTCACAAACT ACACAGACTT CTCTGTTAAG   
  
  
- GTGCTCTACG GGTACTCTCC AAGAACTAAC TCTCGCTCTA AACACCCTTT CTTGACAAGT TACACCAACG   
  
  
- AACACTCCCA CGTCTCTCCC AAGTTTCCGG ACTCTGTATG TTCGTCACCG TTCACTCCTG TTGCGCCCGG   
  
  
- CCCAATTCCG TCCAACGGAA CCTGGTCCTC GAATACTTCC TTCGTTGCCG TTACCACTTC CGTTTAATAG   
  
  
- TATTCCTAAA ATACCACCTA TATTTATCCG TAACCTACGA AGTTCCAACC TTCCCATCTT GGAACACACG   
  
  
- AGAGAGTAAA ACCGTTGGAC GGAC

+     TCA

| Site Name | Organism | Position | Strand | Matrix score. | sequence | function |
| --- | --- | --- | --- | --- | --- | --- |
| TCA | Pisum sativum | 832 | + | 9 | TCATCTTCAT |  |

>HU02G01571.1   
+ +Up\_Stream \_Len000CTAGGG GTTGAGAGAA TCCGATAGCT ATGCAGTCCA AACATTATCT CGTCAAGCAT   
  
  
+ TACTATTTAA ATAATTAATA TTGAACTTGA TAGTTACACT TTTTGAATAC TAATTCCACA CCTCATTGCC   
  
  
+ TTTATTTCCA AACCTCGGGT TGCATTTGGA CCACATACCA GGAAGCTTGG ACGACAAAAA TTTCTTTTCT   
  
  
+ TGAGTATTAT TTTGTTTTAT TATTATTATT TTTTACTTTA TTTGGACCAC TTCACTTGGG TAACAAGATT   
  
  
+ GAATATTTGA CTATTATATT ATATGTCACG TATGGATATG GTAGTTGCTT AGAGCTTTGA CCGTTTGCAG   
  
  
+ GTTTAAGTAT GATTCCCTTG GAAAGGAGCC CCTCTTTATC TTAATTCAGA AGTAACTTTT GTTTTTTTAG   
  
  
+ ATAAATTGAA AAGAAAAATA ATAAATGTCT TAGTTGAGTT TGGTTATTCA ATCCGAAATT TGATCCTGCT   
  
  
+ TAATGTATAG TATATAATAC TAATACGCAT GTAACACAAC ACCTCAGATG GTCATTGTTT TACAATTTTT   
  
  
+ TTGAGTTTAG TATAAAAATA AAATCAATTA TTTTTAGAAG TGATGTTTAA TTTGCATTTT TTTTCCACCT   
  
  
+ AAAATCACGC ACTCGAACAT TAGGGGTTTG GCTAACTCAA AGACTCAAGG TAGTGACTTA TCCAAACTTA   
  
  
+ TTACATCCTA TAGATCTATA AAAACATAAA TCTCTTACAA TCAATCCAAA TTGATTCGGT CTAATTTGAT   
  
  
+ GACCCTGATG AGGGTCCTAC AATTACACAA GTGGGCCTTA GCCCCATGGC CGTTTTTTAA TCTTCATGGG   
  
  
+ TCCAGGCGGA GTTACCATCT CCAGGTCCAT TGAGGGTGTA TCACGTCTCA AATTACGAGT TTGCCTCTTT   
  
  
+ CTACGGGGAT TATAAATATC TACCTGCATA CCTCGAAAAG GAGGAGTTGA GATCAGCGAA ACTTGGCCAT   
  
  
+ CCCAGAGCAT TGAAATGCTC AGAACCCTTT TTCTAGAAAA ATATCGTTTT TCCTGAAAAT ATCCGACCTT   
  
  
+ ATGTCATTTA TTGTCGCTTT CTTGACTCTA ACCCGGGACT CCACACTAAC TTGACTATCG GAAAGGCGTT   
  
  
+ CCCTGGATCA CCATCCAAGA TAACCTCTTT TGCAGGGTTG ACGCTCGTTG GAAGAATCGT CGAATGTAGA   
  
  
+ GCCCCTATCA TCACATAAGC CCGCTCTTAC TTCATGCCAT CCTTGAATAG CAGTTTATCC CGAAGCAAAA   
  
  
+ ATAATTCTAA CTTTCTTATA TTGTAAACCC ACATCTAAAT TTAAATTCAT ACTCCAAAAT TCCGCCACGC   
  
  
+ AAATAAGGCT TTTTAATAGG CTTACATATA ATGGCATGCT ATAGAGACAC TGTTTTGAAG AAACTATTTA   
  
  
+ AACATCAGAC CTCATATTGA GTAGCAAATC AAGACTTCTT TCCTCCAGGC CATATTGAGT AGCAAATTAA   
  
  
+ GAATAACCAG AAGCTTGTGA AAAGAGTTAG AAAATAAAAG GTATAATAGA GACTTATGTT CTTAAGATAT   
  
  
+ CTCCTAGATA TGAATGTGAC CTCCTAGTCG ATATTTGTGC TGGATTAGCG AGTTGTGTGA CTGAGTTTGT   
  
  
+ ATGTGTCTCT TAGCATATGA GTTATTCTAG CTGCTTCTTT AAGCTATGTA GTGACTGTCT TCCTTCTTAT   
  
  
+ TTATAAGTGC TATGTCCAAC TAATAGCCGA AAAAAATGGT TCGCCTTTTC TGGTTTTCTT CTTTTTGAAG   
  
  
+ GTTGATTGGC TAATTTATAT AATGGTTTCA ATACTTATCC TTTATTGTTC CACAAATCCA CCTAACCACG   
  
  
+ TACCATTGAC AATAATTACC CACTAATTTC GGTTGGACTT GATTTCCTTT TGTTTGGTAC CTCAGGCATC   
  
  
+ TTCTGTGGAC CTTCATTCAC AGTTTAATTT TCACATTTTT ATAAGGCTGC TCTTGTGTTG CAAATTTTCC   
  
  
+ TATCCATTCC ACTCTTTGCC CATTCCCAGG CTAATTAGTG TTCTCTGTTC AACCATGGAT TCTGTGCTGG   
  
  
+ TTGATCCTGA GTTCATGAAA AATCTCTACA AATTCAAACC TGAATTGCTC TCAAACTTTT CAATGAATCC   
  
  
+ AAATGACGAC ATCTTTCAAG CCCTTCATTC AGAAAATGAT CCTTTACAGT TCCTCTCATT TGATGAAGGA   
  
  
+ ACCTGTCTTA ACAGCTGCAC TAGTCAACAA GTGCCAGATT TCCCTGATGC TTGTCTCAAG TTCATCAGTG   
  
  
+ ATATTCTTCT CGAAGAGGGT TTAGATGCAA ATCCTGCATC TGCACAGGCT CTCGAAGCCA CCGAGAAGTC   
  
  
+ CTTGTATGAT GCTCTCGGGC TTGGAGAGCC ATACCCCCTT TCATGTGATC ACTTTGCGCC ATCTATCTCT   
  
  
+ ACAAGTATTG AGAGCCCAGA TGACAGTTCT TCCAATAAAA GTTATAGCAG CAATCCCGAG ATAGATGGTT   
  
  
+ CTTATGCTAT CGCTGAGCCC AGTTTCGAGT CCAACCCCAA CTGTGTGCTT GATCAACCCC AGTTGAACTC   
  
  
+ CTTTCCAGCT CTACATGAGA TTTCTCGGTC CTTGGTGGAA CTGGGTTCTC AAGCCTCTGA GTTGAGCTTC   
  
  
+ GATGATGCAG GGAGTGCCCG TGTAGAGAAA AAGGGCAAAT CGATAAAGGG CTCGAGGAGG AAGAAGAGTC   
  
  
+ GTCAAAGAGA GGGTGAAGCG TGTTATGGAG GAAGGAGCCA TAAGGTTCAA GCTTCCTTCA ATGATGATTA   
  
  
+ CTATGAGATG GAACAGTATG ATGATGTAGT ATTGCTCTGT AATAATGAAC TAACGGGCAA TAGCCGTTTC   
  
  
+ AACACCGGGA AATCTTCACC TGAGGAGGGA TGGAGGAGAT TGCAGAGAAG CCGAGGAAAG AAGCAGAACA   
  
  
+ GTTTAGCAGT TGAAGTTGAT CTGATGACCC TGCTGACTCA GTGTGCACAA GCTGTATCGA GCTTTGATCT   
  
  
+ TCGAGGTGCA AACGAGCTAC TTAGGCAAAT CAGGCAGAAT GCTTCGCCCT ATGGTGGCAG CATCCAGAGG   
  
  
+ CTCGCCCATC ATGTAGCCAA TGCTCTCGAG GCACGTATAG CTGGCACAGG CTCTACAGTC TCTACTAACC   
  
  
+ TTGTTGATGC AAAGTTCTCA GCTTCTGACT TCCTAAAGGC TTACAGGTTA TATGTCTCAG CTGTTCCTTA   
  
  
+ CAAAAGGATG TCTTTCTTTC TTGCTAACTG CTCGATTGCA AAGTTGGCAG AGAAAGCAAC AAAGATCCAT   
  
  
+ ATCATTGATT TTGGTGTTTT CCTAGGTTTA CAATGGCCTT GTTTCATACA ACACCTATCA AAAAGGCCAA   
  
  
+ ATGGACCCCC AAAACTCCGA ATCACAGGAA TCGACTACCC CCAGCAGGGT TTCAGACCTG CACAAAGGGT   
  
  
+ TGAAGCTACA GGACACCGAT TATCTGGGTA CTGTGAGCGA TTTGGGGTGC CTTTTTCTTA TCAGGGCATT   
  
  
+ GCTCAGAAGT GGGAAACTAT TCAGCCGGAG GATCTCAAGA TCGAACAAGA CGAGCTGGTG ATTGTCAACT   
  
  
+ GTTTGTTCAG GTCAGGAACA CTGCTCGATG AGACAGTCGA AGCAAACAGT CCAAGAGATG CTTTCTTAGC   
  
  
+ TTTGGTTAGA AAGCTGAATC CCAGCCTATT CATTCACGGG GTTGTCAATG GCACATTCAA CGCTCCATTC   
  
  
+ TTCGTGACTC GATTCAGAGA GGCATTGTTT CATTATTCAT CAGTGTTTGA TGTGTCTGAA GAGACAATTC   
  
  
+ CACGAGATGC CCATGAGAGG TTCTTGATTG AGAGCGAGAT TTGTGGGAAA GAACTGTTCA ATGTGGTTGC   
  
  
+ TTGTGAGGGT GCAGAGAGGG TTCAAAGGCC TGAGACATAC AAGCAGTGGC AAGTGAGGAC AACGCGGGCC   
  
  
+ GGGTTAAGGC AGGTTGCCTT GGACCAGGAG CTTATGAAGG AAGCAACGGC AATGGTGAAG GCAAATTATC   
  
  
+ ATAAGGATTT TATGGTGGAT ATAAATAGGC ATTGGATGCT TCAAGGTTGG AAGGGTAGAA CCTTGTGTGC   
  
  
+ TCTCTCATTT TGGCAACCTG CCTG  

- +Up\_Stream \_Len000GATCCC CAACTCTCTT AGGCTATCGA TACGTCAGGT TTGTAATAGA GCAGTTCGTA   
  
  
- ATGATAAATT TATTAATTAT AACTTGAACT ATCAATGTGA AAAACTTATG ATTAAGGTGT GGAGTAACGG   
  
  
- AAATAAAGGT TTGGAGCCCA ACGTAAACCT GGTGTATGGT CCTTCGAACC TGCTGTTTTT AAAGAAAAGA   
  
  
- ACTCATAATA AAACAAAATA ATAATAATAA AAAATGAAAT AAACCTGGTG AAGTGAACCC ATTGTTCTAA   
  
  
- CTTATAAACT GATAATATAA TATACAGTGC ATACCTATAC CATCAACGAA TCTCGAAACT GGCAAACGTC   
  
  
- CAAATTCATA CTAAGGGAAC CTTTCCTCGG GGAGAAATAG AATTAAGTCT TCATTGAAAA CAAAAAAATC   
  
  
- TATTTAACTT TTCTTTTTAT TATTTACAGA ATCAACTCAA ACCAATAAGT TAGGCTTTAA ACTAGGACGA   
  
  
- ATTACATATC ATATATTATG ATTATGCGTA CATTGTGTTG TGGAGTCTAC CAGTAACAAA ATGTTAAAAA   
  
  
- AACTCAAATC ATATTTTTAT TTTAGTTAAT AAAAATCTTC ACTACAAATT AAACGTAAAA AAAAGGTGGA   
  
  
- TTTTAGTGCG TGAGCTTGTA ATCCCCAAAC CGATTGAGTT TCTGAGTTCC ATCACTGAAT AGGTTTGAAT   
  
  
- AATGTAGGAT ATCTAGATAT TTTTGTATTT AGAGAATGTT AGTTAGGTTT AACTAAGCCA GATTAAACTA   
  
  
- CTGGGACTAC TCCCAGGATG TTAATGTGTT CACCCGGAAT CGGGGTACCG GCAAAAAATT AGAAGTACCC   
  
  
- AGGTCCGCCT CAATGGTAGA GGTCCAGGTA ACTCCCACAT AGTGCAGAGT TTAATGCTCA AACGGAGAAA   
  
  
- GATGCCCCTA ATATTTATAG ATGGACGTAT GGAGCTTTTC CTCCTCAACT CTAGTCGCTT TGAACCGGTA   
  
  
- GGGTCTCGTA ACTTTACGAG TCTTGGGAAA AAGATCTTTT TATAGCAAAA AGGACTTTTA TAGGCTGGAA   
  
  
- TACAGTAAAT AACAGCGAAA GAACTGAGAT TGGGCCCTGA GGTGTGATTG AACTGATAGC CTTTCCGCAA   
  
  
- GGGACCTAGT GGTAGGTTCT ATTGGAGAAA ACGTCCCAAC TGCGAGCAAC CTTCTTAGCA GCTTACATCT   
  
  
- CGGGGATAGT AGTGTATTCG GGCGAGAATG AAGTACGGTA GGAACTTATC GTCAAATAGG GCTTCGTTTT   
  
  
- TATTAAGATT GAAAGAATAT AACATTTGGG TGTAGATTTA AATTTAAGTA TGAGGTTTTA AGGCGGTGCG   
  
  
- TTTATTCCGA AAAATTATCC GAATGTATAT TACCGTACGA TATCTCTGTG ACAAAACTTC TTTGATAAAT   
  
  
- TTGTAGTCTG GAGTATAACT CATCGTTTAG TTCTGAAGAA AGGAGGTCCG GTATAACTCA TCGTTTAATT   
  
  
- CTTATTGGTC TTCGAACACT TTTCTCAATC TTTTATTTTC CATATTATCT CTGAATACAA GAATTCTATA   
  
  
- GAGGATCTAT ACTTACACTG GAGGATCAGC TATAAACACG ACCTAATCGC TCAACACACT GACTCAAACA   
  
  
- TACACAGAGA ATCGTATACT CAATAAGATC GACGAAGAAA TTCGATACAT CACTGACAGA AGGAAGAATA   
  
  
- AATATTCACG ATACAGGTTG ATTATCGGCT TTTTTTACCA AGCGGAAAAG ACCAAAAGAA GAAAAACTTC   
  
  
- CAACTAACCG ATTAAATATA TTACCAAAGT TATGAATAGG AAATAACAAG GTGTTTAGGT GGATTGGTGC   
  
  
- ATGGTAACTG TTATTAATGG GTGATTAAAG CCAACCTGAA CTAAAGGAAA ACAAACCATG GAGTCCGTAG   
  
  
- AAGACACCTG GAAGTAAGTG TCAAATTAAA AGTGTAAAAA TATTCCGACG AGAACACAAC GTTTAAAAGG   
  
  
- ATAGGTAAGG TGAGAAACGG GTAAGGGTCC GATTAATCAC AAGAGACAAG TTGGTACCTA AGACACGACC   
  
  
- AACTAGGACT CAAGTACTTT TTAGAGATGT TTAAGTTTGG ACTTAACGAG AGTTTGAAAA GTTACTTAGG   
  
  
- TTTACTGCTG TAGAAAGTTC GGGAAGTAAG TCTTTTACTA GGAAATGTCA AGGAGAGTAA ACTACTTCCT   
  
  
- TGGACAGAAT TGTCGACGTG ATCAGTTGTT CACGGTCTAA AGGGACTACG AACAGAGTTC AAGTAGTCAC   
  
  
- TATAAGAAGA GCTTCTCCCA AATCTACGTT TAGGACGTAG ACGTGTCCGA GAGCTTCGGT GGCTCTTCAG   
  
  
- GAACATACTA CGAGAGCCCG AACCTCTCGG TATGGGGGAA AGTACACTAG TGAAACGCGG TAGATAGAGA   
  
  
- TGTTCATAAC TCTCGGGTCT ACTGTCAAGA AGGTTATTTT CAATATCGTC GTTAGGGCTC TATCTACCAA   
  
  
- GAATACGATA GCGACTCGGG TCAAAGCTCA GGTTGGGGTT GACACACGAA CTAGTTGGGG TCAACTTGAG   
  
  
- GAAAGGTCGA GATGTACTCT AAAGAGCCAG GAACCACCTT GACCCAAGAG TTCGGAGACT CAACTCGAAG   
  
  
- CTACTACGTC CCTCACGGGC ACATCTCTTT TTCCCGTTTA GCTATTTCCC GAGCTCCTCC TTCTTCTCAG   
  
  
- CAGTTTCTCT CCCACTTCGC ACAATACCTC CTTCCTCGGT ATTCCAAGTT CGAAGGAAGT TACTACTAAT   
  
  
- GATACTCTAC CTTGTCATAC TACTACATCA TAACGAGACA TTATTACTTG ATTGCCCGTT ATCGGCAAAG   
  
  
- TTGTGGCCCT TTAGAAGTGG ACTCCTCCCT ACCTCCTCTA ACGTCTCTTC GGCTCCTTTC TTCGTCTTGT   
  
  
- CAAATCGTCA ACTTCAACTA GACTACTGGG ACGACTGAGT CACACGTGTT CGACATAGCT CGAAACTAGA   
  
  
- AGCTCCACGT TTGCTCGATG AATCCGTTTA GTCCGTCTTA CGAAGCGGGA TACCACCGTC GTAGGTCTCC   
  
  
- GAGCGGGTAG TACATCGGTT ACGAGAGCTC CGTGCATATC GACCGTGTCC GAGATGTCAG AGATGATTGG   
  
  
- AACAACTACG TTTCAAGAGT CGAAGACTGA AGGATTTCCG AATGTCCAAT ATACAGAGTC GACAAGGAAT   
  
  
- GTTTTCCTAC AGAAAGAAAG AACGATTGAC GAGCTAACGT TTCAACCGTC TCTTTCGTTG TTTCTAGGTA   
  
  
- TAGTAACTAA AACCACAAAA GGATCCAAAT GTTACCGGAA CAAAGTATGT TGTGGATAGT TTTTCCGGTT   
  
  
- TACCTGGGGG TTTTGAGGCT TAGTGTCCTT AGCTGATGGG GGTCGTCCCA AAGTCTGGAC GTGTTTCCCA   
  
  
- ACTTCGATGT CCTGTGGCTA ATAGACCCAT GACACTCGCT AAACCCCACG GAAAAAGAAT AGTCCCGTAA   
  
  
- CGAGTCTTCA CCCTTTGATA AGTCGGCCTC CTAGAGTTCT AGCTTGTTCT GCTCGACCAC TAACAGTTGA   
  
  
- CAAACAAGTC CAGTCCTTGT GACGAGCTAC TCTGTCAGCT TCGTTTGTCA GGTTCTCTAC GAAAGAATCG   
  
  
- AAACCAATCT TTCGACTTAG GGTCGGATAA GTAAGTGCCC CAACAGTTAC CGTGTAAGTT GCGAGGTAAG   
  
  
- AAGCACTGAG CTAAGTCTCT CCGTAACAAA GTAATAAGTA GTCACAAACT ACACAGACTT CTCTGTTAAG   
  
  
- GTGCTCTACG GGTACTCTCC AAGAACTAAC TCTCGCTCTA AACACCCTTT CTTGACAAGT TACACCAACG   
  
  
- AACACTCCCA CGTCTCTCCC AAGTTTCCGG ACTCTGTATG TTCGTCACCG TTCACTCCTG TTGCGCCCGG   
  
  
- CCCAATTCCG TCCAACGGAA CCTGGTCCTC GAATACTTCC TTCGTTGCCG TTACCACTTC CGTTTAATAG   
  
  
- TATTCCTAAA ATACCACCTA TATTTATCCG TAACCTACGA AGTTCCAACC TTCCCATCTT GGAACACACG   
  
  
- AGAGAGTAAA ACCGTTGGAC GGAC

+     TCA-element

| Site Name | Organism | Position | Strand | Matrix score. | sequence | function |
| --- | --- | --- | --- | --- | --- | --- |
| TCA-element | Brassica oleracea | 3437 | + | 9 | TCAGAAGAGG | cis-acting element involved in salicylic acid responsiveness |
| TCA-element | Nicotiana tabacum | 1714 | - | 9 | CCATCTTTTT | cis-acting element involved in salicylic acid responsiveness |

>HU02G01571.1   
+ +Up\_Stream \_Len000CTAGGG GTTGAGAGAA TCCGATAGCT ATGCAGTCCA AACATTATCT CGTCAAGCAT   
  
  
+ TACTATTTAA ATAATTAATA TTGAACTTGA TAGTTACACT TTTTGAATAC TAATTCCACA CCTCATTGCC   
  
  
+ TTTATTTCCA AACCTCGGGT TGCATTTGGA CCACATACCA GGAAGCTTGG ACGACAAAAA TTTCTTTTCT   
  
  
+ TGAGTATTAT TTTGTTTTAT TATTATTATT TTTTACTTTA TTTGGACCAC TTCACTTGGG TAACAAGATT   
  
  
+ GAATATTTGA CTATTATATT ATATGTCACG TATGGATATG GTAGTTGCTT AGAGCTTTGA CCGTTTGCAG   
  
  
+ GTTTAAGTAT GATTCCCTTG GAAAGGAGCC CCTCTTTATC TTAATTCAGA AGTAACTTTT GTTTTTTTAG   
  
  
+ ATAAATTGAA AAGAAAAATA ATAAATGTCT TAGTTGAGTT TGGTTATTCA ATCCGAAATT TGATCCTGCT   
  
  
+ TAATGTATAG TATATAATAC TAATACGCAT GTAACACAAC ACCTCAGATG GTCATTGTTT TACAATTTTT   
  
  
+ TTGAGTTTAG TATAAAAATA AAATCAATTA TTTTTAGAAG TGATGTTTAA TTTGCATTTT TTTTCCACCT   
  
  
+ AAAATCACGC ACTCGAACAT TAGGGGTTTG GCTAACTCAA AGACTCAAGG TAGTGACTTA TCCAAACTTA   
  
  
+ TTACATCCTA TAGATCTATA AAAACATAAA TCTCTTACAA TCAATCCAAA TTGATTCGGT CTAATTTGAT   
  
  
+ GACCCTGATG AGGGTCCTAC AATTACACAA GTGGGCCTTA GCCCCATGGC CGTTTTTTAA TCTTCATGGG   
  
  
+ TCCAGGCGGA GTTACCATCT CCAGGTCCAT TGAGGGTGTA TCACGTCTCA AATTACGAGT TTGCCTCTTT   
  
  
+ CTACGGGGAT TATAAATATC TACCTGCATA CCTCGAAAAG GAGGAGTTGA GATCAGCGAA ACTTGGCCAT   
  
  
+ CCCAGAGCAT TGAAATGCTC AGAACCCTTT TTCTAGAAAA ATATCGTTTT TCCTGAAAAT ATCCGACCTT   
  
  
+ ATGTCATTTA TTGTCGCTTT CTTGACTCTA ACCCGGGACT CCACACTAAC TTGACTATCG GAAAGGCGTT   
  
  
+ CCCTGGATCA CCATCCAAGA TAACCTCTTT TGCAGGGTTG ACGCTCGTTG GAAGAATCGT CGAATGTAGA   
  
  
+ GCCCCTATCA TCACATAAGC CCGCTCTTAC TTCATGCCAT CCTTGAATAG CAGTTTATCC CGAAGCAAAA   
  
  
+ ATAATTCTAA CTTTCTTATA TTGTAAACCC ACATCTAAAT TTAAATTCAT ACTCCAAAAT TCCGCCACGC   
  
  
+ AAATAAGGCT TTTTAATAGG CTTACATATA ATGGCATGCT ATAGAGACAC TGTTTTGAAG AAACTATTTA   
  
  
+ AACATCAGAC CTCATATTGA GTAGCAAATC AAGACTTCTT TCCTCCAGGC CATATTGAGT AGCAAATTAA   
  
  
+ GAATAACCAG AAGCTTGTGA AAAGAGTTAG AAAATAAAAG GTATAATAGA GACTTATGTT CTTAAGATAT   
  
  
+ CTCCTAGATA TGAATGTGAC CTCCTAGTCG ATATTTGTGC TGGATTAGCG AGTTGTGTGA CTGAGTTTGT   
  
  
+ ATGTGTCTCT TAGCATATGA GTTATTCTAG CTGCTTCTTT AAGCTATGTA GTGACTGTCT TCCTTCTTAT   
  
  
+ TTATAAGTGC TATGTCCAAC TAATAGCCGA AAAAAATGGT TCGCCTTTTC TGGTTTTCTT CTTTTTGAAG   
  
  
+ GTTGATTGGC TAATTTATAT AATGGTTTCA ATACTTATCC TTTATTGTTC CACAAATCCA CCTAACCACG   
  
  
+ TACCATTGAC AATAATTACC CACTAATTTC GGTTGGACTT GATTTCCTTT TGTTTGGTAC CTCAGGCATC   
  
  
+ TTCTGTGGAC CTTCATTCAC AGTTTAATTT TCACATTTTT ATAAGGCTGC TCTTGTGTTG CAAATTTTCC   
  
  
+ TATCCATTCC ACTCTTTGCC CATTCCCAGG CTAATTAGTG TTCTCTGTTC AACCATGGAT TCTGTGCTGG   
  
  
+ TTGATCCTGA GTTCATGAAA AATCTCTACA AATTCAAACC TGAATTGCTC TCAAACTTTT CAATGAATCC   
  
  
+ AAATGACGAC ATCTTTCAAG CCCTTCATTC AGAAAATGAT CCTTTACAGT TCCTCTCATT TGATGAAGGA   
  
  
+ ACCTGTCTTA ACAGCTGCAC TAGTCAACAA GTGCCAGATT TCCCTGATGC TTGTCTCAAG TTCATCAGTG   
  
  
+ ATATTCTTCT CGAAGAGGGT TTAGATGCAA ATCCTGCATC TGCACAGGCT CTCGAAGCCA CCGAGAAGTC   
  
  
+ CTTGTATGAT GCTCTCGGGC TTGGAGAGCC ATACCCCCTT TCATGTGATC ACTTTGCGCC ATCTATCTCT   
  
  
+ ACAAGTATTG AGAGCCCAGA TGACAGTTCT TCCAATAAAA GTTATAGCAG CAATCCCGAG ATAGATGGTT   
  
  
+ CTTATGCTAT CGCTGAGCCC AGTTTCGAGT CCAACCCCAA CTGTGTGCTT GATCAACCCC AGTTGAACTC   
  
  
+ CTTTCCAGCT CTACATGAGA TTTCTCGGTC CTTGGTGGAA CTGGGTTCTC AAGCCTCTGA GTTGAGCTTC   
  
  
+ GATGATGCAG GGAGTGCCCG TGTAGAGAAA AAGGGCAAAT CGATAAAGGG CTCGAGGAGG AAGAAGAGTC   
  
  
+ GTCAAAGAGA GGGTGAAGCG TGTTATGGAG GAAGGAGCCA TAAGGTTCAA GCTTCCTTCA ATGATGATTA   
  
  
+ CTATGAGATG GAACAGTATG ATGATGTAGT ATTGCTCTGT AATAATGAAC TAACGGGCAA TAGCCGTTTC   
  
  
+ AACACCGGGA AATCTTCACC TGAGGAGGGA TGGAGGAGAT TGCAGAGAAG CCGAGGAAAG AAGCAGAACA   
  
  
+ GTTTAGCAGT TGAAGTTGAT CTGATGACCC TGCTGACTCA GTGTGCACAA GCTGTATCGA GCTTTGATCT   
  
  
+ TCGAGGTGCA AACGAGCTAC TTAGGCAAAT CAGGCAGAAT GCTTCGCCCT ATGGTGGCAG CATCCAGAGG   
  
  
+ CTCGCCCATC ATGTAGCCAA TGCTCTCGAG GCACGTATAG CTGGCACAGG CTCTACAGTC TCTACTAACC   
  
  
+ TTGTTGATGC AAAGTTCTCA GCTTCTGACT TCCTAAAGGC TTACAGGTTA TATGTCTCAG CTGTTCCTTA   
  
  
+ CAAAAGGATG TCTTTCTTTC TTGCTAACTG CTCGATTGCA AAGTTGGCAG AGAAAGCAAC AAAGATCCAT   
  
  
+ ATCATTGATT TTGGTGTTTT CCTAGGTTTA CAATGGCCTT GTTTCATACA ACACCTATCA AAAAGGCCAA   
  
  
+ ATGGACCCCC AAAACTCCGA ATCACAGGAA TCGACTACCC CCAGCAGGGT TTCAGACCTG CACAAAGGGT   
  
  
+ TGAAGCTACA GGACACCGAT TATCTGGGTA CTGTGAGCGA TTTGGGGTGC CTTTTTCTTA TCAGGGCATT   
  
  
+ GCTCAGAAGT GGGAAACTAT TCAGCCGGAG GATCTCAAGA TCGAACAAGA CGAGCTGGTG ATTGTCAACT   
  
  
+ GTTTGTTCAG GTCAGGAACA CTGCTCGATG AGACAGTCGA AGCAAACAGT CCAAGAGATG CTTTCTTAGC   
  
  
+ TTTGGTTAGA AAGCTGAATC CCAGCCTATT CATTCACGGG GTTGTCAATG GCACATTCAA CGCTCCATTC   
  
  
+ TTCGTGACTC GATTCAGAGA GGCATTGTTT CATTATTCAT CAGTGTTTGA TGTGTCTGAA GAGACAATTC   
  
  
+ CACGAGATGC CCATGAGAGG TTCTTGATTG AGAGCGAGAT TTGTGGGAAA GAACTGTTCA ATGTGGTTGC   
  
  
+ TTGTGAGGGT GCAGAGAGGG TTCAAAGGCC TGAGACATAC AAGCAGTGGC AAGTGAGGAC AACGCGGGCC   
  
  
+ GGGTTAAGGC AGGTTGCCTT GGACCAGGAG CTTATGAAGG AAGCAACGGC AATGGTGAAG GCAAATTATC   
  
  
+ ATAAGGATTT TATGGTGGAT ATAAATAGGC ATTGGATGCT TCAAGGTTGG AAGGGTAGAA CCTTGTGTGC   
  
  
+ TCTCTCATTT TGGCAACCTG CCTG  

- +Up\_Stream \_Len000GATCCC CAACTCTCTT AGGCTATCGA TACGTCAGGT TTGTAATAGA GCAGTTCGTA   
  
  
- ATGATAAATT TATTAATTAT AACTTGAACT ATCAATGTGA AAAACTTATG ATTAAGGTGT GGAGTAACGG   
  
  
- AAATAAAGGT TTGGAGCCCA ACGTAAACCT GGTGTATGGT CCTTCGAACC TGCTGTTTTT AAAGAAAAGA   
  
  
- ACTCATAATA AAACAAAATA ATAATAATAA AAAATGAAAT AAACCTGGTG AAGTGAACCC ATTGTTCTAA   
  
  
- CTTATAAACT GATAATATAA TATACAGTGC ATACCTATAC CATCAACGAA TCTCGAAACT GGCAAACGTC   
  
  
- CAAATTCATA CTAAGGGAAC CTTTCCTCGG GGAGAAATAG AATTAAGTCT TCATTGAAAA CAAAAAAATC   
  
  
- TATTTAACTT TTCTTTTTAT TATTTACAGA ATCAACTCAA ACCAATAAGT TAGGCTTTAA ACTAGGACGA   
  
  
- ATTACATATC ATATATTATG ATTATGCGTA CATTGTGTTG TGGAGTCTAC CAGTAACAAA ATGTTAAAAA   
  
  
- AACTCAAATC ATATTTTTAT TTTAGTTAAT AAAAATCTTC ACTACAAATT AAACGTAAAA AAAAGGTGGA   
  
  
- TTTTAGTGCG TGAGCTTGTA ATCCCCAAAC CGATTGAGTT TCTGAGTTCC ATCACTGAAT AGGTTTGAAT   
  
  
- AATGTAGGAT ATCTAGATAT TTTTGTATTT AGAGAATGTT AGTTAGGTTT AACTAAGCCA GATTAAACTA   
  
  
- CTGGGACTAC TCCCAGGATG TTAATGTGTT CACCCGGAAT CGGGGTACCG GCAAAAAATT AGAAGTACCC   
  
  
- AGGTCCGCCT CAATGGTAGA GGTCCAGGTA ACTCCCACAT AGTGCAGAGT TTAATGCTCA AACGGAGAAA   
  
  
- GATGCCCCTA ATATTTATAG ATGGACGTAT GGAGCTTTTC CTCCTCAACT CTAGTCGCTT TGAACCGGTA   
  
  
- GGGTCTCGTA ACTTTACGAG TCTTGGGAAA AAGATCTTTT TATAGCAAAA AGGACTTTTA TAGGCTGGAA   
  
  
- TACAGTAAAT AACAGCGAAA GAACTGAGAT TGGGCCCTGA GGTGTGATTG AACTGATAGC CTTTCCGCAA   
  
  
- GGGACCTAGT GGTAGGTTCT ATTGGAGAAA ACGTCCCAAC TGCGAGCAAC CTTCTTAGCA GCTTACATCT   
  
  
- CGGGGATAGT AGTGTATTCG GGCGAGAATG AAGTACGGTA GGAACTTATC GTCAAATAGG GCTTCGTTTT   
  
  
- TATTAAGATT GAAAGAATAT AACATTTGGG TGTAGATTTA AATTTAAGTA TGAGGTTTTA AGGCGGTGCG   
  
  
- TTTATTCCGA AAAATTATCC GAATGTATAT TACCGTACGA TATCTCTGTG ACAAAACTTC TTTGATAAAT   
  
  
- TTGTAGTCTG GAGTATAACT CATCGTTTAG TTCTGAAGAA AGGAGGTCCG GTATAACTCA TCGTTTAATT   
  
  
- CTTATTGGTC TTCGAACACT TTTCTCAATC TTTTATTTTC CATATTATCT CTGAATACAA GAATTCTATA   
  
  
- GAGGATCTAT ACTTACACTG GAGGATCAGC TATAAACACG ACCTAATCGC TCAACACACT GACTCAAACA   
  
  
- TACACAGAGA ATCGTATACT CAATAAGATC GACGAAGAAA TTCGATACAT CACTGACAGA AGGAAGAATA   
  
  
- AATATTCACG ATACAGGTTG ATTATCGGCT TTTTTTACCA AGCGGAAAAG ACCAAAAGAA GAAAAACTTC   
  
  
- CAACTAACCG ATTAAATATA TTACCAAAGT TATGAATAGG AAATAACAAG GTGTTTAGGT GGATTGGTGC   
  
  
- ATGGTAACTG TTATTAATGG GTGATTAAAG CCAACCTGAA CTAAAGGAAA ACAAACCATG GAGTCCGTAG   
  
  
- AAGACACCTG GAAGTAAGTG TCAAATTAAA AGTGTAAAAA TATTCCGACG AGAACACAAC GTTTAAAAGG   
  
  
- ATAGGTAAGG TGAGAAACGG GTAAGGGTCC GATTAATCAC AAGAGACAAG TTGGTACCTA AGACACGACC   
  
  
- AACTAGGACT CAAGTACTTT TTAGAGATGT TTAAGTTTGG ACTTAACGAG AGTTTGAAAA GTTACTTAGG   
  
  
- TTTACTGCTG TAGAAAGTTC GGGAAGTAAG TCTTTTACTA GGAAATGTCA AGGAGAGTAA ACTACTTCCT   
  
  
- TGGACAGAAT TGTCGACGTG ATCAGTTGTT CACGGTCTAA AGGGACTACG AACAGAGTTC AAGTAGTCAC   
  
  
- TATAAGAAGA GCTTCTCCCA AATCTACGTT TAGGACGTAG ACGTGTCCGA GAGCTTCGGT GGCTCTTCAG   
  
  
- GAACATACTA CGAGAGCCCG AACCTCTCGG TATGGGGGAA AGTACACTAG TGAAACGCGG TAGATAGAGA   
  
  
- TGTTCATAAC TCTCGGGTCT ACTGTCAAGA AGGTTATTTT CAATATCGTC GTTAGGGCTC TATCTACCAA   
  
  
- GAATACGATA GCGACTCGGG TCAAAGCTCA GGTTGGGGTT GACACACGAA CTAGTTGGGG TCAACTTGAG   
  
  
- GAAAGGTCGA GATGTACTCT AAAGAGCCAG GAACCACCTT GACCCAAGAG TTCGGAGACT CAACTCGAAG   
  
  
- CTACTACGTC CCTCACGGGC ACATCTCTTT TTCCCGTTTA GCTATTTCCC GAGCTCCTCC TTCTTCTCAG   
  
  
- CAGTTTCTCT CCCACTTCGC ACAATACCTC CTTCCTCGGT ATTCCAAGTT CGAAGGAAGT TACTACTAAT   
  
  
- GATACTCTAC CTTGTCATAC TACTACATCA TAACGAGACA TTATTACTTG ATTGCCCGTT ATCGGCAAAG   
  
  
- TTGTGGCCCT TTAGAAGTGG ACTCCTCCCT ACCTCCTCTA ACGTCTCTTC GGCTCCTTTC TTCGTCTTGT   
  
  
- CAAATCGTCA ACTTCAACTA GACTACTGGG ACGACTGAGT CACACGTGTT CGACATAGCT CGAAACTAGA   
  
  
- AGCTCCACGT TTGCTCGATG AATCCGTTTA GTCCGTCTTA CGAAGCGGGA TACCACCGTC GTAGGTCTCC   
  
  
- GAGCGGGTAG TACATCGGTT ACGAGAGCTC CGTGCATATC GACCGTGTCC GAGATGTCAG AGATGATTGG   
  
  
- AACAACTACG TTTCAAGAGT CGAAGACTGA AGGATTTCCG AATGTCCAAT ATACAGAGTC GACAAGGAAT   
  
  
- GTTTTCCTAC AGAAAGAAAG AACGATTGAC GAGCTAACGT TTCAACCGTC TCTTTCGTTG TTTCTAGGTA   
  
  
- TAGTAACTAA AACCACAAAA GGATCCAAAT GTTACCGGAA CAAAGTATGT TGTGGATAGT TTTTCCGGTT   
  
  
- TACCTGGGGG TTTTGAGGCT TAGTGTCCTT AGCTGATGGG GGTCGTCCCA AAGTCTGGAC GTGTTTCCCA   
  
  
- ACTTCGATGT CCTGTGGCTA ATAGACCCAT GACACTCGCT AAACCCCACG GAAAAAGAAT AGTCCCGTAA   
  
  
- CGAGTCTTCA CCCTTTGATA AGTCGGCCTC CTAGAGTTCT AGCTTGTTCT GCTCGACCAC TAACAGTTGA   
  
  
- CAAACAAGTC CAGTCCTTGT GACGAGCTAC TCTGTCAGCT TCGTTTGTCA GGTTCTCTAC GAAAGAATCG   
  
  
- AAACCAATCT TTCGACTTAG GGTCGGATAA GTAAGTGCCC CAACAGTTAC CGTGTAAGTT GCGAGGTAAG   
  
  
- AAGCACTGAG CTAAGTCTCT CCGTAACAAA GTAATAAGTA GTCACAAACT ACACAGACTT CTCTGTTAAG   
  
  
- GTGCTCTACG GGTACTCTCC AAGAACTAAC TCTCGCTCTA AACACCCTTT CTTGACAAGT TACACCAACG   
  
  
- AACACTCCCA CGTCTCTCCC AAGTTTCCGG ACTCTGTATG TTCGTCACCG TTCACTCCTG TTGCGCCCGG   
  
  
- CCCAATTCCG TCCAACGGAA CCTGGTCCTC GAATACTTCC TTCGTTGCCG TTACCACTTC CGTTTAATAG   
  
  
- TATTCCTAAA ATACCACCTA TATTTATCCG TAACCTACGA AGTTCCAACC TTCCCATCTT GGAACACACG   
  
  
- AGAGAGTAAA ACCGTTGGAC GGAC

+     TCT-motif

| Site Name | Organism | Position | Strand | Matrix score. | sequence | function |
| --- | --- | --- | --- | --- | --- | --- |
| TCT-motif | Arabidopsis thaliana | 737 | + | 6 | TCTTAC | part of a light responsive element |
| TCT-motif | Arabidopsis thaliana | 1219 | + | 6 | TCTTAC | part of a light responsive element |

>HU02G01571.1   
+ +Up\_Stream \_Len000CTAGGG GTTGAGAGAA TCCGATAGCT ATGCAGTCCA AACATTATCT CGTCAAGCAT   
  
  
+ TACTATTTAA ATAATTAATA TTGAACTTGA TAGTTACACT TTTTGAATAC TAATTCCACA CCTCATTGCC   
  
  
+ TTTATTTCCA AACCTCGGGT TGCATTTGGA CCACATACCA GGAAGCTTGG ACGACAAAAA TTTCTTTTCT   
  
  
+ TGAGTATTAT TTTGTTTTAT TATTATTATT TTTTACTTTA TTTGGACCAC TTCACTTGGG TAACAAGATT   
  
  
+ GAATATTTGA CTATTATATT ATATGTCACG TATGGATATG GTAGTTGCTT AGAGCTTTGA CCGTTTGCAG   
  
  
+ GTTTAAGTAT GATTCCCTTG GAAAGGAGCC CCTCTTTATC TTAATTCAGA AGTAACTTTT GTTTTTTTAG   
  
  
+ ATAAATTGAA AAGAAAAATA ATAAATGTCT TAGTTGAGTT TGGTTATTCA ATCCGAAATT TGATCCTGCT   
  
  
+ TAATGTATAG TATATAATAC TAATACGCAT GTAACACAAC ACCTCAGATG GTCATTGTTT TACAATTTTT   
  
  
+ TTGAGTTTAG TATAAAAATA AAATCAATTA TTTTTAGAAG TGATGTTTAA TTTGCATTTT TTTTCCACCT   
  
  
+ AAAATCACGC ACTCGAACAT TAGGGGTTTG GCTAACTCAA AGACTCAAGG TAGTGACTTA TCCAAACTTA   
  
  
+ TTACATCCTA TAGATCTATA AAAACATAAA TCTCTTACAA TCAATCCAAA TTGATTCGGT CTAATTTGAT   
  
  
+ GACCCTGATG AGGGTCCTAC AATTACACAA GTGGGCCTTA GCCCCATGGC CGTTTTTTAA TCTTCATGGG   
  
  
+ TCCAGGCGGA GTTACCATCT CCAGGTCCAT TGAGGGTGTA TCACGTCTCA AATTACGAGT TTGCCTCTTT   
  
  
+ CTACGGGGAT TATAAATATC TACCTGCATA CCTCGAAAAG GAGGAGTTGA GATCAGCGAA ACTTGGCCAT   
  
  
+ CCCAGAGCAT TGAAATGCTC AGAACCCTTT TTCTAGAAAA ATATCGTTTT TCCTGAAAAT ATCCGACCTT   
  
  
+ ATGTCATTTA TTGTCGCTTT CTTGACTCTA ACCCGGGACT CCACACTAAC TTGACTATCG GAAAGGCGTT   
  
  
+ CCCTGGATCA CCATCCAAGA TAACCTCTTT TGCAGGGTTG ACGCTCGTTG GAAGAATCGT CGAATGTAGA   
  
  
+ GCCCCTATCA TCACATAAGC CCGCTCTTAC TTCATGCCAT CCTTGAATAG CAGTTTATCC CGAAGCAAAA   
  
  
+ ATAATTCTAA CTTTCTTATA TTGTAAACCC ACATCTAAAT TTAAATTCAT ACTCCAAAAT TCCGCCACGC   
  
  
+ AAATAAGGCT TTTTAATAGG CTTACATATA ATGGCATGCT ATAGAGACAC TGTTTTGAAG AAACTATTTA   
  
  
+ AACATCAGAC CTCATATTGA GTAGCAAATC AAGACTTCTT TCCTCCAGGC CATATTGAGT AGCAAATTAA   
  
  
+ GAATAACCAG AAGCTTGTGA AAAGAGTTAG AAAATAAAAG GTATAATAGA GACTTATGTT CTTAAGATAT   
  
  
+ CTCCTAGATA TGAATGTGAC CTCCTAGTCG ATATTTGTGC TGGATTAGCG AGTTGTGTGA CTGAGTTTGT   
  
  
+ ATGTGTCTCT TAGCATATGA GTTATTCTAG CTGCTTCTTT AAGCTATGTA GTGACTGTCT TCCTTCTTAT   
  
  
+ TTATAAGTGC TATGTCCAAC TAATAGCCGA AAAAAATGGT TCGCCTTTTC TGGTTTTCTT CTTTTTGAAG   
  
  
+ GTTGATTGGC TAATTTATAT AATGGTTTCA ATACTTATCC TTTATTGTTC CACAAATCCA CCTAACCACG   
  
  
+ TACCATTGAC AATAATTACC CACTAATTTC GGTTGGACTT GATTTCCTTT TGTTTGGTAC CTCAGGCATC   
  
  
+ TTCTGTGGAC CTTCATTCAC AGTTTAATTT TCACATTTTT ATAAGGCTGC TCTTGTGTTG CAAATTTTCC   
  
  
+ TATCCATTCC ACTCTTTGCC CATTCCCAGG CTAATTAGTG TTCTCTGTTC AACCATGGAT TCTGTGCTGG   
  
  
+ TTGATCCTGA GTTCATGAAA AATCTCTACA AATTCAAACC TGAATTGCTC TCAAACTTTT CAATGAATCC   
  
  
+ AAATGACGAC ATCTTTCAAG CCCTTCATTC AGAAAATGAT CCTTTACAGT TCCTCTCATT TGATGAAGGA   
  
  
+ ACCTGTCTTA ACAGCTGCAC TAGTCAACAA GTGCCAGATT TCCCTGATGC TTGTCTCAAG TTCATCAGTG   
  
  
+ ATATTCTTCT CGAAGAGGGT TTAGATGCAA ATCCTGCATC TGCACAGGCT CTCGAAGCCA CCGAGAAGTC   
  
  
+ CTTGTATGAT GCTCTCGGGC TTGGAGAGCC ATACCCCCTT TCATGTGATC ACTTTGCGCC ATCTATCTCT   
  
  
+ ACAAGTATTG AGAGCCCAGA TGACAGTTCT TCCAATAAAA GTTATAGCAG CAATCCCGAG ATAGATGGTT   
  
  
+ CTTATGCTAT CGCTGAGCCC AGTTTCGAGT CCAACCCCAA CTGTGTGCTT GATCAACCCC AGTTGAACTC   
  
  
+ CTTTCCAGCT CTACATGAGA TTTCTCGGTC CTTGGTGGAA CTGGGTTCTC AAGCCTCTGA GTTGAGCTTC   
  
  
+ GATGATGCAG GGAGTGCCCG TGTAGAGAAA AAGGGCAAAT CGATAAAGGG CTCGAGGAGG AAGAAGAGTC   
  
  
+ GTCAAAGAGA GGGTGAAGCG TGTTATGGAG GAAGGAGCCA TAAGGTTCAA GCTTCCTTCA ATGATGATTA   
  
  
+ CTATGAGATG GAACAGTATG ATGATGTAGT ATTGCTCTGT AATAATGAAC TAACGGGCAA TAGCCGTTTC   
  
  
+ AACACCGGGA AATCTTCACC TGAGGAGGGA TGGAGGAGAT TGCAGAGAAG CCGAGGAAAG AAGCAGAACA   
  
  
+ GTTTAGCAGT TGAAGTTGAT CTGATGACCC TGCTGACTCA GTGTGCACAA GCTGTATCGA GCTTTGATCT   
  
  
+ TCGAGGTGCA AACGAGCTAC TTAGGCAAAT CAGGCAGAAT GCTTCGCCCT ATGGTGGCAG CATCCAGAGG   
  
  
+ CTCGCCCATC ATGTAGCCAA TGCTCTCGAG GCACGTATAG CTGGCACAGG CTCTACAGTC TCTACTAACC   
  
  
+ TTGTTGATGC AAAGTTCTCA GCTTCTGACT TCCTAAAGGC TTACAGGTTA TATGTCTCAG CTGTTCCTTA   
  
  
+ CAAAAGGATG TCTTTCTTTC TTGCTAACTG CTCGATTGCA AAGTTGGCAG AGAAAGCAAC AAAGATCCAT   
  
  
+ ATCATTGATT TTGGTGTTTT CCTAGGTTTA CAATGGCCTT GTTTCATACA ACACCTATCA AAAAGGCCAA   
  
  
+ ATGGACCCCC AAAACTCCGA ATCACAGGAA TCGACTACCC CCAGCAGGGT TTCAGACCTG CACAAAGGGT   
  
  
+ TGAAGCTACA GGACACCGAT TATCTGGGTA CTGTGAGCGA TTTGGGGTGC CTTTTTCTTA TCAGGGCATT   
  
  
+ GCTCAGAAGT GGGAAACTAT TCAGCCGGAG GATCTCAAGA TCGAACAAGA CGAGCTGGTG ATTGTCAACT   
  
  
+ GTTTGTTCAG GTCAGGAACA CTGCTCGATG AGACAGTCGA AGCAAACAGT CCAAGAGATG CTTTCTTAGC   
  
  
+ TTTGGTTAGA AAGCTGAATC CCAGCCTATT CATTCACGGG GTTGTCAATG GCACATTCAA CGCTCCATTC   
  
  
+ TTCGTGACTC GATTCAGAGA GGCATTGTTT CATTATTCAT CAGTGTTTGA TGTGTCTGAA GAGACAATTC   
  
  
+ CACGAGATGC CCATGAGAGG TTCTTGATTG AGAGCGAGAT TTGTGGGAAA GAACTGTTCA ATGTGGTTGC   
  
  
+ TTGTGAGGGT GCAGAGAGGG TTCAAAGGCC TGAGACATAC AAGCAGTGGC AAGTGAGGAC AACGCGGGCC   
  
  
+ GGGTTAAGGC AGGTTGCCTT GGACCAGGAG CTTATGAAGG AAGCAACGGC AATGGTGAAG GCAAATTATC   
  
  
+ ATAAGGATTT TATGGTGGAT ATAAATAGGC ATTGGATGCT TCAAGGTTGG AAGGGTAGAA CCTTGTGTGC   
  
  
+ TCTCTCATTT TGGCAACCTG CCTG  

- +Up\_Stream \_Len000GATCCC CAACTCTCTT AGGCTATCGA TACGTCAGGT TTGTAATAGA GCAGTTCGTA   
  
  
- ATGATAAATT TATTAATTAT AACTTGAACT ATCAATGTGA AAAACTTATG ATTAAGGTGT GGAGTAACGG   
  
  
- AAATAAAGGT TTGGAGCCCA ACGTAAACCT GGTGTATGGT CCTTCGAACC TGCTGTTTTT AAAGAAAAGA   
  
  
- ACTCATAATA AAACAAAATA ATAATAATAA AAAATGAAAT AAACCTGGTG AAGTGAACCC ATTGTTCTAA   
  
  
- CTTATAAACT GATAATATAA TATACAGTGC ATACCTATAC CATCAACGAA TCTCGAAACT GGCAAACGTC   
  
  
- CAAATTCATA CTAAGGGAAC CTTTCCTCGG GGAGAAATAG AATTAAGTCT TCATTGAAAA CAAAAAAATC   
  
  
- TATTTAACTT TTCTTTTTAT TATTTACAGA ATCAACTCAA ACCAATAAGT TAGGCTTTAA ACTAGGACGA   
  
  
- ATTACATATC ATATATTATG ATTATGCGTA CATTGTGTTG TGGAGTCTAC CAGTAACAAA ATGTTAAAAA   
  
  
- AACTCAAATC ATATTTTTAT TTTAGTTAAT AAAAATCTTC ACTACAAATT AAACGTAAAA AAAAGGTGGA   
  
  
- TTTTAGTGCG TGAGCTTGTA ATCCCCAAAC CGATTGAGTT TCTGAGTTCC ATCACTGAAT AGGTTTGAAT   
  
  
- AATGTAGGAT ATCTAGATAT TTTTGTATTT AGAGAATGTT AGTTAGGTTT AACTAAGCCA GATTAAACTA   
  
  
- CTGGGACTAC TCCCAGGATG TTAATGTGTT CACCCGGAAT CGGGGTACCG GCAAAAAATT AGAAGTACCC   
  
  
- AGGTCCGCCT CAATGGTAGA GGTCCAGGTA ACTCCCACAT AGTGCAGAGT TTAATGCTCA AACGGAGAAA   
  
  
- GATGCCCCTA ATATTTATAG ATGGACGTAT GGAGCTTTTC CTCCTCAACT CTAGTCGCTT TGAACCGGTA   
  
  
- GGGTCTCGTA ACTTTACGAG TCTTGGGAAA AAGATCTTTT TATAGCAAAA AGGACTTTTA TAGGCTGGAA   
  
  
- TACAGTAAAT AACAGCGAAA GAACTGAGAT TGGGCCCTGA GGTGTGATTG AACTGATAGC CTTTCCGCAA   
  
  
- GGGACCTAGT GGTAGGTTCT ATTGGAGAAA ACGTCCCAAC TGCGAGCAAC CTTCTTAGCA GCTTACATCT   
  
  
- CGGGGATAGT AGTGTATTCG GGCGAGAATG AAGTACGGTA GGAACTTATC GTCAAATAGG GCTTCGTTTT   
  
  
- TATTAAGATT GAAAGAATAT AACATTTGGG TGTAGATTTA AATTTAAGTA TGAGGTTTTA AGGCGGTGCG   
  
  
- TTTATTCCGA AAAATTATCC GAATGTATAT TACCGTACGA TATCTCTGTG ACAAAACTTC TTTGATAAAT   
  
  
- TTGTAGTCTG GAGTATAACT CATCGTTTAG TTCTGAAGAA AGGAGGTCCG GTATAACTCA TCGTTTAATT   
  
  
- CTTATTGGTC TTCGAACACT TTTCTCAATC TTTTATTTTC CATATTATCT CTGAATACAA GAATTCTATA   
  
  
- GAGGATCTAT ACTTACACTG GAGGATCAGC TATAAACACG ACCTAATCGC TCAACACACT GACTCAAACA   
  
  
- TACACAGAGA ATCGTATACT CAATAAGATC GACGAAGAAA TTCGATACAT CACTGACAGA AGGAAGAATA   
  
  
- AATATTCACG ATACAGGTTG ATTATCGGCT TTTTTTACCA AGCGGAAAAG ACCAAAAGAA GAAAAACTTC   
  
  
- CAACTAACCG ATTAAATATA TTACCAAAGT TATGAATAGG AAATAACAAG GTGTTTAGGT GGATTGGTGC   
  
  
- ATGGTAACTG TTATTAATGG GTGATTAAAG CCAACCTGAA CTAAAGGAAA ACAAACCATG GAGTCCGTAG   
  
  
- AAGACACCTG GAAGTAAGTG TCAAATTAAA AGTGTAAAAA TATTCCGACG AGAACACAAC GTTTAAAAGG   
  
  
- ATAGGTAAGG TGAGAAACGG GTAAGGGTCC GATTAATCAC AAGAGACAAG TTGGTACCTA AGACACGACC   
  
  
- AACTAGGACT CAAGTACTTT TTAGAGATGT TTAAGTTTGG ACTTAACGAG AGTTTGAAAA GTTACTTAGG   
  
  
- TTTACTGCTG TAGAAAGTTC GGGAAGTAAG TCTTTTACTA GGAAATGTCA AGGAGAGTAA ACTACTTCCT   
  
  
- TGGACAGAAT TGTCGACGTG ATCAGTTGTT CACGGTCTAA AGGGACTACG AACAGAGTTC AAGTAGTCAC
[truncated: 138,746 more chars]
