# Supplementary material for: Genome-Wide Identification and Expression Pattern of the GRAS Gene Family in Pitaya (Selenicereus undatus L.)
Source: Biology (Basel). 2022 Dec 21;12(1):11. doi: 10.3390/biology12010011 (PMC9854919; doi:10.3390/biology12010011)
Supplement: Supplementary file 1 [file biology-12-00011-s001.zip › Supplementary file S5/HU02G01572.1_plantcare.html]

Content-Type: text/html; charset=ISO-8859-1


PlantCARE


Webmaster Firefox specific output  
To save the result:
click on the frame with the right mouse button and save the source code as a text file with extension .html  
REFERENCE:PlantCARE: a database of plant cis-acting regulatory elements and a portal to tools for in silico analysis of promoter sequences.  
Lescot, M., Déhais, P., Moreau, Y., De Moor, B., Rouzé ,P.,and Rombauts, S.  
Nucleic Acids Res., Database issue(2002), 30(1):325-327.   


---

>HU02G01572.1   
+ +Up\_Stream \_Len000TTTAAT AAGATCCTTT TATATATATG TGTGTATAAT TCTATGGTAC ATAGGTATGT   
  
  
+ ACCATAGACT TATTGCATTT CTAGGTCCTT CTCTCTCTGC GCTTGTTTTT TGTTTATCTA ATTTATGGAG   
  
  
+ TTGAACATTT TGTTTTATAT ACTTGAACAA TCTATTATAC TAAATTAAGC ATTTTGATTT AATCACTTAT   
  
  
+ GGGTGTGTTT GGTAGGGTTG AAAATAATTT TCCTAGAAAA TAATTTTTAA TATAAAATAA TTTTTCATGT   
  
  
+ AAAATGATTT TCAACTCATA TTTTCAGTTG ATTGGTAAAA AGAAAAATGA ACTTTCTGGA TAATCAGTTT   
  
  
+ GATTAACAAA AAGTGACAAA AAAATTAAAA ATGGTTTTCC GTAATCTTCA AACCATATGT CGAGGAAATA   
  
  
+ AACACGGAAT AAAGAAAGAG TGTCAGAACC CAAGAAATGT AAAATCAACT TACGTTCTAT TTAAAGTGAA   
  
  
+ AAATATTTTT CTTCATTCTA GGAAAATTAA TTGTCCTCTG AAAACAAATT TTCCCTCCTT GTCTAACCAA   
  
  
+ ACACAGTAAA ATTGAAAAAT CATTTTCTAG GAAAATGATT TTCACTCCTA CCAAACACAC CCTTAAACTT   
  
  
+ GTTCCAAATC TATAAACCCG GAATCTTAAT ACGCACATTG TATATTATAT GTCCTACTAT AACTGTATTA   
  
  
+ TCACTGGTTC TTGATTTATC CTTTCAAACT CTTGCTTAGT CAAGGTTGTC CTCGTACAAA CATGATTAAT   
  
  
+ TCAATTAACC ACGTAAGTTA TACGCTGACA AATTTCGGGT ACCAAACATT TGCACTAGAC CTAAAACAGT   
  
  
+ TACTATCATC ATATCGATGT TAAAATTGAC CTGTTCTTAT TTGTCCAGCG TTGAGCAAGG AAATAATCAA   
  
  
+ ATCATGTCTC ATTTAGTCGG TTTTTTGCCT AAGAAGAGCA TAAACAAAGG AAAAAGACAA CGACACATTA   
  
  
+ TACATGCATC TACACAAATG AAATGAGGCC ATATGGAACA TGTTCATGAG AGCTTCACTC GCTAATAATT   
  
  
+ GAACCTACTT TTGACTAAGC CACCAACTAG AATATGGAGA TTGACTGTTA AGAGACTATA TTACATGAAA   
  
  
+ TAGATAAGAG TAGACATTGC TATATATTTT ATCTTTAATT GTCTATTTTT AATGATCATT ACTGTTTATT   
  
  
+ TCAGTGAGTA ATTTTCTCGG ATATAATATT TTATAGCGGT TATTAAATAG ATGTGAAACT ATAATGTTAG   
  
  
+ ACATATCATG CGAAAATAGA GAAATTTTGG GCAAATTAAA AAAAAAAGAA GAAAAACGAG GAAGTATGCC   
  
  
+ CATTGTAAGC AGGTGGATGT ATATCCAGCC TCCATCCAAC AATTTGGGCA AAATTATATC ATCCATTCCA   
  
  
+ AAAAAAAGAT ATAATTTGTG ATATTAATAA CCCGACTCAA TTCAAGCTTA TTTATTTTTT CATATAATTT   
  
  
+ TTTAATATAT AAAAATAAAA AGTGACAACG ACATGCATCC TTTGCGTGTC AAAGGATCCG CCGCGTGGCC   
  
  
+ CCCACATTCA CTGACGGGGT TTTTGGTTTG CGATTAAGAT CAATGGCTAA CGTTGTCTCT CTCAAAACAT   
  
  
+ ATGGACCCCT TTTTCTATCT TTTTCTGCAT TGTCAGCATA GCAATTGTTG AGCACATTTT TTTCGATTTG   
  
  
+ TTGACAATAA GCGTGGTTAT GATTTATGGG CCACTAATAT CAGTCTCTGA TCATTTGCAC ATTTTGGACC   
  
  
+ TCTTTCTCTC TCTAATCTTC CAGGTAACCT GAATTCTTCT CTATTTCTGA CTAGATTAGA TAACAGGATT   
  
  
+ TCAAATATCT GTCAGGTGGC GGGTATAACT TCGTGATGGG CATTCATTTT TGTTCTATCA GGCTGGTGGG   
  
  
+ TTTGAGTTTA TTTTGATTCA TTTCGTGTTC ATCTGTGATA TTAGTAGGAG GGATTCTGTA TACCCACCAA   
  
  
+ CTGCTCGCCC TTTTGCCCGT GTGAGCAATT GGGACTTTTG TTTGTTCTTC GTCGATGCAT CCTCTGGTTG   
  
  
+ CTGATCTGGA ATTGAAACCC CATGTATTCA AATTCAACCC TGATTTGCTG TCAAACTTTC TGAACCATCA   
  
  
+ AAACTCCGCC GAAGTGTTTG AAAAAGATGG CATCTTCCAG ACCCTTCATT TGGCTGATCC CAAAAGCTCT   
  
  
+ TCAATTGCCG AAATCGGGTT TACTAATAGT TCAGATTCTA CACAAGTACC TGATTTTTCA GATGCTTGTC   
  
  
+ TTAAGTTCAT TAGTGATATT CTCCTGGAAG AGGACTTAGA TGAAAGTCCT ACGTCTTTAC ACGATTACAT   
  
  
+ GGCTCTCCTA GCCACTGAGA AGTCTTTGTA TGATGCTCTT GGAAAGGAAT ACTTTCCTTC GTCTACTAGT   
  
  
+ CTTGCCCCAT CTTTAGGCCG AAGTGTTGAC AGCCCAGATA GTGGCTTTGG CCGCGGTTGC TCTGATGGTC   
  
  
+ GCGGGATTGA GGGTTTGGCT AATGATGATG CCGTTTTCAT GTCTAACTGG CAGCTCAACA CCACCCAATT   
  
  
+ GGACCCTTTC CCAATCATGC AAGATATTCC TCGTCCCTAT TTGGAATTGA ATTACCATTC TTCTGGGTCA   
  
  
+ AGCAATGGCA TTGATGATTC GGGGGATGGG TTATCGACCT CTCCTGTAAG TACACTTGCA TCAACCGCCA   
  
  
+ CAGAGGCAGG GAAAAAGTTG GCTGGTAGCT CTAGGAGAAA GAACCGTCAA AGGGATGACT ATGGCCATGA   
  
  
+ AGAGGGAAGG AGTAACAAGC AGCAAGCCTC TTACAATGAC GATTACGTTG AGATGGAGCA GTATGACGAT   
  
  
+ GTACTTCTCT GTAGGGCAGA CAAGGGTAAT ATTTCAACTT GTGCCAATGA ATCCTCGCTT AATGAGGTGC   
  
  
+ GTGAGAAGCT GCAGACGACA GGGTTCAAGG GAAGAACATC CCGTCTTAAG AAGCAATCTA AGGAAGCGAA   
  
  
+ AGAGGTGGAT CTGAGAACTC TTCTTTCTGG TTGTGCACAA GCTGTTTCGA ACTTTGATAT CAGGACTGCT   
  
  
+ AATGAGCTGC TTAAGCAAGT CAGACAGCAT TCTTCACCAT ATGGTGATAG CCTCCAAAGG CTCGCCCATC   
  
  
+ AATTTGCGAA TGGTATTGAG GCACGCTTAG CAGGCACCGG TTCAAGAGTA CCTGCTAATC TCATTGATGC   
  
  
+ ACGGATATCA TCATCTGAAT TTTTAAAAGC TTACAAGTCA TATGTTTCAG CAGTTCCTTT CAGAAGGATG   
  
  
+ TCCTATTTTA TAGCAAACAA CACAATTCTG AAGTTGGCTG AGAAAGCAAC AAAGATTCAC ATAATTGATT   
  
  
+ TTGGTATTCT ATTTGGTCTA CAGTGGCCCT GTCTTATACA AAGTCTCTCA AGGCGAACTG TGGCTCCTCC   
  
  
+ GAAGCTTCGC ATCACTGGGA TAGACTATCC CCAGCATGGT TTCCGGCCAG CAGAAAAGGT TGAGGCAACA   
  
  
+ GGTCGTCGGT TGTCCGGGTA CTGTGAGAGA TTTAATGTAC CCTTTCGATA TGAAACCATT GCAAAGAAGT   
  
  
+ GGGAAACCAT ACGCCCAGAA GATCTAAATA TTGAGAATGA TGAGCTGGTA ATTGTTAATT GTATGTTGCG   
  
  
+ GTCTGTAAAT CTATTGGATG ATACAGTGGC GGTAAATAGT CCAAGGGATG CTTTCTTGAG GTTAATCAAA   
  
  
+ CAGATAAACC CGCGTTTATT CATTCATGCA ATTGTCAATG GAACCTTTAG TACTCCATTC TTCAGCACTC   
  
  
+ GATTCAGGGA AGCCCTATTC CAATACTCTT CTGTATTTGA TATATTTGAA GCGACTATGA CTCGTGAAGA   
  
  
+ TCGTGGAAGG CTGCTGATTG AGAGTCAAAT ATGCGGGCTA GAAGTTTTGA ATGCAATAGC ATGTGAAGGT   
  
  
+ GCAGAGAGGA TTCAAAGGCC TGAAACATAC AAGCAATGGC AGGAGCGGAC AACAAGGGCT GGACTAAGGC   
  
  
+ AGGTTCCAAT AGATGAGGAG CTTGTCAATA GAGCAAAGAC TATAGTGAAA GCAAATTATC ACAAGGATTT   
  
  
+ TGTGGTGGAT GAGGATAGGC GTTGGATGCT TCAAGGTTGG AAAGGAAGGA CACTTAGTGC CCTTTCCGTT   
  
  
+ TGGCAGCCTA ACTA  

- +Up\_Stream \_Len000AAATTA TTCTAGGAAA ATATATATAC ACACATATTA AGATACCATG TATCCATACA   
  
  
- TGGTATCTGA ATAACGTAAA GATCCAGGAA GAGAGAGACG CGAACAAAAA ACAAATAGAT TAAATACCTC   
  
  
- AACTTGTAAA ACAAAATATA TGAACTTGTT AGATAATATG ATTTAATTCG TAAAACTAAA TTAGTGAATA   
  
  
- CCCACACAAA CCATCCCAAC TTTTATTAAA AGGATCTTTT ATTAAAAATT ATATTTTATT AAAAAGTACA   
  
  
- TTTTACTAAA AGTTGAGTAT AAAAGTCAAC TAACCATTTT TCTTTTTACT TGAAAGACCT ATTAGTCAAA   
  
  
- CTAATTGTTT TTCACTGTTT TTTTAATTTT TACCAAAAGG CATTAGAAGT TTGGTATACA GCTCCTTTAT   
  
  
- TTGTGCCTTA TTTCTTTCTC ACAGTCTTGG GTTCTTTACA TTTTAGTTGA ATGCAAGATA AATTTCACTT   
  
  
- TTTATAAAAA GAAGTAAGAT CCTTTTAATT AACAGGAGAC TTTTGTTTAA AAGGGAGGAA CAGATTGGTT   
  
  
- TGTGTCATTT TAACTTTTTA GTAAAAGATC CTTTTACTAA AAGTGAGGAT GGTTTGTGTG GGAATTTGAA   
  
  
- CAAGGTTTAG ATATTTGGGC CTTAGAATTA TGCGTGTAAC ATATAATATA CAGGATGATA TTGACATAAT   
  
  
- AGTGACCAAG AACTAAATAG GAAAGTTTGA GAACGAATCA GTTCCAACAG GAGCATGTTT GTACTAATTA   
  
  
- AGTTAATTGG TGCATTCAAT ATGCGACTGT TTAAAGCCCA TGGTTTGTAA ACGTGATCTG GATTTTGTCA   
  
  
- ATGATAGTAG TATAGCTACA ATTTTAACTG GACAAGAATA AACAGGTCGC AACTCGTTCC TTTATTAGTT   
  
  
- TAGTACAGAG TAAATCAGCC AAAAAACGGA TTCTTCTCGT ATTTGTTTCC TTTTTCTGTT GCTGTGTAAT   
  
  
- ATGTACGTAG ATGTGTTTAC TTTACTCCGG TATACCTTGT ACAAGTACTC TCGAAGTGAG CGATTATTAA   
  
  
- CTTGGATGAA AACTGATTCG GTGGTTGATC TTATACCTCT AACTGACAAT TCTCTGATAT AATGTACTTT   
  
  
- ATCTATTCTC ATCTGTAACG ATATATAAAA TAGAAATTAA CAGATAAAAA TTACTAGTAA TGACAAATAA   
  
  
- AGTCACTCAT TAAAAGAGCC TATATTATAA AATATCGCCA ATAATTTATC TACACTTTGA TATTACAATC   
  
  
- TGTATAGTAC GCTTTTATCT CTTTAAAACC CGTTTAATTT TTTTTTTCTT CTTTTTGCTC CTTCATACGG   
  
  
- GTAACATTCG TCCACCTACA TATAGGTCGG AGGTAGGTTG TTAAACCCGT TTTAATATAG TAGGTAAGGT   
  
  
- TTTTTTTCTA TATTAAACAC TATAATTATT GGGCTGAGTT AAGTTCGAAT AAATAAAAAA GTATATTAAA   
  
  
- AAATTATATA TTTTTATTTT TCACTGTTGC TGTACGTAGG AAACGCACAG TTTCCTAGGC GGCGCACCGG   
  
  
- GGGTGTAAGT GACTGCCCCA AAAACCAAAC GCTAATTCTA GTTACCGATT GCAACAGAGA GAGTTTTGTA   
  
  
- TACCTGGGGA AAAAGATAGA AAAAGACGTA ACAGTCGTAT CGTTAACAAC TCGTGTAAAA AAAGCTAAAC   
  
  
- AACTGTTATT CGCACCAATA CTAAATACCC GGTGATTATA GTCAGAGACT AGTAAACGTG TAAAACCTGG   
  
  
- AGAAAGAGAG AGATTAGAAG GTCCATTGGA CTTAAGAAGA GATAAAGACT GATCTAATCT ATTGTCCTAA   
  
  
- AGTTTATAGA CAGTCCACCG CCCATATTGA AGCACTACCC GTAAGTAAAA ACAAGATAGT CCGACCACCC   
  
  
- AAACTCAAAT AAAACTAAGT AAAGCACAAG TAGACACTAT AATCATCCTC CCTAAGACAT ATGGGTGGTT   
  
  
- GACGAGCGGG AAAACGGGCA CACTCGTTAA CCCTGAAAAC AAACAAGAAG CAGCTACGTA GGAGACCAAC   
  
  
- GACTAGACCT TAACTTTGGG GTACATAAGT TTAAGTTGGG ACTAAACGAC AGTTTGAAAG ACTTGGTAGT   
  
  
- TTTGAGGCGG CTTCACAAAC TTTTTCTACC GTAGAAGGTC TGGGAAGTAA ACCGACTAGG GTTTTCGAGA   
  
  
- AGTTAACGGC TTTAGCCCAA ATGATTATCA AGTCTAAGAT GTGTTCATGG ACTAAAAAGT CTACGAACAG   
  
  
- AATTCAAGTA ATCACTATAA GAGGACCTTC TCCTGAATCT ACTTTCAGGA TGCAGAAATG TGCTAATGTA   
  
  
- CCGAGAGGAT CGGTGACTCT TCAGAAACAT ACTACGAGAA CCTTTCCTTA TGAAAGGAAG CAGATGATCA   
  
  
- GAACGGGGTA GAAATCCGGC TTCACAACTG TCGGGTCTAT CACCGAAACC GGCGCCAACG AGACTACCAG   
  
  
- CGCCCTAACT CCCAAACCGA TTACTACTAC GGCAAAAGTA CAGATTGACC GTCGAGTTGT GGTGGGTTAA   
  
  
- CCTGGGAAAG GGTTAGTACG TTCTATAAGG AGCAGGGATA AACCTTAACT TAATGGTAAG AAGACCCAGT   
  
  
- TCGTTACCGT AACTACTAAG CCCCCTACCC AATAGCTGGA GAGGACATTC ATGTGAACGT AGTTGGCGGT   
  
  
- GTCTCCGTCC CTTTTTCAAC CGACCATCGA GATCCTCTTT CTTGGCAGTT TCCCTACTGA TACCGGTACT   
  
  
- TCTCCCTTCC TCATTGTTCG TCGTTCGGAG AATGTTACTG CTAATGCAAC TCTACCTCGT CATACTGCTA   
  
  
- CATGAAGAGA CATCCCGTCT GTTCCCATTA TAAAGTTGAA CACGGTTACT TAGGAGCGAA TTACTCCACG   
  
  
- CACTCTTCGA CGTCTGCTGT CCCAAGTTCC CTTCTTGTAG GGCAGAATTC TTCGTTAGAT TCCTTCGCTT   
  
  
- TCTCCACCTA GACTCTTGAG AAGAAAGACC AACACGTGTT CGACAAAGCT TGAAACTATA GTCCTGACGA   
  
  
- TTACTCGACG AATTCGTTCA GTCTGTCGTA AGAAGTGGTA TACCACTATC GGAGGTTTCC GAGCGGGTAG   
  
  
- TTAAACGCTT ACCATAACTC CGTGCGAATC GTCCGTGGCC AAGTTCTCAT GGACGATTAG AGTAACTACG   
  
  
- TGCCTATAGT AGTAGACTTA AAAATTTTCG AATGTTCAGT ATACAAAGTC GTCAAGGAAA GTCTTCCTAC   
  
  
- AGGATAAAAT ATCGTTTGTT GTGTTAAGAC TTCAACCGAC TCTTTCGTTG TTTCTAAGTG TATTAACTAA   
  
  
- AACCATAAGA TAAACCAGAT GTCACCGGGA CAGAATATGT TTCAGAGAGT TCCGCTTGAC ACCGAGGAGG   
  
  
- CTTCGAAGCG TAGTGACCCT ATCTGATAGG GGTCGTACCA AAGGCCGGTC GTCTTTTCCA ACTCCGTTGT   
  
  
- CCAGCAGCCA ACAGGCCCAT GACACTCTCT AAATTACATG GGAAAGCTAT ACTTTGGTAA CGTTTCTTCA   
  
  
- CCCTTTGGTA TGCGGGTCTT CTAGATTTAT AACTCTTACT ACTCGACCAT TAACAATTAA CATACAACGC   
  
  
- CAGACATTTA GATAACCTAC TATGTCACCG CCATTTATCA GGTTCCCTAC GAAAGAACTC CAATTAGTTT   
  
  
- GTCTATTTGG GCGCAAATAA GTAAGTACGT TAACAGTTAC CTTGGAAATC ATGAGGTAAG AAGTCGTGAG   
  
  
- CTAAGTCCCT TCGGGATAAG GTTATGAGAA GACATAAACT ATATAAACTT CGCTGATACT GAGCACTTCT   
  
  
- AGCACCTTCC GACGACTAAC TCTCAGTTTA TACGCCCGAT CTTCAAAACT TACGTTATCG TACACTTCCA   
  
  
- CGTCTCTCCT AAGTTTCCGG ACTTTGTATG TTCGTTACCG TCCTCGCCTG TTGTTCCCGA CCTGATTCCG   
  
  
- TCCAAGGTTA TCTACTCCTC GAACAGTTAT CTCGTTTCTG ATATCACTTT CGTTTAATAG TGTTCCTAAA   
  
  
- ACACCACCTA CTCCTATCCG CAACCTACGA AGTTCCAACC TTTCCTTCCT GTGAATCACG GGAAAGGCAA   
  
  
- ACCGTCGGAT TGAT

  
  
Motifs Found  

+   

| Site Name | Organism | Position | Strand | Matrix score. | sequence | function |
| --- | --- | --- | --- | --- | --- | --- |
|  | organism | 3522 | - | 4 | motif\_sequence | short\_function |
|  | organism | 1090 | - | 4 | motif\_sequence | short\_function |
|  | organism | 3222 | + | 4 | motif\_sequence | short\_function |
|  | organism | 2803 | + | 4 | motif\_sequence | short\_function |
|  | organism | 1769 | + | 4 | motif\_sequence | short\_function |
|  | organism | 2136 | + | 4 | motif\_sequence | short\_function |
|  | organism | 992 | + | 4 | motif\_sequence | short\_function |
|  | organism | 947 | - | 4 | motif\_sequence | short\_function |
|  | organism | 3855 | - | 4 | motif\_sequence | short\_function |
|  | organism | 3780 | - | 4 | motif\_sequence | short\_function |
|  | organism | 1020 | - | 4 | motif\_sequence | short\_function |
|  | organism | 1024 | + | 4 | motif\_sequence | short\_function |
|  | organism | 1666 | - | 4 | motif\_sequence | short\_function |
|  | organism | 1741 | - | 4 | motif\_sequence | short\_function |
|  | organism | 2962 | + | 4 | motif\_sequence | short\_function |
|  | organism | 398 | + | 4 | motif\_sequence | short\_function |
|  | organism | 2171 | + | 4 | motif\_sequence | short\_function |
|  | organism | 683 | + | 4 | motif\_sequence | short\_function |
|  | organism | 2908 | - | 4 | motif\_sequence | short\_function |
|  | organism | 3740 | + | 4 | motif\_sequence | short\_function |
|  | organism | 147 | - | 4 | motif\_sequence | short\_function |
|  | organism | 2733 | - | 4 | motif\_sequence | short\_function |
|  | organism | 667 | - | 4 | motif\_sequence | short\_function |
|  | organism | 109 | + | 4 | motif\_sequence | short\_function |
|  | organism | 3469 | + | 4 | motif\_sequence | short\_function |
|  | organism | 2317 | + | 4 | motif\_sequence | short\_function |
|  | organism | 2271 | - | 4 | motif\_sequence | short\_function |
|  | organism | 875 | + | 4 | motif\_sequence | short\_function |
|  | organism | 61 | - | 4 | motif\_sequence | short\_function |
|  | organism | 71 | + | 4 | motif\_sequence | short\_function |
|  | organism | 2633 | + | 4 | motif\_sequence | short\_function |

>HU02G01572.1   
+ +Up\_Stream \_Len000TTTAAT AAGATCCTTT TATATATATG TGTGTATAAT TCTATGGTAC ATAGGTATGT   
  
  
+ ACCATAGACT TATTGCATTT CTAGGTCCTT CTCTCTCTGC GCTTGTTTTT TGTTTATCTA ATTTATGGAG   
  
  
+ TTGAACATTT TGTTTTATAT ACTTGAACAA TCTATTATAC TAAATTAAGC ATTTTGATTT AATCACTTAT   
  
  
+ GGGTGTGTTT GGTAGGGTTG AAAATAATTT TCCTAGAAAA TAATTTTTAA TATAAAATAA TTTTTCATGT   
  
  
+ AAAATGATTT TCAACTCATA TTTTCAGTTG ATTGGTAAAA AGAAAAATGA ACTTTCTGGA TAATCAGTTT   
  
  
+ GATTAACAAA AAGTGACAAA AAAATTAAAA ATGGTTTTCC GTAATCTTCA AACCATATGT CGAGGAAATA   
  
  
+ AACACGGAAT AAAGAAAGAG TGTCAGAACC CAAGAAATGT AAAATCAACT TACGTTCTAT TTAAAGTGAA   
  
  
+ AAATATTTTT CTTCATTCTA GGAAAATTAA TTGTCCTCTG AAAACAAATT TTCCCTCCTT GTCTAACCAA   
  
  
+ ACACAGTAAA ATTGAAAAAT CATTTTCTAG GAAAATGATT TTCACTCCTA CCAAACACAC CCTTAAACTT   
  
  
+ GTTCCAAATC TATAAACCCG GAATCTTAAT ACGCACATTG TATATTATAT GTCCTACTAT AACTGTATTA   
  
  
+ TCACTGGTTC TTGATTTATC CTTTCAAACT CTTGCTTAGT CAAGGTTGTC CTCGTACAAA CATGATTAAT   
  
  
+ TCAATTAACC ACGTAAGTTA TACGCTGACA AATTTCGGGT ACCAAACATT TGCACTAGAC CTAAAACAGT   
  
  
+ TACTATCATC ATATCGATGT TAAAATTGAC CTGTTCTTAT TTGTCCAGCG TTGAGCAAGG AAATAATCAA   
  
  
+ ATCATGTCTC ATTTAGTCGG TTTTTTGCCT AAGAAGAGCA TAAACAAAGG AAAAAGACAA CGACACATTA   
  
  
+ TACATGCATC TACACAAATG AAATGAGGCC ATATGGAACA TGTTCATGAG AGCTTCACTC GCTAATAATT   
  
  
+ GAACCTACTT TTGACTAAGC CACCAACTAG AATATGGAGA TTGACTGTTA AGAGACTATA TTACATGAAA   
  
  
+ TAGATAAGAG TAGACATTGC TATATATTTT ATCTTTAATT GTCTATTTTT AATGATCATT ACTGTTTATT   
  
  
+ TCAGTGAGTA ATTTTCTCGG ATATAATATT TTATAGCGGT TATTAAATAG ATGTGAAACT ATAATGTTAG   
  
  
+ ACATATCATG CGAAAATAGA GAAATTTTGG GCAAATTAAA AAAAAAAGAA GAAAAACGAG GAAGTATGCC   
  
  
+ CATTGTAAGC AGGTGGATGT ATATCCAGCC TCCATCCAAC AATTTGGGCA AAATTATATC ATCCATTCCA   
  
  
+ AAAAAAAGAT ATAATTTGTG ATATTAATAA CCCGACTCAA TTCAAGCTTA TTTATTTTTT CATATAATTT   
  
  
+ TTTAATATAT AAAAATAAAA AGTGACAACG ACATGCATCC TTTGCGTGTC AAAGGATCCG CCGCGTGGCC   
  
  
+ CCCACATTCA CTGACGGGGT TTTTGGTTTG CGATTAAGAT CAATGGCTAA CGTTGTCTCT CTCAAAACAT   
  
  
+ ATGGACCCCT TTTTCTATCT TTTTCTGCAT TGTCAGCATA GCAATTGTTG AGCACATTTT TTTCGATTTG   
  
  
+ TTGACAATAA GCGTGGTTAT GATTTATGGG CCACTAATAT CAGTCTCTGA TCATTTGCAC ATTTTGGACC   
  
  
+ TCTTTCTCTC TCTAATCTTC CAGGTAACCT GAATTCTTCT CTATTTCTGA CTAGATTAGA TAACAGGATT   
  
  
+ TCAAATATCT GTCAGGTGGC GGGTATAACT TCGTGATGGG CATTCATTTT TGTTCTATCA GGCTGGTGGG   
  
  
+ TTTGAGTTTA TTTTGATTCA TTTCGTGTTC ATCTGTGATA TTAGTAGGAG GGATTCTGTA TACCCACCAA   
  
  
+ CTGCTCGCCC TTTTGCCCGT GTGAGCAATT GGGACTTTTG TTTGTTCTTC GTCGATGCAT CCTCTGGTTG   
  
  
+ CTGATCTGGA ATTGAAACCC CATGTATTCA AATTCAACCC TGATTTGCTG TCAAACTTTC TGAACCATCA   
  
  
+ AAACTCCGCC GAAGTGTTTG AAAAAGATGG CATCTTCCAG ACCCTTCATT TGGCTGATCC CAAAAGCTCT   
  
  
+ TCAATTGCCG AAATCGGGTT TACTAATAGT TCAGATTCTA CACAAGTACC TGATTTTTCA GATGCTTGTC   
  
  
+ TTAAGTTCAT TAGTGATATT CTCCTGGAAG AGGACTTAGA TGAAAGTCCT ACGTCTTTAC ACGATTACAT   
  
  
+ GGCTCTCCTA GCCACTGAGA AGTCTTTGTA TGATGCTCTT GGAAAGGAAT ACTTTCCTTC GTCTACTAGT   
  
  
+ CTTGCCCCAT CTTTAGGCCG AAGTGTTGAC AGCCCAGATA GTGGCTTTGG CCGCGGTTGC TCTGATGGTC   
  
  
+ GCGGGATTGA GGGTTTGGCT AATGATGATG CCGTTTTCAT GTCTAACTGG CAGCTCAACA CCACCCAATT   
  
  
+ GGACCCTTTC CCAATCATGC AAGATATTCC TCGTCCCTAT TTGGAATTGA ATTACCATTC TTCTGGGTCA   
  
  
+ AGCAATGGCA TTGATGATTC GGGGGATGGG TTATCGACCT CTCCTGTAAG TACACTTGCA TCAACCGCCA   
  
  
+ CAGAGGCAGG GAAAAAGTTG GCTGGTAGCT CTAGGAGAAA GAACCGTCAA AGGGATGACT ATGGCCATGA   
  
  
+ AGAGGGAAGG AGTAACAAGC AGCAAGCCTC TTACAATGAC GATTACGTTG AGATGGAGCA GTATGACGAT   
  
  
+ GTACTTCTCT GTAGGGCAGA CAAGGGTAAT ATTTCAACTT GTGCCAATGA ATCCTCGCTT AATGAGGTGC   
  
  
+ GTGAGAAGCT GCAGACGACA GGGTTCAAGG GAAGAACATC CCGTCTTAAG AAGCAATCTA AGGAAGCGAA   
  
  
+ AGAGGTGGAT CTGAGAACTC TTCTTTCTGG TTGTGCACAA GCTGTTTCGA ACTTTGATAT CAGGACTGCT   
  
  
+ AATGAGCTGC TTAAGCAAGT CAGACAGCAT TCTTCACCAT ATGGTGATAG CCTCCAAAGG CTCGCCCATC   
  
  
+ AATTTGCGAA TGGTATTGAG GCACGCTTAG CAGGCACCGG TTCAAGAGTA CCTGCTAATC TCATTGATGC   
  
  
+ ACGGATATCA TCATCTGAAT TTTTAAAAGC TTACAAGTCA TATGTTTCAG CAGTTCCTTT CAGAAGGATG   
  
  
+ TCCTATTTTA TAGCAAACAA CACAATTCTG AAGTTGGCTG AGAAAGCAAC AAAGATTCAC ATAATTGATT   
  
  
+ TTGGTATTCT ATTTGGTCTA CAGTGGCCCT GTCTTATACA AAGTCTCTCA AGGCGAACTG TGGCTCCTCC   
  
  
+ GAAGCTTCGC ATCACTGGGA TAGACTATCC CCAGCATGGT TTCCGGCCAG CAGAAAAGGT TGAGGCAACA   
  
  
+ GGTCGTCGGT TGTCCGGGTA CTGTGAGAGA TTTAATGTAC CCTTTCGATA TGAAACCATT GCAAAGAAGT   
  
  
+ GGGAAACCAT ACGCCCAGAA GATCTAAATA TTGAGAATGA TGAGCTGGTA ATTGTTAATT GTATGTTGCG   
  
  
+ GTCTGTAAAT CTATTGGATG ATACAGTGGC GGTAAATAGT CCAAGGGATG CTTTCTTGAG GTTAATCAAA   
  
  
+ CAGATAAACC CGCGTTTATT CATTCATGCA ATTGTCAATG GAACCTTTAG TACTCCATTC TTCAGCACTC   
  
  
+ GATTCAGGGA AGCCCTATTC CAATACTCTT CTGTATTTGA TATATTTGAA GCGACTATGA CTCGTGAAGA   
  
  
+ TCGTGGAAGG CTGCTGATTG AGAGTCAAAT ATGCGGGCTA GAAGTTTTGA ATGCAATAGC ATGTGAAGGT   
  
  
+ GCAGAGAGGA TTCAAAGGCC TGAAACATAC AAGCAATGGC AGGAGCGGAC AACAAGGGCT GGACTAAGGC   
  
  
+ AGGTTCCAAT AGATGAGGAG CTTGTCAATA GAGCAAAGAC TATAGTGAAA GCAAATTATC ACAAGGATTT   
  
  
+ TGTGGTGGAT GAGGATAGGC GTTGGATGCT TCAAGGTTGG AAAGGAAGGA CACTTAGTGC CCTTTCCGTT   
  
  
+ TGGCAGCCTA ACTA  

- +Up\_Stream \_Len000AAATTA TTCTAGGAAA ATATATATAC ACACATATTA AGATACCATG TATCCATACA   
  
  
- TGGTATCTGA ATAACGTAAA GATCCAGGAA GAGAGAGACG CGAACAAAAA ACAAATAGAT TAAATACCTC   
  
  
- AACTTGTAAA ACAAAATATA TGAACTTGTT AGATAATATG ATTTAATTCG TAAAACTAAA TTAGTGAATA   
  
  
- CCCACACAAA CCATCCCAAC TTTTATTAAA AGGATCTTTT ATTAAAAATT ATATTTTATT AAAAAGTACA   
  
  
- TTTTACTAAA AGTTGAGTAT AAAAGTCAAC TAACCATTTT TCTTTTTACT TGAAAGACCT ATTAGTCAAA   
  
  
- CTAATTGTTT TTCACTGTTT TTTTAATTTT TACCAAAAGG CATTAGAAGT TTGGTATACA GCTCCTTTAT   
  
  
- TTGTGCCTTA TTTCTTTCTC ACAGTCTTGG GTTCTTTACA TTTTAGTTGA ATGCAAGATA AATTTCACTT   
  
  
- TTTATAAAAA GAAGTAAGAT CCTTTTAATT AACAGGAGAC TTTTGTTTAA AAGGGAGGAA CAGATTGGTT   
  
  
- TGTGTCATTT TAACTTTTTA GTAAAAGATC CTTTTACTAA AAGTGAGGAT GGTTTGTGTG GGAATTTGAA   
  
  
- CAAGGTTTAG ATATTTGGGC CTTAGAATTA TGCGTGTAAC ATATAATATA CAGGATGATA TTGACATAAT   
  
  
- AGTGACCAAG AACTAAATAG GAAAGTTTGA GAACGAATCA GTTCCAACAG GAGCATGTTT GTACTAATTA   
  
  
- AGTTAATTGG TGCATTCAAT ATGCGACTGT TTAAAGCCCA TGGTTTGTAA ACGTGATCTG GATTTTGTCA   
  
  
- ATGATAGTAG TATAGCTACA ATTTTAACTG GACAAGAATA AACAGGTCGC AACTCGTTCC TTTATTAGTT   
  
  
- TAGTACAGAG TAAATCAGCC AAAAAACGGA TTCTTCTCGT ATTTGTTTCC TTTTTCTGTT GCTGTGTAAT   
  
  
- ATGTACGTAG ATGTGTTTAC TTTACTCCGG TATACCTTGT ACAAGTACTC TCGAAGTGAG CGATTATTAA   
  
  
- CTTGGATGAA AACTGATTCG GTGGTTGATC TTATACCTCT AACTGACAAT TCTCTGATAT AATGTACTTT   
  
  
- ATCTATTCTC ATCTGTAACG ATATATAAAA TAGAAATTAA CAGATAAAAA TTACTAGTAA TGACAAATAA   
  
  
- AGTCACTCAT TAAAAGAGCC TATATTATAA AATATCGCCA ATAATTTATC TACACTTTGA TATTACAATC   
  
  
- TGTATAGTAC GCTTTTATCT CTTTAAAACC CGTTTAATTT TTTTTTTCTT CTTTTTGCTC CTTCATACGG   
  
  
- GTAACATTCG TCCACCTACA TATAGGTCGG AGGTAGGTTG TTAAACCCGT TTTAATATAG TAGGTAAGGT   
  
  
- TTTTTTTCTA TATTAAACAC TATAATTATT GGGCTGAGTT AAGTTCGAAT AAATAAAAAA GTATATTAAA   
  
  
- AAATTATATA TTTTTATTTT TCACTGTTGC TGTACGTAGG AAACGCACAG TTTCCTAGGC GGCGCACCGG   
  
  
- GGGTGTAAGT GACTGCCCCA AAAACCAAAC GCTAATTCTA GTTACCGATT GCAACAGAGA GAGTTTTGTA   
  
  
- TACCTGGGGA AAAAGATAGA AAAAGACGTA ACAGTCGTAT CGTTAACAAC TCGTGTAAAA AAAGCTAAAC   
  
  
- AACTGTTATT CGCACCAATA CTAAATACCC GGTGATTATA GTCAGAGACT AGTAAACGTG TAAAACCTGG   
  
  
- AGAAAGAGAG AGATTAGAAG GTCCATTGGA CTTAAGAAGA GATAAAGACT GATCTAATCT ATTGTCCTAA   
  
  
- AGTTTATAGA CAGTCCACCG CCCATATTGA AGCACTACCC GTAAGTAAAA ACAAGATAGT CCGACCACCC   
  
  
- AAACTCAAAT AAAACTAAGT AAAGCACAAG TAGACACTAT AATCATCCTC CCTAAGACAT ATGGGTGGTT   
  
  
- GACGAGCGGG AAAACGGGCA CACTCGTTAA CCCTGAAAAC AAACAAGAAG CAGCTACGTA GGAGACCAAC   
  
  
- GACTAGACCT TAACTTTGGG GTACATAAGT TTAAGTTGGG ACTAAACGAC AGTTTGAAAG ACTTGGTAGT   
  
  
- TTTGAGGCGG CTTCACAAAC TTTTTCTACC GTAGAAGGTC TGGGAAGTAA ACCGACTAGG GTTTTCGAGA   
  
  
- AGTTAACGGC TTTAGCCCAA ATGATTATCA AGTCTAAGAT GTGTTCATGG ACTAAAAAGT CTACGAACAG   
  
  
- AATTCAAGTA ATCACTATAA GAGGACCTTC TCCTGAATCT ACTTTCAGGA TGCAGAAATG TGCTAATGTA   
  
  
- CCGAGAGGAT CGGTGACTCT TCAGAAACAT ACTACGAGAA CCTTTCCTTA TGAAAGGAAG CAGATGATCA   
  
  
- GAACGGGGTA GAAATCCGGC TTCACAACTG TCGGGTCTAT CACCGAAACC GGCGCCAACG AGACTACCAG   
  
  
- CGCCCTAACT CCCAAACCGA TTACTACTAC GGCAAAAGTA CAGATTGACC GTCGAGTTGT GGTGGGTTAA   
  
  
- CCTGGGAAAG GGTTAGTACG TTCTATAAGG AGCAGGGATA AACCTTAACT TAATGGTAAG AAGACCCAGT   
  
  
- TCGTTACCGT AACTACTAAG CCCCCTACCC AATAGCTGGA GAGGACATTC ATGTGAACGT AGTTGGCGGT   
  
  
- GTCTCCGTCC CTTTTTCAAC CGACCATCGA GATCCTCTTT CTTGGCAGTT TCCCTACTGA TACCGGTACT   
  
  
- TCTCCCTTCC TCATTGTTCG TCGTTCGGAG AATGTTACTG CTAATGCAAC TCTACCTCGT CATACTGCTA   
  
  
- CATGAAGAGA CATCCCGTCT GTTCCCATTA TAAAGTTGAA CACGGTTACT TAGGAGCGAA TTACTCCACG   
  
  
- CACTCTTCGA CGTCTGCTGT CCCAAGTTCC CTTCTTGTAG GGCAGAATTC TTCGTTAGAT TCCTTCGCTT   
  
  
- TCTCCACCTA GACTCTTGAG AAGAAAGACC AACACGTGTT CGACAAAGCT TGAAACTATA GTCCTGACGA   
  
  
- TTACTCGACG AATTCGTTCA GTCTGTCGTA AGAAGTGGTA TACCACTATC GGAGGTTTCC GAGCGGGTAG   
  
  
- TTAAACGCTT ACCATAACTC CGTGCGAATC GTCCGTGGCC AAGTTCTCAT GGACGATTAG AGTAACTACG   
  
  
- TGCCTATAGT AGTAGACTTA AAAATTTTCG AATGTTCAGT ATACAAAGTC GTCAAGGAAA GTCTTCCTAC   
  
  
- AGGATAAAAT ATCGTTTGTT GTGTTAAGAC TTCAACCGAC TCTTTCGTTG TTTCTAAGTG TATTAACTAA   
  
  
- AACCATAAGA TAAACCAGAT GTCACCGGGA CAGAATATGT TTCAGAGAGT TCCGCTTGAC ACCGAGGAGG   
  
  
- CTTCGAAGCG TAGTGACCCT ATCTGATAGG GGTCGTACCA AAGGCCGGTC GTCTTTTCCA ACTCCGTTGT   
  
  
- CCAGCAGCCA ACAGGCCCAT GACACTCTCT AAATTACATG GGAAAGCTAT ACTTTGGTAA CGTTTCTTCA   
  
  
- CCCTTTGGTA TGCGGGTCTT CTAGATTTAT AACTCTTACT ACTCGACCAT TAACAATTAA CATACAACGC   
  
  
- CAGACATTTA GATAACCTAC TATGTCACCG CCATTTATCA GGTTCCCTAC GAAAGAACTC CAATTAGTTT   
  
  
- GTCTATTTGG GCGCAAATAA GTAAGTACGT TAACAGTTAC CTTGGAAATC ATGAGGTAAG AAGTCGTGAG   
  
  
- CTAAGTCCCT TCGGGATAAG GTTATGAGAA GACATAAACT ATATAAACTT CGCTGATACT GAGCACTTCT   
  
  
- AGCACCTTCC GACGACTAAC TCTCAGTTTA TACGCCCGAT CTTCAAAACT TACGTTATCG TACACTTCCA   
  
  
- CGTCTCTCCT AAGTTTCCGG ACTTTGTATG TTCGTTACCG TCCTCGCCTG TTGTTCCCGA CCTGATTCCG   
  
  
- TCCAAGGTTA TCTACTCCTC GAACAGTTAT CTCGTTTCTG ATATCACTTT CGTTTAATAG TGTTCCTAAA   
  
  
- ACACCACCTA CTCCTATCCG CAACCTACGA AGTTCCAACC TTTCCTTCCT GTGAATCACG GGAAAGGCAA   
  
  
- ACCGTCGGAT TGAT

+     AAGAA-motif

| Site Name | Organism | Position | Strand | Matrix score. | sequence | function |
| --- | --- | --- | --- | --- | --- | --- |
| AAGAA-motif | Avena sativa | 2701 | + | 7 | GAAAGAA |  |
| AAGAA-motif | Avena sativa | 2965 | - | 7 | GAAAGAA |  |

>HU02G01572.1   
+ +Up\_Stream \_Len000TTTAAT AAGATCCTTT TATATATATG TGTGTATAAT TCTATGGTAC ATAGGTATGT   
  
  
+ ACCATAGACT TATTGCATTT CTAGGTCCTT CTCTCTCTGC GCTTGTTTTT TGTTTATCTA ATTTATGGAG   
  
  
+ TTGAACATTT TGTTTTATAT ACTTGAACAA TCTATTATAC TAAATTAAGC ATTTTGATTT AATCACTTAT   
  
  
+ GGGTGTGTTT GGTAGGGTTG AAAATAATTT TCCTAGAAAA TAATTTTTAA TATAAAATAA TTTTTCATGT   
  
  
+ AAAATGATTT TCAACTCATA TTTTCAGTTG ATTGGTAAAA AGAAAAATGA ACTTTCTGGA TAATCAGTTT   
  
  
+ GATTAACAAA AAGTGACAAA AAAATTAAAA ATGGTTTTCC GTAATCTTCA AACCATATGT CGAGGAAATA   
  
  
+ AACACGGAAT AAAGAAAGAG TGTCAGAACC CAAGAAATGT AAAATCAACT TACGTTCTAT TTAAAGTGAA   
  
  
+ AAATATTTTT CTTCATTCTA GGAAAATTAA TTGTCCTCTG AAAACAAATT TTCCCTCCTT GTCTAACCAA   
  
  
+ ACACAGTAAA ATTGAAAAAT CATTTTCTAG GAAAATGATT TTCACTCCTA CCAAACACAC CCTTAAACTT   
  
  
+ GTTCCAAATC TATAAACCCG GAATCTTAAT ACGCACATTG TATATTATAT GTCCTACTAT AACTGTATTA   
  
  
+ TCACTGGTTC TTGATTTATC CTTTCAAACT CTTGCTTAGT CAAGGTTGTC CTCGTACAAA CATGATTAAT   
  
  
+ TCAATTAACC ACGTAAGTTA TACGCTGACA AATTTCGGGT ACCAAACATT TGCACTAGAC CTAAAACAGT   
  
  
+ TACTATCATC ATATCGATGT TAAAATTGAC CTGTTCTTAT TTGTCCAGCG TTGAGCAAGG AAATAATCAA   
  
  
+ ATCATGTCTC ATTTAGTCGG TTTTTTGCCT AAGAAGAGCA TAAACAAAGG AAAAAGACAA CGACACATTA   
  
  
+ TACATGCATC TACACAAATG AAATGAGGCC ATATGGAACA TGTTCATGAG AGCTTCACTC GCTAATAATT   
  
  
+ GAACCTACTT TTGACTAAGC CACCAACTAG AATATGGAGA TTGACTGTTA AGAGACTATA TTACATGAAA   
  
  
+ TAGATAAGAG TAGACATTGC TATATATTTT ATCTTTAATT GTCTATTTTT AATGATCATT ACTGTTTATT   
  
  
+ TCAGTGAGTA ATTTTCTCGG ATATAATATT TTATAGCGGT TATTAAATAG ATGTGAAACT ATAATGTTAG   
  
  
+ ACATATCATG CGAAAATAGA GAAATTTTGG GCAAATTAAA AAAAAAAGAA GAAAAACGAG GAAGTATGCC   
  
  
+ CATTGTAAGC AGGTGGATGT ATATCCAGCC TCCATCCAAC AATTTGGGCA AAATTATATC ATCCATTCCA   
  
  
+ AAAAAAAGAT ATAATTTGTG ATATTAATAA CCCGACTCAA TTCAAGCTTA TTTATTTTTT CATATAATTT   
  
  
+ TTTAATATAT AAAAATAAAA AGTGACAACG ACATGCATCC TTTGCGTGTC AAAGGATCCG CCGCGTGGCC   
  
  
+ CCCACATTCA CTGACGGGGT TTTTGGTTTG CGATTAAGAT CAATGGCTAA CGTTGTCTCT CTCAAAACAT   
  
  
+ ATGGACCCCT TTTTCTATCT TTTTCTGCAT TGTCAGCATA GCAATTGTTG AGCACATTTT TTTCGATTTG   
  
  
+ TTGACAATAA GCGTGGTTAT GATTTATGGG CCACTAATAT CAGTCTCTGA TCATTTGCAC ATTTTGGACC   
  
  
+ TCTTTCTCTC TCTAATCTTC CAGGTAACCT GAATTCTTCT CTATTTCTGA CTAGATTAGA TAACAGGATT   
  
  
+ TCAAATATCT GTCAGGTGGC GGGTATAACT TCGTGATGGG CATTCATTTT TGTTCTATCA GGCTGGTGGG   
  
  
+ TTTGAGTTTA TTTTGATTCA TTTCGTGTTC ATCTGTGATA TTAGTAGGAG GGATTCTGTA TACCCACCAA   
  
  
+ CTGCTCGCCC TTTTGCCCGT GTGAGCAATT GGGACTTTTG TTTGTTCTTC GTCGATGCAT CCTCTGGTTG   
  
  
+ CTGATCTGGA ATTGAAACCC CATGTATTCA AATTCAACCC TGATTTGCTG TCAAACTTTC TGAACCATCA   
  
  
+ AAACTCCGCC GAAGTGTTTG AAAAAGATGG CATCTTCCAG ACCCTTCATT TGGCTGATCC CAAAAGCTCT   
  
  
+ TCAATTGCCG AAATCGGGTT TACTAATAGT TCAGATTCTA CACAAGTACC TGATTTTTCA GATGCTTGTC   
  
  
+ TTAAGTTCAT TAGTGATATT CTCCTGGAAG AGGACTTAGA TGAAAGTCCT ACGTCTTTAC ACGATTACAT   
  
  
+ GGCTCTCCTA GCCACTGAGA AGTCTTTGTA TGATGCTCTT GGAAAGGAAT ACTTTCCTTC GTCTACTAGT   
  
  
+ CTTGCCCCAT CTTTAGGCCG AAGTGTTGAC AGCCCAGATA GTGGCTTTGG CCGCGGTTGC TCTGATGGTC   
  
  
+ GCGGGATTGA GGGTTTGGCT AATGATGATG CCGTTTTCAT GTCTAACTGG CAGCTCAACA CCACCCAATT   
  
  
+ GGACCCTTTC CCAATCATGC AAGATATTCC TCGTCCCTAT TTGGAATTGA ATTACCATTC TTCTGGGTCA   
  
  
+ AGCAATGGCA TTGATGATTC GGGGGATGGG TTATCGACCT CTCCTGTAAG TACACTTGCA TCAACCGCCA   
  
  
+ CAGAGGCAGG GAAAAAGTTG GCTGGTAGCT CTAGGAGAAA GAACCGTCAA AGGGATGACT ATGGCCATGA   
  
  
+ AGAGGGAAGG AGTAACAAGC AGCAAGCCTC TTACAATGAC GATTACGTTG AGATGGAGCA GTATGACGAT   
  
  
+ GTACTTCTCT GTAGGGCAGA CAAGGGTAAT ATTTCAACTT GTGCCAATGA ATCCTCGCTT AATGAGGTGC   
  
  
+ GTGAGAAGCT GCAGACGACA GGGTTCAAGG GAAGAACATC CCGTCTTAAG AAGCAATCTA AGGAAGCGAA   
  
  
+ AGAGGTGGAT CTGAGAACTC TTCTTTCTGG TTGTGCACAA GCTGTTTCGA ACTTTGATAT CAGGACTGCT   
  
  
+ AATGAGCTGC TTAAGCAAGT CAGACAGCAT TCTTCACCAT ATGGTGATAG CCTCCAAAGG CTCGCCCATC   
  
  
+ AATTTGCGAA TGGTATTGAG GCACGCTTAG CAGGCACCGG TTCAAGAGTA CCTGCTAATC TCATTGATGC   
  
  
+ ACGGATATCA TCATCTGAAT TTTTAAAAGC TTACAAGTCA TATGTTTCAG CAGTTCCTTT CAGAAGGATG   
  
  
+ TCCTATTTTA TAGCAAACAA CACAATTCTG AAGTTGGCTG AGAAAGCAAC AAAGATTCAC ATAATTGATT   
  
  
+ TTGGTATTCT ATTTGGTCTA CAGTGGCCCT GTCTTATACA AAGTCTCTCA AGGCGAACTG TGGCTCCTCC   
  
  
+ GAAGCTTCGC ATCACTGGGA TAGACTATCC CCAGCATGGT TTCCGGCCAG CAGAAAAGGT TGAGGCAACA   
  
  
+ GGTCGTCGGT TGTCCGGGTA CTGTGAGAGA TTTAATGTAC CCTTTCGATA TGAAACCATT GCAAAGAAGT   
  
  
+ GGGAAACCAT ACGCCCAGAA GATCTAAATA TTGAGAATGA TGAGCTGGTA ATTGTTAATT GTATGTTGCG   
  
  
+ GTCTGTAAAT CTATTGGATG ATACAGTGGC GGTAAATAGT CCAAGGGATG CTTTCTTGAG GTTAATCAAA   
  
  
+ CAGATAAACC CGCGTTTATT CATTCATGCA ATTGTCAATG GAACCTTTAG TACTCCATTC TTCAGCACTC   
  
  
+ GATTCAGGGA AGCCCTATTC CAATACTCTT CTGTATTTGA TATATTTGAA GCGACTATGA CTCGTGAAGA   
  
  
+ TCGTGGAAGG CTGCTGATTG AGAGTCAAAT ATGCGGGCTA GAAGTTTTGA ATGCAATAGC ATGTGAAGGT   
  
  
+ GCAGAGAGGA TTCAAAGGCC TGAAACATAC AAGCAATGGC AGGAGCGGAC AACAAGGGCT GGACTAAGGC   
  
  
+ AGGTTCCAAT AGATGAGGAG CTTGTCAATA GAGCAAAGAC TATAGTGAAA GCAAATTATC ACAAGGATTT   
  
  
+ TGTGGTGGAT GAGGATAGGC GTTGGATGCT TCAAGGTTGG AAAGGAAGGA CACTTAGTGC CCTTTCCGTT   
  
  
+ TGGCAGCCTA ACTA  

- +Up\_Stream \_Len000AAATTA TTCTAGGAAA ATATATATAC ACACATATTA AGATACCATG TATCCATACA   
  
  
- TGGTATCTGA ATAACGTAAA GATCCAGGAA GAGAGAGACG CGAACAAAAA ACAAATAGAT TAAATACCTC   
  
  
- AACTTGTAAA ACAAAATATA TGAACTTGTT AGATAATATG ATTTAATTCG TAAAACTAAA TTAGTGAATA   
  
  
- CCCACACAAA CCATCCCAAC TTTTATTAAA AGGATCTTTT ATTAAAAATT ATATTTTATT AAAAAGTACA   
  
  
- TTTTACTAAA AGTTGAGTAT AAAAGTCAAC TAACCATTTT TCTTTTTACT TGAAAGACCT ATTAGTCAAA   
  
  
- CTAATTGTTT TTCACTGTTT TTTTAATTTT TACCAAAAGG CATTAGAAGT TTGGTATACA GCTCCTTTAT   
  
  
- TTGTGCCTTA TTTCTTTCTC ACAGTCTTGG GTTCTTTACA TTTTAGTTGA ATGCAAGATA AATTTCACTT   
  
  
- TTTATAAAAA GAAGTAAGAT CCTTTTAATT AACAGGAGAC TTTTGTTTAA AAGGGAGGAA CAGATTGGTT   
  
  
- TGTGTCATTT TAACTTTTTA GTAAAAGATC CTTTTACTAA AAGTGAGGAT GGTTTGTGTG GGAATTTGAA   
  
  
- CAAGGTTTAG ATATTTGGGC CTTAGAATTA TGCGTGTAAC ATATAATATA CAGGATGATA TTGACATAAT   
  
  
- AGTGACCAAG AACTAAATAG GAAAGTTTGA GAACGAATCA GTTCCAACAG GAGCATGTTT GTACTAATTA   
  
  
- AGTTAATTGG TGCATTCAAT ATGCGACTGT TTAAAGCCCA TGGTTTGTAA ACGTGATCTG GATTTTGTCA   
  
  
- ATGATAGTAG TATAGCTACA ATTTTAACTG GACAAGAATA AACAGGTCGC AACTCGTTCC TTTATTAGTT   
  
  
- TAGTACAGAG TAAATCAGCC AAAAAACGGA TTCTTCTCGT ATTTGTTTCC TTTTTCTGTT GCTGTGTAAT   
  
  
- ATGTACGTAG ATGTGTTTAC TTTACTCCGG TATACCTTGT ACAAGTACTC TCGAAGTGAG CGATTATTAA   
  
  
- CTTGGATGAA AACTGATTCG GTGGTTGATC TTATACCTCT AACTGACAAT TCTCTGATAT AATGTACTTT   
  
  
- ATCTATTCTC ATCTGTAACG ATATATAAAA TAGAAATTAA CAGATAAAAA TTACTAGTAA TGACAAATAA   
  
  
- AGTCACTCAT TAAAAGAGCC TATATTATAA AATATCGCCA ATAATTTATC TACACTTTGA TATTACAATC   
  
  
- TGTATAGTAC GCTTTTATCT CTTTAAAACC CGTTTAATTT TTTTTTTCTT CTTTTTGCTC CTTCATACGG   
  
  
- GTAACATTCG TCCACCTACA TATAGGTCGG AGGTAGGTTG TTAAACCCGT TTTAATATAG TAGGTAAGGT   
  
  
- TTTTTTTCTA TATTAAACAC TATAATTATT GGGCTGAGTT AAGTTCGAAT AAATAAAAAA GTATATTAAA   
  
  
- AAATTATATA TTTTTATTTT TCACTGTTGC TGTACGTAGG AAACGCACAG TTTCCTAGGC GGCGCACCGG   
  
  
- GGGTGTAAGT GACTGCCCCA AAAACCAAAC GCTAATTCTA GTTACCGATT GCAACAGAGA GAGTTTTGTA   
  
  
- TACCTGGGGA AAAAGATAGA AAAAGACGTA ACAGTCGTAT CGTTAACAAC TCGTGTAAAA AAAGCTAAAC   
  
  
- AACTGTTATT CGCACCAATA CTAAATACCC GGTGATTATA GTCAGAGACT AGTAAACGTG TAAAACCTGG   
  
  
- AGAAAGAGAG AGATTAGAAG GTCCATTGGA CTTAAGAAGA GATAAAGACT GATCTAATCT ATTGTCCTAA   
  
  
- AGTTTATAGA CAGTCCACCG CCCATATTGA AGCACTACCC GTAAGTAAAA ACAAGATAGT CCGACCACCC   
  
  
- AAACTCAAAT AAAACTAAGT AAAGCACAAG TAGACACTAT AATCATCCTC CCTAAGACAT ATGGGTGGTT   
  
  
- GACGAGCGGG AAAACGGGCA CACTCGTTAA CCCTGAAAAC AAACAAGAAG CAGCTACGTA GGAGACCAAC   
  
  
- GACTAGACCT TAACTTTGGG GTACATAAGT TTAAGTTGGG ACTAAACGAC AGTTTGAAAG ACTTGGTAGT   
  
  
- TTTGAGGCGG CTTCACAAAC TTTTTCTACC GTAGAAGGTC TGGGAAGTAA ACCGACTAGG GTTTTCGAGA   
  
  
- AGTTAACGGC TTTAGCCCAA ATGATTATCA AGTCTAAGAT GTGTTCATGG ACTAAAAAGT CTACGAACAG   
  
  
- AATTCAAGTA ATCACTATAA GAGGACCTTC TCCTGAATCT ACTTTCAGGA TGCAGAAATG TGCTAATGTA   
  
  
- CCGAGAGGAT CGGTGACTCT TCAGAAACAT ACTACGAGAA CCTTTCCTTA TGAAAGGAAG CAGATGATCA   
  
  
- GAACGGGGTA GAAATCCGGC TTCACAACTG TCGGGTCTAT CACCGAAACC GGCGCCAACG AGACTACCAG   
  
  
- CGCCCTAACT CCCAAACCGA TTACTACTAC GGCAAAAGTA CAGATTGACC GTCGAGTTGT GGTGGGTTAA   
  
  
- CCTGGGAAAG GGTTAGTACG TTCTATAAGG AGCAGGGATA AACCTTAACT TAATGGTAAG AAGACCCAGT   
  
  
- TCGTTACCGT AACTACTAAG CCCCCTACCC AATAGCTGGA GAGGACATTC ATGTGAACGT AGTTGGCGGT   
  
  
- GTCTCCGTCC CTTTTTCAAC CGACCATCGA GATCCTCTTT CTTGGCAGTT TCCCTACTGA TACCGGTACT   
  
  
- TCTCCCTTCC TCATTGTTCG TCGTTCGGAG AATGTTACTG CTAATGCAAC TCTACCTCGT CATACTGCTA   
  
  
- CATGAAGAGA CATCCCGTCT GTTCCCATTA TAAAGTTGAA CACGGTTACT TAGGAGCGAA TTACTCCACG   
  
  
- CACTCTTCGA CGTCTGCTGT CCCAAGTTCC CTTCTTGTAG GGCAGAATTC TTCGTTAGAT TCCTTCGCTT   
  
  
- TCTCCACCTA GACTCTTGAG AAGAAAGACC AACACGTGTT CGACAAAGCT TGAAACTATA GTCCTGACGA   
  
  
- TTACTCGACG AATTCGTTCA GTCTGTCGTA AGAAGTGGTA TACCACTATC GGAGGTTTCC GAGCGGGTAG   
  
  
- TTAAACGCTT ACCATAACTC CGTGCGAATC GTCCGTGGCC AAGTTCTCAT GGACGATTAG AGTAACTACG   
  
  
- TGCCTATAGT AGTAGACTTA AAAATTTTCG AATGTTCAGT ATACAAAGTC GTCAAGGAAA GTCTTCCTAC   
  
  
- AGGATAAAAT ATCGTTTGTT GTGTTAAGAC TTCAACCGAC TCTTTCGTTG TTTCTAAGTG TATTAACTAA   
  
  
- AACCATAAGA TAAACCAGAT GTCACCGGGA CAGAATATGT TTCAGAGAGT TCCGCTTGAC ACCGAGGAGG   
  
  
- CTTCGAAGCG TAGTGACCCT ATCTGATAGG GGTCGTACCA AAGGCCGGTC GTCTTTTCCA ACTCCGTTGT   
  
  
- CCAGCAGCCA ACAGGCCCAT GACACTCTCT AAATTACATG GGAAAGCTAT ACTTTGGTAA CGTTTCTTCA   
  
  
- CCCTTTGGTA TGCGGGTCTT CTAGATTTAT AACTCTTACT ACTCGACCAT TAACAATTAA CATACAACGC   
  
  
- CAGACATTTA GATAACCTAC TATGTCACCG CCATTTATCA GGTTCCCTAC GAAAGAACTC CAATTAGTTT   
  
  
- GTCTATTTGG GCGCAAATAA GTAAGTACGT TAACAGTTAC CTTGGAAATC ATGAGGTAAG AAGTCGTGAG   
  
  
- CTAAGTCCCT TCGGGATAAG GTTATGAGAA GACATAAACT ATATAAACTT CGCTGATACT GAGCACTTCT   
  
  
- AGCACCTTCC GACGACTAAC TCTCAGTTTA TACGCCCGAT CTTCAAAACT TACGTTATCG TACACTTCCA   
  
  
- CGTCTCTCCT AAGTTTCCGG ACTTTGTATG TTCGTTACCG TCCTCGCCTG TTGTTCCCGA CCTGATTCCG   
  
  
- TCCAAGGTTA TCTACTCCTC GAACAGTTAT CTCGTTTCTG ATATCACTTT CGTTTAATAG TGTTCCTAAA   
  
  
- ACACCACCTA CTCCTATCCG CAACCTACGA AGTTCCAACC TTTCCTTCCT GTGAATCACG GGAAAGGCAA   
  
  
- ACCGTCGGAT TGAT

+     ABRE

| Site Name | Organism | Position | Strand | Matrix score. | sequence | function |
| --- | --- | --- | --- | --- | --- | --- |
| ABRE | Oryza sativa | 1534 | + | 10 | GCCGCGTGGC | cis-acting element involved in the abscisic acid responsiveness |
| ABRE | Arabidopsis thaliana | 649 | + | 7 | AACCCGG | cis-acting element involved in the abscisic acid responsiveness |
| ABRE | Arabidopsis thaliana | 784 | - | 5 | ACGTG | cis-acting element involved in the abscisic acid responsiveness |

>HU02G01572.1   
+ +Up\_Stream \_Len000TTTAAT AAGATCCTTT TATATATATG TGTGTATAAT TCTATGGTAC ATAGGTATGT   
  
  
+ ACCATAGACT TATTGCATTT CTAGGTCCTT CTCTCTCTGC GCTTGTTTTT TGTTTATCTA ATTTATGGAG   
  
  
+ TTGAACATTT TGTTTTATAT ACTTGAACAA TCTATTATAC TAAATTAAGC ATTTTGATTT AATCACTTAT   
  
  
+ GGGTGTGTTT GGTAGGGTTG AAAATAATTT TCCTAGAAAA TAATTTTTAA TATAAAATAA TTTTTCATGT   
  
  
+ AAAATGATTT TCAACTCATA TTTTCAGTTG ATTGGTAAAA AGAAAAATGA ACTTTCTGGA TAATCAGTTT   
  
  
+ GATTAACAAA AAGTGACAAA AAAATTAAAA ATGGTTTTCC GTAATCTTCA AACCATATGT CGAGGAAATA   
  
  
+ AACACGGAAT AAAGAAAGAG TGTCAGAACC CAAGAAATGT AAAATCAACT TACGTTCTAT TTAAAGTGAA   
  
  
+ AAATATTTTT CTTCATTCTA GGAAAATTAA TTGTCCTCTG AAAACAAATT TTCCCTCCTT GTCTAACCAA   
  
  
+ ACACAGTAAA ATTGAAAAAT CATTTTCTAG GAAAATGATT TTCACTCCTA CCAAACACAC CCTTAAACTT   
  
  
+ GTTCCAAATC TATAAACCCG GAATCTTAAT ACGCACATTG TATATTATAT GTCCTACTAT AACTGTATTA   
  
  
+ TCACTGGTTC TTGATTTATC CTTTCAAACT CTTGCTTAGT CAAGGTTGTC CTCGTACAAA CATGATTAAT   
  
  
+ TCAATTAACC ACGTAAGTTA TACGCTGACA AATTTCGGGT ACCAAACATT TGCACTAGAC CTAAAACAGT   
  
  
+ TACTATCATC ATATCGATGT TAAAATTGAC CTGTTCTTAT TTGTCCAGCG TTGAGCAAGG AAATAATCAA   
  
  
+ ATCATGTCTC ATTTAGTCGG TTTTTTGCCT AAGAAGAGCA TAAACAAAGG AAAAAGACAA CGACACATTA   
  
  
+ TACATGCATC TACACAAATG AAATGAGGCC ATATGGAACA TGTTCATGAG AGCTTCACTC GCTAATAATT   
  
  
+ GAACCTACTT TTGACTAAGC CACCAACTAG AATATGGAGA TTGACTGTTA AGAGACTATA TTACATGAAA   
  
  
+ TAGATAAGAG TAGACATTGC TATATATTTT ATCTTTAATT GTCTATTTTT AATGATCATT ACTGTTTATT   
  
  
+ TCAGTGAGTA ATTTTCTCGG ATATAATATT TTATAGCGGT TATTAAATAG ATGTGAAACT ATAATGTTAG   
  
  
+ ACATATCATG CGAAAATAGA GAAATTTTGG GCAAATTAAA AAAAAAAGAA GAAAAACGAG GAAGTATGCC   
  
  
+ CATTGTAAGC AGGTGGATGT ATATCCAGCC TCCATCCAAC AATTTGGGCA AAATTATATC ATCCATTCCA   
  
  
+ AAAAAAAGAT ATAATTTGTG ATATTAATAA CCCGACTCAA TTCAAGCTTA TTTATTTTTT CATATAATTT   
  
  
+ TTTAATATAT AAAAATAAAA AGTGACAACG ACATGCATCC TTTGCGTGTC AAAGGATCCG CCGCGTGGCC   
  
  
+ CCCACATTCA CTGACGGGGT TTTTGGTTTG CGATTAAGAT CAATGGCTAA CGTTGTCTCT CTCAAAACAT   
  
  
+ ATGGACCCCT TTTTCTATCT TTTTCTGCAT TGTCAGCATA GCAATTGTTG AGCACATTTT TTTCGATTTG   
  
  
+ TTGACAATAA GCGTGGTTAT GATTTATGGG CCACTAATAT CAGTCTCTGA TCATTTGCAC ATTTTGGACC   
  
  
+ TCTTTCTCTC TCTAATCTTC CAGGTAACCT GAATTCTTCT CTATTTCTGA CTAGATTAGA TAACAGGATT   
  
  
+ TCAAATATCT GTCAGGTGGC GGGTATAACT TCGTGATGGG CATTCATTTT TGTTCTATCA GGCTGGTGGG   
  
  
+ TTTGAGTTTA TTTTGATTCA TTTCGTGTTC ATCTGTGATA TTAGTAGGAG GGATTCTGTA TACCCACCAA   
  
  
+ CTGCTCGCCC TTTTGCCCGT GTGAGCAATT GGGACTTTTG TTTGTTCTTC GTCGATGCAT CCTCTGGTTG   
  
  
+ CTGATCTGGA ATTGAAACCC CATGTATTCA AATTCAACCC TGATTTGCTG TCAAACTTTC TGAACCATCA   
  
  
+ AAACTCCGCC GAAGTGTTTG AAAAAGATGG CATCTTCCAG ACCCTTCATT TGGCTGATCC CAAAAGCTCT   
  
  
+ TCAATTGCCG AAATCGGGTT TACTAATAGT TCAGATTCTA CACAAGTACC TGATTTTTCA GATGCTTGTC   
  
  
+ TTAAGTTCAT TAGTGATATT CTCCTGGAAG AGGACTTAGA TGAAAGTCCT ACGTCTTTAC ACGATTACAT   
  
  
+ GGCTCTCCTA GCCACTGAGA AGTCTTTGTA TGATGCTCTT GGAAAGGAAT ACTTTCCTTC GTCTACTAGT   
  
  
+ CTTGCCCCAT CTTTAGGCCG AAGTGTTGAC AGCCCAGATA GTGGCTTTGG CCGCGGTTGC TCTGATGGTC   
  
  
+ GCGGGATTGA GGGTTTGGCT AATGATGATG CCGTTTTCAT GTCTAACTGG CAGCTCAACA CCACCCAATT   
  
  
+ GGACCCTTTC CCAATCATGC AAGATATTCC TCGTCCCTAT TTGGAATTGA ATTACCATTC TTCTGGGTCA   
  
  
+ AGCAATGGCA TTGATGATTC GGGGGATGGG TTATCGACCT CTCCTGTAAG TACACTTGCA TCAACCGCCA   
  
  
+ CAGAGGCAGG GAAAAAGTTG GCTGGTAGCT CTAGGAGAAA GAACCGTCAA AGGGATGACT ATGGCCATGA   
  
  
+ AGAGGGAAGG AGTAACAAGC AGCAAGCCTC TTACAATGAC GATTACGTTG AGATGGAGCA GTATGACGAT   
  
  
+ GTACTTCTCT GTAGGGCAGA CAAGGGTAAT ATTTCAACTT GTGCCAATGA ATCCTCGCTT AATGAGGTGC   
  
  
+ GTGAGAAGCT GCAGACGACA GGGTTCAAGG GAAGAACATC CCGTCTTAAG AAGCAATCTA AGGAAGCGAA   
  
  
+ AGAGGTGGAT CTGAGAACTC TTCTTTCTGG TTGTGCACAA GCTGTTTCGA ACTTTGATAT CAGGACTGCT   
  
  
+ AATGAGCTGC TTAAGCAAGT CAGACAGCAT TCTTCACCAT ATGGTGATAG CCTCCAAAGG CTCGCCCATC   
  
  
+ AATTTGCGAA TGGTATTGAG GCACGCTTAG CAGGCACCGG TTCAAGAGTA CCTGCTAATC TCATTGATGC   
  
  
+ ACGGATATCA TCATCTGAAT TTTTAAAAGC TTACAAGTCA TATGTTTCAG CAGTTCCTTT CAGAAGGATG   
  
  
+ TCCTATTTTA TAGCAAACAA CACAATTCTG AAGTTGGCTG AGAAAGCAAC AAAGATTCAC ATAATTGATT   
  
  
+ TTGGTATTCT ATTTGGTCTA CAGTGGCCCT GTCTTATACA AAGTCTCTCA AGGCGAACTG TGGCTCCTCC   
  
  
+ GAAGCTTCGC ATCACTGGGA TAGACTATCC CCAGCATGGT TTCCGGCCAG CAGAAAAGGT TGAGGCAACA   
  
  
+ GGTCGTCGGT TGTCCGGGTA CTGTGAGAGA TTTAATGTAC CCTTTCGATA TGAAACCATT GCAAAGAAGT   
  
  
+ GGGAAACCAT ACGCCCAGAA GATCTAAATA TTGAGAATGA TGAGCTGGTA ATTGTTAATT GTATGTTGCG   
  
  
+ GTCTGTAAAT CTATTGGATG ATACAGTGGC GGTAAATAGT CCAAGGGATG CTTTCTTGAG GTTAATCAAA   
  
  
+ CAGATAAACC CGCGTTTATT CATTCATGCA ATTGTCAATG GAACCTTTAG TACTCCATTC TTCAGCACTC   
  
  
+ GATTCAGGGA AGCCCTATTC CAATACTCTT CTGTATTTGA TATATTTGAA GCGACTATGA CTCGTGAAGA   
  
  
+ TCGTGGAAGG CTGCTGATTG AGAGTCAAAT ATGCGGGCTA GAAGTTTTGA ATGCAATAGC ATGTGAAGGT   
  
  
+ GCAGAGAGGA TTCAAAGGCC TGAAACATAC AAGCAATGGC AGGAGCGGAC AACAAGGGCT GGACTAAGGC   
  
  
+ AGGTTCCAAT AGATGAGGAG CTTGTCAATA GAGCAAAGAC TATAGTGAAA GCAAATTATC ACAAGGATTT   
  
  
+ TGTGGTGGAT GAGGATAGGC GTTGGATGCT TCAAGGTTGG AAAGGAAGGA CACTTAGTGC CCTTTCCGTT   
  
  
+ TGGCAGCCTA ACTA  

- +Up\_Stream \_Len000AAATTA TTCTAGGAAA ATATATATAC ACACATATTA AGATACCATG TATCCATACA   
  
  
- TGGTATCTGA ATAACGTAAA GATCCAGGAA GAGAGAGACG CGAACAAAAA ACAAATAGAT TAAATACCTC   
  
  
- AACTTGTAAA ACAAAATATA TGAACTTGTT AGATAATATG ATTTAATTCG TAAAACTAAA TTAGTGAATA   
  
  
- CCCACACAAA CCATCCCAAC TTTTATTAAA AGGATCTTTT ATTAAAAATT ATATTTTATT AAAAAGTACA   
  
  
- TTTTACTAAA AGTTGAGTAT AAAAGTCAAC TAACCATTTT TCTTTTTACT TGAAAGACCT ATTAGTCAAA   
  
  
- CTAATTGTTT TTCACTGTTT TTTTAATTTT TACCAAAAGG CATTAGAAGT TTGGTATACA GCTCCTTTAT   
  
  
- TTGTGCCTTA TTTCTTTCTC ACAGTCTTGG GTTCTTTACA TTTTAGTTGA ATGCAAGATA AATTTCACTT   
  
  
- TTTATAAAAA GAAGTAAGAT CCTTTTAATT AACAGGAGAC TTTTGTTTAA AAGGGAGGAA CAGATTGGTT   
  
  
- TGTGTCATTT TAACTTTTTA GTAAAAGATC CTTTTACTAA AAGTGAGGAT GGTTTGTGTG GGAATTTGAA   
  
  
- CAAGGTTTAG ATATTTGGGC CTTAGAATTA TGCGTGTAAC ATATAATATA CAGGATGATA TTGACATAAT   
  
  
- AGTGACCAAG AACTAAATAG GAAAGTTTGA GAACGAATCA GTTCCAACAG GAGCATGTTT GTACTAATTA   
  
  
- AGTTAATTGG TGCATTCAAT ATGCGACTGT TTAAAGCCCA TGGTTTGTAA ACGTGATCTG GATTTTGTCA   
  
  
- ATGATAGTAG TATAGCTACA ATTTTAACTG GACAAGAATA AACAGGTCGC AACTCGTTCC TTTATTAGTT   
  
  
- TAGTACAGAG TAAATCAGCC AAAAAACGGA TTCTTCTCGT ATTTGTTTCC TTTTTCTGTT GCTGTGTAAT   
  
  
- ATGTACGTAG ATGTGTTTAC TTTACTCCGG TATACCTTGT ACAAGTACTC TCGAAGTGAG CGATTATTAA   
  
  
- CTTGGATGAA AACTGATTCG GTGGTTGATC TTATACCTCT AACTGACAAT TCTCTGATAT AATGTACTTT   
  
  
- ATCTATTCTC ATCTGTAACG ATATATAAAA TAGAAATTAA CAGATAAAAA TTACTAGTAA TGACAAATAA   
  
  
- AGTCACTCAT TAAAAGAGCC TATATTATAA AATATCGCCA ATAATTTATC TACACTTTGA TATTACAATC   
  
  
- TGTATAGTAC GCTTTTATCT CTTTAAAACC CGTTTAATTT TTTTTTTCTT CTTTTTGCTC CTTCATACGG   
  
  
- GTAACATTCG TCCACCTACA TATAGGTCGG AGGTAGGTTG TTAAACCCGT TTTAATATAG TAGGTAAGGT   
  
  
- TTTTTTTCTA TATTAAACAC TATAATTATT GGGCTGAGTT AAGTTCGAAT AAATAAAAAA GTATATTAAA   
  
  
- AAATTATATA TTTTTATTTT TCACTGTTGC TGTACGTAGG AAACGCACAG TTTCCTAGGC GGCGCACCGG   
  
  
- GGGTGTAAGT GACTGCCCCA AAAACCAAAC GCTAATTCTA GTTACCGATT GCAACAGAGA GAGTTTTGTA   
  
  
- TACCTGGGGA AAAAGATAGA AAAAGACGTA ACAGTCGTAT CGTTAACAAC TCGTGTAAAA AAAGCTAAAC   
  
  
- AACTGTTATT CGCACCAATA CTAAATACCC GGTGATTATA GTCAGAGACT AGTAAACGTG TAAAACCTGG   
  
  
- AGAAAGAGAG AGATTAGAAG GTCCATTGGA CTTAAGAAGA GATAAAGACT GATCTAATCT ATTGTCCTAA   
  
  
- AGTTTATAGA CAGTCCACCG CCCATATTGA AGCACTACCC GTAAGTAAAA ACAAGATAGT CCGACCACCC   
  
  
- AAACTCAAAT AAAACTAAGT AAAGCACAAG TAGACACTAT AATCATCCTC CCTAAGACAT ATGGGTGGTT   
  
  
- GACGAGCGGG AAAACGGGCA CACTCGTTAA CCCTGAAAAC AAACAAGAAG CAGCTACGTA GGAGACCAAC   
  
  
- GACTAGACCT TAACTTTGGG GTACATAAGT TTAAGTTGGG ACTAAACGAC AGTTTGAAAG ACTTGGTAGT   
  
  
- TTTGAGGCGG CTTCACAAAC TTTTTCTACC GTAGAAGGTC TGGGAAGTAA ACCGACTAGG GTTTTCGAGA   
  
  
- AGTTAACGGC TTTAGCCCAA ATGATTATCA AGTCTAAGAT GTGTTCATGG ACTAAAAAGT CTACGAACAG   
  
  
- AATTCAAGTA ATCACTATAA GAGGACCTTC TCCTGAATCT ACTTTCAGGA TGCAGAAATG TGCTAATGTA   
  
  
- CCGAGAGGAT CGGTGACTCT TCAGAAACAT ACTACGAGAA CCTTTCCTTA TGAAAGGAAG CAGATGATCA   
  
  
- GAACGGGGTA GAAATCCGGC TTCACAACTG TCGGGTCTAT CACCGAAACC GGCGCCAACG AGACTACCAG   
  
  
- CGCCCTAACT CCCAAACCGA TTACTACTAC GGCAAAAGTA CAGATTGACC GTCGAGTTGT GGTGGGTTAA   
  
  
- CCTGGGAAAG GGTTAGTACG TTCTATAAGG AGCAGGGATA AACCTTAACT TAATGGTAAG AAGACCCAGT   
  
  
- TCGTTACCGT AACTACTAAG CCCCCTACCC AATAGCTGGA GAGGACATTC ATGTGAACGT AGTTGGCGGT   
  
  
- GTCTCCGTCC CTTTTTCAAC CGACCATCGA GATCCTCTTT CTTGGCAGTT TCCCTACTGA TACCGGTACT   
  
  
- TCTCCCTTCC TCATTGTTCG TCGTTCGGAG AATGTTACTG CTAATGCAAC TCTACCTCGT CATACTGCTA   
  
  
- CATGAAGAGA CATCCCGTCT GTTCCCATTA TAAAGTTGAA CACGGTTACT TAGGAGCGAA TTACTCCACG   
  
  
- CACTCTTCGA CGTCTGCTGT CCCAAGTTCC CTTCTTGTAG GGCAGAATTC TTCGTTAGAT TCCTTCGCTT   
  
  
- TCTCCACCTA GACTCTTGAG AAGAAAGACC AACACGTGTT CGACAAAGCT TGAAACTATA GTCCTGACGA   
  
  
- TTACTCGACG AATTCGTTCA GTCTGTCGTA AGAAGTGGTA TACCACTATC GGAGGTTTCC GAGCGGGTAG   
  
  
- TTAAACGCTT ACCATAACTC CGTGCGAATC GTCCGTGGCC AAGTTCTCAT GGACGATTAG AGTAACTACG   
  
  
- TGCCTATAGT AGTAGACTTA AAAATTTTCG AATGTTCAGT ATACAAAGTC GTCAAGGAAA GTCTTCCTAC   
  
  
- AGGATAAAAT ATCGTTTGTT GTGTTAAGAC TTCAACCGAC TCTTTCGTTG TTTCTAAGTG TATTAACTAA   
  
  
- AACCATAAGA TAAACCAGAT GTCACCGGGA CAGAATATGT TTCAGAGAGT TCCGCTTGAC ACCGAGGAGG   
  
  
- CTTCGAAGCG TAGTGACCCT ATCTGATAGG GGTCGTACCA AAGGCCGGTC GTCTTTTCCA ACTCCGTTGT   
  
  
- CCAGCAGCCA ACAGGCCCAT GACACTCTCT AAATTACATG GGAAAGCTAT ACTTTGGTAA CGTTTCTTCA   
  
  
- CCCTTTGGTA TGCGGGTCTT CTAGATTTAT AACTCTTACT ACTCGACCAT TAACAATTAA CATACAACGC   
  
  
- CAGACATTTA GATAACCTAC TATGTCACCG CCATTTATCA GGTTCCCTAC GAAAGAACTC CAATTAGTTT   
  
  
- GTCTATTTGG GCGCAAATAA GTAAGTACGT TAACAGTTAC CTTGGAAATC ATGAGGTAAG AAGTCGTGAG   
  
  
- CTAAGTCCCT TCGGGATAAG GTTATGAGAA GACATAAACT ATATAAACTT CGCTGATACT GAGCACTTCT   
  
  
- AGCACCTTCC GACGACTAAC TCTCAGTTTA TACGCCCGAT CTTCAAAACT TACGTTATCG TACACTTCCA   
  
  
- CGTCTCTCCT AAGTTTCCGG ACTTTGTATG TTCGTTACCG TCCTCGCCTG TTGTTCCCGA CCTGATTCCG   
  
  
- TCCAAGGTTA TCTACTCCTC GAACAGTTAT CTCGTTTCTG ATATCACTTT CGTTTAATAG TGTTCCTAAA   
  
  
- ACACCACCTA CTCCTATCCG CAACCTACGA AGTTCCAACC TTTCCTTCCT GTGAATCACG GGAAAGGCAA   
  
  
- ACCGTCGGAT TGAT

+     ABRE3a

| Site Name | Organism | Position | Strand | Matrix score. | sequence | function |
| --- | --- | --- | --- | --- | --- | --- |
| ABRE3a | Zea mays | 784 | - | 6 | TACGTG |  |

>HU02G01572.1   
+ +Up\_Stream \_Len000TTTAAT AAGATCCTTT TATATATATG TGTGTATAAT TCTATGGTAC ATAGGTATGT   
  
  
+ ACCATAGACT TATTGCATTT CTAGGTCCTT CTCTCTCTGC GCTTGTTTTT TGTTTATCTA ATTTATGGAG   
  
  
+ TTGAACATTT TGTTTTATAT ACTTGAACAA TCTATTATAC TAAATTAAGC ATTTTGATTT AATCACTTAT   
  
  
+ GGGTGTGTTT GGTAGGGTTG AAAATAATTT TCCTAGAAAA TAATTTTTAA TATAAAATAA TTTTTCATGT   
  
  
+ AAAATGATTT TCAACTCATA TTTTCAGTTG ATTGGTAAAA AGAAAAATGA ACTTTCTGGA TAATCAGTTT   
  
  
+ GATTAACAAA AAGTGACAAA AAAATTAAAA ATGGTTTTCC GTAATCTTCA AACCATATGT CGAGGAAATA   
  
  
+ AACACGGAAT AAAGAAAGAG TGTCAGAACC CAAGAAATGT AAAATCAACT TACGTTCTAT TTAAAGTGAA   
  
  
+ AAATATTTTT CTTCATTCTA GGAAAATTAA TTGTCCTCTG AAAACAAATT TTCCCTCCTT GTCTAACCAA   
  
  
+ ACACAGTAAA ATTGAAAAAT CATTTTCTAG GAAAATGATT TTCACTCCTA CCAAACACAC CCTTAAACTT   
  
  
+ GTTCCAAATC TATAAACCCG GAATCTTAAT ACGCACATTG TATATTATAT GTCCTACTAT AACTGTATTA   
  
  
+ TCACTGGTTC TTGATTTATC CTTTCAAACT CTTGCTTAGT CAAGGTTGTC CTCGTACAAA CATGATTAAT   
  
  
+ TCAATTAACC ACGTAAGTTA TACGCTGACA AATTTCGGGT ACCAAACATT TGCACTAGAC CTAAAACAGT   
  
  
+ TACTATCATC ATATCGATGT TAAAATTGAC CTGTTCTTAT TTGTCCAGCG TTGAGCAAGG AAATAATCAA   
  
  
+ ATCATGTCTC ATTTAGTCGG TTTTTTGCCT AAGAAGAGCA TAAACAAAGG AAAAAGACAA CGACACATTA   
  
  
+ TACATGCATC TACACAAATG AAATGAGGCC ATATGGAACA TGTTCATGAG AGCTTCACTC GCTAATAATT   
  
  
+ GAACCTACTT TTGACTAAGC CACCAACTAG AATATGGAGA TTGACTGTTA AGAGACTATA TTACATGAAA   
  
  
+ TAGATAAGAG TAGACATTGC TATATATTTT ATCTTTAATT GTCTATTTTT AATGATCATT ACTGTTTATT   
  
  
+ TCAGTGAGTA ATTTTCTCGG ATATAATATT TTATAGCGGT TATTAAATAG ATGTGAAACT ATAATGTTAG   
  
  
+ ACATATCATG CGAAAATAGA GAAATTTTGG GCAAATTAAA AAAAAAAGAA GAAAAACGAG GAAGTATGCC   
  
  
+ CATTGTAAGC AGGTGGATGT ATATCCAGCC TCCATCCAAC AATTTGGGCA AAATTATATC ATCCATTCCA   
  
  
+ AAAAAAAGAT ATAATTTGTG ATATTAATAA CCCGACTCAA TTCAAGCTTA TTTATTTTTT CATATAATTT   
  
  
+ TTTAATATAT AAAAATAAAA AGTGACAACG ACATGCATCC TTTGCGTGTC AAAGGATCCG CCGCGTGGCC   
  
  
+ CCCACATTCA CTGACGGGGT TTTTGGTTTG CGATTAAGAT CAATGGCTAA CGTTGTCTCT CTCAAAACAT   
  
  
+ ATGGACCCCT TTTTCTATCT TTTTCTGCAT TGTCAGCATA GCAATTGTTG AGCACATTTT TTTCGATTTG   
  
  
+ TTGACAATAA GCGTGGTTAT GATTTATGGG CCACTAATAT CAGTCTCTGA TCATTTGCAC ATTTTGGACC   
  
  
+ TCTTTCTCTC TCTAATCTTC CAGGTAACCT GAATTCTTCT CTATTTCTGA CTAGATTAGA TAACAGGATT   
  
  
+ TCAAATATCT GTCAGGTGGC GGGTATAACT TCGTGATGGG CATTCATTTT TGTTCTATCA GGCTGGTGGG   
  
  
+ TTTGAGTTTA TTTTGATTCA TTTCGTGTTC ATCTGTGATA TTAGTAGGAG GGATTCTGTA TACCCACCAA   
  
  
+ CTGCTCGCCC TTTTGCCCGT GTGAGCAATT GGGACTTTTG TTTGTTCTTC GTCGATGCAT CCTCTGGTTG   
  
  
+ CTGATCTGGA ATTGAAACCC CATGTATTCA AATTCAACCC TGATTTGCTG TCAAACTTTC TGAACCATCA   
  
  
+ AAACTCCGCC GAAGTGTTTG AAAAAGATGG CATCTTCCAG ACCCTTCATT TGGCTGATCC CAAAAGCTCT   
  
  
+ TCAATTGCCG AAATCGGGTT TACTAATAGT TCAGATTCTA CACAAGTACC TGATTTTTCA GATGCTTGTC   
  
  
+ TTAAGTTCAT TAGTGATATT CTCCTGGAAG AGGACTTAGA TGAAAGTCCT ACGTCTTTAC ACGATTACAT   
  
  
+ GGCTCTCCTA GCCACTGAGA AGTCTTTGTA TGATGCTCTT GGAAAGGAAT ACTTTCCTTC GTCTACTAGT   
  
  
+ CTTGCCCCAT CTTTAGGCCG AAGTGTTGAC AGCCCAGATA GTGGCTTTGG CCGCGGTTGC TCTGATGGTC   
  
  
+ GCGGGATTGA GGGTTTGGCT AATGATGATG CCGTTTTCAT GTCTAACTGG CAGCTCAACA CCACCCAATT   
  
  
+ GGACCCTTTC CCAATCATGC AAGATATTCC TCGTCCCTAT TTGGAATTGA ATTACCATTC TTCTGGGTCA   
  
  
+ AGCAATGGCA TTGATGATTC GGGGGATGGG TTATCGACCT CTCCTGTAAG TACACTTGCA TCAACCGCCA   
  
  
+ CAGAGGCAGG GAAAAAGTTG GCTGGTAGCT CTAGGAGAAA GAACCGTCAA AGGGATGACT ATGGCCATGA   
  
  
+ AGAGGGAAGG AGTAACAAGC AGCAAGCCTC TTACAATGAC GATTACGTTG AGATGGAGCA GTATGACGAT   
  
  
+ GTACTTCTCT GTAGGGCAGA CAAGGGTAAT ATTTCAACTT GTGCCAATGA ATCCTCGCTT AATGAGGTGC   
  
  
+ GTGAGAAGCT GCAGACGACA GGGTTCAAGG GAAGAACATC CCGTCTTAAG AAGCAATCTA AGGAAGCGAA   
  
  
+ AGAGGTGGAT CTGAGAACTC TTCTTTCTGG TTGTGCACAA GCTGTTTCGA ACTTTGATAT CAGGACTGCT   
  
  
+ AATGAGCTGC TTAAGCAAGT CAGACAGCAT TCTTCACCAT ATGGTGATAG CCTCCAAAGG CTCGCCCATC   
  
  
+ AATTTGCGAA TGGTATTGAG GCACGCTTAG CAGGCACCGG TTCAAGAGTA CCTGCTAATC TCATTGATGC   
  
  
+ ACGGATATCA TCATCTGAAT TTTTAAAAGC TTACAAGTCA TATGTTTCAG CAGTTCCTTT CAGAAGGATG   
  
  
+ TCCTATTTTA TAGCAAACAA CACAATTCTG AAGTTGGCTG AGAAAGCAAC AAAGATTCAC ATAATTGATT   
  
  
+ TTGGTATTCT ATTTGGTCTA CAGTGGCCCT GTCTTATACA AAGTCTCTCA AGGCGAACTG TGGCTCCTCC   
  
  
+ GAAGCTTCGC ATCACTGGGA TAGACTATCC CCAGCATGGT TTCCGGCCAG CAGAAAAGGT TGAGGCAACA   
  
  
+ GGTCGTCGGT TGTCCGGGTA CTGTGAGAGA TTTAATGTAC CCTTTCGATA TGAAACCATT GCAAAGAAGT   
  
  
+ GGGAAACCAT ACGCCCAGAA GATCTAAATA TTGAGAATGA TGAGCTGGTA ATTGTTAATT GTATGTTGCG   
  
  
+ GTCTGTAAAT CTATTGGATG ATACAGTGGC GGTAAATAGT CCAAGGGATG CTTTCTTGAG GTTAATCAAA   
  
  
+ CAGATAAACC CGCGTTTATT CATTCATGCA ATTGTCAATG GAACCTTTAG TACTCCATTC TTCAGCACTC   
  
  
+ GATTCAGGGA AGCCCTATTC CAATACTCTT CTGTATTTGA TATATTTGAA GCGACTATGA CTCGTGAAGA   
  
  
+ TCGTGGAAGG CTGCTGATTG AGAGTCAAAT ATGCGGGCTA GAAGTTTTGA ATGCAATAGC ATGTGAAGGT   
  
  
+ GCAGAGAGGA TTCAAAGGCC TGAAACATAC AAGCAATGGC AGGAGCGGAC AACAAGGGCT GGACTAAGGC   
  
  
+ AGGTTCCAAT AGATGAGGAG CTTGTCAATA GAGCAAAGAC TATAGTGAAA GCAAATTATC ACAAGGATTT   
  
  
+ TGTGGTGGAT GAGGATAGGC GTTGGATGCT TCAAGGTTGG AAAGGAAGGA CACTTAGTGC CCTTTCCGTT   
  
  
+ TGGCAGCCTA ACTA  

- +Up\_Stream \_Len000AAATTA TTCTAGGAAA ATATATATAC ACACATATTA AGATACCATG TATCCATACA   
  
  
- TGGTATCTGA ATAACGTAAA GATCCAGGAA GAGAGAGACG CGAACAAAAA ACAAATAGAT TAAATACCTC   
  
  
- AACTTGTAAA ACAAAATATA TGAACTTGTT AGATAATATG ATTTAATTCG TAAAACTAAA TTAGTGAATA   
  
  
- CCCACACAAA CCATCCCAAC TTTTATTAAA AGGATCTTTT ATTAAAAATT ATATTTTATT AAAAAGTACA   
  
  
- TTTTACTAAA AGTTGAGTAT AAAAGTCAAC TAACCATTTT TCTTTTTACT TGAAAGACCT ATTAGTCAAA   
  
  
- CTAATTGTTT TTCACTGTTT TTTTAATTTT TACCAAAAGG CATTAGAAGT TTGGTATACA GCTCCTTTAT   
  
  
- TTGTGCCTTA TTTCTTTCTC ACAGTCTTGG GTTCTTTACA TTTTAGTTGA ATGCAAGATA AATTTCACTT   
  
  
- TTTATAAAAA GAAGTAAGAT CCTTTTAATT AACAGGAGAC TTTTGTTTAA AAGGGAGGAA CAGATTGGTT   
  
  
- TGTGTCATTT TAACTTTTTA GTAAAAGATC CTTTTACTAA AAGTGAGGAT GGTTTGTGTG GGAATTTGAA   
  
  
- CAAGGTTTAG ATATTTGGGC CTTAGAATTA TGCGTGTAAC ATATAATATA CAGGATGATA TTGACATAAT   
  
  
- AGTGACCAAG AACTAAATAG GAAAGTTTGA GAACGAATCA GTTCCAACAG GAGCATGTTT GTACTAATTA   
  
  
- AGTTAATTGG TGCATTCAAT ATGCGACTGT TTAAAGCCCA TGGTTTGTAA ACGTGATCTG GATTTTGTCA   
  
  
- ATGATAGTAG TATAGCTACA ATTTTAACTG GACAAGAATA AACAGGTCGC AACTCGTTCC TTTATTAGTT   
  
  
- TAGTACAGAG TAAATCAGCC AAAAAACGGA TTCTTCTCGT ATTTGTTTCC TTTTTCTGTT GCTGTGTAAT   
  
  
- ATGTACGTAG ATGTGTTTAC TTTACTCCGG TATACCTTGT ACAAGTACTC TCGAAGTGAG CGATTATTAA   
  
  
- CTTGGATGAA AACTGATTCG GTGGTTGATC TTATACCTCT AACTGACAAT TCTCTGATAT AATGTACTTT   
  
  
- ATCTATTCTC ATCTGTAACG ATATATAAAA TAGAAATTAA CAGATAAAAA TTACTAGTAA TGACAAATAA   
  
  
- AGTCACTCAT TAAAAGAGCC TATATTATAA AATATCGCCA ATAATTTATC TACACTTTGA TATTACAATC   
  
  
- TGTATAGTAC GCTTTTATCT CTTTAAAACC CGTTTAATTT TTTTTTTCTT CTTTTTGCTC CTTCATACGG   
  
  
- GTAACATTCG TCCACCTACA TATAGGTCGG AGGTAGGTTG TTAAACCCGT TTTAATATAG TAGGTAAGGT   
  
  
- TTTTTTTCTA TATTAAACAC TATAATTATT GGGCTGAGTT AAGTTCGAAT AAATAAAAAA GTATATTAAA   
  
  
- AAATTATATA TTTTTATTTT TCACTGTTGC TGTACGTAGG AAACGCACAG TTTCCTAGGC GGCGCACCGG   
  
  
- GGGTGTAAGT GACTGCCCCA AAAACCAAAC GCTAATTCTA GTTACCGATT GCAACAGAGA GAGTTTTGTA   
  
  
- TACCTGGGGA AAAAGATAGA AAAAGACGTA ACAGTCGTAT CGTTAACAAC TCGTGTAAAA AAAGCTAAAC   
  
  
- AACTGTTATT CGCACCAATA CTAAATACCC GGTGATTATA GTCAGAGACT AGTAAACGTG TAAAACCTGG   
  
  
- AGAAAGAGAG AGATTAGAAG GTCCATTGGA CTTAAGAAGA GATAAAGACT GATCTAATCT ATTGTCCTAA   
  
  
- AGTTTATAGA CAGTCCACCG CCCATATTGA AGCACTACCC GTAAGTAAAA ACAAGATAGT CCGACCACCC   
  
  
- AAACTCAAAT AAAACTAAGT AAAGCACAAG TAGACACTAT AATCATCCTC CCTAAGACAT ATGGGTGGTT   
  
  
- GACGAGCGGG AAAACGGGCA CACTCGTTAA CCCTGAAAAC AAACAAGAAG CAGCTACGTA GGAGACCAAC   
  
  
- GACTAGACCT TAACTTTGGG GTACATAAGT TTAAGTTGGG ACTAAACGAC AGTTTGAAAG ACTTGGTAGT   
  
  
- TTTGAGGCGG CTTCACAAAC TTTTTCTACC GTAGAAGGTC TGGGAAGTAA ACCGACTAGG GTTTTCGAGA   
  
  
- AGTTAACGGC TTTAGCCCAA ATGATTATCA AGTCTAAGAT GTGTTCATGG ACTAAAAAGT CTACGAACAG   
  
  
- AATTCAAGTA ATCACTATAA GAGGACCTTC TCCTGAATCT ACTTTCAGGA TGCAGAAATG TGCTAATGTA   
  
  
- CCGAGAGGAT CGGTGACTCT TCAGAAACAT ACTACGAGAA CCTTTCCTTA TGAAAGGAAG CAGATGATCA   
  
  
- GAACGGGGTA GAAATCCGGC TTCACAACTG TCGGGTCTAT CACCGAAACC GGCGCCAACG AGACTACCAG   
  
  
- CGCCCTAACT CCCAAACCGA TTACTACTAC GGCAAAAGTA CAGATTGACC GTCGAGTTGT GGTGGGTTAA   
  
  
- CCTGGGAAAG GGTTAGTACG TTCTATAAGG AGCAGGGATA AACCTTAACT TAATGGTAAG AAGACCCAGT   
  
  
- TCGTTACCGT AACTACTAAG CCCCCTACCC AATAGCTGGA GAGGACATTC ATGTGAACGT AGTTGGCGGT   
  
  
- GTCTCCGTCC CTTTTTCAAC CGACCATCGA GATCCTCTTT CTTGGCAGTT TCCCTACTGA TACCGGTACT   
  
  
- TCTCCCTTCC TCATTGTTCG TCGTTCGGAG AATGTTACTG CTAATGCAAC TCTACCTCGT CATACTGCTA   
  
  
- CATGAAGAGA CATCCCGTCT GTTCCCATTA TAAAGTTGAA CACGGTTACT TAGGAGCGAA TTACTCCACG   
  
  
- CACTCTTCGA CGTCTGCTGT CCCAAGTTCC CTTCTTGTAG GGCAGAATTC TTCGTTAGAT TCCTTCGCTT   
  
  
- TCTCCACCTA GACTCTTGAG AAGAAAGACC AACACGTGTT CGACAAAGCT TGAAACTATA GTCCTGACGA   
  
  
- TTACTCGACG AATTCGTTCA GTCTGTCGTA AGAAGTGGTA TACCACTATC GGAGGTTTCC GAGCGGGTAG   
  
  
- TTAAACGCTT ACCATAACTC CGTGCGAATC GTCCGTGGCC AAGTTCTCAT GGACGATTAG AGTAACTACG   
  
  
- TGCCTATAGT AGTAGACTTA AAAATTTTCG AATGTTCAGT ATACAAAGTC GTCAAGGAAA GTCTTCCTAC   
  
  
- AGGATAAAAT ATCGTTTGTT GTGTTAAGAC TTCAACCGAC TCTTTCGTTG TTTCTAAGTG TATTAACTAA   
  
  
- AACCATAAGA TAAACCAGAT GTCACCGGGA CAGAATATGT TTCAGAGAGT TCCGCTTGAC ACCGAGGAGG   
  
  
- CTTCGAAGCG TAGTGACCCT ATCTGATAGG GGTCGTACCA AAGGCCGGTC GTCTTTTCCA ACTCCGTTGT   
  
  
- CCAGCAGCCA ACAGGCCCAT GACACTCTCT AAATTACATG GGAAAGCTAT ACTTTGGTAA CGTTTCTTCA   
  
  
- CCCTTTGGTA TGCGGGTCTT CTAGATTTAT AACTCTTACT ACTCGACCAT TAACAATTAA CATACAACGC   
  
  
- CAGACATTTA GATAACCTAC TATGTCACCG CCATTTATCA GGTTCCCTAC GAAAGAACTC CAATTAGTTT   
  
  
- GTCTATTTGG GCGCAAATAA GTAAGTACGT TAACAGTTAC CTTGGAAATC ATGAGGTAAG AAGTCGTGAG   
  
  
- CTAAGTCCCT TCGGGATAAG GTTATGAGAA GACATAAACT ATATAAACTT CGCTGATACT GAGCACTTCT   
  
  
- AGCACCTTCC GACGACTAAC TCTCAGTTTA TACGCCCGAT CTTCAAAACT TACGTTATCG TACACTTCCA   
  
  
- CGTCTCTCCT AAGTTTCCGG ACTTTGTATG TTCGTTACCG TCCTCGCCTG TTGTTCCCGA CCTGATTCCG   
  
  
- TCCAAGGTTA TCTACTCCTC GAACAGTTAT CTCGTTTCTG ATATCACTTT CGTTTAATAG TGTTCCTAAA   
  
  
- ACACCACCTA CTCCTATCCG CAACCTACGA AGTTCCAACC TTTCCTTCCT GTGAATCACG GGAAAGGCAA   
  
  
- ACCGTCGGAT TGAT

+     ABRE4

| Site Name | Organism | Position | Strand | Matrix score. | sequence | function |
| --- | --- | --- | --- | --- | --- | --- |
| ABRE4 | Zea mays | 784 | + | 6 | CACGTA |  |

>HU02G01572.1   
+ +Up\_Stream \_Len000TTTAAT AAGATCCTTT TATATATATG TGTGTATAAT TCTATGGTAC ATAGGTATGT   
  
  
+ ACCATAGACT TATTGCATTT CTAGGTCCTT CTCTCTCTGC GCTTGTTTTT TGTTTATCTA ATTTATGGAG   
  
  
+ TTGAACATTT TGTTTTATAT ACTTGAACAA TCTATTATAC TAAATTAAGC ATTTTGATTT AATCACTTAT   
  
  
+ GGGTGTGTTT GGTAGGGTTG AAAATAATTT TCCTAGAAAA TAATTTTTAA TATAAAATAA TTTTTCATGT   
  
  
+ AAAATGATTT TCAACTCATA TTTTCAGTTG ATTGGTAAAA AGAAAAATGA ACTTTCTGGA TAATCAGTTT   
  
  
+ GATTAACAAA AAGTGACAAA AAAATTAAAA ATGGTTTTCC GTAATCTTCA AACCATATGT CGAGGAAATA   
  
  
+ AACACGGAAT AAAGAAAGAG TGTCAGAACC CAAGAAATGT AAAATCAACT TACGTTCTAT TTAAAGTGAA   
  
  
+ AAATATTTTT CTTCATTCTA GGAAAATTAA TTGTCCTCTG AAAACAAATT TTCCCTCCTT GTCTAACCAA   
  
  
+ ACACAGTAAA ATTGAAAAAT CATTTTCTAG GAAAATGATT TTCACTCCTA CCAAACACAC CCTTAAACTT   
  
  
+ GTTCCAAATC TATAAACCCG GAATCTTAAT ACGCACATTG TATATTATAT GTCCTACTAT AACTGTATTA   
  
  
+ TCACTGGTTC TTGATTTATC CTTTCAAACT CTTGCTTAGT CAAGGTTGTC CTCGTACAAA CATGATTAAT   
  
  
+ TCAATTAACC ACGTAAGTTA TACGCTGACA AATTTCGGGT ACCAAACATT TGCACTAGAC CTAAAACAGT   
  
  
+ TACTATCATC ATATCGATGT TAAAATTGAC CTGTTCTTAT TTGTCCAGCG TTGAGCAAGG AAATAATCAA   
  
  
+ ATCATGTCTC ATTTAGTCGG TTTTTTGCCT AAGAAGAGCA TAAACAAAGG AAAAAGACAA CGACACATTA   
  
  
+ TACATGCATC TACACAAATG AAATGAGGCC ATATGGAACA TGTTCATGAG AGCTTCACTC GCTAATAATT   
  
  
+ GAACCTACTT TTGACTAAGC CACCAACTAG AATATGGAGA TTGACTGTTA AGAGACTATA TTACATGAAA   
  
  
+ TAGATAAGAG TAGACATTGC TATATATTTT ATCTTTAATT GTCTATTTTT AATGATCATT ACTGTTTATT   
  
  
+ TCAGTGAGTA ATTTTCTCGG ATATAATATT TTATAGCGGT TATTAAATAG ATGTGAAACT ATAATGTTAG   
  
  
+ ACATATCATG CGAAAATAGA GAAATTTTGG GCAAATTAAA AAAAAAAGAA GAAAAACGAG GAAGTATGCC   
  
  
+ CATTGTAAGC AGGTGGATGT ATATCCAGCC TCCATCCAAC AATTTGGGCA AAATTATATC ATCCATTCCA   
  
  
+ AAAAAAAGAT ATAATTTGTG ATATTAATAA CCCGACTCAA TTCAAGCTTA TTTATTTTTT CATATAATTT   
  
  
+ TTTAATATAT AAAAATAAAA AGTGACAACG ACATGCATCC TTTGCGTGTC AAAGGATCCG CCGCGTGGCC   
  
  
+ CCCACATTCA CTGACGGGGT TTTTGGTTTG CGATTAAGAT CAATGGCTAA CGTTGTCTCT CTCAAAACAT   
  
  
+ ATGGACCCCT TTTTCTATCT TTTTCTGCAT TGTCAGCATA GCAATTGTTG AGCACATTTT TTTCGATTTG   
  
  
+ TTGACAATAA GCGTGGTTAT GATTTATGGG CCACTAATAT CAGTCTCTGA TCATTTGCAC ATTTTGGACC   
  
  
+ TCTTTCTCTC TCTAATCTTC CAGGTAACCT GAATTCTTCT CTATTTCTGA CTAGATTAGA TAACAGGATT   
  
  
+ TCAAATATCT GTCAGGTGGC GGGTATAACT TCGTGATGGG CATTCATTTT TGTTCTATCA GGCTGGTGGG   
  
  
+ TTTGAGTTTA TTTTGATTCA TTTCGTGTTC ATCTGTGATA TTAGTAGGAG GGATTCTGTA TACCCACCAA   
  
  
+ CTGCTCGCCC TTTTGCCCGT GTGAGCAATT GGGACTTTTG TTTGTTCTTC GTCGATGCAT CCTCTGGTTG   
  
  
+ CTGATCTGGA ATTGAAACCC CATGTATTCA AATTCAACCC TGATTTGCTG TCAAACTTTC TGAACCATCA   
  
  
+ AAACTCCGCC GAAGTGTTTG AAAAAGATGG CATCTTCCAG ACCCTTCATT TGGCTGATCC CAAAAGCTCT   
  
  
+ TCAATTGCCG AAATCGGGTT TACTAATAGT TCAGATTCTA CACAAGTACC TGATTTTTCA GATGCTTGTC   
  
  
+ TTAAGTTCAT TAGTGATATT CTCCTGGAAG AGGACTTAGA TGAAAGTCCT ACGTCTTTAC ACGATTACAT   
  
  
+ GGCTCTCCTA GCCACTGAGA AGTCTTTGTA TGATGCTCTT GGAAAGGAAT ACTTTCCTTC GTCTACTAGT   
  
  
+ CTTGCCCCAT CTTTAGGCCG AAGTGTTGAC AGCCCAGATA GTGGCTTTGG CCGCGGTTGC TCTGATGGTC   
  
  
+ GCGGGATTGA GGGTTTGGCT AATGATGATG CCGTTTTCAT GTCTAACTGG CAGCTCAACA CCACCCAATT   
  
  
+ GGACCCTTTC CCAATCATGC AAGATATTCC TCGTCCCTAT TTGGAATTGA ATTACCATTC TTCTGGGTCA   
  
  
+ AGCAATGGCA TTGATGATTC GGGGGATGGG TTATCGACCT CTCCTGTAAG TACACTTGCA TCAACCGCCA   
  
  
+ CAGAGGCAGG GAAAAAGTTG GCTGGTAGCT CTAGGAGAAA GAACCGTCAA AGGGATGACT ATGGCCATGA   
  
  
+ AGAGGGAAGG AGTAACAAGC AGCAAGCCTC TTACAATGAC GATTACGTTG AGATGGAGCA GTATGACGAT   
  
  
+ GTACTTCTCT GTAGGGCAGA CAAGGGTAAT ATTTCAACTT GTGCCAATGA ATCCTCGCTT AATGAGGTGC   
  
  
+ GTGAGAAGCT GCAGACGACA GGGTTCAAGG GAAGAACATC CCGTCTTAAG AAGCAATCTA AGGAAGCGAA   
  
  
+ AGAGGTGGAT CTGAGAACTC TTCTTTCTGG TTGTGCACAA GCTGTTTCGA ACTTTGATAT CAGGACTGCT   
  
  
+ AATGAGCTGC TTAAGCAAGT CAGACAGCAT TCTTCACCAT ATGGTGATAG CCTCCAAAGG CTCGCCCATC   
  
  
+ AATTTGCGAA TGGTATTGAG GCACGCTTAG CAGGCACCGG TTCAAGAGTA CCTGCTAATC TCATTGATGC   
  
  
+ ACGGATATCA TCATCTGAAT TTTTAAAAGC TTACAAGTCA TATGTTTCAG CAGTTCCTTT CAGAAGGATG   
  
  
+ TCCTATTTTA TAGCAAACAA CACAATTCTG AAGTTGGCTG AGAAAGCAAC AAAGATTCAC ATAATTGATT   
  
  
+ TTGGTATTCT ATTTGGTCTA CAGTGGCCCT GTCTTATACA AAGTCTCTCA AGGCGAACTG TGGCTCCTCC   
  
  
+ GAAGCTTCGC ATCACTGGGA TAGACTATCC CCAGCATGGT TTCCGGCCAG CAGAAAAGGT TGAGGCAACA   
  
  
+ GGTCGTCGGT TGTCCGGGTA CTGTGAGAGA TTTAATGTAC CCTTTCGATA TGAAACCATT GCAAAGAAGT   
  
  
+ GGGAAACCAT ACGCCCAGAA GATCTAAATA TTGAGAATGA TGAGCTGGTA ATTGTTAATT GTATGTTGCG   
  
  
+ GTCTGTAAAT CTATTGGATG ATACAGTGGC GGTAAATAGT CCAAGGGATG CTTTCTTGAG GTTAATCAAA   
  
  
+ CAGATAAACC CGCGTTTATT CATTCATGCA ATTGTCAATG GAACCTTTAG TACTCCATTC TTCAGCACTC   
  
  
+ GATTCAGGGA AGCCCTATTC CAATACTCTT CTGTATTTGA TATATTTGAA GCGACTATGA CTCGTGAAGA   
  
  
+ TCGTGGAAGG CTGCTGATTG AGAGTCAAAT ATGCGGGCTA GAAGTTTTGA ATGCAATAGC ATGTGAAGGT   
  
  
+ GCAGAGAGGA TTCAAAGGCC TGAAACATAC AAGCAATGGC AGGAGCGGAC AACAAGGGCT GGACTAAGGC   
  
  
+ AGGTTCCAAT AGATGAGGAG CTTGTCAATA GAGCAAAGAC TATAGTGAAA GCAAATTATC ACAAGGATTT   
  
  
+ TGTGGTGGAT GAGGATAGGC GTTGGATGCT TCAAGGTTGG AAAGGAAGGA CACTTAGTGC CCTTTCCGTT   
  
  
+ TGGCAGCCTA ACTA  

- +Up\_Stream \_Len000AAATTA TTCTAGGAAA ATATATATAC ACACATATTA AGATACCATG TATCCATACA   
  
  
- TGGTATCTGA ATAACGTAAA GATCCAGGAA GAGAGAGACG CGAACAAAAA ACAAATAGAT TAAATACCTC   
  
  
- AACTTGTAAA ACAAAATATA TGAACTTGTT AGATAATATG ATTTAATTCG TAAAACTAAA TTAGTGAATA   
  
  
- CCCACACAAA CCATCCCAAC TTTTATTAAA AGGATCTTTT ATTAAAAATT ATATTTTATT AAAAAGTACA   
  
  
- TTTTACTAAA AGTTGAGTAT AAAAGTCAAC TAACCATTTT TCTTTTTACT TGAAAGACCT ATTAGTCAAA   
  
  
- CTAATTGTTT TTCACTGTTT TTTTAATTTT TACCAAAAGG CATTAGAAGT TTGGTATACA GCTCCTTTAT   
  
  
- TTGTGCCTTA TTTCTTTCTC ACAGTCTTGG GTTCTTTACA TTTTAGTTGA ATGCAAGATA AATTTCACTT   
  
  
- TTTATAAAAA GAAGTAAGAT CCTTTTAATT AACAGGAGAC TTTTGTTTAA AAGGGAGGAA CAGATTGGTT   
  
  
- TGTGTCATTT TAACTTTTTA GTAAAAGATC CTTTTACTAA AAGTGAGGAT GGTTTGTGTG GGAATTTGAA   
  
  
- CAAGGTTTAG ATATTTGGGC CTTAGAATTA TGCGTGTAAC ATATAATATA CAGGATGATA TTGACATAAT   
  
  
- AGTGACCAAG AACTAAATAG GAAAGTTTGA GAACGAATCA GTTCCAACAG GAGCATGTTT GTACTAATTA   
  
  
- AGTTAATTGG TGCATTCAAT ATGCGACTGT TTAAAGCCCA TGGTTTGTAA ACGTGATCTG GATTTTGTCA   
  
  
- ATGATAGTAG TATAGCTACA ATTTTAACTG GACAAGAATA AACAGGTCGC AACTCGTTCC TTTATTAGTT   
  
  
- TAGTACAGAG TAAATCAGCC AAAAAACGGA TTCTTCTCGT ATTTGTTTCC TTTTTCTGTT GCTGTGTAAT   
  
  
- ATGTACGTAG ATGTGTTTAC TTTACTCCGG TATACCTTGT ACAAGTACTC TCGAAGTGAG CGATTATTAA   
  
  
- CTTGGATGAA AACTGATTCG GTGGTTGATC TTATACCTCT AACTGACAAT TCTCTGATAT AATGTACTTT   
  
  
- ATCTATTCTC ATCTGTAACG ATATATAAAA TAGAAATTAA CAGATAAAAA TTACTAGTAA TGACAAATAA   
  
  
- AGTCACTCAT TAAAAGAGCC TATATTATAA AATATCGCCA ATAATTTATC TACACTTTGA TATTACAATC   
  
  
- TGTATAGTAC GCTTTTATCT CTTTAAAACC CGTTTAATTT TTTTTTTCTT CTTTTTGCTC CTTCATACGG   
  
  
- GTAACATTCG TCCACCTACA TATAGGTCGG AGGTAGGTTG TTAAACCCGT TTTAATATAG TAGGTAAGGT   
  
  
- TTTTTTTCTA TATTAAACAC TATAATTATT GGGCTGAGTT AAGTTCGAAT AAATAAAAAA GTATATTAAA   
  
  
- AAATTATATA TTTTTATTTT TCACTGTTGC TGTACGTAGG AAACGCACAG TTTCCTAGGC GGCGCACCGG   
  
  
- GGGTGTAAGT GACTGCCCCA AAAACCAAAC GCTAATTCTA GTTACCGATT GCAACAGAGA GAGTTTTGTA   
  
  
- TACCTGGGGA AAAAGATAGA AAAAGACGTA ACAGTCGTAT CGTTAACAAC TCGTGTAAAA AAAGCTAAAC   
  
  
- AACTGTTATT CGCACCAATA CTAAATACCC GGTGATTATA GTCAGAGACT AGTAAACGTG TAAAACCTGG   
  
  
- AGAAAGAGAG AGATTAGAAG GTCCATTGGA CTTAAGAAGA GATAAAGACT GATCTAATCT ATTGTCCTAA   
  
  
- AGTTTATAGA CAGTCCACCG CCCATATTGA AGCACTACCC GTAAGTAAAA ACAAGATAGT CCGACCACCC   
  
  
- AAACTCAAAT AAAACTAAGT AAAGCACAAG TAGACACTAT AATCATCCTC CCTAAGACAT ATGGGTGGTT   
  
  
- GACGAGCGGG AAAACGGGCA CACTCGTTAA CCCTGAAAAC AAACAAGAAG CAGCTACGTA GGAGACCAAC   
  
  
- GACTAGACCT TAACTTTGGG GTACATAAGT TTAAGTTGGG ACTAAACGAC AGTTTGAAAG ACTTGGTAGT   
  
  
- TTTGAGGCGG CTTCACAAAC TTTTTCTACC GTAGAAGGTC TGGGAAGTAA ACCGACTAGG GTTTTCGAGA   
  
  
- AGTTAACGGC TTTAGCCCAA ATGATTATCA AGTCTAAGAT GTGTTCATGG ACTAAAAAGT CTACGAACAG   
  
  
- AATTCAAGTA ATCACTATAA GAGGACCTTC TCCTGAATCT ACTTTCAGGA TGCAGAAATG TGCTAATGTA   
  
  
- CCGAGAGGAT CGGTGACTCT TCAGAAACAT ACTACGAGAA CCTTTCCTTA TGAAAGGAAG CAGATGATCA   
  
  
- GAACGGGGTA GAAATCCGGC TTCACAACTG TCGGGTCTAT CACCGAAACC GGCGCCAACG AGACTACCAG   
  
  
- CGCCCTAACT CCCAAACCGA TTACTACTAC GGCAAAAGTA CAGATTGACC GTCGAGTTGT GGTGGGTTAA   
  
  
- CCTGGGAAAG GGTTAGTACG TTCTATAAGG AGCAGGGATA AACCTTAACT TAATGGTAAG AAGACCCAGT   
  
  
- TCGTTACCGT AACTACTAAG CCCCCTACCC AATAGCTGGA GAGGACATTC ATGTGAACGT AGTTGGCGGT   
  
  
- GTCTCCGTCC CTTTTTCAAC CGACCATCGA GATCCTCTTT CTTGGCAGTT TCCCTACTGA TACCGGTACT   
  
  
- TCTCCCTTCC TCATTGTTCG TCGTTCGGAG AATGTTACTG CTAATGCAAC TCTACCTCGT CATACTGCTA   
  
  
- CATGAAGAGA CATCCCGTCT GTTCCCATTA TAAAGTTGAA CACGGTTACT TAGGAGCGAA TTACTCCACG   
  
  
- CACTCTTCGA CGTCTGCTGT CCCAAGTTCC CTTCTTGTAG GGCAGAATTC TTCGTTAGAT TCCTTCGCTT   
  
  
- TCTCCACCTA GACTCTTGAG AAGAAAGACC AACACGTGTT CGACAAAGCT TGAAACTATA GTCCTGACGA   
  
  
- TTACTCGACG AATTCGTTCA GTCTGTCGTA AGAAGTGGTA TACCACTATC GGAGGTTTCC GAGCGGGTAG   
  
  
- TTAAACGCTT ACCATAACTC CGTGCGAATC GTCCGTGGCC AAGTTCTCAT GGACGATTAG AGTAACTACG   
  
  
- TGCCTATAGT AGTAGACTTA AAAATTTTCG AATGTTCAGT ATACAAAGTC GTCAAGGAAA GTCTTCCTAC   
  
  
- AGGATAAAAT ATCGTTTGTT GTGTTAAGAC TTCAACCGAC TCTTTCGTTG TTTCTAAGTG TATTAACTAA   
  
  
- AACCATAAGA TAAACCAGAT GTCACCGGGA CAGAATATGT TTCAGAGAGT TCCGCTTGAC ACCGAGGAGG   
  
  
- CTTCGAAGCG TAGTGACCCT ATCTGATAGG GGTCGTACCA AAGGCCGGTC GTCTTTTCCA ACTCCGTTGT   
  
  
- CCAGCAGCCA ACAGGCCCAT GACACTCTCT AAATTACATG GGAAAGCTAT ACTTTGGTAA CGTTTCTTCA   
  
  
- CCCTTTGGTA TGCGGGTCTT CTAGATTTAT AACTCTTACT ACTCGACCAT TAACAATTAA CATACAACGC   
  
  
- CAGACATTTA GATAACCTAC TATGTCACCG CCATTTATCA GGTTCCCTAC GAAAGAACTC CAATTAGTTT   
  
  
- GTCTATTTGG GCGCAAATAA GTAAGTACGT TAACAGTTAC CTTGGAAATC ATGAGGTAAG AAGTCGTGAG   
  
  
- CTAAGTCCCT TCGGGATAAG GTTATGAGAA GACATAAACT ATATAAACTT CGCTGATACT GAGCACTTCT   
  
  
- AGCACCTTCC GACGACTAAC TCTCAGTTTA TACGCCCGAT CTTCAAAACT TACGTTATCG TACACTTCCA   
  
  
- CGTCTCTCCT AAGTTTCCGG ACTTTGTATG TTCGTTACCG TCCTCGCCTG TTGTTCCCGA CCTGATTCCG   
  
  
- TCCAAGGTTA TCTACTCCTC GAACAGTTAT CTCGTTTCTG ATATCACTTT CGTTTAATAG TGTTCCTAAA   
  
  
- ACACCACCTA CTCCTATCCG CAACCTACGA AGTTCCAACC TTTCCTTCCT GTGAATCACG GGAAAGGCAA   
  
  
- ACCGTCGGAT TGAT

+     ARE

| Site Name | Organism | Position | Strand | Matrix score. | sequence | function |
| --- | --- | --- | --- | --- | --- | --- |
| ARE | Zea mays | 3487 | + | 6 | AAACCA | cis-acting regulatory element essential for the anaerobic induction |
| ARE | Zea mays | 3508 | + | 6 | AAACCA | cis-acting regulatory element essential for the anaerobic induction |
| ARE | Zea mays | 404 | + | 6 | AAACCA | cis-acting regulatory element essential for the anaerobic induction |
| ARE | Zea mays | 1568 | - | 6 | AAACCA | cis-acting regulatory element essential for the anaerobic induction |
| ARE | Zea mays | 386 | - | 6 | AAACCA | cis-acting regulatory element essential for the anaerobic induction |
| ARE | Zea mays | 3401 | - | 6 | AAACCA | cis-acting regulatory element essential for the anaerobic induction |

>HU02G01572.1   
+ +Up\_Stream \_Len000TTTAAT AAGATCCTTT TATATATATG TGTGTATAAT TCTATGGTAC ATAGGTATGT   
  
  
+ ACCATAGACT TATTGCATTT CTAGGTCCTT CTCTCTCTGC GCTTGTTTTT TGTTTATCTA ATTTATGGAG   
  
  
+ TTGAACATTT TGTTTTATAT ACTTGAACAA TCTATTATAC TAAATTAAGC ATTTTGATTT AATCACTTAT   
  
  
+ GGGTGTGTTT GGTAGGGTTG AAAATAATTT TCCTAGAAAA TAATTTTTAA TATAAAATAA TTTTTCATGT   
  
  
+ AAAATGATTT TCAACTCATA TTTTCAGTTG ATTGGTAAAA AGAAAAATGA ACTTTCTGGA TAATCAGTTT   
  
  
+ GATTAACAAA AAGTGACAAA AAAATTAAAA ATGGTTTTCC GTAATCTTCA AACCATATGT CGAGGAAATA   
  
  
+ AACACGGAAT AAAGAAAGAG TGTCAGAACC CAAGAAATGT AAAATCAACT TACGTTCTAT TTAAAGTGAA   
  
  
+ AAATATTTTT CTTCATTCTA GGAAAATTAA TTGTCCTCTG AAAACAAATT TTCCCTCCTT GTCTAACCAA   
  
  
+ ACACAGTAAA ATTGAAAAAT CATTTTCTAG GAAAATGATT TTCACTCCTA CCAAACACAC CCTTAAACTT   
  
  
+ GTTCCAAATC TATAAACCCG GAATCTTAAT ACGCACATTG TATATTATAT GTCCTACTAT AACTGTATTA   
  
  
+ TCACTGGTTC TTGATTTATC CTTTCAAACT CTTGCTTAGT CAAGGTTGTC CTCGTACAAA CATGATTAAT   
  
  
+ TCAATTAACC ACGTAAGTTA TACGCTGACA AATTTCGGGT ACCAAACATT TGCACTAGAC CTAAAACAGT   
  
  
+ TACTATCATC ATATCGATGT TAAAATTGAC CTGTTCTTAT TTGTCCAGCG TTGAGCAAGG AAATAATCAA   
  
  
+ ATCATGTCTC ATTTAGTCGG TTTTTTGCCT AAGAAGAGCA TAAACAAAGG AAAAAGACAA CGACACATTA   
  
  
+ TACATGCATC TACACAAATG AAATGAGGCC ATATGGAACA TGTTCATGAG AGCTTCACTC GCTAATAATT   
  
  
+ GAACCTACTT TTGACTAAGC CACCAACTAG AATATGGAGA TTGACTGTTA AGAGACTATA TTACATGAAA   
  
  
+ TAGATAAGAG TAGACATTGC TATATATTTT ATCTTTAATT GTCTATTTTT AATGATCATT ACTGTTTATT   
  
  
+ TCAGTGAGTA ATTTTCTCGG ATATAATATT TTATAGCGGT TATTAAATAG ATGTGAAACT ATAATGTTAG   
  
  
+ ACATATCATG CGAAAATAGA GAAATTTTGG GCAAATTAAA AAAAAAAGAA GAAAAACGAG GAAGTATGCC   
  
  
+ CATTGTAAGC AGGTGGATGT ATATCCAGCC TCCATCCAAC AATTTGGGCA AAATTATATC ATCCATTCCA   
  
  
+ AAAAAAAGAT ATAATTTGTG ATATTAATAA CCCGACTCAA TTCAAGCTTA TTTATTTTTT CATATAATTT   
  
  
+ TTTAATATAT AAAAATAAAA AGTGACAACG ACATGCATCC TTTGCGTGTC AAAGGATCCG CCGCGTGGCC   
  
  
+ CCCACATTCA CTGACGGGGT TTTTGGTTTG CGATTAAGAT CAATGGCTAA CGTTGTCTCT CTCAAAACAT   
  
  
+ ATGGACCCCT TTTTCTATCT TTTTCTGCAT TGTCAGCATA GCAATTGTTG AGCACATTTT TTTCGATTTG   
  
  
+ TTGACAATAA GCGTGGTTAT GATTTATGGG CCACTAATAT CAGTCTCTGA TCATTTGCAC ATTTTGGACC   
  
  
+ TCTTTCTCTC TCTAATCTTC CAGGTAACCT GAATTCTTCT CTATTTCTGA CTAGATTAGA TAACAGGATT   
  
  
+ TCAAATATCT GTCAGGTGGC GGGTATAACT TCGTGATGGG CATTCATTTT TGTTCTATCA GGCTGGTGGG   
  
  
+ TTTGAGTTTA TTTTGATTCA TTTCGTGTTC ATCTGTGATA TTAGTAGGAG GGATTCTGTA TACCCACCAA   
  
  
+ CTGCTCGCCC TTTTGCCCGT GTGAGCAATT GGGACTTTTG TTTGTTCTTC GTCGATGCAT CCTCTGGTTG   
  
  
+ CTGATCTGGA ATTGAAACCC CATGTATTCA AATTCAACCC TGATTTGCTG TCAAACTTTC TGAACCATCA   
  
  
+ AAACTCCGCC GAAGTGTTTG AAAAAGATGG CATCTTCCAG ACCCTTCATT TGGCTGATCC CAAAAGCTCT   
  
  
+ TCAATTGCCG AAATCGGGTT TACTAATAGT TCAGATTCTA CACAAGTACC TGATTTTTCA GATGCTTGTC   
  
  
+ TTAAGTTCAT TAGTGATATT CTCCTGGAAG AGGACTTAGA TGAAAGTCCT ACGTCTTTAC ACGATTACAT   
  
  
+ GGCTCTCCTA GCCACTGAGA AGTCTTTGTA TGATGCTCTT GGAAAGGAAT ACTTTCCTTC GTCTACTAGT   
  
  
+ CTTGCCCCAT CTTTAGGCCG AAGTGTTGAC AGCCCAGATA GTGGCTTTGG CCGCGGTTGC TCTGATGGTC   
  
  
+ GCGGGATTGA GGGTTTGGCT AATGATGATG CCGTTTTCAT GTCTAACTGG CAGCTCAACA CCACCCAATT   
  
  
+ GGACCCTTTC CCAATCATGC AAGATATTCC TCGTCCCTAT TTGGAATTGA ATTACCATTC TTCTGGGTCA   
  
  
+ AGCAATGGCA TTGATGATTC GGGGGATGGG TTATCGACCT CTCCTGTAAG TACACTTGCA TCAACCGCCA   
  
  
+ CAGAGGCAGG GAAAAAGTTG GCTGGTAGCT CTAGGAGAAA GAACCGTCAA AGGGATGACT ATGGCCATGA   
  
  
+ AGAGGGAAGG AGTAACAAGC AGCAAGCCTC TTACAATGAC GATTACGTTG AGATGGAGCA GTATGACGAT   
  
  
+ GTACTTCTCT GTAGGGCAGA CAAGGGTAAT ATTTCAACTT GTGCCAATGA ATCCTCGCTT AATGAGGTGC   
  
  
+ GTGAGAAGCT GCAGACGACA GGGTTCAAGG GAAGAACATC CCGTCTTAAG AAGCAATCTA AGGAAGCGAA   
  
  
+ AGAGGTGGAT CTGAGAACTC TTCTTTCTGG TTGTGCACAA GCTGTTTCGA ACTTTGATAT CAGGACTGCT   
  
  
+ AATGAGCTGC TTAAGCAAGT CAGACAGCAT TCTTCACCAT ATGGTGATAG CCTCCAAAGG CTCGCCCATC   
  
  
+ AATTTGCGAA TGGTATTGAG GCACGCTTAG CAGGCACCGG TTCAAGAGTA CCTGCTAATC TCATTGATGC   
  
  
+ ACGGATATCA TCATCTGAAT TTTTAAAAGC TTACAAGTCA TATGTTTCAG CAGTTCCTTT CAGAAGGATG   
  
  
+ TCCTATTTTA TAGCAAACAA CACAATTCTG AAGTTGGCTG AGAAAGCAAC AAAGATTCAC ATAATTGATT   
  
  
+ TTGGTATTCT ATTTGGTCTA CAGTGGCCCT GTCTTATACA AAGTCTCTCA AGGCGAACTG TGGCTCCTCC   
  
  
+ GAAGCTTCGC ATCACTGGGA TAGACTATCC CCAGCATGGT TTCCGGCCAG CAGAAAAGGT TGAGGCAACA   
  
  
+ GGTCGTCGGT TGTCCGGGTA CTGTGAGAGA TTTAATGTAC CCTTTCGATA TGAAACCATT GCAAAGAAGT   
  
  
+ GGGAAACCAT ACGCCCAGAA GATCTAAATA TTGAGAATGA TGAGCTGGTA ATTGTTAATT GTATGTTGCG   
  
  
+ GTCTGTAAAT CTATTGGATG ATACAGTGGC GGTAAATAGT CCAAGGGATG CTTTCTTGAG GTTAATCAAA   
  
  
+ CAGATAAACC CGCGTTTATT CATTCATGCA ATTGTCAATG GAACCTTTAG TACTCCATTC TTCAGCACTC   
  
  
+ GATTCAGGGA AGCCCTATTC CAATACTCTT CTGTATTTGA TATATTTGAA GCGACTATGA CTCGTGAAGA   
  
  
+ TCGTGGAAGG CTGCTGATTG AGAGTCAAAT ATGCGGGCTA GAAGTTTTGA ATGCAATAGC ATGTGAAGGT   
  
  
+ GCAGAGAGGA TTCAAAGGCC TGAAACATAC AAGCAATGGC AGGAGCGGAC AACAAGGGCT GGACTAAGGC   
  
  
+ AGGTTCCAAT AGATGAGGAG CTTGTCAATA GAGCAAAGAC TATAGTGAAA GCAAATTATC ACAAGGATTT   
  
  
+ TGTGGTGGAT GAGGATAGGC GTTGGATGCT TCAAGGTTGG AAAGGAAGGA CACTTAGTGC CCTTTCCGTT   
  
  
+ TGGCAGCCTA ACTA  

- +Up\_Stream \_Len000AAATTA TTCTAGGAAA ATATATATAC ACACATATTA AGATACCATG TATCCATACA   
  
  
- TGGTATCTGA ATAACGTAAA GATCCAGGAA GAGAGAGACG CGAACAAAAA ACAAATAGAT TAAATACCTC   
  
  
- AACTTGTAAA ACAAAATATA TGAACTTGTT AGATAATATG ATTTAATTCG TAAAACTAAA TTAGTGAATA   
  
  
- CCCACACAAA CCATCCCAAC TTTTATTAAA AGGATCTTTT ATTAAAAATT ATATTTTATT AAAAAGTACA   
  
  
- TTTTACTAAA AGTTGAGTAT AAAAGTCAAC TAACCATTTT TCTTTTTACT TGAAAGACCT ATTAGTCAAA   
  
  
- CTAATTGTTT TTCACTGTTT TTTTAATTTT TACCAAAAGG CATTAGAAGT TTGGTATACA GCTCCTTTAT   
  
  
- TTGTGCCTTA TTTCTTTCTC ACAGTCTTGG GTTCTTTACA TTTTAGTTGA ATGCAAGATA AATTTCACTT   
  
  
- TTTATAAAAA GAAGTAAGAT CCTTTTAATT AACAGGAGAC TTTTGTTTAA AAGGGAGGAA CAGATTGGTT   
  
  
- TGTGTCATTT TAACTTTTTA GTAAAAGATC CTTTTACTAA AAGTGAGGAT GGTTTGTGTG GGAATTTGAA   
  
  
- CAAGGTTTAG ATATTTGGGC CTTAGAATTA TGCGTGTAAC ATATAATATA CAGGATGATA TTGACATAAT   
  
  
- AGTGACCAAG AACTAAATAG GAAAGTTTGA GAACGAATCA GTTCCAACAG GAGCATGTTT GTACTAATTA   
  
  
- AGTTAATTGG TGCATTCAAT ATGCGACTGT TTAAAGCCCA TGGTTTGTAA ACGTGATCTG GATTTTGTCA   
  
  
- ATGATAGTAG TATAGCTACA ATTTTAACTG GACAAGAATA AACAGGTCGC AACTCGTTCC TTTATTAGTT   
  
  
- TAGTACAGAG TAAATCAGCC AAAAAACGGA TTCTTCTCGT ATTTGTTTCC TTTTTCTGTT GCTGTGTAAT   
  
  
- ATGTACGTAG ATGTGTTTAC TTTACTCCGG TATACCTTGT ACAAGTACTC TCGAAGTGAG CGATTATTAA   
  
  
- CTTGGATGAA AACTGATTCG GTGGTTGATC TTATACCTCT AACTGACAAT TCTCTGATAT AATGTACTTT   
  
  
- ATCTATTCTC ATCTGTAACG ATATATAAAA TAGAAATTAA CAGATAAAAA TTACTAGTAA TGACAAATAA   
  
  
- AGTCACTCAT TAAAAGAGCC TATATTATAA AATATCGCCA ATAATTTATC TACACTTTGA TATTACAATC   
  
  
- TGTATAGTAC GCTTTTATCT CTTTAAAACC CGTTTAATTT TTTTTTTCTT CTTTTTGCTC CTTCATACGG   
  
  
- GTAACATTCG TCCACCTACA TATAGGTCGG AGGTAGGTTG TTAAACCCGT TTTAATATAG TAGGTAAGGT   
  
  
- TTTTTTTCTA TATTAAACAC TATAATTATT GGGCTGAGTT AAGTTCGAAT AAATAAAAAA GTATATTAAA   
  
  
- AAATTATATA TTTTTATTTT TCACTGTTGC TGTACGTAGG AAACGCACAG TTTCCTAGGC GGCGCACCGG   
  
  
- GGGTGTAAGT GACTGCCCCA AAAACCAAAC GCTAATTCTA GTTACCGATT GCAACAGAGA GAGTTTTGTA   
  
  
- TACCTGGGGA AAAAGATAGA AAAAGACGTA ACAGTCGTAT CGTTAACAAC TCGTGTAAAA AAAGCTAAAC   
  
  
- AACTGTTATT CGCACCAATA CTAAATACCC GGTGATTATA GTCAGAGACT AGTAAACGTG TAAAACCTGG   
  
  
- AGAAAGAGAG AGATTAGAAG GTCCATTGGA CTTAAGAAGA GATAAAGACT GATCTAATCT ATTGTCCTAA   
  
  
- AGTTTATAGA CAGTCCACCG CCCATATTGA AGCACTACCC GTAAGTAAAA ACAAGATAGT CCGACCACCC   
  
  
- AAACTCAAAT AAAACTAAGT AAAGCACAAG TAGACACTAT AATCATCCTC CCTAAGACAT ATGGGTGGTT   
  
  
- GACGAGCGGG AAAACGGGCA CACTCGTTAA CCCTGAAAAC AAACAAGAAG CAGCTACGTA GGAGACCAAC   
  
  
- GACTAGACCT TAACTTTGGG GTACATAAGT TTAAGTTGGG ACTAAACGAC AGTTTGAAAG ACTTGGTAGT   
  
  
- TTTGAGGCGG CTTCACAAAC TTTTTCTACC GTAGAAGGTC TGGGAAGTAA ACCGACTAGG GTTTTCGAGA   
  
  
- AGTTAACGGC TTTAGCCCAA ATGATTATCA AGTCTAAGAT GTGTTCATGG ACTAAAAAGT CTACGAACAG   
  
  
- AATTCAAGTA ATCACTATAA GAGGACCTTC TCCTGAATCT ACTTTCAGGA TGCAGAAATG TGCTAATGTA   
  
  
- CCGAGAGGAT CGGTGACTCT TCAGAAACAT ACTACGAGAA CCTTTCCTTA TGAAAGGAAG CAGATGATCA   
  
  
- GAACGGGGTA GAAATCCGGC TTCACAACTG TCGGGTCTAT CACCGAAACC GGCGCCAACG AGACTACCAG   
  
  
- CGCCCTAACT CCCAAACCGA TTACTACTAC GGCAAAAGTA CAGATTGACC GTCGAGTTGT GGTGGGTTAA   
  
  
- CCTGGGAAAG GGTTAGTACG TTCTATAAGG AGCAGGGATA AACCTTAACT TAATGGTAAG AAGACCCAGT   
  
  
- TCGTTACCGT AACTACTAAG CCCCCTACCC AATAGCTGGA GAGGACATTC ATGTGAACGT AGTTGGCGGT   
  
  
- GTCTCCGTCC CTTTTTCAAC CGACCATCGA GATCCTCTTT CTTGGCAGTT TCCCTACTGA TACCGGTACT   
  
  
- TCTCCCTTCC TCATTGTTCG TCGTTCGGAG AATGTTACTG CTAATGCAAC TCTACCTCGT CATACTGCTA   
  
  
- CATGAAGAGA CATCCCGTCT GTTCCCATTA TAAAGTTGAA CACGGTTACT TAGGAGCGAA TTACTCCACG   
  
  
- CACTCTTCGA CGTCTGCTGT CCCAAGTTCC CTTCTTGTAG GGCAGAATTC TTCGTTAGAT TCCTTCGCTT   
  
  
- TCTCCACCTA GACTCTTGAG AAGAAAGACC AACACGTGTT CGACAAAGCT TGAAACTATA GTCCTGACGA   
  
  
- TTACTCGACG AATTCGTTCA GTCTGTCGTA AGAAGTGGTA TACCACTATC GGAGGTTTCC GAGCGGGTAG   
  
  
- TTAAACGCTT ACCATAACTC CGTGCGAATC GTCCGTGGCC AAGTTCTCAT GGACGATTAG AGTAACTACG   
  
  
- TGCCTATAGT AGTAGACTTA AAAATTTTCG AATGTTCAGT ATACAAAGTC GTCAAGGAAA GTCTTCCTAC   
  
  
- AGGATAAAAT ATCGTTTGTT GTGTTAAGAC TTCAACCGAC TCTTTCGTTG TTTCTAAGTG TATTAACTAA   
  
  
- AACCATAAGA TAAACCAGAT GTCACCGGGA CAGAATATGT TTCAGAGAGT TCCGCTTGAC ACCGAGGAGG   
  
  
- CTTCGAAGCG TAGTGACCCT ATCTGATAGG GGTCGTACCA AAGGCCGGTC GTCTTTTCCA ACTCCGTTGT   
  
  
- CCAGCAGCCA ACAGGCCCAT GACACTCTCT AAATTACATG GGAAAGCTAT ACTTTGGTAA CGTTTCTTCA   
  
  
- CCCTTTGGTA TGCGGGTCTT CTAGATTTAT AACTCTTACT ACTCGACCAT TAACAATTAA CATACAACGC   
  
  
- CAGACATTTA GATAACCTAC TATGTCACCG CCATTTATCA GGTTCCCTAC GAAAGAACTC CAATTAGTTT   
  
  
- GTCTATTTGG GCGCAAATAA GTAAGTACGT TAACAGTTAC CTTGGAAATC ATGAGGTAAG AAGTCGTGAG   
  
  
- CTAAGTCCCT TCGGGATAAG GTTATGAGAA GACATAAACT ATATAAACTT CGCTGATACT GAGCACTTCT   
  
  
- AGCACCTTCC GACGACTAAC TCTCAGTTTA TACGCCCGAT CTTCAAAACT TACGTTATCG TACACTTCCA   
  
  
- CGTCTCTCCT AAGTTTCCGG ACTTTGTATG TTCGTTACCG TCCTCGCCTG TTGTTCCCGA CCTGATTCCG   
  
  
- TCCAAGGTTA TCTACTCCTC GAACAGTTAT CTCGTTTCTG ATATCACTTT CGTTTAATAG TGTTCCTAAA   
  
  
- ACACCACCTA CTCCTATCCG CAACCTACGA AGTTCCAACC TTTCCTTCCT GTGAATCACG GGAAAGGCAA   
  
  
- ACCGTCGGAT TGAT

+     AT1-motif

| Site Name | Organism | Position | Strand | Matrix score. | sequence | function |
| --- | --- | --- | --- | --- | --- | --- |
| AT1-motif | Solanum tuberosum | 263 | - | 13 | AATTATTTTTTATT | part of a light responsive module |

>HU02G01572.1   
+ +Up\_Stream \_Len000TTTAAT AAGATCCTTT TATATATATG TGTGTATAAT TCTATGGTAC ATAGGTATGT   
  
  
+ ACCATAGACT TATTGCATTT CTAGGTCCTT CTCTCTCTGC GCTTGTTTTT TGTTTATCTA ATTTATGGAG   
  
  
+ TTGAACATTT TGTTTTATAT ACTTGAACAA TCTATTATAC TAAATTAAGC ATTTTGATTT AATCACTTAT   
  
  
+ GGGTGTGTTT GGTAGGGTTG AAAATAATTT TCCTAGAAAA TAATTTTTAA TATAAAATAA TTTTTCATGT   
  
  
+ AAAATGATTT TCAACTCATA TTTTCAGTTG ATTGGTAAAA AGAAAAATGA ACTTTCTGGA TAATCAGTTT   
  
  
+ GATTAACAAA AAGTGACAAA AAAATTAAAA ATGGTTTTCC GTAATCTTCA AACCATATGT CGAGGAAATA   
  
  
+ AACACGGAAT AAAGAAAGAG TGTCAGAACC CAAGAAATGT AAAATCAACT TACGTTCTAT TTAAAGTGAA   
  
  
+ AAATATTTTT CTTCATTCTA GGAAAATTAA TTGTCCTCTG AAAACAAATT TTCCCTCCTT GTCTAACCAA   
  
  
+ ACACAGTAAA ATTGAAAAAT CATTTTCTAG GAAAATGATT TTCACTCCTA CCAAACACAC CCTTAAACTT   
  
  
+ GTTCCAAATC TATAAACCCG GAATCTTAAT ACGCACATTG TATATTATAT GTCCTACTAT AACTGTATTA   
  
  
+ TCACTGGTTC TTGATTTATC CTTTCAAACT CTTGCTTAGT CAAGGTTGTC CTCGTACAAA CATGATTAAT   
  
  
+ TCAATTAACC ACGTAAGTTA TACGCTGACA AATTTCGGGT ACCAAACATT TGCACTAGAC CTAAAACAGT   
  
  
+ TACTATCATC ATATCGATGT TAAAATTGAC CTGTTCTTAT TTGTCCAGCG TTGAGCAAGG AAATAATCAA   
  
  
+ ATCATGTCTC ATTTAGTCGG TTTTTTGCCT AAGAAGAGCA TAAACAAAGG AAAAAGACAA CGACACATTA   
  
  
+ TACATGCATC TACACAAATG AAATGAGGCC ATATGGAACA TGTTCATGAG AGCTTCACTC GCTAATAATT   
  
  
+ GAACCTACTT TTGACTAAGC CACCAACTAG AATATGGAGA TTGACTGTTA AGAGACTATA TTACATGAAA   
  
  
+ TAGATAAGAG TAGACATTGC TATATATTTT ATCTTTAATT GTCTATTTTT AATGATCATT ACTGTTTATT   
  
  
+ TCAGTGAGTA ATTTTCTCGG ATATAATATT TTATAGCGGT TATTAAATAG ATGTGAAACT ATAATGTTAG   
  
  
+ ACATATCATG CGAAAATAGA GAAATTTTGG GCAAATTAAA AAAAAAAGAA GAAAAACGAG GAAGTATGCC   
  
  
+ CATTGTAAGC AGGTGGATGT ATATCCAGCC TCCATCCAAC AATTTGGGCA AAATTATATC ATCCATTCCA   
  
  
+ AAAAAAAGAT ATAATTTGTG ATATTAATAA CCCGACTCAA TTCAAGCTTA TTTATTTTTT CATATAATTT   
  
  
+ TTTAATATAT AAAAATAAAA AGTGACAACG ACATGCATCC TTTGCGTGTC AAAGGATCCG CCGCGTGGCC   
  
  
+ CCCACATTCA CTGACGGGGT TTTTGGTTTG CGATTAAGAT CAATGGCTAA CGTTGTCTCT CTCAAAACAT   
  
  
+ ATGGACCCCT TTTTCTATCT TTTTCTGCAT TGTCAGCATA GCAATTGTTG AGCACATTTT TTTCGATTTG   
  
  
+ TTGACAATAA GCGTGGTTAT GATTTATGGG CCACTAATAT CAGTCTCTGA TCATTTGCAC ATTTTGGACC   
  
  
+ TCTTTCTCTC TCTAATCTTC CAGGTAACCT GAATTCTTCT CTATTTCTGA CTAGATTAGA TAACAGGATT   
  
  
+ TCAAATATCT GTCAGGTGGC GGGTATAACT TCGTGATGGG CATTCATTTT TGTTCTATCA GGCTGGTGGG   
  
  
+ TTTGAGTTTA TTTTGATTCA TTTCGTGTTC ATCTGTGATA TTAGTAGGAG GGATTCTGTA TACCCACCAA   
  
  
+ CTGCTCGCCC TTTTGCCCGT GTGAGCAATT GGGACTTTTG TTTGTTCTTC GTCGATGCAT CCTCTGGTTG   
  
  
+ CTGATCTGGA ATTGAAACCC CATGTATTCA AATTCAACCC TGATTTGCTG TCAAACTTTC TGAACCATCA   
  
  
+ AAACTCCGCC GAAGTGTTTG AAAAAGATGG CATCTTCCAG ACCCTTCATT TGGCTGATCC CAAAAGCTCT   
  
  
+ TCAATTGCCG AAATCGGGTT TACTAATAGT TCAGATTCTA CACAAGTACC TGATTTTTCA GATGCTTGTC   
  
  
+ TTAAGTTCAT TAGTGATATT CTCCTGGAAG AGGACTTAGA TGAAAGTCCT ACGTCTTTAC ACGATTACAT   
  
  
+ GGCTCTCCTA GCCACTGAGA AGTCTTTGTA TGATGCTCTT GGAAAGGAAT ACTTTCCTTC GTCTACTAGT   
  
  
+ CTTGCCCCAT CTTTAGGCCG AAGTGTTGAC AGCCCAGATA GTGGCTTTGG CCGCGGTTGC TCTGATGGTC   
  
  
+ GCGGGATTGA GGGTTTGGCT AATGATGATG CCGTTTTCAT GTCTAACTGG CAGCTCAACA CCACCCAATT   
  
  
+ GGACCCTTTC CCAATCATGC AAGATATTCC TCGTCCCTAT TTGGAATTGA ATTACCATTC TTCTGGGTCA   
  
  
+ AGCAATGGCA TTGATGATTC GGGGGATGGG TTATCGACCT CTCCTGTAAG TACACTTGCA TCAACCGCCA   
  
  
+ CAGAGGCAGG GAAAAAGTTG GCTGGTAGCT CTAGGAGAAA GAACCGTCAA AGGGATGACT ATGGCCATGA   
  
  
+ AGAGGGAAGG AGTAACAAGC AGCAAGCCTC TTACAATGAC GATTACGTTG AGATGGAGCA GTATGACGAT   
  
  
+ GTACTTCTCT GTAGGGCAGA CAAGGGTAAT ATTTCAACTT GTGCCAATGA ATCCTCGCTT AATGAGGTGC   
  
  
+ GTGAGAAGCT GCAGACGACA GGGTTCAAGG GAAGAACATC CCGTCTTAAG AAGCAATCTA AGGAAGCGAA   
  
  
+ AGAGGTGGAT CTGAGAACTC TTCTTTCTGG TTGTGCACAA GCTGTTTCGA ACTTTGATAT CAGGACTGCT   
  
  
+ AATGAGCTGC TTAAGCAAGT CAGACAGCAT TCTTCACCAT ATGGTGATAG CCTCCAAAGG CTCGCCCATC   
  
  
+ AATTTGCGAA TGGTATTGAG GCACGCTTAG CAGGCACCGG TTCAAGAGTA CCTGCTAATC TCATTGATGC   
  
  
+ ACGGATATCA TCATCTGAAT TTTTAAAAGC TTACAAGTCA TATGTTTCAG CAGTTCCTTT CAGAAGGATG   
  
  
+ TCCTATTTTA TAGCAAACAA CACAATTCTG AAGTTGGCTG AGAAAGCAAC AAAGATTCAC ATAATTGATT   
  
  
+ TTGGTATTCT ATTTGGTCTA CAGTGGCCCT GTCTTATACA AAGTCTCTCA AGGCGAACTG TGGCTCCTCC   
  
  
+ GAAGCTTCGC ATCACTGGGA TAGACTATCC CCAGCATGGT TTCCGGCCAG CAGAAAAGGT TGAGGCAACA   
  
  
+ GGTCGTCGGT TGTCCGGGTA CTGTGAGAGA TTTAATGTAC CCTTTCGATA TGAAACCATT GCAAAGAAGT   
  
  
+ GGGAAACCAT ACGCCCAGAA GATCTAAATA TTGAGAATGA TGAGCTGGTA ATTGTTAATT GTATGTTGCG   
  
  
+ GTCTGTAAAT CTATTGGATG ATACAGTGGC GGTAAATAGT CCAAGGGATG CTTTCTTGAG GTTAATCAAA   
  
  
+ CAGATAAACC CGCGTTTATT CATTCATGCA ATTGTCAATG GAACCTTTAG TACTCCATTC TTCAGCACTC   
  
  
+ GATTCAGGGA AGCCCTATTC CAATACTCTT CTGTATTTGA TATATTTGAA GCGACTATGA CTCGTGAAGA   
  
  
+ TCGTGGAAGG CTGCTGATTG AGAGTCAAAT ATGCGGGCTA GAAGTTTTGA ATGCAATAGC ATGTGAAGGT   
  
  
+ GCAGAGAGGA TTCAAAGGCC TGAAACATAC AAGCAATGGC AGGAGCGGAC AACAAGGGCT GGACTAAGGC   
  
  
+ AGGTTCCAAT AGATGAGGAG CTTGTCAATA GAGCAAAGAC TATAGTGAAA GCAAATTATC ACAAGGATTT   
  
  
+ TGTGGTGGAT GAGGATAGGC GTTGGATGCT TCAAGGTTGG AAAGGAAGGA CACTTAGTGC CCTTTCCGTT   
  
  
+ TGGCAGCCTA ACTA  

- +Up\_Stream \_Len000AAATTA TTCTAGGAAA ATATATATAC ACACATATTA AGATACCATG TATCCATACA   
  
  
- TGGTATCTGA ATAACGTAAA GATCCAGGAA GAGAGAGACG CGAACAAAAA ACAAATAGAT TAAATACCTC   
  
  
- AACTTGTAAA ACAAAATATA TGAACTTGTT AGATAATATG ATTTAATTCG TAAAACTAAA TTAGTGAATA   
  
  
- CCCACACAAA CCATCCCAAC TTTTATTAAA AGGATCTTTT ATTAAAAATT ATATTTTATT AAAAAGTACA   
  
  
- TTTTACTAAA AGTTGAGTAT AAAAGTCAAC TAACCATTTT TCTTTTTACT TGAAAGACCT ATTAGTCAAA   
  
  
- CTAATTGTTT TTCACTGTTT TTTTAATTTT TACCAAAAGG CATTAGAAGT TTGGTATACA GCTCCTTTAT   
  
  
- TTGTGCCTTA TTTCTTTCTC ACAGTCTTGG GTTCTTTACA TTTTAGTTGA ATGCAAGATA AATTTCACTT   
  
  
- TTTATAAAAA GAAGTAAGAT CCTTTTAATT AACAGGAGAC TTTTGTTTAA AAGGGAGGAA CAGATTGGTT   
  
  
- TGTGTCATTT TAACTTTTTA GTAAAAGATC CTTTTACTAA AAGTGAGGAT GGTTTGTGTG GGAATTTGAA   
  
  
- CAAGGTTTAG ATATTTGGGC CTTAGAATTA TGCGTGTAAC ATATAATATA CAGGATGATA TTGACATAAT   
  
  
- AGTGACCAAG AACTAAATAG GAAAGTTTGA GAACGAATCA GTTCCAACAG GAGCATGTTT GTACTAATTA   
  
  
- AGTTAATTGG TGCATTCAAT ATGCGACTGT TTAAAGCCCA TGGTTTGTAA ACGTGATCTG GATTTTGTCA   
  
  
- ATGATAGTAG TATAGCTACA ATTTTAACTG GACAAGAATA AACAGGTCGC AACTCGTTCC TTTATTAGTT   
  
  
- TAGTACAGAG TAAATCAGCC AAAAAACGGA TTCTTCTCGT ATTTGTTTCC TTTTTCTGTT GCTGTGTAAT   
  
  
- ATGTACGTAG ATGTGTTTAC TTTACTCCGG TATACCTTGT ACAAGTACTC TCGAAGTGAG CGATTATTAA   
  
  
- CTTGGATGAA AACTGATTCG GTGGTTGATC TTATACCTCT AACTGACAAT TCTCTGATAT AATGTACTTT   
  
  
- ATCTATTCTC ATCTGTAACG ATATATAAAA TAGAAATTAA CAGATAAAAA TTACTAGTAA TGACAAATAA   
  
  
- AGTCACTCAT TAAAAGAGCC TATATTATAA AATATCGCCA ATAATTTATC TACACTTTGA TATTACAATC   
  
  
- TGTATAGTAC GCTTTTATCT CTTTAAAACC CGTTTAATTT TTTTTTTCTT CTTTTTGCTC CTTCATACGG   
  
  
- GTAACATTCG TCCACCTACA TATAGGTCGG AGGTAGGTTG TTAAACCCGT TTTAATATAG TAGGTAAGGT   
  
  
- TTTTTTTCTA TATTAAACAC TATAATTATT GGGCTGAGTT AAGTTCGAAT AAATAAAAAA GTATATTAAA   
  
  
- AAATTATATA TTTTTATTTT TCACTGTTGC TGTACGTAGG AAACGCACAG TTTCCTAGGC GGCGCACCGG   
  
  
- GGGTGTAAGT GACTGCCCCA AAAACCAAAC GCTAATTCTA GTTACCGATT GCAACAGAGA GAGTTTTGTA   
  
  
- TACCTGGGGA AAAAGATAGA AAAAGACGTA ACAGTCGTAT CGTTAACAAC TCGTGTAAAA AAAGCTAAAC   
  
  
- AACTGTTATT CGCACCAATA CTAAATACCC GGTGATTATA GTCAGAGACT AGTAAACGTG TAAAACCTGG   
  
  
- AGAAAGAGAG AGATTAGAAG GTCCATTGGA CTTAAGAAGA GATAAAGACT GATCTAATCT ATTGTCCTAA   
  
  
- AGTTTATAGA CAGTCCACCG CCCATATTGA AGCACTACCC GTAAGTAAAA ACAAGATAGT CCGACCACCC   
  
  
- AAACTCAAAT AAAACTAAGT AAAGCACAAG TAGACACTAT AATCATCCTC CCTAAGACAT ATGGGTGGTT   
  
  
- GACGAGCGGG AAAACGGGCA CACTCGTTAA CCCTGAAAAC AAACAAGAAG CAGCTACGTA GGAGACCAAC   
  
  
- GACTAGACCT TAACTTTGGG GTACATAAGT TTAAGTTGGG ACTAAACGAC AGTTTGAAAG ACTTGGTAGT   
  
  
- TTTGAGGCGG CTTCACAAAC TTTTTCTACC GTAGAAGGTC TGGGAAGTAA ACCGACTAGG GTTTTCGAGA   
  
  
- AGTTAACGGC TTTAGCCCAA ATGATTATCA AGTCTAAGAT GTGTTCATGG ACTAAAAAGT CTACGAACAG   
  
  
- AATTCAAGTA ATCACTATAA GAGGACCTTC TCCTGAATCT ACTTTCAGGA TGCAGAAATG TGCTAATGTA   
  
  
- CCGAGAGGAT CGGTGACTCT TCAGAAACAT ACTACGAGAA CCTTTCCTTA TGAAAGGAAG CAGATGATCA   
  
  
- GAACGGGGTA GAAATCCGGC TTCACAACTG TCGGGTCTAT CACCGAAACC GGCGCCAACG AGACTACCAG   
  
  
- CGCCCTAACT CCCAAACCGA TTACTACTAC GGCAAAAGTA CAGATTGACC GTCGAGTTGT GGTGGGTTAA   
  
  
- CCTGGGAAAG GGTTAGTACG TTCTATAAGG AGCAGGGATA AACCTTAACT TAATGGTAAG AAGACCCAGT   
  
  
- TCGTTACCGT AACTACTAAG CCCCCTACCC AATAGCTGGA GAGGACATTC ATGTGAACGT AGTTGGCGGT   
  
  
- GTCTCCGTCC CTTTTTCAAC CGACCATCGA GATCCTCTTT CTTGGCAGTT TCCCTACTGA TACCGGTACT   
  
  
- TCTCCCTTCC TCATTGTTCG TCGTTCGGAG AATGTTACTG CTAATGCAAC TCTACCTCGT CATACTGCTA   
  
  
- CATGAAGAGA CATCCCGTCT GTTCCCATTA TAAAGTTGAA CACGGTTACT TAGGAGCGAA TTACTCCACG   
  
  
- CACTCTTCGA CGTCTGCTGT CCCAAGTTCC CTTCTTGTAG GGCAGAATTC TTCGTTAGAT TCCTTCGCTT   
  
  
- TCTCCACCTA GACTCTTGAG AAGAAAGACC AACACGTGTT CGACAAAGCT TGAAACTATA GTCCTGACGA   
  
  
- TTACTCGACG AATTCGTTCA GTCTGTCGTA AGAAGTGGTA TACCACTATC GGAGGTTTCC GAGCGGGTAG   
  
  
- TTAAACGCTT ACCATAACTC CGTGCGAATC GTCCGTGGCC AAGTTCTCAT GGACGATTAG AGTAACTACG   
  
  
- TGCCTATAGT AGTAGACTTA AAAATTTTCG AATGTTCAGT ATACAAAGTC GTCAAGGAAA GTCTTCCTAC   
  
  
- AGGATAAAAT ATCGTTTGTT GTGTTAAGAC TTCAACCGAC TCTTTCGTTG TTTCTAAGTG TATTAACTAA   
  
  
- AACCATAAGA TAAACCAGAT GTCACCGGGA CAGAATATGT TTCAGAGAGT TCCGCTTGAC ACCGAGGAGG   
  
  
- CTTCGAAGCG TAGTGACCCT ATCTGATAGG GGTCGTACCA AAGGCCGGTC GTCTTTTCCA ACTCCGTTGT   
  
  
- CCAGCAGCCA ACAGGCCCAT GACACTCTCT AAATTACATG GGAAAGCTAT ACTTTGGTAA CGTTTCTTCA   
  
  
- CCCTTTGGTA TGCGGGTCTT CTAGATTTAT AACTCTTACT ACTCGACCAT TAACAATTAA CATACAACGC   
  
  
- CAGACATTTA GATAACCTAC TATGTCACCG CCATTTATCA GGTTCCCTAC GAAAGAACTC CAATTAGTTT   
  
  
- GTCTATTTGG GCGCAAATAA GTAAGTACGT TAACAGTTAC CTTGGAAATC ATGAGGTAAG AAGTCGTGAG   
  
  
- CTAAGTCCCT TCGGGATAAG GTTATGAGAA GACATAAACT ATATAAACTT CGCTGATACT GAGCACTTCT   
  
  
- AGCACCTTCC GACGACTAAC TCTCAGTTTA TACGCCCGAT CTTCAAAACT TACGTTATCG TACACTTCCA   
  
  
- CGTCTCTCCT AAGTTTCCGG ACTTTGTATG TTCGTTACCG TCCTCGCCTG TTGTTCCCGA CCTGATTCCG   
  
  
- TCCAAGGTTA TCTACTCCTC GAACAGTTAT CTCGTTTCTG ATATCACTTT CGTTTAATAG TGTTCCTAAA   
  
  
- ACACCACCTA CTCCTATCCG CAACCTACGA AGTTCCAACC TTTCCTTCCT GTGAATCACG GGAAAGGCAA   
  
  
- ACCGTCGGAT TGAT

+     AT~TATA-box

| Site Name | Organism | Position | Strand | Matrix score. | sequence | function |
| --- | --- | --- | --- | --- | --- | --- |
| AT~TATA-box | Arabidopsis thaliana | 160 | + | 6 | TATATA |  |
| AT~TATA-box | Arabidopsis thaliana | 37 | + | 6 | TATATA |  |
| AT~TATA-box | Arabidopsis thaliana | 1480 | + | 6 | TATATA |  |
| AT~TATA-box | Arabidopsis thaliana | 1145 | + | 6 | TATATA |  |
| AT~TATA-box | Arabidopsis thaliana | 33 | - | 8 | TATATAAA |  |
| AT~TATA-box | Arabidopsis thaliana | 35 | + | 6 | TATATA |  |
| AT~TATA-box | Arabidopsis thaliana | 158 | - | 8 | TATATAAA |  |

>HU02G01572.1   
+ +Up\_Stream \_Len000TTTAAT AAGATCCTTT TATATATATG TGTGTATAAT TCTATGGTAC ATAGGTATGT   
  
  
+ ACCATAGACT TATTGCATTT CTAGGTCCTT CTCTCTCTGC GCTTGTTTTT TGTTTATCTA ATTTATGGAG   
  
  
+ TTGAACATTT TGTTTTATAT ACTTGAACAA TCTATTATAC TAAATTAAGC ATTTTGATTT AATCACTTAT   
  
  
+ GGGTGTGTTT GGTAGGGTTG AAAATAATTT TCCTAGAAAA TAATTTTTAA TATAAAATAA TTTTTCATGT   
  
  
+ AAAATGATTT TCAACTCATA TTTTCAGTTG ATTGGTAAAA AGAAAAATGA ACTTTCTGGA TAATCAGTTT   
  
  
+ GATTAACAAA AAGTGACAAA AAAATTAAAA ATGGTTTTCC GTAATCTTCA AACCATATGT CGAGGAAATA   
  
  
+ AACACGGAAT AAAGAAAGAG TGTCAGAACC CAAGAAATGT AAAATCAACT TACGTTCTAT TTAAAGTGAA   
  
  
+ AAATATTTTT CTTCATTCTA GGAAAATTAA TTGTCCTCTG AAAACAAATT TTCCCTCCTT GTCTAACCAA   
  
  
+ ACACAGTAAA ATTGAAAAAT CATTTTCTAG GAAAATGATT TTCACTCCTA CCAAACACAC CCTTAAACTT   
  
  
+ GTTCCAAATC TATAAACCCG GAATCTTAAT ACGCACATTG TATATTATAT GTCCTACTAT AACTGTATTA   
  
  
+ TCACTGGTTC TTGATTTATC CTTTCAAACT CTTGCTTAGT CAAGGTTGTC CTCGTACAAA CATGATTAAT   
  
  
+ TCAATTAACC ACGTAAGTTA TACGCTGACA AATTTCGGGT ACCAAACATT TGCACTAGAC CTAAAACAGT   
  
  
+ TACTATCATC ATATCGATGT TAAAATTGAC CTGTTCTTAT TTGTCCAGCG TTGAGCAAGG AAATAATCAA   
  
  
+ ATCATGTCTC ATTTAGTCGG TTTTTTGCCT AAGAAGAGCA TAAACAAAGG AAAAAGACAA CGACACATTA   
  
  
+ TACATGCATC TACACAAATG AAATGAGGCC ATATGGAACA TGTTCATGAG AGCTTCACTC GCTAATAATT   
  
  
+ GAACCTACTT TTGACTAAGC CACCAACTAG AATATGGAGA TTGACTGTTA AGAGACTATA TTACATGAAA   
  
  
+ TAGATAAGAG TAGACATTGC TATATATTTT ATCTTTAATT GTCTATTTTT AATGATCATT ACTGTTTATT   
  
  
+ TCAGTGAGTA ATTTTCTCGG ATATAATATT TTATAGCGGT TATTAAATAG ATGTGAAACT ATAATGTTAG   
  
  
+ ACATATCATG CGAAAATAGA GAAATTTTGG GCAAATTAAA AAAAAAAGAA GAAAAACGAG GAAGTATGCC   
  
  
+ CATTGTAAGC AGGTGGATGT ATATCCAGCC TCCATCCAAC AATTTGGGCA AAATTATATC ATCCATTCCA   
  
  
+ AAAAAAAGAT ATAATTTGTG ATATTAATAA CCCGACTCAA TTCAAGCTTA TTTATTTTTT CATATAATTT   
  
  
+ TTTAATATAT AAAAATAAAA AGTGACAACG ACATGCATCC TTTGCGTGTC AAAGGATCCG CCGCGTGGCC   
  
  
+ CCCACATTCA CTGACGGGGT TTTTGGTTTG CGATTAAGAT CAATGGCTAA CGTTGTCTCT CTCAAAACAT   
  
  
+ ATGGACCCCT TTTTCTATCT TTTTCTGCAT TGTCAGCATA GCAATTGTTG AGCACATTTT TTTCGATTTG   
  
  
+ TTGACAATAA GCGTGGTTAT GATTTATGGG CCACTAATAT CAGTCTCTGA TCATTTGCAC ATTTTGGACC   
  
  
+ TCTTTCTCTC TCTAATCTTC CAGGTAACCT GAATTCTTCT CTATTTCTGA CTAGATTAGA TAACAGGATT   
  
  
+ TCAAATATCT GTCAGGTGGC GGGTATAACT TCGTGATGGG CATTCATTTT TGTTCTATCA GGCTGGTGGG   
  
  
+ TTTGAGTTTA TTTTGATTCA TTTCGTGTTC ATCTGTGATA TTAGTAGGAG GGATTCTGTA TACCCACCAA   
  
  
+ CTGCTCGCCC TTTTGCCCGT GTGAGCAATT GGGACTTTTG TTTGTTCTTC GTCGATGCAT CCTCTGGTTG   
  
  
+ CTGATCTGGA ATTGAAACCC CATGTATTCA AATTCAACCC TGATTTGCTG TCAAACTTTC TGAACCATCA   
  
  
+ AAACTCCGCC GAAGTGTTTG AAAAAGATGG CATCTTCCAG ACCCTTCATT TGGCTGATCC CAAAAGCTCT   
  
  
+ TCAATTGCCG AAATCGGGTT TACTAATAGT TCAGATTCTA CACAAGTACC TGATTTTTCA GATGCTTGTC   
  
  
+ TTAAGTTCAT TAGTGATATT CTCCTGGAAG AGGACTTAGA TGAAAGTCCT ACGTCTTTAC ACGATTACAT   
  
  
+ GGCTCTCCTA GCCACTGAGA AGTCTTTGTA TGATGCTCTT GGAAAGGAAT ACTTTCCTTC GTCTACTAGT   
  
  
+ CTTGCCCCAT CTTTAGGCCG AAGTGTTGAC AGCCCAGATA GTGGCTTTGG CCGCGGTTGC TCTGATGGTC   
  
  
+ GCGGGATTGA GGGTTTGGCT AATGATGATG CCGTTTTCAT GTCTAACTGG CAGCTCAACA CCACCCAATT   
  
  
+ GGACCCTTTC CCAATCATGC AAGATATTCC TCGTCCCTAT TTGGAATTGA ATTACCATTC TTCTGGGTCA   
  
  
+ AGCAATGGCA TTGATGATTC GGGGGATGGG TTATCGACCT CTCCTGTAAG TACACTTGCA TCAACCGCCA   
  
  
+ CAGAGGCAGG GAAAAAGTTG GCTGGTAGCT CTAGGAGAAA GAACCGTCAA AGGGATGACT ATGGCCATGA   
  
  
+ AGAGGGAAGG AGTAACAAGC AGCAAGCCTC TTACAATGAC GATTACGTTG AGATGGAGCA GTATGACGAT   
  
  
+ GTACTTCTCT GTAGGGCAGA CAAGGGTAAT ATTTCAACTT GTGCCAATGA ATCCTCGCTT AATGAGGTGC   
  
  
+ GTGAGAAGCT GCAGACGACA GGGTTCAAGG GAAGAACATC CCGTCTTAAG AAGCAATCTA AGGAAGCGAA   
  
  
+ AGAGGTGGAT CTGAGAACTC TTCTTTCTGG TTGTGCACAA GCTGTTTCGA ACTTTGATAT CAGGACTGCT   
  
  
+ AATGAGCTGC TTAAGCAAGT CAGACAGCAT TCTTCACCAT ATGGTGATAG CCTCCAAAGG CTCGCCCATC   
  
  
+ AATTTGCGAA TGGTATTGAG GCACGCTTAG CAGGCACCGG TTCAAGAGTA CCTGCTAATC TCATTGATGC   
  
  
+ ACGGATATCA TCATCTGAAT TTTTAAAAGC TTACAAGTCA TATGTTTCAG CAGTTCCTTT CAGAAGGATG   
  
  
+ TCCTATTTTA TAGCAAACAA CACAATTCTG AAGTTGGCTG AGAAAGCAAC AAAGATTCAC ATAATTGATT   
  
  
+ TTGGTATTCT ATTTGGTCTA CAGTGGCCCT GTCTTATACA AAGTCTCTCA AGGCGAACTG TGGCTCCTCC   
  
  
+ GAAGCTTCGC ATCACTGGGA TAGACTATCC CCAGCATGGT TTCCGGCCAG CAGAAAAGGT TGAGGCAACA   
  
  
+ GGTCGTCGGT TGTCCGGGTA CTGTGAGAGA TTTAATGTAC CCTTTCGATA TGAAACCATT GCAAAGAAGT   
  
  
+ GGGAAACCAT ACGCCCAGAA GATCTAAATA TTGAGAATGA TGAGCTGGTA ATTGTTAATT GTATGTTGCG   
  
  
+ GTCTGTAAAT CTATTGGATG ATACAGTGGC GGTAAATAGT CCAAGGGATG CTTTCTTGAG GTTAATCAAA   
  
  
+ CAGATAAACC CGCGTTTATT CATTCATGCA ATTGTCAATG GAACCTTTAG TACTCCATTC TTCAGCACTC   
  
  
+ GATTCAGGGA AGCCCTATTC CAATACTCTT CTGTATTTGA TATATTTGAA GCGACTATGA CTCGTGAAGA   
  
  
+ TCGTGGAAGG CTGCTGATTG AGAGTCAAAT ATGCGGGCTA GAAGTTTTGA ATGCAATAGC ATGTGAAGGT   
  
  
+ GCAGAGAGGA TTCAAAGGCC TGAAACATAC AAGCAATGGC AGGAGCGGAC AACAAGGGCT GGACTAAGGC   
  
  
+ AGGTTCCAAT AGATGAGGAG CTTGTCAATA GAGCAAAGAC TATAGTGAAA GCAAATTATC ACAAGGATTT   
  
  
+ TGTGGTGGAT GAGGATAGGC GTTGGATGCT TCAAGGTTGG AAAGGAAGGA CACTTAGTGC CCTTTCCGTT   
  
  
+ TGGCAGCCTA ACTA  

- +Up\_Stream \_Len000AAATTA TTCTAGGAAA ATATATATAC ACACATATTA AGATACCATG TATCCATACA   
  
  
- TGGTATCTGA ATAACGTAAA GATCCAGGAA GAGAGAGACG CGAACAAAAA ACAAATAGAT TAAATACCTC   
  
  
- AACTTGTAAA ACAAAATATA TGAACTTGTT AGATAATATG ATTTAATTCG TAAAACTAAA TTAGTGAATA   
  
  
- CCCACACAAA CCATCCCAAC TTTTATTAAA AGGATCTTTT ATTAAAAATT ATATTTTATT AAAAAGTACA   
  
  
- TTTTACTAAA AGTTGAGTAT AAAAGTCAAC TAACCATTTT TCTTTTTACT TGAAAGACCT ATTAGTCAAA   
  
  
- CTAATTGTTT TTCACTGTTT TTTTAATTTT TACCAAAAGG CATTAGAAGT TTGGTATACA GCTCCTTTAT   
  
  
- TTGTGCCTTA TTTCTTTCTC ACAGTCTTGG GTTCTTTACA TTTTAGTTGA ATGCAAGATA AATTTCACTT   
  
  
- TTTATAAAAA GAAGTAAGAT CCTTTTAATT AACAGGAGAC TTTTGTTTAA AAGGGAGGAA CAGATTGGTT   
  
  
- TGTGTCATTT TAACTTTTTA GTAAAAGATC CTTTTACTAA AAGTGAGGAT GGTTTGTGTG GGAATTTGAA   
  
  
- CAAGGTTTAG ATATTTGGGC CTTAGAATTA TGCGTGTAAC ATATAATATA CAGGATGATA TTGACATAAT   
  
  
- AGTGACCAAG AACTAAATAG GAAAGTTTGA GAACGAATCA GTTCCAACAG GAGCATGTTT GTACTAATTA   
  
  
- AGTTAATTGG TGCATTCAAT ATGCGACTGT TTAAAGCCCA TGGTTTGTAA ACGTGATCTG GATTTTGTCA   
  
  
- ATGATAGTAG TATAGCTACA ATTTTAACTG GACAAGAATA AACAGGTCGC AACTCGTTCC TTTATTAGTT   
  
  
- TAGTACAGAG TAAATCAGCC AAAAAACGGA TTCTTCTCGT ATTTGTTTCC TTTTTCTGTT GCTGTGTAAT   
  
  
- ATGTACGTAG ATGTGTTTAC TTTACTCCGG TATACCTTGT ACAAGTACTC TCGAAGTGAG CGATTATTAA   
  
  
- CTTGGATGAA AACTGATTCG GTGGTTGATC TTATACCTCT AACTGACAAT TCTCTGATAT AATGTACTTT   
  
  
- ATCTATTCTC ATCTGTAACG ATATATAAAA TAGAAATTAA CAGATAAAAA TTACTAGTAA TGACAAATAA   
  
  
- AGTCACTCAT TAAAAGAGCC TATATTATAA AATATCGCCA ATAATTTATC TACACTTTGA TATTACAATC   
  
  
- TGTATAGTAC GCTTTTATCT CTTTAAAACC CGTTTAATTT TTTTTTTCTT CTTTTTGCTC CTTCATACGG   
  
  
- GTAACATTCG TCCACCTACA TATAGGTCGG AGGTAGGTTG TTAAACCCGT TTTAATATAG TAGGTAAGGT   
  
  
- TTTTTTTCTA TATTAAACAC TATAATTATT GGGCTGAGTT AAGTTCGAAT AAATAAAAAA GTATATTAAA   
  
  
- AAATTATATA TTTTTATTTT TCACTGTTGC TGTACGTAGG AAACGCACAG TTTCCTAGGC GGCGCACCGG   
  
  
- GGGTGTAAGT GACTGCCCCA AAAACCAAAC GCTAATTCTA GTTACCGATT GCAACAGAGA GAGTTTTGTA   
  
  
- TACCTGGGGA AAAAGATAGA AAAAGACGTA ACAGTCGTAT CGTTAACAAC TCGTGTAAAA AAAGCTAAAC   
  
  
- AACTGTTATT CGCACCAATA CTAAATACCC GGTGATTATA GTCAGAGACT AGTAAACGTG TAAAACCTGG   
  
  
- AGAAAGAGAG AGATTAGAAG GTCCATTGGA CTTAAGAAGA GATAAAGACT GATCTAATCT ATTGTCCTAA   
  
  
- AGTTTATAGA CAGTCCACCG CCCATATTGA AGCACTACCC GTAAGTAAAA ACAAGATAGT CCGACCACCC   
  
  
- AAACTCAAAT AAAACTAAGT AAAGCACAAG TAGACACTAT AATCATCCTC CCTAAGACAT ATGGGTGGTT   
  
  
- GACGAGCGGG AAAACGGGCA CACTCGTTAA CCCTGAAAAC AAACAAGAAG CAGCTACGTA GGAGACCAAC   
  
  
- GACTAGACCT TAACTTTGGG GTACATAAGT TTAAGTTGGG ACTAAACGAC AGTTTGAAAG ACTTGGTAGT   
  
  
- TTTGAGGCGG CTTCACAAAC TTTTTCTACC GTAGAAGGTC TGGGAAGTAA ACCGACTAGG GTTTTCGAGA   
  
  
- AGTTAACGGC TTTAGCCCAA ATGATTATCA AGTCTAAGAT GTGTTCATGG ACTAAAAAGT CTACGAACAG   
  
  
- AATTCAAGTA ATCACTATAA GAGGACCTTC TCCTGAATCT ACTTTCAGGA TGCAGAAATG TGCTAATGTA   
  
  
- CCGAGAGGAT CGGTGACTCT TCAGAAACAT ACTACGAGAA CCTTTCCTTA TGAAAGGAAG CAGATGATCA   
  
  
- GAACGGGGTA GAAATCCGGC TTCACAACTG TCGGGTCTAT CACCGAAACC GGCGCCAACG AGACTACCAG   
  
  
- CGCCCTAACT CCCAAACCGA TTACTACTAC GGCAAAAGTA CAGATTGACC GTCGAGTTGT GGTGGGTTAA   
  
  
- CCTGGGAAAG GGTTAGTACG TTCTATAAGG AGCAGGGATA AACCTTAACT TAATGGTAAG AAGACCCAGT   
  
  
- TCGTTACCGT AACTACTAAG CCCCCTACCC AATAGCTGGA GAGGACATTC ATGTGAACGT AGTTGGCGGT   
  
  
- GTCTCCGTCC CTTTTTCAAC CGACCATCGA GATCCTCTTT CTTGGCAGTT TCCCTACTGA TACCGGTACT   
  
  
- TCTCCCTTCC TCATTGTTCG TCGTTCGGAG AATGTTACTG CTAATGCAAC TCTACCTCGT CATACTGCTA   
  
  
- CATGAAGAGA CATCCCGTCT GTTCCCATTA TAAAGTTGAA CACGGTTACT TAGGAGCGAA TTACTCCACG   
  
  
- CACTCTTCGA CGTCTGCTGT CCCAAGTTCC CTTCTTGTAG GGCAGAATTC TTCGTTAGAT TCCTTCGCTT   
  
  
- TCTCCACCTA GACTCTTGAG AAGAAAGACC AACACGTGTT CGACAAAGCT TGAAACTATA GTCCTGACGA   
  
  
- TTACTCGACG AATTCGTTCA GTCTGTCGTA AGAAGTGGTA TACCACTATC GGAGGTTTCC GAGCGGGTAG   
  
  
- TTAAACGCTT ACCATAACTC CGTGCGAATC GTCCGTGGCC AAGTTCTCAT GGACGATTAG AGTAACTACG   
  
  
- TGCCTATAGT AGTAGACTTA AAAATTTTCG AATGTTCAGT ATACAAAGTC GTCAAGGAAA GTCTTCCTAC   
  
  
- AGGATAAAAT ATCGTTTGTT GTGTTAAGAC TTCAACCGAC TCTTTCGTTG TTTCTAAGTG TATTAACTAA   
  
  
- AACCATAAGA TAAACCAGAT GTCACCGGGA CAGAATATGT TTCAGAGAGT TCCGCTTGAC ACCGAGGAGG   
  
  
- CTTCGAAGCG TAGTGACCCT ATCTGATAGG GGTCGTACCA AAGGCCGGTC GTCTTTTCCA ACTCCGTTGT   
  
  
- CCAGCAGCCA ACAGGCCCAT GACACTCTCT AAATTACATG GGAAAGCTAT ACTTTGGTAA CGTTTCTTCA   
  
  
- CCCTTTGGTA TGCGGGTCTT CTAGATTTAT AACTCTTACT ACTCGACCAT TAACAATTAA CATACAACGC   
  
  
- CAGACATTTA GATAACCTAC TATGTCACCG CCATTTATCA GGTTCCCTAC GAAAGAACTC CAATTAGTTT   
  
  
- GTCTATTTGG GCGCAAATAA GTAAGTACGT TAACAGTTAC CTTGGAAATC ATGAGGTAAG AAGTCGTGAG   
  
  
- CTAAGTCCCT TCGGGATAAG GTTATGAGAA GACATAAACT ATATAAACTT CGCTGATACT GAGCACTTCT   
  
  
- AGCACCTTCC GACGACTAAC TCTCAGTTTA TACGCCCGAT CTTCAAAACT TACGTTATCG TACACTTCCA   
  
  
- CGTCTCTCCT AAGTTTCCGG ACTTTGTATG TTCGTTACCG TCCTCGCCTG TTGTTCCCGA CCTGATTCCG   
  
  
- TCCAAGGTTA TCTACTCCTC GAACAGTTAT CTCGTTTCTG ATATCACTTT CGTTTAATAG TGTTCCTAAA   
  
  
- ACACCACCTA CTCCTATCCG CAACCTACGA AGTTCCAACC TTTCCTTCCT GTGAATCACG GGAAAGGCAA   
  
  
- ACCGTCGGAT TGAT

+     AuxRR-core

| Site Name | Organism | Position | Strand | Matrix score. | sequence | function |
| --- | --- | --- | --- | --- | --- | --- |
| AuxRR-core | Nicotiana tabacum | 1615 | - | 7 | GGTCCAT | cis-acting regulatory element involved in auxin responsiveness |

>HU02G01572.1   
+ +Up\_Stream \_Len000TTTAAT AAGATCCTTT TATATATATG TGTGTATAAT TCTATGGTAC ATAGGTATGT   
  
  
+ ACCATAGACT TATTGCATTT CTAGGTCCTT CTCTCTCTGC GCTTGTTTTT TGTTTATCTA ATTTATGGAG   
  
  
+ TTGAACATTT TGTTTTATAT ACTTGAACAA TCTATTATAC TAAATTAAGC ATTTTGATTT AATCACTTAT   
  
  
+ GGGTGTGTTT GGTAGGGTTG AAAATAATTT TCCTAGAAAA TAATTTTTAA TATAAAATAA TTTTTCATGT   
  
  
+ AAAATGATTT TCAACTCATA TTTTCAGTTG ATTGGTAAAA AGAAAAATGA ACTTTCTGGA TAATCAGTTT   
  
  
+ GATTAACAAA AAGTGACAAA AAAATTAAAA ATGGTTTTCC GTAATCTTCA AACCATATGT CGAGGAAATA   
  
  
+ AACACGGAAT AAAGAAAGAG TGTCAGAACC CAAGAAATGT AAAATCAACT TACGTTCTAT TTAAAGTGAA   
  
  
+ AAATATTTTT CTTCATTCTA GGAAAATTAA TTGTCCTCTG AAAACAAATT TTCCCTCCTT GTCTAACCAA   
  
  
+ ACACAGTAAA ATTGAAAAAT CATTTTCTAG GAAAATGATT TTCACTCCTA CCAAACACAC CCTTAAACTT   
  
  
+ GTTCCAAATC TATAAACCCG GAATCTTAAT ACGCACATTG TATATTATAT GTCCTACTAT AACTGTATTA   
  
  
+ TCACTGGTTC TTGATTTATC CTTTCAAACT CTTGCTTAGT CAAGGTTGTC CTCGTACAAA CATGATTAAT   
  
  
+ TCAATTAACC ACGTAAGTTA TACGCTGACA AATTTCGGGT ACCAAACATT TGCACTAGAC CTAAAACAGT   
  
  
+ TACTATCATC ATATCGATGT TAAAATTGAC CTGTTCTTAT TTGTCCAGCG TTGAGCAAGG AAATAATCAA   
  
  
+ ATCATGTCTC ATTTAGTCGG TTTTTTGCCT AAGAAGAGCA TAAACAAAGG AAAAAGACAA CGACACATTA   
  
  
+ TACATGCATC TACACAAATG AAATGAGGCC ATATGGAACA TGTTCATGAG AGCTTCACTC GCTAATAATT   
  
  
+ GAACCTACTT TTGACTAAGC CACCAACTAG AATATGGAGA TTGACTGTTA AGAGACTATA TTACATGAAA   
  
  
+ TAGATAAGAG TAGACATTGC TATATATTTT ATCTTTAATT GTCTATTTTT AATGATCATT ACTGTTTATT   
  
  
+ TCAGTGAGTA ATTTTCTCGG ATATAATATT TTATAGCGGT TATTAAATAG ATGTGAAACT ATAATGTTAG   
  
  
+ ACATATCATG CGAAAATAGA GAAATTTTGG GCAAATTAAA AAAAAAAGAA GAAAAACGAG GAAGTATGCC   
  
  
+ CATTGTAAGC AGGTGGATGT ATATCCAGCC TCCATCCAAC AATTTGGGCA AAATTATATC ATCCATTCCA   
  
  
+ AAAAAAAGAT ATAATTTGTG ATATTAATAA CCCGACTCAA TTCAAGCTTA TTTATTTTTT CATATAATTT   
  
  
+ TTTAATATAT AAAAATAAAA AGTGACAACG ACATGCATCC TTTGCGTGTC AAAGGATCCG CCGCGTGGCC   
  
  
+ CCCACATTCA CTGACGGGGT TTTTGGTTTG CGATTAAGAT CAATGGCTAA CGTTGTCTCT CTCAAAACAT   
  
  
+ ATGGACCCCT TTTTCTATCT TTTTCTGCAT TGTCAGCATA GCAATTGTTG AGCACATTTT TTTCGATTTG   
  
  
+ TTGACAATAA GCGTGGTTAT GATTTATGGG CCACTAATAT CAGTCTCTGA TCATTTGCAC ATTTTGGACC   
  
  
+ TCTTTCTCTC TCTAATCTTC CAGGTAACCT GAATTCTTCT CTATTTCTGA CTAGATTAGA TAACAGGATT   
  
  
+ TCAAATATCT GTCAGGTGGC GGGTATAACT TCGTGATGGG CATTCATTTT TGTTCTATCA GGCTGGTGGG   
  
  
+ TTTGAGTTTA TTTTGATTCA TTTCGTGTTC ATCTGTGATA TTAGTAGGAG GGATTCTGTA TACCCACCAA   
  
  
+ CTGCTCGCCC TTTTGCCCGT GTGAGCAATT GGGACTTTTG TTTGTTCTTC GTCGATGCAT CCTCTGGTTG   
  
  
+ CTGATCTGGA ATTGAAACCC CATGTATTCA AATTCAACCC TGATTTGCTG TCAAACTTTC TGAACCATCA   
  
  
+ AAACTCCGCC GAAGTGTTTG AAAAAGATGG CATCTTCCAG ACCCTTCATT TGGCTGATCC CAAAAGCTCT   
  
  
+ TCAATTGCCG AAATCGGGTT TACTAATAGT TCAGATTCTA CACAAGTACC TGATTTTTCA GATGCTTGTC   
  
  
+ TTAAGTTCAT TAGTGATATT CTCCTGGAAG AGGACTTAGA TGAAAGTCCT ACGTCTTTAC ACGATTACAT   
  
  
+ GGCTCTCCTA GCCACTGAGA AGTCTTTGTA TGATGCTCTT GGAAAGGAAT ACTTTCCTTC GTCTACTAGT   
  
  
+ CTTGCCCCAT CTTTAGGCCG AAGTGTTGAC AGCCCAGATA GTGGCTTTGG CCGCGGTTGC TCTGATGGTC   
  
  
+ GCGGGATTGA GGGTTTGGCT AATGATGATG CCGTTTTCAT GTCTAACTGG CAGCTCAACA CCACCCAATT   
  
  
+ GGACCCTTTC CCAATCATGC AAGATATTCC TCGTCCCTAT TTGGAATTGA ATTACCATTC TTCTGGGTCA   
  
  
+ AGCAATGGCA TTGATGATTC GGGGGATGGG TTATCGACCT CTCCTGTAAG TACACTTGCA TCAACCGCCA   
  
  
+ CAGAGGCAGG GAAAAAGTTG GCTGGTAGCT CTAGGAGAAA GAACCGTCAA AGGGATGACT ATGGCCATGA   
  
  
+ AGAGGGAAGG AGTAACAAGC AGCAAGCCTC TTACAATGAC GATTACGTTG AGATGGAGCA GTATGACGAT   
  
  
+ GTACTTCTCT GTAGGGCAGA CAAGGGTAAT ATTTCAACTT GTGCCAATGA ATCCTCGCTT AATGAGGTGC   
  
  
+ GTGAGAAGCT GCAGACGACA GGGTTCAAGG GAAGAACATC CCGTCTTAAG AAGCAATCTA AGGAAGCGAA   
  
  
+ AGAGGTGGAT CTGAGAACTC TTCTTTCTGG TTGTGCACAA GCTGTTTCGA ACTTTGATAT CAGGACTGCT   
  
  
+ AATGAGCTGC TTAAGCAAGT CAGACAGCAT TCTTCACCAT ATGGTGATAG CCTCCAAAGG CTCGCCCATC   
  
  
+ AATTTGCGAA TGGTATTGAG GCACGCTTAG CAGGCACCGG TTCAAGAGTA CCTGCTAATC TCATTGATGC   
  
  
+ ACGGATATCA TCATCTGAAT TTTTAAAAGC TTACAAGTCA TATGTTTCAG CAGTTCCTTT CAGAAGGATG   
  
  
+ TCCTATTTTA TAGCAAACAA CACAATTCTG AAGTTGGCTG AGAAAGCAAC AAAGATTCAC ATAATTGATT   
  
  
+ TTGGTATTCT ATTTGGTCTA CAGTGGCCCT GTCTTATACA AAGTCTCTCA AGGCGAACTG TGGCTCCTCC   
  
  
+ GAAGCTTCGC ATCACTGGGA TAGACTATCC CCAGCATGGT TTCCGGCCAG CAGAAAAGGT TGAGGCAACA   
  
  
+ GGTCGTCGGT TGTCCGGGTA CTGTGAGAGA TTTAATGTAC CCTTTCGATA TGAAACCATT GCAAAGAAGT   
  
  
+ GGGAAACCAT ACGCCCAGAA GATCTAAATA TTGAGAATGA TGAGCTGGTA ATTGTTAATT GTATGTTGCG   
  
  
+ GTCTGTAAAT CTATTGGATG ATACAGTGGC GGTAAATAGT CCAAGGGATG CTTTCTTGAG GTTAATCAAA   
  
  
+ CAGATAAACC CGCGTTTATT CATTCATGCA ATTGTCAATG GAACCTTTAG TACTCCATTC TTCAGCACTC   
  
  
+ GATTCAGGGA AGCCCTATTC CAATACTCTT CTGTATTTGA TATATTTGAA GCGACTATGA CTCGTGAAGA   
  
  
+ TCGTGGAAGG CTGCTGATTG AGAGTCAAAT ATGCGGGCTA GAAGTTTTGA ATGCAATAGC ATGTGAAGGT   
  
  
+ GCAGAGAGGA TTCAAAGGCC TGAAACATAC AAGCAATGGC AGGAGCGGAC AACAAGGGCT GGACTAAGGC   
  
  
+ AGGTTCCAAT AGATGAGGAG CTTGTCAATA GAGCAAAGAC TATAGTGAAA GCAAATTATC ACAAGGATTT   
  
  
+ TGTGGTGGAT GAGGATAGGC GTTGGATGCT TCAAGGTTGG AAAGGAAGGA CACTTAGTGC CCTTTCCGTT   
  
  
+ TGGCAGCCTA ACTA  

- +Up\_Stream \_Len000AAATTA TTCTAGGAAA ATATATATAC ACACATATTA AGATACCATG TATCCATACA   
  
  
- TGGTATCTGA ATAACGTAAA GATCCAGGAA GAGAGAGACG CGAACAAAAA ACAAATAGAT TAAATACCTC   
  
  
- AACTTGTAAA ACAAAATATA TGAACTTGTT AGATAATATG ATTTAATTCG TAAAACTAAA TTAGTGAATA   
  
  
- CCCACACAAA CCATCCCAAC TTTTATTAAA AGGATCTTTT ATTAAAAATT ATATTTTATT AAAAAGTACA   
  
  
- TTTTACTAAA AGTTGAGTAT AAAAGTCAAC TAACCATTTT TCTTTTTACT TGAAAGACCT ATTAGTCAAA   
  
  
- CTAATTGTTT TTCACTGTTT TTTTAATTTT TACCAAAAGG CATTAGAAGT TTGGTATACA GCTCCTTTAT   
  
  
- TTGTGCCTTA TTTCTTTCTC ACAGTCTTGG GTTCTTTACA TTTTAGTTGA ATGCAAGATA AATTTCACTT   
  
  
- TTTATAAAAA GAAGTAAGAT CCTTTTAATT AACAGGAGAC TTTTGTTTAA AAGGGAGGAA CAGATTGGTT   
  
  
- TGTGTCATTT TAACTTTTTA GTAAAAGATC CTTTTACTAA AAGTGAGGAT GGTTTGTGTG GGAATTTGAA   
  
  
- CAAGGTTTAG ATATTTGGGC CTTAGAATTA TGCGTGTAAC ATATAATATA CAGGATGATA TTGACATAAT   
  
  
- AGTGACCAAG AACTAAATAG GAAAGTTTGA GAACGAATCA GTTCCAACAG GAGCATGTTT GTACTAATTA   
  
  
- AGTTAATTGG TGCATTCAAT ATGCGACTGT TTAAAGCCCA TGGTTTGTAA ACGTGATCTG GATTTTGTCA   
  
  
- ATGATAGTAG TATAGCTACA ATTTTAACTG GACAAGAATA AACAGGTCGC AACTCGTTCC TTTATTAGTT   
  
  
- TAGTACAGAG TAAATCAGCC AAAAAACGGA TTCTTCTCGT ATTTGTTTCC TTTTTCTGTT GCTGTGTAAT   
  
  
- ATGTACGTAG ATGTGTTTAC TTTACTCCGG TATACCTTGT ACAAGTACTC TCGAAGTGAG CGATTATTAA   
  
  
- CTTGGATGAA AACTGATTCG GTGGTTGATC TTATACCTCT AACTGACAAT TCTCTGATAT AATGTACTTT   
  
  
- ATCTATTCTC ATCTGTAACG ATATATAAAA TAGAAATTAA CAGATAAAAA TTACTAGTAA TGACAAATAA   
  
  
- AGTCACTCAT TAAAAGAGCC TATATTATAA AATATCGCCA ATAATTTATC TACACTTTGA TATTACAATC   
  
  
- TGTATAGTAC GCTTTTATCT CTTTAAAACC CGTTTAATTT TTTTTTTCTT CTTTTTGCTC CTTCATACGG   
  
  
- GTAACATTCG TCCACCTACA TATAGGTCGG AGGTAGGTTG TTAAACCCGT TTTAATATAG TAGGTAAGGT   
  
  
- TTTTTTTCTA TATTAAACAC TATAATTATT GGGCTGAGTT AAGTTCGAAT AAATAAAAAA GTATATTAAA   
  
  
- AAATTATATA TTTTTATTTT TCACTGTTGC TGTACGTAGG AAACGCACAG TTTCCTAGGC GGCGCACCGG   
  
  
- GGGTGTAAGT GACTGCCCCA AAAACCAAAC GCTAATTCTA GTTACCGATT GCAACAGAGA GAGTTTTGTA   
  
  
- TACCTGGGGA AAAAGATAGA AAAAGACGTA ACAGTCGTAT CGTTAACAAC TCGTGTAAAA AAAGCTAAAC   
  
  
- AACTGTTATT CGCACCAATA CTAAATACCC GGTGATTATA GTCAGAGACT AGTAAACGTG TAAAACCTGG   
  
  
- AGAAAGAGAG AGATTAGAAG GTCCATTGGA CTTAAGAAGA GATAAAGACT GATCTAATCT ATTGTCCTAA   
  
  
- AGTTTATAGA CAGTCCACCG CCCATATTGA AGCACTACCC GTAAGTAAAA ACAAGATAGT CCGACCACCC   
  
  
- AAACTCAAAT AAAACTAAGT AAAGCACAAG TAGACACTAT AATCATCCTC CCTAAGACAT ATGGGTGGTT   
  
  
- GACGAGCGGG AAAACGGGCA CACTCGTTAA CCCTGAAAAC AAACAAGAAG CAGCTACGTA GGAGACCAAC   
  
  
- GACTAGACCT TAACTTTGGG GTACATAAGT TTAAGTTGGG ACTAAACGAC AGTTTGAAAG ACTTGGTAGT   
  
  
- TTTGAGGCGG CTTCACAAAC TTTTTCTACC GTAGAAGGTC TGGGAAGTAA ACCGACTAGG GTTTTCGAGA   
  
  
- AGTTAACGGC TTTAGCCCAA ATGATTATCA AGTCTAAGAT GTGTTCATGG ACTAAAAAGT CTACGAACAG   
  
  
- AATTCAAGTA ATCACTATAA GAGGACCTTC TCCTGAATCT ACTTTCAGGA TGCAGAAATG TGCTAATGTA   
  
  
- CCGAGAGGAT CGGTGACTCT TCAGAAACAT ACTACGAGAA CCTTTCCTTA TGAAAGGAAG CAGATGATCA   
  
  
- GAACGGGGTA GAAATCCGGC TTCACAACTG TCGGGTCTAT CACCGAAACC GGCGCCAACG AGACTACCAG   
  
  
- CGCCCTAACT CCCAAACCGA TTACTACTAC GGCAAAAGTA CAGATTGACC GTCGAGTTGT GGTGGGTTAA   
  
  
- CCTGGGAAAG GGTTAGTACG TTCTATAAGG AGCAGGGATA AACCTTAACT TAATGGTAAG AAGACCCAGT   
  
  
- TCGTTACCGT AACTACTAAG CCCCCTACCC AATAGCTGGA GAGGACATTC ATGTGAACGT AGTTGGCGGT   
  
  
- GTCTCCGTCC CTTTTTCAAC CGACCATCGA GATCCTCTTT CTTGGCAGTT TCCCTACTGA TACCGGTACT   
  
  
- TCTCCCTTCC TCATTGTTCG TCGTTCGGAG AATGTTACTG CTAATGCAAC TCTACCTCGT CATACTGCTA   
  
  
- CATGAAGAGA CATCCCGTCT GTTCCCATTA TAAAGTTGAA CACGGTTACT TAGGAGCGAA TTACTCCACG   
  
  
- CACTCTTCGA CGTCTGCTGT CCCAAGTTCC CTTCTTGTAG GGCAGAATTC TTCGTTAGAT TCCTTCGCTT   
  
  
- TCTCCACCTA GACTCTTGAG AAGAAAGACC AACACGTGTT CGACAAAGCT TGAAACTATA GTCCTGACGA   
  
  
- TTACTCGACG AATTCGTTCA GTCTGTCGTA AGAAGTGGTA TACCACTATC GGAGGTTTCC GAGCGGGTAG   
  
  
- TTAAACGCTT ACCATAACTC CGTGCGAATC GTCCGTGGCC AAGTTCTCAT GGACGATTAG AGTAACTACG   
  
  
- TGCCTATAGT AGTAGACTTA AAAATTTTCG AATGTTCAGT ATACAAAGTC GTCAAGGAAA GTCTTCCTAC   
  
  
- AGGATAAAAT ATCGTTTGTT GTGTTAAGAC TTCAACCGAC TCTTTCGTTG TTTCTAAGTG TATTAACTAA   
  
  
- AACCATAAGA TAAACCAGAT GTCACCGGGA CAGAATATGT TTCAGAGAGT TCCGCTTGAC ACCGAGGAGG   
  
  
- CTTCGAAGCG TAGTGACCCT ATCTGATAGG GGTCGTACCA AAGGCCGGTC GTCTTTTCCA ACTCCGTTGT   
  
  
- CCAGCAGCCA ACAGGCCCAT GACACTCTCT AAATTACATG GGAAAGCTAT ACTTTGGTAA CGTTTCTTCA   
  
  
- CCCTTTGGTA TGCGGGTCTT CTAGATTTAT AACTCTTACT ACTCGACCAT TAACAATTAA CATACAACGC   
  
  
- CAGACATTTA GATAACCTAC TATGTCACCG CCATTTATCA GGTTCCCTAC GAAAGAACTC CAATTAGTTT   
  
  
- GTCTATTTGG GCGCAAATAA GTAAGTACGT TAACAGTTAC CTTGGAAATC ATGAGGTAAG AAGTCGTGAG   
  
  
- CTAAGTCCCT TCGGGATAAG GTTATGAGAA GACATAAACT ATATAAACTT CGCTGATACT GAGCACTTCT   
  
  
- AGCACCTTCC GACGACTAAC TCTCAGTTTA TACGCCCGAT CTTCAAAACT TACGTTATCG TACACTTCCA   
  
  
- CGTCTCTCCT AAGTTTCCGG ACTTTGTATG TTCGTTACCG TCCTCGCCTG TTGTTCCCGA CCTGATTCCG   
  
  
- TCCAAGGTTA TCTACTCCTC GAACAGTTAT CTCGTTTCTG ATATCACTTT CGTTTAATAG TGTTCCTAAA   
  
  
- ACACCACCTA CTCCTATCCG CAACCTACGA AGTTCCAACC TTTCCTTCCT GTGAATCACG GGAAAGGCAA   
  
  
- ACCGTCGGAT TGAT

+     Box 4

| Site Name | Organism | Position | Strand | Matrix score. | sequence | function |
| --- | --- | --- | --- | --- | --- | --- |
| Box 4 | Petroselinum crispum | 520 | + | 6 | ATTAAT | part of a conserved DNA module involved in light responsiveness |
| Box 4 | Petroselinum crispum | 1427 | + | 6 | ATTAAT | part of a conserved DNA module involved in light responsiveness |
| Box 4 | Petroselinum crispum | 769 | + | 6 | ATTAAT | part of a conserved DNA module involved in light responsiveness |

>HU02G01572.1   
+ +Up\_Stream \_Len000TTTAAT AAGATCCTTT TATATATATG TGTGTATAAT TCTATGGTAC ATAGGTATGT   
  
  
+ ACCATAGACT TATTGCATTT CTAGGTCCTT CTCTCTCTGC GCTTGTTTTT TGTTTATCTA ATTTATGGAG   
  
  
+ TTGAACATTT TGTTTTATAT ACTTGAACAA TCTATTATAC TAAATTAAGC ATTTTGATTT AATCACTTAT   
  
  
+ GGGTGTGTTT GGTAGGGTTG AAAATAATTT TCCTAGAAAA TAATTTTTAA TATAAAATAA TTTTTCATGT   
  
  
+ AAAATGATTT TCAACTCATA TTTTCAGTTG ATTGGTAAAA AGAAAAATGA ACTTTCTGGA TAATCAGTTT   
  
  
+ GATTAACAAA AAGTGACAAA AAAATTAAAA ATGGTTTTCC GTAATCTTCA AACCATATGT CGAGGAAATA   
  
  
+ AACACGGAAT AAAGAAAGAG TGTCAGAACC CAAGAAATGT AAAATCAACT TACGTTCTAT TTAAAGTGAA   
  
  
+ AAATATTTTT CTTCATTCTA GGAAAATTAA TTGTCCTCTG AAAACAAATT TTCCCTCCTT GTCTAACCAA   
  
  
+ ACACAGTAAA ATTGAAAAAT CATTTTCTAG GAAAATGATT TTCACTCCTA CCAAACACAC CCTTAAACTT   
  
  
+ GTTCCAAATC TATAAACCCG GAATCTTAAT ACGCACATTG TATATTATAT GTCCTACTAT AACTGTATTA   
  
  
+ TCACTGGTTC TTGATTTATC CTTTCAAACT CTTGCTTAGT CAAGGTTGTC CTCGTACAAA CATGATTAAT   
  
  
+ TCAATTAACC ACGTAAGTTA TACGCTGACA AATTTCGGGT ACCAAACATT TGCACTAGAC CTAAAACAGT   
  
  
+ TACTATCATC ATATCGATGT TAAAATTGAC CTGTTCTTAT TTGTCCAGCG TTGAGCAAGG AAATAATCAA   
  
  
+ ATCATGTCTC ATTTAGTCGG TTTTTTGCCT AAGAAGAGCA TAAACAAAGG AAAAAGACAA CGACACATTA   
  
  
+ TACATGCATC TACACAAATG AAATGAGGCC ATATGGAACA TGTTCATGAG AGCTTCACTC GCTAATAATT   
  
  
+ GAACCTACTT TTGACTAAGC CACCAACTAG AATATGGAGA TTGACTGTTA AGAGACTATA TTACATGAAA   
  
  
+ TAGATAAGAG TAGACATTGC TATATATTTT ATCTTTAATT GTCTATTTTT AATGATCATT ACTGTTTATT   
  
  
+ TCAGTGAGTA ATTTTCTCGG ATATAATATT TTATAGCGGT TATTAAATAG ATGTGAAACT ATAATGTTAG   
  
  
+ ACATATCATG CGAAAATAGA GAAATTTTGG GCAAATTAAA AAAAAAAGAA GAAAAACGAG GAAGTATGCC   
  
  
+ CATTGTAAGC AGGTGGATGT ATATCCAGCC TCCATCCAAC AATTTGGGCA AAATTATATC ATCCATTCCA   
  
  
+ AAAAAAAGAT ATAATTTGTG ATATTAATAA CCCGACTCAA TTCAAGCTTA TTTATTTTTT CATATAATTT   
  
  
+ TTTAATATAT AAAAATAAAA AGTGACAACG ACATGCATCC TTTGCGTGTC AAAGGATCCG CCGCGTGGCC   
  
  
+ CCCACATTCA CTGACGGGGT TTTTGGTTTG CGATTAAGAT CAATGGCTAA CGTTGTCTCT CTCAAAACAT   
  
  
+ ATGGACCCCT TTTTCTATCT TTTTCTGCAT TGTCAGCATA GCAATTGTTG AGCACATTTT TTTCGATTTG   
  
  
+ TTGACAATAA GCGTGGTTAT GATTTATGGG CCACTAATAT CAGTCTCTGA TCATTTGCAC ATTTTGGACC   
  
  
+ TCTTTCTCTC TCTAATCTTC CAGGTAACCT GAATTCTTCT CTATTTCTGA CTAGATTAGA TAACAGGATT   
  
  
+ TCAAATATCT GTCAGGTGGC GGGTATAACT TCGTGATGGG CATTCATTTT TGTTCTATCA GGCTGGTGGG   
  
  
+ TTTGAGTTTA TTTTGATTCA TTTCGTGTTC ATCTGTGATA TTAGTAGGAG GGATTCTGTA TACCCACCAA   
  
  
+ CTGCTCGCCC TTTTGCCCGT GTGAGCAATT GGGACTTTTG TTTGTTCTTC GTCGATGCAT CCTCTGGTTG   
  
  
+ CTGATCTGGA ATTGAAACCC CATGTATTCA AATTCAACCC TGATTTGCTG TCAAACTTTC TGAACCATCA   
  
  
+ AAACTCCGCC GAAGTGTTTG AAAAAGATGG CATCTTCCAG ACCCTTCATT TGGCTGATCC CAAAAGCTCT   
  
  
+ TCAATTGCCG AAATCGGGTT TACTAATAGT TCAGATTCTA CACAAGTACC TGATTTTTCA GATGCTTGTC   
  
  
+ TTAAGTTCAT TAGTGATATT CTCCTGGAAG AGGACTTAGA TGAAAGTCCT ACGTCTTTAC ACGATTACAT   
  
  
+ GGCTCTCCTA GCCACTGAGA AGTCTTTGTA TGATGCTCTT GGAAAGGAAT ACTTTCCTTC GTCTACTAGT   
  
  
+ CTTGCCCCAT CTTTAGGCCG AAGTGTTGAC AGCCCAGATA GTGGCTTTGG CCGCGGTTGC TCTGATGGTC   
  
  
+ GCGGGATTGA GGGTTTGGCT AATGATGATG CCGTTTTCAT GTCTAACTGG CAGCTCAACA CCACCCAATT   
  
  
+ GGACCCTTTC CCAATCATGC AAGATATTCC TCGTCCCTAT TTGGAATTGA ATTACCATTC TTCTGGGTCA   
  
  
+ AGCAATGGCA TTGATGATTC GGGGGATGGG TTATCGACCT CTCCTGTAAG TACACTTGCA TCAACCGCCA   
  
  
+ CAGAGGCAGG GAAAAAGTTG GCTGGTAGCT CTAGGAGAAA GAACCGTCAA AGGGATGACT ATGGCCATGA   
  
  
+ AGAGGGAAGG AGTAACAAGC AGCAAGCCTC TTACAATGAC GATTACGTTG AGATGGAGCA GTATGACGAT   
  
  
+ GTACTTCTCT GTAGGGCAGA CAAGGGTAAT ATTTCAACTT GTGCCAATGA ATCCTCGCTT AATGAGGTGC   
  
  
+ GTGAGAAGCT GCAGACGACA GGGTTCAAGG GAAGAACATC CCGTCTTAAG AAGCAATCTA AGGAAGCGAA   
  
  
+ AGAGGTGGAT CTGAGAACTC TTCTTTCTGG TTGTGCACAA GCTGTTTCGA ACTTTGATAT CAGGACTGCT   
  
  
+ AATGAGCTGC TTAAGCAAGT CAGACAGCAT TCTTCACCAT ATGGTGATAG CCTCCAAAGG CTCGCCCATC   
  
  
+ AATTTGCGAA TGGTATTGAG GCACGCTTAG CAGGCACCGG TTCAAGAGTA CCTGCTAATC TCATTGATGC   
  
  
+ ACGGATATCA TCATCTGAAT TTTTAAAAGC TTACAAGTCA TATGTTTCAG CAGTTCCTTT CAGAAGGATG   
  
  
+ TCCTATTTTA TAGCAAACAA CACAATTCTG AAGTTGGCTG AGAAAGCAAC AAAGATTCAC ATAATTGATT   
  
  
+ TTGGTATTCT ATTTGGTCTA CAGTGGCCCT GTCTTATACA AAGTCTCTCA AGGCGAACTG TGGCTCCTCC   
  
  
+ GAAGCTTCGC ATCACTGGGA TAGACTATCC CCAGCATGGT TTCCGGCCAG CAGAAAAGGT TGAGGCAACA   
  
  
+ GGTCGTCGGT TGTCCGGGTA CTGTGAGAGA TTTAATGTAC CCTTTCGATA TGAAACCATT GCAAAGAAGT   
  
  
+ GGGAAACCAT ACGCCCAGAA GATCTAAATA TTGAGAATGA TGAGCTGGTA ATTGTTAATT GTATGTTGCG   
  
  
+ GTCTGTAAAT CTATTGGATG ATACAGTGGC GGTAAATAGT CCAAGGGATG CTTTCTTGAG GTTAATCAAA   
  
  
+ CAGATAAACC CGCGTTTATT CATTCATGCA ATTGTCAATG GAACCTTTAG TACTCCATTC TTCAGCACTC   
  
  
+ GATTCAGGGA AGCCCTATTC CAATACTCTT CTGTATTTGA TATATTTGAA GCGACTATGA CTCGTGAAGA   
  
  
+ TCGTGGAAGG CTGCTGATTG AGAGTCAAAT ATGCGGGCTA GAAGTTTTGA ATGCAATAGC ATGTGAAGGT   
  
  
+ GCAGAGAGGA TTCAAAGGCC TGAAACATAC AAGCAATGGC AGGAGCGGAC AACAAGGGCT GGACTAAGGC   
  
  
+ AGGTTCCAAT AGATGAGGAG CTTGTCAATA GAGCAAAGAC TATAGTGAAA GCAAATTATC ACAAGGATTT   
  
  
+ TGTGGTGGAT GAGGATAGGC GTTGGATGCT TCAAGGTTGG AAAGGAAGGA CACTTAGTGC CCTTTCCGTT   
  
  
+ TGGCAGCCTA ACTA  

- +Up\_Stream \_Len000AAATTA TTCTAGGAAA ATATATATAC ACACATATTA AGATACCATG TATCCATACA   
  
  
- TGGTATCTGA ATAACGTAAA GATCCAGGAA GAGAGAGACG CGAACAAAAA ACAAATAGAT TAAATACCTC   
  
  
- AACTTGTAAA ACAAAATATA TGAACTTGTT AGATAATATG ATTTAATTCG TAAAACTAAA TTAGTGAATA   
  
  
- CCCACACAAA CCATCCCAAC TTTTATTAAA AGGATCTTTT ATTAAAAATT ATATTTTATT AAAAAGTACA   
  
  
- TTTTACTAAA AGTTGAGTAT AAAAGTCAAC TAACCATTTT TCTTTTTACT TGAAAGACCT ATTAGTCAAA   
  
  
- CTAATTGTTT TTCACTGTTT TTTTAATTTT TACCAAAAGG CATTAGAAGT TTGGTATACA GCTCCTTTAT   
  
  
- TTGTGCCTTA TTTCTTTCTC ACAGTCTTGG GTTCTTTACA TTTTAGTTGA ATGCAAGATA AATTTCACTT   
  
  
- TTTATAAAAA GAAGTAAGAT CCTTTTAATT AACAGGAGAC TTTTGTTTAA AAGGGAGGAA CAGATTGGTT   
  
  
- TGTGTCATTT TAACTTTTTA GTAAAAGATC CTTTTACTAA AAGTGAGGAT GGTTTGTGTG GGAATTTGAA   
  
  
- CAAGGTTTAG ATATTTGGGC CTTAGAATTA TGCGTGTAAC ATATAATATA CAGGATGATA TTGACATAAT   
  
  
- AGTGACCAAG AACTAAATAG GAAAGTTTGA GAACGAATCA GTTCCAACAG GAGCATGTTT GTACTAATTA   
  
  
- AGTTAATTGG TGCATTCAAT ATGCGACTGT TTAAAGCCCA TGGTTTGTAA ACGTGATCTG GATTTTGTCA   
  
  
- ATGATAGTAG TATAGCTACA ATTTTAACTG GACAAGAATA AACAGGTCGC AACTCGTTCC TTTATTAGTT   
  
  
- TAGTACAGAG TAAATCAGCC AAAAAACGGA TTCTTCTCGT ATTTGTTTCC TTTTTCTGTT GCTGTGTAAT   
  
  
- ATGTACGTAG ATGTGTTTAC TTTACTCCGG TATACCTTGT ACAAGTACTC TCGAAGTGAG CGATTATTAA   
  
  
- CTTGGATGAA AACTGATTCG GTGGTTGATC TTATACCTCT AACTGACAAT TCTCTGATAT AATGTACTTT   
  
  
- ATCTATTCTC ATCTGTAACG ATATATAAAA TAGAAATTAA CAGATAAAAA TTACTAGTAA TGACAAATAA   
  
  
- AGTCACTCAT TAAAAGAGCC TATATTATAA AATATCGCCA ATAATTTATC TACACTTTGA TATTACAATC   
  
  
- TGTATAGTAC GCTTTTATCT CTTTAAAACC CGTTTAATTT TTTTTTTCTT CTTTTTGCTC CTTCATACGG   
  
  
- GTAACATTCG TCCACCTACA TATAGGTCGG AGGTAGGTTG TTAAACCCGT TTTAATATAG TAGGTAAGGT   
  
  
- TTTTTTTCTA TATTAAACAC TATAATTATT GGGCTGAGTT AAGTTCGAAT AAATAAAAAA GTATATTAAA   
  
  
- AAATTATATA TTTTTATTTT TCACTGTTGC TGTACGTAGG AAACGCACAG TTTCCTAGGC GGCGCACCGG   
  
  
- GGGTGTAAGT GACTGCCCCA AAAACCAAAC GCTAATTCTA GTTACCGATT GCAACAGAGA GAGTTTTGTA   
  
  
- TACCTGGGGA AAAAGATAGA AAAAGACGTA ACAGTCGTAT CGTTAACAAC TCGTGTAAAA AAAGCTAAAC   
  
  
- AACTGTTATT CGCACCAATA CTAAATACCC GGTGATTATA GTCAGAGACT AGTAAACGTG TAAAACCTGG   
  
  
- AGAAAGAGAG AGATTAGAAG GTCCATTGGA CTTAAGAAGA GATAAAGACT GATCTAATCT ATTGTCCTAA   
  
  
- AGTTTATAGA CAGTCCACCG CCCATATTGA AGCACTACCC GTAAGTAAAA ACAAGATAGT CCGACCACCC   
  
  
- AAACTCAAAT AAAACTAAGT AAAGCACAAG TAGACACTAT AATCATCCTC CCTAAGACAT ATGGGTGGTT   
  
  
- GACGAGCGGG AAAACGGGCA CACTCGTTAA CCCTGAAAAC AAACAAGAAG CAGCTACGTA GGAGACCAAC   
  
  
- GACTAGACCT TAACTTTGGG GTACATAAGT TTAAGTTGGG ACTAAACGAC AGTTTGAAAG ACTTGGTAGT   
  
  
- TTTGAGGCGG CTTCACAAAC TTTTTCTACC GTAGAAGGTC TGGGAAGTAA ACCGACTAGG GTTTTCGAGA   
  
  
- AGTTAACGGC TTTAGCCCAA ATGATTATCA AGTCTAAGAT GTGTTCATGG ACTAAAAAGT CTACGAACAG   
  
  
- AATTCAAGTA ATCACTATAA GAGGACCTTC TCCTGAATCT ACTTTCAGGA TGCAGAAATG TGCTAATGTA   
  
  
- CCGAGAGGAT CGGTGACTCT TCAGAAACAT ACTACGAGAA CCTTTCCTTA TGAAAGGAAG CAGATGATCA   
  
  
- GAACGGGGTA GAAATCCGGC TTCACAACTG TCGGGTCTAT CACCGAAACC GGCGCCAACG AGACTACCAG   
  
  
- CGCCCTAACT CCCAAACCGA TTACTACTAC GGCAAAAGTA CAGATTGACC GTCGAGTTGT GGTGGGTTAA   
  
  
- CCTGGGAAAG GGTTAGTACG TTCTATAAGG AGCAGGGATA AACCTTAACT TAATGGTAAG AAGACCCAGT   
  
  
- TCGTTACCGT AACTACTAAG CCCCCTACCC AATAGCTGGA GAGGACATTC ATGTGAACGT AGTTGGCGGT   
  
  
- GTCTCCGTCC CTTTTTCAAC CGACCATCGA GATCCTCTTT CTTGGCAGTT TCCCTACTGA TACCGGTACT   
  
  
- TCTCCCTTCC TCATTGTTCG TCGTTCGGAG AATGTTACTG CTAATGCAAC TCTACCTCGT CATACTGCTA   
  
  
- CATGAAGAGA CATCCCGTCT GTTCCCATTA TAAAGTTGAA CACGGTTACT TAGGAGCGAA TTACTCCACG   
  
  
- CACTCTTCGA CGTCTGCTGT CCCAAGTTCC CTTCTTGTAG GGCAGAATTC TTCGTTAGAT TCCTTCGCTT   
  
  
- TCTCCACCTA GACTCTTGAG AAGAAAGACC AACACGTGTT CGACAAAGCT TGAAACTATA GTCCTGACGA   
  
  
- TTACTCGACG AATTCGTTCA GTCTGTCGTA AGAAGTGGTA TACCACTATC GGAGGTTTCC GAGCGGGTAG   
  
  
- TTAAACGCTT ACCATAACTC CGTGCGAATC GTCCGTGGCC AAGTTCTCAT GGACGATTAG AGTAACTACG   
  
  
- TGCCTATAGT AGTAGACTTA AAAATTTTCG AATGTTCAGT ATACAAAGTC GTCAAGGAAA GTCTTCCTAC   
  
  
- AGGATAAAAT ATCGTTTGTT GTGTTAAGAC TTCAACCGAC TCTTTCGTTG TTTCTAAGTG TATTAACTAA   
  
  
- AACCATAAGA TAAACCAGAT GTCACCGGGA CAGAATATGT TTCAGAGAGT TCCGCTTGAC ACCGAGGAGG   
  
  
- CTTCGAAGCG TAGTGACCCT ATCTGATAGG GGTCGTACCA AAGGCCGGTC GTCTTTTCCA ACTCCGTTGT   
  
  
- CCAGCAGCCA ACAGGCCCAT GACACTCTCT AAATTACATG GGAAAGCTAT ACTTTGGTAA CGTTTCTTCA   
  
  
- CCCTTTGGTA TGCGGGTCTT CTAGATTTAT AACTCTTACT ACTCGACCAT TAACAATTAA CATACAACGC   
  
  
- CAGACATTTA GATAACCTAC TATGTCACCG CCATTTATCA GGTTCCCTAC GAAAGAACTC CAATTAGTTT   
  
  
- GTCTATTTGG GCGCAAATAA GTAAGTACGT TAACAGTTAC CTTGGAAATC ATGAGGTAAG AAGTCGTGAG   
  
  
- CTAAGTCCCT TCGGGATAAG GTTATGAGAA GACATAAACT ATATAAACTT CGCTGATACT GAGCACTTCT   
  
  
- AGCACCTTCC GACGACTAAC TCTCAGTTTA TACGCCCGAT CTTCAAAACT TACGTTATCG TACACTTCCA   
  
  
- CGTCTCTCCT AAGTTTCCGG ACTTTGTATG TTCGTTACCG TCCTCGCCTG TTGTTCCCGA CCTGATTCCG   
  
  
- TCCAAGGTTA TCTACTCCTC GAACAGTTAT CTCGTTTCTG ATATCACTTT CGTTTAATAG TGTTCCTAAA   
  
  
- ACACCACCTA CTCCTATCCG CAACCTACGA AGTTCCAACC TTTCCTTCCT GTGAATCACG GGAAAGGCAA   
  
  
- ACCGTCGGAT TGAT

+     Box III

| Site Name | Organism | Position | Strand | Matrix score. | sequence | function |
| --- | --- | --- | --- | --- | --- | --- |
| Box III | Pisum sativum | 599 | + | 11 | atCATTTTCACt | protein binding site |

>HU02G01572.1   
+ +Up\_Stream \_Len000TTTAAT AAGATCCTTT TATATATATG TGTGTATAAT TCTATGGTAC ATAGGTATGT   
  
  
+ ACCATAGACT TATTGCATTT CTAGGTCCTT CTCTCTCTGC GCTTGTTTTT TGTTTATCTA ATTTATGGAG   
  
  
+ TTGAACATTT TGTTTTATAT ACTTGAACAA TCTATTATAC TAAATTAAGC ATTTTGATTT AATCACTTAT   
  
  
+ GGGTGTGTTT GGTAGGGTTG AAAATAATTT TCCTAGAAAA TAATTTTTAA TATAAAATAA TTTTTCATGT   
  
  
+ AAAATGATTT TCAACTCATA TTTTCAGTTG ATTGGTAAAA AGAAAAATGA ACTTTCTGGA TAATCAGTTT   
  
  
+ GATTAACAAA AAGTGACAAA AAAATTAAAA ATGGTTTTCC GTAATCTTCA AACCATATGT CGAGGAAATA   
  
  
+ AACACGGAAT AAAGAAAGAG TGTCAGAACC CAAGAAATGT AAAATCAACT TACGTTCTAT TTAAAGTGAA   
  
  
+ AAATATTTTT CTTCATTCTA GGAAAATTAA TTGTCCTCTG AAAACAAATT TTCCCTCCTT GTCTAACCAA   
  
  
+ ACACAGTAAA ATTGAAAAAT CATTTTCTAG GAAAATGATT TTCACTCCTA CCAAACACAC CCTTAAACTT   
  
  
+ GTTCCAAATC TATAAACCCG GAATCTTAAT ACGCACATTG TATATTATAT GTCCTACTAT AACTGTATTA   
  
  
+ TCACTGGTTC TTGATTTATC CTTTCAAACT CTTGCTTAGT CAAGGTTGTC CTCGTACAAA CATGATTAAT   
  
  
+ TCAATTAACC ACGTAAGTTA TACGCTGACA AATTTCGGGT ACCAAACATT TGCACTAGAC CTAAAACAGT   
  
  
+ TACTATCATC ATATCGATGT TAAAATTGAC CTGTTCTTAT TTGTCCAGCG TTGAGCAAGG AAATAATCAA   
  
  
+ ATCATGTCTC ATTTAGTCGG TTTTTTGCCT AAGAAGAGCA TAAACAAAGG AAAAAGACAA CGACACATTA   
  
  
+ TACATGCATC TACACAAATG AAATGAGGCC ATATGGAACA TGTTCATGAG AGCTTCACTC GCTAATAATT   
  
  
+ GAACCTACTT TTGACTAAGC CACCAACTAG AATATGGAGA TTGACTGTTA AGAGACTATA TTACATGAAA   
  
  
+ TAGATAAGAG TAGACATTGC TATATATTTT ATCTTTAATT GTCTATTTTT AATGATCATT ACTGTTTATT   
  
  
+ TCAGTGAGTA ATTTTCTCGG ATATAATATT TTATAGCGGT TATTAAATAG ATGTGAAACT ATAATGTTAG   
  
  
+ ACATATCATG CGAAAATAGA GAAATTTTGG GCAAATTAAA AAAAAAAGAA GAAAAACGAG GAAGTATGCC   
  
  
+ CATTGTAAGC AGGTGGATGT ATATCCAGCC TCCATCCAAC AATTTGGGCA AAATTATATC ATCCATTCCA   
  
  
+ AAAAAAAGAT ATAATTTGTG ATATTAATAA CCCGACTCAA TTCAAGCTTA TTTATTTTTT CATATAATTT   
  
  
+ TTTAATATAT AAAAATAAAA AGTGACAACG ACATGCATCC TTTGCGTGTC AAAGGATCCG CCGCGTGGCC   
  
  
+ CCCACATTCA CTGACGGGGT TTTTGGTTTG CGATTAAGAT CAATGGCTAA CGTTGTCTCT CTCAAAACAT   
  
  
+ ATGGACCCCT TTTTCTATCT TTTTCTGCAT TGTCAGCATA GCAATTGTTG AGCACATTTT TTTCGATTTG   
  
  
+ TTGACAATAA GCGTGGTTAT GATTTATGGG CCACTAATAT CAGTCTCTGA TCATTTGCAC ATTTTGGACC   
  
  
+ TCTTTCTCTC TCTAATCTTC CAGGTAACCT GAATTCTTCT CTATTTCTGA CTAGATTAGA TAACAGGATT   
  
  
+ TCAAATATCT GTCAGGTGGC GGGTATAACT TCGTGATGGG CATTCATTTT TGTTCTATCA GGCTGGTGGG   
  
  
+ TTTGAGTTTA TTTTGATTCA TTTCGTGTTC ATCTGTGATA TTAGTAGGAG GGATTCTGTA TACCCACCAA   
  
  
+ CTGCTCGCCC TTTTGCCCGT GTGAGCAATT GGGACTTTTG TTTGTTCTTC GTCGATGCAT CCTCTGGTTG   
  
  
+ CTGATCTGGA ATTGAAACCC CATGTATTCA AATTCAACCC TGATTTGCTG TCAAACTTTC TGAACCATCA   
  
  
+ AAACTCCGCC GAAGTGTTTG AAAAAGATGG CATCTTCCAG ACCCTTCATT TGGCTGATCC CAAAAGCTCT   
  
  
+ TCAATTGCCG AAATCGGGTT TACTAATAGT TCAGATTCTA CACAAGTACC TGATTTTTCA GATGCTTGTC   
  
  
+ TTAAGTTCAT TAGTGATATT CTCCTGGAAG AGGACTTAGA TGAAAGTCCT ACGTCTTTAC ACGATTACAT   
  
  
+ GGCTCTCCTA GCCACTGAGA AGTCTTTGTA TGATGCTCTT GGAAAGGAAT ACTTTCCTTC GTCTACTAGT   
  
  
+ CTTGCCCCAT CTTTAGGCCG AAGTGTTGAC AGCCCAGATA GTGGCTTTGG CCGCGGTTGC TCTGATGGTC   
  
  
+ GCGGGATTGA GGGTTTGGCT AATGATGATG CCGTTTTCAT GTCTAACTGG CAGCTCAACA CCACCCAATT   
  
  
+ GGACCCTTTC CCAATCATGC AAGATATTCC TCGTCCCTAT TTGGAATTGA ATTACCATTC TTCTGGGTCA   
  
  
+ AGCAATGGCA TTGATGATTC GGGGGATGGG TTATCGACCT CTCCTGTAAG TACACTTGCA TCAACCGCCA   
  
  
+ CAGAGGCAGG GAAAAAGTTG GCTGGTAGCT CTAGGAGAAA GAACCGTCAA AGGGATGACT ATGGCCATGA   
  
  
+ AGAGGGAAGG AGTAACAAGC AGCAAGCCTC TTACAATGAC GATTACGTTG AGATGGAGCA GTATGACGAT   
  
  
+ GTACTTCTCT GTAGGGCAGA CAAGGGTAAT ATTTCAACTT GTGCCAATGA ATCCTCGCTT AATGAGGTGC   
  
  
+ GTGAGAAGCT GCAGACGACA GGGTTCAAGG GAAGAACATC CCGTCTTAAG AAGCAATCTA AGGAAGCGAA   
  
  
+ AGAGGTGGAT CTGAGAACTC TTCTTTCTGG TTGTGCACAA GCTGTTTCGA ACTTTGATAT CAGGACTGCT   
  
  
+ AATGAGCTGC TTAAGCAAGT CAGACAGCAT TCTTCACCAT ATGGTGATAG CCTCCAAAGG CTCGCCCATC   
  
  
+ AATTTGCGAA TGGTATTGAG GCACGCTTAG CAGGCACCGG TTCAAGAGTA CCTGCTAATC TCATTGATGC   
  
  
+ ACGGATATCA TCATCTGAAT TTTTAAAAGC TTACAAGTCA TATGTTTCAG CAGTTCCTTT CAGAAGGATG   
  
  
+ TCCTATTTTA TAGCAAACAA CACAATTCTG AAGTTGGCTG AGAAAGCAAC AAAGATTCAC ATAATTGATT   
  
  
+ TTGGTATTCT ATTTGGTCTA CAGTGGCCCT GTCTTATACA AAGTCTCTCA AGGCGAACTG TGGCTCCTCC   
  
  
+ GAAGCTTCGC ATCACTGGGA TAGACTATCC CCAGCATGGT TTCCGGCCAG CAGAAAAGGT TGAGGCAACA   
  
  
+ GGTCGTCGGT TGTCCGGGTA CTGTGAGAGA TTTAATGTAC CCTTTCGATA TGAAACCATT GCAAAGAAGT   
  
  
+ GGGAAACCAT ACGCCCAGAA GATCTAAATA TTGAGAATGA TGAGCTGGTA ATTGTTAATT GTATGTTGCG   
  
  
+ GTCTGTAAAT CTATTGGATG ATACAGTGGC GGTAAATAGT CCAAGGGATG CTTTCTTGAG GTTAATCAAA   
  
  
+ CAGATAAACC CGCGTTTATT CATTCATGCA ATTGTCAATG GAACCTTTAG TACTCCATTC TTCAGCACTC   
  
  
+ GATTCAGGGA AGCCCTATTC CAATACTCTT CTGTATTTGA TATATTTGAA GCGACTATGA CTCGTGAAGA   
  
  
+ TCGTGGAAGG CTGCTGATTG AGAGTCAAAT ATGCGGGCTA GAAGTTTTGA ATGCAATAGC ATGTGAAGGT   
  
  
+ GCAGAGAGGA TTCAAAGGCC TGAAACATAC AAGCAATGGC AGGAGCGGAC AACAAGGGCT GGACTAAGGC   
  
  
+ AGGTTCCAAT AGATGAGGAG CTTGTCAATA GAGCAAAGAC TATAGTGAAA GCAAATTATC ACAAGGATTT   
  
  
+ TGTGGTGGAT GAGGATAGGC GTTGGATGCT TCAAGGTTGG AAAGGAAGGA CACTTAGTGC CCTTTCCGTT   
  
  
+ TGGCAGCCTA ACTA  

- +Up\_Stream \_Len000AAATTA TTCTAGGAAA ATATATATAC ACACATATTA AGATACCATG TATCCATACA   
  
  
- TGGTATCTGA ATAACGTAAA GATCCAGGAA GAGAGAGACG CGAACAAAAA ACAAATAGAT TAAATACCTC   
  
  
- AACTTGTAAA ACAAAATATA TGAACTTGTT AGATAATATG ATTTAATTCG TAAAACTAAA TTAGTGAATA   
  
  
- CCCACACAAA CCATCCCAAC TTTTATTAAA AGGATCTTTT ATTAAAAATT ATATTTTATT AAAAAGTACA   
  
  
- TTTTACTAAA AGTTGAGTAT AAAAGTCAAC TAACCATTTT TCTTTTTACT TGAAAGACCT ATTAGTCAAA   
  
  
- CTAATTGTTT TTCACTGTTT TTTTAATTTT TACCAAAAGG CATTAGAAGT TTGGTATACA GCTCCTTTAT   
  
  
- TTGTGCCTTA TTTCTTTCTC ACAGTCTTGG GTTCTTTACA TTTTAGTTGA ATGCAAGATA AATTTCACTT   
  
  
- TTTATAAAAA GAAGTAAGAT CCTTTTAATT AACAGGAGAC TTTTGTTTAA AAGGGAGGAA CAGATTGGTT   
  
  
- TGTGTCATTT TAACTTTTTA GTAAAAGATC CTTTTACTAA AAGTGAGGAT GGTTTGTGTG GGAATTTGAA   
  
  
- CAAGGTTTAG ATATTTGGGC CTTAGAATTA TGCGTGTAAC ATATAATATA CAGGATGATA TTGACATAAT   
  
  
- AGTGACCAAG AACTAAATAG GAAAGTTTGA GAACGAATCA GTTCCAACAG GAGCATGTTT GTACTAATTA   
  
  
- AGTTAATTGG TGCATTCAAT ATGCGACTGT TTAAAGCCCA TGGTTTGTAA ACGTGATCTG GATTTTGTCA   
  
  
- ATGATAGTAG TATAGCTACA ATTTTAACTG GACAAGAATA AACAGGTCGC AACTCGTTCC TTTATTAGTT   
  
  
- TAGTACAGAG TAAATCAGCC AAAAAACGGA TTCTTCTCGT ATTTGTTTCC TTTTTCTGTT GCTGTGTAAT   
  
  
- ATGTACGTAG ATGTGTTTAC TTTACTCCGG TATACCTTGT ACAAGTACTC TCGAAGTGAG CGATTATTAA   
  
  
- CTTGGATGAA AACTGATTCG GTGGTTGATC TTATACCTCT AACTGACAAT TCTCTGATAT AATGTACTTT   
  
  
- ATCTATTCTC ATCTGTAACG ATATATAAAA TAGAAATTAA CAGATAAAAA TTACTAGTAA TGACAAATAA   
  
  
- AGTCACTCAT TAAAAGAGCC TATATTATAA AATATCGCCA ATAATTTATC TACACTTTGA TATTACAATC   
  
  
- TGTATAGTAC GCTTTTATCT CTTTAAAACC CGTTTAATTT TTTTTTTCTT CTTTTTGCTC CTTCATACGG   
  
  
- GTAACATTCG TCCACCTACA TATAGGTCGG AGGTAGGTTG TTAAACCCGT TTTAATATAG TAGGTAAGGT   
  
  
- TTTTTTTCTA TATTAAACAC TATAATTATT GGGCTGAGTT AAGTTCGAAT AAATAAAAAA GTATATTAAA   
  
  
- AAATTATATA TTTTTATTTT TCACTGTTGC TGTACGTAGG AAACGCACAG TTTCCTAGGC GGCGCACCGG   
  
  
- GGGTGTAAGT GACTGCCCCA AAAACCAAAC GCTAATTCTA GTTACCGATT GCAACAGAGA GAGTTTTGTA   
  
  
- TACCTGGGGA AAAAGATAGA AAAAGACGTA ACAGTCGTAT CGTTAACAAC TCGTGTAAAA AAAGCTAAAC   
  
  
- AACTGTTATT CGCACCAATA CTAAATACCC GGTGATTATA GTCAGAGACT AGTAAACGTG TAAAACCTGG   
  
  
- AGAAAGAGAG AGATTAGAAG GTCCATTGGA CTTAAGAAGA GATAAAGACT GATCTAATCT ATTGTCCTAA   
  
  
- AGTTTATAGA CAGTCCACCG CCCATATTGA AGCACTACCC GTAAGTAAAA ACAAGATAGT CCGACCACCC   
  
  
- AAACTCAAAT AAAACTAAGT AAAGCACAAG TAGACACTAT AATCATCCTC CCTAAGACAT ATGGGTGGTT   
  
  
- GACGAGCGGG AAAACGGGCA CACTCGTTAA CCCTGAAAAC AAACAAGAAG CAGCTACGTA GGAGACCAAC   
  
  
- GACTAGACCT TAACTTTGGG GTACATAAGT TTAAGTTGGG ACTAAACGAC AGTTTGAAAG ACTTGGTAGT   
  
  
- TTTGAGGCGG CTTCACAAAC TTTTTCTACC GTAGAAGGTC TGGGAAGTAA ACCGACTAGG GTTTTCGAGA   
  
  
- AGTTAACGGC TTTAGCCCAA ATGATTATCA AGTCTAAGAT GTGTTCATGG ACTAAAAAGT CTACGAACAG   
  
  
- AATTCAAGTA ATCACTATAA GAGGACCTTC TCCTGAATCT ACTTTCAGGA TGCAGAAATG TGCTAATGTA   
  
  
- CCGAGAGGAT CGGTGACTCT TCAGAAACAT ACTACGAGAA CCTTTCCTTA TGAAAGGAAG CAGATGATCA   
  
  
- GAACGGGGTA GAAATCCGGC TTCACAACTG TCGGGTCTAT CACCGAAACC GGCGCCAACG AGACTACCAG   
  
  
- CGCCCTAACT CCCAAACCGA TTACTACTAC GGCAAAAGTA CAGATTGACC GTCGAGTTGT GGTGGGTTAA   
  
  
- CCTGGGAAAG GGTTAGTACG TTCTATAAGG AGCAGGGATA AACCTTAACT TAATGGTAAG AAGACCCAGT   
  
  
- TCGTTACCGT AACTACTAAG CCCCCTACCC AATAGCTGGA GAGGACATTC ATGTGAACGT AGTTGGCGGT   
  
  
- GTCTCCGTCC CTTTTTCAAC CGACCATCGA GATCCTCTTT CTTGGCAGTT TCCCTACTGA TACCGGTACT   
  
  
- TCTCCCTTCC TCATTGTTCG TCGTTCGGAG AATGTTACTG CTAATGCAAC TCTACCTCGT CATACTGCTA   
  
  
- CATGAAGAGA CATCCCGTCT GTTCCCATTA TAAAGTTGAA CACGGTTACT TAGGAGCGAA TTACTCCACG   
  
  
- CACTCTTCGA CGTCTGCTGT CCCAAGTTCC CTTCTTGTAG GGCAGAATTC TTCGTTAGAT TCCTTCGCTT   
  
  
- TCTCCACCTA GACTCTTGAG AAGAAAGACC AACACGTGTT CGACAAAGCT TGAAACTATA GTCCTGACGA   
  
  
- TTACTCGACG AATTCGTTCA GTCTGTCGTA AGAAGTGGTA TACCACTATC GGAGGTTTCC GAGCGGGTAG   
  
  
- TTAAACGCTT ACCATAACTC CGTGCGAATC GTCCGTGGCC AAGTTCTCAT GGACGATTAG AGTAACTACG   
  
  
- TGCCTATAGT AGTAGACTTA AAAATTTTCG AATGTTCAGT ATACAAAGTC GTCAAGGAAA GTCTTCCTAC   
  
  
- AGGATAAAAT ATCGTTTGTT GTGTTAAGAC TTCAACCGAC TCTTTCGTTG TTTCTAAGTG TATTAACTAA   
  
  
- AACCATAAGA TAAACCAGAT GTCACCGGGA CAGAATATGT TTCAGAGAGT TCCGCTTGAC ACCGAGGAGG   
  
  
- CTTCGAAGCG TAGTGACCCT ATCTGATAGG GGTCGTACCA AAGGCCGGTC GTCTTTTCCA ACTCCGTTGT   
  
  
- CCAGCAGCCA ACAGGCCCAT GACACTCTCT AAATTACATG GGAAAGCTAT ACTTTGGTAA CGTTTCTTCA   
  
  
- CCCTTTGGTA TGCGGGTCTT CTAGATTTAT AACTCTTACT ACTCGACCAT TAACAATTAA CATACAACGC   
  
  
- CAGACATTTA GATAACCTAC TATGTCACCG CCATTTATCA GGTTCCCTAC GAAAGAACTC CAATTAGTTT   
  
  
- GTCTATTTGG GCGCAAATAA GTAAGTACGT TAACAGTTAC CTTGGAAATC ATGAGGTAAG AAGTCGTGAG   
  
  
- CTAAGTCCCT TCGGGATAAG GTTATGAGAA GACATAAACT ATATAAACTT CGCTGATACT GAGCACTTCT   
  
  
- AGCACCTTCC GACGACTAAC TCTCAGTTTA TACGCCCGAT CTTCAAAACT TACGTTATCG TACACTTCCA   
  
  
- CGTCTCTCCT AAGTTTCCGG ACTTTGTATG TTCGTTACCG TCCTCGCCTG TTGTTCCCGA CCTGATTCCG   
  
  
- TCCAAGGTTA TCTACTCCTC GAACAGTTAT CTCGTTTCTG ATATCACTTT CGTTTAATAG TGTTCCTAAA   
  
  
- ACACCACCTA CTCCTATCCG CAACCTACGA AGTTCCAACC TTTCCTTCCT GTGAATCACG GGAAAGGCAA   
  
  
- ACCGTCGGAT TGAT

+     CAAT-box

| Site Name | Organism | Position | Strand | Matrix score. | sequence | function |
| --- | --- | --- | --- | --- | --- | --- |
| CAAT-box | Nicotiana glutinosa | 3735 | + | 4 | CAAT |  |
| CAAT-box | Nicotiana glutinosa | 3673 | + | 4 | CAAT |  |
| CAAT-box | Nicotiana glutinosa | 1643 | - | 4 | CAAT |  |
| CAAT-box | Pisum sativum | 3749 | - | 5 | CAAAT | common cis-acting element in promoter and enhancer regions |
| CAAT-box | Pisum sativum | 1376 | - | 5 | CAAAT | common cis-acting element in promoter and enhancer regions |
| CAAT-box | Pisum sativum | 822 | - | 5 | CAAAT | common cis-acting element in promoter and enhancer regions |
| CAAT-box | Pisum sativum | 2152 | - | 5 | CAAAT | common cis-acting element in promoter and enhancer regions |
| CAAT-box | Pisum sativum | 912 | + | 5 | CAAAT | common cis-acting element in promoter and enhancer regions |
| CAAT-box | Nicotiana glutinosa | 3288 | - | 4 | CAAT |  |
| CAAT-box | Nicotiana glutinosa | 3084 | + | 4 | CAAT |  |
| CAAT-box | Pisum sativum | 1737 | - | 5 | CAAAT | common cis-acting element in promoter and enhancer regions |
| CAAT-box | Pisum sativum | 2063 | + | 5 | CAAAT | common cis-acting element in promoter and enhancer regions |
| CAAT-box | Nicotiana glutinosa | 2604 | - | 4 | CAAT |  |
| CAAT-box | Arabidopsis thaliana | 315 | - | 5 | CCAAT | common cis-acting element in promoter and enhancer regions |
| CAAT-box | Arabidopsis thaliana | 2519 | + | 5 | CCAAT | common cis-acting element in promoter and enhancer regions |
| CAAT-box | Pisum sativum | 2563 | - | 5 | CAAAT | common cis-acting element in promoter and enhancer regions |
| CAAT-box | Nicotiana glutinosa | 1162 | - | 4 | CAAT |  |
| CAAT-box | Arabidopsis thaliana | 3587 | - | 5 | CCAAT | common cis-acting element in promoter and enhancer regions |
| CAAT-box | Nicotiana glutinosa | 3931 | + | 4 | CAAT |  |
| CAAT-box | Pisum sativum | 539 | + | 5 | CAAAT | common cis-acting element in promoter and enhancer regions |
| CAAT-box | Nicotiana glutinosa | 671 | - | 4 | CAAT |  |
| CAAT-box | Pisum sativum | 2077 | - | 5 | CAAAT | common cis-acting element in promoter and enhancer regions |
| CAAT-box | Pisum sativum | 3305 | - | 5 | CAAAT | common cis-acting element in promoter and enhancer regions |
| CAAT-box | Nicotiana glutinosa | 3247 | + | 4 | CAAT |  |
| CAAT-box | Nicotiana glutinosa | 1094 | - | 4 | CAAT |  |
| CAAT-box | Nicotiana glutinosa | 2768 | + | 4 | CAAT |  |
| CAAT-box | Arabidopsis thaliana | 3930 | + | 5 | CCAAT | common cis-acting element in promoter and enhancer regions |
| CAAT-box | Nicotiana glutinosa | 3147 | - | 4 | CAAT |  |
| CAAT-box | Pisum sativum | 3976 | + | 5 | CAAAT | common cis-acting element in promoter and enhancer regions |
| CAAT-box | Nicotiana glutinosa | 1990 | + | 4 | CAAT |  |
| CAAT-box | Arabidopsis thaliana | 1992 | - | 5 | CCAAT | common cis-acting element in promoter and enhancer regions |
| CAAT-box | Nicotiana glutinosa | 3950 | + | 4 | CAAT |  |
| CAAT-box | Nicotiana glutinosa | 1658 | - | 4 | CAAT |  |
| CAAT-box | Nicotiana glutinosa | 3492 | - | 4 | CAAT |  |
| CAAT-box | Nicotiana glutinosa | 2928 | + | 4 | CAAT |  |
| CAAT-box | Nicotiana glutinosa | 1656 | + | 4 | CAAT |  |
| CAAT-box | Pisum sativum | 639 | + | 5 | CAAAT | common cis-acting element in promoter and enhancer regions |
| CAAT-box | Nicotiana glutinosa | 2849 | + | 4 | CAAT |  |
| CAAT-box | Nicotiana glutinosa | 2597 | + | 4 | CAAT |  |
| CAAT-box | Pisum sativum | 1296 | + | 5 | CAAAT | common cis-acting element in promoter and enhancer regions |
| CAAT-box | Pisum sativum | 3086 | - | 5 | CAAAT | common cis-acting element in promoter and enhancer regions |
| CAAT-box | Pisum sativum | 803 | + | 5 | CAAAT | common cis-acting element in promoter and enhancer regions |
| CAAT-box | Nicotiana glutinosa | 2178 | - | 4 | CAAT |  |
| CAAT-box | Nicotiana glutinosa | 3675 | - | 4 | CAAT |  |
| CAAT-box | Nicotiana glutinosa | 1140 | - | 4 | CAAT |  |
| CAAT-box | Arabidopsis thaliana | 2522 | - | 5 | CCAAT | common cis-acting element in promoter and enhancer regions |
| CAAT-box | Nicotiana glutinosa | 3534 | - | 4 | CAAT |  |
| CAAT-box | Nicotiana glutinosa | 1585 | + | 4 | CAAT |  |
| CAAT-box | Nicotiana glutinosa | 1374 | + | 4 | CAAT |  |
| CAAT-box | Pisum sativum | 1826 | + | 5 | CAAAT | common cis-acting element in promoter and enhancer regions |
| CAAT-box | Nicotiana glutinosa | 86 | - | 4 | CAAT |  |
| CAAT-box | Nicotiana glutinosa | 2536 | + | 4 | CAAT |  |
| CAAT-box | Pisum sativum | 1418 | - | 5 | CAAAT | common cis-acting element in promoter and enhancer regions |
| CAAT-box | Nicotiana glutinosa | 3888 | + | 4 | CAAT |  |
| CAAT-box | Nicotiana glutinosa | 3838 | + | 4 | CAAT |  |
| CAAT-box | Nicotiana glutinosa | 1052 | - | 4 | CAAT |  |
| CAAT-box | Nicotiana glutinosa | 2460 | - | 4 | CAAT |  |
| CAAT-box | Nicotiana glutinosa | 3099 | - | 4 | CAAT |  |
| CAAT-box | Nicotiana glutinosa | 2176 | + | 4 | CAAT |  |
| CAAT-box | Nicotiana glutinosa | 776 | + | 4 | CAAT |  |
| CAAT-box | Nicotiana glutinosa | 3680 | + | 4 | CAAT |  |
| CAAT-box | Nicotiana glutinosa | 575 | - | 4 | CAAT |  |
| CAAT-box | Arabidopsis thaliana | 2848 | + | 5 | CCAAT | common cis-acting element in promoter and enhancer regions |
| CAAT-box | Nicotiana glutinosa | 1442 | + | 4 | CAAT |  |
| CAAT-box | Nicotiana glutinosa | 2570 | - | 4 | CAAT |  |
| CAAT-box | Nicotiana glutinosa | 1336 | - | 4 | CAAT |  |
| CAAT-box | Pisum sativum | 3810 | + | 5 | CAAAT | common cis-acting element in promoter and enhancer regions |
| CAAT-box | Nicotiana glutinosa | 869 | - | 4 | CAAT |  |
| CAAT-box | Nicotiana glutinosa | 2520 | + | 4 | CAAT |  |
| CAAT-box | Nicotiana glutinosa | 1689 | + | 4 | CAAT |  |
| CAAT-box | Nicotiana glutinosa | 3555 | - | 4 | CAAT |  |
| CAAT-box | Pisum sativum | 883 | - | 5 | CAAAT | common cis-acting element in promoter and enhancer regions |
| CAAT-box | Arabidopsis thaliana | 2535 | + | 5 | CCAAT | common cis-acting element in promoter and enhancer regions |
| CAAT-box | Nicotiana glutinosa | 172 | + | 4 | CAAT |  |
| CAAT-box | Arabidopsis thaliana | 3734 | + | 5 | CCAAT | common cis-acting element in promoter and enhancer regions |
| CAAT-box | Pisum sativum | 1680 | - | 5 | CAAAT | common cis-acting element in promoter and enhancer regions |
| CAAT-box | Pisum sativum | 3758 | - | 5 | CAAAT | common cis-acting element in promoter and enhancer regions |
| CAAT-box | Nicotiana glutinosa | 3562 | - | 4 | CAAT |  |
| CAAT-box | Nicotiana glutinosa | 524 | - | 4 | CAAT |  |
| CAAT-box | Nicotiana glutinosa | 2045 | - | 4 | CAAT |  |
| CAAT-box | Nicotiana glutinosa | 3801 | - | 4 | CAAT |  |
| CAAT-box | Pisum sativum | 999 | + | 5 | CAAAT | common cis-acting element in promoter and enhancer regions |

>HU02G01572.1   
+ +Up\_Stream \_Len000TTTAAT AAGATCCTTT TATATATATG TGTGTATAAT TCTATGGTAC ATAGGTATGT   
  
  
+ ACCATAGACT TATTGCATTT CTAGGTCCTT CTCTCTCTGC GCTTGTTTTT TGTTTATCTA ATTTATGGAG   
  
  
+ TTGAACATTT TGTTTTATAT ACTTGAACAA TCTATTATAC TAAATTAAGC ATTTTGATTT AATCACTTAT   
  
  
+ GGGTGTGTTT GGTAGGGTTG AAAATAATTT TCCTAGAAAA TAATTTTTAA TATAAAATAA TTTTTCATGT   
  
  
+ AAAATGATTT TCAACTCATA TTTTCAGTTG ATTGGTAAAA AGAAAAATGA ACTTTCTGGA TAATCAGTTT   
  
  
+ GATTAACAAA AAGTGACAAA AAAATTAAAA ATGGTTTTCC GTAATCTTCA AACCATATGT CGAGGAAATA   
  
  
+ AACACGGAAT AAAGAAAGAG TGTCAGAACC CAAGAAATGT AAAATCAACT TACGTTCTAT TTAAAGTGAA   
  
  
+ AAATATTTTT CTTCATTCTA GGAAAATTAA TTGTCCTCTG AAAACAAATT TTCCCTCCTT GTCTAACCAA   
  
  
+ ACACAGTAAA ATTGAAAAAT CATTTTCTAG GAAAATGATT TTCACTCCTA CCAAACACAC CCTTAAACTT   
  
  
+ GTTCCAAATC TATAAACCCG GAATCTTAAT ACGCACATTG TATATTATAT GTCCTACTAT AACTGTATTA   
  
  
+ TCACTGGTTC TTGATTTATC CTTTCAAACT CTTGCTTAGT CAAGGTTGTC CTCGTACAAA CATGATTAAT   
  
  
+ TCAATTAACC ACGTAAGTTA TACGCTGACA AATTTCGGGT ACCAAACATT TGCACTAGAC CTAAAACAGT   
  
  
+ TACTATCATC ATATCGATGT TAAAATTGAC CTGTTCTTAT TTGTCCAGCG TTGAGCAAGG AAATAATCAA   
  
  
+ ATCATGTCTC ATTTAGTCGG TTTTTTGCCT AAGAAGAGCA TAAACAAAGG AAAAAGACAA CGACACATTA   
  
  
+ TACATGCATC TACACAAATG AAATGAGGCC ATATGGAACA TGTTCATGAG AGCTTCACTC GCTAATAATT   
  
  
+ GAACCTACTT TTGACTAAGC CACCAACTAG AATATGGAGA TTGACTGTTA AGAGACTATA TTACATGAAA   
  
  
+ TAGATAAGAG TAGACATTGC TATATATTTT ATCTTTAATT GTCTATTTTT AATGATCATT ACTGTTTATT   
  
  
+ TCAGTGAGTA ATTTTCTCGG ATATAATATT TTATAGCGGT TATTAAATAG ATGTGAAACT ATAATGTTAG   
  
  
+ ACATATCATG CGAAAATAGA GAAATTTTGG GCAAATTAAA AAAAAAAGAA GAAAAACGAG GAAGTATGCC   
  
  
+ CATTGTAAGC AGGTGGATGT ATATCCAGCC TCCATCCAAC AATTTGGGCA AAATTATATC ATCCATTCCA   
  
  
+ AAAAAAAGAT ATAATTTGTG ATATTAATAA CCCGACTCAA TTCAAGCTTA TTTATTTTTT CATATAATTT   
  
  
+ TTTAATATAT AAAAATAAAA AGTGACAACG ACATGCATCC TTTGCGTGTC AAAGGATCCG CCGCGTGGCC   
  
  
+ CCCACATTCA CTGACGGGGT TTTTGGTTTG CGATTAAGAT CAATGGCTAA CGTTGTCTCT CTCAAAACAT   
  
  
+ ATGGACCCCT TTTTCTATCT TTTTCTGCAT TGTCAGCATA GCAATTGTTG AGCACATTTT TTTCGATTTG   
  
  
+ TTGACAATAA GCGTGGTTAT GATTTATGGG CCACTAATAT CAGTCTCTGA TCATTTGCAC ATTTTGGACC   
  
  
+ TCTTTCTCTC TCTAATCTTC CAGGTAACCT GAATTCTTCT CTATTTCTGA CTAGATTAGA TAACAGGATT   
  
  
+ TCAAATATCT GTCAGGTGGC GGGTATAACT TCGTGATGGG CATTCATTTT TGTTCTATCA GGCTGGTGGG   
  
  
+ TTTGAGTTTA TTTTGATTCA TTTCGTGTTC ATCTGTGATA TTAGTAGGAG GGATTCTGTA TACCCACCAA   
  
  
+ CTGCTCGCCC TTTTGCCCGT GTGAGCAATT GGGACTTTTG TTTGTTCTTC GTCGATGCAT CCTCTGGTTG   
  
  
+ CTGATCTGGA ATTGAAACCC CATGTATTCA AATTCAACCC TGATTTGCTG TCAAACTTTC TGAACCATCA   
  
  
+ AAACTCCGCC GAAGTGTTTG AAAAAGATGG CATCTTCCAG ACCCTTCATT TGGCTGATCC CAAAAGCTCT   
  
  
+ TCAATTGCCG AAATCGGGTT TACTAATAGT TCAGATTCTA CACAAGTACC TGATTTTTCA GATGCTTGTC   
  
  
+ TTAAGTTCAT TAGTGATATT CTCCTGGAAG AGGACTTAGA TGAAAGTCCT ACGTCTTTAC ACGATTACAT   
  
  
+ GGCTCTCCTA GCCACTGAGA AGTCTTTGTA TGATGCTCTT GGAAAGGAAT ACTTTCCTTC GTCTACTAGT   
  
  
+ CTTGCCCCAT CTTTAGGCCG AAGTGTTGAC AGCCCAGATA GTGGCTTTGG CCGCGGTTGC TCTGATGGTC   
  
  
+ GCGGGATTGA GGGTTTGGCT AATGATGATG CCGTTTTCAT GTCTAACTGG CAGCTCAACA CCACCCAATT   
  
  
+ GGACCCTTTC CCAATCATGC AAGATATTCC TCGTCCCTAT TTGGAATTGA ATTACCATTC TTCTGGGTCA   
  
  
+ AGCAATGGCA TTGATGATTC GGGGGATGGG TTATCGACCT CTCCTGTAAG TACACTTGCA TCAACCGCCA   
  
  
+ CAGAGGCAGG GAAAAAGTTG GCTGGTAGCT CTAGGAGAAA GAACCGTCAA AGGGATGACT ATGGCCATGA   
  
  
+ AGAGGGAAGG AGTAACAAGC AGCAAGCCTC TTACAATGAC GATTACGTTG AGATGGAGCA GTATGACGAT   
  
  
+ GTACTTCTCT GTAGGGCAGA CAAGGGTAAT ATTTCAACTT GTGCCAATGA ATCCTCGCTT AATGAGGTGC   
  
  
+ GTGAGAAGCT GCAGACGACA GGGTTCAAGG GAAGAACATC CCGTCTTAAG AAGCAATCTA AGGAAGCGAA   
  
  
+ AGAGGTGGAT CTGAGAACTC TTCTTTCTGG TTGTGCACAA GCTGTTTCGA ACTTTGATAT CAGGACTGCT   
  
  
+ AATGAGCTGC TTAAGCAAGT CAGACAGCAT TCTTCACCAT ATGGTGATAG CCTCCAAAGG CTCGCCCATC   
  
  
+ AATTTGCGAA TGGTATTGAG GCACGCTTAG CAGGCACCGG TTCAAGAGTA CCTGCTAATC TCATTGATGC   
  
  
+ ACGGATATCA TCATCTGAAT TTTTAAAAGC TTACAAGTCA TATGTTTCAG CAGTTCCTTT CAGAAGGATG   
  
  
+ TCCTATTTTA TAGCAAACAA CACAATTCTG AAGTTGGCTG AGAAAGCAAC AAAGATTCAC ATAATTGATT   
  
  
+ TTGGTATTCT ATTTGGTCTA CAGTGGCCCT GTCTTATACA AAGTCTCTCA AGGCGAACTG TGGCTCCTCC   
  
  
+ GAAGCTTCGC ATCACTGGGA TAGACTATCC CCAGCATGGT TTCCGGCCAG CAGAAAAGGT TGAGGCAACA   
  
  
+ GGTCGTCGGT TGTCCGGGTA CTGTGAGAGA TTTAATGTAC CCTTTCGATA TGAAACCATT GCAAAGAAGT   
  
  
+ GGGAAACCAT ACGCCCAGAA GATCTAAATA TTGAGAATGA TGAGCTGGTA ATTGTTAATT GTATGTTGCG   
  
  
+ GTCTGTAAAT CTATTGGATG ATACAGTGGC GGTAAATAGT CCAAGGGATG CTTTCTTGAG GTTAATCAAA   
  
  
+ CAGATAAACC CGCGTTTATT CATTCATGCA ATTGTCAATG GAACCTTTAG TACTCCATTC TTCAGCACTC   
  
  
+ GATTCAGGGA AGCCCTATTC CAATACTCTT CTGTATTTGA TATATTTGAA GCGACTATGA CTCGTGAAGA   
  
  
+ TCGTGGAAGG CTGCTGATTG AGAGTCAAAT ATGCGGGCTA GAAGTTTTGA ATGCAATAGC ATGTGAAGGT   
  
  
+ GCAGAGAGGA TTCAAAGGCC TGAAACATAC AAGCAATGGC AGGAGCGGAC AACAAGGGCT GGACTAAGGC   
  
  
+ AGGTTCCAAT AGATGAGGAG CTTGTCAATA GAGCAAAGAC TATAGTGAAA GCAAATTATC ACAAGGATTT   
  
  
+ TGTGGTGGAT GAGGATAGGC GTTGGATGCT TCAAGGTTGG AAAGGAAGGA CACTTAGTGC CCTTTCCGTT   
  
  
+ TGGCAGCCTA ACTA  

- +Up\_Stream \_Len000AAATTA TTCTAGGAAA ATATATATAC ACACATATTA AGATACCATG TATCCATACA   
  
  
- TGGTATCTGA ATAACGTAAA GATCCAGGAA GAGAGAGACG CGAACAAAAA ACAAATAGAT TAAATACCTC   
  
  
- AACTTGTAAA ACAAAATATA TGAACTTGTT AGATAATATG ATTTAATTCG TAAAACTAAA TTAGTGAATA   
  
  
- CCCACACAAA CCATCCCAAC TTTTATTAAA AGGATCTTTT ATTAAAAATT ATATTTTATT AAAAAGTACA   
  
  
- TTTTACTAAA AGTTGAGTAT AAAAGTCAAC TAACCATTTT TCTTTTTACT TGAAAGACCT ATTAGTCAAA   
  
  
- CTAATTGTTT TTCACTGTTT TTTTAATTTT TACCAAAAGG CATTAGAAGT TTGGTATACA GCTCCTTTAT   
  
  
- TTGTGCCTTA TTTCTTTCTC ACAGTCTTGG GTTCTTTACA TTTTAGTTGA ATGCAAGATA AATTTCACTT   
  
  
- TTTATAAAAA GAAGTAAGAT CCTTTTAATT AACAGGAGAC TTTTGTTTAA AAGGGAGGAA CAGATTGGTT   
  
  
- TGTGTCATTT TAACTTTTTA GTAAAAGATC CTTTTACTAA AAGTGAGGAT GGTTTGTGTG GGAATTTGAA   
  
  
- CAAGGTTTAG ATATTTGGGC CTTAGAATTA TGCGTGTAAC ATATAATATA CAGGATGATA TTGACATAAT   
  
  
- AGTGACCAAG AACTAAATAG GAAAGTTTGA GAACGAATCA GTTCCAACAG GAGCATGTTT GTACTAATTA   
  
  
- AGTTAATTGG TGCATTCAAT ATGCGACTGT TTAAAGCCCA TGGTTTGTAA ACGTGATCTG GATTTTGTCA   
  
  
- ATGATAGTAG TATAGCTACA ATTTTAACTG GACAAGAATA AACAGGTCGC AACTCGTTCC TTTATTAGTT   
  
  
- TAGTACAGAG TAAATCAGCC AAAAAACGGA TTCTTCTCGT ATTTGTTTCC TTTTTCTGTT GCTGTGTAAT   
  
  
- ATGTACGTAG ATGTGTTTAC TTTACTCCGG TATACCTTGT ACAAGTACTC TCGAAGTGAG CGATTATTAA   
  
  
- CTTGGATGAA AACTGATTCG GTGGTTGATC TTATACCTCT AACTGACAAT TCTCTGATAT AATGTACTTT   
  
  
- ATCTATTCTC ATCTGTAACG ATATATAAAA TAGAAATTAA CAGATAAAAA TTACTAGTAA TGACAAATAA   
  
  
- AGTCACTCAT TAAAAGAGCC TATATTATAA AATATCGCCA ATAATTTATC TACACTTTGA TATTACAATC   
  
  
- TGTATAGTAC GCTTTTATCT CTTTAAAACC CGTTTAATTT TTTTTTTCTT CTTTTTGCTC CTTCATACGG   
  
  
- GTAACATTCG TCCACCTACA TATAGGTCGG AGGTAGGTTG TTAAACCCGT TTTAATATAG TAGGTAAGGT   
  
  
- TTTTTTTCTA TATTAAACAC TATAATTATT GGGCTGAGTT AAGTTCGAAT AAATAAAAAA GTATATTAAA   
  
  
- AAATTATATA TTTTTATTTT TCACTGTTGC TGTACGTAGG AAACGCACAG TTTCCTAGGC GGCGCACCGG   
  
  
- GGGTGTAAGT GACTGCCCCA AAAACCAAAC GCTAATTCTA GTTACCGATT GCAACAGAGA GAGTTTTGTA   
  
  
- TACCTGGGGA AAAAGATAGA AAAAGACGTA ACAGTCGTAT CGTTAACAAC TCGTGTAAAA AAAGCTAAAC   
  
  
- AACTGTTATT CGCACCAATA CTAAATACCC GGTGATTATA GTCAGAGACT AGTAAACGTG TAAAACCTGG   
  
  
- AGAAAGAGAG AGATTAGAAG GTCCATTGGA CTTAAGAAGA GATAAAGACT GATCTAATCT ATTGTCCTAA   
  
  
- AGTTTATAGA CAGTCCACCG CCCATATTGA AGCACTACCC GTAAGTAAAA ACAAGATAGT CCGACCACCC   
  
  
- AAACTCAAAT AAAACTAAGT AAAGCACAAG TAGACACTAT AATCATCCTC CCTAAGACAT ATGGGTGGTT   
  
  
- GACGAGCGGG AAAACGGGCA CACTCGTTAA CCCTGAAAAC AAACAAGAAG CAGCTACGTA GGAGACCAAC   
  
  
- GACTAGACCT TAACTTTGGG GTACATAAGT TTAAGTTGGG ACTAAACGAC AGTTTGAAAG ACTTGGTAGT   
  
  
- TTTGAGGCGG CTTCACAAAC TTTTTCTACC GTAGAAGGTC TGGGAAGTAA ACCGACTAGG GTTTTCGAGA   
  
  
- AGTTAACGGC TTTAGCCCAA ATGATTATCA AGTCTAAGAT GTGTTCATGG ACTAAAAAGT CTACGAACAG   
  
  
- AATTCAAGTA ATCACTATAA GAGGACCTTC TCCTGAATCT ACTTTCAGGA TGCAGAAATG TGCTAATGTA   
  
  
- CCGAGAGGAT CGGTGACTCT TCAGAAACAT ACTACGAGAA CCTTTCCTTA TGAAAGGAAG CAGATGATCA   
  
  
- GAACGGGGTA GAAATCCGGC TTCACAACTG TCGGGTCTAT CACCGAAACC GGCGCCAACG AGACTACCAG   
  
  
- CGCCCTAACT CCCAAACCGA TTACTACTAC GGCAAAAGTA CAGATTGACC GTCGAGTTGT GGTGGGTTAA   
  
  
- CCTGGGAAAG GGTTAGTACG TTCTATAAGG AGCAGGGATA AACCTTAACT TAATGGTAAG AAGACCCAGT   
  
  
- TCGTTACCGT AACTACTAAG CCCCCTACCC AATAGCTGGA GAGGACATTC ATGTGAACGT AGTTGGCGGT   
  
  
- GTCTCCGTCC CTTTTTCAAC CGACCATCGA GATCCTCTTT CTTGGCAGTT TCCCTACTGA TACCGGTACT   
  
  
- TCTCCCTTCC TCATTGTTCG TCGTTCGGAG AATGTTACTG CTAATGCAAC TCTACCTCGT CATACTGCTA   
  
  
- CATGAAGAGA CATCCCGTCT GTTCCCATTA TAAAGTTGAA CACGGTTACT TAGGAGCGAA TTACTCCACG   
  
  
- CACTCTTCGA CGTCTGCTGT CCCAAGTTCC CTTCTTGTAG GGCAGAATTC TTCGTTAGAT TCCTTCGCTT   
  
  
- TCTCCACCTA GACTCTTGAG AAGAAAGACC AACACGTGTT CGACAAAGCT TGAAACTATA GTCCTGACGA   
  
  
- TTACTCGACG AATTCGTTCA GTCTGTCGTA AGAAGTGGTA TACCACTATC GGAGGTTTCC GAGCGGGTAG   
  
  
- TTAAACGCTT ACCATAACTC CGTGCGAATC GTCCGTGGCC AAGTTCTCAT GGACGATTAG AGTAACTACG   
  
  
- TGCCTATAGT AGTAGACTTA AAAATTTTCG AATGTTCAGT ATACAAAGTC GTCAAGGAAA GTCTTCCTAC   
  
  
- AGGATAAAAT ATCGTTTGTT GTGTTAAGAC TTCAACCGAC TCTTTCGTTG TTTCTAAGTG TATTAACTAA   
  
  
- AACCATAAGA TAAACCAGAT GTCACCGGGA CAGAATATGT TTCAGAGAGT TCCGCTTGAC ACCGAGGAGG   
  
  
- CTTCGAAGCG TAGTGACCCT ATCTGATAGG GGTCGTACCA AAGGCCGGTC GTCTTTTCCA ACTCCGTTGT   
  
  
- CCAGCAGCCA ACAGGCCCAT GACACTCTCT AAATTACATG GGAAAGCTAT ACTTTGGTAA CGTTTCTTCA   
  
  
- CCCTTTGGTA TGCGGGTCTT CTAGATTTAT AACTCTTACT ACTCGACCAT TAACAATTAA CATACAACGC   
  
  
- CAGACATTTA GATAACCTAC TATGTCACCG CCATTTATCA GGTTCCCTAC GAAAGAACTC CAATTAGTTT   
  
  
- GTCTATTTGG GCGCAAATAA GTAAGTACGT TAACAGTTAC CTTGGAAATC ATGAGGTAAG AAGTCGTGAG   
  
  
- CTAAGTCCCT TCGGGATAAG GTTATGAGAA GACATAAACT ATATAAACTT CGCTGATACT GAGCACTTCT   
  
  
- AGCACCTTCC GACGACTAAC TCTCAGTTTA TACGCCCGAT CTTCAAAACT TACGTTATCG TACACTTCCA   
  
  
- CGTCTCTCCT AAGTTTCCGG ACTTTGTATG TTCGTTACCG TCCTCGCCTG TTGTTCCCGA CCTGATTCCG   
  
  
- TCCAAGGTTA TCTACTCCTC GAACAGTTAT CTCGTTTCTG ATATCACTTT CGTTTAATAG TGTTCCTAAA   
  
  
- ACACCACCTA CTCCTATCCG CAACCTACGA AGTTCCAACC TTTCCTTCCT GTGAATCACG GGAAAGGCAA   
  
  
- ACCGTCGGAT TGAT

+     CAT-box

| Site Name | Organism | Position | Strand | Matrix score. | sequence | function |
| --- | --- | --- | --- | --- | --- | --- |
| CAT-box | Arabidopsis thaliana | 3599 | - | 6 | GCCACT | cis-acting regulatory element related to meristem expression |
| CAT-box | Arabidopsis thaliana | 2424 | - | 6 | GCCACT | cis-acting regulatory element related to meristem expression |
| CAT-box | Arabidopsis thaliana | 1714 | + | 6 | GCCACT | cis-acting regulatory element related to meristem expression |
| CAT-box | Arabidopsis thaliana | 2325 | + | 6 | GCCACT | cis-acting regulatory element related to meristem expression |
| CAT-box | Arabidopsis thaliana | 3316 | - | 6 | GCCACT | cis-acting regulatory element related to meristem expression |

>HU02G01572.1   
+ +Up\_Stream \_Len000TTTAAT AAGATCCTTT TATATATATG TGTGTATAAT TCTATGGTAC ATAGGTATGT   
  
  
+ ACCATAGACT TATTGCATTT CTAGGTCCTT CTCTCTCTGC GCTTGTTTTT TGTTTATCTA ATTTATGGAG   
  
  
+ TTGAACATTT TGTTTTATAT ACTTGAACAA TCTATTATAC TAAATTAAGC ATTTTGATTT AATCACTTAT   
  
  
+ GGGTGTGTTT GGTAGGGTTG AAAATAATTT TCCTAGAAAA TAATTTTTAA TATAAAATAA TTTTTCATGT   
  
  
+ AAAATGATTT TCAACTCATA TTTTCAGTTG ATTGGTAAAA AGAAAAATGA ACTTTCTGGA TAATCAGTTT   
  
  
+ GATTAACAAA AAGTGACAAA AAAATTAAAA ATGGTTTTCC GTAATCTTCA AACCATATGT CGAGGAAATA   
  
  
+ AACACGGAAT AAAGAAAGAG TGTCAGAACC CAAGAAATGT AAAATCAACT TACGTTCTAT TTAAAGTGAA   
  
  
+ AAATATTTTT CTTCATTCTA GGAAAATTAA TTGTCCTCTG AAAACAAATT TTCCCTCCTT GTCTAACCAA   
  
  
+ ACACAGTAAA ATTGAAAAAT CATTTTCTAG GAAAATGATT TTCACTCCTA CCAAACACAC CCTTAAACTT   
  
  
+ GTTCCAAATC TATAAACCCG GAATCTTAAT ACGCACATTG TATATTATAT GTCCTACTAT AACTGTATTA   
  
  
+ TCACTGGTTC TTGATTTATC CTTTCAAACT CTTGCTTAGT CAAGGTTGTC CTCGTACAAA CATGATTAAT   
  
  
+ TCAATTAACC ACGTAAGTTA TACGCTGACA AATTTCGGGT ACCAAACATT TGCACTAGAC CTAAAACAGT   
  
  
+ TACTATCATC ATATCGATGT TAAAATTGAC CTGTTCTTAT TTGTCCAGCG TTGAGCAAGG AAATAATCAA   
  
  
+ ATCATGTCTC ATTTAGTCGG TTTTTTGCCT AAGAAGAGCA TAAACAAAGG AAAAAGACAA CGACACATTA   
  
  
+ TACATGCATC TACACAAATG AAATGAGGCC ATATGGAACA TGTTCATGAG AGCTTCACTC GCTAATAATT   
  
  
+ GAACCTACTT TTGACTAAGC CACCAACTAG AATATGGAGA TTGACTGTTA AGAGACTATA TTACATGAAA   
  
  
+ TAGATAAGAG TAGACATTGC TATATATTTT ATCTTTAATT GTCTATTTTT AATGATCATT ACTGTTTATT   
  
  
+ TCAGTGAGTA ATTTTCTCGG ATATAATATT TTATAGCGGT TATTAAATAG ATGTGAAACT ATAATGTTAG   
  
  
+ ACATATCATG CGAAAATAGA GAAATTTTGG GCAAATTAAA AAAAAAAGAA GAAAAACGAG GAAGTATGCC   
  
  
+ CATTGTAAGC AGGTGGATGT ATATCCAGCC TCCATCCAAC AATTTGGGCA AAATTATATC ATCCATTCCA   
  
  
+ AAAAAAAGAT ATAATTTGTG ATATTAATAA CCCGACTCAA TTCAAGCTTA TTTATTTTTT CATATAATTT   
  
  
+ TTTAATATAT AAAAATAAAA AGTGACAACG ACATGCATCC TTTGCGTGTC AAAGGATCCG CCGCGTGGCC   
  
  
+ CCCACATTCA CTGACGGGGT TTTTGGTTTG CGATTAAGAT CAATGGCTAA CGTTGTCTCT CTCAAAACAT   
  
  
+ ATGGACCCCT TTTTCTATCT TTTTCTGCAT TGTCAGCATA GCAATTGTTG AGCACATTTT TTTCGATTTG   
  
  
+ TTGACAATAA GCGTGGTTAT GATTTATGGG CCACTAATAT CAGTCTCTGA TCATTTGCAC ATTTTGGACC   
  
  
+ TCTTTCTCTC TCTAATCTTC CAGGTAACCT GAATTCTTCT CTATTTCTGA CTAGATTAGA TAACAGGATT   
  
  
+ TCAAATATCT GTCAGGTGGC GGGTATAACT TCGTGATGGG CATTCATTTT TGTTCTATCA GGCTGGTGGG   
  
  
+ TTTGAGTTTA TTTTGATTCA TTTCGTGTTC ATCTGTGATA TTAGTAGGAG GGATTCTGTA TACCCACCAA   
  
  
+ CTGCTCGCCC TTTTGCCCGT GTGAGCAATT GGGACTTTTG TTTGTTCTTC GTCGATGCAT CCTCTGGTTG   
  
  
+ CTGATCTGGA ATTGAAACCC CATGTATTCA AATTCAACCC TGATTTGCTG TCAAACTTTC TGAACCATCA   
  
  
+ AAACTCCGCC GAAGTGTTTG AAAAAGATGG CATCTTCCAG ACCCTTCATT TGGCTGATCC CAAAAGCTCT   
  
  
+ TCAATTGCCG AAATCGGGTT TACTAATAGT TCAGATTCTA CACAAGTACC TGATTTTTCA GATGCTTGTC   
  
  
+ TTAAGTTCAT TAGTGATATT CTCCTGGAAG AGGACTTAGA TGAAAGTCCT ACGTCTTTAC ACGATTACAT   
  
  
+ GGCTCTCCTA GCCACTGAGA AGTCTTTGTA TGATGCTCTT GGAAAGGAAT ACTTTCCTTC GTCTACTAGT   
  
  
+ CTTGCCCCAT CTTTAGGCCG AAGTGTTGAC AGCCCAGATA GTGGCTTTGG CCGCGGTTGC TCTGATGGTC   
  
  
+ GCGGGATTGA GGGTTTGGCT AATGATGATG CCGTTTTCAT GTCTAACTGG CAGCTCAACA CCACCCAATT   
  
  
+ GGACCCTTTC CCAATCATGC AAGATATTCC TCGTCCCTAT TTGGAATTGA ATTACCATTC TTCTGGGTCA   
  
  
+ AGCAATGGCA TTGATGATTC GGGGGATGGG TTATCGACCT CTCCTGTAAG TACACTTGCA TCAACCGCCA   
  
  
+ CAGAGGCAGG GAAAAAGTTG GCTGGTAGCT CTAGGAGAAA GAACCGTCAA AGGGATGACT ATGGCCATGA   
  
  
+ AGAGGGAAGG AGTAACAAGC AGCAAGCCTC TTACAATGAC GATTACGTTG AGATGGAGCA GTATGACGAT   
  
  
+ GTACTTCTCT GTAGGGCAGA CAAGGGTAAT ATTTCAACTT GTGCCAATGA ATCCTCGCTT AATGAGGTGC   
  
  
+ GTGAGAAGCT GCAGACGACA GGGTTCAAGG GAAGAACATC CCGTCTTAAG AAGCAATCTA AGGAAGCGAA   
  
  
+ AGAGGTGGAT CTGAGAACTC TTCTTTCTGG TTGTGCACAA GCTGTTTCGA ACTTTGATAT CAGGACTGCT   
  
  
+ AATGAGCTGC TTAAGCAAGT CAGACAGCAT TCTTCACCAT ATGGTGATAG CCTCCAAAGG CTCGCCCATC   
  
  
+ AATTTGCGAA TGGTATTGAG GCACGCTTAG CAGGCACCGG TTCAAGAGTA CCTGCTAATC TCATTGATGC   
  
  
+ ACGGATATCA TCATCTGAAT TTTTAAAAGC TTACAAGTCA TATGTTTCAG CAGTTCCTTT CAGAAGGATG   
  
  
+ TCCTATTTTA TAGCAAACAA CACAATTCTG AAGTTGGCTG AGAAAGCAAC AAAGATTCAC ATAATTGATT   
  
  
+ TTGGTATTCT ATTTGGTCTA CAGTGGCCCT GTCTTATACA AAGTCTCTCA AGGCGAACTG TGGCTCCTCC   
  
  
+ GAAGCTTCGC ATCACTGGGA TAGACTATCC CCAGCATGGT TTCCGGCCAG CAGAAAAGGT TGAGGCAACA   
  
  
+ GGTCGTCGGT TGTCCGGGTA CTGTGAGAGA TTTAATGTAC CCTTTCGATA TGAAACCATT GCAAAGAAGT   
  
  
+ GGGAAACCAT ACGCCCAGAA GATCTAAATA TTGAGAATGA TGAGCTGGTA ATTGTTAATT GTATGTTGCG   
  
  
+ GTCTGTAAAT CTATTGGATG ATACAGTGGC GGTAAATAGT CCAAGGGATG CTTTCTTGAG GTTAATCAAA   
  
  
+ CAGATAAACC CGCGTTTATT CATTCATGCA ATTGTCAATG GAACCTTTAG TACTCCATTC TTCAGCACTC   
  
  
+ GATTCAGGGA AGCCCTATTC CAATACTCTT CTGTATTTGA TATATTTGAA GCGACTATGA CTCGTGAAGA   
  
  
+ TCGTGGAAGG CTGCTGATTG AGAGTCAAAT ATGCGGGCTA GAAGTTTTGA ATGCAATAGC ATGTGAAGGT   
  
  
+ GCAGAGAGGA TTCAAAGGCC TGAAACATAC AAGCAATGGC AGGAGCGGAC AACAAGGGCT GGACTAAGGC   
  
  
+ AGGTTCCAAT AGATGAGGAG CTTGTCAATA GAGCAAAGAC TATAGTGAAA GCAAATTATC ACAAGGATTT   
  
  
+ TGTGGTGGAT GAGGATAGGC GTTGGATGCT TCAAGGTTGG AAAGGAAGGA CACTTAGTGC CCTTTCCGTT   
  
  
+ TGGCAGCCTA ACTA  

- +Up\_Stream \_Len000AAATTA TTCTAGGAAA ATATATATAC ACACATATTA AGATACCATG TATCCATACA   
  
  
- TGGTATCTGA ATAACGTAAA GATCCAGGAA GAGAGAGACG CGAACAAAAA ACAAATAGAT TAAATACCTC   
  
  
- AACTTGTAAA ACAAAATATA TGAACTTGTT AGATAATATG ATTTAATTCG TAAAACTAAA TTAGTGAATA   
  
  
- CCCACACAAA CCATCCCAAC TTTTATTAAA AGGATCTTTT ATTAAAAATT ATATTTTATT AAAAAGTACA   
  
  
- TTTTACTAAA AGTTGAGTAT AAAAGTCAAC TAACCATTTT TCTTTTTACT TGAAAGACCT ATTAGTCAAA   
  
  
- CTAATTGTTT TTCACTGTTT TTTTAATTTT TACCAAAAGG CATTAGAAGT TTGGTATACA GCTCCTTTAT   
  
  
- TTGTGCCTTA TTTCTTTCTC ACAGTCTTGG GTTCTTTACA TTTTAGTTGA ATGCAAGATA AATTTCACTT   
  
  
- TTTATAAAAA GAAGTAAGAT CCTTTTAATT AACAGGAGAC TTTTGTTTAA AAGGGAGGAA CAGATTGGTT   
  
  
- TGTGTCATTT TAACTTTTTA GTAAAAGATC CTTTTACTAA AAGTGAGGAT GGTTTGTGTG GGAATTTGAA   
  
  
- CAAGGTTTAG ATATTTGGGC CTTAGAATTA TGCGTGTAAC ATATAATATA CAGGATGATA TTGACATAAT   
  
  
- AGTGACCAAG AACTAAATAG GAAAGTTTGA GAACGAATCA GTTCCAACAG GAGCATGTTT GTACTAATTA   
  
  
- AGTTAATTGG TGCATTCAAT ATGCGACTGT TTAAAGCCCA TGGTTTGTAA ACGTGATCTG GATTTTGTCA   
  
  
- ATGATAGTAG TATAGCTACA ATTTTAACTG GACAAGAATA AACAGGTCGC AACTCGTTCC TTTATTAGTT   
  
  
- TAGTACAGAG TAAATCAGCC AAAAAACGGA TTCTTCTCGT ATTTGTTTCC TTTTTCTGTT GCTGTGTAAT   
  
  
- ATGTACGTAG ATGTGTTTAC TTTACTCCGG TATACCTTGT ACAAGTACTC TCGAAGTGAG CGATTATTAA   
  
  
- CTTGGATGAA AACTGATTCG GTGGTTGATC TTATACCTCT AACTGACAAT TCTCTGATAT AATGTACTTT   
  
  
- ATCTATTCTC ATCTGTAACG ATATATAAAA TAGAAATTAA CAGATAAAAA TTACTAGTAA TGACAAATAA   
  
  
- AGTCACTCAT TAAAAGAGCC TATATTATAA AATATCGCCA ATAATTTATC TACACTTTGA TATTACAATC   
  
  
- TGTATAGTAC GCTTTTATCT CTTTAAAACC CGTTTAATTT TTTTTTTCTT CTTTTTGCTC CTTCATACGG   
  
  
- GTAACATTCG TCCACCTACA TATAGGTCGG AGGTAGGTTG TTAAACCCGT TTTAATATAG TAGGTAAGGT   
  
  
- TTTTTTTCTA TATTAAACAC TATAATTATT GGGCTGAGTT AAGTTCGAAT AAATAAAAAA GTATATTAAA   
  
  
- AAATTATATA TTTTTATTTT TCACTGTTGC TGTACGTAGG AAACGCACAG TTTCCTAGGC GGCGCACCGG   
  
  
- GGGTGTAAGT GACTGCCCCA AAAACCAAAC GCTAATTCTA GTTACCGATT GCAACAGAGA GAGTTTTGTA   
  
  
- TACCTGGGGA AAAAGATAGA AAAAGACGTA ACAGTCGTAT CGTTAACAAC TCGTGTAAAA AAAGCTAAAC   
  
  
- AACTGTTATT CGCACCAATA CTAAATACCC GGTGATTATA GTCAGAGACT AGTAAACGTG TAAAACCTGG   
  
  
- AGAAAGAGAG AGATTAGAAG GTCCATTGGA CTTAAGAAGA GATAAAGACT GATCTAATCT ATTGTCCTAA   
  
  
- AGTTTATAGA CAGTCCACCG CCCATATTGA AGCACTACCC GTAAGTAAAA ACAAGATAGT CCGACCACCC   
  
  
- AAACTCAAAT AAAACTAAGT AAAGCACAAG TAGACACTAT AATCATCCTC CCTAAGACAT ATGGGTGGTT   
  
  
- GACGAGCGGG AAAACGGGCA CACTCGTTAA CCCTGAAAAC AAACAAGAAG CAGCTACGTA GGAGACCAAC   
  
  
- GACTAGACCT TAACTTTGGG GTACATAAGT TTAAGTTGGG ACTAAACGAC AGTTTGAAAG ACTTGGTAGT   
  
  
- TTTGAGGCGG CTTCACAAAC TTTTTCTACC GTAGAAGGTC TGGGAAGTAA ACCGACTAGG GTTTTCGAGA   
  
  
- AGTTAACGGC TTTAGCCCAA ATGATTATCA AGTCTAAGAT GTGTTCATGG ACTAAAAAGT CTACGAACAG   
  
  
- AATTCAAGTA ATCACTATAA GAGGACCTTC TCCTGAATCT ACTTTCAGGA TGCAGAAATG TGCTAATGTA   
  
  
- CCGAGAGGAT CGGTGACTCT TCAGAAACAT ACTACGAGAA CCTTTCCTTA TGAAAGGAAG CAGATGATCA   
  
  
- GAACGGGGTA GAAATCCGGC TTCACAACTG TCGGGTCTAT CACCGAAACC GGCGCCAACG AGACTACCAG   
  
  
- CGCCCTAACT CCCAAACCGA TTACTACTAC GGCAAAAGTA CAGATTGACC GTCGAGTTGT GGTGGGTTAA   
  
  
- CCTGGGAAAG GGTTAGTACG TTCTATAAGG AGCAGGGATA AACCTTAACT TAATGGTAAG AAGACCCAGT   
  
  
- TCGTTACCGT AACTACTAAG CCCCCTACCC AATAGCTGGA GAGGACATTC ATGTGAACGT AGTTGGCGGT   
  
  
- GTCTCCGTCC CTTTTTCAAC CGACCATCGA GATCCTCTTT CTTGGCAGTT TCCCTACTGA TACCGGTACT   
  
  
- TCTCCCTTCC TCATTGTTCG TCGTTCGGAG AATGTTACTG CTAATGCAAC TCTACCTCGT CATACTGCTA   
  
  
- CATGAAGAGA CATCCCGTCT GTTCCCATTA TAAAGTTGAA CACGGTTACT TAGGAGCGAA TTACTCCACG   
  
  
- CACTCTTCGA CGTCTGCTGT CCCAAGTTCC CTTCTTGTAG GGCAGAATTC TTCGTTAGAT TCCTTCGCTT   
  
  
- TCTCCACCTA GACTCTTGAG AAGAAAGACC AACACGTGTT CGACAAAGCT TGAAACTATA GTCCTGACGA   
  
  
- TTACTCGACG AATTCGTTCA GTCTGTCGTA AGAAGTGGTA TACCACTATC GGAGGTTTCC GAGCGGGTAG   
  
  
- TTAAACGCTT ACCATAACTC CGTGCGAATC GTCCGTGGCC AAGTTCTCAT GGACGATTAG AGTAACTACG   
  
  
- TGCCTATAGT AGTAGACTTA AAAATTTTCG AATGTTCAGT ATACAAAGTC GTCAAGGAAA GTCTTCCTAC   
  
  
- AGGATAAAAT ATCGTTTGTT GTGTTAAGAC TTCAACCGAC TCTTTCGTTG TTTCTAAGTG TATTAACTAA   
  
  
- AACCATAAGA TAAACCAGAT GTCACCGGGA CAGAATATGT TTCAGAGAGT TCCGCTTGAC ACCGAGGAGG   
  
  
- CTTCGAAGCG TAGTGACCCT ATCTGATAGG GGTCGTACCA AAGGCCGGTC GTCTTTTCCA ACTCCGTTGT   
  
  
- CCAGCAGCCA ACAGGCCCAT GACACTCTCT AAATTACATG GGAAAGCTAT ACTTTGGTAA CGTTTCTTCA   
  
  
- CCCTTTGGTA TGCGGGTCTT CTAGATTTAT AACTCTTACT ACTCGACCAT TAACAATTAA CATACAACGC   
  
  
- CAGACATTTA GATAACCTAC TATGTCACCG CCATTTATCA GGTTCCCTAC GAAAGAACTC CAATTAGTTT   
  
  
- GTCTATTTGG GCGCAAATAA GTAAGTACGT TAACAGTTAC CTTGGAAATC ATGAGGTAAG AAGTCGTGAG   
  
  
- CTAAGTCCCT TCGGGATAAG GTTATGAGAA GACATAAACT ATATAAACTT CGCTGATACT GAGCACTTCT   
  
  
- AGCACCTTCC GACGACTAAC TCTCAGTTTA TACGCCCGAT CTTCAAAACT TACGTTATCG TACACTTCCA   
  
  
- CGTCTCTCCT AAGTTTCCGG ACTTTGTATG TTCGTTACCG TCCTCGCCTG TTGTTCCCGA CCTGATTCCG   
  
  
- TCCAAGGTTA TCTACTCCTC GAACAGTTAT CTCGTTTCTG ATATCACTTT CGTTTAATAG TGTTCCTAAA   
  
  
- ACACCACCTA CTCCTATCCG CAACCTACGA AGTTCCAACC TTTCCTTCCT GTGAATCACG GGAAAGGCAA   
  
  
- ACCGTCGGAT TGAT

+     CGTCA-motif

| Site Name | Organism | Position | Strand | Matrix score. | sequence | function |
| --- | --- | --- | --- | --- | --- | --- |
| CGTCA-motif | Hordeum vulgare | 2771 | - | 5 | CGTCA | cis-acting regulatory element involved in the MeJA-responsiveness |
| CGTCA-motif | Hordeum vulgare | 2798 | - | 5 | CGTCA | cis-acting regulatory element involved in the MeJA-responsiveness |
| CGTCA-motif | Hordeum vulgare | 1556 | - | 5 | CGTCA | cis-acting regulatory element involved in the MeJA-responsiveness |
| CGTCA-motif | Hordeum vulgare | 2709 | + | 5 | CGTCA | cis-acting regulatory element involved in the MeJA-responsiveness |

>HU02G01572.1   
+ +Up\_Stream \_Len000TTTAAT AAGATCCTTT TATATATATG TGTGTATAAT TCTATGGTAC ATAGGTATGT   
  
  
+ ACCATAGACT TATTGCATTT CTAGGTCCTT CTCTCTCTGC GCTTGTTTTT TGTTTATCTA ATTTATGGAG   
  
  
+ TTGAACATTT TGTTTTATAT ACTTGAACAA TCTATTATAC TAAATTAAGC ATTTTGATTT AATCACTTAT   
  
  
+ GGGTGTGTTT GGTAGGGTTG AAAATAATTT TCCTAGAAAA TAATTTTTAA TATAAAATAA TTTTTCATGT   
  
  
+ AAAATGATTT TCAACTCATA TTTTCAGTTG ATTGGTAAAA AGAAAAATGA ACTTTCTGGA TAATCAGTTT   
  
  
+ GATTAACAAA AAGTGACAAA AAAATTAAAA ATGGTTTTCC GTAATCTTCA AACCATATGT CGAGGAAATA   
  
  
+ AACACGGAAT AAAGAAAGAG TGTCAGAACC CAAGAAATGT AAAATCAACT TACGTTCTAT TTAAAGTGAA   
  
  
+ AAATATTTTT CTTCATTCTA GGAAAATTAA TTGTCCTCTG AAAACAAATT TTCCCTCCTT GTCTAACCAA   
  
  
+ ACACAGTAAA ATTGAAAAAT CATTTTCTAG GAAAATGATT TTCACTCCTA CCAAACACAC CCTTAAACTT   
  
  
+ GTTCCAAATC TATAAACCCG GAATCTTAAT ACGCACATTG TATATTATAT GTCCTACTAT AACTGTATTA   
  
  
+ TCACTGGTTC TTGATTTATC CTTTCAAACT CTTGCTTAGT CAAGGTTGTC CTCGTACAAA CATGATTAAT   
  
  
+ TCAATTAACC ACGTAAGTTA TACGCTGACA AATTTCGGGT ACCAAACATT TGCACTAGAC CTAAAACAGT   
  
  
+ TACTATCATC ATATCGATGT TAAAATTGAC CTGTTCTTAT TTGTCCAGCG TTGAGCAAGG AAATAATCAA   
  
  
+ ATCATGTCTC ATTTAGTCGG TTTTTTGCCT AAGAAGAGCA TAAACAAAGG AAAAAGACAA CGACACATTA   
  
  
+ TACATGCATC TACACAAATG AAATGAGGCC ATATGGAACA TGTTCATGAG AGCTTCACTC GCTAATAATT   
  
  
+ GAACCTACTT TTGACTAAGC CACCAACTAG AATATGGAGA TTGACTGTTA AGAGACTATA TTACATGAAA   
  
  
+ TAGATAAGAG TAGACATTGC TATATATTTT ATCTTTAATT GTCTATTTTT AATGATCATT ACTGTTTATT   
  
  
+ TCAGTGAGTA ATTTTCTCGG ATATAATATT TTATAGCGGT TATTAAATAG ATGTGAAACT ATAATGTTAG   
  
  
+ ACATATCATG CGAAAATAGA GAAATTTTGG GCAAATTAAA AAAAAAAGAA GAAAAACGAG GAAGTATGCC   
  
  
+ CATTGTAAGC AGGTGGATGT ATATCCAGCC TCCATCCAAC AATTTGGGCA AAATTATATC ATCCATTCCA   
  
  
+ AAAAAAAGAT ATAATTTGTG ATATTAATAA CCCGACTCAA TTCAAGCTTA TTTATTTTTT CATATAATTT   
  
  
+ TTTAATATAT AAAAATAAAA AGTGACAACG ACATGCATCC TTTGCGTGTC AAAGGATCCG CCGCGTGGCC   
  
  
+ CCCACATTCA CTGACGGGGT TTTTGGTTTG CGATTAAGAT CAATGGCTAA CGTTGTCTCT CTCAAAACAT   
  
  
+ ATGGACCCCT TTTTCTATCT TTTTCTGCAT TGTCAGCATA GCAATTGTTG AGCACATTTT TTTCGATTTG   
  
  
+ TTGACAATAA GCGTGGTTAT GATTTATGGG CCACTAATAT CAGTCTCTGA TCATTTGCAC ATTTTGGACC   
  
  
+ TCTTTCTCTC TCTAATCTTC CAGGTAACCT GAATTCTTCT CTATTTCTGA CTAGATTAGA TAACAGGATT   
  
  
+ TCAAATATCT GTCAGGTGGC GGGTATAACT TCGTGATGGG CATTCATTTT TGTTCTATCA GGCTGGTGGG   
  
  
+ TTTGAGTTTA TTTTGATTCA TTTCGTGTTC ATCTGTGATA TTAGTAGGAG GGATTCTGTA TACCCACCAA   
  
  
+ CTGCTCGCCC TTTTGCCCGT GTGAGCAATT GGGACTTTTG TTTGTTCTTC GTCGATGCAT CCTCTGGTTG   
  
  
+ CTGATCTGGA ATTGAAACCC CATGTATTCA AATTCAACCC TGATTTGCTG TCAAACTTTC TGAACCATCA   
  
  
+ AAACTCCGCC GAAGTGTTTG AAAAAGATGG CATCTTCCAG ACCCTTCATT TGGCTGATCC CAAAAGCTCT   
  
  
+ TCAATTGCCG AAATCGGGTT TACTAATAGT TCAGATTCTA CACAAGTACC TGATTTTTCA GATGCTTGTC   
  
  
+ TTAAGTTCAT TAGTGATATT CTCCTGGAAG AGGACTTAGA TGAAAGTCCT ACGTCTTTAC ACGATTACAT   
  
  
+ GGCTCTCCTA GCCACTGAGA AGTCTTTGTA TGATGCTCTT GGAAAGGAAT ACTTTCCTTC GTCTACTAGT   
  
  
+ CTTGCCCCAT CTTTAGGCCG AAGTGTTGAC AGCCCAGATA GTGGCTTTGG CCGCGGTTGC TCTGATGGTC   
  
  
+ GCGGGATTGA GGGTTTGGCT AATGATGATG CCGTTTTCAT GTCTAACTGG CAGCTCAACA CCACCCAATT   
  
  
+ GGACCCTTTC CCAATCATGC AAGATATTCC TCGTCCCTAT TTGGAATTGA ATTACCATTC TTCTGGGTCA   
  
  
+ AGCAATGGCA TTGATGATTC GGGGGATGGG TTATCGACCT CTCCTGTAAG TACACTTGCA TCAACCGCCA   
  
  
+ CAGAGGCAGG GAAAAAGTTG GCTGGTAGCT CTAGGAGAAA GAACCGTCAA AGGGATGACT ATGGCCATGA   
  
  
+ AGAGGGAAGG AGTAACAAGC AGCAAGCCTC TTACAATGAC GATTACGTTG AGATGGAGCA GTATGACGAT   
  
  
+ GTACTTCTCT GTAGGGCAGA CAAGGGTAAT ATTTCAACTT GTGCCAATGA ATCCTCGCTT AATGAGGTGC   
  
  
+ GTGAGAAGCT GCAGACGACA GGGTTCAAGG GAAGAACATC CCGTCTTAAG AAGCAATCTA AGGAAGCGAA   
  
  
+ AGAGGTGGAT CTGAGAACTC TTCTTTCTGG TTGTGCACAA GCTGTTTCGA ACTTTGATAT CAGGACTGCT   
  
  
+ AATGAGCTGC TTAAGCAAGT CAGACAGCAT TCTTCACCAT ATGGTGATAG CCTCCAAAGG CTCGCCCATC   
  
  
+ AATTTGCGAA TGGTATTGAG GCACGCTTAG CAGGCACCGG TTCAAGAGTA CCTGCTAATC TCATTGATGC   
  
  
+ ACGGATATCA TCATCTGAAT TTTTAAAAGC TTACAAGTCA TATGTTTCAG CAGTTCCTTT CAGAAGGATG   
  
  
+ TCCTATTTTA TAGCAAACAA CACAATTCTG AAGTTGGCTG AGAAAGCAAC AAAGATTCAC ATAATTGATT   
  
  
+ TTGGTATTCT ATTTGGTCTA CAGTGGCCCT GTCTTATACA AAGTCTCTCA AGGCGAACTG TGGCTCCTCC   
  
  
+ GAAGCTTCGC ATCACTGGGA TAGACTATCC CCAGCATGGT TTCCGGCCAG CAGAAAAGGT TGAGGCAACA   
  
  
+ GGTCGTCGGT TGTCCGGGTA CTGTGAGAGA TTTAATGTAC CCTTTCGATA TGAAACCATT GCAAAGAAGT   
  
  
+ GGGAAACCAT ACGCCCAGAA GATCTAAATA TTGAGAATGA TGAGCTGGTA ATTGTTAATT GTATGTTGCG   
  
  
+ GTCTGTAAAT CTATTGGATG ATACAGTGGC GGTAAATAGT CCAAGGGATG CTTTCTTGAG GTTAATCAAA   
  
  
+ CAGATAAACC CGCGTTTATT CATTCATGCA ATTGTCAATG GAACCTTTAG TACTCCATTC TTCAGCACTC   
  
  
+ GATTCAGGGA AGCCCTATTC CAATACTCTT CTGTATTTGA TATATTTGAA GCGACTATGA CTCGTGAAGA   
  
  
+ TCGTGGAAGG CTGCTGATTG AGAGTCAAAT ATGCGGGCTA GAAGTTTTGA ATGCAATAGC ATGTGAAGGT   
  
  
+ GCAGAGAGGA TTCAAAGGCC TGAAACATAC AAGCAATGGC AGGAGCGGAC AACAAGGGCT GGACTAAGGC   
  
  
+ AGGTTCCAAT AGATGAGGAG CTTGTCAATA GAGCAAAGAC TATAGTGAAA GCAAATTATC ACAAGGATTT   
  
  
+ TGTGGTGGAT GAGGATAGGC GTTGGATGCT TCAAGGTTGG AAAGGAAGGA CACTTAGTGC CCTTTCCGTT   
  
  
+ TGGCAGCCTA ACTA  

- +Up\_Stream \_Len000AAATTA TTCTAGGAAA ATATATATAC ACACATATTA AGATACCATG TATCCATACA   
  
  
- TGGTATCTGA ATAACGTAAA GATCCAGGAA GAGAGAGACG CGAACAAAAA ACAAATAGAT TAAATACCTC   
  
  
- AACTTGTAAA ACAAAATATA TGAACTTGTT AGATAATATG ATTTAATTCG TAAAACTAAA TTAGTGAATA   
  
  
- CCCACACAAA CCATCCCAAC TTTTATTAAA AGGATCTTTT ATTAAAAATT ATATTTTATT AAAAAGTACA   
  
  
- TTTTACTAAA AGTTGAGTAT AAAAGTCAAC TAACCATTTT TCTTTTTACT TGAAAGACCT ATTAGTCAAA   
  
  
- CTAATTGTTT TTCACTGTTT TTTTAATTTT TACCAAAAGG CATTAGAAGT TTGGTATACA GCTCCTTTAT   
  
  
- TTGTGCCTTA TTTCTTTCTC ACAGTCTTGG GTTCTTTACA TTTTAGTTGA ATGCAAGATA AATTTCACTT   
  
  
- TTTATAAAAA GAAGTAAGAT CCTTTTAATT AACAGGAGAC TTTTGTTTAA AAGGGAGGAA CAGATTGGTT   
  
  
- TGTGTCATTT TAACTTTTTA GTAAAAGATC CTTTTACTAA AAGTGAGGAT GGTTTGTGTG GGAATTTGAA   
  
  
- CAAGGTTTAG ATATTTGGGC CTTAGAATTA TGCGTGTAAC ATATAATATA CAGGATGATA TTGACATAAT   
  
  
- AGTGACCAAG AACTAAATAG GAAAGTTTGA GAACGAATCA GTTCCAACAG GAGCATGTTT GTACTAATTA   
  
  
- AGTTAATTGG TGCATTCAAT ATGCGACTGT TTAAAGCCCA TGGTTTGTAA ACGTGATCTG GATTTTGTCA   
  
  
- ATGATAGTAG TATAGCTACA ATTTTAACTG GACAAGAATA AACAGGTCGC AACTCGTTCC TTTATTAGTT   
  
  
- TAGTACAGAG TAAATCAGCC AAAAAACGGA TTCTTCTCGT ATTTGTTTCC TTTTTCTGTT GCTGTGTAAT   
  
  
- ATGTACGTAG ATGTGTTTAC TTTACTCCGG TATACCTTGT ACAAGTACTC TCGAAGTGAG CGATTATTAA   
  
  
- CTTGGATGAA AACTGATTCG GTGGTTGATC TTATACCTCT AACTGACAAT TCTCTGATAT AATGTACTTT   
  
  
- ATCTATTCTC ATCTGTAACG ATATATAAAA TAGAAATTAA CAGATAAAAA TTACTAGTAA TGACAAATAA   
  
  
- AGTCACTCAT TAAAAGAGCC TATATTATAA AATATCGCCA ATAATTTATC TACACTTTGA TATTACAATC   
  
  
- TGTATAGTAC GCTTTTATCT CTTTAAAACC CGTTTAATTT TTTTTTTCTT CTTTTTGCTC CTTCATACGG   
  
  
- GTAACATTCG TCCACCTACA TATAGGTCGG AGGTAGGTTG TTAAACCCGT TTTAATATAG TAGGTAAGGT   
  
  
- TTTTTTTCTA TATTAAACAC TATAATTATT GGGCTGAGTT AAGTTCGAAT AAATAAAAAA GTATATTAAA   
  
  
- AAATTATATA TTTTTATTTT TCACTGTTGC TGTACGTAGG AAACGCACAG TTTCCTAGGC GGCGCACCGG   
  
  
- GGGTGTAAGT GACTGCCCCA AAAACCAAAC GCTAATTCTA GTTACCGATT GCAACAGAGA GAGTTTTGTA   
  
  
- TACCTGGGGA AAAAGATAGA AAAAGACGTA ACAGTCGTAT CGTTAACAAC TCGTGTAAAA AAAGCTAAAC   
  
  
- AACTGTTATT CGCACCAATA CTAAATACCC GGTGATTATA GTCAGAGACT AGTAAACGTG TAAAACCTGG   
  
  
- AGAAAGAGAG AGATTAGAAG GTCCATTGGA CTTAAGAAGA GATAAAGACT GATCTAATCT ATTGTCCTAA   
  
  
- AGTTTATAGA CAGTCCACCG CCCATATTGA AGCACTACCC GTAAGTAAAA ACAAGATAGT CCGACCACCC   
  
  
- AAACTCAAAT AAAACTAAGT AAAGCACAAG TAGACACTAT AATCATCCTC CCTAAGACAT ATGGGTGGTT   
  
  
- GACGAGCGGG AAAACGGGCA CACTCGTTAA CCCTGAAAAC AAACAAGAAG CAGCTACGTA GGAGACCAAC   
  
  
- GACTAGACCT TAACTTTGGG GTACATAAGT TTAAGTTGGG ACTAAACGAC AGTTTGAAAG ACTTGGTAGT   
  
  
- TTTGAGGCGG CTTCACAAAC TTTTTCTACC GTAGAAGGTC TGGGAAGTAA ACCGACTAGG GTTTTCGAGA   
  
  
- AGTTAACGGC TTTAGCCCAA ATGATTATCA AGTCTAAGAT GTGTTCATGG ACTAAAAAGT CTACGAACAG   
  
  
- AATTCAAGTA ATCACTATAA GAGGACCTTC TCCTGAATCT ACTTTCAGGA TGCAGAAATG TGCTAATGTA   
  
  
- CCGAGAGGAT CGGTGACTCT TCAGAAACAT ACTACGAGAA CCTTTCCTTA TGAAAGGAAG CAGATGATCA   
  
  
- GAACGGGGTA GAAATCCGGC TTCACAACTG TCGGGTCTAT CACCGAAACC GGCGCCAACG AGACTACCAG   
  
  
- CGCCCTAACT CCCAAACCGA TTACTACTAC GGCAAAAGTA CAGATTGACC GTCGAGTTGT GGTGGGTTAA   
  
  
- CCTGGGAAAG GGTTAGTACG TTCTATAAGG AGCAGGGATA AACCTTAACT TAATGGTAAG AAGACCCAGT   
  
  
- TCGTTACCGT AACTACTAAG CCCCCTACCC AATAGCTGGA GAGGACATTC ATGTGAACGT AGTTGGCGGT   
  
  
- GTCTCCGTCC CTTTTTCAAC CGACCATCGA GATCCTCTTT CTTGGCAGTT TCCCTACTGA TACCGGTACT   
  
  
- TCTCCCTTCC TCATTGTTCG TCGTTCGGAG AATGTTACTG CTAATGCAAC TCTACCTCGT CATACTGCTA   
  
  
- CATGAAGAGA CATCCCGTCT GTTCCCATTA TAAAGTTGAA CACGGTTACT TAGGAGCGAA TTACTCCACG   
  
  
- CACTCTTCGA CGTCTGCTGT CCCAAGTTCC CTTCTTGTAG GGCAGAATTC TTCGTTAGAT TCCTTCGCTT   
  
  
- TCTCCACCTA GACTCTTGAG AAGAAAGACC AACACGTGTT CGACAAAGCT TGAAACTATA GTCCTGACGA   
  
  
- TTACTCGACG AATTCGTTCA GTCTGTCGTA AGAAGTGGTA TACCACTATC GGAGGTTTCC GAGCGGGTAG   
  
  
- TTAAACGCTT ACCATAACTC CGTGCGAATC GTCCGTGGCC AAGTTCTCAT GGACGATTAG AGTAACTACG   
  
  
- TGCCTATAGT AGTAGACTTA AAAATTTTCG AATGTTCAGT ATACAAAGTC GTCAAGGAAA GTCTTCCTAC   
  
  
- AGGATAAAAT ATCGTTTGTT GTGTTAAGAC TTCAACCGAC TCTTTCGTTG TTTCTAAGTG TATTAACTAA   
  
  
- AACCATAAGA TAAACCAGAT GTCACCGGGA CAGAATATGT TTCAGAGAGT TCCGCTTGAC ACCGAGGAGG   
  
  
- CTTCGAAGCG TAGTGACCCT ATCTGATAGG GGTCGTACCA AAGGCCGGTC GTCTTTTCCA ACTCCGTTGT   
  
  
- CCAGCAGCCA ACAGGCCCAT GACACTCTCT AAATTACATG GGAAAGCTAT ACTTTGGTAA CGTTTCTTCA   
  
  
- CCCTTTGGTA TGCGGGTCTT CTAGATTTAT AACTCTTACT ACTCGACCAT TAACAATTAA CATACAACGC   
  
  
- CAGACATTTA GATAACCTAC TATGTCACCG CCATTTATCA GGTTCCCTAC GAAAGAACTC CAATTAGTTT   
  
  
- GTCTATTTGG GCGCAAATAA GTAAGTACGT TAACAGTTAC CTTGGAAATC ATGAGGTAAG AAGTCGTGAG   
  
  
- CTAAGTCCCT TCGGGATAAG GTTATGAGAA GACATAAACT ATATAAACTT CGCTGATACT GAGCACTTCT   
  
  
- AGCACCTTCC GACGACTAAC TCTCAGTTTA TACGCCCGAT CTTCAAAACT TACGTTATCG TACACTTCCA   
  
  
- CGTCTCTCCT AAGTTTCCGG ACTTTGTATG TTCGTTACCG TCCTCGCCTG TTGTTCCCGA CCTGATTCCG   
  
  
- TCCAAGGTTA TCTACTCCTC GAACAGTTAT CTCGTTTCTG ATATCACTTT CGTTTAATAG TGTTCCTAAA   
  
  
- ACACCACCTA CTCCTATCCG CAACCTACGA AGTTCCAACC TTTCCTTCCT GTGAATCACG GGAAAGGCAA   
  
  
- ACCGTCGGAT TGAT

+     F-box

| Site Name | Organism | Position | Strand | Matrix score. | sequence | function |
| --- | --- | --- | --- | --- | --- | --- |
| F-box | Lycopersicon esculentum | 3139 | + | 10 | CTATTCTCATT |  |

>HU02G01572.1   
+ +Up\_Stream \_Len000TTTAAT AAGATCCTTT TATATATATG TGTGTATAAT TCTATGGTAC ATAGGTATGT   
  
  
+ ACCATAGACT TATTGCATTT CTAGGTCCTT CTCTCTCTGC GCTTGTTTTT TGTTTATCTA ATTTATGGAG   
  
  
+ TTGAACATTT TGTTTTATAT ACTTGAACAA TCTATTATAC TAAATTAAGC ATTTTGATTT AATCACTTAT   
  
  
+ GGGTGTGTTT GGTAGGGTTG AAAATAATTT TCCTAGAAAA TAATTTTTAA TATAAAATAA TTTTTCATGT   
  
  
+ AAAATGATTT TCAACTCATA TTTTCAGTTG ATTGGTAAAA AGAAAAATGA ACTTTCTGGA TAATCAGTTT   
  
  
+ GATTAACAAA AAGTGACAAA AAAATTAAAA ATGGTTTTCC GTAATCTTCA AACCATATGT CGAGGAAATA   
  
  
+ AACACGGAAT AAAGAAAGAG TGTCAGAACC CAAGAAATGT AAAATCAACT TACGTTCTAT TTAAAGTGAA   
  
  
+ AAATATTTTT CTTCATTCTA GGAAAATTAA TTGTCCTCTG AAAACAAATT TTCCCTCCTT GTCTAACCAA   
  
  
+ ACACAGTAAA ATTGAAAAAT CATTTTCTAG GAAAATGATT TTCACTCCTA CCAAACACAC CCTTAAACTT   
  
  
+ GTTCCAAATC TATAAACCCG GAATCTTAAT ACGCACATTG TATATTATAT GTCCTACTAT AACTGTATTA   
  
  
+ TCACTGGTTC TTGATTTATC CTTTCAAACT CTTGCTTAGT CAAGGTTGTC CTCGTACAAA CATGATTAAT   
  
  
+ TCAATTAACC ACGTAAGTTA TACGCTGACA AATTTCGGGT ACCAAACATT TGCACTAGAC CTAAAACAGT   
  
  
+ TACTATCATC ATATCGATGT TAAAATTGAC CTGTTCTTAT TTGTCCAGCG TTGAGCAAGG AAATAATCAA   
  
  
+ ATCATGTCTC ATTTAGTCGG TTTTTTGCCT AAGAAGAGCA TAAACAAAGG AAAAAGACAA CGACACATTA   
  
  
+ TACATGCATC TACACAAATG AAATGAGGCC ATATGGAACA TGTTCATGAG AGCTTCACTC GCTAATAATT   
  
  
+ GAACCTACTT TTGACTAAGC CACCAACTAG AATATGGAGA TTGACTGTTA AGAGACTATA TTACATGAAA   
  
  
+ TAGATAAGAG TAGACATTGC TATATATTTT ATCTTTAATT GTCTATTTTT AATGATCATT ACTGTTTATT   
  
  
+ TCAGTGAGTA ATTTTCTCGG ATATAATATT TTATAGCGGT TATTAAATAG ATGTGAAACT ATAATGTTAG   
  
  
+ ACATATCATG CGAAAATAGA GAAATTTTGG GCAAATTAAA AAAAAAAGAA GAAAAACGAG GAAGTATGCC   
  
  
+ CATTGTAAGC AGGTGGATGT ATATCCAGCC TCCATCCAAC AATTTGGGCA AAATTATATC ATCCATTCCA   
  
  
+ AAAAAAAGAT ATAATTTGTG ATATTAATAA CCCGACTCAA TTCAAGCTTA TTTATTTTTT CATATAATTT   
  
  
+ TTTAATATAT AAAAATAAAA AGTGACAACG ACATGCATCC TTTGCGTGTC AAAGGATCCG CCGCGTGGCC   
  
  
+ CCCACATTCA CTGACGGGGT TTTTGGTTTG CGATTAAGAT CAATGGCTAA CGTTGTCTCT CTCAAAACAT   
  
  
+ ATGGACCCCT TTTTCTATCT TTTTCTGCAT TGTCAGCATA GCAATTGTTG AGCACATTTT TTTCGATTTG   
  
  
+ TTGACAATAA GCGTGGTTAT GATTTATGGG CCACTAATAT CAGTCTCTGA TCATTTGCAC ATTTTGGACC   
  
  
+ TCTTTCTCTC TCTAATCTTC CAGGTAACCT GAATTCTTCT CTATTTCTGA CTAGATTAGA TAACAGGATT   
  
  
+ TCAAATATCT GTCAGGTGGC GGGTATAACT TCGTGATGGG CATTCATTTT TGTTCTATCA GGCTGGTGGG   
  
  
+ TTTGAGTTTA TTTTGATTCA TTTCGTGTTC ATCTGTGATA TTAGTAGGAG GGATTCTGTA TACCCACCAA   
  
  
+ CTGCTCGCCC TTTTGCCCGT GTGAGCAATT GGGACTTTTG TTTGTTCTTC GTCGATGCAT CCTCTGGTTG   
  
  
+ CTGATCTGGA ATTGAAACCC CATGTATTCA AATTCAACCC TGATTTGCTG TCAAACTTTC TGAACCATCA   
  
  
+ AAACTCCGCC GAAGTGTTTG AAAAAGATGG CATCTTCCAG ACCCTTCATT TGGCTGATCC CAAAAGCTCT   
  
  
+ TCAATTGCCG AAATCGGGTT TACTAATAGT TCAGATTCTA CACAAGTACC TGATTTTTCA GATGCTTGTC   
  
  
+ TTAAGTTCAT TAGTGATATT CTCCTGGAAG AGGACTTAGA TGAAAGTCCT ACGTCTTTAC ACGATTACAT   
  
  
+ GGCTCTCCTA GCCACTGAGA AGTCTTTGTA TGATGCTCTT GGAAAGGAAT ACTTTCCTTC GTCTACTAGT   
  
  
+ CTTGCCCCAT CTTTAGGCCG AAGTGTTGAC AGCCCAGATA GTGGCTTTGG CCGCGGTTGC TCTGATGGTC   
  
  
+ GCGGGATTGA GGGTTTGGCT AATGATGATG CCGTTTTCAT GTCTAACTGG CAGCTCAACA CCACCCAATT   
  
  
+ GGACCCTTTC CCAATCATGC AAGATATTCC TCGTCCCTAT TTGGAATTGA ATTACCATTC TTCTGGGTCA   
  
  
+ AGCAATGGCA TTGATGATTC GGGGGATGGG TTATCGACCT CTCCTGTAAG TACACTTGCA TCAACCGCCA   
  
  
+ CAGAGGCAGG GAAAAAGTTG GCTGGTAGCT CTAGGAGAAA GAACCGTCAA AGGGATGACT ATGGCCATGA   
  
  
+ AGAGGGAAGG AGTAACAAGC AGCAAGCCTC TTACAATGAC GATTACGTTG AGATGGAGCA GTATGACGAT   
  
  
+ GTACTTCTCT GTAGGGCAGA CAAGGGTAAT ATTTCAACTT GTGCCAATGA ATCCTCGCTT AATGAGGTGC   
  
  
+ GTGAGAAGCT GCAGACGACA GGGTTCAAGG GAAGAACATC CCGTCTTAAG AAGCAATCTA AGGAAGCGAA   
  
  
+ AGAGGTGGAT CTGAGAACTC TTCTTTCTGG TTGTGCACAA GCTGTTTCGA ACTTTGATAT CAGGACTGCT   
  
  
+ AATGAGCTGC TTAAGCAAGT CAGACAGCAT TCTTCACCAT ATGGTGATAG CCTCCAAAGG CTCGCCCATC   
  
  
+ AATTTGCGAA TGGTATTGAG GCACGCTTAG CAGGCACCGG TTCAAGAGTA CCTGCTAATC TCATTGATGC   
  
  
+ ACGGATATCA TCATCTGAAT TTTTAAAAGC TTACAAGTCA TATGTTTCAG CAGTTCCTTT CAGAAGGATG   
  
  
+ TCCTATTTTA TAGCAAACAA CACAATTCTG AAGTTGGCTG AGAAAGCAAC AAAGATTCAC ATAATTGATT   
  
  
+ TTGGTATTCT ATTTGGTCTA CAGTGGCCCT GTCTTATACA AAGTCTCTCA AGGCGAACTG TGGCTCCTCC   
  
  
+ GAAGCTTCGC ATCACTGGGA TAGACTATCC CCAGCATGGT TTCCGGCCAG CAGAAAAGGT TGAGGCAACA   
  
  
+ GGTCGTCGGT TGTCCGGGTA CTGTGAGAGA TTTAATGTAC CCTTTCGATA TGAAACCATT GCAAAGAAGT   
  
  
+ GGGAAACCAT ACGCCCAGAA GATCTAAATA TTGAGAATGA TGAGCTGGTA ATTGTTAATT GTATGTTGCG   
  
  
+ GTCTGTAAAT CTATTGGATG ATACAGTGGC GGTAAATAGT CCAAGGGATG CTTTCTTGAG GTTAATCAAA   
  
  
+ CAGATAAACC CGCGTTTATT CATTCATGCA ATTGTCAATG GAACCTTTAG TACTCCATTC TTCAGCACTC   
  
  
+ GATTCAGGGA AGCCCTATTC CAATACTCTT CTGTATTTGA TATATTTGAA GCGACTATGA CTCGTGAAGA   
  
  
+ TCGTGGAAGG CTGCTGATTG AGAGTCAAAT ATGCGGGCTA GAAGTTTTGA ATGCAATAGC ATGTGAAGGT   
  
  
+ GCAGAGAGGA TTCAAAGGCC TGAAACATAC AAGCAATGGC AGGAGCGGAC AACAAGGGCT GGACTAAGGC   
  
  
+ AGGTTCCAAT AGATGAGGAG CTTGTCAATA GAGCAAAGAC TATAGTGAAA GCAAATTATC ACAAGGATTT   
  
  
+ TGTGGTGGAT GAGGATAGGC GTTGGATGCT TCAAGGTTGG AAAGGAAGGA CACTTAGTGC CCTTTCCGTT   
  
  
+ TGGCAGCCTA ACTA  

- +Up\_Stream \_Len000AAATTA TTCTAGGAAA ATATATATAC ACACATATTA AGATACCATG TATCCATACA   
  
  
- TGGTATCTGA ATAACGTAAA GATCCAGGAA GAGAGAGACG CGAACAAAAA ACAAATAGAT TAAATACCTC   
  
  
- AACTTGTAAA ACAAAATATA TGAACTTGTT AGATAATATG ATTTAATTCG TAAAACTAAA TTAGTGAATA   
  
  
- CCCACACAAA CCATCCCAAC TTTTATTAAA AGGATCTTTT ATTAAAAATT ATATTTTATT AAAAAGTACA   
  
  
- TTTTACTAAA AGTTGAGTAT AAAAGTCAAC TAACCATTTT TCTTTTTACT TGAAAGACCT ATTAGTCAAA   
  
  
- CTAATTGTTT TTCACTGTTT TTTTAATTTT TACCAAAAGG CATTAGAAGT TTGGTATACA GCTCCTTTAT   
  
  
- TTGTGCCTTA TTTCTTTCTC ACAGTCTTGG GTTCTTTACA TTTTAGTTGA ATGCAAGATA AATTTCACTT   
  
  
- TTTATAAAAA GAAGTAAGAT CCTTTTAATT AACAGGAGAC TTTTGTTTAA AAGGGAGGAA CAGATTGGTT   
  
  
- TGTGTCATTT TAACTTTTTA GTAAAAGATC CTTTTACTAA AAGTGAGGAT GGTTTGTGTG GGAATTTGAA   
  
  
- CAAGGTTTAG ATATTTGGGC CTTAGAATTA TGCGTGTAAC ATATAATATA CAGGATGATA TTGACATAAT   
  
  
- AGTGACCAAG AACTAAATAG GAAAGTTTGA GAACGAATCA GTTCCAACAG GAGCATGTTT GTACTAATTA   
  
  
- AGTTAATTGG TGCATTCAAT ATGCGACTGT TTAAAGCCCA TGGTTTGTAA ACGTGATCTG GATTTTGTCA   
  
  
- ATGATAGTAG TATAGCTACA ATTTTAACTG GACAAGAATA AACAGGTCGC AACTCGTTCC TTTATTAGTT   
  
  
- TAGTACAGAG TAAATCAGCC AAAAAACGGA TTCTTCTCGT ATTTGTTTCC TTTTTCTGTT GCTGTGTAAT   
  
  
- ATGTACGTAG ATGTGTTTAC TTTACTCCGG TATACCTTGT ACAAGTACTC TCGAAGTGAG CGATTATTAA   
  
  
- CTTGGATGAA AACTGATTCG GTGGTTGATC TTATACCTCT AACTGACAAT TCTCTGATAT AATGTACTTT   
  
  
- ATCTATTCTC ATCTGTAACG ATATATAAAA TAGAAATTAA CAGATAAAAA TTACTAGTAA TGACAAATAA   
  
  
- AGTCACTCAT TAAAAGAGCC TATATTATAA AATATCGCCA ATAATTTATC TACACTTTGA TATTACAATC   
  
  
- TGTATAGTAC GCTTTTATCT CTTTAAAACC CGTTTAATTT TTTTTTTCTT CTTTTTGCTC CTTCATACGG   
  
  
- GTAACATTCG TCCACCTACA TATAGGTCGG AGGTAGGTTG TTAAACCCGT TTTAATATAG TAGGTAAGGT   
  
  
- TTTTTTTCTA TATTAAACAC TATAATTATT GGGCTGAGTT AAGTTCGAAT AAATAAAAAA GTATATTAAA   
  
  
- AAATTATATA TTTTTATTTT TCACTGTTGC TGTACGTAGG AAACGCACAG TTTCCTAGGC GGCGCACCGG   
  
  
- GGGTGTAAGT GACTGCCCCA AAAACCAAAC GCTAATTCTA GTTACCGATT GCAACAGAGA GAGTTTTGTA   
  
  
- TACCTGGGGA AAAAGATAGA AAAAGACGTA ACAGTCGTAT CGTTAACAAC TCGTGTAAAA AAAGCTAAAC   
  
  
- AACTGTTATT CGCACCAATA CTAAATACCC GGTGATTATA GTCAGAGACT AGTAAACGTG TAAAACCTGG   
  
  
- AGAAAGAGAG AGATTAGAAG GTCCATTGGA CTTAAGAAGA GATAAAGACT GATCTAATCT ATTGTCCTAA   
  
  
- AGTTTATAGA CAGTCCACCG CCCATATTGA AGCACTACCC GTAAGTAAAA ACAAGATAGT CCGACCACCC   
  
  
- AAACTCAAAT AAAACTAAGT AAAGCACAAG TAGACACTAT AATCATCCTC CCTAAGACAT ATGGGTGGTT   
  
  
- GACGAGCGGG AAAACGGGCA CACTCGTTAA CCCTGAAAAC AAACAAGAAG CAGCTACGTA GGAGACCAAC   
  
  
- GACTAGACCT TAACTTTGGG GTACATAAGT TTAAGTTGGG ACTAAACGAC AGTTTGAAAG ACTTGGTAGT   
  
  
- TTTGAGGCGG CTTCACAAAC TTTTTCTACC GTAGAAGGTC TGGGAAGTAA ACCGACTAGG GTTTTCGAGA   
  
  
- AGTTAACGGC TTTAGCCCAA ATGATTATCA AGTCTAAGAT GTGTTCATGG ACTAAAAAGT CTACGAACAG   
  
  
- AATTCAAGTA ATCACTATAA GAGGACCTTC TCCTGAATCT ACTTTCAGGA TGCAGAAATG TGCTAATGTA   
  
  
- CCGAGAGGAT CGGTGACTCT TCAGAAACAT ACTACGAGAA CCTTTCCTTA TGAAAGGAAG CAGATGATCA   
  
  
- GAACGGGGTA GAAATCCGGC TTCACAACTG TCGGGTCTAT CACCGAAACC GGCGCCAACG AGACTACCAG   
  
  
- CGCCCTAACT CCCAAACCGA TTACTACTAC GGCAAAAGTA CAGATTGACC GTCGAGTTGT GGTGGGTTAA   
  
  
- CCTGGGAAAG GGTTAGTACG TTCTATAAGG AGCAGGGATA AACCTTAACT TAATGGTAAG AAGACCCAGT   
  
  
- TCGTTACCGT AACTACTAAG CCCCCTACCC AATAGCTGGA GAGGACATTC ATGTGAACGT AGTTGGCGGT   
  
  
- GTCTCCGTCC CTTTTTCAAC CGACCATCGA GATCCTCTTT CTTGGCAGTT TCCCTACTGA TACCGGTACT   
  
  
- TCTCCCTTCC TCATTGTTCG TCGTTCGGAG AATGTTACTG CTAATGCAAC TCTACCTCGT CATACTGCTA   
  
  
- CATGAAGAGA CATCCCGTCT GTTCCCATTA TAAAGTTGAA CACGGTTACT TAGGAGCGAA TTACTCCACG   
  
  
- CACTCTTCGA CGTCTGCTGT CCCAAGTTCC CTTCTTGTAG GGCAGAATTC TTCGTTAGAT TCCTTCGCTT   
  
  
- TCTCCACCTA GACTCTTGAG AAGAAAGACC AACACGTGTT CGACAAAGCT TGAAACTATA GTCCTGACGA   
  
  
- TTACTCGACG AATTCGTTCA GTCTGTCGTA AGAAGTGGTA TACCACTATC GGAGGTTTCC GAGCGGGTAG   
  
  
- TTAAACGCTT ACCATAACTC CGTGCGAATC GTCCGTGGCC AAGTTCTCAT GGACGATTAG AGTAACTACG   
  
  
- TGCCTATAGT AGTAGACTTA AAAATTTTCG AATGTTCAGT ATACAAAGTC GTCAAGGAAA GTCTTCCTAC   
  
  
- AGGATAAAAT ATCGTTTGTT GTGTTAAGAC TTCAACCGAC TCTTTCGTTG TTTCTAAGTG TATTAACTAA   
  
  
- AACCATAAGA TAAACCAGAT GTCACCGGGA CAGAATATGT TTCAGAGAGT TCCGCTTGAC ACCGAGGAGG   
  
  
- CTTCGAAGCG TAGTGACCCT ATCTGATAGG GGTCGTACCA AAGGCCGGTC GTCTTTTCCA ACTCCGTTGT   
  
  
- CCAGCAGCCA ACAGGCCCAT GACACTCTCT AAATTACATG GGAAAGCTAT ACTTTGGTAA CGTTTCTTCA   
  
  
- CCCTTTGGTA TGCGGGTCTT CTAGATTTAT AACTCTTACT ACTCGACCAT TAACAATTAA CATACAACGC   
  
  
- CAGACATTTA GATAACCTAC TATGTCACCG CCATTTATCA GGTTCCCTAC GAAAGAACTC CAATTAGTTT   
  
  
- GTCTATTTGG GCGCAAATAA GTAAGTACGT TAACAGTTAC CTTGGAAATC ATGAGGTAAG AAGTCGTGAG   
  
  
- CTAAGTCCCT TCGGGATAAG GTTATGAGAA GACATAAACT ATATAAACTT CGCTGATACT GAGCACTTCT   
  
  
- AGCACCTTCC GACGACTAAC TCTCAGTTTA TACGCCCGAT CTTCAAAACT TACGTTATCG TACACTTCCA   
  
  
- CGTCTCTCCT AAGTTTCCGG ACTTTGTATG TTCGTTACCG TCCTCGCCTG TTGTTCCCGA CCTGATTCCG   
  
  
- TCCAAGGTTA TCTACTCCTC GAACAGTTAT CTCGTTTCTG ATATCACTTT CGTTTAATAG TGTTCCTAAA   
  
  
- ACACCACCTA CTCCTATCCG CAACCTACGA AGTTCCAACC TTTCCTTCCT GTGAATCACG GGAAAGGCAA   
  
  
- ACCGTCGGAT TGAT

+     G-box

| Site Name | Organism | Position | Strand | Matrix score. | sequence | function |
| --- | --- | --- | --- | --- | --- | --- |
| G-box | Arabidopsis thaliana | 784 | - | 6 | TACGTG | cis-acting regulatory element involved in light responsiveness |
| G-box | Brassica napus | 783 | + | 8 | CCACGTAA | cis-acting regulatory element involved in light responsiveness |

>HU02G01572.1   
+ +Up\_Stream \_Len000TTTAAT AAGATCCTTT TATATATATG TGTGTATAAT TCTATGGTAC ATAGGTATGT   
  
  
+ ACCATAGACT TATTGCATTT CTAGGTCCTT CTCTCTCTGC GCTTGTTTTT TGTTTATCTA ATTTATGGAG   
  
  
+ TTGAACATTT TGTTTTATAT ACTTGAACAA TCTATTATAC TAAATTAAGC ATTTTGATTT AATCACTTAT   
  
  
+ GGGTGTGTTT GGTAGGGTTG AAAATAATTT TCCTAGAAAA TAATTTTTAA TATAAAATAA TTTTTCATGT   
  
  
+ AAAATGATTT TCAACTCATA TTTTCAGTTG ATTGGTAAAA AGAAAAATGA ACTTTCTGGA TAATCAGTTT   
  
  
+ GATTAACAAA AAGTGACAAA AAAATTAAAA ATGGTTTTCC GTAATCTTCA AACCATATGT CGAGGAAATA   
  
  
+ AACACGGAAT AAAGAAAGAG TGTCAGAACC CAAGAAATGT AAAATCAACT TACGTTCTAT TTAAAGTGAA   
  
  
+ AAATATTTTT CTTCATTCTA GGAAAATTAA TTGTCCTCTG AAAACAAATT TTCCCTCCTT GTCTAACCAA   
  
  
+ ACACAGTAAA ATTGAAAAAT CATTTTCTAG GAAAATGATT TTCACTCCTA CCAAACACAC CCTTAAACTT   
  
  
+ GTTCCAAATC TATAAACCCG GAATCTTAAT ACGCACATTG TATATTATAT GTCCTACTAT AACTGTATTA   
  
  
+ TCACTGGTTC TTGATTTATC CTTTCAAACT CTTGCTTAGT CAAGGTTGTC CTCGTACAAA CATGATTAAT   
  
  
+ TCAATTAACC ACGTAAGTTA TACGCTGACA AATTTCGGGT ACCAAACATT TGCACTAGAC CTAAAACAGT   
  
  
+ TACTATCATC ATATCGATGT TAAAATTGAC CTGTTCTTAT TTGTCCAGCG TTGAGCAAGG AAATAATCAA   
  
  
+ ATCATGTCTC ATTTAGTCGG TTTTTTGCCT AAGAAGAGCA TAAACAAAGG AAAAAGACAA CGACACATTA   
  
  
+ TACATGCATC TACACAAATG AAATGAGGCC ATATGGAACA TGTTCATGAG AGCTTCACTC GCTAATAATT   
  
  
+ GAACCTACTT TTGACTAAGC CACCAACTAG AATATGGAGA TTGACTGTTA AGAGACTATA TTACATGAAA   
  
  
+ TAGATAAGAG TAGACATTGC TATATATTTT ATCTTTAATT GTCTATTTTT AATGATCATT ACTGTTTATT   
  
  
+ TCAGTGAGTA ATTTTCTCGG ATATAATATT TTATAGCGGT TATTAAATAG ATGTGAAACT ATAATGTTAG   
  
  
+ ACATATCATG CGAAAATAGA GAAATTTTGG GCAAATTAAA AAAAAAAGAA GAAAAACGAG GAAGTATGCC   
  
  
+ CATTGTAAGC AGGTGGATGT ATATCCAGCC TCCATCCAAC AATTTGGGCA AAATTATATC ATCCATTCCA   
  
  
+ AAAAAAAGAT ATAATTTGTG ATATTAATAA CCCGACTCAA TTCAAGCTTA TTTATTTTTT CATATAATTT   
  
  
+ TTTAATATAT AAAAATAAAA AGTGACAACG ACATGCATCC TTTGCGTGTC AAAGGATCCG CCGCGTGGCC   
  
  
+ CCCACATTCA CTGACGGGGT TTTTGGTTTG CGATTAAGAT CAATGGCTAA CGTTGTCTCT CTCAAAACAT   
  
  
+ ATGGACCCCT TTTTCTATCT TTTTCTGCAT TGTCAGCATA GCAATTGTTG AGCACATTTT TTTCGATTTG   
  
  
+ TTGACAATAA GCGTGGTTAT GATTTATGGG CCACTAATAT CAGTCTCTGA TCATTTGCAC ATTTTGGACC   
  
  
+ TCTTTCTCTC TCTAATCTTC CAGGTAACCT GAATTCTTCT CTATTTCTGA CTAGATTAGA TAACAGGATT   
  
  
+ TCAAATATCT GTCAGGTGGC GGGTATAACT TCGTGATGGG CATTCATTTT TGTTCTATCA GGCTGGTGGG   
  
  
+ TTTGAGTTTA TTTTGATTCA TTTCGTGTTC ATCTGTGATA TTAGTAGGAG GGATTCTGTA TACCCACCAA   
  
  
+ CTGCTCGCCC TTTTGCCCGT GTGAGCAATT GGGACTTTTG TTTGTTCTTC GTCGATGCAT CCTCTGGTTG   
  
  
+ CTGATCTGGA ATTGAAACCC CATGTATTCA AATTCAACCC TGATTTGCTG TCAAACTTTC TGAACCATCA   
  
  
+ AAACTCCGCC GAAGTGTTTG AAAAAGATGG CATCTTCCAG ACCCTTCATT TGGCTGATCC CAAAAGCTCT   
  
  
+ TCAATTGCCG AAATCGGGTT TACTAATAGT TCAGATTCTA CACAAGTACC TGATTTTTCA GATGCTTGTC   
  
  
+ TTAAGTTCAT TAGTGATATT CTCCTGGAAG AGGACTTAGA TGAAAGTCCT ACGTCTTTAC ACGATTACAT   
  
  
+ GGCTCTCCTA GCCACTGAGA AGTCTTTGTA TGATGCTCTT GGAAAGGAAT ACTTTCCTTC GTCTACTAGT   
  
  
+ CTTGCCCCAT CTTTAGGCCG AAGTGTTGAC AGCCCAGATA GTGGCTTTGG CCGCGGTTGC TCTGATGGTC   
  
  
+ GCGGGATTGA GGGTTTGGCT AATGATGATG CCGTTTTCAT GTCTAACTGG CAGCTCAACA CCACCCAATT   
  
  
+ GGACCCTTTC CCAATCATGC AAGATATTCC TCGTCCCTAT TTGGAATTGA ATTACCATTC TTCTGGGTCA   
  
  
+ AGCAATGGCA TTGATGATTC GGGGGATGGG TTATCGACCT CTCCTGTAAG TACACTTGCA TCAACCGCCA   
  
  
+ CAGAGGCAGG GAAAAAGTTG GCTGGTAGCT CTAGGAGAAA GAACCGTCAA AGGGATGACT ATGGCCATGA   
  
  
+ AGAGGGAAGG AGTAACAAGC AGCAAGCCTC TTACAATGAC GATTACGTTG AGATGGAGCA GTATGACGAT   
  
  
+ GTACTTCTCT GTAGGGCAGA CAAGGGTAAT ATTTCAACTT GTGCCAATGA ATCCTCGCTT AATGAGGTGC   
  
  
+ GTGAGAAGCT GCAGACGACA GGGTTCAAGG GAAGAACATC CCGTCTTAAG AAGCAATCTA AGGAAGCGAA   
  
  
+ AGAGGTGGAT CTGAGAACTC TTCTTTCTGG TTGTGCACAA GCTGTTTCGA ACTTTGATAT CAGGACTGCT   
  
  
+ AATGAGCTGC TTAAGCAAGT CAGACAGCAT TCTTCACCAT ATGGTGATAG CCTCCAAAGG CTCGCCCATC   
  
  
+ AATTTGCGAA TGGTATTGAG GCACGCTTAG CAGGCACCGG TTCAAGAGTA CCTGCTAATC TCATTGATGC   
  
  
+ ACGGATATCA TCATCTGAAT TTTTAAAAGC TTACAAGTCA TATGTTTCAG CAGTTCCTTT CAGAAGGATG   
  
  
+ TCCTATTTTA TAGCAAACAA CACAATTCTG AAGTTGGCTG AGAAAGCAAC AAAGATTCAC ATAATTGATT   
  
  
+ TTGGTATTCT ATTTGGTCTA CAGTGGCCCT GTCTTATACA AAGTCTCTCA AGGCGAACTG TGGCTCCTCC   
  
  
+ GAAGCTTCGC ATCACTGGGA TAGACTATCC CCAGCATGGT TTCCGGCCAG CAGAAAAGGT TGAGGCAACA   
  
  
+ GGTCGTCGGT TGTCCGGGTA CTGTGAGAGA TTTAATGTAC CCTTTCGATA TGAAACCATT GCAAAGAAGT   
  
  
+ GGGAAACCAT ACGCCCAGAA GATCTAAATA TTGAGAATGA TGAGCTGGTA ATTGTTAATT GTATGTTGCG   
  
  
+ GTCTGTAAAT CTATTGGATG ATACAGTGGC GGTAAATAGT CCAAGGGATG CTTTCTTGAG GTTAATCAAA   
  
  
+ CAGATAAACC CGCGTTTATT CATTCATGCA ATTGTCAATG GAACCTTTAG TACTCCATTC TTCAGCACTC   
  
  
+ GATTCAGGGA AGCCCTATTC CAATACTCTT CTGTATTTGA TATATTTGAA GCGACTATGA CTCGTGAAGA   
  
  
+ TCGTGGAAGG CTGCTGATTG AGAGTCAAAT ATGCGGGCTA GAAGTTTTGA ATGCAATAGC ATGTGAAGGT   
  
  
+ GCAGAGAGGA TTCAAAGGCC TGAAACATAC AAGCAATGGC AGGAGCGGAC AACAAGGGCT GGACTAAGGC   
  
  
+ AGGTTCCAAT AGATGAGGAG CTTGTCAATA GAGCAAAGAC TATAGTGAAA GCAAATTATC ACAAGGATTT   
  
  
+ TGTGGTGGAT GAGGATAGGC GTTGGATGCT TCAAGGTTGG AAAGGAAGGA CACTTAGTGC CCTTTCCGTT   
  
  
+ TGGCAGCCTA ACTA  

- +Up\_Stream \_Len000AAATTA TTCTAGGAAA ATATATATAC ACACATATTA AGATACCATG TATCCATACA   
  
  
- TGGTATCTGA ATAACGTAAA GATCCAGGAA GAGAGAGACG CGAACAAAAA ACAAATAGAT TAAATACCTC   
  
  
- AACTTGTAAA ACAAAATATA TGAACTTGTT AGATAATATG ATTTAATTCG TAAAACTAAA TTAGTGAATA   
  
  
- CCCACACAAA CCATCCCAAC TTTTATTAAA AGGATCTTTT ATTAAAAATT ATATTTTATT AAAAAGTACA   
  
  
- TTTTACTAAA AGTTGAGTAT AAAAGTCAAC TAACCATTTT TCTTTTTACT TGAAAGACCT ATTAGTCAAA   
  
  
- CTAATTGTTT TTCACTGTTT TTTTAATTTT TACCAAAAGG CATTAGAAGT TTGGTATACA GCTCCTTTAT   
  
  
- TTGTGCCTTA TTTCTTTCTC ACAGTCTTGG GTTCTTTACA TTTTAGTTGA ATGCAAGATA AATTTCACTT   
  
  
- TTTATAAAAA GAAGTAAGAT CCTTTTAATT AACAGGAGAC TTTTGTTTAA AAGGGAGGAA CAGATTGGTT   
  
  
- TGTGTCATTT TAACTTTTTA GTAAAAGATC CTTTTACTAA AAGTGAGGAT GGTTTGTGTG GGAATTTGAA   
  
  
- CAAGGTTTAG ATATTTGGGC CTTAGAATTA TGCGTGTAAC ATATAATATA CAGGATGATA TTGACATAAT   
  
  
- AGTGACCAAG AACTAAATAG GAAAGTTTGA GAACGAATCA GTTCCAACAG GAGCATGTTT GTACTAATTA   
  
  
- AGTTAATTGG TGCATTCAAT ATGCGACTGT TTAAAGCCCA TGGTTTGTAA ACGTGATCTG GATTTTGTCA   
  
  
- ATGATAGTAG TATAGCTACA ATTTTAACTG GACAAGAATA AACAGGTCGC AACTCGTTCC TTTATTAGTT   
  
  
- TAGTACAGAG TAAATCAGCC AAAAAACGGA TTCTTCTCGT ATTTGTTTCC TTTTTCTGTT GCTGTGTAAT   
  
  
- ATGTACGTAG ATGTGTTTAC TTTACTCCGG TATACCTTGT ACAAGTACTC TCGAAGTGAG CGATTATTAA   
  
  
- CTTGGATGAA AACTGATTCG GTGGTTGATC TTATACCTCT AACTGACAAT TCTCTGATAT AATGTACTTT   
  
  
- ATCTATTCTC ATCTGTAACG ATATATAAAA TAGAAATTAA CAGATAAAAA TTACTAGTAA TGACAAATAA   
  
  
- AGTCACTCAT TAAAAGAGCC TATATTATAA AATATCGCCA ATAATTTATC TACACTTTGA TATTACAATC   
  
  
- TGTATAGTAC GCTTTTATCT CTTTAAAACC CGTTTAATTT TTTTTTTCTT CTTTTTGCTC CTTCATACGG   
  
  
- GTAACATTCG TCCACCTACA TATAGGTCGG AGGTAGGTTG TTAAACCCGT TTTAATATAG TAGGTAAGGT   
  
  
- TTTTTTTCTA TATTAAACAC TATAATTATT GGGCTGAGTT AAGTTCGAAT AAATAAAAAA GTATATTAAA   
  
  
- AAATTATATA TTTTTATTTT TCACTGTTGC TGTACGTAGG AAACGCACAG TTTCCTAGGC GGCGCACCGG   
  
  
- GGGTGTAAGT GACTGCCCCA AAAACCAAAC GCTAATTCTA GTTACCGATT GCAACAGAGA GAGTTTTGTA   
  
  
- TACCTGGGGA AAAAGATAGA AAAAGACGTA ACAGTCGTAT CGTTAACAAC TCGTGTAAAA AAAGCTAAAC   
  
  
- AACTGTTATT CGCACCAATA CTAAATACCC GGTGATTATA GTCAGAGACT AGTAAACGTG TAAAACCTGG   
  
  
- AGAAAGAGAG AGATTAGAAG GTCCATTGGA CTTAAGAAGA GATAAAGACT GATCTAATCT ATTGTCCTAA   
  
  
- AGTTTATAGA CAGTCCACCG CCCATATTGA AGCACTACCC GTAAGTAAAA ACAAGATAGT CCGACCACCC   
  
  
- AAACTCAAAT AAAACTAAGT AAAGCACAAG TAGACACTAT AATCATCCTC CCTAAGACAT ATGGGTGGTT   
  
  
- GACGAGCGGG AAAACGGGCA CACTCGTTAA CCCTGAAAAC AAACAAGAAG CAGCTACGTA GGAGACCAAC   
  
  
- GACTAGACCT TAACTTTGGG GTACATAAGT TTAAGTTGGG ACTAAACGAC AGTTTGAAAG ACTTGGTAGT   
  
  
- TTTGAGGCGG CTTCACAAAC TTTTTCTACC GTAGAAGGTC TGGGAAGTAA ACCGACTAGG GTTTTCGAGA   
  
  
- AGTTAACGGC TTTAGCCCAA ATGATTATCA AGTCTAAGAT GTGTTCATGG ACTAAAAAGT CTACGAACAG   
  
  
- AATTCAAGTA ATCACTATAA GAGGACCTTC TCCTGAATCT ACTTTCAGGA TGCAGAAATG TGCTAATGTA   
  
  
- CCGAGAGGAT CGGTGACTCT TCAGAAACAT ACTACGAGAA CCTTTCCTTA TGAAAGGAAG CAGATGATCA   
  
  
- GAACGGGGTA GAAATCCGGC TTCACAACTG TCGGGTCTAT CACCGAAACC GGCGCCAACG AGACTACCAG   
  
  
- CGCCCTAACT CCCAAACCGA TTACTACTAC GGCAAAAGTA CAGATTGACC GTCGAGTTGT GGTGGGTTAA   
  
  
- CCTGGGAAAG GGTTAGTACG TTCTATAAGG AGCAGGGATA AACCTTAACT TAATGGTAAG AAGACCCAGT   
  
  
- TCGTTACCGT AACTACTAAG CCCCCTACCC AATAGCTGGA GAGGACATTC ATGTGAACGT AGTTGGCGGT   
  
  
- GTCTCCGTCC CTTTTTCAAC CGACCATCGA GATCCTCTTT CTTGGCAGTT TCCCTACTGA TACCGGTACT   
  
  
- TCTCCCTTCC TCATTGTTCG TCGTTCGGAG AATGTTACTG CTAATGCAAC TCTACCTCGT CATACTGCTA   
  
  
- CATGAAGAGA CATCCCGTCT GTTCCCATTA TAAAGTTGAA CACGGTTACT TAGGAGCGAA TTACTCCACG   
  
  
- CACTCTTCGA CGTCTGCTGT CCCAAGTTCC CTTCTTGTAG GGCAGAATTC TTCGTTAGAT TCCTTCGCTT   
  
  
- TCTCCACCTA GACTCTTGAG AAGAAAGACC AACACGTGTT CGACAAAGCT TGAAACTATA GTCCTGACGA   
  
  
- TTACTCGACG AATTCGTTCA GTCTGTCGTA AGAAGTGGTA TACCACTATC GGAGGTTTCC GAGCGGGTAG   
  
  
- TTAAACGCTT ACCATAACTC CGTGCGAATC GTCCGTGGCC AAGTTCTCAT GGACGATTAG AGTAACTACG   
  
  
- TGCCTATAGT AGTAGACTTA AAAATTTTCG AATGTTCAGT ATACAAAGTC GTCAAGGAAA GTCTTCCTAC   
  
  
- AGGATAAAAT ATCGTTTGTT GTGTTAAGAC TTCAACCGAC TCTTTCGTTG TTTCTAAGTG TATTAACTAA   
  
  
- AACCATAAGA TAAACCAGAT GTCACCGGGA CAGAATATGT TTCAGAGAGT TCCGCTTGAC ACCGAGGAGG   
  
  
- CTTCGAAGCG TAGTGACCCT ATCTGATAGG GGTCGTACCA AAGGCCGGTC GTCTTTTCCA ACTCCGTTGT   
  
  
- CCAGCAGCCA ACAGGCCCAT GACACTCTCT AAATTACATG GGAAAGCTAT ACTTTGGTAA CGTTTCTTCA   
  
  
- CCCTTTGGTA TGCGGGTCTT CTAGATTTAT AACTCTTACT ACTCGACCAT TAACAATTAA CATACAACGC   
  
  
- CAGACATTTA GATAACCTAC TATGTCACCG CCATTTATCA GGTTCCCTAC GAAAGAACTC CAATTAGTTT   
  
  
- GTCTATTTGG GCGCAAATAA GTAAGTACGT TAACAGTTAC CTTGGAAATC ATGAGGTAAG AAGTCGTGAG   
  
  
- CTAAGTCCCT TCGGGATAAG GTTATGAGAA GACATAAACT ATATAAACTT CGCTGATACT GAGCACTTCT   
  
  
- AGCACCTTCC GACGACTAAC TCTCAGTTTA TACGCCCGAT CTTCAAAACT TACGTTATCG TACACTTCCA   
  
  
- CGTCTCTCCT AAGTTTCCGG ACTTTGTATG TTCGTTACCG TCCTCGCCTG TTGTTCCCGA CCTGATTCCG   
  
  
- TCCAAGGTTA TCTACTCCTC GAACAGTTAT CTCGTTTCTG ATATCACTTT CGTTTAATAG TGTTCCTAAA   
  
  
- ACACCACCTA CTCCTATCCG CAACCTACGA AGTTCCAACC TTTCCTTCCT GTGAATCACG GGAAAGGCAA   
  
  
- ACCGTCGGAT TGAT

+     GA-motif

| Site Name | Organism | Position | Strand | Matrix score. | sequence | function |
| --- | --- | --- | --- | --- | --- | --- |
| GA-motif | Arabidopsis thaliana | 1124 | + | 8 | ATAGATAA | part of a light responsive element |

>HU02G01572.1   
+ +Up\_Stream \_Len000TTTAAT AAGATCCTTT TATATATATG TGTGTATAAT TCTATGGTAC ATAGGTATGT   
  
  
+ ACCATAGACT TATTGCATTT CTAGGTCCTT CTCTCTCTGC GCTTGTTTTT TGTTTATCTA ATTTATGGAG   
  
  
+ TTGAACATTT TGTTTTATAT ACTTGAACAA TCTATTATAC TAAATTAAGC ATTTTGATTT AATCACTTAT   
  
  
+ GGGTGTGTTT GGTAGGGTTG AAAATAATTT TCCTAGAAAA TAATTTTTAA TATAAAATAA TTTTTCATGT   
  
  
+ AAAATGATTT TCAACTCATA TTTTCAGTTG ATTGGTAAAA AGAAAAATGA ACTTTCTGGA TAATCAGTTT   
  
  
+ GATTAACAAA AAGTGACAAA AAAATTAAAA ATGGTTTTCC GTAATCTTCA AACCATATGT CGAGGAAATA   
  
  
+ AACACGGAAT AAAGAAAGAG TGTCAGAACC CAAGAAATGT AAAATCAACT TACGTTCTAT TTAAAGTGAA   
  
  
+ AAATATTTTT CTTCATTCTA GGAAAATTAA TTGTCCTCTG AAAACAAATT TTCCCTCCTT GTCTAACCAA   
  
  
+ ACACAGTAAA ATTGAAAAAT CATTTTCTAG GAAAATGATT TTCACTCCTA CCAAACACAC CCTTAAACTT   
  
  
+ GTTCCAAATC TATAAACCCG GAATCTTAAT ACGCACATTG TATATTATAT GTCCTACTAT AACTGTATTA   
  
  
+ TCACTGGTTC TTGATTTATC CTTTCAAACT CTTGCTTAGT CAAGGTTGTC CTCGTACAAA CATGATTAAT   
  
  
+ TCAATTAACC ACGTAAGTTA TACGCTGACA AATTTCGGGT ACCAAACATT TGCACTAGAC CTAAAACAGT   
  
  
+ TACTATCATC ATATCGATGT TAAAATTGAC CTGTTCTTAT TTGTCCAGCG TTGAGCAAGG AAATAATCAA   
  
  
+ ATCATGTCTC ATTTAGTCGG TTTTTTGCCT AAGAAGAGCA TAAACAAAGG AAAAAGACAA CGACACATTA   
  
  
+ TACATGCATC TACACAAATG AAATGAGGCC ATATGGAACA TGTTCATGAG AGCTTCACTC GCTAATAATT   
  
  
+ GAACCTACTT TTGACTAAGC CACCAACTAG AATATGGAGA TTGACTGTTA AGAGACTATA TTACATGAAA   
  
  
+ TAGATAAGAG TAGACATTGC TATATATTTT ATCTTTAATT GTCTATTTTT AATGATCATT ACTGTTTATT   
  
  
+ TCAGTGAGTA ATTTTCTCGG ATATAATATT TTATAGCGGT TATTAAATAG ATGTGAAACT ATAATGTTAG   
  
  
+ ACATATCATG CGAAAATAGA GAAATTTTGG GCAAATTAAA AAAAAAAGAA GAAAAACGAG GAAGTATGCC   
  
  
+ CATTGTAAGC AGGTGGATGT ATATCCAGCC TCCATCCAAC AATTTGGGCA AAATTATATC ATCCATTCCA   
  
  
+ AAAAAAAGAT ATAATTTGTG ATATTAATAA CCCGACTCAA TTCAAGCTTA TTTATTTTTT CATATAATTT   
  
  
+ TTTAATATAT AAAAATAAAA AGTGACAACG ACATGCATCC TTTGCGTGTC AAAGGATCCG CCGCGTGGCC   
  
  
+ CCCACATTCA CTGACGGGGT TTTTGGTTTG CGATTAAGAT CAATGGCTAA CGTTGTCTCT CTCAAAACAT   
  
  
+ ATGGACCCCT TTTTCTATCT TTTTCTGCAT TGTCAGCATA GCAATTGTTG AGCACATTTT TTTCGATTTG   
  
  
+ TTGACAATAA GCGTGGTTAT GATTTATGGG CCACTAATAT CAGTCTCTGA TCATTTGCAC ATTTTGGACC   
  
  
+ TCTTTCTCTC TCTAATCTTC CAGGTAACCT GAATTCTTCT CTATTTCTGA CTAGATTAGA TAACAGGATT   
  
  
+ TCAAATATCT GTCAGGTGGC GGGTATAACT TCGTGATGGG CATTCATTTT TGTTCTATCA GGCTGGTGGG   
  
  
+ TTTGAGTTTA TTTTGATTCA TTTCGTGTTC ATCTGTGATA TTAGTAGGAG GGATTCTGTA TACCCACCAA   
  
  
+ CTGCTCGCCC TTTTGCCCGT GTGAGCAATT GGGACTTTTG TTTGTTCTTC GTCGATGCAT CCTCTGGTTG   
  
  
+ CTGATCTGGA ATTGAAACCC CATGTATTCA AATTCAACCC TGATTTGCTG TCAAACTTTC TGAACCATCA   
  
  
+ AAACTCCGCC GAAGTGTTTG AAAAAGATGG CATCTTCCAG ACCCTTCATT TGGCTGATCC CAAAAGCTCT   
  
  
+ TCAATTGCCG AAATCGGGTT TACTAATAGT TCAGATTCTA CACAAGTACC TGATTTTTCA GATGCTTGTC   
  
  
+ TTAAGTTCAT TAGTGATATT CTCCTGGAAG AGGACTTAGA TGAAAGTCCT ACGTCTTTAC ACGATTACAT   
  
  
+ GGCTCTCCTA GCCACTGAGA AGTCTTTGTA TGATGCTCTT GGAAAGGAAT ACTTTCCTTC GTCTACTAGT   
  
  
+ CTTGCCCCAT CTTTAGGCCG AAGTGTTGAC AGCCCAGATA GTGGCTTTGG CCGCGGTTGC TCTGATGGTC   
  
  
+ GCGGGATTGA GGGTTTGGCT AATGATGATG CCGTTTTCAT GTCTAACTGG CAGCTCAACA CCACCCAATT   
  
  
+ GGACCCTTTC CCAATCATGC AAGATATTCC TCGTCCCTAT TTGGAATTGA ATTACCATTC TTCTGGGTCA   
  
  
+ AGCAATGGCA TTGATGATTC GGGGGATGGG TTATCGACCT CTCCTGTAAG TACACTTGCA TCAACCGCCA   
  
  
+ CAGAGGCAGG GAAAAAGTTG GCTGGTAGCT CTAGGAGAAA GAACCGTCAA AGGGATGACT ATGGCCATGA   
  
  
+ AGAGGGAAGG AGTAACAAGC AGCAAGCCTC TTACAATGAC GATTACGTTG AGATGGAGCA GTATGACGAT   
  
  
+ GTACTTCTCT GTAGGGCAGA CAAGGGTAAT ATTTCAACTT GTGCCAATGA ATCCTCGCTT AATGAGGTGC   
  
  
+ GTGAGAAGCT GCAGACGACA GGGTTCAAGG GAAGAACATC CCGTCTTAAG AAGCAATCTA AGGAAGCGAA   
  
  
+ AGAGGTGGAT CTGAGAACTC TTCTTTCTGG TTGTGCACAA GCTGTTTCGA ACTTTGATAT CAGGACTGCT   
  
  
+ AATGAGCTGC TTAAGCAAGT CAGACAGCAT TCTTCACCAT ATGGTGATAG CCTCCAAAGG CTCGCCCATC   
  
  
+ AATTTGCGAA TGGTATTGAG GCACGCTTAG CAGGCACCGG TTCAAGAGTA CCTGCTAATC TCATTGATGC   
  
  
+ ACGGATATCA TCATCTGAAT TTTTAAAAGC TTACAAGTCA TATGTTTCAG CAGTTCCTTT CAGAAGGATG   
  
  
+ TCCTATTTTA TAGCAAACAA CACAATTCTG AAGTTGGCTG AGAAAGCAAC AAAGATTCAC ATAATTGATT   
  
  
+ TTGGTATTCT ATTTGGTCTA CAGTGGCCCT GTCTTATACA AAGTCTCTCA AGGCGAACTG TGGCTCCTCC   
  
  
+ GAAGCTTCGC ATCACTGGGA TAGACTATCC CCAGCATGGT TTCCGGCCAG CAGAAAAGGT TGAGGCAACA   
  
  
+ GGTCGTCGGT TGTCCGGGTA CTGTGAGAGA TTTAATGTAC CCTTTCGATA TGAAACCATT GCAAAGAAGT   
  
  
+ GGGAAACCAT ACGCCCAGAA GATCTAAATA TTGAGAATGA TGAGCTGGTA ATTGTTAATT GTATGTTGCG   
  
  
+ GTCTGTAAAT CTATTGGATG ATACAGTGGC GGTAAATAGT CCAAGGGATG CTTTCTTGAG GTTAATCAAA   
  
  
+ CAGATAAACC CGCGTTTATT CATTCATGCA ATTGTCAATG GAACCTTTAG TACTCCATTC TTCAGCACTC   
  
  
+ GATTCAGGGA AGCCCTATTC CAATACTCTT CTGTATTTGA TATATTTGAA GCGACTATGA CTCGTGAAGA   
  
  
+ TCGTGGAAGG CTGCTGATTG AGAGTCAAAT ATGCGGGCTA GAAGTTTTGA ATGCAATAGC ATGTGAAGGT   
  
  
+ GCAGAGAGGA TTCAAAGGCC TGAAACATAC AAGCAATGGC AGGAGCGGAC AACAAGGGCT GGACTAAGGC   
  
  
+ AGGTTCCAAT AGATGAGGAG CTTGTCAATA GAGCAAAGAC TATAGTGAAA GCAAATTATC ACAAGGATTT   
  
  
+ TGTGGTGGAT GAGGATAGGC GTTGGATGCT TCAAGGTTGG AAAGGAAGGA CACTTAGTGC CCTTTCCGTT   
  
  
+ TGGCAGCCTA ACTA  

- +Up\_Stream \_Len000AAATTA TTCTAGGAAA ATATATATAC ACACATATTA AGATACCATG TATCCATACA   
  
  
- TGGTATCTGA ATAACGTAAA GATCCAGGAA GAGAGAGACG CGAACAAAAA ACAAATAGAT TAAATACCTC   
  
  
- AACTTGTAAA ACAAAATATA TGAACTTGTT AGATAATATG ATTTAATTCG TAAAACTAAA TTAGTGAATA   
  
  
- CCCACACAAA CCATCCCAAC TTTTATTAAA AGGATCTTTT ATTAAAAATT ATATTTTATT AAAAAGTACA   
  
  
- TTTTACTAAA AGTTGAGTAT AAAAGTCAAC TAACCATTTT TCTTTTTACT TGAAAGACCT ATTAGTCAAA   
  
  
- CTAATTGTTT TTCACTGTTT TTTTAATTTT TACCAAAAGG CATTAGAAGT TTGGTATACA GCTCCTTTAT   
  
  
- TTGTGCCTTA TTTCTTTCTC ACAGTCTTGG GTTCTTTACA TTTTAGTTGA ATGCAAGATA AATTTCACTT   
  
  
- TTTATAAAAA GAAGTAAGAT CCTTTTAATT AACAGGAGAC TTTTGTTTAA AAGGGAGGAA CAGATTGGTT   
  
  
- TGTGTCATTT TAACTTTTTA GTAAAAGATC CTTTTACTAA AAGTGAGGAT GGTTTGTGTG GGAATTTGAA   
  
  
- CAAGGTTTAG ATATTTGGGC CTTAGAATTA TGCGTGTAAC ATATAATATA CAGGATGATA TTGACATAAT   
  
  
- AGTGACCAAG AACTAAATAG GAAAGTTTGA GAACGAATCA GTTCCAACAG GAGCATGTTT GTACTAATTA   
  
  
- AGTTAATTGG TGCATTCAAT ATGCGACTGT TTAAAGCCCA TGGTTTGTAA ACGTGATCTG GATTTTGTCA   
  
  
- ATGATAGTAG TATAGCTACA ATTTTAACTG GACAAGAATA AACAGGTCGC AACTCGTTCC TTTATTAGTT   
  
  
- TAGTACAGAG TAAATCAGCC AAAAAACGGA TTCTTCTCGT ATTTGTTTCC TTTTTCTGTT GCTGTGTAAT   
  
  
- ATGTACGTAG ATGTGTTTAC TTTACTCCGG TATACCTTGT ACAAGTACTC TCGAAGTGAG CGATTATTAA   
  
  
- CTTGGATGAA AACTGATTCG GTGGTTGATC TTATACCTCT AACTGACAAT TCTCTGATAT AATGTACTTT   
  
  
- ATCTATTCTC ATCTGTAACG ATATATAAAA TAGAAATTAA CAGATAAAAA TTACTAGTAA TGACAAATAA   
  
  
- AGTCACTCAT TAAAAGAGCC TATATTATAA AATATCGCCA ATAATTTATC TACACTTTGA TATTACAATC   
  
  
- TGTATAGTAC GCTTTTATCT CTTTAAAACC CGTTTAATTT TTTTTTTCTT CTTTTTGCTC CTTCATACGG   
  
  
- GTAACATTCG TCCACCTACA TATAGGTCGG AGGTAGGTTG TTAAACCCGT TTTAATATAG TAGGTAAGGT   
  
  
- TTTTTTTCTA TATTAAACAC TATAATTATT GGGCTGAGTT AAGTTCGAAT AAATAAAAAA GTATATTAAA   
  
  
- AAATTATATA TTTTTATTTT TCACTGTTGC TGTACGTAGG AAACGCACAG TTTCCTAGGC GGCGCACCGG   
  
  
- GGGTGTAAGT GACTGCCCCA AAAACCAAAC GCTAATTCTA GTTACCGATT GCAACAGAGA GAGTTTTGTA   
  
  
- TACCTGGGGA AAAAGATAGA AAAAGACGTA ACAGTCGTAT CGTTAACAAC TCGTGTAAAA AAAGCTAAAC   
  
  
- AACTGTTATT CGCACCAATA CTAAATACCC GGTGATTATA GTCAGAGACT AGTAAACGTG TAAAACCTGG   
  
  
- AGAAAGAGAG AGATTAGAAG GTCCATTGGA CTTAAGAAGA GATAAAGACT GATCTAATCT ATTGTCCTAA   
  
  
- AGTTTATAGA CAGTCCACCG CCCATATTGA AGCACTACCC GTAAGTAAAA ACAAGATAGT CCGACCACCC   
  
  
- AAACTCAAAT AAAACTAAGT AAAGCACAAG TAGACACTAT AATCATCCTC CCTAAGACAT ATGGGTGGTT   
  
  
- GACGAGCGGG AAAACGGGCA CACTCGTTAA CCCTGAAAAC AAACAAGAAG CAGCTACGTA GGAGACCAAC   
  
  
- GACTAGACCT TAACTTTGGG GTACATAAGT TTAAGTTGGG ACTAAACGAC AGTTTGAAAG ACTTGGTAGT   
  
  
- TTTGAGGCGG CTTCACAAAC TTTTTCTACC GTAGAAGGTC TGGGAAGTAA ACCGACTAGG GTTTTCGAGA   
  
  
- AGTTAACGGC TTTAGCCCAA ATGATTATCA AGTCTAAGAT GTGTTCATGG ACTAAAAAGT CTACGAACAG   
  
  
- AATTCAAGTA ATCACTATAA GAGGACCTTC TCCTGAATCT ACTTTCAGGA TGCAGAAATG TGCTAATGTA   
  
  
- CCGAGAGGAT CGGTGACTCT TCAGAAACAT ACTACGAGAA CCTTTCCTTA TGAAAGGAAG CAGATGATCA   
  
  
- GAACGGGGTA GAAATCCGGC TTCACAACTG TCGGGTCTAT CACCGAAACC GGCGCCAACG AGACTACCAG   
  
  
- CGCCCTAACT CCCAAACCGA TTACTACTAC GGCAAAAGTA CAGATTGACC GTCGAGTTGT GGTGGGTTAA   
  
  
- CCTGGGAAAG GGTTAGTACG TTCTATAAGG AGCAGGGATA AACCTTAACT TAATGGTAAG AAGACCCAGT   
  
  
- TCGTTACCGT AACTACTAAG CCCCCTACCC AATAGCTGGA GAGGACATTC ATGTGAACGT AGTTGGCGGT   
  
  
- GTCTCCGTCC CTTTTTCAAC CGACCATCGA GATCCTCTTT CTTGGCAGTT TCCCTACTGA TACCGGTACT   
  
  
- TCTCCCTTCC TCATTGTTCG TCGTTCGGAG AATGTTACTG CTAATGCAAC TCTACCTCGT CATACTGCTA   
  
  
- CATGAAGAGA CATCCCGTCT GTTCCCATTA TAAAGTTGAA CACGGTTACT TAGGAGCGAA TTACTCCACG   
  
  
- CACTCTTCGA CGTCTGCTGT CCCAAGTTCC CTTCTTGTAG GGCAGAATTC TTCGTTAGAT TCCTTCGCTT   
  
  
- TCTCCACCTA GACTCTTGAG AAGAAAGACC AACACGTGTT CGACAAAGCT TGAAACTATA GTCCTGACGA   
  
  
- TTACTCGACG AATTCGTTCA GTCTGTCGTA AGAAGTGGTA TACCACTATC GGAGGTTTCC GAGCGGGTAG   
  
  
- TTAAACGCTT ACCATAACTC CGTGCGAATC GTCCGTGGCC AAGTTCTCAT GGACGATTAG AGTAACTACG   
  
  
- TGCCTATAGT AGTAGACTTA AAAATTTTCG AATGTTCAGT ATACAAAGTC GTCAAGGAAA GTCTTCCTAC   
  
  
- AGGATAAAAT ATCGTTTGTT GTGTTAAGAC TTCAACCGAC TCTTTCGTTG TTTCTAAGTG TATTAACTAA   
  
  
- AACCATAAGA TAAACCAGAT GTCACCGGGA CAGAATATGT TTCAGAGAGT TCCGCTTGAC ACCGAGGAGG   
  
  
- CTTCGAAGCG TAGTGACCCT ATCTGATAGG GGTCGTACCA AAGGCCGGTC GTCTTTTCCA ACTCCGTTGT   
  
  
- CCAGCAGCCA ACAGGCCCAT GACACTCTCT AAATTACATG GGAAAGCTAT ACTTTGGTAA CGTTTCTTCA   
  
  
- CCCTTTGGTA TGCGGGTCTT CTAGATTTAT AACTCTTACT ACTCGACCAT TAACAATTAA CATACAACGC   
  
  
- CAGACATTTA GATAACCTAC TATGTCACCG CCATTTATCA GGTTCCCTAC GAAAGAACTC CAATTAGTTT   
  
  
- GTCTATTTGG GCGCAAATAA GTAAGTACGT TAACAGTTAC CTTGGAAATC ATGAGGTAAG AAGTCGTGAG   
  
  
- CTAAGTCCCT TCGGGATAAG GTTATGAGAA GACATAAACT ATATAAACTT CGCTGATACT GAGCACTTCT   
  
  
- AGCACCTTCC GACGACTAAC TCTCAGTTTA TACGCCCGAT CTTCAAAACT TACGTTATCG TACACTTCCA   
  
  
- CGTCTCTCCT AAGTTTCCGG ACTTTGTATG TTCGTTACCG TCCTCGCCTG TTGTTCCCGA CCTGATTCCG   
  
  
- TCCAAGGTTA TCTACTCCTC GAACAGTTAT CTCGTTTCTG ATATCACTTT CGTTTAATAG TGTTCCTAAA   
  
  
- ACACCACCTA CTCCTATCCG CAACCTACGA AGTTCCAACC TTTCCTTCCT GTGAATCACG GGAAAGGCAA   
  
  
- ACCGTCGGAT TGAT

+     GC-motif

| Site Name | Organism | Position | Strand | Matrix score. | sequence | function |
| --- | --- | --- | --- | --- | --- | --- |
| GC-motif | Zea mays | 2614 | - | 6 | CCCCCG | enhancer-like element involved in anoxic specific inducibility |

>HU02G01572.1   
+ +Up\_Stream \_Len000TTTAAT AAGATCCTTT TATATATATG TGTGTATAAT TCTATGGTAC ATAGGTATGT   
  
  
+ ACCATAGACT TATTGCATTT CTAGGTCCTT CTCTCTCTGC GCTTGTTTTT TGTTTATCTA ATTTATGGAG   
  
  
+ TTGAACATTT TGTTTTATAT ACTTGAACAA TCTATTATAC TAAATTAAGC ATTTTGATTT AATCACTTAT   
  
  
+ GGGTGTGTTT GGTAGGGTTG AAAATAATTT TCCTAGAAAA TAATTTTTAA TATAAAATAA TTTTTCATGT   
  
  
+ AAAATGATTT TCAACTCATA TTTTCAGTTG ATTGGTAAAA AGAAAAATGA ACTTTCTGGA TAATCAGTTT   
  
  
+ GATTAACAAA AAGTGACAAA AAAATTAAAA ATGGTTTTCC GTAATCTTCA AACCATATGT CGAGGAAATA   
  
  
+ AACACGGAAT AAAGAAAGAG TGTCAGAACC CAAGAAATGT AAAATCAACT TACGTTCTAT TTAAAGTGAA   
  
  
+ AAATATTTTT CTTCATTCTA GGAAAATTAA TTGTCCTCTG AAAACAAATT TTCCCTCCTT GTCTAACCAA   
  
  
+ ACACAGTAAA ATTGAAAAAT CATTTTCTAG GAAAATGATT TTCACTCCTA CCAAACACAC CCTTAAACTT   
  
  
+ GTTCCAAATC TATAAACCCG GAATCTTAAT ACGCACATTG TATATTATAT GTCCTACTAT AACTGTATTA   
  
  
+ TCACTGGTTC TTGATTTATC CTTTCAAACT CTTGCTTAGT CAAGGTTGTC CTCGTACAAA CATGATTAAT   
  
  
+ TCAATTAACC ACGTAAGTTA TACGCTGACA AATTTCGGGT ACCAAACATT TGCACTAGAC CTAAAACAGT   
  
  
+ TACTATCATC ATATCGATGT TAAAATTGAC CTGTTCTTAT TTGTCCAGCG TTGAGCAAGG AAATAATCAA   
  
  
+ ATCATGTCTC ATTTAGTCGG TTTTTTGCCT AAGAAGAGCA TAAACAAAGG AAAAAGACAA CGACACATTA   
  
  
+ TACATGCATC TACACAAATG AAATGAGGCC ATATGGAACA TGTTCATGAG AGCTTCACTC GCTAATAATT   
  
  
+ GAACCTACTT TTGACTAAGC CACCAACTAG AATATGGAGA TTGACTGTTA AGAGACTATA TTACATGAAA   
  
  
+ TAGATAAGAG TAGACATTGC TATATATTTT ATCTTTAATT GTCTATTTTT AATGATCATT ACTGTTTATT   
  
  
+ TCAGTGAGTA ATTTTCTCGG ATATAATATT TTATAGCGGT TATTAAATAG ATGTGAAACT ATAATGTTAG   
  
  
+ ACATATCATG CGAAAATAGA GAAATTTTGG GCAAATTAAA AAAAAAAGAA GAAAAACGAG GAAGTATGCC   
  
  
+ CATTGTAAGC AGGTGGATGT ATATCCAGCC TCCATCCAAC AATTTGGGCA AAATTATATC ATCCATTCCA   
  
  
+ AAAAAAAGAT ATAATTTGTG ATATTAATAA CCCGACTCAA TTCAAGCTTA TTTATTTTTT CATATAATTT   
  
  
+ TTTAATATAT AAAAATAAAA AGTGACAACG ACATGCATCC TTTGCGTGTC AAAGGATCCG CCGCGTGGCC   
  
  
+ CCCACATTCA CTGACGGGGT TTTTGGTTTG CGATTAAGAT CAATGGCTAA CGTTGTCTCT CTCAAAACAT   
  
  
+ ATGGACCCCT TTTTCTATCT TTTTCTGCAT TGTCAGCATA GCAATTGTTG AGCACATTTT TTTCGATTTG   
  
  
+ TTGACAATAA GCGTGGTTAT GATTTATGGG CCACTAATAT CAGTCTCTGA TCATTTGCAC ATTTTGGACC   
  
  
+ TCTTTCTCTC TCTAATCTTC CAGGTAACCT GAATTCTTCT CTATTTCTGA CTAGATTAGA TAACAGGATT   
  
  
+ TCAAATATCT GTCAGGTGGC GGGTATAACT TCGTGATGGG CATTCATTTT TGTTCTATCA GGCTGGTGGG   
  
  
+ TTTGAGTTTA TTTTGATTCA TTTCGTGTTC ATCTGTGATA TTAGTAGGAG GGATTCTGTA TACCCACCAA   
  
  
+ CTGCTCGCCC TTTTGCCCGT GTGAGCAATT GGGACTTTTG TTTGTTCTTC GTCGATGCAT CCTCTGGTTG   
  
  
+ CTGATCTGGA ATTGAAACCC CATGTATTCA AATTCAACCC TGATTTGCTG TCAAACTTTC TGAACCATCA   
  
  
+ AAACTCCGCC GAAGTGTTTG AAAAAGATGG CATCTTCCAG ACCCTTCATT TGGCTGATCC CAAAAGCTCT   
  
  
+ TCAATTGCCG AAATCGGGTT TACTAATAGT TCAGATTCTA CACAAGTACC TGATTTTTCA GATGCTTGTC   
  
  
+ TTAAGTTCAT TAGTGATATT CTCCTGGAAG AGGACTTAGA TGAAAGTCCT ACGTCTTTAC ACGATTACAT   
  
  
+ GGCTCTCCTA GCCACTGAGA AGTCTTTGTA TGATGCTCTT GGAAAGGAAT ACTTTCCTTC GTCTACTAGT   
  
  
+ CTTGCCCCAT CTTTAGGCCG AAGTGTTGAC AGCCCAGATA GTGGCTTTGG CCGCGGTTGC TCTGATGGTC   
  
  
+ GCGGGATTGA GGGTTTGGCT AATGATGATG CCGTTTTCAT GTCTAACTGG CAGCTCAACA CCACCCAATT   
  
  
+ GGACCCTTTC CCAATCATGC AAGATATTCC TCGTCCCTAT TTGGAATTGA ATTACCATTC TTCTGGGTCA   
  
  
+ AGCAATGGCA TTGATGATTC GGGGGATGGG TTATCGACCT CTCCTGTAAG TACACTTGCA TCAACCGCCA   
  
  
+ CAGAGGCAGG GAAAAAGTTG GCTGGTAGCT CTAGGAGAAA GAACCGTCAA AGGGATGACT ATGGCCATGA   
  
  
+ AGAGGGAAGG AGTAACAAGC AGCAAGCCTC TTACAATGAC GATTACGTTG AGATGGAGCA GTATGACGAT   
  
  
+ GTACTTCTCT GTAGGGCAGA CAAGGGTAAT ATTTCAACTT GTGCCAATGA ATCCTCGCTT AATGAGGTGC   
  
  
+ GTGAGAAGCT GCAGACGACA GGGTTCAAGG GAAGAACATC CCGTCTTAAG AAGCAATCTA AGGAAGCGAA   
  
  
+ AGAGGTGGAT CTGAGAACTC TTCTTTCTGG TTGTGCACAA GCTGTTTCGA ACTTTGATAT CAGGACTGCT   
  
  
+ AATGAGCTGC TTAAGCAAGT CAGACAGCAT TCTTCACCAT ATGGTGATAG CCTCCAAAGG CTCGCCCATC   
  
  
+ AATTTGCGAA TGGTATTGAG GCACGCTTAG CAGGCACCGG TTCAAGAGTA CCTGCTAATC TCATTGATGC   
  
  
+ ACGGATATCA TCATCTGAAT TTTTAAAAGC TTACAAGTCA TATGTTTCAG CAGTTCCTTT CAGAAGGATG   
  
  
+ TCCTATTTTA TAGCAAACAA CACAATTCTG AAGTTGGCTG AGAAAGCAAC AAAGATTCAC ATAATTGATT   
  
  
+ TTGGTATTCT ATTTGGTCTA CAGTGGCCCT GTCTTATACA AAGTCTCTCA AGGCGAACTG TGGCTCCTCC   
  
  
+ GAAGCTTCGC ATCACTGGGA TAGACTATCC CCAGCATGGT TTCCGGCCAG CAGAAAAGGT TGAGGCAACA   
  
  
+ GGTCGTCGGT TGTCCGGGTA CTGTGAGAGA TTTAATGTAC CCTTTCGATA TGAAACCATT GCAAAGAAGT   
  
  
+ GGGAAACCAT ACGCCCAGAA GATCTAAATA TTGAGAATGA TGAGCTGGTA ATTGTTAATT GTATGTTGCG   
  
  
+ GTCTGTAAAT CTATTGGATG ATACAGTGGC GGTAAATAGT CCAAGGGATG CTTTCTTGAG GTTAATCAAA   
  
  
+ CAGATAAACC CGCGTTTATT CATTCATGCA ATTGTCAATG GAACCTTTAG TACTCCATTC TTCAGCACTC   
  
  
+ GATTCAGGGA AGCCCTATTC CAATACTCTT CTGTATTTGA TATATTTGAA GCGACTATGA CTCGTGAAGA   
  
  
+ TCGTGGAAGG CTGCTGATTG AGAGTCAAAT ATGCGGGCTA GAAGTTTTGA ATGCAATAGC ATGTGAAGGT   
  
  
+ GCAGAGAGGA TTCAAAGGCC TGAAACATAC AAGCAATGGC AGGAGCGGAC AACAAGGGCT GGACTAAGGC   
  
  
+ AGGTTCCAAT AGATGAGGAG CTTGTCAATA GAGCAAAGAC TATAGTGAAA GCAAATTATC ACAAGGATTT   
  
  
+ TGTGGTGGAT GAGGATAGGC GTTGGATGCT TCAAGGTTGG AAAGGAAGGA CACTTAGTGC CCTTTCCGTT   
  
  
+ TGGCAGCCTA ACTA  

- +Up\_Stream \_Len000AAATTA TTCTAGGAAA ATATATATAC ACACATATTA AGATACCATG TATCCATACA   
  
  
- TGGTATCTGA ATAACGTAAA GATCCAGGAA GAGAGAGACG CGAACAAAAA ACAAATAGAT TAAATACCTC   
  
  
- AACTTGTAAA ACAAAATATA TGAACTTGTT AGATAATATG ATTTAATTCG TAAAACTAAA TTAGTGAATA   
  
  
- CCCACACAAA CCATCCCAAC TTTTATTAAA AGGATCTTTT ATTAAAAATT ATATTTTATT AAAAAGTACA   
  
  
- TTTTACTAAA AGTTGAGTAT AAAAGTCAAC TAACCATTTT TCTTTTTACT TGAAAGACCT ATTAGTCAAA   
  
  
- CTAATTGTTT TTCACTGTTT TTTTAATTTT TACCAAAAGG CATTAGAAGT TTGGTATACA GCTCCTTTAT   
  
  
- TTGTGCCTTA TTTCTTTCTC ACAGTCTTGG GTTCTTTACA TTTTAGTTGA ATGCAAGATA AATTTCACTT   
  
  
- TTTATAAAAA GAAGTAAGAT CCTTTTAATT AACAGGAGAC TTTTGTTTAA AAGGGAGGAA CAGATTGGTT   
  
  
- TGTGTCATTT TAACTTTTTA GTAAAAGATC CTTTTACTAA AAGTGAGGAT GGTTTGTGTG GGAATTTGAA   
  
  
- CAAGGTTTAG ATATTTGGGC CTTAGAATTA TGCGTGTAAC ATATAATATA CAGGATGATA TTGACATAAT   
  
  
- AGTGACCAAG AACTAAATAG GAAAGTTTGA GAACGAATCA GTTCCAACAG GAGCATGTTT GTACTAATTA   
  
  
- AGTTAATTGG TGCATTCAAT ATGCGACTGT TTAAAGCCCA TGGTTTGTAA ACGTGATCTG GATTTTGTCA   
  
  
- ATGATAGTAG TATAGCTACA ATTTTAACTG GACAAGAATA AACAGGTCGC AACTCGTTCC TTTATTAGTT   
  
  
- TAGTACAGAG TAAATCAGCC AAAAAACGGA TTCTTCTCGT ATTTGTTTCC TTTTTCTGTT GCTGTGTAAT   
  
  
- ATGTACGTAG ATGTGTTTAC TTTACTCCGG TATACCTTGT ACAAGTACTC TCGAAGTGAG CGATTATTAA   
  
  
- CTTGGATGAA AACTGATTCG GTGGTTGATC TTATACCTCT AACTGACAAT TCTCTGATAT AATGTACTTT   
  
  
- ATCTATTCTC ATCTGTAACG ATATATAAAA TAGAAATTAA CAGATAAAAA TTACTAGTAA TGACAAATAA   
  
  
- AGTCACTCAT TAAAAGAGCC TATATTATAA AATATCGCCA ATAATTTATC TACACTTTGA TATTACAATC   
  
  
- TGTATAGTAC GCTTTTATCT CTTTAAAACC CGTTTAATTT TTTTTTTCTT CTTTTTGCTC CTTCATACGG   
  
  
- GTAACATTCG TCCACCTACA TATAGGTCGG AGGTAGGTTG TTAAACCCGT TTTAATATAG TAGGTAAGGT   
  
  
- TTTTTTTCTA TATTAAACAC TATAATTATT GGGCTGAGTT AAGTTCGAAT AAATAAAAAA GTATATTAAA   
  
  
- AAATTATATA TTTTTATTTT TCACTGTTGC TGTACGTAGG AAACGCACAG TTTCCTAGGC GGCGCACCGG   
  
  
- GGGTGTAAGT GACTGCCCCA AAAACCAAAC GCTAATTCTA GTTACCGATT GCAACAGAGA GAGTTTTGTA   
  
  
- TACCTGGGGA AAAAGATAGA AAAAGACGTA ACAGTCGTAT CGTTAACAAC TCGTGTAAAA AAAGCTAAAC   
  
  
- AACTGTTATT CGCACCAATA CTAAATACCC GGTGATTATA GTCAGAGACT AGTAAACGTG TAAAACCTGG   
  
  
- AGAAAGAGAG AGATTAGAAG GTCCATTGGA CTTAAGAAGA GATAAAGACT GATCTAATCT ATTGTCCTAA   
  
  
- AGTTTATAGA CAGTCCACCG CCCATATTGA AGCACTACCC GTAAGTAAAA ACAAGATAGT CCGACCACCC   
  
  
- AAACTCAAAT AAAACTAAGT AAAGCACAAG TAGACACTAT AATCATCCTC CCTAAGACAT ATGGGTGGTT   
  
  
- GACGAGCGGG AAAACGGGCA CACTCGTTAA CCCTGAAAAC AAACAAGAAG CAGCTACGTA GGAGACCAAC   
  
  
- GACTAGACCT TAACTTTGGG GTACATAAGT TTAAGTTGGG ACTAAACGAC AGTTTGAAAG ACTTGGTAGT   
  
  
- TTTGAGGCGG CTTCACAAAC TTTTTCTACC GTAGAAGGTC TGGGAAGTAA ACCGACTAGG GTTTTCGAGA   
  
  
- AGTTAACGGC TTTAGCCCAA ATGATTATCA AGTCTAAGAT GTGTTCATGG ACTAAAAAGT CTACGAACAG   
  
  
- AATTCAAGTA ATCACTATAA GAGGACCTTC TCCTGAATCT ACTTTCAGGA TGCAGAAATG TGCTAATGTA   
  
  
- CCGAGAGGAT CGGTGACTCT TCAGAAACAT ACTACGAGAA CCTTTCCTTA TGAAAGGAAG CAGATGATCA   
  
  
- GAACGGGGTA GAAATCCGGC TTCACAACTG TCGGGTCTAT CACCGAAACC GGCGCCAACG AGACTACCAG   
  
  
- CGCCCTAACT CCCAAACCGA TTACTACTAC GGCAAAAGTA CAGATTGACC GTCGAGTTGT GGTGGGTTAA   
  
  
- CCTGGGAAAG GGTTAGTACG TTCTATAAGG AGCAGGGATA AACCTTAACT TAATGGTAAG AAGACCCAGT   
  
  
- TCGTTACCGT AACTACTAAG CCCCCTACCC AATAGCTGGA GAGGACATTC ATGTGAACGT AGTTGGCGGT   
  
  
- GTCTCCGTCC CTTTTTCAAC CGACCATCGA GATCCTCTTT CTTGGCAGTT TCCCTACTGA TACCGGTACT   
  
  
- TCTCCCTTCC TCATTGTTCG TCGTTCGGAG AATGTTACTG CTAATGCAAC TCTACCTCGT CATACTGCTA   
  
  
- CATGAAGAGA CATCCCGTCT GTTCCCATTA TAAAGTTGAA CACGGTTACT TAGGAGCGAA TTACTCCACG   
  
  
- CACTCTTCGA CGTCTGCTGT CCCAAGTTCC CTTCTTGTAG GGCAGAATTC TTCGTTAGAT TCCTTCGCTT   
  
  
- TCTCCACCTA GACTCTTGAG AAGAAAGACC AACACGTGTT CGACAAAGCT TGAAACTATA GTCCTGACGA   
  
  
- TTACTCGACG AATTCGTTCA GTCTGTCGTA AGAAGTGGTA TACCACTATC GGAGGTTTCC GAGCGGGTAG   
  
  
- TTAAACGCTT ACCATAACTC CGTGCGAATC GTCCGTGGCC AAGTTCTCAT GGACGATTAG AGTAACTACG   
  
  
- TGCCTATAGT AGTAGACTTA AAAATTTTCG AATGTTCAGT ATACAAAGTC GTCAAGGAAA GTCTTCCTAC   
  
  
- AGGATAAAAT ATCGTTTGTT GTGTTAAGAC TTCAACCGAC TCTTTCGTTG TTTCTAAGTG TATTAACTAA   
  
  
- AACCATAAGA TAAACCAGAT GTCACCGGGA CAGAATATGT TTCAGAGAGT TCCGCTTGAC ACCGAGGAGG   
  
  
- CTTCGAAGCG TAGTGACCCT ATCTGATAGG GGTCGTACCA AAGGCCGGTC GTCTTTTCCA ACTCCGTTGT   
  
  
- CCAGCAGCCA ACAGGCCCAT GACACTCTCT AAATTACATG GGAAAGCTAT ACTTTGGTAA CGTTTCTTCA   
  
  
- CCCTTTGGTA TGCGGGTCTT CTAGATTTAT AACTCTTACT ACTCGACCAT TAACAATTAA CATACAACGC   
  
  
- CAGACATTTA GATAACCTAC TATGTCACCG CCATTTATCA GGTTCCCTAC GAAAGAACTC CAATTAGTTT   
  
  
- GTCTATTTGG GCGCAAATAA GTAAGTACGT TAACAGTTAC CTTGGAAATC ATGAGGTAAG AAGTCGTGAG   
  
  
- CTAAGTCCCT TCGGGATAAG GTTATGAGAA GACATAAACT ATATAAACTT CGCTGATACT GAGCACTTCT   
  
  
- AGCACCTTCC GACGACTAAC TCTCAGTTTA TACGCCCGAT CTTCAAAACT TACGTTATCG TACACTTCCA   
  
  
- CGTCTCTCCT AAGTTTCCGG ACTTTGTATG TTCGTTACCG TCCTCGCCTG TTGTTCCCGA CCTGATTCCG   
  
  
- TCCAAGGTTA TCTACTCCTC GAACAGTTAT CTCGTTTCTG ATATCACTTT CGTTTAATAG TGTTCCTAAA   
  
  
- ACACCACCTA CTCCTATCCG CAACCTACGA AGTTCCAACC TTTCCTTCCT GTGAATCACG GGAAAGGCAA   
  
  
- ACCGTCGGAT TGAT

+     GT1-motif

| Site Name | Organism | Position | Strand | Matrix score. | sequence | function |
| --- | --- | --- | --- | --- | --- | --- |
| GT1-motif | Avena sativa | 778 | - | 7 | GGTTAAT | light responsive element |
| GT1-motif | Avena sativa | 3634 | + | 7 | GGTTAAT | light responsive element |
| GT1-motif | Arabidopsis thaliana | 779 | - | 6 | GGTTAA | light responsive element |

>HU02G01572.1   
+ +Up\_Stream \_Len000TTTAAT AAGATCCTTT TATATATATG TGTGTATAAT TCTATGGTAC ATAGGTATGT   
  
  
+ ACCATAGACT TATTGCATTT CTAGGTCCTT CTCTCTCTGC GCTTGTTTTT TGTTTATCTA ATTTATGGAG   
  
  
+ TTGAACATTT TGTTTTATAT ACTTGAACAA TCTATTATAC TAAATTAAGC ATTTTGATTT AATCACTTAT   
  
  
+ GGGTGTGTTT GGTAGGGTTG AAAATAATTT TCCTAGAAAA TAATTTTTAA TATAAAATAA TTTTTCATGT   
  
  
+ AAAATGATTT TCAACTCATA TTTTCAGTTG ATTGGTAAAA AGAAAAATGA ACTTTCTGGA TAATCAGTTT   
  
  
+ GATTAACAAA AAGTGACAAA AAAATTAAAA ATGGTTTTCC GTAATCTTCA AACCATATGT CGAGGAAATA   
  
  
+ AACACGGAAT AAAGAAAGAG TGTCAGAACC CAAGAAATGT AAAATCAACT TACGTTCTAT TTAAAGTGAA   
  
  
+ AAATATTTTT CTTCATTCTA GGAAAATTAA TTGTCCTCTG AAAACAAATT TTCCCTCCTT GTCTAACCAA   
  
  
+ ACACAGTAAA ATTGAAAAAT CATTTTCTAG GAAAATGATT TTCACTCCTA CCAAACACAC CCTTAAACTT   
  
  
+ GTTCCAAATC TATAAACCCG GAATCTTAAT ACGCACATTG TATATTATAT GTCCTACTAT AACTGTATTA   
  
  
+ TCACTGGTTC TTGATTTATC CTTTCAAACT CTTGCTTAGT CAAGGTTGTC CTCGTACAAA CATGATTAAT   
  
  
+ TCAATTAACC ACGTAAGTTA TACGCTGACA AATTTCGGGT ACCAAACATT TGCACTAGAC CTAAAACAGT   
  
  
+ TACTATCATC ATATCGATGT TAAAATTGAC CTGTTCTTAT TTGTCCAGCG TTGAGCAAGG AAATAATCAA   
  
  
+ ATCATGTCTC ATTTAGTCGG TTTTTTGCCT AAGAAGAGCA TAAACAAAGG AAAAAGACAA CGACACATTA   
  
  
+ TACATGCATC TACACAAATG AAATGAGGCC ATATGGAACA TGTTCATGAG AGCTTCACTC GCTAATAATT   
  
  
+ GAACCTACTT TTGACTAAGC CACCAACTAG AATATGGAGA TTGACTGTTA AGAGACTATA TTACATGAAA   
  
  
+ TAGATAAGAG TAGACATTGC TATATATTTT ATCTTTAATT GTCTATTTTT AATGATCATT ACTGTTTATT   
  
  
+ TCAGTGAGTA ATTTTCTCGG ATATAATATT TTATAGCGGT TATTAAATAG ATGTGAAACT ATAATGTTAG   
  
  
+ ACATATCATG CGAAAATAGA GAAATTTTGG GCAAATTAAA AAAAAAAGAA GAAAAACGAG GAAGTATGCC   
  
  
+ CATTGTAAGC AGGTGGATGT ATATCCAGCC TCCATCCAAC AATTTGGGCA AAATTATATC ATCCATTCCA   
  
  
+ AAAAAAAGAT ATAATTTGTG ATATTAATAA CCCGACTCAA TTCAAGCTTA TTTATTTTTT CATATAATTT   
  
  
+ TTTAATATAT AAAAATAAAA AGTGACAACG ACATGCATCC TTTGCGTGTC AAAGGATCCG CCGCGTGGCC   
  
  
+ CCCACATTCA CTGACGGGGT TTTTGGTTTG CGATTAAGAT CAATGGCTAA CGTTGTCTCT CTCAAAACAT   
  
  
+ ATGGACCCCT TTTTCTATCT TTTTCTGCAT TGTCAGCATA GCAATTGTTG AGCACATTTT TTTCGATTTG   
  
  
+ TTGACAATAA GCGTGGTTAT GATTTATGGG CCACTAATAT CAGTCTCTGA TCATTTGCAC ATTTTGGACC   
  
  
+ TCTTTCTCTC TCTAATCTTC CAGGTAACCT GAATTCTTCT CTATTTCTGA CTAGATTAGA TAACAGGATT   
  
  
+ TCAAATATCT GTCAGGTGGC GGGTATAACT TCGTGATGGG CATTCATTTT TGTTCTATCA GGCTGGTGGG   
  
  
+ TTTGAGTTTA TTTTGATTCA TTTCGTGTTC ATCTGTGATA TTAGTAGGAG GGATTCTGTA TACCCACCAA   
  
  
+ CTGCTCGCCC TTTTGCCCGT GTGAGCAATT GGGACTTTTG TTTGTTCTTC GTCGATGCAT CCTCTGGTTG   
  
  
+ CTGATCTGGA ATTGAAACCC CATGTATTCA AATTCAACCC TGATTTGCTG TCAAACTTTC TGAACCATCA   
  
  
+ AAACTCCGCC GAAGTGTTTG AAAAAGATGG CATCTTCCAG ACCCTTCATT TGGCTGATCC CAAAAGCTCT   
  
  
+ TCAATTGCCG AAATCGGGTT TACTAATAGT TCAGATTCTA CACAAGTACC TGATTTTTCA GATGCTTGTC   
  
  
+ TTAAGTTCAT TAGTGATATT CTCCTGGAAG AGGACTTAGA TGAAAGTCCT ACGTCTTTAC ACGATTACAT   
  
  
+ GGCTCTCCTA GCCACTGAGA AGTCTTTGTA TGATGCTCTT GGAAAGGAAT ACTTTCCTTC GTCTACTAGT   
  
  
+ CTTGCCCCAT CTTTAGGCCG AAGTGTTGAC AGCCCAGATA GTGGCTTTGG CCGCGGTTGC TCTGATGGTC   
  
  
+ GCGGGATTGA GGGTTTGGCT AATGATGATG CCGTTTTCAT GTCTAACTGG CAGCTCAACA CCACCCAATT   
  
  
+ GGACCCTTTC CCAATCATGC AAGATATTCC TCGTCCCTAT TTGGAATTGA ATTACCATTC TTCTGGGTCA   
  
  
+ AGCAATGGCA TTGATGATTC GGGGGATGGG TTATCGACCT CTCCTGTAAG TACACTTGCA TCAACCGCCA   
  
  
+ CAGAGGCAGG GAAAAAGTTG GCTGGTAGCT CTAGGAGAAA GAACCGTCAA AGGGATGACT ATGGCCATGA   
  
  
+ AGAGGGAAGG AGTAACAAGC AGCAAGCCTC TTACAATGAC GATTACGTTG AGATGGAGCA GTATGACGAT   
  
  
+ GTACTTCTCT GTAGGGCAGA CAAGGGTAAT ATTTCAACTT GTGCCAATGA ATCCTCGCTT AATGAGGTGC   
  
  
+ GTGAGAAGCT GCAGACGACA GGGTTCAAGG GAAGAACATC CCGTCTTAAG AAGCAATCTA AGGAAGCGAA   
  
  
+ AGAGGTGGAT CTGAGAACTC TTCTTTCTGG TTGTGCACAA GCTGTTTCGA ACTTTGATAT CAGGACTGCT   
  
  
+ AATGAGCTGC TTAAGCAAGT CAGACAGCAT TCTTCACCAT ATGGTGATAG CCTCCAAAGG CTCGCCCATC   
  
  
+ AATTTGCGAA TGGTATTGAG GCACGCTTAG CAGGCACCGG TTCAAGAGTA CCTGCTAATC TCATTGATGC   
  
  
+ ACGGATATCA TCATCTGAAT TTTTAAAAGC TTACAAGTCA TATGTTTCAG CAGTTCCTTT CAGAAGGATG   
  
  
+ TCCTATTTTA TAGCAAACAA CACAATTCTG AAGTTGGCTG AGAAAGCAAC AAAGATTCAC ATAATTGATT   
  
  
+ TTGGTATTCT ATTTGGTCTA CAGTGGCCCT GTCTTATACA AAGTCTCTCA AGGCGAACTG TGGCTCCTCC   
  
  
+ GAAGCTTCGC ATCACTGGGA TAGACTATCC CCAGCATGGT TTCCGGCCAG CAGAAAAGGT TGAGGCAACA   
  
  
+ GGTCGTCGGT TGTCCGGGTA CTGTGAGAGA TTTAATGTAC CCTTTCGATA TGAAACCATT GCAAAGAAGT   
  
  
+ GGGAAACCAT ACGCCCAGAA GATCTAAATA TTGAGAATGA TGAGCTGGTA ATTGTTAATT GTATGTTGCG   
  
  
+ GTCTGTAAAT CTATTGGATG ATACAGTGGC GGTAAATAGT CCAAGGGATG CTTTCTTGAG GTTAATCAAA   
  
  
+ CAGATAAACC CGCGTTTATT CATTCATGCA ATTGTCAATG GAACCTTTAG TACTCCATTC TTCAGCACTC   
  
  
+ GATTCAGGGA AGCCCTATTC CAATACTCTT CTGTATTTGA TATATTTGAA GCGACTATGA CTCGTGAAGA   
  
  
+ TCGTGGAAGG CTGCTGATTG AGAGTCAAAT ATGCGGGCTA GAAGTTTTGA ATGCAATAGC ATGTGAAGGT   
  
  
+ GCAGAGAGGA TTCAAAGGCC TGAAACATAC AAGCAATGGC AGGAGCGGAC AACAAGGGCT GGACTAAGGC   
  
  
+ AGGTTCCAAT AGATGAGGAG CTTGTCAATA GAGCAAAGAC TATAGTGAAA GCAAATTATC ACAAGGATTT   
  
  
+ TGTGGTGGAT GAGGATAGGC GTTGGATGCT TCAAGGTTGG AAAGGAAGGA CACTTAGTGC CCTTTCCGTT   
  
  
+ TGGCAGCCTA ACTA  

- +Up\_Stream \_Len000AAATTA TTCTAGGAAA ATATATATAC ACACATATTA AGATACCATG TATCCATACA   
  
  
- TGGTATCTGA ATAACGTAAA GATCCAGGAA GAGAGAGACG CGAACAAAAA ACAAATAGAT TAAATACCTC   
  
  
- AACTTGTAAA ACAAAATATA TGAACTTGTT AGATAATATG ATTTAATTCG TAAAACTAAA TTAGTGAATA   
  
  
- CCCACACAAA CCATCCCAAC TTTTATTAAA AGGATCTTTT ATTAAAAATT ATATTTTATT AAAAAGTACA   
  
  
- TTTTACTAAA AGTTGAGTAT AAAAGTCAAC TAACCATTTT TCTTTTTACT TGAAAGACCT ATTAGTCAAA   
  
  
- CTAATTGTTT TTCACTGTTT TTTTAATTTT TACCAAAAGG CATTAGAAGT TTGGTATACA GCTCCTTTAT   
  
  
- TTGTGCCTTA TTTCTTTCTC ACAGTCTTGG GTTCTTTACA TTTTAGTTGA ATGCAAGATA AATTTCACTT   
  
  
- TTTATAAAAA GAAGTAAGAT CCTTTTAATT AACAGGAGAC TTTTGTTTAA AAGGGAGGAA CAGATTGGTT   
  
  
- TGTGTCATTT TAACTTTTTA GTAAAAGATC CTTTTACTAA AAGTGAGGAT GGTTTGTGTG GGAATTTGAA   
  
  
- CAAGGTTTAG ATATTTGGGC CTTAGAATTA TGCGTGTAAC ATATAATATA CAGGATGATA TTGACATAAT   
  
  
- AGTGACCAAG AACTAAATAG GAAAGTTTGA GAACGAATCA GTTCCAACAG GAGCATGTTT GTACTAATTA   
  
  
- AGTTAATTGG TGCATTCAAT ATGCGACTGT TTAAAGCCCA TGGTTTGTAA ACGTGATCTG GATTTTGTCA   
  
  
- ATGATAGTAG TATAGCTACA ATTTTAACTG GACAAGAATA AACAGGTCGC AACTCGTTCC TTTATTAGTT   
  
  
- TAGTACAGAG TAAATCAGCC AAAAAACGGA TTCTTCTCGT ATTTGTTTCC TTTTTCTGTT GCTGTGTAAT   
  
  
- ATGTACGTAG ATGTGTTTAC TTTACTCCGG TATACCTTGT ACAAGTACTC TCGAAGTGAG CGATTATTAA   
  
  
- CTTGGATGAA AACTGATTCG GTGGTTGATC TTATACCTCT AACTGACAAT TCTCTGATAT AATGTACTTT   
  
  
- ATCTATTCTC ATCTGTAACG ATATATAAAA TAGAAATTAA CAGATAAAAA TTACTAGTAA TGACAAATAA   
  
  
- AGTCACTCAT TAAAAGAGCC TATATTATAA AATATCGCCA ATAATTTATC TACACTTTGA TATTACAATC   
  
  
- TGTATAGTAC GCTTTTATCT CTTTAAAACC CGTTTAATTT TTTTTTTCTT CTTTTTGCTC CTTCATACGG   
  
  
- GTAACATTCG TCCACCTACA TATAGGTCGG AGGTAGGTTG TTAAACCCGT TTTAATATAG TAGGTAAGGT   
  
  
- TTTTTTTCTA TATTAAACAC TATAATTATT GGGCTGAGTT AAGTTCGAAT AAATAAAAAA GTATATTAAA   
  
  
- AAATTATATA TTTTTATTTT TCACTGTTGC TGTACGTAGG AAACGCACAG TTTCCTAGGC GGCGCACCGG   
  
  
- GGGTGTAAGT GACTGCCCCA AAAACCAAAC GCTAATTCTA GTTACCGATT GCAACAGAGA GAGTTTTGTA   
  
  
- TACCTGGGGA AAAAGATAGA AAAAGACGTA ACAGTCGTAT CGTTAACAAC TCGTGTAAAA AAAGCTAAAC   
  
  
- AACTGTTATT CGCACCAATA CTAAATACCC GGTGATTATA GTCAGAGACT AGTAAACGTG TAAAACCTGG   
  
  
- AGAAAGAGAG AGATTAGAAG GTCCATTGGA CTTAAGAAGA GATAAAGACT GATCTAATCT ATTGTCCTAA   
  
  
- AGTTTATAGA CAGTCCACCG CCCATATTGA AGCACTACCC GTAAGTAAAA ACAAGATAGT CCGACCACCC   
  
  
- AAACTCAAAT AAAACTAAGT AAAGCACAAG TAGACACTAT AATCATCCTC CCTAAGACAT ATGGGTGGTT   
  
  
- GACGAGCGGG AAAACGGGCA CACTCGTTAA CCCTGAAAAC AAACAAGAAG CAGCTACGTA GGAGACCAAC   
  
  
- GACTAGACCT TAACTTTGGG GTACATAAGT TTAAGTTGGG ACTAAACGAC AGTTTGAAAG ACTTGGTAGT   
  
  
- TTTGAGGCGG CTTCACAAAC TTTTTCTACC GTAGAAGGTC TGGGAAGTAA ACCGACTAGG GTTTTCGAGA   
  
  
- AGTTAACGGC TTTAGCCCAA ATGATTATCA AGTCTAAGAT GTGTTCATGG ACTAAAAAGT CTACGAACAG   
  
  
- AATTCAAGTA ATCACTATAA GAGGACCTTC TCCTGAATCT ACTTTCAGGA TGCAGAAATG TGCTAATGTA   
  
  
- CCGAGAGGAT CGGTGACTCT TCAGAAACAT ACTACGAGAA CCTTTCCTTA TGAAAGGAAG CAGATGATCA   
  
  
- GAACGGGGTA GAAATCCGGC TTCACAACTG TCGGGTCTAT CACCGAAACC GGCGCCAACG AGACTACCAG   
  
  
- CGCCCTAACT CCCAAACCGA TTACTACTAC GGCAAAAGTA CAGATTGACC GTCGAGTTGT GGTGGGTTAA   
  
  
- CCTGGGAAAG GGTTAGTACG TTCTATAAGG AGCAGGGATA AACCTTAACT TAATGGTAAG AAGACCCAGT   
  
  
- TCGTTACCGT AACTACTAAG CCCCCTACCC AATAGCTGGA GAGGACATTC ATGTGAACGT AGTTGGCGGT   
  
  
- GTCTCCGTCC CTTTTTCAAC CGACCATCGA GATCCTCTTT CTTGGCAGTT TCCCTACTGA TACCGGTACT   
  
  
- TCTCCCTTCC TCATTGTTCG TCGTTCGGAG AATGTTACTG CTAATGCAAC TCTACCTCGT CATACTGCTA   
  
  
- CATGAAGAGA CATCCCGTCT GTTCCCATTA TAAAGTTGAA CACGGTTACT TAGGAGCGAA TTACTCCACG   
  
  
- CACTCTTCGA CGTCTGCTGT CCCAAGTTCC CTTCTTGTAG GGCAGAATTC TTCGTTAGAT TCCTTCGCTT   
  
  
- TCTCCACCTA GACTCTTGAG AAGAAAGACC AACACGTGTT CGACAAAGCT TGAAACTATA GTCCTGACGA   
  
  
- TTACTCGACG AATTCGTTCA GTCTGTCGTA AGAAGTGGTA TACCACTATC GGAGGTTTCC GAGCGGGTAG   
  
  
- TTAAACGCTT ACCATAACTC CGTGCGAATC GTCCGTGGCC AAGTTCTCAT GGACGATTAG AGTAACTACG   
  
  
- TGCCTATAGT AGTAGACTTA AAAATTTTCG AATGTTCAGT ATACAAAGTC GTCAAGGAAA GTCTTCCTAC   
  
  
- AGGATAAAAT ATCGTTTGTT GTGTTAAGAC TTCAACCGAC TCTTTCGTTG TTTCTAAGTG TATTAACTAA   
  
  
- AACCATAAGA TAAACCAGAT GTCACCGGGA CAGAATATGT TTCAGAGAGT TCCGCTTGAC ACCGAGGAGG   
  
  
- CTTCGAAGCG TAGTGACCCT ATCTGATAGG GGTCGTACCA AAGGCCGGTC GTCTTTTCCA ACTCCGTTGT   
  
  
- CCAGCAGCCA ACAGGCCCAT GACACTCTCT AAATTACATG GGAAAGCTAT ACTTTGGTAA CGTTTCTTCA   
  
  
- CCCTTTGGTA TGCGGGTCTT CTAGATTTAT AACTCTTACT ACTCGACCAT TAACAATTAA CATACAACGC   
  
  
- CAGACATTTA GATAACCTAC TATGTCACCG CCATTTATCA GGTTCCCTAC GAAAGAACTC CAATTAGTTT   
  
  
- GTCTATTTGG GCGCAAATAA GTAAGTACGT TAACAGTTAC CTTGGAAATC ATGAGGTAAG AAGTCGTGAG   
  
  
- CTAAGTCCCT TCGGGATAAG GTTATGAGAA GACATAAACT ATATAAACTT CGCTGATACT GAGCACTTCT   
  
  
- AGCACCTTCC GACGACTAAC TCTCAGTTTA TACGCCCGAT CTTCAAAACT TACGTTATCG TACACTTCCA   
  
  
- CGTCTCTCCT AAGTTTCCGG ACTTTGTATG TTCGTTACCG TCCTCGCCTG TTGTTCCCGA CCTGATTCCG   
  
  
- TCCAAGGTTA TCTACTCCTC GAACAGTTAT CTCGTTTCTG ATATCACTTT CGTTTAATAG TGTTCCTAAA   
  
  
- ACACCACCTA CTCCTATCCG CAACCTACGA AGTTCCAACC TTTCCTTCCT GTGAATCACG GGAAAGGCAA   
  
  
- ACCGTCGGAT TGAT

+     L-box

| Site Name | Organism | Position | Strand | Matrix score. | sequence | function |
| --- | --- | --- | --- | --- | --- | --- |
| L-box | Petroselinum crispum | 1938 | - | 10 | ATCCCACCTAC | part of a light responsive element |

>HU02G01572.1   
+ +Up\_Stream \_Len000TTTAAT AAGATCCTTT TATATATATG TGTGTATAAT TCTATGGTAC ATAGGTATGT   
  
  
+ ACCATAGACT TATTGCATTT CTAGGTCCTT CTCTCTCTGC GCTTGTTTTT TGTTTATCTA ATTTATGGAG   
  
  
+ TTGAACATTT TGTTTTATAT ACTTGAACAA TCTATTATAC TAAATTAAGC ATTTTGATTT AATCACTTAT   
  
  
+ GGGTGTGTTT GGTAGGGTTG AAAATAATTT TCCTAGAAAA TAATTTTTAA TATAAAATAA TTTTTCATGT   
  
  
+ AAAATGATTT TCAACTCATA TTTTCAGTTG ATTGGTAAAA AGAAAAATGA ACTTTCTGGA TAATCAGTTT   
  
  
+ GATTAACAAA AAGTGACAAA AAAATTAAAA ATGGTTTTCC GTAATCTTCA AACCATATGT CGAGGAAATA   
  
  
+ AACACGGAAT AAAGAAAGAG TGTCAGAACC CAAGAAATGT AAAATCAACT TACGTTCTAT TTAAAGTGAA   
  
  
+ AAATATTTTT CTTCATTCTA GGAAAATTAA TTGTCCTCTG AAAACAAATT TTCCCTCCTT GTCTAACCAA   
  
  
+ ACACAGTAAA ATTGAAAAAT CATTTTCTAG GAAAATGATT TTCACTCCTA CCAAACACAC CCTTAAACTT   
  
  
+ GTTCCAAATC TATAAACCCG GAATCTTAAT ACGCACATTG TATATTATAT GTCCTACTAT AACTGTATTA   
  
  
+ TCACTGGTTC TTGATTTATC CTTTCAAACT CTTGCTTAGT CAAGGTTGTC CTCGTACAAA CATGATTAAT   
  
  
+ TCAATTAACC ACGTAAGTTA TACGCTGACA AATTTCGGGT ACCAAACATT TGCACTAGAC CTAAAACAGT   
  
  
+ TACTATCATC ATATCGATGT TAAAATTGAC CTGTTCTTAT TTGTCCAGCG TTGAGCAAGG AAATAATCAA   
  
  
+ ATCATGTCTC ATTTAGTCGG TTTTTTGCCT AAGAAGAGCA TAAACAAAGG AAAAAGACAA CGACACATTA   
  
  
+ TACATGCATC TACACAAATG AAATGAGGCC ATATGGAACA TGTTCATGAG AGCTTCACTC GCTAATAATT   
  
  
+ GAACCTACTT TTGACTAAGC CACCAACTAG AATATGGAGA TTGACTGTTA AGAGACTATA TTACATGAAA   
  
  
+ TAGATAAGAG TAGACATTGC TATATATTTT ATCTTTAATT GTCTATTTTT AATGATCATT ACTGTTTATT   
  
  
+ TCAGTGAGTA ATTTTCTCGG ATATAATATT TTATAGCGGT TATTAAATAG ATGTGAAACT ATAATGTTAG   
  
  
+ ACATATCATG CGAAAATAGA GAAATTTTGG GCAAATTAAA AAAAAAAGAA GAAAAACGAG GAAGTATGCC   
  
  
+ CATTGTAAGC AGGTGGATGT ATATCCAGCC TCCATCCAAC AATTTGGGCA AAATTATATC ATCCATTCCA   
  
  
+ AAAAAAAGAT ATAATTTGTG ATATTAATAA CCCGACTCAA TTCAAGCTTA TTTATTTTTT CATATAATTT   
  
  
+ TTTAATATAT AAAAATAAAA AGTGACAACG ACATGCATCC TTTGCGTGTC AAAGGATCCG CCGCGTGGCC   
  
  
+ CCCACATTCA CTGACGGGGT TTTTGGTTTG CGATTAAGAT CAATGGCTAA CGTTGTCTCT CTCAAAACAT   
  
  
+ ATGGACCCCT TTTTCTATCT TTTTCTGCAT TGTCAGCATA GCAATTGTTG AGCACATTTT TTTCGATTTG   
  
  
+ TTGACAATAA GCGTGGTTAT GATTTATGGG CCACTAATAT CAGTCTCTGA TCATTTGCAC ATTTTGGACC   
  
  
+ TCTTTCTCTC TCTAATCTTC CAGGTAACCT GAATTCTTCT CTATTTCTGA CTAGATTAGA TAACAGGATT   
  
  
+ TCAAATATCT GTCAGGTGGC GGGTATAACT TCGTGATGGG CATTCATTTT TGTTCTATCA GGCTGGTGGG   
  
  
+ TTTGAGTTTA TTTTGATTCA TTTCGTGTTC ATCTGTGATA TTAGTAGGAG GGATTCTGTA TACCCACCAA   
  
  
+ CTGCTCGCCC TTTTGCCCGT GTGAGCAATT GGGACTTTTG TTTGTTCTTC GTCGATGCAT CCTCTGGTTG   
  
  
+ CTGATCTGGA ATTGAAACCC CATGTATTCA AATTCAACCC TGATTTGCTG TCAAACTTTC TGAACCATCA   
  
  
+ AAACTCCGCC GAAGTGTTTG AAAAAGATGG CATCTTCCAG ACCCTTCATT TGGCTGATCC CAAAAGCTCT   
  
  
+ TCAATTGCCG AAATCGGGTT TACTAATAGT TCAGATTCTA CACAAGTACC TGATTTTTCA GATGCTTGTC   
  
  
+ TTAAGTTCAT TAGTGATATT CTCCTGGAAG AGGACTTAGA TGAAAGTCCT ACGTCTTTAC ACGATTACAT   
  
  
+ GGCTCTCCTA GCCACTGAGA AGTCTTTGTA TGATGCTCTT GGAAAGGAAT ACTTTCCTTC GTCTACTAGT   
  
  
+ CTTGCCCCAT CTTTAGGCCG AAGTGTTGAC AGCCCAGATA GTGGCTTTGG CCGCGGTTGC TCTGATGGTC   
  
  
+ GCGGGATTGA GGGTTTGGCT AATGATGATG CCGTTTTCAT GTCTAACTGG CAGCTCAACA CCACCCAATT   
  
  
+ GGACCCTTTC CCAATCATGC AAGATATTCC TCGTCCCTAT TTGGAATTGA ATTACCATTC TTCTGGGTCA   
  
  
+ AGCAATGGCA TTGATGATTC GGGGGATGGG TTATCGACCT CTCCTGTAAG TACACTTGCA TCAACCGCCA   
  
  
+ CAGAGGCAGG GAAAAAGTTG GCTGGTAGCT CTAGGAGAAA GAACCGTCAA AGGGATGACT ATGGCCATGA   
  
  
+ AGAGGGAAGG AGTAACAAGC AGCAAGCCTC TTACAATGAC GATTACGTTG AGATGGAGCA GTATGACGAT   
  
  
+ GTACTTCTCT GTAGGGCAGA CAAGGGTAAT ATTTCAACTT GTGCCAATGA ATCCTCGCTT AATGAGGTGC   
  
  
+ GTGAGAAGCT GCAGACGACA GGGTTCAAGG GAAGAACATC CCGTCTTAAG AAGCAATCTA AGGAAGCGAA   
  
  
+ AGAGGTGGAT CTGAGAACTC TTCTTTCTGG TTGTGCACAA GCTGTTTCGA ACTTTGATAT CAGGACTGCT   
  
  
+ AATGAGCTGC TTAAGCAAGT CAGACAGCAT TCTTCACCAT ATGGTGATAG CCTCCAAAGG CTCGCCCATC   
  
  
+ AATTTGCGAA TGGTATTGAG GCACGCTTAG CAGGCACCGG TTCAAGAGTA CCTGCTAATC TCATTGATGC   
  
  
+ ACGGATATCA TCATCTGAAT TTTTAAAAGC TTACAAGTCA TATGTTTCAG CAGTTCCTTT CAGAAGGATG   
  
  
+ TCCTATTTTA TAGCAAACAA CACAATTCTG AAGTTGGCTG AGAAAGCAAC AAAGATTCAC ATAATTGATT   
  
  
+ TTGGTATTCT ATTTGGTCTA CAGTGGCCCT GTCTTATACA AAGTCTCTCA AGGCGAACTG TGGCTCCTCC   
  
  
+ GAAGCTTCGC ATCACTGGGA TAGACTATCC CCAGCATGGT TTCCGGCCAG CAGAAAAGGT TGAGGCAACA   
  
  
+ GGTCGTCGGT TGTCCGGGTA CTGTGAGAGA TTTAATGTAC CCTTTCGATA TGAAACCATT GCAAAGAAGT   
  
  
+ GGGAAACCAT ACGCCCAGAA GATCTAAATA TTGAGAATGA TGAGCTGGTA ATTGTTAATT GTATGTTGCG   
  
  
+ GTCTGTAAAT CTATTGGATG ATACAGTGGC GGTAAATAGT CCAAGGGATG CTTTCTTGAG GTTAATCAAA   
  
  
+ CAGATAAACC CGCGTTTATT CATTCATGCA ATTGTCAATG GAACCTTTAG TACTCCATTC TTCAGCACTC   
  
  
+ GATTCAGGGA AGCCCTATTC CAATACTCTT CTGTATTTGA TATATTTGAA GCGACTATGA CTCGTGAAGA   
  
  
+ TCGTGGAAGG CTGCTGATTG AGAGTCAAAT ATGCGGGCTA GAAGTTTTGA ATGCAATAGC ATGTGAAGGT   
  
  
+ GCAGAGAGGA TTCAAAGGCC TGAAACATAC AAGCAATGGC AGGAGCGGAC AACAAGGGCT GGACTAAGGC   
  
  
+ AGGTTCCAAT AGATGAGGAG CTTGTCAATA GAGCAAAGAC TATAGTGAAA GCAAATTATC ACAAGGATTT   
  
  
+ TGTGGTGGAT GAGGATAGGC GTTGGATGCT TCAAGGTTGG AAAGGAAGGA CACTTAGTGC CCTTTCCGTT   
  
  
+ TGGCAGCCTA ACTA  

- +Up\_Stream \_Len000AAATTA TTCTAGGAAA ATATATATAC ACACATATTA AGATACCATG TATCCATACA   
  
  
- TGGTATCTGA ATAACGTAAA GATCCAGGAA GAGAGAGACG CGAACAAAAA ACAAATAGAT TAAATACCTC   
  
  
- AACTTGTAAA ACAAAATATA TGAACTTGTT AGATAATATG ATTTAATTCG TAAAACTAAA TTAGTGAATA   
  
  
- CCCACACAAA CCATCCCAAC TTTTATTAAA AGGATCTTTT ATTAAAAATT ATATTTTATT AAAAAGTACA   
  
  
- TTTTACTAAA AGTTGAGTAT AAAAGTCAAC TAACCATTTT TCTTTTTACT TGAAAGACCT ATTAGTCAAA   
  
  
- CTAATTGTTT TTCACTGTTT TTTTAATTTT TACCAAAAGG CATTAGAAGT TTGGTATACA GCTCCTTTAT   
  
  
- TTGTGCCTTA TTTCTTTCTC ACAGTCTTGG GTTCTTTACA TTTTAGTTGA ATGCAAGATA AATTTCACTT   
  
  
- TTTATAAAAA GAAGTAAGAT CCTTTTAATT AACAGGAGAC TTTTGTTTAA AAGGGAGGAA CAGATTGGTT   
  
  
- TGTGTCATTT TAACTTTTTA GTAAAAGATC CTTTTACTAA AAGTGAGGAT GGTTTGTGTG GGAATTTGAA   
  
  
- CAAGGTTTAG ATATTTGGGC CTTAGAATTA TGCGTGTAAC ATATAATATA CAGGATGATA TTGACATAAT   
  
  
- AGTGACCAAG AACTAAATAG GAAAGTTTGA GAACGAATCA GTTCCAACAG GAGCATGTTT GTACTAATTA   
  
  
- AGTTAATTGG TGCATTCAAT ATGCGACTGT TTAAAGCCCA TGGTTTGTAA ACGTGATCTG GATTTTGTCA   
  
  
- ATGATAGTAG TATAGCTACA ATTTTAACTG GACAAGAATA AACAGGTCGC AACTCGTTCC TTTATTAGTT   
  
  
- TAGTACAGAG TAAATCAGCC AAAAAACGGA TTCTTCTCGT ATTTGTTTCC TTTTTCTGTT GCTGTGTAAT   
  
  
- ATGTACGTAG ATGTGTTTAC TTTACTCCGG TATACCTTGT ACAAGTACTC TCGAAGTGAG CGATTATTAA   
  
  
- CTTGGATGAA AACTGATTCG GTGGTTGATC TTATACCTCT AACTGACAAT TCTCTGATAT AATGTACTTT   
  
  
- ATCTATTCTC ATCTGTAACG ATATATAAAA TAGAAATTAA CAGATAAAAA TTACTAGTAA TGACAAATAA   
  
  
- AGTCACTCAT TAAAAGAGCC TATATTATAA AATATCGCCA ATAATTTATC TACACTTTGA TATTACAATC   
  
  
- TGTATAGTAC GCTTTTATCT CTTTAAAACC CGTTTAATTT TTTTTTTCTT CTTTTTGCTC CTTCATACGG   
  
  
- GTAACATTCG TCCACCTACA TATAGGTCGG AGGTAGGTTG TTAAACCCGT TTTAATATAG TAGGTAAGGT   
  
  
- TTTTTTTCTA TATTAAACAC TATAATTATT GGGCTGAGTT AAGTTCGAAT AAATAAAAAA GTATATTAAA   
  
  
- AAATTATATA TTTTTATTTT TCACTGTTGC TGTACGTAGG AAACGCACAG TTTCCTAGGC GGCGCACCGG   
  
  
- GGGTGTAAGT GACTGCCCCA AAAACCAAAC GCTAATTCTA GTTACCGATT GCAACAGAGA GAGTTTTGTA   
  
  
- TACCTGGGGA AAAAGATAGA AAAAGACGTA ACAGTCGTAT CGTTAACAAC TCGTGTAAAA AAAGCTAAAC   
  
  
- AACTGTTATT CGCACCAATA CTAAATACCC GGTGATTATA GTCAGAGACT AGTAAACGTG TAAAACCTGG   
  
  
- AGAAAGAGAG AGATTAGAAG GTCCATTGGA CTTAAGAAGA GATAAAGACT GATCTAATCT ATTGTCCTAA   
  
  
- AGTTTATAGA CAGTCCACCG CCCATATTGA AGCACTACCC GTAAGTAAAA ACAAGATAGT CCGACCACCC   
  
  
- AAACTCAAAT AAAACTAAGT AAAGCACAAG TAGACACTAT AATCATCCTC CCTAAGACAT ATGGGTGGTT   
  
  
- GACGAGCGGG AAAACGGGCA CACTCGTTAA CCCTGAAAAC AAACAAGAAG CAGCTACGTA GGAGACCAAC   
  
  
- GACTAGACCT TAACTTTGGG GTACATAAGT TTAAGTTGGG ACTAAACGAC AGTTTGAAAG ACTTGGTAGT   
  
  
- TTTGAGGCGG CTTCACAAAC TTTTTCTACC GTAGAAGGTC TGGGAAGTAA ACCGACTAGG GTTTTCGAGA   
  
  
- AGTTAACGGC TTTAGCCCAA ATGATTATCA AGTCTAAGAT GTGTTCATGG ACTAAAAAGT CTACGAACAG   
  
  
- AATTCAAGTA ATCACTATAA GAGGACCTTC TCCTGAATCT ACTTTCAGGA TGCAGAAATG TGCTAATGTA   
  
  
- CCGAGAGGAT CGGTGACTCT TCAGAAACAT ACTACGAGAA CCTTTCCTTA TGAAAGGAAG CAGATGATCA   
  
  
- GAACGGGGTA GAAATCCGGC TTCACAACTG TCGGGTCTAT CACCGAAACC GGCGCCAACG AGACTACCAG   
  
  
- CGCCCTAACT CCCAAACCGA TTACTACTAC GGCAAAAGTA CAGATTGACC GTCGAGTTGT GGTGGGTTAA   
  
  
- CCTGGGAAAG GGTTAGTACG TTCTATAAGG AGCAGGGATA AACCTTAACT TAATGGTAAG AAGACCCAGT   
  
  
- TCGTTACCGT AACTACTAAG CCCCCTACCC AATAGCTGGA GAGGACATTC ATGTGAACGT AGTTGGCGGT   
  
  
- GTCTCCGTCC CTTTTTCAAC CGACCATCGA GATCCTCTTT CTTGGCAGTT TCCCTACTGA TACCGGTACT   
  
  
- TCTCCCTTCC TCATTGTTCG TCGTTCGGAG AATGTTACTG CTAATGCAAC TCTACCTCGT CATACTGCTA   
  
  
- CATGAAGAGA CATCCCGTCT GTTCCCATTA TAAAGTTGAA CACGGTTACT TAGGAGCGAA TTACTCCACG   
  
  
- CACTCTTCGA CGTCTGCTGT CCCAAGTTCC CTTCTTGTAG GGCAGAATTC TTCGTTAGAT TCCTTCGCTT   
  
  
- TCTCCACCTA GACTCTTGAG AAGAAAGACC AACACGTGTT CGACAAAGCT TGAAACTATA GTCCTGACGA   
  
  
- TTACTCGACG AATTCGTTCA GTCTGTCGTA AGAAGTGGTA TACCACTATC GGAGGTTTCC GAGCGGGTAG   
  
  
- TTAAACGCTT ACCATAACTC CGTGCGAATC GTCCGTGGCC AAGTTCTCAT GGACGATTAG AGTAACTACG   
  
  
- TGCCTATAGT AGTAGACTTA AAAATTTTCG AATGTTCAGT ATACAAAGTC GTCAAGGAAA GTCTTCCTAC   
  
  
- AGGATAAAAT ATCGTTTGTT GTGTTAAGAC TTCAACCGAC TCTTTCGTTG TTTCTAAGTG TATTAACTAA   
  
  
- AACCATAAGA TAAACCAGAT GTCACCGGGA CAGAATATGT TTCAGAGAGT TCCGCTTGAC ACCGAGGAGG   
  
  
- CTTCGAAGCG TAGTGACCCT ATCTGATAGG GGTCGTACCA AAGGCCGGTC GTCTTTTCCA ACTCCGTTGT   
  
  
- CCAGCAGCCA ACAGGCCCAT GACACTCTCT AAATTACATG GGAAAGCTAT ACTTTGGTAA CGTTTCTTCA   
  
  
- CCCTTTGGTA TGCGGGTCTT CTAGATTTAT AACTCTTACT ACTCGACCAT TAACAATTAA CATACAACGC   
  
  
- CAGACATTTA GATAACCTAC TATGTCACCG CCATTTATCA GGTTCCCTAC GAAAGAACTC CAATTAGTTT   
  
  
- GTCTATTTGG GCGCAAATAA GTAAGTACGT TAACAGTTAC CTTGGAAATC ATGAGGTAAG AAGTCGTGAG   
  
  
- CTAAGTCCCT TCGGGATAAG GTTATGAGAA GACATAAACT ATATAAACTT CGCTGATACT GAGCACTTCT   
  
  
- AGCACCTTCC GACGACTAAC TCTCAGTTTA TACGCCCGAT CTTCAAAACT TACGTTATCG TACACTTCCA   
  
  
- CGTCTCTCCT AAGTTTCCGG ACTTTGTATG TTCGTTACCG TCCTCGCCTG TTGTTCCCGA CCTGATTCCG   
  
  
- TCCAAGGTTA TCTACTCCTC GAACAGTTAT CTCGTTTCTG ATATCACTTT CGTTTAATAG TGTTCCTAAA   
  
  
- ACACCACCTA CTCCTATCCG CAACCTACGA AGTTCCAACC TTTCCTTCCT GTGAATCACG GGAAAGGCAA   
  
  
- ACCGTCGGAT TGAT

+     LTR

| Site Name | Organism | Position | Strand | Matrix score. | sequence | function |
| --- | --- | --- | --- | --- | --- | --- |
| LTR | Hordeum vulgare | 807 | - | 6 | CCGAAA | cis-acting element involved in low-temperature responsiveness |
| LTR | Hordeum vulgare | 2182 | + | 6 | CCGAAA | cis-acting element involved in low-temperature responsiveness |

>HU02G01572.1   
+ +Up\_Stream \_Len000TTTAAT AAGATCCTTT TATATATATG TGTGTATAAT TCTATGGTAC ATAGGTATGT   
  
  
+ ACCATAGACT TATTGCATTT CTAGGTCCTT CTCTCTCTGC GCTTGTTTTT TGTTTATCTA ATTTATGGAG   
  
  
+ TTGAACATTT TGTTTTATAT ACTTGAACAA TCTATTATAC TAAATTAAGC ATTTTGATTT AATCACTTAT   
  
  
+ GGGTGTGTTT GGTAGGGTTG AAAATAATTT TCCTAGAAAA TAATTTTTAA TATAAAATAA TTTTTCATGT   
  
  
+ AAAATGATTT TCAACTCATA TTTTCAGTTG ATTGGTAAAA AGAAAAATGA ACTTTCTGGA TAATCAGTTT   
  
  
+ GATTAACAAA AAGTGACAAA AAAATTAAAA ATGGTTTTCC GTAATCTTCA AACCATATGT CGAGGAAATA   
  
  
+ AACACGGAAT AAAGAAAGAG TGTCAGAACC CAAGAAATGT AAAATCAACT TACGTTCTAT TTAAAGTGAA   
  
  
+ AAATATTTTT CTTCATTCTA GGAAAATTAA TTGTCCTCTG AAAACAAATT TTCCCTCCTT GTCTAACCAA   
  
  
+ ACACAGTAAA ATTGAAAAAT CATTTTCTAG GAAAATGATT TTCACTCCTA CCAAACACAC CCTTAAACTT   
  
  
+ GTTCCAAATC TATAAACCCG GAATCTTAAT ACGCACATTG TATATTATAT GTCCTACTAT AACTGTATTA   
  
  
+ TCACTGGTTC TTGATTTATC CTTTCAAACT CTTGCTTAGT CAAGGTTGTC CTCGTACAAA CATGATTAAT   
  
  
+ TCAATTAACC ACGTAAGTTA TACGCTGACA AATTTCGGGT ACCAAACATT TGCACTAGAC CTAAAACAGT   
  
  
+ TACTATCATC ATATCGATGT TAAAATTGAC CTGTTCTTAT TTGTCCAGCG TTGAGCAAGG AAATAATCAA   
  
  
+ ATCATGTCTC ATTTAGTCGG TTTTTTGCCT AAGAAGAGCA TAAACAAAGG AAAAAGACAA CGACACATTA   
  
  
+ TACATGCATC TACACAAATG AAATGAGGCC ATATGGAACA TGTTCATGAG AGCTTCACTC GCTAATAATT   
  
  
+ GAACCTACTT TTGACTAAGC CACCAACTAG AATATGGAGA TTGACTGTTA AGAGACTATA TTACATGAAA   
  
  
+ TAGATAAGAG TAGACATTGC TATATATTTT ATCTTTAATT GTCTATTTTT AATGATCATT ACTGTTTATT   
  
  
+ TCAGTGAGTA ATTTTCTCGG ATATAATATT TTATAGCGGT TATTAAATAG ATGTGAAACT ATAATGTTAG   
  
  
+ ACATATCATG CGAAAATAGA GAAATTTTGG GCAAATTAAA AAAAAAAGAA GAAAAACGAG GAAGTATGCC   
  
  
+ CATTGTAAGC AGGTGGATGT ATATCCAGCC TCCATCCAAC AATTTGGGCA AAATTATATC ATCCATTCCA   
  
  
+ AAAAAAAGAT ATAATTTGTG ATATTAATAA CCCGACTCAA TTCAAGCTTA TTTATTTTTT CATATAATTT   
  
  
+ TTTAATATAT AAAAATAAAA AGTGACAACG ACATGCATCC TTTGCGTGTC AAAGGATCCG CCGCGTGGCC   
  
  
+ CCCACATTCA CTGACGGGGT TTTTGGTTTG CGATTAAGAT CAATGGCTAA CGTTGTCTCT CTCAAAACAT   
  
  
+ ATGGACCCCT TTTTCTATCT TTTTCTGCAT TGTCAGCATA GCAATTGTTG AGCACATTTT TTTCGATTTG   
  
  
+ TTGACAATAA GCGTGGTTAT GATTTATGGG CCACTAATAT CAGTCTCTGA TCATTTGCAC ATTTTGGACC   
  
  
+ TCTTTCTCTC TCTAATCTTC CAGGTAACCT GAATTCTTCT CTATTTCTGA CTAGATTAGA TAACAGGATT   
  
  
+ TCAAATATCT GTCAGGTGGC GGGTATAACT TCGTGATGGG CATTCATTTT TGTTCTATCA GGCTGGTGGG   
  
  
+ TTTGAGTTTA TTTTGATTCA TTTCGTGTTC ATCTGTGATA TTAGTAGGAG GGATTCTGTA TACCCACCAA   
  
  
+ CTGCTCGCCC TTTTGCCCGT GTGAGCAATT GGGACTTTTG TTTGTTCTTC GTCGATGCAT CCTCTGGTTG   
  
  
+ CTGATCTGGA ATTGAAACCC CATGTATTCA AATTCAACCC TGATTTGCTG TCAAACTTTC TGAACCATCA   
  
  
+ AAACTCCGCC GAAGTGTTTG AAAAAGATGG CATCTTCCAG ACCCTTCATT TGGCTGATCC CAAAAGCTCT   
  
  
+ TCAATTGCCG AAATCGGGTT TACTAATAGT TCAGATTCTA CACAAGTACC TGATTTTTCA GATGCTTGTC   
  
  
+ TTAAGTTCAT TAGTGATATT CTCCTGGAAG AGGACTTAGA TGAAAGTCCT ACGTCTTTAC ACGATTACAT   
  
  
+ GGCTCTCCTA GCCACTGAGA AGTCTTTGTA TGATGCTCTT GGAAAGGAAT ACTTTCCTTC GTCTACTAGT   
  
  
+ CTTGCCCCAT CTTTAGGCCG AAGTGTTGAC AGCCCAGATA GTGGCTTTGG CCGCGGTTGC TCTGATGGTC   
  
  
+ GCGGGATTGA GGGTTTGGCT AATGATGATG CCGTTTTCAT GTCTAACTGG CAGCTCAACA CCACCCAATT   
  
  
+ GGACCCTTTC CCAATCATGC AAGATATTCC TCGTCCCTAT TTGGAATTGA ATTACCATTC TTCTGGGTCA   
  
  
+ AGCAATGGCA TTGATGATTC GGGGGATGGG TTATCGACCT CTCCTGTAAG TACACTTGCA TCAACCGCCA   
  
  
+ CAGAGGCAGG GAAAAAGTTG GCTGGTAGCT CTAGGAGAAA GAACCGTCAA AGGGATGACT ATGGCCATGA   
  
  
+ AGAGGGAAGG AGTAACAAGC AGCAAGCCTC TTACAATGAC GATTACGTTG AGATGGAGCA GTATGACGAT   
  
  
+ GTACTTCTCT GTAGGGCAGA CAAGGGTAAT ATTTCAACTT GTGCCAATGA ATCCTCGCTT AATGAGGTGC   
  
  
+ GTGAGAAGCT GCAGACGACA GGGTTCAAGG GAAGAACATC CCGTCTTAAG AAGCAATCTA AGGAAGCGAA   
  
  
+ AGAGGTGGAT CTGAGAACTC TTCTTTCTGG TTGTGCACAA GCTGTTTCGA ACTTTGATAT CAGGACTGCT   
  
  
+ AATGAGCTGC TTAAGCAAGT CAGACAGCAT TCTTCACCAT ATGGTGATAG CCTCCAAAGG CTCGCCCATC   
  
  
+ AATTTGCGAA TGGTATTGAG GCACGCTTAG CAGGCACCGG TTCAAGAGTA CCTGCTAATC TCATTGATGC   
  
  
+ ACGGATATCA TCATCTGAAT TTTTAAAAGC TTACAAGTCA TATGTTTCAG CAGTTCCTTT CAGAAGGATG   
  
  
+ TCCTATTTTA TAGCAAACAA CACAATTCTG AAGTTGGCTG AGAAAGCAAC AAAGATTCAC ATAATTGATT   
  
  
+ TTGGTATTCT ATTTGGTCTA CAGTGGCCCT GTCTTATACA AAGTCTCTCA AGGCGAACTG TGGCTCCTCC   
  
  
+ GAAGCTTCGC ATCACTGGGA TAGACTATCC CCAGCATGGT TTCCGGCCAG CAGAAAAGGT TGAGGCAACA   
  
  
+ GGTCGTCGGT TGTCCGGGTA CTGTGAGAGA TTTAATGTAC CCTTTCGATA TGAAACCATT GCAAAGAAGT   
  
  
+ GGGAAACCAT ACGCCCAGAA GATCTAAATA TTGAGAATGA TGAGCTGGTA ATTGTTAATT GTATGTTGCG   
  
  
+ GTCTGTAAAT CTATTGGATG ATACAGTGGC GGTAAATAGT CCAAGGGATG CTTTCTTGAG GTTAATCAAA   
  
  
+ CAGATAAACC CGCGTTTATT CATTCATGCA ATTGTCAATG GAACCTTTAG TACTCCATTC TTCAGCACTC   
  
  
+ GATTCAGGGA AGCCCTATTC CAATACTCTT CTGTATTTGA TATATTTGAA GCGACTATGA CTCGTGAAGA   
  
  
+ TCGTGGAAGG CTGCTGATTG AGAGTCAAAT ATGCGGGCTA GAAGTTTTGA ATGCAATAGC ATGTGAAGGT   
  
  
+ GCAGAGAGGA TTCAAAGGCC TGAAACATAC AAGCAATGGC AGGAGCGGAC AACAAGGGCT GGACTAAGGC   
  
  
+ AGGTTCCAAT AGATGAGGAG CTTGTCAATA GAGCAAAGAC TATAGTGAAA GCAAATTATC ACAAGGATTT   
  
  
+ TGTGGTGGAT GAGGATAGGC GTTGGATGCT TCAAGGTTGG AAAGGAAGGA CACTTAGTGC CCTTTCCGTT   
  
  
+ TGGCAGCCTA ACTA  

- +Up\_Stream \_Len000AAATTA TTCTAGGAAA ATATATATAC ACACATATTA AGATACCATG TATCCATACA   
  
  
- TGGTATCTGA ATAACGTAAA GATCCAGGAA GAGAGAGACG CGAACAAAAA ACAAATAGAT TAAATACCTC   
  
  
- AACTTGTAAA ACAAAATATA TGAACTTGTT AGATAATATG ATTTAATTCG TAAAACTAAA TTAGTGAATA   
  
  
- CCCACACAAA CCATCCCAAC TTTTATTAAA AGGATCTTTT ATTAAAAATT ATATTTTATT AAAAAGTACA   
  
  
- TTTTACTAAA AGTTGAGTAT AAAAGTCAAC TAACCATTTT TCTTTTTACT TGAAAGACCT ATTAGTCAAA   
  
  
- CTAATTGTTT TTCACTGTTT TTTTAATTTT TACCAAAAGG CATTAGAAGT TTGGTATACA GCTCCTTTAT   
  
  
- TTGTGCCTTA TTTCTTTCTC ACAGTCTTGG GTTCTTTACA TTTTAGTTGA ATGCAAGATA AATTTCACTT   
  
  
- TTTATAAAAA GAAGTAAGAT CCTTTTAATT AACAGGAGAC TTTTGTTTAA AAGGGAGGAA CAGATTGGTT   
  
  
- TGTGTCATTT TAACTTTTTA GTAAAAGATC CTTTTACTAA AAGTGAGGAT GGTTTGTGTG GGAATTTGAA   
  
  
- CAAGGTTTAG ATATTTGGGC CTTAGAATTA TGCGTGTAAC ATATAATATA CAGGATGATA TTGACATAAT   
  
  
- AGTGACCAAG AACTAAATAG GAAAGTTTGA GAACGAATCA GTTCCAACAG GAGCATGTTT GTACTAATTA   
  
  
- AGTTAATTGG TGCATTCAAT ATGCGACTGT TTAAAGCCCA TGGTTTGTAA ACGTGATCTG GATTTTGTCA   
  
  
- ATGATAGTAG TATAGCTACA ATTTTAACTG GACAAGAATA AACAGGTCGC AACTCGTTCC TTTATTAGTT   
  
  
- TAGTACAGAG TAAATCAGCC AAAAAACGGA TTCTTCTCGT ATTTGTTTCC TTTTTCTGTT GCTGTGTAAT   
  
  
- ATGTACGTAG ATGTGTTTAC TTTACTCCGG TATACCTTGT ACAAGTACTC TCGAAGTGAG CGATTATTAA   
  
  
- CTTGGATGAA AACTGATTCG GTGGTTGATC TTATACCTCT AACTGACAAT TCTCTGATAT AATGTACTTT   
  
  
- ATCTATTCTC ATCTGTAACG ATATATAAAA TAGAAATTAA CAGATAAAAA TTACTAGTAA TGACAAATAA   
  
  
- AGTCACTCAT TAAAAGAGCC TATATTATAA AATATCGCCA ATAATTTATC TACACTTTGA TATTACAATC   
  
  
- TGTATAGTAC GCTTTTATCT CTTTAAAACC CGTTTAATTT TTTTTTTCTT CTTTTTGCTC CTTCATACGG   
  
  
- GTAACATTCG TCCACCTACA TATAGGTCGG AGGTAGGTTG TTAAACCCGT TTTAATATAG TAGGTAAGGT   
  
  
- TTTTTTTCTA TATTAAACAC TATAATTATT GGGCTGAGTT AAGTTCGAAT AAATAAAAAA GTATATTAAA   
  
  
- AAATTATATA TTTTTATTTT TCACTGTTGC TGTACGTAGG AAACGCACAG TTTCCTAGGC GGCGCACCGG   
  
  
- GGGTGTAAGT GACTGCCCCA AAAACCAAAC GCTAATTCTA GTTACCGATT GCAACAGAGA GAGTTTTGTA   
  
  
- TACCTGGGGA AAAAGATAGA AAAAGACGTA ACAGTCGTAT CGTTAACAAC TCGTGTAAAA AAAGCTAAAC   
  
  
- AACTGTTATT CGCACCAATA CTAAATACCC GGTGATTATA GTCAGAGACT AGTAAACGTG TAAAACCTGG   
  
  
- AGAAAGAGAG AGATTAGAAG GTCCATTGGA CTTAAGAAGA GATAAAGACT GATCTAATCT ATTGTCCTAA   
  
  
- AGTTTATAGA CAGTCCACCG CCCATATTGA AGCACTACCC GTAAGTAAAA ACAAGATAGT CCGACCACCC   
  
  
- AAACTCAAAT AAAACTAAGT AAAGCACAAG TAGACACTAT AATCATCCTC CCTAAGACAT ATGGGTGGTT   
  
  
- GACGAGCGGG AAAACGGGCA CACTCGTTAA CCCTGAAAAC AAACAAGAAG CAGCTACGTA GGAGACCAAC   
  
  
- GACTAGACCT TAACTTTGGG GTACATAAGT TTAAGTTGGG ACTAAACGAC AGTTTGAAAG ACTTGGTAGT   
  
  
- TTTGAGGCGG CTTCACAAAC TTTTTCTACC GTAGAAGGTC TGGGAAGTAA ACCGACTAGG GTTTTCGAGA   
  
  
- AGTTAACGGC TTTAGCCCAA ATGATTATCA AGTCTAAGAT GTGTTCATGG ACTAAAAAGT CTACGAACAG   
  
  
- AATTCAAGTA ATCACTATAA GAGGACCTTC TCCTGAATCT ACTTTCAGGA TGCAGAAATG TGCTAATGTA   
  
  
- CCGAGAGGAT CGGTGACTCT TCAGAAACAT ACTACGAGAA CCTTTCCTTA TGAAAGGAAG CAGATGATCA   
  
  
- GAACGGGGTA GAAATCCGGC TTCACAACTG TCGGGTCTAT CACCGAAACC GGCGCCAACG AGACTACCAG   
  
  
- CGCCCTAACT CCCAAACCGA TTACTACTAC GGCAAAAGTA CAGATTGACC GTCGAGTTGT GGTGGGTTAA   
  
  
- CCTGGGAAAG GGTTAGTACG TTCTATAAGG AGCAGGGATA AACCTTAACT TAATGGTAAG AAGACCCAGT   
  
  
- TCGTTACCGT AACTACTAAG CCCCCTACCC AATAGCTGGA GAGGACATTC ATGTGAACGT AGTTGGCGGT   
  
  
- GTCTCCGTCC CTTTTTCAAC CGACCATCGA GATCCTCTTT CTTGGCAGTT TCCCTACTGA TACCGGTACT   
  
  
- TCTCCCTTCC TCATTGTTCG TCGTTCGGAG AATGTTACTG CTAATGCAAC TCTACCTCGT CATACTGCTA   
  
  
- CATGAAGAGA CATCCCGTCT GTTCCCATTA TAAAGTTGAA CACGGTTACT TAGGAGCGAA TTACTCCACG   
  
  
- CACTCTTCGA CGTCTGCTGT CCCAAGTTCC CTTCTTGTAG GGCAGAATTC TTCGTTAGAT TCCTTCGCTT   
  
  
- TCTCCACCTA GACTCTTGAG AAGAAAGACC AACACGTGTT CGACAAAGCT TGAAACTATA GTCCTGACGA   
  
  
- TTACTCGACG AATTCGTTCA GTCTGTCGTA AGAAGTGGTA TACCACTATC GGAGGTTTCC GAGCGGGTAG   
  
  
- TTAAACGCTT ACCATAACTC CGTGCGAATC GTCCGTGGCC AAGTTCTCAT GGACGATTAG AGTAACTACG   
  
  
- TGCCTATAGT AGTAGACTTA AAAATTTTCG AATGTTCAGT ATACAAAGTC GTCAAGGAAA GTCTTCCTAC   
  
  
- AGGATAAAAT ATCGTTTGTT GTGTTAAGAC TTCAACCGAC TCTTTCGTTG TTTCTAAGTG TATTAACTAA   
  
  
- AACCATAAGA TAAACCAGAT GTCACCGGGA CAGAATATGT TTCAGAGAGT TCCGCTTGAC ACCGAGGAGG   
  
  
- CTTCGAAGCG TAGTGACCCT ATCTGATAGG GGTCGTACCA AAGGCCGGTC GTCTTTTCCA ACTCCGTTGT   
  
  
- CCAGCAGCCA ACAGGCCCAT GACACTCTCT AAATTACATG GGAAAGCTAT ACTTTGGTAA CGTTTCTTCA   
  
  
- CCCTTTGGTA TGCGGGTCTT CTAGATTTAT AACTCTTACT ACTCGACCAT TAACAATTAA CATACAACGC   
  
  
- CAGACATTTA GATAACCTAC TATGTCACCG CCATTTATCA GGTTCCCTAC GAAAGAACTC CAATTAGTTT   
  
  
- GTCTATTTGG GCGCAAATAA GTAAGTACGT TAACAGTTAC CTTGGAAATC ATGAGGTAAG AAGTCGTGAG   
  
  
- CTAAGTCCCT TCGGGATAAG GTTATGAGAA GACATAAACT ATATAAACTT CGCTGATACT GAGCACTTCT   
  
  
- AGCACCTTCC GACGACTAAC TCTCAGTTTA TACGCCCGAT CTTCAAAACT TACGTTATCG TACACTTCCA   
  
  
- CGTCTCTCCT AAGTTTCCGG ACTTTGTATG TTCGTTACCG TCCTCGCCTG TTGTTCCCGA CCTGATTCCG   
  
  
- TCCAAGGTTA TCTACTCCTC GAACAGTTAT CTCGTTTCTG ATATCACTTT CGTTTAATAG TGTTCCTAAA   
  
  
- ACACCACCTA CTCCTATCCG CAACCTACGA AGTTCCAACC TTTCCTTCCT GTGAATCACG GGAAAGGCAA   
  
  
- ACCGTCGGAT TGAT

+     MBS

| Site Name | Organism | Position | Strand | Matrix score. | sequence | function |
| --- | --- | --- | --- | --- | --- | --- |
| MBS | Arabidopsis thaliana | 1962 | + | 6 | CAACTG | MYB binding site involved in drought-inducibility |
| MBS | Arabidopsis thaliana | 309 | - | 6 | CAACTG | MYB binding site involved in drought-inducibility |

>HU02G01572.1   
+ +Up\_Stream \_Len000TTTAAT AAGATCCTTT TATATATATG TGTGTATAAT TCTATGGTAC ATAGGTATGT   
  
  
+ ACCATAGACT TATTGCATTT CTAGGTCCTT CTCTCTCTGC GCTTGTTTTT TGTTTATCTA ATTTATGGAG   
  
  
+ TTGAACATTT TGTTTTATAT ACTTGAACAA TCTATTATAC TAAATTAAGC ATTTTGATTT AATCACTTAT   
  
  
+ GGGTGTGTTT GGTAGGGTTG AAAATAATTT TCCTAGAAAA TAATTTTTAA TATAAAATAA TTTTTCATGT   
  
  
+ AAAATGATTT TCAACTCATA TTTTCAGTTG ATTGGTAAAA AGAAAAATGA ACTTTCTGGA TAATCAGTTT   
  
  
+ GATTAACAAA AAGTGACAAA AAAATTAAAA ATGGTTTTCC GTAATCTTCA AACCATATGT CGAGGAAATA   
  
  
+ AACACGGAAT AAAGAAAGAG TGTCAGAACC CAAGAAATGT AAAATCAACT TACGTTCTAT TTAAAGTGAA   
  
  
+ AAATATTTTT CTTCATTCTA GGAAAATTAA TTGTCCTCTG AAAACAAATT TTCCCTCCTT GTCTAACCAA   
  
  
+ ACACAGTAAA ATTGAAAAAT CATTTTCTAG GAAAATGATT TTCACTCCTA CCAAACACAC CCTTAAACTT   
  
  
+ GTTCCAAATC TATAAACCCG GAATCTTAAT ACGCACATTG TATATTATAT GTCCTACTAT AACTGTATTA   
  
  
+ TCACTGGTTC TTGATTTATC CTTTCAAACT CTTGCTTAGT CAAGGTTGTC CTCGTACAAA CATGATTAAT   
  
  
+ TCAATTAACC ACGTAAGTTA TACGCTGACA AATTTCGGGT ACCAAACATT TGCACTAGAC CTAAAACAGT   
  
  
+ TACTATCATC ATATCGATGT TAAAATTGAC CTGTTCTTAT TTGTCCAGCG TTGAGCAAGG AAATAATCAA   
  
  
+ ATCATGTCTC ATTTAGTCGG TTTTTTGCCT AAGAAGAGCA TAAACAAAGG AAAAAGACAA CGACACATTA   
  
  
+ TACATGCATC TACACAAATG AAATGAGGCC ATATGGAACA TGTTCATGAG AGCTTCACTC GCTAATAATT   
  
  
+ GAACCTACTT TTGACTAAGC CACCAACTAG AATATGGAGA TTGACTGTTA AGAGACTATA TTACATGAAA   
  
  
+ TAGATAAGAG TAGACATTGC TATATATTTT ATCTTTAATT GTCTATTTTT AATGATCATT ACTGTTTATT   
  
  
+ TCAGTGAGTA ATTTTCTCGG ATATAATATT TTATAGCGGT TATTAAATAG ATGTGAAACT ATAATGTTAG   
  
  
+ ACATATCATG CGAAAATAGA GAAATTTTGG GCAAATTAAA AAAAAAAGAA GAAAAACGAG GAAGTATGCC   
  
  
+ CATTGTAAGC AGGTGGATGT ATATCCAGCC TCCATCCAAC AATTTGGGCA AAATTATATC ATCCATTCCA   
  
  
+ AAAAAAAGAT ATAATTTGTG ATATTAATAA CCCGACTCAA TTCAAGCTTA TTTATTTTTT CATATAATTT   
  
  
+ TTTAATATAT AAAAATAAAA AGTGACAACG ACATGCATCC TTTGCGTGTC AAAGGATCCG CCGCGTGGCC   
  
  
+ CCCACATTCA CTGACGGGGT TTTTGGTTTG CGATTAAGAT CAATGGCTAA CGTTGTCTCT CTCAAAACAT   
  
  
+ ATGGACCCCT TTTTCTATCT TTTTCTGCAT TGTCAGCATA GCAATTGTTG AGCACATTTT TTTCGATTTG   
  
  
+ TTGACAATAA GCGTGGTTAT GATTTATGGG CCACTAATAT CAGTCTCTGA TCATTTGCAC ATTTTGGACC   
  
  
+ TCTTTCTCTC TCTAATCTTC CAGGTAACCT GAATTCTTCT CTATTTCTGA CTAGATTAGA TAACAGGATT   
  
  
+ TCAAATATCT GTCAGGTGGC GGGTATAACT TCGTGATGGG CATTCATTTT TGTTCTATCA GGCTGGTGGG   
  
  
+ TTTGAGTTTA TTTTGATTCA TTTCGTGTTC ATCTGTGATA TTAGTAGGAG GGATTCTGTA TACCCACCAA   
  
  
+ CTGCTCGCCC TTTTGCCCGT GTGAGCAATT GGGACTTTTG TTTGTTCTTC GTCGATGCAT CCTCTGGTTG   
  
  
+ CTGATCTGGA ATTGAAACCC CATGTATTCA AATTCAACCC TGATTTGCTG TCAAACTTTC TGAACCATCA   
  
  
+ AAACTCCGCC GAAGTGTTTG AAAAAGATGG CATCTTCCAG ACCCTTCATT TGGCTGATCC CAAAAGCTCT   
  
  
+ TCAATTGCCG AAATCGGGTT TACTAATAGT TCAGATTCTA CACAAGTACC TGATTTTTCA GATGCTTGTC   
  
  
+ TTAAGTTCAT TAGTGATATT CTCCTGGAAG AGGACTTAGA TGAAAGTCCT ACGTCTTTAC ACGATTACAT   
  
  
+ GGCTCTCCTA GCCACTGAGA AGTCTTTGTA TGATGCTCTT GGAAAGGAAT ACTTTCCTTC GTCTACTAGT   
  
  
+ CTTGCCCCAT CTTTAGGCCG AAGTGTTGAC AGCCCAGATA GTGGCTTTGG CCGCGGTTGC TCTGATGGTC   
  
  
+ GCGGGATTGA GGGTTTGGCT AATGATGATG CCGTTTTCAT GTCTAACTGG CAGCTCAACA CCACCCAATT   
  
  
+ GGACCCTTTC CCAATCATGC AAGATATTCC TCGTCCCTAT TTGGAATTGA ATTACCATTC TTCTGGGTCA   
  
  
+ AGCAATGGCA TTGATGATTC GGGGGATGGG TTATCGACCT CTCCTGTAAG TACACTTGCA TCAACCGCCA   
  
  
+ CAGAGGCAGG GAAAAAGTTG GCTGGTAGCT CTAGGAGAAA GAACCGTCAA AGGGATGACT ATGGCCATGA   
  
  
+ AGAGGGAAGG AGTAACAAGC AGCAAGCCTC TTACAATGAC GATTACGTTG AGATGGAGCA GTATGACGAT   
  
  
+ GTACTTCTCT GTAGGGCAGA CAAGGGTAAT ATTTCAACTT GTGCCAATGA ATCCTCGCTT AATGAGGTGC   
  
  
+ GTGAGAAGCT GCAGACGACA GGGTTCAAGG GAAGAACATC CCGTCTTAAG AAGCAATCTA AGGAAGCGAA   
  
  
+ AGAGGTGGAT CTGAGAACTC TTCTTTCTGG TTGTGCACAA GCTGTTTCGA ACTTTGATAT CAGGACTGCT   
  
  
+ AATGAGCTGC TTAAGCAAGT CAGACAGCAT TCTTCACCAT ATGGTGATAG CCTCCAAAGG CTCGCCCATC   
  
  
+ AATTTGCGAA TGGTATTGAG GCACGCTTAG CAGGCACCGG TTCAAGAGTA CCTGCTAATC TCATTGATGC   
  
  
+ ACGGATATCA TCATCTGAAT TTTTAAAAGC TTACAAGTCA TATGTTTCAG CAGTTCCTTT CAGAAGGATG   
  
  
+ TCCTATTTTA TAGCAAACAA CACAATTCTG AAGTTGGCTG AGAAAGCAAC AAAGATTCAC ATAATTGATT   
  
  
+ TTGGTATTCT ATTTGGTCTA CAGTGGCCCT GTCTTATACA AAGTCTCTCA AGGCGAACTG TGGCTCCTCC   
  
  
+ GAAGCTTCGC ATCACTGGGA TAGACTATCC CCAGCATGGT TTCCGGCCAG CAGAAAAGGT TGAGGCAACA   
  
  
+ GGTCGTCGGT TGTCCGGGTA CTGTGAGAGA TTTAATGTAC CCTTTCGATA TGAAACCATT GCAAAGAAGT   
  
  
+ GGGAAACCAT ACGCCCAGAA GATCTAAATA TTGAGAATGA TGAGCTGGTA ATTGTTAATT GTATGTTGCG   
  
  
+ GTCTGTAAAT CTATTGGATG ATACAGTGGC GGTAAATAGT CCAAGGGATG CTTTCTTGAG GTTAATCAAA   
  
  
+ CAGATAAACC CGCGTTTATT CATTCATGCA ATTGTCAATG GAACCTTTAG TACTCCATTC TTCAGCACTC   
  
  
+ GATTCAGGGA AGCCCTATTC CAATACTCTT CTGTATTTGA TATATTTGAA GCGACTATGA CTCGTGAAGA   
  
  
+ TCGTGGAAGG CTGCTGATTG AGAGTCAAAT ATGCGGGCTA GAAGTTTTGA ATGCAATAGC ATGTGAAGGT   
  
  
+ GCAGAGAGGA TTCAAAGGCC TGAAACATAC AAGCAATGGC AGGAGCGGAC AACAAGGGCT GGACTAAGGC   
  
  
+ AGGTTCCAAT AGATGAGGAG CTTGTCAATA GAGCAAAGAC TATAGTGAAA GCAAATTATC ACAAGGATTT   
  
  
+ TGTGGTGGAT GAGGATAGGC GTTGGATGCT TCAAGGTTGG AAAGGAAGGA CACTTAGTGC CCTTTCCGTT   
  
  
+ TGGCAGCCTA ACTA  

- +Up\_Stream \_Len000AAATTA TTCTAGGAAA ATATATATAC ACACATATTA AGATACCATG TATCCATACA   
  
  
- TGGTATCTGA ATAACGTAAA GATCCAGGAA GAGAGAGACG CGAACAAAAA ACAAATAGAT TAAATACCTC   
  
  
- AACTTGTAAA ACAAAATATA TGAACTTGTT AGATAATATG ATTTAATTCG TAAAACTAAA TTAGTGAATA   
  
  
- CCCACACAAA CCATCCCAAC TTTTATTAAA AGGATCTTTT ATTAAAAATT ATATTTTATT AAAAAGTACA   
  
  
- TTTTACTAAA AGTTGAGTAT AAAAGTCAAC TAACCATTTT TCTTTTTACT TGAAAGACCT ATTAGTCAAA   
  
  
- CTAATTGTTT TTCACTGTTT TTTTAATTTT TACCAAAAGG CATTAGAAGT TTGGTATACA GCTCCTTTAT   
  
  
- TTGTGCCTTA TTTCTTTCTC ACAGTCTTGG GTTCTTTACA TTTTAGTTGA ATGCAAGATA AATTTCACTT   
  
  
- TTTATAAAAA GAAGTAAGAT CCTTTTAATT AACAGGAGAC TTTTGTTTAA AAGGGAGGAA CAGATTGGTT   
  
  
- TGTGTCATTT TAACTTTTTA GTAAAAGATC CTTTTACTAA AAGTGAGGAT GGTTTGTGTG GGAATTTGAA   
  
  
- CAAGGTTTAG ATATTTGGGC CTTAGAATTA TGCGTGTAAC ATATAATATA CAGGATGATA TTGACATAAT   
  
  
- AGTGACCAAG AACTAAATAG GAAAGTTTGA GAACGAATCA GTTCCAACAG GAGCATGTTT GTACTAATTA   
  
  
- AGTTAATTGG TGCATTCAAT ATGCGACTGT TTAAAGCCCA TGGTTTGTAA ACGTGATCTG GATTTTGTCA   
  
  
- ATGATAGTAG TATAGCTACA ATTTTAACTG GACAAGAATA AACAGGTCGC AACTCGTTCC TTTATTAGTT   
  
  
- TAGTACAGAG TAAATCAGCC AAAAAACGGA TTCTTCTCGT ATTTGTTTCC TTTTTCTGTT GCTGTGTAAT   
  
  
- ATGTACGTAG ATGTGTTTAC TTTACTCCGG TATACCTTGT ACAAGTACTC TCGAAGTGAG CGATTATTAA   
  
  
- CTTGGATGAA AACTGATTCG GTGGTTGATC TTATACCTCT AACTGACAAT TCTCTGATAT AATGTACTTT   
  
  
- ATCTATTCTC ATCTGTAACG ATATATAAAA TAGAAATTAA CAGATAAAAA TTACTAGTAA TGACAAATAA   
  
  
- AGTCACTCAT TAAAAGAGCC TATATTATAA AATATCGCCA ATAATTTATC TACACTTTGA TATTACAATC   
  
  
- TGTATAGTAC GCTTTTATCT CTTTAAAACC CGTTTAATTT TTTTTTTCTT CTTTTTGCTC CTTCATACGG   
  
  
- GTAACATTCG TCCACCTACA TATAGGTCGG AGGTAGGTTG TTAAACCCGT TTTAATATAG TAGGTAAGGT   
  
  
- TTTTTTTCTA TATTAAACAC TATAATTATT GGGCTGAGTT AAGTTCGAAT AAATAAAAAA GTATATTAAA   
  
  
- AAATTATATA TTTTTATTTT TCACTGTTGC TGTACGTAGG AAACGCACAG TTTCCTAGGC GGCGCACCGG   
  
  
- GGGTGTAAGT GACTGCCCCA AAAACCAAAC GCTAATTCTA GTTACCGATT GCAACAGAGA GAGTTTTGTA   
  
  
- TACCTGGGGA AAAAGATAGA AAAAGACGTA ACAGTCGTAT CGTTAACAAC TCGTGTAAAA AAAGCTAAAC   
  
  
- AACTGTTATT CGCACCAATA CTAAATACCC GGTGATTATA GTCAGAGACT AGTAAACGTG TAAAACCTGG   
  
  
- AGAAAGAGAG AGATTAGAAG GTCCATTGGA CTTAAGAAGA GATAAAGACT GATCTAATCT ATTGTCCTAA   
  
  
- AGTTTATAGA CAGTCCACCG CCCATATTGA AGCACTACCC GTAAGTAAAA ACAAGATAGT CCGACCACCC   
  
  
- AAACTCAAAT AAAACTAAGT AAAGCACAAG TAGACACTAT AATCATCCTC CCTAAGACAT ATGGGTGGTT   
  
  
- GACGAGCGGG AAAACGGGCA CACTCGTTAA CCCTGAAAAC AAACAAGAAG CAGCTACGTA GGAGACCAAC   
  
  
- GACTAGACCT TAACTTTGGG GTACATAAGT TTAAGTTGGG ACTAAACGAC AGTTTGAAAG ACTTGGTAGT   
  
  
- TTTGAGGCGG CTTCACAAAC TTTTTCTACC GTAGAAGGTC TGGGAAGTAA ACCGACTAGG GTTTTCGAGA   
  
  
- AGTTAACGGC TTTAGCCCAA ATGATTATCA AGTCTAAGAT GTGTTCATGG ACTAAAAAGT CTACGAACAG   
  
  
- AATTCAAGTA ATCACTATAA GAGGACCTTC TCCTGAATCT ACTTTCAGGA TGCAGAAATG TGCTAATGTA   
  
  
- CCGAGAGGAT CGGTGACTCT TCAGAAACAT ACTACGAGAA CCTTTCCTTA TGAAAGGAAG CAGATGATCA   
  
  
- GAACGGGGTA GAAATCCGGC TTCACAACTG TCGGGTCTAT CACCGAAACC GGCGCCAACG AGACTACCAG   
  
  
- CGCCCTAACT CCCAAACCGA TTACTACTAC GGCAAAAGTA CAGATTGACC GTCGAGTTGT GGTGGGTTAA   
  
  
- CCTGGGAAAG GGTTAGTACG TTCTATAAGG AGCAGGGATA AACCTTAACT TAATGGTAAG AAGACCCAGT   
  
  
- TCGTTACCGT AACTACTAAG CCCCCTACCC AATAGCTGGA GAGGACATTC ATGTGAACGT AGTTGGCGGT   
  
  
- GTCTCCGTCC CTTTTTCAAC CGACCATCGA GATCCTCTTT CTTGGCAGTT TCCCTACTGA TACCGGTACT   
  
  
- TCTCCCTTCC TCATTGTTCG TCGTTCGGAG AATGTTACTG CTAATGCAAC TCTACCTCGT CATACTGCTA   
  
  
- CATGAAGAGA CATCCCGTCT GTTCCCATTA TAAAGTTGAA CACGGTTACT TAGGAGCGAA TTACTCCACG   
  
  
- CACTCTTCGA CGTCTGCTGT CCCAAGTTCC CTTCTTGTAG GGCAGAATTC TTCGTTAGAT TCCTTCGCTT   
  
  
- TCTCCACCTA GACTCTTGAG AAGAAAGACC AACACGTGTT CGACAAAGCT TGAAACTATA GTCCTGACGA   
  
  
- TTACTCGACG AATTCGTTCA GTCTGTCGTA AGAAGTGGTA TACCACTATC GGAGGTTTCC GAGCGGGTAG   
  
  
- TTAAACGCTT ACCATAACTC CGTGCGAATC GTCCGTGGCC AAGTTCTCAT GGACGATTAG AGTAACTACG   
  
  
- TGCCTATAGT AGTAGACTTA AAAATTTTCG AATGTTCAGT ATACAAAGTC GTCAAGGAAA GTCTTCCTAC   
  
  
- AGGATAAAAT ATCGTTTGTT GTGTTAAGAC TTCAACCGAC TCTTTCGTTG TTTCTAAGTG TATTAACTAA   
  
  
- AACCATAAGA TAAACCAGAT GTCACCGGGA CAGAATATGT TTCAGAGAGT TCCGCTTGAC ACCGAGGAGG   
  
  
- CTTCGAAGCG TAGTGACCCT ATCTGATAGG GGTCGTACCA AAGGCCGGTC GTCTTTTCCA ACTCCGTTGT   
  
  
- CCAGCAGCCA ACAGGCCCAT GACACTCTCT AAATTACATG GGAAAGCTAT ACTTTGGTAA CGTTTCTTCA   
  
  
- CCCTTTGGTA TGCGGGTCTT CTAGATTTAT AACTCTTACT ACTCGACCAT TAACAATTAA CATACAACGC   
  
  
- CAGACATTTA GATAACCTAC TATGTCACCG CCATTTATCA GGTTCCCTAC GAAAGAACTC CAATTAGTTT   
  
  
- GTCTATTTGG GCGCAAATAA GTAAGTACGT TAACAGTTAC CTTGGAAATC ATGAGGTAAG AAGTCGTGAG   
  
  
- CTAAGTCCCT TCGGGATAAG GTTATGAGAA GACATAAACT ATATAAACTT CGCTGATACT GAGCACTTCT   
  
  
- AGCACCTTCC GACGACTAAC TCTCAGTTTA TACGCCCGAT CTTCAAAACT TACGTTATCG TACACTTCCA   
  
  
- CGTCTCTCCT AAGTTTCCGG ACTTTGTATG TTCGTTACCG TCCTCGCCTG TTGTTCCCGA CCTGATTCCG   
  
  
- TCCAAGGTTA TCTACTCCTC GAACAGTTAT CTCGTTTCTG ATATCACTTT CGTTTAATAG TGTTCCTAAA   
  
  
- ACACCACCTA CTCCTATCCG CAACCTACGA AGTTCCAACC TTTCCTTCCT GTGAATCACG GGAAAGGCAA   
  
  
- ACCGTCGGAT TGAT

+     MYB

| Site Name | Organism | Position | Strand | Matrix score. | sequence | function |
| --- | --- | --- | --- | --- | --- | --- |
| MYB | Arabidopsis thaliana | 2029 | - | 6 | CAACCA |  |
| MYB | Arabidopsis thaliana | 1698 | - | 6 | TAACCA |  |
| MYB | Arabidopsis thaliana | 780 | + | 6 | TAACCA |  |
| MYB | Arabidopsis thaliana | 2972 | - | 6 | CAACCA |  |
| MYB | Arabidopsis thaliana | 558 | + | 6 | TAACCA |  |
| MYB | Arabidopsis thaliana | 3430 | + | 6 | CAACAG |  |

>HU02G01572.1   
+ +Up\_Stream \_Len000TTTAAT AAGATCCTTT TATATATATG TGTGTATAAT TCTATGGTAC ATAGGTATGT   
  
  
+ ACCATAGACT TATTGCATTT CTAGGTCCTT CTCTCTCTGC GCTTGTTTTT TGTTTATCTA ATTTATGGAG   
  
  
+ TTGAACATTT TGTTTTATAT ACTTGAACAA TCTATTATAC TAAATTAAGC ATTTTGATTT AATCACTTAT   
  
  
+ GGGTGTGTTT GGTAGGGTTG AAAATAATTT TCCTAGAAAA TAATTTTTAA TATAAAATAA TTTTTCATGT   
  
  
+ AAAATGATTT TCAACTCATA TTTTCAGTTG ATTGGTAAAA AGAAAAATGA ACTTTCTGGA TAATCAGTTT   
  
  
+ GATTAACAAA AAGTGACAAA AAAATTAAAA ATGGTTTTCC GTAATCTTCA AACCATATGT CGAGGAAATA   
  
  
+ AACACGGAAT AAAGAAAGAG TGTCAGAACC CAAGAAATGT AAAATCAACT TACGTTCTAT TTAAAGTGAA   
  
  
+ AAATATTTTT CTTCATTCTA GGAAAATTAA TTGTCCTCTG AAAACAAATT TTCCCTCCTT GTCTAACCAA   
  
  
+ ACACAGTAAA ATTGAAAAAT CATTTTCTAG GAAAATGATT TTCACTCCTA CCAAACACAC CCTTAAACTT   
  
  
+ GTTCCAAATC TATAAACCCG GAATCTTAAT ACGCACATTG TATATTATAT GTCCTACTAT AACTGTATTA   
  
  
+ TCACTGGTTC TTGATTTATC CTTTCAAACT CTTGCTTAGT CAAGGTTGTC CTCGTACAAA CATGATTAAT   
  
  
+ TCAATTAACC ACGTAAGTTA TACGCTGACA AATTTCGGGT ACCAAACATT TGCACTAGAC CTAAAACAGT   
  
  
+ TACTATCATC ATATCGATGT TAAAATTGAC CTGTTCTTAT TTGTCCAGCG TTGAGCAAGG AAATAATCAA   
  
  
+ ATCATGTCTC ATTTAGTCGG TTTTTTGCCT AAGAAGAGCA TAAACAAAGG AAAAAGACAA CGACACATTA   
  
  
+ TACATGCATC TACACAAATG AAATGAGGCC ATATGGAACA TGTTCATGAG AGCTTCACTC GCTAATAATT   
  
  
+ GAACCTACTT TTGACTAAGC CACCAACTAG AATATGGAGA TTGACTGTTA AGAGACTATA TTACATGAAA   
  
  
+ TAGATAAGAG TAGACATTGC TATATATTTT ATCTTTAATT GTCTATTTTT AATGATCATT ACTGTTTATT   
  
  
+ TCAGTGAGTA ATTTTCTCGG ATATAATATT TTATAGCGGT TATTAAATAG ATGTGAAACT ATAATGTTAG   
  
  
+ ACATATCATG CGAAAATAGA GAAATTTTGG GCAAATTAAA AAAAAAAGAA GAAAAACGAG GAAGTATGCC   
  
  
+ CATTGTAAGC AGGTGGATGT ATATCCAGCC TCCATCCAAC AATTTGGGCA AAATTATATC ATCCATTCCA   
  
  
+ AAAAAAAGAT ATAATTTGTG ATATTAATAA CCCGACTCAA TTCAAGCTTA TTTATTTTTT CATATAATTT   
  
  
+ TTTAATATAT AAAAATAAAA AGTGACAACG ACATGCATCC TTTGCGTGTC AAAGGATCCG CCGCGTGGCC   
  
  
+ CCCACATTCA CTGACGGGGT TTTTGGTTTG CGATTAAGAT CAATGGCTAA CGTTGTCTCT CTCAAAACAT   
  
  
+ ATGGACCCCT TTTTCTATCT TTTTCTGCAT TGTCAGCATA GCAATTGTTG AGCACATTTT TTTCGATTTG   
  
  
+ TTGACAATAA GCGTGGTTAT GATTTATGGG CCACTAATAT CAGTCTCTGA TCATTTGCAC ATTTTGGACC   
  
  
+ TCTTTCTCTC TCTAATCTTC CAGGTAACCT GAATTCTTCT CTATTTCTGA CTAGATTAGA TAACAGGATT   
  
  
+ TCAAATATCT GTCAGGTGGC GGGTATAACT TCGTGATGGG CATTCATTTT TGTTCTATCA GGCTGGTGGG   
  
  
+ TTTGAGTTTA TTTTGATTCA TTTCGTGTTC ATCTGTGATA TTAGTAGGAG GGATTCTGTA TACCCACCAA   
  
  
+ CTGCTCGCCC TTTTGCCCGT GTGAGCAATT GGGACTTTTG TTTGTTCTTC GTCGATGCAT CCTCTGGTTG   
  
  
+ CTGATCTGGA ATTGAAACCC CATGTATTCA AATTCAACCC TGATTTGCTG TCAAACTTTC TGAACCATCA   
  
  
+ AAACTCCGCC GAAGTGTTTG AAAAAGATGG CATCTTCCAG ACCCTTCATT TGGCTGATCC CAAAAGCTCT   
  
  
+ TCAATTGCCG AAATCGGGTT TACTAATAGT TCAGATTCTA CACAAGTACC TGATTTTTCA GATGCTTGTC   
  
  
+ TTAAGTTCAT TAGTGATATT CTCCTGGAAG AGGACTTAGA TGAAAGTCCT ACGTCTTTAC ACGATTACAT   
  
  
+ GGCTCTCCTA GCCACTGAGA AGTCTTTGTA TGATGCTCTT GGAAAGGAAT ACTTTCCTTC GTCTACTAGT   
  
  
+ CTTGCCCCAT CTTTAGGCCG AAGTGTTGAC AGCCCAGATA GTGGCTTTGG CCGCGGTTGC TCTGATGGTC   
  
  
+ GCGGGATTGA GGGTTTGGCT AATGATGATG CCGTTTTCAT GTCTAACTGG CAGCTCAACA CCACCCAATT   
  
  
+ GGACCCTTTC CCAATCATGC AAGATATTCC TCGTCCCTAT TTGGAATTGA ATTACCATTC TTCTGGGTCA   
  
  
+ AGCAATGGCA TTGATGATTC GGGGGATGGG TTATCGACCT CTCCTGTAAG TACACTTGCA TCAACCGCCA   
  
  
+ CAGAGGCAGG GAAAAAGTTG GCTGGTAGCT CTAGGAGAAA GAACCGTCAA AGGGATGACT ATGGCCATGA   
  
  
+ AGAGGGAAGG AGTAACAAGC AGCAAGCCTC TTACAATGAC GATTACGTTG AGATGGAGCA GTATGACGAT   
  
  
+ GTACTTCTCT GTAGGGCAGA CAAGGGTAAT ATTTCAACTT GTGCCAATGA ATCCTCGCTT AATGAGGTGC   
  
  
+ GTGAGAAGCT GCAGACGACA GGGTTCAAGG GAAGAACATC CCGTCTTAAG AAGCAATCTA AGGAAGCGAA   
  
  
+ AGAGGTGGAT CTGAGAACTC TTCTTTCTGG TTGTGCACAA GCTGTTTCGA ACTTTGATAT CAGGACTGCT   
  
  
+ AATGAGCTGC TTAAGCAAGT CAGACAGCAT TCTTCACCAT ATGGTGATAG CCTCCAAAGG CTCGCCCATC   
  
  
+ AATTTGCGAA TGGTATTGAG GCACGCTTAG CAGGCACCGG TTCAAGAGTA CCTGCTAATC TCATTGATGC   
  
  
+ ACGGATATCA TCATCTGAAT TTTTAAAAGC TTACAAGTCA TATGTTTCAG CAGTTCCTTT CAGAAGGATG   
  
  
+ TCCTATTTTA TAGCAAACAA CACAATTCTG AAGTTGGCTG AGAAAGCAAC AAAGATTCAC ATAATTGATT   
  
  
+ TTGGTATTCT ATTTGGTCTA CAGTGGCCCT GTCTTATACA AAGTCTCTCA AGGCGAACTG TGGCTCCTCC   
  
  
+ GAAGCTTCGC ATCACTGGGA TAGACTATCC CCAGCATGGT TTCCGGCCAG CAGAAAAGGT TGAGGCAACA   
  
  
+ GGTCGTCGGT TGTCCGGGTA CTGTGAGAGA TTTAATGTAC CCTTTCGATA TGAAACCATT GCAAAGAAGT   
  
  
+ GGGAAACCAT ACGCCCAGAA GATCTAAATA TTGAGAATGA TGAGCTGGTA ATTGTTAATT GTATGTTGCG   
  
  
+ GTCTGTAAAT CTATTGGATG ATACAGTGGC GGTAAATAGT CCAAGGGATG CTTTCTTGAG GTTAATCAAA   
  
  
+ CAGATAAACC CGCGTTTATT CATTCATGCA ATTGTCAATG GAACCTTTAG TACTCCATTC TTCAGCACTC   
  
  
+ GATTCAGGGA AGCCCTATTC CAATACTCTT CTGTATTTGA TATATTTGAA GCGACTATGA CTCGTGAAGA   
  
  
+ TCGTGGAAGG CTGCTGATTG AGAGTCAAAT ATGCGGGCTA GAAGTTTTGA ATGCAATAGC ATGTGAAGGT   
  
  
+ GCAGAGAGGA TTCAAAGGCC TGAAACATAC AAGCAATGGC AGGAGCGGAC AACAAGGGCT GGACTAAGGC   
  
  
+ AGGTTCCAAT AGATGAGGAG CTTGTCAATA GAGCAAAGAC TATAGTGAAA GCAAATTATC ACAAGGATTT   
  
  
+ TGTGGTGGAT GAGGATAGGC GTTGGATGCT TCAAGGTTGG AAAGGAAGGA CACTTAGTGC CCTTTCCGTT   
  
  
+ TGGCAGCCTA ACTA  

- +Up\_Stream \_Len000AAATTA TTCTAGGAAA ATATATATAC ACACATATTA AGATACCATG TATCCATACA   
  
  
- TGGTATCTGA ATAACGTAAA GATCCAGGAA GAGAGAGACG CGAACAAAAA ACAAATAGAT TAAATACCTC   
  
  
- AACTTGTAAA ACAAAATATA TGAACTTGTT AGATAATATG ATTTAATTCG TAAAACTAAA TTAGTGAATA   
  
  
- CCCACACAAA CCATCCCAAC TTTTATTAAA AGGATCTTTT ATTAAAAATT ATATTTTATT AAAAAGTACA   
  
  
- TTTTACTAAA AGTTGAGTAT AAAAGTCAAC TAACCATTTT TCTTTTTACT TGAAAGACCT ATTAGTCAAA   
  
  
- CTAATTGTTT TTCACTGTTT TTTTAATTTT TACCAAAAGG CATTAGAAGT TTGGTATACA GCTCCTTTAT   
  
  
- TTGTGCCTTA TTTCTTTCTC ACAGTCTTGG GTTCTTTACA TTTTAGTTGA ATGCAAGATA AATTTCACTT   
  
  
- TTTATAAAAA GAAGTAAGAT CCTTTTAATT AACAGGAGAC TTTTGTTTAA AAGGGAGGAA CAGATTGGTT   
  
  
- TGTGTCATTT TAACTTTTTA GTAAAAGATC CTTTTACTAA AAGTGAGGAT GGTTTGTGTG GGAATTTGAA   
  
  
- CAAGGTTTAG ATATTTGGGC CTTAGAATTA TGCGTGTAAC ATATAATATA CAGGATGATA TTGACATAAT   
  
  
- AGTGACCAAG AACTAAATAG GAAAGTTTGA GAACGAATCA GTTCCAACAG GAGCATGTTT GTACTAATTA   
  
  
- AGTTAATTGG TGCATTCAAT ATGCGACTGT TTAAAGCCCA TGGTTTGTAA ACGTGATCTG GATTTTGTCA   
  
  
- ATGATAGTAG TATAGCTACA ATTTTAACTG GACAAGAATA AACAGGTCGC AACTCGTTCC TTTATTAGTT   
  
  
- TAGTACAGAG TAAATCAGCC AAAAAACGGA TTCTTCTCGT ATTTGTTTCC TTTTTCTGTT GCTGTGTAAT   
  
  
- ATGTACGTAG ATGTGTTTAC TTTACTCCGG TATACCTTGT ACAAGTACTC TCGAAGTGAG CGATTATTAA   
  
  
- CTTGGATGAA AACTGATTCG GTGGTTGATC TTATACCTCT AACTGACAAT TCTCTGATAT AATGTACTTT   
  
  
- ATCTATTCTC ATCTGTAACG ATATATAAAA TAGAAATTAA CAGATAAAAA TTACTAGTAA TGACAAATAA   
  
  
- AGTCACTCAT TAAAAGAGCC TATATTATAA AATATCGCCA ATAATTTATC TACACTTTGA TATTACAATC   
  
  
- TGTATAGTAC GCTTTTATCT CTTTAAAACC CGTTTAATTT TTTTTTTCTT CTTTTTGCTC CTTCATACGG   
  
  
- GTAACATTCG TCCACCTACA TATAGGTCGG AGGTAGGTTG TTAAACCCGT TTTAATATAG TAGGTAAGGT   
  
  
- TTTTTTTCTA TATTAAACAC TATAATTATT GGGCTGAGTT AAGTTCGAAT AAATAAAAAA GTATATTAAA   
  
  
- AAATTATATA TTTTTATTTT TCACTGTTGC TGTACGTAGG AAACGCACAG TTTCCTAGGC GGCGCACCGG   
  
  
- GGGTGTAAGT GACTGCCCCA AAAACCAAAC GCTAATTCTA GTTACCGATT GCAACAGAGA GAGTTTTGTA   
  
  
- TACCTGGGGA AAAAGATAGA AAAAGACGTA ACAGTCGTAT CGTTAACAAC TCGTGTAAAA AAAGCTAAAC   
  
  
- AACTGTTATT CGCACCAATA CTAAATACCC GGTGATTATA GTCAGAGACT AGTAAACGTG TAAAACCTGG   
  
  
- AGAAAGAGAG AGATTAGAAG GTCCATTGGA CTTAAGAAGA GATAAAGACT GATCTAATCT ATTGTCCTAA   
  
  
- AGTTTATAGA CAGTCCACCG CCCATATTGA AGCACTACCC GTAAGTAAAA ACAAGATAGT CCGACCACCC   
  
  
- AAACTCAAAT AAAACTAAGT AAAGCACAAG TAGACACTAT AATCATCCTC CCTAAGACAT ATGGGTGGTT   
  
  
- GACGAGCGGG AAAACGGGCA CACTCGTTAA CCCTGAAAAC AAACAAGAAG CAGCTACGTA GGAGACCAAC   
  
  
- GACTAGACCT TAACTTTGGG GTACATAAGT TTAAGTTGGG ACTAAACGAC AGTTTGAAAG ACTTGGTAGT   
  
  
- TTTGAGGCGG CTTCACAAAC TTTTTCTACC GTAGAAGGTC TGGGAAGTAA ACCGACTAGG GTTTTCGAGA   
  
  
- AGTTAACGGC TTTAGCCCAA ATGATTATCA AGTCTAAGAT GTGTTCATGG ACTAAAAAGT CTACGAACAG   
  
  
- AATTCAAGTA ATCACTATAA GAGGACCTTC TCCTGAATCT ACTTTCAGGA TGCAGAAATG TGCTAATGTA   
  
  
- CCGAGAGGAT CGGTGACTCT TCAGAAACAT ACTACGAGAA CCTTTCCTTA TGAAAGGAAG CAGATGATCA   
  
  
- GAACGGGGTA GAAATCCGGC TTCACAACTG TCGGGTCTAT CACCGAAACC GGCGCCAACG AGACTACCAG   
  
  
- CGCCCTAACT CCCAAACCGA TTACTACTAC GGCAAAAGTA CAGATTGACC GTCGAGTTGT GGTGGGTTAA   
  
  
- CCTGGGAAAG GGTTAGTACG TTCTATAAGG AGCAGGGATA AACCTTAACT TAATGGTAAG AAGACCCAGT   
  
  
- TCGTTACCGT AACTACTAAG CCCCCTACCC AATAGCTGGA GAGGACATTC ATGTGAACGT AGTTGGCGGT   
  
  
- GTCTCCGTCC CTTTTTCAAC CGACCATCGA GATCCTCTTT CTTGGCAGTT TCCCTACTGA TACCGGTACT   
  
  
- TCTCCCTTCC TCATTGTTCG TCGTTCGGAG AATGTTACTG CTAATGCAAC TCTACCTCGT CATACTGCTA   
  
  
- CATGAAGAGA CATCCCGTCT GTTCCCATTA TAAAGTTGAA CACGGTTACT TAGGAGCGAA TTACTCCACG   
  
  
- CACTCTTCGA CGTCTGCTGT CCCAAGTTCC CTTCTTGTAG GGCAGAATTC TTCGTTAGAT TCCTTCGCTT   
  
  
- TCTCCACCTA GACTCTTGAG AAGAAAGACC AACACGTGTT CGACAAAGCT TGAAACTATA GTCCTGACGA   
  
  
- TTACTCGACG AATTCGTTCA GTCTGTCGTA AGAAGTGGTA TACCACTATC GGAGGTTTCC GAGCGGGTAG   
  
  
- TTAAACGCTT ACCATAACTC CGTGCGAATC GTCCGTGGCC AAGTTCTCAT GGACGATTAG AGTAACTACG   
  
  
- TGCCTATAGT AGTAGACTTA AAAATTTTCG AATGTTCAGT ATACAAAGTC GTCAAGGAAA GTCTTCCTAC   
  
  
- AGGATAAAAT ATCGTTTGTT GTGTTAAGAC TTCAACCGAC TCTTTCGTTG TTTCTAAGTG TATTAACTAA   
  
  
- AACCATAAGA TAAACCAGAT GTCACCGGGA CAGAATATGT TTCAGAGAGT TCCGCTTGAC ACCGAGGAGG   
  
  
- CTTCGAAGCG TAGTGACCCT ATCTGATAGG GGTCGTACCA AAGGCCGGTC GTCTTTTCCA ACTCCGTTGT   
  
  
- CCAGCAGCCA ACAGGCCCAT GACACTCTCT AAATTACATG GGAAAGCTAT ACTTTGGTAA CGTTTCTTCA   
  
  
- CCCTTTGGTA TGCGGGTCTT CTAGATTTAT AACTCTTACT ACTCGACCAT TAACAATTAA CATACAACGC   
  
  
- CAGACATTTA GATAACCTAC TATGTCACCG CCATTTATCA GGTTCCCTAC GAAAGAACTC CAATTAGTTT   
  
  
- GTCTATTTGG GCGCAAATAA GTAAGTACGT TAACAGTTAC CTTGGAAATC ATGAGGTAAG AAGTCGTGAG   
  
  
- CTAAGTCCCT TCGGGATAAG GTTATGAGAA GACATAAACT ATATAAACTT CGCTGATACT GAGCACTTCT   
  
  
- AGCACCTTCC GACGACTAAC TCTCAGTTTA TACGCCCGAT CTTCAAAACT TACGTTATCG TACACTTCCA   
  
  
- CGTCTCTCCT AAGTTTCCGG ACTTTGTATG TTCGTTACCG TCCTCGCCTG TTGTTCCCGA CCTGATTCCG   
  
  
- TCCAAGGTTA TCTACTCCTC GAACAGTTAT CTCGTTTCTG ATATCACTTT CGTTTAATAG TGTTCCTAAA   
  
  
- ACACCACCTA CTCCTATCCG CAACCTACGA AGTTCCAACC TTTCCTTCCT GTGAATCACG GGAAAGGCAA   
  
  
- ACCGTCGGAT TGAT

+     MYB-like sequence

| Site Name | Organism | Position | Strand | Matrix score. | sequence | function |
| --- | --- | --- | --- | --- | --- | --- |
| MYB-like sequence | Arabidopsis thaliana | 558 | + | 6 | TAACCA |  |
| MYB-like sequence | Arabidopsis thaliana | 1698 | - | 6 | TAACCA |  |
| MYB-like sequence | Arabidopsis thaliana | 780 | + | 6 | TAACCA |  |

>HU02G01572.1   
+ +Up\_Stream \_Len000TTTAAT AAGATCCTTT TATATATATG TGTGTATAAT TCTATGGTAC ATAGGTATGT   
  
  
+ ACCATAGACT TATTGCATTT CTAGGTCCTT CTCTCTCTGC GCTTGTTTTT TGTTTATCTA ATTTATGGAG   
  
  
+ TTGAACATTT TGTTTTATAT ACTTGAACAA TCTATTATAC TAAATTAAGC ATTTTGATTT AATCACTTAT   
  
  
+ GGGTGTGTTT GGTAGGGTTG AAAATAATTT TCCTAGAAAA TAATTTTTAA TATAAAATAA TTTTTCATGT   
  
  
+ AAAATGATTT TCAACTCATA TTTTCAGTTG ATTGGTAAAA AGAAAAATGA ACTTTCTGGA TAATCAGTTT   
  
  
+ GATTAACAAA AAGTGACAAA AAAATTAAAA ATGGTTTTCC GTAATCTTCA AACCATATGT CGAGGAAATA   
  
  
+ AACACGGAAT AAAGAAAGAG TGTCAGAACC CAAGAAATGT AAAATCAACT TACGTTCTAT TTAAAGTGAA   
  
  
+ AAATATTTTT CTTCATTCTA GGAAAATTAA TTGTCCTCTG AAAACAAATT TTCCCTCCTT GTCTAACCAA   
  
  
+ ACACAGTAAA ATTGAAAAAT CATTTTCTAG GAAAATGATT TTCACTCCTA CCAAACACAC CCTTAAACTT   
  
  
+ GTTCCAAATC TATAAACCCG GAATCTTAAT ACGCACATTG TATATTATAT GTCCTACTAT AACTGTATTA   
  
  
+ TCACTGGTTC TTGATTTATC CTTTCAAACT CTTGCTTAGT CAAGGTTGTC CTCGTACAAA CATGATTAAT   
  
  
+ TCAATTAACC ACGTAAGTTA TACGCTGACA AATTTCGGGT ACCAAACATT TGCACTAGAC CTAAAACAGT   
  
  
+ TACTATCATC ATATCGATGT TAAAATTGAC CTGTTCTTAT TTGTCCAGCG TTGAGCAAGG AAATAATCAA   
  
  
+ ATCATGTCTC ATTTAGTCGG TTTTTTGCCT AAGAAGAGCA TAAACAAAGG AAAAAGACAA CGACACATTA   
  
  
+ TACATGCATC TACACAAATG AAATGAGGCC ATATGGAACA TGTTCATGAG AGCTTCACTC GCTAATAATT   
  
  
+ GAACCTACTT TTGACTAAGC CACCAACTAG AATATGGAGA TTGACTGTTA AGAGACTATA TTACATGAAA   
  
  
+ TAGATAAGAG TAGACATTGC TATATATTTT ATCTTTAATT GTCTATTTTT AATGATCATT ACTGTTTATT   
  
  
+ TCAGTGAGTA ATTTTCTCGG ATATAATATT TTATAGCGGT TATTAAATAG ATGTGAAACT ATAATGTTAG   
  
  
+ ACATATCATG CGAAAATAGA GAAATTTTGG GCAAATTAAA AAAAAAAGAA GAAAAACGAG GAAGTATGCC   
  
  
+ CATTGTAAGC AGGTGGATGT ATATCCAGCC TCCATCCAAC AATTTGGGCA AAATTATATC ATCCATTCCA   
  
  
+ AAAAAAAGAT ATAATTTGTG ATATTAATAA CCCGACTCAA TTCAAGCTTA TTTATTTTTT CATATAATTT   
  
  
+ TTTAATATAT AAAAATAAAA AGTGACAACG ACATGCATCC TTTGCGTGTC AAAGGATCCG CCGCGTGGCC   
  
  
+ CCCACATTCA CTGACGGGGT TTTTGGTTTG CGATTAAGAT CAATGGCTAA CGTTGTCTCT CTCAAAACAT   
  
  
+ ATGGACCCCT TTTTCTATCT TTTTCTGCAT TGTCAGCATA GCAATTGTTG AGCACATTTT TTTCGATTTG   
  
  
+ TTGACAATAA GCGTGGTTAT GATTTATGGG CCACTAATAT CAGTCTCTGA TCATTTGCAC ATTTTGGACC   
  
  
+ TCTTTCTCTC TCTAATCTTC CAGGTAACCT GAATTCTTCT CTATTTCTGA CTAGATTAGA TAACAGGATT   
  
  
+ TCAAATATCT GTCAGGTGGC GGGTATAACT TCGTGATGGG CATTCATTTT TGTTCTATCA GGCTGGTGGG   
  
  
+ TTTGAGTTTA TTTTGATTCA TTTCGTGTTC ATCTGTGATA TTAGTAGGAG GGATTCTGTA TACCCACCAA   
  
  
+ CTGCTCGCCC TTTTGCCCGT GTGAGCAATT GGGACTTTTG TTTGTTCTTC GTCGATGCAT CCTCTGGTTG   
  
  
+ CTGATCTGGA ATTGAAACCC CATGTATTCA AATTCAACCC TGATTTGCTG TCAAACTTTC TGAACCATCA   
  
  
+ AAACTCCGCC GAAGTGTTTG AAAAAGATGG CATCTTCCAG ACCCTTCATT TGGCTGATCC CAAAAGCTCT   
  
  
+ TCAATTGCCG AAATCGGGTT TACTAATAGT TCAGATTCTA CACAAGTACC TGATTTTTCA GATGCTTGTC   
  
  
+ TTAAGTTCAT TAGTGATATT CTCCTGGAAG AGGACTTAGA TGAAAGTCCT ACGTCTTTAC ACGATTACAT   
  
  
+ GGCTCTCCTA GCCACTGAGA AGTCTTTGTA TGATGCTCTT GGAAAGGAAT ACTTTCCTTC GTCTACTAGT   
  
  
+ CTTGCCCCAT CTTTAGGCCG AAGTGTTGAC AGCCCAGATA GTGGCTTTGG CCGCGGTTGC TCTGATGGTC   
  
  
+ GCGGGATTGA GGGTTTGGCT AATGATGATG CCGTTTTCAT GTCTAACTGG CAGCTCAACA CCACCCAATT   
  
  
+ GGACCCTTTC CCAATCATGC AAGATATTCC TCGTCCCTAT TTGGAATTGA ATTACCATTC TTCTGGGTCA   
  
  
+ AGCAATGGCA TTGATGATTC GGGGGATGGG TTATCGACCT CTCCTGTAAG TACACTTGCA TCAACCGCCA   
  
  
+ CAGAGGCAGG GAAAAAGTTG GCTGGTAGCT CTAGGAGAAA GAACCGTCAA AGGGATGACT ATGGCCATGA   
  
  
+ AGAGGGAAGG AGTAACAAGC AGCAAGCCTC TTACAATGAC GATTACGTTG AGATGGAGCA GTATGACGAT   
  
  
+ GTACTTCTCT GTAGGGCAGA CAAGGGTAAT ATTTCAACTT GTGCCAATGA ATCCTCGCTT AATGAGGTGC   
  
  
+ GTGAGAAGCT GCAGACGACA GGGTTCAAGG GAAGAACATC CCGTCTTAAG AAGCAATCTA AGGAAGCGAA   
  
  
+ AGAGGTGGAT CTGAGAACTC TTCTTTCTGG TTGTGCACAA GCTGTTTCGA ACTTTGATAT CAGGACTGCT   
  
  
+ AATGAGCTGC TTAAGCAAGT CAGACAGCAT TCTTCACCAT ATGGTGATAG CCTCCAAAGG CTCGCCCATC   
  
  
+ AATTTGCGAA TGGTATTGAG GCACGCTTAG CAGGCACCGG TTCAAGAGTA CCTGCTAATC TCATTGATGC   
  
  
+ ACGGATATCA TCATCTGAAT TTTTAAAAGC TTACAAGTCA TATGTTTCAG CAGTTCCTTT CAGAAGGATG   
  
  
+ TCCTATTTTA TAGCAAACAA CACAATTCTG AAGTTGGCTG AGAAAGCAAC AAAGATTCAC ATAATTGATT   
  
  
+ TTGGTATTCT ATTTGGTCTA CAGTGGCCCT GTCTTATACA AAGTCTCTCA AGGCGAACTG TGGCTCCTCC   
  
  
+ GAAGCTTCGC ATCACTGGGA TAGACTATCC CCAGCATGGT TTCCGGCCAG CAGAAAAGGT TGAGGCAACA   
  
  
+ GGTCGTCGGT TGTCCGGGTA CTGTGAGAGA TTTAATGTAC CCTTTCGATA TGAAACCATT GCAAAGAAGT   
  
  
+ GGGAAACCAT ACGCCCAGAA GATCTAAATA TTGAGAATGA TGAGCTGGTA ATTGTTAATT GTATGTTGCG   
  
  
+ GTCTGTAAAT CTATTGGATG ATACAGTGGC GGTAAATAGT CCAAGGGATG CTTTCTTGAG GTTAATCAAA   
  
  
+ CAGATAAACC CGCGTTTATT CATTCATGCA ATTGTCAATG GAACCTTTAG TACTCCATTC TTCAGCACTC   
  
  
+ GATTCAGGGA AGCCCTATTC CAATACTCTT CTGTATTTGA TATATTTGAA GCGACTATGA CTCGTGAAGA   
  
  
+ TCGTGGAAGG CTGCTGATTG AGAGTCAAAT ATGCGGGCTA GAAGTTTTGA ATGCAATAGC ATGTGAAGGT   
  
  
+ GCAGAGAGGA TTCAAAGGCC TGAAACATAC AAGCAATGGC AGGAGCGGAC AACAAGGGCT GGACTAAGGC   
  
  
+ AGGTTCCAAT AGATGAGGAG CTTGTCAATA GAGCAAAGAC TATAGTGAAA GCAAATTATC ACAAGGATTT   
  
  
+ TGTGGTGGAT GAGGATAGGC GTTGGATGCT TCAAGGTTGG AAAGGAAGGA CACTTAGTGC CCTTTCCGTT   
  
  
+ TGGCAGCCTA ACTA  

- +Up\_Stream \_Len000AAATTA TTCTAGGAAA ATATATATAC ACACATATTA AGATACCATG TATCCATACA   
  
  
- TGGTATCTGA ATAACGTAAA GATCCAGGAA GAGAGAGACG CGAACAAAAA ACAAATAGAT TAAATACCTC   
  
  
- AACTTGTAAA ACAAAATATA TGAACTTGTT AGATAATATG ATTTAATTCG TAAAACTAAA TTAGTGAATA   
  
  
- CCCACACAAA CCATCCCAAC TTTTATTAAA AGGATCTTTT ATTAAAAATT ATATTTTATT AAAAAGTACA   
  
  
- TTTTACTAAA AGTTGAGTAT AAAAGTCAAC TAACCATTTT TCTTTTTACT TGAAAGACCT ATTAGTCAAA   
  
  
- CTAATTGTTT TTCACTGTTT TTTTAATTTT TACCAAAAGG CATTAGAAGT TTGGTATACA GCTCCTTTAT   
  
  
- TTGTGCCTTA TTTCTTTCTC ACAGTCTTGG GTTCTTTACA TTTTAGTTGA ATGCAAGATA AATTTCACTT   
  
  
- TTTATAAAAA GAAGTAAGAT CCTTTTAATT AACAGGAGAC TTTTGTTTAA AAGGGAGGAA CAGATTGGTT   
  
  
- TGTGTCATTT TAACTTTTTA GTAAAAGATC CTTTTACTAA AAGTGAGGAT GGTTTGTGTG GGAATTTGAA   
  
  
- CAAGGTTTAG ATATTTGGGC CTTAGAATTA TGCGTGTAAC ATATAATATA CAGGATGATA TTGACATAAT   
  
  
- AGTGACCAAG AACTAAATAG GAAAGTTTGA GAACGAATCA GTTCCAACAG GAGCATGTTT GTACTAATTA   
  
  
- AGTTAATTGG TGCATTCAAT ATGCGACTGT TTAAAGCCCA TGGTTTGTAA ACGTGATCTG GATTTTGTCA   
  
  
- ATGATAGTAG TATAGCTACA ATTTTAACTG GACAAGAATA AACAGGTCGC AACTCGTTCC TTTATTAGTT   
  
  
- TAGTACAGAG TAAATCAGCC AAAAAACGGA TTCTTCTCGT ATTTGTTTCC TTTTTCTGTT GCTGTGTAAT   
  
  
- ATGTACGTAG ATGTGTTTAC TTTACTCCGG TATACCTTGT ACAAGTACTC TCGAAGTGAG CGATTATTAA   
  
  
- CTTGGATGAA AACTGATTCG GTGGTTGATC TTATACCTCT AACTGACAAT TCTCTGATAT AATGTACTTT   
  
  
- ATCTATTCTC ATCTGTAACG ATATATAAAA TAGAAATTAA CAGATAAAAA TTACTAGTAA TGACAAATAA   
  
  
- AGTCACTCAT TAAAAGAGCC TATATTATAA AATATCGCCA ATAATTTATC TACACTTTGA TATTACAATC   
  
  
- TGTATAGTAC GCTTTTATCT CTTTAAAACC CGTTTAATTT TTTTTTTCTT CTTTTTGCTC CTTCATACGG   
  
  
- GTAACATTCG TCCACCTACA TATAGGTCGG AGGTAGGTTG TTAAACCCGT TTTAATATAG TAGGTAAGGT   
  
  
- TTTTTTTCTA TATTAAACAC TATAATTATT GGGCTGAGTT AAGTTCGAAT AAATAAAAAA GTATATTAAA   
  
  
- AAATTATATA TTTTTATTTT TCACTGTTGC TGTACGTAGG AAACGCACAG TTTCCTAGGC GGCGCACCGG   
  
  
- GGGTGTAAGT GACTGCCCCA AAAACCAAAC GCTAATTCTA GTTACCGATT GCAACAGAGA GAGTTTTGTA   
  
  
- TACCTGGGGA AAAAGATAGA AAAAGACGTA ACAGTCGTAT CGTTAACAAC TCGTGTAAAA AAAGCTAAAC   
  
  
- AACTGTTATT CGCACCAATA CTAAATACCC GGTGATTATA GTCAGAGACT AGTAAACGTG TAAAACCTGG   
  
  
- AGAAAGAGAG AGATTAGAAG GTCCATTGGA CTTAAGAAGA GATAAAGACT GATCTAATCT ATTGTCCTAA   
  
  
- AGTTTATAGA CAGTCCACCG CCCATATTGA AGCACTACCC GTAAGTAAAA ACAAGATAGT CCGACCACCC   
  
  
- AAACTCAAAT AAAACTAAGT AAAGCACAAG TAGACACTAT AATCATCCTC CCTAAGACAT ATGGGTGGTT   
  
  
- GACGAGCGGG AAAACGGGCA CACTCGTTAA CCCTGAAAAC AAACAAGAAG CAGCTACGTA GGAGACCAAC   
  
  
- GACTAGACCT TAACTTTGGG GTACATAAGT TTAAGTTGGG ACTAAACGAC AGTTTGAAAG ACTTGGTAGT   
  
  
- TTTGAGGCGG CTTCACAAAC TTTTTCTACC GTAGAAGGTC TGGGAAGTAA ACCGACTAGG GTTTTCGAGA   
  
  
- AGTTAACGGC TTTAGCCCAA ATGATTATCA AGTCTAAGAT GTGTTCATGG ACTAAAAAGT CTACGAACAG   
  
  
- AATTCAAGTA ATCACTATAA GAGGACCTTC TCCTGAATCT ACTTTCAGGA TGCAGAAATG TGCTAATGTA   
  
  
- CCGAGAGGAT CGGTGACTCT TCAGAAACAT ACTACGAGAA CCTTTCCTTA TGAAAGGAAG CAGATGATCA   
  
  
- GAACGGGGTA GAAATCCGGC TTCACAACTG TCGGGTCTAT CACCGAAACC GGCGCCAACG AGACTACCAG   
  
  
- CGCCCTAACT CCCAAACCGA TTACTACTAC GGCAAAAGTA CAGATTGACC GTCGAGTTGT GGTGGGTTAA   
  
  
- CCTGGGAAAG GGTTAGTACG TTCTATAAGG AGCAGGGATA AACCTTAACT TAATGGTAAG AAGACCCAGT   
  
  
- TCGTTACCGT AACTACTAAG CCCCCTACCC AATAGCTGGA GAGGACATTC ATGTGAACGT AGTTGGCGGT   
  
  
- GTCTCCGTCC CTTTTTCAAC CGACCATCGA GATCCTCTTT CTTGGCAGTT TCCCTACTGA TACCGGTACT   
  
  
- TCTCCCTTCC TCATTGTTCG TCGTTCGGAG AATGTTACTG CTAATGCAAC TCTACCTCGT CATACTGCTA   
  
  
- CATGAAGAGA CATCCCGTCT GTTCCCATTA TAAAGTTGAA CACGGTTACT TAGGAGCGAA TTACTCCACG   
  
  
- CACTCTTCGA CGTCTGCTGT CCCAAGTTCC CTTCTTGTAG GGCAGAATTC TTCGTTAGAT TCCTTCGCTT   
  
  
- TCTCCACCTA GACTCTTGAG AAGAAAGACC AACACGTGTT CGACAAAGCT TGAAACTATA GTCCTGACGA   
  
  
- TTACTCGACG AATTCGTTCA GTCTGTCGTA AGAAGTGGTA TACCACTATC GGAGGTTTCC GAGCGGGTAG   
  
  
- TTAAACGCTT ACCATAACTC CGTGCGAATC GTCCGTGGCC AAGTTCTCAT GGACGATTAG AGTAACTACG   
  
  
- TGCCTATAGT AGTAGACTTA AAAATTTTCG AATGTTCAGT ATACAAAGTC GTCAAGGAAA GTCTTCCTAC   
  
  
- AGGATAAAAT ATCGTTTGTT GTGTTAAGAC TTCAACCGAC TCTTTCGTTG TTTCTAAGTG TATTAACTAA   
  
  
- AACCATAAGA TAAACCAGAT GTCACCGGGA CAGAATATGT TTCAGAGAGT TCCGCTTGAC ACCGAGGAGG   
  
  
- CTTCGAAGCG TAGTGACCCT ATCTGATAGG GGTCGTACCA AAGGCCGGTC GTCTTTTCCA ACTCCGTTGT   
  
  
- CCAGCAGCCA ACAGGCCCAT GACACTCTCT AAATTACATG GGAAAGCTAT ACTTTGGTAA CGTTTCTTCA   
  
  
- CCCTTTGGTA TGCGGGTCTT CTAGATTTAT AACTCTTACT ACTCGACCAT TAACAATTAA CATACAACGC   
  
  
- CAGACATTTA GATAACCTAC TATGTCACCG CCATTTATCA GGTTCCCTAC GAAAGAACTC CAATTAGTTT   
  
  
- GTCTATTTGG GCGCAAATAA GTAAGTACGT TAACAGTTAC CTTGGAAATC ATGAGGTAAG AAGTCGTGAG   
  
  
- CTAAGTCCCT TCGGGATAAG GTTATGAGAA GACATAAACT ATATAAACTT CGCTGATACT GAGCACTTCT   
  
  
- AGCACCTTCC GACGACTAAC TCTCAGTTTA TACGCCCGAT CTTCAAAACT TACGTTATCG TACACTTCCA   
  
  
- CGTCTCTCCT AAGTTTCCGG ACTTTGTATG TTCGTTACCG TCCTCGCCTG TTGTTCCCGA CCTGATTCCG   
  
  
- TCCAAGGTTA TCTACTCCTC GAACAGTTAT CTCGTTTCTG ATATCACTTT CGTTTAATAG TGTTCCTAAA   
  
  
- ACACCACCTA CTCCTATCCG CAACCTACGA AGTTCCAACC TTTCCTTCCT GTGAATCACG GGAAAGGCAA   
  
  
- ACCGTCGGAT TGAT

+     MYC

| Site Name | Organism | Position | Strand | Matrix score. | sequence | function |
| --- | --- | --- | --- | --- | --- | --- |
| MYC | Arabidopsis thaliana | 2176 | - | 6 | CAATTG |  |
| MYC | Arabidopsis thaliana | 2151 | + | 6 | CATTTG |  |
| MYC | Arabidopsis thaliana | 1990 | + | 6 | CAATTG |  |
| MYC | Arabidopsis thaliana | 3673 | - | 6 | CAATTG |  |
| MYC | Arabidopsis thaliana | 2520 | - | 6 | CAATTG |  |
| MYC | Arabidopsis thaliana | 1656 | + | 6 | CAATTG |  |
| MYC | Arabidopsis thaliana | 999 | - | 6 | CATTTG |  |
| MYC | Arabidopsis thaliana | 3844 | + | 6 | CATGTG |  |
| MYC | Arabidopsis thaliana | 821 | + | 6 | CATTTG |  |
| MYC | Arabidopsis thaliana | 1736 | + | 6 | CATTTG |  |

>HU02G01572.1   
+ +Up\_Stream \_Len000TTTAAT AAGATCCTTT TATATATATG TGTGTATAAT TCTATGGTAC ATAGGTATGT   
  
  
+ ACCATAGACT TATTGCATTT CTAGGTCCTT CTCTCTCTGC GCTTGTTTTT TGTTTATCTA ATTTATGGAG   
  
  
+ TTGAACATTT TGTTTTATAT ACTTGAACAA TCTATTATAC TAAATTAAGC ATTTTGATTT AATCACTTAT   
  
  
+ GGGTGTGTTT GGTAGGGTTG AAAATAATTT TCCTAGAAAA TAATTTTTAA TATAAAATAA TTTTTCATGT   
  
  
+ AAAATGATTT TCAACTCATA TTTTCAGTTG ATTGGTAAAA AGAAAAATGA ACTTTCTGGA TAATCAGTTT   
  
  
+ GATTAACAAA AAGTGACAAA AAAATTAAAA ATGGTTTTCC GTAATCTTCA AACCATATGT CGAGGAAATA   
  
  
+ AACACGGAAT AAAGAAAGAG TGTCAGAACC CAAGAAATGT AAAATCAACT TACGTTCTAT TTAAAGTGAA   
  
  
+ AAATATTTTT CTTCATTCTA GGAAAATTAA TTGTCCTCTG AAAACAAATT TTCCCTCCTT GTCTAACCAA   
  
  
+ ACACAGTAAA ATTGAAAAAT CATTTTCTAG GAAAATGATT TTCACTCCTA CCAAACACAC CCTTAAACTT   
  
  
+ GTTCCAAATC TATAAACCCG GAATCTTAAT ACGCACATTG TATATTATAT GTCCTACTAT AACTGTATTA   
  
  
+ TCACTGGTTC TTGATTTATC CTTTCAAACT CTTGCTTAGT CAAGGTTGTC CTCGTACAAA CATGATTAAT   
  
  
+ TCAATTAACC ACGTAAGTTA TACGCTGACA AATTTCGGGT ACCAAACATT TGCACTAGAC CTAAAACAGT   
  
  
+ TACTATCATC ATATCGATGT TAAAATTGAC CTGTTCTTAT TTGTCCAGCG TTGAGCAAGG AAATAATCAA   
  
  
+ ATCATGTCTC ATTTAGTCGG TTTTTTGCCT AAGAAGAGCA TAAACAAAGG AAAAAGACAA CGACACATTA   
  
  
+ TACATGCATC TACACAAATG AAATGAGGCC ATATGGAACA TGTTCATGAG AGCTTCACTC GCTAATAATT   
  
  
+ GAACCTACTT TTGACTAAGC CACCAACTAG AATATGGAGA TTGACTGTTA AGAGACTATA TTACATGAAA   
  
  
+ TAGATAAGAG TAGACATTGC TATATATTTT ATCTTTAATT GTCTATTTTT AATGATCATT ACTGTTTATT   
  
  
+ TCAGTGAGTA ATTTTCTCGG ATATAATATT TTATAGCGGT TATTAAATAG ATGTGAAACT ATAATGTTAG   
  
  
+ ACATATCATG CGAAAATAGA GAAATTTTGG GCAAATTAAA AAAAAAAGAA GAAAAACGAG GAAGTATGCC   
  
  
+ CATTGTAAGC AGGTGGATGT ATATCCAGCC TCCATCCAAC AATTTGGGCA AAATTATATC ATCCATTCCA   
  
  
+ AAAAAAAGAT ATAATTTGTG ATATTAATAA CCCGACTCAA TTCAAGCTTA TTTATTTTTT CATATAATTT   
  
  
+ TTTAATATAT AAAAATAAAA AGTGACAACG ACATGCATCC TTTGCGTGTC AAAGGATCCG CCGCGTGGCC   
  
  
+ CCCACATTCA CTGACGGGGT TTTTGGTTTG CGATTAAGAT CAATGGCTAA CGTTGTCTCT CTCAAAACAT   
  
  
+ ATGGACCCCT TTTTCTATCT TTTTCTGCAT TGTCAGCATA GCAATTGTTG AGCACATTTT TTTCGATTTG   
  
  
+ TTGACAATAA GCGTGGTTAT GATTTATGGG CCACTAATAT CAGTCTCTGA TCATTTGCAC ATTTTGGACC   
  
  
+ TCTTTCTCTC TCTAATCTTC CAGGTAACCT GAATTCTTCT CTATTTCTGA CTAGATTAGA TAACAGGATT   
  
  
+ TCAAATATCT GTCAGGTGGC GGGTATAACT TCGTGATGGG CATTCATTTT TGTTCTATCA GGCTGGTGGG   
  
  
+ TTTGAGTTTA TTTTGATTCA TTTCGTGTTC ATCTGTGATA TTAGTAGGAG GGATTCTGTA TACCCACCAA   
  
  
+ CTGCTCGCCC TTTTGCCCGT GTGAGCAATT GGGACTTTTG TTTGTTCTTC GTCGATGCAT CCTCTGGTTG   
  
  
+ CTGATCTGGA ATTGAAACCC CATGTATTCA AATTCAACCC TGATTTGCTG TCAAACTTTC TGAACCATCA   
  
  
+ AAACTCCGCC GAAGTGTTTG AAAAAGATGG CATCTTCCAG ACCCTTCATT TGGCTGATCC CAAAAGCTCT   
  
  
+ TCAATTGCCG AAATCGGGTT TACTAATAGT TCAGATTCTA CACAAGTACC TGATTTTTCA GATGCTTGTC   
  
  
+ TTAAGTTCAT TAGTGATATT CTCCTGGAAG AGGACTTAGA TGAAAGTCCT ACGTCTTTAC ACGATTACAT   
  
  
+ GGCTCTCCTA GCCACTGAGA AGTCTTTGTA TGATGCTCTT GGAAAGGAAT ACTTTCCTTC GTCTACTAGT   
  
  
+ CTTGCCCCAT CTTTAGGCCG AAGTGTTGAC AGCCCAGATA GTGGCTTTGG CCGCGGTTGC TCTGATGGTC   
  
  
+ GCGGGATTGA GGGTTTGGCT AATGATGATG CCGTTTTCAT GTCTAACTGG CAGCTCAACA CCACCCAATT   
  
  
+ GGACCCTTTC CCAATCATGC AAGATATTCC TCGTCCCTAT TTGGAATTGA ATTACCATTC TTCTGGGTCA   
  
  
+ AGCAATGGCA TTGATGATTC GGGGGATGGG TTATCGACCT CTCCTGTAAG TACACTTGCA TCAACCGCCA   
  
  
+ CAGAGGCAGG GAAAAAGTTG GCTGGTAGCT CTAGGAGAAA GAACCGTCAA AGGGATGACT ATGGCCATGA   
  
  
+ AGAGGGAAGG AGTAACAAGC AGCAAGCCTC TTACAATGAC GATTACGTTG AGATGGAGCA GTATGACGAT   
  
  
+ GTACTTCTCT GTAGGGCAGA CAAGGGTAAT ATTTCAACTT GTGCCAATGA ATCCTCGCTT AATGAGGTGC   
  
  
+ GTGAGAAGCT GCAGACGACA GGGTTCAAGG GAAGAACATC CCGTCTTAAG AAGCAATCTA AGGAAGCGAA   
  
  
+ AGAGGTGGAT CTGAGAACTC TTCTTTCTGG TTGTGCACAA GCTGTTTCGA ACTTTGATAT CAGGACTGCT   
  
  
+ AATGAGCTGC TTAAGCAAGT CAGACAGCAT TCTTCACCAT ATGGTGATAG CCTCCAAAGG CTCGCCCATC   
  
  
+ AATTTGCGAA TGGTATTGAG GCACGCTTAG CAGGCACCGG TTCAAGAGTA CCTGCTAATC TCATTGATGC   
  
  
+ ACGGATATCA TCATCTGAAT TTTTAAAAGC TTACAAGTCA TATGTTTCAG CAGTTCCTTT CAGAAGGATG   
  
  
+ TCCTATTTTA TAGCAAACAA CACAATTCTG AAGTTGGCTG AGAAAGCAAC AAAGATTCAC ATAATTGATT   
  
  
+ TTGGTATTCT ATTTGGTCTA CAGTGGCCCT GTCTTATACA AAGTCTCTCA AGGCGAACTG TGGCTCCTCC   
  
  
+ GAAGCTTCGC ATCACTGGGA TAGACTATCC CCAGCATGGT TTCCGGCCAG CAGAAAAGGT TGAGGCAACA   
  
  
+ GGTCGTCGGT TGTCCGGGTA CTGTGAGAGA TTTAATGTAC CCTTTCGATA TGAAACCATT GCAAAGAAGT   
  
  
+ GGGAAACCAT ACGCCCAGAA GATCTAAATA TTGAGAATGA TGAGCTGGTA ATTGTTAATT GTATGTTGCG   
  
  
+ GTCTGTAAAT CTATTGGATG ATACAGTGGC GGTAAATAGT CCAAGGGATG CTTTCTTGAG GTTAATCAAA   
  
  
+ CAGATAAACC CGCGTTTATT CATTCATGCA ATTGTCAATG GAACCTTTAG TACTCCATTC TTCAGCACTC   
  
  
+ GATTCAGGGA AGCCCTATTC CAATACTCTT CTGTATTTGA TATATTTGAA GCGACTATGA CTCGTGAAGA   
  
  
+ TCGTGGAAGG CTGCTGATTG AGAGTCAAAT ATGCGGGCTA GAAGTTTTGA ATGCAATAGC ATGTGAAGGT   
  
  
+ GCAGAGAGGA TTCAAAGGCC TGAAACATAC AAGCAATGGC AGGAGCGGAC AACAAGGGCT GGACTAAGGC   
  
  
+ AGGTTCCAAT AGATGAGGAG CTTGTCAATA GAGCAAAGAC TATAGTGAAA GCAAATTATC ACAAGGATTT   
  
  
+ TGTGGTGGAT GAGGATAGGC GTTGGATGCT TCAAGGTTGG AAAGGAAGGA CACTTAGTGC CCTTTCCGTT   
  
  
+ TGGCAGCCTA ACTA  

- +Up\_Stream \_Len000AAATTA TTCTAGGAAA ATATATATAC ACACATATTA AGATACCATG TATCCATACA   
  
  
- TGGTATCTGA ATAACGTAAA GATCCAGGAA GAGAGAGACG CGAACAAAAA ACAAATAGAT TAAATACCTC   
  
  
- AACTTGTAAA ACAAAATATA TGAACTTGTT AGATAATATG ATTTAATTCG TAAAACTAAA TTAGTGAATA   
  
  
- CCCACACAAA CCATCCCAAC TTTTATTAAA AGGATCTTTT ATTAAAAATT ATATTTTATT AAAAAGTACA   
  
  
- TTTTACTAAA AGTTGAGTAT AAAAGTCAAC TAACCATTTT TCTTTTTACT TGAAAGACCT ATTAGTCAAA   
  
  
- CTAATTGTTT TTCACTGTTT TTTTAATTTT TACCAAAAGG CATTAGAAGT TTGGTATACA GCTCCTTTAT   
  
  
- TTGTGCCTTA TTTCTTTCTC ACAGTCTTGG GTTCTTTACA TTTTAGTTGA ATGCAAGATA AATTTCACTT   
  
  
- TTTATAAAAA GAAGTAAGAT CCTTTTAATT AACAGGAGAC TTTTGTTTAA AAGGGAGGAA CAGATTGGTT   
  
  
- TGTGTCATTT TAACTTTTTA GTAAAAGATC CTTTTACTAA AAGTGAGGAT GGTTTGTGTG GGAATTTGAA   
  
  
- CAAGGTTTAG ATATTTGGGC CTTAGAATTA TGCGTGTAAC ATATAATATA CAGGATGATA TTGACATAAT   
  
  
- AGTGACCAAG AACTAAATAG GAAAGTTTGA GAACGAATCA GTTCCAACAG GAGCATGTTT GTACTAATTA   
  
  
- AGTTAATTGG TGCATTCAAT ATGCGACTGT TTAAAGCCCA TGGTTTGTAA ACGTGATCTG GATTTTGTCA   
  
  
- ATGATAGTAG TATAGCTACA ATTTTAACTG GACAAGAATA AACAGGTCGC AACTCGTTCC TTTATTAGTT   
  
  
- TAGTACAGAG TAAATCAGCC AAAAAACGGA TTCTTCTCGT ATTTGTTTCC TTTTTCTGTT GCTGTGTAAT   
  
  
- ATGTACGTAG ATGTGTTTAC TTTACTCCGG TATACCTTGT ACAAGTACTC TCGAAGTGAG CGATTATTAA   
  
  
- CTTGGATGAA AACTGATTCG GTGGTTGATC TTATACCTCT AACTGACAAT TCTCTGATAT AATGTACTTT   
  
  
- ATCTATTCTC ATCTGTAACG ATATATAAAA TAGAAATTAA CAGATAAAAA TTACTAGTAA TGACAAATAA   
  
  
- AGTCACTCAT TAAAAGAGCC TATATTATAA AATATCGCCA ATAATTTATC TACACTTTGA TATTACAATC   
  
  
- TGTATAGTAC GCTTTTATCT CTTTAAAACC CGTTTAATTT TTTTTTTCTT CTTTTTGCTC CTTCATACGG   
  
  
- GTAACATTCG TCCACCTACA TATAGGTCGG AGGTAGGTTG TTAAACCCGT TTTAATATAG TAGGTAAGGT   
  
  
- TTTTTTTCTA TATTAAACAC TATAATTATT GGGCTGAGTT AAGTTCGAAT AAATAAAAAA GTATATTAAA   
  
  
- AAATTATATA TTTTTATTTT TCACTGTTGC TGTACGTAGG AAACGCACAG TTTCCTAGGC GGCGCACCGG   
  
  
- GGGTGTAAGT GACTGCCCCA AAAACCAAAC GCTAATTCTA GTTACCGATT GCAACAGAGA GAGTTTTGTA   
  
  
- TACCTGGGGA AAAAGATAGA AAAAGACGTA ACAGTCGTAT CGTTAACAAC TCGTGTAAAA AAAGCTAAAC   
  
  
- AACTGTTATT CGCACCAATA CTAAATACCC GGTGATTATA GTCAGAGACT AGTAAACGTG TAAAACCTGG   
  
  
- AGAAAGAGAG AGATTAGAAG GTCCATTGGA CTTAAGAAGA GATAAAGACT GATCTAATCT ATTGTCCTAA   
  
  
- AGTTTATAGA CAGTCCACCG CCCATATTGA AGCACTACCC GTAAGTAAAA ACAAGATAGT CCGACCACCC   
  
  
- AAACTCAAAT AAAACTAAGT AAAGCACAAG TAGACACTAT AATCATCCTC CCTAAGACAT ATGGGTGGTT   
  
  
- GACGAGCGGG AAAACGGGCA CACTCGTTAA CCCTGAAAAC AAACAAGAAG CAGCTACGTA GGAGACCAAC   
  
  
- GACTAGACCT TAACTTTGGG GTACATAAGT TTAAGTTGGG ACTAAACGAC AGTTTGAAAG ACTTGGTAGT   
  
  
- TTTGAGGCGG CTTCACAAAC TTTTTCTACC GTAGAAGGTC TGGGAAGTAA ACCGACTAGG GTTTTCGAGA   
  
  
- AGTTAACGGC TTTAGCCCAA ATGATTATCA AGTCTAAGAT GTGTTCATGG ACTAAAAAGT CTACGAACAG   
  
  
- AATTCAAGTA ATCACTATAA GAGGACCTTC TCCTGAATCT ACTTTCAGGA TGCAGAAATG TGCTAATGTA   
  
  
- CCGAGAGGAT CGGTGACTCT TCAGAAACAT ACTACGAGAA CCTTTCCTTA TGAAAGGAAG CAGATGATCA   
  
  
- GAACGGGGTA GAAATCCGGC TTCACAACTG TCGGGTCTAT CACCGAAACC GGCGCCAACG AGACTACCAG   
  
  
- CGCCCTAACT CCCAAACCGA TTACTACTAC GGCAAAAGTA CAGATTGACC GTCGAGTTGT GGTGGGTTAA   
  
  
- CCTGGGAAAG GGTTAGTACG TTCTATAAGG AGCAGGGATA AACCTTAACT TAATGGTAAG AAGACCCAGT   
  
  
- TCGTTACCGT AACTACTAAG CCCCCTACCC AATAGCTGGA GAGGACATTC ATGTGAACGT AGTTGGCGGT   
  
  
- GTCTCCGTCC CTTTTTCAAC CGACCATCGA GATCCTCTTT CTTGGCAGTT TCCCTACTGA TACCGGTACT   
  
  
- TCTCCCTTCC TCATTGTTCG TCGTTCGGAG AATGTTACTG CTAATGCAAC TCTACCTCGT CATACTGCTA   
  
  
- CATGAAGAGA CATCCCGTCT GTTCCCATTA TAAAGTTGAA CACGGTTACT TAGGAGCGAA TTACTCCACG   
  
  
- CACTCTTCGA CGTCTGCTGT CCCAAGTTCC CTTCTTGTAG GGCAGAATTC TTCGTTAGAT TCCTTCGCTT   
  
  
- TCTCCACCTA GACTCTTGAG AAGAAAGACC AACACGTGTT CGACAAAGCT TGAAACTATA GTCCTGACGA   
  
  
- TTACTCGACG AATTCGTTCA GTCTGTCGTA AGAAGTGGTA TACCACTATC GGAGGTTTCC GAGCGGGTAG   
  
  
- TTAAACGCTT ACCATAACTC CGTGCGAATC GTCCGTGGCC AAGTTCTCAT GGACGATTAG AGTAACTACG   
  
  
- TGCCTATAGT AGTAGACTTA AAAATTTTCG AATGTTCAGT ATACAAAGTC GTCAAGGAAA GTCTTCCTAC   
  
  
- AGGATAAAAT ATCGTTTGTT GTGTTAAGAC TTCAACCGAC TCTTTCGTTG TTTCTAAGTG TATTAACTAA   
  
  
- AACCATAAGA TAAACCAGAT GTCACCGGGA CAGAATATGT TTCAGAGAGT TCCGCTTGAC ACCGAGGAGG   
  
  
- CTTCGAAGCG TAGTGACCCT ATCTGATAGG GGTCGTACCA AAGGCCGGTC GTCTTTTCCA ACTCCGTTGT   
  
  
- CCAGCAGCCA ACAGGCCCAT GACACTCTCT AAATTACATG GGAAAGCTAT ACTTTGGTAA CGTTTCTTCA   
  
  
- CCCTTTGGTA TGCGGGTCTT CTAGATTTAT AACTCTTACT ACTCGACCAT TAACAATTAA CATACAACGC   
  
  
- CAGACATTTA GATAACCTAC TATGTCACCG CCATTTATCA GGTTCCCTAC GAAAGAACTC CAATTAGTTT   
  
  
- GTCTATTTGG GCGCAAATAA GTAAGTACGT TAACAGTTAC CTTGGAAATC ATGAGGTAAG AAGTCGTGAG   
  
  
- CTAAGTCCCT TCGGGATAAG GTTATGAGAA GACATAAACT ATATAAACTT CGCTGATACT GAGCACTTCT   
  
  
- AGCACCTTCC GACGACTAAC TCTCAGTTTA TACGCCCGAT CTTCAAAACT TACGTTATCG TACACTTCCA   
  
  
- CGTCTCTCCT AAGTTTCCGG ACTTTGTATG TTCGTTACCG TCCTCGCCTG TTGTTCCCGA CCTGATTCCG   
  
  
- TCCAAGGTTA TCTACTCCTC GAACAGTTAT CTCGTTTCTG ATATCACTTT CGTTTAATAG TGTTCCTAAA   
  
  
- ACACCACCTA CTCCTATCCG CAACCTACGA AGTTCCAACC TTTCCTTCCT GTGAATCACG GGAAAGGCAA   
  
  
- ACCGTCGGAT TGAT

+     Myb

| Site Name | Organism | Position | Strand | Matrix score. | sequence | function |
| --- | --- | --- | --- | --- | --- | --- |
| Myb | Arabidopsis thaliana | 2498 | + | 6 | TAACTG |  |
| Myb | Arabidopsis thaliana | 694 | + | 6 | TAACTG |  |
| Myb | Arabidopsis thaliana | 841 | - | 6 | TAACTG |  |
| Myb | Arabidopsis thaliana | 309 | - | 6 | CAACTG |  |
| Myb | Arabidopsis thaliana | 1962 | + | 6 | CAACTG |  |

>HU02G01572.1   
+ +Up\_Stream \_Len000TTTAAT AAGATCCTTT TATATATATG TGTGTATAAT TCTATGGTAC ATAGGTATGT   
  
  
+ ACCATAGACT TATTGCATTT CTAGGTCCTT CTCTCTCTGC GCTTGTTTTT TGTTTATCTA ATTTATGGAG   
  
  
+ TTGAACATTT TGTTTTATAT ACTTGAACAA TCTATTATAC TAAATTAAGC ATTTTGATTT AATCACTTAT   
  
  
+ GGGTGTGTTT GGTAGGGTTG AAAATAATTT TCCTAGAAAA TAATTTTTAA TATAAAATAA TTTTTCATGT   
  
  
+ AAAATGATTT TCAACTCATA TTTTCAGTTG ATTGGTAAAA AGAAAAATGA ACTTTCTGGA TAATCAGTTT   
  
  
+ GATTAACAAA AAGTGACAAA AAAATTAAAA ATGGTTTTCC GTAATCTTCA AACCATATGT CGAGGAAATA   
  
  
+ AACACGGAAT AAAGAAAGAG TGTCAGAACC CAAGAAATGT AAAATCAACT TACGTTCTAT TTAAAGTGAA   
  
  
+ AAATATTTTT CTTCATTCTA GGAAAATTAA TTGTCCTCTG AAAACAAATT TTCCCTCCTT GTCTAACCAA   
  
  
+ ACACAGTAAA ATTGAAAAAT CATTTTCTAG GAAAATGATT TTCACTCCTA CCAAACACAC CCTTAAACTT   
  
  
+ GTTCCAAATC TATAAACCCG GAATCTTAAT ACGCACATTG TATATTATAT GTCCTACTAT AACTGTATTA   
  
  
+ TCACTGGTTC TTGATTTATC CTTTCAAACT CTTGCTTAGT CAAGGTTGTC CTCGTACAAA CATGATTAAT   
  
  
+ TCAATTAACC ACGTAAGTTA TACGCTGACA AATTTCGGGT ACCAAACATT TGCACTAGAC CTAAAACAGT   
  
  
+ TACTATCATC ATATCGATGT TAAAATTGAC CTGTTCTTAT TTGTCCAGCG TTGAGCAAGG AAATAATCAA   
  
  
+ ATCATGTCTC ATTTAGTCGG TTTTTTGCCT AAGAAGAGCA TAAACAAAGG AAAAAGACAA CGACACATTA   
  
  
+ TACATGCATC TACACAAATG AAATGAGGCC ATATGGAACA TGTTCATGAG AGCTTCACTC GCTAATAATT   
  
  
+ GAACCTACTT TTGACTAAGC CACCAACTAG AATATGGAGA TTGACTGTTA AGAGACTATA TTACATGAAA   
  
  
+ TAGATAAGAG TAGACATTGC TATATATTTT ATCTTTAATT GTCTATTTTT AATGATCATT ACTGTTTATT   
  
  
+ TCAGTGAGTA ATTTTCTCGG ATATAATATT TTATAGCGGT TATTAAATAG ATGTGAAACT ATAATGTTAG   
  
  
+ ACATATCATG CGAAAATAGA GAAATTTTGG GCAAATTAAA AAAAAAAGAA GAAAAACGAG GAAGTATGCC   
  
  
+ CATTGTAAGC AGGTGGATGT ATATCCAGCC TCCATCCAAC AATTTGGGCA AAATTATATC ATCCATTCCA   
  
  
+ AAAAAAAGAT ATAATTTGTG ATATTAATAA CCCGACTCAA TTCAAGCTTA TTTATTTTTT CATATAATTT   
  
  
+ TTTAATATAT AAAAATAAAA AGTGACAACG ACATGCATCC TTTGCGTGTC AAAGGATCCG CCGCGTGGCC   
  
  
+ CCCACATTCA CTGACGGGGT TTTTGGTTTG CGATTAAGAT CAATGGCTAA CGTTGTCTCT CTCAAAACAT   
  
  
+ ATGGACCCCT TTTTCTATCT TTTTCTGCAT TGTCAGCATA GCAATTGTTG AGCACATTTT TTTCGATTTG   
  
  
+ TTGACAATAA GCGTGGTTAT GATTTATGGG CCACTAATAT CAGTCTCTGA TCATTTGCAC ATTTTGGACC   
  
  
+ TCTTTCTCTC TCTAATCTTC CAGGTAACCT GAATTCTTCT CTATTTCTGA CTAGATTAGA TAACAGGATT   
  
  
+ TCAAATATCT GTCAGGTGGC GGGTATAACT TCGTGATGGG CATTCATTTT TGTTCTATCA GGCTGGTGGG   
  
  
+ TTTGAGTTTA TTTTGATTCA TTTCGTGTTC ATCTGTGATA TTAGTAGGAG GGATTCTGTA TACCCACCAA   
  
  
+ CTGCTCGCCC TTTTGCCCGT GTGAGCAATT GGGACTTTTG TTTGTTCTTC GTCGATGCAT CCTCTGGTTG   
  
  
+ CTGATCTGGA ATTGAAACCC CATGTATTCA AATTCAACCC TGATTTGCTG TCAAACTTTC TGAACCATCA   
  
  
+ AAACTCCGCC GAAGTGTTTG AAAAAGATGG CATCTTCCAG ACCCTTCATT TGGCTGATCC CAAAAGCTCT   
  
  
+ TCAATTGCCG AAATCGGGTT TACTAATAGT TCAGATTCTA CACAAGTACC TGATTTTTCA GATGCTTGTC   
  
  
+ TTAAGTTCAT TAGTGATATT CTCCTGGAAG AGGACTTAGA TGAAAGTCCT ACGTCTTTAC ACGATTACAT   
  
  
+ GGCTCTCCTA GCCACTGAGA AGTCTTTGTA TGATGCTCTT GGAAAGGAAT ACTTTCCTTC GTCTACTAGT   
  
  
+ CTTGCCCCAT CTTTAGGCCG AAGTGTTGAC AGCCCAGATA GTGGCTTTGG CCGCGGTTGC TCTGATGGTC   
  
  
+ GCGGGATTGA GGGTTTGGCT AATGATGATG CCGTTTTCAT GTCTAACTGG CAGCTCAACA CCACCCAATT   
  
  
+ GGACCCTTTC CCAATCATGC AAGATATTCC TCGTCCCTAT TTGGAATTGA ATTACCATTC TTCTGGGTCA   
  
  
+ AGCAATGGCA TTGATGATTC GGGGGATGGG TTATCGACCT CTCCTGTAAG TACACTTGCA TCAACCGCCA   
  
  
+ CAGAGGCAGG GAAAAAGTTG GCTGGTAGCT CTAGGAGAAA GAACCGTCAA AGGGATGACT ATGGCCATGA   
  
  
+ AGAGGGAAGG AGTAACAAGC AGCAAGCCTC TTACAATGAC GATTACGTTG AGATGGAGCA GTATGACGAT   
  
  
+ GTACTTCTCT GTAGGGCAGA CAAGGGTAAT ATTTCAACTT GTGCCAATGA ATCCTCGCTT AATGAGGTGC   
  
  
+ GTGAGAAGCT GCAGACGACA GGGTTCAAGG GAAGAACATC CCGTCTTAAG AAGCAATCTA AGGAAGCGAA   
  
  
+ AGAGGTGGAT CTGAGAACTC TTCTTTCTGG TTGTGCACAA GCTGTTTCGA ACTTTGATAT CAGGACTGCT   
  
  
+ AATGAGCTGC TTAAGCAAGT CAGACAGCAT TCTTCACCAT ATGGTGATAG CCTCCAAAGG CTCGCCCATC   
  
  
+ AATTTGCGAA TGGTATTGAG GCACGCTTAG CAGGCACCGG TTCAAGAGTA CCTGCTAATC TCATTGATGC   
  
  
+ ACGGATATCA TCATCTGAAT TTTTAAAAGC TTACAAGTCA TATGTTTCAG CAGTTCCTTT CAGAAGGATG   
  
  
+ TCCTATTTTA TAGCAAACAA CACAATTCTG AAGTTGGCTG AGAAAGCAAC AAAGATTCAC ATAATTGATT   
  
  
+ TTGGTATTCT ATTTGGTCTA CAGTGGCCCT GTCTTATACA AAGTCTCTCA AGGCGAACTG TGGCTCCTCC   
  
  
+ GAAGCTTCGC ATCACTGGGA TAGACTATCC CCAGCATGGT TTCCGGCCAG CAGAAAAGGT TGAGGCAACA   
  
  
+ GGTCGTCGGT TGTCCGGGTA CTGTGAGAGA TTTAATGTAC CCTTTCGATA TGAAACCATT GCAAAGAAGT   
  
  
+ GGGAAACCAT ACGCCCAGAA GATCTAAATA TTGAGAATGA TGAGCTGGTA ATTGTTAATT GTATGTTGCG   
  
  
+ GTCTGTAAAT CTATTGGATG ATACAGTGGC GGTAAATAGT CCAAGGGATG CTTTCTTGAG GTTAATCAAA   
  
  
+ CAGATAAACC CGCGTTTATT CATTCATGCA ATTGTCAATG GAACCTTTAG TACTCCATTC TTCAGCACTC   
  
  
+ GATTCAGGGA AGCCCTATTC CAATACTCTT CTGTATTTGA TATATTTGAA GCGACTATGA CTCGTGAAGA   
  
  
+ TCGTGGAAGG CTGCTGATTG AGAGTCAAAT ATGCGGGCTA GAAGTTTTGA ATGCAATAGC ATGTGAAGGT   
  
  
+ GCAGAGAGGA TTCAAAGGCC TGAAACATAC AAGCAATGGC AGGAGCGGAC AACAAGGGCT GGACTAAGGC   
  
  
+ AGGTTCCAAT AGATGAGGAG CTTGTCAATA GAGCAAAGAC TATAGTGAAA GCAAATTATC ACAAGGATTT   
  
  
+ TGTGGTGGAT GAGGATAGGC GTTGGATGCT TCAAGGTTGG AAAGGAAGGA CACTTAGTGC CCTTTCCGTT   
  
  
+ TGGCAGCCTA ACTA  

- +Up\_Stream \_Len000AAATTA TTCTAGGAAA ATATATATAC ACACATATTA AGATACCATG TATCCATACA   
  
  
- TGGTATCTGA ATAACGTAAA GATCCAGGAA GAGAGAGACG CGAACAAAAA ACAAATAGAT TAAATACCTC   
  
  
- AACTTGTAAA ACAAAATATA TGAACTTGTT AGATAATATG ATTTAATTCG TAAAACTAAA TTAGTGAATA   
  
  
- CCCACACAAA CCATCCCAAC TTTTATTAAA AGGATCTTTT ATTAAAAATT ATATTTTATT AAAAAGTACA   
  
  
- TTTTACTAAA AGTTGAGTAT AAAAGTCAAC TAACCATTTT TCTTTTTACT TGAAAGACCT ATTAGTCAAA   
  
  
- CTAATTGTTT TTCACTGTTT TTTTAATTTT TACCAAAAGG CATTAGAAGT TTGGTATACA GCTCCTTTAT   
  
  
- TTGTGCCTTA TTTCTTTCTC ACAGTCTTGG GTTCTTTACA TTTTAGTTGA ATGCAAGATA AATTTCACTT   
  
  
- TTTATAAAAA GAAGTAAGAT CCTTTTAATT AACAGGAGAC TTTTGTTTAA AAGGGAGGAA CAGATTGGTT   
  
  
- TGTGTCATTT TAACTTTTTA GTAAAAGATC CTTTTACTAA AAGTGAGGAT GGTTTGTGTG GGAATTTGAA   
  
  
- CAAGGTTTAG ATATTTGGGC CTTAGAATTA TGCGTGTAAC ATATAATATA CAGGATGATA TTGACATAAT   
  
  
- AGTGACCAAG AACTAAATAG GAAAGTTTGA GAACGAATCA GTTCCAACAG GAGCATGTTT GTACTAATTA   
  
  
- AGTTAATTGG TGCATTCAAT ATGCGACTGT TTAAAGCCCA TGGTTTGTAA ACGTGATCTG GATTTTGTCA   
  
  
- ATGATAGTAG TATAGCTACA ATTTTAACTG GACAAGAATA AACAGGTCGC AACTCGTTCC TTTATTAGTT   
  
  
- TAGTACAGAG TAAATCAGCC AAAAAACGGA TTCTTCTCGT ATTTGTTTCC TTTTTCTGTT GCTGTGTAAT   
  
  
- ATGTACGTAG ATGTGTTTAC TTTACTCCGG TATACCTTGT ACAAGTACTC TCGAAGTGAG CGATTATTAA   
  
  
- CTTGGATGAA AACTGATTCG GTGGTTGATC TTATACCTCT AACTGACAAT TCTCTGATAT AATGTACTTT   
  
  
- ATCTATTCTC ATCTGTAACG ATATATAAAA TAGAAATTAA CAGATAAAAA TTACTAGTAA TGACAAATAA   
  
  
- AGTCACTCAT TAAAAGAGCC TATATTATAA AATATCGCCA ATAATTTATC TACACTTTGA TATTACAATC   
  
  
- TGTATAGTAC GCTTTTATCT CTTTAAAACC CGTTTAATTT TTTTTTTCTT CTTTTTGCTC CTTCATACGG   
  
  
- GTAACATTCG TCCACCTACA TATAGGTCGG AGGTAGGTTG TTAAACCCGT TTTAATATAG TAGGTAAGGT   
  
  
- TTTTTTTCTA TATTAAACAC TATAATTATT GGGCTGAGTT AAGTTCGAAT AAATAAAAAA GTATATTAAA   
  
  
- AAATTATATA TTTTTATTTT TCACTGTTGC TGTACGTAGG AAACGCACAG TTTCCTAGGC GGCGCACCGG   
  
  
- GGGTGTAAGT GACTGCCCCA AAAACCAAAC GCTAATTCTA GTTACCGATT GCAACAGAGA GAGTTTTGTA   
  
  
- TACCTGGGGA AAAAGATAGA AAAAGACGTA ACAGTCGTAT CGTTAACAAC TCGTGTAAAA AAAGCTAAAC   
  
  
- AACTGTTATT CGCACCAATA CTAAATACCC GGTGATTATA GTCAGAGACT AGTAAACGTG TAAAACCTGG   
  
  
- AGAAAGAGAG AGATTAGAAG GTCCATTGGA CTTAAGAAGA GATAAAGACT GATCTAATCT ATTGTCCTAA   
  
  
- AGTTTATAGA CAGTCCACCG CCCATATTGA AGCACTACCC GTAAGTAAAA ACAAGATAGT CCGACCACCC   
  
  
- AAACTCAAAT AAAACTAAGT AAAGCACAAG TAGACACTAT AATCATCCTC CCTAAGACAT ATGGGTGGTT   
  
  
- GACGAGCGGG AAAACGGGCA CACTCGTTAA CCCTGAAAAC AAACAAGAAG CAGCTACGTA GGAGACCAAC   
  
  
- GACTAGACCT TAACTTTGGG GTACATAAGT TTAAGTTGGG ACTAAACGAC AGTTTGAAAG ACTTGGTAGT   
  
  
- TTTGAGGCGG CTTCACAAAC TTTTTCTACC GTAGAAGGTC TGGGAAGTAA ACCGACTAGG GTTTTCGAGA   
  
  
- AGTTAACGGC TTTAGCCCAA ATGATTATCA AGTCTAAGAT GTGTTCATGG ACTAAAAAGT CTACGAACAG   
  
  
- AATTCAAGTA ATCACTATAA GAGGACCTTC TCCTGAATCT ACTTTCAGGA TGCAGAAATG TGCTAATGTA   
  
  
- CCGAGAGGAT CGGTGACTCT TCAGAAACAT ACTACGAGAA CCTTTCCTTA TGAAAGGAAG CAGATGATCA   
  
  
- GAACGGGGTA GAAATCCGGC TTCACAACTG TCGGGTCTAT CACCGAAACC GGCGCCAACG AGACTACCAG   
  
  
- CGCCCTAACT CCCAAACCGA TTACTACTAC GGCAAAAGTA CAGATTGACC GTCGAGTTGT GGTGGGTTAA   
  
  
- CCTGGGAAAG GGTTAGTACG TTCTATAAGG AGCAGGGATA AACCTTAACT TAATGGTAAG AAGACCCAGT   
  
  
- TCGTTACCGT AACTACTAAG CCCCCTACCC AATAGCTGGA GAGGACATTC ATGTGAACGT AGTTGGCGGT   
  
  
- GTCTCCGTCC CTTTTTCAAC CGACCATCGA GATCCTCTTT CTTGGCAGTT TCCCTACTGA TACCGGTACT   
  
  
- TCTCCCTTCC TCATTGTTCG TCGTTCGGAG AATGTTACTG CTAATGCAAC TCTACCTCGT CATACTGCTA   
  
  
- CATGAAGAGA CATCCCGTCT GTTCCCATTA TAAAGTTGAA CACGGTTACT TAGGAGCGAA TTACTCCACG   
  
  
- CACTCTTCGA CGTCTGCTGT CCCAAGTTCC CTTCTTGTAG GGCAGAATTC TTCGTTAGAT TCCTTCGCTT   
  
  
- TCTCCACCTA GACTCTTGAG AAGAAAGACC AACACGTGTT CGACAAAGCT TGAAACTATA GTCCTGACGA   
  
  
- TTACTCGACG AATTCGTTCA GTCTGTCGTA AGAAGTGGTA TACCACTATC GGAGGTTTCC GAGCGGGTAG   
  
  
- TTAAACGCTT ACCATAACTC CGTGCGAATC GTCCGTGGCC AAGTTCTCAT GGACGATTAG AGTAACTACG   
  
  
- TGCCTATAGT AGTAGACTTA AAAATTTTCG AATGTTCAGT ATACAAAGTC GTCAAGGAAA GTCTTCCTAC   
  
  
- AGGATAAAAT ATCGTTTGTT GTGTTAAGAC TTCAACCGAC TCTTTCGTTG TTTCTAAGTG TATTAACTAA   
  
  
- AACCATAAGA TAAACCAGAT GTCACCGGGA CAGAATATGT TTCAGAGAGT TCCGCTTGAC ACCGAGGAGG   
  
  
- CTTCGAAGCG TAGTGACCCT ATCTGATAGG GGTCGTACCA AAGGCCGGTC GTCTTTTCCA ACTCCGTTGT   
  
  
- CCAGCAGCCA ACAGGCCCAT GACACTCTCT AAATTACATG GGAAAGCTAT ACTTTGGTAA CGTTTCTTCA   
  
  
- CCCTTTGGTA TGCGGGTCTT CTAGATTTAT AACTCTTACT ACTCGACCAT TAACAATTAA CATACAACGC   
  
  
- CAGACATTTA GATAACCTAC TATGTCACCG CCATTTATCA GGTTCCCTAC GAAAGAACTC CAATTAGTTT   
  
  
- GTCTATTTGG GCGCAAATAA GTAAGTACGT TAACAGTTAC CTTGGAAATC ATGAGGTAAG AAGTCGTGAG   
  
  
- CTAAGTCCCT TCGGGATAAG GTTATGAGAA GACATAAACT ATATAAACTT CGCTGATACT GAGCACTTCT   
  
  
- AGCACCTTCC GACGACTAAC TCTCAGTTTA TACGCCCGAT CTTCAAAACT TACGTTATCG TACACTTCCA   
  
  
- CGTCTCTCCT AAGTTTCCGG ACTTTGTATG TTCGTTACCG TCCTCGCCTG TTGTTCCCGA CCTGATTCCG   
  
  
- TCCAAGGTTA TCTACTCCTC GAACAGTTAT CTCGTTTCTG ATATCACTTT CGTTTAATAG TGTTCCTAAA   
  
  
- ACACCACCTA CTCCTATCCG CAACCTACGA AGTTCCAACC TTTCCTTCCT GTGAATCACG GGAAAGGCAA   
  
  
- ACCGTCGGAT TGAT

+     Myb-binding site

| Site Name | Organism | Position | Strand | Matrix score. | sequence | function |
| --- | --- | --- | --- | --- | --- | --- |
| Myb-binding site | Nicotiana tabacum | 3430 | + | 6 | CAACAG |  |

>HU02G01572.1   
+ +Up\_Stream \_Len000TTTAAT AAGATCCTTT TATATATATG TGTGTATAAT TCTATGGTAC ATAGGTATGT   
  
  
+ ACCATAGACT TATTGCATTT CTAGGTCCTT CTCTCTCTGC GCTTGTTTTT TGTTTATCTA ATTTATGGAG   
  
  
+ TTGAACATTT TGTTTTATAT ACTTGAACAA TCTATTATAC TAAATTAAGC ATTTTGATTT AATCACTTAT   
  
  
+ GGGTGTGTTT GGTAGGGTTG AAAATAATTT TCCTAGAAAA TAATTTTTAA TATAAAATAA TTTTTCATGT   
  
  
+ AAAATGATTT TCAACTCATA TTTTCAGTTG ATTGGTAAAA AGAAAAATGA ACTTTCTGGA TAATCAGTTT   
  
  
+ GATTAACAAA AAGTGACAAA AAAATTAAAA ATGGTTTTCC GTAATCTTCA AACCATATGT CGAGGAAATA   
  
  
+ AACACGGAAT AAAGAAAGAG TGTCAGAACC CAAGAAATGT AAAATCAACT TACGTTCTAT TTAAAGTGAA   
  
  
+ AAATATTTTT CTTCATTCTA GGAAAATTAA TTGTCCTCTG AAAACAAATT TTCCCTCCTT GTCTAACCAA   
  
  
+ ACACAGTAAA ATTGAAAAAT CATTTTCTAG GAAAATGATT TTCACTCCTA CCAAACACAC CCTTAAACTT   
  
  
+ GTTCCAAATC TATAAACCCG GAATCTTAAT ACGCACATTG TATATTATAT GTCCTACTAT AACTGTATTA   
  
  
+ TCACTGGTTC TTGATTTATC CTTTCAAACT CTTGCTTAGT CAAGGTTGTC CTCGTACAAA CATGATTAAT   
  
  
+ TCAATTAACC ACGTAAGTTA TACGCTGACA AATTTCGGGT ACCAAACATT TGCACTAGAC CTAAAACAGT   
  
  
+ TACTATCATC ATATCGATGT TAAAATTGAC CTGTTCTTAT TTGTCCAGCG TTGAGCAAGG AAATAATCAA   
  
  
+ ATCATGTCTC ATTTAGTCGG TTTTTTGCCT AAGAAGAGCA TAAACAAAGG AAAAAGACAA CGACACATTA   
  
  
+ TACATGCATC TACACAAATG AAATGAGGCC ATATGGAACA TGTTCATGAG AGCTTCACTC GCTAATAATT   
  
  
+ GAACCTACTT TTGACTAAGC CACCAACTAG AATATGGAGA TTGACTGTTA AGAGACTATA TTACATGAAA   
  
  
+ TAGATAAGAG TAGACATTGC TATATATTTT ATCTTTAATT GTCTATTTTT AATGATCATT ACTGTTTATT   
  
  
+ TCAGTGAGTA ATTTTCTCGG ATATAATATT TTATAGCGGT TATTAAATAG ATGTGAAACT ATAATGTTAG   
  
  
+ ACATATCATG CGAAAATAGA GAAATTTTGG GCAAATTAAA AAAAAAAGAA GAAAAACGAG GAAGTATGCC   
  
  
+ CATTGTAAGC AGGTGGATGT ATATCCAGCC TCCATCCAAC AATTTGGGCA AAATTATATC ATCCATTCCA   
  
  
+ AAAAAAAGAT ATAATTTGTG ATATTAATAA CCCGACTCAA TTCAAGCTTA TTTATTTTTT CATATAATTT   
  
  
+ TTTAATATAT AAAAATAAAA AGTGACAACG ACATGCATCC TTTGCGTGTC AAAGGATCCG CCGCGTGGCC   
  
  
+ CCCACATTCA CTGACGGGGT TTTTGGTTTG CGATTAAGAT CAATGGCTAA CGTTGTCTCT CTCAAAACAT   
  
  
+ ATGGACCCCT TTTTCTATCT TTTTCTGCAT TGTCAGCATA GCAATTGTTG AGCACATTTT TTTCGATTTG   
  
  
+ TTGACAATAA GCGTGGTTAT GATTTATGGG CCACTAATAT CAGTCTCTGA TCATTTGCAC ATTTTGGACC   
  
  
+ TCTTTCTCTC TCTAATCTTC CAGGTAACCT GAATTCTTCT CTATTTCTGA CTAGATTAGA TAACAGGATT   
  
  
+ TCAAATATCT GTCAGGTGGC GGGTATAACT TCGTGATGGG CATTCATTTT TGTTCTATCA GGCTGGTGGG   
  
  
+ TTTGAGTTTA TTTTGATTCA TTTCGTGTTC ATCTGTGATA TTAGTAGGAG GGATTCTGTA TACCCACCAA   
  
  
+ CTGCTCGCCC TTTTGCCCGT GTGAGCAATT GGGACTTTTG TTTGTTCTTC GTCGATGCAT CCTCTGGTTG   
  
  
+ CTGATCTGGA ATTGAAACCC CATGTATTCA AATTCAACCC TGATTTGCTG TCAAACTTTC TGAACCATCA   
  
  
+ AAACTCCGCC GAAGTGTTTG AAAAAGATGG CATCTTCCAG ACCCTTCATT TGGCTGATCC CAAAAGCTCT   
  
  
+ TCAATTGCCG AAATCGGGTT TACTAATAGT TCAGATTCTA CACAAGTACC TGATTTTTCA GATGCTTGTC   
  
  
+ TTAAGTTCAT TAGTGATATT CTCCTGGAAG AGGACTTAGA TGAAAGTCCT ACGTCTTTAC ACGATTACAT   
  
  
+ GGCTCTCCTA GCCACTGAGA AGTCTTTGTA TGATGCTCTT GGAAAGGAAT ACTTTCCTTC GTCTACTAGT   
  
  
+ CTTGCCCCAT CTTTAGGCCG AAGTGTTGAC AGCCCAGATA GTGGCTTTGG CCGCGGTTGC TCTGATGGTC   
  
  
+ GCGGGATTGA GGGTTTGGCT AATGATGATG CCGTTTTCAT GTCTAACTGG CAGCTCAACA CCACCCAATT   
  
  
+ GGACCCTTTC CCAATCATGC AAGATATTCC TCGTCCCTAT TTGGAATTGA ATTACCATTC TTCTGGGTCA   
  
  
+ AGCAATGGCA TTGATGATTC GGGGGATGGG TTATCGACCT CTCCTGTAAG TACACTTGCA TCAACCGCCA   
  
  
+ CAGAGGCAGG GAAAAAGTTG GCTGGTAGCT CTAGGAGAAA GAACCGTCAA AGGGATGACT ATGGCCATGA   
  
  
+ AGAGGGAAGG AGTAACAAGC AGCAAGCCTC TTACAATGAC GATTACGTTG AGATGGAGCA GTATGACGAT   
  
  
+ GTACTTCTCT GTAGGGCAGA CAAGGGTAAT ATTTCAACTT GTGCCAATGA ATCCTCGCTT AATGAGGTGC   
  
  
+ GTGAGAAGCT GCAGACGACA GGGTTCAAGG GAAGAACATC CCGTCTTAAG AAGCAATCTA AGGAAGCGAA   
  
  
+ AGAGGTGGAT CTGAGAACTC TTCTTTCTGG TTGTGCACAA GCTGTTTCGA ACTTTGATAT CAGGACTGCT   
  
  
+ AATGAGCTGC TTAAGCAAGT CAGACAGCAT TCTTCACCAT ATGGTGATAG CCTCCAAAGG CTCGCCCATC   
  
  
+ AATTTGCGAA TGGTATTGAG GCACGCTTAG CAGGCACCGG TTCAAGAGTA CCTGCTAATC TCATTGATGC   
  
  
+ ACGGATATCA TCATCTGAAT TTTTAAAAGC TTACAAGTCA TATGTTTCAG CAGTTCCTTT CAGAAGGATG   
  
  
+ TCCTATTTTA TAGCAAACAA CACAATTCTG AAGTTGGCTG AGAAAGCAAC AAAGATTCAC ATAATTGATT   
  
  
+ TTGGTATTCT ATTTGGTCTA CAGTGGCCCT GTCTTATACA AAGTCTCTCA AGGCGAACTG TGGCTCCTCC   
  
  
+ GAAGCTTCGC ATCACTGGGA TAGACTATCC CCAGCATGGT TTCCGGCCAG CAGAAAAGGT TGAGGCAACA   
  
  
+ GGTCGTCGGT TGTCCGGGTA CTGTGAGAGA TTTAATGTAC CCTTTCGATA TGAAACCATT GCAAAGAAGT   
  
  
+ GGGAAACCAT ACGCCCAGAA GATCTAAATA TTGAGAATGA TGAGCTGGTA ATTGTTAATT GTATGTTGCG   
  
  
+ GTCTGTAAAT CTATTGGATG ATACAGTGGC GGTAAATAGT CCAAGGGATG CTTTCTTGAG GTTAATCAAA   
  
  
+ CAGATAAACC CGCGTTTATT CATTCATGCA ATTGTCAATG GAACCTTTAG TACTCCATTC TTCAGCACTC   
  
  
+ GATTCAGGGA AGCCCTATTC CAATACTCTT CTGTATTTGA TATATTTGAA GCGACTATGA CTCGTGAAGA   
  
  
+ TCGTGGAAGG CTGCTGATTG AGAGTCAAAT ATGCGGGCTA GAAGTTTTGA ATGCAATAGC ATGTGAAGGT   
  
  
+ GCAGAGAGGA TTCAAAGGCC TGAAACATAC AAGCAATGGC AGGAGCGGAC AACAAGGGCT GGACTAAGGC   
  
  
+ AGGTTCCAAT AGATGAGGAG CTTGTCAATA GAGCAAAGAC TATAGTGAAA GCAAATTATC ACAAGGATTT   
  
  
+ TGTGGTGGAT GAGGATAGGC GTTGGATGCT TCAAGGTTGG AAAGGAAGGA CACTTAGTGC CCTTTCCGTT   
  
  
+ TGGCAGCCTA ACTA  

- +Up\_Stream \_Len000AAATTA TTCTAGGAAA ATATATATAC ACACATATTA AGATACCATG TATCCATACA   
  
  
- TGGTATCTGA ATAACGTAAA GATCCAGGAA GAGAGAGACG CGAACAAAAA ACAAATAGAT TAAATACCTC   
  
  
- AACTTGTAAA ACAAAATATA TGAACTTGTT AGATAATATG ATTTAATTCG TAAAACTAAA TTAGTGAATA   
  
  
- CCCACACAAA CCATCCCAAC TTTTATTAAA AGGATCTTTT ATTAAAAATT ATATTTTATT AAAAAGTACA   
  
  
- TTTTACTAAA AGTTGAGTAT AAAAGTCAAC TAACCATTTT TCTTTTTACT TGAAAGACCT ATTAGTCAAA   
  
  
- CTAATTGTTT TTCACTGTTT TTTTAATTTT TACCAAAAGG CATTAGAAGT TTGGTATACA GCTCCTTTAT   
  
  
- TTGTGCCTTA TTTCTTTCTC ACAGTCTTGG GTTCTTTACA TTTTAGTTGA ATGCAAGATA AATTTCACTT   
  
  
- TTTATAAAAA GAAGTAAGAT CCTTTTAATT AACAGGAGAC TTTTGTTTAA AAGGGAGGAA CAGATTGGTT   
  
  
- TGTGTCATTT TAACTTTTTA GTAAAAGATC CTTTTACTAA AAGTGAGGAT GGTTTGTGTG GGAATTTGAA   
  
  
- CAAGGTTTAG ATATTTGGGC CTTAGAATTA TGCGTGTAAC ATATAATATA CAGGATGATA TTGACATAAT   
  
  
- AGTGACCAAG AACTAAATAG GAAAGTTTGA GAACGAATCA GTTCCAACAG GAGCATGTTT GTACTAATTA   
  
  
- AGTTAATTGG TGCATTCAAT ATGCGACTGT TTAAAGCCCA TGGTTTGTAA ACGTGATCTG GATTTTGTCA   
  
  
- ATGATAGTAG TATAGCTACA ATTTTAACTG GACAAGAATA AACAGGTCGC AACTCGTTCC TTTATTAGTT   
  
  
- TAGTACAGAG TAAATCAGCC AAAAAACGGA TTCTTCTCGT ATTTGTTTCC TTTTTCTGTT GCTGTGTAAT   
  
  
- ATGTACGTAG ATGTGTTTAC TTTACTCCGG TATACCTTGT ACAAGTACTC TCGAAGTGAG CGATTATTAA   
  
  
- CTTGGATGAA AACTGATTCG GTGGTTGATC TTATACCTCT AACTGACAAT TCTCTGATAT AATGTACTTT   
  
  
- ATCTATTCTC ATCTGTAACG ATATATAAAA TAGAAATTAA CAGATAAAAA TTACTAGTAA TGACAAATAA   
  
  
- AGTCACTCAT TAAAAGAGCC TATATTATAA AATATCGCCA ATAATTTATC TACACTTTGA TATTACAATC   
  
  
- TGTATAGTAC GCTTTTATCT CTTTAAAACC CGTTTAATTT TTTTTTTCTT CTTTTTGCTC CTTCATACGG   
  
  
- GTAACATTCG TCCACCTACA TATAGGTCGG AGGTAGGTTG TTAAACCCGT TTTAATATAG TAGGTAAGGT   
  
  
- TTTTTTTCTA TATTAAACAC TATAATTATT GGGCTGAGTT AAGTTCGAAT AAATAAAAAA GTATATTAAA   
  
  
- AAATTATATA TTTTTATTTT TCACTGTTGC TGTACGTAGG AAACGCACAG TTTCCTAGGC GGCGCACCGG   
  
  
- GGGTGTAAGT GACTGCCCCA AAAACCAAAC GCTAATTCTA GTTACCGATT GCAACAGAGA GAGTTTTGTA   
  
  
- TACCTGGGGA AAAAGATAGA AAAAGACGTA ACAGTCGTAT CGTTAACAAC TCGTGTAAAA AAAGCTAAAC   
  
  
- AACTGTTATT CGCACCAATA CTAAATACCC GGTGATTATA GTCAGAGACT AGTAAACGTG TAAAACCTGG   
  
  
- AGAAAGAGAG AGATTAGAAG GTCCATTGGA CTTAAGAAGA GATAAAGACT GATCTAATCT ATTGTCCTAA   
  
  
- AGTTTATAGA CAGTCCACCG CCCATATTGA AGCACTACCC GTAAGTAAAA ACAAGATAGT CCGACCACCC   
  
  
- AAACTCAAAT AAAACTAAGT AAAGCACAAG TAGACACTAT AATCATCCTC CCTAAGACAT ATGGGTGGTT   
  
  
- GACGAGCGGG AAAACGGGCA CACTCGTTAA CCCTGAAAAC AAACAAGAAG CAGCTACGTA GGAGACCAAC   
  
  
- GACTAGACCT TAACTTTGGG GTACATAAGT TTAAGTTGGG ACTAAACGAC AGTTTGAAAG ACTTGGTAGT   
  
  
- TTTGAGGCGG CTTCACAAAC TTTTTCTACC GTAGAAGGTC TGGGAAGTAA ACCGACTAGG GTTTTCGAGA   
  
  
- AGTTAACGGC TTTAGCCCAA ATGATTATCA AGTCTAAGAT GTGTTCATGG ACTAAAAAGT CTACGAACAG   
  
  
- AATTCAAGTA ATCACTATAA GAGGACCTTC TCCTGAATCT ACTTTCAGGA TGCAGAAATG TGCTAATGTA   
  
  
- CCGAGAGGAT CGGTGACTCT TCAGAAACAT ACTACGAGAA CCTTTCCTTA TGAAAGGAAG CAGATGATCA   
  
  
- GAACGGGGTA GAAATCCGGC TTCACAACTG TCGGGTCTAT CACCGAAACC GGCGCCAACG AGACTACCAG   
  
  
- CGCCCTAACT CCCAAACCGA TTACTACTAC GGCAAAAGTA CAGATTGACC GTCGAGTTGT GGTGGGTTAA   
  
  
- CCTGGGAAAG GGTTAGTACG TTCTATAAGG AGCAGGGATA AACCTTAACT TAATGGTAAG AAGACCCAGT   
  
  
- TCGTTACCGT AACTACTAAG CCCCCTACCC AATAGCTGGA GAGGACATTC ATGTGAACGT AGTTGGCGGT   
  
  
- GTCTCCGTCC CTTTTTCAAC CGACCATCGA GATCCTCTTT CTTGGCAGTT TCCCTACTGA TACCGGTACT   
  
  
- TCTCCCTTCC TCATTGTTCG TCGTTCGGAG AATGTTACTG CTAATGCAAC TCTACCTCGT CATACTGCTA   
  
  
- CATGAAGAGA CATCCCGTCT GTTCCCATTA TAAAGTTGAA CACGGTTACT TAGGAGCGAA TTACTCCACG   
  
  
- CACTCTTCGA CGTCTGCTGT CCCAAGTTCC CTTCTTGTAG GGCAGAATTC TTCGTTAGAT TCCTTCGCTT   
  
  
- TCTCCACCTA GACTCTTGAG AAGAAAGACC AACACGTGTT CGACAAAGCT TGAAACTATA GTCCTGACGA   
  
  
- TTACTCGACG AATTCGTTCA GTCTGTCGTA AGAAGTGGTA TACCACTATC GGAGGTTTCC GAGCGGGTAG   
  
  
- TTAAACGCTT ACCATAACTC CGTGCGAATC GTCCGTGGCC AAGTTCTCAT GGACGATTAG AGTAACTACG   
  
  
- TGCCTATAGT AGTAGACTTA AAAATTTTCG AATGTTCAGT ATACAAAGTC GTCAAGGAAA GTCTTCCTAC   
  
  
- AGGATAAAAT ATCGTTTGTT GTGTTAAGAC TTCAACCGAC TCTTTCGTTG TTTCTAAGTG TATTAACTAA   
  
  
- AACCATAAGA TAAACCAGAT GTCACCGGGA CAGAATATGT TTCAGAGAGT TCCGCTTGAC ACCGAGGAGG   
  
  
- CTTCGAAGCG TAGTGACCCT ATCTGATAGG GGTCGTACCA AAGGCCGGTC GTCTTTTCCA ACTCCGTTGT   
  
  
- CCAGCAGCCA ACAGGCCCAT GACACTCTCT AAATTACATG GGAAAGCTAT ACTTTGGTAA CGTTTCTTCA   
  
  
- CCCTTTGGTA TGCGGGTCTT CTAGATTTAT AACTCTTACT ACTCGACCAT TAACAATTAA CATACAACGC   
  
  
- CAGACATTTA GATAACCTAC TATGTCACCG CCATTTATCA GGTTCCCTAC GAAAGAACTC CAATTAGTTT   
  
  
- GTCTATTTGG GCGCAAATAA GTAAGTACGT TAACAGTTAC CTTGGAAATC ATGAGGTAAG AAGTCGTGAG   
  
  
- CTAAGTCCCT TCGGGATAAG GTTATGAGAA GACATAAACT ATATAAACTT CGCTGATACT GAGCACTTCT   
  
  
- AGCACCTTCC GACGACTAAC TCTCAGTTTA TACGCCCGAT CTTCAAAACT TACGTTATCG TACACTTCCA   
  
  
- CGTCTCTCCT AAGTTTCCGG ACTTTGTATG TTCGTTACCG TCCTCGCCTG TTGTTCCCGA CCTGATTCCG   
  
  
- TCCAAGGTTA TCTACTCCTC GAACAGTTAT CTCGTTTCTG ATATCACTTT CGTTTAATAG TGTTCCTAAA   
  
  
- ACACCACCTA CTCCTATCCG CAACCTACGA AGTTCCAACC TTTCCTTCCT GTGAATCACG GGAAAGGCAA   
  
  
- ACCGTCGGAT TGAT

+     Myc

| Site Name | Organism | Position | Strand | Matrix score. | sequence | function |
| --- | --- | --- | --- | --- | --- | --- |
| Myc | Arabidopsis thaliana | 1103 | - | 7 | TCTCTTA |  |

>HU02G01572.1   
+ +Up\_Stream \_Len000TTTAAT AAGATCCTTT TATATATATG TGTGTATAAT TCTATGGTAC ATAGGTATGT   
  
  
+ ACCATAGACT TATTGCATTT CTAGGTCCTT CTCTCTCTGC GCTTGTTTTT TGTTTATCTA ATTTATGGAG   
  
  
+ TTGAACATTT TGTTTTATAT ACTTGAACAA TCTATTATAC TAAATTAAGC ATTTTGATTT AATCACTTAT   
  
  
+ GGGTGTGTTT GGTAGGGTTG AAAATAATTT TCCTAGAAAA TAATTTTTAA TATAAAATAA TTTTTCATGT   
  
  
+ AAAATGATTT TCAACTCATA TTTTCAGTTG ATTGGTAAAA AGAAAAATGA ACTTTCTGGA TAATCAGTTT   
  
  
+ GATTAACAAA AAGTGACAAA AAAATTAAAA ATGGTTTTCC GTAATCTTCA AACCATATGT CGAGGAAATA   
  
  
+ AACACGGAAT AAAGAAAGAG TGTCAGAACC CAAGAAATGT AAAATCAACT TACGTTCTAT TTAAAGTGAA   
  
  
+ AAATATTTTT CTTCATTCTA GGAAAATTAA TTGTCCTCTG AAAACAAATT TTCCCTCCTT GTCTAACCAA   
  
  
+ ACACAGTAAA ATTGAAAAAT CATTTTCTAG GAAAATGATT TTCACTCCTA CCAAACACAC CCTTAAACTT   
  
  
+ GTTCCAAATC TATAAACCCG GAATCTTAAT ACGCACATTG TATATTATAT GTCCTACTAT AACTGTATTA   
  
  
+ TCACTGGTTC TTGATTTATC CTTTCAAACT CTTGCTTAGT CAAGGTTGTC CTCGTACAAA CATGATTAAT   
  
  
+ TCAATTAACC ACGTAAGTTA TACGCTGACA AATTTCGGGT ACCAAACATT TGCACTAGAC CTAAAACAGT   
  
  
+ TACTATCATC ATATCGATGT TAAAATTGAC CTGTTCTTAT TTGTCCAGCG TTGAGCAAGG AAATAATCAA   
  
  
+ ATCATGTCTC ATTTAGTCGG TTTTTTGCCT AAGAAGAGCA TAAACAAAGG AAAAAGACAA CGACACATTA   
  
  
+ TACATGCATC TACACAAATG AAATGAGGCC ATATGGAACA TGTTCATGAG AGCTTCACTC GCTAATAATT   
  
  
+ GAACCTACTT TTGACTAAGC CACCAACTAG AATATGGAGA TTGACTGTTA AGAGACTATA TTACATGAAA   
  
  
+ TAGATAAGAG TAGACATTGC TATATATTTT ATCTTTAATT GTCTATTTTT AATGATCATT ACTGTTTATT   
  
  
+ TCAGTGAGTA ATTTTCTCGG ATATAATATT TTATAGCGGT TATTAAATAG ATGTGAAACT ATAATGTTAG   
  
  
+ ACATATCATG CGAAAATAGA GAAATTTTGG GCAAATTAAA AAAAAAAGAA GAAAAACGAG GAAGTATGCC   
  
  
+ CATTGTAAGC AGGTGGATGT ATATCCAGCC TCCATCCAAC AATTTGGGCA AAATTATATC ATCCATTCCA   
  
  
+ AAAAAAAGAT ATAATTTGTG ATATTAATAA CCCGACTCAA TTCAAGCTTA TTTATTTTTT CATATAATTT   
  
  
+ TTTAATATAT AAAAATAAAA AGTGACAACG ACATGCATCC TTTGCGTGTC AAAGGATCCG CCGCGTGGCC   
  
  
+ CCCACATTCA CTGACGGGGT TTTTGGTTTG CGATTAAGAT CAATGGCTAA CGTTGTCTCT CTCAAAACAT   
  
  
+ ATGGACCCCT TTTTCTATCT TTTTCTGCAT TGTCAGCATA GCAATTGTTG AGCACATTTT TTTCGATTTG   
  
  
+ TTGACAATAA GCGTGGTTAT GATTTATGGG CCACTAATAT CAGTCTCTGA TCATTTGCAC ATTTTGGACC   
  
  
+ TCTTTCTCTC TCTAATCTTC CAGGTAACCT GAATTCTTCT CTATTTCTGA CTAGATTAGA TAACAGGATT   
  
  
+ TCAAATATCT GTCAGGTGGC GGGTATAACT TCGTGATGGG CATTCATTTT TGTTCTATCA GGCTGGTGGG   
  
  
+ TTTGAGTTTA TTTTGATTCA TTTCGTGTTC ATCTGTGATA TTAGTAGGAG GGATTCTGTA TACCCACCAA   
  
  
+ CTGCTCGCCC TTTTGCCCGT GTGAGCAATT GGGACTTTTG TTTGTTCTTC GTCGATGCAT CCTCTGGTTG   
  
  
+ CTGATCTGGA ATTGAAACCC CATGTATTCA AATTCAACCC TGATTTGCTG TCAAACTTTC TGAACCATCA   
  
  
+ AAACTCCGCC GAAGTGTTTG AAAAAGATGG CATCTTCCAG ACCCTTCATT TGGCTGATCC CAAAAGCTCT   
  
  
+ TCAATTGCCG AAATCGGGTT TACTAATAGT TCAGATTCTA CACAAGTACC TGATTTTTCA GATGCTTGTC   
  
  
+ TTAAGTTCAT TAGTGATATT CTCCTGGAAG AGGACTTAGA TGAAAGTCCT ACGTCTTTAC ACGATTACAT   
  
  
+ GGCTCTCCTA GCCACTGAGA AGTCTTTGTA TGATGCTCTT GGAAAGGAAT ACTTTCCTTC GTCTACTAGT   
  
  
+ CTTGCCCCAT CTTTAGGCCG AAGTGTTGAC AGCCCAGATA GTGGCTTTGG CCGCGGTTGC TCTGATGGTC   
  
  
+ GCGGGATTGA GGGTTTGGCT AATGATGATG CCGTTTTCAT GTCTAACTGG CAGCTCAACA CCACCCAATT   
  
  
+ GGACCCTTTC CCAATCATGC AAGATATTCC TCGTCCCTAT TTGGAATTGA ATTACCATTC TTCTGGGTCA   
  
  
+ AGCAATGGCA TTGATGATTC GGGGGATGGG TTATCGACCT CTCCTGTAAG TACACTTGCA TCAACCGCCA   
  
  
+ CAGAGGCAGG GAAAAAGTTG GCTGGTAGCT CTAGGAGAAA GAACCGTCAA AGGGATGACT ATGGCCATGA   
  
  
+ AGAGGGAAGG AGTAACAAGC AGCAAGCCTC TTACAATGAC GATTACGTTG AGATGGAGCA GTATGACGAT   
  
  
+ GTACTTCTCT GTAGGGCAGA CAAGGGTAAT ATTTCAACTT GTGCCAATGA ATCCTCGCTT AATGAGGTGC   
  
  
+ GTGAGAAGCT GCAGACGACA GGGTTCAAGG GAAGAACATC CCGTCTTAAG AAGCAATCTA AGGAAGCGAA   
  
  
+ AGAGGTGGAT CTGAGAACTC TTCTTTCTGG TTGTGCACAA GCTGTTTCGA ACTTTGATAT CAGGACTGCT   
  
  
+ AATGAGCTGC TTAAGCAAGT CAGACAGCAT TCTTCACCAT ATGGTGATAG CCTCCAAAGG CTCGCCCATC   
  
  
+ AATTTGCGAA TGGTATTGAG GCACGCTTAG CAGGCACCGG TTCAAGAGTA CCTGCTAATC TCATTGATGC   
  
  
+ ACGGATATCA TCATCTGAAT TTTTAAAAGC TTACAAGTCA TATGTTTCAG CAGTTCCTTT CAGAAGGATG   
  
  
+ TCCTATTTTA TAGCAAACAA CACAATTCTG AAGTTGGCTG AGAAAGCAAC AAAGATTCAC ATAATTGATT   
  
  
+ TTGGTATTCT ATTTGGTCTA CAGTGGCCCT GTCTTATACA AAGTCTCTCA AGGCGAACTG TGGCTCCTCC   
  
  
+ GAAGCTTCGC ATCACTGGGA TAGACTATCC CCAGCATGGT TTCCGGCCAG CAGAAAAGGT TGAGGCAACA   
  
  
+ GGTCGTCGGT TGTCCGGGTA CTGTGAGAGA TTTAATGTAC CCTTTCGATA TGAAACCATT GCAAAGAAGT   
  
  
+ GGGAAACCAT ACGCCCAGAA GATCTAAATA TTGAGAATGA TGAGCTGGTA ATTGTTAATT GTATGTTGCG   
  
  
+ GTCTGTAAAT CTATTGGATG ATACAGTGGC GGTAAATAGT CCAAGGGATG CTTTCTTGAG GTTAATCAAA   
  
  
+ CAGATAAACC CGCGTTTATT CATTCATGCA ATTGTCAATG GAACCTTTAG TACTCCATTC TTCAGCACTC   
  
  
+ GATTCAGGGA AGCCCTATTC CAATACTCTT CTGTATTTGA TATATTTGAA GCGACTATGA CTCGTGAAGA   
  
  
+ TCGTGGAAGG CTGCTGATTG AGAGTCAAAT ATGCGGGCTA GAAGTTTTGA ATGCAATAGC ATGTGAAGGT   
  
  
+ GCAGAGAGGA TTCAAAGGCC TGAAACATAC AAGCAATGGC AGGAGCGGAC AACAAGGGCT GGACTAAGGC   
  
  
+ AGGTTCCAAT AGATGAGGAG CTTGTCAATA GAGCAAAGAC TATAGTGAAA GCAAATTATC ACAAGGATTT   
  
  
+ TGTGGTGGAT GAGGATAGGC GTTGGATGCT TCAAGGTTGG AAAGGAAGGA CACTTAGTGC CCTTTCCGTT   
  
  
+ TGGCAGCCTA ACTA  

- +Up\_Stream \_Len000AAATTA TTCTAGGAAA ATATATATAC ACACATATTA AGATACCATG TATCCATACA   
  
  
- TGGTATCTGA ATAACGTAAA GATCCAGGAA GAGAGAGACG CGAACAAAAA ACAAATAGAT TAAATACCTC   
  
  
- AACTTGTAAA ACAAAATATA TGAACTTGTT AGATAATATG ATTTAATTCG TAAAACTAAA TTAGTGAATA   
  
  
- CCCACACAAA CCATCCCAAC TTTTATTAAA AGGATCTTTT ATTAAAAATT ATATTTTATT AAAAAGTACA   
  
  
- TTTTACTAAA AGTTGAGTAT AAAAGTCAAC TAACCATTTT TCTTTTTACT TGAAAGACCT ATTAGTCAAA   
  
  
- CTAATTGTTT TTCACTGTTT TTTTAATTTT TACCAAAAGG CATTAGAAGT TTGGTATACA GCTCCTTTAT   
  
  
- TTGTGCCTTA TTTCTTTCTC ACAGTCTTGG GTTCTTTACA TTTTAGTTGA ATGCAAGATA AATTTCACTT   
  
  
- TTTATAAAAA GAAGTAAGAT CCTTTTAATT AACAGGAGAC TTTTGTTTAA AAGGGAGGAA CAGATTGGTT   
  
  
- TGTGTCATTT TAACTTTTTA GTAAAAGATC CTTTTACTAA AAGTGAGGAT GGTTTGTGTG GGAATTTGAA   
  
  
- CAAGGTTTAG ATATTTGGGC CTTAGAATTA TGCGTGTAAC ATATAATATA CAGGATGATA TTGACATAAT   
  
  
- AGTGACCAAG AACTAAATAG GAAAGTTTGA GAACGAATCA GTTCCAACAG GAGCATGTTT GTACTAATTA   
  
  
- AGTTAATTGG TGCATTCAAT ATGCGACTGT TTAAAGCCCA TGGTTTGTAA ACGTGATCTG GATTTTGTCA   
  
  
- ATGATAGTAG TATAGCTACA ATTTTAACTG GACAAGAATA AACAGGTCGC AACTCGTTCC TTTATTAGTT   
  
  
- TAGTACAGAG TAAATCAGCC AAAAAACGGA TTCTTCTCGT ATTTGTTTCC TTTTTCTGTT GCTGTGTAAT   
  
  
- ATGTACGTAG ATGTGTTTAC TTTACTCCGG TATACCTTGT ACAAGTACTC TCGAAGTGAG CGATTATTAA   
  
  
- CTTGGATGAA AACTGATTCG GTGGTTGATC TTATACCTCT AACTGACAAT TCTCTGATAT AATGTACTTT   
  
  
- ATCTATTCTC ATCTGTAACG ATATATAAAA TAGAAATTAA CAGATAAAAA TTACTAGTAA TGACAAATAA   
  
  
- AGTCACTCAT TAAAAGAGCC TATATTATAA AATATCGCCA ATAATTTATC TACACTTTGA TATTACAATC   
  
  
- TGTATAGTAC GCTTTTATCT CTTTAAAACC CGTTTAATTT TTTTTTTCTT CTTTTTGCTC CTTCATACGG   
  
  
- GTAACATTCG TCCACCTACA TATAGGTCGG AGGTAGGTTG TTAAACCCGT TTTAATATAG TAGGTAAGGT   
  
  
- TTTTTTTCTA TATTAAACAC TATAATTATT GGGCTGAGTT AAGTTCGAAT AAATAAAAAA GTATATTAAA   
  
  
- AAATTATATA TTTTTATTTT TCACTGTTGC TGTACGTAGG AAACGCACAG TTTCCTAGGC GGCGCACCGG   
  
  
- GGGTGTAAGT GACTGCCCCA AAAACCAAAC GCTAATTCTA GTTACCGATT GCAACAGAGA GAGTTTTGTA   
  
  
- TACCTGGGGA AAAAGATAGA AAAAGACGTA ACAGTCGTAT CGTTAACAAC TCGTGTAAAA AAAGCTAAAC   
  
  
- AACTGTTATT CGCACCAATA CTAAATACCC GGTGATTATA GTCAGAGACT AGTAAACGTG TAAAACCTGG   
  
  
- AGAAAGAGAG AGATTAGAAG GTCCATTGGA CTTAAGAAGA GATAAAGACT GATCTAATCT ATTGTCCTAA   
  
  
- AGTTTATAGA CAGTCCACCG CCCATATTGA AGCACTACCC GTAAGTAAAA ACAAGATAGT CCGACCACCC   
  
  
- AAACTCAAAT AAAACTAAGT AAAGCACAAG TAGACACTAT AATCATCCTC CCTAAGACAT ATGGGTGGTT   
  
  
- GACGAGCGGG AAAACGGGCA CACTCGTTAA CCCTGAAAAC AAACAAGAAG CAGCTACGTA GGAGACCAAC   
  
  
- GACTAGACCT TAACTTTGGG GTACATAAGT TTAAGTTGGG ACTAAACGAC AGTTTGAAAG ACTTGGTAGT   
  
  
- TTTGAGGCGG CTTCACAAAC TTTTTCTACC GTAGAAGGTC TGGGAAGTAA ACCGACTAGG GTTTTCGAGA   
  
  
- AGTTAACGGC TTTAGCCCAA ATGATTATCA AGTCTAAGAT GTGTTCATGG ACTAAAAAGT CTACGAACAG   
  
  
- AATTCAAGTA ATCACTATAA GAGGACCTTC TCCTGAATCT ACTTTCAGGA TGCAGAAATG TGCTAATGTA   
  
  
- CCGAGAGGAT CGGTGACTCT TCAGAAACAT ACTACGAGAA CCTTTCCTTA TGAAAGGAAG CAGATGATCA   
  
  
- GAACGGGGTA GAAATCCGGC TTCACAACTG TCGGGTCTAT CACCGAAACC GGCGCCAACG AGACTACCAG   
  
  
- CGCCCTAACT CCCAAACCGA TTACTACTAC GGCAAAAGTA CAGATTGACC GTCGAGTTGT GGTGGGTTAA   
  
  
- CCTGGGAAAG GGTTAGTACG TTCTATAAGG AGCAGGGATA AACCTTAACT TAATGGTAAG AAGACCCAGT   
  
  
- TCGTTACCGT AACTACTAAG CCCCCTACCC AATAGCTGGA GAGGACATTC ATGTGAACGT AGTTGGCGGT   
  
  
- GTCTCCGTCC CTTTTTCAAC CGACCATCGA GATCCTCTTT CTTGGCAGTT TCCCTACTGA TACCGGTACT   
  
  
- TCTCCCTTCC TCATTGTTCG TCGTTCGGAG AATGTTACTG CTAATGCAAC TCTACCTCGT CATACTGCTA   
  
  
- CATGAAGAGA CATCCCGTCT GTTCCCATTA TAAAGTTGAA CACGGTTACT TAGGAGCGAA TTACTCCACG   
  
  
- CACTCTTCGA CGTCTGCTGT CCCAAGTTCC CTTCTTGTAG GGCAGAATTC TTCGTTAGAT TCCTTCGCTT   
  
  
- TCTCCACCTA GACTCTTGAG AAGAAAGACC AACACGTGTT CGACAAAGCT TGAAACTATA GTCCTGACGA   
  
  
- TTACTCGACG AATTCGTTCA GTCTGTCGTA AGAAGTGGTA TACCACTATC GGAGGTTTCC GAGCGGGTAG   
  
  
- TTAAACGCTT ACCATAACTC CGTGCGAATC GTCCGTGGCC AAGTTCTCAT GGACGATTAG AGTAACTACG   
  
  
- TGCCTATAGT AGTAGACTTA AAAATTTTCG AATGTTCAGT ATACAAAGTC GTCAAGGAAA GTCTTCCTAC   
  
  
- AGGATAAAAT ATCGTTTGTT GTGTTAAGAC TTCAACCGAC TCTTTCGTTG TTTCTAAGTG TATTAACTAA   
  
  
- AACCATAAGA TAAACCAGAT GTCACCGGGA CAGAATATGT TTCAGAGAGT TCCGCTTGAC ACCGAGGAGG   
  
  
- CTTCGAAGCG TAGTGACCCT ATCTGATAGG GGTCGTACCA AAGGCCGGTC GTCTTTTCCA ACTCCGTTGT   
  
  
- CCAGCAGCCA ACAGGCCCAT GACACTCTCT AAATTACATG GGAAAGCTAT ACTTTGGTAA CGTTTCTTCA   
  
  
- CCCTTTGGTA TGCGGGTCTT CTAGATTTAT AACTCTTACT ACTCGACCAT TAACAATTAA CATACAACGC   
  
  
- CAGACATTTA GATAACCTAC TATGTCACCG CCATTTATCA GGTTCCCTAC GAAAGAACTC CAATTAGTTT   
  
  
- GTCTATTTGG GCGCAAATAA GTAAGTACGT TAACAGTTAC CTTGGAAATC ATGAGGTAAG AAGTCGTGAG   
  
  
- CTAAGTCCCT TCGGGATAAG GTTATGAGAA GACATAAACT ATATAAACTT CGCTGATACT GAGCACTTCT   
  
  
- AGCACCTTCC GACGACTAAC TCTCAGTTTA TACGCCCGAT CTTCAAAACT TACGTTATCG TACACTTCCA   
  
  
- CGTCTCTCCT AAGTTTCCGG ACTTTGTATG TTCGTTACCG TCCTCGCCTG TTGTTCCCGA CCTGATTCCG   
  
  
- TCCAAGGTTA TCTACTCCTC GAACAGTTAT CTCGTTTCTG ATATCACTTT CGTTTAATAG TGTTCCTAAA   
  
  
- ACACCACCTA CTCCTATCCG CAACCTACGA AGTTCCAACC TTTCCTTCCT GTGAATCACG GGAAAGGCAA   
  
  
- ACCGTCGGAT TGAT

+     O2-site

| Site Name | Organism | Position | Strand | Matrix score. | sequence | function |
| --- | --- | --- | --- | --- | --- | --- |
| O2-site | Zea mays | 2307 | + | 9 | GATGACATGG | cis-acting regulatory element involved in zein metabolism regulation |

>HU02G01572.1   
+ +Up\_Stream \_Len000TTTAAT AAGATCCTTT TATATATATG TGTGTATAAT TCTATGGTAC ATAGGTATGT   
  
  
+ ACCATAGACT TATTGCATTT CTAGGTCCTT CTCTCTCTGC GCTTGTTTTT TGTTTATCTA ATTTATGGAG   
  
  
+ TTGAACATTT TGTTTTATAT ACTTGAACAA TCTATTATAC TAAATTAAGC ATTTTGATTT AATCACTTAT   
  
  
+ GGGTGTGTTT GGTAGGGTTG AAAATAATTT TCCTAGAAAA TAATTTTTAA TATAAAATAA TTTTTCATGT   
  
  
+ AAAATGATTT TCAACTCATA TTTTCAGTTG ATTGGTAAAA AGAAAAATGA ACTTTCTGGA TAATCAGTTT   
  
  
+ GATTAACAAA AAGTGACAAA AAAATTAAAA ATGGTTTTCC GTAATCTTCA AACCATATGT CGAGGAAATA   
  
  
+ AACACGGAAT AAAGAAAGAG TGTCAGAACC CAAGAAATGT AAAATCAACT TACGTTCTAT TTAAAGTGAA   
  
  
+ AAATATTTTT CTTCATTCTA GGAAAATTAA TTGTCCTCTG AAAACAAATT TTCCCTCCTT GTCTAACCAA   
  
  
+ ACACAGTAAA ATTGAAAAAT CATTTTCTAG GAAAATGATT TTCACTCCTA CCAAACACAC CCTTAAACTT   
  
  
+ GTTCCAAATC TATAAACCCG GAATCTTAAT ACGCACATTG TATATTATAT GTCCTACTAT AACTGTATTA   
  
  
+ TCACTGGTTC TTGATTTATC CTTTCAAACT CTTGCTTAGT CAAGGTTGTC CTCGTACAAA CATGATTAAT   
  
  
+ TCAATTAACC ACGTAAGTTA TACGCTGACA AATTTCGGGT ACCAAACATT TGCACTAGAC CTAAAACAGT   
  
  
+ TACTATCATC ATATCGATGT TAAAATTGAC CTGTTCTTAT TTGTCCAGCG TTGAGCAAGG AAATAATCAA   
  
  
+ ATCATGTCTC ATTTAGTCGG TTTTTTGCCT AAGAAGAGCA TAAACAAAGG AAAAAGACAA CGACACATTA   
  
  
+ TACATGCATC TACACAAATG AAATGAGGCC ATATGGAACA TGTTCATGAG AGCTTCACTC GCTAATAATT   
  
  
+ GAACCTACTT TTGACTAAGC CACCAACTAG AATATGGAGA TTGACTGTTA AGAGACTATA TTACATGAAA   
  
  
+ TAGATAAGAG TAGACATTGC TATATATTTT ATCTTTAATT GTCTATTTTT AATGATCATT ACTGTTTATT   
  
  
+ TCAGTGAGTA ATTTTCTCGG ATATAATATT TTATAGCGGT TATTAAATAG ATGTGAAACT ATAATGTTAG   
  
  
+ ACATATCATG CGAAAATAGA GAAATTTTGG GCAAATTAAA AAAAAAAGAA GAAAAACGAG GAAGTATGCC   
  
  
+ CATTGTAAGC AGGTGGATGT ATATCCAGCC TCCATCCAAC AATTTGGGCA AAATTATATC ATCCATTCCA   
  
  
+ AAAAAAAGAT ATAATTTGTG ATATTAATAA CCCGACTCAA TTCAAGCTTA TTTATTTTTT CATATAATTT   
  
  
+ TTTAATATAT AAAAATAAAA AGTGACAACG ACATGCATCC TTTGCGTGTC AAAGGATCCG CCGCGTGGCC   
  
  
+ CCCACATTCA CTGACGGGGT TTTTGGTTTG CGATTAAGAT CAATGGCTAA CGTTGTCTCT CTCAAAACAT   
  
  
+ ATGGACCCCT TTTTCTATCT TTTTCTGCAT TGTCAGCATA GCAATTGTTG AGCACATTTT TTTCGATTTG   
  
  
+ TTGACAATAA GCGTGGTTAT GATTTATGGG CCACTAATAT CAGTCTCTGA TCATTTGCAC ATTTTGGACC   
  
  
+ TCTTTCTCTC TCTAATCTTC CAGGTAACCT GAATTCTTCT CTATTTCTGA CTAGATTAGA TAACAGGATT   
  
  
+ TCAAATATCT GTCAGGTGGC GGGTATAACT TCGTGATGGG CATTCATTTT TGTTCTATCA GGCTGGTGGG   
  
  
+ TTTGAGTTTA TTTTGATTCA TTTCGTGTTC ATCTGTGATA TTAGTAGGAG GGATTCTGTA TACCCACCAA   
  
  
+ CTGCTCGCCC TTTTGCCCGT GTGAGCAATT GGGACTTTTG TTTGTTCTTC GTCGATGCAT CCTCTGGTTG   
  
  
+ CTGATCTGGA ATTGAAACCC CATGTATTCA AATTCAACCC TGATTTGCTG TCAAACTTTC TGAACCATCA   
  
  
+ AAACTCCGCC GAAGTGTTTG AAAAAGATGG CATCTTCCAG ACCCTTCATT TGGCTGATCC CAAAAGCTCT   
  
  
+ TCAATTGCCG AAATCGGGTT TACTAATAGT TCAGATTCTA CACAAGTACC TGATTTTTCA GATGCTTGTC   
  
  
+ TTAAGTTCAT TAGTGATATT CTCCTGGAAG AGGACTTAGA TGAAAGTCCT ACGTCTTTAC ACGATTACAT   
  
  
+ GGCTCTCCTA GCCACTGAGA AGTCTTTGTA TGATGCTCTT GGAAAGGAAT ACTTTCCTTC GTCTACTAGT   
  
  
+ CTTGCCCCAT CTTTAGGCCG AAGTGTTGAC AGCCCAGATA GTGGCTTTGG CCGCGGTTGC TCTGATGGTC   
  
  
+ GCGGGATTGA GGGTTTGGCT AATGATGATG CCGTTTTCAT GTCTAACTGG CAGCTCAACA CCACCCAATT   
  
  
+ GGACCCTTTC CCAATCATGC AAGATATTCC TCGTCCCTAT TTGGAATTGA ATTACCATTC TTCTGGGTCA   
  
  
+ AGCAATGGCA TTGATGATTC GGGGGATGGG TTATCGACCT CTCCTGTAAG TACACTTGCA TCAACCGCCA   
  
  
+ CAGAGGCAGG GAAAAAGTTG GCTGGTAGCT CTAGGAGAAA GAACCGTCAA AGGGATGACT ATGGCCATGA   
  
  
+ AGAGGGAAGG AGTAACAAGC AGCAAGCCTC TTACAATGAC GATTACGTTG AGATGGAGCA GTATGACGAT   
  
  
+ GTACTTCTCT GTAGGGCAGA CAAGGGTAAT ATTTCAACTT GTGCCAATGA ATCCTCGCTT AATGAGGTGC   
  
  
+ GTGAGAAGCT GCAGACGACA GGGTTCAAGG GAAGAACATC CCGTCTTAAG AAGCAATCTA AGGAAGCGAA   
  
  
+ AGAGGTGGAT CTGAGAACTC TTCTTTCTGG TTGTGCACAA GCTGTTTCGA ACTTTGATAT CAGGACTGCT   
  
  
+ AATGAGCTGC TTAAGCAAGT CAGACAGCAT TCTTCACCAT ATGGTGATAG CCTCCAAAGG CTCGCCCATC   
  
  
+ AATTTGCGAA TGGTATTGAG GCACGCTTAG CAGGCACCGG TTCAAGAGTA CCTGCTAATC TCATTGATGC   
  
  
+ ACGGATATCA TCATCTGAAT TTTTAAAAGC TTACAAGTCA TATGTTTCAG CAGTTCCTTT CAGAAGGATG   
  
  
+ TCCTATTTTA TAGCAAACAA CACAATTCTG AAGTTGGCTG AGAAAGCAAC AAAGATTCAC ATAATTGATT   
  
  
+ TTGGTATTCT ATTTGGTCTA CAGTGGCCCT GTCTTATACA AAGTCTCTCA AGGCGAACTG TGGCTCCTCC   
  
  
+ GAAGCTTCGC ATCACTGGGA TAGACTATCC CCAGCATGGT TTCCGGCCAG CAGAAAAGGT TGAGGCAACA   
  
  
+ GGTCGTCGGT TGTCCGGGTA CTGTGAGAGA TTTAATGTAC CCTTTCGATA TGAAACCATT GCAAAGAAGT   
  
  
+ GGGAAACCAT ACGCCCAGAA GATCTAAATA TTGAGAATGA TGAGCTGGTA ATTGTTAATT GTATGTTGCG   
  
  
+ GTCTGTAAAT CTATTGGATG ATACAGTGGC GGTAAATAGT CCAAGGGATG CTTTCTTGAG GTTAATCAAA   
  
  
+ CAGATAAACC CGCGTTTATT CATTCATGCA ATTGTCAATG GAACCTTTAG TACTCCATTC TTCAGCACTC   
  
  
+ GATTCAGGGA AGCCCTATTC CAATACTCTT CTGTATTTGA TATATTTGAA GCGACTATGA CTCGTGAAGA   
  
  
+ TCGTGGAAGG CTGCTGATTG AGAGTCAAAT ATGCGGGCTA GAAGTTTTGA ATGCAATAGC ATGTGAAGGT   
  
  
+ GCAGAGAGGA TTCAAAGGCC TGAAACATAC AAGCAATGGC AGGAGCGGAC AACAAGGGCT GGACTAAGGC   
  
  
+ AGGTTCCAAT AGATGAGGAG CTTGTCAATA GAGCAAAGAC TATAGTGAAA GCAAATTATC ACAAGGATTT   
  
  
+ TGTGGTGGAT GAGGATAGGC GTTGGATGCT TCAAGGTTGG AAAGGAAGGA CACTTAGTGC CCTTTCCGTT   
  
  
+ TGGCAGCCTA ACTA  

- +Up\_Stream \_Len000AAATTA TTCTAGGAAA ATATATATAC ACACATATTA AGATACCATG TATCCATACA   
  
  
- TGGTATCTGA ATAACGTAAA GATCCAGGAA GAGAGAGACG CGAACAAAAA ACAAATAGAT TAAATACCTC   
  
  
- AACTTGTAAA ACAAAATATA TGAACTTGTT AGATAATATG ATTTAATTCG TAAAACTAAA TTAGTGAATA   
  
  
- CCCACACAAA CCATCCCAAC TTTTATTAAA AGGATCTTTT ATTAAAAATT ATATTTTATT AAAAAGTACA   
  
  
- TTTTACTAAA AGTTGAGTAT AAAAGTCAAC TAACCATTTT TCTTTTTACT TGAAAGACCT ATTAGTCAAA   
  
  
- CTAATTGTTT TTCACTGTTT TTTTAATTTT TACCAAAAGG CATTAGAAGT TTGGTATACA GCTCCTTTAT   
  
  
- TTGTGCCTTA TTTCTTTCTC ACAGTCTTGG GTTCTTTACA TTTTAGTTGA ATGCAAGATA AATTTCACTT   
  
  
- TTTATAAAAA GAAGTAAGAT CCTTTTAATT AACAGGAGAC TTTTGTTTAA AAGGGAGGAA CAGATTGGTT   
  
  
- TGTGTCATTT TAACTTTTTA GTAAAAGATC CTTTTACTAA AAGTGAGGAT GGTTTGTGTG GGAATTTGAA   
  
  
- CAAGGTTTAG ATATTTGGGC CTTAGAATTA TGCGTGTAAC ATATAATATA CAGGATGATA TTGACATAAT   
  
  
- AGTGACCAAG AACTAAATAG GAAAGTTTGA GAACGAATCA GTTCCAACAG GAGCATGTTT GTACTAATTA   
  
  
- AGTTAATTGG TGCATTCAAT ATGCGACTGT TTAAAGCCCA TGGTTTGTAA ACGTGATCTG GATTTTGTCA   
  
  
- ATGATAGTAG TATAGCTACA ATTTTAACTG GACAAGAATA AACAGGTCGC AACTCGTTCC TTTATTAGTT   
  
  
- TAGTACAGAG TAAATCAGCC AAAAAACGGA TTCTTCTCGT ATTTGTTTCC TTTTTCTGTT GCTGTGTAAT   
  
  
- ATGTACGTAG ATGTGTTTAC TTTACTCCGG TATACCTTGT ACAAGTACTC TCGAAGTGAG CGATTATTAA   
  
  
- CTTGGATGAA AACTGATTCG GTGGTTGATC TTATACCTCT AACTGACAAT TCTCTGATAT AATGTACTTT   
  
  
- ATCTATTCTC ATCTGTAACG ATATATAAAA TAGAAATTAA CAGATAAAAA TTACTAGTAA TGACAAATAA   
  
  
- AGTCACTCAT TAAAAGAGCC TATATTATAA AATATCGCCA ATAATTTATC TACACTTTGA TATTACAATC   
  
  
- TGTATAGTAC GCTTTTATCT CTTTAAAACC CGTTTAATTT TTTTTTTCTT CTTTTTGCTC CTTCATACGG   
  
  
- GTAACATTCG TCCACCTACA TATAGGTCGG AGGTAGGTTG TTAAACCCGT TTTAATATAG TAGGTAAGGT   
  
  
- TTTTTTTCTA TATTAAACAC TATAATTATT GGGCTGAGTT AAGTTCGAAT AAATAAAAAA GTATATTAAA   
  
  
- AAATTATATA TTTTTATTTT TCACTGTTGC TGTACGTAGG AAACGCACAG TTTCCTAGGC GGCGCACCGG   
  
  
- GGGTGTAAGT GACTGCCCCA AAAACCAAAC GCTAATTCTA GTTACCGATT GCAACAGAGA GAGTTTTGTA   
  
  
- TACCTGGGGA AAAAGATAGA AAAAGACGTA ACAGTCGTAT CGTTAACAAC TCGTGTAAAA AAAGCTAAAC   
  
  
- AACTGTTATT CGCACCAATA CTAAATACCC GGTGATTATA GTCAGAGACT AGTAAACGTG TAAAACCTGG   
  
  
- AGAAAGAGAG AGATTAGAAG GTCCATTGGA CTTAAGAAGA GATAAAGACT GATCTAATCT ATTGTCCTAA   
  
  
- AGTTTATAGA CAGTCCACCG CCCATATTGA AGCACTACCC GTAAGTAAAA ACAAGATAGT CCGACCACCC   
  
  
- AAACTCAAAT AAAACTAAGT AAAGCACAAG TAGACACTAT AATCATCCTC CCTAAGACAT ATGGGTGGTT   
  
  
- GACGAGCGGG AAAACGGGCA CACTCGTTAA CCCTGAAAAC AAACAAGAAG CAGCTACGTA GGAGACCAAC   
  
  
- GACTAGACCT TAACTTTGGG GTACATAAGT TTAAGTTGGG ACTAAACGAC AGTTTGAAAG ACTTGGTAGT   
  
  
- TTTGAGGCGG CTTCACAAAC TTTTTCTACC GTAGAAGGTC TGGGAAGTAA ACCGACTAGG GTTTTCGAGA   
  
  
- AGTTAACGGC TTTAGCCCAA ATGATTATCA AGTCTAAGAT GTGTTCATGG ACTAAAAAGT CTACGAACAG   
  
  
- AATTCAAGTA ATCACTATAA GAGGACCTTC TCCTGAATCT ACTTTCAGGA TGCAGAAATG TGCTAATGTA   
  
  
- CCGAGAGGAT CGGTGACTCT TCAGAAACAT ACTACGAGAA CCTTTCCTTA TGAAAGGAAG CAGATGATCA   
  
  
- GAACGGGGTA GAAATCCGGC TTCACAACTG TCGGGTCTAT CACCGAAACC GGCGCCAACG AGACTACCAG   
  
  
- CGCCCTAACT CCCAAACCGA TTACTACTAC GGCAAAAGTA CAGATTGACC GTCGAGTTGT GGTGGGTTAA   
  
  
- CCTGGGAAAG GGTTAGTACG TTCTATAAGG AGCAGGGATA AACCTTAACT TAATGGTAAG AAGACCCAGT   
  
  
- TCGTTACCGT AACTACTAAG CCCCCTACCC AATAGCTGGA GAGGACATTC ATGTGAACGT AGTTGGCGGT   
  
  
- GTCTCCGTCC CTTTTTCAAC CGACCATCGA GATCCTCTTT CTTGGCAGTT TCCCTACTGA TACCGGTACT   
  
  
- TCTCCCTTCC TCATTGTTCG TCGTTCGGAG AATGTTACTG CTAATGCAAC TCTACCTCGT CATACTGCTA   
  
  
- CATGAAGAGA CATCCCGTCT GTTCCCATTA TAAAGTTGAA CACGGTTACT TAGGAGCGAA TTACTCCACG   
  
  
- CACTCTTCGA CGTCTGCTGT CCCAAGTTCC CTTCTTGTAG GGCAGAATTC TTCGTTAGAT TCCTTCGCTT   
  
  
- TCTCCACCTA GACTCTTGAG AAGAAAGACC AACACGTGTT CGACAAAGCT TGAAACTATA GTCCTGACGA   
  
  
- TTACTCGACG AATTCGTTCA GTCTGTCGTA AGAAGTGGTA TACCACTATC GGAGGTTTCC GAGCGGGTAG   
  
  
- TTAAACGCTT ACCATAACTC CGTGCGAATC GTCCGTGGCC AAGTTCTCAT GGACGATTAG AGTAACTACG   
  
  
- TGCCTATAGT AGTAGACTTA AAAATTTTCG AATGTTCAGT ATACAAAGTC GTCAAGGAAA GTCTTCCTAC   
  
  
- AGGATAAAAT ATCGTTTGTT GTGTTAAGAC TTCAACCGAC TCTTTCGTTG TTTCTAAGTG TATTAACTAA   
  
  
- AACCATAAGA TAAACCAGAT GTCACCGGGA CAGAATATGT TTCAGAGAGT TCCGCTTGAC ACCGAGGAGG   
  
  
- CTTCGAAGCG TAGTGACCCT ATCTGATAGG GGTCGTACCA AAGGCCGGTC GTCTTTTCCA ACTCCGTTGT   
  
  
- CCAGCAGCCA ACAGGCCCAT GACACTCTCT AAATTACATG GGAAAGCTAT ACTTTGGTAA CGTTTCTTCA   
  
  
- CCCTTTGGTA TGCGGGTCTT CTAGATTTAT AACTCTTACT ACTCGACCAT TAACAATTAA CATACAACGC   
  
  
- CAGACATTTA GATAACCTAC TATGTCACCG CCATTTATCA GGTTCCCTAC GAAAGAACTC CAATTAGTTT   
  
  
- GTCTATTTGG GCGCAAATAA GTAAGTACGT TAACAGTTAC CTTGGAAATC ATGAGGTAAG AAGTCGTGAG   
  
  
- CTAAGTCCCT TCGGGATAAG GTTATGAGAA GACATAAACT ATATAAACTT CGCTGATACT GAGCACTTCT   
  
  
- AGCACCTTCC GACGACTAAC TCTCAGTTTA TACGCCCGAT CTTCAAAACT TACGTTATCG TACACTTCCA   
  
  
- CGTCTCTCCT AAGTTTCCGG ACTTTGTATG TTCGTTACCG TCCTCGCCTG TTGTTCCCGA CCTGATTCCG   
  
  
- TCCAAGGTTA TCTACTCCTC GAACAGTTAT CTCGTTTCTG ATATCACTTT CGTTTAATAG TGTTCCTAAA   
  
  
- ACACCACCTA CTCCTATCCG CAACCTACGA AGTTCCAACC TTTCCTTCCT GTGAATCACG GGAAAGGCAA   
  
  
- ACCGTCGGAT TGAT

+     P-box

| Site Name | Organism | Position | Strand | Matrix score. | sequence | function |
| --- | --- | --- | --- | --- | --- | --- |
| P-box | Oryza sativa | 1973 | + | 7 | CCTTTTG | gibberellin-responsive element |

>HU02G01572.1   
+ +Up\_Stream \_Len000TTTAAT AAGATCCTTT TATATATATG TGTGTATAAT TCTATGGTAC ATAGGTATGT   
  
  
+ ACCATAGACT TATTGCATTT CTAGGTCCTT CTCTCTCTGC GCTTGTTTTT TGTTTATCTA ATTTATGGAG   
  
  
+ TTGAACATTT TGTTTTATAT ACTTGAACAA TCTATTATAC TAAATTAAGC ATTTTGATTT AATCACTTAT   
  
  
+ GGGTGTGTTT GGTAGGGTTG AAAATAATTT TCCTAGAAAA TAATTTTTAA TATAAAATAA TTTTTCATGT   
  
  
+ AAAATGATTT TCAACTCATA TTTTCAGTTG ATTGGTAAAA AGAAAAATGA ACTTTCTGGA TAATCAGTTT   
  
  
+ GATTAACAAA AAGTGACAAA AAAATTAAAA ATGGTTTTCC GTAATCTTCA AACCATATGT CGAGGAAATA   
  
  
+ AACACGGAAT AAAGAAAGAG TGTCAGAACC CAAGAAATGT AAAATCAACT TACGTTCTAT TTAAAGTGAA   
  
  
+ AAATATTTTT CTTCATTCTA GGAAAATTAA TTGTCCTCTG AAAACAAATT TTCCCTCCTT GTCTAACCAA   
  
  
+ ACACAGTAAA ATTGAAAAAT CATTTTCTAG GAAAATGATT TTCACTCCTA CCAAACACAC CCTTAAACTT   
  
  
+ GTTCCAAATC TATAAACCCG GAATCTTAAT ACGCACATTG TATATTATAT GTCCTACTAT AACTGTATTA   
  
  
+ TCACTGGTTC TTGATTTATC CTTTCAAACT CTTGCTTAGT CAAGGTTGTC CTCGTACAAA CATGATTAAT   
  
  
+ TCAATTAACC ACGTAAGTTA TACGCTGACA AATTTCGGGT ACCAAACATT TGCACTAGAC CTAAAACAGT   
  
  
+ TACTATCATC ATATCGATGT TAAAATTGAC CTGTTCTTAT TTGTCCAGCG TTGAGCAAGG AAATAATCAA   
  
  
+ ATCATGTCTC ATTTAGTCGG TTTTTTGCCT AAGAAGAGCA TAAACAAAGG AAAAAGACAA CGACACATTA   
  
  
+ TACATGCATC TACACAAATG AAATGAGGCC ATATGGAACA TGTTCATGAG AGCTTCACTC GCTAATAATT   
  
  
+ GAACCTACTT TTGACTAAGC CACCAACTAG AATATGGAGA TTGACTGTTA AGAGACTATA TTACATGAAA   
  
  
+ TAGATAAGAG TAGACATTGC TATATATTTT ATCTTTAATT GTCTATTTTT AATGATCATT ACTGTTTATT   
  
  
+ TCAGTGAGTA ATTTTCTCGG ATATAATATT TTATAGCGGT TATTAAATAG ATGTGAAACT ATAATGTTAG   
  
  
+ ACATATCATG CGAAAATAGA GAAATTTTGG GCAAATTAAA AAAAAAAGAA GAAAAACGAG GAAGTATGCC   
  
  
+ CATTGTAAGC AGGTGGATGT ATATCCAGCC TCCATCCAAC AATTTGGGCA AAATTATATC ATCCATTCCA   
  
  
+ AAAAAAAGAT ATAATTTGTG ATATTAATAA CCCGACTCAA TTCAAGCTTA TTTATTTTTT CATATAATTT   
  
  
+ TTTAATATAT AAAAATAAAA AGTGACAACG ACATGCATCC TTTGCGTGTC AAAGGATCCG CCGCGTGGCC   
  
  
+ CCCACATTCA CTGACGGGGT TTTTGGTTTG CGATTAAGAT CAATGGCTAA CGTTGTCTCT CTCAAAACAT   
  
  
+ ATGGACCCCT TTTTCTATCT TTTTCTGCAT TGTCAGCATA GCAATTGTTG AGCACATTTT TTTCGATTTG   
  
  
+ TTGACAATAA GCGTGGTTAT GATTTATGGG CCACTAATAT CAGTCTCTGA TCATTTGCAC ATTTTGGACC   
  
  
+ TCTTTCTCTC TCTAATCTTC CAGGTAACCT GAATTCTTCT CTATTTCTGA CTAGATTAGA TAACAGGATT   
  
  
+ TCAAATATCT GTCAGGTGGC GGGTATAACT TCGTGATGGG CATTCATTTT TGTTCTATCA GGCTGGTGGG   
  
  
+ TTTGAGTTTA TTTTGATTCA TTTCGTGTTC ATCTGTGATA TTAGTAGGAG GGATTCTGTA TACCCACCAA   
  
  
+ CTGCTCGCCC TTTTGCCCGT GTGAGCAATT GGGACTTTTG TTTGTTCTTC GTCGATGCAT CCTCTGGTTG   
  
  
+ CTGATCTGGA ATTGAAACCC CATGTATTCA AATTCAACCC TGATTTGCTG TCAAACTTTC TGAACCATCA   
  
  
+ AAACTCCGCC GAAGTGTTTG AAAAAGATGG CATCTTCCAG ACCCTTCATT TGGCTGATCC CAAAAGCTCT   
  
  
+ TCAATTGCCG AAATCGGGTT TACTAATAGT TCAGATTCTA CACAAGTACC TGATTTTTCA GATGCTTGTC   
  
  
+ TTAAGTTCAT TAGTGATATT CTCCTGGAAG AGGACTTAGA TGAAAGTCCT ACGTCTTTAC ACGATTACAT   
  
  
+ GGCTCTCCTA GCCACTGAGA AGTCTTTGTA TGATGCTCTT GGAAAGGAAT ACTTTCCTTC GTCTACTAGT   
  
  
+ CTTGCCCCAT CTTTAGGCCG AAGTGTTGAC AGCCCAGATA GTGGCTTTGG CCGCGGTTGC TCTGATGGTC   
  
  
+ GCGGGATTGA GGGTTTGGCT AATGATGATG CCGTTTTCAT GTCTAACTGG CAGCTCAACA CCACCCAATT   
  
  
+ GGACCCTTTC CCAATCATGC AAGATATTCC TCGTCCCTAT TTGGAATTGA ATTACCATTC TTCTGGGTCA   
  
  
+ AGCAATGGCA TTGATGATTC GGGGGATGGG TTATCGACCT CTCCTGTAAG TACACTTGCA TCAACCGCCA   
  
  
+ CAGAGGCAGG GAAAAAGTTG GCTGGTAGCT CTAGGAGAAA GAACCGTCAA AGGGATGACT ATGGCCATGA   
  
  
+ AGAGGGAAGG AGTAACAAGC AGCAAGCCTC TTACAATGAC GATTACGTTG AGATGGAGCA GTATGACGAT   
  
  
+ GTACTTCTCT GTAGGGCAGA CAAGGGTAAT ATTTCAACTT GTGCCAATGA ATCCTCGCTT AATGAGGTGC   
  
  
+ GTGAGAAGCT GCAGACGACA GGGTTCAAGG GAAGAACATC CCGTCTTAAG AAGCAATCTA AGGAAGCGAA   
  
  
+ AGAGGTGGAT CTGAGAACTC TTCTTTCTGG TTGTGCACAA GCTGTTTCGA ACTTTGATAT CAGGACTGCT   
  
  
+ AATGAGCTGC TTAAGCAAGT CAGACAGCAT TCTTCACCAT ATGGTGATAG CCTCCAAAGG CTCGCCCATC   
  
  
+ AATTTGCGAA TGGTATTGAG GCACGCTTAG CAGGCACCGG TTCAAGAGTA CCTGCTAATC TCATTGATGC   
  
  
+ ACGGATATCA TCATCTGAAT TTTTAAAAGC TTACAAGTCA TATGTTTCAG CAGTTCCTTT CAGAAGGATG   
  
  
+ TCCTATTTTA TAGCAAACAA CACAATTCTG AAGTTGGCTG AGAAAGCAAC AAAGATTCAC ATAATTGATT   
  
  
+ TTGGTATTCT ATTTGGTCTA CAGTGGCCCT GTCTTATACA AAGTCTCTCA AGGCGAACTG TGGCTCCTCC   
  
  
+ GAAGCTTCGC ATCACTGGGA TAGACTATCC CCAGCATGGT TTCCGGCCAG CAGAAAAGGT TGAGGCAACA   
  
  
+ GGTCGTCGGT TGTCCGGGTA CTGTGAGAGA TTTAATGTAC CCTTTCGATA TGAAACCATT GCAAAGAAGT   
  
  
+ GGGAAACCAT ACGCCCAGAA GATCTAAATA TTGAGAATGA TGAGCTGGTA ATTGTTAATT GTATGTTGCG   
  
  
+ GTCTGTAAAT CTATTGGATG ATACAGTGGC GGTAAATAGT CCAAGGGATG CTTTCTTGAG GTTAATCAAA   
  
  
+ CAGATAAACC CGCGTTTATT CATTCATGCA ATTGTCAATG GAACCTTTAG TACTCCATTC TTCAGCACTC   
  
  
+ GATTCAGGGA AGCCCTATTC CAATACTCTT CTGTATTTGA TATATTTGAA GCGACTATGA CTCGTGAAGA   
  
  
+ TCGTGGAAGG CTGCTGATTG AGAGTCAAAT ATGCGGGCTA GAAGTTTTGA ATGCAATAGC ATGTGAAGGT   
  
  
+ GCAGAGAGGA TTCAAAGGCC TGAAACATAC AAGCAATGGC AGGAGCGGAC AACAAGGGCT GGACTAAGGC   
  
  
+ AGGTTCCAAT AGATGAGGAG CTTGTCAATA GAGCAAAGAC TATAGTGAAA GCAAATTATC ACAAGGATTT   
  
  
+ TGTGGTGGAT GAGGATAGGC GTTGGATGCT TCAAGGTTGG AAAGGAAGGA CACTTAGTGC CCTTTCCGTT   
  
  
+ TGGCAGCCTA ACTA  

- +Up\_Stream \_Len000AAATTA TTCTAGGAAA ATATATATAC ACACATATTA AGATACCATG TATCCATACA   
  
  
- TGGTATCTGA ATAACGTAAA GATCCAGGAA GAGAGAGACG CGAACAAAAA ACAAATAGAT TAAATACCTC   
  
  
- AACTTGTAAA ACAAAATATA TGAACTTGTT AGATAATATG ATTTAATTCG TAAAACTAAA TTAGTGAATA   
  
  
- CCCACACAAA CCATCCCAAC TTTTATTAAA AGGATCTTTT ATTAAAAATT ATATTTTATT AAAAAGTACA   
  
  
- TTTTACTAAA AGTTGAGTAT AAAAGTCAAC TAACCATTTT TCTTTTTACT TGAAAGACCT ATTAGTCAAA   
  
  
- CTAATTGTTT TTCACTGTTT TTTTAATTTT TACCAAAAGG CATTAGAAGT TTGGTATACA GCTCCTTTAT   
  
  
- TTGTGCCTTA TTTCTTTCTC ACAGTCTTGG GTTCTTTACA TTTTAGTTGA ATGCAAGATA AATTTCACTT   
  
  
- TTTATAAAAA GAAGTAAGAT CCTTTTAATT AACAGGAGAC TTTTGTTTAA AAGGGAGGAA CAGATTGGTT   
  
  
- TGTGTCATTT TAACTTTTTA GTAAAAGATC CTTTTACTAA AAGTGAGGAT GGTTTGTGTG GGAATTTGAA   
  
  
- CAAGGTTTAG ATATTTGGGC CTTAGAATTA TGCGTGTAAC ATATAATATA CAGGATGATA TTGACATAAT   
  
  
- AGTGACCAAG AACTAAATAG GAAAGTTTGA GAACGAATCA GTTCCAACAG GAGCATGTTT GTACTAATTA   
  
  
- AGTTAATTGG TGCATTCAAT ATGCGACTGT TTAAAGCCCA TGGTTTGTAA ACGTGATCTG GATTTTGTCA   
  
  
- ATGATAGTAG TATAGCTACA ATTTTAACTG GACAAGAATA AACAGGTCGC AACTCGTTCC TTTATTAGTT   
  
  
- TAGTACAGAG TAAATCAGCC AAAAAACGGA TTCTTCTCGT ATTTGTTTCC TTTTTCTGTT GCTGTGTAAT   
  
  
- ATGTACGTAG ATGTGTTTAC TTTACTCCGG TATACCTTGT ACAAGTACTC TCGAAGTGAG CGATTATTAA   
  
  
- CTTGGATGAA AACTGATTCG GTGGTTGATC TTATACCTCT AACTGACAAT TCTCTGATAT AATGTACTTT   
  
  
- ATCTATTCTC ATCTGTAACG ATATATAAAA TAGAAATTAA CAGATAAAAA TTACTAGTAA TGACAAATAA   
  
  
- AGTCACTCAT TAAAAGAGCC TATATTATAA AATATCGCCA ATAATTTATC TACACTTTGA TATTACAATC   
  
  
- TGTATAGTAC GCTTTTATCT CTTTAAAACC CGTTTAATTT TTTTTTTCTT CTTTTTGCTC CTTCATACGG   
  
  
- GTAACATTCG TCCACCTACA TATAGGTCGG AGGTAGGTTG TTAAACCCGT TTTAATATAG TAGGTAAGGT   
  
  
- TTTTTTTCTA TATTAAACAC TATAATTATT GGGCTGAGTT AAGTTCGAAT AAATAAAAAA GTATATTAAA   
  
  
- AAATTATATA TTTTTATTTT TCACTGTTGC TGTACGTAGG AAACGCACAG TTTCCTAGGC GGCGCACCGG   
  
  
- GGGTGTAAGT GACTGCCCCA AAAACCAAAC GCTAATTCTA GTTACCGATT GCAACAGAGA GAGTTTTGTA   
  
  
- TACCTGGGGA AAAAGATAGA AAAAGACGTA ACAGTCGTAT CGTTAACAAC TCGTGTAAAA AAAGCTAAAC   
  
  
- AACTGTTATT CGCACCAATA CTAAATACCC GGTGATTATA GTCAGAGACT AGTAAACGTG TAAAACCTGG   
  
  
- AGAAAGAGAG AGATTAGAAG GTCCATTGGA CTTAAGAAGA GATAAAGACT GATCTAATCT ATTGTCCTAA   
  
  
- AGTTTATAGA CAGTCCACCG CCCATATTGA AGCACTACCC GTAAGTAAAA ACAAGATAGT CCGACCACCC   
  
  
- AAACTCAAAT AAAACTAAGT AAAGCACAAG TAGACACTAT AATCATCCTC CCTAAGACAT ATGGGTGGTT   
  
  
- GACGAGCGGG AAAACGGGCA CACTCGTTAA CCCTGAAAAC AAACAAGAAG CAGCTACGTA GGAGACCAAC   
  
  
- GACTAGACCT TAACTTTGGG GTACATAAGT TTAAGTTGGG ACTAAACGAC AGTTTGAAAG ACTTGGTAGT   
  
  
- TTTGAGGCGG CTTCACAAAC TTTTTCTACC GTAGAAGGTC TGGGAAGTAA ACCGACTAGG GTTTTCGAGA   
  
  
- AGTTAACGGC TTTAGCCCAA ATGATTATCA AGTCTAAGAT GTGTTCATGG ACTAAAAAGT CTACGAACAG   
  
  
- AATTCAAGTA ATCACTATAA GAGGACCTTC TCCTGAATCT ACTTTCAGGA TGCAGAAATG TGCTAATGTA   
  
  
- CCGAGAGGAT CGGTGACTCT TCAGAAACAT ACTACGAGAA CCTTTCCTTA TGAAAGGAAG CAGATGATCA   
  
  
- GAACGGGGTA GAAATCCGGC TTCACAACTG TCGGGTCTAT CACCGAAACC GGCGCCAACG AGACTACCAG   
  
  
- CGCCCTAACT CCCAAACCGA TTACTACTAC GGCAAAAGTA CAGATTGACC GTCGAGTTGT GGTGGGTTAA   
  
  
- CCTGGGAAAG GGTTAGTACG TTCTATAAGG AGCAGGGATA AACCTTAACT TAATGGTAAG AAGACCCAGT   
  
  
- TCGTTACCGT AACTACTAAG CCCCCTACCC AATAGCTGGA GAGGACATTC ATGTGAACGT AGTTGGCGGT   
  
  
- GTCTCCGTCC CTTTTTCAAC CGACCATCGA GATCCTCTTT CTTGGCAGTT TCCCTACTGA TACCGGTACT   
  
  
- TCTCCCTTCC TCATTGTTCG TCGTTCGGAG AATGTTACTG CTAATGCAAC TCTACCTCGT CATACTGCTA   
  
  
- CATGAAGAGA CATCCCGTCT GTTCCCATTA TAAAGTTGAA CACGGTTACT TAGGAGCGAA TTACTCCACG   
  
  
- CACTCTTCGA CGTCTGCTGT CCCAAGTTCC CTTCTTGTAG GGCAGAATTC TTCGTTAGAT TCCTTCGCTT   
  
  
- TCTCCACCTA GACTCTTGAG AAGAAAGACC AACACGTGTT CGACAAAGCT TGAAACTATA GTCCTGACGA   
  
  
- TTACTCGACG AATTCGTTCA GTCTGTCGTA AGAAGTGGTA TACCACTATC GGAGGTTTCC GAGCGGGTAG   
  
  
- TTAAACGCTT ACCATAACTC CGTGCGAATC GTCCGTGGCC AAGTTCTCAT GGACGATTAG AGTAACTACG   
  
  
- TGCCTATAGT AGTAGACTTA AAAATTTTCG AATGTTCAGT ATACAAAGTC GTCAAGGAAA GTCTTCCTAC   
  
  
- AGGATAAAAT ATCGTTTGTT GTGTTAAGAC TTCAACCGAC TCTTTCGTTG TTTCTAAGTG TATTAACTAA   
  
  
- AACCATAAGA TAAACCAGAT GTCACCGGGA CAGAATATGT TTCAGAGAGT TCCGCTTGAC ACCGAGGAGG   
  
  
- CTTCGAAGCG TAGTGACCCT ATCTGATAGG GGTCGTACCA AAGGCCGGTC GTCTTTTCCA ACTCCGTTGT   
  
  
- CCAGCAGCCA ACAGGCCCAT GACACTCTCT AAATTACATG GGAAAGCTAT ACTTTGGTAA CGTTTCTTCA   
  
  
- CCCTTTGGTA TGCGGGTCTT CTAGATTTAT AACTCTTACT ACTCGACCAT TAACAATTAA CATACAACGC   
  
  
- CAGACATTTA GATAACCTAC TATGTCACCG CCATTTATCA GGTTCCCTAC GAAAGAACTC CAATTAGTTT   
  
  
- GTCTATTTGG GCGCAAATAA GTAAGTACGT TAACAGTTAC CTTGGAAATC ATGAGGTAAG AAGTCGTGAG   
  
  
- CTAAGTCCCT TCGGGATAAG GTTATGAGAA GACATAAACT ATATAAACTT CGCTGATACT GAGCACTTCT   
  
  
- AGCACCTTCC GACGACTAAC TCTCAGTTTA TACGCCCGAT CTTCAAAACT TACGTTATCG TACACTTCCA   
  
  
- CGTCTCTCCT AAGTTTCCGG ACTTTGTATG TTCGTTACCG TCCTCGCCTG TTGTTCCCGA CCTGATTCCG   
  
  
- TCCAAGGTTA TCTACTCCTC GAACAGTTAT CTCGTTTCTG ATATCACTTT CGTTTAATAG TGTTCCTAAA   
  
  
- ACACCACCTA CTCCTATCCG CAACCTACGA AGTTCCAACC TTTCCTTCCT GTGAATCACG GGAAAGGCAA   
  
  
- ACCGTCGGAT TGAT

+     STRE

| Site Name | Organism | Position | Strand | Matrix score. | sequence | function |
| --- | --- | --- | --- | --- | --- | --- |
| STRE | Arabidopsis thaliana | 1620 | - | 5 | AGGGG |  |

>HU02G01572.1   
+ +Up\_Stream \_Len000TTTAAT AAGATCCTTT TATATATATG TGTGTATAAT TCTATGGTAC ATAGGTATGT   
  
  
+ ACCATAGACT TATTGCATTT CTAGGTCCTT CTCTCTCTGC GCTTGTTTTT TGTTTATCTA ATTTATGGAG   
  
  
+ TTGAACATTT TGTTTTATAT ACTTGAACAA TCTATTATAC TAAATTAAGC ATTTTGATTT AATCACTTAT   
  
  
+ GGGTGTGTTT GGTAGGGTTG AAAATAATTT TCCTAGAAAA TAATTTTTAA TATAAAATAA TTTTTCATGT   
  
  
+ AAAATGATTT TCAACTCATA TTTTCAGTTG ATTGGTAAAA AGAAAAATGA ACTTTCTGGA TAATCAGTTT   
  
  
+ GATTAACAAA AAGTGACAAA AAAATTAAAA ATGGTTTTCC GTAATCTTCA AACCATATGT CGAGGAAATA   
  
  
+ AACACGGAAT AAAGAAAGAG TGTCAGAACC CAAGAAATGT AAAATCAACT TACGTTCTAT TTAAAGTGAA   
  
  
+ AAATATTTTT CTTCATTCTA GGAAAATTAA TTGTCCTCTG AAAACAAATT TTCCCTCCTT GTCTAACCAA   
  
  
+ ACACAGTAAA ATTGAAAAAT CATTTTCTAG GAAAATGATT TTCACTCCTA CCAAACACAC CCTTAAACTT   
  
  
+ GTTCCAAATC TATAAACCCG GAATCTTAAT ACGCACATTG TATATTATAT GTCCTACTAT AACTGTATTA   
  
  
+ TCACTGGTTC TTGATTTATC CTTTCAAACT CTTGCTTAGT CAAGGTTGTC CTCGTACAAA CATGATTAAT   
  
  
+ TCAATTAACC ACGTAAGTTA TACGCTGACA AATTTCGGGT ACCAAACATT TGCACTAGAC CTAAAACAGT   
  
  
+ TACTATCATC ATATCGATGT TAAAATTGAC CTGTTCTTAT TTGTCCAGCG TTGAGCAAGG AAATAATCAA   
  
  
+ ATCATGTCTC ATTTAGTCGG TTTTTTGCCT AAGAAGAGCA TAAACAAAGG AAAAAGACAA CGACACATTA   
  
  
+ TACATGCATC TACACAAATG AAATGAGGCC ATATGGAACA TGTTCATGAG AGCTTCACTC GCTAATAATT   
  
  
+ GAACCTACTT TTGACTAAGC CACCAACTAG AATATGGAGA TTGACTGTTA AGAGACTATA TTACATGAAA   
  
  
+ TAGATAAGAG TAGACATTGC TATATATTTT ATCTTTAATT GTCTATTTTT AATGATCATT ACTGTTTATT   
  
  
+ TCAGTGAGTA ATTTTCTCGG ATATAATATT TTATAGCGGT TATTAAATAG ATGTGAAACT ATAATGTTAG   
  
  
+ ACATATCATG CGAAAATAGA GAAATTTTGG GCAAATTAAA AAAAAAAGAA GAAAAACGAG GAAGTATGCC   
  
  
+ CATTGTAAGC AGGTGGATGT ATATCCAGCC TCCATCCAAC AATTTGGGCA AAATTATATC ATCCATTCCA   
  
  
+ AAAAAAAGAT ATAATTTGTG ATATTAATAA CCCGACTCAA TTCAAGCTTA TTTATTTTTT CATATAATTT   
  
  
+ TTTAATATAT AAAAATAAAA AGTGACAACG ACATGCATCC TTTGCGTGTC AAAGGATCCG CCGCGTGGCC   
  
  
+ CCCACATTCA CTGACGGGGT TTTTGGTTTG CGATTAAGAT CAATGGCTAA CGTTGTCTCT CTCAAAACAT   
  
  
+ ATGGACCCCT TTTTCTATCT TTTTCTGCAT TGTCAGCATA GCAATTGTTG AGCACATTTT TTTCGATTTG   
  
  
+ TTGACAATAA GCGTGGTTAT GATTTATGGG CCACTAATAT CAGTCTCTGA TCATTTGCAC ATTTTGGACC   
  
  
+ TCTTTCTCTC TCTAATCTTC CAGGTAACCT GAATTCTTCT CTATTTCTGA CTAGATTAGA TAACAGGATT   
  
  
+ TCAAATATCT GTCAGGTGGC GGGTATAACT TCGTGATGGG CATTCATTTT TGTTCTATCA GGCTGGTGGG   
  
  
+ TTTGAGTTTA TTTTGATTCA TTTCGTGTTC ATCTGTGATA TTAGTAGGAG GGATTCTGTA TACCCACCAA   
  
  
+ CTGCTCGCCC TTTTGCCCGT GTGAGCAATT GGGACTTTTG TTTGTTCTTC GTCGATGCAT CCTCTGGTTG   
  
  
+ CTGATCTGGA ATTGAAACCC CATGTATTCA AATTCAACCC TGATTTGCTG TCAAACTTTC TGAACCATCA   
  
  
+ AAACTCCGCC GAAGTGTTTG AAAAAGATGG CATCTTCCAG ACCCTTCATT TGGCTGATCC CAAAAGCTCT   
  
  
+ TCAATTGCCG AAATCGGGTT TACTAATAGT TCAGATTCTA CACAAGTACC TGATTTTTCA GATGCTTGTC   
  
  
+ TTAAGTTCAT TAGTGATATT CTCCTGGAAG AGGACTTAGA TGAAAGTCCT ACGTCTTTAC ACGATTACAT   
  
  
+ GGCTCTCCTA GCCACTGAGA AGTCTTTGTA TGATGCTCTT GGAAAGGAAT ACTTTCCTTC GTCTACTAGT   
  
  
+ CTTGCCCCAT CTTTAGGCCG AAGTGTTGAC AGCCCAGATA GTGGCTTTGG CCGCGGTTGC TCTGATGGTC   
  
  
+ GCGGGATTGA GGGTTTGGCT AATGATGATG CCGTTTTCAT GTCTAACTGG CAGCTCAACA CCACCCAATT   
  
  
+ GGACCCTTTC CCAATCATGC AAGATATTCC TCGTCCCTAT TTGGAATTGA ATTACCATTC TTCTGGGTCA   
  
  
+ AGCAATGGCA TTGATGATTC GGGGGATGGG TTATCGACCT CTCCTGTAAG TACACTTGCA TCAACCGCCA   
  
  
+ CAGAGGCAGG GAAAAAGTTG GCTGGTAGCT CTAGGAGAAA GAACCGTCAA AGGGATGACT ATGGCCATGA   
  
  
+ AGAGGGAAGG AGTAACAAGC AGCAAGCCTC TTACAATGAC GATTACGTTG AGATGGAGCA GTATGACGAT   
  
  
+ GTACTTCTCT GTAGGGCAGA CAAGGGTAAT ATTTCAACTT GTGCCAATGA ATCCTCGCTT AATGAGGTGC   
  
  
+ GTGAGAAGCT GCAGACGACA GGGTTCAAGG GAAGAACATC CCGTCTTAAG AAGCAATCTA AGGAAGCGAA   
  
  
+ AGAGGTGGAT CTGAGAACTC TTCTTTCTGG TTGTGCACAA GCTGTTTCGA ACTTTGATAT CAGGACTGCT   
  
  
+ AATGAGCTGC TTAAGCAAGT CAGACAGCAT TCTTCACCAT ATGGTGATAG CCTCCAAAGG CTCGCCCATC   
  
  
+ AATTTGCGAA TGGTATTGAG GCACGCTTAG CAGGCACCGG TTCAAGAGTA CCTGCTAATC TCATTGATGC   
  
  
+ ACGGATATCA TCATCTGAAT TTTTAAAAGC TTACAAGTCA TATGTTTCAG CAGTTCCTTT CAGAAGGATG   
  
  
+ TCCTATTTTA TAGCAAACAA CACAATTCTG AAGTTGGCTG AGAAAGCAAC AAAGATTCAC ATAATTGATT   
  
  
+ TTGGTATTCT ATTTGGTCTA CAGTGGCCCT GTCTTATACA AAGTCTCTCA AGGCGAACTG TGGCTCCTCC   
  
  
+ GAAGCTTCGC ATCACTGGGA TAGACTATCC CCAGCATGGT TTCCGGCCAG CAGAAAAGGT TGAGGCAACA   
  
  
+ GGTCGTCGGT TGTCCGGGTA CTGTGAGAGA TTTAATGTAC CCTTTCGATA TGAAACCATT GCAAAGAAGT   
  
  
+ GGGAAACCAT ACGCCCAGAA GATCTAAATA TTGAGAATGA TGAGCTGGTA ATTGTTAATT GTATGTTGCG   
  
  
+ GTCTGTAAAT CTATTGGATG ATACAGTGGC GGTAAATAGT CCAAGGGATG CTTTCTTGAG GTTAATCAAA   
  
  
+ CAGATAAACC CGCGTTTATT CATTCATGCA ATTGTCAATG GAACCTTTAG TACTCCATTC TTCAGCACTC   
  
  
+ GATTCAGGGA AGCCCTATTC CAATACTCTT CTGTATTTGA TATATTTGAA GCGACTATGA CTCGTGAAGA   
  
  
+ TCGTGGAAGG CTGCTGATTG AGAGTCAAAT ATGCGGGCTA GAAGTTTTGA ATGCAATAGC ATGTGAAGGT   
  
  
+ GCAGAGAGGA TTCAAAGGCC TGAAACATAC AAGCAATGGC AGGAGCGGAC AACAAGGGCT GGACTAAGGC   
  
  
+ AGGTTCCAAT AGATGAGGAG CTTGTCAATA GAGCAAAGAC TATAGTGAAA GCAAATTATC ACAAGGATTT   
  
  
+ TGTGGTGGAT GAGGATAGGC GTTGGATGCT TCAAGGTTGG AAAGGAAGGA CACTTAGTGC CCTTTCCGTT   
  
  
+ TGGCAGCCTA ACTA  

- +Up\_Stream \_Len000AAATTA TTCTAGGAAA ATATATATAC ACACATATTA AGATACCATG TATCCATACA   
  
  
- TGGTATCTGA ATAACGTAAA GATCCAGGAA GAGAGAGACG CGAACAAAAA ACAAATAGAT TAAATACCTC   
  
  
- AACTTGTAAA ACAAAATATA TGAACTTGTT AGATAATATG ATTTAATTCG TAAAACTAAA TTAGTGAATA   
  
  
- CCCACACAAA CCATCCCAAC TTTTATTAAA AGGATCTTTT ATTAAAAATT ATATTTTATT AAAAAGTACA   
  
  
- TTTTACTAAA AGTTGAGTAT AAAAGTCAAC TAACCATTTT TCTTTTTACT TGAAAGACCT ATTAGTCAAA   
  
  
- CTAATTGTTT TTCACTGTTT TTTTAATTTT TACCAAAAGG CATTAGAAGT TTGGTATACA GCTCCTTTAT   
  
  
- TTGTGCCTTA TTTCTTTCTC ACAGTCTTGG GTTCTTTACA TTTTAGTTGA ATGCAAGATA AATTTCACTT   
  
  
- TTTATAAAAA GAAGTAAGAT CCTTTTAATT AACAGGAGAC TTTTGTTTAA AAGGGAGGAA CAGATTGGTT   
  
  
- TGTGTCATTT TAACTTTTTA GTAAAAGATC CTTTTACTAA AAGTGAGGAT GGTTTGTGTG GGAATTTGAA   
  
  
- CAAGGTTTAG ATATTTGGGC CTTAGAATTA TGCGTGTAAC ATATAATATA CAGGATGATA TTGACATAAT   
  
  
- AGTGACCAAG AACTAAATAG GAAAGTTTGA GAACGAATCA GTTCCAACAG GAGCATGTTT GTACTAATTA   
  
  
- AGTTAATTGG TGCATTCAAT ATGCGACTGT TTAAAGCCCA TGGTTTGTAA ACGTGATCTG GATTTTGTCA   
  
  
- ATGATAGTAG TATAGCTACA ATTTTAACTG GACAAGAATA AACAGGTCGC AACTCGTTCC TTTATTAGTT   
  
  
- TAGTACAGAG TAAATCAGCC AAAAAACGGA TTCTTCTCGT ATTTGTTTCC TTTTTCTGTT GCTGTGTAAT   
  
  
- ATGTACGTAG ATGTGTTTAC TTTACTCCGG TATACCTTGT ACAAGTACTC TCGAAGTGAG CGATTATTAA   
  
  
- CTTGGATGAA AACTGATTCG GTGGTTGATC TTATACCTCT AACTGACAAT TCTCTGATAT AATGTACTTT   
  
  
- ATCTATTCTC ATCTGTAACG ATATATAAAA TAGAAATTAA CAGATAAAAA TTACTAGTAA TGACAAATAA   
  
  
- AGTCACTCAT TAAAAGAGCC TATATTATAA AATATCGCCA ATAATTTATC TACACTTTGA TATTACAATC   
  
  
- TGTATAGTAC GCTTTTATCT CTTTAAAACC CGTTTAATTT TTTTTTTCTT CTTTTTGCTC CTTCATACGG   
  
  
- GTAACATTCG TCCACCTACA TATAGGTCGG AGGTAGGTTG TTAAACCCGT TTTAATATAG TAGGTAAGGT   
  
  
- TTTTTTTCTA TATTAAACAC TATAATTATT GGGCTGAGTT AAGTTCGAAT AAATAAAAAA GTATATTAAA   
  
  
- AAATTATATA TTTTTATTTT TCACTGTTGC TGTACGTAGG AAACGCACAG TTTCCTAGGC GGCGCACCGG   
  
  
- GGGTGTAAGT GACTGCCCCA AAAACCAAAC GCTAATTCTA GTTACCGATT GCAACAGAGA GAGTTTTGTA   
  
  
- TACCTGGGGA AAAAGATAGA AAAAGACGTA ACAGTCGTAT CGTTAACAAC TCGTGTAAAA AAAGCTAAAC   
  
  
- AACTGTTATT CGCACCAATA CTAAATACCC GGTGATTATA GTCAGAGACT AGTAAACGTG TAAAACCTGG   
  
  
- AGAAAGAGAG AGATTAGAAG GTCCATTGGA CTTAAGAAGA GATAAAGACT GATCTAATCT ATTGTCCTAA   
  
  
- AGTTTATAGA CAGTCCACCG CCCATATTGA AGCACTACCC GTAAGTAAAA ACAAGATAGT CCGACCACCC   
  
  
- AAACTCAAAT AAAACTAAGT AAAGCACAAG TAGACACTAT AATCATCCTC CCTAAGACAT ATGGGTGGTT   
  
  
- GACGAGCGGG AAAACGGGCA CACTCGTTAA CCCTGAAAAC AAACAAGAAG CAGCTACGTA GGAGACCAAC   
  
  
- GACTAGACCT TAACTTTGGG GTACATAAGT TTAAGTTGGG ACTAAACGAC AGTTTGAAAG ACTTGGTAGT   
  
  
- TTTGAGGCGG CTTCACAAAC TTTTTCTACC GTAGAAGGTC TGGGAAGTAA ACCGACTAGG GTTTTCGAGA   
  
  
- AGTTAACGGC TTTAGCCCAA ATGATTATCA AGTCTAAGAT GTGTTCATGG ACTAAAAAGT CTACGAACAG   
  
  
- AATTCAAGTA ATCACTATAA GAGGACCTTC TCCTGAATCT ACTTTCAGGA TGCAGAAATG TGCTAATGTA   
  
  
- CCGAGAGGAT CGGTGACTCT TCAGAAACAT ACTACGAGAA CCTTTCCTTA TGAAAGGAAG CAGATGATCA   
  
  
- GAACGGGGTA GAAATCCGGC TTCACAACTG TCGGGTCTAT CACCGAAACC GGCGCCAACG AGACTACCAG   
  
  
- CGCCCTAACT CCCAAACCGA TTACTACTAC GGCAAAAGTA CAGATTGACC GTCGAGTTGT GGTGGGTTAA   
  
  
- CCTGGGAAAG GGTTAGTACG TTCTATAAGG AGCAGGGATA AACCTTAACT TAATGGTAAG AAGACCCAGT   
  
  
- TCGTTACCGT AACTACTAAG CCCCCTACCC AATAGCTGGA GAGGACATTC ATGTGAACGT AGTTGGCGGT   
  
  
- GTCTCCGTCC CTTTTTCAAC CGACCATCGA GATCCTCTTT CTTGGCAGTT TCCCTACTGA TACCGGTACT   
  
  
- TCTCCCTTCC TCATTGTTCG TCGTTCGGAG AATGTTACTG CTAATGCAAC TCTACCTCGT CATACTGCTA   
  
  
- CATGAAGAGA CATCCCGTCT GTTCCCATTA TAAAGTTGAA CACGGTTACT TAGGAGCGAA TTACTCCACG   
  
  
- CACTCTTCGA CGTCTGCTGT CCCAAGTTCC CTTCTTGTAG GGCAGAATTC TTCGTTAGAT TCCTTCGCTT   
  
  
- TCTCCACCTA GACTCTTGAG AAGAAAGACC AACACGTGTT CGACAAAGCT TGAAACTATA GTCCTGACGA   
  
  
- TTACTCGACG AATTCGTTCA GTCTGTCGTA AGAAGTGGTA TACCACTATC GGAGGTTTCC GAGCGGGTAG   
  
  
- TTAAACGCTT ACCATAACTC CGTGCGAATC GTCCGTGGCC AAGTTCTCAT GGACGATTAG AGTAACTACG   
  
  
- TGCCTATAGT AGTAGACTTA AAAATTTTCG AATGTTCAGT ATACAAAGTC GTCAAGGAAA GTCTTCCTAC   
  
  
- AGGATAAAAT ATCGTTTGTT GTGTTAAGAC TTCAACCGAC TCTTTCGTTG TTTCTAAGTG TATTAACTAA   
  
  
- AACCATAAGA TAAACCAGAT GTCACCGGGA CAGAATATGT TTCAGAGAGT TCCGCTTGAC ACCGAGGAGG   
  
  
- CTTCGAAGCG TAGTGACCCT ATCTGATAGG GGTCGTACCA AAGGCCGGTC GTCTTTTCCA ACTCCGTTGT   
  
  
- CCAGCAGCCA ACAGGCCCAT GACACTCTCT AAATTACATG GGAAAGCTAT ACTTTGGTAA CGTTTCTTCA   
  
  
- CCCTTTGGTA TGCGGGTCTT CTAGATTTAT AACTCTTACT ACTCGACCAT TAACAATTAA CATACAACGC   
  
  
- CAGACATTTA GATAACCTAC TATGTCACCG CCATTTATCA GGTTCCCTAC GAAAGAACTC CAATTAGTTT   
  
  
- GTCTATTTGG GCGCAAATAA GTAAGTACGT TAACAGTTAC CTTGGAAATC ATGAGGTAAG AAGTCGTGAG   
  
  
- CTAAGTCCCT TCGGGATAAG GTTATGAGAA GACATAAACT ATATAAACTT CGCTGATACT GAGCACTTCT   
  
  
- AGCACCTTCC GACGACTAAC TCTCAGTTTA TACGCCCGAT CTTCAAAACT TACGTTATCG TACACTTCCA   
  
  
- CGTCTCTCCT AAGTTTCCGG ACTTTGTATG TTCGTTACCG TCCTCGCCTG TTGTTCCCGA CCTGATTCCG   
  
  
- TCCAAGGTTA TCTACTCCTC GAACAGTTAT CTCGTTTCTG ATATCACTTT CGTTTAATAG TGTTCCTAAA   
  
  
- ACACCACCTA CTCCTATCCG CAACCTACGA AGTTCCAACC TTTCCTTCCT GTGAATCACG GGAAAGGCAA   
  
  
- ACCGTCGGAT TGAT

+     TATA

| Site Name | Organism | Position | Strand | Matrix score. | sequence | function |
| --- | --- | --- | --- | --- | --- | --- |
| TATA | Arabidopsis thaliana | 3229 | - | 8 | TATAAAAT |  |
| TATA | Arabidopsis thaliana | 1222 | - | 8 | TATAAAAT |  |
| TATA | Arabidopsis thaliana | 265 | + | 8 | TATAAAAT |  |

>HU02G01572.1   
+ +Up\_Stream \_Len000TTTAAT AAGATCCTTT TATATATATG TGTGTATAAT TCTATGGTAC ATAGGTATGT   
  
  
+ ACCATAGACT TATTGCATTT CTAGGTCCTT CTCTCTCTGC GCTTGTTTTT TGTTTATCTA ATTTATGGAG   
  
  
+ TTGAACATTT TGTTTTATAT ACTTGAACAA TCTATTATAC TAAATTAAGC ATTTTGATTT AATCACTTAT   
  
  
+ GGGTGTGTTT GGTAGGGTTG AAAATAATTT TCCTAGAAAA TAATTTTTAA TATAAAATAA TTTTTCATGT   
  
  
+ AAAATGATTT TCAACTCATA TTTTCAGTTG ATTGGTAAAA AGAAAAATGA ACTTTCTGGA TAATCAGTTT   
  
  
+ GATTAACAAA AAGTGACAAA AAAATTAAAA ATGGTTTTCC GTAATCTTCA AACCATATGT CGAGGAAATA   
  
  
+ AACACGGAAT AAAGAAAGAG TGTCAGAACC CAAGAAATGT AAAATCAACT TACGTTCTAT TTAAAGTGAA   
  
  
+ AAATATTTTT CTTCATTCTA GGAAAATTAA TTGTCCTCTG AAAACAAATT TTCCCTCCTT GTCTAACCAA   
  
  
+ ACACAGTAAA ATTGAAAAAT CATTTTCTAG GAAAATGATT TTCACTCCTA CCAAACACAC CCTTAAACTT   
  
  
+ GTTCCAAATC TATAAACCCG GAATCTTAAT ACGCACATTG TATATTATAT GTCCTACTAT AACTGTATTA   
  
  
+ TCACTGGTTC TTGATTTATC CTTTCAAACT CTTGCTTAGT CAAGGTTGTC CTCGTACAAA CATGATTAAT   
  
  
+ TCAATTAACC ACGTAAGTTA TACGCTGACA AATTTCGGGT ACCAAACATT TGCACTAGAC CTAAAACAGT   
  
  
+ TACTATCATC ATATCGATGT TAAAATTGAC CTGTTCTTAT TTGTCCAGCG TTGAGCAAGG AAATAATCAA   
  
  
+ ATCATGTCTC ATTTAGTCGG TTTTTTGCCT AAGAAGAGCA TAAACAAAGG AAAAAGACAA CGACACATTA   
  
  
+ TACATGCATC TACACAAATG AAATGAGGCC ATATGGAACA TGTTCATGAG AGCTTCACTC GCTAATAATT   
  
  
+ GAACCTACTT TTGACTAAGC CACCAACTAG AATATGGAGA TTGACTGTTA AGAGACTATA TTACATGAAA   
  
  
+ TAGATAAGAG TAGACATTGC TATATATTTT ATCTTTAATT GTCTATTTTT AATGATCATT ACTGTTTATT   
  
  
+ TCAGTGAGTA ATTTTCTCGG ATATAATATT TTATAGCGGT TATTAAATAG ATGTGAAACT ATAATGTTAG   
  
  
+ ACATATCATG CGAAAATAGA GAAATTTTGG GCAAATTAAA AAAAAAAGAA GAAAAACGAG GAAGTATGCC   
  
  
+ CATTGTAAGC AGGTGGATGT ATATCCAGCC TCCATCCAAC AATTTGGGCA AAATTATATC ATCCATTCCA   
  
  
+ AAAAAAAGAT ATAATTTGTG ATATTAATAA CCCGACTCAA TTCAAGCTTA TTTATTTTTT CATATAATTT   
  
  
+ TTTAATATAT AAAAATAAAA AGTGACAACG ACATGCATCC TTTGCGTGTC AAAGGATCCG CCGCGTGGCC   
  
  
+ CCCACATTCA CTGACGGGGT TTTTGGTTTG CGATTAAGAT CAATGGCTAA CGTTGTCTCT CTCAAAACAT   
  
  
+ ATGGACCCCT TTTTCTATCT TTTTCTGCAT TGTCAGCATA GCAATTGTTG AGCACATTTT TTTCGATTTG   
  
  
+ TTGACAATAA GCGTGGTTAT GATTTATGGG CCACTAATAT CAGTCTCTGA TCATTTGCAC ATTTTGGACC   
  
  
+ TCTTTCTCTC TCTAATCTTC CAGGTAACCT GAATTCTTCT CTATTTCTGA CTAGATTAGA TAACAGGATT   
  
  
+ TCAAATATCT GTCAGGTGGC GGGTATAACT TCGTGATGGG CATTCATTTT TGTTCTATCA GGCTGGTGGG   
  
  
+ TTTGAGTTTA TTTTGATTCA TTTCGTGTTC ATCTGTGATA TTAGTAGGAG GGATTCTGTA TACCCACCAA   
  
  
+ CTGCTCGCCC TTTTGCCCGT GTGAGCAATT GGGACTTTTG TTTGTTCTTC GTCGATGCAT CCTCTGGTTG   
  
  
+ CTGATCTGGA ATTGAAACCC CATGTATTCA AATTCAACCC TGATTTGCTG TCAAACTTTC TGAACCATCA   
  
  
+ AAACTCCGCC GAAGTGTTTG AAAAAGATGG CATCTTCCAG ACCCTTCATT TGGCTGATCC CAAAAGCTCT   
  
  
+ TCAATTGCCG AAATCGGGTT TACTAATAGT TCAGATTCTA CACAAGTACC TGATTTTTCA GATGCTTGTC   
  
  
+ TTAAGTTCAT TAGTGATATT CTCCTGGAAG AGGACTTAGA TGAAAGTCCT ACGTCTTTAC ACGATTACAT   
  
  
+ GGCTCTCCTA GCCACTGAGA AGTCTTTGTA TGATGCTCTT GGAAAGGAAT ACTTTCCTTC GTCTACTAGT   
  
  
+ CTTGCCCCAT CTTTAGGCCG AAGTGTTGAC AGCCCAGATA GTGGCTTTGG CCGCGGTTGC TCTGATGGTC   
  
  
+ GCGGGATTGA GGGTTTGGCT AATGATGATG CCGTTTTCAT GTCTAACTGG CAGCTCAACA CCACCCAATT   
  
  
+ GGACCCTTTC CCAATCATGC AAGATATTCC TCGTCCCTAT TTGGAATTGA ATTACCATTC TTCTGGGTCA   
  
  
+ AGCAATGGCA TTGATGATTC GGGGGATGGG TTATCGACCT CTCCTGTAAG TACACTTGCA TCAACCGCCA   
  
  
+ CAGAGGCAGG GAAAAAGTTG GCTGGTAGCT CTAGGAGAAA GAACCGTCAA AGGGATGACT ATGGCCATGA   
  
  
+ AGAGGGAAGG AGTAACAAGC AGCAAGCCTC TTACAATGAC GATTACGTTG AGATGGAGCA GTATGACGAT   
  
  
+ GTACTTCTCT GTAGGGCAGA CAAGGGTAAT ATTTCAACTT GTGCCAATGA ATCCTCGCTT AATGAGGTGC   
  
  
+ GTGAGAAGCT GCAGACGACA GGGTTCAAGG GAAGAACATC CCGTCTTAAG AAGCAATCTA AGGAAGCGAA   
  
  
+ AGAGGTGGAT CTGAGAACTC TTCTTTCTGG TTGTGCACAA GCTGTTTCGA ACTTTGATAT CAGGACTGCT   
  
  
+ AATGAGCTGC TTAAGCAAGT CAGACAGCAT TCTTCACCAT ATGGTGATAG CCTCCAAAGG CTCGCCCATC   
  
  
+ AATTTGCGAA TGGTATTGAG GCACGCTTAG CAGGCACCGG TTCAAGAGTA CCTGCTAATC TCATTGATGC   
  
  
+ ACGGATATCA TCATCTGAAT TTTTAAAAGC TTACAAGTCA TATGTTTCAG CAGTTCCTTT CAGAAGGATG   
  
  
+ TCCTATTTTA TAGCAAACAA CACAATTCTG AAGTTGGCTG AGAAAGCAAC AAAGATTCAC ATAATTGATT   
  
  
+ TTGGTATTCT ATTTGGTCTA CAGTGGCCCT GTCTTATACA AAGTCTCTCA AGGCGAACTG TGGCTCCTCC   
  
  
+ GAAGCTTCGC ATCACTGGGA TAGACTATCC CCAGCATGGT TTCCGGCCAG CAGAAAAGGT TGAGGCAACA   
  
  
+ GGTCGTCGGT TGTCCGGGTA CTGTGAGAGA TTTAATGTAC CCTTTCGATA TGAAACCATT GCAAAGAAGT   
  
  
+ GGGAAACCAT ACGCCCAGAA GATCTAAATA TTGAGAATGA TGAGCTGGTA ATTGTTAATT GTATGTTGCG   
  
  
+ GTCTGTAAAT CTATTGGATG ATACAGTGGC GGTAAATAGT CCAAGGGATG CTTTCTTGAG GTTAATCAAA   
  
  
+ CAGATAAACC CGCGTTTATT CATTCATGCA ATTGTCAATG GAACCTTTAG TACTCCATTC TTCAGCACTC   
  
  
+ GATTCAGGGA AGCCCTATTC CAATACTCTT CTGTATTTGA TATATTTGAA GCGACTATGA CTCGTGAAGA   
  
  
+ TCGTGGAAGG CTGCTGATTG AGAGTCAAAT ATGCGGGCTA GAAGTTTTGA ATGCAATAGC ATGTGAAGGT   
  
  
+ GCAGAGAGGA TTCAAAGGCC TGAAACATAC AAGCAATGGC AGGAGCGGAC AACAAGGGCT GGACTAAGGC   
  
  
+ AGGTTCCAAT AGATGAGGAG CTTGTCAATA GAGCAAAGAC TATAGTGAAA GCAAATTATC ACAAGGATTT   
  
  
+ TGTGGTGGAT GAGGATAGGC GTTGGATGCT TCAAGGTTGG AAAGGAAGGA CACTTAGTGC CCTTTCCGTT   
  
  
+ TGGCAGCCTA ACTA  

- +Up\_Stream \_Len000AAATTA TTCTAGGAAA ATATATATAC ACACATATTA AGATACCATG TATCCATACA   
  
  
- TGGTATCTGA ATAACGTAAA GATCCAGGAA GAGAGAGACG CGAACAAAAA ACAAATAGAT TAAATACCTC   
  
  
- AACTTGTAAA ACAAAATATA TGAACTTGTT AGATAATATG ATTTAATTCG TAAAACTAAA TTAGTGAATA   
  
  
- CCCACACAAA CCATCCCAAC TTTTATTAAA AGGATCTTTT ATTAAAAATT ATATTTTATT AAAAAGTACA   
  
  
- TTTTACTAAA AGTTGAGTAT AAAAGTCAAC TAACCATTTT TCTTTTTACT TGAAAGACCT ATTAGTCAAA   
  
  
- CTAATTGTTT TTCACTGTTT TTTTAATTTT TACCAAAAGG CATTAGAAGT TTGGTATACA GCTCCTTTAT   
  
  
- TTGTGCCTTA TTTCTTTCTC ACAGTCTTGG GTTCTTTACA TTTTAGTTGA ATGCAAGATA AATTTCACTT   
  
  
- TTTATAAAAA GAAGTAAGAT CCTTTTAATT AACAGGAGAC TTTTGTTTAA AAGGGAGGAA CAGATTGGTT   
  
  
- TGTGTCATTT TAACTTTTTA GTAAAAGATC CTTTTACTAA AAGTGAGGAT GGTTTGTGTG GGAATTTGAA   
  
  
- CAAGGTTTAG ATATTTGGGC CTTAGAATTA TGCGTGTAAC ATATAATATA CAGGATGATA TTGACATAAT   
  
  
- AGTGACCAAG AACTAAATAG GAAAGTTTGA GAACGAATCA GTTCCAACAG GAGCATGTTT GTACTAATTA   
  
  
- AGTTAATTGG TGCATTCAAT ATGCGACTGT TTAAAGCCCA TGGTTTGTAA ACGTGATCTG GATTTTGTCA   
  
  
- ATGATAGTAG TATAGCTACA ATTTTAACTG GACAAGAATA AACAGGTCGC AACTCGTTCC TTTATTAGTT   
  
  
- TAGTACAGAG TAAATCAGCC AAAAAACGGA TTCTTCTCGT ATTTGTTTCC TTTTTCTGTT GCTGTGTAAT   
  
  
- ATGTACGTAG ATGTGTTTAC TTTACTCCGG TATACCTTGT ACAAGTACTC TCGAAGTGAG CGATTATTAA   
  
  
- CTTGGATGAA AACTGATTCG GTGGTTGATC TTATACCTCT AACTGACAAT TCTCTGATAT AATGTACTTT   
  
  
- ATCTATTCTC ATCTGTAACG ATATATAAAA TAGAAATTAA CAGATAAAAA TTACTAGTAA TGACAAATAA   
  
  
- AGTCACTCAT TAAAAGAGCC TATATTATAA AATATCGCCA ATAATTTATC TACACTTTGA TATTACAATC   
  
  
- TGTATAGTAC GCTTTTATCT CTTTAAAACC CGTTTAATTT TTTTTTTCTT CTTTTTGCTC CTTCATACGG   
  
  
- GTAACATTCG TCCACCTACA TATAGGTCGG AGGTAGGTTG TTAAACCCGT TTTAATATAG TAGGTAAGGT   
  
  
- TTTTTTTCTA TATTAAACAC TATAATTATT GGGCTGAGTT AAGTTCGAAT AAATAAAAAA GTATATTAAA   
  
  
- AAATTATATA TTTTTATTTT TCACTGTTGC TGTACGTAGG AAACGCACAG TTTCCTAGGC GGCGCACCGG   
  
  
- GGGTGTAAGT GACTGCCCCA AAAACCAAAC GCTAATTCTA GTTACCGATT GCAACAGAGA GAGTTTTGTA   
  
  
- TACCTGGGGA AAAAGATAGA AAAAGACGTA ACAGTCGTAT CGTTAACAAC TCGTGTAAAA AAAGCTAAAC   
  
  
- AACTGTTATT CGCACCAATA CTAAATACCC GGTGATTATA GTCAGAGACT AGTAAACGTG TAAAACCTGG   
  
  
- AGAAAGAGAG AGATTAGAAG GTCCATTGGA CTTAAGAAGA GATAAAGACT GATCTAATCT ATTGTCCTAA   
  
  
- AGTTTATAGA CAGTCCACCG CCCATATTGA AGCACTACCC GTAAGTAAAA ACAAGATAGT CCGACCACCC   
  
  
- AAACTCAAAT AAAACTAAGT AAAGCACAAG TAGACACTAT AATCATCCTC CCTAAGACAT ATGGGTGGTT   
  
  
- GACGAGCGGG AAAACGGGCA CACTCGTTAA CCCTGAAAAC AAACAAGAAG CAGCTACGTA GGAGACCAAC   
  
  
- GACTAGACCT TAACTTTGGG GTACATAAGT TTAAGTTGGG ACTAAACGAC AGTTTGAAAG ACTTGGTAGT   
  
  
- TTTGAGGCGG CTTCACAAAC TTTTTCTACC GTAGAAGGTC TGGGAAGTAA ACCGACTAGG GTTTTCGAGA   
  
  
- AGTTAACGGC TTTAGCCCAA ATGATTATCA AGTCTAAGAT GTGTTCATGG ACTAAAAAGT CTACGAACAG   
  
  
- AATTCAAGTA ATCACTATAA GAGGACCTTC TCCTGAATCT ACTTTCAGGA TGCAGAAATG TGCTAATGTA   
  
  
- CCGAGAGGAT CGGTGACTCT TCAGAAACAT ACTACGAGAA CCTTTCCTTA TGAAAGGAAG CAGATGATCA   
  
  
- GAACGGGGTA GAAATCCGGC TTCACAACTG TCGGGTCTAT CACCGAAACC GGCGCCAACG AGACTACCAG   
  
  
- CGCCCTAACT CCCAAACCGA TTACTACTAC GGCAAAAGTA CAGATTGACC GTCGAGTTGT GGTGGGTTAA   
  
  
- CCTGGGAAAG GGTTAGTACG TTCTATAAGG AGCAGGGATA AACCTTAACT TAATGGTAAG AAGACCCAGT   
  
  
- TCGTTACCGT AACTACTAAG CCCCCTACCC AATAGCTGGA GAGGACATTC ATGTGAACGT AGTTGGCGGT   
  
  
- GTCTCCGTCC CTTTTTCAAC CGACCATCGA GATCCTCTTT CTTGGCAGTT TCCCTACTGA TACCGGTACT   
  
  
- TCTCCCTTCC TCATTGTTCG TCGTTCGGAG AATGTTACTG CTAATGCAAC TCTACCTCGT CATACTGCTA   
  
  
- CATGAAGAGA CATCCCGTCT GTTCCCATTA TAAAGTTGAA CACGGTTACT TAGGAGCGAA TTACTCCACG   
  
  
- CACTCTTCGA CGTCTGCTGT CCCAAGTTCC CTTCTTGTAG GGCAGAATTC TTCGTTAGAT TCCTTCGCTT   
  
  
- TCTCCACCTA GACTCTTGAG AAGAAAGACC AACACGTGTT CGACAAAGCT TGAAACTATA GTCCTGACGA   
  
  
- TTACTCGACG AATTCGTTCA GTCTGTCGTA AGAAGTGGTA TACCACTATC GGAGGTTTCC GAGCGGGTAG   
  
  
- TTAAACGCTT ACCATAACTC CGTGCGAATC GTCCGTGGCC AAGTTCTCAT GGACGATTAG AGTAACTACG   
  
  
- TGCCTATAGT AGTAGACTTA AAAATTTTCG AATGTTCAGT ATACAAAGTC GTCAAGGAAA GTCTTCCTAC   
  
  
- AGGATAAAAT ATCGTTTGTT GTGTTAAGAC TTCAACCGAC TCTTTCGTTG TTTCTAAGTG TATTAACTAA   
  
  
- AACCATAAGA TAAACCAGAT GTCACCGGGA CAGAATATGT TTCAGAGAGT TCCGCTTGAC ACCGAGGAGG   
  
  
- CTTCGAAGCG TAGTGACCCT ATCTGATAGG GGTCGTACCA AAGGCCGGTC GTCTTTTCCA ACTCCGTTGT   
  
  
- CCAGCAGCCA ACAGGCCCAT GACACTCTCT AAATTACATG GGAAAGCTAT ACTTTGGTAA CGTTTCTTCA   
  
  
- CCCTTTGGTA TGCGGGTCTT CTAGATTTAT AACTCTTACT ACTCGACCAT TAACAATTAA CATACAACGC   
  
  
- CAGACATTTA GATAACCTAC TATGTCACCG CCATTTATCA GGTTCCCTAC GAAAGAACTC CAATTAGTTT   
  
  
- GTCTATTTGG GCGCAAATAA GTAAGTACGT TAACAGTTAC CTTGGAAATC ATGAGGTAAG AAGTCGTGAG   
  
  
- CTAAGTCCCT TCGGGATAAG GTTATGAGAA GACATAAACT ATATAAACTT CGCTGATACT GAGCACTTCT   
  
  
- AGCACCTTCC GACGACTAAC TCTCAGTTTA TACGCCCGAT CTTCAAAACT TACGTTATCG TACACTTCCA   
  
  
- CGTCTCTCCT AAGTTTCCGG ACTTTGTATG TTCGTTACCG TCCTCGCCTG TTGTTCCCGA CCTGATTCCG   
  
  
- TCCAAGGTTA TCTACTCCTC GAACAGTTAT CTCGTTTCTG ATATCACTTT CGTTTAATAG TGTTCCTAAA   
  
  
- ACACCACCTA CTCCTATCCG CAACCTACGA AGTTCCAACC TTTCCTTCCT GTGAATCACG GGAAAGGCAA   
  
  
- ACCGTCGGAT TGAT

+     TATA-box

| Site Name | Organism | Position | Strand | Matrix score. | sequence | function |
| --- | --- | --- | --- | --- | --- | --- |
| TATA-box | Arabidopsis thaliana | 3965 | - | 4 | TATA | core promoter element around -30 of transcription start |
| TATA-box | Arabidopsis thaliana | 1254 | + | 4 | TATA | core promoter element around -30 of transcription start |
| TATA-box | Brassica oleracea | 1466 | + | 6 | ATATAA | core promoter element around -30 of transcription start |
| TATA-box | Arabidopsis thaliana | 1389 | + | 4 | TATA | core promoter element around -30 of transcription start |
| TATA-box | Helianthus annuus | 1951 | - | 6 | TATACA | core promoter element around -30 of transcription start |
| TATA-box | Arabidopsis thaliana | 1111 | + | 4 | TATA | core promoter element around -30 of transcription start |
| TATA-box | Brassica napus | 3754 | - | 6 | ATATAT | core promoter element around -30 of transcription start |
| TATA-box | Arabidopsis thaliana | 3329 | - | 4 | TATA | core promoter element around -30 of transcription start |
| TATA-box | Helianthus annuus | 1224 | - | 6 | TATAAA | core promoter element around -30 of transcription start |
| TATA-box | Arabidopsis thaliana | 3233 | - | 4 | TATA | core promoter element around -30 of transcription start |
| TATA-box | Arabidopsis thaliana | 1848 | + | 4 | TATA | core promoter element around -30 of transcription start |
| TATA-box | Arabidopsis thaliana | 983 | + | 4 | TATA | core promoter element around -30 of transcription start |
| TATA-box | Brassica napus | 981 | + | 6 | ATTATA | core promoter element around -30 of transcription start |
| TATA-box | Arabidopsis thaliana | 793 | + | 4 | TATA | core promoter element around -30 of transcription start |
| TATA-box | Arabidopsis thaliana | 1482 | + | 4 | TATA | core promoter element around -30 of transcription start |
| TATA-box | Pisum sativum | 1223 | - | 7 | TATAAAA | core promoter element around -30 of transcription start |
| TATA-box | Arabidopsis thaliana | 162 | + | 4 | TATA | core promoter element around -30 of transcription start |
| TATA-box | Pisum sativum | 157 | - | 7 | TATAAAA | core promoter element around -30 of transcription start |
| TATA-box | Helianthus annuus | 47 | - | 6 | TATACA | core promoter element around -30 of transcription start |
| TATA-box | Arabidopsis thaliana | 482 | + | 8 | TATTTAAA | core promoter element around -30 of transcription start |
| TATA-box | Arabidopsis thaliana | 3755 | - | 4 | TATA | core promoter element around -30 of transcription start |
| TATA-box | Arabidopsis thaliana | 156 | - | 9 | taTATAAAtc | core promoter element around -30 of transcription start |
| TATA-box | Arabidopsis thaliana | 39 | + | 4 | TATA | core promoter element around -30 of transcription start |
| TATA-box | Brassica oleracea | 1215 | + | 6 | ATATAA | core promoter element around -30 of transcription start |
| TATA-box | Arabidopsis thaliana | 675 | + | 4 | TATA | core promoter element around -30 of transcription start |
| TATA-box | Arabidopsis thaliana | 645 | + | 4 | TATA | core promoter element around -30 of transcription start |
| TATA-box | Arabidopsis thaliana | 1354 | + | 4 | TATA | core promoter element around -30 of transcription start |
| TATA-box | Brassica napus | 1479 | + | 6 | ATATAT | core promoter element around -30 of transcription start |
| TATA-box | Pisum sativum | 3230 | - | 7 | TATAAAA | core promoter element around -30 of transcription start |
| TATA-box | Pisum sativum | 32 | - | 7 | TATAAAA | core promoter element around -30 of transcription start |
| TATA-box | Arabidopsis thaliana | 1953 | + | 4 | TATA | core promoter element around -30 of transcription start |
| TATA-box | Arabidopsis thaliana | 1216 | + | 4 | TATA | core promoter element around -30 of transcription start |
| TATA-box | Arabidopsis thaliana | 1147 | + | 4 | TATA | core promoter element around -30 of transcription start |
| TATA-box | Brassica oleracea | 1413 | + | 6 | ATATAA | core promoter element around -30 of transcription start |
| TATA-box | Brassica napus | 38 | + | 6 | ATATAT | core promoter element around -30 of transcription start |
| TATA-box | Helianthus annuus | 158 | - | 6 | TATAAA | core promoter element around -30 of transcription start |
| TATA-box | Arabidopsis thaliana | 982 | - | 5 | TATAA | core promoter element around -30 of transcription start |
| TATA-box | Helianthus annuus | 3231 | - | 6 | TATAAA | core promoter element around -30 of transcription start |
| TATA-box | Arabidopsis thaliana | 34 | - | 7 | TATATAA | core promoter element around -30 of transcription start |
| TATA-box | Brassica napus | 178 | + | 6 | ATTATA | core promoter element around -30 of transcription start |
| TATA-box | Arabidopsis thaliana | 1225 | - | 5 | TATAA | core promoter element around -30 of transcription start |
| TATA-box | Arabidopsis thaliana | 159 | - | 7 | TATATAA | core promoter element around -30 of transcription start |
| TATA-box | Arabidopsis thaliana | 1480 | + | 6 | TATATA | core promoter element around -30 of transcription start |
| TATA-box | Arabidopsis thaliana | 792 | - | 5 | TATAA | core promoter element around -30 of transcription start |
| TATA-box | Brassica napus | 1387 | + | 6 | ATTATA | core promoter element around -30 of transcription start |
| TATA-box | Brassica napus | 36 | + | 6 | ATATAT | core promoter element around -30 of transcription start |
| TATA-box | Arabidopsis thaliana | 160 | + | 6 | TATATA | core promoter element around -30 of transcription start |
| TATA-box | Arabidopsis thaliana | 3232 | - | 5 | TATAA | core promoter element around -30 of transcription start |
| TATA-box | Arabidopsis thaliana | 679 | - | 5 | TATAA | core promoter element around -30 of transcription start |
| TATA-box | Arabidopsis thaliana | 179 | - | 5 | TATAA | core promoter element around -30 of transcription start |
| TATA-box | Arabidopsis thaliana | 265 | + | 4 | TATA | core promoter element around -30 of transcription start |
| TATA-box | Brassica napus | 1146 | + | 6 | ATATAT | core promoter element around -30 of transcription start |
| TATA-box | Arabidopsis thaliana | 35 | + | 6 | TATATA | core promoter element around -30 of transcription start |
| TATA-box | Brassica oleracea | 1481 | + | 6 | ATATAA | core promoter element around -30 of transcription start |
| TATA-box | Arabidopsis thaliana | 49 | + | 4 | TATA | core promoter element around -30 of transcription start |
| TATA-box | Arabidopsis thaliana | 1145 | + | 6 | TATATA | core promoter element around -30 of transcription start |
| TATA-box | Arabidopsis thaliana | 1414 | + | 4 | TATA | core promoter element around -30 of transcription start |
| TATA-box | Arabidopsis thaliana | 680 | + | 4 | TATA | core promoter element around -30 of transcription start |
| TATA-box | Arabidopsis thaliana | 692 | + | 4 | TATA | core promoter element around -30 of transcription start |
| TATA-box | Arabidopsis thaliana | 3328 | - | 5 | TATAA | core promoter element around -30 of transcription start |
| TATA-box | Helianthus annuus | 673 | - | 6 | TATACA | core promoter element around -30 of transcription start |
| TATA-box | Arabidopsis thaliana | 1226 | + | 4 | TATA | core promoter element around -30 of transcription start |
| TATA-box | Brassica napus | 678 | + | 6 | ATTATA | core promoter element around -30 of transcription start |
| TATA-box | Arabidopsis thaliana | 1388 | - | 5 | TATAA | core promoter element around -30 of transcription start |
| TATA-box | Arabidopsis thaliana | 1467 | + | 4 | TATA | core promoter element around -30 of transcription start |
| TATA-box | Arabidopsis thaliana | 37 | + | 6 | TATATA | core promoter element around -30 of transcription start |
| TATA-box | Arabidopsis thaliana | 180 | + | 4 | TATA | core promoter element around -30 of transcription start |
| TATA-box | Helianthus annuus | 1352 | - | 6 | TATACA | core promoter element around -30 of transcription start |
| TATA-box | Brassica oleracea | 264 | + | 6 | ATATAA | core promoter element around -30 of transcription start |
| TATA-box | Helianthus annuus | 33 | - | 6 | TATAAA | core promoter element around -30 of transcription start |

>HU02G01572.1   
+ +Up\_Stream \_Len000TTTAAT AAGATCCTTT TATATATATG TGTGTATAAT TCTATGGTAC ATAGGTATGT   
  
  
+ ACCATAGACT TATTGCATTT CTAGGTCCTT CTCTCTCTGC GCTTGTTTTT TGTTTATCTA ATTTATGGAG   
  
  
+ TTGAACATTT TGTTTTATAT ACTTGAACAA TCTATTATAC TAAATTAAGC ATTTTGATTT AATCACTTAT   
  
  
+ GGGTGTGTTT GGTAGGGTTG AAAATAATTT TCCTAGAAAA TAATTTTTAA TATAAAATAA TTTTTCATGT   
  
  
+ AAAATGATTT TCAACTCATA TTTTCAGTTG ATTGGTAAAA AGAAAAATGA ACTTTCTGGA TAATCAGTTT   
  
  
+ GATTAACAAA AAGTGACAAA AAAATTAAAA ATGGTTTTCC GTAATCTTCA AACCATATGT CGAGGAAATA   
  
  
+ AACACGGAAT AAAGAAAGAG TGTCAGAACC CAAGAAATGT AAAATCAACT TACGTTCTAT TTAAAGTGAA   
  
  
+ AAATATTTTT CTTCATTCTA GGAAAATTAA TTGTCCTCTG AAAACAAATT TTCCCTCCTT GTCTAACCAA   
  
  
+ ACACAGTAAA ATTGAAAAAT CATTTTCTAG GAAAATGATT TTCACTCCTA CCAAACACAC CCTTAAACTT   
  
  
+ GTTCCAAATC TATAAACCCG GAATCTTAAT ACGCACATTG TATATTATAT GTCCTACTAT AACTGTATTA   
  
  
+ TCACTGGTTC TTGATTTATC CTTTCAAACT CTTGCTTAGT CAAGGTTGTC CTCGTACAAA CATGATTAAT   
  
  
+ TCAATTAACC ACGTAAGTTA TACGCTGACA AATTTCGGGT ACCAAACATT TGCACTAGAC CTAAAACAGT   
  
  
+ TACTATCATC ATATCGATGT TAAAATTGAC CTGTTCTTAT TTGTCCAGCG TTGAGCAAGG AAATAATCAA   
  
  
+ ATCATGTCTC ATTTAGTCGG TTTTTTGCCT AAGAAGAGCA TAAACAAAGG AAAAAGACAA CGACACATTA   
  
  
+ TACATGCATC TACACAAATG AAATGAGGCC ATATGGAACA TGTTCATGAG AGCTTCACTC GCTAATAATT   
  
  
+ GAACCTACTT TTGACTAAGC CACCAACTAG AATATGGAGA TTGACTGTTA AGAGACTATA TTACATGAAA   
  
  
+ TAGATAAGAG TAGACATTGC TATATATTTT ATCTTTAATT GTCTATTTTT AATGATCATT ACTGTTTATT   
  
  
+ TCAGTGAGTA ATTTTCTCGG ATATAATATT TTATAGCGGT TATTAAATAG ATGTGAAACT ATAATGTTAG   
  
  
+ ACATATCATG CGAAAATAGA GAAATTTTGG GCAAATTAAA AAAAAAAGAA GAAAAACGAG GAAGTATGCC   
  
  
+ CATTGTAAGC AGGTGGATGT ATATCCAGCC TCCATCCAAC AATTTGGGCA AAATTATATC ATCCATTCCA   
  
  
+ AAAAAAAGAT ATAATTTGTG ATATTAATAA CCCGACTCAA TTCAAGCTTA TTTATTTTTT CATATAATTT   
  
  
+ TTTAATATAT AAAAATAAAA AGTGACAACG ACATGCATCC TTTGCGTGTC AAAGGATCCG CCGCGTGGCC   
  
  
+ CCCACATTCA CTGACGGGGT TTTTGGTTTG CGATTAAGAT CAATGGCTAA CGTTGTCTCT CTCAAAACAT   
  
  
+ ATGGACCCCT TTTTCTATCT TTTTCTGCAT TGTCAGCATA GCAATTGTTG AGCACATTTT TTTCGATTTG   
  
  
+ TTGACAATAA GCGTGGTTAT GATTTATGGG CCACTAATAT CAGTCTCTGA TCATTTGCAC ATTTTGGACC   
  
  
+ TCTTTCTCTC TCTAATCTTC CAGGTAACCT GAATTCTTCT CTATTTCTGA CTAGATTAGA TAACAGGATT   
  
  
+ TCAAATATCT GTCAGGTGGC GGGTATAACT TCGTGATGGG CATTCATTTT TGTTCTATCA GGCTGGTGGG   
  
  
+ TTTGAGTTTA TTTTGATTCA TTTCGTGTTC ATCTGTGATA TTAGTAGGAG GGATTCTGTA TACCCACCAA   
  
  
+ CTGCTCGCCC TTTTGCCCGT GTGAGCAATT GGGACTTTTG TTTGTTCTTC GTCGATGCAT CCTCTGGTTG   
  
  
+ CTGATCTGGA ATTGAAACCC CATGTATTCA AATTCAACCC TGATTTGCTG TCAAACTTTC TGAACCATCA   
  
  
+ AAACTCCGCC GAAGTGTTTG AAAAAGATGG CATCTTCCAG ACCCTTCATT TGGCTGATCC CAAAAGCTCT   
  
  
+ TCAATTGCCG AAATCGGGTT TACTAATAGT TCAGATTCTA CACAAGTACC TGATTTTTCA GATGCTTGTC   
  
  
+ TTAAGTTCAT TAGTGATATT CTCCTGGAAG AGGACTTAGA TGAAAGTCCT ACGTCTTTAC ACGATTACAT   
  
  
+ GGCTCTCCTA GCCACTGAGA AGTCTTTGTA TGATGCTCTT GGAAAGGAAT ACTTTCCTTC GTCTACTAGT   
  
  
+ CTTGCCCCAT CTTTAGGCCG AAGTGTTGAC AGCCCAGATA GTGGCTTTGG CCGCGGTTGC TCTGATGGTC   
  
  
+ GCGGGATTGA GGGTTTGGCT AATGATGATG CCGTTTTCAT GTCTAACTGG CAGCTCAACA CCACCCAATT   
  
  
+ GGACCCTTTC CCAATCATGC AAGATATTCC TCGTCCCTAT TTGGAATTGA ATTACCATTC TTCTGGGTCA   
  
  
+ AGCAATGGCA TTGATGATTC GGGGGATGGG TTATCGACCT CTCCTGTAAG TACACTTGCA TCAACCGCCA   
  
  
+ CAGAGGCAGG GAAAAAGTTG GCTGGTAGCT CTAGGAGAAA GAACCGTCAA AGGGATGACT ATGGCCATGA   
  
  
+ AGAGGGAAGG AGTAACAAGC AGCAAGCCTC TTACAATGAC GATTACGTTG AGATGGAGCA GTATGACGAT   
  
  
+ GTACTTCTCT GTAGGGCAGA CAAGGGTAAT ATTTCAACTT GTGCCAATGA ATCCTCGCTT AATGAGGTGC   
  
  
+ GTGAGAAGCT GCAGACGACA GGGTTCAAGG GAAGAACATC CCGTCTTAAG AAGCAATCTA AGGAAGCGAA   
  
  
+ AGAGGTGGAT CTGAGAACTC TTCTTTCTGG TTGTGCACAA GCTGTTTCGA ACTTTGATAT CAGGACTGCT   
  
  
+ AATGAGCTGC TTAAGCAAGT CAGACAGCAT TCTTCACCAT ATGGTGATAG CCTCCAAAGG CTCGCCCATC   
  
  
+ AATTTGCGAA TGGTATTGAG GCACGCTTAG CAGGCACCGG TTCAAGAGTA CCTGCTAATC TCATTGATGC   
  
  
+ ACGGATATCA TCATCTGAAT TTTTAAAAGC TTACAAGTCA TATGTTTCAG CAGTTCCTTT CAGAAGGATG   
  
  
+ TCCTATTTTA TAGCAAACAA CACAATTCTG AAGTTGGCTG AGAAAGCAAC AAAGATTCAC ATAATTGATT   
  
  
+ TTGGTATTCT ATTTGGTCTA CAGTGGCCCT GTCTTATACA AAGTCTCTCA AGGCGAACTG TGGCTCCTCC   
  
  
+ GAAGCTTCGC ATCACTGGGA TAGACTATCC CCAGCATGGT TTCCGGCCAG CAGAAAAGGT TGAGGCAACA   
  
  
+ GGTCGTCGGT TGTCCGGGTA CTGTGAGAGA TTTAATGTAC CCTTTCGATA TGAAACCATT GCAAAGAAGT   
  
  
+ GGGAAACCAT ACGCCCAGAA GATCTAAATA TTGAGAATGA TGAGCTGGTA ATTGTTAATT GTATGTTGCG   
  
  
+ GTCTGTAAAT CTATTGGATG ATACAGTGGC GGTAAATAGT CCAAGGGATG CTTTCTTGAG GTTAATCAAA   
  
  
+ CAGATAAACC CGCGTTTATT CATTCATGCA ATTGTCAATG GAACCTTTAG TACTCCATTC TTCAGCACTC   
  
  
+ GATTCAGGGA AGCCCTATTC CAATACTCTT CTGTATTTGA TATATTTGAA GCGACTATGA CTCGTGAAGA   
  
  
+ TCGTGGAAGG CTGCTGATTG AGAGTCAAAT ATGCGGGCTA GAAGTTTTGA ATGCAATAGC ATGTGAAGGT   
  
  
+ GCAGAGAGGA TTCAAAGGCC TGAAACATAC AAGCAATGGC AGGAGCGGAC AACAAGGGCT GGACTAAGGC   
  
  
+ AGGTTCCAAT AGATGAGGAG CTTGTCAATA GAGCAAAGAC TATAGTGAAA GCAAATTATC ACAAGGATTT   
  
  
+ TGTGGTGGAT GAGGATAGGC GTTGGATGCT TCAAGGTTGG AAAGGAAGGA CACTTAGTGC CCTTTCCGTT   
  
  
+ TGGCAGCCTA ACTA  

- +Up\_Stream \_Len000AAATTA TTCTAGGAAA ATATATATAC ACACATATTA AGATACCATG TATCCATACA   
  
  
- TGGTATCTGA ATAACGTAAA GATCCAGGAA GAGAGAGACG CGAACAAAAA ACAAATAGAT TAAATACCTC   
  
  
- AACTTGTAAA ACAAAATATA TGAACTTGTT AGATAATATG ATTTAATTCG TAAAACTAAA TTAGTGAATA   
  
  
- CCCACACAAA CCATCCCAAC TTTTATTAAA AGGATCTTTT ATTAAAAATT ATATTTTATT AAAAAGTACA   
  
  
- TTTTACTAAA AGTTGAGTAT AAAAGTCAAC TAACCATTTT TCTTTTTACT TGAAAGACCT ATTAGTCAAA   
  
  
- CTAATTGTTT TTCACTGTTT TTTTAATTTT TACCAAAAGG CATTAGAAGT TTGGTATACA GCTCCTTTAT   
  
  
- TTGTGCCTTA TTTCTTTCTC ACAGTCTTGG GTTCTTTACA TTTTAGTTGA ATGCAAGATA AATTTCACTT   
  
  
- TTTATAAAAA GAAGTAAGAT CCTTTTAATT AACAGGAGAC TTTTGTTTAA AAGGGAGGAA CAGATTGGTT   
  
  
- TGTGTCATTT TAACTTTTTA GTAAAAGATC CTTTTACTAA AAGTGAGGAT GGTTTGTGTG GGAATTTGAA   
  
  
- CAAGGTTTAG ATATTTGGGC CTTAGAATTA TGCGTGTAAC ATATAATATA CAGGATGATA TTGACATAAT   
  
  
- AGTGACCAAG AACTAAATAG GAAAGTTTGA GAACGAATCA GTTCCAACAG GAGCATGTTT GTACTAATTA   
  
  
- AGTTAATTGG TGCATTCAAT ATGCGACTGT TTAAAGCCCA TGGTTTGTAA ACGTGATCTG GATTTTGTCA   
  
  
- ATGATAGTAG TATAGCTACA ATTTTAACTG GACAAGAATA AACAGGTCGC AACTCGTTCC TTTATTAGTT   
  
  
- TAGTACAGAG TAAATCAGCC AAAAAACGGA TTCTTCTCGT ATTTGTTTCC TTTTTCTGTT GCTGTGTAAT   
  
  
- ATGTACGTAG ATGTGTTTAC TTTACTCCGG TATACCTTGT ACAAGTACTC TCGAAGTGAG CGATTATTAA   
  
  
- CTTGGATGAA AACTGATTCG GTGGTTGATC TTATACCTCT AACTGACAAT TCTCTGATAT AATGTACTTT   
  
  
- ATCTATTCTC ATCTGTAACG ATATATAAAA TAGAAATTAA CAGATAAAAA TTACTAGTAA TGACAAATAA   
  
  
- AGTCACTCAT TAAAAGAGCC TATATTATAA AATATCGCCA ATAATTTATC TACACTTTGA TATTACAATC   
  
  
- TGTATAGTAC GCTTTTATCT CTTTAAAACC CGTTTAATTT TTTTTTTCTT CTTTTTGCTC CTTCATACGG   
  
  
- GTAACATTCG TCCACCTACA TATAGGTCGG AGGTAGGTTG TTAAACCCGT TTTAATATAG TAGGTAAGGT   
  
  
- TTTTTTTCTA TATTAAACAC TATAATTATT GGGCTGAGTT AAGTTCGAAT AAATAAAAAA GTATATTAAA   
  
  
- AAATTATATA TTTTTATTTT TCACTGTTGC TGTACGTAGG AAACGCACAG TTTCCTAGGC GGCGCACCGG   
  
  
- GGGTGTAAGT GACTGCCCCA AAAACCAAAC GCTAATTCTA GTTACCGATT GCAACAGAGA GAGTTTTGTA   
  
  
- TACCTGGGGA AAAAGATAGA AAAAGACGTA ACAGTCGTAT CGTTAACAAC TCGTGTAAAA AAAGCTAAAC   
  
  
- AACTGTTATT CGCACCAATA CTAAATACCC GGTGATTATA GTCAGAGACT AGTAAACGTG TAAAACCTGG   
  
  
- AGAAAGAGAG AGATTAGAAG GTCCATTGGA CTTAAGAAGA GATAAAGACT GATCTAATCT ATTGTCCTAA   
  
  
- AGTTTATAGA CAGTCCACCG CCCATATTGA AGCACTACCC GTAAGTAAAA ACAAGATAGT CCGACCACCC   
  
  
- AAACTCAAAT AAAACTAAGT AAAGCACAAG TAGACACTAT AATCATCCTC CCTAAGACAT ATGGGTGGTT   
  
  
- GACGAGCGGG AAAACGGGCA CACTCGTTAA CCCTGAAAAC AAACAAGAAG CAGCTACGTA GGAGACCAAC   
  
  
- GACTAGACCT TAACTTTGGG GTACATAAGT TTAAGTTGGG ACTAAACGAC AGTTTGAAAG ACTTGGTAGT   
  
  
- TTTGAGGCGG CTTCACAAAC TTTTTCTACC GTAGAAGGTC TGGGAAGTAA ACCGACTAGG GTTTTCGAGA   
  
  
- AGTTAACGGC TTTAGCCCAA ATGATTATCA AGTCTAAGAT GTGTTCATGG ACTAAAAAGT CTACGAACAG   
  
  
- AATTCAAGTA ATCACTATAA GAGGACCTTC TCCTGAATCT ACTTTCAGGA TGCAGAAATG TGCTAATGTA   
  
  
- CCGAGAGGAT CGGTGACTCT TCAGAAACAT ACTACGAGAA CCTTTCCTTA TGAAAGGAAG CAGATGATCA   
  
  
- GAACGGGGTA GAAATCCGGC TTCACAACTG TCGGGTCTAT CACCGAAACC GGCGCCAACG AGACTACCAG   
  
  
- CGCCCTAACT CCCAAACCGA TTACTACTAC GGCAAAAGTA CAGATTGACC GTCGAGTTGT GGTGGGTTAA   
  
  
- CCTGGGAAAG GGTTAGTACG TTCTATAAGG AGCAGGGATA AACCTTAACT TAATGGTAAG AAGACCCAGT   
  
  
- TCGTTACCGT AACTACTAAG CCCCCTACCC AATAGCTGGA GAGGACATTC ATGTGAACGT AGTTGGCGGT   
  
  
- GTCTCCGTCC CTTTTTCAAC CGACCATCGA GATCCTCTTT CTTGGCAGTT TCCCTACTGA TACCGGTACT   
  
  
- TCTCCCTTCC TCATTGTTCG TCGTTCGGAG AATGTTACTG CTAATGCAAC TCTACCTCGT CATACTGCTA   
  
  
- CATGAAGAGA CATCCCGTCT GTTCCCATTA TAAAGTTGAA CACGGTTACT TAGGAGCGAA TTACTCCACG   
  
  
- CACTCTTCGA CGTCTGCTGT CCCAAGTTCC CTTCTTGTAG GGCAGAATTC TTCGTTAGAT TCCTTCGCTT   
  
  
- TCTCCACCTA GACTCTTGAG AAGAAAGACC AACACGTGTT CGACAAAGCT TGAAACTATA GTCCTGACGA   
  
  
- TTACTCGACG AATTCGTTCA GTCTGTCGTA AGAAGTGGTA TACCACTATC GGAGGTTTCC GAGCGGGTAG   
  
  
- TTAAACGCTT ACCATAACTC CGTGCGAATC GTCCGTGGCC AAGTTCTCAT GGACGATTAG AGTAACTACG   
  
  
- TGCCTATAGT AGTAGACTTA AAAATTTTCG AATGTTCAGT ATACAAAGTC GTCAAGGAAA GTCTTCCTAC   
  
  
- AGGATAAAAT ATCGTTTGTT GTGTTAAGAC TTCAACCGAC TCTTTCGTTG TTTCTAAGTG TATTAACTAA   
  
  
- AACCATAAGA TAAACCAGAT GTCACCGGGA CAGAATATGT TTCAGAGAGT TCCGCTTGAC ACCGAGGAGG   
  
  
- CTTCGAAGCG TAGTGACCCT ATCTGATAGG GGTCGTACCA AAGGCCGGTC GTCTTTTCCA ACTCCGTTGT   
  
  
- CCAGCAGCCA ACAGGCCCAT GACACTCTCT AAATTACATG GGAAAGCTAT ACTTTGGTAA CGTTTCTTCA   
  
  
- CCCTTTGGTA TGCGGGTCTT CTAGATTTAT AACTCTTACT ACTCGACCAT TAACAATTAA CATACAACGC   
  
  
- CAGACATTTA GATAACCTAC TATGTCACCG CCATTTATCA GGTTCCCTAC GAAAGAACTC CAATTAGTTT   
  
  
- GTCTATTTGG GCGCAAATAA GTAAGTACGT TAACAGTTAC CTTGGAAATC ATGAGGTAAG AAGTCGTGAG   
  
  
- CTAAGTCCCT TCGGGATAAG GTTATGAGAA GACATAAACT ATATAAACTT CGCTGATACT GAGCACTTCT   
  
  
- AGCACCTTCC GACGACTAAC TCTCAGTTTA TACGCCCGAT CTTCAAAACT TACGTTATCG TACACTTCCA   
  
  
- CGTCTCTCCT AAGTTTCCGG ACTTTGTATG TTCGTTACCG TCCTCGCCTG TTGTTCCCGA CCTGATTCCG   
  
  
- TCCAAGGTTA TCTACTCCTC GAACAGTTAT CTCGTTTCTG ATATCACTTT CGTTTAATAG TGTTCCTAAA   
  
  
- ACACCACCTA CTCCTATCCG CAACCTACGA AGTTCCAACC TTTCCTTCCT GTGAATCACG GGAAAGGCAA   
  
  
- ACCGTCGGAT TGAT

+     TATC-box

| Site Name | Organism | Position | Strand | Matrix score. | sequence | function |
| --- | --- | --- | --- | --- | --- | --- |
| TATC-box | Oryza sativa | 3380 | - | 7 | TATCCCA | cis-acting element involved in gibberellin-responsiveness |

>HU02G01572.1   
+ +Up\_Stream \_Len000TTTAAT AAGATCCTTT TATATATATG TGTGTATAAT TCTATGGTAC ATAGGTATGT   
  
  
+ ACCATAGACT TATTGCATTT CTAGGTCCTT CTCTCTCTGC GCTTGTTTTT TGTTTATCTA ATTTATGGAG   
  
  
+ TTGAACATTT TGTTTTATAT ACTTGAACAA TCTATTATAC TAAATTAAGC ATTTTGATTT AATCACTTAT   
  
  
+ GGGTGTGTTT GGTAGGGTTG AAAATAATTT TCCTAGAAAA TAATTTTTAA TATAAAATAA TTTTTCATGT   
  
  
+ AAAATGATTT TCAACTCATA TTTTCAGTTG ATTGGTAAAA AGAAAAATGA ACTTTCTGGA TAATCAGTTT   
  
  
+ GATTAACAAA AAGTGACAAA AAAATTAAAA ATGGTTTTCC GTAATCTTCA AACCATATGT CGAGGAAATA   
  
  
+ AACACGGAAT AAAGAAAGAG TGTCAGAACC CAAGAAATGT AAAATCAACT TACGTTCTAT TTAAAGTGAA   
  
  
+ AAATATTTTT CTTCATTCTA GGAAAATTAA TTGTCCTCTG AAAACAAATT TTCCCTCCTT GTCTAACCAA   
  
  
+ ACACAGTAAA ATTGAAAAAT CATTTTCTAG GAAAATGATT TTCACTCCTA CCAAACACAC CCTTAAACTT   
  
  
+ GTTCCAAATC TATAAACCCG GAATCTTAAT ACGCACATTG TATATTATAT GTCCTACTAT AACTGTATTA   
  
  
+ TCACTGGTTC TTGATTTATC CTTTCAAACT CTTGCTTAGT CAAGGTTGTC CTCGTACAAA CATGATTAAT   
  
  
+ TCAATTAACC ACGTAAGTTA TACGCTGACA AATTTCGGGT ACCAAACATT TGCACTAGAC CTAAAACAGT   
  
  
+ TACTATCATC ATATCGATGT TAAAATTGAC CTGTTCTTAT TTGTCCAGCG TTGAGCAAGG AAATAATCAA   
  
  
+ ATCATGTCTC ATTTAGTCGG TTTTTTGCCT AAGAAGAGCA TAAACAAAGG AAAAAGACAA CGACACATTA   
  
  
+ TACATGCATC TACACAAATG AAATGAGGCC ATATGGAACA TGTTCATGAG AGCTTCACTC GCTAATAATT   
  
  
+ GAACCTACTT TTGACTAAGC CACCAACTAG AATATGGAGA TTGACTGTTA AGAGACTATA TTACATGAAA   
  
  
+ TAGATAAGAG TAGACATTGC TATATATTTT ATCTTTAATT GTCTATTTTT AATGATCATT ACTGTTTATT   
  
  
+ TCAGTGAGTA ATTTTCTCGG ATATAATATT TTATAGCGGT TATTAAATAG ATGTGAAACT ATAATGTTAG   
  
  
+ ACATATCATG CGAAAATAGA GAAATTTTGG GCAAATTAAA AAAAAAAGAA GAAAAACGAG GAAGTATGCC   
  
  
+ CATTGTAAGC AGGTGGATGT ATATCCAGCC TCCATCCAAC AATTTGGGCA AAATTATATC ATCCATTCCA   
  
  
+ AAAAAAAGAT ATAATTTGTG ATATTAATAA CCCGACTCAA TTCAAGCTTA TTTATTTTTT CATATAATTT   
  
  
+ TTTAATATAT AAAAATAAAA AGTGACAACG ACATGCATCC TTTGCGTGTC AAAGGATCCG CCGCGTGGCC   
  
  
+ CCCACATTCA CTGACGGGGT TTTTGGTTTG CGATTAAGAT CAATGGCTAA CGTTGTCTCT CTCAAAACAT   
  
  
+ ATGGACCCCT TTTTCTATCT TTTTCTGCAT TGTCAGCATA GCAATTGTTG AGCACATTTT TTTCGATTTG   
  
  
+ TTGACAATAA GCGTGGTTAT GATTTATGGG CCACTAATAT CAGTCTCTGA TCATTTGCAC ATTTTGGACC   
  
  
+ TCTTTCTCTC TCTAATCTTC CAGGTAACCT GAATTCTTCT CTATTTCTGA CTAGATTAGA TAACAGGATT   
  
  
+ TCAAATATCT GTCAGGTGGC GGGTATAACT TCGTGATGGG CATTCATTTT TGTTCTATCA GGCTGGTGGG   
  
  
+ TTTGAGTTTA TTTTGATTCA TTTCGTGTTC ATCTGTGATA TTAGTAGGAG GGATTCTGTA TACCCACCAA   
  
  
+ CTGCTCGCCC TTTTGCCCGT GTGAGCAATT GGGACTTTTG TTTGTTCTTC GTCGATGCAT CCTCTGGTTG   
  
  
+ CTGATCTGGA ATTGAAACCC CATGTATTCA AATTCAACCC TGATTTGCTG TCAAACTTTC TGAACCATCA   
  
  
+ AAACTCCGCC GAAGTGTTTG AAAAAGATGG CATCTTCCAG ACCCTTCATT TGGCTGATCC CAAAAGCTCT   
  
  
+ TCAATTGCCG AAATCGGGTT TACTAATAGT TCAGATTCTA CACAAGTACC TGATTTTTCA GATGCTTGTC   
  
  
+ TTAAGTTCAT TAGTGATATT CTCCTGGAAG AGGACTTAGA TGAAAGTCCT ACGTCTTTAC ACGATTACAT   
  
  
+ GGCTCTCCTA GCCACTGAGA AGTCTTTGTA TGATGCTCTT GGAAAGGAAT ACTTTCCTTC GTCTACTAGT   
  
  
+ CTTGCCCCAT CTTTAGGCCG AAGTGTTGAC AGCCCAGATA GTGGCTTTGG CCGCGGTTGC TCTGATGGTC   
  
  
+ GCGGGATTGA GGGTTTGGCT AATGATGATG CCGTTTTCAT GTCTAACTGG CAGCTCAACA CCACCCAATT   
  
  
+ GGACCCTTTC CCAATCATGC AAGATATTCC TCGTCCCTAT TTGGAATTGA ATTACCATTC TTCTGGGTCA   
  
  
+ AGCAATGGCA TTGATGATTC GGGGGATGGG TTATCGACCT CTCCTGTAAG TACACTTGCA TCAACCGCCA   
  
  
+ CAGAGGCAGG GAAAAAGTTG GCTGGTAGCT CTAGGAGAAA GAACCGTCAA AGGGATGACT ATGGCCATGA   
  
  
+ AGAGGGAAGG AGTAACAAGC AGCAAGCCTC TTACAATGAC GATTACGTTG AGATGGAGCA GTATGACGAT   
  
  
+ GTACTTCTCT GTAGGGCAGA CAAGGGTAAT ATTTCAACTT GTGCCAATGA ATCCTCGCTT AATGAGGTGC   
  
  
+ GTGAGAAGCT GCAGACGACA GGGTTCAAGG GAAGAACATC CCGTCTTAAG AAGCAATCTA AGGAAGCGAA   
  
  
+ AGAGGTGGAT CTGAGAACTC TTCTTTCTGG TTGTGCACAA GCTGTTTCGA ACTTTGATAT CAGGACTGCT   
  
  
+ AATGAGCTGC TTAAGCAAGT CAGACAGCAT TCTTCACCAT ATGGTGATAG CCTCCAAAGG CTCGCCCATC   
  
  
+ AATTTGCGAA TGGTATTGAG GCACGCTTAG CAGGCACCGG TTCAAGAGTA CCTGCTAATC TCATTGATGC   
  
  
+ ACGGATATCA TCATCTGAAT TTTTAAAAGC TTACAAGTCA TATGTTTCAG CAGTTCCTTT CAGAAGGATG   
  
  
+ TCCTATTTTA TAGCAAACAA CACAATTCTG AAGTTGGCTG AGAAAGCAAC AAAGATTCAC ATAATTGATT   
  
  
+ TTGGTATTCT ATTTGGTCTA CAGTGGCCCT GTCTTATACA AAGTCTCTCA AGGCGAACTG TGGCTCCTCC   
  
  
+ GAAGCTTCGC ATCACTGGGA TAGACTATCC CCAGCATGGT TTCCGGCCAG CAGAAAAGGT TGAGGCAACA   
  
  
+ GGTCGTCGGT TGTCCGGGTA CTGTGAGAGA TTTAATGTAC CCTTTCGATA TGAAACCATT GCAAAGAAGT   
  
  
+ GGGAAACCAT ACGCCCAGAA GATCTAAATA TTGAGAATGA TGAGCTGGTA ATTGTTAATT GTATGTTGCG   
  
  
+ GTCTGTAAAT CTATTGGATG ATACAGTGGC GGTAAATAGT CCAAGGGATG CTTTCTTGAG GTTAATCAAA   
  
  
+ CAGATAAACC CGCGTTTATT CATTCATGCA ATTGTCAATG GAACCTTTAG TACTCCATTC TTCAGCACTC   
  
  
+ GATTCAGGGA AGCCCTATTC CAATACTCTT CTGTATTTGA TATATTTGAA GCGACTATGA CTCGTGAAGA   
  
  
+ TCGTGGAAGG CTGCTGATTG AGAGTCAAAT ATGCGGGCTA GAAGTTTTGA ATGCAATAGC ATGTGAAGGT   
  
  
+ GCAGAGAGGA TTCAAAGGCC TGAAACATAC AAGCAATGGC AGGAGCGGAC AACAAGGGCT GGACTAAGGC   
  
  
+ AGGTTCCAAT AGATGAGGAG CTTGTCAATA GAGCAAAGAC TATAGTGAAA GCAAATTATC ACAAGGATTT   
  
  
+ TGTGGTGGAT GAGGATAGGC GTTGGATGCT TCAAGGTTGG AAAGGAAGGA CACTTAGTGC CCTTTCCGTT   
  
  
+ TGGCAGCCTA ACTA  

- +Up\_Stream \_Len000AAATTA TTCTAGGAAA ATATATATAC ACACATATTA AGATACCATG TATCCATACA   
  
  
- TGGTATCTGA ATAACGTAAA GATCCAGGAA GAGAGAGACG CGAACAAAAA ACAAATAGAT TAAATACCTC   
  
  
- AACTTGTAAA ACAAAATATA TGAACTTGTT AGATAATATG ATTTAATTCG TAAAACTAAA TTAGTGAATA   
  
  
- CCCACACAAA CCATCCCAAC TTTTATTAAA AGGATCTTTT ATTAAAAATT ATATTTTATT AAAAAGTACA   
  
  
- TTTTACTAAA AGTTGAGTAT AAAAGTCAAC TAACCATTTT TCTTTTTACT TGAAAGACCT ATTAGTCAAA   
  
  
- CTAATTGTTT TTCACTGTTT TTTTAATTTT TACCAAAAGG CATTAGAAGT TTGGTATACA GCTCCTTTAT   
  
  
- TTGTGCCTTA TTTCTTTCTC ACAGTCTTGG GTTCTTTACA TTTTAGTTGA ATGCAAGATA AATTTCACTT   
  
  
- TTTATAAAAA GAAGTAAGAT CCTTTTAATT AACAGGAGAC TTTTGTTTAA AAGGGAGGAA CAGATTGGTT   
  
  
- TGTGTCATTT TAACTTTTTA GTAAAAGATC CTTTTACTAA AAGTGAGGAT GGTTTGTGTG GGAATTTGAA   
  
  
- CAAGGTTTAG ATATTTGGGC CTTAGAATTA TGCGTGTAAC ATATAATATA CAGGATGATA TTGACATAAT   
  
  
- AGTGACCAAG AACTAAATAG GAAAGTTTGA GAACGAATCA GTTCCAACAG GAGCATGTTT GTACTAATTA   
  
  
- AGTTAATTGG TGCATTCAAT ATGCGACTGT TTAAAGCCCA TGGTTTGTAA ACGTGATCTG GATTTTGTCA   
  
  
- ATGATAGTAG TATAGCTACA ATTTTAACTG GACAAGAATA AACAGGTCGC AACTCGTTCC TTTATTAGTT   
  
  
- TAGTACAGAG TAAATCAGCC AAAAAACGGA TTCTTCTCGT ATTTGTTTCC TTTTTCTGTT GCTGTGTAAT   
  
  
- ATGTACGTAG ATGTGTTTAC TTTACTCCGG TATACCTTGT ACAAGTACTC TCGAAGTGAG CGATTATTAA   
  
  
- CTTGGATGAA AACTGATTCG GTGGTTGATC TTATACCTCT AACTGACAAT TCTCTGATAT AATGTACTTT   
  
  
- ATCTATTCTC ATCTGTAACG ATATATAAAA TAGAAATTAA CAGATAAAAA TTACTAGTAA TGACAAATAA   
  
  
- AGTCACTCAT TAAAAGAGCC TATATTATAA AATATCGCCA ATAATTTATC TACACTTTGA TATTACAATC   
  
  
- TGTATAGTAC GCTTTTATCT CTTTAAAACC CGTTTAATTT TTTTTTTCTT CTTTTTGCTC CTTCATACGG   
  
  
- GTAACATTCG TCCACCTACA TATAGGTCGG AGGTAGGTTG TTAAACCCGT TTTAATATAG TAGGTAAGGT   
  
  
- TTTTTTTCTA TATTAAACAC TATAATTATT GGGCTGAGTT AAGTTCGAAT AAATAAAAAA GTATATTAAA   
  
  
- AAATTATATA TTTTTATTTT TCACTGTTGC TGTACGTAGG AAACGCACAG TTTCCTAGGC GGCGCACCGG   
  
  
- GGGTGTAAGT GACTGCCCCA AAAACCAAAC GCTAATTCTA GTTACCGATT GCAACAGAGA GAGTTTTGTA   
  
  
- TACCTGGGGA AAAAGATAGA AAAAGACGTA ACAGTCGTAT CGTTAACAAC TCGTGTAAAA AAAGCTAAAC   
  
  
- AACTGTTATT CGCACCAATA CTAAATACCC GGTGATTATA GTCAGAGACT AGTAAACGTG TAAAACCTGG   
  
  
- AGAAAGAGAG AGATTAGAAG GTCCATTGGA CTTAAGAAGA GATAAAGACT GATCTAATCT ATTGTCCTAA   
  
  
- AGTTTATAGA CAGTCCACCG CCCATATTGA AGCACTACCC GTAAGTAAAA ACAAGATAGT CCGACCACCC   
  
  
- AAACTCAAAT AAAACTAAGT AAAGCACAAG TAGACACTAT AATCATCCTC CCTAAGACAT ATGGGTGGTT   
  
  
- GACGAGCGGG AAAACGGGCA CACTCGTTAA CCCTGAAAAC AAACAAGAAG CAGCTACGTA GGAGACCAAC   
  
  
- GACTAGACCT TAACTTTGGG GTACATAAGT TTAAGTTGGG ACTAAACGAC AGTTTGAAAG ACTTGGTAGT   
  
  
- TTTGAGGCGG CTTCACAAAC TTTTTCTACC GTAGAAGGTC TGGGAAGTAA ACCGACTAGG GTTTTCGAGA   
  
  
- AGTTAACGGC TTTAGCCCAA ATGATTATCA AGTCTAAGAT GTGTTCATGG ACTAAAAAGT CTACGAACAG   
  
  
- AATTCAAGTA ATCACTATAA GAGGACCTTC TCCTGAATCT ACTTTCAGGA TGCAGAAATG TGCTAATGTA   
  
  
- CCGAGAGGAT CGGTGACTCT TCAGAAACAT ACTACGAGAA CCTTTCCTTA TGAAAGGAAG CAGATGATCA   
  
  
- GAACGGGGTA GAAATCCGGC TTCACAACTG TCGGGTCTAT CACCGAAACC GGCGCCAACG AGACTACCAG   
  
  
- CGCCCTAACT CCCAAACCGA TTACTACTAC GGCAAAAGTA CAGATTGACC GTCGAGTTGT GGTGGGTTAA   
  
  
- CCTGGGAAAG GGTTAGTACG TTCTATAAGG AGCAGGGATA AACCTTAACT TAATGGTAAG AAGACCCAGT   
  
  
- TCGTTACCGT AACTACTAAG CCCCCTACCC AATAGCTGGA GAGGACATTC ATGTGAACGT AGTTGGCGGT   
  
  
- GTCTCCGTCC CTTTTTCAAC CGACCATCGA GATCCTCTTT CTTGGCAGTT TCCCTACTGA TACCGGTACT   
  
  
- TCTCCCTTCC TCATTGTTCG TCGTTCGGAG AATGTTACTG CTAATGCAAC TCTACCTCGT CATACTGCTA   
  
  
- CATGAAGAGA CATCCCGTCT GTTCCCATTA TAAAGTTGAA CACGGTTACT TAGGAGCGAA TTACTCCACG   
  
  
- CACTCTTCGA CGTCTGCTGT CCCAAGTTCC CTTCTTGTAG GGCAGAATTC TTCGTTAGAT TCCTTCGCTT   
  
  
- TCTCCACCTA GACTCTTGAG AAGAAAGACC AACACGTGTT CGACAAAGCT TGAAACTATA GTCCTGACGA   
  
  
- TTACTCGACG AATTCGTTCA GTCTGTCGTA AGAAGTGGTA TACCACTATC GGAGGTTTCC GAGCGGGTAG   
  
  
- TTAAACGCTT ACCATAACTC CGTGCGAATC GTCCGTGGCC AAGTTCTCAT GGACGATTAG AGTAACTACG   
  
  
- TGCCTATAGT AGTAGACTTA AAAATTTTCG AATGTTCAGT ATACAAAGTC GTCAAGGAAA GTCTTCCTAC   
  
  
- AGGATAAAAT ATCGTTTGTT GTGTTAAGAC TTCAACCGAC TCTTTCGTTG TTTCTAAGTG TATTAACTAA   
  
  
- AACCATAAGA TAAACCAGAT GTCACCGGGA CAGAATATGT TTCAGAGAGT TCCGCTTGAC ACCGAGGAGG   
  
  
- CTTCGAAGCG TAGTGACCCT ATCTGATAGG GGTCGTACCA AAGGCCGGTC GTCTTTTCCA ACTCCGTTGT   
  
  
- CCAGCAGCCA ACAGGCCCAT GACACTCTCT AAATTACATG GGAAAGCTAT ACTTTGGTAA CGTTTCTTCA   
  
  
- CCCTTTGGTA TGCGGGTCTT CTAGATTTAT AACTCTTACT ACTCGACCAT TAACAATTAA CATACAACGC   
  
  
- CAGACATTTA GATAACCTAC TATGTCACCG CCATTTATCA GGTTCCCTAC GAAAGAACTC CAATTAGTTT   
  
  
- GTCTATTTGG GCGCAAATAA GTAAGTACGT TAACAGTTAC CTTGGAAATC ATGAGGTAAG AAGTCGTGAG   
  
  
- CTAAGTCCCT TCGGGATAAG GTTATGAGAA GACATAAACT ATATAAACTT CGCTGATACT GAGCACTTCT   
  
  
- AGCACCTTCC GACGACTAAC TCTCAGTTTA TACGCCCGAT CTTCAAAACT TACGTTATCG TACACTTCCA   
  
  
- CGTCTCTCCT AAGTTTCCGG ACTTTGTATG TTCGTTACCG TCCTCGCCTG TTGTTCCCGA CCTGATTCCG   
  
  
- TCCAAGGTTA TCTACTCCTC GAACAGTTAT CTCGTTTCTG ATATCACTTT CGTTTAATAG TGTTCCTAAA   
  
  
- ACACCACCTA CTCCTATCCG CAACCTACGA AGTTCCAACC TTTCCTTCCT GTGAATCACG GGAAAGGCAA   
  
  
- ACCGTCGGAT TGAT

+     TCA-element

| Site Name | Organism | Position | Strand | Matrix score. | sequence | function |
| --- | --- | --- | --- | --- | --- | --- |
| TCA-element | Nicotiana tabacum | 2125 | - | 10 | CCATCTTTTT | cis-acting element involved in salicylic acid responsiveness |
| TCA-element | Nicotiana tabacum | 1629 | + | 9 | CCATCTTTTT | cis-acting element involved in salicylic acid responsiveness |
| TCA-element | Nicotiana tabacum | 2676 | - | 9 | CCATCTTTTT | cis-acting element involved in salicylic acid responsiveness |

>HU02G01572.1   
+ +Up\_Stream \_Len000TTTAAT AAGATCCTTT TATATATATG TGTGTATAAT TCTATGGTAC ATAGGTATGT   
  
  
+ ACCATAGACT TATTGCATTT CTAGGTCCTT CTCTCTCTGC GCTTGTTTTT TGTTTATCTA ATTTATGGAG   
  
  
+ TTGAACATTT TGTTTTATAT ACTTGAACAA TCTATTATAC TAAATTAAGC ATTTTGATTT AATCACTTAT   
  
  
+ GGGTGTGTTT GGTAGGGTTG AAAATAATTT TCCTAGAAAA TAATTTTTAA TATAAAATAA TTTTTCATGT   
  
  
+ AAAATGATTT TCAACTCATA TTTTCAGTTG ATTGGTAAAA AGAAAAATGA ACTTTCTGGA TAATCAGTTT   
  
  
+ GATTAACAAA AAGTGACAAA AAAATTAAAA ATGGTTTTCC GTAATCTTCA AACCATATGT CGAGGAAATA   
  
  
+ AACACGGAAT AAAGAAAGAG TGTCAGAACC CAAGAAATGT AAAATCAACT TACGTTCTAT TTAAAGTGAA   
  
  
+ AAATATTTTT CTTCATTCTA GGAAAATTAA TTGTCCTCTG AAAACAAATT TTCCCTCCTT GTCTAACCAA   
  
  
+ ACACAGTAAA ATTGAAAAAT CATTTTCTAG GAAAATGATT TTCACTCCTA CCAAACACAC CCTTAAACTT   
  
  
+ GTTCCAAATC TATAAACCCG GAATCTTAAT ACGCACATTG TATATTATAT GTCCTACTAT AACTGTATTA   
  
  
+ TCACTGGTTC TTGATTTATC CTTTCAAACT CTTGCTTAGT CAAGGTTGTC CTCGTACAAA CATGATTAAT   
  
  
+ TCAATTAACC ACGTAAGTTA TACGCTGACA AATTTCGGGT ACCAAACATT TGCACTAGAC CTAAAACAGT   
  
  
+ TACTATCATC ATATCGATGT TAAAATTGAC CTGTTCTTAT TTGTCCAGCG TTGAGCAAGG AAATAATCAA   
  
  
+ ATCATGTCTC ATTTAGTCGG TTTTTTGCCT AAGAAGAGCA TAAACAAAGG AAAAAGACAA CGACACATTA   
  
  
+ TACATGCATC TACACAAATG AAATGAGGCC ATATGGAACA TGTTCATGAG AGCTTCACTC GCTAATAATT   
  
  
+ GAACCTACTT TTGACTAAGC CACCAACTAG AATATGGAGA TTGACTGTTA AGAGACTATA TTACATGAAA   
  
  
+ TAGATAAGAG TAGACATTGC TATATATTTT ATCTTTAATT GTCTATTTTT AATGATCATT ACTGTTTATT   
  
  
+ TCAGTGAGTA ATTTTCTCGG ATATAATATT TTATAGCGGT TATTAAATAG ATGTGAAACT ATAATGTTAG   
  
  
+ ACATATCATG CGAAAATAGA GAAATTTTGG GCAAATTAAA AAAAAAAGAA GAAAAACGAG GAAGTATGCC   
  
  
+ CATTGTAAGC AGGTGGATGT ATATCCAGCC TCCATCCAAC AATTTGGGCA AAATTATATC ATCCATTCCA   
  
  
+ AAAAAAAGAT ATAATTTGTG ATATTAATAA CCCGACTCAA TTCAAGCTTA TTTATTTTTT CATATAATTT   
  
  
+ TTTAATATAT AAAAATAAAA AGTGACAACG ACATGCATCC TTTGCGTGTC AAAGGATCCG CCGCGTGGCC   
  
  
+ CCCACATTCA CTGACGGGGT TTTTGGTTTG CGATTAAGAT CAATGGCTAA CGTTGTCTCT CTCAAAACAT   
  
  
+ ATGGACCCCT TTTTCTATCT TTTTCTGCAT TGTCAGCATA GCAATTGTTG AGCACATTTT TTTCGATTTG   
  
  
+ TTGACAATAA GCGTGGTTAT GATTTATGGG CCACTAATAT CAGTCTCTGA TCATTTGCAC ATTTTGGACC   
  
  
+ TCTTTCTCTC TCTAATCTTC CAGGTAACCT GAATTCTTCT CTATTTCTGA CTAGATTAGA TAACAGGATT   
  
  
+ TCAAATATCT GTCAGGTGGC GGGTATAACT TCGTGATGGG CATTCATTTT TGTTCTATCA GGCTGGTGGG   
  
  
+ TTTGAGTTTA TTTTGATTCA TTTCGTGTTC ATCTGTGATA TTAGTAGGAG GGATTCTGTA TACCCACCAA   
  
  
+ CTGCTCGCCC TTTTGCCCGT GTGAGCAATT GGGACTTTTG TTTGTTCTTC GTCGATGCAT CCTCTGGTTG   
  
  
+ CTGATCTGGA ATTGAAACCC CATGTATTCA AATTCAACCC TGATTTGCTG TCAAACTTTC TGAACCATCA   
  
  
+ AAACTCCGCC GAAGTGTTTG AAAAAGATGG CATCTTCCAG ACCCTTCATT TGGCTGATCC CAAAAGCTCT   
  
  
+ TCAATTGCCG AAATCGGGTT TACTAATAGT TCAGATTCTA CACAAGTACC TGATTTTTCA GATGCTTGTC   
  
  
+ TTAAGTTCAT TAGTGATATT CTCCTGGAAG AGGACTTAGA TGAAAGTCCT ACGTCTTTAC ACGATTACAT   
  
  
+ GGCTCTCCTA GCCACTGAGA AGTCTTTGTA TGATGCTCTT GGAAAGGAAT ACTTTCCTTC GTCTACTAGT   
  
  
+ CTTGCCCCAT CTTTAGGCCG AAGTGTTGAC AGCCCAGATA GTGGCTTTGG CCGCGGTTGC TCTGATGGTC   
  
  
+ GCGGGATTGA GGGTTTGGCT AATGATGATG CCGTTTTCAT GTCTAACTGG CAGCTCAACA CCACCCAATT   
  
  
+ GGACCCTTTC CCAATCATGC AAGATATTCC TCGTCCCTAT TTGGAATTGA ATTACCATTC TTCTGGGTCA   
  
  
+ AGCAATGGCA TTGATGATTC GGGGGATGGG TTATCGACCT CTCCTGTAAG TACACTTGCA TCAACCGCCA   
  
  
+ CAGAGGCAGG GAAAAAGTTG GCTGGTAGCT CTAGGAGAAA GAACCGTCAA AGGGATGACT ATGGCCATGA   
  
  
+ AGAGGGAAGG AGTAACAAGC AGCAAGCCTC TTACAATGAC GATTACGTTG AGATGGAGCA GTATGACGAT   
  
  
+ GTACTTCTCT GTAGGGCAGA CAAGGGTAAT ATTTCAACTT GTGCCAATGA ATCCTCGCTT AATGAGGTGC   
  
  
+ GTGAGAAGCT GCAGACGACA GGGTTCAAGG GAAGAACATC CCGTCTTAAG AAGCAATCTA AGGAAGCGAA   
  
  
+ AGAGGTGGAT CTGAGAACTC TTCTTTCTGG TTGTGCACAA GCTGTTTCGA ACTTTGATAT CAGGACTGCT   
  
  
+ AATGAGCTGC TTAAGCAAGT CAGACAGCAT TCTTCACCAT ATGGTGATAG CCTCCAAAGG CTCGCCCATC   
  
  
+ AATTTGCGAA TGGTATTGAG GCACGCTTAG CAGGCACCGG TTCAAGAGTA CCTGCTAATC TCATTGATGC   
  
  
+ ACGGATATCA TCATCTGAAT TTTTAAAAGC TTACAAGTCA TATGTTTCAG CAGTTCCTTT CAGAAGGATG   
  
  
+ TCCTATTTTA TAGCAAACAA CACAATTCTG AAGTTGGCTG AGAAAGCAAC AAAGATTCAC ATAATTGATT   
  
  
+ TTGGTATTCT ATTTGGTCTA CAGTGGCCCT GTCTTATACA AAGTCTCTCA AGGCGAACTG TGGCTCCTCC   
  
  
+ GAAGCTTCGC ATCACTGGGA TAGACTATCC CCAGCATGGT TTCCGGCCAG CAGAAAAGGT TGAGGCAACA   
  
  
+ GGTCGTCGGT TGTCCGGGTA CTGTGAGAGA TTTAATGTAC CCTTTCGATA TGAAACCATT GCAAAGAAGT   
  
  
+ GGGAAACCAT ACGCCCAGAA GATCTAAATA TTGAGAATGA TGAGCTGGTA ATTGTTAATT GTATGTTGCG   
  
  
+ GTCTGTAAAT CTATTGGATG ATACAGTGGC GGTAAATAGT CCAAGGGATG CTTTCTTGAG GTTAATCAAA   
  
  
+ CAGATAAACC CGCGTTTATT CATTCATGCA ATTGTCAATG GAACCTTTAG TACTCCATTC TTCAGCACTC   
  
  
+ GATTCAGGGA AGCCCTATTC CAATACTCTT CTGTATTTGA TATATTTGAA GCGACTATGA CTCGTGAAGA   
  
  
+ TCGTGGAAGG CTGCTGATTG AGAGTCAAAT ATGCGGGCTA GAAGTTTTGA ATGCAATAGC ATGTGAAGGT   
  
  
+ GCAGAGAGGA TTCAAAGGCC TGAAACATAC AAGCAATGGC AGGAGCGGAC AACAAGGGCT GGACTAAGGC   
  
  
+ AGGTTCCAAT AGATGAGGAG CTTGTCAATA GAGCAAAGAC TATAGTGAAA GCAAATTATC ACAAGGATTT   
  
  
+ TGTGGTGGAT GAGGATAGGC GTTGGATGCT TCAAGGTTGG AAAGGAAGGA CACTTAGTGC CCTTTCCGTT   
  
  
+ TGGCAGCCTA ACTA  

- +Up\_Stream \_Len000AAATTA TTCTAGGAAA ATATATATAC ACACATATTA AGATACCATG TATCCATACA   
  
  
- TGGTATCTGA ATAACGTAAA GATCCAGGAA GAGAGAGACG CGAACAAAAA ACAAATAGAT TAAATACCTC   
  
  
- AACTTGTAAA ACAAAATATA TGAACTTGTT AGATAATATG ATTTAATTCG TAAAACTAAA TTAGTGAATA   
  
  
- CCCACACAAA CCATCCCAAC TTTTATTAAA AGGATCTTTT ATTAAAAATT ATATTTTATT AAAAAGTACA   
  
  
- TTTTACTAAA AGTTGAGTAT AAAAGTCAAC TAACCATTTT TCTTTTTACT TGAAAGACCT ATTAGTCAAA   
  
  
- CTAATTGTTT TTCACTGTTT TTTTAATTTT TACCAAAAGG CATTAGAAGT TTGGTATACA GCTCCTTTAT   
  
  
- TTGTGCCTTA TTTCTTTCTC ACAGTCTTGG GTTCTTTACA TTTTAGTTGA ATGCAAGATA AATTTCACTT   
  
  
- TTTATAAAAA GAAGTAAGAT CCTTTTAATT AACAGGAGAC TTTTGTTTAA AAGGGAGGAA CAGATTGGTT   
  
  
- TGTGTCATTT TAACTTTTTA GTAAAAGATC CTTTTACTAA AAGTGAGGAT GGTTTGTGTG GGAATTTGAA   
  
  
- CAAGGTTTAG ATATTTGGGC CTTAGAATTA TGCGTGTAAC ATATAATATA CAGGATGATA TTGACATAAT   
  
  
- AGTGACCAAG AACTAAATAG GAAAGTTTGA GAACGAATCA GTTCCAACAG GAGCATGTTT GTACTAATTA   
  
  
- AGTTAATTGG TGCATTCAAT ATGCGACTGT TTAAAGCCCA TGGTTTGTAA ACGTGATCTG GATTTTGTCA   
  
  
- ATGATAGTAG TATAGCTACA ATTTTAACTG GACAAGAATA AACAGGTCGC AACTCGTTCC TTTATTAGTT   
  
  
- TAGTACAGAG TAAATCAGCC AAAAAACGGA TTCTTCTCGT ATTTGTTTCC TTTTTCTGTT GCTGTGTAAT   
  
  
- ATGTACGTAG ATGTGTTTAC TTTACTCCGG TATACCTTGT ACAAGTACTC TCGAAGTGAG CGATTATTAA   
  
  
- CTTGGATGAA AACTGATTCG GTGGTTGATC TTATACCTCT AACTGACAAT TCTCTGATAT AATGTACTTT   
  
  
- ATCTATTCTC ATCTGTAACG ATATATAAAA TAGAAATTAA CAGATAAAAA TTACTAGTAA TGACAAATAA   
  
  
- AGTCACTCAT TAAAAGAGCC TATATTATAA AATATCGCCA ATAATTTATC TACACTTTGA TATTACAATC   
  
  
- TGTATAGTAC GCTTTTATCT CTTTAAAACC CGTTTAATTT TTTTTTTCTT CTTTTTGCTC CTTCATACGG   
  
  
- GTAACATTCG TCCACCTACA TATAGGTCGG AGGTAGGTTG TTAAACCCGT TTTAATATAG TAGGTAAGGT   
  
  
- TTTTTTTCTA TATTAAACAC TATAATTATT GGGCTGAGTT AAGTTCGAAT AAATAAAAAA GTATATTAAA   
  
  
- AAATTATATA TTTTTATTTT TCACTGTTGC TGTACGTAGG AAACGCACAG TTTCCTAGGC GGCGCACCGG   
  
  
- GGGTGTAAGT GACTGCCCCA AAAACCAAAC GCTAATTCTA GTTACCGATT GCAACAGAGA GAGTTTTGTA   
  
  
- TACCTGGGGA AAAAGATAGA AAAAGACGTA ACAGTCGTAT CGTTAACAAC TCGTGTAAAA AAAGCTAAAC   
  
  
- AACTGTTATT CGCACCAATA CTAAATACCC GGTGATTATA GTCAGAGACT AGTAAACGTG TAAAACCTGG   
  
  
- AGAAAGAGAG AGATTAGAAG GTCCATTGGA CTTAAGAAGA GATAAAGACT GATCTAATCT ATTGTCCTAA   
  
  
- AGTTTATAGA CAGTCCACCG CCCATATTGA AGCACTACCC GTAAGTAAAA ACAAGATAGT CCGACCACCC   
  
  
- AAACTCAAAT AAAACTAAGT AAAGCACAAG TAGACACTAT AATCATCCTC CCTAAGACAT ATGGGTGGTT   
  
  
- GACGAGCGGG AAAACGGGCA CACTCGTTAA CCCTGAAAAC AAACAAGAAG CAGCTACGTA GGAGACCAAC   
  
  
- GACTAGACCT TAACTTTGGG GTACATAAGT TTAAGTTGGG ACTAAACGAC AGTTTGAAAG ACTTGGTAGT   
  
  
- TTTGAGGCGG CTTCACAAAC TTTTTCTACC GTAGAAGGTC TGGGAAGTAA ACCGACTAGG GTTTTCGAGA   
  
  
- AGTTAACGGC TTTAGCCCAA ATGATTATCA AGTCTAAGAT GTGTTCATGG ACTAAAAAGT CTACGAACAG   
  
  
- AATTCAAGTA ATCACTATAA GAGGACCTTC TCCTGAATCT ACTTTCAGGA TGCAGAAATG TGCTAATGTA   
  
  
- CCGAGAGGAT CGGTGACTCT TCAGAAACAT ACTACGAGAA CCTTTCCTTA TGAAAGGAAG CAGATGATCA   
  
  
- GAACGGGGTA GAAATCCGGC TTCACAACTG TCGGGTCTAT CACCGAAACC GGCGCCAACG AGACTACCAG   
  
  
- CGCCCTAACT CCCAAACCGA TTACTACTAC GGCAAAAGTA CAGATTGACC GTCGAGTTGT GGTGGGTTAA   
  
  
- CCTGGGAAAG GGTTAGTACG TTCTATAAGG AGCAGGGATA AACCTTAACT TAATGGTAAG AAGACCCAGT   
  
  
- TCGTTACCGT AACTACTAAG CCCCCTACCC AATAGCTGGA GAGGACATTC ATGTGAACGT AGTTGGCGGT   
  
  
- GTCTCCGTCC CTTTTTCAAC CGACCATCGA GATCCTCTTT CTTGGCAGTT TCCCTACTGA TACCGGTACT   
  
  
- TCTCCCTTCC TCATTGTTCG TCGTTCGGAG AATGTTACTG CTAATGCAAC TCTACCTCGT CATACTGCTA   
  
  
- CATGAAGAGA CATCCCGTCT GTTCCCATTA TAAAGTTGAA CACGGTTACT TAGGAGCGAA TTACTCCACG   
  
  
- CACTCTTCGA CGTCTGCTGT CCCAAGTTCC CTTCTTGTAG GGCAGAATTC TTCGTTAGAT TCCTTCGCTT   
  
  
- TCTCCACCTA GACTCTTGAG AAGAAAGACC AACACGTGTT CGACAAAGCT TGAAACTATA GTCCTGACGA   
  
  
- TTACTCGACG AATTCGTTCA GTCTGTCGTA AGAAGTGGTA TACCACTATC GGAGGTTTCC GAGCGGGTAG   
  
  
- TTAAACGCTT ACCATAACTC CGTGCGAATC GTCCGTGGCC AAGTTCTCAT GGACGATTAG AGTAACTACG   
  
  
- TGCCTATAGT AGTAGACTTA AAAATTTTCG AATGTTCAGT ATACAAAGTC GTCAAGGAAA GTCTTCCTAC   
  
  
- AGGATAAAAT ATCGTTTGTT GTGTTAAGAC TTCAACCGAC TCTTTCGTTG TTTCTAAGTG TATTAACTAA   
  
  
- AACCATAAGA TAAACCAGAT GTCACCGGGA CAGAATATGT TTCAGAGAGT TCCGCTTGAC ACCGAGGAGG   
  
  
- CTTCGAAGCG TAGTGACCCT ATCTGATAGG GGTCGTACCA AAGGCCGGTC GTCTTTTCCA ACTCCGTTGT   
  
  
- CCAGCAGCCA ACAGGCCCAT GACACTCTCT AAATTACATG GGAAAGCTAT ACTTTGGTAA CGTTTCTTCA   
  
  
- CCCTTTGGTA TGCGGGTCTT CTAGATTTAT AACTCTTACT ACTCGACCAT TAACAATTAA CATACAACGC   
  
  
- CAGACATTTA GATAACCTAC TATGTCACCG CCATTTATCA GGTTCCCTAC GAAAGAACTC CAATTAGTTT   
  
  
- GTCTATTTGG GCGCAAATAA GTAAGTACGT TAACAGTTAC CTTGGAAATC ATGAGGTAAG AAGTCGTGAG   
  
  
- CTAAGTCCCT TCGGGATAAG GTTATGAGAA GACATAAACT ATATAAACTT CGCTGATACT GAGCACTTCT   
  
  
- AGCACCTTCC GACGACTAAC TCTCAGTTTA TACGCCCGAT CTTCAAAACT TACGTTATCG TACACTTCCA   
  
  
- CGTCTCTCCT AAGTTTCCGG ACTTTGTATG TTCGTTACCG TCCTCGCCTG TTGTTCCCGA CCTGATTCCG   
  
  
- TCCAAGGTTA TCTACTCCTC GAACAGTTAT CTCGTTTCTG ATATCACTTT CGTTTAATAG TGTTCCTAAA   
  
  
- ACACCACCTA CTCCTATCCG CAACCTACGA AGTTCCAACC TTTCCTTCCT GTGAATCACG GGAAAGGCAA   
  
  
- ACCGTCGGAT TGAT

+     TCT-motif

| Site Name | Organism | Position | Strand | Matrix score. | sequence | function |
| --- | --- | --- | --- | --- | --- | --- |
| TCT-motif | Arabidopsis thaliana | 2763 | + | 6 | TCTTAC | part of a light responsive element |

>HU02G01572.1   
+ +Up\_Stream \_Len000TTTAAT AAGATCCTTT TATATATATG TGTGTATAAT TCTATGGTAC ATAGGTATGT   
  
  
+ ACCATAGACT TATTGCATTT CTAGGTCCTT CTCTCTCTGC GCTTGTTTTT TGTTTATCTA ATTTATGGAG
[truncated: 117,232 more chars]
