# Supplementary material for: Genome-Wide Identification and Expression Pattern of the GRAS Gene Family in Pitaya (Selenicereus undatus L.)
Source: Biology (Basel). 2022 Dec 21;12(1):11. doi: 10.3390/biology12010011 (PMC9854919; doi:10.3390/biology12010011)
Supplement: Supplementary file 1 [file biology-12-00011-s001.zip › Supplementary file S5/HU02G01573.1_plantcare.html]

Content-Type: text/html; charset=ISO-8859-1


PlantCARE


Webmaster Firefox specific output  
To save the result:
click on the frame with the right mouse button and save the source code as a text file with extension .html  
REFERENCE:PlantCARE: a database of plant cis-acting regulatory elements and a portal to tools for in silico analysis of promoter sequences.  
Lescot, M., Déhais, P., Moreau, Y., De Moor, B., Rouzé ,P.,and Rombauts, S.  
Nucleic Acids Res., Database issue(2002), 30(1):325-327.   


---

>HU02G01573.1   
+ +Up\_Stream \_Len000TGAATT GGAAGTTGAT TCTACCTTTG ATTGATTCGA TTAATTCTAG GTTCTTGGTT   
  
  
+ CTAGTTCTCT CTCCTCTTGT TCTTGATTAT TTAGGTTTTG GATGTGATGT TTATTGAATT GAATGTTGCA   
  
  
+ ATTGTTGTTA TAAAGATGAT GTTAGGCTAA ATATCCTTCA ACTTAGGTTG GGATTGACGT CGAATATGTG   
  
  
+ TAGTTGAATC TTGTACTGGC CTAAATCCCT GCTCCCCCTC TGTTTTGCGA TTATGAATTT CATTATGCAC   
  
  
+ ACCACATGCT CGATGAAATG CTCGAAAGAA CATTCATCCT CTTTGTTTGG GATTTAGGGG AACGTTGAAA   
  
  
+ GACGAAGTCT TTACCTAGTG CGTCTTAGTA GACAATTAGG ATTTTAACAT GAAAATAGTT AAAATTCTAA   
  
  
+ TTGGGATTTG AGATTGTGAA TCTTGTAATC CTTCGACTTC TAGTTCTATG AAAATAGGCG ATTATAGTTC   
  
  
+ TAGGATTAGT CTTCGACCCT TGAGAGAGGG AGGAGATGGG TTTAGTTGAT AATCTATTCA TTCATAACGC   
  
  
+ ATGCCTGGGA GATGTTTGGT AGGGCTAGTA ATTGATTCGC TAACATATTT GTGCTGTTAG TTCCAACCTG   
  
  
+ AGTGTTTACT CTTCTGCTGT TTATTCCGTC TTTTAGTCGT AATTGTCTCC TTTAGTTTAA TTTCAAAACC   
  
  
+ CCAATTGTGA TCGCCTAGAT TGAGCAATCT AATTAGTTCT AATAATTGAA ATCAAGTTCC CTGTGGAATA   
  
  
+ CGACCCGTAC TTGCCGTGTG CTAGCTACTG TTACACCTTG CATTTGCGGT ATATTTATAA CTCAACATAT   
  
  
+ GTCTGCTCAC TAAAAGTACA TATTAGCTCA CCAAAACTGT GTCTCAACGT ACTAAAATAT TTTAGTGAAC   
  
  
+ TATACATTAT TTTTAGTAAG CGAAATAAGA TACACAACAG CACATTTTAA TGAGTTACAG GGCATTTTTA   
  
  
+ GTGAGCCGAA ATCTGATGGT TTTCTTTGTA GATCCGGTTA TATAATTGTA TTTTATACAA CCGAATGGAC   
  
  
+ GATATAACAA CTGAGTTAAA TGAGGAGTCT GAAATTACGA ACATAAAATC CAAAAGAAGA TGCCGATGCC   
  
  
+ ATTGGAAAGA AAGCTGAAGG TGATCGCTTT CTAGTAGTAT TAGGCAGTAG TAATTAAAAA CTAAAATCCA   
  
  
+ GAATTGACAC CGAATTGGGG TTGTGGCACA TGATCGAAAT CAGCATCGAA TCTACACGAT GATCTGGATT   
  
  
+ CTTGGACACT CTGGCTTTAG GCCGTCAATT TCGAGGATTG CCCATAACTT TAATCCTGTT CAAATCCATT   
  
  
+ CAATTTAATA TTACACAAAT CCTTTGATTA AATCACCGTA ATTGTGGTTT GATTAGGGAA GTTTCTTGGG   
  
  
+ GGAAAGGTTC AAGCTTCTGT CTTTCTTAAA GCCTGGATTT TTGGTCCCGC ATGTTCACTT CACATTCCCT   
  
  
+ TTTTCTCTCC CATCGTTACC TGTCTGAATC TGTATGTAAT AAAAGTCATA TTTTGCGAGG TCTTCTTTTG   
  
  
+ ACTGTTCTTG TAGTCACTCT TGATATCTGC ACCATTTTGT TTTTCTCGGC ATTTTCTCCA ACTTGCATTT   
  
  
+ TCTAATGTAA TGTGATCTTT TCTGGACATT CATTTTGTTA ATCTACTCAC TTGGGTCAAG GTTTGCTGAT   
  
  
+ TTTGGCTGGT GTTAAATCCG TTCCTCTTTG GCATTTGTTC ATGTTCCACA ACTGTTTGAT ATCTGGTATC   
  
  
+ TCAGAAACCC CGCCATTCTT TGGTATTTCC TGACTTTCTT GTTCAAGGGT TATTCCTATT TCCCTGAATT   
  
  
+ TTCTTTGATT CTTTAGGTAT TTCAAGCTTG GGGGGTTTTG TTCTTCACTG ATTTTGTCCT CTTCTTCCCT   
  
  
+ TATTGGGTGG TTTGATCTCC TAAATTTCTG TGTATCTGAA TCAGTCGGGG TAGACTTTGT GTTTTTGGTT   
  
  
+ GATTTTTTAT CAATGTATTT TCTTCCTAAT GAAGGGCCTC CCGAACCAAT ACACATGCTT AACTTCAACC   
  
  
+ CTGTTCCTCT TCCAAGCTTT TTGGAGCCTC AAAAGTCTTC GAATGCTGAT AGGCACATAG GGTCCTTCCA   
  
  
+ ATGCTATGGT CTTGATGATG ACCCCGCACC CTTGAATGAC CCAAATTACA TTGTATGTCA ATCTTCATAT   
  
  
+ CCTGATCGAG ATATTACACA GATCCCTGAC TTTCCTGATG ATTGTCTCAA GTTCATCAAT GACATTCTTA   
  
  
+ TGGAAGAGGA TTTGGATAAT CCGCCTATAT CCTTGCAGGA CTATAATGCG CTCCAAGCCA CTGAGAAGTC   
  
  
+ ATTGTATGAT GCCCTTGGAG AGGCTTATCC AGCTTCATCT GATCCATTGC AATCATCAGT TGGTCAGAGC   
  
  
+ ACTGAGAGCT CTGATGATCC TGACAGAAGC AGCGGCATTG ATAAATGGGG TAATAGATAT TACCCCACCA   
  
  
+ GCCTGAATGT GGTTGAAACC AGCCGGCTGA TCAATCAGAG CCAGTTGGAC CCTTCTCAAA TGTTTGATGC   
  
  
+ TGCTGCTCAC CCCTTGGAGT CTAATTGTCA TTCTTTTGTC TCTAGTAATA ACTTTGATCA TTCTATGGAT   
  
  
+ GGGGTTCCAG ATTCTCCTAT CAGTACACTT TCTGTGAATG AAACAAGAAA GGAAGAATCA GTTAGTAGAT   
  
  
+ CAAGGAGCAG GAAGAATCAT CAAAGAGATG GTGCATACCT GGAAGAAAGG AGTACTAAGC AAATAGCATC   
  
  
+ CAGCAATGAG GAATATGGAG AGATGGAGAG GTTTGATGAT GTACTGATCT GCAGGGAAGG AAATGATGAT   
  
  
+ ATTTCACCTT GTACCCGCAA AACCCCAGTT CATGAAGCAG ATGATAACTC GGAACCAAAG GGGAAATCAA   
  
  
+ GAGGATCTAA AAGCAAGAGT AAATCGCGTG TCAAGAAGAA AAGTAACGAA GGACAAGCGG TAGATCTTAG   
  
  
+ GACTCAACTT ATTCAGTGTG CAGAAGCAGT GGCAAGCTTT GAGCTTAAAA GTGCAAATGA GCTACTCAGG   
  
  
+ CGAATTAGGC AGCATGCTTC GCCATTTGGG GATAGTGTTC AAAGGCTAGC ACATTACTTT GCGAATGGGC   
  
  
+ TCGAGGCACG CTTAGCTGGT ACAGGATCAG AATCATACAA AGACTTTGCT GGGAAAAAAT TTTCATCCTA   
  
  
+ TGACATCTTG AGAGGCTACA AGTCATATGT TCAAGCCGTC CCCTTCCAGA GAACAACCAT GTTCTTGACA   
  
  
+ AATCAAACAA TAGTAAAGCT GGCTGAGAAA GCAACAAGGG TTCATATCAT TGATTTTGGC ATCTTTTTTG   
  
  
+ GTTTCCAGTG GCCCTGCCTT GTTCAGAATT TGTCAAGAAG ACCCCAGGGT TCTCCCATAC TCAAAATCAC   
  
  
+ GGGAGTCGAT CATCCCCAGA GTGGGTTCAG ACCAGCCCAG AAAGTTGAAG AGACAGGTCG TCGATTAGCA   
  
  
+ GGCTATTGTG AGAGGTTTGG GGTGCCCTTT GAGTTTCATC CCATTGCTCA AAGGTGGCAT ACCCTAAAGC   
  
  
+ CAGAGCATTT CAAAATTGAA AGCAATGAGC TTGTTGTGGT CACTTGTTTC TACCAATCAG GGAAGCTCCC   
  
  
+ TGATGAGACT GTTGATGTCA ACAGCCCAAG AGACCATGTC TTGAGGCTGA TCAGGGGCTT GAACCCTGAT   
  
  
+ CTATTCATCC ATGGGGTTAT CAATGGCACA TTCAATGCAC CCTTCTTCGT AACTCGGTTT AAAGAGGCAC   
  
  
+ TTTACCACTA CTCTTCTTTG TTCGATGTCT TTGATGCCAC CATAGATCGT GAAAGTCATG AAAGACTGCT   
  
  
+ GACTGAGAAC TATTTGTATG GGAAAGATGC ACTTAACATA GTAGCATGTG AGGGGAACGA GAGGATCGAG   
  
  
+ AGACCCGAGA CTTACAAGCA GTGGCAAGTC CGGAAGCTTA GAGCTGGATT CAAGCAGGTC CCCCTAGACC   
  
  
+ AAGACATTGT AAATGGAGCA AGAGCTATGG TTAAGGCAAA TTACAATAAG GATTTTATGG TAGAACAAGA   
  
  
+ TAGGAATTGG ATGGTCCAGG GCTGGAAAGG AAGGATTTTG AGTGCCATAT CCTGTTGGAA ACCTGCTTA  

- +Up\_Stream \_Len000ACTTAA CCTTCAACTA AGATGGAAAC TAACTAAGCT AATTAAGATC CAAGAACCAA   
  
  
- GATCAAGAGA GAGGAGAACA AGAACTAATA AATCCAAAAC CTACACTACA AATAACTTAA CTTACAACGT   
  
  
- TAACAACAAT ATTTCTACTA CAATCCGATT TATAGGAAGT TGAATCCAAC CCTAACTGCA GCTTATACAC   
  
  
- ATCAACTTAG AACATGACCG GATTTAGGGA CGAGGGGGAG ACAAAACGCT AATACTTAAA GTAATACGTG   
  
  
- TGGTGTACGA GCTACTTTAC GAGCTTTCTT GTAAGTAGGA GAAACAAACC CTAAATCCCC TTGCAACTTT   
  
  
- CTGCTTCAGA AATGGATCAC GCAGAATCAT CTGTTAATCC TAAAATTGTA CTTTTATCAA TTTTAAGATT   
  
  
- AACCCTAAAC TCTAACACTT AGAACATTAG GAAGCTGAAG ATCAAGATAC TTTTATCCGC TAATATCAAG   
  
  
- ATCCTAATCA GAAGCTGGGA ACTCTCTCCC TCCTCTACCC AAATCAACTA TTAGATAAGT AAGTATTGCG   
  
  
- TACGGACCCT CTACAAACCA TCCCGATCAT TAACTAAGCG ATTGTATAAA CACGACAATC AAGGTTGGAC   
  
  
- TCACAAATGA GAAGACGACA AATAAGGCAG AAAATCAGCA TTAACAGAGG AAATCAAATT AAAGTTTTGG   
  
  
- GGTTAACACT AGCGGATCTA ACTCGTTAGA TTAATCAAGA TTATTAACTT TAGTTCAAGG GACACCTTAT   
  
  
- GCTGGGCATG AACGGCACAC GATCGATGAC AATGTGGAAC GTAAACGCCA TATAAATATT GAGTTGTATA   
  
  
- CAGACGAGTG ATTTTCATGT ATAATCGAGT GGTTTTGACA CAGAGTTGCA TGATTTTATA AAATCACTTG   
  
  
- ATATGTAATA AAAATCATTC GCTTTATTCT ATGTGTTGTC GTGTAAAATT ACTCAATGTC CCGTAAAAAT   
  
  
- CACTCGGCTT TAGACTACCA AAAGAAACAT CTAGGCCAAT ATATTAACAT AAAATATGTT GGCTTACCTG   
  
  
- CTATATTGTT GACTCAATTT ACTCCTCAGA CTTTAATGCT TGTATTTTAG GTTTTCTTCT ACGGCTACGG   
  
  
- TAACCTTTCT TTCGACTTCC ACTAGCGAAA GATCATCATA ATCCGTCATC ATTAATTTTT GATTTTAGGT   
  
  
- CTTAACTGTG GCTTAACCCC AACACCGTGT ACTAGCTTTA GTCGTAGCTT AGATGTGCTA CTAGACCTAA   
  
  
- GAACCTGTGA GACCGAAATC CGGCAGTTAA AGCTCCTAAC GGGTATTGAA ATTAGGACAA GTTTAGGTAA   
  
  
- GTTAAATTAT AATGTGTTTA GGAAACTAAT TTAGTGGCAT TAACACCAAA CTAATCCCTT CAAAGAACCC   
  
  
- CCTTTCCAAG TTCGAAGACA GAAAGAATTT CGGACCTAAA AACCAGGGCG TACAAGTGAA GTGTAAGGGA   
  
  
- AAAAGAGAGG GTAGCAATGG ACAGACTTAG ACATACATTA TTTTCAGTAT AAAACGCTCC AGAAGAAAAC   
  
  
- TGACAAGAAC ATCAGTGAGA ACTATAGACG TGGTAAAACA AAAAGAGCCG TAAAAGAGGT TGAACGTAAA   
  
  
- AGATTACATT ACACTAGAAA AGACCTGTAA GTAAAACAAT TAGATGAGTG AACCCAGTTC CAAACGACTA   
  
  
- AAACCGACCA CAATTTAGGC AAGGAGAAAC CGTAAACAAG TACAAGGTGT TGACAAACTA TAGACCATAG   
  
  
- AGTCTTTGGG GCGGTAAGAA ACCATAAAGG ACTGAAAGAA CAAGTTCCCA ATAAGGATAA AGGGACTTAA   
  
  
- AAGAAACTAA GAAATCCATA AAGTTCGAAC CCCCCAAAAC AAGAAGTGAC TAAAACAGGA GAAGAAGGGA   
  
  
- ATAACCCACC AAACTAGAGG ATTTAAAGAC ACATAGACTT AGTCAGCCCC ATCTGAAACA CAAAAACCAA   
  
  
- CTAAAAAATA GTTACATAAA AGAAGGATTA CTTCCCGGAG GGCTTGGTTA TGTGTACGAA TTGAAGTTGG   
  
  
- GACAAGGAGA AGGTTCGAAA AACCTCGGAG TTTTCAGAAG CTTACGACTA TCCGTGTATC CCAGGAAGGT   
  
  
- TACGATACCA GAACTACTAC TGGGGCGTGG GAACTTACTG GGTTTAATGT AACATACAGT TAGAAGTATA   
  
  
- GGACTAGCTC TATAATGTGT CTAGGGACTG AAAGGACTAC TAACAGAGTT CAAGTAGTTA CTGTAAGAAT   
  
  
- ACCTTCTCCT AAACCTATTA GGCGGATATA GGAACGTCCT GATATTACGC GAGGTTCGGT GACTCTTCAG   
  
  
- TAACATACTA CGGGAACCTC TCCGAATAGG TCGAAGTAGA CTAGGTAACG TTAGTAGTCA ACCAGTCTCG   
  
  
- TGACTCTCGA GACTACTAGG ACTGTCTTCG TCGCCGTAAC TATTTACCCC ATTATCTATA ATGGGGTGGT   
  
  
- CGGACTTACA CCAACTTTGG TCGGCCGACT AGTTAGTCTC GGTCAACCTG GGAAGAGTTT ACAAACTACG   
  
  
- ACGACGAGTG GGGAACCTCA GATTAACAGT AAGAAAACAG AGATCATTAT TGAAACTAGT AAGATACCTA   
  
  
- CCCCAAGGTC TAAGAGGATA GTCATGTGAA AGACACTTAC TTTGTTCTTT CCTTCTTAGT CAATCATCTA   
  
  
- GTTCCTCGTC CTTCTTAGTA GTTTCTCTAC CACGTATGGA CCTTCTTTCC TCATGATTCG TTTATCGTAG   
  
  
- GTCGTTACTC CTTATACCTC TCTACCTCTC CAAACTACTA CATGACTAGA CGTCCCTTCC TTTACTACTA   
  
  
- TAAAGTGGAA CATGGGCGTT TTGGGGTCAA GTACTTCGTC TACTATTGAG CCTTGGTTTC CCCTTTAGTT   
  
  
- CTCCTAGATT TTCGTTCTCA TTTAGCGCAC AGTTCTTCTT TTCATTGCTT CCTGTTCGCC ATCTAGAATC   
  
  
- CTGAGTTGAA TAAGTCACAC GTCTTCGTCA CCGTTCGAAA CTCGAATTTT CACGTTTACT CGATGAGTCC   
  
  
- GCTTAATCCG TCGTACGAAG CGGTAAACCC CTATCACAAG TTTCCGATCG TGTAATGAAA CGCTTACCCG   
  
  
- AGCTCCGTGC GAATCGACCA TGTCCTAGTC TTAGTATGTT TCTGAAACGA CCCTTTTTTA AAAGTAGGAT   
  
  
- ACTGTAGAAC TCTCCGATGT TCAGTATACA AGTTCGGCAG GGGAAGGTCT CTTGTTGGTA CAAGAACTGT   
  
  
- TTAGTTTGTT ATCATTTCGA CCGACTCTTT CGTTGTTCCC AAGTATAGTA ACTAAAACCG TAGAAAAAAC   
  
  
- CAAAGGTCAC CGGGACGGAA CAAGTCTTAA ACAGTTCTTC TGGGGTCCCA AGAGGGTATG AGTTTTAGTG   
  
  
- CCCTCAGCTA GTAGGGGTCT CACCCAAGTC TGGTCGGGTC TTTCAACTTC TCTGTCCAGC AGCTAATCGT   
  
  
- CCGATAACAC TCTCCAAACC CCACGGGAAA CTCAAAGTAG GGTAACGAGT TTCCACCGTA TGGGATTTCG   
  
  
- GTCTCGTAAA GTTTTAACTT TCGTTACTCG AACAACACCA GTGAACAAAG ATGGTTAGTC CCTTCGAGGG   
  
  
- ACTACTCTGA CAACTACAGT TGTCGGGTTC TCTGGTACAG AACTCCGACT AGTCCCCGAA CTTGGGACTA   
  
  
- GATAAGTAGG TACCCCAATA GTTACCGTGT AAGTTACGTG GGAAGAAGCA TTGAGCCAAA TTTCTCCGTG   
  
  
- AAATGGTGAT GAGAAGAAAC AAGCTACAGA AACTACGGTG GTATCTAGCA CTTTCAGTAC TTTCTGACGA   
  
  
- CTGACTCTTG ATAAACATAC CCTTTCTACG TGAATTGTAT CATCGTACAC TCCCCTTGCT CTCCTAGCTC   
  
  
- TCTGGGCTCT GAATGTTCGT CACCGTTCAG GCCTTCGAAT CTCGACCTAA GTTCGTCCAG GGGGATCTGG   
  
  
- TTCTGTAACA TTTACCTCGT TCTCGATACC AATTCCGTTT AATGTTATTC CTAAAATACC ATCTTGTTCT   
  
  
- ATCCTTAACC TACCAGGTCC CGACCTTTCC TTCCTAAAAC TCACGGTATA GGACAACCTT TGGACGAAT

  
  
Motifs Found  

+   

| Site Name | Organism | Position | Strand | Matrix score. | sequence | function |
| --- | --- | --- | --- | --- | --- | --- |
|  | organism | 3725 | + | 4 | motif\_sequence | short\_function |
|  | organism | 1909 | + | 4 | motif\_sequence | short\_function |
|  | organism | 3670 | - | 4 | motif\_sequence | short\_function |
|  | organism | 2331 | - | 4 | motif\_sequence | short\_function |
|  | organism | 2659 | - | 4 | motif\_sequence | short\_function |
|  | organism | 1725 | + | 4 | motif\_sequence | short\_function |
|  | organism | 1655 | + | 4 | motif\_sequence | short\_function |
|  | organism | 2247 | - | 4 | motif\_sequence | short\_function |
|  | organism | 1883 | + | 4 | motif\_sequence | short\_function |
|  | organism | 1244 | + | 4 | motif\_sequence | short\_function |
|  | organism | 1110 | - | 4 | motif\_sequence | short\_function |
|  | organism | 1220 | - | 4 | motif\_sequence | short\_function |
|  | organism | 1093 | - | 4 | motif\_sequence | short\_function |
|  | organism | 3103 | - | 4 | motif\_sequence | short\_function |
|  | organism | 643 | + | 4 | motif\_sequence | short\_function |
|  | organism | 2759 | - | 4 | motif\_sequence | short\_function |
|  | organism | 2934 | - | 4 | motif\_sequence | short\_function |
|  | organism | 2841 | - | 4 | motif\_sequence | short\_function |
|  | organism | 1638 | - | 4 | motif\_sequence | short\_function |
|  | organism | 1012 | - | 4 | motif\_sequence | short\_function |
|  | organism | 2751 | - | 4 | motif\_sequence | short\_function |
|  | organism | 1455 | + | 4 | motif\_sequence | short\_function |
|  | organism | 2087 | - | 4 | motif\_sequence | short\_function |
|  | organism | 954 | - | 4 | motif\_sequence | short\_function |
|  | organism | 526 | - | 4 | motif\_sequence | short\_function |
|  | organism | 2773 | + | 4 | motif\_sequence | short\_function |
|  | organism | 2041 | + | 4 | motif\_sequence | short\_function |
|  | organism | 1569 | + | 4 | motif\_sequence | short\_function |
|  | organism | 1320 | + | 4 | motif\_sequence | short\_function |
|  | organism | 3213 | + | 4 | motif\_sequence | short\_function |
|  | organism | 860 | - | 4 | motif\_sequence | short\_function |
|  | organism | 2035 | + | 4 | motif\_sequence | short\_function |
|  | organism | 312 | - | 4 | motif\_sequence | short\_function |
|  | organism | 83 | + | 4 | motif\_sequence | short\_function |
|  | organism | 1546 | + | 4 | motif\_sequence | short\_function |
|  | organism | 1479 | + | 4 | motif\_sequence | short\_function |
|  | organism | 3411 | - | 4 | motif\_sequence | short\_function |
|  | organism | 572 | - | 4 | motif\_sequence | short\_function |
|  | organism | 2165 | + | 4 | motif\_sequence | short\_function |
|  | organism | 3181 | + | 4 | motif\_sequence | short\_function |
|  | organism | 2781 | + | 4 | motif\_sequence | short\_function |
|  | organism | 3063 | - | 4 | motif\_sequence | short\_function |

>HU02G01573.1   
+ +Up\_Stream \_Len000TGAATT GGAAGTTGAT TCTACCTTTG ATTGATTCGA TTAATTCTAG GTTCTTGGTT   
  
  
+ CTAGTTCTCT CTCCTCTTGT TCTTGATTAT TTAGGTTTTG GATGTGATGT TTATTGAATT GAATGTTGCA   
  
  
+ ATTGTTGTTA TAAAGATGAT GTTAGGCTAA ATATCCTTCA ACTTAGGTTG GGATTGACGT CGAATATGTG   
  
  
+ TAGTTGAATC TTGTACTGGC CTAAATCCCT GCTCCCCCTC TGTTTTGCGA TTATGAATTT CATTATGCAC   
  
  
+ ACCACATGCT CGATGAAATG CTCGAAAGAA CATTCATCCT CTTTGTTTGG GATTTAGGGG AACGTTGAAA   
  
  
+ GACGAAGTCT TTACCTAGTG CGTCTTAGTA GACAATTAGG ATTTTAACAT GAAAATAGTT AAAATTCTAA   
  
  
+ TTGGGATTTG AGATTGTGAA TCTTGTAATC CTTCGACTTC TAGTTCTATG AAAATAGGCG ATTATAGTTC   
  
  
+ TAGGATTAGT CTTCGACCCT TGAGAGAGGG AGGAGATGGG TTTAGTTGAT AATCTATTCA TTCATAACGC   
  
  
+ ATGCCTGGGA GATGTTTGGT AGGGCTAGTA ATTGATTCGC TAACATATTT GTGCTGTTAG TTCCAACCTG   
  
  
+ AGTGTTTACT CTTCTGCTGT TTATTCCGTC TTTTAGTCGT AATTGTCTCC TTTAGTTTAA TTTCAAAACC   
  
  
+ CCAATTGTGA TCGCCTAGAT TGAGCAATCT AATTAGTTCT AATAATTGAA ATCAAGTTCC CTGTGGAATA   
  
  
+ CGACCCGTAC TTGCCGTGTG CTAGCTACTG TTACACCTTG CATTTGCGGT ATATTTATAA CTCAACATAT   
  
  
+ GTCTGCTCAC TAAAAGTACA TATTAGCTCA CCAAAACTGT GTCTCAACGT ACTAAAATAT TTTAGTGAAC   
  
  
+ TATACATTAT TTTTAGTAAG CGAAATAAGA TACACAACAG CACATTTTAA TGAGTTACAG GGCATTTTTA   
  
  
+ GTGAGCCGAA ATCTGATGGT TTTCTTTGTA GATCCGGTTA TATAATTGTA TTTTATACAA CCGAATGGAC   
  
  
+ GATATAACAA CTGAGTTAAA TGAGGAGTCT GAAATTACGA ACATAAAATC CAAAAGAAGA TGCCGATGCC   
  
  
+ ATTGGAAAGA AAGCTGAAGG TGATCGCTTT CTAGTAGTAT TAGGCAGTAG TAATTAAAAA CTAAAATCCA   
  
  
+ GAATTGACAC CGAATTGGGG TTGTGGCACA TGATCGAAAT CAGCATCGAA TCTACACGAT GATCTGGATT   
  
  
+ CTTGGACACT CTGGCTTTAG GCCGTCAATT TCGAGGATTG CCCATAACTT TAATCCTGTT CAAATCCATT   
  
  
+ CAATTTAATA TTACACAAAT CCTTTGATTA AATCACCGTA ATTGTGGTTT GATTAGGGAA GTTTCTTGGG   
  
  
+ GGAAAGGTTC AAGCTTCTGT CTTTCTTAAA GCCTGGATTT TTGGTCCCGC ATGTTCACTT CACATTCCCT   
  
  
+ TTTTCTCTCC CATCGTTACC TGTCTGAATC TGTATGTAAT AAAAGTCATA TTTTGCGAGG TCTTCTTTTG   
  
  
+ ACTGTTCTTG TAGTCACTCT TGATATCTGC ACCATTTTGT TTTTCTCGGC ATTTTCTCCA ACTTGCATTT   
  
  
+ TCTAATGTAA TGTGATCTTT TCTGGACATT CATTTTGTTA ATCTACTCAC TTGGGTCAAG GTTTGCTGAT   
  
  
+ TTTGGCTGGT GTTAAATCCG TTCCTCTTTG GCATTTGTTC ATGTTCCACA ACTGTTTGAT ATCTGGTATC   
  
  
+ TCAGAAACCC CGCCATTCTT TGGTATTTCC TGACTTTCTT GTTCAAGGGT TATTCCTATT TCCCTGAATT   
  
  
+ TTCTTTGATT CTTTAGGTAT TTCAAGCTTG GGGGGTTTTG TTCTTCACTG ATTTTGTCCT CTTCTTCCCT   
  
  
+ TATTGGGTGG TTTGATCTCC TAAATTTCTG TGTATCTGAA TCAGTCGGGG TAGACTTTGT GTTTTTGGTT   
  
  
+ GATTTTTTAT CAATGTATTT TCTTCCTAAT GAAGGGCCTC CCGAACCAAT ACACATGCTT AACTTCAACC   
  
  
+ CTGTTCCTCT TCCAAGCTTT TTGGAGCCTC AAAAGTCTTC GAATGCTGAT AGGCACATAG GGTCCTTCCA   
  
  
+ ATGCTATGGT CTTGATGATG ACCCCGCACC CTTGAATGAC CCAAATTACA TTGTATGTCA ATCTTCATAT   
  
  
+ CCTGATCGAG ATATTACACA GATCCCTGAC TTTCCTGATG ATTGTCTCAA GTTCATCAAT GACATTCTTA   
  
  
+ TGGAAGAGGA TTTGGATAAT CCGCCTATAT CCTTGCAGGA CTATAATGCG CTCCAAGCCA CTGAGAAGTC   
  
  
+ ATTGTATGAT GCCCTTGGAG AGGCTTATCC AGCTTCATCT GATCCATTGC AATCATCAGT TGGTCAGAGC   
  
  
+ ACTGAGAGCT CTGATGATCC TGACAGAAGC AGCGGCATTG ATAAATGGGG TAATAGATAT TACCCCACCA   
  
  
+ GCCTGAATGT GGTTGAAACC AGCCGGCTGA TCAATCAGAG CCAGTTGGAC CCTTCTCAAA TGTTTGATGC   
  
  
+ TGCTGCTCAC CCCTTGGAGT CTAATTGTCA TTCTTTTGTC TCTAGTAATA ACTTTGATCA TTCTATGGAT   
  
  
+ GGGGTTCCAG ATTCTCCTAT CAGTACACTT TCTGTGAATG AAACAAGAAA GGAAGAATCA GTTAGTAGAT   
  
  
+ CAAGGAGCAG GAAGAATCAT CAAAGAGATG GTGCATACCT GGAAGAAAGG AGTACTAAGC AAATAGCATC   
  
  
+ CAGCAATGAG GAATATGGAG AGATGGAGAG GTTTGATGAT GTACTGATCT GCAGGGAAGG AAATGATGAT   
  
  
+ ATTTCACCTT GTACCCGCAA AACCCCAGTT CATGAAGCAG ATGATAACTC GGAACCAAAG GGGAAATCAA   
  
  
+ GAGGATCTAA AAGCAAGAGT AAATCGCGTG TCAAGAAGAA AAGTAACGAA GGACAAGCGG TAGATCTTAG   
  
  
+ GACTCAACTT ATTCAGTGTG CAGAAGCAGT GGCAAGCTTT GAGCTTAAAA GTGCAAATGA GCTACTCAGG   
  
  
+ CGAATTAGGC AGCATGCTTC GCCATTTGGG GATAGTGTTC AAAGGCTAGC ACATTACTTT GCGAATGGGC   
  
  
+ TCGAGGCACG CTTAGCTGGT ACAGGATCAG AATCATACAA AGACTTTGCT GGGAAAAAAT TTTCATCCTA   
  
  
+ TGACATCTTG AGAGGCTACA AGTCATATGT TCAAGCCGTC CCCTTCCAGA GAACAACCAT GTTCTTGACA   
  
  
+ AATCAAACAA TAGTAAAGCT GGCTGAGAAA GCAACAAGGG TTCATATCAT TGATTTTGGC ATCTTTTTTG   
  
  
+ GTTTCCAGTG GCCCTGCCTT GTTCAGAATT TGTCAAGAAG ACCCCAGGGT TCTCCCATAC TCAAAATCAC   
  
  
+ GGGAGTCGAT CATCCCCAGA GTGGGTTCAG ACCAGCCCAG AAAGTTGAAG AGACAGGTCG TCGATTAGCA   
  
  
+ GGCTATTGTG AGAGGTTTGG GGTGCCCTTT GAGTTTCATC CCATTGCTCA AAGGTGGCAT ACCCTAAAGC   
  
  
+ CAGAGCATTT CAAAATTGAA AGCAATGAGC TTGTTGTGGT CACTTGTTTC TACCAATCAG GGAAGCTCCC   
  
  
+ TGATGAGACT GTTGATGTCA ACAGCCCAAG AGACCATGTC TTGAGGCTGA TCAGGGGCTT GAACCCTGAT   
  
  
+ CTATTCATCC ATGGGGTTAT CAATGGCACA TTCAATGCAC CCTTCTTCGT AACTCGGTTT AAAGAGGCAC   
  
  
+ TTTACCACTA CTCTTCTTTG TTCGATGTCT TTGATGCCAC CATAGATCGT GAAAGTCATG AAAGACTGCT   
  
  
+ GACTGAGAAC TATTTGTATG GGAAAGATGC ACTTAACATA GTAGCATGTG AGGGGAACGA GAGGATCGAG   
  
  
+ AGACCCGAGA CTTACAAGCA GTGGCAAGTC CGGAAGCTTA GAGCTGGATT CAAGCAGGTC CCCCTAGACC   
  
  
+ AAGACATTGT AAATGGAGCA AGAGCTATGG TTAAGGCAAA TTACAATAAG GATTTTATGG TAGAACAAGA   
  
  
+ TAGGAATTGG ATGGTCCAGG GCTGGAAAGG AAGGATTTTG AGTGCCATAT CCTGTTGGAA ACCTGCTTA  

- +Up\_Stream \_Len000ACTTAA CCTTCAACTA AGATGGAAAC TAACTAAGCT AATTAAGATC CAAGAACCAA   
  
  
- GATCAAGAGA GAGGAGAACA AGAACTAATA AATCCAAAAC CTACACTACA AATAACTTAA CTTACAACGT   
  
  
- TAACAACAAT ATTTCTACTA CAATCCGATT TATAGGAAGT TGAATCCAAC CCTAACTGCA GCTTATACAC   
  
  
- ATCAACTTAG AACATGACCG GATTTAGGGA CGAGGGGGAG ACAAAACGCT AATACTTAAA GTAATACGTG   
  
  
- TGGTGTACGA GCTACTTTAC GAGCTTTCTT GTAAGTAGGA GAAACAAACC CTAAATCCCC TTGCAACTTT   
  
  
- CTGCTTCAGA AATGGATCAC GCAGAATCAT CTGTTAATCC TAAAATTGTA CTTTTATCAA TTTTAAGATT   
  
  
- AACCCTAAAC TCTAACACTT AGAACATTAG GAAGCTGAAG ATCAAGATAC TTTTATCCGC TAATATCAAG   
  
  
- ATCCTAATCA GAAGCTGGGA ACTCTCTCCC TCCTCTACCC AAATCAACTA TTAGATAAGT AAGTATTGCG   
  
  
- TACGGACCCT CTACAAACCA TCCCGATCAT TAACTAAGCG ATTGTATAAA CACGACAATC AAGGTTGGAC   
  
  
- TCACAAATGA GAAGACGACA AATAAGGCAG AAAATCAGCA TTAACAGAGG AAATCAAATT AAAGTTTTGG   
  
  
- GGTTAACACT AGCGGATCTA ACTCGTTAGA TTAATCAAGA TTATTAACTT TAGTTCAAGG GACACCTTAT   
  
  
- GCTGGGCATG AACGGCACAC GATCGATGAC AATGTGGAAC GTAAACGCCA TATAAATATT GAGTTGTATA   
  
  
- CAGACGAGTG ATTTTCATGT ATAATCGAGT GGTTTTGACA CAGAGTTGCA TGATTTTATA AAATCACTTG   
  
  
- ATATGTAATA AAAATCATTC GCTTTATTCT ATGTGTTGTC GTGTAAAATT ACTCAATGTC CCGTAAAAAT   
  
  
- CACTCGGCTT TAGACTACCA AAAGAAACAT CTAGGCCAAT ATATTAACAT AAAATATGTT GGCTTACCTG   
  
  
- CTATATTGTT GACTCAATTT ACTCCTCAGA CTTTAATGCT TGTATTTTAG GTTTTCTTCT ACGGCTACGG   
  
  
- TAACCTTTCT TTCGACTTCC ACTAGCGAAA GATCATCATA ATCCGTCATC ATTAATTTTT GATTTTAGGT   
  
  
- CTTAACTGTG GCTTAACCCC AACACCGTGT ACTAGCTTTA GTCGTAGCTT AGATGTGCTA CTAGACCTAA   
  
  
- GAACCTGTGA GACCGAAATC CGGCAGTTAA AGCTCCTAAC GGGTATTGAA ATTAGGACAA GTTTAGGTAA   
  
  
- GTTAAATTAT AATGTGTTTA GGAAACTAAT TTAGTGGCAT TAACACCAAA CTAATCCCTT CAAAGAACCC   
  
  
- CCTTTCCAAG TTCGAAGACA GAAAGAATTT CGGACCTAAA AACCAGGGCG TACAAGTGAA GTGTAAGGGA   
  
  
- AAAAGAGAGG GTAGCAATGG ACAGACTTAG ACATACATTA TTTTCAGTAT AAAACGCTCC AGAAGAAAAC   
  
  
- TGACAAGAAC ATCAGTGAGA ACTATAGACG TGGTAAAACA AAAAGAGCCG TAAAAGAGGT TGAACGTAAA   
  
  
- AGATTACATT ACACTAGAAA AGACCTGTAA GTAAAACAAT TAGATGAGTG AACCCAGTTC CAAACGACTA   
  
  
- AAACCGACCA CAATTTAGGC AAGGAGAAAC CGTAAACAAG TACAAGGTGT TGACAAACTA TAGACCATAG   
  
  
- AGTCTTTGGG GCGGTAAGAA ACCATAAAGG ACTGAAAGAA CAAGTTCCCA ATAAGGATAA AGGGACTTAA   
  
  
- AAGAAACTAA GAAATCCATA AAGTTCGAAC CCCCCAAAAC AAGAAGTGAC TAAAACAGGA GAAGAAGGGA   
  
  
- ATAACCCACC AAACTAGAGG ATTTAAAGAC ACATAGACTT AGTCAGCCCC ATCTGAAACA CAAAAACCAA   
  
  
- CTAAAAAATA GTTACATAAA AGAAGGATTA CTTCCCGGAG GGCTTGGTTA TGTGTACGAA TTGAAGTTGG   
  
  
- GACAAGGAGA AGGTTCGAAA AACCTCGGAG TTTTCAGAAG CTTACGACTA TCCGTGTATC CCAGGAAGGT   
  
  
- TACGATACCA GAACTACTAC TGGGGCGTGG GAACTTACTG GGTTTAATGT AACATACAGT TAGAAGTATA   
  
  
- GGACTAGCTC TATAATGTGT CTAGGGACTG AAAGGACTAC TAACAGAGTT CAAGTAGTTA CTGTAAGAAT   
  
  
- ACCTTCTCCT AAACCTATTA GGCGGATATA GGAACGTCCT GATATTACGC GAGGTTCGGT GACTCTTCAG   
  
  
- TAACATACTA CGGGAACCTC TCCGAATAGG TCGAAGTAGA CTAGGTAACG TTAGTAGTCA ACCAGTCTCG   
  
  
- TGACTCTCGA GACTACTAGG ACTGTCTTCG TCGCCGTAAC TATTTACCCC ATTATCTATA ATGGGGTGGT   
  
  
- CGGACTTACA CCAACTTTGG TCGGCCGACT AGTTAGTCTC GGTCAACCTG GGAAGAGTTT ACAAACTACG   
  
  
- ACGACGAGTG GGGAACCTCA GATTAACAGT AAGAAAACAG AGATCATTAT TGAAACTAGT AAGATACCTA   
  
  
- CCCCAAGGTC TAAGAGGATA GTCATGTGAA AGACACTTAC TTTGTTCTTT CCTTCTTAGT CAATCATCTA   
  
  
- GTTCCTCGTC CTTCTTAGTA GTTTCTCTAC CACGTATGGA CCTTCTTTCC TCATGATTCG TTTATCGTAG   
  
  
- GTCGTTACTC CTTATACCTC TCTACCTCTC CAAACTACTA CATGACTAGA CGTCCCTTCC TTTACTACTA   
  
  
- TAAAGTGGAA CATGGGCGTT TTGGGGTCAA GTACTTCGTC TACTATTGAG CCTTGGTTTC CCCTTTAGTT   
  
  
- CTCCTAGATT TTCGTTCTCA TTTAGCGCAC AGTTCTTCTT TTCATTGCTT CCTGTTCGCC ATCTAGAATC   
  
  
- CTGAGTTGAA TAAGTCACAC GTCTTCGTCA CCGTTCGAAA CTCGAATTTT CACGTTTACT CGATGAGTCC   
  
  
- GCTTAATCCG TCGTACGAAG CGGTAAACCC CTATCACAAG TTTCCGATCG TGTAATGAAA CGCTTACCCG   
  
  
- AGCTCCGTGC GAATCGACCA TGTCCTAGTC TTAGTATGTT TCTGAAACGA CCCTTTTTTA AAAGTAGGAT   
  
  
- ACTGTAGAAC TCTCCGATGT TCAGTATACA AGTTCGGCAG GGGAAGGTCT CTTGTTGGTA CAAGAACTGT   
  
  
- TTAGTTTGTT ATCATTTCGA CCGACTCTTT CGTTGTTCCC AAGTATAGTA ACTAAAACCG TAGAAAAAAC   
  
  
- CAAAGGTCAC CGGGACGGAA CAAGTCTTAA ACAGTTCTTC TGGGGTCCCA AGAGGGTATG AGTTTTAGTG   
  
  
- CCCTCAGCTA GTAGGGGTCT CACCCAAGTC TGGTCGGGTC TTTCAACTTC TCTGTCCAGC AGCTAATCGT   
  
  
- CCGATAACAC TCTCCAAACC CCACGGGAAA CTCAAAGTAG GGTAACGAGT TTCCACCGTA TGGGATTTCG   
  
  
- GTCTCGTAAA GTTTTAACTT TCGTTACTCG AACAACACCA GTGAACAAAG ATGGTTAGTC CCTTCGAGGG   
  
  
- ACTACTCTGA CAACTACAGT TGTCGGGTTC TCTGGTACAG AACTCCGACT AGTCCCCGAA CTTGGGACTA   
  
  
- GATAAGTAGG TACCCCAATA GTTACCGTGT AAGTTACGTG GGAAGAAGCA TTGAGCCAAA TTTCTCCGTG   
  
  
- AAATGGTGAT GAGAAGAAAC AAGCTACAGA AACTACGGTG GTATCTAGCA CTTTCAGTAC TTTCTGACGA   
  
  
- CTGACTCTTG ATAAACATAC CCTTTCTACG TGAATTGTAT CATCGTACAC TCCCCTTGCT CTCCTAGCTC   
  
  
- TCTGGGCTCT GAATGTTCGT CACCGTTCAG GCCTTCGAAT CTCGACCTAA GTTCGTCCAG GGGGATCTGG   
  
  
- TTCTGTAACA TTTACCTCGT TCTCGATACC AATTCCGTTT AATGTTATTC CTAAAATACC ATCTTGTTCT   
  
  
- ATCCTTAACC TACCAGGTCC CGACCTTTCC TTCCTAAAAC TCACGGTATA GGACAACCTT TGGACGAAT

+     A-box

| Site Name | Organism | Position | Strand | Matrix score. | sequence | function |
| --- | --- | --- | --- | --- | --- | --- |
| A-box | Petroselinum crispum | 3190 | + | 6 | CCGTCC | cis-acting regulatory element |

>HU02G01573.1   
+ +Up\_Stream \_Len000TGAATT GGAAGTTGAT TCTACCTTTG ATTGATTCGA TTAATTCTAG GTTCTTGGTT   
  
  
+ CTAGTTCTCT CTCCTCTTGT TCTTGATTAT TTAGGTTTTG GATGTGATGT TTATTGAATT GAATGTTGCA   
  
  
+ ATTGTTGTTA TAAAGATGAT GTTAGGCTAA ATATCCTTCA ACTTAGGTTG GGATTGACGT CGAATATGTG   
  
  
+ TAGTTGAATC TTGTACTGGC CTAAATCCCT GCTCCCCCTC TGTTTTGCGA TTATGAATTT CATTATGCAC   
  
  
+ ACCACATGCT CGATGAAATG CTCGAAAGAA CATTCATCCT CTTTGTTTGG GATTTAGGGG AACGTTGAAA   
  
  
+ GACGAAGTCT TTACCTAGTG CGTCTTAGTA GACAATTAGG ATTTTAACAT GAAAATAGTT AAAATTCTAA   
  
  
+ TTGGGATTTG AGATTGTGAA TCTTGTAATC CTTCGACTTC TAGTTCTATG AAAATAGGCG ATTATAGTTC   
  
  
+ TAGGATTAGT CTTCGACCCT TGAGAGAGGG AGGAGATGGG TTTAGTTGAT AATCTATTCA TTCATAACGC   
  
  
+ ATGCCTGGGA GATGTTTGGT AGGGCTAGTA ATTGATTCGC TAACATATTT GTGCTGTTAG TTCCAACCTG   
  
  
+ AGTGTTTACT CTTCTGCTGT TTATTCCGTC TTTTAGTCGT AATTGTCTCC TTTAGTTTAA TTTCAAAACC   
  
  
+ CCAATTGTGA TCGCCTAGAT TGAGCAATCT AATTAGTTCT AATAATTGAA ATCAAGTTCC CTGTGGAATA   
  
  
+ CGACCCGTAC TTGCCGTGTG CTAGCTACTG TTACACCTTG CATTTGCGGT ATATTTATAA CTCAACATAT   
  
  
+ GTCTGCTCAC TAAAAGTACA TATTAGCTCA CCAAAACTGT GTCTCAACGT ACTAAAATAT TTTAGTGAAC   
  
  
+ TATACATTAT TTTTAGTAAG CGAAATAAGA TACACAACAG CACATTTTAA TGAGTTACAG GGCATTTTTA   
  
  
+ GTGAGCCGAA ATCTGATGGT TTTCTTTGTA GATCCGGTTA TATAATTGTA TTTTATACAA CCGAATGGAC   
  
  
+ GATATAACAA CTGAGTTAAA TGAGGAGTCT GAAATTACGA ACATAAAATC CAAAAGAAGA TGCCGATGCC   
  
  
+ ATTGGAAAGA AAGCTGAAGG TGATCGCTTT CTAGTAGTAT TAGGCAGTAG TAATTAAAAA CTAAAATCCA   
  
  
+ GAATTGACAC CGAATTGGGG TTGTGGCACA TGATCGAAAT CAGCATCGAA TCTACACGAT GATCTGGATT   
  
  
+ CTTGGACACT CTGGCTTTAG GCCGTCAATT TCGAGGATTG CCCATAACTT TAATCCTGTT CAAATCCATT   
  
  
+ CAATTTAATA TTACACAAAT CCTTTGATTA AATCACCGTA ATTGTGGTTT GATTAGGGAA GTTTCTTGGG   
  
  
+ GGAAAGGTTC AAGCTTCTGT CTTTCTTAAA GCCTGGATTT TTGGTCCCGC ATGTTCACTT CACATTCCCT   
  
  
+ TTTTCTCTCC CATCGTTACC TGTCTGAATC TGTATGTAAT AAAAGTCATA TTTTGCGAGG TCTTCTTTTG   
  
  
+ ACTGTTCTTG TAGTCACTCT TGATATCTGC ACCATTTTGT TTTTCTCGGC ATTTTCTCCA ACTTGCATTT   
  
  
+ TCTAATGTAA TGTGATCTTT TCTGGACATT CATTTTGTTA ATCTACTCAC TTGGGTCAAG GTTTGCTGAT   
  
  
+ TTTGGCTGGT GTTAAATCCG TTCCTCTTTG GCATTTGTTC ATGTTCCACA ACTGTTTGAT ATCTGGTATC   
  
  
+ TCAGAAACCC CGCCATTCTT TGGTATTTCC TGACTTTCTT GTTCAAGGGT TATTCCTATT TCCCTGAATT   
  
  
+ TTCTTTGATT CTTTAGGTAT TTCAAGCTTG GGGGGTTTTG TTCTTCACTG ATTTTGTCCT CTTCTTCCCT   
  
  
+ TATTGGGTGG TTTGATCTCC TAAATTTCTG TGTATCTGAA TCAGTCGGGG TAGACTTTGT GTTTTTGGTT   
  
  
+ GATTTTTTAT CAATGTATTT TCTTCCTAAT GAAGGGCCTC CCGAACCAAT ACACATGCTT AACTTCAACC   
  
  
+ CTGTTCCTCT TCCAAGCTTT TTGGAGCCTC AAAAGTCTTC GAATGCTGAT AGGCACATAG GGTCCTTCCA   
  
  
+ ATGCTATGGT CTTGATGATG ACCCCGCACC CTTGAATGAC CCAAATTACA TTGTATGTCA ATCTTCATAT   
  
  
+ CCTGATCGAG ATATTACACA GATCCCTGAC TTTCCTGATG ATTGTCTCAA GTTCATCAAT GACATTCTTA   
  
  
+ TGGAAGAGGA TTTGGATAAT CCGCCTATAT CCTTGCAGGA CTATAATGCG CTCCAAGCCA CTGAGAAGTC   
  
  
+ ATTGTATGAT GCCCTTGGAG AGGCTTATCC AGCTTCATCT GATCCATTGC AATCATCAGT TGGTCAGAGC   
  
  
+ ACTGAGAGCT CTGATGATCC TGACAGAAGC AGCGGCATTG ATAAATGGGG TAATAGATAT TACCCCACCA   
  
  
+ GCCTGAATGT GGTTGAAACC AGCCGGCTGA TCAATCAGAG CCAGTTGGAC CCTTCTCAAA TGTTTGATGC   
  
  
+ TGCTGCTCAC CCCTTGGAGT CTAATTGTCA TTCTTTTGTC TCTAGTAATA ACTTTGATCA TTCTATGGAT   
  
  
+ GGGGTTCCAG ATTCTCCTAT CAGTACACTT TCTGTGAATG AAACAAGAAA GGAAGAATCA GTTAGTAGAT   
  
  
+ CAAGGAGCAG GAAGAATCAT CAAAGAGATG GTGCATACCT GGAAGAAAGG AGTACTAAGC AAATAGCATC   
  
  
+ CAGCAATGAG GAATATGGAG AGATGGAGAG GTTTGATGAT GTACTGATCT GCAGGGAAGG AAATGATGAT   
  
  
+ ATTTCACCTT GTACCCGCAA AACCCCAGTT CATGAAGCAG ATGATAACTC GGAACCAAAG GGGAAATCAA   
  
  
+ GAGGATCTAA AAGCAAGAGT AAATCGCGTG TCAAGAAGAA AAGTAACGAA GGACAAGCGG TAGATCTTAG   
  
  
+ GACTCAACTT ATTCAGTGTG CAGAAGCAGT GGCAAGCTTT GAGCTTAAAA GTGCAAATGA GCTACTCAGG   
  
  
+ CGAATTAGGC AGCATGCTTC GCCATTTGGG GATAGTGTTC AAAGGCTAGC ACATTACTTT GCGAATGGGC   
  
  
+ TCGAGGCACG CTTAGCTGGT ACAGGATCAG AATCATACAA AGACTTTGCT GGGAAAAAAT TTTCATCCTA   
  
  
+ TGACATCTTG AGAGGCTACA AGTCATATGT TCAAGCCGTC CCCTTCCAGA GAACAACCAT GTTCTTGACA   
  
  
+ AATCAAACAA TAGTAAAGCT GGCTGAGAAA GCAACAAGGG TTCATATCAT TGATTTTGGC ATCTTTTTTG   
  
  
+ GTTTCCAGTG GCCCTGCCTT GTTCAGAATT TGTCAAGAAG ACCCCAGGGT TCTCCCATAC TCAAAATCAC   
  
  
+ GGGAGTCGAT CATCCCCAGA GTGGGTTCAG ACCAGCCCAG AAAGTTGAAG AGACAGGTCG TCGATTAGCA   
  
  
+ GGCTATTGTG AGAGGTTTGG GGTGCCCTTT GAGTTTCATC CCATTGCTCA AAGGTGGCAT ACCCTAAAGC   
  
  
+ CAGAGCATTT CAAAATTGAA AGCAATGAGC TTGTTGTGGT CACTTGTTTC TACCAATCAG GGAAGCTCCC   
  
  
+ TGATGAGACT GTTGATGTCA ACAGCCCAAG AGACCATGTC TTGAGGCTGA TCAGGGGCTT GAACCCTGAT   
  
  
+ CTATTCATCC ATGGGGTTAT CAATGGCACA TTCAATGCAC CCTTCTTCGT AACTCGGTTT AAAGAGGCAC   
  
  
+ TTTACCACTA CTCTTCTTTG TTCGATGTCT TTGATGCCAC CATAGATCGT GAAAGTCATG AAAGACTGCT   
  
  
+ GACTGAGAAC TATTTGTATG GGAAAGATGC ACTTAACATA GTAGCATGTG AGGGGAACGA GAGGATCGAG   
  
  
+ AGACCCGAGA CTTACAAGCA GTGGCAAGTC CGGAAGCTTA GAGCTGGATT CAAGCAGGTC CCCCTAGACC   
  
  
+ AAGACATTGT AAATGGAGCA AGAGCTATGG TTAAGGCAAA TTACAATAAG GATTTTATGG TAGAACAAGA   
  
  
+ TAGGAATTGG ATGGTCCAGG GCTGGAAAGG AAGGATTTTG AGTGCCATAT CCTGTTGGAA ACCTGCTTA  

- +Up\_Stream \_Len000ACTTAA CCTTCAACTA AGATGGAAAC TAACTAAGCT AATTAAGATC CAAGAACCAA   
  
  
- GATCAAGAGA GAGGAGAACA AGAACTAATA AATCCAAAAC CTACACTACA AATAACTTAA CTTACAACGT   
  
  
- TAACAACAAT ATTTCTACTA CAATCCGATT TATAGGAAGT TGAATCCAAC CCTAACTGCA GCTTATACAC   
  
  
- ATCAACTTAG AACATGACCG GATTTAGGGA CGAGGGGGAG ACAAAACGCT AATACTTAAA GTAATACGTG   
  
  
- TGGTGTACGA GCTACTTTAC GAGCTTTCTT GTAAGTAGGA GAAACAAACC CTAAATCCCC TTGCAACTTT   
  
  
- CTGCTTCAGA AATGGATCAC GCAGAATCAT CTGTTAATCC TAAAATTGTA CTTTTATCAA TTTTAAGATT   
  
  
- AACCCTAAAC TCTAACACTT AGAACATTAG GAAGCTGAAG ATCAAGATAC TTTTATCCGC TAATATCAAG   
  
  
- ATCCTAATCA GAAGCTGGGA ACTCTCTCCC TCCTCTACCC AAATCAACTA TTAGATAAGT AAGTATTGCG   
  
  
- TACGGACCCT CTACAAACCA TCCCGATCAT TAACTAAGCG ATTGTATAAA CACGACAATC AAGGTTGGAC   
  
  
- TCACAAATGA GAAGACGACA AATAAGGCAG AAAATCAGCA TTAACAGAGG AAATCAAATT AAAGTTTTGG   
  
  
- GGTTAACACT AGCGGATCTA ACTCGTTAGA TTAATCAAGA TTATTAACTT TAGTTCAAGG GACACCTTAT   
  
  
- GCTGGGCATG AACGGCACAC GATCGATGAC AATGTGGAAC GTAAACGCCA TATAAATATT GAGTTGTATA   
  
  
- CAGACGAGTG ATTTTCATGT ATAATCGAGT GGTTTTGACA CAGAGTTGCA TGATTTTATA AAATCACTTG   
  
  
- ATATGTAATA AAAATCATTC GCTTTATTCT ATGTGTTGTC GTGTAAAATT ACTCAATGTC CCGTAAAAAT   
  
  
- CACTCGGCTT TAGACTACCA AAAGAAACAT CTAGGCCAAT ATATTAACAT AAAATATGTT GGCTTACCTG   
  
  
- CTATATTGTT GACTCAATTT ACTCCTCAGA CTTTAATGCT TGTATTTTAG GTTTTCTTCT ACGGCTACGG   
  
  
- TAACCTTTCT TTCGACTTCC ACTAGCGAAA GATCATCATA ATCCGTCATC ATTAATTTTT GATTTTAGGT   
  
  
- CTTAACTGTG GCTTAACCCC AACACCGTGT ACTAGCTTTA GTCGTAGCTT AGATGTGCTA CTAGACCTAA   
  
  
- GAACCTGTGA GACCGAAATC CGGCAGTTAA AGCTCCTAAC GGGTATTGAA ATTAGGACAA GTTTAGGTAA   
  
  
- GTTAAATTAT AATGTGTTTA GGAAACTAAT TTAGTGGCAT TAACACCAAA CTAATCCCTT CAAAGAACCC   
  
  
- CCTTTCCAAG TTCGAAGACA GAAAGAATTT CGGACCTAAA AACCAGGGCG TACAAGTGAA GTGTAAGGGA   
  
  
- AAAAGAGAGG GTAGCAATGG ACAGACTTAG ACATACATTA TTTTCAGTAT AAAACGCTCC AGAAGAAAAC   
  
  
- TGACAAGAAC ATCAGTGAGA ACTATAGACG TGGTAAAACA AAAAGAGCCG TAAAAGAGGT TGAACGTAAA   
  
  
- AGATTACATT ACACTAGAAA AGACCTGTAA GTAAAACAAT TAGATGAGTG AACCCAGTTC CAAACGACTA   
  
  
- AAACCGACCA CAATTTAGGC AAGGAGAAAC CGTAAACAAG TACAAGGTGT TGACAAACTA TAGACCATAG   
  
  
- AGTCTTTGGG GCGGTAAGAA ACCATAAAGG ACTGAAAGAA CAAGTTCCCA ATAAGGATAA AGGGACTTAA   
  
  
- AAGAAACTAA GAAATCCATA AAGTTCGAAC CCCCCAAAAC AAGAAGTGAC TAAAACAGGA GAAGAAGGGA   
  
  
- ATAACCCACC AAACTAGAGG ATTTAAAGAC ACATAGACTT AGTCAGCCCC ATCTGAAACA CAAAAACCAA   
  
  
- CTAAAAAATA GTTACATAAA AGAAGGATTA CTTCCCGGAG GGCTTGGTTA TGTGTACGAA TTGAAGTTGG   
  
  
- GACAAGGAGA AGGTTCGAAA AACCTCGGAG TTTTCAGAAG CTTACGACTA TCCGTGTATC CCAGGAAGGT   
  
  
- TACGATACCA GAACTACTAC TGGGGCGTGG GAACTTACTG GGTTTAATGT AACATACAGT TAGAAGTATA   
  
  
- GGACTAGCTC TATAATGTGT CTAGGGACTG AAAGGACTAC TAACAGAGTT CAAGTAGTTA CTGTAAGAAT   
  
  
- ACCTTCTCCT AAACCTATTA GGCGGATATA GGAACGTCCT GATATTACGC GAGGTTCGGT GACTCTTCAG   
  
  
- TAACATACTA CGGGAACCTC TCCGAATAGG TCGAAGTAGA CTAGGTAACG TTAGTAGTCA ACCAGTCTCG   
  
  
- TGACTCTCGA GACTACTAGG ACTGTCTTCG TCGCCGTAAC TATTTACCCC ATTATCTATA ATGGGGTGGT   
  
  
- CGGACTTACA CCAACTTTGG TCGGCCGACT AGTTAGTCTC GGTCAACCTG GGAAGAGTTT ACAAACTACG   
  
  
- ACGACGAGTG GGGAACCTCA GATTAACAGT AAGAAAACAG AGATCATTAT TGAAACTAGT AAGATACCTA   
  
  
- CCCCAAGGTC TAAGAGGATA GTCATGTGAA AGACACTTAC TTTGTTCTTT CCTTCTTAGT CAATCATCTA   
  
  
- GTTCCTCGTC CTTCTTAGTA GTTTCTCTAC CACGTATGGA CCTTCTTTCC TCATGATTCG TTTATCGTAG   
  
  
- GTCGTTACTC CTTATACCTC TCTACCTCTC CAAACTACTA CATGACTAGA CGTCCCTTCC TTTACTACTA   
  
  
- TAAAGTGGAA CATGGGCGTT TTGGGGTCAA GTACTTCGTC TACTATTGAG CCTTGGTTTC CCCTTTAGTT   
  
  
- CTCCTAGATT TTCGTTCTCA TTTAGCGCAC AGTTCTTCTT TTCATTGCTT CCTGTTCGCC ATCTAGAATC   
  
  
- CTGAGTTGAA TAAGTCACAC GTCTTCGTCA CCGTTCGAAA CTCGAATTTT CACGTTTACT CGATGAGTCC   
  
  
- GCTTAATCCG TCGTACGAAG CGGTAAACCC CTATCACAAG TTTCCGATCG TGTAATGAAA CGCTTACCCG   
  
  
- AGCTCCGTGC GAATCGACCA TGTCCTAGTC TTAGTATGTT TCTGAAACGA CCCTTTTTTA AAAGTAGGAT   
  
  
- ACTGTAGAAC TCTCCGATGT TCAGTATACA AGTTCGGCAG GGGAAGGTCT CTTGTTGGTA CAAGAACTGT   
  
  
- TTAGTTTGTT ATCATTTCGA CCGACTCTTT CGTTGTTCCC AAGTATAGTA ACTAAAACCG TAGAAAAAAC   
  
  
- CAAAGGTCAC CGGGACGGAA CAAGTCTTAA ACAGTTCTTC TGGGGTCCCA AGAGGGTATG AGTTTTAGTG   
  
  
- CCCTCAGCTA GTAGGGGTCT CACCCAAGTC TGGTCGGGTC TTTCAACTTC TCTGTCCAGC AGCTAATCGT   
  
  
- CCGATAACAC TCTCCAAACC CCACGGGAAA CTCAAAGTAG GGTAACGAGT TTCCACCGTA TGGGATTTCG   
  
  
- GTCTCGTAAA GTTTTAACTT TCGTTACTCG AACAACACCA GTGAACAAAG ATGGTTAGTC CCTTCGAGGG   
  
  
- ACTACTCTGA CAACTACAGT TGTCGGGTTC TCTGGTACAG AACTCCGACT AGTCCCCGAA CTTGGGACTA   
  
  
- GATAAGTAGG TACCCCAATA GTTACCGTGT AAGTTACGTG GGAAGAAGCA TTGAGCCAAA TTTCTCCGTG   
  
  
- AAATGGTGAT GAGAAGAAAC AAGCTACAGA AACTACGGTG GTATCTAGCA CTTTCAGTAC TTTCTGACGA   
  
  
- CTGACTCTTG ATAAACATAC CCTTTCTACG TGAATTGTAT CATCGTACAC TCCCCTTGCT CTCCTAGCTC   
  
  
- TCTGGGCTCT GAATGTTCGT CACCGTTCAG GCCTTCGAAT CTCGACCTAA GTTCGTCCAG GGGGATCTGG   
  
  
- TTCTGTAACA TTTACCTCGT TCTCGATACC AATTCCGTTT AATGTTATTC CTAAAATACC ATCTTGTTCT   
  
  
- ATCCTTAACC TACCAGGTCC CGACCTTTCC TTCCTAAAAC TCACGGTATA GGACAACCTT TGGACGAAT

+     AAGAA-motif

| Site Name | Organism | Position | Strand | Matrix score. | sequence | function |
| --- | --- | --- | --- | --- | --- | --- |
| AAGAA-motif | Avena sativa | 308 | + | 7 | GAAAGAA |  |
| AAGAA-motif | Avena sativa | 1129 | + | 7 | GAAAGAA |  |

>HU02G01573.1   
+ +Up\_Stream \_Len000TGAATT GGAAGTTGAT TCTACCTTTG ATTGATTCGA TTAATTCTAG GTTCTTGGTT   
  
  
+ CTAGTTCTCT CTCCTCTTGT TCTTGATTAT TTAGGTTTTG GATGTGATGT TTATTGAATT GAATGTTGCA   
  
  
+ ATTGTTGTTA TAAAGATGAT GTTAGGCTAA ATATCCTTCA ACTTAGGTTG GGATTGACGT CGAATATGTG   
  
  
+ TAGTTGAATC TTGTACTGGC CTAAATCCCT GCTCCCCCTC TGTTTTGCGA TTATGAATTT CATTATGCAC   
  
  
+ ACCACATGCT CGATGAAATG CTCGAAAGAA CATTCATCCT CTTTGTTTGG GATTTAGGGG AACGTTGAAA   
  
  
+ GACGAAGTCT TTACCTAGTG CGTCTTAGTA GACAATTAGG ATTTTAACAT GAAAATAGTT AAAATTCTAA   
  
  
+ TTGGGATTTG AGATTGTGAA TCTTGTAATC CTTCGACTTC TAGTTCTATG AAAATAGGCG ATTATAGTTC   
  
  
+ TAGGATTAGT CTTCGACCCT TGAGAGAGGG AGGAGATGGG TTTAGTTGAT AATCTATTCA TTCATAACGC   
  
  
+ ATGCCTGGGA GATGTTTGGT AGGGCTAGTA ATTGATTCGC TAACATATTT GTGCTGTTAG TTCCAACCTG   
  
  
+ AGTGTTTACT CTTCTGCTGT TTATTCCGTC TTTTAGTCGT AATTGTCTCC TTTAGTTTAA TTTCAAAACC   
  
  
+ CCAATTGTGA TCGCCTAGAT TGAGCAATCT AATTAGTTCT AATAATTGAA ATCAAGTTCC CTGTGGAATA   
  
  
+ CGACCCGTAC TTGCCGTGTG CTAGCTACTG TTACACCTTG CATTTGCGGT ATATTTATAA CTCAACATAT   
  
  
+ GTCTGCTCAC TAAAAGTACA TATTAGCTCA CCAAAACTGT GTCTCAACGT ACTAAAATAT TTTAGTGAAC   
  
  
+ TATACATTAT TTTTAGTAAG CGAAATAAGA TACACAACAG CACATTTTAA TGAGTTACAG GGCATTTTTA   
  
  
+ GTGAGCCGAA ATCTGATGGT TTTCTTTGTA GATCCGGTTA TATAATTGTA TTTTATACAA CCGAATGGAC   
  
  
+ GATATAACAA CTGAGTTAAA TGAGGAGTCT GAAATTACGA ACATAAAATC CAAAAGAAGA TGCCGATGCC   
  
  
+ ATTGGAAAGA AAGCTGAAGG TGATCGCTTT CTAGTAGTAT TAGGCAGTAG TAATTAAAAA CTAAAATCCA   
  
  
+ GAATTGACAC CGAATTGGGG TTGTGGCACA TGATCGAAAT CAGCATCGAA TCTACACGAT GATCTGGATT   
  
  
+ CTTGGACACT CTGGCTTTAG GCCGTCAATT TCGAGGATTG CCCATAACTT TAATCCTGTT CAAATCCATT   
  
  
+ CAATTTAATA TTACACAAAT CCTTTGATTA AATCACCGTA ATTGTGGTTT GATTAGGGAA GTTTCTTGGG   
  
  
+ GGAAAGGTTC AAGCTTCTGT CTTTCTTAAA GCCTGGATTT TTGGTCCCGC ATGTTCACTT CACATTCCCT   
  
  
+ TTTTCTCTCC CATCGTTACC TGTCTGAATC TGTATGTAAT AAAAGTCATA TTTTGCGAGG TCTTCTTTTG   
  
  
+ ACTGTTCTTG TAGTCACTCT TGATATCTGC ACCATTTTGT TTTTCTCGGC ATTTTCTCCA ACTTGCATTT   
  
  
+ TCTAATGTAA TGTGATCTTT TCTGGACATT CATTTTGTTA ATCTACTCAC TTGGGTCAAG GTTTGCTGAT   
  
  
+ TTTGGCTGGT GTTAAATCCG TTCCTCTTTG GCATTTGTTC ATGTTCCACA ACTGTTTGAT ATCTGGTATC   
  
  
+ TCAGAAACCC CGCCATTCTT TGGTATTTCC TGACTTTCTT GTTCAAGGGT TATTCCTATT TCCCTGAATT   
  
  
+ TTCTTTGATT CTTTAGGTAT TTCAAGCTTG GGGGGTTTTG TTCTTCACTG ATTTTGTCCT CTTCTTCCCT   
  
  
+ TATTGGGTGG TTTGATCTCC TAAATTTCTG TGTATCTGAA TCAGTCGGGG TAGACTTTGT GTTTTTGGTT   
  
  
+ GATTTTTTAT CAATGTATTT TCTTCCTAAT GAAGGGCCTC CCGAACCAAT ACACATGCTT AACTTCAACC   
  
  
+ CTGTTCCTCT TCCAAGCTTT TTGGAGCCTC AAAAGTCTTC GAATGCTGAT AGGCACATAG GGTCCTTCCA   
  
  
+ ATGCTATGGT CTTGATGATG ACCCCGCACC CTTGAATGAC CCAAATTACA TTGTATGTCA ATCTTCATAT   
  
  
+ CCTGATCGAG ATATTACACA GATCCCTGAC TTTCCTGATG ATTGTCTCAA GTTCATCAAT GACATTCTTA   
  
  
+ TGGAAGAGGA TTTGGATAAT CCGCCTATAT CCTTGCAGGA CTATAATGCG CTCCAAGCCA CTGAGAAGTC   
  
  
+ ATTGTATGAT GCCCTTGGAG AGGCTTATCC AGCTTCATCT GATCCATTGC AATCATCAGT TGGTCAGAGC   
  
  
+ ACTGAGAGCT CTGATGATCC TGACAGAAGC AGCGGCATTG ATAAATGGGG TAATAGATAT TACCCCACCA   
  
  
+ GCCTGAATGT GGTTGAAACC AGCCGGCTGA TCAATCAGAG CCAGTTGGAC CCTTCTCAAA TGTTTGATGC   
  
  
+ TGCTGCTCAC CCCTTGGAGT CTAATTGTCA TTCTTTTGTC TCTAGTAATA ACTTTGATCA TTCTATGGAT   
  
  
+ GGGGTTCCAG ATTCTCCTAT CAGTACACTT TCTGTGAATG AAACAAGAAA GGAAGAATCA GTTAGTAGAT   
  
  
+ CAAGGAGCAG GAAGAATCAT CAAAGAGATG GTGCATACCT GGAAGAAAGG AGTACTAAGC AAATAGCATC   
  
  
+ CAGCAATGAG GAATATGGAG AGATGGAGAG GTTTGATGAT GTACTGATCT GCAGGGAAGG AAATGATGAT   
  
  
+ ATTTCACCTT GTACCCGCAA AACCCCAGTT CATGAAGCAG ATGATAACTC GGAACCAAAG GGGAAATCAA   
  
  
+ GAGGATCTAA AAGCAAGAGT AAATCGCGTG TCAAGAAGAA AAGTAACGAA GGACAAGCGG TAGATCTTAG   
  
  
+ GACTCAACTT ATTCAGTGTG CAGAAGCAGT GGCAAGCTTT GAGCTTAAAA GTGCAAATGA GCTACTCAGG   
  
  
+ CGAATTAGGC AGCATGCTTC GCCATTTGGG GATAGTGTTC AAAGGCTAGC ACATTACTTT GCGAATGGGC   
  
  
+ TCGAGGCACG CTTAGCTGGT ACAGGATCAG AATCATACAA AGACTTTGCT GGGAAAAAAT TTTCATCCTA   
  
  
+ TGACATCTTG AGAGGCTACA AGTCATATGT TCAAGCCGTC CCCTTCCAGA GAACAACCAT GTTCTTGACA   
  
  
+ AATCAAACAA TAGTAAAGCT GGCTGAGAAA GCAACAAGGG TTCATATCAT TGATTTTGGC ATCTTTTTTG   
  
  
+ GTTTCCAGTG GCCCTGCCTT GTTCAGAATT TGTCAAGAAG ACCCCAGGGT TCTCCCATAC TCAAAATCAC   
  
  
+ GGGAGTCGAT CATCCCCAGA GTGGGTTCAG ACCAGCCCAG AAAGTTGAAG AGACAGGTCG TCGATTAGCA   
  
  
+ GGCTATTGTG AGAGGTTTGG GGTGCCCTTT GAGTTTCATC CCATTGCTCA AAGGTGGCAT ACCCTAAAGC   
  
  
+ CAGAGCATTT CAAAATTGAA AGCAATGAGC TTGTTGTGGT CACTTGTTTC TACCAATCAG GGAAGCTCCC   
  
  
+ TGATGAGACT GTTGATGTCA ACAGCCCAAG AGACCATGTC TTGAGGCTGA TCAGGGGCTT GAACCCTGAT   
  
  
+ CTATTCATCC ATGGGGTTAT CAATGGCACA TTCAATGCAC CCTTCTTCGT AACTCGGTTT AAAGAGGCAC   
  
  
+ TTTACCACTA CTCTTCTTTG TTCGATGTCT TTGATGCCAC CATAGATCGT GAAAGTCATG AAAGACTGCT   
  
  
+ GACTGAGAAC TATTTGTATG GGAAAGATGC ACTTAACATA GTAGCATGTG AGGGGAACGA GAGGATCGAG   
  
  
+ AGACCCGAGA CTTACAAGCA GTGGCAAGTC CGGAAGCTTA GAGCTGGATT CAAGCAGGTC CCCCTAGACC   
  
  
+ AAGACATTGT AAATGGAGCA AGAGCTATGG TTAAGGCAAA TTACAATAAG GATTTTATGG TAGAACAAGA   
  
  
+ TAGGAATTGG ATGGTCCAGG GCTGGAAAGG AAGGATTTTG AGTGCCATAT CCTGTTGGAA ACCTGCTTA  

- +Up\_Stream \_Len000ACTTAA CCTTCAACTA AGATGGAAAC TAACTAAGCT AATTAAGATC CAAGAACCAA   
  
  
- GATCAAGAGA GAGGAGAACA AGAACTAATA AATCCAAAAC CTACACTACA AATAACTTAA CTTACAACGT   
  
  
- TAACAACAAT ATTTCTACTA CAATCCGATT TATAGGAAGT TGAATCCAAC CCTAACTGCA GCTTATACAC   
  
  
- ATCAACTTAG AACATGACCG GATTTAGGGA CGAGGGGGAG ACAAAACGCT AATACTTAAA GTAATACGTG   
  
  
- TGGTGTACGA GCTACTTTAC GAGCTTTCTT GTAAGTAGGA GAAACAAACC CTAAATCCCC TTGCAACTTT   
  
  
- CTGCTTCAGA AATGGATCAC GCAGAATCAT CTGTTAATCC TAAAATTGTA CTTTTATCAA TTTTAAGATT   
  
  
- AACCCTAAAC TCTAACACTT AGAACATTAG GAAGCTGAAG ATCAAGATAC TTTTATCCGC TAATATCAAG   
  
  
- ATCCTAATCA GAAGCTGGGA ACTCTCTCCC TCCTCTACCC AAATCAACTA TTAGATAAGT AAGTATTGCG   
  
  
- TACGGACCCT CTACAAACCA TCCCGATCAT TAACTAAGCG ATTGTATAAA CACGACAATC AAGGTTGGAC   
  
  
- TCACAAATGA GAAGACGACA AATAAGGCAG AAAATCAGCA TTAACAGAGG AAATCAAATT AAAGTTTTGG   
  
  
- GGTTAACACT AGCGGATCTA ACTCGTTAGA TTAATCAAGA TTATTAACTT TAGTTCAAGG GACACCTTAT   
  
  
- GCTGGGCATG AACGGCACAC GATCGATGAC AATGTGGAAC GTAAACGCCA TATAAATATT GAGTTGTATA   
  
  
- CAGACGAGTG ATTTTCATGT ATAATCGAGT GGTTTTGACA CAGAGTTGCA TGATTTTATA AAATCACTTG   
  
  
- ATATGTAATA AAAATCATTC GCTTTATTCT ATGTGTTGTC GTGTAAAATT ACTCAATGTC CCGTAAAAAT   
  
  
- CACTCGGCTT TAGACTACCA AAAGAAACAT CTAGGCCAAT ATATTAACAT AAAATATGTT GGCTTACCTG   
  
  
- CTATATTGTT GACTCAATTT ACTCCTCAGA CTTTAATGCT TGTATTTTAG GTTTTCTTCT ACGGCTACGG   
  
  
- TAACCTTTCT TTCGACTTCC ACTAGCGAAA GATCATCATA ATCCGTCATC ATTAATTTTT GATTTTAGGT   
  
  
- CTTAACTGTG GCTTAACCCC AACACCGTGT ACTAGCTTTA GTCGTAGCTT AGATGTGCTA CTAGACCTAA   
  
  
- GAACCTGTGA GACCGAAATC CGGCAGTTAA AGCTCCTAAC GGGTATTGAA ATTAGGACAA GTTTAGGTAA   
  
  
- GTTAAATTAT AATGTGTTTA GGAAACTAAT TTAGTGGCAT TAACACCAAA CTAATCCCTT CAAAGAACCC   
  
  
- CCTTTCCAAG TTCGAAGACA GAAAGAATTT CGGACCTAAA AACCAGGGCG TACAAGTGAA GTGTAAGGGA   
  
  
- AAAAGAGAGG GTAGCAATGG ACAGACTTAG ACATACATTA TTTTCAGTAT AAAACGCTCC AGAAGAAAAC   
  
  
- TGACAAGAAC ATCAGTGAGA ACTATAGACG TGGTAAAACA AAAAGAGCCG TAAAAGAGGT TGAACGTAAA   
  
  
- AGATTACATT ACACTAGAAA AGACCTGTAA GTAAAACAAT TAGATGAGTG AACCCAGTTC CAAACGACTA   
  
  
- AAACCGACCA CAATTTAGGC AAGGAGAAAC CGTAAACAAG TACAAGGTGT TGACAAACTA TAGACCATAG   
  
  
- AGTCTTTGGG GCGGTAAGAA ACCATAAAGG ACTGAAAGAA CAAGTTCCCA ATAAGGATAA AGGGACTTAA   
  
  
- AAGAAACTAA GAAATCCATA AAGTTCGAAC CCCCCAAAAC AAGAAGTGAC TAAAACAGGA GAAGAAGGGA   
  
  
- ATAACCCACC AAACTAGAGG ATTTAAAGAC ACATAGACTT AGTCAGCCCC ATCTGAAACA CAAAAACCAA   
  
  
- CTAAAAAATA GTTACATAAA AGAAGGATTA CTTCCCGGAG GGCTTGGTTA TGTGTACGAA TTGAAGTTGG   
  
  
- GACAAGGAGA AGGTTCGAAA AACCTCGGAG TTTTCAGAAG CTTACGACTA TCCGTGTATC CCAGGAAGGT   
  
  
- TACGATACCA GAACTACTAC TGGGGCGTGG GAACTTACTG GGTTTAATGT AACATACAGT TAGAAGTATA   
  
  
- GGACTAGCTC TATAATGTGT CTAGGGACTG AAAGGACTAC TAACAGAGTT CAAGTAGTTA CTGTAAGAAT   
  
  
- ACCTTCTCCT AAACCTATTA GGCGGATATA GGAACGTCCT GATATTACGC GAGGTTCGGT GACTCTTCAG   
  
  
- TAACATACTA CGGGAACCTC TCCGAATAGG TCGAAGTAGA CTAGGTAACG TTAGTAGTCA ACCAGTCTCG   
  
  
- TGACTCTCGA GACTACTAGG ACTGTCTTCG TCGCCGTAAC TATTTACCCC ATTATCTATA ATGGGGTGGT   
  
  
- CGGACTTACA CCAACTTTGG TCGGCCGACT AGTTAGTCTC GGTCAACCTG GGAAGAGTTT ACAAACTACG   
  
  
- ACGACGAGTG GGGAACCTCA GATTAACAGT AAGAAAACAG AGATCATTAT TGAAACTAGT AAGATACCTA   
  
  
- CCCCAAGGTC TAAGAGGATA GTCATGTGAA AGACACTTAC TTTGTTCTTT CCTTCTTAGT CAATCATCTA   
  
  
- GTTCCTCGTC CTTCTTAGTA GTTTCTCTAC CACGTATGGA CCTTCTTTCC TCATGATTCG TTTATCGTAG   
  
  
- GTCGTTACTC CTTATACCTC TCTACCTCTC CAAACTACTA CATGACTAGA CGTCCCTTCC TTTACTACTA   
  
  
- TAAAGTGGAA CATGGGCGTT TTGGGGTCAA GTACTTCGTC TACTATTGAG CCTTGGTTTC CCCTTTAGTT   
  
  
- CTCCTAGATT TTCGTTCTCA TTTAGCGCAC AGTTCTTCTT TTCATTGCTT CCTGTTCGCC ATCTAGAATC   
  
  
- CTGAGTTGAA TAAGTCACAC GTCTTCGTCA CCGTTCGAAA CTCGAATTTT CACGTTTACT CGATGAGTCC   
  
  
- GCTTAATCCG TCGTACGAAG CGGTAAACCC CTATCACAAG TTTCCGATCG TGTAATGAAA CGCTTACCCG   
  
  
- AGCTCCGTGC GAATCGACCA TGTCCTAGTC TTAGTATGTT TCTGAAACGA CCCTTTTTTA AAAGTAGGAT   
  
  
- ACTGTAGAAC TCTCCGATGT TCAGTATACA AGTTCGGCAG GGGAAGGTCT CTTGTTGGTA CAAGAACTGT   
  
  
- TTAGTTTGTT ATCATTTCGA CCGACTCTTT CGTTGTTCCC AAGTATAGTA ACTAAAACCG TAGAAAAAAC   
  
  
- CAAAGGTCAC CGGGACGGAA CAAGTCTTAA ACAGTTCTTC TGGGGTCCCA AGAGGGTATG AGTTTTAGTG   
  
  
- CCCTCAGCTA GTAGGGGTCT CACCCAAGTC TGGTCGGGTC TTTCAACTTC TCTGTCCAGC AGCTAATCGT   
  
  
- CCGATAACAC TCTCCAAACC CCACGGGAAA CTCAAAGTAG GGTAACGAGT TTCCACCGTA TGGGATTTCG   
  
  
- GTCTCGTAAA GTTTTAACTT TCGTTACTCG AACAACACCA GTGAACAAAG ATGGTTAGTC CCTTCGAGGG   
  
  
- ACTACTCTGA CAACTACAGT TGTCGGGTTC TCTGGTACAG AACTCCGACT AGTCCCCGAA CTTGGGACTA   
  
  
- GATAAGTAGG TACCCCAATA GTTACCGTGT AAGTTACGTG GGAAGAAGCA TTGAGCCAAA TTTCTCCGTG   
  
  
- AAATGGTGAT GAGAAGAAAC AAGCTACAGA AACTACGGTG GTATCTAGCA CTTTCAGTAC TTTCTGACGA   
  
  
- CTGACTCTTG ATAAACATAC CCTTTCTACG TGAATTGTAT CATCGTACAC TCCCCTTGCT CTCCTAGCTC   
  
  
- TCTGGGCTCT GAATGTTCGT CACCGTTCAG GCCTTCGAAT CTCGACCTAA GTTCGTCCAG GGGGATCTGG   
  
  
- TTCTGTAACA TTTACCTCGT TCTCGATACC AATTCCGTTT AATGTTATTC CTAAAATACC ATCTTGTTCT   
  
  
- ATCCTTAACC TACCAGGTCC CGACCTTTCC TTCCTAAAAC TCACGGTATA GGACAACCTT TGGACGAAT

+     ACE

| Site Name | Organism | Position | Strand | Matrix score. | sequence | function |
| --- | --- | --- | --- | --- | --- | --- |
| ACE | Petroselinum crispum | 604 | + | 9 | CTAACGTATT | cis-acting element involved in light responsiveness |

>HU02G01573.1   
+ +Up\_Stream \_Len000TGAATT GGAAGTTGAT TCTACCTTTG ATTGATTCGA TTAATTCTAG GTTCTTGGTT   
  
  
+ CTAGTTCTCT CTCCTCTTGT TCTTGATTAT TTAGGTTTTG GATGTGATGT TTATTGAATT GAATGTTGCA   
  
  
+ ATTGTTGTTA TAAAGATGAT GTTAGGCTAA ATATCCTTCA ACTTAGGTTG GGATTGACGT CGAATATGTG   
  
  
+ TAGTTGAATC TTGTACTGGC CTAAATCCCT GCTCCCCCTC TGTTTTGCGA TTATGAATTT CATTATGCAC   
  
  
+ ACCACATGCT CGATGAAATG CTCGAAAGAA CATTCATCCT CTTTGTTTGG GATTTAGGGG AACGTTGAAA   
  
  
+ GACGAAGTCT TTACCTAGTG CGTCTTAGTA GACAATTAGG ATTTTAACAT GAAAATAGTT AAAATTCTAA   
  
  
+ TTGGGATTTG AGATTGTGAA TCTTGTAATC CTTCGACTTC TAGTTCTATG AAAATAGGCG ATTATAGTTC   
  
  
+ TAGGATTAGT CTTCGACCCT TGAGAGAGGG AGGAGATGGG TTTAGTTGAT AATCTATTCA TTCATAACGC   
  
  
+ ATGCCTGGGA GATGTTTGGT AGGGCTAGTA ATTGATTCGC TAACATATTT GTGCTGTTAG TTCCAACCTG   
  
  
+ AGTGTTTACT CTTCTGCTGT TTATTCCGTC TTTTAGTCGT AATTGTCTCC TTTAGTTTAA TTTCAAAACC   
  
  
+ CCAATTGTGA TCGCCTAGAT TGAGCAATCT AATTAGTTCT AATAATTGAA ATCAAGTTCC CTGTGGAATA   
  
  
+ CGACCCGTAC TTGCCGTGTG CTAGCTACTG TTACACCTTG CATTTGCGGT ATATTTATAA CTCAACATAT   
  
  
+ GTCTGCTCAC TAAAAGTACA TATTAGCTCA CCAAAACTGT GTCTCAACGT ACTAAAATAT TTTAGTGAAC   
  
  
+ TATACATTAT TTTTAGTAAG CGAAATAAGA TACACAACAG CACATTTTAA TGAGTTACAG GGCATTTTTA   
  
  
+ GTGAGCCGAA ATCTGATGGT TTTCTTTGTA GATCCGGTTA TATAATTGTA TTTTATACAA CCGAATGGAC   
  
  
+ GATATAACAA CTGAGTTAAA TGAGGAGTCT GAAATTACGA ACATAAAATC CAAAAGAAGA TGCCGATGCC   
  
  
+ ATTGGAAAGA AAGCTGAAGG TGATCGCTTT CTAGTAGTAT TAGGCAGTAG TAATTAAAAA CTAAAATCCA   
  
  
+ GAATTGACAC CGAATTGGGG TTGTGGCACA TGATCGAAAT CAGCATCGAA TCTACACGAT GATCTGGATT   
  
  
+ CTTGGACACT CTGGCTTTAG GCCGTCAATT TCGAGGATTG CCCATAACTT TAATCCTGTT CAAATCCATT   
  
  
+ CAATTTAATA TTACACAAAT CCTTTGATTA AATCACCGTA ATTGTGGTTT GATTAGGGAA GTTTCTTGGG   
  
  
+ GGAAAGGTTC AAGCTTCTGT CTTTCTTAAA GCCTGGATTT TTGGTCCCGC ATGTTCACTT CACATTCCCT   
  
  
+ TTTTCTCTCC CATCGTTACC TGTCTGAATC TGTATGTAAT AAAAGTCATA TTTTGCGAGG TCTTCTTTTG   
  
  
+ ACTGTTCTTG TAGTCACTCT TGATATCTGC ACCATTTTGT TTTTCTCGGC ATTTTCTCCA ACTTGCATTT   
  
  
+ TCTAATGTAA TGTGATCTTT TCTGGACATT CATTTTGTTA ATCTACTCAC TTGGGTCAAG GTTTGCTGAT   
  
  
+ TTTGGCTGGT GTTAAATCCG TTCCTCTTTG GCATTTGTTC ATGTTCCACA ACTGTTTGAT ATCTGGTATC   
  
  
+ TCAGAAACCC CGCCATTCTT TGGTATTTCC TGACTTTCTT GTTCAAGGGT TATTCCTATT TCCCTGAATT   
  
  
+ TTCTTTGATT CTTTAGGTAT TTCAAGCTTG GGGGGTTTTG TTCTTCACTG ATTTTGTCCT CTTCTTCCCT   
  
  
+ TATTGGGTGG TTTGATCTCC TAAATTTCTG TGTATCTGAA TCAGTCGGGG TAGACTTTGT GTTTTTGGTT   
  
  
+ GATTTTTTAT CAATGTATTT TCTTCCTAAT GAAGGGCCTC CCGAACCAAT ACACATGCTT AACTTCAACC   
  
  
+ CTGTTCCTCT TCCAAGCTTT TTGGAGCCTC AAAAGTCTTC GAATGCTGAT AGGCACATAG GGTCCTTCCA   
  
  
+ ATGCTATGGT CTTGATGATG ACCCCGCACC CTTGAATGAC CCAAATTACA TTGTATGTCA ATCTTCATAT   
  
  
+ CCTGATCGAG ATATTACACA GATCCCTGAC TTTCCTGATG ATTGTCTCAA GTTCATCAAT GACATTCTTA   
  
  
+ TGGAAGAGGA TTTGGATAAT CCGCCTATAT CCTTGCAGGA CTATAATGCG CTCCAAGCCA CTGAGAAGTC   
  
  
+ ATTGTATGAT GCCCTTGGAG AGGCTTATCC AGCTTCATCT GATCCATTGC AATCATCAGT TGGTCAGAGC   
  
  
+ ACTGAGAGCT CTGATGATCC TGACAGAAGC AGCGGCATTG ATAAATGGGG TAATAGATAT TACCCCACCA   
  
  
+ GCCTGAATGT GGTTGAAACC AGCCGGCTGA TCAATCAGAG CCAGTTGGAC CCTTCTCAAA TGTTTGATGC   
  
  
+ TGCTGCTCAC CCCTTGGAGT CTAATTGTCA TTCTTTTGTC TCTAGTAATA ACTTTGATCA TTCTATGGAT   
  
  
+ GGGGTTCCAG ATTCTCCTAT CAGTACACTT TCTGTGAATG AAACAAGAAA GGAAGAATCA GTTAGTAGAT   
  
  
+ CAAGGAGCAG GAAGAATCAT CAAAGAGATG GTGCATACCT GGAAGAAAGG AGTACTAAGC AAATAGCATC   
  
  
+ CAGCAATGAG GAATATGGAG AGATGGAGAG GTTTGATGAT GTACTGATCT GCAGGGAAGG AAATGATGAT   
  
  
+ ATTTCACCTT GTACCCGCAA AACCCCAGTT CATGAAGCAG ATGATAACTC GGAACCAAAG GGGAAATCAA   
  
  
+ GAGGATCTAA AAGCAAGAGT AAATCGCGTG TCAAGAAGAA AAGTAACGAA GGACAAGCGG TAGATCTTAG   
  
  
+ GACTCAACTT ATTCAGTGTG CAGAAGCAGT GGCAAGCTTT GAGCTTAAAA GTGCAAATGA GCTACTCAGG   
  
  
+ CGAATTAGGC AGCATGCTTC GCCATTTGGG GATAGTGTTC AAAGGCTAGC ACATTACTTT GCGAATGGGC   
  
  
+ TCGAGGCACG CTTAGCTGGT ACAGGATCAG AATCATACAA AGACTTTGCT GGGAAAAAAT TTTCATCCTA   
  
  
+ TGACATCTTG AGAGGCTACA AGTCATATGT TCAAGCCGTC CCCTTCCAGA GAACAACCAT GTTCTTGACA   
  
  
+ AATCAAACAA TAGTAAAGCT GGCTGAGAAA GCAACAAGGG TTCATATCAT TGATTTTGGC ATCTTTTTTG   
  
  
+ GTTTCCAGTG GCCCTGCCTT GTTCAGAATT TGTCAAGAAG ACCCCAGGGT TCTCCCATAC TCAAAATCAC   
  
  
+ GGGAGTCGAT CATCCCCAGA GTGGGTTCAG ACCAGCCCAG AAAGTTGAAG AGACAGGTCG TCGATTAGCA   
  
  
+ GGCTATTGTG AGAGGTTTGG GGTGCCCTTT GAGTTTCATC CCATTGCTCA AAGGTGGCAT ACCCTAAAGC   
  
  
+ CAGAGCATTT CAAAATTGAA AGCAATGAGC TTGTTGTGGT CACTTGTTTC TACCAATCAG GGAAGCTCCC   
  
  
+ TGATGAGACT GTTGATGTCA ACAGCCCAAG AGACCATGTC TTGAGGCTGA TCAGGGGCTT GAACCCTGAT   
  
  
+ CTATTCATCC ATGGGGTTAT CAATGGCACA TTCAATGCAC CCTTCTTCGT AACTCGGTTT AAAGAGGCAC   
  
  
+ TTTACCACTA CTCTTCTTTG TTCGATGTCT TTGATGCCAC CATAGATCGT GAAAGTCATG AAAGACTGCT   
  
  
+ GACTGAGAAC TATTTGTATG GGAAAGATGC ACTTAACATA GTAGCATGTG AGGGGAACGA GAGGATCGAG   
  
  
+ AGACCCGAGA CTTACAAGCA GTGGCAAGTC CGGAAGCTTA GAGCTGGATT CAAGCAGGTC CCCCTAGACC   
  
  
+ AAGACATTGT AAATGGAGCA AGAGCTATGG TTAAGGCAAA TTACAATAAG GATTTTATGG TAGAACAAGA   
  
  
+ TAGGAATTGG ATGGTCCAGG GCTGGAAAGG AAGGATTTTG AGTGCCATAT CCTGTTGGAA ACCTGCTTA  

- +Up\_Stream \_Len000ACTTAA CCTTCAACTA AGATGGAAAC TAACTAAGCT AATTAAGATC CAAGAACCAA   
  
  
- GATCAAGAGA GAGGAGAACA AGAACTAATA AATCCAAAAC CTACACTACA AATAACTTAA CTTACAACGT   
  
  
- TAACAACAAT ATTTCTACTA CAATCCGATT TATAGGAAGT TGAATCCAAC CCTAACTGCA GCTTATACAC   
  
  
- ATCAACTTAG AACATGACCG GATTTAGGGA CGAGGGGGAG ACAAAACGCT AATACTTAAA GTAATACGTG   
  
  
- TGGTGTACGA GCTACTTTAC GAGCTTTCTT GTAAGTAGGA GAAACAAACC CTAAATCCCC TTGCAACTTT   
  
  
- CTGCTTCAGA AATGGATCAC GCAGAATCAT CTGTTAATCC TAAAATTGTA CTTTTATCAA TTTTAAGATT   
  
  
- AACCCTAAAC TCTAACACTT AGAACATTAG GAAGCTGAAG ATCAAGATAC TTTTATCCGC TAATATCAAG   
  
  
- ATCCTAATCA GAAGCTGGGA ACTCTCTCCC TCCTCTACCC AAATCAACTA TTAGATAAGT AAGTATTGCG   
  
  
- TACGGACCCT CTACAAACCA TCCCGATCAT TAACTAAGCG ATTGTATAAA CACGACAATC AAGGTTGGAC   
  
  
- TCACAAATGA GAAGACGACA AATAAGGCAG AAAATCAGCA TTAACAGAGG AAATCAAATT AAAGTTTTGG   
  
  
- GGTTAACACT AGCGGATCTA ACTCGTTAGA TTAATCAAGA TTATTAACTT TAGTTCAAGG GACACCTTAT   
  
  
- GCTGGGCATG AACGGCACAC GATCGATGAC AATGTGGAAC GTAAACGCCA TATAAATATT GAGTTGTATA   
  
  
- CAGACGAGTG ATTTTCATGT ATAATCGAGT GGTTTTGACA CAGAGTTGCA TGATTTTATA AAATCACTTG   
  
  
- ATATGTAATA AAAATCATTC GCTTTATTCT ATGTGTTGTC GTGTAAAATT ACTCAATGTC CCGTAAAAAT   
  
  
- CACTCGGCTT TAGACTACCA AAAGAAACAT CTAGGCCAAT ATATTAACAT AAAATATGTT GGCTTACCTG   
  
  
- CTATATTGTT GACTCAATTT ACTCCTCAGA CTTTAATGCT TGTATTTTAG GTTTTCTTCT ACGGCTACGG   
  
  
- TAACCTTTCT TTCGACTTCC ACTAGCGAAA GATCATCATA ATCCGTCATC ATTAATTTTT GATTTTAGGT   
  
  
- CTTAACTGTG GCTTAACCCC AACACCGTGT ACTAGCTTTA GTCGTAGCTT AGATGTGCTA CTAGACCTAA   
  
  
- GAACCTGTGA GACCGAAATC CGGCAGTTAA AGCTCCTAAC GGGTATTGAA ATTAGGACAA GTTTAGGTAA   
  
  
- GTTAAATTAT AATGTGTTTA GGAAACTAAT TTAGTGGCAT TAACACCAAA CTAATCCCTT CAAAGAACCC   
  
  
- CCTTTCCAAG TTCGAAGACA GAAAGAATTT CGGACCTAAA AACCAGGGCG TACAAGTGAA GTGTAAGGGA   
  
  
- AAAAGAGAGG GTAGCAATGG ACAGACTTAG ACATACATTA TTTTCAGTAT AAAACGCTCC AGAAGAAAAC   
  
  
- TGACAAGAAC ATCAGTGAGA ACTATAGACG TGGTAAAACA AAAAGAGCCG TAAAAGAGGT TGAACGTAAA   
  
  
- AGATTACATT ACACTAGAAA AGACCTGTAA GTAAAACAAT TAGATGAGTG AACCCAGTTC CAAACGACTA   
  
  
- AAACCGACCA CAATTTAGGC AAGGAGAAAC CGTAAACAAG TACAAGGTGT TGACAAACTA TAGACCATAG   
  
  
- AGTCTTTGGG GCGGTAAGAA ACCATAAAGG ACTGAAAGAA CAAGTTCCCA ATAAGGATAA AGGGACTTAA   
  
  
- AAGAAACTAA GAAATCCATA AAGTTCGAAC CCCCCAAAAC AAGAAGTGAC TAAAACAGGA GAAGAAGGGA   
  
  
- ATAACCCACC AAACTAGAGG ATTTAAAGAC ACATAGACTT AGTCAGCCCC ATCTGAAACA CAAAAACCAA   
  
  
- CTAAAAAATA GTTACATAAA AGAAGGATTA CTTCCCGGAG GGCTTGGTTA TGTGTACGAA TTGAAGTTGG   
  
  
- GACAAGGAGA AGGTTCGAAA AACCTCGGAG TTTTCAGAAG CTTACGACTA TCCGTGTATC CCAGGAAGGT   
  
  
- TACGATACCA GAACTACTAC TGGGGCGTGG GAACTTACTG GGTTTAATGT AACATACAGT TAGAAGTATA   
  
  
- GGACTAGCTC TATAATGTGT CTAGGGACTG AAAGGACTAC TAACAGAGTT CAAGTAGTTA CTGTAAGAAT   
  
  
- ACCTTCTCCT AAACCTATTA GGCGGATATA GGAACGTCCT GATATTACGC GAGGTTCGGT GACTCTTCAG   
  
  
- TAACATACTA CGGGAACCTC TCCGAATAGG TCGAAGTAGA CTAGGTAACG TTAGTAGTCA ACCAGTCTCG   
  
  
- TGACTCTCGA GACTACTAGG ACTGTCTTCG TCGCCGTAAC TATTTACCCC ATTATCTATA ATGGGGTGGT   
  
  
- CGGACTTACA CCAACTTTGG TCGGCCGACT AGTTAGTCTC GGTCAACCTG GGAAGAGTTT ACAAACTACG   
  
  
- ACGACGAGTG GGGAACCTCA GATTAACAGT AAGAAAACAG AGATCATTAT TGAAACTAGT AAGATACCTA   
  
  
- CCCCAAGGTC TAAGAGGATA GTCATGTGAA AGACACTTAC TTTGTTCTTT CCTTCTTAGT CAATCATCTA   
  
  
- GTTCCTCGTC CTTCTTAGTA GTTTCTCTAC CACGTATGGA CCTTCTTTCC TCATGATTCG TTTATCGTAG   
  
  
- GTCGTTACTC CTTATACCTC TCTACCTCTC CAAACTACTA CATGACTAGA CGTCCCTTCC TTTACTACTA   
  
  
- TAAAGTGGAA CATGGGCGTT TTGGGGTCAA GTACTTCGTC TACTATTGAG CCTTGGTTTC CCCTTTAGTT   
  
  
- CTCCTAGATT TTCGTTCTCA TTTAGCGCAC AGTTCTTCTT TTCATTGCTT CCTGTTCGCC ATCTAGAATC   
  
  
- CTGAGTTGAA TAAGTCACAC GTCTTCGTCA CCGTTCGAAA CTCGAATTTT CACGTTTACT CGATGAGTCC   
  
  
- GCTTAATCCG TCGTACGAAG CGGTAAACCC CTATCACAAG TTTCCGATCG TGTAATGAAA CGCTTACCCG   
  
  
- AGCTCCGTGC GAATCGACCA TGTCCTAGTC TTAGTATGTT TCTGAAACGA CCCTTTTTTA AAAGTAGGAT   
  
  
- ACTGTAGAAC TCTCCGATGT TCAGTATACA AGTTCGGCAG GGGAAGGTCT CTTGTTGGTA CAAGAACTGT   
  
  
- TTAGTTTGTT ATCATTTCGA CCGACTCTTT CGTTGTTCCC AAGTATAGTA ACTAAAACCG TAGAAAAAAC   
  
  
- CAAAGGTCAC CGGGACGGAA CAAGTCTTAA ACAGTTCTTC TGGGGTCCCA AGAGGGTATG AGTTTTAGTG   
  
  
- CCCTCAGCTA GTAGGGGTCT CACCCAAGTC TGGTCGGGTC TTTCAACTTC TCTGTCCAGC AGCTAATCGT   
  
  
- CCGATAACAC TCTCCAAACC CCACGGGAAA CTCAAAGTAG GGTAACGAGT TTCCACCGTA TGGGATTTCG   
  
  
- GTCTCGTAAA GTTTTAACTT TCGTTACTCG AACAACACCA GTGAACAAAG ATGGTTAGTC CCTTCGAGGG   
  
  
- ACTACTCTGA CAACTACAGT TGTCGGGTTC TCTGGTACAG AACTCCGACT AGTCCCCGAA CTTGGGACTA   
  
  
- GATAAGTAGG TACCCCAATA GTTACCGTGT AAGTTACGTG GGAAGAAGCA TTGAGCCAAA TTTCTCCGTG   
  
  
- AAATGGTGAT GAGAAGAAAC AAGCTACAGA AACTACGGTG GTATCTAGCA CTTTCAGTAC TTTCTGACGA   
  
  
- CTGACTCTTG ATAAACATAC CCTTTCTACG TGAATTGTAT CATCGTACAC TCCCCTTGCT CTCCTAGCTC   
  
  
- TCTGGGCTCT GAATGTTCGT CACCGTTCAG GCCTTCGAAT CTCGACCTAA GTTCGTCCAG GGGGATCTGG   
  
  
- TTCTGTAACA TTTACCTCGT TCTCGATACC AATTCCGTTT AATGTTATTC CTAAAATACC ATCTTGTTCT   
  
  
- ATCCTTAACC TACCAGGTCC CGACCTTTCC TTCCTAAAAC TCACGGTATA GGACAACCTT TGGACGAAT

+     AE-box

| Site Name | Organism | Position | Strand | Matrix score. | sequence | function |
| --- | --- | --- | --- | --- | --- | --- |
| AE-box | Arabidopsis thaliana | 3548 | - | 8 | AGAAACAA | part of a module for light response |
| AE-box | Arabidopsis thaliana | 1393 | - | 8 | AGAAACTT | part of a module for light response |

>HU02G01573.1   
+ +Up\_Stream \_Len000TGAATT GGAAGTTGAT TCTACCTTTG ATTGATTCGA TTAATTCTAG GTTCTTGGTT   
  
  
+ CTAGTTCTCT CTCCTCTTGT TCTTGATTAT TTAGGTTTTG GATGTGATGT TTATTGAATT GAATGTTGCA   
  
  
+ ATTGTTGTTA TAAAGATGAT GTTAGGCTAA ATATCCTTCA ACTTAGGTTG GGATTGACGT CGAATATGTG   
  
  
+ TAGTTGAATC TTGTACTGGC CTAAATCCCT GCTCCCCCTC TGTTTTGCGA TTATGAATTT CATTATGCAC   
  
  
+ ACCACATGCT CGATGAAATG CTCGAAAGAA CATTCATCCT CTTTGTTTGG GATTTAGGGG AACGTTGAAA   
  
  
+ GACGAAGTCT TTACCTAGTG CGTCTTAGTA GACAATTAGG ATTTTAACAT GAAAATAGTT AAAATTCTAA   
  
  
+ TTGGGATTTG AGATTGTGAA TCTTGTAATC CTTCGACTTC TAGTTCTATG AAAATAGGCG ATTATAGTTC   
  
  
+ TAGGATTAGT CTTCGACCCT TGAGAGAGGG AGGAGATGGG TTTAGTTGAT AATCTATTCA TTCATAACGC   
  
  
+ ATGCCTGGGA GATGTTTGGT AGGGCTAGTA ATTGATTCGC TAACATATTT GTGCTGTTAG TTCCAACCTG   
  
  
+ AGTGTTTACT CTTCTGCTGT TTATTCCGTC TTTTAGTCGT AATTGTCTCC TTTAGTTTAA TTTCAAAACC   
  
  
+ CCAATTGTGA TCGCCTAGAT TGAGCAATCT AATTAGTTCT AATAATTGAA ATCAAGTTCC CTGTGGAATA   
  
  
+ CGACCCGTAC TTGCCGTGTG CTAGCTACTG TTACACCTTG CATTTGCGGT ATATTTATAA CTCAACATAT   
  
  
+ GTCTGCTCAC TAAAAGTACA TATTAGCTCA CCAAAACTGT GTCTCAACGT ACTAAAATAT TTTAGTGAAC   
  
  
+ TATACATTAT TTTTAGTAAG CGAAATAAGA TACACAACAG CACATTTTAA TGAGTTACAG GGCATTTTTA   
  
  
+ GTGAGCCGAA ATCTGATGGT TTTCTTTGTA GATCCGGTTA TATAATTGTA TTTTATACAA CCGAATGGAC   
  
  
+ GATATAACAA CTGAGTTAAA TGAGGAGTCT GAAATTACGA ACATAAAATC CAAAAGAAGA TGCCGATGCC   
  
  
+ ATTGGAAAGA AAGCTGAAGG TGATCGCTTT CTAGTAGTAT TAGGCAGTAG TAATTAAAAA CTAAAATCCA   
  
  
+ GAATTGACAC CGAATTGGGG TTGTGGCACA TGATCGAAAT CAGCATCGAA TCTACACGAT GATCTGGATT   
  
  
+ CTTGGACACT CTGGCTTTAG GCCGTCAATT TCGAGGATTG CCCATAACTT TAATCCTGTT CAAATCCATT   
  
  
+ CAATTTAATA TTACACAAAT CCTTTGATTA AATCACCGTA ATTGTGGTTT GATTAGGGAA GTTTCTTGGG   
  
  
+ GGAAAGGTTC AAGCTTCTGT CTTTCTTAAA GCCTGGATTT TTGGTCCCGC ATGTTCACTT CACATTCCCT   
  
  
+ TTTTCTCTCC CATCGTTACC TGTCTGAATC TGTATGTAAT AAAAGTCATA TTTTGCGAGG TCTTCTTTTG   
  
  
+ ACTGTTCTTG TAGTCACTCT TGATATCTGC ACCATTTTGT TTTTCTCGGC ATTTTCTCCA ACTTGCATTT   
  
  
+ TCTAATGTAA TGTGATCTTT TCTGGACATT CATTTTGTTA ATCTACTCAC TTGGGTCAAG GTTTGCTGAT   
  
  
+ TTTGGCTGGT GTTAAATCCG TTCCTCTTTG GCATTTGTTC ATGTTCCACA ACTGTTTGAT ATCTGGTATC   
  
  
+ TCAGAAACCC CGCCATTCTT TGGTATTTCC TGACTTTCTT GTTCAAGGGT TATTCCTATT TCCCTGAATT   
  
  
+ TTCTTTGATT CTTTAGGTAT TTCAAGCTTG GGGGGTTTTG TTCTTCACTG ATTTTGTCCT CTTCTTCCCT   
  
  
+ TATTGGGTGG TTTGATCTCC TAAATTTCTG TGTATCTGAA TCAGTCGGGG TAGACTTTGT GTTTTTGGTT   
  
  
+ GATTTTTTAT CAATGTATTT TCTTCCTAAT GAAGGGCCTC CCGAACCAAT ACACATGCTT AACTTCAACC   
  
  
+ CTGTTCCTCT TCCAAGCTTT TTGGAGCCTC AAAAGTCTTC GAATGCTGAT AGGCACATAG GGTCCTTCCA   
  
  
+ ATGCTATGGT CTTGATGATG ACCCCGCACC CTTGAATGAC CCAAATTACA TTGTATGTCA ATCTTCATAT   
  
  
+ CCTGATCGAG ATATTACACA GATCCCTGAC TTTCCTGATG ATTGTCTCAA GTTCATCAAT GACATTCTTA   
  
  
+ TGGAAGAGGA TTTGGATAAT CCGCCTATAT CCTTGCAGGA CTATAATGCG CTCCAAGCCA CTGAGAAGTC   
  
  
+ ATTGTATGAT GCCCTTGGAG AGGCTTATCC AGCTTCATCT GATCCATTGC AATCATCAGT TGGTCAGAGC   
  
  
+ ACTGAGAGCT CTGATGATCC TGACAGAAGC AGCGGCATTG ATAAATGGGG TAATAGATAT TACCCCACCA   
  
  
+ GCCTGAATGT GGTTGAAACC AGCCGGCTGA TCAATCAGAG CCAGTTGGAC CCTTCTCAAA TGTTTGATGC   
  
  
+ TGCTGCTCAC CCCTTGGAGT CTAATTGTCA TTCTTTTGTC TCTAGTAATA ACTTTGATCA TTCTATGGAT   
  
  
+ GGGGTTCCAG ATTCTCCTAT CAGTACACTT TCTGTGAATG AAACAAGAAA GGAAGAATCA GTTAGTAGAT   
  
  
+ CAAGGAGCAG GAAGAATCAT CAAAGAGATG GTGCATACCT GGAAGAAAGG AGTACTAAGC AAATAGCATC   
  
  
+ CAGCAATGAG GAATATGGAG AGATGGAGAG GTTTGATGAT GTACTGATCT GCAGGGAAGG AAATGATGAT   
  
  
+ ATTTCACCTT GTACCCGCAA AACCCCAGTT CATGAAGCAG ATGATAACTC GGAACCAAAG GGGAAATCAA   
  
  
+ GAGGATCTAA AAGCAAGAGT AAATCGCGTG TCAAGAAGAA AAGTAACGAA GGACAAGCGG TAGATCTTAG   
  
  
+ GACTCAACTT ATTCAGTGTG CAGAAGCAGT GGCAAGCTTT GAGCTTAAAA GTGCAAATGA GCTACTCAGG   
  
  
+ CGAATTAGGC AGCATGCTTC GCCATTTGGG GATAGTGTTC AAAGGCTAGC ACATTACTTT GCGAATGGGC   
  
  
+ TCGAGGCACG CTTAGCTGGT ACAGGATCAG AATCATACAA AGACTTTGCT GGGAAAAAAT TTTCATCCTA   
  
  
+ TGACATCTTG AGAGGCTACA AGTCATATGT TCAAGCCGTC CCCTTCCAGA GAACAACCAT GTTCTTGACA   
  
  
+ AATCAAACAA TAGTAAAGCT GGCTGAGAAA GCAACAAGGG TTCATATCAT TGATTTTGGC ATCTTTTTTG   
  
  
+ GTTTCCAGTG GCCCTGCCTT GTTCAGAATT TGTCAAGAAG ACCCCAGGGT TCTCCCATAC TCAAAATCAC   
  
  
+ GGGAGTCGAT CATCCCCAGA GTGGGTTCAG ACCAGCCCAG AAAGTTGAAG AGACAGGTCG TCGATTAGCA   
  
  
+ GGCTATTGTG AGAGGTTTGG GGTGCCCTTT GAGTTTCATC CCATTGCTCA AAGGTGGCAT ACCCTAAAGC   
  
  
+ CAGAGCATTT CAAAATTGAA AGCAATGAGC TTGTTGTGGT CACTTGTTTC TACCAATCAG GGAAGCTCCC   
  
  
+ TGATGAGACT GTTGATGTCA ACAGCCCAAG AGACCATGTC TTGAGGCTGA TCAGGGGCTT GAACCCTGAT   
  
  
+ CTATTCATCC ATGGGGTTAT CAATGGCACA TTCAATGCAC CCTTCTTCGT AACTCGGTTT AAAGAGGCAC   
  
  
+ TTTACCACTA CTCTTCTTTG TTCGATGTCT TTGATGCCAC CATAGATCGT GAAAGTCATG AAAGACTGCT   
  
  
+ GACTGAGAAC TATTTGTATG GGAAAGATGC ACTTAACATA GTAGCATGTG AGGGGAACGA GAGGATCGAG   
  
  
+ AGACCCGAGA CTTACAAGCA GTGGCAAGTC CGGAAGCTTA GAGCTGGATT CAAGCAGGTC CCCCTAGACC   
  
  
+ AAGACATTGT AAATGGAGCA AGAGCTATGG TTAAGGCAAA TTACAATAAG GATTTTATGG TAGAACAAGA   
  
  
+ TAGGAATTGG ATGGTCCAGG GCTGGAAAGG AAGGATTTTG AGTGCCATAT CCTGTTGGAA ACCTGCTTA  

- +Up\_Stream \_Len000ACTTAA CCTTCAACTA AGATGGAAAC TAACTAAGCT AATTAAGATC CAAGAACCAA   
  
  
- GATCAAGAGA GAGGAGAACA AGAACTAATA AATCCAAAAC CTACACTACA AATAACTTAA CTTACAACGT   
  
  
- TAACAACAAT ATTTCTACTA CAATCCGATT TATAGGAAGT TGAATCCAAC CCTAACTGCA GCTTATACAC   
  
  
- ATCAACTTAG AACATGACCG GATTTAGGGA CGAGGGGGAG ACAAAACGCT AATACTTAAA GTAATACGTG   
  
  
- TGGTGTACGA GCTACTTTAC GAGCTTTCTT GTAAGTAGGA GAAACAAACC CTAAATCCCC TTGCAACTTT   
  
  
- CTGCTTCAGA AATGGATCAC GCAGAATCAT CTGTTAATCC TAAAATTGTA CTTTTATCAA TTTTAAGATT   
  
  
- AACCCTAAAC TCTAACACTT AGAACATTAG GAAGCTGAAG ATCAAGATAC TTTTATCCGC TAATATCAAG   
  
  
- ATCCTAATCA GAAGCTGGGA ACTCTCTCCC TCCTCTACCC AAATCAACTA TTAGATAAGT AAGTATTGCG   
  
  
- TACGGACCCT CTACAAACCA TCCCGATCAT TAACTAAGCG ATTGTATAAA CACGACAATC AAGGTTGGAC   
  
  
- TCACAAATGA GAAGACGACA AATAAGGCAG AAAATCAGCA TTAACAGAGG AAATCAAATT AAAGTTTTGG   
  
  
- GGTTAACACT AGCGGATCTA ACTCGTTAGA TTAATCAAGA TTATTAACTT TAGTTCAAGG GACACCTTAT   
  
  
- GCTGGGCATG AACGGCACAC GATCGATGAC AATGTGGAAC GTAAACGCCA TATAAATATT GAGTTGTATA   
  
  
- CAGACGAGTG ATTTTCATGT ATAATCGAGT GGTTTTGACA CAGAGTTGCA TGATTTTATA AAATCACTTG   
  
  
- ATATGTAATA AAAATCATTC GCTTTATTCT ATGTGTTGTC GTGTAAAATT ACTCAATGTC CCGTAAAAAT   
  
  
- CACTCGGCTT TAGACTACCA AAAGAAACAT CTAGGCCAAT ATATTAACAT AAAATATGTT GGCTTACCTG   
  
  
- CTATATTGTT GACTCAATTT ACTCCTCAGA CTTTAATGCT TGTATTTTAG GTTTTCTTCT ACGGCTACGG   
  
  
- TAACCTTTCT TTCGACTTCC ACTAGCGAAA GATCATCATA ATCCGTCATC ATTAATTTTT GATTTTAGGT   
  
  
- CTTAACTGTG GCTTAACCCC AACACCGTGT ACTAGCTTTA GTCGTAGCTT AGATGTGCTA CTAGACCTAA   
  
  
- GAACCTGTGA GACCGAAATC CGGCAGTTAA AGCTCCTAAC GGGTATTGAA ATTAGGACAA GTTTAGGTAA   
  
  
- GTTAAATTAT AATGTGTTTA GGAAACTAAT TTAGTGGCAT TAACACCAAA CTAATCCCTT CAAAGAACCC   
  
  
- CCTTTCCAAG TTCGAAGACA GAAAGAATTT CGGACCTAAA AACCAGGGCG TACAAGTGAA GTGTAAGGGA   
  
  
- AAAAGAGAGG GTAGCAATGG ACAGACTTAG ACATACATTA TTTTCAGTAT AAAACGCTCC AGAAGAAAAC   
  
  
- TGACAAGAAC ATCAGTGAGA ACTATAGACG TGGTAAAACA AAAAGAGCCG TAAAAGAGGT TGAACGTAAA   
  
  
- AGATTACATT ACACTAGAAA AGACCTGTAA GTAAAACAAT TAGATGAGTG AACCCAGTTC CAAACGACTA   
  
  
- AAACCGACCA CAATTTAGGC AAGGAGAAAC CGTAAACAAG TACAAGGTGT TGACAAACTA TAGACCATAG   
  
  
- AGTCTTTGGG GCGGTAAGAA ACCATAAAGG ACTGAAAGAA CAAGTTCCCA ATAAGGATAA AGGGACTTAA   
  
  
- AAGAAACTAA GAAATCCATA AAGTTCGAAC CCCCCAAAAC AAGAAGTGAC TAAAACAGGA GAAGAAGGGA   
  
  
- ATAACCCACC AAACTAGAGG ATTTAAAGAC ACATAGACTT AGTCAGCCCC ATCTGAAACA CAAAAACCAA   
  
  
- CTAAAAAATA GTTACATAAA AGAAGGATTA CTTCCCGGAG GGCTTGGTTA TGTGTACGAA TTGAAGTTGG   
  
  
- GACAAGGAGA AGGTTCGAAA AACCTCGGAG TTTTCAGAAG CTTACGACTA TCCGTGTATC CCAGGAAGGT   
  
  
- TACGATACCA GAACTACTAC TGGGGCGTGG GAACTTACTG GGTTTAATGT AACATACAGT TAGAAGTATA   
  
  
- GGACTAGCTC TATAATGTGT CTAGGGACTG AAAGGACTAC TAACAGAGTT CAAGTAGTTA CTGTAAGAAT   
  
  
- ACCTTCTCCT AAACCTATTA GGCGGATATA GGAACGTCCT GATATTACGC GAGGTTCGGT GACTCTTCAG   
  
  
- TAACATACTA CGGGAACCTC TCCGAATAGG TCGAAGTAGA CTAGGTAACG TTAGTAGTCA ACCAGTCTCG   
  
  
- TGACTCTCGA GACTACTAGG ACTGTCTTCG TCGCCGTAAC TATTTACCCC ATTATCTATA ATGGGGTGGT   
  
  
- CGGACTTACA CCAACTTTGG TCGGCCGACT AGTTAGTCTC GGTCAACCTG GGAAGAGTTT ACAAACTACG   
  
  
- ACGACGAGTG GGGAACCTCA GATTAACAGT AAGAAAACAG AGATCATTAT TGAAACTAGT AAGATACCTA   
  
  
- CCCCAAGGTC TAAGAGGATA GTCATGTGAA AGACACTTAC TTTGTTCTTT CCTTCTTAGT CAATCATCTA   
  
  
- GTTCCTCGTC CTTCTTAGTA GTTTCTCTAC CACGTATGGA CCTTCTTTCC TCATGATTCG TTTATCGTAG   
  
  
- GTCGTTACTC CTTATACCTC TCTACCTCTC CAAACTACTA CATGACTAGA CGTCCCTTCC TTTACTACTA   
  
  
- TAAAGTGGAA CATGGGCGTT TTGGGGTCAA GTACTTCGTC TACTATTGAG CCTTGGTTTC CCCTTTAGTT   
  
  
- CTCCTAGATT TTCGTTCTCA TTTAGCGCAC AGTTCTTCTT TTCATTGCTT CCTGTTCGCC ATCTAGAATC   
  
  
- CTGAGTTGAA TAAGTCACAC GTCTTCGTCA CCGTTCGAAA CTCGAATTTT CACGTTTACT CGATGAGTCC   
  
  
- GCTTAATCCG TCGTACGAAG CGGTAAACCC CTATCACAAG TTTCCGATCG TGTAATGAAA CGCTTACCCG   
  
  
- AGCTCCGTGC GAATCGACCA TGTCCTAGTC TTAGTATGTT TCTGAAACGA CCCTTTTTTA AAAGTAGGAT   
  
  
- ACTGTAGAAC TCTCCGATGT TCAGTATACA AGTTCGGCAG GGGAAGGTCT CTTGTTGGTA CAAGAACTGT   
  
  
- TTAGTTTGTT ATCATTTCGA CCGACTCTTT CGTTGTTCCC AAGTATAGTA ACTAAAACCG TAGAAAAAAC   
  
  
- CAAAGGTCAC CGGGACGGAA CAAGTCTTAA ACAGTTCTTC TGGGGTCCCA AGAGGGTATG AGTTTTAGTG   
  
  
- CCCTCAGCTA GTAGGGGTCT CACCCAAGTC TGGTCGGGTC TTTCAACTTC TCTGTCCAGC AGCTAATCGT   
  
  
- CCGATAACAC TCTCCAAACC CCACGGGAAA CTCAAAGTAG GGTAACGAGT TTCCACCGTA TGGGATTTCG   
  
  
- GTCTCGTAAA GTTTTAACTT TCGTTACTCG AACAACACCA GTGAACAAAG ATGGTTAGTC CCTTCGAGGG   
  
  
- ACTACTCTGA CAACTACAGT TGTCGGGTTC TCTGGTACAG AACTCCGACT AGTCCCCGAA CTTGGGACTA   
  
  
- GATAAGTAGG TACCCCAATA GTTACCGTGT AAGTTACGTG GGAAGAAGCA TTGAGCCAAA TTTCTCCGTG   
  
  
- AAATGGTGAT GAGAAGAAAC AAGCTACAGA AACTACGGTG GTATCTAGCA CTTTCAGTAC TTTCTGACGA   
  
  
- CTGACTCTTG ATAAACATAC CCTTTCTACG TGAATTGTAT CATCGTACAC TCCCCTTGCT CTCCTAGCTC   
  
  
- TCTGGGCTCT GAATGTTCGT CACCGTTCAG GCCTTCGAAT CTCGACCTAA GTTCGTCCAG GGGGATCTGG   
  
  
- TTCTGTAACA TTTACCTCGT TCTCGATACC AATTCCGTTT AATGTTATTC CTAAAATACC ATCTTGTTCT   
  
  
- ATCCTTAACC TACCAGGTCC CGACCTTTCC TTCCTAAAAC TCACGGTATA GGACAACCTT TGGACGAAT

+     ARE

| Site Name | Organism | Position | Strand | Matrix score. | sequence | function |
| --- | --- | --- | --- | --- | --- | --- |
| ARE | Zea mays | 3293 | - | 6 | AAACCA | cis-acting regulatory element essential for the anaerobic induction |
| ARE | Zea mays | 1379 | - | 6 | AAACCA | cis-acting regulatory element essential for the anaerobic induction |
| ARE | Zea mays | 2470 | + | 6 | AAACCA | cis-acting regulatory element essential for the anaerobic induction |
| ARE | Zea mays | 1001 | - | 6 | AAACCA | cis-acting regulatory element essential for the anaerobic induction |
| ARE | Zea mays | 1902 | - | 6 | AAACCA | cis-acting regulatory element essential for the anaerobic induction |

>HU02G01573.1   
+ +Up\_Stream \_Len000TGAATT GGAAGTTGAT TCTACCTTTG ATTGATTCGA TTAATTCTAG GTTCTTGGTT   
  
  
+ CTAGTTCTCT CTCCTCTTGT TCTTGATTAT TTAGGTTTTG GATGTGATGT TTATTGAATT GAATGTTGCA   
  
  
+ ATTGTTGTTA TAAAGATGAT GTTAGGCTAA ATATCCTTCA ACTTAGGTTG GGATTGACGT CGAATATGTG   
  
  
+ TAGTTGAATC TTGTACTGGC CTAAATCCCT GCTCCCCCTC TGTTTTGCGA TTATGAATTT CATTATGCAC   
  
  
+ ACCACATGCT CGATGAAATG CTCGAAAGAA CATTCATCCT CTTTGTTTGG GATTTAGGGG AACGTTGAAA   
  
  
+ GACGAAGTCT TTACCTAGTG CGTCTTAGTA GACAATTAGG ATTTTAACAT GAAAATAGTT AAAATTCTAA   
  
  
+ TTGGGATTTG AGATTGTGAA TCTTGTAATC CTTCGACTTC TAGTTCTATG AAAATAGGCG ATTATAGTTC   
  
  
+ TAGGATTAGT CTTCGACCCT TGAGAGAGGG AGGAGATGGG TTTAGTTGAT AATCTATTCA TTCATAACGC   
  
  
+ ATGCCTGGGA GATGTTTGGT AGGGCTAGTA ATTGATTCGC TAACATATTT GTGCTGTTAG TTCCAACCTG   
  
  
+ AGTGTTTACT CTTCTGCTGT TTATTCCGTC TTTTAGTCGT AATTGTCTCC TTTAGTTTAA TTTCAAAACC   
  
  
+ CCAATTGTGA TCGCCTAGAT TGAGCAATCT AATTAGTTCT AATAATTGAA ATCAAGTTCC CTGTGGAATA   
  
  
+ CGACCCGTAC TTGCCGTGTG CTAGCTACTG TTACACCTTG CATTTGCGGT ATATTTATAA CTCAACATAT   
  
  
+ GTCTGCTCAC TAAAAGTACA TATTAGCTCA CCAAAACTGT GTCTCAACGT ACTAAAATAT TTTAGTGAAC   
  
  
+ TATACATTAT TTTTAGTAAG CGAAATAAGA TACACAACAG CACATTTTAA TGAGTTACAG GGCATTTTTA   
  
  
+ GTGAGCCGAA ATCTGATGGT TTTCTTTGTA GATCCGGTTA TATAATTGTA TTTTATACAA CCGAATGGAC   
  
  
+ GATATAACAA CTGAGTTAAA TGAGGAGTCT GAAATTACGA ACATAAAATC CAAAAGAAGA TGCCGATGCC   
  
  
+ ATTGGAAAGA AAGCTGAAGG TGATCGCTTT CTAGTAGTAT TAGGCAGTAG TAATTAAAAA CTAAAATCCA   
  
  
+ GAATTGACAC CGAATTGGGG TTGTGGCACA TGATCGAAAT CAGCATCGAA TCTACACGAT GATCTGGATT   
  
  
+ CTTGGACACT CTGGCTTTAG GCCGTCAATT TCGAGGATTG CCCATAACTT TAATCCTGTT CAAATCCATT   
  
  
+ CAATTTAATA TTACACAAAT CCTTTGATTA AATCACCGTA ATTGTGGTTT GATTAGGGAA GTTTCTTGGG   
  
  
+ GGAAAGGTTC AAGCTTCTGT CTTTCTTAAA GCCTGGATTT TTGGTCCCGC ATGTTCACTT CACATTCCCT   
  
  
+ TTTTCTCTCC CATCGTTACC TGTCTGAATC TGTATGTAAT AAAAGTCATA TTTTGCGAGG TCTTCTTTTG   
  
  
+ ACTGTTCTTG TAGTCACTCT TGATATCTGC ACCATTTTGT TTTTCTCGGC ATTTTCTCCA ACTTGCATTT   
  
  
+ TCTAATGTAA TGTGATCTTT TCTGGACATT CATTTTGTTA ATCTACTCAC TTGGGTCAAG GTTTGCTGAT   
  
  
+ TTTGGCTGGT GTTAAATCCG TTCCTCTTTG GCATTTGTTC ATGTTCCACA ACTGTTTGAT ATCTGGTATC   
  
  
+ TCAGAAACCC CGCCATTCTT TGGTATTTCC TGACTTTCTT GTTCAAGGGT TATTCCTATT TCCCTGAATT   
  
  
+ TTCTTTGATT CTTTAGGTAT TTCAAGCTTG GGGGGTTTTG TTCTTCACTG ATTTTGTCCT CTTCTTCCCT   
  
  
+ TATTGGGTGG TTTGATCTCC TAAATTTCTG TGTATCTGAA TCAGTCGGGG TAGACTTTGT GTTTTTGGTT   
  
  
+ GATTTTTTAT CAATGTATTT TCTTCCTAAT GAAGGGCCTC CCGAACCAAT ACACATGCTT AACTTCAACC   
  
  
+ CTGTTCCTCT TCCAAGCTTT TTGGAGCCTC AAAAGTCTTC GAATGCTGAT AGGCACATAG GGTCCTTCCA   
  
  
+ ATGCTATGGT CTTGATGATG ACCCCGCACC CTTGAATGAC CCAAATTACA TTGTATGTCA ATCTTCATAT   
  
  
+ CCTGATCGAG ATATTACACA GATCCCTGAC TTTCCTGATG ATTGTCTCAA GTTCATCAAT GACATTCTTA   
  
  
+ TGGAAGAGGA TTTGGATAAT CCGCCTATAT CCTTGCAGGA CTATAATGCG CTCCAAGCCA CTGAGAAGTC   
  
  
+ ATTGTATGAT GCCCTTGGAG AGGCTTATCC AGCTTCATCT GATCCATTGC AATCATCAGT TGGTCAGAGC   
  
  
+ ACTGAGAGCT CTGATGATCC TGACAGAAGC AGCGGCATTG ATAAATGGGG TAATAGATAT TACCCCACCA   
  
  
+ GCCTGAATGT GGTTGAAACC AGCCGGCTGA TCAATCAGAG CCAGTTGGAC CCTTCTCAAA TGTTTGATGC   
  
  
+ TGCTGCTCAC CCCTTGGAGT CTAATTGTCA TTCTTTTGTC TCTAGTAATA ACTTTGATCA TTCTATGGAT   
  
  
+ GGGGTTCCAG ATTCTCCTAT CAGTACACTT TCTGTGAATG AAACAAGAAA GGAAGAATCA GTTAGTAGAT   
  
  
+ CAAGGAGCAG GAAGAATCAT CAAAGAGATG GTGCATACCT GGAAGAAAGG AGTACTAAGC AAATAGCATC   
  
  
+ CAGCAATGAG GAATATGGAG AGATGGAGAG GTTTGATGAT GTACTGATCT GCAGGGAAGG AAATGATGAT   
  
  
+ ATTTCACCTT GTACCCGCAA AACCCCAGTT CATGAAGCAG ATGATAACTC GGAACCAAAG GGGAAATCAA   
  
  
+ GAGGATCTAA AAGCAAGAGT AAATCGCGTG TCAAGAAGAA AAGTAACGAA GGACAAGCGG TAGATCTTAG   
  
  
+ GACTCAACTT ATTCAGTGTG CAGAAGCAGT GGCAAGCTTT GAGCTTAAAA GTGCAAATGA GCTACTCAGG   
  
  
+ CGAATTAGGC AGCATGCTTC GCCATTTGGG GATAGTGTTC AAAGGCTAGC ACATTACTTT GCGAATGGGC   
  
  
+ TCGAGGCACG CTTAGCTGGT ACAGGATCAG AATCATACAA AGACTTTGCT GGGAAAAAAT TTTCATCCTA   
  
  
+ TGACATCTTG AGAGGCTACA AGTCATATGT TCAAGCCGTC CCCTTCCAGA GAACAACCAT GTTCTTGACA   
  
  
+ AATCAAACAA TAGTAAAGCT GGCTGAGAAA GCAACAAGGG TTCATATCAT TGATTTTGGC ATCTTTTTTG   
  
  
+ GTTTCCAGTG GCCCTGCCTT GTTCAGAATT TGTCAAGAAG ACCCCAGGGT TCTCCCATAC TCAAAATCAC   
  
  
+ GGGAGTCGAT CATCCCCAGA GTGGGTTCAG ACCAGCCCAG AAAGTTGAAG AGACAGGTCG TCGATTAGCA   
  
  
+ GGCTATTGTG AGAGGTTTGG GGTGCCCTTT GAGTTTCATC CCATTGCTCA AAGGTGGCAT ACCCTAAAGC   
  
  
+ CAGAGCATTT CAAAATTGAA AGCAATGAGC TTGTTGTGGT CACTTGTTTC TACCAATCAG GGAAGCTCCC   
  
  
+ TGATGAGACT GTTGATGTCA ACAGCCCAAG AGACCATGTC TTGAGGCTGA TCAGGGGCTT GAACCCTGAT   
  
  
+ CTATTCATCC ATGGGGTTAT CAATGGCACA TTCAATGCAC CCTTCTTCGT AACTCGGTTT AAAGAGGCAC   
  
  
+ TTTACCACTA CTCTTCTTTG TTCGATGTCT TTGATGCCAC CATAGATCGT GAAAGTCATG AAAGACTGCT   
  
  
+ GACTGAGAAC TATTTGTATG GGAAAGATGC ACTTAACATA GTAGCATGTG AGGGGAACGA GAGGATCGAG   
  
  
+ AGACCCGAGA CTTACAAGCA GTGGCAAGTC CGGAAGCTTA GAGCTGGATT CAAGCAGGTC CCCCTAGACC   
  
  
+ AAGACATTGT AAATGGAGCA AGAGCTATGG TTAAGGCAAA TTACAATAAG GATTTTATGG TAGAACAAGA   
  
  
+ TAGGAATTGG ATGGTCCAGG GCTGGAAAGG AAGGATTTTG AGTGCCATAT CCTGTTGGAA ACCTGCTTA  

- +Up\_Stream \_Len000ACTTAA CCTTCAACTA AGATGGAAAC TAACTAAGCT AATTAAGATC CAAGAACCAA   
  
  
- GATCAAGAGA GAGGAGAACA AGAACTAATA AATCCAAAAC CTACACTACA AATAACTTAA CTTACAACGT   
  
  
- TAACAACAAT ATTTCTACTA CAATCCGATT TATAGGAAGT TGAATCCAAC CCTAACTGCA GCTTATACAC   
  
  
- ATCAACTTAG AACATGACCG GATTTAGGGA CGAGGGGGAG ACAAAACGCT AATACTTAAA GTAATACGTG   
  
  
- TGGTGTACGA GCTACTTTAC GAGCTTTCTT GTAAGTAGGA GAAACAAACC CTAAATCCCC TTGCAACTTT   
  
  
- CTGCTTCAGA AATGGATCAC GCAGAATCAT CTGTTAATCC TAAAATTGTA CTTTTATCAA TTTTAAGATT   
  
  
- AACCCTAAAC TCTAACACTT AGAACATTAG GAAGCTGAAG ATCAAGATAC TTTTATCCGC TAATATCAAG   
  
  
- ATCCTAATCA GAAGCTGGGA ACTCTCTCCC TCCTCTACCC AAATCAACTA TTAGATAAGT AAGTATTGCG   
  
  
- TACGGACCCT CTACAAACCA TCCCGATCAT TAACTAAGCG ATTGTATAAA CACGACAATC AAGGTTGGAC   
  
  
- TCACAAATGA GAAGACGACA AATAAGGCAG AAAATCAGCA TTAACAGAGG AAATCAAATT AAAGTTTTGG   
  
  
- GGTTAACACT AGCGGATCTA ACTCGTTAGA TTAATCAAGA TTATTAACTT TAGTTCAAGG GACACCTTAT   
  
  
- GCTGGGCATG AACGGCACAC GATCGATGAC AATGTGGAAC GTAAACGCCA TATAAATATT GAGTTGTATA   
  
  
- CAGACGAGTG ATTTTCATGT ATAATCGAGT GGTTTTGACA CAGAGTTGCA TGATTTTATA AAATCACTTG   
  
  
- ATATGTAATA AAAATCATTC GCTTTATTCT ATGTGTTGTC GTGTAAAATT ACTCAATGTC CCGTAAAAAT   
  
  
- CACTCGGCTT TAGACTACCA AAAGAAACAT CTAGGCCAAT ATATTAACAT AAAATATGTT GGCTTACCTG   
  
  
- CTATATTGTT GACTCAATTT ACTCCTCAGA CTTTAATGCT TGTATTTTAG GTTTTCTTCT ACGGCTACGG   
  
  
- TAACCTTTCT TTCGACTTCC ACTAGCGAAA GATCATCATA ATCCGTCATC ATTAATTTTT GATTTTAGGT   
  
  
- CTTAACTGTG GCTTAACCCC AACACCGTGT ACTAGCTTTA GTCGTAGCTT AGATGTGCTA CTAGACCTAA   
  
  
- GAACCTGTGA GACCGAAATC CGGCAGTTAA AGCTCCTAAC GGGTATTGAA ATTAGGACAA GTTTAGGTAA   
  
  
- GTTAAATTAT AATGTGTTTA GGAAACTAAT TTAGTGGCAT TAACACCAAA CTAATCCCTT CAAAGAACCC   
  
  
- CCTTTCCAAG TTCGAAGACA GAAAGAATTT CGGACCTAAA AACCAGGGCG TACAAGTGAA GTGTAAGGGA   
  
  
- AAAAGAGAGG GTAGCAATGG ACAGACTTAG ACATACATTA TTTTCAGTAT AAAACGCTCC AGAAGAAAAC   
  
  
- TGACAAGAAC ATCAGTGAGA ACTATAGACG TGGTAAAACA AAAAGAGCCG TAAAAGAGGT TGAACGTAAA   
  
  
- AGATTACATT ACACTAGAAA AGACCTGTAA GTAAAACAAT TAGATGAGTG AACCCAGTTC CAAACGACTA   
  
  
- AAACCGACCA CAATTTAGGC AAGGAGAAAC CGTAAACAAG TACAAGGTGT TGACAAACTA TAGACCATAG   
  
  
- AGTCTTTGGG GCGGTAAGAA ACCATAAAGG ACTGAAAGAA CAAGTTCCCA ATAAGGATAA AGGGACTTAA   
  
  
- AAGAAACTAA GAAATCCATA AAGTTCGAAC CCCCCAAAAC AAGAAGTGAC TAAAACAGGA GAAGAAGGGA   
  
  
- ATAACCCACC AAACTAGAGG ATTTAAAGAC ACATAGACTT AGTCAGCCCC ATCTGAAACA CAAAAACCAA   
  
  
- CTAAAAAATA GTTACATAAA AGAAGGATTA CTTCCCGGAG GGCTTGGTTA TGTGTACGAA TTGAAGTTGG   
  
  
- GACAAGGAGA AGGTTCGAAA AACCTCGGAG TTTTCAGAAG CTTACGACTA TCCGTGTATC CCAGGAAGGT   
  
  
- TACGATACCA GAACTACTAC TGGGGCGTGG GAACTTACTG GGTTTAATGT AACATACAGT TAGAAGTATA   
  
  
- GGACTAGCTC TATAATGTGT CTAGGGACTG AAAGGACTAC TAACAGAGTT CAAGTAGTTA CTGTAAGAAT   
  
  
- ACCTTCTCCT AAACCTATTA GGCGGATATA GGAACGTCCT GATATTACGC GAGGTTCGGT GACTCTTCAG   
  
  
- TAACATACTA CGGGAACCTC TCCGAATAGG TCGAAGTAGA CTAGGTAACG TTAGTAGTCA ACCAGTCTCG   
  
  
- TGACTCTCGA GACTACTAGG ACTGTCTTCG TCGCCGTAAC TATTTACCCC ATTATCTATA ATGGGGTGGT   
  
  
- CGGACTTACA CCAACTTTGG TCGGCCGACT AGTTAGTCTC GGTCAACCTG GGAAGAGTTT ACAAACTACG   
  
  
- ACGACGAGTG GGGAACCTCA GATTAACAGT AAGAAAACAG AGATCATTAT TGAAACTAGT AAGATACCTA   
  
  
- CCCCAAGGTC TAAGAGGATA GTCATGTGAA AGACACTTAC TTTGTTCTTT CCTTCTTAGT CAATCATCTA   
  
  
- GTTCCTCGTC CTTCTTAGTA GTTTCTCTAC CACGTATGGA CCTTCTTTCC TCATGATTCG TTTATCGTAG   
  
  
- GTCGTTACTC CTTATACCTC TCTACCTCTC CAAACTACTA CATGACTAGA CGTCCCTTCC TTTACTACTA   
  
  
- TAAAGTGGAA CATGGGCGTT TTGGGGTCAA GTACTTCGTC TACTATTGAG CCTTGGTTTC CCCTTTAGTT   
  
  
- CTCCTAGATT TTCGTTCTCA TTTAGCGCAC AGTTCTTCTT TTCATTGCTT CCTGTTCGCC ATCTAGAATC   
  
  
- CTGAGTTGAA TAAGTCACAC GTCTTCGTCA CCGTTCGAAA CTCGAATTTT CACGTTTACT CGATGAGTCC   
  
  
- GCTTAATCCG TCGTACGAAG CGGTAAACCC CTATCACAAG TTTCCGATCG TGTAATGAAA CGCTTACCCG   
  
  
- AGCTCCGTGC GAATCGACCA TGTCCTAGTC TTAGTATGTT TCTGAAACGA CCCTTTTTTA AAAGTAGGAT   
  
  
- ACTGTAGAAC TCTCCGATGT TCAGTATACA AGTTCGGCAG GGGAAGGTCT CTTGTTGGTA CAAGAACTGT   
  
  
- TTAGTTTGTT ATCATTTCGA CCGACTCTTT CGTTGTTCCC AAGTATAGTA ACTAAAACCG TAGAAAAAAC   
  
  
- CAAAGGTCAC CGGGACGGAA CAAGTCTTAA ACAGTTCTTC TGGGGTCCCA AGAGGGTATG AGTTTTAGTG   
  
  
- CCCTCAGCTA GTAGGGGTCT CACCCAAGTC TGGTCGGGTC TTTCAACTTC TCTGTCCAGC AGCTAATCGT   
  
  
- CCGATAACAC TCTCCAAACC CCACGGGAAA CTCAAAGTAG GGTAACGAGT TTCCACCGTA TGGGATTTCG   
  
  
- GTCTCGTAAA GTTTTAACTT TCGTTACTCG AACAACACCA GTGAACAAAG ATGGTTAGTC CCTTCGAGGG   
  
  
- ACTACTCTGA CAACTACAGT TGTCGGGTTC TCTGGTACAG AACTCCGACT AGTCCCCGAA CTTGGGACTA   
  
  
- GATAAGTAGG TACCCCAATA GTTACCGTGT AAGTTACGTG GGAAGAAGCA TTGAGCCAAA TTTCTCCGTG   
  
  
- AAATGGTGAT GAGAAGAAAC AAGCTACAGA AACTACGGTG GTATCTAGCA CTTTCAGTAC TTTCTGACGA   
  
  
- CTGACTCTTG ATAAACATAC CCTTTCTACG TGAATTGTAT CATCGTACAC TCCCCTTGCT CTCCTAGCTC   
  
  
- TCTGGGCTCT GAATGTTCGT CACCGTTCAG GCCTTCGAAT CTCGACCTAA GTTCGTCCAG GGGGATCTGG   
  
  
- TTCTGTAACA TTTACCTCGT TCTCGATACC AATTCCGTTT AATGTTATTC CTAAAATACC ATCTTGTTCT   
  
  
- ATCCTTAACC TACCAGGTCC CGACCTTTCC TTCCTAAAAC TCACGGTATA GGACAACCTT TGGACGAAT

+     AT-rich element

| Site Name | Organism | Position | Strand | Matrix score. | sequence | function |
| --- | --- | --- | --- | --- | --- | --- |
| AT-rich element | Glycine max | 749 | + | 10 | ATAGAAATCAA | binding site of AT-rich DNA binding protein (ATBP-1) |

>HU02G01573.1   
+ +Up\_Stream \_Len000TGAATT GGAAGTTGAT TCTACCTTTG ATTGATTCGA TTAATTCTAG GTTCTTGGTT   
  
  
+ CTAGTTCTCT CTCCTCTTGT TCTTGATTAT TTAGGTTTTG GATGTGATGT TTATTGAATT GAATGTTGCA   
  
  
+ ATTGTTGTTA TAAAGATGAT GTTAGGCTAA ATATCCTTCA ACTTAGGTTG GGATTGACGT CGAATATGTG   
  
  
+ TAGTTGAATC TTGTACTGGC CTAAATCCCT GCTCCCCCTC TGTTTTGCGA TTATGAATTT CATTATGCAC   
  
  
+ ACCACATGCT CGATGAAATG CTCGAAAGAA CATTCATCCT CTTTGTTTGG GATTTAGGGG AACGTTGAAA   
  
  
+ GACGAAGTCT TTACCTAGTG CGTCTTAGTA GACAATTAGG ATTTTAACAT GAAAATAGTT AAAATTCTAA   
  
  
+ TTGGGATTTG AGATTGTGAA TCTTGTAATC CTTCGACTTC TAGTTCTATG AAAATAGGCG ATTATAGTTC   
  
  
+ TAGGATTAGT CTTCGACCCT TGAGAGAGGG AGGAGATGGG TTTAGTTGAT AATCTATTCA TTCATAACGC   
  
  
+ ATGCCTGGGA GATGTTTGGT AGGGCTAGTA ATTGATTCGC TAACATATTT GTGCTGTTAG TTCCAACCTG   
  
  
+ AGTGTTTACT CTTCTGCTGT TTATTCCGTC TTTTAGTCGT AATTGTCTCC TTTAGTTTAA TTTCAAAACC   
  
  
+ CCAATTGTGA TCGCCTAGAT TGAGCAATCT AATTAGTTCT AATAATTGAA ATCAAGTTCC CTGTGGAATA   
  
  
+ CGACCCGTAC TTGCCGTGTG CTAGCTACTG TTACACCTTG CATTTGCGGT ATATTTATAA CTCAACATAT   
  
  
+ GTCTGCTCAC TAAAAGTACA TATTAGCTCA CCAAAACTGT GTCTCAACGT ACTAAAATAT TTTAGTGAAC   
  
  
+ TATACATTAT TTTTAGTAAG CGAAATAAGA TACACAACAG CACATTTTAA TGAGTTACAG GGCATTTTTA   
  
  
+ GTGAGCCGAA ATCTGATGGT TTTCTTTGTA GATCCGGTTA TATAATTGTA TTTTATACAA CCGAATGGAC   
  
  
+ GATATAACAA CTGAGTTAAA TGAGGAGTCT GAAATTACGA ACATAAAATC CAAAAGAAGA TGCCGATGCC   
  
  
+ ATTGGAAAGA AAGCTGAAGG TGATCGCTTT CTAGTAGTAT TAGGCAGTAG TAATTAAAAA CTAAAATCCA   
  
  
+ GAATTGACAC CGAATTGGGG TTGTGGCACA TGATCGAAAT CAGCATCGAA TCTACACGAT GATCTGGATT   
  
  
+ CTTGGACACT CTGGCTTTAG GCCGTCAATT TCGAGGATTG CCCATAACTT TAATCCTGTT CAAATCCATT   
  
  
+ CAATTTAATA TTACACAAAT CCTTTGATTA AATCACCGTA ATTGTGGTTT GATTAGGGAA GTTTCTTGGG   
  
  
+ GGAAAGGTTC AAGCTTCTGT CTTTCTTAAA GCCTGGATTT TTGGTCCCGC ATGTTCACTT CACATTCCCT   
  
  
+ TTTTCTCTCC CATCGTTACC TGTCTGAATC TGTATGTAAT AAAAGTCATA TTTTGCGAGG TCTTCTTTTG   
  
  
+ ACTGTTCTTG TAGTCACTCT TGATATCTGC ACCATTTTGT TTTTCTCGGC ATTTTCTCCA ACTTGCATTT   
  
  
+ TCTAATGTAA TGTGATCTTT TCTGGACATT CATTTTGTTA ATCTACTCAC TTGGGTCAAG GTTTGCTGAT   
  
  
+ TTTGGCTGGT GTTAAATCCG TTCCTCTTTG GCATTTGTTC ATGTTCCACA ACTGTTTGAT ATCTGGTATC   
  
  
+ TCAGAAACCC CGCCATTCTT TGGTATTTCC TGACTTTCTT GTTCAAGGGT TATTCCTATT TCCCTGAATT   
  
  
+ TTCTTTGATT CTTTAGGTAT TTCAAGCTTG GGGGGTTTTG TTCTTCACTG ATTTTGTCCT CTTCTTCCCT   
  
  
+ TATTGGGTGG TTTGATCTCC TAAATTTCTG TGTATCTGAA TCAGTCGGGG TAGACTTTGT GTTTTTGGTT   
  
  
+ GATTTTTTAT CAATGTATTT TCTTCCTAAT GAAGGGCCTC CCGAACCAAT ACACATGCTT AACTTCAACC   
  
  
+ CTGTTCCTCT TCCAAGCTTT TTGGAGCCTC AAAAGTCTTC GAATGCTGAT AGGCACATAG GGTCCTTCCA   
  
  
+ ATGCTATGGT CTTGATGATG ACCCCGCACC CTTGAATGAC CCAAATTACA TTGTATGTCA ATCTTCATAT   
  
  
+ CCTGATCGAG ATATTACACA GATCCCTGAC TTTCCTGATG ATTGTCTCAA GTTCATCAAT GACATTCTTA   
  
  
+ TGGAAGAGGA TTTGGATAAT CCGCCTATAT CCTTGCAGGA CTATAATGCG CTCCAAGCCA CTGAGAAGTC   
  
  
+ ATTGTATGAT GCCCTTGGAG AGGCTTATCC AGCTTCATCT GATCCATTGC AATCATCAGT TGGTCAGAGC   
  
  
+ ACTGAGAGCT CTGATGATCC TGACAGAAGC AGCGGCATTG ATAAATGGGG TAATAGATAT TACCCCACCA   
  
  
+ GCCTGAATGT GGTTGAAACC AGCCGGCTGA TCAATCAGAG CCAGTTGGAC CCTTCTCAAA TGTTTGATGC   
  
  
+ TGCTGCTCAC CCCTTGGAGT CTAATTGTCA TTCTTTTGTC TCTAGTAATA ACTTTGATCA TTCTATGGAT   
  
  
+ GGGGTTCCAG ATTCTCCTAT CAGTACACTT TCTGTGAATG AAACAAGAAA GGAAGAATCA GTTAGTAGAT   
  
  
+ CAAGGAGCAG GAAGAATCAT CAAAGAGATG GTGCATACCT GGAAGAAAGG AGTACTAAGC AAATAGCATC   
  
  
+ CAGCAATGAG GAATATGGAG AGATGGAGAG GTTTGATGAT GTACTGATCT GCAGGGAAGG AAATGATGAT   
  
  
+ ATTTCACCTT GTACCCGCAA AACCCCAGTT CATGAAGCAG ATGATAACTC GGAACCAAAG GGGAAATCAA   
  
  
+ GAGGATCTAA AAGCAAGAGT AAATCGCGTG TCAAGAAGAA AAGTAACGAA GGACAAGCGG TAGATCTTAG   
  
  
+ GACTCAACTT ATTCAGTGTG CAGAAGCAGT GGCAAGCTTT GAGCTTAAAA GTGCAAATGA GCTACTCAGG   
  
  
+ CGAATTAGGC AGCATGCTTC GCCATTTGGG GATAGTGTTC AAAGGCTAGC ACATTACTTT GCGAATGGGC   
  
  
+ TCGAGGCACG CTTAGCTGGT ACAGGATCAG AATCATACAA AGACTTTGCT GGGAAAAAAT TTTCATCCTA   
  
  
+ TGACATCTTG AGAGGCTACA AGTCATATGT TCAAGCCGTC CCCTTCCAGA GAACAACCAT GTTCTTGACA   
  
  
+ AATCAAACAA TAGTAAAGCT GGCTGAGAAA GCAACAAGGG TTCATATCAT TGATTTTGGC ATCTTTTTTG   
  
  
+ GTTTCCAGTG GCCCTGCCTT GTTCAGAATT TGTCAAGAAG ACCCCAGGGT TCTCCCATAC TCAAAATCAC   
  
  
+ GGGAGTCGAT CATCCCCAGA GTGGGTTCAG ACCAGCCCAG AAAGTTGAAG AGACAGGTCG TCGATTAGCA   
  
  
+ GGCTATTGTG AGAGGTTTGG GGTGCCCTTT GAGTTTCATC CCATTGCTCA AAGGTGGCAT ACCCTAAAGC   
  
  
+ CAGAGCATTT CAAAATTGAA AGCAATGAGC TTGTTGTGGT CACTTGTTTC TACCAATCAG GGAAGCTCCC   
  
  
+ TGATGAGACT GTTGATGTCA ACAGCCCAAG AGACCATGTC TTGAGGCTGA TCAGGGGCTT GAACCCTGAT   
  
  
+ CTATTCATCC ATGGGGTTAT CAATGGCACA TTCAATGCAC CCTTCTTCGT AACTCGGTTT AAAGAGGCAC   
  
  
+ TTTACCACTA CTCTTCTTTG TTCGATGTCT TTGATGCCAC CATAGATCGT GAAAGTCATG AAAGACTGCT   
  
  
+ GACTGAGAAC TATTTGTATG GGAAAGATGC ACTTAACATA GTAGCATGTG AGGGGAACGA GAGGATCGAG   
  
  
+ AGACCCGAGA CTTACAAGCA GTGGCAAGTC CGGAAGCTTA GAGCTGGATT CAAGCAGGTC CCCCTAGACC   
  
  
+ AAGACATTGT AAATGGAGCA AGAGCTATGG TTAAGGCAAA TTACAATAAG GATTTTATGG TAGAACAAGA   
  
  
+ TAGGAATTGG ATGGTCCAGG GCTGGAAAGG AAGGATTTTG AGTGCCATAT CCTGTTGGAA ACCTGCTTA  

- +Up\_Stream \_Len000ACTTAA CCTTCAACTA AGATGGAAAC TAACTAAGCT AATTAAGATC CAAGAACCAA   
  
  
- GATCAAGAGA GAGGAGAACA AGAACTAATA AATCCAAAAC CTACACTACA AATAACTTAA CTTACAACGT   
  
  
- TAACAACAAT ATTTCTACTA CAATCCGATT TATAGGAAGT TGAATCCAAC CCTAACTGCA GCTTATACAC   
  
  
- ATCAACTTAG AACATGACCG GATTTAGGGA CGAGGGGGAG ACAAAACGCT AATACTTAAA GTAATACGTG   
  
  
- TGGTGTACGA GCTACTTTAC GAGCTTTCTT GTAAGTAGGA GAAACAAACC CTAAATCCCC TTGCAACTTT   
  
  
- CTGCTTCAGA AATGGATCAC GCAGAATCAT CTGTTAATCC TAAAATTGTA CTTTTATCAA TTTTAAGATT   
  
  
- AACCCTAAAC TCTAACACTT AGAACATTAG GAAGCTGAAG ATCAAGATAC TTTTATCCGC TAATATCAAG   
  
  
- ATCCTAATCA GAAGCTGGGA ACTCTCTCCC TCCTCTACCC AAATCAACTA TTAGATAAGT AAGTATTGCG   
  
  
- TACGGACCCT CTACAAACCA TCCCGATCAT TAACTAAGCG ATTGTATAAA CACGACAATC AAGGTTGGAC   
  
  
- TCACAAATGA GAAGACGACA AATAAGGCAG AAAATCAGCA TTAACAGAGG AAATCAAATT AAAGTTTTGG   
  
  
- GGTTAACACT AGCGGATCTA ACTCGTTAGA TTAATCAAGA TTATTAACTT TAGTTCAAGG GACACCTTAT   
  
  
- GCTGGGCATG AACGGCACAC GATCGATGAC AATGTGGAAC GTAAACGCCA TATAAATATT GAGTTGTATA   
  
  
- CAGACGAGTG ATTTTCATGT ATAATCGAGT GGTTTTGACA CAGAGTTGCA TGATTTTATA AAATCACTTG   
  
  
- ATATGTAATA AAAATCATTC GCTTTATTCT ATGTGTTGTC GTGTAAAATT ACTCAATGTC CCGTAAAAAT   
  
  
- CACTCGGCTT TAGACTACCA AAAGAAACAT CTAGGCCAAT ATATTAACAT AAAATATGTT GGCTTACCTG   
  
  
- CTATATTGTT GACTCAATTT ACTCCTCAGA CTTTAATGCT TGTATTTTAG GTTTTCTTCT ACGGCTACGG   
  
  
- TAACCTTTCT TTCGACTTCC ACTAGCGAAA GATCATCATA ATCCGTCATC ATTAATTTTT GATTTTAGGT   
  
  
- CTTAACTGTG GCTTAACCCC AACACCGTGT ACTAGCTTTA GTCGTAGCTT AGATGTGCTA CTAGACCTAA   
  
  
- GAACCTGTGA GACCGAAATC CGGCAGTTAA AGCTCCTAAC GGGTATTGAA ATTAGGACAA GTTTAGGTAA   
  
  
- GTTAAATTAT AATGTGTTTA GGAAACTAAT TTAGTGGCAT TAACACCAAA CTAATCCCTT CAAAGAACCC   
  
  
- CCTTTCCAAG TTCGAAGACA GAAAGAATTT CGGACCTAAA AACCAGGGCG TACAAGTGAA GTGTAAGGGA   
  
  
- AAAAGAGAGG GTAGCAATGG ACAGACTTAG ACATACATTA TTTTCAGTAT AAAACGCTCC AGAAGAAAAC   
  
  
- TGACAAGAAC ATCAGTGAGA ACTATAGACG TGGTAAAACA AAAAGAGCCG TAAAAGAGGT TGAACGTAAA   
  
  
- AGATTACATT ACACTAGAAA AGACCTGTAA GTAAAACAAT TAGATGAGTG AACCCAGTTC CAAACGACTA   
  
  
- AAACCGACCA CAATTTAGGC AAGGAGAAAC CGTAAACAAG TACAAGGTGT TGACAAACTA TAGACCATAG   
  
  
- AGTCTTTGGG GCGGTAAGAA ACCATAAAGG ACTGAAAGAA CAAGTTCCCA ATAAGGATAA AGGGACTTAA   
  
  
- AAGAAACTAA GAAATCCATA AAGTTCGAAC CCCCCAAAAC AAGAAGTGAC TAAAACAGGA GAAGAAGGGA   
  
  
- ATAACCCACC AAACTAGAGG ATTTAAAGAC ACATAGACTT AGTCAGCCCC ATCTGAAACA CAAAAACCAA   
  
  
- CTAAAAAATA GTTACATAAA AGAAGGATTA CTTCCCGGAG GGCTTGGTTA TGTGTACGAA TTGAAGTTGG   
  
  
- GACAAGGAGA AGGTTCGAAA AACCTCGGAG TTTTCAGAAG CTTACGACTA TCCGTGTATC CCAGGAAGGT   
  
  
- TACGATACCA GAACTACTAC TGGGGCGTGG GAACTTACTG GGTTTAATGT AACATACAGT TAGAAGTATA   
  
  
- GGACTAGCTC TATAATGTGT CTAGGGACTG AAAGGACTAC TAACAGAGTT CAAGTAGTTA CTGTAAGAAT   
  
  
- ACCTTCTCCT AAACCTATTA GGCGGATATA GGAACGTCCT GATATTACGC GAGGTTCGGT GACTCTTCAG   
  
  
- TAACATACTA CGGGAACCTC TCCGAATAGG TCGAAGTAGA CTAGGTAACG TTAGTAGTCA ACCAGTCTCG   
  
  
- TGACTCTCGA GACTACTAGG ACTGTCTTCG TCGCCGTAAC TATTTACCCC ATTATCTATA ATGGGGTGGT   
  
  
- CGGACTTACA CCAACTTTGG TCGGCCGACT AGTTAGTCTC GGTCAACCTG GGAAGAGTTT ACAAACTACG   
  
  
- ACGACGAGTG GGGAACCTCA GATTAACAGT AAGAAAACAG AGATCATTAT TGAAACTAGT AAGATACCTA   
  
  
- CCCCAAGGTC TAAGAGGATA GTCATGTGAA AGACACTTAC TTTGTTCTTT CCTTCTTAGT CAATCATCTA   
  
  
- GTTCCTCGTC CTTCTTAGTA GTTTCTCTAC CACGTATGGA CCTTCTTTCC TCATGATTCG TTTATCGTAG   
  
  
- GTCGTTACTC CTTATACCTC TCTACCTCTC CAAACTACTA CATGACTAGA CGTCCCTTCC TTTACTACTA   
  
  
- TAAAGTGGAA CATGGGCGTT TTGGGGTCAA GTACTTCGTC TACTATTGAG CCTTGGTTTC CCCTTTAGTT   
  
  
- CTCCTAGATT TTCGTTCTCA TTTAGCGCAC AGTTCTTCTT TTCATTGCTT CCTGTTCGCC ATCTAGAATC   
  
  
- CTGAGTTGAA TAAGTCACAC GTCTTCGTCA CCGTTCGAAA CTCGAATTTT CACGTTTACT CGATGAGTCC   
  
  
- GCTTAATCCG TCGTACGAAG CGGTAAACCC CTATCACAAG TTTCCGATCG TGTAATGAAA CGCTTACCCG   
  
  
- AGCTCCGTGC GAATCGACCA TGTCCTAGTC TTAGTATGTT TCTGAAACGA CCCTTTTTTA AAAGTAGGAT   
  
  
- ACTGTAGAAC TCTCCGATGT TCAGTATACA AGTTCGGCAG GGGAAGGTCT CTTGTTGGTA CAAGAACTGT   
  
  
- TTAGTTTGTT ATCATTTCGA CCGACTCTTT CGTTGTTCCC AAGTATAGTA ACTAAAACCG TAGAAAAAAC   
  
  
- CAAAGGTCAC CGGGACGGAA CAAGTCTTAA ACAGTTCTTC TGGGGTCCCA AGAGGGTATG AGTTTTAGTG   
  
  
- CCCTCAGCTA GTAGGGGTCT CACCCAAGTC TGGTCGGGTC TTTCAACTTC TCTGTCCAGC AGCTAATCGT   
  
  
- CCGATAACAC TCTCCAAACC CCACGGGAAA CTCAAAGTAG GGTAACGAGT TTCCACCGTA TGGGATTTCG   
  
  
- GTCTCGTAAA GTTTTAACTT TCGTTACTCG AACAACACCA GTGAACAAAG ATGGTTAGTC CCTTCGAGGG   
  
  
- ACTACTCTGA CAACTACAGT TGTCGGGTTC TCTGGTACAG AACTCCGACT AGTCCCCGAA CTTGGGACTA   
  
  
- GATAAGTAGG TACCCCAATA GTTACCGTGT AAGTTACGTG GGAAGAAGCA TTGAGCCAAA TTTCTCCGTG   
  
  
- AAATGGTGAT GAGAAGAAAC AAGCTACAGA AACTACGGTG GTATCTAGCA CTTTCAGTAC TTTCTGACGA   
  
  
- CTGACTCTTG ATAAACATAC CCTTTCTACG TGAATTGTAT CATCGTACAC TCCCCTTGCT CTCCTAGCTC   
  
  
- TCTGGGCTCT GAATGTTCGT CACCGTTCAG GCCTTCGAAT CTCGACCTAA GTTCGTCCAG GGGGATCTGG   
  
  
- TTCTGTAACA TTTACCTCGT TCTCGATACC AATTCCGTTT AATGTTATTC CTAAAATACC ATCTTGTTCT   
  
  
- ATCCTTAACC TACCAGGTCC CGACCTTTCC TTCCTAAAAC TCACGGTATA GGACAACCTT TGGACGAAT

+     AT~TATA-box

| Site Name | Organism | Position | Strand | Matrix score. | sequence | function |
| --- | --- | --- | --- | --- | --- | --- |
| AT~TATA-box | Arabidopsis thaliana | 1023 | + | 6 | TATATA |  |

>HU02G01573.1   
+ +Up\_Stream \_Len000TGAATT GGAAGTTGAT TCTACCTTTG ATTGATTCGA TTAATTCTAG GTTCTTGGTT   
  
  
+ CTAGTTCTCT CTCCTCTTGT TCTTGATTAT TTAGGTTTTG GATGTGATGT TTATTGAATT GAATGTTGCA   
  
  
+ ATTGTTGTTA TAAAGATGAT GTTAGGCTAA ATATCCTTCA ACTTAGGTTG GGATTGACGT CGAATATGTG   
  
  
+ TAGTTGAATC TTGTACTGGC CTAAATCCCT GCTCCCCCTC TGTTTTGCGA TTATGAATTT CATTATGCAC   
  
  
+ ACCACATGCT CGATGAAATG CTCGAAAGAA CATTCATCCT CTTTGTTTGG GATTTAGGGG AACGTTGAAA   
  
  
+ GACGAAGTCT TTACCTAGTG CGTCTTAGTA GACAATTAGG ATTTTAACAT GAAAATAGTT AAAATTCTAA   
  
  
+ TTGGGATTTG AGATTGTGAA TCTTGTAATC CTTCGACTTC TAGTTCTATG AAAATAGGCG ATTATAGTTC   
  
  
+ TAGGATTAGT CTTCGACCCT TGAGAGAGGG AGGAGATGGG TTTAGTTGAT AATCTATTCA TTCATAACGC   
  
  
+ ATGCCTGGGA GATGTTTGGT AGGGCTAGTA ATTGATTCGC TAACATATTT GTGCTGTTAG TTCCAACCTG   
  
  
+ AGTGTTTACT CTTCTGCTGT TTATTCCGTC TTTTAGTCGT AATTGTCTCC TTTAGTTTAA TTTCAAAACC   
  
  
+ CCAATTGTGA TCGCCTAGAT TGAGCAATCT AATTAGTTCT AATAATTGAA ATCAAGTTCC CTGTGGAATA   
  
  
+ CGACCCGTAC TTGCCGTGTG CTAGCTACTG TTACACCTTG CATTTGCGGT ATATTTATAA CTCAACATAT   
  
  
+ GTCTGCTCAC TAAAAGTACA TATTAGCTCA CCAAAACTGT GTCTCAACGT ACTAAAATAT TTTAGTGAAC   
  
  
+ TATACATTAT TTTTAGTAAG CGAAATAAGA TACACAACAG CACATTTTAA TGAGTTACAG GGCATTTTTA   
  
  
+ GTGAGCCGAA ATCTGATGGT TTTCTTTGTA GATCCGGTTA TATAATTGTA TTTTATACAA CCGAATGGAC   
  
  
+ GATATAACAA CTGAGTTAAA TGAGGAGTCT GAAATTACGA ACATAAAATC CAAAAGAAGA TGCCGATGCC   
  
  
+ ATTGGAAAGA AAGCTGAAGG TGATCGCTTT CTAGTAGTAT TAGGCAGTAG TAATTAAAAA CTAAAATCCA   
  
  
+ GAATTGACAC CGAATTGGGG TTGTGGCACA TGATCGAAAT CAGCATCGAA TCTACACGAT GATCTGGATT   
  
  
+ CTTGGACACT CTGGCTTTAG GCCGTCAATT TCGAGGATTG CCCATAACTT TAATCCTGTT CAAATCCATT   
  
  
+ CAATTTAATA TTACACAAAT CCTTTGATTA AATCACCGTA ATTGTGGTTT GATTAGGGAA GTTTCTTGGG   
  
  
+ GGAAAGGTTC AAGCTTCTGT CTTTCTTAAA GCCTGGATTT TTGGTCCCGC ATGTTCACTT CACATTCCCT   
  
  
+ TTTTCTCTCC CATCGTTACC TGTCTGAATC TGTATGTAAT AAAAGTCATA TTTTGCGAGG TCTTCTTTTG   
  
  
+ ACTGTTCTTG TAGTCACTCT TGATATCTGC ACCATTTTGT TTTTCTCGGC ATTTTCTCCA ACTTGCATTT   
  
  
+ TCTAATGTAA TGTGATCTTT TCTGGACATT CATTTTGTTA ATCTACTCAC TTGGGTCAAG GTTTGCTGAT   
  
  
+ TTTGGCTGGT GTTAAATCCG TTCCTCTTTG GCATTTGTTC ATGTTCCACA ACTGTTTGAT ATCTGGTATC   
  
  
+ TCAGAAACCC CGCCATTCTT TGGTATTTCC TGACTTTCTT GTTCAAGGGT TATTCCTATT TCCCTGAATT   
  
  
+ TTCTTTGATT CTTTAGGTAT TTCAAGCTTG GGGGGTTTTG TTCTTCACTG ATTTTGTCCT CTTCTTCCCT   
  
  
+ TATTGGGTGG TTTGATCTCC TAAATTTCTG TGTATCTGAA TCAGTCGGGG TAGACTTTGT GTTTTTGGTT   
  
  
+ GATTTTTTAT CAATGTATTT TCTTCCTAAT GAAGGGCCTC CCGAACCAAT ACACATGCTT AACTTCAACC   
  
  
+ CTGTTCCTCT TCCAAGCTTT TTGGAGCCTC AAAAGTCTTC GAATGCTGAT AGGCACATAG GGTCCTTCCA   
  
  
+ ATGCTATGGT CTTGATGATG ACCCCGCACC CTTGAATGAC CCAAATTACA TTGTATGTCA ATCTTCATAT   
  
  
+ CCTGATCGAG ATATTACACA GATCCCTGAC TTTCCTGATG ATTGTCTCAA GTTCATCAAT GACATTCTTA   
  
  
+ TGGAAGAGGA TTTGGATAAT CCGCCTATAT CCTTGCAGGA CTATAATGCG CTCCAAGCCA CTGAGAAGTC   
  
  
+ ATTGTATGAT GCCCTTGGAG AGGCTTATCC AGCTTCATCT GATCCATTGC AATCATCAGT TGGTCAGAGC   
  
  
+ ACTGAGAGCT CTGATGATCC TGACAGAAGC AGCGGCATTG ATAAATGGGG TAATAGATAT TACCCCACCA   
  
  
+ GCCTGAATGT GGTTGAAACC AGCCGGCTGA TCAATCAGAG CCAGTTGGAC CCTTCTCAAA TGTTTGATGC   
  
  
+ TGCTGCTCAC CCCTTGGAGT CTAATTGTCA TTCTTTTGTC TCTAGTAATA ACTTTGATCA TTCTATGGAT   
  
  
+ GGGGTTCCAG ATTCTCCTAT CAGTACACTT TCTGTGAATG AAACAAGAAA GGAAGAATCA GTTAGTAGAT   
  
  
+ CAAGGAGCAG GAAGAATCAT CAAAGAGATG GTGCATACCT GGAAGAAAGG AGTACTAAGC AAATAGCATC   
  
  
+ CAGCAATGAG GAATATGGAG AGATGGAGAG GTTTGATGAT GTACTGATCT GCAGGGAAGG AAATGATGAT   
  
  
+ ATTTCACCTT GTACCCGCAA AACCCCAGTT CATGAAGCAG ATGATAACTC GGAACCAAAG GGGAAATCAA   
  
  
+ GAGGATCTAA AAGCAAGAGT AAATCGCGTG TCAAGAAGAA AAGTAACGAA GGACAAGCGG TAGATCTTAG   
  
  
+ GACTCAACTT ATTCAGTGTG CAGAAGCAGT GGCAAGCTTT GAGCTTAAAA GTGCAAATGA GCTACTCAGG   
  
  
+ CGAATTAGGC AGCATGCTTC GCCATTTGGG GATAGTGTTC AAAGGCTAGC ACATTACTTT GCGAATGGGC   
  
  
+ TCGAGGCACG CTTAGCTGGT ACAGGATCAG AATCATACAA AGACTTTGCT GGGAAAAAAT TTTCATCCTA   
  
  
+ TGACATCTTG AGAGGCTACA AGTCATATGT TCAAGCCGTC CCCTTCCAGA GAACAACCAT GTTCTTGACA   
  
  
+ AATCAAACAA TAGTAAAGCT GGCTGAGAAA GCAACAAGGG TTCATATCAT TGATTTTGGC ATCTTTTTTG   
  
  
+ GTTTCCAGTG GCCCTGCCTT GTTCAGAATT TGTCAAGAAG ACCCCAGGGT TCTCCCATAC TCAAAATCAC   
  
  
+ GGGAGTCGAT CATCCCCAGA GTGGGTTCAG ACCAGCCCAG AAAGTTGAAG AGACAGGTCG TCGATTAGCA   
  
  
+ GGCTATTGTG AGAGGTTTGG GGTGCCCTTT GAGTTTCATC CCATTGCTCA AAGGTGGCAT ACCCTAAAGC   
  
  
+ CAGAGCATTT CAAAATTGAA AGCAATGAGC TTGTTGTGGT CACTTGTTTC TACCAATCAG GGAAGCTCCC   
  
  
+ TGATGAGACT GTTGATGTCA ACAGCCCAAG AGACCATGTC TTGAGGCTGA TCAGGGGCTT GAACCCTGAT   
  
  
+ CTATTCATCC ATGGGGTTAT CAATGGCACA TTCAATGCAC CCTTCTTCGT AACTCGGTTT AAAGAGGCAC   
  
  
+ TTTACCACTA CTCTTCTTTG TTCGATGTCT TTGATGCCAC CATAGATCGT GAAAGTCATG AAAGACTGCT   
  
  
+ GACTGAGAAC TATTTGTATG GGAAAGATGC ACTTAACATA GTAGCATGTG AGGGGAACGA GAGGATCGAG   
  
  
+ AGACCCGAGA CTTACAAGCA GTGGCAAGTC CGGAAGCTTA GAGCTGGATT CAAGCAGGTC CCCCTAGACC   
  
  
+ AAGACATTGT AAATGGAGCA AGAGCTATGG TTAAGGCAAA TTACAATAAG GATTTTATGG TAGAACAAGA   
  
  
+ TAGGAATTGG ATGGTCCAGG GCTGGAAAGG AAGGATTTTG AGTGCCATAT CCTGTTGGAA ACCTGCTTA  

- +Up\_Stream \_Len000ACTTAA CCTTCAACTA AGATGGAAAC TAACTAAGCT AATTAAGATC CAAGAACCAA   
  
  
- GATCAAGAGA GAGGAGAACA AGAACTAATA AATCCAAAAC CTACACTACA AATAACTTAA CTTACAACGT   
  
  
- TAACAACAAT ATTTCTACTA CAATCCGATT TATAGGAAGT TGAATCCAAC CCTAACTGCA GCTTATACAC   
  
  
- ATCAACTTAG AACATGACCG GATTTAGGGA CGAGGGGGAG ACAAAACGCT AATACTTAAA GTAATACGTG   
  
  
- TGGTGTACGA GCTACTTTAC GAGCTTTCTT GTAAGTAGGA GAAACAAACC CTAAATCCCC TTGCAACTTT   
  
  
- CTGCTTCAGA AATGGATCAC GCAGAATCAT CTGTTAATCC TAAAATTGTA CTTTTATCAA TTTTAAGATT   
  
  
- AACCCTAAAC TCTAACACTT AGAACATTAG GAAGCTGAAG ATCAAGATAC TTTTATCCGC TAATATCAAG   
  
  
- ATCCTAATCA GAAGCTGGGA ACTCTCTCCC TCCTCTACCC AAATCAACTA TTAGATAAGT AAGTATTGCG   
  
  
- TACGGACCCT CTACAAACCA TCCCGATCAT TAACTAAGCG ATTGTATAAA CACGACAATC AAGGTTGGAC   
  
  
- TCACAAATGA GAAGACGACA AATAAGGCAG AAAATCAGCA TTAACAGAGG AAATCAAATT AAAGTTTTGG   
  
  
- GGTTAACACT AGCGGATCTA ACTCGTTAGA TTAATCAAGA TTATTAACTT TAGTTCAAGG GACACCTTAT   
  
  
- GCTGGGCATG AACGGCACAC GATCGATGAC AATGTGGAAC GTAAACGCCA TATAAATATT GAGTTGTATA   
  
  
- CAGACGAGTG ATTTTCATGT ATAATCGAGT GGTTTTGACA CAGAGTTGCA TGATTTTATA AAATCACTTG   
  
  
- ATATGTAATA AAAATCATTC GCTTTATTCT ATGTGTTGTC GTGTAAAATT ACTCAATGTC CCGTAAAAAT   
  
  
- CACTCGGCTT TAGACTACCA AAAGAAACAT CTAGGCCAAT ATATTAACAT AAAATATGTT GGCTTACCTG   
  
  
- CTATATTGTT GACTCAATTT ACTCCTCAGA CTTTAATGCT TGTATTTTAG GTTTTCTTCT ACGGCTACGG   
  
  
- TAACCTTTCT TTCGACTTCC ACTAGCGAAA GATCATCATA ATCCGTCATC ATTAATTTTT GATTTTAGGT   
  
  
- CTTAACTGTG GCTTAACCCC AACACCGTGT ACTAGCTTTA GTCGTAGCTT AGATGTGCTA CTAGACCTAA   
  
  
- GAACCTGTGA GACCGAAATC CGGCAGTTAA AGCTCCTAAC GGGTATTGAA ATTAGGACAA GTTTAGGTAA   
  
  
- GTTAAATTAT AATGTGTTTA GGAAACTAAT TTAGTGGCAT TAACACCAAA CTAATCCCTT CAAAGAACCC   
  
  
- CCTTTCCAAG TTCGAAGACA GAAAGAATTT CGGACCTAAA AACCAGGGCG TACAAGTGAA GTGTAAGGGA   
  
  
- AAAAGAGAGG GTAGCAATGG ACAGACTTAG ACATACATTA TTTTCAGTAT AAAACGCTCC AGAAGAAAAC   
  
  
- TGACAAGAAC ATCAGTGAGA ACTATAGACG TGGTAAAACA AAAAGAGCCG TAAAAGAGGT TGAACGTAAA   
  
  
- AGATTACATT ACACTAGAAA AGACCTGTAA GTAAAACAAT TAGATGAGTG AACCCAGTTC CAAACGACTA   
  
  
- AAACCGACCA CAATTTAGGC AAGGAGAAAC CGTAAACAAG TACAAGGTGT TGACAAACTA TAGACCATAG   
  
  
- AGTCTTTGGG GCGGTAAGAA ACCATAAAGG ACTGAAAGAA CAAGTTCCCA ATAAGGATAA AGGGACTTAA   
  
  
- AAGAAACTAA GAAATCCATA AAGTTCGAAC CCCCCAAAAC AAGAAGTGAC TAAAACAGGA GAAGAAGGGA   
  
  
- ATAACCCACC AAACTAGAGG ATTTAAAGAC ACATAGACTT AGTCAGCCCC ATCTGAAACA CAAAAACCAA   
  
  
- CTAAAAAATA GTTACATAAA AGAAGGATTA CTTCCCGGAG GGCTTGGTTA TGTGTACGAA TTGAAGTTGG   
  
  
- GACAAGGAGA AGGTTCGAAA AACCTCGGAG TTTTCAGAAG CTTACGACTA TCCGTGTATC CCAGGAAGGT   
  
  
- TACGATACCA GAACTACTAC TGGGGCGTGG GAACTTACTG GGTTTAATGT AACATACAGT TAGAAGTATA   
  
  
- GGACTAGCTC TATAATGTGT CTAGGGACTG AAAGGACTAC TAACAGAGTT CAAGTAGTTA CTGTAAGAAT   
  
  
- ACCTTCTCCT AAACCTATTA GGCGGATATA GGAACGTCCT GATATTACGC GAGGTTCGGT GACTCTTCAG   
  
  
- TAACATACTA CGGGAACCTC TCCGAATAGG TCGAAGTAGA CTAGGTAACG TTAGTAGTCA ACCAGTCTCG   
  
  
- TGACTCTCGA GACTACTAGG ACTGTCTTCG TCGCCGTAAC TATTTACCCC ATTATCTATA ATGGGGTGGT   
  
  
- CGGACTTACA CCAACTTTGG TCGGCCGACT AGTTAGTCTC GGTCAACCTG GGAAGAGTTT ACAAACTACG   
  
  
- ACGACGAGTG GGGAACCTCA GATTAACAGT AAGAAAACAG AGATCATTAT TGAAACTAGT AAGATACCTA   
  
  
- CCCCAAGGTC TAAGAGGATA GTCATGTGAA AGACACTTAC TTTGTTCTTT CCTTCTTAGT CAATCATCTA   
  
  
- GTTCCTCGTC CTTCTTAGTA GTTTCTCTAC CACGTATGGA CCTTCTTTCC TCATGATTCG TTTATCGTAG   
  
  
- GTCGTTACTC CTTATACCTC TCTACCTCTC CAAACTACTA CATGACTAGA CGTCCCTTCC TTTACTACTA   
  
  
- TAAAGTGGAA CATGGGCGTT TTGGGGTCAA GTACTTCGTC TACTATTGAG CCTTGGTTTC CCCTTTAGTT   
  
  
- CTCCTAGATT TTCGTTCTCA TTTAGCGCAC AGTTCTTCTT TTCATTGCTT CCTGTTCGCC ATCTAGAATC   
  
  
- CTGAGTTGAA TAAGTCACAC GTCTTCGTCA CCGTTCGAAA CTCGAATTTT CACGTTTACT CGATGAGTCC   
  
  
- GCTTAATCCG TCGTACGAAG CGGTAAACCC CTATCACAAG TTTCCGATCG TGTAATGAAA CGCTTACCCG   
  
  
- AGCTCCGTGC GAATCGACCA TGTCCTAGTC TTAGTATGTT TCTGAAACGA CCCTTTTTTA AAAGTAGGAT   
  
  
- ACTGTAGAAC TCTCCGATGT TCAGTATACA AGTTCGGCAG GGGAAGGTCT CTTGTTGGTA CAAGAACTGT   
  
  
- TTAGTTTGTT ATCATTTCGA CCGACTCTTT CGTTGTTCCC AAGTATAGTA ACTAAAACCG TAGAAAAAAC   
  
  
- CAAAGGTCAC CGGGACGGAA CAAGTCTTAA ACAGTTCTTC TGGGGTCCCA AGAGGGTATG AGTTTTAGTG   
  
  
- CCCTCAGCTA GTAGGGGTCT CACCCAAGTC TGGTCGGGTC TTTCAACTTC TCTGTCCAGC AGCTAATCGT   
  
  
- CCGATAACAC TCTCCAAACC CCACGGGAAA CTCAAAGTAG GGTAACGAGT TTCCACCGTA TGGGATTTCG   
  
  
- GTCTCGTAAA GTTTTAACTT TCGTTACTCG AACAACACCA GTGAACAAAG ATGGTTAGTC CCTTCGAGGG   
  
  
- ACTACTCTGA CAACTACAGT TGTCGGGTTC TCTGGTACAG AACTCCGACT AGTCCCCGAA CTTGGGACTA   
  
  
- GATAAGTAGG TACCCCAATA GTTACCGTGT AAGTTACGTG GGAAGAAGCA TTGAGCCAAA TTTCTCCGTG   
  
  
- AAATGGTGAT GAGAAGAAAC AAGCTACAGA AACTACGGTG GTATCTAGCA CTTTCAGTAC TTTCTGACGA   
  
  
- CTGACTCTTG ATAAACATAC CCTTTCTACG TGAATTGTAT CATCGTACAC TCCCCTTGCT CTCCTAGCTC   
  
  
- TCTGGGCTCT GAATGTTCGT CACCGTTCAG GCCTTCGAAT CTCGACCTAA GTTCGTCCAG GGGGATCTGG   
  
  
- TTCTGTAACA TTTACCTCGT TCTCGATACC AATTCCGTTT AATGTTATTC CTAAAATACC ATCTTGTTCT   
  
  
- ATCCTTAACC TACCAGGTCC CGACCTTTCC TTCCTAAAAC TCACGGTATA GGACAACCTT TGGACGAAT

+     Box 4

| Site Name | Organism | Position | Strand | Matrix score. | sequence | function |
| --- | --- | --- | --- | --- | --- | --- |
| Box 4 | Petroselinum crispum | 54 | + | 6 | ATTAAT | part of a conserved DNA module involved in light responsiveness |

>HU02G01573.1   
+ +Up\_Stream \_Len000TGAATT GGAAGTTGAT TCTACCTTTG ATTGATTCGA TTAATTCTAG GTTCTTGGTT   
  
  
+ CTAGTTCTCT CTCCTCTTGT TCTTGATTAT TTAGGTTTTG GATGTGATGT TTATTGAATT GAATGTTGCA   
  
  
+ ATTGTTGTTA TAAAGATGAT GTTAGGCTAA ATATCCTTCA ACTTAGGTTG GGATTGACGT CGAATATGTG   
  
  
+ TAGTTGAATC TTGTACTGGC CTAAATCCCT GCTCCCCCTC TGTTTTGCGA TTATGAATTT CATTATGCAC   
  
  
+ ACCACATGCT CGATGAAATG CTCGAAAGAA CATTCATCCT CTTTGTTTGG GATTTAGGGG AACGTTGAAA   
  
  
+ GACGAAGTCT TTACCTAGTG CGTCTTAGTA GACAATTAGG ATTTTAACAT GAAAATAGTT AAAATTCTAA   
  
  
+ TTGGGATTTG AGATTGTGAA TCTTGTAATC CTTCGACTTC TAGTTCTATG AAAATAGGCG ATTATAGTTC   
  
  
+ TAGGATTAGT CTTCGACCCT TGAGAGAGGG AGGAGATGGG TTTAGTTGAT AATCTATTCA TTCATAACGC   
  
  
+ ATGCCTGGGA GATGTTTGGT AGGGCTAGTA ATTGATTCGC TAACATATTT GTGCTGTTAG TTCCAACCTG   
  
  
+ AGTGTTTACT CTTCTGCTGT TTATTCCGTC TTTTAGTCGT AATTGTCTCC TTTAGTTTAA TTTCAAAACC   
  
  
+ CCAATTGTGA TCGCCTAGAT TGAGCAATCT AATTAGTTCT AATAATTGAA ATCAAGTTCC CTGTGGAATA   
  
  
+ CGACCCGTAC TTGCCGTGTG CTAGCTACTG TTACACCTTG CATTTGCGGT ATATTTATAA CTCAACATAT   
  
  
+ GTCTGCTCAC TAAAAGTACA TATTAGCTCA CCAAAACTGT GTCTCAACGT ACTAAAATAT TTTAGTGAAC   
  
  
+ TATACATTAT TTTTAGTAAG CGAAATAAGA TACACAACAG CACATTTTAA TGAGTTACAG GGCATTTTTA   
  
  
+ GTGAGCCGAA ATCTGATGGT TTTCTTTGTA GATCCGGTTA TATAATTGTA TTTTATACAA CCGAATGGAC   
  
  
+ GATATAACAA CTGAGTTAAA TGAGGAGTCT GAAATTACGA ACATAAAATC CAAAAGAAGA TGCCGATGCC   
  
  
+ ATTGGAAAGA AAGCTGAAGG TGATCGCTTT CTAGTAGTAT TAGGCAGTAG TAATTAAAAA CTAAAATCCA   
  
  
+ GAATTGACAC CGAATTGGGG TTGTGGCACA TGATCGAAAT CAGCATCGAA TCTACACGAT GATCTGGATT   
  
  
+ CTTGGACACT CTGGCTTTAG GCCGTCAATT TCGAGGATTG CCCATAACTT TAATCCTGTT CAAATCCATT   
  
  
+ CAATTTAATA TTACACAAAT CCTTTGATTA AATCACCGTA ATTGTGGTTT GATTAGGGAA GTTTCTTGGG   
  
  
+ GGAAAGGTTC AAGCTTCTGT CTTTCTTAAA GCCTGGATTT TTGGTCCCGC ATGTTCACTT CACATTCCCT   
  
  
+ TTTTCTCTCC CATCGTTACC TGTCTGAATC TGTATGTAAT AAAAGTCATA TTTTGCGAGG TCTTCTTTTG   
  
  
+ ACTGTTCTTG TAGTCACTCT TGATATCTGC ACCATTTTGT TTTTCTCGGC ATTTTCTCCA ACTTGCATTT   
  
  
+ TCTAATGTAA TGTGATCTTT TCTGGACATT CATTTTGTTA ATCTACTCAC TTGGGTCAAG GTTTGCTGAT   
  
  
+ TTTGGCTGGT GTTAAATCCG TTCCTCTTTG GCATTTGTTC ATGTTCCACA ACTGTTTGAT ATCTGGTATC   
  
  
+ TCAGAAACCC CGCCATTCTT TGGTATTTCC TGACTTTCTT GTTCAAGGGT TATTCCTATT TCCCTGAATT   
  
  
+ TTCTTTGATT CTTTAGGTAT TTCAAGCTTG GGGGGTTTTG TTCTTCACTG ATTTTGTCCT CTTCTTCCCT   
  
  
+ TATTGGGTGG TTTGATCTCC TAAATTTCTG TGTATCTGAA TCAGTCGGGG TAGACTTTGT GTTTTTGGTT   
  
  
+ GATTTTTTAT CAATGTATTT TCTTCCTAAT GAAGGGCCTC CCGAACCAAT ACACATGCTT AACTTCAACC   
  
  
+ CTGTTCCTCT TCCAAGCTTT TTGGAGCCTC AAAAGTCTTC GAATGCTGAT AGGCACATAG GGTCCTTCCA   
  
  
+ ATGCTATGGT CTTGATGATG ACCCCGCACC CTTGAATGAC CCAAATTACA TTGTATGTCA ATCTTCATAT   
  
  
+ CCTGATCGAG ATATTACACA GATCCCTGAC TTTCCTGATG ATTGTCTCAA GTTCATCAAT GACATTCTTA   
  
  
+ TGGAAGAGGA TTTGGATAAT CCGCCTATAT CCTTGCAGGA CTATAATGCG CTCCAAGCCA CTGAGAAGTC   
  
  
+ ATTGTATGAT GCCCTTGGAG AGGCTTATCC AGCTTCATCT GATCCATTGC AATCATCAGT TGGTCAGAGC   
  
  
+ ACTGAGAGCT CTGATGATCC TGACAGAAGC AGCGGCATTG ATAAATGGGG TAATAGATAT TACCCCACCA   
  
  
+ GCCTGAATGT GGTTGAAACC AGCCGGCTGA TCAATCAGAG CCAGTTGGAC CCTTCTCAAA TGTTTGATGC   
  
  
+ TGCTGCTCAC CCCTTGGAGT CTAATTGTCA TTCTTTTGTC TCTAGTAATA ACTTTGATCA TTCTATGGAT   
  
  
+ GGGGTTCCAG ATTCTCCTAT CAGTACACTT TCTGTGAATG AAACAAGAAA GGAAGAATCA GTTAGTAGAT   
  
  
+ CAAGGAGCAG GAAGAATCAT CAAAGAGATG GTGCATACCT GGAAGAAAGG AGTACTAAGC AAATAGCATC   
  
  
+ CAGCAATGAG GAATATGGAG AGATGGAGAG GTTTGATGAT GTACTGATCT GCAGGGAAGG AAATGATGAT   
  
  
+ ATTTCACCTT GTACCCGCAA AACCCCAGTT CATGAAGCAG ATGATAACTC GGAACCAAAG GGGAAATCAA   
  
  
+ GAGGATCTAA AAGCAAGAGT AAATCGCGTG TCAAGAAGAA AAGTAACGAA GGACAAGCGG TAGATCTTAG   
  
  
+ GACTCAACTT ATTCAGTGTG CAGAAGCAGT GGCAAGCTTT GAGCTTAAAA GTGCAAATGA GCTACTCAGG   
  
  
+ CGAATTAGGC AGCATGCTTC GCCATTTGGG GATAGTGTTC AAAGGCTAGC ACATTACTTT GCGAATGGGC   
  
  
+ TCGAGGCACG CTTAGCTGGT ACAGGATCAG AATCATACAA AGACTTTGCT GGGAAAAAAT TTTCATCCTA   
  
  
+ TGACATCTTG AGAGGCTACA AGTCATATGT TCAAGCCGTC CCCTTCCAGA GAACAACCAT GTTCTTGACA   
  
  
+ AATCAAACAA TAGTAAAGCT GGCTGAGAAA GCAACAAGGG TTCATATCAT TGATTTTGGC ATCTTTTTTG   
  
  
+ GTTTCCAGTG GCCCTGCCTT GTTCAGAATT TGTCAAGAAG ACCCCAGGGT TCTCCCATAC TCAAAATCAC   
  
  
+ GGGAGTCGAT CATCCCCAGA GTGGGTTCAG ACCAGCCCAG AAAGTTGAAG AGACAGGTCG TCGATTAGCA   
  
  
+ GGCTATTGTG AGAGGTTTGG GGTGCCCTTT GAGTTTCATC CCATTGCTCA AAGGTGGCAT ACCCTAAAGC   
  
  
+ CAGAGCATTT CAAAATTGAA AGCAATGAGC TTGTTGTGGT CACTTGTTTC TACCAATCAG GGAAGCTCCC   
  
  
+ TGATGAGACT GTTGATGTCA ACAGCCCAAG AGACCATGTC TTGAGGCTGA TCAGGGGCTT GAACCCTGAT   
  
  
+ CTATTCATCC ATGGGGTTAT CAATGGCACA TTCAATGCAC CCTTCTTCGT AACTCGGTTT AAAGAGGCAC   
  
  
+ TTTACCACTA CTCTTCTTTG TTCGATGTCT TTGATGCCAC CATAGATCGT GAAAGTCATG AAAGACTGCT   
  
  
+ GACTGAGAAC TATTTGTATG GGAAAGATGC ACTTAACATA GTAGCATGTG AGGGGAACGA GAGGATCGAG   
  
  
+ AGACCCGAGA CTTACAAGCA GTGGCAAGTC CGGAAGCTTA GAGCTGGATT CAAGCAGGTC CCCCTAGACC   
  
  
+ AAGACATTGT AAATGGAGCA AGAGCTATGG TTAAGGCAAA TTACAATAAG GATTTTATGG TAGAACAAGA   
  
  
+ TAGGAATTGG ATGGTCCAGG GCTGGAAAGG AAGGATTTTG AGTGCCATAT CCTGTTGGAA ACCTGCTTA  

- +Up\_Stream \_Len000ACTTAA CCTTCAACTA AGATGGAAAC TAACTAAGCT AATTAAGATC CAAGAACCAA   
  
  
- GATCAAGAGA GAGGAGAACA AGAACTAATA AATCCAAAAC CTACACTACA AATAACTTAA CTTACAACGT   
  
  
- TAACAACAAT ATTTCTACTA CAATCCGATT TATAGGAAGT TGAATCCAAC CCTAACTGCA GCTTATACAC   
  
  
- ATCAACTTAG AACATGACCG GATTTAGGGA CGAGGGGGAG ACAAAACGCT AATACTTAAA GTAATACGTG   
  
  
- TGGTGTACGA GCTACTTTAC GAGCTTTCTT GTAAGTAGGA GAAACAAACC CTAAATCCCC TTGCAACTTT   
  
  
- CTGCTTCAGA AATGGATCAC GCAGAATCAT CTGTTAATCC TAAAATTGTA CTTTTATCAA TTTTAAGATT   
  
  
- AACCCTAAAC TCTAACACTT AGAACATTAG GAAGCTGAAG ATCAAGATAC TTTTATCCGC TAATATCAAG   
  
  
- ATCCTAATCA GAAGCTGGGA ACTCTCTCCC TCCTCTACCC AAATCAACTA TTAGATAAGT AAGTATTGCG   
  
  
- TACGGACCCT CTACAAACCA TCCCGATCAT TAACTAAGCG ATTGTATAAA CACGACAATC AAGGTTGGAC   
  
  
- TCACAAATGA GAAGACGACA AATAAGGCAG AAAATCAGCA TTAACAGAGG AAATCAAATT AAAGTTTTGG   
  
  
- GGTTAACACT AGCGGATCTA ACTCGTTAGA TTAATCAAGA TTATTAACTT TAGTTCAAGG GACACCTTAT   
  
  
- GCTGGGCATG AACGGCACAC GATCGATGAC AATGTGGAAC GTAAACGCCA TATAAATATT GAGTTGTATA   
  
  
- CAGACGAGTG ATTTTCATGT ATAATCGAGT GGTTTTGACA CAGAGTTGCA TGATTTTATA AAATCACTTG   
  
  
- ATATGTAATA AAAATCATTC GCTTTATTCT ATGTGTTGTC GTGTAAAATT ACTCAATGTC CCGTAAAAAT   
  
  
- CACTCGGCTT TAGACTACCA AAAGAAACAT CTAGGCCAAT ATATTAACAT AAAATATGTT GGCTTACCTG   
  
  
- CTATATTGTT GACTCAATTT ACTCCTCAGA CTTTAATGCT TGTATTTTAG GTTTTCTTCT ACGGCTACGG   
  
  
- TAACCTTTCT TTCGACTTCC ACTAGCGAAA GATCATCATA ATCCGTCATC ATTAATTTTT GATTTTAGGT   
  
  
- CTTAACTGTG GCTTAACCCC AACACCGTGT ACTAGCTTTA GTCGTAGCTT AGATGTGCTA CTAGACCTAA   
  
  
- GAACCTGTGA GACCGAAATC CGGCAGTTAA AGCTCCTAAC GGGTATTGAA ATTAGGACAA GTTTAGGTAA   
  
  
- GTTAAATTAT AATGTGTTTA GGAAACTAAT TTAGTGGCAT TAACACCAAA CTAATCCCTT CAAAGAACCC   
  
  
- CCTTTCCAAG TTCGAAGACA GAAAGAATTT CGGACCTAAA AACCAGGGCG TACAAGTGAA GTGTAAGGGA   
  
  
- AAAAGAGAGG GTAGCAATGG ACAGACTTAG ACATACATTA TTTTCAGTAT AAAACGCTCC AGAAGAAAAC   
  
  
- TGACAAGAAC ATCAGTGAGA ACTATAGACG TGGTAAAACA AAAAGAGCCG TAAAAGAGGT TGAACGTAAA   
  
  
- AGATTACATT ACACTAGAAA AGACCTGTAA GTAAAACAAT TAGATGAGTG AACCCAGTTC CAAACGACTA   
  
  
- AAACCGACCA CAATTTAGGC AAGGAGAAAC CGTAAACAAG TACAAGGTGT TGACAAACTA TAGACCATAG   
  
  
- AGTCTTTGGG GCGGTAAGAA ACCATAAAGG ACTGAAAGAA CAAGTTCCCA ATAAGGATAA AGGGACTTAA   
  
  
- AAGAAACTAA GAAATCCATA AAGTTCGAAC CCCCCAAAAC AAGAAGTGAC TAAAACAGGA GAAGAAGGGA   
  
  
- ATAACCCACC AAACTAGAGG ATTTAAAGAC ACATAGACTT AGTCAGCCCC ATCTGAAACA CAAAAACCAA   
  
  
- CTAAAAAATA GTTACATAAA AGAAGGATTA CTTCCCGGAG GGCTTGGTTA TGTGTACGAA TTGAAGTTGG   
  
  
- GACAAGGAGA AGGTTCGAAA AACCTCGGAG TTTTCAGAAG CTTACGACTA TCCGTGTATC CCAGGAAGGT   
  
  
- TACGATACCA GAACTACTAC TGGGGCGTGG GAACTTACTG GGTTTAATGT AACATACAGT TAGAAGTATA   
  
  
- GGACTAGCTC TATAATGTGT CTAGGGACTG AAAGGACTAC TAACAGAGTT CAAGTAGTTA CTGTAAGAAT   
  
  
- ACCTTCTCCT AAACCTATTA GGCGGATATA GGAACGTCCT GATATTACGC GAGGTTCGGT GACTCTTCAG   
  
  
- TAACATACTA CGGGAACCTC TCCGAATAGG TCGAAGTAGA CTAGGTAACG TTAGTAGTCA ACCAGTCTCG   
  
  
- TGACTCTCGA GACTACTAGG ACTGTCTTCG TCGCCGTAAC TATTTACCCC ATTATCTATA ATGGGGTGGT   
  
  
- CGGACTTACA CCAACTTTGG TCGGCCGACT AGTTAGTCTC GGTCAACCTG GGAAGAGTTT ACAAACTACG   
  
  
- ACGACGAGTG GGGAACCTCA GATTAACAGT AAGAAAACAG AGATCATTAT TGAAACTAGT AAGATACCTA   
  
  
- CCCCAAGGTC TAAGAGGATA GTCATGTGAA AGACACTTAC TTTGTTCTTT CCTTCTTAGT CAATCATCTA   
  
  
- GTTCCTCGTC CTTCTTAGTA GTTTCTCTAC CACGTATGGA CCTTCTTTCC TCATGATTCG TTTATCGTAG   
  
  
- GTCGTTACTC CTTATACCTC TCTACCTCTC CAAACTACTA CATGACTAGA CGTCCCTTCC TTTACTACTA   
  
  
- TAAAGTGGAA CATGGGCGTT TTGGGGTCAA GTACTTCGTC TACTATTGAG CCTTGGTTTC CCCTTTAGTT   
  
  
- CTCCTAGATT TTCGTTCTCA TTTAGCGCAC AGTTCTTCTT TTCATTGCTT CCTGTTCGCC ATCTAGAATC   
  
  
- CTGAGTTGAA TAAGTCACAC GTCTTCGTCA CCGTTCGAAA CTCGAATTTT CACGTTTACT CGATGAGTCC   
  
  
- GCTTAATCCG TCGTACGAAG CGGTAAACCC CTATCACAAG TTTCCGATCG TGTAATGAAA CGCTTACCCG   
  
  
- AGCTCCGTGC GAATCGACCA TGTCCTAGTC TTAGTATGTT TCTGAAACGA CCCTTTTTTA AAAGTAGGAT   
  
  
- ACTGTAGAAC TCTCCGATGT TCAGTATACA AGTTCGGCAG GGGAAGGTCT CTTGTTGGTA CAAGAACTGT   
  
  
- TTAGTTTGTT ATCATTTCGA CCGACTCTTT CGTTGTTCCC AAGTATAGTA ACTAAAACCG TAGAAAAAAC   
  
  
- CAAAGGTCAC CGGGACGGAA CAAGTCTTAA ACAGTTCTTC TGGGGTCCCA AGAGGGTATG AGTTTTAGTG   
  
  
- CCCTCAGCTA GTAGGGGTCT CACCCAAGTC TGGTCGGGTC TTTCAACTTC TCTGTCCAGC AGCTAATCGT   
  
  
- CCGATAACAC TCTCCAAACC CCACGGGAAA CTCAAAGTAG GGTAACGAGT TTCCACCGTA TGGGATTTCG   
  
  
- GTCTCGTAAA GTTTTAACTT TCGTTACTCG AACAACACCA GTGAACAAAG ATGGTTAGTC CCTTCGAGGG   
  
  
- ACTACTCTGA CAACTACAGT TGTCGGGTTC TCTGGTACAG AACTCCGACT AGTCCCCGAA CTTGGGACTA   
  
  
- GATAAGTAGG TACCCCAATA GTTACCGTGT AAGTTACGTG GGAAGAAGCA TTGAGCCAAA TTTCTCCGTG   
  
  
- AAATGGTGAT GAGAAGAAAC AAGCTACAGA AACTACGGTG GTATCTAGCA CTTTCAGTAC TTTCTGACGA   
  
  
- CTGACTCTTG ATAAACATAC CCTTTCTACG TGAATTGTAT CATCGTACAC TCCCCTTGCT CTCCTAGCTC   
  
  
- TCTGGGCTCT GAATGTTCGT CACCGTTCAG GCCTTCGAAT CTCGACCTAA GTTCGTCCAG GGGGATCTGG   
  
  
- TTCTGTAACA TTTACCTCGT TCTCGATACC AATTCCGTTT AATGTTATTC CTAAAATACC ATCTTGTTCT   
  
  
- ATCCTTAACC TACCAGGTCC CGACCTTTCC TTCCTAAAAC TCACGGTATA GGACAACCTT TGGACGAAT

+     CAAT-box

| Site Name | Organism | Position | Strand | Matrix score. | sequence | function |
| --- | --- | --- | --- | --- | --- | --- |
| CAAT-box | Arabidopsis thaliana | 2010 | + | 5 | CCAAT | common cis-acting element in promoter and enhancer regions |
| CAAT-box | Arabidopsis thaliana | 1896 | - | 5 | CCAAT | common cis-acting element in promoter and enhancer regions |
| CAAT-box | Nicotiana glutinosa | 2011 | + | 4 | CAAT |  |
| CAAT-box | Pisum sativum | 1717 | - | 5 | CAAAT | common cis-acting element in promoter and enhancer regions |
| CAAT-box | Pisum sativum | 1325 | + | 5 | CAAAT | common cis-acting element in promoter and enhancer regions |
| CAAT-box | Pisum sativum | 816 | - | 5 | CAAAT | common cis-acting element in promoter and enhancer regions |
| CAAT-box | Pisum sativum | 1350 | + | 5 | CAAAT | common cis-acting element in promoter and enhancer regions |
| CAAT-box | Nicotiana glutinosa | 708 | - | 4 | CAAT |  |
| CAAT-box | Nicotiana glutinosa | 1290 | + | 4 | CAAT |  |
| CAAT-box | Nicotiana glutinosa | 729 | + | 4 | CAAT |  |
| CAAT-box | Nicotiana glutinosa | 706 | + | 4 | CAAT |  |
| CAAT-box | Nicotiana glutinosa | 3527 | + | 4 | CAAT |  |
| CAAT-box | Arabidopsis thaliana | 424 | - | 5 | CCAAT | common cis-acting element in promoter and enhancer regions |
| CAAT-box | Pisum sativum | 3961 | + | 5 | CAAAT | common cis-acting element in promoter and enhancer regions |
| CAAT-box | Pisum sativum | 3796 | - | 5 | CAAAT | common cis-acting element in promoter and enhancer regions |
| CAAT-box | Nicotiana glutinosa | 3930 | - | 4 | CAAT |  |
| CAAT-box | Nicotiana glutinosa | 3677 | + | 4 | CAAT |  |
| CAAT-box | Nicotiana glutinosa | 1975 | + | 4 | CAAT |  |
| CAAT-box | Nicotiana glutinosa | 1301 | - | 4 | CAAT |  |
| CAAT-box | Arabidopsis thaliana | 1208 | - | 5 | CCAAT | common cis-acting element in promoter and enhancer regions |
| CAAT-box | Arabidopsis thaliana | 3557 | + | 5 | CCAAT | common cis-acting element in promoter and enhancer regions |
| CAAT-box | Arabidopsis thaliana | 1125 | - | 5 | CCAAT | common cis-acting element in promoter and enhancer regions |
| CAAT-box | Pisum sativum | 430 | - | 5 | CAAAT | common cis-acting element in promoter and enhancer regions |
| CAAT-box | Nicotiana glutinosa | 3477 | - | 4 | CAAT |  |
| CAAT-box | Nicotiana glutinosa | 2215 | - | 4 | CAAT |  |
| CAAT-box | Pisum sativum | 3038 | - | 5 | CAAAT | common cis-acting element in promoter and enhancer regions |
| CAAT-box | Nicotiana glutinosa | 1375 | - | 4 | CAAT |  |
| CAAT-box | Nicotiana glutinosa | 437 | - | 4 | CAAT |  |
| CAAT-box | Nicotiana glutinosa | 749 | - | 4 | CAAT |  |
| CAAT-box | Pisum sativum | 611 | - | 5 | CAAAT | common cis-acting element in promoter and enhancer regions |
| CAAT-box | Nicotiana glutinosa | 3968 | + | 4 | CAAT |  |
| CAAT-box | Nicotiana glutinosa | 145 | - | 4 | CAAT |  |
| CAAT-box | Nicotiana glutinosa | 3232 | + | 4 | CAAT |  |
| CAAT-box | Nicotiana glutinosa | 1335 | + | 4 | CAAT |  |
| CAAT-box | Nicotiana glutinosa | 2360 | - | 4 | CAAT |  |
| CAAT-box | Nicotiana glutinosa | 197 | - | 4 | CAAT |  |
| CAAT-box | Arabidopsis thaliana | 705 | + | 5 | CCAAT | common cis-acting element in promoter and enhancer regions |
| CAAT-box | Nicotiana glutinosa | 2738 | + | 4 | CAAT |  |
| CAAT-box | Nicotiana glutinosa | 1197 | - | 4 | CAAT |  |
| CAAT-box | Nicotiana glutinosa | 143 | + | 4 | CAAT |  |
| CAAT-box | Nicotiana glutinosa | 3665 | + | 4 | CAAT |  |
| CAAT-box | Nicotiana glutinosa | 2103 | + | 4 | CAAT |  |
| CAAT-box | Nicotiana glutinosa | 1029 | - | 4 | CAAT |  |
| CAAT-box | Nicotiana glutinosa | 595 | - | 4 | CAAT |  |
| CAAT-box | Nicotiana glutinosa | 3519 | - | 4 | CAAT |  |
| CAAT-box | Nicotiana glutinosa | 127 | - | 4 | CAAT |  |
| CAAT-box | Pisum sativum | 2998 | + | 5 | CAAAT | common cis-acting element in promoter and enhancer regions |
| CAAT-box | Pisum sativum | 2724 | + | 5 | CAAAT | common cis-acting element in promoter and enhancer regions |
| CAAT-box | Nicotiana glutinosa | 2548 | - | 4 | CAAT |  |
| CAAT-box | Nicotiana glutinosa | 132 | - | 4 | CAAT |  |
| CAAT-box | Nicotiana glutinosa | 723 | - | 4 | CAAT |  |
| CAAT-box | Nicotiana glutinosa | 45 | - | 4 | CAAT |  |
| CAAT-box | Pisum sativum | 3322 | - | 5 | CAAAT | common cis-acting element in promoter and enhancer regions |
| CAAT-box | Nicotiana glutinosa | 2154 | - | 4 | CAAT |  |
| CAAT-box | Pisum sativum | 2511 | + | 5 | CAAAT | common cis-acting element in promoter and enhancer regions |
| CAAT-box | Pisum sativum | 2146 | + | 5 | CAAAT | common cis-acting element in promoter and enhancer regions |
| CAAT-box | Nicotiana glutinosa | 2315 | - | 4 | CAAT |  |
| CAAT-box | Nicotiana glutinosa | 676 | - | 4 | CAAT |  |
| CAAT-box | Nicotiana glutinosa | 387 | + | 4 | CAAT |  |
| CAAT-box | Arabidopsis thaliana | 22 | - | 5 | CCAAT | common cis-acting element in promoter and enhancer regions |
| CAAT-box | Pisum sativum | 3223 | + | 5 | CAAAT | common cis-acting element in promoter and enhancer regions |
| CAAT-box | Nicotiana glutinosa | 2364 | + | 4 | CAAT |  |
| CAAT-box | Nicotiana glutinosa | 3558 | + | 4 | CAAT |  |
| CAAT-box | Nicotiana glutinosa | 2163 | + | 4 | CAAT |  |
| CAAT-box | Nicotiana glutinosa | 3273 | - | 4 | CAAT |  |
| CAAT-box | Nicotiana glutinosa | 2421 | - | 4 | CAAT |  |
| CAAT-box | Arabidopsis thaliana | 4000 | - | 5 | CCAAT | common cis-acting element in promoter and enhancer regions |
| CAAT-box | Pisum sativum | 2254 | - | 5 | CAAAT | common cis-acting element in promoter and enhancer regions |
| CAAT-box | Nicotiana glutinosa | 3439 | - | 4 | CAAT |  |
| CAAT-box | Nicotiana glutinosa | 2486 | + | 4 | CAAT |  |
| CAAT-box | Arabidopsis thaliana | 2102 | + | 5 | CCAAT | common cis-acting element in promoter and enhancer regions |
| CAAT-box | Nicotiana glutinosa | 2231 | + | 4 | CAAT |  |

>HU02G01573.1   
+ +Up\_Stream \_Len000TGAATT GGAAGTTGAT TCTACCTTTG ATTGATTCGA TTAATTCTAG GTTCTTGGTT   
  
  
+ CTAGTTCTCT CTCCTCTTGT TCTTGATTAT TTAGGTTTTG GATGTGATGT TTATTGAATT GAATGTTGCA   
  
  
+ ATTGTTGTTA TAAAGATGAT GTTAGGCTAA ATATCCTTCA ACTTAGGTTG GGATTGACGT CGAATATGTG   
  
  
+ TAGTTGAATC TTGTACTGGC CTAAATCCCT GCTCCCCCTC TGTTTTGCGA TTATGAATTT CATTATGCAC   
  
  
+ ACCACATGCT CGATGAAATG CTCGAAAGAA CATTCATCCT CTTTGTTTGG GATTTAGGGG AACGTTGAAA   
  
  
+ GACGAAGTCT TTACCTAGTG CGTCTTAGTA GACAATTAGG ATTTTAACAT GAAAATAGTT AAAATTCTAA   
  
  
+ TTGGGATTTG AGATTGTGAA TCTTGTAATC CTTCGACTTC TAGTTCTATG AAAATAGGCG ATTATAGTTC   
  
  
+ TAGGATTAGT CTTCGACCCT TGAGAGAGGG AGGAGATGGG TTTAGTTGAT AATCTATTCA TTCATAACGC   
  
  
+ ATGCCTGGGA GATGTTTGGT AGGGCTAGTA ATTGATTCGC TAACATATTT GTGCTGTTAG TTCCAACCTG   
  
  
+ AGTGTTTACT CTTCTGCTGT TTATTCCGTC TTTTAGTCGT AATTGTCTCC TTTAGTTTAA TTTCAAAACC   
  
  
+ CCAATTGTGA TCGCCTAGAT TGAGCAATCT AATTAGTTCT AATAATTGAA ATCAAGTTCC CTGTGGAATA   
  
  
+ CGACCCGTAC TTGCCGTGTG CTAGCTACTG TTACACCTTG CATTTGCGGT ATATTTATAA CTCAACATAT   
  
  
+ GTCTGCTCAC TAAAAGTACA TATTAGCTCA CCAAAACTGT GTCTCAACGT ACTAAAATAT TTTAGTGAAC   
  
  
+ TATACATTAT TTTTAGTAAG CGAAATAAGA TACACAACAG CACATTTTAA TGAGTTACAG GGCATTTTTA   
  
  
+ GTGAGCCGAA ATCTGATGGT TTTCTTTGTA GATCCGGTTA TATAATTGTA TTTTATACAA CCGAATGGAC   
  
  
+ GATATAACAA CTGAGTTAAA TGAGGAGTCT GAAATTACGA ACATAAAATC CAAAAGAAGA TGCCGATGCC   
  
  
+ ATTGGAAAGA AAGCTGAAGG TGATCGCTTT CTAGTAGTAT TAGGCAGTAG TAATTAAAAA CTAAAATCCA   
  
  
+ GAATTGACAC CGAATTGGGG TTGTGGCACA TGATCGAAAT CAGCATCGAA TCTACACGAT GATCTGGATT   
  
  
+ CTTGGACACT CTGGCTTTAG GCCGTCAATT TCGAGGATTG CCCATAACTT TAATCCTGTT CAAATCCATT   
  
  
+ CAATTTAATA TTACACAAAT CCTTTGATTA AATCACCGTA ATTGTGGTTT GATTAGGGAA GTTTCTTGGG   
  
  
+ GGAAAGGTTC AAGCTTCTGT CTTTCTTAAA GCCTGGATTT TTGGTCCCGC ATGTTCACTT CACATTCCCT   
  
  
+ TTTTCTCTCC CATCGTTACC TGTCTGAATC TGTATGTAAT AAAAGTCATA TTTTGCGAGG TCTTCTTTTG   
  
  
+ ACTGTTCTTG TAGTCACTCT TGATATCTGC ACCATTTTGT TTTTCTCGGC ATTTTCTCCA ACTTGCATTT   
  
  
+ TCTAATGTAA TGTGATCTTT TCTGGACATT CATTTTGTTA ATCTACTCAC TTGGGTCAAG GTTTGCTGAT   
  
  
+ TTTGGCTGGT GTTAAATCCG TTCCTCTTTG GCATTTGTTC ATGTTCCACA ACTGTTTGAT ATCTGGTATC   
  
  
+ TCAGAAACCC CGCCATTCTT TGGTATTTCC TGACTTTCTT GTTCAAGGGT TATTCCTATT TCCCTGAATT   
  
  
+ TTCTTTGATT CTTTAGGTAT TTCAAGCTTG GGGGGTTTTG TTCTTCACTG ATTTTGTCCT CTTCTTCCCT   
  
  
+ TATTGGGTGG TTTGATCTCC TAAATTTCTG TGTATCTGAA TCAGTCGGGG TAGACTTTGT GTTTTTGGTT   
  
  
+ GATTTTTTAT CAATGTATTT TCTTCCTAAT GAAGGGCCTC CCGAACCAAT ACACATGCTT AACTTCAACC   
  
  
+ CTGTTCCTCT TCCAAGCTTT TTGGAGCCTC AAAAGTCTTC GAATGCTGAT AGGCACATAG GGTCCTTCCA   
  
  
+ ATGCTATGGT CTTGATGATG ACCCCGCACC CTTGAATGAC CCAAATTACA TTGTATGTCA ATCTTCATAT   
  
  
+ CCTGATCGAG ATATTACACA GATCCCTGAC TTTCCTGATG ATTGTCTCAA GTTCATCAAT GACATTCTTA   
  
  
+ TGGAAGAGGA TTTGGATAAT CCGCCTATAT CCTTGCAGGA CTATAATGCG CTCCAAGCCA CTGAGAAGTC   
  
  
+ ATTGTATGAT GCCCTTGGAG AGGCTTATCC AGCTTCATCT GATCCATTGC AATCATCAGT TGGTCAGAGC   
  
  
+ ACTGAGAGCT CTGATGATCC TGACAGAAGC AGCGGCATTG ATAAATGGGG TAATAGATAT TACCCCACCA   
  
  
+ GCCTGAATGT GGTTGAAACC AGCCGGCTGA TCAATCAGAG CCAGTTGGAC CCTTCTCAAA TGTTTGATGC   
  
  
+ TGCTGCTCAC CCCTTGGAGT CTAATTGTCA TTCTTTTGTC TCTAGTAATA ACTTTGATCA TTCTATGGAT   
  
  
+ GGGGTTCCAG ATTCTCCTAT CAGTACACTT TCTGTGAATG AAACAAGAAA GGAAGAATCA GTTAGTAGAT   
  
  
+ CAAGGAGCAG GAAGAATCAT CAAAGAGATG GTGCATACCT GGAAGAAAGG AGTACTAAGC AAATAGCATC   
  
  
+ CAGCAATGAG GAATATGGAG AGATGGAGAG GTTTGATGAT GTACTGATCT GCAGGGAAGG AAATGATGAT   
  
  
+ ATTTCACCTT GTACCCGCAA AACCCCAGTT CATGAAGCAG ATGATAACTC GGAACCAAAG GGGAAATCAA   
  
  
+ GAGGATCTAA AAGCAAGAGT AAATCGCGTG TCAAGAAGAA AAGTAACGAA GGACAAGCGG TAGATCTTAG   
  
  
+ GACTCAACTT ATTCAGTGTG CAGAAGCAGT GGCAAGCTTT GAGCTTAAAA GTGCAAATGA GCTACTCAGG   
  
  
+ CGAATTAGGC AGCATGCTTC GCCATTTGGG GATAGTGTTC AAAGGCTAGC ACATTACTTT GCGAATGGGC   
  
  
+ TCGAGGCACG CTTAGCTGGT ACAGGATCAG AATCATACAA AGACTTTGCT GGGAAAAAAT TTTCATCCTA   
  
  
+ TGACATCTTG AGAGGCTACA AGTCATATGT TCAAGCCGTC CCCTTCCAGA GAACAACCAT GTTCTTGACA   
  
  
+ AATCAAACAA TAGTAAAGCT GGCTGAGAAA GCAACAAGGG TTCATATCAT TGATTTTGGC ATCTTTTTTG   
  
  
+ GTTTCCAGTG GCCCTGCCTT GTTCAGAATT TGTCAAGAAG ACCCCAGGGT TCTCCCATAC TCAAAATCAC   
  
  
+ GGGAGTCGAT CATCCCCAGA GTGGGTTCAG ACCAGCCCAG AAAGTTGAAG AGACAGGTCG TCGATTAGCA   
  
  
+ GGCTATTGTG AGAGGTTTGG GGTGCCCTTT GAGTTTCATC CCATTGCTCA AAGGTGGCAT ACCCTAAAGC   
  
  
+ CAGAGCATTT CAAAATTGAA AGCAATGAGC TTGTTGTGGT CACTTGTTTC TACCAATCAG GGAAGCTCCC   
  
  
+ TGATGAGACT GTTGATGTCA ACAGCCCAAG AGACCATGTC TTGAGGCTGA TCAGGGGCTT GAACCCTGAT   
  
  
+ CTATTCATCC ATGGGGTTAT CAATGGCACA TTCAATGCAC CCTTCTTCGT AACTCGGTTT AAAGAGGCAC   
  
  
+ TTTACCACTA CTCTTCTTTG TTCGATGTCT TTGATGCCAC CATAGATCGT GAAAGTCATG AAAGACTGCT   
  
  
+ GACTGAGAAC TATTTGTATG GGAAAGATGC ACTTAACATA GTAGCATGTG AGGGGAACGA GAGGATCGAG   
  
  
+ AGACCCGAGA CTTACAAGCA GTGGCAAGTC CGGAAGCTTA GAGCTGGATT CAAGCAGGTC CCCCTAGACC   
  
  
+ AAGACATTGT AAATGGAGCA AGAGCTATGG TTAAGGCAAA TTACAATAAG GATTTTATGG TAGAACAAGA   
  
  
+ TAGGAATTGG ATGGTCCAGG GCTGGAAAGG AAGGATTTTG AGTGCCATAT CCTGTTGGAA ACCTGCTTA  

- +Up\_Stream \_Len000ACTTAA CCTTCAACTA AGATGGAAAC TAACTAAGCT AATTAAGATC CAAGAACCAA   
  
  
- GATCAAGAGA GAGGAGAACA AGAACTAATA AATCCAAAAC CTACACTACA AATAACTTAA CTTACAACGT   
  
  
- TAACAACAAT ATTTCTACTA CAATCCGATT TATAGGAAGT TGAATCCAAC CCTAACTGCA GCTTATACAC   
  
  
- ATCAACTTAG AACATGACCG GATTTAGGGA CGAGGGGGAG ACAAAACGCT AATACTTAAA GTAATACGTG   
  
  
- TGGTGTACGA GCTACTTTAC GAGCTTTCTT GTAAGTAGGA GAAACAAACC CTAAATCCCC TTGCAACTTT   
  
  
- CTGCTTCAGA AATGGATCAC GCAGAATCAT CTGTTAATCC TAAAATTGTA CTTTTATCAA TTTTAAGATT   
  
  
- AACCCTAAAC TCTAACACTT AGAACATTAG GAAGCTGAAG ATCAAGATAC TTTTATCCGC TAATATCAAG   
  
  
- ATCCTAATCA GAAGCTGGGA ACTCTCTCCC TCCTCTACCC AAATCAACTA TTAGATAAGT AAGTATTGCG   
  
  
- TACGGACCCT CTACAAACCA TCCCGATCAT TAACTAAGCG ATTGTATAAA CACGACAATC AAGGTTGGAC   
  
  
- TCACAAATGA GAAGACGACA AATAAGGCAG AAAATCAGCA TTAACAGAGG AAATCAAATT AAAGTTTTGG   
  
  
- GGTTAACACT AGCGGATCTA ACTCGTTAGA TTAATCAAGA TTATTAACTT TAGTTCAAGG GACACCTTAT   
  
  
- GCTGGGCATG AACGGCACAC GATCGATGAC AATGTGGAAC GTAAACGCCA TATAAATATT GAGTTGTATA   
  
  
- CAGACGAGTG ATTTTCATGT ATAATCGAGT GGTTTTGACA CAGAGTTGCA TGATTTTATA AAATCACTTG   
  
  
- ATATGTAATA AAAATCATTC GCTTTATTCT ATGTGTTGTC GTGTAAAATT ACTCAATGTC CCGTAAAAAT   
  
  
- CACTCGGCTT TAGACTACCA AAAGAAACAT CTAGGCCAAT ATATTAACAT AAAATATGTT GGCTTACCTG   
  
  
- CTATATTGTT GACTCAATTT ACTCCTCAGA CTTTAATGCT TGTATTTTAG GTTTTCTTCT ACGGCTACGG   
  
  
- TAACCTTTCT TTCGACTTCC ACTAGCGAAA GATCATCATA ATCCGTCATC ATTAATTTTT GATTTTAGGT   
  
  
- CTTAACTGTG GCTTAACCCC AACACCGTGT ACTAGCTTTA GTCGTAGCTT AGATGTGCTA CTAGACCTAA   
  
  
- GAACCTGTGA GACCGAAATC CGGCAGTTAA AGCTCCTAAC GGGTATTGAA ATTAGGACAA GTTTAGGTAA   
  
  
- GTTAAATTAT AATGTGTTTA GGAAACTAAT TTAGTGGCAT TAACACCAAA CTAATCCCTT CAAAGAACCC   
  
  
- CCTTTCCAAG TTCGAAGACA GAAAGAATTT CGGACCTAAA AACCAGGGCG TACAAGTGAA GTGTAAGGGA   
  
  
- AAAAGAGAGG GTAGCAATGG ACAGACTTAG ACATACATTA TTTTCAGTAT AAAACGCTCC AGAAGAAAAC   
  
  
- TGACAAGAAC ATCAGTGAGA ACTATAGACG TGGTAAAACA AAAAGAGCCG TAAAAGAGGT TGAACGTAAA   
  
  
- AGATTACATT ACACTAGAAA AGACCTGTAA GTAAAACAAT TAGATGAGTG AACCCAGTTC CAAACGACTA   
  
  
- AAACCGACCA CAATTTAGGC AAGGAGAAAC CGTAAACAAG TACAAGGTGT TGACAAACTA TAGACCATAG   
  
  
- AGTCTTTGGG GCGGTAAGAA ACCATAAAGG ACTGAAAGAA CAAGTTCCCA ATAAGGATAA AGGGACTTAA   
  
  
- AAGAAACTAA GAAATCCATA AAGTTCGAAC CCCCCAAAAC AAGAAGTGAC TAAAACAGGA GAAGAAGGGA   
  
  
- ATAACCCACC AAACTAGAGG ATTTAAAGAC ACATAGACTT AGTCAGCCCC ATCTGAAACA CAAAAACCAA   
  
  
- CTAAAAAATA GTTACATAAA AGAAGGATTA CTTCCCGGAG GGCTTGGTTA TGTGTACGAA TTGAAGTTGG   
  
  
- GACAAGGAGA AGGTTCGAAA AACCTCGGAG TTTTCAGAAG CTTACGACTA TCCGTGTATC CCAGGAAGGT   
  
  
- TACGATACCA GAACTACTAC TGGGGCGTGG GAACTTACTG GGTTTAATGT AACATACAGT TAGAAGTATA   
  
  
- GGACTAGCTC TATAATGTGT CTAGGGACTG AAAGGACTAC TAACAGAGTT CAAGTAGTTA CTGTAAGAAT   
  
  
- ACCTTCTCCT AAACCTATTA GGCGGATATA GGAACGTCCT GATATTACGC GAGGTTCGGT GACTCTTCAG   
  
  
- TAACATACTA CGGGAACCTC TCCGAATAGG TCGAAGTAGA CTAGGTAACG TTAGTAGTCA ACCAGTCTCG   
  
  
- TGACTCTCGA GACTACTAGG ACTGTCTTCG TCGCCGTAAC TATTTACCCC ATTATCTATA ATGGGGTGGT   
  
  
- CGGACTTACA CCAACTTTGG TCGGCCGACT AGTTAGTCTC GGTCAACCTG GGAAGAGTTT ACAAACTACG   
  
  
- ACGACGAGTG GGGAACCTCA GATTAACAGT AAGAAAACAG AGATCATTAT TGAAACTAGT AAGATACCTA   
  
  
- CCCCAAGGTC TAAGAGGATA GTCATGTGAA AGACACTTAC TTTGTTCTTT CCTTCTTAGT CAATCATCTA   
  
  
- GTTCCTCGTC CTTCTTAGTA GTTTCTCTAC CACGTATGGA CCTTCTTTCC TCATGATTCG TTTATCGTAG   
  
  
- GTCGTTACTC CTTATACCTC TCTACCTCTC CAAACTACTA CATGACTAGA CGTCCCTTCC TTTACTACTA   
  
  
- TAAAGTGGAA CATGGGCGTT TTGGGGTCAA GTACTTCGTC TACTATTGAG CCTTGGTTTC CCCTTTAGTT   
  
  
- CTCCTAGATT TTCGTTCTCA TTTAGCGCAC AGTTCTTCTT TTCATTGCTT CCTGTTCGCC ATCTAGAATC   
  
  
- CTGAGTTGAA TAAGTCACAC GTCTTCGTCA CCGTTCGAAA CTCGAATTTT CACGTTTACT CGATGAGTCC   
  
  
- GCTTAATCCG TCGTACGAAG CGGTAAACCC CTATCACAAG TTTCCGATCG TGTAATGAAA CGCTTACCCG   
  
  
- AGCTCCGTGC GAATCGACCA TGTCCTAGTC TTAGTATGTT TCTGAAACGA CCCTTTTTTA AAAGTAGGAT   
  
  
- ACTGTAGAAC TCTCCGATGT TCAGTATACA AGTTCGGCAG GGGAAGGTCT CTTGTTGGTA CAAGAACTGT   
  
  
- TTAGTTTGTT ATCATTTCGA CCGACTCTTT CGTTGTTCCC AAGTATAGTA ACTAAAACCG TAGAAAAAAC   
  
  
- CAAAGGTCAC CGGGACGGAA CAAGTCTTAA ACAGTTCTTC TGGGGTCCCA AGAGGGTATG AGTTTTAGTG   
  
  
- CCCTCAGCTA GTAGGGGTCT CACCCAAGTC TGGTCGGGTC TTTCAACTTC TCTGTCCAGC AGCTAATCGT   
  
  
- CCGATAACAC TCTCCAAACC CCACGGGAAA CTCAAAGTAG GGTAACGAGT TTCCACCGTA TGGGATTTCG   
  
  
- GTCTCGTAAA GTTTTAACTT TCGTTACTCG AACAACACCA GTGAACAAAG ATGGTTAGTC CCTTCGAGGG   
  
  
- ACTACTCTGA CAACTACAGT TGTCGGGTTC TCTGGTACAG AACTCCGACT AGTCCCCGAA CTTGGGACTA   
  
  
- GATAAGTAGG TACCCCAATA GTTACCGTGT AAGTTACGTG GGAAGAAGCA TTGAGCCAAA TTTCTCCGTG   
  
  
- AAATGGTGAT GAGAAGAAAC AAGCTACAGA AACTACGGTG GTATCTAGCA CTTTCAGTAC TTTCTGACGA   
  
  
- CTGACTCTTG ATAAACATAC CCTTTCTACG TGAATTGTAT CATCGTACAC TCCCCTTGCT CTCCTAGCTC   
  
  
- TCTGGGCTCT GAATGTTCGT CACCGTTCAG GCCTTCGAAT CTCGACCTAA GTTCGTCCAG GGGGATCTGG   
  
  
- TTCTGTAACA TTTACCTCGT TCTCGATACC AATTCCGTTT AATGTTATTC CTAAAATACC ATCTTGTTCT   
  
  
- ATCCTTAACC TACCAGGTCC CGACCTTTCC TTCCTAAAAC TCACGGTATA GGACAACCTT TGGACGAAT

+     CAT-box

| Site Name | Organism | Position | Strand | Matrix score. | sequence | function |
| --- | --- | --- | --- | --- | --- | --- |
| CAT-box | Arabidopsis thaliana | 2972 | - | 6 | GCCACT | cis-acting regulatory element related to meristem expression |
| CAT-box | Arabidopsis thaliana | 3874 | - | 6 | GCCACT | cis-acting regulatory element related to meristem expression |
| CAT-box | Arabidopsis thaliana | 3301 | - | 6 | GCCACT | cis-acting regulatory element related to meristem expression |
| CAT-box | Arabidopsis thaliana | 2301 | + | 6 | GCCACT | cis-acting regulatory element related to meristem expression |

>HU02G01573.1   
+ +Up\_Stream \_Len000TGAATT GGAAGTTGAT TCTACCTTTG ATTGATTCGA TTAATTCTAG GTTCTTGGTT   
  
  
+ CTAGTTCTCT CTCCTCTTGT TCTTGATTAT TTAGGTTTTG GATGTGATGT TTATTGAATT GAATGTTGCA   
  
  
+ ATTGTTGTTA TAAAGATGAT GTTAGGCTAA ATATCCTTCA ACTTAGGTTG GGATTGACGT CGAATATGTG   
  
  
+ TAGTTGAATC TTGTACTGGC CTAAATCCCT GCTCCCCCTC TGTTTTGCGA TTATGAATTT CATTATGCAC   
  
  
+ ACCACATGCT CGATGAAATG CTCGAAAGAA CATTCATCCT CTTTGTTTGG GATTTAGGGG AACGTTGAAA   
  
  
+ GACGAAGTCT TTACCTAGTG CGTCTTAGTA GACAATTAGG ATTTTAACAT GAAAATAGTT AAAATTCTAA   
  
  
+ TTGGGATTTG AGATTGTGAA TCTTGTAATC CTTCGACTTC TAGTTCTATG AAAATAGGCG ATTATAGTTC   
  
  
+ TAGGATTAGT CTTCGACCCT TGAGAGAGGG AGGAGATGGG TTTAGTTGAT AATCTATTCA TTCATAACGC   
  
  
+ ATGCCTGGGA GATGTTTGGT AGGGCTAGTA ATTGATTCGC TAACATATTT GTGCTGTTAG TTCCAACCTG   
  
  
+ AGTGTTTACT CTTCTGCTGT TTATTCCGTC TTTTAGTCGT AATTGTCTCC TTTAGTTTAA TTTCAAAACC   
  
  
+ CCAATTGTGA TCGCCTAGAT TGAGCAATCT AATTAGTTCT AATAATTGAA ATCAAGTTCC CTGTGGAATA   
  
  
+ CGACCCGTAC TTGCCGTGTG CTAGCTACTG TTACACCTTG CATTTGCGGT ATATTTATAA CTCAACATAT   
  
  
+ GTCTGCTCAC TAAAAGTACA TATTAGCTCA CCAAAACTGT GTCTCAACGT ACTAAAATAT TTTAGTGAAC   
  
  
+ TATACATTAT TTTTAGTAAG CGAAATAAGA TACACAACAG CACATTTTAA TGAGTTACAG GGCATTTTTA   
  
  
+ GTGAGCCGAA ATCTGATGGT TTTCTTTGTA GATCCGGTTA TATAATTGTA TTTTATACAA CCGAATGGAC   
  
  
+ GATATAACAA CTGAGTTAAA TGAGGAGTCT GAAATTACGA ACATAAAATC CAAAAGAAGA TGCCGATGCC   
  
  
+ ATTGGAAAGA AAGCTGAAGG TGATCGCTTT CTAGTAGTAT TAGGCAGTAG TAATTAAAAA CTAAAATCCA   
  
  
+ GAATTGACAC CGAATTGGGG TTGTGGCACA TGATCGAAAT CAGCATCGAA TCTACACGAT GATCTGGATT   
  
  
+ CTTGGACACT CTGGCTTTAG GCCGTCAATT TCGAGGATTG CCCATAACTT TAATCCTGTT CAAATCCATT   
  
  
+ CAATTTAATA TTACACAAAT CCTTTGATTA AATCACCGTA ATTGTGGTTT GATTAGGGAA GTTTCTTGGG   
  
  
+ GGAAAGGTTC AAGCTTCTGT CTTTCTTAAA GCCTGGATTT TTGGTCCCGC ATGTTCACTT CACATTCCCT   
  
  
+ TTTTCTCTCC CATCGTTACC TGTCTGAATC TGTATGTAAT AAAAGTCATA TTTTGCGAGG TCTTCTTTTG   
  
  
+ ACTGTTCTTG TAGTCACTCT TGATATCTGC ACCATTTTGT TTTTCTCGGC ATTTTCTCCA ACTTGCATTT   
  
  
+ TCTAATGTAA TGTGATCTTT TCTGGACATT CATTTTGTTA ATCTACTCAC TTGGGTCAAG GTTTGCTGAT   
  
  
+ TTTGGCTGGT GTTAAATCCG TTCCTCTTTG GCATTTGTTC ATGTTCCACA ACTGTTTGAT ATCTGGTATC   
  
  
+ TCAGAAACCC CGCCATTCTT TGGTATTTCC TGACTTTCTT GTTCAAGGGT TATTCCTATT TCCCTGAATT   
  
  
+ TTCTTTGATT CTTTAGGTAT TTCAAGCTTG GGGGGTTTTG TTCTTCACTG ATTTTGTCCT CTTCTTCCCT   
  
  
+ TATTGGGTGG TTTGATCTCC TAAATTTCTG TGTATCTGAA TCAGTCGGGG TAGACTTTGT GTTTTTGGTT   
  
  
+ GATTTTTTAT CAATGTATTT TCTTCCTAAT GAAGGGCCTC CCGAACCAAT ACACATGCTT AACTTCAACC   
  
  
+ CTGTTCCTCT TCCAAGCTTT TTGGAGCCTC AAAAGTCTTC GAATGCTGAT AGGCACATAG GGTCCTTCCA   
  
  
+ ATGCTATGGT CTTGATGATG ACCCCGCACC CTTGAATGAC CCAAATTACA TTGTATGTCA ATCTTCATAT   
  
  
+ CCTGATCGAG ATATTACACA GATCCCTGAC TTTCCTGATG ATTGTCTCAA GTTCATCAAT GACATTCTTA   
  
  
+ TGGAAGAGGA TTTGGATAAT CCGCCTATAT CCTTGCAGGA CTATAATGCG CTCCAAGCCA CTGAGAAGTC   
  
  
+ ATTGTATGAT GCCCTTGGAG AGGCTTATCC AGCTTCATCT GATCCATTGC AATCATCAGT TGGTCAGAGC   
  
  
+ ACTGAGAGCT CTGATGATCC TGACAGAAGC AGCGGCATTG ATAAATGGGG TAATAGATAT TACCCCACCA   
  
  
+ GCCTGAATGT GGTTGAAACC AGCCGGCTGA TCAATCAGAG CCAGTTGGAC CCTTCTCAAA TGTTTGATGC   
  
  
+ TGCTGCTCAC CCCTTGGAGT CTAATTGTCA TTCTTTTGTC TCTAGTAATA ACTTTGATCA TTCTATGGAT   
  
  
+ GGGGTTCCAG ATTCTCCTAT CAGTACACTT TCTGTGAATG AAACAAGAAA GGAAGAATCA GTTAGTAGAT   
  
  
+ CAAGGAGCAG GAAGAATCAT CAAAGAGATG GTGCATACCT GGAAGAAAGG AGTACTAAGC AAATAGCATC   
  
  
+ CAGCAATGAG GAATATGGAG AGATGGAGAG GTTTGATGAT GTACTGATCT GCAGGGAAGG AAATGATGAT   
  
  
+ ATTTCACCTT GTACCCGCAA AACCCCAGTT CATGAAGCAG ATGATAACTC GGAACCAAAG GGGAAATCAA   
  
  
+ GAGGATCTAA AAGCAAGAGT AAATCGCGTG TCAAGAAGAA AAGTAACGAA GGACAAGCGG TAGATCTTAG   
  
  
+ GACTCAACTT ATTCAGTGTG CAGAAGCAGT GGCAAGCTTT GAGCTTAAAA GTGCAAATGA GCTACTCAGG   
  
  
+ CGAATTAGGC AGCATGCTTC GCCATTTGGG GATAGTGTTC AAAGGCTAGC ACATTACTTT GCGAATGGGC   
  
  
+ TCGAGGCACG CTTAGCTGGT ACAGGATCAG AATCATACAA AGACTTTGCT GGGAAAAAAT TTTCATCCTA   
  
  
+ TGACATCTTG AGAGGCTACA AGTCATATGT TCAAGCCGTC CCCTTCCAGA GAACAACCAT GTTCTTGACA   
  
  
+ AATCAAACAA TAGTAAAGCT GGCTGAGAAA GCAACAAGGG TTCATATCAT TGATTTTGGC ATCTTTTTTG   
  
  
+ GTTTCCAGTG GCCCTGCCTT GTTCAGAATT TGTCAAGAAG ACCCCAGGGT TCTCCCATAC TCAAAATCAC   
  
  
+ GGGAGTCGAT CATCCCCAGA GTGGGTTCAG ACCAGCCCAG AAAGTTGAAG AGACAGGTCG TCGATTAGCA   
  
  
+ GGCTATTGTG AGAGGTTTGG GGTGCCCTTT GAGTTTCATC CCATTGCTCA AAGGTGGCAT ACCCTAAAGC   
  
  
+ CAGAGCATTT CAAAATTGAA AGCAATGAGC TTGTTGTGGT CACTTGTTTC TACCAATCAG GGAAGCTCCC   
  
  
+ TGATGAGACT GTTGATGTCA ACAGCCCAAG AGACCATGTC TTGAGGCTGA TCAGGGGCTT GAACCCTGAT   
  
  
+ CTATTCATCC ATGGGGTTAT CAATGGCACA TTCAATGCAC CCTTCTTCGT AACTCGGTTT AAAGAGGCAC   
  
  
+ TTTACCACTA CTCTTCTTTG TTCGATGTCT TTGATGCCAC CATAGATCGT GAAAGTCATG AAAGACTGCT   
  
  
+ GACTGAGAAC TATTTGTATG GGAAAGATGC ACTTAACATA GTAGCATGTG AGGGGAACGA GAGGATCGAG   
  
  
+ AGACCCGAGA CTTACAAGCA GTGGCAAGTC CGGAAGCTTA GAGCTGGATT CAAGCAGGTC CCCCTAGACC   
  
  
+ AAGACATTGT AAATGGAGCA AGAGCTATGG TTAAGGCAAA TTACAATAAG GATTTTATGG TAGAACAAGA   
  
  
+ TAGGAATTGG ATGGTCCAGG GCTGGAAAGG AAGGATTTTG AGTGCCATAT CCTGTTGGAA ACCTGCTTA  

- +Up\_Stream \_Len000ACTTAA CCTTCAACTA AGATGGAAAC TAACTAAGCT AATTAAGATC CAAGAACCAA   
  
  
- GATCAAGAGA GAGGAGAACA AGAACTAATA AATCCAAAAC CTACACTACA AATAACTTAA CTTACAACGT   
  
  
- TAACAACAAT ATTTCTACTA CAATCCGATT TATAGGAAGT TGAATCCAAC CCTAACTGCA GCTTATACAC   
  
  
- ATCAACTTAG AACATGACCG GATTTAGGGA CGAGGGGGAG ACAAAACGCT AATACTTAAA GTAATACGTG   
  
  
- TGGTGTACGA GCTACTTTAC GAGCTTTCTT GTAAGTAGGA GAAACAAACC CTAAATCCCC TTGCAACTTT   
  
  
- CTGCTTCAGA AATGGATCAC GCAGAATCAT CTGTTAATCC TAAAATTGTA CTTTTATCAA TTTTAAGATT   
  
  
- AACCCTAAAC TCTAACACTT AGAACATTAG GAAGCTGAAG ATCAAGATAC TTTTATCCGC TAATATCAAG   
  
  
- ATCCTAATCA GAAGCTGGGA ACTCTCTCCC TCCTCTACCC AAATCAACTA TTAGATAAGT AAGTATTGCG   
  
  
- TACGGACCCT CTACAAACCA TCCCGATCAT TAACTAAGCG ATTGTATAAA CACGACAATC AAGGTTGGAC   
  
  
- TCACAAATGA GAAGACGACA AATAAGGCAG AAAATCAGCA TTAACAGAGG AAATCAAATT AAAGTTTTGG   
  
  
- GGTTAACACT AGCGGATCTA ACTCGTTAGA TTAATCAAGA TTATTAACTT TAGTTCAAGG GACACCTTAT   
  
  
- GCTGGGCATG AACGGCACAC GATCGATGAC AATGTGGAAC GTAAACGCCA TATAAATATT GAGTTGTATA   
  
  
- CAGACGAGTG ATTTTCATGT ATAATCGAGT GGTTTTGACA CAGAGTTGCA TGATTTTATA AAATCACTTG   
  
  
- ATATGTAATA AAAATCATTC GCTTTATTCT ATGTGTTGTC GTGTAAAATT ACTCAATGTC CCGTAAAAAT   
  
  
- CACTCGGCTT TAGACTACCA AAAGAAACAT CTAGGCCAAT ATATTAACAT AAAATATGTT GGCTTACCTG   
  
  
- CTATATTGTT GACTCAATTT ACTCCTCAGA CTTTAATGCT TGTATTTTAG GTTTTCTTCT ACGGCTACGG   
  
  
- TAACCTTTCT TTCGACTTCC ACTAGCGAAA GATCATCATA ATCCGTCATC ATTAATTTTT GATTTTAGGT   
  
  
- CTTAACTGTG GCTTAACCCC AACACCGTGT ACTAGCTTTA GTCGTAGCTT AGATGTGCTA CTAGACCTAA   
  
  
- GAACCTGTGA GACCGAAATC CGGCAGTTAA AGCTCCTAAC GGGTATTGAA ATTAGGACAA GTTTAGGTAA   
  
  
- GTTAAATTAT AATGTGTTTA GGAAACTAAT TTAGTGGCAT TAACACCAAA CTAATCCCTT CAAAGAACCC   
  
  
- CCTTTCCAAG TTCGAAGACA GAAAGAATTT CGGACCTAAA AACCAGGGCG TACAAGTGAA GTGTAAGGGA   
  
  
- AAAAGAGAGG GTAGCAATGG ACAGACTTAG ACATACATTA TTTTCAGTAT AAAACGCTCC AGAAGAAAAC   
  
  
- TGACAAGAAC ATCAGTGAGA ACTATAGACG TGGTAAAACA AAAAGAGCCG TAAAAGAGGT TGAACGTAAA   
  
  
- AGATTACATT ACACTAGAAA AGACCTGTAA GTAAAACAAT TAGATGAGTG AACCCAGTTC CAAACGACTA   
  
  
- AAACCGACCA CAATTTAGGC AAGGAGAAAC CGTAAACAAG TACAAGGTGT TGACAAACTA TAGACCATAG   
  
  
- AGTCTTTGGG GCGGTAAGAA ACCATAAAGG ACTGAAAGAA CAAGTTCCCA ATAAGGATAA AGGGACTTAA   
  
  
- AAGAAACTAA GAAATCCATA AAGTTCGAAC CCCCCAAAAC AAGAAGTGAC TAAAACAGGA GAAGAAGGGA   
  
  
- ATAACCCACC AAACTAGAGG ATTTAAAGAC ACATAGACTT AGTCAGCCCC ATCTGAAACA CAAAAACCAA   
  
  
- CTAAAAAATA GTTACATAAA AGAAGGATTA CTTCCCGGAG GGCTTGGTTA TGTGTACGAA TTGAAGTTGG   
  
  
- GACAAGGAGA AGGTTCGAAA AACCTCGGAG TTTTCAGAAG CTTACGACTA TCCGTGTATC CCAGGAAGGT   
  
  
- TACGATACCA GAACTACTAC TGGGGCGTGG GAACTTACTG GGTTTAATGT AACATACAGT TAGAAGTATA   
  
  
- GGACTAGCTC TATAATGTGT CTAGGGACTG AAAGGACTAC TAACAGAGTT CAAGTAGTTA CTGTAAGAAT   
  
  
- ACCTTCTCCT AAACCTATTA GGCGGATATA GGAACGTCCT GATATTACGC GAGGTTCGGT GACTCTTCAG   
  
  
- TAACATACTA CGGGAACCTC TCCGAATAGG TCGAAGTAGA CTAGGTAACG TTAGTAGTCA ACCAGTCTCG   
  
  
- TGACTCTCGA GACTACTAGG ACTGTCTTCG TCGCCGTAAC TATTTACCCC ATTATCTATA ATGGGGTGGT   
  
  
- CGGACTTACA CCAACTTTGG TCGGCCGACT AGTTAGTCTC GGTCAACCTG GGAAGAGTTT ACAAACTACG   
  
  
- ACGACGAGTG GGGAACCTCA GATTAACAGT AAGAAAACAG AGATCATTAT TGAAACTAGT AAGATACCTA   
  
  
- CCCCAAGGTC TAAGAGGATA GTCATGTGAA AGACACTTAC TTTGTTCTTT CCTTCTTAGT CAATCATCTA   
  
  
- GTTCCTCGTC CTTCTTAGTA GTTTCTCTAC CACGTATGGA CCTTCTTTCC TCATGATTCG TTTATCGTAG   
  
  
- GTCGTTACTC CTTATACCTC TCTACCTCTC CAAACTACTA CATGACTAGA CGTCCCTTCC TTTACTACTA   
  
  
- TAAAGTGGAA CATGGGCGTT TTGGGGTCAA GTACTTCGTC TACTATTGAG CCTTGGTTTC CCCTTTAGTT   
  
  
- CTCCTAGATT TTCGTTCTCA TTTAGCGCAC AGTTCTTCTT TTCATTGCTT CCTGTTCGCC ATCTAGAATC   
  
  
- CTGAGTTGAA TAAGTCACAC GTCTTCGTCA CCGTTCGAAA CTCGAATTTT CACGTTTACT CGATGAGTCC   
  
  
- GCTTAATCCG TCGTACGAAG CGGTAAACCC CTATCACAAG TTTCCGATCG TGTAATGAAA CGCTTACCCG   
  
  
- AGCTCCGTGC GAATCGACCA TGTCCTAGTC TTAGTATGTT TCTGAAACGA CCCTTTTTTA AAAGTAGGAT   
  
  
- ACTGTAGAAC TCTCCGATGT TCAGTATACA AGTTCGGCAG GGGAAGGTCT CTTGTTGGTA CAAGAACTGT   
  
  
- TTAGTTTGTT ATCATTTCGA CCGACTCTTT CGTTGTTCCC AAGTATAGTA ACTAAAACCG TAGAAAAAAC   
  
  
- CAAAGGTCAC CGGGACGGAA CAAGTCTTAA ACAGTTCTTC TGGGGTCCCA AGAGGGTATG AGTTTTAGTG   
  
  
- CCCTCAGCTA GTAGGGGTCT CACCCAAGTC TGGTCGGGTC TTTCAACTTC TCTGTCCAGC AGCTAATCGT   
  
  
- CCGATAACAC TCTCCAAACC CCACGGGAAA CTCAAAGTAG GGTAACGAGT TTCCACCGTA TGGGATTTCG   
  
  
- GTCTCGTAAA GTTTTAACTT TCGTTACTCG AACAACACCA GTGAACAAAG ATGGTTAGTC CCTTCGAGGG   
  
  
- ACTACTCTGA CAACTACAGT TGTCGGGTTC TCTGGTACAG AACTCCGACT AGTCCCCGAA CTTGGGACTA   
  
  
- GATAAGTAGG TACCCCAATA GTTACCGTGT AAGTTACGTG GGAAGAAGCA TTGAGCCAAA TTTCTCCGTG   
  
  
- AAATGGTGAT GAGAAGAAAC AAGCTACAGA AACTACGGTG GTATCTAGCA CTTTCAGTAC TTTCTGACGA   
  
  
- CTGACTCTTG ATAAACATAC CCTTTCTACG TGAATTGTAT CATCGTACAC TCCCCTTGCT CTCCTAGCTC   
  
  
- TCTGGGCTCT GAATGTTCGT CACCGTTCAG GCCTTCGAAT CTCGACCTAA GTTCGTCCAG GGGGATCTGG   
  
  
- TTCTGTAACA TTTACCTCGT TCTCGATACC AATTCCGTTT AATGTTATTC CTAAAATACC ATCTTGTTCT   
  
  
- ATCCTTAACC TACCAGGTCC CGACCTTTCC TTCCTAAAAC TCACGGTATA GGACAACCTT TGGACGAAT

+     CCGTCC motif

| Site Name | Organism | Position | Strand | Matrix score. | sequence | function |
| --- | --- | --- | --- | --- | --- | --- |
| CCGTCC motif | Nicotiana tabacum | 3190 | + | 6 | CCGTCC |  |

>HU02G01573.1   
+ +Up\_Stream \_Len000TGAATT GGAAGTTGAT TCTACCTTTG ATTGATTCGA TTAATTCTAG GTTCTTGGTT   
  
  
+ CTAGTTCTCT CTCCTCTTGT TCTTGATTAT TTAGGTTTTG GATGTGATGT TTATTGAATT GAATGTTGCA   
  
  
+ ATTGTTGTTA TAAAGATGAT GTTAGGCTAA ATATCCTTCA ACTTAGGTTG GGATTGACGT CGAATATGTG   
  
  
+ TAGTTGAATC TTGTACTGGC CTAAATCCCT GCTCCCCCTC TGTTTTGCGA TTATGAATTT CATTATGCAC   
  
  
+ ACCACATGCT CGATGAAATG CTCGAAAGAA CATTCATCCT CTTTGTTTGG GATTTAGGGG AACGTTGAAA   
  
  
+ GACGAAGTCT TTACCTAGTG CGTCTTAGTA GACAATTAGG ATTTTAACAT GAAAATAGTT AAAATTCTAA   
  
  
+ TTGGGATTTG AGATTGTGAA TCTTGTAATC CTTCGACTTC TAGTTCTATG AAAATAGGCG ATTATAGTTC   
  
  
+ TAGGATTAGT CTTCGACCCT TGAGAGAGGG AGGAGATGGG TTTAGTTGAT AATCTATTCA TTCATAACGC   
  
  
+ ATGCCTGGGA GATGTTTGGT AGGGCTAGTA ATTGATTCGC TAACATATTT GTGCTGTTAG TTCCAACCTG   
  
  
+ AGTGTTTACT CTTCTGCTGT TTATTCCGTC TTTTAGTCGT AATTGTCTCC TTTAGTTTAA TTTCAAAACC   
  
  
+ CCAATTGTGA TCGCCTAGAT TGAGCAATCT AATTAGTTCT AATAATTGAA ATCAAGTTCC CTGTGGAATA   
  
  
+ CGACCCGTAC TTGCCGTGTG CTAGCTACTG TTACACCTTG CATTTGCGGT ATATTTATAA CTCAACATAT   
  
  
+ GTCTGCTCAC TAAAAGTACA TATTAGCTCA CCAAAACTGT GTCTCAACGT ACTAAAATAT TTTAGTGAAC   
  
  
+ TATACATTAT TTTTAGTAAG CGAAATAAGA TACACAACAG CACATTTTAA TGAGTTACAG GGCATTTTTA   
  
  
+ GTGAGCCGAA ATCTGATGGT TTTCTTTGTA GATCCGGTTA TATAATTGTA TTTTATACAA CCGAATGGAC   
  
  
+ GATATAACAA CTGAGTTAAA TGAGGAGTCT GAAATTACGA ACATAAAATC CAAAAGAAGA TGCCGATGCC   
  
  
+ ATTGGAAAGA AAGCTGAAGG TGATCGCTTT CTAGTAGTAT TAGGCAGTAG TAATTAAAAA CTAAAATCCA   
  
  
+ GAATTGACAC CGAATTGGGG TTGTGGCACA TGATCGAAAT CAGCATCGAA TCTACACGAT GATCTGGATT   
  
  
+ CTTGGACACT CTGGCTTTAG GCCGTCAATT TCGAGGATTG CCCATAACTT TAATCCTGTT CAAATCCATT   
  
  
+ CAATTTAATA TTACACAAAT CCTTTGATTA AATCACCGTA ATTGTGGTTT GATTAGGGAA GTTTCTTGGG   
  
  
+ GGAAAGGTTC AAGCTTCTGT CTTTCTTAAA GCCTGGATTT TTGGTCCCGC ATGTTCACTT CACATTCCCT   
  
  
+ TTTTCTCTCC CATCGTTACC TGTCTGAATC TGTATGTAAT AAAAGTCATA TTTTGCGAGG TCTTCTTTTG   
  
  
+ ACTGTTCTTG TAGTCACTCT TGATATCTGC ACCATTTTGT TTTTCTCGGC ATTTTCTCCA ACTTGCATTT   
  
  
+ TCTAATGTAA TGTGATCTTT TCTGGACATT CATTTTGTTA ATCTACTCAC TTGGGTCAAG GTTTGCTGAT   
  
  
+ TTTGGCTGGT GTTAAATCCG TTCCTCTTTG GCATTTGTTC ATGTTCCACA ACTGTTTGAT ATCTGGTATC   
  
  
+ TCAGAAACCC CGCCATTCTT TGGTATTTCC TGACTTTCTT GTTCAAGGGT TATTCCTATT TCCCTGAATT   
  
  
+ TTCTTTGATT CTTTAGGTAT TTCAAGCTTG GGGGGTTTTG TTCTTCACTG ATTTTGTCCT CTTCTTCCCT   
  
  
+ TATTGGGTGG TTTGATCTCC TAAATTTCTG TGTATCTGAA TCAGTCGGGG TAGACTTTGT GTTTTTGGTT   
  
  
+ GATTTTTTAT CAATGTATTT TCTTCCTAAT GAAGGGCCTC CCGAACCAAT ACACATGCTT AACTTCAACC   
  
  
+ CTGTTCCTCT TCCAAGCTTT TTGGAGCCTC AAAAGTCTTC GAATGCTGAT AGGCACATAG GGTCCTTCCA   
  
  
+ ATGCTATGGT CTTGATGATG ACCCCGCACC CTTGAATGAC CCAAATTACA TTGTATGTCA ATCTTCATAT   
  
  
+ CCTGATCGAG ATATTACACA GATCCCTGAC TTTCCTGATG ATTGTCTCAA GTTCATCAAT GACATTCTTA   
  
  
+ TGGAAGAGGA TTTGGATAAT CCGCCTATAT CCTTGCAGGA CTATAATGCG CTCCAAGCCA CTGAGAAGTC   
  
  
+ ATTGTATGAT GCCCTTGGAG AGGCTTATCC AGCTTCATCT GATCCATTGC AATCATCAGT TGGTCAGAGC   
  
  
+ ACTGAGAGCT CTGATGATCC TGACAGAAGC AGCGGCATTG ATAAATGGGG TAATAGATAT TACCCCACCA   
  
  
+ GCCTGAATGT GGTTGAAACC AGCCGGCTGA TCAATCAGAG CCAGTTGGAC CCTTCTCAAA TGTTTGATGC   
  
  
+ TGCTGCTCAC CCCTTGGAGT CTAATTGTCA TTCTTTTGTC TCTAGTAATA ACTTTGATCA TTCTATGGAT   
  
  
+ GGGGTTCCAG ATTCTCCTAT CAGTACACTT TCTGTGAATG AAACAAGAAA GGAAGAATCA GTTAGTAGAT   
  
  
+ CAAGGAGCAG GAAGAATCAT CAAAGAGATG GTGCATACCT GGAAGAAAGG AGTACTAAGC AAATAGCATC   
  
  
+ CAGCAATGAG GAATATGGAG AGATGGAGAG GTTTGATGAT GTACTGATCT GCAGGGAAGG AAATGATGAT   
  
  
+ ATTTCACCTT GTACCCGCAA AACCCCAGTT CATGAAGCAG ATGATAACTC GGAACCAAAG GGGAAATCAA   
  
  
+ GAGGATCTAA AAGCAAGAGT AAATCGCGTG TCAAGAAGAA AAGTAACGAA GGACAAGCGG TAGATCTTAG   
  
  
+ GACTCAACTT ATTCAGTGTG CAGAAGCAGT GGCAAGCTTT GAGCTTAAAA GTGCAAATGA GCTACTCAGG   
  
  
+ CGAATTAGGC AGCATGCTTC GCCATTTGGG GATAGTGTTC AAAGGCTAGC ACATTACTTT GCGAATGGGC   
  
  
+ TCGAGGCACG CTTAGCTGGT ACAGGATCAG AATCATACAA AGACTTTGCT GGGAAAAAAT TTTCATCCTA   
  
  
+ TGACATCTTG AGAGGCTACA AGTCATATGT TCAAGCCGTC CCCTTCCAGA GAACAACCAT GTTCTTGACA   
  
  
+ AATCAAACAA TAGTAAAGCT GGCTGAGAAA GCAACAAGGG TTCATATCAT TGATTTTGGC ATCTTTTTTG   
  
  
+ GTTTCCAGTG GCCCTGCCTT GTTCAGAATT TGTCAAGAAG ACCCCAGGGT TCTCCCATAC TCAAAATCAC   
  
  
+ GGGAGTCGAT CATCCCCAGA GTGGGTTCAG ACCAGCCCAG AAAGTTGAAG AGACAGGTCG TCGATTAGCA   
  
  
+ GGCTATTGTG AGAGGTTTGG GGTGCCCTTT GAGTTTCATC CCATTGCTCA AAGGTGGCAT ACCCTAAAGC   
  
  
+ CAGAGCATTT CAAAATTGAA AGCAATGAGC TTGTTGTGGT CACTTGTTTC TACCAATCAG GGAAGCTCCC   
  
  
+ TGATGAGACT GTTGATGTCA ACAGCCCAAG AGACCATGTC TTGAGGCTGA TCAGGGGCTT GAACCCTGAT   
  
  
+ CTATTCATCC ATGGGGTTAT CAATGGCACA TTCAATGCAC CCTTCTTCGT AACTCGGTTT AAAGAGGCAC   
  
  
+ TTTACCACTA CTCTTCTTTG TTCGATGTCT TTGATGCCAC CATAGATCGT GAAAGTCATG AAAGACTGCT   
  
  
+ GACTGAGAAC TATTTGTATG GGAAAGATGC ACTTAACATA GTAGCATGTG AGGGGAACGA GAGGATCGAG   
  
  
+ AGACCCGAGA CTTACAAGCA GTGGCAAGTC CGGAAGCTTA GAGCTGGATT CAAGCAGGTC CCCCTAGACC   
  
  
+ AAGACATTGT AAATGGAGCA AGAGCTATGG TTAAGGCAAA TTACAATAAG GATTTTATGG TAGAACAAGA   
  
  
+ TAGGAATTGG ATGGTCCAGG GCTGGAAAGG AAGGATTTTG AGTGCCATAT CCTGTTGGAA ACCTGCTTA  

- +Up\_Stream \_Len000ACTTAA CCTTCAACTA AGATGGAAAC TAACTAAGCT AATTAAGATC CAAGAACCAA   
  
  
- GATCAAGAGA GAGGAGAACA AGAACTAATA AATCCAAAAC CTACACTACA AATAACTTAA CTTACAACGT   
  
  
- TAACAACAAT ATTTCTACTA CAATCCGATT TATAGGAAGT TGAATCCAAC CCTAACTGCA GCTTATACAC   
  
  
- ATCAACTTAG AACATGACCG GATTTAGGGA CGAGGGGGAG ACAAAACGCT AATACTTAAA GTAATACGTG   
  
  
- TGGTGTACGA GCTACTTTAC GAGCTTTCTT GTAAGTAGGA GAAACAAACC CTAAATCCCC TTGCAACTTT   
  
  
- CTGCTTCAGA AATGGATCAC GCAGAATCAT CTGTTAATCC TAAAATTGTA CTTTTATCAA TTTTAAGATT   
  
  
- AACCCTAAAC TCTAACACTT AGAACATTAG GAAGCTGAAG ATCAAGATAC TTTTATCCGC TAATATCAAG   
  
  
- ATCCTAATCA GAAGCTGGGA ACTCTCTCCC TCCTCTACCC AAATCAACTA TTAGATAAGT AAGTATTGCG   
  
  
- TACGGACCCT CTACAAACCA TCCCGATCAT TAACTAAGCG ATTGTATAAA CACGACAATC AAGGTTGGAC   
  
  
- TCACAAATGA GAAGACGACA AATAAGGCAG AAAATCAGCA TTAACAGAGG AAATCAAATT AAAGTTTTGG   
  
  
- GGTTAACACT AGCGGATCTA ACTCGTTAGA TTAATCAAGA TTATTAACTT TAGTTCAAGG GACACCTTAT   
  
  
- GCTGGGCATG AACGGCACAC GATCGATGAC AATGTGGAAC GTAAACGCCA TATAAATATT GAGTTGTATA   
  
  
- CAGACGAGTG ATTTTCATGT ATAATCGAGT GGTTTTGACA CAGAGTTGCA TGATTTTATA AAATCACTTG   
  
  
- ATATGTAATA AAAATCATTC GCTTTATTCT ATGTGTTGTC GTGTAAAATT ACTCAATGTC CCGTAAAAAT   
  
  
- CACTCGGCTT TAGACTACCA AAAGAAACAT CTAGGCCAAT ATATTAACAT AAAATATGTT GGCTTACCTG   
  
  
- CTATATTGTT GACTCAATTT ACTCCTCAGA CTTTAATGCT TGTATTTTAG GTTTTCTTCT ACGGCTACGG   
  
  
- TAACCTTTCT TTCGACTTCC ACTAGCGAAA GATCATCATA ATCCGTCATC ATTAATTTTT GATTTTAGGT   
  
  
- CTTAACTGTG GCTTAACCCC AACACCGTGT ACTAGCTTTA GTCGTAGCTT AGATGTGCTA CTAGACCTAA   
  
  
- GAACCTGTGA GACCGAAATC CGGCAGTTAA AGCTCCTAAC GGGTATTGAA ATTAGGACAA GTTTAGGTAA   
  
  
- GTTAAATTAT AATGTGTTTA GGAAACTAAT TTAGTGGCAT TAACACCAAA CTAATCCCTT CAAAGAACCC   
  
  
- CCTTTCCAAG TTCGAAGACA GAAAGAATTT CGGACCTAAA AACCAGGGCG TACAAGTGAA GTGTAAGGGA   
  
  
- AAAAGAGAGG GTAGCAATGG ACAGACTTAG ACATACATTA TTTTCAGTAT AAAACGCTCC AGAAGAAAAC   
  
  
- TGACAAGAAC ATCAGTGAGA ACTATAGACG TGGTAAAACA AAAAGAGCCG TAAAAGAGGT TGAACGTAAA   
  
  
- AGATTACATT ACACTAGAAA AGACCTGTAA GTAAAACAAT TAGATGAGTG AACCCAGTTC CAAACGACTA   
  
  
- AAACCGACCA CAATTTAGGC AAGGAGAAAC CGTAAACAAG TACAAGGTGT TGACAAACTA TAGACCATAG   
  
  
- AGTCTTTGGG GCGGTAAGAA ACCATAAAGG ACTGAAAGAA CAAGTTCCCA ATAAGGATAA AGGGACTTAA   
  
  
- AAGAAACTAA GAAATCCATA AAGTTCGAAC CCCCCAAAAC AAGAAGTGAC TAAAACAGGA GAAGAAGGGA   
  
  
- ATAACCCACC AAACTAGAGG ATTTAAAGAC ACATAGACTT AGTCAGCCCC ATCTGAAACA CAAAAACCAA   
  
  
- CTAAAAAATA GTTACATAAA AGAAGGATTA CTTCCCGGAG GGCTTGGTTA TGTGTACGAA TTGAAGTTGG   
  
  
- GACAAGGAGA AGGTTCGAAA AACCTCGGAG TTTTCAGAAG CTTACGACTA TCCGTGTATC CCAGGAAGGT   
  
  
- TACGATACCA GAACTACTAC TGGGGCGTGG GAACTTACTG GGTTTAATGT AACATACAGT TAGAAGTATA   
  
  
- GGACTAGCTC TATAATGTGT CTAGGGACTG AAAGGACTAC TAACAGAGTT CAAGTAGTTA CTGTAAGAAT   
  
  
- ACCTTCTCCT AAACCTATTA GGCGGATATA GGAACGTCCT GATATTACGC GAGGTTCGGT GACTCTTCAG   
  
  
- TAACATACTA CGGGAACCTC TCCGAATAGG TCGAAGTAGA CTAGGTAACG TTAGTAGTCA ACCAGTCTCG   
  
  
- TGACTCTCGA GACTACTAGG ACTGTCTTCG TCGCCGTAAC TATTTACCCC ATTATCTATA ATGGGGTGGT   
  
  
- CGGACTTACA CCAACTTTGG TCGGCCGACT AGTTAGTCTC GGTCAACCTG GGAAGAGTTT ACAAACTACG   
  
  
- ACGACGAGTG GGGAACCTCA GATTAACAGT AAGAAAACAG AGATCATTAT TGAAACTAGT AAGATACCTA   
  
  
- CCCCAAGGTC TAAGAGGATA GTCATGTGAA AGACACTTAC TTTGTTCTTT CCTTCTTAGT CAATCATCTA   
  
  
- GTTCCTCGTC CTTCTTAGTA GTTTCTCTAC CACGTATGGA CCTTCTTTCC TCATGATTCG TTTATCGTAG   
  
  
- GTCGTTACTC CTTATACCTC TCTACCTCTC CAAACTACTA CATGACTAGA CGTCCCTTCC TTTACTACTA   
  
  
- TAAAGTGGAA CATGGGCGTT TTGGGGTCAA GTACTTCGTC TACTATTGAG CCTTGGTTTC CCCTTTAGTT   
  
  
- CTCCTAGATT TTCGTTCTCA TTTAGCGCAC AGTTCTTCTT TTCATTGCTT CCTGTTCGCC ATCTAGAATC   
  
  
- CTGAGTTGAA TAAGTCACAC GTCTTCGTCA CCGTTCGAAA CTCGAATTTT CACGTTTACT CGATGAGTCC   
  
  
- GCTTAATCCG TCGTACGAAG CGGTAAACCC CTATCACAAG TTTCCGATCG TGTAATGAAA CGCTTACCCG   
  
  
- AGCTCCGTGC GAATCGACCA TGTCCTAGTC TTAGTATGTT TCTGAAACGA CCCTTTTTTA AAAGTAGGAT   
  
  
- ACTGTAGAAC TCTCCGATGT TCAGTATACA AGTTCGGCAG GGGAAGGTCT CTTGTTGGTA CAAGAACTGT   
  
  
- TTAGTTTGTT ATCATTTCGA CCGACTCTTT CGTTGTTCCC AAGTATAGTA ACTAAAACCG TAGAAAAAAC   
  
  
- CAAAGGTCAC CGGGACGGAA CAAGTCTTAA ACAGTTCTTC TGGGGTCCCA AGAGGGTATG AGTTTTAGTG   
  
  
- CCCTCAGCTA GTAGGGGTCT CACCCAAGTC TGGTCGGGTC TTTCAACTTC TCTGTCCAGC AGCTAATCGT   
  
  
- CCGATAACAC TCTCCAAACC CCACGGGAAA CTCAAAGTAG GGTAACGAGT TTCCACCGTA TGGGATTTCG   
  
  
- GTCTCGTAAA GTTTTAACTT TCGTTACTCG AACAACACCA GTGAACAAAG ATGGTTAGTC CCTTCGAGGG   
  
  
- ACTACTCTGA CAACTACAGT TGTCGGGTTC TCTGGTACAG AACTCCGACT AGTCCCCGAA CTTGGGACTA   
  
  
- GATAAGTAGG TACCCCAATA GTTACCGTGT AAGTTACGTG GGAAGAAGCA TTGAGCCAAA TTTCTCCGTG   
  
  
- AAATGGTGAT GAGAAGAAAC AAGCTACAGA AACTACGGTG GTATCTAGCA CTTTCAGTAC TTTCTGACGA   
  
  
- CTGACTCTTG ATAAACATAC CCTTTCTACG TGAATTGTAT CATCGTACAC TCCCCTTGCT CTCCTAGCTC   
  
  
- TCTGGGCTCT GAATGTTCGT CACCGTTCAG GCCTTCGAAT CTCGACCTAA GTTCGTCCAG GGGGATCTGG   
  
  
- TTCTGTAACA TTTACCTCGT TCTCGATACC AATTCCGTTT AATGTTATTC CTAAAATACC ATCTTGTTCT   
  
  
- ATCCTTAACC TACCAGGTCC CGACCTTTCC TTCCTAAAAC TCACGGTATA GGACAACCTT TGGACGAAT

+     CCGTCC-box

| Site Name | Organism | Position | Strand | Matrix score. | sequence | function |
| --- | --- | --- | --- | --- | --- | --- |
| CCGTCC-box | Petroselinum hortense | 3190 | + | 6 | CCGTCC |  |

>HU02G01573.1   
+ +Up\_Stream \_Len000TGAATT GGAAGTTGAT TCTACCTTTG ATTGATTCGA TTAATTCTAG GTTCTTGGTT   
  
  
+ CTAGTTCTCT CTCCTCTTGT TCTTGATTAT TTAGGTTTTG GATGTGATGT TTATTGAATT GAATGTTGCA   
  
  
+ ATTGTTGTTA TAAAGATGAT GTTAGGCTAA ATATCCTTCA ACTTAGGTTG GGATTGACGT CGAATATGTG   
  
  
+ TAGTTGAATC TTGTACTGGC CTAAATCCCT GCTCCCCCTC TGTTTTGCGA TTATGAATTT CATTATGCAC   
  
  
+ ACCACATGCT CGATGAAATG CTCGAAAGAA CATTCATCCT CTTTGTTTGG GATTTAGGGG AACGTTGAAA   
  
  
+ GACGAAGTCT TTACCTAGTG CGTCTTAGTA GACAATTAGG ATTTTAACAT GAAAATAGTT AAAATTCTAA   
  
  
+ TTGGGATTTG AGATTGTGAA TCTTGTAATC CTTCGACTTC TAGTTCTATG AAAATAGGCG ATTATAGTTC   
  
  
+ TAGGATTAGT CTTCGACCCT TGAGAGAGGG AGGAGATGGG TTTAGTTGAT AATCTATTCA TTCATAACGC   
  
  
+ ATGCCTGGGA GATGTTTGGT AGGGCTAGTA ATTGATTCGC TAACATATTT GTGCTGTTAG TTCCAACCTG   
  
  
+ AGTGTTTACT CTTCTGCTGT TTATTCCGTC TTTTAGTCGT AATTGTCTCC TTTAGTTTAA TTTCAAAACC   
  
  
+ CCAATTGTGA TCGCCTAGAT TGAGCAATCT AATTAGTTCT AATAATTGAA ATCAAGTTCC CTGTGGAATA   
  
  
+ CGACCCGTAC TTGCCGTGTG CTAGCTACTG TTACACCTTG CATTTGCGGT ATATTTATAA CTCAACATAT   
  
  
+ GTCTGCTCAC TAAAAGTACA TATTAGCTCA CCAAAACTGT GTCTCAACGT ACTAAAATAT TTTAGTGAAC   
  
  
+ TATACATTAT TTTTAGTAAG CGAAATAAGA TACACAACAG CACATTTTAA TGAGTTACAG GGCATTTTTA   
  
  
+ GTGAGCCGAA ATCTGATGGT TTTCTTTGTA GATCCGGTTA TATAATTGTA TTTTATACAA CCGAATGGAC   
  
  
+ GATATAACAA CTGAGTTAAA TGAGGAGTCT GAAATTACGA ACATAAAATC CAAAAGAAGA TGCCGATGCC   
  
  
+ ATTGGAAAGA AAGCTGAAGG TGATCGCTTT CTAGTAGTAT TAGGCAGTAG TAATTAAAAA CTAAAATCCA   
  
  
+ GAATTGACAC CGAATTGGGG TTGTGGCACA TGATCGAAAT CAGCATCGAA TCTACACGAT GATCTGGATT   
  
  
+ CTTGGACACT CTGGCTTTAG GCCGTCAATT TCGAGGATTG CCCATAACTT TAATCCTGTT CAAATCCATT   
  
  
+ CAATTTAATA TTACACAAAT CCTTTGATTA AATCACCGTA ATTGTGGTTT GATTAGGGAA GTTTCTTGGG   
  
  
+ GGAAAGGTTC AAGCTTCTGT CTTTCTTAAA GCCTGGATTT TTGGTCCCGC ATGTTCACTT CACATTCCCT   
  
  
+ TTTTCTCTCC CATCGTTACC TGTCTGAATC TGTATGTAAT AAAAGTCATA TTTTGCGAGG TCTTCTTTTG   
  
  
+ ACTGTTCTTG TAGTCACTCT TGATATCTGC ACCATTTTGT TTTTCTCGGC ATTTTCTCCA ACTTGCATTT   
  
  
+ TCTAATGTAA TGTGATCTTT TCTGGACATT CATTTTGTTA ATCTACTCAC TTGGGTCAAG GTTTGCTGAT   
  
  
+ TTTGGCTGGT GTTAAATCCG TTCCTCTTTG GCATTTGTTC ATGTTCCACA ACTGTTTGAT ATCTGGTATC   
  
  
+ TCAGAAACCC CGCCATTCTT TGGTATTTCC TGACTTTCTT GTTCAAGGGT TATTCCTATT TCCCTGAATT   
  
  
+ TTCTTTGATT CTTTAGGTAT TTCAAGCTTG GGGGGTTTTG TTCTTCACTG ATTTTGTCCT CTTCTTCCCT   
  
  
+ TATTGGGTGG TTTGATCTCC TAAATTTCTG TGTATCTGAA TCAGTCGGGG TAGACTTTGT GTTTTTGGTT   
  
  
+ GATTTTTTAT CAATGTATTT TCTTCCTAAT GAAGGGCCTC CCGAACCAAT ACACATGCTT AACTTCAACC   
  
  
+ CTGTTCCTCT TCCAAGCTTT TTGGAGCCTC AAAAGTCTTC GAATGCTGAT AGGCACATAG GGTCCTTCCA   
  
  
+ ATGCTATGGT CTTGATGATG ACCCCGCACC CTTGAATGAC CCAAATTACA TTGTATGTCA ATCTTCATAT   
  
  
+ CCTGATCGAG ATATTACACA GATCCCTGAC TTTCCTGATG ATTGTCTCAA GTTCATCAAT GACATTCTTA   
  
  
+ TGGAAGAGGA TTTGGATAAT CCGCCTATAT CCTTGCAGGA CTATAATGCG CTCCAAGCCA CTGAGAAGTC   
  
  
+ ATTGTATGAT GCCCTTGGAG AGGCTTATCC AGCTTCATCT GATCCATTGC AATCATCAGT TGGTCAGAGC   
  
  
+ ACTGAGAGCT CTGATGATCC TGACAGAAGC AGCGGCATTG ATAAATGGGG TAATAGATAT TACCCCACCA   
  
  
+ GCCTGAATGT GGTTGAAACC AGCCGGCTGA TCAATCAGAG CCAGTTGGAC CCTTCTCAAA TGTTTGATGC   
  
  
+ TGCTGCTCAC CCCTTGGAGT CTAATTGTCA TTCTTTTGTC TCTAGTAATA ACTTTGATCA TTCTATGGAT   
  
  
+ GGGGTTCCAG ATTCTCCTAT CAGTACACTT TCTGTGAATG AAACAAGAAA GGAAGAATCA GTTAGTAGAT   
  
  
+ CAAGGAGCAG GAAGAATCAT CAAAGAGATG GTGCATACCT GGAAGAAAGG AGTACTAAGC AAATAGCATC   
  
  
+ CAGCAATGAG GAATATGGAG AGATGGAGAG GTTTGATGAT GTACTGATCT GCAGGGAAGG AAATGATGAT   
  
  
+ ATTTCACCTT GTACCCGCAA AACCCCAGTT CATGAAGCAG ATGATAACTC GGAACCAAAG GGGAAATCAA   
  
  
+ GAGGATCTAA AAGCAAGAGT AAATCGCGTG TCAAGAAGAA AAGTAACGAA GGACAAGCGG TAGATCTTAG   
  
  
+ GACTCAACTT ATTCAGTGTG CAGAAGCAGT GGCAAGCTTT GAGCTTAAAA GTGCAAATGA GCTACTCAGG   
  
  
+ CGAATTAGGC AGCATGCTTC GCCATTTGGG GATAGTGTTC AAAGGCTAGC ACATTACTTT GCGAATGGGC   
  
  
+ TCGAGGCACG CTTAGCTGGT ACAGGATCAG AATCATACAA AGACTTTGCT GGGAAAAAAT TTTCATCCTA   
  
  
+ TGACATCTTG AGAGGCTACA AGTCATATGT TCAAGCCGTC CCCTTCCAGA GAACAACCAT GTTCTTGACA   
  
  
+ AATCAAACAA TAGTAAAGCT GGCTGAGAAA GCAACAAGGG TTCATATCAT TGATTTTGGC ATCTTTTTTG   
  
  
+ GTTTCCAGTG GCCCTGCCTT GTTCAGAATT TGTCAAGAAG ACCCCAGGGT TCTCCCATAC TCAAAATCAC   
  
  
+ GGGAGTCGAT CATCCCCAGA GTGGGTTCAG ACCAGCCCAG AAAGTTGAAG AGACAGGTCG TCGATTAGCA   
  
  
+ GGCTATTGTG AGAGGTTTGG GGTGCCCTTT GAGTTTCATC CCATTGCTCA AAGGTGGCAT ACCCTAAAGC   
  
  
+ CAGAGCATTT CAAAATTGAA AGCAATGAGC TTGTTGTGGT CACTTGTTTC TACCAATCAG GGAAGCTCCC   
  
  
+ TGATGAGACT GTTGATGTCA ACAGCCCAAG AGACCATGTC TTGAGGCTGA TCAGGGGCTT GAACCCTGAT   
  
  
+ CTATTCATCC ATGGGGTTAT CAATGGCACA TTCAATGCAC CCTTCTTCGT AACTCGGTTT AAAGAGGCAC   
  
  
+ TTTACCACTA CTCTTCTTTG TTCGATGTCT TTGATGCCAC CATAGATCGT GAAAGTCATG AAAGACTGCT   
  
  
+ GACTGAGAAC TATTTGTATG GGAAAGATGC ACTTAACATA GTAGCATGTG AGGGGAACGA GAGGATCGAG   
  
  
+ AGACCCGAGA CTTACAAGCA GTGGCAAGTC CGGAAGCTTA GAGCTGGATT CAAGCAGGTC CCCCTAGACC   
  
  
+ AAGACATTGT AAATGGAGCA AGAGCTATGG TTAAGGCAAA TTACAATAAG GATTTTATGG TAGAACAAGA   
  
  
+ TAGGAATTGG ATGGTCCAGG GCTGGAAAGG AAGGATTTTG AGTGCCATAT CCTGTTGGAA ACCTGCTTA  

- +Up\_Stream \_Len000ACTTAA CCTTCAACTA AGATGGAAAC TAACTAAGCT AATTAAGATC CAAGAACCAA   
  
  
- GATCAAGAGA GAGGAGAACA AGAACTAATA AATCCAAAAC CTACACTACA AATAACTTAA CTTACAACGT   
  
  
- TAACAACAAT ATTTCTACTA CAATCCGATT TATAGGAAGT TGAATCCAAC CCTAACTGCA GCTTATACAC   
  
  
- ATCAACTTAG AACATGACCG GATTTAGGGA CGAGGGGGAG ACAAAACGCT AATACTTAAA GTAATACGTG   
  
  
- TGGTGTACGA GCTACTTTAC GAGCTTTCTT GTAAGTAGGA GAAACAAACC CTAAATCCCC TTGCAACTTT   
  
  
- CTGCTTCAGA AATGGATCAC GCAGAATCAT CTGTTAATCC TAAAATTGTA CTTTTATCAA TTTTAAGATT   
  
  
- AACCCTAAAC TCTAACACTT AGAACATTAG GAAGCTGAAG ATCAAGATAC TTTTATCCGC TAATATCAAG   
  
  
- ATCCTAATCA GAAGCTGGGA ACTCTCTCCC TCCTCTACCC AAATCAACTA TTAGATAAGT AAGTATTGCG   
  
  
- TACGGACCCT CTACAAACCA TCCCGATCAT TAACTAAGCG ATTGTATAAA CACGACAATC AAGGTTGGAC   
  
  
- TCACAAATGA GAAGACGACA AATAAGGCAG AAAATCAGCA TTAACAGAGG AAATCAAATT AAAGTTTTGG   
  
  
- GGTTAACACT AGCGGATCTA ACTCGTTAGA TTAATCAAGA TTATTAACTT TAGTTCAAGG GACACCTTAT   
  
  
- GCTGGGCATG AACGGCACAC GATCGATGAC AATGTGGAAC GTAAACGCCA TATAAATATT GAGTTGTATA   
  
  
- CAGACGAGTG ATTTTCATGT ATAATCGAGT GGTTTTGACA CAGAGTTGCA TGATTTTATA AAATCACTTG   
  
  
- ATATGTAATA AAAATCATTC GCTTTATTCT ATGTGTTGTC GTGTAAAATT ACTCAATGTC CCGTAAAAAT   
  
  
- CACTCGGCTT TAGACTACCA AAAGAAACAT CTAGGCCAAT ATATTAACAT AAAATATGTT GGCTTACCTG   
  
  
- CTATATTGTT GACTCAATTT ACTCCTCAGA CTTTAATGCT TGTATTTTAG GTTTTCTTCT ACGGCTACGG   
  
  
- TAACCTTTCT TTCGACTTCC ACTAGCGAAA GATCATCATA ATCCGTCATC ATTAATTTTT GATTTTAGGT   
  
  
- CTTAACTGTG GCTTAACCCC AACACCGTGT ACTAGCTTTA GTCGTAGCTT AGATGTGCTA CTAGACCTAA   
  
  
- GAACCTGTGA GACCGAAATC CGGCAGTTAA AGCTCCTAAC GGGTATTGAA ATTAGGACAA GTTTAGGTAA   
  
  
- GTTAAATTAT AATGTGTTTA GGAAACTAAT TTAGTGGCAT TAACACCAAA CTAATCCCTT CAAAGAACCC   
  
  
- CCTTTCCAAG TTCGAAGACA GAAAGAATTT CGGACCTAAA AACCAGGGCG TACAAGTGAA GTGTAAGGGA   
  
  
- AAAAGAGAGG GTAGCAATGG ACAGACTTAG ACATACATTA TTTTCAGTAT AAAACGCTCC AGAAGAAAAC   
  
  
- TGACAAGAAC ATCAGTGAGA ACTATAGACG TGGTAAAACA AAAAGAGCCG TAAAAGAGGT TGAACGTAAA   
  
  
- AGATTACATT ACACTAGAAA AGACCTGTAA GTAAAACAAT TAGATGAGTG AACCCAGTTC CAAACGACTA   
  
  
- AAACCGACCA CAATTTAGGC AAGGAGAAAC CGTAAACAAG TACAAGGTGT TGACAAACTA TAGACCATAG   
  
  
- AGTCTTTGGG GCGGTAAGAA ACCATAAAGG ACTGAAAGAA CAAGTTCCCA ATAAGGATAA AGGGACTTAA   
  
  
- AAGAAACTAA GAAATCCATA AAGTTCGAAC CCCCCAAAAC AAGAAGTGAC TAAAACAGGA GAAGAAGGGA   
  
  
- ATAACCCACC AAACTAGAGG ATTTAAAGAC ACATAGACTT AGTCAGCCCC ATCTGAAACA CAAAAACCAA   
  
  
- CTAAAAAATA GTTACATAAA AGAAGGATTA CTTCCCGGAG GGCTTGGTTA TGTGTACGAA TTGAAGTTGG   
  
  
- GACAAGGAGA AGGTTCGAAA AACCTCGGAG TTTTCAGAAG CTTACGACTA TCCGTGTATC CCAGGAAGGT   
  
  
- TACGATACCA GAACTACTAC TGGGGCGTGG GAACTTACTG GGTTTAATGT AACATACAGT TAGAAGTATA   
  
  
- GGACTAGCTC TATAATGTGT CTAGGGACTG AAAGGACTAC TAACAGAGTT CAAGTAGTTA CTGTAAGAAT   
  
  
- ACCTTCTCCT AAACCTATTA GGCGGATATA GGAACGTCCT GATATTACGC GAGGTTCGGT GACTCTTCAG   
  
  
- TAACATACTA CGGGAACCTC TCCGAATAGG TCGAAGTAGA CTAGGTAACG TTAGTAGTCA ACCAGTCTCG   
  
  
- TGACTCTCGA GACTACTAGG ACTGTCTTCG TCGCCGTAAC TATTTACCCC ATTATCTATA ATGGGGTGGT   
  
  
- CGGACTTACA CCAACTTTGG TCGGCCGACT AGTTAGTCTC GGTCAACCTG GGAAGAGTTT ACAAACTACG   
  
  
- ACGACGAGTG GGGAACCTCA GATTAACAGT AAGAAAACAG AGATCATTAT TGAAACTAGT AAGATACCTA   
  
  
- CCCCAAGGTC TAAGAGGATA GTCATGTGAA AGACACTTAC TTTGTTCTTT CCTTCTTAGT CAATCATCTA   
  
  
- GTTCCTCGTC CTTCTTAGTA GTTTCTCTAC CACGTATGGA CCTTCTTTCC TCATGATTCG TTTATCGTAG   
  
  
- GTCGTTACTC CTTATACCTC TCTACCTCTC CAAACTACTA CATGACTAGA CGTCCCTTCC TTTACTACTA   
  
  
- TAAAGTGGAA CATGGGCGTT TTGGGGTCAA GTACTTCGTC TACTATTGAG CCTTGGTTTC CCCTTTAGTT   
  
  
- CTCCTAGATT TTCGTTCTCA TTTAGCGCAC AGTTCTTCTT TTCATTGCTT CCTGTTCGCC ATCTAGAATC   
  
  
- CTGAGTTGAA TAAGTCACAC GTCTTCGTCA CCGTTCGAAA CTCGAATTTT CACGTTTACT CGATGAGTCC   
  
  
- GCTTAATCCG TCGTACGAAG CGGTAAACCC CTATCACAAG TTTCCGATCG TGTAATGAAA CGCTTACCCG   
  
  
- AGCTCCGTGC GAATCGACCA TGTCCTAGTC TTAGTATGTT TCTGAAACGA CCCTTTTTTA AAAGTAGGAT   
  
  
- ACTGTAGAAC TCTCCGATGT TCAGTATACA AGTTCGGCAG GGGAAGGTCT CTTGTTGGTA CAAGAACTGT   
  
  
- TTAGTTTGTT ATCATTTCGA CCGACTCTTT CGTTGTTCCC AAGTATAGTA ACTAAAACCG TAGAAAAAAC   
  
  
- CAAAGGTCAC CGGGACGGAA CAAGTCTTAA ACAGTTCTTC TGGGGTCCCA AGAGGGTATG AGTTTTAGTG   
  
  
- CCCTCAGCTA GTAGGGGTCT CACCCAAGTC TGGTCGGGTC TTTCAACTTC TCTGTCCAGC AGCTAATCGT   
  
  
- CCGATAACAC TCTCCAAACC CCACGGGAAA CTCAAAGTAG GGTAACGAGT TTCCACCGTA TGGGATTTCG   
  
  
- GTCTCGTAAA GTTTTAACTT TCGTTACTCG AACAACACCA GTGAACAAAG ATGGTTAGTC CCTTCGAGGG   
  
  
- ACTACTCTGA CAACTACAGT TGTCGGGTTC TCTGGTACAG AACTCCGACT AGTCCCCGAA CTTGGGACTA   
  
  
- GATAAGTAGG TACCCCAATA GTTACCGTGT AAGTTACGTG GGAAGAAGCA TTGAGCCAAA TTTCTCCGTG   
  
  
- AAATGGTGAT GAGAAGAAAC AAGCTACAGA AACTACGGTG GTATCTAGCA CTTTCAGTAC TTTCTGACGA   
  
  
- CTGACTCTTG ATAAACATAC CCTTTCTACG TGAATTGTAT CATCGTACAC TCCCCTTGCT CTCCTAGCTC   
  
  
- TCTGGGCTCT GAATGTTCGT CACCGTTCAG GCCTTCGAAT CTCGACCTAA GTTCGTCCAG GGGGATCTGG   
  
  
- TTCTGTAACA TTTACCTCGT TCTCGATACC AATTCCGTTT AATGTTATTC CTAAAATACC ATCTTGTTCT   
  
  
- ATCCTTAACC TACCAGGTCC CGACCTTTCC TTCCTAAAAC TCACGGTATA GGACAACCTT TGGACGAAT

+     CGTCA-motif

| Site Name | Organism | Position | Strand | Matrix score. | sequence | function |
| --- | --- | --- | --- | --- | --- | --- |
| CGTCA-motif | Hordeum vulgare | 1287 | + | 5 | CGTCA | cis-acting regulatory element involved in the MeJA-responsiveness |
| CGTCA-motif | Hordeum vulgare | 199 | - | 5 | CGTCA | cis-acting regulatory element involved in the MeJA-responsiveness |

>HU02G01573.1   
+ +Up\_Stream \_Len000TGAATT GGAAGTTGAT TCTACCTTTG ATTGATTCGA TTAATTCTAG GTTCTTGGTT   
  
  
+ CTAGTTCTCT CTCCTCTTGT TCTTGATTAT TTAGGTTTTG GATGTGATGT TTATTGAATT GAATGTTGCA   
  
  
+ ATTGTTGTTA TAAAGATGAT GTTAGGCTAA ATATCCTTCA ACTTAGGTTG GGATTGACGT CGAATATGTG   
  
  
+ TAGTTGAATC TTGTACTGGC CTAAATCCCT GCTCCCCCTC TGTTTTGCGA TTATGAATTT CATTATGCAC   
  
  
+ ACCACATGCT CGATGAAATG CTCGAAAGAA CATTCATCCT CTTTGTTTGG GATTTAGGGG AACGTTGAAA   
  
  
+ GACGAAGTCT TTACCTAGTG CGTCTTAGTA GACAATTAGG ATTTTAACAT GAAAATAGTT AAAATTCTAA   
  
  
+ TTGGGATTTG AGATTGTGAA TCTTGTAATC CTTCGACTTC TAGTTCTATG AAAATAGGCG ATTATAGTTC   
  
  
+ TAGGATTAGT CTTCGACCCT TGAGAGAGGG AGGAGATGGG TTTAGTTGAT AATCTATTCA TTCATAACGC   
  
  
+ ATGCCTGGGA GATGTTTGGT AGGGCTAGTA ATTGATTCGC TAACATATTT GTGCTGTTAG TTCCAACCTG   
  
  
+ AGTGTTTACT CTTCTGCTGT TTATTCCGTC TTTTAGTCGT AATTGTCTCC TTTAGTTTAA TTTCAAAACC   
  
  
+ CCAATTGTGA TCGCCTAGAT TGAGCAATCT AATTAGTTCT AATAATTGAA ATCAAGTTCC CTGTGGAATA   
  
  
+ CGACCCGTAC TTGCCGTGTG CTAGCTACTG TTACACCTTG CATTTGCGGT ATATTTATAA CTCAACATAT   
  
  
+ GTCTGCTCAC TAAAAGTACA TATTAGCTCA CCAAAACTGT GTCTCAACGT ACTAAAATAT TTTAGTGAAC   
  
  
+ TATACATTAT TTTTAGTAAG CGAAATAAGA TACACAACAG CACATTTTAA TGAGTTACAG GGCATTTTTA   
  
  
+ GTGAGCCGAA ATCTGATGGT TTTCTTTGTA GATCCGGTTA TATAATTGTA TTTTATACAA CCGAATGGAC   
  
  
+ GATATAACAA CTGAGTTAAA TGAGGAGTCT GAAATTACGA ACATAAAATC CAAAAGAAGA TGCCGATGCC   
  
  
+ ATTGGAAAGA AAGCTGAAGG TGATCGCTTT CTAGTAGTAT TAGGCAGTAG TAATTAAAAA CTAAAATCCA   
  
  
+ GAATTGACAC CGAATTGGGG TTGTGGCACA TGATCGAAAT CAGCATCGAA TCTACACGAT GATCTGGATT   
  
  
+ CTTGGACACT CTGGCTTTAG GCCGTCAATT TCGAGGATTG CCCATAACTT TAATCCTGTT CAAATCCATT   
  
  
+ CAATTTAATA TTACACAAAT CCTTTGATTA AATCACCGTA ATTGTGGTTT GATTAGGGAA GTTTCTTGGG   
  
  
+ GGAAAGGTTC AAGCTTCTGT CTTTCTTAAA GCCTGGATTT TTGGTCCCGC ATGTTCACTT CACATTCCCT   
  
  
+ TTTTCTCTCC CATCGTTACC TGTCTGAATC TGTATGTAAT AAAAGTCATA TTTTGCGAGG TCTTCTTTTG   
  
  
+ ACTGTTCTTG TAGTCACTCT TGATATCTGC ACCATTTTGT TTTTCTCGGC ATTTTCTCCA ACTTGCATTT   
  
  
+ TCTAATGTAA TGTGATCTTT TCTGGACATT CATTTTGTTA ATCTACTCAC TTGGGTCAAG GTTTGCTGAT   
  
  
+ TTTGGCTGGT GTTAAATCCG TTCCTCTTTG GCATTTGTTC ATGTTCCACA ACTGTTTGAT ATCTGGTATC   
  
  
+ TCAGAAACCC CGCCATTCTT TGGTATTTCC TGACTTTCTT GTTCAAGGGT TATTCCTATT TCCCTGAATT   
  
  
+ TTCTTTGATT CTTTAGGTAT TTCAAGCTTG GGGGGTTTTG TTCTTCACTG ATTTTGTCCT CTTCTTCCCT   
  
  
+ TATTGGGTGG TTTGATCTCC TAAATTTCTG TGTATCTGAA TCAGTCGGGG TAGACTTTGT GTTTTTGGTT   
  
  
+ GATTTTTTAT CAATGTATTT TCTTCCTAAT GAAGGGCCTC CCGAACCAAT ACACATGCTT AACTTCAACC   
  
  
+ CTGTTCCTCT TCCAAGCTTT TTGGAGCCTC AAAAGTCTTC GAATGCTGAT AGGCACATAG GGTCCTTCCA   
  
  
+ ATGCTATGGT CTTGATGATG ACCCCGCACC CTTGAATGAC CCAAATTACA TTGTATGTCA ATCTTCATAT   
  
  
+ CCTGATCGAG ATATTACACA GATCCCTGAC TTTCCTGATG ATTGTCTCAA GTTCATCAAT GACATTCTTA   
  
  
+ TGGAAGAGGA TTTGGATAAT CCGCCTATAT CCTTGCAGGA CTATAATGCG CTCCAAGCCA CTGAGAAGTC   
  
  
+ ATTGTATGAT GCCCTTGGAG AGGCTTATCC AGCTTCATCT GATCCATTGC AATCATCAGT TGGTCAGAGC   
  
  
+ ACTGAGAGCT CTGATGATCC TGACAGAAGC AGCGGCATTG ATAAATGGGG TAATAGATAT TACCCCACCA   
  
  
+ GCCTGAATGT GGTTGAAACC AGCCGGCTGA TCAATCAGAG CCAGTTGGAC CCTTCTCAAA TGTTTGATGC   
  
  
+ TGCTGCTCAC CCCTTGGAGT CTAATTGTCA TTCTTTTGTC TCTAGTAATA ACTTTGATCA TTCTATGGAT   
  
  
+ GGGGTTCCAG ATTCTCCTAT CAGTACACTT TCTGTGAATG AAACAAGAAA GGAAGAATCA GTTAGTAGAT   
  
  
+ CAAGGAGCAG GAAGAATCAT CAAAGAGATG GTGCATACCT GGAAGAAAGG AGTACTAAGC AAATAGCATC   
  
  
+ CAGCAATGAG GAATATGGAG AGATGGAGAG GTTTGATGAT GTACTGATCT GCAGGGAAGG AAATGATGAT   
  
  
+ ATTTCACCTT GTACCCGCAA AACCCCAGTT CATGAAGCAG ATGATAACTC GGAACCAAAG GGGAAATCAA   
  
  
+ GAGGATCTAA AAGCAAGAGT AAATCGCGTG TCAAGAAGAA AAGTAACGAA GGACAAGCGG TAGATCTTAG   
  
  
+ GACTCAACTT ATTCAGTGTG CAGAAGCAGT GGCAAGCTTT GAGCTTAAAA GTGCAAATGA GCTACTCAGG   
  
  
+ CGAATTAGGC AGCATGCTTC GCCATTTGGG GATAGTGTTC AAAGGCTAGC ACATTACTTT GCGAATGGGC   
  
  
+ TCGAGGCACG CTTAGCTGGT ACAGGATCAG AATCATACAA AGACTTTGCT GGGAAAAAAT TTTCATCCTA   
  
  
+ TGACATCTTG AGAGGCTACA AGTCATATGT TCAAGCCGTC CCCTTCCAGA GAACAACCAT GTTCTTGACA   
  
  
+ AATCAAACAA TAGTAAAGCT GGCTGAGAAA GCAACAAGGG TTCATATCAT TGATTTTGGC ATCTTTTTTG   
  
  
+ GTTTCCAGTG GCCCTGCCTT GTTCAGAATT TGTCAAGAAG ACCCCAGGGT TCTCCCATAC TCAAAATCAC   
  
  
+ GGGAGTCGAT CATCCCCAGA GTGGGTTCAG ACCAGCCCAG AAAGTTGAAG AGACAGGTCG TCGATTAGCA   
  
  
+ GGCTATTGTG AGAGGTTTGG GGTGCCCTTT GAGTTTCATC CCATTGCTCA AAGGTGGCAT ACCCTAAAGC   
  
  
+ CAGAGCATTT CAAAATTGAA AGCAATGAGC TTGTTGTGGT CACTTGTTTC TACCAATCAG GGAAGCTCCC   
  
  
+ TGATGAGACT GTTGATGTCA ACAGCCCAAG AGACCATGTC TTGAGGCTGA TCAGGGGCTT GAACCCTGAT   
  
  
+ CTATTCATCC ATGGGGTTAT CAATGGCACA TTCAATGCAC CCTTCTTCGT AACTCGGTTT AAAGAGGCAC   
  
  
+ TTTACCACTA CTCTTCTTTG TTCGATGTCT TTGATGCCAC CATAGATCGT GAAAGTCATG AAAGACTGCT   
  
  
+ GACTGAGAAC TATTTGTATG GGAAAGATGC ACTTAACATA GTAGCATGTG AGGGGAACGA GAGGATCGAG   
  
  
+ AGACCCGAGA CTTACAAGCA GTGGCAAGTC CGGAAGCTTA GAGCTGGATT CAAGCAGGTC CCCCTAGACC   
  
  
+ AAGACATTGT AAATGGAGCA AGAGCTATGG TTAAGGCAAA TTACAATAAG GATTTTATGG TAGAACAAGA   
  
  
+ TAGGAATTGG ATGGTCCAGG GCTGGAAAGG AAGGATTTTG AGTGCCATAT CCTGTTGGAA ACCTGCTTA  

- +Up\_Stream \_Len000ACTTAA CCTTCAACTA AGATGGAAAC TAACTAAGCT AATTAAGATC CAAGAACCAA   
  
  
- GATCAAGAGA GAGGAGAACA AGAACTAATA AATCCAAAAC CTACACTACA AATAACTTAA CTTACAACGT   
  
  
- TAACAACAAT ATTTCTACTA CAATCCGATT TATAGGAAGT TGAATCCAAC CCTAACTGCA GCTTATACAC   
  
  
- ATCAACTTAG AACATGACCG GATTTAGGGA CGAGGGGGAG ACAAAACGCT AATACTTAAA GTAATACGTG   
  
  
- TGGTGTACGA GCTACTTTAC GAGCTTTCTT GTAAGTAGGA GAAACAAACC CTAAATCCCC TTGCAACTTT   
  
  
- CTGCTTCAGA AATGGATCAC GCAGAATCAT CTGTTAATCC TAAAATTGTA CTTTTATCAA TTTTAAGATT   
  
  
- AACCCTAAAC TCTAACACTT AGAACATTAG GAAGCTGAAG ATCAAGATAC TTTTATCCGC TAATATCAAG   
  
  
- ATCCTAATCA GAAGCTGGGA ACTCTCTCCC TCCTCTACCC AAATCAACTA TTAGATAAGT AAGTATTGCG   
  
  
- TACGGACCCT CTACAAACCA TCCCGATCAT TAACTAAGCG ATTGTATAAA CACGACAATC AAGGTTGGAC   
  
  
- TCACAAATGA GAAGACGACA AATAAGGCAG AAAATCAGCA TTAACAGAGG AAATCAAATT AAAGTTTTGG   
  
  
- GGTTAACACT AGCGGATCTA ACTCGTTAGA TTAATCAAGA TTATTAACTT TAGTTCAAGG GACACCTTAT   
  
  
- GCTGGGCATG AACGGCACAC GATCGATGAC AATGTGGAAC GTAAACGCCA TATAAATATT GAGTTGTATA   
  
  
- CAGACGAGTG ATTTTCATGT ATAATCGAGT GGTTTTGACA CAGAGTTGCA TGATTTTATA AAATCACTTG   
  
  
- ATATGTAATA AAAATCATTC GCTTTATTCT ATGTGTTGTC GTGTAAAATT ACTCAATGTC CCGTAAAAAT   
  
  
- CACTCGGCTT TAGACTACCA AAAGAAACAT CTAGGCCAAT ATATTAACAT AAAATATGTT GGCTTACCTG   
  
  
- CTATATTGTT GACTCAATTT ACTCCTCAGA CTTTAATGCT TGTATTTTAG GTTTTCTTCT ACGGCTACGG   
  
  
- TAACCTTTCT TTCGACTTCC ACTAGCGAAA GATCATCATA ATCCGTCATC ATTAATTTTT GATTTTAGGT   
  
  
- CTTAACTGTG GCTTAACCCC AACACCGTGT ACTAGCTTTA GTCGTAGCTT AGATGTGCTA CTAGACCTAA   
  
  
- GAACCTGTGA GACCGAAATC CGGCAGTTAA AGCTCCTAAC GGGTATTGAA ATTAGGACAA GTTTAGGTAA   
  
  
- GTTAAATTAT AATGTGTTTA GGAAACTAAT TTAGTGGCAT TAACACCAAA CTAATCCCTT CAAAGAACCC   
  
  
- CCTTTCCAAG TTCGAAGACA GAAAGAATTT CGGACCTAAA AACCAGGGCG TACAAGTGAA GTGTAAGGGA   
  
  
- AAAAGAGAGG GTAGCAATGG ACAGACTTAG ACATACATTA TTTTCAGTAT AAAACGCTCC AGAAGAAAAC   
  
  
- TGACAAGAAC ATCAGTGAGA ACTATAGACG TGGTAAAACA AAAAGAGCCG TAAAAGAGGT TGAACGTAAA   
  
  
- AGATTACATT ACACTAGAAA AGACCTGTAA GTAAAACAAT TAGATGAGTG AACCCAGTTC CAAACGACTA   
  
  
- AAACCGACCA CAATTTAGGC AAGGAGAAAC CGTAAACAAG TACAAGGTGT TGACAAACTA TAGACCATAG   
  
  
- AGTCTTTGGG GCGGTAAGAA ACCATAAAGG ACTGAAAGAA CAAGTTCCCA ATAAGGATAA AGGGACTTAA   
  
  
- AAGAAACTAA GAAATCCATA AAGTTCGAAC CCCCCAAAAC AAGAAGTGAC TAAAACAGGA GAAGAAGGGA   
  
  
- ATAACCCACC AAACTAGAGG ATTTAAAGAC ACATAGACTT AGTCAGCCCC ATCTGAAACA CAAAAACCAA   
  
  
- CTAAAAAATA GTTACATAAA AGAAGGATTA CTTCCCGGAG GGCTTGGTTA TGTGTACGAA TTGAAGTTGG   
  
  
- GACAAGGAGA AGGTTCGAAA AACCTCGGAG TTTTCAGAAG CTTACGACTA TCCGTGTATC CCAGGAAGGT   
  
  
- TACGATACCA GAACTACTAC TGGGGCGTGG GAACTTACTG GGTTTAATGT AACATACAGT TAGAAGTATA   
  
  
- GGACTAGCTC TATAATGTGT CTAGGGACTG AAAGGACTAC TAACAGAGTT CAAGTAGTTA CTGTAAGAAT   
  
  
- ACCTTCTCCT AAACCTATTA GGCGGATATA GGAACGTCCT GATATTACGC GAGGTTCGGT GACTCTTCAG   
  
  
- TAACATACTA CGGGAACCTC TCCGAATAGG TCGAAGTAGA CTAGGTAACG TTAGTAGTCA ACCAGTCTCG   
  
  
- TGACTCTCGA GACTACTAGG ACTGTCTTCG TCGCCGTAAC TATTTACCCC ATTATCTATA ATGGGGTGGT   
  
  
- CGGACTTACA CCAACTTTGG TCGGCCGACT AGTTAGTCTC GGTCAACCTG GGAAGAGTTT ACAAACTACG   
  
  
- ACGACGAGTG GGGAACCTCA GATTAACAGT AAGAAAACAG AGATCATTAT TGAAACTAGT AAGATACCTA   
  
  
- CCCCAAGGTC TAAGAGGATA GTCATGTGAA AGACACTTAC TTTGTTCTTT CCTTCTTAGT CAATCATCTA   
  
  
- GTTCCTCGTC CTTCTTAGTA GTTTCTCTAC CACGTATGGA CCTTCTTTCC TCATGATTCG TTTATCGTAG   
  
  
- GTCGTTACTC CTTATACCTC TCTACCTCTC CAAACTACTA CATGACTAGA CGTCCCTTCC TTTACTACTA   
  
  
- TAAAGTGGAA CATGGGCGTT TTGGGGTCAA GTACTTCGTC TACTATTGAG CCTTGGTTTC CCCTTTAGTT   
  
  
- CTCCTAGATT TTCGTTCTCA TTTAGCGCAC AGTTCTTCTT TTCATTGCTT CCTGTTCGCC ATCTAGAATC   
  
  
- CTGAGTTGAA TAAGTCACAC GTCTTCGTCA CCGTTCGAAA CTCGAATTTT CACGTTTACT CGATGAGTCC   
  
  
- GCTTAATCCG TCGTACGAAG CGGTAAACCC CTATCACAAG TTTCCGATCG TGTAATGAAA CGCTTACCCG   
  
  
- AGCTCCGTGC GAATCGACCA TGTCCTAGTC TTAGTATGTT TCTGAAACGA CCCTTTTTTA AAAGTAGGAT   
  
  
- ACTGTAGAAC TCTCCGATGT TCAGTATACA AGTTCGGCAG GGGAAGGTCT CTTGTTGGTA CAAGAACTGT   
  
  
- TTAGTTTGTT ATCATTTCGA CCGACTCTTT CGTTGTTCCC AAGTATAGTA ACTAAAACCG TAGAAAAAAC   
  
  
- CAAAGGTCAC CGGGACGGAA CAAGTCTTAA ACAGTTCTTC TGGGGTCCCA AGAGGGTATG AGTTTTAGTG   
  
  
- CCCTCAGCTA GTAGGGGTCT CACCCAAGTC TGGTCGGGTC TTTCAACTTC TCTGTCCAGC AGCTAATCGT   
  
  
- CCGATAACAC TCTCCAAACC CCACGGGAAA CTCAAAGTAG GGTAACGAGT TTCCACCGTA TGGGATTTCG   
  
  
- GTCTCGTAAA GTTTTAACTT TCGTTACTCG AACAACACCA GTGAACAAAG ATGGTTAGTC CCTTCGAGGG   
  
  
- ACTACTCTGA CAACTACAGT TGTCGGGTTC TCTGGTACAG AACTCCGACT AGTCCCCGAA CTTGGGACTA   
  
  
- GATAAGTAGG TACCCCAATA GTTACCGTGT AAGTTACGTG GGAAGAAGCA TTGAGCCAAA TTTCTCCGTG   
  
  
- AAATGGTGAT GAGAAGAAAC AAGCTACAGA AACTACGGTG GTATCTAGCA CTTTCAGTAC TTTCTGACGA   
  
  
- CTGACTCTTG ATAAACATAC CCTTTCTACG TGAATTGTAT CATCGTACAC TCCCCTTGCT CTCCTAGCTC   
  
  
- TCTGGGCTCT GAATGTTCGT CACCGTTCAG GCCTTCGAAT CTCGACCTAA GTTCGTCCAG GGGGATCTGG   
  
  
- TTCTGTAACA TTTACCTCGT TCTCGATACC AATTCCGTTT AATGTTATTC CTAAAATACC ATCTTGTTCT   
  
  
- ATCCTTAACC TACCAGGTCC CGACCTTTCC TTCCTAAAAC TCACGGTATA GGACAACCTT TGGACGAAT

+     GATA-motif

| Site Name | Organism | Position | Strand | Matrix score. | sequence | function |
| --- | --- | --- | --- | --- | --- | --- |
| GATA-motif | Arabidopsis thaliana | 2609 | - | 7 | GATAGGA | part of a light responsive element |
| GATA-motif | Arabidopsis thaliana | 3993 | + | 7 | GATAGGA | part of a light responsive element |

>HU02G01573.1   
+ +Up\_Stream \_Len000TGAATT GGAAGTTGAT TCTACCTTTG ATTGATTCGA TTAATTCTAG GTTCTTGGTT   
  
  
+ CTAGTTCTCT CTCCTCTTGT TCTTGATTAT TTAGGTTTTG GATGTGATGT TTATTGAATT GAATGTTGCA   
  
  
+ ATTGTTGTTA TAAAGATGAT GTTAGGCTAA ATATCCTTCA ACTTAGGTTG GGATTGACGT CGAATATGTG   
  
  
+ TAGTTGAATC TTGTACTGGC CTAAATCCCT GCTCCCCCTC TGTTTTGCGA TTATGAATTT CATTATGCAC   
  
  
+ ACCACATGCT CGATGAAATG CTCGAAAGAA CATTCATCCT CTTTGTTTGG GATTTAGGGG AACGTTGAAA   
  
  
+ GACGAAGTCT TTACCTAGTG CGTCTTAGTA GACAATTAGG ATTTTAACAT GAAAATAGTT AAAATTCTAA   
  
  
+ TTGGGATTTG AGATTGTGAA TCTTGTAATC CTTCGACTTC TAGTTCTATG AAAATAGGCG ATTATAGTTC   
  
  
+ TAGGATTAGT CTTCGACCCT TGAGAGAGGG AGGAGATGGG TTTAGTTGAT AATCTATTCA TTCATAACGC   
  
  
+ ATGCCTGGGA GATGTTTGGT AGGGCTAGTA ATTGATTCGC TAACATATTT GTGCTGTTAG TTCCAACCTG   
  
  
+ AGTGTTTACT CTTCTGCTGT TTATTCCGTC TTTTAGTCGT AATTGTCTCC TTTAGTTTAA TTTCAAAACC   
  
  
+ CCAATTGTGA TCGCCTAGAT TGAGCAATCT AATTAGTTCT AATAATTGAA ATCAAGTTCC CTGTGGAATA   
  
  
+ CGACCCGTAC TTGCCGTGTG CTAGCTACTG TTACACCTTG CATTTGCGGT ATATTTATAA CTCAACATAT   
  
  
+ GTCTGCTCAC TAAAAGTACA TATTAGCTCA CCAAAACTGT GTCTCAACGT ACTAAAATAT TTTAGTGAAC   
  
  
+ TATACATTAT TTTTAGTAAG CGAAATAAGA TACACAACAG CACATTTTAA TGAGTTACAG GGCATTTTTA   
  
  
+ GTGAGCCGAA ATCTGATGGT TTTCTTTGTA GATCCGGTTA TATAATTGTA TTTTATACAA CCGAATGGAC   
  
  
+ GATATAACAA CTGAGTTAAA TGAGGAGTCT GAAATTACGA ACATAAAATC CAAAAGAAGA TGCCGATGCC   
  
  
+ ATTGGAAAGA AAGCTGAAGG TGATCGCTTT CTAGTAGTAT TAGGCAGTAG TAATTAAAAA CTAAAATCCA   
  
  
+ GAATTGACAC CGAATTGGGG TTGTGGCACA TGATCGAAAT CAGCATCGAA TCTACACGAT GATCTGGATT   
  
  
+ CTTGGACACT CTGGCTTTAG GCCGTCAATT TCGAGGATTG CCCATAACTT TAATCCTGTT CAAATCCATT   
  
  
+ CAATTTAATA TTACACAAAT CCTTTGATTA AATCACCGTA ATTGTGGTTT GATTAGGGAA GTTTCTTGGG   
  
  
+ GGAAAGGTTC AAGCTTCTGT CTTTCTTAAA GCCTGGATTT TTGGTCCCGC ATGTTCACTT CACATTCCCT   
  
  
+ TTTTCTCTCC CATCGTTACC TGTCTGAATC TGTATGTAAT AAAAGTCATA TTTTGCGAGG TCTTCTTTTG   
  
  
+ ACTGTTCTTG TAGTCACTCT TGATATCTGC ACCATTTTGT TTTTCTCGGC ATTTTCTCCA ACTTGCATTT   
  
  
+ TCTAATGTAA TGTGATCTTT TCTGGACATT CATTTTGTTA ATCTACTCAC TTGGGTCAAG GTTTGCTGAT   
  
  
+ TTTGGCTGGT GTTAAATCCG TTCCTCTTTG GCATTTGTTC ATGTTCCACA ACTGTTTGAT ATCTGGTATC   
  
  
+ TCAGAAACCC CGCCATTCTT TGGTATTTCC TGACTTTCTT GTTCAAGGGT TATTCCTATT TCCCTGAATT   
  
  
+ TTCTTTGATT CTTTAGGTAT TTCAAGCTTG GGGGGTTTTG TTCTTCACTG ATTTTGTCCT CTTCTTCCCT   
  
  
+ TATTGGGTGG TTTGATCTCC TAAATTTCTG TGTATCTGAA TCAGTCGGGG TAGACTTTGT GTTTTTGGTT   
  
  
+ GATTTTTTAT CAATGTATTT TCTTCCTAAT GAAGGGCCTC CCGAACCAAT ACACATGCTT AACTTCAACC   
  
  
+ CTGTTCCTCT TCCAAGCTTT TTGGAGCCTC AAAAGTCTTC GAATGCTGAT AGGCACATAG GGTCCTTCCA   
  
  
+ ATGCTATGGT CTTGATGATG ACCCCGCACC CTTGAATGAC CCAAATTACA TTGTATGTCA ATCTTCATAT   
  
  
+ CCTGATCGAG ATATTACACA GATCCCTGAC TTTCCTGATG ATTGTCTCAA GTTCATCAAT GACATTCTTA   
  
  
+ TGGAAGAGGA TTTGGATAAT CCGCCTATAT CCTTGCAGGA CTATAATGCG CTCCAAGCCA CTGAGAAGTC   
  
  
+ ATTGTATGAT GCCCTTGGAG AGGCTTATCC AGCTTCATCT GATCCATTGC AATCATCAGT TGGTCAGAGC   
  
  
+ ACTGAGAGCT CTGATGATCC TGACAGAAGC AGCGGCATTG ATAAATGGGG TAATAGATAT TACCCCACCA   
  
  
+ GCCTGAATGT GGTTGAAACC AGCCGGCTGA TCAATCAGAG CCAGTTGGAC CCTTCTCAAA TGTTTGATGC   
  
  
+ TGCTGCTCAC CCCTTGGAGT CTAATTGTCA TTCTTTTGTC TCTAGTAATA ACTTTGATCA TTCTATGGAT   
  
  
+ GGGGTTCCAG ATTCTCCTAT CAGTACACTT TCTGTGAATG AAACAAGAAA GGAAGAATCA GTTAGTAGAT   
  
  
+ CAAGGAGCAG GAAGAATCAT CAAAGAGATG GTGCATACCT GGAAGAAAGG AGTACTAAGC AAATAGCATC   
  
  
+ CAGCAATGAG GAATATGGAG AGATGGAGAG GTTTGATGAT GTACTGATCT GCAGGGAAGG AAATGATGAT   
  
  
+ ATTTCACCTT GTACCCGCAA AACCCCAGTT CATGAAGCAG ATGATAACTC GGAACCAAAG GGGAAATCAA   
  
  
+ GAGGATCTAA AAGCAAGAGT AAATCGCGTG TCAAGAAGAA AAGTAACGAA GGACAAGCGG TAGATCTTAG   
  
  
+ GACTCAACTT ATTCAGTGTG CAGAAGCAGT GGCAAGCTTT GAGCTTAAAA GTGCAAATGA GCTACTCAGG   
  
  
+ CGAATTAGGC AGCATGCTTC GCCATTTGGG GATAGTGTTC AAAGGCTAGC ACATTACTTT GCGAATGGGC   
  
  
+ TCGAGGCACG CTTAGCTGGT ACAGGATCAG AATCATACAA AGACTTTGCT GGGAAAAAAT TTTCATCCTA   
  
  
+ TGACATCTTG AGAGGCTACA AGTCATATGT TCAAGCCGTC CCCTTCCAGA GAACAACCAT GTTCTTGACA   
  
  
+ AATCAAACAA TAGTAAAGCT GGCTGAGAAA GCAACAAGGG TTCATATCAT TGATTTTGGC ATCTTTTTTG   
  
  
+ GTTTCCAGTG GCCCTGCCTT GTTCAGAATT TGTCAAGAAG ACCCCAGGGT TCTCCCATAC TCAAAATCAC   
  
  
+ GGGAGTCGAT CATCCCCAGA GTGGGTTCAG ACCAGCCCAG AAAGTTGAAG AGACAGGTCG TCGATTAGCA   
  
  
+ GGCTATTGTG AGAGGTTTGG GGTGCCCTTT GAGTTTCATC CCATTGCTCA AAGGTGGCAT ACCCTAAAGC   
  
  
+ CAGAGCATTT CAAAATTGAA AGCAATGAGC TTGTTGTGGT CACTTGTTTC TACCAATCAG GGAAGCTCCC   
  
  
+ TGATGAGACT GTTGATGTCA ACAGCCCAAG AGACCATGTC TTGAGGCTGA TCAGGGGCTT GAACCCTGAT   
  
  
+ CTATTCATCC ATGGGGTTAT CAATGGCACA TTCAATGCAC CCTTCTTCGT AACTCGGTTT AAAGAGGCAC   
  
  
+ TTTACCACTA CTCTTCTTTG TTCGATGTCT TTGATGCCAC CATAGATCGT GAAAGTCATG AAAGACTGCT   
  
  
+ GACTGAGAAC TATTTGTATG GGAAAGATGC ACTTAACATA GTAGCATGTG AGGGGAACGA GAGGATCGAG   
  
  
+ AGACCCGAGA CTTACAAGCA GTGGCAAGTC CGGAAGCTTA GAGCTGGATT CAAGCAGGTC CCCCTAGACC   
  
  
+ AAGACATTGT AAATGGAGCA AGAGCTATGG TTAAGGCAAA TTACAATAAG GATTTTATGG TAGAACAAGA   
  
  
+ TAGGAATTGG ATGGTCCAGG GCTGGAAAGG AAGGATTTTG AGTGCCATAT CCTGTTGGAA ACCTGCTTA  

- +Up\_Stream \_Len000ACTTAA CCTTCAACTA AGATGGAAAC TAACTAAGCT AATTAAGATC CAAGAACCAA   
  
  
- GATCAAGAGA GAGGAGAACA AGAACTAATA AATCCAAAAC CTACACTACA AATAACTTAA CTTACAACGT   
  
  
- TAACAACAAT ATTTCTACTA CAATCCGATT TATAGGAAGT TGAATCCAAC CCTAACTGCA GCTTATACAC   
  
  
- ATCAACTTAG AACATGACCG GATTTAGGGA CGAGGGGGAG ACAAAACGCT AATACTTAAA GTAATACGTG   
  
  
- TGGTGTACGA GCTACTTTAC GAGCTTTCTT GTAAGTAGGA GAAACAAACC CTAAATCCCC TTGCAACTTT   
  
  
- CTGCTTCAGA AATGGATCAC GCAGAATCAT CTGTTAATCC TAAAATTGTA CTTTTATCAA TTTTAAGATT   
  
  
- AACCCTAAAC TCTAACACTT AGAACATTAG GAAGCTGAAG ATCAAGATAC TTTTATCCGC TAATATCAAG   
  
  
- ATCCTAATCA GAAGCTGGGA ACTCTCTCCC TCCTCTACCC AAATCAACTA TTAGATAAGT AAGTATTGCG   
  
  
- TACGGACCCT CTACAAACCA TCCCGATCAT TAACTAAGCG ATTGTATAAA CACGACAATC AAGGTTGGAC   
  
  
- TCACAAATGA GAAGACGACA AATAAGGCAG AAAATCAGCA TTAACAGAGG AAATCAAATT AAAGTTTTGG   
  
  
- GGTTAACACT AGCGGATCTA ACTCGTTAGA TTAATCAAGA TTATTAACTT TAGTTCAAGG GACACCTTAT   
  
  
- GCTGGGCATG AACGGCACAC GATCGATGAC AATGTGGAAC GTAAACGCCA TATAAATATT GAGTTGTATA   
  
  
- CAGACGAGTG ATTTTCATGT ATAATCGAGT GGTTTTGACA CAGAGTTGCA TGATTTTATA AAATCACTTG   
  
  
- ATATGTAATA AAAATCATTC GCTTTATTCT ATGTGTTGTC GTGTAAAATT ACTCAATGTC CCGTAAAAAT   
  
  
- CACTCGGCTT TAGACTACCA AAAGAAACAT CTAGGCCAAT ATATTAACAT AAAATATGTT GGCTTACCTG   
  
  
- CTATATTGTT GACTCAATTT ACTCCTCAGA CTTTAATGCT TGTATTTTAG GTTTTCTTCT ACGGCTACGG   
  
  
- TAACCTTTCT TTCGACTTCC ACTAGCGAAA GATCATCATA ATCCGTCATC ATTAATTTTT GATTTTAGGT   
  
  
- CTTAACTGTG GCTTAACCCC AACACCGTGT ACTAGCTTTA GTCGTAGCTT AGATGTGCTA CTAGACCTAA   
  
  
- GAACCTGTGA GACCGAAATC CGGCAGTTAA AGCTCCTAAC GGGTATTGAA ATTAGGACAA GTTTAGGTAA   
  
  
- GTTAAATTAT AATGTGTTTA GGAAACTAAT TTAGTGGCAT TAACACCAAA CTAATCCCTT CAAAGAACCC   
  
  
- CCTTTCCAAG TTCGAAGACA GAAAGAATTT CGGACCTAAA AACCAGGGCG TACAAGTGAA GTGTAAGGGA   
  
  
- AAAAGAGAGG GTAGCAATGG ACAGACTTAG ACATACATTA TTTTCAGTAT AAAACGCTCC AGAAGAAAAC   
  
  
- TGACAAGAAC ATCAGTGAGA ACTATAGACG TGGTAAAACA AAAAGAGCCG TAAAAGAGGT TGAACGTAAA   
  
  
- AGATTACATT ACACTAGAAA AGACCTGTAA GTAAAACAAT TAGATGAGTG AACCCAGTTC CAAACGACTA   
  
  
- AAACCGACCA CAATTTAGGC AAGGAGAAAC CGTAAACAAG TACAAGGTGT TGACAAACTA TAGACCATAG   
  
  
- AGTCTTTGGG GCGGTAAGAA ACCATAAAGG ACTGAAAGAA CAAGTTCCCA ATAAGGATAA AGGGACTTAA   
  
  
- AAGAAACTAA GAAATCCATA AAGTTCGAAC CCCCCAAAAC AAGAAGTGAC TAAAACAGGA GAAGAAGGGA   
  
  
- ATAACCCACC AAACTAGAGG ATTTAAAGAC ACATAGACTT AGTCAGCCCC ATCTGAAACA CAAAAACCAA   
  
  
- CTAAAAAATA GTTACATAAA AGAAGGATTA CTTCCCGGAG GGCTTGGTTA TGTGTACGAA TTGAAGTTGG   
  
  
- GACAAGGAGA AGGTTCGAAA AACCTCGGAG TTTTCAGAAG CTTACGACTA TCCGTGTATC CCAGGAAGGT   
  
  
- TACGATACCA GAACTACTAC TGGGGCGTGG GAACTTACTG GGTTTAATGT AACATACAGT TAGAAGTATA   
  
  
- GGACTAGCTC TATAATGTGT CTAGGGACTG AAAGGACTAC TAACAGAGTT CAAGTAGTTA CTGTAAGAAT   
  
  
- ACCTTCTCCT AAACCTATTA GGCGGATATA GGAACGTCCT GATATTACGC GAGGTTCGGT GACTCTTCAG   
  
  
- TAACATACTA CGGGAACCTC TCCGAATAGG TCGAAGTAGA CTAGGTAACG TTAGTAGTCA ACCAGTCTCG   
  
  
- TGACTCTCGA GACTACTAGG ACTGTCTTCG TCGCCGTAAC TATTTACCCC ATTATCTATA ATGGGGTGGT   
  
  
- CGGACTTACA CCAACTTTGG TCGGCCGACT AGTTAGTCTC GGTCAACCTG GGAAGAGTTT ACAAACTACG   
  
  
- ACGACGAGTG GGGAACCTCA GATTAACAGT AAGAAAACAG AGATCATTAT TGAAACTAGT AAGATACCTA   
  
  
- CCCCAAGGTC TAAGAGGATA GTCATGTGAA AGACACTTAC TTTGTTCTTT CCTTCTTAGT CAATCATCTA   
  
  
- GTTCCTCGTC CTTCTTAGTA GTTTCTCTAC CACGTATGGA CCTTCTTTCC TCATGATTCG TTTATCGTAG   
  
  
- GTCGTTACTC CTTATACCTC TCTACCTCTC CAAACTACTA CATGACTAGA CGTCCCTTCC TTTACTACTA   
  
  
- TAAAGTGGAA CATGGGCGTT TTGGGGTCAA GTACTTCGTC TACTATTGAG CCTTGGTTTC CCCTTTAGTT   
  
  
- CTCCTAGATT TTCGTTCTCA TTTAGCGCAC AGTTCTTCTT TTCATTGCTT CCTGTTCGCC ATCTAGAATC   
  
  
- CTGAGTTGAA TAAGTCACAC GTCTTCGTCA CCGTTCGAAA CTCGAATTTT CACGTTTACT CGATGAGTCC   
  
  
- GCTTAATCCG TCGTACGAAG CGGTAAACCC CTATCACAAG TTTCCGATCG TGTAATGAAA CGCTTACCCG   
  
  
- AGCTCCGTGC GAATCGACCA TGTCCTAGTC TTAGTATGTT TCTGAAACGA CCCTTTTTTA AAAGTAGGAT   
  
  
- ACTGTAGAAC TCTCCGATGT TCAGTATACA AGTTCGGCAG GGGAAGGTCT CTTGTTGGTA CAAGAACTGT   
  
  
- TTAGTTTGTT ATCATTTCGA CCGACTCTTT CGTTGTTCCC AAGTATAGTA ACTAAAACCG TAGAAAAAAC   
  
  
- CAAAGGTCAC CGGGACGGAA CAAGTCTTAA ACAGTTCTTC TGGGGTCCCA AGAGGGTATG AGTTTTAGTG   
  
  
- CCCTCAGCTA GTAGGGGTCT CACCCAAGTC TGGTCGGGTC TTTCAACTTC TCTGTCCAGC AGCTAATCGT   
  
  
- CCGATAACAC TCTCCAAACC CCACGGGAAA CTCAAAGTAG GGTAACGAGT TTCCACCGTA TGGGATTTCG   
  
  
- GTCTCGTAAA GTTTTAACTT TCGTTACTCG AACAACACCA GTGAACAAAG ATGGTTAGTC CCTTCGAGGG   
  
  
- ACTACTCTGA CAACTACAGT TGTCGGGTTC TCTGGTACAG AACTCCGACT AGTCCCCGAA CTTGGGACTA   
  
  
- GATAAGTAGG TACCCCAATA GTTACCGTGT AAGTTACGTG GGAAGAAGCA TTGAGCCAAA TTTCTCCGTG   
  
  
- AAATGGTGAT GAGAAGAAAC AAGCTACAGA AACTACGGTG GTATCTAGCA CTTTCAGTAC TTTCTGACGA   
  
  
- CTGACTCTTG ATAAACATAC CCTTTCTACG TGAATTGTAT CATCGTACAC TCCCCTTGCT CTCCTAGCTC   
  
  
- TCTGGGCTCT GAATGTTCGT CACCGTTCAG GCCTTCGAAT CTCGACCTAA GTTCGTCCAG GGGGATCTGG   
  
  
- TTCTGTAACA TTTACCTCGT TCTCGATACC AATTCCGTTT AATGTTATTC CTAAAATACC ATCTTGTTCT   
  
  
- ATCCTTAACC TACCAGGTCC CGACCTTTCC TTCCTAAAAC TCACGGTATA GGACAACCTT TGGACGAAT

+     GT1-motif

| Site Name | Organism | Position | Strand | Matrix score. | sequence | function |
| --- | --- | --- | --- | --- | --- | --- |
| GT1-motif | Arabidopsis thaliana | 3953 | + | 6 | GGTTAA | light responsive element |

>HU02G01573.1   
+ +Up\_Stream \_Len000TGAATT GGAAGTTGAT TCTACCTTTG ATTGATTCGA TTAATTCTAG GTTCTTGGTT   
  
  
+ CTAGTTCTCT CTCCTCTTGT TCTTGATTAT TTAGGTTTTG GATGTGATGT TTATTGAATT GAATGTTGCA   
  
  
+ ATTGTTGTTA TAAAGATGAT GTTAGGCTAA ATATCCTTCA ACTTAGGTTG GGATTGACGT CGAATATGTG   
  
  
+ TAGTTGAATC TTGTACTGGC CTAAATCCCT GCTCCCCCTC TGTTTTGCGA TTATGAATTT CATTATGCAC   
  
  
+ ACCACATGCT CGATGAAATG CTCGAAAGAA CATTCATCCT CTTTGTTTGG GATTTAGGGG AACGTTGAAA   
  
  
+ GACGAAGTCT TTACCTAGTG CGTCTTAGTA GACAATTAGG ATTTTAACAT GAAAATAGTT AAAATTCTAA   
  
  
+ TTGGGATTTG AGATTGTGAA TCTTGTAATC CTTCGACTTC TAGTTCTATG AAAATAGGCG ATTATAGTTC   
  
  
+ TAGGATTAGT CTTCGACCCT TGAGAGAGGG AGGAGATGGG TTTAGTTGAT AATCTATTCA TTCATAACGC   
  
  
+ ATGCCTGGGA GATGTTTGGT AGGGCTAGTA ATTGATTCGC TAACATATTT GTGCTGTTAG TTCCAACCTG   
  
  
+ AGTGTTTACT CTTCTGCTGT TTATTCCGTC TTTTAGTCGT AATTGTCTCC TTTAGTTTAA TTTCAAAACC   
  
  
+ CCAATTGTGA TCGCCTAGAT TGAGCAATCT AATTAGTTCT AATAATTGAA ATCAAGTTCC CTGTGGAATA   
  
  
+ CGACCCGTAC TTGCCGTGTG CTAGCTACTG TTACACCTTG CATTTGCGGT ATATTTATAA CTCAACATAT   
  
  
+ GTCTGCTCAC TAAAAGTACA TATTAGCTCA CCAAAACTGT GTCTCAACGT ACTAAAATAT TTTAGTGAAC   
  
  
+ TATACATTAT TTTTAGTAAG CGAAATAAGA TACACAACAG CACATTTTAA TGAGTTACAG GGCATTTTTA   
  
  
+ GTGAGCCGAA ATCTGATGGT TTTCTTTGTA GATCCGGTTA TATAATTGTA TTTTATACAA CCGAATGGAC   
  
  
+ GATATAACAA CTGAGTTAAA TGAGGAGTCT GAAATTACGA ACATAAAATC CAAAAGAAGA TGCCGATGCC   
  
  
+ ATTGGAAAGA AAGCTGAAGG TGATCGCTTT CTAGTAGTAT TAGGCAGTAG TAATTAAAAA CTAAAATCCA   
  
  
+ GAATTGACAC CGAATTGGGG TTGTGGCACA TGATCGAAAT CAGCATCGAA TCTACACGAT GATCTGGATT   
  
  
+ CTTGGACACT CTGGCTTTAG GCCGTCAATT TCGAGGATTG CCCATAACTT TAATCCTGTT CAAATCCATT   
  
  
+ CAATTTAATA TTACACAAAT CCTTTGATTA AATCACCGTA ATTGTGGTTT GATTAGGGAA GTTTCTTGGG   
  
  
+ GGAAAGGTTC AAGCTTCTGT CTTTCTTAAA GCCTGGATTT TTGGTCCCGC ATGTTCACTT CACATTCCCT   
  
  
+ TTTTCTCTCC CATCGTTACC TGTCTGAATC TGTATGTAAT AAAAGTCATA TTTTGCGAGG TCTTCTTTTG   
  
  
+ ACTGTTCTTG TAGTCACTCT TGATATCTGC ACCATTTTGT TTTTCTCGGC ATTTTCTCCA ACTTGCATTT   
  
  
+ TCTAATGTAA TGTGATCTTT TCTGGACATT CATTTTGTTA ATCTACTCAC TTGGGTCAAG GTTTGCTGAT   
  
  
+ TTTGGCTGGT GTTAAATCCG TTCCTCTTTG GCATTTGTTC ATGTTCCACA ACTGTTTGAT ATCTGGTATC   
  
  
+ TCAGAAACCC CGCCATTCTT TGGTATTTCC TGACTTTCTT GTTCAAGGGT TATTCCTATT TCCCTGAATT   
  
  
+ TTCTTTGATT CTTTAGGTAT TTCAAGCTTG GGGGGTTTTG TTCTTCACTG ATTTTGTCCT CTTCTTCCCT   
  
  
+ TATTGGGTGG TTTGATCTCC TAAATTTCTG TGTATCTGAA TCAGTCGGGG TAGACTTTGT GTTTTTGGTT   
  
  
+ GATTTTTTAT CAATGTATTT TCTTCCTAAT GAAGGGCCTC CCGAACCAAT ACACATGCTT AACTTCAACC   
  
  
+ CTGTTCCTCT TCCAAGCTTT TTGGAGCCTC AAAAGTCTTC GAATGCTGAT AGGCACATAG GGTCCTTCCA   
  
  
+ ATGCTATGGT CTTGATGATG ACCCCGCACC CTTGAATGAC CCAAATTACA TTGTATGTCA ATCTTCATAT   
  
  
+ CCTGATCGAG ATATTACACA GATCCCTGAC TTTCCTGATG ATTGTCTCAA GTTCATCAAT GACATTCTTA   
  
  
+ TGGAAGAGGA TTTGGATAAT CCGCCTATAT CCTTGCAGGA CTATAATGCG CTCCAAGCCA CTGAGAAGTC   
  
  
+ ATTGTATGAT GCCCTTGGAG AGGCTTATCC AGCTTCATCT GATCCATTGC AATCATCAGT TGGTCAGAGC   
  
  
+ ACTGAGAGCT CTGATGATCC TGACAGAAGC AGCGGCATTG ATAAATGGGG TAATAGATAT TACCCCACCA   
  
  
+ GCCTGAATGT GGTTGAAACC AGCCGGCTGA TCAATCAGAG CCAGTTGGAC CCTTCTCAAA TGTTTGATGC   
  
  
+ TGCTGCTCAC CCCTTGGAGT CTAATTGTCA TTCTTTTGTC TCTAGTAATA ACTTTGATCA TTCTATGGAT   
  
  
+ GGGGTTCCAG ATTCTCCTAT CAGTACACTT TCTGTGAATG AAACAAGAAA GGAAGAATCA GTTAGTAGAT   
  
  
+ CAAGGAGCAG GAAGAATCAT CAAAGAGATG GTGCATACCT GGAAGAAAGG AGTACTAAGC AAATAGCATC   
  
  
+ CAGCAATGAG GAATATGGAG AGATGGAGAG GTTTGATGAT GTACTGATCT GCAGGGAAGG AAATGATGAT   
  
  
+ ATTTCACCTT GTACCCGCAA AACCCCAGTT CATGAAGCAG ATGATAACTC GGAACCAAAG GGGAAATCAA   
  
  
+ GAGGATCTAA AAGCAAGAGT AAATCGCGTG TCAAGAAGAA AAGTAACGAA GGACAAGCGG TAGATCTTAG   
  
  
+ GACTCAACTT ATTCAGTGTG CAGAAGCAGT GGCAAGCTTT GAGCTTAAAA GTGCAAATGA GCTACTCAGG   
  
  
+ CGAATTAGGC AGCATGCTTC GCCATTTGGG GATAGTGTTC AAAGGCTAGC ACATTACTTT GCGAATGGGC   
  
  
+ TCGAGGCACG CTTAGCTGGT ACAGGATCAG AATCATACAA AGACTTTGCT GGGAAAAAAT TTTCATCCTA   
  
  
+ TGACATCTTG AGAGGCTACA AGTCATATGT TCAAGCCGTC CCCTTCCAGA GAACAACCAT GTTCTTGACA   
  
  
+ AATCAAACAA TAGTAAAGCT GGCTGAGAAA GCAACAAGGG TTCATATCAT TGATTTTGGC ATCTTTTTTG   
  
  
+ GTTTCCAGTG GCCCTGCCTT GTTCAGAATT TGTCAAGAAG ACCCCAGGGT TCTCCCATAC TCAAAATCAC   
  
  
+ GGGAGTCGAT CATCCCCAGA GTGGGTTCAG ACCAGCCCAG AAAGTTGAAG AGACAGGTCG TCGATTAGCA   
  
  
+ GGCTATTGTG AGAGGTTTGG GGTGCCCTTT GAGTTTCATC CCATTGCTCA AAGGTGGCAT ACCCTAAAGC   
  
  
+ CAGAGCATTT CAAAATTGAA AGCAATGAGC TTGTTGTGGT CACTTGTTTC TACCAATCAG GGAAGCTCCC   
  
  
+ TGATGAGACT GTTGATGTCA ACAGCCCAAG AGACCATGTC TTGAGGCTGA TCAGGGGCTT GAACCCTGAT   
  
  
+ CTATTCATCC ATGGGGTTAT CAATGGCACA TTCAATGCAC CCTTCTTCGT AACTCGGTTT AAAGAGGCAC   
  
  
+ TTTACCACTA CTCTTCTTTG TTCGATGTCT TTGATGCCAC CATAGATCGT GAAAGTCATG AAAGACTGCT   
  
  
+ GACTGAGAAC TATTTGTATG GGAAAGATGC ACTTAACATA GTAGCATGTG AGGGGAACGA GAGGATCGAG   
  
  
+ AGACCCGAGA CTTACAAGCA GTGGCAAGTC CGGAAGCTTA GAGCTGGATT CAAGCAGGTC CCCCTAGACC   
  
  
+ AAGACATTGT AAATGGAGCA AGAGCTATGG TTAAGGCAAA TTACAATAAG GATTTTATGG TAGAACAAGA   
  
  
+ TAGGAATTGG ATGGTCCAGG GCTGGAAAGG AAGGATTTTG AGTGCCATAT CCTGTTGGAA ACCTGCTTA  

- +Up\_Stream \_Len000ACTTAA CCTTCAACTA AGATGGAAAC TAACTAAGCT AATTAAGATC CAAGAACCAA   
  
  
- GATCAAGAGA GAGGAGAACA AGAACTAATA AATCCAAAAC CTACACTACA AATAACTTAA CTTACAACGT   
  
  
- TAACAACAAT ATTTCTACTA CAATCCGATT TATAGGAAGT TGAATCCAAC CCTAACTGCA GCTTATACAC   
  
  
- ATCAACTTAG AACATGACCG GATTTAGGGA CGAGGGGGAG ACAAAACGCT AATACTTAAA GTAATACGTG   
  
  
- TGGTGTACGA GCTACTTTAC GAGCTTTCTT GTAAGTAGGA GAAACAAACC CTAAATCCCC TTGCAACTTT   
  
  
- CTGCTTCAGA AATGGATCAC GCAGAATCAT CTGTTAATCC TAAAATTGTA CTTTTATCAA TTTTAAGATT   
  
  
- AACCCTAAAC TCTAACACTT AGAACATTAG GAAGCTGAAG ATCAAGATAC TTTTATCCGC TAATATCAAG   
  
  
- ATCCTAATCA GAAGCTGGGA ACTCTCTCCC TCCTCTACCC AAATCAACTA TTAGATAAGT AAGTATTGCG   
  
  
- TACGGACCCT CTACAAACCA TCCCGATCAT TAACTAAGCG ATTGTATAAA CACGACAATC AAGGTTGGAC   
  
  
- TCACAAATGA GAAGACGACA AATAAGGCAG AAAATCAGCA TTAACAGAGG AAATCAAATT AAAGTTTTGG   
  
  
- GGTTAACACT AGCGGATCTA ACTCGTTAGA TTAATCAAGA TTATTAACTT TAGTTCAAGG GACACCTTAT   
  
  
- GCTGGGCATG AACGGCACAC GATCGATGAC AATGTGGAAC GTAAACGCCA TATAAATATT GAGTTGTATA   
  
  
- CAGACGAGTG ATTTTCATGT ATAATCGAGT GGTTTTGACA CAGAGTTGCA TGATTTTATA AAATCACTTG   
  
  
- ATATGTAATA AAAATCATTC GCTTTATTCT ATGTGTTGTC GTGTAAAATT ACTCAATGTC CCGTAAAAAT   
  
  
- CACTCGGCTT TAGACTACCA AAAGAAACAT CTAGGCCAAT ATATTAACAT AAAATATGTT GGCTTACCTG   
  
  
- CTATATTGTT GACTCAATTT ACTCCTCAGA CTTTAATGCT TGTATTTTAG GTTTTCTTCT ACGGCTACGG   
  
  
- TAACCTTTCT TTCGACTTCC ACTAGCGAAA GATCATCATA ATCCGTCATC ATTAATTTTT GATTTTAGGT   
  
  
- CTTAACTGTG GCTTAACCCC AACACCGTGT ACTAGCTTTA GTCGTAGCTT AGATGTGCTA CTAGACCTAA   
  
  
- GAACCTGTGA GACCGAAATC CGGCAGTTAA AGCTCCTAAC GGGTATTGAA ATTAGGACAA GTTTAGGTAA   
  
  
- GTTAAATTAT AATGTGTTTA GGAAACTAAT TTAGTGGCAT TAACACCAAA CTAATCCCTT CAAAGAACCC   
  
  
- CCTTTCCAAG TTCGAAGACA GAAAGAATTT CGGACCTAAA AACCAGGGCG TACAAGTGAA GTGTAAGGGA   
  
  
- AAAAGAGAGG GTAGCAATGG ACAGACTTAG ACATACATTA TTTTCAGTAT AAAACGCTCC AGAAGAAAAC   
  
  
- TGACAAGAAC ATCAGTGAGA ACTATAGACG TGGTAAAACA AAAAGAGCCG TAAAAGAGGT TGAACGTAAA   
  
  
- AGATTACATT ACACTAGAAA AGACCTGTAA GTAAAACAAT TAGATGAGTG AACCCAGTTC CAAACGACTA   
  
  
- AAACCGACCA CAATTTAGGC AAGGAGAAAC CGTAAACAAG TACAAGGTGT TGACAAACTA TAGACCATAG   
  
  
- AGTCTTTGGG GCGGTAAGAA ACCATAAAGG ACTGAAAGAA CAAGTTCCCA ATAAGGATAA AGGGACTTAA   
  
  
- AAGAAACTAA GAAATCCATA AAGTTCGAAC CCCCCAAAAC AAGAAGTGAC TAAAACAGGA GAAGAAGGGA   
  
  
- ATAACCCACC AAACTAGAGG ATTTAAAGAC ACATAGACTT AGTCAGCCCC ATCTGAAACA CAAAAACCAA   
  
  
- CTAAAAAATA GTTACATAAA AGAAGGATTA CTTCCCGGAG GGCTTGGTTA TGTGTACGAA TTGAAGTTGG   
  
  
- GACAAGGAGA AGGTTCGAAA AACCTCGGAG TTTTCAGAAG CTTACGACTA TCCGTGTATC CCAGGAAGGT   
  
  
- TACGATACCA GAACTACTAC TGGGGCGTGG GAACTTACTG GGTTTAATGT AACATACAGT TAGAAGTATA   
  
  
- GGACTAGCTC TATAATGTGT CTAGGGACTG AAAGGACTAC TAACAGAGTT CAAGTAGTTA CTGTAAGAAT   
  
  
- ACCTTCTCCT AAACCTATTA GGCGGATATA GGAACGTCCT GATATTACGC GAGGTTCGGT GACTCTTCAG   
  
  
- TAACATACTA CGGGAACCTC TCCGAATAGG TCGAAGTAGA CTAGGTAACG TTAGTAGTCA ACCAGTCTCG   
  
  
- TGACTCTCGA GACTACTAGG ACTGTCTTCG TCGCCGTAAC TATTTACCCC ATTATCTATA ATGGGGTGGT   
  
  
- CGGACTTACA CCAACTTTGG TCGGCCGACT AGTTAGTCTC GGTCAACCTG GGAAGAGTTT ACAAACTACG   
  
  
- ACGACGAGTG GGGAACCTCA GATTAACAGT AAGAAAACAG AGATCATTAT TGAAACTAGT AAGATACCTA   
  
  
- CCCCAAGGTC TAAGAGGATA GTCATGTGAA AGACACTTAC TTTGTTCTTT CCTTCTTAGT CAATCATCTA   
  
  
- GTTCCTCGTC CTTCTTAGTA GTTTCTCTAC CACGTATGGA CCTTCTTTCC TCATGATTCG TTTATCGTAG   
  
  
- GTCGTTACTC CTTATACCTC TCTACCTCTC CAAACTACTA CATGACTAGA CGTCCCTTCC TTTACTACTA   
  
  
- TAAAGTGGAA CATGGGCGTT TTGGGGTCAA GTACTTCGTC TACTATTGAG CCTTGGTTTC CCCTTTAGTT   
  
  
- CTCCTAGATT TTCGTTCTCA TTTAGCGCAC AGTTCTTCTT TTCATTGCTT CCTGTTCGCC ATCTAGAATC   
  
  
- CTGAGTTGAA TAAGTCACAC GTCTTCGTCA CCGTTCGAAA CTCGAATTTT CACGTTTACT CGATGAGTCC   
  
  
- GCTTAATCCG TCGTACGAAG CGGTAAACCC CTATCACAAG TTTCCGATCG TGTAATGAAA CGCTTACCCG   
  
  
- AGCTCCGTGC GAATCGACCA TGTCCTAGTC TTAGTATGTT TCTGAAACGA CCCTTTTTTA AAAGTAGGAT   
  
  
- ACTGTAGAAC TCTCCGATGT TCAGTATACA AGTTCGGCAG GGGAAGGTCT CTTGTTGGTA CAAGAACTGT   
  
  
- TTAGTTTGTT ATCATTTCGA CCGACTCTTT CGTTGTTCCC AAGTATAGTA ACTAAAACCG TAGAAAAAAC   
  
  
- CAAAGGTCAC CGGGACGGAA CAAGTCTTAA ACAGTTCTTC TGGGGTCCCA AGAGGGTATG AGTTTTAGTG   
  
  
- CCCTCAGCTA GTAGGGGTCT CACCCAAGTC TGGTCGGGTC TTTCAACTTC TCTGTCCAGC AGCTAATCGT   
  
  
- CCGATAACAC TCTCCAAACC CCACGGGAAA CTCAAAGTAG GGTAACGAGT TTCCACCGTA TGGGATTTCG   
  
  
- GTCTCGTAAA GTTTTAACTT TCGTTACTCG AACAACACCA GTGAACAAAG ATGGTTAGTC CCTTCGAGGG   
  
  
- ACTACTCTGA CAACTACAGT TGTCGGGTTC TCTGGTACAG AACTCCGACT AGTCCCCGAA CTTGGGACTA   
  
  
- GATAAGTAGG TACCCCAATA GTTACCGTGT AAGTTACGTG GGAAGAAGCA TTGAGCCAAA TTTCTCCGTG   
  
  
- AAATGGTGAT GAGAAGAAAC AAGCTACAGA AACTACGGTG GTATCTAGCA CTTTCAGTAC TTTCTGACGA   
  
  
- CTGACTCTTG ATAAACATAC CCTTTCTACG TGAATTGTAT CATCGTACAC TCCCCTTGCT CTCCTAGCTC   
  
  
- TCTGGGCTCT GAATGTTCGT CACCGTTCAG GCCTTCGAAT CTCGACCTAA GTTCGTCCAG GGGGATCTGG   
  
  
- TTCTGTAACA TTTACCTCGT TCTCGATACC AATTCCGTTT AATGTTATTC CTAAAATACC ATCTTGTTCT   
  
  
- ATCCTTAACC TACCAGGTCC CGACCTTTCC TTCCTAAAAC TCACGGTATA GGACAACCTT TGGACGAAT

+     LTR

| Site Name | Organism | Position | Strand | Matrix score. | sequence | function |
| --- | --- | --- | --- | --- | --- | --- |
| LTR | Hordeum vulgare | 990 | + | 6 | CCGAAA | cis-acting element involved in low-temperature responsiveness |

>HU02G01573.1   
+ +Up\_Stream \_Len000TGAATT GGAAGTTGAT TCTACCTTTG ATTGATTCGA TTAATTCTAG GTTCTTGGTT   
  
  
+ CTAGTTCTCT CTCCTCTTGT TCTTGATTAT TTAGGTTTTG GATGTGATGT TTATTGAATT GAATGTTGCA   
  
  
+ ATTGTTGTTA TAAAGATGAT GTTAGGCTAA ATATCCTTCA ACTTAGGTTG GGATTGACGT CGAATATGTG   
  
  
+ TAGTTGAATC TTGTACTGGC CTAAATCCCT GCTCCCCCTC TGTTTTGCGA TTATGAATTT CATTATGCAC   
  
  
+ ACCACATGCT CGATGAAATG CTCGAAAGAA CATTCATCCT CTTTGTTTGG GATTTAGGGG AACGTTGAAA   
  
  
+ GACGAAGTCT TTACCTAGTG CGTCTTAGTA GACAATTAGG ATTTTAACAT GAAAATAGTT AAAATTCTAA   
  
  
+ TTGGGATTTG AGATTGTGAA TCTTGTAATC CTTCGACTTC TAGTTCTATG AAAATAGGCG ATTATAGTTC   
  
  
+ TAGGATTAGT CTTCGACCCT TGAGAGAGGG AGGAGATGGG TTTAGTTGAT AATCTATTCA TTCATAACGC   
  
  
+ ATGCCTGGGA GATGTTTGGT AGGGCTAGTA ATTGATTCGC TAACATATTT GTGCTGTTAG TTCCAACCTG   
  
  
+ AGTGTTTACT CTTCTGCTGT TTATTCCGTC TTTTAGTCGT AATTGTCTCC TTTAGTTTAA TTTCAAAACC   
  
  
+ CCAATTGTGA TCGCCTAGAT TGAGCAATCT AATTAGTTCT AATAATTGAA ATCAAGTTCC CTGTGGAATA   
  
  
+ CGACCCGTAC TTGCCGTGTG CTAGCTACTG TTACACCTTG CATTTGCGGT ATATTTATAA CTCAACATAT   
  
  
+ GTCTGCTCAC TAAAAGTACA TATTAGCTCA CCAAAACTGT GTCTCAACGT ACTAAAATAT TTTAGTGAAC   
  
  
+ TATACATTAT TTTTAGTAAG CGAAATAAGA TACACAACAG CACATTTTAA TGAGTTACAG GGCATTTTTA   
  
  
+ GTGAGCCGAA ATCTGATGGT TTTCTTTGTA GATCCGGTTA TATAATTGTA TTTTATACAA CCGAATGGAC   
  
  
+ GATATAACAA CTGAGTTAAA TGAGGAGTCT GAAATTACGA ACATAAAATC CAAAAGAAGA TGCCGATGCC   
  
  
+ ATTGGAAAGA AAGCTGAAGG TGATCGCTTT CTAGTAGTAT TAGGCAGTAG TAATTAAAAA CTAAAATCCA   
  
  
+ GAATTGACAC CGAATTGGGG TTGTGGCACA TGATCGAAAT CAGCATCGAA TCTACACGAT GATCTGGATT   
  
  
+ CTTGGACACT CTGGCTTTAG GCCGTCAATT TCGAGGATTG CCCATAACTT TAATCCTGTT CAAATCCATT   
  
  
+ CAATTTAATA TTACACAAAT CCTTTGATTA AATCACCGTA ATTGTGGTTT GATTAGGGAA GTTTCTTGGG   
  
  
+ GGAAAGGTTC AAGCTTCTGT CTTTCTTAAA GCCTGGATTT TTGGTCCCGC ATGTTCACTT CACATTCCCT   
  
  
+ TTTTCTCTCC CATCGTTACC TGTCTGAATC TGTATGTAAT AAAAGTCATA TTTTGCGAGG TCTTCTTTTG   
  
  
+ ACTGTTCTTG TAGTCACTCT TGATATCTGC ACCATTTTGT TTTTCTCGGC ATTTTCTCCA ACTTGCATTT   
  
  
+ TCTAATGTAA TGTGATCTTT TCTGGACATT CATTTTGTTA ATCTACTCAC TTGGGTCAAG GTTTGCTGAT   
  
  
+ TTTGGCTGGT GTTAAATCCG TTCCTCTTTG GCATTTGTTC ATGTTCCACA ACTGTTTGAT ATCTGGTATC   
  
  
+ TCAGAAACCC CGCCATTCTT TGGTATTTCC TGACTTTCTT GTTCAAGGGT TATTCCTATT TCCCTGAATT   
  
  
+ TTCTTTGATT CTTTAGGTAT TTCAAGCTTG GGGGGTTTTG TTCTTCACTG ATTTTGTCCT CTTCTTCCCT   
  
  
+ TATTGGGTGG TTTGATCTCC TAAATTTCTG TGTATCTGAA TCAGTCGGGG TAGACTTTGT GTTTTTGGTT   
  
  
+ GATTTTTTAT CAATGTATTT TCTTCCTAAT GAAGGGCCTC CCGAACCAAT ACACATGCTT AACTTCAACC   
  
  
+ CTGTTCCTCT TCCAAGCTTT TTGGAGCCTC AAAAGTCTTC GAATGCTGAT AGGCACATAG GGTCCTTCCA   
  
  
+ ATGCTATGGT CTTGATGATG ACCCCGCACC CTTGAATGAC CCAAATTACA TTGTATGTCA ATCTTCATAT   
  
  
+ CCTGATCGAG ATATTACACA GATCCCTGAC TTTCCTGATG ATTGTCTCAA GTTCATCAAT GACATTCTTA   
  
  
+ TGGAAGAGGA TTTGGATAAT CCGCCTATAT CCTTGCAGGA CTATAATGCG CTCCAAGCCA CTGAGAAGTC   
  
  
+ ATTGTATGAT GCCCTTGGAG AGGCTTATCC AGCTTCATCT GATCCATTGC AATCATCAGT TGGTCAGAGC   
  
  
+ ACTGAGAGCT CTGATGATCC TGACAGAAGC AGCGGCATTG ATAAATGGGG TAATAGATAT TACCCCACCA   
  
  
+ GCCTGAATGT GGTTGAAACC AGCCGGCTGA TCAATCAGAG CCAGTTGGAC CCTTCTCAAA TGTTTGATGC   
  
  
+ TGCTGCTCAC CCCTTGGAGT CTAATTGTCA TTCTTTTGTC TCTAGTAATA ACTTTGATCA TTCTATGGAT   
  
  
+ GGGGTTCCAG ATTCTCCTAT CAGTACACTT TCTGTGAATG AAACAAGAAA GGAAGAATCA GTTAGTAGAT   
  
  
+ CAAGGAGCAG GAAGAATCAT CAAAGAGATG GTGCATACCT GGAAGAAAGG AGTACTAAGC AAATAGCATC   
  
  
+ CAGCAATGAG GAATATGGAG AGATGGAGAG GTTTGATGAT GTACTGATCT GCAGGGAAGG AAATGATGAT   
  
  
+ ATTTCACCTT GTACCCGCAA AACCCCAGTT CATGAAGCAG ATGATAACTC GGAACCAAAG GGGAAATCAA   
  
  
+ GAGGATCTAA AAGCAAGAGT AAATCGCGTG TCAAGAAGAA AAGTAACGAA GGACAAGCGG TAGATCTTAG   
  
  
+ GACTCAACTT ATTCAGTGTG CAGAAGCAGT GGCAAGCTTT GAGCTTAAAA GTGCAAATGA GCTACTCAGG   
  
  
+ CGAATTAGGC AGCATGCTTC GCCATTTGGG GATAGTGTTC AAAGGCTAGC ACATTACTTT GCGAATGGGC   
  
  
+ TCGAGGCACG CTTAGCTGGT ACAGGATCAG AATCATACAA AGACTTTGCT GGGAAAAAAT TTTCATCCTA   
  
  
+ TGACATCTTG AGAGGCTACA AGTCATATGT TCAAGCCGTC CCCTTCCAGA GAACAACCAT GTTCTTGACA   
  
  
+ AATCAAACAA TAGTAAAGCT GGCTGAGAAA GCAACAAGGG TTCATATCAT TGATTTTGGC ATCTTTTTTG   
  
  
+ GTTTCCAGTG GCCCTGCCTT GTTCAGAATT TGTCAAGAAG ACCCCAGGGT TCTCCCATAC TCAAAATCAC   
  
  
+ GGGAGTCGAT CATCCCCAGA GTGGGTTCAG ACCAGCCCAG AAAGTTGAAG AGACAGGTCG TCGATTAGCA   
  
  
+ GGCTATTGTG AGAGGTTTGG GGTGCCCTTT GAGTTTCATC CCATTGCTCA AAGGTGGCAT ACCCTAAAGC   
  
  
+ CAGAGCATTT CAAAATTGAA AGCAATGAGC TTGTTGTGGT CACTTGTTTC TACCAATCAG GGAAGCTCCC   
  
  
+ TGATGAGACT GTTGATGTCA ACAGCCCAAG AGACCATGTC TTGAGGCTGA TCAGGGGCTT GAACCCTGAT   
  
  
+ CTATTCATCC ATGGGGTTAT CAATGGCACA TTCAATGCAC CCTTCTTCGT AACTCGGTTT AAAGAGGCAC   
  
  
+ TTTACCACTA CTCTTCTTTG TTCGATGTCT TTGATGCCAC CATAGATCGT GAAAGTCATG AAAGACTGCT   
  
  
+ GACTGAGAAC TATTTGTATG GGAAAGATGC ACTTAACATA GTAGCATGTG AGGGGAACGA GAGGATCGAG   
  
  
+ AGACCCGAGA CTTACAAGCA GTGGCAAGTC CGGAAGCTTA GAGCTGGATT CAAGCAGGTC CCCCTAGACC   
  
  
+ AAGACATTGT AAATGGAGCA AGAGCTATGG TTAAGGCAAA TTACAATAAG GATTTTATGG TAGAACAAGA   
  
  
+ TAGGAATTGG ATGGTCCAGG GCTGGAAAGG AAGGATTTTG AGTGCCATAT CCTGTTGGAA ACCTGCTTA  

- +Up\_Stream \_Len000ACTTAA CCTTCAACTA AGATGGAAAC TAACTAAGCT AATTAAGATC CAAGAACCAA   
  
  
- GATCAAGAGA GAGGAGAACA AGAACTAATA AATCCAAAAC CTACACTACA AATAACTTAA CTTACAACGT   
  
  
- TAACAACAAT ATTTCTACTA CAATCCGATT TATAGGAAGT TGAATCCAAC CCTAACTGCA GCTTATACAC   
  
  
- ATCAACTTAG AACATGACCG GATTTAGGGA CGAGGGGGAG ACAAAACGCT AATACTTAAA GTAATACGTG   
  
  
- TGGTGTACGA GCTACTTTAC GAGCTTTCTT GTAAGTAGGA GAAACAAACC CTAAATCCCC TTGCAACTTT   
  
  
- CTGCTTCAGA AATGGATCAC GCAGAATCAT CTGTTAATCC TAAAATTGTA CTTTTATCAA TTTTAAGATT   
  
  
- AACCCTAAAC TCTAACACTT AGAACATTAG GAAGCTGAAG ATCAAGATAC TTTTATCCGC TAATATCAAG   
  
  
- ATCCTAATCA GAAGCTGGGA ACTCTCTCCC TCCTCTACCC AAATCAACTA TTAGATAAGT AAGTATTGCG   
  
  
- TACGGACCCT CTACAAACCA TCCCGATCAT TAACTAAGCG ATTGTATAAA CACGACAATC AAGGTTGGAC   
  
  
- TCACAAATGA GAAGACGACA AATAAGGCAG AAAATCAGCA TTAACAGAGG AAATCAAATT AAAGTTTTGG   
  
  
- GGTTAACACT AGCGGATCTA ACTCGTTAGA TTAATCAAGA TTATTAACTT TAGTTCAAGG GACACCTTAT   
  
  
- GCTGGGCATG AACGGCACAC GATCGATGAC AATGTGGAAC GTAAACGCCA TATAAATATT GAGTTGTATA   
  
  
- CAGACGAGTG ATTTTCATGT ATAATCGAGT GGTTTTGACA CAGAGTTGCA TGATTTTATA AAATCACTTG   
  
  
- ATATGTAATA AAAATCATTC GCTTTATTCT ATGTGTTGTC GTGTAAAATT ACTCAATGTC CCGTAAAAAT   
  
  
- CACTCGGCTT TAGACTACCA AAAGAAACAT CTAGGCCAAT ATATTAACAT AAAATATGTT GGCTTACCTG   
  
  
- CTATATTGTT GACTCAATTT ACTCCTCAGA CTTTAATGCT TGTATTTTAG GTTTTCTTCT ACGGCTACGG   
  
  
- TAACCTTTCT TTCGACTTCC ACTAGCGAAA GATCATCATA ATCCGTCATC ATTAATTTTT GATTTTAGGT   
  
  
- CTTAACTGTG GCTTAACCCC AACACCGTGT ACTAGCTTTA GTCGTAGCTT AGATGTGCTA CTAGACCTAA   
  
  
- GAACCTGTGA GACCGAAATC CGGCAGTTAA AGCTCCTAAC GGGTATTGAA ATTAGGACAA GTTTAGGTAA   
  
  
- GTTAAATTAT AATGTGTTTA GGAAACTAAT TTAGTGGCAT TAACACCAAA CTAATCCCTT CAAAGAACCC   
  
  
- CCTTTCCAAG TTCGAAGACA GAAAGAATTT CGGACCTAAA AACCAGGGCG TACAAGTGAA GTGTAAGGGA   
  
  
- AAAAGAGAGG GTAGCAATGG ACAGACTTAG ACATACATTA TTTTCAGTAT AAAACGCTCC AGAAGAAAAC   
  
  
- TGACAAGAAC ATCAGTGAGA ACTATAGACG TGGTAAAACA AAAAGAGCCG TAAAAGAGGT TGAACGTAAA   
  
  
- AGATTACATT ACACTAGAAA AGACCTGTAA GTAAAACAAT TAGATGAGTG AACCCAGTTC CAAACGACTA   
  
  
- AAACCGACCA CAATTTAGGC AAGGAGAAAC CGTAAACAAG TACAAGGTGT TGACAAACTA TAGACCATAG   
  
  
- AGTCTTTGGG GCGGTAAGAA ACCATAAAGG ACTGAAAGAA CAAGTTCCCA ATAAGGATAA AGGGACTTAA   
  
  
- AAGAAACTAA GAAATCCATA AAGTTCGAAC CCCCCAAAAC AAGAAGTGAC TAAAACAGGA GAAGAAGGGA   
  
  
- ATAACCCACC AAACTAGAGG ATTTAAAGAC ACATAGACTT AGTCAGCCCC ATCTGAAACA CAAAAACCAA   
  
  
- CTAAAAAATA GTTACATAAA AGAAGGATTA CTTCCCGGAG GGCTTGGTTA TGTGTACGAA TTGAAGTTGG   
  
  
- GACAAGGAGA AGGTTCGAAA AACCTCGGAG TTTTCAGAAG CTTACGACTA TCCGTGTATC CCAGGAAGGT   
  
  
- TACGATACCA GAACTACTAC TGGGGCGTGG GAACTTACTG GGTTTAATGT AACATACAGT TAGAAGTATA   
  
  
- GGACTAGCTC TATAATGTGT CTAGGGACTG AAAGGACTAC TAACAGAGTT CAAGTAGTTA CTGTAAGAAT   
  
  
- ACCTTCTCCT AAACCTATTA GGCGGATATA GGAACGTCCT GATATTACGC GAGGTTCGGT GACTCTTCAG   
  
  
- TAACATACTA CGGGAACCTC TCCGAATAGG TCGAAGTAGA CTAGGTAACG TTAGTAGTCA ACCAGTCTCG   
  
  
- TGACTCTCGA GACTACTAGG ACTGTCTTCG TCGCCGTAAC TATTTACCCC ATTATCTATA ATGGGGTGGT   
  
  
- CGGACTTACA CCAACTTTGG TCGGCCGACT AGTTAGTCTC GGTCAACCTG GGAAGAGTTT ACAAACTACG   
  
  
- ACGACGAGTG GGGAACCTCA GATTAACAGT AAGAAAACAG AGATCATTAT TGAAACTAGT AAGATACCTA   
  
  
- CCCCAAGGTC TAAGAGGATA GTCATGTGAA AGACACTTAC TTTGTTCTTT CCTTCTTAGT CAATCATCTA   
  
  
- GTTCCTCGTC CTTCTTAGTA GTTTCTCTAC CACGTATGGA CCTTCTTTCC TCATGATTCG TTTATCGTAG   
  
  
- GTCGTTACTC CTTATACCTC TCTACCTCTC CAAACTACTA CATGACTAGA CGTCCCTTCC TTTACTACTA   
  
  
- TAAAGTGGAA CATGGGCGTT TTGGGGTCAA GTACTTCGTC TACTATTGAG CCTTGGTTTC CCCTTTAGTT   
  
  
- CTCCTAGATT TTCGTTCTCA TTTAGCGCAC AGTTCTTCTT TTCATTGCTT CCTGTTCGCC ATCTAGAATC   
  
  
- CTGAGTTGAA TAAGTCACAC GTCTTCGTCA CCGTTCGAAA CTCGAATTTT CACGTTTACT CGATGAGTCC   
  
  
- GCTTAATCCG TCGTACGAAG CGGTAAACCC CTATCACAAG TTTCCGATCG TGTAATGAAA CGCTTACCCG   
  
  
- AGCTCCGTGC GAATCGACCA TGTCCTAGTC TTAGTATGTT TCTGAAACGA CCCTTTTTTA AAAGTAGGAT   
  
  
- ACTGTAGAAC TCTCCGATGT TCAGTATACA AGTTCGGCAG GGGAAGGTCT CTTGTTGGTA CAAGAACTGT   
  
  
- TTAGTTTGTT ATCATTTCGA CCGACTCTTT CGTTGTTCCC AAGTATAGTA ACTAAAACCG TAGAAAAAAC   
  
  
- CAAAGGTCAC CGGGACGGAA CAAGTCTTAA ACAGTTCTTC TGGGGTCCCA AGAGGGTATG AGTTTTAGTG   
  
  
- CCCTCAGCTA GTAGGGGTCT CACCCAAGTC TGGTCGGGTC TTTCAACTTC TCTGTCCAGC AGCTAATCGT   
  
  
- CCGATAACAC TCTCCAAACC CCACGGGAAA CTCAAAGTAG GGTAACGAGT TTCCACCGTA TGGGATTTCG   
  
  
- GTCTCGTAAA GTTTTAACTT TCGTTACTCG AACAACACCA GTGAACAAAG ATGGTTAGTC CCTTCGAGGG   
  
  
- ACTACTCTGA CAACTACAGT TGTCGGGTTC TCTGGTACAG AACTCCGACT AGTCCCCGAA CTTGGGACTA   
  
  
- GATAAGTAGG TACCCCAATA GTTACCGTGT AAGTTACGTG GGAAGAAGCA TTGAGCCAAA TTTCTCCGTG   
  
  
- AAATGGTGAT GAGAAGAAAC AAGCTACAGA AACTACGGTG GTATCTAGCA CTTTCAGTAC TTTCTGACGA   
  
  
- CTGACTCTTG ATAAACATAC CCTTTCTACG TGAATTGTAT CATCGTACAC TCCCCTTGCT CTCCTAGCTC   
  
  
- TCTGGGCTCT GAATGTTCGT CACCGTTCAG GCCTTCGAAT CTCGACCTAA GTTCGTCCAG GGGGATCTGG   
  
  
- TTCTGTAACA TTTACCTCGT TCTCGATACC AATTCCGTTT AATGTTATTC CTAAAATACC ATCTTGTTCT   
  
  
- ATCCTTAACC TACCAGGTCC CGACCTTTCC TTCCTAAAAC TCACGGTATA GGACAACCTT TGGACGAAT

+     MBS

| Site Name | Organism | Position | Strand | Matrix score. | sequence | function |
| --- | --- | --- | --- | --- | --- | --- |
| MBS | Arabidopsis thaliana | 2496 | - | 6 | CAACTG | MYB binding site involved in drought-inducibility |
| MBS | Arabidopsis thaliana | 1733 | + | 6 | CAACTG | MYB binding site involved in drought-inducibility |
| MBS | Arabidopsis thaliana | 2371 | - | 6 | CAACTG | MYB binding site involved in drought-inducibility |
| MBS | Arabidopsis thaliana | 1062 | + | 6 | CAACTG | MYB binding site involved in drought-inducibility |

>HU02G01573.1   
+ +Up\_Stream \_Len000TGAATT GGAAGTTGAT TCTACCTTTG ATTGATTCGA TTAATTCTAG GTTCTTGGTT   
  
  
+ CTAGTTCTCT CTCCTCTTGT TCTTGATTAT TTAGGTTTTG GATGTGATGT TTATTGAATT GAATGTTGCA   
  
  
+ ATTGTTGTTA TAAAGATGAT GTTAGGCTAA ATATCCTTCA ACTTAGGTTG GGATTGACGT CGAATATGTG   
  
  
+ TAGTTGAATC TTGTACTGGC CTAAATCCCT GCTCCCCCTC TGTTTTGCGA TTATGAATTT CATTATGCAC   
  
  
+ ACCACATGCT CGATGAAATG CTCGAAAGAA CATTCATCCT CTTTGTTTGG GATTTAGGGG AACGTTGAAA   
  
  
+ GACGAAGTCT TTACCTAGTG CGTCTTAGTA GACAATTAGG ATTTTAACAT GAAAATAGTT AAAATTCTAA   
  
  
+ TTGGGATTTG AGATTGTGAA TCTTGTAATC CTTCGACTTC TAGTTCTATG AAAATAGGCG ATTATAGTTC   
  
  
+ TAGGATTAGT CTTCGACCCT TGAGAGAGGG AGGAGATGGG TTTAGTTGAT AATCTATTCA TTCATAACGC   
  
  
+ ATGCCTGGGA GATGTTTGGT AGGGCTAGTA ATTGATTCGC TAACATATTT GTGCTGTTAG TTCCAACCTG   
  
  
+ AGTGTTTACT CTTCTGCTGT TTATTCCGTC TTTTAGTCGT AATTGTCTCC TTTAGTTTAA TTTCAAAACC   
  
  
+ CCAATTGTGA TCGCCTAGAT TGAGCAATCT AATTAGTTCT AATAATTGAA ATCAAGTTCC CTGTGGAATA   
  
  
+ CGACCCGTAC TTGCCGTGTG CTAGCTACTG TTACACCTTG CATTTGCGGT ATATTTATAA CTCAACATAT   
  
  
+ GTCTGCTCAC TAAAAGTACA TATTAGCTCA CCAAAACTGT GTCTCAACGT ACTAAAATAT TTTAGTGAAC   
  
  
+ TATACATTAT TTTTAGTAAG CGAAATAAGA TACACAACAG CACATTTTAA TGAGTTACAG GGCATTTTTA   
  
  
+ GTGAGCCGAA ATCTGATGGT TTTCTTTGTA GATCCGGTTA TATAATTGTA TTTTATACAA CCGAATGGAC   
  
  
+ GATATAACAA CTGAGTTAAA TGAGGAGTCT GAAATTACGA ACATAAAATC CAAAAGAAGA TGCCGATGCC   
  
  
+ ATTGGAAAGA AAGCTGAAGG TGATCGCTTT CTAGTAGTAT TAGGCAGTAG TAATTAAAAA CTAAAATCCA   
  
  
+ GAATTGACAC CGAATTGGGG TTGTGGCACA TGATCGAAAT CAGCATCGAA TCTACACGAT GATCTGGATT   
  
  
+ CTTGGACACT CTGGCTTTAG GCCGTCAATT TCGAGGATTG CCCATAACTT TAATCCTGTT CAAATCCATT   
  
  
+ CAATTTAATA TTACACAAAT CCTTTGATTA AATCACCGTA ATTGTGGTTT GATTAGGGAA GTTTCTTGGG   
  
  
+ GGAAAGGTTC AAGCTTCTGT CTTTCTTAAA GCCTGGATTT TTGGTCCCGC ATGTTCACTT CACATTCCCT   
  
  
+ TTTTCTCTCC CATCGTTACC TGTCTGAATC TGTATGTAAT AAAAGTCATA TTTTGCGAGG TCTTCTTTTG   
  
  
+ ACTGTTCTTG TAGTCACTCT TGATATCTGC ACCATTTTGT TTTTCTCGGC ATTTTCTCCA ACTTGCATTT   
  
  
+ TCTAATGTAA TGTGATCTTT TCTGGACATT CATTTTGTTA ATCTACTCAC TTGGGTCAAG GTTTGCTGAT   
  
  
+ TTTGGCTGGT GTTAAATCCG TTCCTCTTTG GCATTTGTTC ATGTTCCACA ACTGTTTGAT ATCTGGTATC   
  
  
+ TCAGAAACCC CGCCATTCTT TGGTATTTCC TGACTTTCTT GTTCAAGGGT TATTCCTATT TCCCTGAATT   
  
  
+ TTCTTTGATT CTTTAGGTAT TTCAAGCTTG GGGGGTTTTG TTCTTCACTG ATTTTGTCCT CTTCTTCCCT   
  
  
+ TATTGGGTGG TTTGATCTCC TAAATTTCTG TGTATCTGAA TCAGTCGGGG TAGACTTTGT GTTTTTGGTT   
  
  
+ GATTTTTTAT CAATGTATTT TCTTCCTAAT GAAGGGCCTC CCGAACCAAT ACACATGCTT AACTTCAACC   
  
  
+ CTGTTCCTCT TCCAAGCTTT TTGGAGCCTC AAAAGTCTTC GAATGCTGAT AGGCACATAG GGTCCTTCCA   
  
  
+ ATGCTATGGT CTTGATGATG ACCCCGCACC CTTGAATGAC CCAAATTACA TTGTATGTCA ATCTTCATAT   
  
  
+ CCTGATCGAG ATATTACACA GATCCCTGAC TTTCCTGATG ATTGTCTCAA GTTCATCAAT GACATTCTTA   
  
  
+ TGGAAGAGGA TTTGGATAAT CCGCCTATAT CCTTGCAGGA CTATAATGCG CTCCAAGCCA CTGAGAAGTC   
  
  
+ ATTGTATGAT GCCCTTGGAG AGGCTTATCC AGCTTCATCT GATCCATTGC AATCATCAGT TGGTCAGAGC   
  
  
+ ACTGAGAGCT CTGATGATCC TGACAGAAGC AGCGGCATTG ATAAATGGGG TAATAGATAT TACCCCACCA   
  
  
+ GCCTGAATGT GGTTGAAACC AGCCGGCTGA TCAATCAGAG CCAGTTGGAC CCTTCTCAAA TGTTTGATGC   
  
  
+ TGCTGCTCAC CCCTTGGAGT CTAATTGTCA TTCTTTTGTC TCTAGTAATA ACTTTGATCA TTCTATGGAT   
  
  
+ GGGGTTCCAG ATTCTCCTAT CAGTACACTT TCTGTGAATG AAACAAGAAA GGAAGAATCA GTTAGTAGAT   
  
  
+ CAAGGAGCAG GAAGAATCAT CAAAGAGATG GTGCATACCT GGAAGAAAGG AGTACTAAGC AAATAGCATC   
  
  
+ CAGCAATGAG GAATATGGAG AGATGGAGAG GTTTGATGAT GTACTGATCT GCAGGGAAGG AAATGATGAT   
  
  
+ ATTTCACCTT GTACCCGCAA AACCCCAGTT CATGAAGCAG ATGATAACTC GGAACCAAAG GGGAAATCAA   
  
  
+ GAGGATCTAA AAGCAAGAGT AAATCGCGTG TCAAGAAGAA AAGTAACGAA GGACAAGCGG TAGATCTTAG   
  
  
+ GACTCAACTT ATTCAGTGTG CAGAAGCAGT GGCAAGCTTT GAGCTTAAAA GTGCAAATGA GCTACTCAGG   
  
  
+ CGAATTAGGC AGCATGCTTC GCCATTTGGG GATAGTGTTC AAAGGCTAGC ACATTACTTT GCGAATGGGC   
  
  
+ TCGAGGCACG CTTAGCTGGT ACAGGATCAG AATCATACAA AGACTTTGCT GGGAAAAAAT TTTCATCCTA   
  
  
+ TGACATCTTG AGAGGCTACA AGTCATATGT TCAAGCCGTC CCCTTCCAGA GAACAACCAT GTTCTTGACA   
  
  
+ AATCAAACAA TAGTAAAGCT GGCTGAGAAA GCAACAAGGG TTCATATCAT TGATTTTGGC ATCTTTTTTG   
  
  
+ GTTTCCAGTG GCCCTGCCTT GTTCAGAATT TGTCAAGAAG ACCCCAGGGT TCTCCCATAC TCAAAATCAC   
  
  
+ GGGAGTCGAT CATCCCCAGA GTGGGTTCAG ACCAGCCCAG AAAGTTGAAG AGACAGGTCG TCGATTAGCA   
  
  
+ GGCTATTGTG AGAGGTTTGG GGTGCCCTTT GAGTTTCATC CCATTGCTCA AAGGTGGCAT ACCCTAAAGC   
  
  
+ CAGAGCATTT CAAAATTGAA AGCAATGAGC TTGTTGTGGT CACTTGTTTC TACCAATCAG GGAAGCTCCC   
  
  
+ TGATGAGACT GTTGATGTCA ACAGCCCAAG AGACCATGTC TTGAGGCTGA TCAGGGGCTT GAACCCTGAT   
  
  
+ CTATTCATCC ATGGGGTTAT CAATGGCACA TTCAATGCAC CCTTCTTCGT AACTCGGTTT AAAGAGGCAC   
  
  
+ TTTACCACTA CTCTTCTTTG TTCGATGTCT TTGATGCCAC CATAGATCGT GAAAGTCATG AAAGACTGCT   
  
  
+ GACTGAGAAC TATTTGTATG GGAAAGATGC ACTTAACATA GTAGCATGTG AGGGGAACGA GAGGATCGAG   
  
  
+ AGACCCGAGA CTTACAAGCA GTGGCAAGTC CGGAAGCTTA GAGCTGGATT CAAGCAGGTC CCCCTAGACC   
  
  
+ AAGACATTGT AAATGGAGCA AGAGCTATGG TTAAGGCAAA TTACAATAAG GATTTTATGG TAGAACAAGA   
  
  
+ TAGGAATTGG ATGGTCCAGG GCTGGAAAGG AAGGATTTTG AGTGCCATAT CCTGTTGGAA ACCTGCTTA  

- +Up\_Stream \_Len000ACTTAA CCTTCAACTA AGATGGAAAC TAACTAAGCT AATTAAGATC CAAGAACCAA   
  
  
- GATCAAGAGA GAGGAGAACA AGAACTAATA AATCCAAAAC CTACACTACA AATAACTTAA CTTACAACGT   
  
  
- TAACAACAAT ATTTCTACTA CAATCCGATT TATAGGAAGT TGAATCCAAC CCTAACTGCA GCTTATACAC   
  
  
- ATCAACTTAG AACATGACCG GATTTAGGGA CGAGGGGGAG ACAAAACGCT AATACTTAAA GTAATACGTG   
  
  
- TGGTGTACGA GCTACTTTAC GAGCTTTCTT GTAAGTAGGA GAAACAAACC CTAAATCCCC TTGCAACTTT   
  
  
- CTGCTTCAGA AATGGATCAC GCAGAATCAT CTGTTAATCC TAAAATTGTA CTTTTATCAA TTTTAAGATT   
  
  
- AACCCTAAAC TCTAACACTT AGAACATTAG GAAGCTGAAG ATCAAGATAC TTTTATCCGC TAATATCAAG   
  
  
- ATCCTAATCA GAAGCTGGGA ACTCTCTCCC TCCTCTACCC AAATCAACTA TTAGATAAGT AAGTATTGCG   
  
  
- TACGGACCCT CTACAAACCA TCCCGATCAT TAACTAAGCG ATTGTATAAA CACGACAATC AAGGTTGGAC   
  
  
- TCACAAATGA GAAGACGACA AATAAGGCAG AAAATCAGCA TTAACAGAGG AAATCAAATT AAAGTTTTGG   
  
  
- GGTTAACACT AGCGGATCTA ACTCGTTAGA TTAATCAAGA TTATTAACTT TAGTTCAAGG GACACCTTAT   
  
  
- GCTGGGCATG AACGGCACAC GATCGATGAC AATGTGGAAC GTAAACGCCA TATAAATATT GAGTTGTATA   
  
  
- CAGACGAGTG ATTTTCATGT ATAATCGAGT GGTTTTGACA CAGAGTTGCA TGATTTTATA AAATCACTTG   
  
  
- ATATGTAATA AAAATCATTC GCTTTATTCT ATGTGTTGTC GTGTAAAATT ACTCAATGTC CCGTAAAAAT   
  
  
- CACTCGGCTT TAGACTACCA AAAGAAACAT CTAGGCCAAT ATATTAACAT AAAATATGTT GGCTTACCTG   
  
  
- CTATATTGTT GACTCAATTT ACTCCTCAGA CTTTAATGCT TGTATTTTAG GTTTTCTTCT ACGGCTACGG   
  
  
- TAACCTTTCT TTCGACTTCC ACTAGCGAAA GATCATCATA ATCCGTCATC ATTAATTTTT GATTTTAGGT   
  
  
- CTTAACTGTG GCTTAACCCC AACACCGTGT ACTAGCTTTA GTCGTAGCTT AGATGTGCTA CTAGACCTAA   
  
  
- GAACCTGTGA GACCGAAATC CGGCAGTTAA AGCTCCTAAC GGGTATTGAA ATTAGGACAA GTTTAGGTAA   
  
  
- GTTAAATTAT AATGTGTTTA GGAAACTAAT TTAGTGGCAT TAACACCAAA CTAATCCCTT CAAAGAACCC   
  
  
- CCTTTCCAAG TTCGAAGACA GAAAGAATTT CGGACCTAAA AACCAGGGCG TACAAGTGAA GTGTAAGGGA   
  
  
- AAAAGAGAGG GTAGCAATGG ACAGACTTAG ACATACATTA TTTTCAGTAT AAAACGCTCC AGAAGAAAAC   
  
  
- TGACAAGAAC ATCAGTGAGA ACTATAGACG TGGTAAAACA AAAAGAGCCG TAAAAGAGGT TGAACGTAAA   
  
  
- AGATTACATT ACACTAGAAA AGACCTGTAA GTAAAACAAT TAGATGAGTG AACCCAGTTC CAAACGACTA   
  
  
- AAACCGACCA CAATTTAGGC AAGGAGAAAC CGTAAACAAG TACAAGGTGT TGACAAACTA TAGACCATAG   
  
  
- AGTCTTTGGG GCGGTAAGAA ACCATAAAGG ACTGAAAGAA CAAGTTCCCA ATAAGGATAA AGGGACTTAA   
  
  
- AAGAAACTAA GAAATCCATA AAGTTCGAAC CCCCCAAAAC AAGAAGTGAC TAAAACAGGA GAAGAAGGGA   
  
  
- ATAACCCACC AAACTAGAGG ATTTAAAGAC ACATAGACTT AGTCAGCCCC ATCTGAAACA CAAAAACCAA   
  
  
- CTAAAAAATA GTTACATAAA AGAAGGATTA CTTCCCGGAG GGCTTGGTTA TGTGTACGAA TTGAAGTTGG   
  
  
- GACAAGGAGA AGGTTCGAAA AACCTCGGAG TTTTCAGAAG CTTACGACTA TCCGTGTATC CCAGGAAGGT   
  
  
- TACGATACCA GAACTACTAC TGGGGCGTGG GAACTTACTG GGTTTAATGT AACATACAGT TAGAAGTATA   
  
  
- GGACTAGCTC TATAATGTGT CTAGGGACTG AAAGGACTAC TAACAGAGTT CAAGTAGTTA CTGTAAGAAT   
  
  
- ACCTTCTCCT AAACCTATTA GGCGGATATA GGAACGTCCT GATATTACGC GAGGTTCGGT GACTCTTCAG   
  
  
- TAACATACTA CGGGAACCTC TCCGAATAGG TCGAAGTAGA CTAGGTAACG TTAGTAGTCA ACCAGTCTCG   
  
  
- TGACTCTCGA GACTACTAGG ACTGTCTTCG TCGCCGTAAC TATTTACCCC ATTATCTATA ATGGGGTGGT   
  
  
- CGGACTTACA CCAACTTTGG TCGGCCGACT AGTTAGTCTC GGTCAACCTG GGAAGAGTTT ACAAACTACG   
  
  
- ACGACGAGTG GGGAACCTCA GATTAACAGT AAGAAAACAG AGATCATTAT TGAAACTAGT AAGATACCTA   
  
  
- CCCCAAGGTC TAAGAGGATA GTCATGTGAA AGACACTTAC TTTGTTCTTT CCTTCTTAGT CAATCATCTA   
  
  
- GTTCCTCGTC CTTCTTAGTA GTTTCTCTAC CACGTATGGA CCTTCTTTCC TCATGATTCG TTTATCGTAG   
  
  
- GTCGTTACTC CTTATACCTC TCTACCTCTC CAAACTACTA CATGACTAGA CGTCCCTTCC TTTACTACTA   
  
  
- TAAAGTGGAA CATGGGCGTT TTGGGGTCAA GTACTTCGTC TACTATTGAG CCTTGGTTTC CCCTTTAGTT   
  
  
- CTCCTAGATT TTCGTTCTCA TTTAGCGCAC AGTTCTTCTT TTCATTGCTT CCTGTTCGCC ATCTAGAATC   
  
  
- CTGAGTTGAA TAAGTCACAC GTCTTCGTCA CCGTTCGAAA CTCGAATTTT CACGTTTACT CGATGAGTCC   
  
  
- GCTTAATCCG TCGTACGAAG CGGTAAACCC CTATCACAAG TTTCCGATCG TGTAATGAAA CGCTTACCCG   
  
  
- AGCTCCGTGC GAATCGACCA TGTCCTAGTC TTAGTATGTT TCTGAAACGA CCCTTTTTTA AAAGTAGGAT   
  
  
- ACTGTAGAAC TCTCCGATGT TCAGTATACA AGTTCGGCAG GGGAAGGTCT CTTGTTGGTA CAAGAACTGT   
  
  
- TTAGTTTGTT ATCATTTCGA CCGACTCTTT CGTTGTTCCC AAGTATAGTA ACTAAAACCG TAGAAAAAAC   
  
  
- CAAAGGTCAC CGGGACGGAA CAAGTCTTAA ACAGTTCTTC TGGGGTCCCA AGAGGGTATG AGTTTTAGTG   
  
  
- CCCTCAGCTA GTAGGGGTCT CACCCAAGTC TGGTCGGGTC TTTCAACTTC TCTGTCCAGC AGCTAATCGT   
  
  
- CCGATAACAC TCTCCAAACC CCACGGGAAA CTCAAAGTAG GGTAACGAGT TTCCACCGTA TGGGATTTCG   
  
  
- GTCTCGTAAA GTTTTAACTT TCGTTACTCG AACAACACCA GTGAACAAAG ATGGTTAGTC CCTTCGAGGG   
  
  
- ACTACTCTGA CAACTACAGT TGTCGGGTTC TCTGGTACAG AACTCCGACT AGTCCCCGAA CTTGGGACTA   
  
  
- GATAAGTAGG TACCCCAATA GTTACCGTGT AAGTTACGTG GGAAGAAGCA TTGAGCCAAA TTTCTCCGTG   
  
  
- AAATGGTGAT GAGAAGAAAC AAGCTACAGA AACTACGGTG GTATCTAGCA CTTTCAGTAC TTTCTGACGA   
  
  
- CTGACTCTTG ATAAACATAC CCTTTCTACG TGAATTGTAT CATCGTACAC TCCCCTTGCT CTCCTAGCTC   
  
  
- TCTGGGCTCT GAATGTTCGT CACCGTTCAG GCCTTCGAAT CTCGACCTAA GTTCGTCCAG GGGGATCTGG   
  
  
- TTCTGTAACA TTTACCTCGT TCTCGATACC AATTCCGTTT AATGTTATTC CTAAAATACC ATCTTGTTCT   
  
  
- ATCCTTAACC TACCAGGTCC CGACCTTTCC TTCCTAAAAC TCACGGTATA GGACAACCTT TGGACGAAT

+     MRE

| Site Name | Organism | Position | Strand | Matrix score. | sequence | function |
| --- | --- | --- | --- | --- | --- | --- |
| MRE | Petroselinum crispum | 187 | - | 7 | AACCTAA | MYB binding site involved in light responsiveness |
| MRE | Petroselinum crispum | 105 | - | 7 | AACCTAA | MYB binding site involved in light responsiveness |

>HU02G01573.1   
+ +Up\_Stream \_Len000TGAATT GGAAGTTGAT TCTACCTTTG ATTGATTCGA TTAATTCTAG GTTCTTGGTT   
  
  
+ CTAGTTCTCT CTCCTCTTGT TCTTGATTAT TTAGGTTTTG GATGTGATGT TTATTGAATT GAATGTTGCA   
  
  
+ ATTGTTGTTA TAAAGATGAT GTTAGGCTAA ATATCCTTCA ACTTAGGTTG GGATTGACGT CGAATATGTG   
  
  
+ TAGTTGAATC TTGTACTGGC CTAAATCCCT GCTCCCCCTC TGTTTTGCGA TTATGAATTT CATTATGCAC   
  
  
+ ACCACATGCT CGATGAAATG CTCGAAAGAA CATTCATCCT CTTTGTTTGG GATTTAGGGG AACGTTGAAA   
  
  
+ GACGAAGTCT TTACCTAGTG CGTCTTAGTA GACAATTAGG ATTTTAACAT GAAAATAGTT AAAATTCTAA   
  
  
+ TTGGGATTTG AGATTGTGAA TCTTGTAATC CTTCGACTTC TAGTTCTATG AAAATAGGCG ATTATAGTTC   
  
  
+ TAGGATTAGT CTTCGACCCT TGAGAGAGGG AGGAGATGGG TTTAGTTGAT AATCTATTCA TTCATAACGC   
  
  
+ ATGCCTGGGA GATGTTTGGT AGGGCTAGTA ATTGATTCGC TAACATATTT GTGCTGTTAG TTCCAACCTG   
  
  
+ AGTGTTTACT CTTCTGCTGT TTATTCCGTC TTTTAGTCGT AATTGTCTCC TTTAGTTTAA TTTCAAAACC   
  
  
+ CCAATTGTGA TCGCCTAGAT TGAGCAATCT AATTAGTTCT AATAATTGAA ATCAAGTTCC CTGTGGAATA   
  
  
+ CGACCCGTAC TTGCCGTGTG CTAGCTACTG TTACACCTTG CATTTGCGGT ATATTTATAA CTCAACATAT   
  
  
+ GTCTGCTCAC TAAAAGTACA TATTAGCTCA CCAAAACTGT GTCTCAACGT ACTAAAATAT TTTAGTGAAC   
  
  
+ TATACATTAT TTTTAGTAAG CGAAATAAGA TACACAACAG CACATTTTAA TGAGTTACAG GGCATTTTTA   
  
  
+ GTGAGCCGAA ATCTGATGGT TTTCTTTGTA GATCCGGTTA TATAATTGTA TTTTATACAA CCGAATGGAC   
  
  
+ GATATAACAA CTGAGTTAAA TGAGGAGTCT GAAATTACGA ACATAAAATC CAAAAGAAGA TGCCGATGCC   
  
  
+ ATTGGAAAGA AAGCTGAAGG TGATCGCTTT CTAGTAGTAT TAGGCAGTAG TAATTAAAAA CTAAAATCCA   
  
  
+ GAATTGACAC CGAATTGGGG TTGTGGCACA TGATCGAAAT CAGCATCGAA TCTACACGAT GATCTGGATT   
  
  
+ CTTGGACACT CTGGCTTTAG GCCGTCAATT TCGAGGATTG CCCATAACTT TAATCCTGTT CAAATCCATT   
  
  
+ CAATTTAATA TTACACAAAT CCTTTGATTA AATCACCGTA ATTGTGGTTT GATTAGGGAA GTTTCTTGGG   
  
  
+ GGAAAGGTTC AAGCTTCTGT CTTTCTTAAA GCCTGGATTT TTGGTCCCGC ATGTTCACTT CACATTCCCT   
  
  
+ TTTTCTCTCC CATCGTTACC TGTCTGAATC TGTATGTAAT AAAAGTCATA TTTTGCGAGG TCTTCTTTTG   
  
  
+ ACTGTTCTTG TAGTCACTCT TGATATCTGC ACCATTTTGT TTTTCTCGGC ATTTTCTCCA ACTTGCATTT   
  
  
+ TCTAATGTAA TGTGATCTTT TCTGGACATT CATTTTGTTA ATCTACTCAC TTGGGTCAAG GTTTGCTGAT   
  
  
+ TTTGGCTGGT GTTAAATCCG TTCCTCTTTG GCATTTGTTC ATGTTCCACA ACTGTTTGAT ATCTGGTATC   
  
  
+ TCAGAAACCC CGCCATTCTT TGGTATTTCC TGACTTTCTT GTTCAAGGGT TATTCCTATT TCCCTGAATT   
  
  
+ TTCTTTGATT CTTTAGGTAT TTCAAGCTTG GGGGGTTTTG TTCTTCACTG ATTTTGTCCT CTTCTTCCCT   
  
  
+ TATTGGGTGG TTTGATCTCC TAAATTTCTG TGTATCTGAA TCAGTCGGGG TAGACTTTGT GTTTTTGGTT   
  
  
+ GATTTTTTAT CAATGTATTT TCTTCCTAAT GAAGGGCCTC CCGAACCAAT ACACATGCTT AACTTCAACC   
  
  
+ CTGTTCCTCT TCCAAGCTTT TTGGAGCCTC AAAAGTCTTC GAATGCTGAT AGGCACATAG GGTCCTTCCA   
  
  
+ ATGCTATGGT CTTGATGATG ACCCCGCACC CTTGAATGAC CCAAATTACA TTGTATGTCA ATCTTCATAT   
  
  
+ CCTGATCGAG ATATTACACA GATCCCTGAC TTTCCTGATG ATTGTCTCAA GTTCATCAAT GACATTCTTA   
  
  
+ TGGAAGAGGA TTTGGATAAT CCGCCTATAT CCTTGCAGGA CTATAATGCG CTCCAAGCCA CTGAGAAGTC   
  
  
+ ATTGTATGAT GCCCTTGGAG AGGCTTATCC AGCTTCATCT GATCCATTGC AATCATCAGT TGGTCAGAGC   
  
  
+ ACTGAGAGCT CTGATGATCC TGACAGAAGC AGCGGCATTG ATAAATGGGG TAATAGATAT TACCCCACCA   
  
  
+ GCCTGAATGT GGTTGAAACC AGCCGGCTGA TCAATCAGAG CCAGTTGGAC CCTTCTCAAA TGTTTGATGC   
  
  
+ TGCTGCTCAC CCCTTGGAGT CTAATTGTCA TTCTTTTGTC TCTAGTAATA ACTTTGATCA TTCTATGGAT   
  
  
+ GGGGTTCCAG ATTCTCCTAT CAGTACACTT TCTGTGAATG AAACAAGAAA GGAAGAATCA GTTAGTAGAT   
  
  
+ CAAGGAGCAG GAAGAATCAT CAAAGAGATG GTGCATACCT GGAAGAAAGG AGTACTAAGC AAATAGCATC   
  
  
+ CAGCAATGAG GAATATGGAG AGATGGAGAG GTTTGATGAT GTACTGATCT GCAGGGAAGG AAATGATGAT   
  
  
+ ATTTCACCTT GTACCCGCAA AACCCCAGTT CATGAAGCAG ATGATAACTC GGAACCAAAG GGGAAATCAA   
  
  
+ GAGGATCTAA AAGCAAGAGT AAATCGCGTG TCAAGAAGAA AAGTAACGAA GGACAAGCGG TAGATCTTAG   
  
  
+ GACTCAACTT ATTCAGTGTG CAGAAGCAGT GGCAAGCTTT GAGCTTAAAA GTGCAAATGA GCTACTCAGG   
  
  
+ CGAATTAGGC AGCATGCTTC GCCATTTGGG GATAGTGTTC AAAGGCTAGC ACATTACTTT GCGAATGGGC   
  
  
+ TCGAGGCACG CTTAGCTGGT ACAGGATCAG AATCATACAA AGACTTTGCT GGGAAAAAAT TTTCATCCTA   
  
  
+ TGACATCTTG AGAGGCTACA AGTCATATGT TCAAGCCGTC CCCTTCCAGA GAACAACCAT GTTCTTGACA   
  
  
+ AATCAAACAA TAGTAAAGCT GGCTGAGAAA GCAACAAGGG TTCATATCAT TGATTTTGGC ATCTTTTTTG   
  
  
+ GTTTCCAGTG GCCCTGCCTT GTTCAGAATT TGTCAAGAAG ACCCCAGGGT TCTCCCATAC TCAAAATCAC   
  
  
+ GGGAGTCGAT CATCCCCAGA GTGGGTTCAG ACCAGCCCAG AAAGTTGAAG AGACAGGTCG TCGATTAGCA   
  
  
+ GGCTATTGTG AGAGGTTTGG GGTGCCCTTT GAGTTTCATC CCATTGCTCA AAGGTGGCAT ACCCTAAAGC   
  
  
+ CAGAGCATTT CAAAATTGAA AGCAATGAGC TTGTTGTGGT CACTTGTTTC TACCAATCAG GGAAGCTCCC   
  
  
+ TGATGAGACT GTTGATGTCA ACAGCCCAAG AGACCATGTC TTGAGGCTGA TCAGGGGCTT GAACCCTGAT   
  
  
+ CTATTCATCC ATGGGGTTAT CAATGGCACA TTCAATGCAC CCTTCTTCGT AACTCGGTTT AAAGAGGCAC   
  
  
+ TTTACCACTA CTCTTCTTTG TTCGATGTCT TTGATGCCAC CATAGATCGT GAAAGTCATG AAAGACTGCT   
  
  
+ GACTGAGAAC TATTTGTATG GGAAAGATGC ACTTAACATA GTAGCATGTG AGGGGAACGA GAGGATCGAG   
  
  
+ AGACCCGAGA CTTACAAGCA GTGGCAAGTC CGGAAGCTTA GAGCTGGATT CAAGCAGGTC CCCCTAGACC   
  
  
+ AAGACATTGT AAATGGAGCA AGAGCTATGG TTAAGGCAAA TTACAATAAG GATTTTATGG TAGAACAAGA   
  
  
+ TAGGAATTGG ATGGTCCAGG GCTGGAAAGG AAGGATTTTG AGTGCCATAT CCTGTTGGAA ACCTGCTTA  

- +Up\_Stream \_Len000ACTTAA CCTTCAACTA AGATGGAAAC TAACTAAGCT AATTAAGATC CAAGAACCAA   
  
  
- GATCAAGAGA GAGGAGAACA AGAACTAATA AATCCAAAAC CTACACTACA AATAACTTAA CTTACAACGT   
  
  
- TAACAACAAT ATTTCTACTA CAATCCGATT TATAGGAAGT TGAATCCAAC CCTAACTGCA GCTTATACAC   
  
  
- ATCAACTTAG AACATGACCG GATTTAGGGA CGAGGGGGAG ACAAAACGCT AATACTTAAA GTAATACGTG   
  
  
- TGGTGTACGA GCTACTTTAC GAGCTTTCTT GTAAGTAGGA GAAACAAACC CTAAATCCCC TTGCAACTTT   
  
  
- CTGCTTCAGA AATGGATCAC GCAGAATCAT CTGTTAATCC TAAAATTGTA CTTTTATCAA TTTTAAGATT   
  
  
- AACCCTAAAC TCTAACACTT AGAACATTAG GAAGCTGAAG ATCAAGATAC TTTTATCCGC TAATATCAAG   
  
  
- ATCCTAATCA GAAGCTGGGA ACTCTCTCCC TCCTCTACCC AAATCAACTA TTAGATAAGT AAGTATTGCG   
  
  
- TACGGACCCT CTACAAACCA TCCCGATCAT TAACTAAGCG ATTGTATAAA CACGACAATC AAGGTTGGAC   
  
  
- TCACAAATGA GAAGACGACA AATAAGGCAG AAAATCAGCA TTAACAGAGG AAATCAAATT AAAGTTTTGG   
  
  
- GGTTAACACT AGCGGATCTA ACTCGTTAGA TTAATCAAGA TTATTAACTT TAGTTCAAGG GACACCTTAT   
  
  
- GCTGGGCATG AACGGCACAC GATCGATGAC AATGTGGAAC GTAAACGCCA TATAAATATT GAGTTGTATA   
  
  
- CAGACGAGTG ATTTTCATGT ATAATCGAGT GGTTTTGACA CAGAGTTGCA TGATTTTATA AAATCACTTG   
  
  
- ATATGTAATA AAAATCATTC GCTTTATTCT ATGTGTTGTC GTGTAAAATT ACTCAATGTC CCGTAAAAAT   
  
  
- CACTCGGCTT TAGACTACCA AAAGAAACAT CTAGGCCAAT ATATTAACAT AAAATATGTT GGCTTACCTG   
  
  
- CTATATTGTT GACTCAATTT ACTCCTCAGA CTTTAATGCT TGTATTTTAG GTTTTCTTCT ACGGCTACGG   
  
  
- TAACCTTTCT TTCGACTTCC ACTAGCGAAA GATCATCATA ATCCGTCATC ATTAATTTTT GATTTTAGGT   
  
  
- CTTAACTGTG GCTTAACCCC AACACCGTGT ACTAGCTTTA GTCGTAGCTT AGATGTGCTA CTAGACCTAA   
  
  
- GAACCTGTGA GACCGAAATC CGGCAGTTAA AGCTCCTAAC GGGTATTGAA ATTAGGACAA GTTTAGGTAA   
  
  
- GTTAAATTAT AATGTGTTTA GGAAACTAAT TTAGTGGCAT TAACACCAAA CTAATCCCTT CAAAGAACCC   
  
  
- CCTTTCCAAG TTCGAAGACA GAAAGAATTT CGGACCTAAA AACCAGGGCG TACAAGTGAA GTGTAAGGGA   
  
  
- AAAAGAGAGG GTAGCAATGG ACAGACTTAG ACATACATTA TTTTCAGTAT AAAACGCTCC AGAAGAAAAC   
  
  
- TGACAAGAAC ATCAGTGAGA ACTATAGACG TGGTAAAACA AAAAGAGCCG TAAAAGAGGT TGAACGTAAA   
  
  
- AGATTACATT ACACTAGAAA AGACCTGTAA GTAAAACAAT TAGATGAGTG AACCCAGTTC CAAACGACTA   
  
  
- AAACCGACCA CAATTTAGGC AAGGAGAAAC CGTAAACAAG TACAAGGTGT TGACAAACTA TAGACCATAG   
  
  
- AGTCTTTGGG GCGGTAAGAA ACCATAAAGG ACTGAAAGAA CAAGTTCCCA ATAAGGATAA AGGGACTTAA   
  
  
- AAGAAACTAA GAAATCCATA AAGTTCGAAC CCCCCAAAAC AAGAAGTGAC TAAAACAGGA GAAGAAGGGA   
  
  
- ATAACCCACC AAACTAGAGG ATTTAAAGAC ACATAGACTT AGTCAGCCCC ATCTGAAACA CAAAAACCAA   
  
  
- CTAAAAAATA GTTACATAAA AGAAGGATTA CTTCCCGGAG GGCTTGGTTA TGTGTACGAA TTGAAGTTGG   
  
  
- GACAAGGAGA AGGTTCGAAA AACCTCGGAG TTTTCAGAAG CTTACGACTA TCCGTGTATC CCAGGAAGGT   
  
  
- TACGATACCA GAACTACTAC TGGGGCGTGG GAACTTACTG GGTTTAATGT AACATACAGT TAGAAGTATA   
  
  
- GGACTAGCTC TATAATGTGT CTAGGGACTG AAAGGACTAC TAACAGAGTT CAAGTAGTTA CTGTAAGAAT   
  
  
- ACCTTCTCCT AAACCTATTA GGCGGATATA GGAACGTCCT GATATTACGC GAGGTTCGGT GACTCTTCAG   
  
  
- TAACATACTA CGGGAACCTC TCCGAATAGG TCGAAGTAGA CTAGGTAACG TTAGTAGTCA ACCAGTCTCG   
  
  
- TGACTCTCGA GACTACTAGG ACTGTCTTCG TCGCCGTAAC TATTTACCCC ATTATCTATA ATGGGGTGGT   
  
  
- CGGACTTACA CCAACTTTGG TCGGCCGACT AGTTAGTCTC GGTCAACCTG GGAAGAGTTT ACAAACTACG   
  
  
- ACGACGAGTG GGGAACCTCA GATTAACAGT AAGAAAACAG AGATCATTAT TGAAACTAGT AAGATACCTA   
  
  
- CCCCAAGGTC TAAGAGGATA GTCATGTGAA AGACACTTAC TTTGTTCTTT CCTTCTTAGT CAATCATCTA   
  
  
- GTTCCTCGTC CTTCTTAGTA GTTTCTCTAC CACGTATGGA CCTTCTTTCC TCATGATTCG TTTATCGTAG   
  
  
- GTCGTTACTC CTTATACCTC TCTACCTCTC CAAACTACTA CATGACTAGA CGTCCCTTCC TTTACTACTA   
  
  
- TAAAGTGGAA CATGGGCGTT TTGGGGTCAA GTACTTCGTC TACTATTGAG CCTTGGTTTC CCCTTTAGTT   
  
  
- CTCCTAGATT TTCGTTCTCA TTTAGCGCAC AGTTCTTCTT TTCATTGCTT CCTGTTCGCC ATCTAGAATC   
  
  
- CTGAGTTGAA TAAGTCACAC GTCTTCGTCA CCGTTCGAAA CTCGAATTTT CACGTTTACT CGATGAGTCC   
  
  
- GCTTAATCCG TCGTACGAAG CGGTAAACCC CTATCACAAG TTTCCGATCG TGTAATGAAA CGCTTACCCG   
  
  
- AGCTCCGTGC GAATCGACCA TGTCCTAGTC TTAGTATGTT TCTGAAACGA CCCTTTTTTA AAAGTAGGAT   
  
  
- ACTGTAGAAC TCTCCGATGT TCAGTATACA AGTTCGGCAG GGGAAGGTCT CTTGTTGGTA CAAGAACTGT   
  
  
- TTAGTTTGTT ATCATTTCGA CCGACTCTTT CGTTGTTCCC AAGTATAGTA ACTAAAACCG TAGAAAAAAC   
  
  
- CAAAGGTCAC CGGGACGGAA CAAGTCTTAA ACAGTTCTTC TGGGGTCCCA AGAGGGTATG AGTTTTAGTG   
  
  
- CCCTCAGCTA GTAGGGGTCT CACCCAAGTC TGGTCGGGTC TTTCAACTTC TCTGTCCAGC AGCTAATCGT   
  
  
- CCGATAACAC TCTCCAAACC CCACGGGAAA CTCAAAGTAG GGTAACGAGT TTCCACCGTA TGGGATTTCG   
  
  
- GTCTCGTAAA GTTTTAACTT TCGTTACTCG AACAACACCA GTGAACAAAG ATGGTTAGTC CCTTCGAGGG   
  
  
- ACTACTCTGA CAACTACAGT TGTCGGGTTC TCTGGTACAG AACTCCGACT AGTCCCCGAA CTTGGGACTA   
  
  
- GATAAGTAGG TACCCCAATA GTTACCGTGT AAGTTACGTG GGAAGAAGCA TTGAGCCAAA TTTCTCCGTG   
  
  
- AAATGGTGAT GAGAAGAAAC AAGCTACAGA AACTACGGTG GTATCTAGCA CTTTCAGTAC TTTCTGACGA   
  
  
- CTGACTCTTG ATAAACATAC CCTTTCTACG TGAATTGTAT CATCGTACAC TCCCCTTGCT CTCCTAGCTC   
  
  
- TCTGGGCTCT GAATGTTCGT CACCGTTCAG GCCTTCGAAT CTCGACCTAA GTTCGTCCAG GGGGATCTGG   
  
  
- TTCTGTAACA TTTACCTCGT TCTCGATACC AATTCCGTTT AATGTTATTC CTAAAATACC ATCTTGTTCT   
  
  
- ATCCTTAACC TACCAGGTCC CGACCTTTCC TTCCTAAAAC TCACGGTATA GGACAACCTT TGGACGAAT

+     MYB

| Site Name | Organism | Position | Strand | Matrix score. | sequence | function |
| --- | --- | --- | --- | --- | --- | --- |
| MYB | Arabidopsis thaliana | 3583 | - | 6 | CAACAG |  |
| MYB | Arabidopsis thaliana | 949 | + | 6 | CAACAG |  |
| MYB | Arabidopsis thaliana | 1960 | - | 6 | CAACCA |  |
| MYB | Arabidopsis thaliana | 3952 | - | 6 | TAACCA |  |
| MYB | Arabidopsis thaliana | 3208 | + | 6 | CAACCA |  |
| MYB | Arabidopsis thaliana | 2464 | - | 6 | CAACCA |  |
| MYB | Arabidopsis thaliana | 3593 | + | 6 | CAACAG |  |
| MYB | Arabidopsis thaliana | 4046 | - | 6 | CAACAG |  |

>HU02G01573.1   
+ +Up\_Stream \_Len000TGAATT GGAAGTTGAT TCTACCTTTG ATTGATTCGA TTAATTCTAG GTTCTTGGTT   
  
  
+ CTAGTTCTCT CTCCTCTTGT TCTTGATTAT TTAGGTTTTG GATGTGATGT TTATTGAATT GAATGTTGCA   
  
  
+ ATTGTTGTTA TAAAGATGAT GTTAGGCTAA ATATCCTTCA ACTTAGGTTG GGATTGACGT CGAATATGTG   
  
  
+ TAGTTGAATC TTGTACTGGC CTAAATCCCT GCTCCCCCTC TGTTTTGCGA TTATGAATTT CATTATGCAC   
  
  
+ ACCACATGCT CGATGAAATG CTCGAAAGAA CATTCATCCT CTTTGTTTGG GATTTAGGGG AACGTTGAAA   
  
  
+ GACGAAGTCT TTACCTAGTG CGTCTTAGTA GACAATTAGG ATTTTAACAT GAAAATAGTT AAAATTCTAA   
  
  
+ TTGGGATTTG AGATTGTGAA TCTTGTAATC CTTCGACTTC TAGTTCTATG AAAATAGGCG ATTATAGTTC   
  
  
+ TAGGATTAGT CTTCGACCCT TGAGAGAGGG AGGAGATGGG TTTAGTTGAT AATCTATTCA TTCATAACGC   
  
  
+ ATGCCTGGGA GATGTTTGGT AGGGCTAGTA ATTGATTCGC TAACATATTT GTGCTGTTAG TTCCAACCTG   
  
  
+ AGTGTTTACT CTTCTGCTGT TTATTCCGTC TTTTAGTCGT AATTGTCTCC TTTAGTTTAA TTTCAAAACC   
  
  
+ CCAATTGTGA TCGCCTAGAT TGAGCAATCT AATTAGTTCT AATAATTGAA ATCAAGTTCC CTGTGGAATA   
  
  
+ CGACCCGTAC TTGCCGTGTG CTAGCTACTG TTACACCTTG CATTTGCGGT ATATTTATAA CTCAACATAT   
  
  
+ GTCTGCTCAC TAAAAGTACA TATTAGCTCA CCAAAACTGT GTCTCAACGT ACTAAAATAT TTTAGTGAAC   
  
  
+ TATACATTAT TTTTAGTAAG CGAAATAAGA TACACAACAG CACATTTTAA TGAGTTACAG GGCATTTTTA   
  
  
+ GTGAGCCGAA ATCTGATGGT TTTCTTTGTA GATCCGGTTA TATAATTGTA TTTTATACAA CCGAATGGAC   
  
  
+ GATATAACAA CTGAGTTAAA TGAGGAGTCT GAAATTACGA ACATAAAATC CAAAAGAAGA TGCCGATGCC   
  
  
+ ATTGGAAAGA AAGCTGAAGG TGATCGCTTT CTAGTAGTAT TAGGCAGTAG TAATTAAAAA CTAAAATCCA   
  
  
+ GAATTGACAC CGAATTGGGG TTGTGGCACA TGATCGAAAT CAGCATCGAA TCTACACGAT GATCTGGATT   
  
  
+ CTTGGACACT CTGGCTTTAG GCCGTCAATT TCGAGGATTG CCCATAACTT TAATCCTGTT CAAATCCATT   
  
  
+ CAATTTAATA TTACACAAAT CCTTTGATTA AATCACCGTA ATTGTGGTTT GATTAGGGAA GTTTCTTGGG   
  
  
+ GGAAAGGTTC AAGCTTCTGT CTTTCTTAAA GCCTGGATTT TTGGTCCCGC ATGTTCACTT CACATTCCCT   
  
  
+ TTTTCTCTCC CATCGTTACC TGTCTGAATC TGTATGTAAT AAAAGTCATA TTTTGCGAGG TCTTCTTTTG   
  
  
+ ACTGTTCTTG TAGTCACTCT TGATATCTGC ACCATTTTGT TTTTCTCGGC ATTTTCTCCA ACTTGCATTT   
  
  
+ TCTAATGTAA TGTGATCTTT TCTGGACATT CATTTTGTTA ATCTACTCAC TTGGGTCAAG GTTTGCTGAT   
  
  
+ TTTGGCTGGT GTTAAATCCG TTCCTCTTTG GCATTTGTTC ATGTTCCACA ACTGTTTGAT ATCTGGTATC   
  
  
+ TCAGAAACCC CGCCATTCTT TGGTATTTCC TGACTTTCTT GTTCAAGGGT TATTCCTATT TCCCTGAATT   
  
  
+ TTCTTTGATT CTTTAGGTAT TTCAAGCTTG GGGGGTTTTG TTCTTCACTG ATTTTGTCCT CTTCTTCCCT   
  
  
+ TATTGGGTGG TTTGATCTCC TAAATTTCTG TGTATCTGAA TCAGTCGGGG TAGACTTTGT GTTTTTGGTT   
  
  
+ GATTTTTTAT CAATGTATTT TCTTCCTAAT GAAGGGCCTC CCGAACCAAT ACACATGCTT AACTTCAACC   
  
  
+ CTGTTCCTCT TCCAAGCTTT TTGGAGCCTC AAAAGTCTTC GAATGCTGAT AGGCACATAG GGTCCTTCCA   
  
  
+ ATGCTATGGT CTTGATGATG ACCCCGCACC CTTGAATGAC CCAAATTACA TTGTATGTCA ATCTTCATAT   
  
  
+ CCTGATCGAG ATATTACACA GATCCCTGAC TTTCCTGATG ATTGTCTCAA GTTCATCAAT GACATTCTTA   
  
  
+ TGGAAGAGGA TTTGGATAAT CCGCCTATAT CCTTGCAGGA CTATAATGCG CTCCAAGCCA CTGAGAAGTC   
  
  
+ ATTGTATGAT GCCCTTGGAG AGGCTTATCC AGCTTCATCT GATCCATTGC AATCATCAGT TGGTCAGAGC   
  
  
+ ACTGAGAGCT CTGATGATCC TGACAGAAGC AGCGGCATTG ATAAATGGGG TAATAGATAT TACCCCACCA   
  
  
+ GCCTGAATGT GGTTGAAACC AGCCGGCTGA TCAATCAGAG CCAGTTGGAC CCTTCTCAAA TGTTTGATGC   
  
  
+ TGCTGCTCAC CCCTTGGAGT CTAATTGTCA TTCTTTTGTC TCTAGTAATA ACTTTGATCA TTCTATGGAT   
  
  
+ GGGGTTCCAG ATTCTCCTAT CAGTACACTT TCTGTGAATG AAACAAGAAA GGAAGAATCA GTTAGTAGAT   
  
  
+ CAAGGAGCAG GAAGAATCAT CAAAGAGATG GTGCATACCT GGAAGAAAGG AGTACTAAGC AAATAGCATC   
  
  
+ CAGCAATGAG GAATATGGAG AGATGGAGAG GTTTGATGAT GTACTGATCT GCAGGGAAGG AAATGATGAT   
  
  
+ ATTTCACCTT GTACCCGCAA AACCCCAGTT CATGAAGCAG ATGATAACTC GGAACCAAAG GGGAAATCAA   
  
  
+ GAGGATCTAA AAGCAAGAGT AAATCGCGTG TCAAGAAGAA AAGTAACGAA GGACAAGCGG TAGATCTTAG   
  
  
+ GACTCAACTT ATTCAGTGTG CAGAAGCAGT GGCAAGCTTT GAGCTTAAAA GTGCAAATGA GCTACTCAGG   
  
  
+ CGAATTAGGC AGCATGCTTC GCCATTTGGG GATAGTGTTC AAAGGCTAGC ACATTACTTT GCGAATGGGC   
  
  
+ TCGAGGCACG CTTAGCTGGT ACAGGATCAG AATCATACAA AGACTTTGCT GGGAAAAAAT TTTCATCCTA   
  
  
+ TGACATCTTG AGAGGCTACA AGTCATATGT TCAAGCCGTC CCCTTCCAGA GAACAACCAT GTTCTTGACA   
  
  
+ AATCAAACAA TAGTAAAGCT GGCTGAGAAA GCAACAAGGG TTCATATCAT TGATTTTGGC ATCTTTTTTG   
  
  
+ GTTTCCAGTG GCCCTGCCTT GTTCAGAATT TGTCAAGAAG ACCCCAGGGT TCTCCCATAC TCAAAATCAC   
  
  
+ GGGAGTCGAT CATCCCCAGA GTGGGTTCAG ACCAGCCCAG AAAGTTGAAG AGACAGGTCG TCGATTAGCA   
  
  
+ GGCTATTGTG AGAGGTTTGG GGTGCCCTTT GAGTTTCATC CCATTGCTCA AAGGTGGCAT ACCCTAAAGC   
  
  
+ CAGAGCATTT CAAAATTGAA AGCAATGAGC TTGTTGTGGT CACTTGTTTC TACCAATCAG GGAAGCTCCC   
  
  
+ TGATGAGACT GTTGATGTCA ACAGCCCAAG AGACCATGTC TTGAGGCTGA TCAGGGGCTT GAACCCTGAT   
  
  
+ CTATTCATCC ATGGGGTTAT CAATGGCACA TTCAATGCAC CCTTCTTCGT AACTCGGTTT AAAGAGGCAC   
  
  
+ TTTACCACTA CTCTTCTTTG TTCGATGTCT TTGATGCCAC CATAGATCGT GAAAGTCATG AAAGACTGCT   
  
  
+ GACTGAGAAC TATTTGTATG GGAAAGATGC ACTTAACATA GTAGCATGTG AGGGGAACGA GAGGATCGAG   
  
  
+ AGACCCGAGA CTTACAAGCA GTGGCAAGTC CGGAAGCTTA GAGCTGGATT CAAGCAGGTC CCCCTAGACC   
  
  
+ AAGACATTGT AAATGGAGCA AGAGCTATGG TTAAGGCAAA TTACAATAAG GATTTTATGG TAGAACAAGA   
  
  
+ TAGGAATTGG ATGGTCCAGG GCTGGAAAGG AAGGATTTTG AGTGCCATAT CCTGTTGGAA ACCTGCTTA  

- +Up\_Stream \_Len000ACTTAA CCTTCAACTA AGATGGAAAC TAACTAAGCT AATTAAGATC CAAGAACCAA   
  
  
- GATCAAGAGA GAGGAGAACA AGAACTAATA AATCCAAAAC CTACACTACA AATAACTTAA CTTACAACGT   
  
  
- TAACAACAAT ATTTCTACTA CAATCCGATT TATAGGAAGT TGAATCCAAC CCTAACTGCA GCTTATACAC   
  
  
- ATCAACTTAG AACATGACCG GATTTAGGGA CGAGGGGGAG ACAAAACGCT AATACTTAAA GTAATACGTG   
  
  
- TGGTGTACGA GCTACTTTAC GAGCTTTCTT GTAAGTAGGA GAAACAAACC CTAAATCCCC TTGCAACTTT   
  
  
- CTGCTTCAGA AATGGATCAC GCAGAATCAT CTGTTAATCC TAAAATTGTA CTTTTATCAA TTTTAAGATT   
  
  
- AACCCTAAAC TCTAACACTT AGAACATTAG GAAGCTGAAG ATCAAGATAC TTTTATCCGC TAATATCAAG   
  
  
- ATCCTAATCA GAAGCTGGGA ACTCTCTCCC TCCTCTACCC AAATCAACTA TTAGATAAGT AAGTATTGCG   
  
  
- TACGGACCCT CTACAAACCA TCCCGATCAT TAACTAAGCG ATTGTATAAA CACGACAATC AAGGTTGGAC   
  
  
- TCACAAATGA GAAGACGACA AATAAGGCAG AAAATCAGCA TTAACAGAGG AAATCAAATT AAAGTTTTGG   
  
  
- GGTTAACACT AGCGGATCTA ACTCGTTAGA TTAATCAAGA TTATTAACTT TAGTTCAAGG GACACCTTAT   
  
  
- GCTGGGCATG AACGGCACAC GATCGATGAC AATGTGGAAC GTAAACGCCA TATAAATATT GAGTTGTATA   
  
  
- CAGACGAGTG ATTTTCATGT ATAATCGAGT GGTTTTGACA CAGAGTTGCA TGATTTTATA AAATCACTTG   
  
  
- ATATGTAATA AAAATCATTC GCTTTATTCT ATGTGTTGTC GTGTAAAATT ACTCAATGTC CCGTAAAAAT   
  
  
- CACTCGGCTT TAGACTACCA AAAGAAACAT CTAGGCCAAT ATATTAACAT AAAATATGTT GGCTTACCTG   
  
  
- CTATATTGTT GACTCAATTT ACTCCTCAGA CTTTAATGCT TGTATTTTAG GTTTTCTTCT ACGGCTACGG   
  
  
- TAACCTTTCT TTCGACTTCC ACTAGCGAAA GATCATCATA ATCCGTCATC ATTAATTTTT GATTTTAGGT   
  
  
- CTTAACTGTG GCTTAACCCC AACACCGTGT ACTAGCTTTA GTCGTAGCTT AGATGTGCTA CTAGACCTAA   
  
  
- GAACCTGTGA GACCGAAATC CGGCAGTTAA AGCTCCTAAC GGGTATTGAA ATTAGGACAA GTTTAGGTAA   
  
  
- GTTAAATTAT AATGTGTTTA GGAAACTAAT TTAGTGGCAT TAACACCAAA CTAATCCCTT CAAAGAACCC   
  
  
- CCTTTCCAAG TTCGAAGACA GAAAGAATTT CGGACCTAAA AACCAGGGCG TACAAGTGAA GTGTAAGGGA   
  
  
- AAAAGAGAGG GTAGCAATGG ACAGACTTAG ACATACATTA TTTTCAGTAT AAAACGCTCC AGAAGAAAAC   
  
  
- TGACAAGAAC ATCAGTGAGA ACTATAGACG TGGTAAAACA AAAAGAGCCG TAAAAGAGGT TGAACGTAAA   
  
  
- AGATTACATT ACACTAGAAA AGACCTGTAA GTAAAACAAT TAGATGAGTG AACCCAGTTC CAAACGACTA   
  
  
- AAACCGACCA CAATTTAGGC AAGGAGAAAC CGTAAACAAG TACAAGGTGT TGACAAACTA TAGACCATAG   
  
  
- AGTCTTTGGG GCGGTAAGAA ACCATAAAGG ACTGAAAGAA CAAGTTCCCA ATAAGGATAA AGGGACTTAA   
  
  
- AAGAAACTAA GAAATCCATA AAGTTCGAAC CCCCCAAAAC AAGAAGTGAC TAAAACAGGA GAAGAAGGGA   
  
  
- ATAACCCACC AAACTAGAGG ATTTAAAGAC ACATAGACTT AGTCAGCCCC ATCTGAAACA CAAAAACCAA   
  
  
- CTAAAAAATA GTTACATAAA AGAAGGATTA CTTCCCGGAG GGCTTGGTTA TGTGTACGAA TTGAAGTTGG   
  
  
- GACAAGGAGA AGGTTCGAAA AACCTCGGAG TTTTCAGAAG CTTACGACTA TCCGTGTATC CCAGGAAGGT   
  
  
- TACGATACCA GAACTACTAC TGGGGCGTGG GAACTTACTG GGTTTAATGT AACATACAGT TAGAAGTATA   
  
  
- GGACTAGCTC TATAATGTGT CTAGGGACTG AAAGGACTAC TAACAGAGTT CAAGTAGTTA CTGTAAGAAT   
  
  
- ACCTTCTCCT AAACCTATTA GGCGGATATA GGAACGTCCT GATATTACGC GAGGTTCGGT GACTCTTCAG   
  
  
- TAACATACTA CGGGAACCTC TCCGAATAGG TCGAAGTAGA CTAGGTAACG TTAGTAGTCA ACCAGTCTCG   
  
  
- TGACTCTCGA GACTACTAGG ACTGTCTTCG TCGCCGTAAC TATTTACCCC ATTATCTATA ATGGGGTGGT   
  
  
- CGGACTTACA CCAACTTTGG TCGGCCGACT AGTTAGTCTC GGTCAACCTG GGAAGAGTTT ACAAACTACG   
  
  
- ACGACGAGTG GGGAACCTCA GATTAACAGT AAGAAAACAG AGATCATTAT TGAAACTAGT AAGATACCTA   
  
  
- CCCCAAGGTC TAAGAGGATA GTCATGTGAA AGACACTTAC TTTGTTCTTT CCTTCTTAGT CAATCATCTA   
  
  
- GTTCCTCGTC CTTCTTAGTA GTTTCTCTAC CACGTATGGA CCTTCTTTCC TCATGATTCG TTTATCGTAG   
  
  
- GTCGTTACTC CTTATACCTC TCTACCTCTC CAAACTACTA CATGACTAGA CGTCCCTTCC TTTACTACTA   
  
  
- TAAAGTGGAA CATGGGCGTT TTGGGGTCAA GTACTTCGTC TACTATTGAG CCTTGGTTTC CCCTTTAGTT   
  
  
- CTCCTAGATT TTCGTTCTCA TTTAGCGCAC AGTTCTTCTT TTCATTGCTT CCTGTTCGCC ATCTAGAATC   
  
  
- CTGAGTTGAA TAAGTCACAC GTCTTCGTCA CCGTTCGAAA CTCGAATTTT CACGTTTACT CGATGAGTCC   
  
  
- GCTTAATCCG TCGTACGAAG CGGTAAACCC CTATCACAAG TTTCCGATCG TGTAATGAAA CGCTTACCCG   
  
  
- AGCTCCGTGC GAATCGACCA TGTCCTAGTC TTAGTATGTT TCTGAAACGA CCCTTTTTTA AAAGTAGGAT   
  
  
- ACTGTAGAAC TCTCCGATGT TCAGTATACA AGTTCGGCAG GGGAAGGTCT CTTGTTGGTA CAAGAACTGT   
  
  
- TTAGTTTGTT ATCATTTCGA CCGACTCTTT CGTTGTTCCC AAGTATAGTA ACTAAAACCG TAGAAAAAAC   
  
  
- CAAAGGTCAC CGGGACGGAA CAAGTCTTAA ACAGTTCTTC TGGGGTCCCA AGAGGGTATG AGTTTTAGTG   
  
  
- CCCTCAGCTA GTAGGGGTCT CACCCAAGTC TGGTCGGGTC TTTCAACTTC TCTGTCCAGC AGCTAATCGT   
  
  
- CCGATAACAC TCTCCAAACC CCACGGGAAA CTCAAAGTAG GGTAACGAGT TTCCACCGTA TGGGATTTCG   
  
  
- GTCTCGTAAA GTTTTAACTT TCGTTACTCG AACAACACCA GTGAACAAAG ATGGTTAGTC CCTTCGAGGG   
  
  
- ACTACTCTGA CAACTACAGT TGTCGGGTTC TCTGGTACAG AACTCCGACT AGTCCCCGAA CTTGGGACTA   
  
  
- GATAAGTAGG TACCCCAATA GTTACCGTGT AAGTTACGTG GGAAGAAGCA TTGAGCCAAA TTTCTCCGTG   
  
  
- AAATGGTGAT GAGAAGAAAC AAGCTACAGA AACTACGGTG GTATCTAGCA CTTTCAGTAC TTTCTGACGA   
  
  
- CTGACTCTTG ATAAACATAC CCTTTCTACG TGAATTGTAT CATCGTACAC TCCCCTTGCT CTCCTAGCTC   
  
  
- TCTGGGCTCT GAATGTTCGT CACCGTTCAG GCCTTCGAAT CTCGACCTAA GTTCGTCCAG GGGGATCTGG   
  
  
- TTCTGTAACA TTTACCTCGT TCTCGATACC AATTCCGTTT AATGTTATTC CTAAAATACC ATCTTGTTCT   
  
  
- ATCCTTAACC TACCAGGTCC CGACCTTTCC TTCCTAAAAC TCACGGTATA GGACAACCTT TGGACGAAT

+     MYB-like sequence

| Site Name | Organism | Position | Strand | Matrix score. | sequence | function |
| --- | --- | --- | --- | --- | --- | --- |
| MYB-like sequence | Arabidopsis thaliana | 3952 | - | 6 | TAACCA |  |

>HU02G01573.1   
+ +Up\_Stream \_Len000TGAATT GGAAGTTGAT TCTACCTTTG ATTGATTCGA TTAATTCTAG GTTCTTGGTT   
  
  
+ CTAGTTCTCT CTCCTCTTGT TCTTGATTAT TTAGGTTTTG GATGTGATGT TTATTGAATT GAATGTTGCA   
  
  
+ ATTGTTGTTA TAAAGATGAT GTTAGGCTAA ATATCCTTCA ACTTAGGTTG GGATTGACGT CGAATATGTG   
  
  
+ TAGTTGAATC TTGTACTGGC CTAAATCCCT GCTCCCCCTC TGTTTTGCGA TTATGAATTT CATTATGCAC   
  
  
+ ACCACATGCT CGATGAAATG CTCGAAAGAA CATTCATCCT CTTTGTTTGG GATTTAGGGG AACGTTGAAA   
  
  
+ GACGAAGTCT TTACCTAGTG CGTCTTAGTA GACAATTAGG ATTTTAACAT GAAAATAGTT AAAATTCTAA   
  
  
+ TTGGGATTTG AGATTGTGAA TCTTGTAATC CTTCGACTTC TAGTTCTATG AAAATAGGCG ATTATAGTTC   
  
  
+ TAGGATTAGT CTTCGACCCT TGAGAGAGGG AGGAGATGGG TTTAGTTGAT AATCTATTCA TTCATAACGC   
  
  
+ ATGCCTGGGA GATGTTTGGT AGGGCTAGTA ATTGATTCGC TAACATATTT GTGCTGTTAG TTCCAACCTG   
  
  
+ AGTGTTTACT CTTCTGCTGT TTATTCCGTC TTTTAGTCGT AATTGTCTCC TTTAGTTTAA TTTCAAAACC   
  
  
+ CCAATTGTGA TCGCCTAGAT TGAGCAATCT AATTAGTTCT AATAATTGAA ATCAAGTTCC CTGTGGAATA   
  
  
+ CGACCCGTAC TTGCCGTGTG CTAGCTACTG TTACACCTTG CATTTGCGGT ATATTTATAA CTCAACATAT   
  
  
+ GTCTGCTCAC TAAAAGTACA TATTAGCTCA CCAAAACTGT GTCTCAACGT ACTAAAATAT TTTAGTGAAC   
  
  
+ TATACATTAT TTTTAGTAAG CGAAATAAGA TACACAACAG CACATTTTAA TGAGTTACAG GGCATTTTTA   
  
  
+ GTGAGCCGAA ATCTGATGGT TTTCTTTGTA GATCCGGTTA TATAATTGTA TTTTATACAA CCGAATGGAC   
  
  
+ GATATAACAA CTGAGTTAAA TGAGGAGTCT GAAATTACGA ACATAAAATC CAAAAGAAGA TGCCGATGCC   
  
  
+ ATTGGAAAGA AAGCTGAAGG TGATCGCTTT CTAGTAGTAT TAGGCAGTAG TAATTAAAAA CTAAAATCCA   
  
  
+ GAATTGACAC CGAATTGGGG TTGTGGCACA TGATCGAAAT CAGCATCGAA TCTACACGAT GATCTGGATT   
  
  
+ CTTGGACACT CTGGCTTTAG GCCGTCAATT TCGAGGATTG CCCATAACTT TAATCCTGTT CAAATCCATT   
  
  
+ CAATTTAATA TTACACAAAT CCTTTGATTA AATCACCGTA ATTGTGGTTT GATTAGGGAA GTTTCTTGGG   
  
  
+ GGAAAGGTTC AAGCTTCTGT CTTTCTTAAA GCCTGGATTT TTGGTCCCGC ATGTTCACTT CACATTCCCT   
  
  
+ TTTTCTCTCC CATCGTTACC TGTCTGAATC TGTATGTAAT AAAAGTCATA TTTTGCGAGG TCTTCTTTTG   
  
  
+ ACTGTTCTTG TAGTCACTCT TGATATCTGC ACCATTTTGT TTTTCTCGGC ATTTTCTCCA ACTTGCATTT   
  
  
+ TCTAATGTAA TGTGATCTTT TCTGGACATT CATTTTGTTA ATCTACTCAC TTGGGTCAAG GTTTGCTGAT   
  
  
+ TTTGGCTGGT GTTAAATCCG TTCCTCTTTG GCATTTGTTC ATGTTCCACA ACTGTTTGAT ATCTGGTATC   
  
  
+ TCAGAAACCC CGCCATTCTT TGGTATTTCC TGACTTTCTT GTTCAAGGGT TATTCCTATT TCCCTGAATT   
  
  
+ TTCTTTGATT CTTTAGGTAT TTCAAGCTTG GGGGGTTTTG TTCTTCACTG ATTTTGTCCT CTTCTTCCCT   
  
  
+ TATTGGGTGG TTTGATCTCC TAAATTTCTG TGTATCTGAA TCAGTCGGGG TAGACTTTGT GTTTTTGGTT   
  
  
+ GATTTTTTAT CAATGTATTT TCTTCCTAAT GAAGGGCCTC CCGAACCAAT ACACATGCTT AACTTCAACC   
  
  
+ CTGTTCCTCT TCCAAGCTTT TTGGAGCCTC AAAAGTCTTC GAATGCTGAT AGGCACATAG GGTCCTTCCA   
  
  
+ ATGCTATGGT CTTGATGATG ACCCCGCACC CTTGAATGAC CCAAATTACA TTGTATGTCA ATCTTCATAT   
  
  
+ CCTGATCGAG ATATTACACA GATCCCTGAC TTTCCTGATG ATTGTCTCAA GTTCATCAAT GACATTCTTA   
  
  
+ TGGAAGAGGA TTTGGATAAT CCGCCTATAT CCTTGCAGGA CTATAATGCG CTCCAAGCCA CTGAGAAGTC   
  
  
+ ATTGTATGAT GCCCTTGGAG AGGCTTATCC AGCTTCATCT GATCCATTGC AATCATCAGT TGGTCAGAGC   
  
  
+ ACTGAGAGCT CTGATGATCC TGACAGAAGC AGCGGCATTG ATAAATGGGG TAATAGATAT TACCCCACCA   
  
  
+ GCCTGAATGT GGTTGAAACC AGCCGGCTGA TCAATCAGAG CCAGTTGGAC CCTTCTCAAA TGTTTGATGC   
  
  
+ TGCTGCTCAC CCCTTGGAGT CTAATTGTCA TTCTTTTGTC TCTAGTAATA ACTTTGATCA TTCTATGGAT   
  
  
+ GGGGTTCCAG ATTCTCCTAT CAGTACACTT TCTGTGAATG AAACAAGAAA GGAAGAATCA GTTAGTAGAT   
  
  
+ CAAGGAGCAG GAAGAATCAT CAAAGAGATG GTGCATACCT GGAAGAAAGG AGTACTAAGC AAATAGCATC   
  
  
+ CAGCAATGAG GAATATGGAG AGATGGAGAG GTTTGATGAT GTACTGATCT GCAGGGAAGG AAATGATGAT   
  
  
+ ATTTCACCTT GTACCCGCAA AACCCCAGTT CATGAAGCAG ATGATAACTC GGAACCAAAG GGGAAATCAA   
  
  
+ GAGGATCTAA AAGCAAGAGT AAATCGCGTG TCAAGAAGAA AAGTAACGAA GGACAAGCGG TAGATCTTAG   
  
  
+ GACTCAACTT ATTCAGTGTG CAGAAGCAGT GGCAAGCTTT GAGCTTAAAA GTGCAAATGA GCTACTCAGG   
  
  
+ CGAATTAGGC AGCATGCTTC GCCATTTGGG GATAGTGTTC AAAGGCTAGC ACATTACTTT GCGAATGGGC   
  
  
+ TCGAGGCACG CTTAGCTGGT ACAGGATCAG AATCATACAA AGACTTTGCT GGGAAAAAAT TTTCATCCTA   
  
  
+ TGACATCTTG AGAGGCTACA AGTCATATGT TCAAGCCGTC CCCTTCCAGA GAACAACCAT GTTCTTGACA   
  
  
+ AATCAAACAA TAGTAAAGCT GGCTGAGAAA GCAACAAGGG TTCATATCAT TGATTTTGGC ATCTTTTTTG   
  
  
+ GTTTCCAGTG GCCCTGCCTT GTTCAGAATT TGTCAAGAAG ACCCCAGGGT TCTCCCATAC TCAAAATCAC   
  
  
+ GGGAGTCGAT CATCCCCAGA GTGGGTTCAG ACCAGCCCAG AAAGTTGAAG AGACAGGTCG TCGATTAGCA   
  
  
+ GGCTATTGTG AGAGGTTTGG GGTGCCCTTT GAGTTTCATC CCATTGCTCA AAGGTGGCAT ACCCTAAAGC   
  
  
+ CAGAGCATTT CAAAATTGAA AGCAATGAGC TTGTTGTGGT CACTTGTTTC TACCAATCAG GGAAGCTCCC   
  
  
+ TGATGAGACT GTTGATGTCA ACAGCCCAAG AGACCATGTC TTGAGGCTGA TCAGGGGCTT GAACCCTGAT   
  
  
+ CTATTCATCC ATGGGGTTAT CAATGGCACA TTCAATGCAC CCTTCTTCGT AACTCGGTTT AAAGAGGCAC   
  
  
+ TTTACCACTA CTCTTCTTTG TTCGATGTCT TTGATGCCAC CATAGATCGT GAAAGTCATG AAAGACTGCT   
  
  
+ GACTGAGAAC TATTTGTATG GGAAAGATGC ACTTAACATA GTAGCATGTG AGGGGAACGA GAGGATCGAG   
  
  
+ AGACCCGAGA CTTACAAGCA GTGGCAAGTC CGGAAGCTTA GAGCTGGATT CAAGCAGGTC CCCCTAGACC   
  
  
+ AAGACATTGT AAATGGAGCA AGAGCTATGG TTAAGGCAAA TTACAATAAG GATTTTATGG TAGAACAAGA   
  
  
+ TAGGAATTGG ATGGTCCAGG GCTGGAAAGG AAGGATTTTG AGTGCCATAT CCTGTTGGAA ACCTGCTTA  

- +Up\_Stream \_Len000ACTTAA CCTTCAACTA AGATGGAAAC TAACTAAGCT AATTAAGATC CAAGAACCAA   
  
  
- GATCAAGAGA GAGGAGAACA AGAACTAATA AATCCAAAAC CTACACTACA AATAACTTAA CTTACAACGT   
  
  
- TAACAACAAT ATTTCTACTA CAATCCGATT TATAGGAAGT TGAATCCAAC CCTAACTGCA GCTTATACAC   
  
  
- ATCAACTTAG AACATGACCG GATTTAGGGA CGAGGGGGAG ACAAAACGCT AATACTTAAA GTAATACGTG   
  
  
- TGGTGTACGA GCTACTTTAC GAGCTTTCTT GTAAGTAGGA GAAACAAACC CTAAATCCCC TTGCAACTTT   
  
  
- CTGCTTCAGA AATGGATCAC GCAGAATCAT CTGTTAATCC TAAAATTGTA CTTTTATCAA TTTTAAGATT   
  
  
- AACCCTAAAC TCTAACACTT AGAACATTAG GAAGCTGAAG ATCAAGATAC TTTTATCCGC TAATATCAAG   
  
  
- ATCCTAATCA GAAGCTGGGA ACTCTCTCCC TCCTCTACCC AAATCAACTA TTAGATAAGT AAGTATTGCG   
  
  
- TACGGACCCT CTACAAACCA TCCCGATCAT TAACTAAGCG ATTGTATAAA CACGACAATC AAGGTTGGAC   
  
  
- TCACAAATGA GAAGACGACA AATAAGGCAG AAAATCAGCA TTAACAGAGG AAATCAAATT AAAGTTTTGG   
  
  
- GGTTAACACT AGCGGATCTA ACTCGTTAGA TTAATCAAGA TTATTAACTT TAGTTCAAGG GACACCTTAT   
  
  
- GCTGGGCATG AACGGCACAC GATCGATGAC AATGTGGAAC GTAAACGCCA TATAAATATT GAGTTGTATA   
  
  
- CAGACGAGTG ATTTTCATGT ATAATCGAGT GGTTTTGACA CAGAGTTGCA TGATTTTATA AAATCACTTG   
  
  
- ATATGTAATA AAAATCATTC GCTTTATTCT ATGTGTTGTC GTGTAAAATT ACTCAATGTC CCGTAAAAAT   
  
  
- CACTCGGCTT TAGACTACCA AAAGAAACAT CTAGGCCAAT ATATTAACAT AAAATATGTT GGCTTACCTG   
  
  
- CTATATTGTT GACTCAATTT ACTCCTCAGA CTTTAATGCT TGTATTTTAG GTTTTCTTCT ACGGCTACGG   
  
  
- TAACCTTTCT TTCGACTTCC ACTAGCGAAA GATCATCATA ATCCGTCATC ATTAATTTTT GATTTTAGGT   
  
  
- CTTAACTGTG GCTTAACCCC AACACCGTGT ACTAGCTTTA GTCGTAGCTT AGATGTGCTA CTAGACCTAA   
  
  
- GAACCTGTGA GACCGAAATC CGGCAGTTAA AGCTCCTAAC GGGTATTGAA ATTAGGACAA GTTTAGGTAA   
  
  
- GTTAAATTAT AATGTGTTTA GGAAACTAAT TTAGTGGCAT TAACACCAAA CTAATCCCTT CAAAGAACCC   
  
  
- CCTTTCCAAG TTCGAAGACA GAAAGAATTT CGGACCTAAA AACCAGGGCG TACAAGTGAA GTGTAAGGGA   
  
  
- AAAAGAGAGG GTAGCAATGG ACAGACTTAG ACATACATTA TTTTCAGTAT AAAACGCTCC AGAAGAAAAC   
  
  
- TGACAAGAAC ATCAGTGAGA ACTATAGACG TGGTAAAACA AAAAGAGCCG TAAAAGAGGT TGAACGTAAA   
  
  
- AGATTACATT ACACTAGAAA AGACCTGTAA GTAAAACAAT TAGATGAGTG AACCCAGTTC CAAACGACTA   
  
  
- AAACCGACCA CAATTTAGGC AAGGAGAAAC CGTAAACAAG TACAAGGTGT TGACAAACTA TAGACCATAG   
  
  
- AGTCTTTGGG GCGGTAAGAA ACCATAAAGG ACTGAAAGAA CAAGTTCCCA ATAAGGATAA AGGGACTTAA   
  
  
- AAGAAACTAA GAAATCCATA AAGTTCGAAC CCCCCAAAAC AAGAAGTGAC TAAAACAGGA GAAGAAGGGA   
  
  
- ATAACCCACC AAACTAGAGG ATTTAAAGAC ACATAGACTT AGTCAGCCCC ATCTGAAACA CAAAAACCAA   
  
  
- CTAAAAAATA GTTACATAAA AGAAGGATTA CTTCCCGGAG GGCTTGGTTA TGTGTACGAA TTGAAGTTGG   
  
  
- GACAAGGAGA AGGTTCGAAA AACCTCGGAG TTTTCAGAAG CTTACGACTA TCCGTGTATC CCAGGAAGGT   
  
  
- TACGATACCA GAACTACTAC TGGGGCGTGG GAACTTACTG GGTTTAATGT AACATACAGT TAGAAGTATA   
  
  
- GGACTAGCTC TATAATGTGT CTAGGGACTG AAAGGACTAC TAACAGAGTT CAAGTAGTTA CTGTAAGAAT   
  
  
- ACCTTCTCCT AAACCTATTA GGCGGATATA GGAACGTCCT GATATTACGC GAGGTTCGGT GACTCTTCAG   
  
  
- TAACATACTA CGGGAACCTC TCCGAATAGG TCGAAGTAGA CTAGGTAACG TTAGTAGTCA ACCAGTCTCG   
  
  
- TGACTCTCGA GACTACTAGG ACTGTCTTCG TCGCCGTAAC TATTTACCCC ATTATCTATA ATGGGGTGGT   
  
  
- CGGACTTACA CCAACTTTGG TCGGCCGACT AGTTAGTCTC GGTCAACCTG GGAAGAGTTT ACAAACTACG   
  
  
- ACGACGAGTG GGGAACCTCA GATTAACAGT AAGAAAACAG AGATCATTAT TGAAACTAGT AAGATACCTA   
  
  
- CCCCAAGGTC TAAGAGGATA GTCATGTGAA AGACACTTAC TTTGTTCTTT CCTTCTTAGT CAATCATCTA   
  
  
- GTTCCTCGTC CTTCTTAGTA GTTTCTCTAC CACGTATGGA CCTTCTTTCC TCATGATTCG TTTATCGTAG   
  
  
- GTCGTTACTC CTTATACCTC TCTACCTCTC CAAACTACTA CATGACTAGA CGTCCCTTCC TTTACTACTA   
  
  
- TAAAGTGGAA CATGGGCGTT TTGGGGTCAA GTACTTCGTC TACTATTGAG CCTTGGTTTC CCCTTTAGTT   
  
  
- CTCCTAGATT TTCGTTCTCA TTTAGCGCAC AGTTCTTCTT TTCATTGCTT CCTGTTCGCC ATCTAGAATC   
  
  
- CTGAGTTGAA TAAGTCACAC GTCTTCGTCA CCGTTCGAAA CTCGAATTTT CACGTTTACT CGATGAGTCC   
  
  
- GCTTAATCCG TCGTACGAAG CGGTAAACCC CTATCACAAG TTTCCGATCG TGTAATGAAA CGCTTACCCG   
  
  
- AGCTCCGTGC GAATCGACCA TGTCCTAGTC TTAGTATGTT TCTGAAACGA CCCTTTTTTA AAAGTAGGAT   
  
  
- ACTGTAGAAC TCTCCGATGT TCAGTATACA AGTTCGGCAG GGGAAGGTCT CTTGTTGGTA CAAGAACTGT   
  
  
- TTAGTTTGTT ATCATTTCGA CCGACTCTTT CGTTGTTCCC AAGTATAGTA ACTAAAACCG TAGAAAAAAC   
  
  
- CAAAGGTCAC CGGGACGGAA CAAGTCTTAA ACAGTTCTTC TGGGGTCCCA AGAGGGTATG AGTTTTAGTG   
  
  
- CCCTCAGCTA GTAGGGGTCT CACCCAAGTC TGGTCGGGTC TTTCAACTTC TCTGTCCAGC AGCTAATCGT   
  
  
- CCGATAACAC TCTCCAAACC CCACGGGAAA CTCAAAGTAG GGTAACGAGT TTCCACCGTA TGGGATTTCG   
  
  
- GTCTCGTAAA GTTTTAACTT TCGTTACTCG AACAACACCA GTGAACAAAG ATGGTTAGTC CCTTCGAGGG   
  
  
- ACTACTCTGA CAACTACAGT TGTCGGGTTC TCTGGTACAG AACTCCGACT AGTCCCCGAA CTTGGGACTA   
  
  
- GATAAGTAGG TACCCCAATA GTTACCGTGT AAGTTACGTG GGAAGAAGCA TTGAGCCAAA TTTCTCCGTG   
  
  
- AAATGGTGAT GAGAAGAAAC AAGCTACAGA AACTACGGTG GTATCTAGCA CTTTCAGTAC TTTCTGACGA   
  
  
- CTGACTCTTG ATAAACATAC CCTTTCTACG TGAATTGTAT CATCGTACAC TCCCCTTGCT CTCCTAGCTC   
  
  
- TCTGGGCTCT GAATGTTCGT CACCGTTCAG GCCTTCGAAT CTCGACCTAA GTTCGTCCAG GGGGATCTGG   
  
  
- TTCTGTAACA TTTACCTCGT TCTCGATACC AATTCCGTTT AATGTTATTC CTAAAATACC ATCTTGTTCT   
  
  
- ATCCTTAACC TACCAGGTCC CGACCTTTCC TTCCTAAAAC TCACGGTATA GGACAACCTT TGGACGAAT

+     MYC

| Site Name | Organism | Position | Strand | Matrix score. | sequence | function |
| --- | --- | --- | --- | --- | --- | --- |
| MYC | Arabidopsis thaliana | 1221 | - | 6 | CATGTG |  |
| MYC | Arabidopsis thaliana | 2998 | - | 6 | CATTTG |  |
| MYC | Arabidopsis thaliana | 287 | - | 6 | CATGTG |  |
| MYC | Arabidopsis thaliana | 2016 | - | 6 | CATGTG |  |
| MYC | Arabidopsis thaliana | 815 | + | 6 | CATTTG |  |
| MYC | Arabidopsis thaliana | 3829 | + | 6 | CATGTG |  |
| MYC | Arabidopsis thaliana | 1716 | + | 6 | CATTTG |  |
| MYC | Arabidopsis thaliana | 143 | + | 6 | CAATTG |  |
| MYC | Arabidopsis thaliana | 706 | + | 6 | CAATTG |  |
| MYC | Arabidopsis thaliana | 2511 | - | 6 | CATTTG |  |
| MYC | Arabidopsis thaliana | 3037 | + | 6 | CATTTG |  |

>HU02G01573.1   
+ +Up\_Stream \_Len000TGAATT GGAAGTTGAT TCTACCTTTG ATTGATTCGA TTAATTCTAG GTTCTTGGTT   
  
  
+ CTAGTTCTCT CTCCTCTTGT TCTTGATTAT TTAGGTTTTG GATGTGATGT TTATTGAATT GAATGTTGCA   
  
  
+ ATTGTTGTTA TAAAGATGAT GTTAGGCTAA ATATCCTTCA ACTTAGGTTG GGATTGACGT CGAATATGTG   
  
  
+ TAGTTGAATC TTGTACTGGC CTAAATCCCT GCTCCCCCTC TGTTTTGCGA TTATGAATTT CATTATGCAC   
  
  
+ ACCACATGCT CGATGAAATG CTCGAAAGAA CATTCATCCT CTTTGTTTGG GATTTAGGGG AACGTTGAAA   
  
  
+ GACGAAGTCT TTACCTAGTG CGTCTTAGTA GACAATTAGG ATTTTAACAT GAAAATAGTT AAAATTCTAA   
  
  
+ TTGGGATTTG AGATTGTGAA TCTTGTAATC CTTCGACTTC TAGTTCTATG AAAATAGGCG ATTATAGTTC   
  
  
+ TAGGATTAGT CTTCGACCCT TGAGAGAGGG AGGAGATGGG TTTAGTTGAT AATCTATTCA TTCATAACGC   
  
  
+ ATGCCTGGGA GATGTTTGGT AGGGCTAGTA ATTGATTCGC TAACATATTT GTGCTGTTAG TTCCAACCTG   
  
  
+ AGTGTTTACT CTTCTGCTGT TTATTCCGTC TTTTAGTCGT AATTGTCTCC TTTAGTTTAA TTTCAAAACC   
  
  
+ CCAATTGTGA TCGCCTAGAT TGAGCAATCT AATTAGTTCT AATAATTGAA ATCAAGTTCC CTGTGGAATA   
  
  
+ CGACCCGTAC TTGCCGTGTG CTAGCTACTG TTACACCTTG CATTTGCGGT ATATTTATAA CTCAACATAT   
  
  
+ GTCTGCTCAC TAAAAGTACA TATTAGCTCA CCAAAACTGT GTCTCAACGT ACTAAAATAT TTTAGTGAAC   
  
  
+ TATACATTAT TTTTAGTAAG CGAAATAAGA TACACAACAG CACATTTTAA TGAGTTACAG GGCATTTTTA   
  
  
+ GTGAGCCGAA ATCTGATGGT TTTCTTTGTA GATCCGGTTA TATAATTGTA TTTTATACAA CCGAATGGAC   
  
  
+ GATATAACAA CTGAGTTAAA TGAGGAGTCT GAAATTACGA ACATAAAATC CAAAAGAAGA TGCCGATGCC   
  
  
+ ATTGGAAAGA AAGCTGAAGG TGATCGCTTT CTAGTAGTAT TAGGCAGTAG TAATTAAAAA CTAAAATCCA   
  
  
+ GAATTGACAC CGAATTGGGG TTGTGGCACA TGATCGAAAT CAGCATCGAA TCTACACGAT GATCTGGATT   
  
  
+ CTTGGACACT CTGGCTTTAG GCCGTCAATT TCGAGGATTG CCCATAACTT TAATCCTGTT CAAATCCATT   
  
  
+ CAATTTAATA TTACACAAAT CCTTTGATTA AATCACCGTA ATTGTGGTTT GATTAGGGAA GTTTCTTGGG   
  
  
+ GGAAAGGTTC AAGCTTCTGT CTTTCTTAAA GCCTGGATTT TTGGTCCCGC ATGTTCACTT CACATTCCCT   
  
  
+ TTTTCTCTCC CATCGTTACC TGTCTGAATC TGTATGTAAT AAAAGTCATA TTTTGCGAGG TCTTCTTTTG   
  
  
+ ACTGTTCTTG TAGTCACTCT TGATATCTGC ACCATTTTGT TTTTCTCGGC ATTTTCTCCA ACTTGCATTT   
  
  
+ TCTAATGTAA TGTGATCTTT TCTGGACATT CATTTTGTTA ATCTACTCAC TTGGGTCAAG GTTTGCTGAT   
  
  
+ TTTGGCTGGT GTTAAATCCG TTCCTCTTTG GCATTTGTTC ATGTTCCACA ACTGTTTGAT ATCTGGTATC   
  
  
+ TCAGAAACCC CGCCATTCTT TGGTATTTCC TGACTTTCTT GTTCAAGGGT TATTCCTATT TCCCTGAATT   
  
  
+ TTCTTTGATT CTTTAGGTAT TTCAAGCTTG GGGGGTTTTG TTCTTCACTG ATTTTGTCCT CTTCTTCCCT   
  
  
+ TATTGGGTGG TTTGATCTCC TAAATTTCTG TGTATCTGAA TCAGTCGGGG TAGACTTTGT GTTTTTGGTT   
  
  
+ GATTTTTTAT CAATGTATTT TCTTCCTAAT GAAGGGCCTC CCGAACCAAT ACACATGCTT AACTTCAACC   
  
  
+ CTGTTCCTCT TCCAAGCTTT TTGGAGCCTC AAAAGTCTTC GAATGCTGAT AGGCACATAG GGTCCTTCCA   
  
  
+ ATGCTATGGT CTTGATGATG ACCCCGCACC CTTGAATGAC CCAAATTACA TTGTATGTCA ATCTTCATAT   
  
  
+ CCTGATCGAG ATATTACACA GATCCCTGAC TTTCCTGATG ATTGTCTCAA GTTCATCAAT GACATTCTTA   
  
  
+ TGGAAGAGGA TTTGGATAAT CCGCCTATAT CCTTGCAGGA CTATAATGCG CTCCAAGCCA CTGAGAAGTC   
  
  
+ ATTGTATGAT GCCCTTGGAG AGGCTTATCC AGCTTCATCT GATCCATTGC AATCATCAGT TGGTCAGAGC   
  
  
+ ACTGAGAGCT CTGATGATCC TGACAGAAGC AGCGGCATTG ATAAATGGGG TAATAGATAT TACCCCACCA   
  
  
+ GCCTGAATGT GGTTGAAACC AGCCGGCTGA TCAATCAGAG CCAGTTGGAC CCTTCTCAAA TGTTTGATGC   
  
  
+ TGCTGCTCAC CCCTTGGAGT CTAATTGTCA TTCTTTTGTC TCTAGTAATA ACTTTGATCA TTCTATGGAT   
  
  
+ GGGGTTCCAG ATTCTCCTAT CAGTACACTT TCTGTGAATG AAACAAGAAA GGAAGAATCA GTTAGTAGAT   
  
  
+ CAAGGAGCAG GAAGAATCAT CAAAGAGATG GTGCATACCT GGAAGAAAGG AGTACTAAGC AAATAGCATC   
  
  
+ CAGCAATGAG GAATATGGAG AGATGGAGAG GTTTGATGAT GTACTGATCT GCAGGGAAGG AAATGATGAT   
  
  
+ ATTTCACCTT GTACCCGCAA AACCCCAGTT CATGAAGCAG ATGATAACTC GGAACCAAAG GGGAAATCAA   
  
  
+ GAGGATCTAA AAGCAAGAGT AAATCGCGTG TCAAGAAGAA AAGTAACGAA GGACAAGCGG TAGATCTTAG   
  
  
+ GACTCAACTT ATTCAGTGTG CAGAAGCAGT GGCAAGCTTT GAGCTTAAAA GTGCAAATGA GCTACTCAGG   
  
  
+ CGAATTAGGC AGCATGCTTC GCCATTTGGG GATAGTGTTC AAAGGCTAGC ACATTACTTT GCGAATGGGC   
  
  
+ TCGAGGCACG CTTAGCTGGT ACAGGATCAG AATCATACAA AGACTTTGCT GGGAAAAAAT TTTCATCCTA   
  
  
+ TGACATCTTG AGAGGCTACA AGTCATATGT TCAAGCCGTC CCCTTCCAGA GAACAACCAT GTTCTTGACA   
  
  
+ AATCAAACAA TAGTAAAGCT GGCTGAGAAA GCAACAAGGG TTCATATCAT TGATTTTGGC ATCTTTTTTG   
  
  
+ GTTTCCAGTG GCCCTGCCTT GTTCAGAATT TGTCAAGAAG ACCCCAGGGT TCTCCCATAC TCAAAATCAC   
  
  
+ GGGAGTCGAT CATCCCCAGA GTGGGTTCAG ACCAGCCCAG AAAGTTGAAG AGACAGGTCG TCGATTAGCA   
  
  
+ GGCTATTGTG AGAGGTTTGG GGTGCCCTTT GAGTTTCATC CCATTGCTCA AAGGTGGCAT ACCCTAAAGC   
  
  
+ CAGAGCATTT CAAAATTGAA AGCAATGAGC TTGTTGTGGT CACTTGTTTC TACCAATCAG GGAAGCTCCC   
  
  
+ TGATGAGACT GTTGATGTCA ACAGCCCAAG AGACCATGTC TTGAGGCTGA TCAGGGGCTT GAACCCTGAT   
  
  
+ CTATTCATCC ATGGGGTTAT CAATGGCACA TTCAATGCAC CCTTCTTCGT AACTCGGTTT AAAGAGGCAC   
  
  
+ TTTACCACTA CTCTTCTTTG TTCGATGTCT TTGATGCCAC CATAGATCGT GAAAGTCATG AAAGACTGCT   
  
  
+ GACTGAGAAC TATTTGTATG GGAAAGATGC ACTTAACATA GTAGCATGTG AGGGGAACGA GAGGATCGAG   
  
  
+ AGACCCGAGA CTTACAAGCA GTGGCAAGTC CGGAAGCTTA GAGCTGGATT CAAGCAGGTC CCCCTAGACC   
  
  
+ AAGACATTGT AAATGGAGCA AGAGCTATGG TTAAGGCAAA TTACAATAAG GATTTTATGG TAGAACAAGA   
  
  
+ TAGGAATTGG ATGGTCCAGG GCTGGAAAGG AAGGATTTTG AGTGCCATAT CCTGTTGGAA ACCTGCTTA  

- +Up\_Stream \_Len000ACTTAA CCTTCAACTA AGATGGAAAC TAACTAAGCT AATTAAGATC CAAGAACCAA   
  
  
- GATCAAGAGA GAGGAGAACA AGAACTAATA AATCCAAAAC CTACACTACA AATAACTTAA CTTACAACGT   
  
  
- TAACAACAAT ATTTCTACTA CAATCCGATT TATAGGAAGT TGAATCCAAC CCTAACTGCA GCTTATACAC   
  
  
- ATCAACTTAG AACATGACCG GATTTAGGGA CGAGGGGGAG ACAAAACGCT AATACTTAAA GTAATACGTG   
  
  
- TGGTGTACGA GCTACTTTAC GAGCTTTCTT GTAAGTAGGA GAAACAAACC CTAAATCCCC TTGCAACTTT   
  
  
- CTGCTTCAGA AATGGATCAC GCAGAATCAT CTGTTAATCC TAAAATTGTA CTTTTATCAA TTTTAAGATT   
  
  
- AACCCTAAAC TCTAACACTT AGAACATTAG GAAGCTGAAG ATCAAGATAC TTTTATCCGC TAATATCAAG   
  
  
- ATCCTAATCA GAAGCTGGGA ACTCTCTCCC TCCTCTACCC AAATCAACTA TTAGATAAGT AAGTATTGCG   
  
  
- TACGGACCCT CTACAAACCA TCCCGATCAT TAACTAAGCG ATTGTATAAA CACGACAATC AAGGTTGGAC   
  
  
- TCACAAATGA GAAGACGACA AATAAGGCAG AAAATCAGCA TTAACAGAGG AAATCAAATT AAAGTTTTGG   
  
  
- GGTTAACACT AGCGGATCTA ACTCGTTAGA TTAATCAAGA TTATTAACTT TAGTTCAAGG GACACCTTAT   
  
  
- GCTGGGCATG AACGGCACAC GATCGATGAC AATGTGGAAC GTAAACGCCA TATAAATATT GAGTTGTATA   
  
  
- CAGACGAGTG ATTTTCATGT ATAATCGAGT GGTTTTGACA CAGAGTTGCA TGATTTTATA AAATCACTTG   
  
  
- ATATGTAATA AAAATCATTC GCTTTATTCT ATGTGTTGTC GTGTAAAATT ACTCAATGTC CCGTAAAAAT   
  
  
- CACTCGGCTT TAGACTACCA AAAGAAACAT CTAGGCCAAT ATATTAACAT AAAATATGTT GGCTTACCTG   
  
  
- CTATATTGTT GACTCAATTT ACTCCTCAGA CTTTAATGCT TGTATTTTAG GTTTTCTTCT ACGGCTACGG   
  
  
- TAACCTTTCT TTCGACTTCC ACTAGCGAAA GATCATCATA ATCCGTCATC ATTAATTTTT GATTTTAGGT   
  
  
- CTTAACTGTG GCTTAACCCC AACACCGTGT ACTAGCTTTA GTCGTAGCTT AGATGTGCTA CTAGACCTAA   
  
  
- GAACCTGTGA GACCGAAATC CGGCAGTTAA AGCTCCTAAC GGGTATTGAA ATTAGGACAA GTTTAGGTAA   
  
  
- GTTAAATTAT AATGTGTTTA GGAAACTAAT TTAGTGGCAT TAACACCAAA CTAATCCCTT CAAAGAACCC   
  
  
- CCTTTCCAAG TTCGAAGACA GAAAGAATTT CGGACCTAAA AACCAGGGCG TACAAGTGAA GTGTAAGGGA   
  
  
- AAAAGAGAGG GTAGCAATGG ACAGACTTAG ACATACATTA TTTTCAGTAT AAAACGCTCC AGAAGAAAAC   
  
  
- TGACAAGAAC ATCAGTGAGA ACTATAGACG TGGTAAAACA AAAAGAGCCG TAAAAGAGGT TGAACGTAAA   
  
  
- AGATTACATT ACACTAGAAA AGACCTGTAA GTAAAACAAT TAGATGAGTG AACCCAGTTC CAAACGACTA   
  
  
- AAACCGACCA CAATTTAGGC AAGGAGAAAC CGTAAACAAG TACAAGGTGT TGACAAACTA TAGACCATAG   
  
  
- AGTCTTTGGG GCGGTAAGAA ACCATAAAGG ACTGAAAGAA CAAGTTCCCA ATAAGGATAA AGGGACTTAA   
  
  
- AAGAAACTAA GAAATCCATA AAGTTCGAAC CCCCCAAAAC AAGAAGTGAC TAAAACAGGA GAAGAAGGGA   
  
  
- ATAACCCACC AAACTAGAGG ATTTAAAGAC ACATAGACTT AGTCAGCCCC ATCTGAAACA CAAAAACCAA   
  
  
- CTAAAAAATA GTTACATAAA AGAAGGATTA CTTCCCGGAG GGCTTGGTTA TGTGTACGAA TTGAAGTTGG   
  
  
- GACAAGGAGA AGGTTCGAAA AACCTCGGAG TTTTCAGAAG CTTACGACTA TCCGTGTATC CCAGGAAGGT   
  
  
- TACGATACCA GAACTACTAC TGGGGCGTGG GAACTTACTG GGTTTAATGT AACATACAGT TAGAAGTATA   
  
  
- GGACTAGCTC TATAATGTGT CTAGGGACTG AAAGGACTAC TAACAGAGTT CAAGTAGTTA CTGTAAGAAT   
  
  
- ACCTTCTCCT AAACCTATTA GGCGGATATA GGAACGTCCT GATATTACGC GAGGTTCGGT GACTCTTCAG   
  
  
- TAACATACTA CGGGAACCTC TCCGAATAGG TCGAAGTAGA CTAGGTAACG TTAGTAGTCA ACCAGTCTCG   
  
  
- TGACTCTCGA GACTACTAGG ACTGTCTTCG TCGCCGTAAC TATTTACCCC ATTATCTATA ATGGGGTGGT   
  
  
- CGGACTTACA CCAACTTTGG TCGGCCGACT AGTTAGTCTC GGTCAACCTG GGAAGAGTTT ACAAACTACG   
  
  
- ACGACGAGTG GGGAACCTCA GATTAACAGT AAGAAAACAG AGATCATTAT TGAAACTAGT AAGATACCTA   
  
  
- CCCCAAGGTC TAAGAGGATA GTCATGTGAA AGACACTTAC TTTGTTCTTT CCTTCTTAGT CAATCATCTA   
  
  
- GTTCCTCGTC CTTCTTAGTA GTTTCTCTAC CACGTATGGA CCTTCTTTCC TCATGATTCG TTTATCGTAG   
  
  
- GTCGTTACTC CTTATACCTC TCTACCTCTC CAAACTACTA CATGACTAGA CGTCCCTTCC TTTACTACTA   
  
  
- TAAAGTGGAA CATGGGCGTT TTGGGGTCAA GTACTTCGTC TACTATTGAG CCTTGGTTTC CCCTTTAGTT   
  
  
- CTCCTAGATT TTCGTTCTCA TTTAGCGCAC AGTTCTTCTT TTCATTGCTT CCTGTTCGCC ATCTAGAATC   
  
  
- CTGAGTTGAA TAAGTCACAC GTCTTCGTCA CCGTTCGAAA CTCGAATTTT CACGTTTACT CGATGAGTCC   
  
  
- GCTTAATCCG TCGTACGAAG CGGTAAACCC CTATCACAAG TTTCCGATCG TGTAATGAAA CGCTTACCCG   
  
  
- AGCTCCGTGC GAATCGACCA TGTCCTAGTC TTAGTATGTT TCTGAAACGA CCCTTTTTTA AAAGTAGGAT   
  
  
- ACTGTAGAAC TCTCCGATGT TCAGTATACA AGTTCGGCAG GGGAAGGTCT CTTGTTGGTA CAAGAACTGT   
  
  
- TTAGTTTGTT ATCATTTCGA CCGACTCTTT CGTTGTTCCC AAGTATAGTA ACTAAAACCG TAGAAAAAAC   
  
  
- CAAAGGTCAC CGGGACGGAA CAAGTCTTAA ACAGTTCTTC TGGGGTCCCA AGAGGGTATG AGTTTTAGTG   
  
  
- CCCTCAGCTA GTAGGGGTCT CACCCAAGTC TGGTCGGGTC TTTCAACTTC TCTGTCCAGC AGCTAATCGT   
  
  
- CCGATAACAC TCTCCAAACC CCACGGGAAA CTCAAAGTAG GGTAACGAGT TTCCACCGTA TGGGATTTCG   
  
  
- GTCTCGTAAA GTTTTAACTT TCGTTACTCG AACAACACCA GTGAACAAAG ATGGTTAGTC CCTTCGAGGG   
  
  
- ACTACTCTGA CAACTACAGT TGTCGGGTTC TCTGGTACAG AACTCCGACT AGTCCCCGAA CTTGGGACTA   
  
  
- GATAAGTAGG TACCCCAATA GTTACCGTGT AAGTTACGTG GGAAGAAGCA TTGAGCCAAA TTTCTCCGTG   
  
  
- AAATGGTGAT GAGAAGAAAC AAGCTACAGA AACTACGGTG GTATCTAGCA CTTTCAGTAC TTTCTGACGA   
  
  
- CTGACTCTTG ATAAACATAC CCTTTCTACG TGAATTGTAT CATCGTACAC TCCCCTTGCT CTCCTAGCTC   
  
  
- TCTGGGCTCT GAATGTTCGT CACCGTTCAG GCCTTCGAAT CTCGACCTAA GTTCGTCCAG GGGGATCTGG   
  
  
- TTCTGTAACA TTTACCTCGT TCTCGATACC AATTCCGTTT AATGTTATTC CTAAAATACC ATCTTGTTCT   
  
  
- ATCCTTAACC TACCAGGTCC CGACCTTTCC TTCCTAAAAC TCACGGTATA GGACAACCTT TGGACGAAT

+     Myb

| Site Name | Organism | Position | Strand | Matrix score. | sequence | function |
| --- | --- | --- | --- | --- | --- | --- |
| Myb | Arabidopsis thaliana | 1733 | + | 6 | CAACTG |  |
| Myb | Arabidopsis thaliana | 2653 | - | 6 | TAACTG |  |
| Myb | Arabidopsis thaliana | 2371 | - | 6 | CAACTG |  |
| Myb | Arabidopsis thaliana | 1062 | + | 6 | CAACTG |  |
| Myb | Arabidopsis thaliana | 2496 | - | 6 | CAACTG |  |

>HU02G01573.1   
+ +Up\_Stream \_Len000TGAATT GGAAGTTGAT TCTACCTTTG ATTGATTCGA TTAATTCTAG GTTCTTGGTT   
  
  
+ CTAGTTCTCT CTCCTCTTGT TCTTGATTAT TTAGGTTTTG GATGTGATGT TTATTGAATT GAATGTTGCA   
  
  
+ ATTGTTGTTA TAAAGATGAT GTTAGGCTAA ATATCCTTCA ACTTAGGTTG GGATTGACGT CGAATATGTG   
  
  
+ TAGTTGAATC TTGTACTGGC CTAAATCCCT GCTCCCCCTC TGTTTTGCGA TTATGAATTT CATTATGCAC   
  
  
+ ACCACATGCT CGATGAAATG CTCGAAAGAA CATTCATCCT CTTTGTTTGG GATTTAGGGG AACGTTGAAA   
  
  
+ GACGAAGTCT TTACCTAGTG CGTCTTAGTA GACAATTAGG ATTTTAACAT GAAAATAGTT AAAATTCTAA   
  
  
+ TTGGGATTTG AGATTGTGAA TCTTGTAATC CTTCGACTTC TAGTTCTATG AAAATAGGCG ATTATAGTTC   
  
  
+ TAGGATTAGT CTTCGACCCT TGAGAGAGGG AGGAGATGGG TTTAGTTGAT AATCTATTCA TTCATAACGC   
  
  
+ ATGCCTGGGA GATGTTTGGT AGGGCTAGTA ATTGATTCGC TAACATATTT GTGCTGTTAG TTCCAACCTG   
  
  
+ AGTGTTTACT CTTCTGCTGT TTATTCCGTC TTTTAGTCGT AATTGTCTCC TTTAGTTTAA TTTCAAAACC   
  
  
+ CCAATTGTGA TCGCCTAGAT TGAGCAATCT AATTAGTTCT AATAATTGAA ATCAAGTTCC CTGTGGAATA   
  
  
+ CGACCCGTAC TTGCCGTGTG CTAGCTACTG TTACACCTTG CATTTGCGGT ATATTTATAA CTCAACATAT   
  
  
+ GTCTGCTCAC TAAAAGTACA TATTAGCTCA CCAAAACTGT GTCTCAACGT ACTAAAATAT TTTAGTGAAC   
  
  
+ TATACATTAT TTTTAGTAAG CGAAATAAGA TACACAACAG CACATTTTAA TGAGTTACAG GGCATTTTTA   
  
  
+ GTGAGCCGAA ATCTGATGGT TTTCTTTGTA GATCCGGTTA TATAATTGTA TTTTATACAA CCGAATGGAC   
  
  
+ GATATAACAA CTGAGTTAAA TGAGGAGTCT GAAATTACGA ACATAAAATC CAAAAGAAGA TGCCGATGCC   
  
  
+ ATTGGAAAGA AAGCTGAAGG TGATCGCTTT CTAGTAGTAT TAGGCAGTAG TAATTAAAAA CTAAAATCCA   
  
  
+ GAATTGACAC CGAATTGGGG TTGTGGCACA TGATCGAAAT CAGCATCGAA TCTACACGAT GATCTGGATT   
  
  
+ CTTGGACACT CTGGCTTTAG GCCGTCAATT TCGAGGATTG CCCATAACTT TAATCCTGTT CAAATCCATT   
  
  
+ CAATTTAATA TTACACAAAT CCTTTGATTA AATCACCGTA ATTGTGGTTT GATTAGGGAA GTTTCTTGGG   
  
  
+ GGAAAGGTTC AAGCTTCTGT CTTTCTTAAA GCCTGGATTT TTGGTCCCGC ATGTTCACTT CACATTCCCT   
  
  
+ TTTTCTCTCC CATCGTTACC TGTCTGAATC TGTATGTAAT AAAAGTCATA TTTTGCGAGG TCTTCTTTTG   
  
  
+ ACTGTTCTTG TAGTCACTCT TGATATCTGC ACCATTTTGT TTTTCTCGGC ATTTTCTCCA ACTTGCATTT   
  
  
+ TCTAATGTAA TGTGATCTTT TCTGGACATT CATTTTGTTA ATCTACTCAC TTGGGTCAAG GTTTGCTGAT   
  
  
+ TTTGGCTGGT GTTAAATCCG TTCCTCTTTG GCATTTGTTC ATGTTCCACA ACTGTTTGAT ATCTGGTATC   
  
  
+ TCAGAAACCC CGCCATTCTT TGGTATTTCC TGACTTTCTT GTTCAAGGGT TATTCCTATT TCCCTGAATT   
  
  
+ TTCTTTGATT CTTTAGGTAT TTCAAGCTTG GGGGGTTTTG TTCTTCACTG ATTTTGTCCT CTTCTTCCCT   
  
  
+ TATTGGGTGG TTTGATCTCC TAAATTTCTG TGTATCTGAA TCAGTCGGGG TAGACTTTGT GTTTTTGGTT   
  
  
+ GATTTTTTAT CAATGTATTT TCTTCCTAAT GAAGGGCCTC CCGAACCAAT ACACATGCTT AACTTCAACC   
  
  
+ CTGTTCCTCT TCCAAGCTTT TTGGAGCCTC AAAAGTCTTC GAATGCTGAT AGGCACATAG GGTCCTTCCA   
  
  
+ ATGCTATGGT CTTGATGATG ACCCCGCACC CTTGAATGAC CCAAATTACA TTGTATGTCA ATCTTCATAT   
  
  
+ CCTGATCGAG ATATTACACA GATCCCTGAC TTTCCTGATG ATTGTCTCAA GTTCATCAAT GACATTCTTA   
  
  
+ TGGAAGAGGA TTTGGATAAT CCGCCTATAT CCTTGCAGGA CTATAATGCG CTCCAAGCCA CTGAGAAGTC   
  
  
+ ATTGTATGAT GCCCTTGGAG AGGCTTATCC AGCTTCATCT GATCCATTGC AATCATCAGT TGGTCAGAGC   
  
  
+ ACTGAGAGCT CTGATGATCC TGACAGAAGC AGCGGCATTG ATAAATGGGG TAATAGATAT TACCCCACCA   
  
  
+ GCCTGAATGT GGTTGAAACC AGCCGGCTGA TCAATCAGAG CCAGTTGGAC CCTTCTCAAA TGTTTGATGC   
  
  
+ TGCTGCTCAC CCCTTGGAGT CTAATTGTCA TTCTTTTGTC TCTAGTAATA ACTTTGATCA TTCTATGGAT   
  
  
+ GGGGTTCCAG ATTCTCCTAT CAGTACACTT TCTGTGAATG AAACAAGAAA GGAAGAATCA GTTAGTAGAT   
  
  
+ CAAGGAGCAG GAAGAATCAT CAAAGAGATG GTGCATACCT GGAAGAAAGG AGTACTAAGC AAATAGCATC   
  
  
+ CAGCAATGAG GAATATGGAG AGATGGAGAG GTTTGATGAT GTACTGATCT GCAGGGAAGG AAATGATGAT   
  
  
+ ATTTCACCTT GTACCCGCAA AACCCCAGTT CATGAAGCAG ATGATAACTC GGAACCAAAG GGGAAATCAA   
  
  
+ GAGGATCTAA AAGCAAGAGT AAATCGCGTG TCAAGAAGAA AAGTAACGAA GGACAAGCGG TAGATCTTAG   
  
  
+ GACTCAACTT ATTCAGTGTG CAGAAGCAGT GGCAAGCTTT GAGCTTAAAA GTGCAAATGA GCTACTCAGG   
  
  
+ CGAATTAGGC AGCATGCTTC GCCATTTGGG GATAGTGTTC AAAGGCTAGC ACATTACTTT GCGAATGGGC   
  
  
+ TCGAGGCACG CTTAGCTGGT ACAGGATCAG AATCATACAA AGACTTTGCT GGGAAAAAAT TTTCATCCTA   
  
  
+ TGACATCTTG AGAGGCTACA AGTCATATGT TCAAGCCGTC CCCTTCCAGA GAACAACCAT GTTCTTGACA   
  
  
+ AATCAAACAA TAGTAAAGCT GGCTGAGAAA GCAACAAGGG TTCATATCAT TGATTTTGGC ATCTTTTTTG   
  
  
+ GTTTCCAGTG GCCCTGCCTT GTTCAGAATT TGTCAAGAAG ACCCCAGGGT TCTCCCATAC TCAAAATCAC   
  
  
+ GGGAGTCGAT CATCCCCAGA GTGGGTTCAG ACCAGCCCAG AAAGTTGAAG AGACAGGTCG TCGATTAGCA   
  
  
+ GGCTATTGTG AGAGGTTTGG GGTGCCCTTT GAGTTTCATC CCATTGCTCA AAGGTGGCAT ACCCTAAAGC   
  
  
+ CAGAGCATTT CAAAATTGAA AGCAATGAGC TTGTTGTGGT CACTTGTTTC TACCAATCAG GGAAGCTCCC   
  
  
+ TGATGAGACT GTTGATGTCA ACAGCCCAAG AGACCATGTC TTGAGGCTGA TCAGGGGCTT GAACCCTGAT   
  
  
+ CTATTCATCC ATGGGGTTAT CAATGGCACA TTCAATGCAC CCTTCTTCGT AACTCGGTTT AAAGAGGCAC   
  
  
+ TTTACCACTA CTCTTCTTTG TTCGATGTCT TTGATGCCAC CATAGATCGT GAAAGTCATG AAAGACTGCT   
  
  
+ GACTGAGAAC TATTTGTATG GGAAAGATGC ACTTAACATA GTAGCATGTG AGGGGAACGA GAGGATCGAG   
  
  
+ AGACCCGAGA CTTACAAGCA GTGGCAAGTC CGGAAGCTTA GAGCTGGATT CAAGCAGGTC CCCCTAGACC   
  
  
+ AAGACATTGT AAATGGAGCA AGAGCTATGG TTAAGGCAAA TTACAATAAG GATTTTATGG TAGAACAAGA   
  
  
+ TAGGAATTGG ATGGTCCAGG GCTGGAAAGG AAGGATTTTG AGTGCCATAT CCTGTTGGAA ACCTGCTTA  

- +Up\_Stream \_Len000ACTTAA CCTTCAACTA AGATGGAAAC TAACTAAGCT AATTAAGATC CAAGAACCAA   
  
  
- GATCAAGAGA GAGGAGAACA AGAACTAATA AATCCAAAAC CTACACTACA AATAACTTAA CTTACAACGT   
  
  
- TAACAACAAT ATTTCTACTA CAATCCGATT TATAGGAAGT TGAATCCAAC CCTAACTGCA GCTTATACAC   
  
  
- ATCAACTTAG AACATGACCG GATTTAGGGA CGAGGGGGAG ACAAAACGCT AATACTTAAA GTAATACGTG   
  
  
- TGGTGTACGA GCTACTTTAC GAGCTTTCTT GTAAGTAGGA GAAACAAACC CTAAATCCCC TTGCAACTTT   
  
  
- CTGCTTCAGA AATGGATCAC GCAGAATCAT CTGTTAATCC TAAAATTGTA CTTTTATCAA TTTTAAGATT   
  
  
- AACCCTAAAC TCTAACACTT AGAACATTAG GAAGCTGAAG ATCAAGATAC TTTTATCCGC TAATATCAAG   
  
  
- ATCCTAATCA GAAGCTGGGA ACTCTCTCCC TCCTCTACCC AAATCAACTA TTAGATAAGT AAGTATTGCG   
  
  
- TACGGACCCT CTACAAACCA TCCCGATCAT TAACTAAGCG ATTGTATAAA CACGACAATC AAGGTTGGAC   
  
  
- TCACAAATGA GAAGACGACA AATAAGGCAG AAAATCAGCA TTAACAGAGG AAATCAAATT AAAGTTTTGG   
  
  
- GGTTAACACT AGCGGATCTA ACTCGTTAGA TTAATCAAGA TTATTAACTT TAGTTCAAGG GACACCTTAT   
  
  
- GCTGGGCATG AACGGCACAC GATCGATGAC AATGTGGAAC GTAAACGCCA TATAAATATT GAGTTGTATA   
  
  
- CAGACGAGTG ATTTTCATGT ATAATCGAGT GGTTTTGACA CAGAGTTGCA TGATTTTATA AAATCACTTG   
  
  
- ATATGTAATA AAAATCATTC GCTTTATTCT ATGTGTTGTC GTGTAAAATT ACTCAATGTC CCGTAAAAAT   
  
  
- CACTCGGCTT TAGACTACCA AAAGAAACAT CTAGGCCAAT ATATTAACAT AAAATATGTT GGCTTACCTG   
  
  
- CTATATTGTT GACTCAATTT ACTCCTCAGA CTTTAATGCT TGTATTTTAG GTTTTCTTCT ACGGCTACGG   
  
  
- TAACCTTTCT TTCGACTTCC ACTAGCGAAA GATCATCATA ATCCGTCATC ATTAATTTTT GATTTTAGGT   
  
  
- CTTAACTGTG GCTTAACCCC AACACCGTGT ACTAGCTTTA GTCGTAGCTT AGATGTGCTA CTAGACCTAA   
  
  
- GAACCTGTGA GACCGAAATC CGGCAGTTAA AGCTCCTAAC GGGTATTGAA ATTAGGACAA GTTTAGGTAA   
  
  
- GTTAAATTAT AATGTGTTTA GGAAACTAAT TTAGTGGCAT TAACACCAAA CTAATCCCTT CAAAGAACCC   
  
  
- CCTTTCCAAG TTCGAAGACA GAAAGAATTT CGGACCTAAA AACCAGGGCG TACAAGTGAA GTGTAAGGGA   
  
  
- AAAAGAGAGG GTAGCAATGG ACAGACTTAG ACATACATTA TTTTCAGTAT AAAACGCTCC AGAAGAAAAC   
  
  
- TGACAAGAAC ATCAGTGAGA ACTATAGACG TGGTAAAACA AAAAGAGCCG TAAAAGAGGT TGAACGTAAA   
  
  
- AGATTACATT ACACTAGAAA AGACCTGTAA GTAAAACAAT TAGATGAGTG AACCCAGTTC CAAACGACTA   
  
  
- AAACCGACCA CAATTTAGGC AAGGAGAAAC CGTAAACAAG TACAAGGTGT TGACAAACTA TAGACCATAG   
  
  
- AGTCTTTGGG GCGGTAAGAA ACCATAAAGG ACTGAAAGAA CAAGTTCCCA ATAAGGATAA AGGGACTTAA   
  
  
- AAGAAACTAA GAAATCCATA AAGTTCGAAC CCCCCAAAAC AAGAAGTGAC TAAAACAGGA GAAGAAGGGA   
  
  
- ATAACCCACC AAACTAGAGG ATTTAAAGAC ACATAGACTT AGTCAGCCCC ATCTGAAACA CAAAAACCAA   
  
  
- CTAAAAAATA GTTACATAAA AGAAGGATTA CTTCCCGGAG GGCTTGGTTA TGTGTACGAA TTGAAGTTGG   
  
  
- GACAAGGAGA AGGTTCGAAA AACCTCGGAG TTTTCAGAAG CTTACGACTA TCCGTGTATC CCAGGAAGGT   
  
  
- TACGATACCA GAACTACTAC TGGGGCGTGG GAACTTACTG GGTTTAATGT AACATACAGT TAGAAGTATA   
  
  
- GGACTAGCTC TATAATGTGT CTAGGGACTG AAAGGACTAC TAACAGAGTT CAAGTAGTTA CTGTAAGAAT   
  
  
- ACCTTCTCCT AAACCTATTA GGCGGATATA GGAACGTCCT GATATTACGC GAGGTTCGGT GACTCTTCAG   
  
  
- TAACATACTA CGGGAACCTC TCCGAATAGG TCGAAGTAGA CTAGGTAACG TTAGTAGTCA ACCAGTCTCG   
  
  
- TGACTCTCGA GACTACTAGG ACTGTCTTCG TCGCCGTAAC TATTTACCCC ATTATCTATA ATGGGGTGGT   
  
  
- CGGACTTACA CCAACTTTGG TCGGCCGACT AGTTAGTCTC GGTCAACCTG GGAAGAGTTT ACAAACTACG   
  
  
- ACGACGAGTG GGGAACCTCA GATTAACAGT AAGAAAACAG AGATCATTAT TGAAACTAGT AAGATACCTA   
  
  
- CCCCAAGGTC TAAGAGGATA GTCATGTGAA AGACACTTAC TTTGTTCTTT CCTTCTTAGT CAATCATCTA   
  
  
- GTTCCTCGTC CTTCTTAGTA GTTTCTCTAC CACGTATGGA CCTTCTTTCC TCATGATTCG TTTATCGTAG   
  
  
- GTCGTTACTC CTTATACCTC TCTACCTCTC CAAACTACTA CATGACTAGA CGTCCCTTCC TTTACTACTA   
  
  
- TAAAGTGGAA CATGGGCGTT TTGGGGTCAA GTACTTCGTC TACTATTGAG CCTTGGTTTC CCCTTTAGTT   
  
  
- CTCCTAGATT TTCGTTCTCA TTTAGCGCAC AGTTCTTCTT TTCATTGCTT CCTGTTCGCC ATCTAGAATC   
  
  
- CTGAGTTGAA TAAGTCACAC GTCTTCGTCA CCGTTCGAAA CTCGAATTTT CACGTTTACT CGATGAGTCC   
  
  
- GCTTAATCCG TCGTACGAAG CGGTAAACCC CTATCACAAG TTTCCGATCG TGTAATGAAA CGCTTACCCG   
  
  
- AGCTCCGTGC GAATCGACCA TGTCCTAGTC TTAGTATGTT TCTGAAACGA CCCTTTTTTA AAAGTAGGAT   
  
  
- ACTGTAGAAC TCTCCGATGT TCAGTATACA AGTTCGGCAG GGGAAGGTCT CTTGTTGGTA CAAGAACTGT   
  
  
- TTAGTTTGTT ATCATTTCGA CCGACTCTTT CGTTGTTCCC AAGTATAGTA ACTAAAACCG TAGAAAAAAC   
  
  
- CAAAGGTCAC CGGGACGGAA CAAGTCTTAA ACAGTTCTTC TGGGGTCCCA AGAGGGTATG AGTTTTAGTG   
  
  
- CCCTCAGCTA GTAGGGGTCT CACCCAAGTC TGGTCGGGTC TTTCAACTTC TCTGTCCAGC AGCTAATCGT   
  
  
- CCGATAACAC TCTCCAAACC CCACGGGAAA CTCAAAGTAG GGTAACGAGT TTCCACCGTA TGGGATTTCG   
  
  
- GTCTCGTAAA GTTTTAACTT TCGTTACTCG AACAACACCA GTGAACAAAG ATGGTTAGTC CCTTCGAGGG   
  
  
- ACTACTCTGA CAACTACAGT TGTCGGGTTC TCTGGTACAG AACTCCGACT AGTCCCCGAA CTTGGGACTA   
  
  
- GATAAGTAGG TACCCCAATA GTTACCGTGT AAGTTACGTG GGAAGAAGCA TTGAGCCAAA TTTCTCCGTG   
  
  
- AAATGGTGAT GAGAAGAAAC AAGCTACAGA AACTACGGTG GTATCTAGCA CTTTCAGTAC TTTCTGACGA   
  
  
- CTGACTCTTG ATAAACATAC CCTTTCTACG TGAATTGTAT CATCGTACAC TCCCCTTGCT CTCCTAGCTC   
  
  
- TCTGGGCTCT GAATGTTCGT CACCGTTCAG GCCTTCGAAT CTCGACCTAA GTTCGTCCAG GGGGATCTGG   
  
  
- TTCTGTAACA TTTACCTCGT TCTCGATACC AATTCCGTTT AATGTTATTC CTAAAATACC ATCTTGTTCT   
  
  
- ATCCTTAACC TACCAGGTCC CGACCTTTCC TTCCTAAAAC TCACGGTATA GGACAACCTT TGGACGAAT

+     Myb-binding site

| Site Name | Organism | Position | Strand | Matrix score. | sequence | function |
| --- | --- | --- | --- | --- | --- | --- |
| Myb-binding site | Nicotiana tabacum | 3593 | + | 6 | CAACAG |  |
| Myb-binding site | Nicotiana tabacum | 3583 | - | 6 | CAACAG |  |
| Myb-binding site | Nicotiana tabacum | 4046 | - | 6 | CAACAG |  |
| Myb-binding site | Nicotiana tabacum | 949 | + | 6 | CAACAG |  |

>HU02G01573.1   
+ +Up\_Stream \_Len000TGAATT GGAAGTTGAT TCTACCTTTG ATTGATTCGA TTAATTCTAG GTTCTTGGTT   
  
  
+ CTAGTTCTCT CTCCTCTTGT TCTTGATTAT TTAGGTTTTG GATGTGATGT TTATTGAATT GAATGTTGCA   
  
  
+ ATTGTTGTTA TAAAGATGAT GTTAGGCTAA ATATCCTTCA ACTTAGGTTG GGATTGACGT CGAATATGTG   
  
  
+ TAGTTGAATC TTGTACTGGC CTAAATCCCT GCTCCCCCTC TGTTTTGCGA TTATGAATTT CATTATGCAC   
  
  
+ ACCACATGCT CGATGAAATG CTCGAAAGAA CATTCATCCT CTTTGTTTGG GATTTAGGGG AACGTTGAAA   
  
  
+ GACGAAGTCT TTACCTAGTG CGTCTTAGTA GACAATTAGG ATTTTAACAT GAAAATAGTT AAAATTCTAA   
  
  
+ TTGGGATTTG AGATTGTGAA TCTTGTAATC CTTCGACTTC TAGTTCTATG AAAATAGGCG ATTATAGTTC   
  
  
+ TAGGATTAGT CTTCGACCCT TGAGAGAGGG AGGAGATGGG TTTAGTTGAT AATCTATTCA TTCATAACGC   
  
  
+ ATGCCTGGGA GATGTTTGGT AGGGCTAGTA ATTGATTCGC TAACATATTT GTGCTGTTAG TTCCAACCTG   
  
  
+ AGTGTTTACT CTTCTGCTGT TTATTCCGTC TTTTAGTCGT AATTGTCTCC TTTAGTTTAA TTTCAAAACC   
  
  
+ CCAATTGTGA TCGCCTAGAT TGAGCAATCT AATTAGTTCT AATAATTGAA ATCAAGTTCC CTGTGGAATA   
  
  
+ CGACCCGTAC TTGCCGTGTG CTAGCTACTG TTACACCTTG CATTTGCGGT ATATTTATAA CTCAACATAT   
  
  
+ GTCTGCTCAC TAAAAGTACA TATTAGCTCA CCAAAACTGT GTCTCAACGT ACTAAAATAT TTTAGTGAAC   
  
  
+ TATACATTAT TTTTAGTAAG CGAAATAAGA TACACAACAG CACATTTTAA TGAGTTACAG GGCATTTTTA   
  
  
+ GTGAGCCGAA ATCTGATGGT TTTCTTTGTA GATCCGGTTA TATAATTGTA TTTTATACAA CCGAATGGAC   
  
  
+ GATATAACAA CTGAGTTAAA TGAGGAGTCT GAAATTACGA ACATAAAATC CAAAAGAAGA TGCCGATGCC   
  
  
+ ATTGGAAAGA AAGCTGAAGG TGATCGCTTT CTAGTAGTAT TAGGCAGTAG TAATTAAAAA CTAAAATCCA   
  
  
+ GAATTGACAC CGAATTGGGG TTGTGGCACA TGATCGAAAT CAGCATCGAA TCTACACGAT GATCTGGATT   
  
  
+ CTTGGACACT CTGGCTTTAG GCCGTCAATT TCGAGGATTG CCCATAACTT TAATCCTGTT CAAATCCATT   
  
  
+ CAATTTAATA TTACACAAAT CCTTTGATTA AATCACCGTA ATTGTGGTTT GATTAGGGAA GTTTCTTGGG   
  
  
+ GGAAAGGTTC AAGCTTCTGT CTTTCTTAAA GCCTGGATTT TTGGTCCCGC ATGTTCACTT CACATTCCCT   
  
  
+ TTTTCTCTCC CATCGTTACC TGTCTGAATC TGTATGTAAT AAAAGTCATA TTTTGCGAGG TCTTCTTTTG   
  
  
+ ACTGTTCTTG TAGTCACTCT TGATATCTGC ACCATTTTGT TTTTCTCGGC ATTTTCTCCA ACTTGCATTT   
  
  
+ TCTAATGTAA TGTGATCTTT TCTGGACATT CATTTTGTTA ATCTACTCAC TTGGGTCAAG GTTTGCTGAT   
  
  
+ TTTGGCTGGT GTTAAATCCG TTCCTCTTTG GCATTTGTTC ATGTTCCACA ACTGTTTGAT ATCTGGTATC   
  
  
+ TCAGAAACCC CGCCATTCTT TGGTATTTCC TGACTTTCTT GTTCAAGGGT TATTCCTATT TCCCTGAATT   
  
  
+ TTCTTTGATT CTTTAGGTAT TTCAAGCTTG GGGGGTTTTG TTCTTCACTG ATTTTGTCCT CTTCTTCCCT   
  
  
+ TATTGGGTGG TTTGATCTCC TAAATTTCTG TGTATCTGAA TCAGTCGGGG TAGACTTTGT GTTTTTGGTT   
  
  
+ GATTTTTTAT CAATGTATTT TCTTCCTAAT GAAGGGCCTC CCGAACCAAT ACACATGCTT AACTTCAACC   
  
  
+ CTGTTCCTCT TCCAAGCTTT TTGGAGCCTC AAAAGTCTTC GAATGCTGAT AGGCACATAG GGTCCTTCCA   
  
  
+ ATGCTATGGT CTTGATGATG ACCCCGCACC CTTGAATGAC CCAAATTACA TTGTATGTCA ATCTTCATAT   
  
  
+ CCTGATCGAG ATATTACACA GATCCCTGAC TTTCCTGATG ATTGTCTCAA GTTCATCAAT GACATTCTTA   
  
  
+ TGGAAGAGGA TTTGGATAAT CCGCCTATAT CCTTGCAGGA CTATAATGCG CTCCAAGCCA CTGAGAAGTC   
  
  
+ ATTGTATGAT GCCCTTGGAG AGGCTTATCC AGCTTCATCT GATCCATTGC AATCATCAGT TGGTCAGAGC   
  
  
+ ACTGAGAGCT CTGATGATCC TGACAGAAGC AGCGGCATTG ATAAATGGGG TAATAGATAT TACCCCACCA   
  
  
+ GCCTGAATGT GGTTGAAACC AGCCGGCTGA TCAATCAGAG CCAGTTGGAC CCTTCTCAAA TGTTTGATGC   
  
  
+ TGCTGCTCAC CCCTTGGAGT CTAATTGTCA TTCTTTTGTC TCTAGTAATA ACTTTGATCA TTCTATGGAT   
  
  
+ GGGGTTCCAG ATTCTCCTAT CAGTACACTT TCTGTGAATG AAACAAGAAA GGAAGAATCA GTTAGTAGAT   
  
  
+ CAAGGAGCAG GAAGAATCAT CAAAGAGATG GTGCATACCT GGAAGAAAGG AGTACTAAGC AAATAGCATC   
  
  
+ CAGCAATGAG GAATATGGAG AGATGGAGAG GTTTGATGAT GTACTGATCT GCAGGGAAGG AAATGATGAT   
  
  
+ ATTTCACCTT GTACCCGCAA AACCCCAGTT CATGAAGCAG ATGATAACTC GGAACCAAAG GGGAAATCAA   
  
  
+ GAGGATCTAA AAGCAAGAGT AAATCGCGTG TCAAGAAGAA AAGTAACGAA GGACAAGCGG TAGATCTTAG   
  
  
+ GACTCAACTT ATTCAGTGTG CAGAAGCAGT GGCAAGCTTT GAGCTTAAAA GTGCAAATGA GCTACTCAGG   
  
  
+ CGAATTAGGC AGCATGCTTC GCCATTTGGG GATAGTGTTC AAAGGCTAGC ACATTACTTT GCGAATGGGC   
  
  
+ TCGAGGCACG CTTAGCTGGT ACAGGATCAG AATCATACAA AGACTTTGCT GGGAAAAAAT TTTCATCCTA   
  
  
+ TGACATCTTG AGAGGCTACA AGTCATATGT TCAAGCCGTC CCCTTCCAGA GAACAACCAT GTTCTTGACA   
  
  
+ AATCAAACAA TAGTAAAGCT GGCTGAGAAA GCAACAAGGG TTCATATCAT TGATTTTGGC ATCTTTTTTG   
  
  
+ GTTTCCAGTG GCCCTGCCTT GTTCAGAATT TGTCAAGAAG ACCCCAGGGT TCTCCCATAC TCAAAATCAC   
  
  
+ GGGAGTCGAT CATCCCCAGA GTGGGTTCAG ACCAGCCCAG AAAGTTGAAG AGACAGGTCG TCGATTAGCA   
  
  
+ GGCTATTGTG AGAGGTTTGG GGTGCCCTTT GAGTTTCATC CCATTGCTCA AAGGTGGCAT ACCCTAAAGC   
  
  
+ CAGAGCATTT CAAAATTGAA AGCAATGAGC TTGTTGTGGT CACTTGTTTC TACCAATCAG GGAAGCTCCC   
  
  
+ TGATGAGACT GTTGATGTCA ACAGCCCAAG AGACCATGTC TTGAGGCTGA TCAGGGGCTT GAACCCTGAT   
  
  
+ CTATTCATCC ATGGGGTTAT CAATGGCACA TTCAATGCAC CCTTCTTCGT AACTCGGTTT AAAGAGGCAC   
  
  
+ TTTACCACTA CTCTTCTTTG TTCGATGTCT TTGATGCCAC CATAGATCGT GAAAGTCATG AAAGACTGCT   
  
  
+ GACTGAGAAC TATTTGTATG GGAAAGATGC ACTTAACATA GTAGCATGTG AGGGGAACGA GAGGATCGAG   
  
  
+ AGACCCGAGA CTTACAAGCA GTGGCAAGTC CGGAAGCTTA GAGCTGGATT CAAGCAGGTC CCCCTAGACC   
  
  
+ AAGACATTGT AAATGGAGCA AGAGCTATGG TTAAGGCAAA TTACAATAAG GATTTTATGG TAGAACAAGA   
  
  
+ TAGGAATTGG ATGGTCCAGG GCTGGAAAGG AAGGATTTTG AGTGCCATAT CCTGTTGGAA ACCTGCTTA  

- +Up\_Stream \_Len000ACTTAA CCTTCAACTA AGATGGAAAC TAACTAAGCT AATTAAGATC CAAGAACCAA   
  
  
- GATCAAGAGA GAGGAGAACA AGAACTAATA AATCCAAAAC CTACACTACA AATAACTTAA CTTACAACGT   
  
  
- TAACAACAAT ATTTCTACTA CAATCCGATT TATAGGAAGT TGAATCCAAC CCTAACTGCA GCTTATACAC   
  
  
- ATCAACTTAG AACATGACCG GATTTAGGGA CGAGGGGGAG ACAAAACGCT AATACTTAAA GTAATACGTG   
  
  
- TGGTGTACGA GCTACTTTAC GAGCTTTCTT GTAAGTAGGA GAAACAAACC CTAAATCCCC TTGCAACTTT   
  
  
- CTGCTTCAGA AATGGATCAC GCAGAATCAT CTGTTAATCC TAAAATTGTA CTTTTATCAA TTTTAAGATT   
  
  
- AACCCTAAAC TCTAACACTT AGAACATTAG GAAGCTGAAG ATCAAGATAC TTTTATCCGC TAATATCAAG   
  
  
- ATCCTAATCA GAAGCTGGGA ACTCTCTCCC TCCTCTACCC AAATCAACTA TTAGATAAGT AAGTATTGCG   
  
  
- TACGGACCCT CTACAAACCA TCCCGATCAT TAACTAAGCG ATTGTATAAA CACGACAATC AAGGTTGGAC   
  
  
- TCACAAATGA GAAGACGACA AATAAGGCAG AAAATCAGCA TTAACAGAGG AAATCAAATT AAAGTTTTGG   
  
  
- GGTTAACACT AGCGGATCTA ACTCGTTAGA TTAATCAAGA TTATTAACTT TAGTTCAAGG GACACCTTAT   
  
  
- GCTGGGCATG AACGGCACAC GATCGATGAC AATGTGGAAC GTAAACGCCA TATAAATATT GAGTTGTATA   
  
  
- CAGACGAGTG ATTTTCATGT ATAATCGAGT GGTTTTGACA CAGAGTTGCA TGATTTTATA AAATCACTTG   
  
  
- ATATGTAATA AAAATCATTC GCTTTATTCT ATGTGTTGTC GTGTAAAATT ACTCAATGTC CCGTAAAAAT   
  
  
- CACTCGGCTT TAGACTACCA AAAGAAACAT CTAGGCCAAT ATATTAACAT AAAATATGTT GGCTTACCTG   
  
  
- CTATATTGTT GACTCAATTT ACTCCTCAGA CTTTAATGCT TGTATTTTAG GTTTTCTTCT ACGGCTACGG   
  
  
- TAACCTTTCT TTCGACTTCC ACTAGCGAAA GATCATCATA ATCCGTCATC ATTAATTTTT GATTTTAGGT   
  
  
- CTTAACTGTG GCTTAACCCC AACACCGTGT ACTAGCTTTA GTCGTAGCTT AGATGTGCTA CTAGACCTAA   
  
  
- GAACCTGTGA GACCGAAATC CGGCAGTTAA AGCTCCTAAC GGGTATTGAA ATTAGGACAA GTTTAGGTAA   
  
  
- GTTAAATTAT AATGTGTTTA GGAAACTAAT TTAGTGGCAT TAACACCAAA CTAATCCCTT CAAAGAACCC   
  
  
- CCTTTCCAAG TTCGAAGACA GAAAGAATTT CGGACCTAAA AACCAGGGCG TACAAGTGAA GTGTAAGGGA   
  
  
- AAAAGAGAGG GTAGCAATGG ACAGACTTAG ACATACATTA TTTTCAGTAT AAAACGCTCC AGAAGAAAAC   
  
  
- TGACAAGAAC ATCAGTGAGA ACTATAGACG TGGTAAAACA AAAAGAGCCG TAAAAGAGGT TGAACGTAAA   
  
  
- AGATTACATT ACACTAGAAA AGACCTGTAA GTAAAACAAT TAGATGAGTG AACCCAGTTC CAAACGACTA   
  
  
- AAACCGACCA CAATTTAGGC AAGGAGAAAC CGTAAACAAG TACAAGGTGT TGACAAACTA TAGACCATAG   
  
  
- AGTCTTTGGG GCGGTAAGAA ACCATAAAGG ACTGAAAGAA CAAGTTCCCA ATAAGGATAA AGGGACTTAA   
  
  
- AAGAAACTAA GAAATCCATA AAGTTCGAAC CCCCCAAAAC AAGAAGTGAC TAAAACAGGA GAAGAAGGGA   
  
  
- ATAACCCACC AAACTAGAGG ATTTAAAGAC ACATAGACTT AGTCAGCCCC ATCTGAAACA CAAAAACCAA   
  
  
- CTAAAAAATA GTTACATAAA AGAAGGATTA CTTCCCGGAG GGCTTGGTTA TGTGTACGAA TTGAAGTTGG   
  
  
- GACAAGGAGA AGGTTCGAAA AACCTCGGAG TTTTCAGAAG CTTACGACTA TCCGTGTATC CCAGGAAGGT   
  
  
- TACGATACCA GAACTACTAC TGGGGCGTGG GAACTTACTG GGTTTAATGT AACATACAGT TAGAAGTATA   
  
  
- GGACTAGCTC TATAATGTGT CTAGGGACTG AAAGGACTAC TAACAGAGTT CAAGTAGTTA CTGTAAGAAT   
  
  
- ACCTTCTCCT AAACCTATTA GGCGGATATA GGAACGTCCT GATATTACGC GAGGTTCGGT GACTCTTCAG   
  
  
- TAACATACTA CGGGAACCTC TCCGAATAGG TCGAAGTAGA CTAGGTAACG TTAGTAGTCA ACCAGTCTCG   
  
  
- TGACTCTCGA GACTACTAGG ACTGTCTTCG TCGCCGTAAC TATTTACCCC ATTATCTATA ATGGGGTGGT   
  
  
- CGGACTTACA CCAACTTTGG TCGGCCGACT AGTTAGTCTC GGTCAACCTG GGAAGAGTTT ACAAACTACG   
  
  
- ACGACGAGTG GGGAACCTCA GATTAACAGT AAGAAAACAG AGATCATTAT TGAAACTAGT AAGATACCTA   
  
  
- CCCCAAGGTC TAAGAGGATA GTCATGTGAA AGACACTTAC TTTGTTCTTT CCTTCTTAGT CAATCATCTA   
  
  
- GTTCCTCGTC CTTCTTAGTA GTTTCTCTAC CACGTATGGA CCTTCTTTCC TCATGATTCG TTTATCGTAG   
  
  
- GTCGTTACTC CTTATACCTC TCTACCTCTC CAAACTACTA CATGACTAGA CGTCCCTTCC TTTACTACTA   
  
  
- TAAAGTGGAA CATGGGCGTT TTGGGGTCAA GTACTTCGTC TACTATTGAG CCTTGGTTTC CCCTTTAGTT   
  
  
- CTCCTAGATT TTCGTTCTCA TTTAGCGCAC AGTTCTTCTT TTCATTGCTT CCTGTTCGCC ATCTAGAATC   
  
  
- CTGAGTTGAA TAAGTCACAC GTCTTCGTCA CCGTTCGAAA CTCGAATTTT CACGTTTACT CGATGAGTCC   
  
  
- GCTTAATCCG TCGTACGAAG CGGTAAACCC CTATCACAAG TTTCCGATCG TGTAATGAAA CGCTTACCCG   
  
  
- AGCTCCGTGC GAATCGACCA TGTCCTAGTC TTAGTATGTT TCTGAAACGA CCCTTTTTTA AAAGTAGGAT   
  
  
- ACTGTAGAAC TCTCCGATGT TCAGTATACA AGTTCGGCAG GGGAAGGTCT CTTGTTGGTA CAAGAACTGT   
  
  
- TTAGTTTGTT ATCATTTCGA CCGACTCTTT CGTTGTTCCC AAGTATAGTA ACTAAAACCG TAGAAAAAAC   
  
  
- CAAAGGTCAC CGGGACGGAA CAAGTCTTAA ACAGTTCTTC TGGGGTCCCA AGAGGGTATG AGTTTTAGTG   
  
  
- CCCTCAGCTA GTAGGGGTCT CACCCAAGTC TGGTCGGGTC TTTCAACTTC TCTGTCCAGC AGCTAATCGT   
  
  
- CCGATAACAC TCTCCAAACC CCACGGGAAA CTCAAAGTAG GGTAACGAGT TTCCACCGTA TGGGATTTCG   
  
  
- GTCTCGTAAA GTTTTAACTT TCGTTACTCG AACAACACCA GTGAACAAAG ATGGTTAGTC CCTTCGAGGG   
  
  
- ACTACTCTGA CAACTACAGT TGTCGGGTTC TCTGGTACAG AACTCCGACT AGTCCCCGAA CTTGGGACTA   
  
  
- GATAAGTAGG TACCCCAATA GTTACCGTGT AAGTTACGTG GGAAGAAGCA TTGAGCCAAA TTTCTCCGTG   
  
  
- AAATGGTGAT GAGAAGAAAC AAGCTACAGA AACTACGGTG GTATCTAGCA CTTTCAGTAC TTTCTGACGA   
  
  
- CTGACTCTTG ATAAACATAC CCTTTCTACG TGAATTGTAT CATCGTACAC TCCCCTTGCT CTCCTAGCTC   
  
  
- TCTGGGCTCT GAATGTTCGT CACCGTTCAG GCCTTCGAAT CTCGACCTAA GTTCGTCCAG GGGGATCTGG   
  
  
- TTCTGTAACA TTTACCTCGT TCTCGATACC AATTCCGTTT AATGTTATTC CTAAAATACC ATCTTGTTCT   
  
  
- ATCCTTAACC TACCAGGTCC CGACCTTTCC TTCCTAAAAC TCACGGTATA GGACAACCTT TGGACGAAT

+     O2-site

| Site Name | Organism | Position | Strand | Matrix score. | sequence | function |
| --- | --- | --- | --- | --- | --- | --- |
| O2-site | Zea mays | 1252 | + | 9 | GATGATGTGG | cis-acting regulatory element involved in zein metabolism regulation |

>HU02G01573.1   
+ +Up\_Stream \_Len000TGAATT GGAAGTTGAT TCTACCTTTG ATTGATTCGA TTAATTCTAG GTTCTTGGTT   
  
  
+ CTAGTTCTCT CTCCTCTTGT TCTTGATTAT TTAGGTTTTG GATGTGATGT TTATTGAATT GAATGTTGCA   
  
  
+ ATTGTTGTTA TAAAGATGAT GTTAGGCTAA ATATCCTTCA ACTTAGGTTG GGATTGACGT CGAATATGTG   
  
  
+ TAGTTGAATC TTGTACTGGC CTAAATCCCT GCTCCCCCTC TGTTTTGCGA TTATGAATTT CATTATGCAC   
  
  
+ ACCACATGCT CGATGAAATG CTCGAAAGAA CATTCATCCT CTTTGTTTGG GATTTAGGGG AACGTTGAAA   
  
  
+ GACGAAGTCT TTACCTAGTG CGTCTTAGTA GACAATTAGG ATTTTAACAT GAAAATAGTT AAAATTCTAA   
  
  
+ TTGGGATTTG AGATTGTGAA TCTTGTAATC CTTCGACTTC TAGTTCTATG AAAATAGGCG ATTATAGTTC   
  
  
+ TAGGATTAGT CTTCGACCCT TGAGAGAGGG AGGAGATGGG TTTAGTTGAT AATCTATTCA TTCATAACGC   
  
  
+ ATGCCTGGGA GATGTTTGGT AGGGCTAGTA ATTGATTCGC TAACATATTT GTGCTGTTAG TTCCAACCTG   
  
  
+ AGTGTTTACT CTTCTGCTGT TTATTCCGTC TTTTAGTCGT AATTGTCTCC TTTAGTTTAA TTTCAAAACC   
  
  
+ CCAATTGTGA TCGCCTAGAT TGAGCAATCT AATTAGTTCT AATAATTGAA ATCAAGTTCC CTGTGGAATA   
  
  
+ CGACCCGTAC TTGCCGTGTG CTAGCTACTG TTACACCTTG CATTTGCGGT ATATTTATAA CTCAACATAT   
  
  
+ GTCTGCTCAC TAAAAGTACA TATTAGCTCA CCAAAACTGT GTCTCAACGT ACTAAAATAT TTTAGTGAAC   
  
  
+ TATACATTAT TTTTAGTAAG CGAAATAAGA TACACAACAG CACATTTTAA TGAGTTACAG GGCATTTTTA   
  
  
+ GTGAGCCGAA ATCTGATGGT TTTCTTTGTA GATCCGGTTA TATAATTGTA TTTTATACAA CCGAATGGAC   
  
  
+ GATATAACAA CTGAGTTAAA TGAGGAGTCT GAAATTACGA ACATAAAATC CAAAAGAAGA TGCCGATGCC   
  
  
+ ATTGGAAAGA AAGCTGAAGG TGATCGCTTT CTAGTAGTAT TAGGCAGTAG TAATTAAAAA CTAAAATCCA   
  
  
+ GAATTGACAC CGAATTGGGG TTGTGGCACA TGATCGAAAT CAGCATCGAA TCTACACGAT GATCTGGATT   
  
  
+ CTTGGACACT CTGGCTTTAG GCCGTCAATT TCGAGGATTG CCCATAACTT TAATCCTGTT CAAATCCATT   
  
  
+ CAATTTAATA TTACACAAAT CCTTTGATTA AATCACCGTA ATTGTGGTTT GATTAGGGAA GTTTCTTGGG   
  
  
+ GGAAAGGTTC AAGCTTCTGT CTTTCTTAAA GCCTGGATTT TTGGTCCCGC ATGTTCACTT CACATTCCCT   
  
  
+ TTTTCTCTCC CATCGTTACC TGTCTGAATC TGTATGTAAT AAAAGTCATA TTTTGCGAGG TCTTCTTTTG   
  
  
+ ACTGTTCTTG TAGTCACTCT TGATATCTGC ACCATTTTGT TTTTCTCGGC ATTTTCTCCA ACTTGCATTT   
  
  
+ TCTAATGTAA TGTGATCTTT TCTGGACATT CATTTTGTTA ATCTACTCAC TTGGGTCAAG GTTTGCTGAT   
  
  
+ TTTGGCTGGT GTTAAATCCG TTCCTCTTTG GCATTTGTTC ATGTTCCACA ACTGTTTGAT ATCTGGTATC   
  
  
+ TCAGAAACCC CGCCATTCTT TGGTATTTCC TGACTTTCTT GTTCAAGGGT TATTCCTATT TCCCTGAATT   
  
  
+ TTCTTTGATT CTTTAGGTAT TTCAAGCTTG GGGGGTTTTG TTCTTCACTG ATTTTGTCCT CTTCTTCCCT   
  
  
+ TATTGGGTGG TTTGATCTCC TAAATTTCTG TGTATCTGAA TCAGTCGGGG TAGACTTTGT GTTTTTGGTT   
  
  
+ GATTTTTTAT CAATGTATTT TCTTCCTAAT GAAGGGCCTC CCGAACCAAT ACACATGCTT AACTTCAACC   
  
  
+ CTGTTCCTCT TCCAAGCTTT TTGGAGCCTC AAAAGTCTTC GAATGCTGAT AGGCACATAG GGTCCTTCCA   
  
  
+ ATGCTATGGT CTTGATGATG ACCCCGCACC CTTGAATGAC CCAAATTACA TTGTATGTCA ATCTTCATAT   
  
  
+ CCTGATCGAG ATATTACACA GATCCCTGAC TTTCCTGATG ATTGTCTCAA GTTCATCAAT GACATTCTTA   
  
  
+ TGGAAGAGGA TTTGGATAAT CCGCCTATAT CCTTGCAGGA CTATAATGCG CTCCAAGCCA CTGAGAAGTC   
  
  
+ ATTGTATGAT GCCCTTGGAG AGGCTTATCC AGCTTCATCT GATCCATTGC AATCATCAGT TGGTCAGAGC   
  
  
+ ACTGAGAGCT CTGATGATCC TGACAGAAGC AGCGGCATTG ATAAATGGGG TAATAGATAT TACCCCACCA   
  
  
+ GCCTGAATGT GGTTGAAACC AGCCGGCTGA TCAATCAGAG CCAGTTGGAC CCTTCTCAAA TGTTTGATGC   
  
  
+ TGCTGCTCAC CCCTTGGAGT CTAATTGTCA TTCTTTTGTC TCTAGTAATA ACTTTGATCA TTCTATGGAT   
  
  
+ GGGGTTCCAG ATTCTCCTAT CAGTACACTT TCTGTGAATG AAACAAGAAA GGAAGAATCA GTTAGTAGAT   
  
  
+ CAAGGAGCAG GAAGAATCAT CAAAGAGATG GTGCATACCT GGAAGAAAGG AGTACTAAGC AAATAGCATC   
  
  
+ CAGCAATGAG GAATATGGAG AGATGGAGAG GTTTGATGAT GTACTGATCT GCAGGGAAGG AAATGATGAT   
  
  
+ ATTTCACCTT GTACCCGCAA AACCCCAGTT CATGAAGCAG ATGATAACTC GGAACCAAAG GGGAAATCAA   
  
  
+ GAGGATCTAA AAGCAAGAGT AAATCGCGTG TCAAGAAGAA AAGTAACGAA GGACAAGCGG TAGATCTTAG   
  
  
+ GACTCAACTT ATTCAGTGTG CAGAAGCAGT GGCAAGCTTT GAGCTTAAAA GTGCAAATGA GCTACTCAGG   
  
  
+ CGAATTAGGC AGCATGCTTC GCCATTTGGG GATAGTGTTC AAAGGCTAGC ACATTACTTT GCGAATGGGC   
  
  
+ TCGAGGCACG CTTAGCTGGT ACAGGATCAG AATCATACAA AGACTTTGCT GGGAAAAAAT TTTCATCCTA   
  
  
+ TGACATCTTG AGAGGCTACA AGTCATATGT TCAAGCCGTC CCCTTCCAGA GAACAACCAT GTTCTTGACA   
  
  
+ AATCAAACAA TAGTAAAGCT GGCTGAGAAA GCAACAAGGG TTCATATCAT TGATTTTGGC ATCTTTTTTG   
  
  
+ GTTTCCAGTG GCCCTGCCTT GTTCAGAATT TGTCAAGAAG ACCCCAGGGT TCTCCCATAC TCAAAATCAC   
  
  
+ GGGAGTCGAT CATCCCCAGA GTGGGTTCAG ACCAGCCCAG AAAGTTGAAG AGACAGGTCG TCGATTAGCA   
  
  
+ GGCTATTGTG AGAGGTTTGG GGTGCCCTTT GAGTTTCATC CCATTGCTCA AAGGTGGCAT ACCCTAAAGC   
  
  
+ CAGAGCATTT CAAAATTGAA AGCAATGAGC TTGTTGTGGT CACTTGTTTC TACCAATCAG GGAAGCTCCC   
  
  
+ TGATGAGACT GTTGATGTCA ACAGCCCAAG AGACCATGTC TTGAGGCTGA TCAGGGGCTT GAACCCTGAT   
  
  
+ CTATTCATCC ATGGGGTTAT CAATGGCACA TTCAATGCAC CCTTCTTCGT AACTCGGTTT AAAGAGGCAC   
  
  
+ TTTACCACTA CTCTTCTTTG TTCGATGTCT TTGATGCCAC CATAGATCGT GAAAGTCATG AAAGACTGCT   
  
  
+ GACTGAGAAC TATTTGTATG GGAAAGATGC ACTTAACATA GTAGCATGTG AGGGGAACGA GAGGATCGAG   
  
  
+ AGACCCGAGA CTTACAAGCA GTGGCAAGTC CGGAAGCTTA GAGCTGGATT CAAGCAGGTC CCCCTAGACC   
  
  
+ AAGACATTGT AAATGGAGCA AGAGCTATGG TTAAGGCAAA TTACAATAAG GATTTTATGG TAGAACAAGA   
  
  
+ TAGGAATTGG ATGGTCCAGG GCTGGAAAGG AAGGATTTTG AGTGCCATAT CCTGTTGGAA ACCTGCTTA  

- +Up\_Stream \_Len000ACTTAA CCTTCAACTA AGATGGAAAC TAACTAAGCT AATTAAGATC CAAGAACCAA   
  
  
- GATCAAGAGA GAGGAGAACA AGAACTAATA AATCCAAAAC CTACACTACA AATAACTTAA CTTACAACGT   
  
  
- TAACAACAAT ATTTCTACTA CAATCCGATT TATAGGAAGT TGAATCCAAC CCTAACTGCA GCTTATACAC   
  
  
- ATCAACTTAG AACATGACCG GATTTAGGGA CGAGGGGGAG ACAAAACGCT AATACTTAAA GTAATACGTG   
  
  
- TGGTGTACGA GCTACTTTAC GAGCTTTCTT GTAAGTAGGA GAAACAAACC CTAAATCCCC TTGCAACTTT   
  
  
- CTGCTTCAGA AATGGATCAC GCAGAATCAT CTGTTAATCC TAAAATTGTA CTTTTATCAA TTTTAAGATT   
  
  
- AACCCTAAAC TCTAACACTT AGAACATTAG GAAGCTGAAG ATCAAGATAC TTTTATCCGC TAATATCAAG   
  
  
- ATCCTAATCA GAAGCTGGGA ACTCTCTCCC TCCTCTACCC AAATCAACTA TTAGATAAGT AAGTATTGCG   
  
  
- TACGGACCCT CTACAAACCA TCCCGATCAT TAACTAAGCG ATTGTATAAA CACGACAATC AAGGTTGGAC   
  
  
- TCACAAATGA GAAGACGACA AATAAGGCAG AAAATCAGCA TTAACAGAGG AAATCAAATT AAAGTTTTGG   
  
  
- GGTTAACACT AGCGGATCTA ACTCGTTAGA TTAATCAAGA TTATTAACTT TAGTTCAAGG GACACCTTAT   
  
  
- GCTGGGCATG AACGGCACAC GATCGATGAC AATGTGGAAC GTAAACGCCA TATAAATATT GAGTTGTATA   
  
  
- CAGACGAGTG ATTTTCATGT ATAATCGAGT GGTTTTGACA CAGAGTTGCA TGATTTTATA AAATCACTTG   
  
  
- ATATGTAATA AAAATCATTC GCTTTATTCT ATGTGTTGTC GTGTAAAATT ACTCAATGTC CCGTAAAAAT   
  
  
- CACTCGGCTT TAGACTACCA AAAGAAACAT CTAGGCCAAT ATATTAACAT AAAATATGTT GGCTTACCTG   
  
  
- CTATATTGTT GACTCAATTT ACTCCTCAGA CTTTAATGCT TGTATTTTAG GTTTTCTTCT ACGGCTACGG   
  
  
- TAACCTTTCT TTCGACTTCC ACTAGCGAAA GATCATCATA ATCCGTCATC ATTAATTTTT GATTTTAGGT   
  
  
- CTTAACTGTG GCTTAACCCC AACACCGTGT ACTAGCTTTA GTCGTAGCTT AGATGTGCTA CTAGACCTAA   
  
  
- GAACCTGTGA GACCGAAATC CGGCAGTTAA AGCTCCTAAC GGGTATTGAA ATTAGGACAA GTTTAGGTAA   
  
  
- GTTAAATTAT AATGTGTTTA GGAAACTAAT TTAGTGGCAT TAACACCAAA CTAATCCCTT CAAAGAACCC   
  
  
- CCTTTCCAAG TTCGAAGACA GAAAGAATTT CGGACCTAAA AACCAGGGCG TACAAGTGAA GTGTAAGGGA   
  
  
- AAAAGAGAGG GTAGCAATGG ACAGACTTAG ACATACATTA TTTTCAGTAT AAAACGCTCC AGAAGAAAAC   
  
  
- TGACAAGAAC ATCAGTGAGA ACTATAGACG TGGTAAAACA AAAAGAGCCG TAAAAGAGGT TGAACGTAAA   
  
  
- AGATTACATT ACACTAGAAA AGACCTGTAA GTAAAACAAT TAGATGAGTG AACCCAGTTC CAAACGACTA   
  
  
- AAACCGACCA CAATTTAGGC AAGGAGAAAC CGTAAACAAG TACAAGGTGT TGACAAACTA TAGACCATAG   
  
  
- AGTCTTTGGG GCGGTAAGAA ACCATAAAGG ACTGAAAGAA CAAGTTCCCA ATAAGGATAA AGGGACTTAA   
  
  
- AAGAAACTAA GAAATCCATA AAGTTCGAAC CCCCCAAAAC AAGAAGTGAC TAAAACAGGA GAAGAAGGGA   
  
  
- ATAACCCACC AAACTAGAGG ATTTAAAGAC ACATAGACTT AGTCAGCCCC ATCTGAAACA CAAAAACCAA   
  
  
- CTAAAAAATA GTTACATAAA AGAAGGATTA CTTCCCGGAG GGCTTGGTTA TGTGTACGAA TTGAAGTTGG   
  
  
- GACAAGGAGA AGGTTCGAAA AACCTCGGAG TTTTCAGAAG CTTACGACTA TCCGTGTATC CCAGGAAGGT   
  
  
- TACGATACCA GAACTACTAC TGGGGCGTGG GAACTTACTG GGTTTAATGT AACATACAGT TAGAAGTATA   
  
  
- GGACTAGCTC TATAATGTGT CTAGGGACTG AAAGGACTAC TAACAGAGTT CAAGTAGTTA CTGTAAGAAT   
  
  
- ACCTTCTCCT AAACCTATTA GGCGGATATA GGAACGTCCT GATATTACGC GAGGTTCGGT GACTCTTCAG   
  
  
- TAACATACTA CGGGAACCTC TCCGAATAGG TCGAAGTAGA CTAGGTAACG TTAGTAGTCA ACCAGTCTCG   
  
  
- TGACTCTCGA GACTACTAGG ACTGTCTTCG TCGCCGTAAC TATTTACCCC ATTATCTATA ATGGGGTGGT   
  
  
- CGGACTTACA CCAACTTTGG TCGGCCGACT AGTTAGTCTC GGTCAACCTG GGAAGAGTTT ACAAACTACG   
  
  
- ACGACGAGTG GGGAACCTCA GATTAACAGT AAGAAAACAG AGATCATTAT TGAAACTAGT AAGATACCTA   
  
  
- CCCCAAGGTC TAAGAGGATA GTCATGTGAA AGACACTTAC TTTGTTCTTT CCTTCTTAGT CAATCATCTA   
  
  
- GTTCCTCGTC CTTCTTAGTA GTTTCTCTAC CACGTATGGA CCTTCTTTCC TCATGATTCG TTTATCGTAG   
  
  
- GTCGTTACTC CTTATACCTC TCTACCTCTC CAAACTACTA CATGACTAGA CGTCCCTTCC TTTACTACTA   
  
  
- TAAAGTGGAA CATGGGCGTT TTGGGGTCAA GTACTTCGTC TACTATTGAG CCTTGGTTTC CCCTTTAGTT   
  
  
- CTCCTAGATT TTCGTTCTCA TTTAGCGCAC AGTTCTTCTT TTCATTGCTT CCTGTTCGCC ATCTAGAATC   
  
  
- CTGAGTTGAA TAAGTCACAC GTCTTCGTCA CCGTTCGAAA CTCGAATTTT CACGTTTACT CGATGAGTCC   
  
  
- GCTTAATCCG TCGTACGAAG CGGTAAACCC CTATCACAAG TTTCCGATCG TGTAATGAAA CGCTTACCCG   
  
  
- AGCTCCGTGC GAATCGACCA TGTCCTAGTC TTAGTATGTT TCTGAAACGA CCCTTTTTTA AAAGTAGGAT   
  
  
- ACTGTAGAAC TCTCCGATGT TCAGTATACA AGTTCGGCAG GGGAAGGTCT CTTGTTGGTA CAAGAACTGT   
  
  
- TTAGTTTGTT ATCATTTCGA CCGACTCTTT CGTTGTTCCC AAGTATAGTA ACTAAAACCG TAGAAAAAAC   
  
  
- CAAAGGTCAC CGGGACGGAA CAAGTCTTAA ACAGTTCTTC TGGGGTCCCA AGAGGGTATG AGTTTTAGTG   
  
  
- CCCTCAGCTA GTAGGGGTCT CACCCAAGTC TGGTCGGGTC TTTCAACTTC TCTGTCCAGC AGCTAATCGT   
  
  
- CCGATAACAC TCTCCAAACC CCACGGGAAA CTCAAAGTAG GGTAACGAGT TTCCACCGTA TGGGATTTCG   
  
  
- GTCTCGTAAA GTTTTAACTT TCGTTACTCG AACAACACCA GTGAACAAAG ATGGTTAGTC CCTTCGAGGG   
  
  
- ACTACTCTGA CAACTACAGT TGTCGGGTTC TCTGGTACAG AACTCCGACT AGTCCCCGAA CTTGGGACTA   
  
  
- GATAAGTAGG TACCCCAATA GTTACCGTGT AAGTTACGTG GGAAGAAGCA TTGAGCCAAA TTTCTCCGTG   
  
  
- AAATGGTGAT GAGAAGAAAC AAGCTACAGA AACTACGGTG GTATCTAGCA CTTTCAGTAC TTTCTGACGA   
  
  
- CTGACTCTTG ATAAACATAC CCTTTCTACG TGAATTGTAT CATCGTACAC TCCCCTTGCT CTCCTAGCTC   
  
  
- TCTGGGCTCT GAATGTTCGT CACCGTTCAG GCCTTCGAAT CTCGACCTAA GTTCGTCCAG GGGGATCTGG   
  
  
- TTCTGTAACA TTTACCTCGT TCTCGATACC AATTCCGTTT AATGTTATTC CTAAAATACC ATCTTGTTCT   
  
  
- ATCCTTAACC TACCAGGTCC CGACCTTTCC TTCCTAAAAC TCACGGTATA GGACAACCTT TGGACGAAT

+     STRE

| Site Name | Organism | Position | Strand | Matrix score. | sequence | function |
| --- | --- | --- | --- | --- | --- | --- |
| STRE | Arabidopsis thaliana | 3194 | - | 5 | AGGGG |  |
| STRE | Arabidopsis thaliana | 340 | + | 5 | AGGGG |  |
| STRE | Arabidopsis thaliana | 249 | - | 5 | AGGGG |  |
| STRE | Arabidopsis thaliana | 3915 | - | 5 | AGGGG |  |
| STRE | Arabidopsis thaliana | 3627 | + | 5 | AGGGG |  |
| STRE | Arabidopsis thaliana | 2534 | - | 5 | AGGGG |  |
| STRE | Arabidopsis thaliana | 2863 | + | 5 | AGGGG |  |
| STRE | Arabidopsis thaliana | 3835 | + | 5 | AGGGG |  |

>HU02G01573.1   
+ +Up\_Stream \_Len000TGAATT GGAAGTTGAT TCTACCTTTG ATTGATTCGA TTAATTCTAG GTTCTTGGTT   
  
  
+ CTAGTTCTCT CTCCTCTTGT TCTTGATTAT TTAGGTTTTG GATGTGATGT TTATTGAATT GAATGTTGCA   
  
  
+ ATTGTTGTTA TAAAGATGAT GTTAGGCTAA ATATCCTTCA ACTTAGGTTG GGATTGACGT CGAATATGTG   
  
  
+ TAGTTGAATC TTGTACTGGC CTAAATCCCT GCTCCCCCTC TGTTTTGCGA TTATGAATTT CATTATGCAC   
  
  
+ ACCACATGCT CGATGAAATG CTCGAAAGAA CATTCATCCT CTTTGTTTGG GATTTAGGGG AACGTTGAAA   
  
  
+ GACGAAGTCT TTACCTAGTG CGTCTTAGTA GACAATTAGG ATTTTAACAT GAAAATAGTT AAAATTCTAA   
  
  
+ TTGGGATTTG AGATTGTGAA TCTTGTAATC CTTCGACTTC TAGTTCTATG AAAATAGGCG ATTATAGTTC   
  
  
+ TAGGATTAGT CTTCGACCCT TGAGAGAGGG AGGAGATGGG TTTAGTTGAT AATCTATTCA TTCATAACGC   
  
  
+ ATGCCTGGGA GATGTTTGGT AGGGCTAGTA ATTGATTCGC TAACATATTT GTGCTGTTAG TTCCAACCTG   
  
  
+ AGTGTTTACT CTTCTGCTGT TTATTCCGTC TTTTAGTCGT AATTGTCTCC TTTAGTTTAA TTTCAAAACC   
  
  
+ CCAATTGTGA TCGCCTAGAT TGAGCAATCT AATTAGTTCT AATAATTGAA ATCAAGTTCC CTGTGGAATA   
  
  
+ CGACCCGTAC TTGCCGTGTG CTAGCTACTG TTACACCTTG CATTTGCGGT ATATTTATAA CTCAACATAT   
  
  
+ GTCTGCTCAC TAAAAGTACA TATTAGCTCA CCAAAACTGT GTCTCAACGT ACTAAAATAT TTTAGTGAAC   
  
  
+ TATACATTAT TTTTAGTAAG CGAAATAAGA TACACAACAG CACATTTTAA TGAGTTACAG GGCATTTTTA   
  
  
+ GTGAGCCGAA ATCTGATGGT TTTCTTTGTA GATCCGGTTA TATAATTGTA TTTTATACAA CCGAATGGAC   
  
  
+ GATATAACAA CTGAGTTAAA TGAGGAGTCT GAAATTACGA ACATAAAATC CAAAAGAAGA TGCCGATGCC   
  
  
+ ATTGGAAAGA AAGCTGAAGG TGATCGCTTT CTAGTAGTAT TAGGCAGTAG TAATTAAAAA CTAAAATCCA   
  
  
+ GAATTGACAC CGAATTGGGG TTGTGGCACA TGATCGAAAT CAGCATCGAA TCTACACGAT GATCTGGATT   
  
  
+ CTTGGACACT CTGGCTTTAG GCCGTCAATT TCGAGGATTG CCCATAACTT TAATCCTGTT CAAATCCATT   
  
  
+ CAATTTAATA TTACACAAAT CCTTTGATTA AATCACCGTA ATTGTGGTTT GATTAGGGAA GTTTCTTGGG   
  
  
+ GGAAAGGTTC AAGCTTCTGT CTTTCTTAAA GCCTGGATTT TTGGTCCCGC ATGTTCACTT CACATTCCCT   
  
  
+ TTTTCTCTCC CATCGTTACC TGTCTGAATC TGTATGTAAT AAAAGTCATA TTTTGCGAGG TCTTCTTTTG   
  
  
+ ACTGTTCTTG TAGTCACTCT TGATATCTGC ACCATTTTGT TTTTCTCGGC ATTTTCTCCA ACTTGCATTT   
  
  
+ TCTAATGTAA TGTGATCTTT TCTGGACATT CATTTTGTTA ATCTACTCAC TTGGGTCAAG GTTTGCTGAT   
  
  
+ TTTGGCTGGT GTTAAATCCG TTCCTCTTTG GCATTTGTTC ATGTTCCACA ACTGTTTGAT ATCTGGTATC   
  
  
+ TCAGAAACCC CGCCATTCTT TGGTATTTCC TGACTTTCTT GTTCAAGGGT TATTCCTATT TCCCTGAATT   
  
  
+ TTCTTTGATT CTTTAGGTAT TTCAAGCTTG GGGGGTTTTG TTCTTCACTG ATTTTGTCCT CTTCTTCCCT   
  
  
+ TATTGGGTGG TTTGATCTCC TAAATTTCTG TGTATCTGAA TCAGTCGGGG TAGACTTTGT GTTTTTGGTT   
  
  
+ GATTTTTTAT CAATGTATTT TCTTCCTAAT GAAGGGCCTC CCGAACCAAT ACACATGCTT AACTTCAACC   
  
  
+ CTGTTCCTCT TCCAAGCTTT TTGGAGCCTC AAAAGTCTTC GAATGCTGAT AGGCACATAG GGTCCTTCCA   
  
  
+ ATGCTATGGT CTTGATGATG ACCCCGCACC CTTGAATGAC CCAAATTACA TTGTATGTCA ATCTTCATAT   
  
  
+ CCTGATCGAG ATATTACACA GATCCCTGAC TTTCCTGATG ATTGTCTCAA GTTCATCAAT GACATTCTTA   
  
  
+ TGGAAGAGGA TTTGGATAAT CCGCCTATAT CCTTGCAGGA CTATAATGCG CTCCAAGCCA CTGAGAAGTC   
  
  
+ ATTGTATGAT GCCCTTGGAG AGGCTTATCC AGCTTCATCT GATCCATTGC AATCATCAGT TGGTCAGAGC   
  
  
+ ACTGAGAGCT CTGATGATCC TGACAGAAGC AGCGGCATTG ATAAATGGGG TAATAGATAT TACCCCACCA   
  
  
+ GCCTGAATGT GGTTGAAACC AGCCGGCTGA TCAATCAGAG CCAGTTGGAC CCTTCTCAAA TGTTTGATGC   
  
  
+ TGCTGCTCAC CCCTTGGAGT CTAATTGTCA TTCTTTTGTC TCTAGTAATA ACTTTGATCA TTCTATGGAT   
  
  
+ GGGGTTCCAG ATTCTCCTAT CAGTACACTT TCTGTGAATG AAACAAGAAA GGAAGAATCA GTTAGTAGAT   
  
  
+ CAAGGAGCAG GAAGAATCAT CAAAGAGATG GTGCATACCT GGAAGAAAGG AGTACTAAGC AAATAGCATC   
  
  
+ CAGCAATGAG GAATATGGAG AGATGGAGAG GTTTGATGAT GTACTGATCT GCAGGGAAGG AAATGATGAT   
  
  
+ ATTTCACCTT GTACCCGCAA AACCCCAGTT CATGAAGCAG ATGATAACTC GGAACCAAAG GGGAAATCAA   
  
  
+ GAGGATCTAA AAGCAAGAGT AAATCGCGTG TCAAGAAGAA AAGTAACGAA GGACAAGCGG TAGATCTTAG   
  
  
+ GACTCAACTT ATTCAGTGTG CAGAAGCAGT GGCAAGCTTT GAGCTTAAAA GTGCAAATGA GCTACTCAGG   
  
  
+ CGAATTAGGC AGCATGCTTC GCCATTTGGG GATAGTGTTC AAAGGCTAGC ACATTACTTT GCGAATGGGC   
  
  
+ TCGAGGCACG CTTAGCTGGT ACAGGATCAG AATCATACAA AGACTTTGCT GGGAAAAAAT TTTCATCCTA   
  
  
+ TGACATCTTG AGAGGCTACA AGTCATATGT TCAAGCCGTC CCCTTCCAGA GAACAACCAT GTTCTTGACA   
  
  
+ AATCAAACAA TAGTAAAGCT GGCTGAGAAA GCAACAAGGG TTCATATCAT TGATTTTGGC ATCTTTTTTG   
  
  
+ GTTTCCAGTG GCCCTGCCTT GTTCAGAATT TGTCAAGAAG ACCCCAGGGT TCTCCCATAC TCAAAATCAC   
  
  
+ GGGAGTCGAT CATCCCCAGA GTGGGTTCAG ACCAGCCCAG AAAGTTGAAG AGACAGGTCG TCGATTAGCA   
  
  
+ GGCTATTGTG AGAGGTTTGG GGTGCCCTTT GAGTTTCATC CCATTGCTCA AAGGTGGCAT ACCCTAAAGC   
  
  
+ CAGAGCATTT CAAAATTGAA AGCAATGAGC TTGTTGTGGT CACTTGTTTC TACCAATCAG GGAAGCTCCC   
  
  
+ TGATGAGACT GTTGATGTCA ACAGCCCAAG AGACCATGTC TTGAGGCTGA TCAGGGGCTT GAACCCTGAT   
  
  
+ CTATTCATCC ATGGGGTTAT CAATGGCACA TTCAATGCAC CCTTCTTCGT AACTCGGTTT AAAGAGGCAC   
  
  
+ TTTACCACTA CTCTTCTTTG TTCGATGTCT TTGATGCCAC CATAGATCGT GAAAGTCATG AAAGACTGCT   
  
  
+ GACTGAGAAC TATTTGTATG GGAAAGATGC ACTTAACATA GTAGCATGTG AGGGGAACGA GAGGATCGAG   
  
  
+ AGACCCGAGA CTTACAAGCA GTGGCAAGTC CGGAAGCTTA GAGCTGGATT CAAGCAGGTC CCCCTAGACC   
  
  
+ AAGACATTGT AAATGGAGCA AGAGCTATGG TTAAGGCAAA TTACAATAAG GATTTTATGG TAGAACAAGA   
  
  
+ TAGGAATTGG ATGGTCCAGG GCTGGAAAGG AAGGATTTTG AGTGCCATAT CCTGTTGGAA ACCTGCTTA  

- +Up\_Stream \_Len000ACTTAA CCTTCAACTA AGATGGAAAC TAACTAAGCT AATTAAGATC CAAGAACCAA   
  
  
- GATCAAGAGA GAGGAGAACA AGAACTAATA AATCCAAAAC CTACACTACA AATAACTTAA CTTACAACGT   
  
  
- TAACAACAAT ATTTCTACTA CAATCCGATT TATAGGAAGT TGAATCCAAC CCTAACTGCA GCTTATACAC   
  
  
- ATCAACTTAG AACATGACCG GATTTAGGGA CGAGGGGGAG ACAAAACGCT AATACTTAAA GTAATACGTG   
  
  
- TGGTGTACGA GCTACTTTAC GAGCTTTCTT GTAAGTAGGA GAAACAAACC CTAAATCCCC TTGCAACTTT   
  
  
- CTGCTTCAGA AATGGATCAC GCAGAATCAT CTGTTAATCC TAAAATTGTA CTTTTATCAA TTTTAAGATT   
  
  
- AACCCTAAAC TCTAACACTT AGAACATTAG GAAGCTGAAG ATCAAGATAC TTTTATCCGC TAATATCAAG   
  
  
- ATCCTAATCA GAAGCTGGGA ACTCTCTCCC TCCTCTACCC AAATCAACTA TTAGATAAGT AAGTATTGCG   
  
  
- TACGGACCCT CTACAAACCA TCCCGATCAT TAACTAAGCG ATTGTATAAA CACGACAATC AAGGTTGGAC   
  
  
- TCACAAATGA GAAGACGACA AATAAGGCAG AAAATCAGCA TTAACAGAGG AAATCAAATT AAAGTTTTGG   
  
  
- GGTTAACACT AGCGGATCTA ACTCGTTAGA TTAATCAAGA TTATTAACTT TAGTTCAAGG GACACCTTAT   
  
  
- GCTGGGCATG AACGGCACAC GATCGATGAC AATGTGGAAC GTAAACGCCA TATAAATATT GAGTTGTATA   
  
  
- CAGACGAGTG ATTTTCATGT ATAATCGAGT GGTTTTGACA CAGAGTTGCA TGATTTTATA AAATCACTTG   
  
  
- ATATGTAATA AAAATCATTC GCTTTATTCT ATGTGTTGTC GTGTAAAATT ACTCAATGTC CCGTAAAAAT   
  
  
- CACTCGGCTT TAGACTACCA AAAGAAACAT CTAGGCCAAT ATATTAACAT AAAATATGTT GGCTTACCTG   
  
  
- CTATATTGTT GACTCAATTT ACTCCTCAGA CTTTAATGCT TGTATTTTAG GTTTTCTTCT ACGGCTACGG   
  
  
- TAACCTTTCT TTCGACTTCC ACTAGCGAAA GATCATCATA ATCCGTCATC ATTAATTTTT GATTTTAGGT   
  
  
- CTTAACTGTG GCTTAACCCC AACACCGTGT ACTAGCTTTA GTCGTAGCTT AGATGTGCTA CTAGACCTAA   
  
  
- GAACCTGTGA GACCGAAATC CGGCAGTTAA AGCTCCTAAC GGGTATTGAA ATTAGGACAA GTTTAGGTAA   
  
  
- GTTAAATTAT AATGTGTTTA GGAAACTAAT TTAGTGGCAT TAACACCAAA CTAATCCCTT CAAAGAACCC   
  
  
- CCTTTCCAAG TTCGAAGACA GAAAGAATTT CGGACCTAAA AACCAGGGCG TACAAGTGAA GTGTAAGGGA   
  
  
- AAAAGAGAGG GTAGCAATGG ACAGACTTAG ACATACATTA TTTTCAGTAT AAAACGCTCC AGAAGAAAAC   
  
  
- TGACAAGAAC ATCAGTGAGA ACTATAGACG TGGTAAAACA AAAAGAGCCG TAAAAGAGGT TGAACGTAAA   
  
  
- AGATTACATT ACACTAGAAA AGACCTGTAA GTAAAACAAT TAGATGAGTG AACCCAGTTC CAAACGACTA   
  
  
- AAACCGACCA CAATTTAGGC AAGGAGAAAC CGTAAACAAG TACAAGGTGT TGACAAACTA TAGACCATAG   
  
  
- AGTCTTTGGG GCGGTAAGAA ACCATAAAGG ACTGAAAGAA CAAGTTCCCA ATAAGGATAA AGGGACTTAA   
  
  
- AAGAAACTAA GAAATCCATA AAGTTCGAAC CCCCCAAAAC AAGAAGTGAC TAAAACAGGA GAAGAAGGGA   
  
  
- ATAACCCACC AAACTAGAGG ATTTAAAGAC ACATAGACTT AGTCAGCCCC ATCTGAAACA CAAAAACCAA   
  
  
- CTAAAAAATA GTTACATAAA AGAAGGATTA CTTCCCGGAG GGCTTGGTTA TGTGTACGAA TTGAAGTTGG   
  
  
- GACAAGGAGA AGGTTCGAAA AACCTCGGAG TTTTCAGAAG CTTACGACTA TCCGTGTATC CCAGGAAGGT   
  
  
- TACGATACCA GAACTACTAC TGGGGCGTGG GAACTTACTG GGTTTAATGT AACATACAGT TAGAAGTATA   
  
  
- GGACTAGCTC TATAATGTGT CTAGGGACTG AAAGGACTAC TAACAGAGTT CAAGTAGTTA CTGTAAGAAT   
  
  
- ACCTTCTCCT AAACCTATTA GGCGGATATA GGAACGTCCT GATATTACGC GAGGTTCGGT GACTCTTCAG   
  
  
- TAACATACTA CGGGAACCTC TCCGAATAGG TCGAAGTAGA CTAGGTAACG TTAGTAGTCA ACCAGTCTCG   
  
  
- TGACTCTCGA GACTACTAGG ACTGTCTTCG TCGCCGTAAC TATTTACCCC ATTATCTATA ATGGGGTGGT   
  
  
- CGGACTTACA CCAACTTTGG TCGGCCGACT AGTTAGTCTC GGTCAACCTG GGAAGAGTTT ACAAACTACG   
  
  
- ACGACGAGTG GGGAACCTCA GATTAACAGT AAGAAAACAG AGATCATTAT TGAAACTAGT AAGATACCTA   
  
  
- CCCCAAGGTC TAAGAGGATA GTCATGTGAA AGACACTTAC TTTGTTCTTT CCTTCTTAGT CAATCATCTA   
  
  
- GTTCCTCGTC CTTCTTAGTA GTTTCTCTAC CACGTATGGA CCTTCTTTCC TCATGATTCG TTTATCGTAG   
  
  
- GTCGTTACTC CTTATACCTC TCTACCTCTC CAAACTACTA CATGACTAGA CGTCCCTTCC TTTACTACTA   
  
  
- TAAAGTGGAA CATGGGCGTT TTGGGGTCAA GTACTTCGTC TACTATTGAG CCTTGGTTTC CCCTTTAGTT   
  
  
- CTCCTAGATT TTCGTTCTCA TTTAGCGCAC AGTTCTTCTT TTCATTGCTT CCTGTTCGCC ATCTAGAATC   
  
  
- CTGAGTTGAA TAAGTCACAC GTCTTCGTCA CCGTTCGAAA CTCGAATTTT CACGTTTACT CGATGAGTCC   
  
  
- GCTTAATCCG TCGTACGAAG CGGTAAACCC CTATCACAAG TTTCCGATCG TGTAATGAAA CGCTTACCCG   
  
  
- AGCTCCGTGC GAATCGACCA TGTCCTAGTC TTAGTATGTT TCTGAAACGA CCCTTTTTTA AAAGTAGGAT   
  
  
- ACTGTAGAAC TCTCCGATGT TCAGTATACA AGTTCGGCAG GGGAAGGTCT CTTGTTGGTA CAAGAACTGT   
  
  
- TTAGTTTGTT ATCATTTCGA CCGACTCTTT CGTTGTTCCC AAGTATAGTA ACTAAAACCG TAGAAAAAAC   
  
  
- CAAAGGTCAC CGGGACGGAA CAAGTCTTAA ACAGTTCTTC TGGGGTCCCA AGAGGGTATG AGTTTTAGTG   
  
  
- CCCTCAGCTA GTAGGGGTCT CACCCAAGTC TGGTCGGGTC TTTCAACTTC TCTGTCCAGC AGCTAATCGT   
  
  
- CCGATAACAC TCTCCAAACC CCACGGGAAA CTCAAAGTAG GGTAACGAGT TTCCACCGTA TGGGATTTCG   
  
  
- GTCTCGTAAA GTTTTAACTT TCGTTACTCG AACAACACCA GTGAACAAAG ATGGTTAGTC CCTTCGAGGG   
  
  
- ACTACTCTGA CAACTACAGT TGTCGGGTTC TCTGGTACAG AACTCCGACT AGTCCCCGAA CTTGGGACTA   
  
  
- GATAAGTAGG TACCCCAATA GTTACCGTGT AAGTTACGTG GGAAGAAGCA TTGAGCCAAA TTTCTCCGTG   
  
  
- AAATGGTGAT GAGAAGAAAC AAGCTACAGA AACTACGGTG GTATCTAGCA CTTTCAGTAC TTTCTGACGA   
  
  
- CTGACTCTTG ATAAACATAC CCTTTCTACG TGAATTGTAT CATCGTACAC TCCCCTTGCT CTCCTAGCTC   
  
  
- TCTGGGCTCT GAATGTTCGT CACCGTTCAG GCCTTCGAAT CTCGACCTAA GTTCGTCCAG GGGGATCTGG   
  
  
- TTCTGTAACA TTTACCTCGT TCTCGATACC AATTCCGTTT AATGTTATTC CTAAAATACC ATCTTGTTCT   
  
  
- ATCCTTAACC TACCAGGTCC CGACCTTTCC TTCCTAAAAC TCACGGTATA GGACAACCTT TGGACGAAT

+     TATA

| Site Name | Organism | Position | Strand | Matrix score. | sequence | function |
| --- | --- | --- | --- | --- | --- | --- |
| TATA | Arabidopsis thaliana | 1034 | - | 8 | TATAAAAT |  |

>HU02G01573.1   
+ +Up\_Stream \_Len000TGAATT GGAAGTTGAT TCTACCTTTG ATTGATTCGA TTAATTCTAG GTTCTTGGTT   
  
  
+ CTAGTTCTCT CTCCTCTTGT TCTTGATTAT TTAGGTTTTG GATGTGATGT TTATTGAATT GAATGTTGCA   
  
  
+ ATTGTTGTTA TAAAGATGAT GTTAGGCTAA ATATCCTTCA ACTTAGGTTG GGATTGACGT CGAATATGTG   
  
  
+ TAGTTGAATC TTGTACTGGC CTAAATCCCT GCTCCCCCTC TGTTTTGCGA TTATGAATTT CATTATGCAC   
  
  
+ ACCACATGCT CGATGAAATG CTCGAAAGAA CATTCATCCT CTTTGTTTGG GATTTAGGGG AACGTTGAAA   
  
  
+ GACGAAGTCT TTACCTAGTG CGTCTTAGTA GACAATTAGG ATTTTAACAT GAAAATAGTT AAAATTCTAA   
  
  
+ TTGGGATTTG AGATTGTGAA TCTTGTAATC CTTCGACTTC TAGTTCTATG AAAATAGGCG ATTATAGTTC   
  
  
+ TAGGATTAGT CTTCGACCCT TGAGAGAGGG AGGAGATGGG TTTAGTTGAT AATCTATTCA TTCATAACGC   
  
  
+ ATGCCTGGGA GATGTTTGGT AGGGCTAGTA ATTGATTCGC TAACATATTT GTGCTGTTAG TTCCAACCTG   
  
  
+ AGTGTTTACT CTTCTGCTGT TTATTCCGTC TTTTAGTCGT AATTGTCTCC TTTAGTTTAA TTTCAAAACC   
  
  
+ CCAATTGTGA TCGCCTAGAT TGAGCAATCT AATTAGTTCT AATAATTGAA ATCAAGTTCC CTGTGGAATA   
  
  
+ CGACCCGTAC TTGCCGTGTG CTAGCTACTG TTACACCTTG CATTTGCGGT ATATTTATAA CTCAACATAT   
  
  
+ GTCTGCTCAC TAAAAGTACA TATTAGCTCA CCAAAACTGT GTCTCAACGT ACTAAAATAT TTTAGTGAAC   
  
  
+ TATACATTAT TTTTAGTAAG CGAAATAAGA TACACAACAG CACATTTTAA TGAGTTACAG GGCATTTTTA   
  
  
+ GTGAGCCGAA ATCTGATGGT TTTCTTTGTA GATCCGGTTA TATAATTGTA TTTTATACAA CCGAATGGAC   
  
  
+ GATATAACAA CTGAGTTAAA TGAGGAGTCT GAAATTACGA ACATAAAATC CAAAAGAAGA TGCCGATGCC   
  
  
+ ATTGGAAAGA AAGCTGAAGG TGATCGCTTT CTAGTAGTAT TAGGCAGTAG TAATTAAAAA CTAAAATCCA   
  
  
+ GAATTGACAC CGAATTGGGG TTGTGGCACA TGATCGAAAT CAGCATCGAA TCTACACGAT GATCTGGATT   
  
  
+ CTTGGACACT CTGGCTTTAG GCCGTCAATT TCGAGGATTG CCCATAACTT TAATCCTGTT CAAATCCATT   
  
  
+ CAATTTAATA TTACACAAAT CCTTTGATTA AATCACCGTA ATTGTGGTTT GATTAGGGAA GTTTCTTGGG   
  
  
+ GGAAAGGTTC AAGCTTCTGT CTTTCTTAAA GCCTGGATTT TTGGTCCCGC ATGTTCACTT CACATTCCCT   
  
  
+ TTTTCTCTCC CATCGTTACC TGTCTGAATC TGTATGTAAT AAAAGTCATA TTTTGCGAGG TCTTCTTTTG   
  
  
+ ACTGTTCTTG TAGTCACTCT TGATATCTGC ACCATTTTGT TTTTCTCGGC ATTTTCTCCA ACTTGCATTT   
  
  
+ TCTAATGTAA TGTGATCTTT TCTGGACATT CATTTTGTTA ATCTACTCAC TTGGGTCAAG GTTTGCTGAT   
  
  
+ TTTGGCTGGT GTTAAATCCG TTCCTCTTTG GCATTTGTTC ATGTTCCACA ACTGTTTGAT ATCTGGTATC   
  
  
+ TCAGAAACCC CGCCATTCTT TGGTATTTCC TGACTTTCTT GTTCAAGGGT TATTCCTATT TCCCTGAATT   
  
  
+ TTCTTTGATT CTTTAGGTAT TTCAAGCTTG GGGGGTTTTG TTCTTCACTG ATTTTGTCCT CTTCTTCCCT   
  
  
+ TATTGGGTGG TTTGATCTCC TAAATTTCTG TGTATCTGAA TCAGTCGGGG TAGACTTTGT GTTTTTGGTT   
  
  
+ GATTTTTTAT CAATGTATTT TCTTCCTAAT GAAGGGCCTC CCGAACCAAT ACACATGCTT AACTTCAACC   
  
  
+ CTGTTCCTCT TCCAAGCTTT TTGGAGCCTC AAAAGTCTTC GAATGCTGAT AGGCACATAG GGTCCTTCCA   
  
  
+ ATGCTATGGT CTTGATGATG ACCCCGCACC CTTGAATGAC CCAAATTACA TTGTATGTCA ATCTTCATAT   
  
  
+ CCTGATCGAG ATATTACACA GATCCCTGAC TTTCCTGATG ATTGTCTCAA GTTCATCAAT GACATTCTTA   
  
  
+ TGGAAGAGGA TTTGGATAAT CCGCCTATAT CCTTGCAGGA CTATAATGCG CTCCAAGCCA CTGAGAAGTC   
  
  
+ ATTGTATGAT GCCCTTGGAG AGGCTTATCC AGCTTCATCT GATCCATTGC AATCATCAGT TGGTCAGAGC   
  
  
+ ACTGAGAGCT CTGATGATCC TGACAGAAGC AGCGGCATTG ATAAATGGGG TAATAGATAT TACCCCACCA   
  
  
+ GCCTGAATGT GGTTGAAACC AGCCGGCTGA TCAATCAGAG CCAGTTGGAC CCTTCTCAAA TGTTTGATGC   
  
  
+ TGCTGCTCAC CCCTTGGAGT CTAATTGTCA TTCTTTTGTC TCTAGTAATA ACTTTGATCA TTCTATGGAT   
  
  
+ GGGGTTCCAG ATTCTCCTAT CAGTACACTT TCTGTGAATG AAACAAGAAA GGAAGAATCA GTTAGTAGAT   
  
  
+ CAAGGAGCAG GAAGAATCAT CAAAGAGATG GTGCATACCT GGAAGAAAGG AGTACTAAGC AAATAGCATC   
  
  
+ CAGCAATGAG GAATATGGAG AGATGGAGAG GTTTGATGAT GTACTGATCT GCAGGGAAGG AAATGATGAT   
  
  
+ ATTTCACCTT GTACCCGCAA AACCCCAGTT CATGAAGCAG ATGATAACTC GGAACCAAAG GGGAAATCAA   
  
  
+ GAGGATCTAA AAGCAAGAGT AAATCGCGTG TCAAGAAGAA AAGTAACGAA GGACAAGCGG TAGATCTTAG   
  
  
+ GACTCAACTT ATTCAGTGTG CAGAAGCAGT GGCAAGCTTT GAGCTTAAAA GTGCAAATGA GCTACTCAGG   
  
  
+ CGAATTAGGC AGCATGCTTC GCCATTTGGG GATAGTGTTC AAAGGCTAGC ACATTACTTT GCGAATGGGC   
  
  
+ TCGAGGCACG CTTAGCTGGT ACAGGATCAG AATCATACAA AGACTTTGCT GGGAAAAAAT TTTCATCCTA   
  
  
+ TGACATCTTG AGAGGCTACA AGTCATATGT TCAAGCCGTC CCCTTCCAGA GAACAACCAT GTTCTTGACA   
  
  
+ AATCAAACAA TAGTAAAGCT GGCTGAGAAA GCAACAAGGG TTCATATCAT TGATTTTGGC ATCTTTTTTG   
  
  
+ GTTTCCAGTG GCCCTGCCTT GTTCAGAATT TGTCAAGAAG ACCCCAGGGT TCTCCCATAC TCAAAATCAC   
  
  
+ GGGAGTCGAT CATCCCCAGA GTGGGTTCAG ACCAGCCCAG AAAGTTGAAG AGACAGGTCG TCGATTAGCA   
  
  
+ GGCTATTGTG AGAGGTTTGG GGTGCCCTTT GAGTTTCATC CCATTGCTCA AAGGTGGCAT ACCCTAAAGC   
  
  
+ CAGAGCATTT CAAAATTGAA AGCAATGAGC TTGTTGTGGT CACTTGTTTC TACCAATCAG GGAAGCTCCC   
  
  
+ TGATGAGACT GTTGATGTCA ACAGCCCAAG AGACCATGTC TTGAGGCTGA TCAGGGGCTT GAACCCTGAT   
  
  
+ CTATTCATCC ATGGGGTTAT CAATGGCACA TTCAATGCAC CCTTCTTCGT AACTCGGTTT AAAGAGGCAC   
  
  
+ TTTACCACTA CTCTTCTTTG TTCGATGTCT TTGATGCCAC CATAGATCGT GAAAGTCATG AAAGACTGCT   
  
  
+ GACTGAGAAC TATTTGTATG GGAAAGATGC ACTTAACATA GTAGCATGTG AGGGGAACGA GAGGATCGAG   
  
  
+ AGACCCGAGA CTTACAAGCA GTGGCAAGTC CGGAAGCTTA GAGCTGGATT CAAGCAGGTC CCCCTAGACC   
  
  
+ AAGACATTGT AAATGGAGCA AGAGCTATGG TTAAGGCAAA TTACAATAAG GATTTTATGG TAGAACAAGA   
  
  
+ TAGGAATTGG ATGGTCCAGG GCTGGAAAGG AAGGATTTTG AGTGCCATAT CCTGTTGGAA ACCTGCTTA  

- +Up\_Stream \_Len000ACTTAA CCTTCAACTA AGATGGAAAC TAACTAAGCT AATTAAGATC CAAGAACCAA   
  
  
- GATCAAGAGA GAGGAGAACA AGAACTAATA AATCCAAAAC CTACACTACA AATAACTTAA CTTACAACGT   
  
  
- TAACAACAAT ATTTCTACTA CAATCCGATT TATAGGAAGT TGAATCCAAC CCTAACTGCA GCTTATACAC   
  
  
- ATCAACTTAG AACATGACCG GATTTAGGGA CGAGGGGGAG ACAAAACGCT AATACTTAAA GTAATACGTG   
  
  
- TGGTGTACGA GCTACTTTAC GAGCTTTCTT GTAAGTAGGA GAAACAAACC CTAAATCCCC TTGCAACTTT   
  
  
- CTGCTTCAGA AATGGATCAC GCAGAATCAT CTGTTAATCC TAAAATTGTA CTTTTATCAA TTTTAAGATT   
  
  
- AACCCTAAAC TCTAACACTT AGAACATTAG GAAGCTGAAG ATCAAGATAC TTTTATCCGC TAATATCAAG   
  
  
- ATCCTAATCA GAAGCTGGGA ACTCTCTCCC TCCTCTACCC AAATCAACTA TTAGATAAGT AAGTATTGCG   
  
  
- TACGGACCCT CTACAAACCA TCCCGATCAT TAACTAAGCG ATTGTATAAA CACGACAATC AAGGTTGGAC   
  
  
- TCACAAATGA GAAGACGACA AATAAGGCAG AAAATCAGCA TTAACAGAGG AAATCAAATT AAAGTTTTGG   
  
  
- GGTTAACACT AGCGGATCTA ACTCGTTAGA TTAATCAAGA TTATTAACTT TAGTTCAAGG GACACCTTAT   
  
  
- GCTGGGCATG AACGGCACAC GATCGATGAC AATGTGGAAC GTAAACGCCA TATAAATATT GAGTTGTATA   
  
  
- CAGACGAGTG ATTTTCATGT ATAATCGAGT GGTTTTGACA CAGAGTTGCA TGATTTTATA AAATCACTTG   
  
  
- ATATGTAATA AAAATCATTC GCTTTATTCT ATGTGTTGTC GTGTAAAATT ACTCAATGTC CCGTAAAAAT   
  
  
- CACTCGGCTT TAGACTACCA AAAGAAACAT CTAGGCCAAT ATATTAACAT AAAATATGTT GGCTTACCTG   
  
  
- CTATATTGTT GACTCAATTT ACTCCTCAGA CTTTAATGCT TGTATTTTAG GTTTTCTTCT ACGGCTACGG   
  
  
- TAACCTTTCT TTCGACTTCC ACTAGCGAAA GATCATCATA ATCCGTCATC ATTAATTTTT GATTTTAGGT   
  
  
- CTTAACTGTG GCTTAACCCC AACACCGTGT ACTAGCTTTA GTCGTAGCTT AGATGTGCTA CTAGACCTAA   
  
  
- GAACCTGTGA GACCGAAATC CGGCAGTTAA AGCTCCTAAC GGGTATTGAA ATTAGGACAA GTTTAGGTAA   
  
  
- GTTAAATTAT AATGTGTTTA GGAAACTAAT TTAGTGGCAT TAACACCAAA CTAATCCCTT CAAAGAACCC   
  
  
- CCTTTCCAAG TTCGAAGACA GAAAGAATTT CGGACCTAAA AACCAGGGCG TACAAGTGAA GTGTAAGGGA   
  
  
- AAAAGAGAGG GTAGCAATGG ACAGACTTAG ACATACATTA TTTTCAGTAT AAAACGCTCC AGAAGAAAAC   
  
  
- TGACAAGAAC ATCAGTGAGA ACTATAGACG TGGTAAAACA AAAAGAGCCG TAAAAGAGGT TGAACGTAAA   
  
  
- AGATTACATT ACACTAGAAA AGACCTGTAA GTAAAACAAT TAGATGAGTG AACCCAGTTC CAAACGACTA   
  
  
- AAACCGACCA CAATTTAGGC AAGGAGAAAC CGTAAACAAG TACAAGGTGT TGACAAACTA TAGACCATAG   
  
  
- AGTCTTTGGG GCGGTAAGAA ACCATAAAGG ACTGAAAGAA CAAGTTCCCA ATAAGGATAA AGGGACTTAA   
  
  
- AAGAAACTAA GAAATCCATA AAGTTCGAAC CCCCCAAAAC AAGAAGTGAC TAAAACAGGA GAAGAAGGGA   
  
  
- ATAACCCACC AAACTAGAGG ATTTAAAGAC ACATAGACTT AGTCAGCCCC ATCTGAAACA CAAAAACCAA   
  
  
- CTAAAAAATA GTTACATAAA AGAAGGATTA CTTCCCGGAG GGCTTGGTTA TGTGTACGAA TTGAAGTTGG   
  
  
- GACAAGGAGA AGGTTCGAAA AACCTCGGAG TTTTCAGAAG CTTACGACTA TCCGTGTATC CCAGGAAGGT   
  
  
- TACGATACCA GAACTACTAC TGGGGCGTGG GAACTTACTG GGTTTAATGT AACATACAGT TAGAAGTATA   
  
  
- GGACTAGCTC TATAATGTGT CTAGGGACTG AAAGGACTAC TAACAGAGTT CAAGTAGTTA CTGTAAGAAT   
  
  
- ACCTTCTCCT AAACCTATTA GGCGGATATA GGAACGTCCT GATATTACGC GAGGTTCGGT GACTCTTCAG   
  
  
- TAACATACTA CGGGAACCTC TCCGAATAGG TCGAAGTAGA CTAGGTAACG TTAGTAGTCA ACCAGTCTCG   
  
  
- TGACTCTCGA GACTACTAGG ACTGTCTTCG TCGCCGTAAC TATTTACCCC ATTATCTATA ATGGGGTGGT   
  
  
- CGGACTTACA CCAACTTTGG TCGGCCGACT AGTTAGTCTC GGTCAACCTG GGAAGAGTTT ACAAACTACG   
  
  
- ACGACGAGTG GGGAACCTCA GATTAACAGT AAGAAAACAG AGATCATTAT TGAAACTAGT AAGATACCTA   
  
  
- CCCCAAGGTC TAAGAGGATA GTCATGTGAA AGACACTTAC TTTGTTCTTT CCTTCTTAGT CAATCATCTA   
  
  
- GTTCCTCGTC CTTCTTAGTA GTTTCTCTAC CACGTATGGA CCTTCTTTCC TCATGATTCG TTTATCGTAG   
  
  
- GTCGTTACTC CTTATACCTC TCTACCTCTC CAAACTACTA CATGACTAGA CGTCCCTTCC TTTACTACTA   
  
  
- TAAAGTGGAA CATGGGCGTT TTGGGGTCAA GTACTTCGTC TACTATTGAG CCTTGGTTTC CCCTTTAGTT   
  
  
- CTCCTAGATT TTCGTTCTCA TTTAGCGCAC AGTTCTTCTT TTCATTGCTT CCTGTTCGCC ATCTAGAATC   
  
  
- CTGAGTTGAA TAAGTCACAC GTCTTCGTCA CCGTTCGAAA CTCGAATTTT CACGTTTACT CGATGAGTCC   
  
  
- GCTTAATCCG TCGTACGAAG CGGTAAACCC CTATCACAAG TTTCCGATCG TGTAATGAAA CGCTTACCCG   
  
  
- AGCTCCGTGC GAATCGACCA TGTCCTAGTC TTAGTATGTT TCTGAAACGA CCCTTTTTTA AAAGTAGGAT   
  
  
- ACTGTAGAAC TCTCCGATGT TCAGTATACA AGTTCGGCAG GGGAAGGTCT CTTGTTGGTA CAAGAACTGT   
  
  
- TTAGTTTGTT ATCATTTCGA CCGACTCTTT CGTTGTTCCC AAGTATAGTA ACTAAAACCG TAGAAAAAAC   
  
  
- CAAAGGTCAC CGGGACGGAA CAAGTCTTAA ACAGTTCTTC TGGGGTCCCA AGAGGGTATG AGTTTTAGTG   
  
  
- CCCTCAGCTA GTAGGGGTCT CACCCAAGTC TGGTCGGGTC TTTCAACTTC TCTGTCCAGC AGCTAATCGT   
  
  
- CCGATAACAC TCTCCAAACC CCACGGGAAA CTCAAAGTAG GGTAACGAGT TTCCACCGTA TGGGATTTCG   
  
  
- GTCTCGTAAA GTTTTAACTT TCGTTACTCG AACAACACCA GTGAACAAAG ATGGTTAGTC CCTTCGAGGG   
  
  
- ACTACTCTGA CAACTACAGT TGTCGGGTTC TCTGGTACAG AACTCCGACT AGTCCCCGAA CTTGGGACTA   
  
  
- GATAAGTAGG TACCCCAATA GTTACCGTGT AAGTTACGTG GGAAGAAGCA TTGAGCCAAA TTTCTCCGTG   
  
  
- AAATGGTGAT GAGAAGAAAC AAGCTACAGA AACTACGGTG GTATCTAGCA CTTTCAGTAC TTTCTGACGA   
  
  
- CTGACTCTTG ATAAACATAC CCTTTCTACG TGAATTGTAT CATCGTACAC TCCCCTTGCT CTCCTAGCTC   
  
  
- TCTGGGCTCT GAATGTTCGT CACCGTTCAG GCCTTCGAAT CTCGACCTAA GTTCGTCCAG GGGGATCTGG   
  
  
- TTCTGTAACA TTTACCTCGT TCTCGATACC AATTCCGTTT AATGTTATTC CTAAAATACC ATCTTGTTCT   
  
  
- ATCCTTAACC TACCAGGTCC CGACCTTTCC TTCCTAAAAC TCACGGTATA GGACAACCTT TGGACGAAT

+     TATA-box

| Site Name | Organism | Position | Strand | Matrix score. | sequence | function |
| --- | --- | --- | --- | --- | --- | --- |
| TATA-box | Helianthus annuus | 1036 | - | 6 | TATAAA | core promoter element around -30 of transcription start |
| TATA-box | Arabidopsis thaliana | 1023 | + | 6 | TATATA | core promoter element around -30 of transcription start |
| TATA-box | Arabidopsis thaliana | 152 | - | 5 | TATAA | core promoter element around -30 of transcription start |
| TATA-box | Arabidopsis thaliana | 487 | + | 4 | TATA | core promoter element around -30 of transcription start |
| TATA-box | Brassica juncea | 827 | - | 7 | TATAAAT | core promoter element around -30 of transcription start |
| TATA-box | Arabidopsis thaliana | 915 | + | 4 | TATA | core promoter element around -30 of transcription start |
| TATA-box | Arabidopsis thaliana | 830 | + | 4 | TATA | core promoter element around -30 of transcription start |
| TATA-box | Arabidopsis thaliana | 1022 | - | 7 | TATATAA | core promoter element around -30 of transcription start |
| TATA-box | Arabidopsis thaliana | 824 | + | 4 | TATA | core promoter element around -30 of transcription start |
| TATA-box | Brassica oleracea | 1024 | + | 6 | ATATAA | core promoter element around -30 of transcription start |
| TATA-box | Arabidopsis thaliana | 1025 | + | 4 | TATA | core promoter element around -30 of transcription start |
| TATA-box | Helianthus annuus | 828 | - | 6 | TATAAA | core promoter element around -30 of transcription start |
| TATA-box | Pisum sativum | 1035 | - | 7 | TATAAAA | core promoter element around -30 of transcription start |
| TATA-box | Arabidopsis thaliana | 2286 | - | 4 | TATA | core promoter element around -30 of transcription start |
| TATA-box | Brassica napus | 485 | + | 6 | ATTATA | core promoter element around -30 of transcription start |
| TATA-box | Arabidopsis thaliana | 2270 | - | 4 | TATA | core promoter element around -30 of transcription start |
| TATA-box | Arabidopsis thaliana | 1057 | + | 4 | TATA | core promoter element around -30 of transcription start |
| TATA-box | Brassica oleracea | 1056 | + | 6 | ATATAA | core promoter element around -30 of transcription start |
| TATA-box | Arabidopsis thaliana | 1038 | + | 4 | TATA | core promoter element around -30 of transcription start |
| TATA-box | Arabidopsis thaliana | 1037 | - | 5 | TATAA | core promoter element around -30 of transcription start |
| TATA-box | Arabidopsis thaliana | 153 | + | 4 | TATA | core promoter element around -30 of transcription start |
| TATA-box | Daucus carota | 826 | - | 8 | TATAAATA | core promoter element around -30 of transcription start |
| TATA-box | Arabidopsis thaliana | 829 | - | 5 | TATAA | core promoter element around -30 of transcription start |
| TATA-box | Arabidopsis thaliana | 486 | - | 5 | TATAA | core promoter element around -30 of transcription start |

>HU02G01573.1   
+ +Up\_Stream \_Len000TGAATT GGAAGTTGAT TCTACCTTTG ATTGATTCGA TTAATTCTAG GTTCTTGGTT   
  
  
+ CTAGTTCTCT CTCCTCTTGT TCTTGATTAT TTAGGTTTTG GATGTGATGT TTATTGAATT GAATGTTGCA   
  
  
+ ATTGTTGTTA TAAAGATGAT GTTAGGCTAA ATATCCTTCA ACTTAGGTTG GGATTGACGT CGAATATGTG   
  
  
+ TAGTTGAATC TTGTACTGGC CTAAATCCCT GCTCCCCCTC TGTTTTGCGA TTATGAATTT CATTATGCAC   
  
  
+ ACCACATGCT CGATGAAATG CTCGAAAGAA CATTCATCCT CTTTGTTTGG GATTTAGGGG AACGTTGAAA   
  
  
+ GACGAAGTCT TTACCTAGTG CGTCTTAGTA GACAATTAGG ATTTTAACAT GAAAATAGTT AAAATTCTAA   
  
  
+ TTGGGATTTG AGATTGTGAA TCTTGTAATC CTTCGACTTC TAGTTCTATG AAAATAGGCG ATTATAGTTC   
  
  
+ TAGGATTAGT CTTCGACCCT TGAGAGAGGG AGGAGATGGG TTTAGTTGAT AATCTATTCA TTCATAACGC   
  
  
+ ATGCCTGGGA GATGTTTGGT AGGGCTAGTA ATTGATTCGC TAACATATTT GTGCTGTTAG TTCCAACCTG   
  
  
+ AGTGTTTACT CTTCTGCTGT TTATTCCGTC TTTTAGTCGT AATTGTCTCC TTTAGTTTAA TTTCAAAACC   
  
  
+ CCAATTGTGA TCGCCTAGAT TGAGCAATCT AATTAGTTCT AATAATTGAA ATCAAGTTCC CTGTGGAATA   
  
  
+ CGACCCGTAC TTGCCGTGTG CTAGCTACTG TTACACCTTG CATTTGCGGT ATATTTATAA CTCAACATAT   
  
  
+ GTCTGCTCAC TAAAAGTACA TATTAGCTCA CCAAAACTGT GTCTCAACGT ACTAAAATAT TTTAGTGAAC   
  
  
+ TATACATTAT TTTTAGTAAG CGAAATAAGA TACACAACAG CACATTTTAA TGAGTTACAG GGCATTTTTA   
  
  
+ GTGAGCCGAA ATCTGATGGT TTTCTTTGTA GATCCGGTTA TATAATTGTA TTTTATACAA CCGAATGGAC   
  
  
+ GATATAACAA CTGAGTTAAA TGAGGAGTCT GAAATTACGA ACATAAAATC CAAAAGAAGA TGCCGATGCC   
  
  
+ ATTGGAAAGA AAGCTGAAGG TGATCGCTTT CTAGTAGTAT TAGGCAGTAG TAATTAAAAA CTAAAATCCA   
  
  
+ GAATTGACAC CGAATTGGGG TTGTGGCACA TGATCGAAAT CAGCATCGAA TCTACACGAT GATCTGGATT   
  
  
+ CTTGGACACT CTGGCTTTAG GCCGTCAATT TCGAGGATTG CCCATAACTT TAATCCTGTT CAAATCCATT   
  
  
+ CAATTTAATA TTACACAAAT CCTTTGATTA AATCACCGTA ATTGTGGTTT GATTAGGGAA GTTTCTTGGG   
  
  
+ GGAAAGGTTC AAGCTTCTGT CTTTCTTAAA GCCTGGATTT TTGGTCCCGC ATGTTCACTT CACATTCCCT   
  
  
+ TTTTCTCTCC CATCGTTACC TGTCTGAATC TGTATGTAAT AAAAGTCATA TTTTGCGAGG TCTTCTTTTG   
  
  
+ ACTGTTCTTG TAGTCACTCT TGATATCTGC ACCATTTTGT TTTTCTCGGC ATTTTCTCCA ACTTGCATTT   
  
  
+ TCTAATGTAA TGTGATCTTT TCTGGACATT CATTTTGTTA ATCTACTCAC TTGGGTCAAG GTTTGCTGAT   
  
  
+ TTTGGCTGGT GTTAAATCCG TTCCTCTTTG GCATTTGTTC ATGTTCCACA ACTGTTTGAT ATCTGGTATC   
  
  
+ TCAGAAACCC CGCCATTCTT TGGTATTTCC TGACTTTCTT GTTCAAGGGT TATTCCTATT TCCCTGAATT   
  
  
+ TTCTTTGATT CTTTAGGTAT TTCAAGCTTG GGGGGTTTTG TTCTTCACTG ATTTTGTCCT CTTCTTCCCT   
  
  
+ TATTGGGTGG TTTGATCTCC TAAATTTCTG TGTATCTGAA TCAGTCGGGG TAGACTTTGT GTTTTTGGTT   
  
  
+ GATTTTTTAT CAATGTATTT TCTTCCTAAT GAAGGGCCTC CCGAACCAAT ACACATGCTT AACTTCAACC   
  
  
+ CTGTTCCTCT TCCAAGCTTT TTGGAGCCTC AAAAGTCTTC GAATGCTGAT AGGCACATAG GGTCCTTCCA   
  
  
+ ATGCTATGGT CTTGATGATG ACCCCGCACC CTTGAATGAC CCAAATTACA TTGTATGTCA ATCTTCATAT   
  
  
+ CCTGATCGAG ATATTACACA GATCCCTGAC TTTCCTGATG ATTGTCTCAA GTTCATCAAT GACATTCTTA   
  
  
+ TGGAAGAGGA TTTGGATAAT CCGCCTATAT CCTTGCAGGA CTATAATGCG CTCCAAGCCA CTGAGAAGTC   
  
  
+ ATTGTATGAT GCCCTTGGAG AGGCTTATCC AGCTTCATCT GATCCATTGC AATCATCAGT TGGTCAGAGC   
  
  
+ ACTGAGAGCT CTGATGATCC TGACAGAAGC AGCGGCATTG ATAAATGGGG TAATAGATAT TACCCCACCA   
  
  
+ GCCTGAATGT GGTTGAAACC AGCCGGCTGA TCAATCAGAG CCAGTTGGAC CCTTCTCAAA TGTTTGATGC   
  
  
+ TGCTGCTCAC CCCTTGGAGT CTAATTGTCA TTCTTTTGTC TCTAGTAATA ACTTTGATCA TTCTATGGAT   
  
  
+ GGGGTTCCAG ATTCTCCTAT CAGTACACTT TCTGTGAATG AAACAAGAAA GGAAGAATCA GTTAGTAGAT   
  
  
+ CAAGGAGCAG GAAGAATCAT CAAAGAGATG GTGCATACCT GGAAGAAAGG AGTACTAAGC AAATAGCATC   
  
  
+ CAGCAATGAG GAATATGGAG AGATGGAGAG GTTTGATGAT GTACTGATCT GCAGGGAAGG AAATGATGAT   
  
  
+ ATTTCACCTT GTACCCGCAA AACCCCAGTT CATGAAGCAG ATGATAACTC GGAACCAAAG GGGAAATCAA   
  
  
+ GAGGATCTAA AAGCAAGAGT AAATCGCGTG TCAAGAAGAA AAGTAACGAA GGACAAGCGG TAGATCTTAG   
  
  
+ GACTCAACTT ATTCAGTGTG CAGAAGCAGT GGCAAGCTTT GAGCTTAAAA GTGCAAATGA GCTACTCAGG   
  
  
+ CGAATTAGGC AGCATGCTTC GCCATTTGGG GATAGTGTTC AAAGGCTAGC ACATTACTTT GCGAATGGGC   
  
  
+ TCGAGGCACG CTTAGCTGGT ACAGGATCAG AATCATACAA AGACTTTGCT GGGAAAAAAT TTTCATCCTA   
  
  
+ TGACATCTTG AGAGGCTACA AGTCATATGT TCAAGCCGTC CCCTTCCAGA GAACAACCAT GTTCTTGACA   
  
  
+ AATCAAACAA TAGTAAAGCT GGCTGAGAAA GCAACAAGGG TTCATATCAT TGATTTTGGC ATCTTTTTTG   
  
  
+ GTTTCCAGTG GCCCTGCCTT GTTCAGAATT TGTCAAGAAG ACCCCAGGGT TCTCCCATAC TCAAAATCAC   
  
  
+ GGGAGTCGAT CATCCCCAGA GTGGGTTCAG ACCAGCCCAG AAAGTTGAAG AGACAGGTCG TCGATTAGCA   
  
  
+ GGCTATTGTG AGAGGTTTGG GGTGCCCTTT GAGTTTCATC CCATTGCTCA AAGGTGGCAT ACCCTAAAGC   
  
  
+ CAGAGCATTT CAAAATTGAA AGCAATGAGC TTGTTGTGGT CACTTGTTTC TACCAATCAG GGAAGCTCCC   
  
  
+ TGATGAGACT GTTGATGTCA ACAGCCCAAG AGACCATGTC TTGAGGCTGA TCAGGGGCTT GAACCCTGAT   
  
  
+ CTATTCATCC ATGGGGTTAT CAATGGCACA TTCAATGCAC CCTTCTTCGT AACTCGGTTT AAAGAGGCAC   
  
  
+ TTTACCACTA CTCTTCTTTG TTCGATGTCT TTGATGCCAC CATAGATCGT GAAAGTCATG AAAGACTGCT   
  
  
+ GACTGAGAAC TATTTGTATG GGAAAGATGC ACTTAACATA GTAGCATGTG AGGGGAACGA GAGGATCGAG   
  
  
+ AGACCCGAGA CTTACAAGCA GTGGCAAGTC CGGAAGCTTA GAGCTGGATT CAAGCAGGTC CCCCTAGACC   
  
  
+ AAGACATTGT AAATGGAGCA AGAGCTATGG TTAAGGCAAA TTACAATAAG GATTTTATGG TAGAACAAGA   
  
  
+ TAGGAATTGG ATGGTCCAGG GCTGGAAAGG AAGGATTTTG AGTGCCATAT CCTGTTGGAA ACCTGCTTA  

- +Up\_Stream \_Len000ACTTAA CCTTCAACTA AGATGGAAAC TAACTAAGCT AATTAAGATC CAAGAACCAA   
  
  
- GATCAAGAGA GAGGAGAACA AGAACTAATA AATCCAAAAC CTACACTACA AATAACTTAA CTTACAACGT   
  
  
- TAACAACAAT ATTTCTACTA CAATCCGATT TATAGGAAGT TGAATCCAAC CCTAACTGCA GCTTATACAC   
  
  
- ATCAACTTAG AACATGACCG GATTTAGGGA CGAGGGGGAG ACAAAACGCT AATACTTAAA GTAATACGTG   
  
  
- TGGTGTACGA GCTACTTTAC GAGCTTTCTT GTAAGTAGGA GAAACAAACC CTAAATCCCC TTGCAACTTT   
  
  
- CTGCTTCAGA AATGGATCAC GCAGAATCAT CTGTTAATCC TAAAATTGTA CTTTTATCAA TTTTAAGATT   
  
  
- AACCCTAAAC TCTAACACTT AGAACATTAG GAAGCTGAAG ATCAAGATAC TTTTATCCGC TAATATCAAG   
  
  
- ATCCTAATCA GAAGCTGGGA ACTCTCTCCC TCCTCTACCC AAATCAACTA TTAGATAAGT AAGTATTGCG   
  
  
- TACGGACCCT CTACAAACCA TCCCGATCAT TAACTAAGCG ATTGTATAAA CACGACAATC AAGGTTGGAC   
  
  
- TCACAAATGA GAAGACGACA AATAAGGCAG AAAATCAGCA TTAACAGAGG AAATCAAATT AAAGTTTTGG   
  
  
- GGTTAACACT AGCGGATCTA ACTCGTTAGA TTAATCAAGA TTATTAACTT TAGTTCAAGG GACACCTTAT   
  
  
- GCTGGGCATG AACGGCACAC GATCGATGAC AATGTGGAAC GTAAACGCCA TATAAATATT GAGTTGTATA   
  
  
- CAGACGAGTG ATTTTCATGT ATAATCGAGT GGTTTTGACA CAGAGTTGCA TGATTTTATA AAATCACTTG   
  
  
- ATATGTAATA AAAATCATTC GCTTTATTCT ATGTGTTGTC GTGTAAAATT ACTCAATGTC CCGTAAAAAT   
  
  
- CACTCGGCTT TAGACTACCA AAAGAAACAT CTAGGCCAAT ATATTAACAT AAAATATGTT GGCTTACCTG   
  
  
- CTATATTGTT GACTCAATTT ACTCCTCAGA CTTTAATGCT TGTATTTTAG GTTTTCTTCT ACGGCTACGG   
  
  
- TAACCTTTCT TTCGACTTCC ACTAGCGAAA GATCATCATA ATCCGTCATC ATTAATTTTT GATTTTAGGT   
  
  
- CTTAACTGTG GCTTAACCCC AACACCGTGT ACTAGCTTTA GTCGTAGCTT AGATGTGCTA CTAGACCTAA   
  
  
- GAACCTGTGA GACCGAAATC CGGCAGTTAA AGCTCCTAAC GGGTATTGAA ATTAGGACAA GTTTAGGTAA   
  
  
- GTTAAATTAT AATGTGTTTA GGAAACTAAT TTAGTGGCAT TAACACCAAA CTAATCCCTT CAAAGAACCC   
  
  
- CCTTTCCAAG TTCGAAGACA GAAAGAATTT CGGACCTAAA AACCAGGGCG TACAAGTGAA GTGTAAGGGA   
  
  
- AAAAGAGAGG GTAGCAATGG ACAGACTTAG ACATACATTA TTTTCAGTAT AAAACGCTCC AGAAGAAAAC   
  
  
- TGACAAGAAC ATCAGTGAGA ACTATAGACG TGGTAAAACA AAAAGAGCCG TAAAAGAGGT TGAACGTAAA   
  
  
- AGATTACATT ACACTAGAAA AGACCTGTAA GTAAAACAAT TAGATGAGTG AACCCAGTTC CAAACGACTA   
  
  
- AAACCGACCA CAATTTAGGC AAGGAGAAAC CGTAAACAAG TACAAGGTGT TGACAAACTA TAGACCATAG   
  
  
- AGTCTTTGGG GCGGTAAGAA ACCATAAAGG ACTGAAAGAA CAAGTTCCCA ATAAGGATAA AGGGACTTAA   
  
  
- AAGAAACTAA GAAATCCATA AAGTTCGAAC CCCCCAAAAC AAGAAGTGAC TAAAACAGGA GAAGAAGGGA   
  
  
- ATAACCCACC AAACTAGAGG ATTTAAAGAC ACATAGACTT AGTCAGCCCC ATCTGAAACA CAAAAACCAA   
  
  
- CTAAAAAATA GTTACATAAA AGAAGGATTA CTTCCCGGAG GGCTTGGTTA TGTGTACGAA TTGAAGTTGG   
  
  
- GACAAGGAGA AGGTTCGAAA AACCTCGGAG TTTTCAGAAG CTTACGACTA TCCGTGTATC CCAGGAAGGT   
  
  
- TACGATACCA GAACTACTAC TGGGGCGTGG GAACTTACTG GGTTTAATGT AACATACAGT TAGAAGTATA   
  
  
- GGACTAGCTC TATAATGTGT CTAGGGACTG AAAGGACTAC TAACAGAGTT CAAGTAGTTA CTGTAAGAAT   
  
  
- ACCTTCTCCT AAACCTATTA GGCGGATATA GGAACGTCCT GATATTACGC GAGGTTCGGT GACTCTTCAG   
  
  
- TAACATACTA CGGGAACCTC TCCGAATAGG TCGAAGTAGA CTAGGTAACG TTAGTAGTCA ACCAGTCTCG   
  
  
- TGACTCTCGA GACTACTAGG ACTGTCTTCG TCGCCGTAAC TATTTACCCC ATTATCTATA ATGGGGTGGT   
  
  
- CGGACTTACA CCAACTTTGG TCGGCCGACT AGTTAGTCTC GGTCAACCTG GGAAGAGTTT ACAAACTACG   
  
  
- ACGACGAGTG GGGAACCTCA GATTAACAGT AAGAAAACAG AGATCATTAT TGAAACTAGT AAGATACCTA   
  
  
- CCCCAAGGTC TAAGAGGATA GTCATGTGAA AGACACTTAC TTTGTTCTTT CCTTCTTAGT CAATCATCTA   
  
  
- GTTCCTCGTC CTTCTTAGTA GTTTCTCTAC CACGTATGGA CCTTCTTTCC TCATGATTCG TTTATCGTAG   
  
  
- GTCGTTACTC CTTATACCTC TCTACCTCTC CAAACTACTA CATGACTAGA CGTCCCTTCC TTTACTACTA   
  
  
- TAAAGTGGAA CATGGGCGTT TTGGGGTCAA GTACTTCGTC TACTATTGAG CCTTGGTTTC CCCTTTAGTT   
  
  
- CTCCTAGATT TTCGTTCTCA TTTAGCGCAC AGTTCTTCTT TTCATTGCTT CCTGTTCGCC ATCTAGAATC   
  
  
- CTGAGTTGAA TAAGTCACAC GTCTTCGTCA CCGTTCGAAA CTCGAATTTT CACGTTTACT CGATGAGTCC   
  
  
- GCTTAATCCG TCGTACGAAG CGGTAAACCC CTATCACAAG TTTCCGATCG TGTAATGAAA CGCTTACCCG   
  
  
- AGCTCCGTGC GAATCGACCA TGTCCTAGTC TTAGTATGTT TCTGAAACGA CCCTTTTTTA AAAGTAGGAT   
  
  
- ACTGTAGAAC TCTCCGATGT TCAGTATACA AGTTCGGCAG GGGAAGGTCT CTTGTTGGTA CAAGAACTGT   
  
  
- TTAGTTTGTT ATCATTTCGA CCGACTCTTT CGTTGTTCCC AAGTATAGTA ACTAAAACCG TAGAAAAAAC   
  
  
- CAAAGGTCAC CGGGACGGAA CAAGTCTTAA ACAGTTCTTC TGGGGTCCCA AGAGGGTATG AGTTTTAGTG   
  
  
- CCCTCAGCTA GTAGGGGTCT CACCCAAGTC TGGTCGGGTC TTTCAACTTC TCTGTCCAGC AGCTAATCGT   
  
  
- CCGATAACAC TCTCCAAACC CCACGGGAAA CTCAAAGTAG GGTAACGAGT TTCCACCGTA TGGGATTTCG   
  
  
- GTCTCGTAAA GTTTTAACTT TCGTTACTCG AACAACACCA GTGAACAAAG ATGGTTAGTC CCTTCGAGGG   
  
  
- ACTACTCTGA CAACTACAGT TGTCGGGTTC TCTGGTACAG AACTCCGACT AGTCCCCGAA CTTGGGACTA   
  
  
- GATAAGTAGG TACCCCAATA GTTACCGTGT AAGTTACGTG GGAAGAAGCA TTGAGCCAAA TTTCTCCGTG   
  
  
- AAATGGTGAT GAGAAGAAAC AAGCTACAGA AACTACGGTG GTATCTAGCA CTTTCAGTAC TTTCTGACGA   
  
  
- CTGACTCTTG ATAAACATAC CCTTTCTACG TGAATTGTAT CATCGTACAC TCCCCTTGCT CTCCTAGCTC   
  
  
- TCTGGGCTCT GAATGTTCGT CACCGTTCAG GCCTTCGAAT CTCGACCTAA GTTCGTCCAG GGGGATCTGG   
  
  
- TTCTGTAACA TTTACCTCGT TCTCGATACC AATTCCGTTT AATGTTATTC CTAAAATACC ATCTTGTTCT   
  
  
- ATCCTTAACC TACCAGGTCC CGACCTTTCC TTCCTAAAAC TCACGGTATA GGACAACCTT TGGACGAAT

+     TC-rich repeats

| Site Name | Organism | Position | Strand | Matrix score. | sequence | function |
| --- | --- | --- | --- | --- | --- | --- |
| TC-rich repeats | Nicotiana tabacum | 599 | + | 9 | ATTCTCTAAC | cis-acting element involved in defense and stress responsiveness |

>HU02G01573.1   
+ +Up\_Stream \_Len000TGAATT GGAAGTTGAT TCTACCTTTG ATTGATTCGA TTAATTCTAG GTTCTTGGTT   
  
  
+ CTAGTTCTCT CTCCTCTTGT TCTTGATTAT TTAGGTTTTG GATGTGATGT TTATTGAATT GAATGTTGCA   
  
  
+ ATTGTTGTTA TAAAGATGAT GTTAGGCTAA ATATCCTTCA ACTTAGGTTG GGATTGACGT CGAATATGTG   
  
  
+ TAGTTGAATC TTGTACTGGC CTAAATCCCT GCTCCCCCTC TGTTTTGCGA TTATGAATTT CATTATGCAC   
  
  
+ ACCACATGCT CGATGAAATG CTCGAAAGAA CATTCATCCT CTTTGTTTGG GATTTAGGGG AACGTTGAAA   
  
  
+ GACGAAGTCT TTACCTAGTG CGTCTTAGTA GACAATTAGG ATTTTAACAT GAAAATAGTT AAAATTCTAA   
  
  
+ TTGGGATTTG AGATTGTGAA TCTTGTAATC CTTCGACTTC TAGTTCTATG AAAATAGGCG ATTATAGTTC   
  
  
+ TAGGATTAGT CTTCGACCCT TGAGAGAGGG AGGAGATGGG TTTAGTTGAT AATCTATTCA TTCATAACGC   
  
  
+ ATGCCTGGGA GATGTTTGGT AGGGCTAGTA ATTGATTCGC TAACATATTT GTGCTGTTAG TTCCAACCTG   
  
  
+ AGTGTTTACT CTTCTGCTGT TTATTCCGTC TTTTAGTCGT AATTGTCTCC TTTAGTTTAA TTTCAAAACC   
  
  
+ CCAATTGTGA TCGCCTAGAT TGAGCAATCT AATTAGTTCT AATAATTGAA ATCAAGTTCC CTGTGGAATA   
  
  
+ CGACCCGTAC TTGCCGTGTG CTAGCTACTG TTACACCTTG CATTTGCGGT ATATTTATAA CTCAACATAT   
  
  
+ GTCTGCTCAC TAAAAGTACA TATTAGCTCA CCAAAACTGT GTCTCAACGT ACTAAAATAT TTTAGTGAAC   
  
  
+ TATACATTAT TTTTAGTAAG CGAAATAAGA TACACAACAG CACATTTTAA TGAGTTACAG GGCATTTTTA   
  
  
+ GTGAGCCGAA ATCTGATGGT TTTCTTTGTA GATCCGGTTA TATAATTGTA TTTTATACAA CCGAATGGAC   
  
  
+ GATATAACAA CTGAGTTAAA TGAGGAGTCT GAAATTACGA ACATAAAATC CAAAAGAAGA TGCCGATGCC   
  
  
+ ATTGGAAAGA AAGCTGAAGG TGATCGCTTT CTAGTAGTAT TAGGCAGTAG TAATTAAAAA CTAAAATCCA   
  
  
+ GAATTGACAC CGAATTGGGG TTGTGGCACA TGATCGAAAT CAGCATCGAA TCTACACGAT GATCTGGATT   
  
  
+ CTTGGACACT CTGGCTTTAG GCCGTCAATT TCGAGGATTG CCCATAACTT TAATCCTGTT CAAATCCATT   
  
  
+ CAATTTAATA TTACACAAAT CCTTTGATTA AATCACCGTA ATTGTGGTTT GATTAGGGAA GTTTCTTGGG   
  
  
+ GGAAAGGTTC AAGCTTCTGT CTTTCTTAAA GCCTGGATTT TTGGTCCCGC ATGTTCACTT CACATTCCCT   
  
  
+ TTTTCTCTCC CATCGTTACC TGTCTGAATC TGTATGTAAT AAAAGTCATA TTTTGCGAGG TCTTCTTTTG   
  
  
+ ACTGTTCTTG TAGTCACTCT TGATATCTGC ACCATTTTGT TTTTCTCGGC ATTTTCTCCA ACTTGCATTT   
  
  
+ TCTAATGTAA TGTGATCTTT TCTGGACATT CATTTTGTTA ATCTACTCAC TTGGGTCAAG GTTTGCTGAT   
  
  
+ TTTGGCTGGT GTTAAATCCG TTCCTCTTTG GCATTTGTTC ATGTTCCACA ACTGTTTGAT ATCTGGTATC   
  
  
+ TCAGAAACCC CGCCATTCTT TGGTATTTCC TGACTTTCTT GTTCAAGGGT TATTCCTATT TCCCTGAATT   
  
  
+ TTCTTTGATT CTTTAGGTAT TTCAAGCTTG GGGGGTTTTG TTCTTCACTG ATTTTGTCCT CTTCTTCCCT   
  
  
+ TATTGGGTGG TTTGATCTCC TAAATTTCTG TGTATCTGAA TCAGTCGGGG TAGACTTTGT GTTTTTGGTT   
  
  
+ GATTTTTTAT CAATGTATTT TCTTCCTAAT GAAGGGCCTC CCGAACCAAT ACACATGCTT AACTTCAACC   
  
  
+ CTGTTCCTCT TCCAAGCTTT TTGGAGCCTC AAAAGTCTTC GAATGCTGAT AGGCACATAG GGTCCTTCCA   
  
  
+ ATGCTATGGT CTTGATGATG ACCCCGCACC CTTGAATGAC CCAAATTACA TTGTATGTCA ATCTTCATAT   
  
  
+ CCTGATCGAG ATATTACACA GATCCCTGAC TTTCCTGATG ATTGTCTCAA GTTCATCAAT GACATTCTTA   
  
  
+ TGGAAGAGGA TTTGGATAAT CCGCCTATAT CCTTGCAGGA CTATAATGCG CTCCAAGCCA CTGAGAAGTC   
  
  
+ ATTGTATGAT GCCCTTGGAG AGGCTTATCC AGCTTCATCT GATCCATTGC AATCATCAGT TGGTCAGAGC   
  
  
+ ACTGAGAGCT CTGATGATCC TGACAGAAGC AGCGGCATTG ATAAATGGGG TAATAGATAT TACCCCACCA   
  
  
+ GCCTGAATGT GGTTGAAACC AGCCGGCTGA TCAATCAGAG CCAGTTGGAC CCTTCTCAAA TGTTTGATGC   
  
  
+ TGCTGCTCAC CCCTTGGAGT CTAATTGTCA TTCTTTTGTC TCTAGTAATA ACTTTGATCA TTCTATGGAT   
  
  
+ GGGGTTCCAG ATTCTCCTAT CAGTACACTT TCTGTGAATG AAACAAGAAA GGAAGAATCA GTTAGTAGAT   
  
  
+ CAAGGAGCAG GAAGAATCAT CAAAGAGATG GTGCATACCT GGAAGAAAGG AGTACTAAGC AAATAGCATC   
  
  
+ CAGCAATGAG GAATATGGAG AGATGGAGAG GTTTGATGAT GTACTGATCT GCAGGGAAGG AAATGATGAT   
  
  
+ ATTTCACCTT GTACCCGCAA AACCCCAGTT CATGAAGCAG ATGATAACTC GGAACCAAAG GGGAAATCAA   
  
  
+ GAGGATCTAA AAGCAAGAGT AAATCGCGTG TCAAGAAGAA AAGTAACGAA GGACAAGCGG TAGATCTTAG   
  
  
+ GACTCAACTT ATTCAGTGTG CAGAAGCAGT GGCAAGCTTT GAGCTTAAAA GTGCAAATGA GCTACTCAGG   
  
  
+ CGAATTAGGC AGCATGCTTC GCCATTTGGG GATAGTGTTC AAAGGCTAGC ACATTACTTT GCGAATGGGC   
  
  
+ TCGAGGCACG CTTAGCTGGT ACAGGATCAG AATCATACAA AGACTTTGCT GGGAAAAAAT TTTCATCCTA   
  
  
+ TGACATCTTG AGAGGCTACA AGTCATATGT TCAAGCCGTC CCCTTCCAGA GAACAACCAT GTTCTTGACA   
  
  
+ AATCAAACAA TAGTAAAGCT GGCTGAGAAA GCAACAAGGG TTCATATCAT TGATTTTGGC ATCTTTTTTG   
  
  
+ GTTTCCAGTG GCCCTGCCTT GTTCAGAATT TGTCAAGAAG ACCCCAGGGT TCTCCCATAC TCAAAATCAC   
  
  
+ GGGAGTCGAT CATCCCCAGA GTGGGTTCAG ACCAGCCCAG AAAGTTGAAG AGACAGGTCG TCGATTAGCA   
  
  
+ GGCTATTGTG AGAGGTTTGG GGTGCCCTTT GAGTTTCATC CCATTGCTCA AAGGTGGCAT ACCCTAAAGC   
  
  
+ CAGAGCATTT CAAAATTGAA AGCAATGAGC TTGTTGTGGT CACTTGTTTC TACCAATCAG GGAAGCTCCC   
  
  
+ TGATGAGACT GTTGATGTCA ACAGCCCAAG AGACCATGTC TTGAGGCTGA TCAGGGGCTT GAACCCTGAT   
  
  
+ CTATTCATCC ATGGGGTTAT CAATGGCACA TTCAATGCAC CCTTCTTCGT AACTCGGTTT AAAGAGGCAC   
  
  
+ TTTACCACTA CTCTTCTTTG TTCGATGTCT TTGATGCCAC CATAGATCGT GAAAGTCATG AAAGACTGCT   
  
  
+ GACTGAGAAC TATTTGTATG GGAAAGATGC ACTTAACATA GTAGCATGTG AGGGGAACGA GAGGATCGAG   
  
  
+ AGACCCGAGA CTTACAAGCA GTGGCAAGTC CGGAAGCTTA GAGCTGGATT CAAGCAGGTC CCCCTAGACC   
  
  
+ AAGACATTGT AAATGGAGCA AGAGCTATGG TTAAGGCAAA TTACAATAAG GATTTTATGG TAGAACAAGA   
  
  
+ TAGGAATTGG ATGGTCCAGG GCTGGAAAGG AAGGATTTTG AGTGCCATAT CCTGTTGGAA ACCTGCTTA  

- +Up\_Stream \_Len000ACTTAA CCTTCAACTA AGATGGAAAC TAACTAAGCT AATTAAGATC CAAGAACCAA   
  
  
- GATCAAGAGA GAGGAGAACA AGAACTAATA AATCCAAAAC CTACACTACA AATAACTTAA CTTACAACGT   
  
  
- TAACAACAAT ATTTCTACTA CAATCCGATT TATAGGAAGT TGAATCCAAC CCTAACTGCA GCTTATACAC   
  
  
- ATCAACTTAG AACATGACCG GATTTAGGGA CGAGGGGGAG ACAAAACGCT AATACTTAAA GTAATACGTG   
  
  
- TGGTGTACGA GCTACTTTAC GAGCTTTCTT GTAAGTAGGA GAAACAAACC CTAAATCCCC TTGCAACTTT   
  
  
- CTGCTTCAGA AATGGATCAC GCAGAATCAT CTGTTAATCC TAAAATTGTA CTTTTATCAA TTTTAAGATT   
  
  
- AACCCTAAAC TCTAACACTT AGAACATTAG GAAGCTGAAG ATCAAGATAC TTTTATCCGC TAATATCAAG   
  
  
- ATCCTAATCA GAAGCTGGGA ACTCTCTCCC TCCTCTACCC AAATCAACTA TTAGATAAGT AAGTATTGCG   
  
  
- TACGGACCCT CTACAAACCA TCCCGATCAT TAACTAAGCG ATTGTATAAA CACGACAATC AAGGTTGGAC   
  
  
- TCACAAATGA GAAGACGACA AATAAGGCAG AAAATCAGCA TTAACAGAGG AAATCAAATT AAAGTTTTGG   
  
  
- GGTTAACACT AGCGGATCTA ACTCGTTAGA TTAATCAAGA TTATTAACTT TAGTTCAAGG GACACCTTAT   
  
  
- GCTGGGCATG AACGGCACAC GATCGATGAC AATGTGGAAC GTAAACGCCA TATAAATATT GAGTTGTATA   
  
  
- CAGACGAGTG ATTTTCATGT ATAATCGAGT GGTTTTGACA CAGAGTTGCA TGATTTTATA AAATCACTTG   
  
  
- ATATGTAATA AAAATCATTC GCTTTATTCT ATGTGTTGTC GTGTAAAATT ACTCAATGTC CCGTAAAAAT   
  
  
- CACTCGGCTT TAGACTACCA AAAGAAACAT CTAGGCCAAT ATATTAACAT AAAATATGTT GGCTTACCTG   
  
  
- CTATATTGTT GACTCAATTT ACTCCTCAGA CTTTAATGCT TGTATTTTAG GTTTTCTTCT ACGGCTACGG   
  
  
- TAACCTTTCT TTCGACTTCC ACTAGCGAAA GATCATCATA ATCCGTCATC ATTAATTTTT GATTTTAGGT   
  
  
- CTTAACTGTG GCTTAACCCC AACACCGTGT ACTAGCTTTA GTCGTAGCTT AGATGTGCTA CTAGACCTAA   
  
  
- GAACCTGTGA GACCGAAATC CGGCAGTTAA AGCTCCTAAC GGGTATTGAA ATTAGGACAA GTTTAGGTAA   
  
  
- GTTAAATTAT AATGTGTTTA GGAAACTAAT TTAGTGGCAT TAACACCAAA CTAATCCCTT CAAAGAACCC   
  
  
- CCTTTCCAAG TTCGAAGACA GAAAGAATTT CGGACCTAAA AACCAGGGCG TACAAGTGAA GTGTAAGGGA   
  
  
- AAAAGAGAGG GTAGCAATGG ACAGACTTAG ACATACATTA TTTTCAGTAT AAAACGCTCC AGAAGAAAAC   
  
  
- TGACAAGAAC ATCAGTGAGA ACTATAGACG TGGTAAAACA AAAAGAGCCG TAAAAGAGGT TGAACGTAAA   
  
  
- AGATTACATT ACACTAGAAA AGACCTGTAA GTAAAACAAT TAGATGAGTG AACCCAGTTC CAAACGACTA   
  
  
- AAACCGACCA CAATTTAGGC AAGGAGAAAC CGTAAACAAG TACAAGGTGT TGACAAACTA TAGACCATAG   
  
  
- AGTCTTTGGG GCGGTAAGAA ACCATAAAGG ACTGAAAGAA CAAGTTCCCA ATAAGGATAA AGGGACTTAA   
  
  
- AAGAAACTAA GAAATCCATA AAGTTCGAAC CCCCCAAAAC AAGAAGTGAC TAAAACAGGA GAAGAAGGGA   
  
  
- ATAACCCACC AAACTAGAGG ATTTAAAGAC ACATAGACTT AGTCAGCCCC ATCTGAAACA CAAAAACCAA   
  
  
- CTAAAAAATA GTTACATAAA AGAAGGATTA CTTCCCGGAG GGCTTGGTTA TGTGTACGAA TTGAAGTTGG   
  
  
- GACAAGGAGA AGGTTCGAAA AACCTCGGAG TTTTCAGAAG CTTACGACTA TCCGTGTATC CCAGGAAGGT   
  
  
- TACGATACCA GAACTACTAC TGGGGCGTGG GAACTTACTG GGTTTAATGT AACATACAGT TAGAAGTATA   
  
  
- GGACTAGCTC TATAATGTGT CTAGGGACTG AAAGGACTAC TAACAGAGTT CAAGTAGTTA CTGTAAGAAT   
  
  
- ACCTTCTCCT AAACCTATTA GGCGGATATA GGAACGTCCT GATATTACGC GAGGTTCGGT GACTCTTCAG   
  
  
- TAACATACTA CGGGAACCTC TCCGAATAGG TCGAAGTAGA CTAGGTAACG TTAGTAGTCA ACCAGTCTCG   
  
  
- TGACTCTCGA GACTACTAGG ACTGTCTTCG TCGCCGTAAC TATTTACCCC ATTATCTATA ATGGGGTGGT   
  
  
- CGGACTTACA CCAACTTTGG TCGGCCGACT AGTTAGTCTC GGTCAACCTG GGAAGAGTTT ACAAACTACG   
  
  
- ACGACGAGTG GGGAACCTCA GATTAACAGT AAGAAAACAG AGATCATTAT TGAAACTAGT AAGATACCTA   
  
  
- CCCCAAGGTC TAAGAGGATA GTCATGTGAA AGACACTTAC TTTGTTCTTT CCTTCTTAGT CAATCATCTA   
  
  
- GTTCCTCGTC CTTCTTAGTA GTTTCTCTAC CACGTATGGA CCTTCTTTCC TCATGATTCG TTTATCGTAG   
  
  
- GTCGTTACTC CTTATACCTC TCTACCTCTC CAAACTACTA CATGACTAGA CGTCCCTTCC TTTACTACTA   
  
  
- TAAAGTGGAA CATGGGCGTT TTGGGGTCAA GTACTTCGTC TACTATTGAG CCTTGGTTTC CCCTTTAGTT   
  
  
- CTCCTAGATT TTCGTTCTCA TTTAGCGCAC AGTTCTTCTT TTCATTGCTT CCTGTTCGCC ATCTAGAATC   
  
  
- CTGAGTTGAA TAAGTCACAC GTCTTCGTCA CCGTTCGAAA CTCGAATTTT CACGTTTACT CGATGAGTCC   
  
  
- GCTTAATCCG TCGTACGAAG CGGTAAACCC CTATCACAAG TTTCCGATCG TGTAATGAAA CGCTTACCCG   
  
  
- AGCTCCGTGC GAATCGACCA TGTCCTAGTC TTAGTATGTT TCTGAAACGA CCCTTTTTTA AAAGTAGGAT   
  
  
- ACTGTAGAAC TCTCCGATGT TCAGTATACA AGTTCGGCAG GGGAAGGTCT CTTGTTGGTA CAAGAACTGT   
  
  
- TTAGTTTGTT ATCATTTCGA CCGACTCTTT CGTTGTTCCC AAGTATAGTA ACTAAAACCG TAGAAAAAAC   
  
  
- CAAAGGTCAC CGGGACGGAA CAAGTCTTAA ACAGTTCTTC TGGGGTCCCA AGAGGGTATG AGTTTTAGTG   
  
  
- CCCTCAGCTA GTAGGGGTCT CACCCAAGTC TGGTCGGGTC TTTCAACTTC TCTGTCCAGC AGCTAATCGT   
  
  
- CCGATAACAC TCTCCAAACC CCACGGGAAA CTCAAAGTAG GGTAACGAGT TTCCACCGTA TGGGATTTCG   
  
  
- GTCTCGTAAA GTTTTAACTT TCGTTACTCG AACAACACCA GTGAACAAAG ATGGTTAGTC CCTTCGAGGG   
  
  
- ACTACTCTGA CAACTACAGT TGTCGGGTTC TCTGGTACAG AACTCCGACT AGTCCCCGAA CTTGGGACTA   
  
  
- GATAAGTAGG TACCCCAATA GTTACCGTGT AAGTTACGTG GGAAGAAGCA TTGAGCCAAA TTTCTCCGTG   
  
  
- AAATGGTGAT GAGAAGAAAC AAGCTACAGA AACTACGGTG GTATCTAGCA CTTTCAGTAC TTTCTGACGA   
  
  
- CTGACTCTTG ATAAACATAC CCTTTCTACG TGAATTGTAT CATCGTACAC TCCCCTTGCT CTCCTAGCTC   
  
  
- TCTGGGCTCT GAATGTTCGT CACCGTTCAG GCCTTCGAAT CTCGACCTAA GTTCGTCCAG GGGGATCTGG   
  
  
- TTCTGTAACA TTTACCTCGT TCTCGATACC AATTCCGTTT AATGTTATTC CTAAAATACC ATCTTGTTCT   
  
  
- ATCCTTAACC TACCAGGTCC CGACCTTTCC TTCCTAAAAC TCACGGTATA GGACAACCTT TGGACGAAT

+     TCA

| Site Name | Organism | Position | Strand | Matrix score. | sequence | function |
| --- | --- | --- | --- | --- | --- | --- |
| TCA | Pisum sativum | 154 | - | 9 | TCATCTTCAT |  |

>HU02G01573.1   
+ +Up\_Stream \_Len000TGAATT GGAAGTTGAT TCTACCTTTG ATTGATTCGA TTAATTCTAG GTTCTTGGTT   
  
  
+ CTAGTTCTCT CTCCTCTTGT TCTTGATTAT TTAGGTTTTG GATGTGATGT TTATTGAATT GAATGTTGCA   
  
  
+ ATTGTTGTTA TAAAGATGAT GTTAGGCTAA ATATCCTTCA ACTTAGGTTG GGATTGACGT CGAATATGTG   
  
  
+ TAGTTGAATC TTGTACTGGC CTAAATCCCT GCTCCCCCTC TGTTTTGCGA TTATGAATTT CATTATGCAC   
  
  
+ ACCACATGCT CGATGAAATG CTCGAAAGAA CATTCATCCT CTTTGTTTGG GATTTAGGGG AACGTTGAAA   
  
  
+ GACGAAGTCT TTACCTAGTG CGTCTTAGTA GACAATTAGG ATTTTAACAT GAAAATAGTT AAAATTCTAA   
  
  
+ TTGGGATTTG AGATTGTGAA TCTTGTAATC CTTCGACTTC TAGTTCTATG AAAATAGGCG ATTATAGTTC   
  
  
+ TAGGATTAGT CTTCGACCCT TGAGAGAGGG AGGAGATGGG TTTAGTTGAT AATCTATTCA TTCATAACGC   
  
  
+ ATGCCTGGGA GATGTTTGGT AGGGCTAGTA ATTGATTCGC TAACATATTT GTGCTGTTAG TTCCAACCTG   
  
  
+ AGTGTTTACT CTTCTGCTGT TTATTCCGTC TTTTAGTCGT AATTGTCTCC TTTAGTTTAA TTTCAAAACC   
  
  
+ CCAATTGTGA TCGCCTAGAT TGAGCAATCT AATTAGTTCT AATAATTGAA ATCAAGTTCC CTGTGGAATA   
  
  
+ CGACCCGTAC TTGCCGTGTG CTAGCTACTG TTACACCTTG CATTTGCGGT ATATTTATAA CTCAACATAT   
  
  
+ GTCTGCTCAC TAAAAGTACA TATTAGCTCA CCAAAACTGT GTCTCAACGT ACTAAAATAT TTTAGTGAAC   
  
  
+ TATACATTAT TTTTAGTAAG CGAAATAAGA TACACAACAG CACATTTTAA TGAGTTACAG GGCATTTTTA   
  
  
+ GTGAGCCGAA ATCTGATGGT TTTCTTTGTA GATCCGGTTA TATAATTGTA TTTTATACAA CCGAATGGAC   
  
  
+ GATATAACAA CTGAGTTAAA TGAGGAGTCT GAAATTACGA ACATAAAATC CAAAAGAAGA TGCCGATGCC   
  
  
+ ATTGGAAAGA AAGCTGAAGG TGATCGCTTT CTAGTAGTAT TAGGCAGTAG TAATTAAAAA CTAAAATCCA   
  
  
+ GAATTGACAC CGAATTGGGG TTGTGGCACA TGATCGAAAT CAGCATCGAA TCTACACGAT GATCTGGATT   
  
  
+ CTTGGACACT CTGGCTTTAG GCCGTCAATT TCGAGGATTG CCCATAACTT TAATCCTGTT CAAATCCATT   
  
  
+ CAATTTAATA TTACACAAAT CCTTTGATTA AATCACCGTA ATTGTGGTTT GATTAGGGAA GTTTCTTGGG   
  
  
+ GGAAAGGTTC AAGCTTCTGT CTTTCTTAAA GCCTGGATTT TTGGTCCCGC ATGTTCACTT CACATTCCCT   
  
  
+ TTTTCTCTCC CATCGTTACC TGTCTGAATC TGTATGTAAT AAAAGTCATA TTTTGCGAGG TCTTCTTTTG   
  
  
+ ACTGTTCTTG TAGTCACTCT TGATATCTGC ACCATTTTGT TTTTCTCGGC ATTTTCTCCA ACTTGCATTT   
  
  
+ TCTAATGTAA TGTGATCTTT TCTGGACATT CATTTTGTTA ATCTACTCAC TTGGGTCAAG GTTTGCTGAT   
  
  
+ TTTGGCTGGT GTTAAATCCG TTCCTCTTTG GCATTTGTTC ATGTTCCACA ACTGTTTGAT ATCTGGTATC   
  
  
+ TCAGAAACCC CGCCATTCTT TGGTATTTCC TGACTTTCTT GTTCAAGGGT TATTCCTATT TCCCTGAATT   
  
  
+ TTCTTTGATT CTTTAGGTAT TTCAAGCTTG GGGGGTTTTG TTCTTCACTG ATTTTGTCCT CTTCTTCCCT   
  
  
+ TATTGGGTGG TTTGATCTCC TAAATTTCTG TGTATCTGAA TCAGTCGGGG TAGACTTTGT GTTTTTGGTT   
  
  
+ GATTTTTTAT CAATGTATTT TCTTCCTAAT GAAGGGCCTC CCGAACCAAT ACACATGCTT AACTTCAACC   
  
  
+ CTGTTCCTCT TCCAAGCTTT TTGGAGCCTC AAAAGTCTTC GAATGCTGAT AGGCACATAG GGTCCTTCCA   
  
  
+ ATGCTATGGT CTTGATGATG ACCCCGCACC CTTGAATGAC CCAAATTACA TTGTATGTCA ATCTTCATAT   
  
  
+ CCTGATCGAG ATATTACACA GATCCCTGAC TTTCCTGATG ATTGTCTCAA GTTCATCAAT GACATTCTTA   
  
  
+ TGGAAGAGGA TTTGGATAAT CCGCCTATAT CCTTGCAGGA CTATAATGCG CTCCAAGCCA CTGAGAAGTC   
  
  
+ ATTGTATGAT GCCCTTGGAG AGGCTTATCC AGCTTCATCT GATCCATTGC AATCATCAGT TGGTCAGAGC   
  
  
+ ACTGAGAGCT CTGATGATCC TGACAGAAGC AGCGGCATTG ATAAATGGGG TAATAGATAT TACCCCACCA   
  
  
+ GCCTGAATGT GGTTGAAACC AGCCGGCTGA TCAATCAGAG CCAGTTGGAC CCTTCTCAAA TGTTTGATGC   
  
  
+ TGCTGCTCAC CCCTTGGAGT CTAATTGTCA TTCTTTTGTC TCTAGTAATA ACTTTGATCA TTCTATGGAT   
  
  
+ GGGGTTCCAG ATTCTCCTAT CAGTACACTT TCTGTGAATG AAACAAGAAA GGAAGAATCA GTTAGTAGAT   
  
  
+ CAAGGAGCAG GAAGAATCAT CAAAGAGATG GTGCATACCT GGAAGAAAGG AGTACTAAGC AAATAGCATC   
  
  
+ CAGCAATGAG GAATATGGAG AGATGGAGAG GTTTGATGAT GTACTGATCT GCAGGGAAGG AAATGATGAT   
  
  
+ ATTTCACCTT GTACCCGCAA AACCCCAGTT CATGAAGCAG ATGATAACTC GGAACCAAAG GGGAAATCAA   
  
  
+ GAGGATCTAA AAGCAAGAGT AAATCGCGTG TCAAGAAGAA AAGTAACGAA GGACAAGCGG TAGATCTTAG   
  
  
+ GACTCAACTT ATTCAGTGTG CAGAAGCAGT GGCAAGCTTT GAGCTTAAAA GTGCAAATGA GCTACTCAGG   
  
  
+ CGAATTAGGC AGCATGCTTC GCCATTTGGG GATAGTGTTC AAAGGCTAGC ACATTACTTT GCGAATGGGC   
  
  
+ TCGAGGCACG CTTAGCTGGT ACAGGATCAG AATCATACAA AGACTTTGCT GGGAAAAAAT TTTCATCCTA   
  
  
+ TGACATCTTG AGAGGCTACA AGTCATATGT TCAAGCCGTC CCCTTCCAGA GAACAACCAT GTTCTTGACA   
  
  
+ AATCAAACAA TAGTAAAGCT GGCTGAGAAA GCAACAAGGG TTCATATCAT TGATTTTGGC ATCTTTTTTG   
  
  
+ GTTTCCAGTG GCCCTGCCTT GTTCAGAATT TGTCAAGAAG ACCCCAGGGT TCTCCCATAC TCAAAATCAC   
  
  
+ GGGAGTCGAT CATCCCCAGA GTGGGTTCAG ACCAGCCCAG AAAGTTGAAG AGACAGGTCG TCGATTAGCA   
  
  
+ GGCTATTGTG AGAGGTTTGG GGTGCCCTTT GAGTTTCATC CCATTGCTCA AAGGTGGCAT ACCCTAAAGC   
  
  
+ CAGAGCATTT CAAAATTGAA AGCAATGAGC TTGTTGTGGT CACTTGTTTC TACCAATCAG GGAAGCTCCC   
  
  
+ TGATGAGACT GTTGATGTCA ACAGCCCAAG AGACCATGTC TTGAGGCTGA TCAGGGGCTT GAACCCTGAT   
  
  
+ CTATTCATCC ATGGGGTTAT CAATGGCACA TTCAATGCAC CCTTCTTCGT AACTCGGTTT AAAGAGGCAC   
  
  
+ TTTACCACTA CTCTTCTTTG TTCGATGTCT TTGATGCCAC CATAGATCGT GAAAGTCATG AAAGACTGCT   
  
  
+ GACTGAGAAC TATTTGTATG GGAAAGATGC ACTTAACATA GTAGCATGTG AGGGGAACGA GAGGATCGAG   
  
  
+ AGACCCGAGA CTTACAAGCA GTGGCAAGTC CGGAAGCTTA GAGCTGGATT CAAGCAGGTC CCCCTAGACC   
  
  
+ AAGACATTGT AAATGGAGCA AGAGCTATGG TTAAGGCAAA TTACAATAAG GATTTTATGG TAGAACAAGA   
  
  
+ TAGGAATTGG ATGGTCCAGG GCTGGAAAGG AAGGATTTTG AGTGCCATAT CCTGTTGGAA ACCTGCTTA  

- +Up\_Stream \_Len000ACTTAA CCTTCAACTA AGATGGAAAC TAACTAAGCT AATTAAGATC CAAGAACCAA   
  
  
- GATCAAGAGA GAGGAGAACA AGAACTAATA AATCCAAAAC CTACACTACA AATAACTTAA CTTACAACGT   
  
  
- TAACAACAAT ATTTCTACTA CAATCCGATT TATAGGAAGT TGAATCCAAC CCTAACTGCA GCTTATACAC   
  
  
- ATCAACTTAG AACATGACCG GATTTAGGGA CGAGGGGGAG ACAAAACGCT AATACTTAAA GTAATACGTG   
  
  
- TGGTGTACGA GCTACTTTAC GAGCTTTCTT GTAAGTAGGA GAAACAAACC CTAAATCCCC TTGCAACTTT   
  
  
- CTGCTTCAGA AATGGATCAC GCAGAATCAT CTGTTAATCC TAAAATTGTA CTTTTATCAA TTTTAAGATT   
  
  
- AACCCTAAAC TCTAACACTT AGAACATTAG GAAGCTGAAG ATCAAGATAC TTTTATCCGC TAATATCAAG   
  
  
- ATCCTAATCA GAAGCTGGGA ACTCTCTCCC TCCTCTACCC AAATCAACTA TTAGATAAGT AAGTATTGCG   
  
  
- TACGGACCCT CTACAAACCA TCCCGATCAT TAACTAAGCG ATTGTATAAA CACGACAATC AAGGTTGGAC   
  
  
- TCACAAATGA GAAGACGACA AATAAGGCAG AAAATCAGCA TTAACAGAGG AAATCAAATT AAAGTTTTGG   
  
  
- GGTTAACACT AGCGGATCTA ACTCGTTAGA TTAATCAAGA TTATTAACTT TAGTTCAAGG GACACCTTAT   
  
  
- GCTGGGCATG AACGGCACAC GATCGATGAC AATGTGGAAC GTAAACGCCA TATAAATATT GAGTTGTATA   
  
  
- CAGACGAGTG ATTTTCATGT ATAATCGAGT GGTTTTGACA CAGAGTTGCA TGATTTTATA AAATCACTTG   
  
  
- ATATGTAATA AAAATCATTC GCTTTATTCT ATGTGTTGTC GTGTAAAATT ACTCAATGTC CCGTAAAAAT   
  
  
- CACTCGGCTT TAGACTACCA AAAGAAACAT CTAGGCCAAT ATATTAACAT AAAATATGTT GGCTTACCTG   
  
  
- CTATATTGTT GACTCAATTT ACTCCTCAGA CTTTAATGCT TGTATTTTAG GTTTTCTTCT ACGGCTACGG   
  
  
- TAACCTTTCT TTCGACTTCC ACTAGCGAAA GATCATCATA ATCCGTCATC ATTAATTTTT GATTTTAGGT   
  
  
- CTTAACTGTG GCTTAACCCC AACACCGTGT ACTAGCTTTA GTCGTAGCTT AGATGTGCTA CTAGACCTAA   
  
  
- GAACCTGTGA GACCGAAATC CGGCAGTTAA AGCTCCTAAC GGGTATTGAA ATTAGGACAA GTTTAGGTAA   
  
  
- GTTAAATTAT AATGTGTTTA GGAAACTAAT TTAGTGGCAT TAACACCAAA CTAATCCCTT CAAAGAACCC   
  
  
- CCTTTCCAAG TTCGAAGACA GAAAGAATTT CGGACCTAAA AACCAGGGCG TACAAGTGAA GTGTAAGGGA   
  
  
- AAAAGAGAGG GTAGCAATGG ACAGACTTAG ACATACATTA TTTTCAGTAT AAAACGCTCC AGAAGAAAAC   
  
  
- TGACAAGAAC ATCAGTGAGA ACTATAGACG TGGTAAAACA AAAAGAGCCG TAAAAGAGGT TGAACGTAAA   
  
  
- AGATTACATT ACACTAGAAA AGACCTGTAA GTAAAACAAT TAGATGAGTG AACCCAGTTC CAAACGACTA   
  
  
- AAACCGACCA CAATTTAGGC AAGGAGAAAC CGTAAACAAG TACAAGGTGT TGACAAACTA TAGACCATAG   
  
  
- AGTCTTTGGG GCGGTAAGAA ACCATAAAGG ACTGAAAGAA CAAGTTCCCA ATAAGGATAA AGGGACTTAA   
  
  
- AAGAAACTAA GAAATCCATA AAGTTCGAAC CCCCCAAAAC AAGAAGTGAC TAAAACAGGA GAAGAAGGGA   
  
  
- ATAACCCACC AAACTAGAGG ATTTAAAGAC ACATAGACTT AGTCAGCCCC ATCTGAAACA CAAAAACCAA   
  
  
- CTAAAAAATA GTTACATAAA AGAAGGATTA CTTCCCGGAG GGCTTGGTTA TGTGTACGAA TTGAAGTTGG   
  
  
- GACAAGGAGA AGGTTCGAAA AACCTCGGAG TTTTCAGAAG CTTACGACTA TCCGTGTATC CCAGGAAGGT   
  
  
- TACGATACCA GAACTACTAC TGGGGCGTGG GAACTTACTG GGTTTAATGT AACATACAGT TAGAAGTATA   
  
  
- GGACTAGCTC TATAATGTGT CTAGGGACTG AAAGGACTAC TAACAGAGTT CAAGTAGTTA CTGTAAGAAT   
  
  
- ACCTTCTCCT AAACCTATTA GGCGGATATA GGAACGTCCT GATATTACGC GAGGTTCGGT GACTCTTCAG   
  
  
- TAACATACTA CGGGAACCTC TCCGAATAGG TCGAAGTAGA CTAGGTAACG TTAGTAGTCA ACCAGTCTCG   
  
  
- TGACTCTCGA GACTACTAGG ACTGTCTTCG TCGCCGTAAC TATTTACCCC ATTATCTATA ATGGGGTGGT   
  
  
- CGGACTTACA CCAACTTTGG TCGGCCGACT AGTTAGTCTC GGTCAACCTG GGAAGAGTTT ACAAACTACG   
  
  
- ACGACGAGTG GGGAACCTCA GATTAACAGT AAGAAAACAG AGATCATTAT TGAAACTAGT AAGATACCTA   
  
  
- CCCCAAGGTC TAAGAGGATA GTCATGTGAA AGACACTTAC TTTGTTCTTT CCTTCTTAGT CAATCATCTA   
  
  
- GTTCCTCGTC CTTCTTAGTA GTTTCTCTAC CACGTATGGA CCTTCTTTCC TCATGATTCG TTTATCGTAG   
  
  
- GTCGTTACTC CTTATACCTC TCTACCTCTC CAAACTACTA CATGACTAGA CGTCCCTTCC TTTACTACTA   
  
  
- TAAAGTGGAA CATGGGCGTT TTGGGGTCAA GTACTTCGTC TACTATTGAG CCTTGGTTTC CCCTTTAGTT   
  
  
- CTCCTAGATT TTCGTTCTCA TTTAGCGCAC AGTTCTTCTT TTCATTGCTT CCTGTTCGCC ATCTAGAATC   
  
  
- CTGAGTTGAA TAAGTCACAC GTCTTCGTCA CCGTTCGAAA CTCGAATTTT CACGTTTACT CGATGAGTCC   
  
  
- GCTTAATCCG TCGTACGAAG CGGTAAACCC CTATCACAAG TTTCCGATCG TGTAATGAAA CGCTTACCCG   
  
  
- AGCTCCGTGC GAATCGACCA TGTCCTAGTC TTAGTATGTT TCTGAAACGA CCCTTTTTTA AAAGTAGGAT   
  
  
- ACTGTAGAAC TCTCCGATGT TCAGTATACA AGTTCGGCAG GGGAAGGTCT CTTGTTGGTA CAAGAACTGT   
  
  
- TTAGTTTGTT ATCATTTCGA CCGACTCTTT CGTTGTTCCC AAGTATAGTA ACTAAAACCG TAGAAAAAAC   
  
  
- CAAAGGTCAC CGGGACGGAA CAAGTCTTAA ACAGTTCTTC TGGGGTCCCA AGAGGGTATG AGTTTTAGTG   
  
  
- CCCTCAGCTA GTAGGGGTCT CACCCAAGTC TGGTCGGGTC TTTCAACTTC TCTGTCCAGC AGCTAATCGT   
  
  
- CCGATAACAC TCTCCAAACC CCACGGGAAA CTCAAAGTAG GGTAACGAGT TTCCACCGTA TGGGATTTCG   
  
  
- GTCTCGTAAA GTTTTAACTT TCGTTACTCG AACAACACCA GTGAACAAAG ATGGTTAGTC CCTTCGAGGG   
  
  
- ACTACTCTGA CAACTACAGT TGTCGGGTTC TCTGGTACAG AACTCCGACT AGTCCCCGAA CTTGGGACTA   
  
  
- GATAAGTAGG TACCCCAATA GTTACCGTGT AAGTTACGTG GGAAGAAGCA TTGAGCCAAA TTTCTCCGTG   
  
  
- AAATGGTGAT GAGAAGAAAC AAGCTACAGA AACTACGGTG GTATCTAGCA CTTTCAGTAC TTTCTGACGA   
  
  
- CTGACTCTTG ATAAACATAC CCTTTCTACG TGAATTGTAT CATCGTACAC TCCCCTTGCT CTCCTAGCTC   
  
  
- TCTGGGCTCT GAATGTTCGT CACCGTTCAG GCCTTCGAAT CTCGACCTAA GTTCGTCCAG GGGGATCTGG   
  
  
- TTCTGTAACA TTTACCTCGT TCTCGATACC AATTCCGTTT AATGTTATTC CTAAAATACC ATCTTGTTCT   
  
  
- ATCCTTAACC TACCAGGTCC CGACCTTTCC TTCCTAAAAC TCACGGTATA GGACAACCTT TGGACGAAT

+     TCA-element

| Site Name | Organism | Position | Strand | Matrix score. | sequence | function |
| --- | --- | --- | --- | --- | --- | --- |
| TCA-element | Nicotiana tabacum | 3283 | + | 9 | CCATCTTTTT | cis-acting element involved in salicylic acid responsiveness |
| TCA-element | Nicotiana tabacum | 2686 | - | 9 | CCATCTTTTT | cis-acting element involved in salicylic acid responsiveness |

>HU02G01573.1   
+ +Up\_Stream \_Len000TGAATT GGAAGTTGAT TCTACCTTTG ATTGATTCGA TTAATTCTAG GTTCTTGGTT   
  
  
+ CTAGTTCTCT CTCCTCTTGT TCTTGATTAT TTAGGTTTTG GATGTGATGT TTATTGAATT GAATGTTGCA   
  
  
+ ATTGTTGTTA TAAAGATGAT GTTAGGCTAA ATATCCTTCA ACTTAGGTTG GGATTGACGT CGAATATGTG   
  
  
+ TAGTTGAATC TTGTACTGGC CTAAATCCCT GCTCCCCCTC TGTTTTGCGA TTATGAATTT CATTATGCAC   
  
  
+ ACCACATGCT CGATGAAATG CTCGAAAGAA CATTCATCCT CTTTGTTTGG GATTTAGGGG AACGTTGAAA   
  
  
+ GACGAAGTCT TTACCTAGTG CGTCTTAGTA GACAATTAGG ATTTTAACAT GAAAATAGTT AAAATTCTAA   
  
  
+ TTGGGATTTG AGATTGTGAA TCTTGTAATC CTTCGACTTC TAGTTCTATG AAAATAGGCG ATTATAGTTC   
  
  
+ TAGGATTAGT CTTCGACCCT TGAGAGAGGG AGGAGATGGG TTTAGTTGAT AATCTATTCA TTCATAACGC   
  
  
+ ATGCCTGGGA GATGTTTGGT AGGGCTAGTA ATTGATTCGC TAACATATTT GTGCTGTTAG TTCCAACCTG   
  
  
+ AGTGTTTACT CTTCTGCTGT TTATTCCGTC TTTTAGTCGT AATTGTCTCC TTTAGTTTAA TTTCAAAACC   
  
  
+ CCAATTGTGA TCGCCTAGAT TGAGCAATCT AATTAGTTCT AATAATTGAA ATCAAGTTCC CTGTGGAATA   
  
  
+ CGACCCGTAC TTGCCGTGTG CTAGCTACTG TTACACCTTG CATTTGCGGT ATATTTATAA CTCAACATAT   
  
  
+ GTCTGCTCAC TAAAAGTACA TATTAGCTCA CCAAAACTGT GTCTCAACGT ACTAAAATAT TTTAGTGAAC   
  
  
+ TATACATTAT TTTTAGTAAG CGAAATAAGA TACACAACAG CACATTTTAA TGAGTTACAG GGCATTTTTA   
  
  
+ GTGAGCCGAA ATCTGATGGT TTTCTTTGTA GATCCGGTTA TATAATTGTA TTTTATACAA CCGAATGGAC   
  
  
+ GATATAACAA CTGAGTTAAA TGAGGAGTCT GAAATTACGA ACATAAAATC CAAAAGAAGA TGCCGATGCC   
  
  
+ ATTGGAAAGA AAGCTGAAGG TGATCGCTTT CTAGTAGTAT TAGGCAGTAG TAATTAAAAA CTAAAATCCA   
  
  
+ GAATTGACAC CGAATTGGGG TTGTGGCACA TGATCGAAAT CAGCATCGAA TCTACACGAT GATCTGGATT   
  
  
+ CTTGGACACT CTGGCTTTAG GCCGTCAATT TCGAGGATTG CCCATAACTT TAATCCTGTT CAAATCCATT   
  
  
+ CAATTTAATA TTACACAAAT CCTTTGATTA AATCACCGTA ATTGTGGTTT GATTAGGGAA GTTTCTTGGG   
  
  
+ GGAAAGGTTC AAGCTTCTGT CTTTCTTAAA GCCTGGATTT TTGGTCCCGC ATGTTCACTT CACATTCCCT   
  
  
+ TTTTCTCTCC CATCGTTACC TGTCTGAATC TGTATGTAAT AAAAGTCATA TTTTGCGAGG TCTTCTTTTG   
  
  
+ ACTGTTCTTG TAGTCACTCT TGATATCTGC ACCATTTTGT TTTTCTCGGC ATTTTCTCCA ACTTGCATTT   
  
  
+ TCTAATGTAA TGTGATCTTT TCTGGACATT CATTTTGTTA ATCTACTCAC TTGGGTCAAG GTTTGCTGAT   
  
  
+ TTTGGCTGGT GTTAAATCCG TTCCTCTTTG GCATTTGTTC ATGTTCCACA ACTGTTTGAT ATCTGGTATC   
  
  
+ TCAGAAACCC CGCCATTCTT TGGTATTTCC TGACTTTCTT GTTCAAGGGT TATTCCTATT TCCCTGAATT   
  
  
+ TTCTTTGATT CTTTAGGTAT TTCAAGCTTG GGGGGTTTTG TTCTTCACTG ATTTTGTCCT CTTCTTCCCT   
  
  
+ TATTGGGTGG TTTGATCTCC TAAATTTCTG TGTATCTGAA TCAGTCGGGG TAGACTTTGT GTTTTTGGTT   
  
  
+ GATTTTTTAT CAATGTATTT TCTTCCTAAT GAAGGGCCTC CCGAACCAAT ACACATGCTT AACTTCAACC   
  
  
+ CTGTTCCTCT TCCAAGCTTT TTGGAGCCTC AAAAGTCTTC GAATGCTGAT AGGCACATAG GGTCCTTCCA   
  
  
+ ATGCTATGGT CTTGATGATG ACCCCGCACC CTTGAATGAC CCAAATTACA TTGTATGTCA ATCTTCATAT   
  
  
+ CCTGATCGAG ATATTACACA GATCCCTGAC TTTCCTGATG ATTGTCTCAA GTTCATCAAT GACATTCTTA   
  
  
+ TGGAAGAGGA TTTGGATAAT CCGCCTATAT CCTTGCAGGA CTATAATGCG CTCCAAGCCA CTGAGAAGTC   
  
  
+ ATTGTATGAT GCCCTTGGAG AGGCTTATCC AGCTTCATCT GATCCATTGC AATCATCAGT TGGTCAGAGC   
  
  
+ ACTGAGAGCT CTGATGATCC TGACAGAAGC AGCGGCATTG ATAAATGGGG TAATAGATAT TACCCCACCA   
  
  
+ GCCTGAATGT GGTTGAAACC AGCCGGCTGA TCAATCAGAG CCAGTTGGAC CCTTCTCAAA TGTTTGATGC   
  
  
+ TGCTGCTCAC CCCTTGGAGT CTAATTGTCA TTCTTTTGTC TCTAGTAATA ACTTTGATCA TTCTATGGAT   
  
  
+ GGGGTTCCAG ATTCTCCTAT CAGTACACTT TCTGTGAATG AAACAAGAAA GGAAGAATCA GTTAGTAGAT   
  
  
+ CAAGGAGCAG GAAGAATCAT CAAAGAGATG GTGCATACCT GGAAGAAAGG AGTACTAAGC AAATAGCATC   
  
  
+ CAGCAATGAG GAATATGGAG AGATGGAGAG GTTTGATGAT GTACTGATCT GCAGGGAAGG AAATGATGAT   
  
  
+ ATTTCACCTT GTACCCGCAA AACCCCAGTT CATGAAGCAG ATGATAACTC GGAACCAAAG GGGAAATCAA   
  
  
+ GAGGATCTAA AAGCAAGAGT AAATCGCGTG TCAAGAAGAA AAGTAACGAA GGACAAGCGG TAGATCTTAG   
  
  
+ GACTCAACTT ATTCAGTGTG CAGAAGCAGT GGCAAGCTTT GAGCTTAAAA GTGCAAATGA GCTACTCAGG   
  
  
+ CGAATTAGGC AGCATGCTTC GCCATTTGGG GATAGTGTTC AAAGGCTAGC ACATTACTTT GCGAATGGGC   
  
  
+ TCGAGGCACG CTTAGCTGGT ACAGGATCAG AATCATACAA AGACTTTGCT GGGAAAAAAT TTTCATCCTA   
  
  
+ TGACATCTTG AGAGGCTACA AGTCATATGT TCAAGCCGTC CCCTTCCAGA GAACAACCAT GTTCTTGACA   
  
  
+ AATCAAACAA TAGTAAAGCT GGCTGAGAAA GCAACAAGGG TTCATATCAT TGATTTTGGC ATCTTTTTTG   
  
  
+ GTTTCCAGTG GCCCTGCCTT GTTCAGAATT TGTCAAGAAG ACCCCAGGGT TCTCCCATAC TCAAAATCAC   
  
  
+ GGGAGTCGAT CATCCCCAGA GTGGGTTCAG ACCAGCCCAG AAAGTTGAAG AGACAGGTCG TCGATTAGCA   
  
  
+ GGCTATTGTG AGAGGTTTGG GGTGCCCTTT GAGTTTCATC CCATTGCTCA AAGGTGGCAT ACCCTAAAGC   
  
  
+ CAGAGCATTT CAAAATTGAA AGCAATGAGC TTGTTGTGGT CACTTGTTTC TACCAATCAG GGAAGCTCCC   
  
  
+ TGATGAGACT GTTGATGTCA ACAGCCCAAG AGACCATGTC TTGAGGCTGA TCAGGGGCTT GAACCCTGAT   
  
  
+ CTATTCATCC ATGGGGTTAT CAATGGCACA TTCAATGCAC CCTTCTTCGT AACTCGGTTT AAAGAGGCAC   
  
  
+ TTTACCACTA CTCTTCTTTG TTCGATGTCT TTGATGCCAC CATAGATCGT GAAAGTCATG AAAGACTGCT   
  
  
+ GACTGAGAAC TATTTGTATG GGAAAGATGC ACTTAACATA GTAGCATGTG AGGGGAACGA GAGGATCGAG   
  
  
+ AGACCCGAGA CTTACAAGCA GTGGCAAGTC CGGAAGCTTA GAGCTGGATT CAAGCAGGTC CCCCTAGACC   
  
  
+ AAGACATTGT AAATGGAGCA AGAGCTATGG TTAAGGCAAA TTACAATAAG GATTTTATGG TAGAACAAGA   
  
  
+ TAGGAATTGG ATGGTCCAGG GCTGGAAAGG AAGGATTTTG AGTGCCATAT CCTGTTGGAA ACCTGCTTA  

- +Up\_Stream \_Len000ACTTAA CCTTCAACTA AGATGGAAAC TAACTAAGCT AATTAAGATC CAAGAACCAA   
  
  
- GATCAAGAGA GAGGAGAACA AGAACTAATA AATCCAAAAC CTACACTACA AATAACTTAA CTTACAACGT   
  
  
- TAACAACAAT ATTTCTACTA CAATCCGATT TATAGGAAGT TGAATCCAAC CCTAACTGCA GCTTATACAC   
  
  
- ATCAACTTAG AACATGACCG GATTTAGGGA CGAGGGGGAG ACAAAACGCT AATACTTAAA GTAATACGTG   
  
  
- TGGTGTACGA GCTACTTTAC GAGCTTTCTT GTAAGTAGGA GAAACAAACC CTAAATCCCC TTGCAACTTT   
  
  
- CTGCTTCAGA AATGGATCAC GCAGAATCAT CTGTTAATCC TAAAATTGTA CTTTTATCAA TTTTAAGATT   
  
  
- AACCCTAAAC TCTAACACTT AGAACATTAG GAAGCTGAAG ATCAAGATAC TTTTATCCGC TAATATCAAG   
  
  
- ATCCTAATCA GAAGCTGGGA ACTCTCTCCC TCCTCTACCC AAATCAACTA TTAGATAAGT AAGTATTGCG   
  
  
- TACGGACCCT CTACAAACCA TCCCGATCAT TAACTAAGCG ATTGTATAAA CACGACAATC AAGGTTGGAC   
  
  
- TCACAAATGA GAAGACGACA AATAAGGCAG AAAATCAGCA TTAACAGAGG AAATCAAATT AAAGTTTTGG   
  
  
- GGTTAACACT AGCGGATCTA ACTCGTTAGA TTAATCAAGA TTATTAACTT TAGTTCAAGG GACACCTTAT   
  
  
- GCTGGGCATG AACGGCACAC GATCGATGAC AATGTGGAAC GTAAACGCCA TATAAATATT GAGTTGTATA   
  
  
- CAGACGAGTG ATTTTCATGT ATAATCGAGT GGTTTTGACA CAGAGTTGCA TGATTTTATA AAATCACTTG   
  
  
- ATATGTAATA AAAATCATTC GCTTTATTCT ATGTGTTGTC GTGTAAAATT ACTCAATGTC CCGTAAAAAT   
  
  
- CACTCGGCTT TAGACTACCA AAAGAAACAT CTAGGCCAAT ATATTAACAT AAAATATGTT GGCTTACCTG   
  
  
- CTATATTGTT GACTCAATTT ACTCCTCAGA CTTTAATGCT TGTATTTTAG GTTTTCTTCT ACGGCTACGG   
  
  
- TAACCTTTCT TTCGACTTCC ACTAGCGAAA GATCATCATA ATCCGTCATC ATTAATTTTT GATTTTAGGT   
  
  
- CTTAACTGTG GCTTAACCCC AACACCGTGT ACTAGCTTTA GTCGTAGCTT AGATGTGCTA CTAGACCTAA   
  
  
- GAACCTGTGA GACCGAAATC CGGCAGTTAA AGCTCCTAAC GGGTATTGAA ATTAGGACAA GTTTAGGTAA   
  
  
- GTTAAATTAT AATGTGTTTA GGAAACTAAT TTAGTGGCAT TAACACCAAA CTAATCCCTT CAAAGAACCC   
  
  
- CCTTTCCAAG TTCGAAGACA GAAAGAATTT CGGACCTAAA AACCAGGGCG TACAAGTGAA GTGTAAGGGA   
  
  
- AAAAGAGAGG GTAGCAATGG ACAGACTTAG ACATACATTA TTTTCAGTAT AAAACGCTCC AGAAGAAAAC   
  
  
- TGACAAGAAC ATCAGTGAGA ACTATAGACG TGGTAAAACA AAAAGAGCCG TAAAAGAGGT TGAACGTAAA   
  
  
- AGATTACATT ACACTAGAAA AGACCTGTAA GTAAAACAAT TAGATGAGTG AACCCAGTTC CAAACGACTA   
  
  
- AAACCGACCA CAATTTAGGC AAGGAGAAAC CGTAAACAAG TACAAGGTGT TGACAAACTA TAGACCATAG   
  
  
- AGTCTTTGGG GCGGTAAGAA ACCATAAAGG ACTGAAAGAA CAAGTTCCCA ATAAGGATAA AGGGACTTAA   
  
  
- AAGAAACTAA GAAATCCATA AAGTTCGAAC CCCCCAAAAC AAGAAGTGAC TAAAACAGGA GAAGAAGGGA   
  
  
- ATAACCCACC AAACTAGAGG ATTTAAAGAC ACATAGACTT AGTCAGCCCC ATCTGAAACA CAAAAACCAA   
  
  
- CTAAAAAATA GTTACATAAA AGAAGGATTA CTTCCCGGAG GGCTTGGTTA TGTGTACGAA TTGAAGTTGG   
  
  
- GACAAGGAGA AGGTTCGAAA AACCTCGGAG TTTTCAGAAG CTTACGACTA TCCGTGTATC CCAGGAAGGT   
  
  
- TACGATACCA GAACTACTAC TGGGGCGTGG GAACTTACTG GGTTTAATGT AACATACAGT TAGAAGTATA   
  
  
- GGACTAGCTC TATAATGTGT CTAGGGACTG AAAGGACTAC TAACAGAGTT CAAGTAGTTA CTGTAAGAAT   
  
  
- ACCTTCTCCT AAACCTATTA GGCGGATATA GGAACGTCCT GATATTACGC GAGGTTCGGT GACTCTTCAG   
  
  
- TAACATACTA CGGGAACCTC TCCGAATAGG TCGAAGTAGA CTAGGTAACG TTAGTAGTCA ACCAGTCTCG   
  
  
- TGACTCTCGA GACTACTAGG ACTGTCTTCG TCGCCGTAAC TATTTACCCC ATTATCTATA ATGGGGTGGT   
  
  
- CGGACTTACA CCAACTTTGG TCGGCCGACT AGTTAGTCTC GGTCAACCTG GGAAGAGTTT ACAAACTACG   
  
  
- ACGACGAGTG GGGAACCTCA GATTAACAGT AAGAAAACAG AGATCATTAT TGAAACTAGT AAGATACCTA   
  
  
- CCCCAAGGTC TAAGAGGATA GTCATGTGAA AGACACTTAC TTTGTTCTTT CCTTCTTAGT CAATCATCTA   
  
  
- GTTCCTCGTC CTTCTTAGTA GTTTCTCTAC CACGTATGGA CCTTCTTTCC TCATGATTCG TTTATCGTAG   
  
  
- GTCGTTACTC CTTATACCTC TCTACCTCTC CAAACTACTA CATGACTAGA CGTCCCTTCC TTTACTACTA   
  
  
- TAAAGTGGAA CATGGGCGTT TTGGGGTCAA GTACTTCGTC TACTATTGAG CCTTGGTTTC CCCTTTAGTT   
  
  
- CTCCTAGATT TTCGTTCTCA TTTAGCGCAC AGTTCTTCTT TTCATTGCTT CCTGTTCGCC ATCTAGAATC   
  
  
- CTGAGTTGAA TAAGTCACAC GTCTTCGTCA CCGTTCGAAA CTCGAATTTT CACGTTTACT CGATGAGTCC   
  
  
- GCTTAATCCG TCGTACGAAG CGGTAAACCC CTATCACAAG TTTCCGATCG TGTAATGAAA CGCTTACCCG   
  
  
- AGCTCCGTGC GAATCGACCA TGTCCTAGTC TTAGTATGTT TCTGAAACGA CCCTTTTTTA AAAGTAGGAT   
  
  
- ACTGTAGAAC TCTCCGATGT TCAGTATACA AGTTCGGCAG GGGAAGGTCT CTTGTTGGTA CAAGAACTGT   
  
  
- TTAGTTTGTT ATCATTTCGA CCGACTCTTT CGTTGTTCCC AAGTATAGTA ACTAAAACCG TAGAAAAAAC   
  
  
- CAAAGGTCAC CGGGACGGAA CAAGTCTTAA ACAGTTCTTC TGGGGTCCCA AGAGGGTATG AGTTTTAGTG   
  
  
- CCCTCAGCTA GTAGGGGTCT CACCCAAGTC TGGTCGGGTC TTTCAACTTC TCTGTCCAGC AGCTAATCGT   
  
  
- CCGATAACAC TCTCCAAACC CCACGGGAAA CTCAAAGTAG GGTAACGAGT TTCCACCGTA TGGGATTTCG   
  
  
- GTCTCGTAAA GTTTTAACTT TCGTTACTCG AACAACACCA GTGAACAAAG ATGGTTAGTC CCTTCGAGGG   
  
  
- ACTACTCTGA CAACTACAGT TGTCGGGTTC TCTGGTACAG AACTCCGACT AGTCCCCGAA CTTGGGACTA   
  
  
- GATAAGTAGG TACCCCAATA GTTACCGTGT AAGTTACGTG GGAAGAAGCA TTGAGCCAAA TTTCTCCGTG   
  
  
- AAATGGTGAT GAGAAGAAAC AAGCTACAGA AACTACGGTG GTATCTAGCA CTTTCAGTAC TTTCTGACGA   
  
  
- CTGACTCTTG ATAAACATAC CCTTTCTACG TGAATTGTAT CATCGTACAC TCCCCTTGCT CTCCTAGCTC   
  
  
- TCTGGGCTCT GAATGTTCGT CACCGTTCAG GCCTTCGAAT CTCGACCTAA GTTCGTCCAG GGGGATCTGG   
  
  
- TTCTGTAACA TTTACCTCGT TCTCGATACC AATTCCGTTT AATGTTATTC CTAAAATACC ATCTTGTTCT   
  
  
- ATCCTTAACC TACCAGGTCC CGACCTTTCC TTCCTAAAAC TCACGGTATA GGACAACCTT TGGACGAAT

+     TGACG-motif

| Site Name | Organism | Position | Strand | Matrix score. | sequence | function |
| --- | --- | --- | --- | --- | --- | --- |
| TGACG-motif | Hordeum vulgare | 199 | + | 5 | TGACG | cis-acting regulatory element involved in the MeJA-responsiveness |
| TGACG-motif | Hordeum vulgare | 1287 | - | 5 | TGACG | cis-acting regulatory element involved in the MeJA-responsiveness |

>HU02G01573.1   
+ +Up\_Stream \_Len000TGAATT GGAAGTTGAT TCTACCTTTG ATTGATTCGA TTAATTCTAG GTTCTTGGTT   
  
  
+ CTAGTTCTCT CTCCTCTTGT TCTTGATTAT TTAGGTTTTG GATGTGATGT TTATTGAATT GAATGTTGCA   
  
  
+ ATTGTTGTTA TAAAGATGAT GTTAGGCTAA ATATCCTTCA ACTTAGGTTG GGATTGACGT CGAATATGTG   
  
  
+ TAGTTGAATC TTGTACTGGC CTAAATCCCT GCTCCCCCTC TGTTTTGCGA TTATGAATTT CATTATGCAC   
  
  
+ ACCACATGCT CGATGAAATG CTCGAAAGAA CATTCATCCT CTTTGTTTGG GATTTAGGGG AACGTTGAAA   
  
  
+ GACGAAGTCT TTACCTAGTG CGTCTTAGTA GACAATTAGG ATTTTAACAT GAAAATAGTT AAAATTCTAA   
  
  
+ TTGGGATTTG AGATTGTGAA TCTTGTAATC CTTCGACTTC TAGTTCTATG AAAATAGGCG ATTATAGTTC   
  
  
+ TAGGATTAGT CTTCGACCCT TGAGAGAGGG AGGAGATGGG TTTAGTTGAT AATCTATTCA TTCATAACGC   
  
  
+ ATGCCTGGGA GATGTTTGGT AGGGCTAGTA ATTGATTCGC TAACATATTT GTGCTGTTAG TTCCAACCTG   
  
  
+ AGTGTTTACT CTTCTGCTGT TTATTCCGTC TTTTAGTCGT AATTGTCTCC TTTAGTTTAA TTTCAAAACC   
  
  
+ CCAATTGTGA TCGCCTAGAT TGAGCAATCT AATTAGTTCT AATAATTGAA ATCAAGTTCC CTGTGGAATA   
  
  
+ CGACCCGTAC TTGCCGTGTG CTAGCTACTG TTACACCTTG CATTTGCGGT ATATTTATAA CTCAACATAT   
  
  
+ GTCTGCTCAC TAAAAGTACA TATTAGCTCA CCAAAACTGT GTCTCAACGT ACTAAAATAT TTTAGTGAAC   
  
  
+ TATACATTAT TTTTAGTAAG CGAAATAAGA TACACAACAG CACATTTTAA TGAGTTACAG GGCATTTTTA   
  
  
+ GTGAGCCGAA ATCTGATGGT TTTCTTTGTA GATCCGGTTA TATAATTGTA TTTTATACAA CCGAATGGAC   
  
  
+ GATATAACAA CTGAGTTAAA TGAGGAGTCT GAAATTACGA ACATAAAATC CAAAAGAAGA TGCCGATGCC   
  
  
+ ATTGGAAAGA AAGCTGAAGG TGATCGCTTT CTAGTAGTAT TAGGCAGTAG TAATTAAAAA CTAAAATCCA   
  
  
+ GAATTGACAC CGAATTGGGG TTGTGGCACA TGATCGAAAT CAGCATCGAA TCTACACGAT GATCTGGATT   
  
  
+ CTTGGACACT CTGGCTTTAG GCCGTCAATT TCGAGGATTG CCCATAACTT TAATCCTGTT CAAATCCATT   
  
  
+ CAATTTAATA TTACACAAAT CCTTTGATTA AATCACCGTA ATTGTGGTTT GATTAGGGAA GTTTCTTGGG   
  
  
+ GGAAAGGTTC AAGCTTCTGT CTTTCTTAAA GCCTGGATTT TTGGTCCCGC ATGTTCACTT CACATTCCCT   
  
  
+ TTTTCTCTCC CATCGTTACC TGTCTGAATC TGTATGTAAT AAAAGTCATA TTTTGCGAGG TCTTCTTTTG   
  
  
+ ACTGTTCTTG TAGTCACTCT TGATATCTGC ACCATTTTGT TTTTCTCGGC ATTTTCTCCA ACTTGCATTT   
  
  
+ TCTAATGTAA TGTGATCTTT TCTGGACATT CATTTTGTTA ATCTACTCAC TTGGGTCAAG GTTTGCTGAT   
  
  
+ TTTGGCTGGT GTTAAATCCG TTCCTCTTTG GCATTTGTTC ATGTTCCACA ACTGTTTGAT ATCTGGTATC   
  
  
+ TCAGAAACCC CGCCATTCTT TGGTATTTCC TGACTTTCTT GTTCAAGGGT TATTCCTATT TCCCTGAATT   
  
  
+ TTCTTTGATT CTTTAGGTAT TTCAAGCTTG GGGGGTTTTG TTCTTCACTG ATTTTGTCCT CTTCTTCCCT   
  
  
+ TATTGGGTGG TTTGATCTCC TAAATTTCTG TGTATCTGAA TCAGTCGGGG TAGACTTTGT GTTTTTGGTT   
  
  
+ GATTTTTTAT CAATGTATTT TCTTCCTAAT GAAGGGCCTC CCGAACCAAT ACACATGCTT AACTTCAACC   
  
  
+ CTGTTCCTCT TCCAAGCTTT TTGGAGCCTC AAAAGTCTTC GAATGCTGAT AGGCACATAG GGTCCTTCCA   
  
  
+ ATGCTATGGT CTTGATGATG ACCCCGCACC CTTGAATGAC CCAAATTACA TTGTATGTCA ATCTTCATAT   
  
  
+ CCTGATCGAG ATATTACACA GATCCCTGAC TTTCCTGATG ATTGTCTCAA GTTCATCAAT GACATTCTTA   
  
  
+ TGGAAGAGGA TTTGGATAAT CCGCCTATAT CCTTGCAGGA CTATAATGCG CTCCAAGCCA CTGAGAAGTC   
  
  
+ ATTGTATGAT GCCCTTGGAG AGGCTTATCC AGCTTCATCT GATCCATTGC AATCATCAGT TGGTCAGAGC   
  
  
+ ACTGAGAGCT CTGATGATCC TGACAGAAGC AGCGGCATTG ATAAATGGGG TAATAGATAT TACCCCACCA   
  
  
+ GCCTGAATGT GGTTGAAACC AGCCGGCTGA TCAATCAGAG CCAGTTGGAC CCTTCTCAAA TGTTTGATGC   
  
  
+ TGCTGCTCAC CCCTTGGAGT CTAATTGTCA TTCTTTTGTC TCTAGTAATA ACTTTGATCA TTCTATGGAT   
  
  
+ GGGGTTCCAG ATTCTCCTAT CAGTACACTT TCTGTGAATG AAACAAGAAA GGAAGAATCA GTTAGTAGAT   
  
  
+ CAAGGAGCAG GAAGAATCAT CAAAGAGATG GTGCATACCT GGAAGAAAGG AGTACTAAGC AAATAGCATC   
  
  
+ CAGCAATGAG GAATATGGAG AGATGGAGAG GTTTGATGAT GTACTGATCT GCAGGGAAGG AAATGATGAT   
  
  
+ ATTTCACCTT GTACCCGCAA AACCCCAGTT CATGAAGCAG ATGATAACTC GGAACCAAAG GGGAAATCAA   
  
  
+ GAGGATCTAA AAGCAAGAGT AAATCGCGTG TCAAGAAGAA AAGTAACGAA GGACAAGCGG TAGATCTTAG   
  
  
+ GACTCAACTT ATTCAGTGTG CAGAAGCAGT GGCAAGCTTT GAGCTTAAAA GTGCAAATGA GCTACTCAGG   
  
  
+ CGAATTAGGC AGCATGCTTC GCCATTTGGG GATAGTGTTC AAAGGCTAGC ACATTACTTT GCGAATGGGC   
  
  
+ TCGAGGCACG CTTAGCTGGT ACAGGATCAG AATCATACAA AGACTTTGCT GGGAAAAAAT TTTCATCCTA   
  
  
+ TGACATCTTG AGAGGCTACA AGTCATATGT TCAAGCCGTC CCCTTCCAGA GAACAACCAT GTTCTTGACA   
  
  
+ AATCAAACAA TAGTAAAGCT GGCTGAGAAA GCAACAAGGG TTCATATCAT TGATTTTGGC ATCTTTTTTG   
  
  
+ GTTTCCAGTG GCCCTGCCTT GTTCAGAATT TGTCAAGAAG ACCCCAGGGT TCTCCCATAC TCAAAATCAC   
  
  
+ GGGAGTCGAT CATCCCCAGA GTGGGTTCAG ACCAGCCCAG AAAGTTGAAG AGACAGGTCG TCGATTAGCA   
  
  
+ GGCTATTGTG AGAGGTTTGG GGTGCCCTTT GAGTTTCATC CCATTGCTCA AAGGTGGCAT ACCCTAAAGC   
  
  
+ CAGAGCATTT CAAAATTGAA AGCAATGAGC TTGTTGTGGT CACTTGTTTC TACCAATCAG GGAAGCTCCC   
  
  
+ TGATGAGACT GTTGATGTCA ACAGCCCAAG AGACCATGTC TTGAGGCTGA TCAGGGGCTT GAACCCTGAT   
  
  
+ CTATTCATCC ATGGGGTTAT CAATGGCACA TTCAATGCAC CCTTCTTCGT AACTCGGTTT AAAGAGGCAC   
  
  
+ TTTACCACTA CTCTTCTTTG TTCGATGTCT TTGATGCCAC CATAGATCGT GAAAGTCATG AAAGACTGCT   
  
  
+ GACTGAGAAC TATTTGTATG GGAAAGATGC ACTTAACATA GTAGCATGTG AGGGGAACGA GAGGATCGAG   
  
  
+ AGACCCGAGA CTTACAAGCA GTGGCAAGTC CGGAAGCTTA GAGCTGGATT CAAGCAGGTC CCCCTAGACC   
  
  
+ AAGACATTGT AAATGGAGCA AGAGCTATGG TTAAGGCAAA TTACAATAAG GATTTTATGG TAGAACAAGA   
  
  
+ TAGGAATTGG ATGGTCCAGG GCTGGAAAGG AAGGATTTTG AGTGCCATAT CCTGTTGGAA ACCTGCTTA  

- +Up\_Stream \_Len000ACTTAA CCTTCAACTA AGATGGAAAC TAACTAAGCT AATTAAGATC CAAGAACCAA   
  
  
- GATCAAGAGA GAGGAGAACA AGAACTAATA AATCCAAAAC CTACACTACA AATAACTTAA CTTACAACGT   
  
  
- TAACAACAAT ATTTCTACTA CAATCCGATT TATAGGAAGT TGAATCCAAC CCTAACTGCA GCTTATACAC   
  
  
- ATCAACTTAG AACATGACCG GATTTAGGGA CGAGGGGGAG ACAAAACGCT AATACTTAAA GTAATACGTG   
  
  
- TGGTGTACGA GCTACTTTAC GAGCTTTCTT GTAAGTAGGA GAAACAAACC CTAAATCCCC TTGCAACTTT   
  
  
- CTGCTTCAGA AATGGATCAC GCAGAATCAT CTGTTAATCC TAAAATTGTA CTTTTATCAA TTTTAAGATT   
  
  
- AACCCTAAAC TCTAACACTT AGAACATTAG GAAGCTGAAG ATCAAGATAC TTTTATCCGC TAATATCAAG   
  
  
- ATCCTAATCA GAAGCTGGGA ACTCTCTCCC TCCTCTACCC AAATCAACTA TTAGATAAGT AAGTATTGCG   
  
  
- TACGGACCCT CTACAAACCA TCCCGATCAT TAACTAAGCG ATTGTATAAA CACGACAATC AAGGTTGGAC   
  
  
- TCACAAATGA GAAGACGACA AATAAGGCAG AAAATCAGCA TTAACAGAGG AAATCAAATT AAAGTTTTGG   
  
  
- GGTTAACACT AGCGGATCTA ACTCGTTAGA TTAATCAAGA TTATTAACTT TAGTTCAAGG GACACCTTAT   
  
  
- GCTGGGCATG AACGGCACAC GATCGATGAC AATGTGGAAC GTAAACGCCA TATAAATATT GAGTTGTATA   
  
  
- CAGACGAGTG ATTTTCATGT ATAATCGAGT GGTTTTGACA CAGAGTTGCA TGATTTTATA AAATCACTTG   
  
  
- ATATGTAATA AAAATCATTC GCTTTATTCT ATGTGTTGTC GTGTAAAATT ACTCAATGTC CCGTAAAAAT   
  
  
- CACTCGGCTT TAGACTACCA AAAGAAACAT CTAGGCCAAT ATATTAACAT AAAATATGTT GGCTTACCTG   
  
  
- CTATATTGTT GACTCAATTT ACTCCTCAGA CTTTAATGCT TGTATTTTAG GTTTTCTTCT ACGGCTACGG   
  
  
- TAACCTTTCT TTCGACTTCC ACTAGCGAAA GATCATCATA ATCCGTCATC ATTAATTTTT GATTTTAGGT   
  
  
- CTTAACTGTG GCTTAACCCC AACACCGTGT ACTAGCTTTA GTCGTAGCTT AGATGTGCTA CTAGACCTAA   
  
  
- GAACCTGTGA GACCGAAATC CGGCAGTTAA AGCTCCTAAC GGGTATTGAA ATTAGGACAA GTTTAGGTAA   
  
  
- GTTAAATTAT AATGTGTTTA GGAAACTAAT TTAGTGGCAT TAACACCAAA CTAATCCCTT CAAAGAACCC   
  
  
- CCTTTCCAAG TTCGAAGACA GAAAGAATTT CGGACCTAAA AACCAGGGCG TACAAGTGAA GTGTAAGGGA   
  
  
- AAAAGAGAGG GTAGCAATGG ACAGACTTAG ACATACATTA TTTTCAGTAT AAAACGCTCC AGAAGAAAAC   
  
  
- TGACAAGAAC ATCAGTGAGA ACTATAGACG TGGTAAAACA AAAAGAGCCG TAAAAGAGGT TGAACGTAAA   
  
  
- AGATTACATT ACACTAGAAA AGACCTGTAA GTAAAACAAT TAGATGAGTG AACCCAGTTC CAAACGACTA   
  
  
- AAACCGACCA CAATTTAGGC AAGGAGAAAC CGTAAACAAG TACAAGGTGT TGACAAACTA TAGACCATAG   
  
  
- AGTCTTTGGG GCGGTAAGAA ACCATAAAGG ACTGAAAGAA CAAGTTCCCA ATAAGGATAA AGGGACTTAA   
  
  
- AAGAAACTAA GAAATCCATA AAGTTCGAAC CCCCCAAAAC AAGAAGTGAC TAAAACAGGA GAAGAAGGGA   
  
  
- ATAACCCACC AAACTAGAGG ATTTAAAGAC ACATAGACTT AGTCAGCCCC ATCTGAAACA CAAAAACCAA   
  
  
- CTAAAAAATA GTTACATAAA AGAAGGATTA CTTCCCGGAG GGCTTGGTTA TGTGTACGAA TTGAAGTTGG   
  
  
- GACAAGGAGA AGGTTCGAAA AACCTCGGAG TTTTCAGAAG CTTACGACTA TCCGTGTATC CCAGGAAGGT   
  
  
- TACGATACCA GAACTACTAC TGGGGCGTGG GAACTTACTG GGTTTAATGT AACATACAGT TAGAAGTATA   
  
  
- GGACTAGCTC TATAATGTGT CTAGGGACTG AAAGGACTAC TAACAGAGTT CAAGTAGTTA CTGTAAGAAT   
  
  
- ACCTTCTCCT AAACCTATTA GGCGGATATA GGAACGTCCT GATATTACGC GAGGTTCGGT GACTCTTCAG   
  
  
- TAACATACTA CGGGAACCTC TCCGAATAGG TCGAAGTAGA CTAGGTAACG TTAGTAGTCA ACCAGTCTCG   
  
  
- TGACTCTCGA GACTACTAGG ACTGTCTTCG TCGCCGTAAC TATTTACCCC ATTATCTATA ATGGGGTGGT   
  
  
- CGGACTTACA CCAACTTTGG TCGGCCGACT AGTTAGTCTC GGTCAACCTG GGAAGAGTTT ACAAACTACG   
  
  
- ACGACGAGTG GGGAACCTCA GATTAACAGT AAGAAAACAG AGATCATTAT TGAAACTAGT AAGATACCTA   
  
  
- CCCCAAGGTC TAAGAGGATA GTCATGTGAA AGACACTTAC TTTGTTCTTT CCTTCTTAGT CAATCATCTA   
  
  
- GTTCCTCGTC CTTCTTAGTA GTTTCTCTAC CACGTATGGA CCTTCTTTCC TCATGATTCG TTTATCGTAG   
  
  
- GTCGTTACTC CTTATACCTC TCTACCTCTC CAAACTACTA CATGACTAGA CGTCCCTTCC TTTACTACTA   
  
  
- TAAAGTGGAA CATGGGCGTT TTGGGGTCAA GTACTTCGTC TACTATTGAG CCTTGGTTTC CCCTTTAGTT   
  
  
- CTCCTAGATT TTCGTTCTCA TTTAGCGCAC AGTTCTTCTT TTCATTGCTT CCTGTTCGCC ATCTAGAATC   
  
  
- CTGAGTTGAA TAAGTCACAC GTCTTCGTCA CCGTTCGAAA CTCGAATTTT CACGTTTACT CGATGAGTCC   
  
  
- GCTTAATCCG TCGTACGAAG CGGTAAACCC CTATCACAAG TTTCCGATCG TGTAATGAAA CGCTTACCCG   
  
  
- AGCTCCGTGC GAATCGACCA TGTCCTAGTC TTAGTATGTT TCTGAAACGA CCCTTTTTTA AAAGTAGGAT   
  
  
- ACTGTAGAAC TCTCCGATGT TCAGTATACA AGTTCGGCAG GGGAAGGTCT CTTGTTGGTA CAAGAACTGT   
  
  
- TTAGTTTGTT ATCATTTCGA CCGACTCTTT CGTTGTTCCC AAGTATAGTA ACTAAAACCG TAGAAAAAAC   
  
  
- CAAAGGTCAC CGGGACGGAA CAAGTCTTAA ACAGTTCTTC TGGGGTCCCA AGAGGGTATG AGTTTTAGTG   
  
  
- CCCTCAGCTA GTAGGGGTCT CACCCAAGTC TGGTCGGGTC TTTCAACTTC TCTGTCCAGC AGCTAATCGT   
  
  
- CCGATAACAC TCTCCAAACC CCACGGGAAA CTCAAAGTAG GGTAACGAGT TTCCACCGTA TGGGATTTCG   
  
  
- GTCTCGTAAA GTTTTAACTT TCGTTACTCG AACAACACCA GTGAACAAAG ATGGTTAGTC CCTTCGAGGG   
  
  
- ACTACTCTGA CAACTACAGT TGTCGGGTTC TCTGGTACAG AACTCCGACT AGTCCCCGAA CTTGGGACTA   
  
  
- GATAAGTAGG TACCCCAATA GTTACCGTGT AAGTTACGTG GGAAGAAGCA TTGAGCCAAA TTTCTCCGTG   
  
  
- AAATGGTGAT GAGAAGAAAC AAGCTACAGA AACTACGGTG GTATCTAGCA CTTTCAGTAC TTTCTGACGA   
  
  
- CTGACTCTTG ATAAACATAC CCTTTCTACG TGAATTGTAT CATCGTACAC TCCCCTTGCT CTCCTAGCTC   
  
  
- TCTGGGCTCT GAATGTTCGT CACCGTTCAG GCCTTCGAAT CTCGACCTAA GTTCGTCCAG GGGGATCTGG   
  
  
- TTCTGTAACA TTTACCTCGT TCTCGATACC AATTCCGTTT AATGTTATTC CTAAAATACC ATCTTGTTCT   
  
  
- ATCCTTAACC TACCAGGTCC CGACCTTTCC TTCCTAAAAC TCACGGTATA GGACAACCTT TGGACGAAT

+     Unnamed\_\_4

| Site Name | Organism | Position | Strand | Matrix score. | sequence | function |
| --- | --- | --- | --- | --- | --- | --- |
| Unnamed\_\_4 | Petroselinum hortense | 2002 | + | 4 | CTCC |  |
| Unnamed\_\_4 | Petroselinum hortense | 2057 | - | 4 | CTCC |  |
| Unnamed\_\_4 | Petroselinum hortense | 1911 | + | 4 | CTCC |  |
| Unnamed\_\_4 | Petroselinum hortense | 1600 | + | 4 | CTCC |  |
| Unnamed\_\_4 | Petroselinum hortense | 1481 | + | 4 | CTCC |  |
| Unnamed\_\_4 | Petroselinum hortense | 572 | - | 4 | CTCC |  |
| Unnamed\_\_4 | Petroselinum hortense | 1078 | - | 4 | CTCC |  |
| Unnamed\_\_4 | Petroselinum hortense | 85 | + | 4 | CTCC |  |
| Unnamed\_\_4 | Petroselinum hortense | 2540 | - | 4 | CTCC |  |
| Unnamed\_\_4 | Petroselinum hortense | 3366 | - | 4 | CTCC |  |
| Unnamed\_\_4 | Petroselinum hortense | 3346 | + | 4 | CTCC |  |
| Unnamed\_\_4 | Petroselinum hortense | 2751 | - | 4 | CTCC |  |
| Unnamed\_\_4 | Petroselinum hortense | 2608 | + | 4 | CTCC |  |
| Unnamed\_\_4 | Petroselinum hortense | 3939 | - | 4 | CTCC |  |
| Unnamed\_\_4 | Petroselinum hortense | 3570 | + | 4 | CTCC |  |
| Unnamed\_\_4 | Petroselinum hortense | 523 | - | 4 | CTCC |  |
| Unnamed\_\_4 | Petroselinum hortense | 2759 | - | 4 | CTCC |  |
| Unnamed\_\_4 | Petroselinum hortense | 681 | + | 4 | CTCC |  |
| Unnamed\_\_4 | Petroselinum hortense | 246 | + | 4 | CTCC |  |
| Unnamed\_\_4 | Petroselinum hortense | 526 | - | 4 | CTCC |  |
| Unnamed\_\_4 | Petroselinum hortense | 2295 | + | 4 | CTCC |  |
| Unnamed\_\_4 | Petroselinum hortense | 2331 | - | 4 | CTCC |  |
| Unnamed\_\_4 | Petroselinum hortense | 2713 | - | 4 | CTCC |  |
| Unnamed\_\_4 | Petroselinum hortense | 2668 | - | 4 | CTCC |  |

>HU02G01573.1   
+ +Up\_Stream \_Len000TGAATT GGAAGTTGAT TCTACCTTTG ATTGATTCGA TTAATTCTAG GTTCTTGGTT   
  
  
+ CTAGTTCTCT CTCCTCTTGT TCTTGATTAT TTAGGTTTTG GATGTGATGT TTATTGAATT GAATGTTGCA   
  
  
+ ATTGTTGTTA TAAAGATGAT GTTAGGCTAA ATATCCTTCA ACTTAGGTTG GGATTGACGT CGAATATGTG   
  
  
+ TAGTTGAATC TTGTACTGGC CTAAATCCCT GCTCCCCCTC TGTTTTGCGA TTATGAATTT CATTATGCAC   
  
  
+ ACCACATGCT CGATGAAATG CTCGAAAGAA CATTCATCCT CTTTGTTTGG GATTTAGGGG AACGTTGAAA   
  
  
+ GACGAAGTCT TTACCTAGTG CGTCTTAGTA GACAATTAGG ATTTTAACAT GAAAATAGTT AAAATTCTAA   
  
  
+ TTGGGATTTG AGATTGTGAA TCTTGTAATC CTTCGACTTC TAGTTCTATG AAAATAGGCG ATTATAGTTC   
  
  
+ TAGGATTAGT CTTCGACCCT TGAGAGAGGG AGGAGATGGG TTTAGTTGAT AATCTATTCA TTCATAACGC   
  
  
+ ATGCCTGGGA GATGTTTGGT AGGGCTAGTA ATTGATTCGC TAACATATTT GTGCTGTTAG TTCCAACCTG   
  
  
+ AGTGTTTACT CTTCTGCTGT TTATTCCGTC TTTTAGTCGT AATTGTCTCC TTTAGTTTAA TTTCAAAACC   
  
  
+ CCAATTGTGA TCGCCTAGAT TGAGCAATCT AATTAGTTCT AATAATTGAA ATCAAGTTCC CTGTGGAATA   
  
  
+ CGACCCGTAC TTGCCGTGTG CTAGCTACTG TTACACCTTG CATTTGCGGT ATATTTATAA CTCAACATAT   
  
  
+ GTCTGCTCAC TAAAAGTACA TATTAGCTCA CCAAAACTGT GTCTCAACGT ACTAAAATAT TTTAGTGAAC   
  
  
+ TATACATTAT TTTTAGTAAG CGAAATAAGA TACACAACAG CACATTTTAA TGAGTTACAG GGCATTTTTA   
  
  
+ GTGAGCCGAA ATCTGATGGT TTTCTTTGTA GATCCGGTTA TATAATTGTA TTTTATACAA CCGAATGGAC   
  
  
+ GATATAACAA CTGAGTTAAA TGAGGAGTCT GAAATTACGA ACATAAAATC CAAAAGAAGA TGCCGATGCC   
  
  
+ ATTGGAAAGA AAGCTGAAGG TGATCGCTTT CTAGTAGTAT TAGGCAGTAG TAATTAAAAA CTAAAATCCA   
  
  
+ GAATTGACAC CGAATTGGGG TTGTGGCACA TGATCGAAAT CAGCATCGAA TCTACACGAT GATCTGGATT   
  
  
+ CTTGGACACT CTGGCTTTAG GCCGTCAATT TCGAGGATTG CCCATAACTT TAATCCTGTT CAAATCCATT   
  
  
+ CAATTTAATA TTACACAAAT CCTTTGATTA AATCACCGTA ATTGTGGTTT GATTAGGGAA GTTTCTTGGG   
  
  
+ GGAAAGGTTC AAGCTTCTGT CTTTCTTAAA GCCTGGATTT TTGGTCCCGC ATGTTCACTT CACATTCCCT   
  
  
+ TTTTCTCTCC CATCGTTACC TGTCTGAATC TGTATGTAAT AAAAGTCATA TTTTGCGAGG TCTTCTTTTG   
  
  
+ ACTGTTCTTG TAGTCACTCT TGATATCTGC ACCATTTTGT TTTTCTCGGC ATTTTCTCCA ACTTGCATTT   
  
  
+ TCTAATGTAA TGTGATCTTT TCTGGACATT CATTTTGTTA ATCTACTCAC TTGGGTCAAG GTTTGCTGAT   
  
  
+ TTTGGCTGGT GTTAAATCCG TTCCTCTTTG GCATTTGTTC ATGTTCCACA ACTGTTTGAT ATCTGGTATC   
  
  
+ TCAGAAACCC CGCCATTCTT TGGTATTTCC TGACTTTCTT GTTCAAGGGT TATTCCTATT TCCCTGAATT   
  
  
+ TTCTTTGATT CTTTAGGTAT TTCAAGCTTG GGGGGTTTTG TTCTTCACTG ATTTTGTCCT CTTCTTCCCT   
  
  
+ TATTGGGTGG TTTGATCTCC TAAATTTCTG TGTATCTGAA TCAGTCGGGG TAGACTTTGT GTTTTTGGTT   
  
  
+ GATTTTTTAT CAATGTATTT TCTTCCTAAT GAAGGGCCTC CCGAACCAAT ACACATGCTT AACTTCAACC   
  
  
+ CTGTTCCTCT TCCAAGCTTT TTGGAGCCTC AAAAGTCTTC GAATGCTGAT AGGCACATAG GGTCCTTCCA   
  
  
+ ATGCTATGGT CTTGATGATG ACCCCGCACC CTTGAATGAC CCAAATTACA TTGTATGTCA ATCTTCATAT   
  
  
+ CCTGATCGAG ATATTACACA GATCCCTGAC TTTCCTGATG ATTGTCTCAA GTTCATCAAT GACATTCTTA   
  
  
+ TGGAAGAGGA TTTGGATAAT CCGCCTATAT CCTTGCAGGA CTATAATGCG CTCCAAGCCA CTGAGAAGTC   
  
  
+ ATTGTATGAT GCCCTTGGAG AGGCTTATCC AGCTTCATCT GATCCATTGC AATCATCAGT TGGTCAGAGC   
  
  
+ ACTGAGAGCT CTGATGATCC TGACAGAAGC AGCGGCATTG ATAAATGGGG TAATAGATAT TACCCCACCA   
  
  
+ GCCTGAATGT GGTTGAAACC AGCCGGCTGA TCAATCAGAG CCAGTTGGAC CCTTCTCAAA TGTTTGATGC   
  
  
+ TGCTGCTCAC CCCTTGGAGT CTAATTGTCA TTCTTTTGTC TCTAGTAATA ACTTTGATCA TTCTATGGAT   
  
  
+ GGGGTTCCAG ATTCTCCTAT CAGTACACTT TCTGTGAATG AAACAAGAAA GGAAGAATCA GTTAGTAGAT   
  
  
+ CAAGGAGCAG GAAGAATCAT CAAAGAGATG GTGCATACCT GGAAGAAAGG AGTACTAAGC AAATAGCATC   
  
  
+ CAGCAATGAG GAATATGGAG AGATGGAGAG GTTTGATGAT GTACTGATCT GCAGGGAAGG AAATGATGAT   
  
  
+ ATTTCACCTT GTACCCGCAA AACCCCAGTT CATGAAGCAG ATGATAACTC GGAACCAAAG GGGAAATCAA   
  
  
+ GAGGATCTAA AAGCAAGAGT AAATCGCGTG TCAAGAAGAA AAGTAACGAA GGACAAGCGG TAGATCTTAG   
  
  
+ GACTCAACTT ATTCAGTGTG CAGAAGCAGT GGCAAGCTTT GAGCTTAAAA GTGCAAATGA GCTACTCAGG   
  
  
+ CGAATTAGGC AGCATGCTTC GCCATTTGGG GATAGTGTTC AAAGGCTAGC ACATTACTTT GCGAATGGGC   
  
  
+ TCGAGGCACG CTTAGCTGGT ACAGGATCAG AATCATACAA AGACTTTGCT GGGAAAAAAT TTTCATCCTA   
  
  
+ TGACATCTTG AGAGGCTACA AGTCATATGT TCAAGCCGTC CCCTTCCAGA GAACAACCAT GTTCTTGACA   
  
  
+ AATCAAACAA TAGTAAAGCT GGCTGAGAAA GCAACAAGGG TTCATATCAT TGATTTTGGC ATCTTTTTTG   
  
  
+ GTTTCCAGTG GCCCTGCCTT GTTCAGAATT TGTCAAGAAG ACCCCAGGGT TCTCCCATAC TCAAAATCAC   
  
  
+ GGGAGTCGAT CATCCCCAGA GTGGGTTCAG ACCAGCCCAG AAAGTTGAAG AGACAGGTCG TCGATTAGCA   
  
  
+ GGCTATTGTG AGAGGTTTGG GGTGCCCTTT GAGTTTCATC CCATTGCTCA AAGGTGGCAT ACCCTAAAGC   
  
  
+ CAGAGCATTT CAAAATTGAA AGCAATGAGC TTGTTGTGGT CACTTGTTTC TACCAATCAG GGAAGCTCCC   
  
  
+ TGATGAGACT GTTGATGTCA ACAGCCCAAG AGACCATGTC TTGAGGCTGA TCAGGGGCTT GAACCCTGAT   
  
  
+ CTATTCATCC ATGGGGTTAT CAATGGCACA TTCAATGCAC CCTTCTTCGT AACTCGGTTT AAAGAGGCAC   
  
  
+ TTTACCACTA CTCTTCTTTG TTCGATGTCT TTGATGCCAC CATAGATCGT GAAAGTCATG AAAGACTGCT   
  
  
+ GACTGAGAAC TATTTGTATG GGAAAGATGC ACTTAACATA GTAGCATGTG AGGGGAACGA GAGGATCGAG   
  
  
+ AGACCCGAGA CTTACAAGCA GTGGCAAGTC CGGAAGCTTA GAGCTGGATT CAAGCAGGTC CCCCTAGACC   
  
  
+ AAGACATTGT AAATGGAGCA AGAGCTATGG TTAAGGCAAA TTACAATAAG GATTTTATGG TAGAACAAGA   
  
  
+ TAGGAATTGG ATGGTCCAGG GCTGGAAAGG AAGGATTTTG AGTGCCATAT CCTGTTGGAA ACCTGCTTA  

- +Up\_Stream \_Len000ACTTAA CCTTCAACTA AGATGGAAAC TAACTAAGCT AATTAAGATC CAAGAACCAA   
  
  
- GATCAAGAGA GAGGAGAACA AGAACTAATA AATCCAAAAC CTACACTACA AATAACTTAA CTTACAACGT   
  
  
- TAACAACAAT ATTTCTACTA CAATCCGATT TATAGGAAGT TGAATCCAAC CCTAACTGCA GCTTATACAC   
  
  
- ATCAACTTAG AACATGACCG GATTTAGGGA CGAGGGGGAG ACAAAACGCT AATACTTAAA GTAATACGTG   
  
  
- TGGTGTACGA GCTACTTTAC GAGCTTTCTT GTAAGTAGGA GAAACAAACC CTAAATCCCC TTGCAACTTT   
  
  
- CTGCTTCAGA AATGGATCAC GCAGAATCAT CTGTTAATCC TAAAATTGTA CTTTTATCAA TTTTAAGATT   
  
  
- AACCCTAAAC TCTAACACTT AGAACATTAG GAAGCTGAAG ATCAAGATAC TTTTATCCGC TAATATCAAG   
  
  
- ATCCTAATCA GAAGCTGGGA ACTCTCTCCC TCCTCTACCC AAATCAACTA TTAGATAAGT AAGTATTGCG   
  
  
- TACGGACCCT CTACAAACCA TCCCGATCAT TAACTAAGCG ATTGTATAAA CACGACAATC AAGGTTGGAC   
  
  
- TCACAAATGA GAAGACGACA AATAAGGCAG AAAATCAGCA TTAACAGAGG AAATCAAATT AAAGTTTTGG   
  
  
- GGTTAACACT AGCGGATCTA ACTCGTTAGA TTAATCAAGA TTATTAACTT TAGTTCAAGG GACACCTTAT   
  
  
- GCTGGGCATG AACGGCACAC GATCGATGAC AATGTGGAAC GTAAACGCCA TATAAATATT GAGTTGTATA   
  
  
- CAGACGAGTG ATTTTCATGT ATAATCGAGT GGTTTTGACA CAGAGTTGCA TGATTTTATA AAATCACTTG   
  
  
- ATATGTAATA AAAATCATTC GCTTTATTCT ATGTGTTGTC GTGTAAAATT ACTCAATGTC CCGTAAAAAT   
  
  
- CACTCGGCTT TAGACTACCA AAAGAAACAT CTAGGCCAAT ATATTAACAT AAAATATGTT GGCTTACCTG   
  
  
- CTATATTGTT GACTCAATTT ACTCCTCAGA CTTTAATGCT TGTATTTTAG GTTTTCTTCT ACGGCTACGG   
  
  
- TAACCTTTCT TTCGACTTCC ACTAGCGAAA GATCATCATA ATCCGTCATC ATTAATTTTT GATTTTAGGT   
  
  
- CTTAACTGTG GCTTAACCCC AACACCGTGT ACTAGCTTTA GTCGTAGCTT AGATGTGCTA CTAGACCTAA   
  
  
- GAACCTGTGA GACCGAAATC CGGCAGTTAA AGCTCCTAAC GGGTATTGAA ATTAGGACAA GTTTAGGTAA   
  
  
- GTTAAATTAT AATGTGTTTA GGAAACTAAT TTAGTGGCAT TAACACCAAA CTAATCCCTT CAAAGAACCC   
  
  
- CCTTTCCAAG TTCGAAGACA GAAAGAATTT CGGACCTAAA AACCAGGGCG TACAAGTGAA GTGTAAGGGA   
  
  
- AAAAGAGAGG GTAGCAATGG ACAGACTTAG ACATACATTA TTTTCAGTAT AAAACGCTCC AGAAGAAAAC   
  
  
- TGACAAGAAC ATCAGTGAGA ACTATAGACG TGGTAAAACA AAAAGAGCCG TAAAAGAGGT TGAACGTAAA   
  
  
- AGATTACATT ACACTAGAAA AGACCTGTAA GTAAAACAAT TAGATGAGTG AACCCAGTTC CAAACGACTA   
  
  
- AAACCGACCA CAATTTAGGC AAGGAGAAAC CGTAAACAAG TACAAGGTGT TGACAAACTA TAGACCATAG   
  
  
- AGTCTTTGGG GCGGTAAGAA ACCATAAAGG ACTGAAAGAA CAAGTTCCCA ATAAGGATAA AGGGACTTAA   
  
  
- AAGAAACTAA GAAATCCATA AAGTTCGAAC CCCCCAAAAC AAGAAGTGAC TAAAACAGGA GAAGAAGGGA   
  
  
- ATAACCCACC AAACTAGAGG ATTTAAAGAC ACATAGACTT AGTCAGCCCC ATCTGAAACA CAAAAACCAA   
  
  
- CTAAAAAATA GTTACATAAA AGAAGGATTA CTTCCCGGAG GGCTTGGTTA TGTGTACGAA TTGAAGTTGG   
  
  
- GACAAGGAGA AGGTTCGAAA AACCTCGGAG TTTTCAGAAG CTTACGACTA TCCGTGTATC CCAGGAAGGT   
  
  
- TACGATACCA GAACTACTAC TGGGGCGTGG GAACTTACTG GGTTTAATGT AACATACAGT TAGAAGTATA   
  
  
- GGACTAGCTC TATAATGTGT CTAGGGACTG AAAGGACTAC TAACAGAGTT CAAGTAGTTA CTGTAAGAAT   
  
  
- ACCTTCTCCT AAACCTATTA GGCGGATATA GGAACGTCCT GATATTACGC GAGGTTCGGT GACTCTTCAG   
  
  
- TAACATACTA CGGGAACCTC TCCGAATAGG TCGAAGTAGA CTAGGTAACG TTAGTAGTCA ACCAGTCTCG   
  
  
- TGACTCTCGA GACTACTAGG ACTGTCTTCG TCGCCGTAAC TATTTACCCC ATTATCTATA ATGGGGTGGT   
  
  
- CGGACTTACA CCAACTTTGG TCGGCCGACT AGTTAGTCTC GGTCAACCTG GGAAGAGTTT ACAAACTACG   
  
  
- ACGACGAGTG GGGAACCTCA GATTAACAGT AAGAAAACAG AGATCATTAT TGAAACTAGT AAGATACCTA   
  
  
- CCCCAAGGTC TAAGAGGATA GTCATGTGAA AGACACTTAC TTTGTTCTTT CCTTCTTAGT CAATCATCTA   
  
  
- GTTCCTCGTC CTTCTTAGTA GTTTCTCTAC CACGTATGGA CCTTCTTTCC TCATGATTCG TTTATCGTAG   
  
  
- GTCGTTACTC CTTATACCTC TCTACCTCTC CAAACTACTA CATGACTAGA CGTCCCTTCC TTTACTACTA   
  
  
- TAAAGTGGAA CATGGGCGTT TTGGGGTCAA GTACTTCGTC TACTATTGAG CCTTGGTTTC CCCTTTAGTT   
  
  
- CTCCTAGATT TTCGTTCTCA TTTAGCGCAC AGTTCTTCTT TTCATTGCTT CCTGTTCGCC ATCTAGAATC   
  
  
- CTGAGTTGAA TAAGTCACAC GTCTTCGTCA CCGTTCGAAA CTCGAATTTT CACGTTTACT CGATGAGTCC   
  
  
- GCTTAATCCG TCGTACGAAG CGGTAAACCC CTATCACAAG TTTCCGATCG TGTAATGAAA CGCTTACCCG   
  
  
- AGCTCCGTGC GAATCGACCA TGTCCTAGTC TTAGTATGTT TCTGAAACGA CCCTTTTTTA AAAGTAGGAT   
  
  
- ACTGTAGAAC TCTCCGATGT TCAGTATACA AGTTCGGCAG GGGAAGGTCT CTTGTTGGTA CAAGAACTGT   
  
  
- TTAGTTTGTT ATCATTTCGA CCGACTCTTT CGTTGTTCCC AAGTATAGTA ACTAAAACCG TAGAAAAAAC   
  
  
- CAAAGGTCAC CGGGACGGAA CAAGTCTTAA ACAGTTCTTC TGGGGTCCCA AGAGGGTATG AGTTTTAGTG   
  
  
- CCCTCAGCTA GTAGGGGTCT CACCCAAGTC TGGTCGGGTC TTTCAACTTC TCTGTCCAGC AGCTAATCGT   
  
  
- CCGATAACAC TCTCCAAACC CCACGGGAAA CTCAAAGTAG GGTAACGAGT TTCCACCGTA TGGGATTTCG   
  
  
- GTCTCGTAAA GTTTTAACTT TCGTTACTCG AACAACACCA GTGAACAAAG ATGGTTAGTC CCTTCGAGGG   
  
  
- ACTACTCTGA CAACTACAGT TGTCGGGTTC TCTGGTACAG AACTCCGACT AGTCCCCGAA CTTGGGACTA   
  
  
- GATAAGTAGG TACCCCAATA GTTACCGTGT AAGTTACGTG GGAAGAAGCA TTGAGCCAAA TTTCTCCGTG   
  
  
- AAATGGTGAT GAGAAGAAAC AAGCTACAGA AACTACGGTG GTATCTAGCA CTTTCAGTAC TTTCTGACGA   
  
  
- CTGACTCTTG ATAAACATAC CCTTTCTACG TGAATTGTAT CATCGTACAC TCCCCTTGCT CTCCTAGCTC   
  
  
- TCTGGGCTCT GAATGTTCGT CACCGTTCAG GCCTTCGAAT CTCGACCTAA GTTCGTCCAG GGGGATCTGG   
  
  
- TTCTGTAACA TTTACCTCGT TCTCGATACC AATTCCGTTT AATGTTATTC CTAAAATACC ATCTTGTTCT   
  
  
- ATCCTTAACC TACCAGGTCC CGACCTTTCC TTCCTAAAAC TCACGGTATA GGACAACCTT TGGACGAAT

+     W box

| Site Name | Organism | Position | Strand | Matrix score. | sequence | function |
| --- | --- | --- | --- | --- | --- | --- |
| W box | Arabidopsis thaliana | 1668 | - | 6 | TTGACC |  |

>HU02G01573.1   
+ +Up\_Stream \_Len000TGAATT GGAAGTTGAT TCTACCTTTG ATTGATTCGA TTAATTCTAG GTTCTTGGTT   
  
  
+ CTAGTTCTCT CTCCTCTTGT TCTTGATTAT TTAGGTTTTG GATGTGATGT TTATTGAATT GAATGTTGCA   
  
  
+ ATTGTTGTTA TAAAGATGAT GTTAGGCTAA ATATCCTTCA ACTTAGGTTG GGATTGACGT CGAATATGTG   
  
  
+ TAGTTGAATC TTGTACTGGC CTAAATCCCT GCTCCCCCTC TGTTTTGCGA TTATGAATTT CATTATGCAC   
  
  
+ ACCACATGCT CGATGAAATG CTCGAAAGAA CATTCATCCT CTTTGTTTGG GATTTAGGGG AACGTTGAAA   
  
  
+ GACGAAGTCT TTACCTAGTG CGTCTTAGTA GACAATTAGG ATTTTAACAT GAAAATAGTT AAAATTCTAA   
  
  
+ TTGGGATTTG AGATTGTGAA TCTTGTAATC CTTCGACTTC TAGTTCTATG AAAATAGGCG ATTATAGTTC   
  
  
+ TAGGATTAGT CTTCGACCCT TGAGAGAGGG AGGAGATGGG TTTAGTTGAT AATCTATTCA TTCATAACGC   
  
  
+ ATGCCTGGGA GATGTTTGGT AGGGCTAGTA ATTGATTCGC TAACATATTT GTGCTGTTAG TTCCAACCTG   
  
  
+ AGTGTTTACT CTTCTGCTGT TTATTCCGTC TTTTAGTCGT AATTGTCTCC TTTAGTTTAA TTTCAAAACC   
  
  
+ CCAATTGTGA TCGCCTAGAT TGAGCAATCT AATTAGTTCT AATAATTGAA ATCAAGTTCC CTGTGGAATA   
  
  
+ CGACCCGTAC TTGCCGTGTG CTAGCTACTG TTACACCTTG CATTTGCGGT ATATTTATAA CTCAACATAT   
  
  
+ GTCTGCTCAC TAAAAGTACA TATTAGCTCA CCAAAACTGT GTCTCAACGT ACTAAAATAT TTTAGTGAAC   
  
  
+ TATACATTAT TTTTAGTAAG CGAAATAAGA TACACAACAG CACATTTTAA TGAGTTACAG GGCATTTTTA   
  
  
+ GTGAGCCGAA ATCTGATGGT TTTCTTTGTA GATCCGGTTA TATAATTGTA TTTTATACAA CCGAATGGAC   
  
  
+ GATATAACAA CTGAGTTAAA TGAGGAGTCT GAAATTACGA ACATAAAATC CAAAAGAAGA TGCCGATGCC   
  
  
+ ATTGGAAAGA AAGCTGAAGG TGATCGCTTT CTAGTAGTAT TAGGCAGTAG TAATTAAAAA CTAAAATCCA   
  
  
+ GAATTGACAC CGAATTGGGG TTGTGGCACA TGATCGAAAT CAGCATCGAA TCTACACGAT GATCTGGATT   
  
  
+ CTTGGACACT CTGGCTTTAG GCCGTCAATT TCGAGGATTG CCCATAACTT TAATCCTGTT CAAATCCATT   
  
  
+ CAATTTAATA TTACACAAAT CCTTTGATTA AATCACCGTA ATTGTGGTTT GATTAGGGAA GTTTCTTGGG   
  
  
+ GGAAAGGTTC AAGCTTCTGT CTTTCTTAAA GCCTGGATTT TTGGTCCCGC ATGTTCACTT CACATTCCCT   
  
  
+ TTTTCTCTCC CATCGTTACC TGTCTGAATC TGTATGTAAT AAAAGTCATA TTTTGCGAGG TCTTCTTTTG   
  
  
+ ACTGTTCTTG TAGTCACTCT TGATATCTGC ACCATTTTGT TTTTCTCGGC ATTTTCTCCA ACTTGCATTT   
  
  
+ TCTAATGTAA TGTGATCTTT TCTGGACATT CATTTTGTTA ATCTACTCAC TTGGGTCAAG GTTTGCTGAT   
  
  
+ TTTGGCTGGT GTTAAATCCG TTCCTCTTTG GCATTTGTTC ATGTTCCACA ACTGTTTGAT ATCTGGTATC   
  
  
+ TCAGAAACCC CGCCATTCTT TGGTATTTCC TGACTTTCTT GTTCAAGGGT TATTCCTATT TCCCTGAATT   
  
  
+ TTCTTTGATT CTTTAGGTAT TTCAAGCTTG GGGGGTTTTG TTCTTCACTG ATTTTGTCCT CTTCTTCCCT   
  
  
+ TATTGGGTGG TTTGATCTCC TAAATTTCTG TGTATCTGAA TCAGTCGGGG TAGACTTTGT GTTTTTGGTT   
  
  
+ GATTTTTTAT CAATGTATTT TCTTCCTAAT GAAGGGCCTC CCGAACCAAT ACACATGCTT AACTTCAACC   
  
  
+ CTGTTCCTCT TCCAAGCTTT TTGGAGCCTC AAAAGTCTTC GAATGCTGAT AGGCACATAG GGTCCTTCCA   
  
  
+ ATGCTATGGT CTTGATGATG ACCCCGCACC CTTGAATGAC CCAAATTACA TTGTATGTCA ATCTTCATAT   
  
  
+ CCTGATCGAG ATATTACACA GATCCCTGAC TTTCCTGATG ATTGTCTCAA GTTCATCAAT GACATTCTTA   
  
  
+ TGGAAGAGGA TTTGGATAAT CCGCCTATAT CCTTGCAGGA CTATAATGCG CTCCAAGCCA CTGAGAAGTC   
  
  
+ ATTGTATGAT GCCCTTGGAG AGGCTTATCC AGCTTCATCT GATCCATTGC AATCATCAGT TGGTCAGAGC   
  
  
+ ACTGAGAGCT CTGATGATCC TGACAGAAGC AGCGGCATTG ATAAATGGGG TAATAGATAT TACCCCACCA   
  
  
+ GCCTGAATGT GGTTGAAACC AGCCGGCTGA TCAATCAGAG CCAGTTGGAC CCTTCTCAAA TGTTTGATGC   
  
  
+ TGCTGCTCAC CCCTTGGAGT CTAATTGTCA TTCTTTTGTC TCTAGTAATA ACTTTGATCA TTCTATGGAT   
  
  
+ GGGGTTCCAG ATTCTCCTAT CAGTACACTT TCTGTGAATG AAACAAGAAA GGAAGAATCA GTTAGTAGAT   
  
  
+ CAAGGAGCAG GAAGAATCAT CAAAGAGATG GTGCATACCT GGAAGAAAGG AGTACTAAGC AAATAGCATC   
  
  
+ CAGCAATGAG GAATATGGAG AGATGGAGAG GTTTGATGAT GTACTGATCT GCAGGGAAGG AAATGATGAT   
  
  
+ ATTTCACCTT GTACCCGCAA AACCCCAGTT CATGAAGCAG ATGATAACTC GGAACCAAAG GGGAAATCAA   
  
  
+ GAGGATCTAA AAGCAAGAGT AAATCGCGTG TCAAGAAGAA AAGTAACGAA GGACAAGCGG TAGATCTTAG   
  
  
+ GACTCAACTT ATTCAGTGTG CAGAAGCAGT GGCAAGCTTT GAGCTTAAAA GTGCAAATGA GCTACTCAGG   
  
  
+ CGAATTAGGC AGCATGCTTC GCCATTTGGG GATAGTGTTC AAAGGCTAGC ACATTACTTT GCGAATGGGC   
  
  
+ TCGAGGCACG CTTAGCTGGT ACAGGATCAG AATCATACAA AGACTTTGCT GGGAAAAAAT TTTCATCCTA   
  
  
+ TGACATCTTG AGAGGCTACA AGTCATATGT TCAAGCCGTC CCCTTCCAGA GAACAACCAT GTTCTTGACA   
  
  
+ AATCAAACAA TAGTAAAGCT GGCTGAGAAA GCAACAAGGG TTCATATCAT TGATTTTGGC ATCTTTTTTG   
  
  
+ GTTTCCAGTG GCCCTGCCTT GTTCAGAATT TGTCAAGAAG ACCCCAGGGT TCTCCCATAC TCAAAATCAC   
  
  
+ GGGAGTCGAT CATCCCCAGA GTGGGTTCAG ACCAGCCCAG AAAGTTGAAG AGACAGGTCG TCGATTAGCA   
  
  
+ GGCTATTGTG AGAGGTTTGG GGTGCCCTTT GAGTTTCATC CCATTGCTCA AAGGTGGCAT ACCCTAAAGC   
  
  
+ CAGAGCATTT CAAAATTGAA AGCAATGAGC TTGTTGTGGT CACTTGTTTC TACCAATCAG GGAAGCTCCC   
  
  
+ TGATGAGACT GTTGATGTCA ACAGCCCAAG AGACCATGTC TTGAGGCTGA TCAGGGGCTT GAACCCTGAT   
  
  
+ CTATTCATCC ATGGGGTTAT CAATGGCACA TTCAATGCAC CCTTCTTCGT AACTCGGTTT AAAGAGGCAC   
  
  
+ TTTACCACTA CTCTTCTTTG TTCGATGTCT TTGATGCCAC CATAGATCGT GAAAGTCATG AAAGACTGCT   
  
  
+ GACTGAGAAC TATTTGTATG GGAAAGATGC ACTTAACATA GTAGCATGTG AGGGGAACGA GAGGATCGAG   
  
  
+ AGACCCGAGA CTTACAAGCA GTGGCAAGTC CGGAAGCTTA GAGCTGGATT CAAGCAGGTC CCCCTAGACC   
  
  
+ AAGACATTGT AAATGGAGCA AGAGCTATGG TTAAGGCAAA TTACAATAAG GATTTTATGG TAGAACAAGA   
  
  
+ TAGGAATTGG ATGGTCCAGG GCTGGAAAGG AAGGATTTTG AGTGCCATAT CCTGTTGGAA ACCTGCTTA  

- +Up\_Stream \_Len000ACTTAA CCTTCAACTA AGATGGAAAC TAACTAAGCT AATTAAGATC CAAGAACCAA   
  
  
- GATCAAGAGA GAGGAGAACA AGAACTAATA AATCCAAAAC CTACACTACA AATAACTTAA CTTACAACGT   
  
  
- TAACAACAAT ATTTCTACTA CAATCCGATT TATAGGAAGT TGAATCCAAC CCTAACTGCA GCTTATACAC   
  
  
- ATCAACTTAG AACATGACCG GATTTAGGGA CGAGGGGGAG ACAAAACGCT AATACTTAAA GTAATACGTG   
  
  
- TGGTGTACGA GCTACTTTAC GAGCTTTCTT GTAAGTAGGA GAAACAAACC CTAAATCCCC TTGCAACTTT   
  
  
- CTGCTTCAGA AATGGATCAC GCAGAATCAT CTGTTAATCC TAAAATTGTA CTTTTATCAA TTTTAAGATT   
  
  
- AACCCTAAAC TCTAACACTT AGAACATTAG GAAGCTGAAG ATCAAGATAC TTTTATCCGC TAATATCAAG   
  
  
- ATCCTAATCA GAAGCTGGGA ACTCTCTCCC TCCTCTACCC AAATCAACTA TTAGATAAGT AAGTATTGCG   
  
  
- TACGGACCCT CTACAAACCA TCCCGATCAT TAACTAAGCG ATTGTATAAA CACGACAATC AAGGTTGGAC   
  
  
- TCACAAATGA GAAGACGACA AATAAGGCAG AAAATCAGCA TTAACAGAGG AAATCAAATT AAAGTTTTGG   
  
  
- GGTTAACACT AGCGGATCTA ACTCGTTAGA TTAATCAAGA TTATTAACTT TAGTTCAAGG GACACCTTAT   
  
  
- GCTGGGCATG AACGGCACAC GATCGATGAC AATGTGGAAC GTAAACGCCA TATAAATATT GAGTTGTATA   
  
  
- CAGACGAGTG ATTTTCATGT ATAATCGAGT GGTTTTGACA CAGAGTTGCA TGATTTTATA AAATCACTTG   
  
  
- ATATGTAATA AAAATCATTC GCTTTATTCT ATGTGTTGTC GTGTAAAATT ACTCAATGTC CCGTAAAAAT   
  
  
- CACTCGGCTT TAGACTACCA AAAGAAACAT CTAGGCCAAT ATATTAACAT AAAATATGTT GGCTTACCTG   
  
  
- CTATATTGTT GACTCAATTT ACTCCTCAGA CTTTAATGCT TGTATTTTAG GTTTTCTTCT ACGGCTACGG   
  
  
- TAACCTTTCT TTCGACTTCC ACTAGCGAAA GATCATCATA ATCCGTCATC ATTAATTTTT GATTTTAGGT   
  
  
- CTTAACTGTG GCTTAACCCC AACACCGTGT ACTAGCTTTA GTCGTAGCTT AGATGTGCTA CTAGACCTAA   
  
  
- GAACCTGTGA GACCGAAATC CGGCAGTTAA AGCTCCTAAC GGGTATTGAA ATTAGGACAA GTTTAGGTAA   
  
  
- GTTAAATTAT AATGTGTTTA GGAAACTAAT TTAGTGGCAT TAACACCAAA CTAATCCCTT CAAAGAACCC   
  
  
- CCTTTCCAAG TTCGAAGACA GAAAGAATTT CGGACCTAAA AACCAGGGCG TACAAGTGAA GTGTAAGGGA   
  
  
- AAAAGAGAGG GTAGCAATGG ACAGACTTAG ACATACATTA TTTTCAGTAT AAAACGCTCC AGAAGAAAAC   
  
  
- TGACAAGAAC ATCAGTGAGA ACTATAGACG TGGTAAAACA AAAAGAGCCG TAAAAGAGGT TGAACGTAAA   
  
  
- AGATTACATT ACACTAGAAA AGACCTGTAA GTAAAACAAT TAGATGAGTG AACCCAGTTC CAAACGACTA   
  
  
- AAACCGACCA CAATTTAGGC AAGGAGAAAC CGTAAACAAG TACAAGGTGT TGACAAACTA TAGACCATAG   
  
  
- AGTCTTTGGG GCGGTAAGAA ACCATAAAGG ACTGAAAGAA CAAGTTCCCA ATAAGGATAA AGGGACTTAA   
  
  
- AAGAAACTAA GAAATCCATA AAGTTCGAAC CCCCCAAAAC AAGAAGTGAC TAAAACAGGA GAAGAAGGGA   
  
  
- ATAACCCACC AAACTAGAGG ATTTAAAGAC ACATAGACTT AGTCAGCCCC ATCTGAAACA CAAAAACCAA   
  
  
- CTAAAAAATA GTTACATAAA AGAAGGATTA CTTCCCGGAG GGCTTGGTTA TGTGTACGAA TTGAAGTTGG   
  
  
- GACAAGGAGA AGGTTCGAAA AACCTCGGAG TTTTCAGAAG CTTACGACTA TCCGTGTATC CCAGGAAGGT   
  
  
- TACGATACCA GAACTACTAC TGGGGCGTGG GAACTTACTG GGTTTAATGT AACATACAGT TAGAAGTATA   
  
  
- GGACTAGCTC TATAATGTGT CTAGGGACTG AAAGGACTAC TAACAGAGTT CAAGTAGTTA CTGTAAGAAT   
  
  
- ACCTTCTCCT AAACCTATTA GGCGGATATA GGAACGTCCT GATATTACGC GAGGTTCGGT GACTCTTCAG   
  
  
- TAACATACTA CGGGAACCTC TCCGAATAGG TCGAAGTAGA CTAGGTAACG TTAGTAGTCA ACCAGTCTCG   
  
  
- TGACTCTCGA GACTACTAGG ACTGTCTTCG TCGCCGTAAC TATTTACCCC ATTATCTATA ATGGGGTGGT   
  
  
- CGGACTTACA CCAACTTTGG TCGGCCGACT AGTTAGTCTC GGTCAACCTG GGAAGAGTTT ACAAACTACG   
  
  
- ACGACGAGTG GGGAACCTCA GATTAACAGT AAGAAAACAG AGATCATTAT TGAAACTAGT AAGATACCTA   
  
  
- CCCCAAGGTC TAAGAGGATA GTCATGTGAA AGACACTTAC TTTGTTCTTT CCTTCTTAGT CAATCATCTA   
  
  
- GTTCCTCGTC CTTCTTAGTA GTTTCTCTAC CACGTATGGA CCTTCTTTCC TCATGATTCG TTTATCGTAG   
  
  
- GTCGTTACTC CTTATACCTC TCTACCTCTC CAAACTACTA CATGACTAGA CGTCCCTTCC TTTACTACTA   
  
  
- TAAAGTGGAA CATGGGCGTT TTGGGGTCAA GTACTTCGTC TACTATTGAG CCTTGGTTTC CCCTTTAGTT   
  
  
- CTCCTAGATT TTCGTTCTCA TTTAGCGCAC AGTTCTTCTT TTCATTGCTT CCTGTTCGCC ATCTAGAATC   
  
  
- CTGAGTTGAA TAAGTCACAC GTCTTCGTCA CCGTTCGAAA CTCGAATTTT CACGTTTACT CGATGAGTCC   
  
  
- GCTTAATCCG TCGTACGAAG CGGTAAACCC CTATCACAAG TTTCCGATCG TGTAATGAAA CGCTTACCCG   
  
  
- AGCTCCGTGC GAATCGACCA TGTCCTAGTC TTAGTATGTT TCTGAAACGA CCCTTTTTTA AAAGTAGGAT   
  
  
- ACTGTAGAAC TCTCCGATGT TCAGTATACA AGTTCGGCAG GGGAAGGTCT CTTGTTGGTA CAAGAACTGT   
  
  
- TTAGTTTGTT ATCATTTCGA CCGACTCTTT CGTTGTTCCC AAGTATAGTA ACTAAAACCG TAGAAAAAAC   
  
  
- CAAAGGTCAC CGGGACGGAA CAAGTCTTAA ACAGTTCTTC TGGGGTCCCA AGAGGGTATG AGTTTTAGTG   
  
  
- CCCTCAGCTA GTAGGGGTCT CACCCAAGTC TGGTCGGGTC TTTCAACTTC TCTGTCCAGC AGCTAATCGT   
  
  
- CCGATAACAC TCTCCAAACC CCACGGGAAA CTCAAAGTAG GGTAACGAGT TTCCACCGTA TGGGATTTCG   
  
  
- GTCTCGTAAA GTTTTAACTT TCGTTACTCG AACAACACCA GTGAACAAAG ATGGTTAGTC CCTTCGAGGG   
  
  
- ACTACTCTGA CAACTACAGT TGTCGGGTTC TCTGGTACAG AACTCCGACT AGTCCCCGAA CTTGGGACTA   
  
  
- GATAAGTAGG TACCCCAATA GTTACCGTGT AAGTTACGTG GGAAGAAGCA TTGAGCCAAA TTTCTCCGTG   
  
  
- AAATGGTGAT GAGAAGAAAC AAGCTACAGA AACTACGGTG GTATCTAGCA CTTTCAGTAC TTTCTGACGA   
  
  
- CTGACTCTTG ATAAACATAC CCTTTCTACG TGAATTGTAT CATCGTACAC TCCCCTTGCT CTCCTAGCTC   
  
  
- TCTGGGCTCT GAATGTTCGT CACCGTTCAG GCCTTCGAAT CTCGACCTAA GTTCGTCCAG GGGGATCTGG   
  
  
- TTCTGTAACA TTTACCTCGT TCTCGATACC AATTCCGTTT AATGTTATTC CTAAAATACC ATCTTGTTCT   
  
  
- ATCCTTAACC TACCAGGTCC CGACCTTTCC TTCCTAAAAC TCACGGTATA GGACAACCTT TGGACGAAT

+     WRE3

| Site Name | Organism | Position | Strand | Matrix score. | sequence | function |
| --- | --- | --- | --- | --- | --- | --- |
| WRE3 | Pisum sativum | 3486 | - | 6 | CCACCT |  |

>HU02G01573.1   
+ +Up\_Stream \_Len000TGAATT GGAAGTTGAT TCTACCTTTG ATTGATTCGA TTAATTCTAG GTTCTTGGTT   
  
  
+ CTAGTTCTCT CTCCTCTTGT TCTTGATTAT TTAGGTTTTG GATGTGATGT TTATTGAATT GAATGTTGCA   
  
  
+ ATTGTTGTTA TAAAGATGAT GTTAGGCTAA ATATCCTTCA ACTTAGGTTG GGATTGACGT CGAATATGTG   
  
  
+ TAGTTGAATC TTGTACTGGC CTAAATCCCT GCTCCCCCTC TGTTTTGCGA TTATGAATTT CATTATGCAC   
  
  
+ ACCACATGCT CGATGAAATG CTCGAAAGAA CATTCATCCT CTTTGTTTGG GATTTAGGGG AACGTTGAAA   
  
  
+ GACGAAGTCT TTACCTAGTG CGTCTTAGTA GACAATTAGG ATTTTAACAT GAAAATAGTT AAAATTCTAA   
  
  
+ TTGGGATTTG AGATTGTGAA TCTTGTAATC CTTCGACTTC TAGTTCTATG AAAATAGGCG ATTATAGTTC   
  
  
+ TAGGATTAGT CTTCGACCCT TGAGAGAGGG AGGAGATGGG TTTAGTTGAT AATCTATTCA TTCATAACGC   
  
  
+ ATGCCTGGGA GATGTTTGGT AGGGCTAGTA ATTGATTCGC TAACATATTT GTGCTGTTAG TTCCAACCTG   
  
  
+ AGTGTTTACT CTTCTGCTGT TTATTCCGTC TTTTAGTCGT AATTGTCTCC TTTAGTTTAA TTTCAAAACC   
  
  
+ CCAATTGTGA TCGCCTAGAT TGAGCAATCT AATTAGTTCT AATAATTGAA ATCAAGTTCC CTGTGGAATA   
  
  
+ CGACCCGTAC TTGCCGTGTG CTAGCTACTG TTACACCTTG CATTTGCGGT ATATTTATAA CTCAACATAT   
  
  
+ GTCTGCTCAC TAAAAGTACA TATTAGCTCA CCAAAACTGT GTCTCAACGT ACTAAAATAT TTTAGTGAAC   
  
  
+ TATACATTAT TTTTAGTAAG CGAAATAAGA TACACAACAG CACATTTTAA TGAGTTACAG GGCATTTTTA   
  
  
+ GTGAGCCGAA ATCTGATGGT TTTCTTTGTA GATCCGGTTA TATAATTGTA TTTTATACAA CCGAATGGAC   
  
  
+ GATATAACAA CTGAGTTAAA TGAGGAGTCT GAAATTACGA ACATAAAATC CAAAAGAAGA TGCCGATGCC   
  
  
+ ATTGGAAAGA AAGCTGAAGG TGATCGCTTT CTAGTAGTAT TAGGCAGTAG TAATTAAAAA CTAAAATCCA   
  
  
+ GAATTGACAC CGAATTGGGG TTGTGGCACA TGATCGAAAT CAGCATCGAA TCTACACGAT GATCTGGATT   
  
  
+ CTTGGACACT CTGGCTTTAG GCCGTCAATT TCGAGGATTG CCCATAACTT TAATCCTGTT CAAATCCATT   
  
  
+ CAATTTAATA TTACACAAAT CCTTTGATTA AATCACCGTA ATTGTGGTTT GATTAGGGAA GTTTCTTGGG   
  
  
+ GGAAAGGTTC AAGCTTCTGT CTTTCTTAAA GCCTGGATTT TTGGTCCCGC ATGTTCACTT CACATTCCCT   
  
  
+ TTTTCTCTCC CATCGTTACC TGTCTGAATC TGTATGTAAT AAAAGTCATA TTTTGCGAGG TCTTCTTTTG   
  
  
+ ACTGTTCTTG TAGTCACTCT TGATATCTGC ACCATTTTGT TTTTCTCGGC ATTTTCTCCA ACTTGCATTT   
  
  
+ TCTAATGTAA TGTGATCTTT TCTGGACATT CATTTTGTTA ATCTACTCAC TTGGGTCAAG GTTTGCTGAT   
  
  
+ TTTGGCTGGT GTTAAATCCG TTCCTCTTTG GCATTTGTTC ATGTTCCACA ACTGTTTGAT ATCTGGTATC   
  
  
+ TCAGAAACCC CGCCATTCTT TGGTATTTCC TGACTTTCTT GTTCAAGGGT TATTCCTATT TCCCTGAATT   
  
  
+ TTCTTTGATT CTTTAGGTAT TTCAAGCTTG GGGGGTTTTG TTCTTCACTG ATTTTGTCCT CTTCTTCCCT   
  
  
+ TATTGGGTGG TTTGATCTCC TAAATTTCTG TGTATCTGAA TCAGTCGGGG TAGACTTTGT GTTTTTGGTT   
  
  
+ GATTTTTTAT CAATGTATTT TCTTCCTAAT GAAGGGCCTC CCGAACCAAT ACACATGCTT AACTTCAACC   
  
  
+ CTGTTCCTCT TCCAAGCTTT TTGGAGCCTC AAAAGTCTTC GAATGCTGAT AGGCACATAG GGTCCTTCCA   
  
  
+ ATGCTATGGT CTTGATGATG ACCCCGCACC CTTGAATGAC CCAAATTACA TTGTATGTCA ATCTTCATAT   
  
  
+ CCTGATCGAG ATATTACACA GATCCCTGAC TTTCCTGATG ATTGTCTCAA GTTCATCAAT GACATTCTTA   
  
  
+ TGGAAGAGGA TTTGGATAAT CCGCCTATAT CCTTGCAGGA CTATAATGCG CTCCAAGCCA CTGAGAAGTC   
  
  
+ ATTGTATGAT GCCCTTGGAG AGGCTTATCC AGCTTCATCT GATCCATTGC AATCATCAGT TGGTCAGAGC   
  
  
+ ACTGAGAGCT CTGATGATCC TGACAGAAGC AGCGGCATTG ATAAATGGGG TAATAGATAT TACCCCACCA   
  
  
+ GCCTGAATGT GGTTGAAACC AGCCGGCTGA TCAATCAGAG CCAGTTGGAC CCTTCTCAAA TGTTTGATGC   
  
  
+ TGCTGCTCAC CCCTTGGAGT CTAATTGTCA TTCTTTTGTC TCTAGTAATA ACTTTGATCA TTCTATGGAT   
  
  
+ GGGGTTCCAG ATTCTCCTAT CAGTACACTT TCTGTGAATG AAACAAGAAA GGAAGAATCA GTTAGTAGAT   
  
  
+ CAAGGAGCAG GAAGAATCAT CAAAGAGATG GTGCATACCT GGAAGAAAGG AGTACTAAGC AAATAGCATC   
  
  
+ CAGCAATGAG GAATATGGAG AGATGGAGAG GTTTGATGAT GTACTGATCT GCAGGGAAGG AAATGATGAT   
  
  
+ ATTTCACCTT GTACCCGCAA AACCCCAGTT CATGAAGCAG ATGATAACTC GGAACCAAAG GGGAAATCAA   
  
  
+ GAGGATCTAA AAGCAAGAGT AAATCGCGTG TCAAGAAGAA AAGTAACGAA GGACAAGCGG TAGATCTTAG   
  
  
+ GACTCAACTT ATTCAGTGTG CAGAAGCAGT GGCAAGCTTT GAGCTTAAAA GTGCAAATGA GCTACTCAGG   
  
  
+ CGAATTAGGC AGCATGCTTC GCCATTTGGG GATAGTGTTC AAAGGCTAGC ACATTACTTT GCGAATGGGC   
  
  
+ TCGAGGCACG CTTAGCTGGT ACAGGATCAG AATCATACAA AGACTTTGCT GGGAAAAAAT TTTCATCCTA   
  
  
+ TGACATCTTG AGAGGCTACA AGTCATATGT TCAAGCCGTC CCCTTCCAGA GAACAACCAT GTTCTTGACA   
  
  
+ AATCAAACAA TAGTAAAGCT GGCTGAGAAA GCAACAAGGG TTCATATCAT TGATTTTGGC ATCTTTTTTG   
  
  
+ GTTTCCAGTG GCCCTGCCTT GTTCAGAATT TGTCAAGAAG ACCCCAGGGT TCTCCCATAC TCAAAATCAC   
  
  
+ GGGAGTCGAT CATCCCCAGA GTGGGTTCAG ACCAGCCCAG AAAGTTGAAG AGACAGGTCG TCGATTAGCA   
  
  
+ GGCTATTGTG AGAGGTTTGG GGTGCCCTTT GAGTTTCATC CCATTGCTCA AAGGTGGCAT ACCCTAAAGC   
  
  
+ CAGAGCATTT CAAAATTGAA AGCAATGAGC TTGTTGTGGT CACTTGTTTC TACCAATCAG GGAAGCTCCC   
  
  
+ TGATGAGACT GTTGATGTCA ACAGCCCAAG AGACCATGTC TTGAGGCTGA TCAGGGGCTT GAACCCTGAT   
  
  
+ CTATTCATCC ATGGGGTTAT CAATGGCACA TTCAATGCAC CCTTCTTCGT AACTCGGTTT AAAGAGGCAC   
  
  
+ TTTACCACTA CTCTTCTTTG TTCGATGTCT TTGATGCCAC CATAGATCGT GAAAGTCATG AAAGACTGCT   
  
  
+ GACTGAGAAC TATTTGTATG GGAAAGATGC ACTTAACATA GTAGCATGTG AGGGGAACGA GAGGATCGAG   
  
  
+ AGACCCGAGA CTTACAAGCA GTGGCAAGTC CGGAAGCTTA GAGCTGGATT CAAGCAGGTC CCCCTAGACC   
  
  
+ AAGACATTGT AAATGGAGCA AGAGCTATGG TTAAGGCAAA TTACAATAAG GATTTTATGG TAGAACAAGA   
  
  
+ TAGGAATTGG ATGGTCCAGG GCTGGAAAGG AAGGATTTTG AGTGCCATAT CCTGTTGGAA ACCTGCTTA  

- +Up\_Stream \_Len000ACTTAA CCTTCAACTA AGATGGAAAC TAACTAAGCT AATTAAGATC CAAGAACCAA   
  
  
- GATCAAGAGA GAGGAGAACA AGAACTAATA AATCCAAAAC CTACACTACA AATAACTTAA CTTACAACGT   
  
  
- TAACAACAAT ATTTCTACTA CAATCCGATT TATAGGAAGT TGAATCCAAC CCTAACTGCA GCTTATACAC   
  
  
- ATCAACTTAG AACATGACCG GATTTAGGGA CGAGGGGGAG ACAAAACGCT AATACTTAAA GTAATACGTG   
  
  
- TGGTGTACGA GCTACTTTAC GAGCTTTCTT GTAAGTAGGA GAAACAAACC CTAAATCCCC TTGCAACTTT   
  
  
- CTGCTTCAGA AATGGATCAC GCAGAATCAT CTGTTAATCC TAAAATTGTA CTTTTATCAA TTTTAAGATT   
  
  
- AACCCTAAAC TCTAACACTT AGAACATTAG GAAGCTGAAG ATCAAGATAC TTTTATCCGC TAATATCAAG   
  
  
- ATCCTAATCA GAAGCTGGGA ACTCTCTCCC TCCTCTACCC AAATCAACTA TTAGATAAGT AAGTATTGCG   
  
  
- TACGGACCCT CTACAAACCA TCCCGATCAT TAACTAAGCG ATTGTATAAA CACGACAATC AAGGTTGGAC   
  
  
- TCACAAATGA GAAGACGACA AATAAGGCAG AAAATCAGCA TTAACAGAGG AAATCAAATT AAAGTTTTGG   
  
  
- GGTTAACACT AGCGGATCTA ACTCGTTAGA TTAATCAAGA TTATTAACTT TAGTTCAAGG GACACCTTAT   
  
  
- GCTGGGCATG AACGGCACAC GATCGATGAC AATGTGGAAC GTAAACGCCA TATAAATATT GAGTTGTATA   
  
  
- CAGACGAGTG ATTTTCATGT ATAATCGAGT GGTTTTGACA CAGAGTTGCA TGATTTTATA AAATCACTTG   
  
  
- ATATGTAATA AAAATCATTC GCTTTATTCT ATGTGTTGTC GTGTAAAATT ACTCAATGTC CCGTAAAAAT   
  
  
- CACTCGGCTT TAGACTACCA AAAGAAACAT CTAGGCCAAT ATATTAACAT AAAATATGTT GGCTTACCTG   
  
  
- CTATATTGTT GACTCAATTT ACTCCTCAGA CTTTAATGCT TGTATTTTAG GTTTTCTTCT ACGGCTACGG   
  
  
- TAACCTTTCT TTCGACTTCC ACTAGCGAAA GATCATCATA ATCCGTCATC ATTAATTTTT GATTTTAGGT   
  
  
- CTTAACTGTG GCTTAACCCC AACACCGTGT ACTAGCTTTA GTCGTAGCTT AGATGTGCTA CTAGACCTAA   
  
  
- GAACCTGTGA GACCGAAATC CGGCAGTTAA AGCTCCTAAC GGGTATTGAA ATTAGGACAA GTTTAGGTAA   
  
  
- GTTAAATTAT AATGTGTTTA GGAAACTAAT TTAGTGGCAT TAACACCAAA CTAATCCCTT CAAAGAACCC   
  
  
- CCTTTCCAAG TTCGAAGACA GAAAGAATTT CGGACCTAAA AACCAGGGCG TACAAGTGAA GTGTAAGGGA   
  
  
- AAAAGAGAGG GTAGCAATGG ACAGACTTAG ACATACATTA TTTTCAGTAT AAAACGCTCC AGAAGAAAAC   
  
  
- TGACAAGAAC ATCAGTGAGA ACTATAGACG TGGTAAAACA AAAAGAGCCG TAAAAGAGGT TGAACGTAAA   
  
  
- AGATTACATT ACACTAGAAA AGACCTGTAA GTAAAACAAT TAGATGAGTG AACCCAGTTC CAAACGACTA   
  
  
- AAACCGACCA CAATTTAGGC AAGGAGAAAC CGTAAACAAG TACAAGGTGT TGACAAACTA TAGACCATAG   
  
  
- AGTCTTTGGG GCGGTAAGAA ACCATAAAGG ACTGAAAGAA CAAGTTCCCA ATAAGGATAA AGGGACTTAA   
  
  
- AAGAAACTAA GAAATCCATA AAGTTCGAAC CCCCCAAAAC AAGAAGTGAC TAAAACAGGA GAAGAAGGGA   
  
  
- ATAACCCACC AAACTAGAGG ATTTAAAGAC ACATAGACTT AGTCAGCCCC ATCTGAAACA CAAAAACCAA   
  
  
- CTAAAAAATA GTTACATAAA AGAAGGATTA CTTCCCGGAG GGCTTGGTTA TGTGTACGAA TTGAAGTTGG   
  
  
- GACAAGGAGA AGGTTCGAAA AACCTCGGAG TTTTCAGAAG CTTACGACTA TCCGTGTATC CCAGGAAGGT   
  
  
- TACGATACCA GAACTACTAC TGGGGCGTGG GAACTTACTG GGTTTAATGT AACATACAGT TAGAAGTATA   
  
  
- GGACTAGCTC TATAATGTGT CTAGGGACTG AAAGGACTAC TAACAGAGTT CAAGTAGTTA CTGTAAGAAT   
  
  
- ACCTTCTCCT AAACCTATTA GGCGGATATA GGAACGTCCT GATATTACGC GAGGTTCGGT GACTCTTCAG   
  
  
- TAACATACTA CGGGAACCTC TCCGAATAGG TCGAAGTAGA CTAGGTAACG TTAGTAGTCA ACCAGTCTCG   
  
  
- TGACTCTCGA GACTACTAGG ACTGTCTTCG TCGCCGTAAC TATTTACCCC ATTATCTATA ATGGGGTGGT   
  
  
- CGGACTTACA CCAACTTTGG TCGGCCGACT AGTTAGTCTC GGTCAACCTG GGAAGAGTTT ACAAACTACG   
  
  
- ACGACGAGTG GGGAACCTCA GATTAACAGT AAGAAAACAG AGATCATTAT TGAAACTAGT AAGATACCTA   
  
  
- CCCCAAGGTC TAAGAGGATA GTCATGTGAA AGACACTTAC TTTGTTCTTT CCTTCTTAGT CAATCATCTA   
  
  
- GTTCCTCGTC CTTCTTAGTA GTTTCTCTAC CACGTATGGA CCTTCTTTCC TCATGATTCG TTTATCGTAG   
  
  
- GTCGTTACTC CTTATACCTC TCTACCTCTC CAAACTACTA CATGACTAGA CGTCCCTTCC TTTACTACTA   
  
  
- TAAAGTGGAA CATGGGCGTT TTGGGGTCAA GTACTTCGTC TACTATTGAG CCTTGGTTTC CCCTTTAGTT   
  
  
- CTCCTAGATT TTCGTTCTCA TTTAGCGCAC AGTTCTTCTT TTCATTGCTT CCTGTTCGCC ATCTAGAATC   
  
  
- CTGAGTTGAA TAAGTCACAC GTCTTCGTCA CCGTTCGAAA CTCGAATTTT CACGTTTACT CGATGAGTCC   
  
  
- GCTTAATCCG TCGTACGAAG CGGTAAACCC CTATCACAAG TTTCCGATCG TGTAATGAAA CGCTTACCCG   
  
  
- AGCTCCGTGC GAATCGACCA TGTCCTAGTC TTAGTATGTT TCTGAAACGA CCCTTTTTTA AAAGTAGGAT   
  
  
- ACTGTAGAAC TCTCCGATGT TCAGTATACA AGTTCGGCAG GGGAAGGTCT CTTGTTGGTA CAAGAACTGT   
  
  
- TTAGTTTGTT ATCATTTCGA CCGACTCTTT CGTTGTTCCC AAGTATAGTA ACTAAAACCG TAGAAAAAAC   
  
  
- CAAAGGTCAC CGGGACGGAA CAAGTCTTAA ACAGTTCTTC TGGGGTCCCA AGAGGGTATG AGTTTTAGTG   
  
  
- CCCTCAGCTA GTAGGGGTCT CACCCAAGTC TGGTCGGGTC TTTCAACTTC TCTGTCCAGC AGCTAATCGT   
  
  
- CCGATAACAC TCTCCAAACC CCACGGGAAA CTCAAAGTAG GGTAACGAGT TTCCACCGTA TGGGATTTCG   
  
  
- GTCTCGTAAA GTTTTAACTT TCGTTACTCG AACAACACCA GTGAACAAAG ATGGTTAGTC CCTTCGAGGG   
  
  
- ACTACTCTGA CAACTACAGT TGTCGGGTTC TCTGGTACAG AACTCCGACT AGTCCCCGAA CTTGGGACTA   
  
  
- GATAAGTAGG TACCCCAATA GTTACCGTGT AAGTTACGTG GGAAGAAGCA TTGAGCCAAA TTTCTCCGTG   
  
  
- AAATGGTGAT GAGAAGAAAC AAGCTACAGA AACTACGGTG GTATCTAGCA CTTTCAGTAC TTTCTGACGA   
  
  
- CTGACTCTTG ATAAACATAC CCTTTCTACG TGAATTGTAT CATCGTACAC TCCCCTTGCT CTCCTAGCTC   
  
  
- TCTGGGCTCT GAATGTTCGT CACCGTTCAG GCCTTCGAAT CTCGACCTAA GTTCGTCCAG GGGGATCTGG   
  
  
- TTCTGTAACA TTTACCTCGT TCTCGATACC AATTCCGTTT AATGTTATTC CTAAAATACC ATCTTGTTCT   
  
  
- ATCCTTAACC TACCAGGTCC CGACCTTTCC TTCCTAAAAC TCACGGTATA GGACAACCTT TGGACGAAT

+     WUN-motif

| Site Name | Organism | Position | Strand | Matrix score. | sequence | function |
| --- | --- | --- | --- | --- | --- | --- |
| WUN-motif | Nicotiana glutinosa | 1508 | - | 9 | TTATTACAT |  |

>HU02G01573.1   
+ +Up\_Stream \_Len000TGAATT GGAAGTTGAT TCTACCTTTG ATTGATTCGA TTAATTCTAG GTTCTTGGTT   
  
  
+ CTAGTTCTCT CTCCTCTTGT TCTTGATTAT TTAGGTTTTG GATGTGATGT TTATTGAATT GAATGTTGCA   
  
  
+ ATTGTTGTTA TAAAGATGAT GTTAGGCTAA ATATCCTTCA ACTTAGGTTG GGATTGACGT CGAATATGTG   
  
  
+ TAGTTGAATC TTGTACTGGC CTAAATCCCT GCTCCCCCTC TGTTTTGCGA TTATGAATTT CATTATGCAC   
  
  
+ ACCACATGCT CGATGAAATG CTCGAAAGAA CATTCATCCT CTTTGTTTGG GATTTAGGGG AACGTTGAAA   
  
  
+ GACGAAGTCT TTACCTAGTG CGTCTTAGTA GACAATTAGG ATTTTAACAT GAAAATAGTT AAAATTCTAA   
  
  
+ TTGGGATTTG AGATTGTGAA TCTTGTAATC CTTCGACTTC TAGTTCTATG AAAATAGGCG ATTATAGTTC   
  
  
+ TAGGATTAGT CTTCGACCCT TGAGAGAGGG AGGAGATGGG TTTAGTTGAT AATCTATTCA TTCATAACGC   
  
  
+ ATGCCTGGGA GATGTTTGGT AGGGCTAGTA ATTGATTCGC TAACATATTT GTGCTGTTAG TTCCAACCTG   
  
  
+ AGTGTTTACT CTTCTGCTGT TTATTCCGTC TTTTAGTCGT AATTGTCTCC TTTAGTTTAA TTTCAAAACC   
  
  
+ CCAATTGTGA TCGCCTAGAT TGAGCAATCT AATTAGTTCT AATAATTGAA ATCAAGTTCC CTGTGGAATA   
  
  
+ CGACCCGTAC TTGCCGTGTG CTAGCTACTG TTACACCTTG CATTTGCGGT ATATTTATAA CTCAACATAT   
  
  
+ GTCTGCTCAC TAAAAGTACA TATTAGCTCA CCAAAACTGT GTCTCAACGT ACTAAAATAT TTTAGTGAAC   
  
  
+ TATACATTAT TTTTAGTAAG CGAAATAAGA TACACAACAG CACATTTTAA TGAGTTACAG GGCATTTTTA   
  
  
+ GTGAGCCGAA ATCTGATGGT TTTCTTTGTA GATCCGGTTA TATAATTGTA TTTTATACAA CCGAATGGAC   
  
  
+ GATATAACAA CTGAGTTAAA TGAGGAGTCT GAAATTACGA ACATAAAATC CAAAAGAAGA TGCCGATGCC   
  
  
+ ATTGGAAAGA AAGCTGAAGG TGATCGCTTT CTAGTAGTAT TAGGCAGTAG TAATTAAAAA CTAAAATCCA   
  
  
+ GAATTGACAC CGAATTGGGG TTGTGGCACA TGATCGAAAT CAGCATCGAA TCTACACGAT GATCTGGATT   
  
  
+ CTTGGACACT CTGGCTTTAG GCCGTCAATT TCGAGGATTG CCCATAACTT TAATCCTGTT CAAATCCATT   
  
  
+ CAATTTAATA TTACACAAAT CCTTTGATTA AATCACCGTA ATTGTGGTTT GATTAGGGAA GTTTCTTGGG   
  
  
+ GGAAAGGTTC AAGCTTCTGT CTTTCTTAAA GCCTGGATTT TTGGTCCCGC ATGTTCACTT CACATTCCCT   
  
  
+ TTTTCTCTCC CATCGTTACC TGTCTGAATC TGTATGTAAT AAAAGTCATA TTTTGCGAGG TCTTCTTTTG   
  
  
+ ACTGTTCTTG TAGTCACTCT TGATATCTGC ACCATTTTGT TTTTCTCGGC ATTTTCTCCA ACTTGCATTT   
  
  
+ TCTAATGTAA TGTGATCTTT TCTGGACATT CATTTTGTTA ATCTACTCAC TTGGGTCAAG GTTTGCTGAT   
  
  
+ TTTGGCTGGT GTTAAATCCG TTCCTCTTTG GCATTTGTTC ATGTTCCACA ACTGTTTGAT ATCTGGTATC   
  
  
+ TCAGAAACCC CGCCATTCTT TGGTATTTCC TGACTTTCTT GTTCAAGGGT TATTCCTATT TCCCTGAATT   
  
  
+ TTCTTTGATT CTTTAGGTAT TTCAAGCTTG GGGGGTTTTG TTCTTCACTG ATTTTGTCCT CTTCTTCCCT   
  
  
+ TATTGGGTGG TTTGATCTCC TAAATTTCTG TGTATCTGAA TCAGTCGGGG TAGACTTTGT GTTTTTGGTT   
  
  
+ GATTTTTTAT CAATGTATTT TCTTCCTAAT GAAGGGCCTC CCGAACCAAT ACACATGCTT AACTTCAACC   
  
  
+ CTGTTCCTCT TCCAAGCTTT TTGGAGCCTC AAAAGTCTTC GAATGCTGAT AGGCACATAG GGTCCTTCCA   
  
  
+ ATGCTATGGT CTTGATGATG ACCCCGCACC CTTGAATGAC CCAAATTACA TTGTATGTCA ATCTTCATAT   
  
  
+ CCTGATCGAG ATATTACACA GATCCCTGAC TTTCCTGATG ATTGTCTCAA GTTCATCAAT GACATTCTTA   
  
  
+ TGGAAGAGGA TTTGGATAAT CCGCCTATAT CCTTGCAGGA CTATAATGCG CTCCAAGCCA CTGAGAAGTC   
  
  
+ ATTGTATGAT GCCCTTGGAG AGGCTTATCC AGCTTCATCT GATCCATTGC AATCATCAGT TGGTCAGAGC   
  
  
+ ACTGAGAGCT CTGATGATCC TGACAGAAGC AGCGGCATTG ATAAATGGGG TAATAGATAT TACCCCACCA   
  
  
+ GCCTGAATGT GGTTGAAACC AGCCGGCTGA TCAATCAGAG CCAGTTGGAC CCTTCTCAAA TGTTTGATGC   
  
  
+ TGCTGCTCAC CCCTTGGAGT CTAATTGTCA TTCTTTTGTC TCTAGTAATA ACTTTGATCA TTCTATGGAT   
  
  
+ GGGGTTCCAG ATTCTCCTAT CAGTACACTT TCTGTGAATG AAACAAGAAA GGAAGAATCA GTTAGTAGAT   
  
  
+ CAAGGAGCAG GAAGAATCAT CAAAGAGATG GTGCATACCT GGAAGAAAGG AGTACTAAGC AAATAGCATC   
  
  
+ CAGCAATGAG GAATATGGAG AGATGGAGAG GTTTGATGAT GTACTGATCT GCAGGGAAGG AAATGATGAT   
  
  
+ ATTTCACCTT GTACCCGCAA AACCCCAGTT CATGAAGCAG ATGATAACTC GGAACCAAAG GGGAAATCAA   
  
  
+ GAGGATCTAA AAGCAAGAGT AAATCGCGTG TCAAGAAGAA AAGTAACGAA GGACAAGCGG TAGATCTTAG   
  
  
+ GACTCAACTT ATTCAGTGTG CAGAAGCAGT GGCAAGCTTT GAGCTTAAAA GTGCAAATGA GCTACTCAGG   
  
  
+ CGAATTAGGC AGCATGCTTC GCCATTTGGG GATAGTGTTC AAAGGCTAGC ACATTACTTT GCGAATGGGC   
  
  
+ TCGAGGCACG CTTAGCTGGT ACAGGATCAG AATCATACAA AGACTTTGCT GGGAAAAAAT TTTCATCCTA   
  
  
+ TGACATCTTG AGAGGCTACA AGTCATATGT TCAAGCCGTC CCCTTCCAGA GAACAACCAT GTTCTTGACA   
  
  
+ AATCAAACAA TAGTAAAGCT GGCTGAGAAA GCAACAAGGG TTCATATCAT TGATTTTGGC ATCTTTTTTG   
  
  
+ GTTTCCAGTG GCCCTGCCTT GTTCAGAATT TGTCAAGAAG ACCCCAGGGT TCTCCCATAC TCAAAATCAC   
  
  
+ GGGAGTCGAT CATCCCCAGA GTGGGTTCAG ACCAGCCCAG AAAGTTGAAG AGACAGGTCG TCGATTAGCA   
  
  
+ GGCTATTGTG AGAGGTTTGG GGTGCCCTTT GAGTTTCATC CCATTGCTCA AAGGTGGCAT ACCCTAAAGC   
  
  
+ CAGAGCATTT CAAAATTGAA AGCAATGAGC TTGTTGTGGT CACTTGTTTC TACCAATCAG GGAAGCTCCC   
  
  
+ TGATGAGACT GTTGATGTCA ACAGCCCAAG AGACCATGTC TTGAGGCTGA TCAGGGGCTT GAACCCTGAT   
  
  
+ CTATTCATCC ATGGGGTTAT CAATGGCACA TTCAATGCAC CCTTCTTCGT AACTCGGTTT AAAGAGGCAC   
  
  
+ TTTACCACTA CTCTTCTTTG TTCGATGTCT TTGATGCCAC CATAGATCGT GAAAGTCATG AAAGACTGCT   
  
  
+ GACTGAGAAC TATTTGTATG GGAAAGATGC ACTTAACATA GTAGCATGTG AGGGGAACGA GAGGATCGAG   
  
  
+ AGACCCGAGA CTTACAAGCA GTGGCAAGTC CGGAAGCTTA GAGCTGGATT CAAGCAGGTC CCCCTAGACC   
  
  
+ AAGACATTGT AAATGGAGCA AGAGCTATGG TTAAGGCAAA TTACAATAAG GATTTTATGG TAGAACAAGA   
  
  
+ TAGGAATTGG ATGGTCCAGG GCTGGAAAGG AAGGATTTTG AGTGCCATAT CCTGTTGGAA ACCTGCTTA  

- +Up\_Stream \_Len000ACTTAA CCTTCAACTA AGATGGAAAC TAACTAAGCT AATTAAGATC CAAGAACCAA   
  
  
- GATCAAGAGA GAGGAGAACA AGAACTAATA AATCCAAAAC CTACACTACA AATAACTTAA CTTACAACGT   
  
  
- TAACAACAAT ATTTCTACTA CAATCCGATT TATAGGAAGT TGAATCCAAC CCTAACTGCA GCTTATACAC   
  
  
- ATCAACTTAG AACATGACCG GATTTAGGGA CGAGGGGGAG ACAAAACGCT AATACTTAAA GTAATACGTG   
  
  
- TGGTGTACGA GCTACTTTAC GAGCTTTCTT GTAAGTAGGA GAAACAAACC CTAAATCCCC TTGCAACTTT   
  
  
- CTGCTTCAGA AATGGATCAC GCAGAATCAT CTGTTAATCC TAAAATTGTA CTTTTATCAA TTTTAAGATT   
  
  
- AACCCTAAAC TCTAACACTT AGAACATTAG GAAGCTGAAG ATCAAGATAC TTTTATCCGC TAATATCAAG   
  
  
- ATCCTAATCA GAAGCTGGGA ACTCTCTCCC TCCTCTACCC AAATCAACTA TTAGATAAGT AAGTATTGCG   
  
  
- TACGGACCCT CTACAAACCA TCCCGATCAT TAACTAAGCG ATTGTATAAA CACGACAATC AAGGTTGGAC   
  
  
- TCACAAATGA GAAGACGACA AATAAGGCAG AAAATCAGCA TTAACAGAGG AAATCAAATT AAAGTTTTGG   
  
  
- GGTTAACACT AGCGGATCTA ACTCGTTAGA TTAATCAAGA TTATTAACTT TAGTTCAAGG GACACCTTAT   
  
  
- GCTGGGCATG AACGGCACAC GATCGATGAC AATGTGGAAC GTAAACGCCA TATAAATATT GAGTTGTATA   
  
  
- CAGACGAGTG ATTTTCATGT ATAATCGAGT GGTTTTGACA CAGAGTTGCA TGATTTTATA AAATCACTTG   
  
  
- ATATGTAATA AAAATCATTC GCTTTATTCT ATGTGTTGTC GTGTAAAATT ACTCAATGTC CCGTAAAAAT   
  
  
- CACTCGGCTT TAGACTACCA AAAGAAACAT CTAGGCCAAT ATATTAACAT AAAATATGTT GGCTTACCTG   
  
  
- CTATATTGTT GACTCAATTT ACTCCTCAGA CTTTAATGCT TGTATTTTAG GTTTTCTTCT ACGGCTACGG   
  
  
- TAACCTTTCT TTCGACTTCC ACTAGCGAAA GATCATCATA ATCCGTCATC ATTAATTTTT GATTTTAGGT   
  
  
- CTTAACTGTG GCTTAACCCC AACACCGTGT ACTAGCTTTA GTCGTAGCTT AGATGTGCTA CTAGACCTAA   
  
  
- GAACCTGTGA GACCGAAATC CGGCAGTTAA AGCTCCTAAC GGGTATTGAA ATTAGGACAA GTTTAGGTAA   
  
  
- GTTAAATTAT AATGTGTTTA GGAAACTAAT TTAGTGGCAT TAACACCAAA CTAATCCCTT CAAAGAACCC   
  
  
- CCTTTCCAAG TTCGAAGACA GAAAGAATTT CGGACCTAAA AACCAGGGCG TACAAGTGAA GTGTAAGGGA
[truncated: 24,214 more chars]
