# Supplementary material for: Genome-Wide Identification and Expression Pattern of the GRAS Gene Family in Pitaya (Selenicereus undatus L.)
Source: Biology (Basel). 2022 Dec 21;12(1):11. doi: 10.3390/biology12010011 (PMC9854919; doi:10.3390/biology12010011)
Supplement: Supplementary file 1 [file biology-12-00011-s001.zip › Supplementary file S5/HU02G03005.1_plantcare.html]

Content-Type: text/html; charset=ISO-8859-1


PlantCARE


Webmaster Firefox specific output  
To save the result:
click on the frame with the right mouse button and save the source code as a text file with extension .html  
REFERENCE:PlantCARE: a database of plant cis-acting regulatory elements and a portal to tools for in silico analysis of promoter sequences.  
Lescot, M., Déhais, P., Moreau, Y., De Moor, B., Rouzé ,P.,and Rombauts, S.  
Nucleic Acids Res., Database issue(2002), 30(1):325-327.   


---

>HU02G03005.1   
+ +Up\_Stream \_Len000AAACGG TGCCGTTGAA TTTGCAGGCG ACGAGGTCGG GTTTAAGGCG GATGTCGTAG   
  
  
+ CGCTTTGGAG AAGCAAATTT GGGAAGACGA GGCTTATTCT TGAACTCTTC GTACGCCATT GTTGTTGGTT   
  
  
+ CCAACCTTTC TCTCTCCTCG TGAATTTGAG TTTGGGCAGT AGACTATCCC GGAATAGAGG ACTGATGCGT   
  
  
+ TATATATATA GAAATTGAGA GAGAGAGAGA GCGTGTGTGA AGGATGTTGA AAATGATGGG CGTAGGGAGT   
  
  
+ CTCTCTCTCA CTGTCTGTTA GGAGTACGTG TCGAGAAAGG AAATAGGATT CCCAAGCAGT CTAATTTGGG   
  
  
+ CCGGTCTATA GGCCAGAATG GGCCTATGAT CAGCCCAGAT ATGACCTTGT TTAACCAACT GTATTTGAAT   
  
  
+ TGAAGAAAGG GATATCGCAA TGTCTTGAAC CACATCAAAT AATAAATGTG CCTTTGGTTA GACCATATGA   
  
  
+ AAAATCTTTG AATCAAATCG GTTGGACCTG AACTGCCATT ATTGATATCT TGTTTTTGGT AGAAGCAAAT   
  
  
+ GGTGTCACTT CGTTTTCTAG ATAAAATTAA CTTGAGTTTT TTTGCCACTA TAGATAGTAT TGCAAATATA   
  
  
+ AAAAGTGTGC CACGTGAAAT CAATATTAGG AGGGTGTTTT ATAACAATAG CTGTTCGGGA GAGTTTTAGT   
  
  
+ TATTTTTATT AAATCAAATA GTTAAGAAGT GTTTGGTAAA TAGTTGTATT TGAAAAAGTT ATTCCCATTA   
  
  
+ GCCTTTTAGT AAGAACTTCT TGTGAAAATG ATAATTGTCT AGAAGTTTGA AAAATTCACA CCACATGACA   
  
  
+ TTTAAAAATA ATTTTGTAGA GAAGGGCATA AATGAAAGTT TGCCACCATC TTTTTTAGAA ACATGTAATT   
  
  
+ TACCAAACAC TTTTTCTAAA AACAGTTAAT TCAAATAACT AACAACTAAC AGCTAATATA AATAACTAAC   
  
  
+ AGCTATTTGC TAAACAGGGC CTAAGTAGTT TGAAATTGTT GTTTTCTCTT CAGCTTCACC ATGGCCGATG   
  
  
+ ATGTAAACAA CTATATTCTG AATTTCATTT TTACTATGAT TTGAAAATTT CAAATTTAAA TAAGATTGGA   
  
  
+ TAAAAAAAAG ACCAAAAATC ATAAGTAATA TTAGTCAAGT TCTATTCCAA CCATATACAT AACTGCTTTT   
  
  
+ TCTTTTGGGG GTTTTGAAAC ATGTGTCCTT AGAATAATTG AAACTCGATA TTATTCTCTA AATCTAAGTT   
  
  
+ TACTTTATCA TATTGATTAT TAATTACCTC CTCAACTCCT CCTATACTCT TGTATGCAGA AATAAAAATG   
  
  
+ TTTCCATACC TTATCAGTCG CGATCATAAT AATTAATTTC TTAATTCGGA TTGTATTTAA TTGCGTATCA   
  
  
+ AATGTGAAAT CGTACCAAAT TAGGAAGCAA AACAAACCGA TAACGAAAAA GGTAAGAAAA CTTAGCAAGG   
  
  
+ AAATGAAATC GAAGACCTGT CAACAATATA GCTTCCCTCA CATCCTTCAA CTTAGGGACC ATCCTATACT   
  
  
+ TTCATGTAAT ACAACTAGAG CCACTAGTCA ATAATAGCCT CCTAGTAGTA GCCATAAGCC TTGGTGAGAG   
  
  
+ AATGTTCCGC AAGACTAGTT AACTTGAAAT TCCATTTTGT CGCAGAACTA TTGGAGTGAG AAAGAGAATC   
  
  
+ AAATCATACC ATATCAAATT TGGACCATTT AAGTTCACAA CACACACACA TACACACAAA AGGACGATGT   
  
  
+ ACGGCCCCAC CCCCCACTCT CCTTTCGCCA TCCCCCTACA CCTCTGCCTA CTCTCTCTCT CTCTCTCTCT   
  
  
+ CTCTGGATCA GTCTGTGTAA AAGCTTCCTC TCCAGTACAG CATTTCCTTG TCTGTTCTTG GAGTTTAGTC   
  
  
+ AATTCCTCCG CCCCAAAAAA ACCCACTTTC TCTCTCATCA TGTTCTTCCA GTTCCACCCT TATACGCCAT   
  
  
+ TCATCATCAT CTACATCACC CACATCTTTT TCTCCACAGT TTTCCCCCCT CATTATGCTC TCCGCCTCCA   
  
  
+ CCCCCGCCTT CCCTCTCGCC GTCGCCGGCG ATGACGGGGA TACCACTGAT GCATGCGACG GCACCGCCAC   
  
  
+ TGCCCTCCAC CTCCTCGGGC TGCTGTTGAA GTGCGCGGAA TTCATCTCCA CCGGAGACCT CGCCGGCGCC   
  
  
+ GGAGATATCT TGCCGGAGAT ATTTGAGTTG TCTACACCGT TTGGCTCCCC CGCCGCCCGG GTCGCCGCCT   
  
  
+ TCTTCGGCCA CGCCCTCCAC GCCCGCCTCC TCTCCGCCTC CCTCCGCACA ACTCCGATCG AGAAGCTCAA   
  
  
+ AACCCTGACC CTGGTTTCCC AAATGCGGAA ATTTCACTCC GCCTTGCAAG TATACAACTC CATCACTCCC   
  
  
+ TTCGTGAAAT TCTCTCACTT CACGGCGAAT CAAGCCATCT ACGAGGCGCT GGACGGCGAG GATCGTGTCC   
  
  
+ ACGTCGTCGA CCTTGACATC ATGCAGGGCC TTCAATGGCC GGGATTGTTC CACATCTTAG CCTCTCGACC   
  
  
+ CAGAAAGCCC CTCTCGGTTC GGGTCACCGG GTTCGGGCCA TCCTCCGAGT TGCTCTCCCA AACGGGTAAG   
  
  
+ CGACTCGCTG AGTTCGCCGC TTCACTCGGC CTGCCCTTCG AGTACAACCC GGTGGAGGGC AAAATTGGGA   
  
  
+ ACTTAGTCGA CCTGGGTCGG GTCGGGTCGC TCCCGAATGA AGTGACGGTG GTGCACTGGA TGCACCATAG   
  
  
+ TCTATACGAC ATAACCGGGT CGGATCTTGG GACTTTGAGG GTATTGAGTG CGGTGAGGCC TAGGCTTGTG   
  
  
+ ACTATGGTTG AGCAGGATAT GGACCAAACG GGGTCGTTTT TGGGGAGGTT TGTGGAGGCA TTGCATTATT   
  
  
+ ACTCAGCCTT GTTTGATGCC TTGGGAGAGG GGTTAGATAG GGATAACCTA CAAAGGCATC AAGTGGAGCA   
  
  
+ ACAGCTATTT GGGTGTGAGA TTAGGAACAT CCTGGCCGTT GGTGGGCCCA AGAGGAGGCT CACTGGCGGC   
  
  
+ GATCGGGTCA GAAGGTGGGG CGACGAACTG ACAAGGGTCG GGTTCGAACC AGTTTCGTTG GCGGGTAGCC   
  
  
+ CGGCAACCCA AGCTAGTTTG TTGCTTGGGA TGTTCCCTTG GAAAGGGTAT ACTTTGATGG AGGAAAATGG   
  
  
+ GTGTTTGAGA TTAGGGTGGA AAGATTTGCC CTTGTTAACT GCCTCAGCTT GGCAACCTTG TGAATTTAAC   
  
  
+ AATCCTAGTG CTGGCATTTA   

- +Up\_Stream \_Len000TTTGCC ACGGCAACTT AAACGTCCGC TGCTCCAGCC CAAATTCCGC CTACAGCATC   
  
  
- GCGAAACCTC TTCGTTTAAA CCCTTCTGCT CCGAATAAGA ACTTGAGAAG CATGCGGTAA CAACAACCAA   
  
  
- GGTTGGAAAG AGAGAGGAGC ACTTAAACTC AAACCCGTCA TCTGATAGGG CCTTATCTCC TGACTACGCA   
  
  
- ATATATATAT CTTTAACTCT CTCTCTCTCT CGCACACACT TCCTACAACT TTTACTACCC GCATCCCTCA   
  
  
- GAGAGAGAGT GACAGACAAT CCTCATGCAC AGCTCTTTCC TTTATCCTAA GGGTTCGTCA GATTAAACCC   
  
  
- GGCCAGATAT CCGGTCTTAC CCGGATACTA GTCGGGTCTA TACTGGAACA AATTGGTTGA CATAAACTTA   
  
  
- ACTTCTTTCC CTATAGCGTT ACAGAACTTG GTGTAGTTTA TTATTTACAC GGAAACCAAT CTGGTATACT   
  
  
- TTTTAGAAAC TTAGTTTAGC CAACCTGGAC TTGACGGTAA TAACTATAGA ACAAAAACCA TCTTCGTTTA   
  
  
- CCACAGTGAA GCAAAAGATC TATTTTAATT GAACTCAAAA AAACGGTGAT ATCTATCATA ACGTTTATAT   
  
  
- TTTTCACACG GTGCACTTTA GTTATAATCC TCCCACAAAA TATTGTTATC GACAAGCCCT CTCAAAATCA   
  
  
- ATAAAAATAA TTTAGTTTAT CAATTCTTCA CAAACCATTT ATCAACATAA ACTTTTTCAA TAAGGGTAAT   
  
  
- CGGAAAATCA TTCTTGAAGA ACACTTTTAC TATTAACAGA TCTTCAAACT TTTTAAGTGT GGTGTACTGT   
  
  
- AAATTTTTAT TAAAACATCT CTTCCCGTAT TTACTTTCAA ACGGTGGTAG AAAAAATCTT TGTACATTAA   
  
  
- ATGGTTTGTG AAAAAGATTT TTGTCAATTA AGTTTATTGA TTGTTGATTG TCGATTATAT TTATTGATTG   
  
  
- TCGATAAACG ATTTGTCCCG GATTCATCAA ACTTTAACAA CAAAAGAGAA GTCGAAGTGG TACCGGCTAC   
  
  
- TACATTTGTT GATATAAGAC TTAAAGTAAA AATGATACTA AACTTTTAAA GTTTAAATTT ATTCTAACCT   
  
  
- ATTTTTTTTC TGGTTTTTAG TATTCATTAT AATCAGTTCA AGATAAGGTT GGTATATGTA TTGACGAAAA   
  
  
- AGAAAACCCC CAAAACTTTG TACACAGGAA TCTTATTAAC TTTGAGCTAT AATAAGAGAT TTAGATTCAA   
  
  
- ATGAAATAGT ATAACTAATA ATTAATGGAG GAGTTGAGGA GGATATGAGA ACATACGTCT TTATTTTTAC   
  
  
- AAAGGTATGG AATAGTCAGC GCTAGTATTA TTAATTAAAG AATTAAGCCT AACATAAATT AACGCATAGT   
  
  
- TTACACTTTA GCATGGTTTA ATCCTTCGTT TTGTTTGGCT ATTGCTTTTT CCATTCTTTT GAATCGTTCC   
  
  
- TTTACTTTAG CTTCTGGACA GTTGTTATAT CGAAGGGAGT GTAGGAAGTT GAATCCCTGG TAGGATATGA   
  
  
- AAGTACATTA TGTTGATCTC GGTGATCAGT TATTATCGGA GGATCATCAT CGGTATTCGG AACCACTCTC   
  
  
- TTACAAGGCG TTCTGATCAA TTGAACTTTA AGGTAAAACA GCGTCTTGAT AACCTCACTC TTTCTCTTAG   
  
  
- TTTAGTATGG TATAGTTTAA ACCTGGTAAA TTCAAGTGTT GTGTGTGTGT ATGTGTGTTT TCCTGCTACA   
  
  
- TGCCGGGGTG GGGGGTGAGA GGAAAGCGGT AGGGGGATGT GGAGACGGAT GAGAGAGAGA GAGAGAGAGA   
  
  
- GAGACCTAGT CAGACACATT TTCGAAGGAG AGGTCATGTC GTAAAGGAAC AGACAAGAAC CTCAAATCAG   
  
  
- TTAAGGAGGC GGGGTTTTTT TGGGTGAAAG AGAGAGTAGT ACAAGAAGGT CAAGGTGGGA ATATGCGGTA   
  
  
- AGTAGTAGTA GATGTAGTGG GTGTAGAAAA AGAGGTGTCA AAAGGGGGGA GTAATACGAG AGGCGGAGGT   
  
  
- GGGGGCGGAA GGGAGAGCGG CAGCGGCCGC TACTGCCCCT ATGGTGACTA CGTACGCTGC CGTGGCGGTG   
  
  
- ACGGGAGGTG GAGGAGCCCG ACGACAACTT CACGCGCCTT AAGTAGAGGT GGCCTCTGGA GCGGCCGCGG   
  
  
- CCTCTATAGA ACGGCCTCTA TAAACTCAAC AGATGTGGCA AACCGAGGGG GCGGCGGGCC CAGCGGCGGA   
  
  
- AGAAGCCGGT GCGGGAGGTG CGGGCGGAGG AGAGGCGGAG GGAGGCGTGT TGAGGCTAGC TCTTCGAGTT   
  
  
- TTGGGACTGG GACCAAAGGG TTTACGCCTT TAAAGTGAGG CGGAACGTTC ATATGTTGAG GTAGTGAGGG   
  
  
- AAGCACTTTA AGAGAGTGAA GTGCCGCTTA GTTCGGTAGA TGCTCCGCGA CCTGCCGCTC CTAGCACAGG   
  
  
- TGCAGCAGCT GGAACTGTAG TACGTCCCGG AAGTTACCGG CCCTAACAAG GTGTAGAATC GGAGAGCTGG   
  
  
- GTCTTTCGGG GAGAGCCAAG CCCAGTGGCC CAAGCCCGGT AGGAGGCTCA ACGAGAGGGT TTGCCCATTC   
  
  
- GCTGAGCGAC TCAAGCGGCG AAGTGAGCCG GACGGGAAGC TCATGTTGGG CCACCTCCCG TTTTAACCCT   
  
  
- TGAATCAGCT GGACCCAGCC CAGCCCAGCG AGGGCTTACT TCACTGCCAC CACGTGACCT ACGTGGTATC   
  
  
- AGATATGCTG TATTGGCCCA GCCTAGAACC CTGAAACTCC CATAACTCAC GCCACTCCGG ATCCGAACAC   
  
  
- TGATACCAAC TCGTCCTATA CCTGGTTTGC CCCAGCAAAA ACCCCTCCAA ACACCTCCGT AACGTAATAA   
  
  
- TGAGTCGGAA CAAACTACGG AACCCTCTCC CCAATCTATC CCTATTGGAT GTTTCCGTAG TTCACCTCGT   
  
  
- TGTCGATAAA CCCACACTCT AATCCTTGTA GGACCGGCAA CCACCCGGGT TCTCCTCCGA GTGACCGCCG   
  
  
- CTAGCCCAGT CTTCCACCCC GCTGCTTGAC TGTTCCCAGC CCAAGCTTGG TCAAAGCAAC CGCCCATCGG   
  
  
- GCCGTTGGGT TCGATCAAAC AACGAACCCT ACAAGGGAAC CTTTCCCATA TGAAACTACC TCCTTTTACC   
  
  
- CACAAACTCT AATCCCACCT TTCTAAACGG GAACAATTGA CGGAGTCGAA CCGTTGGAAC ACTTAAATTG   
  
  
- TTAGGATCAC GACCGTAAAT

  
  
Motifs Found  

+   

| Site Name | Organism | Position | Strand | Matrix score. | sequence | function |
| --- | --- | --- | --- | --- | --- | --- |
|  | organism | 1876 | + | 4 | motif\_sequence | short\_function |
|  | organism | 685 | + | 4 | motif\_sequence | short\_function |
|  | organism | 2577 | + | 4 | motif\_sequence | short\_function |
|  | organism | 2189 | - | 4 | motif\_sequence | short\_function |
|  | organism | 1796 | + | 4 | motif\_sequence | short\_function |
|  | organism | 470 | + | 4 | motif\_sequence | short\_function |
|  | organism | 2898 | - | 4 | motif\_sequence | short\_function |
|  | organism | 1030 | + | 4 | motif\_sequence | short\_function |
|  | organism | 2421 | + | 4 | motif\_sequence | short\_function |
|  | organism | 1616 | + | 4 | motif\_sequence | short\_function |
|  | organism | 156 | + | 4 | motif\_sequence | short\_function |
|  | organism | 119 | + | 4 | motif\_sequence | short\_function |
|  | organism | 1859 | - | 4 | motif\_sequence | short\_function |
|  | organism | 2969 | - | 4 | motif\_sequence | short\_function |
|  | organism | 2175 | - | 4 | motif\_sequence | short\_function |
|  | organism | 2274 | + | 4 | motif\_sequence | short\_function |
|  | organism | 2148 | + | 4 | motif\_sequence | short\_function |
|  | organism | 1852 | + | 4 | motif\_sequence | short\_function |
|  | organism | 1771 | + | 4 | motif\_sequence | short\_function |
|  | organism | 2022 | + | 4 | motif\_sequence | short\_function |
|  | organism | 692 | - | 4 | motif\_sequence | short\_function |
|  | organism | 3114 | + | 4 | motif\_sequence | short\_function |
|  | organism | 860 | - | 4 | motif\_sequence | short\_function |
|  | organism | 1751 | + | 4 | motif\_sequence | short\_function |
|  | organism | 1934 | + | 4 | motif\_sequence | short\_function |
|  | organism | 1973 | + | 4 | motif\_sequence | short\_function |

>HU02G03005.1   
+ +Up\_Stream \_Len000AAACGG TGCCGTTGAA TTTGCAGGCG ACGAGGTCGG GTTTAAGGCG GATGTCGTAG   
  
  
+ CGCTTTGGAG AAGCAAATTT GGGAAGACGA GGCTTATTCT TGAACTCTTC GTACGCCATT GTTGTTGGTT   
  
  
+ CCAACCTTTC TCTCTCCTCG TGAATTTGAG TTTGGGCAGT AGACTATCCC GGAATAGAGG ACTGATGCGT   
  
  
+ TATATATATA GAAATTGAGA GAGAGAGAGA GCGTGTGTGA AGGATGTTGA AAATGATGGG CGTAGGGAGT   
  
  
+ CTCTCTCTCA CTGTCTGTTA GGAGTACGTG TCGAGAAAGG AAATAGGATT CCCAAGCAGT CTAATTTGGG   
  
  
+ CCGGTCTATA GGCCAGAATG GGCCTATGAT CAGCCCAGAT ATGACCTTGT TTAACCAACT GTATTTGAAT   
  
  
+ TGAAGAAAGG GATATCGCAA TGTCTTGAAC CACATCAAAT AATAAATGTG CCTTTGGTTA GACCATATGA   
  
  
+ AAAATCTTTG AATCAAATCG GTTGGACCTG AACTGCCATT ATTGATATCT TGTTTTTGGT AGAAGCAAAT   
  
  
+ GGTGTCACTT CGTTTTCTAG ATAAAATTAA CTTGAGTTTT TTTGCCACTA TAGATAGTAT TGCAAATATA   
  
  
+ AAAAGTGTGC CACGTGAAAT CAATATTAGG AGGGTGTTTT ATAACAATAG CTGTTCGGGA GAGTTTTAGT   
  
  
+ TATTTTTATT AAATCAAATA GTTAAGAAGT GTTTGGTAAA TAGTTGTATT TGAAAAAGTT ATTCCCATTA   
  
  
+ GCCTTTTAGT AAGAACTTCT TGTGAAAATG ATAATTGTCT AGAAGTTTGA AAAATTCACA CCACATGACA   
  
  
+ TTTAAAAATA ATTTTGTAGA GAAGGGCATA AATGAAAGTT TGCCACCATC TTTTTTAGAA ACATGTAATT   
  
  
+ TACCAAACAC TTTTTCTAAA AACAGTTAAT TCAAATAACT AACAACTAAC AGCTAATATA AATAACTAAC   
  
  
+ AGCTATTTGC TAAACAGGGC CTAAGTAGTT TGAAATTGTT GTTTTCTCTT CAGCTTCACC ATGGCCGATG   
  
  
+ ATGTAAACAA CTATATTCTG AATTTCATTT TTACTATGAT TTGAAAATTT CAAATTTAAA TAAGATTGGA   
  
  
+ TAAAAAAAAG ACCAAAAATC ATAAGTAATA TTAGTCAAGT TCTATTCCAA CCATATACAT AACTGCTTTT   
  
  
+ TCTTTTGGGG GTTTTGAAAC ATGTGTCCTT AGAATAATTG AAACTCGATA TTATTCTCTA AATCTAAGTT   
  
  
+ TACTTTATCA TATTGATTAT TAATTACCTC CTCAACTCCT CCTATACTCT TGTATGCAGA AATAAAAATG   
  
  
+ TTTCCATACC TTATCAGTCG CGATCATAAT AATTAATTTC TTAATTCGGA TTGTATTTAA TTGCGTATCA   
  
  
+ AATGTGAAAT CGTACCAAAT TAGGAAGCAA AACAAACCGA TAACGAAAAA GGTAAGAAAA CTTAGCAAGG   
  
  
+ AAATGAAATC GAAGACCTGT CAACAATATA GCTTCCCTCA CATCCTTCAA CTTAGGGACC ATCCTATACT   
  
  
+ TTCATGTAAT ACAACTAGAG CCACTAGTCA ATAATAGCCT CCTAGTAGTA GCCATAAGCC TTGGTGAGAG   
  
  
+ AATGTTCCGC AAGACTAGTT AACTTGAAAT TCCATTTTGT CGCAGAACTA TTGGAGTGAG AAAGAGAATC   
  
  
+ AAATCATACC ATATCAAATT TGGACCATTT AAGTTCACAA CACACACACA TACACACAAA AGGACGATGT   
  
  
+ ACGGCCCCAC CCCCCACTCT CCTTTCGCCA TCCCCCTACA CCTCTGCCTA CTCTCTCTCT CTCTCTCTCT   
  
  
+ CTCTGGATCA GTCTGTGTAA AAGCTTCCTC TCCAGTACAG CATTTCCTTG TCTGTTCTTG GAGTTTAGTC   
  
  
+ AATTCCTCCG CCCCAAAAAA ACCCACTTTC TCTCTCATCA TGTTCTTCCA GTTCCACCCT TATACGCCAT   
  
  
+ TCATCATCAT CTACATCACC CACATCTTTT TCTCCACAGT TTTCCCCCCT CATTATGCTC TCCGCCTCCA   
  
  
+ CCCCCGCCTT CCCTCTCGCC GTCGCCGGCG ATGACGGGGA TACCACTGAT GCATGCGACG GCACCGCCAC   
  
  
+ TGCCCTCCAC CTCCTCGGGC TGCTGTTGAA GTGCGCGGAA TTCATCTCCA CCGGAGACCT CGCCGGCGCC   
  
  
+ GGAGATATCT TGCCGGAGAT ATTTGAGTTG TCTACACCGT TTGGCTCCCC CGCCGCCCGG GTCGCCGCCT   
  
  
+ TCTTCGGCCA CGCCCTCCAC GCCCGCCTCC TCTCCGCCTC CCTCCGCACA ACTCCGATCG AGAAGCTCAA   
  
  
+ AACCCTGACC CTGGTTTCCC AAATGCGGAA ATTTCACTCC GCCTTGCAAG TATACAACTC CATCACTCCC   
  
  
+ TTCGTGAAAT TCTCTCACTT CACGGCGAAT CAAGCCATCT ACGAGGCGCT GGACGGCGAG GATCGTGTCC   
  
  
+ ACGTCGTCGA CCTTGACATC ATGCAGGGCC TTCAATGGCC GGGATTGTTC CACATCTTAG CCTCTCGACC   
  
  
+ CAGAAAGCCC CTCTCGGTTC GGGTCACCGG GTTCGGGCCA TCCTCCGAGT TGCTCTCCCA AACGGGTAAG   
  
  
+ CGACTCGCTG AGTTCGCCGC TTCACTCGGC CTGCCCTTCG AGTACAACCC GGTGGAGGGC AAAATTGGGA   
  
  
+ ACTTAGTCGA CCTGGGTCGG GTCGGGTCGC TCCCGAATGA AGTGACGGTG GTGCACTGGA TGCACCATAG   
  
  
+ TCTATACGAC ATAACCGGGT CGGATCTTGG GACTTTGAGG GTATTGAGTG CGGTGAGGCC TAGGCTTGTG   
  
  
+ ACTATGGTTG AGCAGGATAT GGACCAAACG GGGTCGTTTT TGGGGAGGTT TGTGGAGGCA TTGCATTATT   
  
  
+ ACTCAGCCTT GTTTGATGCC TTGGGAGAGG GGTTAGATAG GGATAACCTA CAAAGGCATC AAGTGGAGCA   
  
  
+ ACAGCTATTT GGGTGTGAGA TTAGGAACAT CCTGGCCGTT GGTGGGCCCA AGAGGAGGCT CACTGGCGGC   
  
  
+ GATCGGGTCA GAAGGTGGGG CGACGAACTG ACAAGGGTCG GGTTCGAACC AGTTTCGTTG GCGGGTAGCC   
  
  
+ CGGCAACCCA AGCTAGTTTG TTGCTTGGGA TGTTCCCTTG GAAAGGGTAT ACTTTGATGG AGGAAAATGG   
  
  
+ GTGTTTGAGA TTAGGGTGGA AAGATTTGCC CTTGTTAACT GCCTCAGCTT GGCAACCTTG TGAATTTAAC   
  
  
+ AATCCTAGTG CTGGCATTTA   

- +Up\_Stream \_Len000TTTGCC ACGGCAACTT AAACGTCCGC TGCTCCAGCC CAAATTCCGC CTACAGCATC   
  
  
- GCGAAACCTC TTCGTTTAAA CCCTTCTGCT CCGAATAAGA ACTTGAGAAG CATGCGGTAA CAACAACCAA   
  
  
- GGTTGGAAAG AGAGAGGAGC ACTTAAACTC AAACCCGTCA TCTGATAGGG CCTTATCTCC TGACTACGCA   
  
  
- ATATATATAT CTTTAACTCT CTCTCTCTCT CGCACACACT TCCTACAACT TTTACTACCC GCATCCCTCA   
  
  
- GAGAGAGAGT GACAGACAAT CCTCATGCAC AGCTCTTTCC TTTATCCTAA GGGTTCGTCA GATTAAACCC   
  
  
- GGCCAGATAT CCGGTCTTAC CCGGATACTA GTCGGGTCTA TACTGGAACA AATTGGTTGA CATAAACTTA   
  
  
- ACTTCTTTCC CTATAGCGTT ACAGAACTTG GTGTAGTTTA TTATTTACAC GGAAACCAAT CTGGTATACT   
  
  
- TTTTAGAAAC TTAGTTTAGC CAACCTGGAC TTGACGGTAA TAACTATAGA ACAAAAACCA TCTTCGTTTA   
  
  
- CCACAGTGAA GCAAAAGATC TATTTTAATT GAACTCAAAA AAACGGTGAT ATCTATCATA ACGTTTATAT   
  
  
- TTTTCACACG GTGCACTTTA GTTATAATCC TCCCACAAAA TATTGTTATC GACAAGCCCT CTCAAAATCA   
  
  
- ATAAAAATAA TTTAGTTTAT CAATTCTTCA CAAACCATTT ATCAACATAA ACTTTTTCAA TAAGGGTAAT   
  
  
- CGGAAAATCA TTCTTGAAGA ACACTTTTAC TATTAACAGA TCTTCAAACT TTTTAAGTGT GGTGTACTGT   
  
  
- AAATTTTTAT TAAAACATCT CTTCCCGTAT TTACTTTCAA ACGGTGGTAG AAAAAATCTT TGTACATTAA   
  
  
- ATGGTTTGTG AAAAAGATTT TTGTCAATTA AGTTTATTGA TTGTTGATTG TCGATTATAT TTATTGATTG   
  
  
- TCGATAAACG ATTTGTCCCG GATTCATCAA ACTTTAACAA CAAAAGAGAA GTCGAAGTGG TACCGGCTAC   
  
  
- TACATTTGTT GATATAAGAC TTAAAGTAAA AATGATACTA AACTTTTAAA GTTTAAATTT ATTCTAACCT   
  
  
- ATTTTTTTTC TGGTTTTTAG TATTCATTAT AATCAGTTCA AGATAAGGTT GGTATATGTA TTGACGAAAA   
  
  
- AGAAAACCCC CAAAACTTTG TACACAGGAA TCTTATTAAC TTTGAGCTAT AATAAGAGAT TTAGATTCAA   
  
  
- ATGAAATAGT ATAACTAATA ATTAATGGAG GAGTTGAGGA GGATATGAGA ACATACGTCT TTATTTTTAC   
  
  
- AAAGGTATGG AATAGTCAGC GCTAGTATTA TTAATTAAAG AATTAAGCCT AACATAAATT AACGCATAGT   
  
  
- TTACACTTTA GCATGGTTTA ATCCTTCGTT TTGTTTGGCT ATTGCTTTTT CCATTCTTTT GAATCGTTCC   
  
  
- TTTACTTTAG CTTCTGGACA GTTGTTATAT CGAAGGGAGT GTAGGAAGTT GAATCCCTGG TAGGATATGA   
  
  
- AAGTACATTA TGTTGATCTC GGTGATCAGT TATTATCGGA GGATCATCAT CGGTATTCGG AACCACTCTC   
  
  
- TTACAAGGCG TTCTGATCAA TTGAACTTTA AGGTAAAACA GCGTCTTGAT AACCTCACTC TTTCTCTTAG   
  
  
- TTTAGTATGG TATAGTTTAA ACCTGGTAAA TTCAAGTGTT GTGTGTGTGT ATGTGTGTTT TCCTGCTACA   
  
  
- TGCCGGGGTG GGGGGTGAGA GGAAAGCGGT AGGGGGATGT GGAGACGGAT GAGAGAGAGA GAGAGAGAGA   
  
  
- GAGACCTAGT CAGACACATT TTCGAAGGAG AGGTCATGTC GTAAAGGAAC AGACAAGAAC CTCAAATCAG   
  
  
- TTAAGGAGGC GGGGTTTTTT TGGGTGAAAG AGAGAGTAGT ACAAGAAGGT CAAGGTGGGA ATATGCGGTA   
  
  
- AGTAGTAGTA GATGTAGTGG GTGTAGAAAA AGAGGTGTCA AAAGGGGGGA GTAATACGAG AGGCGGAGGT   
  
  
- GGGGGCGGAA GGGAGAGCGG CAGCGGCCGC TACTGCCCCT ATGGTGACTA CGTACGCTGC CGTGGCGGTG   
  
  
- ACGGGAGGTG GAGGAGCCCG ACGACAACTT CACGCGCCTT AAGTAGAGGT GGCCTCTGGA GCGGCCGCGG   
  
  
- CCTCTATAGA ACGGCCTCTA TAAACTCAAC AGATGTGGCA AACCGAGGGG GCGGCGGGCC CAGCGGCGGA   
  
  
- AGAAGCCGGT GCGGGAGGTG CGGGCGGAGG AGAGGCGGAG GGAGGCGTGT TGAGGCTAGC TCTTCGAGTT   
  
  
- TTGGGACTGG GACCAAAGGG TTTACGCCTT TAAAGTGAGG CGGAACGTTC ATATGTTGAG GTAGTGAGGG   
  
  
- AAGCACTTTA AGAGAGTGAA GTGCCGCTTA GTTCGGTAGA TGCTCCGCGA CCTGCCGCTC CTAGCACAGG   
  
  
- TGCAGCAGCT GGAACTGTAG TACGTCCCGG AAGTTACCGG CCCTAACAAG GTGTAGAATC GGAGAGCTGG   
  
  
- GTCTTTCGGG GAGAGCCAAG CCCAGTGGCC CAAGCCCGGT AGGAGGCTCA ACGAGAGGGT TTGCCCATTC   
  
  
- GCTGAGCGAC TCAAGCGGCG AAGTGAGCCG GACGGGAAGC TCATGTTGGG CCACCTCCCG TTTTAACCCT   
  
  
- TGAATCAGCT GGACCCAGCC CAGCCCAGCG AGGGCTTACT TCACTGCCAC CACGTGACCT ACGTGGTATC   
  
  
- AGATATGCTG TATTGGCCCA GCCTAGAACC CTGAAACTCC CATAACTCAC GCCACTCCGG ATCCGAACAC   
  
  
- TGATACCAAC TCGTCCTATA CCTGGTTTGC CCCAGCAAAA ACCCCTCCAA ACACCTCCGT AACGTAATAA   
  
  
- TGAGTCGGAA CAAACTACGG AACCCTCTCC CCAATCTATC CCTATTGGAT GTTTCCGTAG TTCACCTCGT   
  
  
- TGTCGATAAA CCCACACTCT AATCCTTGTA GGACCGGCAA CCACCCGGGT TCTCCTCCGA GTGACCGCCG   
  
  
- CTAGCCCAGT CTTCCACCCC GCTGCTTGAC TGTTCCCAGC CCAAGCTTGG TCAAAGCAAC CGCCCATCGG   
  
  
- GCCGTTGGGT TCGATCAAAC AACGAACCCT ACAAGGGAAC CTTTCCCATA TGAAACTACC TCCTTTTACC   
  
  
- CACAAACTCT AATCCCACCT TTCTAAACGG GAACAATTGA CGGAGTCGAA CCGTTGGAAC ACTTAAATTG   
  
  
- TTAGGATCAC GACCGTAAAT

+     3-AF3 binding site

| Site Name | Organism | Position | Strand | Matrix score. | sequence | function |
| --- | --- | --- | --- | --- | --- | --- |
| 3-AF3 binding site | Pisum sativum | 2906 | - | 10 | CACTATCTAAC | part of a conserved DNA module array (CMA3) |

>HU02G03005.1   
+ +Up\_Stream \_Len000AAACGG TGCCGTTGAA TTTGCAGGCG ACGAGGTCGG GTTTAAGGCG GATGTCGTAG   
  
  
+ CGCTTTGGAG AAGCAAATTT GGGAAGACGA GGCTTATTCT TGAACTCTTC GTACGCCATT GTTGTTGGTT   
  
  
+ CCAACCTTTC TCTCTCCTCG TGAATTTGAG TTTGGGCAGT AGACTATCCC GGAATAGAGG ACTGATGCGT   
  
  
+ TATATATATA GAAATTGAGA GAGAGAGAGA GCGTGTGTGA AGGATGTTGA AAATGATGGG CGTAGGGAGT   
  
  
+ CTCTCTCTCA CTGTCTGTTA GGAGTACGTG TCGAGAAAGG AAATAGGATT CCCAAGCAGT CTAATTTGGG   
  
  
+ CCGGTCTATA GGCCAGAATG GGCCTATGAT CAGCCCAGAT ATGACCTTGT TTAACCAACT GTATTTGAAT   
  
  
+ TGAAGAAAGG GATATCGCAA TGTCTTGAAC CACATCAAAT AATAAATGTG CCTTTGGTTA GACCATATGA   
  
  
+ AAAATCTTTG AATCAAATCG GTTGGACCTG AACTGCCATT ATTGATATCT TGTTTTTGGT AGAAGCAAAT   
  
  
+ GGTGTCACTT CGTTTTCTAG ATAAAATTAA CTTGAGTTTT TTTGCCACTA TAGATAGTAT TGCAAATATA   
  
  
+ AAAAGTGTGC CACGTGAAAT CAATATTAGG AGGGTGTTTT ATAACAATAG CTGTTCGGGA GAGTTTTAGT   
  
  
+ TATTTTTATT AAATCAAATA GTTAAGAAGT GTTTGGTAAA TAGTTGTATT TGAAAAAGTT ATTCCCATTA   
  
  
+ GCCTTTTAGT AAGAACTTCT TGTGAAAATG ATAATTGTCT AGAAGTTTGA AAAATTCACA CCACATGACA   
  
  
+ TTTAAAAATA ATTTTGTAGA GAAGGGCATA AATGAAAGTT TGCCACCATC TTTTTTAGAA ACATGTAATT   
  
  
+ TACCAAACAC TTTTTCTAAA AACAGTTAAT TCAAATAACT AACAACTAAC AGCTAATATA AATAACTAAC   
  
  
+ AGCTATTTGC TAAACAGGGC CTAAGTAGTT TGAAATTGTT GTTTTCTCTT CAGCTTCACC ATGGCCGATG   
  
  
+ ATGTAAACAA CTATATTCTG AATTTCATTT TTACTATGAT TTGAAAATTT CAAATTTAAA TAAGATTGGA   
  
  
+ TAAAAAAAAG ACCAAAAATC ATAAGTAATA TTAGTCAAGT TCTATTCCAA CCATATACAT AACTGCTTTT   
  
  
+ TCTTTTGGGG GTTTTGAAAC ATGTGTCCTT AGAATAATTG AAACTCGATA TTATTCTCTA AATCTAAGTT   
  
  
+ TACTTTATCA TATTGATTAT TAATTACCTC CTCAACTCCT CCTATACTCT TGTATGCAGA AATAAAAATG   
  
  
+ TTTCCATACC TTATCAGTCG CGATCATAAT AATTAATTTC TTAATTCGGA TTGTATTTAA TTGCGTATCA   
  
  
+ AATGTGAAAT CGTACCAAAT TAGGAAGCAA AACAAACCGA TAACGAAAAA GGTAAGAAAA CTTAGCAAGG   
  
  
+ AAATGAAATC GAAGACCTGT CAACAATATA GCTTCCCTCA CATCCTTCAA CTTAGGGACC ATCCTATACT   
  
  
+ TTCATGTAAT ACAACTAGAG CCACTAGTCA ATAATAGCCT CCTAGTAGTA GCCATAAGCC TTGGTGAGAG   
  
  
+ AATGTTCCGC AAGACTAGTT AACTTGAAAT TCCATTTTGT CGCAGAACTA TTGGAGTGAG AAAGAGAATC   
  
  
+ AAATCATACC ATATCAAATT TGGACCATTT AAGTTCACAA CACACACACA TACACACAAA AGGACGATGT   
  
  
+ ACGGCCCCAC CCCCCACTCT CCTTTCGCCA TCCCCCTACA CCTCTGCCTA CTCTCTCTCT CTCTCTCTCT   
  
  
+ CTCTGGATCA GTCTGTGTAA AAGCTTCCTC TCCAGTACAG CATTTCCTTG TCTGTTCTTG GAGTTTAGTC   
  
  
+ AATTCCTCCG CCCCAAAAAA ACCCACTTTC TCTCTCATCA TGTTCTTCCA GTTCCACCCT TATACGCCAT   
  
  
+ TCATCATCAT CTACATCACC CACATCTTTT TCTCCACAGT TTTCCCCCCT CATTATGCTC TCCGCCTCCA   
  
  
+ CCCCCGCCTT CCCTCTCGCC GTCGCCGGCG ATGACGGGGA TACCACTGAT GCATGCGACG GCACCGCCAC   
  
  
+ TGCCCTCCAC CTCCTCGGGC TGCTGTTGAA GTGCGCGGAA TTCATCTCCA CCGGAGACCT CGCCGGCGCC   
  
  
+ GGAGATATCT TGCCGGAGAT ATTTGAGTTG TCTACACCGT TTGGCTCCCC CGCCGCCCGG GTCGCCGCCT   
  
  
+ TCTTCGGCCA CGCCCTCCAC GCCCGCCTCC TCTCCGCCTC CCTCCGCACA ACTCCGATCG AGAAGCTCAA   
  
  
+ AACCCTGACC CTGGTTTCCC AAATGCGGAA ATTTCACTCC GCCTTGCAAG TATACAACTC CATCACTCCC   
  
  
+ TTCGTGAAAT TCTCTCACTT CACGGCGAAT CAAGCCATCT ACGAGGCGCT GGACGGCGAG GATCGTGTCC   
  
  
+ ACGTCGTCGA CCTTGACATC ATGCAGGGCC TTCAATGGCC GGGATTGTTC CACATCTTAG CCTCTCGACC   
  
  
+ CAGAAAGCCC CTCTCGGTTC GGGTCACCGG GTTCGGGCCA TCCTCCGAGT TGCTCTCCCA AACGGGTAAG   
  
  
+ CGACTCGCTG AGTTCGCCGC TTCACTCGGC CTGCCCTTCG AGTACAACCC GGTGGAGGGC AAAATTGGGA   
  
  
+ ACTTAGTCGA CCTGGGTCGG GTCGGGTCGC TCCCGAATGA AGTGACGGTG GTGCACTGGA TGCACCATAG   
  
  
+ TCTATACGAC ATAACCGGGT CGGATCTTGG GACTTTGAGG GTATTGAGTG CGGTGAGGCC TAGGCTTGTG   
  
  
+ ACTATGGTTG AGCAGGATAT GGACCAAACG GGGTCGTTTT TGGGGAGGTT TGTGGAGGCA TTGCATTATT   
  
  
+ ACTCAGCCTT GTTTGATGCC TTGGGAGAGG GGTTAGATAG GGATAACCTA CAAAGGCATC AAGTGGAGCA   
  
  
+ ACAGCTATTT GGGTGTGAGA TTAGGAACAT CCTGGCCGTT GGTGGGCCCA AGAGGAGGCT CACTGGCGGC   
  
  
+ GATCGGGTCA GAAGGTGGGG CGACGAACTG ACAAGGGTCG GGTTCGAACC AGTTTCGTTG GCGGGTAGCC   
  
  
+ CGGCAACCCA AGCTAGTTTG TTGCTTGGGA TGTTCCCTTG GAAAGGGTAT ACTTTGATGG AGGAAAATGG   
  
  
+ GTGTTTGAGA TTAGGGTGGA AAGATTTGCC CTTGTTAACT GCCTCAGCTT GGCAACCTTG TGAATTTAAC   
  
  
+ AATCCTAGTG CTGGCATTTA   

- +Up\_Stream \_Len000TTTGCC ACGGCAACTT AAACGTCCGC TGCTCCAGCC CAAATTCCGC CTACAGCATC   
  
  
- GCGAAACCTC TTCGTTTAAA CCCTTCTGCT CCGAATAAGA ACTTGAGAAG CATGCGGTAA CAACAACCAA   
  
  
- GGTTGGAAAG AGAGAGGAGC ACTTAAACTC AAACCCGTCA TCTGATAGGG CCTTATCTCC TGACTACGCA   
  
  
- ATATATATAT CTTTAACTCT CTCTCTCTCT CGCACACACT TCCTACAACT TTTACTACCC GCATCCCTCA   
  
  
- GAGAGAGAGT GACAGACAAT CCTCATGCAC AGCTCTTTCC TTTATCCTAA GGGTTCGTCA GATTAAACCC   
  
  
- GGCCAGATAT CCGGTCTTAC CCGGATACTA GTCGGGTCTA TACTGGAACA AATTGGTTGA CATAAACTTA   
  
  
- ACTTCTTTCC CTATAGCGTT ACAGAACTTG GTGTAGTTTA TTATTTACAC GGAAACCAAT CTGGTATACT   
  
  
- TTTTAGAAAC TTAGTTTAGC CAACCTGGAC TTGACGGTAA TAACTATAGA ACAAAAACCA TCTTCGTTTA   
  
  
- CCACAGTGAA GCAAAAGATC TATTTTAATT GAACTCAAAA AAACGGTGAT ATCTATCATA ACGTTTATAT   
  
  
- TTTTCACACG GTGCACTTTA GTTATAATCC TCCCACAAAA TATTGTTATC GACAAGCCCT CTCAAAATCA   
  
  
- ATAAAAATAA TTTAGTTTAT CAATTCTTCA CAAACCATTT ATCAACATAA ACTTTTTCAA TAAGGGTAAT   
  
  
- CGGAAAATCA TTCTTGAAGA ACACTTTTAC TATTAACAGA TCTTCAAACT TTTTAAGTGT GGTGTACTGT   
  
  
- AAATTTTTAT TAAAACATCT CTTCCCGTAT TTACTTTCAA ACGGTGGTAG AAAAAATCTT TGTACATTAA   
  
  
- ATGGTTTGTG AAAAAGATTT TTGTCAATTA AGTTTATTGA TTGTTGATTG TCGATTATAT TTATTGATTG   
  
  
- TCGATAAACG ATTTGTCCCG GATTCATCAA ACTTTAACAA CAAAAGAGAA GTCGAAGTGG TACCGGCTAC   
  
  
- TACATTTGTT GATATAAGAC TTAAAGTAAA AATGATACTA AACTTTTAAA GTTTAAATTT ATTCTAACCT   
  
  
- ATTTTTTTTC TGGTTTTTAG TATTCATTAT AATCAGTTCA AGATAAGGTT GGTATATGTA TTGACGAAAA   
  
  
- AGAAAACCCC CAAAACTTTG TACACAGGAA TCTTATTAAC TTTGAGCTAT AATAAGAGAT TTAGATTCAA   
  
  
- ATGAAATAGT ATAACTAATA ATTAATGGAG GAGTTGAGGA GGATATGAGA ACATACGTCT TTATTTTTAC   
  
  
- AAAGGTATGG AATAGTCAGC GCTAGTATTA TTAATTAAAG AATTAAGCCT AACATAAATT AACGCATAGT   
  
  
- TTACACTTTA GCATGGTTTA ATCCTTCGTT TTGTTTGGCT ATTGCTTTTT CCATTCTTTT GAATCGTTCC   
  
  
- TTTACTTTAG CTTCTGGACA GTTGTTATAT CGAAGGGAGT GTAGGAAGTT GAATCCCTGG TAGGATATGA   
  
  
- AAGTACATTA TGTTGATCTC GGTGATCAGT TATTATCGGA GGATCATCAT CGGTATTCGG AACCACTCTC   
  
  
- TTACAAGGCG TTCTGATCAA TTGAACTTTA AGGTAAAACA GCGTCTTGAT AACCTCACTC TTTCTCTTAG   
  
  
- TTTAGTATGG TATAGTTTAA ACCTGGTAAA TTCAAGTGTT GTGTGTGTGT ATGTGTGTTT TCCTGCTACA   
  
  
- TGCCGGGGTG GGGGGTGAGA GGAAAGCGGT AGGGGGATGT GGAGACGGAT GAGAGAGAGA GAGAGAGAGA   
  
  
- GAGACCTAGT CAGACACATT TTCGAAGGAG AGGTCATGTC GTAAAGGAAC AGACAAGAAC CTCAAATCAG   
  
  
- TTAAGGAGGC GGGGTTTTTT TGGGTGAAAG AGAGAGTAGT ACAAGAAGGT CAAGGTGGGA ATATGCGGTA   
  
  
- AGTAGTAGTA GATGTAGTGG GTGTAGAAAA AGAGGTGTCA AAAGGGGGGA GTAATACGAG AGGCGGAGGT   
  
  
- GGGGGCGGAA GGGAGAGCGG CAGCGGCCGC TACTGCCCCT ATGGTGACTA CGTACGCTGC CGTGGCGGTG   
  
  
- ACGGGAGGTG GAGGAGCCCG ACGACAACTT CACGCGCCTT AAGTAGAGGT GGCCTCTGGA GCGGCCGCGG   
  
  
- CCTCTATAGA ACGGCCTCTA TAAACTCAAC AGATGTGGCA AACCGAGGGG GCGGCGGGCC CAGCGGCGGA   
  
  
- AGAAGCCGGT GCGGGAGGTG CGGGCGGAGG AGAGGCGGAG GGAGGCGTGT TGAGGCTAGC TCTTCGAGTT   
  
  
- TTGGGACTGG GACCAAAGGG TTTACGCCTT TAAAGTGAGG CGGAACGTTC ATATGTTGAG GTAGTGAGGG   
  
  
- AAGCACTTTA AGAGAGTGAA GTGCCGCTTA GTTCGGTAGA TGCTCCGCGA CCTGCCGCTC CTAGCACAGG   
  
  
- TGCAGCAGCT GGAACTGTAG TACGTCCCGG AAGTTACCGG CCCTAACAAG GTGTAGAATC GGAGAGCTGG   
  
  
- GTCTTTCGGG GAGAGCCAAG CCCAGTGGCC CAAGCCCGGT AGGAGGCTCA ACGAGAGGGT TTGCCCATTC   
  
  
- GCTGAGCGAC TCAAGCGGCG AAGTGAGCCG GACGGGAAGC TCATGTTGGG CCACCTCCCG TTTTAACCCT   
  
  
- TGAATCAGCT GGACCCAGCC CAGCCCAGCG AGGGCTTACT TCACTGCCAC CACGTGACCT ACGTGGTATC   
  
  
- AGATATGCTG TATTGGCCCA GCCTAGAACC CTGAAACTCC CATAACTCAC GCCACTCCGG ATCCGAACAC   
  
  
- TGATACCAAC TCGTCCTATA CCTGGTTTGC CCCAGCAAAA ACCCCTCCAA ACACCTCCGT AACGTAATAA   
  
  
- TGAGTCGGAA CAAACTACGG AACCCTCTCC CCAATCTATC CCTATTGGAT GTTTCCGTAG TTCACCTCGT   
  
  
- TGTCGATAAA CCCACACTCT AATCCTTGTA GGACCGGCAA CCACCCGGGT TCTCCTCCGA GTGACCGCCG   
  
  
- CTAGCCCAGT CTTCCACCCC GCTGCTTGAC TGTTCCCAGC CCAAGCTTGG TCAAAGCAAC CGCCCATCGG   
  
  
- GCCGTTGGGT TCGATCAAAC AACGAACCCT ACAAGGGAAC CTTTCCCATA TGAAACTACC TCCTTTTACC   
  
  
- CACAAACTCT AATCCCACCT TTCTAAACGG GAACAATTGA CGGAGTCGAA CCGTTGGAAC ACTTAAATTG   
  
  
- TTAGGATCAC GACCGTAAAT

+     A-box

| Site Name | Organism | Position | Strand | Matrix score. | sequence | function |
| --- | --- | --- | --- | --- | --- | --- |
| A-box | Petroselinum crispum | 2435 | - | 6 | CCGTCC | cis-acting regulatory element |

>HU02G03005.1   
+ +Up\_Stream \_Len000AAACGG TGCCGTTGAA TTTGCAGGCG ACGAGGTCGG GTTTAAGGCG GATGTCGTAG   
  
  
+ CGCTTTGGAG AAGCAAATTT GGGAAGACGA GGCTTATTCT TGAACTCTTC GTACGCCATT GTTGTTGGTT   
  
  
+ CCAACCTTTC TCTCTCCTCG TGAATTTGAG TTTGGGCAGT AGACTATCCC GGAATAGAGG ACTGATGCGT   
  
  
+ TATATATATA GAAATTGAGA GAGAGAGAGA GCGTGTGTGA AGGATGTTGA AAATGATGGG CGTAGGGAGT   
  
  
+ CTCTCTCTCA CTGTCTGTTA GGAGTACGTG TCGAGAAAGG AAATAGGATT CCCAAGCAGT CTAATTTGGG   
  
  
+ CCGGTCTATA GGCCAGAATG GGCCTATGAT CAGCCCAGAT ATGACCTTGT TTAACCAACT GTATTTGAAT   
  
  
+ TGAAGAAAGG GATATCGCAA TGTCTTGAAC CACATCAAAT AATAAATGTG CCTTTGGTTA GACCATATGA   
  
  
+ AAAATCTTTG AATCAAATCG GTTGGACCTG AACTGCCATT ATTGATATCT TGTTTTTGGT AGAAGCAAAT   
  
  
+ GGTGTCACTT CGTTTTCTAG ATAAAATTAA CTTGAGTTTT TTTGCCACTA TAGATAGTAT TGCAAATATA   
  
  
+ AAAAGTGTGC CACGTGAAAT CAATATTAGG AGGGTGTTTT ATAACAATAG CTGTTCGGGA GAGTTTTAGT   
  
  
+ TATTTTTATT AAATCAAATA GTTAAGAAGT GTTTGGTAAA TAGTTGTATT TGAAAAAGTT ATTCCCATTA   
  
  
+ GCCTTTTAGT AAGAACTTCT TGTGAAAATG ATAATTGTCT AGAAGTTTGA AAAATTCACA CCACATGACA   
  
  
+ TTTAAAAATA ATTTTGTAGA GAAGGGCATA AATGAAAGTT TGCCACCATC TTTTTTAGAA ACATGTAATT   
  
  
+ TACCAAACAC TTTTTCTAAA AACAGTTAAT TCAAATAACT AACAACTAAC AGCTAATATA AATAACTAAC   
  
  
+ AGCTATTTGC TAAACAGGGC CTAAGTAGTT TGAAATTGTT GTTTTCTCTT CAGCTTCACC ATGGCCGATG   
  
  
+ ATGTAAACAA CTATATTCTG AATTTCATTT TTACTATGAT TTGAAAATTT CAAATTTAAA TAAGATTGGA   
  
  
+ TAAAAAAAAG ACCAAAAATC ATAAGTAATA TTAGTCAAGT TCTATTCCAA CCATATACAT AACTGCTTTT   
  
  
+ TCTTTTGGGG GTTTTGAAAC ATGTGTCCTT AGAATAATTG AAACTCGATA TTATTCTCTA AATCTAAGTT   
  
  
+ TACTTTATCA TATTGATTAT TAATTACCTC CTCAACTCCT CCTATACTCT TGTATGCAGA AATAAAAATG   
  
  
+ TTTCCATACC TTATCAGTCG CGATCATAAT AATTAATTTC TTAATTCGGA TTGTATTTAA TTGCGTATCA   
  
  
+ AATGTGAAAT CGTACCAAAT TAGGAAGCAA AACAAACCGA TAACGAAAAA GGTAAGAAAA CTTAGCAAGG   
  
  
+ AAATGAAATC GAAGACCTGT CAACAATATA GCTTCCCTCA CATCCTTCAA CTTAGGGACC ATCCTATACT   
  
  
+ TTCATGTAAT ACAACTAGAG CCACTAGTCA ATAATAGCCT CCTAGTAGTA GCCATAAGCC TTGGTGAGAG   
  
  
+ AATGTTCCGC AAGACTAGTT AACTTGAAAT TCCATTTTGT CGCAGAACTA TTGGAGTGAG AAAGAGAATC   
  
  
+ AAATCATACC ATATCAAATT TGGACCATTT AAGTTCACAA CACACACACA TACACACAAA AGGACGATGT   
  
  
+ ACGGCCCCAC CCCCCACTCT CCTTTCGCCA TCCCCCTACA CCTCTGCCTA CTCTCTCTCT CTCTCTCTCT   
  
  
+ CTCTGGATCA GTCTGTGTAA AAGCTTCCTC TCCAGTACAG CATTTCCTTG TCTGTTCTTG GAGTTTAGTC   
  
  
+ AATTCCTCCG CCCCAAAAAA ACCCACTTTC TCTCTCATCA TGTTCTTCCA GTTCCACCCT TATACGCCAT   
  
  
+ TCATCATCAT CTACATCACC CACATCTTTT TCTCCACAGT TTTCCCCCCT CATTATGCTC TCCGCCTCCA   
  
  
+ CCCCCGCCTT CCCTCTCGCC GTCGCCGGCG ATGACGGGGA TACCACTGAT GCATGCGACG GCACCGCCAC   
  
  
+ TGCCCTCCAC CTCCTCGGGC TGCTGTTGAA GTGCGCGGAA TTCATCTCCA CCGGAGACCT CGCCGGCGCC   
  
  
+ GGAGATATCT TGCCGGAGAT ATTTGAGTTG TCTACACCGT TTGGCTCCCC CGCCGCCCGG GTCGCCGCCT   
  
  
+ TCTTCGGCCA CGCCCTCCAC GCCCGCCTCC TCTCCGCCTC CCTCCGCACA ACTCCGATCG AGAAGCTCAA   
  
  
+ AACCCTGACC CTGGTTTCCC AAATGCGGAA ATTTCACTCC GCCTTGCAAG TATACAACTC CATCACTCCC   
  
  
+ TTCGTGAAAT TCTCTCACTT CACGGCGAAT CAAGCCATCT ACGAGGCGCT GGACGGCGAG GATCGTGTCC   
  
  
+ ACGTCGTCGA CCTTGACATC ATGCAGGGCC TTCAATGGCC GGGATTGTTC CACATCTTAG CCTCTCGACC   
  
  
+ CAGAAAGCCC CTCTCGGTTC GGGTCACCGG GTTCGGGCCA TCCTCCGAGT TGCTCTCCCA AACGGGTAAG   
  
  
+ CGACTCGCTG AGTTCGCCGC TTCACTCGGC CTGCCCTTCG AGTACAACCC GGTGGAGGGC AAAATTGGGA   
  
  
+ ACTTAGTCGA CCTGGGTCGG GTCGGGTCGC TCCCGAATGA AGTGACGGTG GTGCACTGGA TGCACCATAG   
  
  
+ TCTATACGAC ATAACCGGGT CGGATCTTGG GACTTTGAGG GTATTGAGTG CGGTGAGGCC TAGGCTTGTG   
  
  
+ ACTATGGTTG AGCAGGATAT GGACCAAACG GGGTCGTTTT TGGGGAGGTT TGTGGAGGCA TTGCATTATT   
  
  
+ ACTCAGCCTT GTTTGATGCC TTGGGAGAGG GGTTAGATAG GGATAACCTA CAAAGGCATC AAGTGGAGCA   
  
  
+ ACAGCTATTT GGGTGTGAGA TTAGGAACAT CCTGGCCGTT GGTGGGCCCA AGAGGAGGCT CACTGGCGGC   
  
  
+ GATCGGGTCA GAAGGTGGGG CGACGAACTG ACAAGGGTCG GGTTCGAACC AGTTTCGTTG GCGGGTAGCC   
  
  
+ CGGCAACCCA AGCTAGTTTG TTGCTTGGGA TGTTCCCTTG GAAAGGGTAT ACTTTGATGG AGGAAAATGG   
  
  
+ GTGTTTGAGA TTAGGGTGGA AAGATTTGCC CTTGTTAACT GCCTCAGCTT GGCAACCTTG TGAATTTAAC   
  
  
+ AATCCTAGTG CTGGCATTTA   

- +Up\_Stream \_Len000TTTGCC ACGGCAACTT AAACGTCCGC TGCTCCAGCC CAAATTCCGC CTACAGCATC   
  
  
- GCGAAACCTC TTCGTTTAAA CCCTTCTGCT CCGAATAAGA ACTTGAGAAG CATGCGGTAA CAACAACCAA   
  
  
- GGTTGGAAAG AGAGAGGAGC ACTTAAACTC AAACCCGTCA TCTGATAGGG CCTTATCTCC TGACTACGCA   
  
  
- ATATATATAT CTTTAACTCT CTCTCTCTCT CGCACACACT TCCTACAACT TTTACTACCC GCATCCCTCA   
  
  
- GAGAGAGAGT GACAGACAAT CCTCATGCAC AGCTCTTTCC TTTATCCTAA GGGTTCGTCA GATTAAACCC   
  
  
- GGCCAGATAT CCGGTCTTAC CCGGATACTA GTCGGGTCTA TACTGGAACA AATTGGTTGA CATAAACTTA   
  
  
- ACTTCTTTCC CTATAGCGTT ACAGAACTTG GTGTAGTTTA TTATTTACAC GGAAACCAAT CTGGTATACT   
  
  
- TTTTAGAAAC TTAGTTTAGC CAACCTGGAC TTGACGGTAA TAACTATAGA ACAAAAACCA TCTTCGTTTA   
  
  
- CCACAGTGAA GCAAAAGATC TATTTTAATT GAACTCAAAA AAACGGTGAT ATCTATCATA ACGTTTATAT   
  
  
- TTTTCACACG GTGCACTTTA GTTATAATCC TCCCACAAAA TATTGTTATC GACAAGCCCT CTCAAAATCA   
  
  
- ATAAAAATAA TTTAGTTTAT CAATTCTTCA CAAACCATTT ATCAACATAA ACTTTTTCAA TAAGGGTAAT   
  
  
- CGGAAAATCA TTCTTGAAGA ACACTTTTAC TATTAACAGA TCTTCAAACT TTTTAAGTGT GGTGTACTGT   
  
  
- AAATTTTTAT TAAAACATCT CTTCCCGTAT TTACTTTCAA ACGGTGGTAG AAAAAATCTT TGTACATTAA   
  
  
- ATGGTTTGTG AAAAAGATTT TTGTCAATTA AGTTTATTGA TTGTTGATTG TCGATTATAT TTATTGATTG   
  
  
- TCGATAAACG ATTTGTCCCG GATTCATCAA ACTTTAACAA CAAAAGAGAA GTCGAAGTGG TACCGGCTAC   
  
  
- TACATTTGTT GATATAAGAC TTAAAGTAAA AATGATACTA AACTTTTAAA GTTTAAATTT ATTCTAACCT   
  
  
- ATTTTTTTTC TGGTTTTTAG TATTCATTAT AATCAGTTCA AGATAAGGTT GGTATATGTA TTGACGAAAA   
  
  
- AGAAAACCCC CAAAACTTTG TACACAGGAA TCTTATTAAC TTTGAGCTAT AATAAGAGAT TTAGATTCAA   
  
  
- ATGAAATAGT ATAACTAATA ATTAATGGAG GAGTTGAGGA GGATATGAGA ACATACGTCT TTATTTTTAC   
  
  
- AAAGGTATGG AATAGTCAGC GCTAGTATTA TTAATTAAAG AATTAAGCCT AACATAAATT AACGCATAGT   
  
  
- TTACACTTTA GCATGGTTTA ATCCTTCGTT TTGTTTGGCT ATTGCTTTTT CCATTCTTTT GAATCGTTCC   
  
  
- TTTACTTTAG CTTCTGGACA GTTGTTATAT CGAAGGGAGT GTAGGAAGTT GAATCCCTGG TAGGATATGA   
  
  
- AAGTACATTA TGTTGATCTC GGTGATCAGT TATTATCGGA GGATCATCAT CGGTATTCGG AACCACTCTC   
  
  
- TTACAAGGCG TTCTGATCAA TTGAACTTTA AGGTAAAACA GCGTCTTGAT AACCTCACTC TTTCTCTTAG   
  
  
- TTTAGTATGG TATAGTTTAA ACCTGGTAAA TTCAAGTGTT GTGTGTGTGT ATGTGTGTTT TCCTGCTACA   
  
  
- TGCCGGGGTG GGGGGTGAGA GGAAAGCGGT AGGGGGATGT GGAGACGGAT GAGAGAGAGA GAGAGAGAGA   
  
  
- GAGACCTAGT CAGACACATT TTCGAAGGAG AGGTCATGTC GTAAAGGAAC AGACAAGAAC CTCAAATCAG   
  
  
- TTAAGGAGGC GGGGTTTTTT TGGGTGAAAG AGAGAGTAGT ACAAGAAGGT CAAGGTGGGA ATATGCGGTA   
  
  
- AGTAGTAGTA GATGTAGTGG GTGTAGAAAA AGAGGTGTCA AAAGGGGGGA GTAATACGAG AGGCGGAGGT   
  
  
- GGGGGCGGAA GGGAGAGCGG CAGCGGCCGC TACTGCCCCT ATGGTGACTA CGTACGCTGC CGTGGCGGTG   
  
  
- ACGGGAGGTG GAGGAGCCCG ACGACAACTT CACGCGCCTT AAGTAGAGGT GGCCTCTGGA GCGGCCGCGG   
  
  
- CCTCTATAGA ACGGCCTCTA TAAACTCAAC AGATGTGGCA AACCGAGGGG GCGGCGGGCC CAGCGGCGGA   
  
  
- AGAAGCCGGT GCGGGAGGTG CGGGCGGAGG AGAGGCGGAG GGAGGCGTGT TGAGGCTAGC TCTTCGAGTT   
  
  
- TTGGGACTGG GACCAAAGGG TTTACGCCTT TAAAGTGAGG CGGAACGTTC ATATGTTGAG GTAGTGAGGG   
  
  
- AAGCACTTTA AGAGAGTGAA GTGCCGCTTA GTTCGGTAGA TGCTCCGCGA CCTGCCGCTC CTAGCACAGG   
  
  
- TGCAGCAGCT GGAACTGTAG TACGTCCCGG AAGTTACCGG CCCTAACAAG GTGTAGAATC GGAGAGCTGG   
  
  
- GTCTTTCGGG GAGAGCCAAG CCCAGTGGCC CAAGCCCGGT AGGAGGCTCA ACGAGAGGGT TTGCCCATTC   
  
  
- GCTGAGCGAC TCAAGCGGCG AAGTGAGCCG GACGGGAAGC TCATGTTGGG CCACCTCCCG TTTTAACCCT   
  
  
- TGAATCAGCT GGACCCAGCC CAGCCCAGCG AGGGCTTACT TCACTGCCAC CACGTGACCT ACGTGGTATC   
  
  
- AGATATGCTG TATTGGCCCA GCCTAGAACC CTGAAACTCC CATAACTCAC GCCACTCCGG ATCCGAACAC   
  
  
- TGATACCAAC TCGTCCTATA CCTGGTTTGC CCCAGCAAAA ACCCCTCCAA ACACCTCCGT AACGTAATAA   
  
  
- TGAGTCGGAA CAAACTACGG AACCCTCTCC CCAATCTATC CCTATTGGAT GTTTCCGTAG TTCACCTCGT   
  
  
- TGTCGATAAA CCCACACTCT AATCCTTGTA GGACCGGCAA CCACCCGGGT TCTCCTCCGA GTGACCGCCG   
  
  
- CTAGCCCAGT CTTCCACCCC GCTGCTTGAC TGTTCCCAGC CCAAGCTTGG TCAAAGCAAC CGCCCATCGG   
  
  
- GCCGTTGGGT TCGATCAAAC AACGAACCCT ACAAGGGAAC CTTTCCCATA TGAAACTACC TCCTTTTACC   
  
  
- CACAAACTCT AATCCCACCT TTCTAAACGG GAACAATTGA CGGAGTCGAA CCGTTGGAAC ACTTAAATTG   
  
  
- TTAGGATCAC GACCGTAAAT

+     ABRE

| Site Name | Organism | Position | Strand | Matrix score. | sequence | function |
| --- | --- | --- | --- | --- | --- | --- |
| ABRE | Arabidopsis thaliana | 2640 | + | 7 | AACCCGG | cis-acting element involved in the abscisic acid responsiveness |
| ABRE | Arabidopsis thaliana | 2454 | - | 5 | ACGTG | cis-acting element involved in the abscisic acid responsiveness |
| ABRE | Arabidopsis thaliana | 310 | + | 5 | ACGTG | cis-acting element involved in the abscisic acid responsiveness |
| ABRE | Arabidopsis thaliana | 646 | + | 5 | ACGTG | cis-acting element involved in the abscisic acid responsiveness |
| ABRE | Arabidopsis thaliana | 645 | + | 6 | CACGTG | cis-acting element involved in the abscisic acid responsiveness |
| ABRE | Oryza sativa | 309 | + | 8 | TACGTGTC | cis-acting element involved in the abscisic acid responsiveness |
| ABRE | Arabidopsis thaliana | 2551 | - | 7 | AACCCGG | cis-acting element involved in the abscisic acid responsiveness |

>HU02G03005.1   
+ +Up\_Stream \_Len000AAACGG TGCCGTTGAA TTTGCAGGCG ACGAGGTCGG GTTTAAGGCG GATGTCGTAG   
  
  
+ CGCTTTGGAG AAGCAAATTT GGGAAGACGA GGCTTATTCT TGAACTCTTC GTACGCCATT GTTGTTGGTT   
  
  
+ CCAACCTTTC TCTCTCCTCG TGAATTTGAG TTTGGGCAGT AGACTATCCC GGAATAGAGG ACTGATGCGT   
  
  
+ TATATATATA GAAATTGAGA GAGAGAGAGA GCGTGTGTGA AGGATGTTGA AAATGATGGG CGTAGGGAGT   
  
  
+ CTCTCTCTCA CTGTCTGTTA GGAGTACGTG TCGAGAAAGG AAATAGGATT CCCAAGCAGT CTAATTTGGG   
  
  
+ CCGGTCTATA GGCCAGAATG GGCCTATGAT CAGCCCAGAT ATGACCTTGT TTAACCAACT GTATTTGAAT   
  
  
+ TGAAGAAAGG GATATCGCAA TGTCTTGAAC CACATCAAAT AATAAATGTG CCTTTGGTTA GACCATATGA   
  
  
+ AAAATCTTTG AATCAAATCG GTTGGACCTG AACTGCCATT ATTGATATCT TGTTTTTGGT AGAAGCAAAT   
  
  
+ GGTGTCACTT CGTTTTCTAG ATAAAATTAA CTTGAGTTTT TTTGCCACTA TAGATAGTAT TGCAAATATA   
  
  
+ AAAAGTGTGC CACGTGAAAT CAATATTAGG AGGGTGTTTT ATAACAATAG CTGTTCGGGA GAGTTTTAGT   
  
  
+ TATTTTTATT AAATCAAATA GTTAAGAAGT GTTTGGTAAA TAGTTGTATT TGAAAAAGTT ATTCCCATTA   
  
  
+ GCCTTTTAGT AAGAACTTCT TGTGAAAATG ATAATTGTCT AGAAGTTTGA AAAATTCACA CCACATGACA   
  
  
+ TTTAAAAATA ATTTTGTAGA GAAGGGCATA AATGAAAGTT TGCCACCATC TTTTTTAGAA ACATGTAATT   
  
  
+ TACCAAACAC TTTTTCTAAA AACAGTTAAT TCAAATAACT AACAACTAAC AGCTAATATA AATAACTAAC   
  
  
+ AGCTATTTGC TAAACAGGGC CTAAGTAGTT TGAAATTGTT GTTTTCTCTT CAGCTTCACC ATGGCCGATG   
  
  
+ ATGTAAACAA CTATATTCTG AATTTCATTT TTACTATGAT TTGAAAATTT CAAATTTAAA TAAGATTGGA   
  
  
+ TAAAAAAAAG ACCAAAAATC ATAAGTAATA TTAGTCAAGT TCTATTCCAA CCATATACAT AACTGCTTTT   
  
  
+ TCTTTTGGGG GTTTTGAAAC ATGTGTCCTT AGAATAATTG AAACTCGATA TTATTCTCTA AATCTAAGTT   
  
  
+ TACTTTATCA TATTGATTAT TAATTACCTC CTCAACTCCT CCTATACTCT TGTATGCAGA AATAAAAATG   
  
  
+ TTTCCATACC TTATCAGTCG CGATCATAAT AATTAATTTC TTAATTCGGA TTGTATTTAA TTGCGTATCA   
  
  
+ AATGTGAAAT CGTACCAAAT TAGGAAGCAA AACAAACCGA TAACGAAAAA GGTAAGAAAA CTTAGCAAGG   
  
  
+ AAATGAAATC GAAGACCTGT CAACAATATA GCTTCCCTCA CATCCTTCAA CTTAGGGACC ATCCTATACT   
  
  
+ TTCATGTAAT ACAACTAGAG CCACTAGTCA ATAATAGCCT CCTAGTAGTA GCCATAAGCC TTGGTGAGAG   
  
  
+ AATGTTCCGC AAGACTAGTT AACTTGAAAT TCCATTTTGT CGCAGAACTA TTGGAGTGAG AAAGAGAATC   
  
  
+ AAATCATACC ATATCAAATT TGGACCATTT AAGTTCACAA CACACACACA TACACACAAA AGGACGATGT   
  
  
+ ACGGCCCCAC CCCCCACTCT CCTTTCGCCA TCCCCCTACA CCTCTGCCTA CTCTCTCTCT CTCTCTCTCT   
  
  
+ CTCTGGATCA GTCTGTGTAA AAGCTTCCTC TCCAGTACAG CATTTCCTTG TCTGTTCTTG GAGTTTAGTC   
  
  
+ AATTCCTCCG CCCCAAAAAA ACCCACTTTC TCTCTCATCA TGTTCTTCCA GTTCCACCCT TATACGCCAT   
  
  
+ TCATCATCAT CTACATCACC CACATCTTTT TCTCCACAGT TTTCCCCCCT CATTATGCTC TCCGCCTCCA   
  
  
+ CCCCCGCCTT CCCTCTCGCC GTCGCCGGCG ATGACGGGGA TACCACTGAT GCATGCGACG GCACCGCCAC   
  
  
+ TGCCCTCCAC CTCCTCGGGC TGCTGTTGAA GTGCGCGGAA TTCATCTCCA CCGGAGACCT CGCCGGCGCC   
  
  
+ GGAGATATCT TGCCGGAGAT ATTTGAGTTG TCTACACCGT TTGGCTCCCC CGCCGCCCGG GTCGCCGCCT   
  
  
+ TCTTCGGCCA CGCCCTCCAC GCCCGCCTCC TCTCCGCCTC CCTCCGCACA ACTCCGATCG AGAAGCTCAA   
  
  
+ AACCCTGACC CTGGTTTCCC AAATGCGGAA ATTTCACTCC GCCTTGCAAG TATACAACTC CATCACTCCC   
  
  
+ TTCGTGAAAT TCTCTCACTT CACGGCGAAT CAAGCCATCT ACGAGGCGCT GGACGGCGAG GATCGTGTCC   
  
  
+ ACGTCGTCGA CCTTGACATC ATGCAGGGCC TTCAATGGCC GGGATTGTTC CACATCTTAG CCTCTCGACC   
  
  
+ CAGAAAGCCC CTCTCGGTTC GGGTCACCGG GTTCGGGCCA TCCTCCGAGT TGCTCTCCCA AACGGGTAAG   
  
  
+ CGACTCGCTG AGTTCGCCGC TTCACTCGGC CTGCCCTTCG AGTACAACCC GGTGGAGGGC AAAATTGGGA   
  
  
+ ACTTAGTCGA CCTGGGTCGG GTCGGGTCGC TCCCGAATGA AGTGACGGTG GTGCACTGGA TGCACCATAG   
  
  
+ TCTATACGAC ATAACCGGGT CGGATCTTGG GACTTTGAGG GTATTGAGTG CGGTGAGGCC TAGGCTTGTG   
  
  
+ ACTATGGTTG AGCAGGATAT GGACCAAACG GGGTCGTTTT TGGGGAGGTT TGTGGAGGCA TTGCATTATT   
  
  
+ ACTCAGCCTT GTTTGATGCC TTGGGAGAGG GGTTAGATAG GGATAACCTA CAAAGGCATC AAGTGGAGCA   
  
  
+ ACAGCTATTT GGGTGTGAGA TTAGGAACAT CCTGGCCGTT GGTGGGCCCA AGAGGAGGCT CACTGGCGGC   
  
  
+ GATCGGGTCA GAAGGTGGGG CGACGAACTG ACAAGGGTCG GGTTCGAACC AGTTTCGTTG GCGGGTAGCC   
  
  
+ CGGCAACCCA AGCTAGTTTG TTGCTTGGGA TGTTCCCTTG GAAAGGGTAT ACTTTGATGG AGGAAAATGG   
  
  
+ GTGTTTGAGA TTAGGGTGGA AAGATTTGCC CTTGTTAACT GCCTCAGCTT GGCAACCTTG TGAATTTAAC   
  
  
+ AATCCTAGTG CTGGCATTTA   

- +Up\_Stream \_Len000TTTGCC ACGGCAACTT AAACGTCCGC TGCTCCAGCC CAAATTCCGC CTACAGCATC   
  
  
- GCGAAACCTC TTCGTTTAAA CCCTTCTGCT CCGAATAAGA ACTTGAGAAG CATGCGGTAA CAACAACCAA   
  
  
- GGTTGGAAAG AGAGAGGAGC ACTTAAACTC AAACCCGTCA TCTGATAGGG CCTTATCTCC TGACTACGCA   
  
  
- ATATATATAT CTTTAACTCT CTCTCTCTCT CGCACACACT TCCTACAACT TTTACTACCC GCATCCCTCA   
  
  
- GAGAGAGAGT GACAGACAAT CCTCATGCAC AGCTCTTTCC TTTATCCTAA GGGTTCGTCA GATTAAACCC   
  
  
- GGCCAGATAT CCGGTCTTAC CCGGATACTA GTCGGGTCTA TACTGGAACA AATTGGTTGA CATAAACTTA   
  
  
- ACTTCTTTCC CTATAGCGTT ACAGAACTTG GTGTAGTTTA TTATTTACAC GGAAACCAAT CTGGTATACT   
  
  
- TTTTAGAAAC TTAGTTTAGC CAACCTGGAC TTGACGGTAA TAACTATAGA ACAAAAACCA TCTTCGTTTA   
  
  
- CCACAGTGAA GCAAAAGATC TATTTTAATT GAACTCAAAA AAACGGTGAT ATCTATCATA ACGTTTATAT   
  
  
- TTTTCACACG GTGCACTTTA GTTATAATCC TCCCACAAAA TATTGTTATC GACAAGCCCT CTCAAAATCA   
  
  
- ATAAAAATAA TTTAGTTTAT CAATTCTTCA CAAACCATTT ATCAACATAA ACTTTTTCAA TAAGGGTAAT   
  
  
- CGGAAAATCA TTCTTGAAGA ACACTTTTAC TATTAACAGA TCTTCAAACT TTTTAAGTGT GGTGTACTGT   
  
  
- AAATTTTTAT TAAAACATCT CTTCCCGTAT TTACTTTCAA ACGGTGGTAG AAAAAATCTT TGTACATTAA   
  
  
- ATGGTTTGTG AAAAAGATTT TTGTCAATTA AGTTTATTGA TTGTTGATTG TCGATTATAT TTATTGATTG   
  
  
- TCGATAAACG ATTTGTCCCG GATTCATCAA ACTTTAACAA CAAAAGAGAA GTCGAAGTGG TACCGGCTAC   
  
  
- TACATTTGTT GATATAAGAC TTAAAGTAAA AATGATACTA AACTTTTAAA GTTTAAATTT ATTCTAACCT   
  
  
- ATTTTTTTTC TGGTTTTTAG TATTCATTAT AATCAGTTCA AGATAAGGTT GGTATATGTA TTGACGAAAA   
  
  
- AGAAAACCCC CAAAACTTTG TACACAGGAA TCTTATTAAC TTTGAGCTAT AATAAGAGAT TTAGATTCAA   
  
  
- ATGAAATAGT ATAACTAATA ATTAATGGAG GAGTTGAGGA GGATATGAGA ACATACGTCT TTATTTTTAC   
  
  
- AAAGGTATGG AATAGTCAGC GCTAGTATTA TTAATTAAAG AATTAAGCCT AACATAAATT AACGCATAGT   
  
  
- TTACACTTTA GCATGGTTTA ATCCTTCGTT TTGTTTGGCT ATTGCTTTTT CCATTCTTTT GAATCGTTCC   
  
  
- TTTACTTTAG CTTCTGGACA GTTGTTATAT CGAAGGGAGT GTAGGAAGTT GAATCCCTGG TAGGATATGA   
  
  
- AAGTACATTA TGTTGATCTC GGTGATCAGT TATTATCGGA GGATCATCAT CGGTATTCGG AACCACTCTC   
  
  
- TTACAAGGCG TTCTGATCAA TTGAACTTTA AGGTAAAACA GCGTCTTGAT AACCTCACTC TTTCTCTTAG   
  
  
- TTTAGTATGG TATAGTTTAA ACCTGGTAAA TTCAAGTGTT GTGTGTGTGT ATGTGTGTTT TCCTGCTACA   
  
  
- TGCCGGGGTG GGGGGTGAGA GGAAAGCGGT AGGGGGATGT GGAGACGGAT GAGAGAGAGA GAGAGAGAGA   
  
  
- GAGACCTAGT CAGACACATT TTCGAAGGAG AGGTCATGTC GTAAAGGAAC AGACAAGAAC CTCAAATCAG   
  
  
- TTAAGGAGGC GGGGTTTTTT TGGGTGAAAG AGAGAGTAGT ACAAGAAGGT CAAGGTGGGA ATATGCGGTA   
  
  
- AGTAGTAGTA GATGTAGTGG GTGTAGAAAA AGAGGTGTCA AAAGGGGGGA GTAATACGAG AGGCGGAGGT   
  
  
- GGGGGCGGAA GGGAGAGCGG CAGCGGCCGC TACTGCCCCT ATGGTGACTA CGTACGCTGC CGTGGCGGTG   
  
  
- ACGGGAGGTG GAGGAGCCCG ACGACAACTT CACGCGCCTT AAGTAGAGGT GGCCTCTGGA GCGGCCGCGG   
  
  
- CCTCTATAGA ACGGCCTCTA TAAACTCAAC AGATGTGGCA AACCGAGGGG GCGGCGGGCC CAGCGGCGGA   
  
  
- AGAAGCCGGT GCGGGAGGTG CGGGCGGAGG AGAGGCGGAG GGAGGCGTGT TGAGGCTAGC TCTTCGAGTT   
  
  
- TTGGGACTGG GACCAAAGGG TTTACGCCTT TAAAGTGAGG CGGAACGTTC ATATGTTGAG GTAGTGAGGG   
  
  
- AAGCACTTTA AGAGAGTGAA GTGCCGCTTA GTTCGGTAGA TGCTCCGCGA CCTGCCGCTC CTAGCACAGG   
  
  
- TGCAGCAGCT GGAACTGTAG TACGTCCCGG AAGTTACCGG CCCTAACAAG GTGTAGAATC GGAGAGCTGG   
  
  
- GTCTTTCGGG GAGAGCCAAG CCCAGTGGCC CAAGCCCGGT AGGAGGCTCA ACGAGAGGGT TTGCCCATTC   
  
  
- GCTGAGCGAC TCAAGCGGCG AAGTGAGCCG GACGGGAAGC TCATGTTGGG CCACCTCCCG TTTTAACCCT   
  
  
- TGAATCAGCT GGACCCAGCC CAGCCCAGCG AGGGCTTACT TCACTGCCAC CACGTGACCT ACGTGGTATC   
  
  
- AGATATGCTG TATTGGCCCA GCCTAGAACC CTGAAACTCC CATAACTCAC GCCACTCCGG ATCCGAACAC   
  
  
- TGATACCAAC TCGTCCTATA CCTGGTTTGC CCCAGCAAAA ACCCCTCCAA ACACCTCCGT AACGTAATAA   
  
  
- TGAGTCGGAA CAAACTACGG AACCCTCTCC CCAATCTATC CCTATTGGAT GTTTCCGTAG TTCACCTCGT   
  
  
- TGTCGATAAA CCCACACTCT AATCCTTGTA GGACCGGCAA CCACCCGGGT TCTCCTCCGA GTGACCGCCG   
  
  
- CTAGCCCAGT CTTCCACCCC GCTGCTTGAC TGTTCCCAGC CCAAGCTTGG TCAAAGCAAC CGCCCATCGG   
  
  
- GCCGTTGGGT TCGATCAAAC AACGAACCCT ACAAGGGAAC CTTTCCCATA TGAAACTACC TCCTTTTACC   
  
  
- CACAAACTCT AATCCCACCT TTCTAAACGG GAACAATTGA CGGAGTCGAA CCGTTGGAAC ACTTAAATTG   
  
  
- TTAGGATCAC GACCGTAAAT

+     ABRE3a

| Site Name | Organism | Position | Strand | Matrix score. | sequence | function |
| --- | --- | --- | --- | --- | --- | --- |
| ABRE3a | Zea mays | 309 | + | 6 | TACGTG |  |

>HU02G03005.1   
+ +Up\_Stream \_Len000AAACGG TGCCGTTGAA TTTGCAGGCG ACGAGGTCGG GTTTAAGGCG GATGTCGTAG   
  
  
+ CGCTTTGGAG AAGCAAATTT GGGAAGACGA GGCTTATTCT TGAACTCTTC GTACGCCATT GTTGTTGGTT   
  
  
+ CCAACCTTTC TCTCTCCTCG TGAATTTGAG TTTGGGCAGT AGACTATCCC GGAATAGAGG ACTGATGCGT   
  
  
+ TATATATATA GAAATTGAGA GAGAGAGAGA GCGTGTGTGA AGGATGTTGA AAATGATGGG CGTAGGGAGT   
  
  
+ CTCTCTCTCA CTGTCTGTTA GGAGTACGTG TCGAGAAAGG AAATAGGATT CCCAAGCAGT CTAATTTGGG   
  
  
+ CCGGTCTATA GGCCAGAATG GGCCTATGAT CAGCCCAGAT ATGACCTTGT TTAACCAACT GTATTTGAAT   
  
  
+ TGAAGAAAGG GATATCGCAA TGTCTTGAAC CACATCAAAT AATAAATGTG CCTTTGGTTA GACCATATGA   
  
  
+ AAAATCTTTG AATCAAATCG GTTGGACCTG AACTGCCATT ATTGATATCT TGTTTTTGGT AGAAGCAAAT   
  
  
+ GGTGTCACTT CGTTTTCTAG ATAAAATTAA CTTGAGTTTT TTTGCCACTA TAGATAGTAT TGCAAATATA   
  
  
+ AAAAGTGTGC CACGTGAAAT CAATATTAGG AGGGTGTTTT ATAACAATAG CTGTTCGGGA GAGTTTTAGT   
  
  
+ TATTTTTATT AAATCAAATA GTTAAGAAGT GTTTGGTAAA TAGTTGTATT TGAAAAAGTT ATTCCCATTA   
  
  
+ GCCTTTTAGT AAGAACTTCT TGTGAAAATG ATAATTGTCT AGAAGTTTGA AAAATTCACA CCACATGACA   
  
  
+ TTTAAAAATA ATTTTGTAGA GAAGGGCATA AATGAAAGTT TGCCACCATC TTTTTTAGAA ACATGTAATT   
  
  
+ TACCAAACAC TTTTTCTAAA AACAGTTAAT TCAAATAACT AACAACTAAC AGCTAATATA AATAACTAAC   
  
  
+ AGCTATTTGC TAAACAGGGC CTAAGTAGTT TGAAATTGTT GTTTTCTCTT CAGCTTCACC ATGGCCGATG   
  
  
+ ATGTAAACAA CTATATTCTG AATTTCATTT TTACTATGAT TTGAAAATTT CAAATTTAAA TAAGATTGGA   
  
  
+ TAAAAAAAAG ACCAAAAATC ATAAGTAATA TTAGTCAAGT TCTATTCCAA CCATATACAT AACTGCTTTT   
  
  
+ TCTTTTGGGG GTTTTGAAAC ATGTGTCCTT AGAATAATTG AAACTCGATA TTATTCTCTA AATCTAAGTT   
  
  
+ TACTTTATCA TATTGATTAT TAATTACCTC CTCAACTCCT CCTATACTCT TGTATGCAGA AATAAAAATG   
  
  
+ TTTCCATACC TTATCAGTCG CGATCATAAT AATTAATTTC TTAATTCGGA TTGTATTTAA TTGCGTATCA   
  
  
+ AATGTGAAAT CGTACCAAAT TAGGAAGCAA AACAAACCGA TAACGAAAAA GGTAAGAAAA CTTAGCAAGG   
  
  
+ AAATGAAATC GAAGACCTGT CAACAATATA GCTTCCCTCA CATCCTTCAA CTTAGGGACC ATCCTATACT   
  
  
+ TTCATGTAAT ACAACTAGAG CCACTAGTCA ATAATAGCCT CCTAGTAGTA GCCATAAGCC TTGGTGAGAG   
  
  
+ AATGTTCCGC AAGACTAGTT AACTTGAAAT TCCATTTTGT CGCAGAACTA TTGGAGTGAG AAAGAGAATC   
  
  
+ AAATCATACC ATATCAAATT TGGACCATTT AAGTTCACAA CACACACACA TACACACAAA AGGACGATGT   
  
  
+ ACGGCCCCAC CCCCCACTCT CCTTTCGCCA TCCCCCTACA CCTCTGCCTA CTCTCTCTCT CTCTCTCTCT   
  
  
+ CTCTGGATCA GTCTGTGTAA AAGCTTCCTC TCCAGTACAG CATTTCCTTG TCTGTTCTTG GAGTTTAGTC   
  
  
+ AATTCCTCCG CCCCAAAAAA ACCCACTTTC TCTCTCATCA TGTTCTTCCA GTTCCACCCT TATACGCCAT   
  
  
+ TCATCATCAT CTACATCACC CACATCTTTT TCTCCACAGT TTTCCCCCCT CATTATGCTC TCCGCCTCCA   
  
  
+ CCCCCGCCTT CCCTCTCGCC GTCGCCGGCG ATGACGGGGA TACCACTGAT GCATGCGACG GCACCGCCAC   
  
  
+ TGCCCTCCAC CTCCTCGGGC TGCTGTTGAA GTGCGCGGAA TTCATCTCCA CCGGAGACCT CGCCGGCGCC   
  
  
+ GGAGATATCT TGCCGGAGAT ATTTGAGTTG TCTACACCGT TTGGCTCCCC CGCCGCCCGG GTCGCCGCCT   
  
  
+ TCTTCGGCCA CGCCCTCCAC GCCCGCCTCC TCTCCGCCTC CCTCCGCACA ACTCCGATCG AGAAGCTCAA   
  
  
+ AACCCTGACC CTGGTTTCCC AAATGCGGAA ATTTCACTCC GCCTTGCAAG TATACAACTC CATCACTCCC   
  
  
+ TTCGTGAAAT TCTCTCACTT CACGGCGAAT CAAGCCATCT ACGAGGCGCT GGACGGCGAG GATCGTGTCC   
  
  
+ ACGTCGTCGA CCTTGACATC ATGCAGGGCC TTCAATGGCC GGGATTGTTC CACATCTTAG CCTCTCGACC   
  
  
+ CAGAAAGCCC CTCTCGGTTC GGGTCACCGG GTTCGGGCCA TCCTCCGAGT TGCTCTCCCA AACGGGTAAG   
  
  
+ CGACTCGCTG AGTTCGCCGC TTCACTCGGC CTGCCCTTCG AGTACAACCC GGTGGAGGGC AAAATTGGGA   
  
  
+ ACTTAGTCGA CCTGGGTCGG GTCGGGTCGC TCCCGAATGA AGTGACGGTG GTGCACTGGA TGCACCATAG   
  
  
+ TCTATACGAC ATAACCGGGT CGGATCTTGG GACTTTGAGG GTATTGAGTG CGGTGAGGCC TAGGCTTGTG   
  
  
+ ACTATGGTTG AGCAGGATAT GGACCAAACG GGGTCGTTTT TGGGGAGGTT TGTGGAGGCA TTGCATTATT   
  
  
+ ACTCAGCCTT GTTTGATGCC TTGGGAGAGG GGTTAGATAG GGATAACCTA CAAAGGCATC AAGTGGAGCA   
  
  
+ ACAGCTATTT GGGTGTGAGA TTAGGAACAT CCTGGCCGTT GGTGGGCCCA AGAGGAGGCT CACTGGCGGC   
  
  
+ GATCGGGTCA GAAGGTGGGG CGACGAACTG ACAAGGGTCG GGTTCGAACC AGTTTCGTTG GCGGGTAGCC   
  
  
+ CGGCAACCCA AGCTAGTTTG TTGCTTGGGA TGTTCCCTTG GAAAGGGTAT ACTTTGATGG AGGAAAATGG   
  
  
+ GTGTTTGAGA TTAGGGTGGA AAGATTTGCC CTTGTTAACT GCCTCAGCTT GGCAACCTTG TGAATTTAAC   
  
  
+ AATCCTAGTG CTGGCATTTA   

- +Up\_Stream \_Len000TTTGCC ACGGCAACTT AAACGTCCGC TGCTCCAGCC CAAATTCCGC CTACAGCATC   
  
  
- GCGAAACCTC TTCGTTTAAA CCCTTCTGCT CCGAATAAGA ACTTGAGAAG CATGCGGTAA CAACAACCAA   
  
  
- GGTTGGAAAG AGAGAGGAGC ACTTAAACTC AAACCCGTCA TCTGATAGGG CCTTATCTCC TGACTACGCA   
  
  
- ATATATATAT CTTTAACTCT CTCTCTCTCT CGCACACACT TCCTACAACT TTTACTACCC GCATCCCTCA   
  
  
- GAGAGAGAGT GACAGACAAT CCTCATGCAC AGCTCTTTCC TTTATCCTAA GGGTTCGTCA GATTAAACCC   
  
  
- GGCCAGATAT CCGGTCTTAC CCGGATACTA GTCGGGTCTA TACTGGAACA AATTGGTTGA CATAAACTTA   
  
  
- ACTTCTTTCC CTATAGCGTT ACAGAACTTG GTGTAGTTTA TTATTTACAC GGAAACCAAT CTGGTATACT   
  
  
- TTTTAGAAAC TTAGTTTAGC CAACCTGGAC TTGACGGTAA TAACTATAGA ACAAAAACCA TCTTCGTTTA   
  
  
- CCACAGTGAA GCAAAAGATC TATTTTAATT GAACTCAAAA AAACGGTGAT ATCTATCATA ACGTTTATAT   
  
  
- TTTTCACACG GTGCACTTTA GTTATAATCC TCCCACAAAA TATTGTTATC GACAAGCCCT CTCAAAATCA   
  
  
- ATAAAAATAA TTTAGTTTAT CAATTCTTCA CAAACCATTT ATCAACATAA ACTTTTTCAA TAAGGGTAAT   
  
  
- CGGAAAATCA TTCTTGAAGA ACACTTTTAC TATTAACAGA TCTTCAAACT TTTTAAGTGT GGTGTACTGT   
  
  
- AAATTTTTAT TAAAACATCT CTTCCCGTAT TTACTTTCAA ACGGTGGTAG AAAAAATCTT TGTACATTAA   
  
  
- ATGGTTTGTG AAAAAGATTT TTGTCAATTA AGTTTATTGA TTGTTGATTG TCGATTATAT TTATTGATTG   
  
  
- TCGATAAACG ATTTGTCCCG GATTCATCAA ACTTTAACAA CAAAAGAGAA GTCGAAGTGG TACCGGCTAC   
  
  
- TACATTTGTT GATATAAGAC TTAAAGTAAA AATGATACTA AACTTTTAAA GTTTAAATTT ATTCTAACCT   
  
  
- ATTTTTTTTC TGGTTTTTAG TATTCATTAT AATCAGTTCA AGATAAGGTT GGTATATGTA TTGACGAAAA   
  
  
- AGAAAACCCC CAAAACTTTG TACACAGGAA TCTTATTAAC TTTGAGCTAT AATAAGAGAT TTAGATTCAA   
  
  
- ATGAAATAGT ATAACTAATA ATTAATGGAG GAGTTGAGGA GGATATGAGA ACATACGTCT TTATTTTTAC   
  
  
- AAAGGTATGG AATAGTCAGC GCTAGTATTA TTAATTAAAG AATTAAGCCT AACATAAATT AACGCATAGT   
  
  
- TTACACTTTA GCATGGTTTA ATCCTTCGTT TTGTTTGGCT ATTGCTTTTT CCATTCTTTT GAATCGTTCC   
  
  
- TTTACTTTAG CTTCTGGACA GTTGTTATAT CGAAGGGAGT GTAGGAAGTT GAATCCCTGG TAGGATATGA   
  
  
- AAGTACATTA TGTTGATCTC GGTGATCAGT TATTATCGGA GGATCATCAT CGGTATTCGG AACCACTCTC   
  
  
- TTACAAGGCG TTCTGATCAA TTGAACTTTA AGGTAAAACA GCGTCTTGAT AACCTCACTC TTTCTCTTAG   
  
  
- TTTAGTATGG TATAGTTTAA ACCTGGTAAA TTCAAGTGTT GTGTGTGTGT ATGTGTGTTT TCCTGCTACA   
  
  
- TGCCGGGGTG GGGGGTGAGA GGAAAGCGGT AGGGGGATGT GGAGACGGAT GAGAGAGAGA GAGAGAGAGA   
  
  
- GAGACCTAGT CAGACACATT TTCGAAGGAG AGGTCATGTC GTAAAGGAAC AGACAAGAAC CTCAAATCAG   
  
  
- TTAAGGAGGC GGGGTTTTTT TGGGTGAAAG AGAGAGTAGT ACAAGAAGGT CAAGGTGGGA ATATGCGGTA   
  
  
- AGTAGTAGTA GATGTAGTGG GTGTAGAAAA AGAGGTGTCA AAAGGGGGGA GTAATACGAG AGGCGGAGGT   
  
  
- GGGGGCGGAA GGGAGAGCGG CAGCGGCCGC TACTGCCCCT ATGGTGACTA CGTACGCTGC CGTGGCGGTG   
  
  
- ACGGGAGGTG GAGGAGCCCG ACGACAACTT CACGCGCCTT AAGTAGAGGT GGCCTCTGGA GCGGCCGCGG   
  
  
- CCTCTATAGA ACGGCCTCTA TAAACTCAAC AGATGTGGCA AACCGAGGGG GCGGCGGGCC CAGCGGCGGA   
  
  
- AGAAGCCGGT GCGGGAGGTG CGGGCGGAGG AGAGGCGGAG GGAGGCGTGT TGAGGCTAGC TCTTCGAGTT   
  
  
- TTGGGACTGG GACCAAAGGG TTTACGCCTT TAAAGTGAGG CGGAACGTTC ATATGTTGAG GTAGTGAGGG   
  
  
- AAGCACTTTA AGAGAGTGAA GTGCCGCTTA GTTCGGTAGA TGCTCCGCGA CCTGCCGCTC CTAGCACAGG   
  
  
- TGCAGCAGCT GGAACTGTAG TACGTCCCGG AAGTTACCGG CCCTAACAAG GTGTAGAATC GGAGAGCTGG   
  
  
- GTCTTTCGGG GAGAGCCAAG CCCAGTGGCC CAAGCCCGGT AGGAGGCTCA ACGAGAGGGT TTGCCCATTC   
  
  
- GCTGAGCGAC TCAAGCGGCG AAGTGAGCCG GACGGGAAGC TCATGTTGGG CCACCTCCCG TTTTAACCCT   
  
  
- TGAATCAGCT GGACCCAGCC CAGCCCAGCG AGGGCTTACT TCACTGCCAC CACGTGACCT ACGTGGTATC   
  
  
- AGATATGCTG TATTGGCCCA GCCTAGAACC CTGAAACTCC CATAACTCAC GCCACTCCGG ATCCGAACAC   
  
  
- TGATACCAAC TCGTCCTATA CCTGGTTTGC CCCAGCAAAA ACCCCTCCAA ACACCTCCGT AACGTAATAA   
  
  
- TGAGTCGGAA CAAACTACGG AACCCTCTCC CCAATCTATC CCTATTGGAT GTTTCCGTAG TTCACCTCGT   
  
  
- TGTCGATAAA CCCACACTCT AATCCTTGTA GGACCGGCAA CCACCCGGGT TCTCCTCCGA GTGACCGCCG   
  
  
- CTAGCCCAGT CTTCCACCCC GCTGCTTGAC TGTTCCCAGC CCAAGCTTGG TCAAAGCAAC CGCCCATCGG   
  
  
- GCCGTTGGGT TCGATCAAAC AACGAACCCT ACAAGGGAAC CTTTCCCATA TGAAACTACC TCCTTTTACC   
  
  
- CACAAACTCT AATCCCACCT TTCTAAACGG GAACAATTGA CGGAGTCGAA CCGTTGGAAC ACTTAAATTG   
  
  
- TTAGGATCAC GACCGTAAAT

+     ABRE4

| Site Name | Organism | Position | Strand | Matrix score. | sequence | function |
| --- | --- | --- | --- | --- | --- | --- |
| ABRE4 | Zea mays | 309 | - | 6 | CACGTA |  |

>HU02G03005.1   
+ +Up\_Stream \_Len000AAACGG TGCCGTTGAA TTTGCAGGCG ACGAGGTCGG GTTTAAGGCG GATGTCGTAG   
  
  
+ CGCTTTGGAG AAGCAAATTT GGGAAGACGA GGCTTATTCT TGAACTCTTC GTACGCCATT GTTGTTGGTT   
  
  
+ CCAACCTTTC TCTCTCCTCG TGAATTTGAG TTTGGGCAGT AGACTATCCC GGAATAGAGG ACTGATGCGT   
  
  
+ TATATATATA GAAATTGAGA GAGAGAGAGA GCGTGTGTGA AGGATGTTGA AAATGATGGG CGTAGGGAGT   
  
  
+ CTCTCTCTCA CTGTCTGTTA GGAGTACGTG TCGAGAAAGG AAATAGGATT CCCAAGCAGT CTAATTTGGG   
  
  
+ CCGGTCTATA GGCCAGAATG GGCCTATGAT CAGCCCAGAT ATGACCTTGT TTAACCAACT GTATTTGAAT   
  
  
+ TGAAGAAAGG GATATCGCAA TGTCTTGAAC CACATCAAAT AATAAATGTG CCTTTGGTTA GACCATATGA   
  
  
+ AAAATCTTTG AATCAAATCG GTTGGACCTG AACTGCCATT ATTGATATCT TGTTTTTGGT AGAAGCAAAT   
  
  
+ GGTGTCACTT CGTTTTCTAG ATAAAATTAA CTTGAGTTTT TTTGCCACTA TAGATAGTAT TGCAAATATA   
  
  
+ AAAAGTGTGC CACGTGAAAT CAATATTAGG AGGGTGTTTT ATAACAATAG CTGTTCGGGA GAGTTTTAGT   
  
  
+ TATTTTTATT AAATCAAATA GTTAAGAAGT GTTTGGTAAA TAGTTGTATT TGAAAAAGTT ATTCCCATTA   
  
  
+ GCCTTTTAGT AAGAACTTCT TGTGAAAATG ATAATTGTCT AGAAGTTTGA AAAATTCACA CCACATGACA   
  
  
+ TTTAAAAATA ATTTTGTAGA GAAGGGCATA AATGAAAGTT TGCCACCATC TTTTTTAGAA ACATGTAATT   
  
  
+ TACCAAACAC TTTTTCTAAA AACAGTTAAT TCAAATAACT AACAACTAAC AGCTAATATA AATAACTAAC   
  
  
+ AGCTATTTGC TAAACAGGGC CTAAGTAGTT TGAAATTGTT GTTTTCTCTT CAGCTTCACC ATGGCCGATG   
  
  
+ ATGTAAACAA CTATATTCTG AATTTCATTT TTACTATGAT TTGAAAATTT CAAATTTAAA TAAGATTGGA   
  
  
+ TAAAAAAAAG ACCAAAAATC ATAAGTAATA TTAGTCAAGT TCTATTCCAA CCATATACAT AACTGCTTTT   
  
  
+ TCTTTTGGGG GTTTTGAAAC ATGTGTCCTT AGAATAATTG AAACTCGATA TTATTCTCTA AATCTAAGTT   
  
  
+ TACTTTATCA TATTGATTAT TAATTACCTC CTCAACTCCT CCTATACTCT TGTATGCAGA AATAAAAATG   
  
  
+ TTTCCATACC TTATCAGTCG CGATCATAAT AATTAATTTC TTAATTCGGA TTGTATTTAA TTGCGTATCA   
  
  
+ AATGTGAAAT CGTACCAAAT TAGGAAGCAA AACAAACCGA TAACGAAAAA GGTAAGAAAA CTTAGCAAGG   
  
  
+ AAATGAAATC GAAGACCTGT CAACAATATA GCTTCCCTCA CATCCTTCAA CTTAGGGACC ATCCTATACT   
  
  
+ TTCATGTAAT ACAACTAGAG CCACTAGTCA ATAATAGCCT CCTAGTAGTA GCCATAAGCC TTGGTGAGAG   
  
  
+ AATGTTCCGC AAGACTAGTT AACTTGAAAT TCCATTTTGT CGCAGAACTA TTGGAGTGAG AAAGAGAATC   
  
  
+ AAATCATACC ATATCAAATT TGGACCATTT AAGTTCACAA CACACACACA TACACACAAA AGGACGATGT   
  
  
+ ACGGCCCCAC CCCCCACTCT CCTTTCGCCA TCCCCCTACA CCTCTGCCTA CTCTCTCTCT CTCTCTCTCT   
  
  
+ CTCTGGATCA GTCTGTGTAA AAGCTTCCTC TCCAGTACAG CATTTCCTTG TCTGTTCTTG GAGTTTAGTC   
  
  
+ AATTCCTCCG CCCCAAAAAA ACCCACTTTC TCTCTCATCA TGTTCTTCCA GTTCCACCCT TATACGCCAT   
  
  
+ TCATCATCAT CTACATCACC CACATCTTTT TCTCCACAGT TTTCCCCCCT CATTATGCTC TCCGCCTCCA   
  
  
+ CCCCCGCCTT CCCTCTCGCC GTCGCCGGCG ATGACGGGGA TACCACTGAT GCATGCGACG GCACCGCCAC   
  
  
+ TGCCCTCCAC CTCCTCGGGC TGCTGTTGAA GTGCGCGGAA TTCATCTCCA CCGGAGACCT CGCCGGCGCC   
  
  
+ GGAGATATCT TGCCGGAGAT ATTTGAGTTG TCTACACCGT TTGGCTCCCC CGCCGCCCGG GTCGCCGCCT   
  
  
+ TCTTCGGCCA CGCCCTCCAC GCCCGCCTCC TCTCCGCCTC CCTCCGCACA ACTCCGATCG AGAAGCTCAA   
  
  
+ AACCCTGACC CTGGTTTCCC AAATGCGGAA ATTTCACTCC GCCTTGCAAG TATACAACTC CATCACTCCC   
  
  
+ TTCGTGAAAT TCTCTCACTT CACGGCGAAT CAAGCCATCT ACGAGGCGCT GGACGGCGAG GATCGTGTCC   
  
  
+ ACGTCGTCGA CCTTGACATC ATGCAGGGCC TTCAATGGCC GGGATTGTTC CACATCTTAG CCTCTCGACC   
  
  
+ CAGAAAGCCC CTCTCGGTTC GGGTCACCGG GTTCGGGCCA TCCTCCGAGT TGCTCTCCCA AACGGGTAAG   
  
  
+ CGACTCGCTG AGTTCGCCGC TTCACTCGGC CTGCCCTTCG AGTACAACCC GGTGGAGGGC AAAATTGGGA   
  
  
+ ACTTAGTCGA CCTGGGTCGG GTCGGGTCGC TCCCGAATGA AGTGACGGTG GTGCACTGGA TGCACCATAG   
  
  
+ TCTATACGAC ATAACCGGGT CGGATCTTGG GACTTTGAGG GTATTGAGTG CGGTGAGGCC TAGGCTTGTG   
  
  
+ ACTATGGTTG AGCAGGATAT GGACCAAACG GGGTCGTTTT TGGGGAGGTT TGTGGAGGCA TTGCATTATT   
  
  
+ ACTCAGCCTT GTTTGATGCC TTGGGAGAGG GGTTAGATAG GGATAACCTA CAAAGGCATC AAGTGGAGCA   
  
  
+ ACAGCTATTT GGGTGTGAGA TTAGGAACAT CCTGGCCGTT GGTGGGCCCA AGAGGAGGCT CACTGGCGGC   
  
  
+ GATCGGGTCA GAAGGTGGGG CGACGAACTG ACAAGGGTCG GGTTCGAACC AGTTTCGTTG GCGGGTAGCC   
  
  
+ CGGCAACCCA AGCTAGTTTG TTGCTTGGGA TGTTCCCTTG GAAAGGGTAT ACTTTGATGG AGGAAAATGG   
  
  
+ GTGTTTGAGA TTAGGGTGGA AAGATTTGCC CTTGTTAACT GCCTCAGCTT GGCAACCTTG TGAATTTAAC   
  
  
+ AATCCTAGTG CTGGCATTTA   

- +Up\_Stream \_Len000TTTGCC ACGGCAACTT AAACGTCCGC TGCTCCAGCC CAAATTCCGC CTACAGCATC   
  
  
- GCGAAACCTC TTCGTTTAAA CCCTTCTGCT CCGAATAAGA ACTTGAGAAG CATGCGGTAA CAACAACCAA   
  
  
- GGTTGGAAAG AGAGAGGAGC ACTTAAACTC AAACCCGTCA TCTGATAGGG CCTTATCTCC TGACTACGCA   
  
  
- ATATATATAT CTTTAACTCT CTCTCTCTCT CGCACACACT TCCTACAACT TTTACTACCC GCATCCCTCA   
  
  
- GAGAGAGAGT GACAGACAAT CCTCATGCAC AGCTCTTTCC TTTATCCTAA GGGTTCGTCA GATTAAACCC   
  
  
- GGCCAGATAT CCGGTCTTAC CCGGATACTA GTCGGGTCTA TACTGGAACA AATTGGTTGA CATAAACTTA   
  
  
- ACTTCTTTCC CTATAGCGTT ACAGAACTTG GTGTAGTTTA TTATTTACAC GGAAACCAAT CTGGTATACT   
  
  
- TTTTAGAAAC TTAGTTTAGC CAACCTGGAC TTGACGGTAA TAACTATAGA ACAAAAACCA TCTTCGTTTA   
  
  
- CCACAGTGAA GCAAAAGATC TATTTTAATT GAACTCAAAA AAACGGTGAT ATCTATCATA ACGTTTATAT   
  
  
- TTTTCACACG GTGCACTTTA GTTATAATCC TCCCACAAAA TATTGTTATC GACAAGCCCT CTCAAAATCA   
  
  
- ATAAAAATAA TTTAGTTTAT CAATTCTTCA CAAACCATTT ATCAACATAA ACTTTTTCAA TAAGGGTAAT   
  
  
- CGGAAAATCA TTCTTGAAGA ACACTTTTAC TATTAACAGA TCTTCAAACT TTTTAAGTGT GGTGTACTGT   
  
  
- AAATTTTTAT TAAAACATCT CTTCCCGTAT TTACTTTCAA ACGGTGGTAG AAAAAATCTT TGTACATTAA   
  
  
- ATGGTTTGTG AAAAAGATTT TTGTCAATTA AGTTTATTGA TTGTTGATTG TCGATTATAT TTATTGATTG   
  
  
- TCGATAAACG ATTTGTCCCG GATTCATCAA ACTTTAACAA CAAAAGAGAA GTCGAAGTGG TACCGGCTAC   
  
  
- TACATTTGTT GATATAAGAC TTAAAGTAAA AATGATACTA AACTTTTAAA GTTTAAATTT ATTCTAACCT   
  
  
- ATTTTTTTTC TGGTTTTTAG TATTCATTAT AATCAGTTCA AGATAAGGTT GGTATATGTA TTGACGAAAA   
  
  
- AGAAAACCCC CAAAACTTTG TACACAGGAA TCTTATTAAC TTTGAGCTAT AATAAGAGAT TTAGATTCAA   
  
  
- ATGAAATAGT ATAACTAATA ATTAATGGAG GAGTTGAGGA GGATATGAGA ACATACGTCT TTATTTTTAC   
  
  
- AAAGGTATGG AATAGTCAGC GCTAGTATTA TTAATTAAAG AATTAAGCCT AACATAAATT AACGCATAGT   
  
  
- TTACACTTTA GCATGGTTTA ATCCTTCGTT TTGTTTGGCT ATTGCTTTTT CCATTCTTTT GAATCGTTCC   
  
  
- TTTACTTTAG CTTCTGGACA GTTGTTATAT CGAAGGGAGT GTAGGAAGTT GAATCCCTGG TAGGATATGA   
  
  
- AAGTACATTA TGTTGATCTC GGTGATCAGT TATTATCGGA GGATCATCAT CGGTATTCGG AACCACTCTC   
  
  
- TTACAAGGCG TTCTGATCAA TTGAACTTTA AGGTAAAACA GCGTCTTGAT AACCTCACTC TTTCTCTTAG   
  
  
- TTTAGTATGG TATAGTTTAA ACCTGGTAAA TTCAAGTGTT GTGTGTGTGT ATGTGTGTTT TCCTGCTACA   
  
  
- TGCCGGGGTG GGGGGTGAGA GGAAAGCGGT AGGGGGATGT GGAGACGGAT GAGAGAGAGA GAGAGAGAGA   
  
  
- GAGACCTAGT CAGACACATT TTCGAAGGAG AGGTCATGTC GTAAAGGAAC AGACAAGAAC CTCAAATCAG   
  
  
- TTAAGGAGGC GGGGTTTTTT TGGGTGAAAG AGAGAGTAGT ACAAGAAGGT CAAGGTGGGA ATATGCGGTA   
  
  
- AGTAGTAGTA GATGTAGTGG GTGTAGAAAA AGAGGTGTCA AAAGGGGGGA GTAATACGAG AGGCGGAGGT   
  
  
- GGGGGCGGAA GGGAGAGCGG CAGCGGCCGC TACTGCCCCT ATGGTGACTA CGTACGCTGC CGTGGCGGTG   
  
  
- ACGGGAGGTG GAGGAGCCCG ACGACAACTT CACGCGCCTT AAGTAGAGGT GGCCTCTGGA GCGGCCGCGG   
  
  
- CCTCTATAGA ACGGCCTCTA TAAACTCAAC AGATGTGGCA AACCGAGGGG GCGGCGGGCC CAGCGGCGGA   
  
  
- AGAAGCCGGT GCGGGAGGTG CGGGCGGAGG AGAGGCGGAG GGAGGCGTGT TGAGGCTAGC TCTTCGAGTT   
  
  
- TTGGGACTGG GACCAAAGGG TTTACGCCTT TAAAGTGAGG CGGAACGTTC ATATGTTGAG GTAGTGAGGG   
  
  
- AAGCACTTTA AGAGAGTGAA GTGCCGCTTA GTTCGGTAGA TGCTCCGCGA CCTGCCGCTC CTAGCACAGG   
  
  
- TGCAGCAGCT GGAACTGTAG TACGTCCCGG AAGTTACCGG CCCTAACAAG GTGTAGAATC GGAGAGCTGG   
  
  
- GTCTTTCGGG GAGAGCCAAG CCCAGTGGCC CAAGCCCGGT AGGAGGCTCA ACGAGAGGGT TTGCCCATTC   
  
  
- GCTGAGCGAC TCAAGCGGCG AAGTGAGCCG GACGGGAAGC TCATGTTGGG CCACCTCCCG TTTTAACCCT   
  
  
- TGAATCAGCT GGACCCAGCC CAGCCCAGCG AGGGCTTACT TCACTGCCAC CACGTGACCT ACGTGGTATC   
  
  
- AGATATGCTG TATTGGCCCA GCCTAGAACC CTGAAACTCC CATAACTCAC GCCACTCCGG ATCCGAACAC   
  
  
- TGATACCAAC TCGTCCTATA CCTGGTTTGC CCCAGCAAAA ACCCCTCCAA ACACCTCCGT AACGTAATAA   
  
  
- TGAGTCGGAA CAAACTACGG AACCCTCTCC CCAATCTATC CCTATTGGAT GTTTCCGTAG TTCACCTCGT   
  
  
- TGTCGATAAA CCCACACTCT AATCCTTGTA GGACCGGCAA CCACCCGGGT TCTCCTCCGA GTGACCGCCG   
  
  
- CTAGCCCAGT CTTCCACCCC GCTGCTTGAC TGTTCCCAGC CCAAGCTTGG TCAAAGCAAC CGCCCATCGG   
  
  
- GCCGTTGGGT TCGATCAAAC AACGAACCCT ACAAGGGAAC CTTTCCCATA TGAAACTACC TCCTTTTACC   
  
  
- CACAAACTCT AATCCCACCT TTCTAAACGG GAACAATTGA CGGAGTCGAA CCGTTGGAAC ACTTAAATTG   
  
  
- TTAGGATCAC GACCGTAAAT

+     ARE

| Site Name | Organism | Position | Strand | Matrix score. | sequence | function |
| --- | --- | --- | --- | --- | --- | --- |
| ARE | Zea mays | 2326 | - | 6 | AAACCA | cis-acting regulatory element essential for the anaerobic induction |

>HU02G03005.1   
+ +Up\_Stream \_Len000AAACGG TGCCGTTGAA TTTGCAGGCG ACGAGGTCGG GTTTAAGGCG GATGTCGTAG   
  
  
+ CGCTTTGGAG AAGCAAATTT GGGAAGACGA GGCTTATTCT TGAACTCTTC GTACGCCATT GTTGTTGGTT   
  
  
+ CCAACCTTTC TCTCTCCTCG TGAATTTGAG TTTGGGCAGT AGACTATCCC GGAATAGAGG ACTGATGCGT   
  
  
+ TATATATATA GAAATTGAGA GAGAGAGAGA GCGTGTGTGA AGGATGTTGA AAATGATGGG CGTAGGGAGT   
  
  
+ CTCTCTCTCA CTGTCTGTTA GGAGTACGTG TCGAGAAAGG AAATAGGATT CCCAAGCAGT CTAATTTGGG   
  
  
+ CCGGTCTATA GGCCAGAATG GGCCTATGAT CAGCCCAGAT ATGACCTTGT TTAACCAACT GTATTTGAAT   
  
  
+ TGAAGAAAGG GATATCGCAA TGTCTTGAAC CACATCAAAT AATAAATGTG CCTTTGGTTA GACCATATGA   
  
  
+ AAAATCTTTG AATCAAATCG GTTGGACCTG AACTGCCATT ATTGATATCT TGTTTTTGGT AGAAGCAAAT   
  
  
+ GGTGTCACTT CGTTTTCTAG ATAAAATTAA CTTGAGTTTT TTTGCCACTA TAGATAGTAT TGCAAATATA   
  
  
+ AAAAGTGTGC CACGTGAAAT CAATATTAGG AGGGTGTTTT ATAACAATAG CTGTTCGGGA GAGTTTTAGT   
  
  
+ TATTTTTATT AAATCAAATA GTTAAGAAGT GTTTGGTAAA TAGTTGTATT TGAAAAAGTT ATTCCCATTA   
  
  
+ GCCTTTTAGT AAGAACTTCT TGTGAAAATG ATAATTGTCT AGAAGTTTGA AAAATTCACA CCACATGACA   
  
  
+ TTTAAAAATA ATTTTGTAGA GAAGGGCATA AATGAAAGTT TGCCACCATC TTTTTTAGAA ACATGTAATT   
  
  
+ TACCAAACAC TTTTTCTAAA AACAGTTAAT TCAAATAACT AACAACTAAC AGCTAATATA AATAACTAAC   
  
  
+ AGCTATTTGC TAAACAGGGC CTAAGTAGTT TGAAATTGTT GTTTTCTCTT CAGCTTCACC ATGGCCGATG   
  
  
+ ATGTAAACAA CTATATTCTG AATTTCATTT TTACTATGAT TTGAAAATTT CAAATTTAAA TAAGATTGGA   
  
  
+ TAAAAAAAAG ACCAAAAATC ATAAGTAATA TTAGTCAAGT TCTATTCCAA CCATATACAT AACTGCTTTT   
  
  
+ TCTTTTGGGG GTTTTGAAAC ATGTGTCCTT AGAATAATTG AAACTCGATA TTATTCTCTA AATCTAAGTT   
  
  
+ TACTTTATCA TATTGATTAT TAATTACCTC CTCAACTCCT CCTATACTCT TGTATGCAGA AATAAAAATG   
  
  
+ TTTCCATACC TTATCAGTCG CGATCATAAT AATTAATTTC TTAATTCGGA TTGTATTTAA TTGCGTATCA   
  
  
+ AATGTGAAAT CGTACCAAAT TAGGAAGCAA AACAAACCGA TAACGAAAAA GGTAAGAAAA CTTAGCAAGG   
  
  
+ AAATGAAATC GAAGACCTGT CAACAATATA GCTTCCCTCA CATCCTTCAA CTTAGGGACC ATCCTATACT   
  
  
+ TTCATGTAAT ACAACTAGAG CCACTAGTCA ATAATAGCCT CCTAGTAGTA GCCATAAGCC TTGGTGAGAG   
  
  
+ AATGTTCCGC AAGACTAGTT AACTTGAAAT TCCATTTTGT CGCAGAACTA TTGGAGTGAG AAAGAGAATC   
  
  
+ AAATCATACC ATATCAAATT TGGACCATTT AAGTTCACAA CACACACACA TACACACAAA AGGACGATGT   
  
  
+ ACGGCCCCAC CCCCCACTCT CCTTTCGCCA TCCCCCTACA CCTCTGCCTA CTCTCTCTCT CTCTCTCTCT   
  
  
+ CTCTGGATCA GTCTGTGTAA AAGCTTCCTC TCCAGTACAG CATTTCCTTG TCTGTTCTTG GAGTTTAGTC   
  
  
+ AATTCCTCCG CCCCAAAAAA ACCCACTTTC TCTCTCATCA TGTTCTTCCA GTTCCACCCT TATACGCCAT   
  
  
+ TCATCATCAT CTACATCACC CACATCTTTT TCTCCACAGT TTTCCCCCCT CATTATGCTC TCCGCCTCCA   
  
  
+ CCCCCGCCTT CCCTCTCGCC GTCGCCGGCG ATGACGGGGA TACCACTGAT GCATGCGACG GCACCGCCAC   
  
  
+ TGCCCTCCAC CTCCTCGGGC TGCTGTTGAA GTGCGCGGAA TTCATCTCCA CCGGAGACCT CGCCGGCGCC   
  
  
+ GGAGATATCT TGCCGGAGAT ATTTGAGTTG TCTACACCGT TTGGCTCCCC CGCCGCCCGG GTCGCCGCCT   
  
  
+ TCTTCGGCCA CGCCCTCCAC GCCCGCCTCC TCTCCGCCTC CCTCCGCACA ACTCCGATCG AGAAGCTCAA   
  
  
+ AACCCTGACC CTGGTTTCCC AAATGCGGAA ATTTCACTCC GCCTTGCAAG TATACAACTC CATCACTCCC   
  
  
+ TTCGTGAAAT TCTCTCACTT CACGGCGAAT CAAGCCATCT ACGAGGCGCT GGACGGCGAG GATCGTGTCC   
  
  
+ ACGTCGTCGA CCTTGACATC ATGCAGGGCC TTCAATGGCC GGGATTGTTC CACATCTTAG CCTCTCGACC   
  
  
+ CAGAAAGCCC CTCTCGGTTC GGGTCACCGG GTTCGGGCCA TCCTCCGAGT TGCTCTCCCA AACGGGTAAG   
  
  
+ CGACTCGCTG AGTTCGCCGC TTCACTCGGC CTGCCCTTCG AGTACAACCC GGTGGAGGGC AAAATTGGGA   
  
  
+ ACTTAGTCGA CCTGGGTCGG GTCGGGTCGC TCCCGAATGA AGTGACGGTG GTGCACTGGA TGCACCATAG   
  
  
+ TCTATACGAC ATAACCGGGT CGGATCTTGG GACTTTGAGG GTATTGAGTG CGGTGAGGCC TAGGCTTGTG   
  
  
+ ACTATGGTTG AGCAGGATAT GGACCAAACG GGGTCGTTTT TGGGGAGGTT TGTGGAGGCA TTGCATTATT   
  
  
+ ACTCAGCCTT GTTTGATGCC TTGGGAGAGG GGTTAGATAG GGATAACCTA CAAAGGCATC AAGTGGAGCA   
  
  
+ ACAGCTATTT GGGTGTGAGA TTAGGAACAT CCTGGCCGTT GGTGGGCCCA AGAGGAGGCT CACTGGCGGC   
  
  
+ GATCGGGTCA GAAGGTGGGG CGACGAACTG ACAAGGGTCG GGTTCGAACC AGTTTCGTTG GCGGGTAGCC   
  
  
+ CGGCAACCCA AGCTAGTTTG TTGCTTGGGA TGTTCCCTTG GAAAGGGTAT ACTTTGATGG AGGAAAATGG   
  
  
+ GTGTTTGAGA TTAGGGTGGA AAGATTTGCC CTTGTTAACT GCCTCAGCTT GGCAACCTTG TGAATTTAAC   
  
  
+ AATCCTAGTG CTGGCATTTA   

- +Up\_Stream \_Len000TTTGCC ACGGCAACTT AAACGTCCGC TGCTCCAGCC CAAATTCCGC CTACAGCATC   
  
  
- GCGAAACCTC TTCGTTTAAA CCCTTCTGCT CCGAATAAGA ACTTGAGAAG CATGCGGTAA CAACAACCAA   
  
  
- GGTTGGAAAG AGAGAGGAGC ACTTAAACTC AAACCCGTCA TCTGATAGGG CCTTATCTCC TGACTACGCA   
  
  
- ATATATATAT CTTTAACTCT CTCTCTCTCT CGCACACACT TCCTACAACT TTTACTACCC GCATCCCTCA   
  
  
- GAGAGAGAGT GACAGACAAT CCTCATGCAC AGCTCTTTCC TTTATCCTAA GGGTTCGTCA GATTAAACCC   
  
  
- GGCCAGATAT CCGGTCTTAC CCGGATACTA GTCGGGTCTA TACTGGAACA AATTGGTTGA CATAAACTTA   
  
  
- ACTTCTTTCC CTATAGCGTT ACAGAACTTG GTGTAGTTTA TTATTTACAC GGAAACCAAT CTGGTATACT   
  
  
- TTTTAGAAAC TTAGTTTAGC CAACCTGGAC TTGACGGTAA TAACTATAGA ACAAAAACCA TCTTCGTTTA   
  
  
- CCACAGTGAA GCAAAAGATC TATTTTAATT GAACTCAAAA AAACGGTGAT ATCTATCATA ACGTTTATAT   
  
  
- TTTTCACACG GTGCACTTTA GTTATAATCC TCCCACAAAA TATTGTTATC GACAAGCCCT CTCAAAATCA   
  
  
- ATAAAAATAA TTTAGTTTAT CAATTCTTCA CAAACCATTT ATCAACATAA ACTTTTTCAA TAAGGGTAAT   
  
  
- CGGAAAATCA TTCTTGAAGA ACACTTTTAC TATTAACAGA TCTTCAAACT TTTTAAGTGT GGTGTACTGT   
  
  
- AAATTTTTAT TAAAACATCT CTTCCCGTAT TTACTTTCAA ACGGTGGTAG AAAAAATCTT TGTACATTAA   
  
  
- ATGGTTTGTG AAAAAGATTT TTGTCAATTA AGTTTATTGA TTGTTGATTG TCGATTATAT TTATTGATTG   
  
  
- TCGATAAACG ATTTGTCCCG GATTCATCAA ACTTTAACAA CAAAAGAGAA GTCGAAGTGG TACCGGCTAC   
  
  
- TACATTTGTT GATATAAGAC TTAAAGTAAA AATGATACTA AACTTTTAAA GTTTAAATTT ATTCTAACCT   
  
  
- ATTTTTTTTC TGGTTTTTAG TATTCATTAT AATCAGTTCA AGATAAGGTT GGTATATGTA TTGACGAAAA   
  
  
- AGAAAACCCC CAAAACTTTG TACACAGGAA TCTTATTAAC TTTGAGCTAT AATAAGAGAT TTAGATTCAA   
  
  
- ATGAAATAGT ATAACTAATA ATTAATGGAG GAGTTGAGGA GGATATGAGA ACATACGTCT TTATTTTTAC   
  
  
- AAAGGTATGG AATAGTCAGC GCTAGTATTA TTAATTAAAG AATTAAGCCT AACATAAATT AACGCATAGT   
  
  
- TTACACTTTA GCATGGTTTA ATCCTTCGTT TTGTTTGGCT ATTGCTTTTT CCATTCTTTT GAATCGTTCC   
  
  
- TTTACTTTAG CTTCTGGACA GTTGTTATAT CGAAGGGAGT GTAGGAAGTT GAATCCCTGG TAGGATATGA   
  
  
- AAGTACATTA TGTTGATCTC GGTGATCAGT TATTATCGGA GGATCATCAT CGGTATTCGG AACCACTCTC   
  
  
- TTACAAGGCG TTCTGATCAA TTGAACTTTA AGGTAAAACA GCGTCTTGAT AACCTCACTC TTTCTCTTAG   
  
  
- TTTAGTATGG TATAGTTTAA ACCTGGTAAA TTCAAGTGTT GTGTGTGTGT ATGTGTGTTT TCCTGCTACA   
  
  
- TGCCGGGGTG GGGGGTGAGA GGAAAGCGGT AGGGGGATGT GGAGACGGAT GAGAGAGAGA GAGAGAGAGA   
  
  
- GAGACCTAGT CAGACACATT TTCGAAGGAG AGGTCATGTC GTAAAGGAAC AGACAAGAAC CTCAAATCAG   
  
  
- TTAAGGAGGC GGGGTTTTTT TGGGTGAAAG AGAGAGTAGT ACAAGAAGGT CAAGGTGGGA ATATGCGGTA   
  
  
- AGTAGTAGTA GATGTAGTGG GTGTAGAAAA AGAGGTGTCA AAAGGGGGGA GTAATACGAG AGGCGGAGGT   
  
  
- GGGGGCGGAA GGGAGAGCGG CAGCGGCCGC TACTGCCCCT ATGGTGACTA CGTACGCTGC CGTGGCGGTG   
  
  
- ACGGGAGGTG GAGGAGCCCG ACGACAACTT CACGCGCCTT AAGTAGAGGT GGCCTCTGGA GCGGCCGCGG   
  
  
- CCTCTATAGA ACGGCCTCTA TAAACTCAAC AGATGTGGCA AACCGAGGGG GCGGCGGGCC CAGCGGCGGA   
  
  
- AGAAGCCGGT GCGGGAGGTG CGGGCGGAGG AGAGGCGGAG GGAGGCGTGT TGAGGCTAGC TCTTCGAGTT   
  
  
- TTGGGACTGG GACCAAAGGG TTTACGCCTT TAAAGTGAGG CGGAACGTTC ATATGTTGAG GTAGTGAGGG   
  
  
- AAGCACTTTA AGAGAGTGAA GTGCCGCTTA GTTCGGTAGA TGCTCCGCGA CCTGCCGCTC CTAGCACAGG   
  
  
- TGCAGCAGCT GGAACTGTAG TACGTCCCGG AAGTTACCGG CCCTAACAAG GTGTAGAATC GGAGAGCTGG   
  
  
- GTCTTTCGGG GAGAGCCAAG CCCAGTGGCC CAAGCCCGGT AGGAGGCTCA ACGAGAGGGT TTGCCCATTC   
  
  
- GCTGAGCGAC TCAAGCGGCG AAGTGAGCCG GACGGGAAGC TCATGTTGGG CCACCTCCCG TTTTAACCCT   
  
  
- TGAATCAGCT GGACCCAGCC CAGCCCAGCG AGGGCTTACT TCACTGCCAC CACGTGACCT ACGTGGTATC   
  
  
- AGATATGCTG TATTGGCCCA GCCTAGAACC CTGAAACTCC CATAACTCAC GCCACTCCGG ATCCGAACAC   
  
  
- TGATACCAAC TCGTCCTATA CCTGGTTTGC CCCAGCAAAA ACCCCTCCAA ACACCTCCGT AACGTAATAA   
  
  
- TGAGTCGGAA CAAACTACGG AACCCTCTCC CCAATCTATC CCTATTGGAT GTTTCCGTAG TTCACCTCGT   
  
  
- TGTCGATAAA CCCACACTCT AATCCTTGTA GGACCGGCAA CCACCCGGGT TCTCCTCCGA GTGACCGCCG   
  
  
- CTAGCCCAGT CTTCCACCCC GCTGCTTGAC TGTTCCCAGC CCAAGCTTGG TCAAAGCAAC CGCCCATCGG   
  
  
- GCCGTTGGGT TCGATCAAAC AACGAACCCT ACAAGGGAAC CTTTCCCATA TGAAACTACC TCCTTTTACC   
  
  
- CACAAACTCT AATCCCACCT TTCTAAACGG GAACAATTGA CGGAGTCGAA CCGTTGGAAC ACTTAAATTG   
  
  
- TTAGGATCAC GACCGTAAAT

+     AT~ABRE

| Site Name | Organism | Position | Strand | Matrix score. | sequence | function |
| --- | --- | --- | --- | --- | --- | --- |
| AT~ABRE | Arabidopsis thaliana | 309 | + | 8 | TACGTGTC |  |

>HU02G03005.1   
+ +Up\_Stream \_Len000AAACGG TGCCGTTGAA TTTGCAGGCG ACGAGGTCGG GTTTAAGGCG GATGTCGTAG   
  
  
+ CGCTTTGGAG AAGCAAATTT GGGAAGACGA GGCTTATTCT TGAACTCTTC GTACGCCATT GTTGTTGGTT   
  
  
+ CCAACCTTTC TCTCTCCTCG TGAATTTGAG TTTGGGCAGT AGACTATCCC GGAATAGAGG ACTGATGCGT   
  
  
+ TATATATATA GAAATTGAGA GAGAGAGAGA GCGTGTGTGA AGGATGTTGA AAATGATGGG CGTAGGGAGT   
  
  
+ CTCTCTCTCA CTGTCTGTTA GGAGTACGTG TCGAGAAAGG AAATAGGATT CCCAAGCAGT CTAATTTGGG   
  
  
+ CCGGTCTATA GGCCAGAATG GGCCTATGAT CAGCCCAGAT ATGACCTTGT TTAACCAACT GTATTTGAAT   
  
  
+ TGAAGAAAGG GATATCGCAA TGTCTTGAAC CACATCAAAT AATAAATGTG CCTTTGGTTA GACCATATGA   
  
  
+ AAAATCTTTG AATCAAATCG GTTGGACCTG AACTGCCATT ATTGATATCT TGTTTTTGGT AGAAGCAAAT   
  
  
+ GGTGTCACTT CGTTTTCTAG ATAAAATTAA CTTGAGTTTT TTTGCCACTA TAGATAGTAT TGCAAATATA   
  
  
+ AAAAGTGTGC CACGTGAAAT CAATATTAGG AGGGTGTTTT ATAACAATAG CTGTTCGGGA GAGTTTTAGT   
  
  
+ TATTTTTATT AAATCAAATA GTTAAGAAGT GTTTGGTAAA TAGTTGTATT TGAAAAAGTT ATTCCCATTA   
  
  
+ GCCTTTTAGT AAGAACTTCT TGTGAAAATG ATAATTGTCT AGAAGTTTGA AAAATTCACA CCACATGACA   
  
  
+ TTTAAAAATA ATTTTGTAGA GAAGGGCATA AATGAAAGTT TGCCACCATC TTTTTTAGAA ACATGTAATT   
  
  
+ TACCAAACAC TTTTTCTAAA AACAGTTAAT TCAAATAACT AACAACTAAC AGCTAATATA AATAACTAAC   
  
  
+ AGCTATTTGC TAAACAGGGC CTAAGTAGTT TGAAATTGTT GTTTTCTCTT CAGCTTCACC ATGGCCGATG   
  
  
+ ATGTAAACAA CTATATTCTG AATTTCATTT TTACTATGAT TTGAAAATTT CAAATTTAAA TAAGATTGGA   
  
  
+ TAAAAAAAAG ACCAAAAATC ATAAGTAATA TTAGTCAAGT TCTATTCCAA CCATATACAT AACTGCTTTT   
  
  
+ TCTTTTGGGG GTTTTGAAAC ATGTGTCCTT AGAATAATTG AAACTCGATA TTATTCTCTA AATCTAAGTT   
  
  
+ TACTTTATCA TATTGATTAT TAATTACCTC CTCAACTCCT CCTATACTCT TGTATGCAGA AATAAAAATG   
  
  
+ TTTCCATACC TTATCAGTCG CGATCATAAT AATTAATTTC TTAATTCGGA TTGTATTTAA TTGCGTATCA   
  
  
+ AATGTGAAAT CGTACCAAAT TAGGAAGCAA AACAAACCGA TAACGAAAAA GGTAAGAAAA CTTAGCAAGG   
  
  
+ AAATGAAATC GAAGACCTGT CAACAATATA GCTTCCCTCA CATCCTTCAA CTTAGGGACC ATCCTATACT   
  
  
+ TTCATGTAAT ACAACTAGAG CCACTAGTCA ATAATAGCCT CCTAGTAGTA GCCATAAGCC TTGGTGAGAG   
  
  
+ AATGTTCCGC AAGACTAGTT AACTTGAAAT TCCATTTTGT CGCAGAACTA TTGGAGTGAG AAAGAGAATC   
  
  
+ AAATCATACC ATATCAAATT TGGACCATTT AAGTTCACAA CACACACACA TACACACAAA AGGACGATGT   
  
  
+ ACGGCCCCAC CCCCCACTCT CCTTTCGCCA TCCCCCTACA CCTCTGCCTA CTCTCTCTCT CTCTCTCTCT   
  
  
+ CTCTGGATCA GTCTGTGTAA AAGCTTCCTC TCCAGTACAG CATTTCCTTG TCTGTTCTTG GAGTTTAGTC   
  
  
+ AATTCCTCCG CCCCAAAAAA ACCCACTTTC TCTCTCATCA TGTTCTTCCA GTTCCACCCT TATACGCCAT   
  
  
+ TCATCATCAT CTACATCACC CACATCTTTT TCTCCACAGT TTTCCCCCCT CATTATGCTC TCCGCCTCCA   
  
  
+ CCCCCGCCTT CCCTCTCGCC GTCGCCGGCG ATGACGGGGA TACCACTGAT GCATGCGACG GCACCGCCAC   
  
  
+ TGCCCTCCAC CTCCTCGGGC TGCTGTTGAA GTGCGCGGAA TTCATCTCCA CCGGAGACCT CGCCGGCGCC   
  
  
+ GGAGATATCT TGCCGGAGAT ATTTGAGTTG TCTACACCGT TTGGCTCCCC CGCCGCCCGG GTCGCCGCCT   
  
  
+ TCTTCGGCCA CGCCCTCCAC GCCCGCCTCC TCTCCGCCTC CCTCCGCACA ACTCCGATCG AGAAGCTCAA   
  
  
+ AACCCTGACC CTGGTTTCCC AAATGCGGAA ATTTCACTCC GCCTTGCAAG TATACAACTC CATCACTCCC   
  
  
+ TTCGTGAAAT TCTCTCACTT CACGGCGAAT CAAGCCATCT ACGAGGCGCT GGACGGCGAG GATCGTGTCC   
  
  
+ ACGTCGTCGA CCTTGACATC ATGCAGGGCC TTCAATGGCC GGGATTGTTC CACATCTTAG CCTCTCGACC   
  
  
+ CAGAAAGCCC CTCTCGGTTC GGGTCACCGG GTTCGGGCCA TCCTCCGAGT TGCTCTCCCA AACGGGTAAG   
  
  
+ CGACTCGCTG AGTTCGCCGC TTCACTCGGC CTGCCCTTCG AGTACAACCC GGTGGAGGGC AAAATTGGGA   
  
  
+ ACTTAGTCGA CCTGGGTCGG GTCGGGTCGC TCCCGAATGA AGTGACGGTG GTGCACTGGA TGCACCATAG   
  
  
+ TCTATACGAC ATAACCGGGT CGGATCTTGG GACTTTGAGG GTATTGAGTG CGGTGAGGCC TAGGCTTGTG   
  
  
+ ACTATGGTTG AGCAGGATAT GGACCAAACG GGGTCGTTTT TGGGGAGGTT TGTGGAGGCA TTGCATTATT   
  
  
+ ACTCAGCCTT GTTTGATGCC TTGGGAGAGG GGTTAGATAG GGATAACCTA CAAAGGCATC AAGTGGAGCA   
  
  
+ ACAGCTATTT GGGTGTGAGA TTAGGAACAT CCTGGCCGTT GGTGGGCCCA AGAGGAGGCT CACTGGCGGC   
  
  
+ GATCGGGTCA GAAGGTGGGG CGACGAACTG ACAAGGGTCG GGTTCGAACC AGTTTCGTTG GCGGGTAGCC   
  
  
+ CGGCAACCCA AGCTAGTTTG TTGCTTGGGA TGTTCCCTTG GAAAGGGTAT ACTTTGATGG AGGAAAATGG   
  
  
+ GTGTTTGAGA TTAGGGTGGA AAGATTTGCC CTTGTTAACT GCCTCAGCTT GGCAACCTTG TGAATTTAAC   
  
  
+ AATCCTAGTG CTGGCATTTA   

- +Up\_Stream \_Len000TTTGCC ACGGCAACTT AAACGTCCGC TGCTCCAGCC CAAATTCCGC CTACAGCATC   
  
  
- GCGAAACCTC TTCGTTTAAA CCCTTCTGCT CCGAATAAGA ACTTGAGAAG CATGCGGTAA CAACAACCAA   
  
  
- GGTTGGAAAG AGAGAGGAGC ACTTAAACTC AAACCCGTCA TCTGATAGGG CCTTATCTCC TGACTACGCA   
  
  
- ATATATATAT CTTTAACTCT CTCTCTCTCT CGCACACACT TCCTACAACT TTTACTACCC GCATCCCTCA   
  
  
- GAGAGAGAGT GACAGACAAT CCTCATGCAC AGCTCTTTCC TTTATCCTAA GGGTTCGTCA GATTAAACCC   
  
  
- GGCCAGATAT CCGGTCTTAC CCGGATACTA GTCGGGTCTA TACTGGAACA AATTGGTTGA CATAAACTTA   
  
  
- ACTTCTTTCC CTATAGCGTT ACAGAACTTG GTGTAGTTTA TTATTTACAC GGAAACCAAT CTGGTATACT   
  
  
- TTTTAGAAAC TTAGTTTAGC CAACCTGGAC TTGACGGTAA TAACTATAGA ACAAAAACCA TCTTCGTTTA   
  
  
- CCACAGTGAA GCAAAAGATC TATTTTAATT GAACTCAAAA AAACGGTGAT ATCTATCATA ACGTTTATAT   
  
  
- TTTTCACACG GTGCACTTTA GTTATAATCC TCCCACAAAA TATTGTTATC GACAAGCCCT CTCAAAATCA   
  
  
- ATAAAAATAA TTTAGTTTAT CAATTCTTCA CAAACCATTT ATCAACATAA ACTTTTTCAA TAAGGGTAAT   
  
  
- CGGAAAATCA TTCTTGAAGA ACACTTTTAC TATTAACAGA TCTTCAAACT TTTTAAGTGT GGTGTACTGT   
  
  
- AAATTTTTAT TAAAACATCT CTTCCCGTAT TTACTTTCAA ACGGTGGTAG AAAAAATCTT TGTACATTAA   
  
  
- ATGGTTTGTG AAAAAGATTT TTGTCAATTA AGTTTATTGA TTGTTGATTG TCGATTATAT TTATTGATTG   
  
  
- TCGATAAACG ATTTGTCCCG GATTCATCAA ACTTTAACAA CAAAAGAGAA GTCGAAGTGG TACCGGCTAC   
  
  
- TACATTTGTT GATATAAGAC TTAAAGTAAA AATGATACTA AACTTTTAAA GTTTAAATTT ATTCTAACCT   
  
  
- ATTTTTTTTC TGGTTTTTAG TATTCATTAT AATCAGTTCA AGATAAGGTT GGTATATGTA TTGACGAAAA   
  
  
- AGAAAACCCC CAAAACTTTG TACACAGGAA TCTTATTAAC TTTGAGCTAT AATAAGAGAT TTAGATTCAA   
  
  
- ATGAAATAGT ATAACTAATA ATTAATGGAG GAGTTGAGGA GGATATGAGA ACATACGTCT TTATTTTTAC   
  
  
- AAAGGTATGG AATAGTCAGC GCTAGTATTA TTAATTAAAG AATTAAGCCT AACATAAATT AACGCATAGT   
  
  
- TTACACTTTA GCATGGTTTA ATCCTTCGTT TTGTTTGGCT ATTGCTTTTT CCATTCTTTT GAATCGTTCC   
  
  
- TTTACTTTAG CTTCTGGACA GTTGTTATAT CGAAGGGAGT GTAGGAAGTT GAATCCCTGG TAGGATATGA   
  
  
- AAGTACATTA TGTTGATCTC GGTGATCAGT TATTATCGGA GGATCATCAT CGGTATTCGG AACCACTCTC   
  
  
- TTACAAGGCG TTCTGATCAA TTGAACTTTA AGGTAAAACA GCGTCTTGAT AACCTCACTC TTTCTCTTAG   
  
  
- TTTAGTATGG TATAGTTTAA ACCTGGTAAA TTCAAGTGTT GTGTGTGTGT ATGTGTGTTT TCCTGCTACA   
  
  
- TGCCGGGGTG GGGGGTGAGA GGAAAGCGGT AGGGGGATGT GGAGACGGAT GAGAGAGAGA GAGAGAGAGA   
  
  
- GAGACCTAGT CAGACACATT TTCGAAGGAG AGGTCATGTC GTAAAGGAAC AGACAAGAAC CTCAAATCAG   
  
  
- TTAAGGAGGC GGGGTTTTTT TGGGTGAAAG AGAGAGTAGT ACAAGAAGGT CAAGGTGGGA ATATGCGGTA   
  
  
- AGTAGTAGTA GATGTAGTGG GTGTAGAAAA AGAGGTGTCA AAAGGGGGGA GTAATACGAG AGGCGGAGGT   
  
  
- GGGGGCGGAA GGGAGAGCGG CAGCGGCCGC TACTGCCCCT ATGGTGACTA CGTACGCTGC CGTGGCGGTG   
  
  
- ACGGGAGGTG GAGGAGCCCG ACGACAACTT CACGCGCCTT AAGTAGAGGT GGCCTCTGGA GCGGCCGCGG   
  
  
- CCTCTATAGA ACGGCCTCTA TAAACTCAAC AGATGTGGCA AACCGAGGGG GCGGCGGGCC CAGCGGCGGA   
  
  
- AGAAGCCGGT GCGGGAGGTG CGGGCGGAGG AGAGGCGGAG GGAGGCGTGT TGAGGCTAGC TCTTCGAGTT   
  
  
- TTGGGACTGG GACCAAAGGG TTTACGCCTT TAAAGTGAGG CGGAACGTTC ATATGTTGAG GTAGTGAGGG   
  
  
- AAGCACTTTA AGAGAGTGAA GTGCCGCTTA GTTCGGTAGA TGCTCCGCGA CCTGCCGCTC CTAGCACAGG   
  
  
- TGCAGCAGCT GGAACTGTAG TACGTCCCGG AAGTTACCGG CCCTAACAAG GTGTAGAATC GGAGAGCTGG   
  
  
- GTCTTTCGGG GAGAGCCAAG CCCAGTGGCC CAAGCCCGGT AGGAGGCTCA ACGAGAGGGT TTGCCCATTC   
  
  
- GCTGAGCGAC TCAAGCGGCG AAGTGAGCCG GACGGGAAGC TCATGTTGGG CCACCTCCCG TTTTAACCCT   
  
  
- TGAATCAGCT GGACCCAGCC CAGCCCAGCG AGGGCTTACT TCACTGCCAC CACGTGACCT ACGTGGTATC   
  
  
- AGATATGCTG TATTGGCCCA GCCTAGAACC CTGAAACTCC CATAACTCAC GCCACTCCGG ATCCGAACAC   
  
  
- TGATACCAAC TCGTCCTATA CCTGGTTTGC CCCAGCAAAA ACCCCTCCAA ACACCTCCGT AACGTAATAA   
  
  
- TGAGTCGGAA CAAACTACGG AACCCTCTCC CCAATCTATC CCTATTGGAT GTTTCCGTAG TTCACCTCGT   
  
  
- TGTCGATAAA CCCACACTCT AATCCTTGTA GGACCGGCAA CCACCCGGGT TCTCCTCCGA GTGACCGCCG   
  
  
- CTAGCCCAGT CTTCCACCCC GCTGCTTGAC TGTTCCCAGC CCAAGCTTGG TCAAAGCAAC CGCCCATCGG   
  
  
- GCCGTTGGGT TCGATCAAAC AACGAACCCT ACAAGGGAAC CTTTCCCATA TGAAACTACC TCCTTTTACC   
  
  
- CACAAACTCT AATCCCACCT TTCTAAACGG GAACAATTGA CGGAGTCGAA CCGTTGGAAC ACTTAAATTG   
  
  
- TTAGGATCAC GACCGTAAAT

+     AT~TATA-box

| Site Name | Organism | Position | Strand | Matrix score. | sequence | function |
| --- | --- | --- | --- | --- | --- | --- |
| AT~TATA-box | Arabidopsis thaliana | 219 | + | 6 | TATATA |  |
| AT~TATA-box | Arabidopsis thaliana | 215 | + | 6 | TATATA |  |
| AT~TATA-box | Arabidopsis thaliana | 217 | + | 6 | TATATA |  |

>HU02G03005.1   
+ +Up\_Stream \_Len000AAACGG TGCCGTTGAA TTTGCAGGCG ACGAGGTCGG GTTTAAGGCG GATGTCGTAG   
  
  
+ CGCTTTGGAG AAGCAAATTT GGGAAGACGA GGCTTATTCT TGAACTCTTC GTACGCCATT GTTGTTGGTT   
  
  
+ CCAACCTTTC TCTCTCCTCG TGAATTTGAG TTTGGGCAGT AGACTATCCC GGAATAGAGG ACTGATGCGT   
  
  
+ TATATATATA GAAATTGAGA GAGAGAGAGA GCGTGTGTGA AGGATGTTGA AAATGATGGG CGTAGGGAGT   
  
  
+ CTCTCTCTCA CTGTCTGTTA GGAGTACGTG TCGAGAAAGG AAATAGGATT CCCAAGCAGT CTAATTTGGG   
  
  
+ CCGGTCTATA GGCCAGAATG GGCCTATGAT CAGCCCAGAT ATGACCTTGT TTAACCAACT GTATTTGAAT   
  
  
+ TGAAGAAAGG GATATCGCAA TGTCTTGAAC CACATCAAAT AATAAATGTG CCTTTGGTTA GACCATATGA   
  
  
+ AAAATCTTTG AATCAAATCG GTTGGACCTG AACTGCCATT ATTGATATCT TGTTTTTGGT AGAAGCAAAT   
  
  
+ GGTGTCACTT CGTTTTCTAG ATAAAATTAA CTTGAGTTTT TTTGCCACTA TAGATAGTAT TGCAAATATA   
  
  
+ AAAAGTGTGC CACGTGAAAT CAATATTAGG AGGGTGTTTT ATAACAATAG CTGTTCGGGA GAGTTTTAGT   
  
  
+ TATTTTTATT AAATCAAATA GTTAAGAAGT GTTTGGTAAA TAGTTGTATT TGAAAAAGTT ATTCCCATTA   
  
  
+ GCCTTTTAGT AAGAACTTCT TGTGAAAATG ATAATTGTCT AGAAGTTTGA AAAATTCACA CCACATGACA   
  
  
+ TTTAAAAATA ATTTTGTAGA GAAGGGCATA AATGAAAGTT TGCCACCATC TTTTTTAGAA ACATGTAATT   
  
  
+ TACCAAACAC TTTTTCTAAA AACAGTTAAT TCAAATAACT AACAACTAAC AGCTAATATA AATAACTAAC   
  
  
+ AGCTATTTGC TAAACAGGGC CTAAGTAGTT TGAAATTGTT GTTTTCTCTT CAGCTTCACC ATGGCCGATG   
  
  
+ ATGTAAACAA CTATATTCTG AATTTCATTT TTACTATGAT TTGAAAATTT CAAATTTAAA TAAGATTGGA   
  
  
+ TAAAAAAAAG ACCAAAAATC ATAAGTAATA TTAGTCAAGT TCTATTCCAA CCATATACAT AACTGCTTTT   
  
  
+ TCTTTTGGGG GTTTTGAAAC ATGTGTCCTT AGAATAATTG AAACTCGATA TTATTCTCTA AATCTAAGTT   
  
  
+ TACTTTATCA TATTGATTAT TAATTACCTC CTCAACTCCT CCTATACTCT TGTATGCAGA AATAAAAATG   
  
  
+ TTTCCATACC TTATCAGTCG CGATCATAAT AATTAATTTC TTAATTCGGA TTGTATTTAA TTGCGTATCA   
  
  
+ AATGTGAAAT CGTACCAAAT TAGGAAGCAA AACAAACCGA TAACGAAAAA GGTAAGAAAA CTTAGCAAGG   
  
  
+ AAATGAAATC GAAGACCTGT CAACAATATA GCTTCCCTCA CATCCTTCAA CTTAGGGACC ATCCTATACT   
  
  
+ TTCATGTAAT ACAACTAGAG CCACTAGTCA ATAATAGCCT CCTAGTAGTA GCCATAAGCC TTGGTGAGAG   
  
  
+ AATGTTCCGC AAGACTAGTT AACTTGAAAT TCCATTTTGT CGCAGAACTA TTGGAGTGAG AAAGAGAATC   
  
  
+ AAATCATACC ATATCAAATT TGGACCATTT AAGTTCACAA CACACACACA TACACACAAA AGGACGATGT   
  
  
+ ACGGCCCCAC CCCCCACTCT CCTTTCGCCA TCCCCCTACA CCTCTGCCTA CTCTCTCTCT CTCTCTCTCT   
  
  
+ CTCTGGATCA GTCTGTGTAA AAGCTTCCTC TCCAGTACAG CATTTCCTTG TCTGTTCTTG GAGTTTAGTC   
  
  
+ AATTCCTCCG CCCCAAAAAA ACCCACTTTC TCTCTCATCA TGTTCTTCCA GTTCCACCCT TATACGCCAT   
  
  
+ TCATCATCAT CTACATCACC CACATCTTTT TCTCCACAGT TTTCCCCCCT CATTATGCTC TCCGCCTCCA   
  
  
+ CCCCCGCCTT CCCTCTCGCC GTCGCCGGCG ATGACGGGGA TACCACTGAT GCATGCGACG GCACCGCCAC   
  
  
+ TGCCCTCCAC CTCCTCGGGC TGCTGTTGAA GTGCGCGGAA TTCATCTCCA CCGGAGACCT CGCCGGCGCC   
  
  
+ GGAGATATCT TGCCGGAGAT ATTTGAGTTG TCTACACCGT TTGGCTCCCC CGCCGCCCGG GTCGCCGCCT   
  
  
+ TCTTCGGCCA CGCCCTCCAC GCCCGCCTCC TCTCCGCCTC CCTCCGCACA ACTCCGATCG AGAAGCTCAA   
  
  
+ AACCCTGACC CTGGTTTCCC AAATGCGGAA ATTTCACTCC GCCTTGCAAG TATACAACTC CATCACTCCC   
  
  
+ TTCGTGAAAT TCTCTCACTT CACGGCGAAT CAAGCCATCT ACGAGGCGCT GGACGGCGAG GATCGTGTCC   
  
  
+ ACGTCGTCGA CCTTGACATC ATGCAGGGCC TTCAATGGCC GGGATTGTTC CACATCTTAG CCTCTCGACC   
  
  
+ CAGAAAGCCC CTCTCGGTTC GGGTCACCGG GTTCGGGCCA TCCTCCGAGT TGCTCTCCCA AACGGGTAAG   
  
  
+ CGACTCGCTG AGTTCGCCGC TTCACTCGGC CTGCCCTTCG AGTACAACCC GGTGGAGGGC AAAATTGGGA   
  
  
+ ACTTAGTCGA CCTGGGTCGG GTCGGGTCGC TCCCGAATGA AGTGACGGTG GTGCACTGGA TGCACCATAG   
  
  
+ TCTATACGAC ATAACCGGGT CGGATCTTGG GACTTTGAGG GTATTGAGTG CGGTGAGGCC TAGGCTTGTG   
  
  
+ ACTATGGTTG AGCAGGATAT GGACCAAACG GGGTCGTTTT TGGGGAGGTT TGTGGAGGCA TTGCATTATT   
  
  
+ ACTCAGCCTT GTTTGATGCC TTGGGAGAGG GGTTAGATAG GGATAACCTA CAAAGGCATC AAGTGGAGCA   
  
  
+ ACAGCTATTT GGGTGTGAGA TTAGGAACAT CCTGGCCGTT GGTGGGCCCA AGAGGAGGCT CACTGGCGGC   
  
  
+ GATCGGGTCA GAAGGTGGGG CGACGAACTG ACAAGGGTCG GGTTCGAACC AGTTTCGTTG GCGGGTAGCC   
  
  
+ CGGCAACCCA AGCTAGTTTG TTGCTTGGGA TGTTCCCTTG GAAAGGGTAT ACTTTGATGG AGGAAAATGG   
  
  
+ GTGTTTGAGA TTAGGGTGGA AAGATTTGCC CTTGTTAACT GCCTCAGCTT GGCAACCTTG TGAATTTAAC   
  
  
+ AATCCTAGTG CTGGCATTTA   

- +Up\_Stream \_Len000TTTGCC ACGGCAACTT AAACGTCCGC TGCTCCAGCC CAAATTCCGC CTACAGCATC   
  
  
- GCGAAACCTC TTCGTTTAAA CCCTTCTGCT CCGAATAAGA ACTTGAGAAG CATGCGGTAA CAACAACCAA   
  
  
- GGTTGGAAAG AGAGAGGAGC ACTTAAACTC AAACCCGTCA TCTGATAGGG CCTTATCTCC TGACTACGCA   
  
  
- ATATATATAT CTTTAACTCT CTCTCTCTCT CGCACACACT TCCTACAACT TTTACTACCC GCATCCCTCA   
  
  
- GAGAGAGAGT GACAGACAAT CCTCATGCAC AGCTCTTTCC TTTATCCTAA GGGTTCGTCA GATTAAACCC   
  
  
- GGCCAGATAT CCGGTCTTAC CCGGATACTA GTCGGGTCTA TACTGGAACA AATTGGTTGA CATAAACTTA   
  
  
- ACTTCTTTCC CTATAGCGTT ACAGAACTTG GTGTAGTTTA TTATTTACAC GGAAACCAAT CTGGTATACT   
  
  
- TTTTAGAAAC TTAGTTTAGC CAACCTGGAC TTGACGGTAA TAACTATAGA ACAAAAACCA TCTTCGTTTA   
  
  
- CCACAGTGAA GCAAAAGATC TATTTTAATT GAACTCAAAA AAACGGTGAT ATCTATCATA ACGTTTATAT   
  
  
- TTTTCACACG GTGCACTTTA GTTATAATCC TCCCACAAAA TATTGTTATC GACAAGCCCT CTCAAAATCA   
  
  
- ATAAAAATAA TTTAGTTTAT CAATTCTTCA CAAACCATTT ATCAACATAA ACTTTTTCAA TAAGGGTAAT   
  
  
- CGGAAAATCA TTCTTGAAGA ACACTTTTAC TATTAACAGA TCTTCAAACT TTTTAAGTGT GGTGTACTGT   
  
  
- AAATTTTTAT TAAAACATCT CTTCCCGTAT TTACTTTCAA ACGGTGGTAG AAAAAATCTT TGTACATTAA   
  
  
- ATGGTTTGTG AAAAAGATTT TTGTCAATTA AGTTTATTGA TTGTTGATTG TCGATTATAT TTATTGATTG   
  
  
- TCGATAAACG ATTTGTCCCG GATTCATCAA ACTTTAACAA CAAAAGAGAA GTCGAAGTGG TACCGGCTAC   
  
  
- TACATTTGTT GATATAAGAC TTAAAGTAAA AATGATACTA AACTTTTAAA GTTTAAATTT ATTCTAACCT   
  
  
- ATTTTTTTTC TGGTTTTTAG TATTCATTAT AATCAGTTCA AGATAAGGTT GGTATATGTA TTGACGAAAA   
  
  
- AGAAAACCCC CAAAACTTTG TACACAGGAA TCTTATTAAC TTTGAGCTAT AATAAGAGAT TTAGATTCAA   
  
  
- ATGAAATAGT ATAACTAATA ATTAATGGAG GAGTTGAGGA GGATATGAGA ACATACGTCT TTATTTTTAC   
  
  
- AAAGGTATGG AATAGTCAGC GCTAGTATTA TTAATTAAAG AATTAAGCCT AACATAAATT AACGCATAGT   
  
  
- TTACACTTTA GCATGGTTTA ATCCTTCGTT TTGTTTGGCT ATTGCTTTTT CCATTCTTTT GAATCGTTCC   
  
  
- TTTACTTTAG CTTCTGGACA GTTGTTATAT CGAAGGGAGT GTAGGAAGTT GAATCCCTGG TAGGATATGA   
  
  
- AAGTACATTA TGTTGATCTC GGTGATCAGT TATTATCGGA GGATCATCAT CGGTATTCGG AACCACTCTC   
  
  
- TTACAAGGCG TTCTGATCAA TTGAACTTTA AGGTAAAACA GCGTCTTGAT AACCTCACTC TTTCTCTTAG   
  
  
- TTTAGTATGG TATAGTTTAA ACCTGGTAAA TTCAAGTGTT GTGTGTGTGT ATGTGTGTTT TCCTGCTACA   
  
  
- TGCCGGGGTG GGGGGTGAGA GGAAAGCGGT AGGGGGATGT GGAGACGGAT GAGAGAGAGA GAGAGAGAGA   
  
  
- GAGACCTAGT CAGACACATT TTCGAAGGAG AGGTCATGTC GTAAAGGAAC AGACAAGAAC CTCAAATCAG   
  
  
- TTAAGGAGGC GGGGTTTTTT TGGGTGAAAG AGAGAGTAGT ACAAGAAGGT CAAGGTGGGA ATATGCGGTA   
  
  
- AGTAGTAGTA GATGTAGTGG GTGTAGAAAA AGAGGTGTCA AAAGGGGGGA GTAATACGAG AGGCGGAGGT   
  
  
- GGGGGCGGAA GGGAGAGCGG CAGCGGCCGC TACTGCCCCT ATGGTGACTA CGTACGCTGC CGTGGCGGTG   
  
  
- ACGGGAGGTG GAGGAGCCCG ACGACAACTT CACGCGCCTT AAGTAGAGGT GGCCTCTGGA GCGGCCGCGG   
  
  
- CCTCTATAGA ACGGCCTCTA TAAACTCAAC AGATGTGGCA AACCGAGGGG GCGGCGGGCC CAGCGGCGGA   
  
  
- AGAAGCCGGT GCGGGAGGTG CGGGCGGAGG AGAGGCGGAG GGAGGCGTGT TGAGGCTAGC TCTTCGAGTT   
  
  
- TTGGGACTGG GACCAAAGGG TTTACGCCTT TAAAGTGAGG CGGAACGTTC ATATGTTGAG GTAGTGAGGG   
  
  
- AAGCACTTTA AGAGAGTGAA GTGCCGCTTA GTTCGGTAGA TGCTCCGCGA CCTGCCGCTC CTAGCACAGG   
  
  
- TGCAGCAGCT GGAACTGTAG TACGTCCCGG AAGTTACCGG CCCTAACAAG GTGTAGAATC GGAGAGCTGG   
  
  
- GTCTTTCGGG GAGAGCCAAG CCCAGTGGCC CAAGCCCGGT AGGAGGCTCA ACGAGAGGGT TTGCCCATTC   
  
  
- GCTGAGCGAC TCAAGCGGCG AAGTGAGCCG GACGGGAAGC TCATGTTGGG CCACCTCCCG TTTTAACCCT   
  
  
- TGAATCAGCT GGACCCAGCC CAGCCCAGCG AGGGCTTACT TCACTGCCAC CACGTGACCT ACGTGGTATC   
  
  
- AGATATGCTG TATTGGCCCA GCCTAGAACC CTGAAACTCC CATAACTCAC GCCACTCCGG ATCCGAACAC   
  
  
- TGATACCAAC TCGTCCTATA CCTGGTTTGC CCCAGCAAAA ACCCCTCCAA ACACCTCCGT AACGTAATAA   
  
  
- TGAGTCGGAA CAAACTACGG AACCCTCTCC CCAATCTATC CCTATTGGAT GTTTCCGTAG TTCACCTCGT   
  
  
- TGTCGATAAA CCCACACTCT AATCCTTGTA GGACCGGCAA CCACCCGGGT TCTCCTCCGA GTGACCGCCG   
  
  
- CTAGCCCAGT CTTCCACCCC GCTGCTTGAC TGTTCCCAGC CCAAGCTTGG TCAAAGCAAC CGCCCATCGG   
  
  
- GCCGTTGGGT TCGATCAAAC AACGAACCCT ACAAGGGAAC CTTTCCCATA TGAAACTACC TCCTTTTACC   
  
  
- CACAAACTCT AATCCCACCT TTCTAAACGG GAACAATTGA CGGAGTCGAA CCGTTGGAAC ACTTAAATTG   
  
  
- TTAGGATCAC GACCGTAAAT

+     AuxRR-core

| Site Name | Organism | Position | Strand | Matrix score. | sequence | function |
| --- | --- | --- | --- | --- | --- | --- |
| AuxRR-core | Nicotiana tabacum | 2823 | - | 7 | GGTCCAT | cis-acting regulatory element involved in auxin responsiveness |

>HU02G03005.1   
+ +Up\_Stream \_Len000AAACGG TGCCGTTGAA TTTGCAGGCG ACGAGGTCGG GTTTAAGGCG GATGTCGTAG   
  
  
+ CGCTTTGGAG AAGCAAATTT GGGAAGACGA GGCTTATTCT TGAACTCTTC GTACGCCATT GTTGTTGGTT   
  
  
+ CCAACCTTTC TCTCTCCTCG TGAATTTGAG TTTGGGCAGT AGACTATCCC GGAATAGAGG ACTGATGCGT   
  
  
+ TATATATATA GAAATTGAGA GAGAGAGAGA GCGTGTGTGA AGGATGTTGA AAATGATGGG CGTAGGGAGT   
  
  
+ CTCTCTCTCA CTGTCTGTTA GGAGTACGTG TCGAGAAAGG AAATAGGATT CCCAAGCAGT CTAATTTGGG   
  
  
+ CCGGTCTATA GGCCAGAATG GGCCTATGAT CAGCCCAGAT ATGACCTTGT TTAACCAACT GTATTTGAAT   
  
  
+ TGAAGAAAGG GATATCGCAA TGTCTTGAAC CACATCAAAT AATAAATGTG CCTTTGGTTA GACCATATGA   
  
  
+ AAAATCTTTG AATCAAATCG GTTGGACCTG AACTGCCATT ATTGATATCT TGTTTTTGGT AGAAGCAAAT   
  
  
+ GGTGTCACTT CGTTTTCTAG ATAAAATTAA CTTGAGTTTT TTTGCCACTA TAGATAGTAT TGCAAATATA   
  
  
+ AAAAGTGTGC CACGTGAAAT CAATATTAGG AGGGTGTTTT ATAACAATAG CTGTTCGGGA GAGTTTTAGT   
  
  
+ TATTTTTATT AAATCAAATA GTTAAGAAGT GTTTGGTAAA TAGTTGTATT TGAAAAAGTT ATTCCCATTA   
  
  
+ GCCTTTTAGT AAGAACTTCT TGTGAAAATG ATAATTGTCT AGAAGTTTGA AAAATTCACA CCACATGACA   
  
  
+ TTTAAAAATA ATTTTGTAGA GAAGGGCATA AATGAAAGTT TGCCACCATC TTTTTTAGAA ACATGTAATT   
  
  
+ TACCAAACAC TTTTTCTAAA AACAGTTAAT TCAAATAACT AACAACTAAC AGCTAATATA AATAACTAAC   
  
  
+ AGCTATTTGC TAAACAGGGC CTAAGTAGTT TGAAATTGTT GTTTTCTCTT CAGCTTCACC ATGGCCGATG   
  
  
+ ATGTAAACAA CTATATTCTG AATTTCATTT TTACTATGAT TTGAAAATTT CAAATTTAAA TAAGATTGGA   
  
  
+ TAAAAAAAAG ACCAAAAATC ATAAGTAATA TTAGTCAAGT TCTATTCCAA CCATATACAT AACTGCTTTT   
  
  
+ TCTTTTGGGG GTTTTGAAAC ATGTGTCCTT AGAATAATTG AAACTCGATA TTATTCTCTA AATCTAAGTT   
  
  
+ TACTTTATCA TATTGATTAT TAATTACCTC CTCAACTCCT CCTATACTCT TGTATGCAGA AATAAAAATG   
  
  
+ TTTCCATACC TTATCAGTCG CGATCATAAT AATTAATTTC TTAATTCGGA TTGTATTTAA TTGCGTATCA   
  
  
+ AATGTGAAAT CGTACCAAAT TAGGAAGCAA AACAAACCGA TAACGAAAAA GGTAAGAAAA CTTAGCAAGG   
  
  
+ AAATGAAATC GAAGACCTGT CAACAATATA GCTTCCCTCA CATCCTTCAA CTTAGGGACC ATCCTATACT   
  
  
+ TTCATGTAAT ACAACTAGAG CCACTAGTCA ATAATAGCCT CCTAGTAGTA GCCATAAGCC TTGGTGAGAG   
  
  
+ AATGTTCCGC AAGACTAGTT AACTTGAAAT TCCATTTTGT CGCAGAACTA TTGGAGTGAG AAAGAGAATC   
  
  
+ AAATCATACC ATATCAAATT TGGACCATTT AAGTTCACAA CACACACACA TACACACAAA AGGACGATGT   
  
  
+ ACGGCCCCAC CCCCCACTCT CCTTTCGCCA TCCCCCTACA CCTCTGCCTA CTCTCTCTCT CTCTCTCTCT   
  
  
+ CTCTGGATCA GTCTGTGTAA AAGCTTCCTC TCCAGTACAG CATTTCCTTG TCTGTTCTTG GAGTTTAGTC   
  
  
+ AATTCCTCCG CCCCAAAAAA ACCCACTTTC TCTCTCATCA TGTTCTTCCA GTTCCACCCT TATACGCCAT   
  
  
+ TCATCATCAT CTACATCACC CACATCTTTT TCTCCACAGT TTTCCCCCCT CATTATGCTC TCCGCCTCCA   
  
  
+ CCCCCGCCTT CCCTCTCGCC GTCGCCGGCG ATGACGGGGA TACCACTGAT GCATGCGACG GCACCGCCAC   
  
  
+ TGCCCTCCAC CTCCTCGGGC TGCTGTTGAA GTGCGCGGAA TTCATCTCCA CCGGAGACCT CGCCGGCGCC   
  
  
+ GGAGATATCT TGCCGGAGAT ATTTGAGTTG TCTACACCGT TTGGCTCCCC CGCCGCCCGG GTCGCCGCCT   
  
  
+ TCTTCGGCCA CGCCCTCCAC GCCCGCCTCC TCTCCGCCTC CCTCCGCACA ACTCCGATCG AGAAGCTCAA   
  
  
+ AACCCTGACC CTGGTTTCCC AAATGCGGAA ATTTCACTCC GCCTTGCAAG TATACAACTC CATCACTCCC   
  
  
+ TTCGTGAAAT TCTCTCACTT CACGGCGAAT CAAGCCATCT ACGAGGCGCT GGACGGCGAG GATCGTGTCC   
  
  
+ ACGTCGTCGA CCTTGACATC ATGCAGGGCC TTCAATGGCC GGGATTGTTC CACATCTTAG CCTCTCGACC   
  
  
+ CAGAAAGCCC CTCTCGGTTC GGGTCACCGG GTTCGGGCCA TCCTCCGAGT TGCTCTCCCA AACGGGTAAG   
  
  
+ CGACTCGCTG AGTTCGCCGC TTCACTCGGC CTGCCCTTCG AGTACAACCC GGTGGAGGGC AAAATTGGGA   
  
  
+ ACTTAGTCGA CCTGGGTCGG GTCGGGTCGC TCCCGAATGA AGTGACGGTG GTGCACTGGA TGCACCATAG   
  
  
+ TCTATACGAC ATAACCGGGT CGGATCTTGG GACTTTGAGG GTATTGAGTG CGGTGAGGCC TAGGCTTGTG   
  
  
+ ACTATGGTTG AGCAGGATAT GGACCAAACG GGGTCGTTTT TGGGGAGGTT TGTGGAGGCA TTGCATTATT   
  
  
+ ACTCAGCCTT GTTTGATGCC TTGGGAGAGG GGTTAGATAG GGATAACCTA CAAAGGCATC AAGTGGAGCA   
  
  
+ ACAGCTATTT GGGTGTGAGA TTAGGAACAT CCTGGCCGTT GGTGGGCCCA AGAGGAGGCT CACTGGCGGC   
  
  
+ GATCGGGTCA GAAGGTGGGG CGACGAACTG ACAAGGGTCG GGTTCGAACC AGTTTCGTTG GCGGGTAGCC   
  
  
+ CGGCAACCCA AGCTAGTTTG TTGCTTGGGA TGTTCCCTTG GAAAGGGTAT ACTTTGATGG AGGAAAATGG   
  
  
+ GTGTTTGAGA TTAGGGTGGA AAGATTTGCC CTTGTTAACT GCCTCAGCTT GGCAACCTTG TGAATTTAAC   
  
  
+ AATCCTAGTG CTGGCATTTA   

- +Up\_Stream \_Len000TTTGCC ACGGCAACTT AAACGTCCGC TGCTCCAGCC CAAATTCCGC CTACAGCATC   
  
  
- GCGAAACCTC TTCGTTTAAA CCCTTCTGCT CCGAATAAGA ACTTGAGAAG CATGCGGTAA CAACAACCAA   
  
  
- GGTTGGAAAG AGAGAGGAGC ACTTAAACTC AAACCCGTCA TCTGATAGGG CCTTATCTCC TGACTACGCA   
  
  
- ATATATATAT CTTTAACTCT CTCTCTCTCT CGCACACACT TCCTACAACT TTTACTACCC GCATCCCTCA   
  
  
- GAGAGAGAGT GACAGACAAT CCTCATGCAC AGCTCTTTCC TTTATCCTAA GGGTTCGTCA GATTAAACCC   
  
  
- GGCCAGATAT CCGGTCTTAC CCGGATACTA GTCGGGTCTA TACTGGAACA AATTGGTTGA CATAAACTTA   
  
  
- ACTTCTTTCC CTATAGCGTT ACAGAACTTG GTGTAGTTTA TTATTTACAC GGAAACCAAT CTGGTATACT   
  
  
- TTTTAGAAAC TTAGTTTAGC CAACCTGGAC TTGACGGTAA TAACTATAGA ACAAAAACCA TCTTCGTTTA   
  
  
- CCACAGTGAA GCAAAAGATC TATTTTAATT GAACTCAAAA AAACGGTGAT ATCTATCATA ACGTTTATAT   
  
  
- TTTTCACACG GTGCACTTTA GTTATAATCC TCCCACAAAA TATTGTTATC GACAAGCCCT CTCAAAATCA   
  
  
- ATAAAAATAA TTTAGTTTAT CAATTCTTCA CAAACCATTT ATCAACATAA ACTTTTTCAA TAAGGGTAAT   
  
  
- CGGAAAATCA TTCTTGAAGA ACACTTTTAC TATTAACAGA TCTTCAAACT TTTTAAGTGT GGTGTACTGT   
  
  
- AAATTTTTAT TAAAACATCT CTTCCCGTAT TTACTTTCAA ACGGTGGTAG AAAAAATCTT TGTACATTAA   
  
  
- ATGGTTTGTG AAAAAGATTT TTGTCAATTA AGTTTATTGA TTGTTGATTG TCGATTATAT TTATTGATTG   
  
  
- TCGATAAACG ATTTGTCCCG GATTCATCAA ACTTTAACAA CAAAAGAGAA GTCGAAGTGG TACCGGCTAC   
  
  
- TACATTTGTT GATATAAGAC TTAAAGTAAA AATGATACTA AACTTTTAAA GTTTAAATTT ATTCTAACCT   
  
  
- ATTTTTTTTC TGGTTTTTAG TATTCATTAT AATCAGTTCA AGATAAGGTT GGTATATGTA TTGACGAAAA   
  
  
- AGAAAACCCC CAAAACTTTG TACACAGGAA TCTTATTAAC TTTGAGCTAT AATAAGAGAT TTAGATTCAA   
  
  
- ATGAAATAGT ATAACTAATA ATTAATGGAG GAGTTGAGGA GGATATGAGA ACATACGTCT TTATTTTTAC   
  
  
- AAAGGTATGG AATAGTCAGC GCTAGTATTA TTAATTAAAG AATTAAGCCT AACATAAATT AACGCATAGT   
  
  
- TTACACTTTA GCATGGTTTA ATCCTTCGTT TTGTTTGGCT ATTGCTTTTT CCATTCTTTT GAATCGTTCC   
  
  
- TTTACTTTAG CTTCTGGACA GTTGTTATAT CGAAGGGAGT GTAGGAAGTT GAATCCCTGG TAGGATATGA   
  
  
- AAGTACATTA TGTTGATCTC GGTGATCAGT TATTATCGGA GGATCATCAT CGGTATTCGG AACCACTCTC   
  
  
- TTACAAGGCG TTCTGATCAA TTGAACTTTA AGGTAAAACA GCGTCTTGAT AACCTCACTC TTTCTCTTAG   
  
  
- TTTAGTATGG TATAGTTTAA ACCTGGTAAA TTCAAGTGTT GTGTGTGTGT ATGTGTGTTT TCCTGCTACA   
  
  
- TGCCGGGGTG GGGGGTGAGA GGAAAGCGGT AGGGGGATGT GGAGACGGAT GAGAGAGAGA GAGAGAGAGA   
  
  
- GAGACCTAGT CAGACACATT TTCGAAGGAG AGGTCATGTC GTAAAGGAAC AGACAAGAAC CTCAAATCAG   
  
  
- TTAAGGAGGC GGGGTTTTTT TGGGTGAAAG AGAGAGTAGT ACAAGAAGGT CAAGGTGGGA ATATGCGGTA   
  
  
- AGTAGTAGTA GATGTAGTGG GTGTAGAAAA AGAGGTGTCA AAAGGGGGGA GTAATACGAG AGGCGGAGGT   
  
  
- GGGGGCGGAA GGGAGAGCGG CAGCGGCCGC TACTGCCCCT ATGGTGACTA CGTACGCTGC CGTGGCGGTG   
  
  
- ACGGGAGGTG GAGGAGCCCG ACGACAACTT CACGCGCCTT AAGTAGAGGT GGCCTCTGGA GCGGCCGCGG   
  
  
- CCTCTATAGA ACGGCCTCTA TAAACTCAAC AGATGTGGCA AACCGAGGGG GCGGCGGGCC CAGCGGCGGA   
  
  
- AGAAGCCGGT GCGGGAGGTG CGGGCGGAGG AGAGGCGGAG GGAGGCGTGT TGAGGCTAGC TCTTCGAGTT   
  
  
- TTGGGACTGG GACCAAAGGG TTTACGCCTT TAAAGTGAGG CGGAACGTTC ATATGTTGAG GTAGTGAGGG   
  
  
- AAGCACTTTA AGAGAGTGAA GTGCCGCTTA GTTCGGTAGA TGCTCCGCGA CCTGCCGCTC CTAGCACAGG   
  
  
- TGCAGCAGCT GGAACTGTAG TACGTCCCGG AAGTTACCGG CCCTAACAAG GTGTAGAATC GGAGAGCTGG   
  
  
- GTCTTTCGGG GAGAGCCAAG CCCAGTGGCC CAAGCCCGGT AGGAGGCTCA ACGAGAGGGT TTGCCCATTC   
  
  
- GCTGAGCGAC TCAAGCGGCG AAGTGAGCCG GACGGGAAGC TCATGTTGGG CCACCTCCCG TTTTAACCCT   
  
  
- TGAATCAGCT GGACCCAGCC CAGCCCAGCG AGGGCTTACT TCACTGCCAC CACGTGACCT ACGTGGTATC   
  
  
- AGATATGCTG TATTGGCCCA GCCTAGAACC CTGAAACTCC CATAACTCAC GCCACTCCGG ATCCGAACAC   
  
  
- TGATACCAAC TCGTCCTATA CCTGGTTTGC CCCAGCAAAA ACCCCTCCAA ACACCTCCGT AACGTAATAA   
  
  
- TGAGTCGGAA CAAACTACGG AACCCTCTCC CCAATCTATC CCTATTGGAT GTTTCCGTAG TTCACCTCGT   
  
  
- TGTCGATAAA CCCACACTCT AATCCTTGTA GGACCGGCAA CCACCCGGGT TCTCCTCCGA GTGACCGCCG   
  
  
- CTAGCCCAGT CTTCCACCCC GCTGCTTGAC TGTTCCCAGC CCAAGCTTGG TCAAAGCAAC CGCCCATCGG   
  
  
- GCCGTTGGGT TCGATCAAAC AACGAACCCT ACAAGGGAAC CTTTCCCATA TGAAACTACC TCCTTTTACC   
  
  
- CACAAACTCT AATCCCACCT TTCTAAACGG GAACAATTGA CGGAGTCGAA CCGTTGGAAC ACTTAAATTG   
  
  
- TTAGGATCAC GACCGTAAAT

+     Box 4

| Site Name | Organism | Position | Strand | Matrix score. | sequence | function |
| --- | --- | --- | --- | --- | --- | --- |
| Box 4 | Petroselinum crispum | 1283 | + | 6 | ATTAAT | part of a conserved DNA module involved in light responsiveness |
| Box 4 | Petroselinum crispum | 1366 | + | 6 | ATTAAT | part of a conserved DNA module involved in light responsiveness |

>HU02G03005.1   
+ +Up\_Stream \_Len000AAACGG TGCCGTTGAA TTTGCAGGCG ACGAGGTCGG GTTTAAGGCG GATGTCGTAG   
  
  
+ CGCTTTGGAG AAGCAAATTT GGGAAGACGA GGCTTATTCT TGAACTCTTC GTACGCCATT GTTGTTGGTT   
  
  
+ CCAACCTTTC TCTCTCCTCG TGAATTTGAG TTTGGGCAGT AGACTATCCC GGAATAGAGG ACTGATGCGT   
  
  
+ TATATATATA GAAATTGAGA GAGAGAGAGA GCGTGTGTGA AGGATGTTGA AAATGATGGG CGTAGGGAGT   
  
  
+ CTCTCTCTCA CTGTCTGTTA GGAGTACGTG TCGAGAAAGG AAATAGGATT CCCAAGCAGT CTAATTTGGG   
  
  
+ CCGGTCTATA GGCCAGAATG GGCCTATGAT CAGCCCAGAT ATGACCTTGT TTAACCAACT GTATTTGAAT   
  
  
+ TGAAGAAAGG GATATCGCAA TGTCTTGAAC CACATCAAAT AATAAATGTG CCTTTGGTTA GACCATATGA   
  
  
+ AAAATCTTTG AATCAAATCG GTTGGACCTG AACTGCCATT ATTGATATCT TGTTTTTGGT AGAAGCAAAT   
  
  
+ GGTGTCACTT CGTTTTCTAG ATAAAATTAA CTTGAGTTTT TTTGCCACTA TAGATAGTAT TGCAAATATA   
  
  
+ AAAAGTGTGC CACGTGAAAT CAATATTAGG AGGGTGTTTT ATAACAATAG CTGTTCGGGA GAGTTTTAGT   
  
  
+ TATTTTTATT AAATCAAATA GTTAAGAAGT GTTTGGTAAA TAGTTGTATT TGAAAAAGTT ATTCCCATTA   
  
  
+ GCCTTTTAGT AAGAACTTCT TGTGAAAATG ATAATTGTCT AGAAGTTTGA AAAATTCACA CCACATGACA   
  
  
+ TTTAAAAATA ATTTTGTAGA GAAGGGCATA AATGAAAGTT TGCCACCATC TTTTTTAGAA ACATGTAATT   
  
  
+ TACCAAACAC TTTTTCTAAA AACAGTTAAT TCAAATAACT AACAACTAAC AGCTAATATA AATAACTAAC   
  
  
+ AGCTATTTGC TAAACAGGGC CTAAGTAGTT TGAAATTGTT GTTTTCTCTT CAGCTTCACC ATGGCCGATG   
  
  
+ ATGTAAACAA CTATATTCTG AATTTCATTT TTACTATGAT TTGAAAATTT CAAATTTAAA TAAGATTGGA   
  
  
+ TAAAAAAAAG ACCAAAAATC ATAAGTAATA TTAGTCAAGT TCTATTCCAA CCATATACAT AACTGCTTTT   
  
  
+ TCTTTTGGGG GTTTTGAAAC ATGTGTCCTT AGAATAATTG AAACTCGATA TTATTCTCTA AATCTAAGTT   
  
  
+ TACTTTATCA TATTGATTAT TAATTACCTC CTCAACTCCT CCTATACTCT TGTATGCAGA AATAAAAATG   
  
  
+ TTTCCATACC TTATCAGTCG CGATCATAAT AATTAATTTC TTAATTCGGA TTGTATTTAA TTGCGTATCA   
  
  
+ AATGTGAAAT CGTACCAAAT TAGGAAGCAA AACAAACCGA TAACGAAAAA GGTAAGAAAA CTTAGCAAGG   
  
  
+ AAATGAAATC GAAGACCTGT CAACAATATA GCTTCCCTCA CATCCTTCAA CTTAGGGACC ATCCTATACT   
  
  
+ TTCATGTAAT ACAACTAGAG CCACTAGTCA ATAATAGCCT CCTAGTAGTA GCCATAAGCC TTGGTGAGAG   
  
  
+ AATGTTCCGC AAGACTAGTT AACTTGAAAT TCCATTTTGT CGCAGAACTA TTGGAGTGAG AAAGAGAATC   
  
  
+ AAATCATACC ATATCAAATT TGGACCATTT AAGTTCACAA CACACACACA TACACACAAA AGGACGATGT   
  
  
+ ACGGCCCCAC CCCCCACTCT CCTTTCGCCA TCCCCCTACA CCTCTGCCTA CTCTCTCTCT CTCTCTCTCT   
  
  
+ CTCTGGATCA GTCTGTGTAA AAGCTTCCTC TCCAGTACAG CATTTCCTTG TCTGTTCTTG GAGTTTAGTC   
  
  
+ AATTCCTCCG CCCCAAAAAA ACCCACTTTC TCTCTCATCA TGTTCTTCCA GTTCCACCCT TATACGCCAT   
  
  
+ TCATCATCAT CTACATCACC CACATCTTTT TCTCCACAGT TTTCCCCCCT CATTATGCTC TCCGCCTCCA   
  
  
+ CCCCCGCCTT CCCTCTCGCC GTCGCCGGCG ATGACGGGGA TACCACTGAT GCATGCGACG GCACCGCCAC   
  
  
+ TGCCCTCCAC CTCCTCGGGC TGCTGTTGAA GTGCGCGGAA TTCATCTCCA CCGGAGACCT CGCCGGCGCC   
  
  
+ GGAGATATCT TGCCGGAGAT ATTTGAGTTG TCTACACCGT TTGGCTCCCC CGCCGCCCGG GTCGCCGCCT   
  
  
+ TCTTCGGCCA CGCCCTCCAC GCCCGCCTCC TCTCCGCCTC CCTCCGCACA ACTCCGATCG AGAAGCTCAA   
  
  
+ AACCCTGACC CTGGTTTCCC AAATGCGGAA ATTTCACTCC GCCTTGCAAG TATACAACTC CATCACTCCC   
  
  
+ TTCGTGAAAT TCTCTCACTT CACGGCGAAT CAAGCCATCT ACGAGGCGCT GGACGGCGAG GATCGTGTCC   
  
  
+ ACGTCGTCGA CCTTGACATC ATGCAGGGCC TTCAATGGCC GGGATTGTTC CACATCTTAG CCTCTCGACC   
  
  
+ CAGAAAGCCC CTCTCGGTTC GGGTCACCGG GTTCGGGCCA TCCTCCGAGT TGCTCTCCCA AACGGGTAAG   
  
  
+ CGACTCGCTG AGTTCGCCGC TTCACTCGGC CTGCCCTTCG AGTACAACCC GGTGGAGGGC AAAATTGGGA   
  
  
+ ACTTAGTCGA CCTGGGTCGG GTCGGGTCGC TCCCGAATGA AGTGACGGTG GTGCACTGGA TGCACCATAG   
  
  
+ TCTATACGAC ATAACCGGGT CGGATCTTGG GACTTTGAGG GTATTGAGTG CGGTGAGGCC TAGGCTTGTG   
  
  
+ ACTATGGTTG AGCAGGATAT GGACCAAACG GGGTCGTTTT TGGGGAGGTT TGTGGAGGCA TTGCATTATT   
  
  
+ ACTCAGCCTT GTTTGATGCC TTGGGAGAGG GGTTAGATAG GGATAACCTA CAAAGGCATC AAGTGGAGCA   
  
  
+ ACAGCTATTT GGGTGTGAGA TTAGGAACAT CCTGGCCGTT GGTGGGCCCA AGAGGAGGCT CACTGGCGGC   
  
  
+ GATCGGGTCA GAAGGTGGGG CGACGAACTG ACAAGGGTCG GGTTCGAACC AGTTTCGTTG GCGGGTAGCC   
  
  
+ CGGCAACCCA AGCTAGTTTG TTGCTTGGGA TGTTCCCTTG GAAAGGGTAT ACTTTGATGG AGGAAAATGG   
  
  
+ GTGTTTGAGA TTAGGGTGGA AAGATTTGCC CTTGTTAACT GCCTCAGCTT GGCAACCTTG TGAATTTAAC   
  
  
+ AATCCTAGTG CTGGCATTTA   

- +Up\_Stream \_Len000TTTGCC ACGGCAACTT AAACGTCCGC TGCTCCAGCC CAAATTCCGC CTACAGCATC   
  
  
- GCGAAACCTC TTCGTTTAAA CCCTTCTGCT CCGAATAAGA ACTTGAGAAG CATGCGGTAA CAACAACCAA   
  
  
- GGTTGGAAAG AGAGAGGAGC ACTTAAACTC AAACCCGTCA TCTGATAGGG CCTTATCTCC TGACTACGCA   
  
  
- ATATATATAT CTTTAACTCT CTCTCTCTCT CGCACACACT TCCTACAACT TTTACTACCC GCATCCCTCA   
  
  
- GAGAGAGAGT GACAGACAAT CCTCATGCAC AGCTCTTTCC TTTATCCTAA GGGTTCGTCA GATTAAACCC   
  
  
- GGCCAGATAT CCGGTCTTAC CCGGATACTA GTCGGGTCTA TACTGGAACA AATTGGTTGA CATAAACTTA   
  
  
- ACTTCTTTCC CTATAGCGTT ACAGAACTTG GTGTAGTTTA TTATTTACAC GGAAACCAAT CTGGTATACT   
  
  
- TTTTAGAAAC TTAGTTTAGC CAACCTGGAC TTGACGGTAA TAACTATAGA ACAAAAACCA TCTTCGTTTA   
  
  
- CCACAGTGAA GCAAAAGATC TATTTTAATT GAACTCAAAA AAACGGTGAT ATCTATCATA ACGTTTATAT   
  
  
- TTTTCACACG GTGCACTTTA GTTATAATCC TCCCACAAAA TATTGTTATC GACAAGCCCT CTCAAAATCA   
  
  
- ATAAAAATAA TTTAGTTTAT CAATTCTTCA CAAACCATTT ATCAACATAA ACTTTTTCAA TAAGGGTAAT   
  
  
- CGGAAAATCA TTCTTGAAGA ACACTTTTAC TATTAACAGA TCTTCAAACT TTTTAAGTGT GGTGTACTGT   
  
  
- AAATTTTTAT TAAAACATCT CTTCCCGTAT TTACTTTCAA ACGGTGGTAG AAAAAATCTT TGTACATTAA   
  
  
- ATGGTTTGTG AAAAAGATTT TTGTCAATTA AGTTTATTGA TTGTTGATTG TCGATTATAT TTATTGATTG   
  
  
- TCGATAAACG ATTTGTCCCG GATTCATCAA ACTTTAACAA CAAAAGAGAA GTCGAAGTGG TACCGGCTAC   
  
  
- TACATTTGTT GATATAAGAC TTAAAGTAAA AATGATACTA AACTTTTAAA GTTTAAATTT ATTCTAACCT   
  
  
- ATTTTTTTTC TGGTTTTTAG TATTCATTAT AATCAGTTCA AGATAAGGTT GGTATATGTA TTGACGAAAA   
  
  
- AGAAAACCCC CAAAACTTTG TACACAGGAA TCTTATTAAC TTTGAGCTAT AATAAGAGAT TTAGATTCAA   
  
  
- ATGAAATAGT ATAACTAATA ATTAATGGAG GAGTTGAGGA GGATATGAGA ACATACGTCT TTATTTTTAC   
  
  
- AAAGGTATGG AATAGTCAGC GCTAGTATTA TTAATTAAAG AATTAAGCCT AACATAAATT AACGCATAGT   
  
  
- TTACACTTTA GCATGGTTTA ATCCTTCGTT TTGTTTGGCT ATTGCTTTTT CCATTCTTTT GAATCGTTCC   
  
  
- TTTACTTTAG CTTCTGGACA GTTGTTATAT CGAAGGGAGT GTAGGAAGTT GAATCCCTGG TAGGATATGA   
  
  
- AAGTACATTA TGTTGATCTC GGTGATCAGT TATTATCGGA GGATCATCAT CGGTATTCGG AACCACTCTC   
  
  
- TTACAAGGCG TTCTGATCAA TTGAACTTTA AGGTAAAACA GCGTCTTGAT AACCTCACTC TTTCTCTTAG   
  
  
- TTTAGTATGG TATAGTTTAA ACCTGGTAAA TTCAAGTGTT GTGTGTGTGT ATGTGTGTTT TCCTGCTACA   
  
  
- TGCCGGGGTG GGGGGTGAGA GGAAAGCGGT AGGGGGATGT GGAGACGGAT GAGAGAGAGA GAGAGAGAGA   
  
  
- GAGACCTAGT CAGACACATT TTCGAAGGAG AGGTCATGTC GTAAAGGAAC AGACAAGAAC CTCAAATCAG   
  
  
- TTAAGGAGGC GGGGTTTTTT TGGGTGAAAG AGAGAGTAGT ACAAGAAGGT CAAGGTGGGA ATATGCGGTA   
  
  
- AGTAGTAGTA GATGTAGTGG GTGTAGAAAA AGAGGTGTCA AAAGGGGGGA GTAATACGAG AGGCGGAGGT   
  
  
- GGGGGCGGAA GGGAGAGCGG CAGCGGCCGC TACTGCCCCT ATGGTGACTA CGTACGCTGC CGTGGCGGTG   
  
  
- ACGGGAGGTG GAGGAGCCCG ACGACAACTT CACGCGCCTT AAGTAGAGGT GGCCTCTGGA GCGGCCGCGG   
  
  
- CCTCTATAGA ACGGCCTCTA TAAACTCAAC AGATGTGGCA AACCGAGGGG GCGGCGGGCC CAGCGGCGGA   
  
  
- AGAAGCCGGT GCGGGAGGTG CGGGCGGAGG AGAGGCGGAG GGAGGCGTGT TGAGGCTAGC TCTTCGAGTT   
  
  
- TTGGGACTGG GACCAAAGGG TTTACGCCTT TAAAGTGAGG CGGAACGTTC ATATGTTGAG GTAGTGAGGG   
  
  
- AAGCACTTTA AGAGAGTGAA GTGCCGCTTA GTTCGGTAGA TGCTCCGCGA CCTGCCGCTC CTAGCACAGG   
  
  
- TGCAGCAGCT GGAACTGTAG TACGTCCCGG AAGTTACCGG CCCTAACAAG GTGTAGAATC GGAGAGCTGG   
  
  
- GTCTTTCGGG GAGAGCCAAG CCCAGTGGCC CAAGCCCGGT AGGAGGCTCA ACGAGAGGGT TTGCCCATTC   
  
  
- GCTGAGCGAC TCAAGCGGCG AAGTGAGCCG GACGGGAAGC TCATGTTGGG CCACCTCCCG TTTTAACCCT   
  
  
- TGAATCAGCT GGACCCAGCC CAGCCCAGCG AGGGCTTACT TCACTGCCAC CACGTGACCT ACGTGGTATC   
  
  
- AGATATGCTG TATTGGCCCA GCCTAGAACC CTGAAACTCC CATAACTCAC GCCACTCCGG ATCCGAACAC   
  
  
- TGATACCAAC TCGTCCTATA CCTGGTTTGC CCCAGCAAAA ACCCCTCCAA ACACCTCCGT AACGTAATAA   
  
  
- TGAGTCGGAA CAAACTACGG AACCCTCTCC CCAATCTATC CCTATTGGAT GTTTCCGTAG TTCACCTCGT   
  
  
- TGTCGATAAA CCCACACTCT AATCCTTGTA GGACCGGCAA CCACCCGGGT TCTCCTCCGA GTGACCGCCG   
  
  
- CTAGCCCAGT CTTCCACCCC GCTGCTTGAC TGTTCCCAGC CCAAGCTTGG TCAAAGCAAC CGCCCATCGG   
  
  
- GCCGTTGGGT TCGATCAAAC AACGAACCCT ACAAGGGAAC CTTTCCCATA TGAAACTACC TCCTTTTACC   
  
  
- CACAAACTCT AATCCCACCT TTCTAAACGG GAACAATTGA CGGAGTCGAA CCGTTGGAAC ACTTAAATTG   
  
  
- TTAGGATCAC GACCGTAAAT

+     Box III

| Site Name | Organism | Position | Strand | Matrix score. | sequence | function |
| --- | --- | --- | --- | --- | --- | --- |
| Box III | Pisum sativum | 795 | - | 11 | atCATTTTCACt | protein binding site |

>HU02G03005.1   
+ +Up\_Stream \_Len000AAACGG TGCCGTTGAA TTTGCAGGCG ACGAGGTCGG GTTTAAGGCG GATGTCGTAG   
  
  
+ CGCTTTGGAG AAGCAAATTT GGGAAGACGA GGCTTATTCT TGAACTCTTC GTACGCCATT GTTGTTGGTT   
  
  
+ CCAACCTTTC TCTCTCCTCG TGAATTTGAG TTTGGGCAGT AGACTATCCC GGAATAGAGG ACTGATGCGT   
  
  
+ TATATATATA GAAATTGAGA GAGAGAGAGA GCGTGTGTGA AGGATGTTGA AAATGATGGG CGTAGGGAGT   
  
  
+ CTCTCTCTCA CTGTCTGTTA GGAGTACGTG TCGAGAAAGG AAATAGGATT CCCAAGCAGT CTAATTTGGG   
  
  
+ CCGGTCTATA GGCCAGAATG GGCCTATGAT CAGCCCAGAT ATGACCTTGT TTAACCAACT GTATTTGAAT   
  
  
+ TGAAGAAAGG GATATCGCAA TGTCTTGAAC CACATCAAAT AATAAATGTG CCTTTGGTTA GACCATATGA   
  
  
+ AAAATCTTTG AATCAAATCG GTTGGACCTG AACTGCCATT ATTGATATCT TGTTTTTGGT AGAAGCAAAT   
  
  
+ GGTGTCACTT CGTTTTCTAG ATAAAATTAA CTTGAGTTTT TTTGCCACTA TAGATAGTAT TGCAAATATA   
  
  
+ AAAAGTGTGC CACGTGAAAT CAATATTAGG AGGGTGTTTT ATAACAATAG CTGTTCGGGA GAGTTTTAGT   
  
  
+ TATTTTTATT AAATCAAATA GTTAAGAAGT GTTTGGTAAA TAGTTGTATT TGAAAAAGTT ATTCCCATTA   
  
  
+ GCCTTTTAGT AAGAACTTCT TGTGAAAATG ATAATTGTCT AGAAGTTTGA AAAATTCACA CCACATGACA   
  
  
+ TTTAAAAATA ATTTTGTAGA GAAGGGCATA AATGAAAGTT TGCCACCATC TTTTTTAGAA ACATGTAATT   
  
  
+ TACCAAACAC TTTTTCTAAA AACAGTTAAT TCAAATAACT AACAACTAAC AGCTAATATA AATAACTAAC   
  
  
+ AGCTATTTGC TAAACAGGGC CTAAGTAGTT TGAAATTGTT GTTTTCTCTT CAGCTTCACC ATGGCCGATG   
  
  
+ ATGTAAACAA CTATATTCTG AATTTCATTT TTACTATGAT TTGAAAATTT CAAATTTAAA TAAGATTGGA   
  
  
+ TAAAAAAAAG ACCAAAAATC ATAAGTAATA TTAGTCAAGT TCTATTCCAA CCATATACAT AACTGCTTTT   
  
  
+ TCTTTTGGGG GTTTTGAAAC ATGTGTCCTT AGAATAATTG AAACTCGATA TTATTCTCTA AATCTAAGTT   
  
  
+ TACTTTATCA TATTGATTAT TAATTACCTC CTCAACTCCT CCTATACTCT TGTATGCAGA AATAAAAATG   
  
  
+ TTTCCATACC TTATCAGTCG CGATCATAAT AATTAATTTC TTAATTCGGA TTGTATTTAA TTGCGTATCA   
  
  
+ AATGTGAAAT CGTACCAAAT TAGGAAGCAA AACAAACCGA TAACGAAAAA GGTAAGAAAA CTTAGCAAGG   
  
  
+ AAATGAAATC GAAGACCTGT CAACAATATA GCTTCCCTCA CATCCTTCAA CTTAGGGACC ATCCTATACT   
  
  
+ TTCATGTAAT ACAACTAGAG CCACTAGTCA ATAATAGCCT CCTAGTAGTA GCCATAAGCC TTGGTGAGAG   
  
  
+ AATGTTCCGC AAGACTAGTT AACTTGAAAT TCCATTTTGT CGCAGAACTA TTGGAGTGAG AAAGAGAATC   
  
  
+ AAATCATACC ATATCAAATT TGGACCATTT AAGTTCACAA CACACACACA TACACACAAA AGGACGATGT   
  
  
+ ACGGCCCCAC CCCCCACTCT CCTTTCGCCA TCCCCCTACA CCTCTGCCTA CTCTCTCTCT CTCTCTCTCT   
  
  
+ CTCTGGATCA GTCTGTGTAA AAGCTTCCTC TCCAGTACAG CATTTCCTTG TCTGTTCTTG GAGTTTAGTC   
  
  
+ AATTCCTCCG CCCCAAAAAA ACCCACTTTC TCTCTCATCA TGTTCTTCCA GTTCCACCCT TATACGCCAT   
  
  
+ TCATCATCAT CTACATCACC CACATCTTTT TCTCCACAGT TTTCCCCCCT CATTATGCTC TCCGCCTCCA   
  
  
+ CCCCCGCCTT CCCTCTCGCC GTCGCCGGCG ATGACGGGGA TACCACTGAT GCATGCGACG GCACCGCCAC   
  
  
+ TGCCCTCCAC CTCCTCGGGC TGCTGTTGAA GTGCGCGGAA TTCATCTCCA CCGGAGACCT CGCCGGCGCC   
  
  
+ GGAGATATCT TGCCGGAGAT ATTTGAGTTG TCTACACCGT TTGGCTCCCC CGCCGCCCGG GTCGCCGCCT   
  
  
+ TCTTCGGCCA CGCCCTCCAC GCCCGCCTCC TCTCCGCCTC CCTCCGCACA ACTCCGATCG AGAAGCTCAA   
  
  
+ AACCCTGACC CTGGTTTCCC AAATGCGGAA ATTTCACTCC GCCTTGCAAG TATACAACTC CATCACTCCC   
  
  
+ TTCGTGAAAT TCTCTCACTT CACGGCGAAT CAAGCCATCT ACGAGGCGCT GGACGGCGAG GATCGTGTCC   
  
  
+ ACGTCGTCGA CCTTGACATC ATGCAGGGCC TTCAATGGCC GGGATTGTTC CACATCTTAG CCTCTCGACC   
  
  
+ CAGAAAGCCC CTCTCGGTTC GGGTCACCGG GTTCGGGCCA TCCTCCGAGT TGCTCTCCCA AACGGGTAAG   
  
  
+ CGACTCGCTG AGTTCGCCGC TTCACTCGGC CTGCCCTTCG AGTACAACCC GGTGGAGGGC AAAATTGGGA   
  
  
+ ACTTAGTCGA CCTGGGTCGG GTCGGGTCGC TCCCGAATGA AGTGACGGTG GTGCACTGGA TGCACCATAG   
  
  
+ TCTATACGAC ATAACCGGGT CGGATCTTGG GACTTTGAGG GTATTGAGTG CGGTGAGGCC TAGGCTTGTG   
  
  
+ ACTATGGTTG AGCAGGATAT GGACCAAACG GGGTCGTTTT TGGGGAGGTT TGTGGAGGCA TTGCATTATT   
  
  
+ ACTCAGCCTT GTTTGATGCC TTGGGAGAGG GGTTAGATAG GGATAACCTA CAAAGGCATC AAGTGGAGCA   
  
  
+ ACAGCTATTT GGGTGTGAGA TTAGGAACAT CCTGGCCGTT GGTGGGCCCA AGAGGAGGCT CACTGGCGGC   
  
  
+ GATCGGGTCA GAAGGTGGGG CGACGAACTG ACAAGGGTCG GGTTCGAACC AGTTTCGTTG GCGGGTAGCC   
  
  
+ CGGCAACCCA AGCTAGTTTG TTGCTTGGGA TGTTCCCTTG GAAAGGGTAT ACTTTGATGG AGGAAAATGG   
  
  
+ GTGTTTGAGA TTAGGGTGGA AAGATTTGCC CTTGTTAACT GCCTCAGCTT GGCAACCTTG TGAATTTAAC   
  
  
+ AATCCTAGTG CTGGCATTTA   

- +Up\_Stream \_Len000TTTGCC ACGGCAACTT AAACGTCCGC TGCTCCAGCC CAAATTCCGC CTACAGCATC   
  
  
- GCGAAACCTC TTCGTTTAAA CCCTTCTGCT CCGAATAAGA ACTTGAGAAG CATGCGGTAA CAACAACCAA   
  
  
- GGTTGGAAAG AGAGAGGAGC ACTTAAACTC AAACCCGTCA TCTGATAGGG CCTTATCTCC TGACTACGCA   
  
  
- ATATATATAT CTTTAACTCT CTCTCTCTCT CGCACACACT TCCTACAACT TTTACTACCC GCATCCCTCA   
  
  
- GAGAGAGAGT GACAGACAAT CCTCATGCAC AGCTCTTTCC TTTATCCTAA GGGTTCGTCA GATTAAACCC   
  
  
- GGCCAGATAT CCGGTCTTAC CCGGATACTA GTCGGGTCTA TACTGGAACA AATTGGTTGA CATAAACTTA   
  
  
- ACTTCTTTCC CTATAGCGTT ACAGAACTTG GTGTAGTTTA TTATTTACAC GGAAACCAAT CTGGTATACT   
  
  
- TTTTAGAAAC TTAGTTTAGC CAACCTGGAC TTGACGGTAA TAACTATAGA ACAAAAACCA TCTTCGTTTA   
  
  
- CCACAGTGAA GCAAAAGATC TATTTTAATT GAACTCAAAA AAACGGTGAT ATCTATCATA ACGTTTATAT   
  
  
- TTTTCACACG GTGCACTTTA GTTATAATCC TCCCACAAAA TATTGTTATC GACAAGCCCT CTCAAAATCA   
  
  
- ATAAAAATAA TTTAGTTTAT CAATTCTTCA CAAACCATTT ATCAACATAA ACTTTTTCAA TAAGGGTAAT   
  
  
- CGGAAAATCA TTCTTGAAGA ACACTTTTAC TATTAACAGA TCTTCAAACT TTTTAAGTGT GGTGTACTGT   
  
  
- AAATTTTTAT TAAAACATCT CTTCCCGTAT TTACTTTCAA ACGGTGGTAG AAAAAATCTT TGTACATTAA   
  
  
- ATGGTTTGTG AAAAAGATTT TTGTCAATTA AGTTTATTGA TTGTTGATTG TCGATTATAT TTATTGATTG   
  
  
- TCGATAAACG ATTTGTCCCG GATTCATCAA ACTTTAACAA CAAAAGAGAA GTCGAAGTGG TACCGGCTAC   
  
  
- TACATTTGTT GATATAAGAC TTAAAGTAAA AATGATACTA AACTTTTAAA GTTTAAATTT ATTCTAACCT   
  
  
- ATTTTTTTTC TGGTTTTTAG TATTCATTAT AATCAGTTCA AGATAAGGTT GGTATATGTA TTGACGAAAA   
  
  
- AGAAAACCCC CAAAACTTTG TACACAGGAA TCTTATTAAC TTTGAGCTAT AATAAGAGAT TTAGATTCAA   
  
  
- ATGAAATAGT ATAACTAATA ATTAATGGAG GAGTTGAGGA GGATATGAGA ACATACGTCT TTATTTTTAC   
  
  
- AAAGGTATGG AATAGTCAGC GCTAGTATTA TTAATTAAAG AATTAAGCCT AACATAAATT AACGCATAGT   
  
  
- TTACACTTTA GCATGGTTTA ATCCTTCGTT TTGTTTGGCT ATTGCTTTTT CCATTCTTTT GAATCGTTCC   
  
  
- TTTACTTTAG CTTCTGGACA GTTGTTATAT CGAAGGGAGT GTAGGAAGTT GAATCCCTGG TAGGATATGA   
  
  
- AAGTACATTA TGTTGATCTC GGTGATCAGT TATTATCGGA GGATCATCAT CGGTATTCGG AACCACTCTC   
  
  
- TTACAAGGCG TTCTGATCAA TTGAACTTTA AGGTAAAACA GCGTCTTGAT AACCTCACTC TTTCTCTTAG   
  
  
- TTTAGTATGG TATAGTTTAA ACCTGGTAAA TTCAAGTGTT GTGTGTGTGT ATGTGTGTTT TCCTGCTACA   
  
  
- TGCCGGGGTG GGGGGTGAGA GGAAAGCGGT AGGGGGATGT GGAGACGGAT GAGAGAGAGA GAGAGAGAGA   
  
  
- GAGACCTAGT CAGACACATT TTCGAAGGAG AGGTCATGTC GTAAAGGAAC AGACAAGAAC CTCAAATCAG   
  
  
- TTAAGGAGGC GGGGTTTTTT TGGGTGAAAG AGAGAGTAGT ACAAGAAGGT CAAGGTGGGA ATATGCGGTA   
  
  
- AGTAGTAGTA GATGTAGTGG GTGTAGAAAA AGAGGTGTCA AAAGGGGGGA GTAATACGAG AGGCGGAGGT   
  
  
- GGGGGCGGAA GGGAGAGCGG CAGCGGCCGC TACTGCCCCT ATGGTGACTA CGTACGCTGC CGTGGCGGTG   
  
  
- ACGGGAGGTG GAGGAGCCCG ACGACAACTT CACGCGCCTT AAGTAGAGGT GGCCTCTGGA GCGGCCGCGG   
  
  
- CCTCTATAGA ACGGCCTCTA TAAACTCAAC AGATGTGGCA AACCGAGGGG GCGGCGGGCC CAGCGGCGGA   
  
  
- AGAAGCCGGT GCGGGAGGTG CGGGCGGAGG AGAGGCGGAG GGAGGCGTGT TGAGGCTAGC TCTTCGAGTT   
  
  
- TTGGGACTGG GACCAAAGGG TTTACGCCTT TAAAGTGAGG CGGAACGTTC ATATGTTGAG GTAGTGAGGG   
  
  
- AAGCACTTTA AGAGAGTGAA GTGCCGCTTA GTTCGGTAGA TGCTCCGCGA CCTGCCGCTC CTAGCACAGG   
  
  
- TGCAGCAGCT GGAACTGTAG TACGTCCCGG AAGTTACCGG CCCTAACAAG GTGTAGAATC GGAGAGCTGG   
  
  
- GTCTTTCGGG GAGAGCCAAG CCCAGTGGCC CAAGCCCGGT AGGAGGCTCA ACGAGAGGGT TTGCCCATTC   
  
  
- GCTGAGCGAC TCAAGCGGCG AAGTGAGCCG GACGGGAAGC TCATGTTGGG CCACCTCCCG TTTTAACCCT   
  
  
- TGAATCAGCT GGACCCAGCC CAGCCCAGCG AGGGCTTACT TCACTGCCAC CACGTGACCT ACGTGGTATC   
  
  
- AGATATGCTG TATTGGCCCA GCCTAGAACC CTGAAACTCC CATAACTCAC GCCACTCCGG ATCCGAACAC   
  
  
- TGATACCAAC TCGTCCTATA CCTGGTTTGC CCCAGCAAAA ACCCCTCCAA ACACCTCCGT AACGTAATAA   
  
  
- TGAGTCGGAA CAAACTACGG AACCCTCTCC CCAATCTATC CCTATTGGAT GTTTCCGTAG TTCACCTCGT   
  
  
- TGTCGATAAA CCCACACTCT AATCCTTGTA GGACCGGCAA CCACCCGGGT TCTCCTCCGA GTGACCGCCG   
  
  
- CTAGCCCAGT CTTCCACCCC GCTGCTTGAC TGTTCCCAGC CCAAGCTTGG TCAAAGCAAC CGCCCATCGG   
  
  
- GCCGTTGGGT TCGATCAAAC AACGAACCCT ACAAGGGAAC CTTTCCCATA TGAAACTACC TCCTTTTACC   
  
  
- CACAAACTCT AATCCCACCT TTCTAAACGG GAACAATTGA CGGAGTCGAA CCGTTGGAAC ACTTAAATTG   
  
  
- TTAGGATCAC GACCGTAAAT

+     CAAT-box

| Site Name | Organism | Position | Strand | Matrix score. | sequence | function |
| --- | --- | --- | --- | --- | --- | --- |
| CAAT-box | Nicotiana glutinosa | 2487 | + | 4 | CAAT |  |
| CAAT-box | Pisum sativum | 2334 | + | 5 | CAAAT | common cis-acting element in promoter and enhancer regions |
| CAAT-box | Pisum sativum | 627 | + | 5 | CAAAT | common cis-acting element in promoter and enhancer regions |
| CAAT-box | Nicotiana glutinosa | 1894 | + | 4 | CAAT |  |
| CAAT-box | Pisum sativum | 989 | - | 5 | CAAAT | common cis-acting element in promoter and enhancer regions |
| CAAT-box | Nicotiana glutinosa | 442 | + | 4 | CAAT |  |
| CAAT-box | Pisum sativum | 1093 | - | 5 | CAAAT | common cis-acting element in promoter and enhancer regions |
| CAAT-box | Nicotiana glutinosa | 535 | - | 4 | CAAT |  |
| CAAT-box | Nicotiana glutinosa | 2498 | - | 4 | CAAT |  |
| CAAT-box | Nicotiana glutinosa | 1231 | - | 4 | CAAT |  |
| CAAT-box | Arabidopsis thaliana | 2656 | - | 8 | CCCAATTT | common cis-acting element in promoter and enhancer regions |
| CAAT-box | Nicotiana glutinosa | 1394 | - | 4 | CAAT |  |
| CAAT-box | Pisum sativum | 719 | + | 5 | CAAAT | common cis-acting element in promoter and enhancer regions |
| CAAT-box | Pisum sativum | 1105 | + | 5 | CAAAT | common cis-acting element in promoter and enhancer regions |
| CAAT-box | Pisum sativum | 2951 | - | 5 | CAAAT | common cis-acting element in promoter and enhancer regions |
| CAAT-box | Nicotiana glutinosa | 1019 | - | 4 | CAAT |  |
| CAAT-box | Nicotiana glutinosa | 808 | - | 4 | CAAT |  |
| CAAT-box | Pisum sativum | 34 | - | 5 | CAAAT | common cis-acting element in promoter and enhancer regions |
| CAAT-box | Pisum sativum | 1702 | - | 5 | CAAAT | common cis-acting element in promoter and enhancer regions |
| CAAT-box | Pisum sativum | 1684 | + | 5 | CAAAT | common cis-acting element in promoter and enhancer regions |
| CAAT-box | Nicotiana glutinosa | 1384 | - | 4 | CAAT |  |
| CAAT-box | Pisum sativum | 2195 | - | 5 | CAAAT | common cis-acting element in promoter and enhancer regions |
| CAAT-box | Nicotiana glutinosa | 423 | - | 4 | CAAT |  |
| CAAT-box | Nicotiana glutinosa | 1276 | - | 4 | CAAT |  |
| CAAT-box | Nicotiana glutinosa | 655 | + | 4 | CAAT |  |
| CAAT-box | Pisum sativum | 168 | - | 5 | CAAAT | common cis-acting element in promoter and enhancer regions |
| CAAT-box | Nicotiana glutinosa | 1498 | + | 4 | CAAT |  |
| CAAT-box | Pisum sativum | 752 | - | 5 | CAAAT | common cis-acting element in promoter and enhancer regions |
| CAAT-box | Nicotiana glutinosa | 623 | - | 4 | CAAT |  |
| CAAT-box | Nicotiana glutinosa | 2864 | - | 4 | CAAT |  |
| CAAT-box | Pisum sativum | 946 | + | 5 | CAAAT | common cis-acting element in promoter and enhancer regions |
| CAAT-box | Arabidopsis thaliana | 2658 | - | 5 | CCAAT | common cis-acting element in promoter and enhancer regions |
| CAAT-box | Pisum sativum | 1699 | + | 5 | CAAAT | common cis-acting element in promoter and enhancer regions |
| CAAT-box | Pisum sativum | 91 | - | 5 | CAAAT | common cis-acting element in promoter and enhancer regions |
| CAAT-box | Nicotiana glutinosa | 679 | + | 4 | CAAT |  |
| CAAT-box | Nicotiana glutinosa | 2777 | - | 4 | CAAT |  |
| CAAT-box | Pisum sativum | 560 | + | 5 | CAAAT | common cis-acting element in promoter and enhancer regions |
| CAAT-box | Nicotiana glutinosa | 132 | - | 4 | CAAT |  |
| CAAT-box | Pisum sativum | 1420 | + | 5 | CAAAT | common cis-acting element in promoter and enhancer regions |
| CAAT-box | Pisum sativum | 88 | + | 5 | CAAAT | common cis-acting element in promoter and enhancer regions |
| CAAT-box | Nicotiana glutinosa | 1573 | + | 4 | CAAT |  |
| CAAT-box | Nicotiana glutinosa | 228 | - | 4 | CAAT |  |
| CAAT-box | Pisum sativum | 1403 | + | 5 | CAAAT | common cis-acting element in promoter and enhancer regions |
| CAAT-box | Nicotiana glutinosa | 3224 | + | 4 | CAAT |  |
| CAAT-box | Pisum sativum | 460 | + | 5 | CAAAT | common cis-acting element in promoter and enhancer regions |
| CAAT-box | Pisum sativum | 348 | - | 5 | CAAAT | common cis-acting element in promoter and enhancer regions |
| CAAT-box | Pisum sativum | 3178 | - | 5 | CAAAT | common cis-acting element in promoter and enhancer regions |
| CAAT-box | Arabidopsis thaliana | 1664 | - | 5 | CCAAT | common cis-acting element in promoter and enhancer regions |
| CAAT-box | Pisum sativum | 508 | + | 5 | CAAAT | common cis-acting element in promoter and enhancer regions |
| CAAT-box | Pisum sativum | 417 | - | 5 | CAAAT | common cis-acting element in promoter and enhancer regions |
| CAAT-box | Arabidopsis thaliana | 1119 | - | 5 | CCAAT | common cis-acting element in promoter and enhancer regions |

>HU02G03005.1   
+ +Up\_Stream \_Len000AAACGG TGCCGTTGAA TTTGCAGGCG ACGAGGTCGG GTTTAAGGCG GATGTCGTAG   
  
  
+ CGCTTTGGAG AAGCAAATTT GGGAAGACGA GGCTTATTCT TGAACTCTTC GTACGCCATT GTTGTTGGTT   
  
  
+ CCAACCTTTC TCTCTCCTCG TGAATTTGAG TTTGGGCAGT AGACTATCCC GGAATAGAGG ACTGATGCGT   
  
  
+ TATATATATA GAAATTGAGA GAGAGAGAGA GCGTGTGTGA AGGATGTTGA AAATGATGGG CGTAGGGAGT   
  
  
+ CTCTCTCTCA CTGTCTGTTA GGAGTACGTG TCGAGAAAGG AAATAGGATT CCCAAGCAGT CTAATTTGGG   
  
  
+ CCGGTCTATA GGCCAGAATG GGCCTATGAT CAGCCCAGAT ATGACCTTGT TTAACCAACT GTATTTGAAT   
  
  
+ TGAAGAAAGG GATATCGCAA TGTCTTGAAC CACATCAAAT AATAAATGTG CCTTTGGTTA GACCATATGA   
  
  
+ AAAATCTTTG AATCAAATCG GTTGGACCTG AACTGCCATT ATTGATATCT TGTTTTTGGT AGAAGCAAAT   
  
  
+ GGTGTCACTT CGTTTTCTAG ATAAAATTAA CTTGAGTTTT TTTGCCACTA TAGATAGTAT TGCAAATATA   
  
  
+ AAAAGTGTGC CACGTGAAAT CAATATTAGG AGGGTGTTTT ATAACAATAG CTGTTCGGGA GAGTTTTAGT   
  
  
+ TATTTTTATT AAATCAAATA GTTAAGAAGT GTTTGGTAAA TAGTTGTATT TGAAAAAGTT ATTCCCATTA   
  
  
+ GCCTTTTAGT AAGAACTTCT TGTGAAAATG ATAATTGTCT AGAAGTTTGA AAAATTCACA CCACATGACA   
  
  
+ TTTAAAAATA ATTTTGTAGA GAAGGGCATA AATGAAAGTT TGCCACCATC TTTTTTAGAA ACATGTAATT   
  
  
+ TACCAAACAC TTTTTCTAAA AACAGTTAAT TCAAATAACT AACAACTAAC AGCTAATATA AATAACTAAC   
  
  
+ AGCTATTTGC TAAACAGGGC CTAAGTAGTT TGAAATTGTT GTTTTCTCTT CAGCTTCACC ATGGCCGATG   
  
  
+ ATGTAAACAA CTATATTCTG AATTTCATTT TTACTATGAT TTGAAAATTT CAAATTTAAA TAAGATTGGA   
  
  
+ TAAAAAAAAG ACCAAAAATC ATAAGTAATA TTAGTCAAGT TCTATTCCAA CCATATACAT AACTGCTTTT   
  
  
+ TCTTTTGGGG GTTTTGAAAC ATGTGTCCTT AGAATAATTG AAACTCGATA TTATTCTCTA AATCTAAGTT   
  
  
+ TACTTTATCA TATTGATTAT TAATTACCTC CTCAACTCCT CCTATACTCT TGTATGCAGA AATAAAAATG   
  
  
+ TTTCCATACC TTATCAGTCG CGATCATAAT AATTAATTTC TTAATTCGGA TTGTATTTAA TTGCGTATCA   
  
  
+ AATGTGAAAT CGTACCAAAT TAGGAAGCAA AACAAACCGA TAACGAAAAA GGTAAGAAAA CTTAGCAAGG   
  
  
+ AAATGAAATC GAAGACCTGT CAACAATATA GCTTCCCTCA CATCCTTCAA CTTAGGGACC ATCCTATACT   
  
  
+ TTCATGTAAT ACAACTAGAG CCACTAGTCA ATAATAGCCT CCTAGTAGTA GCCATAAGCC TTGGTGAGAG   
  
  
+ AATGTTCCGC AAGACTAGTT AACTTGAAAT TCCATTTTGT CGCAGAACTA TTGGAGTGAG AAAGAGAATC   
  
  
+ AAATCATACC ATATCAAATT TGGACCATTT AAGTTCACAA CACACACACA TACACACAAA AGGACGATGT   
  
  
+ ACGGCCCCAC CCCCCACTCT CCTTTCGCCA TCCCCCTACA CCTCTGCCTA CTCTCTCTCT CTCTCTCTCT   
  
  
+ CTCTGGATCA GTCTGTGTAA AAGCTTCCTC TCCAGTACAG CATTTCCTTG TCTGTTCTTG GAGTTTAGTC   
  
  
+ AATTCCTCCG CCCCAAAAAA ACCCACTTTC TCTCTCATCA TGTTCTTCCA GTTCCACCCT TATACGCCAT   
  
  
+ TCATCATCAT CTACATCACC CACATCTTTT TCTCCACAGT TTTCCCCCCT CATTATGCTC TCCGCCTCCA   
  
  
+ CCCCCGCCTT CCCTCTCGCC GTCGCCGGCG ATGACGGGGA TACCACTGAT GCATGCGACG GCACCGCCAC   
  
  
+ TGCCCTCCAC CTCCTCGGGC TGCTGTTGAA GTGCGCGGAA TTCATCTCCA CCGGAGACCT CGCCGGCGCC   
  
  
+ GGAGATATCT TGCCGGAGAT ATTTGAGTTG TCTACACCGT TTGGCTCCCC CGCCGCCCGG GTCGCCGCCT   
  
  
+ TCTTCGGCCA CGCCCTCCAC GCCCGCCTCC TCTCCGCCTC CCTCCGCACA ACTCCGATCG AGAAGCTCAA   
  
  
+ AACCCTGACC CTGGTTTCCC AAATGCGGAA ATTTCACTCC GCCTTGCAAG TATACAACTC CATCACTCCC   
  
  
+ TTCGTGAAAT TCTCTCACTT CACGGCGAAT CAAGCCATCT ACGAGGCGCT GGACGGCGAG GATCGTGTCC   
  
  
+ ACGTCGTCGA CCTTGACATC ATGCAGGGCC TTCAATGGCC GGGATTGTTC CACATCTTAG CCTCTCGACC   
  
  
+ CAGAAAGCCC CTCTCGGTTC GGGTCACCGG GTTCGGGCCA TCCTCCGAGT TGCTCTCCCA AACGGGTAAG   
  
  
+ CGACTCGCTG AGTTCGCCGC TTCACTCGGC CTGCCCTTCG AGTACAACCC GGTGGAGGGC AAAATTGGGA   
  
  
+ ACTTAGTCGA CCTGGGTCGG GTCGGGTCGC TCCCGAATGA AGTGACGGTG GTGCACTGGA TGCACCATAG   
  
  
+ TCTATACGAC ATAACCGGGT CGGATCTTGG GACTTTGAGG GTATTGAGTG CGGTGAGGCC TAGGCTTGTG   
  
  
+ ACTATGGTTG AGCAGGATAT GGACCAAACG GGGTCGTTTT TGGGGAGGTT TGTGGAGGCA TTGCATTATT   
  
  
+ ACTCAGCCTT GTTTGATGCC TTGGGAGAGG GGTTAGATAG GGATAACCTA CAAAGGCATC AAGTGGAGCA   
  
  
+ ACAGCTATTT GGGTGTGAGA TTAGGAACAT CCTGGCCGTT GGTGGGCCCA AGAGGAGGCT CACTGGCGGC   
  
  
+ GATCGGGTCA GAAGGTGGGG CGACGAACTG ACAAGGGTCG GGTTCGAACC AGTTTCGTTG GCGGGTAGCC   
  
  
+ CGGCAACCCA AGCTAGTTTG TTGCTTGGGA TGTTCCCTTG GAAAGGGTAT ACTTTGATGG AGGAAAATGG   
  
  
+ GTGTTTGAGA TTAGGGTGGA AAGATTTGCC CTTGTTAACT GCCTCAGCTT GGCAACCTTG TGAATTTAAC   
  
  
+ AATCCTAGTG CTGGCATTTA   

- +Up\_Stream \_Len000TTTGCC ACGGCAACTT AAACGTCCGC TGCTCCAGCC CAAATTCCGC CTACAGCATC   
  
  
- GCGAAACCTC TTCGTTTAAA CCCTTCTGCT CCGAATAAGA ACTTGAGAAG CATGCGGTAA CAACAACCAA   
  
  
- GGTTGGAAAG AGAGAGGAGC ACTTAAACTC AAACCCGTCA TCTGATAGGG CCTTATCTCC TGACTACGCA   
  
  
- ATATATATAT CTTTAACTCT CTCTCTCTCT CGCACACACT TCCTACAACT TTTACTACCC GCATCCCTCA   
  
  
- GAGAGAGAGT GACAGACAAT CCTCATGCAC AGCTCTTTCC TTTATCCTAA GGGTTCGTCA GATTAAACCC   
  
  
- GGCCAGATAT CCGGTCTTAC CCGGATACTA GTCGGGTCTA TACTGGAACA AATTGGTTGA CATAAACTTA   
  
  
- ACTTCTTTCC CTATAGCGTT ACAGAACTTG GTGTAGTTTA TTATTTACAC GGAAACCAAT CTGGTATACT   
  
  
- TTTTAGAAAC TTAGTTTAGC CAACCTGGAC TTGACGGTAA TAACTATAGA ACAAAAACCA TCTTCGTTTA   
  
  
- CCACAGTGAA GCAAAAGATC TATTTTAATT GAACTCAAAA AAACGGTGAT ATCTATCATA ACGTTTATAT   
  
  
- TTTTCACACG GTGCACTTTA GTTATAATCC TCCCACAAAA TATTGTTATC GACAAGCCCT CTCAAAATCA   
  
  
- ATAAAAATAA TTTAGTTTAT CAATTCTTCA CAAACCATTT ATCAACATAA ACTTTTTCAA TAAGGGTAAT   
  
  
- CGGAAAATCA TTCTTGAAGA ACACTTTTAC TATTAACAGA TCTTCAAACT TTTTAAGTGT GGTGTACTGT   
  
  
- AAATTTTTAT TAAAACATCT CTTCCCGTAT TTACTTTCAA ACGGTGGTAG AAAAAATCTT TGTACATTAA   
  
  
- ATGGTTTGTG AAAAAGATTT TTGTCAATTA AGTTTATTGA TTGTTGATTG TCGATTATAT TTATTGATTG   
  
  
- TCGATAAACG ATTTGTCCCG GATTCATCAA ACTTTAACAA CAAAAGAGAA GTCGAAGTGG TACCGGCTAC   
  
  
- TACATTTGTT GATATAAGAC TTAAAGTAAA AATGATACTA AACTTTTAAA GTTTAAATTT ATTCTAACCT   
  
  
- ATTTTTTTTC TGGTTTTTAG TATTCATTAT AATCAGTTCA AGATAAGGTT GGTATATGTA TTGACGAAAA   
  
  
- AGAAAACCCC CAAAACTTTG TACACAGGAA TCTTATTAAC TTTGAGCTAT AATAAGAGAT TTAGATTCAA   
  
  
- ATGAAATAGT ATAACTAATA ATTAATGGAG GAGTTGAGGA GGATATGAGA ACATACGTCT TTATTTTTAC   
  
  
- AAAGGTATGG AATAGTCAGC GCTAGTATTA TTAATTAAAG AATTAAGCCT AACATAAATT AACGCATAGT   
  
  
- TTACACTTTA GCATGGTTTA ATCCTTCGTT TTGTTTGGCT ATTGCTTTTT CCATTCTTTT GAATCGTTCC   
  
  
- TTTACTTTAG CTTCTGGACA GTTGTTATAT CGAAGGGAGT GTAGGAAGTT GAATCCCTGG TAGGATATGA   
  
  
- AAGTACATTA TGTTGATCTC GGTGATCAGT TATTATCGGA GGATCATCAT CGGTATTCGG AACCACTCTC   
  
  
- TTACAAGGCG TTCTGATCAA TTGAACTTTA AGGTAAAACA GCGTCTTGAT AACCTCACTC TTTCTCTTAG   
  
  
- TTTAGTATGG TATAGTTTAA ACCTGGTAAA TTCAAGTGTT GTGTGTGTGT ATGTGTGTTT TCCTGCTACA   
  
  
- TGCCGGGGTG GGGGGTGAGA GGAAAGCGGT AGGGGGATGT GGAGACGGAT GAGAGAGAGA GAGAGAGAGA   
  
  
- GAGACCTAGT CAGACACATT TTCGAAGGAG AGGTCATGTC GTAAAGGAAC AGACAAGAAC CTCAAATCAG   
  
  
- TTAAGGAGGC GGGGTTTTTT TGGGTGAAAG AGAGAGTAGT ACAAGAAGGT CAAGGTGGGA ATATGCGGTA   
  
  
- AGTAGTAGTA GATGTAGTGG GTGTAGAAAA AGAGGTGTCA AAAGGGGGGA GTAATACGAG AGGCGGAGGT   
  
  
- GGGGGCGGAA GGGAGAGCGG CAGCGGCCGC TACTGCCCCT ATGGTGACTA CGTACGCTGC CGTGGCGGTG   
  
  
- ACGGGAGGTG GAGGAGCCCG ACGACAACTT CACGCGCCTT AAGTAGAGGT GGCCTCTGGA GCGGCCGCGG   
  
  
- CCTCTATAGA ACGGCCTCTA TAAACTCAAC AGATGTGGCA AACCGAGGGG GCGGCGGGCC CAGCGGCGGA   
  
  
- AGAAGCCGGT GCGGGAGGTG CGGGCGGAGG AGAGGCGGAG GGAGGCGTGT TGAGGCTAGC TCTTCGAGTT   
  
  
- TTGGGACTGG GACCAAAGGG TTTACGCCTT TAAAGTGAGG CGGAACGTTC ATATGTTGAG GTAGTGAGGG   
  
  
- AAGCACTTTA AGAGAGTGAA GTGCCGCTTA GTTCGGTAGA TGCTCCGCGA CCTGCCGCTC CTAGCACAGG   
  
  
- TGCAGCAGCT GGAACTGTAG TACGTCCCGG AAGTTACCGG CCCTAACAAG GTGTAGAATC GGAGAGCTGG   
  
  
- GTCTTTCGGG GAGAGCCAAG CCCAGTGGCC CAAGCCCGGT AGGAGGCTCA ACGAGAGGGT TTGCCCATTC   
  
  
- GCTGAGCGAC TCAAGCGGCG AAGTGAGCCG GACGGGAAGC TCATGTTGGG CCACCTCCCG TTTTAACCCT   
  
  
- TGAATCAGCT GGACCCAGCC CAGCCCAGCG AGGGCTTACT TCACTGCCAC CACGTGACCT ACGTGGTATC   
  
  
- AGATATGCTG TATTGGCCCA GCCTAGAACC CTGAAACTCC CATAACTCAC GCCACTCCGG ATCCGAACAC   
  
  
- TGATACCAAC TCGTCCTATA CCTGGTTTGC CCCAGCAAAA ACCCCTCCAA ACACCTCCGT AACGTAATAA   
  
  
- TGAGTCGGAA CAAACTACGG AACCCTCTCC CCAATCTATC CCTATTGGAT GTTTCCGTAG TTCACCTCGT   
  
  
- TGTCGATAAA CCCACACTCT AATCCTTGTA GGACCGGCAA CCACCCGGGT TCTCCTCCGA GTGACCGCCG   
  
  
- CTAGCCCAGT CTTCCACCCC GCTGCTTGAC TGTTCCCAGC CCAAGCTTGG TCAAAGCAAC CGCCCATCGG   
  
  
- GCCGTTGGGT TCGATCAAAC AACGAACCCT ACAAGGGAAC CTTTCCCATA TGAAACTACC TCCTTTTACC   
  
  
- CACAAACTCT AATCCCACCT TTCTAAACGG GAACAATTGA CGGAGTCGAA CCGTTGGAAC ACTTAAATTG   
  
  
- TTAGGATCAC GACCGTAAAT

+     CAT-box

| Site Name | Organism | Position | Strand | Matrix score. | sequence | function |
| --- | --- | --- | --- | --- | --- | --- |
| CAT-box | Arabidopsis thaliana | 608 | + | 6 | GCCACT | cis-acting regulatory element related to meristem expression |
| CAT-box | Arabidopsis thaliana | 2100 | + | 6 | GCCACT | cis-acting regulatory element related to meristem expression |
| CAT-box | Arabidopsis thaliana | 1564 | + | 6 | GCCACT | cis-acting regulatory element related to meristem expression |

>HU02G03005.1   
+ +Up\_Stream \_Len000AAACGG TGCCGTTGAA TTTGCAGGCG ACGAGGTCGG GTTTAAGGCG GATGTCGTAG   
  
  
+ CGCTTTGGAG AAGCAAATTT GGGAAGACGA GGCTTATTCT TGAACTCTTC GTACGCCATT GTTGTTGGTT   
  
  
+ CCAACCTTTC TCTCTCCTCG TGAATTTGAG TTTGGGCAGT AGACTATCCC GGAATAGAGG ACTGATGCGT   
  
  
+ TATATATATA GAAATTGAGA GAGAGAGAGA GCGTGTGTGA AGGATGTTGA AAATGATGGG CGTAGGGAGT   
  
  
+ CTCTCTCTCA CTGTCTGTTA GGAGTACGTG TCGAGAAAGG AAATAGGATT CCCAAGCAGT CTAATTTGGG   
  
  
+ CCGGTCTATA GGCCAGAATG GGCCTATGAT CAGCCCAGAT ATGACCTTGT TTAACCAACT GTATTTGAAT   
  
  
+ TGAAGAAAGG GATATCGCAA TGTCTTGAAC CACATCAAAT AATAAATGTG CCTTTGGTTA GACCATATGA   
  
  
+ AAAATCTTTG AATCAAATCG GTTGGACCTG AACTGCCATT ATTGATATCT TGTTTTTGGT AGAAGCAAAT   
  
  
+ GGTGTCACTT CGTTTTCTAG ATAAAATTAA CTTGAGTTTT TTTGCCACTA TAGATAGTAT TGCAAATATA   
  
  
+ AAAAGTGTGC CACGTGAAAT CAATATTAGG AGGGTGTTTT ATAACAATAG CTGTTCGGGA GAGTTTTAGT   
  
  
+ TATTTTTATT AAATCAAATA GTTAAGAAGT GTTTGGTAAA TAGTTGTATT TGAAAAAGTT ATTCCCATTA   
  
  
+ GCCTTTTAGT AAGAACTTCT TGTGAAAATG ATAATTGTCT AGAAGTTTGA AAAATTCACA CCACATGACA   
  
  
+ TTTAAAAATA ATTTTGTAGA GAAGGGCATA AATGAAAGTT TGCCACCATC TTTTTTAGAA ACATGTAATT   
  
  
+ TACCAAACAC TTTTTCTAAA AACAGTTAAT TCAAATAACT AACAACTAAC AGCTAATATA AATAACTAAC   
  
  
+ AGCTATTTGC TAAACAGGGC CTAAGTAGTT TGAAATTGTT GTTTTCTCTT CAGCTTCACC ATGGCCGATG   
  
  
+ ATGTAAACAA CTATATTCTG AATTTCATTT TTACTATGAT TTGAAAATTT CAAATTTAAA TAAGATTGGA   
  
  
+ TAAAAAAAAG ACCAAAAATC ATAAGTAATA TTAGTCAAGT TCTATTCCAA CCATATACAT AACTGCTTTT   
  
  
+ TCTTTTGGGG GTTTTGAAAC ATGTGTCCTT AGAATAATTG AAACTCGATA TTATTCTCTA AATCTAAGTT   
  
  
+ TACTTTATCA TATTGATTAT TAATTACCTC CTCAACTCCT CCTATACTCT TGTATGCAGA AATAAAAATG   
  
  
+ TTTCCATACC TTATCAGTCG CGATCATAAT AATTAATTTC TTAATTCGGA TTGTATTTAA TTGCGTATCA   
  
  
+ AATGTGAAAT CGTACCAAAT TAGGAAGCAA AACAAACCGA TAACGAAAAA GGTAAGAAAA CTTAGCAAGG   
  
  
+ AAATGAAATC GAAGACCTGT CAACAATATA GCTTCCCTCA CATCCTTCAA CTTAGGGACC ATCCTATACT   
  
  
+ TTCATGTAAT ACAACTAGAG CCACTAGTCA ATAATAGCCT CCTAGTAGTA GCCATAAGCC TTGGTGAGAG   
  
  
+ AATGTTCCGC AAGACTAGTT AACTTGAAAT TCCATTTTGT CGCAGAACTA TTGGAGTGAG AAAGAGAATC   
  
  
+ AAATCATACC ATATCAAATT TGGACCATTT AAGTTCACAA CACACACACA TACACACAAA AGGACGATGT   
  
  
+ ACGGCCCCAC CCCCCACTCT CCTTTCGCCA TCCCCCTACA CCTCTGCCTA CTCTCTCTCT CTCTCTCTCT   
  
  
+ CTCTGGATCA GTCTGTGTAA AAGCTTCCTC TCCAGTACAG CATTTCCTTG TCTGTTCTTG GAGTTTAGTC   
  
  
+ AATTCCTCCG CCCCAAAAAA ACCCACTTTC TCTCTCATCA TGTTCTTCCA GTTCCACCCT TATACGCCAT   
  
  
+ TCATCATCAT CTACATCACC CACATCTTTT TCTCCACAGT TTTCCCCCCT CATTATGCTC TCCGCCTCCA   
  
  
+ CCCCCGCCTT CCCTCTCGCC GTCGCCGGCG ATGACGGGGA TACCACTGAT GCATGCGACG GCACCGCCAC   
  
  
+ TGCCCTCCAC CTCCTCGGGC TGCTGTTGAA GTGCGCGGAA TTCATCTCCA CCGGAGACCT CGCCGGCGCC   
  
  
+ GGAGATATCT TGCCGGAGAT ATTTGAGTTG TCTACACCGT TTGGCTCCCC CGCCGCCCGG GTCGCCGCCT   
  
  
+ TCTTCGGCCA CGCCCTCCAC GCCCGCCTCC TCTCCGCCTC CCTCCGCACA ACTCCGATCG AGAAGCTCAA   
  
  
+ AACCCTGACC CTGGTTTCCC AAATGCGGAA ATTTCACTCC GCCTTGCAAG TATACAACTC CATCACTCCC   
  
  
+ TTCGTGAAAT TCTCTCACTT CACGGCGAAT CAAGCCATCT ACGAGGCGCT GGACGGCGAG GATCGTGTCC   
  
  
+ ACGTCGTCGA CCTTGACATC ATGCAGGGCC TTCAATGGCC GGGATTGTTC CACATCTTAG CCTCTCGACC   
  
  
+ CAGAAAGCCC CTCTCGGTTC GGGTCACCGG GTTCGGGCCA TCCTCCGAGT TGCTCTCCCA AACGGGTAAG   
  
  
+ CGACTCGCTG AGTTCGCCGC TTCACTCGGC CTGCCCTTCG AGTACAACCC GGTGGAGGGC AAAATTGGGA   
  
  
+ ACTTAGTCGA CCTGGGTCGG GTCGGGTCGC TCCCGAATGA AGTGACGGTG GTGCACTGGA TGCACCATAG   
  
  
+ TCTATACGAC ATAACCGGGT CGGATCTTGG GACTTTGAGG GTATTGAGTG CGGTGAGGCC TAGGCTTGTG   
  
  
+ ACTATGGTTG AGCAGGATAT GGACCAAACG GGGTCGTTTT TGGGGAGGTT TGTGGAGGCA TTGCATTATT   
  
  
+ ACTCAGCCTT GTTTGATGCC TTGGGAGAGG GGTTAGATAG GGATAACCTA CAAAGGCATC AAGTGGAGCA   
  
  
+ ACAGCTATTT GGGTGTGAGA TTAGGAACAT CCTGGCCGTT GGTGGGCCCA AGAGGAGGCT CACTGGCGGC   
  
  
+ GATCGGGTCA GAAGGTGGGG CGACGAACTG ACAAGGGTCG GGTTCGAACC AGTTTCGTTG GCGGGTAGCC   
  
  
+ CGGCAACCCA AGCTAGTTTG TTGCTTGGGA TGTTCCCTTG GAAAGGGTAT ACTTTGATGG AGGAAAATGG   
  
  
+ GTGTTTGAGA TTAGGGTGGA AAGATTTGCC CTTGTTAACT GCCTCAGCTT GGCAACCTTG TGAATTTAAC   
  
  
+ AATCCTAGTG CTGGCATTTA   

- +Up\_Stream \_Len000TTTGCC ACGGCAACTT AAACGTCCGC TGCTCCAGCC CAAATTCCGC CTACAGCATC   
  
  
- GCGAAACCTC TTCGTTTAAA CCCTTCTGCT CCGAATAAGA ACTTGAGAAG CATGCGGTAA CAACAACCAA   
  
  
- GGTTGGAAAG AGAGAGGAGC ACTTAAACTC AAACCCGTCA TCTGATAGGG CCTTATCTCC TGACTACGCA   
  
  
- ATATATATAT CTTTAACTCT CTCTCTCTCT CGCACACACT TCCTACAACT TTTACTACCC GCATCCCTCA   
  
  
- GAGAGAGAGT GACAGACAAT CCTCATGCAC AGCTCTTTCC TTTATCCTAA GGGTTCGTCA GATTAAACCC   
  
  
- GGCCAGATAT CCGGTCTTAC CCGGATACTA GTCGGGTCTA TACTGGAACA AATTGGTTGA CATAAACTTA   
  
  
- ACTTCTTTCC CTATAGCGTT ACAGAACTTG GTGTAGTTTA TTATTTACAC GGAAACCAAT CTGGTATACT   
  
  
- TTTTAGAAAC TTAGTTTAGC CAACCTGGAC TTGACGGTAA TAACTATAGA ACAAAAACCA TCTTCGTTTA   
  
  
- CCACAGTGAA GCAAAAGATC TATTTTAATT GAACTCAAAA AAACGGTGAT ATCTATCATA ACGTTTATAT   
  
  
- TTTTCACACG GTGCACTTTA GTTATAATCC TCCCACAAAA TATTGTTATC GACAAGCCCT CTCAAAATCA   
  
  
- ATAAAAATAA TTTAGTTTAT CAATTCTTCA CAAACCATTT ATCAACATAA ACTTTTTCAA TAAGGGTAAT   
  
  
- CGGAAAATCA TTCTTGAAGA ACACTTTTAC TATTAACAGA TCTTCAAACT TTTTAAGTGT GGTGTACTGT   
  
  
- AAATTTTTAT TAAAACATCT CTTCCCGTAT TTACTTTCAA ACGGTGGTAG AAAAAATCTT TGTACATTAA   
  
  
- ATGGTTTGTG AAAAAGATTT TTGTCAATTA AGTTTATTGA TTGTTGATTG TCGATTATAT TTATTGATTG   
  
  
- TCGATAAACG ATTTGTCCCG GATTCATCAA ACTTTAACAA CAAAAGAGAA GTCGAAGTGG TACCGGCTAC   
  
  
- TACATTTGTT GATATAAGAC TTAAAGTAAA AATGATACTA AACTTTTAAA GTTTAAATTT ATTCTAACCT   
  
  
- ATTTTTTTTC TGGTTTTTAG TATTCATTAT AATCAGTTCA AGATAAGGTT GGTATATGTA TTGACGAAAA   
  
  
- AGAAAACCCC CAAAACTTTG TACACAGGAA TCTTATTAAC TTTGAGCTAT AATAAGAGAT TTAGATTCAA   
  
  
- ATGAAATAGT ATAACTAATA ATTAATGGAG GAGTTGAGGA GGATATGAGA ACATACGTCT TTATTTTTAC   
  
  
- AAAGGTATGG AATAGTCAGC GCTAGTATTA TTAATTAAAG AATTAAGCCT AACATAAATT AACGCATAGT   
  
  
- TTACACTTTA GCATGGTTTA ATCCTTCGTT TTGTTTGGCT ATTGCTTTTT CCATTCTTTT GAATCGTTCC   
  
  
- TTTACTTTAG CTTCTGGACA GTTGTTATAT CGAAGGGAGT GTAGGAAGTT GAATCCCTGG TAGGATATGA   
  
  
- AAGTACATTA TGTTGATCTC GGTGATCAGT TATTATCGGA GGATCATCAT CGGTATTCGG AACCACTCTC   
  
  
- TTACAAGGCG TTCTGATCAA TTGAACTTTA AGGTAAAACA GCGTCTTGAT AACCTCACTC TTTCTCTTAG   
  
  
- TTTAGTATGG TATAGTTTAA ACCTGGTAAA TTCAAGTGTT GTGTGTGTGT ATGTGTGTTT TCCTGCTACA   
  
  
- TGCCGGGGTG GGGGGTGAGA GGAAAGCGGT AGGGGGATGT GGAGACGGAT GAGAGAGAGA GAGAGAGAGA   
  
  
- GAGACCTAGT CAGACACATT TTCGAAGGAG AGGTCATGTC GTAAAGGAAC AGACAAGAAC CTCAAATCAG   
  
  
- TTAAGGAGGC GGGGTTTTTT TGGGTGAAAG AGAGAGTAGT ACAAGAAGGT CAAGGTGGGA ATATGCGGTA   
  
  
- AGTAGTAGTA GATGTAGTGG GTGTAGAAAA AGAGGTGTCA AAAGGGGGGA GTAATACGAG AGGCGGAGGT   
  
  
- GGGGGCGGAA GGGAGAGCGG CAGCGGCCGC TACTGCCCCT ATGGTGACTA CGTACGCTGC CGTGGCGGTG   
  
  
- ACGGGAGGTG GAGGAGCCCG ACGACAACTT CACGCGCCTT AAGTAGAGGT GGCCTCTGGA GCGGCCGCGG   
  
  
- CCTCTATAGA ACGGCCTCTA TAAACTCAAC AGATGTGGCA AACCGAGGGG GCGGCGGGCC CAGCGGCGGA   
  
  
- AGAAGCCGGT GCGGGAGGTG CGGGCGGAGG AGAGGCGGAG GGAGGCGTGT TGAGGCTAGC TCTTCGAGTT   
  
  
- TTGGGACTGG GACCAAAGGG TTTACGCCTT TAAAGTGAGG CGGAACGTTC ATATGTTGAG GTAGTGAGGG   
  
  
- AAGCACTTTA AGAGAGTGAA GTGCCGCTTA GTTCGGTAGA TGCTCCGCGA CCTGCCGCTC CTAGCACAGG   
  
  
- TGCAGCAGCT GGAACTGTAG TACGTCCCGG AAGTTACCGG CCCTAACAAG GTGTAGAATC GGAGAGCTGG   
  
  
- GTCTTTCGGG GAGAGCCAAG CCCAGTGGCC CAAGCCCGGT AGGAGGCTCA ACGAGAGGGT TTGCCCATTC   
  
  
- GCTGAGCGAC TCAAGCGGCG AAGTGAGCCG GACGGGAAGC TCATGTTGGG CCACCTCCCG TTTTAACCCT   
  
  
- TGAATCAGCT GGACCCAGCC CAGCCCAGCG AGGGCTTACT TCACTGCCAC CACGTGACCT ACGTGGTATC   
  
  
- AGATATGCTG TATTGGCCCA GCCTAGAACC CTGAAACTCC CATAACTCAC GCCACTCCGG ATCCGAACAC   
  
  
- TGATACCAAC TCGTCCTATA CCTGGTTTGC CCCAGCAAAA ACCCCTCCAA ACACCTCCGT AACGTAATAA   
  
  
- TGAGTCGGAA CAAACTACGG AACCCTCTCC CCAATCTATC CCTATTGGAT GTTTCCGTAG TTCACCTCGT   
  
  
- TGTCGATAAA CCCACACTCT AATCCTTGTA GGACCGGCAA CCACCCGGGT TCTCCTCCGA GTGACCGCCG   
  
  
- CTAGCCCAGT CTTCCACCCC GCTGCTTGAC TGTTCCCAGC CCAAGCTTGG TCAAAGCAAC CGCCCATCGG   
  
  
- GCCGTTGGGT TCGATCAAAC AACGAACCCT ACAAGGGAAC CTTTCCCATA TGAAACTACC TCCTTTTACC   
  
  
- CACAAACTCT AATCCCACCT TTCTAAACGG GAACAATTGA CGGAGTCGAA CCGTTGGAAC ACTTAAATTG   
  
  
- TTAGGATCAC GACCGTAAAT

+     CCAAT-box

| Site Name | Organism | Position | Strand | Matrix score. | sequence | function |
| --- | --- | --- | --- | --- | --- | --- |
| CCAAT-box | Hordeum vulgare | 2980 | - | 6 | CAACGG | MYBHv1 binding site |
| CCAAT-box | Hordeum vulgare | 27 | - | 6 | CAACGG | MYBHv1 binding site |

>HU02G03005.1   
+ +Up\_Stream \_Len000AAACGG TGCCGTTGAA TTTGCAGGCG ACGAGGTCGG GTTTAAGGCG GATGTCGTAG   
  
  
+ CGCTTTGGAG AAGCAAATTT GGGAAGACGA GGCTTATTCT TGAACTCTTC GTACGCCATT GTTGTTGGTT   
  
  
+ CCAACCTTTC TCTCTCCTCG TGAATTTGAG TTTGGGCAGT AGACTATCCC GGAATAGAGG ACTGATGCGT   
  
  
+ TATATATATA GAAATTGAGA GAGAGAGAGA GCGTGTGTGA AGGATGTTGA AAATGATGGG CGTAGGGAGT   
  
  
+ CTCTCTCTCA CTGTCTGTTA GGAGTACGTG TCGAGAAAGG AAATAGGATT CCCAAGCAGT CTAATTTGGG   
  
  
+ CCGGTCTATA GGCCAGAATG GGCCTATGAT CAGCCCAGAT ATGACCTTGT TTAACCAACT GTATTTGAAT   
  
  
+ TGAAGAAAGG GATATCGCAA TGTCTTGAAC CACATCAAAT AATAAATGTG CCTTTGGTTA GACCATATGA   
  
  
+ AAAATCTTTG AATCAAATCG GTTGGACCTG AACTGCCATT ATTGATATCT TGTTTTTGGT AGAAGCAAAT   
  
  
+ GGTGTCACTT CGTTTTCTAG ATAAAATTAA CTTGAGTTTT TTTGCCACTA TAGATAGTAT TGCAAATATA   
  
  
+ AAAAGTGTGC CACGTGAAAT CAATATTAGG AGGGTGTTTT ATAACAATAG CTGTTCGGGA GAGTTTTAGT   
  
  
+ TATTTTTATT AAATCAAATA GTTAAGAAGT GTTTGGTAAA TAGTTGTATT TGAAAAAGTT ATTCCCATTA   
  
  
+ GCCTTTTAGT AAGAACTTCT TGTGAAAATG ATAATTGTCT AGAAGTTTGA AAAATTCACA CCACATGACA   
  
  
+ TTTAAAAATA ATTTTGTAGA GAAGGGCATA AATGAAAGTT TGCCACCATC TTTTTTAGAA ACATGTAATT   
  
  
+ TACCAAACAC TTTTTCTAAA AACAGTTAAT TCAAATAACT AACAACTAAC AGCTAATATA AATAACTAAC   
  
  
+ AGCTATTTGC TAAACAGGGC CTAAGTAGTT TGAAATTGTT GTTTTCTCTT CAGCTTCACC ATGGCCGATG   
  
  
+ ATGTAAACAA CTATATTCTG AATTTCATTT TTACTATGAT TTGAAAATTT CAAATTTAAA TAAGATTGGA   
  
  
+ TAAAAAAAAG ACCAAAAATC ATAAGTAATA TTAGTCAAGT TCTATTCCAA CCATATACAT AACTGCTTTT   
  
  
+ TCTTTTGGGG GTTTTGAAAC ATGTGTCCTT AGAATAATTG AAACTCGATA TTATTCTCTA AATCTAAGTT   
  
  
+ TACTTTATCA TATTGATTAT TAATTACCTC CTCAACTCCT CCTATACTCT TGTATGCAGA AATAAAAATG   
  
  
+ TTTCCATACC TTATCAGTCG CGATCATAAT AATTAATTTC TTAATTCGGA TTGTATTTAA TTGCGTATCA   
  
  
+ AATGTGAAAT CGTACCAAAT TAGGAAGCAA AACAAACCGA TAACGAAAAA GGTAAGAAAA CTTAGCAAGG   
  
  
+ AAATGAAATC GAAGACCTGT CAACAATATA GCTTCCCTCA CATCCTTCAA CTTAGGGACC ATCCTATACT   
  
  
+ TTCATGTAAT ACAACTAGAG CCACTAGTCA ATAATAGCCT CCTAGTAGTA GCCATAAGCC TTGGTGAGAG   
  
  
+ AATGTTCCGC AAGACTAGTT AACTTGAAAT TCCATTTTGT CGCAGAACTA TTGGAGTGAG AAAGAGAATC   
  
  
+ AAATCATACC ATATCAAATT TGGACCATTT AAGTTCACAA CACACACACA TACACACAAA AGGACGATGT   
  
  
+ ACGGCCCCAC CCCCCACTCT CCTTTCGCCA TCCCCCTACA CCTCTGCCTA CTCTCTCTCT CTCTCTCTCT   
  
  
+ CTCTGGATCA GTCTGTGTAA AAGCTTCCTC TCCAGTACAG CATTTCCTTG TCTGTTCTTG GAGTTTAGTC   
  
  
+ AATTCCTCCG CCCCAAAAAA ACCCACTTTC TCTCTCATCA TGTTCTTCCA GTTCCACCCT TATACGCCAT   
  
  
+ TCATCATCAT CTACATCACC CACATCTTTT TCTCCACAGT TTTCCCCCCT CATTATGCTC TCCGCCTCCA   
  
  
+ CCCCCGCCTT CCCTCTCGCC GTCGCCGGCG ATGACGGGGA TACCACTGAT GCATGCGACG GCACCGCCAC   
  
  
+ TGCCCTCCAC CTCCTCGGGC TGCTGTTGAA GTGCGCGGAA TTCATCTCCA CCGGAGACCT CGCCGGCGCC   
  
  
+ GGAGATATCT TGCCGGAGAT ATTTGAGTTG TCTACACCGT TTGGCTCCCC CGCCGCCCGG GTCGCCGCCT   
  
  
+ TCTTCGGCCA CGCCCTCCAC GCCCGCCTCC TCTCCGCCTC CCTCCGCACA ACTCCGATCG AGAAGCTCAA   
  
  
+ AACCCTGACC CTGGTTTCCC AAATGCGGAA ATTTCACTCC GCCTTGCAAG TATACAACTC CATCACTCCC   
  
  
+ TTCGTGAAAT TCTCTCACTT CACGGCGAAT CAAGCCATCT ACGAGGCGCT GGACGGCGAG GATCGTGTCC   
  
  
+ ACGTCGTCGA CCTTGACATC ATGCAGGGCC TTCAATGGCC GGGATTGTTC CACATCTTAG CCTCTCGACC   
  
  
+ CAGAAAGCCC CTCTCGGTTC GGGTCACCGG GTTCGGGCCA TCCTCCGAGT TGCTCTCCCA AACGGGTAAG   
  
  
+ CGACTCGCTG AGTTCGCCGC TTCACTCGGC CTGCCCTTCG AGTACAACCC GGTGGAGGGC AAAATTGGGA   
  
  
+ ACTTAGTCGA CCTGGGTCGG GTCGGGTCGC TCCCGAATGA AGTGACGGTG GTGCACTGGA TGCACCATAG   
  
  
+ TCTATACGAC ATAACCGGGT CGGATCTTGG GACTTTGAGG GTATTGAGTG CGGTGAGGCC TAGGCTTGTG   
  
  
+ ACTATGGTTG AGCAGGATAT GGACCAAACG GGGTCGTTTT TGGGGAGGTT TGTGGAGGCA TTGCATTATT   
  
  
+ ACTCAGCCTT GTTTGATGCC TTGGGAGAGG GGTTAGATAG GGATAACCTA CAAAGGCATC AAGTGGAGCA   
  
  
+ ACAGCTATTT GGGTGTGAGA TTAGGAACAT CCTGGCCGTT GGTGGGCCCA AGAGGAGGCT CACTGGCGGC   
  
  
+ GATCGGGTCA GAAGGTGGGG CGACGAACTG ACAAGGGTCG GGTTCGAACC AGTTTCGTTG GCGGGTAGCC   
  
  
+ CGGCAACCCA AGCTAGTTTG TTGCTTGGGA TGTTCCCTTG GAAAGGGTAT ACTTTGATGG AGGAAAATGG   
  
  
+ GTGTTTGAGA TTAGGGTGGA AAGATTTGCC CTTGTTAACT GCCTCAGCTT GGCAACCTTG TGAATTTAAC   
  
  
+ AATCCTAGTG CTGGCATTTA   

- +Up\_Stream \_Len000TTTGCC ACGGCAACTT AAACGTCCGC TGCTCCAGCC CAAATTCCGC CTACAGCATC   
  
  
- GCGAAACCTC TTCGTTTAAA CCCTTCTGCT CCGAATAAGA ACTTGAGAAG CATGCGGTAA CAACAACCAA   
  
  
- GGTTGGAAAG AGAGAGGAGC ACTTAAACTC AAACCCGTCA TCTGATAGGG CCTTATCTCC TGACTACGCA   
  
  
- ATATATATAT CTTTAACTCT CTCTCTCTCT CGCACACACT TCCTACAACT TTTACTACCC GCATCCCTCA   
  
  
- GAGAGAGAGT GACAGACAAT CCTCATGCAC AGCTCTTTCC TTTATCCTAA GGGTTCGTCA GATTAAACCC   
  
  
- GGCCAGATAT CCGGTCTTAC CCGGATACTA GTCGGGTCTA TACTGGAACA AATTGGTTGA CATAAACTTA   
  
  
- ACTTCTTTCC CTATAGCGTT ACAGAACTTG GTGTAGTTTA TTATTTACAC GGAAACCAAT CTGGTATACT   
  
  
- TTTTAGAAAC TTAGTTTAGC CAACCTGGAC TTGACGGTAA TAACTATAGA ACAAAAACCA TCTTCGTTTA   
  
  
- CCACAGTGAA GCAAAAGATC TATTTTAATT GAACTCAAAA AAACGGTGAT ATCTATCATA ACGTTTATAT   
  
  
- TTTTCACACG GTGCACTTTA GTTATAATCC TCCCACAAAA TATTGTTATC GACAAGCCCT CTCAAAATCA   
  
  
- ATAAAAATAA TTTAGTTTAT CAATTCTTCA CAAACCATTT ATCAACATAA ACTTTTTCAA TAAGGGTAAT   
  
  
- CGGAAAATCA TTCTTGAAGA ACACTTTTAC TATTAACAGA TCTTCAAACT TTTTAAGTGT GGTGTACTGT   
  
  
- AAATTTTTAT TAAAACATCT CTTCCCGTAT TTACTTTCAA ACGGTGGTAG AAAAAATCTT TGTACATTAA   
  
  
- ATGGTTTGTG AAAAAGATTT TTGTCAATTA AGTTTATTGA TTGTTGATTG TCGATTATAT TTATTGATTG   
  
  
- TCGATAAACG ATTTGTCCCG GATTCATCAA ACTTTAACAA CAAAAGAGAA GTCGAAGTGG TACCGGCTAC   
  
  
- TACATTTGTT GATATAAGAC TTAAAGTAAA AATGATACTA AACTTTTAAA GTTTAAATTT ATTCTAACCT   
  
  
- ATTTTTTTTC TGGTTTTTAG TATTCATTAT AATCAGTTCA AGATAAGGTT GGTATATGTA TTGACGAAAA   
  
  
- AGAAAACCCC CAAAACTTTG TACACAGGAA TCTTATTAAC TTTGAGCTAT AATAAGAGAT TTAGATTCAA   
  
  
- ATGAAATAGT ATAACTAATA ATTAATGGAG GAGTTGAGGA GGATATGAGA ACATACGTCT TTATTTTTAC   
  
  
- AAAGGTATGG AATAGTCAGC GCTAGTATTA TTAATTAAAG AATTAAGCCT AACATAAATT AACGCATAGT   
  
  
- TTACACTTTA GCATGGTTTA ATCCTTCGTT TTGTTTGGCT ATTGCTTTTT CCATTCTTTT GAATCGTTCC   
  
  
- TTTACTTTAG CTTCTGGACA GTTGTTATAT CGAAGGGAGT GTAGGAAGTT GAATCCCTGG TAGGATATGA   
  
  
- AAGTACATTA TGTTGATCTC GGTGATCAGT TATTATCGGA GGATCATCAT CGGTATTCGG AACCACTCTC   
  
  
- TTACAAGGCG TTCTGATCAA TTGAACTTTA AGGTAAAACA GCGTCTTGAT AACCTCACTC TTTCTCTTAG   
  
  
- TTTAGTATGG TATAGTTTAA ACCTGGTAAA TTCAAGTGTT GTGTGTGTGT ATGTGTGTTT TCCTGCTACA   
  
  
- TGCCGGGGTG GGGGGTGAGA GGAAAGCGGT AGGGGGATGT GGAGACGGAT GAGAGAGAGA GAGAGAGAGA   
  
  
- GAGACCTAGT CAGACACATT TTCGAAGGAG AGGTCATGTC GTAAAGGAAC AGACAAGAAC CTCAAATCAG   
  
  
- TTAAGGAGGC GGGGTTTTTT TGGGTGAAAG AGAGAGTAGT ACAAGAAGGT CAAGGTGGGA ATATGCGGTA   
  
  
- AGTAGTAGTA GATGTAGTGG GTGTAGAAAA AGAGGTGTCA AAAGGGGGGA GTAATACGAG AGGCGGAGGT   
  
  
- GGGGGCGGAA GGGAGAGCGG CAGCGGCCGC TACTGCCCCT ATGGTGACTA CGTACGCTGC CGTGGCGGTG   
  
  
- ACGGGAGGTG GAGGAGCCCG ACGACAACTT CACGCGCCTT AAGTAGAGGT GGCCTCTGGA GCGGCCGCGG   
  
  
- CCTCTATAGA ACGGCCTCTA TAAACTCAAC AGATGTGGCA AACCGAGGGG GCGGCGGGCC CAGCGGCGGA   
  
  
- AGAAGCCGGT GCGGGAGGTG CGGGCGGAGG AGAGGCGGAG GGAGGCGTGT TGAGGCTAGC TCTTCGAGTT   
  
  
- TTGGGACTGG GACCAAAGGG TTTACGCCTT TAAAGTGAGG CGGAACGTTC ATATGTTGAG GTAGTGAGGG   
  
  
- AAGCACTTTA AGAGAGTGAA GTGCCGCTTA GTTCGGTAGA TGCTCCGCGA CCTGCCGCTC CTAGCACAGG   
  
  
- TGCAGCAGCT GGAACTGTAG TACGTCCCGG AAGTTACCGG CCCTAACAAG GTGTAGAATC GGAGAGCTGG   
  
  
- GTCTTTCGGG GAGAGCCAAG CCCAGTGGCC CAAGCCCGGT AGGAGGCTCA ACGAGAGGGT TTGCCCATTC   
  
  
- GCTGAGCGAC TCAAGCGGCG AAGTGAGCCG GACGGGAAGC TCATGTTGGG CCACCTCCCG TTTTAACCCT   
  
  
- TGAATCAGCT GGACCCAGCC CAGCCCAGCG AGGGCTTACT TCACTGCCAC CACGTGACCT ACGTGGTATC   
  
  
- AGATATGCTG TATTGGCCCA GCCTAGAACC CTGAAACTCC CATAACTCAC GCCACTCCGG ATCCGAACAC   
  
  
- TGATACCAAC TCGTCCTATA CCTGGTTTGC CCCAGCAAAA ACCCCTCCAA ACACCTCCGT AACGTAATAA   
  
  
- TGAGTCGGAA CAAACTACGG AACCCTCTCC CCAATCTATC CCTATTGGAT GTTTCCGTAG TTCACCTCGT   
  
  
- TGTCGATAAA CCCACACTCT AATCCTTGTA GGACCGGCAA CCACCCGGGT TCTCCTCCGA GTGACCGCCG   
  
  
- CTAGCCCAGT CTTCCACCCC GCTGCTTGAC TGTTCCCAGC CCAAGCTTGG TCAAAGCAAC CGCCCATCGG   
  
  
- GCCGTTGGGT TCGATCAAAC AACGAACCCT ACAAGGGAAC CTTTCCCATA TGAAACTACC TCCTTTTACC   
  
  
- CACAAACTCT AATCCCACCT TTCTAAACGG GAACAATTGA CGGAGTCGAA CCGTTGGAAC ACTTAAATTG   
  
  
- TTAGGATCAC GACCGTAAAT

+     CCGTCC motif

| Site Name | Organism | Position | Strand | Matrix score. | sequence | function |
| --- | --- | --- | --- | --- | --- | --- |
| CCGTCC motif | Nicotiana tabacum | 2435 | - | 6 | CCGTCC |  |

>HU02G03005.1   
+ +Up\_Stream \_Len000AAACGG TGCCGTTGAA TTTGCAGGCG ACGAGGTCGG GTTTAAGGCG GATGTCGTAG   
  
  
+ CGCTTTGGAG AAGCAAATTT GGGAAGACGA GGCTTATTCT TGAACTCTTC GTACGCCATT GTTGTTGGTT   
  
  
+ CCAACCTTTC TCTCTCCTCG TGAATTTGAG TTTGGGCAGT AGACTATCCC GGAATAGAGG ACTGATGCGT   
  
  
+ TATATATATA GAAATTGAGA GAGAGAGAGA GCGTGTGTGA AGGATGTTGA AAATGATGGG CGTAGGGAGT   
  
  
+ CTCTCTCTCA CTGTCTGTTA GGAGTACGTG TCGAGAAAGG AAATAGGATT CCCAAGCAGT CTAATTTGGG   
  
  
+ CCGGTCTATA GGCCAGAATG GGCCTATGAT CAGCCCAGAT ATGACCTTGT TTAACCAACT GTATTTGAAT   
  
  
+ TGAAGAAAGG GATATCGCAA TGTCTTGAAC CACATCAAAT AATAAATGTG CCTTTGGTTA GACCATATGA   
  
  
+ AAAATCTTTG AATCAAATCG GTTGGACCTG AACTGCCATT ATTGATATCT TGTTTTTGGT AGAAGCAAAT   
  
  
+ GGTGTCACTT CGTTTTCTAG ATAAAATTAA CTTGAGTTTT TTTGCCACTA TAGATAGTAT TGCAAATATA   
  
  
+ AAAAGTGTGC CACGTGAAAT CAATATTAGG AGGGTGTTTT ATAACAATAG CTGTTCGGGA GAGTTTTAGT   
  
  
+ TATTTTTATT AAATCAAATA GTTAAGAAGT GTTTGGTAAA TAGTTGTATT TGAAAAAGTT ATTCCCATTA   
  
  
+ GCCTTTTAGT AAGAACTTCT TGTGAAAATG ATAATTGTCT AGAAGTTTGA AAAATTCACA CCACATGACA   
  
  
+ TTTAAAAATA ATTTTGTAGA GAAGGGCATA AATGAAAGTT TGCCACCATC TTTTTTAGAA ACATGTAATT   
  
  
+ TACCAAACAC TTTTTCTAAA AACAGTTAAT TCAAATAACT AACAACTAAC AGCTAATATA AATAACTAAC   
  
  
+ AGCTATTTGC TAAACAGGGC CTAAGTAGTT TGAAATTGTT GTTTTCTCTT CAGCTTCACC ATGGCCGATG   
  
  
+ ATGTAAACAA CTATATTCTG AATTTCATTT TTACTATGAT TTGAAAATTT CAAATTTAAA TAAGATTGGA   
  
  
+ TAAAAAAAAG ACCAAAAATC ATAAGTAATA TTAGTCAAGT TCTATTCCAA CCATATACAT AACTGCTTTT   
  
  
+ TCTTTTGGGG GTTTTGAAAC ATGTGTCCTT AGAATAATTG AAACTCGATA TTATTCTCTA AATCTAAGTT   
  
  
+ TACTTTATCA TATTGATTAT TAATTACCTC CTCAACTCCT CCTATACTCT TGTATGCAGA AATAAAAATG   
  
  
+ TTTCCATACC TTATCAGTCG CGATCATAAT AATTAATTTC TTAATTCGGA TTGTATTTAA TTGCGTATCA   
  
  
+ AATGTGAAAT CGTACCAAAT TAGGAAGCAA AACAAACCGA TAACGAAAAA GGTAAGAAAA CTTAGCAAGG   
  
  
+ AAATGAAATC GAAGACCTGT CAACAATATA GCTTCCCTCA CATCCTTCAA CTTAGGGACC ATCCTATACT   
  
  
+ TTCATGTAAT ACAACTAGAG CCACTAGTCA ATAATAGCCT CCTAGTAGTA GCCATAAGCC TTGGTGAGAG   
  
  
+ AATGTTCCGC AAGACTAGTT AACTTGAAAT TCCATTTTGT CGCAGAACTA TTGGAGTGAG AAAGAGAATC   
  
  
+ AAATCATACC ATATCAAATT TGGACCATTT AAGTTCACAA CACACACACA TACACACAAA AGGACGATGT   
  
  
+ ACGGCCCCAC CCCCCACTCT CCTTTCGCCA TCCCCCTACA CCTCTGCCTA CTCTCTCTCT CTCTCTCTCT   
  
  
+ CTCTGGATCA GTCTGTGTAA AAGCTTCCTC TCCAGTACAG CATTTCCTTG TCTGTTCTTG GAGTTTAGTC   
  
  
+ AATTCCTCCG CCCCAAAAAA ACCCACTTTC TCTCTCATCA TGTTCTTCCA GTTCCACCCT TATACGCCAT   
  
  
+ TCATCATCAT CTACATCACC CACATCTTTT TCTCCACAGT TTTCCCCCCT CATTATGCTC TCCGCCTCCA   
  
  
+ CCCCCGCCTT CCCTCTCGCC GTCGCCGGCG ATGACGGGGA TACCACTGAT GCATGCGACG GCACCGCCAC   
  
  
+ TGCCCTCCAC CTCCTCGGGC TGCTGTTGAA GTGCGCGGAA TTCATCTCCA CCGGAGACCT CGCCGGCGCC   
  
  
+ GGAGATATCT TGCCGGAGAT ATTTGAGTTG TCTACACCGT TTGGCTCCCC CGCCGCCCGG GTCGCCGCCT   
  
  
+ TCTTCGGCCA CGCCCTCCAC GCCCGCCTCC TCTCCGCCTC CCTCCGCACA ACTCCGATCG AGAAGCTCAA   
  
  
+ AACCCTGACC CTGGTTTCCC AAATGCGGAA ATTTCACTCC GCCTTGCAAG TATACAACTC CATCACTCCC   
  
  
+ TTCGTGAAAT TCTCTCACTT CACGGCGAAT CAAGCCATCT ACGAGGCGCT GGACGGCGAG GATCGTGTCC   
  
  
+ ACGTCGTCGA CCTTGACATC ATGCAGGGCC TTCAATGGCC GGGATTGTTC CACATCTTAG CCTCTCGACC   
  
  
+ CAGAAAGCCC CTCTCGGTTC GGGTCACCGG GTTCGGGCCA TCCTCCGAGT TGCTCTCCCA AACGGGTAAG   
  
  
+ CGACTCGCTG AGTTCGCCGC TTCACTCGGC CTGCCCTTCG AGTACAACCC GGTGGAGGGC AAAATTGGGA   
  
  
+ ACTTAGTCGA CCTGGGTCGG GTCGGGTCGC TCCCGAATGA AGTGACGGTG GTGCACTGGA TGCACCATAG   
  
  
+ TCTATACGAC ATAACCGGGT CGGATCTTGG GACTTTGAGG GTATTGAGTG CGGTGAGGCC TAGGCTTGTG   
  
  
+ ACTATGGTTG AGCAGGATAT GGACCAAACG GGGTCGTTTT TGGGGAGGTT TGTGGAGGCA TTGCATTATT   
  
  
+ ACTCAGCCTT GTTTGATGCC TTGGGAGAGG GGTTAGATAG GGATAACCTA CAAAGGCATC AAGTGGAGCA   
  
  
+ ACAGCTATTT GGGTGTGAGA TTAGGAACAT CCTGGCCGTT GGTGGGCCCA AGAGGAGGCT CACTGGCGGC   
  
  
+ GATCGGGTCA GAAGGTGGGG CGACGAACTG ACAAGGGTCG GGTTCGAACC AGTTTCGTTG GCGGGTAGCC   
  
  
+ CGGCAACCCA AGCTAGTTTG TTGCTTGGGA TGTTCCCTTG GAAAGGGTAT ACTTTGATGG AGGAAAATGG   
  
  
+ GTGTTTGAGA TTAGGGTGGA AAGATTTGCC CTTGTTAACT GCCTCAGCTT GGCAACCTTG TGAATTTAAC   
  
  
+ AATCCTAGTG CTGGCATTTA   

- +Up\_Stream \_Len000TTTGCC ACGGCAACTT AAACGTCCGC TGCTCCAGCC CAAATTCCGC CTACAGCATC   
  
  
- GCGAAACCTC TTCGTTTAAA CCCTTCTGCT CCGAATAAGA ACTTGAGAAG CATGCGGTAA CAACAACCAA   
  
  
- GGTTGGAAAG AGAGAGGAGC ACTTAAACTC AAACCCGTCA TCTGATAGGG CCTTATCTCC TGACTACGCA   
  
  
- ATATATATAT CTTTAACTCT CTCTCTCTCT CGCACACACT TCCTACAACT TTTACTACCC GCATCCCTCA   
  
  
- GAGAGAGAGT GACAGACAAT CCTCATGCAC AGCTCTTTCC TTTATCCTAA GGGTTCGTCA GATTAAACCC   
  
  
- GGCCAGATAT CCGGTCTTAC CCGGATACTA GTCGGGTCTA TACTGGAACA AATTGGTTGA CATAAACTTA   
  
  
- ACTTCTTTCC CTATAGCGTT ACAGAACTTG GTGTAGTTTA TTATTTACAC GGAAACCAAT CTGGTATACT   
  
  
- TTTTAGAAAC TTAGTTTAGC CAACCTGGAC TTGACGGTAA TAACTATAGA ACAAAAACCA TCTTCGTTTA   
  
  
- CCACAGTGAA GCAAAAGATC TATTTTAATT GAACTCAAAA AAACGGTGAT ATCTATCATA ACGTTTATAT   
  
  
- TTTTCACACG GTGCACTTTA GTTATAATCC TCCCACAAAA TATTGTTATC GACAAGCCCT CTCAAAATCA   
  
  
- ATAAAAATAA TTTAGTTTAT CAATTCTTCA CAAACCATTT ATCAACATAA ACTTTTTCAA TAAGGGTAAT   
  
  
- CGGAAAATCA TTCTTGAAGA ACACTTTTAC TATTAACAGA TCTTCAAACT TTTTAAGTGT GGTGTACTGT   
  
  
- AAATTTTTAT TAAAACATCT CTTCCCGTAT TTACTTTCAA ACGGTGGTAG AAAAAATCTT TGTACATTAA   
  
  
- ATGGTTTGTG AAAAAGATTT TTGTCAATTA AGTTTATTGA TTGTTGATTG TCGATTATAT TTATTGATTG   
  
  
- TCGATAAACG ATTTGTCCCG GATTCATCAA ACTTTAACAA CAAAAGAGAA GTCGAAGTGG TACCGGCTAC   
  
  
- TACATTTGTT GATATAAGAC TTAAAGTAAA AATGATACTA AACTTTTAAA GTTTAAATTT ATTCTAACCT   
  
  
- ATTTTTTTTC TGGTTTTTAG TATTCATTAT AATCAGTTCA AGATAAGGTT GGTATATGTA TTGACGAAAA   
  
  
- AGAAAACCCC CAAAACTTTG TACACAGGAA TCTTATTAAC TTTGAGCTAT AATAAGAGAT TTAGATTCAA   
  
  
- ATGAAATAGT ATAACTAATA ATTAATGGAG GAGTTGAGGA GGATATGAGA ACATACGTCT TTATTTTTAC   
  
  
- AAAGGTATGG AATAGTCAGC GCTAGTATTA TTAATTAAAG AATTAAGCCT AACATAAATT AACGCATAGT   
  
  
- TTACACTTTA GCATGGTTTA ATCCTTCGTT TTGTTTGGCT ATTGCTTTTT CCATTCTTTT GAATCGTTCC   
  
  
- TTTACTTTAG CTTCTGGACA GTTGTTATAT CGAAGGGAGT GTAGGAAGTT GAATCCCTGG TAGGATATGA   
  
  
- AAGTACATTA TGTTGATCTC GGTGATCAGT TATTATCGGA GGATCATCAT CGGTATTCGG AACCACTCTC   
  
  
- TTACAAGGCG TTCTGATCAA TTGAACTTTA AGGTAAAACA GCGTCTTGAT AACCTCACTC TTTCTCTTAG   
  
  
- TTTAGTATGG TATAGTTTAA ACCTGGTAAA TTCAAGTGTT GTGTGTGTGT ATGTGTGTTT TCCTGCTACA   
  
  
- TGCCGGGGTG GGGGGTGAGA GGAAAGCGGT AGGGGGATGT GGAGACGGAT GAGAGAGAGA GAGAGAGAGA   
  
  
- GAGACCTAGT CAGACACATT TTCGAAGGAG AGGTCATGTC GTAAAGGAAC AGACAAGAAC CTCAAATCAG   
  
  
- TTAAGGAGGC GGGGTTTTTT TGGGTGAAAG AGAGAGTAGT ACAAGAAGGT CAAGGTGGGA ATATGCGGTA   
  
  
- AGTAGTAGTA GATGTAGTGG GTGTAGAAAA AGAGGTGTCA AAAGGGGGGA GTAATACGAG AGGCGGAGGT   
  
  
- GGGGGCGGAA GGGAGAGCGG CAGCGGCCGC TACTGCCCCT ATGGTGACTA CGTACGCTGC CGTGGCGGTG   
  
  
- ACGGGAGGTG GAGGAGCCCG ACGACAACTT CACGCGCCTT AAGTAGAGGT GGCCTCTGGA GCGGCCGCGG   
  
  
- CCTCTATAGA ACGGCCTCTA TAAACTCAAC AGATGTGGCA AACCGAGGGG GCGGCGGGCC CAGCGGCGGA   
  
  
- AGAAGCCGGT GCGGGAGGTG CGGGCGGAGG AGAGGCGGAG GGAGGCGTGT TGAGGCTAGC TCTTCGAGTT   
  
  
- TTGGGACTGG GACCAAAGGG TTTACGCCTT TAAAGTGAGG CGGAACGTTC ATATGTTGAG GTAGTGAGGG   
  
  
- AAGCACTTTA AGAGAGTGAA GTGCCGCTTA GTTCGGTAGA TGCTCCGCGA CCTGCCGCTC CTAGCACAGG   
  
  
- TGCAGCAGCT GGAACTGTAG TACGTCCCGG AAGTTACCGG CCCTAACAAG GTGTAGAATC GGAGAGCTGG   
  
  
- GTCTTTCGGG GAGAGCCAAG CCCAGTGGCC CAAGCCCGGT AGGAGGCTCA ACGAGAGGGT TTGCCCATTC   
  
  
- GCTGAGCGAC TCAAGCGGCG AAGTGAGCCG GACGGGAAGC TCATGTTGGG CCACCTCCCG TTTTAACCCT   
  
  
- TGAATCAGCT GGACCCAGCC CAGCCCAGCG AGGGCTTACT TCACTGCCAC CACGTGACCT ACGTGGTATC   
  
  
- AGATATGCTG TATTGGCCCA GCCTAGAACC CTGAAACTCC CATAACTCAC GCCACTCCGG ATCCGAACAC   
  
  
- TGATACCAAC TCGTCCTATA CCTGGTTTGC CCCAGCAAAA ACCCCTCCAA ACACCTCCGT AACGTAATAA   
  
  
- TGAGTCGGAA CAAACTACGG AACCCTCTCC CCAATCTATC CCTATTGGAT GTTTCCGTAG TTCACCTCGT   
  
  
- TGTCGATAAA CCCACACTCT AATCCTTGTA GGACCGGCAA CCACCCGGGT TCTCCTCCGA GTGACCGCCG   
  
  
- CTAGCCCAGT CTTCCACCCC GCTGCTTGAC TGTTCCCAGC CCAAGCTTGG TCAAAGCAAC CGCCCATCGG   
  
  
- GCCGTTGGGT TCGATCAAAC AACGAACCCT ACAAGGGAAC CTTTCCCATA TGAAACTACC TCCTTTTACC   
  
  
- CACAAACTCT AATCCCACCT TTCTAAACGG GAACAATTGA CGGAGTCGAA CCGTTGGAAC ACTTAAATTG   
  
  
- TTAGGATCAC GACCGTAAAT

+     CCGTCC-box

| Site Name | Organism | Position | Strand | Matrix score. | sequence | function |
| --- | --- | --- | --- | --- | --- | --- |
| CCGTCC-box | Petroselinum hortense | 2435 | - | 6 | CCGTCC |  |

>HU02G03005.1   
+ +Up\_Stream \_Len000AAACGG TGCCGTTGAA TTTGCAGGCG ACGAGGTCGG GTTTAAGGCG GATGTCGTAG   
  
  
+ CGCTTTGGAG AAGCAAATTT GGGAAGACGA GGCTTATTCT TGAACTCTTC GTACGCCATT GTTGTTGGTT   
  
  
+ CCAACCTTTC TCTCTCCTCG TGAATTTGAG TTTGGGCAGT AGACTATCCC GGAATAGAGG ACTGATGCGT   
  
  
+ TATATATATA GAAATTGAGA GAGAGAGAGA GCGTGTGTGA AGGATGTTGA AAATGATGGG CGTAGGGAGT   
  
  
+ CTCTCTCTCA CTGTCTGTTA GGAGTACGTG TCGAGAAAGG AAATAGGATT CCCAAGCAGT CTAATTTGGG   
  
  
+ CCGGTCTATA GGCCAGAATG GGCCTATGAT CAGCCCAGAT ATGACCTTGT TTAACCAACT GTATTTGAAT   
  
  
+ TGAAGAAAGG GATATCGCAA TGTCTTGAAC CACATCAAAT AATAAATGTG CCTTTGGTTA GACCATATGA   
  
  
+ AAAATCTTTG AATCAAATCG GTTGGACCTG AACTGCCATT ATTGATATCT TGTTTTTGGT AGAAGCAAAT   
  
  
+ GGTGTCACTT CGTTTTCTAG ATAAAATTAA CTTGAGTTTT TTTGCCACTA TAGATAGTAT TGCAAATATA   
  
  
+ AAAAGTGTGC CACGTGAAAT CAATATTAGG AGGGTGTTTT ATAACAATAG CTGTTCGGGA GAGTTTTAGT   
  
  
+ TATTTTTATT AAATCAAATA GTTAAGAAGT GTTTGGTAAA TAGTTGTATT TGAAAAAGTT ATTCCCATTA   
  
  
+ GCCTTTTAGT AAGAACTTCT TGTGAAAATG ATAATTGTCT AGAAGTTTGA AAAATTCACA CCACATGACA   
  
  
+ TTTAAAAATA ATTTTGTAGA GAAGGGCATA AATGAAAGTT TGCCACCATC TTTTTTAGAA ACATGTAATT   
  
  
+ TACCAAACAC TTTTTCTAAA AACAGTTAAT TCAAATAACT AACAACTAAC AGCTAATATA AATAACTAAC   
  
  
+ AGCTATTTGC TAAACAGGGC CTAAGTAGTT TGAAATTGTT GTTTTCTCTT CAGCTTCACC ATGGCCGATG   
  
  
+ ATGTAAACAA CTATATTCTG AATTTCATTT TTACTATGAT TTGAAAATTT CAAATTTAAA TAAGATTGGA   
  
  
+ TAAAAAAAAG ACCAAAAATC ATAAGTAATA TTAGTCAAGT TCTATTCCAA CCATATACAT AACTGCTTTT   
  
  
+ TCTTTTGGGG GTTTTGAAAC ATGTGTCCTT AGAATAATTG AAACTCGATA TTATTCTCTA AATCTAAGTT   
  
  
+ TACTTTATCA TATTGATTAT TAATTACCTC CTCAACTCCT CCTATACTCT TGTATGCAGA AATAAAAATG   
  
  
+ TTTCCATACC TTATCAGTCG CGATCATAAT AATTAATTTC TTAATTCGGA TTGTATTTAA TTGCGTATCA   
  
  
+ AATGTGAAAT CGTACCAAAT TAGGAAGCAA AACAAACCGA TAACGAAAAA GGTAAGAAAA CTTAGCAAGG   
  
  
+ AAATGAAATC GAAGACCTGT CAACAATATA GCTTCCCTCA CATCCTTCAA CTTAGGGACC ATCCTATACT   
  
  
+ TTCATGTAAT ACAACTAGAG CCACTAGTCA ATAATAGCCT CCTAGTAGTA GCCATAAGCC TTGGTGAGAG   
  
  
+ AATGTTCCGC AAGACTAGTT AACTTGAAAT TCCATTTTGT CGCAGAACTA TTGGAGTGAG AAAGAGAATC   
  
  
+ AAATCATACC ATATCAAATT TGGACCATTT AAGTTCACAA CACACACACA TACACACAAA AGGACGATGT   
  
  
+ ACGGCCCCAC CCCCCACTCT CCTTTCGCCA TCCCCCTACA CCTCTGCCTA CTCTCTCTCT CTCTCTCTCT   
  
  
+ CTCTGGATCA GTCTGTGTAA AAGCTTCCTC TCCAGTACAG CATTTCCTTG TCTGTTCTTG GAGTTTAGTC   
  
  
+ AATTCCTCCG CCCCAAAAAA ACCCACTTTC TCTCTCATCA TGTTCTTCCA GTTCCACCCT TATACGCCAT   
  
  
+ TCATCATCAT CTACATCACC CACATCTTTT TCTCCACAGT TTTCCCCCCT CATTATGCTC TCCGCCTCCA   
  
  
+ CCCCCGCCTT CCCTCTCGCC GTCGCCGGCG ATGACGGGGA TACCACTGAT GCATGCGACG GCACCGCCAC   
  
  
+ TGCCCTCCAC CTCCTCGGGC TGCTGTTGAA GTGCGCGGAA TTCATCTCCA CCGGAGACCT CGCCGGCGCC   
  
  
+ GGAGATATCT TGCCGGAGAT ATTTGAGTTG TCTACACCGT TTGGCTCCCC CGCCGCCCGG GTCGCCGCCT   
  
  
+ TCTTCGGCCA CGCCCTCCAC GCCCGCCTCC TCTCCGCCTC CCTCCGCACA ACTCCGATCG AGAAGCTCAA   
  
  
+ AACCCTGACC CTGGTTTCCC AAATGCGGAA ATTTCACTCC GCCTTGCAAG TATACAACTC CATCACTCCC   
  
  
+ TTCGTGAAAT TCTCTCACTT CACGGCGAAT CAAGCCATCT ACGAGGCGCT GGACGGCGAG GATCGTGTCC   
  
  
+ ACGTCGTCGA CCTTGACATC ATGCAGGGCC TTCAATGGCC GGGATTGTTC CACATCTTAG CCTCTCGACC   
  
  
+ CAGAAAGCCC CTCTCGGTTC GGGTCACCGG GTTCGGGCCA TCCTCCGAGT TGCTCTCCCA AACGGGTAAG   
  
  
+ CGACTCGCTG AGTTCGCCGC TTCACTCGGC CTGCCCTTCG AGTACAACCC GGTGGAGGGC AAAATTGGGA   
  
  
+ ACTTAGTCGA CCTGGGTCGG GTCGGGTCGC TCCCGAATGA AGTGACGGTG GTGCACTGGA TGCACCATAG   
  
  
+ TCTATACGAC ATAACCGGGT CGGATCTTGG GACTTTGAGG GTATTGAGTG CGGTGAGGCC TAGGCTTGTG   
  
  
+ ACTATGGTTG AGCAGGATAT GGACCAAACG GGGTCGTTTT TGGGGAGGTT TGTGGAGGCA TTGCATTATT   
  
  
+ ACTCAGCCTT GTTTGATGCC TTGGGAGAGG GGTTAGATAG GGATAACCTA CAAAGGCATC AAGTGGAGCA   
  
  
+ ACAGCTATTT GGGTGTGAGA TTAGGAACAT CCTGGCCGTT GGTGGGCCCA AGAGGAGGCT CACTGGCGGC   
  
  
+ GATCGGGTCA GAAGGTGGGG CGACGAACTG ACAAGGGTCG GGTTCGAACC AGTTTCGTTG GCGGGTAGCC   
  
  
+ CGGCAACCCA AGCTAGTTTG TTGCTTGGGA TGTTCCCTTG GAAAGGGTAT ACTTTGATGG AGGAAAATGG   
  
  
+ GTGTTTGAGA TTAGGGTGGA AAGATTTGCC CTTGTTAACT GCCTCAGCTT GGCAACCTTG TGAATTTAAC   
  
  
+ AATCCTAGTG CTGGCATTTA   

- +Up\_Stream \_Len000TTTGCC ACGGCAACTT AAACGTCCGC TGCTCCAGCC CAAATTCCGC CTACAGCATC   
  
  
- GCGAAACCTC TTCGTTTAAA CCCTTCTGCT CCGAATAAGA ACTTGAGAAG CATGCGGTAA CAACAACCAA   
  
  
- GGTTGGAAAG AGAGAGGAGC ACTTAAACTC AAACCCGTCA TCTGATAGGG CCTTATCTCC TGACTACGCA   
  
  
- ATATATATAT CTTTAACTCT CTCTCTCTCT CGCACACACT TCCTACAACT TTTACTACCC GCATCCCTCA   
  
  
- GAGAGAGAGT GACAGACAAT CCTCATGCAC AGCTCTTTCC TTTATCCTAA GGGTTCGTCA GATTAAACCC   
  
  
- GGCCAGATAT CCGGTCTTAC CCGGATACTA GTCGGGTCTA TACTGGAACA AATTGGTTGA CATAAACTTA   
  
  
- ACTTCTTTCC CTATAGCGTT ACAGAACTTG GTGTAGTTTA TTATTTACAC GGAAACCAAT CTGGTATACT   
  
  
- TTTTAGAAAC TTAGTTTAGC CAACCTGGAC TTGACGGTAA TAACTATAGA ACAAAAACCA TCTTCGTTTA   
  
  
- CCACAGTGAA GCAAAAGATC TATTTTAATT GAACTCAAAA AAACGGTGAT ATCTATCATA ACGTTTATAT   
  
  
- TTTTCACACG GTGCACTTTA GTTATAATCC TCCCACAAAA TATTGTTATC GACAAGCCCT CTCAAAATCA   
  
  
- ATAAAAATAA TTTAGTTTAT CAATTCTTCA CAAACCATTT ATCAACATAA ACTTTTTCAA TAAGGGTAAT   
  
  
- CGGAAAATCA TTCTTGAAGA ACACTTTTAC TATTAACAGA TCTTCAAACT TTTTAAGTGT GGTGTACTGT   
  
  
- AAATTTTTAT TAAAACATCT CTTCCCGTAT TTACTTTCAA ACGGTGGTAG AAAAAATCTT TGTACATTAA   
  
  
- ATGGTTTGTG AAAAAGATTT TTGTCAATTA AGTTTATTGA TTGTTGATTG TCGATTATAT TTATTGATTG   
  
  
- TCGATAAACG ATTTGTCCCG GATTCATCAA ACTTTAACAA CAAAAGAGAA GTCGAAGTGG TACCGGCTAC   
  
  
- TACATTTGTT GATATAAGAC TTAAAGTAAA AATGATACTA AACTTTTAAA GTTTAAATTT ATTCTAACCT   
  
  
- ATTTTTTTTC TGGTTTTTAG TATTCATTAT AATCAGTTCA AGATAAGGTT GGTATATGTA TTGACGAAAA   
  
  
- AGAAAACCCC CAAAACTTTG TACACAGGAA TCTTATTAAC TTTGAGCTAT AATAAGAGAT TTAGATTCAA   
  
  
- ATGAAATAGT ATAACTAATA ATTAATGGAG GAGTTGAGGA GGATATGAGA ACATACGTCT TTATTTTTAC   
  
  
- AAAGGTATGG AATAGTCAGC GCTAGTATTA TTAATTAAAG AATTAAGCCT AACATAAATT AACGCATAGT   
  
  
- TTACACTTTA GCATGGTTTA ATCCTTCGTT TTGTTTGGCT ATTGCTTTTT CCATTCTTTT GAATCGTTCC   
  
  
- TTTACTTTAG CTTCTGGACA GTTGTTATAT CGAAGGGAGT GTAGGAAGTT GAATCCCTGG TAGGATATGA   
  
  
- AAGTACATTA TGTTGATCTC GGTGATCAGT TATTATCGGA GGATCATCAT CGGTATTCGG AACCACTCTC   
  
  
- TTACAAGGCG TTCTGATCAA TTGAACTTTA AGGTAAAACA GCGTCTTGAT AACCTCACTC TTTCTCTTAG   
  
  
- TTTAGTATGG TATAGTTTAA ACCTGGTAAA TTCAAGTGTT GTGTGTGTGT ATGTGTGTTT TCCTGCTACA   
  
  
- TGCCGGGGTG GGGGGTGAGA GGAAAGCGGT AGGGGGATGT GGAGACGGAT GAGAGAGAGA GAGAGAGAGA   
  
  
- GAGACCTAGT CAGACACATT TTCGAAGGAG AGGTCATGTC GTAAAGGAAC AGACAAGAAC CTCAAATCAG   
  
  
- TTAAGGAGGC GGGGTTTTTT TGGGTGAAAG AGAGAGTAGT ACAAGAAGGT CAAGGTGGGA ATATGCGGTA   
  
  
- AGTAGTAGTA GATGTAGTGG GTGTAGAAAA AGAGGTGTCA AAAGGGGGGA GTAATACGAG AGGCGGAGGT   
  
  
- GGGGGCGGAA GGGAGAGCGG CAGCGGCCGC TACTGCCCCT ATGGTGACTA CGTACGCTGC CGTGGCGGTG   
  
  
- ACGGGAGGTG GAGGAGCCCG ACGACAACTT CACGCGCCTT AAGTAGAGGT GGCCTCTGGA GCGGCCGCGG   
  
  
- CCTCTATAGA ACGGCCTCTA TAAACTCAAC AGATGTGGCA AACCGAGGGG GCGGCGGGCC CAGCGGCGGA   
  
  
- AGAAGCCGGT GCGGGAGGTG CGGGCGGAGG AGAGGCGGAG GGAGGCGTGT TGAGGCTAGC TCTTCGAGTT   
  
  
- TTGGGACTGG GACCAAAGGG TTTACGCCTT TAAAGTGAGG CGGAACGTTC ATATGTTGAG GTAGTGAGGG   
  
  
- AAGCACTTTA AGAGAGTGAA GTGCCGCTTA GTTCGGTAGA TGCTCCGCGA CCTGCCGCTC CTAGCACAGG   
  
  
- TGCAGCAGCT GGAACTGTAG TACGTCCCGG AAGTTACCGG CCCTAACAAG GTGTAGAATC GGAGAGCTGG   
  
  
- GTCTTTCGGG GAGAGCCAAG CCCAGTGGCC CAAGCCCGGT AGGAGGCTCA ACGAGAGGGT TTGCCCATTC   
  
  
- GCTGAGCGAC TCAAGCGGCG AAGTGAGCCG GACGGGAAGC TCATGTTGGG CCACCTCCCG TTTTAACCCT   
  
  
- TGAATCAGCT GGACCCAGCC CAGCCCAGCG AGGGCTTACT TCACTGCCAC CACGTGACCT ACGTGGTATC   
  
  
- AGATATGCTG TATTGGCCCA GCCTAGAACC CTGAAACTCC CATAACTCAC GCCACTCCGG ATCCGAACAC   
  
  
- TGATACCAAC TCGTCCTATA CCTGGTTTGC CCCAGCAAAA ACCCCTCCAA ACACCTCCGT AACGTAATAA   
  
  
- TGAGTCGGAA CAAACTACGG AACCCTCTCC CCAATCTATC CCTATTGGAT GTTTCCGTAG TTCACCTCGT   
  
  
- TGTCGATAAA CCCACACTCT AATCCTTGTA GGACCGGCAA CCACCCGGGT TCTCCTCCGA GTGACCGCCG   
  
  
- CTAGCCCAGT CTTCCACCCC GCTGCTTGAC TGTTCCCAGC CCAAGCTTGG TCAAAGCAAC CGCCCATCGG   
  
  
- GCCGTTGGGT TCGATCAAAC AACGAACCCT ACAAGGGAAC CTTTCCCATA TGAAACTACC TCCTTTTACC   
  
  
- CACAAACTCT AATCCCACCT TTCTAAACGG GAACAATTGA CGGAGTCGAA CCGTTGGAAC ACTTAAATTG   
  
  
- TTAGGATCAC GACCGTAAAT

+     CGTCA-motif

| Site Name | Organism | Position | Strand | Matrix score. | sequence | function |
| --- | --- | --- | --- | --- | --- | --- |
| CGTCA-motif | Hordeum vulgare | 2707 | - | 5 | CGTCA | cis-acting regulatory element involved in the MeJA-responsiveness |
| CGTCA-motif | Hordeum vulgare | 2066 | - | 5 | CGTCA | cis-acting regulatory element involved in the MeJA-responsiveness |

>HU02G03005.1   
+ +Up\_Stream \_Len000AAACGG TGCCGTTGAA TTTGCAGGCG ACGAGGTCGG GTTTAAGGCG GATGTCGTAG   
  
  
+ CGCTTTGGAG AAGCAAATTT GGGAAGACGA GGCTTATTCT TGAACTCTTC GTACGCCATT GTTGTTGGTT   
  
  
+ CCAACCTTTC TCTCTCCTCG TGAATTTGAG TTTGGGCAGT AGACTATCCC GGAATAGAGG ACTGATGCGT   
  
  
+ TATATATATA GAAATTGAGA GAGAGAGAGA GCGTGTGTGA AGGATGTTGA AAATGATGGG CGTAGGGAGT   
  
  
+ CTCTCTCTCA CTGTCTGTTA GGAGTACGTG TCGAGAAAGG AAATAGGATT CCCAAGCAGT CTAATTTGGG   
  
  
+ CCGGTCTATA GGCCAGAATG GGCCTATGAT CAGCCCAGAT ATGACCTTGT TTAACCAACT GTATTTGAAT   
  
  
+ TGAAGAAAGG GATATCGCAA TGTCTTGAAC CACATCAAAT AATAAATGTG CCTTTGGTTA GACCATATGA   
  
  
+ AAAATCTTTG AATCAAATCG GTTGGACCTG AACTGCCATT ATTGATATCT TGTTTTTGGT AGAAGCAAAT   
  
  
+ GGTGTCACTT CGTTTTCTAG ATAAAATTAA CTTGAGTTTT TTTGCCACTA TAGATAGTAT TGCAAATATA   
  
  
+ AAAAGTGTGC CACGTGAAAT CAATATTAGG AGGGTGTTTT ATAACAATAG CTGTTCGGGA GAGTTTTAGT   
  
  
+ TATTTTTATT AAATCAAATA GTTAAGAAGT GTTTGGTAAA TAGTTGTATT TGAAAAAGTT ATTCCCATTA   
  
  
+ GCCTTTTAGT AAGAACTTCT TGTGAAAATG ATAATTGTCT AGAAGTTTGA AAAATTCACA CCACATGACA   
  
  
+ TTTAAAAATA ATTTTGTAGA GAAGGGCATA AATGAAAGTT TGCCACCATC TTTTTTAGAA ACATGTAATT   
  
  
+ TACCAAACAC TTTTTCTAAA AACAGTTAAT TCAAATAACT AACAACTAAC AGCTAATATA AATAACTAAC   
  
  
+ AGCTATTTGC TAAACAGGGC CTAAGTAGTT TGAAATTGTT GTTTTCTCTT CAGCTTCACC ATGGCCGATG   
  
  
+ ATGTAAACAA CTATATTCTG AATTTCATTT TTACTATGAT TTGAAAATTT CAAATTTAAA TAAGATTGGA   
  
  
+ TAAAAAAAAG ACCAAAAATC ATAAGTAATA TTAGTCAAGT TCTATTCCAA CCATATACAT AACTGCTTTT   
  
  
+ TCTTTTGGGG GTTTTGAAAC ATGTGTCCTT AGAATAATTG AAACTCGATA TTATTCTCTA AATCTAAGTT   
  
  
+ TACTTTATCA TATTGATTAT TAATTACCTC CTCAACTCCT CCTATACTCT TGTATGCAGA AATAAAAATG   
  
  
+ TTTCCATACC TTATCAGTCG CGATCATAAT AATTAATTTC TTAATTCGGA TTGTATTTAA TTGCGTATCA   
  
  
+ AATGTGAAAT CGTACCAAAT TAGGAAGCAA AACAAACCGA TAACGAAAAA GGTAAGAAAA CTTAGCAAGG   
  
  
+ AAATGAAATC GAAGACCTGT CAACAATATA GCTTCCCTCA CATCCTTCAA CTTAGGGACC ATCCTATACT   
  
  
+ TTCATGTAAT ACAACTAGAG CCACTAGTCA ATAATAGCCT CCTAGTAGTA GCCATAAGCC TTGGTGAGAG   
  
  
+ AATGTTCCGC AAGACTAGTT AACTTGAAAT TCCATTTTGT CGCAGAACTA TTGGAGTGAG AAAGAGAATC   
  
  
+ AAATCATACC ATATCAAATT TGGACCATTT AAGTTCACAA CACACACACA TACACACAAA AGGACGATGT   
  
  
+ ACGGCCCCAC CCCCCACTCT CCTTTCGCCA TCCCCCTACA CCTCTGCCTA CTCTCTCTCT CTCTCTCTCT   
  
  
+ CTCTGGATCA GTCTGTGTAA AAGCTTCCTC TCCAGTACAG CATTTCCTTG TCTGTTCTTG GAGTTTAGTC   
  
  
+ AATTCCTCCG CCCCAAAAAA ACCCACTTTC TCTCTCATCA TGTTCTTCCA GTTCCACCCT TATACGCCAT   
  
  
+ TCATCATCAT CTACATCACC CACATCTTTT TCTCCACAGT TTTCCCCCCT CATTATGCTC TCCGCCTCCA   
  
  
+ CCCCCGCCTT CCCTCTCGCC GTCGCCGGCG ATGACGGGGA TACCACTGAT GCATGCGACG GCACCGCCAC   
  
  
+ TGCCCTCCAC CTCCTCGGGC TGCTGTTGAA GTGCGCGGAA TTCATCTCCA CCGGAGACCT CGCCGGCGCC   
  
  
+ GGAGATATCT TGCCGGAGAT ATTTGAGTTG TCTACACCGT TTGGCTCCCC CGCCGCCCGG GTCGCCGCCT   
  
  
+ TCTTCGGCCA CGCCCTCCAC GCCCGCCTCC TCTCCGCCTC CCTCCGCACA ACTCCGATCG AGAAGCTCAA   
  
  
+ AACCCTGACC CTGGTTTCCC AAATGCGGAA ATTTCACTCC GCCTTGCAAG TATACAACTC CATCACTCCC   
  
  
+ TTCGTGAAAT TCTCTCACTT CACGGCGAAT CAAGCCATCT ACGAGGCGCT GGACGGCGAG GATCGTGTCC   
  
  
+ ACGTCGTCGA CCTTGACATC ATGCAGGGCC TTCAATGGCC GGGATTGTTC CACATCTTAG CCTCTCGACC   
  
  
+ CAGAAAGCCC CTCTCGGTTC GGGTCACCGG GTTCGGGCCA TCCTCCGAGT TGCTCTCCCA AACGGGTAAG   
  
  
+ CGACTCGCTG AGTTCGCCGC TTCACTCGGC CTGCCCTTCG AGTACAACCC GGTGGAGGGC AAAATTGGGA   
  
  
+ ACTTAGTCGA CCTGGGTCGG GTCGGGTCGC TCCCGAATGA AGTGACGGTG GTGCACTGGA TGCACCATAG   
  
  
+ TCTATACGAC ATAACCGGGT CGGATCTTGG GACTTTGAGG GTATTGAGTG CGGTGAGGCC TAGGCTTGTG   
  
  
+ ACTATGGTTG AGCAGGATAT GGACCAAACG GGGTCGTTTT TGGGGAGGTT TGTGGAGGCA TTGCATTATT   
  
  
+ ACTCAGCCTT GTTTGATGCC TTGGGAGAGG GGTTAGATAG GGATAACCTA CAAAGGCATC AAGTGGAGCA   
  
  
+ ACAGCTATTT GGGTGTGAGA TTAGGAACAT CCTGGCCGTT GGTGGGCCCA AGAGGAGGCT CACTGGCGGC   
  
  
+ GATCGGGTCA GAAGGTGGGG CGACGAACTG ACAAGGGTCG GGTTCGAACC AGTTTCGTTG GCGGGTAGCC   
  
  
+ CGGCAACCCA AGCTAGTTTG TTGCTTGGGA TGTTCCCTTG GAAAGGGTAT ACTTTGATGG AGGAAAATGG   
  
  
+ GTGTTTGAGA TTAGGGTGGA AAGATTTGCC CTTGTTAACT GCCTCAGCTT GGCAACCTTG TGAATTTAAC   
  
  
+ AATCCTAGTG CTGGCATTTA   

- +Up\_Stream \_Len000TTTGCC ACGGCAACTT AAACGTCCGC TGCTCCAGCC CAAATTCCGC CTACAGCATC   
  
  
- GCGAAACCTC TTCGTTTAAA CCCTTCTGCT CCGAATAAGA ACTTGAGAAG CATGCGGTAA CAACAACCAA   
  
  
- GGTTGGAAAG AGAGAGGAGC ACTTAAACTC AAACCCGTCA TCTGATAGGG CCTTATCTCC TGACTACGCA   
  
  
- ATATATATAT CTTTAACTCT CTCTCTCTCT CGCACACACT TCCTACAACT TTTACTACCC GCATCCCTCA   
  
  
- GAGAGAGAGT GACAGACAAT CCTCATGCAC AGCTCTTTCC TTTATCCTAA GGGTTCGTCA GATTAAACCC   
  
  
- GGCCAGATAT CCGGTCTTAC CCGGATACTA GTCGGGTCTA TACTGGAACA AATTGGTTGA CATAAACTTA   
  
  
- ACTTCTTTCC CTATAGCGTT ACAGAACTTG GTGTAGTTTA TTATTTACAC GGAAACCAAT CTGGTATACT   
  
  
- TTTTAGAAAC TTAGTTTAGC CAACCTGGAC TTGACGGTAA TAACTATAGA ACAAAAACCA TCTTCGTTTA   
  
  
- CCACAGTGAA GCAAAAGATC TATTTTAATT GAACTCAAAA AAACGGTGAT ATCTATCATA ACGTTTATAT   
  
  
- TTTTCACACG GTGCACTTTA GTTATAATCC TCCCACAAAA TATTGTTATC GACAAGCCCT CTCAAAATCA   
  
  
- ATAAAAATAA TTTAGTTTAT CAATTCTTCA CAAACCATTT ATCAACATAA ACTTTTTCAA TAAGGGTAAT   
  
  
- CGGAAAATCA TTCTTGAAGA ACACTTTTAC TATTAACAGA TCTTCAAACT TTTTAAGTGT GGTGTACTGT   
  
  
- AAATTTTTAT TAAAACATCT CTTCCCGTAT TTACTTTCAA ACGGTGGTAG AAAAAATCTT TGTACATTAA   
  
  
- ATGGTTTGTG AAAAAGATTT TTGTCAATTA AGTTTATTGA TTGTTGATTG TCGATTATAT TTATTGATTG   
  
  
- TCGATAAACG ATTTGTCCCG GATTCATCAA ACTTTAACAA CAAAAGAGAA GTCGAAGTGG TACCGGCTAC   
  
  
- TACATTTGTT GATATAAGAC TTAAAGTAAA AATGATACTA AACTTTTAAA GTTTAAATTT ATTCTAACCT   
  
  
- ATTTTTTTTC TGGTTTTTAG TATTCATTAT AATCAGTTCA AGATAAGGTT GGTATATGTA TTGACGAAAA   
  
  
- AGAAAACCCC CAAAACTTTG TACACAGGAA TCTTATTAAC TTTGAGCTAT AATAAGAGAT TTAGATTCAA   
  
  
- ATGAAATAGT ATAACTAATA ATTAATGGAG GAGTTGAGGA GGATATGAGA ACATACGTCT TTATTTTTAC   
  
  
- AAAGGTATGG AATAGTCAGC GCTAGTATTA TTAATTAAAG AATTAAGCCT AACATAAATT AACGCATAGT   
  
  
- TTACACTTTA GCATGGTTTA ATCCTTCGTT TTGTTTGGCT ATTGCTTTTT CCATTCTTTT GAATCGTTCC   
  
  
- TTTACTTTAG CTTCTGGACA GTTGTTATAT CGAAGGGAGT GTAGGAAGTT GAATCCCTGG TAGGATATGA   
  
  
- AAGTACATTA TGTTGATCTC GGTGATCAGT TATTATCGGA GGATCATCAT CGGTATTCGG AACCACTCTC   
  
  
- TTACAAGGCG TTCTGATCAA TTGAACTTTA AGGTAAAACA GCGTCTTGAT AACCTCACTC TTTCTCTTAG   
  
  
- TTTAGTATGG TATAGTTTAA ACCTGGTAAA TTCAAGTGTT GTGTGTGTGT ATGTGTGTTT TCCTGCTACA   
  
  
- TGCCGGGGTG GGGGGTGAGA GGAAAGCGGT AGGGGGATGT GGAGACGGAT GAGAGAGAGA GAGAGAGAGA   
  
  
- GAGACCTAGT CAGACACATT TTCGAAGGAG AGGTCATGTC GTAAAGGAAC AGACAAGAAC CTCAAATCAG   
  
  
- TTAAGGAGGC GGGGTTTTTT TGGGTGAAAG AGAGAGTAGT ACAAGAAGGT CAAGGTGGGA ATATGCGGTA   
  
  
- AGTAGTAGTA GATGTAGTGG GTGTAGAAAA AGAGGTGTCA AAAGGGGGGA GTAATACGAG AGGCGGAGGT   
  
  
- GGGGGCGGAA GGGAGAGCGG CAGCGGCCGC TACTGCCCCT ATGGTGACTA CGTACGCTGC CGTGGCGGTG   
  
  
- ACGGGAGGTG GAGGAGCCCG ACGACAACTT CACGCGCCTT AAGTAGAGGT GGCCTCTGGA GCGGCCGCGG   
  
  
- CCTCTATAGA ACGGCCTCTA TAAACTCAAC AGATGTGGCA AACCGAGGGG GCGGCGGGCC CAGCGGCGGA   
  
  
- AGAAGCCGGT GCGGGAGGTG CGGGCGGAGG AGAGGCGGAG GGAGGCGTGT TGAGGCTAGC TCTTCGAGTT   
  
  
- TTGGGACTGG GACCAAAGGG TTTACGCCTT TAAAGTGAGG CGGAACGTTC ATATGTTGAG GTAGTGAGGG   
  
  
- AAGCACTTTA AGAGAGTGAA GTGCCGCTTA GTTCGGTAGA TGCTCCGCGA CCTGCCGCTC CTAGCACAGG   
  
  
- TGCAGCAGCT GGAACTGTAG TACGTCCCGG AAGTTACCGG CCCTAACAAG GTGTAGAATC GGAGAGCTGG   
  
  
- GTCTTTCGGG GAGAGCCAAG CCCAGTGGCC CAAGCCCGGT AGGAGGCTCA ACGAGAGGGT TTGCCCATTC   
  
  
- GCTGAGCGAC TCAAGCGGCG AAGTGAGCCG GACGGGAAGC TCATGTTGGG CCACCTCCCG TTTTAACCCT   
  
  
- TGAATCAGCT GGACCCAGCC CAGCCCAGCG AGGGCTTACT TCACTGCCAC CACGTGACCT ACGTGGTATC   
  
  
- AGATATGCTG TATTGGCCCA GCCTAGAACC CTGAAACTCC CATAACTCAC GCCACTCCGG ATCCGAACAC   
  
  
- TGATACCAAC TCGTCCTATA CCTGGTTTGC CCCAGCAAAA ACCCCTCCAA ACACCTCCGT AACGTAATAA   
  
  
- TGAGTCGGAA CAAACTACGG AACCCTCTCC CCAATCTATC CCTATTGGAT GTTTCCGTAG TTCACCTCGT   
  
  
- TGTCGATAAA CCCACACTCT AATCCTTGTA GGACCGGCAA CCACCCGGGT TCTCCTCCGA GTGACCGCCG   
  
  
- CTAGCCCAGT CTTCCACCCC GCTGCTTGAC TGTTCCCAGC CCAAGCTTGG TCAAAGCAAC CGCCCATCGG   
  
  
- GCCGTTGGGT TCGATCAAAC AACGAACCCT ACAAGGGAAC CTTTCCCATA TGAAACTACC TCCTTTTACC   
  
  
- CACAAACTCT AATCCCACCT TTCTAAACGG GAACAATTGA CGGAGTCGAA CCGTTGGAAC ACTTAAATTG   
  
  
- TTAGGATCAC GACCGTAAAT

+     DRE1

| Site Name | Organism | Position | Strand | Matrix score. | sequence | function |
| --- | --- | --- | --- | --- | --- | --- |
| DRE1 | Zea mays | 2536 | - | 7 | ACCGAGA |  |

>HU02G03005.1   
+ +Up\_Stream \_Len000AAACGG TGCCGTTGAA TTTGCAGGCG ACGAGGTCGG GTTTAAGGCG GATGTCGTAG   
  
  
+ CGCTTTGGAG AAGCAAATTT GGGAAGACGA GGCTTATTCT TGAACTCTTC GTACGCCATT GTTGTTGGTT   
  
  
+ CCAACCTTTC TCTCTCCTCG TGAATTTGAG TTTGGGCAGT AGACTATCCC GGAATAGAGG ACTGATGCGT   
  
  
+ TATATATATA GAAATTGAGA GAGAGAGAGA GCGTGTGTGA AGGATGTTGA AAATGATGGG CGTAGGGAGT   
  
  
+ CTCTCTCTCA CTGTCTGTTA GGAGTACGTG TCGAGAAAGG AAATAGGATT CCCAAGCAGT CTAATTTGGG   
  
  
+ CCGGTCTATA GGCCAGAATG GGCCTATGAT CAGCCCAGAT ATGACCTTGT TTAACCAACT GTATTTGAAT   
  
  
+ TGAAGAAAGG GATATCGCAA TGTCTTGAAC CACATCAAAT AATAAATGTG CCTTTGGTTA GACCATATGA   
  
  
+ AAAATCTTTG AATCAAATCG GTTGGACCTG AACTGCCATT ATTGATATCT TGTTTTTGGT AGAAGCAAAT   
  
  
+ GGTGTCACTT CGTTTTCTAG ATAAAATTAA CTTGAGTTTT TTTGCCACTA TAGATAGTAT TGCAAATATA   
  
  
+ AAAAGTGTGC CACGTGAAAT CAATATTAGG AGGGTGTTTT ATAACAATAG CTGTTCGGGA GAGTTTTAGT   
  
  
+ TATTTTTATT AAATCAAATA GTTAAGAAGT GTTTGGTAAA TAGTTGTATT TGAAAAAGTT ATTCCCATTA   
  
  
+ GCCTTTTAGT AAGAACTTCT TGTGAAAATG ATAATTGTCT AGAAGTTTGA AAAATTCACA CCACATGACA   
  
  
+ TTTAAAAATA ATTTTGTAGA GAAGGGCATA AATGAAAGTT TGCCACCATC TTTTTTAGAA ACATGTAATT   
  
  
+ TACCAAACAC TTTTTCTAAA AACAGTTAAT TCAAATAACT AACAACTAAC AGCTAATATA AATAACTAAC   
  
  
+ AGCTATTTGC TAAACAGGGC CTAAGTAGTT TGAAATTGTT GTTTTCTCTT CAGCTTCACC ATGGCCGATG   
  
  
+ ATGTAAACAA CTATATTCTG AATTTCATTT TTACTATGAT TTGAAAATTT CAAATTTAAA TAAGATTGGA   
  
  
+ TAAAAAAAAG ACCAAAAATC ATAAGTAATA TTAGTCAAGT TCTATTCCAA CCATATACAT AACTGCTTTT   
  
  
+ TCTTTTGGGG GTTTTGAAAC ATGTGTCCTT AGAATAATTG AAACTCGATA TTATTCTCTA AATCTAAGTT   
  
  
+ TACTTTATCA TATTGATTAT TAATTACCTC CTCAACTCCT CCTATACTCT TGTATGCAGA AATAAAAATG   
  
  
+ TTTCCATACC TTATCAGTCG CGATCATAAT AATTAATTTC TTAATTCGGA TTGTATTTAA TTGCGTATCA   
  
  
+ AATGTGAAAT CGTACCAAAT TAGGAAGCAA AACAAACCGA TAACGAAAAA GGTAAGAAAA CTTAGCAAGG   
  
  
+ AAATGAAATC GAAGACCTGT CAACAATATA GCTTCCCTCA CATCCTTCAA CTTAGGGACC ATCCTATACT   
  
  
+ TTCATGTAAT ACAACTAGAG CCACTAGTCA ATAATAGCCT CCTAGTAGTA GCCATAAGCC TTGGTGAGAG   
  
  
+ AATGTTCCGC AAGACTAGTT AACTTGAAAT TCCATTTTGT CGCAGAACTA TTGGAGTGAG AAAGAGAATC   
  
  
+ AAATCATACC ATATCAAATT TGGACCATTT AAGTTCACAA CACACACACA TACACACAAA AGGACGATGT   
  
  
+ ACGGCCCCAC CCCCCACTCT CCTTTCGCCA TCCCCCTACA CCTCTGCCTA CTCTCTCTCT CTCTCTCTCT   
  
  
+ CTCTGGATCA GTCTGTGTAA AAGCTTCCTC TCCAGTACAG CATTTCCTTG TCTGTTCTTG GAGTTTAGTC   
  
  
+ AATTCCTCCG CCCCAAAAAA ACCCACTTTC TCTCTCATCA TGTTCTTCCA GTTCCACCCT TATACGCCAT   
  
  
+ TCATCATCAT CTACATCACC CACATCTTTT TCTCCACAGT TTTCCCCCCT CATTATGCTC TCCGCCTCCA   
  
  
+ CCCCCGCCTT CCCTCTCGCC GTCGCCGGCG ATGACGGGGA TACCACTGAT GCATGCGACG GCACCGCCAC   
  
  
+ TGCCCTCCAC CTCCTCGGGC TGCTGTTGAA GTGCGCGGAA TTCATCTCCA CCGGAGACCT CGCCGGCGCC   
  
  
+ GGAGATATCT TGCCGGAGAT ATTTGAGTTG TCTACACCGT TTGGCTCCCC CGCCGCCCGG GTCGCCGCCT   
  
  
+ TCTTCGGCCA CGCCCTCCAC GCCCGCCTCC TCTCCGCCTC CCTCCGCACA ACTCCGATCG AGAAGCTCAA   
  
  
+ AACCCTGACC CTGGTTTCCC AAATGCGGAA ATTTCACTCC GCCTTGCAAG TATACAACTC CATCACTCCC   
  
  
+ TTCGTGAAAT TCTCTCACTT CACGGCGAAT CAAGCCATCT ACGAGGCGCT GGACGGCGAG GATCGTGTCC   
  
  
+ ACGTCGTCGA CCTTGACATC ATGCAGGGCC TTCAATGGCC GGGATTGTTC CACATCTTAG CCTCTCGACC   
  
  
+ CAGAAAGCCC CTCTCGGTTC GGGTCACCGG GTTCGGGCCA TCCTCCGAGT TGCTCTCCCA AACGGGTAAG   
  
  
+ CGACTCGCTG AGTTCGCCGC TTCACTCGGC CTGCCCTTCG AGTACAACCC GGTGGAGGGC AAAATTGGGA   
  
  
+ ACTTAGTCGA CCTGGGTCGG GTCGGGTCGC TCCCGAATGA AGTGACGGTG GTGCACTGGA TGCACCATAG   
  
  
+ TCTATACGAC ATAACCGGGT CGGATCTTGG GACTTTGAGG GTATTGAGTG CGGTGAGGCC TAGGCTTGTG   
  
  
+ ACTATGGTTG AGCAGGATAT GGACCAAACG GGGTCGTTTT TGGGGAGGTT TGTGGAGGCA TTGCATTATT   
  
  
+ ACTCAGCCTT GTTTGATGCC TTGGGAGAGG GGTTAGATAG GGATAACCTA CAAAGGCATC AAGTGGAGCA   
  
  
+ ACAGCTATTT GGGTGTGAGA TTAGGAACAT CCTGGCCGTT GGTGGGCCCA AGAGGAGGCT CACTGGCGGC   
  
  
+ GATCGGGTCA GAAGGTGGGG CGACGAACTG ACAAGGGTCG GGTTCGAACC AGTTTCGTTG GCGGGTAGCC   
  
  
+ CGGCAACCCA AGCTAGTTTG TTGCTTGGGA TGTTCCCTTG GAAAGGGTAT ACTTTGATGG AGGAAAATGG   
  
  
+ GTGTTTGAGA TTAGGGTGGA AAGATTTGCC CTTGTTAACT GCCTCAGCTT GGCAACCTTG TGAATTTAAC   
  
  
+ AATCCTAGTG CTGGCATTTA   

- +Up\_Stream \_Len000TTTGCC ACGGCAACTT AAACGTCCGC TGCTCCAGCC CAAATTCCGC CTACAGCATC   
  
  
- GCGAAACCTC TTCGTTTAAA CCCTTCTGCT CCGAATAAGA ACTTGAGAAG CATGCGGTAA CAACAACCAA   
  
  
- GGTTGGAAAG AGAGAGGAGC ACTTAAACTC AAACCCGTCA TCTGATAGGG CCTTATCTCC TGACTACGCA   
  
  
- ATATATATAT CTTTAACTCT CTCTCTCTCT CGCACACACT TCCTACAACT TTTACTACCC GCATCCCTCA   
  
  
- GAGAGAGAGT GACAGACAAT CCTCATGCAC AGCTCTTTCC TTTATCCTAA GGGTTCGTCA GATTAAACCC   
  
  
- GGCCAGATAT CCGGTCTTAC CCGGATACTA GTCGGGTCTA TACTGGAACA AATTGGTTGA CATAAACTTA   
  
  
- ACTTCTTTCC CTATAGCGTT ACAGAACTTG GTGTAGTTTA TTATTTACAC GGAAACCAAT CTGGTATACT   
  
  
- TTTTAGAAAC TTAGTTTAGC CAACCTGGAC TTGACGGTAA TAACTATAGA ACAAAAACCA TCTTCGTTTA   
  
  
- CCACAGTGAA GCAAAAGATC TATTTTAATT GAACTCAAAA AAACGGTGAT ATCTATCATA ACGTTTATAT   
  
  
- TTTTCACACG GTGCACTTTA GTTATAATCC TCCCACAAAA TATTGTTATC GACAAGCCCT CTCAAAATCA   
  
  
- ATAAAAATAA TTTAGTTTAT CAATTCTTCA CAAACCATTT ATCAACATAA ACTTTTTCAA TAAGGGTAAT   
  
  
- CGGAAAATCA TTCTTGAAGA ACACTTTTAC TATTAACAGA TCTTCAAACT TTTTAAGTGT GGTGTACTGT   
  
  
- AAATTTTTAT TAAAACATCT CTTCCCGTAT TTACTTTCAA ACGGTGGTAG AAAAAATCTT TGTACATTAA   
  
  
- ATGGTTTGTG AAAAAGATTT TTGTCAATTA AGTTTATTGA TTGTTGATTG TCGATTATAT TTATTGATTG   
  
  
- TCGATAAACG ATTTGTCCCG GATTCATCAA ACTTTAACAA CAAAAGAGAA GTCGAAGTGG TACCGGCTAC   
  
  
- TACATTTGTT GATATAAGAC TTAAAGTAAA AATGATACTA AACTTTTAAA GTTTAAATTT ATTCTAACCT   
  
  
- ATTTTTTTTC TGGTTTTTAG TATTCATTAT AATCAGTTCA AGATAAGGTT GGTATATGTA TTGACGAAAA   
  
  
- AGAAAACCCC CAAAACTTTG TACACAGGAA TCTTATTAAC TTTGAGCTAT AATAAGAGAT TTAGATTCAA   
  
  
- ATGAAATAGT ATAACTAATA ATTAATGGAG GAGTTGAGGA GGATATGAGA ACATACGTCT TTATTTTTAC   
  
  
- AAAGGTATGG AATAGTCAGC GCTAGTATTA TTAATTAAAG AATTAAGCCT AACATAAATT AACGCATAGT   
  
  
- TTACACTTTA GCATGGTTTA ATCCTTCGTT TTGTTTGGCT ATTGCTTTTT CCATTCTTTT GAATCGTTCC   
  
  
- TTTACTTTAG CTTCTGGACA GTTGTTATAT CGAAGGGAGT GTAGGAAGTT GAATCCCTGG TAGGATATGA   
  
  
- AAGTACATTA TGTTGATCTC GGTGATCAGT TATTATCGGA GGATCATCAT CGGTATTCGG AACCACTCTC   
  
  
- TTACAAGGCG TTCTGATCAA TTGAACTTTA AGGTAAAACA GCGTCTTGAT AACCTCACTC TTTCTCTTAG   
  
  
- TTTAGTATGG TATAGTTTAA ACCTGGTAAA TTCAAGTGTT GTGTGTGTGT ATGTGTGTTT TCCTGCTACA   
  
  
- TGCCGGGGTG GGGGGTGAGA GGAAAGCGGT AGGGGGATGT GGAGACGGAT GAGAGAGAGA GAGAGAGAGA   
  
  
- GAGACCTAGT CAGACACATT TTCGAAGGAG AGGTCATGTC GTAAAGGAAC AGACAAGAAC CTCAAATCAG   
  
  
- TTAAGGAGGC GGGGTTTTTT TGGGTGAAAG AGAGAGTAGT ACAAGAAGGT CAAGGTGGGA ATATGCGGTA   
  
  
- AGTAGTAGTA GATGTAGTGG GTGTAGAAAA AGAGGTGTCA AAAGGGGGGA GTAATACGAG AGGCGGAGGT   
  
  
- GGGGGCGGAA GGGAGAGCGG CAGCGGCCGC TACTGCCCCT ATGGTGACTA CGTACGCTGC CGTGGCGGTG   
  
  
- ACGGGAGGTG GAGGAGCCCG ACGACAACTT CACGCGCCTT AAGTAGAGGT GGCCTCTGGA GCGGCCGCGG   
  
  
- CCTCTATAGA ACGGCCTCTA TAAACTCAAC AGATGTGGCA AACCGAGGGG GCGGCGGGCC CAGCGGCGGA   
  
  
- AGAAGCCGGT GCGGGAGGTG CGGGCGGAGG AGAGGCGGAG GGAGGCGTGT TGAGGCTAGC TCTTCGAGTT   
  
  
- TTGGGACTGG GACCAAAGGG TTTACGCCTT TAAAGTGAGG CGGAACGTTC ATATGTTGAG GTAGTGAGGG   
  
  
- AAGCACTTTA AGAGAGTGAA GTGCCGCTTA GTTCGGTAGA TGCTCCGCGA CCTGCCGCTC CTAGCACAGG   
  
  
- TGCAGCAGCT GGAACTGTAG TACGTCCCGG AAGTTACCGG CCCTAACAAG GTGTAGAATC GGAGAGCTGG   
  
  
- GTCTTTCGGG GAGAGCCAAG CCCAGTGGCC CAAGCCCGGT AGGAGGCTCA ACGAGAGGGT TTGCCCATTC   
  
  
- GCTGAGCGAC TCAAGCGGCG AAGTGAGCCG GACGGGAAGC TCATGTTGGG CCACCTCCCG TTTTAACCCT   
  
  
- TGAATCAGCT GGACCCAGCC CAGCCCAGCG AGGGCTTACT TCACTGCCAC CACGTGACCT ACGTGGTATC   
  
  
- AGATATGCTG TATTGGCCCA GCCTAGAACC CTGAAACTCC CATAACTCAC GCCACTCCGG ATCCGAACAC   
  
  
- TGATACCAAC TCGTCCTATA CCTGGTTTGC CCCAGCAAAA ACCCCTCCAA ACACCTCCGT AACGTAATAA   
  
  
- TGAGTCGGAA CAAACTACGG AACCCTCTCC CCAATCTATC CCTATTGGAT GTTTCCGTAG TTCACCTCGT   
  
  
- TGTCGATAAA CCCACACTCT AATCCTTGTA GGACCGGCAA CCACCCGGGT TCTCCTCCGA GTGACCGCCG   
  
  
- CTAGCCCAGT CTTCCACCCC GCTGCTTGAC TGTTCCCAGC CCAAGCTTGG TCAAAGCAAC CGCCCATCGG   
  
  
- GCCGTTGGGT TCGATCAAAC AACGAACCCT ACAAGGGAAC CTTTCCCATA TGAAACTACC TCCTTTTACC   
  
  
- CACAAACTCT AATCCCACCT TTCTAAACGG GAACAATTGA CGGAGTCGAA CCGTTGGAAC ACTTAAATTG   
  
  
- TTAGGATCAC GACCGTAAAT

+     G-Box

| Site Name | Organism | Position | Strand | Matrix score. | sequence | function |
| --- | --- | --- | --- | --- | --- | --- |
| G-Box | Pisum sativum | 645 | + | 6 | CACGTG | cis-acting regulatory element involved in light responsiveness |

>HU02G03005.1   
+ +Up\_Stream \_Len000AAACGG TGCCGTTGAA TTTGCAGGCG ACGAGGTCGG GTTTAAGGCG GATGTCGTAG   
  
  
+ CGCTTTGGAG AAGCAAATTT GGGAAGACGA GGCTTATTCT TGAACTCTTC GTACGCCATT GTTGTTGGTT   
  
  
+ CCAACCTTTC TCTCTCCTCG TGAATTTGAG TTTGGGCAGT AGACTATCCC GGAATAGAGG ACTGATGCGT   
  
  
+ TATATATATA GAAATTGAGA GAGAGAGAGA GCGTGTGTGA AGGATGTTGA AAATGATGGG CGTAGGGAGT   
  
  
+ CTCTCTCTCA CTGTCTGTTA GGAGTACGTG TCGAGAAAGG AAATAGGATT CCCAAGCAGT CTAATTTGGG   
  
  
+ CCGGTCTATA GGCCAGAATG GGCCTATGAT CAGCCCAGAT ATGACCTTGT TTAACCAACT GTATTTGAAT   
  
  
+ TGAAGAAAGG GATATCGCAA TGTCTTGAAC CACATCAAAT AATAAATGTG CCTTTGGTTA GACCATATGA   
  
  
+ AAAATCTTTG AATCAAATCG GTTGGACCTG AACTGCCATT ATTGATATCT TGTTTTTGGT AGAAGCAAAT   
  
  
+ GGTGTCACTT CGTTTTCTAG ATAAAATTAA CTTGAGTTTT TTTGCCACTA TAGATAGTAT TGCAAATATA   
  
  
+ AAAAGTGTGC CACGTGAAAT CAATATTAGG AGGGTGTTTT ATAACAATAG CTGTTCGGGA GAGTTTTAGT   
  
  
+ TATTTTTATT AAATCAAATA GTTAAGAAGT GTTTGGTAAA TAGTTGTATT TGAAAAAGTT ATTCCCATTA   
  
  
+ GCCTTTTAGT AAGAACTTCT TGTGAAAATG ATAATTGTCT AGAAGTTTGA AAAATTCACA CCACATGACA   
  
  
+ TTTAAAAATA ATTTTGTAGA GAAGGGCATA AATGAAAGTT TGCCACCATC TTTTTTAGAA ACATGTAATT   
  
  
+ TACCAAACAC TTTTTCTAAA AACAGTTAAT TCAAATAACT AACAACTAAC AGCTAATATA AATAACTAAC   
  
  
+ AGCTATTTGC TAAACAGGGC CTAAGTAGTT TGAAATTGTT GTTTTCTCTT CAGCTTCACC ATGGCCGATG   
  
  
+ ATGTAAACAA CTATATTCTG AATTTCATTT TTACTATGAT TTGAAAATTT CAAATTTAAA TAAGATTGGA   
  
  
+ TAAAAAAAAG ACCAAAAATC ATAAGTAATA TTAGTCAAGT TCTATTCCAA CCATATACAT AACTGCTTTT   
  
  
+ TCTTTTGGGG GTTTTGAAAC ATGTGTCCTT AGAATAATTG AAACTCGATA TTATTCTCTA AATCTAAGTT   
  
  
+ TACTTTATCA TATTGATTAT TAATTACCTC CTCAACTCCT CCTATACTCT TGTATGCAGA AATAAAAATG   
  
  
+ TTTCCATACC TTATCAGTCG CGATCATAAT AATTAATTTC TTAATTCGGA TTGTATTTAA TTGCGTATCA   
  
  
+ AATGTGAAAT CGTACCAAAT TAGGAAGCAA AACAAACCGA TAACGAAAAA GGTAAGAAAA CTTAGCAAGG   
  
  
+ AAATGAAATC GAAGACCTGT CAACAATATA GCTTCCCTCA CATCCTTCAA CTTAGGGACC ATCCTATACT   
  
  
+ TTCATGTAAT ACAACTAGAG CCACTAGTCA ATAATAGCCT CCTAGTAGTA GCCATAAGCC TTGGTGAGAG   
  
  
+ AATGTTCCGC AAGACTAGTT AACTTGAAAT TCCATTTTGT CGCAGAACTA TTGGAGTGAG AAAGAGAATC   
  
  
+ AAATCATACC ATATCAAATT TGGACCATTT AAGTTCACAA CACACACACA TACACACAAA AGGACGATGT   
  
  
+ ACGGCCCCAC CCCCCACTCT CCTTTCGCCA TCCCCCTACA CCTCTGCCTA CTCTCTCTCT CTCTCTCTCT   
  
  
+ CTCTGGATCA GTCTGTGTAA AAGCTTCCTC TCCAGTACAG CATTTCCTTG TCTGTTCTTG GAGTTTAGTC   
  
  
+ AATTCCTCCG CCCCAAAAAA ACCCACTTTC TCTCTCATCA TGTTCTTCCA GTTCCACCCT TATACGCCAT   
  
  
+ TCATCATCAT CTACATCACC CACATCTTTT TCTCCACAGT TTTCCCCCCT CATTATGCTC TCCGCCTCCA   
  
  
+ CCCCCGCCTT CCCTCTCGCC GTCGCCGGCG ATGACGGGGA TACCACTGAT GCATGCGACG GCACCGCCAC   
  
  
+ TGCCCTCCAC CTCCTCGGGC TGCTGTTGAA GTGCGCGGAA TTCATCTCCA CCGGAGACCT CGCCGGCGCC   
  
  
+ GGAGATATCT TGCCGGAGAT ATTTGAGTTG TCTACACCGT TTGGCTCCCC CGCCGCCCGG GTCGCCGCCT   
  
  
+ TCTTCGGCCA CGCCCTCCAC GCCCGCCTCC TCTCCGCCTC CCTCCGCACA ACTCCGATCG AGAAGCTCAA   
  
  
+ AACCCTGACC CTGGTTTCCC AAATGCGGAA ATTTCACTCC GCCTTGCAAG TATACAACTC CATCACTCCC   
  
  
+ TTCGTGAAAT TCTCTCACTT CACGGCGAAT CAAGCCATCT ACGAGGCGCT GGACGGCGAG GATCGTGTCC   
  
  
+ ACGTCGTCGA CCTTGACATC ATGCAGGGCC TTCAATGGCC GGGATTGTTC CACATCTTAG CCTCTCGACC   
  
  
+ CAGAAAGCCC CTCTCGGTTC GGGTCACCGG GTTCGGGCCA TCCTCCGAGT TGCTCTCCCA AACGGGTAAG   
  
  
+ CGACTCGCTG AGTTCGCCGC TTCACTCGGC CTGCCCTTCG AGTACAACCC GGTGGAGGGC AAAATTGGGA   
  
  
+ ACTTAGTCGA CCTGGGTCGG GTCGGGTCGC TCCCGAATGA AGTGACGGTG GTGCACTGGA TGCACCATAG   
  
  
+ TCTATACGAC ATAACCGGGT CGGATCTTGG GACTTTGAGG GTATTGAGTG CGGTGAGGCC TAGGCTTGTG   
  
  
+ ACTATGGTTG AGCAGGATAT GGACCAAACG GGGTCGTTTT TGGGGAGGTT TGTGGAGGCA TTGCATTATT   
  
  
+ ACTCAGCCTT GTTTGATGCC TTGGGAGAGG GGTTAGATAG GGATAACCTA CAAAGGCATC AAGTGGAGCA   
  
  
+ ACAGCTATTT GGGTGTGAGA TTAGGAACAT CCTGGCCGTT GGTGGGCCCA AGAGGAGGCT CACTGGCGGC   
  
  
+ GATCGGGTCA GAAGGTGGGG CGACGAACTG ACAAGGGTCG GGTTCGAACC AGTTTCGTTG GCGGGTAGCC   
  
  
+ CGGCAACCCA AGCTAGTTTG TTGCTTGGGA TGTTCCCTTG GAAAGGGTAT ACTTTGATGG AGGAAAATGG   
  
  
+ GTGTTTGAGA TTAGGGTGGA AAGATTTGCC CTTGTTAACT GCCTCAGCTT GGCAACCTTG TGAATTTAAC   
  
  
+ AATCCTAGTG CTGGCATTTA   

- +Up\_Stream \_Len000TTTGCC ACGGCAACTT AAACGTCCGC TGCTCCAGCC CAAATTCCGC CTACAGCATC   
  
  
- GCGAAACCTC TTCGTTTAAA CCCTTCTGCT CCGAATAAGA ACTTGAGAAG CATGCGGTAA CAACAACCAA   
  
  
- GGTTGGAAAG AGAGAGGAGC ACTTAAACTC AAACCCGTCA TCTGATAGGG CCTTATCTCC TGACTACGCA   
  
  
- ATATATATAT CTTTAACTCT CTCTCTCTCT CGCACACACT TCCTACAACT TTTACTACCC GCATCCCTCA   
  
  
- GAGAGAGAGT GACAGACAAT CCTCATGCAC AGCTCTTTCC TTTATCCTAA GGGTTCGTCA GATTAAACCC   
  
  
- GGCCAGATAT CCGGTCTTAC CCGGATACTA GTCGGGTCTA TACTGGAACA AATTGGTTGA CATAAACTTA   
  
  
- ACTTCTTTCC CTATAGCGTT ACAGAACTTG GTGTAGTTTA TTATTTACAC GGAAACCAAT CTGGTATACT   
  
  
- TTTTAGAAAC TTAGTTTAGC CAACCTGGAC TTGACGGTAA TAACTATAGA ACAAAAACCA TCTTCGTTTA   
  
  
- CCACAGTGAA GCAAAAGATC TATTTTAATT GAACTCAAAA AAACGGTGAT ATCTATCATA ACGTTTATAT   
  
  
- TTTTCACACG GTGCACTTTA GTTATAATCC TCCCACAAAA TATTGTTATC GACAAGCCCT CTCAAAATCA   
  
  
- ATAAAAATAA TTTAGTTTAT CAATTCTTCA CAAACCATTT ATCAACATAA ACTTTTTCAA TAAGGGTAAT   
  
  
- CGGAAAATCA TTCTTGAAGA ACACTTTTAC TATTAACAGA TCTTCAAACT TTTTAAGTGT GGTGTACTGT   
  
  
- AAATTTTTAT TAAAACATCT CTTCCCGTAT TTACTTTCAA ACGGTGGTAG AAAAAATCTT TGTACATTAA   
  
  
- ATGGTTTGTG AAAAAGATTT TTGTCAATTA AGTTTATTGA TTGTTGATTG TCGATTATAT TTATTGATTG   
  
  
- TCGATAAACG ATTTGTCCCG GATTCATCAA ACTTTAACAA CAAAAGAGAA GTCGAAGTGG TACCGGCTAC   
  
  
- TACATTTGTT GATATAAGAC TTAAAGTAAA AATGATACTA AACTTTTAAA GTTTAAATTT ATTCTAACCT   
  
  
- ATTTTTTTTC TGGTTTTTAG TATTCATTAT AATCAGTTCA AGATAAGGTT GGTATATGTA TTGACGAAAA   
  
  
- AGAAAACCCC CAAAACTTTG TACACAGGAA TCTTATTAAC TTTGAGCTAT AATAAGAGAT TTAGATTCAA   
  
  
- ATGAAATAGT ATAACTAATA ATTAATGGAG GAGTTGAGGA GGATATGAGA ACATACGTCT TTATTTTTAC   
  
  
- AAAGGTATGG AATAGTCAGC GCTAGTATTA TTAATTAAAG AATTAAGCCT AACATAAATT AACGCATAGT   
  
  
- TTACACTTTA GCATGGTTTA ATCCTTCGTT TTGTTTGGCT ATTGCTTTTT CCATTCTTTT GAATCGTTCC   
  
  
- TTTACTTTAG CTTCTGGACA GTTGTTATAT CGAAGGGAGT GTAGGAAGTT GAATCCCTGG TAGGATATGA   
  
  
- AAGTACATTA TGTTGATCTC GGTGATCAGT TATTATCGGA GGATCATCAT CGGTATTCGG AACCACTCTC   
  
  
- TTACAAGGCG TTCTGATCAA TTGAACTTTA AGGTAAAACA GCGTCTTGAT AACCTCACTC TTTCTCTTAG   
  
  
- TTTAGTATGG TATAGTTTAA ACCTGGTAAA TTCAAGTGTT GTGTGTGTGT ATGTGTGTTT TCCTGCTACA   
  
  
- TGCCGGGGTG GGGGGTGAGA GGAAAGCGGT AGGGGGATGT GGAGACGGAT GAGAGAGAGA GAGAGAGAGA   
  
  
- GAGACCTAGT CAGACACATT TTCGAAGGAG AGGTCATGTC GTAAAGGAAC AGACAAGAAC CTCAAATCAG   
  
  
- TTAAGGAGGC GGGGTTTTTT TGGGTGAAAG AGAGAGTAGT ACAAGAAGGT CAAGGTGGGA ATATGCGGTA   
  
  
- AGTAGTAGTA GATGTAGTGG GTGTAGAAAA AGAGGTGTCA AAAGGGGGGA GTAATACGAG AGGCGGAGGT   
  
  
- GGGGGCGGAA GGGAGAGCGG CAGCGGCCGC TACTGCCCCT ATGGTGACTA CGTACGCTGC CGTGGCGGTG   
  
  
- ACGGGAGGTG GAGGAGCCCG ACGACAACTT CACGCGCCTT AAGTAGAGGT GGCCTCTGGA GCGGCCGCGG   
  
  
- CCTCTATAGA ACGGCCTCTA TAAACTCAAC AGATGTGGCA AACCGAGGGG GCGGCGGGCC CAGCGGCGGA   
  
  
- AGAAGCCGGT GCGGGAGGTG CGGGCGGAGG AGAGGCGGAG GGAGGCGTGT TGAGGCTAGC TCTTCGAGTT   
  
  
- TTGGGACTGG GACCAAAGGG TTTACGCCTT TAAAGTGAGG CGGAACGTTC ATATGTTGAG GTAGTGAGGG   
  
  
- AAGCACTTTA AGAGAGTGAA GTGCCGCTTA GTTCGGTAGA TGCTCCGCGA CCTGCCGCTC CTAGCACAGG   
  
  
- TGCAGCAGCT GGAACTGTAG TACGTCCCGG AAGTTACCGG CCCTAACAAG GTGTAGAATC GGAGAGCTGG   
  
  
- GTCTTTCGGG GAGAGCCAAG CCCAGTGGCC CAAGCCCGGT AGGAGGCTCA ACGAGAGGGT TTGCCCATTC   
  
  
- GCTGAGCGAC TCAAGCGGCG AAGTGAGCCG GACGGGAAGC TCATGTTGGG CCACCTCCCG TTTTAACCCT   
  
  
- TGAATCAGCT GGACCCAGCC CAGCCCAGCG AGGGCTTACT TCACTGCCAC CACGTGACCT ACGTGGTATC   
  
  
- AGATATGCTG TATTGGCCCA GCCTAGAACC CTGAAACTCC CATAACTCAC GCCACTCCGG ATCCGAACAC   
  
  
- TGATACCAAC TCGTCCTATA CCTGGTTTGC CCCAGCAAAA ACCCCTCCAA ACACCTCCGT AACGTAATAA   
  
  
- TGAGTCGGAA CAAACTACGG AACCCTCTCC CCAATCTATC CCTATTGGAT GTTTCCGTAG TTCACCTCGT   
  
  
- TGTCGATAAA CCCACACTCT AATCCTTGTA GGACCGGCAA CCACCCGGGT TCTCCTCCGA GTGACCGCCG   
  
  
- CTAGCCCAGT CTTCCACCCC GCTGCTTGAC TGTTCCCAGC CCAAGCTTGG TCAAAGCAAC CGCCCATCGG   
  
  
- GCCGTTGGGT TCGATCAAAC AACGAACCCT ACAAGGGAAC CTTTCCCATA TGAAACTACC TCCTTTTACC   
  
  
- CACAAACTCT AATCCCACCT TTCTAAACGG GAACAATTGA CGGAGTCGAA CCGTTGGAAC ACTTAAATTG   
  
  
- TTAGGATCAC GACCGTAAAT

+     G-box

| Site Name | Organism | Position | Strand | Matrix score. | sequence | function |
| --- | --- | --- | --- | --- | --- | --- |
| G-box | Arabidopsis thaliana | 645 | + | 6 | CACGTG | cis-acting regulatory element involved in light responsiveness |
| G-box | Arabidopsis thaliana | 643 | + | 9 | GCCACGTGGA | cis-acting regulatory element involved in light responsiveness |
| G-box | Arabidopsis thaliana | 309 | + | 6 | TACGTG | cis-acting regulatory element involved in light responsiveness |
| G-box | Zea mays | 2454 | + | 6 | CACGTC | cis-acting regulatory element involved in light responsiveness |

>HU02G03005.1   
+ +Up\_Stream \_Len000AAACGG TGCCGTTGAA TTTGCAGGCG ACGAGGTCGG GTTTAAGGCG GATGTCGTAG   
  
  
+ CGCTTTGGAG AAGCAAATTT GGGAAGACGA GGCTTATTCT TGAACTCTTC GTACGCCATT GTTGTTGGTT   
  
  
+ CCAACCTTTC TCTCTCCTCG TGAATTTGAG TTTGGGCAGT AGACTATCCC GGAATAGAGG ACTGATGCGT   
  
  
+ TATATATATA GAAATTGAGA GAGAGAGAGA GCGTGTGTGA AGGATGTTGA AAATGATGGG CGTAGGGAGT   
  
  
+ CTCTCTCTCA CTGTCTGTTA GGAGTACGTG TCGAGAAAGG AAATAGGATT CCCAAGCAGT CTAATTTGGG   
  
  
+ CCGGTCTATA GGCCAGAATG GGCCTATGAT CAGCCCAGAT ATGACCTTGT TTAACCAACT GTATTTGAAT   
  
  
+ TGAAGAAAGG GATATCGCAA TGTCTTGAAC CACATCAAAT AATAAATGTG CCTTTGGTTA GACCATATGA   
  
  
+ AAAATCTTTG AATCAAATCG GTTGGACCTG AACTGCCATT ATTGATATCT TGTTTTTGGT AGAAGCAAAT   
  
  
+ GGTGTCACTT CGTTTTCTAG ATAAAATTAA CTTGAGTTTT TTTGCCACTA TAGATAGTAT TGCAAATATA   
  
  
+ AAAAGTGTGC CACGTGAAAT CAATATTAGG AGGGTGTTTT ATAACAATAG CTGTTCGGGA GAGTTTTAGT   
  
  
+ TATTTTTATT AAATCAAATA GTTAAGAAGT GTTTGGTAAA TAGTTGTATT TGAAAAAGTT ATTCCCATTA   
  
  
+ GCCTTTTAGT AAGAACTTCT TGTGAAAATG ATAATTGTCT AGAAGTTTGA AAAATTCACA CCACATGACA   
  
  
+ TTTAAAAATA ATTTTGTAGA GAAGGGCATA AATGAAAGTT TGCCACCATC TTTTTTAGAA ACATGTAATT   
  
  
+ TACCAAACAC TTTTTCTAAA AACAGTTAAT TCAAATAACT AACAACTAAC AGCTAATATA AATAACTAAC   
  
  
+ AGCTATTTGC TAAACAGGGC CTAAGTAGTT TGAAATTGTT GTTTTCTCTT CAGCTTCACC ATGGCCGATG   
  
  
+ ATGTAAACAA CTATATTCTG AATTTCATTT TTACTATGAT TTGAAAATTT CAAATTTAAA TAAGATTGGA   
  
  
+ TAAAAAAAAG ACCAAAAATC ATAAGTAATA TTAGTCAAGT TCTATTCCAA CCATATACAT AACTGCTTTT   
  
  
+ TCTTTTGGGG GTTTTGAAAC ATGTGTCCTT AGAATAATTG AAACTCGATA TTATTCTCTA AATCTAAGTT   
  
  
+ TACTTTATCA TATTGATTAT TAATTACCTC CTCAACTCCT CCTATACTCT TGTATGCAGA AATAAAAATG   
  
  
+ TTTCCATACC TTATCAGTCG CGATCATAAT AATTAATTTC TTAATTCGGA TTGTATTTAA TTGCGTATCA   
  
  
+ AATGTGAAAT CGTACCAAAT TAGGAAGCAA AACAAACCGA TAACGAAAAA GGTAAGAAAA CTTAGCAAGG   
  
  
+ AAATGAAATC GAAGACCTGT CAACAATATA GCTTCCCTCA CATCCTTCAA CTTAGGGACC ATCCTATACT   
  
  
+ TTCATGTAAT ACAACTAGAG CCACTAGTCA ATAATAGCCT CCTAGTAGTA GCCATAAGCC TTGGTGAGAG   
  
  
+ AATGTTCCGC AAGACTAGTT AACTTGAAAT TCCATTTTGT CGCAGAACTA TTGGAGTGAG AAAGAGAATC   
  
  
+ AAATCATACC ATATCAAATT TGGACCATTT AAGTTCACAA CACACACACA TACACACAAA AGGACGATGT   
  
  
+ ACGGCCCCAC CCCCCACTCT CCTTTCGCCA TCCCCCTACA CCTCTGCCTA CTCTCTCTCT CTCTCTCTCT   
  
  
+ CTCTGGATCA GTCTGTGTAA AAGCTTCCTC TCCAGTACAG CATTTCCTTG TCTGTTCTTG GAGTTTAGTC   
  
  
+ AATTCCTCCG CCCCAAAAAA ACCCACTTTC TCTCTCATCA TGTTCTTCCA GTTCCACCCT TATACGCCAT   
  
  
+ TCATCATCAT CTACATCACC CACATCTTTT TCTCCACAGT TTTCCCCCCT CATTATGCTC TCCGCCTCCA   
  
  
+ CCCCCGCCTT CCCTCTCGCC GTCGCCGGCG ATGACGGGGA TACCACTGAT GCATGCGACG GCACCGCCAC   
  
  
+ TGCCCTCCAC CTCCTCGGGC TGCTGTTGAA GTGCGCGGAA TTCATCTCCA CCGGAGACCT CGCCGGCGCC   
  
  
+ GGAGATATCT TGCCGGAGAT ATTTGAGTTG TCTACACCGT TTGGCTCCCC CGCCGCCCGG GTCGCCGCCT   
  
  
+ TCTTCGGCCA CGCCCTCCAC GCCCGCCTCC TCTCCGCCTC CCTCCGCACA ACTCCGATCG AGAAGCTCAA   
  
  
+ AACCCTGACC CTGGTTTCCC AAATGCGGAA ATTTCACTCC GCCTTGCAAG TATACAACTC CATCACTCCC   
  
  
+ TTCGTGAAAT TCTCTCACTT CACGGCGAAT CAAGCCATCT ACGAGGCGCT GGACGGCGAG GATCGTGTCC   
  
  
+ ACGTCGTCGA CCTTGACATC ATGCAGGGCC TTCAATGGCC GGGATTGTTC CACATCTTAG CCTCTCGACC   
  
  
+ CAGAAAGCCC CTCTCGGTTC GGGTCACCGG GTTCGGGCCA TCCTCCGAGT TGCTCTCCCA AACGGGTAAG   
  
  
+ CGACTCGCTG AGTTCGCCGC TTCACTCGGC CTGCCCTTCG AGTACAACCC GGTGGAGGGC AAAATTGGGA   
  
  
+ ACTTAGTCGA CCTGGGTCGG GTCGGGTCGC TCCCGAATGA AGTGACGGTG GTGCACTGGA TGCACCATAG   
  
  
+ TCTATACGAC ATAACCGGGT CGGATCTTGG GACTTTGAGG GTATTGAGTG CGGTGAGGCC TAGGCTTGTG   
  
  
+ ACTATGGTTG AGCAGGATAT GGACCAAACG GGGTCGTTTT TGGGGAGGTT TGTGGAGGCA TTGCATTATT   
  
  
+ ACTCAGCCTT GTTTGATGCC TTGGGAGAGG GGTTAGATAG GGATAACCTA CAAAGGCATC AAGTGGAGCA   
  
  
+ ACAGCTATTT GGGTGTGAGA TTAGGAACAT CCTGGCCGTT GGTGGGCCCA AGAGGAGGCT CACTGGCGGC   
  
  
+ GATCGGGTCA GAAGGTGGGG CGACGAACTG ACAAGGGTCG GGTTCGAACC AGTTTCGTTG GCGGGTAGCC   
  
  
+ CGGCAACCCA AGCTAGTTTG TTGCTTGGGA TGTTCCCTTG GAAAGGGTAT ACTTTGATGG AGGAAAATGG   
  
  
+ GTGTTTGAGA TTAGGGTGGA AAGATTTGCC CTTGTTAACT GCCTCAGCTT GGCAACCTTG TGAATTTAAC   
  
  
+ AATCCTAGTG CTGGCATTTA   

- +Up\_Stream \_Len000TTTGCC ACGGCAACTT AAACGTCCGC TGCTCCAGCC CAAATTCCGC CTACAGCATC   
  
  
- GCGAAACCTC TTCGTTTAAA CCCTTCTGCT CCGAATAAGA ACTTGAGAAG CATGCGGTAA CAACAACCAA   
  
  
- GGTTGGAAAG AGAGAGGAGC ACTTAAACTC AAACCCGTCA TCTGATAGGG CCTTATCTCC TGACTACGCA   
  
  
- ATATATATAT CTTTAACTCT CTCTCTCTCT CGCACACACT TCCTACAACT TTTACTACCC GCATCCCTCA   
  
  
- GAGAGAGAGT GACAGACAAT CCTCATGCAC AGCTCTTTCC TTTATCCTAA GGGTTCGTCA GATTAAACCC   
  
  
- GGCCAGATAT CCGGTCTTAC CCGGATACTA GTCGGGTCTA TACTGGAACA AATTGGTTGA CATAAACTTA   
  
  
- ACTTCTTTCC CTATAGCGTT ACAGAACTTG GTGTAGTTTA TTATTTACAC GGAAACCAAT CTGGTATACT   
  
  
- TTTTAGAAAC TTAGTTTAGC CAACCTGGAC TTGACGGTAA TAACTATAGA ACAAAAACCA TCTTCGTTTA   
  
  
- CCACAGTGAA GCAAAAGATC TATTTTAATT GAACTCAAAA AAACGGTGAT ATCTATCATA ACGTTTATAT   
  
  
- TTTTCACACG GTGCACTTTA GTTATAATCC TCCCACAAAA TATTGTTATC GACAAGCCCT CTCAAAATCA   
  
  
- ATAAAAATAA TTTAGTTTAT CAATTCTTCA CAAACCATTT ATCAACATAA ACTTTTTCAA TAAGGGTAAT   
  
  
- CGGAAAATCA TTCTTGAAGA ACACTTTTAC TATTAACAGA TCTTCAAACT TTTTAAGTGT GGTGTACTGT   
  
  
- AAATTTTTAT TAAAACATCT CTTCCCGTAT TTACTTTCAA ACGGTGGTAG AAAAAATCTT TGTACATTAA   
  
  
- ATGGTTTGTG AAAAAGATTT TTGTCAATTA AGTTTATTGA TTGTTGATTG TCGATTATAT TTATTGATTG   
  
  
- TCGATAAACG ATTTGTCCCG GATTCATCAA ACTTTAACAA CAAAAGAGAA GTCGAAGTGG TACCGGCTAC   
  
  
- TACATTTGTT GATATAAGAC TTAAAGTAAA AATGATACTA AACTTTTAAA GTTTAAATTT ATTCTAACCT   
  
  
- ATTTTTTTTC TGGTTTTTAG TATTCATTAT AATCAGTTCA AGATAAGGTT GGTATATGTA TTGACGAAAA   
  
  
- AGAAAACCCC CAAAACTTTG TACACAGGAA TCTTATTAAC TTTGAGCTAT AATAAGAGAT TTAGATTCAA   
  
  
- ATGAAATAGT ATAACTAATA ATTAATGGAG GAGTTGAGGA GGATATGAGA ACATACGTCT TTATTTTTAC   
  
  
- AAAGGTATGG AATAGTCAGC GCTAGTATTA TTAATTAAAG AATTAAGCCT AACATAAATT AACGCATAGT   
  
  
- TTACACTTTA GCATGGTTTA ATCCTTCGTT TTGTTTGGCT ATTGCTTTTT CCATTCTTTT GAATCGTTCC   
  
  
- TTTACTTTAG CTTCTGGACA GTTGTTATAT CGAAGGGAGT GTAGGAAGTT GAATCCCTGG TAGGATATGA   
  
  
- AAGTACATTA TGTTGATCTC GGTGATCAGT TATTATCGGA GGATCATCAT CGGTATTCGG AACCACTCTC   
  
  
- TTACAAGGCG TTCTGATCAA TTGAACTTTA AGGTAAAACA GCGTCTTGAT AACCTCACTC TTTCTCTTAG   
  
  
- TTTAGTATGG TATAGTTTAA ACCTGGTAAA TTCAAGTGTT GTGTGTGTGT ATGTGTGTTT TCCTGCTACA   
  
  
- TGCCGGGGTG GGGGGTGAGA GGAAAGCGGT AGGGGGATGT GGAGACGGAT GAGAGAGAGA GAGAGAGAGA   
  
  
- GAGACCTAGT CAGACACATT TTCGAAGGAG AGGTCATGTC GTAAAGGAAC AGACAAGAAC CTCAAATCAG   
  
  
- TTAAGGAGGC GGGGTTTTTT TGGGTGAAAG AGAGAGTAGT ACAAGAAGGT CAAGGTGGGA ATATGCGGTA   
  
  
- AGTAGTAGTA GATGTAGTGG GTGTAGAAAA AGAGGTGTCA AAAGGGGGGA GTAATACGAG AGGCGGAGGT   
  
  
- GGGGGCGGAA GGGAGAGCGG CAGCGGCCGC TACTGCCCCT ATGGTGACTA CGTACGCTGC CGTGGCGGTG   
  
  
- ACGGGAGGTG GAGGAGCCCG ACGACAACTT CACGCGCCTT AAGTAGAGGT GGCCTCTGGA GCGGCCGCGG   
  
  
- CCTCTATAGA ACGGCCTCTA TAAACTCAAC AGATGTGGCA AACCGAGGGG GCGGCGGGCC CAGCGGCGGA   
  
  
- AGAAGCCGGT GCGGGAGGTG CGGGCGGAGG AGAGGCGGAG GGAGGCGTGT TGAGGCTAGC TCTTCGAGTT   
  
  
- TTGGGACTGG GACCAAAGGG TTTACGCCTT TAAAGTGAGG CGGAACGTTC ATATGTTGAG GTAGTGAGGG   
  
  
- AAGCACTTTA AGAGAGTGAA GTGCCGCTTA GTTCGGTAGA TGCTCCGCGA CCTGCCGCTC CTAGCACAGG   
  
  
- TGCAGCAGCT GGAACTGTAG TACGTCCCGG AAGTTACCGG CCCTAACAAG GTGTAGAATC GGAGAGCTGG   
  
  
- GTCTTTCGGG GAGAGCCAAG CCCAGTGGCC CAAGCCCGGT AGGAGGCTCA ACGAGAGGGT TTGCCCATTC   
  
  
- GCTGAGCGAC TCAAGCGGCG AAGTGAGCCG GACGGGAAGC TCATGTTGGG CCACCTCCCG TTTTAACCCT   
  
  
- TGAATCAGCT GGACCCAGCC CAGCCCAGCG AGGGCTTACT TCACTGCCAC CACGTGACCT ACGTGGTATC   
  
  
- AGATATGCTG TATTGGCCCA GCCTAGAACC CTGAAACTCC CATAACTCAC GCCACTCCGG ATCCGAACAC   
  
  
- TGATACCAAC TCGTCCTATA CCTGGTTTGC CCCAGCAAAA ACCCCTCCAA ACACCTCCGT AACGTAATAA   
  
  
- TGAGTCGGAA CAAACTACGG AACCCTCTCC CCAATCTATC CCTATTGGAT GTTTCCGTAG TTCACCTCGT   
  
  
- TGTCGATAAA CCCACACTCT AATCCTTGTA GGACCGGCAA CCACCCGGGT TCTCCTCCGA GTGACCGCCG   
  
  
- CTAGCCCAGT CTTCCACCCC GCTGCTTGAC TGTTCCCAGC CCAAGCTTGG TCAAAGCAAC CGCCCATCGG   
  
  
- GCCGTTGGGT TCGATCAAAC AACGAACCCT ACAAGGGAAC CTTTCCCATA TGAAACTACC TCCTTTTACC   
  
  
- CACAAACTCT AATCCCACCT TTCTAAACGG GAACAATTGA CGGAGTCGAA CCGTTGGAAC ACTTAAATTG   
  
  
- TTAGGATCAC GACCGTAAAT

+     GATA-motif

| Site Name | Organism | Position | Strand | Matrix score. | sequence | function |
| --- | --- | --- | --- | --- | --- | --- |
| GATA-motif | Pisum sativum | 2910 | + | 7 | GATAGGG | part of a light responsive element |

>HU02G03005.1   
+ +Up\_Stream \_Len000AAACGG TGCCGTTGAA TTTGCAGGCG ACGAGGTCGG GTTTAAGGCG GATGTCGTAG   
  
  
+ CGCTTTGGAG AAGCAAATTT GGGAAGACGA GGCTTATTCT TGAACTCTTC GTACGCCATT GTTGTTGGTT   
  
  
+ CCAACCTTTC TCTCTCCTCG TGAATTTGAG TTTGGGCAGT AGACTATCCC GGAATAGAGG ACTGATGCGT   
  
  
+ TATATATATA GAAATTGAGA GAGAGAGAGA GCGTGTGTGA AGGATGTTGA AAATGATGGG CGTAGGGAGT   
  
  
+ CTCTCTCTCA CTGTCTGTTA GGAGTACGTG TCGAGAAAGG AAATAGGATT CCCAAGCAGT CTAATTTGGG   
  
  
+ CCGGTCTATA GGCCAGAATG GGCCTATGAT CAGCCCAGAT ATGACCTTGT TTAACCAACT GTATTTGAAT   
  
  
+ TGAAGAAAGG GATATCGCAA TGTCTTGAAC CACATCAAAT AATAAATGTG CCTTTGGTTA GACCATATGA   
  
  
+ AAAATCTTTG AATCAAATCG GTTGGACCTG AACTGCCATT ATTGATATCT TGTTTTTGGT AGAAGCAAAT   
  
  
+ GGTGTCACTT CGTTTTCTAG ATAAAATTAA CTTGAGTTTT TTTGCCACTA TAGATAGTAT TGCAAATATA   
  
  
+ AAAAGTGTGC CACGTGAAAT CAATATTAGG AGGGTGTTTT ATAACAATAG CTGTTCGGGA GAGTTTTAGT   
  
  
+ TATTTTTATT AAATCAAATA GTTAAGAAGT GTTTGGTAAA TAGTTGTATT TGAAAAAGTT ATTCCCATTA   
  
  
+ GCCTTTTAGT AAGAACTTCT TGTGAAAATG ATAATTGTCT AGAAGTTTGA AAAATTCACA CCACATGACA   
  
  
+ TTTAAAAATA ATTTTGTAGA GAAGGGCATA AATGAAAGTT TGCCACCATC TTTTTTAGAA ACATGTAATT   
  
  
+ TACCAAACAC TTTTTCTAAA AACAGTTAAT TCAAATAACT AACAACTAAC AGCTAATATA AATAACTAAC   
  
  
+ AGCTATTTGC TAAACAGGGC CTAAGTAGTT TGAAATTGTT GTTTTCTCTT CAGCTTCACC ATGGCCGATG   
  
  
+ ATGTAAACAA CTATATTCTG AATTTCATTT TTACTATGAT TTGAAAATTT CAAATTTAAA TAAGATTGGA   
  
  
+ TAAAAAAAAG ACCAAAAATC ATAAGTAATA TTAGTCAAGT TCTATTCCAA CCATATACAT AACTGCTTTT   
  
  
+ TCTTTTGGGG GTTTTGAAAC ATGTGTCCTT AGAATAATTG AAACTCGATA TTATTCTCTA AATCTAAGTT   
  
  
+ TACTTTATCA TATTGATTAT TAATTACCTC CTCAACTCCT CCTATACTCT TGTATGCAGA AATAAAAATG   
  
  
+ TTTCCATACC TTATCAGTCG CGATCATAAT AATTAATTTC TTAATTCGGA TTGTATTTAA TTGCGTATCA   
  
  
+ AATGTGAAAT CGTACCAAAT TAGGAAGCAA AACAAACCGA TAACGAAAAA GGTAAGAAAA CTTAGCAAGG   
  
  
+ AAATGAAATC GAAGACCTGT CAACAATATA GCTTCCCTCA CATCCTTCAA CTTAGGGACC ATCCTATACT   
  
  
+ TTCATGTAAT ACAACTAGAG CCACTAGTCA ATAATAGCCT CCTAGTAGTA GCCATAAGCC TTGGTGAGAG   
  
  
+ AATGTTCCGC AAGACTAGTT AACTTGAAAT TCCATTTTGT CGCAGAACTA TTGGAGTGAG AAAGAGAATC   
  
  
+ AAATCATACC ATATCAAATT TGGACCATTT AAGTTCACAA CACACACACA TACACACAAA AGGACGATGT   
  
  
+ ACGGCCCCAC CCCCCACTCT CCTTTCGCCA TCCCCCTACA CCTCTGCCTA CTCTCTCTCT CTCTCTCTCT   
  
  
+ CTCTGGATCA GTCTGTGTAA AAGCTTCCTC TCCAGTACAG CATTTCCTTG TCTGTTCTTG GAGTTTAGTC   
  
  
+ AATTCCTCCG CCCCAAAAAA ACCCACTTTC TCTCTCATCA TGTTCTTCCA GTTCCACCCT TATACGCCAT   
  
  
+ TCATCATCAT CTACATCACC CACATCTTTT TCTCCACAGT TTTCCCCCCT CATTATGCTC TCCGCCTCCA   
  
  
+ CCCCCGCCTT CCCTCTCGCC GTCGCCGGCG ATGACGGGGA TACCACTGAT GCATGCGACG GCACCGCCAC   
  
  
+ TGCCCTCCAC CTCCTCGGGC TGCTGTTGAA GTGCGCGGAA TTCATCTCCA CCGGAGACCT CGCCGGCGCC   
  
  
+ GGAGATATCT TGCCGGAGAT ATTTGAGTTG TCTACACCGT TTGGCTCCCC CGCCGCCCGG GTCGCCGCCT   
  
  
+ TCTTCGGCCA CGCCCTCCAC GCCCGCCTCC TCTCCGCCTC CCTCCGCACA ACTCCGATCG AGAAGCTCAA   
  
  
+ AACCCTGACC CTGGTTTCCC AAATGCGGAA ATTTCACTCC GCCTTGCAAG TATACAACTC CATCACTCCC   
  
  
+ TTCGTGAAAT TCTCTCACTT CACGGCGAAT CAAGCCATCT ACGAGGCGCT GGACGGCGAG GATCGTGTCC   
  
  
+ ACGTCGTCGA CCTTGACATC ATGCAGGGCC TTCAATGGCC GGGATTGTTC CACATCTTAG CCTCTCGACC   
  
  
+ CAGAAAGCCC CTCTCGGTTC GGGTCACCGG GTTCGGGCCA TCCTCCGAGT TGCTCTCCCA AACGGGTAAG   
  
  
+ CGACTCGCTG AGTTCGCCGC TTCACTCGGC CTGCCCTTCG AGTACAACCC GGTGGAGGGC AAAATTGGGA   
  
  
+ ACTTAGTCGA CCTGGGTCGG GTCGGGTCGC TCCCGAATGA AGTGACGGTG GTGCACTGGA TGCACCATAG   
  
  
+ TCTATACGAC ATAACCGGGT CGGATCTTGG GACTTTGAGG GTATTGAGTG CGGTGAGGCC TAGGCTTGTG   
  
  
+ ACTATGGTTG AGCAGGATAT GGACCAAACG GGGTCGTTTT TGGGGAGGTT TGTGGAGGCA TTGCATTATT   
  
  
+ ACTCAGCCTT GTTTGATGCC TTGGGAGAGG GGTTAGATAG GGATAACCTA CAAAGGCATC AAGTGGAGCA   
  
  
+ ACAGCTATTT GGGTGTGAGA TTAGGAACAT CCTGGCCGTT GGTGGGCCCA AGAGGAGGCT CACTGGCGGC   
  
  
+ GATCGGGTCA GAAGGTGGGG CGACGAACTG ACAAGGGTCG GGTTCGAACC AGTTTCGTTG GCGGGTAGCC   
  
  
+ CGGCAACCCA AGCTAGTTTG TTGCTTGGGA TGTTCCCTTG GAAAGGGTAT ACTTTGATGG AGGAAAATGG   
  
  
+ GTGTTTGAGA TTAGGGTGGA AAGATTTGCC CTTGTTAACT GCCTCAGCTT GGCAACCTTG TGAATTTAAC   
  
  
+ AATCCTAGTG CTGGCATTTA   

- +Up\_Stream \_Len000TTTGCC ACGGCAACTT AAACGTCCGC TGCTCCAGCC CAAATTCCGC CTACAGCATC   
  
  
- GCGAAACCTC TTCGTTTAAA CCCTTCTGCT CCGAATAAGA ACTTGAGAAG CATGCGGTAA CAACAACCAA   
  
  
- GGTTGGAAAG AGAGAGGAGC ACTTAAACTC AAACCCGTCA TCTGATAGGG CCTTATCTCC TGACTACGCA   
  
  
- ATATATATAT CTTTAACTCT CTCTCTCTCT CGCACACACT TCCTACAACT TTTACTACCC GCATCCCTCA   
  
  
- GAGAGAGAGT GACAGACAAT CCTCATGCAC AGCTCTTTCC TTTATCCTAA GGGTTCGTCA GATTAAACCC   
  
  
- GGCCAGATAT CCGGTCTTAC CCGGATACTA GTCGGGTCTA TACTGGAACA AATTGGTTGA CATAAACTTA   
  
  
- ACTTCTTTCC CTATAGCGTT ACAGAACTTG GTGTAGTTTA TTATTTACAC GGAAACCAAT CTGGTATACT   
  
  
- TTTTAGAAAC TTAGTTTAGC CAACCTGGAC TTGACGGTAA TAACTATAGA ACAAAAACCA TCTTCGTTTA   
  
  
- CCACAGTGAA GCAAAAGATC TATTTTAATT GAACTCAAAA AAACGGTGAT ATCTATCATA ACGTTTATAT   
  
  
- TTTTCACACG GTGCACTTTA GTTATAATCC TCCCACAAAA TATTGTTATC GACAAGCCCT CTCAAAATCA   
  
  
- ATAAAAATAA TTTAGTTTAT CAATTCTTCA CAAACCATTT ATCAACATAA ACTTTTTCAA TAAGGGTAAT   
  
  
- CGGAAAATCA TTCTTGAAGA ACACTTTTAC TATTAACAGA TCTTCAAACT TTTTAAGTGT GGTGTACTGT   
  
  
- AAATTTTTAT TAAAACATCT CTTCCCGTAT TTACTTTCAA ACGGTGGTAG AAAAAATCTT TGTACATTAA   
  
  
- ATGGTTTGTG AAAAAGATTT TTGTCAATTA AGTTTATTGA TTGTTGATTG TCGATTATAT TTATTGATTG   
  
  
- TCGATAAACG ATTTGTCCCG GATTCATCAA ACTTTAACAA CAAAAGAGAA GTCGAAGTGG TACCGGCTAC   
  
  
- TACATTTGTT GATATAAGAC TTAAAGTAAA AATGATACTA AACTTTTAAA GTTTAAATTT ATTCTAACCT   
  
  
- ATTTTTTTTC TGGTTTTTAG TATTCATTAT AATCAGTTCA AGATAAGGTT GGTATATGTA TTGACGAAAA   
  
  
- AGAAAACCCC CAAAACTTTG TACACAGGAA TCTTATTAAC TTTGAGCTAT AATAAGAGAT TTAGATTCAA   
  
  
- ATGAAATAGT ATAACTAATA ATTAATGGAG GAGTTGAGGA GGATATGAGA ACATACGTCT TTATTTTTAC   
  
  
- AAAGGTATGG AATAGTCAGC GCTAGTATTA TTAATTAAAG AATTAAGCCT AACATAAATT AACGCATAGT   
  
  
- TTACACTTTA GCATGGTTTA ATCCTTCGTT TTGTTTGGCT ATTGCTTTTT CCATTCTTTT GAATCGTTCC   
  
  
- TTTACTTTAG CTTCTGGACA GTTGTTATAT CGAAGGGAGT GTAGGAAGTT GAATCCCTGG TAGGATATGA   
  
  
- AAGTACATTA TGTTGATCTC GGTGATCAGT TATTATCGGA GGATCATCAT CGGTATTCGG AACCACTCTC   
  
  
- TTACAAGGCG TTCTGATCAA TTGAACTTTA AGGTAAAACA GCGTCTTGAT AACCTCACTC TTTCTCTTAG   
  
  
- TTTAGTATGG TATAGTTTAA ACCTGGTAAA TTCAAGTGTT GTGTGTGTGT ATGTGTGTTT TCCTGCTACA   
  
  
- TGCCGGGGTG GGGGGTGAGA GGAAAGCGGT AGGGGGATGT GGAGACGGAT GAGAGAGAGA GAGAGAGAGA   
  
  
- GAGACCTAGT CAGACACATT TTCGAAGGAG AGGTCATGTC GTAAAGGAAC AGACAAGAAC CTCAAATCAG   
  
  
- TTAAGGAGGC GGGGTTTTTT TGGGTGAAAG AGAGAGTAGT ACAAGAAGGT CAAGGTGGGA ATATGCGGTA   
  
  
- AGTAGTAGTA GATGTAGTGG GTGTAGAAAA AGAGGTGTCA AAAGGGGGGA GTAATACGAG AGGCGGAGGT   
  
  
- GGGGGCGGAA GGGAGAGCGG CAGCGGCCGC TACTGCCCCT ATGGTGACTA CGTACGCTGC CGTGGCGGTG   
  
  
- ACGGGAGGTG GAGGAGCCCG ACGACAACTT CACGCGCCTT AAGTAGAGGT GGCCTCTGGA GCGGCCGCGG   
  
  
- CCTCTATAGA ACGGCCTCTA TAAACTCAAC AGATGTGGCA AACCGAGGGG GCGGCGGGCC CAGCGGCGGA   
  
  
- AGAAGCCGGT GCGGGAGGTG CGGGCGGAGG AGAGGCGGAG GGAGGCGTGT TGAGGCTAGC TCTTCGAGTT   
  
  
- TTGGGACTGG GACCAAAGGG TTTACGCCTT TAAAGTGAGG CGGAACGTTC ATATGTTGAG GTAGTGAGGG   
  
  
- AAGCACTTTA AGAGAGTGAA GTGCCGCTTA GTTCGGTAGA TGCTCCGCGA CCTGCCGCTC CTAGCACAGG   
  
  
- TGCAGCAGCT GGAACTGTAG TACGTCCCGG AAGTTACCGG CCCTAACAAG GTGTAGAATC GGAGAGCTGG   
  
  
- GTCTTTCGGG GAGAGCCAAG CCCAGTGGCC CAAGCCCGGT AGGAGGCTCA ACGAGAGGGT TTGCCCATTC   
  
  
- GCTGAGCGAC TCAAGCGGCG AAGTGAGCCG GACGGGAAGC TCATGTTGGG CCACCTCCCG TTTTAACCCT   
  
  
- TGAATCAGCT GGACCCAGCC CAGCCCAGCG AGGGCTTACT TCACTGCCAC CACGTGACCT ACGTGGTATC   
  
  
- AGATATGCTG TATTGGCCCA GCCTAGAACC CTGAAACTCC CATAACTCAC GCCACTCCGG ATCCGAACAC   
  
  
- TGATACCAAC TCGTCCTATA CCTGGTTTGC CCCAGCAAAA ACCCCTCCAA ACACCTCCGT AACGTAATAA   
  
  
- TGAGTCGGAA CAAACTACGG AACCCTCTCC CCAATCTATC CCTATTGGAT GTTTCCGTAG TTCACCTCGT   
  
  
- TGTCGATAAA CCCACACTCT AATCCTTGTA GGACCGGCAA CCACCCGGGT TCTCCTCCGA GTGACCGCCG   
  
  
- CTAGCCCAGT CTTCCACCCC GCTGCTTGAC TGTTCCCAGC CCAAGCTTGG TCAAAGCAAC CGCCCATCGG   
  
  
- GCCGTTGGGT TCGATCAAAC AACGAACCCT ACAAGGGAAC CTTTCCCATA TGAAACTACC TCCTTTTACC   
  
  
- CACAAACTCT AATCCCACCT TTCTAAACGG GAACAATTGA CGGAGTCGAA CCGTTGGAAC ACTTAAATTG   
  
  
- TTAGGATCAC GACCGTAAAT

+     GC-motif

| Site Name | Organism | Position | Strand | Matrix score. | sequence | function |
| --- | --- | --- | --- | --- | --- | --- |
| GC-motif | Zea mays | 2035 | + | 6 | CCCCCG | enhancer-like element involved in anoxic specific inducibility |
| GC-motif | Zea mays | 2221 | + | 6 | CCCCCG | enhancer-like element involved in anoxic specific inducibility |

>HU02G03005.1   
+ +Up\_Stream \_Len000AAACGG TGCCGTTGAA TTTGCAGGCG ACGAGGTCGG GTTTAAGGCG GATGTCGTAG   
  
  
+ CGCTTTGGAG AAGCAAATTT GGGAAGACGA GGCTTATTCT TGAACTCTTC GTACGCCATT GTTGTTGGTT   
  
  
+ CCAACCTTTC TCTCTCCTCG TGAATTTGAG TTTGGGCAGT AGACTATCCC GGAATAGAGG ACTGATGCGT   
  
  
+ TATATATATA GAAATTGAGA GAGAGAGAGA GCGTGTGTGA AGGATGTTGA AAATGATGGG CGTAGGGAGT   
  
  
+ CTCTCTCTCA CTGTCTGTTA GGAGTACGTG TCGAGAAAGG AAATAGGATT CCCAAGCAGT CTAATTTGGG   
  
  
+ CCGGTCTATA GGCCAGAATG GGCCTATGAT CAGCCCAGAT ATGACCTTGT TTAACCAACT GTATTTGAAT   
  
  
+ TGAAGAAAGG GATATCGCAA TGTCTTGAAC CACATCAAAT AATAAATGTG CCTTTGGTTA GACCATATGA   
  
  
+ AAAATCTTTG AATCAAATCG GTTGGACCTG AACTGCCATT ATTGATATCT TGTTTTTGGT AGAAGCAAAT   
  
  
+ GGTGTCACTT CGTTTTCTAG ATAAAATTAA CTTGAGTTTT TTTGCCACTA TAGATAGTAT TGCAAATATA   
  
  
+ AAAAGTGTGC CACGTGAAAT CAATATTAGG AGGGTGTTTT ATAACAATAG CTGTTCGGGA GAGTTTTAGT   
  
  
+ TATTTTTATT AAATCAAATA GTTAAGAAGT GTTTGGTAAA TAGTTGTATT TGAAAAAGTT ATTCCCATTA   
  
  
+ GCCTTTTAGT AAGAACTTCT TGTGAAAATG ATAATTGTCT AGAAGTTTGA AAAATTCACA CCACATGACA   
  
  
+ TTTAAAAATA ATTTTGTAGA GAAGGGCATA AATGAAAGTT TGCCACCATC TTTTTTAGAA ACATGTAATT   
  
  
+ TACCAAACAC TTTTTCTAAA AACAGTTAAT TCAAATAACT AACAACTAAC AGCTAATATA AATAACTAAC   
  
  
+ AGCTATTTGC TAAACAGGGC CTAAGTAGTT TGAAATTGTT GTTTTCTCTT CAGCTTCACC ATGGCCGATG   
  
  
+ ATGTAAACAA CTATATTCTG AATTTCATTT TTACTATGAT TTGAAAATTT CAAATTTAAA TAAGATTGGA   
  
  
+ TAAAAAAAAG ACCAAAAATC ATAAGTAATA TTAGTCAAGT TCTATTCCAA CCATATACAT AACTGCTTTT   
  
  
+ TCTTTTGGGG GTTTTGAAAC ATGTGTCCTT AGAATAATTG AAACTCGATA TTATTCTCTA AATCTAAGTT   
  
  
+ TACTTTATCA TATTGATTAT TAATTACCTC CTCAACTCCT CCTATACTCT TGTATGCAGA AATAAAAATG   
  
  
+ TTTCCATACC TTATCAGTCG CGATCATAAT AATTAATTTC TTAATTCGGA TTGTATTTAA TTGCGTATCA   
  
  
+ AATGTGAAAT CGTACCAAAT TAGGAAGCAA AACAAACCGA TAACGAAAAA GGTAAGAAAA CTTAGCAAGG   
  
  
+ AAATGAAATC GAAGACCTGT CAACAATATA GCTTCCCTCA CATCCTTCAA CTTAGGGACC ATCCTATACT   
  
  
+ TTCATGTAAT ACAACTAGAG CCACTAGTCA ATAATAGCCT CCTAGTAGTA GCCATAAGCC TTGGTGAGAG   
  
  
+ AATGTTCCGC AAGACTAGTT AACTTGAAAT TCCATTTTGT CGCAGAACTA TTGGAGTGAG AAAGAGAATC   
  
  
+ AAATCATACC ATATCAAATT TGGACCATTT AAGTTCACAA CACACACACA TACACACAAA AGGACGATGT   
  
  
+ ACGGCCCCAC CCCCCACTCT CCTTTCGCCA TCCCCCTACA CCTCTGCCTA CTCTCTCTCT CTCTCTCTCT   
  
  
+ CTCTGGATCA GTCTGTGTAA AAGCTTCCTC TCCAGTACAG CATTTCCTTG TCTGTTCTTG GAGTTTAGTC   
  
  
+ AATTCCTCCG CCCCAAAAAA ACCCACTTTC TCTCTCATCA TGTTCTTCCA GTTCCACCCT TATACGCCAT   
  
  
+ TCATCATCAT CTACATCACC CACATCTTTT TCTCCACAGT TTTCCCCCCT CATTATGCTC TCCGCCTCCA   
  
  
+ CCCCCGCCTT CCCTCTCGCC GTCGCCGGCG ATGACGGGGA TACCACTGAT GCATGCGACG GCACCGCCAC   
  
  
+ TGCCCTCCAC CTCCTCGGGC TGCTGTTGAA GTGCGCGGAA TTCATCTCCA CCGGAGACCT CGCCGGCGCC   
  
  
+ GGAGATATCT TGCCGGAGAT ATTTGAGTTG TCTACACCGT TTGGCTCCCC CGCCGCCCGG GTCGCCGCCT   
  
  
+ TCTTCGGCCA CGCCCTCCAC GCCCGCCTCC TCTCCGCCTC CCTCCGCACA ACTCCGATCG AGAAGCTCAA   
  
  
+ AACCCTGACC CTGGTTTCCC AAATGCGGAA ATTTCACTCC GCCTTGCAAG TATACAACTC CATCACTCCC   
  
  
+ TTCGTGAAAT TCTCTCACTT CACGGCGAAT CAAGCCATCT ACGAGGCGCT GGACGGCGAG GATCGTGTCC   
  
  
+ ACGTCGTCGA CCTTGACATC ATGCAGGGCC TTCAATGGCC GGGATTGTTC CACATCTTAG CCTCTCGACC   
  
  
+ CAGAAAGCCC CTCTCGGTTC GGGTCACCGG GTTCGGGCCA TCCTCCGAGT TGCTCTCCCA AACGGGTAAG   
  
  
+ CGACTCGCTG AGTTCGCCGC TTCACTCGGC CTGCCCTTCG AGTACAACCC GGTGGAGGGC AAAATTGGGA   
  
  
+ ACTTAGTCGA CCTGGGTCGG GTCGGGTCGC TCCCGAATGA AGTGACGGTG GTGCACTGGA TGCACCATAG   
  
  
+ TCTATACGAC ATAACCGGGT CGGATCTTGG GACTTTGAGG GTATTGAGTG CGGTGAGGCC TAGGCTTGTG   
  
  
+ ACTATGGTTG AGCAGGATAT GGACCAAACG GGGTCGTTTT TGGGGAGGTT TGTGGAGGCA TTGCATTATT   
  
  
+ ACTCAGCCTT GTTTGATGCC TTGGGAGAGG GGTTAGATAG GGATAACCTA CAAAGGCATC AAGTGGAGCA   
  
  
+ ACAGCTATTT GGGTGTGAGA TTAGGAACAT CCTGGCCGTT GGTGGGCCCA AGAGGAGGCT CACTGGCGGC   
  
  
+ GATCGGGTCA GAAGGTGGGG CGACGAACTG ACAAGGGTCG GGTTCGAACC AGTTTCGTTG GCGGGTAGCC   
  
  
+ CGGCAACCCA AGCTAGTTTG TTGCTTGGGA TGTTCCCTTG GAAAGGGTAT ACTTTGATGG AGGAAAATGG   
  
  
+ GTGTTTGAGA TTAGGGTGGA AAGATTTGCC CTTGTTAACT GCCTCAGCTT GGCAACCTTG TGAATTTAAC   
  
  
+ AATCCTAGTG CTGGCATTTA   

- +Up\_Stream \_Len000TTTGCC ACGGCAACTT AAACGTCCGC TGCTCCAGCC CAAATTCCGC CTACAGCATC   
  
  
- GCGAAACCTC TTCGTTTAAA CCCTTCTGCT CCGAATAAGA ACTTGAGAAG CATGCGGTAA CAACAACCAA   
  
  
- GGTTGGAAAG AGAGAGGAGC ACTTAAACTC AAACCCGTCA TCTGATAGGG CCTTATCTCC TGACTACGCA   
  
  
- ATATATATAT CTTTAACTCT CTCTCTCTCT CGCACACACT TCCTACAACT TTTACTACCC GCATCCCTCA   
  
  
- GAGAGAGAGT GACAGACAAT CCTCATGCAC AGCTCTTTCC TTTATCCTAA GGGTTCGTCA GATTAAACCC   
  
  
- GGCCAGATAT CCGGTCTTAC CCGGATACTA GTCGGGTCTA TACTGGAACA AATTGGTTGA CATAAACTTA   
  
  
- ACTTCTTTCC CTATAGCGTT ACAGAACTTG GTGTAGTTTA TTATTTACAC GGAAACCAAT CTGGTATACT   
  
  
- TTTTAGAAAC TTAGTTTAGC CAACCTGGAC TTGACGGTAA TAACTATAGA ACAAAAACCA TCTTCGTTTA   
  
  
- CCACAGTGAA GCAAAAGATC TATTTTAATT GAACTCAAAA AAACGGTGAT ATCTATCATA ACGTTTATAT   
  
  
- TTTTCACACG GTGCACTTTA GTTATAATCC TCCCACAAAA TATTGTTATC GACAAGCCCT CTCAAAATCA   
  
  
- ATAAAAATAA TTTAGTTTAT CAATTCTTCA CAAACCATTT ATCAACATAA ACTTTTTCAA TAAGGGTAAT   
  
  
- CGGAAAATCA TTCTTGAAGA ACACTTTTAC TATTAACAGA TCTTCAAACT TTTTAAGTGT GGTGTACTGT   
  
  
- AAATTTTTAT TAAAACATCT CTTCCCGTAT TTACTTTCAA ACGGTGGTAG AAAAAATCTT TGTACATTAA   
  
  
- ATGGTTTGTG AAAAAGATTT TTGTCAATTA AGTTTATTGA TTGTTGATTG TCGATTATAT TTATTGATTG   
  
  
- TCGATAAACG ATTTGTCCCG GATTCATCAA ACTTTAACAA CAAAAGAGAA GTCGAAGTGG TACCGGCTAC   
  
  
- TACATTTGTT GATATAAGAC TTAAAGTAAA AATGATACTA AACTTTTAAA GTTTAAATTT ATTCTAACCT   
  
  
- ATTTTTTTTC TGGTTTTTAG TATTCATTAT AATCAGTTCA AGATAAGGTT GGTATATGTA TTGACGAAAA   
  
  
- AGAAAACCCC CAAAACTTTG TACACAGGAA TCTTATTAAC TTTGAGCTAT AATAAGAGAT TTAGATTCAA   
  
  
- ATGAAATAGT ATAACTAATA ATTAATGGAG GAGTTGAGGA GGATATGAGA ACATACGTCT TTATTTTTAC   
  
  
- AAAGGTATGG AATAGTCAGC GCTAGTATTA TTAATTAAAG AATTAAGCCT AACATAAATT AACGCATAGT   
  
  
- TTACACTTTA GCATGGTTTA ATCCTTCGTT TTGTTTGGCT ATTGCTTTTT CCATTCTTTT GAATCGTTCC   
  
  
- TTTACTTTAG CTTCTGGACA GTTGTTATAT CGAAGGGAGT GTAGGAAGTT GAATCCCTGG TAGGATATGA   
  
  
- AAGTACATTA TGTTGATCTC GGTGATCAGT TATTATCGGA GGATCATCAT CGGTATTCGG AACCACTCTC   
  
  
- TTACAAGGCG TTCTGATCAA TTGAACTTTA AGGTAAAACA GCGTCTTGAT AACCTCACTC TTTCTCTTAG   
  
  
- TTTAGTATGG TATAGTTTAA ACCTGGTAAA TTCAAGTGTT GTGTGTGTGT ATGTGTGTTT TCCTGCTACA   
  
  
- TGCCGGGGTG GGGGGTGAGA GGAAAGCGGT AGGGGGATGT GGAGACGGAT GAGAGAGAGA GAGAGAGAGA   
  
  
- GAGACCTAGT CAGACACATT TTCGAAGGAG AGGTCATGTC GTAAAGGAAC AGACAAGAAC CTCAAATCAG   
  
  
- TTAAGGAGGC GGGGTTTTTT TGGGTGAAAG AGAGAGTAGT ACAAGAAGGT CAAGGTGGGA ATATGCGGTA   
  
  
- AGTAGTAGTA GATGTAGTGG GTGTAGAAAA AGAGGTGTCA AAAGGGGGGA GTAATACGAG AGGCGGAGGT   
  
  
- GGGGGCGGAA GGGAGAGCGG CAGCGGCCGC TACTGCCCCT ATGGTGACTA CGTACGCTGC CGTGGCGGTG   
  
  
- ACGGGAGGTG GAGGAGCCCG ACGACAACTT CACGCGCCTT AAGTAGAGGT GGCCTCTGGA GCGGCCGCGG   
  
  
- CCTCTATAGA ACGGCCTCTA TAAACTCAAC AGATGTGGCA AACCGAGGGG GCGGCGGGCC CAGCGGCGGA   
  
  
- AGAAGCCGGT GCGGGAGGTG CGGGCGGAGG AGAGGCGGAG GGAGGCGTGT TGAGGCTAGC TCTTCGAGTT   
  
  
- TTGGGACTGG GACCAAAGGG TTTACGCCTT TAAAGTGAGG CGGAACGTTC ATATGTTGAG GTAGTGAGGG   
  
  
- AAGCACTTTA AGAGAGTGAA GTGCCGCTTA GTTCGGTAGA TGCTCCGCGA CCTGCCGCTC CTAGCACAGG   
  
  
- TGCAGCAGCT GGAACTGTAG TACGTCCCGG AAGTTACCGG CCCTAACAAG GTGTAGAATC GGAGAGCTGG   
  
  
- GTCTTTCGGG GAGAGCCAAG CCCAGTGGCC CAAGCCCGGT AGGAGGCTCA ACGAGAGGGT TTGCCCATTC   
  
  
- GCTGAGCGAC TCAAGCGGCG AAGTGAGCCG GACGGGAAGC TCATGTTGGG CCACCTCCCG TTTTAACCCT   
  
  
- TGAATCAGCT GGACCCAGCC CAGCCCAGCG AGGGCTTACT TCACTGCCAC CACGTGACCT ACGTGGTATC   
  
  
- AGATATGCTG TATTGGCCCA GCCTAGAACC CTGAAACTCC CATAACTCAC GCCACTCCGG ATCCGAACAC   
  
  
- TGATACCAAC TCGTCCTATA CCTGGTTTGC CCCAGCAAAA ACCCCTCCAA ACACCTCCGT AACGTAATAA   
  
  
- TGAGTCGGAA CAAACTACGG AACCCTCTCC CCAATCTATC CCTATTGGAT GTTTCCGTAG TTCACCTCGT   
  
  
- TGTCGATAAA CCCACACTCT AATCCTTGTA GGACCGGCAA CCACCCGGGT TCTCCTCCGA GTGACCGCCG   
  
  
- CTAGCCCAGT CTTCCACCCC GCTGCTTGAC TGTTCCCAGC CCAAGCTTGG TCAAAGCAAC CGCCCATCGG   
  
  
- GCCGTTGGGT TCGATCAAAC AACGAACCCT ACAAGGGAAC CTTTCCCATA TGAAACTACC TCCTTTTACC   
  
  
- CACAAACTCT AATCCCACCT TTCTAAACGG GAACAATTGA CGGAGTCGAA CCGTTGGAAC ACTTAAATTG   
  
  
- TTAGGATCAC GACCGTAAAT

+     GT1-motif

| Site Name | Organism | Position | Strand | Matrix score. | sequence | function |
| --- | --- | --- | --- | --- | --- | --- |
| GT1-motif | Solanum tuberosum | 247 | + | 9 | GTGTGTGAA | light responsive element |
| GT1-motif | Arabidopsis thaliana | 405 | - | 6 | GGTTAA | light responsive element |

>HU02G03005.1   
+ +Up\_Stream \_Len000AAACGG TGCCGTTGAA TTTGCAGGCG ACGAGGTCGG GTTTAAGGCG GATGTCGTAG   
  
  
+ CGCTTTGGAG AAGCAAATTT GGGAAGACGA GGCTTATTCT TGAACTCTTC GTACGCCATT GTTGTTGGTT   
  
  
+ CCAACCTTTC TCTCTCCTCG TGAATTTGAG TTTGGGCAGT AGACTATCCC GGAATAGAGG ACTGATGCGT   
  
  
+ TATATATATA GAAATTGAGA GAGAGAGAGA GCGTGTGTGA AGGATGTTGA AAATGATGGG CGTAGGGAGT   
  
  
+ CTCTCTCTCA CTGTCTGTTA GGAGTACGTG TCGAGAAAGG AAATAGGATT CCCAAGCAGT CTAATTTGGG   
  
  
+ CCGGTCTATA GGCCAGAATG GGCCTATGAT CAGCCCAGAT ATGACCTTGT TTAACCAACT GTATTTGAAT   
  
  
+ TGAAGAAAGG GATATCGCAA TGTCTTGAAC CACATCAAAT AATAAATGTG CCTTTGGTTA GACCATATGA   
  
  
+ AAAATCTTTG AATCAAATCG GTTGGACCTG AACTGCCATT ATTGATATCT TGTTTTTGGT AGAAGCAAAT   
  
  
+ GGTGTCACTT CGTTTTCTAG ATAAAATTAA CTTGAGTTTT TTTGCCACTA TAGATAGTAT TGCAAATATA   
  
  
+ AAAAGTGTGC CACGTGAAAT CAATATTAGG AGGGTGTTTT ATAACAATAG CTGTTCGGGA GAGTTTTAGT   
  
  
+ TATTTTTATT AAATCAAATA GTTAAGAAGT GTTTGGTAAA TAGTTGTATT TGAAAAAGTT ATTCCCATTA   
  
  
+ GCCTTTTAGT AAGAACTTCT TGTGAAAATG ATAATTGTCT AGAAGTTTGA AAAATTCACA CCACATGACA   
  
  
+ TTTAAAAATA ATTTTGTAGA GAAGGGCATA AATGAAAGTT TGCCACCATC TTTTTTAGAA ACATGTAATT   
  
  
+ TACCAAACAC TTTTTCTAAA AACAGTTAAT TCAAATAACT AACAACTAAC AGCTAATATA AATAACTAAC   
  
  
+ AGCTATTTGC TAAACAGGGC CTAAGTAGTT TGAAATTGTT GTTTTCTCTT CAGCTTCACC ATGGCCGATG   
  
  
+ ATGTAAACAA CTATATTCTG AATTTCATTT TTACTATGAT TTGAAAATTT CAAATTTAAA TAAGATTGGA   
  
  
+ TAAAAAAAAG ACCAAAAATC ATAAGTAATA TTAGTCAAGT TCTATTCCAA CCATATACAT AACTGCTTTT   
  
  
+ TCTTTTGGGG GTTTTGAAAC ATGTGTCCTT AGAATAATTG AAACTCGATA TTATTCTCTA AATCTAAGTT   
  
  
+ TACTTTATCA TATTGATTAT TAATTACCTC CTCAACTCCT CCTATACTCT TGTATGCAGA AATAAAAATG   
  
  
+ TTTCCATACC TTATCAGTCG CGATCATAAT AATTAATTTC TTAATTCGGA TTGTATTTAA TTGCGTATCA   
  
  
+ AATGTGAAAT CGTACCAAAT TAGGAAGCAA AACAAACCGA TAACGAAAAA GGTAAGAAAA CTTAGCAAGG   
  
  
+ AAATGAAATC GAAGACCTGT CAACAATATA GCTTCCCTCA CATCCTTCAA CTTAGGGACC ATCCTATACT   
  
  
+ TTCATGTAAT ACAACTAGAG CCACTAGTCA ATAATAGCCT CCTAGTAGTA GCCATAAGCC TTGGTGAGAG   
  
  
+ AATGTTCCGC AAGACTAGTT AACTTGAAAT TCCATTTTGT CGCAGAACTA TTGGAGTGAG AAAGAGAATC   
  
  
+ AAATCATACC ATATCAAATT TGGACCATTT AAGTTCACAA CACACACACA TACACACAAA AGGACGATGT   
  
  
+ ACGGCCCCAC CCCCCACTCT CCTTTCGCCA TCCCCCTACA CCTCTGCCTA CTCTCTCTCT CTCTCTCTCT   
  
  
+ CTCTGGATCA GTCTGTGTAA AAGCTTCCTC TCCAGTACAG CATTTCCTTG TCTGTTCTTG GAGTTTAGTC   
  
  
+ AATTCCTCCG CCCCAAAAAA ACCCACTTTC TCTCTCATCA TGTTCTTCCA GTTCCACCCT TATACGCCAT   
  
  
+ TCATCATCAT CTACATCACC CACATCTTTT TCTCCACAGT TTTCCCCCCT CATTATGCTC TCCGCCTCCA   
  
  
+ CCCCCGCCTT CCCTCTCGCC GTCGCCGGCG ATGACGGGGA TACCACTGAT GCATGCGACG GCACCGCCAC   
  
  
+ TGCCCTCCAC CTCCTCGGGC TGCTGTTGAA GTGCGCGGAA TTCATCTCCA CCGGAGACCT CGCCGGCGCC   
  
  
+ GGAGATATCT TGCCGGAGAT ATTTGAGTTG TCTACACCGT TTGGCTCCCC CGCCGCCCGG GTCGCCGCCT   
  
  
+ TCTTCGGCCA CGCCCTCCAC GCCCGCCTCC TCTCCGCCTC CCTCCGCACA ACTCCGATCG AGAAGCTCAA   
  
  
+ AACCCTGACC CTGGTTTCCC AAATGCGGAA ATTTCACTCC GCCTTGCAAG TATACAACTC CATCACTCCC   
  
  
+ TTCGTGAAAT TCTCTCACTT CACGGCGAAT CAAGCCATCT ACGAGGCGCT GGACGGCGAG GATCGTGTCC   
  
  
+ ACGTCGTCGA CCTTGACATC ATGCAGGGCC TTCAATGGCC GGGATTGTTC CACATCTTAG CCTCTCGACC   
  
  
+ CAGAAAGCCC CTCTCGGTTC GGGTCACCGG GTTCGGGCCA TCCTCCGAGT TGCTCTCCCA AACGGGTAAG   
  
  
+ CGACTCGCTG AGTTCGCCGC TTCACTCGGC CTGCCCTTCG AGTACAACCC GGTGGAGGGC AAAATTGGGA   
  
  
+ ACTTAGTCGA CCTGGGTCGG GTCGGGTCGC TCCCGAATGA AGTGACGGTG GTGCACTGGA TGCACCATAG   
  
  
+ TCTATACGAC ATAACCGGGT CGGATCTTGG GACTTTGAGG GTATTGAGTG CGGTGAGGCC TAGGCTTGTG   
  
  
+ ACTATGGTTG AGCAGGATAT GGACCAAACG GGGTCGTTTT TGGGGAGGTT TGTGGAGGCA TTGCATTATT   
  
  
+ ACTCAGCCTT GTTTGATGCC TTGGGAGAGG GGTTAGATAG GGATAACCTA CAAAGGCATC AAGTGGAGCA   
  
  
+ ACAGCTATTT GGGTGTGAGA TTAGGAACAT CCTGGCCGTT GGTGGGCCCA AGAGGAGGCT CACTGGCGGC   
  
  
+ GATCGGGTCA GAAGGTGGGG CGACGAACTG ACAAGGGTCG GGTTCGAACC AGTTTCGTTG GCGGGTAGCC   
  
  
+ CGGCAACCCA AGCTAGTTTG TTGCTTGGGA TGTTCCCTTG GAAAGGGTAT ACTTTGATGG AGGAAAATGG   
  
  
+ GTGTTTGAGA TTAGGGTGGA AAGATTTGCC CTTGTTAACT GCCTCAGCTT GGCAACCTTG TGAATTTAAC   
  
  
+ AATCCTAGTG CTGGCATTTA   

- +Up\_Stream \_Len000TTTGCC ACGGCAACTT AAACGTCCGC TGCTCCAGCC CAAATTCCGC CTACAGCATC   
  
  
- GCGAAACCTC TTCGTTTAAA CCCTTCTGCT CCGAATAAGA ACTTGAGAAG CATGCGGTAA CAACAACCAA   
  
  
- GGTTGGAAAG AGAGAGGAGC ACTTAAACTC AAACCCGTCA TCTGATAGGG CCTTATCTCC TGACTACGCA   
  
  
- ATATATATAT CTTTAACTCT CTCTCTCTCT CGCACACACT TCCTACAACT TTTACTACCC GCATCCCTCA   
  
  
- GAGAGAGAGT GACAGACAAT CCTCATGCAC AGCTCTTTCC TTTATCCTAA GGGTTCGTCA GATTAAACCC   
  
  
- GGCCAGATAT CCGGTCTTAC CCGGATACTA GTCGGGTCTA TACTGGAACA AATTGGTTGA CATAAACTTA   
  
  
- ACTTCTTTCC CTATAGCGTT ACAGAACTTG GTGTAGTTTA TTATTTACAC GGAAACCAAT CTGGTATACT   
  
  
- TTTTAGAAAC TTAGTTTAGC CAACCTGGAC TTGACGGTAA TAACTATAGA ACAAAAACCA TCTTCGTTTA   
  
  
- CCACAGTGAA GCAAAAGATC TATTTTAATT GAACTCAAAA AAACGGTGAT ATCTATCATA ACGTTTATAT   
  
  
- TTTTCACACG GTGCACTTTA GTTATAATCC TCCCACAAAA TATTGTTATC GACAAGCCCT CTCAAAATCA   
  
  
- ATAAAAATAA TTTAGTTTAT CAATTCTTCA CAAACCATTT ATCAACATAA ACTTTTTCAA TAAGGGTAAT   
  
  
- CGGAAAATCA TTCTTGAAGA ACACTTTTAC TATTAACAGA TCTTCAAACT TTTTAAGTGT GGTGTACTGT   
  
  
- AAATTTTTAT TAAAACATCT CTTCCCGTAT TTACTTTCAA ACGGTGGTAG AAAAAATCTT TGTACATTAA   
  
  
- ATGGTTTGTG AAAAAGATTT TTGTCAATTA AGTTTATTGA TTGTTGATTG TCGATTATAT TTATTGATTG   
  
  
- TCGATAAACG ATTTGTCCCG GATTCATCAA ACTTTAACAA CAAAAGAGAA GTCGAAGTGG TACCGGCTAC   
  
  
- TACATTTGTT GATATAAGAC TTAAAGTAAA AATGATACTA AACTTTTAAA GTTTAAATTT ATTCTAACCT   
  
  
- ATTTTTTTTC TGGTTTTTAG TATTCATTAT AATCAGTTCA AGATAAGGTT GGTATATGTA TTGACGAAAA   
  
  
- AGAAAACCCC CAAAACTTTG TACACAGGAA TCTTATTAAC TTTGAGCTAT AATAAGAGAT TTAGATTCAA   
  
  
- ATGAAATAGT ATAACTAATA ATTAATGGAG GAGTTGAGGA GGATATGAGA ACATACGTCT TTATTTTTAC   
  
  
- AAAGGTATGG AATAGTCAGC GCTAGTATTA TTAATTAAAG AATTAAGCCT AACATAAATT AACGCATAGT   
  
  
- TTACACTTTA GCATGGTTTA ATCCTTCGTT TTGTTTGGCT ATTGCTTTTT CCATTCTTTT GAATCGTTCC   
  
  
- TTTACTTTAG CTTCTGGACA GTTGTTATAT CGAAGGGAGT GTAGGAAGTT GAATCCCTGG TAGGATATGA   
  
  
- AAGTACATTA TGTTGATCTC GGTGATCAGT TATTATCGGA GGATCATCAT CGGTATTCGG AACCACTCTC   
  
  
- TTACAAGGCG TTCTGATCAA TTGAACTTTA AGGTAAAACA GCGTCTTGAT AACCTCACTC TTTCTCTTAG   
  
  
- TTTAGTATGG TATAGTTTAA ACCTGGTAAA TTCAAGTGTT GTGTGTGTGT ATGTGTGTTT TCCTGCTACA   
  
  
- TGCCGGGGTG GGGGGTGAGA GGAAAGCGGT AGGGGGATGT GGAGACGGAT GAGAGAGAGA GAGAGAGAGA   
  
  
- GAGACCTAGT CAGACACATT TTCGAAGGAG AGGTCATGTC GTAAAGGAAC AGACAAGAAC CTCAAATCAG   
  
  
- TTAAGGAGGC GGGGTTTTTT TGGGTGAAAG AGAGAGTAGT ACAAGAAGGT CAAGGTGGGA ATATGCGGTA   
  
  
- AGTAGTAGTA GATGTAGTGG GTGTAGAAAA AGAGGTGTCA AAAGGGGGGA GTAATACGAG AGGCGGAGGT   
  
  
- GGGGGCGGAA GGGAGAGCGG CAGCGGCCGC TACTGCCCCT ATGGTGACTA CGTACGCTGC CGTGGCGGTG   
  
  
- ACGGGAGGTG GAGGAGCCCG ACGACAACTT CACGCGCCTT AAGTAGAGGT GGCCTCTGGA GCGGCCGCGG   
  
  
- CCTCTATAGA ACGGCCTCTA TAAACTCAAC AGATGTGGCA AACCGAGGGG GCGGCGGGCC CAGCGGCGGA   
  
  
- AGAAGCCGGT GCGGGAGGTG CGGGCGGAGG AGAGGCGGAG GGAGGCGTGT TGAGGCTAGC TCTTCGAGTT   
  
  
- TTGGGACTGG GACCAAAGGG TTTACGCCTT TAAAGTGAGG CGGAACGTTC ATATGTTGAG GTAGTGAGGG   
  
  
- AAGCACTTTA AGAGAGTGAA GTGCCGCTTA GTTCGGTAGA TGCTCCGCGA CCTGCCGCTC CTAGCACAGG   
  
  
- TGCAGCAGCT GGAACTGTAG TACGTCCCGG AAGTTACCGG CCCTAACAAG GTGTAGAATC GGAGAGCTGG   
  
  
- GTCTTTCGGG GAGAGCCAAG CCCAGTGGCC CAAGCCCGGT AGGAGGCTCA ACGAGAGGGT TTGCCCATTC   
  
  
- GCTGAGCGAC TCAAGCGGCG AAGTGAGCCG GACGGGAAGC TCATGTTGGG CCACCTCCCG TTTTAACCCT   
  
  
- TGAATCAGCT GGACCCAGCC CAGCCCAGCG AGGGCTTACT TCACTGCCAC CACGTGACCT ACGTGGTATC   
  
  
- AGATATGCTG TATTGGCCCA GCCTAGAACC CTGAAACTCC CATAACTCAC GCCACTCCGG ATCCGAACAC   
  
  
- TGATACCAAC TCGTCCTATA CCTGGTTTGC CCCAGCAAAA ACCCCTCCAA ACACCTCCGT AACGTAATAA   
  
  
- TGAGTCGGAA CAAACTACGG AACCCTCTCC CCAATCTATC CCTATTGGAT GTTTCCGTAG TTCACCTCGT   
  
  
- TGTCGATAAA CCCACACTCT AATCCTTGTA GGACCGGCAA CCACCCGGGT TCTCCTCCGA GTGACCGCCG   
  
  
- CTAGCCCAGT CTTCCACCCC GCTGCTTGAC TGTTCCCAGC CCAAGCTTGG TCAAAGCAAC CGCCCATCGG   
  
  
- GCCGTTGGGT TCGATCAAAC AACGAACCCT ACAAGGGAAC CTTTCCCATA TGAAACTACC TCCTTTTACC   
  
  
- CACAAACTCT AATCCCACCT TTCTAAACGG GAACAATTGA CGGAGTCGAA CCGTTGGAAC ACTTAAATTG   
  
  
- TTAGGATCAC GACCGTAAAT

+     I-box

| Site Name | Organism | Position | Strand | Matrix score. | sequence | function |
| --- | --- | --- | --- | --- | --- | --- |
| I-box | Flaveria trinervia | 1693 | + | 10 | cCATATCCAAT | part of a light responsive element |

>HU02G03005.1   
+ +Up\_Stream \_Len000AAACGG TGCCGTTGAA TTTGCAGGCG ACGAGGTCGG GTTTAAGGCG GATGTCGTAG   
  
  
+ CGCTTTGGAG AAGCAAATTT GGGAAGACGA GGCTTATTCT TGAACTCTTC GTACGCCATT GTTGTTGGTT   
  
  
+ CCAACCTTTC TCTCTCCTCG TGAATTTGAG TTTGGGCAGT AGACTATCCC GGAATAGAGG ACTGATGCGT   
  
  
+ TATATATATA GAAATTGAGA GAGAGAGAGA GCGTGTGTGA AGGATGTTGA AAATGATGGG CGTAGGGAGT   
  
  
+ CTCTCTCTCA CTGTCTGTTA GGAGTACGTG TCGAGAAAGG AAATAGGATT CCCAAGCAGT CTAATTTGGG   
  
  
+ CCGGTCTATA GGCCAGAATG GGCCTATGAT CAGCCCAGAT ATGACCTTGT TTAACCAACT GTATTTGAAT   
  
  
+ TGAAGAAAGG GATATCGCAA TGTCTTGAAC CACATCAAAT AATAAATGTG CCTTTGGTTA GACCATATGA   
  
  
+ AAAATCTTTG AATCAAATCG GTTGGACCTG AACTGCCATT ATTGATATCT TGTTTTTGGT AGAAGCAAAT   
  
  
+ GGTGTCACTT CGTTTTCTAG ATAAAATTAA CTTGAGTTTT TTTGCCACTA TAGATAGTAT TGCAAATATA   
  
  
+ AAAAGTGTGC CACGTGAAAT CAATATTAGG AGGGTGTTTT ATAACAATAG CTGTTCGGGA GAGTTTTAGT   
  
  
+ TATTTTTATT AAATCAAATA GTTAAGAAGT GTTTGGTAAA TAGTTGTATT TGAAAAAGTT ATTCCCATTA   
  
  
+ GCCTTTTAGT AAGAACTTCT TGTGAAAATG ATAATTGTCT AGAAGTTTGA AAAATTCACA CCACATGACA   
  
  
+ TTTAAAAATA ATTTTGTAGA GAAGGGCATA AATGAAAGTT TGCCACCATC TTTTTTAGAA ACATGTAATT   
  
  
+ TACCAAACAC TTTTTCTAAA AACAGTTAAT TCAAATAACT AACAACTAAC AGCTAATATA AATAACTAAC   
  
  
+ AGCTATTTGC TAAACAGGGC CTAAGTAGTT TGAAATTGTT GTTTTCTCTT CAGCTTCACC ATGGCCGATG   
  
  
+ ATGTAAACAA CTATATTCTG AATTTCATTT TTACTATGAT TTGAAAATTT CAAATTTAAA TAAGATTGGA   
  
  
+ TAAAAAAAAG ACCAAAAATC ATAAGTAATA TTAGTCAAGT TCTATTCCAA CCATATACAT AACTGCTTTT   
  
  
+ TCTTTTGGGG GTTTTGAAAC ATGTGTCCTT AGAATAATTG AAACTCGATA TTATTCTCTA AATCTAAGTT   
  
  
+ TACTTTATCA TATTGATTAT TAATTACCTC CTCAACTCCT CCTATACTCT TGTATGCAGA AATAAAAATG   
  
  
+ TTTCCATACC TTATCAGTCG CGATCATAAT AATTAATTTC TTAATTCGGA TTGTATTTAA TTGCGTATCA   
  
  
+ AATGTGAAAT CGTACCAAAT TAGGAAGCAA AACAAACCGA TAACGAAAAA GGTAAGAAAA CTTAGCAAGG   
  
  
+ AAATGAAATC GAAGACCTGT CAACAATATA GCTTCCCTCA CATCCTTCAA CTTAGGGACC ATCCTATACT   
  
  
+ TTCATGTAAT ACAACTAGAG CCACTAGTCA ATAATAGCCT CCTAGTAGTA GCCATAAGCC TTGGTGAGAG   
  
  
+ AATGTTCCGC AAGACTAGTT AACTTGAAAT TCCATTTTGT CGCAGAACTA TTGGAGTGAG AAAGAGAATC   
  
  
+ AAATCATACC ATATCAAATT TGGACCATTT AAGTTCACAA CACACACACA TACACACAAA AGGACGATGT   
  
  
+ ACGGCCCCAC CCCCCACTCT CCTTTCGCCA TCCCCCTACA CCTCTGCCTA CTCTCTCTCT CTCTCTCTCT   
  
  
+ CTCTGGATCA GTCTGTGTAA AAGCTTCCTC TCCAGTACAG CATTTCCTTG TCTGTTCTTG GAGTTTAGTC   
  
  
+ AATTCCTCCG CCCCAAAAAA ACCCACTTTC TCTCTCATCA TGTTCTTCCA GTTCCACCCT TATACGCCAT   
  
  
+ TCATCATCAT CTACATCACC CACATCTTTT TCTCCACAGT TTTCCCCCCT CATTATGCTC TCCGCCTCCA   
  
  
+ CCCCCGCCTT CCCTCTCGCC GTCGCCGGCG ATGACGGGGA TACCACTGAT GCATGCGACG GCACCGCCAC   
  
  
+ TGCCCTCCAC CTCCTCGGGC TGCTGTTGAA GTGCGCGGAA TTCATCTCCA CCGGAGACCT CGCCGGCGCC   
  
  
+ GGAGATATCT TGCCGGAGAT ATTTGAGTTG TCTACACCGT TTGGCTCCCC CGCCGCCCGG GTCGCCGCCT   
  
  
+ TCTTCGGCCA CGCCCTCCAC GCCCGCCTCC TCTCCGCCTC CCTCCGCACA ACTCCGATCG AGAAGCTCAA   
  
  
+ AACCCTGACC CTGGTTTCCC AAATGCGGAA ATTTCACTCC GCCTTGCAAG TATACAACTC CATCACTCCC   
  
  
+ TTCGTGAAAT TCTCTCACTT CACGGCGAAT CAAGCCATCT ACGAGGCGCT GGACGGCGAG GATCGTGTCC   
  
  
+ ACGTCGTCGA CCTTGACATC ATGCAGGGCC TTCAATGGCC GGGATTGTTC CACATCTTAG CCTCTCGACC   
  
  
+ CAGAAAGCCC CTCTCGGTTC GGGTCACCGG GTTCGGGCCA TCCTCCGAGT TGCTCTCCCA AACGGGTAAG   
  
  
+ CGACTCGCTG AGTTCGCCGC TTCACTCGGC CTGCCCTTCG AGTACAACCC GGTGGAGGGC AAAATTGGGA   
  
  
+ ACTTAGTCGA CCTGGGTCGG GTCGGGTCGC TCCCGAATGA AGTGACGGTG GTGCACTGGA TGCACCATAG   
  
  
+ TCTATACGAC ATAACCGGGT CGGATCTTGG GACTTTGAGG GTATTGAGTG CGGTGAGGCC TAGGCTTGTG   
  
  
+ ACTATGGTTG AGCAGGATAT GGACCAAACG GGGTCGTTTT TGGGGAGGTT TGTGGAGGCA TTGCATTATT   
  
  
+ ACTCAGCCTT GTTTGATGCC TTGGGAGAGG GGTTAGATAG GGATAACCTA CAAAGGCATC AAGTGGAGCA   
  
  
+ ACAGCTATTT GGGTGTGAGA TTAGGAACAT CCTGGCCGTT GGTGGGCCCA AGAGGAGGCT CACTGGCGGC   
  
  
+ GATCGGGTCA GAAGGTGGGG CGACGAACTG ACAAGGGTCG GGTTCGAACC AGTTTCGTTG GCGGGTAGCC   
  
  
+ CGGCAACCCA AGCTAGTTTG TTGCTTGGGA TGTTCCCTTG GAAAGGGTAT ACTTTGATGG AGGAAAATGG   
  
  
+ GTGTTTGAGA TTAGGGTGGA AAGATTTGCC CTTGTTAACT GCCTCAGCTT GGCAACCTTG TGAATTTAAC   
  
  
+ AATCCTAGTG CTGGCATTTA   

- +Up\_Stream \_Len000TTTGCC ACGGCAACTT AAACGTCCGC TGCTCCAGCC CAAATTCCGC CTACAGCATC   
  
  
- GCGAAACCTC TTCGTTTAAA CCCTTCTGCT CCGAATAAGA ACTTGAGAAG CATGCGGTAA CAACAACCAA   
  
  
- GGTTGGAAAG AGAGAGGAGC ACTTAAACTC AAACCCGTCA TCTGATAGGG CCTTATCTCC TGACTACGCA   
  
  
- ATATATATAT CTTTAACTCT CTCTCTCTCT CGCACACACT TCCTACAACT TTTACTACCC GCATCCCTCA   
  
  
- GAGAGAGAGT GACAGACAAT CCTCATGCAC AGCTCTTTCC TTTATCCTAA GGGTTCGTCA GATTAAACCC   
  
  
- GGCCAGATAT CCGGTCTTAC CCGGATACTA GTCGGGTCTA TACTGGAACA AATTGGTTGA CATAAACTTA   
  
  
- ACTTCTTTCC CTATAGCGTT ACAGAACTTG GTGTAGTTTA TTATTTACAC GGAAACCAAT CTGGTATACT   
  
  
- TTTTAGAAAC TTAGTTTAGC CAACCTGGAC TTGACGGTAA TAACTATAGA ACAAAAACCA TCTTCGTTTA   
  
  
- CCACAGTGAA GCAAAAGATC TATTTTAATT GAACTCAAAA AAACGGTGAT ATCTATCATA ACGTTTATAT   
  
  
- TTTTCACACG GTGCACTTTA GTTATAATCC TCCCACAAAA TATTGTTATC GACAAGCCCT CTCAAAATCA   
  
  
- ATAAAAATAA TTTAGTTTAT CAATTCTTCA CAAACCATTT ATCAACATAA ACTTTTTCAA TAAGGGTAAT   
  
  
- CGGAAAATCA TTCTTGAAGA ACACTTTTAC TATTAACAGA TCTTCAAACT TTTTAAGTGT GGTGTACTGT   
  
  
- AAATTTTTAT TAAAACATCT CTTCCCGTAT TTACTTTCAA ACGGTGGTAG AAAAAATCTT TGTACATTAA   
  
  
- ATGGTTTGTG AAAAAGATTT TTGTCAATTA AGTTTATTGA TTGTTGATTG TCGATTATAT TTATTGATTG   
  
  
- TCGATAAACG ATTTGTCCCG GATTCATCAA ACTTTAACAA CAAAAGAGAA GTCGAAGTGG TACCGGCTAC   
  
  
- TACATTTGTT GATATAAGAC TTAAAGTAAA AATGATACTA AACTTTTAAA GTTTAAATTT ATTCTAACCT   
  
  
- ATTTTTTTTC TGGTTTTTAG TATTCATTAT AATCAGTTCA AGATAAGGTT GGTATATGTA TTGACGAAAA   
  
  
- AGAAAACCCC CAAAACTTTG TACACAGGAA TCTTATTAAC TTTGAGCTAT AATAAGAGAT TTAGATTCAA   
  
  
- ATGAAATAGT ATAACTAATA ATTAATGGAG GAGTTGAGGA GGATATGAGA ACATACGTCT TTATTTTTAC   
  
  
- AAAGGTATGG AATAGTCAGC GCTAGTATTA TTAATTAAAG AATTAAGCCT AACATAAATT AACGCATAGT   
  
  
- TTACACTTTA GCATGGTTTA ATCCTTCGTT TTGTTTGGCT ATTGCTTTTT CCATTCTTTT GAATCGTTCC   
  
  
- TTTACTTTAG CTTCTGGACA GTTGTTATAT CGAAGGGAGT GTAGGAAGTT GAATCCCTGG TAGGATATGA   
  
  
- AAGTACATTA TGTTGATCTC GGTGATCAGT TATTATCGGA GGATCATCAT CGGTATTCGG AACCACTCTC   
  
  
- TTACAAGGCG TTCTGATCAA TTGAACTTTA AGGTAAAACA GCGTCTTGAT AACCTCACTC TTTCTCTTAG   
  
  
- TTTAGTATGG TATAGTTTAA ACCTGGTAAA TTCAAGTGTT GTGTGTGTGT ATGTGTGTTT TCCTGCTACA   
  
  
- TGCCGGGGTG GGGGGTGAGA GGAAAGCGGT AGGGGGATGT GGAGACGGAT GAGAGAGAGA GAGAGAGAGA   
  
  
- GAGACCTAGT CAGACACATT TTCGAAGGAG AGGTCATGTC GTAAAGGAAC AGACAAGAAC CTCAAATCAG   
  
  
- TTAAGGAGGC GGGGTTTTTT TGGGTGAAAG AGAGAGTAGT ACAAGAAGGT CAAGGTGGGA ATATGCGGTA   
  
  
- AGTAGTAGTA GATGTAGTGG GTGTAGAAAA AGAGGTGTCA AAAGGGGGGA GTAATACGAG AGGCGGAGGT   
  
  
- GGGGGCGGAA GGGAGAGCGG CAGCGGCCGC TACTGCCCCT ATGGTGACTA CGTACGCTGC CGTGGCGGTG   
  
  
- ACGGGAGGTG GAGGAGCCCG ACGACAACTT CACGCGCCTT AAGTAGAGGT GGCCTCTGGA GCGGCCGCGG   
  
  
- CCTCTATAGA ACGGCCTCTA TAAACTCAAC AGATGTGGCA AACCGAGGGG GCGGCGGGCC CAGCGGCGGA   
  
  
- AGAAGCCGGT GCGGGAGGTG CGGGCGGAGG AGAGGCGGAG GGAGGCGTGT TGAGGCTAGC TCTTCGAGTT   
  
  
- TTGGGACTGG GACCAAAGGG TTTACGCCTT TAAAGTGAGG CGGAACGTTC ATATGTTGAG GTAGTGAGGG   
  
  
- AAGCACTTTA AGAGAGTGAA GTGCCGCTTA GTTCGGTAGA TGCTCCGCGA CCTGCCGCTC CTAGCACAGG   
  
  
- TGCAGCAGCT GGAACTGTAG TACGTCCCGG AAGTTACCGG CCCTAACAAG GTGTAGAATC GGAGAGCTGG   
  
  
- GTCTTTCGGG GAGAGCCAAG CCCAGTGGCC CAAGCCCGGT AGGAGGCTCA ACGAGAGGGT TTGCCCATTC   
  
  
- GCTGAGCGAC TCAAGCGGCG AAGTGAGCCG GACGGGAAGC TCATGTTGGG CCACCTCCCG TTTTAACCCT   
  
  
- TGAATCAGCT GGACCCAGCC CAGCCCAGCG AGGGCTTACT TCACTGCCAC CACGTGACCT ACGTGGTATC   
  
  
- AGATATGCTG TATTGGCCCA GCCTAGAACC CTGAAACTCC CATAACTCAC GCCACTCCGG ATCCGAACAC   
  
  
- TGATACCAAC TCGTCCTATA CCTGGTTTGC CCCAGCAAAA ACCCCTCCAA ACACCTCCGT AACGTAATAA   
  
  
- TGAGTCGGAA CAAACTACGG AACCCTCTCC CCAATCTATC CCTATTGGAT GTTTCCGTAG TTCACCTCGT   
  
  
- TGTCGATAAA CCCACACTCT AATCCTTGTA GGACCGGCAA CCACCCGGGT TCTCCTCCGA GTGACCGCCG   
  
  
- CTAGCCCAGT CTTCCACCCC GCTGCTTGAC TGTTCCCAGC CCAAGCTTGG TCAAAGCAAC CGCCCATCGG   
  
  
- GCCGTTGGGT TCGATCAAAC AACGAACCCT ACAAGGGAAC CTTTCCCATA TGAAACTACC TCCTTTTACC   
  
  
- CACAAACTCT AATCCCACCT TTCTAAACGG GAACAATTGA CGGAGTCGAA CCGTTGGAAC ACTTAAATTG   
  
  
- TTAGGATCAC GACCGTAAAT

+     LAMP-element

| Site Name | Organism | Position | Strand | Matrix score. | sequence | function |
| --- | --- | --- | --- | --- | --- | --- |
| LAMP-element | Pisum sativum | 1267 | + | 8 | CTTTATCA | part of a light responsive element |

>HU02G03005.1   
+ +Up\_Stream \_Len000AAACGG TGCCGTTGAA TTTGCAGGCG ACGAGGTCGG GTTTAAGGCG GATGTCGTAG   
  
  
+ CGCTTTGGAG AAGCAAATTT GGGAAGACGA GGCTTATTCT TGAACTCTTC GTACGCCATT GTTGTTGGTT   
  
  
+ CCAACCTTTC TCTCTCCTCG TGAATTTGAG TTTGGGCAGT AGACTATCCC GGAATAGAGG ACTGATGCGT   
  
  
+ TATATATATA GAAATTGAGA GAGAGAGAGA GCGTGTGTGA AGGATGTTGA AAATGATGGG CGTAGGGAGT   
  
  
+ CTCTCTCTCA CTGTCTGTTA GGAGTACGTG TCGAGAAAGG AAATAGGATT CCCAAGCAGT CTAATTTGGG   
  
  
+ CCGGTCTATA GGCCAGAATG GGCCTATGAT CAGCCCAGAT ATGACCTTGT TTAACCAACT GTATTTGAAT   
  
  
+ TGAAGAAAGG GATATCGCAA TGTCTTGAAC CACATCAAAT AATAAATGTG CCTTTGGTTA GACCATATGA   
  
  
+ AAAATCTTTG AATCAAATCG GTTGGACCTG AACTGCCATT ATTGATATCT TGTTTTTGGT AGAAGCAAAT   
  
  
+ GGTGTCACTT CGTTTTCTAG ATAAAATTAA CTTGAGTTTT TTTGCCACTA TAGATAGTAT TGCAAATATA   
  
  
+ AAAAGTGTGC CACGTGAAAT CAATATTAGG AGGGTGTTTT ATAACAATAG CTGTTCGGGA GAGTTTTAGT   
  
  
+ TATTTTTATT AAATCAAATA GTTAAGAAGT GTTTGGTAAA TAGTTGTATT TGAAAAAGTT ATTCCCATTA   
  
  
+ GCCTTTTAGT AAGAACTTCT TGTGAAAATG ATAATTGTCT AGAAGTTTGA AAAATTCACA CCACATGACA   
  
  
+ TTTAAAAATA ATTTTGTAGA GAAGGGCATA AATGAAAGTT TGCCACCATC TTTTTTAGAA ACATGTAATT   
  
  
+ TACCAAACAC TTTTTCTAAA AACAGTTAAT TCAAATAACT AACAACTAAC AGCTAATATA AATAACTAAC   
  
  
+ AGCTATTTGC TAAACAGGGC CTAAGTAGTT TGAAATTGTT GTTTTCTCTT CAGCTTCACC ATGGCCGATG   
  
  
+ ATGTAAACAA CTATATTCTG AATTTCATTT TTACTATGAT TTGAAAATTT CAAATTTAAA TAAGATTGGA   
  
  
+ TAAAAAAAAG ACCAAAAATC ATAAGTAATA TTAGTCAAGT TCTATTCCAA CCATATACAT AACTGCTTTT   
  
  
+ TCTTTTGGGG GTTTTGAAAC ATGTGTCCTT AGAATAATTG AAACTCGATA TTATTCTCTA AATCTAAGTT   
  
  
+ TACTTTATCA TATTGATTAT TAATTACCTC CTCAACTCCT CCTATACTCT TGTATGCAGA AATAAAAATG   
  
  
+ TTTCCATACC TTATCAGTCG CGATCATAAT AATTAATTTC TTAATTCGGA TTGTATTTAA TTGCGTATCA   
  
  
+ AATGTGAAAT CGTACCAAAT TAGGAAGCAA AACAAACCGA TAACGAAAAA GGTAAGAAAA CTTAGCAAGG   
  
  
+ AAATGAAATC GAAGACCTGT CAACAATATA GCTTCCCTCA CATCCTTCAA CTTAGGGACC ATCCTATACT   
  
  
+ TTCATGTAAT ACAACTAGAG CCACTAGTCA ATAATAGCCT CCTAGTAGTA GCCATAAGCC TTGGTGAGAG   
  
  
+ AATGTTCCGC AAGACTAGTT AACTTGAAAT TCCATTTTGT CGCAGAACTA TTGGAGTGAG AAAGAGAATC   
  
  
+ AAATCATACC ATATCAAATT TGGACCATTT AAGTTCACAA CACACACACA TACACACAAA AGGACGATGT   
  
  
+ ACGGCCCCAC CCCCCACTCT CCTTTCGCCA TCCCCCTACA CCTCTGCCTA CTCTCTCTCT CTCTCTCTCT   
  
  
+ CTCTGGATCA GTCTGTGTAA AAGCTTCCTC TCCAGTACAG CATTTCCTTG TCTGTTCTTG GAGTTTAGTC   
  
  
+ AATTCCTCCG CCCCAAAAAA ACCCACTTTC TCTCTCATCA TGTTCTTCCA GTTCCACCCT TATACGCCAT   
  
  
+ TCATCATCAT CTACATCACC CACATCTTTT TCTCCACAGT TTTCCCCCCT CATTATGCTC TCCGCCTCCA   
  
  
+ CCCCCGCCTT CCCTCTCGCC GTCGCCGGCG ATGACGGGGA TACCACTGAT GCATGCGACG GCACCGCCAC   
  
  
+ TGCCCTCCAC CTCCTCGGGC TGCTGTTGAA GTGCGCGGAA TTCATCTCCA CCGGAGACCT CGCCGGCGCC   
  
  
+ GGAGATATCT TGCCGGAGAT ATTTGAGTTG TCTACACCGT TTGGCTCCCC CGCCGCCCGG GTCGCCGCCT   
  
  
+ TCTTCGGCCA CGCCCTCCAC GCCCGCCTCC TCTCCGCCTC CCTCCGCACA ACTCCGATCG AGAAGCTCAA   
  
  
+ AACCCTGACC CTGGTTTCCC AAATGCGGAA ATTTCACTCC GCCTTGCAAG TATACAACTC CATCACTCCC   
  
  
+ TTCGTGAAAT TCTCTCACTT CACGGCGAAT CAAGCCATCT ACGAGGCGCT GGACGGCGAG GATCGTGTCC   
  
  
+ ACGTCGTCGA CCTTGACATC ATGCAGGGCC TTCAATGGCC GGGATTGTTC CACATCTTAG CCTCTCGACC   
  
  
+ CAGAAAGCCC CTCTCGGTTC GGGTCACCGG GTTCGGGCCA TCCTCCGAGT TGCTCTCCCA AACGGGTAAG   
  
  
+ CGACTCGCTG AGTTCGCCGC TTCACTCGGC CTGCCCTTCG AGTACAACCC GGTGGAGGGC AAAATTGGGA   
  
  
+ ACTTAGTCGA CCTGGGTCGG GTCGGGTCGC TCCCGAATGA AGTGACGGTG GTGCACTGGA TGCACCATAG   
  
  
+ TCTATACGAC ATAACCGGGT CGGATCTTGG GACTTTGAGG GTATTGAGTG CGGTGAGGCC TAGGCTTGTG   
  
  
+ ACTATGGTTG AGCAGGATAT GGACCAAACG GGGTCGTTTT TGGGGAGGTT TGTGGAGGCA TTGCATTATT   
  
  
+ ACTCAGCCTT GTTTGATGCC TTGGGAGAGG GGTTAGATAG GGATAACCTA CAAAGGCATC AAGTGGAGCA   
  
  
+ ACAGCTATTT GGGTGTGAGA TTAGGAACAT CCTGGCCGTT GGTGGGCCCA AGAGGAGGCT CACTGGCGGC   
  
  
+ GATCGGGTCA GAAGGTGGGG CGACGAACTG ACAAGGGTCG GGTTCGAACC AGTTTCGTTG GCGGGTAGCC   
  
  
+ CGGCAACCCA AGCTAGTTTG TTGCTTGGGA TGTTCCCTTG GAAAGGGTAT ACTTTGATGG AGGAAAATGG   
  
  
+ GTGTTTGAGA TTAGGGTGGA AAGATTTGCC CTTGTTAACT GCCTCAGCTT GGCAACCTTG TGAATTTAAC   
  
  
+ AATCCTAGTG CTGGCATTTA   

- +Up\_Stream \_Len000TTTGCC ACGGCAACTT AAACGTCCGC TGCTCCAGCC CAAATTCCGC CTACAGCATC   
  
  
- GCGAAACCTC TTCGTTTAAA CCCTTCTGCT CCGAATAAGA ACTTGAGAAG CATGCGGTAA CAACAACCAA   
  
  
- GGTTGGAAAG AGAGAGGAGC ACTTAAACTC AAACCCGTCA TCTGATAGGG CCTTATCTCC TGACTACGCA   
  
  
- ATATATATAT CTTTAACTCT CTCTCTCTCT CGCACACACT TCCTACAACT TTTACTACCC GCATCCCTCA   
  
  
- GAGAGAGAGT GACAGACAAT CCTCATGCAC AGCTCTTTCC TTTATCCTAA GGGTTCGTCA GATTAAACCC   
  
  
- GGCCAGATAT CCGGTCTTAC CCGGATACTA GTCGGGTCTA TACTGGAACA AATTGGTTGA CATAAACTTA   
  
  
- ACTTCTTTCC CTATAGCGTT ACAGAACTTG GTGTAGTTTA TTATTTACAC GGAAACCAAT CTGGTATACT   
  
  
- TTTTAGAAAC TTAGTTTAGC CAACCTGGAC TTGACGGTAA TAACTATAGA ACAAAAACCA TCTTCGTTTA   
  
  
- CCACAGTGAA GCAAAAGATC TATTTTAATT GAACTCAAAA AAACGGTGAT ATCTATCATA ACGTTTATAT   
  
  
- TTTTCACACG GTGCACTTTA GTTATAATCC TCCCACAAAA TATTGTTATC GACAAGCCCT CTCAAAATCA   
  
  
- ATAAAAATAA TTTAGTTTAT CAATTCTTCA CAAACCATTT ATCAACATAA ACTTTTTCAA TAAGGGTAAT   
  
  
- CGGAAAATCA TTCTTGAAGA ACACTTTTAC TATTAACAGA TCTTCAAACT TTTTAAGTGT GGTGTACTGT   
  
  
- AAATTTTTAT TAAAACATCT CTTCCCGTAT TTACTTTCAA ACGGTGGTAG AAAAAATCTT TGTACATTAA   
  
  
- ATGGTTTGTG AAAAAGATTT TTGTCAATTA AGTTTATTGA TTGTTGATTG TCGATTATAT TTATTGATTG   
  
  
- TCGATAAACG ATTTGTCCCG GATTCATCAA ACTTTAACAA CAAAAGAGAA GTCGAAGTGG TACCGGCTAC   
  
  
- TACATTTGTT GATATAAGAC TTAAAGTAAA AATGATACTA AACTTTTAAA GTTTAAATTT ATTCTAACCT   
  
  
- ATTTTTTTTC TGGTTTTTAG TATTCATTAT AATCAGTTCA AGATAAGGTT GGTATATGTA TTGACGAAAA   
  
  
- AGAAAACCCC CAAAACTTTG TACACAGGAA TCTTATTAAC TTTGAGCTAT AATAAGAGAT TTAGATTCAA   
  
  
- ATGAAATAGT ATAACTAATA ATTAATGGAG GAGTTGAGGA GGATATGAGA ACATACGTCT TTATTTTTAC   
  
  
- AAAGGTATGG AATAGTCAGC GCTAGTATTA TTAATTAAAG AATTAAGCCT AACATAAATT AACGCATAGT   
  
  
- TTACACTTTA GCATGGTTTA ATCCTTCGTT TTGTTTGGCT ATTGCTTTTT CCATTCTTTT GAATCGTTCC   
  
  
- TTTACTTTAG CTTCTGGACA GTTGTTATAT CGAAGGGAGT GTAGGAAGTT GAATCCCTGG TAGGATATGA   
  
  
- AAGTACATTA TGTTGATCTC GGTGATCAGT TATTATCGGA GGATCATCAT CGGTATTCGG AACCACTCTC   
  
  
- TTACAAGGCG TTCTGATCAA TTGAACTTTA AGGTAAAACA GCGTCTTGAT AACCTCACTC TTTCTCTTAG   
  
  
- TTTAGTATGG TATAGTTTAA ACCTGGTAAA TTCAAGTGTT GTGTGTGTGT ATGTGTGTTT TCCTGCTACA   
  
  
- TGCCGGGGTG GGGGGTGAGA GGAAAGCGGT AGGGGGATGT GGAGACGGAT GAGAGAGAGA GAGAGAGAGA   
  
  
- GAGACCTAGT CAGACACATT TTCGAAGGAG AGGTCATGTC GTAAAGGAAC AGACAAGAAC CTCAAATCAG   
  
  
- TTAAGGAGGC GGGGTTTTTT TGGGTGAAAG AGAGAGTAGT ACAAGAAGGT CAAGGTGGGA ATATGCGGTA   
  
  
- AGTAGTAGTA GATGTAGTGG GTGTAGAAAA AGAGGTGTCA AAAGGGGGGA GTAATACGAG AGGCGGAGGT   
  
  
- GGGGGCGGAA GGGAGAGCGG CAGCGGCCGC TACTGCCCCT ATGGTGACTA CGTACGCTGC CGTGGCGGTG   
  
  
- ACGGGAGGTG GAGGAGCCCG ACGACAACTT CACGCGCCTT AAGTAGAGGT GGCCTCTGGA GCGGCCGCGG   
  
  
- CCTCTATAGA ACGGCCTCTA TAAACTCAAC AGATGTGGCA AACCGAGGGG GCGGCGGGCC CAGCGGCGGA   
  
  
- AGAAGCCGGT GCGGGAGGTG CGGGCGGAGG AGAGGCGGAG GGAGGCGTGT TGAGGCTAGC TCTTCGAGTT   
  
  
- TTGGGACTGG GACCAAAGGG TTTACGCCTT TAAAGTGAGG CGGAACGTTC ATATGTTGAG GTAGTGAGGG   
  
  
- AAGCACTTTA AGAGAGTGAA GTGCCGCTTA GTTCGGTAGA TGCTCCGCGA CCTGCCGCTC CTAGCACAGG   
  
  
- TGCAGCAGCT GGAACTGTAG TACGTCCCGG AAGTTACCGG CCCTAACAAG GTGTAGAATC GGAGAGCTGG   
  
  
- GTCTTTCGGG GAGAGCCAAG CCCAGTGGCC CAAGCCCGGT AGGAGGCTCA ACGAGAGGGT TTGCCCATTC   
  
  
- GCTGAGCGAC TCAAGCGGCG AAGTGAGCCG GACGGGAAGC TCATGTTGGG CCACCTCCCG TTTTAACCCT   
  
  
- TGAATCAGCT GGACCCAGCC CAGCCCAGCG AGGGCTTACT TCACTGCCAC CACGTGACCT ACGTGGTATC   
  
  
- AGATATGCTG TATTGGCCCA GCCTAGAACC CTGAAACTCC CATAACTCAC GCCACTCCGG ATCCGAACAC   
  
  
- TGATACCAAC TCGTCCTATA CCTGGTTTGC CCCAGCAAAA ACCCCTCCAA ACACCTCCGT AACGTAATAA   
  
  
- TGAGTCGGAA CAAACTACGG AACCCTCTCC CCAATCTATC CCTATTGGAT GTTTCCGTAG TTCACCTCGT   
  
  
- TGTCGATAAA CCCACACTCT AATCCTTGTA GGACCGGCAA CCACCCGGGT TCTCCTCCGA GTGACCGCCG   
  
  
- CTAGCCCAGT CTTCCACCCC GCTGCTTGAC TGTTCCCAGC CCAAGCTTGG TCAAAGCAAC CGCCCATCGG   
  
  
- GCCGTTGGGT TCGATCAAAC AACGAACCCT ACAAGGGAAC CTTTCCCATA TGAAACTACC TCCTTTTACC   
  
  
- CACAAACTCT AATCCCACCT TTCTAAACGG GAACAATTGA CGGAGTCGAA CCGTTGGAAC ACTTAAATTG   
  
  
- TTAGGATCAC GACCGTAAAT

+     MBS

| Site Name | Organism | Position | Strand | Matrix score. | sequence | function |
| --- | --- | --- | --- | --- | --- | --- |
| MBS | Arabidopsis thaliana | 410 | + | 6 | CAACTG | MYB binding site involved in drought-inducibility |

>HU02G03005.1   
+ +Up\_Stream \_Len000AAACGG TGCCGTTGAA TTTGCAGGCG ACGAGGTCGG GTTTAAGGCG GATGTCGTAG   
  
  
+ CGCTTTGGAG AAGCAAATTT GGGAAGACGA GGCTTATTCT TGAACTCTTC GTACGCCATT GTTGTTGGTT   
  
  
+ CCAACCTTTC TCTCTCCTCG TGAATTTGAG TTTGGGCAGT AGACTATCCC GGAATAGAGG ACTGATGCGT   
  
  
+ TATATATATA GAAATTGAGA GAGAGAGAGA GCGTGTGTGA AGGATGTTGA AAATGATGGG CGTAGGGAGT   
  
  
+ CTCTCTCTCA CTGTCTGTTA GGAGTACGTG TCGAGAAAGG AAATAGGATT CCCAAGCAGT CTAATTTGGG   
  
  
+ CCGGTCTATA GGCCAGAATG GGCCTATGAT CAGCCCAGAT ATGACCTTGT TTAACCAACT GTATTTGAAT   
  
  
+ TGAAGAAAGG GATATCGCAA TGTCTTGAAC CACATCAAAT AATAAATGTG CCTTTGGTTA GACCATATGA   
  
  
+ AAAATCTTTG AATCAAATCG GTTGGACCTG AACTGCCATT ATTGATATCT TGTTTTTGGT AGAAGCAAAT   
  
  
+ GGTGTCACTT CGTTTTCTAG ATAAAATTAA CTTGAGTTTT TTTGCCACTA TAGATAGTAT TGCAAATATA   
  
  
+ AAAAGTGTGC CACGTGAAAT CAATATTAGG AGGGTGTTTT ATAACAATAG CTGTTCGGGA GAGTTTTAGT   
  
  
+ TATTTTTATT AAATCAAATA GTTAAGAAGT GTTTGGTAAA TAGTTGTATT TGAAAAAGTT ATTCCCATTA   
  
  
+ GCCTTTTAGT AAGAACTTCT TGTGAAAATG ATAATTGTCT AGAAGTTTGA AAAATTCACA CCACATGACA   
  
  
+ TTTAAAAATA ATTTTGTAGA GAAGGGCATA AATGAAAGTT TGCCACCATC TTTTTTAGAA ACATGTAATT   
  
  
+ TACCAAACAC TTTTTCTAAA AACAGTTAAT TCAAATAACT AACAACTAAC AGCTAATATA AATAACTAAC   
  
  
+ AGCTATTTGC TAAACAGGGC CTAAGTAGTT TGAAATTGTT GTTTTCTCTT CAGCTTCACC ATGGCCGATG   
  
  
+ ATGTAAACAA CTATATTCTG AATTTCATTT TTACTATGAT TTGAAAATTT CAAATTTAAA TAAGATTGGA   
  
  
+ TAAAAAAAAG ACCAAAAATC ATAAGTAATA TTAGTCAAGT TCTATTCCAA CCATATACAT AACTGCTTTT   
  
  
+ TCTTTTGGGG GTTTTGAAAC ATGTGTCCTT AGAATAATTG AAACTCGATA TTATTCTCTA AATCTAAGTT   
  
  
+ TACTTTATCA TATTGATTAT TAATTACCTC CTCAACTCCT CCTATACTCT TGTATGCAGA AATAAAAATG   
  
  
+ TTTCCATACC TTATCAGTCG CGATCATAAT AATTAATTTC TTAATTCGGA TTGTATTTAA TTGCGTATCA   
  
  
+ AATGTGAAAT CGTACCAAAT TAGGAAGCAA AACAAACCGA TAACGAAAAA GGTAAGAAAA CTTAGCAAGG   
  
  
+ AAATGAAATC GAAGACCTGT CAACAATATA GCTTCCCTCA CATCCTTCAA CTTAGGGACC ATCCTATACT   
  
  
+ TTCATGTAAT ACAACTAGAG CCACTAGTCA ATAATAGCCT CCTAGTAGTA GCCATAAGCC TTGGTGAGAG   
  
  
+ AATGTTCCGC AAGACTAGTT AACTTGAAAT TCCATTTTGT CGCAGAACTA TTGGAGTGAG AAAGAGAATC   
  
  
+ AAATCATACC ATATCAAATT TGGACCATTT AAGTTCACAA CACACACACA TACACACAAA AGGACGATGT   
  
  
+ ACGGCCCCAC CCCCCACTCT CCTTTCGCCA TCCCCCTACA CCTCTGCCTA CTCTCTCTCT CTCTCTCTCT   
  
  
+ CTCTGGATCA GTCTGTGTAA AAGCTTCCTC TCCAGTACAG CATTTCCTTG TCTGTTCTTG GAGTTTAGTC   
  
  
+ AATTCCTCCG CCCCAAAAAA ACCCACTTTC TCTCTCATCA TGTTCTTCCA GTTCCACCCT TATACGCCAT   
  
  
+ TCATCATCAT CTACATCACC CACATCTTTT TCTCCACAGT TTTCCCCCCT CATTATGCTC TCCGCCTCCA   
  
  
+ CCCCCGCCTT CCCTCTCGCC GTCGCCGGCG ATGACGGGGA TACCACTGAT GCATGCGACG GCACCGCCAC   
  
  
+ TGCCCTCCAC CTCCTCGGGC TGCTGTTGAA GTGCGCGGAA TTCATCTCCA CCGGAGACCT CGCCGGCGCC   
  
  
+ GGAGATATCT TGCCGGAGAT ATTTGAGTTG TCTACACCGT TTGGCTCCCC CGCCGCCCGG GTCGCCGCCT   
  
  
+ TCTTCGGCCA CGCCCTCCAC GCCCGCCTCC TCTCCGCCTC CCTCCGCACA ACTCCGATCG AGAAGCTCAA   
  
  
+ AACCCTGACC CTGGTTTCCC AAATGCGGAA ATTTCACTCC GCCTTGCAAG TATACAACTC CATCACTCCC   
  
  
+ TTCGTGAAAT TCTCTCACTT CACGGCGAAT CAAGCCATCT ACGAGGCGCT GGACGGCGAG GATCGTGTCC   
  
  
+ ACGTCGTCGA CCTTGACATC ATGCAGGGCC TTCAATGGCC GGGATTGTTC CACATCTTAG CCTCTCGACC   
  
  
+ CAGAAAGCCC CTCTCGGTTC GGGTCACCGG GTTCGGGCCA TCCTCCGAGT TGCTCTCCCA AACGGGTAAG   
  
  
+ CGACTCGCTG AGTTCGCCGC TTCACTCGGC CTGCCCTTCG AGTACAACCC GGTGGAGGGC AAAATTGGGA   
  
  
+ ACTTAGTCGA CCTGGGTCGG GTCGGGTCGC TCCCGAATGA AGTGACGGTG GTGCACTGGA TGCACCATAG   
  
  
+ TCTATACGAC ATAACCGGGT CGGATCTTGG GACTTTGAGG GTATTGAGTG CGGTGAGGCC TAGGCTTGTG   
  
  
+ ACTATGGTTG AGCAGGATAT GGACCAAACG GGGTCGTTTT TGGGGAGGTT TGTGGAGGCA TTGCATTATT   
  
  
+ ACTCAGCCTT GTTTGATGCC TTGGGAGAGG GGTTAGATAG GGATAACCTA CAAAGGCATC AAGTGGAGCA   
  
  
+ ACAGCTATTT GGGTGTGAGA TTAGGAACAT CCTGGCCGTT GGTGGGCCCA AGAGGAGGCT CACTGGCGGC   
  
  
+ GATCGGGTCA GAAGGTGGGG CGACGAACTG ACAAGGGTCG GGTTCGAACC AGTTTCGTTG GCGGGTAGCC   
  
  
+ CGGCAACCCA AGCTAGTTTG TTGCTTGGGA TGTTCCCTTG GAAAGGGTAT ACTTTGATGG AGGAAAATGG   
  
  
+ GTGTTTGAGA TTAGGGTGGA AAGATTTGCC CTTGTTAACT GCCTCAGCTT GGCAACCTTG TGAATTTAAC   
  
  
+ AATCCTAGTG CTGGCATTTA   

- +Up\_Stream \_Len000TTTGCC ACGGCAACTT AAACGTCCGC TGCTCCAGCC CAAATTCCGC CTACAGCATC   
  
  
- GCGAAACCTC TTCGTTTAAA CCCTTCTGCT CCGAATAAGA ACTTGAGAAG CATGCGGTAA CAACAACCAA   
  
  
- GGTTGGAAAG AGAGAGGAGC ACTTAAACTC AAACCCGTCA TCTGATAGGG CCTTATCTCC TGACTACGCA   
  
  
- ATATATATAT CTTTAACTCT CTCTCTCTCT CGCACACACT TCCTACAACT TTTACTACCC GCATCCCTCA   
  
  
- GAGAGAGAGT GACAGACAAT CCTCATGCAC AGCTCTTTCC TTTATCCTAA GGGTTCGTCA GATTAAACCC   
  
  
- GGCCAGATAT CCGGTCTTAC CCGGATACTA GTCGGGTCTA TACTGGAACA AATTGGTTGA CATAAACTTA   
  
  
- ACTTCTTTCC CTATAGCGTT ACAGAACTTG GTGTAGTTTA TTATTTACAC GGAAACCAAT CTGGTATACT   
  
  
- TTTTAGAAAC TTAGTTTAGC CAACCTGGAC TTGACGGTAA TAACTATAGA ACAAAAACCA TCTTCGTTTA   
  
  
- CCACAGTGAA GCAAAAGATC TATTTTAATT GAACTCAAAA AAACGGTGAT ATCTATCATA ACGTTTATAT   
  
  
- TTTTCACACG GTGCACTTTA GTTATAATCC TCCCACAAAA TATTGTTATC GACAAGCCCT CTCAAAATCA   
  
  
- ATAAAAATAA TTTAGTTTAT CAATTCTTCA CAAACCATTT ATCAACATAA ACTTTTTCAA TAAGGGTAAT   
  
  
- CGGAAAATCA TTCTTGAAGA ACACTTTTAC TATTAACAGA TCTTCAAACT TTTTAAGTGT GGTGTACTGT   
  
  
- AAATTTTTAT TAAAACATCT CTTCCCGTAT TTACTTTCAA ACGGTGGTAG AAAAAATCTT TGTACATTAA   
  
  
- ATGGTTTGTG AAAAAGATTT TTGTCAATTA AGTTTATTGA TTGTTGATTG TCGATTATAT TTATTGATTG   
  
  
- TCGATAAACG ATTTGTCCCG GATTCATCAA ACTTTAACAA CAAAAGAGAA GTCGAAGTGG TACCGGCTAC   
  
  
- TACATTTGTT GATATAAGAC TTAAAGTAAA AATGATACTA AACTTTTAAA GTTTAAATTT ATTCTAACCT   
  
  
- ATTTTTTTTC TGGTTTTTAG TATTCATTAT AATCAGTTCA AGATAAGGTT GGTATATGTA TTGACGAAAA   
  
  
- AGAAAACCCC CAAAACTTTG TACACAGGAA TCTTATTAAC TTTGAGCTAT AATAAGAGAT TTAGATTCAA   
  
  
- ATGAAATAGT ATAACTAATA ATTAATGGAG GAGTTGAGGA GGATATGAGA ACATACGTCT TTATTTTTAC   
  
  
- AAAGGTATGG AATAGTCAGC GCTAGTATTA TTAATTAAAG AATTAAGCCT AACATAAATT AACGCATAGT   
  
  
- TTACACTTTA GCATGGTTTA ATCCTTCGTT TTGTTTGGCT ATTGCTTTTT CCATTCTTTT GAATCGTTCC   
  
  
- TTTACTTTAG CTTCTGGACA GTTGTTATAT CGAAGGGAGT GTAGGAAGTT GAATCCCTGG TAGGATATGA   
  
  
- AAGTACATTA TGTTGATCTC GGTGATCAGT TATTATCGGA GGATCATCAT CGGTATTCGG AACCACTCTC   
  
  
- TTACAAGGCG TTCTGATCAA TTGAACTTTA AGGTAAAACA GCGTCTTGAT AACCTCACTC TTTCTCTTAG   
  
  
- TTTAGTATGG TATAGTTTAA ACCTGGTAAA TTCAAGTGTT GTGTGTGTGT ATGTGTGTTT TCCTGCTACA   
  
  
- TGCCGGGGTG GGGGGTGAGA GGAAAGCGGT AGGGGGATGT GGAGACGGAT GAGAGAGAGA GAGAGAGAGA   
  
  
- GAGACCTAGT CAGACACATT TTCGAAGGAG AGGTCATGTC GTAAAGGAAC AGACAAGAAC CTCAAATCAG   
  
  
- TTAAGGAGGC GGGGTTTTTT TGGGTGAAAG AGAGAGTAGT ACAAGAAGGT CAAGGTGGGA ATATGCGGTA   
  
  
- AGTAGTAGTA GATGTAGTGG GTGTAGAAAA AGAGGTGTCA AAAGGGGGGA GTAATACGAG AGGCGGAGGT   
  
  
- GGGGGCGGAA GGGAGAGCGG CAGCGGCCGC TACTGCCCCT ATGGTGACTA CGTACGCTGC CGTGGCGGTG   
  
  
- ACGGGAGGTG GAGGAGCCCG ACGACAACTT CACGCGCCTT AAGTAGAGGT GGCCTCTGGA GCGGCCGCGG   
  
  
- CCTCTATAGA ACGGCCTCTA TAAACTCAAC AGATGTGGCA AACCGAGGGG GCGGCGGGCC CAGCGGCGGA   
  
  
- AGAAGCCGGT GCGGGAGGTG CGGGCGGAGG AGAGGCGGAG GGAGGCGTGT TGAGGCTAGC TCTTCGAGTT   
  
  
- TTGGGACTGG GACCAAAGGG TTTACGCCTT TAAAGTGAGG CGGAACGTTC ATATGTTGAG GTAGTGAGGG   
  
  
- AAGCACTTTA AGAGAGTGAA GTGCCGCTTA GTTCGGTAGA TGCTCCGCGA CCTGCCGCTC CTAGCACAGG   
  
  
- TGCAGCAGCT GGAACTGTAG TACGTCCCGG AAGTTACCGG CCCTAACAAG GTGTAGAATC GGAGAGCTGG   
  
  
- GTCTTTCGGG GAGAGCCAAG CCCAGTGGCC CAAGCCCGGT AGGAGGCTCA ACGAGAGGGT TTGCCCATTC   
  
  
- GCTGAGCGAC TCAAGCGGCG AAGTGAGCCG GACGGGAAGC TCATGTTGGG CCACCTCCCG TTTTAACCCT   
  
  
- TGAATCAGCT GGACCCAGCC CAGCCCAGCG AGGGCTTACT TCACTGCCAC CACGTGACCT ACGTGGTATC   
  
  
- AGATATGCTG TATTGGCCCA GCCTAGAACC CTGAAACTCC CATAACTCAC GCCACTCCGG ATCCGAACAC   
  
  
- TGATACCAAC TCGTCCTATA CCTGGTTTGC CCCAGCAAAA ACCCCTCCAA ACACCTCCGT AACGTAATAA   
  
  
- TGAGTCGGAA CAAACTACGG AACCCTCTCC CCAATCTATC CCTATTGGAT GTTTCCGTAG TTCACCTCGT   
  
  
- TGTCGATAAA CCCACACTCT AATCCTTGTA GGACCGGCAA CCACCCGGGT TCTCCTCCGA GTGACCGCCG   
  
  
- CTAGCCCAGT CTTCCACCCC GCTGCTTGAC TGTTCCCAGC CCAAGCTTGG TCAAAGCAAC CGCCCATCGG   
  
  
- GCCGTTGGGT TCGATCAAAC AACGAACCCT ACAAGGGAAC CTTTCCCATA TGAAACTACC TCCTTTTACC   
  
  
- CACAAACTCT AATCCCACCT TTCTAAACGG GAACAATTGA CGGAGTCGAA CCGTTGGAAC ACTTAAATTG   
  
  
- TTAGGATCAC GACCGTAAAT

+     MSA-like

| Site Name | Organism | Position | Strand | Matrix score. | sequence | function |
| --- | --- | --- | --- | --- | --- | --- |
| MSA-like | Catharanthus roseus | 2977 | - | 8.5 | (T/C)C(T/C)AACGG(T/C)(T/C)A | cis-acting element involved in cell cycle regulation |

>HU02G03005.1   
+ +Up\_Stream \_Len000AAACGG TGCCGTTGAA TTTGCAGGCG ACGAGGTCGG GTTTAAGGCG GATGTCGTAG   
  
  
+ CGCTTTGGAG AAGCAAATTT GGGAAGACGA GGCTTATTCT TGAACTCTTC GTACGCCATT GTTGTTGGTT   
  
  
+ CCAACCTTTC TCTCTCCTCG TGAATTTGAG TTTGGGCAGT AGACTATCCC GGAATAGAGG ACTGATGCGT   
  
  
+ TATATATATA GAAATTGAGA GAGAGAGAGA GCGTGTGTGA AGGATGTTGA AAATGATGGG CGTAGGGAGT   
  
  
+ CTCTCTCTCA CTGTCTGTTA GGAGTACGTG TCGAGAAAGG AAATAGGATT CCCAAGCAGT CTAATTTGGG   
  
  
+ CCGGTCTATA GGCCAGAATG GGCCTATGAT CAGCCCAGAT ATGACCTTGT TTAACCAACT GTATTTGAAT   
  
  
+ TGAAGAAAGG GATATCGCAA TGTCTTGAAC CACATCAAAT AATAAATGTG CCTTTGGTTA GACCATATGA   
  
  
+ AAAATCTTTG AATCAAATCG GTTGGACCTG AACTGCCATT ATTGATATCT TGTTTTTGGT AGAAGCAAAT   
  
  
+ GGTGTCACTT CGTTTTCTAG ATAAAATTAA CTTGAGTTTT TTTGCCACTA TAGATAGTAT TGCAAATATA   
  
  
+ AAAAGTGTGC CACGTGAAAT CAATATTAGG AGGGTGTTTT ATAACAATAG CTGTTCGGGA GAGTTTTAGT   
  
  
+ TATTTTTATT AAATCAAATA GTTAAGAAGT GTTTGGTAAA TAGTTGTATT TGAAAAAGTT ATTCCCATTA   
  
  
+ GCCTTTTAGT AAGAACTTCT TGTGAAAATG ATAATTGTCT AGAAGTTTGA AAAATTCACA CCACATGACA   
  
  
+ TTTAAAAATA ATTTTGTAGA GAAGGGCATA AATGAAAGTT TGCCACCATC TTTTTTAGAA ACATGTAATT   
  
  
+ TACCAAACAC TTTTTCTAAA AACAGTTAAT TCAAATAACT AACAACTAAC AGCTAATATA AATAACTAAC   
  
  
+ AGCTATTTGC TAAACAGGGC CTAAGTAGTT TGAAATTGTT GTTTTCTCTT CAGCTTCACC ATGGCCGATG   
  
  
+ ATGTAAACAA CTATATTCTG AATTTCATTT TTACTATGAT TTGAAAATTT CAAATTTAAA TAAGATTGGA   
  
  
+ TAAAAAAAAG ACCAAAAATC ATAAGTAATA TTAGTCAAGT TCTATTCCAA CCATATACAT AACTGCTTTT   
  
  
+ TCTTTTGGGG GTTTTGAAAC ATGTGTCCTT AGAATAATTG AAACTCGATA TTATTCTCTA AATCTAAGTT   
  
  
+ TACTTTATCA TATTGATTAT TAATTACCTC CTCAACTCCT CCTATACTCT TGTATGCAGA AATAAAAATG   
  
  
+ TTTCCATACC TTATCAGTCG CGATCATAAT AATTAATTTC TTAATTCGGA TTGTATTTAA TTGCGTATCA   
  
  
+ AATGTGAAAT CGTACCAAAT TAGGAAGCAA AACAAACCGA TAACGAAAAA GGTAAGAAAA CTTAGCAAGG   
  
  
+ AAATGAAATC GAAGACCTGT CAACAATATA GCTTCCCTCA CATCCTTCAA CTTAGGGACC ATCCTATACT   
  
  
+ TTCATGTAAT ACAACTAGAG CCACTAGTCA ATAATAGCCT CCTAGTAGTA GCCATAAGCC TTGGTGAGAG   
  
  
+ AATGTTCCGC AAGACTAGTT AACTTGAAAT TCCATTTTGT CGCAGAACTA TTGGAGTGAG AAAGAGAATC   
  
  
+ AAATCATACC ATATCAAATT TGGACCATTT AAGTTCACAA CACACACACA TACACACAAA AGGACGATGT   
  
  
+ ACGGCCCCAC CCCCCACTCT CCTTTCGCCA TCCCCCTACA CCTCTGCCTA CTCTCTCTCT CTCTCTCTCT   
  
  
+ CTCTGGATCA GTCTGTGTAA AAGCTTCCTC TCCAGTACAG CATTTCCTTG TCTGTTCTTG GAGTTTAGTC   
  
  
+ AATTCCTCCG CCCCAAAAAA ACCCACTTTC TCTCTCATCA TGTTCTTCCA GTTCCACCCT TATACGCCAT   
  
  
+ TCATCATCAT CTACATCACC CACATCTTTT TCTCCACAGT TTTCCCCCCT CATTATGCTC TCCGCCTCCA   
  
  
+ CCCCCGCCTT CCCTCTCGCC GTCGCCGGCG ATGACGGGGA TACCACTGAT GCATGCGACG GCACCGCCAC   
  
  
+ TGCCCTCCAC CTCCTCGGGC TGCTGTTGAA GTGCGCGGAA TTCATCTCCA CCGGAGACCT CGCCGGCGCC   
  
  
+ GGAGATATCT TGCCGGAGAT ATTTGAGTTG TCTACACCGT TTGGCTCCCC CGCCGCCCGG GTCGCCGCCT   
  
  
+ TCTTCGGCCA CGCCCTCCAC GCCCGCCTCC TCTCCGCCTC CCTCCGCACA ACTCCGATCG AGAAGCTCAA   
  
  
+ AACCCTGACC CTGGTTTCCC AAATGCGGAA ATTTCACTCC GCCTTGCAAG TATACAACTC CATCACTCCC   
  
  
+ TTCGTGAAAT TCTCTCACTT CACGGCGAAT CAAGCCATCT ACGAGGCGCT GGACGGCGAG GATCGTGTCC   
  
  
+ ACGTCGTCGA CCTTGACATC ATGCAGGGCC TTCAATGGCC GGGATTGTTC CACATCTTAG CCTCTCGACC   
  
  
+ CAGAAAGCCC CTCTCGGTTC GGGTCACCGG GTTCGGGCCA TCCTCCGAGT TGCTCTCCCA AACGGGTAAG   
  
  
+ CGACTCGCTG AGTTCGCCGC TTCACTCGGC CTGCCCTTCG AGTACAACCC GGTGGAGGGC AAAATTGGGA   
  
  
+ ACTTAGTCGA CCTGGGTCGG GTCGGGTCGC TCCCGAATGA AGTGACGGTG GTGCACTGGA TGCACCATAG   
  
  
+ TCTATACGAC ATAACCGGGT CGGATCTTGG GACTTTGAGG GTATTGAGTG CGGTGAGGCC TAGGCTTGTG   
  
  
+ ACTATGGTTG AGCAGGATAT GGACCAAACG GGGTCGTTTT TGGGGAGGTT TGTGGAGGCA TTGCATTATT   
  
  
+ ACTCAGCCTT GTTTGATGCC TTGGGAGAGG GGTTAGATAG GGATAACCTA CAAAGGCATC AAGTGGAGCA   
  
  
+ ACAGCTATTT GGGTGTGAGA TTAGGAACAT CCTGGCCGTT GGTGGGCCCA AGAGGAGGCT CACTGGCGGC   
  
  
+ GATCGGGTCA GAAGGTGGGG CGACGAACTG ACAAGGGTCG GGTTCGAACC AGTTTCGTTG GCGGGTAGCC   
  
  
+ CGGCAACCCA AGCTAGTTTG TTGCTTGGGA TGTTCCCTTG GAAAGGGTAT ACTTTGATGG AGGAAAATGG   
  
  
+ GTGTTTGAGA TTAGGGTGGA AAGATTTGCC CTTGTTAACT GCCTCAGCTT GGCAACCTTG TGAATTTAAC   
  
  
+ AATCCTAGTG CTGGCATTTA   

- +Up\_Stream \_Len000TTTGCC ACGGCAACTT AAACGTCCGC TGCTCCAGCC CAAATTCCGC CTACAGCATC   
  
  
- GCGAAACCTC TTCGTTTAAA CCCTTCTGCT CCGAATAAGA ACTTGAGAAG CATGCGGTAA CAACAACCAA   
  
  
- GGTTGGAAAG AGAGAGGAGC ACTTAAACTC AAACCCGTCA TCTGATAGGG CCTTATCTCC TGACTACGCA   
  
  
- ATATATATAT CTTTAACTCT CTCTCTCTCT CGCACACACT TCCTACAACT TTTACTACCC GCATCCCTCA   
  
  
- GAGAGAGAGT GACAGACAAT CCTCATGCAC AGCTCTTTCC TTTATCCTAA GGGTTCGTCA GATTAAACCC   
  
  
- GGCCAGATAT CCGGTCTTAC CCGGATACTA GTCGGGTCTA TACTGGAACA AATTGGTTGA CATAAACTTA   
  
  
- ACTTCTTTCC CTATAGCGTT ACAGAACTTG GTGTAGTTTA TTATTTACAC GGAAACCAAT CTGGTATACT   
  
  
- TTTTAGAAAC TTAGTTTAGC CAACCTGGAC TTGACGGTAA TAACTATAGA ACAAAAACCA TCTTCGTTTA   
  
  
- CCACAGTGAA GCAAAAGATC TATTTTAATT GAACTCAAAA AAACGGTGAT ATCTATCATA ACGTTTATAT   
  
  
- TTTTCACACG GTGCACTTTA GTTATAATCC TCCCACAAAA TATTGTTATC GACAAGCCCT CTCAAAATCA   
  
  
- ATAAAAATAA TTTAGTTTAT CAATTCTTCA CAAACCATTT ATCAACATAA ACTTTTTCAA TAAGGGTAAT   
  
  
- CGGAAAATCA TTCTTGAAGA ACACTTTTAC TATTAACAGA TCTTCAAACT TTTTAAGTGT GGTGTACTGT   
  
  
- AAATTTTTAT TAAAACATCT CTTCCCGTAT TTACTTTCAA ACGGTGGTAG AAAAAATCTT TGTACATTAA   
  
  
- ATGGTTTGTG AAAAAGATTT TTGTCAATTA AGTTTATTGA TTGTTGATTG TCGATTATAT TTATTGATTG   
  
  
- TCGATAAACG ATTTGTCCCG GATTCATCAA ACTTTAACAA CAAAAGAGAA GTCGAAGTGG TACCGGCTAC   
  
  
- TACATTTGTT GATATAAGAC TTAAAGTAAA AATGATACTA AACTTTTAAA GTTTAAATTT ATTCTAACCT   
  
  
- ATTTTTTTTC TGGTTTTTAG TATTCATTAT AATCAGTTCA AGATAAGGTT GGTATATGTA TTGACGAAAA   
  
  
- AGAAAACCCC CAAAACTTTG TACACAGGAA TCTTATTAAC TTTGAGCTAT AATAAGAGAT TTAGATTCAA   
  
  
- ATGAAATAGT ATAACTAATA ATTAATGGAG GAGTTGAGGA GGATATGAGA ACATACGTCT TTATTTTTAC   
  
  
- AAAGGTATGG AATAGTCAGC GCTAGTATTA TTAATTAAAG AATTAAGCCT AACATAAATT AACGCATAGT   
  
  
- TTACACTTTA GCATGGTTTA ATCCTTCGTT TTGTTTGGCT ATTGCTTTTT CCATTCTTTT GAATCGTTCC   
  
  
- TTTACTTTAG CTTCTGGACA GTTGTTATAT CGAAGGGAGT GTAGGAAGTT GAATCCCTGG TAGGATATGA   
  
  
- AAGTACATTA TGTTGATCTC GGTGATCAGT TATTATCGGA GGATCATCAT CGGTATTCGG AACCACTCTC   
  
  
- TTACAAGGCG TTCTGATCAA TTGAACTTTA AGGTAAAACA GCGTCTTGAT AACCTCACTC TTTCTCTTAG   
  
  
- TTTAGTATGG TATAGTTTAA ACCTGGTAAA TTCAAGTGTT GTGTGTGTGT ATGTGTGTTT TCCTGCTACA   
  
  
- TGCCGGGGTG GGGGGTGAGA GGAAAGCGGT AGGGGGATGT GGAGACGGAT GAGAGAGAGA GAGAGAGAGA   
  
  
- GAGACCTAGT CAGACACATT TTCGAAGGAG AGGTCATGTC GTAAAGGAAC AGACAAGAAC CTCAAATCAG   
  
  
- TTAAGGAGGC GGGGTTTTTT TGGGTGAAAG AGAGAGTAGT ACAAGAAGGT CAAGGTGGGA ATATGCGGTA   
  
  
- AGTAGTAGTA GATGTAGTGG GTGTAGAAAA AGAGGTGTCA AAAGGGGGGA GTAATACGAG AGGCGGAGGT   
  
  
- GGGGGCGGAA GGGAGAGCGG CAGCGGCCGC TACTGCCCCT ATGGTGACTA CGTACGCTGC CGTGGCGGTG   
  
  
- ACGGGAGGTG GAGGAGCCCG ACGACAACTT CACGCGCCTT AAGTAGAGGT GGCCTCTGGA GCGGCCGCGG   
  
  
- CCTCTATAGA ACGGCCTCTA TAAACTCAAC AGATGTGGCA AACCGAGGGG GCGGCGGGCC CAGCGGCGGA   
  
  
- AGAAGCCGGT GCGGGAGGTG CGGGCGGAGG AGAGGCGGAG GGAGGCGTGT TGAGGCTAGC TCTTCGAGTT   
  
  
- TTGGGACTGG GACCAAAGGG TTTACGCCTT TAAAGTGAGG CGGAACGTTC ATATGTTGAG GTAGTGAGGG   
  
  
- AAGCACTTTA AGAGAGTGAA GTGCCGCTTA GTTCGGTAGA TGCTCCGCGA CCTGCCGCTC CTAGCACAGG   
  
  
- TGCAGCAGCT GGAACTGTAG TACGTCCCGG AAGTTACCGG CCCTAACAAG GTGTAGAATC GGAGAGCTGG   
  
  
- GTCTTTCGGG GAGAGCCAAG CCCAGTGGCC CAAGCCCGGT AGGAGGCTCA ACGAGAGGGT TTGCCCATTC   
  
  
- GCTGAGCGAC TCAAGCGGCG AAGTGAGCCG GACGGGAAGC TCATGTTGGG CCACCTCCCG TTTTAACCCT   
  
  
- TGAATCAGCT GGACCCAGCC CAGCCCAGCG AGGGCTTACT TCACTGCCAC CACGTGACCT ACGTGGTATC   
  
  
- AGATATGCTG TATTGGCCCA GCCTAGAACC CTGAAACTCC CATAACTCAC GCCACTCCGG ATCCGAACAC   
  
  
- TGATACCAAC TCGTCCTATA CCTGGTTTGC CCCAGCAAAA ACCCCTCCAA ACACCTCCGT AACGTAATAA   
  
  
- TGAGTCGGAA CAAACTACGG AACCCTCTCC CCAATCTATC CCTATTGGAT GTTTCCGTAG TTCACCTCGT   
  
  
- TGTCGATAAA CCCACACTCT AATCCTTGTA GGACCGGCAA CCACCCGGGT TCTCCTCCGA GTGACCGCCG   
  
  
- CTAGCCCAGT CTTCCACCCC GCTGCTTGAC TGTTCCCAGC CCAAGCTTGG TCAAAGCAAC CGCCCATCGG   
  
  
- GCCGTTGGGT TCGATCAAAC AACGAACCCT ACAAGGGAAC CTTTCCCATA TGAAACTACC TCCTTTTACC   
  
  
- CACAAACTCT AATCCCACCT TTCTAAACGG GAACAATTGA CGGAGTCGAA CCGTTGGAAC ACTTAAATTG   
  
  
- TTAGGATCAC GACCGTAAAT

+     MYB

| Site Name | Organism | Position | Strand | Matrix score. | sequence | function |
| --- | --- | --- | --- | --- | --- | --- |
| MYB | Arabidopsis thaliana | 2809 | - | 6 | CAACCA |  |
| MYB | Arabidopsis thaliana | 2943 | + | 6 | CAACAG |  |
| MYB | Arabidopsis thaliana | 406 | + | 6 | TAACCA |  |
| MYB | Arabidopsis thaliana | 1172 | + | 6 | CAACCA |  |
| MYB | Arabidopsis thaliana | 479 | - | 6 | TAACCA |  |
| MYB | Arabidopsis thaliana | 2127 | - | 6 | CAACAG |  |

>HU02G03005.1   
+ +Up\_Stream \_Len000AAACGG TGCCGTTGAA TTTGCAGGCG ACGAGGTCGG GTTTAAGGCG GATGTCGTAG   
  
  
+ CGCTTTGGAG AAGCAAATTT GGGAAGACGA GGCTTATTCT TGAACTCTTC GTACGCCATT GTTGTTGGTT   
  
  
+ CCAACCTTTC TCTCTCCTCG TGAATTTGAG TTTGGGCAGT AGACTATCCC GGAATAGAGG ACTGATGCGT   
  
  
+ TATATATATA GAAATTGAGA GAGAGAGAGA GCGTGTGTGA AGGATGTTGA AAATGATGGG CGTAGGGAGT   
  
  
+ CTCTCTCTCA CTGTCTGTTA GGAGTACGTG TCGAGAAAGG AAATAGGATT CCCAAGCAGT CTAATTTGGG   
  
  
+ CCGGTCTATA GGCCAGAATG GGCCTATGAT CAGCCCAGAT ATGACCTTGT TTAACCAACT GTATTTGAAT   
  
  
+ TGAAGAAAGG GATATCGCAA TGTCTTGAAC CACATCAAAT AATAAATGTG CCTTTGGTTA GACCATATGA   
  
  
+ AAAATCTTTG AATCAAATCG GTTGGACCTG AACTGCCATT ATTGATATCT TGTTTTTGGT AGAAGCAAAT   
  
  
+ GGTGTCACTT CGTTTTCTAG ATAAAATTAA CTTGAGTTTT TTTGCCACTA TAGATAGTAT TGCAAATATA   
  
  
+ AAAAGTGTGC CACGTGAAAT CAATATTAGG AGGGTGTTTT ATAACAATAG CTGTTCGGGA GAGTTTTAGT   
  
  
+ TATTTTTATT AAATCAAATA GTTAAGAAGT GTTTGGTAAA TAGTTGTATT TGAAAAAGTT ATTCCCATTA   
  
  
+ GCCTTTTAGT AAGAACTTCT TGTGAAAATG ATAATTGTCT AGAAGTTTGA AAAATTCACA CCACATGACA   
  
  
+ TTTAAAAATA ATTTTGTAGA GAAGGGCATA AATGAAAGTT TGCCACCATC TTTTTTAGAA ACATGTAATT   
  
  
+ TACCAAACAC TTTTTCTAAA AACAGTTAAT TCAAATAACT AACAACTAAC AGCTAATATA AATAACTAAC   
  
  
+ AGCTATTTGC TAAACAGGGC CTAAGTAGTT TGAAATTGTT GTTTTCTCTT CAGCTTCACC ATGGCCGATG   
  
  
+ ATGTAAACAA CTATATTCTG AATTTCATTT TTACTATGAT TTGAAAATTT CAAATTTAAA TAAGATTGGA   
  
  
+ TAAAAAAAAG ACCAAAAATC ATAAGTAATA TTAGTCAAGT TCTATTCCAA CCATATACAT AACTGCTTTT   
  
  
+ TCTTTTGGGG GTTTTGAAAC ATGTGTCCTT AGAATAATTG AAACTCGATA TTATTCTCTA AATCTAAGTT   
  
  
+ TACTTTATCA TATTGATTAT TAATTACCTC CTCAACTCCT CCTATACTCT TGTATGCAGA AATAAAAATG   
  
  
+ TTTCCATACC TTATCAGTCG CGATCATAAT AATTAATTTC TTAATTCGGA TTGTATTTAA TTGCGTATCA   
  
  
+ AATGTGAAAT CGTACCAAAT TAGGAAGCAA AACAAACCGA TAACGAAAAA GGTAAGAAAA CTTAGCAAGG   
  
  
+ AAATGAAATC GAAGACCTGT CAACAATATA GCTTCCCTCA CATCCTTCAA CTTAGGGACC ATCCTATACT   
  
  
+ TTCATGTAAT ACAACTAGAG CCACTAGTCA ATAATAGCCT CCTAGTAGTA GCCATAAGCC TTGGTGAGAG   
  
  
+ AATGTTCCGC AAGACTAGTT AACTTGAAAT TCCATTTTGT CGCAGAACTA TTGGAGTGAG AAAGAGAATC   
  
  
+ AAATCATACC ATATCAAATT TGGACCATTT AAGTTCACAA CACACACACA TACACACAAA AGGACGATGT   
  
  
+ ACGGCCCCAC CCCCCACTCT CCTTTCGCCA TCCCCCTACA CCTCTGCCTA CTCTCTCTCT CTCTCTCTCT   
  
  
+ CTCTGGATCA GTCTGTGTAA AAGCTTCCTC TCCAGTACAG CATTTCCTTG TCTGTTCTTG GAGTTTAGTC   
  
  
+ AATTCCTCCG CCCCAAAAAA ACCCACTTTC TCTCTCATCA TGTTCTTCCA GTTCCACCCT TATACGCCAT   
  
  
+ TCATCATCAT CTACATCACC CACATCTTTT TCTCCACAGT TTTCCCCCCT CATTATGCTC TCCGCCTCCA   
  
  
+ CCCCCGCCTT CCCTCTCGCC GTCGCCGGCG ATGACGGGGA TACCACTGAT GCATGCGACG GCACCGCCAC   
  
  
+ TGCCCTCCAC CTCCTCGGGC TGCTGTTGAA GTGCGCGGAA TTCATCTCCA CCGGAGACCT CGCCGGCGCC   
  
  
+ GGAGATATCT TGCCGGAGAT ATTTGAGTTG TCTACACCGT TTGGCTCCCC CGCCGCCCGG GTCGCCGCCT   
  
  
+ TCTTCGGCCA CGCCCTCCAC GCCCGCCTCC TCTCCGCCTC CCTCCGCACA ACTCCGATCG AGAAGCTCAA   
  
  
+ AACCCTGACC CTGGTTTCCC AAATGCGGAA ATTTCACTCC GCCTTGCAAG TATACAACTC CATCACTCCC   
  
  
+ TTCGTGAAAT TCTCTCACTT CACGGCGAAT CAAGCCATCT ACGAGGCGCT GGACGGCGAG GATCGTGTCC   
  
  
+ ACGTCGTCGA CCTTGACATC ATGCAGGGCC TTCAATGGCC GGGATTGTTC CACATCTTAG CCTCTCGACC   
  
  
+ CAGAAAGCCC CTCTCGGTTC GGGTCACCGG GTTCGGGCCA TCCTCCGAGT TGCTCTCCCA AACGGGTAAG   
  
  
+ CGACTCGCTG AGTTCGCCGC TTCACTCGGC CTGCCCTTCG AGTACAACCC GGTGGAGGGC AAAATTGGGA   
  
  
+ ACTTAGTCGA CCTGGGTCGG GTCGGGTCGC TCCCGAATGA AGTGACGGTG GTGCACTGGA TGCACCATAG   
  
  
+ TCTATACGAC ATAACCGGGT CGGATCTTGG GACTTTGAGG GTATTGAGTG CGGTGAGGCC TAGGCTTGTG   
  
  
+ ACTATGGTTG AGCAGGATAT GGACCAAACG GGGTCGTTTT TGGGGAGGTT TGTGGAGGCA TTGCATTATT   
  
  
+ ACTCAGCCTT GTTTGATGCC TTGGGAGAGG GGTTAGATAG GGATAACCTA CAAAGGCATC AAGTGGAGCA   
  
  
+ ACAGCTATTT GGGTGTGAGA TTAGGAACAT CCTGGCCGTT GGTGGGCCCA AGAGGAGGCT CACTGGCGGC   
  
  
+ GATCGGGTCA GAAGGTGGGG CGACGAACTG ACAAGGGTCG GGTTCGAACC AGTTTCGTTG GCGGGTAGCC   
  
  
+ CGGCAACCCA AGCTAGTTTG TTGCTTGGGA TGTTCCCTTG GAAAGGGTAT ACTTTGATGG AGGAAAATGG   
  
  
+ GTGTTTGAGA TTAGGGTGGA AAGATTTGCC CTTGTTAACT GCCTCAGCTT GGCAACCTTG TGAATTTAAC   
  
  
+ AATCCTAGTG CTGGCATTTA   

- +Up\_Stream \_Len000TTTGCC ACGGCAACTT AAACGTCCGC TGCTCCAGCC CAAATTCCGC CTACAGCATC   
  
  
- GCGAAACCTC TTCGTTTAAA CCCTTCTGCT CCGAATAAGA ACTTGAGAAG CATGCGGTAA CAACAACCAA   
  
  
- GGTTGGAAAG AGAGAGGAGC ACTTAAACTC AAACCCGTCA TCTGATAGGG CCTTATCTCC TGACTACGCA   
  
  
- ATATATATAT CTTTAACTCT CTCTCTCTCT CGCACACACT TCCTACAACT TTTACTACCC GCATCCCTCA   
  
  
- GAGAGAGAGT GACAGACAAT CCTCATGCAC AGCTCTTTCC TTTATCCTAA GGGTTCGTCA GATTAAACCC   
  
  
- GGCCAGATAT CCGGTCTTAC CCGGATACTA GTCGGGTCTA TACTGGAACA AATTGGTTGA CATAAACTTA   
  
  
- ACTTCTTTCC CTATAGCGTT ACAGAACTTG GTGTAGTTTA TTATTTACAC GGAAACCAAT CTGGTATACT   
  
  
- TTTTAGAAAC TTAGTTTAGC CAACCTGGAC TTGACGGTAA TAACTATAGA ACAAAAACCA TCTTCGTTTA   
  
  
- CCACAGTGAA GCAAAAGATC TATTTTAATT GAACTCAAAA AAACGGTGAT ATCTATCATA ACGTTTATAT   
  
  
- TTTTCACACG GTGCACTTTA GTTATAATCC TCCCACAAAA TATTGTTATC GACAAGCCCT CTCAAAATCA   
  
  
- ATAAAAATAA TTTAGTTTAT CAATTCTTCA CAAACCATTT ATCAACATAA ACTTTTTCAA TAAGGGTAAT   
  
  
- CGGAAAATCA TTCTTGAAGA ACACTTTTAC TATTAACAGA TCTTCAAACT TTTTAAGTGT GGTGTACTGT   
  
  
- AAATTTTTAT TAAAACATCT CTTCCCGTAT TTACTTTCAA ACGGTGGTAG AAAAAATCTT TGTACATTAA   
  
  
- ATGGTTTGTG AAAAAGATTT TTGTCAATTA AGTTTATTGA TTGTTGATTG TCGATTATAT TTATTGATTG   
  
  
- TCGATAAACG ATTTGTCCCG GATTCATCAA ACTTTAACAA CAAAAGAGAA GTCGAAGTGG TACCGGCTAC   
  
  
- TACATTTGTT GATATAAGAC TTAAAGTAAA AATGATACTA AACTTTTAAA GTTTAAATTT ATTCTAACCT   
  
  
- ATTTTTTTTC TGGTTTTTAG TATTCATTAT AATCAGTTCA AGATAAGGTT GGTATATGTA TTGACGAAAA   
  
  
- AGAAAACCCC CAAAACTTTG TACACAGGAA TCTTATTAAC TTTGAGCTAT AATAAGAGAT TTAGATTCAA   
  
  
- ATGAAATAGT ATAACTAATA ATTAATGGAG GAGTTGAGGA GGATATGAGA ACATACGTCT TTATTTTTAC   
  
  
- AAAGGTATGG AATAGTCAGC GCTAGTATTA TTAATTAAAG AATTAAGCCT AACATAAATT AACGCATAGT   
  
  
- TTACACTTTA GCATGGTTTA ATCCTTCGTT TTGTTTGGCT ATTGCTTTTT CCATTCTTTT GAATCGTTCC   
  
  
- TTTACTTTAG CTTCTGGACA GTTGTTATAT CGAAGGGAGT GTAGGAAGTT GAATCCCTGG TAGGATATGA   
  
  
- AAGTACATTA TGTTGATCTC GGTGATCAGT TATTATCGGA GGATCATCAT CGGTATTCGG AACCACTCTC   
  
  
- TTACAAGGCG TTCTGATCAA TTGAACTTTA AGGTAAAACA GCGTCTTGAT AACCTCACTC TTTCTCTTAG   
  
  
- TTTAGTATGG TATAGTTTAA ACCTGGTAAA TTCAAGTGTT GTGTGTGTGT ATGTGTGTTT TCCTGCTACA   
  
  
- TGCCGGGGTG GGGGGTGAGA GGAAAGCGGT AGGGGGATGT GGAGACGGAT GAGAGAGAGA GAGAGAGAGA   
  
  
- GAGACCTAGT CAGACACATT TTCGAAGGAG AGGTCATGTC GTAAAGGAAC AGACAAGAAC CTCAAATCAG   
  
  
- TTAAGGAGGC GGGGTTTTTT TGGGTGAAAG AGAGAGTAGT ACAAGAAGGT CAAGGTGGGA ATATGCGGTA   
  
  
- AGTAGTAGTA GATGTAGTGG GTGTAGAAAA AGAGGTGTCA AAAGGGGGGA GTAATACGAG AGGCGGAGGT   
  
  
- GGGGGCGGAA GGGAGAGCGG CAGCGGCCGC TACTGCCCCT ATGGTGACTA CGTACGCTGC CGTGGCGGTG   
  
  
- ACGGGAGGTG GAGGAGCCCG ACGACAACTT CACGCGCCTT AAGTAGAGGT GGCCTCTGGA GCGGCCGCGG   
  
  
- CCTCTATAGA ACGGCCTCTA TAAACTCAAC AGATGTGGCA AACCGAGGGG GCGGCGGGCC CAGCGGCGGA   
  
  
- AGAAGCCGGT GCGGGAGGTG CGGGCGGAGG AGAGGCGGAG GGAGGCGTGT TGAGGCTAGC TCTTCGAGTT   
  
  
- TTGGGACTGG GACCAAAGGG TTTACGCCTT TAAAGTGAGG CGGAACGTTC ATATGTTGAG GTAGTGAGGG   
  
  
- AAGCACTTTA AGAGAGTGAA GTGCCGCTTA GTTCGGTAGA TGCTCCGCGA CCTGCCGCTC CTAGCACAGG   
  
  
- TGCAGCAGCT GGAACTGTAG TACGTCCCGG AAGTTACCGG CCCTAACAAG GTGTAGAATC GGAGAGCTGG   
  
  
- GTCTTTCGGG GAGAGCCAAG CCCAGTGGCC CAAGCCCGGT AGGAGGCTCA ACGAGAGGGT TTGCCCATTC   
  
  
- GCTGAGCGAC TCAAGCGGCG AAGTGAGCCG GACGGGAAGC TCATGTTGGG CCACCTCCCG TTTTAACCCT   
  
  
- TGAATCAGCT GGACCCAGCC CAGCCCAGCG AGGGCTTACT TCACTGCCAC CACGTGACCT ACGTGGTATC   
  
  
- AGATATGCTG TATTGGCCCA GCCTAGAACC CTGAAACTCC CATAACTCAC GCCACTCCGG ATCCGAACAC   
  
  
- TGATACCAAC TCGTCCTATA CCTGGTTTGC CCCAGCAAAA ACCCCTCCAA ACACCTCCGT AACGTAATAA   
  
  
- TGAGTCGGAA CAAACTACGG AACCCTCTCC CCAATCTATC CCTATTGGAT GTTTCCGTAG TTCACCTCGT   
  
  
- TGTCGATAAA CCCACACTCT AATCCTTGTA GGACCGGCAA CCACCCGGGT TCTCCTCCGA GTGACCGCCG   
  
  
- CTAGCCCAGT CTTCCACCCC GCTGCTTGAC TGTTCCCAGC CCAAGCTTGG TCAAAGCAAC CGCCCATCGG   
  
  
- GCCGTTGGGT TCGATCAAAC AACGAACCCT ACAAGGGAAC CTTTCCCATA TGAAACTACC TCCTTTTACC   
  
  
- CACAAACTCT AATCCCACCT TTCTAAACGG GAACAATTGA CGGAGTCGAA CCGTTGGAAC ACTTAAATTG   
  
  
- TTAGGATCAC GACCGTAAAT

+     MYB recognition site

| Site Name | Organism | Position | Strand | Matrix score. | sequence | function |
| --- | --- | --- | --- | --- | --- | --- |
| MYB recognition site | Arabidopsis thaliana | 2980 | + | 6 | CCGTTG |  |
| MYB recognition site | Arabidopsis thaliana | 27 | + | 6 | CCGTTG |  |

>HU02G03005.1   
+ +Up\_Stream \_Len000AAACGG TGCCGTTGAA TTTGCAGGCG ACGAGGTCGG GTTTAAGGCG GATGTCGTAG   
  
  
+ CGCTTTGGAG AAGCAAATTT GGGAAGACGA GGCTTATTCT TGAACTCTTC GTACGCCATT GTTGTTGGTT   
  
  
+ CCAACCTTTC TCTCTCCTCG TGAATTTGAG TTTGGGCAGT AGACTATCCC GGAATAGAGG ACTGATGCGT   
  
  
+ TATATATATA GAAATTGAGA GAGAGAGAGA GCGTGTGTGA AGGATGTTGA AAATGATGGG CGTAGGGAGT   
  
  
+ CTCTCTCTCA CTGTCTGTTA GGAGTACGTG TCGAGAAAGG AAATAGGATT CCCAAGCAGT CTAATTTGGG   
  
  
+ CCGGTCTATA GGCCAGAATG GGCCTATGAT CAGCCCAGAT ATGACCTTGT TTAACCAACT GTATTTGAAT   
  
  
+ TGAAGAAAGG GATATCGCAA TGTCTTGAAC CACATCAAAT AATAAATGTG CCTTTGGTTA GACCATATGA   
  
  
+ AAAATCTTTG AATCAAATCG GTTGGACCTG AACTGCCATT ATTGATATCT TGTTTTTGGT AGAAGCAAAT   
  
  
+ GGTGTCACTT CGTTTTCTAG ATAAAATTAA CTTGAGTTTT TTTGCCACTA TAGATAGTAT TGCAAATATA   
  
  
+ AAAAGTGTGC CACGTGAAAT CAATATTAGG AGGGTGTTTT ATAACAATAG CTGTTCGGGA GAGTTTTAGT   
  
  
+ TATTTTTATT AAATCAAATA GTTAAGAAGT GTTTGGTAAA TAGTTGTATT TGAAAAAGTT ATTCCCATTA   
  
  
+ GCCTTTTAGT AAGAACTTCT TGTGAAAATG ATAATTGTCT AGAAGTTTGA AAAATTCACA CCACATGACA   
  
  
+ TTTAAAAATA ATTTTGTAGA GAAGGGCATA AATGAAAGTT TGCCACCATC TTTTTTAGAA ACATGTAATT   
  
  
+ TACCAAACAC TTTTTCTAAA AACAGTTAAT TCAAATAACT AACAACTAAC AGCTAATATA AATAACTAAC   
  
  
+ AGCTATTTGC TAAACAGGGC CTAAGTAGTT TGAAATTGTT GTTTTCTCTT CAGCTTCACC ATGGCCGATG   
  
  
+ ATGTAAACAA CTATATTCTG AATTTCATTT TTACTATGAT TTGAAAATTT CAAATTTAAA TAAGATTGGA   
  
  
+ TAAAAAAAAG ACCAAAAATC ATAAGTAATA TTAGTCAAGT TCTATTCCAA CCATATACAT AACTGCTTTT   
  
  
+ TCTTTTGGGG GTTTTGAAAC ATGTGTCCTT AGAATAATTG AAACTCGATA TTATTCTCTA AATCTAAGTT   
  
  
+ TACTTTATCA TATTGATTAT TAATTACCTC CTCAACTCCT CCTATACTCT TGTATGCAGA AATAAAAATG   
  
  
+ TTTCCATACC TTATCAGTCG CGATCATAAT AATTAATTTC TTAATTCGGA TTGTATTTAA TTGCGTATCA   
  
  
+ AATGTGAAAT CGTACCAAAT TAGGAAGCAA AACAAACCGA TAACGAAAAA GGTAAGAAAA CTTAGCAAGG   
  
  
+ AAATGAAATC GAAGACCTGT CAACAATATA GCTTCCCTCA CATCCTTCAA CTTAGGGACC ATCCTATACT   
  
  
+ TTCATGTAAT ACAACTAGAG CCACTAGTCA ATAATAGCCT CCTAGTAGTA GCCATAAGCC TTGGTGAGAG   
  
  
+ AATGTTCCGC AAGACTAGTT AACTTGAAAT TCCATTTTGT CGCAGAACTA TTGGAGTGAG AAAGAGAATC   
  
  
+ AAATCATACC ATATCAAATT TGGACCATTT AAGTTCACAA CACACACACA TACACACAAA AGGACGATGT   
  
  
+ ACGGCCCCAC CCCCCACTCT CCTTTCGCCA TCCCCCTACA CCTCTGCCTA CTCTCTCTCT CTCTCTCTCT   
  
  
+ CTCTGGATCA GTCTGTGTAA AAGCTTCCTC TCCAGTACAG CATTTCCTTG TCTGTTCTTG GAGTTTAGTC   
  
  
+ AATTCCTCCG CCCCAAAAAA ACCCACTTTC TCTCTCATCA TGTTCTTCCA GTTCCACCCT TATACGCCAT   
  
  
+ TCATCATCAT CTACATCACC CACATCTTTT TCTCCACAGT TTTCCCCCCT CATTATGCTC TCCGCCTCCA   
  
  
+ CCCCCGCCTT CCCTCTCGCC GTCGCCGGCG ATGACGGGGA TACCACTGAT GCATGCGACG GCACCGCCAC   
  
  
+ TGCCCTCCAC CTCCTCGGGC TGCTGTTGAA GTGCGCGGAA TTCATCTCCA CCGGAGACCT CGCCGGCGCC   
  
  
+ GGAGATATCT TGCCGGAGAT ATTTGAGTTG TCTACACCGT TTGGCTCCCC CGCCGCCCGG GTCGCCGCCT   
  
  
+ TCTTCGGCCA CGCCCTCCAC GCCCGCCTCC TCTCCGCCTC CCTCCGCACA ACTCCGATCG AGAAGCTCAA   
  
  
+ AACCCTGACC CTGGTTTCCC AAATGCGGAA ATTTCACTCC GCCTTGCAAG TATACAACTC CATCACTCCC   
  
  
+ TTCGTGAAAT TCTCTCACTT CACGGCGAAT CAAGCCATCT ACGAGGCGCT GGACGGCGAG GATCGTGTCC   
  
  
+ ACGTCGTCGA CCTTGACATC ATGCAGGGCC TTCAATGGCC GGGATTGTTC CACATCTTAG CCTCTCGACC   
  
  
+ CAGAAAGCCC CTCTCGGTTC GGGTCACCGG GTTCGGGCCA TCCTCCGAGT TGCTCTCCCA AACGGGTAAG   
  
  
+ CGACTCGCTG AGTTCGCCGC TTCACTCGGC CTGCCCTTCG AGTACAACCC GGTGGAGGGC AAAATTGGGA   
  
  
+ ACTTAGTCGA CCTGGGTCGG GTCGGGTCGC TCCCGAATGA AGTGACGGTG GTGCACTGGA TGCACCATAG   
  
  
+ TCTATACGAC ATAACCGGGT CGGATCTTGG GACTTTGAGG GTATTGAGTG CGGTGAGGCC TAGGCTTGTG   
  
  
+ ACTATGGTTG AGCAGGATAT GGACCAAACG GGGTCGTTTT TGGGGAGGTT TGTGGAGGCA TTGCATTATT   
  
  
+ ACTCAGCCTT GTTTGATGCC TTGGGAGAGG GGTTAGATAG GGATAACCTA CAAAGGCATC AAGTGGAGCA   
  
  
+ ACAGCTATTT GGGTGTGAGA TTAGGAACAT CCTGGCCGTT GGTGGGCCCA AGAGGAGGCT CACTGGCGGC   
  
  
+ GATCGGGTCA GAAGGTGGGG CGACGAACTG ACAAGGGTCG GGTTCGAACC AGTTTCGTTG GCGGGTAGCC   
  
  
+ CGGCAACCCA AGCTAGTTTG TTGCTTGGGA TGTTCCCTTG GAAAGGGTAT ACTTTGATGG AGGAAAATGG   
  
  
+ GTGTTTGAGA TTAGGGTGGA AAGATTTGCC CTTGTTAACT GCCTCAGCTT GGCAACCTTG TGAATTTAAC   
  
  
+ AATCCTAGTG CTGGCATTTA   

- +Up\_Stream \_Len000TTTGCC ACGGCAACTT AAACGTCCGC TGCTCCAGCC CAAATTCCGC CTACAGCATC   
  
  
- GCGAAACCTC TTCGTTTAAA CCCTTCTGCT CCGAATAAGA ACTTGAGAAG CATGCGGTAA CAACAACCAA   
  
  
- GGTTGGAAAG AGAGAGGAGC ACTTAAACTC AAACCCGTCA TCTGATAGGG CCTTATCTCC TGACTACGCA   
  
  
- ATATATATAT CTTTAACTCT CTCTCTCTCT CGCACACACT TCCTACAACT TTTACTACCC GCATCCCTCA   
  
  
- GAGAGAGAGT GACAGACAAT CCTCATGCAC AGCTCTTTCC TTTATCCTAA GGGTTCGTCA GATTAAACCC   
  
  
- GGCCAGATAT CCGGTCTTAC CCGGATACTA GTCGGGTCTA TACTGGAACA AATTGGTTGA CATAAACTTA   
  
  
- ACTTCTTTCC CTATAGCGTT ACAGAACTTG GTGTAGTTTA TTATTTACAC GGAAACCAAT CTGGTATACT   
  
  
- TTTTAGAAAC TTAGTTTAGC CAACCTGGAC TTGACGGTAA TAACTATAGA ACAAAAACCA TCTTCGTTTA   
  
  
- CCACAGTGAA GCAAAAGATC TATTTTAATT GAACTCAAAA AAACGGTGAT ATCTATCATA ACGTTTATAT   
  
  
- TTTTCACACG GTGCACTTTA GTTATAATCC TCCCACAAAA TATTGTTATC GACAAGCCCT CTCAAAATCA   
  
  
- ATAAAAATAA TTTAGTTTAT CAATTCTTCA CAAACCATTT ATCAACATAA ACTTTTTCAA TAAGGGTAAT   
  
  
- CGGAAAATCA TTCTTGAAGA ACACTTTTAC TATTAACAGA TCTTCAAACT TTTTAAGTGT GGTGTACTGT   
  
  
- AAATTTTTAT TAAAACATCT CTTCCCGTAT TTACTTTCAA ACGGTGGTAG AAAAAATCTT TGTACATTAA   
  
  
- ATGGTTTGTG AAAAAGATTT TTGTCAATTA AGTTTATTGA TTGTTGATTG TCGATTATAT TTATTGATTG   
  
  
- TCGATAAACG ATTTGTCCCG GATTCATCAA ACTTTAACAA CAAAAGAGAA GTCGAAGTGG TACCGGCTAC   
  
  
- TACATTTGTT GATATAAGAC TTAAAGTAAA AATGATACTA AACTTTTAAA GTTTAAATTT ATTCTAACCT   
  
  
- ATTTTTTTTC TGGTTTTTAG TATTCATTAT AATCAGTTCA AGATAAGGTT GGTATATGTA TTGACGAAAA   
  
  
- AGAAAACCCC CAAAACTTTG TACACAGGAA TCTTATTAAC TTTGAGCTAT AATAAGAGAT TTAGATTCAA   
  
  
- ATGAAATAGT ATAACTAATA ATTAATGGAG GAGTTGAGGA GGATATGAGA ACATACGTCT TTATTTTTAC   
  
  
- AAAGGTATGG AATAGTCAGC GCTAGTATTA TTAATTAAAG AATTAAGCCT AACATAAATT AACGCATAGT   
  
  
- TTACACTTTA GCATGGTTTA ATCCTTCGTT TTGTTTGGCT ATTGCTTTTT CCATTCTTTT GAATCGTTCC   
  
  
- TTTACTTTAG CTTCTGGACA GTTGTTATAT CGAAGGGAGT GTAGGAAGTT GAATCCCTGG TAGGATATGA   
  
  
- AAGTACATTA TGTTGATCTC GGTGATCAGT TATTATCGGA GGATCATCAT CGGTATTCGG AACCACTCTC   
  
  
- TTACAAGGCG TTCTGATCAA TTGAACTTTA AGGTAAAACA GCGTCTTGAT AACCTCACTC TTTCTCTTAG   
  
  
- TTTAGTATGG TATAGTTTAA ACCTGGTAAA TTCAAGTGTT GTGTGTGTGT ATGTGTGTTT TCCTGCTACA   
  
  
- TGCCGGGGTG GGGGGTGAGA GGAAAGCGGT AGGGGGATGT GGAGACGGAT GAGAGAGAGA GAGAGAGAGA   
  
  
- GAGACCTAGT CAGACACATT TTCGAAGGAG AGGTCATGTC GTAAAGGAAC AGACAAGAAC CTCAAATCAG   
  
  
- TTAAGGAGGC GGGGTTTTTT TGGGTGAAAG AGAGAGTAGT ACAAGAAGGT CAAGGTGGGA ATATGCGGTA   
  
  
- AGTAGTAGTA GATGTAGTGG GTGTAGAAAA AGAGGTGTCA AAAGGGGGGA GTAATACGAG AGGCGGAGGT   
  
  
- GGGGGCGGAA GGGAGAGCGG CAGCGGCCGC TACTGCCCCT ATGGTGACTA CGTACGCTGC CGTGGCGGTG   
  
  
- ACGGGAGGTG GAGGAGCCCG ACGACAACTT CACGCGCCTT AAGTAGAGGT GGCCTCTGGA GCGGCCGCGG   
  
  
- CCTCTATAGA ACGGCCTCTA TAAACTCAAC AGATGTGGCA AACCGAGGGG GCGGCGGGCC CAGCGGCGGA   
  
  
- AGAAGCCGGT GCGGGAGGTG CGGGCGGAGG AGAGGCGGAG GGAGGCGTGT TGAGGCTAGC TCTTCGAGTT   
  
  
- TTGGGACTGG GACCAAAGGG TTTACGCCTT TAAAGTGAGG CGGAACGTTC ATATGTTGAG GTAGTGAGGG   
  
  
- AAGCACTTTA AGAGAGTGAA GTGCCGCTTA GTTCGGTAGA TGCTCCGCGA CCTGCCGCTC CTAGCACAGG   
  
  
- TGCAGCAGCT GGAACTGTAG TACGTCCCGG AAGTTACCGG CCCTAACAAG GTGTAGAATC GGAGAGCTGG   
  
  
- GTCTTTCGGG GAGAGCCAAG CCCAGTGGCC CAAGCCCGGT AGGAGGCTCA ACGAGAGGGT TTGCCCATTC   
  
  
- GCTGAGCGAC TCAAGCGGCG AAGTGAGCCG GACGGGAAGC TCATGTTGGG CCACCTCCCG TTTTAACCCT   
  
  
- TGAATCAGCT GGACCCAGCC CAGCCCAGCG AGGGCTTACT TCACTGCCAC CACGTGACCT ACGTGGTATC   
  
  
- AGATATGCTG TATTGGCCCA GCCTAGAACC CTGAAACTCC CATAACTCAC GCCACTCCGG ATCCGAACAC   
  
  
- TGATACCAAC TCGTCCTATA CCTGGTTTGC CCCAGCAAAA ACCCCTCCAA ACACCTCCGT AACGTAATAA   
  
  
- TGAGTCGGAA CAAACTACGG AACCCTCTCC CCAATCTATC CCTATTGGAT GTTTCCGTAG TTCACCTCGT   
  
  
- TGTCGATAAA CCCACACTCT AATCCTTGTA GGACCGGCAA CCACCCGGGT TCTCCTCCGA GTGACCGCCG   
  
  
- CTAGCCCAGT CTTCCACCCC GCTGCTTGAC TGTTCCCAGC CCAAGCTTGG TCAAAGCAAC CGCCCATCGG   
  
  
- GCCGTTGGGT TCGATCAAAC AACGAACCCT ACAAGGGAAC CTTTCCCATA TGAAACTACC TCCTTTTACC   
  
  
- CACAAACTCT AATCCCACCT TTCTAAACGG GAACAATTGA CGGAGTCGAA CCGTTGGAAC ACTTAAATTG   
  
  
- TTAGGATCAC GACCGTAAAT

+     MYB-like sequence

| Site Name | Organism | Position | Strand | Matrix score. | sequence | function |
| --- | --- | --- | --- | --- | --- | --- |
| MYB-like sequence | Arabidopsis thaliana | 406 | + | 6 | TAACCA |  |
| MYB-like sequence | Arabidopsis thaliana | 479 | - | 6 | TAACCA |  |

>HU02G03005.1   
+ +Up\_Stream \_Len000AAACGG TGCCGTTGAA TTTGCAGGCG ACGAGGTCGG GTTTAAGGCG GATGTCGTAG   
  
  
+ CGCTTTGGAG AAGCAAATTT GGGAAGACGA GGCTTATTCT TGAACTCTTC GTACGCCATT GTTGTTGGTT   
  
  
+ CCAACCTTTC TCTCTCCTCG TGAATTTGAG TTTGGGCAGT AGACTATCCC GGAATAGAGG ACTGATGCGT   
  
  
+ TATATATATA GAAATTGAGA GAGAGAGAGA GCGTGTGTGA AGGATGTTGA AAATGATGGG CGTAGGGAGT   
  
  
+ CTCTCTCTCA CTGTCTGTTA GGAGTACGTG TCGAGAAAGG AAATAGGATT CCCAAGCAGT CTAATTTGGG   
  
  
+ CCGGTCTATA GGCCAGAATG GGCCTATGAT CAGCCCAGAT ATGACCTTGT TTAACCAACT GTATTTGAAT   
  
  
+ TGAAGAAAGG GATATCGCAA TGTCTTGAAC CACATCAAAT AATAAATGTG CCTTTGGTTA GACCATATGA   
  
  
+ AAAATCTTTG AATCAAATCG GTTGGACCTG AACTGCCATT ATTGATATCT TGTTTTTGGT AGAAGCAAAT   
  
  
+ GGTGTCACTT CGTTTTCTAG ATAAAATTAA CTTGAGTTTT TTTGCCACTA TAGATAGTAT TGCAAATATA   
  
  
+ AAAAGTGTGC CACGTGAAAT CAATATTAGG AGGGTGTTTT ATAACAATAG CTGTTCGGGA GAGTTTTAGT   
  
  
+ TATTTTTATT AAATCAAATA GTTAAGAAGT GTTTGGTAAA TAGTTGTATT TGAAAAAGTT ATTCCCATTA   
  
  
+ GCCTTTTAGT AAGAACTTCT TGTGAAAATG ATAATTGTCT AGAAGTTTGA AAAATTCACA CCACATGACA   
  
  
+ TTTAAAAATA ATTTTGTAGA GAAGGGCATA AATGAAAGTT TGCCACCATC TTTTTTAGAA ACATGTAATT   
  
  
+ TACCAAACAC TTTTTCTAAA AACAGTTAAT TCAAATAACT AACAACTAAC AGCTAATATA AATAACTAAC   
  
  
+ AGCTATTTGC TAAACAGGGC CTAAGTAGTT TGAAATTGTT GTTTTCTCTT CAGCTTCACC ATGGCCGATG   
  
  
+ ATGTAAACAA CTATATTCTG AATTTCATTT TTACTATGAT TTGAAAATTT CAAATTTAAA TAAGATTGGA   
  
  
+ TAAAAAAAAG ACCAAAAATC ATAAGTAATA TTAGTCAAGT TCTATTCCAA CCATATACAT AACTGCTTTT   
  
  
+ TCTTTTGGGG GTTTTGAAAC ATGTGTCCTT AGAATAATTG AAACTCGATA TTATTCTCTA AATCTAAGTT   
  
  
+ TACTTTATCA TATTGATTAT TAATTACCTC CTCAACTCCT CCTATACTCT TGTATGCAGA AATAAAAATG   
  
  
+ TTTCCATACC TTATCAGTCG CGATCATAAT AATTAATTTC TTAATTCGGA TTGTATTTAA TTGCGTATCA   
  
  
+ AATGTGAAAT CGTACCAAAT TAGGAAGCAA AACAAACCGA TAACGAAAAA GGTAAGAAAA CTTAGCAAGG   
  
  
+ AAATGAAATC GAAGACCTGT CAACAATATA GCTTCCCTCA CATCCTTCAA CTTAGGGACC ATCCTATACT   
  
  
+ TTCATGTAAT ACAACTAGAG CCACTAGTCA ATAATAGCCT CCTAGTAGTA GCCATAAGCC TTGGTGAGAG   
  
  
+ AATGTTCCGC AAGACTAGTT AACTTGAAAT TCCATTTTGT CGCAGAACTA TTGGAGTGAG AAAGAGAATC   
  
  
+ AAATCATACC ATATCAAATT TGGACCATTT AAGTTCACAA CACACACACA TACACACAAA AGGACGATGT   
  
  
+ ACGGCCCCAC CCCCCACTCT CCTTTCGCCA TCCCCCTACA CCTCTGCCTA CTCTCTCTCT CTCTCTCTCT   
  
  
+ CTCTGGATCA GTCTGTGTAA AAGCTTCCTC TCCAGTACAG CATTTCCTTG TCTGTTCTTG GAGTTTAGTC   
  
  
+ AATTCCTCCG CCCCAAAAAA ACCCACTTTC TCTCTCATCA TGTTCTTCCA GTTCCACCCT TATACGCCAT   
  
  
+ TCATCATCAT CTACATCACC CACATCTTTT TCTCCACAGT TTTCCCCCCT CATTATGCTC TCCGCCTCCA   
  
  
+ CCCCCGCCTT CCCTCTCGCC GTCGCCGGCG ATGACGGGGA TACCACTGAT GCATGCGACG GCACCGCCAC   
  
  
+ TGCCCTCCAC CTCCTCGGGC TGCTGTTGAA GTGCGCGGAA TTCATCTCCA CCGGAGACCT CGCCGGCGCC   
  
  
+ GGAGATATCT TGCCGGAGAT ATTTGAGTTG TCTACACCGT TTGGCTCCCC CGCCGCCCGG GTCGCCGCCT   
  
  
+ TCTTCGGCCA CGCCCTCCAC GCCCGCCTCC TCTCCGCCTC CCTCCGCACA ACTCCGATCG AGAAGCTCAA   
  
  
+ AACCCTGACC CTGGTTTCCC AAATGCGGAA ATTTCACTCC GCCTTGCAAG TATACAACTC CATCACTCCC   
  
  
+ TTCGTGAAAT TCTCTCACTT CACGGCGAAT CAAGCCATCT ACGAGGCGCT GGACGGCGAG GATCGTGTCC   
  
  
+ ACGTCGTCGA CCTTGACATC ATGCAGGGCC TTCAATGGCC GGGATTGTTC CACATCTTAG CCTCTCGACC   
  
  
+ CAGAAAGCCC CTCTCGGTTC GGGTCACCGG GTTCGGGCCA TCCTCCGAGT TGCTCTCCCA AACGGGTAAG   
  
  
+ CGACTCGCTG AGTTCGCCGC TTCACTCGGC CTGCCCTTCG AGTACAACCC GGTGGAGGGC AAAATTGGGA   
  
  
+ ACTTAGTCGA CCTGGGTCGG GTCGGGTCGC TCCCGAATGA AGTGACGGTG GTGCACTGGA TGCACCATAG   
  
  
+ TCTATACGAC ATAACCGGGT CGGATCTTGG GACTTTGAGG GTATTGAGTG CGGTGAGGCC TAGGCTTGTG   
  
  
+ ACTATGGTTG AGCAGGATAT GGACCAAACG GGGTCGTTTT TGGGGAGGTT TGTGGAGGCA TTGCATTATT   
  
  
+ ACTCAGCCTT GTTTGATGCC TTGGGAGAGG GGTTAGATAG GGATAACCTA CAAAGGCATC AAGTGGAGCA   
  
  
+ ACAGCTATTT GGGTGTGAGA TTAGGAACAT CCTGGCCGTT GGTGGGCCCA AGAGGAGGCT CACTGGCGGC   
  
  
+ GATCGGGTCA GAAGGTGGGG CGACGAACTG ACAAGGGTCG GGTTCGAACC AGTTTCGTTG GCGGGTAGCC   
  
  
+ CGGCAACCCA AGCTAGTTTG TTGCTTGGGA TGTTCCCTTG GAAAGGGTAT ACTTTGATGG AGGAAAATGG   
  
  
+ GTGTTTGAGA TTAGGGTGGA AAGATTTGCC CTTGTTAACT GCCTCAGCTT GGCAACCTTG TGAATTTAAC   
  
  
+ AATCCTAGTG CTGGCATTTA   

- +Up\_Stream \_Len000TTTGCC ACGGCAACTT AAACGTCCGC TGCTCCAGCC CAAATTCCGC CTACAGCATC   
  
  
- GCGAAACCTC TTCGTTTAAA CCCTTCTGCT CCGAATAAGA ACTTGAGAAG CATGCGGTAA CAACAACCAA   
  
  
- GGTTGGAAAG AGAGAGGAGC ACTTAAACTC AAACCCGTCA TCTGATAGGG CCTTATCTCC TGACTACGCA   
  
  
- ATATATATAT CTTTAACTCT CTCTCTCTCT CGCACACACT TCCTACAACT TTTACTACCC GCATCCCTCA   
  
  
- GAGAGAGAGT GACAGACAAT CCTCATGCAC AGCTCTTTCC TTTATCCTAA GGGTTCGTCA GATTAAACCC   
  
  
- GGCCAGATAT CCGGTCTTAC CCGGATACTA GTCGGGTCTA TACTGGAACA AATTGGTTGA CATAAACTTA   
  
  
- ACTTCTTTCC CTATAGCGTT ACAGAACTTG GTGTAGTTTA TTATTTACAC GGAAACCAAT CTGGTATACT   
  
  
- TTTTAGAAAC TTAGTTTAGC CAACCTGGAC TTGACGGTAA TAACTATAGA ACAAAAACCA TCTTCGTTTA   
  
  
- CCACAGTGAA GCAAAAGATC TATTTTAATT GAACTCAAAA AAACGGTGAT ATCTATCATA ACGTTTATAT   
  
  
- TTTTCACACG GTGCACTTTA GTTATAATCC TCCCACAAAA TATTGTTATC GACAAGCCCT CTCAAAATCA   
  
  
- ATAAAAATAA TTTAGTTTAT CAATTCTTCA CAAACCATTT ATCAACATAA ACTTTTTCAA TAAGGGTAAT   
  
  
- CGGAAAATCA TTCTTGAAGA ACACTTTTAC TATTAACAGA TCTTCAAACT TTTTAAGTGT GGTGTACTGT   
  
  
- AAATTTTTAT TAAAACATCT CTTCCCGTAT TTACTTTCAA ACGGTGGTAG AAAAAATCTT TGTACATTAA   
  
  
- ATGGTTTGTG AAAAAGATTT TTGTCAATTA AGTTTATTGA TTGTTGATTG TCGATTATAT TTATTGATTG   
  
  
- TCGATAAACG ATTTGTCCCG GATTCATCAA ACTTTAACAA CAAAAGAGAA GTCGAAGTGG TACCGGCTAC   
  
  
- TACATTTGTT GATATAAGAC TTAAAGTAAA AATGATACTA AACTTTTAAA GTTTAAATTT ATTCTAACCT   
  
  
- ATTTTTTTTC TGGTTTTTAG TATTCATTAT AATCAGTTCA AGATAAGGTT GGTATATGTA TTGACGAAAA   
  
  
- AGAAAACCCC CAAAACTTTG TACACAGGAA TCTTATTAAC TTTGAGCTAT AATAAGAGAT TTAGATTCAA   
  
  
- ATGAAATAGT ATAACTAATA ATTAATGGAG GAGTTGAGGA GGATATGAGA ACATACGTCT TTATTTTTAC   
  
  
- AAAGGTATGG AATAGTCAGC GCTAGTATTA TTAATTAAAG AATTAAGCCT AACATAAATT AACGCATAGT   
  
  
- TTACACTTTA GCATGGTTTA ATCCTTCGTT TTGTTTGGCT ATTGCTTTTT CCATTCTTTT GAATCGTTCC   
  
  
- TTTACTTTAG CTTCTGGACA GTTGTTATAT CGAAGGGAGT GTAGGAAGTT GAATCCCTGG TAGGATATGA   
  
  
- AAGTACATTA TGTTGATCTC GGTGATCAGT TATTATCGGA GGATCATCAT CGGTATTCGG AACCACTCTC   
  
  
- TTACAAGGCG TTCTGATCAA TTGAACTTTA AGGTAAAACA GCGTCTTGAT AACCTCACTC TTTCTCTTAG   
  
  
- TTTAGTATGG TATAGTTTAA ACCTGGTAAA TTCAAGTGTT GTGTGTGTGT ATGTGTGTTT TCCTGCTACA   
  
  
- TGCCGGGGTG GGGGGTGAGA GGAAAGCGGT AGGGGGATGT GGAGACGGAT GAGAGAGAGA GAGAGAGAGA   
  
  
- GAGACCTAGT CAGACACATT TTCGAAGGAG AGGTCATGTC GTAAAGGAAC AGACAAGAAC CTCAAATCAG   
  
  
- TTAAGGAGGC GGGGTTTTTT TGGGTGAAAG AGAGAGTAGT ACAAGAAGGT CAAGGTGGGA ATATGCGGTA   
  
  
- AGTAGTAGTA GATGTAGTGG GTGTAGAAAA AGAGGTGTCA AAAGGGGGGA GTAATACGAG AGGCGGAGGT   
  
  
- GGGGGCGGAA GGGAGAGCGG CAGCGGCCGC TACTGCCCCT ATGGTGACTA CGTACGCTGC CGTGGCGGTG   
  
  
- ACGGGAGGTG GAGGAGCCCG ACGACAACTT CACGCGCCTT AAGTAGAGGT GGCCTCTGGA GCGGCCGCGG   
  
  
- CCTCTATAGA ACGGCCTCTA TAAACTCAAC AGATGTGGCA AACCGAGGGG GCGGCGGGCC CAGCGGCGGA   
  
  
- AGAAGCCGGT GCGGGAGGTG CGGGCGGAGG AGAGGCGGAG GGAGGCGTGT TGAGGCTAGC TCTTCGAGTT   
  
  
- TTGGGACTGG GACCAAAGGG TTTACGCCTT TAAAGTGAGG CGGAACGTTC ATATGTTGAG GTAGTGAGGG   
  
  
- AAGCACTTTA AGAGAGTGAA GTGCCGCTTA GTTCGGTAGA TGCTCCGCGA CCTGCCGCTC CTAGCACAGG   
  
  
- TGCAGCAGCT GGAACTGTAG TACGTCCCGG AAGTTACCGG CCCTAACAAG GTGTAGAATC GGAGAGCTGG   
  
  
- GTCTTTCGGG GAGAGCCAAG CCCAGTGGCC CAAGCCCGGT AGGAGGCTCA ACGAGAGGGT TTGCCCATTC   
  
  
- GCTGAGCGAC TCAAGCGGCG AAGTGAGCCG GACGGGAAGC TCATGTTGGG CCACCTCCCG TTTTAACCCT   
  
  
- TGAATCAGCT GGACCCAGCC CAGCCCAGCG AGGGCTTACT TCACTGCCAC CACGTGACCT ACGTGGTATC   
  
  
- AGATATGCTG TATTGGCCCA GCCTAGAACC CTGAAACTCC CATAACTCAC GCCACTCCGG ATCCGAACAC   
  
  
- TGATACCAAC TCGTCCTATA CCTGGTTTGC CCCAGCAAAA ACCCCTCCAA ACACCTCCGT AACGTAATAA   
  
  
- TGAGTCGGAA CAAACTACGG AACCCTCTCC CCAATCTATC CCTATTGGAT GTTTCCGTAG TTCACCTCGT   
  
  
- TGTCGATAAA CCCACACTCT AATCCTTGTA GGACCGGCAA CCACCCGGGT TCTCCTCCGA GTGACCGCCG   
  
  
- CTAGCCCAGT CTTCCACCCC GCTGCTTGAC TGTTCCCAGC CCAAGCTTGG TCAAAGCAAC CGCCCATCGG   
  
  
- GCCGTTGGGT TCGATCAAAC AACGAACCCT ACAAGGGAAC CTTTCCCATA TGAAACTACC TCCTTTTACC   
  
  
- CACAAACTCT AATCCCACCT TTCTAAACGG GAACAATTGA CGGAGTCGAA CCGTTGGAAC ACTTAAATTG   
  
  
- TTAGGATCAC GACCGTAAAT

+     MYC

| Site Name | Organism | Position | Strand | Matrix score. | sequence | function |
| --- | --- | --- | --- | --- | --- | --- |
| MYC | Arabidopsis thaliana | 2334 | - | 6 | CATTTG |  |
| MYC | Arabidopsis thaliana | 560 | - | 6 | CATTTG |  |
| MYC | Arabidopsis thaliana | 836 | - | 6 | CATGTG |  |
| MYC | Arabidopsis thaliana | 1214 | + | 6 | CATGTG |  |
| MYC | Arabidopsis thaliana | 1403 | - | 6 | CATTTG |  |

>HU02G03005.1   
+ +Up\_Stream \_Len000AAACGG TGCCGTTGAA TTTGCAGGCG ACGAGGTCGG GTTTAAGGCG GATGTCGTAG   
  
  
+ CGCTTTGGAG AAGCAAATTT GGGAAGACGA GGCTTATTCT TGAACTCTTC GTACGCCATT GTTGTTGGTT   
  
  
+ CCAACCTTTC TCTCTCCTCG TGAATTTGAG TTTGGGCAGT AGACTATCCC GGAATAGAGG ACTGATGCGT   
  
  
+ TATATATATA GAAATTGAGA GAGAGAGAGA GCGTGTGTGA AGGATGTTGA AAATGATGGG CGTAGGGAGT   
  
  
+ CTCTCTCTCA CTGTCTGTTA GGAGTACGTG TCGAGAAAGG AAATAGGATT CCCAAGCAGT CTAATTTGGG   
  
  
+ CCGGTCTATA GGCCAGAATG GGCCTATGAT CAGCCCAGAT ATGACCTTGT TTAACCAACT GTATTTGAAT   
  
  
+ TGAAGAAAGG GATATCGCAA TGTCTTGAAC CACATCAAAT AATAAATGTG CCTTTGGTTA GACCATATGA   
  
  
+ AAAATCTTTG AATCAAATCG GTTGGACCTG AACTGCCATT ATTGATATCT TGTTTTTGGT AGAAGCAAAT   
  
  
+ GGTGTCACTT CGTTTTCTAG ATAAAATTAA CTTGAGTTTT TTTGCCACTA TAGATAGTAT TGCAAATATA   
  
  
+ AAAAGTGTGC CACGTGAAAT CAATATTAGG AGGGTGTTTT ATAACAATAG CTGTTCGGGA GAGTTTTAGT   
  
  
+ TATTTTTATT AAATCAAATA GTTAAGAAGT GTTTGGTAAA TAGTTGTATT TGAAAAAGTT ATTCCCATTA   
  
  
+ GCCTTTTAGT AAGAACTTCT TGTGAAAATG ATAATTGTCT AGAAGTTTGA AAAATTCACA CCACATGACA   
  
  
+ TTTAAAAATA ATTTTGTAGA GAAGGGCATA AATGAAAGTT TGCCACCATC TTTTTTAGAA ACATGTAATT   
  
  
+ TACCAAACAC TTTTTCTAAA AACAGTTAAT TCAAATAACT AACAACTAAC AGCTAATATA AATAACTAAC   
  
  
+ AGCTATTTGC TAAACAGGGC CTAAGTAGTT TGAAATTGTT GTTTTCTCTT CAGCTTCACC ATGGCCGATG   
  
  
+ ATGTAAACAA CTATATTCTG AATTTCATTT TTACTATGAT TTGAAAATTT CAAATTTAAA TAAGATTGGA   
  
  
+ TAAAAAAAAG ACCAAAAATC ATAAGTAATA TTAGTCAAGT TCTATTCCAA CCATATACAT AACTGCTTTT   
  
  
+ TCTTTTGGGG GTTTTGAAAC ATGTGTCCTT AGAATAATTG AAACTCGATA TTATTCTCTA AATCTAAGTT   
  
  
+ TACTTTATCA TATTGATTAT TAATTACCTC CTCAACTCCT CCTATACTCT TGTATGCAGA AATAAAAATG   
  
  
+ TTTCCATACC TTATCAGTCG CGATCATAAT AATTAATTTC TTAATTCGGA TTGTATTTAA TTGCGTATCA   
  
  
+ AATGTGAAAT CGTACCAAAT TAGGAAGCAA AACAAACCGA TAACGAAAAA GGTAAGAAAA CTTAGCAAGG   
  
  
+ AAATGAAATC GAAGACCTGT CAACAATATA GCTTCCCTCA CATCCTTCAA CTTAGGGACC ATCCTATACT   
  
  
+ TTCATGTAAT ACAACTAGAG CCACTAGTCA ATAATAGCCT CCTAGTAGTA GCCATAAGCC TTGGTGAGAG   
  
  
+ AATGTTCCGC AAGACTAGTT AACTTGAAAT TCCATTTTGT CGCAGAACTA TTGGAGTGAG AAAGAGAATC   
  
  
+ AAATCATACC ATATCAAATT TGGACCATTT AAGTTCACAA CACACACACA TACACACAAA AGGACGATGT   
  
  
+ ACGGCCCCAC CCCCCACTCT CCTTTCGCCA TCCCCCTACA CCTCTGCCTA CTCTCTCTCT CTCTCTCTCT   
  
  
+ CTCTGGATCA GTCTGTGTAA AAGCTTCCTC TCCAGTACAG CATTTCCTTG TCTGTTCTTG GAGTTTAGTC   
  
  
+ AATTCCTCCG CCCCAAAAAA ACCCACTTTC TCTCTCATCA TGTTCTTCCA GTTCCACCCT TATACGCCAT   
  
  
+ TCATCATCAT CTACATCACC CACATCTTTT TCTCCACAGT TTTCCCCCCT CATTATGCTC TCCGCCTCCA   
  
  
+ CCCCCGCCTT CCCTCTCGCC GTCGCCGGCG ATGACGGGGA TACCACTGAT GCATGCGACG GCACCGCCAC   
  
  
+ TGCCCTCCAC CTCCTCGGGC TGCTGTTGAA GTGCGCGGAA TTCATCTCCA CCGGAGACCT CGCCGGCGCC   
  
  
+ GGAGATATCT TGCCGGAGAT ATTTGAGTTG TCTACACCGT TTGGCTCCCC CGCCGCCCGG GTCGCCGCCT   
  
  
+ TCTTCGGCCA CGCCCTCCAC GCCCGCCTCC TCTCCGCCTC CCTCCGCACA ACTCCGATCG AGAAGCTCAA   
  
  
+ AACCCTGACC CTGGTTTCCC AAATGCGGAA ATTTCACTCC GCCTTGCAAG TATACAACTC CATCACTCCC   
  
  
+ TTCGTGAAAT TCTCTCACTT CACGGCGAAT CAAGCCATCT ACGAGGCGCT GGACGGCGAG GATCGTGTCC   
  
  
+ ACGTCGTCGA CCTTGACATC ATGCAGGGCC TTCAATGGCC GGGATTGTTC CACATCTTAG CCTCTCGACC   
  
  
+ CAGAAAGCCC CTCTCGGTTC GGGTCACCGG GTTCGGGCCA TCCTCCGAGT TGCTCTCCCA AACGGGTAAG   
  
  
+ CGACTCGCTG AGTTCGCCGC TTCACTCGGC CTGCCCTTCG AGTACAACCC GGTGGAGGGC AAAATTGGGA   
  
  
+ ACTTAGTCGA CCTGGGTCGG GTCGGGTCGC TCCCGAATGA AGTGACGGTG GTGCACTGGA TGCACCATAG   
  
  
+ TCTATACGAC ATAACCGGGT CGGATCTTGG GACTTTGAGG GTATTGAGTG CGGTGAGGCC TAGGCTTGTG   
  
  
+ ACTATGGTTG AGCAGGATAT GGACCAAACG GGGTCGTTTT TGGGGAGGTT TGTGGAGGCA TTGCATTATT   
  
  
+ ACTCAGCCTT GTTTGATGCC TTGGGAGAGG GGTTAGATAG GGATAACCTA CAAAGGCATC AAGTGGAGCA   
  
  
+ ACAGCTATTT GGGTGTGAGA TTAGGAACAT CCTGGCCGTT GGTGGGCCCA AGAGGAGGCT CACTGGCGGC   
  
  
+ GATCGGGTCA GAAGGTGGGG CGACGAACTG ACAAGGGTCG GGTTCGAACC AGTTTCGTTG GCGGGTAGCC   
  
  
+ CGGCAACCCA AGCTAGTTTG TTGCTTGGGA TGTTCCCTTG GAAAGGGTAT ACTTTGATGG AGGAAAATGG   
  
  
+ GTGTTTGAGA TTAGGGTGGA AAGATTTGCC CTTGTTAACT GCCTCAGCTT GGCAACCTTG TGAATTTAAC   
  
  
+ AATCCTAGTG CTGGCATTTA   

- +Up\_Stream \_Len000TTTGCC ACGGCAACTT AAACGTCCGC TGCTCCAGCC CAAATTCCGC CTACAGCATC   
  
  
- GCGAAACCTC TTCGTTTAAA CCCTTCTGCT CCGAATAAGA ACTTGAGAAG CATGCGGTAA CAACAACCAA   
  
  
- GGTTGGAAAG AGAGAGGAGC ACTTAAACTC AAACCCGTCA TCTGATAGGG CCTTATCTCC TGACTACGCA   
  
  
- ATATATATAT CTTTAACTCT CTCTCTCTCT CGCACACACT TCCTACAACT TTTACTACCC GCATCCCTCA   
  
  
- GAGAGAGAGT GACAGACAAT CCTCATGCAC AGCTCTTTCC TTTATCCTAA GGGTTCGTCA GATTAAACCC   
  
  
- GGCCAGATAT CCGGTCTTAC CCGGATACTA GTCGGGTCTA TACTGGAACA AATTGGTTGA CATAAACTTA   
  
  
- ACTTCTTTCC CTATAGCGTT ACAGAACTTG GTGTAGTTTA TTATTTACAC GGAAACCAAT CTGGTATACT   
  
  
- TTTTAGAAAC TTAGTTTAGC CAACCTGGAC TTGACGGTAA TAACTATAGA ACAAAAACCA TCTTCGTTTA   
  
  
- CCACAGTGAA GCAAAAGATC TATTTTAATT GAACTCAAAA AAACGGTGAT ATCTATCATA ACGTTTATAT   
  
  
- TTTTCACACG GTGCACTTTA GTTATAATCC TCCCACAAAA TATTGTTATC GACAAGCCCT CTCAAAATCA   
  
  
- ATAAAAATAA TTTAGTTTAT CAATTCTTCA CAAACCATTT ATCAACATAA ACTTTTTCAA TAAGGGTAAT   
  
  
- CGGAAAATCA TTCTTGAAGA ACACTTTTAC TATTAACAGA TCTTCAAACT TTTTAAGTGT GGTGTACTGT   
  
  
- AAATTTTTAT TAAAACATCT CTTCCCGTAT TTACTTTCAA ACGGTGGTAG AAAAAATCTT TGTACATTAA   
  
  
- ATGGTTTGTG AAAAAGATTT TTGTCAATTA AGTTTATTGA TTGTTGATTG TCGATTATAT TTATTGATTG   
  
  
- TCGATAAACG ATTTGTCCCG GATTCATCAA ACTTTAACAA CAAAAGAGAA GTCGAAGTGG TACCGGCTAC   
  
  
- TACATTTGTT GATATAAGAC TTAAAGTAAA AATGATACTA AACTTTTAAA GTTTAAATTT ATTCTAACCT   
  
  
- ATTTTTTTTC TGGTTTTTAG TATTCATTAT AATCAGTTCA AGATAAGGTT GGTATATGTA TTGACGAAAA   
  
  
- AGAAAACCCC CAAAACTTTG TACACAGGAA TCTTATTAAC TTTGAGCTAT AATAAGAGAT TTAGATTCAA   
  
  
- ATGAAATAGT ATAACTAATA ATTAATGGAG GAGTTGAGGA GGATATGAGA ACATACGTCT TTATTTTTAC   
  
  
- AAAGGTATGG AATAGTCAGC GCTAGTATTA TTAATTAAAG AATTAAGCCT AACATAAATT AACGCATAGT   
  
  
- TTACACTTTA GCATGGTTTA ATCCTTCGTT TTGTTTGGCT ATTGCTTTTT CCATTCTTTT GAATCGTTCC   
  
  
- TTTACTTTAG CTTCTGGACA GTTGTTATAT CGAAGGGAGT GTAGGAAGTT GAATCCCTGG TAGGATATGA   
  
  
- AAGTACATTA TGTTGATCTC GGTGATCAGT TATTATCGGA GGATCATCAT CGGTATTCGG AACCACTCTC   
  
  
- TTACAAGGCG TTCTGATCAA TTGAACTTTA AGGTAAAACA GCGTCTTGAT AACCTCACTC TTTCTCTTAG   
  
  
- TTTAGTATGG TATAGTTTAA ACCTGGTAAA TTCAAGTGTT GTGTGTGTGT ATGTGTGTTT TCCTGCTACA   
  
  
- TGCCGGGGTG GGGGGTGAGA GGAAAGCGGT AGGGGGATGT GGAGACGGAT GAGAGAGAGA GAGAGAGAGA   
  
  
- GAGACCTAGT CAGACACATT TTCGAAGGAG AGGTCATGTC GTAAAGGAAC AGACAAGAAC CTCAAATCAG   
  
  
- TTAAGGAGGC GGGGTTTTTT TGGGTGAAAG AGAGAGTAGT ACAAGAAGGT CAAGGTGGGA ATATGCGGTA   
  
  
- AGTAGTAGTA GATGTAGTGG GTGTAGAAAA AGAGGTGTCA AAAGGGGGGA GTAATACGAG AGGCGGAGGT   
  
  
- GGGGGCGGAA GGGAGAGCGG CAGCGGCCGC TACTGCCCCT ATGGTGACTA CGTACGCTGC CGTGGCGGTG   
  
  
- ACGGGAGGTG GAGGAGCCCG ACGACAACTT CACGCGCCTT AAGTAGAGGT GGCCTCTGGA GCGGCCGCGG   
  
  
- CCTCTATAGA ACGGCCTCTA TAAACTCAAC AGATGTGGCA AACCGAGGGG GCGGCGGGCC CAGCGGCGGA   
  
  
- AGAAGCCGGT GCGGGAGGTG CGGGCGGAGG AGAGGCGGAG GGAGGCGTGT TGAGGCTAGC TCTTCGAGTT   
  
  
- TTGGGACTGG GACCAAAGGG TTTACGCCTT TAAAGTGAGG CGGAACGTTC ATATGTTGAG GTAGTGAGGG   
  
  
- AAGCACTTTA AGAGAGTGAA GTGCCGCTTA GTTCGGTAGA TGCTCCGCGA CCTGCCGCTC CTAGCACAGG   
  
  
- TGCAGCAGCT GGAACTGTAG TACGTCCCGG AAGTTACCGG CCCTAACAAG GTGTAGAATC GGAGAGCTGG   
  
  
- GTCTTTCGGG GAGAGCCAAG CCCAGTGGCC CAAGCCCGGT AGGAGGCTCA ACGAGAGGGT TTGCCCATTC   
  
  
- GCTGAGCGAC TCAAGCGGCG AAGTGAGCCG GACGGGAAGC TCATGTTGGG CCACCTCCCG TTTTAACCCT   
  
  
- TGAATCAGCT GGACCCAGCC CAGCCCAGCG AGGGCTTACT TCACTGCCAC CACGTGACCT ACGTGGTATC   
  
  
- AGATATGCTG TATTGGCCCA GCCTAGAACC CTGAAACTCC CATAACTCAC GCCACTCCGG ATCCGAACAC   
  
  
- TGATACCAAC TCGTCCTATA CCTGGTTTGC CCCAGCAAAA ACCCCTCCAA ACACCTCCGT AACGTAATAA   
  
  
- TGAGTCGGAA CAAACTACGG AACCCTCTCC CCAATCTATC CCTATTGGAT GTTTCCGTAG TTCACCTCGT   
  
  
- TGTCGATAAA CCCACACTCT AATCCTTGTA GGACCGGCAA CCACCCGGGT TCTCCTCCGA GTGACCGCCG   
  
  
- CTAGCCCAGT CTTCCACCCC GCTGCTTGAC TGTTCCCAGC CCAAGCTTGG TCAAAGCAAC CGCCCATCGG   
  
  
- GCCGTTGGGT TCGATCAAAC AACGAACCCT ACAAGGGAAC CTTTCCCATA TGAAACTACC TCCTTTTACC   
  
  
- CACAAACTCT AATCCCACCT TTCTAAACGG GAACAATTGA CGGAGTCGAA CCGTTGGAAC ACTTAAATTG   
  
  
- TTAGGATCAC GACCGTAAAT

+     Myb

| Site Name | Organism | Position | Strand | Matrix score. | sequence | function |
| --- | --- | --- | --- | --- | --- | --- |
| Myb | Arabidopsis thaliana | 410 | + | 6 | CAACTG |  |
| Myb | Arabidopsis thaliana | 1184 | + | 6 | TAACTG |  |
| Myb | Arabidopsis thaliana | 937 | - | 6 | TAACTG |  |
| Myb | Arabidopsis thaliana | 3190 | + | 6 | TAACTG |  |

>HU02G03005.1   
+ +Up\_Stream \_Len000AAACGG TGCCGTTGAA TTTGCAGGCG ACGAGGTCGG GTTTAAGGCG GATGTCGTAG   
  
  
+ CGCTTTGGAG AAGCAAATTT GGGAAGACGA GGCTTATTCT TGAACTCTTC GTACGCCATT GTTGTTGGTT   
  
  
+ CCAACCTTTC TCTCTCCTCG TGAATTTGAG TTTGGGCAGT AGACTATCCC GGAATAGAGG ACTGATGCGT   
  
  
+ TATATATATA GAAATTGAGA GAGAGAGAGA GCGTGTGTGA AGGATGTTGA AAATGATGGG CGTAGGGAGT   
  
  
+ CTCTCTCTCA CTGTCTGTTA GGAGTACGTG TCGAGAAAGG AAATAGGATT CCCAAGCAGT CTAATTTGGG   
  
  
+ CCGGTCTATA GGCCAGAATG GGCCTATGAT CAGCCCAGAT ATGACCTTGT TTAACCAACT GTATTTGAAT   
  
  
+ TGAAGAAAGG GATATCGCAA TGTCTTGAAC CACATCAAAT AATAAATGTG CCTTTGGTTA GACCATATGA   
  
  
+ AAAATCTTTG AATCAAATCG GTTGGACCTG AACTGCCATT ATTGATATCT TGTTTTTGGT AGAAGCAAAT   
  
  
+ GGTGTCACTT CGTTTTCTAG ATAAAATTAA CTTGAGTTTT TTTGCCACTA TAGATAGTAT TGCAAATATA   
  
  
+ AAAAGTGTGC CACGTGAAAT CAATATTAGG AGGGTGTTTT ATAACAATAG CTGTTCGGGA GAGTTTTAGT   
  
  
+ TATTTTTATT AAATCAAATA GTTAAGAAGT GTTTGGTAAA TAGTTGTATT TGAAAAAGTT ATTCCCATTA   
  
  
+ GCCTTTTAGT AAGAACTTCT TGTGAAAATG ATAATTGTCT AGAAGTTTGA AAAATTCACA CCACATGACA   
  
  
+ TTTAAAAATA ATTTTGTAGA GAAGGGCATA AATGAAAGTT TGCCACCATC TTTTTTAGAA ACATGTAATT   
  
  
+ TACCAAACAC TTTTTCTAAA AACAGTTAAT TCAAATAACT AACAACTAAC AGCTAATATA AATAACTAAC   
  
  
+ AGCTATTTGC TAAACAGGGC CTAAGTAGTT TGAAATTGTT GTTTTCTCTT CAGCTTCACC ATGGCCGATG   
  
  
+ ATGTAAACAA CTATATTCTG AATTTCATTT TTACTATGAT TTGAAAATTT CAAATTTAAA TAAGATTGGA   
  
  
+ TAAAAAAAAG ACCAAAAATC ATAAGTAATA TTAGTCAAGT TCTATTCCAA CCATATACAT AACTGCTTTT   
  
  
+ TCTTTTGGGG GTTTTGAAAC ATGTGTCCTT AGAATAATTG AAACTCGATA TTATTCTCTA AATCTAAGTT   
  
  
+ TACTTTATCA TATTGATTAT TAATTACCTC CTCAACTCCT CCTATACTCT TGTATGCAGA AATAAAAATG   
  
  
+ TTTCCATACC TTATCAGTCG CGATCATAAT AATTAATTTC TTAATTCGGA TTGTATTTAA TTGCGTATCA   
  
  
+ AATGTGAAAT CGTACCAAAT TAGGAAGCAA AACAAACCGA TAACGAAAAA GGTAAGAAAA CTTAGCAAGG   
  
  
+ AAATGAAATC GAAGACCTGT CAACAATATA GCTTCCCTCA CATCCTTCAA CTTAGGGACC ATCCTATACT   
  
  
+ TTCATGTAAT ACAACTAGAG CCACTAGTCA ATAATAGCCT CCTAGTAGTA GCCATAAGCC TTGGTGAGAG   
  
  
+ AATGTTCCGC AAGACTAGTT AACTTGAAAT TCCATTTTGT CGCAGAACTA TTGGAGTGAG AAAGAGAATC   
  
  
+ AAATCATACC ATATCAAATT TGGACCATTT AAGTTCACAA CACACACACA TACACACAAA AGGACGATGT   
  
  
+ ACGGCCCCAC CCCCCACTCT CCTTTCGCCA TCCCCCTACA CCTCTGCCTA CTCTCTCTCT CTCTCTCTCT   
  
  
+ CTCTGGATCA GTCTGTGTAA AAGCTTCCTC TCCAGTACAG CATTTCCTTG TCTGTTCTTG GAGTTTAGTC   
  
  
+ AATTCCTCCG CCCCAAAAAA ACCCACTTTC TCTCTCATCA TGTTCTTCCA GTTCCACCCT TATACGCCAT   
  
  
+ TCATCATCAT CTACATCACC CACATCTTTT TCTCCACAGT TTTCCCCCCT CATTATGCTC TCCGCCTCCA   
  
  
+ CCCCCGCCTT CCCTCTCGCC GTCGCCGGCG ATGACGGGGA TACCACTGAT GCATGCGACG GCACCGCCAC   
  
  
+ TGCCCTCCAC CTCCTCGGGC TGCTGTTGAA GTGCGCGGAA TTCATCTCCA CCGGAGACCT CGCCGGCGCC   
  
  
+ GGAGATATCT TGCCGGAGAT ATTTGAGTTG TCTACACCGT TTGGCTCCCC CGCCGCCCGG GTCGCCGCCT   
  
  
+ TCTTCGGCCA CGCCCTCCAC GCCCGCCTCC TCTCCGCCTC CCTCCGCACA ACTCCGATCG AGAAGCTCAA   
  
  
+ AACCCTGACC CTGGTTTCCC AAATGCGGAA ATTTCACTCC GCCTTGCAAG TATACAACTC CATCACTCCC   
  
  
+ TTCGTGAAAT TCTCTCACTT CACGGCGAAT CAAGCCATCT ACGAGGCGCT GGACGGCGAG GATCGTGTCC   
  
  
+ ACGTCGTCGA CCTTGACATC ATGCAGGGCC TTCAATGGCC GGGATTGTTC CACATCTTAG CCTCTCGACC   
  
  
+ CAGAAAGCCC CTCTCGGTTC GGGTCACCGG GTTCGGGCCA TCCTCCGAGT TGCTCTCCCA AACGGGTAAG   
  
  
+ CGACTCGCTG AGTTCGCCGC TTCACTCGGC CTGCCCTTCG AGTACAACCC GGTGGAGGGC AAAATTGGGA   
  
  
+ ACTTAGTCGA CCTGGGTCGG GTCGGGTCGC TCCCGAATGA AGTGACGGTG GTGCACTGGA TGCACCATAG   
  
  
+ TCTATACGAC ATAACCGGGT CGGATCTTGG GACTTTGAGG GTATTGAGTG CGGTGAGGCC TAGGCTTGTG   
  
  
+ ACTATGGTTG AGCAGGATAT GGACCAAACG GGGTCGTTTT TGGGGAGGTT TGTGGAGGCA TTGCATTATT   
  
  
+ ACTCAGCCTT GTTTGATGCC TTGGGAGAGG GGTTAGATAG GGATAACCTA CAAAGGCATC AAGTGGAGCA   
  
  
+ ACAGCTATTT GGGTGTGAGA TTAGGAACAT CCTGGCCGTT GGTGGGCCCA AGAGGAGGCT CACTGGCGGC   
  
  
+ GATCGGGTCA GAAGGTGGGG CGACGAACTG ACAAGGGTCG GGTTCGAACC AGTTTCGTTG GCGGGTAGCC   
  
  
+ CGGCAACCCA AGCTAGTTTG TTGCTTGGGA TGTTCCCTTG GAAAGGGTAT ACTTTGATGG AGGAAAATGG   
  
  
+ GTGTTTGAGA TTAGGGTGGA AAGATTTGCC CTTGTTAACT GCCTCAGCTT GGCAACCTTG TGAATTTAAC   
  
  
+ AATCCTAGTG CTGGCATTTA   

- +Up\_Stream \_Len000TTTGCC ACGGCAACTT AAACGTCCGC TGCTCCAGCC CAAATTCCGC CTACAGCATC   
  
  
- GCGAAACCTC TTCGTTTAAA CCCTTCTGCT CCGAATAAGA ACTTGAGAAG CATGCGGTAA CAACAACCAA   
  
  
- GGTTGGAAAG AGAGAGGAGC ACTTAAACTC AAACCCGTCA TCTGATAGGG CCTTATCTCC TGACTACGCA   
  
  
- ATATATATAT CTTTAACTCT CTCTCTCTCT CGCACACACT TCCTACAACT TTTACTACCC GCATCCCTCA   
  
  
- GAGAGAGAGT GACAGACAAT CCTCATGCAC AGCTCTTTCC TTTATCCTAA GGGTTCGTCA GATTAAACCC   
  
  
- GGCCAGATAT CCGGTCTTAC CCGGATACTA GTCGGGTCTA TACTGGAACA AATTGGTTGA CATAAACTTA   
  
  
- ACTTCTTTCC CTATAGCGTT ACAGAACTTG GTGTAGTTTA TTATTTACAC GGAAACCAAT CTGGTATACT   
  
  
- TTTTAGAAAC TTAGTTTAGC CAACCTGGAC TTGACGGTAA TAACTATAGA ACAAAAACCA TCTTCGTTTA   
  
  
- CCACAGTGAA GCAAAAGATC TATTTTAATT GAACTCAAAA AAACGGTGAT ATCTATCATA ACGTTTATAT   
  
  
- TTTTCACACG GTGCACTTTA GTTATAATCC TCCCACAAAA TATTGTTATC GACAAGCCCT CTCAAAATCA   
  
  
- ATAAAAATAA TTTAGTTTAT CAATTCTTCA CAAACCATTT ATCAACATAA ACTTTTTCAA TAAGGGTAAT   
  
  
- CGGAAAATCA TTCTTGAAGA ACACTTTTAC TATTAACAGA TCTTCAAACT TTTTAAGTGT GGTGTACTGT   
  
  
- AAATTTTTAT TAAAACATCT CTTCCCGTAT TTACTTTCAA ACGGTGGTAG AAAAAATCTT TGTACATTAA   
  
  
- ATGGTTTGTG AAAAAGATTT TTGTCAATTA AGTTTATTGA TTGTTGATTG TCGATTATAT TTATTGATTG   
  
  
- TCGATAAACG ATTTGTCCCG GATTCATCAA ACTTTAACAA CAAAAGAGAA GTCGAAGTGG TACCGGCTAC   
  
  
- TACATTTGTT GATATAAGAC TTAAAGTAAA AATGATACTA AACTTTTAAA GTTTAAATTT ATTCTAACCT   
  
  
- ATTTTTTTTC TGGTTTTTAG TATTCATTAT AATCAGTTCA AGATAAGGTT GGTATATGTA TTGACGAAAA   
  
  
- AGAAAACCCC CAAAACTTTG TACACAGGAA TCTTATTAAC TTTGAGCTAT AATAAGAGAT TTAGATTCAA   
  
  
- ATGAAATAGT ATAACTAATA ATTAATGGAG GAGTTGAGGA GGATATGAGA ACATACGTCT TTATTTTTAC   
  
  
- AAAGGTATGG AATAGTCAGC GCTAGTATTA TTAATTAAAG AATTAAGCCT AACATAAATT AACGCATAGT   
  
  
- TTACACTTTA GCATGGTTTA ATCCTTCGTT TTGTTTGGCT ATTGCTTTTT CCATTCTTTT GAATCGTTCC   
  
  
- TTTACTTTAG CTTCTGGACA GTTGTTATAT CGAAGGGAGT GTAGGAAGTT GAATCCCTGG TAGGATATGA   
  
  
- AAGTACATTA TGTTGATCTC GGTGATCAGT TATTATCGGA GGATCATCAT CGGTATTCGG AACCACTCTC   
  
  
- TTACAAGGCG TTCTGATCAA TTGAACTTTA AGGTAAAACA GCGTCTTGAT AACCTCACTC TTTCTCTTAG   
  
  
- TTTAGTATGG TATAGTTTAA ACCTGGTAAA TTCAAGTGTT GTGTGTGTGT ATGTGTGTTT TCCTGCTACA   
  
  
- TGCCGGGGTG GGGGGTGAGA GGAAAGCGGT AGGGGGATGT GGAGACGGAT GAGAGAGAGA GAGAGAGAGA   
  
  
- GAGACCTAGT CAGACACATT TTCGAAGGAG AGGTCATGTC GTAAAGGAAC AGACAAGAAC CTCAAATCAG   
  
  
- TTAAGGAGGC GGGGTTTTTT TGGGTGAAAG AGAGAGTAGT ACAAGAAGGT CAAGGTGGGA ATATGCGGTA   
  
  
- AGTAGTAGTA GATGTAGTGG GTGTAGAAAA AGAGGTGTCA AAAGGGGGGA GTAATACGAG AGGCGGAGGT   
  
  
- GGGGGCGGAA GGGAGAGCGG CAGCGGCCGC TACTGCCCCT ATGGTGACTA CGTACGCTGC CGTGGCGGTG   
  
  
- ACGGGAGGTG GAGGAGCCCG ACGACAACTT CACGCGCCTT AAGTAGAGGT GGCCTCTGGA GCGGCCGCGG   
  
  
- CCTCTATAGA ACGGCCTCTA TAAACTCAAC AGATGTGGCA AACCGAGGGG GCGGCGGGCC CAGCGGCGGA   
  
  
- AGAAGCCGGT GCGGGAGGTG CGGGCGGAGG AGAGGCGGAG GGAGGCGTGT TGAGGCTAGC TCTTCGAGTT   
  
  
- TTGGGACTGG GACCAAAGGG TTTACGCCTT TAAAGTGAGG CGGAACGTTC ATATGTTGAG GTAGTGAGGG   
  
  
- AAGCACTTTA AGAGAGTGAA GTGCCGCTTA GTTCGGTAGA TGCTCCGCGA CCTGCCGCTC CTAGCACAGG   
  
  
- TGCAGCAGCT GGAACTGTAG TACGTCCCGG AAGTTACCGG CCCTAACAAG GTGTAGAATC GGAGAGCTGG   
  
  
- GTCTTTCGGG GAGAGCCAAG CCCAGTGGCC CAAGCCCGGT AGGAGGCTCA ACGAGAGGGT TTGCCCATTC   
  
  
- GCTGAGCGAC TCAAGCGGCG AAGTGAGCCG GACGGGAAGC TCATGTTGGG CCACCTCCCG TTTTAACCCT   
  
  
- TGAATCAGCT GGACCCAGCC CAGCCCAGCG AGGGCTTACT TCACTGCCAC CACGTGACCT ACGTGGTATC   
  
  
- AGATATGCTG TATTGGCCCA GCCTAGAACC CTGAAACTCC CATAACTCAC GCCACTCCGG ATCCGAACAC   
  
  
- TGATACCAAC TCGTCCTATA CCTGGTTTGC CCCAGCAAAA ACCCCTCCAA ACACCTCCGT AACGTAATAA   
  
  
- TGAGTCGGAA CAAACTACGG AACCCTCTCC CCAATCTATC CCTATTGGAT GTTTCCGTAG TTCACCTCGT   
  
  
- TGTCGATAAA CCCACACTCT AATCCTTGTA GGACCGGCAA CCACCCGGGT TCTCCTCCGA GTGACCGCCG   
  
  
- CTAGCCCAGT CTTCCACCCC GCTGCTTGAC TGTTCCCAGC CCAAGCTTGG TCAAAGCAAC CGCCCATCGG   
  
  
- GCCGTTGGGT TCGATCAAAC AACGAACCCT ACAAGGGAAC CTTTCCCATA TGAAACTACC TCCTTTTACC   
  
  
- CACAAACTCT AATCCCACCT TTCTAAACGG GAACAATTGA CGGAGTCGAA CCGTTGGAAC ACTTAAATTG   
  
  
- TTAGGATCAC GACCGTAAAT

+     Myb-binding site

| Site Name | Organism | Position | Strand | Matrix score. | sequence | function |
| --- | --- | --- | --- | --- | --- | --- |
| Myb-binding site | Nicotiana tabacum | 2127 | - | 6 | CAACAG |  |
| Myb-binding site | Nicotiana tabacum | 2943 | + | 6 | CAACAG |  |

>HU02G03005.1   
+ +Up\_Stream \_Len000AAACGG TGCCGTTGAA TTTGCAGGCG ACGAGGTCGG GTTTAAGGCG GATGTCGTAG   
  
  
+ CGCTTTGGAG AAGCAAATTT GGGAAGACGA GGCTTATTCT TGAACTCTTC GTACGCCATT GTTGTTGGTT   
  
  
+ CCAACCTTTC TCTCTCCTCG TGAATTTGAG TTTGGGCAGT AGACTATCCC GGAATAGAGG ACTGATGCGT   
  
  
+ TATATATATA GAAATTGAGA GAGAGAGAGA GCGTGTGTGA AGGATGTTGA AAATGATGGG CGTAGGGAGT   
  
  
+ CTCTCTCTCA CTGTCTGTTA GGAGTACGTG TCGAGAAAGG AAATAGGATT CCCAAGCAGT CTAATTTGGG   
  
  
+ CCGGTCTATA GGCCAGAATG GGCCTATGAT CAGCCCAGAT ATGACCTTGT TTAACCAACT GTATTTGAAT   
  
  
+ TGAAGAAAGG GATATCGCAA TGTCTTGAAC CACATCAAAT AATAAATGTG CCTTTGGTTA GACCATATGA   
  
  
+ AAAATCTTTG AATCAAATCG GTTGGACCTG AACTGCCATT ATTGATATCT TGTTTTTGGT AGAAGCAAAT   
  
  
+ GGTGTCACTT CGTTTTCTAG ATAAAATTAA CTTGAGTTTT TTTGCCACTA TAGATAGTAT TGCAAATATA   
  
  
+ AAAAGTGTGC CACGTGAAAT CAATATTAGG AGGGTGTTTT ATAACAATAG CTGTTCGGGA GAGTTTTAGT   
  
  
+ TATTTTTATT AAATCAAATA GTTAAGAAGT GTTTGGTAAA TAGTTGTATT TGAAAAAGTT ATTCCCATTA   
  
  
+ GCCTTTTAGT AAGAACTTCT TGTGAAAATG ATAATTGTCT AGAAGTTTGA AAAATTCACA CCACATGACA   
  
  
+ TTTAAAAATA ATTTTGTAGA GAAGGGCATA AATGAAAGTT TGCCACCATC TTTTTTAGAA ACATGTAATT   
  
  
+ TACCAAACAC TTTTTCTAAA AACAGTTAAT TCAAATAACT AACAACTAAC AGCTAATATA AATAACTAAC   
  
  
+ AGCTATTTGC TAAACAGGGC CTAAGTAGTT TGAAATTGTT GTTTTCTCTT CAGCTTCACC ATGGCCGATG   
  
  
+ ATGTAAACAA CTATATTCTG AATTTCATTT TTACTATGAT TTGAAAATTT CAAATTTAAA TAAGATTGGA   
  
  
+ TAAAAAAAAG ACCAAAAATC ATAAGTAATA TTAGTCAAGT TCTATTCCAA CCATATACAT AACTGCTTTT   
  
  
+ TCTTTTGGGG GTTTTGAAAC ATGTGTCCTT AGAATAATTG AAACTCGATA TTATTCTCTA AATCTAAGTT   
  
  
+ TACTTTATCA TATTGATTAT TAATTACCTC CTCAACTCCT CCTATACTCT TGTATGCAGA AATAAAAATG   
  
  
+ TTTCCATACC TTATCAGTCG CGATCATAAT AATTAATTTC TTAATTCGGA TTGTATTTAA TTGCGTATCA   
  
  
+ AATGTGAAAT CGTACCAAAT TAGGAAGCAA AACAAACCGA TAACGAAAAA GGTAAGAAAA CTTAGCAAGG   
  
  
+ AAATGAAATC GAAGACCTGT CAACAATATA GCTTCCCTCA CATCCTTCAA CTTAGGGACC ATCCTATACT   
  
  
+ TTCATGTAAT ACAACTAGAG CCACTAGTCA ATAATAGCCT CCTAGTAGTA GCCATAAGCC TTGGTGAGAG   
  
  
+ AATGTTCCGC AAGACTAGTT AACTTGAAAT TCCATTTTGT CGCAGAACTA TTGGAGTGAG AAAGAGAATC   
  
  
+ AAATCATACC ATATCAAATT TGGACCATTT AAGTTCACAA CACACACACA TACACACAAA AGGACGATGT   
  
  
+ ACGGCCCCAC CCCCCACTCT CCTTTCGCCA TCCCCCTACA CCTCTGCCTA CTCTCTCTCT CTCTCTCTCT   
  
  
+ CTCTGGATCA GTCTGTGTAA AAGCTTCCTC TCCAGTACAG CATTTCCTTG TCTGTTCTTG GAGTTTAGTC   
  
  
+ AATTCCTCCG CCCCAAAAAA ACCCACTTTC TCTCTCATCA TGTTCTTCCA GTTCCACCCT TATACGCCAT   
  
  
+ TCATCATCAT CTACATCACC CACATCTTTT TCTCCACAGT TTTCCCCCCT CATTATGCTC TCCGCCTCCA   
  
  
+ CCCCCGCCTT CCCTCTCGCC GTCGCCGGCG ATGACGGGGA TACCACTGAT GCATGCGACG GCACCGCCAC   
  
  
+ TGCCCTCCAC CTCCTCGGGC TGCTGTTGAA GTGCGCGGAA TTCATCTCCA CCGGAGACCT CGCCGGCGCC   
  
  
+ GGAGATATCT TGCCGGAGAT ATTTGAGTTG TCTACACCGT TTGGCTCCCC CGCCGCCCGG GTCGCCGCCT   
  
  
+ TCTTCGGCCA CGCCCTCCAC GCCCGCCTCC TCTCCGCCTC CCTCCGCACA ACTCCGATCG AGAAGCTCAA   
  
  
+ AACCCTGACC CTGGTTTCCC AAATGCGGAA ATTTCACTCC GCCTTGCAAG TATACAACTC CATCACTCCC   
  
  
+ TTCGTGAAAT TCTCTCACTT CACGGCGAAT CAAGCCATCT ACGAGGCGCT GGACGGCGAG GATCGTGTCC   
  
  
+ ACGTCGTCGA CCTTGACATC ATGCAGGGCC TTCAATGGCC GGGATTGTTC CACATCTTAG CCTCTCGACC   
  
  
+ CAGAAAGCCC CTCTCGGTTC GGGTCACCGG GTTCGGGCCA TCCTCCGAGT TGCTCTCCCA AACGGGTAAG   
  
  
+ CGACTCGCTG AGTTCGCCGC TTCACTCGGC CTGCCCTTCG AGTACAACCC GGTGGAGGGC AAAATTGGGA   
  
  
+ ACTTAGTCGA CCTGGGTCGG GTCGGGTCGC TCCCGAATGA AGTGACGGTG GTGCACTGGA TGCACCATAG   
  
  
+ TCTATACGAC ATAACCGGGT CGGATCTTGG GACTTTGAGG GTATTGAGTG CGGTGAGGCC TAGGCTTGTG   
  
  
+ ACTATGGTTG AGCAGGATAT GGACCAAACG GGGTCGTTTT TGGGGAGGTT TGTGGAGGCA TTGCATTATT   
  
  
+ ACTCAGCCTT GTTTGATGCC TTGGGAGAGG GGTTAGATAG GGATAACCTA CAAAGGCATC AAGTGGAGCA   
  
  
+ ACAGCTATTT GGGTGTGAGA TTAGGAACAT CCTGGCCGTT GGTGGGCCCA AGAGGAGGCT CACTGGCGGC   
  
  
+ GATCGGGTCA GAAGGTGGGG CGACGAACTG ACAAGGGTCG GGTTCGAACC AGTTTCGTTG GCGGGTAGCC   
  
  
+ CGGCAACCCA AGCTAGTTTG TTGCTTGGGA TGTTCCCTTG GAAAGGGTAT ACTTTGATGG AGGAAAATGG   
  
  
+ GTGTTTGAGA TTAGGGTGGA AAGATTTGCC CTTGTTAACT GCCTCAGCTT GGCAACCTTG TGAATTTAAC   
  
  
+ AATCCTAGTG CTGGCATTTA   

- +Up\_Stream \_Len000TTTGCC ACGGCAACTT AAACGTCCGC TGCTCCAGCC CAAATTCCGC CTACAGCATC   
  
  
- GCGAAACCTC TTCGTTTAAA CCCTTCTGCT CCGAATAAGA ACTTGAGAAG CATGCGGTAA CAACAACCAA   
  
  
- GGTTGGAAAG AGAGAGGAGC ACTTAAACTC AAACCCGTCA TCTGATAGGG CCTTATCTCC TGACTACGCA   
  
  
- ATATATATAT CTTTAACTCT CTCTCTCTCT CGCACACACT TCCTACAACT TTTACTACCC GCATCCCTCA   
  
  
- GAGAGAGAGT GACAGACAAT CCTCATGCAC AGCTCTTTCC TTTATCCTAA GGGTTCGTCA GATTAAACCC   
  
  
- GGCCAGATAT CCGGTCTTAC CCGGATACTA GTCGGGTCTA TACTGGAACA AATTGGTTGA CATAAACTTA   
  
  
- ACTTCTTTCC CTATAGCGTT ACAGAACTTG GTGTAGTTTA TTATTTACAC GGAAACCAAT CTGGTATACT   
  
  
- TTTTAGAAAC TTAGTTTAGC CAACCTGGAC TTGACGGTAA TAACTATAGA ACAAAAACCA TCTTCGTTTA   
  
  
- CCACAGTGAA GCAAAAGATC TATTTTAATT GAACTCAAAA AAACGGTGAT ATCTATCATA ACGTTTATAT   
  
  
- TTTTCACACG GTGCACTTTA GTTATAATCC TCCCACAAAA TATTGTTATC GACAAGCCCT CTCAAAATCA   
  
  
- ATAAAAATAA TTTAGTTTAT CAATTCTTCA CAAACCATTT ATCAACATAA ACTTTTTCAA TAAGGGTAAT   
  
  
- CGGAAAATCA TTCTTGAAGA ACACTTTTAC TATTAACAGA TCTTCAAACT TTTTAAGTGT GGTGTACTGT   
  
  
- AAATTTTTAT TAAAACATCT CTTCCCGTAT TTACTTTCAA ACGGTGGTAG AAAAAATCTT TGTACATTAA   
  
  
- ATGGTTTGTG AAAAAGATTT TTGTCAATTA AGTTTATTGA TTGTTGATTG TCGATTATAT TTATTGATTG   
  
  
- TCGATAAACG ATTTGTCCCG GATTCATCAA ACTTTAACAA CAAAAGAGAA GTCGAAGTGG TACCGGCTAC   
  
  
- TACATTTGTT GATATAAGAC TTAAAGTAAA AATGATACTA AACTTTTAAA GTTTAAATTT ATTCTAACCT   
  
  
- ATTTTTTTTC TGGTTTTTAG TATTCATTAT AATCAGTTCA AGATAAGGTT GGTATATGTA TTGACGAAAA   
  
  
- AGAAAACCCC CAAAACTTTG TACACAGGAA TCTTATTAAC TTTGAGCTAT AATAAGAGAT TTAGATTCAA   
  
  
- ATGAAATAGT ATAACTAATA ATTAATGGAG GAGTTGAGGA GGATATGAGA ACATACGTCT TTATTTTTAC   
  
  
- AAAGGTATGG AATAGTCAGC GCTAGTATTA TTAATTAAAG AATTAAGCCT AACATAAATT AACGCATAGT   
  
  
- TTACACTTTA GCATGGTTTA ATCCTTCGTT TTGTTTGGCT ATTGCTTTTT CCATTCTTTT GAATCGTTCC   
  
  
- TTTACTTTAG CTTCTGGACA GTTGTTATAT CGAAGGGAGT GTAGGAAGTT GAATCCCTGG TAGGATATGA   
  
  
- AAGTACATTA TGTTGATCTC GGTGATCAGT TATTATCGGA GGATCATCAT CGGTATTCGG AACCACTCTC   
  
  
- TTACAAGGCG TTCTGATCAA TTGAACTTTA AGGTAAAACA GCGTCTTGAT AACCTCACTC TTTCTCTTAG   
  
  
- TTTAGTATGG TATAGTTTAA ACCTGGTAAA TTCAAGTGTT GTGTGTGTGT ATGTGTGTTT TCCTGCTACA   
  
  
- TGCCGGGGTG GGGGGTGAGA GGAAAGCGGT AGGGGGATGT GGAGACGGAT GAGAGAGAGA GAGAGAGAGA   
  
  
- GAGACCTAGT CAGACACATT TTCGAAGGAG AGGTCATGTC GTAAAGGAAC AGACAAGAAC CTCAAATCAG   
  
  
- TTAAGGAGGC GGGGTTTTTT TGGGTGAAAG AGAGAGTAGT ACAAGAAGGT CAAGGTGGGA ATATGCGGTA   
  
  
- AGTAGTAGTA GATGTAGTGG GTGTAGAAAA AGAGGTGTCA AAAGGGGGGA GTAATACGAG AGGCGGAGGT   
  
  
- GGGGGCGGAA GGGAGAGCGG CAGCGGCCGC TACTGCCCCT ATGGTGACTA CGTACGCTGC CGTGGCGGTG   
  
  
- ACGGGAGGTG GAGGAGCCCG ACGACAACTT CACGCGCCTT AAGTAGAGGT GGCCTCTGGA GCGGCCGCGG   
  
  
- CCTCTATAGA ACGGCCTCTA TAAACTCAAC AGATGTGGCA AACCGAGGGG GCGGCGGGCC CAGCGGCGGA   
  
  
- AGAAGCCGGT GCGGGAGGTG CGGGCGGAGG AGAGGCGGAG GGAGGCGTGT TGAGGCTAGC TCTTCGAGTT   
  
  
- TTGGGACTGG GACCAAAGGG TTTACGCCTT TAAAGTGAGG CGGAACGTTC ATATGTTGAG GTAGTGAGGG   
  
  
- AAGCACTTTA AGAGAGTGAA GTGCCGCTTA GTTCGGTAGA TGCTCCGCGA CCTGCCGCTC CTAGCACAGG   
  
  
- TGCAGCAGCT GGAACTGTAG TACGTCCCGG AAGTTACCGG CCCTAACAAG GTGTAGAATC GGAGAGCTGG   
  
  
- GTCTTTCGGG GAGAGCCAAG CCCAGTGGCC CAAGCCCGGT AGGAGGCTCA ACGAGAGGGT TTGCCCATTC   
  
  
- GCTGAGCGAC TCAAGCGGCG AAGTGAGCCG GACGGGAAGC TCATGTTGGG CCACCTCCCG TTTTAACCCT   
  
  
- TGAATCAGCT GGACCCAGCC CAGCCCAGCG AGGGCTTACT TCACTGCCAC CACGTGACCT ACGTGGTATC   
  
  
- AGATATGCTG TATTGGCCCA GCCTAGAACC CTGAAACTCC CATAACTCAC GCCACTCCGG ATCCGAACAC   
  
  
- TGATACCAAC TCGTCCTATA CCTGGTTTGC CCCAGCAAAA ACCCCTCCAA ACACCTCCGT AACGTAATAA   
  
  
- TGAGTCGGAA CAAACTACGG AACCCTCTCC CCAATCTATC CCTATTGGAT GTTTCCGTAG TTCACCTCGT   
  
  
- TGTCGATAAA CCCACACTCT AATCCTTGTA GGACCGGCAA CCACCCGGGT TCTCCTCCGA GTGACCGCCG   
  
  
- CTAGCCCAGT CTTCCACCCC GCTGCTTGAC TGTTCCCAGC CCAAGCTTGG TCAAAGCAAC CGCCCATCGG   
  
  
- GCCGTTGGGT TCGATCAAAC AACGAACCCT ACAAGGGAAC CTTTCCCATA TGAAACTACC TCCTTTTACC   
  
  
- CACAAACTCT AATCCCACCT TTCTAAACGG GAACAATTGA CGGAGTCGAA CCGTTGGAAC ACTTAAATTG   
  
  
- TTAGGATCAC GACCGTAAAT

+     NON

| Site Name | Organism | Position | Strand | Matrix score. | sequence | function |
| --- | --- | --- | --- | --- | --- | --- |
| NON | Nicotiana tabacum | 2975 | - | 10 | CAACGGCCACG |  |

>HU02G03005.1   
+ +Up\_Stream \_Len000AAACGG TGCCGTTGAA TTTGCAGGCG ACGAGGTCGG GTTTAAGGCG GATGTCGTAG   
  
  
+ CGCTTTGGAG AAGCAAATTT GGGAAGACGA GGCTTATTCT TGAACTCTTC GTACGCCATT GTTGTTGGTT   
  
  
+ CCAACCTTTC TCTCTCCTCG TGAATTTGAG TTTGGGCAGT AGACTATCCC GGAATAGAGG ACTGATGCGT   
  
  
+ TATATATATA GAAATTGAGA GAGAGAGAGA GCGTGTGTGA AGGATGTTGA AAATGATGGG CGTAGGGAGT   
  
  
+ CTCTCTCTCA CTGTCTGTTA GGAGTACGTG TCGAGAAAGG AAATAGGATT CCCAAGCAGT CTAATTTGGG   
  
  
+ CCGGTCTATA GGCCAGAATG GGCCTATGAT CAGCCCAGAT ATGACCTTGT TTAACCAACT GTATTTGAAT   
  
  
+ TGAAGAAAGG GATATCGCAA TGTCTTGAAC CACATCAAAT AATAAATGTG CCTTTGGTTA GACCATATGA   
  
  
+ AAAATCTTTG AATCAAATCG GTTGGACCTG AACTGCCATT ATTGATATCT TGTTTTTGGT AGAAGCAAAT   
  
  
+ GGTGTCACTT CGTTTTCTAG ATAAAATTAA CTTGAGTTTT TTTGCCACTA TAGATAGTAT TGCAAATATA   
  
  
+ AAAAGTGTGC CACGTGAAAT CAATATTAGG AGGGTGTTTT ATAACAATAG CTGTTCGGGA GAGTTTTAGT   
  
  
+ TATTTTTATT AAATCAAATA GTTAAGAAGT GTTTGGTAAA TAGTTGTATT TGAAAAAGTT ATTCCCATTA   
  
  
+ GCCTTTTAGT AAGAACTTCT TGTGAAAATG ATAATTGTCT AGAAGTTTGA AAAATTCACA CCACATGACA   
  
  
+ TTTAAAAATA ATTTTGTAGA GAAGGGCATA AATGAAAGTT TGCCACCATC TTTTTTAGAA ACATGTAATT   
  
  
+ TACCAAACAC TTTTTCTAAA AACAGTTAAT TCAAATAACT AACAACTAAC AGCTAATATA AATAACTAAC   
  
  
+ AGCTATTTGC TAAACAGGGC CTAAGTAGTT TGAAATTGTT GTTTTCTCTT CAGCTTCACC ATGGCCGATG   
  
  
+ ATGTAAACAA CTATATTCTG AATTTCATTT TTACTATGAT TTGAAAATTT CAAATTTAAA TAAGATTGGA   
  
  
+ TAAAAAAAAG ACCAAAAATC ATAAGTAATA TTAGTCAAGT TCTATTCCAA CCATATACAT AACTGCTTTT   
  
  
+ TCTTTTGGGG GTTTTGAAAC ATGTGTCCTT AGAATAATTG AAACTCGATA TTATTCTCTA AATCTAAGTT   
  
  
+ TACTTTATCA TATTGATTAT TAATTACCTC CTCAACTCCT CCTATACTCT TGTATGCAGA AATAAAAATG   
  
  
+ TTTCCATACC TTATCAGTCG CGATCATAAT AATTAATTTC TTAATTCGGA TTGTATTTAA TTGCGTATCA   
  
  
+ AATGTGAAAT CGTACCAAAT TAGGAAGCAA AACAAACCGA TAACGAAAAA GGTAAGAAAA CTTAGCAAGG   
  
  
+ AAATGAAATC GAAGACCTGT CAACAATATA GCTTCCCTCA CATCCTTCAA CTTAGGGACC ATCCTATACT   
  
  
+ TTCATGTAAT ACAACTAGAG CCACTAGTCA ATAATAGCCT CCTAGTAGTA GCCATAAGCC TTGGTGAGAG   
  
  
+ AATGTTCCGC AAGACTAGTT AACTTGAAAT TCCATTTTGT CGCAGAACTA TTGGAGTGAG AAAGAGAATC   
  
  
+ AAATCATACC ATATCAAATT TGGACCATTT AAGTTCACAA CACACACACA TACACACAAA AGGACGATGT   
  
  
+ ACGGCCCCAC CCCCCACTCT CCTTTCGCCA TCCCCCTACA CCTCTGCCTA CTCTCTCTCT CTCTCTCTCT   
  
  
+ CTCTGGATCA GTCTGTGTAA AAGCTTCCTC TCCAGTACAG CATTTCCTTG TCTGTTCTTG GAGTTTAGTC   
  
  
+ AATTCCTCCG CCCCAAAAAA ACCCACTTTC TCTCTCATCA TGTTCTTCCA GTTCCACCCT TATACGCCAT   
  
  
+ TCATCATCAT CTACATCACC CACATCTTTT TCTCCACAGT TTTCCCCCCT CATTATGCTC TCCGCCTCCA   
  
  
+ CCCCCGCCTT CCCTCTCGCC GTCGCCGGCG ATGACGGGGA TACCACTGAT GCATGCGACG GCACCGCCAC   
  
  
+ TGCCCTCCAC CTCCTCGGGC TGCTGTTGAA GTGCGCGGAA TTCATCTCCA CCGGAGACCT CGCCGGCGCC   
  
  
+ GGAGATATCT TGCCGGAGAT ATTTGAGTTG TCTACACCGT TTGGCTCCCC CGCCGCCCGG GTCGCCGCCT   
  
  
+ TCTTCGGCCA CGCCCTCCAC GCCCGCCTCC TCTCCGCCTC CCTCCGCACA ACTCCGATCG AGAAGCTCAA   
  
  
+ AACCCTGACC CTGGTTTCCC AAATGCGGAA ATTTCACTCC GCCTTGCAAG TATACAACTC CATCACTCCC   
  
  
+ TTCGTGAAAT TCTCTCACTT CACGGCGAAT CAAGCCATCT ACGAGGCGCT GGACGGCGAG GATCGTGTCC   
  
  
+ ACGTCGTCGA CCTTGACATC ATGCAGGGCC TTCAATGGCC GGGATTGTTC CACATCTTAG CCTCTCGACC   
  
  
+ CAGAAAGCCC CTCTCGGTTC GGGTCACCGG GTTCGGGCCA TCCTCCGAGT TGCTCTCCCA AACGGGTAAG   
  
  
+ CGACTCGCTG AGTTCGCCGC TTCACTCGGC CTGCCCTTCG AGTACAACCC GGTGGAGGGC AAAATTGGGA   
  
  
+ ACTTAGTCGA CCTGGGTCGG GTCGGGTCGC TCCCGAATGA AGTGACGGTG GTGCACTGGA TGCACCATAG   
  
  
+ TCTATACGAC ATAACCGGGT CGGATCTTGG GACTTTGAGG GTATTGAGTG CGGTGAGGCC TAGGCTTGTG   
  
  
+ ACTATGGTTG AGCAGGATAT GGACCAAACG GGGTCGTTTT TGGGGAGGTT TGTGGAGGCA TTGCATTATT   
  
  
+ ACTCAGCCTT GTTTGATGCC TTGGGAGAGG GGTTAGATAG GGATAACCTA CAAAGGCATC AAGTGGAGCA   
  
  
+ ACAGCTATTT GGGTGTGAGA TTAGGAACAT CCTGGCCGTT GGTGGGCCCA AGAGGAGGCT CACTGGCGGC   
  
  
+ GATCGGGTCA GAAGGTGGGG CGACGAACTG ACAAGGGTCG GGTTCGAACC AGTTTCGTTG GCGGGTAGCC   
  
  
+ CGGCAACCCA AGCTAGTTTG TTGCTTGGGA TGTTCCCTTG GAAAGGGTAT ACTTTGATGG AGGAAAATGG   
  
  
+ GTGTTTGAGA TTAGGGTGGA AAGATTTGCC CTTGTTAACT GCCTCAGCTT GGCAACCTTG TGAATTTAAC   
  
  
+ AATCCTAGTG CTGGCATTTA   

- +Up\_Stream \_Len000TTTGCC ACGGCAACTT AAACGTCCGC TGCTCCAGCC CAAATTCCGC CTACAGCATC   
  
  
- GCGAAACCTC TTCGTTTAAA CCCTTCTGCT CCGAATAAGA ACTTGAGAAG CATGCGGTAA CAACAACCAA   
  
  
- GGTTGGAAAG AGAGAGGAGC ACTTAAACTC AAACCCGTCA TCTGATAGGG CCTTATCTCC TGACTACGCA   
  
  
- ATATATATAT CTTTAACTCT CTCTCTCTCT CGCACACACT TCCTACAACT TTTACTACCC GCATCCCTCA   
  
  
- GAGAGAGAGT GACAGACAAT CCTCATGCAC AGCTCTTTCC TTTATCCTAA GGGTTCGTCA GATTAAACCC   
  
  
- GGCCAGATAT CCGGTCTTAC CCGGATACTA GTCGGGTCTA TACTGGAACA AATTGGTTGA CATAAACTTA   
  
  
- ACTTCTTTCC CTATAGCGTT ACAGAACTTG GTGTAGTTTA TTATTTACAC GGAAACCAAT CTGGTATACT   
  
  
- TTTTAGAAAC TTAGTTTAGC CAACCTGGAC TTGACGGTAA TAACTATAGA ACAAAAACCA TCTTCGTTTA   
  
  
- CCACAGTGAA GCAAAAGATC TATTTTAATT GAACTCAAAA AAACGGTGAT ATCTATCATA ACGTTTATAT   
  
  
- TTTTCACACG GTGCACTTTA GTTATAATCC TCCCACAAAA TATTGTTATC GACAAGCCCT CTCAAAATCA   
  
  
- ATAAAAATAA TTTAGTTTAT CAATTCTTCA CAAACCATTT ATCAACATAA ACTTTTTCAA TAAGGGTAAT   
  
  
- CGGAAAATCA TTCTTGAAGA ACACTTTTAC TATTAACAGA TCTTCAAACT TTTTAAGTGT GGTGTACTGT   
  
  
- AAATTTTTAT TAAAACATCT CTTCCCGTAT TTACTTTCAA ACGGTGGTAG AAAAAATCTT TGTACATTAA   
  
  
- ATGGTTTGTG AAAAAGATTT TTGTCAATTA AGTTTATTGA TTGTTGATTG TCGATTATAT TTATTGATTG   
  
  
- TCGATAAACG ATTTGTCCCG GATTCATCAA ACTTTAACAA CAAAAGAGAA GTCGAAGTGG TACCGGCTAC   
  
  
- TACATTTGTT GATATAAGAC TTAAAGTAAA AATGATACTA AACTTTTAAA GTTTAAATTT ATTCTAACCT   
  
  
- ATTTTTTTTC TGGTTTTTAG TATTCATTAT AATCAGTTCA AGATAAGGTT GGTATATGTA TTGACGAAAA   
  
  
- AGAAAACCCC CAAAACTTTG TACACAGGAA TCTTATTAAC TTTGAGCTAT AATAAGAGAT TTAGATTCAA   
  
  
- ATGAAATAGT ATAACTAATA ATTAATGGAG GAGTTGAGGA GGATATGAGA ACATACGTCT TTATTTTTAC   
  
  
- AAAGGTATGG AATAGTCAGC GCTAGTATTA TTAATTAAAG AATTAAGCCT AACATAAATT AACGCATAGT   
  
  
- TTACACTTTA GCATGGTTTA ATCCTTCGTT TTGTTTGGCT ATTGCTTTTT CCATTCTTTT GAATCGTTCC   
  
  
- TTTACTTTAG CTTCTGGACA GTTGTTATAT CGAAGGGAGT GTAGGAAGTT GAATCCCTGG TAGGATATGA   
  
  
- AAGTACATTA TGTTGATCTC GGTGATCAGT TATTATCGGA GGATCATCAT CGGTATTCGG AACCACTCTC   
  
  
- TTACAAGGCG TTCTGATCAA TTGAACTTTA AGGTAAAACA GCGTCTTGAT AACCTCACTC TTTCTCTTAG   
  
  
- TTTAGTATGG TATAGTTTAA ACCTGGTAAA TTCAAGTGTT GTGTGTGTGT ATGTGTGTTT TCCTGCTACA   
  
  
- TGCCGGGGTG GGGGGTGAGA GGAAAGCGGT AGGGGGATGT GGAGACGGAT GAGAGAGAGA GAGAGAGAGA   
  
  
- GAGACCTAGT CAGACACATT TTCGAAGGAG AGGTCATGTC GTAAAGGAAC AGACAAGAAC CTCAAATCAG   
  
  
- TTAAGGAGGC GGGGTTTTTT TGGGTGAAAG AGAGAGTAGT ACAAGAAGGT CAAGGTGGGA ATATGCGGTA   
  
  
- AGTAGTAGTA GATGTAGTGG GTGTAGAAAA AGAGGTGTCA AAAGGGGGGA GTAATACGAG AGGCGGAGGT   
  
  
- GGGGGCGGAA GGGAGAGCGG CAGCGGCCGC TACTGCCCCT ATGGTGACTA CGTACGCTGC CGTGGCGGTG   
  
  
- ACGGGAGGTG GAGGAGCCCG ACGACAACTT CACGCGCCTT AAGTAGAGGT GGCCTCTGGA GCGGCCGCGG   
  
  
- CCTCTATAGA ACGGCCTCTA TAAACTCAAC AGATGTGGCA AACCGAGGGG GCGGCGGGCC CAGCGGCGGA   
  
  
- AGAAGCCGGT GCGGGAGGTG CGGGCGGAGG AGAGGCGGAG GGAGGCGTGT TGAGGCTAGC TCTTCGAGTT   
  
  
- TTGGGACTGG GACCAAAGGG TTTACGCCTT TAAAGTGAGG CGGAACGTTC ATATGTTGAG GTAGTGAGGG   
  
  
- AAGCACTTTA AGAGAGTGAA GTGCCGCTTA GTTCGGTAGA TGCTCCGCGA CCTGCCGCTC CTAGCACAGG   
  
  
- TGCAGCAGCT GGAACTGTAG TACGTCCCGG AAGTTACCGG CCCTAACAAG GTGTAGAATC GGAGAGCTGG   
  
  
- GTCTTTCGGG GAGAGCCAAG CCCAGTGGCC CAAGCCCGGT AGGAGGCTCA ACGAGAGGGT TTGCCCATTC   
  
  
- GCTGAGCGAC TCAAGCGGCG AAGTGAGCCG GACGGGAAGC TCATGTTGGG CCACCTCCCG TTTTAACCCT   
  
  
- TGAATCAGCT GGACCCAGCC CAGCCCAGCG AGGGCTTACT TCACTGCCAC CACGTGACCT ACGTGGTATC   
  
  
- AGATATGCTG TATTGGCCCA GCCTAGAACC CTGAAACTCC CATAACTCAC GCCACTCCGG ATCCGAACAC   
  
  
- TGATACCAAC TCGTCCTATA CCTGGTTTGC CCCAGCAAAA ACCCCTCCAA ACACCTCCGT AACGTAATAA   
  
  
- TGAGTCGGAA CAAACTACGG AACCCTCTCC CCAATCTATC CCTATTGGAT GTTTCCGTAG TTCACCTCGT   
  
  
- TGTCGATAAA CCCACACTCT AATCCTTGTA GGACCGGCAA CCACCCGGGT TCTCCTCCGA GTGACCGCCG   
  
  
- CTAGCCCAGT CTTCCACCCC GCTGCTTGAC TGTTCCCAGC CCAAGCTTGG TCAAAGCAAC CGCCCATCGG   
  
  
- GCCGTTGGGT TCGATCAAAC AACGAACCCT ACAAGGGAAC CTTTCCCATA TGAAACTACC TCCTTTTACC   
  
  
- CACAAACTCT AATCCCACCT TTCTAAACGG GAACAATTGA CGGAGTCGAA CCGTTGGAAC ACTTAAATTG   
  
  
- TTAGGATCAC GACCGTAAAT

+     P-box

| Site Name | Organism | Position | Strand | Matrix score. | sequence | function |
| --- | --- | --- | --- | --- | --- | --- |
| P-box | Oryza sativa | 1741 | - | 7 | CCTTTTG | gibberellin-responsive element |

>HU02G03005.1   
+ +Up\_Stream \_Len000AAACGG TGCCGTTGAA TTTGCAGGCG ACGAGGTCGG GTTTAAGGCG GATGTCGTAG   
  
  
+ CGCTTTGGAG AAGCAAATTT GGGAAGACGA GGCTTATTCT TGAACTCTTC GTACGCCATT GTTGTTGGTT   
  
  
+ CCAACCTTTC TCTCTCCTCG TGAATTTGAG TTTGGGCAGT AGACTATCCC GGAATAGAGG ACTGATGCGT   
  
  
+ TATATATATA GAAATTGAGA GAGAGAGAGA GCGTGTGTGA AGGATGTTGA AAATGATGGG CGTAGGGAGT   
  
  
+ CTCTCTCTCA CTGTCTGTTA GGAGTACGTG TCGAGAAAGG AAATAGGATT CCCAAGCAGT CTAATTTGGG   
  
  
+ CCGGTCTATA GGCCAGAATG GGCCTATGAT CAGCCCAGAT ATGACCTTGT TTAACCAACT GTATTTGAAT   
  
  
+ TGAAGAAAGG GATATCGCAA TGTCTTGAAC CACATCAAAT AATAAATGTG CCTTTGGTTA GACCATATGA   
  
  
+ AAAATCTTTG AATCAAATCG GTTGGACCTG AACTGCCATT ATTGATATCT TGTTTTTGGT AGAAGCAAAT   
  
  
+ GGTGTCACTT CGTTTTCTAG ATAAAATTAA CTTGAGTTTT TTTGCCACTA TAGATAGTAT TGCAAATATA   
  
  
+ AAAAGTGTGC CACGTGAAAT CAATATTAGG AGGGTGTTTT ATAACAATAG CTGTTCGGGA GAGTTTTAGT   
  
  
+ TATTTTTATT AAATCAAATA GTTAAGAAGT GTTTGGTAAA TAGTTGTATT TGAAAAAGTT ATTCCCATTA   
  
  
+ GCCTTTTAGT AAGAACTTCT TGTGAAAATG ATAATTGTCT AGAAGTTTGA AAAATTCACA CCACATGACA   
  
  
+ TTTAAAAATA ATTTTGTAGA GAAGGGCATA AATGAAAGTT TGCCACCATC TTTTTTAGAA ACATGTAATT   
  
  
+ TACCAAACAC TTTTTCTAAA AACAGTTAAT TCAAATAACT AACAACTAAC AGCTAATATA AATAACTAAC   
  
  
+ AGCTATTTGC TAAACAGGGC CTAAGTAGTT TGAAATTGTT GTTTTCTCTT CAGCTTCACC ATGGCCGATG   
  
  
+ ATGTAAACAA CTATATTCTG AATTTCATTT TTACTATGAT TTGAAAATTT CAAATTTAAA TAAGATTGGA   
  
  
+ TAAAAAAAAG ACCAAAAATC ATAAGTAATA TTAGTCAAGT TCTATTCCAA CCATATACAT AACTGCTTTT   
  
  
+ TCTTTTGGGG GTTTTGAAAC ATGTGTCCTT AGAATAATTG AAACTCGATA TTATTCTCTA AATCTAAGTT   
  
  
+ TACTTTATCA TATTGATTAT TAATTACCTC CTCAACTCCT CCTATACTCT TGTATGCAGA AATAAAAATG   
  
  
+ TTTCCATACC TTATCAGTCG CGATCATAAT AATTAATTTC TTAATTCGGA TTGTATTTAA TTGCGTATCA   
  
  
+ AATGTGAAAT CGTACCAAAT TAGGAAGCAA AACAAACCGA TAACGAAAAA GGTAAGAAAA CTTAGCAAGG   
  
  
+ AAATGAAATC GAAGACCTGT CAACAATATA GCTTCCCTCA CATCCTTCAA CTTAGGGACC ATCCTATACT   
  
  
+ TTCATGTAAT ACAACTAGAG CCACTAGTCA ATAATAGCCT CCTAGTAGTA GCCATAAGCC TTGGTGAGAG   
  
  
+ AATGTTCCGC AAGACTAGTT AACTTGAAAT TCCATTTTGT CGCAGAACTA TTGGAGTGAG AAAGAGAATC   
  
  
+ AAATCATACC ATATCAAATT TGGACCATTT AAGTTCACAA CACACACACA TACACACAAA AGGACGATGT   
  
  
+ ACGGCCCCAC CCCCCACTCT CCTTTCGCCA TCCCCCTACA CCTCTGCCTA CTCTCTCTCT CTCTCTCTCT   
  
  
+ CTCTGGATCA GTCTGTGTAA AAGCTTCCTC TCCAGTACAG CATTTCCTTG TCTGTTCTTG GAGTTTAGTC   
  
  
+ AATTCCTCCG CCCCAAAAAA ACCCACTTTC TCTCTCATCA TGTTCTTCCA GTTCCACCCT TATACGCCAT   
  
  
+ TCATCATCAT CTACATCACC CACATCTTTT TCTCCACAGT TTTCCCCCCT CATTATGCTC TCCGCCTCCA   
  
  
+ CCCCCGCCTT CCCTCTCGCC GTCGCCGGCG ATGACGGGGA TACCACTGAT GCATGCGACG GCACCGCCAC   
  
  
+ TGCCCTCCAC CTCCTCGGGC TGCTGTTGAA GTGCGCGGAA TTCATCTCCA CCGGAGACCT CGCCGGCGCC   
  
  
+ GGAGATATCT TGCCGGAGAT ATTTGAGTTG TCTACACCGT TTGGCTCCCC CGCCGCCCGG GTCGCCGCCT   
  
  
+ TCTTCGGCCA CGCCCTCCAC GCCCGCCTCC TCTCCGCCTC CCTCCGCACA ACTCCGATCG AGAAGCTCAA   
  
  
+ AACCCTGACC CTGGTTTCCC AAATGCGGAA ATTTCACTCC GCCTTGCAAG TATACAACTC CATCACTCCC   
  
  
+ TTCGTGAAAT TCTCTCACTT CACGGCGAAT CAAGCCATCT ACGAGGCGCT GGACGGCGAG GATCGTGTCC   
  
  
+ ACGTCGTCGA CCTTGACATC ATGCAGGGCC TTCAATGGCC GGGATTGTTC CACATCTTAG CCTCTCGACC   
  
  
+ CAGAAAGCCC CTCTCGGTTC GGGTCACCGG GTTCGGGCCA TCCTCCGAGT TGCTCTCCCA AACGGGTAAG   
  
  
+ CGACTCGCTG AGTTCGCCGC TTCACTCGGC CTGCCCTTCG AGTACAACCC GGTGGAGGGC AAAATTGGGA   
  
  
+ ACTTAGTCGA CCTGGGTCGG GTCGGGTCGC TCCCGAATGA AGTGACGGTG GTGCACTGGA TGCACCATAG   
  
  
+ TCTATACGAC ATAACCGGGT CGGATCTTGG GACTTTGAGG GTATTGAGTG CGGTGAGGCC TAGGCTTGTG   
  
  
+ ACTATGGTTG AGCAGGATAT GGACCAAACG GGGTCGTTTT TGGGGAGGTT TGTGGAGGCA TTGCATTATT   
  
  
+ ACTCAGCCTT GTTTGATGCC TTGGGAGAGG GGTTAGATAG GGATAACCTA CAAAGGCATC AAGTGGAGCA   
  
  
+ ACAGCTATTT GGGTGTGAGA TTAGGAACAT CCTGGCCGTT GGTGGGCCCA AGAGGAGGCT CACTGGCGGC   
  
  
+ GATCGGGTCA GAAGGTGGGG CGACGAACTG ACAAGGGTCG GGTTCGAACC AGTTTCGTTG GCGGGTAGCC   
  
  
+ CGGCAACCCA AGCTAGTTTG TTGCTTGGGA TGTTCCCTTG GAAAGGGTAT ACTTTGATGG AGGAAAATGG   
  
  
+ GTGTTTGAGA TTAGGGTGGA AAGATTTGCC CTTGTTAACT GCCTCAGCTT GGCAACCTTG TGAATTTAAC   
  
  
+ AATCCTAGTG CTGGCATTTA   

- +Up\_Stream \_Len000TTTGCC ACGGCAACTT AAACGTCCGC TGCTCCAGCC CAAATTCCGC CTACAGCATC   
  
  
- GCGAAACCTC TTCGTTTAAA CCCTTCTGCT CCGAATAAGA ACTTGAGAAG CATGCGGTAA CAACAACCAA   
  
  
- GGTTGGAAAG AGAGAGGAGC ACTTAAACTC AAACCCGTCA TCTGATAGGG CCTTATCTCC TGACTACGCA   
  
  
- ATATATATAT CTTTAACTCT CTCTCTCTCT CGCACACACT TCCTACAACT TTTACTACCC GCATCCCTCA   
  
  
- GAGAGAGAGT GACAGACAAT CCTCATGCAC AGCTCTTTCC TTTATCCTAA GGGTTCGTCA GATTAAACCC   
  
  
- GGCCAGATAT CCGGTCTTAC CCGGATACTA GTCGGGTCTA TACTGGAACA AATTGGTTGA CATAAACTTA   
  
  
- ACTTCTTTCC CTATAGCGTT ACAGAACTTG GTGTAGTTTA TTATTTACAC GGAAACCAAT CTGGTATACT   
  
  
- TTTTAGAAAC TTAGTTTAGC CAACCTGGAC TTGACGGTAA TAACTATAGA ACAAAAACCA TCTTCGTTTA   
  
  
- CCACAGTGAA GCAAAAGATC TATTTTAATT GAACTCAAAA AAACGGTGAT ATCTATCATA ACGTTTATAT   
  
  
- TTTTCACACG GTGCACTTTA GTTATAATCC TCCCACAAAA TATTGTTATC GACAAGCCCT CTCAAAATCA   
  
  
- ATAAAAATAA TTTAGTTTAT CAATTCTTCA CAAACCATTT ATCAACATAA ACTTTTTCAA TAAGGGTAAT   
  
  
- CGGAAAATCA TTCTTGAAGA ACACTTTTAC TATTAACAGA TCTTCAAACT TTTTAAGTGT GGTGTACTGT   
  
  
- AAATTTTTAT TAAAACATCT CTTCCCGTAT TTACTTTCAA ACGGTGGTAG AAAAAATCTT TGTACATTAA   
  
  
- ATGGTTTGTG AAAAAGATTT TTGTCAATTA AGTTTATTGA TTGTTGATTG TCGATTATAT TTATTGATTG   
  
  
- TCGATAAACG ATTTGTCCCG GATTCATCAA ACTTTAACAA CAAAAGAGAA GTCGAAGTGG TACCGGCTAC   
  
  
- TACATTTGTT GATATAAGAC TTAAAGTAAA AATGATACTA AACTTTTAAA GTTTAAATTT ATTCTAACCT   
  
  
- ATTTTTTTTC TGGTTTTTAG TATTCATTAT AATCAGTTCA AGATAAGGTT GGTATATGTA TTGACGAAAA   
  
  
- AGAAAACCCC CAAAACTTTG TACACAGGAA TCTTATTAAC TTTGAGCTAT AATAAGAGAT TTAGATTCAA   
  
  
- ATGAAATAGT ATAACTAATA ATTAATGGAG GAGTTGAGGA GGATATGAGA ACATACGTCT TTATTTTTAC   
  
  
- AAAGGTATGG AATAGTCAGC GCTAGTATTA TTAATTAAAG AATTAAGCCT AACATAAATT AACGCATAGT   
  
  
- TTACACTTTA GCATGGTTTA ATCCTTCGTT TTGTTTGGCT ATTGCTTTTT CCATTCTTTT GAATCGTTCC   
  
  
- TTTACTTTAG CTTCTGGACA GTTGTTATAT CGAAGGGAGT GTAGGAAGTT GAATCCCTGG TAGGATATGA   
  
  
- AAGTACATTA TGTTGATCTC GGTGATCAGT TATTATCGGA GGATCATCAT CGGTATTCGG AACCACTCTC   
  
  
- TTACAAGGCG TTCTGATCAA TTGAACTTTA AGGTAAAACA GCGTCTTGAT AACCTCACTC TTTCTCTTAG   
  
  
- TTTAGTATGG TATAGTTTAA ACCTGGTAAA TTCAAGTGTT GTGTGTGTGT ATGTGTGTTT TCCTGCTACA   
  
  
- TGCCGGGGTG GGGGGTGAGA GGAAAGCGGT AGGGGGATGT GGAGACGGAT GAGAGAGAGA GAGAGAGAGA   
  
  
- GAGACCTAGT CAGACACATT TTCGAAGGAG AGGTCATGTC GTAAAGGAAC AGACAAGAAC CTCAAATCAG   
  
  
- TTAAGGAGGC GGGGTTTTTT TGGGTGAAAG AGAGAGTAGT ACAAGAAGGT CAAGGTGGGA ATATGCGGTA   
  
  
- AGTAGTAGTA GATGTAGTGG GTGTAGAAAA AGAGGTGTCA AAAGGGGGGA GTAATACGAG AGGCGGAGGT   
  
  
- GGGGGCGGAA GGGAGAGCGG CAGCGGCCGC TACTGCCCCT ATGGTGACTA CGTACGCTGC CGTGGCGGTG   
  
  
- ACGGGAGGTG GAGGAGCCCG ACGACAACTT CACGCGCCTT AAGTAGAGGT GGCCTCTGGA GCGGCCGCGG   
  
  
- CCTCTATAGA ACGGCCTCTA TAAACTCAAC AGATGTGGCA AACCGAGGGG GCGGCGGGCC CAGCGGCGGA   
  
  
- AGAAGCCGGT GCGGGAGGTG CGGGCGGAGG AGAGGCGGAG GGAGGCGTGT TGAGGCTAGC TCTTCGAGTT   
  
  
- TTGGGACTGG GACCAAAGGG TTTACGCCTT TAAAGTGAGG CGGAACGTTC ATATGTTGAG GTAGTGAGGG   
  
  
- AAGCACTTTA AGAGAGTGAA GTGCCGCTTA GTTCGGTAGA TGCTCCGCGA CCTGCCGCTC CTAGCACAGG   
  
  
- TGCAGCAGCT GGAACTGTAG TACGTCCCGG AAGTTACCGG CCCTAACAAG GTGTAGAATC GGAGAGCTGG   
  
  
- GTCTTTCGGG GAGAGCCAAG CCCAGTGGCC CAAGCCCGGT AGGAGGCTCA ACGAGAGGGT TTGCCCATTC   
  
  
- GCTGAGCGAC TCAAGCGGCG AAGTGAGCCG GACGGGAAGC TCATGTTGGG CCACCTCCCG TTTTAACCCT   
  
  
- TGAATCAGCT GGACCCAGCC CAGCCCAGCG AGGGCTTACT TCACTGCCAC CACGTGACCT ACGTGGTATC   
  
  
- AGATATGCTG TATTGGCCCA GCCTAGAACC CTGAAACTCC CATAACTCAC GCCACTCCGG ATCCGAACAC   
  
  
- TGATACCAAC TCGTCCTATA CCTGGTTTGC CCCAGCAAAA ACCCCTCCAA ACACCTCCGT AACGTAATAA   
  
  
- TGAGTCGGAA CAAACTACGG AACCCTCTCC CCAATCTATC CCTATTGGAT GTTTCCGTAG TTCACCTCGT   
  
  
- TGTCGATAAA CCCACACTCT AATCCTTGTA GGACCGGCAA CCACCCGGGT TCTCCTCCGA GTGACCGCCG   
  
  
- CTAGCCCAGT CTTCCACCCC GCTGCTTGAC TGTTCCCAGC CCAAGCTTGG TCAAAGCAAC CGCCCATCGG   
  
  
- GCCGTTGGGT TCGATCAAAC AACGAACCCT ACAAGGGAAC CTTTCCCATA TGAAACTACC TCCTTTTACC   
  
  
- CACAAACTCT AATCCCACCT TTCTAAACGG GAACAATTGA CGGAGTCGAA CCGTTGGAAC ACTTAAATTG   
  
  
- TTAGGATCAC GACCGTAAAT

+     STRE

| Site Name | Organism | Position | Strand | Matrix score. | sequence | function |
| --- | --- | --- | --- | --- | --- | --- |
| STRE | Arabidopsis thaliana | 2532 | - | 5 | AGGGG |  |
| STRE | Arabidopsis thaliana | 1787 | - | 5 | AGGGG |  |
| STRE | Arabidopsis thaliana | 2010 | - | 5 | AGGGG |  |
| STRE | Arabidopsis thaliana | 2902 | + | 5 | AGGGG |  |

>HU02G03005.1   
+ +Up\_Stream \_Len000AAACGG TGCCGTTGAA TTTGCAGGCG ACGAGGTCGG GTTTAAGGCG GATGTCGTAG   
  
  
+ CGCTTTGGAG AAGCAAATTT GGGAAGACGA GGCTTATTCT TGAACTCTTC GTACGCCATT GTTGTTGGTT   
  
  
+ CCAACCTTTC TCTCTCCTCG TGAATTTGAG TTTGGGCAGT AGACTATCCC GGAATAGAGG ACTGATGCGT   
  
  
+ TATATATATA GAAATTGAGA GAGAGAGAGA GCGTGTGTGA AGGATGTTGA AAATGATGGG CGTAGGGAGT   
  
  
+ CTCTCTCTCA CTGTCTGTTA GGAGTACGTG TCGAGAAAGG AAATAGGATT CCCAAGCAGT CTAATTTGGG   
  
  
+ CCGGTCTATA GGCCAGAATG GGCCTATGAT CAGCCCAGAT ATGACCTTGT TTAACCAACT GTATTTGAAT   
  
  
+ TGAAGAAAGG GATATCGCAA TGTCTTGAAC CACATCAAAT AATAAATGTG CCTTTGGTTA GACCATATGA   
  
  
+ AAAATCTTTG AATCAAATCG GTTGGACCTG AACTGCCATT ATTGATATCT TGTTTTTGGT AGAAGCAAAT   
  
  
+ GGTGTCACTT CGTTTTCTAG ATAAAATTAA CTTGAGTTTT TTTGCCACTA TAGATAGTAT TGCAAATATA   
  
  
+ AAAAGTGTGC CACGTGAAAT CAATATTAGG AGGGTGTTTT ATAACAATAG CTGTTCGGGA GAGTTTTAGT   
  
  
+ TATTTTTATT AAATCAAATA GTTAAGAAGT GTTTGGTAAA TAGTTGTATT TGAAAAAGTT ATTCCCATTA   
  
  
+ GCCTTTTAGT AAGAACTTCT TGTGAAAATG ATAATTGTCT AGAAGTTTGA AAAATTCACA CCACATGACA   
  
  
+ TTTAAAAATA ATTTTGTAGA GAAGGGCATA AATGAAAGTT TGCCACCATC TTTTTTAGAA ACATGTAATT   
  
  
+ TACCAAACAC TTTTTCTAAA AACAGTTAAT TCAAATAACT AACAACTAAC AGCTAATATA AATAACTAAC   
  
  
+ AGCTATTTGC TAAACAGGGC CTAAGTAGTT TGAAATTGTT GTTTTCTCTT CAGCTTCACC ATGGCCGATG   
  
  
+ ATGTAAACAA CTATATTCTG AATTTCATTT TTACTATGAT TTGAAAATTT CAAATTTAAA TAAGATTGGA   
  
  
+ TAAAAAAAAG ACCAAAAATC ATAAGTAATA TTAGTCAAGT TCTATTCCAA CCATATACAT AACTGCTTTT   
  
  
+ TCTTTTGGGG GTTTTGAAAC ATGTGTCCTT AGAATAATTG AAACTCGATA TTATTCTCTA AATCTAAGTT   
  
  
+ TACTTTATCA TATTGATTAT TAATTACCTC CTCAACTCCT CCTATACTCT TGTATGCAGA AATAAAAATG   
  
  
+ TTTCCATACC TTATCAGTCG CGATCATAAT AATTAATTTC TTAATTCGGA TTGTATTTAA TTGCGTATCA   
  
  
+ AATGTGAAAT CGTACCAAAT TAGGAAGCAA AACAAACCGA TAACGAAAAA GGTAAGAAAA CTTAGCAAGG   
  
  
+ AAATGAAATC GAAGACCTGT CAACAATATA GCTTCCCTCA CATCCTTCAA CTTAGGGACC ATCCTATACT   
  
  
+ TTCATGTAAT ACAACTAGAG CCACTAGTCA ATAATAGCCT CCTAGTAGTA GCCATAAGCC TTGGTGAGAG   
  
  
+ AATGTTCCGC AAGACTAGTT AACTTGAAAT TCCATTTTGT CGCAGAACTA TTGGAGTGAG AAAGAGAATC   
  
  
+ AAATCATACC ATATCAAATT TGGACCATTT AAGTTCACAA CACACACACA TACACACAAA AGGACGATGT   
  
  
+ ACGGCCCCAC CCCCCACTCT CCTTTCGCCA TCCCCCTACA CCTCTGCCTA CTCTCTCTCT CTCTCTCTCT   
  
  
+ CTCTGGATCA GTCTGTGTAA AAGCTTCCTC TCCAGTACAG CATTTCCTTG TCTGTTCTTG GAGTTTAGTC   
  
  
+ AATTCCTCCG CCCCAAAAAA ACCCACTTTC TCTCTCATCA TGTTCTTCCA GTTCCACCCT TATACGCCAT   
  
  
+ TCATCATCAT CTACATCACC CACATCTTTT TCTCCACAGT TTTCCCCCCT CATTATGCTC TCCGCCTCCA   
  
  
+ CCCCCGCCTT CCCTCTCGCC GTCGCCGGCG ATGACGGGGA TACCACTGAT GCATGCGACG GCACCGCCAC   
  
  
+ TGCCCTCCAC CTCCTCGGGC TGCTGTTGAA GTGCGCGGAA TTCATCTCCA CCGGAGACCT CGCCGGCGCC   
  
  
+ GGAGATATCT TGCCGGAGAT ATTTGAGTTG TCTACACCGT TTGGCTCCCC CGCCGCCCGG GTCGCCGCCT   
  
  
+ TCTTCGGCCA CGCCCTCCAC GCCCGCCTCC TCTCCGCCTC CCTCCGCACA ACTCCGATCG AGAAGCTCAA   
  
  
+ AACCCTGACC CTGGTTTCCC AAATGCGGAA ATTTCACTCC GCCTTGCAAG TATACAACTC CATCACTCCC   
  
  
+ TTCGTGAAAT TCTCTCACTT CACGGCGAAT CAAGCCATCT ACGAGGCGCT GGACGGCGAG GATCGTGTCC   
  
  
+ ACGTCGTCGA CCTTGACATC ATGCAGGGCC TTCAATGGCC GGGATTGTTC CACATCTTAG CCTCTCGACC   
  
  
+ CAGAAAGCCC CTCTCGGTTC GGGTCACCGG GTTCGGGCCA TCCTCCGAGT TGCTCTCCCA AACGGGTAAG   
  
  
+ CGACTCGCTG AGTTCGCCGC TTCACTCGGC CTGCCCTTCG AGTACAACCC GGTGGAGGGC AAAATTGGGA   
  
  
+ ACTTAGTCGA CCTGGGTCGG GTCGGGTCGC TCCCGAATGA AGTGACGGTG GTGCACTGGA TGCACCATAG   
  
  
+ TCTATACGAC ATAACCGGGT CGGATCTTGG GACTTTGAGG GTATTGAGTG CGGTGAGGCC TAGGCTTGTG   
  
  
+ ACTATGGTTG AGCAGGATAT GGACCAAACG GGGTCGTTTT TGGGGAGGTT TGTGGAGGCA TTGCATTATT   
  
  
+ ACTCAGCCTT GTTTGATGCC TTGGGAGAGG GGTTAGATAG GGATAACCTA CAAAGGCATC AAGTGGAGCA   
  
  
+ ACAGCTATTT GGGTGTGAGA TTAGGAACAT CCTGGCCGTT GGTGGGCCCA AGAGGAGGCT CACTGGCGGC   
  
  
+ GATCGGGTCA GAAGGTGGGG CGACGAACTG ACAAGGGTCG GGTTCGAACC AGTTTCGTTG GCGGGTAGCC   
  
  
+ CGGCAACCCA AGCTAGTTTG TTGCTTGGGA TGTTCCCTTG GAAAGGGTAT ACTTTGATGG AGGAAAATGG   
  
  
+ GTGTTTGAGA TTAGGGTGGA AAGATTTGCC CTTGTTAACT GCCTCAGCTT GGCAACCTTG TGAATTTAAC   
  
  
+ AATCCTAGTG CTGGCATTTA   

- +Up\_Stream \_Len000TTTGCC ACGGCAACTT AAACGTCCGC TGCTCCAGCC CAAATTCCGC CTACAGCATC   
  
  
- GCGAAACCTC TTCGTTTAAA CCCTTCTGCT CCGAATAAGA ACTTGAGAAG CATGCGGTAA CAACAACCAA   
  
  
- GGTTGGAAAG AGAGAGGAGC ACTTAAACTC AAACCCGTCA TCTGATAGGG CCTTATCTCC TGACTACGCA   
  
  
- ATATATATAT CTTTAACTCT CTCTCTCTCT CGCACACACT TCCTACAACT TTTACTACCC GCATCCCTCA   
  
  
- GAGAGAGAGT GACAGACAAT CCTCATGCAC AGCTCTTTCC TTTATCCTAA GGGTTCGTCA GATTAAACCC   
  
  
- GGCCAGATAT CCGGTCTTAC CCGGATACTA GTCGGGTCTA TACTGGAACA AATTGGTTGA CATAAACTTA   
  
  
- ACTTCTTTCC CTATAGCGTT ACAGAACTTG GTGTAGTTTA TTATTTACAC GGAAACCAAT CTGGTATACT   
  
  
- TTTTAGAAAC TTAGTTTAGC CAACCTGGAC TTGACGGTAA TAACTATAGA ACAAAAACCA TCTTCGTTTA   
  
  
- CCACAGTGAA GCAAAAGATC TATTTTAATT GAACTCAAAA AAACGGTGAT ATCTATCATA ACGTTTATAT   
  
  
- TTTTCACACG GTGCACTTTA GTTATAATCC TCCCACAAAA TATTGTTATC GACAAGCCCT CTCAAAATCA   
  
  
- ATAAAAATAA TTTAGTTTAT CAATTCTTCA CAAACCATTT ATCAACATAA ACTTTTTCAA TAAGGGTAAT   
  
  
- CGGAAAATCA TTCTTGAAGA ACACTTTTAC TATTAACAGA TCTTCAAACT TTTTAAGTGT GGTGTACTGT   
  
  
- AAATTTTTAT TAAAACATCT CTTCCCGTAT TTACTTTCAA ACGGTGGTAG AAAAAATCTT TGTACATTAA   
  
  
- ATGGTTTGTG AAAAAGATTT TTGTCAATTA AGTTTATTGA TTGTTGATTG TCGATTATAT TTATTGATTG   
  
  
- TCGATAAACG ATTTGTCCCG GATTCATCAA ACTTTAACAA CAAAAGAGAA GTCGAAGTGG TACCGGCTAC   
  
  
- TACATTTGTT GATATAAGAC TTAAAGTAAA AATGATACTA AACTTTTAAA GTTTAAATTT ATTCTAACCT   
  
  
- ATTTTTTTTC TGGTTTTTAG TATTCATTAT AATCAGTTCA AGATAAGGTT GGTATATGTA TTGACGAAAA   
  
  
- AGAAAACCCC CAAAACTTTG TACACAGGAA TCTTATTAAC TTTGAGCTAT AATAAGAGAT TTAGATTCAA   
  
  
- ATGAAATAGT ATAACTAATA ATTAATGGAG GAGTTGAGGA GGATATGAGA ACATACGTCT TTATTTTTAC   
  
  
- AAAGGTATGG AATAGTCAGC GCTAGTATTA TTAATTAAAG AATTAAGCCT AACATAAATT AACGCATAGT   
  
  
- TTACACTTTA GCATGGTTTA ATCCTTCGTT TTGTTTGGCT ATTGCTTTTT CCATTCTTTT GAATCGTTCC   
  
  
- TTTACTTTAG CTTCTGGACA GTTGTTATAT CGAAGGGAGT GTAGGAAGTT GAATCCCTGG TAGGATATGA   
  
  
- AAGTACATTA TGTTGATCTC GGTGATCAGT TATTATCGGA GGATCATCAT CGGTATTCGG AACCACTCTC   
  
  
- TTACAAGGCG TTCTGATCAA TTGAACTTTA AGGTAAAACA GCGTCTTGAT AACCTCACTC TTTCTCTTAG   
  
  
- TTTAGTATGG TATAGTTTAA ACCTGGTAAA TTCAAGTGTT GTGTGTGTGT ATGTGTGTTT TCCTGCTACA   
  
  
- TGCCGGGGTG GGGGGTGAGA GGAAAGCGGT AGGGGGATGT GGAGACGGAT GAGAGAGAGA GAGAGAGAGA   
  
  
- GAGACCTAGT CAGACACATT TTCGAAGGAG AGGTCATGTC GTAAAGGAAC AGACAAGAAC CTCAAATCAG   
  
  
- TTAAGGAGGC GGGGTTTTTT TGGGTGAAAG AGAGAGTAGT ACAAGAAGGT CAAGGTGGGA ATATGCGGTA   
  
  
- AGTAGTAGTA GATGTAGTGG GTGTAGAAAA AGAGGTGTCA AAAGGGGGGA GTAATACGAG AGGCGGAGGT   
  
  
- GGGGGCGGAA GGGAGAGCGG CAGCGGCCGC TACTGCCCCT ATGGTGACTA CGTACGCTGC CGTGGCGGTG   
  
  
- ACGGGAGGTG GAGGAGCCCG ACGACAACTT CACGCGCCTT AAGTAGAGGT GGCCTCTGGA GCGGCCGCGG   
  
  
- CCTCTATAGA ACGGCCTCTA TAAACTCAAC AGATGTGGCA AACCGAGGGG GCGGCGGGCC CAGCGGCGGA   
  
  
- AGAAGCCGGT GCGGGAGGTG CGGGCGGAGG AGAGGCGGAG GGAGGCGTGT TGAGGCTAGC TCTTCGAGTT   
  
  
- TTGGGACTGG GACCAAAGGG TTTACGCCTT TAAAGTGAGG CGGAACGTTC ATATGTTGAG GTAGTGAGGG   
  
  
- AAGCACTTTA AGAGAGTGAA GTGCCGCTTA GTTCGGTAGA TGCTCCGCGA CCTGCCGCTC CTAGCACAGG   
  
  
- TGCAGCAGCT GGAACTGTAG TACGTCCCGG AAGTTACCGG CCCTAACAAG GTGTAGAATC GGAGAGCTGG   
  
  
- GTCTTTCGGG GAGAGCCAAG CCCAGTGGCC CAAGCCCGGT AGGAGGCTCA ACGAGAGGGT TTGCCCATTC   
  
  
- GCTGAGCGAC TCAAGCGGCG AAGTGAGCCG GACGGGAAGC TCATGTTGGG CCACCTCCCG TTTTAACCCT   
  
  
- TGAATCAGCT GGACCCAGCC CAGCCCAGCG AGGGCTTACT TCACTGCCAC CACGTGACCT ACGTGGTATC   
  
  
- AGATATGCTG TATTGGCCCA GCCTAGAACC CTGAAACTCC CATAACTCAC GCCACTCCGG ATCCGAACAC   
  
  
- TGATACCAAC TCGTCCTATA CCTGGTTTGC CCCAGCAAAA ACCCCTCCAA ACACCTCCGT AACGTAATAA   
  
  
- TGAGTCGGAA CAAACTACGG AACCCTCTCC CCAATCTATC CCTATTGGAT GTTTCCGTAG TTCACCTCGT   
  
  
- TGTCGATAAA CCCACACTCT AATCCTTGTA GGACCGGCAA CCACCCGGGT TCTCCTCCGA GTGACCGCCG   
  
  
- CTAGCCCAGT CTTCCACCCC GCTGCTTGAC TGTTCCCAGC CCAAGCTTGG TCAAAGCAAC CGCCCATCGG   
  
  
- GCCGTTGGGT TCGATCAAAC AACGAACCCT ACAAGGGAAC CTTTCCCATA TGAAACTACC TCCTTTTACC   
  
  
- CACAAACTCT AATCCCACCT TTCTAAACGG GAACAATTGA CGGAGTCGAA CCGTTGGAAC ACTTAAATTG   
  
  
- TTAGGATCAC GACCGTAAAT

+     Sp1

| Site Name | Organism | Position | Strand | Matrix score. | sequence | function |
| --- | --- | --- | --- | --- | --- | --- |
| Sp1 | Oryza sativa | 2227 | - | 6 | GGGCGG | light responsive element |
| Sp1 | Oryza sativa | 1902 | - | 6 | GGGCGG | light responsive element |

>HU02G03005.1   
+ +Up\_Stream \_Len000AAACGG TGCCGTTGAA TTTGCAGGCG ACGAGGTCGG GTTTAAGGCG GATGTCGTAG   
  
  
+ CGCTTTGGAG AAGCAAATTT GGGAAGACGA GGCTTATTCT TGAACTCTTC GTACGCCATT GTTGTTGGTT   
  
  
+ CCAACCTTTC TCTCTCCTCG TGAATTTGAG TTTGGGCAGT AGACTATCCC GGAATAGAGG ACTGATGCGT   
  
  
+ TATATATATA GAAATTGAGA GAGAGAGAGA GCGTGTGTGA AGGATGTTGA AAATGATGGG CGTAGGGAGT   
  
  
+ CTCTCTCTCA CTGTCTGTTA GGAGTACGTG TCGAGAAAGG AAATAGGATT CCCAAGCAGT CTAATTTGGG   
  
  
+ CCGGTCTATA GGCCAGAATG GGCCTATGAT CAGCCCAGAT ATGACCTTGT TTAACCAACT GTATTTGAAT   
  
  
+ TGAAGAAAGG GATATCGCAA TGTCTTGAAC CACATCAAAT AATAAATGTG CCTTTGGTTA GACCATATGA   
  
  
+ AAAATCTTTG AATCAAATCG GTTGGACCTG AACTGCCATT ATTGATATCT TGTTTTTGGT AGAAGCAAAT   
  
  
+ GGTGTCACTT CGTTTTCTAG ATAAAATTAA CTTGAGTTTT TTTGCCACTA TAGATAGTAT TGCAAATATA   
  
  
+ AAAAGTGTGC CACGTGAAAT CAATATTAGG AGGGTGTTTT ATAACAATAG CTGTTCGGGA GAGTTTTAGT   
  
  
+ TATTTTTATT AAATCAAATA GTTAAGAAGT GTTTGGTAAA TAGTTGTATT TGAAAAAGTT ATTCCCATTA   
  
  
+ GCCTTTTAGT AAGAACTTCT TGTGAAAATG ATAATTGTCT AGAAGTTTGA AAAATTCACA CCACATGACA   
  
  
+ TTTAAAAATA ATTTTGTAGA GAAGGGCATA AATGAAAGTT TGCCACCATC TTTTTTAGAA ACATGTAATT   
  
  
+ TACCAAACAC TTTTTCTAAA AACAGTTAAT TCAAATAACT AACAACTAAC AGCTAATATA AATAACTAAC   
  
  
+ AGCTATTTGC TAAACAGGGC CTAAGTAGTT TGAAATTGTT GTTTTCTCTT CAGCTTCACC ATGGCCGATG   
  
  
+ ATGTAAACAA CTATATTCTG AATTTCATTT TTACTATGAT TTGAAAATTT CAAATTTAAA TAAGATTGGA   
  
  
+ TAAAAAAAAG ACCAAAAATC ATAAGTAATA TTAGTCAAGT TCTATTCCAA CCATATACAT AACTGCTTTT   
  
  
+ TCTTTTGGGG GTTTTGAAAC ATGTGTCCTT AGAATAATTG AAACTCGATA TTATTCTCTA AATCTAAGTT   
  
  
+ TACTTTATCA TATTGATTAT TAATTACCTC CTCAACTCCT CCTATACTCT TGTATGCAGA AATAAAAATG   
  
  
+ TTTCCATACC TTATCAGTCG CGATCATAAT AATTAATTTC TTAATTCGGA TTGTATTTAA TTGCGTATCA   
  
  
+ AATGTGAAAT CGTACCAAAT TAGGAAGCAA AACAAACCGA TAACGAAAAA GGTAAGAAAA CTTAGCAAGG   
  
  
+ AAATGAAATC GAAGACCTGT CAACAATATA GCTTCCCTCA CATCCTTCAA CTTAGGGACC ATCCTATACT   
  
  
+ TTCATGTAAT ACAACTAGAG CCACTAGTCA ATAATAGCCT CCTAGTAGTA GCCATAAGCC TTGGTGAGAG   
  
  
+ AATGTTCCGC AAGACTAGTT AACTTGAAAT TCCATTTTGT CGCAGAACTA TTGGAGTGAG AAAGAGAATC   
  
  
+ AAATCATACC ATATCAAATT TGGACCATTT AAGTTCACAA CACACACACA TACACACAAA AGGACGATGT   
  
  
+ ACGGCCCCAC CCCCCACTCT CCTTTCGCCA TCCCCCTACA CCTCTGCCTA CTCTCTCTCT CTCTCTCTCT   
  
  
+ CTCTGGATCA GTCTGTGTAA AAGCTTCCTC TCCAGTACAG CATTTCCTTG TCTGTTCTTG GAGTTTAGTC   
  
  
+ AATTCCTCCG CCCCAAAAAA ACCCACTTTC TCTCTCATCA TGTTCTTCCA GTTCCACCCT TATACGCCAT   
  
  
+ TCATCATCAT CTACATCACC CACATCTTTT TCTCCACAGT TTTCCCCCCT CATTATGCTC TCCGCCTCCA   
  
  
+ CCCCCGCCTT CCCTCTCGCC GTCGCCGGCG ATGACGGGGA TACCACTGAT GCATGCGACG GCACCGCCAC   
  
  
+ TGCCCTCCAC CTCCTCGGGC TGCTGTTGAA GTGCGCGGAA TTCATCTCCA CCGGAGACCT CGCCGGCGCC   
  
  
+ GGAGATATCT TGCCGGAGAT ATTTGAGTTG TCTACACCGT TTGGCTCCCC CGCCGCCCGG GTCGCCGCCT   
  
  
+ TCTTCGGCCA CGCCCTCCAC GCCCGCCTCC TCTCCGCCTC CCTCCGCACA ACTCCGATCG AGAAGCTCAA   
  
  
+ AACCCTGACC CTGGTTTCCC AAATGCGGAA ATTTCACTCC GCCTTGCAAG TATACAACTC CATCACTCCC   
  
  
+ TTCGTGAAAT TCTCTCACTT CACGGCGAAT CAAGCCATCT ACGAGGCGCT GGACGGCGAG GATCGTGTCC   
  
  
+ ACGTCGTCGA CCTTGACATC ATGCAGGGCC TTCAATGGCC GGGATTGTTC CACATCTTAG CCTCTCGACC   
  
  
+ CAGAAAGCCC CTCTCGGTTC GGGTCACCGG GTTCGGGCCA TCCTCCGAGT TGCTCTCCCA AACGGGTAAG   
  
  
+ CGACTCGCTG AGTTCGCCGC TTCACTCGGC CTGCCCTTCG AGTACAACCC GGTGGAGGGC AAAATTGGGA   
  
  
+ ACTTAGTCGA CCTGGGTCGG GTCGGGTCGC TCCCGAATGA AGTGACGGTG GTGCACTGGA TGCACCATAG   
  
  
+ TCTATACGAC ATAACCGGGT CGGATCTTGG GACTTTGAGG GTATTGAGTG CGGTGAGGCC TAGGCTTGTG   
  
  
+ ACTATGGTTG AGCAGGATAT GGACCAAACG GGGTCGTTTT TGGGGAGGTT TGTGGAGGCA TTGCATTATT   
  
  
+ ACTCAGCCTT GTTTGATGCC TTGGGAGAGG GGTTAGATAG GGATAACCTA CAAAGGCATC AAGTGGAGCA   
  
  
+ ACAGCTATTT GGGTGTGAGA TTAGGAACAT CCTGGCCGTT GGTGGGCCCA AGAGGAGGCT CACTGGCGGC   
  
  
+ GATCGGGTCA GAAGGTGGGG CGACGAACTG ACAAGGGTCG GGTTCGAACC AGTTTCGTTG GCGGGTAGCC   
  
  
+ CGGCAACCCA AGCTAGTTTG TTGCTTGGGA TGTTCCCTTG GAAAGGGTAT ACTTTGATGG AGGAAAATGG   
  
  
+ GTGTTTGAGA TTAGGGTGGA AAGATTTGCC CTTGTTAACT GCCTCAGCTT GGCAACCTTG TGAATTTAAC   
  
  
+ AATCCTAGTG CTGGCATTTA   

- +Up\_Stream \_Len000TTTGCC ACGGCAACTT AAACGTCCGC TGCTCCAGCC CAAATTCCGC CTACAGCATC   
  
  
- GCGAAACCTC TTCGTTTAAA CCCTTCTGCT CCGAATAAGA ACTTGAGAAG CATGCGGTAA CAACAACCAA   
  
  
- GGTTGGAAAG AGAGAGGAGC ACTTAAACTC AAACCCGTCA TCTGATAGGG CCTTATCTCC TGACTACGCA   
  
  
- ATATATATAT CTTTAACTCT CTCTCTCTCT CGCACACACT TCCTACAACT TTTACTACCC GCATCCCTCA   
  
  
- GAGAGAGAGT GACAGACAAT CCTCATGCAC AGCTCTTTCC TTTATCCTAA GGGTTCGTCA GATTAAACCC   
  
  
- GGCCAGATAT CCGGTCTTAC CCGGATACTA GTCGGGTCTA TACTGGAACA AATTGGTTGA CATAAACTTA   
  
  
- ACTTCTTTCC CTATAGCGTT ACAGAACTTG GTGTAGTTTA TTATTTACAC GGAAACCAAT CTGGTATACT   
  
  
- TTTTAGAAAC TTAGTTTAGC CAACCTGGAC TTGACGGTAA TAACTATAGA ACAAAAACCA TCTTCGTTTA   
  
  
- CCACAGTGAA GCAAAAGATC TATTTTAATT GAACTCAAAA AAACGGTGAT ATCTATCATA ACGTTTATAT   
  
  
- TTTTCACACG GTGCACTTTA GTTATAATCC TCCCACAAAA TATTGTTATC GACAAGCCCT CTCAAAATCA   
  
  
- ATAAAAATAA TTTAGTTTAT CAATTCTTCA CAAACCATTT ATCAACATAA ACTTTTTCAA TAAGGGTAAT   
  
  
- CGGAAAATCA TTCTTGAAGA ACACTTTTAC TATTAACAGA TCTTCAAACT TTTTAAGTGT GGTGTACTGT   
  
  
- AAATTTTTAT TAAAACATCT CTTCCCGTAT TTACTTTCAA ACGGTGGTAG AAAAAATCTT TGTACATTAA   
  
  
- ATGGTTTGTG AAAAAGATTT TTGTCAATTA AGTTTATTGA TTGTTGATTG TCGATTATAT TTATTGATTG   
  
  
- TCGATAAACG ATTTGTCCCG GATTCATCAA ACTTTAACAA CAAAAGAGAA GTCGAAGTGG TACCGGCTAC   
  
  
- TACATTTGTT GATATAAGAC TTAAAGTAAA AATGATACTA AACTTTTAAA GTTTAAATTT ATTCTAACCT   
  
  
- ATTTTTTTTC TGGTTTTTAG TATTCATTAT AATCAGTTCA AGATAAGGTT GGTATATGTA TTGACGAAAA   
  
  
- AGAAAACCCC CAAAACTTTG TACACAGGAA TCTTATTAAC TTTGAGCTAT AATAAGAGAT TTAGATTCAA   
  
  
- ATGAAATAGT ATAACTAATA ATTAATGGAG GAGTTGAGGA GGATATGAGA ACATACGTCT TTATTTTTAC   
  
  
- AAAGGTATGG AATAGTCAGC GCTAGTATTA TTAATTAAAG AATTAAGCCT AACATAAATT AACGCATAGT   
  
  
- TTACACTTTA GCATGGTTTA ATCCTTCGTT TTGTTTGGCT ATTGCTTTTT CCATTCTTTT GAATCGTTCC   
  
  
- TTTACTTTAG CTTCTGGACA GTTGTTATAT CGAAGGGAGT GTAGGAAGTT GAATCCCTGG TAGGATATGA   
  
  
- AAGTACATTA TGTTGATCTC GGTGATCAGT TATTATCGGA GGATCATCAT CGGTATTCGG AACCACTCTC   
  
  
- TTACAAGGCG TTCTGATCAA TTGAACTTTA AGGTAAAACA GCGTCTTGAT AACCTCACTC TTTCTCTTAG   
  
  
- TTTAGTATGG TATAGTTTAA ACCTGGTAAA TTCAAGTGTT GTGTGTGTGT ATGTGTGTTT TCCTGCTACA   
  
  
- TGCCGGGGTG GGGGGTGAGA GGAAAGCGGT AGGGGGATGT GGAGACGGAT GAGAGAGAGA GAGAGAGAGA   
  
  
- GAGACCTAGT CAGACACATT TTCGAAGGAG AGGTCATGTC GTAAAGGAAC AGACAAGAAC CTCAAATCAG   
  
  
- TTAAGGAGGC GGGGTTTTTT TGGGTGAAAG AGAGAGTAGT ACAAGAAGGT CAAGGTGGGA ATATGCGGTA   
  
  
- AGTAGTAGTA GATGTAGTGG GTGTAGAAAA AGAGGTGTCA AAAGGGGGGA GTAATACGAG AGGCGGAGGT   
  
  
- GGGGGCGGAA GGGAGAGCGG CAGCGGCCGC TACTGCCCCT ATGGTGACTA CGTACGCTGC CGTGGCGGTG   
  
  
- ACGGGAGGTG GAGGAGCCCG ACGACAACTT CACGCGCCTT AAGTAGAGGT GGCCTCTGGA GCGGCCGCGG   
  
  
- CCTCTATAGA ACGGCCTCTA TAAACTCAAC AGATGTGGCA AACCGAGGGG GCGGCGGGCC CAGCGGCGGA   
  
  
- AGAAGCCGGT GCGGGAGGTG CGGGCGGAGG AGAGGCGGAG GGAGGCGTGT TGAGGCTAGC TCTTCGAGTT   
  
  
- TTGGGACTGG GACCAAAGGG TTTACGCCTT TAAAGTGAGG CGGAACGTTC ATATGTTGAG GTAGTGAGGG   
  
  
- AAGCACTTTA AGAGAGTGAA GTGCCGCTTA GTTCGGTAGA TGCTCCGCGA CCTGCCGCTC CTAGCACAGG   
  
  
- TGCAGCAGCT GGAACTGTAG TACGTCCCGG AAGTTACCGG CCCTAACAAG GTGTAGAATC GGAGAGCTGG   
  
  
- GTCTTTCGGG GAGAGCCAAG CCCAGTGGCC CAAGCCCGGT AGGAGGCTCA ACGAGAGGGT TTGCCCATTC   
  
  
- GCTGAGCGAC TCAAGCGGCG AAGTGAGCCG GACGGGAAGC TCATGTTGGG CCACCTCCCG TTTTAACCCT   
  
  
- TGAATCAGCT GGACCCAGCC CAGCCCAGCG AGGGCTTACT TCACTGCCAC CACGTGACCT ACGTGGTATC   
  
  
- AGATATGCTG TATTGGCCCA GCCTAGAACC CTGAAACTCC CATAACTCAC GCCACTCCGG ATCCGAACAC   
  
  
- TGATACCAAC TCGTCCTATA CCTGGTTTGC CCCAGCAAAA ACCCCTCCAA ACACCTCCGT AACGTAATAA   
  
  
- TGAGTCGGAA CAAACTACGG AACCCTCTCC CCAATCTATC CCTATTGGAT GTTTCCGTAG TTCACCTCGT   
  
  
- TGTCGATAAA CCCACACTCT AATCCTTGTA GGACCGGCAA CCACCCGGGT TCTCCTCCGA GTGACCGCCG   
  
  
- CTAGCCCAGT CTTCCACCCC GCTGCTTGAC TGTTCCCAGC CCAAGCTTGG TCAAAGCAAC CGCCCATCGG   
  
  
- GCCGTTGGGT TCGATCAAAC AACGAACCCT ACAAGGGAAC CTTTCCCATA TGAAACTACC TCCTTTTACC   
  
  
- CACAAACTCT AATCCCACCT TTCTAAACGG GAACAATTGA CGGAGTCGAA CCGTTGGAAC ACTTAAATTG   
  
  
- TTAGGATCAC GACCGTAAAT

+     TATA-box

| Site Name | Organism | Position | Strand | Matrix score. | sequence | function |
| --- | --- | --- | --- | --- | --- | --- |
| TATA-box | Pisum sativum | 671 | - | 7 | TATAAAA | core promoter element around -30 of transcription start |
| TATA-box | Arabidopsis thaliana | 631 | + | 4 | TATA | core promoter element around -30 of transcription start |
| TATA-box | Arabidopsis thaliana | 2737 | - | 4 | TATA | core promoter element around -30 of transcription start |
| TATA-box | Arabidopsis thaliana | 1066 | + | 4 | TATA | core promoter element around -30 of transcription start |
| TATA-box | Arabidopsis thaliana | 1501 | + | 4 | TATA | core promoter element around -30 of transcription start |
| TATA-box | Arabidopsis thaliana | 1539 | + | 4 | TATA | core promoter element around -30 of transcription start |
| TATA-box | Arabidopsis thaliana | 1307 | + | 4 | TATA | core promoter element around -30 of transcription start |
| TATA-box | Brassica napus | 218 | + | 6 | ATATAT | core promoter element around -30 of transcription start |
| TATA-box | Helianthus annuus | 672 | - | 6 | TATAAA | core promoter element around -30 of transcription start |
| TATA-box | Arabidopsis thaliana | 3132 | - | 4 | TATA | core promoter element around -30 of transcription start |
| TATA-box | Arabidopsis thaliana | 221 | + | 4 | TATA | core promoter element around -30 of transcription start |
| TATA-box | Arabidopsis thaliana | 1109 | - | 8 | TATTTAAA | core promoter element around -30 of transcription start |
| TATA-box | Arabidopsis thaliana | 2365 | - | 4 | TATA | core promoter element around -30 of transcription start |
| TATA-box | Arabidopsis thaliana | 1954 | - | 5 | TATAA | core promoter element around -30 of transcription start |
| TATA-box | Arabidopsis thaliana | 673 | - | 5 | TATAA | core promoter element around -30 of transcription start |
| TATA-box | Arabidopsis thaliana | 1955 | - | 4 | TATA | core promoter element around -30 of transcription start |
| TATA-box | Brassica oleracea | 970 | + | 6 | ATATAA | core promoter element around -30 of transcription start |
| TATA-box | Arabidopsis thaliana | 613 | + | 4 | TATA | core promoter element around -30 of transcription start |
| TATA-box | Arabidopsis thaliana | 674 | + | 4 | TATA | core promoter element around -30 of transcription start |
| TATA-box | Brassica oleracea | 630 | + | 6 | ATATAA | core promoter element around -30 of transcription start |
| TATA-box | Arabidopsis thaliana | 361 | + | 4 | TATA | core promoter element around -30 of transcription start |
| TATA-box | Arabidopsis thaliana | 1178 | + | 4 | TATA | core promoter element around -30 of transcription start |
| TATA-box | Arabidopsis thaliana | 971 | + | 4 | TATA | core promoter element around -30 of transcription start |
| TATA-box | Oryza sativa | 856 | - | 7 | TACAAAA | core promoter element around -30 of transcription start |
| TATA-box | Arabidopsis thaliana | 214 | - | 7 | TATATAA | core promoter element around -30 of transcription start |
| TATA-box | Arabidopsis thaliana | 217 | + | 6 | TATATA | core promoter element around -30 of transcription start |
| TATA-box | Arabidopsis thaliana | 219 | + | 6 | TATATA | core promoter element around -30 of transcription start |
| TATA-box | Arabidopsis thaliana | 215 | + | 6 | TATATA | core promoter element around -30 of transcription start |
| TATA-box | Brassica napus | 216 | + | 6 | ATATAT | core promoter element around -30 of transcription start |

>HU02G03005.1   
+ +Up\_Stream \_Len000AAACGG TGCCGTTGAA TTTGCAGGCG ACGAGGTCGG GTTTAAGGCG GATGTCGTAG   
  
  
+ CGCTTTGGAG AAGCAAATTT GGGAAGACGA GGCTTATTCT TGAACTCTTC GTACGCCATT GTTGTTGGTT   
  
  
+ CCAACCTTTC TCTCTCCTCG TGAATTTGAG TTTGGGCAGT AGACTATCCC GGAATAGAGG ACTGATGCGT   
  
  
+ TATATATATA GAAATTGAGA GAGAGAGAGA GCGTGTGTGA AGGATGTTGA AAATGATGGG CGTAGGGAGT   
  
  
+ CTCTCTCTCA CTGTCTGTTA GGAGTACGTG TCGAGAAAGG AAATAGGATT CCCAAGCAGT CTAATTTGGG   
  
  
+ CCGGTCTATA GGCCAGAATG GGCCTATGAT CAGCCCAGAT ATGACCTTGT TTAACCAACT GTATTTGAAT   
  
  
+ TGAAGAAAGG GATATCGCAA TGTCTTGAAC CACATCAAAT AATAAATGTG CCTTTGGTTA GACCATATGA   
  
  
+ AAAATCTTTG AATCAAATCG GTTGGACCTG AACTGCCATT ATTGATATCT TGTTTTTGGT AGAAGCAAAT   
  
  
+ GGTGTCACTT CGTTTTCTAG ATAAAATTAA CTTGAGTTTT TTTGCCACTA TAGATAGTAT TGCAAATATA   
  
  
+ AAAAGTGTGC CACGTGAAAT CAATATTAGG AGGGTGTTTT ATAACAATAG CTGTTCGGGA GAGTTTTAGT   
  
  
+ TATTTTTATT AAATCAAATA GTTAAGAAGT GTTTGGTAAA TAGTTGTATT TGAAAAAGTT ATTCCCATTA   
  
  
+ GCCTTTTAGT AAGAACTTCT TGTGAAAATG ATAATTGTCT AGAAGTTTGA AAAATTCACA CCACATGACA   
  
  
+ TTTAAAAATA ATTTTGTAGA GAAGGGCATA AATGAAAGTT TGCCACCATC TTTTTTAGAA ACATGTAATT   
  
  
+ TACCAAACAC TTTTTCTAAA AACAGTTAAT TCAAATAACT AACAACTAAC AGCTAATATA AATAACTAAC   
  
  
+ AGCTATTTGC TAAACAGGGC CTAAGTAGTT TGAAATTGTT GTTTTCTCTT CAGCTTCACC ATGGCCGATG   
  
  
+ ATGTAAACAA CTATATTCTG AATTTCATTT TTACTATGAT TTGAAAATTT CAAATTTAAA TAAGATTGGA   
  
  
+ TAAAAAAAAG ACCAAAAATC ATAAGTAATA TTAGTCAAGT TCTATTCCAA CCATATACAT AACTGCTTTT   
  
  
+ TCTTTTGGGG GTTTTGAAAC ATGTGTCCTT AGAATAATTG AAACTCGATA TTATTCTCTA AATCTAAGTT   
  
  
+ TACTTTATCA TATTGATTAT TAATTACCTC CTCAACTCCT CCTATACTCT TGTATGCAGA AATAAAAATG   
  
  
+ TTTCCATACC TTATCAGTCG CGATCATAAT AATTAATTTC TTAATTCGGA TTGTATTTAA TTGCGTATCA   
  
  
+ AATGTGAAAT CGTACCAAAT TAGGAAGCAA AACAAACCGA TAACGAAAAA GGTAAGAAAA CTTAGCAAGG   
  
  
+ AAATGAAATC GAAGACCTGT CAACAATATA GCTTCCCTCA CATCCTTCAA CTTAGGGACC ATCCTATACT   
  
  
+ TTCATGTAAT ACAACTAGAG CCACTAGTCA ATAATAGCCT CCTAGTAGTA GCCATAAGCC TTGGTGAGAG   
  
  
+ AATGTTCCGC AAGACTAGTT AACTTGAAAT TCCATTTTGT CGCAGAACTA TTGGAGTGAG AAAGAGAATC   
  
  
+ AAATCATACC ATATCAAATT TGGACCATTT AAGTTCACAA CACACACACA TACACACAAA AGGACGATGT   
  
  
+ ACGGCCCCAC CCCCCACTCT CCTTTCGCCA TCCCCCTACA CCTCTGCCTA CTCTCTCTCT CTCTCTCTCT   
  
  
+ CTCTGGATCA GTCTGTGTAA AAGCTTCCTC TCCAGTACAG CATTTCCTTG TCTGTTCTTG GAGTTTAGTC   
  
  
+ AATTCCTCCG CCCCAAAAAA ACCCACTTTC TCTCTCATCA TGTTCTTCCA GTTCCACCCT TATACGCCAT   
  
  
+ TCATCATCAT CTACATCACC CACATCTTTT TCTCCACAGT TTTCCCCCCT CATTATGCTC TCCGCCTCCA   
  
  
+ CCCCCGCCTT CCCTCTCGCC GTCGCCGGCG ATGACGGGGA TACCACTGAT GCATGCGACG GCACCGCCAC   
  
  
+ TGCCCTCCAC CTCCTCGGGC TGCTGTTGAA GTGCGCGGAA TTCATCTCCA CCGGAGACCT CGCCGGCGCC   
  
  
+ GGAGATATCT TGCCGGAGAT ATTTGAGTTG TCTACACCGT TTGGCTCCCC CGCCGCCCGG GTCGCCGCCT   
  
  
+ TCTTCGGCCA CGCCCTCCAC GCCCGCCTCC TCTCCGCCTC CCTCCGCACA ACTCCGATCG AGAAGCTCAA   
  
  
+ AACCCTGACC CTGGTTTCCC AAATGCGGAA ATTTCACTCC GCCTTGCAAG TATACAACTC CATCACTCCC   
  
  
+ TTCGTGAAAT TCTCTCACTT CACGGCGAAT CAAGCCATCT ACGAGGCGCT GGACGGCGAG GATCGTGTCC   
  
  
+ ACGTCGTCGA CCTTGACATC ATGCAGGGCC TTCAATGGCC GGGATTGTTC CACATCTTAG CCTCTCGACC   
  
  
+ CAGAAAGCCC CTCTCGGTTC GGGTCACCGG GTTCGGGCCA TCCTCCGAGT TGCTCTCCCA AACGGGTAAG   
  
  
+ CGACTCGCTG AGTTCGCCGC TTCACTCGGC CTGCCCTTCG AGTACAACCC GGTGGAGGGC AAAATTGGGA   
  
  
+ ACTTAGTCGA CCTGGGTCGG GTCGGGTCGC TCCCGAATGA AGTGACGGTG GTGCACTGGA TGCACCATAG   
  
  
+ TCTATACGAC ATAACCGGGT CGGATCTTGG GACTTTGAGG GTATTGAGTG CGGTGAGGCC TAGGCTTGTG   
  
  
+ ACTATGGTTG AGCAGGATAT GGACCAAACG GGGTCGTTTT TGGGGAGGTT TGTGGAGGCA TTGCATTATT   
  
  
+ ACTCAGCCTT GTTTGATGCC TTGGGAGAGG GGTTAGATAG GGATAACCTA CAAAGGCATC AAGTGGAGCA   
  
  
+ ACAGCTATTT GGGTGTGAGA TTAGGAACAT CCTGGCCGTT GGTGGGCCCA AGAGGAGGCT CACTGGCGGC   
  
  
+ GATCGGGTCA GAAGGTGGGG CGACGAACTG ACAAGGGTCG GGTTCGAACC AGTTTCGTTG GCGGGTAGCC   
  
  
+ CGGCAACCCA AGCTAGTTTG TTGCTTGGGA TGTTCCCTTG GAAAGGGTAT ACTTTGATGG AGGAAAATGG   
  
  
+ GTGTTTGAGA TTAGGGTGGA AAGATTTGCC CTTGTTAACT GCCTCAGCTT GGCAACCTTG TGAATTTAAC   
  
  
+ AATCCTAGTG CTGGCATTTA   

- +Up\_Stream \_Len000TTTGCC ACGGCAACTT AAACGTCCGC TGCTCCAGCC CAAATTCCGC CTACAGCATC   
  
  
- GCGAAACCTC TTCGTTTAAA CCCTTCTGCT CCGAATAAGA ACTTGAGAAG CATGCGGTAA CAACAACCAA   
  
  
- GGTTGGAAAG AGAGAGGAGC ACTTAAACTC AAACCCGTCA TCTGATAGGG CCTTATCTCC TGACTACGCA   
  
  
- ATATATATAT CTTTAACTCT CTCTCTCTCT CGCACACACT TCCTACAACT TTTACTACCC GCATCCCTCA   
  
  
- GAGAGAGAGT GACAGACAAT CCTCATGCAC AGCTCTTTCC TTTATCCTAA GGGTTCGTCA GATTAAACCC   
  
  
- GGCCAGATAT CCGGTCTTAC CCGGATACTA GTCGGGTCTA TACTGGAACA AATTGGTTGA CATAAACTTA   
  
  
- ACTTCTTTCC CTATAGCGTT ACAGAACTTG GTGTAGTTTA TTATTTACAC GGAAACCAAT CTGGTATACT   
  
  
- TTTTAGAAAC TTAGTTTAGC CAACCTGGAC TTGACGGTAA TAACTATAGA ACAAAAACCA TCTTCGTTTA   
  
  
- CCACAGTGAA GCAAAAGATC TATTTTAATT GAACTCAAAA AAACGGTGAT ATCTATCATA ACGTTTATAT   
  
  
- TTTTCACACG GTGCACTTTA GTTATAATCC TCCCACAAAA TATTGTTATC GACAAGCCCT CTCAAAATCA   
  
  
- ATAAAAATAA TTTAGTTTAT CAATTCTTCA CAAACCATTT ATCAACATAA ACTTTTTCAA TAAGGGTAAT   
  
  
- CGGAAAATCA TTCTTGAAGA ACACTTTTAC TATTAACAGA TCTTCAAACT TTTTAAGTGT GGTGTACTGT   
  
  
- AAATTTTTAT TAAAACATCT CTTCCCGTAT TTACTTTCAA ACGGTGGTAG AAAAAATCTT TGTACATTAA   
  
  
- ATGGTTTGTG AAAAAGATTT TTGTCAATTA AGTTTATTGA TTGTTGATTG TCGATTATAT TTATTGATTG   
  
  
- TCGATAAACG ATTTGTCCCG GATTCATCAA ACTTTAACAA CAAAAGAGAA GTCGAAGTGG TACCGGCTAC   
  
  
- TACATTTGTT GATATAAGAC TTAAAGTAAA AATGATACTA AACTTTTAAA GTTTAAATTT ATTCTAACCT   
  
  
- ATTTTTTTTC TGGTTTTTAG TATTCATTAT AATCAGTTCA AGATAAGGTT GGTATATGTA TTGACGAAAA   
  
  
- AGAAAACCCC CAAAACTTTG TACACAGGAA TCTTATTAAC TTTGAGCTAT AATAAGAGAT TTAGATTCAA   
  
  
- ATGAAATAGT ATAACTAATA ATTAATGGAG GAGTTGAGGA GGATATGAGA ACATACGTCT TTATTTTTAC   
  
  
- AAAGGTATGG AATAGTCAGC GCTAGTATTA TTAATTAAAG AATTAAGCCT AACATAAATT AACGCATAGT   
  
  
- TTACACTTTA GCATGGTTTA ATCCTTCGTT TTGTTTGGCT ATTGCTTTTT CCATTCTTTT GAATCGTTCC   
  
  
- TTTACTTTAG CTTCTGGACA GTTGTTATAT CGAAGGGAGT GTAGGAAGTT GAATCCCTGG TAGGATATGA   
  
  
- AAGTACATTA TGTTGATCTC GGTGATCAGT TATTATCGGA GGATCATCAT CGGTATTCGG AACCACTCTC   
  
  
- TTACAAGGCG TTCTGATCAA TTGAACTTTA AGGTAAAACA GCGTCTTGAT AACCTCACTC TTTCTCTTAG   
  
  
- TTTAGTATGG TATAGTTTAA ACCTGGTAAA TTCAAGTGTT GTGTGTGTGT ATGTGTGTTT TCCTGCTACA   
  
  
- TGCCGGGGTG GGGGGTGAGA GGAAAGCGGT AGGGGGATGT GGAGACGGAT GAGAGAGAGA GAGAGAGAGA   
  
  
- GAGACCTAGT CAGACACATT TTCGAAGGAG AGGTCATGTC GTAAAGGAAC AGACAAGAAC CTCAAATCAG   
  
  
- TTAAGGAGGC GGGGTTTTTT TGGGTGAAAG AGAGAGTAGT ACAAGAAGGT CAAGGTGGGA ATATGCGGTA   
  
  
- AGTAGTAGTA GATGTAGTGG GTGTAGAAAA AGAGGTGTCA AAAGGGGGGA GTAATACGAG AGGCGGAGGT   
  
  
- GGGGGCGGAA GGGAGAGCGG CAGCGGCCGC TACTGCCCCT ATGGTGACTA CGTACGCTGC CGTGGCGGTG   
  
  
- ACGGGAGGTG GAGGAGCCCG ACGACAACTT CACGCGCCTT AAGTAGAGGT GGCCTCTGGA GCGGCCGCGG   
  
  
- CCTCTATAGA ACGGCCTCTA TAAACTCAAC AGATGTGGCA AACCGAGGGG GCGGCGGGCC CAGCGGCGGA   
  
  
- AGAAGCCGGT GCGGGAGGTG CGGGCGGAGG AGAGGCGGAG GGAGGCGTGT TGAGGCTAGC TCTTCGAGTT   
  
  
- TTGGGACTGG GACCAAAGGG TTTACGCCTT TAAAGTGAGG CGGAACGTTC ATATGTTGAG GTAGTGAGGG   
  
  
- AAGCACTTTA AGAGAGTGAA GTGCCGCTTA GTTCGGTAGA TGCTCCGCGA CCTGCCGCTC CTAGCACAGG   
  
  
- TGCAGCAGCT GGAACTGTAG TACGTCCCGG AAGTTACCGG CCCTAACAAG GTGTAGAATC GGAGAGCTGG   
  
  
- GTCTTTCGGG GAGAGCCAAG CCCAGTGGCC CAAGCCCGGT AGGAGGCTCA ACGAGAGGGT TTGCCCATTC   
  
  
- GCTGAGCGAC TCAAGCGGCG AAGTGAGCCG GACGGGAAGC TCATGTTGGG CCACCTCCCG TTTTAACCCT   
  
  
- TGAATCAGCT GGACCCAGCC CAGCCCAGCG AGGGCTTACT TCACTGCCAC CACGTGACCT ACGTGGTATC   
  
  
- AGATATGCTG TATTGGCCCA GCCTAGAACC CTGAAACTCC CATAACTCAC GCCACTCCGG ATCCGAACAC   
  
  
- TGATACCAAC TCGTCCTATA CCTGGTTTGC CCCAGCAAAA ACCCCTCCAA ACACCTCCGT AACGTAATAA   
  
  
- TGAGTCGGAA CAAACTACGG AACCCTCTCC CCAATCTATC CCTATTGGAT GTTTCCGTAG TTCACCTCGT   
  
  
- TGTCGATAAA CCCACACTCT AATCCTTGTA GGACCGGCAA CCACCCGGGT TCTCCTCCGA GTGACCGCCG   
  
  
- CTAGCCCAGT CTTCCACCCC GCTGCTTGAC TGTTCCCAGC CCAAGCTTGG TCAAAGCAAC CGCCCATCGG   
  
  
- GCCGTTGGGT TCGATCAAAC AACGAACCCT ACAAGGGAAC CTTTCCCATA TGAAACTACC TCCTTTTACC   
  
  
- CACAAACTCT AATCCCACCT TTCTAAACGG GAACAATTGA CGGAGTCGAA CCGTTGGAAC ACTTAAATTG   
  
  
- TTAGGATCAC GACCGTAAAT

+     TC-rich repeats

| Site Name | Organism | Position | Strand | Matrix score. | sequence | function |
| --- | --- | --- | --- | --- | --- | --- |
| TC-rich repeats | Nicotiana tabacum | 1608 | - | 9 | ATTCTCTAAC | cis-acting element involved in defense and stress responsiveness |
| TC-rich repeats | Nicotiana tabacum | 1247 | + | 9 | ATTCTCTAAC | cis-acting element involved in defense and stress responsiveness |
| TC-rich repeats | Nicotiana tabacum | 1456 | - | 10 | GTTTTCTTAC | cis-acting element involved in defense and stress responsiveness |
| TC-rich repeats | Nicotiana tabacum | 2393 | + | 9 | ATTCTCTAAC | cis-acting element involved in defense and stress responsiveness |

>HU02G03005.1   
+ +Up\_Stream \_Len000AAACGG TGCCGTTGAA TTTGCAGGCG ACGAGGTCGG GTTTAAGGCG GATGTCGTAG   
  
  
+ CGCTTTGGAG AAGCAAATTT GGGAAGACGA GGCTTATTCT TGAACTCTTC GTACGCCATT GTTGTTGGTT   
  
  
+ CCAACCTTTC TCTCTCCTCG TGAATTTGAG TTTGGGCAGT AGACTATCCC GGAATAGAGG ACTGATGCGT   
  
  
+ TATATATATA GAAATTGAGA GAGAGAGAGA GCGTGTGTGA AGGATGTTGA AAATGATGGG CGTAGGGAGT   
  
  
+ CTCTCTCTCA CTGTCTGTTA GGAGTACGTG TCGAGAAAGG AAATAGGATT CCCAAGCAGT CTAATTTGGG   
  
  
+ CCGGTCTATA GGCCAGAATG GGCCTATGAT CAGCCCAGAT ATGACCTTGT TTAACCAACT GTATTTGAAT   
  
  
+ TGAAGAAAGG GATATCGCAA TGTCTTGAAC CACATCAAAT AATAAATGTG CCTTTGGTTA GACCATATGA   
  
  
+ AAAATCTTTG AATCAAATCG GTTGGACCTG AACTGCCATT ATTGATATCT TGTTTTTGGT AGAAGCAAAT   
  
  
+ GGTGTCACTT CGTTTTCTAG ATAAAATTAA CTTGAGTTTT TTTGCCACTA TAGATAGTAT TGCAAATATA   
  
  
+ AAAAGTGTGC CACGTGAAAT CAATATTAGG AGGGTGTTTT ATAACAATAG CTGTTCGGGA GAGTTTTAGT   
  
  
+ TATTTTTATT AAATCAAATA GTTAAGAAGT GTTTGGTAAA TAGTTGTATT TGAAAAAGTT ATTCCCATTA   
  
  
+ GCCTTTTAGT AAGAACTTCT TGTGAAAATG ATAATTGTCT AGAAGTTTGA AAAATTCACA CCACATGACA   
  
  
+ TTTAAAAATA ATTTTGTAGA GAAGGGCATA AATGAAAGTT TGCCACCATC TTTTTTAGAA ACATGTAATT   
  
  
+ TACCAAACAC TTTTTCTAAA AACAGTTAAT TCAAATAACT AACAACTAAC AGCTAATATA AATAACTAAC   
  
  
+ AGCTATTTGC TAAACAGGGC CTAAGTAGTT TGAAATTGTT GTTTTCTCTT CAGCTTCACC ATGGCCGATG   
  
  
+ ATGTAAACAA CTATATTCTG AATTTCATTT TTACTATGAT TTGAAAATTT CAAATTTAAA TAAGATTGGA   
  
  
+ TAAAAAAAAG ACCAAAAATC ATAAGTAATA TTAGTCAAGT TCTATTCCAA CCATATACAT AACTGCTTTT   
  
  
+ TCTTTTGGGG GTTTTGAAAC ATGTGTCCTT AGAATAATTG AAACTCGATA TTATTCTCTA AATCTAAGTT   
  
  
+ TACTTTATCA TATTGATTAT TAATTACCTC CTCAACTCCT CCTATACTCT TGTATGCAGA AATAAAAATG   
  
  
+ TTTCCATACC TTATCAGTCG CGATCATAAT AATTAATTTC TTAATTCGGA TTGTATTTAA TTGCGTATCA   
  
  
+ AATGTGAAAT CGTACCAAAT TAGGAAGCAA AACAAACCGA TAACGAAAAA GGTAAGAAAA CTTAGCAAGG   
  
  
+ AAATGAAATC GAAGACCTGT CAACAATATA GCTTCCCTCA CATCCTTCAA CTTAGGGACC ATCCTATACT   
  
  
+ TTCATGTAAT ACAACTAGAG CCACTAGTCA ATAATAGCCT CCTAGTAGTA GCCATAAGCC TTGGTGAGAG   
  
  
+ AATGTTCCGC AAGACTAGTT AACTTGAAAT TCCATTTTGT CGCAGAACTA TTGGAGTGAG AAAGAGAATC   
  
  
+ AAATCATACC ATATCAAATT TGGACCATTT AAGTTCACAA CACACACACA TACACACAAA AGGACGATGT   
  
  
+ ACGGCCCCAC CCCCCACTCT CCTTTCGCCA TCCCCCTACA CCTCTGCCTA CTCTCTCTCT CTCTCTCTCT   
  
  
+ CTCTGGATCA GTCTGTGTAA AAGCTTCCTC TCCAGTACAG CATTTCCTTG TCTGTTCTTG GAGTTTAGTC   
  
  
+ AATTCCTCCG CCCCAAAAAA ACCCACTTTC TCTCTCATCA TGTTCTTCCA GTTCCACCCT TATACGCCAT   
  
  
+ TCATCATCAT CTACATCACC CACATCTTTT TCTCCACAGT TTTCCCCCCT CATTATGCTC TCCGCCTCCA   
  
  
+ CCCCCGCCTT CCCTCTCGCC GTCGCCGGCG ATGACGGGGA TACCACTGAT GCATGCGACG GCACCGCCAC   
  
  
+ TGCCCTCCAC CTCCTCGGGC TGCTGTTGAA GTGCGCGGAA TTCATCTCCA CCGGAGACCT CGCCGGCGCC   
  
  
+ GGAGATATCT TGCCGGAGAT ATTTGAGTTG TCTACACCGT TTGGCTCCCC CGCCGCCCGG GTCGCCGCCT   
  
  
+ TCTTCGGCCA CGCCCTCCAC GCCCGCCTCC TCTCCGCCTC CCTCCGCACA ACTCCGATCG AGAAGCTCAA   
  
  
+ AACCCTGACC CTGGTTTCCC AAATGCGGAA ATTTCACTCC GCCTTGCAAG TATACAACTC CATCACTCCC   
  
  
+ TTCGTGAAAT TCTCTCACTT CACGGCGAAT CAAGCCATCT ACGAGGCGCT GGACGGCGAG GATCGTGTCC   
  
  
+ ACGTCGTCGA CCTTGACATC ATGCAGGGCC TTCAATGGCC GGGATTGTTC CACATCTTAG CCTCTCGACC   
  
  
+ CAGAAAGCCC CTCTCGGTTC GGGTCACCGG GTTCGGGCCA TCCTCCGAGT TGCTCTCCCA AACGGGTAAG   
  
  
+ CGACTCGCTG AGTTCGCCGC TTCACTCGGC CTGCCCTTCG AGTACAACCC GGTGGAGGGC AAAATTGGGA   
  
  
+ ACTTAGTCGA CCTGGGTCGG GTCGGGTCGC TCCCGAATGA AGTGACGGTG GTGCACTGGA TGCACCATAG   
  
  
+ TCTATACGAC ATAACCGGGT CGGATCTTGG GACTTTGAGG GTATTGAGTG CGGTGAGGCC TAGGCTTGTG   
  
  
+ ACTATGGTTG AGCAGGATAT GGACCAAACG GGGTCGTTTT TGGGGAGGTT TGTGGAGGCA TTGCATTATT   
  
  
+ ACTCAGCCTT GTTTGATGCC TTGGGAGAGG GGTTAGATAG GGATAACCTA CAAAGGCATC AAGTGGAGCA   
  
  
+ ACAGCTATTT GGGTGTGAGA TTAGGAACAT CCTGGCCGTT GGTGGGCCCA AGAGGAGGCT CACTGGCGGC   
  
  
+ GATCGGGTCA GAAGGTGGGG CGACGAACTG ACAAGGGTCG GGTTCGAACC AGTTTCGTTG GCGGGTAGCC   
  
  
+ CGGCAACCCA AGCTAGTTTG TTGCTTGGGA TGTTCCCTTG GAAAGGGTAT ACTTTGATGG AGGAAAATGG   
  
  
+ GTGTTTGAGA TTAGGGTGGA AAGATTTGCC CTTGTTAACT GCCTCAGCTT GGCAACCTTG TGAATTTAAC   
  
  
+ AATCCTAGTG CTGGCATTTA   

- +Up\_Stream \_Len000TTTGCC ACGGCAACTT AAACGTCCGC TGCTCCAGCC CAAATTCCGC CTACAGCATC   
  
  
- GCGAAACCTC TTCGTTTAAA CCCTTCTGCT CCGAATAAGA ACTTGAGAAG CATGCGGTAA CAACAACCAA   
  
  
- GGTTGGAAAG AGAGAGGAGC ACTTAAACTC AAACCCGTCA TCTGATAGGG CCTTATCTCC TGACTACGCA   
  
  
- ATATATATAT CTTTAACTCT CTCTCTCTCT CGCACACACT TCCTACAACT TTTACTACCC GCATCCCTCA   
  
  
- GAGAGAGAGT GACAGACAAT CCTCATGCAC AGCTCTTTCC TTTATCCTAA GGGTTCGTCA GATTAAACCC   
  
  
- GGCCAGATAT CCGGTCTTAC CCGGATACTA GTCGGGTCTA TACTGGAACA AATTGGTTGA CATAAACTTA   
  
  
- ACTTCTTTCC CTATAGCGTT ACAGAACTTG GTGTAGTTTA TTATTTACAC GGAAACCAAT CTGGTATACT   
  
  
- TTTTAGAAAC TTAGTTTAGC CAACCTGGAC TTGACGGTAA TAACTATAGA ACAAAAACCA TCTTCGTTTA   
  
  
- CCACAGTGAA GCAAAAGATC TATTTTAATT GAACTCAAAA AAACGGTGAT ATCTATCATA ACGTTTATAT   
  
  
- TTTTCACACG GTGCACTTTA GTTATAATCC TCCCACAAAA TATTGTTATC GACAAGCCCT CTCAAAATCA   
  
  
- ATAAAAATAA TTTAGTTTAT CAATTCTTCA CAAACCATTT ATCAACATAA ACTTTTTCAA TAAGGGTAAT   
  
  
- CGGAAAATCA TTCTTGAAGA ACACTTTTAC TATTAACAGA TCTTCAAACT TTTTAAGTGT GGTGTACTGT   
  
  
- AAATTTTTAT TAAAACATCT CTTCCCGTAT TTACTTTCAA ACGGTGGTAG AAAAAATCTT TGTACATTAA   
  
  
- ATGGTTTGTG AAAAAGATTT TTGTCAATTA AGTTTATTGA TTGTTGATTG TCGATTATAT TTATTGATTG   
  
  
- TCGATAAACG ATTTGTCCCG GATTCATCAA ACTTTAACAA CAAAAGAGAA GTCGAAGTGG TACCGGCTAC   
  
  
- TACATTTGTT GATATAAGAC TTAAAGTAAA AATGATACTA AACTTTTAAA GTTTAAATTT ATTCTAACCT   
  
  
- ATTTTTTTTC TGGTTTTTAG TATTCATTAT AATCAGTTCA AGATAAGGTT GGTATATGTA TTGACGAAAA   
  
  
- AGAAAACCCC CAAAACTTTG TACACAGGAA TCTTATTAAC TTTGAGCTAT AATAAGAGAT TTAGATTCAA   
  
  
- ATGAAATAGT ATAACTAATA ATTAATGGAG GAGTTGAGGA GGATATGAGA ACATACGTCT TTATTTTTAC   
  
  
- AAAGGTATGG AATAGTCAGC GCTAGTATTA TTAATTAAAG AATTAAGCCT AACATAAATT AACGCATAGT   
  
  
- TTACACTTTA GCATGGTTTA ATCCTTCGTT TTGTTTGGCT ATTGCTTTTT CCATTCTTTT GAATCGTTCC   
  
  
- TTTACTTTAG CTTCTGGACA GTTGTTATAT CGAAGGGAGT GTAGGAAGTT GAATCCCTGG TAGGATATGA   
  
  
- AAGTACATTA TGTTGATCTC GGTGATCAGT TATTATCGGA GGATCATCAT CGGTATTCGG AACCACTCTC   
  
  
- TTACAAGGCG TTCTGATCAA TTGAACTTTA AGGTAAAACA GCGTCTTGAT AACCTCACTC TTTCTCTTAG   
  
  
- TTTAGTATGG TATAGTTTAA ACCTGGTAAA TTCAAGTGTT GTGTGTGTGT ATGTGTGTTT TCCTGCTACA   
  
  
- TGCCGGGGTG GGGGGTGAGA GGAAAGCGGT AGGGGGATGT GGAGACGGAT GAGAGAGAGA GAGAGAGAGA   
  
  
- GAGACCTAGT CAGACACATT TTCGAAGGAG AGGTCATGTC GTAAAGGAAC AGACAAGAAC CTCAAATCAG   
  
  
- TTAAGGAGGC GGGGTTTTTT TGGGTGAAAG AGAGAGTAGT ACAAGAAGGT CAAGGTGGGA ATATGCGGTA   
  
  
- AGTAGTAGTA GATGTAGTGG GTGTAGAAAA AGAGGTGTCA AAAGGGGGGA GTAATACGAG AGGCGGAGGT   
  
  
- GGGGGCGGAA GGGAGAGCGG CAGCGGCCGC TACTGCCCCT ATGGTGACTA CGTACGCTGC CGTGGCGGTG   
  
  
- ACGGGAGGTG GAGGAGCCCG ACGACAACTT CACGCGCCTT AAGTAGAGGT GGCCTCTGGA GCGGCCGCGG   
  
  
- CCTCTATAGA ACGGCCTCTA TAAACTCAAC AGATGTGGCA AACCGAGGGG GCGGCGGGCC CAGCGGCGGA   
  
  
- AGAAGCCGGT GCGGGAGGTG CGGGCGGAGG AGAGGCGGAG GGAGGCGTGT TGAGGCTAGC TCTTCGAGTT   
  
  
- TTGGGACTGG GACCAAAGGG TTTACGCCTT TAAAGTGAGG CGGAACGTTC ATATGTTGAG GTAGTGAGGG   
  
  
- AAGCACTTTA AGAGAGTGAA GTGCCGCTTA GTTCGGTAGA TGCTCCGCGA CCTGCCGCTC CTAGCACAGG   
  
  
- TGCAGCAGCT GGAACTGTAG TACGTCCCGG AAGTTACCGG CCCTAACAAG GTGTAGAATC GGAGAGCTGG   
  
  
- GTCTTTCGGG GAGAGCCAAG CCCAGTGGCC CAAGCCCGGT AGGAGGCTCA ACGAGAGGGT TTGCCCATTC   
  
  
- GCTGAGCGAC TCAAGCGGCG AAGTGAGCCG GACGGGAAGC TCATGTTGGG CCACCTCCCG TTTTAACCCT   
  
  
- TGAATCAGCT GGACCCAGCC CAGCCCAGCG AGGGCTTACT TCACTGCCAC CACGTGACCT ACGTGGTATC   
  
  
- AGATATGCTG TATTGGCCCA GCCTAGAACC CTGAAACTCC CATAACTCAC GCCACTCCGG ATCCGAACAC   
  
  
- TGATACCAAC TCGTCCTATA CCTGGTTTGC CCCAGCAAAA ACCCCTCCAA ACACCTCCGT AACGTAATAA   
  
  
- TGAGTCGGAA CAAACTACGG AACCCTCTCC CCAATCTATC CCTATTGGAT GTTTCCGTAG TTCACCTCGT   
  
  
- TGTCGATAAA CCCACACTCT AATCCTTGTA GGACCGGCAA CCACCCGGGT TCTCCTCCGA GTGACCGCCG   
  
  
- CTAGCCCAGT CTTCCACCCC GCTGCTTGAC TGTTCCCAGC CCAAGCTTGG TCAAAGCAAC CGCCCATCGG   
  
  
- GCCGTTGGGT TCGATCAAAC AACGAACCCT ACAAGGGAAC CTTTCCCATA TGAAACTACC TCCTTTTACC   
  
  
- CACAAACTCT AATCCCACCT TTCTAAACGG GAACAATTGA CGGAGTCGAA CCGTTGGAAC ACTTAAATTG   
  
  
- TTAGGATCAC GACCGTAAAT

+     TCA

| Site Name | Organism | Position | Strand | Matrix score. | sequence | function |
| --- | --- | --- | --- | --- | --- | --- |
| TCA | Pisum sativum | 1965 | + | 9 | TCATCTTCAT |  |
| TCA | Pisum sativum | 1971 | + | 9 | TCATCTTCAT |  |

>HU02G03005.1   
+ +Up\_Stream \_Len000AAACGG TGCCGTTGAA TTTGCAGGCG ACGAGGTCGG GTTTAAGGCG GATGTCGTAG   
  
  
+ CGCTTTGGAG AAGCAAATTT GGGAAGACGA GGCTTATTCT TGAACTCTTC GTACGCCATT GTTGTTGGTT   
  
  
+ CCAACCTTTC TCTCTCCTCG TGAATTTGAG TTTGGGCAGT AGACTATCCC GGAATAGAGG ACTGATGCGT   
  
  
+ TATATATATA GAAATTGAGA GAGAGAGAGA GCGTGTGTGA AGGATGTTGA AAATGATGGG CGTAGGGAGT   
  
  
+ CTCTCTCTCA CTGTCTGTTA GGAGTACGTG TCGAGAAAGG AAATAGGATT CCCAAGCAGT CTAATTTGGG   
  
  
+ CCGGTCTATA GGCCAGAATG GGCCTATGAT CAGCCCAGAT ATGACCTTGT TTAACCAACT GTATTTGAAT   
  
  
+ TGAAGAAAGG GATATCGCAA TGTCTTGAAC CACATCAAAT AATAAATGTG CCTTTGGTTA GACCATATGA   
  
  
+ AAAATCTTTG AATCAAATCG GTTGGACCTG AACTGCCATT ATTGATATCT TGTTTTTGGT AGAAGCAAAT   
  
  
+ GGTGTCACTT CGTTTTCTAG ATAAAATTAA CTTGAGTTTT TTTGCCACTA TAGATAGTAT TGCAAATATA   
  
  
+ AAAAGTGTGC CACGTGAAAT CAATATTAGG AGGGTGTTTT ATAACAATAG CTGTTCGGGA GAGTTTTAGT   
  
  
+ TATTTTTATT AAATCAAATA GTTAAGAAGT GTTTGGTAAA TAGTTGTATT TGAAAAAGTT ATTCCCATTA   
  
  
+ GCCTTTTAGT AAGAACTTCT TGTGAAAATG ATAATTGTCT AGAAGTTTGA AAAATTCACA CCACATGACA   
  
  
+ TTTAAAAATA ATTTTGTAGA GAAGGGCATA AATGAAAGTT TGCCACCATC TTTTTTAGAA ACATGTAATT   
  
  
+ TACCAAACAC TTTTTCTAAA AACAGTTAAT TCAAATAACT AACAACTAAC AGCTAATATA AATAACTAAC   
  
  
+ AGCTATTTGC TAAACAGGGC CTAAGTAGTT TGAAATTGTT GTTTTCTCTT CAGCTTCACC ATGGCCGATG   
  
  
+ ATGTAAACAA CTATATTCTG AATTTCATTT TTACTATGAT TTGAAAATTT CAAATTTAAA TAAGATTGGA   
  
  
+ TAAAAAAAAG ACCAAAAATC ATAAGTAATA TTAGTCAAGT TCTATTCCAA CCATATACAT AACTGCTTTT   
  
  
+ TCTTTTGGGG GTTTTGAAAC ATGTGTCCTT AGAATAATTG AAACTCGATA TTATTCTCTA AATCTAAGTT   
  
  
+ TACTTTATCA TATTGATTAT TAATTACCTC CTCAACTCCT CCTATACTCT TGTATGCAGA AATAAAAATG   
  
  
+ TTTCCATACC TTATCAGTCG CGATCATAAT AATTAATTTC TTAATTCGGA TTGTATTTAA TTGCGTATCA   
  
  
+ AATGTGAAAT CGTACCAAAT TAGGAAGCAA AACAAACCGA TAACGAAAAA GGTAAGAAAA CTTAGCAAGG   
  
  
+ AAATGAAATC GAAGACCTGT CAACAATATA GCTTCCCTCA CATCCTTCAA CTTAGGGACC ATCCTATACT   
  
  
+ TTCATGTAAT ACAACTAGAG CCACTAGTCA ATAATAGCCT CCTAGTAGTA GCCATAAGCC TTGGTGAGAG   
  
  
+ AATGTTCCGC AAGACTAGTT AACTTGAAAT TCCATTTTGT CGCAGAACTA TTGGAGTGAG AAAGAGAATC   
  
  
+ AAATCATACC ATATCAAATT TGGACCATTT AAGTTCACAA CACACACACA TACACACAAA AGGACGATGT   
  
  
+ ACGGCCCCAC CCCCCACTCT CCTTTCGCCA TCCCCCTACA CCTCTGCCTA CTCTCTCTCT CTCTCTCTCT   
  
  
+ CTCTGGATCA GTCTGTGTAA AAGCTTCCTC TCCAGTACAG CATTTCCTTG TCTGTTCTTG GAGTTTAGTC   
  
  
+ AATTCCTCCG CCCCAAAAAA ACCCACTTTC TCTCTCATCA TGTTCTTCCA GTTCCACCCT TATACGCCAT   
  
  
+ TCATCATCAT CTACATCACC CACATCTTTT TCTCCACAGT TTTCCCCCCT CATTATGCTC TCCGCCTCCA   
  
  
+ CCCCCGCCTT CCCTCTCGCC GTCGCCGGCG ATGACGGGGA TACCACTGAT GCATGCGACG GCACCGCCAC   
  
  
+ TGCCCTCCAC CTCCTCGGGC TGCTGTTGAA GTGCGCGGAA TTCATCTCCA CCGGAGACCT CGCCGGCGCC   
  
  
+ GGAGATATCT TGCCGGAGAT ATTTGAGTTG TCTACACCGT TTGGCTCCCC CGCCGCCCGG GTCGCCGCCT   
  
  
+ TCTTCGGCCA CGCCCTCCAC GCCCGCCTCC TCTCCGCCTC CCTCCGCACA ACTCCGATCG AGAAGCTCAA   
  
  
+ AACCCTGACC CTGGTTTCCC AAATGCGGAA ATTTCACTCC GCCTTGCAAG TATACAACTC CATCACTCCC   
  
  
+ TTCGTGAAAT TCTCTCACTT CACGGCGAAT CAAGCCATCT ACGAGGCGCT GGACGGCGAG GATCGTGTCC   
  
  
+ ACGTCGTCGA CCTTGACATC ATGCAGGGCC TTCAATGGCC GGGATTGTTC CACATCTTAG CCTCTCGACC   
  
  
+ CAGAAAGCCC CTCTCGGTTC GGGTCACCGG GTTCGGGCCA TCCTCCGAGT TGCTCTCCCA AACGGGTAAG   
  
  
+ CGACTCGCTG AGTTCGCCGC TTCACTCGGC CTGCCCTTCG AGTACAACCC GGTGGAGGGC AAAATTGGGA   
  
  
+ ACTTAGTCGA CCTGGGTCGG GTCGGGTCGC TCCCGAATGA AGTGACGGTG GTGCACTGGA TGCACCATAG   
  
  
+ TCTATACGAC ATAACCGGGT CGGATCTTGG GACTTTGAGG GTATTGAGTG CGGTGAGGCC TAGGCTTGTG   
  
  
+ ACTATGGTTG AGCAGGATAT GGACCAAACG GGGTCGTTTT TGGGGAGGTT TGTGGAGGCA TTGCATTATT   
  
  
+ ACTCAGCCTT GTTTGATGCC TTGGGAGAGG GGTTAGATAG GGATAACCTA CAAAGGCATC AAGTGGAGCA   
  
  
+ ACAGCTATTT GGGTGTGAGA TTAGGAACAT CCTGGCCGTT GGTGGGCCCA AGAGGAGGCT CACTGGCGGC   
  
  
+ GATCGGGTCA GAAGGTGGGG CGACGAACTG ACAAGGGTCG GGTTCGAACC AGTTTCGTTG GCGGGTAGCC   
  
  
+ CGGCAACCCA AGCTAGTTTG TTGCTTGGGA TGTTCCCTTG GAAAGGGTAT ACTTTGATGG AGGAAAATGG   
  
  
+ GTGTTTGAGA TTAGGGTGGA AAGATTTGCC CTTGTTAACT GCCTCAGCTT GGCAACCTTG TGAATTTAAC   
  
  
+ AATCCTAGTG CTGGCATTTA   

- +Up\_Stream \_Len000TTTGCC ACGGCAACTT AAACGTCCGC TGCTCCAGCC CAAATTCCGC CTACAGCATC   
  
  
- GCGAAACCTC TTCGTTTAAA CCCTTCTGCT CCGAATAAGA ACTTGAGAAG CATGCGGTAA CAACAACCAA   
  
  
- GGTTGGAAAG AGAGAGGAGC ACTTAAACTC AAACCCGTCA TCTGATAGGG CCTTATCTCC TGACTACGCA   
  
  
- ATATATATAT CTTTAACTCT CTCTCTCTCT CGCACACACT TCCTACAACT TTTACTACCC GCATCCCTCA   
  
  
- GAGAGAGAGT GACAGACAAT CCTCATGCAC AGCTCTTTCC TTTATCCTAA GGGTTCGTCA GATTAAACCC   
  
  
- GGCCAGATAT CCGGTCTTAC CCGGATACTA GTCGGGTCTA TACTGGAACA AATTGGTTGA CATAAACTTA   
  
  
- ACTTCTTTCC CTATAGCGTT ACAGAACTTG GTGTAGTTTA TTATTTACAC GGAAACCAAT CTGGTATACT   
  
  
- TTTTAGAAAC TTAGTTTAGC CAACCTGGAC TTGACGGTAA TAACTATAGA ACAAAAACCA TCTTCGTTTA   
  
  
- CCACAGTGAA GCAAAAGATC TATTTTAATT GAACTCAAAA AAACGGTGAT ATCTATCATA ACGTTTATAT   
  
  
- TTTTCACACG GTGCACTTTA GTTATAATCC TCCCACAAAA TATTGTTATC GACAAGCCCT CTCAAAATCA   
  
  
- ATAAAAATAA TTTAGTTTAT CAATTCTTCA CAAACCATTT ATCAACATAA ACTTTTTCAA TAAGGGTAAT   
  
  
- CGGAAAATCA TTCTTGAAGA ACACTTTTAC TATTAACAGA TCTTCAAACT TTTTAAGTGT GGTGTACTGT   
  
  
- AAATTTTTAT TAAAACATCT CTTCCCGTAT TTACTTTCAA ACGGTGGTAG AAAAAATCTT TGTACATTAA   
  
  
- ATGGTTTGTG AAAAAGATTT TTGTCAATTA AGTTTATTGA TTGTTGATTG TCGATTATAT TTATTGATTG   
  
  
- TCGATAAACG ATTTGTCCCG GATTCATCAA ACTTTAACAA CAAAAGAGAA GTCGAAGTGG TACCGGCTAC   
  
  
- TACATTTGTT GATATAAGAC TTAAAGTAAA AATGATACTA AACTTTTAAA GTTTAAATTT ATTCTAACCT   
  
  
- ATTTTTTTTC TGGTTTTTAG TATTCATTAT AATCAGTTCA AGATAAGGTT GGTATATGTA TTGACGAAAA   
  
  
- AGAAAACCCC CAAAACTTTG TACACAGGAA TCTTATTAAC TTTGAGCTAT AATAAGAGAT TTAGATTCAA   
  
  
- ATGAAATAGT ATAACTAATA ATTAATGGAG GAGTTGAGGA GGATATGAGA ACATACGTCT TTATTTTTAC   
  
  
- AAAGGTATGG AATAGTCAGC GCTAGTATTA TTAATTAAAG AATTAAGCCT AACATAAATT AACGCATAGT   
  
  
- TTACACTTTA GCATGGTTTA ATCCTTCGTT TTGTTTGGCT ATTGCTTTTT CCATTCTTTT GAATCGTTCC   
  
  
- TTTACTTTAG CTTCTGGACA GTTGTTATAT CGAAGGGAGT GTAGGAAGTT GAATCCCTGG TAGGATATGA   
  
  
- AAGTACATTA TGTTGATCTC GGTGATCAGT TATTATCGGA GGATCATCAT CGGTATTCGG AACCACTCTC   
  
  
- TTACAAGGCG TTCTGATCAA TTGAACTTTA AGGTAAAACA GCGTCTTGAT AACCTCACTC TTTCTCTTAG   
  
  
- TTTAGTATGG TATAGTTTAA ACCTGGTAAA TTCAAGTGTT GTGTGTGTGT ATGTGTGTTT TCCTGCTACA   
  
  
- TGCCGGGGTG GGGGGTGAGA GGAAAGCGGT AGGGGGATGT GGAGACGGAT GAGAGAGAGA GAGAGAGAGA   
  
  
- GAGACCTAGT CAGACACATT TTCGAAGGAG AGGTCATGTC GTAAAGGAAC AGACAAGAAC CTCAAATCAG   
  
  
- TTAAGGAGGC GGGGTTTTTT TGGGTGAAAG AGAGAGTAGT ACAAGAAGGT CAAGGTGGGA ATATGCGGTA   
  
  
- AGTAGTAGTA GATGTAGTGG GTGTAGAAAA AGAGGTGTCA AAAGGGGGGA GTAATACGAG AGGCGGAGGT   
  
  
- GGGGGCGGAA GGGAGAGCGG CAGCGGCCGC TACTGCCCCT ATGGTGACTA CGTACGCTGC CGTGGCGGTG   
  
  
- ACGGGAGGTG GAGGAGCCCG ACGACAACTT CACGCGCCTT AAGTAGAGGT GGCCTCTGGA GCGGCCGCGG   
  
  
- CCTCTATAGA ACGGCCTCTA TAAACTCAAC AGATGTGGCA AACCGAGGGG GCGGCGGGCC CAGCGGCGGA   
  
  
- AGAAGCCGGT GCGGGAGGTG CGGGCGGAGG AGAGGCGGAG GGAGGCGTGT TGAGGCTAGC TCTTCGAGTT   
  
  
- TTGGGACTGG GACCAAAGGG TTTACGCCTT TAAAGTGAGG CGGAACGTTC ATATGTTGAG GTAGTGAGGG   
  
  
- AAGCACTTTA AGAGAGTGAA GTGCCGCTTA GTTCGGTAGA TGCTCCGCGA CCTGCCGCTC CTAGCACAGG   
  
  
- TGCAGCAGCT GGAACTGTAG TACGTCCCGG AAGTTACCGG CCCTAACAAG GTGTAGAATC GGAGAGCTGG   
  
  
- GTCTTTCGGG GAGAGCCAAG CCCAGTGGCC CAAGCCCGGT AGGAGGCTCA ACGAGAGGGT TTGCCCATTC   
  
  
- GCTGAGCGAC TCAAGCGGCG AAGTGAGCCG GACGGGAAGC TCATGTTGGG CCACCTCCCG TTTTAACCCT   
  
  
- TGAATCAGCT GGACCCAGCC CAGCCCAGCG AGGGCTTACT TCACTGCCAC CACGTGACCT ACGTGGTATC   
  
  
- AGATATGCTG TATTGGCCCA GCCTAGAACC CTGAAACTCC CATAACTCAC GCCACTCCGG ATCCGAACAC   
  
  
- TGATACCAAC TCGTCCTATA CCTGGTTTGC CCCAGCAAAA ACCCCTCCAA ACACCTCCGT AACGTAATAA   
  
  
- TGAGTCGGAA CAAACTACGG AACCCTCTCC CCAATCTATC CCTATTGGAT GTTTCCGTAG TTCACCTCGT   
  
  
- TGTCGATAAA CCCACACTCT AATCCTTGTA GGACCGGCAA CCACCCGGGT TCTCCTCCGA GTGACCGCCG   
  
  
- CTAGCCCAGT CTTCCACCCC GCTGCTTGAC TGTTCCCAGC CCAAGCTTGG TCAAAGCAAC CGCCCATCGG   
  
  
- GCCGTTGGGT TCGATCAAAC AACGAACCCT ACAAGGGAAC CTTTCCCATA TGAAACTACC TCCTTTTACC   
  
  
- CACAAACTCT AATCCCACCT TTCTAAACGG GAACAATTGA CGGAGTCGAA CCGTTGGAAC ACTTAAATTG   
  
  
- TTAGGATCAC GACCGTAAAT

+     TCA-element

| Site Name | Organism | Position | Strand | Matrix score. | sequence | function |
| --- | --- | --- | --- | --- | --- | --- |
| TCA-element | Nicotiana tabacum | 264 | - | 9 | CCATCTTTTT | cis-acting element involved in salicylic acid responsiveness |
| TCA-element | Nicotiana tabacum | 1986 | + | 9 | CCATCTTTTT | cis-acting element involved in salicylic acid responsiveness |
| TCA-element | Nicotiana tabacum | 890 | + | 10 | CCATCTTTTT | cis-acting element involved in salicylic acid responsiveness |

>HU02G03005.1   
+ +Up\_Stream \_Len000AAACGG TGCCGTTGAA TTTGCAGGCG ACGAGGTCGG GTTTAAGGCG GATGTCGTAG   
  
  
+ CGCTTTGGAG AAGCAAATTT GGGAAGACGA GGCTTATTCT TGAACTCTTC GTACGCCATT GTTGTTGGTT   
  
  
+ CCAACCTTTC TCTCTCCTCG TGAATTTGAG TTTGGGCAGT AGACTATCCC GGAATAGAGG ACTGATGCGT   
  
  
+ TATATATATA GAAATTGAGA GAGAGAGAGA GCGTGTGTGA AGGATGTTGA AAATGATGGG CGTAGGGAGT   
  
  
+ CTCTCTCTCA CTGTCTGTTA GGAGTACGTG TCGAGAAAGG AAATAGGATT CCCAAGCAGT CTAATTTGGG   
  
  
+ CCGGTCTATA GGCCAGAATG GGCCTATGAT CAGCCCAGAT ATGACCTTGT TTAACCAACT GTATTTGAAT   
  
  
+ TGAAGAAAGG GATATCGCAA TGTCTTGAAC CACATCAAAT AATAAATGTG CCTTTGGTTA GACCATATGA   
  
  
+ AAAATCTTTG AATCAAATCG GTTGGACCTG AACTGCCATT ATTGATATCT TGTTTTTGGT AGAAGCAAAT   
  
  
+ GGTGTCACTT CGTTTTCTAG ATAAAATTAA CTTGAGTTTT TTTGCCACTA TAGATAGTAT TGCAAATATA   
  
  
+ AAAAGTGTGC CACGTGAAAT CAATATTAGG AGGGTGTTTT ATAACAATAG CTGTTCGGGA GAGTTTTAGT   
  
  
+ TATTTTTATT AAATCAAATA GTTAAGAAGT GTTTGGTAAA TAGTTGTATT TGAAAAAGTT ATTCCCATTA   
  
  
+ GCCTTTTAGT AAGAACTTCT TGTGAAAATG ATAATTGTCT AGAAGTTTGA AAAATTCACA CCACATGACA   
  
  
+ TTTAAAAATA ATTTTGTAGA GAAGGGCATA AATGAAAGTT TGCCACCATC TTTTTTAGAA ACATGTAATT   
  
  
+ TACCAAACAC TTTTTCTAAA AACAGTTAAT TCAAATAACT AACAACTAAC AGCTAATATA AATAACTAAC   
  
  
+ AGCTATTTGC TAAACAGGGC CTAAGTAGTT TGAAATTGTT GTTTTCTCTT CAGCTTCACC ATGGCCGATG   
  
  
+ ATGTAAACAA CTATATTCTG AATTTCATTT TTACTATGAT TTGAAAATTT CAAATTTAAA TAAGATTGGA   
  
  
+ TAAAAAAAAG ACCAAAAATC ATAAGTAATA TTAGTCAAGT TCTATTCCAA CCATATACAT AACTGCTTTT   
  
  
+ TCTTTTGGGG GTTTTGAAAC ATGTGTCCTT AGAATAATTG AAACTCGATA TTATTCTCTA AATCTAAGTT   
  
  
+ TACTTTATCA TATTGATTAT TAATTACCTC CTCAACTCCT CCTATACTCT TGTATGCAGA AATAAAAATG   
  
  
+ TTTCCATACC TTATCAGTCG CGATCATAAT AATTAATTTC TTAATTCGGA TTGTATTTAA TTGCGTATCA   
  
  
+ AATGTGAAAT CGTACCAAAT TAGGAAGCAA AACAAACCGA TAACGAAAAA GGTAAGAAAA CTTAGCAAGG   
  
  
+ AAATGAAATC GAAGACCTGT CAACAATATA GCTTCCCTCA CATCCTTCAA CTTAGGGACC ATCCTATACT   
  
  
+ TTCATGTAAT ACAACTAGAG CCACTAGTCA ATAATAGCCT CCTAGTAGTA GCCATAAGCC TTGGTGAGAG   
  
  
+ AATGTTCCGC AAGACTAGTT AACTTGAAAT TCCATTTTGT CGCAGAACTA TTGGAGTGAG AAAGAGAATC   
  
  
+ AAATCATACC ATATCAAATT TGGACCATTT AAGTTCACAA CACACACACA TACACACAAA AGGACGATGT   
  
  
+ ACGGCCCCAC CCCCCACTCT CCTTTCGCCA TCCCCCTACA CCTCTGCCTA CTCTCTCTCT CTCTCTCTCT   
  
  
+ CTCTGGATCA GTCTGTGTAA AAGCTTCCTC TCCAGTACAG CATTTCCTTG TCTGTTCTTG GAGTTTAGTC   
  
  
+ AATTCCTCCG CCCCAAAAAA ACCCACTTTC TCTCTCATCA TGTTCTTCCA GTTCCACCCT TATACGCCAT   
  
  
+ TCATCATCAT CTACATCACC CACATCTTTT TCTCCACAGT TTTCCCCCCT CATTATGCTC TCCGCCTCCA   
  
  
+ CCCCCGCCTT CCCTCTCGCC GTCGCCGGCG ATGACGGGGA TACCACTGAT GCATGCGACG GCACCGCCAC   
  
  
+ TGCCCTCCAC CTCCTCGGGC TGCTGTTGAA GTGCGCGGAA TTCATCTCCA CCGGAGACCT CGCCGGCGCC   
  
  
+ GGAGATATCT TGCCGGAGAT ATTTGAGTTG TCTACACCGT TTGGCTCCCC CGCCGCCCGG GTCGCCGCCT   
  
  
+ TCTTCGGCCA CGCCCTCCAC GCCCGCCTCC TCTCCGCCTC CCTCCGCACA ACTCCGATCG AGAAGCTCAA   
  
  
+ AACCCTGACC CTGGTTTCCC AAATGCGGAA ATTTCACTCC GCCTTGCAAG TATACAACTC CATCACTCCC   
  
  
+ TTCGTGAAAT TCTCTCACTT CACGGCGAAT CAAGCCATCT ACGAGGCGCT GGACGGCGAG GATCGTGTCC   
  
  
+ ACGTCGTCGA CCTTGACATC ATGCAGGGCC TTCAATGGCC GGGATTGTTC CACATCTTAG CCTCTCGACC   
  
  
+ CAGAAAGCCC CTCTCGGTTC GGGTCACCGG GTTCGGGCCA TCCTCCGAGT TGCTCTCCCA AACGGGTAAG   
  
  
+ CGACTCGCTG AGTTCGCCGC TTCACTCGGC CTGCCCTTCG AGTACAACCC GGTGGAGGGC AAAATTGGGA   
  
  
+ ACTTAGTCGA CCTGGGTCGG GTCGGGTCGC TCCCGAATGA AGTGACGGTG GTGCACTGGA TGCACCATAG   
  
  
+ TCTATACGAC ATAACCGGGT CGGATCTTGG GACTTTGAGG GTATTGAGTG CGGTGAGGCC TAGGCTTGTG   
  
  
+ ACTATGGTTG AGCAGGATAT GGACCAAACG GGGTCGTTTT TGGGGAGGTT TGTGGAGGCA TTGCATTATT   
  
  
+ ACTCAGCCTT GTTTGATGCC TTGGGAGAGG GGTTAGATAG GGATAACCTA CAAAGGCATC AAGTGGAGCA   
  
  
+ ACAGCTATTT GGGTGTGAGA TTAGGAACAT CCTGGCCGTT GGTGGGCCCA AGAGGAGGCT CACTGGCGGC   
  
  
+ GATCGGGTCA GAAGGTGGGG CGACGAACTG ACAAGGGTCG GGTTCGAACC AGTTTCGTTG GCGGGTAGCC   
  
  
+ CGGCAACCCA AGCTAGTTTG TTGCTTGGGA TGTTCCCTTG GAAAGGGTAT ACTTTGATGG AGGAAAATGG   
  
  
+ GTGTTTGAGA TTAGGGTGGA AAGATTTGCC CTTGTTAACT GCCTCAGCTT GGCAACCTTG TGAATTTAAC   
  
  
+ AATCCTAGTG CTGGCATTTA   

- +Up\_Stream \_Len000TTTGCC ACGGCAACTT AAACGTCCGC TGCTCCAGCC CAAATTCCGC CTACAGCATC   
  
  
- GCGAAACCTC TTCGTTTAAA CCCTTCTGCT CCGAATAAGA ACTTGAGAAG CATGCGGTAA CAACAACCAA   
  
  
- GGTTGGAAAG AGAGAGGAGC ACTTAAACTC AAACCCGTCA TCTGATAGGG CCTTATCTCC TGACTACGCA   
  
  
- ATATATATAT CTTTAACTCT CTCTCTCTCT CGCACACACT TCCTACAACT TTTACTACCC GCATCCCTCA   
  
  
- GAGAGAGAGT GACAGACAAT CCTCATGCAC AGCTCTTTCC TTTATCCTAA GGGTTCGTCA GATTAAACCC   
  
  
- GGCCAGATAT CCGGTCTTAC CCGGATACTA GTCGGGTCTA TACTGGAACA AATTGGTTGA CATAAACTTA   
  
  
- ACTTCTTTCC CTATAGCGTT ACAGAACTTG GTGTAGTTTA TTATTTACAC GGAAACCAAT CTGGTATACT   
  
  
- TTTTAGAAAC TTAGTTTAGC CAACCTGGAC TTGACGGTAA TAACTATAGA ACAAAAACCA TCTTCGTTTA   
  
  
- CCACAGTGAA GCAAAAGATC TATTTTAATT GAACTCAAAA AAACGGTGAT ATCTATCATA ACGTTTATAT   
  
  
- TTTTCACACG GTGCACTTTA GTTATAATCC TCCCACAAAA TATTGTTATC GACAAGCCCT CTCAAAATCA   
  
  
- ATAAAAATAA TTTAGTTTAT CAATTCTTCA CAAACCATTT ATCAACATAA ACTTTTTCAA TAAGGGTAAT   
  
  
- CGGAAAATCA TTCTTGAAGA ACACTTTTAC TATTAACAGA TCTTCAAACT TTTTAAGTGT GGTGTACTGT   
  
  
- AAATTTTTAT TAAAACATCT CTTCCCGTAT TTACTTTCAA ACGGTGGTAG AAAAAATCTT TGTACATTAA   
  
  
- ATGGTTTGTG AAAAAGATTT TTGTCAATTA AGTTTATTGA TTGTTGATTG TCGATTATAT TTATTGATTG   
  
  
- TCGATAAACG ATTTGTCCCG GATTCATCAA ACTTTAACAA CAAAAGAGAA GTCGAAGTGG TACCGGCTAC   
  
  
- TACATTTGTT GATATAAGAC TTAAAGTAAA AATGATACTA AACTTTTAAA GTTTAAATTT ATTCTAACCT   
  
  
- ATTTTTTTTC TGGTTTTTAG TATTCATTAT AATCAGTTCA AGATAAGGTT GGTATATGTA TTGACGAAAA   
  
  
- AGAAAACCCC CAAAACTTTG TACACAGGAA TCTTATTAAC TTTGAGCTAT AATAAGAGAT TTAGATTCAA   
  
  
- ATGAAATAGT ATAACTAATA ATTAATGGAG GAGTTGAGGA GGATATGAGA ACATACGTCT TTATTTTTAC   
  
  
- AAAGGTATGG AATAGTCAGC GCTAGTATTA TTAATTAAAG AATTAAGCCT AACATAAATT AACGCATAGT   
  
  
- TTACACTTTA GCATGGTTTA ATCCTTCGTT TTGTTTGGCT ATTGCTTTTT CCATTCTTTT GAATCGTTCC   
  
  
- TTTACTTTAG CTTCTGGACA GTTGTTATAT CGAAGGGAGT GTAGGAAGTT GAATCCCTGG TAGGATATGA   
  
  
- AAGTACATTA TGTTGATCTC GGTGATCAGT TATTATCGGA GGATCATCAT CGGTATTCGG AACCACTCTC   
  
  
- TTACAAGGCG TTCTGATCAA TTGAACTTTA AGGTAAAACA GCGTCTTGAT AACCTCACTC TTTCTCTTAG   
  
  
- TTTAGTATGG TATAGTTTAA ACCTGGTAAA TTCAAGTGTT GTGTGTGTGT ATGTGTGTTT TCCTGCTACA   
  
  
- TGCCGGGGTG GGGGGTGAGA GGAAAGCGGT AGGGGGATGT GGAGACGGAT GAGAGAGAGA GAGAGAGAGA   
  
  
- GAGACCTAGT CAGACACATT TTCGAAGGAG AGGTCATGTC GTAAAGGAAC AGACAAGAAC CTCAAATCAG   
  
  
- TTAAGGAGGC GGGGTTTTTT TGGGTGAAAG AGAGAGTAGT ACAAGAAGGT CAAGGTGGGA ATATGCGGTA   
  
  
- AGTAGTAGTA GATGTAGTGG GTGTAGAAAA AGAGGTGTCA AAAGGGGGGA GTAATACGAG AGGCGGAGGT   
  
  
- GGGGGCGGAA GGGAGAGCGG CAGCGGCCGC TACTGCCCCT ATGGTGACTA CGTACGCTGC CGTGGCGGTG   
  
  
- ACGGGAGGTG GAGGAGCCCG ACGACAACTT CACGCGCCTT AAGTAGAGGT GGCCTCTGGA GCGGCCGCGG   
  
  
- CCTCTATAGA ACGGCCTCTA TAAACTCAAC AGATGTGGCA AACCGAGGGG GCGGCGGGCC CAGCGGCGGA   
  
  
- AGAAGCCGGT GCGGGAGGTG CGGGCGGAGG AGAGGCGGAG GGAGGCGTGT TGAGGCTAGC TCTTCGAGTT   
  
  
- TTGGGACTGG GACCAAAGGG TTTACGCCTT TAAAGTGAGG CGGAACGTTC ATATGTTGAG GTAGTGAGGG   
  
  
- AAGCACTTTA AGAGAGTGAA GTGCCGCTTA GTTCGGTAGA TGCTCCGCGA CCTGCCGCTC CTAGCACAGG   
  
  
- TGCAGCAGCT GGAACTGTAG TACGTCCCGG AAGTTACCGG CCCTAACAAG GTGTAGAATC GGAGAGCTGG   
  
  
- GTCTTTCGGG GAGAGCCAAG CCCAGTGGCC CAAGCCCGGT AGGAGGCTCA ACGAGAGGGT TTGCCCATTC   
  
  
- GCTGAGCGAC TCAAGCGGCG AAGTGAGCCG GACGGGAAGC TCATGTTGGG CCACCTCCCG TTTTAACCCT   
  
  
- TGAATCAGCT GGACCCAGCC CAGCCCAGCG AGGGCTTACT TCACTGCCAC CACGTGACCT ACGTGGTATC   
  
  
- AGATATGCTG TATTGGCCCA GCCTAGAACC CTGAAACTCC CATAACTCAC GCCACTCCGG ATCCGAACAC   
  
  
- TGATACCAAC TCGTCCTATA CCTGGTTTGC CCCAGCAAAA ACCCCTCCAA ACACCTCCGT AACGTAATAA   
  
  
- TGAGTCGGAA CAAACTACGG AACCCTCTCC CCAATCTATC CCTATTGGAT GTTTCCGTAG TTCACCTCGT   
  
  
- TGTCGATAAA CCCACACTCT AATCCTTGTA GGACCGGCAA CCACCCGGGT TCTCCTCCGA GTGACCGCCG   
  
  
- CTAGCCCAGT CTTCCACCCC GCTGCTTGAC TGTTCCCAGC CCAAGCTTGG TCAAAGCAAC CGCCCATCGG   
  
  
- GCCGTTGGGT TCGATCAAAC AACGAACCCT ACAAGGGAAC CTTTCCCATA TGAAACTACC TCCTTTTACC   
  
  
- CACAAACTCT AATCCCACCT TTCTAAACGG GAACAATTGA CGGAGTCGAA CCGTTGGAAC ACTTAAATTG   
  
  
- TTAGGATCAC GACCGTAAAT

+     TCT-motif

| Site Name | Organism | Position | Strand | Matrix score. | sequence | function |
| --- | --- | --- | --- | --- | --- | --- |
| TCT-motif | Arabidopsis thaliana | 1456 | - | 6 | TCTTAC | part of a light responsive element |
| TCT-motif | Arabidopsis thaliana | 783 | - | 6 | TCTTAC | part of a light responsive element |

>HU02G03005.1   
+ +Up\_Stream \_Len000AAACGG TGCCGTTGAA TTTGCAGGCG ACGAGGTCGG GTTTAAGGCG GATGTCGTAG   
  
  
+ CGCTTTGGAG AAGCAAATTT GGGAAGACGA GGCTTATTCT TGAACTCTTC GTACGCCATT GTTGTTGGTT   
  
  
+ CCAACCTTTC TCTCTCCTCG TGAATTTGAG TTTGGGCAGT AGACTATCCC GGAATAGAGG ACTGATGCGT   
  
  
+ TATATATATA GAAATTGAGA GAGAGAGAGA GCGTGTGTGA AGGATGTTGA AAATGATGGG CGTAGGGAGT   
  
  
+ CTCTCTCTCA CTGTCTGTTA GGAGTACGTG TCGAGAAAGG AAATAGGATT CCCAAGCAGT CTAATTTGGG   
  
  
+ CCGGTCTATA GGCCAGAATG GGCCTATGAT CAGCCCAGAT ATGACCTTGT TTAACCAACT GTATTTGAAT   
  
  
+ TGAAGAAAGG GATATCGCAA TGTCTTGAAC CACATCAAAT AATAAATGTG CCTTTGGTTA GACCATATGA   
  
  
+ AAAATCTTTG AATCAAATCG GTTGGACCTG AACTGCCATT ATTGATATCT TGTTTTTGGT AGAAGCAAAT   
  
  
+ GGTGTCACTT CGTTTTCTAG ATAAAATTAA CTTGAGTTTT TTTGCCACTA TAGATAGTAT TGCAAATATA   
  
  
+ AAAAGTGTGC CACGTGAAAT CAATATTAGG AGGGTGTTTT ATAACAATAG CTGTTCGGGA GAGTTTTAGT   
  
  
+ TATTTTTATT AAATCAAATA GTTAAGAAGT GTTTGGTAAA TAGTTGTATT TGAAAAAGTT ATTCCCATTA   
  
  
+ GCCTTTTAGT AAGAACTTCT TGTGAAAATG ATAATTGTCT AGAAGTTTGA AAAATTCACA CCACATGACA   
  
  
+ TTTAAAAATA ATTTTGTAGA GAAGGGCATA AATGAAAGTT TGCCACCATC TTTTTTAGAA ACATGTAATT   
  
  
+ TACCAAACAC TTTTTCTAAA AACAGTTAAT TCAAATAACT AACAACTAAC AGCTAATATA AATAACTAAC   
  
  
+ AGCTATTTGC TAAACAGGGC CTAAGTAGTT TGAAATTGTT GTTTTCTCTT CAGCTTCACC ATGGCCGATG   
  
  
+ ATGTAAACAA CTATATTCTG AATTTCATTT TTACTATGAT TTGAAAATTT CAAATTTAAA TAAGATTGGA   
  
  
+ TAAAAAAAAG ACCAAAAATC ATAAGTAATA TTAGTCAAGT TCTATTCCAA CCATATACAT AACTGCTTTT   
  
  
+ TCTTTTGGGG GTTTTGAAAC ATGTGTCCTT AGAATAATTG AAACTCGATA TTATTCTCTA AATCTAAGTT   
  
  
+ TACTTTATCA TATTGATTAT TAATTACCTC CTCAACTCCT CCTATACTCT TGTATGCAGA AATAAAAATG   
  
  
+ TTTCCATACC TTATCAGTCG CGATCATAAT AATTAATTTC TTAATTCGGA TTGTATTTAA TTGCGTATCA   
  
  
+ AATGTGAAAT CGTACCAAAT TAGGAAGCAA AACAAACCGA TAACGAAAAA GGTAAGAAAA CTTAGCAAGG   
  
  
+ AAATGAAATC GAAGACCTGT CAACAATATA GCTTCCCTCA CATCCTTCAA CTTAGGGACC ATCCTATACT   
  
  
+ TTCATGTAAT ACAACTAGAG CCACTAGTCA ATAATAGCCT CCTAGTAGTA GCCATAAGCC TTGGTGAGAG   
  
  
+ AATGTTCCGC AAGACTAGTT AACTTGAAAT TCCATTTTGT CGCAGAACTA TTGGAGTGAG AAAGAGAATC   
  
  
+ AAATCATACC ATATCAAATT TGGACCATTT AAGTTCACAA CACACACACA TACACACAAA AGGACGATGT   
  
  
+ ACGGCCCCAC CCCCCACTCT CCTTTCGCCA TCCCCCTACA CCTCTGCCTA CTCTCTCTCT CTCTCTCTCT   
  
  
+ CTCTGGATCA GTCTGTGTAA AAGCTTCCTC TCCAGTACAG CATTTCCTTG TCTGTTCTTG GAGTTTAGTC   
  
  
+ AATTCCTCCG CCCCAAAAAA ACCCACTTTC TCTCTCATCA TGTTCTTCCA GTTCCACCCT TATACGCCAT   
  
  
+ TCATCATCAT CTACATCACC CACATCTTTT TCTCCACAGT TTTCCCCCCT CATTATGCTC TCCGCCTCCA   
  
  
+ CCCCCGCCTT CCCTCTCGCC GTCGCCGGCG ATGACGGGGA TACCACTGAT GCATGCGACG GCACCGCCAC   
  
  
+ TGCCCTCCAC CTCCTCGGGC TGCTGTTGAA GTGCGCGGAA TTCATCTCCA CCGGAGACCT CGCCGGCGCC   
  
  
+ GGAGATATCT TGCCGGAGAT ATTTGAGTTG TCTACACCGT TTGGCTCCCC CGCCGCCCGG GTCGCCGCCT   
  
  
+ TCTTCGGCCA CGCCCTCCAC GCCCGCCTCC TCTCCGCCTC CCTCCGCACA ACTCCGATCG AGAAGCTCAA   
  
  
+ AACCCTGACC CTGGTTTCCC AAATGCGGAA ATTTCACTCC GCCTTGCAAG TATACAACTC CATCACTCCC   
  
  
+ TTCGTGAAAT TCTCTCACTT CACGGCGAAT CAAGCCATCT ACGAGGCGCT GGACGGCGAG GATCGTGTCC   
  
  
+ ACGTCGTCGA CCTTGACATC ATGCAGGGCC TTCAATGGCC GGGATTGTTC CACATCTTAG CCTCTCGACC   
  
  
+ CAGAAAGCCC CTCTCGGTTC GGGTCACCGG GTTCGGGCCA TCCTCCGAGT TGCTCTCCCA AACGGGTAAG   
  
  
+ CGACTCGCTG AGTTCGCCGC TTCACTCGGC CTGCCCTTCG AGTACAACCC GGTGGAGGGC AAAATTGGGA   
  
  
+ ACTTAGTCGA CCTGGGTCGG GTCGGGTCGC TCCCGAATGA AGTGACGGTG GTGCACTGGA TGCACCATAG   
  
  
+ TCTATACGAC ATAACCGGGT CGGATCTTGG GACTTTGAGG GTATTGAGTG CGGTGAGGCC TAGGCTTGTG   
  
  
+ ACTATGGTTG AGCAGGATAT GGACCAAACG GGGTCGTTTT TGGGGAGGTT TGTGGAGGCA TTGCATTATT   
  
  
+ ACTCAGCCTT GTTTGATGCC TTGGGAGAGG GGTTAGATAG GGATAACCTA CAAAGGCATC AAGTGGAGCA   
  
  
+ ACAGCTATTT GGGTGTGAGA TTAGGAACAT CCTGGCCGTT GGTGGGCCCA AGAGGAGGCT CACTGGCGGC   
  
  
+ GATCGGGTCA GAAGGTGGGG CGACGAACTG ACAAGGGTCG GGTTCGAACC AGTTTCGTTG GCGGGTAGCC   
  
  
+ CGGCAACCCA AGCTAGTTTG TTGCTTGGGA TGTTCCCTTG GAAAGGGTAT ACTTTGATGG AGGAAAATGG   
  
  
+ GTGTTTGAGA TTAGGGTGGA AAGATTTGCC CTTGTTAACT GCCTCAGCTT GGCAACCTTG TGAATTTAAC   
  
  
+ AATCCTAGTG CTGGCATTTA   

- +Up\_Stream \_Len000TTTGCC ACGGCAACTT AAACGTCCGC TGCTCCAGCC CAAATTCCGC CTACAGCATC   
  
  
- GCGAAACCTC TTCGTTTAAA CCCTTCTGCT CCGAATAAGA ACTTGAGAAG CATGCGGTAA CAACAACCAA   
  
  
- GGTTGGAAAG AGAGAGGAGC ACTTAAACTC AAACCCGTCA TCTGATAGGG CCTTATCTCC TGACTACGCA   
  
  
- ATATATATAT CTTTAACTCT CTCTCTCTCT CGCACACACT TCCTACAACT TTTACTACCC GCATCCCTCA   
  
  
- GAGAGAGAGT GACAGACAAT CCTCATGCAC AGCTCTTTCC TTTATCCTAA GGGTTCGTCA GATTAAACCC   
  
  
- GGCCAGATAT CCGGTCTTAC CCGGATACTA GTCGGGTCTA TACTGGAACA AATTGGTTGA CATAAACTTA   
  
  
- ACTTCTTTCC CTATAGCGTT ACAGAACTTG GTGTAGTTTA TTATTTACAC GGAAACCAAT CTGGTATACT   
  
  
- TTTTAGAAAC TTAGTTTAGC CAACCTGGAC TTGACGGTAA TAACTATAGA ACAAAAACCA TCTTCGTTTA   
  
  
- CCACAGTGAA GCAAAAGATC TATTTTAATT GAACTCAAAA AAACGGTGAT ATCTATCATA ACGTTTATAT   
  
  
- TTTTCACACG GTGCACTTTA GTTATAATCC TCCCACAAAA TATTGTTATC GACAAGCCCT CTCAAAATCA   
  
  
- ATAAAAATAA TTTAGTTTAT CAATTCTTCA CAAACCATTT ATCAACATAA ACTTTTTCAA TAAGGGTAAT   
  
  
- CGGAAAATCA TTCTTGAAGA ACACTTTTAC TATTAACAGA TCTTCAAACT TTTTAAGTGT GGTGTACTGT   
  
  
- AAATTTTTAT TAAAACATCT CTTCCCGTAT TTACTTTCAA ACGGTGGTAG AAAAAATCTT TGTACATTAA   
  
  
- ATGGTTTGTG AAAAAGATTT TTGTCAATTA AGTTTATTGA TTGTTGATTG TCGATTATAT TTATTGATTG   
  
  
- TCGATAAACG ATTTGTCCCG GATTCATCAA ACTTTAACAA CAAAAGAGAA GTCGAAGTGG TACCGGCTAC   
  
  
- TACATTTGTT GATATAAGAC TTAAAGTAAA AATGATACTA AACTTTTAAA GTTTAAATTT ATTCTAACCT   
  
  
- ATTTTTTTTC TGGTTTTTAG TATTCATTAT AATCAGTTCA AGATAAGGTT GGTATATGTA TTGACGAAAA   
  
  
- AGAAAACCCC CAAAACTTTG TACACAGGAA TCTTATTAAC TTTGAGCTAT AATAAGAGAT TTAGATTCAA   
  
  
- ATGAAATAGT ATAACTAATA ATTAATGGAG GAGTTGAGGA GGATATGAGA ACATACGTCT TTATTTTTAC   
  
  
- AAAGGTATGG AATAGTCAGC GCTAGTATTA TTAATTAAAG AATTAAGCCT AACATAAATT AACGCATAGT   
  
  
- TTACACTTTA GCATGGTTTA ATCCTTCGTT TTGTTTGGCT ATTGCTTTTT CCATTCTTTT GAATCGTTCC   
  
  
- TTTACTTTAG CTTCTGGACA GTTGTTATAT CGAAGGGAGT GTAGGAAGTT GAATCCCTGG TAGGATATGA   
  
  
- AAGTACATTA TGTTGATCTC GGTGATCAGT TATTATCGGA GGATCATCAT CGGTATTCGG AACCACTCTC   
  
  
- TTACAAGGCG TTCTGATCAA TTGAACTTTA AGGTAAAACA GCGTCTTGAT AACCTCACTC TTTCTCTTAG   
  
  
- TTTAGTATGG TATAGTTTAA ACCTGGTAAA TTCAAGTGTT GTGTGTGTGT ATGTGTGTTT TCCTGCTACA   
  
  
- TGCCGGGGTG GGGGGTGAGA GGAAAGCGGT AGGGGGATGT GGAGACGGAT GAGAGAGAGA GAGAGAGAGA   
  
  
- GAGACCTAGT CAGACACATT TTCGAAGGAG AGGTCATGTC GTAAAGGAAC AGACAAGAAC CTCAAATCAG   
  
  
- TTAAGGAGGC GGGGTTTTTT TGGGTGAAAG AGAGAGTAGT ACAAGAAGGT CAAGGTGGGA ATATGCGGTA   
  
  
- AGTAGTAGTA GATGTAGTGG GTGTAGAAAA AGAGGTGTCA AAAGGGGGGA GTAATACGAG AGGCGGAGGT   
  
  
- GGGGGCGGAA GGGAGAGCGG CAGCGGCCGC TACTGCCCCT ATGGTGACTA CGTACGCTGC CGTGGCGGTG   
  
  
- ACGGGAGGTG GAGGAGCCCG ACGACAACTT CACGCGCCTT AAGTAGAGGT GGCCTCTGGA GCGGCCGCGG   
  
  
- CCTCTATAGA ACGGCCTCTA TAAACTCAAC AGATGTGGCA AACCGAGGGG GCGGCGGGCC CAGCGGCGGA   
  
  
- AGAAGCCGGT GCGGGAGGTG CGGGCGGAGG AGAGGCGGAG GGAGGCGTGT TGAGGCTAGC TCTTCGAGTT   
  
  
- TTGGGACTGG GACCAAAGGG TTTACGCCTT TAAAGTGAGG CGGAACGTTC ATATGTTGAG GTAGTGAGGG   
  
  
- AAGCACTTTA AGAGAGTGAA GTGCCGCTTA GTTCGGTAGA TGCTCCGCGA CCTGCCGCTC CTAGCACAGG   
  
  
- TGCAGCAGCT GGAACTGTAG TACGTCCCGG AAGTTACCGG CCCTAACAAG GTGTAGAATC GGAGAGCTGG   
  
  
- GTCTTTCGGG GAGAGCCAAG CCCAGTGGCC CAAGCCCGGT AGGAGGCTCA ACGAGAGGGT TTGCCCATTC   
  
  
- GCTGAGCGAC TCAAGCGGCG AAGTGAGCCG GACGGGAAGC TCATGTTGGG CCACCTCCCG TTTTAACCCT   
  
  
- TGAATCAGCT GGACCCAGCC CAGCCCAGCG AGGGCTTACT TCACTGCCAC CACGTGACCT ACGTGGTATC   
  
  
- AGATATGCTG TATTGGCCCA GCCTAGAACC CTGAAACTCC CATAACTCAC GCCACTCCGG ATCCGAACAC   
  
  
- TGATACCAAC TCGTCCTATA CCTGGTTTGC CCCAGCAAAA ACCCCTCCAA ACACCTCCGT AACGTAATAA   
  
  
- TGAGTCGGAA CAAACTACGG AACCCTCTCC CCAATCTATC CCTATTGGAT GTTTCCGTAG TTCACCTCGT   
  
  
- TGTCGATAAA CCCACACTCT AATCCTTGTA GGACCGGCAA CCACCCGGGT TCTCCTCCGA GTGACCGCCG   
  
  
- CTAGCCCAGT CTTCCACCCC GCTGCTTGAC TGTTCCCAGC CCAAGCTTGG TCAAAGCAAC CGCCCATCGG   
  
  
- GCCGTTGGGT TCGATCAAAC AACGAACCCT ACAAGGGAAC CTTTCCCATA TGAAACTACC TCCTTTTACC   
  
  
- CACAAACTCT AATCCCACCT TTCTAAACGG GAACAATTGA CGGAGTCGAA CCGTTGGAAC ACTTAAATTG   
  
  
- TTAGGATCAC GACCGTAAAT

+     TGA-element

| Site Name | Organism | Position | Strand | Matrix score. | sequence | function |
| --- | --- | --- | --- | --- | --- | --- |
| TGA-element | Brassica oleracea | 2837 | - | 6 | AACGAC | auxin-responsive element |

>HU02G03005.1   
+ +Up\_Stream \_Len000AAACGG TGCCGTTGAA TTTGCAGGCG ACGAGGTCGG GTTTAAGGCG GATGTCGTAG   
  
  
+ CGCTTTGGAG AAGCAAATTT GGGAAGACGA GGCTTATTCT TGAACTCTTC GTACGCCATT GTTGTTGGTT   
  
  
+ CCAACCTTTC TCTCTCCTCG TGAATTTGAG TTTGGGCAGT AGACTATCCC GGAATAGAGG ACTGATGCGT   
  
  
+ TATATATATA GAAATTGAGA GAGAGAGAGA GCGTGTGTGA AGGATGTTGA AAATGATGGG CGTAGGGAGT   
  
  
+ CTCTCTCTCA CTGTCTGTTA GGAGTACGTG TCGAGAAAGG AAATAGGATT CCCAAGCAGT CTAATTTGGG   
  
  
+ CCGGTCTATA GGCCAGAATG GGCCTATGAT CAGCCCAGAT ATGACCTTGT TTAACCAACT GTATTTGAAT   
  
  
+ TGAAGAAAGG GATATCGCAA TGTCTTGAAC CACATCAAAT AATAAATGTG CCTTTGGTTA GACCATATGA   
  
  
+ AAAATCTTTG AATCAAATCG GTTGGACCTG AACTGCCATT ATTGATATCT TGTTTTTGGT AGAAGCAAAT   
  
  
+ GGTGTCACTT CGTTTTCTAG ATAAAATTAA CTTGAGTTTT TTTGCCACTA TAGATAGTAT TGCAAATATA   
  
  
+ AAAAGTGTGC CACGTGAAAT CAATATTAGG AGGGTGTTTT ATAACAATAG CTGTTCGGGA GAGTTTTAGT   
  
  
+ TATTTTTATT AAATCAAATA GTTAAGAAGT GTTTGGTAAA TAGTTGTATT TGAAAAAGTT ATTCCCATTA   
  
  
+ GCCTTTTAGT AAGAACTTCT TGTGAAAATG ATAATTGTCT AGAAGTTTGA AAAATTCACA CCACATGACA   
  
  
+ TTTAAAAATA ATTTTGTAGA GAAGGGCATA AATGAAAGTT TGCCACCATC TTTTTTAGAA ACATGTAATT   
  
  
+ TACCAAACAC TTTTTCTAAA AACAGTTAAT TCAAATAACT AACAACTAAC AGCTAATATA AATAACTAAC   
  
  
+ AGCTATTTGC TAAACAGGGC CTAAGTAGTT TGAAATTGTT GTTTTCTCTT CAGCTTCACC ATGGCCGATG   
  
  
+ ATGTAAACAA CTATATTCTG AATTTCATTT TTACTATGAT TTGAAAATTT CAAATTTAAA TAAGATTGGA   
  
  
+ TAAAAAAAAG ACCAAAAATC ATAAGTAATA TTAGTCAAGT TCTATTCCAA CCATATACAT AACTGCTTTT   
  
  
+ TCTTTTGGGG GTTTTGAAAC ATGTGTCCTT AGAATAATTG AAACTCGATA TTATTCTCTA AATCTAAGTT   
  
  
+ TACTTTATCA TATTGATTAT TAATTACCTC CTCAACTCCT CCTATACTCT TGTATGCAGA AATAAAAATG   
  
  
+ TTTCCATACC TTATCAGTCG CGATCATAAT AATTAATTTC TTAATTCGGA TTGTATTTAA TTGCGTATCA   
  
  
+ AATGTGAAAT CGTACCAAAT TAGGAAGCAA AACAAACCGA TAACGAAAAA GGTAAGAAAA CTTAGCAAGG   
  
  
+ AAATGAAATC GAAGACCTGT CAACAATATA GCTTCCCTCA CATCCTTCAA CTTAGGGACC ATCCTATACT   
  
  
+ TTCATGTAAT ACAACTAGAG CCACTAGTCA ATAATAGCCT CCTAGTAGTA GCCATAAGCC TTGGTGAGAG   
  
  
+ AATGTTCCGC AAGACTAGTT AACTTGAAAT TCCATTTTGT CGCAGAACTA TTGGAGTGAG AAAGAGAATC   
  
  
+ AAATCATACC ATATCAAATT TGGACCATTT AAGTTCACAA CACACACACA TACACACAAA AGGACGATGT   
  
  
+ ACGGCCCCAC CCCCCACTCT CCTTTCGCCA TCCCCCTACA CCTCTGCCTA CTCTCTCTCT CTCTCTCTCT   
  
  
+ CTCTGGATCA GTCTGTGTAA AAGCTTCCTC TCCAGTACAG CATTTCCTTG TCTGTTCTTG GAGTTTAGTC   
  
  
+ AATTCCTCCG CCCCAAAAAA ACCCACTTTC TCTCTCATCA TGTTCTTCCA GTTCCACCCT TATACGCCAT   
  
  
+ TCATCATCAT CTACATCACC CACATCTTTT TCTCCACAGT TTTCCCCCCT CATTATGCTC TCCGCCTCCA   
  
  
+ CCCCCGCCTT CCCTCTCGCC GTCGCCGGCG ATGACGGGGA TACCACTGAT GCATGCGACG GCACCGCCAC   
  
  
+ TGCCCTCCAC CTCCTCGGGC TGCTGTTGAA GTGCGCGGAA TTCATCTCCA CCGGAGACCT CGCCGGCGCC   
  
  
+ GGAGATATCT TGCCGGAGAT ATTTGAGTTG TCTACACCGT TTGGCTCCCC CGCCGCCCGG GTCGCCGCCT   
  
  
+ TCTTCGGCCA CGCCCTCCAC GCCCGCCTCC TCTCCGCCTC CCTCCGCACA ACTCCGATCG AGAAGCTCAA   
  
  
+ AACCCTGACC CTGGTTTCCC AAATGCGGAA ATTTCACTCC GCCTTGCAAG TATACAACTC CATCACTCCC   
  
  
+ TTCGTGAAAT TCTCTCACTT CACGGCGAAT CAAGCCATCT ACGAGGCGCT GGACGGCGAG GATCGTGTCC   
  
  
+ ACGTCGTCGA CCTTGACATC ATGCAGGGCC TTCAATGGCC GGGATTGTTC CACATCTTAG CCTCTCGACC   
  
  
+ CAGAAAGCCC CTCTCGGTTC GGGTCACCGG GTTCGGGCCA TCCTCCGAGT TGCTCTCCCA AACGGGTAAG   
  
  
+ CGACTCGCTG AGTTCGCCGC TTCACTCGGC CTGCCCTTCG AGTACAACCC GGTGGAGGGC AAAATTGGGA   
  
  
+ ACTTAGTCGA CCTGGGTCGG GTCGGGTCGC TCCCGAATGA AGTGACGGTG GTGCACTGGA TGCACCATAG   
  
  
+ TCTATACGAC ATAACCGGGT CGGATCTTGG GACTTTGAGG GTATTGAGTG CGGTGAGGCC TAGGCTTGTG   
  
  
+ ACTATGGTTG AGCAGGATAT GGACCAAACG GGGTCGTTTT TGGGGAGGTT TGTGGAGGCA TTGCATTATT   
  
  
+ ACTCAGCCTT GTTTGATGCC TTGGGAGAGG GGTTAGATAG GGATAACCTA CAAAGGCATC AAGTGGAGCA   
  
  
+ ACAGCTATTT GGGTGTGAGA TTAGGAACAT CCTGGCCGTT GGTGGGCCCA AGAGGAGGCT CACTGGCGGC   
  
  
+ GATCGGGTCA GAAGGTGGGG CGACGAACTG ACAAGGGTCG GGTTCGAACC AGTTTCGTTG GCGGGTAGCC   
  
  
+ CGGCAACCCA AGCTAGTTTG TTGCTTGGGA TGTTCCCTTG GAAAGGGTAT ACTTTGATGG AGGAAAATGG   
  
  
+ GTGTTTGAGA TTAGGGTGGA AAGATTTGCC CTTGTTAACT GCCTCAGCTT GGCAACCTTG TGAATTTAAC   
  
  
+ AATCCTAGTG CTGGCATTTA   

- +Up\_Stream \_Len000TTTGCC ACGGCAACTT AAACGTCCGC TGCTCCAGCC CAAATTCCGC CTACAGCATC   
  
  
- GCGAAACCTC TTCGTTTAAA CCCTTCTGCT CCGAATAAGA ACTTGAGAAG CATGCGGTAA CAACAACCAA   
  
  
- GGTTGGAAAG AGAGAGGAGC ACTTAAACTC AAACCCGTCA TCTGATAGGG CCTTATCTCC TGACTACGCA   
  
  
- ATATATATAT CTTTAACTCT CTCTCTCTCT CGCACACACT TCCTACAACT TTTACTACCC GCATCCCTCA   
  
  
- GAGAGAGAGT GACAGACAAT CCTCATGCAC AGCTCTTTCC TTTATCCTAA GGGTTCGTCA GATTAAACCC   
  
  
- GGCCAGATAT CCGGTCTTAC CCGGATACTA GTCGGGTCTA TACTGGAACA AATTGGTTGA CATAAACTTA   
  
  
- ACTTCTTTCC CTATAGCGTT ACAGAACTTG GTGTAGTTTA TTATTTACAC GGAAACCAAT CTGGTATACT   
  
  
- TTTTAGAAAC TTAGTTTAGC CAACCTGGAC TTGACGGTAA TAACTATAGA ACAAAAACCA TCTTCGTTTA   
  
  
- CCACAGTGAA GCAAAAGATC TATTTTAATT GAACTCAAAA AAACGGTGAT ATCTATCATA ACGTTTATAT   
  
  
- TTTTCACACG GTGCACTTTA GTTATAATCC TCCCACAAAA TATTGTTATC GACAAGCCCT CTCAAAATCA   
  
  
- ATAAAAATAA TTTAGTTTAT CAATTCTTCA CAAACCATTT ATCAACATAA ACTTTTTCAA TAAGGGTAAT   
  
  
- CGGAAAATCA TTCTTGAAGA ACACTTTTAC TATTAACAGA TCTTCAAACT TTTTAAGTGT GGTGTACTGT   
  
  
- AAATTTTTAT TAAAACATCT CTTCCCGTAT TTACTTTCAA ACGGTGGTAG AAAAAATCTT TGTACATTAA   
  
  
- ATGGTTTGTG AAAAAGATTT TTGTCAATTA AGTTTATTGA TTGTTGATTG TCGATTATAT TTATTGATTG   
  
  
- TCGATAAACG ATTTGTCCCG GATTCATCAA ACTTTAACAA CAAAAGAGAA GTCGAAGTGG TACCGGCTAC   
  
  
- TACATTTGTT GATATAAGAC TTAAAGTAAA AATGATACTA AACTTTTAAA GTTTAAATTT ATTCTAACCT   
  
  
- ATTTTTTTTC TGGTTTTTAG TATTCATTAT AATCAGTTCA AGATAAGGTT GGTATATGTA TTGACGAAAA   
  
  
- AGAAAACCCC CAAAACTTTG TACACAGGAA TCTTATTAAC TTTGAGCTAT AATAAGAGAT TTAGATTCAA   
  
  
- ATGAAATAGT ATAACTAATA ATTAATGGAG GAGTTGAGGA GGATATGAGA ACATACGTCT TTATTTTTAC   
  
  
- AAAGGTATGG AATAGTCAGC GCTAGTATTA TTAATTAAAG AATTAAGCCT AACATAAATT AACGCATAGT   
  
  
- TTACACTTTA GCATGGTTTA ATCCTTCGTT TTGTTTGGCT ATTGCTTTTT CCATTCTTTT GAATCGTTCC   
  
  
- TTTACTTTAG CTTCTGGACA GTTGTTATAT CGAAGGGAGT GTAGGAAGTT GAATCCCTGG TAGGATATGA   
  
  
- AAGTACATTA TGTTGATCTC GGTGATCAGT TATTATCGGA GGATCATCAT CGGTATTCGG AACCACTCTC   
  
  
- TTACAAGGCG TTCTGATCAA TTGAACTTTA AGGTAAAACA GCGTCTTGAT AACCTCACTC TTTCTCTTAG   
  
  
- TTTAGTATGG TATAGTTTAA ACCTGGTAAA TTCAAGTGTT GTGTGTGTGT ATGTGTGTTT TCCTGCTACA   
  
  
- TGCCGGGGTG GGGGGTGAGA GGAAAGCGGT AGGGGGATGT GGAGACGGAT GAGAGAGAGA GAGAGAGAGA   
  
  
- GAGACCTAGT CAGACACATT TTCGAAGGAG AGGTCATGTC GTAAAGGAAC AGACAAGAAC CTCAAATCAG   
  
  
- TTAAGGAGGC GGGGTTTTTT TGGGTGAAAG AGAGAGTAGT ACAAGAAGGT CAAGGTGGGA ATATGCGGTA   
  
  
- AGTAGTAGTA GATGTAGTGG GTGTAGAAAA AGAGGTGTCA AAAGGGGGGA GTAATACGAG AGGCGGAGGT   
  
  
- GGGGGCGGAA GGGAGAGCGG CAGCGGCCGC TACTGCCCCT ATGGTGACTA CGTACGCTGC CGTGGCGGTG   
  
  
- ACGGGAGGTG GAGGAGCCCG ACGACAACTT CACGCGCCTT AAGTAGAGGT GGCCTCTGGA GCGGCCGCGG   
  
  
- CCTCTATAGA ACGGCCTCTA TAAACTCAAC AGATGTGGCA AACCGAGGGG GCGGCGGGCC CAGCGGCGGA   
  
  
- AGAAGCCGGT GCGGGAGGTG CGGGCGGAGG AGAGGCGGAG GGAGGCGTGT TGAGGCTAGC TCTTCGAGTT   
  
  
- TTGGGACTGG GACCAAAGGG TTTACGCCTT TAAAGTGAGG CGGAACGTTC ATATGTTGAG GTAGTGAGGG   
  
  
- AAGCACTTTA AGAGAGTGAA GTGCCGCTTA GTTCGGTAGA TGCTCCGCGA CCTGCCGCTC CTAGCACAGG   
  
  
- TGCAGCAGCT GGAACTGTAG TACGTCCCGG AAGTTACCGG CCCTAACAAG GTGTAGAATC GGAGAGCTGG   
  
  
- GTCTTTCGGG GAGAGCCAAG CCCAGTGGCC CAAGCCCGGT AGGAGGCTCA ACGAGAGGGT TTGCCCATTC   
  
  
- GCTGAGCGAC TCAAGCGGCG AAGTGAGCCG GACGGGAAGC TCATGTTGGG CCACCTCCCG TTTTAACCCT   
  
  
- TGAATCAGCT GGACCCAGCC CAGCCCAGCG AGGGCTTACT TCACTGCCAC CACGTGACCT ACGTGGTATC   
  
  
- AGATATGCTG TATTGGCCCA GCCTAGAACC CTGAAACTCC CATAACTCAC GCCACTCCGG ATCCGAACAC   
  
  
- TGATACCAAC TCGTCCTATA CCTGGTTTGC CCCAGCAAAA ACCCCTCCAA ACACCTCCGT AACGTAATAA   
  
  
- TGAGTCGGAA CAAACTACGG AACCCTCTCC CCAATCTATC CCTATTGGAT GTTTCCGTAG TTCACCTCGT   
  
  
- TGTCGATAAA CCCACACTCT AATCCTTGTA GGACCGGCAA CCACCCGGGT TCTCCTCCGA GTGACCGCCG   
  
  
- CTAGCCCAGT CTTCCACCCC GCTGCTTGAC TGTTCCCAGC CCAAGCTTGG TCAAAGCAAC CGCCCATCGG   
  
  
- GCCGTTGGGT TCGATCAAAC AACGAACCCT ACAAGGGAAC CTTTCCCATA TGAAACTACC TCCTTTTACC   
  
  
- CACAAACTCT AATCCCACCT TTCTAAACGG GAACAATTGA CGGAGTCGAA CCGTTGGAAC ACTTAAATTG   
  
  
- TTAGGATCAC GACCGTAAAT

+     TGACG-motif

| Site Name | Organism | Position | Strand | Matrix score. | sequence | function |
| --- | --- | --- | --- | --- | --- | --- |
| TGACG-motif | Hordeum vulgare | 2707 | + | 5 | TGACG | cis-acting regulatory element involved in the MeJA-responsiveness |
| TGACG-motif | Hordeum vulgare | 2066 | + | 5 | TGACG | cis-acting regulatory element involved in the MeJA-responsiveness |

>HU02G03005.1   
+ +Up\_Stream \_Len000AAACGG TGCCGTTGAA TTTGCAGGCG ACGAGGTCGG GTTTAAGGCG GATGTCGTAG   
  
  
+ CGCTTTGGAG AAGCAAATTT GGGAAGACGA GGCTTATTCT TGAACTCTTC GTACGCCATT GTTGTTGGTT   
  
  
+ CCAACCTTTC TCTCTCCTCG TGAATTTGAG TTTGGGCAGT AGACTATCCC GGAATAGAGG ACTGATGCGT   
  
  
+ TATATATATA GAAATTGAGA GAGAGAGAGA GCGTGTGTGA AGGATGTTGA AAATGATGGG CGTAGGGAGT   
  
  
+ CTCTCTCTCA CTGTCTGTTA GGAGTACGTG TCGAGAAAGG AAATAGGATT CCCAAGCAGT CTAATTTGGG   
  
  
+ CCGGTCTATA GGCCAGAATG GGCCTATGAT CAGCCCAGAT ATGACCTTGT TTAACCAACT GTATTTGAAT   
  
  
+ TGAAGAAAGG GATATCGCAA TGTCTTGAAC CACATCAAAT AATAAATGTG CCTTTGGTTA GACCATATGA   
  
  
+ AAAATCTTTG AATCAAATCG GTTGGACCTG AACTGCCATT ATTGATATCT TGTTTTTGGT AGAAGCAAAT   
  
  
+ GGTGTCACTT CGTTTTCTAG ATAAAATTAA CTTGAGTTTT TTTGCCACTA TAGATAGTAT TGCAAATATA   
  
  
+ AAAAGTGTGC CACGTGAAAT CAATATTAGG AGGGTGTTTT ATAACAATAG CTGTTCGGGA GAGTTTTAGT   
  
  
+ TATTTTTATT AAATCAAATA GTTAAGAAGT GTTTGGTAAA TAGTTGTATT TGAAAAAGTT ATTCCCATTA   
  
  
+ GCCTTTTAGT AAGAACTTCT TGTGAAAATG ATAATTGTCT AGAAGTTTGA AAAATTCACA CCACATGACA   
  
  
+ TTTAAAAATA ATTTTGTAGA GAAGGGCATA AATGAAAGTT TGCCACCATC TTTTTTAGAA ACATGTAATT   
  
  
+ TACCAAACAC TTTTTCTAAA AACAGTTAAT TCAAATAACT AACAACTAAC AGCTAATATA AATAACTAAC   
  
  
+ AGCTATTTGC TAAACAGGGC CTAAGTAGTT TGAAATTGTT GTTTTCTCTT CAGCTTCACC ATGGCCGATG   
  
  
+ ATGTAAACAA CTATATTCTG AATTTCATTT TTACTATGAT TTGAAAATTT CAAATTTAAA TAAGATTGGA   
  
  
+ TAAAAAAAAG ACCAAAAATC ATAAGTAATA TTAGTCAAGT TCTATTCCAA CCATATACAT AACTGCTTTT   
  
  
+ TCTTTTGGGG GTTTTGAAAC ATGTGTCCTT AGAATAATTG AAACTCGATA TTATTCTCTA AATCTAAGTT   
  
  
+ TACTTTATCA TATTGATTAT TAATTACCTC CTCAACTCCT CCTATACTCT TGTATGCAGA AATAAAAATG   
  
  
+ TTTCCATACC TTATCAGTCG CGATCATAAT AATTAATTTC TTAATTCGGA TTGTATTTAA TTGCGTATCA   
  
  
+ AATGTGAAAT CGTACCAAAT TAGGAAGCAA AACAAACCGA TAACGAAAAA GGTAAGAAAA CTTAGCAAGG   
  
  
+ AAATGAAATC GAAGACCTGT CAACAATATA GCTTCCCTCA CATCCTTCAA CTTAGGGACC ATCCTATACT   
  
  
+ TTCATGTAAT ACAACTAGAG CCACTAGTCA ATAATAGCCT CCTAGTAGTA GCCATAAGCC TTGGTGAGAG   
  
  
+ AATGTTCCGC AAGACTAGTT AACTTGAAAT TCCATTTTGT CGCAGAACTA TTGGAGTGAG AAAGAGAATC   
  
  
+ AAATCATACC ATATCAAATT TGGACCATTT AAGTTCACAA CACACACACA TACACACAAA AGGACGATGT   
  
  
+ ACGGCCCCAC CCCCCACTCT CCTTTCGCCA TCCCCCTACA CCTCTGCCTA CTCTCTCTCT CTCTCTCTCT   
  
  
+ CTCTGGATCA GTCTGTGTAA AAGCTTCCTC TCCAGTACAG CATTTCCTTG TCTGTTCTTG GAGTTTAGTC   
  
  
+ AATTCCTCCG CCCCAAAAAA ACCCACTTTC TCTCTCATCA TGTTCTTCCA GTTCCACCCT TATACGCCAT   
  
  
+ TCATCATCAT CTACATCACC CACATCTTTT TCTCCACAGT TTTCCCCCCT CATTATGCTC TCCGCCTCCA   
  
  
+ CCCCCGCCTT CCCTCTCGCC GTCGCCGGCG ATGACGGGGA TACCACTGAT GCATGCGACG GCACCGCCAC   
  
  
+ TGCCCTCCAC CTCCTCGGGC TGCTGTTGAA GTGCGCGGAA TTCATCTCCA CCGGAGACCT CGCCGGCGCC   
  
  
+ GGAGATATCT TGCCGGAGAT ATTTGAGTTG TCTACACCGT TTGGCTCCCC CGCCGCCCGG GTCGCCGCCT   
  
  
+ TCTTCGGCCA CGCCCTCCAC GCCCGCCTCC TCTCCGCCTC CCTCCGCACA ACTCCGATCG AGAAGCTCAA   
  
  
+ AACCCTGACC CTGGTTTCCC AAATGCGGAA ATTTCACTCC GCCTTGCAAG TATACAACTC CATCACTCCC   
  
  
+ TTCGTGAAAT TCTCTCACTT CACGGCGAAT CAAGCCATCT ACGAGGCGCT GGACGGCGAG GATCGTGTCC   
  
  
+ ACGTCGTCGA CCTTGACATC ATGCAGGGCC TTCAATGGCC GGGATTGTTC CACATCTTAG CCTCTCGACC   
  
  
+ CAGAAAGCCC CTCTCGGTTC GGGTCACCGG GTTCGGGCCA TCCTCCGAGT TGCTCTCCCA AACGGGTAAG   
  
  
+ CGACTCGCTG AGTTCGCCGC TTCACTCGGC CTGCCCTTCG AGTACAACCC GGTGGAGGGC AAAATTGGGA   
  
  
+ ACTTAGTCGA CCTGGGTCGG GTCGGGTCGC TCCCGAATGA AGTGACGGTG GTGCACTGGA TGCACCATAG   
  
  
+ TCTATACGAC ATAACCGGGT CGGATCTTGG GACTTTGAGG GTATTGAGTG CGGTGAGGCC TAGGCTTGTG   
  
  
+ ACTATGGTTG AGCAGGATAT GGACCAAACG GGGTCGTTTT TGGGGAGGTT TGTGGAGGCA TTGCATTATT   
  
  
+ ACTCAGCCTT GTTTGATGCC TTGGGAGAGG GGTTAGATAG GGATAACCTA CAAAGGCATC AAGTGGAGCA   
  
  
+ ACAGCTATTT GGGTGTGAGA TTAGGAACAT CCTGGCCGTT GGTGGGCCCA AGAGGAGGCT CACTGGCGGC   
  
  
+ GATCGGGTCA GAAGGTGGGG CGACGAACTG ACAAGGGTCG GGTTCGAACC AGTTTCGTTG GCGGGTAGCC   
  
  
+ CGGCAACCCA AGCTAGTTTG TTGCTTGGGA TGTTCCCTTG GAAAGGGTAT ACTTTGATGG AGGAAAATGG   
  
  
+ GTGTTTGAGA TTAGGGTGGA AAGATTTGCC CTTGTTAACT GCCTCAGCTT GGCAACCTTG TGAATTTAAC   
  
  
+ AATCCTAGTG CTGGCATTTA   

- +Up\_Stream \_Len000TTTGCC ACGGCAACTT AAACGTCCGC TGCTCCAGCC CAAATTCCGC CTACAGCATC   
  
  
- GCGAAACCTC TTCGTTTAAA CCCTTCTGCT CCGAATAAGA ACTTGAGAAG CATGCGGTAA CAACAACCAA   
  
  
- GGTTGGAAAG AGAGAGGAGC ACTTAAACTC AAACCCGTCA TCTGATAGGG CCTTATCTCC TGACTACGCA   
  
  
- ATATATATAT CTTTAACTCT CTCTCTCTCT CGCACACACT TCCTACAACT TTTACTACCC GCATCCCTCA   
  
  
- GAGAGAGAGT GACAGACAAT CCTCATGCAC AGCTCTTTCC TTTATCCTAA GGGTTCGTCA GATTAAACCC   
  
  
- GGCCAGATAT CCGGTCTTAC CCGGATACTA GTCGGGTCTA TACTGGAACA AATTGGTTGA CATAAACTTA   
  
  
- ACTTCTTTCC CTATAGCGTT ACAGAACTTG GTGTAGTTTA TTATTTACAC GGAAACCAAT CTGGTATACT   
  
  
- TTTTAGAAAC TTAGTTTAGC CAACCTGGAC TTGACGGTAA TAACTATAGA ACAAAAACCA TCTTCGTTTA   
  
  
- CCACAGTGAA GCAAAAGATC TATTTTAATT GAACTCAAAA AAACGGTGAT ATCTATCATA ACGTTTATAT   
  
  
- TTTTCACACG GTGCACTTTA GTTATAATCC TCCCACAAAA TATTGTTATC GACAAGCCCT CTCAAAATCA   
  
  
- ATAAAAATAA TTTAGTTTAT CAATTCTTCA CAAACCATTT ATCAACATAA ACTTTTTCAA TAAGGGTAAT   
  
  
- CGGAAAATCA TTCTTGAAGA ACACTTTTAC TATTAACAGA TCTTCAAACT TTTTAAGTGT GGTGTACTGT   
  
  
- AAATTTTTAT TAAAACATCT CTTCCCGTAT TTACTTTCAA ACGGTGGTAG AAAAAATCTT TGTACATTAA   
  
  
- ATGGTTTGTG AAAAAGATTT TTGTCAATTA AGTTTATTGA TTGTTGATTG TCGATTATAT TTATTGATTG   
  
  
- TCGATAAACG ATTTGTCCCG GATTCATCAA ACTTTAACAA CAAAAGAGAA GTCGAAGTGG TACCGGCTAC   
  
  
- TACATTTGTT GATATAAGAC TTAAAGTAAA AATGATACTA AACTTTTAAA GTTTAAATTT ATTCTAACCT   
  
  
- ATTTTTTTTC TGGTTTTTAG TATTCATTAT AATCAGTTCA AGATAAGGTT GGTATATGTA TTGACGAAAA   
  
  
- AGAAAACCCC CAAAACTTTG TACACAGGAA TCTTATTAAC TTTGAGCTAT AATAAGAGAT TTAGATTCAA   
  
  
- ATGAAATAGT ATAACTAATA ATTAATGGAG GAGTTGAGGA GGATATGAGA ACATACGTCT TTATTTTTAC   
  
  
- AAAGGTATGG AATAGTCAGC GCTAGTATTA TTAATTAAAG AATTAAGCCT AACATAAATT AACGCATAGT   
  
  
- TTACACTTTA GCATGGTTTA ATCCTTCGTT TTGTTTGGCT ATTGCTTTTT CCATTCTTTT GAATCGTTCC   
  
  
- TTTACTTTAG CTTCTGGACA GTTGTTATAT CGAAGGGAGT GTAGGAAGTT GAATCCCTGG TAGGATATGA   
  
  
- AAGTACATTA TGTTGATCTC GGTGATCAGT TATTATCGGA GGATCATCAT CGGTATTCGG AACCACTCTC   
  
  
- TTACAAGGCG TTCTGATCAA TTGAACTTTA AGGTAAAACA GCGTCTTGAT AACCTCACTC TTTCTCTTAG   
  
  
- TTTAGTATGG TATAGTTTAA ACCTGGTAAA TTCAAGTGTT GTGTGTGTGT ATGTGTGTTT TCCTGCTACA   
  
  
- TGCCGGGGTG GGGGGTGAGA GGAAAGCGGT AGGGGGATGT GGAGACGGAT GAGAGAGAGA GAGAGAGAGA   
  
  
- GAGACCTAGT CAGACACATT TTCGAAGGAG AGGTCATGTC GTAAAGGAAC AGACAAGAAC CTCAAATCAG   
  
  
- TTAAGGAGGC GGGGTTTTTT TGGGTGAAAG AGAGAGTAGT ACAAGAAGGT CAAGGTGGGA ATATGCGGTA   
  
  
- AGTAGTAGTA GATGTAGTGG GTGTAGAAAA AGAGGTGTCA AAAGGGGGGA GTAATACGAG AGGCGGAGGT   
  
  
- GGGGGCGGAA GGGAGAGCGG CAGCGGCCGC TACTGCCCCT ATGGTGACTA CGTACGCTGC CGTGGCGGTG   
  
  
- ACGGGAGGTG GAGGAGCCCG ACGACAACTT CACGCGCCTT AAGTAGAGGT GGCCTCTGGA GCGGCCGCGG   
  
  
- CCTCTATAGA ACGGCCTCTA TAAACTCAAC AGATGTGGCA AACCGAGGGG GCGGCGGGCC CAGCGGCGGA   
  
  
- AGAAGCCGGT GCGGGAGGTG CGGGCGGAGG AGAGGCGGAG GGAGGCGTGT TGAGGCTAGC TCTTCGAGTT   
  
  
- TTGGGACTGG GACCAAAGGG TTTACGCCTT TAAAGTGAGG CGGAACGTTC ATATGTTGAG GTAGTGAGGG   
  
  
- AAGCACTTTA AGAGAGTGAA GTGCCGCTTA GTTCGGTAGA TGCTCCGCGA CCTGCCGCTC CTAGCACAGG   
  
  
- TGCAGCAGCT GGAACTGTAG TACGTCCCGG AAGTTACCGG CCCTAACAAG GTGTAGAATC GGAGAGCTGG   
  
  
- GTCTTTCGGG GAGAGCCAAG CCCAGTGGCC CAAGCCCGGT AGGAGGCTCA ACGAGAGGGT TTGCCCATTC   
  
  
- GCTGAGCGAC TCAAGCGGCG AAGTGAGCCG GACGGGAAGC TCATGTTGGG CCACCTCCCG TTTTAACCCT   
  
  
- TGAATCAGCT GGACCCAGCC CAGCCCAGCG AGGGCTTACT TCACTGCCAC CACGTGACCT ACGTGGTATC
[truncated: 45,636 more chars]
