# Supplementary material for: Genome-Wide Identification and Expression Pattern of the GRAS Gene Family in Pitaya (Selenicereus undatus L.)
Source: Biology (Basel). 2022 Dec 21;12(1):11. doi: 10.3390/biology12010011 (PMC9854919; doi:10.3390/biology12010011)
Supplement: Supplementary file 1 [file biology-12-00011-s001.zip › Supplementary file S5/HU02G03154.1_plantcare.html]

Content-Type: text/html; charset=ISO-8859-1


PlantCARE


Webmaster Firefox specific output  
To save the result:
click on the frame with the right mouse button and save the source code as a text file with extension .html  
REFERENCE:PlantCARE: a database of plant cis-acting regulatory elements and a portal to tools for in silico analysis of promoter sequences.  
Lescot, M., Déhais, P., Moreau, Y., De Moor, B., Rouzé ,P.,and Rombauts, S.  
Nucleic Acids Res., Database issue(2002), 30(1):325-327.   


---

>HU02G03154.1   
+ +Up\_Stream \_Len000ATGATA AAAGATATTT ATTAAGCTCA AGCTATTTTA ACTTTTAAAA AAATGAATGT   
  
  
+ GGCTTTTTTT GCTATTCAGG TACAACACAA TATGAAGCAT TAACTCTAGA AAATGTGGTA ATGTATGTCA   
  
  
+ TATAGGAGAG TATATACTCT CACTTGCCAT GCGATGCGTG CATGTGTGTG TGTGTGTGTA TCTGTTTCTA   
  
  
+ TGCATATTTT ATGTGGGGTT TTGTCATCTA GACATCTATT AATATGTTAG CATTCACATG ACTTATAGGA   
  
  
+ ATGCACTATT ATGAGTATGA TATAGAGTTT TAAAAATGCA TATGCCTTGT GTGTTCATTA GCTTAAAGAA   
  
  
+ GAGTTTTTGA ATATACATAT TTAGGTAATT CTTGCTAATG TCGTAATTAG CAGTTGTATC TATCTTATTG   
  
  
+ ATTTGTATTT TTAATTGCTT GTTATACTGT TACACTTAGA AAATTCATGA ATACTCTTAA AATATGCAAA   
  
  
+ TTATATATAT ATATATATAT ATATATATAT ATATATATAT ATAAAAACTG AATAAGTTAA TATGGTGATG   
  
  
+ ACATTTGTCA TGTTATCATT GGTCGCTTAA TTTTTAAATA AAAAATATGA TTTTGTGACA CCTAATAAAT   
  
  
+ GATATTAGCC ATATTATTAA ATTCAGCATT TATTCATCAA TGTAATATAC TAAATCATGC AATTATCATT   
  
  
+ GGTTCTCTTT TATGTAATAA AGTTAATTGA CAATTCAAGG GTTACTAATT AGTATAATGT TGAATTGGAA   
  
  
+ CGTAAAAATC AAATCGTATG TAAAGTAATT TTATTCTGAA TTTAAAAAAT GGAGCAAATA TCTCTATTGT   
  
  
+ TCTTTAATAA AATTTTAATT GATTTTTATT TCTAATTAAG TGACGGAACC AAATTTTTTT CCTCCTTTTT   
  
  
+ TGGTTTGGTA AGTTATCATT AGCCAATCTT TTATTTACAT TTTGTCAGTT TTTCTTTTTT AAGATGGTTA   
  
  
+ AATGGCTTCA ATTAAACAAA TTTTTTTACT TATTTATATT TTTAATAAAT TTTCAAAATG TATATTAACG   
  
  
+ CATGACCGTG CGAAGCACGG AATCTACCCT AGTTAATCTA TGATTCTTAA ATATGCAAGT AGGCTGTTAT   
  
  
+ TCATTTGTTG TTTTTTACTT ATTTATATTT TTTCCCTCCC GGTATGATAG GGTTTGTCAT ATTGTTATTC   
  
  
+ ATTAGCTGTT AAACTGTATT CATTATAGCA CAAGTAGGCT TAATCAAAGA TAGTTAGTCT CCAATTTCAT   
  
  
+ CAGAAAATTT AAGAAAAAGA CACCTTAGGA TCAAAAGTAT GAAATTTAGA GACCAGACAA CGATAGAATT   
  
  
+ GAAAAGATGA GTTTTCAATT TGGAGAAGAC GGAAACTCAG ATGAATGAAA GTATAGTACA TTGTACTCCT   
  
  
+ TGGAGTATAA TCTTATCTTC ACTATTGATA GGCTCGAAGC ATAATCAGTG ATTGGAAACT TTTGGTTGCA   
  
  
+ ATAATTCAAA ATTTGATACT CTATGATTTA CTTTTTTAAT TGAAGGTCAT GATTTAATTA ACTAAATGAA   
  
  
+ AAGAATTTAT GATCGAAAAA TTTTAACTCT TATTGACTTA TAGTTGACTC CAGTTAAATG GAAAATTGAA   
  
  
+ GTGATTAATC TAAAAGTGGC CCGCTCTAAT ACCATTTGAG AAAAATTATT CTTACTGCAA AGTTTAAATC   
  
  
+ AATATGTGAA GATAGTTTAT AGTTTTATCC TAAAAGAAAT TAAATAGATT GTTAAAAATA ATTTTTAAAT   
  
  
+ TCAGATATAC GTGTGATGGC CATAACCGTA AATGATATGC ATGTCGACAA ATTCCAAAGA AGGCTAATAA   
  
  
+ TTAGACTTAA ATAATACATG CATACTAACC AAAAAAAAAA AATTAAGCAA GATTCTATCC AACCTATCAT   
  
  
+ AATAAAGTTT GGCCATACTT CAAACCATCA CATAACATCA CTAATATATA AAGCTAGGTT TTGGGAAACC   
  
  
+ TAACTTTGAG GACCACATAT TTGTTAAGGC CTAGGAGGCC ATGAGTTTGT CAAGATGCCT CATAGTATGG   
  
  
+ AGAGGTCTAT ATCCTTGCAA AATGATCATT CCCTTCTACC TAAAAGAACC ACTTCATTTC CACTTTTACT   
  
  
+ CCCTGGCAAA TGGTTGATAA ACCAAACCAT AACCAAGCCC TTGAAGAACT ATAAAGACAC GAATCGATCA   
  
  
+ TTGAATTCGA ACAAGCCTTG TGTGATAGGA GGATGGGCCT CAACACTTCT CCGCGAGTGT GCAAGAGCAA   
  
  
+ TCTCGGAGAA AAATCCTAAG AGCCAACAAC TTCTTTGGGT GTTAAATGAA CTTGTTTCTC CTTATGGCGA   
  
  
+ TTGCGAACAA AGATTGGCAT ATTACTTCTT ACAAGTGTTG TTGGCCAAAG CCAACAATTT GGGACCTCAC   
  
  
+ TTTCATGAGA GCCTAAAACT TGCCATGGAG AAAAACTGCT GCTTTGATAC CTACATGAAG CTTATATTGA   
  
  
+ AGTTCCAAGA GGTCAGTCCA TGGACAACCT TTGGTCATGT GGCTTCAAAT GGTGCAATAT TGGAGGACCT   
  
  
+ TAGAAGTTTA CAAAAGTCGA TCATCAAAGA AACAAGGCAA AGGATGGAGA AGTTTTCAAG GCTAATGGGT   
  
  
+ GTTCCCTTCA AGTTTCATGT CATAAACGAG TTAGATAACC TAGGAGAGCT TCGAAAAGAG GATTTAGACA   
  
  
+ TTGAAGATGG TGAGGCCATC GCTGTGAACT GTGTTCAAGC CTTGCAACGG GTTCATGTGG AGAAGAGGGA   
  
  
+ GCATGTGCTT GATGTGATTC GATCTATTAG GCCTTGTATC ATAACACTGG TGGAGGAAGA AGCAGATCTC   
  
  
+ ACTTCTACAA GAAACGACTT CTTCAAGTGC TTCGATGAGT GTTTGAGATT TTCTAAGTCA TATTTCGATA   
  
  
+ TGTTAGAAGA AAGCTTCCCT CCAATAAGCA ACGAACGAAT CAAGCTAGAA AGGGAACAAT GGATGAATAT   
  
  
+ CTCCAGAGCC CTAGCTTGTC ATGGTGAAAG TGGAGGAGAA TATAGGCCAA AGAAAGGAAC TCAATGGAAT   
  
  
+ GAGATGCTCG AACAAGCATT TTGCCCATCT CAATTTAGTG ATGATGTACT AAGTGATGTT AGGGCATTGT   
  
  
+ TGAAAAGACA CAAAAGTGGT TGGGATCTCA CCTTACCACA AAGTGACCAT GAAATAGGCA TACACTTAAA   
  
  
+ TTGGAAGGGT GAAAATGTTG TTTGGGCTTC TGCATGGAGA CCTAGCTA  

- +Up\_Stream \_Len000TACTAT TTTCTATAAA TAATTCGAGT TCGATAAAAT TGAAAATTTT TTTACTTACA   
  
  
- CCGAAAAAAA CGATAAGTCC ATGTTGTGTT ATACTTCGTA ATTGAGATCT TTTACACCAT TACATACAGT   
  
  
- ATATCCTCTC ATATATGAGA GTGAACGGTA CGCTACGCAC GTACACACAC ACACACACAT AGACAAAGAT   
  
  
- ACGTATAAAA TACACCCCAA AACAGTAGAT CTGTAGATAA TTATACAATC GTAAGTGTAC TGAATATCCT   
  
  
- TACGTGATAA TACTCATACT ATATCTCAAA ATTTTTACGT ATACGGAACA CACAAGTAAT CGAATTTCTT   
  
  
- CTCAAAAACT TATATGTATA AATCCATTAA GAACGATTAC AGCATTAATC GTCAACATAG ATAGAATAAC   
  
  
- TAAACATAAA AATTAACGAA CAATATGACA ATGTGAATCT TTTAAGTACT TATGAGAATT TTATACGTTT   
  
  
- AATATATATA TATATATATA TATATATATA TATATATATA TATTTTTGAC TTATTCAATT ATACCACTAC   
  
  
- TGTAAACAGT ACAATAGTAA CCAGCGAATT AAAAATTTAT TTTTTATACT AAAACACTGT GGATTATTTA   
  
  
- CTATAATCGG TATAATAATT TAAGTCGTAA ATAAGTAGTT ACATTATATG ATTTAGTACG TTAATAGTAA   
  
  
- CCAAGAGAAA ATACATTATT TCAATTAACT GTTAAGTTCC CAATGATTAA TCATATTACA ACTTAACCTT   
  
  
- GCATTTTTAG TTTAGCATAC ATTTCATTAA AATAAGACTT AAATTTTTTA CCTCGTTTAT AGAGATAACA   
  
  
- AGAAATTATT TTAAAATTAA CTAAAAATAA AGATTAATTC ACTGCCTTGG TTTAAAAAAA GGAGGAAAAA   
  
  
- ACCAAACCAT TCAATAGTAA TCGGTTAGAA AATAAATGTA AAACAGTCAA AAAGAAAAAA TTCTACCAAT   
  
  
- TTACCGAAGT TAATTTGTTT AAAAAAATGA ATAAATATAA AAATTATTTA AAAGTTTTAC ATATAATTGC   
  
  
- GTACTGGCAC GCTTCGTGCC TTAGATGGGA TCAATTAGAT ACTAAGAATT TATACGTTCA TCCGACAATA   
  
  
- AGTAAACAAC AAAAAATGAA TAAATATAAA AAAGGGAGGG CCATACTATC CCAAACAGTA TAACAATAAG   
  
  
- TAATCGACAA TTTGACATAA GTAATATCGT GTTCATCCGA ATTAGTTTCT ATCAATCAGA GGTTAAAGTA   
  
  
- GTCTTTTAAA TTCTTTTTCT GTGGAATCCT AGTTTTCATA CTTTAAATCT CTGGTCTGTT GCTATCTTAA   
  
  
- CTTTTCTACT CAAAAGTTAA ACCTCTTCTG CCTTTGAGTC TACTTACTTT CATATCATGT AACATGAGGA   
  
  
- ACCTCATATT AGAATAGAAG TGATAACTAT CCGAGCTTCG TATTAGTCAC TAACCTTTGA AAACCAACGT   
  
  
- TATTAAGTTT TAAACTATGA GATACTAAAT GAAAAAATTA ACTTCCAGTA CTAAATTAAT TGATTTACTT   
  
  
- TTCTTAAATA CTAGCTTTTT AAAATTGAGA ATAACTGAAT ATCAACTGAG GTCAATTTAC CTTTTAACTT   
  
  
- CACTAATTAG ATTTTCACCG GGCGAGATTA TGGTAAACTC TTTTTAATAA GAATGACGTT TCAAATTTAG   
  
  
- TTATACACTT CTATCAAATA TCAAAATAGG ATTTTCTTTA ATTTATCTAA CAATTTTTAT TAAAAATTTA   
  
  
- AGTCTATATG CACACTACCG GTATTGGCAT TTACTATACG TACAGCTGTT TAAGGTTTCT TCCGATTATT   
  
  
- AATCTGAATT TATTATGTAC GTATGATTGG TTTTTTTTTT TTAATTCGTT CTAAGATAGG TTGGATAGTA   
  
  
- TTATTTCAAA CCGGTATGAA GTTTGGTAGT GTATTGTAGT GATTATATAT TTCGATCCAA AACCCTTTGG   
  
  
- ATTGAAACTC CTGGTGTATA AACAATTCCG GATCCTCCGG TACTCAAACA GTTCTACGGA GTATCATACC   
  
  
- TCTCCAGATA TAGGAACGTT TTACTAGTAA GGGAAGATGG ATTTTCTTGG TGAAGTAAAG GTGAAAATGA   
  
  
- GGGACCGTTT ACCAACTATT TGGTTTGGTA TTGGTTCGGG AACTTCTTGA TATTTCTGTG CTTAGCTAGT   
  
  
- AACTTAAGCT TGTTCGGAAC ACACTATCCT CCTACCCGGA GTTGTGAAGA GGCGCTCACA CGTTCTCGTT   
  
  
- AGAGCCTCTT TTTAGGATTC TCGGTTGTTG AAGAAACCCA CAATTTACTT GAACAAAGAG GAATACCGCT   
  
  
- AACGCTTGTT TCTAACCGTA TAATGAAGAA TGTTCACAAC AACCGGTTTC GGTTGTTAAA CCCTGGAGTG   
  
  
- AAAGTACTCT CGGATTTTGA ACGGTACCTC TTTTTGACGA CGAAACTATG GATGTACTTC GAATATAACT   
  
  
- TCAAGGTTCT CCAGTCAGGT ACCTGTTGGA AACCAGTACA CCGAAGTTTA CCACGTTATA ACCTCCTGGA   
  
  
- ATCTTCAAAT GTTTTCAGCT AGTAGTTTCT TTGTTCCGTT TCCTACCTCT TCAAAAGTTC CGATTACCCA   
  
  
- CAAGGGAAGT TCAAAGTACA GTATTTGCTC AATCTATTGG ATCCTCTCGA AGCTTTTCTC CTAAATCTGT   
  
  
- AACTTCTACC ACTCCGGTAG CGACACTTGA CACAAGTTCG GAACGTTGCC CAAGTACACC TCTTCTCCCT   
  
  
- CGTACACGAA CTACACTAAG CTAGATAATC CGGAACATAG TATTGTGACC ACCTCCTTCT TCGTCTAGAG   
  
  
- TGAAGATGTT CTTTGCTGAA GAAGTTCACG AAGCTACTCA CAAACTCTAA AAGATTCAGT ATAAAGCTAT   
  
  
- ACAATCTTCT TTCGAAGGGA GGTTATTCGT TGCTTGCTTA GTTCGATCTT TCCCTTGTTA CCTACTTATA   
  
  
- GAGGTCTCGG GATCGAACAG TACCACTTTC ACCTCCTCTT ATATCCGGTT TCTTTCCTTG AGTTACCTTA   
  
  
- CTCTACGAGC TTGTTCGTAA AACGGGTAGA GTTAAATCAC TACTACATGA TTCACTACAA TCCCGTAACA   
  
  
- ACTTTTCTGT GTTTTCACCA ACCCTAGAGT GGAATGGTGT TTCACTGGTA CTTTATCCGT ATGTGAATTT   
  
  
- AACCTTCCCA CTTTTACAAC AAACCCGAAG ACGTACCTCT GGATCGAT

  
  
Motifs Found  

+   

| Site Name | Organism | Position | Strand | Matrix score. | sequence | function |
| --- | --- | --- | --- | --- | --- | --- |
|  | organism | 2796 | - | 4 | motif\_sequence | short\_function |
|  | organism | 1390 | - | 4 | motif\_sequence | short\_function |
|  | organism | 2033 | - | 4 | motif\_sequence | short\_function |
|  | organism | 352 | - | 4 | motif\_sequence | short\_function |
|  | organism | 149 | - | 4 | motif\_sequence | short\_function |
|  | organism | 2737 | + | 4 | motif\_sequence | short\_function |
|  | organism | 1419 | + | 4 | motif\_sequence | short\_function |
|  | organism | 1076 | + | 4 | motif\_sequence | short\_function |
|  | organism | 2667 | - | 4 | motif\_sequence | short\_function |
|  | organism | 2726 | - | 4 | motif\_sequence | short\_function |
|  | organism | 2637 | - | 4 | motif\_sequence | short\_function |
|  | organism | 2943 | + | 4 | motif\_sequence | short\_function |
|  | organism | 1692 | - | 4 | motif\_sequence | short\_function |
|  | organism | 3058 | + | 4 | motif\_sequence | short\_function |

>HU02G03154.1   
+ +Up\_Stream \_Len000ATGATA AAAGATATTT ATTAAGCTCA AGCTATTTTA ACTTTTAAAA AAATGAATGT   
  
  
+ GGCTTTTTTT GCTATTCAGG TACAACACAA TATGAAGCAT TAACTCTAGA AAATGTGGTA ATGTATGTCA   
  
  
+ TATAGGAGAG TATATACTCT CACTTGCCAT GCGATGCGTG CATGTGTGTG TGTGTGTGTA TCTGTTTCTA   
  
  
+ TGCATATTTT ATGTGGGGTT TTGTCATCTA GACATCTATT AATATGTTAG CATTCACATG ACTTATAGGA   
  
  
+ ATGCACTATT ATGAGTATGA TATAGAGTTT TAAAAATGCA TATGCCTTGT GTGTTCATTA GCTTAAAGAA   
  
  
+ GAGTTTTTGA ATATACATAT TTAGGTAATT CTTGCTAATG TCGTAATTAG CAGTTGTATC TATCTTATTG   
  
  
+ ATTTGTATTT TTAATTGCTT GTTATACTGT TACACTTAGA AAATTCATGA ATACTCTTAA AATATGCAAA   
  
  
+ TTATATATAT ATATATATAT ATATATATAT ATATATATAT ATAAAAACTG AATAAGTTAA TATGGTGATG   
  
  
+ ACATTTGTCA TGTTATCATT GGTCGCTTAA TTTTTAAATA AAAAATATGA TTTTGTGACA CCTAATAAAT   
  
  
+ GATATTAGCC ATATTATTAA ATTCAGCATT TATTCATCAA TGTAATATAC TAAATCATGC AATTATCATT   
  
  
+ GGTTCTCTTT TATGTAATAA AGTTAATTGA CAATTCAAGG GTTACTAATT AGTATAATGT TGAATTGGAA   
  
  
+ CGTAAAAATC AAATCGTATG TAAAGTAATT TTATTCTGAA TTTAAAAAAT GGAGCAAATA TCTCTATTGT   
  
  
+ TCTTTAATAA AATTTTAATT GATTTTTATT TCTAATTAAG TGACGGAACC AAATTTTTTT CCTCCTTTTT   
  
  
+ TGGTTTGGTA AGTTATCATT AGCCAATCTT TTATTTACAT TTTGTCAGTT TTTCTTTTTT AAGATGGTTA   
  
  
+ AATGGCTTCA ATTAAACAAA TTTTTTTACT TATTTATATT TTTAATAAAT TTTCAAAATG TATATTAACG   
  
  
+ CATGACCGTG CGAAGCACGG AATCTACCCT AGTTAATCTA TGATTCTTAA ATATGCAAGT AGGCTGTTAT   
  
  
+ TCATTTGTTG TTTTTTACTT ATTTATATTT TTTCCCTCCC GGTATGATAG GGTTTGTCAT ATTGTTATTC   
  
  
+ ATTAGCTGTT AAACTGTATT CATTATAGCA CAAGTAGGCT TAATCAAAGA TAGTTAGTCT CCAATTTCAT   
  
  
+ CAGAAAATTT AAGAAAAAGA CACCTTAGGA TCAAAAGTAT GAAATTTAGA GACCAGACAA CGATAGAATT   
  
  
+ GAAAAGATGA GTTTTCAATT TGGAGAAGAC GGAAACTCAG ATGAATGAAA GTATAGTACA TTGTACTCCT   
  
  
+ TGGAGTATAA TCTTATCTTC ACTATTGATA GGCTCGAAGC ATAATCAGTG ATTGGAAACT TTTGGTTGCA   
  
  
+ ATAATTCAAA ATTTGATACT CTATGATTTA CTTTTTTAAT TGAAGGTCAT GATTTAATTA ACTAAATGAA   
  
  
+ AAGAATTTAT GATCGAAAAA TTTTAACTCT TATTGACTTA TAGTTGACTC CAGTTAAATG GAAAATTGAA   
  
  
+ GTGATTAATC TAAAAGTGGC CCGCTCTAAT ACCATTTGAG AAAAATTATT CTTACTGCAA AGTTTAAATC   
  
  
+ AATATGTGAA GATAGTTTAT AGTTTTATCC TAAAAGAAAT TAAATAGATT GTTAAAAATA ATTTTTAAAT   
  
  
+ TCAGATATAC GTGTGATGGC CATAACCGTA AATGATATGC ATGTCGACAA ATTCCAAAGA AGGCTAATAA   
  
  
+ TTAGACTTAA ATAATACATG CATACTAACC AAAAAAAAAA AATTAAGCAA GATTCTATCC AACCTATCAT   
  
  
+ AATAAAGTTT GGCCATACTT CAAACCATCA CATAACATCA CTAATATATA AAGCTAGGTT TTGGGAAACC   
  
  
+ TAACTTTGAG GACCACATAT TTGTTAAGGC CTAGGAGGCC ATGAGTTTGT CAAGATGCCT CATAGTATGG   
  
  
+ AGAGGTCTAT ATCCTTGCAA AATGATCATT CCCTTCTACC TAAAAGAACC ACTTCATTTC CACTTTTACT   
  
  
+ CCCTGGCAAA TGGTTGATAA ACCAAACCAT AACCAAGCCC TTGAAGAACT ATAAAGACAC GAATCGATCA   
  
  
+ TTGAATTCGA ACAAGCCTTG TGTGATAGGA GGATGGGCCT CAACACTTCT CCGCGAGTGT GCAAGAGCAA   
  
  
+ TCTCGGAGAA AAATCCTAAG AGCCAACAAC TTCTTTGGGT GTTAAATGAA CTTGTTTCTC CTTATGGCGA   
  
  
+ TTGCGAACAA AGATTGGCAT ATTACTTCTT ACAAGTGTTG TTGGCCAAAG CCAACAATTT GGGACCTCAC   
  
  
+ TTTCATGAGA GCCTAAAACT TGCCATGGAG AAAAACTGCT GCTTTGATAC CTACATGAAG CTTATATTGA   
  
  
+ AGTTCCAAGA GGTCAGTCCA TGGACAACCT TTGGTCATGT GGCTTCAAAT GGTGCAATAT TGGAGGACCT   
  
  
+ TAGAAGTTTA CAAAAGTCGA TCATCAAAGA AACAAGGCAA AGGATGGAGA AGTTTTCAAG GCTAATGGGT   
  
  
+ GTTCCCTTCA AGTTTCATGT CATAAACGAG TTAGATAACC TAGGAGAGCT TCGAAAAGAG GATTTAGACA   
  
  
+ TTGAAGATGG TGAGGCCATC GCTGTGAACT GTGTTCAAGC CTTGCAACGG GTTCATGTGG AGAAGAGGGA   
  
  
+ GCATGTGCTT GATGTGATTC GATCTATTAG GCCTTGTATC ATAACACTGG TGGAGGAAGA AGCAGATCTC   
  
  
+ ACTTCTACAA GAAACGACTT CTTCAAGTGC TTCGATGAGT GTTTGAGATT TTCTAAGTCA TATTTCGATA   
  
  
+ TGTTAGAAGA AAGCTTCCCT CCAATAAGCA ACGAACGAAT CAAGCTAGAA AGGGAACAAT GGATGAATAT   
  
  
+ CTCCAGAGCC CTAGCTTGTC ATGGTGAAAG TGGAGGAGAA TATAGGCCAA AGAAAGGAAC TCAATGGAAT   
  
  
+ GAGATGCTCG AACAAGCATT TTGCCCATCT CAATTTAGTG ATGATGTACT AAGTGATGTT AGGGCATTGT   
  
  
+ TGAAAAGACA CAAAAGTGGT TGGGATCTCA CCTTACCACA AAGTGACCAT GAAATAGGCA TACACTTAAA   
  
  
+ TTGGAAGGGT GAAAATGTTG TTTGGGCTTC TGCATGGAGA CCTAGCTA  

- +Up\_Stream \_Len000TACTAT TTTCTATAAA TAATTCGAGT TCGATAAAAT TGAAAATTTT TTTACTTACA   
  
  
- CCGAAAAAAA CGATAAGTCC ATGTTGTGTT ATACTTCGTA ATTGAGATCT TTTACACCAT TACATACAGT   
  
  
- ATATCCTCTC ATATATGAGA GTGAACGGTA CGCTACGCAC GTACACACAC ACACACACAT AGACAAAGAT   
  
  
- ACGTATAAAA TACACCCCAA AACAGTAGAT CTGTAGATAA TTATACAATC GTAAGTGTAC TGAATATCCT   
  
  
- TACGTGATAA TACTCATACT ATATCTCAAA ATTTTTACGT ATACGGAACA CACAAGTAAT CGAATTTCTT   
  
  
- CTCAAAAACT TATATGTATA AATCCATTAA GAACGATTAC AGCATTAATC GTCAACATAG ATAGAATAAC   
  
  
- TAAACATAAA AATTAACGAA CAATATGACA ATGTGAATCT TTTAAGTACT TATGAGAATT TTATACGTTT   
  
  
- AATATATATA TATATATATA TATATATATA TATATATATA TATTTTTGAC TTATTCAATT ATACCACTAC   
  
  
- TGTAAACAGT ACAATAGTAA CCAGCGAATT AAAAATTTAT TTTTTATACT AAAACACTGT GGATTATTTA   
  
  
- CTATAATCGG TATAATAATT TAAGTCGTAA ATAAGTAGTT ACATTATATG ATTTAGTACG TTAATAGTAA   
  
  
- CCAAGAGAAA ATACATTATT TCAATTAACT GTTAAGTTCC CAATGATTAA TCATATTACA ACTTAACCTT   
  
  
- GCATTTTTAG TTTAGCATAC ATTTCATTAA AATAAGACTT AAATTTTTTA CCTCGTTTAT AGAGATAACA   
  
  
- AGAAATTATT TTAAAATTAA CTAAAAATAA AGATTAATTC ACTGCCTTGG TTTAAAAAAA GGAGGAAAAA   
  
  
- ACCAAACCAT TCAATAGTAA TCGGTTAGAA AATAAATGTA AAACAGTCAA AAAGAAAAAA TTCTACCAAT   
  
  
- TTACCGAAGT TAATTTGTTT AAAAAAATGA ATAAATATAA AAATTATTTA AAAGTTTTAC ATATAATTGC   
  
  
- GTACTGGCAC GCTTCGTGCC TTAGATGGGA TCAATTAGAT ACTAAGAATT TATACGTTCA TCCGACAATA   
  
  
- AGTAAACAAC AAAAAATGAA TAAATATAAA AAAGGGAGGG CCATACTATC CCAAACAGTA TAACAATAAG   
  
  
- TAATCGACAA TTTGACATAA GTAATATCGT GTTCATCCGA ATTAGTTTCT ATCAATCAGA GGTTAAAGTA   
  
  
- GTCTTTTAAA TTCTTTTTCT GTGGAATCCT AGTTTTCATA CTTTAAATCT CTGGTCTGTT GCTATCTTAA   
  
  
- CTTTTCTACT CAAAAGTTAA ACCTCTTCTG CCTTTGAGTC TACTTACTTT CATATCATGT AACATGAGGA   
  
  
- ACCTCATATT AGAATAGAAG TGATAACTAT CCGAGCTTCG TATTAGTCAC TAACCTTTGA AAACCAACGT   
  
  
- TATTAAGTTT TAAACTATGA GATACTAAAT GAAAAAATTA ACTTCCAGTA CTAAATTAAT TGATTTACTT   
  
  
- TTCTTAAATA CTAGCTTTTT AAAATTGAGA ATAACTGAAT ATCAACTGAG GTCAATTTAC CTTTTAACTT   
  
  
- CACTAATTAG ATTTTCACCG GGCGAGATTA TGGTAAACTC TTTTTAATAA GAATGACGTT TCAAATTTAG   
  
  
- TTATACACTT CTATCAAATA TCAAAATAGG ATTTTCTTTA ATTTATCTAA CAATTTTTAT TAAAAATTTA   
  
  
- AGTCTATATG CACACTACCG GTATTGGCAT TTACTATACG TACAGCTGTT TAAGGTTTCT TCCGATTATT   
  
  
- AATCTGAATT TATTATGTAC GTATGATTGG TTTTTTTTTT TTAATTCGTT CTAAGATAGG TTGGATAGTA   
  
  
- TTATTTCAAA CCGGTATGAA GTTTGGTAGT GTATTGTAGT GATTATATAT TTCGATCCAA AACCCTTTGG   
  
  
- ATTGAAACTC CTGGTGTATA AACAATTCCG GATCCTCCGG TACTCAAACA GTTCTACGGA GTATCATACC   
  
  
- TCTCCAGATA TAGGAACGTT TTACTAGTAA GGGAAGATGG ATTTTCTTGG TGAAGTAAAG GTGAAAATGA   
  
  
- GGGACCGTTT ACCAACTATT TGGTTTGGTA TTGGTTCGGG AACTTCTTGA TATTTCTGTG CTTAGCTAGT   
  
  
- AACTTAAGCT TGTTCGGAAC ACACTATCCT CCTACCCGGA GTTGTGAAGA GGCGCTCACA CGTTCTCGTT   
  
  
- AGAGCCTCTT TTTAGGATTC TCGGTTGTTG AAGAAACCCA CAATTTACTT GAACAAAGAG GAATACCGCT   
  
  
- AACGCTTGTT TCTAACCGTA TAATGAAGAA TGTTCACAAC AACCGGTTTC GGTTGTTAAA CCCTGGAGTG   
  
  
- AAAGTACTCT CGGATTTTGA ACGGTACCTC TTTTTGACGA CGAAACTATG GATGTACTTC GAATATAACT   
  
  
- TCAAGGTTCT CCAGTCAGGT ACCTGTTGGA AACCAGTACA CCGAAGTTTA CCACGTTATA ACCTCCTGGA   
  
  
- ATCTTCAAAT GTTTTCAGCT AGTAGTTTCT TTGTTCCGTT TCCTACCTCT TCAAAAGTTC CGATTACCCA   
  
  
- CAAGGGAAGT TCAAAGTACA GTATTTGCTC AATCTATTGG ATCCTCTCGA AGCTTTTCTC CTAAATCTGT   
  
  
- AACTTCTACC ACTCCGGTAG CGACACTTGA CACAAGTTCG GAACGTTGCC CAAGTACACC TCTTCTCCCT   
  
  
- CGTACACGAA CTACACTAAG CTAGATAATC CGGAACATAG TATTGTGACC ACCTCCTTCT TCGTCTAGAG   
  
  
- TGAAGATGTT CTTTGCTGAA GAAGTTCACG AAGCTACTCA CAAACTCTAA AAGATTCAGT ATAAAGCTAT   
  
  
- ACAATCTTCT TTCGAAGGGA GGTTATTCGT TGCTTGCTTA GTTCGATCTT TCCCTTGTTA CCTACTTATA   
  
  
- GAGGTCTCGG GATCGAACAG TACCACTTTC ACCTCCTCTT ATATCCGGTT TCTTTCCTTG AGTTACCTTA   
  
  
- CTCTACGAGC TTGTTCGTAA AACGGGTAGA GTTAAATCAC TACTACATGA TTCACTACAA TCCCGTAACA   
  
  
- ACTTTTCTGT GTTTTCACCA ACCCTAGAGT GGAATGGTGT TTCACTGGTA CTTTATCCGT ATGTGAATTT   
  
  
- AACCTTCCCA CTTTTACAAC AAACCCGAAG ACGTACCTCT GGATCGAT

+     ABRE

| Site Name | Organism | Position | Strand | Matrix score. | sequence | function |
| --- | --- | --- | --- | --- | --- | --- |
| ABRE | Arabidopsis thaliana | 1763 | + | 5 | ACGTG | cis-acting element involved in the abscisic acid responsiveness |

>HU02G03154.1   
+ +Up\_Stream \_Len000ATGATA AAAGATATTT ATTAAGCTCA AGCTATTTTA ACTTTTAAAA AAATGAATGT   
  
  
+ GGCTTTTTTT GCTATTCAGG TACAACACAA TATGAAGCAT TAACTCTAGA AAATGTGGTA ATGTATGTCA   
  
  
+ TATAGGAGAG TATATACTCT CACTTGCCAT GCGATGCGTG CATGTGTGTG TGTGTGTGTA TCTGTTTCTA   
  
  
+ TGCATATTTT ATGTGGGGTT TTGTCATCTA GACATCTATT AATATGTTAG CATTCACATG ACTTATAGGA   
  
  
+ ATGCACTATT ATGAGTATGA TATAGAGTTT TAAAAATGCA TATGCCTTGT GTGTTCATTA GCTTAAAGAA   
  
  
+ GAGTTTTTGA ATATACATAT TTAGGTAATT CTTGCTAATG TCGTAATTAG CAGTTGTATC TATCTTATTG   
  
  
+ ATTTGTATTT TTAATTGCTT GTTATACTGT TACACTTAGA AAATTCATGA ATACTCTTAA AATATGCAAA   
  
  
+ TTATATATAT ATATATATAT ATATATATAT ATATATATAT ATAAAAACTG AATAAGTTAA TATGGTGATG   
  
  
+ ACATTTGTCA TGTTATCATT GGTCGCTTAA TTTTTAAATA AAAAATATGA TTTTGTGACA CCTAATAAAT   
  
  
+ GATATTAGCC ATATTATTAA ATTCAGCATT TATTCATCAA TGTAATATAC TAAATCATGC AATTATCATT   
  
  
+ GGTTCTCTTT TATGTAATAA AGTTAATTGA CAATTCAAGG GTTACTAATT AGTATAATGT TGAATTGGAA   
  
  
+ CGTAAAAATC AAATCGTATG TAAAGTAATT TTATTCTGAA TTTAAAAAAT GGAGCAAATA TCTCTATTGT   
  
  
+ TCTTTAATAA AATTTTAATT GATTTTTATT TCTAATTAAG TGACGGAACC AAATTTTTTT CCTCCTTTTT   
  
  
+ TGGTTTGGTA AGTTATCATT AGCCAATCTT TTATTTACAT TTTGTCAGTT TTTCTTTTTT AAGATGGTTA   
  
  
+ AATGGCTTCA ATTAAACAAA TTTTTTTACT TATTTATATT TTTAATAAAT TTTCAAAATG TATATTAACG   
  
  
+ CATGACCGTG CGAAGCACGG AATCTACCCT AGTTAATCTA TGATTCTTAA ATATGCAAGT AGGCTGTTAT   
  
  
+ TCATTTGTTG TTTTTTACTT ATTTATATTT TTTCCCTCCC GGTATGATAG GGTTTGTCAT ATTGTTATTC   
  
  
+ ATTAGCTGTT AAACTGTATT CATTATAGCA CAAGTAGGCT TAATCAAAGA TAGTTAGTCT CCAATTTCAT   
  
  
+ CAGAAAATTT AAGAAAAAGA CACCTTAGGA TCAAAAGTAT GAAATTTAGA GACCAGACAA CGATAGAATT   
  
  
+ GAAAAGATGA GTTTTCAATT TGGAGAAGAC GGAAACTCAG ATGAATGAAA GTATAGTACA TTGTACTCCT   
  
  
+ TGGAGTATAA TCTTATCTTC ACTATTGATA GGCTCGAAGC ATAATCAGTG ATTGGAAACT TTTGGTTGCA   
  
  
+ ATAATTCAAA ATTTGATACT CTATGATTTA CTTTTTTAAT TGAAGGTCAT GATTTAATTA ACTAAATGAA   
  
  
+ AAGAATTTAT GATCGAAAAA TTTTAACTCT TATTGACTTA TAGTTGACTC CAGTTAAATG GAAAATTGAA   
  
  
+ GTGATTAATC TAAAAGTGGC CCGCTCTAAT ACCATTTGAG AAAAATTATT CTTACTGCAA AGTTTAAATC   
  
  
+ AATATGTGAA GATAGTTTAT AGTTTTATCC TAAAAGAAAT TAAATAGATT GTTAAAAATA ATTTTTAAAT   
  
  
+ TCAGATATAC GTGTGATGGC CATAACCGTA AATGATATGC ATGTCGACAA ATTCCAAAGA AGGCTAATAA   
  
  
+ TTAGACTTAA ATAATACATG CATACTAACC AAAAAAAAAA AATTAAGCAA GATTCTATCC AACCTATCAT   
  
  
+ AATAAAGTTT GGCCATACTT CAAACCATCA CATAACATCA CTAATATATA AAGCTAGGTT TTGGGAAACC   
  
  
+ TAACTTTGAG GACCACATAT TTGTTAAGGC CTAGGAGGCC ATGAGTTTGT CAAGATGCCT CATAGTATGG   
  
  
+ AGAGGTCTAT ATCCTTGCAA AATGATCATT CCCTTCTACC TAAAAGAACC ACTTCATTTC CACTTTTACT   
  
  
+ CCCTGGCAAA TGGTTGATAA ACCAAACCAT AACCAAGCCC TTGAAGAACT ATAAAGACAC GAATCGATCA   
  
  
+ TTGAATTCGA ACAAGCCTTG TGTGATAGGA GGATGGGCCT CAACACTTCT CCGCGAGTGT GCAAGAGCAA   
  
  
+ TCTCGGAGAA AAATCCTAAG AGCCAACAAC TTCTTTGGGT GTTAAATGAA CTTGTTTCTC CTTATGGCGA   
  
  
+ TTGCGAACAA AGATTGGCAT ATTACTTCTT ACAAGTGTTG TTGGCCAAAG CCAACAATTT GGGACCTCAC   
  
  
+ TTTCATGAGA GCCTAAAACT TGCCATGGAG AAAAACTGCT GCTTTGATAC CTACATGAAG CTTATATTGA   
  
  
+ AGTTCCAAGA GGTCAGTCCA TGGACAACCT TTGGTCATGT GGCTTCAAAT GGTGCAATAT TGGAGGACCT   
  
  
+ TAGAAGTTTA CAAAAGTCGA TCATCAAAGA AACAAGGCAA AGGATGGAGA AGTTTTCAAG GCTAATGGGT   
  
  
+ GTTCCCTTCA AGTTTCATGT CATAAACGAG TTAGATAACC TAGGAGAGCT TCGAAAAGAG GATTTAGACA   
  
  
+ TTGAAGATGG TGAGGCCATC GCTGTGAACT GTGTTCAAGC CTTGCAACGG GTTCATGTGG AGAAGAGGGA   
  
  
+ GCATGTGCTT GATGTGATTC GATCTATTAG GCCTTGTATC ATAACACTGG TGGAGGAAGA AGCAGATCTC   
  
  
+ ACTTCTACAA GAAACGACTT CTTCAAGTGC TTCGATGAGT GTTTGAGATT TTCTAAGTCA TATTTCGATA   
  
  
+ TGTTAGAAGA AAGCTTCCCT CCAATAAGCA ACGAACGAAT CAAGCTAGAA AGGGAACAAT GGATGAATAT   
  
  
+ CTCCAGAGCC CTAGCTTGTC ATGGTGAAAG TGGAGGAGAA TATAGGCCAA AGAAAGGAAC TCAATGGAAT   
  
  
+ GAGATGCTCG AACAAGCATT TTGCCCATCT CAATTTAGTG ATGATGTACT AAGTGATGTT AGGGCATTGT   
  
  
+ TGAAAAGACA CAAAAGTGGT TGGGATCTCA CCTTACCACA AAGTGACCAT GAAATAGGCA TACACTTAAA   
  
  
+ TTGGAAGGGT GAAAATGTTG TTTGGGCTTC TGCATGGAGA CCTAGCTA  

- +Up\_Stream \_Len000TACTAT TTTCTATAAA TAATTCGAGT TCGATAAAAT TGAAAATTTT TTTACTTACA   
  
  
- CCGAAAAAAA CGATAAGTCC ATGTTGTGTT ATACTTCGTA ATTGAGATCT TTTACACCAT TACATACAGT   
  
  
- ATATCCTCTC ATATATGAGA GTGAACGGTA CGCTACGCAC GTACACACAC ACACACACAT AGACAAAGAT   
  
  
- ACGTATAAAA TACACCCCAA AACAGTAGAT CTGTAGATAA TTATACAATC GTAAGTGTAC TGAATATCCT   
  
  
- TACGTGATAA TACTCATACT ATATCTCAAA ATTTTTACGT ATACGGAACA CACAAGTAAT CGAATTTCTT   
  
  
- CTCAAAAACT TATATGTATA AATCCATTAA GAACGATTAC AGCATTAATC GTCAACATAG ATAGAATAAC   
  
  
- TAAACATAAA AATTAACGAA CAATATGACA ATGTGAATCT TTTAAGTACT TATGAGAATT TTATACGTTT   
  
  
- AATATATATA TATATATATA TATATATATA TATATATATA TATTTTTGAC TTATTCAATT ATACCACTAC   
  
  
- TGTAAACAGT ACAATAGTAA CCAGCGAATT AAAAATTTAT TTTTTATACT AAAACACTGT GGATTATTTA   
  
  
- CTATAATCGG TATAATAATT TAAGTCGTAA ATAAGTAGTT ACATTATATG ATTTAGTACG TTAATAGTAA   
  
  
- CCAAGAGAAA ATACATTATT TCAATTAACT GTTAAGTTCC CAATGATTAA TCATATTACA ACTTAACCTT   
  
  
- GCATTTTTAG TTTAGCATAC ATTTCATTAA AATAAGACTT AAATTTTTTA CCTCGTTTAT AGAGATAACA   
  
  
- AGAAATTATT TTAAAATTAA CTAAAAATAA AGATTAATTC ACTGCCTTGG TTTAAAAAAA GGAGGAAAAA   
  
  
- ACCAAACCAT TCAATAGTAA TCGGTTAGAA AATAAATGTA AAACAGTCAA AAAGAAAAAA TTCTACCAAT   
  
  
- TTACCGAAGT TAATTTGTTT AAAAAAATGA ATAAATATAA AAATTATTTA AAAGTTTTAC ATATAATTGC   
  
  
- GTACTGGCAC GCTTCGTGCC TTAGATGGGA TCAATTAGAT ACTAAGAATT TATACGTTCA TCCGACAATA   
  
  
- AGTAAACAAC AAAAAATGAA TAAATATAAA AAAGGGAGGG CCATACTATC CCAAACAGTA TAACAATAAG   
  
  
- TAATCGACAA TTTGACATAA GTAATATCGT GTTCATCCGA ATTAGTTTCT ATCAATCAGA GGTTAAAGTA   
  
  
- GTCTTTTAAA TTCTTTTTCT GTGGAATCCT AGTTTTCATA CTTTAAATCT CTGGTCTGTT GCTATCTTAA   
  
  
- CTTTTCTACT CAAAAGTTAA ACCTCTTCTG CCTTTGAGTC TACTTACTTT CATATCATGT AACATGAGGA   
  
  
- ACCTCATATT AGAATAGAAG TGATAACTAT CCGAGCTTCG TATTAGTCAC TAACCTTTGA AAACCAACGT   
  
  
- TATTAAGTTT TAAACTATGA GATACTAAAT GAAAAAATTA ACTTCCAGTA CTAAATTAAT TGATTTACTT   
  
  
- TTCTTAAATA CTAGCTTTTT AAAATTGAGA ATAACTGAAT ATCAACTGAG GTCAATTTAC CTTTTAACTT   
  
  
- CACTAATTAG ATTTTCACCG GGCGAGATTA TGGTAAACTC TTTTTAATAA GAATGACGTT TCAAATTTAG   
  
  
- TTATACACTT CTATCAAATA TCAAAATAGG ATTTTCTTTA ATTTATCTAA CAATTTTTAT TAAAAATTTA   
  
  
- AGTCTATATG CACACTACCG GTATTGGCAT TTACTATACG TACAGCTGTT TAAGGTTTCT TCCGATTATT   
  
  
- AATCTGAATT TATTATGTAC GTATGATTGG TTTTTTTTTT TTAATTCGTT CTAAGATAGG TTGGATAGTA   
  
  
- TTATTTCAAA CCGGTATGAA GTTTGGTAGT GTATTGTAGT GATTATATAT TTCGATCCAA AACCCTTTGG   
  
  
- ATTGAAACTC CTGGTGTATA AACAATTCCG GATCCTCCGG TACTCAAACA GTTCTACGGA GTATCATACC   
  
  
- TCTCCAGATA TAGGAACGTT TTACTAGTAA GGGAAGATGG ATTTTCTTGG TGAAGTAAAG GTGAAAATGA   
  
  
- GGGACCGTTT ACCAACTATT TGGTTTGGTA TTGGTTCGGG AACTTCTTGA TATTTCTGTG CTTAGCTAGT   
  
  
- AACTTAAGCT TGTTCGGAAC ACACTATCCT CCTACCCGGA GTTGTGAAGA GGCGCTCACA CGTTCTCGTT   
  
  
- AGAGCCTCTT TTTAGGATTC TCGGTTGTTG AAGAAACCCA CAATTTACTT GAACAAAGAG GAATACCGCT   
  
  
- AACGCTTGTT TCTAACCGTA TAATGAAGAA TGTTCACAAC AACCGGTTTC GGTTGTTAAA CCCTGGAGTG   
  
  
- AAAGTACTCT CGGATTTTGA ACGGTACCTC TTTTTGACGA CGAAACTATG GATGTACTTC GAATATAACT   
  
  
- TCAAGGTTCT CCAGTCAGGT ACCTGTTGGA AACCAGTACA CCGAAGTTTA CCACGTTATA ACCTCCTGGA   
  
  
- ATCTTCAAAT GTTTTCAGCT AGTAGTTTCT TTGTTCCGTT TCCTACCTCT TCAAAAGTTC CGATTACCCA   
  
  
- CAAGGGAAGT TCAAAGTACA GTATTTGCTC AATCTATTGG ATCCTCTCGA AGCTTTTCTC CTAAATCTGT   
  
  
- AACTTCTACC ACTCCGGTAG CGACACTTGA CACAAGTTCG GAACGTTGCC CAAGTACACC TCTTCTCCCT   
  
  
- CGTACACGAA CTACACTAAG CTAGATAATC CGGAACATAG TATTGTGACC ACCTCCTTCT TCGTCTAGAG   
  
  
- TGAAGATGTT CTTTGCTGAA GAAGTTCACG AAGCTACTCA CAAACTCTAA AAGATTCAGT ATAAAGCTAT   
  
  
- ACAATCTTCT TTCGAAGGGA GGTTATTCGT TGCTTGCTTA GTTCGATCTT TCCCTTGTTA CCTACTTATA   
  
  
- GAGGTCTCGG GATCGAACAG TACCACTTTC ACCTCCTCTT ATATCCGGTT TCTTTCCTTG AGTTACCTTA   
  
  
- CTCTACGAGC TTGTTCGTAA AACGGGTAGA GTTAAATCAC TACTACATGA TTCACTACAA TCCCGTAACA   
  
  
- ACTTTTCTGT GTTTTCACCA ACCCTAGAGT GGAATGGTGT TTCACTGGTA CTTTATCCGT ATGTGAATTT   
  
  
- AACCTTCCCA CTTTTACAAC AAACCCGAAG ACGTACCTCT GGATCGAT

+     ABRE3a

| Site Name | Organism | Position | Strand | Matrix score. | sequence | function |
| --- | --- | --- | --- | --- | --- | --- |
| ABRE3a | Zea mays | 1762 | + | 6 | TACGTG |  |

>HU02G03154.1   
+ +Up\_Stream \_Len000ATGATA AAAGATATTT ATTAAGCTCA AGCTATTTTA ACTTTTAAAA AAATGAATGT   
  
  
+ GGCTTTTTTT GCTATTCAGG TACAACACAA TATGAAGCAT TAACTCTAGA AAATGTGGTA ATGTATGTCA   
  
  
+ TATAGGAGAG TATATACTCT CACTTGCCAT GCGATGCGTG CATGTGTGTG TGTGTGTGTA TCTGTTTCTA   
  
  
+ TGCATATTTT ATGTGGGGTT TTGTCATCTA GACATCTATT AATATGTTAG CATTCACATG ACTTATAGGA   
  
  
+ ATGCACTATT ATGAGTATGA TATAGAGTTT TAAAAATGCA TATGCCTTGT GTGTTCATTA GCTTAAAGAA   
  
  
+ GAGTTTTTGA ATATACATAT TTAGGTAATT CTTGCTAATG TCGTAATTAG CAGTTGTATC TATCTTATTG   
  
  
+ ATTTGTATTT TTAATTGCTT GTTATACTGT TACACTTAGA AAATTCATGA ATACTCTTAA AATATGCAAA   
  
  
+ TTATATATAT ATATATATAT ATATATATAT ATATATATAT ATAAAAACTG AATAAGTTAA TATGGTGATG   
  
  
+ ACATTTGTCA TGTTATCATT GGTCGCTTAA TTTTTAAATA AAAAATATGA TTTTGTGACA CCTAATAAAT   
  
  
+ GATATTAGCC ATATTATTAA ATTCAGCATT TATTCATCAA TGTAATATAC TAAATCATGC AATTATCATT   
  
  
+ GGTTCTCTTT TATGTAATAA AGTTAATTGA CAATTCAAGG GTTACTAATT AGTATAATGT TGAATTGGAA   
  
  
+ CGTAAAAATC AAATCGTATG TAAAGTAATT TTATTCTGAA TTTAAAAAAT GGAGCAAATA TCTCTATTGT   
  
  
+ TCTTTAATAA AATTTTAATT GATTTTTATT TCTAATTAAG TGACGGAACC AAATTTTTTT CCTCCTTTTT   
  
  
+ TGGTTTGGTA AGTTATCATT AGCCAATCTT TTATTTACAT TTTGTCAGTT TTTCTTTTTT AAGATGGTTA   
  
  
+ AATGGCTTCA ATTAAACAAA TTTTTTTACT TATTTATATT TTTAATAAAT TTTCAAAATG TATATTAACG   
  
  
+ CATGACCGTG CGAAGCACGG AATCTACCCT AGTTAATCTA TGATTCTTAA ATATGCAAGT AGGCTGTTAT   
  
  
+ TCATTTGTTG TTTTTTACTT ATTTATATTT TTTCCCTCCC GGTATGATAG GGTTTGTCAT ATTGTTATTC   
  
  
+ ATTAGCTGTT AAACTGTATT CATTATAGCA CAAGTAGGCT TAATCAAAGA TAGTTAGTCT CCAATTTCAT   
  
  
+ CAGAAAATTT AAGAAAAAGA CACCTTAGGA TCAAAAGTAT GAAATTTAGA GACCAGACAA CGATAGAATT   
  
  
+ GAAAAGATGA GTTTTCAATT TGGAGAAGAC GGAAACTCAG ATGAATGAAA GTATAGTACA TTGTACTCCT   
  
  
+ TGGAGTATAA TCTTATCTTC ACTATTGATA GGCTCGAAGC ATAATCAGTG ATTGGAAACT TTTGGTTGCA   
  
  
+ ATAATTCAAA ATTTGATACT CTATGATTTA CTTTTTTAAT TGAAGGTCAT GATTTAATTA ACTAAATGAA   
  
  
+ AAGAATTTAT GATCGAAAAA TTTTAACTCT TATTGACTTA TAGTTGACTC CAGTTAAATG GAAAATTGAA   
  
  
+ GTGATTAATC TAAAAGTGGC CCGCTCTAAT ACCATTTGAG AAAAATTATT CTTACTGCAA AGTTTAAATC   
  
  
+ AATATGTGAA GATAGTTTAT AGTTTTATCC TAAAAGAAAT TAAATAGATT GTTAAAAATA ATTTTTAAAT   
  
  
+ TCAGATATAC GTGTGATGGC CATAACCGTA AATGATATGC ATGTCGACAA ATTCCAAAGA AGGCTAATAA   
  
  
+ TTAGACTTAA ATAATACATG CATACTAACC AAAAAAAAAA AATTAAGCAA GATTCTATCC AACCTATCAT   
  
  
+ AATAAAGTTT GGCCATACTT CAAACCATCA CATAACATCA CTAATATATA AAGCTAGGTT TTGGGAAACC   
  
  
+ TAACTTTGAG GACCACATAT TTGTTAAGGC CTAGGAGGCC ATGAGTTTGT CAAGATGCCT CATAGTATGG   
  
  
+ AGAGGTCTAT ATCCTTGCAA AATGATCATT CCCTTCTACC TAAAAGAACC ACTTCATTTC CACTTTTACT   
  
  
+ CCCTGGCAAA TGGTTGATAA ACCAAACCAT AACCAAGCCC TTGAAGAACT ATAAAGACAC GAATCGATCA   
  
  
+ TTGAATTCGA ACAAGCCTTG TGTGATAGGA GGATGGGCCT CAACACTTCT CCGCGAGTGT GCAAGAGCAA   
  
  
+ TCTCGGAGAA AAATCCTAAG AGCCAACAAC TTCTTTGGGT GTTAAATGAA CTTGTTTCTC CTTATGGCGA   
  
  
+ TTGCGAACAA AGATTGGCAT ATTACTTCTT ACAAGTGTTG TTGGCCAAAG CCAACAATTT GGGACCTCAC   
  
  
+ TTTCATGAGA GCCTAAAACT TGCCATGGAG AAAAACTGCT GCTTTGATAC CTACATGAAG CTTATATTGA   
  
  
+ AGTTCCAAGA GGTCAGTCCA TGGACAACCT TTGGTCATGT GGCTTCAAAT GGTGCAATAT TGGAGGACCT   
  
  
+ TAGAAGTTTA CAAAAGTCGA TCATCAAAGA AACAAGGCAA AGGATGGAGA AGTTTTCAAG GCTAATGGGT   
  
  
+ GTTCCCTTCA AGTTTCATGT CATAAACGAG TTAGATAACC TAGGAGAGCT TCGAAAAGAG GATTTAGACA   
  
  
+ TTGAAGATGG TGAGGCCATC GCTGTGAACT GTGTTCAAGC CTTGCAACGG GTTCATGTGG AGAAGAGGGA   
  
  
+ GCATGTGCTT GATGTGATTC GATCTATTAG GCCTTGTATC ATAACACTGG TGGAGGAAGA AGCAGATCTC   
  
  
+ ACTTCTACAA GAAACGACTT CTTCAAGTGC TTCGATGAGT GTTTGAGATT TTCTAAGTCA TATTTCGATA   
  
  
+ TGTTAGAAGA AAGCTTCCCT CCAATAAGCA ACGAACGAAT CAAGCTAGAA AGGGAACAAT GGATGAATAT   
  
  
+ CTCCAGAGCC CTAGCTTGTC ATGGTGAAAG TGGAGGAGAA TATAGGCCAA AGAAAGGAAC TCAATGGAAT   
  
  
+ GAGATGCTCG AACAAGCATT TTGCCCATCT CAATTTAGTG ATGATGTACT AAGTGATGTT AGGGCATTGT   
  
  
+ TGAAAAGACA CAAAAGTGGT TGGGATCTCA CCTTACCACA AAGTGACCAT GAAATAGGCA TACACTTAAA   
  
  
+ TTGGAAGGGT GAAAATGTTG TTTGGGCTTC TGCATGGAGA CCTAGCTA  

- +Up\_Stream \_Len000TACTAT TTTCTATAAA TAATTCGAGT TCGATAAAAT TGAAAATTTT TTTACTTACA   
  
  
- CCGAAAAAAA CGATAAGTCC ATGTTGTGTT ATACTTCGTA ATTGAGATCT TTTACACCAT TACATACAGT   
  
  
- ATATCCTCTC ATATATGAGA GTGAACGGTA CGCTACGCAC GTACACACAC ACACACACAT AGACAAAGAT   
  
  
- ACGTATAAAA TACACCCCAA AACAGTAGAT CTGTAGATAA TTATACAATC GTAAGTGTAC TGAATATCCT   
  
  
- TACGTGATAA TACTCATACT ATATCTCAAA ATTTTTACGT ATACGGAACA CACAAGTAAT CGAATTTCTT   
  
  
- CTCAAAAACT TATATGTATA AATCCATTAA GAACGATTAC AGCATTAATC GTCAACATAG ATAGAATAAC   
  
  
- TAAACATAAA AATTAACGAA CAATATGACA ATGTGAATCT TTTAAGTACT TATGAGAATT TTATACGTTT   
  
  
- AATATATATA TATATATATA TATATATATA TATATATATA TATTTTTGAC TTATTCAATT ATACCACTAC   
  
  
- TGTAAACAGT ACAATAGTAA CCAGCGAATT AAAAATTTAT TTTTTATACT AAAACACTGT GGATTATTTA   
  
  
- CTATAATCGG TATAATAATT TAAGTCGTAA ATAAGTAGTT ACATTATATG ATTTAGTACG TTAATAGTAA   
  
  
- CCAAGAGAAA ATACATTATT TCAATTAACT GTTAAGTTCC CAATGATTAA TCATATTACA ACTTAACCTT   
  
  
- GCATTTTTAG TTTAGCATAC ATTTCATTAA AATAAGACTT AAATTTTTTA CCTCGTTTAT AGAGATAACA   
  
  
- AGAAATTATT TTAAAATTAA CTAAAAATAA AGATTAATTC ACTGCCTTGG TTTAAAAAAA GGAGGAAAAA   
  
  
- ACCAAACCAT TCAATAGTAA TCGGTTAGAA AATAAATGTA AAACAGTCAA AAAGAAAAAA TTCTACCAAT   
  
  
- TTACCGAAGT TAATTTGTTT AAAAAAATGA ATAAATATAA AAATTATTTA AAAGTTTTAC ATATAATTGC   
  
  
- GTACTGGCAC GCTTCGTGCC TTAGATGGGA TCAATTAGAT ACTAAGAATT TATACGTTCA TCCGACAATA   
  
  
- AGTAAACAAC AAAAAATGAA TAAATATAAA AAAGGGAGGG CCATACTATC CCAAACAGTA TAACAATAAG   
  
  
- TAATCGACAA TTTGACATAA GTAATATCGT GTTCATCCGA ATTAGTTTCT ATCAATCAGA GGTTAAAGTA   
  
  
- GTCTTTTAAA TTCTTTTTCT GTGGAATCCT AGTTTTCATA CTTTAAATCT CTGGTCTGTT GCTATCTTAA   
  
  
- CTTTTCTACT CAAAAGTTAA ACCTCTTCTG CCTTTGAGTC TACTTACTTT CATATCATGT AACATGAGGA   
  
  
- ACCTCATATT AGAATAGAAG TGATAACTAT CCGAGCTTCG TATTAGTCAC TAACCTTTGA AAACCAACGT   
  
  
- TATTAAGTTT TAAACTATGA GATACTAAAT GAAAAAATTA ACTTCCAGTA CTAAATTAAT TGATTTACTT   
  
  
- TTCTTAAATA CTAGCTTTTT AAAATTGAGA ATAACTGAAT ATCAACTGAG GTCAATTTAC CTTTTAACTT   
  
  
- CACTAATTAG ATTTTCACCG GGCGAGATTA TGGTAAACTC TTTTTAATAA GAATGACGTT TCAAATTTAG   
  
  
- TTATACACTT CTATCAAATA TCAAAATAGG ATTTTCTTTA ATTTATCTAA CAATTTTTAT TAAAAATTTA   
  
  
- AGTCTATATG CACACTACCG GTATTGGCAT TTACTATACG TACAGCTGTT TAAGGTTTCT TCCGATTATT   
  
  
- AATCTGAATT TATTATGTAC GTATGATTGG TTTTTTTTTT TTAATTCGTT CTAAGATAGG TTGGATAGTA   
  
  
- TTATTTCAAA CCGGTATGAA GTTTGGTAGT GTATTGTAGT GATTATATAT TTCGATCCAA AACCCTTTGG   
  
  
- ATTGAAACTC CTGGTGTATA AACAATTCCG GATCCTCCGG TACTCAAACA GTTCTACGGA GTATCATACC   
  
  
- TCTCCAGATA TAGGAACGTT TTACTAGTAA GGGAAGATGG ATTTTCTTGG TGAAGTAAAG GTGAAAATGA   
  
  
- GGGACCGTTT ACCAACTATT TGGTTTGGTA TTGGTTCGGG AACTTCTTGA TATTTCTGTG CTTAGCTAGT   
  
  
- AACTTAAGCT TGTTCGGAAC ACACTATCCT CCTACCCGGA GTTGTGAAGA GGCGCTCACA CGTTCTCGTT   
  
  
- AGAGCCTCTT TTTAGGATTC TCGGTTGTTG AAGAAACCCA CAATTTACTT GAACAAAGAG GAATACCGCT   
  
  
- AACGCTTGTT TCTAACCGTA TAATGAAGAA TGTTCACAAC AACCGGTTTC GGTTGTTAAA CCCTGGAGTG   
  
  
- AAAGTACTCT CGGATTTTGA ACGGTACCTC TTTTTGACGA CGAAACTATG GATGTACTTC GAATATAACT   
  
  
- TCAAGGTTCT CCAGTCAGGT ACCTGTTGGA AACCAGTACA CCGAAGTTTA CCACGTTATA ACCTCCTGGA   
  
  
- ATCTTCAAAT GTTTTCAGCT AGTAGTTTCT TTGTTCCGTT TCCTACCTCT TCAAAAGTTC CGATTACCCA   
  
  
- CAAGGGAAGT TCAAAGTACA GTATTTGCTC AATCTATTGG ATCCTCTCGA AGCTTTTCTC CTAAATCTGT   
  
  
- AACTTCTACC ACTCCGGTAG CGACACTTGA CACAAGTTCG GAACGTTGCC CAAGTACACC TCTTCTCCCT   
  
  
- CGTACACGAA CTACACTAAG CTAGATAATC CGGAACATAG TATTGTGACC ACCTCCTTCT TCGTCTAGAG   
  
  
- TGAAGATGTT CTTTGCTGAA GAAGTTCACG AAGCTACTCA CAAACTCTAA AAGATTCAGT ATAAAGCTAT   
  
  
- ACAATCTTCT TTCGAAGGGA GGTTATTCGT TGCTTGCTTA GTTCGATCTT TCCCTTGTTA CCTACTTATA   
  
  
- GAGGTCTCGG GATCGAACAG TACCACTTTC ACCTCCTCTT ATATCCGGTT TCTTTCCTTG AGTTACCTTA   
  
  
- CTCTACGAGC TTGTTCGTAA AACGGGTAGA GTTAAATCAC TACTACATGA TTCACTACAA TCCCGTAACA   
  
  
- ACTTTTCTGT GTTTTCACCA ACCCTAGAGT GGAATGGTGT TTCACTGGTA CTTTATCCGT ATGTGAATTT   
  
  
- AACCTTCCCA CTTTTACAAC AAACCCGAAG ACGTACCTCT GGATCGAT

+     ABRE4

| Site Name | Organism | Position | Strand | Matrix score. | sequence | function |
| --- | --- | --- | --- | --- | --- | --- |
| ABRE4 | Zea mays | 1762 | - | 6 | CACGTA |  |

>HU02G03154.1   
+ +Up\_Stream \_Len000ATGATA AAAGATATTT ATTAAGCTCA AGCTATTTTA ACTTTTAAAA AAATGAATGT   
  
  
+ GGCTTTTTTT GCTATTCAGG TACAACACAA TATGAAGCAT TAACTCTAGA AAATGTGGTA ATGTATGTCA   
  
  
+ TATAGGAGAG TATATACTCT CACTTGCCAT GCGATGCGTG CATGTGTGTG TGTGTGTGTA TCTGTTTCTA   
  
  
+ TGCATATTTT ATGTGGGGTT TTGTCATCTA GACATCTATT AATATGTTAG CATTCACATG ACTTATAGGA   
  
  
+ ATGCACTATT ATGAGTATGA TATAGAGTTT TAAAAATGCA TATGCCTTGT GTGTTCATTA GCTTAAAGAA   
  
  
+ GAGTTTTTGA ATATACATAT TTAGGTAATT CTTGCTAATG TCGTAATTAG CAGTTGTATC TATCTTATTG   
  
  
+ ATTTGTATTT TTAATTGCTT GTTATACTGT TACACTTAGA AAATTCATGA ATACTCTTAA AATATGCAAA   
  
  
+ TTATATATAT ATATATATAT ATATATATAT ATATATATAT ATAAAAACTG AATAAGTTAA TATGGTGATG   
  
  
+ ACATTTGTCA TGTTATCATT GGTCGCTTAA TTTTTAAATA AAAAATATGA TTTTGTGACA CCTAATAAAT   
  
  
+ GATATTAGCC ATATTATTAA ATTCAGCATT TATTCATCAA TGTAATATAC TAAATCATGC AATTATCATT   
  
  
+ GGTTCTCTTT TATGTAATAA AGTTAATTGA CAATTCAAGG GTTACTAATT AGTATAATGT TGAATTGGAA   
  
  
+ CGTAAAAATC AAATCGTATG TAAAGTAATT TTATTCTGAA TTTAAAAAAT GGAGCAAATA TCTCTATTGT   
  
  
+ TCTTTAATAA AATTTTAATT GATTTTTATT TCTAATTAAG TGACGGAACC AAATTTTTTT CCTCCTTTTT   
  
  
+ TGGTTTGGTA AGTTATCATT AGCCAATCTT TTATTTACAT TTTGTCAGTT TTTCTTTTTT AAGATGGTTA   
  
  
+ AATGGCTTCA ATTAAACAAA TTTTTTTACT TATTTATATT TTTAATAAAT TTTCAAAATG TATATTAACG   
  
  
+ CATGACCGTG CGAAGCACGG AATCTACCCT AGTTAATCTA TGATTCTTAA ATATGCAAGT AGGCTGTTAT   
  
  
+ TCATTTGTTG TTTTTTACTT ATTTATATTT TTTCCCTCCC GGTATGATAG GGTTTGTCAT ATTGTTATTC   
  
  
+ ATTAGCTGTT AAACTGTATT CATTATAGCA CAAGTAGGCT TAATCAAAGA TAGTTAGTCT CCAATTTCAT   
  
  
+ CAGAAAATTT AAGAAAAAGA CACCTTAGGA TCAAAAGTAT GAAATTTAGA GACCAGACAA CGATAGAATT   
  
  
+ GAAAAGATGA GTTTTCAATT TGGAGAAGAC GGAAACTCAG ATGAATGAAA GTATAGTACA TTGTACTCCT   
  
  
+ TGGAGTATAA TCTTATCTTC ACTATTGATA GGCTCGAAGC ATAATCAGTG ATTGGAAACT TTTGGTTGCA   
  
  
+ ATAATTCAAA ATTTGATACT CTATGATTTA CTTTTTTAAT TGAAGGTCAT GATTTAATTA ACTAAATGAA   
  
  
+ AAGAATTTAT GATCGAAAAA TTTTAACTCT TATTGACTTA TAGTTGACTC CAGTTAAATG GAAAATTGAA   
  
  
+ GTGATTAATC TAAAAGTGGC CCGCTCTAAT ACCATTTGAG AAAAATTATT CTTACTGCAA AGTTTAAATC   
  
  
+ AATATGTGAA GATAGTTTAT AGTTTTATCC TAAAAGAAAT TAAATAGATT GTTAAAAATA ATTTTTAAAT   
  
  
+ TCAGATATAC GTGTGATGGC CATAACCGTA AATGATATGC ATGTCGACAA ATTCCAAAGA AGGCTAATAA   
  
  
+ TTAGACTTAA ATAATACATG CATACTAACC AAAAAAAAAA AATTAAGCAA GATTCTATCC AACCTATCAT   
  
  
+ AATAAAGTTT GGCCATACTT CAAACCATCA CATAACATCA CTAATATATA AAGCTAGGTT TTGGGAAACC   
  
  
+ TAACTTTGAG GACCACATAT TTGTTAAGGC CTAGGAGGCC ATGAGTTTGT CAAGATGCCT CATAGTATGG   
  
  
+ AGAGGTCTAT ATCCTTGCAA AATGATCATT CCCTTCTACC TAAAAGAACC ACTTCATTTC CACTTTTACT   
  
  
+ CCCTGGCAAA TGGTTGATAA ACCAAACCAT AACCAAGCCC TTGAAGAACT ATAAAGACAC GAATCGATCA   
  
  
+ TTGAATTCGA ACAAGCCTTG TGTGATAGGA GGATGGGCCT CAACACTTCT CCGCGAGTGT GCAAGAGCAA   
  
  
+ TCTCGGAGAA AAATCCTAAG AGCCAACAAC TTCTTTGGGT GTTAAATGAA CTTGTTTCTC CTTATGGCGA   
  
  
+ TTGCGAACAA AGATTGGCAT ATTACTTCTT ACAAGTGTTG TTGGCCAAAG CCAACAATTT GGGACCTCAC   
  
  
+ TTTCATGAGA GCCTAAAACT TGCCATGGAG AAAAACTGCT GCTTTGATAC CTACATGAAG CTTATATTGA   
  
  
+ AGTTCCAAGA GGTCAGTCCA TGGACAACCT TTGGTCATGT GGCTTCAAAT GGTGCAATAT TGGAGGACCT   
  
  
+ TAGAAGTTTA CAAAAGTCGA TCATCAAAGA AACAAGGCAA AGGATGGAGA AGTTTTCAAG GCTAATGGGT   
  
  
+ GTTCCCTTCA AGTTTCATGT CATAAACGAG TTAGATAACC TAGGAGAGCT TCGAAAAGAG GATTTAGACA   
  
  
+ TTGAAGATGG TGAGGCCATC GCTGTGAACT GTGTTCAAGC CTTGCAACGG GTTCATGTGG AGAAGAGGGA   
  
  
+ GCATGTGCTT GATGTGATTC GATCTATTAG GCCTTGTATC ATAACACTGG TGGAGGAAGA AGCAGATCTC   
  
  
+ ACTTCTACAA GAAACGACTT CTTCAAGTGC TTCGATGAGT GTTTGAGATT TTCTAAGTCA TATTTCGATA   
  
  
+ TGTTAGAAGA AAGCTTCCCT CCAATAAGCA ACGAACGAAT CAAGCTAGAA AGGGAACAAT GGATGAATAT   
  
  
+ CTCCAGAGCC CTAGCTTGTC ATGGTGAAAG TGGAGGAGAA TATAGGCCAA AGAAAGGAAC TCAATGGAAT   
  
  
+ GAGATGCTCG AACAAGCATT TTGCCCATCT CAATTTAGTG ATGATGTACT AAGTGATGTT AGGGCATTGT   
  
  
+ TGAAAAGACA CAAAAGTGGT TGGGATCTCA CCTTACCACA AAGTGACCAT GAAATAGGCA TACACTTAAA   
  
  
+ TTGGAAGGGT GAAAATGTTG TTTGGGCTTC TGCATGGAGA CCTAGCTA  

- +Up\_Stream \_Len000TACTAT TTTCTATAAA TAATTCGAGT TCGATAAAAT TGAAAATTTT TTTACTTACA   
  
  
- CCGAAAAAAA CGATAAGTCC ATGTTGTGTT ATACTTCGTA ATTGAGATCT TTTACACCAT TACATACAGT   
  
  
- ATATCCTCTC ATATATGAGA GTGAACGGTA CGCTACGCAC GTACACACAC ACACACACAT AGACAAAGAT   
  
  
- ACGTATAAAA TACACCCCAA AACAGTAGAT CTGTAGATAA TTATACAATC GTAAGTGTAC TGAATATCCT   
  
  
- TACGTGATAA TACTCATACT ATATCTCAAA ATTTTTACGT ATACGGAACA CACAAGTAAT CGAATTTCTT   
  
  
- CTCAAAAACT TATATGTATA AATCCATTAA GAACGATTAC AGCATTAATC GTCAACATAG ATAGAATAAC   
  
  
- TAAACATAAA AATTAACGAA CAATATGACA ATGTGAATCT TTTAAGTACT TATGAGAATT TTATACGTTT   
  
  
- AATATATATA TATATATATA TATATATATA TATATATATA TATTTTTGAC TTATTCAATT ATACCACTAC   
  
  
- TGTAAACAGT ACAATAGTAA CCAGCGAATT AAAAATTTAT TTTTTATACT AAAACACTGT GGATTATTTA   
  
  
- CTATAATCGG TATAATAATT TAAGTCGTAA ATAAGTAGTT ACATTATATG ATTTAGTACG TTAATAGTAA   
  
  
- CCAAGAGAAA ATACATTATT TCAATTAACT GTTAAGTTCC CAATGATTAA TCATATTACA ACTTAACCTT   
  
  
- GCATTTTTAG TTTAGCATAC ATTTCATTAA AATAAGACTT AAATTTTTTA CCTCGTTTAT AGAGATAACA   
  
  
- AGAAATTATT TTAAAATTAA CTAAAAATAA AGATTAATTC ACTGCCTTGG TTTAAAAAAA GGAGGAAAAA   
  
  
- ACCAAACCAT TCAATAGTAA TCGGTTAGAA AATAAATGTA AAACAGTCAA AAAGAAAAAA TTCTACCAAT   
  
  
- TTACCGAAGT TAATTTGTTT AAAAAAATGA ATAAATATAA AAATTATTTA AAAGTTTTAC ATATAATTGC   
  
  
- GTACTGGCAC GCTTCGTGCC TTAGATGGGA TCAATTAGAT ACTAAGAATT TATACGTTCA TCCGACAATA   
  
  
- AGTAAACAAC AAAAAATGAA TAAATATAAA AAAGGGAGGG CCATACTATC CCAAACAGTA TAACAATAAG   
  
  
- TAATCGACAA TTTGACATAA GTAATATCGT GTTCATCCGA ATTAGTTTCT ATCAATCAGA GGTTAAAGTA   
  
  
- GTCTTTTAAA TTCTTTTTCT GTGGAATCCT AGTTTTCATA CTTTAAATCT CTGGTCTGTT GCTATCTTAA   
  
  
- CTTTTCTACT CAAAAGTTAA ACCTCTTCTG CCTTTGAGTC TACTTACTTT CATATCATGT AACATGAGGA   
  
  
- ACCTCATATT AGAATAGAAG TGATAACTAT CCGAGCTTCG TATTAGTCAC TAACCTTTGA AAACCAACGT   
  
  
- TATTAAGTTT TAAACTATGA GATACTAAAT GAAAAAATTA ACTTCCAGTA CTAAATTAAT TGATTTACTT   
  
  
- TTCTTAAATA CTAGCTTTTT AAAATTGAGA ATAACTGAAT ATCAACTGAG GTCAATTTAC CTTTTAACTT   
  
  
- CACTAATTAG ATTTTCACCG GGCGAGATTA TGGTAAACTC TTTTTAATAA GAATGACGTT TCAAATTTAG   
  
  
- TTATACACTT CTATCAAATA TCAAAATAGG ATTTTCTTTA ATTTATCTAA CAATTTTTAT TAAAAATTTA   
  
  
- AGTCTATATG CACACTACCG GTATTGGCAT TTACTATACG TACAGCTGTT TAAGGTTTCT TCCGATTATT   
  
  
- AATCTGAATT TATTATGTAC GTATGATTGG TTTTTTTTTT TTAATTCGTT CTAAGATAGG TTGGATAGTA   
  
  
- TTATTTCAAA CCGGTATGAA GTTTGGTAGT GTATTGTAGT GATTATATAT TTCGATCCAA AACCCTTTGG   
  
  
- ATTGAAACTC CTGGTGTATA AACAATTCCG GATCCTCCGG TACTCAAACA GTTCTACGGA GTATCATACC   
  
  
- TCTCCAGATA TAGGAACGTT TTACTAGTAA GGGAAGATGG ATTTTCTTGG TGAAGTAAAG GTGAAAATGA   
  
  
- GGGACCGTTT ACCAACTATT TGGTTTGGTA TTGGTTCGGG AACTTCTTGA TATTTCTGTG CTTAGCTAGT   
  
  
- AACTTAAGCT TGTTCGGAAC ACACTATCCT CCTACCCGGA GTTGTGAAGA GGCGCTCACA CGTTCTCGTT   
  
  
- AGAGCCTCTT TTTAGGATTC TCGGTTGTTG AAGAAACCCA CAATTTACTT GAACAAAGAG GAATACCGCT   
  
  
- AACGCTTGTT TCTAACCGTA TAATGAAGAA TGTTCACAAC AACCGGTTTC GGTTGTTAAA CCCTGGAGTG   
  
  
- AAAGTACTCT CGGATTTTGA ACGGTACCTC TTTTTGACGA CGAAACTATG GATGTACTTC GAATATAACT   
  
  
- TCAAGGTTCT CCAGTCAGGT ACCTGTTGGA AACCAGTACA CCGAAGTTTA CCACGTTATA ACCTCCTGGA   
  
  
- ATCTTCAAAT GTTTTCAGCT AGTAGTTTCT TTGTTCCGTT TCCTACCTCT TCAAAAGTTC CGATTACCCA   
  
  
- CAAGGGAAGT TCAAAGTACA GTATTTGCTC AATCTATTGG ATCCTCTCGA AGCTTTTCTC CTAAATCTGT   
  
  
- AACTTCTACC ACTCCGGTAG CGACACTTGA CACAAGTTCG GAACGTTGCC CAAGTACACC TCTTCTCCCT   
  
  
- CGTACACGAA CTACACTAAG CTAGATAATC CGGAACATAG TATTGTGACC ACCTCCTTCT TCGTCTAGAG   
  
  
- TGAAGATGTT CTTTGCTGAA GAAGTTCACG AAGCTACTCA CAAACTCTAA AAGATTCAGT ATAAAGCTAT   
  
  
- ACAATCTTCT TTCGAAGGGA GGTTATTCGT TGCTTGCTTA GTTCGATCTT TCCCTTGTTA CCTACTTATA   
  
  
- GAGGTCTCGG GATCGAACAG TACCACTTTC ACCTCCTCTT ATATCCGGTT TCTTTCCTTG AGTTACCTTA   
  
  
- CTCTACGAGC TTGTTCGTAA AACGGGTAGA GTTAAATCAC TACTACATGA TTCACTACAA TCCCGTAACA   
  
  
- ACTTTTCTGT GTTTTCACCA ACCCTAGAGT GGAATGGTGT TTCACTGGTA CTTTATCCGT ATGTGAATTT   
  
  
- AACCTTCCCA CTTTTACAAC AAACCCGAAG ACGTACCTCT GGATCGAT

+     ACE

| Site Name | Organism | Position | Strand | Matrix score. | sequence | function |
| --- | --- | --- | --- | --- | --- | --- |
| ACE | Petroselinum crispum | 255 | - | 9 | CTAACGTATT | cis-acting element involved in light responsiveness |

>HU02G03154.1   
+ +Up\_Stream \_Len000ATGATA AAAGATATTT ATTAAGCTCA AGCTATTTTA ACTTTTAAAA AAATGAATGT   
  
  
+ GGCTTTTTTT GCTATTCAGG TACAACACAA TATGAAGCAT TAACTCTAGA AAATGTGGTA ATGTATGTCA   
  
  
+ TATAGGAGAG TATATACTCT CACTTGCCAT GCGATGCGTG CATGTGTGTG TGTGTGTGTA TCTGTTTCTA   
  
  
+ TGCATATTTT ATGTGGGGTT TTGTCATCTA GACATCTATT AATATGTTAG CATTCACATG ACTTATAGGA   
  
  
+ ATGCACTATT ATGAGTATGA TATAGAGTTT TAAAAATGCA TATGCCTTGT GTGTTCATTA GCTTAAAGAA   
  
  
+ GAGTTTTTGA ATATACATAT TTAGGTAATT CTTGCTAATG TCGTAATTAG CAGTTGTATC TATCTTATTG   
  
  
+ ATTTGTATTT TTAATTGCTT GTTATACTGT TACACTTAGA AAATTCATGA ATACTCTTAA AATATGCAAA   
  
  
+ TTATATATAT ATATATATAT ATATATATAT ATATATATAT ATAAAAACTG AATAAGTTAA TATGGTGATG   
  
  
+ ACATTTGTCA TGTTATCATT GGTCGCTTAA TTTTTAAATA AAAAATATGA TTTTGTGACA CCTAATAAAT   
  
  
+ GATATTAGCC ATATTATTAA ATTCAGCATT TATTCATCAA TGTAATATAC TAAATCATGC AATTATCATT   
  
  
+ GGTTCTCTTT TATGTAATAA AGTTAATTGA CAATTCAAGG GTTACTAATT AGTATAATGT TGAATTGGAA   
  
  
+ CGTAAAAATC AAATCGTATG TAAAGTAATT TTATTCTGAA TTTAAAAAAT GGAGCAAATA TCTCTATTGT   
  
  
+ TCTTTAATAA AATTTTAATT GATTTTTATT TCTAATTAAG TGACGGAACC AAATTTTTTT CCTCCTTTTT   
  
  
+ TGGTTTGGTA AGTTATCATT AGCCAATCTT TTATTTACAT TTTGTCAGTT TTTCTTTTTT AAGATGGTTA   
  
  
+ AATGGCTTCA ATTAAACAAA TTTTTTTACT TATTTATATT TTTAATAAAT TTTCAAAATG TATATTAACG   
  
  
+ CATGACCGTG CGAAGCACGG AATCTACCCT AGTTAATCTA TGATTCTTAA ATATGCAAGT AGGCTGTTAT   
  
  
+ TCATTTGTTG TTTTTTACTT ATTTATATTT TTTCCCTCCC GGTATGATAG GGTTTGTCAT ATTGTTATTC   
  
  
+ ATTAGCTGTT AAACTGTATT CATTATAGCA CAAGTAGGCT TAATCAAAGA TAGTTAGTCT CCAATTTCAT   
  
  
+ CAGAAAATTT AAGAAAAAGA CACCTTAGGA TCAAAAGTAT GAAATTTAGA GACCAGACAA CGATAGAATT   
  
  
+ GAAAAGATGA GTTTTCAATT TGGAGAAGAC GGAAACTCAG ATGAATGAAA GTATAGTACA TTGTACTCCT   
  
  
+ TGGAGTATAA TCTTATCTTC ACTATTGATA GGCTCGAAGC ATAATCAGTG ATTGGAAACT TTTGGTTGCA   
  
  
+ ATAATTCAAA ATTTGATACT CTATGATTTA CTTTTTTAAT TGAAGGTCAT GATTTAATTA ACTAAATGAA   
  
  
+ AAGAATTTAT GATCGAAAAA TTTTAACTCT TATTGACTTA TAGTTGACTC CAGTTAAATG GAAAATTGAA   
  
  
+ GTGATTAATC TAAAAGTGGC CCGCTCTAAT ACCATTTGAG AAAAATTATT CTTACTGCAA AGTTTAAATC   
  
  
+ AATATGTGAA GATAGTTTAT AGTTTTATCC TAAAAGAAAT TAAATAGATT GTTAAAAATA ATTTTTAAAT   
  
  
+ TCAGATATAC GTGTGATGGC CATAACCGTA AATGATATGC ATGTCGACAA ATTCCAAAGA AGGCTAATAA   
  
  
+ TTAGACTTAA ATAATACATG CATACTAACC AAAAAAAAAA AATTAAGCAA GATTCTATCC AACCTATCAT   
  
  
+ AATAAAGTTT GGCCATACTT CAAACCATCA CATAACATCA CTAATATATA AAGCTAGGTT TTGGGAAACC   
  
  
+ TAACTTTGAG GACCACATAT TTGTTAAGGC CTAGGAGGCC ATGAGTTTGT CAAGATGCCT CATAGTATGG   
  
  
+ AGAGGTCTAT ATCCTTGCAA AATGATCATT CCCTTCTACC TAAAAGAACC ACTTCATTTC CACTTTTACT   
  
  
+ CCCTGGCAAA TGGTTGATAA ACCAAACCAT AACCAAGCCC TTGAAGAACT ATAAAGACAC GAATCGATCA   
  
  
+ TTGAATTCGA ACAAGCCTTG TGTGATAGGA GGATGGGCCT CAACACTTCT CCGCGAGTGT GCAAGAGCAA   
  
  
+ TCTCGGAGAA AAATCCTAAG AGCCAACAAC TTCTTTGGGT GTTAAATGAA CTTGTTTCTC CTTATGGCGA   
  
  
+ TTGCGAACAA AGATTGGCAT ATTACTTCTT ACAAGTGTTG TTGGCCAAAG CCAACAATTT GGGACCTCAC   
  
  
+ TTTCATGAGA GCCTAAAACT TGCCATGGAG AAAAACTGCT GCTTTGATAC CTACATGAAG CTTATATTGA   
  
  
+ AGTTCCAAGA GGTCAGTCCA TGGACAACCT TTGGTCATGT GGCTTCAAAT GGTGCAATAT TGGAGGACCT   
  
  
+ TAGAAGTTTA CAAAAGTCGA TCATCAAAGA AACAAGGCAA AGGATGGAGA AGTTTTCAAG GCTAATGGGT   
  
  
+ GTTCCCTTCA AGTTTCATGT CATAAACGAG TTAGATAACC TAGGAGAGCT TCGAAAAGAG GATTTAGACA   
  
  
+ TTGAAGATGG TGAGGCCATC GCTGTGAACT GTGTTCAAGC CTTGCAACGG GTTCATGTGG AGAAGAGGGA   
  
  
+ GCATGTGCTT GATGTGATTC GATCTATTAG GCCTTGTATC ATAACACTGG TGGAGGAAGA AGCAGATCTC   
  
  
+ ACTTCTACAA GAAACGACTT CTTCAAGTGC TTCGATGAGT GTTTGAGATT TTCTAAGTCA TATTTCGATA   
  
  
+ TGTTAGAAGA AAGCTTCCCT CCAATAAGCA ACGAACGAAT CAAGCTAGAA AGGGAACAAT GGATGAATAT   
  
  
+ CTCCAGAGCC CTAGCTTGTC ATGGTGAAAG TGGAGGAGAA TATAGGCCAA AGAAAGGAAC TCAATGGAAT   
  
  
+ GAGATGCTCG AACAAGCATT TTGCCCATCT CAATTTAGTG ATGATGTACT AAGTGATGTT AGGGCATTGT   
  
  
+ TGAAAAGACA CAAAAGTGGT TGGGATCTCA CCTTACCACA AAGTGACCAT GAAATAGGCA TACACTTAAA   
  
  
+ TTGGAAGGGT GAAAATGTTG TTTGGGCTTC TGCATGGAGA CCTAGCTA  

- +Up\_Stream \_Len000TACTAT TTTCTATAAA TAATTCGAGT TCGATAAAAT TGAAAATTTT TTTACTTACA   
  
  
- CCGAAAAAAA CGATAAGTCC ATGTTGTGTT ATACTTCGTA ATTGAGATCT TTTACACCAT TACATACAGT   
  
  
- ATATCCTCTC ATATATGAGA GTGAACGGTA CGCTACGCAC GTACACACAC ACACACACAT AGACAAAGAT   
  
  
- ACGTATAAAA TACACCCCAA AACAGTAGAT CTGTAGATAA TTATACAATC GTAAGTGTAC TGAATATCCT   
  
  
- TACGTGATAA TACTCATACT ATATCTCAAA ATTTTTACGT ATACGGAACA CACAAGTAAT CGAATTTCTT   
  
  
- CTCAAAAACT TATATGTATA AATCCATTAA GAACGATTAC AGCATTAATC GTCAACATAG ATAGAATAAC   
  
  
- TAAACATAAA AATTAACGAA CAATATGACA ATGTGAATCT TTTAAGTACT TATGAGAATT TTATACGTTT   
  
  
- AATATATATA TATATATATA TATATATATA TATATATATA TATTTTTGAC TTATTCAATT ATACCACTAC   
  
  
- TGTAAACAGT ACAATAGTAA CCAGCGAATT AAAAATTTAT TTTTTATACT AAAACACTGT GGATTATTTA   
  
  
- CTATAATCGG TATAATAATT TAAGTCGTAA ATAAGTAGTT ACATTATATG ATTTAGTACG TTAATAGTAA   
  
  
- CCAAGAGAAA ATACATTATT TCAATTAACT GTTAAGTTCC CAATGATTAA TCATATTACA ACTTAACCTT   
  
  
- GCATTTTTAG TTTAGCATAC ATTTCATTAA AATAAGACTT AAATTTTTTA CCTCGTTTAT AGAGATAACA   
  
  
- AGAAATTATT TTAAAATTAA CTAAAAATAA AGATTAATTC ACTGCCTTGG TTTAAAAAAA GGAGGAAAAA   
  
  
- ACCAAACCAT TCAATAGTAA TCGGTTAGAA AATAAATGTA AAACAGTCAA AAAGAAAAAA TTCTACCAAT   
  
  
- TTACCGAAGT TAATTTGTTT AAAAAAATGA ATAAATATAA AAATTATTTA AAAGTTTTAC ATATAATTGC   
  
  
- GTACTGGCAC GCTTCGTGCC TTAGATGGGA TCAATTAGAT ACTAAGAATT TATACGTTCA TCCGACAATA   
  
  
- AGTAAACAAC AAAAAATGAA TAAATATAAA AAAGGGAGGG CCATACTATC CCAAACAGTA TAACAATAAG   
  
  
- TAATCGACAA TTTGACATAA GTAATATCGT GTTCATCCGA ATTAGTTTCT ATCAATCAGA GGTTAAAGTA   
  
  
- GTCTTTTAAA TTCTTTTTCT GTGGAATCCT AGTTTTCATA CTTTAAATCT CTGGTCTGTT GCTATCTTAA   
  
  
- CTTTTCTACT CAAAAGTTAA ACCTCTTCTG CCTTTGAGTC TACTTACTTT CATATCATGT AACATGAGGA   
  
  
- ACCTCATATT AGAATAGAAG TGATAACTAT CCGAGCTTCG TATTAGTCAC TAACCTTTGA AAACCAACGT   
  
  
- TATTAAGTTT TAAACTATGA GATACTAAAT GAAAAAATTA ACTTCCAGTA CTAAATTAAT TGATTTACTT   
  
  
- TTCTTAAATA CTAGCTTTTT AAAATTGAGA ATAACTGAAT ATCAACTGAG GTCAATTTAC CTTTTAACTT   
  
  
- CACTAATTAG ATTTTCACCG GGCGAGATTA TGGTAAACTC TTTTTAATAA GAATGACGTT TCAAATTTAG   
  
  
- TTATACACTT CTATCAAATA TCAAAATAGG ATTTTCTTTA ATTTATCTAA CAATTTTTAT TAAAAATTTA   
  
  
- AGTCTATATG CACACTACCG GTATTGGCAT TTACTATACG TACAGCTGTT TAAGGTTTCT TCCGATTATT   
  
  
- AATCTGAATT TATTATGTAC GTATGATTGG TTTTTTTTTT TTAATTCGTT CTAAGATAGG TTGGATAGTA   
  
  
- TTATTTCAAA CCGGTATGAA GTTTGGTAGT GTATTGTAGT GATTATATAT TTCGATCCAA AACCCTTTGG   
  
  
- ATTGAAACTC CTGGTGTATA AACAATTCCG GATCCTCCGG TACTCAAACA GTTCTACGGA GTATCATACC   
  
  
- TCTCCAGATA TAGGAACGTT TTACTAGTAA GGGAAGATGG ATTTTCTTGG TGAAGTAAAG GTGAAAATGA   
  
  
- GGGACCGTTT ACCAACTATT TGGTTTGGTA TTGGTTCGGG AACTTCTTGA TATTTCTGTG CTTAGCTAGT   
  
  
- AACTTAAGCT TGTTCGGAAC ACACTATCCT CCTACCCGGA GTTGTGAAGA GGCGCTCACA CGTTCTCGTT   
  
  
- AGAGCCTCTT TTTAGGATTC TCGGTTGTTG AAGAAACCCA CAATTTACTT GAACAAAGAG GAATACCGCT   
  
  
- AACGCTTGTT TCTAACCGTA TAATGAAGAA TGTTCACAAC AACCGGTTTC GGTTGTTAAA CCCTGGAGTG   
  
  
- AAAGTACTCT CGGATTTTGA ACGGTACCTC TTTTTGACGA CGAAACTATG GATGTACTTC GAATATAACT   
  
  
- TCAAGGTTCT CCAGTCAGGT ACCTGTTGGA AACCAGTACA CCGAAGTTTA CCACGTTATA ACCTCCTGGA   
  
  
- ATCTTCAAAT GTTTTCAGCT AGTAGTTTCT TTGTTCCGTT TCCTACCTCT TCAAAAGTTC CGATTACCCA   
  
  
- CAAGGGAAGT TCAAAGTACA GTATTTGCTC AATCTATTGG ATCCTCTCGA AGCTTTTCTC CTAAATCTGT   
  
  
- AACTTCTACC ACTCCGGTAG CGACACTTGA CACAAGTTCG GAACGTTGCC CAAGTACACC TCTTCTCCCT   
  
  
- CGTACACGAA CTACACTAAG CTAGATAATC CGGAACATAG TATTGTGACC ACCTCCTTCT TCGTCTAGAG   
  
  
- TGAAGATGTT CTTTGCTGAA GAAGTTCACG AAGCTACTCA CAAACTCTAA AAGATTCAGT ATAAAGCTAT   
  
  
- ACAATCTTCT TTCGAAGGGA GGTTATTCGT TGCTTGCTTA GTTCGATCTT TCCCTTGTTA CCTACTTATA   
  
  
- GAGGTCTCGG GATCGAACAG TACCACTTTC ACCTCCTCTT ATATCCGGTT TCTTTCCTTG AGTTACCTTA   
  
  
- CTCTACGAGC TTGTTCGTAA AACGGGTAGA GTTAAATCAC TACTACATGA TTCACTACAA TCCCGTAACA   
  
  
- ACTTTTCTGT GTTTTCACCA ACCCTAGAGT GGAATGGTGT TTCACTGGTA CTTTATCCGT ATGTGAATTT   
  
  
- AACCTTCCCA CTTTTACAAC AAACCCGAAG ACGTACCTCT GGATCGAT

+     AE-box

| Site Name | Organism | Position | Strand | Matrix score. | sequence | function |
| --- | --- | --- | --- | --- | --- | --- |
| AE-box | Arabidopsis thaliana | 2296 | - | 8 | AGAAACAA | part of a module for light response |
| AE-box | Arabidopsis thaliana | 2552 | + | 8 | AGAAACAA | part of a module for light response |

>HU02G03154.1   
+ +Up\_Stream \_Len000ATGATA AAAGATATTT ATTAAGCTCA AGCTATTTTA ACTTTTAAAA AAATGAATGT   
  
  
+ GGCTTTTTTT GCTATTCAGG TACAACACAA TATGAAGCAT TAACTCTAGA AAATGTGGTA ATGTATGTCA   
  
  
+ TATAGGAGAG TATATACTCT CACTTGCCAT GCGATGCGTG CATGTGTGTG TGTGTGTGTA TCTGTTTCTA   
  
  
+ TGCATATTTT ATGTGGGGTT TTGTCATCTA GACATCTATT AATATGTTAG CATTCACATG ACTTATAGGA   
  
  
+ ATGCACTATT ATGAGTATGA TATAGAGTTT TAAAAATGCA TATGCCTTGT GTGTTCATTA GCTTAAAGAA   
  
  
+ GAGTTTTTGA ATATACATAT TTAGGTAATT CTTGCTAATG TCGTAATTAG CAGTTGTATC TATCTTATTG   
  
  
+ ATTTGTATTT TTAATTGCTT GTTATACTGT TACACTTAGA AAATTCATGA ATACTCTTAA AATATGCAAA   
  
  
+ TTATATATAT ATATATATAT ATATATATAT ATATATATAT ATAAAAACTG AATAAGTTAA TATGGTGATG   
  
  
+ ACATTTGTCA TGTTATCATT GGTCGCTTAA TTTTTAAATA AAAAATATGA TTTTGTGACA CCTAATAAAT   
  
  
+ GATATTAGCC ATATTATTAA ATTCAGCATT TATTCATCAA TGTAATATAC TAAATCATGC AATTATCATT   
  
  
+ GGTTCTCTTT TATGTAATAA AGTTAATTGA CAATTCAAGG GTTACTAATT AGTATAATGT TGAATTGGAA   
  
  
+ CGTAAAAATC AAATCGTATG TAAAGTAATT TTATTCTGAA TTTAAAAAAT GGAGCAAATA TCTCTATTGT   
  
  
+ TCTTTAATAA AATTTTAATT GATTTTTATT TCTAATTAAG TGACGGAACC AAATTTTTTT CCTCCTTTTT   
  
  
+ TGGTTTGGTA AGTTATCATT AGCCAATCTT TTATTTACAT TTTGTCAGTT TTTCTTTTTT AAGATGGTTA   
  
  
+ AATGGCTTCA ATTAAACAAA TTTTTTTACT TATTTATATT TTTAATAAAT TTTCAAAATG TATATTAACG   
  
  
+ CATGACCGTG CGAAGCACGG AATCTACCCT AGTTAATCTA TGATTCTTAA ATATGCAAGT AGGCTGTTAT   
  
  
+ TCATTTGTTG TTTTTTACTT ATTTATATTT TTTCCCTCCC GGTATGATAG GGTTTGTCAT ATTGTTATTC   
  
  
+ ATTAGCTGTT AAACTGTATT CATTATAGCA CAAGTAGGCT TAATCAAAGA TAGTTAGTCT CCAATTTCAT   
  
  
+ CAGAAAATTT AAGAAAAAGA CACCTTAGGA TCAAAAGTAT GAAATTTAGA GACCAGACAA CGATAGAATT   
  
  
+ GAAAAGATGA GTTTTCAATT TGGAGAAGAC GGAAACTCAG ATGAATGAAA GTATAGTACA TTGTACTCCT   
  
  
+ TGGAGTATAA TCTTATCTTC ACTATTGATA GGCTCGAAGC ATAATCAGTG ATTGGAAACT TTTGGTTGCA   
  
  
+ ATAATTCAAA ATTTGATACT CTATGATTTA CTTTTTTAAT TGAAGGTCAT GATTTAATTA ACTAAATGAA   
  
  
+ AAGAATTTAT GATCGAAAAA TTTTAACTCT TATTGACTTA TAGTTGACTC CAGTTAAATG GAAAATTGAA   
  
  
+ GTGATTAATC TAAAAGTGGC CCGCTCTAAT ACCATTTGAG AAAAATTATT CTTACTGCAA AGTTTAAATC   
  
  
+ AATATGTGAA GATAGTTTAT AGTTTTATCC TAAAAGAAAT TAAATAGATT GTTAAAAATA ATTTTTAAAT   
  
  
+ TCAGATATAC GTGTGATGGC CATAACCGTA AATGATATGC ATGTCGACAA ATTCCAAAGA AGGCTAATAA   
  
  
+ TTAGACTTAA ATAATACATG CATACTAACC AAAAAAAAAA AATTAAGCAA GATTCTATCC AACCTATCAT   
  
  
+ AATAAAGTTT GGCCATACTT CAAACCATCA CATAACATCA CTAATATATA AAGCTAGGTT TTGGGAAACC   
  
  
+ TAACTTTGAG GACCACATAT TTGTTAAGGC CTAGGAGGCC ATGAGTTTGT CAAGATGCCT CATAGTATGG   
  
  
+ AGAGGTCTAT ATCCTTGCAA AATGATCATT CCCTTCTACC TAAAAGAACC ACTTCATTTC CACTTTTACT   
  
  
+ CCCTGGCAAA TGGTTGATAA ACCAAACCAT AACCAAGCCC TTGAAGAACT ATAAAGACAC GAATCGATCA   
  
  
+ TTGAATTCGA ACAAGCCTTG TGTGATAGGA GGATGGGCCT CAACACTTCT CCGCGAGTGT GCAAGAGCAA   
  
  
+ TCTCGGAGAA AAATCCTAAG AGCCAACAAC TTCTTTGGGT GTTAAATGAA CTTGTTTCTC CTTATGGCGA   
  
  
+ TTGCGAACAA AGATTGGCAT ATTACTTCTT ACAAGTGTTG TTGGCCAAAG CCAACAATTT GGGACCTCAC   
  
  
+ TTTCATGAGA GCCTAAAACT TGCCATGGAG AAAAACTGCT GCTTTGATAC CTACATGAAG CTTATATTGA   
  
  
+ AGTTCCAAGA GGTCAGTCCA TGGACAACCT TTGGTCATGT GGCTTCAAAT GGTGCAATAT TGGAGGACCT   
  
  
+ TAGAAGTTTA CAAAAGTCGA TCATCAAAGA AACAAGGCAA AGGATGGAGA AGTTTTCAAG GCTAATGGGT   
  
  
+ GTTCCCTTCA AGTTTCATGT CATAAACGAG TTAGATAACC TAGGAGAGCT TCGAAAAGAG GATTTAGACA   
  
  
+ TTGAAGATGG TGAGGCCATC GCTGTGAACT GTGTTCAAGC CTTGCAACGG GTTCATGTGG AGAAGAGGGA   
  
  
+ GCATGTGCTT GATGTGATTC GATCTATTAG GCCTTGTATC ATAACACTGG TGGAGGAAGA AGCAGATCTC   
  
  
+ ACTTCTACAA GAAACGACTT CTTCAAGTGC TTCGATGAGT GTTTGAGATT TTCTAAGTCA TATTTCGATA   
  
  
+ TGTTAGAAGA AAGCTTCCCT CCAATAAGCA ACGAACGAAT CAAGCTAGAA AGGGAACAAT GGATGAATAT   
  
  
+ CTCCAGAGCC CTAGCTTGTC ATGGTGAAAG TGGAGGAGAA TATAGGCCAA AGAAAGGAAC TCAATGGAAT   
  
  
+ GAGATGCTCG AACAAGCATT TTGCCCATCT CAATTTAGTG ATGATGTACT AAGTGATGTT AGGGCATTGT   
  
  
+ TGAAAAGACA CAAAAGTGGT TGGGATCTCA CCTTACCACA AAGTGACCAT GAAATAGGCA TACACTTAAA   
  
  
+ TTGGAAGGGT GAAAATGTTG TTTGGGCTTC TGCATGGAGA CCTAGCTA  

- +Up\_Stream \_Len000TACTAT TTTCTATAAA TAATTCGAGT TCGATAAAAT TGAAAATTTT TTTACTTACA   
  
  
- CCGAAAAAAA CGATAAGTCC ATGTTGTGTT ATACTTCGTA ATTGAGATCT TTTACACCAT TACATACAGT   
  
  
- ATATCCTCTC ATATATGAGA GTGAACGGTA CGCTACGCAC GTACACACAC ACACACACAT AGACAAAGAT   
  
  
- ACGTATAAAA TACACCCCAA AACAGTAGAT CTGTAGATAA TTATACAATC GTAAGTGTAC TGAATATCCT   
  
  
- TACGTGATAA TACTCATACT ATATCTCAAA ATTTTTACGT ATACGGAACA CACAAGTAAT CGAATTTCTT   
  
  
- CTCAAAAACT TATATGTATA AATCCATTAA GAACGATTAC AGCATTAATC GTCAACATAG ATAGAATAAC   
  
  
- TAAACATAAA AATTAACGAA CAATATGACA ATGTGAATCT TTTAAGTACT TATGAGAATT TTATACGTTT   
  
  
- AATATATATA TATATATATA TATATATATA TATATATATA TATTTTTGAC TTATTCAATT ATACCACTAC   
  
  
- TGTAAACAGT ACAATAGTAA CCAGCGAATT AAAAATTTAT TTTTTATACT AAAACACTGT GGATTATTTA   
  
  
- CTATAATCGG TATAATAATT TAAGTCGTAA ATAAGTAGTT ACATTATATG ATTTAGTACG TTAATAGTAA   
  
  
- CCAAGAGAAA ATACATTATT TCAATTAACT GTTAAGTTCC CAATGATTAA TCATATTACA ACTTAACCTT   
  
  
- GCATTTTTAG TTTAGCATAC ATTTCATTAA AATAAGACTT AAATTTTTTA CCTCGTTTAT AGAGATAACA   
  
  
- AGAAATTATT TTAAAATTAA CTAAAAATAA AGATTAATTC ACTGCCTTGG TTTAAAAAAA GGAGGAAAAA   
  
  
- ACCAAACCAT TCAATAGTAA TCGGTTAGAA AATAAATGTA AAACAGTCAA AAAGAAAAAA TTCTACCAAT   
  
  
- TTACCGAAGT TAATTTGTTT AAAAAAATGA ATAAATATAA AAATTATTTA AAAGTTTTAC ATATAATTGC   
  
  
- GTACTGGCAC GCTTCGTGCC TTAGATGGGA TCAATTAGAT ACTAAGAATT TATACGTTCA TCCGACAATA   
  
  
- AGTAAACAAC AAAAAATGAA TAAATATAAA AAAGGGAGGG CCATACTATC CCAAACAGTA TAACAATAAG   
  
  
- TAATCGACAA TTTGACATAA GTAATATCGT GTTCATCCGA ATTAGTTTCT ATCAATCAGA GGTTAAAGTA   
  
  
- GTCTTTTAAA TTCTTTTTCT GTGGAATCCT AGTTTTCATA CTTTAAATCT CTGGTCTGTT GCTATCTTAA   
  
  
- CTTTTCTACT CAAAAGTTAA ACCTCTTCTG CCTTTGAGTC TACTTACTTT CATATCATGT AACATGAGGA   
  
  
- ACCTCATATT AGAATAGAAG TGATAACTAT CCGAGCTTCG TATTAGTCAC TAACCTTTGA AAACCAACGT   
  
  
- TATTAAGTTT TAAACTATGA GATACTAAAT GAAAAAATTA ACTTCCAGTA CTAAATTAAT TGATTTACTT   
  
  
- TTCTTAAATA CTAGCTTTTT AAAATTGAGA ATAACTGAAT ATCAACTGAG GTCAATTTAC CTTTTAACTT   
  
  
- CACTAATTAG ATTTTCACCG GGCGAGATTA TGGTAAACTC TTTTTAATAA GAATGACGTT TCAAATTTAG   
  
  
- TTATACACTT CTATCAAATA TCAAAATAGG ATTTTCTTTA ATTTATCTAA CAATTTTTAT TAAAAATTTA   
  
  
- AGTCTATATG CACACTACCG GTATTGGCAT TTACTATACG TACAGCTGTT TAAGGTTTCT TCCGATTATT   
  
  
- AATCTGAATT TATTATGTAC GTATGATTGG TTTTTTTTTT TTAATTCGTT CTAAGATAGG TTGGATAGTA   
  
  
- TTATTTCAAA CCGGTATGAA GTTTGGTAGT GTATTGTAGT GATTATATAT TTCGATCCAA AACCCTTTGG   
  
  
- ATTGAAACTC CTGGTGTATA AACAATTCCG GATCCTCCGG TACTCAAACA GTTCTACGGA GTATCATACC   
  
  
- TCTCCAGATA TAGGAACGTT TTACTAGTAA GGGAAGATGG ATTTTCTTGG TGAAGTAAAG GTGAAAATGA   
  
  
- GGGACCGTTT ACCAACTATT TGGTTTGGTA TTGGTTCGGG AACTTCTTGA TATTTCTGTG CTTAGCTAGT   
  
  
- AACTTAAGCT TGTTCGGAAC ACACTATCCT CCTACCCGGA GTTGTGAAGA GGCGCTCACA CGTTCTCGTT   
  
  
- AGAGCCTCTT TTTAGGATTC TCGGTTGTTG AAGAAACCCA CAATTTACTT GAACAAAGAG GAATACCGCT   
  
  
- AACGCTTGTT TCTAACCGTA TAATGAAGAA TGTTCACAAC AACCGGTTTC GGTTGTTAAA CCCTGGAGTG   
  
  
- AAAGTACTCT CGGATTTTGA ACGGTACCTC TTTTTGACGA CGAAACTATG GATGTACTTC GAATATAACT   
  
  
- TCAAGGTTCT CCAGTCAGGT ACCTGTTGGA AACCAGTACA CCGAAGTTTA CCACGTTATA ACCTCCTGGA   
  
  
- ATCTTCAAAT GTTTTCAGCT AGTAGTTTCT TTGTTCCGTT TCCTACCTCT TCAAAAGTTC CGATTACCCA   
  
  
- CAAGGGAAGT TCAAAGTACA GTATTTGCTC AATCTATTGG ATCCTCTCGA AGCTTTTCTC CTAAATCTGT   
  
  
- AACTTCTACC ACTCCGGTAG CGACACTTGA CACAAGTTCG GAACGTTGCC CAAGTACACC TCTTCTCCCT   
  
  
- CGTACACGAA CTACACTAAG CTAGATAATC CGGAACATAG TATTGTGACC ACCTCCTTCT TCGTCTAGAG   
  
  
- TGAAGATGTT CTTTGCTGAA GAAGTTCACG AAGCTACTCA CAAACTCTAA AAGATTCAGT ATAAAGCTAT   
  
  
- ACAATCTTCT TTCGAAGGGA GGTTATTCGT TGCTTGCTTA GTTCGATCTT TCCCTTGTTA CCTACTTATA   
  
  
- GAGGTCTCGG GATCGAACAG TACCACTTTC ACCTCCTCTT ATATCCGGTT TCTTTCCTTG AGTTACCTTA   
  
  
- CTCTACGAGC TTGTTCGTAA AACGGGTAGA GTTAAATCAC TACTACATGA TTCACTACAA TCCCGTAACA   
  
  
- ACTTTTCTGT GTTTTCACCA ACCCTAGAGT GGAATGGTGT TTCACTGGTA CTTTATCCGT ATGTGAATTT   
  
  
- AACCTTCCCA CTTTTACAAC AAACCCGAAG ACGTACCTCT GGATCGAT

+     ARE

| Site Name | Organism | Position | Strand | Matrix score. | sequence | function |
| --- | --- | --- | --- | --- | --- | --- |
| ARE | Zea mays | 2123 | + | 6 | AAACCA | cis-acting regulatory element essential for the anaerobic induction |
| ARE | Zea mays | 2128 | + | 6 | AAACCA | cis-acting regulatory element essential for the anaerobic induction |
| ARE | Zea mays | 915 | - | 6 | AAACCA | cis-acting regulatory element essential for the anaerobic induction |
| ARE | Zea mays | 1916 | + | 6 | AAACCA | cis-acting regulatory element essential for the anaerobic induction |

>HU02G03154.1   
+ +Up\_Stream \_Len000ATGATA AAAGATATTT ATTAAGCTCA AGCTATTTTA ACTTTTAAAA AAATGAATGT   
  
  
+ GGCTTTTTTT GCTATTCAGG TACAACACAA TATGAAGCAT TAACTCTAGA AAATGTGGTA ATGTATGTCA   
  
  
+ TATAGGAGAG TATATACTCT CACTTGCCAT GCGATGCGTG CATGTGTGTG TGTGTGTGTA TCTGTTTCTA   
  
  
+ TGCATATTTT ATGTGGGGTT TTGTCATCTA GACATCTATT AATATGTTAG CATTCACATG ACTTATAGGA   
  
  
+ ATGCACTATT ATGAGTATGA TATAGAGTTT TAAAAATGCA TATGCCTTGT GTGTTCATTA GCTTAAAGAA   
  
  
+ GAGTTTTTGA ATATACATAT TTAGGTAATT CTTGCTAATG TCGTAATTAG CAGTTGTATC TATCTTATTG   
  
  
+ ATTTGTATTT TTAATTGCTT GTTATACTGT TACACTTAGA AAATTCATGA ATACTCTTAA AATATGCAAA   
  
  
+ TTATATATAT ATATATATAT ATATATATAT ATATATATAT ATAAAAACTG AATAAGTTAA TATGGTGATG   
  
  
+ ACATTTGTCA TGTTATCATT GGTCGCTTAA TTTTTAAATA AAAAATATGA TTTTGTGACA CCTAATAAAT   
  
  
+ GATATTAGCC ATATTATTAA ATTCAGCATT TATTCATCAA TGTAATATAC TAAATCATGC AATTATCATT   
  
  
+ GGTTCTCTTT TATGTAATAA AGTTAATTGA CAATTCAAGG GTTACTAATT AGTATAATGT TGAATTGGAA   
  
  
+ CGTAAAAATC AAATCGTATG TAAAGTAATT TTATTCTGAA TTTAAAAAAT GGAGCAAATA TCTCTATTGT   
  
  
+ TCTTTAATAA AATTTTAATT GATTTTTATT TCTAATTAAG TGACGGAACC AAATTTTTTT CCTCCTTTTT   
  
  
+ TGGTTTGGTA AGTTATCATT AGCCAATCTT TTATTTACAT TTTGTCAGTT TTTCTTTTTT AAGATGGTTA   
  
  
+ AATGGCTTCA ATTAAACAAA TTTTTTTACT TATTTATATT TTTAATAAAT TTTCAAAATG TATATTAACG   
  
  
+ CATGACCGTG CGAAGCACGG AATCTACCCT AGTTAATCTA TGATTCTTAA ATATGCAAGT AGGCTGTTAT   
  
  
+ TCATTTGTTG TTTTTTACTT ATTTATATTT TTTCCCTCCC GGTATGATAG GGTTTGTCAT ATTGTTATTC   
  
  
+ ATTAGCTGTT AAACTGTATT CATTATAGCA CAAGTAGGCT TAATCAAAGA TAGTTAGTCT CCAATTTCAT   
  
  
+ CAGAAAATTT AAGAAAAAGA CACCTTAGGA TCAAAAGTAT GAAATTTAGA GACCAGACAA CGATAGAATT   
  
  
+ GAAAAGATGA GTTTTCAATT TGGAGAAGAC GGAAACTCAG ATGAATGAAA GTATAGTACA TTGTACTCCT   
  
  
+ TGGAGTATAA TCTTATCTTC ACTATTGATA GGCTCGAAGC ATAATCAGTG ATTGGAAACT TTTGGTTGCA   
  
  
+ ATAATTCAAA ATTTGATACT CTATGATTTA CTTTTTTAAT TGAAGGTCAT GATTTAATTA ACTAAATGAA   
  
  
+ AAGAATTTAT GATCGAAAAA TTTTAACTCT TATTGACTTA TAGTTGACTC CAGTTAAATG GAAAATTGAA   
  
  
+ GTGATTAATC TAAAAGTGGC CCGCTCTAAT ACCATTTGAG AAAAATTATT CTTACTGCAA AGTTTAAATC   
  
  
+ AATATGTGAA GATAGTTTAT AGTTTTATCC TAAAAGAAAT TAAATAGATT GTTAAAAATA ATTTTTAAAT   
  
  
+ TCAGATATAC GTGTGATGGC CATAACCGTA AATGATATGC ATGTCGACAA ATTCCAAAGA AGGCTAATAA   
  
  
+ TTAGACTTAA ATAATACATG CATACTAACC AAAAAAAAAA AATTAAGCAA GATTCTATCC AACCTATCAT   
  
  
+ AATAAAGTTT GGCCATACTT CAAACCATCA CATAACATCA CTAATATATA AAGCTAGGTT TTGGGAAACC   
  
  
+ TAACTTTGAG GACCACATAT TTGTTAAGGC CTAGGAGGCC ATGAGTTTGT CAAGATGCCT CATAGTATGG   
  
  
+ AGAGGTCTAT ATCCTTGCAA AATGATCATT CCCTTCTACC TAAAAGAACC ACTTCATTTC CACTTTTACT   
  
  
+ CCCTGGCAAA TGGTTGATAA ACCAAACCAT AACCAAGCCC TTGAAGAACT ATAAAGACAC GAATCGATCA   
  
  
+ TTGAATTCGA ACAAGCCTTG TGTGATAGGA GGATGGGCCT CAACACTTCT CCGCGAGTGT GCAAGAGCAA   
  
  
+ TCTCGGAGAA AAATCCTAAG AGCCAACAAC TTCTTTGGGT GTTAAATGAA CTTGTTTCTC CTTATGGCGA   
  
  
+ TTGCGAACAA AGATTGGCAT ATTACTTCTT ACAAGTGTTG TTGGCCAAAG CCAACAATTT GGGACCTCAC   
  
  
+ TTTCATGAGA GCCTAAAACT TGCCATGGAG AAAAACTGCT GCTTTGATAC CTACATGAAG CTTATATTGA   
  
  
+ AGTTCCAAGA GGTCAGTCCA TGGACAACCT TTGGTCATGT GGCTTCAAAT GGTGCAATAT TGGAGGACCT   
  
  
+ TAGAAGTTTA CAAAAGTCGA TCATCAAAGA AACAAGGCAA AGGATGGAGA AGTTTTCAAG GCTAATGGGT   
  
  
+ GTTCCCTTCA AGTTTCATGT CATAAACGAG TTAGATAACC TAGGAGAGCT TCGAAAAGAG GATTTAGACA   
  
  
+ TTGAAGATGG TGAGGCCATC GCTGTGAACT GTGTTCAAGC CTTGCAACGG GTTCATGTGG AGAAGAGGGA   
  
  
+ GCATGTGCTT GATGTGATTC GATCTATTAG GCCTTGTATC ATAACACTGG TGGAGGAAGA AGCAGATCTC   
  
  
+ ACTTCTACAA GAAACGACTT CTTCAAGTGC TTCGATGAGT GTTTGAGATT TTCTAAGTCA TATTTCGATA   
  
  
+ TGTTAGAAGA AAGCTTCCCT CCAATAAGCA ACGAACGAAT CAAGCTAGAA AGGGAACAAT GGATGAATAT   
  
  
+ CTCCAGAGCC CTAGCTTGTC ATGGTGAAAG TGGAGGAGAA TATAGGCCAA AGAAAGGAAC TCAATGGAAT   
  
  
+ GAGATGCTCG AACAAGCATT TTGCCCATCT CAATTTAGTG ATGATGTACT AAGTGATGTT AGGGCATTGT   
  
  
+ TGAAAAGACA CAAAAGTGGT TGGGATCTCA CCTTACCACA AAGTGACCAT GAAATAGGCA TACACTTAAA   
  
  
+ TTGGAAGGGT GAAAATGTTG TTTGGGCTTC TGCATGGAGA CCTAGCTA  

- +Up\_Stream \_Len000TACTAT TTTCTATAAA TAATTCGAGT TCGATAAAAT TGAAAATTTT TTTACTTACA   
  
  
- CCGAAAAAAA CGATAAGTCC ATGTTGTGTT ATACTTCGTA ATTGAGATCT TTTACACCAT TACATACAGT   
  
  
- ATATCCTCTC ATATATGAGA GTGAACGGTA CGCTACGCAC GTACACACAC ACACACACAT AGACAAAGAT   
  
  
- ACGTATAAAA TACACCCCAA AACAGTAGAT CTGTAGATAA TTATACAATC GTAAGTGTAC TGAATATCCT   
  
  
- TACGTGATAA TACTCATACT ATATCTCAAA ATTTTTACGT ATACGGAACA CACAAGTAAT CGAATTTCTT   
  
  
- CTCAAAAACT TATATGTATA AATCCATTAA GAACGATTAC AGCATTAATC GTCAACATAG ATAGAATAAC   
  
  
- TAAACATAAA AATTAACGAA CAATATGACA ATGTGAATCT TTTAAGTACT TATGAGAATT TTATACGTTT   
  
  
- AATATATATA TATATATATA TATATATATA TATATATATA TATTTTTGAC TTATTCAATT ATACCACTAC   
  
  
- TGTAAACAGT ACAATAGTAA CCAGCGAATT AAAAATTTAT TTTTTATACT AAAACACTGT GGATTATTTA   
  
  
- CTATAATCGG TATAATAATT TAAGTCGTAA ATAAGTAGTT ACATTATATG ATTTAGTACG TTAATAGTAA   
  
  
- CCAAGAGAAA ATACATTATT TCAATTAACT GTTAAGTTCC CAATGATTAA TCATATTACA ACTTAACCTT   
  
  
- GCATTTTTAG TTTAGCATAC ATTTCATTAA AATAAGACTT AAATTTTTTA CCTCGTTTAT AGAGATAACA   
  
  
- AGAAATTATT TTAAAATTAA CTAAAAATAA AGATTAATTC ACTGCCTTGG TTTAAAAAAA GGAGGAAAAA   
  
  
- ACCAAACCAT TCAATAGTAA TCGGTTAGAA AATAAATGTA AAACAGTCAA AAAGAAAAAA TTCTACCAAT   
  
  
- TTACCGAAGT TAATTTGTTT AAAAAAATGA ATAAATATAA AAATTATTTA AAAGTTTTAC ATATAATTGC   
  
  
- GTACTGGCAC GCTTCGTGCC TTAGATGGGA TCAATTAGAT ACTAAGAATT TATACGTTCA TCCGACAATA   
  
  
- AGTAAACAAC AAAAAATGAA TAAATATAAA AAAGGGAGGG CCATACTATC CCAAACAGTA TAACAATAAG   
  
  
- TAATCGACAA TTTGACATAA GTAATATCGT GTTCATCCGA ATTAGTTTCT ATCAATCAGA GGTTAAAGTA   
  
  
- GTCTTTTAAA TTCTTTTTCT GTGGAATCCT AGTTTTCATA CTTTAAATCT CTGGTCTGTT GCTATCTTAA   
  
  
- CTTTTCTACT CAAAAGTTAA ACCTCTTCTG CCTTTGAGTC TACTTACTTT CATATCATGT AACATGAGGA   
  
  
- ACCTCATATT AGAATAGAAG TGATAACTAT CCGAGCTTCG TATTAGTCAC TAACCTTTGA AAACCAACGT   
  
  
- TATTAAGTTT TAAACTATGA GATACTAAAT GAAAAAATTA ACTTCCAGTA CTAAATTAAT TGATTTACTT   
  
  
- TTCTTAAATA CTAGCTTTTT AAAATTGAGA ATAACTGAAT ATCAACTGAG GTCAATTTAC CTTTTAACTT   
  
  
- CACTAATTAG ATTTTCACCG GGCGAGATTA TGGTAAACTC TTTTTAATAA GAATGACGTT TCAAATTTAG   
  
  
- TTATACACTT CTATCAAATA TCAAAATAGG ATTTTCTTTA ATTTATCTAA CAATTTTTAT TAAAAATTTA   
  
  
- AGTCTATATG CACACTACCG GTATTGGCAT TTACTATACG TACAGCTGTT TAAGGTTTCT TCCGATTATT   
  
  
- AATCTGAATT TATTATGTAC GTATGATTGG TTTTTTTTTT TTAATTCGTT CTAAGATAGG TTGGATAGTA   
  
  
- TTATTTCAAA CCGGTATGAA GTTTGGTAGT GTATTGTAGT GATTATATAT TTCGATCCAA AACCCTTTGG   
  
  
- ATTGAAACTC CTGGTGTATA AACAATTCCG GATCCTCCGG TACTCAAACA GTTCTACGGA GTATCATACC   
  
  
- TCTCCAGATA TAGGAACGTT TTACTAGTAA GGGAAGATGG ATTTTCTTGG TGAAGTAAAG GTGAAAATGA   
  
  
- GGGACCGTTT ACCAACTATT TGGTTTGGTA TTGGTTCGGG AACTTCTTGA TATTTCTGTG CTTAGCTAGT   
  
  
- AACTTAAGCT TGTTCGGAAC ACACTATCCT CCTACCCGGA GTTGTGAAGA GGCGCTCACA CGTTCTCGTT   
  
  
- AGAGCCTCTT TTTAGGATTC TCGGTTGTTG AAGAAACCCA CAATTTACTT GAACAAAGAG GAATACCGCT   
  
  
- AACGCTTGTT TCTAACCGTA TAATGAAGAA TGTTCACAAC AACCGGTTTC GGTTGTTAAA CCCTGGAGTG   
  
  
- AAAGTACTCT CGGATTTTGA ACGGTACCTC TTTTTGACGA CGAAACTATG GATGTACTTC GAATATAACT   
  
  
- TCAAGGTTCT CCAGTCAGGT ACCTGTTGGA AACCAGTACA CCGAAGTTTA CCACGTTATA ACCTCCTGGA   
  
  
- ATCTTCAAAT GTTTTCAGCT AGTAGTTTCT TTGTTCCGTT TCCTACCTCT TCAAAAGTTC CGATTACCCA   
  
  
- CAAGGGAAGT TCAAAGTACA GTATTTGCTC AATCTATTGG ATCCTCTCGA AGCTTTTCTC CTAAATCTGT   
  
  
- AACTTCTACC ACTCCGGTAG CGACACTTGA CACAAGTTCG GAACGTTGCC CAAGTACACC TCTTCTCCCT   
  
  
- CGTACACGAA CTACACTAAG CTAGATAATC CGGAACATAG TATTGTGACC ACCTCCTTCT TCGTCTAGAG   
  
  
- TGAAGATGTT CTTTGCTGAA GAAGTTCACG AAGCTACTCA CAAACTCTAA AAGATTCAGT ATAAAGCTAT   
  
  
- ACAATCTTCT TTCGAAGGGA GGTTATTCGT TGCTTGCTTA GTTCGATCTT TCCCTTGTTA CCTACTTATA   
  
  
- GAGGTCTCGG GATCGAACAG TACCACTTTC ACCTCCTCTT ATATCCGGTT TCTTTCCTTG AGTTACCTTA   
  
  
- CTCTACGAGC TTGTTCGTAA AACGGGTAGA GTTAAATCAC TACTACATGA TTCACTACAA TCCCGTAACA   
  
  
- ACTTTTCTGT GTTTTCACCA ACCCTAGAGT GGAATGGTGT TTCACTGGTA CTTTATCCGT ATGTGAATTT   
  
  
- AACCTTCCCA CTTTTACAAC AAACCCGAAG ACGTACCTCT GGATCGAT

+     AT-rich element

| Site Name | Organism | Position | Strand | Matrix score. | sequence | function |
| --- | --- | --- | --- | --- | --- | --- |
| AT-rich element | Glycine max | 863 | - | 10 | ATAGAAATCAA | binding site of AT-rich DNA binding protein (ATBP-1) |
| AT-rich element | Glycine max | 422 | - | 10 | ATAGAAATCAA | binding site of AT-rich DNA binding protein (ATBP-1) |

>HU02G03154.1   
+ +Up\_Stream \_Len000ATGATA AAAGATATTT ATTAAGCTCA AGCTATTTTA ACTTTTAAAA AAATGAATGT   
  
  
+ GGCTTTTTTT GCTATTCAGG TACAACACAA TATGAAGCAT TAACTCTAGA AAATGTGGTA ATGTATGTCA   
  
  
+ TATAGGAGAG TATATACTCT CACTTGCCAT GCGATGCGTG CATGTGTGTG TGTGTGTGTA TCTGTTTCTA   
  
  
+ TGCATATTTT ATGTGGGGTT TTGTCATCTA GACATCTATT AATATGTTAG CATTCACATG ACTTATAGGA   
  
  
+ ATGCACTATT ATGAGTATGA TATAGAGTTT TAAAAATGCA TATGCCTTGT GTGTTCATTA GCTTAAAGAA   
  
  
+ GAGTTTTTGA ATATACATAT TTAGGTAATT CTTGCTAATG TCGTAATTAG CAGTTGTATC TATCTTATTG   
  
  
+ ATTTGTATTT TTAATTGCTT GTTATACTGT TACACTTAGA AAATTCATGA ATACTCTTAA AATATGCAAA   
  
  
+ TTATATATAT ATATATATAT ATATATATAT ATATATATAT ATAAAAACTG AATAAGTTAA TATGGTGATG   
  
  
+ ACATTTGTCA TGTTATCATT GGTCGCTTAA TTTTTAAATA AAAAATATGA TTTTGTGACA CCTAATAAAT   
  
  
+ GATATTAGCC ATATTATTAA ATTCAGCATT TATTCATCAA TGTAATATAC TAAATCATGC AATTATCATT   
  
  
+ GGTTCTCTTT TATGTAATAA AGTTAATTGA CAATTCAAGG GTTACTAATT AGTATAATGT TGAATTGGAA   
  
  
+ CGTAAAAATC AAATCGTATG TAAAGTAATT TTATTCTGAA TTTAAAAAAT GGAGCAAATA TCTCTATTGT   
  
  
+ TCTTTAATAA AATTTTAATT GATTTTTATT TCTAATTAAG TGACGGAACC AAATTTTTTT CCTCCTTTTT   
  
  
+ TGGTTTGGTA AGTTATCATT AGCCAATCTT TTATTTACAT TTTGTCAGTT TTTCTTTTTT AAGATGGTTA   
  
  
+ AATGGCTTCA ATTAAACAAA TTTTTTTACT TATTTATATT TTTAATAAAT TTTCAAAATG TATATTAACG   
  
  
+ CATGACCGTG CGAAGCACGG AATCTACCCT AGTTAATCTA TGATTCTTAA ATATGCAAGT AGGCTGTTAT   
  
  
+ TCATTTGTTG TTTTTTACTT ATTTATATTT TTTCCCTCCC GGTATGATAG GGTTTGTCAT ATTGTTATTC   
  
  
+ ATTAGCTGTT AAACTGTATT CATTATAGCA CAAGTAGGCT TAATCAAAGA TAGTTAGTCT CCAATTTCAT   
  
  
+ CAGAAAATTT AAGAAAAAGA CACCTTAGGA TCAAAAGTAT GAAATTTAGA GACCAGACAA CGATAGAATT   
  
  
+ GAAAAGATGA GTTTTCAATT TGGAGAAGAC GGAAACTCAG ATGAATGAAA GTATAGTACA TTGTACTCCT   
  
  
+ TGGAGTATAA TCTTATCTTC ACTATTGATA GGCTCGAAGC ATAATCAGTG ATTGGAAACT TTTGGTTGCA   
  
  
+ ATAATTCAAA ATTTGATACT CTATGATTTA CTTTTTTAAT TGAAGGTCAT GATTTAATTA ACTAAATGAA   
  
  
+ AAGAATTTAT GATCGAAAAA TTTTAACTCT TATTGACTTA TAGTTGACTC CAGTTAAATG GAAAATTGAA   
  
  
+ GTGATTAATC TAAAAGTGGC CCGCTCTAAT ACCATTTGAG AAAAATTATT CTTACTGCAA AGTTTAAATC   
  
  
+ AATATGTGAA GATAGTTTAT AGTTTTATCC TAAAAGAAAT TAAATAGATT GTTAAAAATA ATTTTTAAAT   
  
  
+ TCAGATATAC GTGTGATGGC CATAACCGTA AATGATATGC ATGTCGACAA ATTCCAAAGA AGGCTAATAA   
  
  
+ TTAGACTTAA ATAATACATG CATACTAACC AAAAAAAAAA AATTAAGCAA GATTCTATCC AACCTATCAT   
  
  
+ AATAAAGTTT GGCCATACTT CAAACCATCA CATAACATCA CTAATATATA AAGCTAGGTT TTGGGAAACC   
  
  
+ TAACTTTGAG GACCACATAT TTGTTAAGGC CTAGGAGGCC ATGAGTTTGT CAAGATGCCT CATAGTATGG   
  
  
+ AGAGGTCTAT ATCCTTGCAA AATGATCATT CCCTTCTACC TAAAAGAACC ACTTCATTTC CACTTTTACT   
  
  
+ CCCTGGCAAA TGGTTGATAA ACCAAACCAT AACCAAGCCC TTGAAGAACT ATAAAGACAC GAATCGATCA   
  
  
+ TTGAATTCGA ACAAGCCTTG TGTGATAGGA GGATGGGCCT CAACACTTCT CCGCGAGTGT GCAAGAGCAA   
  
  
+ TCTCGGAGAA AAATCCTAAG AGCCAACAAC TTCTTTGGGT GTTAAATGAA CTTGTTTCTC CTTATGGCGA   
  
  
+ TTGCGAACAA AGATTGGCAT ATTACTTCTT ACAAGTGTTG TTGGCCAAAG CCAACAATTT GGGACCTCAC   
  
  
+ TTTCATGAGA GCCTAAAACT TGCCATGGAG AAAAACTGCT GCTTTGATAC CTACATGAAG CTTATATTGA   
  
  
+ AGTTCCAAGA GGTCAGTCCA TGGACAACCT TTGGTCATGT GGCTTCAAAT GGTGCAATAT TGGAGGACCT   
  
  
+ TAGAAGTTTA CAAAAGTCGA TCATCAAAGA AACAAGGCAA AGGATGGAGA AGTTTTCAAG GCTAATGGGT   
  
  
+ GTTCCCTTCA AGTTTCATGT CATAAACGAG TTAGATAACC TAGGAGAGCT TCGAAAAGAG GATTTAGACA   
  
  
+ TTGAAGATGG TGAGGCCATC GCTGTGAACT GTGTTCAAGC CTTGCAACGG GTTCATGTGG AGAAGAGGGA   
  
  
+ GCATGTGCTT GATGTGATTC GATCTATTAG GCCTTGTATC ATAACACTGG TGGAGGAAGA AGCAGATCTC   
  
  
+ ACTTCTACAA GAAACGACTT CTTCAAGTGC TTCGATGAGT GTTTGAGATT TTCTAAGTCA TATTTCGATA   
  
  
+ TGTTAGAAGA AAGCTTCCCT CCAATAAGCA ACGAACGAAT CAAGCTAGAA AGGGAACAAT GGATGAATAT   
  
  
+ CTCCAGAGCC CTAGCTTGTC ATGGTGAAAG TGGAGGAGAA TATAGGCCAA AGAAAGGAAC TCAATGGAAT   
  
  
+ GAGATGCTCG AACAAGCATT TTGCCCATCT CAATTTAGTG ATGATGTACT AAGTGATGTT AGGGCATTGT   
  
  
+ TGAAAAGACA CAAAAGTGGT TGGGATCTCA CCTTACCACA AAGTGACCAT GAAATAGGCA TACACTTAAA   
  
  
+ TTGGAAGGGT GAAAATGTTG TTTGGGCTTC TGCATGGAGA CCTAGCTA  

- +Up\_Stream \_Len000TACTAT TTTCTATAAA TAATTCGAGT TCGATAAAAT TGAAAATTTT TTTACTTACA   
  
  
- CCGAAAAAAA CGATAAGTCC ATGTTGTGTT ATACTTCGTA ATTGAGATCT TTTACACCAT TACATACAGT   
  
  
- ATATCCTCTC ATATATGAGA GTGAACGGTA CGCTACGCAC GTACACACAC ACACACACAT AGACAAAGAT   
  
  
- ACGTATAAAA TACACCCCAA AACAGTAGAT CTGTAGATAA TTATACAATC GTAAGTGTAC TGAATATCCT   
  
  
- TACGTGATAA TACTCATACT ATATCTCAAA ATTTTTACGT ATACGGAACA CACAAGTAAT CGAATTTCTT   
  
  
- CTCAAAAACT TATATGTATA AATCCATTAA GAACGATTAC AGCATTAATC GTCAACATAG ATAGAATAAC   
  
  
- TAAACATAAA AATTAACGAA CAATATGACA ATGTGAATCT TTTAAGTACT TATGAGAATT TTATACGTTT   
  
  
- AATATATATA TATATATATA TATATATATA TATATATATA TATTTTTGAC TTATTCAATT ATACCACTAC   
  
  
- TGTAAACAGT ACAATAGTAA CCAGCGAATT AAAAATTTAT TTTTTATACT AAAACACTGT GGATTATTTA   
  
  
- CTATAATCGG TATAATAATT TAAGTCGTAA ATAAGTAGTT ACATTATATG ATTTAGTACG TTAATAGTAA   
  
  
- CCAAGAGAAA ATACATTATT TCAATTAACT GTTAAGTTCC CAATGATTAA TCATATTACA ACTTAACCTT   
  
  
- GCATTTTTAG TTTAGCATAC ATTTCATTAA AATAAGACTT AAATTTTTTA CCTCGTTTAT AGAGATAACA   
  
  
- AGAAATTATT TTAAAATTAA CTAAAAATAA AGATTAATTC ACTGCCTTGG TTTAAAAAAA GGAGGAAAAA   
  
  
- ACCAAACCAT TCAATAGTAA TCGGTTAGAA AATAAATGTA AAACAGTCAA AAAGAAAAAA TTCTACCAAT   
  
  
- TTACCGAAGT TAATTTGTTT AAAAAAATGA ATAAATATAA AAATTATTTA AAAGTTTTAC ATATAATTGC   
  
  
- GTACTGGCAC GCTTCGTGCC TTAGATGGGA TCAATTAGAT ACTAAGAATT TATACGTTCA TCCGACAATA   
  
  
- AGTAAACAAC AAAAAATGAA TAAATATAAA AAAGGGAGGG CCATACTATC CCAAACAGTA TAACAATAAG   
  
  
- TAATCGACAA TTTGACATAA GTAATATCGT GTTCATCCGA ATTAGTTTCT ATCAATCAGA GGTTAAAGTA   
  
  
- GTCTTTTAAA TTCTTTTTCT GTGGAATCCT AGTTTTCATA CTTTAAATCT CTGGTCTGTT GCTATCTTAA   
  
  
- CTTTTCTACT CAAAAGTTAA ACCTCTTCTG CCTTTGAGTC TACTTACTTT CATATCATGT AACATGAGGA   
  
  
- ACCTCATATT AGAATAGAAG TGATAACTAT CCGAGCTTCG TATTAGTCAC TAACCTTTGA AAACCAACGT   
  
  
- TATTAAGTTT TAAACTATGA GATACTAAAT GAAAAAATTA ACTTCCAGTA CTAAATTAAT TGATTTACTT   
  
  
- TTCTTAAATA CTAGCTTTTT AAAATTGAGA ATAACTGAAT ATCAACTGAG GTCAATTTAC CTTTTAACTT   
  
  
- CACTAATTAG ATTTTCACCG GGCGAGATTA TGGTAAACTC TTTTTAATAA GAATGACGTT TCAAATTTAG   
  
  
- TTATACACTT CTATCAAATA TCAAAATAGG ATTTTCTTTA ATTTATCTAA CAATTTTTAT TAAAAATTTA   
  
  
- AGTCTATATG CACACTACCG GTATTGGCAT TTACTATACG TACAGCTGTT TAAGGTTTCT TCCGATTATT   
  
  
- AATCTGAATT TATTATGTAC GTATGATTGG TTTTTTTTTT TTAATTCGTT CTAAGATAGG TTGGATAGTA   
  
  
- TTATTTCAAA CCGGTATGAA GTTTGGTAGT GTATTGTAGT GATTATATAT TTCGATCCAA AACCCTTTGG   
  
  
- ATTGAAACTC CTGGTGTATA AACAATTCCG GATCCTCCGG TACTCAAACA GTTCTACGGA GTATCATACC   
  
  
- TCTCCAGATA TAGGAACGTT TTACTAGTAA GGGAAGATGG ATTTTCTTGG TGAAGTAAAG GTGAAAATGA   
  
  
- GGGACCGTTT ACCAACTATT TGGTTTGGTA TTGGTTCGGG AACTTCTTGA TATTTCTGTG CTTAGCTAGT   
  
  
- AACTTAAGCT TGTTCGGAAC ACACTATCCT CCTACCCGGA GTTGTGAAGA GGCGCTCACA CGTTCTCGTT   
  
  
- AGAGCCTCTT TTTAGGATTC TCGGTTGTTG AAGAAACCCA CAATTTACTT GAACAAAGAG GAATACCGCT   
  
  
- AACGCTTGTT TCTAACCGTA TAATGAAGAA TGTTCACAAC AACCGGTTTC GGTTGTTAAA CCCTGGAGTG   
  
  
- AAAGTACTCT CGGATTTTGA ACGGTACCTC TTTTTGACGA CGAAACTATG GATGTACTTC GAATATAACT   
  
  
- TCAAGGTTCT CCAGTCAGGT ACCTGTTGGA AACCAGTACA CCGAAGTTTA CCACGTTATA ACCTCCTGGA   
  
  
- ATCTTCAAAT GTTTTCAGCT AGTAGTTTCT TTGTTCCGTT TCCTACCTCT TCAAAAGTTC CGATTACCCA   
  
  
- CAAGGGAAGT TCAAAGTACA GTATTTGCTC AATCTATTGG ATCCTCTCGA AGCTTTTCTC CTAAATCTGT   
  
  
- AACTTCTACC ACTCCGGTAG CGACACTTGA CACAAGTTCG GAACGTTGCC CAAGTACACC TCTTCTCCCT   
  
  
- CGTACACGAA CTACACTAAG CTAGATAATC CGGAACATAG TATTGTGACC ACCTCCTTCT TCGTCTAGAG   
  
  
- TGAAGATGTT CTTTGCTGAA GAAGTTCACG AAGCTACTCA CAAACTCTAA AAGATTCAGT ATAAAGCTAT   
  
  
- ACAATCTTCT TTCGAAGGGA GGTTATTCGT TGCTTGCTTA GTTCGATCTT TCCCTTGTTA CCTACTTATA   
  
  
- GAGGTCTCGG GATCGAACAG TACCACTTTC ACCTCCTCTT ATATCCGGTT TCTTTCCTTG AGTTACCTTA   
  
  
- CTCTACGAGC TTGTTCGTAA AACGGGTAGA GTTAAATCAC TACTACATGA TTCACTACAA TCCCGTAACA   
  
  
- ACTTTTCTGT GTTTTCACCA ACCCTAGAGT GGAATGGTGT TTCACTGGTA CTTTATCCGT ATGTGAATTT   
  
  
- AACCTTCCCA CTTTTACAAC AAACCCGAAG ACGTACCTCT GGATCGAT

+     AT~TATA-box

| Site Name | Organism | Position | Strand | Matrix score. | sequence | function |
| --- | --- | --- | --- | --- | --- | --- |
| AT~TATA-box | Arabidopsis thaliana | 1939 | - | 6 | TATATA |  |
| AT~TATA-box | Arabidopsis thaliana | 526 | + | 6 | TATATA |  |
| AT~TATA-box | Arabidopsis thaliana | 532 | + | 6 | TATATA |  |
| AT~TATA-box | Arabidopsis thaliana | 528 | + | 6 | TATATA |  |
| AT~TATA-box | Arabidopsis thaliana | 530 | + | 6 | TATATA |  |
| AT~TATA-box | Arabidopsis thaliana | 512 | + | 6 | TATATA |  |
| AT~TATA-box | Arabidopsis thaliana | 502 | + | 6 | TATATA |  |
| AT~TATA-box | Arabidopsis thaliana | 522 | + | 6 | TATATA |  |
| AT~TATA-box | Arabidopsis thaliana | 520 | + | 6 | TATATA |  |
| AT~TATA-box | Arabidopsis thaliana | 504 | + | 6 | TATATA |  |
| AT~TATA-box | Arabidopsis thaliana | 500 | + | 6 | TATATA |  |
| AT~TATA-box | Arabidopsis thaliana | 518 | + | 6 | TATATA |  |
| AT~TATA-box | Arabidopsis thaliana | 524 | + | 6 | TATATA |  |
| AT~TATA-box | Arabidopsis thaliana | 498 | + | 6 | TATATA |  |
| AT~TATA-box | Arabidopsis thaliana | 155 | + | 6 | TATATA |  |
| AT~TATA-box | Arabidopsis thaliana | 496 | + | 6 | TATATA |  |
| AT~TATA-box | Arabidopsis thaliana | 514 | + | 6 | TATATA |  |
| AT~TATA-box | Arabidopsis thaliana | 510 | + | 6 | TATATA |  |
| AT~TATA-box | Arabidopsis thaliana | 516 | + | 6 | TATATA |  |
| AT~TATA-box | Arabidopsis thaliana | 508 | + | 6 | TATATA |  |
| AT~TATA-box | Arabidopsis thaliana | 506 | + | 6 | TATATA |  |

>HU02G03154.1   
+ +Up\_Stream \_Len000ATGATA AAAGATATTT ATTAAGCTCA AGCTATTTTA ACTTTTAAAA AAATGAATGT   
  
  
+ GGCTTTTTTT GCTATTCAGG TACAACACAA TATGAAGCAT TAACTCTAGA AAATGTGGTA ATGTATGTCA   
  
  
+ TATAGGAGAG TATATACTCT CACTTGCCAT GCGATGCGTG CATGTGTGTG TGTGTGTGTA TCTGTTTCTA   
  
  
+ TGCATATTTT ATGTGGGGTT TTGTCATCTA GACATCTATT AATATGTTAG CATTCACATG ACTTATAGGA   
  
  
+ ATGCACTATT ATGAGTATGA TATAGAGTTT TAAAAATGCA TATGCCTTGT GTGTTCATTA GCTTAAAGAA   
  
  
+ GAGTTTTTGA ATATACATAT TTAGGTAATT CTTGCTAATG TCGTAATTAG CAGTTGTATC TATCTTATTG   
  
  
+ ATTTGTATTT TTAATTGCTT GTTATACTGT TACACTTAGA AAATTCATGA ATACTCTTAA AATATGCAAA   
  
  
+ TTATATATAT ATATATATAT ATATATATAT ATATATATAT ATAAAAACTG AATAAGTTAA TATGGTGATG   
  
  
+ ACATTTGTCA TGTTATCATT GGTCGCTTAA TTTTTAAATA AAAAATATGA TTTTGTGACA CCTAATAAAT   
  
  
+ GATATTAGCC ATATTATTAA ATTCAGCATT TATTCATCAA TGTAATATAC TAAATCATGC AATTATCATT   
  
  
+ GGTTCTCTTT TATGTAATAA AGTTAATTGA CAATTCAAGG GTTACTAATT AGTATAATGT TGAATTGGAA   
  
  
+ CGTAAAAATC AAATCGTATG TAAAGTAATT TTATTCTGAA TTTAAAAAAT GGAGCAAATA TCTCTATTGT   
  
  
+ TCTTTAATAA AATTTTAATT GATTTTTATT TCTAATTAAG TGACGGAACC AAATTTTTTT CCTCCTTTTT   
  
  
+ TGGTTTGGTA AGTTATCATT AGCCAATCTT TTATTTACAT TTTGTCAGTT TTTCTTTTTT AAGATGGTTA   
  
  
+ AATGGCTTCA ATTAAACAAA TTTTTTTACT TATTTATATT TTTAATAAAT TTTCAAAATG TATATTAACG   
  
  
+ CATGACCGTG CGAAGCACGG AATCTACCCT AGTTAATCTA TGATTCTTAA ATATGCAAGT AGGCTGTTAT   
  
  
+ TCATTTGTTG TTTTTTACTT ATTTATATTT TTTCCCTCCC GGTATGATAG GGTTTGTCAT ATTGTTATTC   
  
  
+ ATTAGCTGTT AAACTGTATT CATTATAGCA CAAGTAGGCT TAATCAAAGA TAGTTAGTCT CCAATTTCAT   
  
  
+ CAGAAAATTT AAGAAAAAGA CACCTTAGGA TCAAAAGTAT GAAATTTAGA GACCAGACAA CGATAGAATT   
  
  
+ GAAAAGATGA GTTTTCAATT TGGAGAAGAC GGAAACTCAG ATGAATGAAA GTATAGTACA TTGTACTCCT   
  
  
+ TGGAGTATAA TCTTATCTTC ACTATTGATA GGCTCGAAGC ATAATCAGTG ATTGGAAACT TTTGGTTGCA   
  
  
+ ATAATTCAAA ATTTGATACT CTATGATTTA CTTTTTTAAT TGAAGGTCAT GATTTAATTA ACTAAATGAA   
  
  
+ AAGAATTTAT GATCGAAAAA TTTTAACTCT TATTGACTTA TAGTTGACTC CAGTTAAATG GAAAATTGAA   
  
  
+ GTGATTAATC TAAAAGTGGC CCGCTCTAAT ACCATTTGAG AAAAATTATT CTTACTGCAA AGTTTAAATC   
  
  
+ AATATGTGAA GATAGTTTAT AGTTTTATCC TAAAAGAAAT TAAATAGATT GTTAAAAATA ATTTTTAAAT   
  
  
+ TCAGATATAC GTGTGATGGC CATAACCGTA AATGATATGC ATGTCGACAA ATTCCAAAGA AGGCTAATAA   
  
  
+ TTAGACTTAA ATAATACATG CATACTAACC AAAAAAAAAA AATTAAGCAA GATTCTATCC AACCTATCAT   
  
  
+ AATAAAGTTT GGCCATACTT CAAACCATCA CATAACATCA CTAATATATA AAGCTAGGTT TTGGGAAACC   
  
  
+ TAACTTTGAG GACCACATAT TTGTTAAGGC CTAGGAGGCC ATGAGTTTGT CAAGATGCCT CATAGTATGG   
  
  
+ AGAGGTCTAT ATCCTTGCAA AATGATCATT CCCTTCTACC TAAAAGAACC ACTTCATTTC CACTTTTACT   
  
  
+ CCCTGGCAAA TGGTTGATAA ACCAAACCAT AACCAAGCCC TTGAAGAACT ATAAAGACAC GAATCGATCA   
  
  
+ TTGAATTCGA ACAAGCCTTG TGTGATAGGA GGATGGGCCT CAACACTTCT CCGCGAGTGT GCAAGAGCAA   
  
  
+ TCTCGGAGAA AAATCCTAAG AGCCAACAAC TTCTTTGGGT GTTAAATGAA CTTGTTTCTC CTTATGGCGA   
  
  
+ TTGCGAACAA AGATTGGCAT ATTACTTCTT ACAAGTGTTG TTGGCCAAAG CCAACAATTT GGGACCTCAC   
  
  
+ TTTCATGAGA GCCTAAAACT TGCCATGGAG AAAAACTGCT GCTTTGATAC CTACATGAAG CTTATATTGA   
  
  
+ AGTTCCAAGA GGTCAGTCCA TGGACAACCT TTGGTCATGT GGCTTCAAAT GGTGCAATAT TGGAGGACCT   
  
  
+ TAGAAGTTTA CAAAAGTCGA TCATCAAAGA AACAAGGCAA AGGATGGAGA AGTTTTCAAG GCTAATGGGT   
  
  
+ GTTCCCTTCA AGTTTCATGT CATAAACGAG TTAGATAACC TAGGAGAGCT TCGAAAAGAG GATTTAGACA   
  
  
+ TTGAAGATGG TGAGGCCATC GCTGTGAACT GTGTTCAAGC CTTGCAACGG GTTCATGTGG AGAAGAGGGA   
  
  
+ GCATGTGCTT GATGTGATTC GATCTATTAG GCCTTGTATC ATAACACTGG TGGAGGAAGA AGCAGATCTC   
  
  
+ ACTTCTACAA GAAACGACTT CTTCAAGTGC TTCGATGAGT GTTTGAGATT TTCTAAGTCA TATTTCGATA   
  
  
+ TGTTAGAAGA AAGCTTCCCT CCAATAAGCA ACGAACGAAT CAAGCTAGAA AGGGAACAAT GGATGAATAT   
  
  
+ CTCCAGAGCC CTAGCTTGTC ATGGTGAAAG TGGAGGAGAA TATAGGCCAA AGAAAGGAAC TCAATGGAAT   
  
  
+ GAGATGCTCG AACAAGCATT TTGCCCATCT CAATTTAGTG ATGATGTACT AAGTGATGTT AGGGCATTGT   
  
  
+ TGAAAAGACA CAAAAGTGGT TGGGATCTCA CCTTACCACA AAGTGACCAT GAAATAGGCA TACACTTAAA   
  
  
+ TTGGAAGGGT GAAAATGTTG TTTGGGCTTC TGCATGGAGA CCTAGCTA  

- +Up\_Stream \_Len000TACTAT TTTCTATAAA TAATTCGAGT TCGATAAAAT TGAAAATTTT TTTACTTACA   
  
  
- CCGAAAAAAA CGATAAGTCC ATGTTGTGTT ATACTTCGTA ATTGAGATCT TTTACACCAT TACATACAGT   
  
  
- ATATCCTCTC ATATATGAGA GTGAACGGTA CGCTACGCAC GTACACACAC ACACACACAT AGACAAAGAT   
  
  
- ACGTATAAAA TACACCCCAA AACAGTAGAT CTGTAGATAA TTATACAATC GTAAGTGTAC TGAATATCCT   
  
  
- TACGTGATAA TACTCATACT ATATCTCAAA ATTTTTACGT ATACGGAACA CACAAGTAAT CGAATTTCTT   
  
  
- CTCAAAAACT TATATGTATA AATCCATTAA GAACGATTAC AGCATTAATC GTCAACATAG ATAGAATAAC   
  
  
- TAAACATAAA AATTAACGAA CAATATGACA ATGTGAATCT TTTAAGTACT TATGAGAATT TTATACGTTT   
  
  
- AATATATATA TATATATATA TATATATATA TATATATATA TATTTTTGAC TTATTCAATT ATACCACTAC   
  
  
- TGTAAACAGT ACAATAGTAA CCAGCGAATT AAAAATTTAT TTTTTATACT AAAACACTGT GGATTATTTA   
  
  
- CTATAATCGG TATAATAATT TAAGTCGTAA ATAAGTAGTT ACATTATATG ATTTAGTACG TTAATAGTAA   
  
  
- CCAAGAGAAA ATACATTATT TCAATTAACT GTTAAGTTCC CAATGATTAA TCATATTACA ACTTAACCTT   
  
  
- GCATTTTTAG TTTAGCATAC ATTTCATTAA AATAAGACTT AAATTTTTTA CCTCGTTTAT AGAGATAACA   
  
  
- AGAAATTATT TTAAAATTAA CTAAAAATAA AGATTAATTC ACTGCCTTGG TTTAAAAAAA GGAGGAAAAA   
  
  
- ACCAAACCAT TCAATAGTAA TCGGTTAGAA AATAAATGTA AAACAGTCAA AAAGAAAAAA TTCTACCAAT   
  
  
- TTACCGAAGT TAATTTGTTT AAAAAAATGA ATAAATATAA AAATTATTTA AAAGTTTTAC ATATAATTGC   
  
  
- GTACTGGCAC GCTTCGTGCC TTAGATGGGA TCAATTAGAT ACTAAGAATT TATACGTTCA TCCGACAATA   
  
  
- AGTAAACAAC AAAAAATGAA TAAATATAAA AAAGGGAGGG CCATACTATC CCAAACAGTA TAACAATAAG   
  
  
- TAATCGACAA TTTGACATAA GTAATATCGT GTTCATCCGA ATTAGTTTCT ATCAATCAGA GGTTAAAGTA   
  
  
- GTCTTTTAAA TTCTTTTTCT GTGGAATCCT AGTTTTCATA CTTTAAATCT CTGGTCTGTT GCTATCTTAA   
  
  
- CTTTTCTACT CAAAAGTTAA ACCTCTTCTG CCTTTGAGTC TACTTACTTT CATATCATGT AACATGAGGA   
  
  
- ACCTCATATT AGAATAGAAG TGATAACTAT CCGAGCTTCG TATTAGTCAC TAACCTTTGA AAACCAACGT   
  
  
- TATTAAGTTT TAAACTATGA GATACTAAAT GAAAAAATTA ACTTCCAGTA CTAAATTAAT TGATTTACTT   
  
  
- TTCTTAAATA CTAGCTTTTT AAAATTGAGA ATAACTGAAT ATCAACTGAG GTCAATTTAC CTTTTAACTT   
  
  
- CACTAATTAG ATTTTCACCG GGCGAGATTA TGGTAAACTC TTTTTAATAA GAATGACGTT TCAAATTTAG   
  
  
- TTATACACTT CTATCAAATA TCAAAATAGG ATTTTCTTTA ATTTATCTAA CAATTTTTAT TAAAAATTTA   
  
  
- AGTCTATATG CACACTACCG GTATTGGCAT TTACTATACG TACAGCTGTT TAAGGTTTCT TCCGATTATT   
  
  
- AATCTGAATT TATTATGTAC GTATGATTGG TTTTTTTTTT TTAATTCGTT CTAAGATAGG TTGGATAGTA   
  
  
- TTATTTCAAA CCGGTATGAA GTTTGGTAGT GTATTGTAGT GATTATATAT TTCGATCCAA AACCCTTTGG   
  
  
- ATTGAAACTC CTGGTGTATA AACAATTCCG GATCCTCCGG TACTCAAACA GTTCTACGGA GTATCATACC   
  
  
- TCTCCAGATA TAGGAACGTT TTACTAGTAA GGGAAGATGG ATTTTCTTGG TGAAGTAAAG GTGAAAATGA   
  
  
- GGGACCGTTT ACCAACTATT TGGTTTGGTA TTGGTTCGGG AACTTCTTGA TATTTCTGTG CTTAGCTAGT   
  
  
- AACTTAAGCT TGTTCGGAAC ACACTATCCT CCTACCCGGA GTTGTGAAGA GGCGCTCACA CGTTCTCGTT   
  
  
- AGAGCCTCTT TTTAGGATTC TCGGTTGTTG AAGAAACCCA CAATTTACTT GAACAAAGAG GAATACCGCT   
  
  
- AACGCTTGTT TCTAACCGTA TAATGAAGAA TGTTCACAAC AACCGGTTTC GGTTGTTAAA CCCTGGAGTG   
  
  
- AAAGTACTCT CGGATTTTGA ACGGTACCTC TTTTTGACGA CGAAACTATG GATGTACTTC GAATATAACT   
  
  
- TCAAGGTTCT CCAGTCAGGT ACCTGTTGGA AACCAGTACA CCGAAGTTTA CCACGTTATA ACCTCCTGGA   
  
  
- ATCTTCAAAT GTTTTCAGCT AGTAGTTTCT TTGTTCCGTT TCCTACCTCT TCAAAAGTTC CGATTACCCA   
  
  
- CAAGGGAAGT TCAAAGTACA GTATTTGCTC AATCTATTGG ATCCTCTCGA AGCTTTTCTC CTAAATCTGT   
  
  
- AACTTCTACC ACTCCGGTAG CGACACTTGA CACAAGTTCG GAACGTTGCC CAAGTACACC TCTTCTCCCT   
  
  
- CGTACACGAA CTACACTAAG CTAGATAATC CGGAACATAG TATTGTGACC ACCTCCTTCT TCGTCTAGAG   
  
  
- TGAAGATGTT CTTTGCTGAA GAAGTTCACG AAGCTACTCA CAAACTCTAA AAGATTCAGT ATAAAGCTAT   
  
  
- ACAATCTTCT TTCGAAGGGA GGTTATTCGT TGCTTGCTTA GTTCGATCTT TCCCTTGTTA CCTACTTATA   
  
  
- GAGGTCTCGG GATCGAACAG TACCACTTTC ACCTCCTCTT ATATCCGGTT TCTTTCCTTG AGTTACCTTA   
  
  
- CTCTACGAGC TTGTTCGTAA AACGGGTAGA GTTAAATCAC TACTACATGA TTCACTACAA TCCCGTAACA   
  
  
- ACTTTTCTGT GTTTTCACCA ACCCTAGAGT GGAATGGTGT TTCACTGGTA CTTTATCCGT ATGTGAATTT   
  
  
- AACCTTCCCA CTTTTACAAC AAACCCGAAG ACGTACCTCT GGATCGAT

+     Box 4

| Site Name | Organism | Position | Strand | Matrix score. | sequence | function |
| --- | --- | --- | --- | --- | --- | --- |
| Box 4 | Petroselinum crispum | 1618 | - | 6 | ATTAAT | part of a conserved DNA module involved in light responsiveness |
| Box 4 | Petroselinum crispum | 252 | + | 6 | ATTAAT | part of a conserved DNA module involved in light responsiveness |

>HU02G03154.1   
+ +Up\_Stream \_Len000ATGATA AAAGATATTT ATTAAGCTCA AGCTATTTTA ACTTTTAAAA AAATGAATGT   
  
  
+ GGCTTTTTTT GCTATTCAGG TACAACACAA TATGAAGCAT TAACTCTAGA AAATGTGGTA ATGTATGTCA   
  
  
+ TATAGGAGAG TATATACTCT CACTTGCCAT GCGATGCGTG CATGTGTGTG TGTGTGTGTA TCTGTTTCTA   
  
  
+ TGCATATTTT ATGTGGGGTT TTGTCATCTA GACATCTATT AATATGTTAG CATTCACATG ACTTATAGGA   
  
  
+ ATGCACTATT ATGAGTATGA TATAGAGTTT TAAAAATGCA TATGCCTTGT GTGTTCATTA GCTTAAAGAA   
  
  
+ GAGTTTTTGA ATATACATAT TTAGGTAATT CTTGCTAATG TCGTAATTAG CAGTTGTATC TATCTTATTG   
  
  
+ ATTTGTATTT TTAATTGCTT GTTATACTGT TACACTTAGA AAATTCATGA ATACTCTTAA AATATGCAAA   
  
  
+ TTATATATAT ATATATATAT ATATATATAT ATATATATAT ATAAAAACTG AATAAGTTAA TATGGTGATG   
  
  
+ ACATTTGTCA TGTTATCATT GGTCGCTTAA TTTTTAAATA AAAAATATGA TTTTGTGACA CCTAATAAAT   
  
  
+ GATATTAGCC ATATTATTAA ATTCAGCATT TATTCATCAA TGTAATATAC TAAATCATGC AATTATCATT   
  
  
+ GGTTCTCTTT TATGTAATAA AGTTAATTGA CAATTCAAGG GTTACTAATT AGTATAATGT TGAATTGGAA   
  
  
+ CGTAAAAATC AAATCGTATG TAAAGTAATT TTATTCTGAA TTTAAAAAAT GGAGCAAATA TCTCTATTGT   
  
  
+ TCTTTAATAA AATTTTAATT GATTTTTATT TCTAATTAAG TGACGGAACC AAATTTTTTT CCTCCTTTTT   
  
  
+ TGGTTTGGTA AGTTATCATT AGCCAATCTT TTATTTACAT TTTGTCAGTT TTTCTTTTTT AAGATGGTTA   
  
  
+ AATGGCTTCA ATTAAACAAA TTTTTTTACT TATTTATATT TTTAATAAAT TTTCAAAATG TATATTAACG   
  
  
+ CATGACCGTG CGAAGCACGG AATCTACCCT AGTTAATCTA TGATTCTTAA ATATGCAAGT AGGCTGTTAT   
  
  
+ TCATTTGTTG TTTTTTACTT ATTTATATTT TTTCCCTCCC GGTATGATAG GGTTTGTCAT ATTGTTATTC   
  
  
+ ATTAGCTGTT AAACTGTATT CATTATAGCA CAAGTAGGCT TAATCAAAGA TAGTTAGTCT CCAATTTCAT   
  
  
+ CAGAAAATTT AAGAAAAAGA CACCTTAGGA TCAAAAGTAT GAAATTTAGA GACCAGACAA CGATAGAATT   
  
  
+ GAAAAGATGA GTTTTCAATT TGGAGAAGAC GGAAACTCAG ATGAATGAAA GTATAGTACA TTGTACTCCT   
  
  
+ TGGAGTATAA TCTTATCTTC ACTATTGATA GGCTCGAAGC ATAATCAGTG ATTGGAAACT TTTGGTTGCA   
  
  
+ ATAATTCAAA ATTTGATACT CTATGATTTA CTTTTTTAAT TGAAGGTCAT GATTTAATTA ACTAAATGAA   
  
  
+ AAGAATTTAT GATCGAAAAA TTTTAACTCT TATTGACTTA TAGTTGACTC CAGTTAAATG GAAAATTGAA   
  
  
+ GTGATTAATC TAAAAGTGGC CCGCTCTAAT ACCATTTGAG AAAAATTATT CTTACTGCAA AGTTTAAATC   
  
  
+ AATATGTGAA GATAGTTTAT AGTTTTATCC TAAAAGAAAT TAAATAGATT GTTAAAAATA ATTTTTAAAT   
  
  
+ TCAGATATAC GTGTGATGGC CATAACCGTA AATGATATGC ATGTCGACAA ATTCCAAAGA AGGCTAATAA   
  
  
+ TTAGACTTAA ATAATACATG CATACTAACC AAAAAAAAAA AATTAAGCAA GATTCTATCC AACCTATCAT   
  
  
+ AATAAAGTTT GGCCATACTT CAAACCATCA CATAACATCA CTAATATATA AAGCTAGGTT TTGGGAAACC   
  
  
+ TAACTTTGAG GACCACATAT TTGTTAAGGC CTAGGAGGCC ATGAGTTTGT CAAGATGCCT CATAGTATGG   
  
  
+ AGAGGTCTAT ATCCTTGCAA AATGATCATT CCCTTCTACC TAAAAGAACC ACTTCATTTC CACTTTTACT   
  
  
+ CCCTGGCAAA TGGTTGATAA ACCAAACCAT AACCAAGCCC TTGAAGAACT ATAAAGACAC GAATCGATCA   
  
  
+ TTGAATTCGA ACAAGCCTTG TGTGATAGGA GGATGGGCCT CAACACTTCT CCGCGAGTGT GCAAGAGCAA   
  
  
+ TCTCGGAGAA AAATCCTAAG AGCCAACAAC TTCTTTGGGT GTTAAATGAA CTTGTTTCTC CTTATGGCGA   
  
  
+ TTGCGAACAA AGATTGGCAT ATTACTTCTT ACAAGTGTTG TTGGCCAAAG CCAACAATTT GGGACCTCAC   
  
  
+ TTTCATGAGA GCCTAAAACT TGCCATGGAG AAAAACTGCT GCTTTGATAC CTACATGAAG CTTATATTGA   
  
  
+ AGTTCCAAGA GGTCAGTCCA TGGACAACCT TTGGTCATGT GGCTTCAAAT GGTGCAATAT TGGAGGACCT   
  
  
+ TAGAAGTTTA CAAAAGTCGA TCATCAAAGA AACAAGGCAA AGGATGGAGA AGTTTTCAAG GCTAATGGGT   
  
  
+ GTTCCCTTCA AGTTTCATGT CATAAACGAG TTAGATAACC TAGGAGAGCT TCGAAAAGAG GATTTAGACA   
  
  
+ TTGAAGATGG TGAGGCCATC GCTGTGAACT GTGTTCAAGC CTTGCAACGG GTTCATGTGG AGAAGAGGGA   
  
  
+ GCATGTGCTT GATGTGATTC GATCTATTAG GCCTTGTATC ATAACACTGG TGGAGGAAGA AGCAGATCTC   
  
  
+ ACTTCTACAA GAAACGACTT CTTCAAGTGC TTCGATGAGT GTTTGAGATT TTCTAAGTCA TATTTCGATA   
  
  
+ TGTTAGAAGA AAGCTTCCCT CCAATAAGCA ACGAACGAAT CAAGCTAGAA AGGGAACAAT GGATGAATAT   
  
  
+ CTCCAGAGCC CTAGCTTGTC ATGGTGAAAG TGGAGGAGAA TATAGGCCAA AGAAAGGAAC TCAATGGAAT   
  
  
+ GAGATGCTCG AACAAGCATT TTGCCCATCT CAATTTAGTG ATGATGTACT AAGTGATGTT AGGGCATTGT   
  
  
+ TGAAAAGACA CAAAAGTGGT TGGGATCTCA CCTTACCACA AAGTGACCAT GAAATAGGCA TACACTTAAA   
  
  
+ TTGGAAGGGT GAAAATGTTG TTTGGGCTTC TGCATGGAGA CCTAGCTA  

- +Up\_Stream \_Len000TACTAT TTTCTATAAA TAATTCGAGT TCGATAAAAT TGAAAATTTT TTTACTTACA   
  
  
- CCGAAAAAAA CGATAAGTCC ATGTTGTGTT ATACTTCGTA ATTGAGATCT TTTACACCAT TACATACAGT   
  
  
- ATATCCTCTC ATATATGAGA GTGAACGGTA CGCTACGCAC GTACACACAC ACACACACAT AGACAAAGAT   
  
  
- ACGTATAAAA TACACCCCAA AACAGTAGAT CTGTAGATAA TTATACAATC GTAAGTGTAC TGAATATCCT   
  
  
- TACGTGATAA TACTCATACT ATATCTCAAA ATTTTTACGT ATACGGAACA CACAAGTAAT CGAATTTCTT   
  
  
- CTCAAAAACT TATATGTATA AATCCATTAA GAACGATTAC AGCATTAATC GTCAACATAG ATAGAATAAC   
  
  
- TAAACATAAA AATTAACGAA CAATATGACA ATGTGAATCT TTTAAGTACT TATGAGAATT TTATACGTTT   
  
  
- AATATATATA TATATATATA TATATATATA TATATATATA TATTTTTGAC TTATTCAATT ATACCACTAC   
  
  
- TGTAAACAGT ACAATAGTAA CCAGCGAATT AAAAATTTAT TTTTTATACT AAAACACTGT GGATTATTTA   
  
  
- CTATAATCGG TATAATAATT TAAGTCGTAA ATAAGTAGTT ACATTATATG ATTTAGTACG TTAATAGTAA   
  
  
- CCAAGAGAAA ATACATTATT TCAATTAACT GTTAAGTTCC CAATGATTAA TCATATTACA ACTTAACCTT   
  
  
- GCATTTTTAG TTTAGCATAC ATTTCATTAA AATAAGACTT AAATTTTTTA CCTCGTTTAT AGAGATAACA   
  
  
- AGAAATTATT TTAAAATTAA CTAAAAATAA AGATTAATTC ACTGCCTTGG TTTAAAAAAA GGAGGAAAAA   
  
  
- ACCAAACCAT TCAATAGTAA TCGGTTAGAA AATAAATGTA AAACAGTCAA AAAGAAAAAA TTCTACCAAT   
  
  
- TTACCGAAGT TAATTTGTTT AAAAAAATGA ATAAATATAA AAATTATTTA AAAGTTTTAC ATATAATTGC   
  
  
- GTACTGGCAC GCTTCGTGCC TTAGATGGGA TCAATTAGAT ACTAAGAATT TATACGTTCA TCCGACAATA   
  
  
- AGTAAACAAC AAAAAATGAA TAAATATAAA AAAGGGAGGG CCATACTATC CCAAACAGTA TAACAATAAG   
  
  
- TAATCGACAA TTTGACATAA GTAATATCGT GTTCATCCGA ATTAGTTTCT ATCAATCAGA GGTTAAAGTA   
  
  
- GTCTTTTAAA TTCTTTTTCT GTGGAATCCT AGTTTTCATA CTTTAAATCT CTGGTCTGTT GCTATCTTAA   
  
  
- CTTTTCTACT CAAAAGTTAA ACCTCTTCTG CCTTTGAGTC TACTTACTTT CATATCATGT AACATGAGGA   
  
  
- ACCTCATATT AGAATAGAAG TGATAACTAT CCGAGCTTCG TATTAGTCAC TAACCTTTGA AAACCAACGT   
  
  
- TATTAAGTTT TAAACTATGA GATACTAAAT GAAAAAATTA ACTTCCAGTA CTAAATTAAT TGATTTACTT   
  
  
- TTCTTAAATA CTAGCTTTTT AAAATTGAGA ATAACTGAAT ATCAACTGAG GTCAATTTAC CTTTTAACTT   
  
  
- CACTAATTAG ATTTTCACCG GGCGAGATTA TGGTAAACTC TTTTTAATAA GAATGACGTT TCAAATTTAG   
  
  
- TTATACACTT CTATCAAATA TCAAAATAGG ATTTTCTTTA ATTTATCTAA CAATTTTTAT TAAAAATTTA   
  
  
- AGTCTATATG CACACTACCG GTATTGGCAT TTACTATACG TACAGCTGTT TAAGGTTTCT TCCGATTATT   
  
  
- AATCTGAATT TATTATGTAC GTATGATTGG TTTTTTTTTT TTAATTCGTT CTAAGATAGG TTGGATAGTA   
  
  
- TTATTTCAAA CCGGTATGAA GTTTGGTAGT GTATTGTAGT GATTATATAT TTCGATCCAA AACCCTTTGG   
  
  
- ATTGAAACTC CTGGTGTATA AACAATTCCG GATCCTCCGG TACTCAAACA GTTCTACGGA GTATCATACC   
  
  
- TCTCCAGATA TAGGAACGTT TTACTAGTAA GGGAAGATGG ATTTTCTTGG TGAAGTAAAG GTGAAAATGA   
  
  
- GGGACCGTTT ACCAACTATT TGGTTTGGTA TTGGTTCGGG AACTTCTTGA TATTTCTGTG CTTAGCTAGT   
  
  
- AACTTAAGCT TGTTCGGAAC ACACTATCCT CCTACCCGGA GTTGTGAAGA GGCGCTCACA CGTTCTCGTT   
  
  
- AGAGCCTCTT TTTAGGATTC TCGGTTGTTG AAGAAACCCA CAATTTACTT GAACAAAGAG GAATACCGCT   
  
  
- AACGCTTGTT TCTAACCGTA TAATGAAGAA TGTTCACAAC AACCGGTTTC GGTTGTTAAA CCCTGGAGTG   
  
  
- AAAGTACTCT CGGATTTTGA ACGGTACCTC TTTTTGACGA CGAAACTATG GATGTACTTC GAATATAACT   
  
  
- TCAAGGTTCT CCAGTCAGGT ACCTGTTGGA AACCAGTACA CCGAAGTTTA CCACGTTATA ACCTCCTGGA   
  
  
- ATCTTCAAAT GTTTTCAGCT AGTAGTTTCT TTGTTCCGTT TCCTACCTCT TCAAAAGTTC CGATTACCCA   
  
  
- CAAGGGAAGT TCAAAGTACA GTATTTGCTC AATCTATTGG ATCCTCTCGA AGCTTTTCTC CTAAATCTGT   
  
  
- AACTTCTACC ACTCCGGTAG CGACACTTGA CACAAGTTCG GAACGTTGCC CAAGTACACC TCTTCTCCCT   
  
  
- CGTACACGAA CTACACTAAG CTAGATAATC CGGAACATAG TATTGTGACC ACCTCCTTCT TCGTCTAGAG   
  
  
- TGAAGATGTT CTTTGCTGAA GAAGTTCACG AAGCTACTCA CAAACTCTAA AAGATTCAGT ATAAAGCTAT   
  
  
- ACAATCTTCT TTCGAAGGGA GGTTATTCGT TGCTTGCTTA GTTCGATCTT TCCCTTGTTA CCTACTTATA   
  
  
- GAGGTCTCGG GATCGAACAG TACCACTTTC ACCTCCTCTT ATATCCGGTT TCTTTCCTTG AGTTACCTTA   
  
  
- CTCTACGAGC TTGTTCGTAA AACGGGTAGA GTTAAATCAC TACTACATGA TTCACTACAA TCCCGTAACA   
  
  
- ACTTTTCTGT GTTTTCACCA ACCCTAGAGT GGAATGGTGT TTCACTGGTA CTTTATCCGT ATGTGAATTT   
  
  
- AACCTTCCCA CTTTTACAAC AAACCCGAAG ACGTACCTCT GGATCGAT

+     CAAT-box

| Site Name | Organism | Position | Strand | Matrix score. | sequence | function |
| --- | --- | --- | --- | --- | --- | --- |
| CAAT-box | Nicotiana glutinosa | 2369 | + | 4 | CAAT |  |
| CAAT-box | Nicotiana glutinosa | 1332 | - | 4 | CAAT |  |
| CAAT-box | Arabidopsis thaliana | 2327 | - | 5 | CCAAT | common cis-acting element in promoter and enhancer regions |
| CAAT-box | Nicotiana glutinosa | 3045 | + | 4 | CAAT |  |
| CAAT-box | Nicotiana glutinosa | 1185 | - | 4 | CAAT |  |
| CAAT-box | Nicotiana glutinosa | 1256 | + | 4 | CAAT |  |
| CAAT-box | Nicotiana glutinosa | 730 | - | 4 | CAAT |  |
| CAAT-box | Pisum sativum | 1983 | - | 5 | CAAAT | common cis-acting element in promoter and enhancer regions |
| CAAT-box | Pisum sativum | 1802 | + | 5 | CAAAT | common cis-acting element in promoter and enhancer regions |
| CAAT-box | Pisum sativum | 1485 | - | 5 | CAAAT | common cis-acting element in promoter and enhancer regions |
| CAAT-box | Nicotiana glutinosa | 938 | + | 4 | CAAT |  |
| CAAT-box | Nicotiana glutinosa | 735 | + | 4 | CAAT |  |
| CAAT-box | Pisum sativum | 491 | + | 5 | CAAAT | common cis-acting element in promoter and enhancer regions |
| CAAT-box | Pisum sativum | 1352 | - | 5 | CAAAT | common cis-acting element in promoter and enhancer regions |
| CAAT-box | Arabidopsis thaliana | 937 | + | 5 | CCAAT | common cis-acting element in promoter and enhancer regions |
| CAAT-box | Nicotiana glutinosa | 1576 | - | 4 | CAAT |  |
| CAAT-box | Arabidopsis thaliana | 1255 | + | 5 | CCAAT | common cis-acting element in promoter and enhancer regions |
| CAAT-box | Pisum sativum | 1127 | - | 5 | CAAAT | common cis-acting element in promoter and enhancer regions |
| CAAT-box | Nicotiana glutinosa | 3006 | + | 4 | CAAT |  |
| CAAT-box | Nicotiana glutinosa | 2896 | + | 4 | CAAT |  |
| CAAT-box | Arabidopsis thaliana | 582 | - | 5 | CCAAT | common cis-acting element in promoter and enhancer regions |
| CAAT-box | Nicotiana glutinosa | 672 | + | 4 | CAAT |  |
| CAAT-box | Nicotiana glutinosa | 2242 | + | 4 | CAAT |  |
| CAAT-box | Nicotiana glutinosa | 2509 | + | 4 | CAAT |  |
| CAAT-box | Nicotiana glutinosa | 1428 | - | 4 | CAAT |  |
| CAAT-box | Nicotiana glutinosa | 862 | - | 4 | CAAT |  |
| CAAT-box | Nicotiana glutinosa | 2931 | + | 4 | CAAT |  |
| CAAT-box | Arabidopsis thaliana | 3154 | - | 5 | CCAAT | common cis-acting element in promoter and enhancer regions |
| CAAT-box | Nicotiana glutinosa | 1350 | + | 4 | CAAT |  |
| CAAT-box | Pisum sativum | 567 | - | 5 | CAAAT | common cis-acting element in promoter and enhancer regions |
| CAAT-box | Pisum sativum | 1001 | + | 5 | CAAAT | common cis-acting element in promoter and enhancer regions |
| CAAT-box | Pisum sativum | 425 | - | 5 | CAAAT | common cis-acting element in promoter and enhancer regions |
| CAAT-box | Nicotiana glutinosa | 421 | - | 4 | CAAT |  |
| CAAT-box | Pisum sativum | 2111 | + | 5 | CAAAT | common cis-acting element in promoter and enhancer regions |
| CAAT-box | Pisum sativum | 894 | + | 5 | CAAAT | common cis-acting element in promoter and enhancer regions |
| CAAT-box | Nicotiana glutinosa | 2450 | - | 4 | CAAT |  |
| CAAT-box | Nicotiana glutinosa | 1394 | - | 4 | CAAT |  |
| CAAT-box | Nicotiana glutinosa | 1609 | - | 4 | CAAT |  |
| CAAT-box | Nicotiana glutinosa | 694 | + | 4 | CAAT |  |
| CAAT-box | Arabidopsis thaliana | 702 | - | 5 | CCAAT | common cis-acting element in promoter and enhancer regions |
| CAAT-box | Nicotiana glutinosa | 993 | + | 4 | CAAT |  |
| CAAT-box | Nicotiana glutinosa | 102 | + | 4 | CAAT |  |
| CAAT-box | Arabidopsis thaliana | 1455 | - | 5 | CCAAT | common cis-acting element in promoter and enhancer regions |
| CAAT-box | Pisum sativum | 2500 | + | 5 | CAAAT | common cis-acting element in promoter and enhancer regions |
| CAAT-box | Pisum sativum | 784 | + | 5 | CAAAT | common cis-acting element in promoter and enhancer regions |
| CAAT-box | Arabidopsis thaliana | 768 | - | 5 | CCAAT | common cis-acting element in promoter and enhancer regions |
| CAAT-box | Nicotiana glutinosa | 438 | - | 4 | CAAT |  |
| CAAT-box | Nicotiana glutinosa | 1513 | - | 4 | CAAT |  |
| CAAT-box | Nicotiana glutinosa | 840 | - | 4 | CAAT |  |
| CAAT-box | Nicotiana glutinosa | 3080 | - | 4 | CAAT |  |
| CAAT-box | Pisum sativum | 829 | + | 5 | CAAAT | common cis-acting element in promoter and enhancer regions |
| CAAT-box | Arabidopsis thaliana | 2513 | - | 5 | CCAAT | common cis-acting element in promoter and enhancer regions |
| CAAT-box | Nicotiana glutinosa | 2174 | - | 4 | CAAT |  |
| CAAT-box | Nicotiana glutinosa | 2314 | - | 4 | CAAT |  |
| CAAT-box | Pisum sativum | 2371 | - | 5 | CAAAT | common cis-acting element in promoter and enhancer regions |
| CAAT-box | Pisum sativum | 1648 | - | 5 | CAAAT | common cis-acting element in promoter and enhancer regions |
| CAAT-box | Arabidopsis thaliana | 2895 | + | 5 | CCAAT | common cis-acting element in promoter and enhancer regions |
| CAAT-box | Nicotiana glutinosa | 1473 | + | 4 | CAAT |  |
| CAAT-box | Nicotiana glutinosa | 2664 | - | 4 | CAAT |  |
| CAAT-box | Nicotiana glutinosa | 1732 | - | 4 | CAAT |  |
| CAAT-box | Nicotiana glutinosa | 1684 | + | 4 | CAAT |  |

>HU02G03154.1   
+ +Up\_Stream \_Len000ATGATA AAAGATATTT ATTAAGCTCA AGCTATTTTA ACTTTTAAAA AAATGAATGT   
  
  
+ GGCTTTTTTT GCTATTCAGG TACAACACAA TATGAAGCAT TAACTCTAGA AAATGTGGTA ATGTATGTCA   
  
  
+ TATAGGAGAG TATATACTCT CACTTGCCAT GCGATGCGTG CATGTGTGTG TGTGTGTGTA TCTGTTTCTA   
  
  
+ TGCATATTTT ATGTGGGGTT TTGTCATCTA GACATCTATT AATATGTTAG CATTCACATG ACTTATAGGA   
  
  
+ ATGCACTATT ATGAGTATGA TATAGAGTTT TAAAAATGCA TATGCCTTGT GTGTTCATTA GCTTAAAGAA   
  
  
+ GAGTTTTTGA ATATACATAT TTAGGTAATT CTTGCTAATG TCGTAATTAG CAGTTGTATC TATCTTATTG   
  
  
+ ATTTGTATTT TTAATTGCTT GTTATACTGT TACACTTAGA AAATTCATGA ATACTCTTAA AATATGCAAA   
  
  
+ TTATATATAT ATATATATAT ATATATATAT ATATATATAT ATAAAAACTG AATAAGTTAA TATGGTGATG   
  
  
+ ACATTTGTCA TGTTATCATT GGTCGCTTAA TTTTTAAATA AAAAATATGA TTTTGTGACA CCTAATAAAT   
  
  
+ GATATTAGCC ATATTATTAA ATTCAGCATT TATTCATCAA TGTAATATAC TAAATCATGC AATTATCATT   
  
  
+ GGTTCTCTTT TATGTAATAA AGTTAATTGA CAATTCAAGG GTTACTAATT AGTATAATGT TGAATTGGAA   
  
  
+ CGTAAAAATC AAATCGTATG TAAAGTAATT TTATTCTGAA TTTAAAAAAT GGAGCAAATA TCTCTATTGT   
  
  
+ TCTTTAATAA AATTTTAATT GATTTTTATT TCTAATTAAG TGACGGAACC AAATTTTTTT CCTCCTTTTT   
  
  
+ TGGTTTGGTA AGTTATCATT AGCCAATCTT TTATTTACAT TTTGTCAGTT TTTCTTTTTT AAGATGGTTA   
  
  
+ AATGGCTTCA ATTAAACAAA TTTTTTTACT TATTTATATT TTTAATAAAT TTTCAAAATG TATATTAACG   
  
  
+ CATGACCGTG CGAAGCACGG AATCTACCCT AGTTAATCTA TGATTCTTAA ATATGCAAGT AGGCTGTTAT   
  
  
+ TCATTTGTTG TTTTTTACTT ATTTATATTT TTTCCCTCCC GGTATGATAG GGTTTGTCAT ATTGTTATTC   
  
  
+ ATTAGCTGTT AAACTGTATT CATTATAGCA CAAGTAGGCT TAATCAAAGA TAGTTAGTCT CCAATTTCAT   
  
  
+ CAGAAAATTT AAGAAAAAGA CACCTTAGGA TCAAAAGTAT GAAATTTAGA GACCAGACAA CGATAGAATT   
  
  
+ GAAAAGATGA GTTTTCAATT TGGAGAAGAC GGAAACTCAG ATGAATGAAA GTATAGTACA TTGTACTCCT   
  
  
+ TGGAGTATAA TCTTATCTTC ACTATTGATA GGCTCGAAGC ATAATCAGTG ATTGGAAACT TTTGGTTGCA   
  
  
+ ATAATTCAAA ATTTGATACT CTATGATTTA CTTTTTTAAT TGAAGGTCAT GATTTAATTA ACTAAATGAA   
  
  
+ AAGAATTTAT GATCGAAAAA TTTTAACTCT TATTGACTTA TAGTTGACTC CAGTTAAATG GAAAATTGAA   
  
  
+ GTGATTAATC TAAAAGTGGC CCGCTCTAAT ACCATTTGAG AAAAATTATT CTTACTGCAA AGTTTAAATC   
  
  
+ AATATGTGAA GATAGTTTAT AGTTTTATCC TAAAAGAAAT TAAATAGATT GTTAAAAATA ATTTTTAAAT   
  
  
+ TCAGATATAC GTGTGATGGC CATAACCGTA AATGATATGC ATGTCGACAA ATTCCAAAGA AGGCTAATAA   
  
  
+ TTAGACTTAA ATAATACATG CATACTAACC AAAAAAAAAA AATTAAGCAA GATTCTATCC AACCTATCAT   
  
  
+ AATAAAGTTT GGCCATACTT CAAACCATCA CATAACATCA CTAATATATA AAGCTAGGTT TTGGGAAACC   
  
  
+ TAACTTTGAG GACCACATAT TTGTTAAGGC CTAGGAGGCC ATGAGTTTGT CAAGATGCCT CATAGTATGG   
  
  
+ AGAGGTCTAT ATCCTTGCAA AATGATCATT CCCTTCTACC TAAAAGAACC ACTTCATTTC CACTTTTACT   
  
  
+ CCCTGGCAAA TGGTTGATAA ACCAAACCAT AACCAAGCCC TTGAAGAACT ATAAAGACAC GAATCGATCA   
  
  
+ TTGAATTCGA ACAAGCCTTG TGTGATAGGA GGATGGGCCT CAACACTTCT CCGCGAGTGT GCAAGAGCAA   
  
  
+ TCTCGGAGAA AAATCCTAAG AGCCAACAAC TTCTTTGGGT GTTAAATGAA CTTGTTTCTC CTTATGGCGA   
  
  
+ TTGCGAACAA AGATTGGCAT ATTACTTCTT ACAAGTGTTG TTGGCCAAAG CCAACAATTT GGGACCTCAC   
  
  
+ TTTCATGAGA GCCTAAAACT TGCCATGGAG AAAAACTGCT GCTTTGATAC CTACATGAAG CTTATATTGA   
  
  
+ AGTTCCAAGA GGTCAGTCCA TGGACAACCT TTGGTCATGT GGCTTCAAAT GGTGCAATAT TGGAGGACCT   
  
  
+ TAGAAGTTTA CAAAAGTCGA TCATCAAAGA AACAAGGCAA AGGATGGAGA AGTTTTCAAG GCTAATGGGT   
  
  
+ GTTCCCTTCA AGTTTCATGT CATAAACGAG TTAGATAACC TAGGAGAGCT TCGAAAAGAG GATTTAGACA   
  
  
+ TTGAAGATGG TGAGGCCATC GCTGTGAACT GTGTTCAAGC CTTGCAACGG GTTCATGTGG AGAAGAGGGA   
  
  
+ GCATGTGCTT GATGTGATTC GATCTATTAG GCCTTGTATC ATAACACTGG TGGAGGAAGA AGCAGATCTC   
  
  
+ ACTTCTACAA GAAACGACTT CTTCAAGTGC TTCGATGAGT GTTTGAGATT TTCTAAGTCA TATTTCGATA   
  
  
+ TGTTAGAAGA AAGCTTCCCT CCAATAAGCA ACGAACGAAT CAAGCTAGAA AGGGAACAAT GGATGAATAT   
  
  
+ CTCCAGAGCC CTAGCTTGTC ATGGTGAAAG TGGAGGAGAA TATAGGCCAA AGAAAGGAAC TCAATGGAAT   
  
  
+ GAGATGCTCG AACAAGCATT TTGCCCATCT CAATTTAGTG ATGATGTACT AAGTGATGTT AGGGCATTGT   
  
  
+ TGAAAAGACA CAAAAGTGGT TGGGATCTCA CCTTACCACA AAGTGACCAT GAAATAGGCA TACACTTAAA   
  
  
+ TTGGAAGGGT GAAAATGTTG TTTGGGCTTC TGCATGGAGA CCTAGCTA  

- +Up\_Stream \_Len000TACTAT TTTCTATAAA TAATTCGAGT TCGATAAAAT TGAAAATTTT TTTACTTACA   
  
  
- CCGAAAAAAA CGATAAGTCC ATGTTGTGTT ATACTTCGTA ATTGAGATCT TTTACACCAT TACATACAGT   
  
  
- ATATCCTCTC ATATATGAGA GTGAACGGTA CGCTACGCAC GTACACACAC ACACACACAT AGACAAAGAT   
  
  
- ACGTATAAAA TACACCCCAA AACAGTAGAT CTGTAGATAA TTATACAATC GTAAGTGTAC TGAATATCCT   
  
  
- TACGTGATAA TACTCATACT ATATCTCAAA ATTTTTACGT ATACGGAACA CACAAGTAAT CGAATTTCTT   
  
  
- CTCAAAAACT TATATGTATA AATCCATTAA GAACGATTAC AGCATTAATC GTCAACATAG ATAGAATAAC   
  
  
- TAAACATAAA AATTAACGAA CAATATGACA ATGTGAATCT TTTAAGTACT TATGAGAATT TTATACGTTT   
  
  
- AATATATATA TATATATATA TATATATATA TATATATATA TATTTTTGAC TTATTCAATT ATACCACTAC   
  
  
- TGTAAACAGT ACAATAGTAA CCAGCGAATT AAAAATTTAT TTTTTATACT AAAACACTGT GGATTATTTA   
  
  
- CTATAATCGG TATAATAATT TAAGTCGTAA ATAAGTAGTT ACATTATATG ATTTAGTACG TTAATAGTAA   
  
  
- CCAAGAGAAA ATACATTATT TCAATTAACT GTTAAGTTCC CAATGATTAA TCATATTACA ACTTAACCTT   
  
  
- GCATTTTTAG TTTAGCATAC ATTTCATTAA AATAAGACTT AAATTTTTTA CCTCGTTTAT AGAGATAACA   
  
  
- AGAAATTATT TTAAAATTAA CTAAAAATAA AGATTAATTC ACTGCCTTGG TTTAAAAAAA GGAGGAAAAA   
  
  
- ACCAAACCAT TCAATAGTAA TCGGTTAGAA AATAAATGTA AAACAGTCAA AAAGAAAAAA TTCTACCAAT   
  
  
- TTACCGAAGT TAATTTGTTT AAAAAAATGA ATAAATATAA AAATTATTTA AAAGTTTTAC ATATAATTGC   
  
  
- GTACTGGCAC GCTTCGTGCC TTAGATGGGA TCAATTAGAT ACTAAGAATT TATACGTTCA TCCGACAATA   
  
  
- AGTAAACAAC AAAAAATGAA TAAATATAAA AAAGGGAGGG CCATACTATC CCAAACAGTA TAACAATAAG   
  
  
- TAATCGACAA TTTGACATAA GTAATATCGT GTTCATCCGA ATTAGTTTCT ATCAATCAGA GGTTAAAGTA   
  
  
- GTCTTTTAAA TTCTTTTTCT GTGGAATCCT AGTTTTCATA CTTTAAATCT CTGGTCTGTT GCTATCTTAA   
  
  
- CTTTTCTACT CAAAAGTTAA ACCTCTTCTG CCTTTGAGTC TACTTACTTT CATATCATGT AACATGAGGA   
  
  
- ACCTCATATT AGAATAGAAG TGATAACTAT CCGAGCTTCG TATTAGTCAC TAACCTTTGA AAACCAACGT   
  
  
- TATTAAGTTT TAAACTATGA GATACTAAAT GAAAAAATTA ACTTCCAGTA CTAAATTAAT TGATTTACTT   
  
  
- TTCTTAAATA CTAGCTTTTT AAAATTGAGA ATAACTGAAT ATCAACTGAG GTCAATTTAC CTTTTAACTT   
  
  
- CACTAATTAG ATTTTCACCG GGCGAGATTA TGGTAAACTC TTTTTAATAA GAATGACGTT TCAAATTTAG   
  
  
- TTATACACTT CTATCAAATA TCAAAATAGG ATTTTCTTTA ATTTATCTAA CAATTTTTAT TAAAAATTTA   
  
  
- AGTCTATATG CACACTACCG GTATTGGCAT TTACTATACG TACAGCTGTT TAAGGTTTCT TCCGATTATT   
  
  
- AATCTGAATT TATTATGTAC GTATGATTGG TTTTTTTTTT TTAATTCGTT CTAAGATAGG TTGGATAGTA   
  
  
- TTATTTCAAA CCGGTATGAA GTTTGGTAGT GTATTGTAGT GATTATATAT TTCGATCCAA AACCCTTTGG   
  
  
- ATTGAAACTC CTGGTGTATA AACAATTCCG GATCCTCCGG TACTCAAACA GTTCTACGGA GTATCATACC   
  
  
- TCTCCAGATA TAGGAACGTT TTACTAGTAA GGGAAGATGG ATTTTCTTGG TGAAGTAAAG GTGAAAATGA   
  
  
- GGGACCGTTT ACCAACTATT TGGTTTGGTA TTGGTTCGGG AACTTCTTGA TATTTCTGTG CTTAGCTAGT   
  
  
- AACTTAAGCT TGTTCGGAAC ACACTATCCT CCTACCCGGA GTTGTGAAGA GGCGCTCACA CGTTCTCGTT   
  
  
- AGAGCCTCTT TTTAGGATTC TCGGTTGTTG AAGAAACCCA CAATTTACTT GAACAAAGAG GAATACCGCT   
  
  
- AACGCTTGTT TCTAACCGTA TAATGAAGAA TGTTCACAAC AACCGGTTTC GGTTGTTAAA CCCTGGAGTG   
  
  
- AAAGTACTCT CGGATTTTGA ACGGTACCTC TTTTTGACGA CGAAACTATG GATGTACTTC GAATATAACT   
  
  
- TCAAGGTTCT CCAGTCAGGT ACCTGTTGGA AACCAGTACA CCGAAGTTTA CCACGTTATA ACCTCCTGGA   
  
  
- ATCTTCAAAT GTTTTCAGCT AGTAGTTTCT TTGTTCCGTT TCCTACCTCT TCAAAAGTTC CGATTACCCA   
  
  
- CAAGGGAAGT TCAAAGTACA GTATTTGCTC AATCTATTGG ATCCTCTCGA AGCTTTTCTC CTAAATCTGT   
  
  
- AACTTCTACC ACTCCGGTAG CGACACTTGA CACAAGTTCG GAACGTTGCC CAAGTACACC TCTTCTCCCT   
  
  
- CGTACACGAA CTACACTAAG CTAGATAATC CGGAACATAG TATTGTGACC ACCTCCTTCT TCGTCTAGAG   
  
  
- TGAAGATGTT CTTTGCTGAA GAAGTTCACG AAGCTACTCA CAAACTCTAA AAGATTCAGT ATAAAGCTAT   
  
  
- ACAATCTTCT TTCGAAGGGA GGTTATTCGT TGCTTGCTTA GTTCGATCTT TCCCTTGTTA CCTACTTATA   
  
  
- GAGGTCTCGG GATCGAACAG TACCACTTTC ACCTCCTCTT ATATCCGGTT TCTTTCCTTG AGTTACCTTA   
  
  
- CTCTACGAGC TTGTTCGTAA AACGGGTAGA GTTAAATCAC TACTACATGA TTCACTACAA TCCCGTAACA   
  
  
- ACTTTTCTGT GTTTTCACCA ACCCTAGAGT GGAATGGTGT TTCACTGGTA CTTTATCCGT ATGTGAATTT   
  
  
- AACCTTCCCA CTTTTACAAC AAACCCGAAG ACGTACCTCT GGATCGAT

+     CAT-box

| Site Name | Organism | Position | Strand | Matrix score. | sequence | function |
| --- | --- | --- | --- | --- | --- | --- |
| CAT-box | Arabidopsis thaliana | 1629 | - | 6 | GCCACT | cis-acting regulatory element related to meristem expression |

>HU02G03154.1   
+ +Up\_Stream \_Len000ATGATA AAAGATATTT ATTAAGCTCA AGCTATTTTA ACTTTTAAAA AAATGAATGT   
  
  
+ GGCTTTTTTT GCTATTCAGG TACAACACAA TATGAAGCAT TAACTCTAGA AAATGTGGTA ATGTATGTCA   
  
  
+ TATAGGAGAG TATATACTCT CACTTGCCAT GCGATGCGTG CATGTGTGTG TGTGTGTGTA TCTGTTTCTA   
  
  
+ TGCATATTTT ATGTGGGGTT TTGTCATCTA GACATCTATT AATATGTTAG CATTCACATG ACTTATAGGA   
  
  
+ ATGCACTATT ATGAGTATGA TATAGAGTTT TAAAAATGCA TATGCCTTGT GTGTTCATTA GCTTAAAGAA   
  
  
+ GAGTTTTTGA ATATACATAT TTAGGTAATT CTTGCTAATG TCGTAATTAG CAGTTGTATC TATCTTATTG   
  
  
+ ATTTGTATTT TTAATTGCTT GTTATACTGT TACACTTAGA AAATTCATGA ATACTCTTAA AATATGCAAA   
  
  
+ TTATATATAT ATATATATAT ATATATATAT ATATATATAT ATAAAAACTG AATAAGTTAA TATGGTGATG   
  
  
+ ACATTTGTCA TGTTATCATT GGTCGCTTAA TTTTTAAATA AAAAATATGA TTTTGTGACA CCTAATAAAT   
  
  
+ GATATTAGCC ATATTATTAA ATTCAGCATT TATTCATCAA TGTAATATAC TAAATCATGC AATTATCATT   
  
  
+ GGTTCTCTTT TATGTAATAA AGTTAATTGA CAATTCAAGG GTTACTAATT AGTATAATGT TGAATTGGAA   
  
  
+ CGTAAAAATC AAATCGTATG TAAAGTAATT TTATTCTGAA TTTAAAAAAT GGAGCAAATA TCTCTATTGT   
  
  
+ TCTTTAATAA AATTTTAATT GATTTTTATT TCTAATTAAG TGACGGAACC AAATTTTTTT CCTCCTTTTT   
  
  
+ TGGTTTGGTA AGTTATCATT AGCCAATCTT TTATTTACAT TTTGTCAGTT TTTCTTTTTT AAGATGGTTA   
  
  
+ AATGGCTTCA ATTAAACAAA TTTTTTTACT TATTTATATT TTTAATAAAT TTTCAAAATG TATATTAACG   
  
  
+ CATGACCGTG CGAAGCACGG AATCTACCCT AGTTAATCTA TGATTCTTAA ATATGCAAGT AGGCTGTTAT   
  
  
+ TCATTTGTTG TTTTTTACTT ATTTATATTT TTTCCCTCCC GGTATGATAG GGTTTGTCAT ATTGTTATTC   
  
  
+ ATTAGCTGTT AAACTGTATT CATTATAGCA CAAGTAGGCT TAATCAAAGA TAGTTAGTCT CCAATTTCAT   
  
  
+ CAGAAAATTT AAGAAAAAGA CACCTTAGGA TCAAAAGTAT GAAATTTAGA GACCAGACAA CGATAGAATT   
  
  
+ GAAAAGATGA GTTTTCAATT TGGAGAAGAC GGAAACTCAG ATGAATGAAA GTATAGTACA TTGTACTCCT   
  
  
+ TGGAGTATAA TCTTATCTTC ACTATTGATA GGCTCGAAGC ATAATCAGTG ATTGGAAACT TTTGGTTGCA   
  
  
+ ATAATTCAAA ATTTGATACT CTATGATTTA CTTTTTTAAT TGAAGGTCAT GATTTAATTA ACTAAATGAA   
  
  
+ AAGAATTTAT GATCGAAAAA TTTTAACTCT TATTGACTTA TAGTTGACTC CAGTTAAATG GAAAATTGAA   
  
  
+ GTGATTAATC TAAAAGTGGC CCGCTCTAAT ACCATTTGAG AAAAATTATT CTTACTGCAA AGTTTAAATC   
  
  
+ AATATGTGAA GATAGTTTAT AGTTTTATCC TAAAAGAAAT TAAATAGATT GTTAAAAATA ATTTTTAAAT   
  
  
+ TCAGATATAC GTGTGATGGC CATAACCGTA AATGATATGC ATGTCGACAA ATTCCAAAGA AGGCTAATAA   
  
  
+ TTAGACTTAA ATAATACATG CATACTAACC AAAAAAAAAA AATTAAGCAA GATTCTATCC AACCTATCAT   
  
  
+ AATAAAGTTT GGCCATACTT CAAACCATCA CATAACATCA CTAATATATA AAGCTAGGTT TTGGGAAACC   
  
  
+ TAACTTTGAG GACCACATAT TTGTTAAGGC CTAGGAGGCC ATGAGTTTGT CAAGATGCCT CATAGTATGG   
  
  
+ AGAGGTCTAT ATCCTTGCAA AATGATCATT CCCTTCTACC TAAAAGAACC ACTTCATTTC CACTTTTACT   
  
  
+ CCCTGGCAAA TGGTTGATAA ACCAAACCAT AACCAAGCCC TTGAAGAACT ATAAAGACAC GAATCGATCA   
  
  
+ TTGAATTCGA ACAAGCCTTG TGTGATAGGA GGATGGGCCT CAACACTTCT CCGCGAGTGT GCAAGAGCAA   
  
  
+ TCTCGGAGAA AAATCCTAAG AGCCAACAAC TTCTTTGGGT GTTAAATGAA CTTGTTTCTC CTTATGGCGA   
  
  
+ TTGCGAACAA AGATTGGCAT ATTACTTCTT ACAAGTGTTG TTGGCCAAAG CCAACAATTT GGGACCTCAC   
  
  
+ TTTCATGAGA GCCTAAAACT TGCCATGGAG AAAAACTGCT GCTTTGATAC CTACATGAAG CTTATATTGA   
  
  
+ AGTTCCAAGA GGTCAGTCCA TGGACAACCT TTGGTCATGT GGCTTCAAAT GGTGCAATAT TGGAGGACCT   
  
  
+ TAGAAGTTTA CAAAAGTCGA TCATCAAAGA AACAAGGCAA AGGATGGAGA AGTTTTCAAG GCTAATGGGT   
  
  
+ GTTCCCTTCA AGTTTCATGT CATAAACGAG TTAGATAACC TAGGAGAGCT TCGAAAAGAG GATTTAGACA   
  
  
+ TTGAAGATGG TGAGGCCATC GCTGTGAACT GTGTTCAAGC CTTGCAACGG GTTCATGTGG AGAAGAGGGA   
  
  
+ GCATGTGCTT GATGTGATTC GATCTATTAG GCCTTGTATC ATAACACTGG TGGAGGAAGA AGCAGATCTC   
  
  
+ ACTTCTACAA GAAACGACTT CTTCAAGTGC TTCGATGAGT GTTTGAGATT TTCTAAGTCA TATTTCGATA   
  
  
+ TGTTAGAAGA AAGCTTCCCT CCAATAAGCA ACGAACGAAT CAAGCTAGAA AGGGAACAAT GGATGAATAT   
  
  
+ CTCCAGAGCC CTAGCTTGTC ATGGTGAAAG TGGAGGAGAA TATAGGCCAA AGAAAGGAAC TCAATGGAAT   
  
  
+ GAGATGCTCG AACAAGCATT TTGCCCATCT CAATTTAGTG ATGATGTACT AAGTGATGTT AGGGCATTGT   
  
  
+ TGAAAAGACA CAAAAGTGGT TGGGATCTCA CCTTACCACA AAGTGACCAT GAAATAGGCA TACACTTAAA   
  
  
+ TTGGAAGGGT GAAAATGTTG TTTGGGCTTC TGCATGGAGA CCTAGCTA  

- +Up\_Stream \_Len000TACTAT TTTCTATAAA TAATTCGAGT TCGATAAAAT TGAAAATTTT TTTACTTACA   
  
  
- CCGAAAAAAA CGATAAGTCC ATGTTGTGTT ATACTTCGTA ATTGAGATCT TTTACACCAT TACATACAGT   
  
  
- ATATCCTCTC ATATATGAGA GTGAACGGTA CGCTACGCAC GTACACACAC ACACACACAT AGACAAAGAT   
  
  
- ACGTATAAAA TACACCCCAA AACAGTAGAT CTGTAGATAA TTATACAATC GTAAGTGTAC TGAATATCCT   
  
  
- TACGTGATAA TACTCATACT ATATCTCAAA ATTTTTACGT ATACGGAACA CACAAGTAAT CGAATTTCTT   
  
  
- CTCAAAAACT TATATGTATA AATCCATTAA GAACGATTAC AGCATTAATC GTCAACATAG ATAGAATAAC   
  
  
- TAAACATAAA AATTAACGAA CAATATGACA ATGTGAATCT TTTAAGTACT TATGAGAATT TTATACGTTT   
  
  
- AATATATATA TATATATATA TATATATATA TATATATATA TATTTTTGAC TTATTCAATT ATACCACTAC   
  
  
- TGTAAACAGT ACAATAGTAA CCAGCGAATT AAAAATTTAT TTTTTATACT AAAACACTGT GGATTATTTA   
  
  
- CTATAATCGG TATAATAATT TAAGTCGTAA ATAAGTAGTT ACATTATATG ATTTAGTACG TTAATAGTAA   
  
  
- CCAAGAGAAA ATACATTATT TCAATTAACT GTTAAGTTCC CAATGATTAA TCATATTACA ACTTAACCTT   
  
  
- GCATTTTTAG TTTAGCATAC ATTTCATTAA AATAAGACTT AAATTTTTTA CCTCGTTTAT AGAGATAACA   
  
  
- AGAAATTATT TTAAAATTAA CTAAAAATAA AGATTAATTC ACTGCCTTGG TTTAAAAAAA GGAGGAAAAA   
  
  
- ACCAAACCAT TCAATAGTAA TCGGTTAGAA AATAAATGTA AAACAGTCAA AAAGAAAAAA TTCTACCAAT   
  
  
- TTACCGAAGT TAATTTGTTT AAAAAAATGA ATAAATATAA AAATTATTTA AAAGTTTTAC ATATAATTGC   
  
  
- GTACTGGCAC GCTTCGTGCC TTAGATGGGA TCAATTAGAT ACTAAGAATT TATACGTTCA TCCGACAATA   
  
  
- AGTAAACAAC AAAAAATGAA TAAATATAAA AAAGGGAGGG CCATACTATC CCAAACAGTA TAACAATAAG   
  
  
- TAATCGACAA TTTGACATAA GTAATATCGT GTTCATCCGA ATTAGTTTCT ATCAATCAGA GGTTAAAGTA   
  
  
- GTCTTTTAAA TTCTTTTTCT GTGGAATCCT AGTTTTCATA CTTTAAATCT CTGGTCTGTT GCTATCTTAA   
  
  
- CTTTTCTACT CAAAAGTTAA ACCTCTTCTG CCTTTGAGTC TACTTACTTT CATATCATGT AACATGAGGA   
  
  
- ACCTCATATT AGAATAGAAG TGATAACTAT CCGAGCTTCG TATTAGTCAC TAACCTTTGA AAACCAACGT   
  
  
- TATTAAGTTT TAAACTATGA GATACTAAAT GAAAAAATTA ACTTCCAGTA CTAAATTAAT TGATTTACTT   
  
  
- TTCTTAAATA CTAGCTTTTT AAAATTGAGA ATAACTGAAT ATCAACTGAG GTCAATTTAC CTTTTAACTT   
  
  
- CACTAATTAG ATTTTCACCG GGCGAGATTA TGGTAAACTC TTTTTAATAA GAATGACGTT TCAAATTTAG   
  
  
- TTATACACTT CTATCAAATA TCAAAATAGG ATTTTCTTTA ATTTATCTAA CAATTTTTAT TAAAAATTTA   
  
  
- AGTCTATATG CACACTACCG GTATTGGCAT TTACTATACG TACAGCTGTT TAAGGTTTCT TCCGATTATT   
  
  
- AATCTGAATT TATTATGTAC GTATGATTGG TTTTTTTTTT TTAATTCGTT CTAAGATAGG TTGGATAGTA   
  
  
- TTATTTCAAA CCGGTATGAA GTTTGGTAGT GTATTGTAGT GATTATATAT TTCGATCCAA AACCCTTTGG   
  
  
- ATTGAAACTC CTGGTGTATA AACAATTCCG GATCCTCCGG TACTCAAACA GTTCTACGGA GTATCATACC   
  
  
- TCTCCAGATA TAGGAACGTT TTACTAGTAA GGGAAGATGG ATTTTCTTGG TGAAGTAAAG GTGAAAATGA   
  
  
- GGGACCGTTT ACCAACTATT TGGTTTGGTA TTGGTTCGGG AACTTCTTGA TATTTCTGTG CTTAGCTAGT   
  
  
- AACTTAAGCT TGTTCGGAAC ACACTATCCT CCTACCCGGA GTTGTGAAGA GGCGCTCACA CGTTCTCGTT   
  
  
- AGAGCCTCTT TTTAGGATTC TCGGTTGTTG AAGAAACCCA CAATTTACTT GAACAAAGAG GAATACCGCT   
  
  
- AACGCTTGTT TCTAACCGTA TAATGAAGAA TGTTCACAAC AACCGGTTTC GGTTGTTAAA CCCTGGAGTG   
  
  
- AAAGTACTCT CGGATTTTGA ACGGTACCTC TTTTTGACGA CGAAACTATG GATGTACTTC GAATATAACT   
  
  
- TCAAGGTTCT CCAGTCAGGT ACCTGTTGGA AACCAGTACA CCGAAGTTTA CCACGTTATA ACCTCCTGGA   
  
  
- ATCTTCAAAT GTTTTCAGCT AGTAGTTTCT TTGTTCCGTT TCCTACCTCT TCAAAAGTTC CGATTACCCA   
  
  
- CAAGGGAAGT TCAAAGTACA GTATTTGCTC AATCTATTGG ATCCTCTCGA AGCTTTTCTC CTAAATCTGT   
  
  
- AACTTCTACC ACTCCGGTAG CGACACTTGA CACAAGTTCG GAACGTTGCC CAAGTACACC TCTTCTCCCT   
  
  
- CGTACACGAA CTACACTAAG CTAGATAATC CGGAACATAG TATTGTGACC ACCTCCTTCT TCGTCTAGAG   
  
  
- TGAAGATGTT CTTTGCTGAA GAAGTTCACG AAGCTACTCA CAAACTCTAA AAGATTCAGT ATAAAGCTAT   
  
  
- ACAATCTTCT TTCGAAGGGA GGTTATTCGT TGCTTGCTTA GTTCGATCTT TCCCTTGTTA CCTACTTATA   
  
  
- GAGGTCTCGG GATCGAACAG TACCACTTTC ACCTCCTCTT ATATCCGGTT TCTTTCCTTG AGTTACCTTA   
  
  
- CTCTACGAGC TTGTTCGTAA AACGGGTAGA GTTAAATCAC TACTACATGA TTCACTACAA TCCCGTAACA   
  
  
- ACTTTTCTGT GTTTTCACCA ACCCTAGAGT GGAATGGTGT TTCACTGGTA CTTTATCCGT ATGTGAATTT   
  
  
- AACCTTCCCA CTTTTACAAC AAACCCGAAG ACGTACCTCT GGATCGAT

+     CCAAT-box

| Site Name | Organism | Position | Strand | Matrix score. | sequence | function |
| --- | --- | --- | --- | --- | --- | --- |
| CCAAT-box | Hordeum vulgare | 2709 | + | 6 | CAACGG | MYBHv1 binding site |

>HU02G03154.1   
+ +Up\_Stream \_Len000ATGATA AAAGATATTT ATTAAGCTCA AGCTATTTTA ACTTTTAAAA AAATGAATGT   
  
  
+ GGCTTTTTTT GCTATTCAGG TACAACACAA TATGAAGCAT TAACTCTAGA AAATGTGGTA ATGTATGTCA   
  
  
+ TATAGGAGAG TATATACTCT CACTTGCCAT GCGATGCGTG CATGTGTGTG TGTGTGTGTA TCTGTTTCTA   
  
  
+ TGCATATTTT ATGTGGGGTT TTGTCATCTA GACATCTATT AATATGTTAG CATTCACATG ACTTATAGGA   
  
  
+ ATGCACTATT ATGAGTATGA TATAGAGTTT TAAAAATGCA TATGCCTTGT GTGTTCATTA GCTTAAAGAA   
  
  
+ GAGTTTTTGA ATATACATAT TTAGGTAATT CTTGCTAATG TCGTAATTAG CAGTTGTATC TATCTTATTG   
  
  
+ ATTTGTATTT TTAATTGCTT GTTATACTGT TACACTTAGA AAATTCATGA ATACTCTTAA AATATGCAAA   
  
  
+ TTATATATAT ATATATATAT ATATATATAT ATATATATAT ATAAAAACTG AATAAGTTAA TATGGTGATG   
  
  
+ ACATTTGTCA TGTTATCATT GGTCGCTTAA TTTTTAAATA AAAAATATGA TTTTGTGACA CCTAATAAAT   
  
  
+ GATATTAGCC ATATTATTAA ATTCAGCATT TATTCATCAA TGTAATATAC TAAATCATGC AATTATCATT   
  
  
+ GGTTCTCTTT TATGTAATAA AGTTAATTGA CAATTCAAGG GTTACTAATT AGTATAATGT TGAATTGGAA   
  
  
+ CGTAAAAATC AAATCGTATG TAAAGTAATT TTATTCTGAA TTTAAAAAAT GGAGCAAATA TCTCTATTGT   
  
  
+ TCTTTAATAA AATTTTAATT GATTTTTATT TCTAATTAAG TGACGGAACC AAATTTTTTT CCTCCTTTTT   
  
  
+ TGGTTTGGTA AGTTATCATT AGCCAATCTT TTATTTACAT TTTGTCAGTT TTTCTTTTTT AAGATGGTTA   
  
  
+ AATGGCTTCA ATTAAACAAA TTTTTTTACT TATTTATATT TTTAATAAAT TTTCAAAATG TATATTAACG   
  
  
+ CATGACCGTG CGAAGCACGG AATCTACCCT AGTTAATCTA TGATTCTTAA ATATGCAAGT AGGCTGTTAT   
  
  
+ TCATTTGTTG TTTTTTACTT ATTTATATTT TTTCCCTCCC GGTATGATAG GGTTTGTCAT ATTGTTATTC   
  
  
+ ATTAGCTGTT AAACTGTATT CATTATAGCA CAAGTAGGCT TAATCAAAGA TAGTTAGTCT CCAATTTCAT   
  
  
+ CAGAAAATTT AAGAAAAAGA CACCTTAGGA TCAAAAGTAT GAAATTTAGA GACCAGACAA CGATAGAATT   
  
  
+ GAAAAGATGA GTTTTCAATT TGGAGAAGAC GGAAACTCAG ATGAATGAAA GTATAGTACA TTGTACTCCT   
  
  
+ TGGAGTATAA TCTTATCTTC ACTATTGATA GGCTCGAAGC ATAATCAGTG ATTGGAAACT TTTGGTTGCA   
  
  
+ ATAATTCAAA ATTTGATACT CTATGATTTA CTTTTTTAAT TGAAGGTCAT GATTTAATTA ACTAAATGAA   
  
  
+ AAGAATTTAT GATCGAAAAA TTTTAACTCT TATTGACTTA TAGTTGACTC CAGTTAAATG GAAAATTGAA   
  
  
+ GTGATTAATC TAAAAGTGGC CCGCTCTAAT ACCATTTGAG AAAAATTATT CTTACTGCAA AGTTTAAATC   
  
  
+ AATATGTGAA GATAGTTTAT AGTTTTATCC TAAAAGAAAT TAAATAGATT GTTAAAAATA ATTTTTAAAT   
  
  
+ TCAGATATAC GTGTGATGGC CATAACCGTA AATGATATGC ATGTCGACAA ATTCCAAAGA AGGCTAATAA   
  
  
+ TTAGACTTAA ATAATACATG CATACTAACC AAAAAAAAAA AATTAAGCAA GATTCTATCC AACCTATCAT   
  
  
+ AATAAAGTTT GGCCATACTT CAAACCATCA CATAACATCA CTAATATATA AAGCTAGGTT TTGGGAAACC   
  
  
+ TAACTTTGAG GACCACATAT TTGTTAAGGC CTAGGAGGCC ATGAGTTTGT CAAGATGCCT CATAGTATGG   
  
  
+ AGAGGTCTAT ATCCTTGCAA AATGATCATT CCCTTCTACC TAAAAGAACC ACTTCATTTC CACTTTTACT   
  
  
+ CCCTGGCAAA TGGTTGATAA ACCAAACCAT AACCAAGCCC TTGAAGAACT ATAAAGACAC GAATCGATCA   
  
  
+ TTGAATTCGA ACAAGCCTTG TGTGATAGGA GGATGGGCCT CAACACTTCT CCGCGAGTGT GCAAGAGCAA   
  
  
+ TCTCGGAGAA AAATCCTAAG AGCCAACAAC TTCTTTGGGT GTTAAATGAA CTTGTTTCTC CTTATGGCGA   
  
  
+ TTGCGAACAA AGATTGGCAT ATTACTTCTT ACAAGTGTTG TTGGCCAAAG CCAACAATTT GGGACCTCAC   
  
  
+ TTTCATGAGA GCCTAAAACT TGCCATGGAG AAAAACTGCT GCTTTGATAC CTACATGAAG CTTATATTGA   
  
  
+ AGTTCCAAGA GGTCAGTCCA TGGACAACCT TTGGTCATGT GGCTTCAAAT GGTGCAATAT TGGAGGACCT   
  
  
+ TAGAAGTTTA CAAAAGTCGA TCATCAAAGA AACAAGGCAA AGGATGGAGA AGTTTTCAAG GCTAATGGGT   
  
  
+ GTTCCCTTCA AGTTTCATGT CATAAACGAG TTAGATAACC TAGGAGAGCT TCGAAAAGAG GATTTAGACA   
  
  
+ TTGAAGATGG TGAGGCCATC GCTGTGAACT GTGTTCAAGC CTTGCAACGG GTTCATGTGG AGAAGAGGGA   
  
  
+ GCATGTGCTT GATGTGATTC GATCTATTAG GCCTTGTATC ATAACACTGG TGGAGGAAGA AGCAGATCTC   
  
  
+ ACTTCTACAA GAAACGACTT CTTCAAGTGC TTCGATGAGT GTTTGAGATT TTCTAAGTCA TATTTCGATA   
  
  
+ TGTTAGAAGA AAGCTTCCCT CCAATAAGCA ACGAACGAAT CAAGCTAGAA AGGGAACAAT GGATGAATAT   
  
  
+ CTCCAGAGCC CTAGCTTGTC ATGGTGAAAG TGGAGGAGAA TATAGGCCAA AGAAAGGAAC TCAATGGAAT   
  
  
+ GAGATGCTCG AACAAGCATT TTGCCCATCT CAATTTAGTG ATGATGTACT AAGTGATGTT AGGGCATTGT   
  
  
+ TGAAAAGACA CAAAAGTGGT TGGGATCTCA CCTTACCACA AAGTGACCAT GAAATAGGCA TACACTTAAA   
  
  
+ TTGGAAGGGT GAAAATGTTG TTTGGGCTTC TGCATGGAGA CCTAGCTA  

- +Up\_Stream \_Len000TACTAT TTTCTATAAA TAATTCGAGT TCGATAAAAT TGAAAATTTT TTTACTTACA   
  
  
- CCGAAAAAAA CGATAAGTCC ATGTTGTGTT ATACTTCGTA ATTGAGATCT TTTACACCAT TACATACAGT   
  
  
- ATATCCTCTC ATATATGAGA GTGAACGGTA CGCTACGCAC GTACACACAC ACACACACAT AGACAAAGAT   
  
  
- ACGTATAAAA TACACCCCAA AACAGTAGAT CTGTAGATAA TTATACAATC GTAAGTGTAC TGAATATCCT   
  
  
- TACGTGATAA TACTCATACT ATATCTCAAA ATTTTTACGT ATACGGAACA CACAAGTAAT CGAATTTCTT   
  
  
- CTCAAAAACT TATATGTATA AATCCATTAA GAACGATTAC AGCATTAATC GTCAACATAG ATAGAATAAC   
  
  
- TAAACATAAA AATTAACGAA CAATATGACA ATGTGAATCT TTTAAGTACT TATGAGAATT TTATACGTTT   
  
  
- AATATATATA TATATATATA TATATATATA TATATATATA TATTTTTGAC TTATTCAATT ATACCACTAC   
  
  
- TGTAAACAGT ACAATAGTAA CCAGCGAATT AAAAATTTAT TTTTTATACT AAAACACTGT GGATTATTTA   
  
  
- CTATAATCGG TATAATAATT TAAGTCGTAA ATAAGTAGTT ACATTATATG ATTTAGTACG TTAATAGTAA   
  
  
- CCAAGAGAAA ATACATTATT TCAATTAACT GTTAAGTTCC CAATGATTAA TCATATTACA ACTTAACCTT   
  
  
- GCATTTTTAG TTTAGCATAC ATTTCATTAA AATAAGACTT AAATTTTTTA CCTCGTTTAT AGAGATAACA   
  
  
- AGAAATTATT TTAAAATTAA CTAAAAATAA AGATTAATTC ACTGCCTTGG TTTAAAAAAA GGAGGAAAAA   
  
  
- ACCAAACCAT TCAATAGTAA TCGGTTAGAA AATAAATGTA AAACAGTCAA AAAGAAAAAA TTCTACCAAT   
  
  
- TTACCGAAGT TAATTTGTTT AAAAAAATGA ATAAATATAA AAATTATTTA AAAGTTTTAC ATATAATTGC   
  
  
- GTACTGGCAC GCTTCGTGCC TTAGATGGGA TCAATTAGAT ACTAAGAATT TATACGTTCA TCCGACAATA   
  
  
- AGTAAACAAC AAAAAATGAA TAAATATAAA AAAGGGAGGG CCATACTATC CCAAACAGTA TAACAATAAG   
  
  
- TAATCGACAA TTTGACATAA GTAATATCGT GTTCATCCGA ATTAGTTTCT ATCAATCAGA GGTTAAAGTA   
  
  
- GTCTTTTAAA TTCTTTTTCT GTGGAATCCT AGTTTTCATA CTTTAAATCT CTGGTCTGTT GCTATCTTAA   
  
  
- CTTTTCTACT CAAAAGTTAA ACCTCTTCTG CCTTTGAGTC TACTTACTTT CATATCATGT AACATGAGGA   
  
  
- ACCTCATATT AGAATAGAAG TGATAACTAT CCGAGCTTCG TATTAGTCAC TAACCTTTGA AAACCAACGT   
  
  
- TATTAAGTTT TAAACTATGA GATACTAAAT GAAAAAATTA ACTTCCAGTA CTAAATTAAT TGATTTACTT   
  
  
- TTCTTAAATA CTAGCTTTTT AAAATTGAGA ATAACTGAAT ATCAACTGAG GTCAATTTAC CTTTTAACTT   
  
  
- CACTAATTAG ATTTTCACCG GGCGAGATTA TGGTAAACTC TTTTTAATAA GAATGACGTT TCAAATTTAG   
  
  
- TTATACACTT CTATCAAATA TCAAAATAGG ATTTTCTTTA ATTTATCTAA CAATTTTTAT TAAAAATTTA   
  
  
- AGTCTATATG CACACTACCG GTATTGGCAT TTACTATACG TACAGCTGTT TAAGGTTTCT TCCGATTATT   
  
  
- AATCTGAATT TATTATGTAC GTATGATTGG TTTTTTTTTT TTAATTCGTT CTAAGATAGG TTGGATAGTA   
  
  
- TTATTTCAAA CCGGTATGAA GTTTGGTAGT GTATTGTAGT GATTATATAT TTCGATCCAA AACCCTTTGG   
  
  
- ATTGAAACTC CTGGTGTATA AACAATTCCG GATCCTCCGG TACTCAAACA GTTCTACGGA GTATCATACC   
  
  
- TCTCCAGATA TAGGAACGTT TTACTAGTAA GGGAAGATGG ATTTTCTTGG TGAAGTAAAG GTGAAAATGA   
  
  
- GGGACCGTTT ACCAACTATT TGGTTTGGTA TTGGTTCGGG AACTTCTTGA TATTTCTGTG CTTAGCTAGT   
  
  
- AACTTAAGCT TGTTCGGAAC ACACTATCCT CCTACCCGGA GTTGTGAAGA GGCGCTCACA CGTTCTCGTT   
  
  
- AGAGCCTCTT TTTAGGATTC TCGGTTGTTG AAGAAACCCA CAATTTACTT GAACAAAGAG GAATACCGCT   
  
  
- AACGCTTGTT TCTAACCGTA TAATGAAGAA TGTTCACAAC AACCGGTTTC GGTTGTTAAA CCCTGGAGTG   
  
  
- AAAGTACTCT CGGATTTTGA ACGGTACCTC TTTTTGACGA CGAAACTATG GATGTACTTC GAATATAACT   
  
  
- TCAAGGTTCT CCAGTCAGGT ACCTGTTGGA AACCAGTACA CCGAAGTTTA CCACGTTATA ACCTCCTGGA   
  
  
- ATCTTCAAAT GTTTTCAGCT AGTAGTTTCT TTGTTCCGTT TCCTACCTCT TCAAAAGTTC CGATTACCCA   
  
  
- CAAGGGAAGT TCAAAGTACA GTATTTGCTC AATCTATTGG ATCCTCTCGA AGCTTTTCTC CTAAATCTGT   
  
  
- AACTTCTACC ACTCCGGTAG CGACACTTGA CACAAGTTCG GAACGTTGCC CAAGTACACC TCTTCTCCCT   
  
  
- CGTACACGAA CTACACTAAG CTAGATAATC CGGAACATAG TATTGTGACC ACCTCCTTCT TCGTCTAGAG   
  
  
- TGAAGATGTT CTTTGCTGAA GAAGTTCACG AAGCTACTCA CAAACTCTAA AAGATTCAGT ATAAAGCTAT   
  
  
- ACAATCTTCT TTCGAAGGGA GGTTATTCGT TGCTTGCTTA GTTCGATCTT TCCCTTGTTA CCTACTTATA   
  
  
- GAGGTCTCGG GATCGAACAG TACCACTTTC ACCTCCTCTT ATATCCGGTT TCTTTCCTTG AGTTACCTTA   
  
  
- CTCTACGAGC TTGTTCGTAA AACGGGTAGA GTTAAATCAC TACTACATGA TTCACTACAA TCCCGTAACA   
  
  
- ACTTTTCTGT GTTTTCACCA ACCCTAGAGT GGAATGGTGT TTCACTGGTA CTTTATCCGT ATGTGAATTT   
  
  
- AACCTTCCCA CTTTTACAAC AAACCCGAAG ACGTACCTCT GGATCGAT

+     CGTCA-motif

| Site Name | Organism | Position | Strand | Matrix score. | sequence | function |
| --- | --- | --- | --- | --- | --- | --- |
| CGTCA-motif | Hordeum vulgare | 885 | - | 5 | CGTCA | cis-acting regulatory element involved in the MeJA-responsiveness |

>HU02G03154.1   
+ +Up\_Stream \_Len000ATGATA AAAGATATTT ATTAAGCTCA AGCTATTTTA ACTTTTAAAA AAATGAATGT   
  
  
+ GGCTTTTTTT GCTATTCAGG TACAACACAA TATGAAGCAT TAACTCTAGA AAATGTGGTA ATGTATGTCA   
  
  
+ TATAGGAGAG TATATACTCT CACTTGCCAT GCGATGCGTG CATGTGTGTG TGTGTGTGTA TCTGTTTCTA   
  
  
+ TGCATATTTT ATGTGGGGTT TTGTCATCTA GACATCTATT AATATGTTAG CATTCACATG ACTTATAGGA   
  
  
+ ATGCACTATT ATGAGTATGA TATAGAGTTT TAAAAATGCA TATGCCTTGT GTGTTCATTA GCTTAAAGAA   
  
  
+ GAGTTTTTGA ATATACATAT TTAGGTAATT CTTGCTAATG TCGTAATTAG CAGTTGTATC TATCTTATTG   
  
  
+ ATTTGTATTT TTAATTGCTT GTTATACTGT TACACTTAGA AAATTCATGA ATACTCTTAA AATATGCAAA   
  
  
+ TTATATATAT ATATATATAT ATATATATAT ATATATATAT ATAAAAACTG AATAAGTTAA TATGGTGATG   
  
  
+ ACATTTGTCA TGTTATCATT GGTCGCTTAA TTTTTAAATA AAAAATATGA TTTTGTGACA CCTAATAAAT   
  
  
+ GATATTAGCC ATATTATTAA ATTCAGCATT TATTCATCAA TGTAATATAC TAAATCATGC AATTATCATT   
  
  
+ GGTTCTCTTT TATGTAATAA AGTTAATTGA CAATTCAAGG GTTACTAATT AGTATAATGT TGAATTGGAA   
  
  
+ CGTAAAAATC AAATCGTATG TAAAGTAATT TTATTCTGAA TTTAAAAAAT GGAGCAAATA TCTCTATTGT   
  
  
+ TCTTTAATAA AATTTTAATT GATTTTTATT TCTAATTAAG TGACGGAACC AAATTTTTTT CCTCCTTTTT   
  
  
+ TGGTTTGGTA AGTTATCATT AGCCAATCTT TTATTTACAT TTTGTCAGTT TTTCTTTTTT AAGATGGTTA   
  
  
+ AATGGCTTCA ATTAAACAAA TTTTTTTACT TATTTATATT TTTAATAAAT TTTCAAAATG TATATTAACG   
  
  
+ CATGACCGTG CGAAGCACGG AATCTACCCT AGTTAATCTA TGATTCTTAA ATATGCAAGT AGGCTGTTAT   
  
  
+ TCATTTGTTG TTTTTTACTT ATTTATATTT TTTCCCTCCC GGTATGATAG GGTTTGTCAT ATTGTTATTC   
  
  
+ ATTAGCTGTT AAACTGTATT CATTATAGCA CAAGTAGGCT TAATCAAAGA TAGTTAGTCT CCAATTTCAT   
  
  
+ CAGAAAATTT AAGAAAAAGA CACCTTAGGA TCAAAAGTAT GAAATTTAGA GACCAGACAA CGATAGAATT   
  
  
+ GAAAAGATGA GTTTTCAATT TGGAGAAGAC GGAAACTCAG ATGAATGAAA GTATAGTACA TTGTACTCCT   
  
  
+ TGGAGTATAA TCTTATCTTC ACTATTGATA GGCTCGAAGC ATAATCAGTG ATTGGAAACT TTTGGTTGCA   
  
  
+ ATAATTCAAA ATTTGATACT CTATGATTTA CTTTTTTAAT TGAAGGTCAT GATTTAATTA ACTAAATGAA   
  
  
+ AAGAATTTAT GATCGAAAAA TTTTAACTCT TATTGACTTA TAGTTGACTC CAGTTAAATG GAAAATTGAA   
  
  
+ GTGATTAATC TAAAAGTGGC CCGCTCTAAT ACCATTTGAG AAAAATTATT CTTACTGCAA AGTTTAAATC   
  
  
+ AATATGTGAA GATAGTTTAT AGTTTTATCC TAAAAGAAAT TAAATAGATT GTTAAAAATA ATTTTTAAAT   
  
  
+ TCAGATATAC GTGTGATGGC CATAACCGTA AATGATATGC ATGTCGACAA ATTCCAAAGA AGGCTAATAA   
  
  
+ TTAGACTTAA ATAATACATG CATACTAACC AAAAAAAAAA AATTAAGCAA GATTCTATCC AACCTATCAT   
  
  
+ AATAAAGTTT GGCCATACTT CAAACCATCA CATAACATCA CTAATATATA AAGCTAGGTT TTGGGAAACC   
  
  
+ TAACTTTGAG GACCACATAT TTGTTAAGGC CTAGGAGGCC ATGAGTTTGT CAAGATGCCT CATAGTATGG   
  
  
+ AGAGGTCTAT ATCCTTGCAA AATGATCATT CCCTTCTACC TAAAAGAACC ACTTCATTTC CACTTTTACT   
  
  
+ CCCTGGCAAA TGGTTGATAA ACCAAACCAT AACCAAGCCC TTGAAGAACT ATAAAGACAC GAATCGATCA   
  
  
+ TTGAATTCGA ACAAGCCTTG TGTGATAGGA GGATGGGCCT CAACACTTCT CCGCGAGTGT GCAAGAGCAA   
  
  
+ TCTCGGAGAA AAATCCTAAG AGCCAACAAC TTCTTTGGGT GTTAAATGAA CTTGTTTCTC CTTATGGCGA   
  
  
+ TTGCGAACAA AGATTGGCAT ATTACTTCTT ACAAGTGTTG TTGGCCAAAG CCAACAATTT GGGACCTCAC   
  
  
+ TTTCATGAGA GCCTAAAACT TGCCATGGAG AAAAACTGCT GCTTTGATAC CTACATGAAG CTTATATTGA   
  
  
+ AGTTCCAAGA GGTCAGTCCA TGGACAACCT TTGGTCATGT GGCTTCAAAT GGTGCAATAT TGGAGGACCT   
  
  
+ TAGAAGTTTA CAAAAGTCGA TCATCAAAGA AACAAGGCAA AGGATGGAGA AGTTTTCAAG GCTAATGGGT   
  
  
+ GTTCCCTTCA AGTTTCATGT CATAAACGAG TTAGATAACC TAGGAGAGCT TCGAAAAGAG GATTTAGACA   
  
  
+ TTGAAGATGG TGAGGCCATC GCTGTGAACT GTGTTCAAGC CTTGCAACGG GTTCATGTGG AGAAGAGGGA   
  
  
+ GCATGTGCTT GATGTGATTC GATCTATTAG GCCTTGTATC ATAACACTGG TGGAGGAAGA AGCAGATCTC   
  
  
+ ACTTCTACAA GAAACGACTT CTTCAAGTGC TTCGATGAGT GTTTGAGATT TTCTAAGTCA TATTTCGATA   
  
  
+ TGTTAGAAGA AAGCTTCCCT CCAATAAGCA ACGAACGAAT CAAGCTAGAA AGGGAACAAT GGATGAATAT   
  
  
+ CTCCAGAGCC CTAGCTTGTC ATGGTGAAAG TGGAGGAGAA TATAGGCCAA AGAAAGGAAC TCAATGGAAT   
  
  
+ GAGATGCTCG AACAAGCATT TTGCCCATCT CAATTTAGTG ATGATGTACT AAGTGATGTT AGGGCATTGT   
  
  
+ TGAAAAGACA CAAAAGTGGT TGGGATCTCA CCTTACCACA AAGTGACCAT GAAATAGGCA TACACTTAAA   
  
  
+ TTGGAAGGGT GAAAATGTTG TTTGGGCTTC TGCATGGAGA CCTAGCTA  

- +Up\_Stream \_Len000TACTAT TTTCTATAAA TAATTCGAGT TCGATAAAAT TGAAAATTTT TTTACTTACA   
  
  
- CCGAAAAAAA CGATAAGTCC ATGTTGTGTT ATACTTCGTA ATTGAGATCT TTTACACCAT TACATACAGT   
  
  
- ATATCCTCTC ATATATGAGA GTGAACGGTA CGCTACGCAC GTACACACAC ACACACACAT AGACAAAGAT   
  
  
- ACGTATAAAA TACACCCCAA AACAGTAGAT CTGTAGATAA TTATACAATC GTAAGTGTAC TGAATATCCT   
  
  
- TACGTGATAA TACTCATACT ATATCTCAAA ATTTTTACGT ATACGGAACA CACAAGTAAT CGAATTTCTT   
  
  
- CTCAAAAACT TATATGTATA AATCCATTAA GAACGATTAC AGCATTAATC GTCAACATAG ATAGAATAAC   
  
  
- TAAACATAAA AATTAACGAA CAATATGACA ATGTGAATCT TTTAAGTACT TATGAGAATT TTATACGTTT   
  
  
- AATATATATA TATATATATA TATATATATA TATATATATA TATTTTTGAC TTATTCAATT ATACCACTAC   
  
  
- TGTAAACAGT ACAATAGTAA CCAGCGAATT AAAAATTTAT TTTTTATACT AAAACACTGT GGATTATTTA   
  
  
- CTATAATCGG TATAATAATT TAAGTCGTAA ATAAGTAGTT ACATTATATG ATTTAGTACG TTAATAGTAA   
  
  
- CCAAGAGAAA ATACATTATT TCAATTAACT GTTAAGTTCC CAATGATTAA TCATATTACA ACTTAACCTT   
  
  
- GCATTTTTAG TTTAGCATAC ATTTCATTAA AATAAGACTT AAATTTTTTA CCTCGTTTAT AGAGATAACA   
  
  
- AGAAATTATT TTAAAATTAA CTAAAAATAA AGATTAATTC ACTGCCTTGG TTTAAAAAAA GGAGGAAAAA   
  
  
- ACCAAACCAT TCAATAGTAA TCGGTTAGAA AATAAATGTA AAACAGTCAA AAAGAAAAAA TTCTACCAAT   
  
  
- TTACCGAAGT TAATTTGTTT AAAAAAATGA ATAAATATAA AAATTATTTA AAAGTTTTAC ATATAATTGC   
  
  
- GTACTGGCAC GCTTCGTGCC TTAGATGGGA TCAATTAGAT ACTAAGAATT TATACGTTCA TCCGACAATA   
  
  
- AGTAAACAAC AAAAAATGAA TAAATATAAA AAAGGGAGGG CCATACTATC CCAAACAGTA TAACAATAAG   
  
  
- TAATCGACAA TTTGACATAA GTAATATCGT GTTCATCCGA ATTAGTTTCT ATCAATCAGA GGTTAAAGTA   
  
  
- GTCTTTTAAA TTCTTTTTCT GTGGAATCCT AGTTTTCATA CTTTAAATCT CTGGTCTGTT GCTATCTTAA   
  
  
- CTTTTCTACT CAAAAGTTAA ACCTCTTCTG CCTTTGAGTC TACTTACTTT CATATCATGT AACATGAGGA   
  
  
- ACCTCATATT AGAATAGAAG TGATAACTAT CCGAGCTTCG TATTAGTCAC TAACCTTTGA AAACCAACGT   
  
  
- TATTAAGTTT TAAACTATGA GATACTAAAT GAAAAAATTA ACTTCCAGTA CTAAATTAAT TGATTTACTT   
  
  
- TTCTTAAATA CTAGCTTTTT AAAATTGAGA ATAACTGAAT ATCAACTGAG GTCAATTTAC CTTTTAACTT   
  
  
- CACTAATTAG ATTTTCACCG GGCGAGATTA TGGTAAACTC TTTTTAATAA GAATGACGTT TCAAATTTAG   
  
  
- TTATACACTT CTATCAAATA TCAAAATAGG ATTTTCTTTA ATTTATCTAA CAATTTTTAT TAAAAATTTA   
  
  
- AGTCTATATG CACACTACCG GTATTGGCAT TTACTATACG TACAGCTGTT TAAGGTTTCT TCCGATTATT   
  
  
- AATCTGAATT TATTATGTAC GTATGATTGG TTTTTTTTTT TTAATTCGTT CTAAGATAGG TTGGATAGTA   
  
  
- TTATTTCAAA CCGGTATGAA GTTTGGTAGT GTATTGTAGT GATTATATAT TTCGATCCAA AACCCTTTGG   
  
  
- ATTGAAACTC CTGGTGTATA AACAATTCCG GATCCTCCGG TACTCAAACA GTTCTACGGA GTATCATACC   
  
  
- TCTCCAGATA TAGGAACGTT TTACTAGTAA GGGAAGATGG ATTTTCTTGG TGAAGTAAAG GTGAAAATGA   
  
  
- GGGACCGTTT ACCAACTATT TGGTTTGGTA TTGGTTCGGG AACTTCTTGA TATTTCTGTG CTTAGCTAGT   
  
  
- AACTTAAGCT TGTTCGGAAC ACACTATCCT CCTACCCGGA GTTGTGAAGA GGCGCTCACA CGTTCTCGTT   
  
  
- AGAGCCTCTT TTTAGGATTC TCGGTTGTTG AAGAAACCCA CAATTTACTT GAACAAAGAG GAATACCGCT   
  
  
- AACGCTTGTT TCTAACCGTA TAATGAAGAA TGTTCACAAC AACCGGTTTC GGTTGTTAAA CCCTGGAGTG   
  
  
- AAAGTACTCT CGGATTTTGA ACGGTACCTC TTTTTGACGA CGAAACTATG GATGTACTTC GAATATAACT   
  
  
- TCAAGGTTCT CCAGTCAGGT ACCTGTTGGA AACCAGTACA CCGAAGTTTA CCACGTTATA ACCTCCTGGA   
  
  
- ATCTTCAAAT GTTTTCAGCT AGTAGTTTCT TTGTTCCGTT TCCTACCTCT TCAAAAGTTC CGATTACCCA   
  
  
- CAAGGGAAGT TCAAAGTACA GTATTTGCTC AATCTATTGG ATCCTCTCGA AGCTTTTCTC CTAAATCTGT   
  
  
- AACTTCTACC ACTCCGGTAG CGACACTTGA CACAAGTTCG GAACGTTGCC CAAGTACACC TCTTCTCCCT   
  
  
- CGTACACGAA CTACACTAAG CTAGATAATC CGGAACATAG TATTGTGACC ACCTCCTTCT TCGTCTAGAG   
  
  
- TGAAGATGTT CTTTGCTGAA GAAGTTCACG AAGCTACTCA CAAACTCTAA AAGATTCAGT ATAAAGCTAT   
  
  
- ACAATCTTCT TTCGAAGGGA GGTTATTCGT TGCTTGCTTA GTTCGATCTT TCCCTTGTTA CCTACTTATA   
  
  
- GAGGTCTCGG GATCGAACAG TACCACTTTC ACCTCCTCTT ATATCCGGTT TCTTTCCTTG AGTTACCTTA   
  
  
- CTCTACGAGC TTGTTCGTAA AACGGGTAGA GTTAAATCAC TACTACATGA TTCACTACAA TCCCGTAACA   
  
  
- ACTTTTCTGT GTTTTCACCA ACCCTAGAGT GGAATGGTGT TTCACTGGTA CTTTATCCGT ATGTGAATTT   
  
  
- AACCTTCCCA CTTTTACAAC AAACCCGAAG ACGTACCTCT GGATCGAT

+     ERE

| Site Name | Organism | Position | Strand | Matrix score. | sequence | function |
| --- | --- | --- | --- | --- | --- | --- |
| ERE | Nicotiana glutinos | 1302 | - | 8 | ATTTCATA |  |

>HU02G03154.1   
+ +Up\_Stream \_Len000ATGATA AAAGATATTT ATTAAGCTCA AGCTATTTTA ACTTTTAAAA AAATGAATGT   
  
  
+ GGCTTTTTTT GCTATTCAGG TACAACACAA TATGAAGCAT TAACTCTAGA AAATGTGGTA ATGTATGTCA   
  
  
+ TATAGGAGAG TATATACTCT CACTTGCCAT GCGATGCGTG CATGTGTGTG TGTGTGTGTA TCTGTTTCTA   
  
  
+ TGCATATTTT ATGTGGGGTT TTGTCATCTA GACATCTATT AATATGTTAG CATTCACATG ACTTATAGGA   
  
  
+ ATGCACTATT ATGAGTATGA TATAGAGTTT TAAAAATGCA TATGCCTTGT GTGTTCATTA GCTTAAAGAA   
  
  
+ GAGTTTTTGA ATATACATAT TTAGGTAATT CTTGCTAATG TCGTAATTAG CAGTTGTATC TATCTTATTG   
  
  
+ ATTTGTATTT TTAATTGCTT GTTATACTGT TACACTTAGA AAATTCATGA ATACTCTTAA AATATGCAAA   
  
  
+ TTATATATAT ATATATATAT ATATATATAT ATATATATAT ATAAAAACTG AATAAGTTAA TATGGTGATG   
  
  
+ ACATTTGTCA TGTTATCATT GGTCGCTTAA TTTTTAAATA AAAAATATGA TTTTGTGACA CCTAATAAAT   
  
  
+ GATATTAGCC ATATTATTAA ATTCAGCATT TATTCATCAA TGTAATATAC TAAATCATGC AATTATCATT   
  
  
+ GGTTCTCTTT TATGTAATAA AGTTAATTGA CAATTCAAGG GTTACTAATT AGTATAATGT TGAATTGGAA   
  
  
+ CGTAAAAATC AAATCGTATG TAAAGTAATT TTATTCTGAA TTTAAAAAAT GGAGCAAATA TCTCTATTGT   
  
  
+ TCTTTAATAA AATTTTAATT GATTTTTATT TCTAATTAAG TGACGGAACC AAATTTTTTT CCTCCTTTTT   
  
  
+ TGGTTTGGTA AGTTATCATT AGCCAATCTT TTATTTACAT TTTGTCAGTT TTTCTTTTTT AAGATGGTTA   
  
  
+ AATGGCTTCA ATTAAACAAA TTTTTTTACT TATTTATATT TTTAATAAAT TTTCAAAATG TATATTAACG   
  
  
+ CATGACCGTG CGAAGCACGG AATCTACCCT AGTTAATCTA TGATTCTTAA ATATGCAAGT AGGCTGTTAT   
  
  
+ TCATTTGTTG TTTTTTACTT ATTTATATTT TTTCCCTCCC GGTATGATAG GGTTTGTCAT ATTGTTATTC   
  
  
+ ATTAGCTGTT AAACTGTATT CATTATAGCA CAAGTAGGCT TAATCAAAGA TAGTTAGTCT CCAATTTCAT   
  
  
+ CAGAAAATTT AAGAAAAAGA CACCTTAGGA TCAAAAGTAT GAAATTTAGA GACCAGACAA CGATAGAATT   
  
  
+ GAAAAGATGA GTTTTCAATT TGGAGAAGAC GGAAACTCAG ATGAATGAAA GTATAGTACA TTGTACTCCT   
  
  
+ TGGAGTATAA TCTTATCTTC ACTATTGATA GGCTCGAAGC ATAATCAGTG ATTGGAAACT TTTGGTTGCA   
  
  
+ ATAATTCAAA ATTTGATACT CTATGATTTA CTTTTTTAAT TGAAGGTCAT GATTTAATTA ACTAAATGAA   
  
  
+ AAGAATTTAT GATCGAAAAA TTTTAACTCT TATTGACTTA TAGTTGACTC CAGTTAAATG GAAAATTGAA   
  
  
+ GTGATTAATC TAAAAGTGGC CCGCTCTAAT ACCATTTGAG AAAAATTATT CTTACTGCAA AGTTTAAATC   
  
  
+ AATATGTGAA GATAGTTTAT AGTTTTATCC TAAAAGAAAT TAAATAGATT GTTAAAAATA ATTTTTAAAT   
  
  
+ TCAGATATAC GTGTGATGGC CATAACCGTA AATGATATGC ATGTCGACAA ATTCCAAAGA AGGCTAATAA   
  
  
+ TTAGACTTAA ATAATACATG CATACTAACC AAAAAAAAAA AATTAAGCAA GATTCTATCC AACCTATCAT   
  
  
+ AATAAAGTTT GGCCATACTT CAAACCATCA CATAACATCA CTAATATATA AAGCTAGGTT TTGGGAAACC   
  
  
+ TAACTTTGAG GACCACATAT TTGTTAAGGC CTAGGAGGCC ATGAGTTTGT CAAGATGCCT CATAGTATGG   
  
  
+ AGAGGTCTAT ATCCTTGCAA AATGATCATT CCCTTCTACC TAAAAGAACC ACTTCATTTC CACTTTTACT   
  
  
+ CCCTGGCAAA TGGTTGATAA ACCAAACCAT AACCAAGCCC TTGAAGAACT ATAAAGACAC GAATCGATCA   
  
  
+ TTGAATTCGA ACAAGCCTTG TGTGATAGGA GGATGGGCCT CAACACTTCT CCGCGAGTGT GCAAGAGCAA   
  
  
+ TCTCGGAGAA AAATCCTAAG AGCCAACAAC TTCTTTGGGT GTTAAATGAA CTTGTTTCTC CTTATGGCGA   
  
  
+ TTGCGAACAA AGATTGGCAT ATTACTTCTT ACAAGTGTTG TTGGCCAAAG CCAACAATTT GGGACCTCAC   
  
  
+ TTTCATGAGA GCCTAAAACT TGCCATGGAG AAAAACTGCT GCTTTGATAC CTACATGAAG CTTATATTGA   
  
  
+ AGTTCCAAGA GGTCAGTCCA TGGACAACCT TTGGTCATGT GGCTTCAAAT GGTGCAATAT TGGAGGACCT   
  
  
+ TAGAAGTTTA CAAAAGTCGA TCATCAAAGA AACAAGGCAA AGGATGGAGA AGTTTTCAAG GCTAATGGGT   
  
  
+ GTTCCCTTCA AGTTTCATGT CATAAACGAG TTAGATAACC TAGGAGAGCT TCGAAAAGAG GATTTAGACA   
  
  
+ TTGAAGATGG TGAGGCCATC GCTGTGAACT GTGTTCAAGC CTTGCAACGG GTTCATGTGG AGAAGAGGGA   
  
  
+ GCATGTGCTT GATGTGATTC GATCTATTAG GCCTTGTATC ATAACACTGG TGGAGGAAGA AGCAGATCTC   
  
  
+ ACTTCTACAA GAAACGACTT CTTCAAGTGC TTCGATGAGT GTTTGAGATT TTCTAAGTCA TATTTCGATA   
  
  
+ TGTTAGAAGA AAGCTTCCCT CCAATAAGCA ACGAACGAAT CAAGCTAGAA AGGGAACAAT GGATGAATAT   
  
  
+ CTCCAGAGCC CTAGCTTGTC ATGGTGAAAG TGGAGGAGAA TATAGGCCAA AGAAAGGAAC TCAATGGAAT   
  
  
+ GAGATGCTCG AACAAGCATT TTGCCCATCT CAATTTAGTG ATGATGTACT AAGTGATGTT AGGGCATTGT   
  
  
+ TGAAAAGACA CAAAAGTGGT TGGGATCTCA CCTTACCACA AAGTGACCAT GAAATAGGCA TACACTTAAA   
  
  
+ TTGGAAGGGT GAAAATGTTG TTTGGGCTTC TGCATGGAGA CCTAGCTA  

- +Up\_Stream \_Len000TACTAT TTTCTATAAA TAATTCGAGT TCGATAAAAT TGAAAATTTT TTTACTTACA   
  
  
- CCGAAAAAAA CGATAAGTCC ATGTTGTGTT ATACTTCGTA ATTGAGATCT TTTACACCAT TACATACAGT   
  
  
- ATATCCTCTC ATATATGAGA GTGAACGGTA CGCTACGCAC GTACACACAC ACACACACAT AGACAAAGAT   
  
  
- ACGTATAAAA TACACCCCAA AACAGTAGAT CTGTAGATAA TTATACAATC GTAAGTGTAC TGAATATCCT   
  
  
- TACGTGATAA TACTCATACT ATATCTCAAA ATTTTTACGT ATACGGAACA CACAAGTAAT CGAATTTCTT   
  
  
- CTCAAAAACT TATATGTATA AATCCATTAA GAACGATTAC AGCATTAATC GTCAACATAG ATAGAATAAC   
  
  
- TAAACATAAA AATTAACGAA CAATATGACA ATGTGAATCT TTTAAGTACT TATGAGAATT TTATACGTTT   
  
  
- AATATATATA TATATATATA TATATATATA TATATATATA TATTTTTGAC TTATTCAATT ATACCACTAC   
  
  
- TGTAAACAGT ACAATAGTAA CCAGCGAATT AAAAATTTAT TTTTTATACT AAAACACTGT GGATTATTTA   
  
  
- CTATAATCGG TATAATAATT TAAGTCGTAA ATAAGTAGTT ACATTATATG ATTTAGTACG TTAATAGTAA   
  
  
- CCAAGAGAAA ATACATTATT TCAATTAACT GTTAAGTTCC CAATGATTAA TCATATTACA ACTTAACCTT   
  
  
- GCATTTTTAG TTTAGCATAC ATTTCATTAA AATAAGACTT AAATTTTTTA CCTCGTTTAT AGAGATAACA   
  
  
- AGAAATTATT TTAAAATTAA CTAAAAATAA AGATTAATTC ACTGCCTTGG TTTAAAAAAA GGAGGAAAAA   
  
  
- ACCAAACCAT TCAATAGTAA TCGGTTAGAA AATAAATGTA AAACAGTCAA AAAGAAAAAA TTCTACCAAT   
  
  
- TTACCGAAGT TAATTTGTTT AAAAAAATGA ATAAATATAA AAATTATTTA AAAGTTTTAC ATATAATTGC   
  
  
- GTACTGGCAC GCTTCGTGCC TTAGATGGGA TCAATTAGAT ACTAAGAATT TATACGTTCA TCCGACAATA   
  
  
- AGTAAACAAC AAAAAATGAA TAAATATAAA AAAGGGAGGG CCATACTATC CCAAACAGTA TAACAATAAG   
  
  
- TAATCGACAA TTTGACATAA GTAATATCGT GTTCATCCGA ATTAGTTTCT ATCAATCAGA GGTTAAAGTA   
  
  
- GTCTTTTAAA TTCTTTTTCT GTGGAATCCT AGTTTTCATA CTTTAAATCT CTGGTCTGTT GCTATCTTAA   
  
  
- CTTTTCTACT CAAAAGTTAA ACCTCTTCTG CCTTTGAGTC TACTTACTTT CATATCATGT AACATGAGGA   
  
  
- ACCTCATATT AGAATAGAAG TGATAACTAT CCGAGCTTCG TATTAGTCAC TAACCTTTGA AAACCAACGT   
  
  
- TATTAAGTTT TAAACTATGA GATACTAAAT GAAAAAATTA ACTTCCAGTA CTAAATTAAT TGATTTACTT   
  
  
- TTCTTAAATA CTAGCTTTTT AAAATTGAGA ATAACTGAAT ATCAACTGAG GTCAATTTAC CTTTTAACTT   
  
  
- CACTAATTAG ATTTTCACCG GGCGAGATTA TGGTAAACTC TTTTTAATAA GAATGACGTT TCAAATTTAG   
  
  
- TTATACACTT CTATCAAATA TCAAAATAGG ATTTTCTTTA ATTTATCTAA CAATTTTTAT TAAAAATTTA   
  
  
- AGTCTATATG CACACTACCG GTATTGGCAT TTACTATACG TACAGCTGTT TAAGGTTTCT TCCGATTATT   
  
  
- AATCTGAATT TATTATGTAC GTATGATTGG TTTTTTTTTT TTAATTCGTT CTAAGATAGG TTGGATAGTA   
  
  
- TTATTTCAAA CCGGTATGAA GTTTGGTAGT GTATTGTAGT GATTATATAT TTCGATCCAA AACCCTTTGG   
  
  
- ATTGAAACTC CTGGTGTATA AACAATTCCG GATCCTCCGG TACTCAAACA GTTCTACGGA GTATCATACC   
  
  
- TCTCCAGATA TAGGAACGTT TTACTAGTAA GGGAAGATGG ATTTTCTTGG TGAAGTAAAG GTGAAAATGA   
  
  
- GGGACCGTTT ACCAACTATT TGGTTTGGTA TTGGTTCGGG AACTTCTTGA TATTTCTGTG CTTAGCTAGT   
  
  
- AACTTAAGCT TGTTCGGAAC ACACTATCCT CCTACCCGGA GTTGTGAAGA GGCGCTCACA CGTTCTCGTT   
  
  
- AGAGCCTCTT TTTAGGATTC TCGGTTGTTG AAGAAACCCA CAATTTACTT GAACAAAGAG GAATACCGCT   
  
  
- AACGCTTGTT TCTAACCGTA TAATGAAGAA TGTTCACAAC AACCGGTTTC GGTTGTTAAA CCCTGGAGTG   
  
  
- AAAGTACTCT CGGATTTTGA ACGGTACCTC TTTTTGACGA CGAAACTATG GATGTACTTC GAATATAACT   
  
  
- TCAAGGTTCT CCAGTCAGGT ACCTGTTGGA AACCAGTACA CCGAAGTTTA CCACGTTATA ACCTCCTGGA   
  
  
- ATCTTCAAAT GTTTTCAGCT AGTAGTTTCT TTGTTCCGTT TCCTACCTCT TCAAAAGTTC CGATTACCCA   
  
  
- CAAGGGAAGT TCAAAGTACA GTATTTGCTC AATCTATTGG ATCCTCTCGA AGCTTTTCTC CTAAATCTGT   
  
  
- AACTTCTACC ACTCCGGTAG CGACACTTGA CACAAGTTCG GAACGTTGCC CAAGTACACC TCTTCTCCCT   
  
  
- CGTACACGAA CTACACTAAG CTAGATAATC CGGAACATAG TATTGTGACC ACCTCCTTCT TCGTCTAGAG   
  
  
- TGAAGATGTT CTTTGCTGAA GAAGTTCACG AAGCTACTCA CAAACTCTAA AAGATTCAGT ATAAAGCTAT   
  
  
- ACAATCTTCT TTCGAAGGGA GGTTATTCGT TGCTTGCTTA GTTCGATCTT TCCCTTGTTA CCTACTTATA   
  
  
- GAGGTCTCGG GATCGAACAG TACCACTTTC ACCTCCTCTT ATATCCGGTT TCTTTCCTTG AGTTACCTTA   
  
  
- CTCTACGAGC TTGTTCGTAA AACGGGTAGA GTTAAATCAC TACTACATGA TTCACTACAA TCCCGTAACA   
  
  
- ACTTTTCTGT GTTTTCACCA ACCCTAGAGT GGAATGGTGT TTCACTGGTA CTTTATCCGT ATGTGAATTT   
  
  
- AACCTTCCCA CTTTTACAAC AAACCCGAAG ACGTACCTCT GGATCGAT

+     G-box

| Site Name | Organism | Position | Strand | Matrix score. | sequence | function |
| --- | --- | --- | --- | --- | --- | --- |
| G-box | Arabidopsis thaliana | 1762 | + | 6 | TACGTG | cis-acting regulatory element involved in light responsiveness |

>HU02G03154.1   
+ +Up\_Stream \_Len000ATGATA AAAGATATTT ATTAAGCTCA AGCTATTTTA ACTTTTAAAA AAATGAATGT   
  
  
+ GGCTTTTTTT GCTATTCAGG TACAACACAA TATGAAGCAT TAACTCTAGA AAATGTGGTA ATGTATGTCA   
  
  
+ TATAGGAGAG TATATACTCT CACTTGCCAT GCGATGCGTG CATGTGTGTG TGTGTGTGTA TCTGTTTCTA   
  
  
+ TGCATATTTT ATGTGGGGTT TTGTCATCTA GACATCTATT AATATGTTAG CATTCACATG ACTTATAGGA   
  
  
+ ATGCACTATT ATGAGTATGA TATAGAGTTT TAAAAATGCA TATGCCTTGT GTGTTCATTA GCTTAAAGAA   
  
  
+ GAGTTTTTGA ATATACATAT TTAGGTAATT CTTGCTAATG TCGTAATTAG CAGTTGTATC TATCTTATTG   
  
  
+ ATTTGTATTT TTAATTGCTT GTTATACTGT TACACTTAGA AAATTCATGA ATACTCTTAA AATATGCAAA   
  
  
+ TTATATATAT ATATATATAT ATATATATAT ATATATATAT ATAAAAACTG AATAAGTTAA TATGGTGATG   
  
  
+ ACATTTGTCA TGTTATCATT GGTCGCTTAA TTTTTAAATA AAAAATATGA TTTTGTGACA CCTAATAAAT   
  
  
+ GATATTAGCC ATATTATTAA ATTCAGCATT TATTCATCAA TGTAATATAC TAAATCATGC AATTATCATT   
  
  
+ GGTTCTCTTT TATGTAATAA AGTTAATTGA CAATTCAAGG GTTACTAATT AGTATAATGT TGAATTGGAA   
  
  
+ CGTAAAAATC AAATCGTATG TAAAGTAATT TTATTCTGAA TTTAAAAAAT GGAGCAAATA TCTCTATTGT   
  
  
+ TCTTTAATAA AATTTTAATT GATTTTTATT TCTAATTAAG TGACGGAACC AAATTTTTTT CCTCCTTTTT   
  
  
+ TGGTTTGGTA AGTTATCATT AGCCAATCTT TTATTTACAT TTTGTCAGTT TTTCTTTTTT AAGATGGTTA   
  
  
+ AATGGCTTCA ATTAAACAAA TTTTTTTACT TATTTATATT TTTAATAAAT TTTCAAAATG TATATTAACG   
  
  
+ CATGACCGTG CGAAGCACGG AATCTACCCT AGTTAATCTA TGATTCTTAA ATATGCAAGT AGGCTGTTAT   
  
  
+ TCATTTGTTG TTTTTTACTT ATTTATATTT TTTCCCTCCC GGTATGATAG GGTTTGTCAT ATTGTTATTC   
  
  
+ ATTAGCTGTT AAACTGTATT CATTATAGCA CAAGTAGGCT TAATCAAAGA TAGTTAGTCT CCAATTTCAT   
  
  
+ CAGAAAATTT AAGAAAAAGA CACCTTAGGA TCAAAAGTAT GAAATTTAGA GACCAGACAA CGATAGAATT   
  
  
+ GAAAAGATGA GTTTTCAATT TGGAGAAGAC GGAAACTCAG ATGAATGAAA GTATAGTACA TTGTACTCCT   
  
  
+ TGGAGTATAA TCTTATCTTC ACTATTGATA GGCTCGAAGC ATAATCAGTG ATTGGAAACT TTTGGTTGCA   
  
  
+ ATAATTCAAA ATTTGATACT CTATGATTTA CTTTTTTAAT TGAAGGTCAT GATTTAATTA ACTAAATGAA   
  
  
+ AAGAATTTAT GATCGAAAAA TTTTAACTCT TATTGACTTA TAGTTGACTC CAGTTAAATG GAAAATTGAA   
  
  
+ GTGATTAATC TAAAAGTGGC CCGCTCTAAT ACCATTTGAG AAAAATTATT CTTACTGCAA AGTTTAAATC   
  
  
+ AATATGTGAA GATAGTTTAT AGTTTTATCC TAAAAGAAAT TAAATAGATT GTTAAAAATA ATTTTTAAAT   
  
  
+ TCAGATATAC GTGTGATGGC CATAACCGTA AATGATATGC ATGTCGACAA ATTCCAAAGA AGGCTAATAA   
  
  
+ TTAGACTTAA ATAATACATG CATACTAACC AAAAAAAAAA AATTAAGCAA GATTCTATCC AACCTATCAT   
  
  
+ AATAAAGTTT GGCCATACTT CAAACCATCA CATAACATCA CTAATATATA AAGCTAGGTT TTGGGAAACC   
  
  
+ TAACTTTGAG GACCACATAT TTGTTAAGGC CTAGGAGGCC ATGAGTTTGT CAAGATGCCT CATAGTATGG   
  
  
+ AGAGGTCTAT ATCCTTGCAA AATGATCATT CCCTTCTACC TAAAAGAACC ACTTCATTTC CACTTTTACT   
  
  
+ CCCTGGCAAA TGGTTGATAA ACCAAACCAT AACCAAGCCC TTGAAGAACT ATAAAGACAC GAATCGATCA   
  
  
+ TTGAATTCGA ACAAGCCTTG TGTGATAGGA GGATGGGCCT CAACACTTCT CCGCGAGTGT GCAAGAGCAA   
  
  
+ TCTCGGAGAA AAATCCTAAG AGCCAACAAC TTCTTTGGGT GTTAAATGAA CTTGTTTCTC CTTATGGCGA   
  
  
+ TTGCGAACAA AGATTGGCAT ATTACTTCTT ACAAGTGTTG TTGGCCAAAG CCAACAATTT GGGACCTCAC   
  
  
+ TTTCATGAGA GCCTAAAACT TGCCATGGAG AAAAACTGCT GCTTTGATAC CTACATGAAG CTTATATTGA   
  
  
+ AGTTCCAAGA GGTCAGTCCA TGGACAACCT TTGGTCATGT GGCTTCAAAT GGTGCAATAT TGGAGGACCT   
  
  
+ TAGAAGTTTA CAAAAGTCGA TCATCAAAGA AACAAGGCAA AGGATGGAGA AGTTTTCAAG GCTAATGGGT   
  
  
+ GTTCCCTTCA AGTTTCATGT CATAAACGAG TTAGATAACC TAGGAGAGCT TCGAAAAGAG GATTTAGACA   
  
  
+ TTGAAGATGG TGAGGCCATC GCTGTGAACT GTGTTCAAGC CTTGCAACGG GTTCATGTGG AGAAGAGGGA   
  
  
+ GCATGTGCTT GATGTGATTC GATCTATTAG GCCTTGTATC ATAACACTGG TGGAGGAAGA AGCAGATCTC   
  
  
+ ACTTCTACAA GAAACGACTT CTTCAAGTGC TTCGATGAGT GTTTGAGATT TTCTAAGTCA TATTTCGATA   
  
  
+ TGTTAGAAGA AAGCTTCCCT CCAATAAGCA ACGAACGAAT CAAGCTAGAA AGGGAACAAT GGATGAATAT   
  
  
+ CTCCAGAGCC CTAGCTTGTC ATGGTGAAAG TGGAGGAGAA TATAGGCCAA AGAAAGGAAC TCAATGGAAT   
  
  
+ GAGATGCTCG AACAAGCATT TTGCCCATCT CAATTTAGTG ATGATGTACT AAGTGATGTT AGGGCATTGT   
  
  
+ TGAAAAGACA CAAAAGTGGT TGGGATCTCA CCTTACCACA AAGTGACCAT GAAATAGGCA TACACTTAAA   
  
  
+ TTGGAAGGGT GAAAATGTTG TTTGGGCTTC TGCATGGAGA CCTAGCTA  

- +Up\_Stream \_Len000TACTAT TTTCTATAAA TAATTCGAGT TCGATAAAAT TGAAAATTTT TTTACTTACA   
  
  
- CCGAAAAAAA CGATAAGTCC ATGTTGTGTT ATACTTCGTA ATTGAGATCT TTTACACCAT TACATACAGT   
  
  
- ATATCCTCTC ATATATGAGA GTGAACGGTA CGCTACGCAC GTACACACAC ACACACACAT AGACAAAGAT   
  
  
- ACGTATAAAA TACACCCCAA AACAGTAGAT CTGTAGATAA TTATACAATC GTAAGTGTAC TGAATATCCT   
  
  
- TACGTGATAA TACTCATACT ATATCTCAAA ATTTTTACGT ATACGGAACA CACAAGTAAT CGAATTTCTT   
  
  
- CTCAAAAACT TATATGTATA AATCCATTAA GAACGATTAC AGCATTAATC GTCAACATAG ATAGAATAAC   
  
  
- TAAACATAAA AATTAACGAA CAATATGACA ATGTGAATCT TTTAAGTACT TATGAGAATT TTATACGTTT   
  
  
- AATATATATA TATATATATA TATATATATA TATATATATA TATTTTTGAC TTATTCAATT ATACCACTAC   
  
  
- TGTAAACAGT ACAATAGTAA CCAGCGAATT AAAAATTTAT TTTTTATACT AAAACACTGT GGATTATTTA   
  
  
- CTATAATCGG TATAATAATT TAAGTCGTAA ATAAGTAGTT ACATTATATG ATTTAGTACG TTAATAGTAA   
  
  
- CCAAGAGAAA ATACATTATT TCAATTAACT GTTAAGTTCC CAATGATTAA TCATATTACA ACTTAACCTT   
  
  
- GCATTTTTAG TTTAGCATAC ATTTCATTAA AATAAGACTT AAATTTTTTA CCTCGTTTAT AGAGATAACA   
  
  
- AGAAATTATT TTAAAATTAA CTAAAAATAA AGATTAATTC ACTGCCTTGG TTTAAAAAAA GGAGGAAAAA   
  
  
- ACCAAACCAT TCAATAGTAA TCGGTTAGAA AATAAATGTA AAACAGTCAA AAAGAAAAAA TTCTACCAAT   
  
  
- TTACCGAAGT TAATTTGTTT AAAAAAATGA ATAAATATAA AAATTATTTA AAAGTTTTAC ATATAATTGC   
  
  
- GTACTGGCAC GCTTCGTGCC TTAGATGGGA TCAATTAGAT ACTAAGAATT TATACGTTCA TCCGACAATA   
  
  
- AGTAAACAAC AAAAAATGAA TAAATATAAA AAAGGGAGGG CCATACTATC CCAAACAGTA TAACAATAAG   
  
  
- TAATCGACAA TTTGACATAA GTAATATCGT GTTCATCCGA ATTAGTTTCT ATCAATCAGA GGTTAAAGTA   
  
  
- GTCTTTTAAA TTCTTTTTCT GTGGAATCCT AGTTTTCATA CTTTAAATCT CTGGTCTGTT GCTATCTTAA   
  
  
- CTTTTCTACT CAAAAGTTAA ACCTCTTCTG CCTTTGAGTC TACTTACTTT CATATCATGT AACATGAGGA   
  
  
- ACCTCATATT AGAATAGAAG TGATAACTAT CCGAGCTTCG TATTAGTCAC TAACCTTTGA AAACCAACGT   
  
  
- TATTAAGTTT TAAACTATGA GATACTAAAT GAAAAAATTA ACTTCCAGTA CTAAATTAAT TGATTTACTT   
  
  
- TTCTTAAATA CTAGCTTTTT AAAATTGAGA ATAACTGAAT ATCAACTGAG GTCAATTTAC CTTTTAACTT   
  
  
- CACTAATTAG ATTTTCACCG GGCGAGATTA TGGTAAACTC TTTTTAATAA GAATGACGTT TCAAATTTAG   
  
  
- TTATACACTT CTATCAAATA TCAAAATAGG ATTTTCTTTA ATTTATCTAA CAATTTTTAT TAAAAATTTA   
  
  
- AGTCTATATG CACACTACCG GTATTGGCAT TTACTATACG TACAGCTGTT TAAGGTTTCT TCCGATTATT   
  
  
- AATCTGAATT TATTATGTAC GTATGATTGG TTTTTTTTTT TTAATTCGTT CTAAGATAGG TTGGATAGTA   
  
  
- TTATTTCAAA CCGGTATGAA GTTTGGTAGT GTATTGTAGT GATTATATAT TTCGATCCAA AACCCTTTGG   
  
  
- ATTGAAACTC CTGGTGTATA AACAATTCCG GATCCTCCGG TACTCAAACA GTTCTACGGA GTATCATACC   
  
  
- TCTCCAGATA TAGGAACGTT TTACTAGTAA GGGAAGATGG ATTTTCTTGG TGAAGTAAAG GTGAAAATGA   
  
  
- GGGACCGTTT ACCAACTATT TGGTTTGGTA TTGGTTCGGG AACTTCTTGA TATTTCTGTG CTTAGCTAGT   
  
  
- AACTTAAGCT TGTTCGGAAC ACACTATCCT CCTACCCGGA GTTGTGAAGA GGCGCTCACA CGTTCTCGTT   
  
  
- AGAGCCTCTT TTTAGGATTC TCGGTTGTTG AAGAAACCCA CAATTTACTT GAACAAAGAG GAATACCGCT   
  
  
- AACGCTTGTT TCTAACCGTA TAATGAAGAA TGTTCACAAC AACCGGTTTC GGTTGTTAAA CCCTGGAGTG   
  
  
- AAAGTACTCT CGGATTTTGA ACGGTACCTC TTTTTGACGA CGAAACTATG GATGTACTTC GAATATAACT   
  
  
- TCAAGGTTCT CCAGTCAGGT ACCTGTTGGA AACCAGTACA CCGAAGTTTA CCACGTTATA ACCTCCTGGA   
  
  
- ATCTTCAAAT GTTTTCAGCT AGTAGTTTCT TTGTTCCGTT TCCTACCTCT TCAAAAGTTC CGATTACCCA   
  
  
- CAAGGGAAGT TCAAAGTACA GTATTTGCTC AATCTATTGG ATCCTCTCGA AGCTTTTCTC CTAAATCTGT   
  
  
- AACTTCTACC ACTCCGGTAG CGACACTTGA CACAAGTTCG GAACGTTGCC CAAGTACACC TCTTCTCCCT   
  
  
- CGTACACGAA CTACACTAAG CTAGATAATC CGGAACATAG TATTGTGACC ACCTCCTTCT TCGTCTAGAG   
  
  
- TGAAGATGTT CTTTGCTGAA GAAGTTCACG AAGCTACTCA CAAACTCTAA AAGATTCAGT ATAAAGCTAT   
  
  
- ACAATCTTCT TTCGAAGGGA GGTTATTCGT TGCTTGCTTA GTTCGATCTT TCCCTTGTTA CCTACTTATA   
  
  
- GAGGTCTCGG GATCGAACAG TACCACTTTC ACCTCCTCTT ATATCCGGTT TCTTTCCTTG AGTTACCTTA   
  
  
- CTCTACGAGC TTGTTCGTAA AACGGGTAGA GTTAAATCAC TACTACATGA TTCACTACAA TCCCGTAACA   
  
  
- ACTTTTCTGT GTTTTCACCA ACCCTAGAGT GGAATGGTGT TTCACTGGTA CTTTATCCGT ATGTGAATTT   
  
  
- AACCTTCCCA CTTTTACAAC AAACCCGAAG ACGTACCTCT GGATCGAT

+     GATA-motif

| Site Name | Organism | Position | Strand | Matrix score. | sequence | function |
| --- | --- | --- | --- | --- | --- | --- |
| GATA-motif | Arabidopsis thaliana | 2198 | + | 7 | GATAGGA | part of a light responsive element |
| GATA-motif | Arabidopsis thaliana | 1413 | - | 11 | AAGATAAGATT | part of a light responsive element |
| GATA-motif | Pisum sativum | 1170 | + | 7 | GATAGGG | part of a light responsive element |

>HU02G03154.1   
+ +Up\_Stream \_Len000ATGATA AAAGATATTT ATTAAGCTCA AGCTATTTTA ACTTTTAAAA AAATGAATGT   
  
  
+ GGCTTTTTTT GCTATTCAGG TACAACACAA TATGAAGCAT TAACTCTAGA AAATGTGGTA ATGTATGTCA   
  
  
+ TATAGGAGAG TATATACTCT CACTTGCCAT GCGATGCGTG CATGTGTGTG TGTGTGTGTA TCTGTTTCTA   
  
  
+ TGCATATTTT ATGTGGGGTT TTGTCATCTA GACATCTATT AATATGTTAG CATTCACATG ACTTATAGGA   
  
  
+ ATGCACTATT ATGAGTATGA TATAGAGTTT TAAAAATGCA TATGCCTTGT GTGTTCATTA GCTTAAAGAA   
  
  
+ GAGTTTTTGA ATATACATAT TTAGGTAATT CTTGCTAATG TCGTAATTAG CAGTTGTATC TATCTTATTG   
  
  
+ ATTTGTATTT TTAATTGCTT GTTATACTGT TACACTTAGA AAATTCATGA ATACTCTTAA AATATGCAAA   
  
  
+ TTATATATAT ATATATATAT ATATATATAT ATATATATAT ATAAAAACTG AATAAGTTAA TATGGTGATG   
  
  
+ ACATTTGTCA TGTTATCATT GGTCGCTTAA TTTTTAAATA AAAAATATGA TTTTGTGACA CCTAATAAAT   
  
  
+ GATATTAGCC ATATTATTAA ATTCAGCATT TATTCATCAA TGTAATATAC TAAATCATGC AATTATCATT   
  
  
+ GGTTCTCTTT TATGTAATAA AGTTAATTGA CAATTCAAGG GTTACTAATT AGTATAATGT TGAATTGGAA   
  
  
+ CGTAAAAATC AAATCGTATG TAAAGTAATT TTATTCTGAA TTTAAAAAAT GGAGCAAATA TCTCTATTGT   
  
  
+ TCTTTAATAA AATTTTAATT GATTTTTATT TCTAATTAAG TGACGGAACC AAATTTTTTT CCTCCTTTTT   
  
  
+ TGGTTTGGTA AGTTATCATT AGCCAATCTT TTATTTACAT TTTGTCAGTT TTTCTTTTTT AAGATGGTTA   
  
  
+ AATGGCTTCA ATTAAACAAA TTTTTTTACT TATTTATATT TTTAATAAAT TTTCAAAATG TATATTAACG   
  
  
+ CATGACCGTG CGAAGCACGG AATCTACCCT AGTTAATCTA TGATTCTTAA ATATGCAAGT AGGCTGTTAT   
  
  
+ TCATTTGTTG TTTTTTACTT ATTTATATTT TTTCCCTCCC GGTATGATAG GGTTTGTCAT ATTGTTATTC   
  
  
+ ATTAGCTGTT AAACTGTATT CATTATAGCA CAAGTAGGCT TAATCAAAGA TAGTTAGTCT CCAATTTCAT   
  
  
+ CAGAAAATTT AAGAAAAAGA CACCTTAGGA TCAAAAGTAT GAAATTTAGA GACCAGACAA CGATAGAATT   
  
  
+ GAAAAGATGA GTTTTCAATT TGGAGAAGAC GGAAACTCAG ATGAATGAAA GTATAGTACA TTGTACTCCT   
  
  
+ TGGAGTATAA TCTTATCTTC ACTATTGATA GGCTCGAAGC ATAATCAGTG ATTGGAAACT TTTGGTTGCA   
  
  
+ ATAATTCAAA ATTTGATACT CTATGATTTA CTTTTTTAAT TGAAGGTCAT GATTTAATTA ACTAAATGAA   
  
  
+ AAGAATTTAT GATCGAAAAA TTTTAACTCT TATTGACTTA TAGTTGACTC CAGTTAAATG GAAAATTGAA   
  
  
+ GTGATTAATC TAAAAGTGGC CCGCTCTAAT ACCATTTGAG AAAAATTATT CTTACTGCAA AGTTTAAATC   
  
  
+ AATATGTGAA GATAGTTTAT AGTTTTATCC TAAAAGAAAT TAAATAGATT GTTAAAAATA ATTTTTAAAT   
  
  
+ TCAGATATAC GTGTGATGGC CATAACCGTA AATGATATGC ATGTCGACAA ATTCCAAAGA AGGCTAATAA   
  
  
+ TTAGACTTAA ATAATACATG CATACTAACC AAAAAAAAAA AATTAAGCAA GATTCTATCC AACCTATCAT   
  
  
+ AATAAAGTTT GGCCATACTT CAAACCATCA CATAACATCA CTAATATATA AAGCTAGGTT TTGGGAAACC   
  
  
+ TAACTTTGAG GACCACATAT TTGTTAAGGC CTAGGAGGCC ATGAGTTTGT CAAGATGCCT CATAGTATGG   
  
  
+ AGAGGTCTAT ATCCTTGCAA AATGATCATT CCCTTCTACC TAAAAGAACC ACTTCATTTC CACTTTTACT   
  
  
+ CCCTGGCAAA TGGTTGATAA ACCAAACCAT AACCAAGCCC TTGAAGAACT ATAAAGACAC GAATCGATCA   
  
  
+ TTGAATTCGA ACAAGCCTTG TGTGATAGGA GGATGGGCCT CAACACTTCT CCGCGAGTGT GCAAGAGCAA   
  
  
+ TCTCGGAGAA AAATCCTAAG AGCCAACAAC TTCTTTGGGT GTTAAATGAA CTTGTTTCTC CTTATGGCGA   
  
  
+ TTGCGAACAA AGATTGGCAT ATTACTTCTT ACAAGTGTTG TTGGCCAAAG CCAACAATTT GGGACCTCAC   
  
  
+ TTTCATGAGA GCCTAAAACT TGCCATGGAG AAAAACTGCT GCTTTGATAC CTACATGAAG CTTATATTGA   
  
  
+ AGTTCCAAGA GGTCAGTCCA TGGACAACCT TTGGTCATGT GGCTTCAAAT GGTGCAATAT TGGAGGACCT   
  
  
+ TAGAAGTTTA CAAAAGTCGA TCATCAAAGA AACAAGGCAA AGGATGGAGA AGTTTTCAAG GCTAATGGGT   
  
  
+ GTTCCCTTCA AGTTTCATGT CATAAACGAG TTAGATAACC TAGGAGAGCT TCGAAAAGAG GATTTAGACA   
  
  
+ TTGAAGATGG TGAGGCCATC GCTGTGAACT GTGTTCAAGC CTTGCAACGG GTTCATGTGG AGAAGAGGGA   
  
  
+ GCATGTGCTT GATGTGATTC GATCTATTAG GCCTTGTATC ATAACACTGG TGGAGGAAGA AGCAGATCTC   
  
  
+ ACTTCTACAA GAAACGACTT CTTCAAGTGC TTCGATGAGT GTTTGAGATT TTCTAAGTCA TATTTCGATA   
  
  
+ TGTTAGAAGA AAGCTTCCCT CCAATAAGCA ACGAACGAAT CAAGCTAGAA AGGGAACAAT GGATGAATAT   
  
  
+ CTCCAGAGCC CTAGCTTGTC ATGGTGAAAG TGGAGGAGAA TATAGGCCAA AGAAAGGAAC TCAATGGAAT   
  
  
+ GAGATGCTCG AACAAGCATT TTGCCCATCT CAATTTAGTG ATGATGTACT AAGTGATGTT AGGGCATTGT   
  
  
+ TGAAAAGACA CAAAAGTGGT TGGGATCTCA CCTTACCACA AAGTGACCAT GAAATAGGCA TACACTTAAA   
  
  
+ TTGGAAGGGT GAAAATGTTG TTTGGGCTTC TGCATGGAGA CCTAGCTA  

- +Up\_Stream \_Len000TACTAT TTTCTATAAA TAATTCGAGT TCGATAAAAT TGAAAATTTT TTTACTTACA   
  
  
- CCGAAAAAAA CGATAAGTCC ATGTTGTGTT ATACTTCGTA ATTGAGATCT TTTACACCAT TACATACAGT   
  
  
- ATATCCTCTC ATATATGAGA GTGAACGGTA CGCTACGCAC GTACACACAC ACACACACAT AGACAAAGAT   
  
  
- ACGTATAAAA TACACCCCAA AACAGTAGAT CTGTAGATAA TTATACAATC GTAAGTGTAC TGAATATCCT   
  
  
- TACGTGATAA TACTCATACT ATATCTCAAA ATTTTTACGT ATACGGAACA CACAAGTAAT CGAATTTCTT   
  
  
- CTCAAAAACT TATATGTATA AATCCATTAA GAACGATTAC AGCATTAATC GTCAACATAG ATAGAATAAC   
  
  
- TAAACATAAA AATTAACGAA CAATATGACA ATGTGAATCT TTTAAGTACT TATGAGAATT TTATACGTTT   
  
  
- AATATATATA TATATATATA TATATATATA TATATATATA TATTTTTGAC TTATTCAATT ATACCACTAC   
  
  
- TGTAAACAGT ACAATAGTAA CCAGCGAATT AAAAATTTAT TTTTTATACT AAAACACTGT GGATTATTTA   
  
  
- CTATAATCGG TATAATAATT TAAGTCGTAA ATAAGTAGTT ACATTATATG ATTTAGTACG TTAATAGTAA   
  
  
- CCAAGAGAAA ATACATTATT TCAATTAACT GTTAAGTTCC CAATGATTAA TCATATTACA ACTTAACCTT   
  
  
- GCATTTTTAG TTTAGCATAC ATTTCATTAA AATAAGACTT AAATTTTTTA CCTCGTTTAT AGAGATAACA   
  
  
- AGAAATTATT TTAAAATTAA CTAAAAATAA AGATTAATTC ACTGCCTTGG TTTAAAAAAA GGAGGAAAAA   
  
  
- ACCAAACCAT TCAATAGTAA TCGGTTAGAA AATAAATGTA AAACAGTCAA AAAGAAAAAA TTCTACCAAT   
  
  
- TTACCGAAGT TAATTTGTTT AAAAAAATGA ATAAATATAA AAATTATTTA AAAGTTTTAC ATATAATTGC   
  
  
- GTACTGGCAC GCTTCGTGCC TTAGATGGGA TCAATTAGAT ACTAAGAATT TATACGTTCA TCCGACAATA   
  
  
- AGTAAACAAC AAAAAATGAA TAAATATAAA AAAGGGAGGG CCATACTATC CCAAACAGTA TAACAATAAG   
  
  
- TAATCGACAA TTTGACATAA GTAATATCGT GTTCATCCGA ATTAGTTTCT ATCAATCAGA GGTTAAAGTA   
  
  
- GTCTTTTAAA TTCTTTTTCT GTGGAATCCT AGTTTTCATA CTTTAAATCT CTGGTCTGTT GCTATCTTAA   
  
  
- CTTTTCTACT CAAAAGTTAA ACCTCTTCTG CCTTTGAGTC TACTTACTTT CATATCATGT AACATGAGGA   
  
  
- ACCTCATATT AGAATAGAAG TGATAACTAT CCGAGCTTCG TATTAGTCAC TAACCTTTGA AAACCAACGT   
  
  
- TATTAAGTTT TAAACTATGA GATACTAAAT GAAAAAATTA ACTTCCAGTA CTAAATTAAT TGATTTACTT   
  
  
- TTCTTAAATA CTAGCTTTTT AAAATTGAGA ATAACTGAAT ATCAACTGAG GTCAATTTAC CTTTTAACTT   
  
  
- CACTAATTAG ATTTTCACCG GGCGAGATTA TGGTAAACTC TTTTTAATAA GAATGACGTT TCAAATTTAG   
  
  
- TTATACACTT CTATCAAATA TCAAAATAGG ATTTTCTTTA ATTTATCTAA CAATTTTTAT TAAAAATTTA   
  
  
- AGTCTATATG CACACTACCG GTATTGGCAT TTACTATACG TACAGCTGTT TAAGGTTTCT TCCGATTATT   
  
  
- AATCTGAATT TATTATGTAC GTATGATTGG TTTTTTTTTT TTAATTCGTT CTAAGATAGG TTGGATAGTA   
  
  
- TTATTTCAAA CCGGTATGAA GTTTGGTAGT GTATTGTAGT GATTATATAT TTCGATCCAA AACCCTTTGG   
  
  
- ATTGAAACTC CTGGTGTATA AACAATTCCG GATCCTCCGG TACTCAAACA GTTCTACGGA GTATCATACC   
  
  
- TCTCCAGATA TAGGAACGTT TTACTAGTAA GGGAAGATGG ATTTTCTTGG TGAAGTAAAG GTGAAAATGA   
  
  
- GGGACCGTTT ACCAACTATT TGGTTTGGTA TTGGTTCGGG AACTTCTTGA TATTTCTGTG CTTAGCTAGT   
  
  
- AACTTAAGCT TGTTCGGAAC ACACTATCCT CCTACCCGGA GTTGTGAAGA GGCGCTCACA CGTTCTCGTT   
  
  
- AGAGCCTCTT TTTAGGATTC TCGGTTGTTG AAGAAACCCA CAATTTACTT GAACAAAGAG GAATACCGCT   
  
  
- AACGCTTGTT TCTAACCGTA TAATGAAGAA TGTTCACAAC AACCGGTTTC GGTTGTTAAA CCCTGGAGTG   
  
  
- AAAGTACTCT CGGATTTTGA ACGGTACCTC TTTTTGACGA CGAAACTATG GATGTACTTC GAATATAACT   
  
  
- TCAAGGTTCT CCAGTCAGGT ACCTGTTGGA AACCAGTACA CCGAAGTTTA CCACGTTATA ACCTCCTGGA   
  
  
- ATCTTCAAAT GTTTTCAGCT AGTAGTTTCT TTGTTCCGTT TCCTACCTCT TCAAAAGTTC CGATTACCCA   
  
  
- CAAGGGAAGT TCAAAGTACA GTATTTGCTC AATCTATTGG ATCCTCTCGA AGCTTTTCTC CTAAATCTGT   
  
  
- AACTTCTACC ACTCCGGTAG CGACACTTGA CACAAGTTCG GAACGTTGCC CAAGTACACC TCTTCTCCCT   
  
  
- CGTACACGAA CTACACTAAG CTAGATAATC CGGAACATAG TATTGTGACC ACCTCCTTCT TCGTCTAGAG   
  
  
- TGAAGATGTT CTTTGCTGAA GAAGTTCACG AAGCTACTCA CAAACTCTAA AAGATTCAGT ATAAAGCTAT   
  
  
- ACAATCTTCT TTCGAAGGGA GGTTATTCGT TGCTTGCTTA GTTCGATCTT TCCCTTGTTA CCTACTTATA   
  
  
- GAGGTCTCGG GATCGAACAG TACCACTTTC ACCTCCTCTT ATATCCGGTT TCTTTCCTTG AGTTACCTTA   
  
  
- CTCTACGAGC TTGTTCGTAA AACGGGTAGA GTTAAATCAC TACTACATGA TTCACTACAA TCCCGTAACA   
  
  
- ACTTTTCTGT GTTTTCACCA ACCCTAGAGT GGAATGGTGT TTCACTGGTA CTTTATCCGT ATGTGAATTT   
  
  
- AACCTTCCCA CTTTTACAAC AAACCCGAAG ACGTACCTCT GGATCGAT

+     GT1-motif

| Site Name | Organism | Position | Strand | Matrix score. | sequence | function |
| --- | --- | --- | --- | --- | --- | --- |
| GT1-motif | Arabidopsis thaliana | 980 | + | 6 | GGTTAA | light responsive element |

>HU02G03154.1   
+ +Up\_Stream \_Len000ATGATA AAAGATATTT ATTAAGCTCA AGCTATTTTA ACTTTTAAAA AAATGAATGT   
  
  
+ GGCTTTTTTT GCTATTCAGG TACAACACAA TATGAAGCAT TAACTCTAGA AAATGTGGTA ATGTATGTCA   
  
  
+ TATAGGAGAG TATATACTCT CACTTGCCAT GCGATGCGTG CATGTGTGTG TGTGTGTGTA TCTGTTTCTA   
  
  
+ TGCATATTTT ATGTGGGGTT TTGTCATCTA GACATCTATT AATATGTTAG CATTCACATG ACTTATAGGA   
  
  
+ ATGCACTATT ATGAGTATGA TATAGAGTTT TAAAAATGCA TATGCCTTGT GTGTTCATTA GCTTAAAGAA   
  
  
+ GAGTTTTTGA ATATACATAT TTAGGTAATT CTTGCTAATG TCGTAATTAG CAGTTGTATC TATCTTATTG   
  
  
+ ATTTGTATTT TTAATTGCTT GTTATACTGT TACACTTAGA AAATTCATGA ATACTCTTAA AATATGCAAA   
  
  
+ TTATATATAT ATATATATAT ATATATATAT ATATATATAT ATAAAAACTG AATAAGTTAA TATGGTGATG   
  
  
+ ACATTTGTCA TGTTATCATT GGTCGCTTAA TTTTTAAATA AAAAATATGA TTTTGTGACA CCTAATAAAT   
  
  
+ GATATTAGCC ATATTATTAA ATTCAGCATT TATTCATCAA TGTAATATAC TAAATCATGC AATTATCATT   
  
  
+ GGTTCTCTTT TATGTAATAA AGTTAATTGA CAATTCAAGG GTTACTAATT AGTATAATGT TGAATTGGAA   
  
  
+ CGTAAAAATC AAATCGTATG TAAAGTAATT TTATTCTGAA TTTAAAAAAT GGAGCAAATA TCTCTATTGT   
  
  
+ TCTTTAATAA AATTTTAATT GATTTTTATT TCTAATTAAG TGACGGAACC AAATTTTTTT CCTCCTTTTT   
  
  
+ TGGTTTGGTA AGTTATCATT AGCCAATCTT TTATTTACAT TTTGTCAGTT TTTCTTTTTT AAGATGGTTA   
  
  
+ AATGGCTTCA ATTAAACAAA TTTTTTTACT TATTTATATT TTTAATAAAT TTTCAAAATG TATATTAACG   
  
  
+ CATGACCGTG CGAAGCACGG AATCTACCCT AGTTAATCTA TGATTCTTAA ATATGCAAGT AGGCTGTTAT   
  
  
+ TCATTTGTTG TTTTTTACTT ATTTATATTT TTTCCCTCCC GGTATGATAG GGTTTGTCAT ATTGTTATTC   
  
  
+ ATTAGCTGTT AAACTGTATT CATTATAGCA CAAGTAGGCT TAATCAAAGA TAGTTAGTCT CCAATTTCAT   
  
  
+ CAGAAAATTT AAGAAAAAGA CACCTTAGGA TCAAAAGTAT GAAATTTAGA GACCAGACAA CGATAGAATT   
  
  
+ GAAAAGATGA GTTTTCAATT TGGAGAAGAC GGAAACTCAG ATGAATGAAA GTATAGTACA TTGTACTCCT   
  
  
+ TGGAGTATAA TCTTATCTTC ACTATTGATA GGCTCGAAGC ATAATCAGTG ATTGGAAACT TTTGGTTGCA   
  
  
+ ATAATTCAAA ATTTGATACT CTATGATTTA CTTTTTTAAT TGAAGGTCAT GATTTAATTA ACTAAATGAA   
  
  
+ AAGAATTTAT GATCGAAAAA TTTTAACTCT TATTGACTTA TAGTTGACTC CAGTTAAATG GAAAATTGAA   
  
  
+ GTGATTAATC TAAAAGTGGC CCGCTCTAAT ACCATTTGAG AAAAATTATT CTTACTGCAA AGTTTAAATC   
  
  
+ AATATGTGAA GATAGTTTAT AGTTTTATCC TAAAAGAAAT TAAATAGATT GTTAAAAATA ATTTTTAAAT   
  
  
+ TCAGATATAC GTGTGATGGC CATAACCGTA AATGATATGC ATGTCGACAA ATTCCAAAGA AGGCTAATAA   
  
  
+ TTAGACTTAA ATAATACATG CATACTAACC AAAAAAAAAA AATTAAGCAA GATTCTATCC AACCTATCAT   
  
  
+ AATAAAGTTT GGCCATACTT CAAACCATCA CATAACATCA CTAATATATA AAGCTAGGTT TTGGGAAACC   
  
  
+ TAACTTTGAG GACCACATAT TTGTTAAGGC CTAGGAGGCC ATGAGTTTGT CAAGATGCCT CATAGTATGG   
  
  
+ AGAGGTCTAT ATCCTTGCAA AATGATCATT CCCTTCTACC TAAAAGAACC ACTTCATTTC CACTTTTACT   
  
  
+ CCCTGGCAAA TGGTTGATAA ACCAAACCAT AACCAAGCCC TTGAAGAACT ATAAAGACAC GAATCGATCA   
  
  
+ TTGAATTCGA ACAAGCCTTG TGTGATAGGA GGATGGGCCT CAACACTTCT CCGCGAGTGT GCAAGAGCAA   
  
  
+ TCTCGGAGAA AAATCCTAAG AGCCAACAAC TTCTTTGGGT GTTAAATGAA CTTGTTTCTC CTTATGGCGA   
  
  
+ TTGCGAACAA AGATTGGCAT ATTACTTCTT ACAAGTGTTG TTGGCCAAAG CCAACAATTT GGGACCTCAC   
  
  
+ TTTCATGAGA GCCTAAAACT TGCCATGGAG AAAAACTGCT GCTTTGATAC CTACATGAAG CTTATATTGA   
  
  
+ AGTTCCAAGA GGTCAGTCCA TGGACAACCT TTGGTCATGT GGCTTCAAAT GGTGCAATAT TGGAGGACCT   
  
  
+ TAGAAGTTTA CAAAAGTCGA TCATCAAAGA AACAAGGCAA AGGATGGAGA AGTTTTCAAG GCTAATGGGT   
  
  
+ GTTCCCTTCA AGTTTCATGT CATAAACGAG TTAGATAACC TAGGAGAGCT TCGAAAAGAG GATTTAGACA   
  
  
+ TTGAAGATGG TGAGGCCATC GCTGTGAACT GTGTTCAAGC CTTGCAACGG GTTCATGTGG AGAAGAGGGA   
  
  
+ GCATGTGCTT GATGTGATTC GATCTATTAG GCCTTGTATC ATAACACTGG TGGAGGAAGA AGCAGATCTC   
  
  
+ ACTTCTACAA GAAACGACTT CTTCAAGTGC TTCGATGAGT GTTTGAGATT TTCTAAGTCA TATTTCGATA   
  
  
+ TGTTAGAAGA AAGCTTCCCT CCAATAAGCA ACGAACGAAT CAAGCTAGAA AGGGAACAAT GGATGAATAT   
  
  
+ CTCCAGAGCC CTAGCTTGTC ATGGTGAAAG TGGAGGAGAA TATAGGCCAA AGAAAGGAAC TCAATGGAAT   
  
  
+ GAGATGCTCG AACAAGCATT TTGCCCATCT CAATTTAGTG ATGATGTACT AAGTGATGTT AGGGCATTGT   
  
  
+ TGAAAAGACA CAAAAGTGGT TGGGATCTCA CCTTACCACA AAGTGACCAT GAAATAGGCA TACACTTAAA   
  
  
+ TTGGAAGGGT GAAAATGTTG TTTGGGCTTC TGCATGGAGA CCTAGCTA  

- +Up\_Stream \_Len000TACTAT TTTCTATAAA TAATTCGAGT TCGATAAAAT TGAAAATTTT TTTACTTACA   
  
  
- CCGAAAAAAA CGATAAGTCC ATGTTGTGTT ATACTTCGTA ATTGAGATCT TTTACACCAT TACATACAGT   
  
  
- ATATCCTCTC ATATATGAGA GTGAACGGTA CGCTACGCAC GTACACACAC ACACACACAT AGACAAAGAT   
  
  
- ACGTATAAAA TACACCCCAA AACAGTAGAT CTGTAGATAA TTATACAATC GTAAGTGTAC TGAATATCCT   
  
  
- TACGTGATAA TACTCATACT ATATCTCAAA ATTTTTACGT ATACGGAACA CACAAGTAAT CGAATTTCTT   
  
  
- CTCAAAAACT TATATGTATA AATCCATTAA GAACGATTAC AGCATTAATC GTCAACATAG ATAGAATAAC   
  
  
- TAAACATAAA AATTAACGAA CAATATGACA ATGTGAATCT TTTAAGTACT TATGAGAATT TTATACGTTT   
  
  
- AATATATATA TATATATATA TATATATATA TATATATATA TATTTTTGAC TTATTCAATT ATACCACTAC   
  
  
- TGTAAACAGT ACAATAGTAA CCAGCGAATT AAAAATTTAT TTTTTATACT AAAACACTGT GGATTATTTA   
  
  
- CTATAATCGG TATAATAATT TAAGTCGTAA ATAAGTAGTT ACATTATATG ATTTAGTACG TTAATAGTAA   
  
  
- CCAAGAGAAA ATACATTATT TCAATTAACT GTTAAGTTCC CAATGATTAA TCATATTACA ACTTAACCTT   
  
  
- GCATTTTTAG TTTAGCATAC ATTTCATTAA AATAAGACTT AAATTTTTTA CCTCGTTTAT AGAGATAACA   
  
  
- AGAAATTATT TTAAAATTAA CTAAAAATAA AGATTAATTC ACTGCCTTGG TTTAAAAAAA GGAGGAAAAA   
  
  
- ACCAAACCAT TCAATAGTAA TCGGTTAGAA AATAAATGTA AAACAGTCAA AAAGAAAAAA TTCTACCAAT   
  
  
- TTACCGAAGT TAATTTGTTT AAAAAAATGA ATAAATATAA AAATTATTTA AAAGTTTTAC ATATAATTGC   
  
  
- GTACTGGCAC GCTTCGTGCC TTAGATGGGA TCAATTAGAT ACTAAGAATT TATACGTTCA TCCGACAATA   
  
  
- AGTAAACAAC AAAAAATGAA TAAATATAAA AAAGGGAGGG CCATACTATC CCAAACAGTA TAACAATAAG   
  
  
- TAATCGACAA TTTGACATAA GTAATATCGT GTTCATCCGA ATTAGTTTCT ATCAATCAGA GGTTAAAGTA   
  
  
- GTCTTTTAAA TTCTTTTTCT GTGGAATCCT AGTTTTCATA CTTTAAATCT CTGGTCTGTT GCTATCTTAA   
  
  
- CTTTTCTACT CAAAAGTTAA ACCTCTTCTG CCTTTGAGTC TACTTACTTT CATATCATGT AACATGAGGA   
  
  
- ACCTCATATT AGAATAGAAG TGATAACTAT CCGAGCTTCG TATTAGTCAC TAACCTTTGA AAACCAACGT   
  
  
- TATTAAGTTT TAAACTATGA GATACTAAAT GAAAAAATTA ACTTCCAGTA CTAAATTAAT TGATTTACTT   
  
  
- TTCTTAAATA CTAGCTTTTT AAAATTGAGA ATAACTGAAT ATCAACTGAG GTCAATTTAC CTTTTAACTT   
  
  
- CACTAATTAG ATTTTCACCG GGCGAGATTA TGGTAAACTC TTTTTAATAA GAATGACGTT TCAAATTTAG   
  
  
- TTATACACTT CTATCAAATA TCAAAATAGG ATTTTCTTTA ATTTATCTAA CAATTTTTAT TAAAAATTTA   
  
  
- AGTCTATATG CACACTACCG GTATTGGCAT TTACTATACG TACAGCTGTT TAAGGTTTCT TCCGATTATT   
  
  
- AATCTGAATT TATTATGTAC GTATGATTGG TTTTTTTTTT TTAATTCGTT CTAAGATAGG TTGGATAGTA   
  
  
- TTATTTCAAA CCGGTATGAA GTTTGGTAGT GTATTGTAGT GATTATATAT TTCGATCCAA AACCCTTTGG   
  
  
- ATTGAAACTC CTGGTGTATA AACAATTCCG GATCCTCCGG TACTCAAACA GTTCTACGGA GTATCATACC   
  
  
- TCTCCAGATA TAGGAACGTT TTACTAGTAA GGGAAGATGG ATTTTCTTGG TGAAGTAAAG GTGAAAATGA   
  
  
- GGGACCGTTT ACCAACTATT TGGTTTGGTA TTGGTTCGGG AACTTCTTGA TATTTCTGTG CTTAGCTAGT   
  
  
- AACTTAAGCT TGTTCGGAAC ACACTATCCT CCTACCCGGA GTTGTGAAGA GGCGCTCACA CGTTCTCGTT   
  
  
- AGAGCCTCTT TTTAGGATTC TCGGTTGTTG AAGAAACCCA CAATTTACTT GAACAAAGAG GAATACCGCT   
  
  
- AACGCTTGTT TCTAACCGTA TAATGAAGAA TGTTCACAAC AACCGGTTTC GGTTGTTAAA CCCTGGAGTG   
  
  
- AAAGTACTCT CGGATTTTGA ACGGTACCTC TTTTTGACGA CGAAACTATG GATGTACTTC GAATATAACT   
  
  
- TCAAGGTTCT CCAGTCAGGT ACCTGTTGGA AACCAGTACA CCGAAGTTTA CCACGTTATA ACCTCCTGGA   
  
  
- ATCTTCAAAT GTTTTCAGCT AGTAGTTTCT TTGTTCCGTT TCCTACCTCT TCAAAAGTTC CGATTACCCA   
  
  
- CAAGGGAAGT TCAAAGTACA GTATTTGCTC AATCTATTGG ATCCTCTCGA AGCTTTTCTC CTAAATCTGT   
  
  
- AACTTCTACC ACTCCGGTAG CGACACTTGA CACAAGTTCG GAACGTTGCC CAAGTACACC TCTTCTCCCT   
  
  
- CGTACACGAA CTACACTAAG CTAGATAATC CGGAACATAG TATTGTGACC ACCTCCTTCT TCGTCTAGAG   
  
  
- TGAAGATGTT CTTTGCTGAA GAAGTTCACG AAGCTACTCA CAAACTCTAA AAGATTCAGT ATAAAGCTAT   
  
  
- ACAATCTTCT TTCGAAGGGA GGTTATTCGT TGCTTGCTTA GTTCGATCTT TCCCTTGTTA CCTACTTATA   
  
  
- GAGGTCTCGG GATCGAACAG TACCACTTTC ACCTCCTCTT ATATCCGGTT TCTTTCCTTG AGTTACCTTA   
  
  
- CTCTACGAGC TTGTTCGTAA AACGGGTAGA GTTAAATCAC TACTACATGA TTCACTACAA TCCCGTAACA   
  
  
- ACTTTTCTGT GTTTTCACCA ACCCTAGAGT GGAATGGTGT TTCACTGGTA CTTTATCCGT ATGTGAATTT   
  
  
- AACCTTCCCA CTTTTACAAC AAACCCGAAG ACGTACCTCT GGATCGAT

+     Gap-box

| Site Name | Organism | Position | Strand | Matrix score. | sequence | function |
| --- | --- | --- | --- | --- | --- | --- |
| Gap-box | Arabidopsis thaliana | 1122 | - | 9 | CAAATGAA(A/G)A | part of a light responsive element |

>HU02G03154.1   
+ +Up\_Stream \_Len000ATGATA AAAGATATTT ATTAAGCTCA AGCTATTTTA ACTTTTAAAA AAATGAATGT   
  
  
+ GGCTTTTTTT GCTATTCAGG TACAACACAA TATGAAGCAT TAACTCTAGA AAATGTGGTA ATGTATGTCA   
  
  
+ TATAGGAGAG TATATACTCT CACTTGCCAT GCGATGCGTG CATGTGTGTG TGTGTGTGTA TCTGTTTCTA   
  
  
+ TGCATATTTT ATGTGGGGTT TTGTCATCTA GACATCTATT AATATGTTAG CATTCACATG ACTTATAGGA   
  
  
+ ATGCACTATT ATGAGTATGA TATAGAGTTT TAAAAATGCA TATGCCTTGT GTGTTCATTA GCTTAAAGAA   
  
  
+ GAGTTTTTGA ATATACATAT TTAGGTAATT CTTGCTAATG TCGTAATTAG CAGTTGTATC TATCTTATTG   
  
  
+ ATTTGTATTT TTAATTGCTT GTTATACTGT TACACTTAGA AAATTCATGA ATACTCTTAA AATATGCAAA   
  
  
+ TTATATATAT ATATATATAT ATATATATAT ATATATATAT ATAAAAACTG AATAAGTTAA TATGGTGATG   
  
  
+ ACATTTGTCA TGTTATCATT GGTCGCTTAA TTTTTAAATA AAAAATATGA TTTTGTGACA CCTAATAAAT   
  
  
+ GATATTAGCC ATATTATTAA ATTCAGCATT TATTCATCAA TGTAATATAC TAAATCATGC AATTATCATT   
  
  
+ GGTTCTCTTT TATGTAATAA AGTTAATTGA CAATTCAAGG GTTACTAATT AGTATAATGT TGAATTGGAA   
  
  
+ CGTAAAAATC AAATCGTATG TAAAGTAATT TTATTCTGAA TTTAAAAAAT GGAGCAAATA TCTCTATTGT   
  
  
+ TCTTTAATAA AATTTTAATT GATTTTTATT TCTAATTAAG TGACGGAACC AAATTTTTTT CCTCCTTTTT   
  
  
+ TGGTTTGGTA AGTTATCATT AGCCAATCTT TTATTTACAT TTTGTCAGTT TTTCTTTTTT AAGATGGTTA   
  
  
+ AATGGCTTCA ATTAAACAAA TTTTTTTACT TATTTATATT TTTAATAAAT TTTCAAAATG TATATTAACG   
  
  
+ CATGACCGTG CGAAGCACGG AATCTACCCT AGTTAATCTA TGATTCTTAA ATATGCAAGT AGGCTGTTAT   
  
  
+ TCATTTGTTG TTTTTTACTT ATTTATATTT TTTCCCTCCC GGTATGATAG GGTTTGTCAT ATTGTTATTC   
  
  
+ ATTAGCTGTT AAACTGTATT CATTATAGCA CAAGTAGGCT TAATCAAAGA TAGTTAGTCT CCAATTTCAT   
  
  
+ CAGAAAATTT AAGAAAAAGA CACCTTAGGA TCAAAAGTAT GAAATTTAGA GACCAGACAA CGATAGAATT   
  
  
+ GAAAAGATGA GTTTTCAATT TGGAGAAGAC GGAAACTCAG ATGAATGAAA GTATAGTACA TTGTACTCCT   
  
  
+ TGGAGTATAA TCTTATCTTC ACTATTGATA GGCTCGAAGC ATAATCAGTG ATTGGAAACT TTTGGTTGCA   
  
  
+ ATAATTCAAA ATTTGATACT CTATGATTTA CTTTTTTAAT TGAAGGTCAT GATTTAATTA ACTAAATGAA   
  
  
+ AAGAATTTAT GATCGAAAAA TTTTAACTCT TATTGACTTA TAGTTGACTC CAGTTAAATG GAAAATTGAA   
  
  
+ GTGATTAATC TAAAAGTGGC CCGCTCTAAT ACCATTTGAG AAAAATTATT CTTACTGCAA AGTTTAAATC   
  
  
+ AATATGTGAA GATAGTTTAT AGTTTTATCC TAAAAGAAAT TAAATAGATT GTTAAAAATA ATTTTTAAAT   
  
  
+ TCAGATATAC GTGTGATGGC CATAACCGTA AATGATATGC ATGTCGACAA ATTCCAAAGA AGGCTAATAA   
  
  
+ TTAGACTTAA ATAATACATG CATACTAACC AAAAAAAAAA AATTAAGCAA GATTCTATCC AACCTATCAT   
  
  
+ AATAAAGTTT GGCCATACTT CAAACCATCA CATAACATCA CTAATATATA AAGCTAGGTT TTGGGAAACC   
  
  
+ TAACTTTGAG GACCACATAT TTGTTAAGGC CTAGGAGGCC ATGAGTTTGT CAAGATGCCT CATAGTATGG   
  
  
+ AGAGGTCTAT ATCCTTGCAA AATGATCATT CCCTTCTACC TAAAAGAACC ACTTCATTTC CACTTTTACT   
  
  
+ CCCTGGCAAA TGGTTGATAA ACCAAACCAT AACCAAGCCC TTGAAGAACT ATAAAGACAC GAATCGATCA   
  
  
+ TTGAATTCGA ACAAGCCTTG TGTGATAGGA GGATGGGCCT CAACACTTCT CCGCGAGTGT GCAAGAGCAA   
  
  
+ TCTCGGAGAA AAATCCTAAG AGCCAACAAC TTCTTTGGGT GTTAAATGAA CTTGTTTCTC CTTATGGCGA   
  
  
+ TTGCGAACAA AGATTGGCAT ATTACTTCTT ACAAGTGTTG TTGGCCAAAG CCAACAATTT GGGACCTCAC   
  
  
+ TTTCATGAGA GCCTAAAACT TGCCATGGAG AAAAACTGCT GCTTTGATAC CTACATGAAG CTTATATTGA   
  
  
+ AGTTCCAAGA GGTCAGTCCA TGGACAACCT TTGGTCATGT GGCTTCAAAT GGTGCAATAT TGGAGGACCT   
  
  
+ TAGAAGTTTA CAAAAGTCGA TCATCAAAGA AACAAGGCAA AGGATGGAGA AGTTTTCAAG GCTAATGGGT   
  
  
+ GTTCCCTTCA AGTTTCATGT CATAAACGAG TTAGATAACC TAGGAGAGCT TCGAAAAGAG GATTTAGACA   
  
  
+ TTGAAGATGG TGAGGCCATC GCTGTGAACT GTGTTCAAGC CTTGCAACGG GTTCATGTGG AGAAGAGGGA   
  
  
+ GCATGTGCTT GATGTGATTC GATCTATTAG GCCTTGTATC ATAACACTGG TGGAGGAAGA AGCAGATCTC   
  
  
+ ACTTCTACAA GAAACGACTT CTTCAAGTGC TTCGATGAGT GTTTGAGATT TTCTAAGTCA TATTTCGATA   
  
  
+ TGTTAGAAGA AAGCTTCCCT CCAATAAGCA ACGAACGAAT CAAGCTAGAA AGGGAACAAT GGATGAATAT   
  
  
+ CTCCAGAGCC CTAGCTTGTC ATGGTGAAAG TGGAGGAGAA TATAGGCCAA AGAAAGGAAC TCAATGGAAT   
  
  
+ GAGATGCTCG AACAAGCATT TTGCCCATCT CAATTTAGTG ATGATGTACT AAGTGATGTT AGGGCATTGT   
  
  
+ TGAAAAGACA CAAAAGTGGT TGGGATCTCA CCTTACCACA AAGTGACCAT GAAATAGGCA TACACTTAAA   
  
  
+ TTGGAAGGGT GAAAATGTTG TTTGGGCTTC TGCATGGAGA CCTAGCTA  

- +Up\_Stream \_Len000TACTAT TTTCTATAAA TAATTCGAGT TCGATAAAAT TGAAAATTTT TTTACTTACA   
  
  
- CCGAAAAAAA CGATAAGTCC ATGTTGTGTT ATACTTCGTA ATTGAGATCT TTTACACCAT TACATACAGT   
  
  
- ATATCCTCTC ATATATGAGA GTGAACGGTA CGCTACGCAC GTACACACAC ACACACACAT AGACAAAGAT   
  
  
- ACGTATAAAA TACACCCCAA AACAGTAGAT CTGTAGATAA TTATACAATC GTAAGTGTAC TGAATATCCT   
  
  
- TACGTGATAA TACTCATACT ATATCTCAAA ATTTTTACGT ATACGGAACA CACAAGTAAT CGAATTTCTT   
  
  
- CTCAAAAACT TATATGTATA AATCCATTAA GAACGATTAC AGCATTAATC GTCAACATAG ATAGAATAAC   
  
  
- TAAACATAAA AATTAACGAA CAATATGACA ATGTGAATCT TTTAAGTACT TATGAGAATT TTATACGTTT   
  
  
- AATATATATA TATATATATA TATATATATA TATATATATA TATTTTTGAC TTATTCAATT ATACCACTAC   
  
  
- TGTAAACAGT ACAATAGTAA CCAGCGAATT AAAAATTTAT TTTTTATACT AAAACACTGT GGATTATTTA   
  
  
- CTATAATCGG TATAATAATT TAAGTCGTAA ATAAGTAGTT ACATTATATG ATTTAGTACG TTAATAGTAA   
  
  
- CCAAGAGAAA ATACATTATT TCAATTAACT GTTAAGTTCC CAATGATTAA TCATATTACA ACTTAACCTT   
  
  
- GCATTTTTAG TTTAGCATAC ATTTCATTAA AATAAGACTT AAATTTTTTA CCTCGTTTAT AGAGATAACA   
  
  
- AGAAATTATT TTAAAATTAA CTAAAAATAA AGATTAATTC ACTGCCTTGG TTTAAAAAAA GGAGGAAAAA   
  
  
- ACCAAACCAT TCAATAGTAA TCGGTTAGAA AATAAATGTA AAACAGTCAA AAAGAAAAAA TTCTACCAAT   
  
  
- TTACCGAAGT TAATTTGTTT AAAAAAATGA ATAAATATAA AAATTATTTA AAAGTTTTAC ATATAATTGC   
  
  
- GTACTGGCAC GCTTCGTGCC TTAGATGGGA TCAATTAGAT ACTAAGAATT TATACGTTCA TCCGACAATA   
  
  
- AGTAAACAAC AAAAAATGAA TAAATATAAA AAAGGGAGGG CCATACTATC CCAAACAGTA TAACAATAAG   
  
  
- TAATCGACAA TTTGACATAA GTAATATCGT GTTCATCCGA ATTAGTTTCT ATCAATCAGA GGTTAAAGTA   
  
  
- GTCTTTTAAA TTCTTTTTCT GTGGAATCCT AGTTTTCATA CTTTAAATCT CTGGTCTGTT GCTATCTTAA   
  
  
- CTTTTCTACT CAAAAGTTAA ACCTCTTCTG CCTTTGAGTC TACTTACTTT CATATCATGT AACATGAGGA   
  
  
- ACCTCATATT AGAATAGAAG TGATAACTAT CCGAGCTTCG TATTAGTCAC TAACCTTTGA AAACCAACGT   
  
  
- TATTAAGTTT TAAACTATGA GATACTAAAT GAAAAAATTA ACTTCCAGTA CTAAATTAAT TGATTTACTT   
  
  
- TTCTTAAATA CTAGCTTTTT AAAATTGAGA ATAACTGAAT ATCAACTGAG GTCAATTTAC CTTTTAACTT   
  
  
- CACTAATTAG ATTTTCACCG GGCGAGATTA TGGTAAACTC TTTTTAATAA GAATGACGTT TCAAATTTAG   
  
  
- TTATACACTT CTATCAAATA TCAAAATAGG ATTTTCTTTA ATTTATCTAA CAATTTTTAT TAAAAATTTA   
  
  
- AGTCTATATG CACACTACCG GTATTGGCAT TTACTATACG TACAGCTGTT TAAGGTTTCT TCCGATTATT   
  
  
- AATCTGAATT TATTATGTAC GTATGATTGG TTTTTTTTTT TTAATTCGTT CTAAGATAGG TTGGATAGTA   
  
  
- TTATTTCAAA CCGGTATGAA GTTTGGTAGT GTATTGTAGT GATTATATAT TTCGATCCAA AACCCTTTGG   
  
  
- ATTGAAACTC CTGGTGTATA AACAATTCCG GATCCTCCGG TACTCAAACA GTTCTACGGA GTATCATACC   
  
  
- TCTCCAGATA TAGGAACGTT TTACTAGTAA GGGAAGATGG ATTTTCTTGG TGAAGTAAAG GTGAAAATGA   
  
  
- GGGACCGTTT ACCAACTATT TGGTTTGGTA TTGGTTCGGG AACTTCTTGA TATTTCTGTG CTTAGCTAGT   
  
  
- AACTTAAGCT TGTTCGGAAC ACACTATCCT CCTACCCGGA GTTGTGAAGA GGCGCTCACA CGTTCTCGTT   
  
  
- AGAGCCTCTT TTTAGGATTC TCGGTTGTTG AAGAAACCCA CAATTTACTT GAACAAAGAG GAATACCGCT   
  
  
- AACGCTTGTT TCTAACCGTA TAATGAAGAA TGTTCACAAC AACCGGTTTC GGTTGTTAAA CCCTGGAGTG   
  
  
- AAAGTACTCT CGGATTTTGA ACGGTACCTC TTTTTGACGA CGAAACTATG GATGTACTTC GAATATAACT   
  
  
- TCAAGGTTCT CCAGTCAGGT ACCTGTTGGA AACCAGTACA CCGAAGTTTA CCACGTTATA ACCTCCTGGA   
  
  
- ATCTTCAAAT GTTTTCAGCT AGTAGTTTCT TTGTTCCGTT TCCTACCTCT TCAAAAGTTC CGATTACCCA   
  
  
- CAAGGGAAGT TCAAAGTACA GTATTTGCTC AATCTATTGG ATCCTCTCGA AGCTTTTCTC CTAAATCTGT   
  
  
- AACTTCTACC ACTCCGGTAG CGACACTTGA CACAAGTTCG GAACGTTGCC CAAGTACACC TCTTCTCCCT   
  
  
- CGTACACGAA CTACACTAAG CTAGATAATC CGGAACATAG TATTGTGACC ACCTCCTTCT TCGTCTAGAG   
  
  
- TGAAGATGTT CTTTGCTGAA GAAGTTCACG AAGCTACTCA CAAACTCTAA AAGATTCAGT ATAAAGCTAT   
  
  
- ACAATCTTCT TTCGAAGGGA GGTTATTCGT TGCTTGCTTA GTTCGATCTT TCCCTTGTTA CCTACTTATA   
  
  
- GAGGTCTCGG GATCGAACAG TACCACTTTC ACCTCCTCTT ATATCCGGTT TCTTTCCTTG AGTTACCTTA   
  
  
- CTCTACGAGC TTGTTCGTAA AACGGGTAGA GTTAAATCAC TACTACATGA TTCACTACAA TCCCGTAACA   
  
  
- ACTTTTCTGT GTTTTCACCA ACCCTAGAGT GGAATGGTGT TTCACTGGTA CTTTATCCGT ATGTGAATTT   
  
  
- AACCTTCCCA CTTTTACAAC AAACCCGAAG ACGTACCTCT GGATCGAT

+     I-box

| Site Name | Organism | Position | Strand | Matrix score. | sequence | function |
| --- | --- | --- | --- | --- | --- | --- |
| I-box | Pisum sativum | 2626 | + | 9 | TAGATAACC | part of a light responsive element |

>HU02G03154.1   
+ +Up\_Stream \_Len000ATGATA AAAGATATTT ATTAAGCTCA AGCTATTTTA ACTTTTAAAA AAATGAATGT   
  
  
+ GGCTTTTTTT GCTATTCAGG TACAACACAA TATGAAGCAT TAACTCTAGA AAATGTGGTA ATGTATGTCA   
  
  
+ TATAGGAGAG TATATACTCT CACTTGCCAT GCGATGCGTG CATGTGTGTG TGTGTGTGTA TCTGTTTCTA   
  
  
+ TGCATATTTT ATGTGGGGTT TTGTCATCTA GACATCTATT AATATGTTAG CATTCACATG ACTTATAGGA   
  
  
+ ATGCACTATT ATGAGTATGA TATAGAGTTT TAAAAATGCA TATGCCTTGT GTGTTCATTA GCTTAAAGAA   
  
  
+ GAGTTTTTGA ATATACATAT TTAGGTAATT CTTGCTAATG TCGTAATTAG CAGTTGTATC TATCTTATTG   
  
  
+ ATTTGTATTT TTAATTGCTT GTTATACTGT TACACTTAGA AAATTCATGA ATACTCTTAA AATATGCAAA   
  
  
+ TTATATATAT ATATATATAT ATATATATAT ATATATATAT ATAAAAACTG AATAAGTTAA TATGGTGATG   
  
  
+ ACATTTGTCA TGTTATCATT GGTCGCTTAA TTTTTAAATA AAAAATATGA TTTTGTGACA CCTAATAAAT   
  
  
+ GATATTAGCC ATATTATTAA ATTCAGCATT TATTCATCAA TGTAATATAC TAAATCATGC AATTATCATT   
  
  
+ GGTTCTCTTT TATGTAATAA AGTTAATTGA CAATTCAAGG GTTACTAATT AGTATAATGT TGAATTGGAA   
  
  
+ CGTAAAAATC AAATCGTATG TAAAGTAATT TTATTCTGAA TTTAAAAAAT GGAGCAAATA TCTCTATTGT   
  
  
+ TCTTTAATAA AATTTTAATT GATTTTTATT TCTAATTAAG TGACGGAACC AAATTTTTTT CCTCCTTTTT   
  
  
+ TGGTTTGGTA AGTTATCATT AGCCAATCTT TTATTTACAT TTTGTCAGTT TTTCTTTTTT AAGATGGTTA   
  
  
+ AATGGCTTCA ATTAAACAAA TTTTTTTACT TATTTATATT TTTAATAAAT TTTCAAAATG TATATTAACG   
  
  
+ CATGACCGTG CGAAGCACGG AATCTACCCT AGTTAATCTA TGATTCTTAA ATATGCAAGT AGGCTGTTAT   
  
  
+ TCATTTGTTG TTTTTTACTT ATTTATATTT TTTCCCTCCC GGTATGATAG GGTTTGTCAT ATTGTTATTC   
  
  
+ ATTAGCTGTT AAACTGTATT CATTATAGCA CAAGTAGGCT TAATCAAAGA TAGTTAGTCT CCAATTTCAT   
  
  
+ CAGAAAATTT AAGAAAAAGA CACCTTAGGA TCAAAAGTAT GAAATTTAGA GACCAGACAA CGATAGAATT   
  
  
+ GAAAAGATGA GTTTTCAATT TGGAGAAGAC GGAAACTCAG ATGAATGAAA GTATAGTACA TTGTACTCCT   
  
  
+ TGGAGTATAA TCTTATCTTC ACTATTGATA GGCTCGAAGC ATAATCAGTG ATTGGAAACT TTTGGTTGCA   
  
  
+ ATAATTCAAA ATTTGATACT CTATGATTTA CTTTTTTAAT TGAAGGTCAT GATTTAATTA ACTAAATGAA   
  
  
+ AAGAATTTAT GATCGAAAAA TTTTAACTCT TATTGACTTA TAGTTGACTC CAGTTAAATG GAAAATTGAA   
  
  
+ GTGATTAATC TAAAAGTGGC CCGCTCTAAT ACCATTTGAG AAAAATTATT CTTACTGCAA AGTTTAAATC   
  
  
+ AATATGTGAA GATAGTTTAT AGTTTTATCC TAAAAGAAAT TAAATAGATT GTTAAAAATA ATTTTTAAAT   
  
  
+ TCAGATATAC GTGTGATGGC CATAACCGTA AATGATATGC ATGTCGACAA ATTCCAAAGA AGGCTAATAA   
  
  
+ TTAGACTTAA ATAATACATG CATACTAACC AAAAAAAAAA AATTAAGCAA GATTCTATCC AACCTATCAT   
  
  
+ AATAAAGTTT GGCCATACTT CAAACCATCA CATAACATCA CTAATATATA AAGCTAGGTT TTGGGAAACC   
  
  
+ TAACTTTGAG GACCACATAT TTGTTAAGGC CTAGGAGGCC ATGAGTTTGT CAAGATGCCT CATAGTATGG   
  
  
+ AGAGGTCTAT ATCCTTGCAA AATGATCATT CCCTTCTACC TAAAAGAACC ACTTCATTTC CACTTTTACT   
  
  
+ CCCTGGCAAA TGGTTGATAA ACCAAACCAT AACCAAGCCC TTGAAGAACT ATAAAGACAC GAATCGATCA   
  
  
+ TTGAATTCGA ACAAGCCTTG TGTGATAGGA GGATGGGCCT CAACACTTCT CCGCGAGTGT GCAAGAGCAA   
  
  
+ TCTCGGAGAA AAATCCTAAG AGCCAACAAC TTCTTTGGGT GTTAAATGAA CTTGTTTCTC CTTATGGCGA   
  
  
+ TTGCGAACAA AGATTGGCAT ATTACTTCTT ACAAGTGTTG TTGGCCAAAG CCAACAATTT GGGACCTCAC   
  
  
+ TTTCATGAGA GCCTAAAACT TGCCATGGAG AAAAACTGCT GCTTTGATAC CTACATGAAG CTTATATTGA   
  
  
+ AGTTCCAAGA GGTCAGTCCA TGGACAACCT TTGGTCATGT GGCTTCAAAT GGTGCAATAT TGGAGGACCT   
  
  
+ TAGAAGTTTA CAAAAGTCGA TCATCAAAGA AACAAGGCAA AGGATGGAGA AGTTTTCAAG GCTAATGGGT   
  
  
+ GTTCCCTTCA AGTTTCATGT CATAAACGAG TTAGATAACC TAGGAGAGCT TCGAAAAGAG GATTTAGACA   
  
  
+ TTGAAGATGG TGAGGCCATC GCTGTGAACT GTGTTCAAGC CTTGCAACGG GTTCATGTGG AGAAGAGGGA   
  
  
+ GCATGTGCTT GATGTGATTC GATCTATTAG GCCTTGTATC ATAACACTGG TGGAGGAAGA AGCAGATCTC   
  
  
+ ACTTCTACAA GAAACGACTT CTTCAAGTGC TTCGATGAGT GTTTGAGATT TTCTAAGTCA TATTTCGATA   
  
  
+ TGTTAGAAGA AAGCTTCCCT CCAATAAGCA ACGAACGAAT CAAGCTAGAA AGGGAACAAT GGATGAATAT   
  
  
+ CTCCAGAGCC CTAGCTTGTC ATGGTGAAAG TGGAGGAGAA TATAGGCCAA AGAAAGGAAC TCAATGGAAT   
  
  
+ GAGATGCTCG AACAAGCATT TTGCCCATCT CAATTTAGTG ATGATGTACT AAGTGATGTT AGGGCATTGT   
  
  
+ TGAAAAGACA CAAAAGTGGT TGGGATCTCA CCTTACCACA AAGTGACCAT GAAATAGGCA TACACTTAAA   
  
  
+ TTGGAAGGGT GAAAATGTTG TTTGGGCTTC TGCATGGAGA CCTAGCTA  

- +Up\_Stream \_Len000TACTAT TTTCTATAAA TAATTCGAGT TCGATAAAAT TGAAAATTTT TTTACTTACA   
  
  
- CCGAAAAAAA CGATAAGTCC ATGTTGTGTT ATACTTCGTA ATTGAGATCT TTTACACCAT TACATACAGT   
  
  
- ATATCCTCTC ATATATGAGA GTGAACGGTA CGCTACGCAC GTACACACAC ACACACACAT AGACAAAGAT   
  
  
- ACGTATAAAA TACACCCCAA AACAGTAGAT CTGTAGATAA TTATACAATC GTAAGTGTAC TGAATATCCT   
  
  
- TACGTGATAA TACTCATACT ATATCTCAAA ATTTTTACGT ATACGGAACA CACAAGTAAT CGAATTTCTT   
  
  
- CTCAAAAACT TATATGTATA AATCCATTAA GAACGATTAC AGCATTAATC GTCAACATAG ATAGAATAAC   
  
  
- TAAACATAAA AATTAACGAA CAATATGACA ATGTGAATCT TTTAAGTACT TATGAGAATT TTATACGTTT   
  
  
- AATATATATA TATATATATA TATATATATA TATATATATA TATTTTTGAC TTATTCAATT ATACCACTAC   
  
  
- TGTAAACAGT ACAATAGTAA CCAGCGAATT AAAAATTTAT TTTTTATACT AAAACACTGT GGATTATTTA   
  
  
- CTATAATCGG TATAATAATT TAAGTCGTAA ATAAGTAGTT ACATTATATG ATTTAGTACG TTAATAGTAA   
  
  
- CCAAGAGAAA ATACATTATT TCAATTAACT GTTAAGTTCC CAATGATTAA TCATATTACA ACTTAACCTT   
  
  
- GCATTTTTAG TTTAGCATAC ATTTCATTAA AATAAGACTT AAATTTTTTA CCTCGTTTAT AGAGATAACA   
  
  
- AGAAATTATT TTAAAATTAA CTAAAAATAA AGATTAATTC ACTGCCTTGG TTTAAAAAAA GGAGGAAAAA   
  
  
- ACCAAACCAT TCAATAGTAA TCGGTTAGAA AATAAATGTA AAACAGTCAA AAAGAAAAAA TTCTACCAAT   
  
  
- TTACCGAAGT TAATTTGTTT AAAAAAATGA ATAAATATAA AAATTATTTA AAAGTTTTAC ATATAATTGC   
  
  
- GTACTGGCAC GCTTCGTGCC TTAGATGGGA TCAATTAGAT ACTAAGAATT TATACGTTCA TCCGACAATA   
  
  
- AGTAAACAAC AAAAAATGAA TAAATATAAA AAAGGGAGGG CCATACTATC CCAAACAGTA TAACAATAAG   
  
  
- TAATCGACAA TTTGACATAA GTAATATCGT GTTCATCCGA ATTAGTTTCT ATCAATCAGA GGTTAAAGTA   
  
  
- GTCTTTTAAA TTCTTTTTCT GTGGAATCCT AGTTTTCATA CTTTAAATCT CTGGTCTGTT GCTATCTTAA   
  
  
- CTTTTCTACT CAAAAGTTAA ACCTCTTCTG CCTTTGAGTC TACTTACTTT CATATCATGT AACATGAGGA   
  
  
- ACCTCATATT AGAATAGAAG TGATAACTAT CCGAGCTTCG TATTAGTCAC TAACCTTTGA AAACCAACGT   
  
  
- TATTAAGTTT TAAACTATGA GATACTAAAT GAAAAAATTA ACTTCCAGTA CTAAATTAAT TGATTTACTT   
  
  
- TTCTTAAATA CTAGCTTTTT AAAATTGAGA ATAACTGAAT ATCAACTGAG GTCAATTTAC CTTTTAACTT   
  
  
- CACTAATTAG ATTTTCACCG GGCGAGATTA TGGTAAACTC TTTTTAATAA GAATGACGTT TCAAATTTAG   
  
  
- TTATACACTT CTATCAAATA TCAAAATAGG ATTTTCTTTA ATTTATCTAA CAATTTTTAT TAAAAATTTA   
  
  
- AGTCTATATG CACACTACCG GTATTGGCAT TTACTATACG TACAGCTGTT TAAGGTTTCT TCCGATTATT   
  
  
- AATCTGAATT TATTATGTAC GTATGATTGG TTTTTTTTTT TTAATTCGTT CTAAGATAGG TTGGATAGTA   
  
  
- TTATTTCAAA CCGGTATGAA GTTTGGTAGT GTATTGTAGT GATTATATAT TTCGATCCAA AACCCTTTGG   
  
  
- ATTGAAACTC CTGGTGTATA AACAATTCCG GATCCTCCGG TACTCAAACA GTTCTACGGA GTATCATACC   
  
  
- TCTCCAGATA TAGGAACGTT TTACTAGTAA GGGAAGATGG ATTTTCTTGG TGAAGTAAAG GTGAAAATGA   
  
  
- GGGACCGTTT ACCAACTATT TGGTTTGGTA TTGGTTCGGG AACTTCTTGA TATTTCTGTG CTTAGCTAGT   
  
  
- AACTTAAGCT TGTTCGGAAC ACACTATCCT CCTACCCGGA GTTGTGAAGA GGCGCTCACA CGTTCTCGTT   
  
  
- AGAGCCTCTT TTTAGGATTC TCGGTTGTTG AAGAAACCCA CAATTTACTT GAACAAAGAG GAATACCGCT   
  
  
- AACGCTTGTT TCTAACCGTA TAATGAAGAA TGTTCACAAC AACCGGTTTC GGTTGTTAAA CCCTGGAGTG   
  
  
- AAAGTACTCT CGGATTTTGA ACGGTACCTC TTTTTGACGA CGAAACTATG GATGTACTTC GAATATAACT   
  
  
- TCAAGGTTCT CCAGTCAGGT ACCTGTTGGA AACCAGTACA CCGAAGTTTA CCACGTTATA ACCTCCTGGA   
  
  
- ATCTTCAAAT GTTTTCAGCT AGTAGTTTCT TTGTTCCGTT TCCTACCTCT TCAAAAGTTC CGATTACCCA   
  
  
- CAAGGGAAGT TCAAAGTACA GTATTTGCTC AATCTATTGG ATCCTCTCGA AGCTTTTCTC CTAAATCTGT   
  
  
- AACTTCTACC ACTCCGGTAG CGACACTTGA CACAAGTTCG GAACGTTGCC CAAGTACACC TCTTCTCCCT   
  
  
- CGTACACGAA CTACACTAAG CTAGATAATC CGGAACATAG TATTGTGACC ACCTCCTTCT TCGTCTAGAG   
  
  
- TGAAGATGTT CTTTGCTGAA GAAGTTCACG AAGCTACTCA CAAACTCTAA AAGATTCAGT ATAAAGCTAT   
  
  
- ACAATCTTCT TTCGAAGGGA GGTTATTCGT TGCTTGCTTA GTTCGATCTT TCCCTTGTTA CCTACTTATA   
  
  
- GAGGTCTCGG GATCGAACAG TACCACTTTC ACCTCCTCTT ATATCCGGTT TCTTTCCTTG AGTTACCTTA   
  
  
- CTCTACGAGC TTGTTCGTAA AACGGGTAGA GTTAAATCAC TACTACATGA TTCACTACAA TCCCGTAACA   
  
  
- ACTTTTCTGT GTTTTCACCA ACCCTAGAGT GGAATGGTGT TTCACTGGTA CTTTATCCGT ATGTGAATTT   
  
  
- AACCTTCCCA CTTTTACAAC AAACCCGAAG ACGTACCTCT GGATCGAT

+     MBS

| Site Name | Organism | Position | Strand | Matrix score. | sequence | function |
| --- | --- | --- | --- | --- | --- | --- |
| MBS | Arabidopsis thaliana | 405 | - | 6 | CAACTG | MYB binding site involved in drought-inducibility |

>HU02G03154.1   
+ +Up\_Stream \_Len000ATGATA AAAGATATTT ATTAAGCTCA AGCTATTTTA ACTTTTAAAA AAATGAATGT   
  
  
+ GGCTTTTTTT GCTATTCAGG TACAACACAA TATGAAGCAT TAACTCTAGA AAATGTGGTA ATGTATGTCA   
  
  
+ TATAGGAGAG TATATACTCT CACTTGCCAT GCGATGCGTG CATGTGTGTG TGTGTGTGTA TCTGTTTCTA   
  
  
+ TGCATATTTT ATGTGGGGTT TTGTCATCTA GACATCTATT AATATGTTAG CATTCACATG ACTTATAGGA   
  
  
+ ATGCACTATT ATGAGTATGA TATAGAGTTT TAAAAATGCA TATGCCTTGT GTGTTCATTA GCTTAAAGAA   
  
  
+ GAGTTTTTGA ATATACATAT TTAGGTAATT CTTGCTAATG TCGTAATTAG CAGTTGTATC TATCTTATTG   
  
  
+ ATTTGTATTT TTAATTGCTT GTTATACTGT TACACTTAGA AAATTCATGA ATACTCTTAA AATATGCAAA   
  
  
+ TTATATATAT ATATATATAT ATATATATAT ATATATATAT ATAAAAACTG AATAAGTTAA TATGGTGATG   
  
  
+ ACATTTGTCA TGTTATCATT GGTCGCTTAA TTTTTAAATA AAAAATATGA TTTTGTGACA CCTAATAAAT   
  
  
+ GATATTAGCC ATATTATTAA ATTCAGCATT TATTCATCAA TGTAATATAC TAAATCATGC AATTATCATT   
  
  
+ GGTTCTCTTT TATGTAATAA AGTTAATTGA CAATTCAAGG GTTACTAATT AGTATAATGT TGAATTGGAA   
  
  
+ CGTAAAAATC AAATCGTATG TAAAGTAATT TTATTCTGAA TTTAAAAAAT GGAGCAAATA TCTCTATTGT   
  
  
+ TCTTTAATAA AATTTTAATT GATTTTTATT TCTAATTAAG TGACGGAACC AAATTTTTTT CCTCCTTTTT   
  
  
+ TGGTTTGGTA AGTTATCATT AGCCAATCTT TTATTTACAT TTTGTCAGTT TTTCTTTTTT AAGATGGTTA   
  
  
+ AATGGCTTCA ATTAAACAAA TTTTTTTACT TATTTATATT TTTAATAAAT TTTCAAAATG TATATTAACG   
  
  
+ CATGACCGTG CGAAGCACGG AATCTACCCT AGTTAATCTA TGATTCTTAA ATATGCAAGT AGGCTGTTAT   
  
  
+ TCATTTGTTG TTTTTTACTT ATTTATATTT TTTCCCTCCC GGTATGATAG GGTTTGTCAT ATTGTTATTC   
  
  
+ ATTAGCTGTT AAACTGTATT CATTATAGCA CAAGTAGGCT TAATCAAAGA TAGTTAGTCT CCAATTTCAT   
  
  
+ CAGAAAATTT AAGAAAAAGA CACCTTAGGA TCAAAAGTAT GAAATTTAGA GACCAGACAA CGATAGAATT   
  
  
+ GAAAAGATGA GTTTTCAATT TGGAGAAGAC GGAAACTCAG ATGAATGAAA GTATAGTACA TTGTACTCCT   
  
  
+ TGGAGTATAA TCTTATCTTC ACTATTGATA GGCTCGAAGC ATAATCAGTG ATTGGAAACT TTTGGTTGCA   
  
  
+ ATAATTCAAA ATTTGATACT CTATGATTTA CTTTTTTAAT TGAAGGTCAT GATTTAATTA ACTAAATGAA   
  
  
+ AAGAATTTAT GATCGAAAAA TTTTAACTCT TATTGACTTA TAGTTGACTC CAGTTAAATG GAAAATTGAA   
  
  
+ GTGATTAATC TAAAAGTGGC CCGCTCTAAT ACCATTTGAG AAAAATTATT CTTACTGCAA AGTTTAAATC   
  
  
+ AATATGTGAA GATAGTTTAT AGTTTTATCC TAAAAGAAAT TAAATAGATT GTTAAAAATA ATTTTTAAAT   
  
  
+ TCAGATATAC GTGTGATGGC CATAACCGTA AATGATATGC ATGTCGACAA ATTCCAAAGA AGGCTAATAA   
  
  
+ TTAGACTTAA ATAATACATG CATACTAACC AAAAAAAAAA AATTAAGCAA GATTCTATCC AACCTATCAT   
  
  
+ AATAAAGTTT GGCCATACTT CAAACCATCA CATAACATCA CTAATATATA AAGCTAGGTT TTGGGAAACC   
  
  
+ TAACTTTGAG GACCACATAT TTGTTAAGGC CTAGGAGGCC ATGAGTTTGT CAAGATGCCT CATAGTATGG   
  
  
+ AGAGGTCTAT ATCCTTGCAA AATGATCATT CCCTTCTACC TAAAAGAACC ACTTCATTTC CACTTTTACT   
  
  
+ CCCTGGCAAA TGGTTGATAA ACCAAACCAT AACCAAGCCC TTGAAGAACT ATAAAGACAC GAATCGATCA   
  
  
+ TTGAATTCGA ACAAGCCTTG TGTGATAGGA GGATGGGCCT CAACACTTCT CCGCGAGTGT GCAAGAGCAA   
  
  
+ TCTCGGAGAA AAATCCTAAG AGCCAACAAC TTCTTTGGGT GTTAAATGAA CTTGTTTCTC CTTATGGCGA   
  
  
+ TTGCGAACAA AGATTGGCAT ATTACTTCTT ACAAGTGTTG TTGGCCAAAG CCAACAATTT GGGACCTCAC   
  
  
+ TTTCATGAGA GCCTAAAACT TGCCATGGAG AAAAACTGCT GCTTTGATAC CTACATGAAG CTTATATTGA   
  
  
+ AGTTCCAAGA GGTCAGTCCA TGGACAACCT TTGGTCATGT GGCTTCAAAT GGTGCAATAT TGGAGGACCT   
  
  
+ TAGAAGTTTA CAAAAGTCGA TCATCAAAGA AACAAGGCAA AGGATGGAGA AGTTTTCAAG GCTAATGGGT   
  
  
+ GTTCCCTTCA AGTTTCATGT CATAAACGAG TTAGATAACC TAGGAGAGCT TCGAAAAGAG GATTTAGACA   
  
  
+ TTGAAGATGG TGAGGCCATC GCTGTGAACT GTGTTCAAGC CTTGCAACGG GTTCATGTGG AGAAGAGGGA   
  
  
+ GCATGTGCTT GATGTGATTC GATCTATTAG GCCTTGTATC ATAACACTGG TGGAGGAAGA AGCAGATCTC   
  
  
+ ACTTCTACAA GAAACGACTT CTTCAAGTGC TTCGATGAGT GTTTGAGATT TTCTAAGTCA TATTTCGATA   
  
  
+ TGTTAGAAGA AAGCTTCCCT CCAATAAGCA ACGAACGAAT CAAGCTAGAA AGGGAACAAT GGATGAATAT   
  
  
+ CTCCAGAGCC CTAGCTTGTC ATGGTGAAAG TGGAGGAGAA TATAGGCCAA AGAAAGGAAC TCAATGGAAT   
  
  
+ GAGATGCTCG AACAAGCATT TTGCCCATCT CAATTTAGTG ATGATGTACT AAGTGATGTT AGGGCATTGT   
  
  
+ TGAAAAGACA CAAAAGTGGT TGGGATCTCA CCTTACCACA AAGTGACCAT GAAATAGGCA TACACTTAAA   
  
  
+ TTGGAAGGGT GAAAATGTTG TTTGGGCTTC TGCATGGAGA CCTAGCTA  

- +Up\_Stream \_Len000TACTAT TTTCTATAAA TAATTCGAGT TCGATAAAAT TGAAAATTTT TTTACTTACA   
  
  
- CCGAAAAAAA CGATAAGTCC ATGTTGTGTT ATACTTCGTA ATTGAGATCT TTTACACCAT TACATACAGT   
  
  
- ATATCCTCTC ATATATGAGA GTGAACGGTA CGCTACGCAC GTACACACAC ACACACACAT AGACAAAGAT   
  
  
- ACGTATAAAA TACACCCCAA AACAGTAGAT CTGTAGATAA TTATACAATC GTAAGTGTAC TGAATATCCT   
  
  
- TACGTGATAA TACTCATACT ATATCTCAAA ATTTTTACGT ATACGGAACA CACAAGTAAT CGAATTTCTT   
  
  
- CTCAAAAACT TATATGTATA AATCCATTAA GAACGATTAC AGCATTAATC GTCAACATAG ATAGAATAAC   
  
  
- TAAACATAAA AATTAACGAA CAATATGACA ATGTGAATCT TTTAAGTACT TATGAGAATT TTATACGTTT   
  
  
- AATATATATA TATATATATA TATATATATA TATATATATA TATTTTTGAC TTATTCAATT ATACCACTAC   
  
  
- TGTAAACAGT ACAATAGTAA CCAGCGAATT AAAAATTTAT TTTTTATACT AAAACACTGT GGATTATTTA   
  
  
- CTATAATCGG TATAATAATT TAAGTCGTAA ATAAGTAGTT ACATTATATG ATTTAGTACG TTAATAGTAA   
  
  
- CCAAGAGAAA ATACATTATT TCAATTAACT GTTAAGTTCC CAATGATTAA TCATATTACA ACTTAACCTT   
  
  
- GCATTTTTAG TTTAGCATAC ATTTCATTAA AATAAGACTT AAATTTTTTA CCTCGTTTAT AGAGATAACA   
  
  
- AGAAATTATT TTAAAATTAA CTAAAAATAA AGATTAATTC ACTGCCTTGG TTTAAAAAAA GGAGGAAAAA   
  
  
- ACCAAACCAT TCAATAGTAA TCGGTTAGAA AATAAATGTA AAACAGTCAA AAAGAAAAAA TTCTACCAAT   
  
  
- TTACCGAAGT TAATTTGTTT AAAAAAATGA ATAAATATAA AAATTATTTA AAAGTTTTAC ATATAATTGC   
  
  
- GTACTGGCAC GCTTCGTGCC TTAGATGGGA TCAATTAGAT ACTAAGAATT TATACGTTCA TCCGACAATA   
  
  
- AGTAAACAAC AAAAAATGAA TAAATATAAA AAAGGGAGGG CCATACTATC CCAAACAGTA TAACAATAAG   
  
  
- TAATCGACAA TTTGACATAA GTAATATCGT GTTCATCCGA ATTAGTTTCT ATCAATCAGA GGTTAAAGTA   
  
  
- GTCTTTTAAA TTCTTTTTCT GTGGAATCCT AGTTTTCATA CTTTAAATCT CTGGTCTGTT GCTATCTTAA   
  
  
- CTTTTCTACT CAAAAGTTAA ACCTCTTCTG CCTTTGAGTC TACTTACTTT CATATCATGT AACATGAGGA   
  
  
- ACCTCATATT AGAATAGAAG TGATAACTAT CCGAGCTTCG TATTAGTCAC TAACCTTTGA AAACCAACGT   
  
  
- TATTAAGTTT TAAACTATGA GATACTAAAT GAAAAAATTA ACTTCCAGTA CTAAATTAAT TGATTTACTT   
  
  
- TTCTTAAATA CTAGCTTTTT AAAATTGAGA ATAACTGAAT ATCAACTGAG GTCAATTTAC CTTTTAACTT   
  
  
- CACTAATTAG ATTTTCACCG GGCGAGATTA TGGTAAACTC TTTTTAATAA GAATGACGTT TCAAATTTAG   
  
  
- TTATACACTT CTATCAAATA TCAAAATAGG ATTTTCTTTA ATTTATCTAA CAATTTTTAT TAAAAATTTA   
  
  
- AGTCTATATG CACACTACCG GTATTGGCAT TTACTATACG TACAGCTGTT TAAGGTTTCT TCCGATTATT   
  
  
- AATCTGAATT TATTATGTAC GTATGATTGG TTTTTTTTTT TTAATTCGTT CTAAGATAGG TTGGATAGTA   
  
  
- TTATTTCAAA CCGGTATGAA GTTTGGTAGT GTATTGTAGT GATTATATAT TTCGATCCAA AACCCTTTGG   
  
  
- ATTGAAACTC CTGGTGTATA AACAATTCCG GATCCTCCGG TACTCAAACA GTTCTACGGA GTATCATACC   
  
  
- TCTCCAGATA TAGGAACGTT TTACTAGTAA GGGAAGATGG ATTTTCTTGG TGAAGTAAAG GTGAAAATGA   
  
  
- GGGACCGTTT ACCAACTATT TGGTTTGGTA TTGGTTCGGG AACTTCTTGA TATTTCTGTG CTTAGCTAGT   
  
  
- AACTTAAGCT TGTTCGGAAC ACACTATCCT CCTACCCGGA GTTGTGAAGA GGCGCTCACA CGTTCTCGTT   
  
  
- AGAGCCTCTT TTTAGGATTC TCGGTTGTTG AAGAAACCCA CAATTTACTT GAACAAAGAG GAATACCGCT   
  
  
- AACGCTTGTT TCTAACCGTA TAATGAAGAA TGTTCACAAC AACCGGTTTC GGTTGTTAAA CCCTGGAGTG   
  
  
- AAAGTACTCT CGGATTTTGA ACGGTACCTC TTTTTGACGA CGAAACTATG GATGTACTTC GAATATAACT   
  
  
- TCAAGGTTCT CCAGTCAGGT ACCTGTTGGA AACCAGTACA CCGAAGTTTA CCACGTTATA ACCTCCTGGA   
  
  
- ATCTTCAAAT GTTTTCAGCT AGTAGTTTCT TTGTTCCGTT TCCTACCTCT TCAAAAGTTC CGATTACCCA   
  
  
- CAAGGGAAGT TCAAAGTACA GTATTTGCTC AATCTATTGG ATCCTCTCGA AGCTTTTCTC CTAAATCTGT   
  
  
- AACTTCTACC ACTCCGGTAG CGACACTTGA CACAAGTTCG GAACGTTGCC CAAGTACACC TCTTCTCCCT   
  
  
- CGTACACGAA CTACACTAAG CTAGATAATC CGGAACATAG TATTGTGACC ACCTCCTTCT TCGTCTAGAG   
  
  
- TGAAGATGTT CTTTGCTGAA GAAGTTCACG AAGCTACTCA CAAACTCTAA AAGATTCAGT ATAAAGCTAT   
  
  
- ACAATCTTCT TTCGAAGGGA GGTTATTCGT TGCTTGCTTA GTTCGATCTT TCCCTTGTTA CCTACTTATA   
  
  
- GAGGTCTCGG GATCGAACAG TACCACTTTC ACCTCCTCTT ATATCCGGTT TCTTTCCTTG AGTTACCTTA   
  
  
- CTCTACGAGC TTGTTCGTAA AACGGGTAGA GTTAAATCAC TACTACATGA TTCACTACAA TCCCGTAACA   
  
  
- ACTTTTCTGT GTTTTCACCA ACCCTAGAGT GGAATGGTGT TTCACTGGTA CTTTATCCGT ATGTGAATTT   
  
  
- AACCTTCCCA CTTTTACAAC AAACCCGAAG ACGTACCTCT GGATCGAT

+     MRE

| Site Name | Organism | Position | Strand | Matrix score. | sequence | function |
| --- | --- | --- | --- | --- | --- | --- |
| MRE | Petroselinum crispum | 1961 | + | 7 | AACCTAA | MYB binding site involved in light responsiveness |

>HU02G03154.1   
+ +Up\_Stream \_Len000ATGATA AAAGATATTT ATTAAGCTCA AGCTATTTTA ACTTTTAAAA AAATGAATGT   
  
  
+ GGCTTTTTTT GCTATTCAGG TACAACACAA TATGAAGCAT TAACTCTAGA AAATGTGGTA ATGTATGTCA   
  
  
+ TATAGGAGAG TATATACTCT CACTTGCCAT GCGATGCGTG CATGTGTGTG TGTGTGTGTA TCTGTTTCTA   
  
  
+ TGCATATTTT ATGTGGGGTT TTGTCATCTA GACATCTATT AATATGTTAG CATTCACATG ACTTATAGGA   
  
  
+ ATGCACTATT ATGAGTATGA TATAGAGTTT TAAAAATGCA TATGCCTTGT GTGTTCATTA GCTTAAAGAA   
  
  
+ GAGTTTTTGA ATATACATAT TTAGGTAATT CTTGCTAATG TCGTAATTAG CAGTTGTATC TATCTTATTG   
  
  
+ ATTTGTATTT TTAATTGCTT GTTATACTGT TACACTTAGA AAATTCATGA ATACTCTTAA AATATGCAAA   
  
  
+ TTATATATAT ATATATATAT ATATATATAT ATATATATAT ATAAAAACTG AATAAGTTAA TATGGTGATG   
  
  
+ ACATTTGTCA TGTTATCATT GGTCGCTTAA TTTTTAAATA AAAAATATGA TTTTGTGACA CCTAATAAAT   
  
  
+ GATATTAGCC ATATTATTAA ATTCAGCATT TATTCATCAA TGTAATATAC TAAATCATGC AATTATCATT   
  
  
+ GGTTCTCTTT TATGTAATAA AGTTAATTGA CAATTCAAGG GTTACTAATT AGTATAATGT TGAATTGGAA   
  
  
+ CGTAAAAATC AAATCGTATG TAAAGTAATT TTATTCTGAA TTTAAAAAAT GGAGCAAATA TCTCTATTGT   
  
  
+ TCTTTAATAA AATTTTAATT GATTTTTATT TCTAATTAAG TGACGGAACC AAATTTTTTT CCTCCTTTTT   
  
  
+ TGGTTTGGTA AGTTATCATT AGCCAATCTT TTATTTACAT TTTGTCAGTT TTTCTTTTTT AAGATGGTTA   
  
  
+ AATGGCTTCA ATTAAACAAA TTTTTTTACT TATTTATATT TTTAATAAAT TTTCAAAATG TATATTAACG   
  
  
+ CATGACCGTG CGAAGCACGG AATCTACCCT AGTTAATCTA TGATTCTTAA ATATGCAAGT AGGCTGTTAT   
  
  
+ TCATTTGTTG TTTTTTACTT ATTTATATTT TTTCCCTCCC GGTATGATAG GGTTTGTCAT ATTGTTATTC   
  
  
+ ATTAGCTGTT AAACTGTATT CATTATAGCA CAAGTAGGCT TAATCAAAGA TAGTTAGTCT CCAATTTCAT   
  
  
+ CAGAAAATTT AAGAAAAAGA CACCTTAGGA TCAAAAGTAT GAAATTTAGA GACCAGACAA CGATAGAATT   
  
  
+ GAAAAGATGA GTTTTCAATT TGGAGAAGAC GGAAACTCAG ATGAATGAAA GTATAGTACA TTGTACTCCT   
  
  
+ TGGAGTATAA TCTTATCTTC ACTATTGATA GGCTCGAAGC ATAATCAGTG ATTGGAAACT TTTGGTTGCA   
  
  
+ ATAATTCAAA ATTTGATACT CTATGATTTA CTTTTTTAAT TGAAGGTCAT GATTTAATTA ACTAAATGAA   
  
  
+ AAGAATTTAT GATCGAAAAA TTTTAACTCT TATTGACTTA TAGTTGACTC CAGTTAAATG GAAAATTGAA   
  
  
+ GTGATTAATC TAAAAGTGGC CCGCTCTAAT ACCATTTGAG AAAAATTATT CTTACTGCAA AGTTTAAATC   
  
  
+ AATATGTGAA GATAGTTTAT AGTTTTATCC TAAAAGAAAT TAAATAGATT GTTAAAAATA ATTTTTAAAT   
  
  
+ TCAGATATAC GTGTGATGGC CATAACCGTA AATGATATGC ATGTCGACAA ATTCCAAAGA AGGCTAATAA   
  
  
+ TTAGACTTAA ATAATACATG CATACTAACC AAAAAAAAAA AATTAAGCAA GATTCTATCC AACCTATCAT   
  
  
+ AATAAAGTTT GGCCATACTT CAAACCATCA CATAACATCA CTAATATATA AAGCTAGGTT TTGGGAAACC   
  
  
+ TAACTTTGAG GACCACATAT TTGTTAAGGC CTAGGAGGCC ATGAGTTTGT CAAGATGCCT CATAGTATGG   
  
  
+ AGAGGTCTAT ATCCTTGCAA AATGATCATT CCCTTCTACC TAAAAGAACC ACTTCATTTC CACTTTTACT   
  
  
+ CCCTGGCAAA TGGTTGATAA ACCAAACCAT AACCAAGCCC TTGAAGAACT ATAAAGACAC GAATCGATCA   
  
  
+ TTGAATTCGA ACAAGCCTTG TGTGATAGGA GGATGGGCCT CAACACTTCT CCGCGAGTGT GCAAGAGCAA   
  
  
+ TCTCGGAGAA AAATCCTAAG AGCCAACAAC TTCTTTGGGT GTTAAATGAA CTTGTTTCTC CTTATGGCGA   
  
  
+ TTGCGAACAA AGATTGGCAT ATTACTTCTT ACAAGTGTTG TTGGCCAAAG CCAACAATTT GGGACCTCAC   
  
  
+ TTTCATGAGA GCCTAAAACT TGCCATGGAG AAAAACTGCT GCTTTGATAC CTACATGAAG CTTATATTGA   
  
  
+ AGTTCCAAGA GGTCAGTCCA TGGACAACCT TTGGTCATGT GGCTTCAAAT GGTGCAATAT TGGAGGACCT   
  
  
+ TAGAAGTTTA CAAAAGTCGA TCATCAAAGA AACAAGGCAA AGGATGGAGA AGTTTTCAAG GCTAATGGGT   
  
  
+ GTTCCCTTCA AGTTTCATGT CATAAACGAG TTAGATAACC TAGGAGAGCT TCGAAAAGAG GATTTAGACA   
  
  
+ TTGAAGATGG TGAGGCCATC GCTGTGAACT GTGTTCAAGC CTTGCAACGG GTTCATGTGG AGAAGAGGGA   
  
  
+ GCATGTGCTT GATGTGATTC GATCTATTAG GCCTTGTATC ATAACACTGG TGGAGGAAGA AGCAGATCTC   
  
  
+ ACTTCTACAA GAAACGACTT CTTCAAGTGC TTCGATGAGT GTTTGAGATT TTCTAAGTCA TATTTCGATA   
  
  
+ TGTTAGAAGA AAGCTTCCCT CCAATAAGCA ACGAACGAAT CAAGCTAGAA AGGGAACAAT GGATGAATAT   
  
  
+ CTCCAGAGCC CTAGCTTGTC ATGGTGAAAG TGGAGGAGAA TATAGGCCAA AGAAAGGAAC TCAATGGAAT   
  
  
+ GAGATGCTCG AACAAGCATT TTGCCCATCT CAATTTAGTG ATGATGTACT AAGTGATGTT AGGGCATTGT   
  
  
+ TGAAAAGACA CAAAAGTGGT TGGGATCTCA CCTTACCACA AAGTGACCAT GAAATAGGCA TACACTTAAA   
  
  
+ TTGGAAGGGT GAAAATGTTG TTTGGGCTTC TGCATGGAGA CCTAGCTA  

- +Up\_Stream \_Len000TACTAT TTTCTATAAA TAATTCGAGT TCGATAAAAT TGAAAATTTT TTTACTTACA   
  
  
- CCGAAAAAAA CGATAAGTCC ATGTTGTGTT ATACTTCGTA ATTGAGATCT TTTACACCAT TACATACAGT   
  
  
- ATATCCTCTC ATATATGAGA GTGAACGGTA CGCTACGCAC GTACACACAC ACACACACAT AGACAAAGAT   
  
  
- ACGTATAAAA TACACCCCAA AACAGTAGAT CTGTAGATAA TTATACAATC GTAAGTGTAC TGAATATCCT   
  
  
- TACGTGATAA TACTCATACT ATATCTCAAA ATTTTTACGT ATACGGAACA CACAAGTAAT CGAATTTCTT   
  
  
- CTCAAAAACT TATATGTATA AATCCATTAA GAACGATTAC AGCATTAATC GTCAACATAG ATAGAATAAC   
  
  
- TAAACATAAA AATTAACGAA CAATATGACA ATGTGAATCT TTTAAGTACT TATGAGAATT TTATACGTTT   
  
  
- AATATATATA TATATATATA TATATATATA TATATATATA TATTTTTGAC TTATTCAATT ATACCACTAC   
  
  
- TGTAAACAGT ACAATAGTAA CCAGCGAATT AAAAATTTAT TTTTTATACT AAAACACTGT GGATTATTTA   
  
  
- CTATAATCGG TATAATAATT TAAGTCGTAA ATAAGTAGTT ACATTATATG ATTTAGTACG TTAATAGTAA   
  
  
- CCAAGAGAAA ATACATTATT TCAATTAACT GTTAAGTTCC CAATGATTAA TCATATTACA ACTTAACCTT   
  
  
- GCATTTTTAG TTTAGCATAC ATTTCATTAA AATAAGACTT AAATTTTTTA CCTCGTTTAT AGAGATAACA   
  
  
- AGAAATTATT TTAAAATTAA CTAAAAATAA AGATTAATTC ACTGCCTTGG TTTAAAAAAA GGAGGAAAAA   
  
  
- ACCAAACCAT TCAATAGTAA TCGGTTAGAA AATAAATGTA AAACAGTCAA AAAGAAAAAA TTCTACCAAT   
  
  
- TTACCGAAGT TAATTTGTTT AAAAAAATGA ATAAATATAA AAATTATTTA AAAGTTTTAC ATATAATTGC   
  
  
- GTACTGGCAC GCTTCGTGCC TTAGATGGGA TCAATTAGAT ACTAAGAATT TATACGTTCA TCCGACAATA   
  
  
- AGTAAACAAC AAAAAATGAA TAAATATAAA AAAGGGAGGG CCATACTATC CCAAACAGTA TAACAATAAG   
  
  
- TAATCGACAA TTTGACATAA GTAATATCGT GTTCATCCGA ATTAGTTTCT ATCAATCAGA GGTTAAAGTA   
  
  
- GTCTTTTAAA TTCTTTTTCT GTGGAATCCT AGTTTTCATA CTTTAAATCT CTGGTCTGTT GCTATCTTAA   
  
  
- CTTTTCTACT CAAAAGTTAA ACCTCTTCTG CCTTTGAGTC TACTTACTTT CATATCATGT AACATGAGGA   
  
  
- ACCTCATATT AGAATAGAAG TGATAACTAT CCGAGCTTCG TATTAGTCAC TAACCTTTGA AAACCAACGT   
  
  
- TATTAAGTTT TAAACTATGA GATACTAAAT GAAAAAATTA ACTTCCAGTA CTAAATTAAT TGATTTACTT   
  
  
- TTCTTAAATA CTAGCTTTTT AAAATTGAGA ATAACTGAAT ATCAACTGAG GTCAATTTAC CTTTTAACTT   
  
  
- CACTAATTAG ATTTTCACCG GGCGAGATTA TGGTAAACTC TTTTTAATAA GAATGACGTT TCAAATTTAG   
  
  
- TTATACACTT CTATCAAATA TCAAAATAGG ATTTTCTTTA ATTTATCTAA CAATTTTTAT TAAAAATTTA   
  
  
- AGTCTATATG CACACTACCG GTATTGGCAT TTACTATACG TACAGCTGTT TAAGGTTTCT TCCGATTATT   
  
  
- AATCTGAATT TATTATGTAC GTATGATTGG TTTTTTTTTT TTAATTCGTT CTAAGATAGG TTGGATAGTA   
  
  
- TTATTTCAAA CCGGTATGAA GTTTGGTAGT GTATTGTAGT GATTATATAT TTCGATCCAA AACCCTTTGG   
  
  
- ATTGAAACTC CTGGTGTATA AACAATTCCG GATCCTCCGG TACTCAAACA GTTCTACGGA GTATCATACC   
  
  
- TCTCCAGATA TAGGAACGTT TTACTAGTAA GGGAAGATGG ATTTTCTTGG TGAAGTAAAG GTGAAAATGA   
  
  
- GGGACCGTTT ACCAACTATT TGGTTTGGTA TTGGTTCGGG AACTTCTTGA TATTTCTGTG CTTAGCTAGT   
  
  
- AACTTAAGCT TGTTCGGAAC ACACTATCCT CCTACCCGGA GTTGTGAAGA GGCGCTCACA CGTTCTCGTT   
  
  
- AGAGCCTCTT TTTAGGATTC TCGGTTGTTG AAGAAACCCA CAATTTACTT GAACAAAGAG GAATACCGCT   
  
  
- AACGCTTGTT TCTAACCGTA TAATGAAGAA TGTTCACAAC AACCGGTTTC GGTTGTTAAA CCCTGGAGTG   
  
  
- AAAGTACTCT CGGATTTTGA ACGGTACCTC TTTTTGACGA CGAAACTATG GATGTACTTC GAATATAACT   
  
  
- TCAAGGTTCT CCAGTCAGGT ACCTGTTGGA AACCAGTACA CCGAAGTTTA CCACGTTATA ACCTCCTGGA   
  
  
- ATCTTCAAAT GTTTTCAGCT AGTAGTTTCT TTGTTCCGTT TCCTACCTCT TCAAAAGTTC CGATTACCCA   
  
  
- CAAGGGAAGT TCAAAGTACA GTATTTGCTC AATCTATTGG ATCCTCTCGA AGCTTTTCTC CTAAATCTGT   
  
  
- AACTTCTACC ACTCCGGTAG CGACACTTGA CACAAGTTCG GAACGTTGCC CAAGTACACC TCTTCTCCCT   
  
  
- CGTACACGAA CTACACTAAG CTAGATAATC CGGAACATAG TATTGTGACC ACCTCCTTCT TCGTCTAGAG   
  
  
- TGAAGATGTT CTTTGCTGAA GAAGTTCACG AAGCTACTCA CAAACTCTAA AAGATTCAGT ATAAAGCTAT   
  
  
- ACAATCTTCT TTCGAAGGGA GGTTATTCGT TGCTTGCTTA GTTCGATCTT TCCCTTGTTA CCTACTTATA   
  
  
- GAGGTCTCGG GATCGAACAG TACCACTTTC ACCTCCTCTT ATATCCGGTT TCTTTCCTTG AGTTACCTTA   
  
  
- CTCTACGAGC TTGTTCGTAA AACGGGTAGA GTTAAATCAC TACTACATGA TTCACTACAA TCCCGTAACA   
  
  
- ACTTTTCTGT GTTTTCACCA ACCCTAGAGT GGAATGGTGT TTCACTGGTA CTTTATCCGT ATGTGAATTT   
  
  
- AACCTTCCCA CTTTTACAAC AAACCCGAAG ACGTACCTCT GGATCGAT

+     MYB

| Site Name | Organism | Position | Strand | Matrix score. | sequence | function |
| --- | --- | --- | --- | --- | --- | --- |
| MYB | Arabidopsis thaliana | 2134 | + | 6 | TAACCA |  |
| MYB | Arabidopsis thaliana | 3101 | - | 6 | CAACCA |  |
| MYB | Arabidopsis thaliana | 979 | - | 6 | TAACCA |  |
| MYB | Arabidopsis thaliana | 2115 | - | 6 | CAACCA |  |
| MYB | Arabidopsis thaliana | 1467 | - | 6 | CAACCA |  |
| MYB | Arabidopsis thaliana | 1850 | + | 6 | TAACCA |  |

>HU02G03154.1   
+ +Up\_Stream \_Len000ATGATA AAAGATATTT ATTAAGCTCA AGCTATTTTA ACTTTTAAAA AAATGAATGT   
  
  
+ GGCTTTTTTT GCTATTCAGG TACAACACAA TATGAAGCAT TAACTCTAGA AAATGTGGTA ATGTATGTCA   
  
  
+ TATAGGAGAG TATATACTCT CACTTGCCAT GCGATGCGTG CATGTGTGTG TGTGTGTGTA TCTGTTTCTA   
  
  
+ TGCATATTTT ATGTGGGGTT TTGTCATCTA GACATCTATT AATATGTTAG CATTCACATG ACTTATAGGA   
  
  
+ ATGCACTATT ATGAGTATGA TATAGAGTTT TAAAAATGCA TATGCCTTGT GTGTTCATTA GCTTAAAGAA   
  
  
+ GAGTTTTTGA ATATACATAT TTAGGTAATT CTTGCTAATG TCGTAATTAG CAGTTGTATC TATCTTATTG   
  
  
+ ATTTGTATTT TTAATTGCTT GTTATACTGT TACACTTAGA AAATTCATGA ATACTCTTAA AATATGCAAA   
  
  
+ TTATATATAT ATATATATAT ATATATATAT ATATATATAT ATAAAAACTG AATAAGTTAA TATGGTGATG   
  
  
+ ACATTTGTCA TGTTATCATT GGTCGCTTAA TTTTTAAATA AAAAATATGA TTTTGTGACA CCTAATAAAT   
  
  
+ GATATTAGCC ATATTATTAA ATTCAGCATT TATTCATCAA TGTAATATAC TAAATCATGC AATTATCATT   
  
  
+ GGTTCTCTTT TATGTAATAA AGTTAATTGA CAATTCAAGG GTTACTAATT AGTATAATGT TGAATTGGAA   
  
  
+ CGTAAAAATC AAATCGTATG TAAAGTAATT TTATTCTGAA TTTAAAAAAT GGAGCAAATA TCTCTATTGT   
  
  
+ TCTTTAATAA AATTTTAATT GATTTTTATT TCTAATTAAG TGACGGAACC AAATTTTTTT CCTCCTTTTT   
  
  
+ TGGTTTGGTA AGTTATCATT AGCCAATCTT TTATTTACAT TTTGTCAGTT TTTCTTTTTT AAGATGGTTA   
  
  
+ AATGGCTTCA ATTAAACAAA TTTTTTTACT TATTTATATT TTTAATAAAT TTTCAAAATG TATATTAACG   
  
  
+ CATGACCGTG CGAAGCACGG AATCTACCCT AGTTAATCTA TGATTCTTAA ATATGCAAGT AGGCTGTTAT   
  
  
+ TCATTTGTTG TTTTTTACTT ATTTATATTT TTTCCCTCCC GGTATGATAG GGTTTGTCAT ATTGTTATTC   
  
  
+ ATTAGCTGTT AAACTGTATT CATTATAGCA CAAGTAGGCT TAATCAAAGA TAGTTAGTCT CCAATTTCAT   
  
  
+ CAGAAAATTT AAGAAAAAGA CACCTTAGGA TCAAAAGTAT GAAATTTAGA GACCAGACAA CGATAGAATT   
  
  
+ GAAAAGATGA GTTTTCAATT TGGAGAAGAC GGAAACTCAG ATGAATGAAA GTATAGTACA TTGTACTCCT   
  
  
+ TGGAGTATAA TCTTATCTTC ACTATTGATA GGCTCGAAGC ATAATCAGTG ATTGGAAACT TTTGGTTGCA   
  
  
+ ATAATTCAAA ATTTGATACT CTATGATTTA CTTTTTTAAT TGAAGGTCAT GATTTAATTA ACTAAATGAA   
  
  
+ AAGAATTTAT GATCGAAAAA TTTTAACTCT TATTGACTTA TAGTTGACTC CAGTTAAATG GAAAATTGAA   
  
  
+ GTGATTAATC TAAAAGTGGC CCGCTCTAAT ACCATTTGAG AAAAATTATT CTTACTGCAA AGTTTAAATC   
  
  
+ AATATGTGAA GATAGTTTAT AGTTTTATCC TAAAAGAAAT TAAATAGATT GTTAAAAATA ATTTTTAAAT   
  
  
+ TCAGATATAC GTGTGATGGC CATAACCGTA AATGATATGC ATGTCGACAA ATTCCAAAGA AGGCTAATAA   
  
  
+ TTAGACTTAA ATAATACATG CATACTAACC AAAAAAAAAA AATTAAGCAA GATTCTATCC AACCTATCAT   
  
  
+ AATAAAGTTT GGCCATACTT CAAACCATCA CATAACATCA CTAATATATA AAGCTAGGTT TTGGGAAACC   
  
  
+ TAACTTTGAG GACCACATAT TTGTTAAGGC CTAGGAGGCC ATGAGTTTGT CAAGATGCCT CATAGTATGG   
  
  
+ AGAGGTCTAT ATCCTTGCAA AATGATCATT CCCTTCTACC TAAAAGAACC ACTTCATTTC CACTTTTACT   
  
  
+ CCCTGGCAAA TGGTTGATAA ACCAAACCAT AACCAAGCCC TTGAAGAACT ATAAAGACAC GAATCGATCA   
  
  
+ TTGAATTCGA ACAAGCCTTG TGTGATAGGA GGATGGGCCT CAACACTTCT CCGCGAGTGT GCAAGAGCAA   
  
  
+ TCTCGGAGAA AAATCCTAAG AGCCAACAAC TTCTTTGGGT GTTAAATGAA CTTGTTTCTC CTTATGGCGA   
  
  
+ TTGCGAACAA AGATTGGCAT ATTACTTCTT ACAAGTGTTG TTGGCCAAAG CCAACAATTT GGGACCTCAC   
  
  
+ TTTCATGAGA GCCTAAAACT TGCCATGGAG AAAAACTGCT GCTTTGATAC CTACATGAAG CTTATATTGA   
  
  
+ AGTTCCAAGA GGTCAGTCCA TGGACAACCT TTGGTCATGT GGCTTCAAAT GGTGCAATAT TGGAGGACCT   
  
  
+ TAGAAGTTTA CAAAAGTCGA TCATCAAAGA AACAAGGCAA AGGATGGAGA AGTTTTCAAG GCTAATGGGT   
  
  
+ GTTCCCTTCA AGTTTCATGT CATAAACGAG TTAGATAACC TAGGAGAGCT TCGAAAAGAG GATTTAGACA   
  
  
+ TTGAAGATGG TGAGGCCATC GCTGTGAACT GTGTTCAAGC CTTGCAACGG GTTCATGTGG AGAAGAGGGA   
  
  
+ GCATGTGCTT GATGTGATTC GATCTATTAG GCCTTGTATC ATAACACTGG TGGAGGAAGA AGCAGATCTC   
  
  
+ ACTTCTACAA GAAACGACTT CTTCAAGTGC TTCGATGAGT GTTTGAGATT TTCTAAGTCA TATTTCGATA   
  
  
+ TGTTAGAAGA AAGCTTCCCT CCAATAAGCA ACGAACGAAT CAAGCTAGAA AGGGAACAAT GGATGAATAT   
  
  
+ CTCCAGAGCC CTAGCTTGTC ATGGTGAAAG TGGAGGAGAA TATAGGCCAA AGAAAGGAAC TCAATGGAAT   
  
  
+ GAGATGCTCG AACAAGCATT TTGCCCATCT CAATTTAGTG ATGATGTACT AAGTGATGTT AGGGCATTGT   
  
  
+ TGAAAAGACA CAAAAGTGGT TGGGATCTCA CCTTACCACA AAGTGACCAT GAAATAGGCA TACACTTAAA   
  
  
+ TTGGAAGGGT GAAAATGTTG TTTGGGCTTC TGCATGGAGA CCTAGCTA  

- +Up\_Stream \_Len000TACTAT TTTCTATAAA TAATTCGAGT TCGATAAAAT TGAAAATTTT TTTACTTACA   
  
  
- CCGAAAAAAA CGATAAGTCC ATGTTGTGTT ATACTTCGTA ATTGAGATCT TTTACACCAT TACATACAGT   
  
  
- ATATCCTCTC ATATATGAGA GTGAACGGTA CGCTACGCAC GTACACACAC ACACACACAT AGACAAAGAT   
  
  
- ACGTATAAAA TACACCCCAA AACAGTAGAT CTGTAGATAA TTATACAATC GTAAGTGTAC TGAATATCCT   
  
  
- TACGTGATAA TACTCATACT ATATCTCAAA ATTTTTACGT ATACGGAACA CACAAGTAAT CGAATTTCTT   
  
  
- CTCAAAAACT TATATGTATA AATCCATTAA GAACGATTAC AGCATTAATC GTCAACATAG ATAGAATAAC   
  
  
- TAAACATAAA AATTAACGAA CAATATGACA ATGTGAATCT TTTAAGTACT TATGAGAATT TTATACGTTT   
  
  
- AATATATATA TATATATATA TATATATATA TATATATATA TATTTTTGAC TTATTCAATT ATACCACTAC   
  
  
- TGTAAACAGT ACAATAGTAA CCAGCGAATT AAAAATTTAT TTTTTATACT AAAACACTGT GGATTATTTA   
  
  
- CTATAATCGG TATAATAATT TAAGTCGTAA ATAAGTAGTT ACATTATATG ATTTAGTACG TTAATAGTAA   
  
  
- CCAAGAGAAA ATACATTATT TCAATTAACT GTTAAGTTCC CAATGATTAA TCATATTACA ACTTAACCTT   
  
  
- GCATTTTTAG TTTAGCATAC ATTTCATTAA AATAAGACTT AAATTTTTTA CCTCGTTTAT AGAGATAACA   
  
  
- AGAAATTATT TTAAAATTAA CTAAAAATAA AGATTAATTC ACTGCCTTGG TTTAAAAAAA GGAGGAAAAA   
  
  
- ACCAAACCAT TCAATAGTAA TCGGTTAGAA AATAAATGTA AAACAGTCAA AAAGAAAAAA TTCTACCAAT   
  
  
- TTACCGAAGT TAATTTGTTT AAAAAAATGA ATAAATATAA AAATTATTTA AAAGTTTTAC ATATAATTGC   
  
  
- GTACTGGCAC GCTTCGTGCC TTAGATGGGA TCAATTAGAT ACTAAGAATT TATACGTTCA TCCGACAATA   
  
  
- AGTAAACAAC AAAAAATGAA TAAATATAAA AAAGGGAGGG CCATACTATC CCAAACAGTA TAACAATAAG   
  
  
- TAATCGACAA TTTGACATAA GTAATATCGT GTTCATCCGA ATTAGTTTCT ATCAATCAGA GGTTAAAGTA   
  
  
- GTCTTTTAAA TTCTTTTTCT GTGGAATCCT AGTTTTCATA CTTTAAATCT CTGGTCTGTT GCTATCTTAA   
  
  
- CTTTTCTACT CAAAAGTTAA ACCTCTTCTG CCTTTGAGTC TACTTACTTT CATATCATGT AACATGAGGA   
  
  
- ACCTCATATT AGAATAGAAG TGATAACTAT CCGAGCTTCG TATTAGTCAC TAACCTTTGA AAACCAACGT   
  
  
- TATTAAGTTT TAAACTATGA GATACTAAAT GAAAAAATTA ACTTCCAGTA CTAAATTAAT TGATTTACTT   
  
  
- TTCTTAAATA CTAGCTTTTT AAAATTGAGA ATAACTGAAT ATCAACTGAG GTCAATTTAC CTTTTAACTT   
  
  
- CACTAATTAG ATTTTCACCG GGCGAGATTA TGGTAAACTC TTTTTAATAA GAATGACGTT TCAAATTTAG   
  
  
- TTATACACTT CTATCAAATA TCAAAATAGG ATTTTCTTTA ATTTATCTAA CAATTTTTAT TAAAAATTTA   
  
  
- AGTCTATATG CACACTACCG GTATTGGCAT TTACTATACG TACAGCTGTT TAAGGTTTCT TCCGATTATT   
  
  
- AATCTGAATT TATTATGTAC GTATGATTGG TTTTTTTTTT TTAATTCGTT CTAAGATAGG TTGGATAGTA   
  
  
- TTATTTCAAA CCGGTATGAA GTTTGGTAGT GTATTGTAGT GATTATATAT TTCGATCCAA AACCCTTTGG   
  
  
- ATTGAAACTC CTGGTGTATA AACAATTCCG GATCCTCCGG TACTCAAACA GTTCTACGGA GTATCATACC   
  
  
- TCTCCAGATA TAGGAACGTT TTACTAGTAA GGGAAGATGG ATTTTCTTGG TGAAGTAAAG GTGAAAATGA   
  
  
- GGGACCGTTT ACCAACTATT TGGTTTGGTA TTGGTTCGGG AACTTCTTGA TATTTCTGTG CTTAGCTAGT   
  
  
- AACTTAAGCT TGTTCGGAAC ACACTATCCT CCTACCCGGA GTTGTGAAGA GGCGCTCACA CGTTCTCGTT   
  
  
- AGAGCCTCTT TTTAGGATTC TCGGTTGTTG AAGAAACCCA CAATTTACTT GAACAAAGAG GAATACCGCT   
  
  
- AACGCTTGTT TCTAACCGTA TAATGAAGAA TGTTCACAAC AACCGGTTTC GGTTGTTAAA CCCTGGAGTG   
  
  
- AAAGTACTCT CGGATTTTGA ACGGTACCTC TTTTTGACGA CGAAACTATG GATGTACTTC GAATATAACT   
  
  
- TCAAGGTTCT CCAGTCAGGT ACCTGTTGGA AACCAGTACA CCGAAGTTTA CCACGTTATA ACCTCCTGGA   
  
  
- ATCTTCAAAT GTTTTCAGCT AGTAGTTTCT TTGTTCCGTT TCCTACCTCT TCAAAAGTTC CGATTACCCA   
  
  
- CAAGGGAAGT TCAAAGTACA GTATTTGCTC AATCTATTGG ATCCTCTCGA AGCTTTTCTC CTAAATCTGT   
  
  
- AACTTCTACC ACTCCGGTAG CGACACTTGA CACAAGTTCG GAACGTTGCC CAAGTACACC TCTTCTCCCT   
  
  
- CGTACACGAA CTACACTAAG CTAGATAATC CGGAACATAG TATTGTGACC ACCTCCTTCT TCGTCTAGAG   
  
  
- TGAAGATGTT CTTTGCTGAA GAAGTTCACG AAGCTACTCA CAAACTCTAA AAGATTCAGT ATAAAGCTAT   
  
  
- ACAATCTTCT TTCGAAGGGA GGTTATTCGT TGCTTGCTTA GTTCGATCTT TCCCTTGTTA CCTACTTATA   
  
  
- GAGGTCTCGG GATCGAACAG TACCACTTTC ACCTCCTCTT ATATCCGGTT TCTTTCCTTG AGTTACCTTA   
  
  
- CTCTACGAGC TTGTTCGTAA AACGGGTAGA GTTAAATCAC TACTACATGA TTCACTACAA TCCCGTAACA   
  
  
- ACTTTTCTGT GTTTTCACCA ACCCTAGAGT GGAATGGTGT TTCACTGGTA CTTTATCCGT ATGTGAATTT   
  
  
- AACCTTCCCA CTTTTACAAC AAACCCGAAG ACGTACCTCT GGATCGAT

+     MYB recognition site

| Site Name | Organism | Position | Strand | Matrix score. | sequence | function |
| --- | --- | --- | --- | --- | --- | --- |
| MYB recognition site | Arabidopsis thaliana | 2709 | - | 6 | CCGTTG |  |

>HU02G03154.1   
+ +Up\_Stream \_Len000ATGATA AAAGATATTT ATTAAGCTCA AGCTATTTTA ACTTTTAAAA AAATGAATGT   
  
  
+ GGCTTTTTTT GCTATTCAGG TACAACACAA TATGAAGCAT TAACTCTAGA AAATGTGGTA ATGTATGTCA   
  
  
+ TATAGGAGAG TATATACTCT CACTTGCCAT GCGATGCGTG CATGTGTGTG TGTGTGTGTA TCTGTTTCTA   
  
  
+ TGCATATTTT ATGTGGGGTT TTGTCATCTA GACATCTATT AATATGTTAG CATTCACATG ACTTATAGGA   
  
  
+ ATGCACTATT ATGAGTATGA TATAGAGTTT TAAAAATGCA TATGCCTTGT GTGTTCATTA GCTTAAAGAA   
  
  
+ GAGTTTTTGA ATATACATAT TTAGGTAATT CTTGCTAATG TCGTAATTAG CAGTTGTATC TATCTTATTG   
  
  
+ ATTTGTATTT TTAATTGCTT GTTATACTGT TACACTTAGA AAATTCATGA ATACTCTTAA AATATGCAAA   
  
  
+ TTATATATAT ATATATATAT ATATATATAT ATATATATAT ATAAAAACTG AATAAGTTAA TATGGTGATG   
  
  
+ ACATTTGTCA TGTTATCATT GGTCGCTTAA TTTTTAAATA AAAAATATGA TTTTGTGACA CCTAATAAAT   
  
  
+ GATATTAGCC ATATTATTAA ATTCAGCATT TATTCATCAA TGTAATATAC TAAATCATGC AATTATCATT   
  
  
+ GGTTCTCTTT TATGTAATAA AGTTAATTGA CAATTCAAGG GTTACTAATT AGTATAATGT TGAATTGGAA   
  
  
+ CGTAAAAATC AAATCGTATG TAAAGTAATT TTATTCTGAA TTTAAAAAAT GGAGCAAATA TCTCTATTGT   
  
  
+ TCTTTAATAA AATTTTAATT GATTTTTATT TCTAATTAAG TGACGGAACC AAATTTTTTT CCTCCTTTTT   
  
  
+ TGGTTTGGTA AGTTATCATT AGCCAATCTT TTATTTACAT TTTGTCAGTT TTTCTTTTTT AAGATGGTTA   
  
  
+ AATGGCTTCA ATTAAACAAA TTTTTTTACT TATTTATATT TTTAATAAAT TTTCAAAATG TATATTAACG   
  
  
+ CATGACCGTG CGAAGCACGG AATCTACCCT AGTTAATCTA TGATTCTTAA ATATGCAAGT AGGCTGTTAT   
  
  
+ TCATTTGTTG TTTTTTACTT ATTTATATTT TTTCCCTCCC GGTATGATAG GGTTTGTCAT ATTGTTATTC   
  
  
+ ATTAGCTGTT AAACTGTATT CATTATAGCA CAAGTAGGCT TAATCAAAGA TAGTTAGTCT CCAATTTCAT   
  
  
+ CAGAAAATTT AAGAAAAAGA CACCTTAGGA TCAAAAGTAT GAAATTTAGA GACCAGACAA CGATAGAATT   
  
  
+ GAAAAGATGA GTTTTCAATT TGGAGAAGAC GGAAACTCAG ATGAATGAAA GTATAGTACA TTGTACTCCT   
  
  
+ TGGAGTATAA TCTTATCTTC ACTATTGATA GGCTCGAAGC ATAATCAGTG ATTGGAAACT TTTGGTTGCA   
  
  
+ ATAATTCAAA ATTTGATACT CTATGATTTA CTTTTTTAAT TGAAGGTCAT GATTTAATTA ACTAAATGAA   
  
  
+ AAGAATTTAT GATCGAAAAA TTTTAACTCT TATTGACTTA TAGTTGACTC CAGTTAAATG GAAAATTGAA   
  
  
+ GTGATTAATC TAAAAGTGGC CCGCTCTAAT ACCATTTGAG AAAAATTATT CTTACTGCAA AGTTTAAATC   
  
  
+ AATATGTGAA GATAGTTTAT AGTTTTATCC TAAAAGAAAT TAAATAGATT GTTAAAAATA ATTTTTAAAT   
  
  
+ TCAGATATAC GTGTGATGGC CATAACCGTA AATGATATGC ATGTCGACAA ATTCCAAAGA AGGCTAATAA   
  
  
+ TTAGACTTAA ATAATACATG CATACTAACC AAAAAAAAAA AATTAAGCAA GATTCTATCC AACCTATCAT   
  
  
+ AATAAAGTTT GGCCATACTT CAAACCATCA CATAACATCA CTAATATATA AAGCTAGGTT TTGGGAAACC   
  
  
+ TAACTTTGAG GACCACATAT TTGTTAAGGC CTAGGAGGCC ATGAGTTTGT CAAGATGCCT CATAGTATGG   
  
  
+ AGAGGTCTAT ATCCTTGCAA AATGATCATT CCCTTCTACC TAAAAGAACC ACTTCATTTC CACTTTTACT   
  
  
+ CCCTGGCAAA TGGTTGATAA ACCAAACCAT AACCAAGCCC TTGAAGAACT ATAAAGACAC GAATCGATCA   
  
  
+ TTGAATTCGA ACAAGCCTTG TGTGATAGGA GGATGGGCCT CAACACTTCT CCGCGAGTGT GCAAGAGCAA   
  
  
+ TCTCGGAGAA AAATCCTAAG AGCCAACAAC TTCTTTGGGT GTTAAATGAA CTTGTTTCTC CTTATGGCGA   
  
  
+ TTGCGAACAA AGATTGGCAT ATTACTTCTT ACAAGTGTTG TTGGCCAAAG CCAACAATTT GGGACCTCAC   
  
  
+ TTTCATGAGA GCCTAAAACT TGCCATGGAG AAAAACTGCT GCTTTGATAC CTACATGAAG CTTATATTGA   
  
  
+ AGTTCCAAGA GGTCAGTCCA TGGACAACCT TTGGTCATGT GGCTTCAAAT GGTGCAATAT TGGAGGACCT   
  
  
+ TAGAAGTTTA CAAAAGTCGA TCATCAAAGA AACAAGGCAA AGGATGGAGA AGTTTTCAAG GCTAATGGGT   
  
  
+ GTTCCCTTCA AGTTTCATGT CATAAACGAG TTAGATAACC TAGGAGAGCT TCGAAAAGAG GATTTAGACA   
  
  
+ TTGAAGATGG TGAGGCCATC GCTGTGAACT GTGTTCAAGC CTTGCAACGG GTTCATGTGG AGAAGAGGGA   
  
  
+ GCATGTGCTT GATGTGATTC GATCTATTAG GCCTTGTATC ATAACACTGG TGGAGGAAGA AGCAGATCTC   
  
  
+ ACTTCTACAA GAAACGACTT CTTCAAGTGC TTCGATGAGT GTTTGAGATT TTCTAAGTCA TATTTCGATA   
  
  
+ TGTTAGAAGA AAGCTTCCCT CCAATAAGCA ACGAACGAAT CAAGCTAGAA AGGGAACAAT GGATGAATAT   
  
  
+ CTCCAGAGCC CTAGCTTGTC ATGGTGAAAG TGGAGGAGAA TATAGGCCAA AGAAAGGAAC TCAATGGAAT   
  
  
+ GAGATGCTCG AACAAGCATT TTGCCCATCT CAATTTAGTG ATGATGTACT AAGTGATGTT AGGGCATTGT   
  
  
+ TGAAAAGACA CAAAAGTGGT TGGGATCTCA CCTTACCACA AAGTGACCAT GAAATAGGCA TACACTTAAA   
  
  
+ TTGGAAGGGT GAAAATGTTG TTTGGGCTTC TGCATGGAGA CCTAGCTA  

- +Up\_Stream \_Len000TACTAT TTTCTATAAA TAATTCGAGT TCGATAAAAT TGAAAATTTT TTTACTTACA   
  
  
- CCGAAAAAAA CGATAAGTCC ATGTTGTGTT ATACTTCGTA ATTGAGATCT TTTACACCAT TACATACAGT   
  
  
- ATATCCTCTC ATATATGAGA GTGAACGGTA CGCTACGCAC GTACACACAC ACACACACAT AGACAAAGAT   
  
  
- ACGTATAAAA TACACCCCAA AACAGTAGAT CTGTAGATAA TTATACAATC GTAAGTGTAC TGAATATCCT   
  
  
- TACGTGATAA TACTCATACT ATATCTCAAA ATTTTTACGT ATACGGAACA CACAAGTAAT CGAATTTCTT   
  
  
- CTCAAAAACT TATATGTATA AATCCATTAA GAACGATTAC AGCATTAATC GTCAACATAG ATAGAATAAC   
  
  
- TAAACATAAA AATTAACGAA CAATATGACA ATGTGAATCT TTTAAGTACT TATGAGAATT TTATACGTTT   
  
  
- AATATATATA TATATATATA TATATATATA TATATATATA TATTTTTGAC TTATTCAATT ATACCACTAC   
  
  
- TGTAAACAGT ACAATAGTAA CCAGCGAATT AAAAATTTAT TTTTTATACT AAAACACTGT GGATTATTTA   
  
  
- CTATAATCGG TATAATAATT TAAGTCGTAA ATAAGTAGTT ACATTATATG ATTTAGTACG TTAATAGTAA   
  
  
- CCAAGAGAAA ATACATTATT TCAATTAACT GTTAAGTTCC CAATGATTAA TCATATTACA ACTTAACCTT   
  
  
- GCATTTTTAG TTTAGCATAC ATTTCATTAA AATAAGACTT AAATTTTTTA CCTCGTTTAT AGAGATAACA   
  
  
- AGAAATTATT TTAAAATTAA CTAAAAATAA AGATTAATTC ACTGCCTTGG TTTAAAAAAA GGAGGAAAAA   
  
  
- ACCAAACCAT TCAATAGTAA TCGGTTAGAA AATAAATGTA AAACAGTCAA AAAGAAAAAA TTCTACCAAT   
  
  
- TTACCGAAGT TAATTTGTTT AAAAAAATGA ATAAATATAA AAATTATTTA AAAGTTTTAC ATATAATTGC   
  
  
- GTACTGGCAC GCTTCGTGCC TTAGATGGGA TCAATTAGAT ACTAAGAATT TATACGTTCA TCCGACAATA   
  
  
- AGTAAACAAC AAAAAATGAA TAAATATAAA AAAGGGAGGG CCATACTATC CCAAACAGTA TAACAATAAG   
  
  
- TAATCGACAA TTTGACATAA GTAATATCGT GTTCATCCGA ATTAGTTTCT ATCAATCAGA GGTTAAAGTA   
  
  
- GTCTTTTAAA TTCTTTTTCT GTGGAATCCT AGTTTTCATA CTTTAAATCT CTGGTCTGTT GCTATCTTAA   
  
  
- CTTTTCTACT CAAAAGTTAA ACCTCTTCTG CCTTTGAGTC TACTTACTTT CATATCATGT AACATGAGGA   
  
  
- ACCTCATATT AGAATAGAAG TGATAACTAT CCGAGCTTCG TATTAGTCAC TAACCTTTGA AAACCAACGT   
  
  
- TATTAAGTTT TAAACTATGA GATACTAAAT GAAAAAATTA ACTTCCAGTA CTAAATTAAT TGATTTACTT   
  
  
- TTCTTAAATA CTAGCTTTTT AAAATTGAGA ATAACTGAAT ATCAACTGAG GTCAATTTAC CTTTTAACTT   
  
  
- CACTAATTAG ATTTTCACCG GGCGAGATTA TGGTAAACTC TTTTTAATAA GAATGACGTT TCAAATTTAG   
  
  
- TTATACACTT CTATCAAATA TCAAAATAGG ATTTTCTTTA ATTTATCTAA CAATTTTTAT TAAAAATTTA   
  
  
- AGTCTATATG CACACTACCG GTATTGGCAT TTACTATACG TACAGCTGTT TAAGGTTTCT TCCGATTATT   
  
  
- AATCTGAATT TATTATGTAC GTATGATTGG TTTTTTTTTT TTAATTCGTT CTAAGATAGG TTGGATAGTA   
  
  
- TTATTTCAAA CCGGTATGAA GTTTGGTAGT GTATTGTAGT GATTATATAT TTCGATCCAA AACCCTTTGG   
  
  
- ATTGAAACTC CTGGTGTATA AACAATTCCG GATCCTCCGG TACTCAAACA GTTCTACGGA GTATCATACC   
  
  
- TCTCCAGATA TAGGAACGTT TTACTAGTAA GGGAAGATGG ATTTTCTTGG TGAAGTAAAG GTGAAAATGA   
  
  
- GGGACCGTTT ACCAACTATT TGGTTTGGTA TTGGTTCGGG AACTTCTTGA TATTTCTGTG CTTAGCTAGT   
  
  
- AACTTAAGCT TGTTCGGAAC ACACTATCCT CCTACCCGGA GTTGTGAAGA GGCGCTCACA CGTTCTCGTT   
  
  
- AGAGCCTCTT TTTAGGATTC TCGGTTGTTG AAGAAACCCA CAATTTACTT GAACAAAGAG GAATACCGCT   
  
  
- AACGCTTGTT TCTAACCGTA TAATGAAGAA TGTTCACAAC AACCGGTTTC GGTTGTTAAA CCCTGGAGTG   
  
  
- AAAGTACTCT CGGATTTTGA ACGGTACCTC TTTTTGACGA CGAAACTATG GATGTACTTC GAATATAACT   
  
  
- TCAAGGTTCT CCAGTCAGGT ACCTGTTGGA AACCAGTACA CCGAAGTTTA CCACGTTATA ACCTCCTGGA   
  
  
- ATCTTCAAAT GTTTTCAGCT AGTAGTTTCT TTGTTCCGTT TCCTACCTCT TCAAAAGTTC CGATTACCCA   
  
  
- CAAGGGAAGT TCAAAGTACA GTATTTGCTC AATCTATTGG ATCCTCTCGA AGCTTTTCTC CTAAATCTGT   
  
  
- AACTTCTACC ACTCCGGTAG CGACACTTGA CACAAGTTCG GAACGTTGCC CAAGTACACC TCTTCTCCCT   
  
  
- CGTACACGAA CTACACTAAG CTAGATAATC CGGAACATAG TATTGTGACC ACCTCCTTCT TCGTCTAGAG   
  
  
- TGAAGATGTT CTTTGCTGAA GAAGTTCACG AAGCTACTCA CAAACTCTAA AAGATTCAGT ATAAAGCTAT   
  
  
- ACAATCTTCT TTCGAAGGGA GGTTATTCGT TGCTTGCTTA GTTCGATCTT TCCCTTGTTA CCTACTTATA   
  
  
- GAGGTCTCGG GATCGAACAG TACCACTTTC ACCTCCTCTT ATATCCGGTT TCTTTCCTTG AGTTACCTTA   
  
  
- CTCTACGAGC TTGTTCGTAA AACGGGTAGA GTTAAATCAC TACTACATGA TTCACTACAA TCCCGTAACA   
  
  
- ACTTTTCTGT GTTTTCACCA ACCCTAGAGT GGAATGGTGT TTCACTGGTA CTTTATCCGT ATGTGAATTT   
  
  
- AACCTTCCCA CTTTTACAAC AAACCCGAAG ACGTACCTCT GGATCGAT

+     MYB-like sequence

| Site Name | Organism | Position | Strand | Matrix score. | sequence | function |
| --- | --- | --- | --- | --- | --- | --- |
| MYB-like sequence | Arabidopsis thaliana | 1850 | + | 6 | TAACCA |  |
| MYB-like sequence | Arabidopsis thaliana | 2134 | + | 6 | TAACCA |  |
| MYB-like sequence | Arabidopsis thaliana | 979 | - | 6 | TAACCA |  |

>HU02G03154.1   
+ +Up\_Stream \_Len000ATGATA AAAGATATTT ATTAAGCTCA AGCTATTTTA ACTTTTAAAA AAATGAATGT   
  
  
+ GGCTTTTTTT GCTATTCAGG TACAACACAA TATGAAGCAT TAACTCTAGA AAATGTGGTA ATGTATGTCA   
  
  
+ TATAGGAGAG TATATACTCT CACTTGCCAT GCGATGCGTG CATGTGTGTG TGTGTGTGTA TCTGTTTCTA   
  
  
+ TGCATATTTT ATGTGGGGTT TTGTCATCTA GACATCTATT AATATGTTAG CATTCACATG ACTTATAGGA   
  
  
+ ATGCACTATT ATGAGTATGA TATAGAGTTT TAAAAATGCA TATGCCTTGT GTGTTCATTA GCTTAAAGAA   
  
  
+ GAGTTTTTGA ATATACATAT TTAGGTAATT CTTGCTAATG TCGTAATTAG CAGTTGTATC TATCTTATTG   
  
  
+ ATTTGTATTT TTAATTGCTT GTTATACTGT TACACTTAGA AAATTCATGA ATACTCTTAA AATATGCAAA   
  
  
+ TTATATATAT ATATATATAT ATATATATAT ATATATATAT ATAAAAACTG AATAAGTTAA TATGGTGATG   
  
  
+ ACATTTGTCA TGTTATCATT GGTCGCTTAA TTTTTAAATA AAAAATATGA TTTTGTGACA CCTAATAAAT   
  
  
+ GATATTAGCC ATATTATTAA ATTCAGCATT TATTCATCAA TGTAATATAC TAAATCATGC AATTATCATT   
  
  
+ GGTTCTCTTT TATGTAATAA AGTTAATTGA CAATTCAAGG GTTACTAATT AGTATAATGT TGAATTGGAA   
  
  
+ CGTAAAAATC AAATCGTATG TAAAGTAATT TTATTCTGAA TTTAAAAAAT GGAGCAAATA TCTCTATTGT   
  
  
+ TCTTTAATAA AATTTTAATT GATTTTTATT TCTAATTAAG TGACGGAACC AAATTTTTTT CCTCCTTTTT   
  
  
+ TGGTTTGGTA AGTTATCATT AGCCAATCTT TTATTTACAT TTTGTCAGTT TTTCTTTTTT AAGATGGTTA   
  
  
+ AATGGCTTCA ATTAAACAAA TTTTTTTACT TATTTATATT TTTAATAAAT TTTCAAAATG TATATTAACG   
  
  
+ CATGACCGTG CGAAGCACGG AATCTACCCT AGTTAATCTA TGATTCTTAA ATATGCAAGT AGGCTGTTAT   
  
  
+ TCATTTGTTG TTTTTTACTT ATTTATATTT TTTCCCTCCC GGTATGATAG GGTTTGTCAT ATTGTTATTC   
  
  
+ ATTAGCTGTT AAACTGTATT CATTATAGCA CAAGTAGGCT TAATCAAAGA TAGTTAGTCT CCAATTTCAT   
  
  
+ CAGAAAATTT AAGAAAAAGA CACCTTAGGA TCAAAAGTAT GAAATTTAGA GACCAGACAA CGATAGAATT   
  
  
+ GAAAAGATGA GTTTTCAATT TGGAGAAGAC GGAAACTCAG ATGAATGAAA GTATAGTACA TTGTACTCCT   
  
  
+ TGGAGTATAA TCTTATCTTC ACTATTGATA GGCTCGAAGC ATAATCAGTG ATTGGAAACT TTTGGTTGCA   
  
  
+ ATAATTCAAA ATTTGATACT CTATGATTTA CTTTTTTAAT TGAAGGTCAT GATTTAATTA ACTAAATGAA   
  
  
+ AAGAATTTAT GATCGAAAAA TTTTAACTCT TATTGACTTA TAGTTGACTC CAGTTAAATG GAAAATTGAA   
  
  
+ GTGATTAATC TAAAAGTGGC CCGCTCTAAT ACCATTTGAG AAAAATTATT CTTACTGCAA AGTTTAAATC   
  
  
+ AATATGTGAA GATAGTTTAT AGTTTTATCC TAAAAGAAAT TAAATAGATT GTTAAAAATA ATTTTTAAAT   
  
  
+ TCAGATATAC GTGTGATGGC CATAACCGTA AATGATATGC ATGTCGACAA ATTCCAAAGA AGGCTAATAA   
  
  
+ TTAGACTTAA ATAATACATG CATACTAACC AAAAAAAAAA AATTAAGCAA GATTCTATCC AACCTATCAT   
  
  
+ AATAAAGTTT GGCCATACTT CAAACCATCA CATAACATCA CTAATATATA AAGCTAGGTT TTGGGAAACC   
  
  
+ TAACTTTGAG GACCACATAT TTGTTAAGGC CTAGGAGGCC ATGAGTTTGT CAAGATGCCT CATAGTATGG   
  
  
+ AGAGGTCTAT ATCCTTGCAA AATGATCATT CCCTTCTACC TAAAAGAACC ACTTCATTTC CACTTTTACT   
  
  
+ CCCTGGCAAA TGGTTGATAA ACCAAACCAT AACCAAGCCC TTGAAGAACT ATAAAGACAC GAATCGATCA   
  
  
+ TTGAATTCGA ACAAGCCTTG TGTGATAGGA GGATGGGCCT CAACACTTCT CCGCGAGTGT GCAAGAGCAA   
  
  
+ TCTCGGAGAA AAATCCTAAG AGCCAACAAC TTCTTTGGGT GTTAAATGAA CTTGTTTCTC CTTATGGCGA   
  
  
+ TTGCGAACAA AGATTGGCAT ATTACTTCTT ACAAGTGTTG TTGGCCAAAG CCAACAATTT GGGACCTCAC   
  
  
+ TTTCATGAGA GCCTAAAACT TGCCATGGAG AAAAACTGCT GCTTTGATAC CTACATGAAG CTTATATTGA   
  
  
+ AGTTCCAAGA GGTCAGTCCA TGGACAACCT TTGGTCATGT GGCTTCAAAT GGTGCAATAT TGGAGGACCT   
  
  
+ TAGAAGTTTA CAAAAGTCGA TCATCAAAGA AACAAGGCAA AGGATGGAGA AGTTTTCAAG GCTAATGGGT   
  
  
+ GTTCCCTTCA AGTTTCATGT CATAAACGAG TTAGATAACC TAGGAGAGCT TCGAAAAGAG GATTTAGACA   
  
  
+ TTGAAGATGG TGAGGCCATC GCTGTGAACT GTGTTCAAGC CTTGCAACGG GTTCATGTGG AGAAGAGGGA   
  
  
+ GCATGTGCTT GATGTGATTC GATCTATTAG GCCTTGTATC ATAACACTGG TGGAGGAAGA AGCAGATCTC   
  
  
+ ACTTCTACAA GAAACGACTT CTTCAAGTGC TTCGATGAGT GTTTGAGATT TTCTAAGTCA TATTTCGATA   
  
  
+ TGTTAGAAGA AAGCTTCCCT CCAATAAGCA ACGAACGAAT CAAGCTAGAA AGGGAACAAT GGATGAATAT   
  
  
+ CTCCAGAGCC CTAGCTTGTC ATGGTGAAAG TGGAGGAGAA TATAGGCCAA AGAAAGGAAC TCAATGGAAT   
  
  
+ GAGATGCTCG AACAAGCATT TTGCCCATCT CAATTTAGTG ATGATGTACT AAGTGATGTT AGGGCATTGT   
  
  
+ TGAAAAGACA CAAAAGTGGT TGGGATCTCA CCTTACCACA AAGTGACCAT GAAATAGGCA TACACTTAAA   
  
  
+ TTGGAAGGGT GAAAATGTTG TTTGGGCTTC TGCATGGAGA CCTAGCTA  

- +Up\_Stream \_Len000TACTAT TTTCTATAAA TAATTCGAGT TCGATAAAAT TGAAAATTTT TTTACTTACA   
  
  
- CCGAAAAAAA CGATAAGTCC ATGTTGTGTT ATACTTCGTA ATTGAGATCT TTTACACCAT TACATACAGT   
  
  
- ATATCCTCTC ATATATGAGA GTGAACGGTA CGCTACGCAC GTACACACAC ACACACACAT AGACAAAGAT   
  
  
- ACGTATAAAA TACACCCCAA AACAGTAGAT CTGTAGATAA TTATACAATC GTAAGTGTAC TGAATATCCT   
  
  
- TACGTGATAA TACTCATACT ATATCTCAAA ATTTTTACGT ATACGGAACA CACAAGTAAT CGAATTTCTT   
  
  
- CTCAAAAACT TATATGTATA AATCCATTAA GAACGATTAC AGCATTAATC GTCAACATAG ATAGAATAAC   
  
  
- TAAACATAAA AATTAACGAA CAATATGACA ATGTGAATCT TTTAAGTACT TATGAGAATT TTATACGTTT   
  
  
- AATATATATA TATATATATA TATATATATA TATATATATA TATTTTTGAC TTATTCAATT ATACCACTAC   
  
  
- TGTAAACAGT ACAATAGTAA CCAGCGAATT AAAAATTTAT TTTTTATACT AAAACACTGT GGATTATTTA   
  
  
- CTATAATCGG TATAATAATT TAAGTCGTAA ATAAGTAGTT ACATTATATG ATTTAGTACG TTAATAGTAA   
  
  
- CCAAGAGAAA ATACATTATT TCAATTAACT GTTAAGTTCC CAATGATTAA TCATATTACA ACTTAACCTT   
  
  
- GCATTTTTAG TTTAGCATAC ATTTCATTAA AATAAGACTT AAATTTTTTA CCTCGTTTAT AGAGATAACA   
  
  
- AGAAATTATT TTAAAATTAA CTAAAAATAA AGATTAATTC ACTGCCTTGG TTTAAAAAAA GGAGGAAAAA   
  
  
- ACCAAACCAT TCAATAGTAA TCGGTTAGAA AATAAATGTA AAACAGTCAA AAAGAAAAAA TTCTACCAAT   
  
  
- TTACCGAAGT TAATTTGTTT AAAAAAATGA ATAAATATAA AAATTATTTA AAAGTTTTAC ATATAATTGC   
  
  
- GTACTGGCAC GCTTCGTGCC TTAGATGGGA TCAATTAGAT ACTAAGAATT TATACGTTCA TCCGACAATA   
  
  
- AGTAAACAAC AAAAAATGAA TAAATATAAA AAAGGGAGGG CCATACTATC CCAAACAGTA TAACAATAAG   
  
  
- TAATCGACAA TTTGACATAA GTAATATCGT GTTCATCCGA ATTAGTTTCT ATCAATCAGA GGTTAAAGTA   
  
  
- GTCTTTTAAA TTCTTTTTCT GTGGAATCCT AGTTTTCATA CTTTAAATCT CTGGTCTGTT GCTATCTTAA   
  
  
- CTTTTCTACT CAAAAGTTAA ACCTCTTCTG CCTTTGAGTC TACTTACTTT CATATCATGT AACATGAGGA   
  
  
- ACCTCATATT AGAATAGAAG TGATAACTAT CCGAGCTTCG TATTAGTCAC TAACCTTTGA AAACCAACGT   
  
  
- TATTAAGTTT TAAACTATGA GATACTAAAT GAAAAAATTA ACTTCCAGTA CTAAATTAAT TGATTTACTT   
  
  
- TTCTTAAATA CTAGCTTTTT AAAATTGAGA ATAACTGAAT ATCAACTGAG GTCAATTTAC CTTTTAACTT   
  
  
- CACTAATTAG ATTTTCACCG GGCGAGATTA TGGTAAACTC TTTTTAATAA GAATGACGTT TCAAATTTAG   
  
  
- TTATACACTT CTATCAAATA TCAAAATAGG ATTTTCTTTA ATTTATCTAA CAATTTTTAT TAAAAATTTA   
  
  
- AGTCTATATG CACACTACCG GTATTGGCAT TTACTATACG TACAGCTGTT TAAGGTTTCT TCCGATTATT   
  
  
- AATCTGAATT TATTATGTAC GTATGATTGG TTTTTTTTTT TTAATTCGTT CTAAGATAGG TTGGATAGTA   
  
  
- TTATTTCAAA CCGGTATGAA GTTTGGTAGT GTATTGTAGT GATTATATAT TTCGATCCAA AACCCTTTGG   
  
  
- ATTGAAACTC CTGGTGTATA AACAATTCCG GATCCTCCGG TACTCAAACA GTTCTACGGA GTATCATACC   
  
  
- TCTCCAGATA TAGGAACGTT TTACTAGTAA GGGAAGATGG ATTTTCTTGG TGAAGTAAAG GTGAAAATGA   
  
  
- GGGACCGTTT ACCAACTATT TGGTTTGGTA TTGGTTCGGG AACTTCTTGA TATTTCTGTG CTTAGCTAGT   
  
  
- AACTTAAGCT TGTTCGGAAC ACACTATCCT CCTACCCGGA GTTGTGAAGA GGCGCTCACA CGTTCTCGTT   
  
  
- AGAGCCTCTT TTTAGGATTC TCGGTTGTTG AAGAAACCCA CAATTTACTT GAACAAAGAG GAATACCGCT   
  
  
- AACGCTTGTT TCTAACCGTA TAATGAAGAA TGTTCACAAC AACCGGTTTC GGTTGTTAAA CCCTGGAGTG   
  
  
- AAAGTACTCT CGGATTTTGA ACGGTACCTC TTTTTGACGA CGAAACTATG GATGTACTTC GAATATAACT   
  
  
- TCAAGGTTCT CCAGTCAGGT ACCTGTTGGA AACCAGTACA CCGAAGTTTA CCACGTTATA ACCTCCTGGA   
  
  
- ATCTTCAAAT GTTTTCAGCT AGTAGTTTCT TTGTTCCGTT TCCTACCTCT TCAAAAGTTC CGATTACCCA   
  
  
- CAAGGGAAGT TCAAAGTACA GTATTTGCTC AATCTATTGG ATCCTCTCGA AGCTTTTCTC CTAAATCTGT   
  
  
- AACTTCTACC ACTCCGGTAG CGACACTTGA CACAAGTTCG GAACGTTGCC CAAGTACACC TCTTCTCCCT   
  
  
- CGTACACGAA CTACACTAAG CTAGATAATC CGGAACATAG TATTGTGACC ACCTCCTTCT TCGTCTAGAG   
  
  
- TGAAGATGTT CTTTGCTGAA GAAGTTCACG AAGCTACTCA CAAACTCTAA AAGATTCAGT ATAAAGCTAT   
  
  
- ACAATCTTCT TTCGAAGGGA GGTTATTCGT TGCTTGCTTA GTTCGATCTT TCCCTTGTTA CCTACTTATA   
  
  
- GAGGTCTCGG GATCGAACAG TACCACTTTC ACCTCCTCTT ATATCCGGTT TCTTTCCTTG AGTTACCTTA   
  
  
- CTCTACGAGC TTGTTCGTAA AACGGGTAGA GTTAAATCAC TACTACATGA TTCACTACAA TCCCGTAACA   
  
  
- ACTTTTCTGT GTTTTCACCA ACCCTAGAGT GGAATGGTGT TTCACTGGTA CTTTATCCGT ATGTGAATTT   
  
  
- AACCTTCCCA CTTTTACAAC AAACCCGAAG ACGTACCTCT GGATCGAT

+     MYC

| Site Name | Organism | Position | Strand | Matrix score. | sequence | function |
| --- | --- | --- | --- | --- | --- | --- |
| MYC | Arabidopsis thaliana | 2500 | - | 6 | CATTTG |  |
| MYC | Arabidopsis thaliana | 2111 | - | 6 | CATTTG |  |
| MYC | Arabidopsis thaliana | 2490 | + | 6 | CATGTG |  |
| MYC | Arabidopsis thaliana | 2736 | + | 6 | CATGTG |  |
| MYC | Arabidopsis thaliana | 2718 | + | 6 | CATGTG |  |
| MYC | Arabidopsis thaliana | 1647 | + | 6 | CATTTG |  |
| MYC | Arabidopsis thaliana | 269 | - | 6 | CATGTG |  |
| MYC | Arabidopsis thaliana | 566 | + | 6 | CATTTG |  |
| MYC | Arabidopsis thaliana | 1126 | + | 6 | CATTTG |  |
| MYC | Arabidopsis thaliana | 185 | + | 6 | CATGTG |  |

>HU02G03154.1   
+ +Up\_Stream \_Len000ATGATA AAAGATATTT ATTAAGCTCA AGCTATTTTA ACTTTTAAAA AAATGAATGT   
  
  
+ GGCTTTTTTT GCTATTCAGG TACAACACAA TATGAAGCAT TAACTCTAGA AAATGTGGTA ATGTATGTCA   
  
  
+ TATAGGAGAG TATATACTCT CACTTGCCAT GCGATGCGTG CATGTGTGTG TGTGTGTGTA TCTGTTTCTA   
  
  
+ TGCATATTTT ATGTGGGGTT TTGTCATCTA GACATCTATT AATATGTTAG CATTCACATG ACTTATAGGA   
  
  
+ ATGCACTATT ATGAGTATGA TATAGAGTTT TAAAAATGCA TATGCCTTGT GTGTTCATTA GCTTAAAGAA   
  
  
+ GAGTTTTTGA ATATACATAT TTAGGTAATT CTTGCTAATG TCGTAATTAG CAGTTGTATC TATCTTATTG   
  
  
+ ATTTGTATTT TTAATTGCTT GTTATACTGT TACACTTAGA AAATTCATGA ATACTCTTAA AATATGCAAA   
  
  
+ TTATATATAT ATATATATAT ATATATATAT ATATATATAT ATAAAAACTG AATAAGTTAA TATGGTGATG   
  
  
+ ACATTTGTCA TGTTATCATT GGTCGCTTAA TTTTTAAATA AAAAATATGA TTTTGTGACA CCTAATAAAT   
  
  
+ GATATTAGCC ATATTATTAA ATTCAGCATT TATTCATCAA TGTAATATAC TAAATCATGC AATTATCATT   
  
  
+ GGTTCTCTTT TATGTAATAA AGTTAATTGA CAATTCAAGG GTTACTAATT AGTATAATGT TGAATTGGAA   
  
  
+ CGTAAAAATC AAATCGTATG TAAAGTAATT TTATTCTGAA TTTAAAAAAT GGAGCAAATA TCTCTATTGT   
  
  
+ TCTTTAATAA AATTTTAATT GATTTTTATT TCTAATTAAG TGACGGAACC AAATTTTTTT CCTCCTTTTT   
  
  
+ TGGTTTGGTA AGTTATCATT AGCCAATCTT TTATTTACAT TTTGTCAGTT TTTCTTTTTT AAGATGGTTA   
  
  
+ AATGGCTTCA ATTAAACAAA TTTTTTTACT TATTTATATT TTTAATAAAT TTTCAAAATG TATATTAACG   
  
  
+ CATGACCGTG CGAAGCACGG AATCTACCCT AGTTAATCTA TGATTCTTAA ATATGCAAGT AGGCTGTTAT   
  
  
+ TCATTTGTTG TTTTTTACTT ATTTATATTT TTTCCCTCCC GGTATGATAG GGTTTGTCAT ATTGTTATTC   
  
  
+ ATTAGCTGTT AAACTGTATT CATTATAGCA CAAGTAGGCT TAATCAAAGA TAGTTAGTCT CCAATTTCAT   
  
  
+ CAGAAAATTT AAGAAAAAGA CACCTTAGGA TCAAAAGTAT GAAATTTAGA GACCAGACAA CGATAGAATT   
  
  
+ GAAAAGATGA GTTTTCAATT TGGAGAAGAC GGAAACTCAG ATGAATGAAA GTATAGTACA TTGTACTCCT   
  
  
+ TGGAGTATAA TCTTATCTTC ACTATTGATA GGCTCGAAGC ATAATCAGTG ATTGGAAACT TTTGGTTGCA   
  
  
+ ATAATTCAAA ATTTGATACT CTATGATTTA CTTTTTTAAT TGAAGGTCAT GATTTAATTA ACTAAATGAA   
  
  
+ AAGAATTTAT GATCGAAAAA TTTTAACTCT TATTGACTTA TAGTTGACTC CAGTTAAATG GAAAATTGAA   
  
  
+ GTGATTAATC TAAAAGTGGC CCGCTCTAAT ACCATTTGAG AAAAATTATT CTTACTGCAA AGTTTAAATC   
  
  
+ AATATGTGAA GATAGTTTAT AGTTTTATCC TAAAAGAAAT TAAATAGATT GTTAAAAATA ATTTTTAAAT   
  
  
+ TCAGATATAC GTGTGATGGC CATAACCGTA AATGATATGC ATGTCGACAA ATTCCAAAGA AGGCTAATAA   
  
  
+ TTAGACTTAA ATAATACATG CATACTAACC AAAAAAAAAA AATTAAGCAA GATTCTATCC AACCTATCAT   
  
  
+ AATAAAGTTT GGCCATACTT CAAACCATCA CATAACATCA CTAATATATA AAGCTAGGTT TTGGGAAACC   
  
  
+ TAACTTTGAG GACCACATAT TTGTTAAGGC CTAGGAGGCC ATGAGTTTGT CAAGATGCCT CATAGTATGG   
  
  
+ AGAGGTCTAT ATCCTTGCAA AATGATCATT CCCTTCTACC TAAAAGAACC ACTTCATTTC CACTTTTACT   
  
  
+ CCCTGGCAAA TGGTTGATAA ACCAAACCAT AACCAAGCCC TTGAAGAACT ATAAAGACAC GAATCGATCA   
  
  
+ TTGAATTCGA ACAAGCCTTG TGTGATAGGA GGATGGGCCT CAACACTTCT CCGCGAGTGT GCAAGAGCAA   
  
  
+ TCTCGGAGAA AAATCCTAAG AGCCAACAAC TTCTTTGGGT GTTAAATGAA CTTGTTTCTC CTTATGGCGA   
  
  
+ TTGCGAACAA AGATTGGCAT ATTACTTCTT ACAAGTGTTG TTGGCCAAAG CCAACAATTT GGGACCTCAC   
  
  
+ TTTCATGAGA GCCTAAAACT TGCCATGGAG AAAAACTGCT GCTTTGATAC CTACATGAAG CTTATATTGA   
  
  
+ AGTTCCAAGA GGTCAGTCCA TGGACAACCT TTGGTCATGT GGCTTCAAAT GGTGCAATAT TGGAGGACCT   
  
  
+ TAGAAGTTTA CAAAAGTCGA TCATCAAAGA AACAAGGCAA AGGATGGAGA AGTTTTCAAG GCTAATGGGT   
  
  
+ GTTCCCTTCA AGTTTCATGT CATAAACGAG TTAGATAACC TAGGAGAGCT TCGAAAAGAG GATTTAGACA   
  
  
+ TTGAAGATGG TGAGGCCATC GCTGTGAACT GTGTTCAAGC CTTGCAACGG GTTCATGTGG AGAAGAGGGA   
  
  
+ GCATGTGCTT GATGTGATTC GATCTATTAG GCCTTGTATC ATAACACTGG TGGAGGAAGA AGCAGATCTC   
  
  
+ ACTTCTACAA GAAACGACTT CTTCAAGTGC TTCGATGAGT GTTTGAGATT TTCTAAGTCA TATTTCGATA   
  
  
+ TGTTAGAAGA AAGCTTCCCT CCAATAAGCA ACGAACGAAT CAAGCTAGAA AGGGAACAAT GGATGAATAT   
  
  
+ CTCCAGAGCC CTAGCTTGTC ATGGTGAAAG TGGAGGAGAA TATAGGCCAA AGAAAGGAAC TCAATGGAAT   
  
  
+ GAGATGCTCG AACAAGCATT TTGCCCATCT CAATTTAGTG ATGATGTACT AAGTGATGTT AGGGCATTGT   
  
  
+ TGAAAAGACA CAAAAGTGGT TGGGATCTCA CCTTACCACA AAGTGACCAT GAAATAGGCA TACACTTAAA   
  
  
+ TTGGAAGGGT GAAAATGTTG TTTGGGCTTC TGCATGGAGA CCTAGCTA  

- +Up\_Stream \_Len000TACTAT TTTCTATAAA TAATTCGAGT TCGATAAAAT TGAAAATTTT TTTACTTACA   
  
  
- CCGAAAAAAA CGATAAGTCC ATGTTGTGTT ATACTTCGTA ATTGAGATCT TTTACACCAT TACATACAGT   
  
  
- ATATCCTCTC ATATATGAGA GTGAACGGTA CGCTACGCAC GTACACACAC ACACACACAT AGACAAAGAT   
  
  
- ACGTATAAAA TACACCCCAA AACAGTAGAT CTGTAGATAA TTATACAATC GTAAGTGTAC TGAATATCCT   
  
  
- TACGTGATAA TACTCATACT ATATCTCAAA ATTTTTACGT ATACGGAACA CACAAGTAAT CGAATTTCTT   
  
  
- CTCAAAAACT TATATGTATA AATCCATTAA GAACGATTAC AGCATTAATC GTCAACATAG ATAGAATAAC   
  
  
- TAAACATAAA AATTAACGAA CAATATGACA ATGTGAATCT TTTAAGTACT TATGAGAATT TTATACGTTT   
  
  
- AATATATATA TATATATATA TATATATATA TATATATATA TATTTTTGAC TTATTCAATT ATACCACTAC   
  
  
- TGTAAACAGT ACAATAGTAA CCAGCGAATT AAAAATTTAT TTTTTATACT AAAACACTGT GGATTATTTA   
  
  
- CTATAATCGG TATAATAATT TAAGTCGTAA ATAAGTAGTT ACATTATATG ATTTAGTACG TTAATAGTAA   
  
  
- CCAAGAGAAA ATACATTATT TCAATTAACT GTTAAGTTCC CAATGATTAA TCATATTACA ACTTAACCTT   
  
  
- GCATTTTTAG TTTAGCATAC ATTTCATTAA AATAAGACTT AAATTTTTTA CCTCGTTTAT AGAGATAACA   
  
  
- AGAAATTATT TTAAAATTAA CTAAAAATAA AGATTAATTC ACTGCCTTGG TTTAAAAAAA GGAGGAAAAA   
  
  
- ACCAAACCAT TCAATAGTAA TCGGTTAGAA AATAAATGTA AAACAGTCAA AAAGAAAAAA TTCTACCAAT   
  
  
- TTACCGAAGT TAATTTGTTT AAAAAAATGA ATAAATATAA AAATTATTTA AAAGTTTTAC ATATAATTGC   
  
  
- GTACTGGCAC GCTTCGTGCC TTAGATGGGA TCAATTAGAT ACTAAGAATT TATACGTTCA TCCGACAATA   
  
  
- AGTAAACAAC AAAAAATGAA TAAATATAAA AAAGGGAGGG CCATACTATC CCAAACAGTA TAACAATAAG   
  
  
- TAATCGACAA TTTGACATAA GTAATATCGT GTTCATCCGA ATTAGTTTCT ATCAATCAGA GGTTAAAGTA   
  
  
- GTCTTTTAAA TTCTTTTTCT GTGGAATCCT AGTTTTCATA CTTTAAATCT CTGGTCTGTT GCTATCTTAA   
  
  
- CTTTTCTACT CAAAAGTTAA ACCTCTTCTG CCTTTGAGTC TACTTACTTT CATATCATGT AACATGAGGA   
  
  
- ACCTCATATT AGAATAGAAG TGATAACTAT CCGAGCTTCG TATTAGTCAC TAACCTTTGA AAACCAACGT   
  
  
- TATTAAGTTT TAAACTATGA GATACTAAAT GAAAAAATTA ACTTCCAGTA CTAAATTAAT TGATTTACTT   
  
  
- TTCTTAAATA CTAGCTTTTT AAAATTGAGA ATAACTGAAT ATCAACTGAG GTCAATTTAC CTTTTAACTT   
  
  
- CACTAATTAG ATTTTCACCG GGCGAGATTA TGGTAAACTC TTTTTAATAA GAATGACGTT TCAAATTTAG   
  
  
- TTATACACTT CTATCAAATA TCAAAATAGG ATTTTCTTTA ATTTATCTAA CAATTTTTAT TAAAAATTTA   
  
  
- AGTCTATATG CACACTACCG GTATTGGCAT TTACTATACG TACAGCTGTT TAAGGTTTCT TCCGATTATT   
  
  
- AATCTGAATT TATTATGTAC GTATGATTGG TTTTTTTTTT TTAATTCGTT CTAAGATAGG TTGGATAGTA   
  
  
- TTATTTCAAA CCGGTATGAA GTTTGGTAGT GTATTGTAGT GATTATATAT TTCGATCCAA AACCCTTTGG   
  
  
- ATTGAAACTC CTGGTGTATA AACAATTCCG GATCCTCCGG TACTCAAACA GTTCTACGGA GTATCATACC   
  
  
- TCTCCAGATA TAGGAACGTT TTACTAGTAA GGGAAGATGG ATTTTCTTGG TGAAGTAAAG GTGAAAATGA   
  
  
- GGGACCGTTT ACCAACTATT TGGTTTGGTA TTGGTTCGGG AACTTCTTGA TATTTCTGTG CTTAGCTAGT   
  
  
- AACTTAAGCT TGTTCGGAAC ACACTATCCT CCTACCCGGA GTTGTGAAGA GGCGCTCACA CGTTCTCGTT   
  
  
- AGAGCCTCTT TTTAGGATTC TCGGTTGTTG AAGAAACCCA CAATTTACTT GAACAAAGAG GAATACCGCT   
  
  
- AACGCTTGTT TCTAACCGTA TAATGAAGAA TGTTCACAAC AACCGGTTTC GGTTGTTAAA CCCTGGAGTG   
  
  
- AAAGTACTCT CGGATTTTGA ACGGTACCTC TTTTTGACGA CGAAACTATG GATGTACTTC GAATATAACT   
  
  
- TCAAGGTTCT CCAGTCAGGT ACCTGTTGGA AACCAGTACA CCGAAGTTTA CCACGTTATA ACCTCCTGGA   
  
  
- ATCTTCAAAT GTTTTCAGCT AGTAGTTTCT TTGTTCCGTT TCCTACCTCT TCAAAAGTTC CGATTACCCA   
  
  
- CAAGGGAAGT TCAAAGTACA GTATTTGCTC AATCTATTGG ATCCTCTCGA AGCTTTTCTC CTAAATCTGT   
  
  
- AACTTCTACC ACTCCGGTAG CGACACTTGA CACAAGTTCG GAACGTTGCC CAAGTACACC TCTTCTCCCT   
  
  
- CGTACACGAA CTACACTAAG CTAGATAATC CGGAACATAG TATTGTGACC ACCTCCTTCT TCGTCTAGAG   
  
  
- TGAAGATGTT CTTTGCTGAA GAAGTTCACG AAGCTACTCA CAAACTCTAA AAGATTCAGT ATAAAGCTAT   
  
  
- ACAATCTTCT TTCGAAGGGA GGTTATTCGT TGCTTGCTTA GTTCGATCTT TCCCTTGTTA CCTACTTATA   
  
  
- GAGGTCTCGG GATCGAACAG TACCACTTTC ACCTCCTCTT ATATCCGGTT TCTTTCCTTG AGTTACCTTA   
  
  
- CTCTACGAGC TTGTTCGTAA AACGGGTAGA GTTAAATCAC TACTACATGA TTCACTACAA TCCCGTAACA   
  
  
- ACTTTTCTGT GTTTTCACCA ACCCTAGAGT GGAATGGTGT TTCACTGGTA CTTTATCCGT ATGTGAATTT   
  
  
- AACCTTCCCA CTTTTACAAC AAACCCGAAG ACGTACCTCT GGATCGAT

+     Myb

| Site Name | Organism | Position | Strand | Matrix score. | sequence | function |
| --- | --- | --- | --- | --- | --- | --- |
| Myb | Arabidopsis thaliana | 1595 | - | 6 | TAACTG |  |
| Myb | Arabidopsis thaliana | 405 | - | 6 | CAACTG |  |

>HU02G03154.1   
+ +Up\_Stream \_Len000ATGATA AAAGATATTT ATTAAGCTCA AGCTATTTTA ACTTTTAAAA AAATGAATGT   
  
  
+ GGCTTTTTTT GCTATTCAGG TACAACACAA TATGAAGCAT TAACTCTAGA AAATGTGGTA ATGTATGTCA   
  
  
+ TATAGGAGAG TATATACTCT CACTTGCCAT GCGATGCGTG CATGTGTGTG TGTGTGTGTA TCTGTTTCTA   
  
  
+ TGCATATTTT ATGTGGGGTT TTGTCATCTA GACATCTATT AATATGTTAG CATTCACATG ACTTATAGGA   
  
  
+ ATGCACTATT ATGAGTATGA TATAGAGTTT TAAAAATGCA TATGCCTTGT GTGTTCATTA GCTTAAAGAA   
  
  
+ GAGTTTTTGA ATATACATAT TTAGGTAATT CTTGCTAATG TCGTAATTAG CAGTTGTATC TATCTTATTG   
  
  
+ ATTTGTATTT TTAATTGCTT GTTATACTGT TACACTTAGA AAATTCATGA ATACTCTTAA AATATGCAAA   
  
  
+ TTATATATAT ATATATATAT ATATATATAT ATATATATAT ATAAAAACTG AATAAGTTAA TATGGTGATG   
  
  
+ ACATTTGTCA TGTTATCATT GGTCGCTTAA TTTTTAAATA AAAAATATGA TTTTGTGACA CCTAATAAAT   
  
  
+ GATATTAGCC ATATTATTAA ATTCAGCATT TATTCATCAA TGTAATATAC TAAATCATGC AATTATCATT   
  
  
+ GGTTCTCTTT TATGTAATAA AGTTAATTGA CAATTCAAGG GTTACTAATT AGTATAATGT TGAATTGGAA   
  
  
+ CGTAAAAATC AAATCGTATG TAAAGTAATT TTATTCTGAA TTTAAAAAAT GGAGCAAATA TCTCTATTGT   
  
  
+ TCTTTAATAA AATTTTAATT GATTTTTATT TCTAATTAAG TGACGGAACC AAATTTTTTT CCTCCTTTTT   
  
  
+ TGGTTTGGTA AGTTATCATT AGCCAATCTT TTATTTACAT TTTGTCAGTT TTTCTTTTTT AAGATGGTTA   
  
  
+ AATGGCTTCA ATTAAACAAA TTTTTTTACT TATTTATATT TTTAATAAAT TTTCAAAATG TATATTAACG   
  
  
+ CATGACCGTG CGAAGCACGG AATCTACCCT AGTTAATCTA TGATTCTTAA ATATGCAAGT AGGCTGTTAT   
  
  
+ TCATTTGTTG TTTTTTACTT ATTTATATTT TTTCCCTCCC GGTATGATAG GGTTTGTCAT ATTGTTATTC   
  
  
+ ATTAGCTGTT AAACTGTATT CATTATAGCA CAAGTAGGCT TAATCAAAGA TAGTTAGTCT CCAATTTCAT   
  
  
+ CAGAAAATTT AAGAAAAAGA CACCTTAGGA TCAAAAGTAT GAAATTTAGA GACCAGACAA CGATAGAATT   
  
  
+ GAAAAGATGA GTTTTCAATT TGGAGAAGAC GGAAACTCAG ATGAATGAAA GTATAGTACA TTGTACTCCT   
  
  
+ TGGAGTATAA TCTTATCTTC ACTATTGATA GGCTCGAAGC ATAATCAGTG ATTGGAAACT TTTGGTTGCA   
  
  
+ ATAATTCAAA ATTTGATACT CTATGATTTA CTTTTTTAAT TGAAGGTCAT GATTTAATTA ACTAAATGAA   
  
  
+ AAGAATTTAT GATCGAAAAA TTTTAACTCT TATTGACTTA TAGTTGACTC CAGTTAAATG GAAAATTGAA   
  
  
+ GTGATTAATC TAAAAGTGGC CCGCTCTAAT ACCATTTGAG AAAAATTATT CTTACTGCAA AGTTTAAATC   
  
  
+ AATATGTGAA GATAGTTTAT AGTTTTATCC TAAAAGAAAT TAAATAGATT GTTAAAAATA ATTTTTAAAT   
  
  
+ TCAGATATAC GTGTGATGGC CATAACCGTA AATGATATGC ATGTCGACAA ATTCCAAAGA AGGCTAATAA   
  
  
+ TTAGACTTAA ATAATACATG CATACTAACC AAAAAAAAAA AATTAAGCAA GATTCTATCC AACCTATCAT   
  
  
+ AATAAAGTTT GGCCATACTT CAAACCATCA CATAACATCA CTAATATATA AAGCTAGGTT TTGGGAAACC   
  
  
+ TAACTTTGAG GACCACATAT TTGTTAAGGC CTAGGAGGCC ATGAGTTTGT CAAGATGCCT CATAGTATGG   
  
  
+ AGAGGTCTAT ATCCTTGCAA AATGATCATT CCCTTCTACC TAAAAGAACC ACTTCATTTC CACTTTTACT   
  
  
+ CCCTGGCAAA TGGTTGATAA ACCAAACCAT AACCAAGCCC TTGAAGAACT ATAAAGACAC GAATCGATCA   
  
  
+ TTGAATTCGA ACAAGCCTTG TGTGATAGGA GGATGGGCCT CAACACTTCT CCGCGAGTGT GCAAGAGCAA   
  
  
+ TCTCGGAGAA AAATCCTAAG AGCCAACAAC TTCTTTGGGT GTTAAATGAA CTTGTTTCTC CTTATGGCGA   
  
  
+ TTGCGAACAA AGATTGGCAT ATTACTTCTT ACAAGTGTTG TTGGCCAAAG CCAACAATTT GGGACCTCAC   
  
  
+ TTTCATGAGA GCCTAAAACT TGCCATGGAG AAAAACTGCT GCTTTGATAC CTACATGAAG CTTATATTGA   
  
  
+ AGTTCCAAGA GGTCAGTCCA TGGACAACCT TTGGTCATGT GGCTTCAAAT GGTGCAATAT TGGAGGACCT   
  
  
+ TAGAAGTTTA CAAAAGTCGA TCATCAAAGA AACAAGGCAA AGGATGGAGA AGTTTTCAAG GCTAATGGGT   
  
  
+ GTTCCCTTCA AGTTTCATGT CATAAACGAG TTAGATAACC TAGGAGAGCT TCGAAAAGAG GATTTAGACA   
  
  
+ TTGAAGATGG TGAGGCCATC GCTGTGAACT GTGTTCAAGC CTTGCAACGG GTTCATGTGG AGAAGAGGGA   
  
  
+ GCATGTGCTT GATGTGATTC GATCTATTAG GCCTTGTATC ATAACACTGG TGGAGGAAGA AGCAGATCTC   
  
  
+ ACTTCTACAA GAAACGACTT CTTCAAGTGC TTCGATGAGT GTTTGAGATT TTCTAAGTCA TATTTCGATA   
  
  
+ TGTTAGAAGA AAGCTTCCCT CCAATAAGCA ACGAACGAAT CAAGCTAGAA AGGGAACAAT GGATGAATAT   
  
  
+ CTCCAGAGCC CTAGCTTGTC ATGGTGAAAG TGGAGGAGAA TATAGGCCAA AGAAAGGAAC TCAATGGAAT   
  
  
+ GAGATGCTCG AACAAGCATT TTGCCCATCT CAATTTAGTG ATGATGTACT AAGTGATGTT AGGGCATTGT   
  
  
+ TGAAAAGACA CAAAAGTGGT TGGGATCTCA CCTTACCACA AAGTGACCAT GAAATAGGCA TACACTTAAA   
  
  
+ TTGGAAGGGT GAAAATGTTG TTTGGGCTTC TGCATGGAGA CCTAGCTA  

- +Up\_Stream \_Len000TACTAT TTTCTATAAA TAATTCGAGT TCGATAAAAT TGAAAATTTT TTTACTTACA   
  
  
- CCGAAAAAAA CGATAAGTCC ATGTTGTGTT ATACTTCGTA ATTGAGATCT TTTACACCAT TACATACAGT   
  
  
- ATATCCTCTC ATATATGAGA GTGAACGGTA CGCTACGCAC GTACACACAC ACACACACAT AGACAAAGAT   
  
  
- ACGTATAAAA TACACCCCAA AACAGTAGAT CTGTAGATAA TTATACAATC GTAAGTGTAC TGAATATCCT   
  
  
- TACGTGATAA TACTCATACT ATATCTCAAA ATTTTTACGT ATACGGAACA CACAAGTAAT CGAATTTCTT   
  
  
- CTCAAAAACT TATATGTATA AATCCATTAA GAACGATTAC AGCATTAATC GTCAACATAG ATAGAATAAC   
  
  
- TAAACATAAA AATTAACGAA CAATATGACA ATGTGAATCT TTTAAGTACT TATGAGAATT TTATACGTTT   
  
  
- AATATATATA TATATATATA TATATATATA TATATATATA TATTTTTGAC TTATTCAATT ATACCACTAC   
  
  
- TGTAAACAGT ACAATAGTAA CCAGCGAATT AAAAATTTAT TTTTTATACT AAAACACTGT GGATTATTTA   
  
  
- CTATAATCGG TATAATAATT TAAGTCGTAA ATAAGTAGTT ACATTATATG ATTTAGTACG TTAATAGTAA   
  
  
- CCAAGAGAAA ATACATTATT TCAATTAACT GTTAAGTTCC CAATGATTAA TCATATTACA ACTTAACCTT   
  
  
- GCATTTTTAG TTTAGCATAC ATTTCATTAA AATAAGACTT AAATTTTTTA CCTCGTTTAT AGAGATAACA   
  
  
- AGAAATTATT TTAAAATTAA CTAAAAATAA AGATTAATTC ACTGCCTTGG TTTAAAAAAA GGAGGAAAAA   
  
  
- ACCAAACCAT TCAATAGTAA TCGGTTAGAA AATAAATGTA AAACAGTCAA AAAGAAAAAA TTCTACCAAT   
  
  
- TTACCGAAGT TAATTTGTTT AAAAAAATGA ATAAATATAA AAATTATTTA AAAGTTTTAC ATATAATTGC   
  
  
- GTACTGGCAC GCTTCGTGCC TTAGATGGGA TCAATTAGAT ACTAAGAATT TATACGTTCA TCCGACAATA   
  
  
- AGTAAACAAC AAAAAATGAA TAAATATAAA AAAGGGAGGG CCATACTATC CCAAACAGTA TAACAATAAG   
  
  
- TAATCGACAA TTTGACATAA GTAATATCGT GTTCATCCGA ATTAGTTTCT ATCAATCAGA GGTTAAAGTA   
  
  
- GTCTTTTAAA TTCTTTTTCT GTGGAATCCT AGTTTTCATA CTTTAAATCT CTGGTCTGTT GCTATCTTAA   
  
  
- CTTTTCTACT CAAAAGTTAA ACCTCTTCTG CCTTTGAGTC TACTTACTTT CATATCATGT AACATGAGGA   
  
  
- ACCTCATATT AGAATAGAAG TGATAACTAT CCGAGCTTCG TATTAGTCAC TAACCTTTGA AAACCAACGT   
  
  
- TATTAAGTTT TAAACTATGA GATACTAAAT GAAAAAATTA ACTTCCAGTA CTAAATTAAT TGATTTACTT   
  
  
- TTCTTAAATA CTAGCTTTTT AAAATTGAGA ATAACTGAAT ATCAACTGAG GTCAATTTAC CTTTTAACTT   
  
  
- CACTAATTAG ATTTTCACCG GGCGAGATTA TGGTAAACTC TTTTTAATAA GAATGACGTT TCAAATTTAG   
  
  
- TTATACACTT CTATCAAATA TCAAAATAGG ATTTTCTTTA ATTTATCTAA CAATTTTTAT TAAAAATTTA   
  
  
- AGTCTATATG CACACTACCG GTATTGGCAT TTACTATACG TACAGCTGTT TAAGGTTTCT TCCGATTATT   
  
  
- AATCTGAATT TATTATGTAC GTATGATTGG TTTTTTTTTT TTAATTCGTT CTAAGATAGG TTGGATAGTA   
  
  
- TTATTTCAAA CCGGTATGAA GTTTGGTAGT GTATTGTAGT GATTATATAT TTCGATCCAA AACCCTTTGG   
  
  
- ATTGAAACTC CTGGTGTATA AACAATTCCG GATCCTCCGG TACTCAAACA GTTCTACGGA GTATCATACC   
  
  
- TCTCCAGATA TAGGAACGTT TTACTAGTAA GGGAAGATGG ATTTTCTTGG TGAAGTAAAG GTGAAAATGA   
  
  
- GGGACCGTTT ACCAACTATT TGGTTTGGTA TTGGTTCGGG AACTTCTTGA TATTTCTGTG CTTAGCTAGT   
  
  
- AACTTAAGCT TGTTCGGAAC ACACTATCCT CCTACCCGGA GTTGTGAAGA GGCGCTCACA CGTTCTCGTT   
  
  
- AGAGCCTCTT TTTAGGATTC TCGGTTGTTG AAGAAACCCA CAATTTACTT GAACAAAGAG GAATACCGCT   
  
  
- AACGCTTGTT TCTAACCGTA TAATGAAGAA TGTTCACAAC AACCGGTTTC GGTTGTTAAA CCCTGGAGTG   
  
  
- AAAGTACTCT CGGATTTTGA ACGGTACCTC TTTTTGACGA CGAAACTATG GATGTACTTC GAATATAACT   
  
  
- TCAAGGTTCT CCAGTCAGGT ACCTGTTGGA AACCAGTACA CCGAAGTTTA CCACGTTATA ACCTCCTGGA   
  
  
- ATCTTCAAAT GTTTTCAGCT AGTAGTTTCT TTGTTCCGTT TCCTACCTCT TCAAAAGTTC CGATTACCCA   
  
  
- CAAGGGAAGT TCAAAGTACA GTATTTGCTC AATCTATTGG ATCCTCTCGA AGCTTTTCTC CTAAATCTGT   
  
  
- AACTTCTACC ACTCCGGTAG CGACACTTGA CACAAGTTCG GAACGTTGCC CAAGTACACC TCTTCTCCCT   
  
  
- CGTACACGAA CTACACTAAG CTAGATAATC CGGAACATAG TATTGTGACC ACCTCCTTCT TCGTCTAGAG   
  
  
- TGAAGATGTT CTTTGCTGAA GAAGTTCACG AAGCTACTCA CAAACTCTAA AAGATTCAGT ATAAAGCTAT   
  
  
- ACAATCTTCT TTCGAAGGGA GGTTATTCGT TGCTTGCTTA GTTCGATCTT TCCCTTGTTA CCTACTTATA   
  
  
- GAGGTCTCGG GATCGAACAG TACCACTTTC ACCTCCTCTT ATATCCGGTT TCTTTCCTTG AGTTACCTTA   
  
  
- CTCTACGAGC TTGTTCGTAA AACGGGTAGA GTTAAATCAC TACTACATGA TTCACTACAA TCCCGTAACA   
  
  
- ACTTTTCTGT GTTTTCACCA ACCCTAGAGT GGAATGGTGT TTCACTGGTA CTTTATCCGT ATGTGAATTT   
  
  
- AACCTTCCCA CTTTTACAAC AAACCCGAAG ACGTACCTCT GGATCGAT

+     TATA-box

| Site Name | Organism | Position | Strand | Matrix score. | sequence | function |
| --- | --- | --- | --- | --- | --- | --- |
| TATA-box | Oryza sativa | 2533 | + | 7 | TACAAAA | core promoter element around -30 of transcription start |
| TATA-box | Arabidopsis thaliana | 524 | + | 6 | TATATA | core promoter element around -30 of transcription start |
| TATA-box | Arabidopsis thaliana | 534 | + | 4 | TATA | core promoter element around -30 of transcription start |
| TATA-box | Arabidopsis thaliana | 518 | + | 6 | TATATA | core promoter element around -30 of transcription start |
| TATA-box | Arabidopsis thaliana | 2447 | - | 4 | TATA | core promoter element around -30 of transcription start |
| TATA-box | Arabidopsis thaliana | 1147 | - | 5 | TATAA | core promoter element around -30 of transcription start |
| TATA-box | Brassica napus | 499 | + | 6 | ATATAT | core promoter element around -30 of transcription start |
| TATA-box | Brassica napus | 505 | + | 6 | ATATAT | core promoter element around -30 of transcription start |
| TATA-box | Brassica napus | 494 | + | 6 | ATTATA | core promoter element around -30 of transcription start |
| TATA-box | Daucus carota | 1015 | - | 8 | TATAAATA | core promoter element around -30 of transcription start |
| TATA-box | Helianthus annuus | 1146 | - | 6 | TATAAA | core promoter element around -30 of transcription start |
| TATA-box | Helianthus annuus | 1017 | - | 6 | TATAAA | core promoter element around -30 of transcription start |
| TATA-box | Brassica napus | 517 | + | 6 | ATATAT | core promoter element around -30 of transcription start |
| TATA-box | Arabidopsis thaliana | 157 | + | 4 | TATA | core promoter element around -30 of transcription start |
| TATA-box | Brassica napus | 529 | + | 6 | ATATAT | core promoter element around -30 of transcription start |
| TATA-box | Brassica juncea | 1145 | - | 7 | TATAAAT | core promoter element around -30 of transcription start |
| TATA-box | Arabidopsis thaliana | 155 | + | 6 | TATATA | core promoter element around -30 of transcription start |
| TATA-box | Arabidopsis thaliana | 514 | + | 6 | TATATA | core promoter element around -30 of transcription start |
| TATA-box | Arabidopsis thaliana | 510 | + | 6 | TATATA | core promoter element around -30 of transcription start |
| TATA-box | Arabidopsis thaliana | 1217 | - | 5 | TATAA | core promoter element around -30 of transcription start |
| TATA-box | Brassica napus | 1216 | + | 6 | ATTATA | core promoter element around -30 of transcription start |
| TATA-box | Arabidopsis thaliana | 1410 | + | 4 | TATA | core promoter element around -30 of transcription start |
| TATA-box | Brassica napus | 501 | + | 6 | ATATAT | core promoter element around -30 of transcription start |
| TATA-box | Arabidopsis thaliana | 1218 | + | 4 | TATA | core promoter element around -30 of transcription start |
| TATA-box | Arabidopsis thaliana | 1582 | - | 5 | TATAA | core promoter element around -30 of transcription start |
| TATA-box | Arabidopsis thaliana | 495 | - | 7 | TATATAA | core promoter element around -30 of transcription start |
| TATA-box | Brassica napus | 497 | + | 6 | ATATAT | core promoter element around -30 of transcription start |
| TATA-box | Arabidopsis thaliana | 504 | + | 6 | TATATA | core promoter element around -30 of transcription start |
| TATA-box | Arabidopsis thaliana | 530 | + | 6 | TATATA | core promoter element around -30 of transcription start |
| TATA-box | Arabidopsis thaliana | 680 | + | 4 | TATA | core promoter element around -30 of transcription start |
| TATA-box | Arabidopsis thaliana | 597 | - | 8 | TATTTAAA | core promoter element around -30 of transcription start |
| TATA-box | Arabidopsis thaliana | 1386 | + | 4 | TATA | core promoter element around -30 of transcription start |
| TATA-box | Arabidopsis thaliana | 1018 | - | 5 | TATAA | core promoter element around -30 of transcription start |
| TATA-box | Brassica juncea | 1016 | - | 7 | TATAAAT | core promoter element around -30 of transcription start |
| TATA-box | Helianthus annuus | 1043 | - | 6 | TATACA | core promoter element around -30 of transcription start |
| TATA-box | Arabidopsis thaliana | 2985 | - | 4 | TATA | core promoter element around -30 of transcription start |
| TATA-box | Brassica napus | 1938 | - | 6 | ATATAT | core promoter element around -30 of transcription start |
| TATA-box | Arabidopsis thaliana | 526 | + | 6 | TATATA | core promoter element around -30 of transcription start |
| TATA-box | Brassica napus | 523 | + | 6 | ATATAT | core promoter element around -30 of transcription start |
| TATA-box | Brassica oleracea | 1940 | + | 6 | ATATAA | core promoter element around -30 of transcription start |
| TATA-box | Arabidopsis thaliana | 1939 | - | 6 | TATATA | core promoter element around -30 of transcription start |
| TATA-box | Brassica napus | 525 | + | 6 | ATATAT | core promoter element around -30 of transcription start |
| TATA-box | Brassica oleracea | 533 | + | 6 | ATATAA | core promoter element around -30 of transcription start |
| TATA-box | Arabidopsis thaliana | 528 | + | 6 | TATATA | core promoter element around -30 of transcription start |
| TATA-box | Arabidopsis thaliana | 512 | + | 6 | TATATA | core promoter element around -30 of transcription start |
| TATA-box | Brassica napus | 527 | + | 6 | ATATAT | core promoter element around -30 of transcription start |
| TATA-box | Brassica napus | 521 | + | 6 | ATATAT | core promoter element around -30 of transcription start |
| TATA-box | Brassica napus | 511 | + | 6 | ATATAT | core promoter element around -30 of transcription start |
| TATA-box | Arabidopsis thaliana | 1019 | + | 4 | TATA | core promoter element around -30 of transcription start |
| TATA-box | Arabidopsis thaliana | 522 | + | 6 | TATATA | core promoter element around -30 of transcription start |
| TATA-box | Brassica napus | 519 | + | 6 | ATATAT | core promoter element around -30 of transcription start |
| TATA-box | Arabidopsis thaliana | 508 | + | 6 | TATATA | core promoter element around -30 of transcription start |
| TATA-box | Arabidopsis thaliana | 520 | + | 6 | TATATA | core promoter element around -30 of transcription start |
| TATA-box | Arabidopsis thaliana | 1760 | - | 4 | TATA | core promoter element around -30 of transcription start |
| TATA-box | Arabidopsis thaliana | 532 | + | 6 | TATATA | core promoter element around -30 of transcription start |
| TATA-box | Arabidopsis thaliana | 2154 | - | 4 | TATA | core promoter element around -30 of transcription start |
| TATA-box | Brassica napus | 515 | + | 6 | ATATAT | core promoter element around -30 of transcription start |
| TATA-box | Arabidopsis thaliana | 2446 | - | 5 | TATAA | core promoter element around -30 of transcription start |
| TATA-box | Helianthus annuus | 1700 | - | 6 | TATAAA | core promoter element around -30 of transcription start |
| TATA-box | Brassica napus | 507 | + | 6 | ATATAT | core promoter element around -30 of transcription start |
| TATA-box | Brassica napus | 509 | + | 6 | ATATAT | core promoter element around -30 of transcription start |
| TATA-box | Arabidopsis thaliana | 1148 | + | 4 | TATA | core promoter element around -30 of transcription start |
| TATA-box | Arabidopsis thaliana | 498 | + | 6 | TATATA | core promoter element around -30 of transcription start |
| TATA-box | Brassica napus | 503 | + | 6 | ATATAT | core promoter element around -30 of transcription start |
| TATA-box | Brassica napus | 531 | + | 6 | ATATAT | core promoter element around -30 of transcription start |
| TATA-box | Brassica napus | 513 | + | 6 | ATATAT | core promoter element around -30 of transcription start |
| TATA-box | Arabidopsis thaliana | 1701 | - | 5 | TATAA | core promoter element around -30 of transcription start |
| TATA-box | Arabidopsis thaliana | 1583 | + | 4 | TATA | core promoter element around -30 of transcription start |
| TATA-box | Arabidopsis thaliana | 447 | + | 4 | TATA | core promoter element around -30 of transcription start |
| TATA-box | Arabidopsis thaliana | 446 | - | 5 | TATAA | core promoter element around -30 of transcription start |
| TATA-box | Daucus carota | 1144 | - | 8 | TATAAATA | core promoter element around -30 of transcription start |
| TATA-box | Arabidopsis thaliana | 1045 | + | 4 | TATA | core promoter element around -30 of transcription start |
| TATA-box | Arabidopsis thaliana | 500 | + | 6 | TATATA | core promoter element around -30 of transcription start |
| TATA-box | Arabidopsis thaliana | 516 | + | 6 | TATATA | core promoter element around -30 of transcription start |
| TATA-box | Arabidopsis thaliana | 2042 | - | 4 | TATA | core promoter element around -30 of transcription start |
| TATA-box | Arabidopsis thaliana | 1941 | - | 4 | TATA | core promoter element around -30 of transcription start |
| TATA-box | Oryza sativa | 713 | - | 8 | TACATAAA | core promoter element around -30 of transcription start |
| TATA-box | Arabidopsis thaliana | 145 | + | 4 | TATA | core promoter element around -30 of transcription start |
| TATA-box | Arabidopsis thaliana | 757 | + | 4 | TATA | core promoter element around -30 of transcription start |
| TATA-box | Arabidopsis thaliana | 305 | + | 4 | TATA | core promoter element around -30 of transcription start |
| TATA-box | Arabidopsis thaliana | 366 | + | 4 | TATA | core promoter element around -30 of transcription start |
| TATA-box | Arabidopsis thaliana | 1702 | - | 4 | TATA | core promoter element around -30 of transcription start |
| TATA-box | Arabidopsis thaliana | 496 | + | 6 | TATATA | core promoter element around -30 of transcription start |
| TATA-box | Arabidopsis thaliana | 506 | + | 6 | TATATA | core promoter element around -30 of transcription start |
| TATA-box | Arabidopsis thaliana | 278 | + | 4 | TATA | core promoter element around -30 of transcription start |
| TATA-box | Arabidopsis thaliana | 277 | - | 5 | TATAA | core promoter element around -30 of transcription start |
| TATA-box | Arabidopsis thaliana | 502 | + | 6 | TATATA | core promoter element around -30 of transcription start |

>HU02G03154.1   
+ +Up\_Stream \_Len000ATGATA AAAGATATTT ATTAAGCTCA AGCTATTTTA ACTTTTAAAA AAATGAATGT   
  
  
+ GGCTTTTTTT GCTATTCAGG TACAACACAA TATGAAGCAT TAACTCTAGA AAATGTGGTA ATGTATGTCA   
  
  
+ TATAGGAGAG TATATACTCT CACTTGCCAT GCGATGCGTG CATGTGTGTG TGTGTGTGTA TCTGTTTCTA   
  
  
+ TGCATATTTT ATGTGGGGTT TTGTCATCTA GACATCTATT AATATGTTAG CATTCACATG ACTTATAGGA   
  
  
+ ATGCACTATT ATGAGTATGA TATAGAGTTT TAAAAATGCA TATGCCTTGT GTGTTCATTA GCTTAAAGAA   
  
  
+ GAGTTTTTGA ATATACATAT TTAGGTAATT CTTGCTAATG TCGTAATTAG CAGTTGTATC TATCTTATTG   
  
  
+ ATTTGTATTT TTAATTGCTT GTTATACTGT TACACTTAGA AAATTCATGA ATACTCTTAA AATATGCAAA   
  
  
+ TTATATATAT ATATATATAT ATATATATAT ATATATATAT ATAAAAACTG AATAAGTTAA TATGGTGATG   
  
  
+ ACATTTGTCA TGTTATCATT GGTCGCTTAA TTTTTAAATA AAAAATATGA TTTTGTGACA CCTAATAAAT   
  
  
+ GATATTAGCC ATATTATTAA ATTCAGCATT TATTCATCAA TGTAATATAC TAAATCATGC AATTATCATT   
  
  
+ GGTTCTCTTT TATGTAATAA AGTTAATTGA CAATTCAAGG GTTACTAATT AGTATAATGT TGAATTGGAA   
  
  
+ CGTAAAAATC AAATCGTATG TAAAGTAATT TTATTCTGAA TTTAAAAAAT GGAGCAAATA TCTCTATTGT   
  
  
+ TCTTTAATAA AATTTTAATT GATTTTTATT TCTAATTAAG TGACGGAACC AAATTTTTTT CCTCCTTTTT   
  
  
+ TGGTTTGGTA AGTTATCATT AGCCAATCTT TTATTTACAT TTTGTCAGTT TTTCTTTTTT AAGATGGTTA   
  
  
+ AATGGCTTCA ATTAAACAAA TTTTTTTACT TATTTATATT TTTAATAAAT TTTCAAAATG TATATTAACG   
  
  
+ CATGACCGTG CGAAGCACGG AATCTACCCT AGTTAATCTA TGATTCTTAA ATATGCAAGT AGGCTGTTAT   
  
  
+ TCATTTGTTG TTTTTTACTT ATTTATATTT TTTCCCTCCC GGTATGATAG GGTTTGTCAT ATTGTTATTC   
  
  
+ ATTAGCTGTT AAACTGTATT CATTATAGCA CAAGTAGGCT TAATCAAAGA TAGTTAGTCT CCAATTTCAT   
  
  
+ CAGAAAATTT AAGAAAAAGA CACCTTAGGA TCAAAAGTAT GAAATTTAGA GACCAGACAA CGATAGAATT   
  
  
+ GAAAAGATGA GTTTTCAATT TGGAGAAGAC GGAAACTCAG ATGAATGAAA GTATAGTACA TTGTACTCCT   
  
  
+ TGGAGTATAA TCTTATCTTC ACTATTGATA GGCTCGAAGC ATAATCAGTG ATTGGAAACT TTTGGTTGCA   
  
  
+ ATAATTCAAA ATTTGATACT CTATGATTTA CTTTTTTAAT TGAAGGTCAT GATTTAATTA ACTAAATGAA   
  
  
+ AAGAATTTAT GATCGAAAAA TTTTAACTCT TATTGACTTA TAGTTGACTC CAGTTAAATG GAAAATTGAA   
  
  
+ GTGATTAATC TAAAAGTGGC CCGCTCTAAT ACCATTTGAG AAAAATTATT CTTACTGCAA AGTTTAAATC   
  
  
+ AATATGTGAA GATAGTTTAT AGTTTTATCC TAAAAGAAAT TAAATAGATT GTTAAAAATA ATTTTTAAAT   
  
  
+ TCAGATATAC GTGTGATGGC CATAACCGTA AATGATATGC ATGTCGACAA ATTCCAAAGA AGGCTAATAA   
  
  
+ TTAGACTTAA ATAATACATG CATACTAACC AAAAAAAAAA AATTAAGCAA GATTCTATCC AACCTATCAT   
  
  
+ AATAAAGTTT GGCCATACTT CAAACCATCA CATAACATCA CTAATATATA AAGCTAGGTT TTGGGAAACC   
  
  
+ TAACTTTGAG GACCACATAT TTGTTAAGGC CTAGGAGGCC ATGAGTTTGT CAAGATGCCT CATAGTATGG   
  
  
+ AGAGGTCTAT ATCCTTGCAA AATGATCATT CCCTTCTACC TAAAAGAACC ACTTCATTTC CACTTTTACT   
  
  
+ CCCTGGCAAA TGGTTGATAA ACCAAACCAT AACCAAGCCC TTGAAGAACT ATAAAGACAC GAATCGATCA   
  
  
+ TTGAATTCGA ACAAGCCTTG TGTGATAGGA GGATGGGCCT CAACACTTCT CCGCGAGTGT GCAAGAGCAA   
  
  
+ TCTCGGAGAA AAATCCTAAG AGCCAACAAC TTCTTTGGGT GTTAAATGAA CTTGTTTCTC CTTATGGCGA   
  
  
+ TTGCGAACAA AGATTGGCAT ATTACTTCTT ACAAGTGTTG TTGGCCAAAG CCAACAATTT GGGACCTCAC   
  
  
+ TTTCATGAGA GCCTAAAACT TGCCATGGAG AAAAACTGCT GCTTTGATAC CTACATGAAG CTTATATTGA   
  
  
+ AGTTCCAAGA GGTCAGTCCA TGGACAACCT TTGGTCATGT GGCTTCAAAT GGTGCAATAT TGGAGGACCT   
  
  
+ TAGAAGTTTA CAAAAGTCGA TCATCAAAGA AACAAGGCAA AGGATGGAGA AGTTTTCAAG GCTAATGGGT   
  
  
+ GTTCCCTTCA AGTTTCATGT CATAAACGAG TTAGATAACC TAGGAGAGCT TCGAAAAGAG GATTTAGACA   
  
  
+ TTGAAGATGG TGAGGCCATC GCTGTGAACT GTGTTCAAGC CTTGCAACGG GTTCATGTGG AGAAGAGGGA   
  
  
+ GCATGTGCTT GATGTGATTC GATCTATTAG GCCTTGTATC ATAACACTGG TGGAGGAAGA AGCAGATCTC   
  
  
+ ACTTCTACAA GAAACGACTT CTTCAAGTGC TTCGATGAGT GTTTGAGATT TTCTAAGTCA TATTTCGATA   
  
  
+ TGTTAGAAGA AAGCTTCCCT CCAATAAGCA ACGAACGAAT CAAGCTAGAA AGGGAACAAT GGATGAATAT   
  
  
+ CTCCAGAGCC CTAGCTTGTC ATGGTGAAAG TGGAGGAGAA TATAGGCCAA AGAAAGGAAC TCAATGGAAT   
  
  
+ GAGATGCTCG AACAAGCATT TTGCCCATCT CAATTTAGTG ATGATGTACT AAGTGATGTT AGGGCATTGT   
  
  
+ TGAAAAGACA CAAAAGTGGT TGGGATCTCA CCTTACCACA AAGTGACCAT GAAATAGGCA TACACTTAAA   
  
  
+ TTGGAAGGGT GAAAATGTTG TTTGGGCTTC TGCATGGAGA CCTAGCTA  

- +Up\_Stream \_Len000TACTAT TTTCTATAAA TAATTCGAGT TCGATAAAAT TGAAAATTTT TTTACTTACA   
  
  
- CCGAAAAAAA CGATAAGTCC ATGTTGTGTT ATACTTCGTA ATTGAGATCT TTTACACCAT TACATACAGT   
  
  
- ATATCCTCTC ATATATGAGA GTGAACGGTA CGCTACGCAC GTACACACAC ACACACACAT AGACAAAGAT   
  
  
- ACGTATAAAA TACACCCCAA AACAGTAGAT CTGTAGATAA TTATACAATC GTAAGTGTAC TGAATATCCT   
  
  
- TACGTGATAA TACTCATACT ATATCTCAAA ATTTTTACGT ATACGGAACA CACAAGTAAT CGAATTTCTT   
  
  
- CTCAAAAACT TATATGTATA AATCCATTAA GAACGATTAC AGCATTAATC GTCAACATAG ATAGAATAAC   
  
  
- TAAACATAAA AATTAACGAA CAATATGACA ATGTGAATCT TTTAAGTACT TATGAGAATT TTATACGTTT   
  
  
- AATATATATA TATATATATA TATATATATA TATATATATA TATTTTTGAC TTATTCAATT ATACCACTAC   
  
  
- TGTAAACAGT ACAATAGTAA CCAGCGAATT AAAAATTTAT TTTTTATACT AAAACACTGT GGATTATTTA   
  
  
- CTATAATCGG TATAATAATT TAAGTCGTAA ATAAGTAGTT ACATTATATG ATTTAGTACG TTAATAGTAA   
  
  
- CCAAGAGAAA ATACATTATT TCAATTAACT GTTAAGTTCC CAATGATTAA TCATATTACA ACTTAACCTT   
  
  
- GCATTTTTAG TTTAGCATAC ATTTCATTAA AATAAGACTT AAATTTTTTA CCTCGTTTAT AGAGATAACA   
  
  
- AGAAATTATT TTAAAATTAA CTAAAAATAA AGATTAATTC ACTGCCTTGG TTTAAAAAAA GGAGGAAAAA   
  
  
- ACCAAACCAT TCAATAGTAA TCGGTTAGAA AATAAATGTA AAACAGTCAA AAAGAAAAAA TTCTACCAAT   
  
  
- TTACCGAAGT TAATTTGTTT AAAAAAATGA ATAAATATAA AAATTATTTA AAAGTTTTAC ATATAATTGC   
  
  
- GTACTGGCAC GCTTCGTGCC TTAGATGGGA TCAATTAGAT ACTAAGAATT TATACGTTCA TCCGACAATA   
  
  
- AGTAAACAAC AAAAAATGAA TAAATATAAA AAAGGGAGGG CCATACTATC CCAAACAGTA TAACAATAAG   
  
  
- TAATCGACAA TTTGACATAA GTAATATCGT GTTCATCCGA ATTAGTTTCT ATCAATCAGA GGTTAAAGTA   
  
  
- GTCTTTTAAA TTCTTTTTCT GTGGAATCCT AGTTTTCATA CTTTAAATCT CTGGTCTGTT GCTATCTTAA   
  
  
- CTTTTCTACT CAAAAGTTAA ACCTCTTCTG CCTTTGAGTC TACTTACTTT CATATCATGT AACATGAGGA   
  
  
- ACCTCATATT AGAATAGAAG TGATAACTAT CCGAGCTTCG TATTAGTCAC TAACCTTTGA AAACCAACGT   
  
  
- TATTAAGTTT TAAACTATGA GATACTAAAT GAAAAAATTA ACTTCCAGTA CTAAATTAAT TGATTTACTT   
  
  
- TTCTTAAATA CTAGCTTTTT AAAATTGAGA ATAACTGAAT ATCAACTGAG GTCAATTTAC CTTTTAACTT   
  
  
- CACTAATTAG ATTTTCACCG GGCGAGATTA TGGTAAACTC TTTTTAATAA GAATGACGTT TCAAATTTAG   
  
  
- TTATACACTT CTATCAAATA TCAAAATAGG ATTTTCTTTA ATTTATCTAA CAATTTTTAT TAAAAATTTA   
  
  
- AGTCTATATG CACACTACCG GTATTGGCAT TTACTATACG TACAGCTGTT TAAGGTTTCT TCCGATTATT   
  
  
- AATCTGAATT TATTATGTAC GTATGATTGG TTTTTTTTTT TTAATTCGTT CTAAGATAGG TTGGATAGTA   
  
  
- TTATTTCAAA CCGGTATGAA GTTTGGTAGT GTATTGTAGT GATTATATAT TTCGATCCAA AACCCTTTGG   
  
  
- ATTGAAACTC CTGGTGTATA AACAATTCCG GATCCTCCGG TACTCAAACA GTTCTACGGA GTATCATACC   
  
  
- TCTCCAGATA TAGGAACGTT TTACTAGTAA GGGAAGATGG ATTTTCTTGG TGAAGTAAAG GTGAAAATGA   
  
  
- GGGACCGTTT ACCAACTATT TGGTTTGGTA TTGGTTCGGG AACTTCTTGA TATTTCTGTG CTTAGCTAGT   
  
  
- AACTTAAGCT TGTTCGGAAC ACACTATCCT CCTACCCGGA GTTGTGAAGA GGCGCTCACA CGTTCTCGTT   
  
  
- AGAGCCTCTT TTTAGGATTC TCGGTTGTTG AAGAAACCCA CAATTTACTT GAACAAAGAG GAATACCGCT   
  
  
- AACGCTTGTT TCTAACCGTA TAATGAAGAA TGTTCACAAC AACCGGTTTC GGTTGTTAAA CCCTGGAGTG   
  
  
- AAAGTACTCT CGGATTTTGA ACGGTACCTC TTTTTGACGA CGAAACTATG GATGTACTTC GAATATAACT   
  
  
- TCAAGGTTCT CCAGTCAGGT ACCTGTTGGA AACCAGTACA CCGAAGTTTA CCACGTTATA ACCTCCTGGA   
  
  
- ATCTTCAAAT GTTTTCAGCT AGTAGTTTCT TTGTTCCGTT TCCTACCTCT TCAAAAGTTC CGATTACCCA   
  
  
- CAAGGGAAGT TCAAAGTACA GTATTTGCTC AATCTATTGG ATCCTCTCGA AGCTTTTCTC CTAAATCTGT   
  
  
- AACTTCTACC ACTCCGGTAG CGACACTTGA CACAAGTTCG GAACGTTGCC CAAGTACACC TCTTCTCCCT   
  
  
- CGTACACGAA CTACACTAAG CTAGATAATC CGGAACATAG TATTGTGACC ACCTCCTTCT TCGTCTAGAG   
  
  
- TGAAGATGTT CTTTGCTGAA GAAGTTCACG AAGCTACTCA CAAACTCTAA AAGATTCAGT ATAAAGCTAT   
  
  
- ACAATCTTCT TTCGAAGGGA GGTTATTCGT TGCTTGCTTA GTTCGATCTT TCCCTTGTTA CCTACTTATA   
  
  
- GAGGTCTCGG GATCGAACAG TACCACTTTC ACCTCCTCTT ATATCCGGTT TCTTTCCTTG AGTTACCTTA   
  
  
- CTCTACGAGC TTGTTCGTAA AACGGGTAGA GTTAAATCAC TACTACATGA TTCACTACAA TCCCGTAACA   
  
  
- ACTTTTCTGT GTTTTCACCA ACCCTAGAGT GGAATGGTGT TTCACTGGTA CTTTATCCGT ATGTGAATTT   
  
  
- AACCTTCCCA CTTTTACAAC AAACCCGAAG ACGTACCTCT GGATCGAT

+     TCT-motif

| Site Name | Organism | Position | Strand | Matrix score. | sequence | function |
| --- | --- | --- | --- | --- | --- | --- |
| TCT-motif | Arabidopsis thaliana | 2341 | + | 6 | TCTTAC | part of a light responsive element |
| TCT-motif | Arabidopsis thaliana | 1664 | + | 6 | TCTTAC | part of a light responsive element |

>HU02G03154.1   
+ +Up\_Stream \_Len000ATGATA AAAGATATTT ATTAAGCTCA AGCTATTTTA ACTTTTAAAA AAATGAATGT   
  
  
+ GGCTTTTTTT GCTATTCAGG TACAACACAA TATGAAGCAT TAACTCTAGA AAATGTGGTA ATGTATGTCA   
  
  
+ TATAGGAGAG TATATACTCT CACTTGCCAT GCGATGCGTG CATGTGTGTG TGTGTGTGTA TCTGTTTCTA   
  
  
+ TGCATATTTT ATGTGGGGTT TTGTCATCTA GACATCTATT AATATGTTAG CATTCACATG ACTTATAGGA   
  
  
+ ATGCACTATT ATGAGTATGA TATAGAGTTT TAAAAATGCA TATGCCTTGT GTGTTCATTA GCTTAAAGAA   
  
  
+ GAGTTTTTGA ATATACATAT TTAGGTAATT CTTGCTAATG TCGTAATTAG CAGTTGTATC TATCTTATTG   
  
  
+ ATTTGTATTT TTAATTGCTT GTTATACTGT TACACTTAGA AAATTCATGA ATACTCTTAA AATATGCAAA   
  
  
+ TTATATATAT ATATATATAT ATATATATAT ATATATATAT ATAAAAACTG AATAAGTTAA TATGGTGATG   
  
  
+ ACATTTGTCA TGTTATCATT GGTCGCTTAA TTTTTAAATA AAAAATATGA TTTTGTGACA CCTAATAAAT   
  
  
+ GATATTAGCC ATATTATTAA ATTCAGCATT TATTCATCAA TGTAATATAC TAAATCATGC AATTATCATT   
  
  
+ GGTTCTCTTT TATGTAATAA AGTTAATTGA CAATTCAAGG GTTACTAATT AGTATAATGT TGAATTGGAA   
  
  
+ CGTAAAAATC AAATCGTATG TAAAGTAATT TTATTCTGAA TTTAAAAAAT GGAGCAAATA TCTCTATTGT   
  
  
+ TCTTTAATAA AATTTTAATT GATTTTTATT TCTAATTAAG TGACGGAACC AAATTTTTTT CCTCCTTTTT   
  
  
+ TGGTTTGGTA AGTTATCATT AGCCAATCTT TTATTTACAT TTTGTCAGTT TTTCTTTTTT AAGATGGTTA   
  
  
+ AATGGCTTCA ATTAAACAAA TTTTTTTACT TATTTATATT TTTAATAAAT TTTCAAAATG TATATTAACG   
  
  
+ CATGACCGTG CGAAGCACGG AATCTACCCT AGTTAATCTA TGATTCTTAA ATATGCAAGT AGGCTGTTAT   
  
  
+ TCATTTGTTG TTTTTTACTT ATTTATATTT TTTCCCTCCC GGTATGATAG GGTTTGTCAT ATTGTTATTC   
  
  
+ ATTAGCTGTT AAACTGTATT CATTATAGCA CAAGTAGGCT TAATCAAAGA TAGTTAGTCT CCAATTTCAT   
  
  
+ CAGAAAATTT AAGAAAAAGA CACCTTAGGA TCAAAAGTAT GAAATTTAGA GACCAGACAA CGATAGAATT   
  
  
+ GAAAAGATGA GTTTTCAATT TGGAGAAGAC GGAAACTCAG ATGAATGAAA GTATAGTACA TTGTACTCCT   
  
  
+ TGGAGTATAA TCTTATCTTC ACTATTGATA GGCTCGAAGC ATAATCAGTG ATTGGAAACT TTTGGTTGCA   
  
  
+ ATAATTCAAA ATTTGATACT CTATGATTTA CTTTTTTAAT TGAAGGTCAT GATTTAATTA ACTAAATGAA   
  
  
+ AAGAATTTAT GATCGAAAAA TTTTAACTCT TATTGACTTA TAGTTGACTC CAGTTAAATG GAAAATTGAA   
  
  
+ GTGATTAATC TAAAAGTGGC CCGCTCTAAT ACCATTTGAG AAAAATTATT CTTACTGCAA AGTTTAAATC   
  
  
+ AATATGTGAA GATAGTTTAT AGTTTTATCC TAAAAGAAAT TAAATAGATT GTTAAAAATA ATTTTTAAAT   
  
  
+ TCAGATATAC GTGTGATGGC CATAACCGTA AATGATATGC ATGTCGACAA ATTCCAAAGA AGGCTAATAA   
  
  
+ TTAGACTTAA ATAATACATG CATACTAACC AAAAAAAAAA AATTAAGCAA GATTCTATCC AACCTATCAT   
  
  
+ AATAAAGTTT GGCCATACTT CAAACCATCA CATAACATCA CTAATATATA AAGCTAGGTT TTGGGAAACC   
  
  
+ TAACTTTGAG GACCACATAT TTGTTAAGGC CTAGGAGGCC ATGAGTTTGT CAAGATGCCT CATAGTATGG   
  
  
+ AGAGGTCTAT ATCCTTGCAA AATGATCATT CCCTTCTACC TAAAAGAACC ACTTCATTTC CACTTTTACT   
  
  
+ CCCTGGCAAA TGGTTGATAA ACCAAACCAT AACCAAGCCC TTGAAGAACT ATAAAGACAC GAATCGATCA   
  
  
+ TTGAATTCGA ACAAGCCTTG TGTGATAGGA GGATGGGCCT CAACACTTCT CCGCGAGTGT GCAAGAGCAA   
  
  
+ TCTCGGAGAA AAATCCTAAG AGCCAACAAC TTCTTTGGGT GTTAAATGAA CTTGTTTCTC CTTATGGCGA   
  
  
+ TTGCGAACAA AGATTGGCAT ATTACTTCTT ACAAGTGTTG TTGGCCAAAG CCAACAATTT GGGACCTCAC   
  
  
+ TTTCATGAGA GCCTAAAACT TGCCATGGAG AAAAACTGCT GCTTTGATAC CTACATGAAG CTTATATTGA   
  
  
+ AGTTCCAAGA GGTCAGTCCA TGGACAACCT TTGGTCATGT GGCTTCAAAT GGTGCAATAT TGGAGGACCT   
  
  
+ TAGAAGTTTA CAAAAGTCGA TCATCAAAGA AACAAGGCAA AGGATGGAGA AGTTTTCAAG GCTAATGGGT   
  
  
+ GTTCCCTTCA AGTTTCATGT CATAAACGAG TTAGATAACC TAGGAGAGCT TCGAAAAGAG GATTTAGACA   
  
  
+ TTGAAGATGG TGAGGCCATC GCTGTGAACT GTGTTCAAGC CTTGCAACGG GTTCATGTGG AGAAGAGGGA   
  
  
+ GCATGTGCTT GATGTGATTC GATCTATTAG GCCTTGTATC ATAACACTGG TGGAGGAAGA AGCAGATCTC   
  
  
+ ACTTCTACAA GAAACGACTT CTTCAAGTGC TTCGATGAGT GTTTGAGATT TTCTAAGTCA TATTTCGATA   
  
  
+ TGTTAGAAGA AAGCTTCCCT CCAATAAGCA ACGAACGAAT CAAGCTAGAA AGGGAACAAT GGATGAATAT   
  
  
+ CTCCAGAGCC CTAGCTTGTC ATGGTGAAAG TGGAGGAGAA TATAGGCCAA AGAAAGGAAC TCAATGGAAT   
  
  
+ GAGATGCTCG AACAAGCATT TTGCCCATCT CAATTTAGTG ATGATGTACT AAGTGATGTT AGGGCATTGT   
  
  
+ TGAAAAGACA CAAAAGTGGT TGGGATCTCA CCTTACCACA AAGTGACCAT GAAATAGGCA TACACTTAAA   
  
  
+ TTGGAAGGGT GAAAATGTTG TTTGGGCTTC TGCATGGAGA CCTAGCTA  

- +Up\_Stream \_Len000TACTAT TTTCTATAAA TAATTCGAGT TCGATAAAAT TGAAAATTTT TTTACTTACA   
  
  
- CCGAAAAAAA CGATAAGTCC ATGTTGTGTT ATACTTCGTA ATTGAGATCT TTTACACCAT TACATACAGT   
  
  
- ATATCCTCTC ATATATGAGA GTGAACGGTA CGCTACGCAC GTACACACAC ACACACACAT AGACAAAGAT   
  
  
- ACGTATAAAA TACACCCCAA AACAGTAGAT CTGTAGATAA TTATACAATC GTAAGTGTAC TGAATATCCT   
  
  
- TACGTGATAA TACTCATACT ATATCTCAAA ATTTTTACGT ATACGGAACA CACAAGTAAT CGAATTTCTT   
  
  
- CTCAAAAACT TATATGTATA AATCCATTAA GAACGATTAC AGCATTAATC GTCAACATAG ATAGAATAAC   
  
  
- TAAACATAAA AATTAACGAA CAATATGACA ATGTGAATCT TTTAAGTACT TATGAGAATT TTATACGTTT   
  
  
- AATATATATA TATATATATA TATATATATA TATATATATA TATTTTTGAC TTATTCAATT ATACCACTAC   
  
  
- TGTAAACAGT ACAATAGTAA CCAGCGAATT AAAAATTTAT TTTTTATACT AAAACACTGT GGATTATTTA   
  
  
- CTATAATCGG TATAATAATT TAAGTCGTAA ATAAGTAGTT ACATTATATG ATTTAGTACG TTAATAGTAA   
  
  
- CCAAGAGAAA ATACATTATT TCAATTAACT GTTAAGTTCC CAATGATTAA TCATATTACA ACTTAACCTT   
  
  
- GCATTTTTAG TTTAGCATAC ATTTCATTAA AATAAGACTT AAATTTTTTA CCTCGTTTAT AGAGATAACA   
  
  
- AGAAATTATT TTAAAATTAA CTAAAAATAA AGATTAATTC ACTGCCTTGG TTTAAAAAAA GGAGGAAAAA   
  
  
- ACCAAACCAT TCAATAGTAA TCGGTTAGAA AATAAATGTA AAACAGTCAA AAAGAAAAAA TTCTACCAAT   
  
  
- TTACCGAAGT TAATTTGTTT AAAAAAATGA ATAAATATAA AAATTATTTA AAAGTTTTAC ATATAATTGC   
  
  
- GTACTGGCAC GCTTCGTGCC TTAGATGGGA TCAATTAGAT ACTAAGAATT TATACGTTCA TCCGACAATA   
  
  
- AGTAAACAAC AAAAAATGAA TAAATATAAA AAAGGGAGGG CCATACTATC CCAAACAGTA TAACAATAAG   
  
  
- TAATCGACAA TTTGACATAA GTAATATCGT GTTCATCCGA ATTAGTTTCT ATCAATCAGA GGTTAAAGTA   
  
  
- GTCTTTTAAA TTCTTTTTCT GTGGAATCCT AGTTTTCATA CTTTAAATCT CTGGTCTGTT GCTATCTTAA   
  
  
- CTTTTCTACT CAAAAGTTAA ACCTCTTCTG CCTTTGAGTC TACTTACTTT CATATCATGT AACATGAGGA   
  
  
- ACCTCATATT AGAATAGAAG TGATAACTAT CCGAGCTTCG TATTAGTCAC TAACCTTTGA AAACCAACGT   
  
  
- TATTAAGTTT TAAACTATGA GATACTAAAT GAAAAAATTA ACTTCCAGTA CTAAATTAAT TGATTTACTT   
  
  
- TTCTTAAATA CTAGCTTTTT AAAATTGAGA ATAACTGAAT ATCAACTGAG GTCAATTTAC CTTTTAACTT   
  
  
- CACTAATTAG ATTTTCACCG GGCGAGATTA TGGTAAACTC TTTTTAATAA GAATGACGTT TCAAATTTAG   
  
  
- TTATACACTT CTATCAAATA TCAAAATAGG ATTTTCTTTA ATTTATCTAA CAATTTTTAT TAAAAATTTA   
  
  
- AGTCTATATG CACACTACCG GTATTGGCAT TTACTATACG TACAGCTGTT TAAGGTTTCT TCCGATTATT   
  
  
- AATCTGAATT TATTATGTAC GTATGATTGG TTTTTTTTTT TTAATTCGTT CTAAGATAGG TTGGATAGTA   
  
  
- TTATTTCAAA CCGGTATGAA GTTTGGTAGT GTATTGTAGT GATTATATAT TTCGATCCAA AACCCTTTGG   
  
  
- ATTGAAACTC CTGGTGTATA AACAATTCCG GATCCTCCGG TACTCAAACA GTTCTACGGA GTATCATACC   
  
  
- TCTCCAGATA TAGGAACGTT TTACTAGTAA GGGAAGATGG ATTTTCTTGG TGAAGTAAAG GTGAAAATGA   
  
  
- GGGACCGTTT ACCAACTATT TGGTTTGGTA TTGGTTCGGG AACTTCTTGA TATTTCTGTG CTTAGCTAGT   
  
  
- AACTTAAGCT TGTTCGGAAC ACACTATCCT CCTACCCGGA GTTGTGAAGA GGCGCTCACA CGTTCTCGTT   
  
  
- AGAGCCTCTT TTTAGGATTC TCGGTTGTTG AAGAAACCCA CAATTTACTT GAACAAAGAG GAATACCGCT   
  
  
- AACGCTTGTT TCTAACCGTA TAATGAAGAA TGTTCACAAC AACCGGTTTC GGTTGTTAAA CCCTGGAGTG   
  
  
- AAAGTACTCT CGGATTTTGA ACGGTACCTC TTTTTGACGA CGAAACTATG GATGTACTTC GAATATAACT   
  
  
- TCAAGGTTCT CCAGTCAGGT ACCTGTTGGA AACCAGTACA CCGAAGTTTA CCACGTTATA ACCTCCTGGA   
  
  
- ATCTTCAAAT GTTTTCAGCT AGTAGTTTCT TTGTTCCGTT TCCTACCTCT TCAAAAGTTC CGATTACCCA   
  
  
- CAAGGGAAGT TCAAAGTACA GTATTTGCTC AATCTATTGG ATCCTCTCGA AGCTTTTCTC CTAAATCTGT   
  
  
- AACTTCTACC ACTCCGGTAG CGACACTTGA CACAAGTTCG GAACGTTGCC CAAGTACACC TCTTCTCCCT   
  
  
- CGTACACGAA CTACACTAAG CTAGATAATC CGGAACATAG TATTGTGACC ACCTCCTTCT TCGTCTAGAG   
  
  
- TGAAGATGTT CTTTGCTGAA GAAGTTCACG AAGCTACTCA CAAACTCTAA AAGATTCAGT ATAAAGCTAT   
  
  
- ACAATCTTCT TTCGAAGGGA GGTTATTCGT TGCTTGCTTA GTTCGATCTT TCCCTTGTTA CCTACTTATA   
  
  
- GAGGTCTCGG GATCGAACAG TACCACTTTC ACCTCCTCTT ATATCCGGTT TCTTTCCTTG AGTTACCTTA   
  
  
- CTCTACGAGC TTGTTCGTAA AACGGGTAGA GTTAAATCAC TACTACATGA TTCACTACAA TCCCGTAACA   
  
  
- ACTTTTCTGT GTTTTCACCA ACCCTAGAGT GGAATGGTGT TTCACTGGTA CTTTATCCGT ATGTGAATTT   
  
  
- AACCTTCCCA CTTTTACAAC AAACCCGAAG ACGTACCTCT GGATCGAT

+     TGA-element

| Site Name | Organism | Position | Strand | Matrix score. | sequence | function |
| --- | --- | --- | --- | --- | --- | --- |
| TGA-element | Brassica oleracea | 2817 | + | 6 | AACGAC | auxin-responsive element |

>HU02G03154.1   
+ +Up\_Stream \_Len000ATGATA AAAGATATTT ATTAAGCTCA AGCTATTTTA ACTTTTAAAA AAATGAATGT   
  
  
+ GGCTTTTTTT GCTATTCAGG TACAACACAA TATGAAGCAT TAACTCTAGA AAATGTGGTA ATGTATGTCA   
  
  
+ TATAGGAGAG TATATACTCT CACTTGCCAT GCGATGCGTG CATGTGTGTG TGTGTGTGTA TCTGTTTCTA   
  
  
+ TGCATATTTT ATGTGGGGTT TTGTCATCTA GACATCTATT AATATGTTAG CATTCACATG ACTTATAGGA   
  
  
+ ATGCACTATT ATGAGTATGA TATAGAGTTT TAAAAATGCA TATGCCTTGT GTGTTCATTA GCTTAAAGAA   
  
  
+ GAGTTTTTGA ATATACATAT TTAGGTAATT CTTGCTAATG TCGTAATTAG CAGTTGTATC TATCTTATTG   
  
  
+ ATTTGTATTT TTAATTGCTT GTTATACTGT TACACTTAGA AAATTCATGA ATACTCTTAA AATATGCAAA   
  
  
+ TTATATATAT ATATATATAT ATATATATAT ATATATATAT ATAAAAACTG AATAAGTTAA TATGGTGATG   
  
  
+ ACATTTGTCA TGTTATCATT GGTCGCTTAA TTTTTAAATA AAAAATATGA TTTTGTGACA CCTAATAAAT   
  
  
+ GATATTAGCC ATATTATTAA ATTCAGCATT TATTCATCAA TGTAATATAC TAAATCATGC AATTATCATT   
  
  
+ GGTTCTCTTT TATGTAATAA AGTTAATTGA CAATTCAAGG GTTACTAATT AGTATAATGT TGAATTGGAA   
  
  
+ CGTAAAAATC AAATCGTATG TAAAGTAATT TTATTCTGAA TTTAAAAAAT GGAGCAAATA TCTCTATTGT   
  
  
+ TCTTTAATAA AATTTTAATT GATTTTTATT TCTAATTAAG TGACGGAACC AAATTTTTTT CCTCCTTTTT   
  
  
+ TGGTTTGGTA AGTTATCATT AGCCAATCTT TTATTTACAT TTTGTCAGTT TTTCTTTTTT AAGATGGTTA   
  
  
+ AATGGCTTCA ATTAAACAAA TTTTTTTACT TATTTATATT TTTAATAAAT TTTCAAAATG TATATTAACG   
  
  
+ CATGACCGTG CGAAGCACGG AATCTACCCT AGTTAATCTA TGATTCTTAA ATATGCAAGT AGGCTGTTAT   
  
  
+ TCATTTGTTG TTTTTTACTT ATTTATATTT TTTCCCTCCC GGTATGATAG GGTTTGTCAT ATTGTTATTC   
  
  
+ ATTAGCTGTT AAACTGTATT CATTATAGCA CAAGTAGGCT TAATCAAAGA TAGTTAGTCT CCAATTTCAT   
  
  
+ CAGAAAATTT AAGAAAAAGA CACCTTAGGA TCAAAAGTAT GAAATTTAGA GACCAGACAA CGATAGAATT   
  
  
+ GAAAAGATGA GTTTTCAATT TGGAGAAGAC GGAAACTCAG ATGAATGAAA GTATAGTACA TTGTACTCCT   
  
  
+ TGGAGTATAA TCTTATCTTC ACTATTGATA GGCTCGAAGC ATAATCAGTG ATTGGAAACT TTTGGTTGCA   
  
  
+ ATAATTCAAA ATTTGATACT CTATGATTTA CTTTTTTAAT TGAAGGTCAT GATTTAATTA ACTAAATGAA   
  
  
+ AAGAATTTAT GATCGAAAAA TTTTAACTCT TATTGACTTA TAGTTGACTC CAGTTAAATG GAAAATTGAA   
  
  
+ GTGATTAATC TAAAAGTGGC CCGCTCTAAT ACCATTTGAG AAAAATTATT CTTACTGCAA AGTTTAAATC   
  
  
+ AATATGTGAA GATAGTTTAT AGTTTTATCC TAAAAGAAAT TAAATAGATT GTTAAAAATA ATTTTTAAAT   
  
  
+ TCAGATATAC GTGTGATGGC CATAACCGTA AATGATATGC ATGTCGACAA ATTCCAAAGA AGGCTAATAA   
  
  
+ TTAGACTTAA ATAATACATG CATACTAACC AAAAAAAAAA AATTAAGCAA GATTCTATCC AACCTATCAT   
  
  
+ AATAAAGTTT GGCCATACTT CAAACCATCA CATAACATCA CTAATATATA AAGCTAGGTT TTGGGAAACC   
  
  
+ TAACTTTGAG GACCACATAT TTGTTAAGGC CTAGGAGGCC ATGAGTTTGT CAAGATGCCT CATAGTATGG   
  
  
+ AGAGGTCTAT ATCCTTGCAA AATGATCATT CCCTTCTACC TAAAAGAACC ACTTCATTTC CACTTTTACT   
  
  
+ CCCTGGCAAA TGGTTGATAA ACCAAACCAT AACCAAGCCC TTGAAGAACT ATAAAGACAC GAATCGATCA   
  
  
+ TTGAATTCGA ACAAGCCTTG TGTGATAGGA GGATGGGCCT CAACACTTCT CCGCGAGTGT GCAAGAGCAA   
  
  
+ TCTCGGAGAA AAATCCTAAG AGCCAACAAC TTCTTTGGGT GTTAAATGAA CTTGTTTCTC CTTATGGCGA   
  
  
+ TTGCGAACAA AGATTGGCAT ATTACTTCTT ACAAGTGTTG TTGGCCAAAG CCAACAATTT GGGACCTCAC   
  
  
+ TTTCATGAGA GCCTAAAACT TGCCATGGAG AAAAACTGCT GCTTTGATAC CTACATGAAG CTTATATTGA   
  
  
+ AGTTCCAAGA GGTCAGTCCA TGGACAACCT TTGGTCATGT GGCTTCAAAT GGTGCAATAT TGGAGGACCT   
  
  
+ TAGAAGTTTA CAAAAGTCGA TCATCAAAGA AACAAGGCAA AGGATGGAGA AGTTTTCAAG GCTAATGGGT   
  
  
+ GTTCCCTTCA AGTTTCATGT CATAAACGAG TTAGATAACC TAGGAGAGCT TCGAAAAGAG GATTTAGACA   
  
  
+ TTGAAGATGG TGAGGCCATC GCTGTGAACT GTGTTCAAGC CTTGCAACGG GTTCATGTGG AGAAGAGGGA   
  
  
+ GCATGTGCTT GATGTGATTC GATCTATTAG GCCTTGTATC ATAACACTGG TGGAGGAAGA AGCAGATCTC   
  
  
+ ACTTCTACAA GAAACGACTT CTTCAAGTGC TTCGATGAGT GTTTGAGATT TTCTAAGTCA TATTTCGATA   
  
  
+ TGTTAGAAGA AAGCTTCCCT CCAATAAGCA ACGAACGAAT CAAGCTAGAA AGGGAACAAT GGATGAATAT   
  
  
+ CTCCAGAGCC CTAGCTTGTC ATGGTGAAAG TGGAGGAGAA TATAGGCCAA AGAAAGGAAC TCAATGGAAT   
  
  
+ GAGATGCTCG AACAAGCATT TTGCCCATCT CAATTTAGTG ATGATGTACT AAGTGATGTT AGGGCATTGT   
  
  
+ TGAAAAGACA CAAAAGTGGT TGGGATCTCA CCTTACCACA AAGTGACCAT GAAATAGGCA TACACTTAAA   
  
  
+ TTGGAAGGGT GAAAATGTTG TTTGGGCTTC TGCATGGAGA CCTAGCTA  

- +Up\_Stream \_Len000TACTAT TTTCTATAAA TAATTCGAGT TCGATAAAAT TGAAAATTTT TTTACTTACA   
  
  
- CCGAAAAAAA CGATAAGTCC ATGTTGTGTT ATACTTCGTA ATTGAGATCT TTTACACCAT TACATACAGT   
  
  
- ATATCCTCTC ATATATGAGA GTGAACGGTA CGCTACGCAC GTACACACAC ACACACACAT AGACAAAGAT   
  
  
- ACGTATAAAA TACACCCCAA AACAGTAGAT CTGTAGATAA TTATACAATC GTAAGTGTAC TGAATATCCT   
  
  
- TACGTGATAA TACTCATACT ATATCTCAAA ATTTTTACGT ATACGGAACA CACAAGTAAT CGAATTTCTT   
  
  
- CTCAAAAACT TATATGTATA AATCCATTAA GAACGATTAC AGCATTAATC GTCAACATAG ATAGAATAAC   
  
  
- TAAACATAAA AATTAACGAA CAATATGACA ATGTGAATCT TTTAAGTACT TATGAGAATT TTATACGTTT   
  
  
- AATATATATA TATATATATA TATATATATA TATATATATA TATTTTTGAC TTATTCAATT ATACCACTAC   
  
  
- TGTAAACAGT ACAATAGTAA CCAGCGAATT AAAAATTTAT TTTTTATACT AAAACACTGT GGATTATTTA   
  
  
- CTATAATCGG TATAATAATT TAAGTCGTAA ATAAGTAGTT ACATTATATG ATTTAGTACG TTAATAGTAA   
  
  
- CCAAGAGAAA ATACATTATT TCAATTAACT GTTAAGTTCC CAATGATTAA TCATATTACA ACTTAACCTT   
  
  
- GCATTTTTAG TTTAGCATAC ATTTCATTAA AATAAGACTT AAATTTTTTA CCTCGTTTAT AGAGATAACA   
  
  
- AGAAATTATT TTAAAATTAA CTAAAAATAA AGATTAATTC ACTGCCTTGG TTTAAAAAAA GGAGGAAAAA   
  
  
- ACCAAACCAT TCAATAGTAA TCGGTTAGAA AATAAATGTA AAACAGTCAA AAAGAAAAAA TTCTACCAAT   
  
  
- TTACCGAAGT TAATTTGTTT AAAAAAATGA ATAAATATAA AAATTATTTA AAAGTTTTAC ATATAATTGC   
  
  
- GTACTGGCAC GCTTCGTGCC TTAGATGGGA TCAATTAGAT ACTAAGAATT TATACGTTCA TCCGACAATA   
  
  
- AGTAAACAAC AAAAAATGAA TAAATATAAA AAAGGGAGGG CCATACTATC CCAAACAGTA TAACAATAAG   
  
  
- TAATCGACAA TTTGACATAA GTAATATCGT GTTCATCCGA ATTAGTTTCT ATCAATCAGA GGTTAAAGTA   
  
  
- GTCTTTTAAA TTCTTTTTCT GTGGAATCCT AGTTTTCATA CTTTAAATCT CTGGTCTGTT GCTATCTTAA   
  
  
- CTTTTCTACT CAAAAGTTAA ACCTCTTCTG CCTTTGAGTC TACTTACTTT CATATCATGT AACATGAGGA   
  
  
- ACCTCATATT AGAATAGAAG TGATAACTAT CCGAGCTTCG TATTAGTCAC TAACCTTTGA AAACCAACGT   
  
  
- TATTAAGTTT TAAACTATGA GATACTAAAT GAAAAAATTA ACTTCCAGTA CTAAATTAAT TGATTTACTT   
  
  
- TTCTTAAATA CTAGCTTTTT AAAATTGAGA ATAACTGAAT ATCAACTGAG GTCAATTTAC CTTTTAACTT   
  
  
- CACTAATTAG ATTTTCACCG GGCGAGATTA TGGTAAACTC TTTTTAATAA GAATGACGTT TCAAATTTAG   
  
  
- TTATACACTT CTATCAAATA TCAAAATAGG ATTTTCTTTA ATTTATCTAA CAATTTTTAT TAAAAATTTA   
  
  
- AGTCTATATG CACACTACCG GTATTGGCAT TTACTATACG TACAGCTGTT TAAGGTTTCT TCCGATTATT   
  
  
- AATCTGAATT TATTATGTAC GTATGATTGG TTTTTTTTTT TTAATTCGTT CTAAGATAGG TTGGATAGTA   
  
  
- TTATTTCAAA CCGGTATGAA GTTTGGTAGT GTATTGTAGT GATTATATAT TTCGATCCAA AACCCTTTGG   
  
  
- ATTGAAACTC CTGGTGTATA AACAATTCCG GATCCTCCGG TACTCAAACA GTTCTACGGA GTATCATACC   
  
  
- TCTCCAGATA TAGGAACGTT TTACTAGTAA GGGAAGATGG ATTTTCTTGG TGAAGTAAAG GTGAAAATGA   
  
  
- GGGACCGTTT ACCAACTATT TGGTTTGGTA TTGGTTCGGG AACTTCTTGA TATTTCTGTG CTTAGCTAGT   
  
  
- AACTTAAGCT TGTTCGGAAC ACACTATCCT CCTACCCGGA GTTGTGAAGA GGCGCTCACA CGTTCTCGTT   
  
  
- AGAGCCTCTT TTTAGGATTC TCGGTTGTTG AAGAAACCCA CAATTTACTT GAACAAAGAG GAATACCGCT   
  
  
- AACGCTTGTT TCTAACCGTA TAATGAAGAA TGTTCACAAC AACCGGTTTC GGTTGTTAAA CCCTGGAGTG   
  
  
- AAAGTACTCT CGGATTTTGA ACGGTACCTC TTTTTGACGA CGAAACTATG GATGTACTTC GAATATAACT   
  
  
- TCAAGGTTCT CCAGTCAGGT ACCTGTTGGA AACCAGTACA CCGAAGTTTA CCACGTTATA ACCTCCTGGA   
  
  
- ATCTTCAAAT GTTTTCAGCT AGTAGTTTCT TTGTTCCGTT TCCTACCTCT TCAAAAGTTC CGATTACCCA   
  
  
- CAAGGGAAGT TCAAAGTACA GTATTTGCTC AATCTATTGG ATCCTCTCGA AGCTTTTCTC CTAAATCTGT   
  
  
- AACTTCTACC ACTCCGGTAG CGACACTTGA CACAAGTTCG GAACGTTGCC CAAGTACACC TCTTCTCCCT   
  
  
- CGTACACGAA CTACACTAAG CTAGATAATC CGGAACATAG TATTGTGACC ACCTCCTTCT TCGTCTAGAG   
  
  
- TGAAGATGTT CTTTGCTGAA GAAGTTCACG AAGCTACTCA CAAACTCTAA AAGATTCAGT ATAAAGCTAT   
  
  
- ACAATCTTCT TTCGAAGGGA GGTTATTCGT TGCTTGCTTA GTTCGATCTT TCCCTTGTTA CCTACTTATA   
  
  
- GAGGTCTCGG GATCGAACAG TACCACTTTC ACCTCCTCTT ATATCCGGTT TCTTTCCTTG AGTTACCTTA   
  
  
- CTCTACGAGC TTGTTCGTAA AACGGGTAGA GTTAAATCAC TACTACATGA TTCACTACAA TCCCGTAACA   
  
  
- ACTTTTCTGT GTTTTCACCA ACCCTAGAGT GGAATGGTGT TTCACTGGTA CTTTATCCGT ATGTGAATTT   
  
  
- AACCTTCCCA CTTTTACAAC AAACCCGAAG ACGTACCTCT GGATCGAT

+     TGACG-motif

| Site Name | Organism | Position | Strand | Matrix score. | sequence | function |
| --- | --- | --- | --- | --- | --- | --- |
| TGACG-motif | Hordeum vulgare | 885 | + | 5 | TGACG | cis-acting regulatory element involved in the MeJA-responsiveness |

>HU02G03154.1   
+ +Up\_Stream \_Len000ATGATA AAAGATATTT ATTAAGCTCA AGCTATTTTA ACTTTTAAAA AAATGAATGT   
  
  
+ GGCTTTTTTT GCTATTCAGG TACAACACAA TATGAAGCAT TAACTCTAGA AAATGTGGTA ATGTATGTCA   
  
  
+ TATAGGAGAG TATATACTCT CACTTGCCAT GCGATGCGTG CATGTGTGTG TGTGTGTGTA TCTGTTTCTA   
  
  
+ TGCATATTTT ATGTGGGGTT TTGTCATCTA GACATCTATT AATATGTTAG CATTCACATG ACTTATAGGA   
  
  
+ ATGCACTATT ATGAGTATGA TATAGAGTTT TAAAAATGCA TATGCCTTGT GTGTTCATTA GCTTAAAGAA   
  
  
+ GAGTTTTTGA ATATACATAT TTAGGTAATT CTTGCTAATG TCGTAATTAG CAGTTGTATC TATCTTATTG   
  
  
+ ATTTGTATTT TTAATTGCTT GTTATACTGT TACACTTAGA AAATTCATGA ATACTCTTAA AATATGCAAA   
  
  
+ TTATATATAT ATATATATAT ATATATATAT ATATATATAT ATAAAAACTG AATAAGTTAA TATGGTGATG   
  
  
+ ACATTTGTCA TGTTATCATT GGTCGCTTAA TTTTTAAATA AAAAATATGA TTTTGTGACA CCTAATAAAT   
  
  
+ GATATTAGCC ATATTATTAA ATTCAGCATT TATTCATCAA TGTAATATAC TAAATCATGC AATTATCATT   
  
  
+ GGTTCTCTTT TATGTAATAA AGTTAATTGA CAATTCAAGG GTTACTAATT AGTATAATGT TGAATTGGAA   
  
  
+ CGTAAAAATC AAATCGTATG TAAAGTAATT TTATTCTGAA TTTAAAAAAT GGAGCAAATA TCTCTATTGT   
  
  
+ TCTTTAATAA AATTTTAATT GATTTTTATT TCTAATTAAG TGACGGAACC AAATTTTTTT CCTCCTTTTT   
  
  
+ TGGTTTGGTA AGTTATCATT AGCCAATCTT TTATTTACAT TTTGTCAGTT TTTCTTTTTT AAGATGGTTA   
  
  
+ AATGGCTTCA ATTAAACAAA TTTTTTTACT TATTTATATT TTTAATAAAT TTTCAAAATG TATATTAACG   
  
  
+ CATGACCGTG CGAAGCACGG AATCTACCCT AGTTAATCTA TGATTCTTAA ATATGCAAGT AGGCTGTTAT   
  
  
+ TCATTTGTTG TTTTTTACTT ATTTATATTT TTTCCCTCCC GGTATGATAG GGTTTGTCAT ATTGTTATTC   
  
  
+ ATTAGCTGTT AAACTGTATT CATTATAGCA CAAGTAGGCT TAATCAAAGA TAGTTAGTCT CCAATTTCAT   
  
  
+ CAGAAAATTT AAGAAAAAGA CACCTTAGGA TCAAAAGTAT GAAATTTAGA GACCAGACAA CGATAGAATT   
  
  
+ GAAAAGATGA GTTTTCAATT TGGAGAAGAC GGAAACTCAG ATGAATGAAA GTATAGTACA TTGTACTCCT   
  
  
+ TGGAGTATAA TCTTATCTTC ACTATTGATA GGCTCGAAGC ATAATCAGTG ATTGGAAACT TTTGGTTGCA   
  
  
+ ATAATTCAAA ATTTGATACT CTATGATTTA CTTTTTTAAT TGAAGGTCAT GATTTAATTA ACTAAATGAA   
  
  
+ AAGAATTTAT GATCGAAAAA TTTTAACTCT TATTGACTTA TAGTTGACTC CAGTTAAATG GAAAATTGAA   
  
  
+ GTGATTAATC TAAAAGTGGC CCGCTCTAAT ACCATTTGAG AAAAATTATT CTTACTGCAA AGTTTAAATC   
  
  
+ AATATGTGAA GATAGTTTAT AGTTTTATCC TAAAAGAAAT TAAATAGATT GTTAAAAATA ATTTTTAAAT   
  
  
+ TCAGATATAC GTGTGATGGC CATAACCGTA AATGATATGC ATGTCGACAA ATTCCAAAGA AGGCTAATAA   
  
  
+ TTAGACTTAA ATAATACATG CATACTAACC AAAAAAAAAA AATTAAGCAA GATTCTATCC AACCTATCAT   
  
  
+ AATAAAGTTT GGCCATACTT CAAACCATCA CATAACATCA CTAATATATA AAGCTAGGTT TTGGGAAACC   
  
  
+ TAACTTTGAG GACCACATAT TTGTTAAGGC CTAGGAGGCC ATGAGTTTGT CAAGATGCCT CATAGTATGG   
  
  
+ AGAGGTCTAT ATCCTTGCAA AATGATCATT CCCTTCTACC TAAAAGAACC ACTTCATTTC CACTTTTACT   
  
  
+ CCCTGGCAAA TGGTTGATAA ACCAAACCAT AACCAAGCCC TTGAAGAACT ATAAAGACAC GAATCGATCA   
  
  
+ TTGAATTCGA ACAAGCCTTG TGTGATAGGA GGATGGGCCT CAACACTTCT CCGCGAGTGT GCAAGAGCAA   
  
  
+ TCTCGGAGAA AAATCCTAAG AGCCAACAAC TTCTTTGGGT GTTAAATGAA CTTGTTTCTC CTTATGGCGA   
  
  
+ TTGCGAACAA AGATTGGCAT ATTACTTCTT ACAAGTGTTG TTGGCCAAAG CCAACAATTT GGGACCTCAC   
  
  
+ TTTCATGAGA GCCTAAAACT TGCCATGGAG AAAAACTGCT GCTTTGATAC CTACATGAAG CTTATATTGA   
  
  
+ AGTTCCAAGA GGTCAGTCCA TGGACAACCT TTGGTCATGT GGCTTCAAAT GGTGCAATAT TGGAGGACCT   
  
  
+ TAGAAGTTTA CAAAAGTCGA TCATCAAAGA AACAAGGCAA AGGATGGAGA AGTTTTCAAG GCTAATGGGT   
  
  
+ GTTCCCTTCA AGTTTCATGT CATAAACGAG TTAGATAACC TAGGAGAGCT TCGAAAAGAG GATTTAGACA   
  
  
+ TTGAAGATGG TGAGGCCATC GCTGTGAACT GTGTTCAAGC CTTGCAACGG GTTCATGTGG AGAAGAGGGA   
  
  
+ GCATGTGCTT GATGTGATTC GATCTATTAG GCCTTGTATC ATAACACTGG TGGAGGAAGA AGCAGATCTC   
  
  
+ ACTTCTACAA GAAACGACTT CTTCAAGTGC TTCGATGAGT GTTTGAGATT TTCTAAGTCA TATTTCGATA   
  
  
+ TGTTAGAAGA AAGCTTCCCT CCAATAAGCA ACGAACGAAT CAAGCTAGAA AGGGAACAAT GGATGAATAT   
  
  
+ CTCCAGAGCC CTAGCTTGTC ATGGTGAAAG TGGAGGAGAA TATAGGCCAA AGAAAGGAAC TCAATGGAAT   
  
  
+ GAGATGCTCG AACAAGCATT TTGCCCATCT CAATTTAGTG ATGATGTACT AAGTGATGTT AGGGCATTGT   
  
  
+ TGAAAAGACA CAAAAGTGGT TGGGATCTCA CCTTACCACA AAGTGACCAT GAAATAGGCA TACACTTAAA   
  
  
+ TTGGAAGGGT GAAAATGTTG TTTGGGCTTC TGCATGGAGA CCTAGCTA  

- +Up\_Stream \_Len000TACTAT TTTCTATAAA TAATTCGAGT TCGATAAAAT TGAAAATTTT TTTACTTACA   
  
  
- CCGAAAAAAA CGATAAGTCC ATGTTGTGTT ATACTTCGTA ATTGAGATCT TTTACACCAT TACATACAGT   
  
  
- ATATCCTCTC ATATATGAGA GTGAACGGTA CGCTACGCAC GTACACACAC ACACACACAT AGACAAAGAT   
  
  
- ACGTATAAAA TACACCCCAA AACAGTAGAT CTGTAGATAA TTATACAATC GTAAGTGTAC TGAATATCCT   
  
  
- TACGTGATAA TACTCATACT ATATCTCAAA ATTTTTACGT ATACGGAACA CACAAGTAAT CGAATTTCTT   
  
  
- CTCAAAAACT TATATGTATA AATCCATTAA GAACGATTAC AGCATTAATC GTCAACATAG ATAGAATAAC   
  
  
- TAAACATAAA AATTAACGAA CAATATGACA ATGTGAATCT TTTAAGTACT TATGAGAATT TTATACGTTT   
  
  
- AATATATATA TATATATATA TATATATATA TATATATATA TATTTTTGAC TTATTCAATT ATACCACTAC   
  
  
- TGTAAACAGT ACAATAGTAA CCAGCGAATT AAAAATTTAT TTTTTATACT AAAACACTGT GGATTATTTA   
  
  
- CTATAATCGG TATAATAATT TAAGTCGTAA ATAAGTAGTT ACATTATATG ATTTAGTACG TTAATAGTAA   
  
  
- CCAAGAGAAA ATACATTATT TCAATTAACT GTTAAGTTCC CAATGATTAA TCATATTACA ACTTAACCTT   
  
  
- GCATTTTTAG TTTAGCATAC ATTTCATTAA AATAAGACTT AAATTTTTTA CCTCGTTTAT AGAGATAACA   
  
  
- AGAAATTATT TTAAAATTAA CTAAAAATAA AGATTAATTC ACTGCCTTGG TTTAAAAAAA GGAGGAAAAA   
  
  
- ACCAAACCAT TCAATAGTAA TCGGTTAGAA AATAAATGTA AAACAGTCAA AAAGAAAAAA TTCTACCAAT   
  
  
- TTACCGAAGT TAATTTGTTT AAAAAAATGA ATAAATATAA AAATTATTTA AAAGTTTTAC ATATAATTGC   
  
  
- GTACTGGCAC GCTTCGTGCC TTAGATGGGA TCAATTAGAT ACTAAGAATT TATACGTTCA TCCGACAATA   
  
  
- AGTAAACAAC AAAAAATGAA TAAATATAAA AAAGGGAGGG CCATACTATC CCAAACAGTA TAACAATAAG   
  
  
- TAATCGACAA TTTGACATAA GTAATATCGT GTTCATCCGA ATTAGTTTCT ATCAATCAGA GGTTAAAGTA   
  
  
- GTCTTTTAAA TTCTTTTTCT GTGGAATCCT AGTTTTCATA CTTTAAATCT CTGGTCTGTT GCTATCTTAA   
  
  
- CTTTTCTACT CAAAAGTTAA ACCTCTTCTG CCTTTGAGTC TACTTACTTT CATATCATGT AACATGAGGA   
  
  
- ACCTCATATT AGAATAGAAG TGATAACTAT CCGAGCTTCG TATTAGTCAC TAACCTTTGA AAACCAACGT   
  
  
- TATTAAGTTT TAAACTATGA GATACTAAAT GAAAAAATTA ACTTCCAGTA CTAAATTAAT TGATTTACTT   
  
  
- TTCTTAAATA CTAGCTTTTT AAAATTGAGA ATAACTGAAT ATCAACTGAG GTCAATTTAC CTTTTAACTT   
  
  
- CACTAATTAG ATTTTCACCG GGCGAGATTA TGGTAAACTC TTTTTAATAA GAATGACGTT TCAAATTTAG   
  
  
- TTATACACTT CTATCAAATA TCAAAATAGG ATTTTCTTTA ATTTATCTAA CAATTTTTAT TAAAAATTTA   
  
  
- AGTCTATATG CACACTACCG GTATTGGCAT TTACTATACG TACAGCTGTT TAAGGTTTCT TCCGATTATT   
  
  
- AATCTGAATT TATTATGTAC GTATGATTGG TTTTTTTTTT TTAATTCGTT CTAAGATAGG TTGGATAGTA   
  
  
- TTATTTCAAA CCGGTATGAA GTTTGGTAGT GTATTGTAGT GATTATATAT TTCGATCCAA AACCCTTTGG   
  
  
- ATTGAAACTC CTGGTGTATA AACAATTCCG GATCCTCCGG TACTCAAACA GTTCTACGGA GTATCATACC   
  
  
- TCTCCAGATA TAGGAACGTT TTACTAGTAA GGGAAGATGG ATTTTCTTGG TGAAGTAAAG GTGAAAATGA   
  
  
- GGGACCGTTT ACCAACTATT TGGTTTGGTA TTGGTTCGGG AACTTCTTGA TATTTCTGTG CTTAGCTAGT   
  
  
- AACTTAAGCT TGTTCGGAAC ACACTATCCT CCTACCCGGA GTTGTGAAGA GGCGCTCACA CGTTCTCGTT   
  
  
- AGAGCCTCTT TTTAGGATTC TCGGTTGTTG AAGAAACCCA CAATTTACTT GAACAAAGAG GAATACCGCT   
  
  
- AACGCTTGTT TCTAACCGTA TAATGAAGAA TGTTCACAAC AACCGGTTTC GGTTGTTAAA CCCTGGAGTG   
  
  
- AAAGTACTCT CGGATTTTGA ACGGTACCTC TTTTTGACGA CGAAACTATG GATGTACTTC GAATATAACT   
  
  
- TCAAGGTTCT CCAGTCAGGT ACCTGTTGGA AACCAGTACA CCGAAGTTTA CCACGTTATA ACCTCCTGGA   
  
  
- ATCTTCAAAT GTTTTCAGCT AGTAGTTTCT TTGTTCCGTT TCCTACCTCT TCAAAAGTTC CGATTACCCA   
  
  
- CAAGGGAAGT TCAAAGTACA GTATTTGCTC AATCTATTGG ATCCTCTCGA AGCTTTTCTC CTAAATCTGT   
  
  
- AACTTCTACC ACTCCGGTAG CGACACTTGA CACAAGTTCG GAACGTTGCC CAAGTACACC TCTTCTCCCT   
  
  
- CGTACACGAA CTACACTAAG CTAGATAATC CGGAACATAG TATTGTGACC ACCTCCTTCT TCGTCTAGAG   
  
  
- TGAAGATGTT CTTTGCTGAA GAAGTTCACG AAGCTACTCA CAAACTCTAA AAGATTCAGT ATAAAGCTAT   
  
  
- ACAATCTTCT TTCGAAGGGA GGTTATTCGT TGCTTGCTTA GTTCGATCTT TCCCTTGTTA CCTACTTATA   
  
  
- GAGGTCTCGG GATCGAACAG TACCACTTTC ACCTCCTCTT ATATCCGGTT TCTTTCCTTG AGTTACCTTA   
  
  
- CTCTACGAGC TTGTTCGTAA AACGGGTAGA GTTAAATCAC TACTACATGA TTCACTACAA TCCCGTAACA   
  
  
- ACTTTTCTGT GTTTTCACCA ACCCTAGAGT GGAATGGTGT TTCACTGGTA CTTTATCCGT ATGTGAATTT   
  
  
- AACCTTCCCA CTTTTACAAC AAACCCGAAG ACGTACCTCT GGATCGAT

+     Unnamed\_\_2

| Site Name | Organism | Position | Strand | Matrix score. | sequence | function |
| --- | --- | --- | --- | --- | --- | --- |
| Unnamed\_\_2 | Petroselinum hortense | 1961 | + | 9 | AACCTAACCT |  |

>HU02G03154.1   
+ +Up\_Stream \_Len000ATGATA AAAGATATTT ATTAAGCTCA AGCTATTTTA ACTTTTAAAA AAATGAATGT   
  
  
+ GGCTTTTTTT GCTATTCAGG TACAACACAA TATGAAGCAT TAACTCTAGA AAATGTGGTA ATGTATGTCA   
  
  
+ TATAGGAGAG TATATACTCT CACTTGCCAT GCGATGCGTG CATGTGTGTG TGTGTGTGTA TCTGTTTCTA   
  
  
+ TGCATATTTT ATGTGGGGTT TTGTCATCTA GACATCTATT AATATGTTAG CATTCACATG ACTTATAGGA   
  
  
+ ATGCACTATT ATGAGTATGA TATAGAGTTT TAAAAATGCA TATGCCTTGT GTGTTCATTA GCTTAAAGAA   
  
  
+ GAGTTTTTGA ATATACATAT TTAGGTAATT CTTGCTAATG TCGTAATTAG CAGTTGTATC TATCTTATTG   
  
  
+ ATTTGTATTT TTAATTGCTT GTTATACTGT TACACTTAGA AAATTCATGA ATACTCTTAA AATATGCAAA   
  
  
+ TTATATATAT ATATATATAT ATATATATAT ATATATATAT ATAAAAACTG AATAAGTTAA TATGGTGATG   
  
  
+ ACATTTGTCA TGTTATCATT GGTCGCTTAA TTTTTAAATA AAAAATATGA TTTTGTGACA CCTAATAAAT   
  
  
+ GATATTAGCC ATATTATTAA ATTCAGCATT TATTCATCAA TGTAATATAC TAAATCATGC AATTATCATT   
  
  
+ GGTTCTCTTT TATGTAATAA AGTTAATTGA CAATTCAAGG GTTACTAATT AGTATAATGT TGAATTGGAA   
  
  
+ CGTAAAAATC AAATCGTATG TAAAGTAATT TTATTCTGAA TTTAAAAAAT GGAGCAAATA TCTCTATTGT   
  
  
+ TCTTTAATAA AATTTTAATT GATTTTTATT TCTAATTAAG TGACGGAACC AAATTTTTTT CCTCCTTTTT   
  
  
+ TGGTTTGGTA AGTTATCATT AGCCAATCTT TTATTTACAT TTTGTCAGTT TTTCTTTTTT AAGATGGTTA   
  
  
+ AATGGCTTCA ATTAAACAAA TTTTTTTACT TATTTATATT TTTAATAAAT TTTCAAAATG TATATTAACG   
  
  
+ CATGACCGTG CGAAGCACGG AATCTACCCT AGTTAATCTA TGATTCTTAA ATATGCAAGT AGGCTGTTAT   
  
  
+ TCATTTGTTG TTTTTTACTT ATTTATATTT TTTCCCTCCC GGTATGATAG GGTTTGTCAT ATTGTTATTC   
  
  
+ ATTAGCTGTT AAACTGTATT CATTATAGCA CAAGTAGGCT TAATCAAAGA TAGTTAGTCT CCAATTTCAT   
  
  
+ CAGAAAATTT AAGAAAAAGA CACCTTAGGA TCAAAAGTAT GAAATTTAGA GACCAGACAA CGATAGAATT   
  
  
+ GAAAAGATGA GTTTTCAATT TGGAGAAGAC GGAAACTCAG ATGAATGAAA GTATAGTACA TTGTACTCCT   
  
  
+ TGGAGTATAA TCTTATCTTC ACTATTGATA GGCTCGAAGC ATAATCAGTG ATTGGAAACT TTTGGTTGCA   
  
  
+ ATAATTCAAA ATTTGATACT CTATGATTTA CTTTTTTAAT TGAAGGTCAT GATTTAATTA ACTAAATGAA   
  
  
+ AAGAATTTAT GATCGAAAAA TTTTAACTCT TATTGACTTA TAGTTGACTC CAGTTAAATG GAAAATTGAA   
  
  
+ GTGATTAATC TAAAAGTGGC CCGCTCTAAT ACCATTTGAG AAAAATTATT CTTACTGCAA AGTTTAAATC   
  
  
+ AATATGTGAA GATAGTTTAT AGTTTTATCC TAAAAGAAAT TAAATAGATT GTTAAAAATA ATTTTTAAAT   
  
  
+ TCAGATATAC GTGTGATGGC CATAACCGTA AATGATATGC ATGTCGACAA ATTCCAAAGA AGGCTAATAA   
  
  
+ TTAGACTTAA ATAATACATG CATACTAACC AAAAAAAAAA AATTAAGCAA GATTCTATCC AACCTATCAT   
  
  
+ AATAAAGTTT GGCCATACTT CAAACCATCA CATAACATCA CTAATATATA AAGCTAGGTT TTGGGAAACC   
  
  
+ TAACTTTGAG GACCACATAT TTGTTAAGGC CTAGGAGGCC ATGAGTTTGT CAAGATGCCT CATAGTATGG   
  
  
+ AGAGGTCTAT ATCCTTGCAA AATGATCATT CCCTTCTACC TAAAAGAACC ACTTCATTTC CACTTTTACT   
  
  
+ CCCTGGCAAA TGGTTGATAA ACCAAACCAT AACCAAGCCC TTGAAGAACT ATAAAGACAC GAATCGATCA   
  
  
+ TTGAATTCGA ACAAGCCTTG TGTGATAGGA GGATGGGCCT CAACACTTCT CCGCGAGTGT GCAAGAGCAA   
  
  
+ TCTCGGAGAA AAATCCTAAG AGCCAACAAC TTCTTTGGGT GTTAAATGAA CTTGTTTCTC CTTATGGCGA   
  
  
+ TTGCGAACAA AGATTGGCAT ATTACTTCTT ACAAGTGTTG TTGGCCAAAG CCAACAATTT GGGACCTCAC   
  
  
+ TTTCATGAGA GCCTAAAACT TGCCATGGAG AAAAACTGCT GCTTTGATAC CTACATGAAG CTTATATTGA   
  
  
+ AGTTCCAAGA GGTCAGTCCA TGGACAACCT TTGGTCATGT GGCTTCAAAT GGTGCAATAT TGGAGGACCT   
  
  
+ TAGAAGTTTA CAAAAGTCGA TCATCAAAGA AACAAGGCAA AGGATGGAGA AGTTTTCAAG GCTAATGGGT   
  
  
+ GTTCCCTTCA AGTTTCATGT CATAAACGAG TTAGATAACC TAGGAGAGCT TCGAAAAGAG GATTTAGACA   
  
  
+ TTGAAGATGG TGAGGCCATC GCTGTGAACT GTGTTCAAGC CTTGCAACGG GTTCATGTGG AGAAGAGGGA   
  
  
+ GCATGTGCTT GATGTGATTC GATCTATTAG GCCTTGTATC ATAACACTGG TGGAGGAAGA AGCAGATCTC   
  
  
+ ACTTCTACAA GAAACGACTT CTTCAAGTGC TTCGATGAGT GTTTGAGATT TTCTAAGTCA TATTTCGATA   
  
  
+ TGTTAGAAGA AAGCTTCCCT CCAATAAGCA ACGAACGAAT CAAGCTAGAA AGGGAACAAT GGATGAATAT   
  
  
+ CTCCAGAGCC CTAGCTTGTC ATGGTGAAAG TGGAGGAGAA TATAGGCCAA AGAAAGGAAC TCAATGGAAT   
  
  
+ GAGATGCTCG AACAAGCATT TTGCCCATCT CAATTTAGTG ATGATGTACT AAGTGATGTT AGGGCATTGT   
  
  
+ TGAAAAGACA CAAAAGTGGT TGGGATCTCA CCTTACCACA AAGTGACCAT GAAATAGGCA TACACTTAAA   
  
  
+ TTGGAAGGGT GAAAATGTTG TTTGGGCTTC TGCATGGAGA CCTAGCTA  

- +Up\_Stream \_Len000TACTAT TTTCTATAAA TAATTCGAGT TCGATAAAAT TGAAAATTTT TTTACTTACA   
  
  
- CCGAAAAAAA CGATAAGTCC ATGTTGTGTT ATACTTCGTA ATTGAGATCT TTTACACCAT TACATACAGT   
  
  
- ATATCCTCTC ATATATGAGA GTGAACGGTA CGCTACGCAC GTACACACAC ACACACACAT AGACAAAGAT   
  
  
- ACGTATAAAA TACACCCCAA AACAGTAGAT CTGTAGATAA TTATACAATC GTAAGTGTAC TGAATATCCT   
  
  
- TACGTGATAA TACTCATACT ATATCTCAAA ATTTTTACGT ATACGGAACA CACAAGTAAT CGAATTTCTT   
  
  
- CTCAAAAACT TATATGTATA AATCCATTAA GAACGATTAC AGCATTAATC GTCAACATAG ATAGAATAAC   
  
  
- TAAACATAAA AATTAACGAA CAATATGACA ATGTGAATCT TTTAAGTACT TATGAGAATT TTATACGTTT   
  
  
- AATATATATA TATATATATA TATATATATA TATATATATA TATTTTTGAC TTATTCAATT ATACCACTAC   
  
  
- TGTAAACAGT ACAATAGTAA CCAGCGAATT AAAAATTTAT TTTTTATACT AAAACACTGT GGATTATTTA   
  
  
- CTATAATCGG TATAATAATT TAAGTCGTAA ATAAGTAGTT ACATTATATG ATTTAGTACG TTAATAGTAA   
  
  
- CCAAGAGAAA ATACATTATT TCAATTAACT GTTAAGTTCC CAATGATTAA TCATATTACA ACTTAACCTT   
  
  
- GCATTTTTAG TTTAGCATAC ATTTCATTAA AATAAGACTT AAATTTTTTA CCTCGTTTAT AGAGATAACA   
  
  
- AGAAATTATT TTAAAATTAA CTAAAAATAA AGATTAATTC ACTGCCTTGG TTTAAAAAAA GGAGGAAAAA   
  
  
- ACCAAACCAT TCAATAGTAA TCGGTTAGAA AATAAATGTA AAACAGTCAA AAAGAAAAAA TTCTACCAAT   
  
  
- TTACCGAAGT TAATTTGTTT AAAAAAATGA ATAAATATAA AAATTATTTA AAAGTTTTAC ATATAATTGC   
  
  
- GTACTGGCAC GCTTCGTGCC TTAGATGGGA TCAATTAGAT ACTAAGAATT TATACGTTCA TCCGACAATA   
  
  
- AGTAAACAAC AAAAAATGAA TAAATATAAA AAAGGGAGGG CCATACTATC CCAAACAGTA TAACAATAAG   
  
  
- TAATCGACAA TTTGACATAA GTAATATCGT GTTCATCCGA ATTAGTTTCT ATCAATCAGA GGTTAAAGTA   
  
  
- GTCTTTTAAA TTCTTTTTCT GTGGAATCCT AGTTTTCATA CTTTAAATCT CTGGTCTGTT GCTATCTTAA   
  
  
- CTTTTCTACT CAAAAGTTAA ACCTCTTCTG CCTTTGAGTC TACTTACTTT CATATCATGT AACATGAGGA   
  
  
- ACCTCATATT AGAATAGAAG TGATAACTAT CCGAGCTTCG TATTAGTCAC TAACCTTTGA AAACCAACGT   
  
  
- TATTAAGTTT TAAACTATGA GATACTAAAT GAAAAAATTA ACTTCCAGTA CTAAATTAAT TGATTTACTT   
  
  
- TTCTTAAATA CTAGCTTTTT AAAATTGAGA ATAACTGAAT ATCAACTGAG GTCAATTTAC CTTTTAACTT   
  
  
- CACTAATTAG ATTTTCACCG GGCGAGATTA TGGTAAACTC TTTTTAATAA GAATGACGTT TCAAATTTAG   
  
  
- TTATACACTT CTATCAAATA TCAAAATAGG ATTTTCTTTA ATTTATCTAA CAATTTTTAT TAAAAATTTA   
  
  
- AGTCTATATG CACACTACCG GTATTGGCAT TTACTATACG TACAGCTGTT TAAGGTTTCT TCCGATTATT   
  
  
- AATCTGAATT TATTATGTAC GTATGATTGG TTTTTTTTTT TTAATTCGTT CTAAGATAGG TTGGATAGTA   
  
  
- TTATTTCAAA CCGGTATGAA GTTTGGTAGT GTATTGTAGT GATTATATAT TTCGATCCAA AACCCTTTGG   
  
  
- ATTGAAACTC CTGGTGTATA AACAATTCCG GATCCTCCGG TACTCAAACA GTTCTACGGA GTATCATACC   
  
  
- TCTCCAGATA TAGGAACGTT TTACTAGTAA GGGAAGATGG ATTTTCTTGG TGAAGTAAAG GTGAAAATGA   
  
  
- GGGACCGTTT ACCAACTATT TGGTTTGGTA TTGGTTCGGG AACTTCTTGA TATTTCTGTG CTTAGCTAGT   
  
  
- AACTTAAGCT TGTTCGGAAC ACACTATCCT CCTACCCGGA GTTGTGAAGA GGCGCTCACA CGTTCTCGTT   
  
  
- AGAGCCTCTT TTTAGGATTC TCGGTTGTTG AAGAAACCCA CAATTTACTT GAACAAAGAG GAATACCGCT   
  
  
- AACGCTTGTT TCTAACCGTA TAATGAAGAA TGTTCACAAC AACCGGTTTC GGTTGTTAAA CCCTGGAGTG   
  
  
- AAAGTACTCT CGGATTTTGA ACGGTACCTC TTTTTGACGA CGAAACTATG GATGTACTTC GAATATAACT   
  
  
- TCAAGGTTCT CCAGTCAGGT ACCTGTTGGA AACCAGTACA CCGAAGTTTA CCACGTTATA ACCTCCTGGA   
  
  
- ATCTTCAAAT GTTTTCAGCT AGTAGTTTCT TTGTTCCGTT TCCTACCTCT TCAAAAGTTC CGATTACCCA   
  
  
- CAAGGGAAGT TCAAAGTACA GTATTTGCTC AATCTATTGG ATCCTCTCGA AGCTTTTCTC CTAAATCTGT   
  
  
- AACTTCTACC ACTCCGGTAG CGACACTTGA CACAAGTTCG GAACGTTGCC CAAGTACACC TCTTCTCCCT   
  
  
- CGTACACGAA CTACACTAAG CTAGATAATC CGGAACATAG TATTGTGACC ACCTCCTTCT TCGTCTAGAG   
  
  
- TGAAGATGTT CTTTGCTGAA GAAGTTCACG AAGCTACTCA CAAACTCTAA AAGATTCAGT ATAAAGCTAT   
  
  
- ACAATCTTCT TTCGAAGGGA GGTTATTCGT TGCTTGCTTA GTTCGATCTT TCCCTTGTTA CCTACTTATA   
  
  
- GAGGTCTCGG GATCGAACAG TACCACTTTC ACCTCCTCTT ATATCCGGTT TCTTTCCTTG AGTTACCTTA   
  
  
- CTCTACGAGC TTGTTCGTAA AACGGGTAGA GTTAAATCAC TACTACATGA TTCACTACAA TCCCGTAACA   
  
  
- ACTTTTCTGT GTTTTCACCA ACCCTAGAGT GGAATGGTGT TTCACTGGTA CTTTATCCGT ATGTGAATTT   
  
  
- AACCTTCCCA CTTTTACAAC AAACCCGAAG ACGTACCTCT GGATCGAT

+     Unnamed\_\_4

| Site Name | Organism | Position | Strand | Matrix score. | sequence | function |
| --- | --- | --- | --- | --- | --- | --- |
| Unnamed\_\_4 | Petroselinum hortense | 3190 | - | 4 | CTCC |  |
| Unnamed\_\_4 | Petroselinum hortense | 2637 | - | 4 | CTCC |  |
| Unnamed\_\_4 | Petroselinum hortense | 2570 | - | 4 | CTCC |  |
| Unnamed\_\_4 | Petroselinum hortense | 149 | - | 4 | CTCC |  |
| Unnamed\_\_4 | Petroselinum hortense | 1592 | + | 4 | CTCC |  |
| Unnamed\_\_4 | Petroselinum hortense | 2786 | - | 4 | CTCC |  |
| Unnamed\_\_4 | Petroselinum hortense | 2202 | - | 4 | CTCC |  |
| Unnamed\_\_4 | Petroselinum hortense | 2723 | - | 4 | CTCC |  |
| Unnamed\_\_4 | Petroselinum hortense | 2732 | - | 4 | CTCC |  |
| Unnamed\_\_4 | Petroselinum hortense | 2411 | - | 4 | CTCC |  |
| Unnamed\_\_4 | Petroselinum hortense | 1253 | + | 4 | CTCC |  |
| Unnamed\_\_4 | Petroselinum hortense | 906 | + | 4 | CTCC |  |
| Unnamed\_\_4 | Petroselinum hortense | 2945 | + | 4 | CTCC |  |
| Unnamed\_\_4 | Petroselinum hortense | 2033 | - | 4 | CTCC |  |
| Unnamed\_\_4 | Petroselinum hortense | 2249 | - | 4 | CTCC |  |
| Unnamed\_\_4 | Petroselinum hortense | 1400 | + | 4 | CTCC |  |
| Unnamed\_\_4 | Petroselinum hortense | 2979 | - | 4 | CTCC |  |
| Unnamed\_\_4 | Petroselinum hortense | 2223 | + | 4 | CTCC |  |
| Unnamed\_\_4 | Petroselinum hortense | 2302 | + | 4 | CTCC |  |
| Unnamed\_\_4 | Petroselinum hortense | 1160 | + | 4 | CTCC |  |
| Unnamed\_\_4 | Petroselinum hortense | 2976 | - | 4 | CTCC |  |
| Unnamed\_\_4 | Petroselinum hortense | 1998 | - | 4 | CTCC |  |
| Unnamed\_\_4 | Petroselinum hortense | 2516 | - | 4 | CTCC |  |
| Unnamed\_\_4 | Petroselinum hortense | 1356 | - | 4 | CTCC |  |
| Unnamed\_\_4 | Petroselinum hortense | 2893 | + | 4 | CTCC |  |
| Unnamed\_\_4 | Petroselinum hortense | 2103 | + | 4 | CTCC |  |
| Unnamed\_\_4 | Petroselinum hortense | 825 | - | 4 | CTCC |  |
| Unnamed\_\_4 | Petroselinum hortense | 1406 | - | 4 | CTCC |  |

>HU02G03154.1   
+ +Up\_Stream \_Len000ATGATA AAAGATATTT ATTAAGCTCA AGCTATTTTA ACTTTTAAAA AAATGAATGT   
  
  
+ GGCTTTTTTT GCTATTCAGG TACAACACAA TATGAAGCAT TAACTCTAGA AAATGTGGTA ATGTATGTCA   
  
  
+ TATAGGAGAG TATATACTCT CACTTGCCAT GCGATGCGTG CATGTGTGTG TGTGTGTGTA TCTGTTTCTA   
  
  
+ TGCATATTTT ATGTGGGGTT TTGTCATCTA GACATCTATT AATATGTTAG CATTCACATG ACTTATAGGA   
  
  
+ ATGCACTATT ATGAGTATGA TATAGAGTTT TAAAAATGCA TATGCCTTGT GTGTTCATTA GCTTAAAGAA   
  
  
+ GAGTTTTTGA ATATACATAT TTAGGTAATT CTTGCTAATG TCGTAATTAG CAGTTGTATC TATCTTATTG   
  
  
+ ATTTGTATTT TTAATTGCTT GTTATACTGT TACACTTAGA AAATTCATGA ATACTCTTAA AATATGCAAA   
  
  
+ TTATATATAT ATATATATAT ATATATATAT ATATATATAT ATAAAAACTG AATAAGTTAA TATGGTGATG   
  
  
+ ACATTTGTCA TGTTATCATT GGTCGCTTAA TTTTTAAATA AAAAATATGA TTTTGTGACA CCTAATAAAT   
  
  
+ GATATTAGCC ATATTATTAA ATTCAGCATT TATTCATCAA TGTAATATAC TAAATCATGC AATTATCATT   
  
  
+ GGTTCTCTTT TATGTAATAA AGTTAATTGA CAATTCAAGG GTTACTAATT AGTATAATGT TGAATTGGAA   
  
  
+ CGTAAAAATC AAATCGTATG TAAAGTAATT TTATTCTGAA TTTAAAAAAT GGAGCAAATA TCTCTATTGT   
  
  
+ TCTTTAATAA AATTTTAATT GATTTTTATT TCTAATTAAG TGACGGAACC AAATTTTTTT CCTCCTTTTT   
  
  
+ TGGTTTGGTA AGTTATCATT AGCCAATCTT TTATTTACAT TTTGTCAGTT TTTCTTTTTT AAGATGGTTA   
  
  
+ AATGGCTTCA ATTAAACAAA TTTTTTTACT TATTTATATT TTTAATAAAT TTTCAAAATG TATATTAACG   
  
  
+ CATGACCGTG CGAAGCACGG AATCTACCCT AGTTAATCTA TGATTCTTAA ATATGCAAGT AGGCTGTTAT   
  
  
+ TCATTTGTTG TTTTTTACTT ATTTATATTT TTTCCCTCCC GGTATGATAG GGTTTGTCAT ATTGTTATTC   
  
  
+ ATTAGCTGTT AAACTGTATT CATTATAGCA CAAGTAGGCT TAATCAAAGA TAGTTAGTCT CCAATTTCAT   
  
  
+ CAGAAAATTT AAGAAAAAGA CACCTTAGGA TCAAAAGTAT GAAATTTAGA GACCAGACAA CGATAGAATT   
  
  
+ GAAAAGATGA GTTTTCAATT TGGAGAAGAC GGAAACTCAG ATGAATGAAA GTATAGTACA TTGTACTCCT   
  
  
+ TGGAGTATAA TCTTATCTTC ACTATTGATA GGCTCGAAGC ATAATCAGTG ATTGGAAACT TTTGGTTGCA   
  
  
+ ATAATTCAAA ATTTGATACT CTATGATTTA CTTTTTTAAT TGAAGGTCAT GATTTAATTA ACTAAATGAA   
  
  
+ AAGAATTTAT GATCGAAAAA TTTTAACTCT TATTGACTTA TAGTTGACTC CAGTTAAATG GAAAATTGAA   
  
  
+ GTGATTAATC TAAAAGTGGC CCGCTCTAAT ACCATTTGAG AAAAATTATT CTTACTGCAA AGTTTAAATC   
  
  
+ AATATGTGAA GATAGTTTAT AGTTTTATCC TAAAAGAAAT TAAATAGATT GTTAAAAATA ATTTTTAAAT   
  
  
+ TCAGATATAC GTGTGATGGC CATAACCGTA AATGATATGC ATGTCGACAA ATTCCAAAGA AGGCTAATAA   
  
  
+ TTAGACTTAA ATAATACATG CATACTAACC AAAAAAAAAA AATTAAGCAA GATTCTATCC AACCTATCAT   
  
  
+ AATAAAGTTT GGCCATACTT CAAACCATCA CATAACATCA CTAATATATA AAGCTAGGTT TTGGGAAACC   
  
  
+ TAACTTTGAG GACCACATAT TTGTTAAGGC CTAGGAGGCC ATGAGTTTGT CAAGATGCCT CATAGTATGG   
  
  
+ AGAGGTCTAT ATCCTTGCAA AATGATCATT CCCTTCTACC TAAAAGAACC ACTTCATTTC CACTTTTACT   
  
  
+ CCCTGGCAAA TGGTTGATAA ACCAAACCAT AACCAAGCCC TTGAAGAACT ATAAAGACAC GAATCGATCA   
  
  
+ TTGAATTCGA ACAAGCCTTG TGTGATAGGA GGATGGGCCT CAACACTTCT CCGCGAGTGT GCAAGAGCAA   
  
  
+ TCTCGGAGAA AAATCCTAAG AGCCAACAAC TTCTTTGGGT GTTAAATGAA CTTGTTTCTC CTTATGGCGA   
  
  
+ TTGCGAACAA AGATTGGCAT ATTACTTCTT ACAAGTGTTG TTGGCCAAAG CCAACAATTT GGGACCTCAC   
  
  
+ TTTCATGAGA GCCTAAAACT TGCCATGGAG AAAAACTGCT GCTTTGATAC CTACATGAAG CTTATATTGA   
  
  
+ AGTTCCAAGA GGTCAGTCCA TGGACAACCT TTGGTCATGT GGCTTCAAAT GGTGCAATAT TGGAGGACCT   
  
  
+ TAGAAGTTTA CAAAAGTCGA TCATCAAAGA AACAAGGCAA AGGATGGAGA AGTTTTCAAG GCTAATGGGT   
  
  
+ GTTCCCTTCA AGTTTCATGT CATAAACGAG TTAGATAACC TAGGAGAGCT TCGAAAAGAG GATTTAGACA   
  
  
+ TTGAAGATGG TGAGGCCATC GCTGTGAACT GTGTTCAAGC CTTGCAACGG GTTCATGTGG AGAAGAGGGA   
  
  
+ GCATGTGCTT GATGTGATTC GATCTATTAG GCCTTGTATC ATAACACTGG TGGAGGAAGA AGCAGATCTC   
  
  
+ ACTTCTACAA GAAACGACTT CTTCAAGTGC TTCGATGAGT GTTTGAGATT TTCTAAGTCA TATTTCGATA   
  
  
+ TGTTAGAAGA AAGCTTCCCT CCAATAAGCA ACGAACGAAT CAAGCTAGAA AGGGAACAAT GGATGAATAT   
  
  
+ CTCCAGAGCC CTAGCTTGTC ATGGTGAAAG TGGAGGAGAA TATAGGCCAA AGAAAGGAAC TCAATGGAAT   
  
  
+ GAGATGCTCG AACAAGCATT TTGCCCATCT CAATTTAGTG ATGATGTACT AAGTGATGTT AGGGCATTGT   
  
  
+ TGAAAAGACA CAAAAGTGGT TGGGATCTCA CCTTACCACA AAGTGACCAT GAAATAGGCA TACACTTAAA   
  
  
+ TTGGAAGGGT GAAAATGTTG TTTGGGCTTC TGCATGGAGA CCTAGCTA  

- +Up\_Stream \_Len000TACTAT TTTCTATAAA TAATTCGAGT TCGATAAAAT TGAAAATTTT TTTACTTACA   
  
  
- CCGAAAAAAA CGATAAGTCC ATGTTGTGTT ATACTTCGTA ATTGAGATCT TTTACACCAT TACATACAGT   
  
  
- ATATCCTCTC ATATATGAGA GTGAACGGTA CGCTACGCAC GTACACACAC ACACACACAT AGACAAAGAT   
  
  
- ACGTATAAAA TACACCCCAA AACAGTAGAT CTGTAGATAA TTATACAATC GTAAGTGTAC TGAATATCCT   
  
  
- TACGTGATAA TACTCATACT ATATCTCAAA ATTTTTACGT ATACGGAACA CACAAGTAAT CGAATTTCTT   
  
  
- CTCAAAAACT TATATGTATA AATCCATTAA GAACGATTAC AGCATTAATC GTCAACATAG ATAGAATAAC   
  
  
- TAAACATAAA AATTAACGAA CAATATGACA ATGTGAATCT TTTAAGTACT TATGAGAATT TTATACGTTT   
  
  
- AATATATATA TATATATATA TATATATATA TATATATATA TATTTTTGAC TTATTCAATT ATACCACTAC   
  
  
- TGTAAACAGT ACAATAGTAA CCAGCGAATT AAAAATTTAT TTTTTATACT AAAACACTGT GGATTATTTA   
  
  
- CTATAATCGG TATAATAATT TAAGTCGTAA ATAAGTAGTT ACATTATATG ATTTAGTACG TTAATAGTAA   
  
  
- CCAAGAGAAA ATACATTATT TCAATTAACT GTTAAGTTCC CAATGATTAA TCATATTACA ACTTAACCTT   
  
  
- GCATTTTTAG TTTAGCATAC ATTTCATTAA AATAAGACTT AAATTTTTTA CCTCGTTTAT AGAGATAACA   
  
  
- AGAAATTATT TTAAAATTAA CTAAAAATAA AGATTAATTC ACTGCCTTGG TTTAAAAAAA GGAGGAAAAA   
  
  
- ACCAAACCAT TCAATAGTAA TCGGTTAGAA AATAAATGTA AAACAGTCAA AAAGAAAAAA TTCTACCAAT   
  
  
- TTACCGAAGT TAATTTGTTT AAAAAAATGA ATAAATATAA AAATTATTTA AAAGTTTTAC ATATAATTGC   
  
  
- GTACTGGCAC GCTTCGTGCC TTAGATGGGA TCAATTAGAT ACTAAGAATT TATACGTTCA TCCGACAATA   
  
  
- AGTAAACAAC AAAAAATGAA TAAATATAAA AAAGGGAGGG CCATACTATC CCAAACAGTA TAACAATAAG   
  
  
- TAATCGACAA TTTGACATAA GTAATATCGT GTTCATCCGA ATTAGTTTCT ATCAATCAGA GGTTAAAGTA   
  
  
- GTCTTTTAAA TTCTTTTTCT GTGGAATCCT AGTTTTCATA CTTTAAATCT CTGGTCTGTT GCTATCTTAA   
  
  
- CTTTTCTACT CAAAAGTTAA ACCTCTTCTG CCTTTGAGTC TACTTACTTT CATATCATGT AACATGAGGA   
  
  
- ACCTCATATT AGAATAGAAG TGATAACTAT CCGAGCTTCG TATTAGTCAC TAACCTTTGA AAACCAACGT   
  
  
- TATTAAGTTT TAAACTATGA GATACTAAAT GAAAAAATTA ACTTCCAGTA CTAAATTAAT TGATTTACTT   
  
  
- TTCTTAAATA CTAGCTTTTT AAAATTGAGA ATAACTGAAT ATCAACTGAG GTCAATTTAC CTTTTAACTT   
  
  
- CACTAATTAG ATTTTCACCG GGCGAGATTA TGGTAAACTC TTTTTAATAA GAATGACGTT TCAAATTTAG   
  
  
- TTATACACTT CTATCAAATA TCAAAATAGG ATTTTCTTTA ATTTATCTAA CAATTTTTAT TAAAAATTTA   
  
  
- AGTCTATATG CACACTACCG GTATTGGCAT TTACTATACG TACAGCTGTT TAAGGTTTCT TCCGATTATT   
  
  
- AATCTGAATT TATTATGTAC GTATGATTGG TTTTTTTTTT TTAATTCGTT CTAAGATAGG TTGGATAGTA   
  
  
- TTATTTCAAA CCGGTATGAA GTTTGGTAGT GTATTGTAGT GATTATATAT TTCGATCCAA AACCCTTTGG   
  
  
- ATTGAAACTC CTGGTGTATA AACAATTCCG GATCCTCCGG TACTCAAACA GTTCTACGGA GTATCATACC   
  
  
- TCTCCAGATA TAGGAACGTT TTACTAGTAA GGGAAGATGG ATTTTCTTGG TGAAGTAAAG GTGAAAATGA   
  
  
- GGGACCGTTT ACCAACTATT TGGTTTGGTA TTGGTTCGGG AACTTCTTGA TATTTCTGTG CTTAGCTAGT   
  
  
- AACTTAAGCT TGTTCGGAAC ACACTATCCT CCTACCCGGA GTTGTGAAGA GGCGCTCACA CGTTCTCGTT   
  
  
- AGAGCCTCTT TTTAGGATTC TCGGTTGTTG AAGAAACCCA CAATTTACTT GAACAAAGAG GAATACCGCT   
  
  
- AACGCTTGTT TCTAACCGTA TAATGAAGAA TGTTCACAAC AACCGGTTTC GGTTGTTAAA CCCTGGAGTG   
  
  
- AAAGTACTCT CGGATTTTGA ACGGTACCTC TTTTTGACGA CGAAACTATG GATGTACTTC GAATATAACT   
  
  
- TCAAGGTTCT CCAGTCAGGT ACCTGTTGGA AACCAGTACA CCGAAGTTTA CCACGTTATA ACCTCCTGGA   
  
  
- ATCTTCAAAT GTTTTCAGCT AGTAGTTTCT TTGTTCCGTT TCCTACCTCT TCAAAAGTTC CGATTACCCA   
  
  
- CAAGGGAAGT TCAAAGTACA GTATTTGCTC AATCTATTGG ATCCTCTCGA AGCTTTTCTC CTAAATCTGT   
  
  
- AACTTCTACC ACTCCGGTAG CGACACTTGA CACAAGTTCG GAACGTTGCC CAAGTACACC TCTTCTCCCT   
  
  
- CGTACACGAA CTACACTAAG CTAGATAATC CGGAACATAG TATTGTGACC ACCTCCTTCT TCGTCTAGAG   
  
  
- TGAAGATGTT CTTTGCTGAA GAAGTTCACG AAGCTACTCA CAAACTCTAA AAGATTCAGT ATAAAGCTAT   
  
  
- ACAATCTTCT TTCGAAGGGA GGTTATTCGT TGCTTGCTTA GTTCGATCTT TCCCTTGTTA CCTACTTATA   
  
  
- GAGGTCTCGG GATCGAACAG TACCACTTTC ACCTCCTCTT ATATCCGGTT TCTTTCCTTG AGTTACCTTA   
  
  
- CTCTACGAGC TTGTTCGTAA AACGGGTAGA GTTAAATCAC TACTACATGA TTCACTACAA TCCCGTAACA   
  
  
- ACTTTTCTGT GTTTTCACCA ACCCTAGAGT GGAATGGTGT TTCACTGGTA CTTTATCCGT ATGTGAATTT   
  
  
- AACCTTCCCA CTTTTACAAC AAACCCGAAG ACGTACCTCT GGATCGAT

+     Unnamed\_\_6

| Site Name | Organism | Position | Strand | Matrix score. | sequence | function |
| --- | --- | --- | --- | --- | --- | --- |
| Unnamed\_\_6 | Zea mays | 27 | - | 10 | taTAAATATct |  |

>HU02G03154.1   
+ +Up\_Stream \_Len000ATGATA AAAGATATTT ATTAAGCTCA AGCTATTTTA ACTTTTAAAA AAATGAATGT   
  
  
+ GGCTTTTTTT GCTATTCAGG TACAACACAA TATGAAGCAT TAACTCTAGA AAATGTGGTA ATGTATGTCA   
  
  
+ TATAGGAGAG TATATACTCT CACTTGCCAT GCGATGCGTG CATGTGTGTG TGTGTGTGTA TCTGTTTCTA   
  
  
+ TGCATATTTT ATGTGGGGTT TTGTCATCTA GACATCTATT AATATGTTAG CATTCACATG ACTTATAGGA   
  
  
+ ATGCACTATT ATGAGTATGA TATAGAGTTT TAAAAATGCA TATGCCTTGT GTGTTCATTA GCTTAAAGAA   
  
  
+ GAGTTTTTGA ATATACATAT TTAGGTAATT CTTGCTAATG TCGTAATTAG CAGTTGTATC TATCTTATTG   
  
  
+ ATTTGTATTT TTAATTGCTT GTTATACTGT TACACTTAGA AAATTCATGA ATACTCTTAA AATATGCAAA   
  
  
+ TTATATATAT ATATATATAT ATATATATAT ATATATATAT ATAAAAACTG AATAAGTTAA TATGGTGATG   
  
  
+ ACATTTGTCA TGTTATCATT GGTCGCTTAA TTTTTAAATA AAAAATATGA TTTTGTGACA CCTAATAAAT   
  
  
+ GATATTAGCC ATATTATTAA ATTCAGCATT TATTCATCAA TGTAATATAC TAAATCATGC AATTATCATT   
  
  
+ GGTTCTCTTT TATGTAATAA AGTTAATTGA CAATTCAAGG GTTACTAATT AGTATAATGT TGAATTGGAA   
  
  
+ CGTAAAAATC AAATCGTATG TAAAGTAATT TTATTCTGAA TTTAAAAAAT GGAGCAAATA TCTCTATTGT   
  
  
+ TCTTTAATAA AATTTTAATT GATTTTTATT TCTAATTAAG TGACGGAACC AAATTTTTTT CCTCCTTTTT   
  
  
+ TGGTTTGGTA AGTTATCATT AGCCAATCTT TTATTTACAT TTTGTCAGTT TTTCTTTTTT AAGATGGTTA   
  
  
+ AATGGCTTCA ATTAAACAAA TTTTTTTACT TATTTATATT TTTAATAAAT TTTCAAAATG TATATTAACG   
  
  
+ CATGACCGTG CGAAGCACGG AATCTACCCT AGTTAATCTA TGATTCTTAA ATATGCAAGT AGGCTGTTAT   
  
  
+ TCATTTGTTG TTTTTTACTT ATTTATATTT TTTCCCTCCC GGTATGATAG GGTTTGTCAT ATTGTTATTC   
  
  
+ ATTAGCTGTT AAACTGTATT CATTATAGCA CAAGTAGGCT TAATCAAAGA TAGTTAGTCT CCAATTTCAT   
  
  
+ CAGAAAATTT AAGAAAAAGA CACCTTAGGA TCAAAAGTAT GAAATTTAGA GACCAGACAA CGATAGAATT   
  
  
+ GAAAAGATGA GTTTTCAATT TGGAGAAGAC GGAAACTCAG ATGAATGAAA GTATAGTACA TTGTACTCCT   
  
  
+ TGGAGTATAA TCTTATCTTC ACTATTGATA GGCTCGAAGC ATAATCAGTG ATTGGAAACT TTTGGTTGCA   
  
  
+ ATAATTCAAA ATTTGATACT CTATGATTTA CTTTTTTAAT TGAAGGTCAT GATTTAATTA ACTAAATGAA   
  
  
+ AAGAATTTAT GATCGAAAAA TTTTAACTCT TATTGACTTA TAGTTGACTC CAGTTAAATG GAAAATTGAA   
  
  
+ GTGATTAATC TAAAAGTGGC CCGCTCTAAT ACCATTTGAG AAAAATTATT CTTACTGCAA AGTTTAAATC   
  
  
+ AATATGTGAA GATAGTTTAT AGTTTTATCC TAAAAGAAAT TAAATAGATT GTTAAAAATA ATTTTTAAAT   
  
  
+ TCAGATATAC GTGTGATGGC CATAACCGTA AATGATATGC ATGTCGACAA ATTCCAAAGA AGGCTAATAA   
  
  
+ TTAGACTTAA ATAATACATG CATACTAACC AAAAAAAAAA AATTAAGCAA GATTCTATCC AACCTATCAT   
  
  
+ AATAAAGTTT GGCCATACTT CAAACCATCA CATAACATCA CTAATATATA AAGCTAGGTT TTGGGAAACC   
  
  
+ TAACTTTGAG GACCACATAT TTGTTAAGGC CTAGGAGGCC ATGAGTTTGT CAAGATGCCT CATAGTATGG   
  
  
+ AGAGGTCTAT ATCCTTGCAA AATGATCATT CCCTTCTACC TAAAAGAACC ACTTCATTTC CACTTTTACT   
  
  
+ CCCTGGCAAA TGGTTGATAA ACCAAACCAT AACCAAGCCC TTGAAGAACT ATAAAGACAC GAATCGATCA   
  
  
+ TTGAATTCGA ACAAGCCTTG TGTGATAGGA GGATGGGCCT CAACACTTCT CCGCGAGTGT GCAAGAGCAA   
  
  
+ TCTCGGAGAA AAATCCTAAG AGCCAACAAC TTCTTTGGGT GTTAAATGAA CTTGTTTCTC CTTATGGCGA   
  
  
+ TTGCGAACAA AGATTGGCAT ATTACTTCTT ACAAGTGTTG TTGGCCAAAG CCAACAATTT GGGACCTCAC   
  
  
+ TTTCATGAGA GCCTAAAACT TGCCATGGAG AAAAACTGCT GCTTTGATAC CTACATGAAG CTTATATTGA   
  
  
+ AGTTCCAAGA GGTCAGTCCA TGGACAACCT TTGGTCATGT GGCTTCAAAT GGTGCAATAT TGGAGGACCT   
  
  
+ TAGAAGTTTA CAAAAGTCGA TCATCAAAGA AACAAGGCAA AGGATGGAGA AGTTTTCAAG GCTAATGGGT   
  
  
+ GTTCCCTTCA AGTTTCATGT CATAAACGAG TTAGATAACC TAGGAGAGCT TCGAAAAGAG GATTTAGACA   
  
  
+ TTGAAGATGG TGAGGCCATC GCTGTGAACT GTGTTCAAGC CTTGCAACGG GTTCATGTGG AGAAGAGGGA   
  
  
+ GCATGTGCTT GATGTGATTC GATCTATTAG GCCTTGTATC ATAACACTGG TGGAGGAAGA AGCAGATCTC   
  
  
+ ACTTCTACAA GAAACGACTT CTTCAAGTGC TTCGATGAGT GTTTGAGATT TTCTAAGTCA TATTTCGATA   
  
  
+ TGTTAGAAGA AAGCTTCCCT CCAATAAGCA ACGAACGAAT CAAGCTAGAA AGGGAACAAT GGATGAATAT   
  
  
+ CTCCAGAGCC CTAGCTTGTC ATGGTGAAAG TGGAGGAGAA TATAGGCCAA AGAAAGGAAC TCAATGGAAT   
  
  
+ GAGATGCTCG AACAAGCATT TTGCCCATCT CAATTTAGTG ATGATGTACT AAGTGATGTT AGGGCATTGT   
  
  
+ TGAAAAGACA CAAAAGTGGT TGGGATCTCA CCTTACCACA AAGTGACCAT GAAATAGGCA TACACTTAAA   
  
  
+ TTGGAAGGGT GAAAATGTTG TTTGGGCTTC TGCATGGAGA CCTAGCTA  

- +Up\_Stream \_Len000TACTAT TTTCTATAAA TAATTCGAGT TCGATAAAAT TGAAAATTTT TTTACTTACA   
  
  
- CCGAAAAAAA CGATAAGTCC ATGTTGTGTT ATACTTCGTA ATTGAGATCT TTTACACCAT TACATACAGT   
  
  
- ATATCCTCTC ATATATGAGA GTGAACGGTA CGCTACGCAC GTACACACAC ACACACACAT AGACAAAGAT   
  
  
- ACGTATAAAA TACACCCCAA AACAGTAGAT CTGTAGATAA TTATACAATC GTAAGTGTAC TGAATATCCT   
  
  
- TACGTGATAA TACTCATACT ATATCTCAAA ATTTTTACGT ATACGGAACA CACAAGTAAT CGAATTTCTT   
  
  
- CTCAAAAACT TATATGTATA AATCCATTAA GAACGATTAC AGCATTAATC GTCAACATAG ATAGAATAAC   
  
  
- TAAACATAAA AATTAACGAA CAATATGACA ATGTGAATCT TTTAAGTACT TATGAGAATT TTATACGTTT   
  
  
- AATATATATA TATATATATA TATATATATA TATATATATA TATTTTTGAC TTATTCAATT ATACCACTAC   
  
  
- TGTAAACAGT ACAATAGTAA CCAGCGAATT AAAAATTTAT TTTTTATACT AAAACACTGT GGATTATTTA   
  
  
- CTATAATCGG TATAATAATT TAAGTCGTAA ATAAGTAGTT ACATTATATG ATTTAGTACG TTAATAGTAA   
  
  
- CCAAGAGAAA ATACATTATT TCAATTAACT GTTAAGTTCC CAATGATTAA TCATATTACA ACTTAACCTT   
  
  
- GCATTTTTAG TTTAGCATAC ATTTCATTAA AATAAGACTT AAATTTTTTA CCTCGTTTAT AGAGATAACA   
  
  
- AGAAATTATT TTAAAATTAA CTAAAAATAA AGATTAATTC ACTGCCTTGG TTTAAAAAAA GGAGGAAAAA   
  
  
- ACCAAACCAT TCAATAGTAA TCGGTTAGAA AATAAATGTA AAACAGTCAA AAAGAAAAAA TTCTACCAAT   
  
  
- TTACCGAAGT TAATTTGTTT AAAAAAATGA ATAAATATAA AAATTATTTA AAAGTTTTAC ATATAATTGC   
  
  
- GTACTGGCAC GCTTCGTGCC TTAGATGGGA TCAATTAGAT ACTAAGAATT TATACGTTCA TCCGACAATA   
  
  
- AGTAAACAAC AAAAAATGAA TAAATATAAA AAAGGGAGGG CCATACTATC CCAAACAGTA TAACAATAAG   
  
  
- TAATCGACAA TTTGACATAA GTAATATCGT GTTCATCCGA ATTAGTTTCT ATCAATCAGA GGTTAAAGTA   
  
  
- GTCTTTTAAA TTCTTTTTCT GTGGAATCCT AGTTTTCATA CTTTAAATCT CTGGTCTGTT GCTATCTTAA   
  
  
- CTTTTCTACT CAAAAGTTAA ACCTCTTCTG CCTTTGAGTC TACTTACTTT CATATCATGT AACATGAGGA   
  
  
- ACCTCATATT AGAATAGAAG TGATAACTAT CCGAGCTTCG TATTAGTCAC TAACCTTTGA AAACCAACGT   
  
  
- TATTAAGTTT TAAACTATGA GATACTAAAT GAAAAAATTA ACTTCCAGTA CTAAATTAAT TGATTTACTT   
  
  
- TTCTTAAATA CTAGCTTTTT AAAATTGAGA ATAACTGAAT ATCAACTGAG GTCAATTTAC CTTTTAACTT   
  
  
- CACTAATTAG ATTTTCACCG GGCGAGATTA TGGTAAACTC TTTTTAATAA GAATGACGTT TCAAATTTAG   
  
  
- TTATACACTT CTATCAAATA TCAAAATAGG ATTTTCTTTA ATTTATCTAA CAATTTTTAT TAAAAATTTA   
  
  
- AGTCTATATG CACACTACCG GTATTGGCAT TTACTATACG TACAGCTGTT TAAGGTTTCT TCCGATTATT   
  
  
- AATCTGAATT TATTATGTAC GTATGATTGG TTTTTTTTTT TTAATTCGTT CTAAGATAGG TTGGATAGTA   
  
  
- TTATTTCAAA CCGGTATGAA GTTTGGTAGT GTATTGTAGT GATTATATAT TTCGATCCAA AACCCTTTGG   
  
  
- ATTGAAACTC CTGGTGTATA AACAATTCCG GATCCTCCGG TACTCAAACA GTTCTACGGA GTATCATACC   
  
  
- TCTCCAGATA TAGGAACGTT TTACTAGTAA GGGAAGATGG ATTTTCTTGG TGAAGTAAAG GTGAAAATGA   
  
  
- GGGACCGTTT ACCAACTATT TGGTTTGGTA TTGGTTCGGG AACTTCTTGA TATTTCTGTG CTTAGCTAGT   
  
  
- AACTTAAGCT TGTTCGGAAC ACACTATCCT CCTACCCGGA GTTGTGAAGA GGCGCTCACA CGTTCTCGTT   
  
  
- AGAGCCTCTT TTTAGGATTC TCGGTTGTTG AAGAAACCCA CAATTTACTT GAACAAAGAG GAATACCGCT   
  
  
- AACGCTTGTT TCTAACCGTA TAATGAAGAA TGTTCACAAC AACCGGTTTC GGTTGTTAAA CCCTGGAGTG   
  
  
- AAAGTACTCT CGGATTTTGA ACGGTACCTC TTTTTGACGA CGAAACTATG GATGTACTTC GAATATAACT   
  
  
- TCAAGGTTCT CCAGTCAGGT ACCTGTTGGA AACCAGTACA CCGAAGTTTA CCACGTTATA ACCTCCTGGA   
  
  
- ATCTTCAAAT GTTTTCAGCT AGTAGTTTCT TTGTTCCGTT TCCTACCTCT TCAAAAGTTC CGATTACCCA   
  
  
- CAAGGGAAGT TCAAAGTACA GTATTTGCTC AATCTATTGG ATCCTCTCGA AGCTTTTCTC CTAAATCTGT   
  
  
- AACTTCTACC ACTCCGGTAG CGACACTTGA CACAAGTTCG GAACGTTGCC CAAGTACACC TCTTCTCCCT   
  
  
- CGTACACGAA CTACACTAAG CTAGATAATC CGGAACATAG TATTGTGACC ACCTCCTTCT TCGTCTAGAG   
  
  
- TGAAGATGTT CTTTGCTGAA GAAGTTCACG AAGCTACTCA CAAACTCTAA AAGATTCAGT ATAAAGCTAT   
  
  
- ACAATCTTCT TTCGAAGGGA GGTTATTCGT TGCTTGCTTA GTTCGATCTT TCCCTTGTTA CCTACTTATA   
  
  
- GAGGTCTCGG GATCGAACAG TACCACTTTC ACCTCCTCTT ATATCCGGTT TCTTTCCTTG AGTTACCTTA   
  
  
- CTCTACGAGC TTGTTCGTAA AACGGGTAGA GTTAAATCAC TACTACATGA TTCACTACAA TCCCGTAACA   
  
  
- ACTTTTCTGT GTTTTCACCA ACCCTAGAGT GGAATGGTGT TTCACTGGTA CTTTATCCGT ATGTGAATTT   
  
  
- AACCTTCCCA CTTTTACAAC AAACCCGAAG ACGTACCTCT GGATCGAT

+     WUN-motif

| Site Name | Organism | Position | Strand | Matrix score. | sequence | function |
| --- | --- | --- | --- | --- | --- | --- |
| WUN-motif | Nicotiana glutinosa | 716 | - | 9 | TTATTACAT |  |
| WUN-motif | Nicotiana glutinosa | 798 | - | 8 | AAATTACT |  |

>HU02G03154.1   
+ +Up\_Stream \_Len000ATGATA AAAGATATTT ATTAAGCTCA AGCTATTTTA ACTTTTAAAA AAATGAATGT   
  
  
+ GGCTTTTTTT GCTATTCAGG TACAACACAA TATGAAGCAT TAACTCTAGA AAATGTGGTA ATGTATGTCA   
  
  
+ TATAGGAGAG TATATACTCT CACTTGCCAT GCGATGCGTG CATGTGTGTG TGTGTGTGTA TCTGTTTCTA   
  
  
+ TGCATATTTT ATGTGGGGTT TTGTCATCTA GACATCTATT AATATGTTAG CATTCACATG ACTTATAGGA   
  
  
+ ATGCACTATT ATGAGTATGA TATAGAGTTT TAAAAATGCA TATGCCTTGT GTGTTCATTA GCTTAAAGAA   
  
  
+ GAGTTTTTGA ATATACATAT TTAGGTAATT CTTGCTAATG TCGTAATTAG CAGTTGTATC TATCTTATTG   
  
  
+ ATTTGTATTT TTAATTGCTT GTTATACTGT TACACTTAGA AAATTCATGA ATACTCTTAA AATATGCAAA   
  
  
+ TTATATATAT ATATATATAT ATATATATAT ATATATATAT ATAAAAACTG AATAAGTTAA TATGGTGATG   
  
  
+ ACATTTGTCA TGTTATCATT GGTCGCTTAA TTTTTAAATA AAAAATATGA TTTTGTGACA CCTAATAAAT   
  
  
+ GATATTAGCC ATATTATTAA ATTCAGCATT TATTCATCAA TGTAATATAC TAAATCATGC AATTATCATT   
  
  
+ GGTTCTCTTT TATGTAATAA AGTTAATTGA CAATTCAAGG GTTACTAATT AGTATAATGT TGAATTGGAA   
  
  
+ CGTAAAAATC AAATCGTATG TAAAGTAATT TTATTCTGAA TTTAAAAAAT GGAGCAAATA TCTCTATTGT   
  
  
+ TCTTTAATAA AATTTTAATT GATTTTTATT TCTAATTAAG TGACGGAACC AAATTTTTTT CCTCCTTTTT   
  
  
+ TGGTTTGGTA AGTTATCATT AGCCAATCTT TTATTTACAT TTTGTCAGTT TTTCTTTTTT AAGATGGTTA   
  
  
+ AATGGCTTCA ATTAAACAAA TTTTTTTACT TATTTATATT TTTAATAAAT TTTCAAAATG TATATTAACG   
  
  
+ CATGACCGTG CGAAGCACGG AATCTACCCT AGTTAATCTA TGATTCTTAA ATATGCAAGT AGGCTGTTAT   
  
  
+ TCATTTGTTG TTTTTTACTT ATTTATATTT TTTCCCTCCC GGTATGATAG GGTTTGTCAT ATTGTTATTC   
  
  
+ ATTAGCTGTT AAACTGTATT CATTATAGCA CAAGTAGGCT TAATCAAAGA TAGTTAGTCT CCAATTTCAT   
  
  
+ CAGAAAATTT AAGAAAAAGA CACCTTAGGA TCAAAAGTAT GAAATTTAGA GACCAGACAA CGATAGAATT   
  
  
+ GAAAAGATGA GTTTTCAATT TGGAGAAGAC GGAAACTCAG ATGAATGAAA GTATAGTACA TTGTACTCCT   
  
  
+ TGGAGTATAA TCTTATCTTC ACTATTGATA GGCTCGAAGC ATAATCAGTG ATTGGAAACT TTTGGTTGCA   
  
  
+ ATAATTCAAA ATTTGATACT CTATGATTTA CTTTTTTAAT TGAAGGTCAT GATTTAATTA ACTAAATGAA   
  
  
+ AAGAATTTAT GATCGAAAAA TTTTAACTCT TATTGACTTA TAGTTGACTC CAGTTAAATG GAAAATTGAA   
  
  
+ GTGATTAATC TAAAAGTGGC CCGCTCTAAT ACCATTTGAG AAAAATTATT CTTACTGCAA AGTTTAAATC   
  
  
+ AATATGTGAA GATAGTTTAT AGTTTTATCC TAAAAGAAAT TAAATAGATT GTTAAAAATA ATTTTTAAAT   
  
  
+ TCAGATATAC GTGTGATGGC CATAACCGTA AATGATATGC ATGTCGACAA ATTCCAAAGA AGGCTAATAA   
  
  
+ TTAGACTTAA ATAATACATG CATACTAACC AAAAAAAAAA AATTAAGCAA GATTCTATCC AACCTATCAT   
  
  
+ AATAAAGTTT GGCCATACTT CAAACCATCA CATAACATCA CTAATATATA AAGCTAGGTT TTGGGAAACC   
  
  
+ TAACTTTGAG GACCACATAT TTGTTAAGGC CTAGGAGGCC ATGAGTTTGT CAAGATGCCT CATAGTATGG   
  
  
+ AGAGGTCTAT ATCCTTGCAA AATGATCATT CCCTTCTACC TAAAAGAACC ACTTCATTTC CACTTTTACT   
  
  
+ CCCTGGCAAA TGGTTGATAA ACCAAACCAT AACCAAGCCC TTGAAGAACT ATAAAGACAC GAATCGATCA   
  
  
+ TTGAATTCGA ACAAGCCTTG TGTGATAGGA GGATGGGCCT CAACACTTCT CCGCGAGTGT GCAAGAGCAA   
  
  
+ TCTCGGAGAA AAATCCTAAG AGCCAACAAC TTCTTTGGGT GTTAAATGAA CTTGTTTCTC CTTATGGCGA   
  
  
+ TTGCGAACAA AGATTGGCAT ATTACTTCTT ACAAGTGTTG TTGGCCAAAG CCAACAATTT GGGACCTCAC   
  
  
+ TTTCATGAGA GCCTAAAACT TGCCATGGAG AAAAACTGCT GCTTTGATAC CTACATGAAG CTTATATTGA   
  
  
+ AGTTCCAAGA GGTCAGTCCA TGGACAACCT TTGGTCATGT GGCTTCAAAT GGTGCAATAT TGGAGGACCT   
  
  
+ TAGAAGTTTA CAAAAGTCGA TCATCAAAGA AACAAGGCAA AGGATGGAGA AGTTTTCAAG GCTAATGGGT   
  
  
+ GTTCCCTTCA AGTTTCATGT CATAAACGAG TTAGATAACC TAGGAGAGCT TCGAAAAGAG GATTTAGACA   
  
  
+ TTGAAGATGG TGAGGCCATC GCTGTGAACT GTGTTCAAGC CTTGCAACGG GTTCATGTGG AGAAGAGGGA   
  
  
+ GCATGTGCTT GATGTGATTC GATCTATTAG GCCTTGTATC ATAACACTGG TGGAGGAAGA AGCAGATCTC   
  
  
+ ACTTCTACAA GAAACGACTT CTTCAAGTGC TTCGATGAGT GTTTGAGATT TTCTAAGTCA TATTTCGATA   
  
  
+ TGTTAGAAGA AAGCTTCCCT CCAATAAGCA ACGAACGAAT CAAGCTAGAA AGGGAACAAT GGATGAATAT   
  
  
+ CTCCAGAGCC CTAGCTTGTC ATGGTGAAAG TGGAGGAGAA TATAGGCCAA AGAAAGGAAC TCAATGGAAT   
  
  
+ GAGATGCTCG AACAAGCATT TTGCCCATCT CAATTTAGTG ATGATGTACT AAGTGATGTT AGGGCATTGT   
  
  
+ TGAAAAGACA CAAAAGTGGT TGGGATCTCA CCTTACCACA AAGTGACCAT GAAATAGGCA TACACTTAAA   
  
  
+ TTGGAAGGGT GAAAATGTTG TTTGGGCTTC TGCATGGAGA CCTAGCTA  

- +Up\_Stream \_Len000TACTAT TTTCTATAAA TAATTCGAGT TCGATAAAAT TGAAAATTTT TTTACTTACA   
  
  
- CCGAAAAAAA CGATAAGTCC ATGTTGTGTT ATACTTCGTA ATTGAGATCT TTTACACCAT TACATACAGT   
  
  
- ATATCCTCTC ATATATGAGA GTGAACGGTA CGCTACGCAC GTACACACAC ACACACACAT AGACAAAGAT   
  
  
- ACGTATAAAA TACACCCCAA AACAGTAGAT CTGTAGATAA TTATACAATC GTAAGTGTAC TGAATATCCT   
  
  
- TACGTGATAA TACTCATACT ATATCTCAAA ATTTTTACGT ATACGGAACA CACAAGTAAT CGAATTTCTT   
  
  
- CTCAAAAACT TATATGTATA AATCCATTAA GAACGATTAC AGCATTAATC GTCAACATAG ATAGAATAAC   
  
  
- TAAACATAAA AATTAACGAA CAATATGACA ATGTGAATCT TTTAAGTACT TATGAGAATT TTATACGTTT   
  
  
- AATATATATA TATATATATA TATATATATA TATATATATA TATTTTTGAC TTATTCAATT ATACCACTAC   
  
  
- TGTAAACAGT ACAATAGTAA CCAGCGAATT AAAAATTTAT TTTTTATACT AAAACACTGT GGATTATTTA   
  
  
- CTATAATCGG TATAATAATT TAAGTCGTAA ATAAGTAGTT ACATTATATG ATTTAGTACG TTAATAGTAA   
  
  
- CCAAGAGAAA ATACATTATT TCAATTAACT GTTAAGTTCC CAATGATTAA TCATATTACA ACTTAACCTT   
  
  
- GCATTTTTAG TTTAGCATAC ATTTCATTAA AATAAGACTT AAATTTTTTA CCTCGTTTAT AGAGATAACA   
  
  
- AGAAATTATT TTAAAATTAA CTAAAAATAA AGATTAATTC ACTGCCTTGG TTTAAAAAAA GGAGGAAAAA   
  
  
- ACCAAACCAT TCAATAGTAA TCGGTTAGAA AATAAATGTA AAACAGTCAA AAAGAAAAAA TTCTACCAAT   
  
  
- TTACCGAAGT TAATTTGTTT AAAAAAATGA ATAAATATAA AAATTATTTA AAAGTTTTAC ATATAATTGC   
  
  
- GTACTGGCAC GCTTCGTGCC TTAGATGGGA TCAATTAGAT ACTAAGAATT TATACGTTCA TCCGACAATA   
  
  
- AGTAAACAAC AAAAAATGAA TAAATATAAA AAAGGGAGGG CCATACTATC CCAAACAGTA TAACAATAAG   
  
  
- TAATCGACAA TTTGACATAA GTAATATCGT GTTCATCCGA ATTAGTTTCT ATCAATCAGA GGTTAAAGTA   
  
  
- GTCTTTTAAA TTCTTTTTCT GTGGAATCCT AGTTTTCATA CTTTAAATCT CTGGTCTGTT GCTATCTTAA   
  
  
- CTTTTCTACT CAAAAGTTAA ACCTCTTCTG CCTTTGAGTC TACTTACTTT CATATCATGT AACATGAGGA   
  
  
- ACCTCATATT AGAATAGAAG TGATAACTAT CCGAGCTTCG TATTAGTCAC TAACCTTTGA AAACCAACGT   
  
  
- TATTAAGTTT TAAACTATGA GATACTAAAT GAAAAAATTA ACTTCCAGTA CTAAATTAAT TGATTTACTT   
  
  
- TTCTTAAATA CTAGCTTTTT AAAATTGAGA ATAACTGAAT ATCAACTGAG GTCAATTTAC CTTTTAACTT   
  
  
- CACTAATTAG ATTTTCACCG GGCGAGATTA TGGTAAACTC TTTTTAATAA GAATGACGTT TCAAATTTAG   
  
  
- TTATACACTT CTATCAAATA TCAAAATAGG ATTTTCTTTA ATTTATCTAA CAATTTTTAT TAAAAATTTA   
  
  
- AGTCTATATG CACACTACCG GTATTGGCAT TTACTATACG TACAGCTGTT TAAGGTTTCT TCCGATTATT   
  
  
- AATCTGAATT TATTATGTAC GTATGATTGG TTTTTTTTTT TTAATTCGTT CTAAGATAGG TTGGATAGTA   
  
  
- TTATTTCAAA CCGGTATGAA GTTTGGTAGT GTATTGTAGT GATTATATAT TTCGATCCAA AACCCTTTGG   
  
  
- ATTGAAACTC CTGGTGTATA AACAATTCCG GATCCTCCGG TACTCAAACA GTTCTACGGA GTATCATACC   
  
  
- TCTCCAGATA TAGGAACGTT TTACTAGTAA GGGAAGATGG ATTTTCTTGG TGAAGTAAAG GTGAAAATGA   
  
  
- GGGACCGTTT ACCAACTATT TGGTTTGGTA TTGGTTCGGG AACTTCTTGA TATTTCTGTG CTTAGCTAGT   
  
  
- AACTTAAGCT TGTTCGGAAC ACACTATCCT CCTACCCGGA GTTGTGAAGA GGCGCTCACA CGTTCTCGTT   
  
  
- AGAGCCTCTT TTTAGGATTC TCGGTTGTTG AAGAAACCCA CAATTTACTT GAACAAAGAG GAATACCGCT   
  
  
- AACGCTTGTT TCTAACCGTA TAATGAAGAA TGTTCACAAC AACCGGTTTC GGTTGTTAAA CCCTGGAGTG   
  
  
- AAAGTACTCT CGGATTTTGA ACGGTACCTC TTTTTGACGA CGAAACTATG GATGTACTTC GAATATAACT   
  
  
- TCAAGGTTCT CCAGTCAGGT ACCTGTTGGA AACCAGTACA CCGAAGTTTA CCACGTTATA ACCTCCTGGA   
  
  
- ATCTTCAAAT GTTTTCAGCT AGTAGTTTCT TTGTTCCGTT TCCTACCTCT TCAAAAGTTC CGATTACCCA   
  
  
- CAAGGGAAGT TCAAAGTACA GTATTTGCTC AATCTATTGG ATCCTCTCGA AGCTTTTCTC CTAAATCTGT   
  
  
- AACTTCTACC ACTCCGGTAG CGACACTTGA CACAAGTTCG GAACGTTGCC CAAGTACACC TCTTCTCCCT   
  
  
- CGTACACGAA CTACACTAAG CTAGATAATC CGGAACATAG TATTGTGACC ACCTCCTTCT TCGTCTAGAG   
  
  
- TGAAGATGTT CTTTGCTGAA GAAGTTCACG AAGCTACTCA CAAACTCTAA AAGATTCAGT ATAAAGCTAT   
  
  
- ACAATCTTCT TTCGAAGGGA GGTTATTCGT TGCTTGCTTA GTTCGATCTT TCCCTTGTTA CCTACTTATA   
  
  
- GAGGTCTCGG GATCGAACAG TACCACTTTC ACCTCCTCTT ATATCCGGTT TCTTTCCTTG AGTTACCTTA   
  
  
- CTCTACGAGC TTGTTCGTAA AACGGGTAGA GTTAAATCAC TACTACATGA TTCACTACAA TCCCGTAACA   
  
  
- ACTTTTCTGT GTTTTCACCA ACCCTAGAGT GGAATGGTGT TTCACTGGTA CTTTATCCGT ATGTGAATTT   
  
  
- AACCTTCCCA CTTTTACAAC AAACCCGAAG ACGTACCTCT GGATCGAT

+     as-1

| Site Name | Organism | Position | Strand | Matrix score. | sequence | function |
| --- | --- | --- | --- | --- | --- | --- |
| as-1 | Arabidopsis thaliana | 885 | + | 5 | TGACG |  |

>HU02G03154.1   
+ +Up\_Stream \_Len000ATGATA AAAGATATTT ATTAAGCTCA AGCTATTTTA ACTTTTAAAA AAATGAATGT   
  
  
+ GGCTTTTTTT GCTATTCAGG TACAACACAA TATGAAGCAT TAACTCTAGA AAATGTGGTA ATGTATGTCA   
  
  
+ TATAGGAGAG TATATACTCT CACTTGCCAT GCGATGCGTG CATGTGTGTG TGTGTGTGTA TCTGTTTCTA   
  
  
+ TGCATATTTT ATGTGGGGTT TTGTCATCTA GACATCTATT AATATGTTAG CATTCACATG ACTTATAGGA   
  
  
+ ATGCACTATT ATGAGTATGA TATAGAGTTT TAAAAATGCA TATGCCTTGT GTGTTCATTA GCTTAAAGAA   
  
  
+ GAGTTTTTGA ATATACATAT TTAGGTAATT CTTGCTAATG TCGTAATTAG CAGTTGTATC TATCTTATTG   
  
  
+ ATTTGTATTT TTAATTGCTT GTTATACTGT TACACTTAGA AAATTCATGA ATACTCTTAA AATATGCAAA   
  
  
+ TTATATATAT ATATATATAT ATATATATAT ATATATATAT ATAAAAACTG AATAAGTTAA TATGGTGATG   
  
  
+ ACATTTGTCA TGTTATCATT GGTCGCTTAA TTTTTAAATA AAAAATATGA TTTTGTGACA CCTAATAAAT   
  
  
+ GATATTAGCC ATATTATTAA ATTCAGCATT TATTCATCAA TGTAATATAC TAAATCATGC AATTATCATT   
  
  
+ GGTTCTCTTT TATGTAATAA AGTTAATTGA CAATTCAAGG GTTACTAATT AGTATAATGT TGAATTGGAA   
  
  
+ CGTAAAAATC AAATCGTATG TAAAGTAATT TTATTCTGAA TTTAAAAAAT GGAGCAAATA TCTCTATTGT   
  
  
+ TCTTTAATAA AATTTTAATT GATTTTTATT TCTAATTAAG TGACGGAACC AAATTTTTTT CCTCCTTTTT   
  
  
+ TGGTTTGGTA AGTTATCATT AGCCAATCTT TTATTTACAT TTTGTCAGTT TTTCTTTTTT AAGATGGTTA   
  
  
+ AATGGCTTCA ATTAAACAAA TTTTTTTACT TATTTATATT TTTAATAAAT TTTCAAAATG TATATTAACG   
  
  
+ CATGACCGTG CGAAGCACGG AATCTACCCT AGTTAATCTA TGATTCTTAA ATATGCAAGT AGGCTGTTAT   
  
  
+ TCATTTGTTG TTTTTTACTT ATTTATATTT TTTCCCTCCC GGTATGATAG GGTTTGTCAT ATTGTTATTC   
  
  
+ ATTAGCTGTT AAACTGTATT CATTATAGCA CAAGTAGGCT TAATCAAAGA TAGTTAGTCT CCAATTTCAT   
  
  
+ CAGAAAATTT AAGAAAAAGA CACCTTAGGA TCAAAAGTAT GAAATTTAGA GACCAGACAA CGATAGAATT   
  
  
+ GAAAAGATGA GTTTTCAATT TGGAGAAGAC GGAAACTCAG ATGAATGAAA GTATAGTACA TTGTACTCCT   
  
  
+ TGGAGTATAA TCTTATCTTC ACTATTGATA GGCTCGAAGC ATAATCAGTG ATTGGAAACT TTTGGTTGCA   
  
  
+ ATAATTCAAA ATTTGATACT CTATGATTTA CTTTTTTAAT TGAAGGTCAT GATTTAATTA ACTAAATGAA   
  
  
+ AAGAATTTAT GATCGAAAAA TTTTAACTCT TATTGACTTA TAGTTGACTC CAGTTAAATG GAAAATTGAA   
  
  
+ GTGATTAATC TAAAAGTGGC CCGCTCTAAT ACCATTTGAG AAAAATTATT CTTACTGCAA AGTTTAAATC   
  
  
+ AATATGTGAA GATAGTTTAT AGTTTTATCC TAAAAGAAAT TAAATAGATT GTTAAAAATA ATTTTTAAAT   
  
  
+ TCAGATATAC GTGTGATGGC CATAACCGTA AATGATATGC ATGTCGACAA ATTCCAAAGA AGGCTAATAA   
  
  
+ TTAGACTTAA ATAATACATG CATACTAACC AAAAAAAAAA AATTAAGCAA GATTCTATCC AACCTATCAT   
  
  
+ AATAAAGTTT GGCCATACTT CAAACCATCA CATAACATCA CTAATATATA AAGCTAGGTT TTGGGAAACC   
  
  
+ TAACTTTGAG GACCACATAT TTGTTAAGGC CTAGGAGGCC ATGAGTTTGT CAAGATGCCT CATAGTATGG   
  
  
+ AGAGGTCTAT ATCCTTGCAA AATGATCATT CCCTTCTACC TAAAAGAACC ACTTCATTTC CACTTTTACT   
  
  
+ CCCTGGCAAA TGGTTGATAA ACCAAACCAT AACCAAGCCC TTGAAGAACT ATAAAGACAC GAATCGATCA   
  
  
+ TTGAATTCGA ACAAGCCTTG TGTGATAGGA GGATGGGCCT CAACACTTCT CCGCGAGTGT GCAAGAGCAA   
  
  
+ TCTCGGAGAA AAATCCTAAG AGCCAACAAC TTCTTTGGGT GTTAAATGAA CTTGTTTCTC CTTATGGCGA   
  
  
+ TTGCGAACAA AGATTGGCAT ATTACTTCTT ACAAGTGTTG TTGGCCAAAG CCAACAATTT GGGACCTCAC   
  
  
+ TTTCATGAGA GCCTAAAACT TGCCATGGAG AAAAACTGCT GCTTTGATAC CTACATGAAG CTTATATTGA   
  
  
+ AGTTCCAAGA GGTCAGTCCA TGGACAACCT TTGGTCATGT GGCTTCAAAT GGTGCAATAT TGGAGGACCT   
  
  
+ TAGAAGTTTA CAAAAGTCGA TCATCAAAGA AACAAGGCAA AGGATGGAGA AGTTTTCAAG GCTAATGGGT   
  
  
+ GTTCCCTTCA AGTTTCATGT CATAAACGAG TTAGATAACC TAGGAGAGCT TCGAAAAGAG GATTTAGACA   
  
  
+ TTGAAGATGG TGAGGCCATC GCTGTGAACT GTGTTCAAGC CTTGCAACGG GTTCATGTGG AGAAGAGGGA   
  
  
+ GCATGTGCTT GATGTGATTC GATCTATTAG GCCTTGTATC ATAACACTGG TGGAGGAAGA AGCAGATCTC   
  
  
+ ACTTCTACAA GAAACGACTT CTTCAAGTGC TTCGATGAGT GTTTGAGATT TTCTAAGTCA TATTTCGATA   
  
  
+ TGTTAGAAGA AAGCTTCCCT CCAATAAGCA ACGAACGAAT CAAGCTAGAA AGGGAACAAT GGATGAATAT   
  
  
+ CTCCAGAGCC CTAGCTTGTC ATGGTGAAAG TGGAGGAGAA TATAGGCCAA AGAAAGGAAC TCAATGGAAT   
  
  
+ GAGATGCTCG AACAAGCATT TTGCCCATCT CAATTTAGTG ATGATGTACT AAGTGATGTT AGGGCATTGT   
  
  
+ TGAAAAGACA CAAAAGTGGT TGGGATCTCA CCTTACCACA AAGTGACCAT GAAATAGGCA TACACTTAAA   
  
  
+ TTGGAAGGGT GAAAATGTTG TTTGGGCTTC TGCATGGAGA CCTAGCTA  

- +Up\_Stream \_Len000TACTAT TTTCTATAAA TAATTCGAGT TCGATAAAAT TGAAAATTTT TTTACTTACA   
  
  
- CCGAAAAAAA CGATAAGTCC ATGTTGTGTT ATACTTCGTA ATTGAGATCT TTTACACCAT TACATACAGT   
  
  
- ATATCCTCTC ATATATGAGA GTGAACGGTA CGCTACGCAC GTACACACAC ACACACACAT AGACAAAGAT   
  
  
- ACGTATAAAA TACACCCCAA AACAGTAGAT CTGTAGATAA TTATACAATC GTAAGTGTAC TGAATATCCT   
  
  
- TACGTGATAA TACTCATACT ATATCTCAAA ATTTTTACGT ATACGGAACA CACAAGTAAT CGAATTTCTT   
  
  
- CTCAAAAACT TATATGTATA AATCCATTAA GAACGATTAC AGCATTAATC GTCAACATAG ATAGAATAAC   
  
  
- TAAACATAAA AATTAACGAA CAATATGACA ATGTGAATCT TTTAAGTACT TATGAGAATT TTATACGTTT   
  
  
- AATATATATA TATATATATA TATATATATA TATATATATA TATTTTTGAC TTATTCAATT ATACCACTAC   
  
  
- TGTAAACAGT ACAATAGTAA CCAGCGAATT AAAAATTTAT TTTTTATACT AAAACACTGT GGATTATTTA   
  
  
- CTATAATCGG TATAATAATT TAAGTCGTAA ATAAGTAGTT ACATTATATG ATTTAGTACG TTAATAGTAA   
  
  
- CCAAGAGAAA ATACATTATT TCAATTAACT GTTAAGTTCC CAATGATTAA TCATATTACA ACTTAACCTT   
  
  
- GCATTTTTAG TTTAGCATAC ATTTCATTAA AATAAGACTT AAATTTTTTA CCTCGTTTAT AGAGATAACA   
  
  
- AGAAATTATT TTAAAATTAA CTAAAAATAA AGATTAATTC ACTGCCTTGG TTTAAAAAAA GGAGGAAAAA   
  
  
- ACCAAACCAT TCAATAGTAA TCGGTTAGAA AATAAATGTA AAACAGTCAA AAAGAAAAAA TTCTACCAAT   
  
  
- TTACCGAAGT TAATTTGTTT AAAAAAATGA ATAAATATAA AAATTATTTA AAAGTTTTAC ATATAATTGC   
  
  
- GTACTGGCAC GCTTCGTGCC TTAGATGGGA TCAATTAGAT ACTAAGAATT TATACGTTCA TCCGACAATA   
  
  
- AGTAAACAAC AAAAAATGAA TAAATATAAA AAAGGGAGGG CCATACTATC CCAAACAGTA TAACAATAAG   
  
  
- TAATCGACAA TTTGACATAA GTAATATCGT GTTCATCCGA ATTAGTTTCT ATCAATCAGA GGTTAAAGTA   
  
  
- GTCTTTTAAA TTCTTTTTCT GTGGAATCCT AGTTTTCATA CTTTAAATCT CTGGTCTGTT GCTATCTTAA   
  
  
- CTTTTCTACT CAAAAGTTAA ACCTCTTCTG CCTTTGAGTC TACTTACTTT CATATCATGT AACATGAGGA   
  
  
- ACCTCATATT AGAATAGAAG TGATAACTAT CCGAGCTTCG TATTAGTCAC TAACCTTTGA AAACCAACGT   
  
  
- TATTAAGTTT TAAACTATGA GATACTAAAT GAAAAAATTA ACTTCCAGTA CTAAATTAAT TGATTTACTT   
  
  
- TTCTTAAATA CTAGCTTTTT AAAATTGAGA ATAACTGAAT ATCAACTGAG GTCAATTTAC CTTTTAACTT   
  
  
- CACTAATTAG ATTTTCACCG GGCGAGATTA TGGTAAACTC TTTTTAATAA GAATGACGTT TCAAATTTAG   
  
  
- TTATACACTT CTATCAAATA TCAAAATAGG ATTTTCTTTA ATTTATCTAA CAATTTTTAT TAAAAATTTA   
  
  
- AGTCTATATG CACACTACCG GTATTGGCAT TTACTATACG TACAGCTGTT TAAGGTTTCT TCCGATTATT   
  
  
- AATCTGAATT TATTATGTAC GTATGATTGG TTTTTTTTTT TTAATTCGTT CTAAGATAGG TTGGATAGTA   
  
  
- TTATTTCAAA CCGGTATGAA GTTTGGTAGT GTATTGTAGT GATTATATAT TTCGATCCAA AACCCTTTGG   
  
  
- ATTGAAACTC CTGGTGTATA AACAATTCCG GATCCTCCGG TACTCAAACA GTTCTACGGA GTATCATACC   
  
  
- TCTCCAGATA TAGGAACGTT TTACTAGTAA GGGAAGATGG ATTTTCTTGG TGAAGTAAAG GTGAAAATGA   
  
  
- GGGACCGTTT ACCAACTATT TGGTTTGGTA TTGGTTCGGG AACTTCTTGA TATTTCTGTG CTTAGCTAGT   
  
  
- AACTTAAGCT TGTTCGGAAC ACACTATCCT CCTACCCGGA GTTGTGAAGA GGCGCTCACA CGTTCTCGTT   
  
  
- AGAGCCTCTT TTTAGGATTC TCGGTTGTTG AAGAAACCCA CAATTTACTT GAACAAAGAG GAATACCGCT   
  
  
- AACGCTTGTT TCTAACCGTA TAATGAAGAA TGTTCACAAC AACCGGTTTC GGTTGTTAAA CCCTGGAGTG   
  
  
- AAAGTACTCT CGGATTTTGA ACGGTACCTC TTTTTGACGA CGAAACTATG GATGTACTTC GAATATAACT   
  
  
- TCAAGGTTCT CCAGTCAGGT ACCTGTTGGA AACCAGTACA CCGAAGTTTA CCACGTTATA ACCTCCTGGA   
  
  
- ATCTTCAAAT GTTTTCAGCT AGTAGTTTCT TTGTTCCGTT TCCTACCTCT TCAAAAGTTC CGATTACCCA   
  
  
- CAAGGGAAGT TCAAAGTACA GTATTTGCTC AATCTATTGG ATCCTCTCGA AGCTTTTCTC CTAAATCTGT   
  
  
- AACTTCTACC ACTCCGGTAG CGACACTTGA CACAAGTTCG GAACGTTGCC CAAGTACACC TCTTCTCCCT   
  
  
- CGTACACGAA CTACACTAAG CTAGATAATC CGGAACATAG TATTGTGACC ACCTCCTTCT TCGTCTAGAG   
  
  
- TGAAGATGTT CTTTGCTGAA GAAGTTCACG AAGCTACTCA CAAACTCTAA AAGATTCAGT ATAAAGCTAT   
  
  
- ACAATCTTCT TTCGAAGGGA GGTTATTCGT TGCTTGCTTA GTTCGATCTT TCCCTTGTTA CCTACTTATA   
  
  
- GAGGTCTCGG GATCGAACAG TACCACTTTC ACCTCCTCTT ATATCCGGTT TCTTTCCTTG AGTTACCTTA   
  
  
- CTCTACGAGC TTGTTCGTAA AACGGGTAGA GTTAAATCAC TACTACATGA TTCACTACAA TCCCGTAACA   
  
  
- ACTTTTCTGT GTTTTCACCA ACCCTAGAGT GGAATGGTGT TTCACTGGTA CTTTATCCGT ATGTGAATTT   
  
  
- AACCTTCCCA CTTTTACAAC AAACCCGAAG ACGTACCTCT GGATCGAT
