# Supplementary material for: Genome-Wide Identification and Expression Pattern of the GRAS Gene Family in Pitaya (Selenicereus undatus L.)
Source: Biology (Basel). 2022 Dec 21;12(1):11. doi: 10.3390/biology12010011 (PMC9854919; doi:10.3390/biology12010011)
Supplement: Supplementary file 1 [file biology-12-00011-s001.zip › Supplementary file S5/HU03G01737.1_plantcare.html]

Content-Type: text/html; charset=ISO-8859-1


PlantCARE


Webmaster Firefox specific output  
To save the result:
click on the frame with the right mouse button and save the source code as a text file with extension .html  
REFERENCE:PlantCARE: a database of plant cis-acting regulatory elements and a portal to tools for in silico analysis of promoter sequences.  
Lescot, M., Déhais, P., Moreau, Y., De Moor, B., Rouzé ,P.,and Rombauts, S.  
Nucleic Acids Res., Database issue(2002), 30(1):325-327.   


---

>HU03G01737.1   
+ +Up\_Stream \_Len000TTTTTT TTAAGTTTTC GTACTTGGTG TTGATGATTT GTGCTTTGTA ACCAATGGTT   
  
  
+ TTAGTAAATA GTAATTAGTC GTAACATTCT GCGATTTCGC TCAATTTATA TATCAACTTT AGAATATGAG   
  
  
+ TAACCGTAAA GATCAAACGT AGTAGTAGAG GTGTCATAGG TTTTTTTTTA AAAAAATTCG GAGTAAGAAA   
  
  
+ GTAGGGTACA TTATTTTTGC TTGTTAAATT CCGCTAGTAT AGCAATACGA GATGGAACAT TTGTAAAAAC   
  
  
+ TACGAGACGA AGCGTTTGTA CAAAACAAAC TACCATACTG CAGATCATAT AATTTTTTGG GGGGTTATGT   
  
  
+ ATCTTAGTGA TGACATTAAT TGATGGATAA CCCTATTCTA AATATCTGGA TCCATCATTA ACTGACTCGT   
  
  
+ TTCAAATGTT AGGATTATGG GTCTTGACAT GGTATACATG TCAATAGATT TATGTTAGAT GAAAAGTTAT   
  
  
+ ATGACGCTTT TTATGATTTT TGTACTAGCA TATAGTGCTT AATTGGACTA TATATTTTTA CAAGTTGTGC   
  
  
+ TTAACACCTT TTAATCAGAA ATATTTATTA TATGGAAAAA ATTTTATTAA TATGTGTTTA TGTGTTTAAA   
  
  
+ AAACATACCC AGCCTTGAAA TAGACCGTGA TATAGAAATT GGACCCATAA TACTCACATT ATATATGCTT   
  
  
+ TATTGTTATA ATTAAGGATC ATTGTTGTTT TGCAATTATG TGGCAAGATA TAATCCACAA AGTTTTACAT   
  
  
+ TTATATTGCA ATTACTCTGG TAAATGCTAT TAATGTACAT TTGAGATTAA GACATATATA TAACCCTTTT   
  
  
+ TTTTCCCTTC TTTTTTCCTT TTTCTATTTT GAATGGCATC TTAAACCAAA GGAGCTGTAC AAGTTACGTT   
  
  
+ ATAGCACTCA TGGTTGACTA AACAAGACAG ATGAGAGTTT AAGTCAAGTC CTCCTGGCAA TGATGTTTGC   
  
  
+ CATTTGGACC AATAAATACA TCATGTATAT TACCATAATA GTGTGGTTGA CTTAACAATA CATAGGGGAT   
  
  
+ TGCATACAAA GGTAAGCCTC AAAAACTTGT GCATATGCAT TGAAATCCAT GATCAAAAAA ATTCAAGAGC   
  
  
+ AGGTTGCACA AAAACACGGA GATGAGATGA AAGAGACAGA AAACACATAT ACATTCACAG TAGTAGGGGT   
  
  
+ ACTGGTAGAG CTATGGCTAG GCTAAGATGA TGTGAAAGAA ACAATATAAT GGTAGTAACT GGTAATATTT   
  
  
+ ATAGGGCACA TATAGGTGCG GGCATAGGGT CGTGTCTCTC AGTCTCAGGT ACGGTTCGAT GGGACTCTGC   
  
  
+ GATAGATGTT ATCAAATTCA ACCATTCGAA CATCTCCCAG AGAGAGAGAG AGAGACTGAG TGAGTGAGTG   
  
  
+ AGTGGGTGGG TGTATGTGTG TGAGTTGTTT TGTTCATTTA TTATAGGTTC TCTGCTGCAT ATATCTCCAC   
  
  
+ CATTAAATTT ACCAAAACTT GTACCAGCTG CTTTACTTGC TTTCCTCATT TTCCCCCTTT TTTGTCTCTC   
  
  
+ TCTCTCTCTC TCCCCCCCCC CCCCCCCCTC TCTCTCTGTG TCTGAATCAG TATCAGCCTA TCAGGATGCT   
  
  
+ TCGCCTCCAG AAAGGCATCA CTCACACACT TATTAGCTTA AAACTTTCTT TTATTCCTTT TGCTCTTTCA   
  
  
+ CTGCTCTCTT TTACCCAAAT ACGCCCGTCT TCAATTCTTC TCTCATTAAA TATGAAAAAG AAAGAGAGAA   
  
  
+ ATAGAGAGAG ATTTAAACCA GAAGGGGTGA TTCCTTTTTC TTGGTGGGCT TATTAATGCT CTGTCTACCC   
  
  
+ CATCTAGCCG AAAAATAGTA GCTTAAGACT CAACTGCTTA TTAGCATCTC TCCTCTAGCA ATAACCGAGG   
  
  
+ TAATAGCATA TTTTTCTCCC CTTAGCTTCT ACCTTGCTTT TTGTGACACT CAAACCTAAA CTGAGAGAGA   
  
  
+ GAAAGAGAAG ATAGAGGAGA GATAAATATT AGCGGGATTA GGGCAAATTT TGATATGCTG GGCTCTTCCT   
  
  
+ CATTCAACTC TGAGGAAGAA GATCAAGATC ATGCCAACCC ACCTCCGCCG CAACAACCAC CACCGTTACA   
  
  
+ GCTGCCACTT CCACGGCCAA GATTCAGCCA GTTAATATCA TCATCACCTC CAATAATATC AGCATCTTCT   
  
  
+ TCATCATTAA TCCATAATAA CCCTCGTCAG CTGCTAATAA GTTGCGGTGA ACTCATCTCC CGCTGTGACT   
  
  
+ TCTCCTCAGC TAACCGCCTT CTCTCTCTTC TCTCCGGCGA CTTCGCTTCC TCCTCCGGCG ACTCCACTGA   
  
  
+ ACGCCTTGTT TATTACTTCT CCAAGGCTTT GTTCCTCCGC CTTCGCCGAC AGTTTTCTCT ATCTTCTTCA   
  
  
+ ACTTTTACTG GTTATAGTTG TAACCCTAGT TGTTCTGCGT CTATTGCTCC ACTTTTTCTA ACCAACCCTA   
  
  
+ ATCCTAGACC ACCACAAGAA CTGCCGGTAC GAGTAACTAG CAGGGTTACT CCAAGTAGCT GCTCGTCTTA   
  
  
+ CCTAACCCTA AACCAGATCA CTCCATTTAT CCGGTTCACC CACCTGACGG CCAACCAGGC AATTTTGGAG   
  
  
+ GCTGTGGAGG GCTATAGGGC AGTCCACATC ATCGACATGG ACATCATGCA TGGAGTGCAG TGGCCTCCGC   
  
  
+ TCCTCCAAGC AATCGTGGAG CGGTCGGCCA CCCTTAGCCA ACCTCCCCCT GTGGTCCGTC TCAAAGGGGG   
  
  
+ AGGGCCGGAC CCGGACCTCT TACAGAGGAC GGGGGACCGA CTATGGAACT TTGCCCAATC ATTAGGACTA   
  
  
+ GAGTTCCATT TCCAGGGCCT CATGATCCCC GCCGAGTCAA CAGGTTTTGT TGAACCTGCT GAGGCAGCGG   
  
  
+ TCAATGCCCT AGCCTTGCAG GAGGCGGTGG GGTGTCAAGA AGGGGAGGCC CTTGCAGTTC ACTGCGGGGA   
  
  
+ CTATCTCCAC CGCCTCTTAA AAGAGTACGA CACAAGGCCT CTTAGACGGT TCCTCCACAA GGTCAAGACT   
  
  
+ CTGAACCCCA GGGTCTTGAC CTTGGGAGAG AGGGAAGCCG ACCACAACCA CCCTCTCTTC CTGCGGCGGT   
  
  
+ TCACCGAGGC AGTCAACCAC TACGGGGCAG TGTTTGACTC CCTGGAAGCA ACCCTTCCGC CGCATAGCCA   
  
  
+ AGAGAGGGTA GCTGTGGAGG AAGGGTGGTT CGGAGAGGAG ATTAAGGACG TGGTAGGAGA AGAAAGCGAG   
  
  
+ AGGAGAATAG AGAGGCACCA GAAGTTCGAG TCGTGGGAAG CATTGTTGAG GAGCTCAGGG TTCAAGAGTG   
  
  
+ TGCCCCTCAG CCCATTCTCG CTGTCCCAAG CTAAGCTGCT GCTGCGCCTC CATTACCCTT CCGAAGGGTA   
  
  
+ CCAACTCCAG GTTTTGCATA GTAATTGCTT GTTGCTTGGG TGGAAGAATC GCCCTCTTTT TTCTGTCTCT   
  
  
+ TCTTGGCAAT A  

- +Up\_Stream \_Len000AAAAAA AATTCAAAAG CATGAACCAC AACTACTAAA CACGAAACAT TGGTTACCAA   
  
  
- AATCATTTAT CATTAATCAG CATTGTAAGA CGCTAAAGCG AGTTAAATAT ATAGTTGAAA TCTTATACTC   
  
  
- ATTGGCATTT CTAGTTTGCA TCATCATCTC CACAGTATCC AAAAAAAAAT TTTTTTAAGC CTCATTCTTT   
  
  
- CATCCCATGT AATAAAAACG AACAATTTAA GGCGATCATA TCGTTATGCT CTACCTTGTA AACATTTTTG   
  
  
- ATGCTCTGCT TCGCAAACAT GTTTTGTTTG ATGGTATGAC GTCTAGTATA TTAAAAAACC CCCCAATACA   
  
  
- TAGAATCACT ACTGTAATTA ACTACCTATT GGGATAAGAT TTATAGACCT AGGTAGTAAT TGACTGAGCA   
  
  
- AAGTTTACAA TCCTAATACC CAGAACTGTA CCATATGTAC AGTTATCTAA ATACAATCTA CTTTTCAATA   
  
  
- TACTGCGAAA AATACTAAAA ACATGATCGT ATATCACGAA TTAACCTGAT ATATAAAAAT GTTCAACACG   
  
  
- AATTGTGGAA AATTAGTCTT TATAAATAAT ATACCTTTTT TAAAATAATT ATACACAAAT ACACAAATTT   
  
  
- TTTGTATGGG TCGGAACTTT ATCTGGCACT ATATCTTTAA CCTGGGTATT ATGAGTGTAA TATATACGAA   
  
  
- ATAACAATAT TAATTCCTAG TAACAACAAA ACGTTAATAC ACCGTTCTAT ATTAGGTGTT TCAAAATGTA   
  
  
- AATATAACGT TAATGAGACC ATTTACGATA ATTACATGTA AACTCTAATT CTGTATATAT ATTGGGAAAA   
  
  
- AAAAGGGAAG AAAAAAGGAA AAAGATAAAA CTTACCGTAG AATTTGGTTT CCTCGACATG TTCAATGCAA   
  
  
- TATCGTGAGT ACCAACTGAT TTGTTCTGTC TACTCTCAAA TTCAGTTCAG GAGGACCGTT ACTACAAACG   
  
  
- GTAAACCTGG TTATTTATGT AGTACATATA ATGGTATTAT CACACCAACT GAATTGTTAT GTATCCCCTA   
  
  
- ACGTATGTTT CCATTCGGAG TTTTTGAACA CGTATACGTA ACTTTAGGTA CTAGTTTTTT TAAGTTCTCG   
  
  
- TCCAACGTGT TTTTGTGCCT CTACTCTACT TTCTCTGTCT TTTGTGTATA TGTAAGTGTC ATCATCCCCA   
  
  
- TGACCATCTC GATACCGATC CGATTCTACT ACACTTTCTT TGTTATATTA CCATCATTGA CCATTATAAA   
  
  
- TATCCCGTGT ATATCCACGC CCGTATCCCA GCACAGAGAG TCAGAGTCCA TGCCAAGCTA CCCTGAGACG   
  
  
- CTATCTACAA TAGTTTAAGT TGGTAAGCTT GTAGAGGGTC TCTCTCTCTC TCTCTGACTC ACTCACTCAC   
  
  
- TCACCCACCC ACATACACAC ACTCAACAAA ACAAGTAAAT AATATCCAAG AGACGACGTA TATAGAGGTG   
  
  
- GTAATTTAAA TGGTTTTGAA CATGGTCGAC GAAATGAACG AAAGGAGTAA AAGGGGGAAA AAACAGAGAG   
  
  
- AGAGAGAGAG AGGGGGGGGG GGGGGGGGAG AGAGAGACAC AGACTTAGTC ATAGTCGGAT AGTCCTACGA   
  
  
- AGCGGAGGTC TTTCCGTAGT GAGTGTGTGA ATAATCGAAT TTTGAAAGAA AATAAGGAAA ACGAGAAAGT   
  
  
- GACGAGAGAA AATGGGTTTA TGCGGGCAGA AGTTAAGAAG AGAGTAATTT ATACTTTTTC TTTCTCTCTT   
  
  
- TATCTCTCTC TAAATTTGGT CTTCCCCACT AAGGAAAAAG AACCACCCGA ATAATTACGA GACAGATGGG   
  
  
- GTAGATCGGC TTTTTATCAT CGAATTCTGA GTTGACGAAT AATCGTAGAG AGGAGATCGT TATTGGCTCC   
  
  
- ATTATCGTAT AAAAAGAGGG GAATCGAAGA TGGAACGAAA AACACTGTGA GTTTGGATTT GACTCTCTCT   
  
  
- CTTTCTCTTC TATCTCCTCT CTATTTATAA TCGCCCTAAT CCCGTTTAAA ACTATACGAC CCGAGAAGGA   
  
  
- GTAAGTTGAG ACTCCTTCTT CTAGTTCTAG TACGGTTGGG TGGAGGCGGC GTTGTTGGTG GTGGCAATGT   
  
  
- CGACGGTGAA GGTGCCGGTT CTAAGTCGGT CAATTATAGT AGTAGTGGAG GTTATTATAG TCGTAGAAGA   
  
  
- AGTAGTAATT AGGTATTATT GGGAGCAGTC GACGATTATT CAACGCCACT TGAGTAGAGG GCGACACTGA   
  
  
- AGAGGAGTCG ATTGGCGGAA GAGAGAGAAG AGAGGCCGCT GAAGCGAAGG AGGAGGCCGC TGAGGTGACT   
  
  
- TGCGGAACAA ATAATGAAGA GGTTCCGAAA CAAGGAGGCG GAAGCGGCTG TCAAAAGAGA TAGAAGAAGT   
  
  
- TGAAAATGAC CAATATCAAC ATTGGGATCA ACAAGACGCA GATAACGAGG TGAAAAAGAT TGGTTGGGAT   
  
  
- TAGGATCTGG TGGTGTTCTT GACGGCCATG CTCATTGATC GTCCCAATGA GGTTCATCGA CGAGCAGAAT   
  
  
- GGATTGGGAT TTGGTCTAGT GAGGTAAATA GGCCAAGTGG GTGGACTGCC GGTTGGTCCG TTAAAACCTC   
  
  
- CGACACCTCC CGATATCCCG TCAGGTGTAG TAGCTGTACC TGTAGTACGT ACCTCACGTC ACCGGAGGCG   
  
  
- AGGAGGTTCG TTAGCACCTC GCCAGCCGGT GGGAATCGGT TGGAGGGGGA CACCAGGCAG AGTTTCCCCC   
  
  
- TCCCGGCCTG GGCCTGGAGA ATGTCTCCTG CCCCCTGGCT GATACCTTGA AACGGGTTAG TAATCCTGAT   
  
  
- CTCAAGGTAA AGGTCCCGGA GTACTAGGGG CGGCTCAGTT GTCCAAAACA ACTTGGACGA CTCCGTCGCC   
  
  
- AGTTACGGGA TCGGAACGTC CTCCGCCACC CCACAGTTCT TCCCCTCCGG GAACGTCAAG TGACGCCCCT   
  
  
- GATAGAGGTG GCGGAGAATT TTCTCATGCT GTGTTCCGGA GAATCTGCCA AGGAGGTGTT CCAGTTCTGA   
  
  
- GACTTGGGGT CCCAGAACTG GAACCCTCTC TCCCTTCGGC TGGTGTTGGT GGGAGAGAAG GACGCCGCCA   
  
  
- AGTGGCTCCG TCAGTTGGTG ATGCCCCGTC ACAAACTGAG GGACCTTCGT TGGGAAGGCG GCGTATCGGT   
  
  
- TCTCTCCCAT CGACACCTCC TTCCCACCAA GCCTCTCCTC TAATTCCTGC ACCATCCTCT TCTTTCGCTC   
  
  
- TCCTCTTATC TCTCCGTGGT CTTCAAGCTC AGCACCCTTC GTAACAACTC CTCGAGTCCC AAGTTCTCAC   
  
  
- ACGGGGAGTC GGGTAAGAGC GACAGGGTTC GATTCGACGA CGACGCGGAG GTAATGGGAA GGCTTCCCAT   
  
  
- GGTTGAGGTC CAAAACGTAT CATTAACGAA CAACGAACCC ACCTTCTTAG CGGGAGAAAA AAGACAGAGA   
  
  
- AGAACCGTTA T

  
  
Motifs Found  

+   

| Site Name | Organism | Position | Strand | Matrix score. | sequence | function |
| --- | --- | --- | --- | --- | --- | --- |
|  | organism | 3315 | + | 4 | motif\_sequence | short\_function |
|  | organism | 3431 | + | 4 | motif\_sequence | short\_function |
|  | organism | 3191 | - | 4 | motif\_sequence | short\_function |
|  | organism | 2274 | + | 4 | motif\_sequence | short\_function |
|  | organism | 1366 | + | 4 | motif\_sequence | short\_function |
|  | organism | 1142 | - | 4 | motif\_sequence | short\_function |
|  | organism | 3186 | - | 4 | motif\_sequence | short\_function |
|  | organism | 3069 | + | 4 | motif\_sequence | short\_function |
|  | organism | 2229 | + | 4 | motif\_sequence | short\_function |
|  | organism | 1872 | + | 4 | motif\_sequence | short\_function |
|  | organism | 1270 | - | 4 | motif\_sequence | short\_function |
|  | organism | 899 | + | 4 | motif\_sequence | short\_function |
|  | organism | 3039 | - | 4 | motif\_sequence | short\_function |
|  | organism | 269 | - | 4 | motif\_sequence | short\_function |
|  | organism | 220 | - | 4 | motif\_sequence | short\_function |
|  | organism | 1980 | - | 4 | motif\_sequence | short\_function |
|  | organism | 1467 | + | 4 | motif\_sequence | short\_function |
|  | organism | 807 | + | 4 | motif\_sequence | short\_function |
|  | organism | 2375 | + | 4 | motif\_sequence | short\_function |
|  | organism | 1329 | + | 4 | motif\_sequence | short\_function |
|  | organism | 2027 | + | 4 | motif\_sequence | short\_function |
|  | organism | 1362 | - | 4 | motif\_sequence | short\_function |
|  | organism | 169 | - | 4 | motif\_sequence | short\_function |
|  | organism | 2633 | - | 4 | motif\_sequence | short\_function |
|  | organism | 2947 | + | 4 | motif\_sequence | short\_function |
|  | organism | 809 | - | 4 | motif\_sequence | short\_function |
|  | organism | 1454 | + | 4 | motif\_sequence | short\_function |
|  | organism | 2052 | - | 4 | motif\_sequence | short\_function |
|  | organism | 324 | - | 4 | motif\_sequence | short\_function |
|  | organism | 1971 | - | 4 | motif\_sequence | short\_function |
|  | organism | 2168 | + | 4 | motif\_sequence | short\_function |
|  | organism | 1199 | - | 4 | motif\_sequence | short\_function |
|  | organism | 1552 | + | 4 | motif\_sequence | short\_function |
|  | organism | 2269 | + | 4 | motif\_sequence | short\_function |

>HU03G01737.1   
+ +Up\_Stream \_Len000TTTTTT TTAAGTTTTC GTACTTGGTG TTGATGATTT GTGCTTTGTA ACCAATGGTT   
  
  
+ TTAGTAAATA GTAATTAGTC GTAACATTCT GCGATTTCGC TCAATTTATA TATCAACTTT AGAATATGAG   
  
  
+ TAACCGTAAA GATCAAACGT AGTAGTAGAG GTGTCATAGG TTTTTTTTTA AAAAAATTCG GAGTAAGAAA   
  
  
+ GTAGGGTACA TTATTTTTGC TTGTTAAATT CCGCTAGTAT AGCAATACGA GATGGAACAT TTGTAAAAAC   
  
  
+ TACGAGACGA AGCGTTTGTA CAAAACAAAC TACCATACTG CAGATCATAT AATTTTTTGG GGGGTTATGT   
  
  
+ ATCTTAGTGA TGACATTAAT TGATGGATAA CCCTATTCTA AATATCTGGA TCCATCATTA ACTGACTCGT   
  
  
+ TTCAAATGTT AGGATTATGG GTCTTGACAT GGTATACATG TCAATAGATT TATGTTAGAT GAAAAGTTAT   
  
  
+ ATGACGCTTT TTATGATTTT TGTACTAGCA TATAGTGCTT AATTGGACTA TATATTTTTA CAAGTTGTGC   
  
  
+ TTAACACCTT TTAATCAGAA ATATTTATTA TATGGAAAAA ATTTTATTAA TATGTGTTTA TGTGTTTAAA   
  
  
+ AAACATACCC AGCCTTGAAA TAGACCGTGA TATAGAAATT GGACCCATAA TACTCACATT ATATATGCTT   
  
  
+ TATTGTTATA ATTAAGGATC ATTGTTGTTT TGCAATTATG TGGCAAGATA TAATCCACAA AGTTTTACAT   
  
  
+ TTATATTGCA ATTACTCTGG TAAATGCTAT TAATGTACAT TTGAGATTAA GACATATATA TAACCCTTTT   
  
  
+ TTTTCCCTTC TTTTTTCCTT TTTCTATTTT GAATGGCATC TTAAACCAAA GGAGCTGTAC AAGTTACGTT   
  
  
+ ATAGCACTCA TGGTTGACTA AACAAGACAG ATGAGAGTTT AAGTCAAGTC CTCCTGGCAA TGATGTTTGC   
  
  
+ CATTTGGACC AATAAATACA TCATGTATAT TACCATAATA GTGTGGTTGA CTTAACAATA CATAGGGGAT   
  
  
+ TGCATACAAA GGTAAGCCTC AAAAACTTGT GCATATGCAT TGAAATCCAT GATCAAAAAA ATTCAAGAGC   
  
  
+ AGGTTGCACA AAAACACGGA GATGAGATGA AAGAGACAGA AAACACATAT ACATTCACAG TAGTAGGGGT   
  
  
+ ACTGGTAGAG CTATGGCTAG GCTAAGATGA TGTGAAAGAA ACAATATAAT GGTAGTAACT GGTAATATTT   
  
  
+ ATAGGGCACA TATAGGTGCG GGCATAGGGT CGTGTCTCTC AGTCTCAGGT ACGGTTCGAT GGGACTCTGC   
  
  
+ GATAGATGTT ATCAAATTCA ACCATTCGAA CATCTCCCAG AGAGAGAGAG AGAGACTGAG TGAGTGAGTG   
  
  
+ AGTGGGTGGG TGTATGTGTG TGAGTTGTTT TGTTCATTTA TTATAGGTTC TCTGCTGCAT ATATCTCCAC   
  
  
+ CATTAAATTT ACCAAAACTT GTACCAGCTG CTTTACTTGC TTTCCTCATT TTCCCCCTTT TTTGTCTCTC   
  
  
+ TCTCTCTCTC TCCCCCCCCC CCCCCCCCTC TCTCTCTGTG TCTGAATCAG TATCAGCCTA TCAGGATGCT   
  
  
+ TCGCCTCCAG AAAGGCATCA CTCACACACT TATTAGCTTA AAACTTTCTT TTATTCCTTT TGCTCTTTCA   
  
  
+ CTGCTCTCTT TTACCCAAAT ACGCCCGTCT TCAATTCTTC TCTCATTAAA TATGAAAAAG AAAGAGAGAA   
  
  
+ ATAGAGAGAG ATTTAAACCA GAAGGGGTGA TTCCTTTTTC TTGGTGGGCT TATTAATGCT CTGTCTACCC   
  
  
+ CATCTAGCCG AAAAATAGTA GCTTAAGACT CAACTGCTTA TTAGCATCTC TCCTCTAGCA ATAACCGAGG   
  
  
+ TAATAGCATA TTTTTCTCCC CTTAGCTTCT ACCTTGCTTT TTGTGACACT CAAACCTAAA CTGAGAGAGA   
  
  
+ GAAAGAGAAG ATAGAGGAGA GATAAATATT AGCGGGATTA GGGCAAATTT TGATATGCTG GGCTCTTCCT   
  
  
+ CATTCAACTC TGAGGAAGAA GATCAAGATC ATGCCAACCC ACCTCCGCCG CAACAACCAC CACCGTTACA   
  
  
+ GCTGCCACTT CCACGGCCAA GATTCAGCCA GTTAATATCA TCATCACCTC CAATAATATC AGCATCTTCT   
  
  
+ TCATCATTAA TCCATAATAA CCCTCGTCAG CTGCTAATAA GTTGCGGTGA ACTCATCTCC CGCTGTGACT   
  
  
+ TCTCCTCAGC TAACCGCCTT CTCTCTCTTC TCTCCGGCGA CTTCGCTTCC TCCTCCGGCG ACTCCACTGA   
  
  
+ ACGCCTTGTT TATTACTTCT CCAAGGCTTT GTTCCTCCGC CTTCGCCGAC AGTTTTCTCT ATCTTCTTCA   
  
  
+ ACTTTTACTG GTTATAGTTG TAACCCTAGT TGTTCTGCGT CTATTGCTCC ACTTTTTCTA ACCAACCCTA   
  
  
+ ATCCTAGACC ACCACAAGAA CTGCCGGTAC GAGTAACTAG CAGGGTTACT CCAAGTAGCT GCTCGTCTTA   
  
  
+ CCTAACCCTA AACCAGATCA CTCCATTTAT CCGGTTCACC CACCTGACGG CCAACCAGGC AATTTTGGAG   
  
  
+ GCTGTGGAGG GCTATAGGGC AGTCCACATC ATCGACATGG ACATCATGCA TGGAGTGCAG TGGCCTCCGC   
  
  
+ TCCTCCAAGC AATCGTGGAG CGGTCGGCCA CCCTTAGCCA ACCTCCCCCT GTGGTCCGTC TCAAAGGGGG   
  
  
+ AGGGCCGGAC CCGGACCTCT TACAGAGGAC GGGGGACCGA CTATGGAACT TTGCCCAATC ATTAGGACTA   
  
  
+ GAGTTCCATT TCCAGGGCCT CATGATCCCC GCCGAGTCAA CAGGTTTTGT TGAACCTGCT GAGGCAGCGG   
  
  
+ TCAATGCCCT AGCCTTGCAG GAGGCGGTGG GGTGTCAAGA AGGGGAGGCC CTTGCAGTTC ACTGCGGGGA   
  
  
+ CTATCTCCAC CGCCTCTTAA AAGAGTACGA CACAAGGCCT CTTAGACGGT TCCTCCACAA GGTCAAGACT   
  
  
+ CTGAACCCCA GGGTCTTGAC CTTGGGAGAG AGGGAAGCCG ACCACAACCA CCCTCTCTTC CTGCGGCGGT   
  
  
+ TCACCGAGGC AGTCAACCAC TACGGGGCAG TGTTTGACTC CCTGGAAGCA ACCCTTCCGC CGCATAGCCA   
  
  
+ AGAGAGGGTA GCTGTGGAGG AAGGGTGGTT CGGAGAGGAG ATTAAGGACG TGGTAGGAGA AGAAAGCGAG   
  
  
+ AGGAGAATAG AGAGGCACCA GAAGTTCGAG TCGTGGGAAG CATTGTTGAG GAGCTCAGGG TTCAAGAGTG   
  
  
+ TGCCCCTCAG CCCATTCTCG CTGTCCCAAG CTAAGCTGCT GCTGCGCCTC CATTACCCTT CCGAAGGGTA   
  
  
+ CCAACTCCAG GTTTTGCATA GTAATTGCTT GTTGCTTGGG TGGAAGAATC GCCCTCTTTT TTCTGTCTCT   
  
  
+ TCTTGGCAAT A  

- +Up\_Stream \_Len000AAAAAA AATTCAAAAG CATGAACCAC AACTACTAAA CACGAAACAT TGGTTACCAA   
  
  
- AATCATTTAT CATTAATCAG CATTGTAAGA CGCTAAAGCG AGTTAAATAT ATAGTTGAAA TCTTATACTC   
  
  
- ATTGGCATTT CTAGTTTGCA TCATCATCTC CACAGTATCC AAAAAAAAAT TTTTTTAAGC CTCATTCTTT   
  
  
- CATCCCATGT AATAAAAACG AACAATTTAA GGCGATCATA TCGTTATGCT CTACCTTGTA AACATTTTTG   
  
  
- ATGCTCTGCT TCGCAAACAT GTTTTGTTTG ATGGTATGAC GTCTAGTATA TTAAAAAACC CCCCAATACA   
  
  
- TAGAATCACT ACTGTAATTA ACTACCTATT GGGATAAGAT TTATAGACCT AGGTAGTAAT TGACTGAGCA   
  
  
- AAGTTTACAA TCCTAATACC CAGAACTGTA CCATATGTAC AGTTATCTAA ATACAATCTA CTTTTCAATA   
  
  
- TACTGCGAAA AATACTAAAA ACATGATCGT ATATCACGAA TTAACCTGAT ATATAAAAAT GTTCAACACG   
  
  
- AATTGTGGAA AATTAGTCTT TATAAATAAT ATACCTTTTT TAAAATAATT ATACACAAAT ACACAAATTT   
  
  
- TTTGTATGGG TCGGAACTTT ATCTGGCACT ATATCTTTAA CCTGGGTATT ATGAGTGTAA TATATACGAA   
  
  
- ATAACAATAT TAATTCCTAG TAACAACAAA ACGTTAATAC ACCGTTCTAT ATTAGGTGTT TCAAAATGTA   
  
  
- AATATAACGT TAATGAGACC ATTTACGATA ATTACATGTA AACTCTAATT CTGTATATAT ATTGGGAAAA   
  
  
- AAAAGGGAAG AAAAAAGGAA AAAGATAAAA CTTACCGTAG AATTTGGTTT CCTCGACATG TTCAATGCAA   
  
  
- TATCGTGAGT ACCAACTGAT TTGTTCTGTC TACTCTCAAA TTCAGTTCAG GAGGACCGTT ACTACAAACG   
  
  
- GTAAACCTGG TTATTTATGT AGTACATATA ATGGTATTAT CACACCAACT GAATTGTTAT GTATCCCCTA   
  
  
- ACGTATGTTT CCATTCGGAG TTTTTGAACA CGTATACGTA ACTTTAGGTA CTAGTTTTTT TAAGTTCTCG   
  
  
- TCCAACGTGT TTTTGTGCCT CTACTCTACT TTCTCTGTCT TTTGTGTATA TGTAAGTGTC ATCATCCCCA   
  
  
- TGACCATCTC GATACCGATC CGATTCTACT ACACTTTCTT TGTTATATTA CCATCATTGA CCATTATAAA   
  
  
- TATCCCGTGT ATATCCACGC CCGTATCCCA GCACAGAGAG TCAGAGTCCA TGCCAAGCTA CCCTGAGACG   
  
  
- CTATCTACAA TAGTTTAAGT TGGTAAGCTT GTAGAGGGTC TCTCTCTCTC TCTCTGACTC ACTCACTCAC   
  
  
- TCACCCACCC ACATACACAC ACTCAACAAA ACAAGTAAAT AATATCCAAG AGACGACGTA TATAGAGGTG   
  
  
- GTAATTTAAA TGGTTTTGAA CATGGTCGAC GAAATGAACG AAAGGAGTAA AAGGGGGAAA AAACAGAGAG   
  
  
- AGAGAGAGAG AGGGGGGGGG GGGGGGGGAG AGAGAGACAC AGACTTAGTC ATAGTCGGAT AGTCCTACGA   
  
  
- AGCGGAGGTC TTTCCGTAGT GAGTGTGTGA ATAATCGAAT TTTGAAAGAA AATAAGGAAA ACGAGAAAGT   
  
  
- GACGAGAGAA AATGGGTTTA TGCGGGCAGA AGTTAAGAAG AGAGTAATTT ATACTTTTTC TTTCTCTCTT   
  
  
- TATCTCTCTC TAAATTTGGT CTTCCCCACT AAGGAAAAAG AACCACCCGA ATAATTACGA GACAGATGGG   
  
  
- GTAGATCGGC TTTTTATCAT CGAATTCTGA GTTGACGAAT AATCGTAGAG AGGAGATCGT TATTGGCTCC   
  
  
- ATTATCGTAT AAAAAGAGGG GAATCGAAGA TGGAACGAAA AACACTGTGA GTTTGGATTT GACTCTCTCT   
  
  
- CTTTCTCTTC TATCTCCTCT CTATTTATAA TCGCCCTAAT CCCGTTTAAA ACTATACGAC CCGAGAAGGA   
  
  
- GTAAGTTGAG ACTCCTTCTT CTAGTTCTAG TACGGTTGGG TGGAGGCGGC GTTGTTGGTG GTGGCAATGT   
  
  
- CGACGGTGAA GGTGCCGGTT CTAAGTCGGT CAATTATAGT AGTAGTGGAG GTTATTATAG TCGTAGAAGA   
  
  
- AGTAGTAATT AGGTATTATT GGGAGCAGTC GACGATTATT CAACGCCACT TGAGTAGAGG GCGACACTGA   
  
  
- AGAGGAGTCG ATTGGCGGAA GAGAGAGAAG AGAGGCCGCT GAAGCGAAGG AGGAGGCCGC TGAGGTGACT   
  
  
- TGCGGAACAA ATAATGAAGA GGTTCCGAAA CAAGGAGGCG GAAGCGGCTG TCAAAAGAGA TAGAAGAAGT   
  
  
- TGAAAATGAC CAATATCAAC ATTGGGATCA ACAAGACGCA GATAACGAGG TGAAAAAGAT TGGTTGGGAT   
  
  
- TAGGATCTGG TGGTGTTCTT GACGGCCATG CTCATTGATC GTCCCAATGA GGTTCATCGA CGAGCAGAAT   
  
  
- GGATTGGGAT TTGGTCTAGT GAGGTAAATA GGCCAAGTGG GTGGACTGCC GGTTGGTCCG TTAAAACCTC   
  
  
- CGACACCTCC CGATATCCCG TCAGGTGTAG TAGCTGTACC TGTAGTACGT ACCTCACGTC ACCGGAGGCG   
  
  
- AGGAGGTTCG TTAGCACCTC GCCAGCCGGT GGGAATCGGT TGGAGGGGGA CACCAGGCAG AGTTTCCCCC   
  
  
- TCCCGGCCTG GGCCTGGAGA ATGTCTCCTG CCCCCTGGCT GATACCTTGA AACGGGTTAG TAATCCTGAT   
  
  
- CTCAAGGTAA AGGTCCCGGA GTACTAGGGG CGGCTCAGTT GTCCAAAACA ACTTGGACGA CTCCGTCGCC   
  
  
- AGTTACGGGA TCGGAACGTC CTCCGCCACC CCACAGTTCT TCCCCTCCGG GAACGTCAAG TGACGCCCCT   
  
  
- GATAGAGGTG GCGGAGAATT TTCTCATGCT GTGTTCCGGA GAATCTGCCA AGGAGGTGTT CCAGTTCTGA   
  
  
- GACTTGGGGT CCCAGAACTG GAACCCTCTC TCCCTTCGGC TGGTGTTGGT GGGAGAGAAG GACGCCGCCA   
  
  
- AGTGGCTCCG TCAGTTGGTG ATGCCCCGTC ACAAACTGAG GGACCTTCGT TGGGAAGGCG GCGTATCGGT   
  
  
- TCTCTCCCAT CGACACCTCC TTCCCACCAA GCCTCTCCTC TAATTCCTGC ACCATCCTCT TCTTTCGCTC   
  
  
- TCCTCTTATC TCTCCGTGGT CTTCAAGCTC AGCACCCTTC GTAACAACTC CTCGAGTCCC AAGTTCTCAC   
  
  
- ACGGGGAGTC GGGTAAGAGC GACAGGGTTC GATTCGACGA CGACGCGGAG GTAATGGGAA GGCTTCCCAT   
  
  
- GGTTGAGGTC CAAAACGTAT CATTAACGAA CAACGAACCC ACCTTCTTAG CGGGAGAAAA AAGACAGAGA   
  
  
- AGAACCGTTA T

+     A-box

| Site Name | Organism | Position | Strand | Matrix score. | sequence | function |
| --- | --- | --- | --- | --- | --- | --- |
| A-box | Petroselinum crispum | 2761 | - | 6 | CCGTCC | cis-acting regulatory element |

>HU03G01737.1   
+ +Up\_Stream \_Len000TTTTTT TTAAGTTTTC GTACTTGGTG TTGATGATTT GTGCTTTGTA ACCAATGGTT   
  
  
+ TTAGTAAATA GTAATTAGTC GTAACATTCT GCGATTTCGC TCAATTTATA TATCAACTTT AGAATATGAG   
  
  
+ TAACCGTAAA GATCAAACGT AGTAGTAGAG GTGTCATAGG TTTTTTTTTA AAAAAATTCG GAGTAAGAAA   
  
  
+ GTAGGGTACA TTATTTTTGC TTGTTAAATT CCGCTAGTAT AGCAATACGA GATGGAACAT TTGTAAAAAC   
  
  
+ TACGAGACGA AGCGTTTGTA CAAAACAAAC TACCATACTG CAGATCATAT AATTTTTTGG GGGGTTATGT   
  
  
+ ATCTTAGTGA TGACATTAAT TGATGGATAA CCCTATTCTA AATATCTGGA TCCATCATTA ACTGACTCGT   
  
  
+ TTCAAATGTT AGGATTATGG GTCTTGACAT GGTATACATG TCAATAGATT TATGTTAGAT GAAAAGTTAT   
  
  
+ ATGACGCTTT TTATGATTTT TGTACTAGCA TATAGTGCTT AATTGGACTA TATATTTTTA CAAGTTGTGC   
  
  
+ TTAACACCTT TTAATCAGAA ATATTTATTA TATGGAAAAA ATTTTATTAA TATGTGTTTA TGTGTTTAAA   
  
  
+ AAACATACCC AGCCTTGAAA TAGACCGTGA TATAGAAATT GGACCCATAA TACTCACATT ATATATGCTT   
  
  
+ TATTGTTATA ATTAAGGATC ATTGTTGTTT TGCAATTATG TGGCAAGATA TAATCCACAA AGTTTTACAT   
  
  
+ TTATATTGCA ATTACTCTGG TAAATGCTAT TAATGTACAT TTGAGATTAA GACATATATA TAACCCTTTT   
  
  
+ TTTTCCCTTC TTTTTTCCTT TTTCTATTTT GAATGGCATC TTAAACCAAA GGAGCTGTAC AAGTTACGTT   
  
  
+ ATAGCACTCA TGGTTGACTA AACAAGACAG ATGAGAGTTT AAGTCAAGTC CTCCTGGCAA TGATGTTTGC   
  
  
+ CATTTGGACC AATAAATACA TCATGTATAT TACCATAATA GTGTGGTTGA CTTAACAATA CATAGGGGAT   
  
  
+ TGCATACAAA GGTAAGCCTC AAAAACTTGT GCATATGCAT TGAAATCCAT GATCAAAAAA ATTCAAGAGC   
  
  
+ AGGTTGCACA AAAACACGGA GATGAGATGA AAGAGACAGA AAACACATAT ACATTCACAG TAGTAGGGGT   
  
  
+ ACTGGTAGAG CTATGGCTAG GCTAAGATGA TGTGAAAGAA ACAATATAAT GGTAGTAACT GGTAATATTT   
  
  
+ ATAGGGCACA TATAGGTGCG GGCATAGGGT CGTGTCTCTC AGTCTCAGGT ACGGTTCGAT GGGACTCTGC   
  
  
+ GATAGATGTT ATCAAATTCA ACCATTCGAA CATCTCCCAG AGAGAGAGAG AGAGACTGAG TGAGTGAGTG   
  
  
+ AGTGGGTGGG TGTATGTGTG TGAGTTGTTT TGTTCATTTA TTATAGGTTC TCTGCTGCAT ATATCTCCAC   
  
  
+ CATTAAATTT ACCAAAACTT GTACCAGCTG CTTTACTTGC TTTCCTCATT TTCCCCCTTT TTTGTCTCTC   
  
  
+ TCTCTCTCTC TCCCCCCCCC CCCCCCCCTC TCTCTCTGTG TCTGAATCAG TATCAGCCTA TCAGGATGCT   
  
  
+ TCGCCTCCAG AAAGGCATCA CTCACACACT TATTAGCTTA AAACTTTCTT TTATTCCTTT TGCTCTTTCA   
  
  
+ CTGCTCTCTT TTACCCAAAT ACGCCCGTCT TCAATTCTTC TCTCATTAAA TATGAAAAAG AAAGAGAGAA   
  
  
+ ATAGAGAGAG ATTTAAACCA GAAGGGGTGA TTCCTTTTTC TTGGTGGGCT TATTAATGCT CTGTCTACCC   
  
  
+ CATCTAGCCG AAAAATAGTA GCTTAAGACT CAACTGCTTA TTAGCATCTC TCCTCTAGCA ATAACCGAGG   
  
  
+ TAATAGCATA TTTTTCTCCC CTTAGCTTCT ACCTTGCTTT TTGTGACACT CAAACCTAAA CTGAGAGAGA   
  
  
+ GAAAGAGAAG ATAGAGGAGA GATAAATATT AGCGGGATTA GGGCAAATTT TGATATGCTG GGCTCTTCCT   
  
  
+ CATTCAACTC TGAGGAAGAA GATCAAGATC ATGCCAACCC ACCTCCGCCG CAACAACCAC CACCGTTACA   
  
  
+ GCTGCCACTT CCACGGCCAA GATTCAGCCA GTTAATATCA TCATCACCTC CAATAATATC AGCATCTTCT   
  
  
+ TCATCATTAA TCCATAATAA CCCTCGTCAG CTGCTAATAA GTTGCGGTGA ACTCATCTCC CGCTGTGACT   
  
  
+ TCTCCTCAGC TAACCGCCTT CTCTCTCTTC TCTCCGGCGA CTTCGCTTCC TCCTCCGGCG ACTCCACTGA   
  
  
+ ACGCCTTGTT TATTACTTCT CCAAGGCTTT GTTCCTCCGC CTTCGCCGAC AGTTTTCTCT ATCTTCTTCA   
  
  
+ ACTTTTACTG GTTATAGTTG TAACCCTAGT TGTTCTGCGT CTATTGCTCC ACTTTTTCTA ACCAACCCTA   
  
  
+ ATCCTAGACC ACCACAAGAA CTGCCGGTAC GAGTAACTAG CAGGGTTACT CCAAGTAGCT GCTCGTCTTA   
  
  
+ CCTAACCCTA AACCAGATCA CTCCATTTAT CCGGTTCACC CACCTGACGG CCAACCAGGC AATTTTGGAG   
  
  
+ GCTGTGGAGG GCTATAGGGC AGTCCACATC ATCGACATGG ACATCATGCA TGGAGTGCAG TGGCCTCCGC   
  
  
+ TCCTCCAAGC AATCGTGGAG CGGTCGGCCA CCCTTAGCCA ACCTCCCCCT GTGGTCCGTC TCAAAGGGGG   
  
  
+ AGGGCCGGAC CCGGACCTCT TACAGAGGAC GGGGGACCGA CTATGGAACT TTGCCCAATC ATTAGGACTA   
  
  
+ GAGTTCCATT TCCAGGGCCT CATGATCCCC GCCGAGTCAA CAGGTTTTGT TGAACCTGCT GAGGCAGCGG   
  
  
+ TCAATGCCCT AGCCTTGCAG GAGGCGGTGG GGTGTCAAGA AGGGGAGGCC CTTGCAGTTC ACTGCGGGGA   
  
  
+ CTATCTCCAC CGCCTCTTAA AAGAGTACGA CACAAGGCCT CTTAGACGGT TCCTCCACAA GGTCAAGACT   
  
  
+ CTGAACCCCA GGGTCTTGAC CTTGGGAGAG AGGGAAGCCG ACCACAACCA CCCTCTCTTC CTGCGGCGGT   
  
  
+ TCACCGAGGC AGTCAACCAC TACGGGGCAG TGTTTGACTC CCTGGAAGCA ACCCTTCCGC CGCATAGCCA   
  
  
+ AGAGAGGGTA GCTGTGGAGG AAGGGTGGTT CGGAGAGGAG ATTAAGGACG TGGTAGGAGA AGAAAGCGAG   
  
  
+ AGGAGAATAG AGAGGCACCA GAAGTTCGAG TCGTGGGAAG CATTGTTGAG GAGCTCAGGG TTCAAGAGTG   
  
  
+ TGCCCCTCAG CCCATTCTCG CTGTCCCAAG CTAAGCTGCT GCTGCGCCTC CATTACCCTT CCGAAGGGTA   
  
  
+ CCAACTCCAG GTTTTGCATA GTAATTGCTT GTTGCTTGGG TGGAAGAATC GCCCTCTTTT TTCTGTCTCT   
  
  
+ TCTTGGCAAT A  

- +Up\_Stream \_Len000AAAAAA AATTCAAAAG CATGAACCAC AACTACTAAA CACGAAACAT TGGTTACCAA   
  
  
- AATCATTTAT CATTAATCAG CATTGTAAGA CGCTAAAGCG AGTTAAATAT ATAGTTGAAA TCTTATACTC   
  
  
- ATTGGCATTT CTAGTTTGCA TCATCATCTC CACAGTATCC AAAAAAAAAT TTTTTTAAGC CTCATTCTTT   
  
  
- CATCCCATGT AATAAAAACG AACAATTTAA GGCGATCATA TCGTTATGCT CTACCTTGTA AACATTTTTG   
  
  
- ATGCTCTGCT TCGCAAACAT GTTTTGTTTG ATGGTATGAC GTCTAGTATA TTAAAAAACC CCCCAATACA   
  
  
- TAGAATCACT ACTGTAATTA ACTACCTATT GGGATAAGAT TTATAGACCT AGGTAGTAAT TGACTGAGCA   
  
  
- AAGTTTACAA TCCTAATACC CAGAACTGTA CCATATGTAC AGTTATCTAA ATACAATCTA CTTTTCAATA   
  
  
- TACTGCGAAA AATACTAAAA ACATGATCGT ATATCACGAA TTAACCTGAT ATATAAAAAT GTTCAACACG   
  
  
- AATTGTGGAA AATTAGTCTT TATAAATAAT ATACCTTTTT TAAAATAATT ATACACAAAT ACACAAATTT   
  
  
- TTTGTATGGG TCGGAACTTT ATCTGGCACT ATATCTTTAA CCTGGGTATT ATGAGTGTAA TATATACGAA   
  
  
- ATAACAATAT TAATTCCTAG TAACAACAAA ACGTTAATAC ACCGTTCTAT ATTAGGTGTT TCAAAATGTA   
  
  
- AATATAACGT TAATGAGACC ATTTACGATA ATTACATGTA AACTCTAATT CTGTATATAT ATTGGGAAAA   
  
  
- AAAAGGGAAG AAAAAAGGAA AAAGATAAAA CTTACCGTAG AATTTGGTTT CCTCGACATG TTCAATGCAA   
  
  
- TATCGTGAGT ACCAACTGAT TTGTTCTGTC TACTCTCAAA TTCAGTTCAG GAGGACCGTT ACTACAAACG   
  
  
- GTAAACCTGG TTATTTATGT AGTACATATA ATGGTATTAT CACACCAACT GAATTGTTAT GTATCCCCTA   
  
  
- ACGTATGTTT CCATTCGGAG TTTTTGAACA CGTATACGTA ACTTTAGGTA CTAGTTTTTT TAAGTTCTCG   
  
  
- TCCAACGTGT TTTTGTGCCT CTACTCTACT TTCTCTGTCT TTTGTGTATA TGTAAGTGTC ATCATCCCCA   
  
  
- TGACCATCTC GATACCGATC CGATTCTACT ACACTTTCTT TGTTATATTA CCATCATTGA CCATTATAAA   
  
  
- TATCCCGTGT ATATCCACGC CCGTATCCCA GCACAGAGAG TCAGAGTCCA TGCCAAGCTA CCCTGAGACG   
  
  
- CTATCTACAA TAGTTTAAGT TGGTAAGCTT GTAGAGGGTC TCTCTCTCTC TCTCTGACTC ACTCACTCAC   
  
  
- TCACCCACCC ACATACACAC ACTCAACAAA ACAAGTAAAT AATATCCAAG AGACGACGTA TATAGAGGTG   
  
  
- GTAATTTAAA TGGTTTTGAA CATGGTCGAC GAAATGAACG AAAGGAGTAA AAGGGGGAAA AAACAGAGAG   
  
  
- AGAGAGAGAG AGGGGGGGGG GGGGGGGGAG AGAGAGACAC AGACTTAGTC ATAGTCGGAT AGTCCTACGA   
  
  
- AGCGGAGGTC TTTCCGTAGT GAGTGTGTGA ATAATCGAAT TTTGAAAGAA AATAAGGAAA ACGAGAAAGT   
  
  
- GACGAGAGAA AATGGGTTTA TGCGGGCAGA AGTTAAGAAG AGAGTAATTT ATACTTTTTC TTTCTCTCTT   
  
  
- TATCTCTCTC TAAATTTGGT CTTCCCCACT AAGGAAAAAG AACCACCCGA ATAATTACGA GACAGATGGG   
  
  
- GTAGATCGGC TTTTTATCAT CGAATTCTGA GTTGACGAAT AATCGTAGAG AGGAGATCGT TATTGGCTCC   
  
  
- ATTATCGTAT AAAAAGAGGG GAATCGAAGA TGGAACGAAA AACACTGTGA GTTTGGATTT GACTCTCTCT   
  
  
- CTTTCTCTTC TATCTCCTCT CTATTTATAA TCGCCCTAAT CCCGTTTAAA ACTATACGAC CCGAGAAGGA   
  
  
- GTAAGTTGAG ACTCCTTCTT CTAGTTCTAG TACGGTTGGG TGGAGGCGGC GTTGTTGGTG GTGGCAATGT   
  
  
- CGACGGTGAA GGTGCCGGTT CTAAGTCGGT CAATTATAGT AGTAGTGGAG GTTATTATAG TCGTAGAAGA   
  
  
- AGTAGTAATT AGGTATTATT GGGAGCAGTC GACGATTATT CAACGCCACT TGAGTAGAGG GCGACACTGA   
  
  
- AGAGGAGTCG ATTGGCGGAA GAGAGAGAAG AGAGGCCGCT GAAGCGAAGG AGGAGGCCGC TGAGGTGACT   
  
  
- TGCGGAACAA ATAATGAAGA GGTTCCGAAA CAAGGAGGCG GAAGCGGCTG TCAAAAGAGA TAGAAGAAGT   
  
  
- TGAAAATGAC CAATATCAAC ATTGGGATCA ACAAGACGCA GATAACGAGG TGAAAAAGAT TGGTTGGGAT   
  
  
- TAGGATCTGG TGGTGTTCTT GACGGCCATG CTCATTGATC GTCCCAATGA GGTTCATCGA CGAGCAGAAT   
  
  
- GGATTGGGAT TTGGTCTAGT GAGGTAAATA GGCCAAGTGG GTGGACTGCC GGTTGGTCCG TTAAAACCTC   
  
  
- CGACACCTCC CGATATCCCG TCAGGTGTAG TAGCTGTACC TGTAGTACGT ACCTCACGTC ACCGGAGGCG   
  
  
- AGGAGGTTCG TTAGCACCTC GCCAGCCGGT GGGAATCGGT TGGAGGGGGA CACCAGGCAG AGTTTCCCCC   
  
  
- TCCCGGCCTG GGCCTGGAGA ATGTCTCCTG CCCCCTGGCT GATACCTTGA AACGGGTTAG TAATCCTGAT   
  
  
- CTCAAGGTAA AGGTCCCGGA GTACTAGGGG CGGCTCAGTT GTCCAAAACA ACTTGGACGA CTCCGTCGCC   
  
  
- AGTTACGGGA TCGGAACGTC CTCCGCCACC CCACAGTTCT TCCCCTCCGG GAACGTCAAG TGACGCCCCT   
  
  
- GATAGAGGTG GCGGAGAATT TTCTCATGCT GTGTTCCGGA GAATCTGCCA AGGAGGTGTT CCAGTTCTGA   
  
  
- GACTTGGGGT CCCAGAACTG GAACCCTCTC TCCCTTCGGC TGGTGTTGGT GGGAGAGAAG GACGCCGCCA   
  
  
- AGTGGCTCCG TCAGTTGGTG ATGCCCCGTC ACAAACTGAG GGACCTTCGT TGGGAAGGCG GCGTATCGGT   
  
  
- TCTCTCCCAT CGACACCTCC TTCCCACCAA GCCTCTCCTC TAATTCCTGC ACCATCCTCT TCTTTCGCTC   
  
  
- TCCTCTTATC TCTCCGTGGT CTTCAAGCTC AGCACCCTTC GTAACAACTC CTCGAGTCCC AAGTTCTCAC   
  
  
- ACGGGGAGTC GGGTAAGAGC GACAGGGTTC GATTCGACGA CGACGCGGAG GTAATGGGAA GGCTTCCCAT   
  
  
- GGTTGAGGTC CAAAACGTAT CATTAACGAA CAACGAACCC ACCTTCTTAG CGGGAGAAAA AAGACAGAGA   
  
  
- AGAACCGTTA T

+     AAGAA-motif

| Site Name | Organism | Position | Strand | Matrix score. | sequence | function |
| --- | --- | --- | --- | --- | --- | --- |
| AAGAA-motif | Avena sativa | 1228 | + | 7 | GAAAGAA |  |

>HU03G01737.1   
+ +Up\_Stream \_Len000TTTTTT TTAAGTTTTC GTACTTGGTG TTGATGATTT GTGCTTTGTA ACCAATGGTT   
  
  
+ TTAGTAAATA GTAATTAGTC GTAACATTCT GCGATTTCGC TCAATTTATA TATCAACTTT AGAATATGAG   
  
  
+ TAACCGTAAA GATCAAACGT AGTAGTAGAG GTGTCATAGG TTTTTTTTTA AAAAAATTCG GAGTAAGAAA   
  
  
+ GTAGGGTACA TTATTTTTGC TTGTTAAATT CCGCTAGTAT AGCAATACGA GATGGAACAT TTGTAAAAAC   
  
  
+ TACGAGACGA AGCGTTTGTA CAAAACAAAC TACCATACTG CAGATCATAT AATTTTTTGG GGGGTTATGT   
  
  
+ ATCTTAGTGA TGACATTAAT TGATGGATAA CCCTATTCTA AATATCTGGA TCCATCATTA ACTGACTCGT   
  
  
+ TTCAAATGTT AGGATTATGG GTCTTGACAT GGTATACATG TCAATAGATT TATGTTAGAT GAAAAGTTAT   
  
  
+ ATGACGCTTT TTATGATTTT TGTACTAGCA TATAGTGCTT AATTGGACTA TATATTTTTA CAAGTTGTGC   
  
  
+ TTAACACCTT TTAATCAGAA ATATTTATTA TATGGAAAAA ATTTTATTAA TATGTGTTTA TGTGTTTAAA   
  
  
+ AAACATACCC AGCCTTGAAA TAGACCGTGA TATAGAAATT GGACCCATAA TACTCACATT ATATATGCTT   
  
  
+ TATTGTTATA ATTAAGGATC ATTGTTGTTT TGCAATTATG TGGCAAGATA TAATCCACAA AGTTTTACAT   
  
  
+ TTATATTGCA ATTACTCTGG TAAATGCTAT TAATGTACAT TTGAGATTAA GACATATATA TAACCCTTTT   
  
  
+ TTTTCCCTTC TTTTTTCCTT TTTCTATTTT GAATGGCATC TTAAACCAAA GGAGCTGTAC AAGTTACGTT   
  
  
+ ATAGCACTCA TGGTTGACTA AACAAGACAG ATGAGAGTTT AAGTCAAGTC CTCCTGGCAA TGATGTTTGC   
  
  
+ CATTTGGACC AATAAATACA TCATGTATAT TACCATAATA GTGTGGTTGA CTTAACAATA CATAGGGGAT   
  
  
+ TGCATACAAA GGTAAGCCTC AAAAACTTGT GCATATGCAT TGAAATCCAT GATCAAAAAA ATTCAAGAGC   
  
  
+ AGGTTGCACA AAAACACGGA GATGAGATGA AAGAGACAGA AAACACATAT ACATTCACAG TAGTAGGGGT   
  
  
+ ACTGGTAGAG CTATGGCTAG GCTAAGATGA TGTGAAAGAA ACAATATAAT GGTAGTAACT GGTAATATTT   
  
  
+ ATAGGGCACA TATAGGTGCG GGCATAGGGT CGTGTCTCTC AGTCTCAGGT ACGGTTCGAT GGGACTCTGC   
  
  
+ GATAGATGTT ATCAAATTCA ACCATTCGAA CATCTCCCAG AGAGAGAGAG AGAGACTGAG TGAGTGAGTG   
  
  
+ AGTGGGTGGG TGTATGTGTG TGAGTTGTTT TGTTCATTTA TTATAGGTTC TCTGCTGCAT ATATCTCCAC   
  
  
+ CATTAAATTT ACCAAAACTT GTACCAGCTG CTTTACTTGC TTTCCTCATT TTCCCCCTTT TTTGTCTCTC   
  
  
+ TCTCTCTCTC TCCCCCCCCC CCCCCCCCTC TCTCTCTGTG TCTGAATCAG TATCAGCCTA TCAGGATGCT   
  
  
+ TCGCCTCCAG AAAGGCATCA CTCACACACT TATTAGCTTA AAACTTTCTT TTATTCCTTT TGCTCTTTCA   
  
  
+ CTGCTCTCTT TTACCCAAAT ACGCCCGTCT TCAATTCTTC TCTCATTAAA TATGAAAAAG AAAGAGAGAA   
  
  
+ ATAGAGAGAG ATTTAAACCA GAAGGGGTGA TTCCTTTTTC TTGGTGGGCT TATTAATGCT CTGTCTACCC   
  
  
+ CATCTAGCCG AAAAATAGTA GCTTAAGACT CAACTGCTTA TTAGCATCTC TCCTCTAGCA ATAACCGAGG   
  
  
+ TAATAGCATA TTTTTCTCCC CTTAGCTTCT ACCTTGCTTT TTGTGACACT CAAACCTAAA CTGAGAGAGA   
  
  
+ GAAAGAGAAG ATAGAGGAGA GATAAATATT AGCGGGATTA GGGCAAATTT TGATATGCTG GGCTCTTCCT   
  
  
+ CATTCAACTC TGAGGAAGAA GATCAAGATC ATGCCAACCC ACCTCCGCCG CAACAACCAC CACCGTTACA   
  
  
+ GCTGCCACTT CCACGGCCAA GATTCAGCCA GTTAATATCA TCATCACCTC CAATAATATC AGCATCTTCT   
  
  
+ TCATCATTAA TCCATAATAA CCCTCGTCAG CTGCTAATAA GTTGCGGTGA ACTCATCTCC CGCTGTGACT   
  
  
+ TCTCCTCAGC TAACCGCCTT CTCTCTCTTC TCTCCGGCGA CTTCGCTTCC TCCTCCGGCG ACTCCACTGA   
  
  
+ ACGCCTTGTT TATTACTTCT CCAAGGCTTT GTTCCTCCGC CTTCGCCGAC AGTTTTCTCT ATCTTCTTCA   
  
  
+ ACTTTTACTG GTTATAGTTG TAACCCTAGT TGTTCTGCGT CTATTGCTCC ACTTTTTCTA ACCAACCCTA   
  
  
+ ATCCTAGACC ACCACAAGAA CTGCCGGTAC GAGTAACTAG CAGGGTTACT CCAAGTAGCT GCTCGTCTTA   
  
  
+ CCTAACCCTA AACCAGATCA CTCCATTTAT CCGGTTCACC CACCTGACGG CCAACCAGGC AATTTTGGAG   
  
  
+ GCTGTGGAGG GCTATAGGGC AGTCCACATC ATCGACATGG ACATCATGCA TGGAGTGCAG TGGCCTCCGC   
  
  
+ TCCTCCAAGC AATCGTGGAG CGGTCGGCCA CCCTTAGCCA ACCTCCCCCT GTGGTCCGTC TCAAAGGGGG   
  
  
+ AGGGCCGGAC CCGGACCTCT TACAGAGGAC GGGGGACCGA CTATGGAACT TTGCCCAATC ATTAGGACTA   
  
  
+ GAGTTCCATT TCCAGGGCCT CATGATCCCC GCCGAGTCAA CAGGTTTTGT TGAACCTGCT GAGGCAGCGG   
  
  
+ TCAATGCCCT AGCCTTGCAG GAGGCGGTGG GGTGTCAAGA AGGGGAGGCC CTTGCAGTTC ACTGCGGGGA   
  
  
+ CTATCTCCAC CGCCTCTTAA AAGAGTACGA CACAAGGCCT CTTAGACGGT TCCTCCACAA GGTCAAGACT   
  
  
+ CTGAACCCCA GGGTCTTGAC CTTGGGAGAG AGGGAAGCCG ACCACAACCA CCCTCTCTTC CTGCGGCGGT   
  
  
+ TCACCGAGGC AGTCAACCAC TACGGGGCAG TGTTTGACTC CCTGGAAGCA ACCCTTCCGC CGCATAGCCA   
  
  
+ AGAGAGGGTA GCTGTGGAGG AAGGGTGGTT CGGAGAGGAG ATTAAGGACG TGGTAGGAGA AGAAAGCGAG   
  
  
+ AGGAGAATAG AGAGGCACCA GAAGTTCGAG TCGTGGGAAG CATTGTTGAG GAGCTCAGGG TTCAAGAGTG   
  
  
+ TGCCCCTCAG CCCATTCTCG CTGTCCCAAG CTAAGCTGCT GCTGCGCCTC CATTACCCTT CCGAAGGGTA   
  
  
+ CCAACTCCAG GTTTTGCATA GTAATTGCTT GTTGCTTGGG TGGAAGAATC GCCCTCTTTT TTCTGTCTCT   
  
  
+ TCTTGGCAAT A  

- +Up\_Stream \_Len000AAAAAA AATTCAAAAG CATGAACCAC AACTACTAAA CACGAAACAT TGGTTACCAA   
  
  
- AATCATTTAT CATTAATCAG CATTGTAAGA CGCTAAAGCG AGTTAAATAT ATAGTTGAAA TCTTATACTC   
  
  
- ATTGGCATTT CTAGTTTGCA TCATCATCTC CACAGTATCC AAAAAAAAAT TTTTTTAAGC CTCATTCTTT   
  
  
- CATCCCATGT AATAAAAACG AACAATTTAA GGCGATCATA TCGTTATGCT CTACCTTGTA AACATTTTTG   
  
  
- ATGCTCTGCT TCGCAAACAT GTTTTGTTTG ATGGTATGAC GTCTAGTATA TTAAAAAACC CCCCAATACA   
  
  
- TAGAATCACT ACTGTAATTA ACTACCTATT GGGATAAGAT TTATAGACCT AGGTAGTAAT TGACTGAGCA   
  
  
- AAGTTTACAA TCCTAATACC CAGAACTGTA CCATATGTAC AGTTATCTAA ATACAATCTA CTTTTCAATA   
  
  
- TACTGCGAAA AATACTAAAA ACATGATCGT ATATCACGAA TTAACCTGAT ATATAAAAAT GTTCAACACG   
  
  
- AATTGTGGAA AATTAGTCTT TATAAATAAT ATACCTTTTT TAAAATAATT ATACACAAAT ACACAAATTT   
  
  
- TTTGTATGGG TCGGAACTTT ATCTGGCACT ATATCTTTAA CCTGGGTATT ATGAGTGTAA TATATACGAA   
  
  
- ATAACAATAT TAATTCCTAG TAACAACAAA ACGTTAATAC ACCGTTCTAT ATTAGGTGTT TCAAAATGTA   
  
  
- AATATAACGT TAATGAGACC ATTTACGATA ATTACATGTA AACTCTAATT CTGTATATAT ATTGGGAAAA   
  
  
- AAAAGGGAAG AAAAAAGGAA AAAGATAAAA CTTACCGTAG AATTTGGTTT CCTCGACATG TTCAATGCAA   
  
  
- TATCGTGAGT ACCAACTGAT TTGTTCTGTC TACTCTCAAA TTCAGTTCAG GAGGACCGTT ACTACAAACG   
  
  
- GTAAACCTGG TTATTTATGT AGTACATATA ATGGTATTAT CACACCAACT GAATTGTTAT GTATCCCCTA   
  
  
- ACGTATGTTT CCATTCGGAG TTTTTGAACA CGTATACGTA ACTTTAGGTA CTAGTTTTTT TAAGTTCTCG   
  
  
- TCCAACGTGT TTTTGTGCCT CTACTCTACT TTCTCTGTCT TTTGTGTATA TGTAAGTGTC ATCATCCCCA   
  
  
- TGACCATCTC GATACCGATC CGATTCTACT ACACTTTCTT TGTTATATTA CCATCATTGA CCATTATAAA   
  
  
- TATCCCGTGT ATATCCACGC CCGTATCCCA GCACAGAGAG TCAGAGTCCA TGCCAAGCTA CCCTGAGACG   
  
  
- CTATCTACAA TAGTTTAAGT TGGTAAGCTT GTAGAGGGTC TCTCTCTCTC TCTCTGACTC ACTCACTCAC   
  
  
- TCACCCACCC ACATACACAC ACTCAACAAA ACAAGTAAAT AATATCCAAG AGACGACGTA TATAGAGGTG   
  
  
- GTAATTTAAA TGGTTTTGAA CATGGTCGAC GAAATGAACG AAAGGAGTAA AAGGGGGAAA AAACAGAGAG   
  
  
- AGAGAGAGAG AGGGGGGGGG GGGGGGGGAG AGAGAGACAC AGACTTAGTC ATAGTCGGAT AGTCCTACGA   
  
  
- AGCGGAGGTC TTTCCGTAGT GAGTGTGTGA ATAATCGAAT TTTGAAAGAA AATAAGGAAA ACGAGAAAGT   
  
  
- GACGAGAGAA AATGGGTTTA TGCGGGCAGA AGTTAAGAAG AGAGTAATTT ATACTTTTTC TTTCTCTCTT   
  
  
- TATCTCTCTC TAAATTTGGT CTTCCCCACT AAGGAAAAAG AACCACCCGA ATAATTACGA GACAGATGGG   
  
  
- GTAGATCGGC TTTTTATCAT CGAATTCTGA GTTGACGAAT AATCGTAGAG AGGAGATCGT TATTGGCTCC   
  
  
- ATTATCGTAT AAAAAGAGGG GAATCGAAGA TGGAACGAAA AACACTGTGA GTTTGGATTT GACTCTCTCT   
  
  
- CTTTCTCTTC TATCTCCTCT CTATTTATAA TCGCCCTAAT CCCGTTTAAA ACTATACGAC CCGAGAAGGA   
  
  
- GTAAGTTGAG ACTCCTTCTT CTAGTTCTAG TACGGTTGGG TGGAGGCGGC GTTGTTGGTG GTGGCAATGT   
  
  
- CGACGGTGAA GGTGCCGGTT CTAAGTCGGT CAATTATAGT AGTAGTGGAG GTTATTATAG TCGTAGAAGA   
  
  
- AGTAGTAATT AGGTATTATT GGGAGCAGTC GACGATTATT CAACGCCACT TGAGTAGAGG GCGACACTGA   
  
  
- AGAGGAGTCG ATTGGCGGAA GAGAGAGAAG AGAGGCCGCT GAAGCGAAGG AGGAGGCCGC TGAGGTGACT   
  
  
- TGCGGAACAA ATAATGAAGA GGTTCCGAAA CAAGGAGGCG GAAGCGGCTG TCAAAAGAGA TAGAAGAAGT   
  
  
- TGAAAATGAC CAATATCAAC ATTGGGATCA ACAAGACGCA GATAACGAGG TGAAAAAGAT TGGTTGGGAT   
  
  
- TAGGATCTGG TGGTGTTCTT GACGGCCATG CTCATTGATC GTCCCAATGA GGTTCATCGA CGAGCAGAAT   
  
  
- GGATTGGGAT TTGGTCTAGT GAGGTAAATA GGCCAAGTGG GTGGACTGCC GGTTGGTCCG TTAAAACCTC   
  
  
- CGACACCTCC CGATATCCCG TCAGGTGTAG TAGCTGTACC TGTAGTACGT ACCTCACGTC ACCGGAGGCG   
  
  
- AGGAGGTTCG TTAGCACCTC GCCAGCCGGT GGGAATCGGT TGGAGGGGGA CACCAGGCAG AGTTTCCCCC   
  
  
- TCCCGGCCTG GGCCTGGAGA ATGTCTCCTG CCCCCTGGCT GATACCTTGA AACGGGTTAG TAATCCTGAT   
  
  
- CTCAAGGTAA AGGTCCCGGA GTACTAGGGG CGGCTCAGTT GTCCAAAACA ACTTGGACGA CTCCGTCGCC   
  
  
- AGTTACGGGA TCGGAACGTC CTCCGCCACC CCACAGTTCT TCCCCTCCGG GAACGTCAAG TGACGCCCCT   
  
  
- GATAGAGGTG GCGGAGAATT TTCTCATGCT GTGTTCCGGA GAATCTGCCA AGGAGGTGTT CCAGTTCTGA   
  
  
- GACTTGGGGT CCCAGAACTG GAACCCTCTC TCCCTTCGGC TGGTGTTGGT GGGAGAGAAG GACGCCGCCA   
  
  
- AGTGGCTCCG TCAGTTGGTG ATGCCCCGTC ACAAACTGAG GGACCTTCGT TGGGAAGGCG GCGTATCGGT   
  
  
- TCTCTCCCAT CGACACCTCC TTCCCACCAA GCCTCTCCTC TAATTCCTGC ACCATCCTCT TCTTTCGCTC   
  
  
- TCCTCTTATC TCTCCGTGGT CTTCAAGCTC AGCACCCTTC GTAACAACTC CTCGAGTCCC AAGTTCTCAC   
  
  
- ACGGGGAGTC GGGTAAGAGC GACAGGGTTC GATTCGACGA CGACGCGGAG GTAATGGGAA GGCTTCCCAT   
  
  
- GGTTGAGGTC CAAAACGTAT CATTAACGAA CAACGAACCC ACCTTCTTAG CGGGAGAAAA AAGACAGAGA   
  
  
- AGAACCGTTA T

+     ABRE

| Site Name | Organism | Position | Strand | Matrix score. | sequence | function |
| --- | --- | --- | --- | --- | --- | --- |
| ABRE | Arabidopsis thaliana | 3202 | + | 5 | ACGTG | cis-acting element involved in the abscisic acid responsiveness |

>HU03G01737.1   
+ +Up\_Stream \_Len000TTTTTT TTAAGTTTTC GTACTTGGTG TTGATGATTT GTGCTTTGTA ACCAATGGTT   
  
  
+ TTAGTAAATA GTAATTAGTC GTAACATTCT GCGATTTCGC TCAATTTATA TATCAACTTT AGAATATGAG   
  
  
+ TAACCGTAAA GATCAAACGT AGTAGTAGAG GTGTCATAGG TTTTTTTTTA AAAAAATTCG GAGTAAGAAA   
  
  
+ GTAGGGTACA TTATTTTTGC TTGTTAAATT CCGCTAGTAT AGCAATACGA GATGGAACAT TTGTAAAAAC   
  
  
+ TACGAGACGA AGCGTTTGTA CAAAACAAAC TACCATACTG CAGATCATAT AATTTTTTGG GGGGTTATGT   
  
  
+ ATCTTAGTGA TGACATTAAT TGATGGATAA CCCTATTCTA AATATCTGGA TCCATCATTA ACTGACTCGT   
  
  
+ TTCAAATGTT AGGATTATGG GTCTTGACAT GGTATACATG TCAATAGATT TATGTTAGAT GAAAAGTTAT   
  
  
+ ATGACGCTTT TTATGATTTT TGTACTAGCA TATAGTGCTT AATTGGACTA TATATTTTTA CAAGTTGTGC   
  
  
+ TTAACACCTT TTAATCAGAA ATATTTATTA TATGGAAAAA ATTTTATTAA TATGTGTTTA TGTGTTTAAA   
  
  
+ AAACATACCC AGCCTTGAAA TAGACCGTGA TATAGAAATT GGACCCATAA TACTCACATT ATATATGCTT   
  
  
+ TATTGTTATA ATTAAGGATC ATTGTTGTTT TGCAATTATG TGGCAAGATA TAATCCACAA AGTTTTACAT   
  
  
+ TTATATTGCA ATTACTCTGG TAAATGCTAT TAATGTACAT TTGAGATTAA GACATATATA TAACCCTTTT   
  
  
+ TTTTCCCTTC TTTTTTCCTT TTTCTATTTT GAATGGCATC TTAAACCAAA GGAGCTGTAC AAGTTACGTT   
  
  
+ ATAGCACTCA TGGTTGACTA AACAAGACAG ATGAGAGTTT AAGTCAAGTC CTCCTGGCAA TGATGTTTGC   
  
  
+ CATTTGGACC AATAAATACA TCATGTATAT TACCATAATA GTGTGGTTGA CTTAACAATA CATAGGGGAT   
  
  
+ TGCATACAAA GGTAAGCCTC AAAAACTTGT GCATATGCAT TGAAATCCAT GATCAAAAAA ATTCAAGAGC   
  
  
+ AGGTTGCACA AAAACACGGA GATGAGATGA AAGAGACAGA AAACACATAT ACATTCACAG TAGTAGGGGT   
  
  
+ ACTGGTAGAG CTATGGCTAG GCTAAGATGA TGTGAAAGAA ACAATATAAT GGTAGTAACT GGTAATATTT   
  
  
+ ATAGGGCACA TATAGGTGCG GGCATAGGGT CGTGTCTCTC AGTCTCAGGT ACGGTTCGAT GGGACTCTGC   
  
  
+ GATAGATGTT ATCAAATTCA ACCATTCGAA CATCTCCCAG AGAGAGAGAG AGAGACTGAG TGAGTGAGTG   
  
  
+ AGTGGGTGGG TGTATGTGTG TGAGTTGTTT TGTTCATTTA TTATAGGTTC TCTGCTGCAT ATATCTCCAC   
  
  
+ CATTAAATTT ACCAAAACTT GTACCAGCTG CTTTACTTGC TTTCCTCATT TTCCCCCTTT TTTGTCTCTC   
  
  
+ TCTCTCTCTC TCCCCCCCCC CCCCCCCCTC TCTCTCTGTG TCTGAATCAG TATCAGCCTA TCAGGATGCT   
  
  
+ TCGCCTCCAG AAAGGCATCA CTCACACACT TATTAGCTTA AAACTTTCTT TTATTCCTTT TGCTCTTTCA   
  
  
+ CTGCTCTCTT TTACCCAAAT ACGCCCGTCT TCAATTCTTC TCTCATTAAA TATGAAAAAG AAAGAGAGAA   
  
  
+ ATAGAGAGAG ATTTAAACCA GAAGGGGTGA TTCCTTTTTC TTGGTGGGCT TATTAATGCT CTGTCTACCC   
  
  
+ CATCTAGCCG AAAAATAGTA GCTTAAGACT CAACTGCTTA TTAGCATCTC TCCTCTAGCA ATAACCGAGG   
  
  
+ TAATAGCATA TTTTTCTCCC CTTAGCTTCT ACCTTGCTTT TTGTGACACT CAAACCTAAA CTGAGAGAGA   
  
  
+ GAAAGAGAAG ATAGAGGAGA GATAAATATT AGCGGGATTA GGGCAAATTT TGATATGCTG GGCTCTTCCT   
  
  
+ CATTCAACTC TGAGGAAGAA GATCAAGATC ATGCCAACCC ACCTCCGCCG CAACAACCAC CACCGTTACA   
  
  
+ GCTGCCACTT CCACGGCCAA GATTCAGCCA GTTAATATCA TCATCACCTC CAATAATATC AGCATCTTCT   
  
  
+ TCATCATTAA TCCATAATAA CCCTCGTCAG CTGCTAATAA GTTGCGGTGA ACTCATCTCC CGCTGTGACT   
  
  
+ TCTCCTCAGC TAACCGCCTT CTCTCTCTTC TCTCCGGCGA CTTCGCTTCC TCCTCCGGCG ACTCCACTGA   
  
  
+ ACGCCTTGTT TATTACTTCT CCAAGGCTTT GTTCCTCCGC CTTCGCCGAC AGTTTTCTCT ATCTTCTTCA   
  
  
+ ACTTTTACTG GTTATAGTTG TAACCCTAGT TGTTCTGCGT CTATTGCTCC ACTTTTTCTA ACCAACCCTA   
  
  
+ ATCCTAGACC ACCACAAGAA CTGCCGGTAC GAGTAACTAG CAGGGTTACT CCAAGTAGCT GCTCGTCTTA   
  
  
+ CCTAACCCTA AACCAGATCA CTCCATTTAT CCGGTTCACC CACCTGACGG CCAACCAGGC AATTTTGGAG   
  
  
+ GCTGTGGAGG GCTATAGGGC AGTCCACATC ATCGACATGG ACATCATGCA TGGAGTGCAG TGGCCTCCGC   
  
  
+ TCCTCCAAGC AATCGTGGAG CGGTCGGCCA CCCTTAGCCA ACCTCCCCCT GTGGTCCGTC TCAAAGGGGG   
  
  
+ AGGGCCGGAC CCGGACCTCT TACAGAGGAC GGGGGACCGA CTATGGAACT TTGCCCAATC ATTAGGACTA   
  
  
+ GAGTTCCATT TCCAGGGCCT CATGATCCCC GCCGAGTCAA CAGGTTTTGT TGAACCTGCT GAGGCAGCGG   
  
  
+ TCAATGCCCT AGCCTTGCAG GAGGCGGTGG GGTGTCAAGA AGGGGAGGCC CTTGCAGTTC ACTGCGGGGA   
  
  
+ CTATCTCCAC CGCCTCTTAA AAGAGTACGA CACAAGGCCT CTTAGACGGT TCCTCCACAA GGTCAAGACT   
  
  
+ CTGAACCCCA GGGTCTTGAC CTTGGGAGAG AGGGAAGCCG ACCACAACCA CCCTCTCTTC CTGCGGCGGT   
  
  
+ TCACCGAGGC AGTCAACCAC TACGGGGCAG TGTTTGACTC CCTGGAAGCA ACCCTTCCGC CGCATAGCCA   
  
  
+ AGAGAGGGTA GCTGTGGAGG AAGGGTGGTT CGGAGAGGAG ATTAAGGACG TGGTAGGAGA AGAAAGCGAG   
  
  
+ AGGAGAATAG AGAGGCACCA GAAGTTCGAG TCGTGGGAAG CATTGTTGAG GAGCTCAGGG TTCAAGAGTG   
  
  
+ TGCCCCTCAG CCCATTCTCG CTGTCCCAAG CTAAGCTGCT GCTGCGCCTC CATTACCCTT CCGAAGGGTA   
  
  
+ CCAACTCCAG GTTTTGCATA GTAATTGCTT GTTGCTTGGG TGGAAGAATC GCCCTCTTTT TTCTGTCTCT   
  
  
+ TCTTGGCAAT A  

- +Up\_Stream \_Len000AAAAAA AATTCAAAAG CATGAACCAC AACTACTAAA CACGAAACAT TGGTTACCAA   
  
  
- AATCATTTAT CATTAATCAG CATTGTAAGA CGCTAAAGCG AGTTAAATAT ATAGTTGAAA TCTTATACTC   
  
  
- ATTGGCATTT CTAGTTTGCA TCATCATCTC CACAGTATCC AAAAAAAAAT TTTTTTAAGC CTCATTCTTT   
  
  
- CATCCCATGT AATAAAAACG AACAATTTAA GGCGATCATA TCGTTATGCT CTACCTTGTA AACATTTTTG   
  
  
- ATGCTCTGCT TCGCAAACAT GTTTTGTTTG ATGGTATGAC GTCTAGTATA TTAAAAAACC CCCCAATACA   
  
  
- TAGAATCACT ACTGTAATTA ACTACCTATT GGGATAAGAT TTATAGACCT AGGTAGTAAT TGACTGAGCA   
  
  
- AAGTTTACAA TCCTAATACC CAGAACTGTA CCATATGTAC AGTTATCTAA ATACAATCTA CTTTTCAATA   
  
  
- TACTGCGAAA AATACTAAAA ACATGATCGT ATATCACGAA TTAACCTGAT ATATAAAAAT GTTCAACACG   
  
  
- AATTGTGGAA AATTAGTCTT TATAAATAAT ATACCTTTTT TAAAATAATT ATACACAAAT ACACAAATTT   
  
  
- TTTGTATGGG TCGGAACTTT ATCTGGCACT ATATCTTTAA CCTGGGTATT ATGAGTGTAA TATATACGAA   
  
  
- ATAACAATAT TAATTCCTAG TAACAACAAA ACGTTAATAC ACCGTTCTAT ATTAGGTGTT TCAAAATGTA   
  
  
- AATATAACGT TAATGAGACC ATTTACGATA ATTACATGTA AACTCTAATT CTGTATATAT ATTGGGAAAA   
  
  
- AAAAGGGAAG AAAAAAGGAA AAAGATAAAA CTTACCGTAG AATTTGGTTT CCTCGACATG TTCAATGCAA   
  
  
- TATCGTGAGT ACCAACTGAT TTGTTCTGTC TACTCTCAAA TTCAGTTCAG GAGGACCGTT ACTACAAACG   
  
  
- GTAAACCTGG TTATTTATGT AGTACATATA ATGGTATTAT CACACCAACT GAATTGTTAT GTATCCCCTA   
  
  
- ACGTATGTTT CCATTCGGAG TTTTTGAACA CGTATACGTA ACTTTAGGTA CTAGTTTTTT TAAGTTCTCG   
  
  
- TCCAACGTGT TTTTGTGCCT CTACTCTACT TTCTCTGTCT TTTGTGTATA TGTAAGTGTC ATCATCCCCA   
  
  
- TGACCATCTC GATACCGATC CGATTCTACT ACACTTTCTT TGTTATATTA CCATCATTGA CCATTATAAA   
  
  
- TATCCCGTGT ATATCCACGC CCGTATCCCA GCACAGAGAG TCAGAGTCCA TGCCAAGCTA CCCTGAGACG   
  
  
- CTATCTACAA TAGTTTAAGT TGGTAAGCTT GTAGAGGGTC TCTCTCTCTC TCTCTGACTC ACTCACTCAC   
  
  
- TCACCCACCC ACATACACAC ACTCAACAAA ACAAGTAAAT AATATCCAAG AGACGACGTA TATAGAGGTG   
  
  
- GTAATTTAAA TGGTTTTGAA CATGGTCGAC GAAATGAACG AAAGGAGTAA AAGGGGGAAA AAACAGAGAG   
  
  
- AGAGAGAGAG AGGGGGGGGG GGGGGGGGAG AGAGAGACAC AGACTTAGTC ATAGTCGGAT AGTCCTACGA   
  
  
- AGCGGAGGTC TTTCCGTAGT GAGTGTGTGA ATAATCGAAT TTTGAAAGAA AATAAGGAAA ACGAGAAAGT   
  
  
- GACGAGAGAA AATGGGTTTA TGCGGGCAGA AGTTAAGAAG AGAGTAATTT ATACTTTTTC TTTCTCTCTT   
  
  
- TATCTCTCTC TAAATTTGGT CTTCCCCACT AAGGAAAAAG AACCACCCGA ATAATTACGA GACAGATGGG   
  
  
- GTAGATCGGC TTTTTATCAT CGAATTCTGA GTTGACGAAT AATCGTAGAG AGGAGATCGT TATTGGCTCC   
  
  
- ATTATCGTAT AAAAAGAGGG GAATCGAAGA TGGAACGAAA AACACTGTGA GTTTGGATTT GACTCTCTCT   
  
  
- CTTTCTCTTC TATCTCCTCT CTATTTATAA TCGCCCTAAT CCCGTTTAAA ACTATACGAC CCGAGAAGGA   
  
  
- GTAAGTTGAG ACTCCTTCTT CTAGTTCTAG TACGGTTGGG TGGAGGCGGC GTTGTTGGTG GTGGCAATGT   
  
  
- CGACGGTGAA GGTGCCGGTT CTAAGTCGGT CAATTATAGT AGTAGTGGAG GTTATTATAG TCGTAGAAGA   
  
  
- AGTAGTAATT AGGTATTATT GGGAGCAGTC GACGATTATT CAACGCCACT TGAGTAGAGG GCGACACTGA   
  
  
- AGAGGAGTCG ATTGGCGGAA GAGAGAGAAG AGAGGCCGCT GAAGCGAAGG AGGAGGCCGC TGAGGTGACT   
  
  
- TGCGGAACAA ATAATGAAGA GGTTCCGAAA CAAGGAGGCG GAAGCGGCTG TCAAAAGAGA TAGAAGAAGT   
  
  
- TGAAAATGAC CAATATCAAC ATTGGGATCA ACAAGACGCA GATAACGAGG TGAAAAAGAT TGGTTGGGAT   
  
  
- TAGGATCTGG TGGTGTTCTT GACGGCCATG CTCATTGATC GTCCCAATGA GGTTCATCGA CGAGCAGAAT   
  
  
- GGATTGGGAT TTGGTCTAGT GAGGTAAATA GGCCAAGTGG GTGGACTGCC GGTTGGTCCG TTAAAACCTC   
  
  
- CGACACCTCC CGATATCCCG TCAGGTGTAG TAGCTGTACC TGTAGTACGT ACCTCACGTC ACCGGAGGCG   
  
  
- AGGAGGTTCG TTAGCACCTC GCCAGCCGGT GGGAATCGGT TGGAGGGGGA CACCAGGCAG AGTTTCCCCC   
  
  
- TCCCGGCCTG GGCCTGGAGA ATGTCTCCTG CCCCCTGGCT GATACCTTGA AACGGGTTAG TAATCCTGAT   
  
  
- CTCAAGGTAA AGGTCCCGGA GTACTAGGGG CGGCTCAGTT GTCCAAAACA ACTTGGACGA CTCCGTCGCC   
  
  
- AGTTACGGGA TCGGAACGTC CTCCGCCACC CCACAGTTCT TCCCCTCCGG GAACGTCAAG TGACGCCCCT   
  
  
- GATAGAGGTG GCGGAGAATT TTCTCATGCT GTGTTCCGGA GAATCTGCCA AGGAGGTGTT CCAGTTCTGA   
  
  
- GACTTGGGGT CCCAGAACTG GAACCCTCTC TCCCTTCGGC TGGTGTTGGT GGGAGAGAAG GACGCCGCCA   
  
  
- AGTGGCTCCG TCAGTTGGTG ATGCCCCGTC ACAAACTGAG GGACCTTCGT TGGGAAGGCG GCGTATCGGT   
  
  
- TCTCTCCCAT CGACACCTCC TTCCCACCAA GCCTCTCCTC TAATTCCTGC ACCATCCTCT TCTTTCGCTC   
  
  
- TCCTCTTATC TCTCCGTGGT CTTCAAGCTC AGCACCCTTC GTAACAACTC CTCGAGTCCC AAGTTCTCAC   
  
  
- ACGGGGAGTC GGGTAAGAGC GACAGGGTTC GATTCGACGA CGACGCGGAG GTAATGGGAA GGCTTCCCAT   
  
  
- GGTTGAGGTC CAAAACGTAT CATTAACGAA CAACGAACCC ACCTTCTTAG CGGGAGAAAA AAGACAGAGA   
  
  
- AGAACCGTTA T

+     AE-box

| Site Name | Organism | Position | Strand | Matrix score. | sequence | function |
| --- | --- | --- | --- | --- | --- | --- |
| AE-box | Arabidopsis thaliana | 1231 | + | 8 | AGAAACAA | part of a module for light response |

>HU03G01737.1   
+ +Up\_Stream \_Len000TTTTTT TTAAGTTTTC GTACTTGGTG TTGATGATTT GTGCTTTGTA ACCAATGGTT   
  
  
+ TTAGTAAATA GTAATTAGTC GTAACATTCT GCGATTTCGC TCAATTTATA TATCAACTTT AGAATATGAG   
  
  
+ TAACCGTAAA GATCAAACGT AGTAGTAGAG GTGTCATAGG TTTTTTTTTA AAAAAATTCG GAGTAAGAAA   
  
  
+ GTAGGGTACA TTATTTTTGC TTGTTAAATT CCGCTAGTAT AGCAATACGA GATGGAACAT TTGTAAAAAC   
  
  
+ TACGAGACGA AGCGTTTGTA CAAAACAAAC TACCATACTG CAGATCATAT AATTTTTTGG GGGGTTATGT   
  
  
+ ATCTTAGTGA TGACATTAAT TGATGGATAA CCCTATTCTA AATATCTGGA TCCATCATTA ACTGACTCGT   
  
  
+ TTCAAATGTT AGGATTATGG GTCTTGACAT GGTATACATG TCAATAGATT TATGTTAGAT GAAAAGTTAT   
  
  
+ ATGACGCTTT TTATGATTTT TGTACTAGCA TATAGTGCTT AATTGGACTA TATATTTTTA CAAGTTGTGC   
  
  
+ TTAACACCTT TTAATCAGAA ATATTTATTA TATGGAAAAA ATTTTATTAA TATGTGTTTA TGTGTTTAAA   
  
  
+ AAACATACCC AGCCTTGAAA TAGACCGTGA TATAGAAATT GGACCCATAA TACTCACATT ATATATGCTT   
  
  
+ TATTGTTATA ATTAAGGATC ATTGTTGTTT TGCAATTATG TGGCAAGATA TAATCCACAA AGTTTTACAT   
  
  
+ TTATATTGCA ATTACTCTGG TAAATGCTAT TAATGTACAT TTGAGATTAA GACATATATA TAACCCTTTT   
  
  
+ TTTTCCCTTC TTTTTTCCTT TTTCTATTTT GAATGGCATC TTAAACCAAA GGAGCTGTAC AAGTTACGTT   
  
  
+ ATAGCACTCA TGGTTGACTA AACAAGACAG ATGAGAGTTT AAGTCAAGTC CTCCTGGCAA TGATGTTTGC   
  
  
+ CATTTGGACC AATAAATACA TCATGTATAT TACCATAATA GTGTGGTTGA CTTAACAATA CATAGGGGAT   
  
  
+ TGCATACAAA GGTAAGCCTC AAAAACTTGT GCATATGCAT TGAAATCCAT GATCAAAAAA ATTCAAGAGC   
  
  
+ AGGTTGCACA AAAACACGGA GATGAGATGA AAGAGACAGA AAACACATAT ACATTCACAG TAGTAGGGGT   
  
  
+ ACTGGTAGAG CTATGGCTAG GCTAAGATGA TGTGAAAGAA ACAATATAAT GGTAGTAACT GGTAATATTT   
  
  
+ ATAGGGCACA TATAGGTGCG GGCATAGGGT CGTGTCTCTC AGTCTCAGGT ACGGTTCGAT GGGACTCTGC   
  
  
+ GATAGATGTT ATCAAATTCA ACCATTCGAA CATCTCCCAG AGAGAGAGAG AGAGACTGAG TGAGTGAGTG   
  
  
+ AGTGGGTGGG TGTATGTGTG TGAGTTGTTT TGTTCATTTA TTATAGGTTC TCTGCTGCAT ATATCTCCAC   
  
  
+ CATTAAATTT ACCAAAACTT GTACCAGCTG CTTTACTTGC TTTCCTCATT TTCCCCCTTT TTTGTCTCTC   
  
  
+ TCTCTCTCTC TCCCCCCCCC CCCCCCCCTC TCTCTCTGTG TCTGAATCAG TATCAGCCTA TCAGGATGCT   
  
  
+ TCGCCTCCAG AAAGGCATCA CTCACACACT TATTAGCTTA AAACTTTCTT TTATTCCTTT TGCTCTTTCA   
  
  
+ CTGCTCTCTT TTACCCAAAT ACGCCCGTCT TCAATTCTTC TCTCATTAAA TATGAAAAAG AAAGAGAGAA   
  
  
+ ATAGAGAGAG ATTTAAACCA GAAGGGGTGA TTCCTTTTTC TTGGTGGGCT TATTAATGCT CTGTCTACCC   
  
  
+ CATCTAGCCG AAAAATAGTA GCTTAAGACT CAACTGCTTA TTAGCATCTC TCCTCTAGCA ATAACCGAGG   
  
  
+ TAATAGCATA TTTTTCTCCC CTTAGCTTCT ACCTTGCTTT TTGTGACACT CAAACCTAAA CTGAGAGAGA   
  
  
+ GAAAGAGAAG ATAGAGGAGA GATAAATATT AGCGGGATTA GGGCAAATTT TGATATGCTG GGCTCTTCCT   
  
  
+ CATTCAACTC TGAGGAAGAA GATCAAGATC ATGCCAACCC ACCTCCGCCG CAACAACCAC CACCGTTACA   
  
  
+ GCTGCCACTT CCACGGCCAA GATTCAGCCA GTTAATATCA TCATCACCTC CAATAATATC AGCATCTTCT   
  
  
+ TCATCATTAA TCCATAATAA CCCTCGTCAG CTGCTAATAA GTTGCGGTGA ACTCATCTCC CGCTGTGACT   
  
  
+ TCTCCTCAGC TAACCGCCTT CTCTCTCTTC TCTCCGGCGA CTTCGCTTCC TCCTCCGGCG ACTCCACTGA   
  
  
+ ACGCCTTGTT TATTACTTCT CCAAGGCTTT GTTCCTCCGC CTTCGCCGAC AGTTTTCTCT ATCTTCTTCA   
  
  
+ ACTTTTACTG GTTATAGTTG TAACCCTAGT TGTTCTGCGT CTATTGCTCC ACTTTTTCTA ACCAACCCTA   
  
  
+ ATCCTAGACC ACCACAAGAA CTGCCGGTAC GAGTAACTAG CAGGGTTACT CCAAGTAGCT GCTCGTCTTA   
  
  
+ CCTAACCCTA AACCAGATCA CTCCATTTAT CCGGTTCACC CACCTGACGG CCAACCAGGC AATTTTGGAG   
  
  
+ GCTGTGGAGG GCTATAGGGC AGTCCACATC ATCGACATGG ACATCATGCA TGGAGTGCAG TGGCCTCCGC   
  
  
+ TCCTCCAAGC AATCGTGGAG CGGTCGGCCA CCCTTAGCCA ACCTCCCCCT GTGGTCCGTC TCAAAGGGGG   
  
  
+ AGGGCCGGAC CCGGACCTCT TACAGAGGAC GGGGGACCGA CTATGGAACT TTGCCCAATC ATTAGGACTA   
  
  
+ GAGTTCCATT TCCAGGGCCT CATGATCCCC GCCGAGTCAA CAGGTTTTGT TGAACCTGCT GAGGCAGCGG   
  
  
+ TCAATGCCCT AGCCTTGCAG GAGGCGGTGG GGTGTCAAGA AGGGGAGGCC CTTGCAGTTC ACTGCGGGGA   
  
  
+ CTATCTCCAC CGCCTCTTAA AAGAGTACGA CACAAGGCCT CTTAGACGGT TCCTCCACAA GGTCAAGACT   
  
  
+ CTGAACCCCA GGGTCTTGAC CTTGGGAGAG AGGGAAGCCG ACCACAACCA CCCTCTCTTC CTGCGGCGGT   
  
  
+ TCACCGAGGC AGTCAACCAC TACGGGGCAG TGTTTGACTC CCTGGAAGCA ACCCTTCCGC CGCATAGCCA   
  
  
+ AGAGAGGGTA GCTGTGGAGG AAGGGTGGTT CGGAGAGGAG ATTAAGGACG TGGTAGGAGA AGAAAGCGAG   
  
  
+ AGGAGAATAG AGAGGCACCA GAAGTTCGAG TCGTGGGAAG CATTGTTGAG GAGCTCAGGG TTCAAGAGTG   
  
  
+ TGCCCCTCAG CCCATTCTCG CTGTCCCAAG CTAAGCTGCT GCTGCGCCTC CATTACCCTT CCGAAGGGTA   
  
  
+ CCAACTCCAG GTTTTGCATA GTAATTGCTT GTTGCTTGGG TGGAAGAATC GCCCTCTTTT TTCTGTCTCT   
  
  
+ TCTTGGCAAT A  

- +Up\_Stream \_Len000AAAAAA AATTCAAAAG CATGAACCAC AACTACTAAA CACGAAACAT TGGTTACCAA   
  
  
- AATCATTTAT CATTAATCAG CATTGTAAGA CGCTAAAGCG AGTTAAATAT ATAGTTGAAA TCTTATACTC   
  
  
- ATTGGCATTT CTAGTTTGCA TCATCATCTC CACAGTATCC AAAAAAAAAT TTTTTTAAGC CTCATTCTTT   
  
  
- CATCCCATGT AATAAAAACG AACAATTTAA GGCGATCATA TCGTTATGCT CTACCTTGTA AACATTTTTG   
  
  
- ATGCTCTGCT TCGCAAACAT GTTTTGTTTG ATGGTATGAC GTCTAGTATA TTAAAAAACC CCCCAATACA   
  
  
- TAGAATCACT ACTGTAATTA ACTACCTATT GGGATAAGAT TTATAGACCT AGGTAGTAAT TGACTGAGCA   
  
  
- AAGTTTACAA TCCTAATACC CAGAACTGTA CCATATGTAC AGTTATCTAA ATACAATCTA CTTTTCAATA   
  
  
- TACTGCGAAA AATACTAAAA ACATGATCGT ATATCACGAA TTAACCTGAT ATATAAAAAT GTTCAACACG   
  
  
- AATTGTGGAA AATTAGTCTT TATAAATAAT ATACCTTTTT TAAAATAATT ATACACAAAT ACACAAATTT   
  
  
- TTTGTATGGG TCGGAACTTT ATCTGGCACT ATATCTTTAA CCTGGGTATT ATGAGTGTAA TATATACGAA   
  
  
- ATAACAATAT TAATTCCTAG TAACAACAAA ACGTTAATAC ACCGTTCTAT ATTAGGTGTT TCAAAATGTA   
  
  
- AATATAACGT TAATGAGACC ATTTACGATA ATTACATGTA AACTCTAATT CTGTATATAT ATTGGGAAAA   
  
  
- AAAAGGGAAG AAAAAAGGAA AAAGATAAAA CTTACCGTAG AATTTGGTTT CCTCGACATG TTCAATGCAA   
  
  
- TATCGTGAGT ACCAACTGAT TTGTTCTGTC TACTCTCAAA TTCAGTTCAG GAGGACCGTT ACTACAAACG   
  
  
- GTAAACCTGG TTATTTATGT AGTACATATA ATGGTATTAT CACACCAACT GAATTGTTAT GTATCCCCTA   
  
  
- ACGTATGTTT CCATTCGGAG TTTTTGAACA CGTATACGTA ACTTTAGGTA CTAGTTTTTT TAAGTTCTCG   
  
  
- TCCAACGTGT TTTTGTGCCT CTACTCTACT TTCTCTGTCT TTTGTGTATA TGTAAGTGTC ATCATCCCCA   
  
  
- TGACCATCTC GATACCGATC CGATTCTACT ACACTTTCTT TGTTATATTA CCATCATTGA CCATTATAAA   
  
  
- TATCCCGTGT ATATCCACGC CCGTATCCCA GCACAGAGAG TCAGAGTCCA TGCCAAGCTA CCCTGAGACG   
  
  
- CTATCTACAA TAGTTTAAGT TGGTAAGCTT GTAGAGGGTC TCTCTCTCTC TCTCTGACTC ACTCACTCAC   
  
  
- TCACCCACCC ACATACACAC ACTCAACAAA ACAAGTAAAT AATATCCAAG AGACGACGTA TATAGAGGTG   
  
  
- GTAATTTAAA TGGTTTTGAA CATGGTCGAC GAAATGAACG AAAGGAGTAA AAGGGGGAAA AAACAGAGAG   
  
  
- AGAGAGAGAG AGGGGGGGGG GGGGGGGGAG AGAGAGACAC AGACTTAGTC ATAGTCGGAT AGTCCTACGA   
  
  
- AGCGGAGGTC TTTCCGTAGT GAGTGTGTGA ATAATCGAAT TTTGAAAGAA AATAAGGAAA ACGAGAAAGT   
  
  
- GACGAGAGAA AATGGGTTTA TGCGGGCAGA AGTTAAGAAG AGAGTAATTT ATACTTTTTC TTTCTCTCTT   
  
  
- TATCTCTCTC TAAATTTGGT CTTCCCCACT AAGGAAAAAG AACCACCCGA ATAATTACGA GACAGATGGG   
  
  
- GTAGATCGGC TTTTTATCAT CGAATTCTGA GTTGACGAAT AATCGTAGAG AGGAGATCGT TATTGGCTCC   
  
  
- ATTATCGTAT AAAAAGAGGG GAATCGAAGA TGGAACGAAA AACACTGTGA GTTTGGATTT GACTCTCTCT   
  
  
- CTTTCTCTTC TATCTCCTCT CTATTTATAA TCGCCCTAAT CCCGTTTAAA ACTATACGAC CCGAGAAGGA   
  
  
- GTAAGTTGAG ACTCCTTCTT CTAGTTCTAG TACGGTTGGG TGGAGGCGGC GTTGTTGGTG GTGGCAATGT   
  
  
- CGACGGTGAA GGTGCCGGTT CTAAGTCGGT CAATTATAGT AGTAGTGGAG GTTATTATAG TCGTAGAAGA   
  
  
- AGTAGTAATT AGGTATTATT GGGAGCAGTC GACGATTATT CAACGCCACT TGAGTAGAGG GCGACACTGA   
  
  
- AGAGGAGTCG ATTGGCGGAA GAGAGAGAAG AGAGGCCGCT GAAGCGAAGG AGGAGGCCGC TGAGGTGACT   
  
  
- TGCGGAACAA ATAATGAAGA GGTTCCGAAA CAAGGAGGCG GAAGCGGCTG TCAAAAGAGA TAGAAGAAGT   
  
  
- TGAAAATGAC CAATATCAAC ATTGGGATCA ACAAGACGCA GATAACGAGG TGAAAAAGAT TGGTTGGGAT   
  
  
- TAGGATCTGG TGGTGTTCTT GACGGCCATG CTCATTGATC GTCCCAATGA GGTTCATCGA CGAGCAGAAT   
  
  
- GGATTGGGAT TTGGTCTAGT GAGGTAAATA GGCCAAGTGG GTGGACTGCC GGTTGGTCCG TTAAAACCTC   
  
  
- CGACACCTCC CGATATCCCG TCAGGTGTAG TAGCTGTACC TGTAGTACGT ACCTCACGTC ACCGGAGGCG   
  
  
- AGGAGGTTCG TTAGCACCTC GCCAGCCGGT GGGAATCGGT TGGAGGGGGA CACCAGGCAG AGTTTCCCCC   
  
  
- TCCCGGCCTG GGCCTGGAGA ATGTCTCCTG CCCCCTGGCT GATACCTTGA AACGGGTTAG TAATCCTGAT   
  
  
- CTCAAGGTAA AGGTCCCGGA GTACTAGGGG CGGCTCAGTT GTCCAAAACA ACTTGGACGA CTCCGTCGCC   
  
  
- AGTTACGGGA TCGGAACGTC CTCCGCCACC CCACAGTTCT TCCCCTCCGG GAACGTCAAG TGACGCCCCT   
  
  
- GATAGAGGTG GCGGAGAATT TTCTCATGCT GTGTTCCGGA GAATCTGCCA AGGAGGTGTT CCAGTTCTGA   
  
  
- GACTTGGGGT CCCAGAACTG GAACCCTCTC TCCCTTCGGC TGGTGTTGGT GGGAGAGAAG GACGCCGCCA   
  
  
- AGTGGCTCCG TCAGTTGGTG ATGCCCCGTC ACAAACTGAG GGACCTTCGT TGGGAAGGCG GCGTATCGGT   
  
  
- TCTCTCCCAT CGACACCTCC TTCCCACCAA GCCTCTCCTC TAATTCCTGC ACCATCCTCT TCTTTCGCTC   
  
  
- TCCTCTTATC TCTCCGTGGT CTTCAAGCTC AGCACCCTTC GTAACAACTC CTCGAGTCCC AAGTTCTCAC   
  
  
- ACGGGGAGTC GGGTAAGAGC GACAGGGTTC GATTCGACGA CGACGCGGAG GTAATGGGAA GGCTTCCCAT   
  
  
- GGTTGAGGTC CAAAACGTAT CATTAACGAA CAACGAACCC ACCTTCTTAG CGGGAGAAAA AAGACAGAGA   
  
  
- AGAACCGTTA T

+     ARE

| Site Name | Organism | Position | Strand | Matrix score. | sequence | function |
| --- | --- | --- | --- | --- | --- | --- |
| ARE | Zea mays | 70 | - | 6 | AAACCA | cis-acting regulatory element essential for the anaerobic induction |
| ARE | Zea mays | 887 | + | 6 | AAACCA | cis-acting regulatory element essential for the anaerobic induction |
| ARE | Zea mays | 2534 | + | 6 | AAACCA | cis-acting regulatory element essential for the anaerobic induction |
| ARE | Zea mays | 1769 | + | 6 | AAACCA | cis-acting regulatory element essential for the anaerobic induction |

>HU03G01737.1   
+ +Up\_Stream \_Len000TTTTTT TTAAGTTTTC GTACTTGGTG TTGATGATTT GTGCTTTGTA ACCAATGGTT   
  
  
+ TTAGTAAATA GTAATTAGTC GTAACATTCT GCGATTTCGC TCAATTTATA TATCAACTTT AGAATATGAG   
  
  
+ TAACCGTAAA GATCAAACGT AGTAGTAGAG GTGTCATAGG TTTTTTTTTA AAAAAATTCG GAGTAAGAAA   
  
  
+ GTAGGGTACA TTATTTTTGC TTGTTAAATT CCGCTAGTAT AGCAATACGA GATGGAACAT TTGTAAAAAC   
  
  
+ TACGAGACGA AGCGTTTGTA CAAAACAAAC TACCATACTG CAGATCATAT AATTTTTTGG GGGGTTATGT   
  
  
+ ATCTTAGTGA TGACATTAAT TGATGGATAA CCCTATTCTA AATATCTGGA TCCATCATTA ACTGACTCGT   
  
  
+ TTCAAATGTT AGGATTATGG GTCTTGACAT GGTATACATG TCAATAGATT TATGTTAGAT GAAAAGTTAT   
  
  
+ ATGACGCTTT TTATGATTTT TGTACTAGCA TATAGTGCTT AATTGGACTA TATATTTTTA CAAGTTGTGC   
  
  
+ TTAACACCTT TTAATCAGAA ATATTTATTA TATGGAAAAA ATTTTATTAA TATGTGTTTA TGTGTTTAAA   
  
  
+ AAACATACCC AGCCTTGAAA TAGACCGTGA TATAGAAATT GGACCCATAA TACTCACATT ATATATGCTT   
  
  
+ TATTGTTATA ATTAAGGATC ATTGTTGTTT TGCAATTATG TGGCAAGATA TAATCCACAA AGTTTTACAT   
  
  
+ TTATATTGCA ATTACTCTGG TAAATGCTAT TAATGTACAT TTGAGATTAA GACATATATA TAACCCTTTT   
  
  
+ TTTTCCCTTC TTTTTTCCTT TTTCTATTTT GAATGGCATC TTAAACCAAA GGAGCTGTAC AAGTTACGTT   
  
  
+ ATAGCACTCA TGGTTGACTA AACAAGACAG ATGAGAGTTT AAGTCAAGTC CTCCTGGCAA TGATGTTTGC   
  
  
+ CATTTGGACC AATAAATACA TCATGTATAT TACCATAATA GTGTGGTTGA CTTAACAATA CATAGGGGAT   
  
  
+ TGCATACAAA GGTAAGCCTC AAAAACTTGT GCATATGCAT TGAAATCCAT GATCAAAAAA ATTCAAGAGC   
  
  
+ AGGTTGCACA AAAACACGGA GATGAGATGA AAGAGACAGA AAACACATAT ACATTCACAG TAGTAGGGGT   
  
  
+ ACTGGTAGAG CTATGGCTAG GCTAAGATGA TGTGAAAGAA ACAATATAAT GGTAGTAACT GGTAATATTT   
  
  
+ ATAGGGCACA TATAGGTGCG GGCATAGGGT CGTGTCTCTC AGTCTCAGGT ACGGTTCGAT GGGACTCTGC   
  
  
+ GATAGATGTT ATCAAATTCA ACCATTCGAA CATCTCCCAG AGAGAGAGAG AGAGACTGAG TGAGTGAGTG   
  
  
+ AGTGGGTGGG TGTATGTGTG TGAGTTGTTT TGTTCATTTA TTATAGGTTC TCTGCTGCAT ATATCTCCAC   
  
  
+ CATTAAATTT ACCAAAACTT GTACCAGCTG CTTTACTTGC TTTCCTCATT TTCCCCCTTT TTTGTCTCTC   
  
  
+ TCTCTCTCTC TCCCCCCCCC CCCCCCCCTC TCTCTCTGTG TCTGAATCAG TATCAGCCTA TCAGGATGCT   
  
  
+ TCGCCTCCAG AAAGGCATCA CTCACACACT TATTAGCTTA AAACTTTCTT TTATTCCTTT TGCTCTTTCA   
  
  
+ CTGCTCTCTT TTACCCAAAT ACGCCCGTCT TCAATTCTTC TCTCATTAAA TATGAAAAAG AAAGAGAGAA   
  
  
+ ATAGAGAGAG ATTTAAACCA GAAGGGGTGA TTCCTTTTTC TTGGTGGGCT TATTAATGCT CTGTCTACCC   
  
  
+ CATCTAGCCG AAAAATAGTA GCTTAAGACT CAACTGCTTA TTAGCATCTC TCCTCTAGCA ATAACCGAGG   
  
  
+ TAATAGCATA TTTTTCTCCC CTTAGCTTCT ACCTTGCTTT TTGTGACACT CAAACCTAAA CTGAGAGAGA   
  
  
+ GAAAGAGAAG ATAGAGGAGA GATAAATATT AGCGGGATTA GGGCAAATTT TGATATGCTG GGCTCTTCCT   
  
  
+ CATTCAACTC TGAGGAAGAA GATCAAGATC ATGCCAACCC ACCTCCGCCG CAACAACCAC CACCGTTACA   
  
  
+ GCTGCCACTT CCACGGCCAA GATTCAGCCA GTTAATATCA TCATCACCTC CAATAATATC AGCATCTTCT   
  
  
+ TCATCATTAA TCCATAATAA CCCTCGTCAG CTGCTAATAA GTTGCGGTGA ACTCATCTCC CGCTGTGACT   
  
  
+ TCTCCTCAGC TAACCGCCTT CTCTCTCTTC TCTCCGGCGA CTTCGCTTCC TCCTCCGGCG ACTCCACTGA   
  
  
+ ACGCCTTGTT TATTACTTCT CCAAGGCTTT GTTCCTCCGC CTTCGCCGAC AGTTTTCTCT ATCTTCTTCA   
  
  
+ ACTTTTACTG GTTATAGTTG TAACCCTAGT TGTTCTGCGT CTATTGCTCC ACTTTTTCTA ACCAACCCTA   
  
  
+ ATCCTAGACC ACCACAAGAA CTGCCGGTAC GAGTAACTAG CAGGGTTACT CCAAGTAGCT GCTCGTCTTA   
  
  
+ CCTAACCCTA AACCAGATCA CTCCATTTAT CCGGTTCACC CACCTGACGG CCAACCAGGC AATTTTGGAG   
  
  
+ GCTGTGGAGG GCTATAGGGC AGTCCACATC ATCGACATGG ACATCATGCA TGGAGTGCAG TGGCCTCCGC   
  
  
+ TCCTCCAAGC AATCGTGGAG CGGTCGGCCA CCCTTAGCCA ACCTCCCCCT GTGGTCCGTC TCAAAGGGGG   
  
  
+ AGGGCCGGAC CCGGACCTCT TACAGAGGAC GGGGGACCGA CTATGGAACT TTGCCCAATC ATTAGGACTA   
  
  
+ GAGTTCCATT TCCAGGGCCT CATGATCCCC GCCGAGTCAA CAGGTTTTGT TGAACCTGCT GAGGCAGCGG   
  
  
+ TCAATGCCCT AGCCTTGCAG GAGGCGGTGG GGTGTCAAGA AGGGGAGGCC CTTGCAGTTC ACTGCGGGGA   
  
  
+ CTATCTCCAC CGCCTCTTAA AAGAGTACGA CACAAGGCCT CTTAGACGGT TCCTCCACAA GGTCAAGACT   
  
  
+ CTGAACCCCA GGGTCTTGAC CTTGGGAGAG AGGGAAGCCG ACCACAACCA CCCTCTCTTC CTGCGGCGGT   
  
  
+ TCACCGAGGC AGTCAACCAC TACGGGGCAG TGTTTGACTC CCTGGAAGCA ACCCTTCCGC CGCATAGCCA   
  
  
+ AGAGAGGGTA GCTGTGGAGG AAGGGTGGTT CGGAGAGGAG ATTAAGGACG TGGTAGGAGA AGAAAGCGAG   
  
  
+ AGGAGAATAG AGAGGCACCA GAAGTTCGAG TCGTGGGAAG CATTGTTGAG GAGCTCAGGG TTCAAGAGTG   
  
  
+ TGCCCCTCAG CCCATTCTCG CTGTCCCAAG CTAAGCTGCT GCTGCGCCTC CATTACCCTT CCGAAGGGTA   
  
  
+ CCAACTCCAG GTTTTGCATA GTAATTGCTT GTTGCTTGGG TGGAAGAATC GCCCTCTTTT TTCTGTCTCT   
  
  
+ TCTTGGCAAT A  

- +Up\_Stream \_Len000AAAAAA AATTCAAAAG CATGAACCAC AACTACTAAA CACGAAACAT TGGTTACCAA   
  
  
- AATCATTTAT CATTAATCAG CATTGTAAGA CGCTAAAGCG AGTTAAATAT ATAGTTGAAA TCTTATACTC   
  
  
- ATTGGCATTT CTAGTTTGCA TCATCATCTC CACAGTATCC AAAAAAAAAT TTTTTTAAGC CTCATTCTTT   
  
  
- CATCCCATGT AATAAAAACG AACAATTTAA GGCGATCATA TCGTTATGCT CTACCTTGTA AACATTTTTG   
  
  
- ATGCTCTGCT TCGCAAACAT GTTTTGTTTG ATGGTATGAC GTCTAGTATA TTAAAAAACC CCCCAATACA   
  
  
- TAGAATCACT ACTGTAATTA ACTACCTATT GGGATAAGAT TTATAGACCT AGGTAGTAAT TGACTGAGCA   
  
  
- AAGTTTACAA TCCTAATACC CAGAACTGTA CCATATGTAC AGTTATCTAA ATACAATCTA CTTTTCAATA   
  
  
- TACTGCGAAA AATACTAAAA ACATGATCGT ATATCACGAA TTAACCTGAT ATATAAAAAT GTTCAACACG   
  
  
- AATTGTGGAA AATTAGTCTT TATAAATAAT ATACCTTTTT TAAAATAATT ATACACAAAT ACACAAATTT   
  
  
- TTTGTATGGG TCGGAACTTT ATCTGGCACT ATATCTTTAA CCTGGGTATT ATGAGTGTAA TATATACGAA   
  
  
- ATAACAATAT TAATTCCTAG TAACAACAAA ACGTTAATAC ACCGTTCTAT ATTAGGTGTT TCAAAATGTA   
  
  
- AATATAACGT TAATGAGACC ATTTACGATA ATTACATGTA AACTCTAATT CTGTATATAT ATTGGGAAAA   
  
  
- AAAAGGGAAG AAAAAAGGAA AAAGATAAAA CTTACCGTAG AATTTGGTTT CCTCGACATG TTCAATGCAA   
  
  
- TATCGTGAGT ACCAACTGAT TTGTTCTGTC TACTCTCAAA TTCAGTTCAG GAGGACCGTT ACTACAAACG   
  
  
- GTAAACCTGG TTATTTATGT AGTACATATA ATGGTATTAT CACACCAACT GAATTGTTAT GTATCCCCTA   
  
  
- ACGTATGTTT CCATTCGGAG TTTTTGAACA CGTATACGTA ACTTTAGGTA CTAGTTTTTT TAAGTTCTCG   
  
  
- TCCAACGTGT TTTTGTGCCT CTACTCTACT TTCTCTGTCT TTTGTGTATA TGTAAGTGTC ATCATCCCCA   
  
  
- TGACCATCTC GATACCGATC CGATTCTACT ACACTTTCTT TGTTATATTA CCATCATTGA CCATTATAAA   
  
  
- TATCCCGTGT ATATCCACGC CCGTATCCCA GCACAGAGAG TCAGAGTCCA TGCCAAGCTA CCCTGAGACG   
  
  
- CTATCTACAA TAGTTTAAGT TGGTAAGCTT GTAGAGGGTC TCTCTCTCTC TCTCTGACTC ACTCACTCAC   
  
  
- TCACCCACCC ACATACACAC ACTCAACAAA ACAAGTAAAT AATATCCAAG AGACGACGTA TATAGAGGTG   
  
  
- GTAATTTAAA TGGTTTTGAA CATGGTCGAC GAAATGAACG AAAGGAGTAA AAGGGGGAAA AAACAGAGAG   
  
  
- AGAGAGAGAG AGGGGGGGGG GGGGGGGGAG AGAGAGACAC AGACTTAGTC ATAGTCGGAT AGTCCTACGA   
  
  
- AGCGGAGGTC TTTCCGTAGT GAGTGTGTGA ATAATCGAAT TTTGAAAGAA AATAAGGAAA ACGAGAAAGT   
  
  
- GACGAGAGAA AATGGGTTTA TGCGGGCAGA AGTTAAGAAG AGAGTAATTT ATACTTTTTC TTTCTCTCTT   
  
  
- TATCTCTCTC TAAATTTGGT CTTCCCCACT AAGGAAAAAG AACCACCCGA ATAATTACGA GACAGATGGG   
  
  
- GTAGATCGGC TTTTTATCAT CGAATTCTGA GTTGACGAAT AATCGTAGAG AGGAGATCGT TATTGGCTCC   
  
  
- ATTATCGTAT AAAAAGAGGG GAATCGAAGA TGGAACGAAA AACACTGTGA GTTTGGATTT GACTCTCTCT   
  
  
- CTTTCTCTTC TATCTCCTCT CTATTTATAA TCGCCCTAAT CCCGTTTAAA ACTATACGAC CCGAGAAGGA   
  
  
- GTAAGTTGAG ACTCCTTCTT CTAGTTCTAG TACGGTTGGG TGGAGGCGGC GTTGTTGGTG GTGGCAATGT   
  
  
- CGACGGTGAA GGTGCCGGTT CTAAGTCGGT CAATTATAGT AGTAGTGGAG GTTATTATAG TCGTAGAAGA   
  
  
- AGTAGTAATT AGGTATTATT GGGAGCAGTC GACGATTATT CAACGCCACT TGAGTAGAGG GCGACACTGA   
  
  
- AGAGGAGTCG ATTGGCGGAA GAGAGAGAAG AGAGGCCGCT GAAGCGAAGG AGGAGGCCGC TGAGGTGACT   
  
  
- TGCGGAACAA ATAATGAAGA GGTTCCGAAA CAAGGAGGCG GAAGCGGCTG TCAAAAGAGA TAGAAGAAGT   
  
  
- TGAAAATGAC CAATATCAAC ATTGGGATCA ACAAGACGCA GATAACGAGG TGAAAAAGAT TGGTTGGGAT   
  
  
- TAGGATCTGG TGGTGTTCTT GACGGCCATG CTCATTGATC GTCCCAATGA GGTTCATCGA CGAGCAGAAT   
  
  
- GGATTGGGAT TTGGTCTAGT GAGGTAAATA GGCCAAGTGG GTGGACTGCC GGTTGGTCCG TTAAAACCTC   
  
  
- CGACACCTCC CGATATCCCG TCAGGTGTAG TAGCTGTACC TGTAGTACGT ACCTCACGTC ACCGGAGGCG   
  
  
- AGGAGGTTCG TTAGCACCTC GCCAGCCGGT GGGAATCGGT TGGAGGGGGA CACCAGGCAG AGTTTCCCCC   
  
  
- TCCCGGCCTG GGCCTGGAGA ATGTCTCCTG CCCCCTGGCT GATACCTTGA AACGGGTTAG TAATCCTGAT   
  
  
- CTCAAGGTAA AGGTCCCGGA GTACTAGGGG CGGCTCAGTT GTCCAAAACA ACTTGGACGA CTCCGTCGCC   
  
  
- AGTTACGGGA TCGGAACGTC CTCCGCCACC CCACAGTTCT TCCCCTCCGG GAACGTCAAG TGACGCCCCT   
  
  
- GATAGAGGTG GCGGAGAATT TTCTCATGCT GTGTTCCGGA GAATCTGCCA AGGAGGTGTT CCAGTTCTGA   
  
  
- GACTTGGGGT CCCAGAACTG GAACCCTCTC TCCCTTCGGC TGGTGTTGGT GGGAGAGAAG GACGCCGCCA   
  
  
- AGTGGCTCCG TCAGTTGGTG ATGCCCCGTC ACAAACTGAG GGACCTTCGT TGGGAAGGCG GCGTATCGGT   
  
  
- TCTCTCCCAT CGACACCTCC TTCCCACCAA GCCTCTCCTC TAATTCCTGC ACCATCCTCT TCTTTCGCTC   
  
  
- TCCTCTTATC TCTCCGTGGT CTTCAAGCTC AGCACCCTTC GTAACAACTC CTCGAGTCCC AAGTTCTCAC   
  
  
- ACGGGGAGTC GGGTAAGAGC GACAGGGTTC GATTCGACGA CGACGCGGAG GTAATGGGAA GGCTTCCCAT   
  
  
- GGTTGAGGTC CAAAACGTAT CATTAACGAA CAACGAACCC ACCTTCTTAG CGGGAGAAAA AAGACAGAGA   
  
  
- AGAACCGTTA T

+     AT~TATA-box

| Site Name | Organism | Position | Strand | Matrix score. | sequence | function |
| --- | --- | --- | --- | --- | --- | --- |
| AT~TATA-box | Arabidopsis thaliana | 831 | + | 6 | TATATA |  |
| AT~TATA-box | Arabidopsis thaliana | 829 | + | 6 | TATATA |  |
| AT~TATA-box | Arabidopsis thaliana | 694 | + | 6 | TATATA |  |
| AT~TATA-box | Arabidopsis thaliana | 543 | + | 6 | TATATA |  |
| AT~TATA-box | Arabidopsis thaliana | 121 | + | 6 | TATATA |  |
| AT~TATA-box | Arabidopsis thaliana | 119 | - | 8 | TATATAAA |  |

>HU03G01737.1   
+ +Up\_Stream \_Len000TTTTTT TTAAGTTTTC GTACTTGGTG TTGATGATTT GTGCTTTGTA ACCAATGGTT   
  
  
+ TTAGTAAATA GTAATTAGTC GTAACATTCT GCGATTTCGC TCAATTTATA TATCAACTTT AGAATATGAG   
  
  
+ TAACCGTAAA GATCAAACGT AGTAGTAGAG GTGTCATAGG TTTTTTTTTA AAAAAATTCG GAGTAAGAAA   
  
  
+ GTAGGGTACA TTATTTTTGC TTGTTAAATT CCGCTAGTAT AGCAATACGA GATGGAACAT TTGTAAAAAC   
  
  
+ TACGAGACGA AGCGTTTGTA CAAAACAAAC TACCATACTG CAGATCATAT AATTTTTTGG GGGGTTATGT   
  
  
+ ATCTTAGTGA TGACATTAAT TGATGGATAA CCCTATTCTA AATATCTGGA TCCATCATTA ACTGACTCGT   
  
  
+ TTCAAATGTT AGGATTATGG GTCTTGACAT GGTATACATG TCAATAGATT TATGTTAGAT GAAAAGTTAT   
  
  
+ ATGACGCTTT TTATGATTTT TGTACTAGCA TATAGTGCTT AATTGGACTA TATATTTTTA CAAGTTGTGC   
  
  
+ TTAACACCTT TTAATCAGAA ATATTTATTA TATGGAAAAA ATTTTATTAA TATGTGTTTA TGTGTTTAAA   
  
  
+ AAACATACCC AGCCTTGAAA TAGACCGTGA TATAGAAATT GGACCCATAA TACTCACATT ATATATGCTT   
  
  
+ TATTGTTATA ATTAAGGATC ATTGTTGTTT TGCAATTATG TGGCAAGATA TAATCCACAA AGTTTTACAT   
  
  
+ TTATATTGCA ATTACTCTGG TAAATGCTAT TAATGTACAT TTGAGATTAA GACATATATA TAACCCTTTT   
  
  
+ TTTTCCCTTC TTTTTTCCTT TTTCTATTTT GAATGGCATC TTAAACCAAA GGAGCTGTAC AAGTTACGTT   
  
  
+ ATAGCACTCA TGGTTGACTA AACAAGACAG ATGAGAGTTT AAGTCAAGTC CTCCTGGCAA TGATGTTTGC   
  
  
+ CATTTGGACC AATAAATACA TCATGTATAT TACCATAATA GTGTGGTTGA CTTAACAATA CATAGGGGAT   
  
  
+ TGCATACAAA GGTAAGCCTC AAAAACTTGT GCATATGCAT TGAAATCCAT GATCAAAAAA ATTCAAGAGC   
  
  
+ AGGTTGCACA AAAACACGGA GATGAGATGA AAGAGACAGA AAACACATAT ACATTCACAG TAGTAGGGGT   
  
  
+ ACTGGTAGAG CTATGGCTAG GCTAAGATGA TGTGAAAGAA ACAATATAAT GGTAGTAACT GGTAATATTT   
  
  
+ ATAGGGCACA TATAGGTGCG GGCATAGGGT CGTGTCTCTC AGTCTCAGGT ACGGTTCGAT GGGACTCTGC   
  
  
+ GATAGATGTT ATCAAATTCA ACCATTCGAA CATCTCCCAG AGAGAGAGAG AGAGACTGAG TGAGTGAGTG   
  
  
+ AGTGGGTGGG TGTATGTGTG TGAGTTGTTT TGTTCATTTA TTATAGGTTC TCTGCTGCAT ATATCTCCAC   
  
  
+ CATTAAATTT ACCAAAACTT GTACCAGCTG CTTTACTTGC TTTCCTCATT TTCCCCCTTT TTTGTCTCTC   
  
  
+ TCTCTCTCTC TCCCCCCCCC CCCCCCCCTC TCTCTCTGTG TCTGAATCAG TATCAGCCTA TCAGGATGCT   
  
  
+ TCGCCTCCAG AAAGGCATCA CTCACACACT TATTAGCTTA AAACTTTCTT TTATTCCTTT TGCTCTTTCA   
  
  
+ CTGCTCTCTT TTACCCAAAT ACGCCCGTCT TCAATTCTTC TCTCATTAAA TATGAAAAAG AAAGAGAGAA   
  
  
+ ATAGAGAGAG ATTTAAACCA GAAGGGGTGA TTCCTTTTTC TTGGTGGGCT TATTAATGCT CTGTCTACCC   
  
  
+ CATCTAGCCG AAAAATAGTA GCTTAAGACT CAACTGCTTA TTAGCATCTC TCCTCTAGCA ATAACCGAGG   
  
  
+ TAATAGCATA TTTTTCTCCC CTTAGCTTCT ACCTTGCTTT TTGTGACACT CAAACCTAAA CTGAGAGAGA   
  
  
+ GAAAGAGAAG ATAGAGGAGA GATAAATATT AGCGGGATTA GGGCAAATTT TGATATGCTG GGCTCTTCCT   
  
  
+ CATTCAACTC TGAGGAAGAA GATCAAGATC ATGCCAACCC ACCTCCGCCG CAACAACCAC CACCGTTACA   
  
  
+ GCTGCCACTT CCACGGCCAA GATTCAGCCA GTTAATATCA TCATCACCTC CAATAATATC AGCATCTTCT   
  
  
+ TCATCATTAA TCCATAATAA CCCTCGTCAG CTGCTAATAA GTTGCGGTGA ACTCATCTCC CGCTGTGACT   
  
  
+ TCTCCTCAGC TAACCGCCTT CTCTCTCTTC TCTCCGGCGA CTTCGCTTCC TCCTCCGGCG ACTCCACTGA   
  
  
+ ACGCCTTGTT TATTACTTCT CCAAGGCTTT GTTCCTCCGC CTTCGCCGAC AGTTTTCTCT ATCTTCTTCA   
  
  
+ ACTTTTACTG GTTATAGTTG TAACCCTAGT TGTTCTGCGT CTATTGCTCC ACTTTTTCTA ACCAACCCTA   
  
  
+ ATCCTAGACC ACCACAAGAA CTGCCGGTAC GAGTAACTAG CAGGGTTACT CCAAGTAGCT GCTCGTCTTA   
  
  
+ CCTAACCCTA AACCAGATCA CTCCATTTAT CCGGTTCACC CACCTGACGG CCAACCAGGC AATTTTGGAG   
  
  
+ GCTGTGGAGG GCTATAGGGC AGTCCACATC ATCGACATGG ACATCATGCA TGGAGTGCAG TGGCCTCCGC   
  
  
+ TCCTCCAAGC AATCGTGGAG CGGTCGGCCA CCCTTAGCCA ACCTCCCCCT GTGGTCCGTC TCAAAGGGGG   
  
  
+ AGGGCCGGAC CCGGACCTCT TACAGAGGAC GGGGGACCGA CTATGGAACT TTGCCCAATC ATTAGGACTA   
  
  
+ GAGTTCCATT TCCAGGGCCT CATGATCCCC GCCGAGTCAA CAGGTTTTGT TGAACCTGCT GAGGCAGCGG   
  
  
+ TCAATGCCCT AGCCTTGCAG GAGGCGGTGG GGTGTCAAGA AGGGGAGGCC CTTGCAGTTC ACTGCGGGGA   
  
  
+ CTATCTCCAC CGCCTCTTAA AAGAGTACGA CACAAGGCCT CTTAGACGGT TCCTCCACAA GGTCAAGACT   
  
  
+ CTGAACCCCA GGGTCTTGAC CTTGGGAGAG AGGGAAGCCG ACCACAACCA CCCTCTCTTC CTGCGGCGGT   
  
  
+ TCACCGAGGC AGTCAACCAC TACGGGGCAG TGTTTGACTC CCTGGAAGCA ACCCTTCCGC CGCATAGCCA   
  
  
+ AGAGAGGGTA GCTGTGGAGG AAGGGTGGTT CGGAGAGGAG ATTAAGGACG TGGTAGGAGA AGAAAGCGAG   
  
  
+ AGGAGAATAG AGAGGCACCA GAAGTTCGAG TCGTGGGAAG CATTGTTGAG GAGCTCAGGG TTCAAGAGTG   
  
  
+ TGCCCCTCAG CCCATTCTCG CTGTCCCAAG CTAAGCTGCT GCTGCGCCTC CATTACCCTT CCGAAGGGTA   
  
  
+ CCAACTCCAG GTTTTGCATA GTAATTGCTT GTTGCTTGGG TGGAAGAATC GCCCTCTTTT TTCTGTCTCT   
  
  
+ TCTTGGCAAT A  

- +Up\_Stream \_Len000AAAAAA AATTCAAAAG CATGAACCAC AACTACTAAA CACGAAACAT TGGTTACCAA   
  
  
- AATCATTTAT CATTAATCAG CATTGTAAGA CGCTAAAGCG AGTTAAATAT ATAGTTGAAA TCTTATACTC   
  
  
- ATTGGCATTT CTAGTTTGCA TCATCATCTC CACAGTATCC AAAAAAAAAT TTTTTTAAGC CTCATTCTTT   
  
  
- CATCCCATGT AATAAAAACG AACAATTTAA GGCGATCATA TCGTTATGCT CTACCTTGTA AACATTTTTG   
  
  
- ATGCTCTGCT TCGCAAACAT GTTTTGTTTG ATGGTATGAC GTCTAGTATA TTAAAAAACC CCCCAATACA   
  
  
- TAGAATCACT ACTGTAATTA ACTACCTATT GGGATAAGAT TTATAGACCT AGGTAGTAAT TGACTGAGCA   
  
  
- AAGTTTACAA TCCTAATACC CAGAACTGTA CCATATGTAC AGTTATCTAA ATACAATCTA CTTTTCAATA   
  
  
- TACTGCGAAA AATACTAAAA ACATGATCGT ATATCACGAA TTAACCTGAT ATATAAAAAT GTTCAACACG   
  
  
- AATTGTGGAA AATTAGTCTT TATAAATAAT ATACCTTTTT TAAAATAATT ATACACAAAT ACACAAATTT   
  
  
- TTTGTATGGG TCGGAACTTT ATCTGGCACT ATATCTTTAA CCTGGGTATT ATGAGTGTAA TATATACGAA   
  
  
- ATAACAATAT TAATTCCTAG TAACAACAAA ACGTTAATAC ACCGTTCTAT ATTAGGTGTT TCAAAATGTA   
  
  
- AATATAACGT TAATGAGACC ATTTACGATA ATTACATGTA AACTCTAATT CTGTATATAT ATTGGGAAAA   
  
  
- AAAAGGGAAG AAAAAAGGAA AAAGATAAAA CTTACCGTAG AATTTGGTTT CCTCGACATG TTCAATGCAA   
  
  
- TATCGTGAGT ACCAACTGAT TTGTTCTGTC TACTCTCAAA TTCAGTTCAG GAGGACCGTT ACTACAAACG   
  
  
- GTAAACCTGG TTATTTATGT AGTACATATA ATGGTATTAT CACACCAACT GAATTGTTAT GTATCCCCTA   
  
  
- ACGTATGTTT CCATTCGGAG TTTTTGAACA CGTATACGTA ACTTTAGGTA CTAGTTTTTT TAAGTTCTCG   
  
  
- TCCAACGTGT TTTTGTGCCT CTACTCTACT TTCTCTGTCT TTTGTGTATA TGTAAGTGTC ATCATCCCCA   
  
  
- TGACCATCTC GATACCGATC CGATTCTACT ACACTTTCTT TGTTATATTA CCATCATTGA CCATTATAAA   
  
  
- TATCCCGTGT ATATCCACGC CCGTATCCCA GCACAGAGAG TCAGAGTCCA TGCCAAGCTA CCCTGAGACG   
  
  
- CTATCTACAA TAGTTTAAGT TGGTAAGCTT GTAGAGGGTC TCTCTCTCTC TCTCTGACTC ACTCACTCAC   
  
  
- TCACCCACCC ACATACACAC ACTCAACAAA ACAAGTAAAT AATATCCAAG AGACGACGTA TATAGAGGTG   
  
  
- GTAATTTAAA TGGTTTTGAA CATGGTCGAC GAAATGAACG AAAGGAGTAA AAGGGGGAAA AAACAGAGAG   
  
  
- AGAGAGAGAG AGGGGGGGGG GGGGGGGGAG AGAGAGACAC AGACTTAGTC ATAGTCGGAT AGTCCTACGA   
  
  
- AGCGGAGGTC TTTCCGTAGT GAGTGTGTGA ATAATCGAAT TTTGAAAGAA AATAAGGAAA ACGAGAAAGT   
  
  
- GACGAGAGAA AATGGGTTTA TGCGGGCAGA AGTTAAGAAG AGAGTAATTT ATACTTTTTC TTTCTCTCTT   
  
  
- TATCTCTCTC TAAATTTGGT CTTCCCCACT AAGGAAAAAG AACCACCCGA ATAATTACGA GACAGATGGG   
  
  
- GTAGATCGGC TTTTTATCAT CGAATTCTGA GTTGACGAAT AATCGTAGAG AGGAGATCGT TATTGGCTCC   
  
  
- ATTATCGTAT AAAAAGAGGG GAATCGAAGA TGGAACGAAA AACACTGTGA GTTTGGATTT GACTCTCTCT   
  
  
- CTTTCTCTTC TATCTCCTCT CTATTTATAA TCGCCCTAAT CCCGTTTAAA ACTATACGAC CCGAGAAGGA   
  
  
- GTAAGTTGAG ACTCCTTCTT CTAGTTCTAG TACGGTTGGG TGGAGGCGGC GTTGTTGGTG GTGGCAATGT   
  
  
- CGACGGTGAA GGTGCCGGTT CTAAGTCGGT CAATTATAGT AGTAGTGGAG GTTATTATAG TCGTAGAAGA   
  
  
- AGTAGTAATT AGGTATTATT GGGAGCAGTC GACGATTATT CAACGCCACT TGAGTAGAGG GCGACACTGA   
  
  
- AGAGGAGTCG ATTGGCGGAA GAGAGAGAAG AGAGGCCGCT GAAGCGAAGG AGGAGGCCGC TGAGGTGACT   
  
  
- TGCGGAACAA ATAATGAAGA GGTTCCGAAA CAAGGAGGCG GAAGCGGCTG TCAAAAGAGA TAGAAGAAGT   
  
  
- TGAAAATGAC CAATATCAAC ATTGGGATCA ACAAGACGCA GATAACGAGG TGAAAAAGAT TGGTTGGGAT   
  
  
- TAGGATCTGG TGGTGTTCTT GACGGCCATG CTCATTGATC GTCCCAATGA GGTTCATCGA CGAGCAGAAT   
  
  
- GGATTGGGAT TTGGTCTAGT GAGGTAAATA GGCCAAGTGG GTGGACTGCC GGTTGGTCCG TTAAAACCTC   
  
  
- CGACACCTCC CGATATCCCG TCAGGTGTAG TAGCTGTACC TGTAGTACGT ACCTCACGTC ACCGGAGGCG   
  
  
- AGGAGGTTCG TTAGCACCTC GCCAGCCGGT GGGAATCGGT TGGAGGGGGA CACCAGGCAG AGTTTCCCCC   
  
  
- TCCCGGCCTG GGCCTGGAGA ATGTCTCCTG CCCCCTGGCT GATACCTTGA AACGGGTTAG TAATCCTGAT   
  
  
- CTCAAGGTAA AGGTCCCGGA GTACTAGGGG CGGCTCAGTT GTCCAAAACA ACTTGGACGA CTCCGTCGCC   
  
  
- AGTTACGGGA TCGGAACGTC CTCCGCCACC CCACAGTTCT TCCCCTCCGG GAACGTCAAG TGACGCCCCT   
  
  
- GATAGAGGTG GCGGAGAATT TTCTCATGCT GTGTTCCGGA GAATCTGCCA AGGAGGTGTT CCAGTTCTGA   
  
  
- GACTTGGGGT CCCAGAACTG GAACCCTCTC TCCCTTCGGC TGGTGTTGGT GGGAGAGAAG GACGCCGCCA   
  
  
- AGTGGCTCCG TCAGTTGGTG ATGCCCCGTC ACAAACTGAG GGACCTTCGT TGGGAAGGCG GCGTATCGGT   
  
  
- TCTCTCCCAT CGACACCTCC TTCCCACCAA GCCTCTCCTC TAATTCCTGC ACCATCCTCT TCTTTCGCTC   
  
  
- TCCTCTTATC TCTCCGTGGT CTTCAAGCTC AGCACCCTTC GTAACAACTC CTCGAGTCCC AAGTTCTCAC   
  
  
- ACGGGGAGTC GGGTAAGAGC GACAGGGTTC GATTCGACGA CGACGCGGAG GTAATGGGAA GGCTTCCCAT   
  
  
- GGTTGAGGTC CAAAACGTAT CATTAACGAA CAACGAACCC ACCTTCTTAG CGGGAGAAAA AAGACAGAGA   
  
  
- AGAACCGTTA T

+     Box 4

| Site Name | Organism | Position | Strand | Matrix score. | sequence | function |
| --- | --- | --- | --- | --- | --- | --- |
| Box 4 | Petroselinum crispum | 2180 | - | 6 | ATTAAT | part of a conserved DNA module involved in light responsiveness |
| Box 4 | Petroselinum crispum | 369 | + | 6 | ATTAAT | part of a conserved DNA module involved in light responsiveness |
| Box 4 | Petroselinum crispum | 1806 | - | 6 | ATTAAT | part of a conserved DNA module involved in light responsiveness |
| Box 4 | Petroselinum crispum | 803 | + | 6 | ATTAAT | part of a conserved DNA module involved in light responsiveness |
| Box 4 | Petroselinum crispum | 610 | + | 6 | ATTAAT | part of a conserved DNA module involved in light responsiveness |

>HU03G01737.1   
+ +Up\_Stream \_Len000TTTTTT TTAAGTTTTC GTACTTGGTG TTGATGATTT GTGCTTTGTA ACCAATGGTT   
  
  
+ TTAGTAAATA GTAATTAGTC GTAACATTCT GCGATTTCGC TCAATTTATA TATCAACTTT AGAATATGAG   
  
  
+ TAACCGTAAA GATCAAACGT AGTAGTAGAG GTGTCATAGG TTTTTTTTTA AAAAAATTCG GAGTAAGAAA   
  
  
+ GTAGGGTACA TTATTTTTGC TTGTTAAATT CCGCTAGTAT AGCAATACGA GATGGAACAT TTGTAAAAAC   
  
  
+ TACGAGACGA AGCGTTTGTA CAAAACAAAC TACCATACTG CAGATCATAT AATTTTTTGG GGGGTTATGT   
  
  
+ ATCTTAGTGA TGACATTAAT TGATGGATAA CCCTATTCTA AATATCTGGA TCCATCATTA ACTGACTCGT   
  
  
+ TTCAAATGTT AGGATTATGG GTCTTGACAT GGTATACATG TCAATAGATT TATGTTAGAT GAAAAGTTAT   
  
  
+ ATGACGCTTT TTATGATTTT TGTACTAGCA TATAGTGCTT AATTGGACTA TATATTTTTA CAAGTTGTGC   
  
  
+ TTAACACCTT TTAATCAGAA ATATTTATTA TATGGAAAAA ATTTTATTAA TATGTGTTTA TGTGTTTAAA   
  
  
+ AAACATACCC AGCCTTGAAA TAGACCGTGA TATAGAAATT GGACCCATAA TACTCACATT ATATATGCTT   
  
  
+ TATTGTTATA ATTAAGGATC ATTGTTGTTT TGCAATTATG TGGCAAGATA TAATCCACAA AGTTTTACAT   
  
  
+ TTATATTGCA ATTACTCTGG TAAATGCTAT TAATGTACAT TTGAGATTAA GACATATATA TAACCCTTTT   
  
  
+ TTTTCCCTTC TTTTTTCCTT TTTCTATTTT GAATGGCATC TTAAACCAAA GGAGCTGTAC AAGTTACGTT   
  
  
+ ATAGCACTCA TGGTTGACTA AACAAGACAG ATGAGAGTTT AAGTCAAGTC CTCCTGGCAA TGATGTTTGC   
  
  
+ CATTTGGACC AATAAATACA TCATGTATAT TACCATAATA GTGTGGTTGA CTTAACAATA CATAGGGGAT   
  
  
+ TGCATACAAA GGTAAGCCTC AAAAACTTGT GCATATGCAT TGAAATCCAT GATCAAAAAA ATTCAAGAGC   
  
  
+ AGGTTGCACA AAAACACGGA GATGAGATGA AAGAGACAGA AAACACATAT ACATTCACAG TAGTAGGGGT   
  
  
+ ACTGGTAGAG CTATGGCTAG GCTAAGATGA TGTGAAAGAA ACAATATAAT GGTAGTAACT GGTAATATTT   
  
  
+ ATAGGGCACA TATAGGTGCG GGCATAGGGT CGTGTCTCTC AGTCTCAGGT ACGGTTCGAT GGGACTCTGC   
  
  
+ GATAGATGTT ATCAAATTCA ACCATTCGAA CATCTCCCAG AGAGAGAGAG AGAGACTGAG TGAGTGAGTG   
  
  
+ AGTGGGTGGG TGTATGTGTG TGAGTTGTTT TGTTCATTTA TTATAGGTTC TCTGCTGCAT ATATCTCCAC   
  
  
+ CATTAAATTT ACCAAAACTT GTACCAGCTG CTTTACTTGC TTTCCTCATT TTCCCCCTTT TTTGTCTCTC   
  
  
+ TCTCTCTCTC TCCCCCCCCC CCCCCCCCTC TCTCTCTGTG TCTGAATCAG TATCAGCCTA TCAGGATGCT   
  
  
+ TCGCCTCCAG AAAGGCATCA CTCACACACT TATTAGCTTA AAACTTTCTT TTATTCCTTT TGCTCTTTCA   
  
  
+ CTGCTCTCTT TTACCCAAAT ACGCCCGTCT TCAATTCTTC TCTCATTAAA TATGAAAAAG AAAGAGAGAA   
  
  
+ ATAGAGAGAG ATTTAAACCA GAAGGGGTGA TTCCTTTTTC TTGGTGGGCT TATTAATGCT CTGTCTACCC   
  
  
+ CATCTAGCCG AAAAATAGTA GCTTAAGACT CAACTGCTTA TTAGCATCTC TCCTCTAGCA ATAACCGAGG   
  
  
+ TAATAGCATA TTTTTCTCCC CTTAGCTTCT ACCTTGCTTT TTGTGACACT CAAACCTAAA CTGAGAGAGA   
  
  
+ GAAAGAGAAG ATAGAGGAGA GATAAATATT AGCGGGATTA GGGCAAATTT TGATATGCTG GGCTCTTCCT   
  
  
+ CATTCAACTC TGAGGAAGAA GATCAAGATC ATGCCAACCC ACCTCCGCCG CAACAACCAC CACCGTTACA   
  
  
+ GCTGCCACTT CCACGGCCAA GATTCAGCCA GTTAATATCA TCATCACCTC CAATAATATC AGCATCTTCT   
  
  
+ TCATCATTAA TCCATAATAA CCCTCGTCAG CTGCTAATAA GTTGCGGTGA ACTCATCTCC CGCTGTGACT   
  
  
+ TCTCCTCAGC TAACCGCCTT CTCTCTCTTC TCTCCGGCGA CTTCGCTTCC TCCTCCGGCG ACTCCACTGA   
  
  
+ ACGCCTTGTT TATTACTTCT CCAAGGCTTT GTTCCTCCGC CTTCGCCGAC AGTTTTCTCT ATCTTCTTCA   
  
  
+ ACTTTTACTG GTTATAGTTG TAACCCTAGT TGTTCTGCGT CTATTGCTCC ACTTTTTCTA ACCAACCCTA   
  
  
+ ATCCTAGACC ACCACAAGAA CTGCCGGTAC GAGTAACTAG CAGGGTTACT CCAAGTAGCT GCTCGTCTTA   
  
  
+ CCTAACCCTA AACCAGATCA CTCCATTTAT CCGGTTCACC CACCTGACGG CCAACCAGGC AATTTTGGAG   
  
  
+ GCTGTGGAGG GCTATAGGGC AGTCCACATC ATCGACATGG ACATCATGCA TGGAGTGCAG TGGCCTCCGC   
  
  
+ TCCTCCAAGC AATCGTGGAG CGGTCGGCCA CCCTTAGCCA ACCTCCCCCT GTGGTCCGTC TCAAAGGGGG   
  
  
+ AGGGCCGGAC CCGGACCTCT TACAGAGGAC GGGGGACCGA CTATGGAACT TTGCCCAATC ATTAGGACTA   
  
  
+ GAGTTCCATT TCCAGGGCCT CATGATCCCC GCCGAGTCAA CAGGTTTTGT TGAACCTGCT GAGGCAGCGG   
  
  
+ TCAATGCCCT AGCCTTGCAG GAGGCGGTGG GGTGTCAAGA AGGGGAGGCC CTTGCAGTTC ACTGCGGGGA   
  
  
+ CTATCTCCAC CGCCTCTTAA AAGAGTACGA CACAAGGCCT CTTAGACGGT TCCTCCACAA GGTCAAGACT   
  
  
+ CTGAACCCCA GGGTCTTGAC CTTGGGAGAG AGGGAAGCCG ACCACAACCA CCCTCTCTTC CTGCGGCGGT   
  
  
+ TCACCGAGGC AGTCAACCAC TACGGGGCAG TGTTTGACTC CCTGGAAGCA ACCCTTCCGC CGCATAGCCA   
  
  
+ AGAGAGGGTA GCTGTGGAGG AAGGGTGGTT CGGAGAGGAG ATTAAGGACG TGGTAGGAGA AGAAAGCGAG   
  
  
+ AGGAGAATAG AGAGGCACCA GAAGTTCGAG TCGTGGGAAG CATTGTTGAG GAGCTCAGGG TTCAAGAGTG   
  
  
+ TGCCCCTCAG CCCATTCTCG CTGTCCCAAG CTAAGCTGCT GCTGCGCCTC CATTACCCTT CCGAAGGGTA   
  
  
+ CCAACTCCAG GTTTTGCATA GTAATTGCTT GTTGCTTGGG TGGAAGAATC GCCCTCTTTT TTCTGTCTCT   
  
  
+ TCTTGGCAAT A  

- +Up\_Stream \_Len000AAAAAA AATTCAAAAG CATGAACCAC AACTACTAAA CACGAAACAT TGGTTACCAA   
  
  
- AATCATTTAT CATTAATCAG CATTGTAAGA CGCTAAAGCG AGTTAAATAT ATAGTTGAAA TCTTATACTC   
  
  
- ATTGGCATTT CTAGTTTGCA TCATCATCTC CACAGTATCC AAAAAAAAAT TTTTTTAAGC CTCATTCTTT   
  
  
- CATCCCATGT AATAAAAACG AACAATTTAA GGCGATCATA TCGTTATGCT CTACCTTGTA AACATTTTTG   
  
  
- ATGCTCTGCT TCGCAAACAT GTTTTGTTTG ATGGTATGAC GTCTAGTATA TTAAAAAACC CCCCAATACA   
  
  
- TAGAATCACT ACTGTAATTA ACTACCTATT GGGATAAGAT TTATAGACCT AGGTAGTAAT TGACTGAGCA   
  
  
- AAGTTTACAA TCCTAATACC CAGAACTGTA CCATATGTAC AGTTATCTAA ATACAATCTA CTTTTCAATA   
  
  
- TACTGCGAAA AATACTAAAA ACATGATCGT ATATCACGAA TTAACCTGAT ATATAAAAAT GTTCAACACG   
  
  
- AATTGTGGAA AATTAGTCTT TATAAATAAT ATACCTTTTT TAAAATAATT ATACACAAAT ACACAAATTT   
  
  
- TTTGTATGGG TCGGAACTTT ATCTGGCACT ATATCTTTAA CCTGGGTATT ATGAGTGTAA TATATACGAA   
  
  
- ATAACAATAT TAATTCCTAG TAACAACAAA ACGTTAATAC ACCGTTCTAT ATTAGGTGTT TCAAAATGTA   
  
  
- AATATAACGT TAATGAGACC ATTTACGATA ATTACATGTA AACTCTAATT CTGTATATAT ATTGGGAAAA   
  
  
- AAAAGGGAAG AAAAAAGGAA AAAGATAAAA CTTACCGTAG AATTTGGTTT CCTCGACATG TTCAATGCAA   
  
  
- TATCGTGAGT ACCAACTGAT TTGTTCTGTC TACTCTCAAA TTCAGTTCAG GAGGACCGTT ACTACAAACG   
  
  
- GTAAACCTGG TTATTTATGT AGTACATATA ATGGTATTAT CACACCAACT GAATTGTTAT GTATCCCCTA   
  
  
- ACGTATGTTT CCATTCGGAG TTTTTGAACA CGTATACGTA ACTTTAGGTA CTAGTTTTTT TAAGTTCTCG   
  
  
- TCCAACGTGT TTTTGTGCCT CTACTCTACT TTCTCTGTCT TTTGTGTATA TGTAAGTGTC ATCATCCCCA   
  
  
- TGACCATCTC GATACCGATC CGATTCTACT ACACTTTCTT TGTTATATTA CCATCATTGA CCATTATAAA   
  
  
- TATCCCGTGT ATATCCACGC CCGTATCCCA GCACAGAGAG TCAGAGTCCA TGCCAAGCTA CCCTGAGACG   
  
  
- CTATCTACAA TAGTTTAAGT TGGTAAGCTT GTAGAGGGTC TCTCTCTCTC TCTCTGACTC ACTCACTCAC   
  
  
- TCACCCACCC ACATACACAC ACTCAACAAA ACAAGTAAAT AATATCCAAG AGACGACGTA TATAGAGGTG   
  
  
- GTAATTTAAA TGGTTTTGAA CATGGTCGAC GAAATGAACG AAAGGAGTAA AAGGGGGAAA AAACAGAGAG   
  
  
- AGAGAGAGAG AGGGGGGGGG GGGGGGGGAG AGAGAGACAC AGACTTAGTC ATAGTCGGAT AGTCCTACGA   
  
  
- AGCGGAGGTC TTTCCGTAGT GAGTGTGTGA ATAATCGAAT TTTGAAAGAA AATAAGGAAA ACGAGAAAGT   
  
  
- GACGAGAGAA AATGGGTTTA TGCGGGCAGA AGTTAAGAAG AGAGTAATTT ATACTTTTTC TTTCTCTCTT   
  
  
- TATCTCTCTC TAAATTTGGT CTTCCCCACT AAGGAAAAAG AACCACCCGA ATAATTACGA GACAGATGGG   
  
  
- GTAGATCGGC TTTTTATCAT CGAATTCTGA GTTGACGAAT AATCGTAGAG AGGAGATCGT TATTGGCTCC   
  
  
- ATTATCGTAT AAAAAGAGGG GAATCGAAGA TGGAACGAAA AACACTGTGA GTTTGGATTT GACTCTCTCT   
  
  
- CTTTCTCTTC TATCTCCTCT CTATTTATAA TCGCCCTAAT CCCGTTTAAA ACTATACGAC CCGAGAAGGA   
  
  
- GTAAGTTGAG ACTCCTTCTT CTAGTTCTAG TACGGTTGGG TGGAGGCGGC GTTGTTGGTG GTGGCAATGT   
  
  
- CGACGGTGAA GGTGCCGGTT CTAAGTCGGT CAATTATAGT AGTAGTGGAG GTTATTATAG TCGTAGAAGA   
  
  
- AGTAGTAATT AGGTATTATT GGGAGCAGTC GACGATTATT CAACGCCACT TGAGTAGAGG GCGACACTGA   
  
  
- AGAGGAGTCG ATTGGCGGAA GAGAGAGAAG AGAGGCCGCT GAAGCGAAGG AGGAGGCCGC TGAGGTGACT   
  
  
- TGCGGAACAA ATAATGAAGA GGTTCCGAAA CAAGGAGGCG GAAGCGGCTG TCAAAAGAGA TAGAAGAAGT   
  
  
- TGAAAATGAC CAATATCAAC ATTGGGATCA ACAAGACGCA GATAACGAGG TGAAAAAGAT TGGTTGGGAT   
  
  
- TAGGATCTGG TGGTGTTCTT GACGGCCATG CTCATTGATC GTCCCAATGA GGTTCATCGA CGAGCAGAAT   
  
  
- GGATTGGGAT TTGGTCTAGT GAGGTAAATA GGCCAAGTGG GTGGACTGCC GGTTGGTCCG TTAAAACCTC   
  
  
- CGACACCTCC CGATATCCCG TCAGGTGTAG TAGCTGTACC TGTAGTACGT ACCTCACGTC ACCGGAGGCG   
  
  
- AGGAGGTTCG TTAGCACCTC GCCAGCCGGT GGGAATCGGT TGGAGGGGGA CACCAGGCAG AGTTTCCCCC   
  
  
- TCCCGGCCTG GGCCTGGAGA ATGTCTCCTG CCCCCTGGCT GATACCTTGA AACGGGTTAG TAATCCTGAT   
  
  
- CTCAAGGTAA AGGTCCCGGA GTACTAGGGG CGGCTCAGTT GTCCAAAACA ACTTGGACGA CTCCGTCGCC   
  
  
- AGTTACGGGA TCGGAACGTC CTCCGCCACC CCACAGTTCT TCCCCTCCGG GAACGTCAAG TGACGCCCCT   
  
  
- GATAGAGGTG GCGGAGAATT TTCTCATGCT GTGTTCCGGA GAATCTGCCA AGGAGGTGTT CCAGTTCTGA   
  
  
- GACTTGGGGT CCCAGAACTG GAACCCTCTC TCCCTTCGGC TGGTGTTGGT GGGAGAGAAG GACGCCGCCA   
  
  
- AGTGGCTCCG TCAGTTGGTG ATGCCCCGTC ACAAACTGAG GGACCTTCGT TGGGAAGGCG GCGTATCGGT   
  
  
- TCTCTCCCAT CGACACCTCC TTCCCACCAA GCCTCTCCTC TAATTCCTGC ACCATCCTCT TCTTTCGCTC   
  
  
- TCCTCTTATC TCTCCGTGGT CTTCAAGCTC AGCACCCTTC GTAACAACTC CTCGAGTCCC AAGTTCTCAC   
  
  
- ACGGGGAGTC GGGTAAGAGC GACAGGGTTC GATTCGACGA CGACGCGGAG GTAATGGGAA GGCTTCCCAT   
  
  
- GGTTGAGGTC CAAAACGTAT CATTAACGAA CAACGAACCC ACCTTCTTAG CGGGAGAAAA AAGACAGAGA   
  
  
- AGAACCGTTA T

+     CAAT-box

| Site Name | Organism | Position | Strand | Matrix score. | sequence | function |
| --- | --- | --- | --- | --- | --- | --- |
| CAAT-box | Arabidopsis thaliana | 2154 | + | 5 | CCAAT | common cis-acting element in promoter and enhancer regions |
| CAAT-box | Pisum sativum | 1347 | + | 5 | CAAAT | common cis-acting element in promoter and enhancer regions |
| CAAT-box | Nicotiana glutinosa | 2876 | + | 4 | CAAT |  |
| CAAT-box | Nicotiana glutinosa | 3388 | - | 4 | CAAT |  |
| CAAT-box | Pisum sativum | 2008 | + | 5 | CAAAT | common cis-acting element in promoter and enhancer regions |
| CAAT-box | Pisum sativum | 813 | - | 5 | CAAAT | common cis-acting element in promoter and enhancer regions |
| CAAT-box | Nicotiana glutinosa | 1883 | + | 4 | CAAT |  |
| CAAT-box | Petunia hybrida | 2066 | + | 7 | TGCCAAC | common cis-acting element in promoter and enhancer regions |
| CAAT-box | Nicotiana glutinosa | 1236 | + | 4 | CAAT |  |
| CAAT-box | Nicotiana glutinosa | 972 | + | 4 | CAAT |  |
| CAAT-box | Nicotiana glutinosa | 3266 | - | 4 | CAAT |  |
| CAAT-box | Nicotiana glutinosa | 779 | - | 4 | CAAT |  |
| CAAT-box | Nicotiana glutinosa | 737 | + | 4 | CAAT |  |
| CAAT-box | Pisum sativum | 1700 | + | 5 | CAAAT | common cis-acting element in promoter and enhancer regions |
| CAAT-box | Arabidopsis thaliana | 2789 | + | 5 | CCAAT | common cis-acting element in promoter and enhancer regions |
| CAAT-box | Arabidopsis thaliana | 672 | - | 5 | CCAAT | common cis-acting element in promoter and enhancer regions |
| CAAT-box | Nicotiana glutinosa | 2584 | + | 4 | CAAT |  |
| CAAT-box | Nicotiana glutinosa | 2155 | + | 4 | CAAT |  |
| CAAT-box | Nicotiana glutinosa | 1093 | - | 4 | CAAT |  |
| CAAT-box | Nicotiana glutinosa | 706 | - | 4 | CAAT |  |
| CAAT-box | Nicotiana glutinosa | 725 | - | 4 | CAAT |  |
| CAAT-box | Arabidopsis thaliana | 993 | + | 5 | CCAAT | common cis-acting element in promoter and enhancer regions |
| CAAT-box | Nicotiana glutinosa | 257 | + | 4 | CAAT |  |
| CAAT-box | Arabidopsis thaliana | 536 | - | 5 | CCAAT | common cis-acting element in promoter and enhancer regions |
| CAAT-box | Nicotiana glutinosa | 466 | + | 4 | CAAT |  |
| CAAT-box | Nicotiana glutinosa | 373 | - | 4 | CAAT |  |
| CAAT-box | Pisum sativum | 427 | + | 5 | CAAAT | common cis-acting element in promoter and enhancer regions |
| CAAT-box | Nicotiana glutinosa | 116 | + | 4 | CAAT |  |
| CAAT-box | Nicotiana glutinosa | 67 | + | 4 | CAAT |  |
| CAAT-box | Pisum sativum | 273 | - | 5 | CAAAT | common cis-acting element in promoter and enhancer regions |
| CAAT-box | Pisum sativum | 986 | - | 5 | CAAAT | common cis-acting element in promoter and enhancer regions |
| CAAT-box | Pisum sativum | 51 | - | 5 | CAAAT | common cis-acting element in promoter and enhancer regions |
| CAAT-box | Arabidopsis thaliana | 66 | + | 5 | CCAAT | common cis-acting element in promoter and enhancer regions |
| CAAT-box | Nicotiana glutinosa | 1716 | + | 4 | CAAT |  |
| CAAT-box | Nicotiana glutinosa | 783 | + | 4 | CAAT |  |
| CAAT-box | Nicotiana glutinosa | 1040 | + | 4 | CAAT |  |
| CAAT-box | Nicotiana glutinosa | 2790 | + | 4 | CAAT |  |
| CAAT-box | Nicotiana glutinosa | 2427 | - | 4 | CAAT |  |
| CAAT-box | Nicotiana glutinosa | 1053 | - | 4 | CAAT |  |
| CAAT-box | Nicotiana glutinosa | 994 | + | 4 | CAAT |  |
| CAAT-box | Nicotiana glutinosa | 3441 | + | 4 | CAAT |  |
| CAAT-box | Nicotiana glutinosa | 2674 | + | 4 | CAAT |  |

>HU03G01737.1   
+ +Up\_Stream \_Len000TTTTTT TTAAGTTTTC GTACTTGGTG TTGATGATTT GTGCTTTGTA ACCAATGGTT   
  
  
+ TTAGTAAATA GTAATTAGTC GTAACATTCT GCGATTTCGC TCAATTTATA TATCAACTTT AGAATATGAG   
  
  
+ TAACCGTAAA GATCAAACGT AGTAGTAGAG GTGTCATAGG TTTTTTTTTA AAAAAATTCG GAGTAAGAAA   
  
  
+ GTAGGGTACA TTATTTTTGC TTGTTAAATT CCGCTAGTAT AGCAATACGA GATGGAACAT TTGTAAAAAC   
  
  
+ TACGAGACGA AGCGTTTGTA CAAAACAAAC TACCATACTG CAGATCATAT AATTTTTTGG GGGGTTATGT   
  
  
+ ATCTTAGTGA TGACATTAAT TGATGGATAA CCCTATTCTA AATATCTGGA TCCATCATTA ACTGACTCGT   
  
  
+ TTCAAATGTT AGGATTATGG GTCTTGACAT GGTATACATG TCAATAGATT TATGTTAGAT GAAAAGTTAT   
  
  
+ ATGACGCTTT TTATGATTTT TGTACTAGCA TATAGTGCTT AATTGGACTA TATATTTTTA CAAGTTGTGC   
  
  
+ TTAACACCTT TTAATCAGAA ATATTTATTA TATGGAAAAA ATTTTATTAA TATGTGTTTA TGTGTTTAAA   
  
  
+ AAACATACCC AGCCTTGAAA TAGACCGTGA TATAGAAATT GGACCCATAA TACTCACATT ATATATGCTT   
  
  
+ TATTGTTATA ATTAAGGATC ATTGTTGTTT TGCAATTATG TGGCAAGATA TAATCCACAA AGTTTTACAT   
  
  
+ TTATATTGCA ATTACTCTGG TAAATGCTAT TAATGTACAT TTGAGATTAA GACATATATA TAACCCTTTT   
  
  
+ TTTTCCCTTC TTTTTTCCTT TTTCTATTTT GAATGGCATC TTAAACCAAA GGAGCTGTAC AAGTTACGTT   
  
  
+ ATAGCACTCA TGGTTGACTA AACAAGACAG ATGAGAGTTT AAGTCAAGTC CTCCTGGCAA TGATGTTTGC   
  
  
+ CATTTGGACC AATAAATACA TCATGTATAT TACCATAATA GTGTGGTTGA CTTAACAATA CATAGGGGAT   
  
  
+ TGCATACAAA GGTAAGCCTC AAAAACTTGT GCATATGCAT TGAAATCCAT GATCAAAAAA ATTCAAGAGC   
  
  
+ AGGTTGCACA AAAACACGGA GATGAGATGA AAGAGACAGA AAACACATAT ACATTCACAG TAGTAGGGGT   
  
  
+ ACTGGTAGAG CTATGGCTAG GCTAAGATGA TGTGAAAGAA ACAATATAAT GGTAGTAACT GGTAATATTT   
  
  
+ ATAGGGCACA TATAGGTGCG GGCATAGGGT CGTGTCTCTC AGTCTCAGGT ACGGTTCGAT GGGACTCTGC   
  
  
+ GATAGATGTT ATCAAATTCA ACCATTCGAA CATCTCCCAG AGAGAGAGAG AGAGACTGAG TGAGTGAGTG   
  
  
+ AGTGGGTGGG TGTATGTGTG TGAGTTGTTT TGTTCATTTA TTATAGGTTC TCTGCTGCAT ATATCTCCAC   
  
  
+ CATTAAATTT ACCAAAACTT GTACCAGCTG CTTTACTTGC TTTCCTCATT TTCCCCCTTT TTTGTCTCTC   
  
  
+ TCTCTCTCTC TCCCCCCCCC CCCCCCCCTC TCTCTCTGTG TCTGAATCAG TATCAGCCTA TCAGGATGCT   
  
  
+ TCGCCTCCAG AAAGGCATCA CTCACACACT TATTAGCTTA AAACTTTCTT TTATTCCTTT TGCTCTTTCA   
  
  
+ CTGCTCTCTT TTACCCAAAT ACGCCCGTCT TCAATTCTTC TCTCATTAAA TATGAAAAAG AAAGAGAGAA   
  
  
+ ATAGAGAGAG ATTTAAACCA GAAGGGGTGA TTCCTTTTTC TTGGTGGGCT TATTAATGCT CTGTCTACCC   
  
  
+ CATCTAGCCG AAAAATAGTA GCTTAAGACT CAACTGCTTA TTAGCATCTC TCCTCTAGCA ATAACCGAGG   
  
  
+ TAATAGCATA TTTTTCTCCC CTTAGCTTCT ACCTTGCTTT TTGTGACACT CAAACCTAAA CTGAGAGAGA   
  
  
+ GAAAGAGAAG ATAGAGGAGA GATAAATATT AGCGGGATTA GGGCAAATTT TGATATGCTG GGCTCTTCCT   
  
  
+ CATTCAACTC TGAGGAAGAA GATCAAGATC ATGCCAACCC ACCTCCGCCG CAACAACCAC CACCGTTACA   
  
  
+ GCTGCCACTT CCACGGCCAA GATTCAGCCA GTTAATATCA TCATCACCTC CAATAATATC AGCATCTTCT   
  
  
+ TCATCATTAA TCCATAATAA CCCTCGTCAG CTGCTAATAA GTTGCGGTGA ACTCATCTCC CGCTGTGACT   
  
  
+ TCTCCTCAGC TAACCGCCTT CTCTCTCTTC TCTCCGGCGA CTTCGCTTCC TCCTCCGGCG ACTCCACTGA   
  
  
+ ACGCCTTGTT TATTACTTCT CCAAGGCTTT GTTCCTCCGC CTTCGCCGAC AGTTTTCTCT ATCTTCTTCA   
  
  
+ ACTTTTACTG GTTATAGTTG TAACCCTAGT TGTTCTGCGT CTATTGCTCC ACTTTTTCTA ACCAACCCTA   
  
  
+ ATCCTAGACC ACCACAAGAA CTGCCGGTAC GAGTAACTAG CAGGGTTACT CCAAGTAGCT GCTCGTCTTA   
  
  
+ CCTAACCCTA AACCAGATCA CTCCATTTAT CCGGTTCACC CACCTGACGG CCAACCAGGC AATTTTGGAG   
  
  
+ GCTGTGGAGG GCTATAGGGC AGTCCACATC ATCGACATGG ACATCATGCA TGGAGTGCAG TGGCCTCCGC   
  
  
+ TCCTCCAAGC AATCGTGGAG CGGTCGGCCA CCCTTAGCCA ACCTCCCCCT GTGGTCCGTC TCAAAGGGGG   
  
  
+ AGGGCCGGAC CCGGACCTCT TACAGAGGAC GGGGGACCGA CTATGGAACT TTGCCCAATC ATTAGGACTA   
  
  
+ GAGTTCCATT TCCAGGGCCT CATGATCCCC GCCGAGTCAA CAGGTTTTGT TGAACCTGCT GAGGCAGCGG   
  
  
+ TCAATGCCCT AGCCTTGCAG GAGGCGGTGG GGTGTCAAGA AGGGGAGGCC CTTGCAGTTC ACTGCGGGGA   
  
  
+ CTATCTCCAC CGCCTCTTAA AAGAGTACGA CACAAGGCCT CTTAGACGGT TCCTCCACAA GGTCAAGACT   
  
  
+ CTGAACCCCA GGGTCTTGAC CTTGGGAGAG AGGGAAGCCG ACCACAACCA CCCTCTCTTC CTGCGGCGGT   
  
  
+ TCACCGAGGC AGTCAACCAC TACGGGGCAG TGTTTGACTC CCTGGAAGCA ACCCTTCCGC CGCATAGCCA   
  
  
+ AGAGAGGGTA GCTGTGGAGG AAGGGTGGTT CGGAGAGGAG ATTAAGGACG TGGTAGGAGA AGAAAGCGAG   
  
  
+ AGGAGAATAG AGAGGCACCA GAAGTTCGAG TCGTGGGAAG CATTGTTGAG GAGCTCAGGG TTCAAGAGTG   
  
  
+ TGCCCCTCAG CCCATTCTCG CTGTCCCAAG CTAAGCTGCT GCTGCGCCTC CATTACCCTT CCGAAGGGTA   
  
  
+ CCAACTCCAG GTTTTGCATA GTAATTGCTT GTTGCTTGGG TGGAAGAATC GCCCTCTTTT TTCTGTCTCT   
  
  
+ TCTTGGCAAT A  

- +Up\_Stream \_Len000AAAAAA AATTCAAAAG CATGAACCAC AACTACTAAA CACGAAACAT TGGTTACCAA   
  
  
- AATCATTTAT CATTAATCAG CATTGTAAGA CGCTAAAGCG AGTTAAATAT ATAGTTGAAA TCTTATACTC   
  
  
- ATTGGCATTT CTAGTTTGCA TCATCATCTC CACAGTATCC AAAAAAAAAT TTTTTTAAGC CTCATTCTTT   
  
  
- CATCCCATGT AATAAAAACG AACAATTTAA GGCGATCATA TCGTTATGCT CTACCTTGTA AACATTTTTG   
  
  
- ATGCTCTGCT TCGCAAACAT GTTTTGTTTG ATGGTATGAC GTCTAGTATA TTAAAAAACC CCCCAATACA   
  
  
- TAGAATCACT ACTGTAATTA ACTACCTATT GGGATAAGAT TTATAGACCT AGGTAGTAAT TGACTGAGCA   
  
  
- AAGTTTACAA TCCTAATACC CAGAACTGTA CCATATGTAC AGTTATCTAA ATACAATCTA CTTTTCAATA   
  
  
- TACTGCGAAA AATACTAAAA ACATGATCGT ATATCACGAA TTAACCTGAT ATATAAAAAT GTTCAACACG   
  
  
- AATTGTGGAA AATTAGTCTT TATAAATAAT ATACCTTTTT TAAAATAATT ATACACAAAT ACACAAATTT   
  
  
- TTTGTATGGG TCGGAACTTT ATCTGGCACT ATATCTTTAA CCTGGGTATT ATGAGTGTAA TATATACGAA   
  
  
- ATAACAATAT TAATTCCTAG TAACAACAAA ACGTTAATAC ACCGTTCTAT ATTAGGTGTT TCAAAATGTA   
  
  
- AATATAACGT TAATGAGACC ATTTACGATA ATTACATGTA AACTCTAATT CTGTATATAT ATTGGGAAAA   
  
  
- AAAAGGGAAG AAAAAAGGAA AAAGATAAAA CTTACCGTAG AATTTGGTTT CCTCGACATG TTCAATGCAA   
  
  
- TATCGTGAGT ACCAACTGAT TTGTTCTGTC TACTCTCAAA TTCAGTTCAG GAGGACCGTT ACTACAAACG   
  
  
- GTAAACCTGG TTATTTATGT AGTACATATA ATGGTATTAT CACACCAACT GAATTGTTAT GTATCCCCTA   
  
  
- ACGTATGTTT CCATTCGGAG TTTTTGAACA CGTATACGTA ACTTTAGGTA CTAGTTTTTT TAAGTTCTCG   
  
  
- TCCAACGTGT TTTTGTGCCT CTACTCTACT TTCTCTGTCT TTTGTGTATA TGTAAGTGTC ATCATCCCCA   
  
  
- TGACCATCTC GATACCGATC CGATTCTACT ACACTTTCTT TGTTATATTA CCATCATTGA CCATTATAAA   
  
  
- TATCCCGTGT ATATCCACGC CCGTATCCCA GCACAGAGAG TCAGAGTCCA TGCCAAGCTA CCCTGAGACG   
  
  
- CTATCTACAA TAGTTTAAGT TGGTAAGCTT GTAGAGGGTC TCTCTCTCTC TCTCTGACTC ACTCACTCAC   
  
  
- TCACCCACCC ACATACACAC ACTCAACAAA ACAAGTAAAT AATATCCAAG AGACGACGTA TATAGAGGTG   
  
  
- GTAATTTAAA TGGTTTTGAA CATGGTCGAC GAAATGAACG AAAGGAGTAA AAGGGGGAAA AAACAGAGAG   
  
  
- AGAGAGAGAG AGGGGGGGGG GGGGGGGGAG AGAGAGACAC AGACTTAGTC ATAGTCGGAT AGTCCTACGA   
  
  
- AGCGGAGGTC TTTCCGTAGT GAGTGTGTGA ATAATCGAAT TTTGAAAGAA AATAAGGAAA ACGAGAAAGT   
  
  
- GACGAGAGAA AATGGGTTTA TGCGGGCAGA AGTTAAGAAG AGAGTAATTT ATACTTTTTC TTTCTCTCTT   
  
  
- TATCTCTCTC TAAATTTGGT CTTCCCCACT AAGGAAAAAG AACCACCCGA ATAATTACGA GACAGATGGG   
  
  
- GTAGATCGGC TTTTTATCAT CGAATTCTGA GTTGACGAAT AATCGTAGAG AGGAGATCGT TATTGGCTCC   
  
  
- ATTATCGTAT AAAAAGAGGG GAATCGAAGA TGGAACGAAA AACACTGTGA GTTTGGATTT GACTCTCTCT   
  
  
- CTTTCTCTTC TATCTCCTCT CTATTTATAA TCGCCCTAAT CCCGTTTAAA ACTATACGAC CCGAGAAGGA   
  
  
- GTAAGTTGAG ACTCCTTCTT CTAGTTCTAG TACGGTTGGG TGGAGGCGGC GTTGTTGGTG GTGGCAATGT   
  
  
- CGACGGTGAA GGTGCCGGTT CTAAGTCGGT CAATTATAGT AGTAGTGGAG GTTATTATAG TCGTAGAAGA   
  
  
- AGTAGTAATT AGGTATTATT GGGAGCAGTC GACGATTATT CAACGCCACT TGAGTAGAGG GCGACACTGA   
  
  
- AGAGGAGTCG ATTGGCGGAA GAGAGAGAAG AGAGGCCGCT GAAGCGAAGG AGGAGGCCGC TGAGGTGACT   
  
  
- TGCGGAACAA ATAATGAAGA GGTTCCGAAA CAAGGAGGCG GAAGCGGCTG TCAAAAGAGA TAGAAGAAGT   
  
  
- TGAAAATGAC CAATATCAAC ATTGGGATCA ACAAGACGCA GATAACGAGG TGAAAAAGAT TGGTTGGGAT   
  
  
- TAGGATCTGG TGGTGTTCTT GACGGCCATG CTCATTGATC GTCCCAATGA GGTTCATCGA CGAGCAGAAT   
  
  
- GGATTGGGAT TTGGTCTAGT GAGGTAAATA GGCCAAGTGG GTGGACTGCC GGTTGGTCCG TTAAAACCTC   
  
  
- CGACACCTCC CGATATCCCG TCAGGTGTAG TAGCTGTACC TGTAGTACGT ACCTCACGTC ACCGGAGGCG   
  
  
- AGGAGGTTCG TTAGCACCTC GCCAGCCGGT GGGAATCGGT TGGAGGGGGA CACCAGGCAG AGTTTCCCCC   
  
  
- TCCCGGCCTG GGCCTGGAGA ATGTCTCCTG CCCCCTGGCT GATACCTTGA AACGGGTTAG TAATCCTGAT   
  
  
- CTCAAGGTAA AGGTCCCGGA GTACTAGGGG CGGCTCAGTT GTCCAAAACA ACTTGGACGA CTCCGTCGCC   
  
  
- AGTTACGGGA TCGGAACGTC CTCCGCCACC CCACAGTTCT TCCCCTCCGG GAACGTCAAG TGACGCCCCT   
  
  
- GATAGAGGTG GCGGAGAATT TTCTCATGCT GTGTTCCGGA GAATCTGCCA AGGAGGTGTT CCAGTTCTGA   
  
  
- GACTTGGGGT CCCAGAACTG GAACCCTCTC TCCCTTCGGC TGGTGTTGGT GGGAGAGAAG GACGCCGCCA   
  
  
- AGTGGCTCCG TCAGTTGGTG ATGCCCCGTC ACAAACTGAG GGACCTTCGT TGGGAAGGCG GCGTATCGGT   
  
  
- TCTCTCCCAT CGACACCTCC TTCCCACCAA GCCTCTCCTC TAATTCCTGC ACCATCCTCT TCTTTCGCTC   
  
  
- TCCTCTTATC TCTCCGTGGT CTTCAAGCTC AGCACCCTTC GTAACAACTC CTCGAGTCCC AAGTTCTCAC   
  
  
- ACGGGGAGTC GGGTAAGAGC GACAGGGTTC GATTCGACGA CGACGCGGAG GTAATGGGAA GGCTTCCCAT   
  
  
- GGTTGAGGTC CAAAACGTAT CATTAACGAA CAACGAACCC ACCTTCTTAG CGGGAGAAAA AAGACAGAGA   
  
  
- AGAACCGTTA T

+     CARE

| Site Name | Organism | Position | Strand | Matrix score. | sequence | function |
| --- | --- | --- | --- | --- | --- | --- |
| CARE | Oryza sativa | 1424 | - | 8 | CAACTCAC |  |

>HU03G01737.1   
+ +Up\_Stream \_Len000TTTTTT TTAAGTTTTC GTACTTGGTG TTGATGATTT GTGCTTTGTA ACCAATGGTT   
  
  
+ TTAGTAAATA GTAATTAGTC GTAACATTCT GCGATTTCGC TCAATTTATA TATCAACTTT AGAATATGAG   
  
  
+ TAACCGTAAA GATCAAACGT AGTAGTAGAG GTGTCATAGG TTTTTTTTTA AAAAAATTCG GAGTAAGAAA   
  
  
+ GTAGGGTACA TTATTTTTGC TTGTTAAATT CCGCTAGTAT AGCAATACGA GATGGAACAT TTGTAAAAAC   
  
  
+ TACGAGACGA AGCGTTTGTA CAAAACAAAC TACCATACTG CAGATCATAT AATTTTTTGG GGGGTTATGT   
  
  
+ ATCTTAGTGA TGACATTAAT TGATGGATAA CCCTATTCTA AATATCTGGA TCCATCATTA ACTGACTCGT   
  
  
+ TTCAAATGTT AGGATTATGG GTCTTGACAT GGTATACATG TCAATAGATT TATGTTAGAT GAAAAGTTAT   
  
  
+ ATGACGCTTT TTATGATTTT TGTACTAGCA TATAGTGCTT AATTGGACTA TATATTTTTA CAAGTTGTGC   
  
  
+ TTAACACCTT TTAATCAGAA ATATTTATTA TATGGAAAAA ATTTTATTAA TATGTGTTTA TGTGTTTAAA   
  
  
+ AAACATACCC AGCCTTGAAA TAGACCGTGA TATAGAAATT GGACCCATAA TACTCACATT ATATATGCTT   
  
  
+ TATTGTTATA ATTAAGGATC ATTGTTGTTT TGCAATTATG TGGCAAGATA TAATCCACAA AGTTTTACAT   
  
  
+ TTATATTGCA ATTACTCTGG TAAATGCTAT TAATGTACAT TTGAGATTAA GACATATATA TAACCCTTTT   
  
  
+ TTTTCCCTTC TTTTTTCCTT TTTCTATTTT GAATGGCATC TTAAACCAAA GGAGCTGTAC AAGTTACGTT   
  
  
+ ATAGCACTCA TGGTTGACTA AACAAGACAG ATGAGAGTTT AAGTCAAGTC CTCCTGGCAA TGATGTTTGC   
  
  
+ CATTTGGACC AATAAATACA TCATGTATAT TACCATAATA GTGTGGTTGA CTTAACAATA CATAGGGGAT   
  
  
+ TGCATACAAA GGTAAGCCTC AAAAACTTGT GCATATGCAT TGAAATCCAT GATCAAAAAA ATTCAAGAGC   
  
  
+ AGGTTGCACA AAAACACGGA GATGAGATGA AAGAGACAGA AAACACATAT ACATTCACAG TAGTAGGGGT   
  
  
+ ACTGGTAGAG CTATGGCTAG GCTAAGATGA TGTGAAAGAA ACAATATAAT GGTAGTAACT GGTAATATTT   
  
  
+ ATAGGGCACA TATAGGTGCG GGCATAGGGT CGTGTCTCTC AGTCTCAGGT ACGGTTCGAT GGGACTCTGC   
  
  
+ GATAGATGTT ATCAAATTCA ACCATTCGAA CATCTCCCAG AGAGAGAGAG AGAGACTGAG TGAGTGAGTG   
  
  
+ AGTGGGTGGG TGTATGTGTG TGAGTTGTTT TGTTCATTTA TTATAGGTTC TCTGCTGCAT ATATCTCCAC   
  
  
+ CATTAAATTT ACCAAAACTT GTACCAGCTG CTTTACTTGC TTTCCTCATT TTCCCCCTTT TTTGTCTCTC   
  
  
+ TCTCTCTCTC TCCCCCCCCC CCCCCCCCTC TCTCTCTGTG TCTGAATCAG TATCAGCCTA TCAGGATGCT   
  
  
+ TCGCCTCCAG AAAGGCATCA CTCACACACT TATTAGCTTA AAACTTTCTT TTATTCCTTT TGCTCTTTCA   
  
  
+ CTGCTCTCTT TTACCCAAAT ACGCCCGTCT TCAATTCTTC TCTCATTAAA TATGAAAAAG AAAGAGAGAA   
  
  
+ ATAGAGAGAG ATTTAAACCA GAAGGGGTGA TTCCTTTTTC TTGGTGGGCT TATTAATGCT CTGTCTACCC   
  
  
+ CATCTAGCCG AAAAATAGTA GCTTAAGACT CAACTGCTTA TTAGCATCTC TCCTCTAGCA ATAACCGAGG   
  
  
+ TAATAGCATA TTTTTCTCCC CTTAGCTTCT ACCTTGCTTT TTGTGACACT CAAACCTAAA CTGAGAGAGA   
  
  
+ GAAAGAGAAG ATAGAGGAGA GATAAATATT AGCGGGATTA GGGCAAATTT TGATATGCTG GGCTCTTCCT   
  
  
+ CATTCAACTC TGAGGAAGAA GATCAAGATC ATGCCAACCC ACCTCCGCCG CAACAACCAC CACCGTTACA   
  
  
+ GCTGCCACTT CCACGGCCAA GATTCAGCCA GTTAATATCA TCATCACCTC CAATAATATC AGCATCTTCT   
  
  
+ TCATCATTAA TCCATAATAA CCCTCGTCAG CTGCTAATAA GTTGCGGTGA ACTCATCTCC CGCTGTGACT   
  
  
+ TCTCCTCAGC TAACCGCCTT CTCTCTCTTC TCTCCGGCGA CTTCGCTTCC TCCTCCGGCG ACTCCACTGA   
  
  
+ ACGCCTTGTT TATTACTTCT CCAAGGCTTT GTTCCTCCGC CTTCGCCGAC AGTTTTCTCT ATCTTCTTCA   
  
  
+ ACTTTTACTG GTTATAGTTG TAACCCTAGT TGTTCTGCGT CTATTGCTCC ACTTTTTCTA ACCAACCCTA   
  
  
+ ATCCTAGACC ACCACAAGAA CTGCCGGTAC GAGTAACTAG CAGGGTTACT CCAAGTAGCT GCTCGTCTTA   
  
  
+ CCTAACCCTA AACCAGATCA CTCCATTTAT CCGGTTCACC CACCTGACGG CCAACCAGGC AATTTTGGAG   
  
  
+ GCTGTGGAGG GCTATAGGGC AGTCCACATC ATCGACATGG ACATCATGCA TGGAGTGCAG TGGCCTCCGC   
  
  
+ TCCTCCAAGC AATCGTGGAG CGGTCGGCCA CCCTTAGCCA ACCTCCCCCT GTGGTCCGTC TCAAAGGGGG   
  
  
+ AGGGCCGGAC CCGGACCTCT TACAGAGGAC GGGGGACCGA CTATGGAACT TTGCCCAATC ATTAGGACTA   
  
  
+ GAGTTCCATT TCCAGGGCCT CATGATCCCC GCCGAGTCAA CAGGTTTTGT TGAACCTGCT GAGGCAGCGG   
  
  
+ TCAATGCCCT AGCCTTGCAG GAGGCGGTGG GGTGTCAAGA AGGGGAGGCC CTTGCAGTTC ACTGCGGGGA   
  
  
+ CTATCTCCAC CGCCTCTTAA AAGAGTACGA CACAAGGCCT CTTAGACGGT TCCTCCACAA GGTCAAGACT   
  
  
+ CTGAACCCCA GGGTCTTGAC CTTGGGAGAG AGGGAAGCCG ACCACAACCA CCCTCTCTTC CTGCGGCGGT   
  
  
+ TCACCGAGGC AGTCAACCAC TACGGGGCAG TGTTTGACTC CCTGGAAGCA ACCCTTCCGC CGCATAGCCA   
  
  
+ AGAGAGGGTA GCTGTGGAGG AAGGGTGGTT CGGAGAGGAG ATTAAGGACG TGGTAGGAGA AGAAAGCGAG   
  
  
+ AGGAGAATAG AGAGGCACCA GAAGTTCGAG TCGTGGGAAG CATTGTTGAG GAGCTCAGGG TTCAAGAGTG   
  
  
+ TGCCCCTCAG CCCATTCTCG CTGTCCCAAG CTAAGCTGCT GCTGCGCCTC CATTACCCTT CCGAAGGGTA   
  
  
+ CCAACTCCAG GTTTTGCATA GTAATTGCTT GTTGCTTGGG TGGAAGAATC GCCCTCTTTT TTCTGTCTCT   
  
  
+ TCTTGGCAAT A  

- +Up\_Stream \_Len000AAAAAA AATTCAAAAG CATGAACCAC AACTACTAAA CACGAAACAT TGGTTACCAA   
  
  
- AATCATTTAT CATTAATCAG CATTGTAAGA CGCTAAAGCG AGTTAAATAT ATAGTTGAAA TCTTATACTC   
  
  
- ATTGGCATTT CTAGTTTGCA TCATCATCTC CACAGTATCC AAAAAAAAAT TTTTTTAAGC CTCATTCTTT   
  
  
- CATCCCATGT AATAAAAACG AACAATTTAA GGCGATCATA TCGTTATGCT CTACCTTGTA AACATTTTTG   
  
  
- ATGCTCTGCT TCGCAAACAT GTTTTGTTTG ATGGTATGAC GTCTAGTATA TTAAAAAACC CCCCAATACA   
  
  
- TAGAATCACT ACTGTAATTA ACTACCTATT GGGATAAGAT TTATAGACCT AGGTAGTAAT TGACTGAGCA   
  
  
- AAGTTTACAA TCCTAATACC CAGAACTGTA CCATATGTAC AGTTATCTAA ATACAATCTA CTTTTCAATA   
  
  
- TACTGCGAAA AATACTAAAA ACATGATCGT ATATCACGAA TTAACCTGAT ATATAAAAAT GTTCAACACG   
  
  
- AATTGTGGAA AATTAGTCTT TATAAATAAT ATACCTTTTT TAAAATAATT ATACACAAAT ACACAAATTT   
  
  
- TTTGTATGGG TCGGAACTTT ATCTGGCACT ATATCTTTAA CCTGGGTATT ATGAGTGTAA TATATACGAA   
  
  
- ATAACAATAT TAATTCCTAG TAACAACAAA ACGTTAATAC ACCGTTCTAT ATTAGGTGTT TCAAAATGTA   
  
  
- AATATAACGT TAATGAGACC ATTTACGATA ATTACATGTA AACTCTAATT CTGTATATAT ATTGGGAAAA   
  
  
- AAAAGGGAAG AAAAAAGGAA AAAGATAAAA CTTACCGTAG AATTTGGTTT CCTCGACATG TTCAATGCAA   
  
  
- TATCGTGAGT ACCAACTGAT TTGTTCTGTC TACTCTCAAA TTCAGTTCAG GAGGACCGTT ACTACAAACG   
  
  
- GTAAACCTGG TTATTTATGT AGTACATATA ATGGTATTAT CACACCAACT GAATTGTTAT GTATCCCCTA   
  
  
- ACGTATGTTT CCATTCGGAG TTTTTGAACA CGTATACGTA ACTTTAGGTA CTAGTTTTTT TAAGTTCTCG   
  
  
- TCCAACGTGT TTTTGTGCCT CTACTCTACT TTCTCTGTCT TTTGTGTATA TGTAAGTGTC ATCATCCCCA   
  
  
- TGACCATCTC GATACCGATC CGATTCTACT ACACTTTCTT TGTTATATTA CCATCATTGA CCATTATAAA   
  
  
- TATCCCGTGT ATATCCACGC CCGTATCCCA GCACAGAGAG TCAGAGTCCA TGCCAAGCTA CCCTGAGACG   
  
  
- CTATCTACAA TAGTTTAAGT TGGTAAGCTT GTAGAGGGTC TCTCTCTCTC TCTCTGACTC ACTCACTCAC   
  
  
- TCACCCACCC ACATACACAC ACTCAACAAA ACAAGTAAAT AATATCCAAG AGACGACGTA TATAGAGGTG   
  
  
- GTAATTTAAA TGGTTTTGAA CATGGTCGAC GAAATGAACG AAAGGAGTAA AAGGGGGAAA AAACAGAGAG   
  
  
- AGAGAGAGAG AGGGGGGGGG GGGGGGGGAG AGAGAGACAC AGACTTAGTC ATAGTCGGAT AGTCCTACGA   
  
  
- AGCGGAGGTC TTTCCGTAGT GAGTGTGTGA ATAATCGAAT TTTGAAAGAA AATAAGGAAA ACGAGAAAGT   
  
  
- GACGAGAGAA AATGGGTTTA TGCGGGCAGA AGTTAAGAAG AGAGTAATTT ATACTTTTTC TTTCTCTCTT   
  
  
- TATCTCTCTC TAAATTTGGT CTTCCCCACT AAGGAAAAAG AACCACCCGA ATAATTACGA GACAGATGGG   
  
  
- GTAGATCGGC TTTTTATCAT CGAATTCTGA GTTGACGAAT AATCGTAGAG AGGAGATCGT TATTGGCTCC   
  
  
- ATTATCGTAT AAAAAGAGGG GAATCGAAGA TGGAACGAAA AACACTGTGA GTTTGGATTT GACTCTCTCT   
  
  
- CTTTCTCTTC TATCTCCTCT CTATTTATAA TCGCCCTAAT CCCGTTTAAA ACTATACGAC CCGAGAAGGA   
  
  
- GTAAGTTGAG ACTCCTTCTT CTAGTTCTAG TACGGTTGGG TGGAGGCGGC GTTGTTGGTG GTGGCAATGT   
  
  
- CGACGGTGAA GGTGCCGGTT CTAAGTCGGT CAATTATAGT AGTAGTGGAG GTTATTATAG TCGTAGAAGA   
  
  
- AGTAGTAATT AGGTATTATT GGGAGCAGTC GACGATTATT CAACGCCACT TGAGTAGAGG GCGACACTGA   
  
  
- AGAGGAGTCG ATTGGCGGAA GAGAGAGAAG AGAGGCCGCT GAAGCGAAGG AGGAGGCCGC TGAGGTGACT   
  
  
- TGCGGAACAA ATAATGAAGA GGTTCCGAAA CAAGGAGGCG GAAGCGGCTG TCAAAAGAGA TAGAAGAAGT   
  
  
- TGAAAATGAC CAATATCAAC ATTGGGATCA ACAAGACGCA GATAACGAGG TGAAAAAGAT TGGTTGGGAT   
  
  
- TAGGATCTGG TGGTGTTCTT GACGGCCATG CTCATTGATC GTCCCAATGA GGTTCATCGA CGAGCAGAAT   
  
  
- GGATTGGGAT TTGGTCTAGT GAGGTAAATA GGCCAAGTGG GTGGACTGCC GGTTGGTCCG TTAAAACCTC   
  
  
- CGACACCTCC CGATATCCCG TCAGGTGTAG TAGCTGTACC TGTAGTACGT ACCTCACGTC ACCGGAGGCG   
  
  
- AGGAGGTTCG TTAGCACCTC GCCAGCCGGT GGGAATCGGT TGGAGGGGGA CACCAGGCAG AGTTTCCCCC   
  
  
- TCCCGGCCTG GGCCTGGAGA ATGTCTCCTG CCCCCTGGCT GATACCTTGA AACGGGTTAG TAATCCTGAT   
  
  
- CTCAAGGTAA AGGTCCCGGA GTACTAGGGG CGGCTCAGTT GTCCAAAACA ACTTGGACGA CTCCGTCGCC   
  
  
- AGTTACGGGA TCGGAACGTC CTCCGCCACC CCACAGTTCT TCCCCTCCGG GAACGTCAAG TGACGCCCCT   
  
  
- GATAGAGGTG GCGGAGAATT TTCTCATGCT GTGTTCCGGA GAATCTGCCA AGGAGGTGTT CCAGTTCTGA   
  
  
- GACTTGGGGT CCCAGAACTG GAACCCTCTC TCCCTTCGGC TGGTGTTGGT GGGAGAGAAG GACGCCGCCA   
  
  
- AGTGGCTCCG TCAGTTGGTG ATGCCCCGTC ACAAACTGAG GGACCTTCGT TGGGAAGGCG GCGTATCGGT   
  
  
- TCTCTCCCAT CGACACCTCC TTCCCACCAA GCCTCTCCTC TAATTCCTGC ACCATCCTCT TCTTTCGCTC   
  
  
- TCCTCTTATC TCTCCGTGGT CTTCAAGCTC AGCACCCTTC GTAACAACTC CTCGAGTCCC AAGTTCTCAC   
  
  
- ACGGGGAGTC GGGTAAGAGC GACAGGGTTC GATTCGACGA CGACGCGGAG GTAATGGGAA GGCTTCCCAT   
  
  
- GGTTGAGGTC CAAAACGTAT CATTAACGAA CAACGAACCC ACCTTCTTAG CGGGAGAAAA AAGACAGAGA   
  
  
- AGAACCGTTA T

+     CAT-box

| Site Name | Organism | Position | Strand | Matrix score. | sequence | function |
| --- | --- | --- | --- | --- | --- | --- |
| CAT-box | Arabidopsis thaliana | 2108 | + | 6 | GCCACT | cis-acting regulatory element related to meristem expression |
| CAT-box | Arabidopsis thaliana | 2653 | - | 6 | GCCACT | cis-acting regulatory element related to meristem expression |

>HU03G01737.1   
+ +Up\_Stream \_Len000TTTTTT TTAAGTTTTC GTACTTGGTG TTGATGATTT GTGCTTTGTA ACCAATGGTT   
  
  
+ TTAGTAAATA GTAATTAGTC GTAACATTCT GCGATTTCGC TCAATTTATA TATCAACTTT AGAATATGAG   
  
  
+ TAACCGTAAA GATCAAACGT AGTAGTAGAG GTGTCATAGG TTTTTTTTTA AAAAAATTCG GAGTAAGAAA   
  
  
+ GTAGGGTACA TTATTTTTGC TTGTTAAATT CCGCTAGTAT AGCAATACGA GATGGAACAT TTGTAAAAAC   
  
  
+ TACGAGACGA AGCGTTTGTA CAAAACAAAC TACCATACTG CAGATCATAT AATTTTTTGG GGGGTTATGT   
  
  
+ ATCTTAGTGA TGACATTAAT TGATGGATAA CCCTATTCTA AATATCTGGA TCCATCATTA ACTGACTCGT   
  
  
+ TTCAAATGTT AGGATTATGG GTCTTGACAT GGTATACATG TCAATAGATT TATGTTAGAT GAAAAGTTAT   
  
  
+ ATGACGCTTT TTATGATTTT TGTACTAGCA TATAGTGCTT AATTGGACTA TATATTTTTA CAAGTTGTGC   
  
  
+ TTAACACCTT TTAATCAGAA ATATTTATTA TATGGAAAAA ATTTTATTAA TATGTGTTTA TGTGTTTAAA   
  
  
+ AAACATACCC AGCCTTGAAA TAGACCGTGA TATAGAAATT GGACCCATAA TACTCACATT ATATATGCTT   
  
  
+ TATTGTTATA ATTAAGGATC ATTGTTGTTT TGCAATTATG TGGCAAGATA TAATCCACAA AGTTTTACAT   
  
  
+ TTATATTGCA ATTACTCTGG TAAATGCTAT TAATGTACAT TTGAGATTAA GACATATATA TAACCCTTTT   
  
  
+ TTTTCCCTTC TTTTTTCCTT TTTCTATTTT GAATGGCATC TTAAACCAAA GGAGCTGTAC AAGTTACGTT   
  
  
+ ATAGCACTCA TGGTTGACTA AACAAGACAG ATGAGAGTTT AAGTCAAGTC CTCCTGGCAA TGATGTTTGC   
  
  
+ CATTTGGACC AATAAATACA TCATGTATAT TACCATAATA GTGTGGTTGA CTTAACAATA CATAGGGGAT   
  
  
+ TGCATACAAA GGTAAGCCTC AAAAACTTGT GCATATGCAT TGAAATCCAT GATCAAAAAA ATTCAAGAGC   
  
  
+ AGGTTGCACA AAAACACGGA GATGAGATGA AAGAGACAGA AAACACATAT ACATTCACAG TAGTAGGGGT   
  
  
+ ACTGGTAGAG CTATGGCTAG GCTAAGATGA TGTGAAAGAA ACAATATAAT GGTAGTAACT GGTAATATTT   
  
  
+ ATAGGGCACA TATAGGTGCG GGCATAGGGT CGTGTCTCTC AGTCTCAGGT ACGGTTCGAT GGGACTCTGC   
  
  
+ GATAGATGTT ATCAAATTCA ACCATTCGAA CATCTCCCAG AGAGAGAGAG AGAGACTGAG TGAGTGAGTG   
  
  
+ AGTGGGTGGG TGTATGTGTG TGAGTTGTTT TGTTCATTTA TTATAGGTTC TCTGCTGCAT ATATCTCCAC   
  
  
+ CATTAAATTT ACCAAAACTT GTACCAGCTG CTTTACTTGC TTTCCTCATT TTCCCCCTTT TTTGTCTCTC   
  
  
+ TCTCTCTCTC TCCCCCCCCC CCCCCCCCTC TCTCTCTGTG TCTGAATCAG TATCAGCCTA TCAGGATGCT   
  
  
+ TCGCCTCCAG AAAGGCATCA CTCACACACT TATTAGCTTA AAACTTTCTT TTATTCCTTT TGCTCTTTCA   
  
  
+ CTGCTCTCTT TTACCCAAAT ACGCCCGTCT TCAATTCTTC TCTCATTAAA TATGAAAAAG AAAGAGAGAA   
  
  
+ ATAGAGAGAG ATTTAAACCA GAAGGGGTGA TTCCTTTTTC TTGGTGGGCT TATTAATGCT CTGTCTACCC   
  
  
+ CATCTAGCCG AAAAATAGTA GCTTAAGACT CAACTGCTTA TTAGCATCTC TCCTCTAGCA ATAACCGAGG   
  
  
+ TAATAGCATA TTTTTCTCCC CTTAGCTTCT ACCTTGCTTT TTGTGACACT CAAACCTAAA CTGAGAGAGA   
  
  
+ GAAAGAGAAG ATAGAGGAGA GATAAATATT AGCGGGATTA GGGCAAATTT TGATATGCTG GGCTCTTCCT   
  
  
+ CATTCAACTC TGAGGAAGAA GATCAAGATC ATGCCAACCC ACCTCCGCCG CAACAACCAC CACCGTTACA   
  
  
+ GCTGCCACTT CCACGGCCAA GATTCAGCCA GTTAATATCA TCATCACCTC CAATAATATC AGCATCTTCT   
  
  
+ TCATCATTAA TCCATAATAA CCCTCGTCAG CTGCTAATAA GTTGCGGTGA ACTCATCTCC CGCTGTGACT   
  
  
+ TCTCCTCAGC TAACCGCCTT CTCTCTCTTC TCTCCGGCGA CTTCGCTTCC TCCTCCGGCG ACTCCACTGA   
  
  
+ ACGCCTTGTT TATTACTTCT CCAAGGCTTT GTTCCTCCGC CTTCGCCGAC AGTTTTCTCT ATCTTCTTCA   
  
  
+ ACTTTTACTG GTTATAGTTG TAACCCTAGT TGTTCTGCGT CTATTGCTCC ACTTTTTCTA ACCAACCCTA   
  
  
+ ATCCTAGACC ACCACAAGAA CTGCCGGTAC GAGTAACTAG CAGGGTTACT CCAAGTAGCT GCTCGTCTTA   
  
  
+ CCTAACCCTA AACCAGATCA CTCCATTTAT CCGGTTCACC CACCTGACGG CCAACCAGGC AATTTTGGAG   
  
  
+ GCTGTGGAGG GCTATAGGGC AGTCCACATC ATCGACATGG ACATCATGCA TGGAGTGCAG TGGCCTCCGC   
  
  
+ TCCTCCAAGC AATCGTGGAG CGGTCGGCCA CCCTTAGCCA ACCTCCCCCT GTGGTCCGTC TCAAAGGGGG   
  
  
+ AGGGCCGGAC CCGGACCTCT TACAGAGGAC GGGGGACCGA CTATGGAACT TTGCCCAATC ATTAGGACTA   
  
  
+ GAGTTCCATT TCCAGGGCCT CATGATCCCC GCCGAGTCAA CAGGTTTTGT TGAACCTGCT GAGGCAGCGG   
  
  
+ TCAATGCCCT AGCCTTGCAG GAGGCGGTGG GGTGTCAAGA AGGGGAGGCC CTTGCAGTTC ACTGCGGGGA   
  
  
+ CTATCTCCAC CGCCTCTTAA AAGAGTACGA CACAAGGCCT CTTAGACGGT TCCTCCACAA GGTCAAGACT   
  
  
+ CTGAACCCCA GGGTCTTGAC CTTGGGAGAG AGGGAAGCCG ACCACAACCA CCCTCTCTTC CTGCGGCGGT   
  
  
+ TCACCGAGGC AGTCAACCAC TACGGGGCAG TGTTTGACTC CCTGGAAGCA ACCCTTCCGC CGCATAGCCA   
  
  
+ AGAGAGGGTA GCTGTGGAGG AAGGGTGGTT CGGAGAGGAG ATTAAGGACG TGGTAGGAGA AGAAAGCGAG   
  
  
+ AGGAGAATAG AGAGGCACCA GAAGTTCGAG TCGTGGGAAG CATTGTTGAG GAGCTCAGGG TTCAAGAGTG   
  
  
+ TGCCCCTCAG CCCATTCTCG CTGTCCCAAG CTAAGCTGCT GCTGCGCCTC CATTACCCTT CCGAAGGGTA   
  
  
+ CCAACTCCAG GTTTTGCATA GTAATTGCTT GTTGCTTGGG TGGAAGAATC GCCCTCTTTT TTCTGTCTCT   
  
  
+ TCTTGGCAAT A  

- +Up\_Stream \_Len000AAAAAA AATTCAAAAG CATGAACCAC AACTACTAAA CACGAAACAT TGGTTACCAA   
  
  
- AATCATTTAT CATTAATCAG CATTGTAAGA CGCTAAAGCG AGTTAAATAT ATAGTTGAAA TCTTATACTC   
  
  
- ATTGGCATTT CTAGTTTGCA TCATCATCTC CACAGTATCC AAAAAAAAAT TTTTTTAAGC CTCATTCTTT   
  
  
- CATCCCATGT AATAAAAACG AACAATTTAA GGCGATCATA TCGTTATGCT CTACCTTGTA AACATTTTTG   
  
  
- ATGCTCTGCT TCGCAAACAT GTTTTGTTTG ATGGTATGAC GTCTAGTATA TTAAAAAACC CCCCAATACA   
  
  
- TAGAATCACT ACTGTAATTA ACTACCTATT GGGATAAGAT TTATAGACCT AGGTAGTAAT TGACTGAGCA   
  
  
- AAGTTTACAA TCCTAATACC CAGAACTGTA CCATATGTAC AGTTATCTAA ATACAATCTA CTTTTCAATA   
  
  
- TACTGCGAAA AATACTAAAA ACATGATCGT ATATCACGAA TTAACCTGAT ATATAAAAAT GTTCAACACG   
  
  
- AATTGTGGAA AATTAGTCTT TATAAATAAT ATACCTTTTT TAAAATAATT ATACACAAAT ACACAAATTT   
  
  
- TTTGTATGGG TCGGAACTTT ATCTGGCACT ATATCTTTAA CCTGGGTATT ATGAGTGTAA TATATACGAA   
  
  
- ATAACAATAT TAATTCCTAG TAACAACAAA ACGTTAATAC ACCGTTCTAT ATTAGGTGTT TCAAAATGTA   
  
  
- AATATAACGT TAATGAGACC ATTTACGATA ATTACATGTA AACTCTAATT CTGTATATAT ATTGGGAAAA   
  
  
- AAAAGGGAAG AAAAAAGGAA AAAGATAAAA CTTACCGTAG AATTTGGTTT CCTCGACATG TTCAATGCAA   
  
  
- TATCGTGAGT ACCAACTGAT TTGTTCTGTC TACTCTCAAA TTCAGTTCAG GAGGACCGTT ACTACAAACG   
  
  
- GTAAACCTGG TTATTTATGT AGTACATATA ATGGTATTAT CACACCAACT GAATTGTTAT GTATCCCCTA   
  
  
- ACGTATGTTT CCATTCGGAG TTTTTGAACA CGTATACGTA ACTTTAGGTA CTAGTTTTTT TAAGTTCTCG   
  
  
- TCCAACGTGT TTTTGTGCCT CTACTCTACT TTCTCTGTCT TTTGTGTATA TGTAAGTGTC ATCATCCCCA   
  
  
- TGACCATCTC GATACCGATC CGATTCTACT ACACTTTCTT TGTTATATTA CCATCATTGA CCATTATAAA   
  
  
- TATCCCGTGT ATATCCACGC CCGTATCCCA GCACAGAGAG TCAGAGTCCA TGCCAAGCTA CCCTGAGACG   
  
  
- CTATCTACAA TAGTTTAAGT TGGTAAGCTT GTAGAGGGTC TCTCTCTCTC TCTCTGACTC ACTCACTCAC   
  
  
- TCACCCACCC ACATACACAC ACTCAACAAA ACAAGTAAAT AATATCCAAG AGACGACGTA TATAGAGGTG   
  
  
- GTAATTTAAA TGGTTTTGAA CATGGTCGAC GAAATGAACG AAAGGAGTAA AAGGGGGAAA AAACAGAGAG   
  
  
- AGAGAGAGAG AGGGGGGGGG GGGGGGGGAG AGAGAGACAC AGACTTAGTC ATAGTCGGAT AGTCCTACGA   
  
  
- AGCGGAGGTC TTTCCGTAGT GAGTGTGTGA ATAATCGAAT TTTGAAAGAA AATAAGGAAA ACGAGAAAGT   
  
  
- GACGAGAGAA AATGGGTTTA TGCGGGCAGA AGTTAAGAAG AGAGTAATTT ATACTTTTTC TTTCTCTCTT   
  
  
- TATCTCTCTC TAAATTTGGT CTTCCCCACT AAGGAAAAAG AACCACCCGA ATAATTACGA GACAGATGGG   
  
  
- GTAGATCGGC TTTTTATCAT CGAATTCTGA GTTGACGAAT AATCGTAGAG AGGAGATCGT TATTGGCTCC   
  
  
- ATTATCGTAT AAAAAGAGGG GAATCGAAGA TGGAACGAAA AACACTGTGA GTTTGGATTT GACTCTCTCT   
  
  
- CTTTCTCTTC TATCTCCTCT CTATTTATAA TCGCCCTAAT CCCGTTTAAA ACTATACGAC CCGAGAAGGA   
  
  
- GTAAGTTGAG ACTCCTTCTT CTAGTTCTAG TACGGTTGGG TGGAGGCGGC GTTGTTGGTG GTGGCAATGT   
  
  
- CGACGGTGAA GGTGCCGGTT CTAAGTCGGT CAATTATAGT AGTAGTGGAG GTTATTATAG TCGTAGAAGA   
  
  
- AGTAGTAATT AGGTATTATT GGGAGCAGTC GACGATTATT CAACGCCACT TGAGTAGAGG GCGACACTGA   
  
  
- AGAGGAGTCG ATTGGCGGAA GAGAGAGAAG AGAGGCCGCT GAAGCGAAGG AGGAGGCCGC TGAGGTGACT   
  
  
- TGCGGAACAA ATAATGAAGA GGTTCCGAAA CAAGGAGGCG GAAGCGGCTG TCAAAAGAGA TAGAAGAAGT   
  
  
- TGAAAATGAC CAATATCAAC ATTGGGATCA ACAAGACGCA GATAACGAGG TGAAAAAGAT TGGTTGGGAT   
  
  
- TAGGATCTGG TGGTGTTCTT GACGGCCATG CTCATTGATC GTCCCAATGA GGTTCATCGA CGAGCAGAAT   
  
  
- GGATTGGGAT TTGGTCTAGT GAGGTAAATA GGCCAAGTGG GTGGACTGCC GGTTGGTCCG TTAAAACCTC   
  
  
- CGACACCTCC CGATATCCCG TCAGGTGTAG TAGCTGTACC TGTAGTACGT ACCTCACGTC ACCGGAGGCG   
  
  
- AGGAGGTTCG TTAGCACCTC GCCAGCCGGT GGGAATCGGT TGGAGGGGGA CACCAGGCAG AGTTTCCCCC   
  
  
- TCCCGGCCTG GGCCTGGAGA ATGTCTCCTG CCCCCTGGCT GATACCTTGA AACGGGTTAG TAATCCTGAT   
  
  
- CTCAAGGTAA AGGTCCCGGA GTACTAGGGG CGGCTCAGTT GTCCAAAACA ACTTGGACGA CTCCGTCGCC   
  
  
- AGTTACGGGA TCGGAACGTC CTCCGCCACC CCACAGTTCT TCCCCTCCGG GAACGTCAAG TGACGCCCCT   
  
  
- GATAGAGGTG GCGGAGAATT TTCTCATGCT GTGTTCCGGA GAATCTGCCA AGGAGGTGTT CCAGTTCTGA   
  
  
- GACTTGGGGT CCCAGAACTG GAACCCTCTC TCCCTTCGGC TGGTGTTGGT GGGAGAGAAG GACGCCGCCA   
  
  
- AGTGGCTCCG TCAGTTGGTG ATGCCCCGTC ACAAACTGAG GGACCTTCGT TGGGAAGGCG GCGTATCGGT   
  
  
- TCTCTCCCAT CGACACCTCC TTCCCACCAA GCCTCTCCTC TAATTCCTGC ACCATCCTCT TCTTTCGCTC   
  
  
- TCCTCTTATC TCTCCGTGGT CTTCAAGCTC AGCACCCTTC GTAACAACTC CTCGAGTCCC AAGTTCTCAC   
  
  
- ACGGGGAGTC GGGTAAGAGC GACAGGGTTC GATTCGACGA CGACGCGGAG GTAATGGGAA GGCTTCCCAT   
  
  
- GGTTGAGGTC CAAAACGTAT CATTAACGAA CAACGAACCC ACCTTCTTAG CGGGAGAAAA AAGACAGAGA   
  
  
- AGAACCGTTA T

+     CCGTCC motif

| Site Name | Organism | Position | Strand | Matrix score. | sequence | function |
| --- | --- | --- | --- | --- | --- | --- |
| CCGTCC motif | Nicotiana tabacum | 2761 | - | 6 | CCGTCC |  |

>HU03G01737.1   
+ +Up\_Stream \_Len000TTTTTT TTAAGTTTTC GTACTTGGTG TTGATGATTT GTGCTTTGTA ACCAATGGTT   
  
  
+ TTAGTAAATA GTAATTAGTC GTAACATTCT GCGATTTCGC TCAATTTATA TATCAACTTT AGAATATGAG   
  
  
+ TAACCGTAAA GATCAAACGT AGTAGTAGAG GTGTCATAGG TTTTTTTTTA AAAAAATTCG GAGTAAGAAA   
  
  
+ GTAGGGTACA TTATTTTTGC TTGTTAAATT CCGCTAGTAT AGCAATACGA GATGGAACAT TTGTAAAAAC   
  
  
+ TACGAGACGA AGCGTTTGTA CAAAACAAAC TACCATACTG CAGATCATAT AATTTTTTGG GGGGTTATGT   
  
  
+ ATCTTAGTGA TGACATTAAT TGATGGATAA CCCTATTCTA AATATCTGGA TCCATCATTA ACTGACTCGT   
  
  
+ TTCAAATGTT AGGATTATGG GTCTTGACAT GGTATACATG TCAATAGATT TATGTTAGAT GAAAAGTTAT   
  
  
+ ATGACGCTTT TTATGATTTT TGTACTAGCA TATAGTGCTT AATTGGACTA TATATTTTTA CAAGTTGTGC   
  
  
+ TTAACACCTT TTAATCAGAA ATATTTATTA TATGGAAAAA ATTTTATTAA TATGTGTTTA TGTGTTTAAA   
  
  
+ AAACATACCC AGCCTTGAAA TAGACCGTGA TATAGAAATT GGACCCATAA TACTCACATT ATATATGCTT   
  
  
+ TATTGTTATA ATTAAGGATC ATTGTTGTTT TGCAATTATG TGGCAAGATA TAATCCACAA AGTTTTACAT   
  
  
+ TTATATTGCA ATTACTCTGG TAAATGCTAT TAATGTACAT TTGAGATTAA GACATATATA TAACCCTTTT   
  
  
+ TTTTCCCTTC TTTTTTCCTT TTTCTATTTT GAATGGCATC TTAAACCAAA GGAGCTGTAC AAGTTACGTT   
  
  
+ ATAGCACTCA TGGTTGACTA AACAAGACAG ATGAGAGTTT AAGTCAAGTC CTCCTGGCAA TGATGTTTGC   
  
  
+ CATTTGGACC AATAAATACA TCATGTATAT TACCATAATA GTGTGGTTGA CTTAACAATA CATAGGGGAT   
  
  
+ TGCATACAAA GGTAAGCCTC AAAAACTTGT GCATATGCAT TGAAATCCAT GATCAAAAAA ATTCAAGAGC   
  
  
+ AGGTTGCACA AAAACACGGA GATGAGATGA AAGAGACAGA AAACACATAT ACATTCACAG TAGTAGGGGT   
  
  
+ ACTGGTAGAG CTATGGCTAG GCTAAGATGA TGTGAAAGAA ACAATATAAT GGTAGTAACT GGTAATATTT   
  
  
+ ATAGGGCACA TATAGGTGCG GGCATAGGGT CGTGTCTCTC AGTCTCAGGT ACGGTTCGAT GGGACTCTGC   
  
  
+ GATAGATGTT ATCAAATTCA ACCATTCGAA CATCTCCCAG AGAGAGAGAG AGAGACTGAG TGAGTGAGTG   
  
  
+ AGTGGGTGGG TGTATGTGTG TGAGTTGTTT TGTTCATTTA TTATAGGTTC TCTGCTGCAT ATATCTCCAC   
  
  
+ CATTAAATTT ACCAAAACTT GTACCAGCTG CTTTACTTGC TTTCCTCATT TTCCCCCTTT TTTGTCTCTC   
  
  
+ TCTCTCTCTC TCCCCCCCCC CCCCCCCCTC TCTCTCTGTG TCTGAATCAG TATCAGCCTA TCAGGATGCT   
  
  
+ TCGCCTCCAG AAAGGCATCA CTCACACACT TATTAGCTTA AAACTTTCTT TTATTCCTTT TGCTCTTTCA   
  
  
+ CTGCTCTCTT TTACCCAAAT ACGCCCGTCT TCAATTCTTC TCTCATTAAA TATGAAAAAG AAAGAGAGAA   
  
  
+ ATAGAGAGAG ATTTAAACCA GAAGGGGTGA TTCCTTTTTC TTGGTGGGCT TATTAATGCT CTGTCTACCC   
  
  
+ CATCTAGCCG AAAAATAGTA GCTTAAGACT CAACTGCTTA TTAGCATCTC TCCTCTAGCA ATAACCGAGG   
  
  
+ TAATAGCATA TTTTTCTCCC CTTAGCTTCT ACCTTGCTTT TTGTGACACT CAAACCTAAA CTGAGAGAGA   
  
  
+ GAAAGAGAAG ATAGAGGAGA GATAAATATT AGCGGGATTA GGGCAAATTT TGATATGCTG GGCTCTTCCT   
  
  
+ CATTCAACTC TGAGGAAGAA GATCAAGATC ATGCCAACCC ACCTCCGCCG CAACAACCAC CACCGTTACA   
  
  
+ GCTGCCACTT CCACGGCCAA GATTCAGCCA GTTAATATCA TCATCACCTC CAATAATATC AGCATCTTCT   
  
  
+ TCATCATTAA TCCATAATAA CCCTCGTCAG CTGCTAATAA GTTGCGGTGA ACTCATCTCC CGCTGTGACT   
  
  
+ TCTCCTCAGC TAACCGCCTT CTCTCTCTTC TCTCCGGCGA CTTCGCTTCC TCCTCCGGCG ACTCCACTGA   
  
  
+ ACGCCTTGTT TATTACTTCT CCAAGGCTTT GTTCCTCCGC CTTCGCCGAC AGTTTTCTCT ATCTTCTTCA   
  
  
+ ACTTTTACTG GTTATAGTTG TAACCCTAGT TGTTCTGCGT CTATTGCTCC ACTTTTTCTA ACCAACCCTA   
  
  
+ ATCCTAGACC ACCACAAGAA CTGCCGGTAC GAGTAACTAG CAGGGTTACT CCAAGTAGCT GCTCGTCTTA   
  
  
+ CCTAACCCTA AACCAGATCA CTCCATTTAT CCGGTTCACC CACCTGACGG CCAACCAGGC AATTTTGGAG   
  
  
+ GCTGTGGAGG GCTATAGGGC AGTCCACATC ATCGACATGG ACATCATGCA TGGAGTGCAG TGGCCTCCGC   
  
  
+ TCCTCCAAGC AATCGTGGAG CGGTCGGCCA CCCTTAGCCA ACCTCCCCCT GTGGTCCGTC TCAAAGGGGG   
  
  
+ AGGGCCGGAC CCGGACCTCT TACAGAGGAC GGGGGACCGA CTATGGAACT TTGCCCAATC ATTAGGACTA   
  
  
+ GAGTTCCATT TCCAGGGCCT CATGATCCCC GCCGAGTCAA CAGGTTTTGT TGAACCTGCT GAGGCAGCGG   
  
  
+ TCAATGCCCT AGCCTTGCAG GAGGCGGTGG GGTGTCAAGA AGGGGAGGCC CTTGCAGTTC ACTGCGGGGA   
  
  
+ CTATCTCCAC CGCCTCTTAA AAGAGTACGA CACAAGGCCT CTTAGACGGT TCCTCCACAA GGTCAAGACT   
  
  
+ CTGAACCCCA GGGTCTTGAC CTTGGGAGAG AGGGAAGCCG ACCACAACCA CCCTCTCTTC CTGCGGCGGT   
  
  
+ TCACCGAGGC AGTCAACCAC TACGGGGCAG TGTTTGACTC CCTGGAAGCA ACCCTTCCGC CGCATAGCCA   
  
  
+ AGAGAGGGTA GCTGTGGAGG AAGGGTGGTT CGGAGAGGAG ATTAAGGACG TGGTAGGAGA AGAAAGCGAG   
  
  
+ AGGAGAATAG AGAGGCACCA GAAGTTCGAG TCGTGGGAAG CATTGTTGAG GAGCTCAGGG TTCAAGAGTG   
  
  
+ TGCCCCTCAG CCCATTCTCG CTGTCCCAAG CTAAGCTGCT GCTGCGCCTC CATTACCCTT CCGAAGGGTA   
  
  
+ CCAACTCCAG GTTTTGCATA GTAATTGCTT GTTGCTTGGG TGGAAGAATC GCCCTCTTTT TTCTGTCTCT   
  
  
+ TCTTGGCAAT A  

- +Up\_Stream \_Len000AAAAAA AATTCAAAAG CATGAACCAC AACTACTAAA CACGAAACAT TGGTTACCAA   
  
  
- AATCATTTAT CATTAATCAG CATTGTAAGA CGCTAAAGCG AGTTAAATAT ATAGTTGAAA TCTTATACTC   
  
  
- ATTGGCATTT CTAGTTTGCA TCATCATCTC CACAGTATCC AAAAAAAAAT TTTTTTAAGC CTCATTCTTT   
  
  
- CATCCCATGT AATAAAAACG AACAATTTAA GGCGATCATA TCGTTATGCT CTACCTTGTA AACATTTTTG   
  
  
- ATGCTCTGCT TCGCAAACAT GTTTTGTTTG ATGGTATGAC GTCTAGTATA TTAAAAAACC CCCCAATACA   
  
  
- TAGAATCACT ACTGTAATTA ACTACCTATT GGGATAAGAT TTATAGACCT AGGTAGTAAT TGACTGAGCA   
  
  
- AAGTTTACAA TCCTAATACC CAGAACTGTA CCATATGTAC AGTTATCTAA ATACAATCTA CTTTTCAATA   
  
  
- TACTGCGAAA AATACTAAAA ACATGATCGT ATATCACGAA TTAACCTGAT ATATAAAAAT GTTCAACACG   
  
  
- AATTGTGGAA AATTAGTCTT TATAAATAAT ATACCTTTTT TAAAATAATT ATACACAAAT ACACAAATTT   
  
  
- TTTGTATGGG TCGGAACTTT ATCTGGCACT ATATCTTTAA CCTGGGTATT ATGAGTGTAA TATATACGAA   
  
  
- ATAACAATAT TAATTCCTAG TAACAACAAA ACGTTAATAC ACCGTTCTAT ATTAGGTGTT TCAAAATGTA   
  
  
- AATATAACGT TAATGAGACC ATTTACGATA ATTACATGTA AACTCTAATT CTGTATATAT ATTGGGAAAA   
  
  
- AAAAGGGAAG AAAAAAGGAA AAAGATAAAA CTTACCGTAG AATTTGGTTT CCTCGACATG TTCAATGCAA   
  
  
- TATCGTGAGT ACCAACTGAT TTGTTCTGTC TACTCTCAAA TTCAGTTCAG GAGGACCGTT ACTACAAACG   
  
  
- GTAAACCTGG TTATTTATGT AGTACATATA ATGGTATTAT CACACCAACT GAATTGTTAT GTATCCCCTA   
  
  
- ACGTATGTTT CCATTCGGAG TTTTTGAACA CGTATACGTA ACTTTAGGTA CTAGTTTTTT TAAGTTCTCG   
  
  
- TCCAACGTGT TTTTGTGCCT CTACTCTACT TTCTCTGTCT TTTGTGTATA TGTAAGTGTC ATCATCCCCA   
  
  
- TGACCATCTC GATACCGATC CGATTCTACT ACACTTTCTT TGTTATATTA CCATCATTGA CCATTATAAA   
  
  
- TATCCCGTGT ATATCCACGC CCGTATCCCA GCACAGAGAG TCAGAGTCCA TGCCAAGCTA CCCTGAGACG   
  
  
- CTATCTACAA TAGTTTAAGT TGGTAAGCTT GTAGAGGGTC TCTCTCTCTC TCTCTGACTC ACTCACTCAC   
  
  
- TCACCCACCC ACATACACAC ACTCAACAAA ACAAGTAAAT AATATCCAAG AGACGACGTA TATAGAGGTG   
  
  
- GTAATTTAAA TGGTTTTGAA CATGGTCGAC GAAATGAACG AAAGGAGTAA AAGGGGGAAA AAACAGAGAG   
  
  
- AGAGAGAGAG AGGGGGGGGG GGGGGGGGAG AGAGAGACAC AGACTTAGTC ATAGTCGGAT AGTCCTACGA   
  
  
- AGCGGAGGTC TTTCCGTAGT GAGTGTGTGA ATAATCGAAT TTTGAAAGAA AATAAGGAAA ACGAGAAAGT   
  
  
- GACGAGAGAA AATGGGTTTA TGCGGGCAGA AGTTAAGAAG AGAGTAATTT ATACTTTTTC TTTCTCTCTT   
  
  
- TATCTCTCTC TAAATTTGGT CTTCCCCACT AAGGAAAAAG AACCACCCGA ATAATTACGA GACAGATGGG   
  
  
- GTAGATCGGC TTTTTATCAT CGAATTCTGA GTTGACGAAT AATCGTAGAG AGGAGATCGT TATTGGCTCC   
  
  
- ATTATCGTAT AAAAAGAGGG GAATCGAAGA TGGAACGAAA AACACTGTGA GTTTGGATTT GACTCTCTCT   
  
  
- CTTTCTCTTC TATCTCCTCT CTATTTATAA TCGCCCTAAT CCCGTTTAAA ACTATACGAC CCGAGAAGGA   
  
  
- GTAAGTTGAG ACTCCTTCTT CTAGTTCTAG TACGGTTGGG TGGAGGCGGC GTTGTTGGTG GTGGCAATGT   
  
  
- CGACGGTGAA GGTGCCGGTT CTAAGTCGGT CAATTATAGT AGTAGTGGAG GTTATTATAG TCGTAGAAGA   
  
  
- AGTAGTAATT AGGTATTATT GGGAGCAGTC GACGATTATT CAACGCCACT TGAGTAGAGG GCGACACTGA   
  
  
- AGAGGAGTCG ATTGGCGGAA GAGAGAGAAG AGAGGCCGCT GAAGCGAAGG AGGAGGCCGC TGAGGTGACT   
  
  
- TGCGGAACAA ATAATGAAGA GGTTCCGAAA CAAGGAGGCG GAAGCGGCTG TCAAAAGAGA TAGAAGAAGT   
  
  
- TGAAAATGAC CAATATCAAC ATTGGGATCA ACAAGACGCA GATAACGAGG TGAAAAAGAT TGGTTGGGAT   
  
  
- TAGGATCTGG TGGTGTTCTT GACGGCCATG CTCATTGATC GTCCCAATGA GGTTCATCGA CGAGCAGAAT   
  
  
- GGATTGGGAT TTGGTCTAGT GAGGTAAATA GGCCAAGTGG GTGGACTGCC GGTTGGTCCG TTAAAACCTC   
  
  
- CGACACCTCC CGATATCCCG TCAGGTGTAG TAGCTGTACC TGTAGTACGT ACCTCACGTC ACCGGAGGCG   
  
  
- AGGAGGTTCG TTAGCACCTC GCCAGCCGGT GGGAATCGGT TGGAGGGGGA CACCAGGCAG AGTTTCCCCC   
  
  
- TCCCGGCCTG GGCCTGGAGA ATGTCTCCTG CCCCCTGGCT GATACCTTGA AACGGGTTAG TAATCCTGAT   
  
  
- CTCAAGGTAA AGGTCCCGGA GTACTAGGGG CGGCTCAGTT GTCCAAAACA ACTTGGACGA CTCCGTCGCC   
  
  
- AGTTACGGGA TCGGAACGTC CTCCGCCACC CCACAGTTCT TCCCCTCCGG GAACGTCAAG TGACGCCCCT   
  
  
- GATAGAGGTG GCGGAGAATT TTCTCATGCT GTGTTCCGGA GAATCTGCCA AGGAGGTGTT CCAGTTCTGA   
  
  
- GACTTGGGGT CCCAGAACTG GAACCCTCTC TCCCTTCGGC TGGTGTTGGT GGGAGAGAAG GACGCCGCCA   
  
  
- AGTGGCTCCG TCAGTTGGTG ATGCCCCGTC ACAAACTGAG GGACCTTCGT TGGGAAGGCG GCGTATCGGT   
  
  
- TCTCTCCCAT CGACACCTCC TTCCCACCAA GCCTCTCCTC TAATTCCTGC ACCATCCTCT TCTTTCGCTC   
  
  
- TCCTCTTATC TCTCCGTGGT CTTCAAGCTC AGCACCCTTC GTAACAACTC CTCGAGTCCC AAGTTCTCAC   
  
  
- ACGGGGAGTC GGGTAAGAGC GACAGGGTTC GATTCGACGA CGACGCGGAG GTAATGGGAA GGCTTCCCAT   
  
  
- GGTTGAGGTC CAAAACGTAT CATTAACGAA CAACGAACCC ACCTTCTTAG CGGGAGAAAA AAGACAGAGA   
  
  
- AGAACCGTTA T

+     CCGTCC-box

| Site Name | Organism | Position | Strand | Matrix score. | sequence | function |
| --- | --- | --- | --- | --- | --- | --- |
| CCGTCC-box | Petroselinum hortense | 2761 | - | 6 | CCGTCC |  |

>HU03G01737.1   
+ +Up\_Stream \_Len000TTTTTT TTAAGTTTTC GTACTTGGTG TTGATGATTT GTGCTTTGTA ACCAATGGTT   
  
  
+ TTAGTAAATA GTAATTAGTC GTAACATTCT GCGATTTCGC TCAATTTATA TATCAACTTT AGAATATGAG   
  
  
+ TAACCGTAAA GATCAAACGT AGTAGTAGAG GTGTCATAGG TTTTTTTTTA AAAAAATTCG GAGTAAGAAA   
  
  
+ GTAGGGTACA TTATTTTTGC TTGTTAAATT CCGCTAGTAT AGCAATACGA GATGGAACAT TTGTAAAAAC   
  
  
+ TACGAGACGA AGCGTTTGTA CAAAACAAAC TACCATACTG CAGATCATAT AATTTTTTGG GGGGTTATGT   
  
  
+ ATCTTAGTGA TGACATTAAT TGATGGATAA CCCTATTCTA AATATCTGGA TCCATCATTA ACTGACTCGT   
  
  
+ TTCAAATGTT AGGATTATGG GTCTTGACAT GGTATACATG TCAATAGATT TATGTTAGAT GAAAAGTTAT   
  
  
+ ATGACGCTTT TTATGATTTT TGTACTAGCA TATAGTGCTT AATTGGACTA TATATTTTTA CAAGTTGTGC   
  
  
+ TTAACACCTT TTAATCAGAA ATATTTATTA TATGGAAAAA ATTTTATTAA TATGTGTTTA TGTGTTTAAA   
  
  
+ AAACATACCC AGCCTTGAAA TAGACCGTGA TATAGAAATT GGACCCATAA TACTCACATT ATATATGCTT   
  
  
+ TATTGTTATA ATTAAGGATC ATTGTTGTTT TGCAATTATG TGGCAAGATA TAATCCACAA AGTTTTACAT   
  
  
+ TTATATTGCA ATTACTCTGG TAAATGCTAT TAATGTACAT TTGAGATTAA GACATATATA TAACCCTTTT   
  
  
+ TTTTCCCTTC TTTTTTCCTT TTTCTATTTT GAATGGCATC TTAAACCAAA GGAGCTGTAC AAGTTACGTT   
  
  
+ ATAGCACTCA TGGTTGACTA AACAAGACAG ATGAGAGTTT AAGTCAAGTC CTCCTGGCAA TGATGTTTGC   
  
  
+ CATTTGGACC AATAAATACA TCATGTATAT TACCATAATA GTGTGGTTGA CTTAACAATA CATAGGGGAT   
  
  
+ TGCATACAAA GGTAAGCCTC AAAAACTTGT GCATATGCAT TGAAATCCAT GATCAAAAAA ATTCAAGAGC   
  
  
+ AGGTTGCACA AAAACACGGA GATGAGATGA AAGAGACAGA AAACACATAT ACATTCACAG TAGTAGGGGT   
  
  
+ ACTGGTAGAG CTATGGCTAG GCTAAGATGA TGTGAAAGAA ACAATATAAT GGTAGTAACT GGTAATATTT   
  
  
+ ATAGGGCACA TATAGGTGCG GGCATAGGGT CGTGTCTCTC AGTCTCAGGT ACGGTTCGAT GGGACTCTGC   
  
  
+ GATAGATGTT ATCAAATTCA ACCATTCGAA CATCTCCCAG AGAGAGAGAG AGAGACTGAG TGAGTGAGTG   
  
  
+ AGTGGGTGGG TGTATGTGTG TGAGTTGTTT TGTTCATTTA TTATAGGTTC TCTGCTGCAT ATATCTCCAC   
  
  
+ CATTAAATTT ACCAAAACTT GTACCAGCTG CTTTACTTGC TTTCCTCATT TTCCCCCTTT TTTGTCTCTC   
  
  
+ TCTCTCTCTC TCCCCCCCCC CCCCCCCCTC TCTCTCTGTG TCTGAATCAG TATCAGCCTA TCAGGATGCT   
  
  
+ TCGCCTCCAG AAAGGCATCA CTCACACACT TATTAGCTTA AAACTTTCTT TTATTCCTTT TGCTCTTTCA   
  
  
+ CTGCTCTCTT TTACCCAAAT ACGCCCGTCT TCAATTCTTC TCTCATTAAA TATGAAAAAG AAAGAGAGAA   
  
  
+ ATAGAGAGAG ATTTAAACCA GAAGGGGTGA TTCCTTTTTC TTGGTGGGCT TATTAATGCT CTGTCTACCC   
  
  
+ CATCTAGCCG AAAAATAGTA GCTTAAGACT CAACTGCTTA TTAGCATCTC TCCTCTAGCA ATAACCGAGG   
  
  
+ TAATAGCATA TTTTTCTCCC CTTAGCTTCT ACCTTGCTTT TTGTGACACT CAAACCTAAA CTGAGAGAGA   
  
  
+ GAAAGAGAAG ATAGAGGAGA GATAAATATT AGCGGGATTA GGGCAAATTT TGATATGCTG GGCTCTTCCT   
  
  
+ CATTCAACTC TGAGGAAGAA GATCAAGATC ATGCCAACCC ACCTCCGCCG CAACAACCAC CACCGTTACA   
  
  
+ GCTGCCACTT CCACGGCCAA GATTCAGCCA GTTAATATCA TCATCACCTC CAATAATATC AGCATCTTCT   
  
  
+ TCATCATTAA TCCATAATAA CCCTCGTCAG CTGCTAATAA GTTGCGGTGA ACTCATCTCC CGCTGTGACT   
  
  
+ TCTCCTCAGC TAACCGCCTT CTCTCTCTTC TCTCCGGCGA CTTCGCTTCC TCCTCCGGCG ACTCCACTGA   
  
  
+ ACGCCTTGTT TATTACTTCT CCAAGGCTTT GTTCCTCCGC CTTCGCCGAC AGTTTTCTCT ATCTTCTTCA   
  
  
+ ACTTTTACTG GTTATAGTTG TAACCCTAGT TGTTCTGCGT CTATTGCTCC ACTTTTTCTA ACCAACCCTA   
  
  
+ ATCCTAGACC ACCACAAGAA CTGCCGGTAC GAGTAACTAG CAGGGTTACT CCAAGTAGCT GCTCGTCTTA   
  
  
+ CCTAACCCTA AACCAGATCA CTCCATTTAT CCGGTTCACC CACCTGACGG CCAACCAGGC AATTTTGGAG   
  
  
+ GCTGTGGAGG GCTATAGGGC AGTCCACATC ATCGACATGG ACATCATGCA TGGAGTGCAG TGGCCTCCGC   
  
  
+ TCCTCCAAGC AATCGTGGAG CGGTCGGCCA CCCTTAGCCA ACCTCCCCCT GTGGTCCGTC TCAAAGGGGG   
  
  
+ AGGGCCGGAC CCGGACCTCT TACAGAGGAC GGGGGACCGA CTATGGAACT TTGCCCAATC ATTAGGACTA   
  
  
+ GAGTTCCATT TCCAGGGCCT CATGATCCCC GCCGAGTCAA CAGGTTTTGT TGAACCTGCT GAGGCAGCGG   
  
  
+ TCAATGCCCT AGCCTTGCAG GAGGCGGTGG GGTGTCAAGA AGGGGAGGCC CTTGCAGTTC ACTGCGGGGA   
  
  
+ CTATCTCCAC CGCCTCTTAA AAGAGTACGA CACAAGGCCT CTTAGACGGT TCCTCCACAA GGTCAAGACT   
  
  
+ CTGAACCCCA GGGTCTTGAC CTTGGGAGAG AGGGAAGCCG ACCACAACCA CCCTCTCTTC CTGCGGCGGT   
  
  
+ TCACCGAGGC AGTCAACCAC TACGGGGCAG TGTTTGACTC CCTGGAAGCA ACCCTTCCGC CGCATAGCCA   
  
  
+ AGAGAGGGTA GCTGTGGAGG AAGGGTGGTT CGGAGAGGAG ATTAAGGACG TGGTAGGAGA AGAAAGCGAG   
  
  
+ AGGAGAATAG AGAGGCACCA GAAGTTCGAG TCGTGGGAAG CATTGTTGAG GAGCTCAGGG TTCAAGAGTG   
  
  
+ TGCCCCTCAG CCCATTCTCG CTGTCCCAAG CTAAGCTGCT GCTGCGCCTC CATTACCCTT CCGAAGGGTA   
  
  
+ CCAACTCCAG GTTTTGCATA GTAATTGCTT GTTGCTTGGG TGGAAGAATC GCCCTCTTTT TTCTGTCTCT   
  
  
+ TCTTGGCAAT A  

- +Up\_Stream \_Len000AAAAAA AATTCAAAAG CATGAACCAC AACTACTAAA CACGAAACAT TGGTTACCAA   
  
  
- AATCATTTAT CATTAATCAG CATTGTAAGA CGCTAAAGCG AGTTAAATAT ATAGTTGAAA TCTTATACTC   
  
  
- ATTGGCATTT CTAGTTTGCA TCATCATCTC CACAGTATCC AAAAAAAAAT TTTTTTAAGC CTCATTCTTT   
  
  
- CATCCCATGT AATAAAAACG AACAATTTAA GGCGATCATA TCGTTATGCT CTACCTTGTA AACATTTTTG   
  
  
- ATGCTCTGCT TCGCAAACAT GTTTTGTTTG ATGGTATGAC GTCTAGTATA TTAAAAAACC CCCCAATACA   
  
  
- TAGAATCACT ACTGTAATTA ACTACCTATT GGGATAAGAT TTATAGACCT AGGTAGTAAT TGACTGAGCA   
  
  
- AAGTTTACAA TCCTAATACC CAGAACTGTA CCATATGTAC AGTTATCTAA ATACAATCTA CTTTTCAATA   
  
  
- TACTGCGAAA AATACTAAAA ACATGATCGT ATATCACGAA TTAACCTGAT ATATAAAAAT GTTCAACACG   
  
  
- AATTGTGGAA AATTAGTCTT TATAAATAAT ATACCTTTTT TAAAATAATT ATACACAAAT ACACAAATTT   
  
  
- TTTGTATGGG TCGGAACTTT ATCTGGCACT ATATCTTTAA CCTGGGTATT ATGAGTGTAA TATATACGAA   
  
  
- ATAACAATAT TAATTCCTAG TAACAACAAA ACGTTAATAC ACCGTTCTAT ATTAGGTGTT TCAAAATGTA   
  
  
- AATATAACGT TAATGAGACC ATTTACGATA ATTACATGTA AACTCTAATT CTGTATATAT ATTGGGAAAA   
  
  
- AAAAGGGAAG AAAAAAGGAA AAAGATAAAA CTTACCGTAG AATTTGGTTT CCTCGACATG TTCAATGCAA   
  
  
- TATCGTGAGT ACCAACTGAT TTGTTCTGTC TACTCTCAAA TTCAGTTCAG GAGGACCGTT ACTACAAACG   
  
  
- GTAAACCTGG TTATTTATGT AGTACATATA ATGGTATTAT CACACCAACT GAATTGTTAT GTATCCCCTA   
  
  
- ACGTATGTTT CCATTCGGAG TTTTTGAACA CGTATACGTA ACTTTAGGTA CTAGTTTTTT TAAGTTCTCG   
  
  
- TCCAACGTGT TTTTGTGCCT CTACTCTACT TTCTCTGTCT TTTGTGTATA TGTAAGTGTC ATCATCCCCA   
  
  
- TGACCATCTC GATACCGATC CGATTCTACT ACACTTTCTT TGTTATATTA CCATCATTGA CCATTATAAA   
  
  
- TATCCCGTGT ATATCCACGC CCGTATCCCA GCACAGAGAG TCAGAGTCCA TGCCAAGCTA CCCTGAGACG   
  
  
- CTATCTACAA TAGTTTAAGT TGGTAAGCTT GTAGAGGGTC TCTCTCTCTC TCTCTGACTC ACTCACTCAC   
  
  
- TCACCCACCC ACATACACAC ACTCAACAAA ACAAGTAAAT AATATCCAAG AGACGACGTA TATAGAGGTG   
  
  
- GTAATTTAAA TGGTTTTGAA CATGGTCGAC GAAATGAACG AAAGGAGTAA AAGGGGGAAA AAACAGAGAG   
  
  
- AGAGAGAGAG AGGGGGGGGG GGGGGGGGAG AGAGAGACAC AGACTTAGTC ATAGTCGGAT AGTCCTACGA   
  
  
- AGCGGAGGTC TTTCCGTAGT GAGTGTGTGA ATAATCGAAT TTTGAAAGAA AATAAGGAAA ACGAGAAAGT   
  
  
- GACGAGAGAA AATGGGTTTA TGCGGGCAGA AGTTAAGAAG AGAGTAATTT ATACTTTTTC TTTCTCTCTT   
  
  
- TATCTCTCTC TAAATTTGGT CTTCCCCACT AAGGAAAAAG AACCACCCGA ATAATTACGA GACAGATGGG   
  
  
- GTAGATCGGC TTTTTATCAT CGAATTCTGA GTTGACGAAT AATCGTAGAG AGGAGATCGT TATTGGCTCC   
  
  
- ATTATCGTAT AAAAAGAGGG GAATCGAAGA TGGAACGAAA AACACTGTGA GTTTGGATTT GACTCTCTCT   
  
  
- CTTTCTCTTC TATCTCCTCT CTATTTATAA TCGCCCTAAT CCCGTTTAAA ACTATACGAC CCGAGAAGGA   
  
  
- GTAAGTTGAG ACTCCTTCTT CTAGTTCTAG TACGGTTGGG TGGAGGCGGC GTTGTTGGTG GTGGCAATGT   
  
  
- CGACGGTGAA GGTGCCGGTT CTAAGTCGGT CAATTATAGT AGTAGTGGAG GTTATTATAG TCGTAGAAGA   
  
  
- AGTAGTAATT AGGTATTATT GGGAGCAGTC GACGATTATT CAACGCCACT TGAGTAGAGG GCGACACTGA   
  
  
- AGAGGAGTCG ATTGGCGGAA GAGAGAGAAG AGAGGCCGCT GAAGCGAAGG AGGAGGCCGC TGAGGTGACT   
  
  
- TGCGGAACAA ATAATGAAGA GGTTCCGAAA CAAGGAGGCG GAAGCGGCTG TCAAAAGAGA TAGAAGAAGT   
  
  
- TGAAAATGAC CAATATCAAC ATTGGGATCA ACAAGACGCA GATAACGAGG TGAAAAAGAT TGGTTGGGAT   
  
  
- TAGGATCTGG TGGTGTTCTT GACGGCCATG CTCATTGATC GTCCCAATGA GGTTCATCGA CGAGCAGAAT   
  
  
- GGATTGGGAT TTGGTCTAGT GAGGTAAATA GGCCAAGTGG GTGGACTGCC GGTTGGTCCG TTAAAACCTC   
  
  
- CGACACCTCC CGATATCCCG TCAGGTGTAG TAGCTGTACC TGTAGTACGT ACCTCACGTC ACCGGAGGCG   
  
  
- AGGAGGTTCG TTAGCACCTC GCCAGCCGGT GGGAATCGGT TGGAGGGGGA CACCAGGCAG AGTTTCCCCC   
  
  
- TCCCGGCCTG GGCCTGGAGA ATGTCTCCTG CCCCCTGGCT GATACCTTGA AACGGGTTAG TAATCCTGAT   
  
  
- CTCAAGGTAA AGGTCCCGGA GTACTAGGGG CGGCTCAGTT GTCCAAAACA ACTTGGACGA CTCCGTCGCC   
  
  
- AGTTACGGGA TCGGAACGTC CTCCGCCACC CCACAGTTCT TCCCCTCCGG GAACGTCAAG TGACGCCCCT   
  
  
- GATAGAGGTG GCGGAGAATT TTCTCATGCT GTGTTCCGGA GAATCTGCCA AGGAGGTGTT CCAGTTCTGA   
  
  
- GACTTGGGGT CCCAGAACTG GAACCCTCTC TCCCTTCGGC TGGTGTTGGT GGGAGAGAAG GACGCCGCCA   
  
  
- AGTGGCTCCG TCAGTTGGTG ATGCCCCGTC ACAAACTGAG GGACCTTCGT TGGGAAGGCG GCGTATCGGT   
  
  
- TCTCTCCCAT CGACACCTCC TTCCCACCAA GCCTCTCCTC TAATTCCTGC ACCATCCTCT TCTTTCGCTC   
  
  
- TCCTCTTATC TCTCCGTGGT CTTCAAGCTC AGCACCCTTC GTAACAACTC CTCGAGTCCC AAGTTCTCAC   
  
  
- ACGGGGAGTC GGGTAAGAGC GACAGGGTTC GATTCGACGA CGACGCGGAG GTAATGGGAA GGCTTCCCAT   
  
  
- GGTTGAGGTC CAAAACGTAT CATTAACGAA CAACGAACCC ACCTTCTTAG CGGGAGAAAA AAGACAGAGA   
  
  
- AGAACCGTTA T

+     CGTCA-motif

| Site Name | Organism | Position | Strand | Matrix score. | sequence | function |
| --- | --- | --- | --- | --- | --- | --- |
| CGTCA-motif | Hordeum vulgare | 2569 | - | 5 | CGTCA | cis-acting regulatory element involved in the MeJA-responsiveness |
| CGTCA-motif | Hordeum vulgare | 496 | - | 5 | CGTCA | cis-acting regulatory element involved in the MeJA-responsiveness |
| CGTCA-motif | Hordeum vulgare | 2199 | + | 5 | CGTCA | cis-acting regulatory element involved in the MeJA-responsiveness |

>HU03G01737.1   
+ +Up\_Stream \_Len000TTTTTT TTAAGTTTTC GTACTTGGTG TTGATGATTT GTGCTTTGTA ACCAATGGTT   
  
  
+ TTAGTAAATA GTAATTAGTC GTAACATTCT GCGATTTCGC TCAATTTATA TATCAACTTT AGAATATGAG   
  
  
+ TAACCGTAAA GATCAAACGT AGTAGTAGAG GTGTCATAGG TTTTTTTTTA AAAAAATTCG GAGTAAGAAA   
  
  
+ GTAGGGTACA TTATTTTTGC TTGTTAAATT CCGCTAGTAT AGCAATACGA GATGGAACAT TTGTAAAAAC   
  
  
+ TACGAGACGA AGCGTTTGTA CAAAACAAAC TACCATACTG CAGATCATAT AATTTTTTGG GGGGTTATGT   
  
  
+ ATCTTAGTGA TGACATTAAT TGATGGATAA CCCTATTCTA AATATCTGGA TCCATCATTA ACTGACTCGT   
  
  
+ TTCAAATGTT AGGATTATGG GTCTTGACAT GGTATACATG TCAATAGATT TATGTTAGAT GAAAAGTTAT   
  
  
+ ATGACGCTTT TTATGATTTT TGTACTAGCA TATAGTGCTT AATTGGACTA TATATTTTTA CAAGTTGTGC   
  
  
+ TTAACACCTT TTAATCAGAA ATATTTATTA TATGGAAAAA ATTTTATTAA TATGTGTTTA TGTGTTTAAA   
  
  
+ AAACATACCC AGCCTTGAAA TAGACCGTGA TATAGAAATT GGACCCATAA TACTCACATT ATATATGCTT   
  
  
+ TATTGTTATA ATTAAGGATC ATTGTTGTTT TGCAATTATG TGGCAAGATA TAATCCACAA AGTTTTACAT   
  
  
+ TTATATTGCA ATTACTCTGG TAAATGCTAT TAATGTACAT TTGAGATTAA GACATATATA TAACCCTTTT   
  
  
+ TTTTCCCTTC TTTTTTCCTT TTTCTATTTT GAATGGCATC TTAAACCAAA GGAGCTGTAC AAGTTACGTT   
  
  
+ ATAGCACTCA TGGTTGACTA AACAAGACAG ATGAGAGTTT AAGTCAAGTC CTCCTGGCAA TGATGTTTGC   
  
  
+ CATTTGGACC AATAAATACA TCATGTATAT TACCATAATA GTGTGGTTGA CTTAACAATA CATAGGGGAT   
  
  
+ TGCATACAAA GGTAAGCCTC AAAAACTTGT GCATATGCAT TGAAATCCAT GATCAAAAAA ATTCAAGAGC   
  
  
+ AGGTTGCACA AAAACACGGA GATGAGATGA AAGAGACAGA AAACACATAT ACATTCACAG TAGTAGGGGT   
  
  
+ ACTGGTAGAG CTATGGCTAG GCTAAGATGA TGTGAAAGAA ACAATATAAT GGTAGTAACT GGTAATATTT   
  
  
+ ATAGGGCACA TATAGGTGCG GGCATAGGGT CGTGTCTCTC AGTCTCAGGT ACGGTTCGAT GGGACTCTGC   
  
  
+ GATAGATGTT ATCAAATTCA ACCATTCGAA CATCTCCCAG AGAGAGAGAG AGAGACTGAG TGAGTGAGTG   
  
  
+ AGTGGGTGGG TGTATGTGTG TGAGTTGTTT TGTTCATTTA TTATAGGTTC TCTGCTGCAT ATATCTCCAC   
  
  
+ CATTAAATTT ACCAAAACTT GTACCAGCTG CTTTACTTGC TTTCCTCATT TTCCCCCTTT TTTGTCTCTC   
  
  
+ TCTCTCTCTC TCCCCCCCCC CCCCCCCCTC TCTCTCTGTG TCTGAATCAG TATCAGCCTA TCAGGATGCT   
  
  
+ TCGCCTCCAG AAAGGCATCA CTCACACACT TATTAGCTTA AAACTTTCTT TTATTCCTTT TGCTCTTTCA   
  
  
+ CTGCTCTCTT TTACCCAAAT ACGCCCGTCT TCAATTCTTC TCTCATTAAA TATGAAAAAG AAAGAGAGAA   
  
  
+ ATAGAGAGAG ATTTAAACCA GAAGGGGTGA TTCCTTTTTC TTGGTGGGCT TATTAATGCT CTGTCTACCC   
  
  
+ CATCTAGCCG AAAAATAGTA GCTTAAGACT CAACTGCTTA TTAGCATCTC TCCTCTAGCA ATAACCGAGG   
  
  
+ TAATAGCATA TTTTTCTCCC CTTAGCTTCT ACCTTGCTTT TTGTGACACT CAAACCTAAA CTGAGAGAGA   
  
  
+ GAAAGAGAAG ATAGAGGAGA GATAAATATT AGCGGGATTA GGGCAAATTT TGATATGCTG GGCTCTTCCT   
  
  
+ CATTCAACTC TGAGGAAGAA GATCAAGATC ATGCCAACCC ACCTCCGCCG CAACAACCAC CACCGTTACA   
  
  
+ GCTGCCACTT CCACGGCCAA GATTCAGCCA GTTAATATCA TCATCACCTC CAATAATATC AGCATCTTCT   
  
  
+ TCATCATTAA TCCATAATAA CCCTCGTCAG CTGCTAATAA GTTGCGGTGA ACTCATCTCC CGCTGTGACT   
  
  
+ TCTCCTCAGC TAACCGCCTT CTCTCTCTTC TCTCCGGCGA CTTCGCTTCC TCCTCCGGCG ACTCCACTGA   
  
  
+ ACGCCTTGTT TATTACTTCT CCAAGGCTTT GTTCCTCCGC CTTCGCCGAC AGTTTTCTCT ATCTTCTTCA   
  
  
+ ACTTTTACTG GTTATAGTTG TAACCCTAGT TGTTCTGCGT CTATTGCTCC ACTTTTTCTA ACCAACCCTA   
  
  
+ ATCCTAGACC ACCACAAGAA CTGCCGGTAC GAGTAACTAG CAGGGTTACT CCAAGTAGCT GCTCGTCTTA   
  
  
+ CCTAACCCTA AACCAGATCA CTCCATTTAT CCGGTTCACC CACCTGACGG CCAACCAGGC AATTTTGGAG   
  
  
+ GCTGTGGAGG GCTATAGGGC AGTCCACATC ATCGACATGG ACATCATGCA TGGAGTGCAG TGGCCTCCGC   
  
  
+ TCCTCCAAGC AATCGTGGAG CGGTCGGCCA CCCTTAGCCA ACCTCCCCCT GTGGTCCGTC TCAAAGGGGG   
  
  
+ AGGGCCGGAC CCGGACCTCT TACAGAGGAC GGGGGACCGA CTATGGAACT TTGCCCAATC ATTAGGACTA   
  
  
+ GAGTTCCATT TCCAGGGCCT CATGATCCCC GCCGAGTCAA CAGGTTTTGT TGAACCTGCT GAGGCAGCGG   
  
  
+ TCAATGCCCT AGCCTTGCAG GAGGCGGTGG GGTGTCAAGA AGGGGAGGCC CTTGCAGTTC ACTGCGGGGA   
  
  
+ CTATCTCCAC CGCCTCTTAA AAGAGTACGA CACAAGGCCT CTTAGACGGT TCCTCCACAA GGTCAAGACT   
  
  
+ CTGAACCCCA GGGTCTTGAC CTTGGGAGAG AGGGAAGCCG ACCACAACCA CCCTCTCTTC CTGCGGCGGT   
  
  
+ TCACCGAGGC AGTCAACCAC TACGGGGCAG TGTTTGACTC CCTGGAAGCA ACCCTTCCGC CGCATAGCCA   
  
  
+ AGAGAGGGTA GCTGTGGAGG AAGGGTGGTT CGGAGAGGAG ATTAAGGACG TGGTAGGAGA AGAAAGCGAG   
  
  
+ AGGAGAATAG AGAGGCACCA GAAGTTCGAG TCGTGGGAAG CATTGTTGAG GAGCTCAGGG TTCAAGAGTG   
  
  
+ TGCCCCTCAG CCCATTCTCG CTGTCCCAAG CTAAGCTGCT GCTGCGCCTC CATTACCCTT CCGAAGGGTA   
  
  
+ CCAACTCCAG GTTTTGCATA GTAATTGCTT GTTGCTTGGG TGGAAGAATC GCCCTCTTTT TTCTGTCTCT   
  
  
+ TCTTGGCAAT A  

- +Up\_Stream \_Len000AAAAAA AATTCAAAAG CATGAACCAC AACTACTAAA CACGAAACAT TGGTTACCAA   
  
  
- AATCATTTAT CATTAATCAG CATTGTAAGA CGCTAAAGCG AGTTAAATAT ATAGTTGAAA TCTTATACTC   
  
  
- ATTGGCATTT CTAGTTTGCA TCATCATCTC CACAGTATCC AAAAAAAAAT TTTTTTAAGC CTCATTCTTT   
  
  
- CATCCCATGT AATAAAAACG AACAATTTAA GGCGATCATA TCGTTATGCT CTACCTTGTA AACATTTTTG   
  
  
- ATGCTCTGCT TCGCAAACAT GTTTTGTTTG ATGGTATGAC GTCTAGTATA TTAAAAAACC CCCCAATACA   
  
  
- TAGAATCACT ACTGTAATTA ACTACCTATT GGGATAAGAT TTATAGACCT AGGTAGTAAT TGACTGAGCA   
  
  
- AAGTTTACAA TCCTAATACC CAGAACTGTA CCATATGTAC AGTTATCTAA ATACAATCTA CTTTTCAATA   
  
  
- TACTGCGAAA AATACTAAAA ACATGATCGT ATATCACGAA TTAACCTGAT ATATAAAAAT GTTCAACACG   
  
  
- AATTGTGGAA AATTAGTCTT TATAAATAAT ATACCTTTTT TAAAATAATT ATACACAAAT ACACAAATTT   
  
  
- TTTGTATGGG TCGGAACTTT ATCTGGCACT ATATCTTTAA CCTGGGTATT ATGAGTGTAA TATATACGAA   
  
  
- ATAACAATAT TAATTCCTAG TAACAACAAA ACGTTAATAC ACCGTTCTAT ATTAGGTGTT TCAAAATGTA   
  
  
- AATATAACGT TAATGAGACC ATTTACGATA ATTACATGTA AACTCTAATT CTGTATATAT ATTGGGAAAA   
  
  
- AAAAGGGAAG AAAAAAGGAA AAAGATAAAA CTTACCGTAG AATTTGGTTT CCTCGACATG TTCAATGCAA   
  
  
- TATCGTGAGT ACCAACTGAT TTGTTCTGTC TACTCTCAAA TTCAGTTCAG GAGGACCGTT ACTACAAACG   
  
  
- GTAAACCTGG TTATTTATGT AGTACATATA ATGGTATTAT CACACCAACT GAATTGTTAT GTATCCCCTA   
  
  
- ACGTATGTTT CCATTCGGAG TTTTTGAACA CGTATACGTA ACTTTAGGTA CTAGTTTTTT TAAGTTCTCG   
  
  
- TCCAACGTGT TTTTGTGCCT CTACTCTACT TTCTCTGTCT TTTGTGTATA TGTAAGTGTC ATCATCCCCA   
  
  
- TGACCATCTC GATACCGATC CGATTCTACT ACACTTTCTT TGTTATATTA CCATCATTGA CCATTATAAA   
  
  
- TATCCCGTGT ATATCCACGC CCGTATCCCA GCACAGAGAG TCAGAGTCCA TGCCAAGCTA CCCTGAGACG   
  
  
- CTATCTACAA TAGTTTAAGT TGGTAAGCTT GTAGAGGGTC TCTCTCTCTC TCTCTGACTC ACTCACTCAC   
  
  
- TCACCCACCC ACATACACAC ACTCAACAAA ACAAGTAAAT AATATCCAAG AGACGACGTA TATAGAGGTG   
  
  
- GTAATTTAAA TGGTTTTGAA CATGGTCGAC GAAATGAACG AAAGGAGTAA AAGGGGGAAA AAACAGAGAG   
  
  
- AGAGAGAGAG AGGGGGGGGG GGGGGGGGAG AGAGAGACAC AGACTTAGTC ATAGTCGGAT AGTCCTACGA   
  
  
- AGCGGAGGTC TTTCCGTAGT GAGTGTGTGA ATAATCGAAT TTTGAAAGAA AATAAGGAAA ACGAGAAAGT   
  
  
- GACGAGAGAA AATGGGTTTA TGCGGGCAGA AGTTAAGAAG AGAGTAATTT ATACTTTTTC TTTCTCTCTT   
  
  
- TATCTCTCTC TAAATTTGGT CTTCCCCACT AAGGAAAAAG AACCACCCGA ATAATTACGA GACAGATGGG   
  
  
- GTAGATCGGC TTTTTATCAT CGAATTCTGA GTTGACGAAT AATCGTAGAG AGGAGATCGT TATTGGCTCC   
  
  
- ATTATCGTAT AAAAAGAGGG GAATCGAAGA TGGAACGAAA AACACTGTGA GTTTGGATTT GACTCTCTCT   
  
  
- CTTTCTCTTC TATCTCCTCT CTATTTATAA TCGCCCTAAT CCCGTTTAAA ACTATACGAC CCGAGAAGGA   
  
  
- GTAAGTTGAG ACTCCTTCTT CTAGTTCTAG TACGGTTGGG TGGAGGCGGC GTTGTTGGTG GTGGCAATGT   
  
  
- CGACGGTGAA GGTGCCGGTT CTAAGTCGGT CAATTATAGT AGTAGTGGAG GTTATTATAG TCGTAGAAGA   
  
  
- AGTAGTAATT AGGTATTATT GGGAGCAGTC GACGATTATT CAACGCCACT TGAGTAGAGG GCGACACTGA   
  
  
- AGAGGAGTCG ATTGGCGGAA GAGAGAGAAG AGAGGCCGCT GAAGCGAAGG AGGAGGCCGC TGAGGTGACT   
  
  
- TGCGGAACAA ATAATGAAGA GGTTCCGAAA CAAGGAGGCG GAAGCGGCTG TCAAAAGAGA TAGAAGAAGT   
  
  
- TGAAAATGAC CAATATCAAC ATTGGGATCA ACAAGACGCA GATAACGAGG TGAAAAAGAT TGGTTGGGAT   
  
  
- TAGGATCTGG TGGTGTTCTT GACGGCCATG CTCATTGATC GTCCCAATGA GGTTCATCGA CGAGCAGAAT   
  
  
- GGATTGGGAT TTGGTCTAGT GAGGTAAATA GGCCAAGTGG GTGGACTGCC GGTTGGTCCG TTAAAACCTC   
  
  
- CGACACCTCC CGATATCCCG TCAGGTGTAG TAGCTGTACC TGTAGTACGT ACCTCACGTC ACCGGAGGCG   
  
  
- AGGAGGTTCG TTAGCACCTC GCCAGCCGGT GGGAATCGGT TGGAGGGGGA CACCAGGCAG AGTTTCCCCC   
  
  
- TCCCGGCCTG GGCCTGGAGA ATGTCTCCTG CCCCCTGGCT GATACCTTGA AACGGGTTAG TAATCCTGAT   
  
  
- CTCAAGGTAA AGGTCCCGGA GTACTAGGGG CGGCTCAGTT GTCCAAAACA ACTTGGACGA CTCCGTCGCC   
  
  
- AGTTACGGGA TCGGAACGTC CTCCGCCACC CCACAGTTCT TCCCCTCCGG GAACGTCAAG TGACGCCCCT   
  
  
- GATAGAGGTG GCGGAGAATT TTCTCATGCT GTGTTCCGGA GAATCTGCCA AGGAGGTGTT CCAGTTCTGA   
  
  
- GACTTGGGGT CCCAGAACTG GAACCCTCTC TCCCTTCGGC TGGTGTTGGT GGGAGAGAAG GACGCCGCCA   
  
  
- AGTGGCTCCG TCAGTTGGTG ATGCCCCGTC ACAAACTGAG GGACCTTCGT TGGGAAGGCG GCGTATCGGT   
  
  
- TCTCTCCCAT CGACACCTCC TTCCCACCAA GCCTCTCCTC TAATTCCTGC ACCATCCTCT TCTTTCGCTC   
  
  
- TCCTCTTATC TCTCCGTGGT CTTCAAGCTC AGCACCCTTC GTAACAACTC CTCGAGTCCC AAGTTCTCAC   
  
  
- ACGGGGAGTC GGGTAAGAGC GACAGGGTTC GATTCGACGA CGACGCGGAG GTAATGGGAA GGCTTCCCAT   
  
  
- GGTTGAGGTC CAAAACGTAT CATTAACGAA CAACGAACCC ACCTTCTTAG CGGGAGAAAA AAGACAGAGA   
  
  
- AGAACCGTTA T

+     CTAG-motif

| Site Name | Organism | Position | Strand | Matrix score. | sequence | function |
| --- | --- | --- | --- | --- | --- | --- |
| CTAG-motif | Avena sativa | 243 | - | 9 | ACTAGCAGAA |  |

>HU03G01737.1   
+ +Up\_Stream \_Len000TTTTTT TTAAGTTTTC GTACTTGGTG TTGATGATTT GTGCTTTGTA ACCAATGGTT   
  
  
+ TTAGTAAATA GTAATTAGTC GTAACATTCT GCGATTTCGC TCAATTTATA TATCAACTTT AGAATATGAG   
  
  
+ TAACCGTAAA GATCAAACGT AGTAGTAGAG GTGTCATAGG TTTTTTTTTA AAAAAATTCG GAGTAAGAAA   
  
  
+ GTAGGGTACA TTATTTTTGC TTGTTAAATT CCGCTAGTAT AGCAATACGA GATGGAACAT TTGTAAAAAC   
  
  
+ TACGAGACGA AGCGTTTGTA CAAAACAAAC TACCATACTG CAGATCATAT AATTTTTTGG GGGGTTATGT   
  
  
+ ATCTTAGTGA TGACATTAAT TGATGGATAA CCCTATTCTA AATATCTGGA TCCATCATTA ACTGACTCGT   
  
  
+ TTCAAATGTT AGGATTATGG GTCTTGACAT GGTATACATG TCAATAGATT TATGTTAGAT GAAAAGTTAT   
  
  
+ ATGACGCTTT TTATGATTTT TGTACTAGCA TATAGTGCTT AATTGGACTA TATATTTTTA CAAGTTGTGC   
  
  
+ TTAACACCTT TTAATCAGAA ATATTTATTA TATGGAAAAA ATTTTATTAA TATGTGTTTA TGTGTTTAAA   
  
  
+ AAACATACCC AGCCTTGAAA TAGACCGTGA TATAGAAATT GGACCCATAA TACTCACATT ATATATGCTT   
  
  
+ TATTGTTATA ATTAAGGATC ATTGTTGTTT TGCAATTATG TGGCAAGATA TAATCCACAA AGTTTTACAT   
  
  
+ TTATATTGCA ATTACTCTGG TAAATGCTAT TAATGTACAT TTGAGATTAA GACATATATA TAACCCTTTT   
  
  
+ TTTTCCCTTC TTTTTTCCTT TTTCTATTTT GAATGGCATC TTAAACCAAA GGAGCTGTAC AAGTTACGTT   
  
  
+ ATAGCACTCA TGGTTGACTA AACAAGACAG ATGAGAGTTT AAGTCAAGTC CTCCTGGCAA TGATGTTTGC   
  
  
+ CATTTGGACC AATAAATACA TCATGTATAT TACCATAATA GTGTGGTTGA CTTAACAATA CATAGGGGAT   
  
  
+ TGCATACAAA GGTAAGCCTC AAAAACTTGT GCATATGCAT TGAAATCCAT GATCAAAAAA ATTCAAGAGC   
  
  
+ AGGTTGCACA AAAACACGGA GATGAGATGA AAGAGACAGA AAACACATAT ACATTCACAG TAGTAGGGGT   
  
  
+ ACTGGTAGAG CTATGGCTAG GCTAAGATGA TGTGAAAGAA ACAATATAAT GGTAGTAACT GGTAATATTT   
  
  
+ ATAGGGCACA TATAGGTGCG GGCATAGGGT CGTGTCTCTC AGTCTCAGGT ACGGTTCGAT GGGACTCTGC   
  
  
+ GATAGATGTT ATCAAATTCA ACCATTCGAA CATCTCCCAG AGAGAGAGAG AGAGACTGAG TGAGTGAGTG   
  
  
+ AGTGGGTGGG TGTATGTGTG TGAGTTGTTT TGTTCATTTA TTATAGGTTC TCTGCTGCAT ATATCTCCAC   
  
  
+ CATTAAATTT ACCAAAACTT GTACCAGCTG CTTTACTTGC TTTCCTCATT TTCCCCCTTT TTTGTCTCTC   
  
  
+ TCTCTCTCTC TCCCCCCCCC CCCCCCCCTC TCTCTCTGTG TCTGAATCAG TATCAGCCTA TCAGGATGCT   
  
  
+ TCGCCTCCAG AAAGGCATCA CTCACACACT TATTAGCTTA AAACTTTCTT TTATTCCTTT TGCTCTTTCA   
  
  
+ CTGCTCTCTT TTACCCAAAT ACGCCCGTCT TCAATTCTTC TCTCATTAAA TATGAAAAAG AAAGAGAGAA   
  
  
+ ATAGAGAGAG ATTTAAACCA GAAGGGGTGA TTCCTTTTTC TTGGTGGGCT TATTAATGCT CTGTCTACCC   
  
  
+ CATCTAGCCG AAAAATAGTA GCTTAAGACT CAACTGCTTA TTAGCATCTC TCCTCTAGCA ATAACCGAGG   
  
  
+ TAATAGCATA TTTTTCTCCC CTTAGCTTCT ACCTTGCTTT TTGTGACACT CAAACCTAAA CTGAGAGAGA   
  
  
+ GAAAGAGAAG ATAGAGGAGA GATAAATATT AGCGGGATTA GGGCAAATTT TGATATGCTG GGCTCTTCCT   
  
  
+ CATTCAACTC TGAGGAAGAA GATCAAGATC ATGCCAACCC ACCTCCGCCG CAACAACCAC CACCGTTACA   
  
  
+ GCTGCCACTT CCACGGCCAA GATTCAGCCA GTTAATATCA TCATCACCTC CAATAATATC AGCATCTTCT   
  
  
+ TCATCATTAA TCCATAATAA CCCTCGTCAG CTGCTAATAA GTTGCGGTGA ACTCATCTCC CGCTGTGACT   
  
  
+ TCTCCTCAGC TAACCGCCTT CTCTCTCTTC TCTCCGGCGA CTTCGCTTCC TCCTCCGGCG ACTCCACTGA   
  
  
+ ACGCCTTGTT TATTACTTCT CCAAGGCTTT GTTCCTCCGC CTTCGCCGAC AGTTTTCTCT ATCTTCTTCA   
  
  
+ ACTTTTACTG GTTATAGTTG TAACCCTAGT TGTTCTGCGT CTATTGCTCC ACTTTTTCTA ACCAACCCTA   
  
  
+ ATCCTAGACC ACCACAAGAA CTGCCGGTAC GAGTAACTAG CAGGGTTACT CCAAGTAGCT GCTCGTCTTA   
  
  
+ CCTAACCCTA AACCAGATCA CTCCATTTAT CCGGTTCACC CACCTGACGG CCAACCAGGC AATTTTGGAG   
  
  
+ GCTGTGGAGG GCTATAGGGC AGTCCACATC ATCGACATGG ACATCATGCA TGGAGTGCAG TGGCCTCCGC   
  
  
+ TCCTCCAAGC AATCGTGGAG CGGTCGGCCA CCCTTAGCCA ACCTCCCCCT GTGGTCCGTC TCAAAGGGGG   
  
  
+ AGGGCCGGAC CCGGACCTCT TACAGAGGAC GGGGGACCGA CTATGGAACT TTGCCCAATC ATTAGGACTA   
  
  
+ GAGTTCCATT TCCAGGGCCT CATGATCCCC GCCGAGTCAA CAGGTTTTGT TGAACCTGCT GAGGCAGCGG   
  
  
+ TCAATGCCCT AGCCTTGCAG GAGGCGGTGG GGTGTCAAGA AGGGGAGGCC CTTGCAGTTC ACTGCGGGGA   
  
  
+ CTATCTCCAC CGCCTCTTAA AAGAGTACGA CACAAGGCCT CTTAGACGGT TCCTCCACAA GGTCAAGACT   
  
  
+ CTGAACCCCA GGGTCTTGAC CTTGGGAGAG AGGGAAGCCG ACCACAACCA CCCTCTCTTC CTGCGGCGGT   
  
  
+ TCACCGAGGC AGTCAACCAC TACGGGGCAG TGTTTGACTC CCTGGAAGCA ACCCTTCCGC CGCATAGCCA   
  
  
+ AGAGAGGGTA GCTGTGGAGG AAGGGTGGTT CGGAGAGGAG ATTAAGGACG TGGTAGGAGA AGAAAGCGAG   
  
  
+ AGGAGAATAG AGAGGCACCA GAAGTTCGAG TCGTGGGAAG CATTGTTGAG GAGCTCAGGG TTCAAGAGTG   
  
  
+ TGCCCCTCAG CCCATTCTCG CTGTCCCAAG CTAAGCTGCT GCTGCGCCTC CATTACCCTT CCGAAGGGTA   
  
  
+ CCAACTCCAG GTTTTGCATA GTAATTGCTT GTTGCTTGGG TGGAAGAATC GCCCTCTTTT TTCTGTCTCT   
  
  
+ TCTTGGCAAT A  

- +Up\_Stream \_Len000AAAAAA AATTCAAAAG CATGAACCAC AACTACTAAA CACGAAACAT TGGTTACCAA   
  
  
- AATCATTTAT CATTAATCAG CATTGTAAGA CGCTAAAGCG AGTTAAATAT ATAGTTGAAA TCTTATACTC   
  
  
- ATTGGCATTT CTAGTTTGCA TCATCATCTC CACAGTATCC AAAAAAAAAT TTTTTTAAGC CTCATTCTTT   
  
  
- CATCCCATGT AATAAAAACG AACAATTTAA GGCGATCATA TCGTTATGCT CTACCTTGTA AACATTTTTG   
  
  
- ATGCTCTGCT TCGCAAACAT GTTTTGTTTG ATGGTATGAC GTCTAGTATA TTAAAAAACC CCCCAATACA   
  
  
- TAGAATCACT ACTGTAATTA ACTACCTATT GGGATAAGAT TTATAGACCT AGGTAGTAAT TGACTGAGCA   
  
  
- AAGTTTACAA TCCTAATACC CAGAACTGTA CCATATGTAC AGTTATCTAA ATACAATCTA CTTTTCAATA   
  
  
- TACTGCGAAA AATACTAAAA ACATGATCGT ATATCACGAA TTAACCTGAT ATATAAAAAT GTTCAACACG   
  
  
- AATTGTGGAA AATTAGTCTT TATAAATAAT ATACCTTTTT TAAAATAATT ATACACAAAT ACACAAATTT   
  
  
- TTTGTATGGG TCGGAACTTT ATCTGGCACT ATATCTTTAA CCTGGGTATT ATGAGTGTAA TATATACGAA   
  
  
- ATAACAATAT TAATTCCTAG TAACAACAAA ACGTTAATAC ACCGTTCTAT ATTAGGTGTT TCAAAATGTA   
  
  
- AATATAACGT TAATGAGACC ATTTACGATA ATTACATGTA AACTCTAATT CTGTATATAT ATTGGGAAAA   
  
  
- AAAAGGGAAG AAAAAAGGAA AAAGATAAAA CTTACCGTAG AATTTGGTTT CCTCGACATG TTCAATGCAA   
  
  
- TATCGTGAGT ACCAACTGAT TTGTTCTGTC TACTCTCAAA TTCAGTTCAG GAGGACCGTT ACTACAAACG   
  
  
- GTAAACCTGG TTATTTATGT AGTACATATA ATGGTATTAT CACACCAACT GAATTGTTAT GTATCCCCTA   
  
  
- ACGTATGTTT CCATTCGGAG TTTTTGAACA CGTATACGTA ACTTTAGGTA CTAGTTTTTT TAAGTTCTCG   
  
  
- TCCAACGTGT TTTTGTGCCT CTACTCTACT TTCTCTGTCT TTTGTGTATA TGTAAGTGTC ATCATCCCCA   
  
  
- TGACCATCTC GATACCGATC CGATTCTACT ACACTTTCTT TGTTATATTA CCATCATTGA CCATTATAAA   
  
  
- TATCCCGTGT ATATCCACGC CCGTATCCCA GCACAGAGAG TCAGAGTCCA TGCCAAGCTA CCCTGAGACG   
  
  
- CTATCTACAA TAGTTTAAGT TGGTAAGCTT GTAGAGGGTC TCTCTCTCTC TCTCTGACTC ACTCACTCAC   
  
  
- TCACCCACCC ACATACACAC ACTCAACAAA ACAAGTAAAT AATATCCAAG AGACGACGTA TATAGAGGTG   
  
  
- GTAATTTAAA TGGTTTTGAA CATGGTCGAC GAAATGAACG AAAGGAGTAA AAGGGGGAAA AAACAGAGAG   
  
  
- AGAGAGAGAG AGGGGGGGGG GGGGGGGGAG AGAGAGACAC AGACTTAGTC ATAGTCGGAT AGTCCTACGA   
  
  
- AGCGGAGGTC TTTCCGTAGT GAGTGTGTGA ATAATCGAAT TTTGAAAGAA AATAAGGAAA ACGAGAAAGT   
  
  
- GACGAGAGAA AATGGGTTTA TGCGGGCAGA AGTTAAGAAG AGAGTAATTT ATACTTTTTC TTTCTCTCTT   
  
  
- TATCTCTCTC TAAATTTGGT CTTCCCCACT AAGGAAAAAG AACCACCCGA ATAATTACGA GACAGATGGG   
  
  
- GTAGATCGGC TTTTTATCAT CGAATTCTGA GTTGACGAAT AATCGTAGAG AGGAGATCGT TATTGGCTCC   
  
  
- ATTATCGTAT AAAAAGAGGG GAATCGAAGA TGGAACGAAA AACACTGTGA GTTTGGATTT GACTCTCTCT   
  
  
- CTTTCTCTTC TATCTCCTCT CTATTTATAA TCGCCCTAAT CCCGTTTAAA ACTATACGAC CCGAGAAGGA   
  
  
- GTAAGTTGAG ACTCCTTCTT CTAGTTCTAG TACGGTTGGG TGGAGGCGGC GTTGTTGGTG GTGGCAATGT   
  
  
- CGACGGTGAA GGTGCCGGTT CTAAGTCGGT CAATTATAGT AGTAGTGGAG GTTATTATAG TCGTAGAAGA   
  
  
- AGTAGTAATT AGGTATTATT GGGAGCAGTC GACGATTATT CAACGCCACT TGAGTAGAGG GCGACACTGA   
  
  
- AGAGGAGTCG ATTGGCGGAA GAGAGAGAAG AGAGGCCGCT GAAGCGAAGG AGGAGGCCGC TGAGGTGACT   
  
  
- TGCGGAACAA ATAATGAAGA GGTTCCGAAA CAAGGAGGCG GAAGCGGCTG TCAAAAGAGA TAGAAGAAGT   
  
  
- TGAAAATGAC CAATATCAAC ATTGGGATCA ACAAGACGCA GATAACGAGG TGAAAAAGAT TGGTTGGGAT   
  
  
- TAGGATCTGG TGGTGTTCTT GACGGCCATG CTCATTGATC GTCCCAATGA GGTTCATCGA CGAGCAGAAT   
  
  
- GGATTGGGAT TTGGTCTAGT GAGGTAAATA GGCCAAGTGG GTGGACTGCC GGTTGGTCCG TTAAAACCTC   
  
  
- CGACACCTCC CGATATCCCG TCAGGTGTAG TAGCTGTACC TGTAGTACGT ACCTCACGTC ACCGGAGGCG   
  
  
- AGGAGGTTCG TTAGCACCTC GCCAGCCGGT GGGAATCGGT TGGAGGGGGA CACCAGGCAG AGTTTCCCCC   
  
  
- TCCCGGCCTG GGCCTGGAGA ATGTCTCCTG CCCCCTGGCT GATACCTTGA AACGGGTTAG TAATCCTGAT   
  
  
- CTCAAGGTAA AGGTCCCGGA GTACTAGGGG CGGCTCAGTT GTCCAAAACA ACTTGGACGA CTCCGTCGCC   
  
  
- AGTTACGGGA TCGGAACGTC CTCCGCCACC CCACAGTTCT TCCCCTCCGG GAACGTCAAG TGACGCCCCT   
  
  
- GATAGAGGTG GCGGAGAATT TTCTCATGCT GTGTTCCGGA GAATCTGCCA AGGAGGTGTT CCAGTTCTGA   
  
  
- GACTTGGGGT CCCAGAACTG GAACCCTCTC TCCCTTCGGC TGGTGTTGGT GGGAGAGAAG GACGCCGCCA   
  
  
- AGTGGCTCCG TCAGTTGGTG ATGCCCCGTC ACAAACTGAG GGACCTTCGT TGGGAAGGCG GCGTATCGGT   
  
  
- TCTCTCCCAT CGACACCTCC TTCCCACCAA GCCTCTCCTC TAATTCCTGC ACCATCCTCT TCTTTCGCTC   
  
  
- TCCTCTTATC TCTCCGTGGT CTTCAAGCTC AGCACCCTTC GTAACAACTC CTCGAGTCCC AAGTTCTCAC   
  
  
- ACGGGGAGTC GGGTAAGAGC GACAGGGTTC GATTCGACGA CGACGCGGAG GTAATGGGAA GGCTTCCCAT   
  
  
- GGTTGAGGTC CAAAACGTAT CATTAACGAA CAACGAACCC ACCTTCTTAG CGGGAGAAAA AAGACAGAGA   
  
  
- AGAACCGTTA T

+     DRE core

| Site Name | Organism | Position | Strand | Matrix score. | sequence | function |
| --- | --- | --- | --- | --- | --- | --- |
| DRE core | Arabidopsis thaliana | 3051 | + | 6 | GCCGAC |  |
| DRE core | Arabidopsis thaliana | 2687 | - | 6 | GCCGAC |  |
| DRE core | Arabidopsis thaliana | 2359 | + | 6 | GCCGAC |  |

>HU03G01737.1   
+ +Up\_Stream \_Len000TTTTTT TTAAGTTTTC GTACTTGGTG TTGATGATTT GTGCTTTGTA ACCAATGGTT   
  
  
+ TTAGTAAATA GTAATTAGTC GTAACATTCT GCGATTTCGC TCAATTTATA TATCAACTTT AGAATATGAG   
  
  
+ TAACCGTAAA GATCAAACGT AGTAGTAGAG GTGTCATAGG TTTTTTTTTA AAAAAATTCG GAGTAAGAAA   
  
  
+ GTAGGGTACA TTATTTTTGC TTGTTAAATT CCGCTAGTAT AGCAATACGA GATGGAACAT TTGTAAAAAC   
  
  
+ TACGAGACGA AGCGTTTGTA CAAAACAAAC TACCATACTG CAGATCATAT AATTTTTTGG GGGGTTATGT   
  
  
+ ATCTTAGTGA TGACATTAAT TGATGGATAA CCCTATTCTA AATATCTGGA TCCATCATTA ACTGACTCGT   
  
  
+ TTCAAATGTT AGGATTATGG GTCTTGACAT GGTATACATG TCAATAGATT TATGTTAGAT GAAAAGTTAT   
  
  
+ ATGACGCTTT TTATGATTTT TGTACTAGCA TATAGTGCTT AATTGGACTA TATATTTTTA CAAGTTGTGC   
  
  
+ TTAACACCTT TTAATCAGAA ATATTTATTA TATGGAAAAA ATTTTATTAA TATGTGTTTA TGTGTTTAAA   
  
  
+ AAACATACCC AGCCTTGAAA TAGACCGTGA TATAGAAATT GGACCCATAA TACTCACATT ATATATGCTT   
  
  
+ TATTGTTATA ATTAAGGATC ATTGTTGTTT TGCAATTATG TGGCAAGATA TAATCCACAA AGTTTTACAT   
  
  
+ TTATATTGCA ATTACTCTGG TAAATGCTAT TAATGTACAT TTGAGATTAA GACATATATA TAACCCTTTT   
  
  
+ TTTTCCCTTC TTTTTTCCTT TTTCTATTTT GAATGGCATC TTAAACCAAA GGAGCTGTAC AAGTTACGTT   
  
  
+ ATAGCACTCA TGGTTGACTA AACAAGACAG ATGAGAGTTT AAGTCAAGTC CTCCTGGCAA TGATGTTTGC   
  
  
+ CATTTGGACC AATAAATACA TCATGTATAT TACCATAATA GTGTGGTTGA CTTAACAATA CATAGGGGAT   
  
  
+ TGCATACAAA GGTAAGCCTC AAAAACTTGT GCATATGCAT TGAAATCCAT GATCAAAAAA ATTCAAGAGC   
  
  
+ AGGTTGCACA AAAACACGGA GATGAGATGA AAGAGACAGA AAACACATAT ACATTCACAG TAGTAGGGGT   
  
  
+ ACTGGTAGAG CTATGGCTAG GCTAAGATGA TGTGAAAGAA ACAATATAAT GGTAGTAACT GGTAATATTT   
  
  
+ ATAGGGCACA TATAGGTGCG GGCATAGGGT CGTGTCTCTC AGTCTCAGGT ACGGTTCGAT GGGACTCTGC   
  
  
+ GATAGATGTT ATCAAATTCA ACCATTCGAA CATCTCCCAG AGAGAGAGAG AGAGACTGAG TGAGTGAGTG   
  
  
+ AGTGGGTGGG TGTATGTGTG TGAGTTGTTT TGTTCATTTA TTATAGGTTC TCTGCTGCAT ATATCTCCAC   
  
  
+ CATTAAATTT ACCAAAACTT GTACCAGCTG CTTTACTTGC TTTCCTCATT TTCCCCCTTT TTTGTCTCTC   
  
  
+ TCTCTCTCTC TCCCCCCCCC CCCCCCCCTC TCTCTCTGTG TCTGAATCAG TATCAGCCTA TCAGGATGCT   
  
  
+ TCGCCTCCAG AAAGGCATCA CTCACACACT TATTAGCTTA AAACTTTCTT TTATTCCTTT TGCTCTTTCA   
  
  
+ CTGCTCTCTT TTACCCAAAT ACGCCCGTCT TCAATTCTTC TCTCATTAAA TATGAAAAAG AAAGAGAGAA   
  
  
+ ATAGAGAGAG ATTTAAACCA GAAGGGGTGA TTCCTTTTTC TTGGTGGGCT TATTAATGCT CTGTCTACCC   
  
  
+ CATCTAGCCG AAAAATAGTA GCTTAAGACT CAACTGCTTA TTAGCATCTC TCCTCTAGCA ATAACCGAGG   
  
  
+ TAATAGCATA TTTTTCTCCC CTTAGCTTCT ACCTTGCTTT TTGTGACACT CAAACCTAAA CTGAGAGAGA   
  
  
+ GAAAGAGAAG ATAGAGGAGA GATAAATATT AGCGGGATTA GGGCAAATTT TGATATGCTG GGCTCTTCCT   
  
  
+ CATTCAACTC TGAGGAAGAA GATCAAGATC ATGCCAACCC ACCTCCGCCG CAACAACCAC CACCGTTACA   
  
  
+ GCTGCCACTT CCACGGCCAA GATTCAGCCA GTTAATATCA TCATCACCTC CAATAATATC AGCATCTTCT   
  
  
+ TCATCATTAA TCCATAATAA CCCTCGTCAG CTGCTAATAA GTTGCGGTGA ACTCATCTCC CGCTGTGACT   
  
  
+ TCTCCTCAGC TAACCGCCTT CTCTCTCTTC TCTCCGGCGA CTTCGCTTCC TCCTCCGGCG ACTCCACTGA   
  
  
+ ACGCCTTGTT TATTACTTCT CCAAGGCTTT GTTCCTCCGC CTTCGCCGAC AGTTTTCTCT ATCTTCTTCA   
  
  
+ ACTTTTACTG GTTATAGTTG TAACCCTAGT TGTTCTGCGT CTATTGCTCC ACTTTTTCTA ACCAACCCTA   
  
  
+ ATCCTAGACC ACCACAAGAA CTGCCGGTAC GAGTAACTAG CAGGGTTACT CCAAGTAGCT GCTCGTCTTA   
  
  
+ CCTAACCCTA AACCAGATCA CTCCATTTAT CCGGTTCACC CACCTGACGG CCAACCAGGC AATTTTGGAG   
  
  
+ GCTGTGGAGG GCTATAGGGC AGTCCACATC ATCGACATGG ACATCATGCA TGGAGTGCAG TGGCCTCCGC   
  
  
+ TCCTCCAAGC AATCGTGGAG CGGTCGGCCA CCCTTAGCCA ACCTCCCCCT GTGGTCCGTC TCAAAGGGGG   
  
  
+ AGGGCCGGAC CCGGACCTCT TACAGAGGAC GGGGGACCGA CTATGGAACT TTGCCCAATC ATTAGGACTA   
  
  
+ GAGTTCCATT TCCAGGGCCT CATGATCCCC GCCGAGTCAA CAGGTTTTGT TGAACCTGCT GAGGCAGCGG   
  
  
+ TCAATGCCCT AGCCTTGCAG GAGGCGGTGG GGTGTCAAGA AGGGGAGGCC CTTGCAGTTC ACTGCGGGGA   
  
  
+ CTATCTCCAC CGCCTCTTAA AAGAGTACGA CACAAGGCCT CTTAGACGGT TCCTCCACAA GGTCAAGACT   
  
  
+ CTGAACCCCA GGGTCTTGAC CTTGGGAGAG AGGGAAGCCG ACCACAACCA CCCTCTCTTC CTGCGGCGGT   
  
  
+ TCACCGAGGC AGTCAACCAC TACGGGGCAG TGTTTGACTC CCTGGAAGCA ACCCTTCCGC CGCATAGCCA   
  
  
+ AGAGAGGGTA GCTGTGGAGG AAGGGTGGTT CGGAGAGGAG ATTAAGGACG TGGTAGGAGA AGAAAGCGAG   
  
  
+ AGGAGAATAG AGAGGCACCA GAAGTTCGAG TCGTGGGAAG CATTGTTGAG GAGCTCAGGG TTCAAGAGTG   
  
  
+ TGCCCCTCAG CCCATTCTCG CTGTCCCAAG CTAAGCTGCT GCTGCGCCTC CATTACCCTT CCGAAGGGTA   
  
  
+ CCAACTCCAG GTTTTGCATA GTAATTGCTT GTTGCTTGGG TGGAAGAATC GCCCTCTTTT TTCTGTCTCT   
  
  
+ TCTTGGCAAT A  

- +Up\_Stream \_Len000AAAAAA AATTCAAAAG CATGAACCAC AACTACTAAA CACGAAACAT TGGTTACCAA   
  
  
- AATCATTTAT CATTAATCAG CATTGTAAGA CGCTAAAGCG AGTTAAATAT ATAGTTGAAA TCTTATACTC   
  
  
- ATTGGCATTT CTAGTTTGCA TCATCATCTC CACAGTATCC AAAAAAAAAT TTTTTTAAGC CTCATTCTTT   
  
  
- CATCCCATGT AATAAAAACG AACAATTTAA GGCGATCATA TCGTTATGCT CTACCTTGTA AACATTTTTG   
  
  
- ATGCTCTGCT TCGCAAACAT GTTTTGTTTG ATGGTATGAC GTCTAGTATA TTAAAAAACC CCCCAATACA   
  
  
- TAGAATCACT ACTGTAATTA ACTACCTATT GGGATAAGAT TTATAGACCT AGGTAGTAAT TGACTGAGCA   
  
  
- AAGTTTACAA TCCTAATACC CAGAACTGTA CCATATGTAC AGTTATCTAA ATACAATCTA CTTTTCAATA   
  
  
- TACTGCGAAA AATACTAAAA ACATGATCGT ATATCACGAA TTAACCTGAT ATATAAAAAT GTTCAACACG   
  
  
- AATTGTGGAA AATTAGTCTT TATAAATAAT ATACCTTTTT TAAAATAATT ATACACAAAT ACACAAATTT   
  
  
- TTTGTATGGG TCGGAACTTT ATCTGGCACT ATATCTTTAA CCTGGGTATT ATGAGTGTAA TATATACGAA   
  
  
- ATAACAATAT TAATTCCTAG TAACAACAAA ACGTTAATAC ACCGTTCTAT ATTAGGTGTT TCAAAATGTA   
  
  
- AATATAACGT TAATGAGACC ATTTACGATA ATTACATGTA AACTCTAATT CTGTATATAT ATTGGGAAAA   
  
  
- AAAAGGGAAG AAAAAAGGAA AAAGATAAAA CTTACCGTAG AATTTGGTTT CCTCGACATG TTCAATGCAA   
  
  
- TATCGTGAGT ACCAACTGAT TTGTTCTGTC TACTCTCAAA TTCAGTTCAG GAGGACCGTT ACTACAAACG   
  
  
- GTAAACCTGG TTATTTATGT AGTACATATA ATGGTATTAT CACACCAACT GAATTGTTAT GTATCCCCTA   
  
  
- ACGTATGTTT CCATTCGGAG TTTTTGAACA CGTATACGTA ACTTTAGGTA CTAGTTTTTT TAAGTTCTCG   
  
  
- TCCAACGTGT TTTTGTGCCT CTACTCTACT TTCTCTGTCT TTTGTGTATA TGTAAGTGTC ATCATCCCCA   
  
  
- TGACCATCTC GATACCGATC CGATTCTACT ACACTTTCTT TGTTATATTA CCATCATTGA CCATTATAAA   
  
  
- TATCCCGTGT ATATCCACGC CCGTATCCCA GCACAGAGAG TCAGAGTCCA TGCCAAGCTA CCCTGAGACG   
  
  
- CTATCTACAA TAGTTTAAGT TGGTAAGCTT GTAGAGGGTC TCTCTCTCTC TCTCTGACTC ACTCACTCAC   
  
  
- TCACCCACCC ACATACACAC ACTCAACAAA ACAAGTAAAT AATATCCAAG AGACGACGTA TATAGAGGTG   
  
  
- GTAATTTAAA TGGTTTTGAA CATGGTCGAC GAAATGAACG AAAGGAGTAA AAGGGGGAAA AAACAGAGAG   
  
  
- AGAGAGAGAG AGGGGGGGGG GGGGGGGGAG AGAGAGACAC AGACTTAGTC ATAGTCGGAT AGTCCTACGA   
  
  
- AGCGGAGGTC TTTCCGTAGT GAGTGTGTGA ATAATCGAAT TTTGAAAGAA AATAAGGAAA ACGAGAAAGT   
  
  
- GACGAGAGAA AATGGGTTTA TGCGGGCAGA AGTTAAGAAG AGAGTAATTT ATACTTTTTC TTTCTCTCTT   
  
  
- TATCTCTCTC TAAATTTGGT CTTCCCCACT AAGGAAAAAG AACCACCCGA ATAATTACGA GACAGATGGG   
  
  
- GTAGATCGGC TTTTTATCAT CGAATTCTGA GTTGACGAAT AATCGTAGAG AGGAGATCGT TATTGGCTCC   
  
  
- ATTATCGTAT AAAAAGAGGG GAATCGAAGA TGGAACGAAA AACACTGTGA GTTTGGATTT GACTCTCTCT   
  
  
- CTTTCTCTTC TATCTCCTCT CTATTTATAA TCGCCCTAAT CCCGTTTAAA ACTATACGAC CCGAGAAGGA   
  
  
- GTAAGTTGAG ACTCCTTCTT CTAGTTCTAG TACGGTTGGG TGGAGGCGGC GTTGTTGGTG GTGGCAATGT   
  
  
- CGACGGTGAA GGTGCCGGTT CTAAGTCGGT CAATTATAGT AGTAGTGGAG GTTATTATAG TCGTAGAAGA   
  
  
- AGTAGTAATT AGGTATTATT GGGAGCAGTC GACGATTATT CAACGCCACT TGAGTAGAGG GCGACACTGA   
  
  
- AGAGGAGTCG ATTGGCGGAA GAGAGAGAAG AGAGGCCGCT GAAGCGAAGG AGGAGGCCGC TGAGGTGACT   
  
  
- TGCGGAACAA ATAATGAAGA GGTTCCGAAA CAAGGAGGCG GAAGCGGCTG TCAAAAGAGA TAGAAGAAGT   
  
  
- TGAAAATGAC CAATATCAAC ATTGGGATCA ACAAGACGCA GATAACGAGG TGAAAAAGAT TGGTTGGGAT   
  
  
- TAGGATCTGG TGGTGTTCTT GACGGCCATG CTCATTGATC GTCCCAATGA GGTTCATCGA CGAGCAGAAT   
  
  
- GGATTGGGAT TTGGTCTAGT GAGGTAAATA GGCCAAGTGG GTGGACTGCC GGTTGGTCCG TTAAAACCTC   
  
  
- CGACACCTCC CGATATCCCG TCAGGTGTAG TAGCTGTACC TGTAGTACGT ACCTCACGTC ACCGGAGGCG   
  
  
- AGGAGGTTCG TTAGCACCTC GCCAGCCGGT GGGAATCGGT TGGAGGGGGA CACCAGGCAG AGTTTCCCCC   
  
  
- TCCCGGCCTG GGCCTGGAGA ATGTCTCCTG CCCCCTGGCT GATACCTTGA AACGGGTTAG TAATCCTGAT   
  
  
- CTCAAGGTAA AGGTCCCGGA GTACTAGGGG CGGCTCAGTT GTCCAAAACA ACTTGGACGA CTCCGTCGCC   
  
  
- AGTTACGGGA TCGGAACGTC CTCCGCCACC CCACAGTTCT TCCCCTCCGG GAACGTCAAG TGACGCCCCT   
  
  
- GATAGAGGTG GCGGAGAATT TTCTCATGCT GTGTTCCGGA GAATCTGCCA AGGAGGTGTT CCAGTTCTGA   
  
  
- GACTTGGGGT CCCAGAACTG GAACCCTCTC TCCCTTCGGC TGGTGTTGGT GGGAGAGAAG GACGCCGCCA   
  
  
- AGTGGCTCCG TCAGTTGGTG ATGCCCCGTC ACAAACTGAG GGACCTTCGT TGGGAAGGCG GCGTATCGGT   
  
  
- TCTCTCCCAT CGACACCTCC TTCCCACCAA GCCTCTCCTC TAATTCCTGC ACCATCCTCT TCTTTCGCTC   
  
  
- TCCTCTTATC TCTCCGTGGT CTTCAAGCTC AGCACCCTTC GTAACAACTC CTCGAGTCCC AAGTTCTCAC   
  
  
- ACGGGGAGTC GGGTAAGAGC GACAGGGTTC GATTCGACGA CGACGCGGAG GTAATGGGAA GGCTTCCCAT   
  
  
- GGTTGAGGTC CAAAACGTAT CATTAACGAA CAACGAACCC ACCTTCTTAG CGGGAGAAAA AAGACAGAGA   
  
  
- AGAACCGTTA T

+     G-Box

| Site Name | Organism | Position | Strand | Matrix score. | sequence | function |
| --- | --- | --- | --- | --- | --- | --- |
| G-Box | Triticum aestivum | 982 | - | 10 | TCCACATGGCA | cis-acting regulatory element involved in light responsiveness |

>HU03G01737.1   
+ +Up\_Stream \_Len000TTTTTT TTAAGTTTTC GTACTTGGTG TTGATGATTT GTGCTTTGTA ACCAATGGTT   
  
  
+ TTAGTAAATA GTAATTAGTC GTAACATTCT GCGATTTCGC TCAATTTATA TATCAACTTT AGAATATGAG   
  
  
+ TAACCGTAAA GATCAAACGT AGTAGTAGAG GTGTCATAGG TTTTTTTTTA AAAAAATTCG GAGTAAGAAA   
  
  
+ GTAGGGTACA TTATTTTTGC TTGTTAAATT CCGCTAGTAT AGCAATACGA GATGGAACAT TTGTAAAAAC   
  
  
+ TACGAGACGA AGCGTTTGTA CAAAACAAAC TACCATACTG CAGATCATAT AATTTTTTGG GGGGTTATGT   
  
  
+ ATCTTAGTGA TGACATTAAT TGATGGATAA CCCTATTCTA AATATCTGGA TCCATCATTA ACTGACTCGT   
  
  
+ TTCAAATGTT AGGATTATGG GTCTTGACAT GGTATACATG TCAATAGATT TATGTTAGAT GAAAAGTTAT   
  
  
+ ATGACGCTTT TTATGATTTT TGTACTAGCA TATAGTGCTT AATTGGACTA TATATTTTTA CAAGTTGTGC   
  
  
+ TTAACACCTT TTAATCAGAA ATATTTATTA TATGGAAAAA ATTTTATTAA TATGTGTTTA TGTGTTTAAA   
  
  
+ AAACATACCC AGCCTTGAAA TAGACCGTGA TATAGAAATT GGACCCATAA TACTCACATT ATATATGCTT   
  
  
+ TATTGTTATA ATTAAGGATC ATTGTTGTTT TGCAATTATG TGGCAAGATA TAATCCACAA AGTTTTACAT   
  
  
+ TTATATTGCA ATTACTCTGG TAAATGCTAT TAATGTACAT TTGAGATTAA GACATATATA TAACCCTTTT   
  
  
+ TTTTCCCTTC TTTTTTCCTT TTTCTATTTT GAATGGCATC TTAAACCAAA GGAGCTGTAC AAGTTACGTT   
  
  
+ ATAGCACTCA TGGTTGACTA AACAAGACAG ATGAGAGTTT AAGTCAAGTC CTCCTGGCAA TGATGTTTGC   
  
  
+ CATTTGGACC AATAAATACA TCATGTATAT TACCATAATA GTGTGGTTGA CTTAACAATA CATAGGGGAT   
  
  
+ TGCATACAAA GGTAAGCCTC AAAAACTTGT GCATATGCAT TGAAATCCAT GATCAAAAAA ATTCAAGAGC   
  
  
+ AGGTTGCACA AAAACACGGA GATGAGATGA AAGAGACAGA AAACACATAT ACATTCACAG TAGTAGGGGT   
  
  
+ ACTGGTAGAG CTATGGCTAG GCTAAGATGA TGTGAAAGAA ACAATATAAT GGTAGTAACT GGTAATATTT   
  
  
+ ATAGGGCACA TATAGGTGCG GGCATAGGGT CGTGTCTCTC AGTCTCAGGT ACGGTTCGAT GGGACTCTGC   
  
  
+ GATAGATGTT ATCAAATTCA ACCATTCGAA CATCTCCCAG AGAGAGAGAG AGAGACTGAG TGAGTGAGTG   
  
  
+ AGTGGGTGGG TGTATGTGTG TGAGTTGTTT TGTTCATTTA TTATAGGTTC TCTGCTGCAT ATATCTCCAC   
  
  
+ CATTAAATTT ACCAAAACTT GTACCAGCTG CTTTACTTGC TTTCCTCATT TTCCCCCTTT TTTGTCTCTC   
  
  
+ TCTCTCTCTC TCCCCCCCCC CCCCCCCCTC TCTCTCTGTG TCTGAATCAG TATCAGCCTA TCAGGATGCT   
  
  
+ TCGCCTCCAG AAAGGCATCA CTCACACACT TATTAGCTTA AAACTTTCTT TTATTCCTTT TGCTCTTTCA   
  
  
+ CTGCTCTCTT TTACCCAAAT ACGCCCGTCT TCAATTCTTC TCTCATTAAA TATGAAAAAG AAAGAGAGAA   
  
  
+ ATAGAGAGAG ATTTAAACCA GAAGGGGTGA TTCCTTTTTC TTGGTGGGCT TATTAATGCT CTGTCTACCC   
  
  
+ CATCTAGCCG AAAAATAGTA GCTTAAGACT CAACTGCTTA TTAGCATCTC TCCTCTAGCA ATAACCGAGG   
  
  
+ TAATAGCATA TTTTTCTCCC CTTAGCTTCT ACCTTGCTTT TTGTGACACT CAAACCTAAA CTGAGAGAGA   
  
  
+ GAAAGAGAAG ATAGAGGAGA GATAAATATT AGCGGGATTA GGGCAAATTT TGATATGCTG GGCTCTTCCT   
  
  
+ CATTCAACTC TGAGGAAGAA GATCAAGATC ATGCCAACCC ACCTCCGCCG CAACAACCAC CACCGTTACA   
  
  
+ GCTGCCACTT CCACGGCCAA GATTCAGCCA GTTAATATCA TCATCACCTC CAATAATATC AGCATCTTCT   
  
  
+ TCATCATTAA TCCATAATAA CCCTCGTCAG CTGCTAATAA GTTGCGGTGA ACTCATCTCC CGCTGTGACT   
  
  
+ TCTCCTCAGC TAACCGCCTT CTCTCTCTTC TCTCCGGCGA CTTCGCTTCC TCCTCCGGCG ACTCCACTGA   
  
  
+ ACGCCTTGTT TATTACTTCT CCAAGGCTTT GTTCCTCCGC CTTCGCCGAC AGTTTTCTCT ATCTTCTTCA   
  
  
+ ACTTTTACTG GTTATAGTTG TAACCCTAGT TGTTCTGCGT CTATTGCTCC ACTTTTTCTA ACCAACCCTA   
  
  
+ ATCCTAGACC ACCACAAGAA CTGCCGGTAC GAGTAACTAG CAGGGTTACT CCAAGTAGCT GCTCGTCTTA   
  
  
+ CCTAACCCTA AACCAGATCA CTCCATTTAT CCGGTTCACC CACCTGACGG CCAACCAGGC AATTTTGGAG   
  
  
+ GCTGTGGAGG GCTATAGGGC AGTCCACATC ATCGACATGG ACATCATGCA TGGAGTGCAG TGGCCTCCGC   
  
  
+ TCCTCCAAGC AATCGTGGAG CGGTCGGCCA CCCTTAGCCA ACCTCCCCCT GTGGTCCGTC TCAAAGGGGG   
  
  
+ AGGGCCGGAC CCGGACCTCT TACAGAGGAC GGGGGACCGA CTATGGAACT TTGCCCAATC ATTAGGACTA   
  
  
+ GAGTTCCATT TCCAGGGCCT CATGATCCCC GCCGAGTCAA CAGGTTTTGT TGAACCTGCT GAGGCAGCGG   
  
  
+ TCAATGCCCT AGCCTTGCAG GAGGCGGTGG GGTGTCAAGA AGGGGAGGCC CTTGCAGTTC ACTGCGGGGA   
  
  
+ CTATCTCCAC CGCCTCTTAA AAGAGTACGA CACAAGGCCT CTTAGACGGT TCCTCCACAA GGTCAAGACT   
  
  
+ CTGAACCCCA GGGTCTTGAC CTTGGGAGAG AGGGAAGCCG ACCACAACCA CCCTCTCTTC CTGCGGCGGT   
  
  
+ TCACCGAGGC AGTCAACCAC TACGGGGCAG TGTTTGACTC CCTGGAAGCA ACCCTTCCGC CGCATAGCCA   
  
  
+ AGAGAGGGTA GCTGTGGAGG AAGGGTGGTT CGGAGAGGAG ATTAAGGACG TGGTAGGAGA AGAAAGCGAG   
  
  
+ AGGAGAATAG AGAGGCACCA GAAGTTCGAG TCGTGGGAAG CATTGTTGAG GAGCTCAGGG TTCAAGAGTG   
  
  
+ TGCCCCTCAG CCCATTCTCG CTGTCCCAAG CTAAGCTGCT GCTGCGCCTC CATTACCCTT CCGAAGGGTA   
  
  
+ CCAACTCCAG GTTTTGCATA GTAATTGCTT GTTGCTTGGG TGGAAGAATC GCCCTCTTTT TTCTGTCTCT   
  
  
+ TCTTGGCAAT A  

- +Up\_Stream \_Len000AAAAAA AATTCAAAAG CATGAACCAC AACTACTAAA CACGAAACAT TGGTTACCAA   
  
  
- AATCATTTAT CATTAATCAG CATTGTAAGA CGCTAAAGCG AGTTAAATAT ATAGTTGAAA TCTTATACTC   
  
  
- ATTGGCATTT CTAGTTTGCA TCATCATCTC CACAGTATCC AAAAAAAAAT TTTTTTAAGC CTCATTCTTT   
  
  
- CATCCCATGT AATAAAAACG AACAATTTAA GGCGATCATA TCGTTATGCT CTACCTTGTA AACATTTTTG   
  
  
- ATGCTCTGCT TCGCAAACAT GTTTTGTTTG ATGGTATGAC GTCTAGTATA TTAAAAAACC CCCCAATACA   
  
  
- TAGAATCACT ACTGTAATTA ACTACCTATT GGGATAAGAT TTATAGACCT AGGTAGTAAT TGACTGAGCA   
  
  
- AAGTTTACAA TCCTAATACC CAGAACTGTA CCATATGTAC AGTTATCTAA ATACAATCTA CTTTTCAATA   
  
  
- TACTGCGAAA AATACTAAAA ACATGATCGT ATATCACGAA TTAACCTGAT ATATAAAAAT GTTCAACACG   
  
  
- AATTGTGGAA AATTAGTCTT TATAAATAAT ATACCTTTTT TAAAATAATT ATACACAAAT ACACAAATTT   
  
  
- TTTGTATGGG TCGGAACTTT ATCTGGCACT ATATCTTTAA CCTGGGTATT ATGAGTGTAA TATATACGAA   
  
  
- ATAACAATAT TAATTCCTAG TAACAACAAA ACGTTAATAC ACCGTTCTAT ATTAGGTGTT TCAAAATGTA   
  
  
- AATATAACGT TAATGAGACC ATTTACGATA ATTACATGTA AACTCTAATT CTGTATATAT ATTGGGAAAA   
  
  
- AAAAGGGAAG AAAAAAGGAA AAAGATAAAA CTTACCGTAG AATTTGGTTT CCTCGACATG TTCAATGCAA   
  
  
- TATCGTGAGT ACCAACTGAT TTGTTCTGTC TACTCTCAAA TTCAGTTCAG GAGGACCGTT ACTACAAACG   
  
  
- GTAAACCTGG TTATTTATGT AGTACATATA ATGGTATTAT CACACCAACT GAATTGTTAT GTATCCCCTA   
  
  
- ACGTATGTTT CCATTCGGAG TTTTTGAACA CGTATACGTA ACTTTAGGTA CTAGTTTTTT TAAGTTCTCG   
  
  
- TCCAACGTGT TTTTGTGCCT CTACTCTACT TTCTCTGTCT TTTGTGTATA TGTAAGTGTC ATCATCCCCA   
  
  
- TGACCATCTC GATACCGATC CGATTCTACT ACACTTTCTT TGTTATATTA CCATCATTGA CCATTATAAA   
  
  
- TATCCCGTGT ATATCCACGC CCGTATCCCA GCACAGAGAG TCAGAGTCCA TGCCAAGCTA CCCTGAGACG   
  
  
- CTATCTACAA TAGTTTAAGT TGGTAAGCTT GTAGAGGGTC TCTCTCTCTC TCTCTGACTC ACTCACTCAC   
  
  
- TCACCCACCC ACATACACAC ACTCAACAAA ACAAGTAAAT AATATCCAAG AGACGACGTA TATAGAGGTG   
  
  
- GTAATTTAAA TGGTTTTGAA CATGGTCGAC GAAATGAACG AAAGGAGTAA AAGGGGGAAA AAACAGAGAG   
  
  
- AGAGAGAGAG AGGGGGGGGG GGGGGGGGAG AGAGAGACAC AGACTTAGTC ATAGTCGGAT AGTCCTACGA   
  
  
- AGCGGAGGTC TTTCCGTAGT GAGTGTGTGA ATAATCGAAT TTTGAAAGAA AATAAGGAAA ACGAGAAAGT   
  
  
- GACGAGAGAA AATGGGTTTA TGCGGGCAGA AGTTAAGAAG AGAGTAATTT ATACTTTTTC TTTCTCTCTT   
  
  
- TATCTCTCTC TAAATTTGGT CTTCCCCACT AAGGAAAAAG AACCACCCGA ATAATTACGA GACAGATGGG   
  
  
- GTAGATCGGC TTTTTATCAT CGAATTCTGA GTTGACGAAT AATCGTAGAG AGGAGATCGT TATTGGCTCC   
  
  
- ATTATCGTAT AAAAAGAGGG GAATCGAAGA TGGAACGAAA AACACTGTGA GTTTGGATTT GACTCTCTCT   
  
  
- CTTTCTCTTC TATCTCCTCT CTATTTATAA TCGCCCTAAT CCCGTTTAAA ACTATACGAC CCGAGAAGGA   
  
  
- GTAAGTTGAG ACTCCTTCTT CTAGTTCTAG TACGGTTGGG TGGAGGCGGC GTTGTTGGTG GTGGCAATGT   
  
  
- CGACGGTGAA GGTGCCGGTT CTAAGTCGGT CAATTATAGT AGTAGTGGAG GTTATTATAG TCGTAGAAGA   
  
  
- AGTAGTAATT AGGTATTATT GGGAGCAGTC GACGATTATT CAACGCCACT TGAGTAGAGG GCGACACTGA   
  
  
- AGAGGAGTCG ATTGGCGGAA GAGAGAGAAG AGAGGCCGCT GAAGCGAAGG AGGAGGCCGC TGAGGTGACT   
  
  
- TGCGGAACAA ATAATGAAGA GGTTCCGAAA CAAGGAGGCG GAAGCGGCTG TCAAAAGAGA TAGAAGAAGT   
  
  
- TGAAAATGAC CAATATCAAC ATTGGGATCA ACAAGACGCA GATAACGAGG TGAAAAAGAT TGGTTGGGAT   
  
  
- TAGGATCTGG TGGTGTTCTT GACGGCCATG CTCATTGATC GTCCCAATGA GGTTCATCGA CGAGCAGAAT   
  
  
- GGATTGGGAT TTGGTCTAGT GAGGTAAATA GGCCAAGTGG GTGGACTGCC GGTTGGTCCG TTAAAACCTC   
  
  
- CGACACCTCC CGATATCCCG TCAGGTGTAG TAGCTGTACC TGTAGTACGT ACCTCACGTC ACCGGAGGCG   
  
  
- AGGAGGTTCG TTAGCACCTC GCCAGCCGGT GGGAATCGGT TGGAGGGGGA CACCAGGCAG AGTTTCCCCC   
  
  
- TCCCGGCCTG GGCCTGGAGA ATGTCTCCTG CCCCCTGGCT GATACCTTGA AACGGGTTAG TAATCCTGAT   
  
  
- CTCAAGGTAA AGGTCCCGGA GTACTAGGGG CGGCTCAGTT GTCCAAAACA ACTTGGACGA CTCCGTCGCC   
  
  
- AGTTACGGGA TCGGAACGTC CTCCGCCACC CCACAGTTCT TCCCCTCCGG GAACGTCAAG TGACGCCCCT   
  
  
- GATAGAGGTG GCGGAGAATT TTCTCATGCT GTGTTCCGGA GAATCTGCCA AGGAGGTGTT CCAGTTCTGA   
  
  
- GACTTGGGGT CCCAGAACTG GAACCCTCTC TCCCTTCGGC TGGTGTTGGT GGGAGAGAAG GACGCCGCCA   
  
  
- AGTGGCTCCG TCAGTTGGTG ATGCCCCGTC ACAAACTGAG GGACCTTCGT TGGGAAGGCG GCGTATCGGT   
  
  
- TCTCTCCCAT CGACACCTCC TTCCCACCAA GCCTCTCCTC TAATTCCTGC ACCATCCTCT TCTTTCGCTC   
  
  
- TCCTCTTATC TCTCCGTGGT CTTCAAGCTC AGCACCCTTC GTAACAACTC CTCGAGTCCC AAGTTCTCAC   
  
  
- ACGGGGAGTC GGGTAAGAGC GACAGGGTTC GATTCGACGA CGACGCGGAG GTAATGGGAA GGCTTCCCAT   
  
  
- GGTTGAGGTC CAAAACGTAT CATTAACGAA CAACGAACCC ACCTTCTTAG CGGGAGAAAA AAGACAGAGA   
  
  
- AGAACCGTTA T

+     G-box

| Site Name | Organism | Position | Strand | Matrix score. | sequence | function |
| --- | --- | --- | --- | --- | --- | --- |
| G-box | Zea mays | 3201 | - | 6 | CACGTC | cis-acting regulatory element involved in light responsiveness |
| G-box | Zea mays | 3254 | - | 6 | CACGAC | cis-acting regulatory element involved in light responsiveness |
| G-box | Zea mays | 1293 | - | 6 | CACGAC | cis-acting regulatory element involved in light responsiveness |

>HU03G01737.1   
+ +Up\_Stream \_Len000TTTTTT TTAAGTTTTC GTACTTGGTG TTGATGATTT GTGCTTTGTA ACCAATGGTT   
  
  
+ TTAGTAAATA GTAATTAGTC GTAACATTCT GCGATTTCGC TCAATTTATA TATCAACTTT AGAATATGAG   
  
  
+ TAACCGTAAA GATCAAACGT AGTAGTAGAG GTGTCATAGG TTTTTTTTTA AAAAAATTCG GAGTAAGAAA   
  
  
+ GTAGGGTACA TTATTTTTGC TTGTTAAATT CCGCTAGTAT AGCAATACGA GATGGAACAT TTGTAAAAAC   
  
  
+ TACGAGACGA AGCGTTTGTA CAAAACAAAC TACCATACTG CAGATCATAT AATTTTTTGG GGGGTTATGT   
  
  
+ ATCTTAGTGA TGACATTAAT TGATGGATAA CCCTATTCTA AATATCTGGA TCCATCATTA ACTGACTCGT   
  
  
+ TTCAAATGTT AGGATTATGG GTCTTGACAT GGTATACATG TCAATAGATT TATGTTAGAT GAAAAGTTAT   
  
  
+ ATGACGCTTT TTATGATTTT TGTACTAGCA TATAGTGCTT AATTGGACTA TATATTTTTA CAAGTTGTGC   
  
  
+ TTAACACCTT TTAATCAGAA ATATTTATTA TATGGAAAAA ATTTTATTAA TATGTGTTTA TGTGTTTAAA   
  
  
+ AAACATACCC AGCCTTGAAA TAGACCGTGA TATAGAAATT GGACCCATAA TACTCACATT ATATATGCTT   
  
  
+ TATTGTTATA ATTAAGGATC ATTGTTGTTT TGCAATTATG TGGCAAGATA TAATCCACAA AGTTTTACAT   
  
  
+ TTATATTGCA ATTACTCTGG TAAATGCTAT TAATGTACAT TTGAGATTAA GACATATATA TAACCCTTTT   
  
  
+ TTTTCCCTTC TTTTTTCCTT TTTCTATTTT GAATGGCATC TTAAACCAAA GGAGCTGTAC AAGTTACGTT   
  
  
+ ATAGCACTCA TGGTTGACTA AACAAGACAG ATGAGAGTTT AAGTCAAGTC CTCCTGGCAA TGATGTTTGC   
  
  
+ CATTTGGACC AATAAATACA TCATGTATAT TACCATAATA GTGTGGTTGA CTTAACAATA CATAGGGGAT   
  
  
+ TGCATACAAA GGTAAGCCTC AAAAACTTGT GCATATGCAT TGAAATCCAT GATCAAAAAA ATTCAAGAGC   
  
  
+ AGGTTGCACA AAAACACGGA GATGAGATGA AAGAGACAGA AAACACATAT ACATTCACAG TAGTAGGGGT   
  
  
+ ACTGGTAGAG CTATGGCTAG GCTAAGATGA TGTGAAAGAA ACAATATAAT GGTAGTAACT GGTAATATTT   
  
  
+ ATAGGGCACA TATAGGTGCG GGCATAGGGT CGTGTCTCTC AGTCTCAGGT ACGGTTCGAT GGGACTCTGC   
  
  
+ GATAGATGTT ATCAAATTCA ACCATTCGAA CATCTCCCAG AGAGAGAGAG AGAGACTGAG TGAGTGAGTG   
  
  
+ AGTGGGTGGG TGTATGTGTG TGAGTTGTTT TGTTCATTTA TTATAGGTTC TCTGCTGCAT ATATCTCCAC   
  
  
+ CATTAAATTT ACCAAAACTT GTACCAGCTG CTTTACTTGC TTTCCTCATT TTCCCCCTTT TTTGTCTCTC   
  
  
+ TCTCTCTCTC TCCCCCCCCC CCCCCCCCTC TCTCTCTGTG TCTGAATCAG TATCAGCCTA TCAGGATGCT   
  
  
+ TCGCCTCCAG AAAGGCATCA CTCACACACT TATTAGCTTA AAACTTTCTT TTATTCCTTT TGCTCTTTCA   
  
  
+ CTGCTCTCTT TTACCCAAAT ACGCCCGTCT TCAATTCTTC TCTCATTAAA TATGAAAAAG AAAGAGAGAA   
  
  
+ ATAGAGAGAG ATTTAAACCA GAAGGGGTGA TTCCTTTTTC TTGGTGGGCT TATTAATGCT CTGTCTACCC   
  
  
+ CATCTAGCCG AAAAATAGTA GCTTAAGACT CAACTGCTTA TTAGCATCTC TCCTCTAGCA ATAACCGAGG   
  
  
+ TAATAGCATA TTTTTCTCCC CTTAGCTTCT ACCTTGCTTT TTGTGACACT CAAACCTAAA CTGAGAGAGA   
  
  
+ GAAAGAGAAG ATAGAGGAGA GATAAATATT AGCGGGATTA GGGCAAATTT TGATATGCTG GGCTCTTCCT   
  
  
+ CATTCAACTC TGAGGAAGAA GATCAAGATC ATGCCAACCC ACCTCCGCCG CAACAACCAC CACCGTTACA   
  
  
+ GCTGCCACTT CCACGGCCAA GATTCAGCCA GTTAATATCA TCATCACCTC CAATAATATC AGCATCTTCT   
  
  
+ TCATCATTAA TCCATAATAA CCCTCGTCAG CTGCTAATAA GTTGCGGTGA ACTCATCTCC CGCTGTGACT   
  
  
+ TCTCCTCAGC TAACCGCCTT CTCTCTCTTC TCTCCGGCGA CTTCGCTTCC TCCTCCGGCG ACTCCACTGA   
  
  
+ ACGCCTTGTT TATTACTTCT CCAAGGCTTT GTTCCTCCGC CTTCGCCGAC AGTTTTCTCT ATCTTCTTCA   
  
  
+ ACTTTTACTG GTTATAGTTG TAACCCTAGT TGTTCTGCGT CTATTGCTCC ACTTTTTCTA ACCAACCCTA   
  
  
+ ATCCTAGACC ACCACAAGAA CTGCCGGTAC GAGTAACTAG CAGGGTTACT CCAAGTAGCT GCTCGTCTTA   
  
  
+ CCTAACCCTA AACCAGATCA CTCCATTTAT CCGGTTCACC CACCTGACGG CCAACCAGGC AATTTTGGAG   
  
  
+ GCTGTGGAGG GCTATAGGGC AGTCCACATC ATCGACATGG ACATCATGCA TGGAGTGCAG TGGCCTCCGC   
  
  
+ TCCTCCAAGC AATCGTGGAG CGGTCGGCCA CCCTTAGCCA ACCTCCCCCT GTGGTCCGTC TCAAAGGGGG   
  
  
+ AGGGCCGGAC CCGGACCTCT TACAGAGGAC GGGGGACCGA CTATGGAACT TTGCCCAATC ATTAGGACTA   
  
  
+ GAGTTCCATT TCCAGGGCCT CATGATCCCC GCCGAGTCAA CAGGTTTTGT TGAACCTGCT GAGGCAGCGG   
  
  
+ TCAATGCCCT AGCCTTGCAG GAGGCGGTGG GGTGTCAAGA AGGGGAGGCC CTTGCAGTTC ACTGCGGGGA   
  
  
+ CTATCTCCAC CGCCTCTTAA AAGAGTACGA CACAAGGCCT CTTAGACGGT TCCTCCACAA GGTCAAGACT   
  
  
+ CTGAACCCCA GGGTCTTGAC CTTGGGAGAG AGGGAAGCCG ACCACAACCA CCCTCTCTTC CTGCGGCGGT   
  
  
+ TCACCGAGGC AGTCAACCAC TACGGGGCAG TGTTTGACTC CCTGGAAGCA ACCCTTCCGC CGCATAGCCA   
  
  
+ AGAGAGGGTA GCTGTGGAGG AAGGGTGGTT CGGAGAGGAG ATTAAGGACG TGGTAGGAGA AGAAAGCGAG   
  
  
+ AGGAGAATAG AGAGGCACCA GAAGTTCGAG TCGTGGGAAG CATTGTTGAG GAGCTCAGGG TTCAAGAGTG   
  
  
+ TGCCCCTCAG CCCATTCTCG CTGTCCCAAG CTAAGCTGCT GCTGCGCCTC CATTACCCTT CCGAAGGGTA   
  
  
+ CCAACTCCAG GTTTTGCATA GTAATTGCTT GTTGCTTGGG TGGAAGAATC GCCCTCTTTT TTCTGTCTCT   
  
  
+ TCTTGGCAAT A  

- +Up\_Stream \_Len000AAAAAA AATTCAAAAG CATGAACCAC AACTACTAAA CACGAAACAT TGGTTACCAA   
  
  
- AATCATTTAT CATTAATCAG CATTGTAAGA CGCTAAAGCG AGTTAAATAT ATAGTTGAAA TCTTATACTC   
  
  
- ATTGGCATTT CTAGTTTGCA TCATCATCTC CACAGTATCC AAAAAAAAAT TTTTTTAAGC CTCATTCTTT   
  
  
- CATCCCATGT AATAAAAACG AACAATTTAA GGCGATCATA TCGTTATGCT CTACCTTGTA AACATTTTTG   
  
  
- ATGCTCTGCT TCGCAAACAT GTTTTGTTTG ATGGTATGAC GTCTAGTATA TTAAAAAACC CCCCAATACA   
  
  
- TAGAATCACT ACTGTAATTA ACTACCTATT GGGATAAGAT TTATAGACCT AGGTAGTAAT TGACTGAGCA   
  
  
- AAGTTTACAA TCCTAATACC CAGAACTGTA CCATATGTAC AGTTATCTAA ATACAATCTA CTTTTCAATA   
  
  
- TACTGCGAAA AATACTAAAA ACATGATCGT ATATCACGAA TTAACCTGAT ATATAAAAAT GTTCAACACG   
  
  
- AATTGTGGAA AATTAGTCTT TATAAATAAT ATACCTTTTT TAAAATAATT ATACACAAAT ACACAAATTT   
  
  
- TTTGTATGGG TCGGAACTTT ATCTGGCACT ATATCTTTAA CCTGGGTATT ATGAGTGTAA TATATACGAA   
  
  
- ATAACAATAT TAATTCCTAG TAACAACAAA ACGTTAATAC ACCGTTCTAT ATTAGGTGTT TCAAAATGTA   
  
  
- AATATAACGT TAATGAGACC ATTTACGATA ATTACATGTA AACTCTAATT CTGTATATAT ATTGGGAAAA   
  
  
- AAAAGGGAAG AAAAAAGGAA AAAGATAAAA CTTACCGTAG AATTTGGTTT CCTCGACATG TTCAATGCAA   
  
  
- TATCGTGAGT ACCAACTGAT TTGTTCTGTC TACTCTCAAA TTCAGTTCAG GAGGACCGTT ACTACAAACG   
  
  
- GTAAACCTGG TTATTTATGT AGTACATATA ATGGTATTAT CACACCAACT GAATTGTTAT GTATCCCCTA   
  
  
- ACGTATGTTT CCATTCGGAG TTTTTGAACA CGTATACGTA ACTTTAGGTA CTAGTTTTTT TAAGTTCTCG   
  
  
- TCCAACGTGT TTTTGTGCCT CTACTCTACT TTCTCTGTCT TTTGTGTATA TGTAAGTGTC ATCATCCCCA   
  
  
- TGACCATCTC GATACCGATC CGATTCTACT ACACTTTCTT TGTTATATTA CCATCATTGA CCATTATAAA   
  
  
- TATCCCGTGT ATATCCACGC CCGTATCCCA GCACAGAGAG TCAGAGTCCA TGCCAAGCTA CCCTGAGACG   
  
  
- CTATCTACAA TAGTTTAAGT TGGTAAGCTT GTAGAGGGTC TCTCTCTCTC TCTCTGACTC ACTCACTCAC   
  
  
- TCACCCACCC ACATACACAC ACTCAACAAA ACAAGTAAAT AATATCCAAG AGACGACGTA TATAGAGGTG   
  
  
- GTAATTTAAA TGGTTTTGAA CATGGTCGAC GAAATGAACG AAAGGAGTAA AAGGGGGAAA AAACAGAGAG   
  
  
- AGAGAGAGAG AGGGGGGGGG GGGGGGGGAG AGAGAGACAC AGACTTAGTC ATAGTCGGAT AGTCCTACGA   
  
  
- AGCGGAGGTC TTTCCGTAGT GAGTGTGTGA ATAATCGAAT TTTGAAAGAA AATAAGGAAA ACGAGAAAGT   
  
  
- GACGAGAGAA AATGGGTTTA TGCGGGCAGA AGTTAAGAAG AGAGTAATTT ATACTTTTTC TTTCTCTCTT   
  
  
- TATCTCTCTC TAAATTTGGT CTTCCCCACT AAGGAAAAAG AACCACCCGA ATAATTACGA GACAGATGGG   
  
  
- GTAGATCGGC TTTTTATCAT CGAATTCTGA GTTGACGAAT AATCGTAGAG AGGAGATCGT TATTGGCTCC   
  
  
- ATTATCGTAT AAAAAGAGGG GAATCGAAGA TGGAACGAAA AACACTGTGA GTTTGGATTT GACTCTCTCT   
  
  
- CTTTCTCTTC TATCTCCTCT CTATTTATAA TCGCCCTAAT CCCGTTTAAA ACTATACGAC CCGAGAAGGA   
  
  
- GTAAGTTGAG ACTCCTTCTT CTAGTTCTAG TACGGTTGGG TGGAGGCGGC GTTGTTGGTG GTGGCAATGT   
  
  
- CGACGGTGAA GGTGCCGGTT CTAAGTCGGT CAATTATAGT AGTAGTGGAG GTTATTATAG TCGTAGAAGA   
  
  
- AGTAGTAATT AGGTATTATT GGGAGCAGTC GACGATTATT CAACGCCACT TGAGTAGAGG GCGACACTGA   
  
  
- AGAGGAGTCG ATTGGCGGAA GAGAGAGAAG AGAGGCCGCT GAAGCGAAGG AGGAGGCCGC TGAGGTGACT   
  
  
- TGCGGAACAA ATAATGAAGA GGTTCCGAAA CAAGGAGGCG GAAGCGGCTG TCAAAAGAGA TAGAAGAAGT   
  
  
- TGAAAATGAC CAATATCAAC ATTGGGATCA ACAAGACGCA GATAACGAGG TGAAAAAGAT TGGTTGGGAT   
  
  
- TAGGATCTGG TGGTGTTCTT GACGGCCATG CTCATTGATC GTCCCAATGA GGTTCATCGA CGAGCAGAAT   
  
  
- GGATTGGGAT TTGGTCTAGT GAGGTAAATA GGCCAAGTGG GTGGACTGCC GGTTGGTCCG TTAAAACCTC   
  
  
- CGACACCTCC CGATATCCCG TCAGGTGTAG TAGCTGTACC TGTAGTACGT ACCTCACGTC ACCGGAGGCG   
  
  
- AGGAGGTTCG TTAGCACCTC GCCAGCCGGT GGGAATCGGT TGGAGGGGGA CACCAGGCAG AGTTTCCCCC   
  
  
- TCCCGGCCTG GGCCTGGAGA ATGTCTCCTG CCCCCTGGCT GATACCTTGA AACGGGTTAG TAATCCTGAT   
  
  
- CTCAAGGTAA AGGTCCCGGA GTACTAGGGG CGGCTCAGTT GTCCAAAACA ACTTGGACGA CTCCGTCGCC   
  
  
- AGTTACGGGA TCGGAACGTC CTCCGCCACC CCACAGTTCT TCCCCTCCGG GAACGTCAAG TGACGCCCCT   
  
  
- GATAGAGGTG GCGGAGAATT TTCTCATGCT GTGTTCCGGA GAATCTGCCA AGGAGGTGTT CCAGTTCTGA   
  
  
- GACTTGGGGT CCCAGAACTG GAACCCTCTC TCCCTTCGGC TGGTGTTGGT GGGAGAGAAG GACGCCGCCA   
  
  
- AGTGGCTCCG TCAGTTGGTG ATGCCCCGTC ACAAACTGAG GGACCTTCGT TGGGAAGGCG GCGTATCGGT   
  
  
- TCTCTCCCAT CGACACCTCC TTCCCACCAA GCCTCTCCTC TAATTCCTGC ACCATCCTCT TCTTTCGCTC   
  
  
- TCCTCTTATC TCTCCGTGGT CTTCAAGCTC AGCACCCTTC GTAACAACTC CTCGAGTCCC AAGTTCTCAC   
  
  
- ACGGGGAGTC GGGTAAGAGC GACAGGGTTC GATTCGACGA CGACGCGGAG GTAATGGGAA GGCTTCCCAT   
  
  
- GGTTGAGGTC CAAAACGTAT CATTAACGAA CAACGAACCC ACCTTCTTAG CGGGAGAAAA AAGACAGAGA   
  
  
- AGAACCGTTA T

+     GC-motif

| Site Name | Organism | Position | Strand | Matrix score. | sequence | function |
| --- | --- | --- | --- | --- | --- | --- |
| GC-motif | Zea mays | 2764 | - | 6 | CCCCCG | enhancer-like element involved in anoxic specific inducibility |

>HU03G01737.1   
+ +Up\_Stream \_Len000TTTTTT TTAAGTTTTC GTACTTGGTG TTGATGATTT GTGCTTTGTA ACCAATGGTT   
  
  
+ TTAGTAAATA GTAATTAGTC GTAACATTCT GCGATTTCGC TCAATTTATA TATCAACTTT AGAATATGAG   
  
  
+ TAACCGTAAA GATCAAACGT AGTAGTAGAG GTGTCATAGG TTTTTTTTTA AAAAAATTCG GAGTAAGAAA   
  
  
+ GTAGGGTACA TTATTTTTGC TTGTTAAATT CCGCTAGTAT AGCAATACGA GATGGAACAT TTGTAAAAAC   
  
  
+ TACGAGACGA AGCGTTTGTA CAAAACAAAC TACCATACTG CAGATCATAT AATTTTTTGG GGGGTTATGT   
  
  
+ ATCTTAGTGA TGACATTAAT TGATGGATAA CCCTATTCTA AATATCTGGA TCCATCATTA ACTGACTCGT   
  
  
+ TTCAAATGTT AGGATTATGG GTCTTGACAT GGTATACATG TCAATAGATT TATGTTAGAT GAAAAGTTAT   
  
  
+ ATGACGCTTT TTATGATTTT TGTACTAGCA TATAGTGCTT AATTGGACTA TATATTTTTA CAAGTTGTGC   
  
  
+ TTAACACCTT TTAATCAGAA ATATTTATTA TATGGAAAAA ATTTTATTAA TATGTGTTTA TGTGTTTAAA   
  
  
+ AAACATACCC AGCCTTGAAA TAGACCGTGA TATAGAAATT GGACCCATAA TACTCACATT ATATATGCTT   
  
  
+ TATTGTTATA ATTAAGGATC ATTGTTGTTT TGCAATTATG TGGCAAGATA TAATCCACAA AGTTTTACAT   
  
  
+ TTATATTGCA ATTACTCTGG TAAATGCTAT TAATGTACAT TTGAGATTAA GACATATATA TAACCCTTTT   
  
  
+ TTTTCCCTTC TTTTTTCCTT TTTCTATTTT GAATGGCATC TTAAACCAAA GGAGCTGTAC AAGTTACGTT   
  
  
+ ATAGCACTCA TGGTTGACTA AACAAGACAG ATGAGAGTTT AAGTCAAGTC CTCCTGGCAA TGATGTTTGC   
  
  
+ CATTTGGACC AATAAATACA TCATGTATAT TACCATAATA GTGTGGTTGA CTTAACAATA CATAGGGGAT   
  
  
+ TGCATACAAA GGTAAGCCTC AAAAACTTGT GCATATGCAT TGAAATCCAT GATCAAAAAA ATTCAAGAGC   
  
  
+ AGGTTGCACA AAAACACGGA GATGAGATGA AAGAGACAGA AAACACATAT ACATTCACAG TAGTAGGGGT   
  
  
+ ACTGGTAGAG CTATGGCTAG GCTAAGATGA TGTGAAAGAA ACAATATAAT GGTAGTAACT GGTAATATTT   
  
  
+ ATAGGGCACA TATAGGTGCG GGCATAGGGT CGTGTCTCTC AGTCTCAGGT ACGGTTCGAT GGGACTCTGC   
  
  
+ GATAGATGTT ATCAAATTCA ACCATTCGAA CATCTCCCAG AGAGAGAGAG AGAGACTGAG TGAGTGAGTG   
  
  
+ AGTGGGTGGG TGTATGTGTG TGAGTTGTTT TGTTCATTTA TTATAGGTTC TCTGCTGCAT ATATCTCCAC   
  
  
+ CATTAAATTT ACCAAAACTT GTACCAGCTG CTTTACTTGC TTTCCTCATT TTCCCCCTTT TTTGTCTCTC   
  
  
+ TCTCTCTCTC TCCCCCCCCC CCCCCCCCTC TCTCTCTGTG TCTGAATCAG TATCAGCCTA TCAGGATGCT   
  
  
+ TCGCCTCCAG AAAGGCATCA CTCACACACT TATTAGCTTA AAACTTTCTT TTATTCCTTT TGCTCTTTCA   
  
  
+ CTGCTCTCTT TTACCCAAAT ACGCCCGTCT TCAATTCTTC TCTCATTAAA TATGAAAAAG AAAGAGAGAA   
  
  
+ ATAGAGAGAG ATTTAAACCA GAAGGGGTGA TTCCTTTTTC TTGGTGGGCT TATTAATGCT CTGTCTACCC   
  
  
+ CATCTAGCCG AAAAATAGTA GCTTAAGACT CAACTGCTTA TTAGCATCTC TCCTCTAGCA ATAACCGAGG   
  
  
+ TAATAGCATA TTTTTCTCCC CTTAGCTTCT ACCTTGCTTT TTGTGACACT CAAACCTAAA CTGAGAGAGA   
  
  
+ GAAAGAGAAG ATAGAGGAGA GATAAATATT AGCGGGATTA GGGCAAATTT TGATATGCTG GGCTCTTCCT   
  
  
+ CATTCAACTC TGAGGAAGAA GATCAAGATC ATGCCAACCC ACCTCCGCCG CAACAACCAC CACCGTTACA   
  
  
+ GCTGCCACTT CCACGGCCAA GATTCAGCCA GTTAATATCA TCATCACCTC CAATAATATC AGCATCTTCT   
  
  
+ TCATCATTAA TCCATAATAA CCCTCGTCAG CTGCTAATAA GTTGCGGTGA ACTCATCTCC CGCTGTGACT   
  
  
+ TCTCCTCAGC TAACCGCCTT CTCTCTCTTC TCTCCGGCGA CTTCGCTTCC TCCTCCGGCG ACTCCACTGA   
  
  
+ ACGCCTTGTT TATTACTTCT CCAAGGCTTT GTTCCTCCGC CTTCGCCGAC AGTTTTCTCT ATCTTCTTCA   
  
  
+ ACTTTTACTG GTTATAGTTG TAACCCTAGT TGTTCTGCGT CTATTGCTCC ACTTTTTCTA ACCAACCCTA   
  
  
+ ATCCTAGACC ACCACAAGAA CTGCCGGTAC GAGTAACTAG CAGGGTTACT CCAAGTAGCT GCTCGTCTTA   
  
  
+ CCTAACCCTA AACCAGATCA CTCCATTTAT CCGGTTCACC CACCTGACGG CCAACCAGGC AATTTTGGAG   
  
  
+ GCTGTGGAGG GCTATAGGGC AGTCCACATC ATCGACATGG ACATCATGCA TGGAGTGCAG TGGCCTCCGC   
  
  
+ TCCTCCAAGC AATCGTGGAG CGGTCGGCCA CCCTTAGCCA ACCTCCCCCT GTGGTCCGTC TCAAAGGGGG   
  
  
+ AGGGCCGGAC CCGGACCTCT TACAGAGGAC GGGGGACCGA CTATGGAACT TTGCCCAATC ATTAGGACTA   
  
  
+ GAGTTCCATT TCCAGGGCCT CATGATCCCC GCCGAGTCAA CAGGTTTTGT TGAACCTGCT GAGGCAGCGG   
  
  
+ TCAATGCCCT AGCCTTGCAG GAGGCGGTGG GGTGTCAAGA AGGGGAGGCC CTTGCAGTTC ACTGCGGGGA   
  
  
+ CTATCTCCAC CGCCTCTTAA AAGAGTACGA CACAAGGCCT CTTAGACGGT TCCTCCACAA GGTCAAGACT   
  
  
+ CTGAACCCCA GGGTCTTGAC CTTGGGAGAG AGGGAAGCCG ACCACAACCA CCCTCTCTTC CTGCGGCGGT   
  
  
+ TCACCGAGGC AGTCAACCAC TACGGGGCAG TGTTTGACTC CCTGGAAGCA ACCCTTCCGC CGCATAGCCA   
  
  
+ AGAGAGGGTA GCTGTGGAGG AAGGGTGGTT CGGAGAGGAG ATTAAGGACG TGGTAGGAGA AGAAAGCGAG   
  
  
+ AGGAGAATAG AGAGGCACCA GAAGTTCGAG TCGTGGGAAG CATTGTTGAG GAGCTCAGGG TTCAAGAGTG   
  
  
+ TGCCCCTCAG CCCATTCTCG CTGTCCCAAG CTAAGCTGCT GCTGCGCCTC CATTACCCTT CCGAAGGGTA   
  
  
+ CCAACTCCAG GTTTTGCATA GTAATTGCTT GTTGCTTGGG TGGAAGAATC GCCCTCTTTT TTCTGTCTCT   
  
  
+ TCTTGGCAAT A  

- +Up\_Stream \_Len000AAAAAA AATTCAAAAG CATGAACCAC AACTACTAAA CACGAAACAT TGGTTACCAA   
  
  
- AATCATTTAT CATTAATCAG CATTGTAAGA CGCTAAAGCG AGTTAAATAT ATAGTTGAAA TCTTATACTC   
  
  
- ATTGGCATTT CTAGTTTGCA TCATCATCTC CACAGTATCC AAAAAAAAAT TTTTTTAAGC CTCATTCTTT   
  
  
- CATCCCATGT AATAAAAACG AACAATTTAA GGCGATCATA TCGTTATGCT CTACCTTGTA AACATTTTTG   
  
  
- ATGCTCTGCT TCGCAAACAT GTTTTGTTTG ATGGTATGAC GTCTAGTATA TTAAAAAACC CCCCAATACA   
  
  
- TAGAATCACT ACTGTAATTA ACTACCTATT GGGATAAGAT TTATAGACCT AGGTAGTAAT TGACTGAGCA   
  
  
- AAGTTTACAA TCCTAATACC CAGAACTGTA CCATATGTAC AGTTATCTAA ATACAATCTA CTTTTCAATA   
  
  
- TACTGCGAAA AATACTAAAA ACATGATCGT ATATCACGAA TTAACCTGAT ATATAAAAAT GTTCAACACG   
  
  
- AATTGTGGAA AATTAGTCTT TATAAATAAT ATACCTTTTT TAAAATAATT ATACACAAAT ACACAAATTT   
  
  
- TTTGTATGGG TCGGAACTTT ATCTGGCACT ATATCTTTAA CCTGGGTATT ATGAGTGTAA TATATACGAA   
  
  
- ATAACAATAT TAATTCCTAG TAACAACAAA ACGTTAATAC ACCGTTCTAT ATTAGGTGTT TCAAAATGTA   
  
  
- AATATAACGT TAATGAGACC ATTTACGATA ATTACATGTA AACTCTAATT CTGTATATAT ATTGGGAAAA   
  
  
- AAAAGGGAAG AAAAAAGGAA AAAGATAAAA CTTACCGTAG AATTTGGTTT CCTCGACATG TTCAATGCAA   
  
  
- TATCGTGAGT ACCAACTGAT TTGTTCTGTC TACTCTCAAA TTCAGTTCAG GAGGACCGTT ACTACAAACG   
  
  
- GTAAACCTGG TTATTTATGT AGTACATATA ATGGTATTAT CACACCAACT GAATTGTTAT GTATCCCCTA   
  
  
- ACGTATGTTT CCATTCGGAG TTTTTGAACA CGTATACGTA ACTTTAGGTA CTAGTTTTTT TAAGTTCTCG   
  
  
- TCCAACGTGT TTTTGTGCCT CTACTCTACT TTCTCTGTCT TTTGTGTATA TGTAAGTGTC ATCATCCCCA   
  
  
- TGACCATCTC GATACCGATC CGATTCTACT ACACTTTCTT TGTTATATTA CCATCATTGA CCATTATAAA   
  
  
- TATCCCGTGT ATATCCACGC CCGTATCCCA GCACAGAGAG TCAGAGTCCA TGCCAAGCTA CCCTGAGACG   
  
  
- CTATCTACAA TAGTTTAAGT TGGTAAGCTT GTAGAGGGTC TCTCTCTCTC TCTCTGACTC ACTCACTCAC   
  
  
- TCACCCACCC ACATACACAC ACTCAACAAA ACAAGTAAAT AATATCCAAG AGACGACGTA TATAGAGGTG   
  
  
- GTAATTTAAA TGGTTTTGAA CATGGTCGAC GAAATGAACG AAAGGAGTAA AAGGGGGAAA AAACAGAGAG   
  
  
- AGAGAGAGAG AGGGGGGGGG GGGGGGGGAG AGAGAGACAC AGACTTAGTC ATAGTCGGAT AGTCCTACGA   
  
  
- AGCGGAGGTC TTTCCGTAGT GAGTGTGTGA ATAATCGAAT TTTGAAAGAA AATAAGGAAA ACGAGAAAGT   
  
  
- GACGAGAGAA AATGGGTTTA TGCGGGCAGA AGTTAAGAAG AGAGTAATTT ATACTTTTTC TTTCTCTCTT   
  
  
- TATCTCTCTC TAAATTTGGT CTTCCCCACT AAGGAAAAAG AACCACCCGA ATAATTACGA GACAGATGGG   
  
  
- GTAGATCGGC TTTTTATCAT CGAATTCTGA GTTGACGAAT AATCGTAGAG AGGAGATCGT TATTGGCTCC   
  
  
- ATTATCGTAT AAAAAGAGGG GAATCGAAGA TGGAACGAAA AACACTGTGA GTTTGGATTT GACTCTCTCT   
  
  
- CTTTCTCTTC TATCTCCTCT CTATTTATAA TCGCCCTAAT CCCGTTTAAA ACTATACGAC CCGAGAAGGA   
  
  
- GTAAGTTGAG ACTCCTTCTT CTAGTTCTAG TACGGTTGGG TGGAGGCGGC GTTGTTGGTG GTGGCAATGT   
  
  
- CGACGGTGAA GGTGCCGGTT CTAAGTCGGT CAATTATAGT AGTAGTGGAG GTTATTATAG TCGTAGAAGA   
  
  
- AGTAGTAATT AGGTATTATT GGGAGCAGTC GACGATTATT CAACGCCACT TGAGTAGAGG GCGACACTGA   
  
  
- AGAGGAGTCG ATTGGCGGAA GAGAGAGAAG AGAGGCCGCT GAAGCGAAGG AGGAGGCCGC TGAGGTGACT   
  
  
- TGCGGAACAA ATAATGAAGA GGTTCCGAAA CAAGGAGGCG GAAGCGGCTG TCAAAAGAGA TAGAAGAAGT   
  
  
- TGAAAATGAC CAATATCAAC ATTGGGATCA ACAAGACGCA GATAACGAGG TGAAAAAGAT TGGTTGGGAT   
  
  
- TAGGATCTGG TGGTGTTCTT GACGGCCATG CTCATTGATC GTCCCAATGA GGTTCATCGA CGAGCAGAAT   
  
  
- GGATTGGGAT TTGGTCTAGT GAGGTAAATA GGCCAAGTGG GTGGACTGCC GGTTGGTCCG TTAAAACCTC   
  
  
- CGACACCTCC CGATATCCCG TCAGGTGTAG TAGCTGTACC TGTAGTACGT ACCTCACGTC ACCGGAGGCG   
  
  
- AGGAGGTTCG TTAGCACCTC GCCAGCCGGT GGGAATCGGT TGGAGGGGGA CACCAGGCAG AGTTTCCCCC   
  
  
- TCCCGGCCTG GGCCTGGAGA ATGTCTCCTG CCCCCTGGCT GATACCTTGA AACGGGTTAG TAATCCTGAT   
  
  
- CTCAAGGTAA AGGTCCCGGA GTACTAGGGG CGGCTCAGTT GTCCAAAACA ACTTGGACGA CTCCGTCGCC   
  
  
- AGTTACGGGA TCGGAACGTC CTCCGCCACC CCACAGTTCT TCCCCTCCGG GAACGTCAAG TGACGCCCCT   
  
  
- GATAGAGGTG GCGGAGAATT TTCTCATGCT GTGTTCCGGA GAATCTGCCA AGGAGGTGTT CCAGTTCTGA   
  
  
- GACTTGGGGT CCCAGAACTG GAACCCTCTC TCCCTTCGGC TGGTGTTGGT GGGAGAGAAG GACGCCGCCA   
  
  
- AGTGGCTCCG TCAGTTGGTG ATGCCCCGTC ACAAACTGAG GGACCTTCGT TGGGAAGGCG GCGTATCGGT   
  
  
- TCTCTCCCAT CGACACCTCC TTCCCACCAA GCCTCTCCTC TAATTCCTGC ACCATCCTCT TCTTTCGCTC   
  
  
- TCCTCTTATC TCTCCGTGGT CTTCAAGCTC AGCACCCTTC GTAACAACTC CTCGAGTCCC AAGTTCTCAC   
  
  
- ACGGGGAGTC GGGTAAGAGC GACAGGGTTC GATTCGACGA CGACGCGGAG GTAATGGGAA GGCTTCCCAT   
  
  
- GGTTGAGGTC CAAAACGTAT CATTAACGAA CAACGAACCC ACCTTCTTAG CGGGAGAAAA AAGACAGAGA   
  
  
- AGAACCGTTA T

+     LTR

| Site Name | Organism | Position | Strand | Matrix score. | sequence | function |
| --- | --- | --- | --- | --- | --- | --- |
| LTR | Hordeum vulgare | 1832 | + | 6 | CCGAAA | cis-acting element involved in low-temperature responsiveness |

>HU03G01737.1   
+ +Up\_Stream \_Len000TTTTTT TTAAGTTTTC GTACTTGGTG TTGATGATTT GTGCTTTGTA ACCAATGGTT   
  
  
+ TTAGTAAATA GTAATTAGTC GTAACATTCT GCGATTTCGC TCAATTTATA TATCAACTTT AGAATATGAG   
  
  
+ TAACCGTAAA GATCAAACGT AGTAGTAGAG GTGTCATAGG TTTTTTTTTA AAAAAATTCG GAGTAAGAAA   
  
  
+ GTAGGGTACA TTATTTTTGC TTGTTAAATT CCGCTAGTAT AGCAATACGA GATGGAACAT TTGTAAAAAC   
  
  
+ TACGAGACGA AGCGTTTGTA CAAAACAAAC TACCATACTG CAGATCATAT AATTTTTTGG GGGGTTATGT   
  
  
+ ATCTTAGTGA TGACATTAAT TGATGGATAA CCCTATTCTA AATATCTGGA TCCATCATTA ACTGACTCGT   
  
  
+ TTCAAATGTT AGGATTATGG GTCTTGACAT GGTATACATG TCAATAGATT TATGTTAGAT GAAAAGTTAT   
  
  
+ ATGACGCTTT TTATGATTTT TGTACTAGCA TATAGTGCTT AATTGGACTA TATATTTTTA CAAGTTGTGC   
  
  
+ TTAACACCTT TTAATCAGAA ATATTTATTA TATGGAAAAA ATTTTATTAA TATGTGTTTA TGTGTTTAAA   
  
  
+ AAACATACCC AGCCTTGAAA TAGACCGTGA TATAGAAATT GGACCCATAA TACTCACATT ATATATGCTT   
  
  
+ TATTGTTATA ATTAAGGATC ATTGTTGTTT TGCAATTATG TGGCAAGATA TAATCCACAA AGTTTTACAT   
  
  
+ TTATATTGCA ATTACTCTGG TAAATGCTAT TAATGTACAT TTGAGATTAA GACATATATA TAACCCTTTT   
  
  
+ TTTTCCCTTC TTTTTTCCTT TTTCTATTTT GAATGGCATC TTAAACCAAA GGAGCTGTAC AAGTTACGTT   
  
  
+ ATAGCACTCA TGGTTGACTA AACAAGACAG ATGAGAGTTT AAGTCAAGTC CTCCTGGCAA TGATGTTTGC   
  
  
+ CATTTGGACC AATAAATACA TCATGTATAT TACCATAATA GTGTGGTTGA CTTAACAATA CATAGGGGAT   
  
  
+ TGCATACAAA GGTAAGCCTC AAAAACTTGT GCATATGCAT TGAAATCCAT GATCAAAAAA ATTCAAGAGC   
  
  
+ AGGTTGCACA AAAACACGGA GATGAGATGA AAGAGACAGA AAACACATAT ACATTCACAG TAGTAGGGGT   
  
  
+ ACTGGTAGAG CTATGGCTAG GCTAAGATGA TGTGAAAGAA ACAATATAAT GGTAGTAACT GGTAATATTT   
  
  
+ ATAGGGCACA TATAGGTGCG GGCATAGGGT CGTGTCTCTC AGTCTCAGGT ACGGTTCGAT GGGACTCTGC   
  
  
+ GATAGATGTT ATCAAATTCA ACCATTCGAA CATCTCCCAG AGAGAGAGAG AGAGACTGAG TGAGTGAGTG   
  
  
+ AGTGGGTGGG TGTATGTGTG TGAGTTGTTT TGTTCATTTA TTATAGGTTC TCTGCTGCAT ATATCTCCAC   
  
  
+ CATTAAATTT ACCAAAACTT GTACCAGCTG CTTTACTTGC TTTCCTCATT TTCCCCCTTT TTTGTCTCTC   
  
  
+ TCTCTCTCTC TCCCCCCCCC CCCCCCCCTC TCTCTCTGTG TCTGAATCAG TATCAGCCTA TCAGGATGCT   
  
  
+ TCGCCTCCAG AAAGGCATCA CTCACACACT TATTAGCTTA AAACTTTCTT TTATTCCTTT TGCTCTTTCA   
  
  
+ CTGCTCTCTT TTACCCAAAT ACGCCCGTCT TCAATTCTTC TCTCATTAAA TATGAAAAAG AAAGAGAGAA   
  
  
+ ATAGAGAGAG ATTTAAACCA GAAGGGGTGA TTCCTTTTTC TTGGTGGGCT TATTAATGCT CTGTCTACCC   
  
  
+ CATCTAGCCG AAAAATAGTA GCTTAAGACT CAACTGCTTA TTAGCATCTC TCCTCTAGCA ATAACCGAGG   
  
  
+ TAATAGCATA TTTTTCTCCC CTTAGCTTCT ACCTTGCTTT TTGTGACACT CAAACCTAAA CTGAGAGAGA   
  
  
+ GAAAGAGAAG ATAGAGGAGA GATAAATATT AGCGGGATTA GGGCAAATTT TGATATGCTG GGCTCTTCCT   
  
  
+ CATTCAACTC TGAGGAAGAA GATCAAGATC ATGCCAACCC ACCTCCGCCG CAACAACCAC CACCGTTACA   
  
  
+ GCTGCCACTT CCACGGCCAA GATTCAGCCA GTTAATATCA TCATCACCTC CAATAATATC AGCATCTTCT   
  
  
+ TCATCATTAA TCCATAATAA CCCTCGTCAG CTGCTAATAA GTTGCGGTGA ACTCATCTCC CGCTGTGACT   
  
  
+ TCTCCTCAGC TAACCGCCTT CTCTCTCTTC TCTCCGGCGA CTTCGCTTCC TCCTCCGGCG ACTCCACTGA   
  
  
+ ACGCCTTGTT TATTACTTCT CCAAGGCTTT GTTCCTCCGC CTTCGCCGAC AGTTTTCTCT ATCTTCTTCA   
  
  
+ ACTTTTACTG GTTATAGTTG TAACCCTAGT TGTTCTGCGT CTATTGCTCC ACTTTTTCTA ACCAACCCTA   
  
  
+ ATCCTAGACC ACCACAAGAA CTGCCGGTAC GAGTAACTAG CAGGGTTACT CCAAGTAGCT GCTCGTCTTA   
  
  
+ CCTAACCCTA AACCAGATCA CTCCATTTAT CCGGTTCACC CACCTGACGG CCAACCAGGC AATTTTGGAG   
  
  
+ GCTGTGGAGG GCTATAGGGC AGTCCACATC ATCGACATGG ACATCATGCA TGGAGTGCAG TGGCCTCCGC   
  
  
+ TCCTCCAAGC AATCGTGGAG CGGTCGGCCA CCCTTAGCCA ACCTCCCCCT GTGGTCCGTC TCAAAGGGGG   
  
  
+ AGGGCCGGAC CCGGACCTCT TACAGAGGAC GGGGGACCGA CTATGGAACT TTGCCCAATC ATTAGGACTA   
  
  
+ GAGTTCCATT TCCAGGGCCT CATGATCCCC GCCGAGTCAA CAGGTTTTGT TGAACCTGCT GAGGCAGCGG   
  
  
+ TCAATGCCCT AGCCTTGCAG GAGGCGGTGG GGTGTCAAGA AGGGGAGGCC CTTGCAGTTC ACTGCGGGGA   
  
  
+ CTATCTCCAC CGCCTCTTAA AAGAGTACGA CACAAGGCCT CTTAGACGGT TCCTCCACAA GGTCAAGACT   
  
  
+ CTGAACCCCA GGGTCTTGAC CTTGGGAGAG AGGGAAGCCG ACCACAACCA CCCTCTCTTC CTGCGGCGGT   
  
  
+ TCACCGAGGC AGTCAACCAC TACGGGGCAG TGTTTGACTC CCTGGAAGCA ACCCTTCCGC CGCATAGCCA   
  
  
+ AGAGAGGGTA GCTGTGGAGG AAGGGTGGTT CGGAGAGGAG ATTAAGGACG TGGTAGGAGA AGAAAGCGAG   
  
  
+ AGGAGAATAG AGAGGCACCA GAAGTTCGAG TCGTGGGAAG CATTGTTGAG GAGCTCAGGG TTCAAGAGTG   
  
  
+ TGCCCCTCAG CCCATTCTCG CTGTCCCAAG CTAAGCTGCT GCTGCGCCTC CATTACCCTT CCGAAGGGTA   
  
  
+ CCAACTCCAG GTTTTGCATA GTAATTGCTT GTTGCTTGGG TGGAAGAATC GCCCTCTTTT TTCTGTCTCT   
  
  
+ TCTTGGCAAT A  

- +Up\_Stream \_Len000AAAAAA AATTCAAAAG CATGAACCAC AACTACTAAA CACGAAACAT TGGTTACCAA   
  
  
- AATCATTTAT CATTAATCAG CATTGTAAGA CGCTAAAGCG AGTTAAATAT ATAGTTGAAA TCTTATACTC   
  
  
- ATTGGCATTT CTAGTTTGCA TCATCATCTC CACAGTATCC AAAAAAAAAT TTTTTTAAGC CTCATTCTTT   
  
  
- CATCCCATGT AATAAAAACG AACAATTTAA GGCGATCATA TCGTTATGCT CTACCTTGTA AACATTTTTG   
  
  
- ATGCTCTGCT TCGCAAACAT GTTTTGTTTG ATGGTATGAC GTCTAGTATA TTAAAAAACC CCCCAATACA   
  
  
- TAGAATCACT ACTGTAATTA ACTACCTATT GGGATAAGAT TTATAGACCT AGGTAGTAAT TGACTGAGCA   
  
  
- AAGTTTACAA TCCTAATACC CAGAACTGTA CCATATGTAC AGTTATCTAA ATACAATCTA CTTTTCAATA   
  
  
- TACTGCGAAA AATACTAAAA ACATGATCGT ATATCACGAA TTAACCTGAT ATATAAAAAT GTTCAACACG   
  
  
- AATTGTGGAA AATTAGTCTT TATAAATAAT ATACCTTTTT TAAAATAATT ATACACAAAT ACACAAATTT   
  
  
- TTTGTATGGG TCGGAACTTT ATCTGGCACT ATATCTTTAA CCTGGGTATT ATGAGTGTAA TATATACGAA   
  
  
- ATAACAATAT TAATTCCTAG TAACAACAAA ACGTTAATAC ACCGTTCTAT ATTAGGTGTT TCAAAATGTA   
  
  
- AATATAACGT TAATGAGACC ATTTACGATA ATTACATGTA AACTCTAATT CTGTATATAT ATTGGGAAAA   
  
  
- AAAAGGGAAG AAAAAAGGAA AAAGATAAAA CTTACCGTAG AATTTGGTTT CCTCGACATG TTCAATGCAA   
  
  
- TATCGTGAGT ACCAACTGAT TTGTTCTGTC TACTCTCAAA TTCAGTTCAG GAGGACCGTT ACTACAAACG   
  
  
- GTAAACCTGG TTATTTATGT AGTACATATA ATGGTATTAT CACACCAACT GAATTGTTAT GTATCCCCTA   
  
  
- ACGTATGTTT CCATTCGGAG TTTTTGAACA CGTATACGTA ACTTTAGGTA CTAGTTTTTT TAAGTTCTCG   
  
  
- TCCAACGTGT TTTTGTGCCT CTACTCTACT TTCTCTGTCT TTTGTGTATA TGTAAGTGTC ATCATCCCCA   
  
  
- TGACCATCTC GATACCGATC CGATTCTACT ACACTTTCTT TGTTATATTA CCATCATTGA CCATTATAAA   
  
  
- TATCCCGTGT ATATCCACGC CCGTATCCCA GCACAGAGAG TCAGAGTCCA TGCCAAGCTA CCCTGAGACG   
  
  
- CTATCTACAA TAGTTTAAGT TGGTAAGCTT GTAGAGGGTC TCTCTCTCTC TCTCTGACTC ACTCACTCAC   
  
  
- TCACCCACCC ACATACACAC ACTCAACAAA ACAAGTAAAT AATATCCAAG AGACGACGTA TATAGAGGTG   
  
  
- GTAATTTAAA TGGTTTTGAA CATGGTCGAC GAAATGAACG AAAGGAGTAA AAGGGGGAAA AAACAGAGAG   
  
  
- AGAGAGAGAG AGGGGGGGGG GGGGGGGGAG AGAGAGACAC AGACTTAGTC ATAGTCGGAT AGTCCTACGA   
  
  
- AGCGGAGGTC TTTCCGTAGT GAGTGTGTGA ATAATCGAAT TTTGAAAGAA AATAAGGAAA ACGAGAAAGT   
  
  
- GACGAGAGAA AATGGGTTTA TGCGGGCAGA AGTTAAGAAG AGAGTAATTT ATACTTTTTC TTTCTCTCTT   
  
  
- TATCTCTCTC TAAATTTGGT CTTCCCCACT AAGGAAAAAG AACCACCCGA ATAATTACGA GACAGATGGG   
  
  
- GTAGATCGGC TTTTTATCAT CGAATTCTGA GTTGACGAAT AATCGTAGAG AGGAGATCGT TATTGGCTCC   
  
  
- ATTATCGTAT AAAAAGAGGG GAATCGAAGA TGGAACGAAA AACACTGTGA GTTTGGATTT GACTCTCTCT   
  
  
- CTTTCTCTTC TATCTCCTCT CTATTTATAA TCGCCCTAAT CCCGTTTAAA ACTATACGAC CCGAGAAGGA   
  
  
- GTAAGTTGAG ACTCCTTCTT CTAGTTCTAG TACGGTTGGG TGGAGGCGGC GTTGTTGGTG GTGGCAATGT   
  
  
- CGACGGTGAA GGTGCCGGTT CTAAGTCGGT CAATTATAGT AGTAGTGGAG GTTATTATAG TCGTAGAAGA   
  
  
- AGTAGTAATT AGGTATTATT GGGAGCAGTC GACGATTATT CAACGCCACT TGAGTAGAGG GCGACACTGA   
  
  
- AGAGGAGTCG ATTGGCGGAA GAGAGAGAAG AGAGGCCGCT GAAGCGAAGG AGGAGGCCGC TGAGGTGACT   
  
  
- TGCGGAACAA ATAATGAAGA GGTTCCGAAA CAAGGAGGCG GAAGCGGCTG TCAAAAGAGA TAGAAGAAGT   
  
  
- TGAAAATGAC CAATATCAAC ATTGGGATCA ACAAGACGCA GATAACGAGG TGAAAAAGAT TGGTTGGGAT   
  
  
- TAGGATCTGG TGGTGTTCTT GACGGCCATG CTCATTGATC GTCCCAATGA GGTTCATCGA CGAGCAGAAT   
  
  
- GGATTGGGAT TTGGTCTAGT GAGGTAAATA GGCCAAGTGG GTGGACTGCC GGTTGGTCCG TTAAAACCTC   
  
  
- CGACACCTCC CGATATCCCG TCAGGTGTAG TAGCTGTACC TGTAGTACGT ACCTCACGTC ACCGGAGGCG   
  
  
- AGGAGGTTCG TTAGCACCTC GCCAGCCGGT GGGAATCGGT TGGAGGGGGA CACCAGGCAG AGTTTCCCCC   
  
  
- TCCCGGCCTG GGCCTGGAGA ATGTCTCCTG CCCCCTGGCT GATACCTTGA AACGGGTTAG TAATCCTGAT   
  
  
- CTCAAGGTAA AGGTCCCGGA GTACTAGGGG CGGCTCAGTT GTCCAAAACA ACTTGGACGA CTCCGTCGCC   
  
  
- AGTTACGGGA TCGGAACGTC CTCCGCCACC CCACAGTTCT TCCCCTCCGG GAACGTCAAG TGACGCCCCT   
  
  
- GATAGAGGTG GCGGAGAATT TTCTCATGCT GTGTTCCGGA GAATCTGCCA AGGAGGTGTT CCAGTTCTGA   
  
  
- GACTTGGGGT CCCAGAACTG GAACCCTCTC TCCCTTCGGC TGGTGTTGGT GGGAGAGAAG GACGCCGCCA   
  
  
- AGTGGCTCCG TCAGTTGGTG ATGCCCCGTC ACAAACTGAG GGACCTTCGT TGGGAAGGCG GCGTATCGGT   
  
  
- TCTCTCCCAT CGACACCTCC TTCCCACCAA GCCTCTCCTC TAATTCCTGC ACCATCCTCT TCTTTCGCTC   
  
  
- TCCTCTTATC TCTCCGTGGT CTTCAAGCTC AGCACCCTTC GTAACAACTC CTCGAGTCCC AAGTTCTCAC   
  
  
- ACGGGGAGTC GGGTAAGAGC GACAGGGTTC GATTCGACGA CGACGCGGAG GTAATGGGAA GGCTTCCCAT   
  
  
- GGTTGAGGTC CAAAACGTAT CATTAACGAA CAACGAACCC ACCTTCTTAG CGGGAGAAAA AAGACAGAGA   
  
  
- AGAACCGTTA T

+     MBS

| Site Name | Organism | Position | Strand | Matrix score. | sequence | function |
| --- | --- | --- | --- | --- | --- | --- |
| MBS | Arabidopsis thaliana | 1855 | + | 6 | CAACTG | MYB binding site involved in drought-inducibility |

>HU03G01737.1   
+ +Up\_Stream \_Len000TTTTTT TTAAGTTTTC GTACTTGGTG TTGATGATTT GTGCTTTGTA ACCAATGGTT   
  
  
+ TTAGTAAATA GTAATTAGTC GTAACATTCT GCGATTTCGC TCAATTTATA TATCAACTTT AGAATATGAG   
  
  
+ TAACCGTAAA GATCAAACGT AGTAGTAGAG GTGTCATAGG TTTTTTTTTA AAAAAATTCG GAGTAAGAAA   
  
  
+ GTAGGGTACA TTATTTTTGC TTGTTAAATT CCGCTAGTAT AGCAATACGA GATGGAACAT TTGTAAAAAC   
  
  
+ TACGAGACGA AGCGTTTGTA CAAAACAAAC TACCATACTG CAGATCATAT AATTTTTTGG GGGGTTATGT   
  
  
+ ATCTTAGTGA TGACATTAAT TGATGGATAA CCCTATTCTA AATATCTGGA TCCATCATTA ACTGACTCGT   
  
  
+ TTCAAATGTT AGGATTATGG GTCTTGACAT GGTATACATG TCAATAGATT TATGTTAGAT GAAAAGTTAT   
  
  
+ ATGACGCTTT TTATGATTTT TGTACTAGCA TATAGTGCTT AATTGGACTA TATATTTTTA CAAGTTGTGC   
  
  
+ TTAACACCTT TTAATCAGAA ATATTTATTA TATGGAAAAA ATTTTATTAA TATGTGTTTA TGTGTTTAAA   
  
  
+ AAACATACCC AGCCTTGAAA TAGACCGTGA TATAGAAATT GGACCCATAA TACTCACATT ATATATGCTT   
  
  
+ TATTGTTATA ATTAAGGATC ATTGTTGTTT TGCAATTATG TGGCAAGATA TAATCCACAA AGTTTTACAT   
  
  
+ TTATATTGCA ATTACTCTGG TAAATGCTAT TAATGTACAT TTGAGATTAA GACATATATA TAACCCTTTT   
  
  
+ TTTTCCCTTC TTTTTTCCTT TTTCTATTTT GAATGGCATC TTAAACCAAA GGAGCTGTAC AAGTTACGTT   
  
  
+ ATAGCACTCA TGGTTGACTA AACAAGACAG ATGAGAGTTT AAGTCAAGTC CTCCTGGCAA TGATGTTTGC   
  
  
+ CATTTGGACC AATAAATACA TCATGTATAT TACCATAATA GTGTGGTTGA CTTAACAATA CATAGGGGAT   
  
  
+ TGCATACAAA GGTAAGCCTC AAAAACTTGT GCATATGCAT TGAAATCCAT GATCAAAAAA ATTCAAGAGC   
  
  
+ AGGTTGCACA AAAACACGGA GATGAGATGA AAGAGACAGA AAACACATAT ACATTCACAG TAGTAGGGGT   
  
  
+ ACTGGTAGAG CTATGGCTAG GCTAAGATGA TGTGAAAGAA ACAATATAAT GGTAGTAACT GGTAATATTT   
  
  
+ ATAGGGCACA TATAGGTGCG GGCATAGGGT CGTGTCTCTC AGTCTCAGGT ACGGTTCGAT GGGACTCTGC   
  
  
+ GATAGATGTT ATCAAATTCA ACCATTCGAA CATCTCCCAG AGAGAGAGAG AGAGACTGAG TGAGTGAGTG   
  
  
+ AGTGGGTGGG TGTATGTGTG TGAGTTGTTT TGTTCATTTA TTATAGGTTC TCTGCTGCAT ATATCTCCAC   
  
  
+ CATTAAATTT ACCAAAACTT GTACCAGCTG CTTTACTTGC TTTCCTCATT TTCCCCCTTT TTTGTCTCTC   
  
  
+ TCTCTCTCTC TCCCCCCCCC CCCCCCCCTC TCTCTCTGTG TCTGAATCAG TATCAGCCTA TCAGGATGCT   
  
  
+ TCGCCTCCAG AAAGGCATCA CTCACACACT TATTAGCTTA AAACTTTCTT TTATTCCTTT TGCTCTTTCA   
  
  
+ CTGCTCTCTT TTACCCAAAT ACGCCCGTCT TCAATTCTTC TCTCATTAAA TATGAAAAAG AAAGAGAGAA   
  
  
+ ATAGAGAGAG ATTTAAACCA GAAGGGGTGA TTCCTTTTTC TTGGTGGGCT TATTAATGCT CTGTCTACCC   
  
  
+ CATCTAGCCG AAAAATAGTA GCTTAAGACT CAACTGCTTA TTAGCATCTC TCCTCTAGCA ATAACCGAGG   
  
  
+ TAATAGCATA TTTTTCTCCC CTTAGCTTCT ACCTTGCTTT TTGTGACACT CAAACCTAAA CTGAGAGAGA   
  
  
+ GAAAGAGAAG ATAGAGGAGA GATAAATATT AGCGGGATTA GGGCAAATTT TGATATGCTG GGCTCTTCCT   
  
  
+ CATTCAACTC TGAGGAAGAA GATCAAGATC ATGCCAACCC ACCTCCGCCG CAACAACCAC CACCGTTACA   
  
  
+ GCTGCCACTT CCACGGCCAA GATTCAGCCA GTTAATATCA TCATCACCTC CAATAATATC AGCATCTTCT   
  
  
+ TCATCATTAA TCCATAATAA CCCTCGTCAG CTGCTAATAA GTTGCGGTGA ACTCATCTCC CGCTGTGACT   
  
  
+ TCTCCTCAGC TAACCGCCTT CTCTCTCTTC TCTCCGGCGA CTTCGCTTCC TCCTCCGGCG ACTCCACTGA   
  
  
+ ACGCCTTGTT TATTACTTCT CCAAGGCTTT GTTCCTCCGC CTTCGCCGAC AGTTTTCTCT ATCTTCTTCA   
  
  
+ ACTTTTACTG GTTATAGTTG TAACCCTAGT TGTTCTGCGT CTATTGCTCC ACTTTTTCTA ACCAACCCTA   
  
  
+ ATCCTAGACC ACCACAAGAA CTGCCGGTAC GAGTAACTAG CAGGGTTACT CCAAGTAGCT GCTCGTCTTA   
  
  
+ CCTAACCCTA AACCAGATCA CTCCATTTAT CCGGTTCACC CACCTGACGG CCAACCAGGC AATTTTGGAG   
  
  
+ GCTGTGGAGG GCTATAGGGC AGTCCACATC ATCGACATGG ACATCATGCA TGGAGTGCAG TGGCCTCCGC   
  
  
+ TCCTCCAAGC AATCGTGGAG CGGTCGGCCA CCCTTAGCCA ACCTCCCCCT GTGGTCCGTC TCAAAGGGGG   
  
  
+ AGGGCCGGAC CCGGACCTCT TACAGAGGAC GGGGGACCGA CTATGGAACT TTGCCCAATC ATTAGGACTA   
  
  
+ GAGTTCCATT TCCAGGGCCT CATGATCCCC GCCGAGTCAA CAGGTTTTGT TGAACCTGCT GAGGCAGCGG   
  
  
+ TCAATGCCCT AGCCTTGCAG GAGGCGGTGG GGTGTCAAGA AGGGGAGGCC CTTGCAGTTC ACTGCGGGGA   
  
  
+ CTATCTCCAC CGCCTCTTAA AAGAGTACGA CACAAGGCCT CTTAGACGGT TCCTCCACAA GGTCAAGACT   
  
  
+ CTGAACCCCA GGGTCTTGAC CTTGGGAGAG AGGGAAGCCG ACCACAACCA CCCTCTCTTC CTGCGGCGGT   
  
  
+ TCACCGAGGC AGTCAACCAC TACGGGGCAG TGTTTGACTC CCTGGAAGCA ACCCTTCCGC CGCATAGCCA   
  
  
+ AGAGAGGGTA GCTGTGGAGG AAGGGTGGTT CGGAGAGGAG ATTAAGGACG TGGTAGGAGA AGAAAGCGAG   
  
  
+ AGGAGAATAG AGAGGCACCA GAAGTTCGAG TCGTGGGAAG CATTGTTGAG GAGCTCAGGG TTCAAGAGTG   
  
  
+ TGCCCCTCAG CCCATTCTCG CTGTCCCAAG CTAAGCTGCT GCTGCGCCTC CATTACCCTT CCGAAGGGTA   
  
  
+ CCAACTCCAG GTTTTGCATA GTAATTGCTT GTTGCTTGGG TGGAAGAATC GCCCTCTTTT TTCTGTCTCT   
  
  
+ TCTTGGCAAT A  

- +Up\_Stream \_Len000AAAAAA AATTCAAAAG CATGAACCAC AACTACTAAA CACGAAACAT TGGTTACCAA   
  
  
- AATCATTTAT CATTAATCAG CATTGTAAGA CGCTAAAGCG AGTTAAATAT ATAGTTGAAA TCTTATACTC   
  
  
- ATTGGCATTT CTAGTTTGCA TCATCATCTC CACAGTATCC AAAAAAAAAT TTTTTTAAGC CTCATTCTTT   
  
  
- CATCCCATGT AATAAAAACG AACAATTTAA GGCGATCATA TCGTTATGCT CTACCTTGTA AACATTTTTG   
  
  
- ATGCTCTGCT TCGCAAACAT GTTTTGTTTG ATGGTATGAC GTCTAGTATA TTAAAAAACC CCCCAATACA   
  
  
- TAGAATCACT ACTGTAATTA ACTACCTATT GGGATAAGAT TTATAGACCT AGGTAGTAAT TGACTGAGCA   
  
  
- AAGTTTACAA TCCTAATACC CAGAACTGTA CCATATGTAC AGTTATCTAA ATACAATCTA CTTTTCAATA   
  
  
- TACTGCGAAA AATACTAAAA ACATGATCGT ATATCACGAA TTAACCTGAT ATATAAAAAT GTTCAACACG   
  
  
- AATTGTGGAA AATTAGTCTT TATAAATAAT ATACCTTTTT TAAAATAATT ATACACAAAT ACACAAATTT   
  
  
- TTTGTATGGG TCGGAACTTT ATCTGGCACT ATATCTTTAA CCTGGGTATT ATGAGTGTAA TATATACGAA   
  
  
- ATAACAATAT TAATTCCTAG TAACAACAAA ACGTTAATAC ACCGTTCTAT ATTAGGTGTT TCAAAATGTA   
  
  
- AATATAACGT TAATGAGACC ATTTACGATA ATTACATGTA AACTCTAATT CTGTATATAT ATTGGGAAAA   
  
  
- AAAAGGGAAG AAAAAAGGAA AAAGATAAAA CTTACCGTAG AATTTGGTTT CCTCGACATG TTCAATGCAA   
  
  
- TATCGTGAGT ACCAACTGAT TTGTTCTGTC TACTCTCAAA TTCAGTTCAG GAGGACCGTT ACTACAAACG   
  
  
- GTAAACCTGG TTATTTATGT AGTACATATA ATGGTATTAT CACACCAACT GAATTGTTAT GTATCCCCTA   
  
  
- ACGTATGTTT CCATTCGGAG TTTTTGAACA CGTATACGTA ACTTTAGGTA CTAGTTTTTT TAAGTTCTCG   
  
  
- TCCAACGTGT TTTTGTGCCT CTACTCTACT TTCTCTGTCT TTTGTGTATA TGTAAGTGTC ATCATCCCCA   
  
  
- TGACCATCTC GATACCGATC CGATTCTACT ACACTTTCTT TGTTATATTA CCATCATTGA CCATTATAAA   
  
  
- TATCCCGTGT ATATCCACGC CCGTATCCCA GCACAGAGAG TCAGAGTCCA TGCCAAGCTA CCCTGAGACG   
  
  
- CTATCTACAA TAGTTTAAGT TGGTAAGCTT GTAGAGGGTC TCTCTCTCTC TCTCTGACTC ACTCACTCAC   
  
  
- TCACCCACCC ACATACACAC ACTCAACAAA ACAAGTAAAT AATATCCAAG AGACGACGTA TATAGAGGTG   
  
  
- GTAATTTAAA TGGTTTTGAA CATGGTCGAC GAAATGAACG AAAGGAGTAA AAGGGGGAAA AAACAGAGAG   
  
  
- AGAGAGAGAG AGGGGGGGGG GGGGGGGGAG AGAGAGACAC AGACTTAGTC ATAGTCGGAT AGTCCTACGA   
  
  
- AGCGGAGGTC TTTCCGTAGT GAGTGTGTGA ATAATCGAAT TTTGAAAGAA AATAAGGAAA ACGAGAAAGT   
  
  
- GACGAGAGAA AATGGGTTTA TGCGGGCAGA AGTTAAGAAG AGAGTAATTT ATACTTTTTC TTTCTCTCTT   
  
  
- TATCTCTCTC TAAATTTGGT CTTCCCCACT AAGGAAAAAG AACCACCCGA ATAATTACGA GACAGATGGG   
  
  
- GTAGATCGGC TTTTTATCAT CGAATTCTGA GTTGACGAAT AATCGTAGAG AGGAGATCGT TATTGGCTCC   
  
  
- ATTATCGTAT AAAAAGAGGG GAATCGAAGA TGGAACGAAA AACACTGTGA GTTTGGATTT GACTCTCTCT   
  
  
- CTTTCTCTTC TATCTCCTCT CTATTTATAA TCGCCCTAAT CCCGTTTAAA ACTATACGAC CCGAGAAGGA   
  
  
- GTAAGTTGAG ACTCCTTCTT CTAGTTCTAG TACGGTTGGG TGGAGGCGGC GTTGTTGGTG GTGGCAATGT   
  
  
- CGACGGTGAA GGTGCCGGTT CTAAGTCGGT CAATTATAGT AGTAGTGGAG GTTATTATAG TCGTAGAAGA   
  
  
- AGTAGTAATT AGGTATTATT GGGAGCAGTC GACGATTATT CAACGCCACT TGAGTAGAGG GCGACACTGA   
  
  
- AGAGGAGTCG ATTGGCGGAA GAGAGAGAAG AGAGGCCGCT GAAGCGAAGG AGGAGGCCGC TGAGGTGACT   
  
  
- TGCGGAACAA ATAATGAAGA GGTTCCGAAA CAAGGAGGCG GAAGCGGCTG TCAAAAGAGA TAGAAGAAGT   
  
  
- TGAAAATGAC CAATATCAAC ATTGGGATCA ACAAGACGCA GATAACGAGG TGAAAAAGAT TGGTTGGGAT   
  
  
- TAGGATCTGG TGGTGTTCTT GACGGCCATG CTCATTGATC GTCCCAATGA GGTTCATCGA CGAGCAGAAT   
  
  
- GGATTGGGAT TTGGTCTAGT GAGGTAAATA GGCCAAGTGG GTGGACTGCC GGTTGGTCCG TTAAAACCTC   
  
  
- CGACACCTCC CGATATCCCG TCAGGTGTAG TAGCTGTACC TGTAGTACGT ACCTCACGTC ACCGGAGGCG   
  
  
- AGGAGGTTCG TTAGCACCTC GCCAGCCGGT GGGAATCGGT TGGAGGGGGA CACCAGGCAG AGTTTCCCCC   
  
  
- TCCCGGCCTG GGCCTGGAGA ATGTCTCCTG CCCCCTGGCT GATACCTTGA AACGGGTTAG TAATCCTGAT   
  
  
- CTCAAGGTAA AGGTCCCGGA GTACTAGGGG CGGCTCAGTT GTCCAAAACA ACTTGGACGA CTCCGTCGCC   
  
  
- AGTTACGGGA TCGGAACGTC CTCCGCCACC CCACAGTTCT TCCCCTCCGG GAACGTCAAG TGACGCCCCT   
  
  
- GATAGAGGTG GCGGAGAATT TTCTCATGCT GTGTTCCGGA GAATCTGCCA AGGAGGTGTT CCAGTTCTGA   
  
  
- GACTTGGGGT CCCAGAACTG GAACCCTCTC TCCCTTCGGC TGGTGTTGGT GGGAGAGAAG GACGCCGCCA   
  
  
- AGTGGCTCCG TCAGTTGGTG ATGCCCCGTC ACAAACTGAG GGACCTTCGT TGGGAAGGCG GCGTATCGGT   
  
  
- TCTCTCCCAT CGACACCTCC TTCCCACCAA GCCTCTCCTC TAATTCCTGC ACCATCCTCT TCTTTCGCTC   
  
  
- TCCTCTTATC TCTCCGTGGT CTTCAAGCTC AGCACCCTTC GTAACAACTC CTCGAGTCCC AAGTTCTCAC   
  
  
- ACGGGGAGTC GGGTAAGAGC GACAGGGTTC GATTCGACGA CGACGCGGAG GTAATGGGAA GGCTTCCCAT   
  
  
- GGTTGAGGTC CAAAACGTAT CATTAACGAA CAACGAACCC ACCTTCTTAG CGGGAGAAAA AAGACAGAGA   
  
  
- AGAACCGTTA T

+     MBSI

| Site Name | Organism | Position | Strand | Matrix score. | sequence | function |
| --- | --- | --- | --- | --- | --- | --- |
| MBSI | Petunia hybrida | 835 | - | 10.5 | aaaAaaC(G/C)GTTA | MYB binding site involved in flavonoid biosynthetic genes regulation |
| MBSI | Petunia hybrida | 145 | - | 11 | TTTTTACGGTTA | MYB binding site involved in flavonoid biosynthetic genes regulation |

>HU03G01737.1   
+ +Up\_Stream \_Len000TTTTTT TTAAGTTTTC GTACTTGGTG TTGATGATTT GTGCTTTGTA ACCAATGGTT   
  
  
+ TTAGTAAATA GTAATTAGTC GTAACATTCT GCGATTTCGC TCAATTTATA TATCAACTTT AGAATATGAG   
  
  
+ TAACCGTAAA GATCAAACGT AGTAGTAGAG GTGTCATAGG TTTTTTTTTA AAAAAATTCG GAGTAAGAAA   
  
  
+ GTAGGGTACA TTATTTTTGC TTGTTAAATT CCGCTAGTAT AGCAATACGA GATGGAACAT TTGTAAAAAC   
  
  
+ TACGAGACGA AGCGTTTGTA CAAAACAAAC TACCATACTG CAGATCATAT AATTTTTTGG GGGGTTATGT   
  
  
+ ATCTTAGTGA TGACATTAAT TGATGGATAA CCCTATTCTA AATATCTGGA TCCATCATTA ACTGACTCGT   
  
  
+ TTCAAATGTT AGGATTATGG GTCTTGACAT GGTATACATG TCAATAGATT TATGTTAGAT GAAAAGTTAT   
  
  
+ ATGACGCTTT TTATGATTTT TGTACTAGCA TATAGTGCTT AATTGGACTA TATATTTTTA CAAGTTGTGC   
  
  
+ TTAACACCTT TTAATCAGAA ATATTTATTA TATGGAAAAA ATTTTATTAA TATGTGTTTA TGTGTTTAAA   
  
  
+ AAACATACCC AGCCTTGAAA TAGACCGTGA TATAGAAATT GGACCCATAA TACTCACATT ATATATGCTT   
  
  
+ TATTGTTATA ATTAAGGATC ATTGTTGTTT TGCAATTATG TGGCAAGATA TAATCCACAA AGTTTTACAT   
  
  
+ TTATATTGCA ATTACTCTGG TAAATGCTAT TAATGTACAT TTGAGATTAA GACATATATA TAACCCTTTT   
  
  
+ TTTTCCCTTC TTTTTTCCTT TTTCTATTTT GAATGGCATC TTAAACCAAA GGAGCTGTAC AAGTTACGTT   
  
  
+ ATAGCACTCA TGGTTGACTA AACAAGACAG ATGAGAGTTT AAGTCAAGTC CTCCTGGCAA TGATGTTTGC   
  
  
+ CATTTGGACC AATAAATACA TCATGTATAT TACCATAATA GTGTGGTTGA CTTAACAATA CATAGGGGAT   
  
  
+ TGCATACAAA GGTAAGCCTC AAAAACTTGT GCATATGCAT TGAAATCCAT GATCAAAAAA ATTCAAGAGC   
  
  
+ AGGTTGCACA AAAACACGGA GATGAGATGA AAGAGACAGA AAACACATAT ACATTCACAG TAGTAGGGGT   
  
  
+ ACTGGTAGAG CTATGGCTAG GCTAAGATGA TGTGAAAGAA ACAATATAAT GGTAGTAACT GGTAATATTT   
  
  
+ ATAGGGCACA TATAGGTGCG GGCATAGGGT CGTGTCTCTC AGTCTCAGGT ACGGTTCGAT GGGACTCTGC   
  
  
+ GATAGATGTT ATCAAATTCA ACCATTCGAA CATCTCCCAG AGAGAGAGAG AGAGACTGAG TGAGTGAGTG   
  
  
+ AGTGGGTGGG TGTATGTGTG TGAGTTGTTT TGTTCATTTA TTATAGGTTC TCTGCTGCAT ATATCTCCAC   
  
  
+ CATTAAATTT ACCAAAACTT GTACCAGCTG CTTTACTTGC TTTCCTCATT TTCCCCCTTT TTTGTCTCTC   
  
  
+ TCTCTCTCTC TCCCCCCCCC CCCCCCCCTC TCTCTCTGTG TCTGAATCAG TATCAGCCTA TCAGGATGCT   
  
  
+ TCGCCTCCAG AAAGGCATCA CTCACACACT TATTAGCTTA AAACTTTCTT TTATTCCTTT TGCTCTTTCA   
  
  
+ CTGCTCTCTT TTACCCAAAT ACGCCCGTCT TCAATTCTTC TCTCATTAAA TATGAAAAAG AAAGAGAGAA   
  
  
+ ATAGAGAGAG ATTTAAACCA GAAGGGGTGA TTCCTTTTTC TTGGTGGGCT TATTAATGCT CTGTCTACCC   
  
  
+ CATCTAGCCG AAAAATAGTA GCTTAAGACT CAACTGCTTA TTAGCATCTC TCCTCTAGCA ATAACCGAGG   
  
  
+ TAATAGCATA TTTTTCTCCC CTTAGCTTCT ACCTTGCTTT TTGTGACACT CAAACCTAAA CTGAGAGAGA   
  
  
+ GAAAGAGAAG ATAGAGGAGA GATAAATATT AGCGGGATTA GGGCAAATTT TGATATGCTG GGCTCTTCCT   
  
  
+ CATTCAACTC TGAGGAAGAA GATCAAGATC ATGCCAACCC ACCTCCGCCG CAACAACCAC CACCGTTACA   
  
  
+ GCTGCCACTT CCACGGCCAA GATTCAGCCA GTTAATATCA TCATCACCTC CAATAATATC AGCATCTTCT   
  
  
+ TCATCATTAA TCCATAATAA CCCTCGTCAG CTGCTAATAA GTTGCGGTGA ACTCATCTCC CGCTGTGACT   
  
  
+ TCTCCTCAGC TAACCGCCTT CTCTCTCTTC TCTCCGGCGA CTTCGCTTCC TCCTCCGGCG ACTCCACTGA   
  
  
+ ACGCCTTGTT TATTACTTCT CCAAGGCTTT GTTCCTCCGC CTTCGCCGAC AGTTTTCTCT ATCTTCTTCA   
  
  
+ ACTTTTACTG GTTATAGTTG TAACCCTAGT TGTTCTGCGT CTATTGCTCC ACTTTTTCTA ACCAACCCTA   
  
  
+ ATCCTAGACC ACCACAAGAA CTGCCGGTAC GAGTAACTAG CAGGGTTACT CCAAGTAGCT GCTCGTCTTA   
  
  
+ CCTAACCCTA AACCAGATCA CTCCATTTAT CCGGTTCACC CACCTGACGG CCAACCAGGC AATTTTGGAG   
  
  
+ GCTGTGGAGG GCTATAGGGC AGTCCACATC ATCGACATGG ACATCATGCA TGGAGTGCAG TGGCCTCCGC   
  
  
+ TCCTCCAAGC AATCGTGGAG CGGTCGGCCA CCCTTAGCCA ACCTCCCCCT GTGGTCCGTC TCAAAGGGGG   
  
  
+ AGGGCCGGAC CCGGACCTCT TACAGAGGAC GGGGGACCGA CTATGGAACT TTGCCCAATC ATTAGGACTA   
  
  
+ GAGTTCCATT TCCAGGGCCT CATGATCCCC GCCGAGTCAA CAGGTTTTGT TGAACCTGCT GAGGCAGCGG   
  
  
+ TCAATGCCCT AGCCTTGCAG GAGGCGGTGG GGTGTCAAGA AGGGGAGGCC CTTGCAGTTC ACTGCGGGGA   
  
  
+ CTATCTCCAC CGCCTCTTAA AAGAGTACGA CACAAGGCCT CTTAGACGGT TCCTCCACAA GGTCAAGACT   
  
  
+ CTGAACCCCA GGGTCTTGAC CTTGGGAGAG AGGGAAGCCG ACCACAACCA CCCTCTCTTC CTGCGGCGGT   
  
  
+ TCACCGAGGC AGTCAACCAC TACGGGGCAG TGTTTGACTC CCTGGAAGCA ACCCTTCCGC CGCATAGCCA   
  
  
+ AGAGAGGGTA GCTGTGGAGG AAGGGTGGTT CGGAGAGGAG ATTAAGGACG TGGTAGGAGA AGAAAGCGAG   
  
  
+ AGGAGAATAG AGAGGCACCA GAAGTTCGAG TCGTGGGAAG CATTGTTGAG GAGCTCAGGG TTCAAGAGTG   
  
  
+ TGCCCCTCAG CCCATTCTCG CTGTCCCAAG CTAAGCTGCT GCTGCGCCTC CATTACCCTT CCGAAGGGTA   
  
  
+ CCAACTCCAG GTTTTGCATA GTAATTGCTT GTTGCTTGGG TGGAAGAATC GCCCTCTTTT TTCTGTCTCT   
  
  
+ TCTTGGCAAT A  

- +Up\_Stream \_Len000AAAAAA AATTCAAAAG CATGAACCAC AACTACTAAA CACGAAACAT TGGTTACCAA   
  
  
- AATCATTTAT CATTAATCAG CATTGTAAGA CGCTAAAGCG AGTTAAATAT ATAGTTGAAA TCTTATACTC   
  
  
- ATTGGCATTT CTAGTTTGCA TCATCATCTC CACAGTATCC AAAAAAAAAT TTTTTTAAGC CTCATTCTTT   
  
  
- CATCCCATGT AATAAAAACG AACAATTTAA GGCGATCATA TCGTTATGCT CTACCTTGTA AACATTTTTG   
  
  
- ATGCTCTGCT TCGCAAACAT GTTTTGTTTG ATGGTATGAC GTCTAGTATA TTAAAAAACC CCCCAATACA   
  
  
- TAGAATCACT ACTGTAATTA ACTACCTATT GGGATAAGAT TTATAGACCT AGGTAGTAAT TGACTGAGCA   
  
  
- AAGTTTACAA TCCTAATACC CAGAACTGTA CCATATGTAC AGTTATCTAA ATACAATCTA CTTTTCAATA   
  
  
- TACTGCGAAA AATACTAAAA ACATGATCGT ATATCACGAA TTAACCTGAT ATATAAAAAT GTTCAACACG   
  
  
- AATTGTGGAA AATTAGTCTT TATAAATAAT ATACCTTTTT TAAAATAATT ATACACAAAT ACACAAATTT   
  
  
- TTTGTATGGG TCGGAACTTT ATCTGGCACT ATATCTTTAA CCTGGGTATT ATGAGTGTAA TATATACGAA   
  
  
- ATAACAATAT TAATTCCTAG TAACAACAAA ACGTTAATAC ACCGTTCTAT ATTAGGTGTT TCAAAATGTA   
  
  
- AATATAACGT TAATGAGACC ATTTACGATA ATTACATGTA AACTCTAATT CTGTATATAT ATTGGGAAAA   
  
  
- AAAAGGGAAG AAAAAAGGAA AAAGATAAAA CTTACCGTAG AATTTGGTTT CCTCGACATG TTCAATGCAA   
  
  
- TATCGTGAGT ACCAACTGAT TTGTTCTGTC TACTCTCAAA TTCAGTTCAG GAGGACCGTT ACTACAAACG   
  
  
- GTAAACCTGG TTATTTATGT AGTACATATA ATGGTATTAT CACACCAACT GAATTGTTAT GTATCCCCTA   
  
  
- ACGTATGTTT CCATTCGGAG TTTTTGAACA CGTATACGTA ACTTTAGGTA CTAGTTTTTT TAAGTTCTCG   
  
  
- TCCAACGTGT TTTTGTGCCT CTACTCTACT TTCTCTGTCT TTTGTGTATA TGTAAGTGTC ATCATCCCCA   
  
  
- TGACCATCTC GATACCGATC CGATTCTACT ACACTTTCTT TGTTATATTA CCATCATTGA CCATTATAAA   
  
  
- TATCCCGTGT ATATCCACGC CCGTATCCCA GCACAGAGAG TCAGAGTCCA TGCCAAGCTA CCCTGAGACG   
  
  
- CTATCTACAA TAGTTTAAGT TGGTAAGCTT GTAGAGGGTC TCTCTCTCTC TCTCTGACTC ACTCACTCAC   
  
  
- TCACCCACCC ACATACACAC ACTCAACAAA ACAAGTAAAT AATATCCAAG AGACGACGTA TATAGAGGTG   
  
  
- GTAATTTAAA TGGTTTTGAA CATGGTCGAC GAAATGAACG AAAGGAGTAA AAGGGGGAAA AAACAGAGAG   
  
  
- AGAGAGAGAG AGGGGGGGGG GGGGGGGGAG AGAGAGACAC AGACTTAGTC ATAGTCGGAT AGTCCTACGA   
  
  
- AGCGGAGGTC TTTCCGTAGT GAGTGTGTGA ATAATCGAAT TTTGAAAGAA AATAAGGAAA ACGAGAAAGT   
  
  
- GACGAGAGAA AATGGGTTTA TGCGGGCAGA AGTTAAGAAG AGAGTAATTT ATACTTTTTC TTTCTCTCTT   
  
  
- TATCTCTCTC TAAATTTGGT CTTCCCCACT AAGGAAAAAG AACCACCCGA ATAATTACGA GACAGATGGG   
  
  
- GTAGATCGGC TTTTTATCAT CGAATTCTGA GTTGACGAAT AATCGTAGAG AGGAGATCGT TATTGGCTCC   
  
  
- ATTATCGTAT AAAAAGAGGG GAATCGAAGA TGGAACGAAA AACACTGTGA GTTTGGATTT GACTCTCTCT   
  
  
- CTTTCTCTTC TATCTCCTCT CTATTTATAA TCGCCCTAAT CCCGTTTAAA ACTATACGAC CCGAGAAGGA   
  
  
- GTAAGTTGAG ACTCCTTCTT CTAGTTCTAG TACGGTTGGG TGGAGGCGGC GTTGTTGGTG GTGGCAATGT   
  
  
- CGACGGTGAA GGTGCCGGTT CTAAGTCGGT CAATTATAGT AGTAGTGGAG GTTATTATAG TCGTAGAAGA   
  
  
- AGTAGTAATT AGGTATTATT GGGAGCAGTC GACGATTATT CAACGCCACT TGAGTAGAGG GCGACACTGA   
  
  
- AGAGGAGTCG ATTGGCGGAA GAGAGAGAAG AGAGGCCGCT GAAGCGAAGG AGGAGGCCGC TGAGGTGACT   
  
  
- TGCGGAACAA ATAATGAAGA GGTTCCGAAA CAAGGAGGCG GAAGCGGCTG TCAAAAGAGA TAGAAGAAGT   
  
  
- TGAAAATGAC CAATATCAAC ATTGGGATCA ACAAGACGCA GATAACGAGG TGAAAAAGAT TGGTTGGGAT   
  
  
- TAGGATCTGG TGGTGTTCTT GACGGCCATG CTCATTGATC GTCCCAATGA GGTTCATCGA CGAGCAGAAT   
  
  
- GGATTGGGAT TTGGTCTAGT GAGGTAAATA GGCCAAGTGG GTGGACTGCC GGTTGGTCCG TTAAAACCTC   
  
  
- CGACACCTCC CGATATCCCG TCAGGTGTAG TAGCTGTACC TGTAGTACGT ACCTCACGTC ACCGGAGGCG   
  
  
- AGGAGGTTCG TTAGCACCTC GCCAGCCGGT GGGAATCGGT TGGAGGGGGA CACCAGGCAG AGTTTCCCCC   
  
  
- TCCCGGCCTG GGCCTGGAGA ATGTCTCCTG CCCCCTGGCT GATACCTTGA AACGGGTTAG TAATCCTGAT   
  
  
- CTCAAGGTAA AGGTCCCGGA GTACTAGGGG CGGCTCAGTT GTCCAAAACA ACTTGGACGA CTCCGTCGCC   
  
  
- AGTTACGGGA TCGGAACGTC CTCCGCCACC CCACAGTTCT TCCCCTCCGG GAACGTCAAG TGACGCCCCT   
  
  
- GATAGAGGTG GCGGAGAATT TTCTCATGCT GTGTTCCGGA GAATCTGCCA AGGAGGTGTT CCAGTTCTGA   
  
  
- GACTTGGGGT CCCAGAACTG GAACCCTCTC TCCCTTCGGC TGGTGTTGGT GGGAGAGAAG GACGCCGCCA   
  
  
- AGTGGCTCCG TCAGTTGGTG ATGCCCCGTC ACAAACTGAG GGACCTTCGT TGGGAAGGCG GCGTATCGGT   
  
  
- TCTCTCCCAT CGACACCTCC TTCCCACCAA GCCTCTCCTC TAATTCCTGC ACCATCCTCT TCTTTCGCTC   
  
  
- TCCTCTTATC TCTCCGTGGT CTTCAAGCTC AGCACCCTTC GTAACAACTC CTCGAGTCCC AAGTTCTCAC   
  
  
- ACGGGGAGTC GGGTAAGAGC GACAGGGTTC GATTCGACGA CGACGCGGAG GTAATGGGAA GGCTTCCCAT   
  
  
- GGTTGAGGTC CAAAACGTAT CATTAACGAA CAACGAACCC ACCTTCTTAG CGGGAGAAAA AAGACAGAGA   
  
  
- AGAACCGTTA T

+     MRE

| Site Name | Organism | Position | Strand | Matrix score. | sequence | function |
| --- | --- | --- | --- | --- | --- | --- |
| MRE | Petroselinum crispum | 1947 | + | 7 | AACCTAA | MYB binding site involved in light responsiveness |

>HU03G01737.1   
+ +Up\_Stream \_Len000TTTTTT TTAAGTTTTC GTACTTGGTG TTGATGATTT GTGCTTTGTA ACCAATGGTT   
  
  
+ TTAGTAAATA GTAATTAGTC GTAACATTCT GCGATTTCGC TCAATTTATA TATCAACTTT AGAATATGAG   
  
  
+ TAACCGTAAA GATCAAACGT AGTAGTAGAG GTGTCATAGG TTTTTTTTTA AAAAAATTCG GAGTAAGAAA   
  
  
+ GTAGGGTACA TTATTTTTGC TTGTTAAATT CCGCTAGTAT AGCAATACGA GATGGAACAT TTGTAAAAAC   
  
  
+ TACGAGACGA AGCGTTTGTA CAAAACAAAC TACCATACTG CAGATCATAT AATTTTTTGG GGGGTTATGT   
  
  
+ ATCTTAGTGA TGACATTAAT TGATGGATAA CCCTATTCTA AATATCTGGA TCCATCATTA ACTGACTCGT   
  
  
+ TTCAAATGTT AGGATTATGG GTCTTGACAT GGTATACATG TCAATAGATT TATGTTAGAT GAAAAGTTAT   
  
  
+ ATGACGCTTT TTATGATTTT TGTACTAGCA TATAGTGCTT AATTGGACTA TATATTTTTA CAAGTTGTGC   
  
  
+ TTAACACCTT TTAATCAGAA ATATTTATTA TATGGAAAAA ATTTTATTAA TATGTGTTTA TGTGTTTAAA   
  
  
+ AAACATACCC AGCCTTGAAA TAGACCGTGA TATAGAAATT GGACCCATAA TACTCACATT ATATATGCTT   
  
  
+ TATTGTTATA ATTAAGGATC ATTGTTGTTT TGCAATTATG TGGCAAGATA TAATCCACAA AGTTTTACAT   
  
  
+ TTATATTGCA ATTACTCTGG TAAATGCTAT TAATGTACAT TTGAGATTAA GACATATATA TAACCCTTTT   
  
  
+ TTTTCCCTTC TTTTTTCCTT TTTCTATTTT GAATGGCATC TTAAACCAAA GGAGCTGTAC AAGTTACGTT   
  
  
+ ATAGCACTCA TGGTTGACTA AACAAGACAG ATGAGAGTTT AAGTCAAGTC CTCCTGGCAA TGATGTTTGC   
  
  
+ CATTTGGACC AATAAATACA TCATGTATAT TACCATAATA GTGTGGTTGA CTTAACAATA CATAGGGGAT   
  
  
+ TGCATACAAA GGTAAGCCTC AAAAACTTGT GCATATGCAT TGAAATCCAT GATCAAAAAA ATTCAAGAGC   
  
  
+ AGGTTGCACA AAAACACGGA GATGAGATGA AAGAGACAGA AAACACATAT ACATTCACAG TAGTAGGGGT   
  
  
+ ACTGGTAGAG CTATGGCTAG GCTAAGATGA TGTGAAAGAA ACAATATAAT GGTAGTAACT GGTAATATTT   
  
  
+ ATAGGGCACA TATAGGTGCG GGCATAGGGT CGTGTCTCTC AGTCTCAGGT ACGGTTCGAT GGGACTCTGC   
  
  
+ GATAGATGTT ATCAAATTCA ACCATTCGAA CATCTCCCAG AGAGAGAGAG AGAGACTGAG TGAGTGAGTG   
  
  
+ AGTGGGTGGG TGTATGTGTG TGAGTTGTTT TGTTCATTTA TTATAGGTTC TCTGCTGCAT ATATCTCCAC   
  
  
+ CATTAAATTT ACCAAAACTT GTACCAGCTG CTTTACTTGC TTTCCTCATT TTCCCCCTTT TTTGTCTCTC   
  
  
+ TCTCTCTCTC TCCCCCCCCC CCCCCCCCTC TCTCTCTGTG TCTGAATCAG TATCAGCCTA TCAGGATGCT   
  
  
+ TCGCCTCCAG AAAGGCATCA CTCACACACT TATTAGCTTA AAACTTTCTT TTATTCCTTT TGCTCTTTCA   
  
  
+ CTGCTCTCTT TTACCCAAAT ACGCCCGTCT TCAATTCTTC TCTCATTAAA TATGAAAAAG AAAGAGAGAA   
  
  
+ ATAGAGAGAG ATTTAAACCA GAAGGGGTGA TTCCTTTTTC TTGGTGGGCT TATTAATGCT CTGTCTACCC   
  
  
+ CATCTAGCCG AAAAATAGTA GCTTAAGACT CAACTGCTTA TTAGCATCTC TCCTCTAGCA ATAACCGAGG   
  
  
+ TAATAGCATA TTTTTCTCCC CTTAGCTTCT ACCTTGCTTT TTGTGACACT CAAACCTAAA CTGAGAGAGA   
  
  
+ GAAAGAGAAG ATAGAGGAGA GATAAATATT AGCGGGATTA GGGCAAATTT TGATATGCTG GGCTCTTCCT   
  
  
+ CATTCAACTC TGAGGAAGAA GATCAAGATC ATGCCAACCC ACCTCCGCCG CAACAACCAC CACCGTTACA   
  
  
+ GCTGCCACTT CCACGGCCAA GATTCAGCCA GTTAATATCA TCATCACCTC CAATAATATC AGCATCTTCT   
  
  
+ TCATCATTAA TCCATAATAA CCCTCGTCAG CTGCTAATAA GTTGCGGTGA ACTCATCTCC CGCTGTGACT   
  
  
+ TCTCCTCAGC TAACCGCCTT CTCTCTCTTC TCTCCGGCGA CTTCGCTTCC TCCTCCGGCG ACTCCACTGA   
  
  
+ ACGCCTTGTT TATTACTTCT CCAAGGCTTT GTTCCTCCGC CTTCGCCGAC AGTTTTCTCT ATCTTCTTCA   
  
  
+ ACTTTTACTG GTTATAGTTG TAACCCTAGT TGTTCTGCGT CTATTGCTCC ACTTTTTCTA ACCAACCCTA   
  
  
+ ATCCTAGACC ACCACAAGAA CTGCCGGTAC GAGTAACTAG CAGGGTTACT CCAAGTAGCT GCTCGTCTTA   
  
  
+ CCTAACCCTA AACCAGATCA CTCCATTTAT CCGGTTCACC CACCTGACGG CCAACCAGGC AATTTTGGAG   
  
  
+ GCTGTGGAGG GCTATAGGGC AGTCCACATC ATCGACATGG ACATCATGCA TGGAGTGCAG TGGCCTCCGC   
  
  
+ TCCTCCAAGC AATCGTGGAG CGGTCGGCCA CCCTTAGCCA ACCTCCCCCT GTGGTCCGTC TCAAAGGGGG   
  
  
+ AGGGCCGGAC CCGGACCTCT TACAGAGGAC GGGGGACCGA CTATGGAACT TTGCCCAATC ATTAGGACTA   
  
  
+ GAGTTCCATT TCCAGGGCCT CATGATCCCC GCCGAGTCAA CAGGTTTTGT TGAACCTGCT GAGGCAGCGG   
  
  
+ TCAATGCCCT AGCCTTGCAG GAGGCGGTGG GGTGTCAAGA AGGGGAGGCC CTTGCAGTTC ACTGCGGGGA   
  
  
+ CTATCTCCAC CGCCTCTTAA AAGAGTACGA CACAAGGCCT CTTAGACGGT TCCTCCACAA GGTCAAGACT   
  
  
+ CTGAACCCCA GGGTCTTGAC CTTGGGAGAG AGGGAAGCCG ACCACAACCA CCCTCTCTTC CTGCGGCGGT   
  
  
+ TCACCGAGGC AGTCAACCAC TACGGGGCAG TGTTTGACTC CCTGGAAGCA ACCCTTCCGC CGCATAGCCA   
  
  
+ AGAGAGGGTA GCTGTGGAGG AAGGGTGGTT CGGAGAGGAG ATTAAGGACG TGGTAGGAGA AGAAAGCGAG   
  
  
+ AGGAGAATAG AGAGGCACCA GAAGTTCGAG TCGTGGGAAG CATTGTTGAG GAGCTCAGGG TTCAAGAGTG   
  
  
+ TGCCCCTCAG CCCATTCTCG CTGTCCCAAG CTAAGCTGCT GCTGCGCCTC CATTACCCTT CCGAAGGGTA   
  
  
+ CCAACTCCAG GTTTTGCATA GTAATTGCTT GTTGCTTGGG TGGAAGAATC GCCCTCTTTT TTCTGTCTCT   
  
  
+ TCTTGGCAAT A  

- +Up\_Stream \_Len000AAAAAA AATTCAAAAG CATGAACCAC AACTACTAAA CACGAAACAT TGGTTACCAA   
  
  
- AATCATTTAT CATTAATCAG CATTGTAAGA CGCTAAAGCG AGTTAAATAT ATAGTTGAAA TCTTATACTC   
  
  
- ATTGGCATTT CTAGTTTGCA TCATCATCTC CACAGTATCC AAAAAAAAAT TTTTTTAAGC CTCATTCTTT   
  
  
- CATCCCATGT AATAAAAACG AACAATTTAA GGCGATCATA TCGTTATGCT CTACCTTGTA AACATTTTTG   
  
  
- ATGCTCTGCT TCGCAAACAT GTTTTGTTTG ATGGTATGAC GTCTAGTATA TTAAAAAACC CCCCAATACA   
  
  
- TAGAATCACT ACTGTAATTA ACTACCTATT GGGATAAGAT TTATAGACCT AGGTAGTAAT TGACTGAGCA   
  
  
- AAGTTTACAA TCCTAATACC CAGAACTGTA CCATATGTAC AGTTATCTAA ATACAATCTA CTTTTCAATA   
  
  
- TACTGCGAAA AATACTAAAA ACATGATCGT ATATCACGAA TTAACCTGAT ATATAAAAAT GTTCAACACG   
  
  
- AATTGTGGAA AATTAGTCTT TATAAATAAT ATACCTTTTT TAAAATAATT ATACACAAAT ACACAAATTT   
  
  
- TTTGTATGGG TCGGAACTTT ATCTGGCACT ATATCTTTAA CCTGGGTATT ATGAGTGTAA TATATACGAA   
  
  
- ATAACAATAT TAATTCCTAG TAACAACAAA ACGTTAATAC ACCGTTCTAT ATTAGGTGTT TCAAAATGTA   
  
  
- AATATAACGT TAATGAGACC ATTTACGATA ATTACATGTA AACTCTAATT CTGTATATAT ATTGGGAAAA   
  
  
- AAAAGGGAAG AAAAAAGGAA AAAGATAAAA CTTACCGTAG AATTTGGTTT CCTCGACATG TTCAATGCAA   
  
  
- TATCGTGAGT ACCAACTGAT TTGTTCTGTC TACTCTCAAA TTCAGTTCAG GAGGACCGTT ACTACAAACG   
  
  
- GTAAACCTGG TTATTTATGT AGTACATATA ATGGTATTAT CACACCAACT GAATTGTTAT GTATCCCCTA   
  
  
- ACGTATGTTT CCATTCGGAG TTTTTGAACA CGTATACGTA ACTTTAGGTA CTAGTTTTTT TAAGTTCTCG   
  
  
- TCCAACGTGT TTTTGTGCCT CTACTCTACT TTCTCTGTCT TTTGTGTATA TGTAAGTGTC ATCATCCCCA   
  
  
- TGACCATCTC GATACCGATC CGATTCTACT ACACTTTCTT TGTTATATTA CCATCATTGA CCATTATAAA   
  
  
- TATCCCGTGT ATATCCACGC CCGTATCCCA GCACAGAGAG TCAGAGTCCA TGCCAAGCTA CCCTGAGACG   
  
  
- CTATCTACAA TAGTTTAAGT TGGTAAGCTT GTAGAGGGTC TCTCTCTCTC TCTCTGACTC ACTCACTCAC   
  
  
- TCACCCACCC ACATACACAC ACTCAACAAA ACAAGTAAAT AATATCCAAG AGACGACGTA TATAGAGGTG   
  
  
- GTAATTTAAA TGGTTTTGAA CATGGTCGAC GAAATGAACG AAAGGAGTAA AAGGGGGAAA AAACAGAGAG   
  
  
- AGAGAGAGAG AGGGGGGGGG GGGGGGGGAG AGAGAGACAC AGACTTAGTC ATAGTCGGAT AGTCCTACGA   
  
  
- AGCGGAGGTC TTTCCGTAGT GAGTGTGTGA ATAATCGAAT TTTGAAAGAA AATAAGGAAA ACGAGAAAGT   
  
  
- GACGAGAGAA AATGGGTTTA TGCGGGCAGA AGTTAAGAAG AGAGTAATTT ATACTTTTTC TTTCTCTCTT   
  
  
- TATCTCTCTC TAAATTTGGT CTTCCCCACT AAGGAAAAAG AACCACCCGA ATAATTACGA GACAGATGGG   
  
  
- GTAGATCGGC TTTTTATCAT CGAATTCTGA GTTGACGAAT AATCGTAGAG AGGAGATCGT TATTGGCTCC   
  
  
- ATTATCGTAT AAAAAGAGGG GAATCGAAGA TGGAACGAAA AACACTGTGA GTTTGGATTT GACTCTCTCT   
  
  
- CTTTCTCTTC TATCTCCTCT CTATTTATAA TCGCCCTAAT CCCGTTTAAA ACTATACGAC CCGAGAAGGA   
  
  
- GTAAGTTGAG ACTCCTTCTT CTAGTTCTAG TACGGTTGGG TGGAGGCGGC GTTGTTGGTG GTGGCAATGT   
  
  
- CGACGGTGAA GGTGCCGGTT CTAAGTCGGT CAATTATAGT AGTAGTGGAG GTTATTATAG TCGTAGAAGA   
  
  
- AGTAGTAATT AGGTATTATT GGGAGCAGTC GACGATTATT CAACGCCACT TGAGTAGAGG GCGACACTGA   
  
  
- AGAGGAGTCG ATTGGCGGAA GAGAGAGAAG AGAGGCCGCT GAAGCGAAGG AGGAGGCCGC TGAGGTGACT   
  
  
- TGCGGAACAA ATAATGAAGA GGTTCCGAAA CAAGGAGGCG GAAGCGGCTG TCAAAAGAGA TAGAAGAAGT   
  
  
- TGAAAATGAC CAATATCAAC ATTGGGATCA ACAAGACGCA GATAACGAGG TGAAAAAGAT TGGTTGGGAT   
  
  
- TAGGATCTGG TGGTGTTCTT GACGGCCATG CTCATTGATC GTCCCAATGA GGTTCATCGA CGAGCAGAAT   
  
  
- GGATTGGGAT TTGGTCTAGT GAGGTAAATA GGCCAAGTGG GTGGACTGCC GGTTGGTCCG TTAAAACCTC   
  
  
- CGACACCTCC CGATATCCCG TCAGGTGTAG TAGCTGTACC TGTAGTACGT ACCTCACGTC ACCGGAGGCG   
  
  
- AGGAGGTTCG TTAGCACCTC GCCAGCCGGT GGGAATCGGT TGGAGGGGGA CACCAGGCAG AGTTTCCCCC   
  
  
- TCCCGGCCTG GGCCTGGAGA ATGTCTCCTG CCCCCTGGCT GATACCTTGA AACGGGTTAG TAATCCTGAT   
  
  
- CTCAAGGTAA AGGTCCCGGA GTACTAGGGG CGGCTCAGTT GTCCAAAACA ACTTGGACGA CTCCGTCGCC   
  
  
- AGTTACGGGA TCGGAACGTC CTCCGCCACC CCACAGTTCT TCCCCTCCGG GAACGTCAAG TGACGCCCCT   
  
  
- GATAGAGGTG GCGGAGAATT TTCTCATGCT GTGTTCCGGA GAATCTGCCA AGGAGGTGTT CCAGTTCTGA   
  
  
- GACTTGGGGT CCCAGAACTG GAACCCTCTC TCCCTTCGGC TGGTGTTGGT GGGAGAGAAG GACGCCGCCA   
  
  
- AGTGGCTCCG TCAGTTGGTG ATGCCCCGTC ACAAACTGAG GGACCTTCGT TGGGAAGGCG GCGTATCGGT   
  
  
- TCTCTCCCAT CGACACCTCC TTCCCACCAA GCCTCTCCTC TAATTCCTGC ACCATCCTCT TCTTTCGCTC   
  
  
- TCCTCTTATC TCTCCGTGGT CTTCAAGCTC AGCACCCTTC GTAACAACTC CTCGAGTCCC AAGTTCTCAC   
  
  
- ACGGGGAGTC GGGTAAGAGC GACAGGGTTC GATTCGACGA CGACGCGGAG GTAATGGGAA GGCTTCCCAT   
  
  
- GGTTGAGGTC CAAAACGTAT CATTAACGAA CAACGAACCC ACCTTCTTAG CGGGAGAAAA AAGACAGAGA   
  
  
- AGAACCGTTA T

+     MYB

| Site Name | Organism | Position | Strand | Matrix score. | sequence | function |
| --- | --- | --- | --- | --- | --- | --- |
| MYB | Arabidopsis thaliana | 2443 | + | 6 | TAACCA |  |
| MYB | Arabidopsis thaliana | 2393 | - | 6 | TAACCA |  |
| MYB | Arabidopsis thaliana | 3098 | + | 6 | CAACCA |  |
| MYB | Arabidopsis thaliana | 2088 | + | 6 | CAACCA |  |
| MYB | Arabidopsis thaliana | 1353 | + | 6 | CAACCA |  |
| MYB | Arabidopsis thaliana | 925 | - | 6 | CAACCA |  |
| MYB | Arabidopsis thaliana | 1028 | - | 6 | CAACCA |  |
| MYB | Arabidopsis thaliana | 3059 | + | 6 | CAACCA |  |
| MYB | Arabidopsis thaliana | 2842 | + | 6 | CAACAG |  |
| MYB | Arabidopsis thaliana | 2576 | + | 6 | CAACCA |  |
| MYB | Arabidopsis thaliana | 63 | + | 6 | TAACCA |  |

>HU03G01737.1   
+ +Up\_Stream \_Len000TTTTTT TTAAGTTTTC GTACTTGGTG TTGATGATTT GTGCTTTGTA ACCAATGGTT   
  
  
+ TTAGTAAATA GTAATTAGTC GTAACATTCT GCGATTTCGC TCAATTTATA TATCAACTTT AGAATATGAG   
  
  
+ TAACCGTAAA GATCAAACGT AGTAGTAGAG GTGTCATAGG TTTTTTTTTA AAAAAATTCG GAGTAAGAAA   
  
  
+ GTAGGGTACA TTATTTTTGC TTGTTAAATT CCGCTAGTAT AGCAATACGA GATGGAACAT TTGTAAAAAC   
  
  
+ TACGAGACGA AGCGTTTGTA CAAAACAAAC TACCATACTG CAGATCATAT AATTTTTTGG GGGGTTATGT   
  
  
+ ATCTTAGTGA TGACATTAAT TGATGGATAA CCCTATTCTA AATATCTGGA TCCATCATTA ACTGACTCGT   
  
  
+ TTCAAATGTT AGGATTATGG GTCTTGACAT GGTATACATG TCAATAGATT TATGTTAGAT GAAAAGTTAT   
  
  
+ ATGACGCTTT TTATGATTTT TGTACTAGCA TATAGTGCTT AATTGGACTA TATATTTTTA CAAGTTGTGC   
  
  
+ TTAACACCTT TTAATCAGAA ATATTTATTA TATGGAAAAA ATTTTATTAA TATGTGTTTA TGTGTTTAAA   
  
  
+ AAACATACCC AGCCTTGAAA TAGACCGTGA TATAGAAATT GGACCCATAA TACTCACATT ATATATGCTT   
  
  
+ TATTGTTATA ATTAAGGATC ATTGTTGTTT TGCAATTATG TGGCAAGATA TAATCCACAA AGTTTTACAT   
  
  
+ TTATATTGCA ATTACTCTGG TAAATGCTAT TAATGTACAT TTGAGATTAA GACATATATA TAACCCTTTT   
  
  
+ TTTTCCCTTC TTTTTTCCTT TTTCTATTTT GAATGGCATC TTAAACCAAA GGAGCTGTAC AAGTTACGTT   
  
  
+ ATAGCACTCA TGGTTGACTA AACAAGACAG ATGAGAGTTT AAGTCAAGTC CTCCTGGCAA TGATGTTTGC   
  
  
+ CATTTGGACC AATAAATACA TCATGTATAT TACCATAATA GTGTGGTTGA CTTAACAATA CATAGGGGAT   
  
  
+ TGCATACAAA GGTAAGCCTC AAAAACTTGT GCATATGCAT TGAAATCCAT GATCAAAAAA ATTCAAGAGC   
  
  
+ AGGTTGCACA AAAACACGGA GATGAGATGA AAGAGACAGA AAACACATAT ACATTCACAG TAGTAGGGGT   
  
  
+ ACTGGTAGAG CTATGGCTAG GCTAAGATGA TGTGAAAGAA ACAATATAAT GGTAGTAACT GGTAATATTT   
  
  
+ ATAGGGCACA TATAGGTGCG GGCATAGGGT CGTGTCTCTC AGTCTCAGGT ACGGTTCGAT GGGACTCTGC   
  
  
+ GATAGATGTT ATCAAATTCA ACCATTCGAA CATCTCCCAG AGAGAGAGAG AGAGACTGAG TGAGTGAGTG   
  
  
+ AGTGGGTGGG TGTATGTGTG TGAGTTGTTT TGTTCATTTA TTATAGGTTC TCTGCTGCAT ATATCTCCAC   
  
  
+ CATTAAATTT ACCAAAACTT GTACCAGCTG CTTTACTTGC TTTCCTCATT TTCCCCCTTT TTTGTCTCTC   
  
  
+ TCTCTCTCTC TCCCCCCCCC CCCCCCCCTC TCTCTCTGTG TCTGAATCAG TATCAGCCTA TCAGGATGCT   
  
  
+ TCGCCTCCAG AAAGGCATCA CTCACACACT TATTAGCTTA AAACTTTCTT TTATTCCTTT TGCTCTTTCA   
  
  
+ CTGCTCTCTT TTACCCAAAT ACGCCCGTCT TCAATTCTTC TCTCATTAAA TATGAAAAAG AAAGAGAGAA   
  
  
+ ATAGAGAGAG ATTTAAACCA GAAGGGGTGA TTCCTTTTTC TTGGTGGGCT TATTAATGCT CTGTCTACCC   
  
  
+ CATCTAGCCG AAAAATAGTA GCTTAAGACT CAACTGCTTA TTAGCATCTC TCCTCTAGCA ATAACCGAGG   
  
  
+ TAATAGCATA TTTTTCTCCC CTTAGCTTCT ACCTTGCTTT TTGTGACACT CAAACCTAAA CTGAGAGAGA   
  
  
+ GAAAGAGAAG ATAGAGGAGA GATAAATATT AGCGGGATTA GGGCAAATTT TGATATGCTG GGCTCTTCCT   
  
  
+ CATTCAACTC TGAGGAAGAA GATCAAGATC ATGCCAACCC ACCTCCGCCG CAACAACCAC CACCGTTACA   
  
  
+ GCTGCCACTT CCACGGCCAA GATTCAGCCA GTTAATATCA TCATCACCTC CAATAATATC AGCATCTTCT   
  
  
+ TCATCATTAA TCCATAATAA CCCTCGTCAG CTGCTAATAA GTTGCGGTGA ACTCATCTCC CGCTGTGACT   
  
  
+ TCTCCTCAGC TAACCGCCTT CTCTCTCTTC TCTCCGGCGA CTTCGCTTCC TCCTCCGGCG ACTCCACTGA   
  
  
+ ACGCCTTGTT TATTACTTCT CCAAGGCTTT GTTCCTCCGC CTTCGCCGAC AGTTTTCTCT ATCTTCTTCA   
  
  
+ ACTTTTACTG GTTATAGTTG TAACCCTAGT TGTTCTGCGT CTATTGCTCC ACTTTTTCTA ACCAACCCTA   
  
  
+ ATCCTAGACC ACCACAAGAA CTGCCGGTAC GAGTAACTAG CAGGGTTACT CCAAGTAGCT GCTCGTCTTA   
  
  
+ CCTAACCCTA AACCAGATCA CTCCATTTAT CCGGTTCACC CACCTGACGG CCAACCAGGC AATTTTGGAG   
  
  
+ GCTGTGGAGG GCTATAGGGC AGTCCACATC ATCGACATGG ACATCATGCA TGGAGTGCAG TGGCCTCCGC   
  
  
+ TCCTCCAAGC AATCGTGGAG CGGTCGGCCA CCCTTAGCCA ACCTCCCCCT GTGGTCCGTC TCAAAGGGGG   
  
  
+ AGGGCCGGAC CCGGACCTCT TACAGAGGAC GGGGGACCGA CTATGGAACT TTGCCCAATC ATTAGGACTA   
  
  
+ GAGTTCCATT TCCAGGGCCT CATGATCCCC GCCGAGTCAA CAGGTTTTGT TGAACCTGCT GAGGCAGCGG   
  
  
+ TCAATGCCCT AGCCTTGCAG GAGGCGGTGG GGTGTCAAGA AGGGGAGGCC CTTGCAGTTC ACTGCGGGGA   
  
  
+ CTATCTCCAC CGCCTCTTAA AAGAGTACGA CACAAGGCCT CTTAGACGGT TCCTCCACAA GGTCAAGACT   
  
  
+ CTGAACCCCA GGGTCTTGAC CTTGGGAGAG AGGGAAGCCG ACCACAACCA CCCTCTCTTC CTGCGGCGGT   
  
  
+ TCACCGAGGC AGTCAACCAC TACGGGGCAG TGTTTGACTC CCTGGAAGCA ACCCTTCCGC CGCATAGCCA   
  
  
+ AGAGAGGGTA GCTGTGGAGG AAGGGTGGTT CGGAGAGGAG ATTAAGGACG TGGTAGGAGA AGAAAGCGAG   
  
  
+ AGGAGAATAG AGAGGCACCA GAAGTTCGAG TCGTGGGAAG CATTGTTGAG GAGCTCAGGG TTCAAGAGTG   
  
  
+ TGCCCCTCAG CCCATTCTCG CTGTCCCAAG CTAAGCTGCT GCTGCGCCTC CATTACCCTT CCGAAGGGTA   
  
  
+ CCAACTCCAG GTTTTGCATA GTAATTGCTT GTTGCTTGGG TGGAAGAATC GCCCTCTTTT TTCTGTCTCT   
  
  
+ TCTTGGCAAT A  

- +Up\_Stream \_Len000AAAAAA AATTCAAAAG CATGAACCAC AACTACTAAA CACGAAACAT TGGTTACCAA   
  
  
- AATCATTTAT CATTAATCAG CATTGTAAGA CGCTAAAGCG AGTTAAATAT ATAGTTGAAA TCTTATACTC   
  
  
- ATTGGCATTT CTAGTTTGCA TCATCATCTC CACAGTATCC AAAAAAAAAT TTTTTTAAGC CTCATTCTTT   
  
  
- CATCCCATGT AATAAAAACG AACAATTTAA GGCGATCATA TCGTTATGCT CTACCTTGTA AACATTTTTG   
  
  
- ATGCTCTGCT TCGCAAACAT GTTTTGTTTG ATGGTATGAC GTCTAGTATA TTAAAAAACC CCCCAATACA   
  
  
- TAGAATCACT ACTGTAATTA ACTACCTATT GGGATAAGAT TTATAGACCT AGGTAGTAAT TGACTGAGCA   
  
  
- AAGTTTACAA TCCTAATACC CAGAACTGTA CCATATGTAC AGTTATCTAA ATACAATCTA CTTTTCAATA   
  
  
- TACTGCGAAA AATACTAAAA ACATGATCGT ATATCACGAA TTAACCTGAT ATATAAAAAT GTTCAACACG   
  
  
- AATTGTGGAA AATTAGTCTT TATAAATAAT ATACCTTTTT TAAAATAATT ATACACAAAT ACACAAATTT   
  
  
- TTTGTATGGG TCGGAACTTT ATCTGGCACT ATATCTTTAA CCTGGGTATT ATGAGTGTAA TATATACGAA   
  
  
- ATAACAATAT TAATTCCTAG TAACAACAAA ACGTTAATAC ACCGTTCTAT ATTAGGTGTT TCAAAATGTA   
  
  
- AATATAACGT TAATGAGACC ATTTACGATA ATTACATGTA AACTCTAATT CTGTATATAT ATTGGGAAAA   
  
  
- AAAAGGGAAG AAAAAAGGAA AAAGATAAAA CTTACCGTAG AATTTGGTTT CCTCGACATG TTCAATGCAA   
  
  
- TATCGTGAGT ACCAACTGAT TTGTTCTGTC TACTCTCAAA TTCAGTTCAG GAGGACCGTT ACTACAAACG   
  
  
- GTAAACCTGG TTATTTATGT AGTACATATA ATGGTATTAT CACACCAACT GAATTGTTAT GTATCCCCTA   
  
  
- ACGTATGTTT CCATTCGGAG TTTTTGAACA CGTATACGTA ACTTTAGGTA CTAGTTTTTT TAAGTTCTCG   
  
  
- TCCAACGTGT TTTTGTGCCT CTACTCTACT TTCTCTGTCT TTTGTGTATA TGTAAGTGTC ATCATCCCCA   
  
  
- TGACCATCTC GATACCGATC CGATTCTACT ACACTTTCTT TGTTATATTA CCATCATTGA CCATTATAAA   
  
  
- TATCCCGTGT ATATCCACGC CCGTATCCCA GCACAGAGAG TCAGAGTCCA TGCCAAGCTA CCCTGAGACG   
  
  
- CTATCTACAA TAGTTTAAGT TGGTAAGCTT GTAGAGGGTC TCTCTCTCTC TCTCTGACTC ACTCACTCAC   
  
  
- TCACCCACCC ACATACACAC ACTCAACAAA ACAAGTAAAT AATATCCAAG AGACGACGTA TATAGAGGTG   
  
  
- GTAATTTAAA TGGTTTTGAA CATGGTCGAC GAAATGAACG AAAGGAGTAA AAGGGGGAAA AAACAGAGAG   
  
  
- AGAGAGAGAG AGGGGGGGGG GGGGGGGGAG AGAGAGACAC AGACTTAGTC ATAGTCGGAT AGTCCTACGA   
  
  
- AGCGGAGGTC TTTCCGTAGT GAGTGTGTGA ATAATCGAAT TTTGAAAGAA AATAAGGAAA ACGAGAAAGT   
  
  
- GACGAGAGAA AATGGGTTTA TGCGGGCAGA AGTTAAGAAG AGAGTAATTT ATACTTTTTC TTTCTCTCTT   
  
  
- TATCTCTCTC TAAATTTGGT CTTCCCCACT AAGGAAAAAG AACCACCCGA ATAATTACGA GACAGATGGG   
  
  
- GTAGATCGGC TTTTTATCAT CGAATTCTGA GTTGACGAAT AATCGTAGAG AGGAGATCGT TATTGGCTCC   
  
  
- ATTATCGTAT AAAAAGAGGG GAATCGAAGA TGGAACGAAA AACACTGTGA GTTTGGATTT GACTCTCTCT   
  
  
- CTTTCTCTTC TATCTCCTCT CTATTTATAA TCGCCCTAAT CCCGTTTAAA ACTATACGAC CCGAGAAGGA   
  
  
- GTAAGTTGAG ACTCCTTCTT CTAGTTCTAG TACGGTTGGG TGGAGGCGGC GTTGTTGGTG GTGGCAATGT   
  
  
- CGACGGTGAA GGTGCCGGTT CTAAGTCGGT CAATTATAGT AGTAGTGGAG GTTATTATAG TCGTAGAAGA   
  
  
- AGTAGTAATT AGGTATTATT GGGAGCAGTC GACGATTATT CAACGCCACT TGAGTAGAGG GCGACACTGA   
  
  
- AGAGGAGTCG ATTGGCGGAA GAGAGAGAAG AGAGGCCGCT GAAGCGAAGG AGGAGGCCGC TGAGGTGACT   
  
  
- TGCGGAACAA ATAATGAAGA GGTTCCGAAA CAAGGAGGCG GAAGCGGCTG TCAAAAGAGA TAGAAGAAGT   
  
  
- TGAAAATGAC CAATATCAAC ATTGGGATCA ACAAGACGCA GATAACGAGG TGAAAAAGAT TGGTTGGGAT   
  
  
- TAGGATCTGG TGGTGTTCTT GACGGCCATG CTCATTGATC GTCCCAATGA GGTTCATCGA CGAGCAGAAT   
  
  
- GGATTGGGAT TTGGTCTAGT GAGGTAAATA GGCCAAGTGG GTGGACTGCC GGTTGGTCCG TTAAAACCTC   
  
  
- CGACACCTCC CGATATCCCG TCAGGTGTAG TAGCTGTACC TGTAGTACGT ACCTCACGTC ACCGGAGGCG   
  
  
- AGGAGGTTCG TTAGCACCTC GCCAGCCGGT GGGAATCGGT TGGAGGGGGA CACCAGGCAG AGTTTCCCCC   
  
  
- TCCCGGCCTG GGCCTGGAGA ATGTCTCCTG CCCCCTGGCT GATACCTTGA AACGGGTTAG TAATCCTGAT   
  
  
- CTCAAGGTAA AGGTCCCGGA GTACTAGGGG CGGCTCAGTT GTCCAAAACA ACTTGGACGA CTCCGTCGCC   
  
  
- AGTTACGGGA TCGGAACGTC CTCCGCCACC CCACAGTTCT TCCCCTCCGG GAACGTCAAG TGACGCCCCT   
  
  
- GATAGAGGTG GCGGAGAATT TTCTCATGCT GTGTTCCGGA GAATCTGCCA AGGAGGTGTT CCAGTTCTGA   
  
  
- GACTTGGGGT CCCAGAACTG GAACCCTCTC TCCCTTCGGC TGGTGTTGGT GGGAGAGAAG GACGCCGCCA   
  
  
- AGTGGCTCCG TCAGTTGGTG ATGCCCCGTC ACAAACTGAG GGACCTTCGT TGGGAAGGCG GCGTATCGGT   
  
  
- TCTCTCCCAT CGACACCTCC TTCCCACCAA GCCTCTCCTC TAATTCCTGC ACCATCCTCT TCTTTCGCTC   
  
  
- TCCTCTTATC TCTCCGTGGT CTTCAAGCTC AGCACCCTTC GTAACAACTC CTCGAGTCCC AAGTTCTCAC   
  
  
- ACGGGGAGTC GGGTAAGAGC GACAGGGTTC GATTCGACGA CGACGCGGAG GTAATGGGAA GGCTTCCCAT   
  
  
- GGTTGAGGTC CAAAACGTAT CATTAACGAA CAACGAACCC ACCTTCTTAG CGGGAGAAAA AAGACAGAGA   
  
  
- AGAACCGTTA T

+     MYB-like sequence

| Site Name | Organism | Position | Strand | Matrix score. | sequence | function |
| --- | --- | --- | --- | --- | --- | --- |
| MYB-like sequence | Arabidopsis thaliana | 2443 | + | 6 | TAACCA |  |
| MYB-like sequence | Arabidopsis thaliana | 63 | + | 6 | TAACCA |  |
| MYB-like sequence | Arabidopsis thaliana | 2393 | - | 6 | TAACCA |  |

>HU03G01737.1   
+ +Up\_Stream \_Len000TTTTTT TTAAGTTTTC GTACTTGGTG TTGATGATTT GTGCTTTGTA ACCAATGGTT   
  
  
+ TTAGTAAATA GTAATTAGTC GTAACATTCT GCGATTTCGC TCAATTTATA TATCAACTTT AGAATATGAG   
  
  
+ TAACCGTAAA GATCAAACGT AGTAGTAGAG GTGTCATAGG TTTTTTTTTA AAAAAATTCG GAGTAAGAAA   
  
  
+ GTAGGGTACA TTATTTTTGC TTGTTAAATT CCGCTAGTAT AGCAATACGA GATGGAACAT TTGTAAAAAC   
  
  
+ TACGAGACGA AGCGTTTGTA CAAAACAAAC TACCATACTG CAGATCATAT AATTTTTTGG GGGGTTATGT   
  
  
+ ATCTTAGTGA TGACATTAAT TGATGGATAA CCCTATTCTA AATATCTGGA TCCATCATTA ACTGACTCGT   
  
  
+ TTCAAATGTT AGGATTATGG GTCTTGACAT GGTATACATG TCAATAGATT TATGTTAGAT GAAAAGTTAT   
  
  
+ ATGACGCTTT TTATGATTTT TGTACTAGCA TATAGTGCTT AATTGGACTA TATATTTTTA CAAGTTGTGC   
  
  
+ TTAACACCTT TTAATCAGAA ATATTTATTA TATGGAAAAA ATTTTATTAA TATGTGTTTA TGTGTTTAAA   
  
  
+ AAACATACCC AGCCTTGAAA TAGACCGTGA TATAGAAATT GGACCCATAA TACTCACATT ATATATGCTT   
  
  
+ TATTGTTATA ATTAAGGATC ATTGTTGTTT TGCAATTATG TGGCAAGATA TAATCCACAA AGTTTTACAT   
  
  
+ TTATATTGCA ATTACTCTGG TAAATGCTAT TAATGTACAT TTGAGATTAA GACATATATA TAACCCTTTT   
  
  
+ TTTTCCCTTC TTTTTTCCTT TTTCTATTTT GAATGGCATC TTAAACCAAA GGAGCTGTAC AAGTTACGTT   
  
  
+ ATAGCACTCA TGGTTGACTA AACAAGACAG ATGAGAGTTT AAGTCAAGTC CTCCTGGCAA TGATGTTTGC   
  
  
+ CATTTGGACC AATAAATACA TCATGTATAT TACCATAATA GTGTGGTTGA CTTAACAATA CATAGGGGAT   
  
  
+ TGCATACAAA GGTAAGCCTC AAAAACTTGT GCATATGCAT TGAAATCCAT GATCAAAAAA ATTCAAGAGC   
  
  
+ AGGTTGCACA AAAACACGGA GATGAGATGA AAGAGACAGA AAACACATAT ACATTCACAG TAGTAGGGGT   
  
  
+ ACTGGTAGAG CTATGGCTAG GCTAAGATGA TGTGAAAGAA ACAATATAAT GGTAGTAACT GGTAATATTT   
  
  
+ ATAGGGCACA TATAGGTGCG GGCATAGGGT CGTGTCTCTC AGTCTCAGGT ACGGTTCGAT GGGACTCTGC   
  
  
+ GATAGATGTT ATCAAATTCA ACCATTCGAA CATCTCCCAG AGAGAGAGAG AGAGACTGAG TGAGTGAGTG   
  
  
+ AGTGGGTGGG TGTATGTGTG TGAGTTGTTT TGTTCATTTA TTATAGGTTC TCTGCTGCAT ATATCTCCAC   
  
  
+ CATTAAATTT ACCAAAACTT GTACCAGCTG CTTTACTTGC TTTCCTCATT TTCCCCCTTT TTTGTCTCTC   
  
  
+ TCTCTCTCTC TCCCCCCCCC CCCCCCCCTC TCTCTCTGTG TCTGAATCAG TATCAGCCTA TCAGGATGCT   
  
  
+ TCGCCTCCAG AAAGGCATCA CTCACACACT TATTAGCTTA AAACTTTCTT TTATTCCTTT TGCTCTTTCA   
  
  
+ CTGCTCTCTT TTACCCAAAT ACGCCCGTCT TCAATTCTTC TCTCATTAAA TATGAAAAAG AAAGAGAGAA   
  
  
+ ATAGAGAGAG ATTTAAACCA GAAGGGGTGA TTCCTTTTTC TTGGTGGGCT TATTAATGCT CTGTCTACCC   
  
  
+ CATCTAGCCG AAAAATAGTA GCTTAAGACT CAACTGCTTA TTAGCATCTC TCCTCTAGCA ATAACCGAGG   
  
  
+ TAATAGCATA TTTTTCTCCC CTTAGCTTCT ACCTTGCTTT TTGTGACACT CAAACCTAAA CTGAGAGAGA   
  
  
+ GAAAGAGAAG ATAGAGGAGA GATAAATATT AGCGGGATTA GGGCAAATTT TGATATGCTG GGCTCTTCCT   
  
  
+ CATTCAACTC TGAGGAAGAA GATCAAGATC ATGCCAACCC ACCTCCGCCG CAACAACCAC CACCGTTACA   
  
  
+ GCTGCCACTT CCACGGCCAA GATTCAGCCA GTTAATATCA TCATCACCTC CAATAATATC AGCATCTTCT   
  
  
+ TCATCATTAA TCCATAATAA CCCTCGTCAG CTGCTAATAA GTTGCGGTGA ACTCATCTCC CGCTGTGACT   
  
  
+ TCTCCTCAGC TAACCGCCTT CTCTCTCTTC TCTCCGGCGA CTTCGCTTCC TCCTCCGGCG ACTCCACTGA   
  
  
+ ACGCCTTGTT TATTACTTCT CCAAGGCTTT GTTCCTCCGC CTTCGCCGAC AGTTTTCTCT ATCTTCTTCA   
  
  
+ ACTTTTACTG GTTATAGTTG TAACCCTAGT TGTTCTGCGT CTATTGCTCC ACTTTTTCTA ACCAACCCTA   
  
  
+ ATCCTAGACC ACCACAAGAA CTGCCGGTAC GAGTAACTAG CAGGGTTACT CCAAGTAGCT GCTCGTCTTA   
  
  
+ CCTAACCCTA AACCAGATCA CTCCATTTAT CCGGTTCACC CACCTGACGG CCAACCAGGC AATTTTGGAG   
  
  
+ GCTGTGGAGG GCTATAGGGC AGTCCACATC ATCGACATGG ACATCATGCA TGGAGTGCAG TGGCCTCCGC   
  
  
+ TCCTCCAAGC AATCGTGGAG CGGTCGGCCA CCCTTAGCCA ACCTCCCCCT GTGGTCCGTC TCAAAGGGGG   
  
  
+ AGGGCCGGAC CCGGACCTCT TACAGAGGAC GGGGGACCGA CTATGGAACT TTGCCCAATC ATTAGGACTA   
  
  
+ GAGTTCCATT TCCAGGGCCT CATGATCCCC GCCGAGTCAA CAGGTTTTGT TGAACCTGCT GAGGCAGCGG   
  
  
+ TCAATGCCCT AGCCTTGCAG GAGGCGGTGG GGTGTCAAGA AGGGGAGGCC CTTGCAGTTC ACTGCGGGGA   
  
  
+ CTATCTCCAC CGCCTCTTAA AAGAGTACGA CACAAGGCCT CTTAGACGGT TCCTCCACAA GGTCAAGACT   
  
  
+ CTGAACCCCA GGGTCTTGAC CTTGGGAGAG AGGGAAGCCG ACCACAACCA CCCTCTCTTC CTGCGGCGGT   
  
  
+ TCACCGAGGC AGTCAACCAC TACGGGGCAG TGTTTGACTC CCTGGAAGCA ACCCTTCCGC CGCATAGCCA   
  
  
+ AGAGAGGGTA GCTGTGGAGG AAGGGTGGTT CGGAGAGGAG ATTAAGGACG TGGTAGGAGA AGAAAGCGAG   
  
  
+ AGGAGAATAG AGAGGCACCA GAAGTTCGAG TCGTGGGAAG CATTGTTGAG GAGCTCAGGG TTCAAGAGTG   
  
  
+ TGCCCCTCAG CCCATTCTCG CTGTCCCAAG CTAAGCTGCT GCTGCGCCTC CATTACCCTT CCGAAGGGTA   
  
  
+ CCAACTCCAG GTTTTGCATA GTAATTGCTT GTTGCTTGGG TGGAAGAATC GCCCTCTTTT TTCTGTCTCT   
  
  
+ TCTTGGCAAT A  

- +Up\_Stream \_Len000AAAAAA AATTCAAAAG CATGAACCAC AACTACTAAA CACGAAACAT TGGTTACCAA   
  
  
- AATCATTTAT CATTAATCAG CATTGTAAGA CGCTAAAGCG AGTTAAATAT ATAGTTGAAA TCTTATACTC   
  
  
- ATTGGCATTT CTAGTTTGCA TCATCATCTC CACAGTATCC AAAAAAAAAT TTTTTTAAGC CTCATTCTTT   
  
  
- CATCCCATGT AATAAAAACG AACAATTTAA GGCGATCATA TCGTTATGCT CTACCTTGTA AACATTTTTG   
  
  
- ATGCTCTGCT TCGCAAACAT GTTTTGTTTG ATGGTATGAC GTCTAGTATA TTAAAAAACC CCCCAATACA   
  
  
- TAGAATCACT ACTGTAATTA ACTACCTATT GGGATAAGAT TTATAGACCT AGGTAGTAAT TGACTGAGCA   
  
  
- AAGTTTACAA TCCTAATACC CAGAACTGTA CCATATGTAC AGTTATCTAA ATACAATCTA CTTTTCAATA   
  
  
- TACTGCGAAA AATACTAAAA ACATGATCGT ATATCACGAA TTAACCTGAT ATATAAAAAT GTTCAACACG   
  
  
- AATTGTGGAA AATTAGTCTT TATAAATAAT ATACCTTTTT TAAAATAATT ATACACAAAT ACACAAATTT   
  
  
- TTTGTATGGG TCGGAACTTT ATCTGGCACT ATATCTTTAA CCTGGGTATT ATGAGTGTAA TATATACGAA   
  
  
- ATAACAATAT TAATTCCTAG TAACAACAAA ACGTTAATAC ACCGTTCTAT ATTAGGTGTT TCAAAATGTA   
  
  
- AATATAACGT TAATGAGACC ATTTACGATA ATTACATGTA AACTCTAATT CTGTATATAT ATTGGGAAAA   
  
  
- AAAAGGGAAG AAAAAAGGAA AAAGATAAAA CTTACCGTAG AATTTGGTTT CCTCGACATG TTCAATGCAA   
  
  
- TATCGTGAGT ACCAACTGAT TTGTTCTGTC TACTCTCAAA TTCAGTTCAG GAGGACCGTT ACTACAAACG   
  
  
- GTAAACCTGG TTATTTATGT AGTACATATA ATGGTATTAT CACACCAACT GAATTGTTAT GTATCCCCTA   
  
  
- ACGTATGTTT CCATTCGGAG TTTTTGAACA CGTATACGTA ACTTTAGGTA CTAGTTTTTT TAAGTTCTCG   
  
  
- TCCAACGTGT TTTTGTGCCT CTACTCTACT TTCTCTGTCT TTTGTGTATA TGTAAGTGTC ATCATCCCCA   
  
  
- TGACCATCTC GATACCGATC CGATTCTACT ACACTTTCTT TGTTATATTA CCATCATTGA CCATTATAAA   
  
  
- TATCCCGTGT ATATCCACGC CCGTATCCCA GCACAGAGAG TCAGAGTCCA TGCCAAGCTA CCCTGAGACG   
  
  
- CTATCTACAA TAGTTTAAGT TGGTAAGCTT GTAGAGGGTC TCTCTCTCTC TCTCTGACTC ACTCACTCAC   
  
  
- TCACCCACCC ACATACACAC ACTCAACAAA ACAAGTAAAT AATATCCAAG AGACGACGTA TATAGAGGTG   
  
  
- GTAATTTAAA TGGTTTTGAA CATGGTCGAC GAAATGAACG AAAGGAGTAA AAGGGGGAAA AAACAGAGAG   
  
  
- AGAGAGAGAG AGGGGGGGGG GGGGGGGGAG AGAGAGACAC AGACTTAGTC ATAGTCGGAT AGTCCTACGA   
  
  
- AGCGGAGGTC TTTCCGTAGT GAGTGTGTGA ATAATCGAAT TTTGAAAGAA AATAAGGAAA ACGAGAAAGT   
  
  
- GACGAGAGAA AATGGGTTTA TGCGGGCAGA AGTTAAGAAG AGAGTAATTT ATACTTTTTC TTTCTCTCTT   
  
  
- TATCTCTCTC TAAATTTGGT CTTCCCCACT AAGGAAAAAG AACCACCCGA ATAATTACGA GACAGATGGG   
  
  
- GTAGATCGGC TTTTTATCAT CGAATTCTGA GTTGACGAAT AATCGTAGAG AGGAGATCGT TATTGGCTCC   
  
  
- ATTATCGTAT AAAAAGAGGG GAATCGAAGA TGGAACGAAA AACACTGTGA GTTTGGATTT GACTCTCTCT   
  
  
- CTTTCTCTTC TATCTCCTCT CTATTTATAA TCGCCCTAAT CCCGTTTAAA ACTATACGAC CCGAGAAGGA   
  
  
- GTAAGTTGAG ACTCCTTCTT CTAGTTCTAG TACGGTTGGG TGGAGGCGGC GTTGTTGGTG GTGGCAATGT   
  
  
- CGACGGTGAA GGTGCCGGTT CTAAGTCGGT CAATTATAGT AGTAGTGGAG GTTATTATAG TCGTAGAAGA   
  
  
- AGTAGTAATT AGGTATTATT GGGAGCAGTC GACGATTATT CAACGCCACT TGAGTAGAGG GCGACACTGA   
  
  
- AGAGGAGTCG ATTGGCGGAA GAGAGAGAAG AGAGGCCGCT GAAGCGAAGG AGGAGGCCGC TGAGGTGACT   
  
  
- TGCGGAACAA ATAATGAAGA GGTTCCGAAA CAAGGAGGCG GAAGCGGCTG TCAAAAGAGA TAGAAGAAGT   
  
  
- TGAAAATGAC CAATATCAAC ATTGGGATCA ACAAGACGCA GATAACGAGG TGAAAAAGAT TGGTTGGGAT   
  
  
- TAGGATCTGG TGGTGTTCTT GACGGCCATG CTCATTGATC GTCCCAATGA GGTTCATCGA CGAGCAGAAT   
  
  
- GGATTGGGAT TTGGTCTAGT GAGGTAAATA GGCCAAGTGG GTGGACTGCC GGTTGGTCCG TTAAAACCTC   
  
  
- CGACACCTCC CGATATCCCG TCAGGTGTAG TAGCTGTACC TGTAGTACGT ACCTCACGTC ACCGGAGGCG   
  
  
- AGGAGGTTCG TTAGCACCTC GCCAGCCGGT GGGAATCGGT TGGAGGGGGA CACCAGGCAG AGTTTCCCCC   
  
  
- TCCCGGCCTG GGCCTGGAGA ATGTCTCCTG CCCCCTGGCT GATACCTTGA AACGGGTTAG TAATCCTGAT   
  
  
- CTCAAGGTAA AGGTCCCGGA GTACTAGGGG CGGCTCAGTT GTCCAAAACA ACTTGGACGA CTCCGTCGCC   
  
  
- AGTTACGGGA TCGGAACGTC CTCCGCCACC CCACAGTTCT TCCCCTCCGG GAACGTCAAG TGACGCCCCT   
  
  
- GATAGAGGTG GCGGAGAATT TTCTCATGCT GTGTTCCGGA GAATCTGCCA AGGAGGTGTT CCAGTTCTGA   
  
  
- GACTTGGGGT CCCAGAACTG GAACCCTCTC TCCCTTCGGC TGGTGTTGGT GGGAGAGAAG GACGCCGCCA   
  
  
- AGTGGCTCCG TCAGTTGGTG ATGCCCCGTC ACAAACTGAG GGACCTTCGT TGGGAAGGCG GCGTATCGGT   
  
  
- TCTCTCCCAT CGACACCTCC TTCCCACCAA GCCTCTCCTC TAATTCCTGC ACCATCCTCT TCTTTCGCTC   
  
  
- TCCTCTTATC TCTCCGTGGT CTTCAAGCTC AGCACCCTTC GTAACAACTC CTCGAGTCCC AAGTTCTCAC   
  
  
- ACGGGGAGTC GGGTAAGAGC GACAGGGTTC GATTCGACGA CGACGCGGAG GTAATGGGAA GGCTTCCCAT   
  
  
- GGTTGAGGTC CAAAACGTAT CATTAACGAA CAACGAACCC ACCTTCTTAG CGGGAGAAAA AAGACAGAGA   
  
  
- AGAACCGTTA T

+     MYC

| Site Name | Organism | Position | Strand | Matrix score. | sequence | function |
| --- | --- | --- | --- | --- | --- | --- |
| MYC | Arabidopsis thaliana | 427 | - | 6 | CATTTG |  |
| MYC | Arabidopsis thaliana | 272 | + | 6 | CATTTG |  |
| MYC | Arabidopsis thaliana | 985 | + | 6 | CATTTG |  |
| MYC | Arabidopsis thaliana | 812 | + | 6 | CATTTG |  |

>HU03G01737.1   
+ +Up\_Stream \_Len000TTTTTT TTAAGTTTTC GTACTTGGTG TTGATGATTT GTGCTTTGTA ACCAATGGTT   
  
  
+ TTAGTAAATA GTAATTAGTC GTAACATTCT GCGATTTCGC TCAATTTATA TATCAACTTT AGAATATGAG   
  
  
+ TAACCGTAAA GATCAAACGT AGTAGTAGAG GTGTCATAGG TTTTTTTTTA AAAAAATTCG GAGTAAGAAA   
  
  
+ GTAGGGTACA TTATTTTTGC TTGTTAAATT CCGCTAGTAT AGCAATACGA GATGGAACAT TTGTAAAAAC   
  
  
+ TACGAGACGA AGCGTTTGTA CAAAACAAAC TACCATACTG CAGATCATAT AATTTTTTGG GGGGTTATGT   
  
  
+ ATCTTAGTGA TGACATTAAT TGATGGATAA CCCTATTCTA AATATCTGGA TCCATCATTA ACTGACTCGT   
  
  
+ TTCAAATGTT AGGATTATGG GTCTTGACAT GGTATACATG TCAATAGATT TATGTTAGAT GAAAAGTTAT   
  
  
+ ATGACGCTTT TTATGATTTT TGTACTAGCA TATAGTGCTT AATTGGACTA TATATTTTTA CAAGTTGTGC   
  
  
+ TTAACACCTT TTAATCAGAA ATATTTATTA TATGGAAAAA ATTTTATTAA TATGTGTTTA TGTGTTTAAA   
  
  
+ AAACATACCC AGCCTTGAAA TAGACCGTGA TATAGAAATT GGACCCATAA TACTCACATT ATATATGCTT   
  
  
+ TATTGTTATA ATTAAGGATC ATTGTTGTTT TGCAATTATG TGGCAAGATA TAATCCACAA AGTTTTACAT   
  
  
+ TTATATTGCA ATTACTCTGG TAAATGCTAT TAATGTACAT TTGAGATTAA GACATATATA TAACCCTTTT   
  
  
+ TTTTCCCTTC TTTTTTCCTT TTTCTATTTT GAATGGCATC TTAAACCAAA GGAGCTGTAC AAGTTACGTT   
  
  
+ ATAGCACTCA TGGTTGACTA AACAAGACAG ATGAGAGTTT AAGTCAAGTC CTCCTGGCAA TGATGTTTGC   
  
  
+ CATTTGGACC AATAAATACA TCATGTATAT TACCATAATA GTGTGGTTGA CTTAACAATA CATAGGGGAT   
  
  
+ TGCATACAAA GGTAAGCCTC AAAAACTTGT GCATATGCAT TGAAATCCAT GATCAAAAAA ATTCAAGAGC   
  
  
+ AGGTTGCACA AAAACACGGA GATGAGATGA AAGAGACAGA AAACACATAT ACATTCACAG TAGTAGGGGT   
  
  
+ ACTGGTAGAG CTATGGCTAG GCTAAGATGA TGTGAAAGAA ACAATATAAT GGTAGTAACT GGTAATATTT   
  
  
+ ATAGGGCACA TATAGGTGCG GGCATAGGGT CGTGTCTCTC AGTCTCAGGT ACGGTTCGAT GGGACTCTGC   
  
  
+ GATAGATGTT ATCAAATTCA ACCATTCGAA CATCTCCCAG AGAGAGAGAG AGAGACTGAG TGAGTGAGTG   
  
  
+ AGTGGGTGGG TGTATGTGTG TGAGTTGTTT TGTTCATTTA TTATAGGTTC TCTGCTGCAT ATATCTCCAC   
  
  
+ CATTAAATTT ACCAAAACTT GTACCAGCTG CTTTACTTGC TTTCCTCATT TTCCCCCTTT TTTGTCTCTC   
  
  
+ TCTCTCTCTC TCCCCCCCCC CCCCCCCCTC TCTCTCTGTG TCTGAATCAG TATCAGCCTA TCAGGATGCT   
  
  
+ TCGCCTCCAG AAAGGCATCA CTCACACACT TATTAGCTTA AAACTTTCTT TTATTCCTTT TGCTCTTTCA   
  
  
+ CTGCTCTCTT TTACCCAAAT ACGCCCGTCT TCAATTCTTC TCTCATTAAA TATGAAAAAG AAAGAGAGAA   
  
  
+ ATAGAGAGAG ATTTAAACCA GAAGGGGTGA TTCCTTTTTC TTGGTGGGCT TATTAATGCT CTGTCTACCC   
  
  
+ CATCTAGCCG AAAAATAGTA GCTTAAGACT CAACTGCTTA TTAGCATCTC TCCTCTAGCA ATAACCGAGG   
  
  
+ TAATAGCATA TTTTTCTCCC CTTAGCTTCT ACCTTGCTTT TTGTGACACT CAAACCTAAA CTGAGAGAGA   
  
  
+ GAAAGAGAAG ATAGAGGAGA GATAAATATT AGCGGGATTA GGGCAAATTT TGATATGCTG GGCTCTTCCT   
  
  
+ CATTCAACTC TGAGGAAGAA GATCAAGATC ATGCCAACCC ACCTCCGCCG CAACAACCAC CACCGTTACA   
  
  
+ GCTGCCACTT CCACGGCCAA GATTCAGCCA GTTAATATCA TCATCACCTC CAATAATATC AGCATCTTCT   
  
  
+ TCATCATTAA TCCATAATAA CCCTCGTCAG CTGCTAATAA GTTGCGGTGA ACTCATCTCC CGCTGTGACT   
  
  
+ TCTCCTCAGC TAACCGCCTT CTCTCTCTTC TCTCCGGCGA CTTCGCTTCC TCCTCCGGCG ACTCCACTGA   
  
  
+ ACGCCTTGTT TATTACTTCT CCAAGGCTTT GTTCCTCCGC CTTCGCCGAC AGTTTTCTCT ATCTTCTTCA   
  
  
+ ACTTTTACTG GTTATAGTTG TAACCCTAGT TGTTCTGCGT CTATTGCTCC ACTTTTTCTA ACCAACCCTA   
  
  
+ ATCCTAGACC ACCACAAGAA CTGCCGGTAC GAGTAACTAG CAGGGTTACT CCAAGTAGCT GCTCGTCTTA   
  
  
+ CCTAACCCTA AACCAGATCA CTCCATTTAT CCGGTTCACC CACCTGACGG CCAACCAGGC AATTTTGGAG   
  
  
+ GCTGTGGAGG GCTATAGGGC AGTCCACATC ATCGACATGG ACATCATGCA TGGAGTGCAG TGGCCTCCGC   
  
  
+ TCCTCCAAGC AATCGTGGAG CGGTCGGCCA CCCTTAGCCA ACCTCCCCCT GTGGTCCGTC TCAAAGGGGG   
  
  
+ AGGGCCGGAC CCGGACCTCT TACAGAGGAC GGGGGACCGA CTATGGAACT TTGCCCAATC ATTAGGACTA   
  
  
+ GAGTTCCATT TCCAGGGCCT CATGATCCCC GCCGAGTCAA CAGGTTTTGT TGAACCTGCT GAGGCAGCGG   
  
  
+ TCAATGCCCT AGCCTTGCAG GAGGCGGTGG GGTGTCAAGA AGGGGAGGCC CTTGCAGTTC ACTGCGGGGA   
  
  
+ CTATCTCCAC CGCCTCTTAA AAGAGTACGA CACAAGGCCT CTTAGACGGT TCCTCCACAA GGTCAAGACT   
  
  
+ CTGAACCCCA GGGTCTTGAC CTTGGGAGAG AGGGAAGCCG ACCACAACCA CCCTCTCTTC CTGCGGCGGT   
  
  
+ TCACCGAGGC AGTCAACCAC TACGGGGCAG TGTTTGACTC CCTGGAAGCA ACCCTTCCGC CGCATAGCCA   
  
  
+ AGAGAGGGTA GCTGTGGAGG AAGGGTGGTT CGGAGAGGAG ATTAAGGACG TGGTAGGAGA AGAAAGCGAG   
  
  
+ AGGAGAATAG AGAGGCACCA GAAGTTCGAG TCGTGGGAAG CATTGTTGAG GAGCTCAGGG TTCAAGAGTG   
  
  
+ TGCCCCTCAG CCCATTCTCG CTGTCCCAAG CTAAGCTGCT GCTGCGCCTC CATTACCCTT CCGAAGGGTA   
  
  
+ CCAACTCCAG GTTTTGCATA GTAATTGCTT GTTGCTTGGG TGGAAGAATC GCCCTCTTTT TTCTGTCTCT   
  
  
+ TCTTGGCAAT A  

- +Up\_Stream \_Len000AAAAAA AATTCAAAAG CATGAACCAC AACTACTAAA CACGAAACAT TGGTTACCAA   
  
  
- AATCATTTAT CATTAATCAG CATTGTAAGA CGCTAAAGCG AGTTAAATAT ATAGTTGAAA TCTTATACTC   
  
  
- ATTGGCATTT CTAGTTTGCA TCATCATCTC CACAGTATCC AAAAAAAAAT TTTTTTAAGC CTCATTCTTT   
  
  
- CATCCCATGT AATAAAAACG AACAATTTAA GGCGATCATA TCGTTATGCT CTACCTTGTA AACATTTTTG   
  
  
- ATGCTCTGCT TCGCAAACAT GTTTTGTTTG ATGGTATGAC GTCTAGTATA TTAAAAAACC CCCCAATACA   
  
  
- TAGAATCACT ACTGTAATTA ACTACCTATT GGGATAAGAT TTATAGACCT AGGTAGTAAT TGACTGAGCA   
  
  
- AAGTTTACAA TCCTAATACC CAGAACTGTA CCATATGTAC AGTTATCTAA ATACAATCTA CTTTTCAATA   
  
  
- TACTGCGAAA AATACTAAAA ACATGATCGT ATATCACGAA TTAACCTGAT ATATAAAAAT GTTCAACACG   
  
  
- AATTGTGGAA AATTAGTCTT TATAAATAAT ATACCTTTTT TAAAATAATT ATACACAAAT ACACAAATTT   
  
  
- TTTGTATGGG TCGGAACTTT ATCTGGCACT ATATCTTTAA CCTGGGTATT ATGAGTGTAA TATATACGAA   
  
  
- ATAACAATAT TAATTCCTAG TAACAACAAA ACGTTAATAC ACCGTTCTAT ATTAGGTGTT TCAAAATGTA   
  
  
- AATATAACGT TAATGAGACC ATTTACGATA ATTACATGTA AACTCTAATT CTGTATATAT ATTGGGAAAA   
  
  
- AAAAGGGAAG AAAAAAGGAA AAAGATAAAA CTTACCGTAG AATTTGGTTT CCTCGACATG TTCAATGCAA   
  
  
- TATCGTGAGT ACCAACTGAT TTGTTCTGTC TACTCTCAAA TTCAGTTCAG GAGGACCGTT ACTACAAACG   
  
  
- GTAAACCTGG TTATTTATGT AGTACATATA ATGGTATTAT CACACCAACT GAATTGTTAT GTATCCCCTA   
  
  
- ACGTATGTTT CCATTCGGAG TTTTTGAACA CGTATACGTA ACTTTAGGTA CTAGTTTTTT TAAGTTCTCG   
  
  
- TCCAACGTGT TTTTGTGCCT CTACTCTACT TTCTCTGTCT TTTGTGTATA TGTAAGTGTC ATCATCCCCA   
  
  
- TGACCATCTC GATACCGATC CGATTCTACT ACACTTTCTT TGTTATATTA CCATCATTGA CCATTATAAA   
  
  
- TATCCCGTGT ATATCCACGC CCGTATCCCA GCACAGAGAG TCAGAGTCCA TGCCAAGCTA CCCTGAGACG   
  
  
- CTATCTACAA TAGTTTAAGT TGGTAAGCTT GTAGAGGGTC TCTCTCTCTC TCTCTGACTC ACTCACTCAC   
  
  
- TCACCCACCC ACATACACAC ACTCAACAAA ACAAGTAAAT AATATCCAAG AGACGACGTA TATAGAGGTG   
  
  
- GTAATTTAAA TGGTTTTGAA CATGGTCGAC GAAATGAACG AAAGGAGTAA AAGGGGGAAA AAACAGAGAG   
  
  
- AGAGAGAGAG AGGGGGGGGG GGGGGGGGAG AGAGAGACAC AGACTTAGTC ATAGTCGGAT AGTCCTACGA   
  
  
- AGCGGAGGTC TTTCCGTAGT GAGTGTGTGA ATAATCGAAT TTTGAAAGAA AATAAGGAAA ACGAGAAAGT   
  
  
- GACGAGAGAA AATGGGTTTA TGCGGGCAGA AGTTAAGAAG AGAGTAATTT ATACTTTTTC TTTCTCTCTT   
  
  
- TATCTCTCTC TAAATTTGGT CTTCCCCACT AAGGAAAAAG AACCACCCGA ATAATTACGA GACAGATGGG   
  
  
- GTAGATCGGC TTTTTATCAT CGAATTCTGA GTTGACGAAT AATCGTAGAG AGGAGATCGT TATTGGCTCC   
  
  
- ATTATCGTAT AAAAAGAGGG GAATCGAAGA TGGAACGAAA AACACTGTGA GTTTGGATTT GACTCTCTCT   
  
  
- CTTTCTCTTC TATCTCCTCT CTATTTATAA TCGCCCTAAT CCCGTTTAAA ACTATACGAC CCGAGAAGGA   
  
  
- GTAAGTTGAG ACTCCTTCTT CTAGTTCTAG TACGGTTGGG TGGAGGCGGC GTTGTTGGTG GTGGCAATGT   
  
  
- CGACGGTGAA GGTGCCGGTT CTAAGTCGGT CAATTATAGT AGTAGTGGAG GTTATTATAG TCGTAGAAGA   
  
  
- AGTAGTAATT AGGTATTATT GGGAGCAGTC GACGATTATT CAACGCCACT TGAGTAGAGG GCGACACTGA   
  
  
- AGAGGAGTCG ATTGGCGGAA GAGAGAGAAG AGAGGCCGCT GAAGCGAAGG AGGAGGCCGC TGAGGTGACT   
  
  
- TGCGGAACAA ATAATGAAGA GGTTCCGAAA CAAGGAGGCG GAAGCGGCTG TCAAAAGAGA TAGAAGAAGT   
  
  
- TGAAAATGAC CAATATCAAC ATTGGGATCA ACAAGACGCA GATAACGAGG TGAAAAAGAT TGGTTGGGAT   
  
  
- TAGGATCTGG TGGTGTTCTT GACGGCCATG CTCATTGATC GTCCCAATGA GGTTCATCGA CGAGCAGAAT   
  
  
- GGATTGGGAT TTGGTCTAGT GAGGTAAATA GGCCAAGTGG GTGGACTGCC GGTTGGTCCG TTAAAACCTC   
  
  
- CGACACCTCC CGATATCCCG TCAGGTGTAG TAGCTGTACC TGTAGTACGT ACCTCACGTC ACCGGAGGCG   
  
  
- AGGAGGTTCG TTAGCACCTC GCCAGCCGGT GGGAATCGGT TGGAGGGGGA CACCAGGCAG AGTTTCCCCC   
  
  
- TCCCGGCCTG GGCCTGGAGA ATGTCTCCTG CCCCCTGGCT GATACCTTGA AACGGGTTAG TAATCCTGAT   
  
  
- CTCAAGGTAA AGGTCCCGGA GTACTAGGGG CGGCTCAGTT GTCCAAAACA ACTTGGACGA CTCCGTCGCC   
  
  
- AGTTACGGGA TCGGAACGTC CTCCGCCACC CCACAGTTCT TCCCCTCCGG GAACGTCAAG TGACGCCCCT   
  
  
- GATAGAGGTG GCGGAGAATT TTCTCATGCT GTGTTCCGGA GAATCTGCCA AGGAGGTGTT CCAGTTCTGA   
  
  
- GACTTGGGGT CCCAGAACTG GAACCCTCTC TCCCTTCGGC TGGTGTTGGT GGGAGAGAAG GACGCCGCCA   
  
  
- AGTGGCTCCG TCAGTTGGTG ATGCCCCGTC ACAAACTGAG GGACCTTCGT TGGGAAGGCG GCGTATCGGT   
  
  
- TCTCTCCCAT CGACACCTCC TTCCCACCAA GCCTCTCCTC TAATTCCTGC ACCATCCTCT TCTTTCGCTC   
  
  
- TCCTCTTATC TCTCCGTGGT CTTCAAGCTC AGCACCCTTC GTAACAACTC CTCGAGTCCC AAGTTCTCAC   
  
  
- ACGGGGAGTC GGGTAAGAGC GACAGGGTTC GATTCGACGA CGACGCGGAG GTAATGGGAA GGCTTCCCAT   
  
  
- GGTTGAGGTC CAAAACGTAT CATTAACGAA CAACGAACCC ACCTTCTTAG CGGGAGAAAA AAGACAGAGA   
  
  
- AGAACCGTTA T

+     Myb

| Site Name | Organism | Position | Strand | Matrix score. | sequence | function |
| --- | --- | --- | --- | --- | --- | --- |
| Myb | Arabidopsis thaliana | 2133 | - | 6 | TAACTG |  |
| Myb | Arabidopsis thaliana | 1855 | + | 6 | CAACTG |  |
| Myb | Arabidopsis thaliana | 1250 | + | 6 | TAACTG |  |
| Myb | Arabidopsis thaliana | 413 | + | 6 | TAACTG |  |

>HU03G01737.1   
+ +Up\_Stream \_Len000TTTTTT TTAAGTTTTC GTACTTGGTG TTGATGATTT GTGCTTTGTA ACCAATGGTT   
  
  
+ TTAGTAAATA GTAATTAGTC GTAACATTCT GCGATTTCGC TCAATTTATA TATCAACTTT AGAATATGAG   
  
  
+ TAACCGTAAA GATCAAACGT AGTAGTAGAG GTGTCATAGG TTTTTTTTTA AAAAAATTCG GAGTAAGAAA   
  
  
+ GTAGGGTACA TTATTTTTGC TTGTTAAATT CCGCTAGTAT AGCAATACGA GATGGAACAT TTGTAAAAAC   
  
  
+ TACGAGACGA AGCGTTTGTA CAAAACAAAC TACCATACTG CAGATCATAT AATTTTTTGG GGGGTTATGT   
  
  
+ ATCTTAGTGA TGACATTAAT TGATGGATAA CCCTATTCTA AATATCTGGA TCCATCATTA ACTGACTCGT   
  
  
+ TTCAAATGTT AGGATTATGG GTCTTGACAT GGTATACATG TCAATAGATT TATGTTAGAT GAAAAGTTAT   
  
  
+ ATGACGCTTT TTATGATTTT TGTACTAGCA TATAGTGCTT AATTGGACTA TATATTTTTA CAAGTTGTGC   
  
  
+ TTAACACCTT TTAATCAGAA ATATTTATTA TATGGAAAAA ATTTTATTAA TATGTGTTTA TGTGTTTAAA   
  
  
+ AAACATACCC AGCCTTGAAA TAGACCGTGA TATAGAAATT GGACCCATAA TACTCACATT ATATATGCTT   
  
  
+ TATTGTTATA ATTAAGGATC ATTGTTGTTT TGCAATTATG TGGCAAGATA TAATCCACAA AGTTTTACAT   
  
  
+ TTATATTGCA ATTACTCTGG TAAATGCTAT TAATGTACAT TTGAGATTAA GACATATATA TAACCCTTTT   
  
  
+ TTTTCCCTTC TTTTTTCCTT TTTCTATTTT GAATGGCATC TTAAACCAAA GGAGCTGTAC AAGTTACGTT   
  
  
+ ATAGCACTCA TGGTTGACTA AACAAGACAG ATGAGAGTTT AAGTCAAGTC CTCCTGGCAA TGATGTTTGC   
  
  
+ CATTTGGACC AATAAATACA TCATGTATAT TACCATAATA GTGTGGTTGA CTTAACAATA CATAGGGGAT   
  
  
+ TGCATACAAA GGTAAGCCTC AAAAACTTGT GCATATGCAT TGAAATCCAT GATCAAAAAA ATTCAAGAGC   
  
  
+ AGGTTGCACA AAAACACGGA GATGAGATGA AAGAGACAGA AAACACATAT ACATTCACAG TAGTAGGGGT   
  
  
+ ACTGGTAGAG CTATGGCTAG GCTAAGATGA TGTGAAAGAA ACAATATAAT GGTAGTAACT GGTAATATTT   
  
  
+ ATAGGGCACA TATAGGTGCG GGCATAGGGT CGTGTCTCTC AGTCTCAGGT ACGGTTCGAT GGGACTCTGC   
  
  
+ GATAGATGTT ATCAAATTCA ACCATTCGAA CATCTCCCAG AGAGAGAGAG AGAGACTGAG TGAGTGAGTG   
  
  
+ AGTGGGTGGG TGTATGTGTG TGAGTTGTTT TGTTCATTTA TTATAGGTTC TCTGCTGCAT ATATCTCCAC   
  
  
+ CATTAAATTT ACCAAAACTT GTACCAGCTG CTTTACTTGC TTTCCTCATT TTCCCCCTTT TTTGTCTCTC   
  
  
+ TCTCTCTCTC TCCCCCCCCC CCCCCCCCTC TCTCTCTGTG TCTGAATCAG TATCAGCCTA TCAGGATGCT   
  
  
+ TCGCCTCCAG AAAGGCATCA CTCACACACT TATTAGCTTA AAACTTTCTT TTATTCCTTT TGCTCTTTCA   
  
  
+ CTGCTCTCTT TTACCCAAAT ACGCCCGTCT TCAATTCTTC TCTCATTAAA TATGAAAAAG AAAGAGAGAA   
  
  
+ ATAGAGAGAG ATTTAAACCA GAAGGGGTGA TTCCTTTTTC TTGGTGGGCT TATTAATGCT CTGTCTACCC   
  
  
+ CATCTAGCCG AAAAATAGTA GCTTAAGACT CAACTGCTTA TTAGCATCTC TCCTCTAGCA ATAACCGAGG   
  
  
+ TAATAGCATA TTTTTCTCCC CTTAGCTTCT ACCTTGCTTT TTGTGACACT CAAACCTAAA CTGAGAGAGA   
  
  
+ GAAAGAGAAG ATAGAGGAGA GATAAATATT AGCGGGATTA GGGCAAATTT TGATATGCTG GGCTCTTCCT   
  
  
+ CATTCAACTC TGAGGAAGAA GATCAAGATC ATGCCAACCC ACCTCCGCCG CAACAACCAC CACCGTTACA   
  
  
+ GCTGCCACTT CCACGGCCAA GATTCAGCCA GTTAATATCA TCATCACCTC CAATAATATC AGCATCTTCT   
  
  
+ TCATCATTAA TCCATAATAA CCCTCGTCAG CTGCTAATAA GTTGCGGTGA ACTCATCTCC CGCTGTGACT   
  
  
+ TCTCCTCAGC TAACCGCCTT CTCTCTCTTC TCTCCGGCGA CTTCGCTTCC TCCTCCGGCG ACTCCACTGA   
  
  
+ ACGCCTTGTT TATTACTTCT CCAAGGCTTT GTTCCTCCGC CTTCGCCGAC AGTTTTCTCT ATCTTCTTCA   
  
  
+ ACTTTTACTG GTTATAGTTG TAACCCTAGT TGTTCTGCGT CTATTGCTCC ACTTTTTCTA ACCAACCCTA   
  
  
+ ATCCTAGACC ACCACAAGAA CTGCCGGTAC GAGTAACTAG CAGGGTTACT CCAAGTAGCT GCTCGTCTTA   
  
  
+ CCTAACCCTA AACCAGATCA CTCCATTTAT CCGGTTCACC CACCTGACGG CCAACCAGGC AATTTTGGAG   
  
  
+ GCTGTGGAGG GCTATAGGGC AGTCCACATC ATCGACATGG ACATCATGCA TGGAGTGCAG TGGCCTCCGC   
  
  
+ TCCTCCAAGC AATCGTGGAG CGGTCGGCCA CCCTTAGCCA ACCTCCCCCT GTGGTCCGTC TCAAAGGGGG   
  
  
+ AGGGCCGGAC CCGGACCTCT TACAGAGGAC GGGGGACCGA CTATGGAACT TTGCCCAATC ATTAGGACTA   
  
  
+ GAGTTCCATT TCCAGGGCCT CATGATCCCC GCCGAGTCAA CAGGTTTTGT TGAACCTGCT GAGGCAGCGG   
  
  
+ TCAATGCCCT AGCCTTGCAG GAGGCGGTGG GGTGTCAAGA AGGGGAGGCC CTTGCAGTTC ACTGCGGGGA   
  
  
+ CTATCTCCAC CGCCTCTTAA AAGAGTACGA CACAAGGCCT CTTAGACGGT TCCTCCACAA GGTCAAGACT   
  
  
+ CTGAACCCCA GGGTCTTGAC CTTGGGAGAG AGGGAAGCCG ACCACAACCA CCCTCTCTTC CTGCGGCGGT   
  
  
+ TCACCGAGGC AGTCAACCAC TACGGGGCAG TGTTTGACTC CCTGGAAGCA ACCCTTCCGC CGCATAGCCA   
  
  
+ AGAGAGGGTA GCTGTGGAGG AAGGGTGGTT CGGAGAGGAG ATTAAGGACG TGGTAGGAGA AGAAAGCGAG   
  
  
+ AGGAGAATAG AGAGGCACCA GAAGTTCGAG TCGTGGGAAG CATTGTTGAG GAGCTCAGGG TTCAAGAGTG   
  
  
+ TGCCCCTCAG CCCATTCTCG CTGTCCCAAG CTAAGCTGCT GCTGCGCCTC CATTACCCTT CCGAAGGGTA   
  
  
+ CCAACTCCAG GTTTTGCATA GTAATTGCTT GTTGCTTGGG TGGAAGAATC GCCCTCTTTT TTCTGTCTCT   
  
  
+ TCTTGGCAAT A  

- +Up\_Stream \_Len000AAAAAA AATTCAAAAG CATGAACCAC AACTACTAAA CACGAAACAT TGGTTACCAA   
  
  
- AATCATTTAT CATTAATCAG CATTGTAAGA CGCTAAAGCG AGTTAAATAT ATAGTTGAAA TCTTATACTC   
  
  
- ATTGGCATTT CTAGTTTGCA TCATCATCTC CACAGTATCC AAAAAAAAAT TTTTTTAAGC CTCATTCTTT   
  
  
- CATCCCATGT AATAAAAACG AACAATTTAA GGCGATCATA TCGTTATGCT CTACCTTGTA AACATTTTTG   
  
  
- ATGCTCTGCT TCGCAAACAT GTTTTGTTTG ATGGTATGAC GTCTAGTATA TTAAAAAACC CCCCAATACA   
  
  
- TAGAATCACT ACTGTAATTA ACTACCTATT GGGATAAGAT TTATAGACCT AGGTAGTAAT TGACTGAGCA   
  
  
- AAGTTTACAA TCCTAATACC CAGAACTGTA CCATATGTAC AGTTATCTAA ATACAATCTA CTTTTCAATA   
  
  
- TACTGCGAAA AATACTAAAA ACATGATCGT ATATCACGAA TTAACCTGAT ATATAAAAAT GTTCAACACG   
  
  
- AATTGTGGAA AATTAGTCTT TATAAATAAT ATACCTTTTT TAAAATAATT ATACACAAAT ACACAAATTT   
  
  
- TTTGTATGGG TCGGAACTTT ATCTGGCACT ATATCTTTAA CCTGGGTATT ATGAGTGTAA TATATACGAA   
  
  
- ATAACAATAT TAATTCCTAG TAACAACAAA ACGTTAATAC ACCGTTCTAT ATTAGGTGTT TCAAAATGTA   
  
  
- AATATAACGT TAATGAGACC ATTTACGATA ATTACATGTA AACTCTAATT CTGTATATAT ATTGGGAAAA   
  
  
- AAAAGGGAAG AAAAAAGGAA AAAGATAAAA CTTACCGTAG AATTTGGTTT CCTCGACATG TTCAATGCAA   
  
  
- TATCGTGAGT ACCAACTGAT TTGTTCTGTC TACTCTCAAA TTCAGTTCAG GAGGACCGTT ACTACAAACG   
  
  
- GTAAACCTGG TTATTTATGT AGTACATATA ATGGTATTAT CACACCAACT GAATTGTTAT GTATCCCCTA   
  
  
- ACGTATGTTT CCATTCGGAG TTTTTGAACA CGTATACGTA ACTTTAGGTA CTAGTTTTTT TAAGTTCTCG   
  
  
- TCCAACGTGT TTTTGTGCCT CTACTCTACT TTCTCTGTCT TTTGTGTATA TGTAAGTGTC ATCATCCCCA   
  
  
- TGACCATCTC GATACCGATC CGATTCTACT ACACTTTCTT TGTTATATTA CCATCATTGA CCATTATAAA   
  
  
- TATCCCGTGT ATATCCACGC CCGTATCCCA GCACAGAGAG TCAGAGTCCA TGCCAAGCTA CCCTGAGACG   
  
  
- CTATCTACAA TAGTTTAAGT TGGTAAGCTT GTAGAGGGTC TCTCTCTCTC TCTCTGACTC ACTCACTCAC   
  
  
- TCACCCACCC ACATACACAC ACTCAACAAA ACAAGTAAAT AATATCCAAG AGACGACGTA TATAGAGGTG   
  
  
- GTAATTTAAA TGGTTTTGAA CATGGTCGAC GAAATGAACG AAAGGAGTAA AAGGGGGAAA AAACAGAGAG   
  
  
- AGAGAGAGAG AGGGGGGGGG GGGGGGGGAG AGAGAGACAC AGACTTAGTC ATAGTCGGAT AGTCCTACGA   
  
  
- AGCGGAGGTC TTTCCGTAGT GAGTGTGTGA ATAATCGAAT TTTGAAAGAA AATAAGGAAA ACGAGAAAGT   
  
  
- GACGAGAGAA AATGGGTTTA TGCGGGCAGA AGTTAAGAAG AGAGTAATTT ATACTTTTTC TTTCTCTCTT   
  
  
- TATCTCTCTC TAAATTTGGT CTTCCCCACT AAGGAAAAAG AACCACCCGA ATAATTACGA GACAGATGGG   
  
  
- GTAGATCGGC TTTTTATCAT CGAATTCTGA GTTGACGAAT AATCGTAGAG AGGAGATCGT TATTGGCTCC   
  
  
- ATTATCGTAT AAAAAGAGGG GAATCGAAGA TGGAACGAAA AACACTGTGA GTTTGGATTT GACTCTCTCT   
  
  
- CTTTCTCTTC TATCTCCTCT CTATTTATAA TCGCCCTAAT CCCGTTTAAA ACTATACGAC CCGAGAAGGA   
  
  
- GTAAGTTGAG ACTCCTTCTT CTAGTTCTAG TACGGTTGGG TGGAGGCGGC GTTGTTGGTG GTGGCAATGT   
  
  
- CGACGGTGAA GGTGCCGGTT CTAAGTCGGT CAATTATAGT AGTAGTGGAG GTTATTATAG TCGTAGAAGA   
  
  
- AGTAGTAATT AGGTATTATT GGGAGCAGTC GACGATTATT CAACGCCACT TGAGTAGAGG GCGACACTGA   
  
  
- AGAGGAGTCG ATTGGCGGAA GAGAGAGAAG AGAGGCCGCT GAAGCGAAGG AGGAGGCCGC TGAGGTGACT   
  
  
- TGCGGAACAA ATAATGAAGA GGTTCCGAAA CAAGGAGGCG GAAGCGGCTG TCAAAAGAGA TAGAAGAAGT   
  
  
- TGAAAATGAC CAATATCAAC ATTGGGATCA ACAAGACGCA GATAACGAGG TGAAAAAGAT TGGTTGGGAT   
  
  
- TAGGATCTGG TGGTGTTCTT GACGGCCATG CTCATTGATC GTCCCAATGA GGTTCATCGA CGAGCAGAAT   
  
  
- GGATTGGGAT TTGGTCTAGT GAGGTAAATA GGCCAAGTGG GTGGACTGCC GGTTGGTCCG TTAAAACCTC   
  
  
- CGACACCTCC CGATATCCCG TCAGGTGTAG TAGCTGTACC TGTAGTACGT ACCTCACGTC ACCGGAGGCG   
  
  
- AGGAGGTTCG TTAGCACCTC GCCAGCCGGT GGGAATCGGT TGGAGGGGGA CACCAGGCAG AGTTTCCCCC   
  
  
- TCCCGGCCTG GGCCTGGAGA ATGTCTCCTG CCCCCTGGCT GATACCTTGA AACGGGTTAG TAATCCTGAT   
  
  
- CTCAAGGTAA AGGTCCCGGA GTACTAGGGG CGGCTCAGTT GTCCAAAACA ACTTGGACGA CTCCGTCGCC   
  
  
- AGTTACGGGA TCGGAACGTC CTCCGCCACC CCACAGTTCT TCCCCTCCGG GAACGTCAAG TGACGCCCCT   
  
  
- GATAGAGGTG GCGGAGAATT TTCTCATGCT GTGTTCCGGA GAATCTGCCA AGGAGGTGTT CCAGTTCTGA   
  
  
- GACTTGGGGT CCCAGAACTG GAACCCTCTC TCCCTTCGGC TGGTGTTGGT GGGAGAGAAG GACGCCGCCA   
  
  
- AGTGGCTCCG TCAGTTGGTG ATGCCCCGTC ACAAACTGAG GGACCTTCGT TGGGAAGGCG GCGTATCGGT   
  
  
- TCTCTCCCAT CGACACCTCC TTCCCACCAA GCCTCTCCTC TAATTCCTGC ACCATCCTCT TCTTTCGCTC   
  
  
- TCCTCTTATC TCTCCGTGGT CTTCAAGCTC AGCACCCTTC GTAACAACTC CTCGAGTCCC AAGTTCTCAC   
  
  
- ACGGGGAGTC GGGTAAGAGC GACAGGGTTC GATTCGACGA CGACGCGGAG GTAATGGGAA GGCTTCCCAT   
  
  
- GGTTGAGGTC CAAAACGTAT CATTAACGAA CAACGAACCC ACCTTCTTAG CGGGAGAAAA AAGACAGAGA   
  
  
- AGAACCGTTA T

+     Myb-binding site

| Site Name | Organism | Position | Strand | Matrix score. | sequence | function |
| --- | --- | --- | --- | --- | --- | --- |
| Myb-binding site | Nicotiana tabacum | 2842 | + | 6 | CAACAG |  |

>HU03G01737.1   
+ +Up\_Stream \_Len000TTTTTT TTAAGTTTTC GTACTTGGTG TTGATGATTT GTGCTTTGTA ACCAATGGTT   
  
  
+ TTAGTAAATA GTAATTAGTC GTAACATTCT GCGATTTCGC TCAATTTATA TATCAACTTT AGAATATGAG   
  
  
+ TAACCGTAAA GATCAAACGT AGTAGTAGAG GTGTCATAGG TTTTTTTTTA AAAAAATTCG GAGTAAGAAA   
  
  
+ GTAGGGTACA TTATTTTTGC TTGTTAAATT CCGCTAGTAT AGCAATACGA GATGGAACAT TTGTAAAAAC   
  
  
+ TACGAGACGA AGCGTTTGTA CAAAACAAAC TACCATACTG CAGATCATAT AATTTTTTGG GGGGTTATGT   
  
  
+ ATCTTAGTGA TGACATTAAT TGATGGATAA CCCTATTCTA AATATCTGGA TCCATCATTA ACTGACTCGT   
  
  
+ TTCAAATGTT AGGATTATGG GTCTTGACAT GGTATACATG TCAATAGATT TATGTTAGAT GAAAAGTTAT   
  
  
+ ATGACGCTTT TTATGATTTT TGTACTAGCA TATAGTGCTT AATTGGACTA TATATTTTTA CAAGTTGTGC   
  
  
+ TTAACACCTT TTAATCAGAA ATATTTATTA TATGGAAAAA ATTTTATTAA TATGTGTTTA TGTGTTTAAA   
  
  
+ AAACATACCC AGCCTTGAAA TAGACCGTGA TATAGAAATT GGACCCATAA TACTCACATT ATATATGCTT   
  
  
+ TATTGTTATA ATTAAGGATC ATTGTTGTTT TGCAATTATG TGGCAAGATA TAATCCACAA AGTTTTACAT   
  
  
+ TTATATTGCA ATTACTCTGG TAAATGCTAT TAATGTACAT TTGAGATTAA GACATATATA TAACCCTTTT   
  
  
+ TTTTCCCTTC TTTTTTCCTT TTTCTATTTT GAATGGCATC TTAAACCAAA GGAGCTGTAC AAGTTACGTT   
  
  
+ ATAGCACTCA TGGTTGACTA AACAAGACAG ATGAGAGTTT AAGTCAAGTC CTCCTGGCAA TGATGTTTGC   
  
  
+ CATTTGGACC AATAAATACA TCATGTATAT TACCATAATA GTGTGGTTGA CTTAACAATA CATAGGGGAT   
  
  
+ TGCATACAAA GGTAAGCCTC AAAAACTTGT GCATATGCAT TGAAATCCAT GATCAAAAAA ATTCAAGAGC   
  
  
+ AGGTTGCACA AAAACACGGA GATGAGATGA AAGAGACAGA AAACACATAT ACATTCACAG TAGTAGGGGT   
  
  
+ ACTGGTAGAG CTATGGCTAG GCTAAGATGA TGTGAAAGAA ACAATATAAT GGTAGTAACT GGTAATATTT   
  
  
+ ATAGGGCACA TATAGGTGCG GGCATAGGGT CGTGTCTCTC AGTCTCAGGT ACGGTTCGAT GGGACTCTGC   
  
  
+ GATAGATGTT ATCAAATTCA ACCATTCGAA CATCTCCCAG AGAGAGAGAG AGAGACTGAG TGAGTGAGTG   
  
  
+ AGTGGGTGGG TGTATGTGTG TGAGTTGTTT TGTTCATTTA TTATAGGTTC TCTGCTGCAT ATATCTCCAC   
  
  
+ CATTAAATTT ACCAAAACTT GTACCAGCTG CTTTACTTGC TTTCCTCATT TTCCCCCTTT TTTGTCTCTC   
  
  
+ TCTCTCTCTC TCCCCCCCCC CCCCCCCCTC TCTCTCTGTG TCTGAATCAG TATCAGCCTA TCAGGATGCT   
  
  
+ TCGCCTCCAG AAAGGCATCA CTCACACACT TATTAGCTTA AAACTTTCTT TTATTCCTTT TGCTCTTTCA   
  
  
+ CTGCTCTCTT TTACCCAAAT ACGCCCGTCT TCAATTCTTC TCTCATTAAA TATGAAAAAG AAAGAGAGAA   
  
  
+ ATAGAGAGAG ATTTAAACCA GAAGGGGTGA TTCCTTTTTC TTGGTGGGCT TATTAATGCT CTGTCTACCC   
  
  
+ CATCTAGCCG AAAAATAGTA GCTTAAGACT CAACTGCTTA TTAGCATCTC TCCTCTAGCA ATAACCGAGG   
  
  
+ TAATAGCATA TTTTTCTCCC CTTAGCTTCT ACCTTGCTTT TTGTGACACT CAAACCTAAA CTGAGAGAGA   
  
  
+ GAAAGAGAAG ATAGAGGAGA GATAAATATT AGCGGGATTA GGGCAAATTT TGATATGCTG GGCTCTTCCT   
  
  
+ CATTCAACTC TGAGGAAGAA GATCAAGATC ATGCCAACCC ACCTCCGCCG CAACAACCAC CACCGTTACA   
  
  
+ GCTGCCACTT CCACGGCCAA GATTCAGCCA GTTAATATCA TCATCACCTC CAATAATATC AGCATCTTCT   
  
  
+ TCATCATTAA TCCATAATAA CCCTCGTCAG CTGCTAATAA GTTGCGGTGA ACTCATCTCC CGCTGTGACT   
  
  
+ TCTCCTCAGC TAACCGCCTT CTCTCTCTTC TCTCCGGCGA CTTCGCTTCC TCCTCCGGCG ACTCCACTGA   
  
  
+ ACGCCTTGTT TATTACTTCT CCAAGGCTTT GTTCCTCCGC CTTCGCCGAC AGTTTTCTCT ATCTTCTTCA   
  
  
+ ACTTTTACTG GTTATAGTTG TAACCCTAGT TGTTCTGCGT CTATTGCTCC ACTTTTTCTA ACCAACCCTA   
  
  
+ ATCCTAGACC ACCACAAGAA CTGCCGGTAC GAGTAACTAG CAGGGTTACT CCAAGTAGCT GCTCGTCTTA   
  
  
+ CCTAACCCTA AACCAGATCA CTCCATTTAT CCGGTTCACC CACCTGACGG CCAACCAGGC AATTTTGGAG   
  
  
+ GCTGTGGAGG GCTATAGGGC AGTCCACATC ATCGACATGG ACATCATGCA TGGAGTGCAG TGGCCTCCGC   
  
  
+ TCCTCCAAGC AATCGTGGAG CGGTCGGCCA CCCTTAGCCA ACCTCCCCCT GTGGTCCGTC TCAAAGGGGG   
  
  
+ AGGGCCGGAC CCGGACCTCT TACAGAGGAC GGGGGACCGA CTATGGAACT TTGCCCAATC ATTAGGACTA   
  
  
+ GAGTTCCATT TCCAGGGCCT CATGATCCCC GCCGAGTCAA CAGGTTTTGT TGAACCTGCT GAGGCAGCGG   
  
  
+ TCAATGCCCT AGCCTTGCAG GAGGCGGTGG GGTGTCAAGA AGGGGAGGCC CTTGCAGTTC ACTGCGGGGA   
  
  
+ CTATCTCCAC CGCCTCTTAA AAGAGTACGA CACAAGGCCT CTTAGACGGT TCCTCCACAA GGTCAAGACT   
  
  
+ CTGAACCCCA GGGTCTTGAC CTTGGGAGAG AGGGAAGCCG ACCACAACCA CCCTCTCTTC CTGCGGCGGT   
  
  
+ TCACCGAGGC AGTCAACCAC TACGGGGCAG TGTTTGACTC CCTGGAAGCA ACCCTTCCGC CGCATAGCCA   
  
  
+ AGAGAGGGTA GCTGTGGAGG AAGGGTGGTT CGGAGAGGAG ATTAAGGACG TGGTAGGAGA AGAAAGCGAG   
  
  
+ AGGAGAATAG AGAGGCACCA GAAGTTCGAG TCGTGGGAAG CATTGTTGAG GAGCTCAGGG TTCAAGAGTG   
  
  
+ TGCCCCTCAG CCCATTCTCG CTGTCCCAAG CTAAGCTGCT GCTGCGCCTC CATTACCCTT CCGAAGGGTA   
  
  
+ CCAACTCCAG GTTTTGCATA GTAATTGCTT GTTGCTTGGG TGGAAGAATC GCCCTCTTTT TTCTGTCTCT   
  
  
+ TCTTGGCAAT A  

- +Up\_Stream \_Len000AAAAAA AATTCAAAAG CATGAACCAC AACTACTAAA CACGAAACAT TGGTTACCAA   
  
  
- AATCATTTAT CATTAATCAG CATTGTAAGA CGCTAAAGCG AGTTAAATAT ATAGTTGAAA TCTTATACTC   
  
  
- ATTGGCATTT CTAGTTTGCA TCATCATCTC CACAGTATCC AAAAAAAAAT TTTTTTAAGC CTCATTCTTT   
  
  
- CATCCCATGT AATAAAAACG AACAATTTAA GGCGATCATA TCGTTATGCT CTACCTTGTA AACATTTTTG   
  
  
- ATGCTCTGCT TCGCAAACAT GTTTTGTTTG ATGGTATGAC GTCTAGTATA TTAAAAAACC CCCCAATACA   
  
  
- TAGAATCACT ACTGTAATTA ACTACCTATT GGGATAAGAT TTATAGACCT AGGTAGTAAT TGACTGAGCA   
  
  
- AAGTTTACAA TCCTAATACC CAGAACTGTA CCATATGTAC AGTTATCTAA ATACAATCTA CTTTTCAATA   
  
  
- TACTGCGAAA AATACTAAAA ACATGATCGT ATATCACGAA TTAACCTGAT ATATAAAAAT GTTCAACACG   
  
  
- AATTGTGGAA AATTAGTCTT TATAAATAAT ATACCTTTTT TAAAATAATT ATACACAAAT ACACAAATTT   
  
  
- TTTGTATGGG TCGGAACTTT ATCTGGCACT ATATCTTTAA CCTGGGTATT ATGAGTGTAA TATATACGAA   
  
  
- ATAACAATAT TAATTCCTAG TAACAACAAA ACGTTAATAC ACCGTTCTAT ATTAGGTGTT TCAAAATGTA   
  
  
- AATATAACGT TAATGAGACC ATTTACGATA ATTACATGTA AACTCTAATT CTGTATATAT ATTGGGAAAA   
  
  
- AAAAGGGAAG AAAAAAGGAA AAAGATAAAA CTTACCGTAG AATTTGGTTT CCTCGACATG TTCAATGCAA   
  
  
- TATCGTGAGT ACCAACTGAT TTGTTCTGTC TACTCTCAAA TTCAGTTCAG GAGGACCGTT ACTACAAACG   
  
  
- GTAAACCTGG TTATTTATGT AGTACATATA ATGGTATTAT CACACCAACT GAATTGTTAT GTATCCCCTA   
  
  
- ACGTATGTTT CCATTCGGAG TTTTTGAACA CGTATACGTA ACTTTAGGTA CTAGTTTTTT TAAGTTCTCG   
  
  
- TCCAACGTGT TTTTGTGCCT CTACTCTACT TTCTCTGTCT TTTGTGTATA TGTAAGTGTC ATCATCCCCA   
  
  
- TGACCATCTC GATACCGATC CGATTCTACT ACACTTTCTT TGTTATATTA CCATCATTGA CCATTATAAA   
  
  
- TATCCCGTGT ATATCCACGC CCGTATCCCA GCACAGAGAG TCAGAGTCCA TGCCAAGCTA CCCTGAGACG   
  
  
- CTATCTACAA TAGTTTAAGT TGGTAAGCTT GTAGAGGGTC TCTCTCTCTC TCTCTGACTC ACTCACTCAC   
  
  
- TCACCCACCC ACATACACAC ACTCAACAAA ACAAGTAAAT AATATCCAAG AGACGACGTA TATAGAGGTG   
  
  
- GTAATTTAAA TGGTTTTGAA CATGGTCGAC GAAATGAACG AAAGGAGTAA AAGGGGGAAA AAACAGAGAG   
  
  
- AGAGAGAGAG AGGGGGGGGG GGGGGGGGAG AGAGAGACAC AGACTTAGTC ATAGTCGGAT AGTCCTACGA   
  
  
- AGCGGAGGTC TTTCCGTAGT GAGTGTGTGA ATAATCGAAT TTTGAAAGAA AATAAGGAAA ACGAGAAAGT   
  
  
- GACGAGAGAA AATGGGTTTA TGCGGGCAGA AGTTAAGAAG AGAGTAATTT ATACTTTTTC TTTCTCTCTT   
  
  
- TATCTCTCTC TAAATTTGGT CTTCCCCACT AAGGAAAAAG AACCACCCGA ATAATTACGA GACAGATGGG   
  
  
- GTAGATCGGC TTTTTATCAT CGAATTCTGA GTTGACGAAT AATCGTAGAG AGGAGATCGT TATTGGCTCC   
  
  
- ATTATCGTAT AAAAAGAGGG GAATCGAAGA TGGAACGAAA AACACTGTGA GTTTGGATTT GACTCTCTCT   
  
  
- CTTTCTCTTC TATCTCCTCT CTATTTATAA TCGCCCTAAT CCCGTTTAAA ACTATACGAC CCGAGAAGGA   
  
  
- GTAAGTTGAG ACTCCTTCTT CTAGTTCTAG TACGGTTGGG TGGAGGCGGC GTTGTTGGTG GTGGCAATGT   
  
  
- CGACGGTGAA GGTGCCGGTT CTAAGTCGGT CAATTATAGT AGTAGTGGAG GTTATTATAG TCGTAGAAGA   
  
  
- AGTAGTAATT AGGTATTATT GGGAGCAGTC GACGATTATT CAACGCCACT TGAGTAGAGG GCGACACTGA   
  
  
- AGAGGAGTCG ATTGGCGGAA GAGAGAGAAG AGAGGCCGCT GAAGCGAAGG AGGAGGCCGC TGAGGTGACT   
  
  
- TGCGGAACAA ATAATGAAGA GGTTCCGAAA CAAGGAGGCG GAAGCGGCTG TCAAAAGAGA TAGAAGAAGT   
  
  
- TGAAAATGAC CAATATCAAC ATTGGGATCA ACAAGACGCA GATAACGAGG TGAAAAAGAT TGGTTGGGAT   
  
  
- TAGGATCTGG TGGTGTTCTT GACGGCCATG CTCATTGATC GTCCCAATGA GGTTCATCGA CGAGCAGAAT   
  
  
- GGATTGGGAT TTGGTCTAGT GAGGTAAATA GGCCAAGTGG GTGGACTGCC GGTTGGTCCG TTAAAACCTC   
  
  
- CGACACCTCC CGATATCCCG TCAGGTGTAG TAGCTGTACC TGTAGTACGT ACCTCACGTC ACCGGAGGCG   
  
  
- AGGAGGTTCG TTAGCACCTC GCCAGCCGGT GGGAATCGGT TGGAGGGGGA CACCAGGCAG AGTTTCCCCC   
  
  
- TCCCGGCCTG GGCCTGGAGA ATGTCTCCTG CCCCCTGGCT GATACCTTGA AACGGGTTAG TAATCCTGAT   
  
  
- CTCAAGGTAA AGGTCCCGGA GTACTAGGGG CGGCTCAGTT GTCCAAAACA ACTTGGACGA CTCCGTCGCC   
  
  
- AGTTACGGGA TCGGAACGTC CTCCGCCACC CCACAGTTCT TCCCCTCCGG GAACGTCAAG TGACGCCCCT   
  
  
- GATAGAGGTG GCGGAGAATT TTCTCATGCT GTGTTCCGGA GAATCTGCCA AGGAGGTGTT CCAGTTCTGA   
  
  
- GACTTGGGGT CCCAGAACTG GAACCCTCTC TCCCTTCGGC TGGTGTTGGT GGGAGAGAAG GACGCCGCCA   
  
  
- AGTGGCTCCG TCAGTTGGTG ATGCCCCGTC ACAAACTGAG GGACCTTCGT TGGGAAGGCG GCGTATCGGT   
  
  
- TCTCTCCCAT CGACACCTCC TTCCCACCAA GCCTCTCCTC TAATTCCTGC ACCATCCTCT TCTTTCGCTC   
  
  
- TCCTCTTATC TCTCCGTGGT CTTCAAGCTC AGCACCCTTC GTAACAACTC CTCGAGTCCC AAGTTCTCAC   
  
  
- ACGGGGAGTC GGGTAAGAGC GACAGGGTTC GATTCGACGA CGACGCGGAG GTAATGGGAA GGCTTCCCAT   
  
  
- GGTTGAGGTC CAAAACGTAT CATTAACGAA CAACGAACCC ACCTTCTTAG CGGGAGAAAA AAGACAGAGA   
  
  
- AGAACCGTTA T

+     O2-site

| Site Name | Organism | Position | Strand | Matrix score. | sequence | function |
| --- | --- | --- | --- | --- | --- | --- |
| O2-site | Zea mays | 2618 | - | 10 | GATGATGTGG | cis-acting regulatory element involved in zein metabolism regulation |
| O2-site | Zea mays | 1220 | + | 9 | GATGATGTGG | cis-acting regulatory element involved in zein metabolism regulation |
| O2-site | Zea mays | 1145 | + | 8 | GATGA(C/T)(A/G)TG(A/G) | cis-acting regulatory element involved in zein metabolism regulation |

>HU03G01737.1   
+ +Up\_Stream \_Len000TTTTTT TTAAGTTTTC GTACTTGGTG TTGATGATTT GTGCTTTGTA ACCAATGGTT   
  
  
+ TTAGTAAATA GTAATTAGTC GTAACATTCT GCGATTTCGC TCAATTTATA TATCAACTTT AGAATATGAG   
  
  
+ TAACCGTAAA GATCAAACGT AGTAGTAGAG GTGTCATAGG TTTTTTTTTA AAAAAATTCG GAGTAAGAAA   
  
  
+ GTAGGGTACA TTATTTTTGC TTGTTAAATT CCGCTAGTAT AGCAATACGA GATGGAACAT TTGTAAAAAC   
  
  
+ TACGAGACGA AGCGTTTGTA CAAAACAAAC TACCATACTG CAGATCATAT AATTTTTTGG GGGGTTATGT   
  
  
+ ATCTTAGTGA TGACATTAAT TGATGGATAA CCCTATTCTA AATATCTGGA TCCATCATTA ACTGACTCGT   
  
  
+ TTCAAATGTT AGGATTATGG GTCTTGACAT GGTATACATG TCAATAGATT TATGTTAGAT GAAAAGTTAT   
  
  
+ ATGACGCTTT TTATGATTTT TGTACTAGCA TATAGTGCTT AATTGGACTA TATATTTTTA CAAGTTGTGC   
  
  
+ TTAACACCTT TTAATCAGAA ATATTTATTA TATGGAAAAA ATTTTATTAA TATGTGTTTA TGTGTTTAAA   
  
  
+ AAACATACCC AGCCTTGAAA TAGACCGTGA TATAGAAATT GGACCCATAA TACTCACATT ATATATGCTT   
  
  
+ TATTGTTATA ATTAAGGATC ATTGTTGTTT TGCAATTATG TGGCAAGATA TAATCCACAA AGTTTTACAT   
  
  
+ TTATATTGCA ATTACTCTGG TAAATGCTAT TAATGTACAT TTGAGATTAA GACATATATA TAACCCTTTT   
  
  
+ TTTTCCCTTC TTTTTTCCTT TTTCTATTTT GAATGGCATC TTAAACCAAA GGAGCTGTAC AAGTTACGTT   
  
  
+ ATAGCACTCA TGGTTGACTA AACAAGACAG ATGAGAGTTT AAGTCAAGTC CTCCTGGCAA TGATGTTTGC   
  
  
+ CATTTGGACC AATAAATACA TCATGTATAT TACCATAATA GTGTGGTTGA CTTAACAATA CATAGGGGAT   
  
  
+ TGCATACAAA GGTAAGCCTC AAAAACTTGT GCATATGCAT TGAAATCCAT GATCAAAAAA ATTCAAGAGC   
  
  
+ AGGTTGCACA AAAACACGGA GATGAGATGA AAGAGACAGA AAACACATAT ACATTCACAG TAGTAGGGGT   
  
  
+ ACTGGTAGAG CTATGGCTAG GCTAAGATGA TGTGAAAGAA ACAATATAAT GGTAGTAACT GGTAATATTT   
  
  
+ ATAGGGCACA TATAGGTGCG GGCATAGGGT CGTGTCTCTC AGTCTCAGGT ACGGTTCGAT GGGACTCTGC   
  
  
+ GATAGATGTT ATCAAATTCA ACCATTCGAA CATCTCCCAG AGAGAGAGAG AGAGACTGAG TGAGTGAGTG   
  
  
+ AGTGGGTGGG TGTATGTGTG TGAGTTGTTT TGTTCATTTA TTATAGGTTC TCTGCTGCAT ATATCTCCAC   
  
  
+ CATTAAATTT ACCAAAACTT GTACCAGCTG CTTTACTTGC TTTCCTCATT TTCCCCCTTT TTTGTCTCTC   
  
  
+ TCTCTCTCTC TCCCCCCCCC CCCCCCCCTC TCTCTCTGTG TCTGAATCAG TATCAGCCTA TCAGGATGCT   
  
  
+ TCGCCTCCAG AAAGGCATCA CTCACACACT TATTAGCTTA AAACTTTCTT TTATTCCTTT TGCTCTTTCA   
  
  
+ CTGCTCTCTT TTACCCAAAT ACGCCCGTCT TCAATTCTTC TCTCATTAAA TATGAAAAAG AAAGAGAGAA   
  
  
+ ATAGAGAGAG ATTTAAACCA GAAGGGGTGA TTCCTTTTTC TTGGTGGGCT TATTAATGCT CTGTCTACCC   
  
  
+ CATCTAGCCG AAAAATAGTA GCTTAAGACT CAACTGCTTA TTAGCATCTC TCCTCTAGCA ATAACCGAGG   
  
  
+ TAATAGCATA TTTTTCTCCC CTTAGCTTCT ACCTTGCTTT TTGTGACACT CAAACCTAAA CTGAGAGAGA   
  
  
+ GAAAGAGAAG ATAGAGGAGA GATAAATATT AGCGGGATTA GGGCAAATTT TGATATGCTG GGCTCTTCCT   
  
  
+ CATTCAACTC TGAGGAAGAA GATCAAGATC ATGCCAACCC ACCTCCGCCG CAACAACCAC CACCGTTACA   
  
  
+ GCTGCCACTT CCACGGCCAA GATTCAGCCA GTTAATATCA TCATCACCTC CAATAATATC AGCATCTTCT   
  
  
+ TCATCATTAA TCCATAATAA CCCTCGTCAG CTGCTAATAA GTTGCGGTGA ACTCATCTCC CGCTGTGACT   
  
  
+ TCTCCTCAGC TAACCGCCTT CTCTCTCTTC TCTCCGGCGA CTTCGCTTCC TCCTCCGGCG ACTCCACTGA   
  
  
+ ACGCCTTGTT TATTACTTCT CCAAGGCTTT GTTCCTCCGC CTTCGCCGAC AGTTTTCTCT ATCTTCTTCA   
  
  
+ ACTTTTACTG GTTATAGTTG TAACCCTAGT TGTTCTGCGT CTATTGCTCC ACTTTTTCTA ACCAACCCTA   
  
  
+ ATCCTAGACC ACCACAAGAA CTGCCGGTAC GAGTAACTAG CAGGGTTACT CCAAGTAGCT GCTCGTCTTA   
  
  
+ CCTAACCCTA AACCAGATCA CTCCATTTAT CCGGTTCACC CACCTGACGG CCAACCAGGC AATTTTGGAG   
  
  
+ GCTGTGGAGG GCTATAGGGC AGTCCACATC ATCGACATGG ACATCATGCA TGGAGTGCAG TGGCCTCCGC   
  
  
+ TCCTCCAAGC AATCGTGGAG CGGTCGGCCA CCCTTAGCCA ACCTCCCCCT GTGGTCCGTC TCAAAGGGGG   
  
  
+ AGGGCCGGAC CCGGACCTCT TACAGAGGAC GGGGGACCGA CTATGGAACT TTGCCCAATC ATTAGGACTA   
  
  
+ GAGTTCCATT TCCAGGGCCT CATGATCCCC GCCGAGTCAA CAGGTTTTGT TGAACCTGCT GAGGCAGCGG   
  
  
+ TCAATGCCCT AGCCTTGCAG GAGGCGGTGG GGTGTCAAGA AGGGGAGGCC CTTGCAGTTC ACTGCGGGGA   
  
  
+ CTATCTCCAC CGCCTCTTAA AAGAGTACGA CACAAGGCCT CTTAGACGGT TCCTCCACAA GGTCAAGACT   
  
  
+ CTGAACCCCA GGGTCTTGAC CTTGGGAGAG AGGGAAGCCG ACCACAACCA CCCTCTCTTC CTGCGGCGGT   
  
  
+ TCACCGAGGC AGTCAACCAC TACGGGGCAG TGTTTGACTC CCTGGAAGCA ACCCTTCCGC CGCATAGCCA   
  
  
+ AGAGAGGGTA GCTGTGGAGG AAGGGTGGTT CGGAGAGGAG ATTAAGGACG TGGTAGGAGA AGAAAGCGAG   
  
  
+ AGGAGAATAG AGAGGCACCA GAAGTTCGAG TCGTGGGAAG CATTGTTGAG GAGCTCAGGG TTCAAGAGTG   
  
  
+ TGCCCCTCAG CCCATTCTCG CTGTCCCAAG CTAAGCTGCT GCTGCGCCTC CATTACCCTT CCGAAGGGTA   
  
  
+ CCAACTCCAG GTTTTGCATA GTAATTGCTT GTTGCTTGGG TGGAAGAATC GCCCTCTTTT TTCTGTCTCT   
  
  
+ TCTTGGCAAT A  

- +Up\_Stream \_Len000AAAAAA AATTCAAAAG CATGAACCAC AACTACTAAA CACGAAACAT TGGTTACCAA   
  
  
- AATCATTTAT CATTAATCAG CATTGTAAGA CGCTAAAGCG AGTTAAATAT ATAGTTGAAA TCTTATACTC   
  
  
- ATTGGCATTT CTAGTTTGCA TCATCATCTC CACAGTATCC AAAAAAAAAT TTTTTTAAGC CTCATTCTTT   
  
  
- CATCCCATGT AATAAAAACG AACAATTTAA GGCGATCATA TCGTTATGCT CTACCTTGTA AACATTTTTG   
  
  
- ATGCTCTGCT TCGCAAACAT GTTTTGTTTG ATGGTATGAC GTCTAGTATA TTAAAAAACC CCCCAATACA   
  
  
- TAGAATCACT ACTGTAATTA ACTACCTATT GGGATAAGAT TTATAGACCT AGGTAGTAAT TGACTGAGCA   
  
  
- AAGTTTACAA TCCTAATACC CAGAACTGTA CCATATGTAC AGTTATCTAA ATACAATCTA CTTTTCAATA   
  
  
- TACTGCGAAA AATACTAAAA ACATGATCGT ATATCACGAA TTAACCTGAT ATATAAAAAT GTTCAACACG   
  
  
- AATTGTGGAA AATTAGTCTT TATAAATAAT ATACCTTTTT TAAAATAATT ATACACAAAT ACACAAATTT   
  
  
- TTTGTATGGG TCGGAACTTT ATCTGGCACT ATATCTTTAA CCTGGGTATT ATGAGTGTAA TATATACGAA   
  
  
- ATAACAATAT TAATTCCTAG TAACAACAAA ACGTTAATAC ACCGTTCTAT ATTAGGTGTT TCAAAATGTA   
  
  
- AATATAACGT TAATGAGACC ATTTACGATA ATTACATGTA AACTCTAATT CTGTATATAT ATTGGGAAAA   
  
  
- AAAAGGGAAG AAAAAAGGAA AAAGATAAAA CTTACCGTAG AATTTGGTTT CCTCGACATG TTCAATGCAA   
  
  
- TATCGTGAGT ACCAACTGAT TTGTTCTGTC TACTCTCAAA TTCAGTTCAG GAGGACCGTT ACTACAAACG   
  
  
- GTAAACCTGG TTATTTATGT AGTACATATA ATGGTATTAT CACACCAACT GAATTGTTAT GTATCCCCTA   
  
  
- ACGTATGTTT CCATTCGGAG TTTTTGAACA CGTATACGTA ACTTTAGGTA CTAGTTTTTT TAAGTTCTCG   
  
  
- TCCAACGTGT TTTTGTGCCT CTACTCTACT TTCTCTGTCT TTTGTGTATA TGTAAGTGTC ATCATCCCCA   
  
  
- TGACCATCTC GATACCGATC CGATTCTACT ACACTTTCTT TGTTATATTA CCATCATTGA CCATTATAAA   
  
  
- TATCCCGTGT ATATCCACGC CCGTATCCCA GCACAGAGAG TCAGAGTCCA TGCCAAGCTA CCCTGAGACG   
  
  
- CTATCTACAA TAGTTTAAGT TGGTAAGCTT GTAGAGGGTC TCTCTCTCTC TCTCTGACTC ACTCACTCAC   
  
  
- TCACCCACCC ACATACACAC ACTCAACAAA ACAAGTAAAT AATATCCAAG AGACGACGTA TATAGAGGTG   
  
  
- GTAATTTAAA TGGTTTTGAA CATGGTCGAC GAAATGAACG AAAGGAGTAA AAGGGGGAAA AAACAGAGAG   
  
  
- AGAGAGAGAG AGGGGGGGGG GGGGGGGGAG AGAGAGACAC AGACTTAGTC ATAGTCGGAT AGTCCTACGA   
  
  
- AGCGGAGGTC TTTCCGTAGT GAGTGTGTGA ATAATCGAAT TTTGAAAGAA AATAAGGAAA ACGAGAAAGT   
  
  
- GACGAGAGAA AATGGGTTTA TGCGGGCAGA AGTTAAGAAG AGAGTAATTT ATACTTTTTC TTTCTCTCTT   
  
  
- TATCTCTCTC TAAATTTGGT CTTCCCCACT AAGGAAAAAG AACCACCCGA ATAATTACGA GACAGATGGG   
  
  
- GTAGATCGGC TTTTTATCAT CGAATTCTGA GTTGACGAAT AATCGTAGAG AGGAGATCGT TATTGGCTCC   
  
  
- ATTATCGTAT AAAAAGAGGG GAATCGAAGA TGGAACGAAA AACACTGTGA GTTTGGATTT GACTCTCTCT   
  
  
- CTTTCTCTTC TATCTCCTCT CTATTTATAA TCGCCCTAAT CCCGTTTAAA ACTATACGAC CCGAGAAGGA   
  
  
- GTAAGTTGAG ACTCCTTCTT CTAGTTCTAG TACGGTTGGG TGGAGGCGGC GTTGTTGGTG GTGGCAATGT   
  
  
- CGACGGTGAA GGTGCCGGTT CTAAGTCGGT CAATTATAGT AGTAGTGGAG GTTATTATAG TCGTAGAAGA   
  
  
- AGTAGTAATT AGGTATTATT GGGAGCAGTC GACGATTATT CAACGCCACT TGAGTAGAGG GCGACACTGA   
  
  
- AGAGGAGTCG ATTGGCGGAA GAGAGAGAAG AGAGGCCGCT GAAGCGAAGG AGGAGGCCGC TGAGGTGACT   
  
  
- TGCGGAACAA ATAATGAAGA GGTTCCGAAA CAAGGAGGCG GAAGCGGCTG TCAAAAGAGA TAGAAGAAGT   
  
  
- TGAAAATGAC CAATATCAAC ATTGGGATCA ACAAGACGCA GATAACGAGG TGAAAAAGAT TGGTTGGGAT   
  
  
- TAGGATCTGG TGGTGTTCTT GACGGCCATG CTCATTGATC GTCCCAATGA GGTTCATCGA CGAGCAGAAT   
  
  
- GGATTGGGAT TTGGTCTAGT GAGGTAAATA GGCCAAGTGG GTGGACTGCC GGTTGGTCCG TTAAAACCTC   
  
  
- CGACACCTCC CGATATCCCG TCAGGTGTAG TAGCTGTACC TGTAGTACGT ACCTCACGTC ACCGGAGGCG   
  
  
- AGGAGGTTCG TTAGCACCTC GCCAGCCGGT GGGAATCGGT TGGAGGGGGA CACCAGGCAG AGTTTCCCCC   
  
  
- TCCCGGCCTG GGCCTGGAGA ATGTCTCCTG CCCCCTGGCT GATACCTTGA AACGGGTTAG TAATCCTGAT   
  
  
- CTCAAGGTAA AGGTCCCGGA GTACTAGGGG CGGCTCAGTT GTCCAAAACA ACTTGGACGA CTCCGTCGCC   
  
  
- AGTTACGGGA TCGGAACGTC CTCCGCCACC CCACAGTTCT TCCCCTCCGG GAACGTCAAG TGACGCCCCT   
  
  
- GATAGAGGTG GCGGAGAATT TTCTCATGCT GTGTTCCGGA GAATCTGCCA AGGAGGTGTT CCAGTTCTGA   
  
  
- GACTTGGGGT CCCAGAACTG GAACCCTCTC TCCCTTCGGC TGGTGTTGGT GGGAGAGAAG GACGCCGCCA   
  
  
- AGTGGCTCCG TCAGTTGGTG ATGCCCCGTC ACAAACTGAG GGACCTTCGT TGGGAAGGCG GCGTATCGGT   
  
  
- TCTCTCCCAT CGACACCTCC TTCCCACCAA GCCTCTCCTC TAATTCCTGC ACCATCCTCT TCTTTCGCTC   
  
  
- TCCTCTTATC TCTCCGTGGT CTTCAAGCTC AGCACCCTTC GTAACAACTC CTCGAGTCCC AAGTTCTCAC   
  
  
- ACGGGGAGTC GGGTAAGAGC GACAGGGTTC GATTCGACGA CGACGCGGAG GTAATGGGAA GGCTTCCCAT   
  
  
- GGTTGAGGTC CAAAACGTAT CATTAACGAA CAACGAACCC ACCTTCTTAG CGGGAGAAAA AAGACAGAGA   
  
  
- AGAACCGTTA T

+     P-box

| Site Name | Organism | Position | Strand | Matrix score. | sequence | function |
| --- | --- | --- | --- | --- | --- | --- |
| P-box | Oryza sativa | 1670 | + | 7 | CCTTTTG | gibberellin-responsive element |

>HU03G01737.1   
+ +Up\_Stream \_Len000TTTTTT TTAAGTTTTC GTACTTGGTG TTGATGATTT GTGCTTTGTA ACCAATGGTT   
  
  
+ TTAGTAAATA GTAATTAGTC GTAACATTCT GCGATTTCGC TCAATTTATA TATCAACTTT AGAATATGAG   
  
  
+ TAACCGTAAA GATCAAACGT AGTAGTAGAG GTGTCATAGG TTTTTTTTTA AAAAAATTCG GAGTAAGAAA   
  
  
+ GTAGGGTACA TTATTTTTGC TTGTTAAATT CCGCTAGTAT AGCAATACGA GATGGAACAT TTGTAAAAAC   
  
  
+ TACGAGACGA AGCGTTTGTA CAAAACAAAC TACCATACTG CAGATCATAT AATTTTTTGG GGGGTTATGT   
  
  
+ ATCTTAGTGA TGACATTAAT TGATGGATAA CCCTATTCTA AATATCTGGA TCCATCATTA ACTGACTCGT   
  
  
+ TTCAAATGTT AGGATTATGG GTCTTGACAT GGTATACATG TCAATAGATT TATGTTAGAT GAAAAGTTAT   
  
  
+ ATGACGCTTT TTATGATTTT TGTACTAGCA TATAGTGCTT AATTGGACTA TATATTTTTA CAAGTTGTGC   
  
  
+ TTAACACCTT TTAATCAGAA ATATTTATTA TATGGAAAAA ATTTTATTAA TATGTGTTTA TGTGTTTAAA   
  
  
+ AAACATACCC AGCCTTGAAA TAGACCGTGA TATAGAAATT GGACCCATAA TACTCACATT ATATATGCTT   
  
  
+ TATTGTTATA ATTAAGGATC ATTGTTGTTT TGCAATTATG TGGCAAGATA TAATCCACAA AGTTTTACAT   
  
  
+ TTATATTGCA ATTACTCTGG TAAATGCTAT TAATGTACAT TTGAGATTAA GACATATATA TAACCCTTTT   
  
  
+ TTTTCCCTTC TTTTTTCCTT TTTCTATTTT GAATGGCATC TTAAACCAAA GGAGCTGTAC AAGTTACGTT   
  
  
+ ATAGCACTCA TGGTTGACTA AACAAGACAG ATGAGAGTTT AAGTCAAGTC CTCCTGGCAA TGATGTTTGC   
  
  
+ CATTTGGACC AATAAATACA TCATGTATAT TACCATAATA GTGTGGTTGA CTTAACAATA CATAGGGGAT   
  
  
+ TGCATACAAA GGTAAGCCTC AAAAACTTGT GCATATGCAT TGAAATCCAT GATCAAAAAA ATTCAAGAGC   
  
  
+ AGGTTGCACA AAAACACGGA GATGAGATGA AAGAGACAGA AAACACATAT ACATTCACAG TAGTAGGGGT   
  
  
+ ACTGGTAGAG CTATGGCTAG GCTAAGATGA TGTGAAAGAA ACAATATAAT GGTAGTAACT GGTAATATTT   
  
  
+ ATAGGGCACA TATAGGTGCG GGCATAGGGT CGTGTCTCTC AGTCTCAGGT ACGGTTCGAT GGGACTCTGC   
  
  
+ GATAGATGTT ATCAAATTCA ACCATTCGAA CATCTCCCAG AGAGAGAGAG AGAGACTGAG TGAGTGAGTG   
  
  
+ AGTGGGTGGG TGTATGTGTG TGAGTTGTTT TGTTCATTTA TTATAGGTTC TCTGCTGCAT ATATCTCCAC   
  
  
+ CATTAAATTT ACCAAAACTT GTACCAGCTG CTTTACTTGC TTTCCTCATT TTCCCCCTTT TTTGTCTCTC   
  
  
+ TCTCTCTCTC TCCCCCCCCC CCCCCCCCTC TCTCTCTGTG TCTGAATCAG TATCAGCCTA TCAGGATGCT   
  
  
+ TCGCCTCCAG AAAGGCATCA CTCACACACT TATTAGCTTA AAACTTTCTT TTATTCCTTT TGCTCTTTCA   
  
  
+ CTGCTCTCTT TTACCCAAAT ACGCCCGTCT TCAATTCTTC TCTCATTAAA TATGAAAAAG AAAGAGAGAA   
  
  
+ ATAGAGAGAG ATTTAAACCA GAAGGGGTGA TTCCTTTTTC TTGGTGGGCT TATTAATGCT CTGTCTACCC   
  
  
+ CATCTAGCCG AAAAATAGTA GCTTAAGACT CAACTGCTTA TTAGCATCTC TCCTCTAGCA ATAACCGAGG   
  
  
+ TAATAGCATA TTTTTCTCCC CTTAGCTTCT ACCTTGCTTT TTGTGACACT CAAACCTAAA CTGAGAGAGA   
  
  
+ GAAAGAGAAG ATAGAGGAGA GATAAATATT AGCGGGATTA GGGCAAATTT TGATATGCTG GGCTCTTCCT   
  
  
+ CATTCAACTC TGAGGAAGAA GATCAAGATC ATGCCAACCC ACCTCCGCCG CAACAACCAC CACCGTTACA   
  
  
+ GCTGCCACTT CCACGGCCAA GATTCAGCCA GTTAATATCA TCATCACCTC CAATAATATC AGCATCTTCT   
  
  
+ TCATCATTAA TCCATAATAA CCCTCGTCAG CTGCTAATAA GTTGCGGTGA ACTCATCTCC CGCTGTGACT   
  
  
+ TCTCCTCAGC TAACCGCCTT CTCTCTCTTC TCTCCGGCGA CTTCGCTTCC TCCTCCGGCG ACTCCACTGA   
  
  
+ ACGCCTTGTT TATTACTTCT CCAAGGCTTT GTTCCTCCGC CTTCGCCGAC AGTTTTCTCT ATCTTCTTCA   
  
  
+ ACTTTTACTG GTTATAGTTG TAACCCTAGT TGTTCTGCGT CTATTGCTCC ACTTTTTCTA ACCAACCCTA   
  
  
+ ATCCTAGACC ACCACAAGAA CTGCCGGTAC GAGTAACTAG CAGGGTTACT CCAAGTAGCT GCTCGTCTTA   
  
  
+ CCTAACCCTA AACCAGATCA CTCCATTTAT CCGGTTCACC CACCTGACGG CCAACCAGGC AATTTTGGAG   
  
  
+ GCTGTGGAGG GCTATAGGGC AGTCCACATC ATCGACATGG ACATCATGCA TGGAGTGCAG TGGCCTCCGC   
  
  
+ TCCTCCAAGC AATCGTGGAG CGGTCGGCCA CCCTTAGCCA ACCTCCCCCT GTGGTCCGTC TCAAAGGGGG   
  
  
+ AGGGCCGGAC CCGGACCTCT TACAGAGGAC GGGGGACCGA CTATGGAACT TTGCCCAATC ATTAGGACTA   
  
  
+ GAGTTCCATT TCCAGGGCCT CATGATCCCC GCCGAGTCAA CAGGTTTTGT TGAACCTGCT GAGGCAGCGG   
  
  
+ TCAATGCCCT AGCCTTGCAG GAGGCGGTGG GGTGTCAAGA AGGGGAGGCC CTTGCAGTTC ACTGCGGGGA   
  
  
+ CTATCTCCAC CGCCTCTTAA AAGAGTACGA CACAAGGCCT CTTAGACGGT TCCTCCACAA GGTCAAGACT   
  
  
+ CTGAACCCCA GGGTCTTGAC CTTGGGAGAG AGGGAAGCCG ACCACAACCA CCCTCTCTTC CTGCGGCGGT   
  
  
+ TCACCGAGGC AGTCAACCAC TACGGGGCAG TGTTTGACTC CCTGGAAGCA ACCCTTCCGC CGCATAGCCA   
  
  
+ AGAGAGGGTA GCTGTGGAGG AAGGGTGGTT CGGAGAGGAG ATTAAGGACG TGGTAGGAGA AGAAAGCGAG   
  
  
+ AGGAGAATAG AGAGGCACCA GAAGTTCGAG TCGTGGGAAG CATTGTTGAG GAGCTCAGGG TTCAAGAGTG   
  
  
+ TGCCCCTCAG CCCATTCTCG CTGTCCCAAG CTAAGCTGCT GCTGCGCCTC CATTACCCTT CCGAAGGGTA   
  
  
+ CCAACTCCAG GTTTTGCATA GTAATTGCTT GTTGCTTGGG TGGAAGAATC GCCCTCTTTT TTCTGTCTCT   
  
  
+ TCTTGGCAAT A  

- +Up\_Stream \_Len000AAAAAA AATTCAAAAG CATGAACCAC AACTACTAAA CACGAAACAT TGGTTACCAA   
  
  
- AATCATTTAT CATTAATCAG CATTGTAAGA CGCTAAAGCG AGTTAAATAT ATAGTTGAAA TCTTATACTC   
  
  
- ATTGGCATTT CTAGTTTGCA TCATCATCTC CACAGTATCC AAAAAAAAAT TTTTTTAAGC CTCATTCTTT   
  
  
- CATCCCATGT AATAAAAACG AACAATTTAA GGCGATCATA TCGTTATGCT CTACCTTGTA AACATTTTTG   
  
  
- ATGCTCTGCT TCGCAAACAT GTTTTGTTTG ATGGTATGAC GTCTAGTATA TTAAAAAACC CCCCAATACA   
  
  
- TAGAATCACT ACTGTAATTA ACTACCTATT GGGATAAGAT TTATAGACCT AGGTAGTAAT TGACTGAGCA   
  
  
- AAGTTTACAA TCCTAATACC CAGAACTGTA CCATATGTAC AGTTATCTAA ATACAATCTA CTTTTCAATA   
  
  
- TACTGCGAAA AATACTAAAA ACATGATCGT ATATCACGAA TTAACCTGAT ATATAAAAAT GTTCAACACG   
  
  
- AATTGTGGAA AATTAGTCTT TATAAATAAT ATACCTTTTT TAAAATAATT ATACACAAAT ACACAAATTT   
  
  
- TTTGTATGGG TCGGAACTTT ATCTGGCACT ATATCTTTAA CCTGGGTATT ATGAGTGTAA TATATACGAA   
  
  
- ATAACAATAT TAATTCCTAG TAACAACAAA ACGTTAATAC ACCGTTCTAT ATTAGGTGTT TCAAAATGTA   
  
  
- AATATAACGT TAATGAGACC ATTTACGATA ATTACATGTA AACTCTAATT CTGTATATAT ATTGGGAAAA   
  
  
- AAAAGGGAAG AAAAAAGGAA AAAGATAAAA CTTACCGTAG AATTTGGTTT CCTCGACATG TTCAATGCAA   
  
  
- TATCGTGAGT ACCAACTGAT TTGTTCTGTC TACTCTCAAA TTCAGTTCAG GAGGACCGTT ACTACAAACG   
  
  
- GTAAACCTGG TTATTTATGT AGTACATATA ATGGTATTAT CACACCAACT GAATTGTTAT GTATCCCCTA   
  
  
- ACGTATGTTT CCATTCGGAG TTTTTGAACA CGTATACGTA ACTTTAGGTA CTAGTTTTTT TAAGTTCTCG   
  
  
- TCCAACGTGT TTTTGTGCCT CTACTCTACT TTCTCTGTCT TTTGTGTATA TGTAAGTGTC ATCATCCCCA   
  
  
- TGACCATCTC GATACCGATC CGATTCTACT ACACTTTCTT TGTTATATTA CCATCATTGA CCATTATAAA   
  
  
- TATCCCGTGT ATATCCACGC CCGTATCCCA GCACAGAGAG TCAGAGTCCA TGCCAAGCTA CCCTGAGACG   
  
  
- CTATCTACAA TAGTTTAAGT TGGTAAGCTT GTAGAGGGTC TCTCTCTCTC TCTCTGACTC ACTCACTCAC   
  
  
- TCACCCACCC ACATACACAC ACTCAACAAA ACAAGTAAAT AATATCCAAG AGACGACGTA TATAGAGGTG   
  
  
- GTAATTTAAA TGGTTTTGAA CATGGTCGAC GAAATGAACG AAAGGAGTAA AAGGGGGAAA AAACAGAGAG   
  
  
- AGAGAGAGAG AGGGGGGGGG GGGGGGGGAG AGAGAGACAC AGACTTAGTC ATAGTCGGAT AGTCCTACGA   
  
  
- AGCGGAGGTC TTTCCGTAGT GAGTGTGTGA ATAATCGAAT TTTGAAAGAA AATAAGGAAA ACGAGAAAGT   
  
  
- GACGAGAGAA AATGGGTTTA TGCGGGCAGA AGTTAAGAAG AGAGTAATTT ATACTTTTTC TTTCTCTCTT   
  
  
- TATCTCTCTC TAAATTTGGT CTTCCCCACT AAGGAAAAAG AACCACCCGA ATAATTACGA GACAGATGGG   
  
  
- GTAGATCGGC TTTTTATCAT CGAATTCTGA GTTGACGAAT AATCGTAGAG AGGAGATCGT TATTGGCTCC   
  
  
- ATTATCGTAT AAAAAGAGGG GAATCGAAGA TGGAACGAAA AACACTGTGA GTTTGGATTT GACTCTCTCT   
  
  
- CTTTCTCTTC TATCTCCTCT CTATTTATAA TCGCCCTAAT CCCGTTTAAA ACTATACGAC CCGAGAAGGA   
  
  
- GTAAGTTGAG ACTCCTTCTT CTAGTTCTAG TACGGTTGGG TGGAGGCGGC GTTGTTGGTG GTGGCAATGT   
  
  
- CGACGGTGAA GGTGCCGGTT CTAAGTCGGT CAATTATAGT AGTAGTGGAG GTTATTATAG TCGTAGAAGA   
  
  
- AGTAGTAATT AGGTATTATT GGGAGCAGTC GACGATTATT CAACGCCACT TGAGTAGAGG GCGACACTGA   
  
  
- AGAGGAGTCG ATTGGCGGAA GAGAGAGAAG AGAGGCCGCT GAAGCGAAGG AGGAGGCCGC TGAGGTGACT   
  
  
- TGCGGAACAA ATAATGAAGA GGTTCCGAAA CAAGGAGGCG GAAGCGGCTG TCAAAAGAGA TAGAAGAAGT   
  
  
- TGAAAATGAC CAATATCAAC ATTGGGATCA ACAAGACGCA GATAACGAGG TGAAAAAGAT TGGTTGGGAT   
  
  
- TAGGATCTGG TGGTGTTCTT GACGGCCATG CTCATTGATC GTCCCAATGA GGTTCATCGA CGAGCAGAAT   
  
  
- GGATTGGGAT TTGGTCTAGT GAGGTAAATA GGCCAAGTGG GTGGACTGCC GGTTGGTCCG TTAAAACCTC   
  
  
- CGACACCTCC CGATATCCCG TCAGGTGTAG TAGCTGTACC TGTAGTACGT ACCTCACGTC ACCGGAGGCG   
  
  
- AGGAGGTTCG TTAGCACCTC GCCAGCCGGT GGGAATCGGT TGGAGGGGGA CACCAGGCAG AGTTTCCCCC   
  
  
- TCCCGGCCTG GGCCTGGAGA ATGTCTCCTG CCCCCTGGCT GATACCTTGA AACGGGTTAG TAATCCTGAT   
  
  
- CTCAAGGTAA AGGTCCCGGA GTACTAGGGG CGGCTCAGTT GTCCAAAACA ACTTGGACGA CTCCGTCGCC   
  
  
- AGTTACGGGA TCGGAACGTC CTCCGCCACC CCACAGTTCT TCCCCTCCGG GAACGTCAAG TGACGCCCCT   
  
  
- GATAGAGGTG GCGGAGAATT TTCTCATGCT GTGTTCCGGA GAATCTGCCA AGGAGGTGTT CCAGTTCTGA   
  
  
- GACTTGGGGT CCCAGAACTG GAACCCTCTC TCCCTTCGGC TGGTGTTGGT GGGAGAGAAG GACGCCGCCA   
  
  
- AGTGGCTCCG TCAGTTGGTG ATGCCCCGTC ACAAACTGAG GGACCTTCGT TGGGAAGGCG GCGTATCGGT   
  
  
- TCTCTCCCAT CGACACCTCC TTCCCACCAA GCCTCTCCTC TAATTCCTGC ACCATCCTCT TCTTTCGCTC   
  
  
- TCCTCTTATC TCTCCGTGGT CTTCAAGCTC AGCACCCTTC GTAACAACTC CTCGAGTCCC AAGTTCTCAC   
  
  
- ACGGGGAGTC GGGTAAGAGC GACAGGGTTC GATTCGACGA CGACGCGGAG GTAATGGGAA GGCTTCCCAT   
  
  
- GGTTGAGGTC CAAAACGTAT CATTAACGAA CAACGAACCC ACCTTCTTAG CGGGAGAAAA AAGACAGAGA   
  
  
- AGAACCGTTA T

+     RY-element

| Site Name | Organism | Position | Strand | Matrix score. | sequence | function |
| --- | --- | --- | --- | --- | --- | --- |
| RY-element | Helianthus annuus | 2639 | - | 8 | CATGCATG | cis-acting regulatory element involved in seed-specific regulation |

>HU03G01737.1   
+ +Up\_Stream \_Len000TTTTTT TTAAGTTTTC GTACTTGGTG TTGATGATTT GTGCTTTGTA ACCAATGGTT   
  
  
+ TTAGTAAATA GTAATTAGTC GTAACATTCT GCGATTTCGC TCAATTTATA TATCAACTTT AGAATATGAG   
  
  
+ TAACCGTAAA GATCAAACGT AGTAGTAGAG GTGTCATAGG TTTTTTTTTA AAAAAATTCG GAGTAAGAAA   
  
  
+ GTAGGGTACA TTATTTTTGC TTGTTAAATT CCGCTAGTAT AGCAATACGA GATGGAACAT TTGTAAAAAC   
  
  
+ TACGAGACGA AGCGTTTGTA CAAAACAAAC TACCATACTG CAGATCATAT AATTTTTTGG GGGGTTATGT   
  
  
+ ATCTTAGTGA TGACATTAAT TGATGGATAA CCCTATTCTA AATATCTGGA TCCATCATTA ACTGACTCGT   
  
  
+ TTCAAATGTT AGGATTATGG GTCTTGACAT GGTATACATG TCAATAGATT TATGTTAGAT GAAAAGTTAT   
  
  
+ ATGACGCTTT TTATGATTTT TGTACTAGCA TATAGTGCTT AATTGGACTA TATATTTTTA CAAGTTGTGC   
  
  
+ TTAACACCTT TTAATCAGAA ATATTTATTA TATGGAAAAA ATTTTATTAA TATGTGTTTA TGTGTTTAAA   
  
  
+ AAACATACCC AGCCTTGAAA TAGACCGTGA TATAGAAATT GGACCCATAA TACTCACATT ATATATGCTT   
  
  
+ TATTGTTATA ATTAAGGATC ATTGTTGTTT TGCAATTATG TGGCAAGATA TAATCCACAA AGTTTTACAT   
  
  
+ TTATATTGCA ATTACTCTGG TAAATGCTAT TAATGTACAT TTGAGATTAA GACATATATA TAACCCTTTT   
  
  
+ TTTTCCCTTC TTTTTTCCTT TTTCTATTTT GAATGGCATC TTAAACCAAA GGAGCTGTAC AAGTTACGTT   
  
  
+ ATAGCACTCA TGGTTGACTA AACAAGACAG ATGAGAGTTT AAGTCAAGTC CTCCTGGCAA TGATGTTTGC   
  
  
+ CATTTGGACC AATAAATACA TCATGTATAT TACCATAATA GTGTGGTTGA CTTAACAATA CATAGGGGAT   
  
  
+ TGCATACAAA GGTAAGCCTC AAAAACTTGT GCATATGCAT TGAAATCCAT GATCAAAAAA ATTCAAGAGC   
  
  
+ AGGTTGCACA AAAACACGGA GATGAGATGA AAGAGACAGA AAACACATAT ACATTCACAG TAGTAGGGGT   
  
  
+ ACTGGTAGAG CTATGGCTAG GCTAAGATGA TGTGAAAGAA ACAATATAAT GGTAGTAACT GGTAATATTT   
  
  
+ ATAGGGCACA TATAGGTGCG GGCATAGGGT CGTGTCTCTC AGTCTCAGGT ACGGTTCGAT GGGACTCTGC   
  
  
+ GATAGATGTT ATCAAATTCA ACCATTCGAA CATCTCCCAG AGAGAGAGAG AGAGACTGAG TGAGTGAGTG   
  
  
+ AGTGGGTGGG TGTATGTGTG TGAGTTGTTT TGTTCATTTA TTATAGGTTC TCTGCTGCAT ATATCTCCAC   
  
  
+ CATTAAATTT ACCAAAACTT GTACCAGCTG CTTTACTTGC TTTCCTCATT TTCCCCCTTT TTTGTCTCTC   
  
  
+ TCTCTCTCTC TCCCCCCCCC CCCCCCCCTC TCTCTCTGTG TCTGAATCAG TATCAGCCTA TCAGGATGCT   
  
  
+ TCGCCTCCAG AAAGGCATCA CTCACACACT TATTAGCTTA AAACTTTCTT TTATTCCTTT TGCTCTTTCA   
  
  
+ CTGCTCTCTT TTACCCAAAT ACGCCCGTCT TCAATTCTTC TCTCATTAAA TATGAAAAAG AAAGAGAGAA   
  
  
+ ATAGAGAGAG ATTTAAACCA GAAGGGGTGA TTCCTTTTTC TTGGTGGGCT TATTAATGCT CTGTCTACCC   
  
  
+ CATCTAGCCG AAAAATAGTA GCTTAAGACT CAACTGCTTA TTAGCATCTC TCCTCTAGCA ATAACCGAGG   
  
  
+ TAATAGCATA TTTTTCTCCC CTTAGCTTCT ACCTTGCTTT TTGTGACACT CAAACCTAAA CTGAGAGAGA   
  
  
+ GAAAGAGAAG ATAGAGGAGA GATAAATATT AGCGGGATTA GGGCAAATTT TGATATGCTG GGCTCTTCCT   
  
  
+ CATTCAACTC TGAGGAAGAA GATCAAGATC ATGCCAACCC ACCTCCGCCG CAACAACCAC CACCGTTACA   
  
  
+ GCTGCCACTT CCACGGCCAA GATTCAGCCA GTTAATATCA TCATCACCTC CAATAATATC AGCATCTTCT   
  
  
+ TCATCATTAA TCCATAATAA CCCTCGTCAG CTGCTAATAA GTTGCGGTGA ACTCATCTCC CGCTGTGACT   
  
  
+ TCTCCTCAGC TAACCGCCTT CTCTCTCTTC TCTCCGGCGA CTTCGCTTCC TCCTCCGGCG ACTCCACTGA   
  
  
+ ACGCCTTGTT TATTACTTCT CCAAGGCTTT GTTCCTCCGC CTTCGCCGAC AGTTTTCTCT ATCTTCTTCA   
  
  
+ ACTTTTACTG GTTATAGTTG TAACCCTAGT TGTTCTGCGT CTATTGCTCC ACTTTTTCTA ACCAACCCTA   
  
  
+ ATCCTAGACC ACCACAAGAA CTGCCGGTAC GAGTAACTAG CAGGGTTACT CCAAGTAGCT GCTCGTCTTA   
  
  
+ CCTAACCCTA AACCAGATCA CTCCATTTAT CCGGTTCACC CACCTGACGG CCAACCAGGC AATTTTGGAG   
  
  
+ GCTGTGGAGG GCTATAGGGC AGTCCACATC ATCGACATGG ACATCATGCA TGGAGTGCAG TGGCCTCCGC   
  
  
+ TCCTCCAAGC AATCGTGGAG CGGTCGGCCA CCCTTAGCCA ACCTCCCCCT GTGGTCCGTC TCAAAGGGGG   
  
  
+ AGGGCCGGAC CCGGACCTCT TACAGAGGAC GGGGGACCGA CTATGGAACT TTGCCCAATC ATTAGGACTA   
  
  
+ GAGTTCCATT TCCAGGGCCT CATGATCCCC GCCGAGTCAA CAGGTTTTGT TGAACCTGCT GAGGCAGCGG   
  
  
+ TCAATGCCCT AGCCTTGCAG GAGGCGGTGG GGTGTCAAGA AGGGGAGGCC CTTGCAGTTC ACTGCGGGGA   
  
  
+ CTATCTCCAC CGCCTCTTAA AAGAGTACGA CACAAGGCCT CTTAGACGGT TCCTCCACAA GGTCAAGACT   
  
  
+ CTGAACCCCA GGGTCTTGAC CTTGGGAGAG AGGGAAGCCG ACCACAACCA CCCTCTCTTC CTGCGGCGGT   
  
  
+ TCACCGAGGC AGTCAACCAC TACGGGGCAG TGTTTGACTC CCTGGAAGCA ACCCTTCCGC CGCATAGCCA   
  
  
+ AGAGAGGGTA GCTGTGGAGG AAGGGTGGTT CGGAGAGGAG ATTAAGGACG TGGTAGGAGA AGAAAGCGAG   
  
  
+ AGGAGAATAG AGAGGCACCA GAAGTTCGAG TCGTGGGAAG CATTGTTGAG GAGCTCAGGG TTCAAGAGTG   
  
  
+ TGCCCCTCAG CCCATTCTCG CTGTCCCAAG CTAAGCTGCT GCTGCGCCTC CATTACCCTT CCGAAGGGTA   
  
  
+ CCAACTCCAG GTTTTGCATA GTAATTGCTT GTTGCTTGGG TGGAAGAATC GCCCTCTTTT TTCTGTCTCT   
  
  
+ TCTTGGCAAT A  

- +Up\_Stream \_Len000AAAAAA AATTCAAAAG CATGAACCAC AACTACTAAA CACGAAACAT TGGTTACCAA   
  
  
- AATCATTTAT CATTAATCAG CATTGTAAGA CGCTAAAGCG AGTTAAATAT ATAGTTGAAA TCTTATACTC   
  
  
- ATTGGCATTT CTAGTTTGCA TCATCATCTC CACAGTATCC AAAAAAAAAT TTTTTTAAGC CTCATTCTTT   
  
  
- CATCCCATGT AATAAAAACG AACAATTTAA GGCGATCATA TCGTTATGCT CTACCTTGTA AACATTTTTG   
  
  
- ATGCTCTGCT TCGCAAACAT GTTTTGTTTG ATGGTATGAC GTCTAGTATA TTAAAAAACC CCCCAATACA   
  
  
- TAGAATCACT ACTGTAATTA ACTACCTATT GGGATAAGAT TTATAGACCT AGGTAGTAAT TGACTGAGCA   
  
  
- AAGTTTACAA TCCTAATACC CAGAACTGTA CCATATGTAC AGTTATCTAA ATACAATCTA CTTTTCAATA   
  
  
- TACTGCGAAA AATACTAAAA ACATGATCGT ATATCACGAA TTAACCTGAT ATATAAAAAT GTTCAACACG   
  
  
- AATTGTGGAA AATTAGTCTT TATAAATAAT ATACCTTTTT TAAAATAATT ATACACAAAT ACACAAATTT   
  
  
- TTTGTATGGG TCGGAACTTT ATCTGGCACT ATATCTTTAA CCTGGGTATT ATGAGTGTAA TATATACGAA   
  
  
- ATAACAATAT TAATTCCTAG TAACAACAAA ACGTTAATAC ACCGTTCTAT ATTAGGTGTT TCAAAATGTA   
  
  
- AATATAACGT TAATGAGACC ATTTACGATA ATTACATGTA AACTCTAATT CTGTATATAT ATTGGGAAAA   
  
  
- AAAAGGGAAG AAAAAAGGAA AAAGATAAAA CTTACCGTAG AATTTGGTTT CCTCGACATG TTCAATGCAA   
  
  
- TATCGTGAGT ACCAACTGAT TTGTTCTGTC TACTCTCAAA TTCAGTTCAG GAGGACCGTT ACTACAAACG   
  
  
- GTAAACCTGG TTATTTATGT AGTACATATA ATGGTATTAT CACACCAACT GAATTGTTAT GTATCCCCTA   
  
  
- ACGTATGTTT CCATTCGGAG TTTTTGAACA CGTATACGTA ACTTTAGGTA CTAGTTTTTT TAAGTTCTCG   
  
  
- TCCAACGTGT TTTTGTGCCT CTACTCTACT TTCTCTGTCT TTTGTGTATA TGTAAGTGTC ATCATCCCCA   
  
  
- TGACCATCTC GATACCGATC CGATTCTACT ACACTTTCTT TGTTATATTA CCATCATTGA CCATTATAAA   
  
  
- TATCCCGTGT ATATCCACGC CCGTATCCCA GCACAGAGAG TCAGAGTCCA TGCCAAGCTA CCCTGAGACG   
  
  
- CTATCTACAA TAGTTTAAGT TGGTAAGCTT GTAGAGGGTC TCTCTCTCTC TCTCTGACTC ACTCACTCAC   
  
  
- TCACCCACCC ACATACACAC ACTCAACAAA ACAAGTAAAT AATATCCAAG AGACGACGTA TATAGAGGTG   
  
  
- GTAATTTAAA TGGTTTTGAA CATGGTCGAC GAAATGAACG AAAGGAGTAA AAGGGGGAAA AAACAGAGAG   
  
  
- AGAGAGAGAG AGGGGGGGGG GGGGGGGGAG AGAGAGACAC AGACTTAGTC ATAGTCGGAT AGTCCTACGA   
  
  
- AGCGGAGGTC TTTCCGTAGT GAGTGTGTGA ATAATCGAAT TTTGAAAGAA AATAAGGAAA ACGAGAAAGT   
  
  
- GACGAGAGAA AATGGGTTTA TGCGGGCAGA AGTTAAGAAG AGAGTAATTT ATACTTTTTC TTTCTCTCTT   
  
  
- TATCTCTCTC TAAATTTGGT CTTCCCCACT AAGGAAAAAG AACCACCCGA ATAATTACGA GACAGATGGG   
  
  
- GTAGATCGGC TTTTTATCAT CGAATTCTGA GTTGACGAAT AATCGTAGAG AGGAGATCGT TATTGGCTCC   
  
  
- ATTATCGTAT AAAAAGAGGG GAATCGAAGA TGGAACGAAA AACACTGTGA GTTTGGATTT GACTCTCTCT   
  
  
- CTTTCTCTTC TATCTCCTCT CTATTTATAA TCGCCCTAAT CCCGTTTAAA ACTATACGAC CCGAGAAGGA   
  
  
- GTAAGTTGAG ACTCCTTCTT CTAGTTCTAG TACGGTTGGG TGGAGGCGGC GTTGTTGGTG GTGGCAATGT   
  
  
- CGACGGTGAA GGTGCCGGTT CTAAGTCGGT CAATTATAGT AGTAGTGGAG GTTATTATAG TCGTAGAAGA   
  
  
- AGTAGTAATT AGGTATTATT GGGAGCAGTC GACGATTATT CAACGCCACT TGAGTAGAGG GCGACACTGA   
  
  
- AGAGGAGTCG ATTGGCGGAA GAGAGAGAAG AGAGGCCGCT GAAGCGAAGG AGGAGGCCGC TGAGGTGACT   
  
  
- TGCGGAACAA ATAATGAAGA GGTTCCGAAA CAAGGAGGCG GAAGCGGCTG TCAAAAGAGA TAGAAGAAGT   
  
  
- TGAAAATGAC CAATATCAAC ATTGGGATCA ACAAGACGCA GATAACGAGG TGAAAAAGAT TGGTTGGGAT   
  
  
- TAGGATCTGG TGGTGTTCTT GACGGCCATG CTCATTGATC GTCCCAATGA GGTTCATCGA CGAGCAGAAT   
  
  
- GGATTGGGAT TTGGTCTAGT GAGGTAAATA GGCCAAGTGG GTGGACTGCC GGTTGGTCCG TTAAAACCTC   
  
  
- CGACACCTCC CGATATCCCG TCAGGTGTAG TAGCTGTACC TGTAGTACGT ACCTCACGTC ACCGGAGGCG   
  
  
- AGGAGGTTCG TTAGCACCTC GCCAGCCGGT GGGAATCGGT TGGAGGGGGA CACCAGGCAG AGTTTCCCCC   
  
  
- TCCCGGCCTG GGCCTGGAGA ATGTCTCCTG CCCCCTGGCT GATACCTTGA AACGGGTTAG TAATCCTGAT   
  
  
- CTCAAGGTAA AGGTCCCGGA GTACTAGGGG CGGCTCAGTT GTCCAAAACA ACTTGGACGA CTCCGTCGCC   
  
  
- AGTTACGGGA TCGGAACGTC CTCCGCCACC CCACAGTTCT TCCCCTCCGG GAACGTCAAG TGACGCCCCT   
  
  
- GATAGAGGTG GCGGAGAATT TTCTCATGCT GTGTTCCGGA GAATCTGCCA AGGAGGTGTT CCAGTTCTGA   
  
  
- GACTTGGGGT CCCAGAACTG GAACCCTCTC TCCCTTCGGC TGGTGTTGGT GGGAGAGAAG GACGCCGCCA   
  
  
- AGTGGCTCCG TCAGTTGGTG ATGCCCCGTC ACAAACTGAG GGACCTTCGT TGGGAAGGCG GCGTATCGGT   
  
  
- TCTCTCCCAT CGACACCTCC TTCCCACCAA GCCTCTCCTC TAATTCCTGC ACCATCCTCT TCTTTCGCTC   
  
  
- TCCTCTTATC TCTCCGTGGT CTTCAAGCTC AGCACCCTTC GTAACAACTC CTCGAGTCCC AAGTTCTCAC   
  
  
- ACGGGGAGTC GGGTAAGAGC GACAGGGTTC GATTCGACGA CGACGCGGAG GTAATGGGAA GGCTTCCCAT   
  
  
- GGTTGAGGTC CAAAACGTAT CATTAACGAA CAACGAACCC ACCTTCTTAG CGGGAGAAAA AAGACAGAGA   
  
  
- AGAACCGTTA T

+     STRE

| Site Name | Organism | Position | Strand | Matrix score. | sequence | function |
| --- | --- | --- | --- | --- | --- | --- |
| STRE | Arabidopsis thaliana | 1912 | - | 5 | AGGGG |  |
| STRE | Arabidopsis thaliana | 2729 | + | 5 | AGGGG |  |
| STRE | Arabidopsis thaliana | 1189 | + | 5 | AGGGG |  |
| STRE | Arabidopsis thaliana | 1048 | + | 5 | AGGGG |  |
| STRE | Arabidopsis thaliana | 1777 | + | 5 | AGGGG |  |
| STRE | Arabidopsis thaliana | 2915 | + | 5 | AGGGG |  |
| STRE | Arabidopsis thaliana | 1528 | - | 5 | AGGGG |  |
| STRE | Arabidopsis thaliana | 2710 | - | 5 | AGGGG |  |
| STRE | Arabidopsis thaliana | 1569 | - | 5 | AGGGG |  |
| STRE | Arabidopsis thaliana | 3297 | - | 5 | AGGGG |  |

>HU03G01737.1   
+ +Up\_Stream \_Len000TTTTTT TTAAGTTTTC GTACTTGGTG TTGATGATTT GTGCTTTGTA ACCAATGGTT   
  
  
+ TTAGTAAATA GTAATTAGTC GTAACATTCT GCGATTTCGC TCAATTTATA TATCAACTTT AGAATATGAG   
  
  
+ TAACCGTAAA GATCAAACGT AGTAGTAGAG GTGTCATAGG TTTTTTTTTA AAAAAATTCG GAGTAAGAAA   
  
  
+ GTAGGGTACA TTATTTTTGC TTGTTAAATT CCGCTAGTAT AGCAATACGA GATGGAACAT TTGTAAAAAC   
  
  
+ TACGAGACGA AGCGTTTGTA CAAAACAAAC TACCATACTG CAGATCATAT AATTTTTTGG GGGGTTATGT   
  
  
+ ATCTTAGTGA TGACATTAAT TGATGGATAA CCCTATTCTA AATATCTGGA TCCATCATTA ACTGACTCGT   
  
  
+ TTCAAATGTT AGGATTATGG GTCTTGACAT GGTATACATG TCAATAGATT TATGTTAGAT GAAAAGTTAT   
  
  
+ ATGACGCTTT TTATGATTTT TGTACTAGCA TATAGTGCTT AATTGGACTA TATATTTTTA CAAGTTGTGC   
  
  
+ TTAACACCTT TTAATCAGAA ATATTTATTA TATGGAAAAA ATTTTATTAA TATGTGTTTA TGTGTTTAAA   
  
  
+ AAACATACCC AGCCTTGAAA TAGACCGTGA TATAGAAATT GGACCCATAA TACTCACATT ATATATGCTT   
  
  
+ TATTGTTATA ATTAAGGATC ATTGTTGTTT TGCAATTATG TGGCAAGATA TAATCCACAA AGTTTTACAT   
  
  
+ TTATATTGCA ATTACTCTGG TAAATGCTAT TAATGTACAT TTGAGATTAA GACATATATA TAACCCTTTT   
  
  
+ TTTTCCCTTC TTTTTTCCTT TTTCTATTTT GAATGGCATC TTAAACCAAA GGAGCTGTAC AAGTTACGTT   
  
  
+ ATAGCACTCA TGGTTGACTA AACAAGACAG ATGAGAGTTT AAGTCAAGTC CTCCTGGCAA TGATGTTTGC   
  
  
+ CATTTGGACC AATAAATACA TCATGTATAT TACCATAATA GTGTGGTTGA CTTAACAATA CATAGGGGAT   
  
  
+ TGCATACAAA GGTAAGCCTC AAAAACTTGT GCATATGCAT TGAAATCCAT GATCAAAAAA ATTCAAGAGC   
  
  
+ AGGTTGCACA AAAACACGGA GATGAGATGA AAGAGACAGA AAACACATAT ACATTCACAG TAGTAGGGGT   
  
  
+ ACTGGTAGAG CTATGGCTAG GCTAAGATGA TGTGAAAGAA ACAATATAAT GGTAGTAACT GGTAATATTT   
  
  
+ ATAGGGCACA TATAGGTGCG GGCATAGGGT CGTGTCTCTC AGTCTCAGGT ACGGTTCGAT GGGACTCTGC   
  
  
+ GATAGATGTT ATCAAATTCA ACCATTCGAA CATCTCCCAG AGAGAGAGAG AGAGACTGAG TGAGTGAGTG   
  
  
+ AGTGGGTGGG TGTATGTGTG TGAGTTGTTT TGTTCATTTA TTATAGGTTC TCTGCTGCAT ATATCTCCAC   
  
  
+ CATTAAATTT ACCAAAACTT GTACCAGCTG CTTTACTTGC TTTCCTCATT TTCCCCCTTT TTTGTCTCTC   
  
  
+ TCTCTCTCTC TCCCCCCCCC CCCCCCCCTC TCTCTCTGTG TCTGAATCAG TATCAGCCTA TCAGGATGCT   
  
  
+ TCGCCTCCAG AAAGGCATCA CTCACACACT TATTAGCTTA AAACTTTCTT TTATTCCTTT TGCTCTTTCA   
  
  
+ CTGCTCTCTT TTACCCAAAT ACGCCCGTCT TCAATTCTTC TCTCATTAAA TATGAAAAAG AAAGAGAGAA   
  
  
+ ATAGAGAGAG ATTTAAACCA GAAGGGGTGA TTCCTTTTTC TTGGTGGGCT TATTAATGCT CTGTCTACCC   
  
  
+ CATCTAGCCG AAAAATAGTA GCTTAAGACT CAACTGCTTA TTAGCATCTC TCCTCTAGCA ATAACCGAGG   
  
  
+ TAATAGCATA TTTTTCTCCC CTTAGCTTCT ACCTTGCTTT TTGTGACACT CAAACCTAAA CTGAGAGAGA   
  
  
+ GAAAGAGAAG ATAGAGGAGA GATAAATATT AGCGGGATTA GGGCAAATTT TGATATGCTG GGCTCTTCCT   
  
  
+ CATTCAACTC TGAGGAAGAA GATCAAGATC ATGCCAACCC ACCTCCGCCG CAACAACCAC CACCGTTACA   
  
  
+ GCTGCCACTT CCACGGCCAA GATTCAGCCA GTTAATATCA TCATCACCTC CAATAATATC AGCATCTTCT   
  
  
+ TCATCATTAA TCCATAATAA CCCTCGTCAG CTGCTAATAA GTTGCGGTGA ACTCATCTCC CGCTGTGACT   
  
  
+ TCTCCTCAGC TAACCGCCTT CTCTCTCTTC TCTCCGGCGA CTTCGCTTCC TCCTCCGGCG ACTCCACTGA   
  
  
+ ACGCCTTGTT TATTACTTCT CCAAGGCTTT GTTCCTCCGC CTTCGCCGAC AGTTTTCTCT ATCTTCTTCA   
  
  
+ ACTTTTACTG GTTATAGTTG TAACCCTAGT TGTTCTGCGT CTATTGCTCC ACTTTTTCTA ACCAACCCTA   
  
  
+ ATCCTAGACC ACCACAAGAA CTGCCGGTAC GAGTAACTAG CAGGGTTACT CCAAGTAGCT GCTCGTCTTA   
  
  
+ CCTAACCCTA AACCAGATCA CTCCATTTAT CCGGTTCACC CACCTGACGG CCAACCAGGC AATTTTGGAG   
  
  
+ GCTGTGGAGG GCTATAGGGC AGTCCACATC ATCGACATGG ACATCATGCA TGGAGTGCAG TGGCCTCCGC   
  
  
+ TCCTCCAAGC AATCGTGGAG CGGTCGGCCA CCCTTAGCCA ACCTCCCCCT GTGGTCCGTC TCAAAGGGGG   
  
  
+ AGGGCCGGAC CCGGACCTCT TACAGAGGAC GGGGGACCGA CTATGGAACT TTGCCCAATC ATTAGGACTA   
  
  
+ GAGTTCCATT TCCAGGGCCT CATGATCCCC GCCGAGTCAA CAGGTTTTGT TGAACCTGCT GAGGCAGCGG   
  
  
+ TCAATGCCCT AGCCTTGCAG GAGGCGGTGG GGTGTCAAGA AGGGGAGGCC CTTGCAGTTC ACTGCGGGGA   
  
  
+ CTATCTCCAC CGCCTCTTAA AAGAGTACGA CACAAGGCCT CTTAGACGGT TCCTCCACAA GGTCAAGACT   
  
  
+ CTGAACCCCA GGGTCTTGAC CTTGGGAGAG AGGGAAGCCG ACCACAACCA CCCTCTCTTC CTGCGGCGGT   
  
  
+ TCACCGAGGC AGTCAACCAC TACGGGGCAG TGTTTGACTC CCTGGAAGCA ACCCTTCCGC CGCATAGCCA   
  
  
+ AGAGAGGGTA GCTGTGGAGG AAGGGTGGTT CGGAGAGGAG ATTAAGGACG TGGTAGGAGA AGAAAGCGAG   
  
  
+ AGGAGAATAG AGAGGCACCA GAAGTTCGAG TCGTGGGAAG CATTGTTGAG GAGCTCAGGG TTCAAGAGTG   
  
  
+ TGCCCCTCAG CCCATTCTCG CTGTCCCAAG CTAAGCTGCT GCTGCGCCTC CATTACCCTT CCGAAGGGTA   
  
  
+ CCAACTCCAG GTTTTGCATA GTAATTGCTT GTTGCTTGGG TGGAAGAATC GCCCTCTTTT TTCTGTCTCT   
  
  
+ TCTTGGCAAT A  

- +Up\_Stream \_Len000AAAAAA AATTCAAAAG CATGAACCAC AACTACTAAA CACGAAACAT TGGTTACCAA   
  
  
- AATCATTTAT CATTAATCAG CATTGTAAGA CGCTAAAGCG AGTTAAATAT ATAGTTGAAA TCTTATACTC   
  
  
- ATTGGCATTT CTAGTTTGCA TCATCATCTC CACAGTATCC AAAAAAAAAT TTTTTTAAGC CTCATTCTTT   
  
  
- CATCCCATGT AATAAAAACG AACAATTTAA GGCGATCATA TCGTTATGCT CTACCTTGTA AACATTTTTG   
  
  
- ATGCTCTGCT TCGCAAACAT GTTTTGTTTG ATGGTATGAC GTCTAGTATA TTAAAAAACC CCCCAATACA   
  
  
- TAGAATCACT ACTGTAATTA ACTACCTATT GGGATAAGAT TTATAGACCT AGGTAGTAAT TGACTGAGCA   
  
  
- AAGTTTACAA TCCTAATACC CAGAACTGTA CCATATGTAC AGTTATCTAA ATACAATCTA CTTTTCAATA   
  
  
- TACTGCGAAA AATACTAAAA ACATGATCGT ATATCACGAA TTAACCTGAT ATATAAAAAT GTTCAACACG   
  
  
- AATTGTGGAA AATTAGTCTT TATAAATAAT ATACCTTTTT TAAAATAATT ATACACAAAT ACACAAATTT   
  
  
- TTTGTATGGG TCGGAACTTT ATCTGGCACT ATATCTTTAA CCTGGGTATT ATGAGTGTAA TATATACGAA   
  
  
- ATAACAATAT TAATTCCTAG TAACAACAAA ACGTTAATAC ACCGTTCTAT ATTAGGTGTT TCAAAATGTA   
  
  
- AATATAACGT TAATGAGACC ATTTACGATA ATTACATGTA AACTCTAATT CTGTATATAT ATTGGGAAAA   
  
  
- AAAAGGGAAG AAAAAAGGAA AAAGATAAAA CTTACCGTAG AATTTGGTTT CCTCGACATG TTCAATGCAA   
  
  
- TATCGTGAGT ACCAACTGAT TTGTTCTGTC TACTCTCAAA TTCAGTTCAG GAGGACCGTT ACTACAAACG   
  
  
- GTAAACCTGG TTATTTATGT AGTACATATA ATGGTATTAT CACACCAACT GAATTGTTAT GTATCCCCTA   
  
  
- ACGTATGTTT CCATTCGGAG TTTTTGAACA CGTATACGTA ACTTTAGGTA CTAGTTTTTT TAAGTTCTCG   
  
  
- TCCAACGTGT TTTTGTGCCT CTACTCTACT TTCTCTGTCT TTTGTGTATA TGTAAGTGTC ATCATCCCCA   
  
  
- TGACCATCTC GATACCGATC CGATTCTACT ACACTTTCTT TGTTATATTA CCATCATTGA CCATTATAAA   
  
  
- TATCCCGTGT ATATCCACGC CCGTATCCCA GCACAGAGAG TCAGAGTCCA TGCCAAGCTA CCCTGAGACG   
  
  
- CTATCTACAA TAGTTTAAGT TGGTAAGCTT GTAGAGGGTC TCTCTCTCTC TCTCTGACTC ACTCACTCAC   
  
  
- TCACCCACCC ACATACACAC ACTCAACAAA ACAAGTAAAT AATATCCAAG AGACGACGTA TATAGAGGTG   
  
  
- GTAATTTAAA TGGTTTTGAA CATGGTCGAC GAAATGAACG AAAGGAGTAA AAGGGGGAAA AAACAGAGAG   
  
  
- AGAGAGAGAG AGGGGGGGGG GGGGGGGGAG AGAGAGACAC AGACTTAGTC ATAGTCGGAT AGTCCTACGA   
  
  
- AGCGGAGGTC TTTCCGTAGT GAGTGTGTGA ATAATCGAAT TTTGAAAGAA AATAAGGAAA ACGAGAAAGT   
  
  
- GACGAGAGAA AATGGGTTTA TGCGGGCAGA AGTTAAGAAG AGAGTAATTT ATACTTTTTC TTTCTCTCTT   
  
  
- TATCTCTCTC TAAATTTGGT CTTCCCCACT AAGGAAAAAG AACCACCCGA ATAATTACGA GACAGATGGG   
  
  
- GTAGATCGGC TTTTTATCAT CGAATTCTGA GTTGACGAAT AATCGTAGAG AGGAGATCGT TATTGGCTCC   
  
  
- ATTATCGTAT AAAAAGAGGG GAATCGAAGA TGGAACGAAA AACACTGTGA GTTTGGATTT GACTCTCTCT   
  
  
- CTTTCTCTTC TATCTCCTCT CTATTTATAA TCGCCCTAAT CCCGTTTAAA ACTATACGAC CCGAGAAGGA   
  
  
- GTAAGTTGAG ACTCCTTCTT CTAGTTCTAG TACGGTTGGG TGGAGGCGGC GTTGTTGGTG GTGGCAATGT   
  
  
- CGACGGTGAA GGTGCCGGTT CTAAGTCGGT CAATTATAGT AGTAGTGGAG GTTATTATAG TCGTAGAAGA   
  
  
- AGTAGTAATT AGGTATTATT GGGAGCAGTC GACGATTATT CAACGCCACT TGAGTAGAGG GCGACACTGA   
  
  
- AGAGGAGTCG ATTGGCGGAA GAGAGAGAAG AGAGGCCGCT GAAGCGAAGG AGGAGGCCGC TGAGGTGACT   
  
  
- TGCGGAACAA ATAATGAAGA GGTTCCGAAA CAAGGAGGCG GAAGCGGCTG TCAAAAGAGA TAGAAGAAGT   
  
  
- TGAAAATGAC CAATATCAAC ATTGGGATCA ACAAGACGCA GATAACGAGG TGAAAAAGAT TGGTTGGGAT   
  
  
- TAGGATCTGG TGGTGTTCTT GACGGCCATG CTCATTGATC GTCCCAATGA GGTTCATCGA CGAGCAGAAT   
  
  
- GGATTGGGAT TTGGTCTAGT GAGGTAAATA GGCCAAGTGG GTGGACTGCC GGTTGGTCCG TTAAAACCTC   
  
  
- CGACACCTCC CGATATCCCG TCAGGTGTAG TAGCTGTACC TGTAGTACGT ACCTCACGTC ACCGGAGGCG   
  
  
- AGGAGGTTCG TTAGCACCTC GCCAGCCGGT GGGAATCGGT TGGAGGGGGA CACCAGGCAG AGTTTCCCCC   
  
  
- TCCCGGCCTG GGCCTGGAGA ATGTCTCCTG CCCCCTGGCT GATACCTTGA AACGGGTTAG TAATCCTGAT   
  
  
- CTCAAGGTAA AGGTCCCGGA GTACTAGGGG CGGCTCAGTT GTCCAAAACA ACTTGGACGA CTCCGTCGCC   
  
  
- AGTTACGGGA TCGGAACGTC CTCCGCCACC CCACAGTTCT TCCCCTCCGG GAACGTCAAG TGACGCCCCT   
  
  
- GATAGAGGTG GCGGAGAATT TTCTCATGCT GTGTTCCGGA GAATCTGCCA AGGAGGTGTT CCAGTTCTGA   
  
  
- GACTTGGGGT CCCAGAACTG GAACCCTCTC TCCCTTCGGC TGGTGTTGGT GGGAGAGAAG GACGCCGCCA   
  
  
- AGTGGCTCCG TCAGTTGGTG ATGCCCCGTC ACAAACTGAG GGACCTTCGT TGGGAAGGCG GCGTATCGGT   
  
  
- TCTCTCCCAT CGACACCTCC TTCCCACCAA GCCTCTCCTC TAATTCCTGC ACCATCCTCT TCTTTCGCTC   
  
  
- TCCTCTTATC TCTCCGTGGT CTTCAAGCTC AGCACCCTTC GTAACAACTC CTCGAGTCCC AAGTTCTCAC   
  
  
- ACGGGGAGTC GGGTAAGAGC GACAGGGTTC GATTCGACGA CGACGCGGAG GTAATGGGAA GGCTTCCCAT   
  
  
- GGTTGAGGTC CAAAACGTAT CATTAACGAA CAACGAACCC ACCTTCTTAG CGGGAGAAAA AAGACAGAGA   
  
  
- AGAACCGTTA T

+     TATA-box

| Site Name | Organism | Position | Strand | Matrix score. | sequence | function |
| --- | --- | --- | --- | --- | --- | --- |
| TATA-box | Arabidopsis thaliana | 123 | + | 4 | TATA | core promoter element around -30 of transcription start |
| TATA-box | Brassica napus | 122 | + | 6 | ATATAT | core promoter element around -30 of transcription start |
| TATA-box | Arabidopsis thaliana | 753 | + | 4 | TATA | core promoter element around -30 of transcription start |
| TATA-box | Brassica oleracea | 752 | + | 6 | ATATAA | core promoter element around -30 of transcription start |
| TATA-box | Arabidopsis thaliana | 2396 | - | 5 | TATAA | core promoter element around -30 of transcription start |
| TATA-box | Brassica napus | 1463 | + | 6 | ATATAT | core promoter element around -30 of transcription start |
| TATA-box | Arabidopsis thaliana | 1464 | + | 4 | TATA | core promoter element around -30 of transcription start |
| TATA-box | Arabidopsis thaliana | 1446 | + | 4 | TATA | core promoter element around -30 of transcription start |
| TATA-box | Arabidopsis thaliana | 1442 | - | 9 | ccTATAAAaa | core promoter element around -30 of transcription start |
| TATA-box | Arabidopsis thaliana | 1275 | + | 4 | TATA | core promoter element around -30 of transcription start |
| TATA-box | Helianthus annuus | 119 | - | 6 | TATAAA | core promoter element around -30 of transcription start |
| TATA-box | Arabidopsis thaliana | 1263 | - | 5 | TATAA | core promoter element around -30 of transcription start |
| TATA-box | Arabidopsis thaliana | 1239 | + | 4 | TATA | core promoter element around -30 of transcription start |
| TATA-box | Brassica oleracea | 1238 | + | 6 | ATATAA | core promoter element around -30 of transcription start |
| TATA-box | Arabidopsis thaliana | 913 | - | 5 | TATAA | core promoter element around -30 of transcription start |
| TATA-box | Arabidopsis thaliana | 831 | + | 6 | TATATA | core promoter element around -30 of transcription start |
| TATA-box | Arabidopsis thaliana | 775 | - | 5 | TATAA | core promoter element around -30 of transcription start |
| TATA-box | Brassica napus | 695 | + | 6 | ATATAT | core promoter element around -30 of transcription start |
| TATA-box | Brassica napus | 692 | + | 6 | ATTATA | core promoter element around -30 of transcription start |
| TATA-box | Arabidopsis thaliana | 117 | - | 9 | taTATAAAtc | core promoter element around -30 of transcription start |
| TATA-box | Arabidopsis thaliana | 665 | + | 4 | TATA | core promoter element around -30 of transcription start |
| TATA-box | Arabidopsis thaliana | 914 | + | 4 | TATA | core promoter element around -30 of transcription start |
| TATA-box | Arabidopsis thaliana | 833 | + | 4 | TATA | core promoter element around -30 of transcription start |
| TATA-box | Arabidopsis thaliana | 491 | - | 5 | TATAA | core promoter element around -30 of transcription start |
| TATA-box | Arabidopsis thaliana | 457 | + | 4 | TATA | core promoter element around -30 of transcription start |
| TATA-box | Arabidopsis thaliana | 332 | + | 4 | TATA | core promoter element around -30 of transcription start |
| TATA-box | Brassica oleracea | 331 | + | 6 | ATATAA | core promoter element around -30 of transcription start |
| TATA-box | Brassica oleracea | 832 | + | 6 | ATATAA | core promoter element around -30 of transcription start |
| TATA-box | Arabidopsis thaliana | 1260 | - | 9 | ccTATAAAaa | core promoter element around -30 of transcription start |
| TATA-box | Brassica juncea | 118 | - | 7 | TATAAAT | core promoter element around -30 of transcription start |
| TATA-box | Arabidopsis thaliana | 693 | - | 7 | TATATAA | core promoter element around -30 of transcription start |
| TATA-box | Arabidopsis thaliana | 121 | + | 6 | TATATA | core promoter element around -30 of transcription start |
| TATA-box | Arabidopsis thaliana | 710 | - | 5 | TATAA | core promoter element around -30 of transcription start |
| TATA-box | Helianthus annuus | 1008 | - | 6 | TATACA | core promoter element around -30 of transcription start |
| TATA-box | Oryza sativa | 512 | - | 7 | TACAAAA | core promoter element around -30 of transcription start |
| TATA-box | Arabidopsis thaliana | 696 | + | 4 | TATA | core promoter element around -30 of transcription start |
| TATA-box | Brassica napus | 1444 | + | 6 | ATTATA | core promoter element around -30 of transcription start |
| TATA-box | Arabidopsis thaliana | 120 | - | 7 | TATATAA | core promoter element around -30 of transcription start |
| TATA-box | Arabidopsis thaliana | 1445 | - | 5 | TATAA | core promoter element around -30 of transcription start |
| TATA-box | Arabidopsis thaliana | 711 | + | 4 | TATA | core promoter element around -30 of transcription start |
| TATA-box | Arabidopsis thaliana | 776 | + | 4 | TATA | core promoter element around -30 of transcription start |
| TATA-box | Oryza sativa | 303 | + | 7 | TACAAAA | core promoter element around -30 of transcription start |
| TATA-box | Arabidopsis thaliana | 1010 | + | 4 | TATA | core promoter element around -30 of transcription start |
| TATA-box | Arabidopsis thaliana | 252 | + | 4 | TATA | core promoter element around -30 of transcription start |
| TATA-box | Arabidopsis thaliana | 592 | - | 5 | TATAA | core promoter element around -30 of transcription start |
| TATA-box | Arabidopsis thaliana | 545 | + | 4 | TATA | core promoter element around -30 of transcription start |
| TATA-box | Brassica juncea | 1261 | - | 7 | TATAAAT | core promoter element around -30 of transcription start |
| TATA-box | Arabidopsis thaliana | 492 | + | 4 | TATA | core promoter element around -30 of transcription start |
| TATA-box | Helianthus annuus | 774 | - | 6 | TATAAA | core promoter element around -30 of transcription start |
| TATA-box | Arabidopsis thaliana | 525 | + | 4 | TATA | core promoter element around -30 of transcription start |
| TATA-box | Arabidopsis thaliana | 1264 | + | 4 | TATA | core promoter element around -30 of transcription start |
| TATA-box | Arabidopsis thaliana | 694 | + | 6 | TATATA | core promoter element around -30 of transcription start |
| TATA-box | Brassica napus | 830 | + | 6 | ATATAT | core promoter element around -30 of transcription start |
| TATA-box | Arabidopsis thaliana | 829 | + | 6 | TATATA | core promoter element around -30 of transcription start |
| TATA-box | Helianthus annuus | 1262 | - | 6 | TATAAA | core promoter element around -30 of transcription start |
| TATA-box | Arabidopsis thaliana | 1172 | + | 4 | TATA | core promoter element around -30 of transcription start |
| TATA-box | Arabidopsis thaliana | 543 | + | 6 | TATATA | core promoter element around -30 of transcription start |
| TATA-box | Arabidopsis thaliana | 593 | + | 4 | TATA | core promoter element around -30 of transcription start |
| TATA-box | Arabidopsis thaliana | 2397 | - | 4 | TATA | core promoter element around -30 of transcription start |
| TATA-box | Brassica juncea | 773 | - | 7 | TATAAAT | core promoter element around -30 of transcription start |
| TATA-box | Arabidopsis thaliana | 2607 | - | 4 | TATA | core promoter element around -30 of transcription start |
| TATA-box | Brassica napus | 544 | + | 6 | ATATAT | core promoter element around -30 of transcription start |
| TATA-box | Brassica napus | 591 | + | 6 | ATTATA | core promoter element around -30 of transcription start |
| TATA-box | Brassica napus | 828 | + | 6 | ATATAT | core promoter element around -30 of transcription start |

>HU03G01737.1   
+ +Up\_Stream \_Len000TTTTTT TTAAGTTTTC GTACTTGGTG TTGATGATTT GTGCTTTGTA ACCAATGGTT   
  
  
+ TTAGTAAATA GTAATTAGTC GTAACATTCT GCGATTTCGC TCAATTTATA TATCAACTTT AGAATATGAG   
  
  
+ TAACCGTAAA GATCAAACGT AGTAGTAGAG GTGTCATAGG TTTTTTTTTA AAAAAATTCG GAGTAAGAAA   
  
  
+ GTAGGGTACA TTATTTTTGC TTGTTAAATT CCGCTAGTAT AGCAATACGA GATGGAACAT TTGTAAAAAC   
  
  
+ TACGAGACGA AGCGTTTGTA CAAAACAAAC TACCATACTG CAGATCATAT AATTTTTTGG GGGGTTATGT   
  
  
+ ATCTTAGTGA TGACATTAAT TGATGGATAA CCCTATTCTA AATATCTGGA TCCATCATTA ACTGACTCGT   
  
  
+ TTCAAATGTT AGGATTATGG GTCTTGACAT GGTATACATG TCAATAGATT TATGTTAGAT GAAAAGTTAT   
  
  
+ ATGACGCTTT TTATGATTTT TGTACTAGCA TATAGTGCTT AATTGGACTA TATATTTTTA CAAGTTGTGC   
  
  
+ TTAACACCTT TTAATCAGAA ATATTTATTA TATGGAAAAA ATTTTATTAA TATGTGTTTA TGTGTTTAAA   
  
  
+ AAACATACCC AGCCTTGAAA TAGACCGTGA TATAGAAATT GGACCCATAA TACTCACATT ATATATGCTT   
  
  
+ TATTGTTATA ATTAAGGATC ATTGTTGTTT TGCAATTATG TGGCAAGATA TAATCCACAA AGTTTTACAT   
  
  
+ TTATATTGCA ATTACTCTGG TAAATGCTAT TAATGTACAT TTGAGATTAA GACATATATA TAACCCTTTT   
  
  
+ TTTTCCCTTC TTTTTTCCTT TTTCTATTTT GAATGGCATC TTAAACCAAA GGAGCTGTAC AAGTTACGTT   
  
  
+ ATAGCACTCA TGGTTGACTA AACAAGACAG ATGAGAGTTT AAGTCAAGTC CTCCTGGCAA TGATGTTTGC   
  
  
+ CATTTGGACC AATAAATACA TCATGTATAT TACCATAATA GTGTGGTTGA CTTAACAATA CATAGGGGAT   
  
  
+ TGCATACAAA GGTAAGCCTC AAAAACTTGT GCATATGCAT TGAAATCCAT GATCAAAAAA ATTCAAGAGC   
  
  
+ AGGTTGCACA AAAACACGGA GATGAGATGA AAGAGACAGA AAACACATAT ACATTCACAG TAGTAGGGGT   
  
  
+ ACTGGTAGAG CTATGGCTAG GCTAAGATGA TGTGAAAGAA ACAATATAAT GGTAGTAACT GGTAATATTT   
  
  
+ ATAGGGCACA TATAGGTGCG GGCATAGGGT CGTGTCTCTC AGTCTCAGGT ACGGTTCGAT GGGACTCTGC   
  
  
+ GATAGATGTT ATCAAATTCA ACCATTCGAA CATCTCCCAG AGAGAGAGAG AGAGACTGAG TGAGTGAGTG   
  
  
+ AGTGGGTGGG TGTATGTGTG TGAGTTGTTT TGTTCATTTA TTATAGGTTC TCTGCTGCAT ATATCTCCAC   
  
  
+ CATTAAATTT ACCAAAACTT GTACCAGCTG CTTTACTTGC TTTCCTCATT TTCCCCCTTT TTTGTCTCTC   
  
  
+ TCTCTCTCTC TCCCCCCCCC CCCCCCCCTC TCTCTCTGTG TCTGAATCAG TATCAGCCTA TCAGGATGCT   
  
  
+ TCGCCTCCAG AAAGGCATCA CTCACACACT TATTAGCTTA AAACTTTCTT TTATTCCTTT TGCTCTTTCA   
  
  
+ CTGCTCTCTT TTACCCAAAT ACGCCCGTCT TCAATTCTTC TCTCATTAAA TATGAAAAAG AAAGAGAGAA   
  
  
+ ATAGAGAGAG ATTTAAACCA GAAGGGGTGA TTCCTTTTTC TTGGTGGGCT TATTAATGCT CTGTCTACCC   
  
  
+ CATCTAGCCG AAAAATAGTA GCTTAAGACT CAACTGCTTA TTAGCATCTC TCCTCTAGCA ATAACCGAGG   
  
  
+ TAATAGCATA TTTTTCTCCC CTTAGCTTCT ACCTTGCTTT TTGTGACACT CAAACCTAAA CTGAGAGAGA   
  
  
+ GAAAGAGAAG ATAGAGGAGA GATAAATATT AGCGGGATTA GGGCAAATTT TGATATGCTG GGCTCTTCCT   
  
  
+ CATTCAACTC TGAGGAAGAA GATCAAGATC ATGCCAACCC ACCTCCGCCG CAACAACCAC CACCGTTACA   
  
  
+ GCTGCCACTT CCACGGCCAA GATTCAGCCA GTTAATATCA TCATCACCTC CAATAATATC AGCATCTTCT   
  
  
+ TCATCATTAA TCCATAATAA CCCTCGTCAG CTGCTAATAA GTTGCGGTGA ACTCATCTCC CGCTGTGACT   
  
  
+ TCTCCTCAGC TAACCGCCTT CTCTCTCTTC TCTCCGGCGA CTTCGCTTCC TCCTCCGGCG ACTCCACTGA   
  
  
+ ACGCCTTGTT TATTACTTCT CCAAGGCTTT GTTCCTCCGC CTTCGCCGAC AGTTTTCTCT ATCTTCTTCA   
  
  
+ ACTTTTACTG GTTATAGTTG TAACCCTAGT TGTTCTGCGT CTATTGCTCC ACTTTTTCTA ACCAACCCTA   
  
  
+ ATCCTAGACC ACCACAAGAA CTGCCGGTAC GAGTAACTAG CAGGGTTACT CCAAGTAGCT GCTCGTCTTA   
  
  
+ CCTAACCCTA AACCAGATCA CTCCATTTAT CCGGTTCACC CACCTGACGG CCAACCAGGC AATTTTGGAG   
  
  
+ GCTGTGGAGG GCTATAGGGC AGTCCACATC ATCGACATGG ACATCATGCA TGGAGTGCAG TGGCCTCCGC   
  
  
+ TCCTCCAAGC AATCGTGGAG CGGTCGGCCA CCCTTAGCCA ACCTCCCCCT GTGGTCCGTC TCAAAGGGGG   
  
  
+ AGGGCCGGAC CCGGACCTCT TACAGAGGAC GGGGGACCGA CTATGGAACT TTGCCCAATC ATTAGGACTA   
  
  
+ GAGTTCCATT TCCAGGGCCT CATGATCCCC GCCGAGTCAA CAGGTTTTGT TGAACCTGCT GAGGCAGCGG   
  
  
+ TCAATGCCCT AGCCTTGCAG GAGGCGGTGG GGTGTCAAGA AGGGGAGGCC CTTGCAGTTC ACTGCGGGGA   
  
  
+ CTATCTCCAC CGCCTCTTAA AAGAGTACGA CACAAGGCCT CTTAGACGGT TCCTCCACAA GGTCAAGACT   
  
  
+ CTGAACCCCA GGGTCTTGAC CTTGGGAGAG AGGGAAGCCG ACCACAACCA CCCTCTCTTC CTGCGGCGGT   
  
  
+ TCACCGAGGC AGTCAACCAC TACGGGGCAG TGTTTGACTC CCTGGAAGCA ACCCTTCCGC CGCATAGCCA   
  
  
+ AGAGAGGGTA GCTGTGGAGG AAGGGTGGTT CGGAGAGGAG ATTAAGGACG TGGTAGGAGA AGAAAGCGAG   
  
  
+ AGGAGAATAG AGAGGCACCA GAAGTTCGAG TCGTGGGAAG CATTGTTGAG GAGCTCAGGG TTCAAGAGTG   
  
  
+ TGCCCCTCAG CCCATTCTCG CTGTCCCAAG CTAAGCTGCT GCTGCGCCTC CATTACCCTT CCGAAGGGTA   
  
  
+ CCAACTCCAG GTTTTGCATA GTAATTGCTT GTTGCTTGGG TGGAAGAATC GCCCTCTTTT TTCTGTCTCT   
  
  
+ TCTTGGCAAT A  

- +Up\_Stream \_Len000AAAAAA AATTCAAAAG CATGAACCAC AACTACTAAA CACGAAACAT TGGTTACCAA   
  
  
- AATCATTTAT CATTAATCAG CATTGTAAGA CGCTAAAGCG AGTTAAATAT ATAGTTGAAA TCTTATACTC   
  
  
- ATTGGCATTT CTAGTTTGCA TCATCATCTC CACAGTATCC AAAAAAAAAT TTTTTTAAGC CTCATTCTTT   
  
  
- CATCCCATGT AATAAAAACG AACAATTTAA GGCGATCATA TCGTTATGCT CTACCTTGTA AACATTTTTG   
  
  
- ATGCTCTGCT TCGCAAACAT GTTTTGTTTG ATGGTATGAC GTCTAGTATA TTAAAAAACC CCCCAATACA   
  
  
- TAGAATCACT ACTGTAATTA ACTACCTATT GGGATAAGAT TTATAGACCT AGGTAGTAAT TGACTGAGCA   
  
  
- AAGTTTACAA TCCTAATACC CAGAACTGTA CCATATGTAC AGTTATCTAA ATACAATCTA CTTTTCAATA   
  
  
- TACTGCGAAA AATACTAAAA ACATGATCGT ATATCACGAA TTAACCTGAT ATATAAAAAT GTTCAACACG   
  
  
- AATTGTGGAA AATTAGTCTT TATAAATAAT ATACCTTTTT TAAAATAATT ATACACAAAT ACACAAATTT   
  
  
- TTTGTATGGG TCGGAACTTT ATCTGGCACT ATATCTTTAA CCTGGGTATT ATGAGTGTAA TATATACGAA   
  
  
- ATAACAATAT TAATTCCTAG TAACAACAAA ACGTTAATAC ACCGTTCTAT ATTAGGTGTT TCAAAATGTA   
  
  
- AATATAACGT TAATGAGACC ATTTACGATA ATTACATGTA AACTCTAATT CTGTATATAT ATTGGGAAAA   
  
  
- AAAAGGGAAG AAAAAAGGAA AAAGATAAAA CTTACCGTAG AATTTGGTTT CCTCGACATG TTCAATGCAA   
  
  
- TATCGTGAGT ACCAACTGAT TTGTTCTGTC TACTCTCAAA TTCAGTTCAG GAGGACCGTT ACTACAAACG   
  
  
- GTAAACCTGG TTATTTATGT AGTACATATA ATGGTATTAT CACACCAACT GAATTGTTAT GTATCCCCTA   
  
  
- ACGTATGTTT CCATTCGGAG TTTTTGAACA CGTATACGTA ACTTTAGGTA CTAGTTTTTT TAAGTTCTCG   
  
  
- TCCAACGTGT TTTTGTGCCT CTACTCTACT TTCTCTGTCT TTTGTGTATA TGTAAGTGTC ATCATCCCCA   
  
  
- TGACCATCTC GATACCGATC CGATTCTACT ACACTTTCTT TGTTATATTA CCATCATTGA CCATTATAAA   
  
  
- TATCCCGTGT ATATCCACGC CCGTATCCCA GCACAGAGAG TCAGAGTCCA TGCCAAGCTA CCCTGAGACG   
  
  
- CTATCTACAA TAGTTTAAGT TGGTAAGCTT GTAGAGGGTC TCTCTCTCTC TCTCTGACTC ACTCACTCAC   
  
  
- TCACCCACCC ACATACACAC ACTCAACAAA ACAAGTAAAT AATATCCAAG AGACGACGTA TATAGAGGTG   
  
  
- GTAATTTAAA TGGTTTTGAA CATGGTCGAC GAAATGAACG AAAGGAGTAA AAGGGGGAAA AAACAGAGAG   
  
  
- AGAGAGAGAG AGGGGGGGGG GGGGGGGGAG AGAGAGACAC AGACTTAGTC ATAGTCGGAT AGTCCTACGA   
  
  
- AGCGGAGGTC TTTCCGTAGT GAGTGTGTGA ATAATCGAAT TTTGAAAGAA AATAAGGAAA ACGAGAAAGT   
  
  
- GACGAGAGAA AATGGGTTTA TGCGGGCAGA AGTTAAGAAG AGAGTAATTT ATACTTTTTC TTTCTCTCTT   
  
  
- TATCTCTCTC TAAATTTGGT CTTCCCCACT AAGGAAAAAG AACCACCCGA ATAATTACGA GACAGATGGG   
  
  
- GTAGATCGGC TTTTTATCAT CGAATTCTGA GTTGACGAAT AATCGTAGAG AGGAGATCGT TATTGGCTCC   
  
  
- ATTATCGTAT AAAAAGAGGG GAATCGAAGA TGGAACGAAA AACACTGTGA GTTTGGATTT GACTCTCTCT   
  
  
- CTTTCTCTTC TATCTCCTCT CTATTTATAA TCGCCCTAAT CCCGTTTAAA ACTATACGAC CCGAGAAGGA   
  
  
- GTAAGTTGAG ACTCCTTCTT CTAGTTCTAG TACGGTTGGG TGGAGGCGGC GTTGTTGGTG GTGGCAATGT   
  
  
- CGACGGTGAA GGTGCCGGTT CTAAGTCGGT CAATTATAGT AGTAGTGGAG GTTATTATAG TCGTAGAAGA   
  
  
- AGTAGTAATT AGGTATTATT GGGAGCAGTC GACGATTATT CAACGCCACT TGAGTAGAGG GCGACACTGA   
  
  
- AGAGGAGTCG ATTGGCGGAA GAGAGAGAAG AGAGGCCGCT GAAGCGAAGG AGGAGGCCGC TGAGGTGACT   
  
  
- TGCGGAACAA ATAATGAAGA GGTTCCGAAA CAAGGAGGCG GAAGCGGCTG TCAAAAGAGA TAGAAGAAGT   
  
  
- TGAAAATGAC CAATATCAAC ATTGGGATCA ACAAGACGCA GATAACGAGG TGAAAAAGAT TGGTTGGGAT   
  
  
- TAGGATCTGG TGGTGTTCTT GACGGCCATG CTCATTGATC GTCCCAATGA GGTTCATCGA CGAGCAGAAT   
  
  
- GGATTGGGAT TTGGTCTAGT GAGGTAAATA GGCCAAGTGG GTGGACTGCC GGTTGGTCCG TTAAAACCTC   
  
  
- CGACACCTCC CGATATCCCG TCAGGTGTAG TAGCTGTACC TGTAGTACGT ACCTCACGTC ACCGGAGGCG   
  
  
- AGGAGGTTCG TTAGCACCTC GCCAGCCGGT GGGAATCGGT TGGAGGGGGA CACCAGGCAG AGTTTCCCCC   
  
  
- TCCCGGCCTG GGCCTGGAGA ATGTCTCCTG CCCCCTGGCT GATACCTTGA AACGGGTTAG TAATCCTGAT   
  
  
- CTCAAGGTAA AGGTCCCGGA GTACTAGGGG CGGCTCAGTT GTCCAAAACA ACTTGGACGA CTCCGTCGCC   
  
  
- AGTTACGGGA TCGGAACGTC CTCCGCCACC CCACAGTTCT TCCCCTCCGG GAACGTCAAG TGACGCCCCT   
  
  
- GATAGAGGTG GCGGAGAATT TTCTCATGCT GTGTTCCGGA GAATCTGCCA AGGAGGTGTT CCAGTTCTGA   
  
  
- GACTTGGGGT CCCAGAACTG GAACCCTCTC TCCCTTCGGC TGGTGTTGGT GGGAGAGAAG GACGCCGCCA   
  
  
- AGTGGCTCCG TCAGTTGGTG ATGCCCCGTC ACAAACTGAG GGACCTTCGT TGGGAAGGCG GCGTATCGGT   
  
  
- TCTCTCCCAT CGACACCTCC TTCCCACCAA GCCTCTCCTC TAATTCCTGC ACCATCCTCT TCTTTCGCTC   
  
  
- TCCTCTTATC TCTCCGTGGT CTTCAAGCTC AGCACCCTTC GTAACAACTC CTCGAGTCCC AAGTTCTCAC   
  
  
- ACGGGGAGTC GGGTAAGAGC GACAGGGTTC GATTCGACGA CGACGCGGAG GTAATGGGAA GGCTTCCCAT   
  
  
- GGTTGAGGTC CAAAACGTAT CATTAACGAA CAACGAACCC ACCTTCTTAG CGGGAGAAAA AAGACAGAGA   
  
  
- AGAACCGTTA T

+     TC-rich repeats

| Site Name | Organism | Position | Strand | Matrix score. | sequence | function |
| --- | --- | --- | --- | --- | --- | --- |
| TC-rich repeats | Nicotiana tabacum | 29 | + | 9 | GTTTTCTTAC | cis-acting element involved in defense and stress responsiveness |

>HU03G01737.1   
+ +Up\_Stream \_Len000TTTTTT TTAAGTTTTC GTACTTGGTG TTGATGATTT GTGCTTTGTA ACCAATGGTT   
  
  
+ TTAGTAAATA GTAATTAGTC GTAACATTCT GCGATTTCGC TCAATTTATA TATCAACTTT AGAATATGAG   
  
  
+ TAACCGTAAA GATCAAACGT AGTAGTAGAG GTGTCATAGG TTTTTTTTTA AAAAAATTCG GAGTAAGAAA   
  
  
+ GTAGGGTACA TTATTTTTGC TTGTTAAATT CCGCTAGTAT AGCAATACGA GATGGAACAT TTGTAAAAAC   
  
  
+ TACGAGACGA AGCGTTTGTA CAAAACAAAC TACCATACTG CAGATCATAT AATTTTTTGG GGGGTTATGT   
  
  
+ ATCTTAGTGA TGACATTAAT TGATGGATAA CCCTATTCTA AATATCTGGA TCCATCATTA ACTGACTCGT   
  
  
+ TTCAAATGTT AGGATTATGG GTCTTGACAT GGTATACATG TCAATAGATT TATGTTAGAT GAAAAGTTAT   
  
  
+ ATGACGCTTT TTATGATTTT TGTACTAGCA TATAGTGCTT AATTGGACTA TATATTTTTA CAAGTTGTGC   
  
  
+ TTAACACCTT TTAATCAGAA ATATTTATTA TATGGAAAAA ATTTTATTAA TATGTGTTTA TGTGTTTAAA   
  
  
+ AAACATACCC AGCCTTGAAA TAGACCGTGA TATAGAAATT GGACCCATAA TACTCACATT ATATATGCTT   
  
  
+ TATTGTTATA ATTAAGGATC ATTGTTGTTT TGCAATTATG TGGCAAGATA TAATCCACAA AGTTTTACAT   
  
  
+ TTATATTGCA ATTACTCTGG TAAATGCTAT TAATGTACAT TTGAGATTAA GACATATATA TAACCCTTTT   
  
  
+ TTTTCCCTTC TTTTTTCCTT TTTCTATTTT GAATGGCATC TTAAACCAAA GGAGCTGTAC AAGTTACGTT   
  
  
+ ATAGCACTCA TGGTTGACTA AACAAGACAG ATGAGAGTTT AAGTCAAGTC CTCCTGGCAA TGATGTTTGC   
  
  
+ CATTTGGACC AATAAATACA TCATGTATAT TACCATAATA GTGTGGTTGA CTTAACAATA CATAGGGGAT   
  
  
+ TGCATACAAA GGTAAGCCTC AAAAACTTGT GCATATGCAT TGAAATCCAT GATCAAAAAA ATTCAAGAGC   
  
  
+ AGGTTGCACA AAAACACGGA GATGAGATGA AAGAGACAGA AAACACATAT ACATTCACAG TAGTAGGGGT   
  
  
+ ACTGGTAGAG CTATGGCTAG GCTAAGATGA TGTGAAAGAA ACAATATAAT GGTAGTAACT GGTAATATTT   
  
  
+ ATAGGGCACA TATAGGTGCG GGCATAGGGT CGTGTCTCTC AGTCTCAGGT ACGGTTCGAT GGGACTCTGC   
  
  
+ GATAGATGTT ATCAAATTCA ACCATTCGAA CATCTCCCAG AGAGAGAGAG AGAGACTGAG TGAGTGAGTG   
  
  
+ AGTGGGTGGG TGTATGTGTG TGAGTTGTTT TGTTCATTTA TTATAGGTTC TCTGCTGCAT ATATCTCCAC   
  
  
+ CATTAAATTT ACCAAAACTT GTACCAGCTG CTTTACTTGC TTTCCTCATT TTCCCCCTTT TTTGTCTCTC   
  
  
+ TCTCTCTCTC TCCCCCCCCC CCCCCCCCTC TCTCTCTGTG TCTGAATCAG TATCAGCCTA TCAGGATGCT   
  
  
+ TCGCCTCCAG AAAGGCATCA CTCACACACT TATTAGCTTA AAACTTTCTT TTATTCCTTT TGCTCTTTCA   
  
  
+ CTGCTCTCTT TTACCCAAAT ACGCCCGTCT TCAATTCTTC TCTCATTAAA TATGAAAAAG AAAGAGAGAA   
  
  
+ ATAGAGAGAG ATTTAAACCA GAAGGGGTGA TTCCTTTTTC TTGGTGGGCT TATTAATGCT CTGTCTACCC   
  
  
+ CATCTAGCCG AAAAATAGTA GCTTAAGACT CAACTGCTTA TTAGCATCTC TCCTCTAGCA ATAACCGAGG   
  
  
+ TAATAGCATA TTTTTCTCCC CTTAGCTTCT ACCTTGCTTT TTGTGACACT CAAACCTAAA CTGAGAGAGA   
  
  
+ GAAAGAGAAG ATAGAGGAGA GATAAATATT AGCGGGATTA GGGCAAATTT TGATATGCTG GGCTCTTCCT   
  
  
+ CATTCAACTC TGAGGAAGAA GATCAAGATC ATGCCAACCC ACCTCCGCCG CAACAACCAC CACCGTTACA   
  
  
+ GCTGCCACTT CCACGGCCAA GATTCAGCCA GTTAATATCA TCATCACCTC CAATAATATC AGCATCTTCT   
  
  
+ TCATCATTAA TCCATAATAA CCCTCGTCAG CTGCTAATAA GTTGCGGTGA ACTCATCTCC CGCTGTGACT   
  
  
+ TCTCCTCAGC TAACCGCCTT CTCTCTCTTC TCTCCGGCGA CTTCGCTTCC TCCTCCGGCG ACTCCACTGA   
  
  
+ ACGCCTTGTT TATTACTTCT CCAAGGCTTT GTTCCTCCGC CTTCGCCGAC AGTTTTCTCT ATCTTCTTCA   
  
  
+ ACTTTTACTG GTTATAGTTG TAACCCTAGT TGTTCTGCGT CTATTGCTCC ACTTTTTCTA ACCAACCCTA   
  
  
+ ATCCTAGACC ACCACAAGAA CTGCCGGTAC GAGTAACTAG CAGGGTTACT CCAAGTAGCT GCTCGTCTTA   
  
  
+ CCTAACCCTA AACCAGATCA CTCCATTTAT CCGGTTCACC CACCTGACGG CCAACCAGGC AATTTTGGAG   
  
  
+ GCTGTGGAGG GCTATAGGGC AGTCCACATC ATCGACATGG ACATCATGCA TGGAGTGCAG TGGCCTCCGC   
  
  
+ TCCTCCAAGC AATCGTGGAG CGGTCGGCCA CCCTTAGCCA ACCTCCCCCT GTGGTCCGTC TCAAAGGGGG   
  
  
+ AGGGCCGGAC CCGGACCTCT TACAGAGGAC GGGGGACCGA CTATGGAACT TTGCCCAATC ATTAGGACTA   
  
  
+ GAGTTCCATT TCCAGGGCCT CATGATCCCC GCCGAGTCAA CAGGTTTTGT TGAACCTGCT GAGGCAGCGG   
  
  
+ TCAATGCCCT AGCCTTGCAG GAGGCGGTGG GGTGTCAAGA AGGGGAGGCC CTTGCAGTTC ACTGCGGGGA   
  
  
+ CTATCTCCAC CGCCTCTTAA AAGAGTACGA CACAAGGCCT CTTAGACGGT TCCTCCACAA GGTCAAGACT   
  
  
+ CTGAACCCCA GGGTCTTGAC CTTGGGAGAG AGGGAAGCCG ACCACAACCA CCCTCTCTTC CTGCGGCGGT   
  
  
+ TCACCGAGGC AGTCAACCAC TACGGGGCAG TGTTTGACTC CCTGGAAGCA ACCCTTCCGC CGCATAGCCA   
  
  
+ AGAGAGGGTA GCTGTGGAGG AAGGGTGGTT CGGAGAGGAG ATTAAGGACG TGGTAGGAGA AGAAAGCGAG   
  
  
+ AGGAGAATAG AGAGGCACCA GAAGTTCGAG TCGTGGGAAG CATTGTTGAG GAGCTCAGGG TTCAAGAGTG   
  
  
+ TGCCCCTCAG CCCATTCTCG CTGTCCCAAG CTAAGCTGCT GCTGCGCCTC CATTACCCTT CCGAAGGGTA   
  
  
+ CCAACTCCAG GTTTTGCATA GTAATTGCTT GTTGCTTGGG TGGAAGAATC GCCCTCTTTT TTCTGTCTCT   
  
  
+ TCTTGGCAAT A  

- +Up\_Stream \_Len000AAAAAA AATTCAAAAG CATGAACCAC AACTACTAAA CACGAAACAT TGGTTACCAA   
  
  
- AATCATTTAT CATTAATCAG CATTGTAAGA CGCTAAAGCG AGTTAAATAT ATAGTTGAAA TCTTATACTC   
  
  
- ATTGGCATTT CTAGTTTGCA TCATCATCTC CACAGTATCC AAAAAAAAAT TTTTTTAAGC CTCATTCTTT   
  
  
- CATCCCATGT AATAAAAACG AACAATTTAA GGCGATCATA TCGTTATGCT CTACCTTGTA AACATTTTTG   
  
  
- ATGCTCTGCT TCGCAAACAT GTTTTGTTTG ATGGTATGAC GTCTAGTATA TTAAAAAACC CCCCAATACA   
  
  
- TAGAATCACT ACTGTAATTA ACTACCTATT GGGATAAGAT TTATAGACCT AGGTAGTAAT TGACTGAGCA   
  
  
- AAGTTTACAA TCCTAATACC CAGAACTGTA CCATATGTAC AGTTATCTAA ATACAATCTA CTTTTCAATA   
  
  
- TACTGCGAAA AATACTAAAA ACATGATCGT ATATCACGAA TTAACCTGAT ATATAAAAAT GTTCAACACG   
  
  
- AATTGTGGAA AATTAGTCTT TATAAATAAT ATACCTTTTT TAAAATAATT ATACACAAAT ACACAAATTT   
  
  
- TTTGTATGGG TCGGAACTTT ATCTGGCACT ATATCTTTAA CCTGGGTATT ATGAGTGTAA TATATACGAA   
  
  
- ATAACAATAT TAATTCCTAG TAACAACAAA ACGTTAATAC ACCGTTCTAT ATTAGGTGTT TCAAAATGTA   
  
  
- AATATAACGT TAATGAGACC ATTTACGATA ATTACATGTA AACTCTAATT CTGTATATAT ATTGGGAAAA   
  
  
- AAAAGGGAAG AAAAAAGGAA AAAGATAAAA CTTACCGTAG AATTTGGTTT CCTCGACATG TTCAATGCAA   
  
  
- TATCGTGAGT ACCAACTGAT TTGTTCTGTC TACTCTCAAA TTCAGTTCAG GAGGACCGTT ACTACAAACG   
  
  
- GTAAACCTGG TTATTTATGT AGTACATATA ATGGTATTAT CACACCAACT GAATTGTTAT GTATCCCCTA   
  
  
- ACGTATGTTT CCATTCGGAG TTTTTGAACA CGTATACGTA ACTTTAGGTA CTAGTTTTTT TAAGTTCTCG   
  
  
- TCCAACGTGT TTTTGTGCCT CTACTCTACT TTCTCTGTCT TTTGTGTATA TGTAAGTGTC ATCATCCCCA   
  
  
- TGACCATCTC GATACCGATC CGATTCTACT ACACTTTCTT TGTTATATTA CCATCATTGA CCATTATAAA   
  
  
- TATCCCGTGT ATATCCACGC CCGTATCCCA GCACAGAGAG TCAGAGTCCA TGCCAAGCTA CCCTGAGACG   
  
  
- CTATCTACAA TAGTTTAAGT TGGTAAGCTT GTAGAGGGTC TCTCTCTCTC TCTCTGACTC ACTCACTCAC   
  
  
- TCACCCACCC ACATACACAC ACTCAACAAA ACAAGTAAAT AATATCCAAG AGACGACGTA TATAGAGGTG   
  
  
- GTAATTTAAA TGGTTTTGAA CATGGTCGAC GAAATGAACG AAAGGAGTAA AAGGGGGAAA AAACAGAGAG   
  
  
- AGAGAGAGAG AGGGGGGGGG GGGGGGGGAG AGAGAGACAC AGACTTAGTC ATAGTCGGAT AGTCCTACGA   
  
  
- AGCGGAGGTC TTTCCGTAGT GAGTGTGTGA ATAATCGAAT TTTGAAAGAA AATAAGGAAA ACGAGAAAGT   
  
  
- GACGAGAGAA AATGGGTTTA TGCGGGCAGA AGTTAAGAAG AGAGTAATTT ATACTTTTTC TTTCTCTCTT   
  
  
- TATCTCTCTC TAAATTTGGT CTTCCCCACT AAGGAAAAAG AACCACCCGA ATAATTACGA GACAGATGGG   
  
  
- GTAGATCGGC TTTTTATCAT CGAATTCTGA GTTGACGAAT AATCGTAGAG AGGAGATCGT TATTGGCTCC   
  
  
- ATTATCGTAT AAAAAGAGGG GAATCGAAGA TGGAACGAAA AACACTGTGA GTTTGGATTT GACTCTCTCT   
  
  
- CTTTCTCTTC TATCTCCTCT CTATTTATAA TCGCCCTAAT CCCGTTTAAA ACTATACGAC CCGAGAAGGA   
  
  
- GTAAGTTGAG ACTCCTTCTT CTAGTTCTAG TACGGTTGGG TGGAGGCGGC GTTGTTGGTG GTGGCAATGT   
  
  
- CGACGGTGAA GGTGCCGGTT CTAAGTCGGT CAATTATAGT AGTAGTGGAG GTTATTATAG TCGTAGAAGA   
  
  
- AGTAGTAATT AGGTATTATT GGGAGCAGTC GACGATTATT CAACGCCACT TGAGTAGAGG GCGACACTGA   
  
  
- AGAGGAGTCG ATTGGCGGAA GAGAGAGAAG AGAGGCCGCT GAAGCGAAGG AGGAGGCCGC TGAGGTGACT   
  
  
- TGCGGAACAA ATAATGAAGA GGTTCCGAAA CAAGGAGGCG GAAGCGGCTG TCAAAAGAGA TAGAAGAAGT   
  
  
- TGAAAATGAC CAATATCAAC ATTGGGATCA ACAAGACGCA GATAACGAGG TGAAAAAGAT TGGTTGGGAT   
  
  
- TAGGATCTGG TGGTGTTCTT GACGGCCATG CTCATTGATC GTCCCAATGA GGTTCATCGA CGAGCAGAAT   
  
  
- GGATTGGGAT TTGGTCTAGT GAGGTAAATA GGCCAAGTGG GTGGACTGCC GGTTGGTCCG TTAAAACCTC   
  
  
- CGACACCTCC CGATATCCCG TCAGGTGTAG TAGCTGTACC TGTAGTACGT ACCTCACGTC ACCGGAGGCG   
  
  
- AGGAGGTTCG TTAGCACCTC GCCAGCCGGT GGGAATCGGT TGGAGGGGGA CACCAGGCAG AGTTTCCCCC   
  
  
- TCCCGGCCTG GGCCTGGAGA ATGTCTCCTG CCCCCTGGCT GATACCTTGA AACGGGTTAG TAATCCTGAT   
  
  
- CTCAAGGTAA AGGTCCCGGA GTACTAGGGG CGGCTCAGTT GTCCAAAACA ACTTGGACGA CTCCGTCGCC   
  
  
- AGTTACGGGA TCGGAACGTC CTCCGCCACC CCACAGTTCT TCCCCTCCGG GAACGTCAAG TGACGCCCCT   
  
  
- GATAGAGGTG GCGGAGAATT TTCTCATGCT GTGTTCCGGA GAATCTGCCA AGGAGGTGTT CCAGTTCTGA   
  
  
- GACTTGGGGT CCCAGAACTG GAACCCTCTC TCCCTTCGGC TGGTGTTGGT GGGAGAGAAG GACGCCGCCA   
  
  
- AGTGGCTCCG TCAGTTGGTG ATGCCCCGTC ACAAACTGAG GGACCTTCGT TGGGAAGGCG GCGTATCGGT   
  
  
- TCTCTCCCAT CGACACCTCC TTCCCACCAA GCCTCTCCTC TAATTCCTGC ACCATCCTCT TCTTTCGCTC   
  
  
- TCCTCTTATC TCTCCGTGGT CTTCAAGCTC AGCACCCTTC GTAACAACTC CTCGAGTCCC AAGTTCTCAC   
  
  
- ACGGGGAGTC GGGTAAGAGC GACAGGGTTC GATTCGACGA CGACGCGGAG GTAATGGGAA GGCTTCCCAT   
  
  
- GGTTGAGGTC CAAAACGTAT CATTAACGAA CAACGAACCC ACCTTCTTAG CGGGAGAAAA AAGACAGAGA   
  
  
- AGAACCGTTA T

+     TCA

| Site Name | Organism | Position | Strand | Matrix score. | sequence | function |
| --- | --- | --- | --- | --- | --- | --- |
| TCA | Pisum sativum | 2169 | + | 9 | TCATCTTCAT |  |

>HU03G01737.1   
+ +Up\_Stream \_Len000TTTTTT TTAAGTTTTC GTACTTGGTG TTGATGATTT GTGCTTTGTA ACCAATGGTT   
  
  
+ TTAGTAAATA GTAATTAGTC GTAACATTCT GCGATTTCGC TCAATTTATA TATCAACTTT AGAATATGAG   
  
  
+ TAACCGTAAA GATCAAACGT AGTAGTAGAG GTGTCATAGG TTTTTTTTTA AAAAAATTCG GAGTAAGAAA   
  
  
+ GTAGGGTACA TTATTTTTGC TTGTTAAATT CCGCTAGTAT AGCAATACGA GATGGAACAT TTGTAAAAAC   
  
  
+ TACGAGACGA AGCGTTTGTA CAAAACAAAC TACCATACTG CAGATCATAT AATTTTTTGG GGGGTTATGT   
  
  
+ ATCTTAGTGA TGACATTAAT TGATGGATAA CCCTATTCTA AATATCTGGA TCCATCATTA ACTGACTCGT   
  
  
+ TTCAAATGTT AGGATTATGG GTCTTGACAT GGTATACATG TCAATAGATT TATGTTAGAT GAAAAGTTAT   
  
  
+ ATGACGCTTT TTATGATTTT TGTACTAGCA TATAGTGCTT AATTGGACTA TATATTTTTA CAAGTTGTGC   
  
  
+ TTAACACCTT TTAATCAGAA ATATTTATTA TATGGAAAAA ATTTTATTAA TATGTGTTTA TGTGTTTAAA   
  
  
+ AAACATACCC AGCCTTGAAA TAGACCGTGA TATAGAAATT GGACCCATAA TACTCACATT ATATATGCTT   
  
  
+ TATTGTTATA ATTAAGGATC ATTGTTGTTT TGCAATTATG TGGCAAGATA TAATCCACAA AGTTTTACAT   
  
  
+ TTATATTGCA ATTACTCTGG TAAATGCTAT TAATGTACAT TTGAGATTAA GACATATATA TAACCCTTTT   
  
  
+ TTTTCCCTTC TTTTTTCCTT TTTCTATTTT GAATGGCATC TTAAACCAAA GGAGCTGTAC AAGTTACGTT   
  
  
+ ATAGCACTCA TGGTTGACTA AACAAGACAG ATGAGAGTTT AAGTCAAGTC CTCCTGGCAA TGATGTTTGC   
  
  
+ CATTTGGACC AATAAATACA TCATGTATAT TACCATAATA GTGTGGTTGA CTTAACAATA CATAGGGGAT   
  
  
+ TGCATACAAA GGTAAGCCTC AAAAACTTGT GCATATGCAT TGAAATCCAT GATCAAAAAA ATTCAAGAGC   
  
  
+ AGGTTGCACA AAAACACGGA GATGAGATGA AAGAGACAGA AAACACATAT ACATTCACAG TAGTAGGGGT   
  
  
+ ACTGGTAGAG CTATGGCTAG GCTAAGATGA TGTGAAAGAA ACAATATAAT GGTAGTAACT GGTAATATTT   
  
  
+ ATAGGGCACA TATAGGTGCG GGCATAGGGT CGTGTCTCTC AGTCTCAGGT ACGGTTCGAT GGGACTCTGC   
  
  
+ GATAGATGTT ATCAAATTCA ACCATTCGAA CATCTCCCAG AGAGAGAGAG AGAGACTGAG TGAGTGAGTG   
  
  
+ AGTGGGTGGG TGTATGTGTG TGAGTTGTTT TGTTCATTTA TTATAGGTTC TCTGCTGCAT ATATCTCCAC   
  
  
+ CATTAAATTT ACCAAAACTT GTACCAGCTG CTTTACTTGC TTTCCTCATT TTCCCCCTTT TTTGTCTCTC   
  
  
+ TCTCTCTCTC TCCCCCCCCC CCCCCCCCTC TCTCTCTGTG TCTGAATCAG TATCAGCCTA TCAGGATGCT   
  
  
+ TCGCCTCCAG AAAGGCATCA CTCACACACT TATTAGCTTA AAACTTTCTT TTATTCCTTT TGCTCTTTCA   
  
  
+ CTGCTCTCTT TTACCCAAAT ACGCCCGTCT TCAATTCTTC TCTCATTAAA TATGAAAAAG AAAGAGAGAA   
  
  
+ ATAGAGAGAG ATTTAAACCA GAAGGGGTGA TTCCTTTTTC TTGGTGGGCT TATTAATGCT CTGTCTACCC   
  
  
+ CATCTAGCCG AAAAATAGTA GCTTAAGACT CAACTGCTTA TTAGCATCTC TCCTCTAGCA ATAACCGAGG   
  
  
+ TAATAGCATA TTTTTCTCCC CTTAGCTTCT ACCTTGCTTT TTGTGACACT CAAACCTAAA CTGAGAGAGA   
  
  
+ GAAAGAGAAG ATAGAGGAGA GATAAATATT AGCGGGATTA GGGCAAATTT TGATATGCTG GGCTCTTCCT   
  
  
+ CATTCAACTC TGAGGAAGAA GATCAAGATC ATGCCAACCC ACCTCCGCCG CAACAACCAC CACCGTTACA   
  
  
+ GCTGCCACTT CCACGGCCAA GATTCAGCCA GTTAATATCA TCATCACCTC CAATAATATC AGCATCTTCT   
  
  
+ TCATCATTAA TCCATAATAA CCCTCGTCAG CTGCTAATAA GTTGCGGTGA ACTCATCTCC CGCTGTGACT   
  
  
+ TCTCCTCAGC TAACCGCCTT CTCTCTCTTC TCTCCGGCGA CTTCGCTTCC TCCTCCGGCG ACTCCACTGA   
  
  
+ ACGCCTTGTT TATTACTTCT CCAAGGCTTT GTTCCTCCGC CTTCGCCGAC AGTTTTCTCT ATCTTCTTCA   
  
  
+ ACTTTTACTG GTTATAGTTG TAACCCTAGT TGTTCTGCGT CTATTGCTCC ACTTTTTCTA ACCAACCCTA   
  
  
+ ATCCTAGACC ACCACAAGAA CTGCCGGTAC GAGTAACTAG CAGGGTTACT CCAAGTAGCT GCTCGTCTTA   
  
  
+ CCTAACCCTA AACCAGATCA CTCCATTTAT CCGGTTCACC CACCTGACGG CCAACCAGGC AATTTTGGAG   
  
  
+ GCTGTGGAGG GCTATAGGGC AGTCCACATC ATCGACATGG ACATCATGCA TGGAGTGCAG TGGCCTCCGC   
  
  
+ TCCTCCAAGC AATCGTGGAG CGGTCGGCCA CCCTTAGCCA ACCTCCCCCT GTGGTCCGTC TCAAAGGGGG   
  
  
+ AGGGCCGGAC CCGGACCTCT TACAGAGGAC GGGGGACCGA CTATGGAACT TTGCCCAATC ATTAGGACTA   
  
  
+ GAGTTCCATT TCCAGGGCCT CATGATCCCC GCCGAGTCAA CAGGTTTTGT TGAACCTGCT GAGGCAGCGG   
  
  
+ TCAATGCCCT AGCCTTGCAG GAGGCGGTGG GGTGTCAAGA AGGGGAGGCC CTTGCAGTTC ACTGCGGGGA   
  
  
+ CTATCTCCAC CGCCTCTTAA AAGAGTACGA CACAAGGCCT CTTAGACGGT TCCTCCACAA GGTCAAGACT   
  
  
+ CTGAACCCCA GGGTCTTGAC CTTGGGAGAG AGGGAAGCCG ACCACAACCA CCCTCTCTTC CTGCGGCGGT   
  
  
+ TCACCGAGGC AGTCAACCAC TACGGGGCAG TGTTTGACTC CCTGGAAGCA ACCCTTCCGC CGCATAGCCA   
  
  
+ AGAGAGGGTA GCTGTGGAGG AAGGGTGGTT CGGAGAGGAG ATTAAGGACG TGGTAGGAGA AGAAAGCGAG   
  
  
+ AGGAGAATAG AGAGGCACCA GAAGTTCGAG TCGTGGGAAG CATTGTTGAG GAGCTCAGGG TTCAAGAGTG   
  
  
+ TGCCCCTCAG CCCATTCTCG CTGTCCCAAG CTAAGCTGCT GCTGCGCCTC CATTACCCTT CCGAAGGGTA   
  
  
+ CCAACTCCAG GTTTTGCATA GTAATTGCTT GTTGCTTGGG TGGAAGAATC GCCCTCTTTT TTCTGTCTCT   
  
  
+ TCTTGGCAAT A  

- +Up\_Stream \_Len000AAAAAA AATTCAAAAG CATGAACCAC AACTACTAAA CACGAAACAT TGGTTACCAA   
  
  
- AATCATTTAT CATTAATCAG CATTGTAAGA CGCTAAAGCG AGTTAAATAT ATAGTTGAAA TCTTATACTC   
  
  
- ATTGGCATTT CTAGTTTGCA TCATCATCTC CACAGTATCC AAAAAAAAAT TTTTTTAAGC CTCATTCTTT   
  
  
- CATCCCATGT AATAAAAACG AACAATTTAA GGCGATCATA TCGTTATGCT CTACCTTGTA AACATTTTTG   
  
  
- ATGCTCTGCT TCGCAAACAT GTTTTGTTTG ATGGTATGAC GTCTAGTATA TTAAAAAACC CCCCAATACA   
  
  
- TAGAATCACT ACTGTAATTA ACTACCTATT GGGATAAGAT TTATAGACCT AGGTAGTAAT TGACTGAGCA   
  
  
- AAGTTTACAA TCCTAATACC CAGAACTGTA CCATATGTAC AGTTATCTAA ATACAATCTA CTTTTCAATA   
  
  
- TACTGCGAAA AATACTAAAA ACATGATCGT ATATCACGAA TTAACCTGAT ATATAAAAAT GTTCAACACG   
  
  
- AATTGTGGAA AATTAGTCTT TATAAATAAT ATACCTTTTT TAAAATAATT ATACACAAAT ACACAAATTT   
  
  
- TTTGTATGGG TCGGAACTTT ATCTGGCACT ATATCTTTAA CCTGGGTATT ATGAGTGTAA TATATACGAA   
  
  
- ATAACAATAT TAATTCCTAG TAACAACAAA ACGTTAATAC ACCGTTCTAT ATTAGGTGTT TCAAAATGTA   
  
  
- AATATAACGT TAATGAGACC ATTTACGATA ATTACATGTA AACTCTAATT CTGTATATAT ATTGGGAAAA   
  
  
- AAAAGGGAAG AAAAAAGGAA AAAGATAAAA CTTACCGTAG AATTTGGTTT CCTCGACATG TTCAATGCAA   
  
  
- TATCGTGAGT ACCAACTGAT TTGTTCTGTC TACTCTCAAA TTCAGTTCAG GAGGACCGTT ACTACAAACG   
  
  
- GTAAACCTGG TTATTTATGT AGTACATATA ATGGTATTAT CACACCAACT GAATTGTTAT GTATCCCCTA   
  
  
- ACGTATGTTT CCATTCGGAG TTTTTGAACA CGTATACGTA ACTTTAGGTA CTAGTTTTTT TAAGTTCTCG   
  
  
- TCCAACGTGT TTTTGTGCCT CTACTCTACT TTCTCTGTCT TTTGTGTATA TGTAAGTGTC ATCATCCCCA   
  
  
- TGACCATCTC GATACCGATC CGATTCTACT ACACTTTCTT TGTTATATTA CCATCATTGA CCATTATAAA   
  
  
- TATCCCGTGT ATATCCACGC CCGTATCCCA GCACAGAGAG TCAGAGTCCA TGCCAAGCTA CCCTGAGACG   
  
  
- CTATCTACAA TAGTTTAAGT TGGTAAGCTT GTAGAGGGTC TCTCTCTCTC TCTCTGACTC ACTCACTCAC   
  
  
- TCACCCACCC ACATACACAC ACTCAACAAA ACAAGTAAAT AATATCCAAG AGACGACGTA TATAGAGGTG   
  
  
- GTAATTTAAA TGGTTTTGAA CATGGTCGAC GAAATGAACG AAAGGAGTAA AAGGGGGAAA AAACAGAGAG   
  
  
- AGAGAGAGAG AGGGGGGGGG GGGGGGGGAG AGAGAGACAC AGACTTAGTC ATAGTCGGAT AGTCCTACGA   
  
  
- AGCGGAGGTC TTTCCGTAGT GAGTGTGTGA ATAATCGAAT TTTGAAAGAA AATAAGGAAA ACGAGAAAGT   
  
  
- GACGAGAGAA AATGGGTTTA TGCGGGCAGA AGTTAAGAAG AGAGTAATTT ATACTTTTTC TTTCTCTCTT   
  
  
- TATCTCTCTC TAAATTTGGT CTTCCCCACT AAGGAAAAAG AACCACCCGA ATAATTACGA GACAGATGGG   
  
  
- GTAGATCGGC TTTTTATCAT CGAATTCTGA GTTGACGAAT AATCGTAGAG AGGAGATCGT TATTGGCTCC   
  
  
- ATTATCGTAT AAAAAGAGGG GAATCGAAGA TGGAACGAAA AACACTGTGA GTTTGGATTT GACTCTCTCT   
  
  
- CTTTCTCTTC TATCTCCTCT CTATTTATAA TCGCCCTAAT CCCGTTTAAA ACTATACGAC CCGAGAAGGA   
  
  
- GTAAGTTGAG ACTCCTTCTT CTAGTTCTAG TACGGTTGGG TGGAGGCGGC GTTGTTGGTG GTGGCAATGT   
  
  
- CGACGGTGAA GGTGCCGGTT CTAAGTCGGT CAATTATAGT AGTAGTGGAG GTTATTATAG TCGTAGAAGA   
  
  
- AGTAGTAATT AGGTATTATT GGGAGCAGTC GACGATTATT CAACGCCACT TGAGTAGAGG GCGACACTGA   
  
  
- AGAGGAGTCG ATTGGCGGAA GAGAGAGAAG AGAGGCCGCT GAAGCGAAGG AGGAGGCCGC TGAGGTGACT   
  
  
- TGCGGAACAA ATAATGAAGA GGTTCCGAAA CAAGGAGGCG GAAGCGGCTG TCAAAAGAGA TAGAAGAAGT   
  
  
- TGAAAATGAC CAATATCAAC ATTGGGATCA ACAAGACGCA GATAACGAGG TGAAAAAGAT TGGTTGGGAT   
  
  
- TAGGATCTGG TGGTGTTCTT GACGGCCATG CTCATTGATC GTCCCAATGA GGTTCATCGA CGAGCAGAAT   
  
  
- GGATTGGGAT TTGGTCTAGT GAGGTAAATA GGCCAAGTGG GTGGACTGCC GGTTGGTCCG TTAAAACCTC   
  
  
- CGACACCTCC CGATATCCCG TCAGGTGTAG TAGCTGTACC TGTAGTACGT ACCTCACGTC ACCGGAGGCG   
  
  
- AGGAGGTTCG TTAGCACCTC GCCAGCCGGT GGGAATCGGT TGGAGGGGGA CACCAGGCAG AGTTTCCCCC   
  
  
- TCCCGGCCTG GGCCTGGAGA ATGTCTCCTG CCCCCTGGCT GATACCTTGA AACGGGTTAG TAATCCTGAT   
  
  
- CTCAAGGTAA AGGTCCCGGA GTACTAGGGG CGGCTCAGTT GTCCAAAACA ACTTGGACGA CTCCGTCGCC   
  
  
- AGTTACGGGA TCGGAACGTC CTCCGCCACC CCACAGTTCT TCCCCTCCGG GAACGTCAAG TGACGCCCCT   
  
  
- GATAGAGGTG GCGGAGAATT TTCTCATGCT GTGTTCCGGA GAATCTGCCA AGGAGGTGTT CCAGTTCTGA   
  
  
- GACTTGGGGT CCCAGAACTG GAACCCTCTC TCCCTTCGGC TGGTGTTGGT GGGAGAGAAG GACGCCGCCA   
  
  
- AGTGGCTCCG TCAGTTGGTG ATGCCCCGTC ACAAACTGAG GGACCTTCGT TGGGAAGGCG GCGTATCGGT   
  
  
- TCTCTCCCAT CGACACCTCC TTCCCACCAA GCCTCTCCTC TAATTCCTGC ACCATCCTCT TCTTTCGCTC   
  
  
- TCCTCTTATC TCTCCGTGGT CTTCAAGCTC AGCACCCTTC GTAACAACTC CTCGAGTCCC AAGTTCTCAC   
  
  
- ACGGGGAGTC GGGTAAGAGC GACAGGGTTC GATTCGACGA CGACGCGGAG GTAATGGGAA GGCTTCCCAT   
  
  
- GGTTGAGGTC CAAAACGTAT CATTAACGAA CAACGAACCC ACCTTCTTAG CGGGAGAAAA AAGACAGAGA   
  
  
- AGAACCGTTA T

+     TCA-element

| Site Name | Organism | Position | Strand | Matrix score. | sequence | function |
| --- | --- | --- | --- | --- | --- | --- |
| TCA-element | Nicotiana tabacum | 3416 | + | 9 | CCATCTTTTT | cis-acting element involved in salicylic acid responsiveness |
| TCA-element | Nicotiana tabacum | 850 | + | 9 | CCATCTTTTT | cis-acting element involved in salicylic acid responsiveness |

>HU03G01737.1   
+ +Up\_Stream \_Len000TTTTTT TTAAGTTTTC GTACTTGGTG TTGATGATTT GTGCTTTGTA ACCAATGGTT   
  
  
+ TTAGTAAATA GTAATTAGTC GTAACATTCT GCGATTTCGC TCAATTTATA TATCAACTTT AGAATATGAG   
  
  
+ TAACCGTAAA GATCAAACGT AGTAGTAGAG GTGTCATAGG TTTTTTTTTA AAAAAATTCG GAGTAAGAAA   
  
  
+ GTAGGGTACA TTATTTTTGC TTGTTAAATT CCGCTAGTAT AGCAATACGA GATGGAACAT TTGTAAAAAC   
  
  
+ TACGAGACGA AGCGTTTGTA CAAAACAAAC TACCATACTG CAGATCATAT AATTTTTTGG GGGGTTATGT   
  
  
+ ATCTTAGTGA TGACATTAAT TGATGGATAA CCCTATTCTA AATATCTGGA TCCATCATTA ACTGACTCGT   
  
  
+ TTCAAATGTT AGGATTATGG GTCTTGACAT GGTATACATG TCAATAGATT TATGTTAGAT GAAAAGTTAT   
  
  
+ ATGACGCTTT TTATGATTTT TGTACTAGCA TATAGTGCTT AATTGGACTA TATATTTTTA CAAGTTGTGC   
  
  
+ TTAACACCTT TTAATCAGAA ATATTTATTA TATGGAAAAA ATTTTATTAA TATGTGTTTA TGTGTTTAAA   
  
  
+ AAACATACCC AGCCTTGAAA TAGACCGTGA TATAGAAATT GGACCCATAA TACTCACATT ATATATGCTT   
  
  
+ TATTGTTATA ATTAAGGATC ATTGTTGTTT TGCAATTATG TGGCAAGATA TAATCCACAA AGTTTTACAT   
  
  
+ TTATATTGCA ATTACTCTGG TAAATGCTAT TAATGTACAT TTGAGATTAA GACATATATA TAACCCTTTT   
  
  
+ TTTTCCCTTC TTTTTTCCTT TTTCTATTTT GAATGGCATC TTAAACCAAA GGAGCTGTAC AAGTTACGTT   
  
  
+ ATAGCACTCA TGGTTGACTA AACAAGACAG ATGAGAGTTT AAGTCAAGTC CTCCTGGCAA TGATGTTTGC   
  
  
+ CATTTGGACC AATAAATACA TCATGTATAT TACCATAATA GTGTGGTTGA CTTAACAATA CATAGGGGAT   
  
  
+ TGCATACAAA GGTAAGCCTC AAAAACTTGT GCATATGCAT TGAAATCCAT GATCAAAAAA ATTCAAGAGC   
  
  
+ AGGTTGCACA AAAACACGGA GATGAGATGA AAGAGACAGA AAACACATAT ACATTCACAG TAGTAGGGGT   
  
  
+ ACTGGTAGAG CTATGGCTAG GCTAAGATGA TGTGAAAGAA ACAATATAAT GGTAGTAACT GGTAATATTT   
  
  
+ ATAGGGCACA TATAGGTGCG GGCATAGGGT CGTGTCTCTC AGTCTCAGGT ACGGTTCGAT GGGACTCTGC   
  
  
+ GATAGATGTT ATCAAATTCA ACCATTCGAA CATCTCCCAG AGAGAGAGAG AGAGACTGAG TGAGTGAGTG   
  
  
+ AGTGGGTGGG TGTATGTGTG TGAGTTGTTT TGTTCATTTA TTATAGGTTC TCTGCTGCAT ATATCTCCAC   
  
  
+ CATTAAATTT ACCAAAACTT GTACCAGCTG CTTTACTTGC TTTCCTCATT TTCCCCCTTT TTTGTCTCTC   
  
  
+ TCTCTCTCTC TCCCCCCCCC CCCCCCCCTC TCTCTCTGTG TCTGAATCAG TATCAGCCTA TCAGGATGCT   
  
  
+ TCGCCTCCAG AAAGGCATCA CTCACACACT TATTAGCTTA AAACTTTCTT TTATTCCTTT TGCTCTTTCA   
  
  
+ CTGCTCTCTT TTACCCAAAT ACGCCCGTCT TCAATTCTTC TCTCATTAAA TATGAAAAAG AAAGAGAGAA   
  
  
+ ATAGAGAGAG ATTTAAACCA GAAGGGGTGA TTCCTTTTTC TTGGTGGGCT TATTAATGCT CTGTCTACCC   
  
  
+ CATCTAGCCG AAAAATAGTA GCTTAAGACT CAACTGCTTA TTAGCATCTC TCCTCTAGCA ATAACCGAGG   
  
  
+ TAATAGCATA TTTTTCTCCC CTTAGCTTCT ACCTTGCTTT TTGTGACACT CAAACCTAAA CTGAGAGAGA   
  
  
+ GAAAGAGAAG ATAGAGGAGA GATAAATATT AGCGGGATTA GGGCAAATTT TGATATGCTG GGCTCTTCCT   
  
  
+ CATTCAACTC TGAGGAAGAA GATCAAGATC ATGCCAACCC ACCTCCGCCG CAACAACCAC CACCGTTACA   
  
  
+ GCTGCCACTT CCACGGCCAA GATTCAGCCA GTTAATATCA TCATCACCTC CAATAATATC AGCATCTTCT   
  
  
+ TCATCATTAA TCCATAATAA CCCTCGTCAG CTGCTAATAA GTTGCGGTGA ACTCATCTCC CGCTGTGACT   
  
  
+ TCTCCTCAGC TAACCGCCTT CTCTCTCTTC TCTCCGGCGA CTTCGCTTCC TCCTCCGGCG ACTCCACTGA   
  
  
+ ACGCCTTGTT TATTACTTCT CCAAGGCTTT GTTCCTCCGC CTTCGCCGAC AGTTTTCTCT ATCTTCTTCA   
  
  
+ ACTTTTACTG GTTATAGTTG TAACCCTAGT TGTTCTGCGT CTATTGCTCC ACTTTTTCTA ACCAACCCTA   
  
  
+ ATCCTAGACC ACCACAAGAA CTGCCGGTAC GAGTAACTAG CAGGGTTACT CCAAGTAGCT GCTCGTCTTA   
  
  
+ CCTAACCCTA AACCAGATCA CTCCATTTAT CCGGTTCACC CACCTGACGG CCAACCAGGC AATTTTGGAG   
  
  
+ GCTGTGGAGG GCTATAGGGC AGTCCACATC ATCGACATGG ACATCATGCA TGGAGTGCAG TGGCCTCCGC   
  
  
+ TCCTCCAAGC AATCGTGGAG CGGTCGGCCA CCCTTAGCCA ACCTCCCCCT GTGGTCCGTC TCAAAGGGGG   
  
  
+ AGGGCCGGAC CCGGACCTCT TACAGAGGAC GGGGGACCGA CTATGGAACT TTGCCCAATC ATTAGGACTA   
  
  
+ GAGTTCCATT TCCAGGGCCT CATGATCCCC GCCGAGTCAA CAGGTTTTGT TGAACCTGCT GAGGCAGCGG   
  
  
+ TCAATGCCCT AGCCTTGCAG GAGGCGGTGG GGTGTCAAGA AGGGGAGGCC CTTGCAGTTC ACTGCGGGGA   
  
  
+ CTATCTCCAC CGCCTCTTAA AAGAGTACGA CACAAGGCCT CTTAGACGGT TCCTCCACAA GGTCAAGACT   
  
  
+ CTGAACCCCA GGGTCTTGAC CTTGGGAGAG AGGGAAGCCG ACCACAACCA CCCTCTCTTC CTGCGGCGGT   
  
  
+ TCACCGAGGC AGTCAACCAC TACGGGGCAG TGTTTGACTC CCTGGAAGCA ACCCTTCCGC CGCATAGCCA   
  
  
+ AGAGAGGGTA GCTGTGGAGG AAGGGTGGTT CGGAGAGGAG ATTAAGGACG TGGTAGGAGA AGAAAGCGAG   
  
  
+ AGGAGAATAG AGAGGCACCA GAAGTTCGAG TCGTGGGAAG CATTGTTGAG GAGCTCAGGG TTCAAGAGTG   
  
  
+ TGCCCCTCAG CCCATTCTCG CTGTCCCAAG CTAAGCTGCT GCTGCGCCTC CATTACCCTT CCGAAGGGTA   
  
  
+ CCAACTCCAG GTTTTGCATA GTAATTGCTT GTTGCTTGGG TGGAAGAATC GCCCTCTTTT TTCTGTCTCT   
  
  
+ TCTTGGCAAT A  

- +Up\_Stream \_Len000AAAAAA AATTCAAAAG CATGAACCAC AACTACTAAA CACGAAACAT TGGTTACCAA   
  
  
- AATCATTTAT CATTAATCAG CATTGTAAGA CGCTAAAGCG AGTTAAATAT ATAGTTGAAA TCTTATACTC   
  
  
- ATTGGCATTT CTAGTTTGCA TCATCATCTC CACAGTATCC AAAAAAAAAT TTTTTTAAGC CTCATTCTTT   
  
  
- CATCCCATGT AATAAAAACG AACAATTTAA GGCGATCATA TCGTTATGCT CTACCTTGTA AACATTTTTG   
  
  
- ATGCTCTGCT TCGCAAACAT GTTTTGTTTG ATGGTATGAC GTCTAGTATA TTAAAAAACC CCCCAATACA   
  
  
- TAGAATCACT ACTGTAATTA ACTACCTATT GGGATAAGAT TTATAGACCT AGGTAGTAAT TGACTGAGCA   
  
  
- AAGTTTACAA TCCTAATACC CAGAACTGTA CCATATGTAC AGTTATCTAA ATACAATCTA CTTTTCAATA   
  
  
- TACTGCGAAA AATACTAAAA ACATGATCGT ATATCACGAA TTAACCTGAT ATATAAAAAT GTTCAACACG   
  
  
- AATTGTGGAA AATTAGTCTT TATAAATAAT ATACCTTTTT TAAAATAATT ATACACAAAT ACACAAATTT   
  
  
- TTTGTATGGG TCGGAACTTT ATCTGGCACT ATATCTTTAA CCTGGGTATT ATGAGTGTAA TATATACGAA   
  
  
- ATAACAATAT TAATTCCTAG TAACAACAAA ACGTTAATAC ACCGTTCTAT ATTAGGTGTT TCAAAATGTA   
  
  
- AATATAACGT TAATGAGACC ATTTACGATA ATTACATGTA AACTCTAATT CTGTATATAT ATTGGGAAAA   
  
  
- AAAAGGGAAG AAAAAAGGAA AAAGATAAAA CTTACCGTAG AATTTGGTTT CCTCGACATG TTCAATGCAA   
  
  
- TATCGTGAGT ACCAACTGAT TTGTTCTGTC TACTCTCAAA TTCAGTTCAG GAGGACCGTT ACTACAAACG   
  
  
- GTAAACCTGG TTATTTATGT AGTACATATA ATGGTATTAT CACACCAACT GAATTGTTAT GTATCCCCTA   
  
  
- ACGTATGTTT CCATTCGGAG TTTTTGAACA CGTATACGTA ACTTTAGGTA CTAGTTTTTT TAAGTTCTCG   
  
  
- TCCAACGTGT TTTTGTGCCT CTACTCTACT TTCTCTGTCT TTTGTGTATA TGTAAGTGTC ATCATCCCCA   
  
  
- TGACCATCTC GATACCGATC CGATTCTACT ACACTTTCTT TGTTATATTA CCATCATTGA CCATTATAAA   
  
  
- TATCCCGTGT ATATCCACGC CCGTATCCCA GCACAGAGAG TCAGAGTCCA TGCCAAGCTA CCCTGAGACG   
  
  
- CTATCTACAA TAGTTTAAGT TGGTAAGCTT GTAGAGGGTC TCTCTCTCTC TCTCTGACTC ACTCACTCAC   
  
  
- TCACCCACCC ACATACACAC ACTCAACAAA ACAAGTAAAT AATATCCAAG AGACGACGTA TATAGAGGTG   
  
  
- GTAATTTAAA TGGTTTTGAA CATGGTCGAC GAAATGAACG AAAGGAGTAA AAGGGGGAAA AAACAGAGAG   
  
  
- AGAGAGAGAG AGGGGGGGGG GGGGGGGGAG AGAGAGACAC AGACTTAGTC ATAGTCGGAT AGTCCTACGA   
  
  
- AGCGGAGGTC TTTCCGTAGT GAGTGTGTGA ATAATCGAAT TTTGAAAGAA AATAAGGAAA ACGAGAAAGT   
  
  
- GACGAGAGAA AATGGGTTTA TGCGGGCAGA AGTTAAGAAG AGAGTAATTT ATACTTTTTC TTTCTCTCTT   
  
  
- TATCTCTCTC TAAATTTGGT CTTCCCCACT AAGGAAAAAG AACCACCCGA ATAATTACGA GACAGATGGG   
  
  
- GTAGATCGGC TTTTTATCAT CGAATTCTGA GTTGACGAAT AATCGTAGAG AGGAGATCGT TATTGGCTCC   
  
  
- ATTATCGTAT AAAAAGAGGG GAATCGAAGA TGGAACGAAA AACACTGTGA GTTTGGATTT GACTCTCTCT   
  
  
- CTTTCTCTTC TATCTCCTCT CTATTTATAA TCGCCCTAAT CCCGTTTAAA ACTATACGAC CCGAGAAGGA   
  
  
- GTAAGTTGAG ACTCCTTCTT CTAGTTCTAG TACGGTTGGG TGGAGGCGGC GTTGTTGGTG GTGGCAATGT   
  
  
- CGACGGTGAA GGTGCCGGTT CTAAGTCGGT CAATTATAGT AGTAGTGGAG GTTATTATAG TCGTAGAAGA   
  
  
- AGTAGTAATT AGGTATTATT GGGAGCAGTC GACGATTATT CAACGCCACT TGAGTAGAGG GCGACACTGA   
  
  
- AGAGGAGTCG ATTGGCGGAA GAGAGAGAAG AGAGGCCGCT GAAGCGAAGG AGGAGGCCGC TGAGGTGACT   
  
  
- TGCGGAACAA ATAATGAAGA GGTTCCGAAA CAAGGAGGCG GAAGCGGCTG TCAAAAGAGA TAGAAGAAGT   
  
  
- TGAAAATGAC CAATATCAAC ATTGGGATCA ACAAGACGCA GATAACGAGG TGAAAAAGAT TGGTTGGGAT   
  
  
- TAGGATCTGG TGGTGTTCTT GACGGCCATG CTCATTGATC GTCCCAATGA GGTTCATCGA CGAGCAGAAT   
  
  
- GGATTGGGAT TTGGTCTAGT GAGGTAAATA GGCCAAGTGG GTGGACTGCC GGTTGGTCCG TTAAAACCTC   
  
  
- CGACACCTCC CGATATCCCG TCAGGTGTAG TAGCTGTACC TGTAGTACGT ACCTCACGTC ACCGGAGGCG   
  
  
- AGGAGGTTCG TTAGCACCTC GCCAGCCGGT GGGAATCGGT TGGAGGGGGA CACCAGGCAG AGTTTCCCCC   
  
  
- TCCCGGCCTG GGCCTGGAGA ATGTCTCCTG CCCCCTGGCT GATACCTTGA AACGGGTTAG TAATCCTGAT   
  
  
- CTCAAGGTAA AGGTCCCGGA GTACTAGGGG CGGCTCAGTT GTCCAAAACA ACTTGGACGA CTCCGTCGCC   
  
  
- AGTTACGGGA TCGGAACGTC CTCCGCCACC CCACAGTTCT TCCCCTCCGG GAACGTCAAG TGACGCCCCT   
  
  
- GATAGAGGTG GCGGAGAATT TTCTCATGCT GTGTTCCGGA GAATCTGCCA AGGAGGTGTT CCAGTTCTGA   
  
  
- GACTTGGGGT CCCAGAACTG GAACCCTCTC TCCCTTCGGC TGGTGTTGGT GGGAGAGAAG GACGCCGCCA   
  
  
- AGTGGCTCCG TCAGTTGGTG ATGCCCCGTC ACAAACTGAG GGACCTTCGT TGGGAAGGCG GCGTATCGGT   
  
  
- TCTCTCCCAT CGACACCTCC TTCCCACCAA GCCTCTCCTC TAATTCCTGC ACCATCCTCT TCTTTCGCTC   
  
  
- TCCTCTTATC TCTCCGTGGT CTTCAAGCTC AGCACCCTTC GTAACAACTC CTCGAGTCCC AAGTTCTCAC   
  
  
- ACGGGGAGTC GGGTAAGAGC GACAGGGTTC GATTCGACGA CGACGCGGAG GTAATGGGAA GGCTTCCCAT   
  
  
- GGTTGAGGTC CAAAACGTAT CATTAACGAA CAACGAACCC ACCTTCTTAG CGGGAGAAAA AAGACAGAGA   
  
  
- AGAACCGTTA T

+     TCT-motif

| Site Name | Organism | Position | Strand | Matrix score. | sequence | function |
| --- | --- | --- | --- | --- | --- | --- |
| TCT-motif | Arabidopsis thaliana | 2752 | + | 6 | TCTTAC | part of a light responsive element |
| TCT-motif | Arabidopsis thaliana | 2520 | + | 6 | TCTTAC | part of a light responsive element |
| TCT-motif | Arabidopsis thaliana | 207 | - | 6 | TCTTAC | part of a light responsive element |

>HU03G01737.1   
+ +Up\_Stream \_Len000TTTTTT TTAAGTTTTC GTACTTGGTG TTGATGATTT GTGCTTTGTA ACCAATGGTT   
  
  
+ TTAGTAAATA GTAATTAGTC GTAACATTCT GCGATTTCGC TCAATTTATA TATCAACTTT AGAATATGAG   
  
  
+ TAACCGTAAA GATCAAACGT AGTAGTAGAG GTGTCATAGG TTTTTTTTTA AAAAAATTCG GAGTAAGAAA   
  
  
+ GTAGGGTACA TTATTTTTGC TTGTTAAATT CCGCTAGTAT AGCAATACGA GATGGAACAT TTGTAAAAAC   
  
  
+ TACGAGACGA AGCGTTTGTA CAAAACAAAC TACCATACTG CAGATCATAT AATTTTTTGG GGGGTTATGT   
  
  
+ ATCTTAGTGA TGACATTAAT TGATGGATAA CCCTATTCTA AATATCTGGA TCCATCATTA ACTGACTCGT   
  
  
+ TTCAAATGTT AGGATTATGG GTCTTGACAT GGTATACATG TCAATAGATT TATGTTAGAT GAAAAGTTAT   
  
  
+ ATGACGCTTT TTATGATTTT TGTACTAGCA TATAGTGCTT AATTGGACTA TATATTTTTA CAAGTTGTGC   
  
  
+ TTAACACCTT TTAATCAGAA ATATTTATTA TATGGAAAAA ATTTTATTAA TATGTGTTTA TGTGTTTAAA   
  
  
+ AAACATACCC AGCCTTGAAA TAGACCGTGA TATAGAAATT GGACCCATAA TACTCACATT ATATATGCTT   
  
  
+ TATTGTTATA ATTAAGGATC ATTGTTGTTT TGCAATTATG TGGCAAGATA TAATCCACAA AGTTTTACAT   
  
  
+ TTATATTGCA ATTACTCTGG TAAATGCTAT TAATGTACAT TTGAGATTAA GACATATATA TAACCCTTTT   
  
  
+ TTTTCCCTTC TTTTTTCCTT TTTCTATTTT GAATGGCATC TTAAACCAAA GGAGCTGTAC AAGTTACGTT   
  
  
+ ATAGCACTCA TGGTTGACTA AACAAGACAG ATGAGAGTTT AAGTCAAGTC CTCCTGGCAA TGATGTTTGC   
  
  
+ CATTTGGACC AATAAATACA TCATGTATAT TACCATAATA GTGTGGTTGA CTTAACAATA CATAGGGGAT   
  
  
+ TGCATACAAA GGTAAGCCTC AAAAACTTGT GCATATGCAT TGAAATCCAT GATCAAAAAA ATTCAAGAGC   
  
  
+ AGGTTGCACA AAAACACGGA GATGAGATGA AAGAGACAGA AAACACATAT ACATTCACAG TAGTAGGGGT   
  
  
+ ACTGGTAGAG CTATGGCTAG GCTAAGATGA TGTGAAAGAA ACAATATAAT GGTAGTAACT GGTAATATTT   
  
  
+ ATAGGGCACA TATAGGTGCG GGCATAGGGT CGTGTCTCTC AGTCTCAGGT ACGGTTCGAT GGGACTCTGC   
  
  
+ GATAGATGTT ATCAAATTCA ACCATTCGAA CATCTCCCAG AGAGAGAGAG AGAGACTGAG TGAGTGAGTG   
  
  
+ AGTGGGTGGG TGTATGTGTG TGAGTTGTTT TGTTCATTTA TTATAGGTTC TCTGCTGCAT ATATCTCCAC   
  
  
+ CATTAAATTT ACCAAAACTT GTACCAGCTG CTTTACTTGC TTTCCTCATT TTCCCCCTTT TTTGTCTCTC   
  
  
+ TCTCTCTCTC TCCCCCCCCC CCCCCCCCTC TCTCTCTGTG TCTGAATCAG TATCAGCCTA TCAGGATGCT   
  
  
+ TCGCCTCCAG AAAGGCATCA CTCACACACT TATTAGCTTA AAACTTTCTT TTATTCCTTT TGCTCTTTCA   
  
  
+ CTGCTCTCTT TTACCCAAAT ACGCCCGTCT TCAATTCTTC TCTCATTAAA TATGAAAAAG AAAGAGAGAA   
  
  
+ ATAGAGAGAG ATTTAAACCA GAAGGGGTGA TTCCTTTTTC TTGGTGGGCT TATTAATGCT CTGTCTACCC   
  
  
+ CATCTAGCCG AAAAATAGTA GCTTAAGACT CAACTGCTTA TTAGCATCTC TCCTCTAGCA ATAACCGAGG   
  
  
+ TAATAGCATA TTTTTCTCCC CTTAGCTTCT ACCTTGCTTT TTGTGACACT CAAACCTAAA CTGAGAGAGA   
  
  
+ GAAAGAGAAG ATAGAGGAGA GATAAATATT AGCGGGATTA GGGCAAATTT TGATATGCTG GGCTCTTCCT   
  
  
+ CATTCAACTC TGAGGAAGAA GATCAAGATC ATGCCAACCC ACCTCCGCCG CAACAACCAC CACCGTTACA   
  
  
+ GCTGCCACTT CCACGGCCAA GATTCAGCCA GTTAATATCA TCATCACCTC CAATAATATC AGCATCTTCT   
  
  
+ TCATCATTAA TCCATAATAA CCCTCGTCAG CTGCTAATAA GTTGCGGTGA ACTCATCTCC CGCTGTGACT   
  
  
+ TCTCCTCAGC TAACCGCCTT CTCTCTCTTC TCTCCGGCGA CTTCGCTTCC TCCTCCGGCG ACTCCACTGA   
  
  
+ ACGCCTTGTT TATTACTTCT CCAAGGCTTT GTTCCTCCGC CTTCGCCGAC AGTTTTCTCT ATCTTCTTCA   
  
  
+ ACTTTTACTG GTTATAGTTG TAACCCTAGT TGTTCTGCGT CTATTGCTCC ACTTTTTCTA ACCAACCCTA   
  
  
+ ATCCTAGACC ACCACAAGAA CTGCCGGTAC GAGTAACTAG CAGGGTTACT CCAAGTAGCT GCTCGTCTTA   
  
  
+ CCTAACCCTA AACCAGATCA CTCCATTTAT CCGGTTCACC CACCTGACGG CCAACCAGGC AATTTTGGAG   
  
  
+ GCTGTGGAGG GCTATAGGGC AGTCCACATC ATCGACATGG ACATCATGCA TGGAGTGCAG TGGCCTCCGC   
  
  
+ TCCTCCAAGC AATCGTGGAG CGGTCGGCCA CCCTTAGCCA ACCTCCCCCT GTGGTCCGTC TCAAAGGGGG   
  
  
+ AGGGCCGGAC CCGGACCTCT TACAGAGGAC GGGGGACCGA CTATGGAACT TTGCCCAATC ATTAGGACTA   
  
  
+ GAGTTCCATT TCCAGGGCCT CATGATCCCC GCCGAGTCAA CAGGTTTTGT TGAACCTGCT GAGGCAGCGG   
  
  
+ TCAATGCCCT AGCCTTGCAG GAGGCGGTGG GGTGTCAAGA AGGGGAGGCC CTTGCAGTTC ACTGCGGGGA   
  
  
+ CTATCTCCAC CGCCTCTTAA AAGAGTACGA CACAAGGCCT CTTAGACGGT TCCTCCACAA GGTCAAGACT   
  
  
+ CTGAACCCCA GGGTCTTGAC CTTGGGAGAG AGGGAAGCCG ACCACAACCA CCCTCTCTTC CTGCGGCGGT   
  
  
+ TCACCGAGGC AGTCAACCAC TACGGGGCAG TGTTTGACTC CCTGGAAGCA ACCCTTCCGC CGCATAGCCA   
  
  
+ AGAGAGGGTA GCTGTGGAGG AAGGGTGGTT CGGAGAGGAG ATTAAGGACG TGGTAGGAGA AGAAAGCGAG   
  
  
+ AGGAGAATAG AGAGGCACCA GAAGTTCGAG TCGTGGGAAG CATTGTTGAG GAGCTCAGGG TTCAAGAGTG   
  
  
+ TGCCCCTCAG CCCATTCTCG CTGTCCCAAG CTAAGCTGCT GCTGCGCCTC CATTACCCTT CCGAAGGGTA   
  
  
+ CCAACTCCAG GTTTTGCATA GTAATTGCTT GTTGCTTGGG TGGAAGAATC GCCCTCTTTT TTCTGTCTCT   
  
  
+ TCTTGGCAAT A  

- +Up\_Stream \_Len000AAAAAA AATTCAAAAG CATGAACCAC AACTACTAAA CACGAAACAT TGGTTACCAA   
  
  
- AATCATTTAT CATTAATCAG CATTGTAAGA CGCTAAAGCG AGTTAAATAT ATAGTTGAAA TCTTATACTC   
  
  
- ATTGGCATTT CTAGTTTGCA TCATCATCTC CACAGTATCC AAAAAAAAAT TTTTTTAAGC CTCATTCTTT   
  
  
- CATCCCATGT AATAAAAACG AACAATTTAA GGCGATCATA TCGTTATGCT CTACCTTGTA AACATTTTTG   
  
  
- ATGCTCTGCT TCGCAAACAT GTTTTGTTTG ATGGTATGAC GTCTAGTATA TTAAAAAACC CCCCAATACA   
  
  
- TAGAATCACT ACTGTAATTA ACTACCTATT GGGATAAGAT TTATAGACCT AGGTAGTAAT TGACTGAGCA   
  
  
- AAGTTTACAA TCCTAATACC CAGAACTGTA CCATATGTAC AGTTATCTAA ATACAATCTA CTTTTCAATA   
  
  
- TACTGCGAAA AATACTAAAA ACATGATCGT ATATCACGAA TTAACCTGAT ATATAAAAAT GTTCAACACG   
  
  
- AATTGTGGAA AATTAGTCTT TATAAATAAT ATACCTTTTT TAAAATAATT ATACACAAAT ACACAAATTT   
  
  
- TTTGTATGGG TCGGAACTTT ATCTGGCACT ATATCTTTAA CCTGGGTATT ATGAGTGTAA TATATACGAA   
  
  
- ATAACAATAT TAATTCCTAG TAACAACAAA ACGTTAATAC ACCGTTCTAT ATTAGGTGTT TCAAAATGTA   
  
  
- AATATAACGT TAATGAGACC ATTTACGATA ATTACATGTA AACTCTAATT CTGTATATAT ATTGGGAAAA   
  
  
- AAAAGGGAAG AAAAAAGGAA AAAGATAAAA CTTACCGTAG AATTTGGTTT CCTCGACATG TTCAATGCAA   
  
  
- TATCGTGAGT ACCAACTGAT TTGTTCTGTC TACTCTCAAA TTCAGTTCAG GAGGACCGTT ACTACAAACG   
  
  
- GTAAACCTGG TTATTTATGT AGTACATATA ATGGTATTAT CACACCAACT GAATTGTTAT GTATCCCCTA   
  
  
- ACGTATGTTT CCATTCGGAG TTTTTGAACA CGTATACGTA ACTTTAGGTA CTAGTTTTTT TAAGTTCTCG   
  
  
- TCCAACGTGT TTTTGTGCCT CTACTCTACT TTCTCTGTCT TTTGTGTATA TGTAAGTGTC ATCATCCCCA   
  
  
- TGACCATCTC GATACCGATC CGATTCTACT ACACTTTCTT TGTTATATTA CCATCATTGA CCATTATAAA   
  
  
- TATCCCGTGT ATATCCACGC CCGTATCCCA GCACAGAGAG TCAGAGTCCA TGCCAAGCTA CCCTGAGACG   
  
  
- CTATCTACAA TAGTTTAAGT TGGTAAGCTT GTAGAGGGTC TCTCTCTCTC TCTCTGACTC ACTCACTCAC   
  
  
- TCACCCACCC ACATACACAC ACTCAACAAA ACAAGTAAAT AATATCCAAG AGACGACGTA TATAGAGGTG   
  
  
- GTAATTTAAA TGGTTTTGAA CATGGTCGAC GAAATGAACG AAAGGAGTAA AAGGGGGAAA AAACAGAGAG   
  
  
- AGAGAGAGAG AGGGGGGGGG GGGGGGGGAG AGAGAGACAC AGACTTAGTC ATAGTCGGAT AGTCCTACGA   
  
  
- AGCGGAGGTC TTTCCGTAGT GAGTGTGTGA ATAATCGAAT TTTGAAAGAA AATAAGGAAA ACGAGAAAGT   
  
  
- GACGAGAGAA AATGGGTTTA TGCGGGCAGA AGTTAAGAAG AGAGTAATTT ATACTTTTTC TTTCTCTCTT   
  
  
- TATCTCTCTC TAAATTTGGT CTTCCCCACT AAGGAAAAAG AACCACCCGA ATAATTACGA GACAGATGGG   
  
  
- GTAGATCGGC TTTTTATCAT CGAATTCTGA GTTGACGAAT AATCGTAGAG AGGAGATCGT TATTGGCTCC   
  
  
- ATTATCGTAT AAAAAGAGGG GAATCGAAGA TGGAACGAAA AACACTGTGA GTTTGGATTT GACTCTCTCT   
  
  
- CTTTCTCTTC TATCTCCTCT CTATTTATAA TCGCCCTAAT CCCGTTTAAA ACTATACGAC CCGAGAAGGA   
  
  
- GTAAGTTGAG ACTCCTTCTT CTAGTTCTAG TACGGTTGGG TGGAGGCGGC GTTGTTGGTG GTGGCAATGT   
  
  
- CGACGGTGAA GGTGCCGGTT CTAAGTCGGT CAATTATAGT AGTAGTGGAG GTTATTATAG TCGTAGAAGA   
  
  
- AGTAGTAATT AGGTATTATT GGGAGCAGTC GACGATTATT CAACGCCACT TGAGTAGAGG GCGACACTGA   
  
  
- AGAGGAGTCG ATTGGCGGAA GAGAGAGAAG AGAGGCCGCT GAAGCGAAGG AGGAGGCCGC TGAGGTGACT   
  
  
- TGCGGAACAA ATAATGAAGA GGTTCCGAAA CAAGGAGGCG GAAGCGGCTG TCAAAAGAGA TAGAAGAAGT   
  
  
- TGAAAATGAC CAATATCAAC ATTGGGATCA ACAAGACGCA GATAACGAGG TGAAAAAGAT TGGTTGGGAT   
  
  
- TAGGATCTGG TGGTGTTCTT GACGGCCATG CTCATTGATC GTCCCAATGA GGTTCATCGA CGAGCAGAAT   
  
  
- GGATTGGGAT TTGGTCTAGT GAGGTAAATA GGCCAAGTGG GTGGACTGCC GGTTGGTCCG TTAAAACCTC   
  
  
- CGACACCTCC CGATATCCCG TCAGGTGTAG TAGCTGTACC TGTAGTACGT ACCTCACGTC ACCGGAGGCG   
  
  
- AGGAGGTTCG TTAGCACCTC GCCAGCCGGT GGGAATCGGT TGGAGGGGGA CACCAGGCAG AGTTTCCCCC   
  
  
- TCCCGGCCTG GGCCTGGAGA ATGTCTCCTG CCCCCTGGCT GATACCTTGA AACGGGTTAG TAATCCTGAT   
  
  
- CTCAAGGTAA AGGTCCCGGA GTACTAGGGG CGGCTCAGTT GTCCAAAACA ACTTGGACGA CTCCGTCGCC   
  
  
- AGTTACGGGA TCGGAACGTC CTCCGCCACC CCACAGTTCT TCCCCTCCGG GAACGTCAAG TGACGCCCCT   
  
  
- GATAGAGGTG GCGGAGAATT TTCTCATGCT GTGTTCCGGA GAATCTGCCA AGGAGGTGTT CCAGTTCTGA   
  
  
- GACTTGGGGT CCCAGAACTG GAACCCTCTC TCCCTTCGGC TGGTGTTGGT GGGAGAGAAG GACGCCGCCA   
  
  
- AGTGGCTCCG TCAGTTGGTG ATGCCCCGTC ACAAACTGAG GGACCTTCGT TGGGAAGGCG GCGTATCGGT   
  
  
- TCTCTCCCAT CGACACCTCC TTCCCACCAA GCCTCTCCTC TAATTCCTGC ACCATCCTCT TCTTTCGCTC   
  
  
- TCCTCTTATC TCTCCGTGGT CTTCAAGCTC AGCACCCTTC GTAACAACTC CTCGAGTCCC AAGTTCTCAC   
  
  
- ACGGGGAGTC GGGTAAGAGC GACAGGGTTC GATTCGACGA CGACGCGGAG GTAATGGGAA GGCTTCCCAT   
  
  
- GGTTGAGGTC CAAAACGTAT CATTAACGAA CAACGAACCC ACCTTCTTAG CGGGAGAAAA AAGACAGAGA   
  
  
- AGAACCGTTA T

+     TGACG-motif

| Site Name | Organism | Position | Strand | Matrix score. | sequence | function |
| --- | --- | --- | --- | --- | --- | --- |
| TGACG-motif | Hordeum vulgare | 2569 | + | 5 | TGACG | cis-acting regulatory element involved in the MeJA-responsiveness |
| TGACG-motif | Hordeum vulgare | 2199 | - | 5 | TGACG | cis-acting regulatory element involved in the MeJA-responsiveness |
| TGACG-motif | Hordeum vulgare | 496 | + | 5 | TGACG | cis-acting regulatory element involved in the MeJA-responsiveness |

>HU03G01737.1   
+ +Up\_Stream \_Len000TTTTTT TTAAGTTTTC GTACTTGGTG TTGATGATTT GTGCTTTGTA ACCAATGGTT   
  
  
+ TTAGTAAATA GTAATTAGTC GTAACATTCT GCGATTTCGC TCAATTTATA TATCAACTTT AGAATATGAG   
  
  
+ TAACCGTAAA GATCAAACGT AGTAGTAGAG GTGTCATAGG TTTTTTTTTA AAAAAATTCG GAGTAAGAAA   
  
  
+ GTAGGGTACA TTATTTTTGC TTGTTAAATT CCGCTAGTAT AGCAATACGA GATGGAACAT TTGTAAAAAC   
  
  
+ TACGAGACGA AGCGTTTGTA CAAAACAAAC TACCATACTG CAGATCATAT AATTTTTTGG GGGGTTATGT   
  
  
+ ATCTTAGTGA TGACATTAAT TGATGGATAA CCCTATTCTA AATATCTGGA TCCATCATTA ACTGACTCGT   
  
  
+ TTCAAATGTT AGGATTATGG GTCTTGACAT GGTATACATG TCAATAGATT TATGTTAGAT GAAAAGTTAT   
  
  
+ ATGACGCTTT TTATGATTTT TGTACTAGCA TATAGTGCTT AATTGGACTA TATATTTTTA CAAGTTGTGC   
  
  
+ TTAACACCTT TTAATCAGAA ATATTTATTA TATGGAAAAA ATTTTATTAA TATGTGTTTA TGTGTTTAAA   
  
  
+ AAACATACCC AGCCTTGAAA TAGACCGTGA TATAGAAATT GGACCCATAA TACTCACATT ATATATGCTT   
  
  
+ TATTGTTATA ATTAAGGATC ATTGTTGTTT TGCAATTATG TGGCAAGATA TAATCCACAA AGTTTTACAT   
  
  
+ TTATATTGCA ATTACTCTGG TAAATGCTAT TAATGTACAT TTGAGATTAA GACATATATA TAACCCTTTT   
  
  
+ TTTTCCCTTC TTTTTTCCTT TTTCTATTTT GAATGGCATC TTAAACCAAA GGAGCTGTAC AAGTTACGTT   
  
  
+ ATAGCACTCA TGGTTGACTA AACAAGACAG ATGAGAGTTT AAGTCAAGTC CTCCTGGCAA TGATGTTTGC   
  
  
+ CATTTGGACC AATAAATACA TCATGTATAT TACCATAATA GTGTGGTTGA CTTAACAATA CATAGGGGAT   
  
  
+ TGCATACAAA GGTAAGCCTC AAAAACTTGT GCATATGCAT TGAAATCCAT GATCAAAAAA ATTCAAGAGC   
  
  
+ AGGTTGCACA AAAACACGGA GATGAGATGA AAGAGACAGA AAACACATAT ACATTCACAG TAGTAGGGGT   
  
  
+ ACTGGTAGAG CTATGGCTAG GCTAAGATGA TGTGAAAGAA ACAATATAAT GGTAGTAACT GGTAATATTT   
  
  
+ ATAGGGCACA TATAGGTGCG GGCATAGGGT CGTGTCTCTC AGTCTCAGGT ACGGTTCGAT GGGACTCTGC   
  
  
+ GATAGATGTT ATCAAATTCA ACCATTCGAA CATCTCCCAG AGAGAGAGAG AGAGACTGAG TGAGTGAGTG   
  
  
+ AGTGGGTGGG TGTATGTGTG TGAGTTGTTT TGTTCATTTA TTATAGGTTC TCTGCTGCAT ATATCTCCAC   
  
  
+ CATTAAATTT ACCAAAACTT GTACCAGCTG CTTTACTTGC TTTCCTCATT TTCCCCCTTT TTTGTCTCTC   
  
  
+ TCTCTCTCTC TCCCCCCCCC CCCCCCCCTC TCTCTCTGTG TCTGAATCAG TATCAGCCTA TCAGGATGCT   
  
  
+ TCGCCTCCAG AAAGGCATCA CTCACACACT TATTAGCTTA AAACTTTCTT TTATTCCTTT TGCTCTTTCA   
  
  
+ CTGCTCTCTT TTACCCAAAT ACGCCCGTCT TCAATTCTTC TCTCATTAAA TATGAAAAAG AAAGAGAGAA   
  
  
+ ATAGAGAGAG ATTTAAACCA GAAGGGGTGA TTCCTTTTTC TTGGTGGGCT TATTAATGCT CTGTCTACCC   
  
  
+ CATCTAGCCG AAAAATAGTA GCTTAAGACT CAACTGCTTA TTAGCATCTC TCCTCTAGCA ATAACCGAGG   
  
  
+ TAATAGCATA TTTTTCTCCC CTTAGCTTCT ACCTTGCTTT TTGTGACACT CAAACCTAAA CTGAGAGAGA   
  
  
+ GAAAGAGAAG ATAGAGGAGA GATAAATATT AGCGGGATTA GGGCAAATTT TGATATGCTG GGCTCTTCCT   
  
  
+ CATTCAACTC TGAGGAAGAA GATCAAGATC ATGCCAACCC ACCTCCGCCG CAACAACCAC CACCGTTACA   
  
  
+ GCTGCCACTT CCACGGCCAA GATTCAGCCA GTTAATATCA TCATCACCTC CAATAATATC AGCATCTTCT   
  
  
+ TCATCATTAA TCCATAATAA CCCTCGTCAG CTGCTAATAA GTTGCGGTGA ACTCATCTCC CGCTGTGACT   
  
  
+ TCTCCTCAGC TAACCGCCTT CTCTCTCTTC TCTCCGGCGA CTTCGCTTCC TCCTCCGGCG ACTCCACTGA   
  
  
+ ACGCCTTGTT TATTACTTCT CCAAGGCTTT GTTCCTCCGC CTTCGCCGAC AGTTTTCTCT ATCTTCTTCA   
  
  
+ ACTTTTACTG GTTATAGTTG TAACCCTAGT TGTTCTGCGT CTATTGCTCC ACTTTTTCTA ACCAACCCTA   
  
  
+ ATCCTAGACC ACCACAAGAA CTGCCGGTAC GAGTAACTAG CAGGGTTACT CCAAGTAGCT GCTCGTCTTA   
  
  
+ CCTAACCCTA AACCAGATCA CTCCATTTAT CCGGTTCACC CACCTGACGG CCAACCAGGC AATTTTGGAG   
  
  
+ GCTGTGGAGG GCTATAGGGC AGTCCACATC ATCGACATGG ACATCATGCA TGGAGTGCAG TGGCCTCCGC   
  
  
+ TCCTCCAAGC AATCGTGGAG CGGTCGGCCA CCCTTAGCCA ACCTCCCCCT GTGGTCCGTC TCAAAGGGGG   
  
  
+ AGGGCCGGAC CCGGACCTCT TACAGAGGAC GGGGGACCGA CTATGGAACT TTGCCCAATC ATTAGGACTA   
  
  
+ GAGTTCCATT TCCAGGGCCT CATGATCCCC GCCGAGTCAA CAGGTTTTGT TGAACCTGCT GAGGCAGCGG   
  
  
+ TCAATGCCCT AGCCTTGCAG GAGGCGGTGG GGTGTCAAGA AGGGGAGGCC CTTGCAGTTC ACTGCGGGGA   
  
  
+ CTATCTCCAC CGCCTCTTAA AAGAGTACGA CACAAGGCCT CTTAGACGGT TCCTCCACAA GGTCAAGACT   
  
  
+ CTGAACCCCA GGGTCTTGAC CTTGGGAGAG AGGGAAGCCG ACCACAACCA CCCTCTCTTC CTGCGGCGGT   
  
  
+ TCACCGAGGC AGTCAACCAC TACGGGGCAG TGTTTGACTC CCTGGAAGCA ACCCTTCCGC CGCATAGCCA   
  
  
+ AGAGAGGGTA GCTGTGGAGG AAGGGTGGTT CGGAGAGGAG ATTAAGGACG TGGTAGGAGA AGAAAGCGAG   
  
  
+ AGGAGAATAG AGAGGCACCA GAAGTTCGAG TCGTGGGAAG CATTGTTGAG GAGCTCAGGG TTCAAGAGTG   
  
  
+ TGCCCCTCAG CCCATTCTCG CTGTCCCAAG CTAAGCTGCT GCTGCGCCTC CATTACCCTT CCGAAGGGTA   
  
  
+ CCAACTCCAG GTTTTGCATA GTAATTGCTT GTTGCTTGGG TGGAAGAATC GCCCTCTTTT TTCTGTCTCT   
  
  
+ TCTTGGCAAT A  

- +Up\_Stream \_Len000AAAAAA AATTCAAAAG CATGAACCAC AACTACTAAA CACGAAACAT TGGTTACCAA   
  
  
- AATCATTTAT CATTAATCAG CATTGTAAGA CGCTAAAGCG AGTTAAATAT ATAGTTGAAA TCTTATACTC   
  
  
- ATTGGCATTT CTAGTTTGCA TCATCATCTC CACAGTATCC AAAAAAAAAT TTTTTTAAGC CTCATTCTTT   
  
  
- CATCCCATGT AATAAAAACG AACAATTTAA GGCGATCATA TCGTTATGCT CTACCTTGTA AACATTTTTG   
  
  
- ATGCTCTGCT TCGCAAACAT GTTTTGTTTG ATGGTATGAC GTCTAGTATA TTAAAAAACC CCCCAATACA   
  
  
- TAGAATCACT ACTGTAATTA ACTACCTATT GGGATAAGAT TTATAGACCT AGGTAGTAAT TGACTGAGCA   
  
  
- AAGTTTACAA TCCTAATACC CAGAACTGTA CCATATGTAC AGTTATCTAA ATACAATCTA CTTTTCAATA   
  
  
- TACTGCGAAA AATACTAAAA ACATGATCGT ATATCACGAA TTAACCTGAT ATATAAAAAT GTTCAACACG   
  
  
- AATTGTGGAA AATTAGTCTT TATAAATAAT ATACCTTTTT TAAAATAATT ATACACAAAT ACACAAATTT   
  
  
- TTTGTATGGG TCGGAACTTT ATCTGGCACT ATATCTTTAA CCTGGGTATT ATGAGTGTAA TATATACGAA   
  
  
- ATAACAATAT TAATTCCTAG TAACAACAAA ACGTTAATAC ACCGTTCTAT ATTAGGTGTT TCAAAATGTA   
  
  
- AATATAACGT TAATGAGACC ATTTACGATA ATTACATGTA AACTCTAATT CTGTATATAT ATTGGGAAAA   
  
  
- AAAAGGGAAG AAAAAAGGAA AAAGATAAAA CTTACCGTAG AATTTGGTTT CCTCGACATG TTCAATGCAA   
  
  
- TATCGTGAGT ACCAACTGAT TTGTTCTGTC TACTCTCAAA TTCAGTTCAG GAGGACCGTT ACTACAAACG   
  
  
- GTAAACCTGG TTATTTATGT AGTACATATA ATGGTATTAT CACACCAACT GAATTGTTAT GTATCCCCTA   
  
  
- ACGTATGTTT CCATTCGGAG TTTTTGAACA CGTATACGTA ACTTTAGGTA CTAGTTTTTT TAAGTTCTCG   
  
  
- TCCAACGTGT TTTTGTGCCT CTACTCTACT TTCTCTGTCT TTTGTGTATA TGTAAGTGTC ATCATCCCCA   
  
  
- TGACCATCTC GATACCGATC CGATTCTACT ACACTTTCTT TGTTATATTA CCATCATTGA CCATTATAAA   
  
  
- TATCCCGTGT ATATCCACGC CCGTATCCCA GCACAGAGAG TCAGAGTCCA TGCCAAGCTA CCCTGAGACG   
  
  
- CTATCTACAA TAGTTTAAGT TGGTAAGCTT GTAGAGGGTC TCTCTCTCTC TCTCTGACTC ACTCACTCAC   
  
  
- TCACCCACCC ACATACACAC ACTCAACAAA ACAAGTAAAT AATATCCAAG AGACGACGTA TATAGAGGTG   
  
  
- GTAATTTAAA TGGTTTTGAA CATGGTCGAC GAAATGAACG AAAGGAGTAA AAGGGGGAAA AAACAGAGAG   
  
  
- AGAGAGAGAG AGGGGGGGGG GGGGGGGGAG AGAGAGACAC AGACTTAGTC ATAGTCGGAT AGTCCTACGA   
  
  
- AGCGGAGGTC TTTCCGTAGT GAGTGTGTGA ATAATCGAAT TTTGAAAGAA AATAAGGAAA ACGAGAAAGT   
  
  
- GACGAGAGAA AATGGGTTTA TGCGGGCAGA AGTTAAGAAG AGAGTAATTT ATACTTTTTC TTTCTCTCTT   
  
  
- TATCTCTCTC TAAATTTGGT CTTCCCCACT AAGGAAAAAG AACCACCCGA ATAATTACGA GACAGATGGG   
  
  
- GTAGATCGGC TTTTTATCAT CGAATTCTGA GTTGACGAAT AATCGTAGAG AGGAGATCGT TATTGGCTCC   
  
  
- ATTATCGTAT AAAAAGAGGG GAATCGAAGA TGGAACGAAA AACACTGTGA GTTTGGATTT GACTCTCTCT   
  
  
- CTTTCTCTTC TATCTCCTCT CTATTTATAA TCGCCCTAAT CCCGTTTAAA ACTATACGAC CCGAGAAGGA   
  
  
- GTAAGTTGAG ACTCCTTCTT CTAGTTCTAG TACGGTTGGG TGGAGGCGGC GTTGTTGGTG GTGGCAATGT   
  
  
- CGACGGTGAA GGTGCCGGTT CTAAGTCGGT CAATTATAGT AGTAGTGGAG GTTATTATAG TCGTAGAAGA   
  
  
- AGTAGTAATT AGGTATTATT GGGAGCAGTC GACGATTATT CAACGCCACT TGAGTAGAGG GCGACACTGA   
  
  
- AGAGGAGTCG ATTGGCGGAA GAGAGAGAAG AGAGGCCGCT GAAGCGAAGG AGGAGGCCGC TGAGGTGACT   
  
  
- TGCGGAACAA ATAATGAAGA GGTTCCGAAA CAAGGAGGCG GAAGCGGCTG TCAAAAGAGA TAGAAGAAGT   
  
  
- TGAAAATGAC CAATATCAAC ATTGGGATCA ACAAGACGCA GATAACGAGG TGAAAAAGAT TGGTTGGGAT   
  
  
- TAGGATCTGG TGGTGTTCTT GACGGCCATG CTCATTGATC GTCCCAATGA GGTTCATCGA CGAGCAGAAT   
  
  
- GGATTGGGAT TTGGTCTAGT GAGGTAAATA GGCCAAGTGG GTGGACTGCC GGTTGGTCCG TTAAAACCTC   
  
  
- CGACACCTCC CGATATCCCG TCAGGTGTAG TAGCTGTACC TGTAGTACGT ACCTCACGTC ACCGGAGGCG   
  
  
- AGGAGGTTCG TTAGCACCTC GCCAGCCGGT GGGAATCGGT TGGAGGGGGA CACCAGGCAG AGTTTCCCCC   
  
  
- TCCCGGCCTG GGCCTGGAGA ATGTCTCCTG CCCCCTGGCT GATACCTTGA AACGGGTTAG TAATCCTGAT   
  
  
- CTCAAGGTAA AGGTCCCGGA GTACTAGGGG CGGCTCAGTT GTCCAAAACA ACTTGGACGA CTCCGTCGCC   
  
  
- AGTTACGGGA TCGGAACGTC CTCCGCCACC CCACAGTTCT TCCCCTCCGG GAACGTCAAG TGACGCCCCT   
  
  
- GATAGAGGTG GCGGAGAATT TTCTCATGCT GTGTTCCGGA GAATCTGCCA AGGAGGTGTT CCAGTTCTGA   
  
  
- GACTTGGGGT CCCAGAACTG GAACCCTCTC TCCCTTCGGC TGGTGTTGGT GGGAGAGAAG GACGCCGCCA   
  
  
- AGTGGCTCCG TCAGTTGGTG ATGCCCCGTC ACAAACTGAG GGACCTTCGT TGGGAAGGCG GCGTATCGGT   
  
  
- TCTCTCCCAT CGACACCTCC TTCCCACCAA GCCTCTCCTC TAATTCCTGC ACCATCCTCT TCTTTCGCTC   
  
  
- TCCTCTTATC TCTCCGTGGT CTTCAAGCTC AGCACCCTTC GTAACAACTC CTCGAGTCCC AAGTTCTCAC   
  
  
- ACGGGGAGTC GGGTAAGAGC GACAGGGTTC GATTCGACGA CGACGCGGAG GTAATGGGAA GGCTTCCCAT   
  
  
- GGTTGAGGTC CAAAACGTAT CATTAACGAA CAACGAACCC ACCTTCTTAG CGGGAGAAAA AAGACAGAGA   
  
  
- AGAACCGTTA T

+     Unnamed\_\_1

| Site Name | Organism | Position | Strand | Matrix score. | sequence | function |
| --- | --- | --- | --- | --- | --- | --- |
| Unnamed\_\_1 | Zea mays | 2115 | - | 5 | CGTGG |  |
| Unnamed\_\_1 | Zea mays | 3256 | + | 5 | CGTGG |  |
| Unnamed\_\_1 | Zea mays | 2678 | + | 5 | CGTGG |  |
| Unnamed\_\_1 | Zea mays | 3203 | + | 5 | CGTGG |  |

>HU03G01737.1   
+ +Up\_Stream \_Len000TTTTTT TTAAGTTTTC GTACTTGGTG TTGATGATTT GTGCTTTGTA ACCAATGGTT   
  
  
+ TTAGTAAATA GTAATTAGTC GTAACATTCT GCGATTTCGC TCAATTTATA TATCAACTTT AGAATATGAG   
  
  
+ TAACCGTAAA GATCAAACGT AGTAGTAGAG GTGTCATAGG TTTTTTTTTA AAAAAATTCG GAGTAAGAAA   
  
  
+ GTAGGGTACA TTATTTTTGC TTGTTAAATT CCGCTAGTAT AGCAATACGA GATGGAACAT TTGTAAAAAC   
  
  
+ TACGAGACGA AGCGTTTGTA CAAAACAAAC TACCATACTG CAGATCATAT AATTTTTTGG GGGGTTATGT   
  
  
+ ATCTTAGTGA TGACATTAAT TGATGGATAA CCCTATTCTA AATATCTGGA TCCATCATTA ACTGACTCGT   
  
  
+ TTCAAATGTT AGGATTATGG GTCTTGACAT GGTATACATG TCAATAGATT TATGTTAGAT GAAAAGTTAT   
  
  
+ ATGACGCTTT TTATGATTTT TGTACTAGCA TATAGTGCTT AATTGGACTA TATATTTTTA CAAGTTGTGC   
  
  
+ TTAACACCTT TTAATCAGAA ATATTTATTA TATGGAAAAA ATTTTATTAA TATGTGTTTA TGTGTTTAAA   
  
  
+ AAACATACCC AGCCTTGAAA TAGACCGTGA TATAGAAATT GGACCCATAA TACTCACATT ATATATGCTT   
  
  
+ TATTGTTATA ATTAAGGATC ATTGTTGTTT TGCAATTATG TGGCAAGATA TAATCCACAA AGTTTTACAT   
  
  
+ TTATATTGCA ATTACTCTGG TAAATGCTAT TAATGTACAT TTGAGATTAA GACATATATA TAACCCTTTT   
  
  
+ TTTTCCCTTC TTTTTTCCTT TTTCTATTTT GAATGGCATC TTAAACCAAA GGAGCTGTAC AAGTTACGTT   
  
  
+ ATAGCACTCA TGGTTGACTA AACAAGACAG ATGAGAGTTT AAGTCAAGTC CTCCTGGCAA TGATGTTTGC   
  
  
+ CATTTGGACC AATAAATACA TCATGTATAT TACCATAATA GTGTGGTTGA CTTAACAATA CATAGGGGAT   
  
  
+ TGCATACAAA GGTAAGCCTC AAAAACTTGT GCATATGCAT TGAAATCCAT GATCAAAAAA ATTCAAGAGC   
  
  
+ AGGTTGCACA AAAACACGGA GATGAGATGA AAGAGACAGA AAACACATAT ACATTCACAG TAGTAGGGGT   
  
  
+ ACTGGTAGAG CTATGGCTAG GCTAAGATGA TGTGAAAGAA ACAATATAAT GGTAGTAACT GGTAATATTT   
  
  
+ ATAGGGCACA TATAGGTGCG GGCATAGGGT CGTGTCTCTC AGTCTCAGGT ACGGTTCGAT GGGACTCTGC   
  
  
+ GATAGATGTT ATCAAATTCA ACCATTCGAA CATCTCCCAG AGAGAGAGAG AGAGACTGAG TGAGTGAGTG   
  
  
+ AGTGGGTGGG TGTATGTGTG TGAGTTGTTT TGTTCATTTA TTATAGGTTC TCTGCTGCAT ATATCTCCAC   
  
  
+ CATTAAATTT ACCAAAACTT GTACCAGCTG CTTTACTTGC TTTCCTCATT TTCCCCCTTT TTTGTCTCTC   
  
  
+ TCTCTCTCTC TCCCCCCCCC CCCCCCCCTC TCTCTCTGTG TCTGAATCAG TATCAGCCTA TCAGGATGCT   
  
  
+ TCGCCTCCAG AAAGGCATCA CTCACACACT TATTAGCTTA AAACTTTCTT TTATTCCTTT TGCTCTTTCA   
  
  
+ CTGCTCTCTT TTACCCAAAT ACGCCCGTCT TCAATTCTTC TCTCATTAAA TATGAAAAAG AAAGAGAGAA   
  
  
+ ATAGAGAGAG ATTTAAACCA GAAGGGGTGA TTCCTTTTTC TTGGTGGGCT TATTAATGCT CTGTCTACCC   
  
  
+ CATCTAGCCG AAAAATAGTA GCTTAAGACT CAACTGCTTA TTAGCATCTC TCCTCTAGCA ATAACCGAGG   
  
  
+ TAATAGCATA TTTTTCTCCC CTTAGCTTCT ACCTTGCTTT TTGTGACACT CAAACCTAAA CTGAGAGAGA   
  
  
+ GAAAGAGAAG ATAGAGGAGA GATAAATATT AGCGGGATTA GGGCAAATTT TGATATGCTG GGCTCTTCCT   
  
  
+ CATTCAACTC TGAGGAAGAA GATCAAGATC ATGCCAACCC ACCTCCGCCG CAACAACCAC CACCGTTACA   
  
  
+ GCTGCCACTT CCACGGCCAA GATTCAGCCA GTTAATATCA TCATCACCTC CAATAATATC AGCATCTTCT   
  
  
+ TCATCATTAA TCCATAATAA CCCTCGTCAG CTGCTAATAA GTTGCGGTGA ACTCATCTCC CGCTGTGACT   
  
  
+ TCTCCTCAGC TAACCGCCTT CTCTCTCTTC TCTCCGGCGA CTTCGCTTCC TCCTCCGGCG ACTCCACTGA   
  
  
+ ACGCCTTGTT TATTACTTCT CCAAGGCTTT GTTCCTCCGC CTTCGCCGAC AGTTTTCTCT ATCTTCTTCA   
  
  
+ ACTTTTACTG GTTATAGTTG TAACCCTAGT TGTTCTGCGT CTATTGCTCC ACTTTTTCTA ACCAACCCTA   
  
  
+ ATCCTAGACC ACCACAAGAA CTGCCGGTAC GAGTAACTAG CAGGGTTACT CCAAGTAGCT GCTCGTCTTA   
  
  
+ CCTAACCCTA AACCAGATCA CTCCATTTAT CCGGTTCACC CACCTGACGG CCAACCAGGC AATTTTGGAG   
  
  
+ GCTGTGGAGG GCTATAGGGC AGTCCACATC ATCGACATGG ACATCATGCA TGGAGTGCAG TGGCCTCCGC   
  
  
+ TCCTCCAAGC AATCGTGGAG CGGTCGGCCA CCCTTAGCCA ACCTCCCCCT GTGGTCCGTC TCAAAGGGGG   
  
  
+ AGGGCCGGAC CCGGACCTCT TACAGAGGAC GGGGGACCGA CTATGGAACT TTGCCCAATC ATTAGGACTA   
  
  
+ GAGTTCCATT TCCAGGGCCT CATGATCCCC GCCGAGTCAA CAGGTTTTGT TGAACCTGCT GAGGCAGCGG   
  
  
+ TCAATGCCCT AGCCTTGCAG GAGGCGGTGG GGTGTCAAGA AGGGGAGGCC CTTGCAGTTC ACTGCGGGGA   
  
  
+ CTATCTCCAC CGCCTCTTAA AAGAGTACGA CACAAGGCCT CTTAGACGGT TCCTCCACAA GGTCAAGACT   
  
  
+ CTGAACCCCA GGGTCTTGAC CTTGGGAGAG AGGGAAGCCG ACCACAACCA CCCTCTCTTC CTGCGGCGGT   
  
  
+ TCACCGAGGC AGTCAACCAC TACGGGGCAG TGTTTGACTC CCTGGAAGCA ACCCTTCCGC CGCATAGCCA   
  
  
+ AGAGAGGGTA GCTGTGGAGG AAGGGTGGTT CGGAGAGGAG ATTAAGGACG TGGTAGGAGA AGAAAGCGAG   
  
  
+ AGGAGAATAG AGAGGCACCA GAAGTTCGAG TCGTGGGAAG CATTGTTGAG GAGCTCAGGG TTCAAGAGTG   
  
  
+ TGCCCCTCAG CCCATTCTCG CTGTCCCAAG CTAAGCTGCT GCTGCGCCTC CATTACCCTT CCGAAGGGTA   
  
  
+ CCAACTCCAG GTTTTGCATA GTAATTGCTT GTTGCTTGGG TGGAAGAATC GCCCTCTTTT TTCTGTCTCT   
  
  
+ TCTTGGCAAT A  

- +Up\_Stream \_Len000AAAAAA AATTCAAAAG CATGAACCAC AACTACTAAA CACGAAACAT TGGTTACCAA   
  
  
- AATCATTTAT CATTAATCAG CATTGTAAGA CGCTAAAGCG AGTTAAATAT ATAGTTGAAA TCTTATACTC   
  
  
- ATTGGCATTT CTAGTTTGCA TCATCATCTC CACAGTATCC AAAAAAAAAT TTTTTTAAGC CTCATTCTTT   
  
  
- CATCCCATGT AATAAAAACG AACAATTTAA GGCGATCATA TCGTTATGCT CTACCTTGTA AACATTTTTG   
  
  
- ATGCTCTGCT TCGCAAACAT GTTTTGTTTG ATGGTATGAC GTCTAGTATA TTAAAAAACC CCCCAATACA   
  
  
- TAGAATCACT ACTGTAATTA ACTACCTATT GGGATAAGAT TTATAGACCT AGGTAGTAAT TGACTGAGCA   
  
  
- AAGTTTACAA TCCTAATACC CAGAACTGTA CCATATGTAC AGTTATCTAA ATACAATCTA CTTTTCAATA   
  
  
- TACTGCGAAA AATACTAAAA ACATGATCGT ATATCACGAA TTAACCTGAT ATATAAAAAT GTTCAACACG   
  
  
- AATTGTGGAA AATTAGTCTT TATAAATAAT ATACCTTTTT TAAAATAATT ATACACAAAT ACACAAATTT   
  
  
- TTTGTATGGG TCGGAACTTT ATCTGGCACT ATATCTTTAA CCTGGGTATT ATGAGTGTAA TATATACGAA   
  
  
- ATAACAATAT TAATTCCTAG TAACAACAAA ACGTTAATAC ACCGTTCTAT ATTAGGTGTT TCAAAATGTA   
  
  
- AATATAACGT TAATGAGACC ATTTACGATA ATTACATGTA AACTCTAATT CTGTATATAT ATTGGGAAAA   
  
  
- AAAAGGGAAG AAAAAAGGAA AAAGATAAAA CTTACCGTAG AATTTGGTTT CCTCGACATG TTCAATGCAA   
  
  
- TATCGTGAGT ACCAACTGAT TTGTTCTGTC TACTCTCAAA TTCAGTTCAG GAGGACCGTT ACTACAAACG   
  
  
- GTAAACCTGG TTATTTATGT AGTACATATA ATGGTATTAT CACACCAACT GAATTGTTAT GTATCCCCTA   
  
  
- ACGTATGTTT CCATTCGGAG TTTTTGAACA CGTATACGTA ACTTTAGGTA CTAGTTTTTT TAAGTTCTCG   
  
  
- TCCAACGTGT TTTTGTGCCT CTACTCTACT TTCTCTGTCT TTTGTGTATA TGTAAGTGTC ATCATCCCCA   
  
  
- TGACCATCTC GATACCGATC CGATTCTACT ACACTTTCTT TGTTATATTA CCATCATTGA CCATTATAAA   
  
  
- TATCCCGTGT ATATCCACGC CCGTATCCCA GCACAGAGAG TCAGAGTCCA TGCCAAGCTA CCCTGAGACG   
  
  
- CTATCTACAA TAGTTTAAGT TGGTAAGCTT GTAGAGGGTC TCTCTCTCTC TCTCTGACTC ACTCACTCAC   
  
  
- TCACCCACCC ACATACACAC ACTCAACAAA ACAAGTAAAT AATATCCAAG AGACGACGTA TATAGAGGTG   
  
  
- GTAATTTAAA TGGTTTTGAA CATGGTCGAC GAAATGAACG AAAGGAGTAA AAGGGGGAAA AAACAGAGAG   
  
  
- AGAGAGAGAG AGGGGGGGGG GGGGGGGGAG AGAGAGACAC AGACTTAGTC ATAGTCGGAT AGTCCTACGA   
  
  
- AGCGGAGGTC TTTCCGTAGT GAGTGTGTGA ATAATCGAAT TTTGAAAGAA AATAAGGAAA ACGAGAAAGT   
  
  
- GACGAGAGAA AATGGGTTTA TGCGGGCAGA AGTTAAGAAG AGAGTAATTT ATACTTTTTC TTTCTCTCTT   
  
  
- TATCTCTCTC TAAATTTGGT CTTCCCCACT AAGGAAAAAG AACCACCCGA ATAATTACGA GACAGATGGG   
  
  
- GTAGATCGGC TTTTTATCAT CGAATTCTGA GTTGACGAAT AATCGTAGAG AGGAGATCGT TATTGGCTCC   
  
  
- ATTATCGTAT AAAAAGAGGG GAATCGAAGA TGGAACGAAA AACACTGTGA GTTTGGATTT GACTCTCTCT   
  
  
- CTTTCTCTTC TATCTCCTCT CTATTTATAA TCGCCCTAAT CCCGTTTAAA ACTATACGAC CCGAGAAGGA   
  
  
- GTAAGTTGAG ACTCCTTCTT CTAGTTCTAG TACGGTTGGG TGGAGGCGGC GTTGTTGGTG GTGGCAATGT   
  
  
- CGACGGTGAA GGTGCCGGTT CTAAGTCGGT CAATTATAGT AGTAGTGGAG GTTATTATAG TCGTAGAAGA   
  
  
- AGTAGTAATT AGGTATTATT GGGAGCAGTC GACGATTATT CAACGCCACT TGAGTAGAGG GCGACACTGA   
  
  
- AGAGGAGTCG ATTGGCGGAA GAGAGAGAAG AGAGGCCGCT GAAGCGAAGG AGGAGGCCGC TGAGGTGACT   
  
  
- TGCGGAACAA ATAATGAAGA GGTTCCGAAA CAAGGAGGCG GAAGCGGCTG TCAAAAGAGA TAGAAGAAGT   
  
  
- TGAAAATGAC CAATATCAAC ATTGGGATCA ACAAGACGCA GATAACGAGG TGAAAAAGAT TGGTTGGGAT   
  
  
- TAGGATCTGG TGGTGTTCTT GACGGCCATG CTCATTGATC GTCCCAATGA GGTTCATCGA CGAGCAGAAT   
  
  
- GGATTGGGAT TTGGTCTAGT GAGGTAAATA GGCCAAGTGG GTGGACTGCC GGTTGGTCCG TTAAAACCTC   
  
  
- CGACACCTCC CGATATCCCG TCAGGTGTAG TAGCTGTACC TGTAGTACGT ACCTCACGTC ACCGGAGGCG   
  
  
- AGGAGGTTCG TTAGCACCTC GCCAGCCGGT GGGAATCGGT TGGAGGGGGA CACCAGGCAG AGTTTCCCCC   
  
  
- TCCCGGCCTG GGCCTGGAGA ATGTCTCCTG CCCCCTGGCT GATACCTTGA AACGGGTTAG TAATCCTGAT   
  
  
- CTCAAGGTAA AGGTCCCGGA GTACTAGGGG CGGCTCAGTT GTCCAAAACA ACTTGGACGA CTCCGTCGCC   
  
  
- AGTTACGGGA TCGGAACGTC CTCCGCCACC CCACAGTTCT TCCCCTCCGG GAACGTCAAG TGACGCCCCT   
  
  
- GATAGAGGTG GCGGAGAATT TTCTCATGCT GTGTTCCGGA GAATCTGCCA AGGAGGTGTT CCAGTTCTGA   
  
  
- GACTTGGGGT CCCAGAACTG GAACCCTCTC TCCCTTCGGC TGGTGTTGGT GGGAGAGAAG GACGCCGCCA   
  
  
- AGTGGCTCCG TCAGTTGGTG ATGCCCCGTC ACAAACTGAG GGACCTTCGT TGGGAAGGCG GCGTATCGGT   
  
  
- TCTCTCCCAT CGACACCTCC TTCCCACCAA GCCTCTCCTC TAATTCCTGC ACCATCCTCT TCTTTCGCTC   
  
  
- TCCTCTTATC TCTCCGTGGT CTTCAAGCTC AGCACCCTTC GTAACAACTC CTCGAGTCCC AAGTTCTCAC   
  
  
- ACGGGGAGTC GGGTAAGAGC GACAGGGTTC GATTCGACGA CGACGCGGAG GTAATGGGAA GGCTTCCCAT   
  
  
- GGTTGAGGTC CAAAACGTAT CATTAACGAA CAACGAACCC ACCTTCTTAG CGGGAGAAAA AAGACAGAGA   
  
  
- AGAACCGTTA T

+     Unnamed\_\_16

| Site Name | Organism | Position | Strand | Matrix score. | sequence | function |
| --- | --- | --- | --- | --- | --- | --- |
| Unnamed\_\_16 | Zea mays | 2512 | + | 9 | GCTGCCCGTC |  |

>HU03G01737.1   
+ +Up\_Stream \_Len000TTTTTT TTAAGTTTTC GTACTTGGTG TTGATGATTT GTGCTTTGTA ACCAATGGTT   
  
  
+ TTAGTAAATA GTAATTAGTC GTAACATTCT GCGATTTCGC TCAATTTATA TATCAACTTT AGAATATGAG   
  
  
+ TAACCGTAAA GATCAAACGT AGTAGTAGAG GTGTCATAGG TTTTTTTTTA AAAAAATTCG GAGTAAGAAA   
  
  
+ GTAGGGTACA TTATTTTTGC TTGTTAAATT CCGCTAGTAT AGCAATACGA GATGGAACAT TTGTAAAAAC   
  
  
+ TACGAGACGA AGCGTTTGTA CAAAACAAAC TACCATACTG CAGATCATAT AATTTTTTGG GGGGTTATGT   
  
  
+ ATCTTAGTGA TGACATTAAT TGATGGATAA CCCTATTCTA AATATCTGGA TCCATCATTA ACTGACTCGT   
  
  
+ TTCAAATGTT AGGATTATGG GTCTTGACAT GGTATACATG TCAATAGATT TATGTTAGAT GAAAAGTTAT   
  
  
+ ATGACGCTTT TTATGATTTT TGTACTAGCA TATAGTGCTT AATTGGACTA TATATTTTTA CAAGTTGTGC   
  
  
+ TTAACACCTT TTAATCAGAA ATATTTATTA TATGGAAAAA ATTTTATTAA TATGTGTTTA TGTGTTTAAA   
  
  
+ AAACATACCC AGCCTTGAAA TAGACCGTGA TATAGAAATT GGACCCATAA TACTCACATT ATATATGCTT   
  
  
+ TATTGTTATA ATTAAGGATC ATTGTTGTTT TGCAATTATG TGGCAAGATA TAATCCACAA AGTTTTACAT   
  
  
+ TTATATTGCA ATTACTCTGG TAAATGCTAT TAATGTACAT TTGAGATTAA GACATATATA TAACCCTTTT   
  
  
+ TTTTCCCTTC TTTTTTCCTT TTTCTATTTT GAATGGCATC TTAAACCAAA GGAGCTGTAC AAGTTACGTT   
  
  
+ ATAGCACTCA TGGTTGACTA AACAAGACAG ATGAGAGTTT AAGTCAAGTC CTCCTGGCAA TGATGTTTGC   
  
  
+ CATTTGGACC AATAAATACA TCATGTATAT TACCATAATA GTGTGGTTGA CTTAACAATA CATAGGGGAT   
  
  
+ TGCATACAAA GGTAAGCCTC AAAAACTTGT GCATATGCAT TGAAATCCAT GATCAAAAAA ATTCAAGAGC   
  
  
+ AGGTTGCACA AAAACACGGA GATGAGATGA AAGAGACAGA AAACACATAT ACATTCACAG TAGTAGGGGT   
  
  
+ ACTGGTAGAG CTATGGCTAG GCTAAGATGA TGTGAAAGAA ACAATATAAT GGTAGTAACT GGTAATATTT   
  
  
+ ATAGGGCACA TATAGGTGCG GGCATAGGGT CGTGTCTCTC AGTCTCAGGT ACGGTTCGAT GGGACTCTGC   
  
  
+ GATAGATGTT ATCAAATTCA ACCATTCGAA CATCTCCCAG AGAGAGAGAG AGAGACTGAG TGAGTGAGTG   
  
  
+ AGTGGGTGGG TGTATGTGTG TGAGTTGTTT TGTTCATTTA TTATAGGTTC TCTGCTGCAT ATATCTCCAC   
  
  
+ CATTAAATTT ACCAAAACTT GTACCAGCTG CTTTACTTGC TTTCCTCATT TTCCCCCTTT TTTGTCTCTC   
  
  
+ TCTCTCTCTC TCCCCCCCCC CCCCCCCCTC TCTCTCTGTG TCTGAATCAG TATCAGCCTA TCAGGATGCT   
  
  
+ TCGCCTCCAG AAAGGCATCA CTCACACACT TATTAGCTTA AAACTTTCTT TTATTCCTTT TGCTCTTTCA   
  
  
+ CTGCTCTCTT TTACCCAAAT ACGCCCGTCT TCAATTCTTC TCTCATTAAA TATGAAAAAG AAAGAGAGAA   
  
  
+ ATAGAGAGAG ATTTAAACCA GAAGGGGTGA TTCCTTTTTC TTGGTGGGCT TATTAATGCT CTGTCTACCC   
  
  
+ CATCTAGCCG AAAAATAGTA GCTTAAGACT CAACTGCTTA TTAGCATCTC TCCTCTAGCA ATAACCGAGG   
  
  
+ TAATAGCATA TTTTTCTCCC CTTAGCTTCT ACCTTGCTTT TTGTGACACT CAAACCTAAA CTGAGAGAGA   
  
  
+ GAAAGAGAAG ATAGAGGAGA GATAAATATT AGCGGGATTA GGGCAAATTT TGATATGCTG GGCTCTTCCT   
  
  
+ CATTCAACTC TGAGGAAGAA GATCAAGATC ATGCCAACCC ACCTCCGCCG CAACAACCAC CACCGTTACA   
  
  
+ GCTGCCACTT CCACGGCCAA GATTCAGCCA GTTAATATCA TCATCACCTC CAATAATATC AGCATCTTCT   
  
  
+ TCATCATTAA TCCATAATAA CCCTCGTCAG CTGCTAATAA GTTGCGGTGA ACTCATCTCC CGCTGTGACT   
  
  
+ TCTCCTCAGC TAACCGCCTT CTCTCTCTTC TCTCCGGCGA CTTCGCTTCC TCCTCCGGCG ACTCCACTGA   
  
  
+ ACGCCTTGTT TATTACTTCT CCAAGGCTTT GTTCCTCCGC CTTCGCCGAC AGTTTTCTCT ATCTTCTTCA   
  
  
+ ACTTTTACTG GTTATAGTTG TAACCCTAGT TGTTCTGCGT CTATTGCTCC ACTTTTTCTA ACCAACCCTA   
  
  
+ ATCCTAGACC ACCACAAGAA CTGCCGGTAC GAGTAACTAG CAGGGTTACT CCAAGTAGCT GCTCGTCTTA   
  
  
+ CCTAACCCTA AACCAGATCA CTCCATTTAT CCGGTTCACC CACCTGACGG CCAACCAGGC AATTTTGGAG   
  
  
+ GCTGTGGAGG GCTATAGGGC AGTCCACATC ATCGACATGG ACATCATGCA TGGAGTGCAG TGGCCTCCGC   
  
  
+ TCCTCCAAGC AATCGTGGAG CGGTCGGCCA CCCTTAGCCA ACCTCCCCCT GTGGTCCGTC TCAAAGGGGG   
  
  
+ AGGGCCGGAC CCGGACCTCT TACAGAGGAC GGGGGACCGA CTATGGAACT TTGCCCAATC ATTAGGACTA   
  
  
+ GAGTTCCATT TCCAGGGCCT CATGATCCCC GCCGAGTCAA CAGGTTTTGT TGAACCTGCT GAGGCAGCGG   
  
  
+ TCAATGCCCT AGCCTTGCAG GAGGCGGTGG GGTGTCAAGA AGGGGAGGCC CTTGCAGTTC ACTGCGGGGA   
  
  
+ CTATCTCCAC CGCCTCTTAA AAGAGTACGA CACAAGGCCT CTTAGACGGT TCCTCCACAA GGTCAAGACT   
  
  
+ CTGAACCCCA GGGTCTTGAC CTTGGGAGAG AGGGAAGCCG ACCACAACCA CCCTCTCTTC CTGCGGCGGT   
  
  
+ TCACCGAGGC AGTCAACCAC TACGGGGCAG TGTTTGACTC CCTGGAAGCA ACCCTTCCGC CGCATAGCCA   
  
  
+ AGAGAGGGTA GCTGTGGAGG AAGGGTGGTT CGGAGAGGAG ATTAAGGACG TGGTAGGAGA AGAAAGCGAG   
  
  
+ AGGAGAATAG AGAGGCACCA GAAGTTCGAG TCGTGGGAAG CATTGTTGAG GAGCTCAGGG TTCAAGAGTG   
  
  
+ TGCCCCTCAG CCCATTCTCG CTGTCCCAAG CTAAGCTGCT GCTGCGCCTC CATTACCCTT CCGAAGGGTA   
  
  
+ CCAACTCCAG GTTTTGCATA GTAATTGCTT GTTGCTTGGG TGGAAGAATC GCCCTCTTTT TTCTGTCTCT   
  
  
+ TCTTGGCAAT A  

- +Up\_Stream \_Len000AAAAAA AATTCAAAAG CATGAACCAC AACTACTAAA CACGAAACAT TGGTTACCAA   
  
  
- AATCATTTAT CATTAATCAG CATTGTAAGA CGCTAAAGCG AGTTAAATAT ATAGTTGAAA TCTTATACTC   
  
  
- ATTGGCATTT CTAGTTTGCA TCATCATCTC CACAGTATCC AAAAAAAAAT TTTTTTAAGC CTCATTCTTT   
  
  
- CATCCCATGT AATAAAAACG AACAATTTAA GGCGATCATA TCGTTATGCT CTACCTTGTA AACATTTTTG   
  
  
- ATGCTCTGCT TCGCAAACAT GTTTTGTTTG ATGGTATGAC GTCTAGTATA TTAAAAAACC CCCCAATACA   
  
  
- TAGAATCACT ACTGTAATTA ACTACCTATT GGGATAAGAT TTATAGACCT AGGTAGTAAT TGACTGAGCA   
  
  
- AAGTTTACAA TCCTAATACC CAGAACTGTA CCATATGTAC AGTTATCTAA ATACAATCTA CTTTTCAATA   
  
  
- TACTGCGAAA AATACTAAAA ACATGATCGT ATATCACGAA TTAACCTGAT ATATAAAAAT GTTCAACACG   
  
  
- AATTGTGGAA AATTAGTCTT TATAAATAAT ATACCTTTTT TAAAATAATT ATACACAAAT ACACAAATTT   
  
  
- TTTGTATGGG TCGGAACTTT ATCTGGCACT ATATCTTTAA CCTGGGTATT ATGAGTGTAA TATATACGAA   
  
  
- ATAACAATAT TAATTCCTAG TAACAACAAA ACGTTAATAC ACCGTTCTAT ATTAGGTGTT TCAAAATGTA   
  
  
- AATATAACGT TAATGAGACC ATTTACGATA ATTACATGTA AACTCTAATT CTGTATATAT ATTGGGAAAA   
  
  
- AAAAGGGAAG AAAAAAGGAA AAAGATAAAA CTTACCGTAG AATTTGGTTT CCTCGACATG TTCAATGCAA   
  
  
- TATCGTGAGT ACCAACTGAT TTGTTCTGTC TACTCTCAAA TTCAGTTCAG GAGGACCGTT ACTACAAACG   
  
  
- GTAAACCTGG TTATTTATGT AGTACATATA ATGGTATTAT CACACCAACT GAATTGTTAT GTATCCCCTA   
  
  
- ACGTATGTTT CCATTCGGAG TTTTTGAACA CGTATACGTA ACTTTAGGTA CTAGTTTTTT TAAGTTCTCG   
  
  
- TCCAACGTGT TTTTGTGCCT CTACTCTACT TTCTCTGTCT TTTGTGTATA TGTAAGTGTC ATCATCCCCA   
  
  
- TGACCATCTC GATACCGATC CGATTCTACT ACACTTTCTT TGTTATATTA CCATCATTGA CCATTATAAA   
  
  
- TATCCCGTGT ATATCCACGC CCGTATCCCA GCACAGAGAG TCAGAGTCCA TGCCAAGCTA CCCTGAGACG   
  
  
- CTATCTACAA TAGTTTAAGT TGGTAAGCTT GTAGAGGGTC TCTCTCTCTC TCTCTGACTC ACTCACTCAC   
  
  
- TCACCCACCC ACATACACAC ACTCAACAAA ACAAGTAAAT AATATCCAAG AGACGACGTA TATAGAGGTG   
  
  
- GTAATTTAAA TGGTTTTGAA CATGGTCGAC GAAATGAACG AAAGGAGTAA AAGGGGGAAA AAACAGAGAG   
  
  
- AGAGAGAGAG AGGGGGGGGG GGGGGGGGAG AGAGAGACAC AGACTTAGTC ATAGTCGGAT AGTCCTACGA   
  
  
- AGCGGAGGTC TTTCCGTAGT GAGTGTGTGA ATAATCGAAT TTTGAAAGAA AATAAGGAAA ACGAGAAAGT   
  
  
- GACGAGAGAA AATGGGTTTA TGCGGGCAGA AGTTAAGAAG AGAGTAATTT ATACTTTTTC TTTCTCTCTT   
  
  
- TATCTCTCTC TAAATTTGGT CTTCCCCACT AAGGAAAAAG AACCACCCGA ATAATTACGA GACAGATGGG   
  
  
- GTAGATCGGC TTTTTATCAT CGAATTCTGA GTTGACGAAT AATCGTAGAG AGGAGATCGT TATTGGCTCC   
  
  
- ATTATCGTAT AAAAAGAGGG GAATCGAAGA TGGAACGAAA AACACTGTGA GTTTGGATTT GACTCTCTCT   
  
  
- CTTTCTCTTC TATCTCCTCT CTATTTATAA TCGCCCTAAT CCCGTTTAAA ACTATACGAC CCGAGAAGGA   
  
  
- GTAAGTTGAG ACTCCTTCTT CTAGTTCTAG TACGGTTGGG TGGAGGCGGC GTTGTTGGTG GTGGCAATGT   
  
  
- CGACGGTGAA GGTGCCGGTT CTAAGTCGGT CAATTATAGT AGTAGTGGAG GTTATTATAG TCGTAGAAGA   
  
  
- AGTAGTAATT AGGTATTATT GGGAGCAGTC GACGATTATT CAACGCCACT TGAGTAGAGG GCGACACTGA   
  
  
- AGAGGAGTCG ATTGGCGGAA GAGAGAGAAG AGAGGCCGCT GAAGCGAAGG AGGAGGCCGC TGAGGTGACT   
  
  
- TGCGGAACAA ATAATGAAGA GGTTCCGAAA CAAGGAGGCG GAAGCGGCTG TCAAAAGAGA TAGAAGAAGT   
  
  
- TGAAAATGAC CAATATCAAC ATTGGGATCA ACAAGACGCA GATAACGAGG TGAAAAAGAT TGGTTGGGAT   
  
  
- TAGGATCTGG TGGTGTTCTT GACGGCCATG CTCATTGATC GTCCCAATGA GGTTCATCGA CGAGCAGAAT   
  
  
- GGATTGGGAT TTGGTCTAGT GAGGTAAATA GGCCAAGTGG GTGGACTGCC GGTTGGTCCG TTAAAACCTC   
  
  
- CGACACCTCC CGATATCCCG TCAGGTGTAG TAGCTGTACC TGTAGTACGT ACCTCACGTC ACCGGAGGCG   
  
  
- AGGAGGTTCG TTAGCACCTC GCCAGCCGGT GGGAATCGGT TGGAGGGGGA CACCAGGCAG AGTTTCCCCC   
  
  
- TCCCGGCCTG GGCCTGGAGA ATGTCTCCTG CCCCCTGGCT GATACCTTGA AACGGGTTAG TAATCCTGAT   
  
  
- CTCAAGGTAA AGGTCCCGGA GTACTAGGGG CGGCTCAGTT GTCCAAAACA ACTTGGACGA CTCCGTCGCC   
  
  
- AGTTACGGGA TCGGAACGTC CTCCGCCACC CCACAGTTCT TCCCCTCCGG GAACGTCAAG TGACGCCCCT   
  
  
- GATAGAGGTG GCGGAGAATT TTCTCATGCT GTGTTCCGGA GAATCTGCCA AGGAGGTGTT CCAGTTCTGA   
  
  
- GACTTGGGGT CCCAGAACTG GAACCCTCTC TCCCTTCGGC TGGTGTTGGT GGGAGAGAAG GACGCCGCCA   
  
  
- AGTGGCTCCG TCAGTTGGTG ATGCCCCGTC ACAAACTGAG GGACCTTCGT TGGGAAGGCG GCGTATCGGT   
  
  
- TCTCTCCCAT CGACACCTCC TTCCCACCAA GCCTCTCCTC TAATTCCTGC ACCATCCTCT TCTTTCGCTC   
  
  
- TCCTCTTATC TCTCCGTGGT CTTCAAGCTC AGCACCCTTC GTAACAACTC CTCGAGTCCC AAGTTCTCAC   
  
  
- ACGGGGAGTC GGGTAAGAGC GACAGGGTTC GATTCGACGA CGACGCGGAG GTAATGGGAA GGCTTCCCAT   
  
  
- GGTTGAGGTC CAAAACGTAT CATTAACGAA CAACGAACCC ACCTTCTTAG CGGGAGAAAA AAGACAGAGA   
  
  
- AGAACCGTTA T

+     Unnamed\_\_2

| Site Name | Organism | Position | Strand | Matrix score. | sequence | function |
| --- | --- | --- | --- | --- | --- | --- |
| Unnamed\_\_2 | Petroselinum hortense | 1947 | + | 9 | AACCTAACCT |  |

>HU03G01737.1   
+ +Up\_Stream \_Len000TTTTTT TTAAGTTTTC GTACTTGGTG TTGATGATTT GTGCTTTGTA ACCAATGGTT   
  
  
+ TTAGTAAATA GTAATTAGTC GTAACATTCT GCGATTTCGC TCAATTTATA TATCAACTTT AGAATATGAG   
  
  
+ TAACCGTAAA GATCAAACGT AGTAGTAGAG GTGTCATAGG TTTTTTTTTA AAAAAATTCG GAGTAAGAAA   
  
  
+ GTAGGGTACA TTATTTTTGC TTGTTAAATT CCGCTAGTAT AGCAATACGA GATGGAACAT TTGTAAAAAC   
  
  
+ TACGAGACGA AGCGTTTGTA CAAAACAAAC TACCATACTG CAGATCATAT AATTTTTTGG GGGGTTATGT   
  
  
+ ATCTTAGTGA TGACATTAAT TGATGGATAA CCCTATTCTA AATATCTGGA TCCATCATTA ACTGACTCGT   
  
  
+ TTCAAATGTT AGGATTATGG GTCTTGACAT GGTATACATG TCAATAGATT TATGTTAGAT GAAAAGTTAT   
  
  
+ ATGACGCTTT TTATGATTTT TGTACTAGCA TATAGTGCTT AATTGGACTA TATATTTTTA CAAGTTGTGC   
  
  
+ TTAACACCTT TTAATCAGAA ATATTTATTA TATGGAAAAA ATTTTATTAA TATGTGTTTA TGTGTTTAAA   
  
  
+ AAACATACCC AGCCTTGAAA TAGACCGTGA TATAGAAATT GGACCCATAA TACTCACATT ATATATGCTT   
  
  
+ TATTGTTATA ATTAAGGATC ATTGTTGTTT TGCAATTATG TGGCAAGATA TAATCCACAA AGTTTTACAT   
  
  
+ TTATATTGCA ATTACTCTGG TAAATGCTAT TAATGTACAT TTGAGATTAA GACATATATA TAACCCTTTT   
  
  
+ TTTTCCCTTC TTTTTTCCTT TTTCTATTTT GAATGGCATC TTAAACCAAA GGAGCTGTAC AAGTTACGTT   
  
  
+ ATAGCACTCA TGGTTGACTA AACAAGACAG ATGAGAGTTT AAGTCAAGTC CTCCTGGCAA TGATGTTTGC   
  
  
+ CATTTGGACC AATAAATACA TCATGTATAT TACCATAATA GTGTGGTTGA CTTAACAATA CATAGGGGAT   
  
  
+ TGCATACAAA GGTAAGCCTC AAAAACTTGT GCATATGCAT TGAAATCCAT GATCAAAAAA ATTCAAGAGC   
  
  
+ AGGTTGCACA AAAACACGGA GATGAGATGA AAGAGACAGA AAACACATAT ACATTCACAG TAGTAGGGGT   
  
  
+ ACTGGTAGAG CTATGGCTAG GCTAAGATGA TGTGAAAGAA ACAATATAAT GGTAGTAACT GGTAATATTT   
  
  
+ ATAGGGCACA TATAGGTGCG GGCATAGGGT CGTGTCTCTC AGTCTCAGGT ACGGTTCGAT GGGACTCTGC   
  
  
+ GATAGATGTT ATCAAATTCA ACCATTCGAA CATCTCCCAG AGAGAGAGAG AGAGACTGAG TGAGTGAGTG   
  
  
+ AGTGGGTGGG TGTATGTGTG TGAGTTGTTT TGTTCATTTA TTATAGGTTC TCTGCTGCAT ATATCTCCAC   
  
  
+ CATTAAATTT ACCAAAACTT GTACCAGCTG CTTTACTTGC TTTCCTCATT TTCCCCCTTT TTTGTCTCTC   
  
  
+ TCTCTCTCTC TCCCCCCCCC CCCCCCCCTC TCTCTCTGTG TCTGAATCAG TATCAGCCTA TCAGGATGCT   
  
  
+ TCGCCTCCAG AAAGGCATCA CTCACACACT TATTAGCTTA AAACTTTCTT TTATTCCTTT TGCTCTTTCA   
  
  
+ CTGCTCTCTT TTACCCAAAT ACGCCCGTCT TCAATTCTTC TCTCATTAAA TATGAAAAAG AAAGAGAGAA   
  
  
+ ATAGAGAGAG ATTTAAACCA GAAGGGGTGA TTCCTTTTTC TTGGTGGGCT TATTAATGCT CTGTCTACCC   
  
  
+ CATCTAGCCG AAAAATAGTA GCTTAAGACT CAACTGCTTA TTAGCATCTC TCCTCTAGCA ATAACCGAGG   
  
  
+ TAATAGCATA TTTTTCTCCC CTTAGCTTCT ACCTTGCTTT TTGTGACACT CAAACCTAAA CTGAGAGAGA   
  
  
+ GAAAGAGAAG ATAGAGGAGA GATAAATATT AGCGGGATTA GGGCAAATTT TGATATGCTG GGCTCTTCCT   
  
  
+ CATTCAACTC TGAGGAAGAA GATCAAGATC ATGCCAACCC ACCTCCGCCG CAACAACCAC CACCGTTACA   
  
  
+ GCTGCCACTT CCACGGCCAA GATTCAGCCA GTTAATATCA TCATCACCTC CAATAATATC AGCATCTTCT   
  
  
+ TCATCATTAA TCCATAATAA CCCTCGTCAG CTGCTAATAA GTTGCGGTGA ACTCATCTCC CGCTGTGACT   
  
  
+ TCTCCTCAGC TAACCGCCTT CTCTCTCTTC TCTCCGGCGA CTTCGCTTCC TCCTCCGGCG ACTCCACTGA   
  
  
+ ACGCCTTGTT TATTACTTCT CCAAGGCTTT GTTCCTCCGC CTTCGCCGAC AGTTTTCTCT ATCTTCTTCA   
  
  
+ ACTTTTACTG GTTATAGTTG TAACCCTAGT TGTTCTGCGT CTATTGCTCC ACTTTTTCTA ACCAACCCTA   
  
  
+ ATCCTAGACC ACCACAAGAA CTGCCGGTAC GAGTAACTAG CAGGGTTACT CCAAGTAGCT GCTCGTCTTA   
  
  
+ CCTAACCCTA AACCAGATCA CTCCATTTAT CCGGTTCACC CACCTGACGG CCAACCAGGC AATTTTGGAG   
  
  
+ GCTGTGGAGG GCTATAGGGC AGTCCACATC ATCGACATGG ACATCATGCA TGGAGTGCAG TGGCCTCCGC   
  
  
+ TCCTCCAAGC AATCGTGGAG CGGTCGGCCA CCCTTAGCCA ACCTCCCCCT GTGGTCCGTC TCAAAGGGGG   
  
  
+ AGGGCCGGAC CCGGACCTCT TACAGAGGAC GGGGGACCGA CTATGGAACT TTGCCCAATC ATTAGGACTA   
  
  
+ GAGTTCCATT TCCAGGGCCT CATGATCCCC GCCGAGTCAA CAGGTTTTGT TGAACCTGCT GAGGCAGCGG   
  
  
+ TCAATGCCCT AGCCTTGCAG GAGGCGGTGG GGTGTCAAGA AGGGGAGGCC CTTGCAGTTC ACTGCGGGGA   
  
  
+ CTATCTCCAC CGCCTCTTAA AAGAGTACGA CACAAGGCCT CTTAGACGGT TCCTCCACAA GGTCAAGACT   
  
  
+ CTGAACCCCA GGGTCTTGAC CTTGGGAGAG AGGGAAGCCG ACCACAACCA CCCTCTCTTC CTGCGGCGGT   
  
  
+ TCACCGAGGC AGTCAACCAC TACGGGGCAG TGTTTGACTC CCTGGAAGCA ACCCTTCCGC CGCATAGCCA   
  
  
+ AGAGAGGGTA GCTGTGGAGG AAGGGTGGTT CGGAGAGGAG ATTAAGGACG TGGTAGGAGA AGAAAGCGAG   
  
  
+ AGGAGAATAG AGAGGCACCA GAAGTTCGAG TCGTGGGAAG CATTGTTGAG GAGCTCAGGG TTCAAGAGTG   
  
  
+ TGCCCCTCAG CCCATTCTCG CTGTCCCAAG CTAAGCTGCT GCTGCGCCTC CATTACCCTT CCGAAGGGTA   
  
  
+ CCAACTCCAG GTTTTGCATA GTAATTGCTT GTTGCTTGGG TGGAAGAATC GCCCTCTTTT TTCTGTCTCT   
  
  
+ TCTTGGCAAT A  

- +Up\_Stream \_Len000AAAAAA AATTCAAAAG CATGAACCAC AACTACTAAA CACGAAACAT TGGTTACCAA   
  
  
- AATCATTTAT CATTAATCAG CATTGTAAGA CGCTAAAGCG AGTTAAATAT ATAGTTGAAA TCTTATACTC   
  
  
- ATTGGCATTT CTAGTTTGCA TCATCATCTC CACAGTATCC AAAAAAAAAT TTTTTTAAGC CTCATTCTTT   
  
  
- CATCCCATGT AATAAAAACG AACAATTTAA GGCGATCATA TCGTTATGCT CTACCTTGTA AACATTTTTG   
  
  
- ATGCTCTGCT TCGCAAACAT GTTTTGTTTG ATGGTATGAC GTCTAGTATA TTAAAAAACC CCCCAATACA   
  
  
- TAGAATCACT ACTGTAATTA ACTACCTATT GGGATAAGAT TTATAGACCT AGGTAGTAAT TGACTGAGCA   
  
  
- AAGTTTACAA TCCTAATACC CAGAACTGTA CCATATGTAC AGTTATCTAA ATACAATCTA CTTTTCAATA   
  
  
- TACTGCGAAA AATACTAAAA ACATGATCGT ATATCACGAA TTAACCTGAT ATATAAAAAT GTTCAACACG   
  
  
- AATTGTGGAA AATTAGTCTT TATAAATAAT ATACCTTTTT TAAAATAATT ATACACAAAT ACACAAATTT   
  
  
- TTTGTATGGG TCGGAACTTT ATCTGGCACT ATATCTTTAA CCTGGGTATT ATGAGTGTAA TATATACGAA   
  
  
- ATAACAATAT TAATTCCTAG TAACAACAAA ACGTTAATAC ACCGTTCTAT ATTAGGTGTT TCAAAATGTA   
  
  
- AATATAACGT TAATGAGACC ATTTACGATA ATTACATGTA AACTCTAATT CTGTATATAT ATTGGGAAAA   
  
  
- AAAAGGGAAG AAAAAAGGAA AAAGATAAAA CTTACCGTAG AATTTGGTTT CCTCGACATG TTCAATGCAA   
  
  
- TATCGTGAGT ACCAACTGAT TTGTTCTGTC TACTCTCAAA TTCAGTTCAG GAGGACCGTT ACTACAAACG   
  
  
- GTAAACCTGG TTATTTATGT AGTACATATA ATGGTATTAT CACACCAACT GAATTGTTAT GTATCCCCTA   
  
  
- ACGTATGTTT CCATTCGGAG TTTTTGAACA CGTATACGTA ACTTTAGGTA CTAGTTTTTT TAAGTTCTCG   
  
  
- TCCAACGTGT TTTTGTGCCT CTACTCTACT TTCTCTGTCT TTTGTGTATA TGTAAGTGTC ATCATCCCCA   
  
  
- TGACCATCTC GATACCGATC CGATTCTACT ACACTTTCTT TGTTATATTA CCATCATTGA CCATTATAAA   
  
  
- TATCCCGTGT ATATCCACGC CCGTATCCCA GCACAGAGAG TCAGAGTCCA TGCCAAGCTA CCCTGAGACG   
  
  
- CTATCTACAA TAGTTTAAGT TGGTAAGCTT GTAGAGGGTC TCTCTCTCTC TCTCTGACTC ACTCACTCAC   
  
  
- TCACCCACCC ACATACACAC ACTCAACAAA ACAAGTAAAT AATATCCAAG AGACGACGTA TATAGAGGTG   
  
  
- GTAATTTAAA TGGTTTTGAA CATGGTCGAC GAAATGAACG AAAGGAGTAA AAGGGGGAAA AAACAGAGAG   
  
  
- AGAGAGAGAG AGGGGGGGGG GGGGGGGGAG AGAGAGACAC AGACTTAGTC ATAGTCGGAT AGTCCTACGA   
  
  
- AGCGGAGGTC TTTCCGTAGT GAGTGTGTGA ATAATCGAAT TTTGAAAGAA AATAAGGAAA ACGAGAAAGT   
  
  
- GACGAGAGAA AATGGGTTTA TGCGGGCAGA AGTTAAGAAG AGAGTAATTT ATACTTTTTC TTTCTCTCTT   
  
  
- TATCTCTCTC TAAATTTGGT CTTCCCCACT AAGGAAAAAG AACCACCCGA ATAATTACGA GACAGATGGG   
  
  
- GTAGATCGGC TTTTTATCAT CGAATTCTGA GTTGACGAAT AATCGTAGAG AGGAGATCGT TATTGGCTCC   
  
  
- ATTATCGTAT AAAAAGAGGG GAATCGAAGA TGGAACGAAA AACACTGTGA GTTTGGATTT GACTCTCTCT   
  
  
- CTTTCTCTTC TATCTCCTCT CTATTTATAA TCGCCCTAAT CCCGTTTAAA ACTATACGAC CCGAGAAGGA   
  
  
- GTAAGTTGAG ACTCCTTCTT CTAGTTCTAG TACGGTTGGG TGGAGGCGGC GTTGTTGGTG GTGGCAATGT   
  
  
- CGACGGTGAA GGTGCCGGTT CTAAGTCGGT CAATTATAGT AGTAGTGGAG GTTATTATAG TCGTAGAAGA   
  
  
- AGTAGTAATT AGGTATTATT GGGAGCAGTC GACGATTATT CAACGCCACT TGAGTAGAGG GCGACACTGA   
  
  
- AGAGGAGTCG ATTGGCGGAA GAGAGAGAAG AGAGGCCGCT GAAGCGAAGG AGGAGGCCGC TGAGGTGACT   
  
  
- TGCGGAACAA ATAATGAAGA GGTTCCGAAA CAAGGAGGCG GAAGCGGCTG TCAAAAGAGA TAGAAGAAGT   
  
  
- TGAAAATGAC CAATATCAAC ATTGGGATCA ACAAGACGCA GATAACGAGG TGAAAAAGAT TGGTTGGGAT   
  
  
- TAGGATCTGG TGGTGTTCTT GACGGCCATG CTCATTGATC GTCCCAATGA GGTTCATCGA CGAGCAGAAT   
  
  
- GGATTGGGAT TTGGTCTAGT GAGGTAAATA GGCCAAGTGG GTGGACTGCC GGTTGGTCCG TTAAAACCTC   
  
  
- CGACACCTCC CGATATCCCG TCAGGTGTAG TAGCTGTACC TGTAGTACGT ACCTCACGTC ACCGGAGGCG   
  
  
- AGGAGGTTCG TTAGCACCTC GCCAGCCGGT GGGAATCGGT TGGAGGGGGA CACCAGGCAG AGTTTCCCCC   
  
  
- TCCCGGCCTG GGCCTGGAGA ATGTCTCCTG CCCCCTGGCT GATACCTTGA AACGGGTTAG TAATCCTGAT   
  
  
- CTCAAGGTAA AGGTCCCGGA GTACTAGGGG CGGCTCAGTT GTCCAAAACA ACTTGGACGA CTCCGTCGCC   
  
  
- AGTTACGGGA TCGGAACGTC CTCCGCCACC CCACAGTTCT TCCCCTCCGG GAACGTCAAG TGACGCCCCT   
  
  
- GATAGAGGTG GCGGAGAATT TTCTCATGCT GTGTTCCGGA GAATCTGCCA AGGAGGTGTT CCAGTTCTGA   
  
  
- GACTTGGGGT CCCAGAACTG GAACCCTCTC TCCCTTCGGC TGGTGTTGGT GGGAGAGAAG GACGCCGCCA   
  
  
- AGTGGCTCCG TCAGTTGGTG ATGCCCCGTC ACAAACTGAG GGACCTTCGT TGGGAAGGCG GCGTATCGGT   
  
  
- TCTCTCCCAT CGACACCTCC TTCCCACCAA GCCTCTCCTC TAATTCCTGC ACCATCCTCT TCTTTCGCTC   
  
  
- TCCTCTTATC TCTCCGTGGT CTTCAAGCTC AGCACCCTTC GTAACAACTC CTCGAGTCCC AAGTTCTCAC   
  
  
- ACGGGGAGTC GGGTAAGAGC GACAGGGTTC GATTCGACGA CGACGCGGAG GTAATGGGAA GGCTTCCCAT   
  
  
- GGTTGAGGTC CAAAACGTAT CATTAACGAA CAACGAACCC ACCTTCTTAG CGGGAGAAAA AAGACAGAGA   
  
  
- AGAACCGTTA T

+     Unnamed\_\_4

| Site Name | Organism | Position | Strand | Matrix score. | sequence | function |
| --- | --- | --- | --- | --- | --- | --- |
| Unnamed\_\_4 | Petroselinum hortense | 2667 | + | 4 | CTCC |  |
| Unnamed\_\_4 | Petroselinum hortense | 2664 | + | 4 | CTCC |  |
| Unnamed\_\_4 | Petroselinum hortense | 2646 | - | 4 | CTCC |  |
| Unnamed\_\_4 | Petroselinum hortense | 2591 | - | 4 | CTCC |  |
| Unnamed\_\_4 | Petroselinum hortense | 2918 | - | 4 | CTCC |  |
| Unnamed\_\_4 | Petroselinum hortense | 1469 | + | 4 | CTCC |  |
| Unnamed\_\_4 | Petroselinum hortense | 3122 | + | 4 | CTCC |  |
| Unnamed\_\_4 | Petroselinum hortense | 1980 | - | 4 | CTCC |  |
| Unnamed\_\_4 | Petroselinum hortense | 1368 | + | 4 | CTCC |  |
| Unnamed\_\_4 | Petroselinum hortense | 895 | - | 4 | CTCC |  |
| Unnamed\_\_4 | Petroselinum hortense | 2294 | + | 4 | CTCC |  |
| Unnamed\_\_4 | Petroselinum hortense | 2276 | + | 4 | CTCC |  |
| Unnamed\_\_4 | Petroselinum hortense | 2545 | + | 4 | CTCC |  |
| Unnamed\_\_4 | Petroselinum hortense | 1874 | + | 4 | CTCC |  |
| Unnamed\_\_4 | Petroselinum hortense | 1910 | + | 4 | CTCC |  |
| Unnamed\_\_4 | Petroselinum hortense | 2681 | - | 4 | CTCC |  |
| Unnamed\_\_4 | Petroselinum hortense | 204 | - | 4 | CTCC |  |
| Unnamed\_\_4 | Petroselinum hortense | 2600 | - | 4 | CTCC |  |
| Unnamed\_\_4 | Petroselinum hortense | 2997 | + | 4 | CTCC |  |
| Unnamed\_\_4 | Petroselinum hortense | 3186 | - | 4 | CTCC |  |
| Unnamed\_\_4 | Petroselinum hortense | 3226 | - | 4 | CTCC |  |
| Unnamed\_\_4 | Petroselinum hortense | 3039 | - | 4 | CTCC |  |
| Unnamed\_\_4 | Petroselinum hortense | 2152 | + | 4 | CTCC |  |
| Unnamed\_\_4 | Petroselinum hortense | 2349 | + | 4 | CTCC |  |
| Unnamed\_\_4 | Petroselinum hortense | 2659 | + | 4 | CTCC |  |
| Unnamed\_\_4 | Petroselinum hortense | 965 | + | 4 | CTCC |  |
| Unnamed\_\_4 | Petroselinum hortense | 1554 | + | 4 | CTCC |  |
| Unnamed\_\_4 | Petroselinum hortense | 2306 | + | 4 | CTCC |  |
| Unnamed\_\_4 | Petroselinum hortense | 2077 | + | 4 | CTCC |  |
| Unnamed\_\_4 | Petroselinum hortense | 2246 | + | 4 | CTCC |  |
| Unnamed\_\_4 | Petroselinum hortense | 2231 | + | 4 | CTCC |  |
| Unnamed\_\_4 | Petroselinum hortense | 2503 | + | 4 | CTCC |  |
| Unnamed\_\_4 | Petroselinum hortense | 3191 | - | 4 | CTCC |  |
| Unnamed\_\_4 | Petroselinum hortense | 2297 | + | 4 | CTCC |  |
| Unnamed\_\_4 | Petroselinum hortense | 2733 | - | 4 | CTCC |  |
| Unnamed\_\_4 | Petroselinum hortense | 1142 | - | 4 | CTCC |  |
| Unnamed\_\_4 | Petroselinum hortense | 3369 | + | 4 | CTCC |  |
| Unnamed\_\_4 | Petroselinum hortense | 3170 | - | 4 | CTCC |  |
| Unnamed\_\_4 | Petroselinum hortense | 1619 | + | 4 | CTCC |  |
| Unnamed\_\_4 | Petroselinum hortense | 2431 | + | 4 | CTCC |  |
| Unnamed\_\_4 | Petroselinum hortense | 2949 | + | 4 | CTCC |  |
| Unnamed\_\_4 | Petroselinum hortense | 2333 | + | 4 | CTCC |  |
| Unnamed\_\_4 | Petroselinum hortense | 2707 | + | 4 | CTCC |  |
| Unnamed\_\_4 | Petroselinum hortense | 2894 | - | 4 | CTCC |  |
| Unnamed\_\_4 | Petroselinum hortense | 3274 | - | 4 | CTCC |  |
| Unnamed\_\_4 | Petroselinum hortense | 3210 | - | 4 | CTCC |  |
| Unnamed\_\_4 | Petroselinum hortense | 3342 | + | 4 | CTCC |  |

>HU03G01737.1   
+ +Up\_Stream \_Len000TTTTTT TTAAGTTTTC GTACTTGGTG TTGATGATTT GTGCTTTGTA ACCAATGGTT   
  
  
+ TTAGTAAATA GTAATTAGTC GTAACATTCT GCGATTTCGC TCAATTTATA TATCAACTTT AGAATATGAG   
  
  
+ TAACCGTAAA GATCAAACGT AGTAGTAGAG GTGTCATAGG TTTTTTTTTA AAAAAATTCG GAGTAAGAAA   
  
  
+ GTAGGGTACA TTATTTTTGC TTGTTAAATT CCGCTAGTAT AGCAATACGA GATGGAACAT TTGTAAAAAC   
  
  
+ TACGAGACGA AGCGTTTGTA CAAAACAAAC TACCATACTG CAGATCATAT AATTTTTTGG GGGGTTATGT   
  
  
+ ATCTTAGTGA TGACATTAAT TGATGGATAA CCCTATTCTA AATATCTGGA TCCATCATTA ACTGACTCGT   
  
  
+ TTCAAATGTT AGGATTATGG GTCTTGACAT GGTATACATG TCAATAGATT TATGTTAGAT GAAAAGTTAT   
  
  
+ ATGACGCTTT TTATGATTTT TGTACTAGCA TATAGTGCTT AATTGGACTA TATATTTTTA CAAGTTGTGC   
  
  
+ TTAACACCTT TTAATCAGAA ATATTTATTA TATGGAAAAA ATTTTATTAA TATGTGTTTA TGTGTTTAAA   
  
  
+ AAACATACCC AGCCTTGAAA TAGACCGTGA TATAGAAATT GGACCCATAA TACTCACATT ATATATGCTT   
  
  
+ TATTGTTATA ATTAAGGATC ATTGTTGTTT TGCAATTATG TGGCAAGATA TAATCCACAA AGTTTTACAT   
  
  
+ TTATATTGCA ATTACTCTGG TAAATGCTAT TAATGTACAT TTGAGATTAA GACATATATA TAACCCTTTT   
  
  
+ TTTTCCCTTC TTTTTTCCTT TTTCTATTTT GAATGGCATC TTAAACCAAA GGAGCTGTAC AAGTTACGTT   
  
  
+ ATAGCACTCA TGGTTGACTA AACAAGACAG ATGAGAGTTT AAGTCAAGTC CTCCTGGCAA TGATGTTTGC   
  
  
+ CATTTGGACC AATAAATACA TCATGTATAT TACCATAATA GTGTGGTTGA CTTAACAATA CATAGGGGAT   
  
  
+ TGCATACAAA GGTAAGCCTC AAAAACTTGT GCATATGCAT TGAAATCCAT GATCAAAAAA ATTCAAGAGC   
  
  
+ AGGTTGCACA AAAACACGGA GATGAGATGA AAGAGACAGA AAACACATAT ACATTCACAG TAGTAGGGGT   
  
  
+ ACTGGTAGAG CTATGGCTAG GCTAAGATGA TGTGAAAGAA ACAATATAAT GGTAGTAACT GGTAATATTT   
  
  
+ ATAGGGCACA TATAGGTGCG GGCATAGGGT CGTGTCTCTC AGTCTCAGGT ACGGTTCGAT GGGACTCTGC   
  
  
+ GATAGATGTT ATCAAATTCA ACCATTCGAA CATCTCCCAG AGAGAGAGAG AGAGACTGAG TGAGTGAGTG   
  
  
+ AGTGGGTGGG TGTATGTGTG TGAGTTGTTT TGTTCATTTA TTATAGGTTC TCTGCTGCAT ATATCTCCAC   
  
  
+ CATTAAATTT ACCAAAACTT GTACCAGCTG CTTTACTTGC TTTCCTCATT TTCCCCCTTT TTTGTCTCTC   
  
  
+ TCTCTCTCTC TCCCCCCCCC CCCCCCCCTC TCTCTCTGTG TCTGAATCAG TATCAGCCTA TCAGGATGCT   
  
  
+ TCGCCTCCAG AAAGGCATCA CTCACACACT TATTAGCTTA AAACTTTCTT TTATTCCTTT TGCTCTTTCA   
  
  
+ CTGCTCTCTT TTACCCAAAT ACGCCCGTCT TCAATTCTTC TCTCATTAAA TATGAAAAAG AAAGAGAGAA   
  
  
+ ATAGAGAGAG ATTTAAACCA GAAGGGGTGA TTCCTTTTTC TTGGTGGGCT TATTAATGCT CTGTCTACCC   
  
  
+ CATCTAGCCG AAAAATAGTA GCTTAAGACT CAACTGCTTA TTAGCATCTC TCCTCTAGCA ATAACCGAGG   
  
  
+ TAATAGCATA TTTTTCTCCC CTTAGCTTCT ACCTTGCTTT TTGTGACACT CAAACCTAAA CTGAGAGAGA   
  
  
+ GAAAGAGAAG ATAGAGGAGA GATAAATATT AGCGGGATTA GGGCAAATTT TGATATGCTG GGCTCTTCCT   
  
  
+ CATTCAACTC TGAGGAAGAA GATCAAGATC ATGCCAACCC ACCTCCGCCG CAACAACCAC CACCGTTACA   
  
  
+ GCTGCCACTT CCACGGCCAA GATTCAGCCA GTTAATATCA TCATCACCTC CAATAATATC AGCATCTTCT   
  
  
+ TCATCATTAA TCCATAATAA CCCTCGTCAG CTGCTAATAA GTTGCGGTGA ACTCATCTCC CGCTGTGACT   
  
  
+ TCTCCTCAGC TAACCGCCTT CTCTCTCTTC TCTCCGGCGA CTTCGCTTCC TCCTCCGGCG ACTCCACTGA   
  
  
+ ACGCCTTGTT TATTACTTCT CCAAGGCTTT GTTCCTCCGC CTTCGCCGAC AGTTTTCTCT ATCTTCTTCA   
  
  
+ ACTTTTACTG GTTATAGTTG TAACCCTAGT TGTTCTGCGT CTATTGCTCC ACTTTTTCTA ACCAACCCTA   
  
  
+ ATCCTAGACC ACCACAAGAA CTGCCGGTAC GAGTAACTAG CAGGGTTACT CCAAGTAGCT GCTCGTCTTA   
  
  
+ CCTAACCCTA AACCAGATCA CTCCATTTAT CCGGTTCACC CACCTGACGG CCAACCAGGC AATTTTGGAG   
  
  
+ GCTGTGGAGG GCTATAGGGC AGTCCACATC ATCGACATGG ACATCATGCA TGGAGTGCAG TGGCCTCCGC   
  
  
+ TCCTCCAAGC AATCGTGGAG CGGTCGGCCA CCCTTAGCCA ACCTCCCCCT GTGGTCCGTC TCAAAGGGGG   
  
  
+ AGGGCCGGAC CCGGACCTCT TACAGAGGAC GGGGGACCGA CTATGGAACT TTGCCCAATC ATTAGGACTA   
  
  
+ GAGTTCCATT TCCAGGGCCT CATGATCCCC GCCGAGTCAA CAGGTTTTGT TGAACCTGCT GAGGCAGCGG   
  
  
+ TCAATGCCCT AGCCTTGCAG GAGGCGGTGG GGTGTCAAGA AGGGGAGGCC CTTGCAGTTC ACTGCGGGGA   
  
  
+ CTATCTCCAC CGCCTCTTAA AAGAGTACGA CACAAGGCCT CTTAGACGGT TCCTCCACAA GGTCAAGACT   
  
  
+ CTGAACCCCA GGGTCTTGAC CTTGGGAGAG AGGGAAGCCG ACCACAACCA CCCTCTCTTC CTGCGGCGGT   
  
  
+ TCACCGAGGC AGTCAACCAC TACGGGGCAG TGTTTGACTC CCTGGAAGCA ACCCTTCCGC CGCATAGCCA   
  
  
+ AGAGAGGGTA GCTGTGGAGG AAGGGTGGTT CGGAGAGGAG ATTAAGGACG TGGTAGGAGA AGAAAGCGAG   
  
  
+ AGGAGAATAG AGAGGCACCA GAAGTTCGAG TCGTGGGAAG CATTGTTGAG GAGCTCAGGG TTCAAGAGTG   
  
  
+ TGCCCCTCAG CCCATTCTCG CTGTCCCAAG CTAAGCTGCT GCTGCGCCTC CATTACCCTT CCGAAGGGTA   
  
  
+ CCAACTCCAG GTTTTGCATA GTAATTGCTT GTTGCTTGGG TGGAAGAATC GCCCTCTTTT TTCTGTCTCT   
  
  
+ TCTTGGCAAT A  

- +Up\_Stream \_Len000AAAAAA AATTCAAAAG CATGAACCAC AACTACTAAA CACGAAACAT TGGTTACCAA   
  
  
- AATCATTTAT CATTAATCAG CATTGTAAGA CGCTAAAGCG AGTTAAATAT ATAGTTGAAA TCTTATACTC
[truncated: 39,977 more chars]
