# Supplementary material for: Genome-Wide Identification and Expression Pattern of the GRAS Gene Family in Pitaya (Selenicereus undatus L.)
Source: Biology (Basel). 2022 Dec 21;12(1):11. doi: 10.3390/biology12010011 (PMC9854919; doi:10.3390/biology12010011)
Supplement: Supplementary file 1 [file biology-12-00011-s001.zip › Supplementary file S5/HU03G02797.1_plantcare.html]

Content-Type: text/html; charset=ISO-8859-1


PlantCARE


Webmaster Firefox specific output  
To save the result:
click on the frame with the right mouse button and save the source code as a text file with extension .html  
REFERENCE:PlantCARE: a database of plant cis-acting regulatory elements and a portal to tools for in silico analysis of promoter sequences.  
Lescot, M., Déhais, P., Moreau, Y., De Moor, B., Rouzé ,P.,and Rombauts, S.  
Nucleic Acids Res., Database issue(2002), 30(1):325-327.   


---

>HU03G02797.1   
+ +Up\_Stream \_Len000CATTTT CAATTAGAAA TTTCTTCAAT TATATACCAT CTCCAAAAAT AGTCCTCATA   
  
  
+ AGTTGGCAGG TGAATGATCC TAATGGAAAA TTACTTTTAA CTGTTGAATT TGCAATAGGG TACCGAATTT   
  
  
+ GTGAAGTTTA TTGCCGCACG TGCCTAGCTT GTGACTTCTC GATAATCAAA AATTAATTAG AGAGAGACAG   
  
  
+ CTAAAGTTTT GAGAATAATA CTGAAAAATT ACATTAAATT TTATTTATGT TTATTTTCAT ACGCTATGTT   
  
  
+ TAACTGCTAT ACGCTGTGTT TAACTGCATC TCCTTCTATA TTTGATAAAT TGGTAAAAAA AAATAATTCA   
  
  
+ AAATTAAAGA CTAGTAATAT TTTCACTTAT CTGATAGATT TATAAGAAAT ATTTAGATAG CTAAATATAT   
  
  
+ TCTCTCCTGA AAGAGGAGAC TGATAGTTGA ATTTCATAGG ATATAAACAT GTATGTACAT TGAACGAATC   
  
  
+ TTTTTTCTAT GGTGATCGTT ATAAAGTTAT TTATTACTGT AAACATTAAG TGATAATTAG GATATGTTCT   
  
  
+ ATTTCACGTT TTAGATAGAT AGATAGATGT AAGGAGTTGT AAGAAGCTGT AAGAATATGT AAGGAGCTGT   
  
  
+ AAGGAGATGT AAGGAGCTGT AAGAAGATGT AAGAGAAACT GTAAGAAGCT ACAAGGATAG ATGTAACGAG   
  
  
+ TTGTTGAATT GTTGAAGAGT TGTATAGTGT AATTTGTTTT ACGATGAACG TTTTGGATGA AAAATAATAA   
  
  
+ TATTAAAATA ATATGCTTAT TAAAAATAAA TAAATAATTA AGTTAATTAC ACAACAATAA AATATATTAA   
  
  
+ TCCCCATAAA TTTAGTGAAT AAAACTTCGT CCACTTGAGA TGATTTTTTA TATGAACACT AGTTTGATAG   
  
  
+ AATAAACAAA GTATAAAAAA AAATAAAGTT TTTTGACAAA ATTGAATACA ACTCTTCAAA TAAATTCCTA   
  
  
+ TTTTAAGTAA ATAATTTTAA ATATAAATAA ATTTATGGTC ATCTATAATA ATATATATTT TATGTCGAAA   
  
  
+ CTCAAATATA ATTTACATCG TTATATTATA TTTTTATGTT TATGTAAATG TTGTGTTCAT AAGATATTTA   
  
  
+ TAAATTTTGT ATATTGGTAG TATCATTAAA AAAAATATAC ATATTTATGT AACTAAAATA AATGAATGTT   
  
  
+ ATAGAAAATC TATAAGATGT AAGGAGATGT ATGAAGATGT AAGGAGATGT AAGAAGATAT AAGGAGATGT   
  
  
+ AGAAAATGTA AGAAGATGTG AGAAGCTGTA AGGAGATGTA AGTTATTCAC TAAATCCTAA AAAATGCGAA   
  
  
+ ATAGAACAGG GGCTTAATTG AGCACTCAAG ATGCATAATT TGTGACGGAC CAAATGGATA AATATAGACC   
  
  
+ TGGCAAATGG GTTATTCGAT TTAGGTACGA GTATGGTTAA GTTTGGGTTT GGTTATGTTT CAGGATAAGT   
  
  
+ GACATTTCGA CTCAATTTGG TTTTGGTTGT ATGAACTTCG GCTCGAGATT TTTGGTTTGG CCAATACACT   
  
  
+ TATCATGCAA AAATAAGTAA CTTTATAGAA AATTTTGGAT CGGATATGAT GCGGTTCGGA TCAATTCGGA   
  
  
+ TTTTGGTTCA CATTCTGGTA AACCTATTTC GGATGTCAAG TAGGATATGA GTCCAGATCG TTGGATTTCG   
  
  
+ GGTATCAGCT CAATTTTACC AGATCTAGAT AAATCATAAA TGTACGGGTT TATTTTTTCC AGCTTGAATG   
  
  
+ ATTTTCTTTA TTAAAAAAAG ATAGATTTTA GATAAAACAC AACATTTCAT TTTCATTTGT TCCCCCCTAT   
  
  
+ AAATTTCTGT GACCACCTTC ACTTTCTTGC CCCATTCCCC CCCCCCCCCC GCTCTCTCTC TCTCTCTCTC   
  
  
+ TCTTCCCTTT ACAAAGGCAC CATTCAAATT CAAACCACTC TTTTTAAAAC CCCAAAAATT ATTCTCAAAA   
  
  
+ TAATACCTCA TAATATAAAT GGGTTCGTAT CACCATATAT GGGCCTCATC TCTCATGGGA ACATACGAAT   
  
  
+ TCAATGGCTT GCCCGATAGT TATCGGGTCA TGCCATCGGA GCCCCGGCAA CTAATCAATG GTCAAGCCTC   
  
  
+ AGATTTGGCC TCTTTGAGCC ATTATAGTAA CACCTTTCTC TCTGAACTCC CCCCTCTACC CACTCTGGGA   
  
  
+ CCCGGATCCG ACCCGGGTCT ATCGTGTCCG GGTCCTGCTC TGAGTCCAAT GGAGTGGATT GGTGACGCGT   
  
  
+ GGACACACCA GATTGAACAT GATCTGCCAC GTCAGCAGAG TCAACGGCTG TTGACTGATG GTGACATGGG   
  
  
+ GGAGGAGGAT TCCGGTATAA GGTTGGTGCA TGCCTTGCTG ACGTGCGCAG AGGCCGTCCA ATGCGGCGAG   
  
  
+ CTCCGACTGG CTGCTTCGCT AGTTGATGAG ATGACCAATG GGCTGTTGCC ACGTGTTAGT ACGGTGTGTG   
  
  
+ GGATCGGCAA GGTAGCCGGC TATTTTGTTG AGGCTTTGAG CCGGCGCCTG TTTCAGCCCG GCCCGGAAAC   
  
  
+ TGGCCCGACC TGGCCGGGCT CGGAGTTCGA GGCTGAGGTG TTGTATCGTC ACTTCTATGA GGCTTGCCCT   
  
  
+ TATCTCAAAT TTGCTCACTT CACGGCTAAT CAAGCCATCT TGGAGGCATT TGAGGGCCAT GATTATGTCC   
  
  
+ ACGTCATCGA CTTCGGCTTC ATGACCGGCT TGCAATGGCC GGCTTTGATT CAAGCCCTGG CTTTAAGGCC   
  
  
+ CGGTGGGCCA CCATCTCTTA GGCTCACTGG GATTGGCCCG TCTTTAGGAA GTGGGCCCGA ATCGATACGT   
  
  
+ GAAATCGGGT TGAGGCTAGC CCAATTGGCC CGATCCATGA ACATGAGGTT CGCATTTCGG GCTGTAGCAA   
  
  
+ CTTCTCGGCT CGAGGACGTG AAGCCGTCAA TGCTCGGAAT GGACCCTAGA GAAGCCGTCG CTATGAATTC   
  
  
+ CGTCATGCAT CTTCATCGAC TTTTAGGATT AGACATCAAC TCAGTTCTAA ACTGGATTCG AAACCTAAAC   
  
  
+ CCGAAGATCG TAACTTTAGT AGAACAAGAG GCGAACCATA ACCAACCTGA ATTTCTCCCC CGATTCACTG   
  
  
+ AAGCACTATG TTACTACTCA ACTATGTTCG ACTCATTAGA GACTTGTCAA GTCCAACCGC TGAAAGTCCT   
  
  
+ AGCCGAGATG TATATACAAA AGGAGATATC CAACCTAGTG TGCTACGAAG GCTCAGCCCG GGTCCAGAGG   
  
  
+ CACGAGCCGC TCGCCAAATG GCAAGCTCGG ATGGGCCAAG CCGGGTTCAA GCCGCTAGGC CTGAGTCAAA   
  
  
+ ATGCCCTTAG GCAAGCTAAC ATGTTGGTAA CATTGTTCTC CTCACAAGGG TATGACGTGG AGGAGAGAGA   
  
  
+ AGGGTGTTTG ACACTGGGCT GGCATAACCG CCCTCTCATA GTGACTTCGA CTTGGCAAGT GGGGCCCAAA   
  
  
+ ATGGACAGTC CAAGTGTGAC TCATGAGATG TCATCATCCT CAGCTTCTTC ATCTTA  

- +Up\_Stream \_Len000GTAAAA GTTAATCTTT AAAGAAGTTA ATATATGGTA GAGGTTTTTA TCAGGAGTAT   
  
  
- TCAACCGTCC ACTTACTAGG ATTACCTTTT AATGAAAATT GACAACTTAA ACGTTATCCC ATGGCTTAAA   
  
  
- CACTTCAAAT AACGGCGTGC ACGGATCGAA CACTGAAGAG CTATTAGTTT TTAATTAATC TCTCTCTGTC   
  
  
- GATTTCAAAA CTCTTATTAT GACTTTTTAA TGTAATTTAA AATAAATACA AATAAAAGTA TGCGATACAA   
  
  
- ATTGACGATA TGCGACACAA ATTGACGTAG AGGAAGATAT AAACTATTTA ACCATTTTTT TTTATTAAGT   
  
  
- TTTAATTTCT GATCATTATA AAAGTGAATA GACTATCTAA ATATTCTTTA TAAATCTATC GATTTATATA   
  
  
- AGAGAGGACT TTCTCCTCTG ACTATCAACT TAAAGTATCC TATATTTGTA CATACATGTA ACTTGCTTAG   
  
  
- AAAAAAGATA CCACTAGCAA TATTTCAATA AATAATGACA TTTGTAATTC ACTATTAATC CTATACAAGA   
  
  
- TAAAGTGCAA AATCTATCTA TCTATCTACA TTCCTCAACA TTCTTCGACA TTCTTATACA TTCCTCGACA   
  
  
- TTCCTCTACA TTCCTCGACA TTCTTCTACA TTCTCTTTGA CATTCTTCGA TGTTCCTATC TACATTGCTC   
  
  
- AACAACTTAA CAACTTCTCA ACATATCACA TTAAACAAAA TGCTACTTGC AAAACCTACT TTTTATTATT   
  
  
- ATAATTTTAT TATACGAATA ATTTTTATTT ATTTATTAAT TCAATTAATG TGTTGTTATT TTATATAATT   
  
  
- AGGGGTATTT AAATCACTTA TTTTGAAGCA GGTGAACTCT ACTAAAAAAT ATACTTGTGA TCAAACTATC   
  
  
- TTATTTGTTT CATATTTTTT TTTATTTCAA AAAACTGTTT TAACTTATGT TGAGAAGTTT ATTTAAGGAT   
  
  
- AAAATTCATT TATTAAAATT TATATTTATT TAAATACCAG TAGATATTAT TATATATAAA ATACAGCTTT   
  
  
- GAGTTTATAT TAAATGTAGC AATATAATAT AAAAATACAA ATACATTTAC AACACAAGTA TTCTATAAAT   
  
  
- ATTTAAAACA TATAACCATC ATAGTAATTT TTTTTATATG TATAAATACA TTGATTTTAT TTACTTACAA   
  
  
- TATCTTTTAG ATATTCTACA TTCCTCTACA TACTTCTACA TTCCTCTACA TTCTTCTATA TTCCTCTACA   
  
  
- TCTTTTACAT TCTTCTACAC TCTTCGACAT TCCTCTACAT TCAATAAGTG ATTTAGGATT TTTTACGCTT   
  
  
- TATCTTGTCC CCGAATTAAC TCGTGAGTTC TACGTATTAA ACACTGCCTG GTTTACCTAT TTATATCTGG   
  
  
- ACCGTTTACC CAATAAGCTA AATCCATGCT CATACCAATT CAAACCCAAA CCAATACAAA GTCCTATTCA   
  
  
- CTGTAAAGCT GAGTTAAACC AAAACCAACA TACTTGAAGC CGAGCTCTAA AAACCAAACC GGTTATGTGA   
  
  
- ATAGTACGTT TTTATTCATT GAAATATCTT TTAAAACCTA GCCTATACTA CGCCAAGCCT AGTTAAGCCT   
  
  
- AAAACCAAGT GTAAGACCAT TTGGATAAAG CCTACAGTTC ATCCTATACT CAGGTCTAGC AACCTAAAGC   
  
  
- CCATAGTCGA GTTAAAATGG TCTAGATCTA TTTAGTATTT ACATGCCCAA ATAAAAAAGG TCGAACTTAC   
  
  
- TAAAAGAAAT AATTTTTTTC TATCTAAAAT CTATTTTGTG TTGTAAAGTA AAAGTAAACA AGGGGGGATA   
  
  
- TTTAAAGACA CTGGTGGAAG TGAAAGAACG GGGTAAGGGG GGGGGGGGGG CGAGAGAGAG AGAGAGAGAG   
  
  
- AGAAGGGAAA TGTTTCCGTG GTAAGTTTAA GTTTGGTGAG AAAAATTTTG GGGTTTTTAA TAAGAGTTTT   
  
  
- ATTATGGAGT ATTATATTTA CCCAAGCATA GTGGTATATA CCCGGAGTAG AGAGTACCCT TGTATGCTTA   
  
  
- AGTTACCGAA CGGGCTATCA ATAGCCCAGT ACGGTAGCCT CGGGGCCGTT GATTAGTTAC CAGTTCGGAG   
  
  
- TCTAAACCGG AGAAACTCGG TAATATCATT GTGGAAAGAG AGACTTGAGG GGGGAGATGG GTGAGACCCT   
  
  
- GGGCCTAGGC TGGGCCCAGA TAGCACAGGC CCAGGACGAG ACTCAGGTTA CCTCACCTAA CCACTGCGCA   
  
  
- CCTGTGTGGT CTAACTTGTA CTAGACGGTG CAGTCGTCTC AGTTGCCGAC AACTGACTAC CACTGTACCC   
  
  
- CCTCCTCCTA AGGCCATATT CCAACCACGT ACGGAACGAC TGCACGCGTC TCCGGCAGGT TACGCCGCTC   
  
  
- GAGGCTGACC GACGAAGCGA TCAACTACTC TACTGGTTAC CCGACAACGG TGCACAATCA TGCCACACAC   
  
  
- CCTAGCCGTT CCATCGGCCG ATAAAACAAC TCCGAAACTC GGCCGCGGAC AAAGTCGGGC CGGGCCTTTG   
  
  
- ACCGGGCTGG ACCGGCCCGA GCCTCAAGCT CCGACTCCAC AACATAGCAG TGAAGATACT CCGAACGGGA   
  
  
- ATAGAGTTTA AACGAGTGAA GTGCCGATTA GTTCGGTAGA ACCTCCGTAA ACTCCCGGTA CTAATACAGG   
  
  
- TGCAGTAGCT GAAGCCGAAG TACTGGCCGA ACGTTACCGG CCGAAACTAA GTTCGGGACC GAAATTCCGG   
  
  
- GCCACCCGGT GGTAGAGAAT CCGAGTGACC CTAACCGGGC AGAAATCCTT CACCCGGGCT TAGCTATGCA   
  
  
- CTTTAGCCCA ACTCCGATCG GGTTAACCGG GCTAGGTACT TGTACTCCAA GCGTAAAGCC CGACATCGTT   
  
  
- GAAGAGCCGA GCTCCTGCAC TTCGGCAGTT ACGAGCCTTA CCTGGGATCT CTTCGGCAGC GATACTTAAG   
  
  
- GCAGTACGTA GAAGTAGCTG AAAATCCTAA TCTGTAGTTG AGTCAAGATT TGACCTAAGC TTTGGATTTG   
  
  
- GGCTTCTAGC ATTGAAATCA TCTTGTTCTC CGCTTGGTAT TGGTTGGACT TAAAGAGGGG GCTAAGTGAC   
  
  
- TTCGTGATAC AATGATGAGT TGATACAAGC TGAGTAATCT CTGAACAGTT CAGGTTGGCG ACTTTCAGGA   
  
  
- TCGGCTCTAC ATATATGTTT TCCTCTATAG GTTGGATCAC ACGATGCTTC CGAGTCGGGC CCAGGTCTCC   
  
  
- GTGCTCGGCG AGCGGTTTAC CGTTCGAGCC TACCCGGTTC GGCCCAAGTT CGGCGATCCG GACTCAGTTT   
  
  
- TACGGGAATC CGTTCGATTG TACAACCATT GTAACAAGAG GAGTGTTCCC ATACTGCACC TCCTCTCTCT   
  
  
- TCCCACAAAC TGTGACCCGA CCGTATTGGC GGGAGAGTAT CACTGAAGCT GAACCGTTCA CCCCGGGTTT   
  
  
- TACCTGTCAG GTTCACACTG AGTACTCTAC AGTAGTAGGA GTCGAAGAAG TAGAAT

  
  
Motifs Found  

+   

| Site Name | Organism | Position | Strand | Matrix score. | sequence | function |
| --- | --- | --- | --- | --- | --- | --- |
|  | organism | 3437 | - | 4 | motif\_sequence | short\_function |
|  | organism | 2361 | - | 4 | motif\_sequence | short\_function |
|  | organism | 2953 | + | 4 | motif\_sequence | short\_function |
|  | organism | 1257 | - | 4 | motif\_sequence | short\_function |
|  | organism | 3356 | - | 4 | motif\_sequence | short\_function |
|  | organism | 3176 | - | 4 | motif\_sequence | short\_function |
|  | organism | 3108 | + | 4 | motif\_sequence | short\_function |
|  | organism | 3017 | - | 4 | motif\_sequence | short\_function |
|  | organism | 2659 | + | 4 | motif\_sequence | short\_function |
|  | organism | 1338 | - | 4 | motif\_sequence | short\_function |
|  | organism | 1247 | - | 4 | motif\_sequence | short\_function |
|  | organism | 2279 | - | 4 | motif\_sequence | short\_function |
|  | organism | 2266 | + | 4 | motif\_sequence | short\_function |
|  | organism | 2158 | + | 4 | motif\_sequence | short\_function |
|  | organism | 2023 | - | 4 | motif\_sequence | short\_function |
|  | organism | 1894 | + | 4 | motif\_sequence | short\_function |
|  | organism | 1724 | + | 4 | motif\_sequence | short\_function |
|  | organism | 2259 | - | 4 | motif\_sequence | short\_function |
|  | organism | 1227 | - | 4 | motif\_sequence | short\_function |
|  | organism | 477 | + | 4 | motif\_sequence | short\_function |
|  | organism | 657 | - | 4 | motif\_sequence | short\_function |
|  | organism | 637 | - | 4 | motif\_sequence | short\_function |
|  | organism | 2843 | - | 4 | motif\_sequence | short\_function |
|  | organism | 1276 | - | 4 | motif\_sequence | short\_function |
|  | organism | 1296 | - | 4 | motif\_sequence | short\_function |
|  | organism | 718 | - | 4 | motif\_sequence | short\_function |
|  | organism | 1217 | - | 4 | motif\_sequence | short\_function |
|  | organism | 426 | + | 4 | motif\_sequence | short\_function |
|  | organism | 558 | + | 4 | motif\_sequence | short\_function |
|  | organism | 966 | + | 4 | motif\_sequence | short\_function |
|  | organism | 312 | + | 4 | motif\_sequence | short\_function |
|  | organism | 1237 | - | 4 | motif\_sequence | short\_function |
|  | organism | 479 | - | 4 | motif\_sequence | short\_function |
|  | organism | 53 | + | 4 | motif\_sequence | short\_function |

>HU03G02797.1   
+ +Up\_Stream \_Len000CATTTT CAATTAGAAA TTTCTTCAAT TATATACCAT CTCCAAAAAT AGTCCTCATA   
  
  
+ AGTTGGCAGG TGAATGATCC TAATGGAAAA TTACTTTTAA CTGTTGAATT TGCAATAGGG TACCGAATTT   
  
  
+ GTGAAGTTTA TTGCCGCACG TGCCTAGCTT GTGACTTCTC GATAATCAAA AATTAATTAG AGAGAGACAG   
  
  
+ CTAAAGTTTT GAGAATAATA CTGAAAAATT ACATTAAATT TTATTTATGT TTATTTTCAT ACGCTATGTT   
  
  
+ TAACTGCTAT ACGCTGTGTT TAACTGCATC TCCTTCTATA TTTGATAAAT TGGTAAAAAA AAATAATTCA   
  
  
+ AAATTAAAGA CTAGTAATAT TTTCACTTAT CTGATAGATT TATAAGAAAT ATTTAGATAG CTAAATATAT   
  
  
+ TCTCTCCTGA AAGAGGAGAC TGATAGTTGA ATTTCATAGG ATATAAACAT GTATGTACAT TGAACGAATC   
  
  
+ TTTTTTCTAT GGTGATCGTT ATAAAGTTAT TTATTACTGT AAACATTAAG TGATAATTAG GATATGTTCT   
  
  
+ ATTTCACGTT TTAGATAGAT AGATAGATGT AAGGAGTTGT AAGAAGCTGT AAGAATATGT AAGGAGCTGT   
  
  
+ AAGGAGATGT AAGGAGCTGT AAGAAGATGT AAGAGAAACT GTAAGAAGCT ACAAGGATAG ATGTAACGAG   
  
  
+ TTGTTGAATT GTTGAAGAGT TGTATAGTGT AATTTGTTTT ACGATGAACG TTTTGGATGA AAAATAATAA   
  
  
+ TATTAAAATA ATATGCTTAT TAAAAATAAA TAAATAATTA AGTTAATTAC ACAACAATAA AATATATTAA   
  
  
+ TCCCCATAAA TTTAGTGAAT AAAACTTCGT CCACTTGAGA TGATTTTTTA TATGAACACT AGTTTGATAG   
  
  
+ AATAAACAAA GTATAAAAAA AAATAAAGTT TTTTGACAAA ATTGAATACA ACTCTTCAAA TAAATTCCTA   
  
  
+ TTTTAAGTAA ATAATTTTAA ATATAAATAA ATTTATGGTC ATCTATAATA ATATATATTT TATGTCGAAA   
  
  
+ CTCAAATATA ATTTACATCG TTATATTATA TTTTTATGTT TATGTAAATG TTGTGTTCAT AAGATATTTA   
  
  
+ TAAATTTTGT ATATTGGTAG TATCATTAAA AAAAATATAC ATATTTATGT AACTAAAATA AATGAATGTT   
  
  
+ ATAGAAAATC TATAAGATGT AAGGAGATGT ATGAAGATGT AAGGAGATGT AAGAAGATAT AAGGAGATGT   
  
  
+ AGAAAATGTA AGAAGATGTG AGAAGCTGTA AGGAGATGTA AGTTATTCAC TAAATCCTAA AAAATGCGAA   
  
  
+ ATAGAACAGG GGCTTAATTG AGCACTCAAG ATGCATAATT TGTGACGGAC CAAATGGATA AATATAGACC   
  
  
+ TGGCAAATGG GTTATTCGAT TTAGGTACGA GTATGGTTAA GTTTGGGTTT GGTTATGTTT CAGGATAAGT   
  
  
+ GACATTTCGA CTCAATTTGG TTTTGGTTGT ATGAACTTCG GCTCGAGATT TTTGGTTTGG CCAATACACT   
  
  
+ TATCATGCAA AAATAAGTAA CTTTATAGAA AATTTTGGAT CGGATATGAT GCGGTTCGGA TCAATTCGGA   
  
  
+ TTTTGGTTCA CATTCTGGTA AACCTATTTC GGATGTCAAG TAGGATATGA GTCCAGATCG TTGGATTTCG   
  
  
+ GGTATCAGCT CAATTTTACC AGATCTAGAT AAATCATAAA TGTACGGGTT TATTTTTTCC AGCTTGAATG   
  
  
+ ATTTTCTTTA TTAAAAAAAG ATAGATTTTA GATAAAACAC AACATTTCAT TTTCATTTGT TCCCCCCTAT   
  
  
+ AAATTTCTGT GACCACCTTC ACTTTCTTGC CCCATTCCCC CCCCCCCCCC GCTCTCTCTC TCTCTCTCTC   
  
  
+ TCTTCCCTTT ACAAAGGCAC CATTCAAATT CAAACCACTC TTTTTAAAAC CCCAAAAATT ATTCTCAAAA   
  
  
+ TAATACCTCA TAATATAAAT GGGTTCGTAT CACCATATAT GGGCCTCATC TCTCATGGGA ACATACGAAT   
  
  
+ TCAATGGCTT GCCCGATAGT TATCGGGTCA TGCCATCGGA GCCCCGGCAA CTAATCAATG GTCAAGCCTC   
  
  
+ AGATTTGGCC TCTTTGAGCC ATTATAGTAA CACCTTTCTC TCTGAACTCC CCCCTCTACC CACTCTGGGA   
  
  
+ CCCGGATCCG ACCCGGGTCT ATCGTGTCCG GGTCCTGCTC TGAGTCCAAT GGAGTGGATT GGTGACGCGT   
  
  
+ GGACACACCA GATTGAACAT GATCTGCCAC GTCAGCAGAG TCAACGGCTG TTGACTGATG GTGACATGGG   
  
  
+ GGAGGAGGAT TCCGGTATAA GGTTGGTGCA TGCCTTGCTG ACGTGCGCAG AGGCCGTCCA ATGCGGCGAG   
  
  
+ CTCCGACTGG CTGCTTCGCT AGTTGATGAG ATGACCAATG GGCTGTTGCC ACGTGTTAGT ACGGTGTGTG   
  
  
+ GGATCGGCAA GGTAGCCGGC TATTTTGTTG AGGCTTTGAG CCGGCGCCTG TTTCAGCCCG GCCCGGAAAC   
  
  
+ TGGCCCGACC TGGCCGGGCT CGGAGTTCGA GGCTGAGGTG TTGTATCGTC ACTTCTATGA GGCTTGCCCT   
  
  
+ TATCTCAAAT TTGCTCACTT CACGGCTAAT CAAGCCATCT TGGAGGCATT TGAGGGCCAT GATTATGTCC   
  
  
+ ACGTCATCGA CTTCGGCTTC ATGACCGGCT TGCAATGGCC GGCTTTGATT CAAGCCCTGG CTTTAAGGCC   
  
  
+ CGGTGGGCCA CCATCTCTTA GGCTCACTGG GATTGGCCCG TCTTTAGGAA GTGGGCCCGA ATCGATACGT   
  
  
+ GAAATCGGGT TGAGGCTAGC CCAATTGGCC CGATCCATGA ACATGAGGTT CGCATTTCGG GCTGTAGCAA   
  
  
+ CTTCTCGGCT CGAGGACGTG AAGCCGTCAA TGCTCGGAAT GGACCCTAGA GAAGCCGTCG CTATGAATTC   
  
  
+ CGTCATGCAT CTTCATCGAC TTTTAGGATT AGACATCAAC TCAGTTCTAA ACTGGATTCG AAACCTAAAC   
  
  
+ CCGAAGATCG TAACTTTAGT AGAACAAGAG GCGAACCATA ACCAACCTGA ATTTCTCCCC CGATTCACTG   
  
  
+ AAGCACTATG TTACTACTCA ACTATGTTCG ACTCATTAGA GACTTGTCAA GTCCAACCGC TGAAAGTCCT   
  
  
+ AGCCGAGATG TATATACAAA AGGAGATATC CAACCTAGTG TGCTACGAAG GCTCAGCCCG GGTCCAGAGG   
  
  
+ CACGAGCCGC TCGCCAAATG GCAAGCTCGG ATGGGCCAAG CCGGGTTCAA GCCGCTAGGC CTGAGTCAAA   
  
  
+ ATGCCCTTAG GCAAGCTAAC ATGTTGGTAA CATTGTTCTC CTCACAAGGG TATGACGTGG AGGAGAGAGA   
  
  
+ AGGGTGTTTG ACACTGGGCT GGCATAACCG CCCTCTCATA GTGACTTCGA CTTGGCAAGT GGGGCCCAAA   
  
  
+ ATGGACAGTC CAAGTGTGAC TCATGAGATG TCATCATCCT CAGCTTCTTC ATCTTA  

- +Up\_Stream \_Len000GTAAAA GTTAATCTTT AAAGAAGTTA ATATATGGTA GAGGTTTTTA TCAGGAGTAT   
  
  
- TCAACCGTCC ACTTACTAGG ATTACCTTTT AATGAAAATT GACAACTTAA ACGTTATCCC ATGGCTTAAA   
  
  
- CACTTCAAAT AACGGCGTGC ACGGATCGAA CACTGAAGAG CTATTAGTTT TTAATTAATC TCTCTCTGTC   
  
  
- GATTTCAAAA CTCTTATTAT GACTTTTTAA TGTAATTTAA AATAAATACA AATAAAAGTA TGCGATACAA   
  
  
- ATTGACGATA TGCGACACAA ATTGACGTAG AGGAAGATAT AAACTATTTA ACCATTTTTT TTTATTAAGT   
  
  
- TTTAATTTCT GATCATTATA AAAGTGAATA GACTATCTAA ATATTCTTTA TAAATCTATC GATTTATATA   
  
  
- AGAGAGGACT TTCTCCTCTG ACTATCAACT TAAAGTATCC TATATTTGTA CATACATGTA ACTTGCTTAG   
  
  
- AAAAAAGATA CCACTAGCAA TATTTCAATA AATAATGACA TTTGTAATTC ACTATTAATC CTATACAAGA   
  
  
- TAAAGTGCAA AATCTATCTA TCTATCTACA TTCCTCAACA TTCTTCGACA TTCTTATACA TTCCTCGACA   
  
  
- TTCCTCTACA TTCCTCGACA TTCTTCTACA TTCTCTTTGA CATTCTTCGA TGTTCCTATC TACATTGCTC   
  
  
- AACAACTTAA CAACTTCTCA ACATATCACA TTAAACAAAA TGCTACTTGC AAAACCTACT TTTTATTATT   
  
  
- ATAATTTTAT TATACGAATA ATTTTTATTT ATTTATTAAT TCAATTAATG TGTTGTTATT TTATATAATT   
  
  
- AGGGGTATTT AAATCACTTA TTTTGAAGCA GGTGAACTCT ACTAAAAAAT ATACTTGTGA TCAAACTATC   
  
  
- TTATTTGTTT CATATTTTTT TTTATTTCAA AAAACTGTTT TAACTTATGT TGAGAAGTTT ATTTAAGGAT   
  
  
- AAAATTCATT TATTAAAATT TATATTTATT TAAATACCAG TAGATATTAT TATATATAAA ATACAGCTTT   
  
  
- GAGTTTATAT TAAATGTAGC AATATAATAT AAAAATACAA ATACATTTAC AACACAAGTA TTCTATAAAT   
  
  
- ATTTAAAACA TATAACCATC ATAGTAATTT TTTTTATATG TATAAATACA TTGATTTTAT TTACTTACAA   
  
  
- TATCTTTTAG ATATTCTACA TTCCTCTACA TACTTCTACA TTCCTCTACA TTCTTCTATA TTCCTCTACA   
  
  
- TCTTTTACAT TCTTCTACAC TCTTCGACAT TCCTCTACAT TCAATAAGTG ATTTAGGATT TTTTACGCTT   
  
  
- TATCTTGTCC CCGAATTAAC TCGTGAGTTC TACGTATTAA ACACTGCCTG GTTTACCTAT TTATATCTGG   
  
  
- ACCGTTTACC CAATAAGCTA AATCCATGCT CATACCAATT CAAACCCAAA CCAATACAAA GTCCTATTCA   
  
  
- CTGTAAAGCT GAGTTAAACC AAAACCAACA TACTTGAAGC CGAGCTCTAA AAACCAAACC GGTTATGTGA   
  
  
- ATAGTACGTT TTTATTCATT GAAATATCTT TTAAAACCTA GCCTATACTA CGCCAAGCCT AGTTAAGCCT   
  
  
- AAAACCAAGT GTAAGACCAT TTGGATAAAG CCTACAGTTC ATCCTATACT CAGGTCTAGC AACCTAAAGC   
  
  
- CCATAGTCGA GTTAAAATGG TCTAGATCTA TTTAGTATTT ACATGCCCAA ATAAAAAAGG TCGAACTTAC   
  
  
- TAAAAGAAAT AATTTTTTTC TATCTAAAAT CTATTTTGTG TTGTAAAGTA AAAGTAAACA AGGGGGGATA   
  
  
- TTTAAAGACA CTGGTGGAAG TGAAAGAACG GGGTAAGGGG GGGGGGGGGG CGAGAGAGAG AGAGAGAGAG   
  
  
- AGAAGGGAAA TGTTTCCGTG GTAAGTTTAA GTTTGGTGAG AAAAATTTTG GGGTTTTTAA TAAGAGTTTT   
  
  
- ATTATGGAGT ATTATATTTA CCCAAGCATA GTGGTATATA CCCGGAGTAG AGAGTACCCT TGTATGCTTA   
  
  
- AGTTACCGAA CGGGCTATCA ATAGCCCAGT ACGGTAGCCT CGGGGCCGTT GATTAGTTAC CAGTTCGGAG   
  
  
- TCTAAACCGG AGAAACTCGG TAATATCATT GTGGAAAGAG AGACTTGAGG GGGGAGATGG GTGAGACCCT   
  
  
- GGGCCTAGGC TGGGCCCAGA TAGCACAGGC CCAGGACGAG ACTCAGGTTA CCTCACCTAA CCACTGCGCA   
  
  
- CCTGTGTGGT CTAACTTGTA CTAGACGGTG CAGTCGTCTC AGTTGCCGAC AACTGACTAC CACTGTACCC   
  
  
- CCTCCTCCTA AGGCCATATT CCAACCACGT ACGGAACGAC TGCACGCGTC TCCGGCAGGT TACGCCGCTC   
  
  
- GAGGCTGACC GACGAAGCGA TCAACTACTC TACTGGTTAC CCGACAACGG TGCACAATCA TGCCACACAC   
  
  
- CCTAGCCGTT CCATCGGCCG ATAAAACAAC TCCGAAACTC GGCCGCGGAC AAAGTCGGGC CGGGCCTTTG   
  
  
- ACCGGGCTGG ACCGGCCCGA GCCTCAAGCT CCGACTCCAC AACATAGCAG TGAAGATACT CCGAACGGGA   
  
  
- ATAGAGTTTA AACGAGTGAA GTGCCGATTA GTTCGGTAGA ACCTCCGTAA ACTCCCGGTA CTAATACAGG   
  
  
- TGCAGTAGCT GAAGCCGAAG TACTGGCCGA ACGTTACCGG CCGAAACTAA GTTCGGGACC GAAATTCCGG   
  
  
- GCCACCCGGT GGTAGAGAAT CCGAGTGACC CTAACCGGGC AGAAATCCTT CACCCGGGCT TAGCTATGCA   
  
  
- CTTTAGCCCA ACTCCGATCG GGTTAACCGG GCTAGGTACT TGTACTCCAA GCGTAAAGCC CGACATCGTT   
  
  
- GAAGAGCCGA GCTCCTGCAC TTCGGCAGTT ACGAGCCTTA CCTGGGATCT CTTCGGCAGC GATACTTAAG   
  
  
- GCAGTACGTA GAAGTAGCTG AAAATCCTAA TCTGTAGTTG AGTCAAGATT TGACCTAAGC TTTGGATTTG   
  
  
- GGCTTCTAGC ATTGAAATCA TCTTGTTCTC CGCTTGGTAT TGGTTGGACT TAAAGAGGGG GCTAAGTGAC   
  
  
- TTCGTGATAC AATGATGAGT TGATACAAGC TGAGTAATCT CTGAACAGTT CAGGTTGGCG ACTTTCAGGA   
  
  
- TCGGCTCTAC ATATATGTTT TCCTCTATAG GTTGGATCAC ACGATGCTTC CGAGTCGGGC CCAGGTCTCC   
  
  
- GTGCTCGGCG AGCGGTTTAC CGTTCGAGCC TACCCGGTTC GGCCCAAGTT CGGCGATCCG GACTCAGTTT   
  
  
- TACGGGAATC CGTTCGATTG TACAACCATT GTAACAAGAG GAGTGTTCCC ATACTGCACC TCCTCTCTCT   
  
  
- TCCCACAAAC TGTGACCCGA CCGTATTGGC GGGAGAGTAT CACTGAAGCT GAACCGTTCA CCCCGGGTTT   
  
  
- TACCTGTCAG GTTCACACTG AGTACTCTAC AGTAGTAGGA GTCGAAGAAG TAGAAT

+     A-box

| Site Name | Organism | Position | Strand | Matrix score. | sequence | function |
| --- | --- | --- | --- | --- | --- | --- |
| A-box | Petroselinum crispum | 2368 | + | 6 | CCGTCC | cis-acting regulatory element |

>HU03G02797.1   
+ +Up\_Stream \_Len000CATTTT CAATTAGAAA TTTCTTCAAT TATATACCAT CTCCAAAAAT AGTCCTCATA   
  
  
+ AGTTGGCAGG TGAATGATCC TAATGGAAAA TTACTTTTAA CTGTTGAATT TGCAATAGGG TACCGAATTT   
  
  
+ GTGAAGTTTA TTGCCGCACG TGCCTAGCTT GTGACTTCTC GATAATCAAA AATTAATTAG AGAGAGACAG   
  
  
+ CTAAAGTTTT GAGAATAATA CTGAAAAATT ACATTAAATT TTATTTATGT TTATTTTCAT ACGCTATGTT   
  
  
+ TAACTGCTAT ACGCTGTGTT TAACTGCATC TCCTTCTATA TTTGATAAAT TGGTAAAAAA AAATAATTCA   
  
  
+ AAATTAAAGA CTAGTAATAT TTTCACTTAT CTGATAGATT TATAAGAAAT ATTTAGATAG CTAAATATAT   
  
  
+ TCTCTCCTGA AAGAGGAGAC TGATAGTTGA ATTTCATAGG ATATAAACAT GTATGTACAT TGAACGAATC   
  
  
+ TTTTTTCTAT GGTGATCGTT ATAAAGTTAT TTATTACTGT AAACATTAAG TGATAATTAG GATATGTTCT   
  
  
+ ATTTCACGTT TTAGATAGAT AGATAGATGT AAGGAGTTGT AAGAAGCTGT AAGAATATGT AAGGAGCTGT   
  
  
+ AAGGAGATGT AAGGAGCTGT AAGAAGATGT AAGAGAAACT GTAAGAAGCT ACAAGGATAG ATGTAACGAG   
  
  
+ TTGTTGAATT GTTGAAGAGT TGTATAGTGT AATTTGTTTT ACGATGAACG TTTTGGATGA AAAATAATAA   
  
  
+ TATTAAAATA ATATGCTTAT TAAAAATAAA TAAATAATTA AGTTAATTAC ACAACAATAA AATATATTAA   
  
  
+ TCCCCATAAA TTTAGTGAAT AAAACTTCGT CCACTTGAGA TGATTTTTTA TATGAACACT AGTTTGATAG   
  
  
+ AATAAACAAA GTATAAAAAA AAATAAAGTT TTTTGACAAA ATTGAATACA ACTCTTCAAA TAAATTCCTA   
  
  
+ TTTTAAGTAA ATAATTTTAA ATATAAATAA ATTTATGGTC ATCTATAATA ATATATATTT TATGTCGAAA   
  
  
+ CTCAAATATA ATTTACATCG TTATATTATA TTTTTATGTT TATGTAAATG TTGTGTTCAT AAGATATTTA   
  
  
+ TAAATTTTGT ATATTGGTAG TATCATTAAA AAAAATATAC ATATTTATGT AACTAAAATA AATGAATGTT   
  
  
+ ATAGAAAATC TATAAGATGT AAGGAGATGT ATGAAGATGT AAGGAGATGT AAGAAGATAT AAGGAGATGT   
  
  
+ AGAAAATGTA AGAAGATGTG AGAAGCTGTA AGGAGATGTA AGTTATTCAC TAAATCCTAA AAAATGCGAA   
  
  
+ ATAGAACAGG GGCTTAATTG AGCACTCAAG ATGCATAATT TGTGACGGAC CAAATGGATA AATATAGACC   
  
  
+ TGGCAAATGG GTTATTCGAT TTAGGTACGA GTATGGTTAA GTTTGGGTTT GGTTATGTTT CAGGATAAGT   
  
  
+ GACATTTCGA CTCAATTTGG TTTTGGTTGT ATGAACTTCG GCTCGAGATT TTTGGTTTGG CCAATACACT   
  
  
+ TATCATGCAA AAATAAGTAA CTTTATAGAA AATTTTGGAT CGGATATGAT GCGGTTCGGA TCAATTCGGA   
  
  
+ TTTTGGTTCA CATTCTGGTA AACCTATTTC GGATGTCAAG TAGGATATGA GTCCAGATCG TTGGATTTCG   
  
  
+ GGTATCAGCT CAATTTTACC AGATCTAGAT AAATCATAAA TGTACGGGTT TATTTTTTCC AGCTTGAATG   
  
  
+ ATTTTCTTTA TTAAAAAAAG ATAGATTTTA GATAAAACAC AACATTTCAT TTTCATTTGT TCCCCCCTAT   
  
  
+ AAATTTCTGT GACCACCTTC ACTTTCTTGC CCCATTCCCC CCCCCCCCCC GCTCTCTCTC TCTCTCTCTC   
  
  
+ TCTTCCCTTT ACAAAGGCAC CATTCAAATT CAAACCACTC TTTTTAAAAC CCCAAAAATT ATTCTCAAAA   
  
  
+ TAATACCTCA TAATATAAAT GGGTTCGTAT CACCATATAT GGGCCTCATC TCTCATGGGA ACATACGAAT   
  
  
+ TCAATGGCTT GCCCGATAGT TATCGGGTCA TGCCATCGGA GCCCCGGCAA CTAATCAATG GTCAAGCCTC   
  
  
+ AGATTTGGCC TCTTTGAGCC ATTATAGTAA CACCTTTCTC TCTGAACTCC CCCCTCTACC CACTCTGGGA   
  
  
+ CCCGGATCCG ACCCGGGTCT ATCGTGTCCG GGTCCTGCTC TGAGTCCAAT GGAGTGGATT GGTGACGCGT   
  
  
+ GGACACACCA GATTGAACAT GATCTGCCAC GTCAGCAGAG TCAACGGCTG TTGACTGATG GTGACATGGG   
  
  
+ GGAGGAGGAT TCCGGTATAA GGTTGGTGCA TGCCTTGCTG ACGTGCGCAG AGGCCGTCCA ATGCGGCGAG   
  
  
+ CTCCGACTGG CTGCTTCGCT AGTTGATGAG ATGACCAATG GGCTGTTGCC ACGTGTTAGT ACGGTGTGTG   
  
  
+ GGATCGGCAA GGTAGCCGGC TATTTTGTTG AGGCTTTGAG CCGGCGCCTG TTTCAGCCCG GCCCGGAAAC   
  
  
+ TGGCCCGACC TGGCCGGGCT CGGAGTTCGA GGCTGAGGTG TTGTATCGTC ACTTCTATGA GGCTTGCCCT   
  
  
+ TATCTCAAAT TTGCTCACTT CACGGCTAAT CAAGCCATCT TGGAGGCATT TGAGGGCCAT GATTATGTCC   
  
  
+ ACGTCATCGA CTTCGGCTTC ATGACCGGCT TGCAATGGCC GGCTTTGATT CAAGCCCTGG CTTTAAGGCC   
  
  
+ CGGTGGGCCA CCATCTCTTA GGCTCACTGG GATTGGCCCG TCTTTAGGAA GTGGGCCCGA ATCGATACGT   
  
  
+ GAAATCGGGT TGAGGCTAGC CCAATTGGCC CGATCCATGA ACATGAGGTT CGCATTTCGG GCTGTAGCAA   
  
  
+ CTTCTCGGCT CGAGGACGTG AAGCCGTCAA TGCTCGGAAT GGACCCTAGA GAAGCCGTCG CTATGAATTC   
  
  
+ CGTCATGCAT CTTCATCGAC TTTTAGGATT AGACATCAAC TCAGTTCTAA ACTGGATTCG AAACCTAAAC   
  
  
+ CCGAAGATCG TAACTTTAGT AGAACAAGAG GCGAACCATA ACCAACCTGA ATTTCTCCCC CGATTCACTG   
  
  
+ AAGCACTATG TTACTACTCA ACTATGTTCG ACTCATTAGA GACTTGTCAA GTCCAACCGC TGAAAGTCCT   
  
  
+ AGCCGAGATG TATATACAAA AGGAGATATC CAACCTAGTG TGCTACGAAG GCTCAGCCCG GGTCCAGAGG   
  
  
+ CACGAGCCGC TCGCCAAATG GCAAGCTCGG ATGGGCCAAG CCGGGTTCAA GCCGCTAGGC CTGAGTCAAA   
  
  
+ ATGCCCTTAG GCAAGCTAAC ATGTTGGTAA CATTGTTCTC CTCACAAGGG TATGACGTGG AGGAGAGAGA   
  
  
+ AGGGTGTTTG ACACTGGGCT GGCATAACCG CCCTCTCATA GTGACTTCGA CTTGGCAAGT GGGGCCCAAA   
  
  
+ ATGGACAGTC CAAGTGTGAC TCATGAGATG TCATCATCCT CAGCTTCTTC ATCTTA  

- +Up\_Stream \_Len000GTAAAA GTTAATCTTT AAAGAAGTTA ATATATGGTA GAGGTTTTTA TCAGGAGTAT   
  
  
- TCAACCGTCC ACTTACTAGG ATTACCTTTT AATGAAAATT GACAACTTAA ACGTTATCCC ATGGCTTAAA   
  
  
- CACTTCAAAT AACGGCGTGC ACGGATCGAA CACTGAAGAG CTATTAGTTT TTAATTAATC TCTCTCTGTC   
  
  
- GATTTCAAAA CTCTTATTAT GACTTTTTAA TGTAATTTAA AATAAATACA AATAAAAGTA TGCGATACAA   
  
  
- ATTGACGATA TGCGACACAA ATTGACGTAG AGGAAGATAT AAACTATTTA ACCATTTTTT TTTATTAAGT   
  
  
- TTTAATTTCT GATCATTATA AAAGTGAATA GACTATCTAA ATATTCTTTA TAAATCTATC GATTTATATA   
  
  
- AGAGAGGACT TTCTCCTCTG ACTATCAACT TAAAGTATCC TATATTTGTA CATACATGTA ACTTGCTTAG   
  
  
- AAAAAAGATA CCACTAGCAA TATTTCAATA AATAATGACA TTTGTAATTC ACTATTAATC CTATACAAGA   
  
  
- TAAAGTGCAA AATCTATCTA TCTATCTACA TTCCTCAACA TTCTTCGACA TTCTTATACA TTCCTCGACA   
  
  
- TTCCTCTACA TTCCTCGACA TTCTTCTACA TTCTCTTTGA CATTCTTCGA TGTTCCTATC TACATTGCTC   
  
  
- AACAACTTAA CAACTTCTCA ACATATCACA TTAAACAAAA TGCTACTTGC AAAACCTACT TTTTATTATT   
  
  
- ATAATTTTAT TATACGAATA ATTTTTATTT ATTTATTAAT TCAATTAATG TGTTGTTATT TTATATAATT   
  
  
- AGGGGTATTT AAATCACTTA TTTTGAAGCA GGTGAACTCT ACTAAAAAAT ATACTTGTGA TCAAACTATC   
  
  
- TTATTTGTTT CATATTTTTT TTTATTTCAA AAAACTGTTT TAACTTATGT TGAGAAGTTT ATTTAAGGAT   
  
  
- AAAATTCATT TATTAAAATT TATATTTATT TAAATACCAG TAGATATTAT TATATATAAA ATACAGCTTT   
  
  
- GAGTTTATAT TAAATGTAGC AATATAATAT AAAAATACAA ATACATTTAC AACACAAGTA TTCTATAAAT   
  
  
- ATTTAAAACA TATAACCATC ATAGTAATTT TTTTTATATG TATAAATACA TTGATTTTAT TTACTTACAA   
  
  
- TATCTTTTAG ATATTCTACA TTCCTCTACA TACTTCTACA TTCCTCTACA TTCTTCTATA TTCCTCTACA   
  
  
- TCTTTTACAT TCTTCTACAC TCTTCGACAT TCCTCTACAT TCAATAAGTG ATTTAGGATT TTTTACGCTT   
  
  
- TATCTTGTCC CCGAATTAAC TCGTGAGTTC TACGTATTAA ACACTGCCTG GTTTACCTAT TTATATCTGG   
  
  
- ACCGTTTACC CAATAAGCTA AATCCATGCT CATACCAATT CAAACCCAAA CCAATACAAA GTCCTATTCA   
  
  
- CTGTAAAGCT GAGTTAAACC AAAACCAACA TACTTGAAGC CGAGCTCTAA AAACCAAACC GGTTATGTGA   
  
  
- ATAGTACGTT TTTATTCATT GAAATATCTT TTAAAACCTA GCCTATACTA CGCCAAGCCT AGTTAAGCCT   
  
  
- AAAACCAAGT GTAAGACCAT TTGGATAAAG CCTACAGTTC ATCCTATACT CAGGTCTAGC AACCTAAAGC   
  
  
- CCATAGTCGA GTTAAAATGG TCTAGATCTA TTTAGTATTT ACATGCCCAA ATAAAAAAGG TCGAACTTAC   
  
  
- TAAAAGAAAT AATTTTTTTC TATCTAAAAT CTATTTTGTG TTGTAAAGTA AAAGTAAACA AGGGGGGATA   
  
  
- TTTAAAGACA CTGGTGGAAG TGAAAGAACG GGGTAAGGGG GGGGGGGGGG CGAGAGAGAG AGAGAGAGAG   
  
  
- AGAAGGGAAA TGTTTCCGTG GTAAGTTTAA GTTTGGTGAG AAAAATTTTG GGGTTTTTAA TAAGAGTTTT   
  
  
- ATTATGGAGT ATTATATTTA CCCAAGCATA GTGGTATATA CCCGGAGTAG AGAGTACCCT TGTATGCTTA   
  
  
- AGTTACCGAA CGGGCTATCA ATAGCCCAGT ACGGTAGCCT CGGGGCCGTT GATTAGTTAC CAGTTCGGAG   
  
  
- TCTAAACCGG AGAAACTCGG TAATATCATT GTGGAAAGAG AGACTTGAGG GGGGAGATGG GTGAGACCCT   
  
  
- GGGCCTAGGC TGGGCCCAGA TAGCACAGGC CCAGGACGAG ACTCAGGTTA CCTCACCTAA CCACTGCGCA   
  
  
- CCTGTGTGGT CTAACTTGTA CTAGACGGTG CAGTCGTCTC AGTTGCCGAC AACTGACTAC CACTGTACCC   
  
  
- CCTCCTCCTA AGGCCATATT CCAACCACGT ACGGAACGAC TGCACGCGTC TCCGGCAGGT TACGCCGCTC   
  
  
- GAGGCTGACC GACGAAGCGA TCAACTACTC TACTGGTTAC CCGACAACGG TGCACAATCA TGCCACACAC   
  
  
- CCTAGCCGTT CCATCGGCCG ATAAAACAAC TCCGAAACTC GGCCGCGGAC AAAGTCGGGC CGGGCCTTTG   
  
  
- ACCGGGCTGG ACCGGCCCGA GCCTCAAGCT CCGACTCCAC AACATAGCAG TGAAGATACT CCGAACGGGA   
  
  
- ATAGAGTTTA AACGAGTGAA GTGCCGATTA GTTCGGTAGA ACCTCCGTAA ACTCCCGGTA CTAATACAGG   
  
  
- TGCAGTAGCT GAAGCCGAAG TACTGGCCGA ACGTTACCGG CCGAAACTAA GTTCGGGACC GAAATTCCGG   
  
  
- GCCACCCGGT GGTAGAGAAT CCGAGTGACC CTAACCGGGC AGAAATCCTT CACCCGGGCT TAGCTATGCA   
  
  
- CTTTAGCCCA ACTCCGATCG GGTTAACCGG GCTAGGTACT TGTACTCCAA GCGTAAAGCC CGACATCGTT   
  
  
- GAAGAGCCGA GCTCCTGCAC TTCGGCAGTT ACGAGCCTTA CCTGGGATCT CTTCGGCAGC GATACTTAAG   
  
  
- GCAGTACGTA GAAGTAGCTG AAAATCCTAA TCTGTAGTTG AGTCAAGATT TGACCTAAGC TTTGGATTTG   
  
  
- GGCTTCTAGC ATTGAAATCA TCTTGTTCTC CGCTTGGTAT TGGTTGGACT TAAAGAGGGG GCTAAGTGAC   
  
  
- TTCGTGATAC AATGATGAGT TGATACAAGC TGAGTAATCT CTGAACAGTT CAGGTTGGCG ACTTTCAGGA   
  
  
- TCGGCTCTAC ATATATGTTT TCCTCTATAG GTTGGATCAC ACGATGCTTC CGAGTCGGGC CCAGGTCTCC   
  
  
- GTGCTCGGCG AGCGGTTTAC CGTTCGAGCC TACCCGGTTC GGCCCAAGTT CGGCGATCCG GACTCAGTTT   
  
  
- TACGGGAATC CGTTCGATTG TACAACCATT GTAACAAGAG GAGTGTTCCC ATACTGCACC TCCTCTCTCT   
  
  
- TCCCACAAAC TGTGACCCGA CCGTATTGGC GGGAGAGTAT CACTGAAGCT GAACCGTTCA CCCCGGGTTT   
  
  
- TACCTGTCAG GTTCACACTG AGTACTCTAC AGTAGTAGGA GTCGAAGAAG TAGAAT

+     AAGAA-motif

| Site Name | Organism | Position | Strand | Matrix score. | sequence | function |
| --- | --- | --- | --- | --- | --- | --- |
| AAGAA-motif | Avena sativa | 336 | + | 9 | gGTAAAGAAA |  |

>HU03G02797.1   
+ +Up\_Stream \_Len000CATTTT CAATTAGAAA TTTCTTCAAT TATATACCAT CTCCAAAAAT AGTCCTCATA   
  
  
+ AGTTGGCAGG TGAATGATCC TAATGGAAAA TTACTTTTAA CTGTTGAATT TGCAATAGGG TACCGAATTT   
  
  
+ GTGAAGTTTA TTGCCGCACG TGCCTAGCTT GTGACTTCTC GATAATCAAA AATTAATTAG AGAGAGACAG   
  
  
+ CTAAAGTTTT GAGAATAATA CTGAAAAATT ACATTAAATT TTATTTATGT TTATTTTCAT ACGCTATGTT   
  
  
+ TAACTGCTAT ACGCTGTGTT TAACTGCATC TCCTTCTATA TTTGATAAAT TGGTAAAAAA AAATAATTCA   
  
  
+ AAATTAAAGA CTAGTAATAT TTTCACTTAT CTGATAGATT TATAAGAAAT ATTTAGATAG CTAAATATAT   
  
  
+ TCTCTCCTGA AAGAGGAGAC TGATAGTTGA ATTTCATAGG ATATAAACAT GTATGTACAT TGAACGAATC   
  
  
+ TTTTTTCTAT GGTGATCGTT ATAAAGTTAT TTATTACTGT AAACATTAAG TGATAATTAG GATATGTTCT   
  
  
+ ATTTCACGTT TTAGATAGAT AGATAGATGT AAGGAGTTGT AAGAAGCTGT AAGAATATGT AAGGAGCTGT   
  
  
+ AAGGAGATGT AAGGAGCTGT AAGAAGATGT AAGAGAAACT GTAAGAAGCT ACAAGGATAG ATGTAACGAG   
  
  
+ TTGTTGAATT GTTGAAGAGT TGTATAGTGT AATTTGTTTT ACGATGAACG TTTTGGATGA AAAATAATAA   
  
  
+ TATTAAAATA ATATGCTTAT TAAAAATAAA TAAATAATTA AGTTAATTAC ACAACAATAA AATATATTAA   
  
  
+ TCCCCATAAA TTTAGTGAAT AAAACTTCGT CCACTTGAGA TGATTTTTTA TATGAACACT AGTTTGATAG   
  
  
+ AATAAACAAA GTATAAAAAA AAATAAAGTT TTTTGACAAA ATTGAATACA ACTCTTCAAA TAAATTCCTA   
  
  
+ TTTTAAGTAA ATAATTTTAA ATATAAATAA ATTTATGGTC ATCTATAATA ATATATATTT TATGTCGAAA   
  
  
+ CTCAAATATA ATTTACATCG TTATATTATA TTTTTATGTT TATGTAAATG TTGTGTTCAT AAGATATTTA   
  
  
+ TAAATTTTGT ATATTGGTAG TATCATTAAA AAAAATATAC ATATTTATGT AACTAAAATA AATGAATGTT   
  
  
+ ATAGAAAATC TATAAGATGT AAGGAGATGT ATGAAGATGT AAGGAGATGT AAGAAGATAT AAGGAGATGT   
  
  
+ AGAAAATGTA AGAAGATGTG AGAAGCTGTA AGGAGATGTA AGTTATTCAC TAAATCCTAA AAAATGCGAA   
  
  
+ ATAGAACAGG GGCTTAATTG AGCACTCAAG ATGCATAATT TGTGACGGAC CAAATGGATA AATATAGACC   
  
  
+ TGGCAAATGG GTTATTCGAT TTAGGTACGA GTATGGTTAA GTTTGGGTTT GGTTATGTTT CAGGATAAGT   
  
  
+ GACATTTCGA CTCAATTTGG TTTTGGTTGT ATGAACTTCG GCTCGAGATT TTTGGTTTGG CCAATACACT   
  
  
+ TATCATGCAA AAATAAGTAA CTTTATAGAA AATTTTGGAT CGGATATGAT GCGGTTCGGA TCAATTCGGA   
  
  
+ TTTTGGTTCA CATTCTGGTA AACCTATTTC GGATGTCAAG TAGGATATGA GTCCAGATCG TTGGATTTCG   
  
  
+ GGTATCAGCT CAATTTTACC AGATCTAGAT AAATCATAAA TGTACGGGTT TATTTTTTCC AGCTTGAATG   
  
  
+ ATTTTCTTTA TTAAAAAAAG ATAGATTTTA GATAAAACAC AACATTTCAT TTTCATTTGT TCCCCCCTAT   
  
  
+ AAATTTCTGT GACCACCTTC ACTTTCTTGC CCCATTCCCC CCCCCCCCCC GCTCTCTCTC TCTCTCTCTC   
  
  
+ TCTTCCCTTT ACAAAGGCAC CATTCAAATT CAAACCACTC TTTTTAAAAC CCCAAAAATT ATTCTCAAAA   
  
  
+ TAATACCTCA TAATATAAAT GGGTTCGTAT CACCATATAT GGGCCTCATC TCTCATGGGA ACATACGAAT   
  
  
+ TCAATGGCTT GCCCGATAGT TATCGGGTCA TGCCATCGGA GCCCCGGCAA CTAATCAATG GTCAAGCCTC   
  
  
+ AGATTTGGCC TCTTTGAGCC ATTATAGTAA CACCTTTCTC TCTGAACTCC CCCCTCTACC CACTCTGGGA   
  
  
+ CCCGGATCCG ACCCGGGTCT ATCGTGTCCG GGTCCTGCTC TGAGTCCAAT GGAGTGGATT GGTGACGCGT   
  
  
+ GGACACACCA GATTGAACAT GATCTGCCAC GTCAGCAGAG TCAACGGCTG TTGACTGATG GTGACATGGG   
  
  
+ GGAGGAGGAT TCCGGTATAA GGTTGGTGCA TGCCTTGCTG ACGTGCGCAG AGGCCGTCCA ATGCGGCGAG   
  
  
+ CTCCGACTGG CTGCTTCGCT AGTTGATGAG ATGACCAATG GGCTGTTGCC ACGTGTTAGT ACGGTGTGTG   
  
  
+ GGATCGGCAA GGTAGCCGGC TATTTTGTTG AGGCTTTGAG CCGGCGCCTG TTTCAGCCCG GCCCGGAAAC   
  
  
+ TGGCCCGACC TGGCCGGGCT CGGAGTTCGA GGCTGAGGTG TTGTATCGTC ACTTCTATGA GGCTTGCCCT   
  
  
+ TATCTCAAAT TTGCTCACTT CACGGCTAAT CAAGCCATCT TGGAGGCATT TGAGGGCCAT GATTATGTCC   
  
  
+ ACGTCATCGA CTTCGGCTTC ATGACCGGCT TGCAATGGCC GGCTTTGATT CAAGCCCTGG CTTTAAGGCC   
  
  
+ CGGTGGGCCA CCATCTCTTA GGCTCACTGG GATTGGCCCG TCTTTAGGAA GTGGGCCCGA ATCGATACGT   
  
  
+ GAAATCGGGT TGAGGCTAGC CCAATTGGCC CGATCCATGA ACATGAGGTT CGCATTTCGG GCTGTAGCAA   
  
  
+ CTTCTCGGCT CGAGGACGTG AAGCCGTCAA TGCTCGGAAT GGACCCTAGA GAAGCCGTCG CTATGAATTC   
  
  
+ CGTCATGCAT CTTCATCGAC TTTTAGGATT AGACATCAAC TCAGTTCTAA ACTGGATTCG AAACCTAAAC   
  
  
+ CCGAAGATCG TAACTTTAGT AGAACAAGAG GCGAACCATA ACCAACCTGA ATTTCTCCCC CGATTCACTG   
  
  
+ AAGCACTATG TTACTACTCA ACTATGTTCG ACTCATTAGA GACTTGTCAA GTCCAACCGC TGAAAGTCCT   
  
  
+ AGCCGAGATG TATATACAAA AGGAGATATC CAACCTAGTG TGCTACGAAG GCTCAGCCCG GGTCCAGAGG   
  
  
+ CACGAGCCGC TCGCCAAATG GCAAGCTCGG ATGGGCCAAG CCGGGTTCAA GCCGCTAGGC CTGAGTCAAA   
  
  
+ ATGCCCTTAG GCAAGCTAAC ATGTTGGTAA CATTGTTCTC CTCACAAGGG TATGACGTGG AGGAGAGAGA   
  
  
+ AGGGTGTTTG ACACTGGGCT GGCATAACCG CCCTCTCATA GTGACTTCGA CTTGGCAAGT GGGGCCCAAA   
  
  
+ ATGGACAGTC CAAGTGTGAC TCATGAGATG TCATCATCCT CAGCTTCTTC ATCTTA  

- +Up\_Stream \_Len000GTAAAA GTTAATCTTT AAAGAAGTTA ATATATGGTA GAGGTTTTTA TCAGGAGTAT   
  
  
- TCAACCGTCC ACTTACTAGG ATTACCTTTT AATGAAAATT GACAACTTAA ACGTTATCCC ATGGCTTAAA   
  
  
- CACTTCAAAT AACGGCGTGC ACGGATCGAA CACTGAAGAG CTATTAGTTT TTAATTAATC TCTCTCTGTC   
  
  
- GATTTCAAAA CTCTTATTAT GACTTTTTAA TGTAATTTAA AATAAATACA AATAAAAGTA TGCGATACAA   
  
  
- ATTGACGATA TGCGACACAA ATTGACGTAG AGGAAGATAT AAACTATTTA ACCATTTTTT TTTATTAAGT   
  
  
- TTTAATTTCT GATCATTATA AAAGTGAATA GACTATCTAA ATATTCTTTA TAAATCTATC GATTTATATA   
  
  
- AGAGAGGACT TTCTCCTCTG ACTATCAACT TAAAGTATCC TATATTTGTA CATACATGTA ACTTGCTTAG   
  
  
- AAAAAAGATA CCACTAGCAA TATTTCAATA AATAATGACA TTTGTAATTC ACTATTAATC CTATACAAGA   
  
  
- TAAAGTGCAA AATCTATCTA TCTATCTACA TTCCTCAACA TTCTTCGACA TTCTTATACA TTCCTCGACA   
  
  
- TTCCTCTACA TTCCTCGACA TTCTTCTACA TTCTCTTTGA CATTCTTCGA TGTTCCTATC TACATTGCTC   
  
  
- AACAACTTAA CAACTTCTCA ACATATCACA TTAAACAAAA TGCTACTTGC AAAACCTACT TTTTATTATT   
  
  
- ATAATTTTAT TATACGAATA ATTTTTATTT ATTTATTAAT TCAATTAATG TGTTGTTATT TTATATAATT   
  
  
- AGGGGTATTT AAATCACTTA TTTTGAAGCA GGTGAACTCT ACTAAAAAAT ATACTTGTGA TCAAACTATC   
  
  
- TTATTTGTTT CATATTTTTT TTTATTTCAA AAAACTGTTT TAACTTATGT TGAGAAGTTT ATTTAAGGAT   
  
  
- AAAATTCATT TATTAAAATT TATATTTATT TAAATACCAG TAGATATTAT TATATATAAA ATACAGCTTT   
  
  
- GAGTTTATAT TAAATGTAGC AATATAATAT AAAAATACAA ATACATTTAC AACACAAGTA TTCTATAAAT   
  
  
- ATTTAAAACA TATAACCATC ATAGTAATTT TTTTTATATG TATAAATACA TTGATTTTAT TTACTTACAA   
  
  
- TATCTTTTAG ATATTCTACA TTCCTCTACA TACTTCTACA TTCCTCTACA TTCTTCTATA TTCCTCTACA   
  
  
- TCTTTTACAT TCTTCTACAC TCTTCGACAT TCCTCTACAT TCAATAAGTG ATTTAGGATT TTTTACGCTT   
  
  
- TATCTTGTCC CCGAATTAAC TCGTGAGTTC TACGTATTAA ACACTGCCTG GTTTACCTAT TTATATCTGG   
  
  
- ACCGTTTACC CAATAAGCTA AATCCATGCT CATACCAATT CAAACCCAAA CCAATACAAA GTCCTATTCA   
  
  
- CTGTAAAGCT GAGTTAAACC AAAACCAACA TACTTGAAGC CGAGCTCTAA AAACCAAACC GGTTATGTGA   
  
  
- ATAGTACGTT TTTATTCATT GAAATATCTT TTAAAACCTA GCCTATACTA CGCCAAGCCT AGTTAAGCCT   
  
  
- AAAACCAAGT GTAAGACCAT TTGGATAAAG CCTACAGTTC ATCCTATACT CAGGTCTAGC AACCTAAAGC   
  
  
- CCATAGTCGA GTTAAAATGG TCTAGATCTA TTTAGTATTT ACATGCCCAA ATAAAAAAGG TCGAACTTAC   
  
  
- TAAAAGAAAT AATTTTTTTC TATCTAAAAT CTATTTTGTG TTGTAAAGTA AAAGTAAACA AGGGGGGATA   
  
  
- TTTAAAGACA CTGGTGGAAG TGAAAGAACG GGGTAAGGGG GGGGGGGGGG CGAGAGAGAG AGAGAGAGAG   
  
  
- AGAAGGGAAA TGTTTCCGTG GTAAGTTTAA GTTTGGTGAG AAAAATTTTG GGGTTTTTAA TAAGAGTTTT   
  
  
- ATTATGGAGT ATTATATTTA CCCAAGCATA GTGGTATATA CCCGGAGTAG AGAGTACCCT TGTATGCTTA   
  
  
- AGTTACCGAA CGGGCTATCA ATAGCCCAGT ACGGTAGCCT CGGGGCCGTT GATTAGTTAC CAGTTCGGAG   
  
  
- TCTAAACCGG AGAAACTCGG TAATATCATT GTGGAAAGAG AGACTTGAGG GGGGAGATGG GTGAGACCCT   
  
  
- GGGCCTAGGC TGGGCCCAGA TAGCACAGGC CCAGGACGAG ACTCAGGTTA CCTCACCTAA CCACTGCGCA   
  
  
- CCTGTGTGGT CTAACTTGTA CTAGACGGTG CAGTCGTCTC AGTTGCCGAC AACTGACTAC CACTGTACCC   
  
  
- CCTCCTCCTA AGGCCATATT CCAACCACGT ACGGAACGAC TGCACGCGTC TCCGGCAGGT TACGCCGCTC   
  
  
- GAGGCTGACC GACGAAGCGA TCAACTACTC TACTGGTTAC CCGACAACGG TGCACAATCA TGCCACACAC   
  
  
- CCTAGCCGTT CCATCGGCCG ATAAAACAAC TCCGAAACTC GGCCGCGGAC AAAGTCGGGC CGGGCCTTTG   
  
  
- ACCGGGCTGG ACCGGCCCGA GCCTCAAGCT CCGACTCCAC AACATAGCAG TGAAGATACT CCGAACGGGA   
  
  
- ATAGAGTTTA AACGAGTGAA GTGCCGATTA GTTCGGTAGA ACCTCCGTAA ACTCCCGGTA CTAATACAGG   
  
  
- TGCAGTAGCT GAAGCCGAAG TACTGGCCGA ACGTTACCGG CCGAAACTAA GTTCGGGACC GAAATTCCGG   
  
  
- GCCACCCGGT GGTAGAGAAT CCGAGTGACC CTAACCGGGC AGAAATCCTT CACCCGGGCT TAGCTATGCA   
  
  
- CTTTAGCCCA ACTCCGATCG GGTTAACCGG GCTAGGTACT TGTACTCCAA GCGTAAAGCC CGACATCGTT   
  
  
- GAAGAGCCGA GCTCCTGCAC TTCGGCAGTT ACGAGCCTTA CCTGGGATCT CTTCGGCAGC GATACTTAAG   
  
  
- GCAGTACGTA GAAGTAGCTG AAAATCCTAA TCTGTAGTTG AGTCAAGATT TGACCTAAGC TTTGGATTTG   
  
  
- GGCTTCTAGC ATTGAAATCA TCTTGTTCTC CGCTTGGTAT TGGTTGGACT TAAAGAGGGG GCTAAGTGAC   
  
  
- TTCGTGATAC AATGATGAGT TGATACAAGC TGAGTAATCT CTGAACAGTT CAGGTTGGCG ACTTTCAGGA   
  
  
- TCGGCTCTAC ATATATGTTT TCCTCTATAG GTTGGATCAC ACGATGCTTC CGAGTCGGGC CCAGGTCTCC   
  
  
- GTGCTCGGCG AGCGGTTTAC CGTTCGAGCC TACCCGGTTC GGCCCAAGTT CGGCGATCCG GACTCAGTTT   
  
  
- TACGGGAATC CGTTCGATTG TACAACCATT GTAACAAGAG GAGTGTTCCC ATACTGCACC TCCTCTCTCT   
  
  
- TCCCACAAAC TGTGACCCGA CCGTATTGGC GGGAGAGTAT CACTGAAGCT GAACCGTTCA CCCCGGGTTT   
  
  
- TACCTGTCAG GTTCACACTG AGTACTCTAC AGTAGTAGGA GTCGAAGAAG TAGAAT

+     ABRE

| Site Name | Organism | Position | Strand | Matrix score. | sequence | function |
| --- | --- | --- | --- | --- | --- | --- |
| ABRE | Arabidopsis thaliana | 3349 | + | 5 | ACGTG | cis-acting element involved in the abscisic acid responsiveness |
| ABRE | Arabidopsis thaliana | 2890 | + | 5 | ACGTG | cis-acting element involved in the abscisic acid responsiveness |
| ABRE | Arabidopsis thaliana | 3265 | - | 7 | AACCCGG | cis-acting element involved in the abscisic acid responsiveness |
| ABRE | Arabidopsis thaliana | 2434 | - | 6 | CACGTG | cis-acting element involved in the abscisic acid responsiveness |
| ABRE | Arabidopsis thaliana | 2355 | + | 5 | ACGTG | cis-acting element involved in the abscisic acid responsiveness |
| ABRE | Arabidopsis thaliana | 2664 | - | 5 | ACGTG | cis-acting element involved in the abscisic acid responsiveness |
| ABRE | Arabidopsis thaliana | 2435 | + | 5 | ACGTG | cis-acting element involved in the abscisic acid responsiveness |
| ABRE | Triticum aestivum | 2432 | - | 9 | GACACGTGGC | cis-acting element involved in the abscisic acid responsiveness |
| ABRE | Arabidopsis thaliana | 2272 | - | 5 | ACGTG | cis-acting element involved in the abscisic acid responsiveness |
| ABRE | Arabidopsis thaliana | 2801 | + | 5 | ACGTG | cis-acting element involved in the abscisic acid responsiveness |
| ABRE | Arabidopsis thaliana | 162 | + | 5 | ACGTG | cis-acting element involved in the abscisic acid responsiveness |
| ABRE | Arabidopsis thaliana | 161 | + | 6 | CACGTG | cis-acting element involved in the abscisic acid responsiveness |
| ABRE | Hordeum vulgare | 159 | + | 9 | CGCACGTGTC | cis-acting element involved in the abscisic acid responsiveness |
| ABRE | Arabidopsis thaliana | 569 | - | 5 | ACGTG | cis-acting element involved in the abscisic acid responsiveness |

>HU03G02797.1   
+ +Up\_Stream \_Len000CATTTT CAATTAGAAA TTTCTTCAAT TATATACCAT CTCCAAAAAT AGTCCTCATA   
  
  
+ AGTTGGCAGG TGAATGATCC TAATGGAAAA TTACTTTTAA CTGTTGAATT TGCAATAGGG TACCGAATTT   
  
  
+ GTGAAGTTTA TTGCCGCACG TGCCTAGCTT GTGACTTCTC GATAATCAAA AATTAATTAG AGAGAGACAG   
  
  
+ CTAAAGTTTT GAGAATAATA CTGAAAAATT ACATTAAATT TTATTTATGT TTATTTTCAT ACGCTATGTT   
  
  
+ TAACTGCTAT ACGCTGTGTT TAACTGCATC TCCTTCTATA TTTGATAAAT TGGTAAAAAA AAATAATTCA   
  
  
+ AAATTAAAGA CTAGTAATAT TTTCACTTAT CTGATAGATT TATAAGAAAT ATTTAGATAG CTAAATATAT   
  
  
+ TCTCTCCTGA AAGAGGAGAC TGATAGTTGA ATTTCATAGG ATATAAACAT GTATGTACAT TGAACGAATC   
  
  
+ TTTTTTCTAT GGTGATCGTT ATAAAGTTAT TTATTACTGT AAACATTAAG TGATAATTAG GATATGTTCT   
  
  
+ ATTTCACGTT TTAGATAGAT AGATAGATGT AAGGAGTTGT AAGAAGCTGT AAGAATATGT AAGGAGCTGT   
  
  
+ AAGGAGATGT AAGGAGCTGT AAGAAGATGT AAGAGAAACT GTAAGAAGCT ACAAGGATAG ATGTAACGAG   
  
  
+ TTGTTGAATT GTTGAAGAGT TGTATAGTGT AATTTGTTTT ACGATGAACG TTTTGGATGA AAAATAATAA   
  
  
+ TATTAAAATA ATATGCTTAT TAAAAATAAA TAAATAATTA AGTTAATTAC ACAACAATAA AATATATTAA   
  
  
+ TCCCCATAAA TTTAGTGAAT AAAACTTCGT CCACTTGAGA TGATTTTTTA TATGAACACT AGTTTGATAG   
  
  
+ AATAAACAAA GTATAAAAAA AAATAAAGTT TTTTGACAAA ATTGAATACA ACTCTTCAAA TAAATTCCTA   
  
  
+ TTTTAAGTAA ATAATTTTAA ATATAAATAA ATTTATGGTC ATCTATAATA ATATATATTT TATGTCGAAA   
  
  
+ CTCAAATATA ATTTACATCG TTATATTATA TTTTTATGTT TATGTAAATG TTGTGTTCAT AAGATATTTA   
  
  
+ TAAATTTTGT ATATTGGTAG TATCATTAAA AAAAATATAC ATATTTATGT AACTAAAATA AATGAATGTT   
  
  
+ ATAGAAAATC TATAAGATGT AAGGAGATGT ATGAAGATGT AAGGAGATGT AAGAAGATAT AAGGAGATGT   
  
  
+ AGAAAATGTA AGAAGATGTG AGAAGCTGTA AGGAGATGTA AGTTATTCAC TAAATCCTAA AAAATGCGAA   
  
  
+ ATAGAACAGG GGCTTAATTG AGCACTCAAG ATGCATAATT TGTGACGGAC CAAATGGATA AATATAGACC   
  
  
+ TGGCAAATGG GTTATTCGAT TTAGGTACGA GTATGGTTAA GTTTGGGTTT GGTTATGTTT CAGGATAAGT   
  
  
+ GACATTTCGA CTCAATTTGG TTTTGGTTGT ATGAACTTCG GCTCGAGATT TTTGGTTTGG CCAATACACT   
  
  
+ TATCATGCAA AAATAAGTAA CTTTATAGAA AATTTTGGAT CGGATATGAT GCGGTTCGGA TCAATTCGGA   
  
  
+ TTTTGGTTCA CATTCTGGTA AACCTATTTC GGATGTCAAG TAGGATATGA GTCCAGATCG TTGGATTTCG   
  
  
+ GGTATCAGCT CAATTTTACC AGATCTAGAT AAATCATAAA TGTACGGGTT TATTTTTTCC AGCTTGAATG   
  
  
+ ATTTTCTTTA TTAAAAAAAG ATAGATTTTA GATAAAACAC AACATTTCAT TTTCATTTGT TCCCCCCTAT   
  
  
+ AAATTTCTGT GACCACCTTC ACTTTCTTGC CCCATTCCCC CCCCCCCCCC GCTCTCTCTC TCTCTCTCTC   
  
  
+ TCTTCCCTTT ACAAAGGCAC CATTCAAATT CAAACCACTC TTTTTAAAAC CCCAAAAATT ATTCTCAAAA   
  
  
+ TAATACCTCA TAATATAAAT GGGTTCGTAT CACCATATAT GGGCCTCATC TCTCATGGGA ACATACGAAT   
  
  
+ TCAATGGCTT GCCCGATAGT TATCGGGTCA TGCCATCGGA GCCCCGGCAA CTAATCAATG GTCAAGCCTC   
  
  
+ AGATTTGGCC TCTTTGAGCC ATTATAGTAA CACCTTTCTC TCTGAACTCC CCCCTCTACC CACTCTGGGA   
  
  
+ CCCGGATCCG ACCCGGGTCT ATCGTGTCCG GGTCCTGCTC TGAGTCCAAT GGAGTGGATT GGTGACGCGT   
  
  
+ GGACACACCA GATTGAACAT GATCTGCCAC GTCAGCAGAG TCAACGGCTG TTGACTGATG GTGACATGGG   
  
  
+ GGAGGAGGAT TCCGGTATAA GGTTGGTGCA TGCCTTGCTG ACGTGCGCAG AGGCCGTCCA ATGCGGCGAG   
  
  
+ CTCCGACTGG CTGCTTCGCT AGTTGATGAG ATGACCAATG GGCTGTTGCC ACGTGTTAGT ACGGTGTGTG   
  
  
+ GGATCGGCAA GGTAGCCGGC TATTTTGTTG AGGCTTTGAG CCGGCGCCTG TTTCAGCCCG GCCCGGAAAC   
  
  
+ TGGCCCGACC TGGCCGGGCT CGGAGTTCGA GGCTGAGGTG TTGTATCGTC ACTTCTATGA GGCTTGCCCT   
  
  
+ TATCTCAAAT TTGCTCACTT CACGGCTAAT CAAGCCATCT TGGAGGCATT TGAGGGCCAT GATTATGTCC   
  
  
+ ACGTCATCGA CTTCGGCTTC ATGACCGGCT TGCAATGGCC GGCTTTGATT CAAGCCCTGG CTTTAAGGCC   
  
  
+ CGGTGGGCCA CCATCTCTTA GGCTCACTGG GATTGGCCCG TCTTTAGGAA GTGGGCCCGA ATCGATACGT   
  
  
+ GAAATCGGGT TGAGGCTAGC CCAATTGGCC CGATCCATGA ACATGAGGTT CGCATTTCGG GCTGTAGCAA   
  
  
+ CTTCTCGGCT CGAGGACGTG AAGCCGTCAA TGCTCGGAAT GGACCCTAGA GAAGCCGTCG CTATGAATTC   
  
  
+ CGTCATGCAT CTTCATCGAC TTTTAGGATT AGACATCAAC TCAGTTCTAA ACTGGATTCG AAACCTAAAC   
  
  
+ CCGAAGATCG TAACTTTAGT AGAACAAGAG GCGAACCATA ACCAACCTGA ATTTCTCCCC CGATTCACTG   
  
  
+ AAGCACTATG TTACTACTCA ACTATGTTCG ACTCATTAGA GACTTGTCAA GTCCAACCGC TGAAAGTCCT   
  
  
+ AGCCGAGATG TATATACAAA AGGAGATATC CAACCTAGTG TGCTACGAAG GCTCAGCCCG GGTCCAGAGG   
  
  
+ CACGAGCCGC TCGCCAAATG GCAAGCTCGG ATGGGCCAAG CCGGGTTCAA GCCGCTAGGC CTGAGTCAAA   
  
  
+ ATGCCCTTAG GCAAGCTAAC ATGTTGGTAA CATTGTTCTC CTCACAAGGG TATGACGTGG AGGAGAGAGA   
  
  
+ AGGGTGTTTG ACACTGGGCT GGCATAACCG CCCTCTCATA GTGACTTCGA CTTGGCAAGT GGGGCCCAAA   
  
  
+ ATGGACAGTC CAAGTGTGAC TCATGAGATG TCATCATCCT CAGCTTCTTC ATCTTA  

- +Up\_Stream \_Len000GTAAAA GTTAATCTTT AAAGAAGTTA ATATATGGTA GAGGTTTTTA TCAGGAGTAT   
  
  
- TCAACCGTCC ACTTACTAGG ATTACCTTTT AATGAAAATT GACAACTTAA ACGTTATCCC ATGGCTTAAA   
  
  
- CACTTCAAAT AACGGCGTGC ACGGATCGAA CACTGAAGAG CTATTAGTTT TTAATTAATC TCTCTCTGTC   
  
  
- GATTTCAAAA CTCTTATTAT GACTTTTTAA TGTAATTTAA AATAAATACA AATAAAAGTA TGCGATACAA   
  
  
- ATTGACGATA TGCGACACAA ATTGACGTAG AGGAAGATAT AAACTATTTA ACCATTTTTT TTTATTAAGT   
  
  
- TTTAATTTCT GATCATTATA AAAGTGAATA GACTATCTAA ATATTCTTTA TAAATCTATC GATTTATATA   
  
  
- AGAGAGGACT TTCTCCTCTG ACTATCAACT TAAAGTATCC TATATTTGTA CATACATGTA ACTTGCTTAG   
  
  
- AAAAAAGATA CCACTAGCAA TATTTCAATA AATAATGACA TTTGTAATTC ACTATTAATC CTATACAAGA   
  
  
- TAAAGTGCAA AATCTATCTA TCTATCTACA TTCCTCAACA TTCTTCGACA TTCTTATACA TTCCTCGACA   
  
  
- TTCCTCTACA TTCCTCGACA TTCTTCTACA TTCTCTTTGA CATTCTTCGA TGTTCCTATC TACATTGCTC   
  
  
- AACAACTTAA CAACTTCTCA ACATATCACA TTAAACAAAA TGCTACTTGC AAAACCTACT TTTTATTATT   
  
  
- ATAATTTTAT TATACGAATA ATTTTTATTT ATTTATTAAT TCAATTAATG TGTTGTTATT TTATATAATT   
  
  
- AGGGGTATTT AAATCACTTA TTTTGAAGCA GGTGAACTCT ACTAAAAAAT ATACTTGTGA TCAAACTATC   
  
  
- TTATTTGTTT CATATTTTTT TTTATTTCAA AAAACTGTTT TAACTTATGT TGAGAAGTTT ATTTAAGGAT   
  
  
- AAAATTCATT TATTAAAATT TATATTTATT TAAATACCAG TAGATATTAT TATATATAAA ATACAGCTTT   
  
  
- GAGTTTATAT TAAATGTAGC AATATAATAT AAAAATACAA ATACATTTAC AACACAAGTA TTCTATAAAT   
  
  
- ATTTAAAACA TATAACCATC ATAGTAATTT TTTTTATATG TATAAATACA TTGATTTTAT TTACTTACAA   
  
  
- TATCTTTTAG ATATTCTACA TTCCTCTACA TACTTCTACA TTCCTCTACA TTCTTCTATA TTCCTCTACA   
  
  
- TCTTTTACAT TCTTCTACAC TCTTCGACAT TCCTCTACAT TCAATAAGTG ATTTAGGATT TTTTACGCTT   
  
  
- TATCTTGTCC CCGAATTAAC TCGTGAGTTC TACGTATTAA ACACTGCCTG GTTTACCTAT TTATATCTGG   
  
  
- ACCGTTTACC CAATAAGCTA AATCCATGCT CATACCAATT CAAACCCAAA CCAATACAAA GTCCTATTCA   
  
  
- CTGTAAAGCT GAGTTAAACC AAAACCAACA TACTTGAAGC CGAGCTCTAA AAACCAAACC GGTTATGTGA   
  
  
- ATAGTACGTT TTTATTCATT GAAATATCTT TTAAAACCTA GCCTATACTA CGCCAAGCCT AGTTAAGCCT   
  
  
- AAAACCAAGT GTAAGACCAT TTGGATAAAG CCTACAGTTC ATCCTATACT CAGGTCTAGC AACCTAAAGC   
  
  
- CCATAGTCGA GTTAAAATGG TCTAGATCTA TTTAGTATTT ACATGCCCAA ATAAAAAAGG TCGAACTTAC   
  
  
- TAAAAGAAAT AATTTTTTTC TATCTAAAAT CTATTTTGTG TTGTAAAGTA AAAGTAAACA AGGGGGGATA   
  
  
- TTTAAAGACA CTGGTGGAAG TGAAAGAACG GGGTAAGGGG GGGGGGGGGG CGAGAGAGAG AGAGAGAGAG   
  
  
- AGAAGGGAAA TGTTTCCGTG GTAAGTTTAA GTTTGGTGAG AAAAATTTTG GGGTTTTTAA TAAGAGTTTT   
  
  
- ATTATGGAGT ATTATATTTA CCCAAGCATA GTGGTATATA CCCGGAGTAG AGAGTACCCT TGTATGCTTA   
  
  
- AGTTACCGAA CGGGCTATCA ATAGCCCAGT ACGGTAGCCT CGGGGCCGTT GATTAGTTAC CAGTTCGGAG   
  
  
- TCTAAACCGG AGAAACTCGG TAATATCATT GTGGAAAGAG AGACTTGAGG GGGGAGATGG GTGAGACCCT   
  
  
- GGGCCTAGGC TGGGCCCAGA TAGCACAGGC CCAGGACGAG ACTCAGGTTA CCTCACCTAA CCACTGCGCA   
  
  
- CCTGTGTGGT CTAACTTGTA CTAGACGGTG CAGTCGTCTC AGTTGCCGAC AACTGACTAC CACTGTACCC   
  
  
- CCTCCTCCTA AGGCCATATT CCAACCACGT ACGGAACGAC TGCACGCGTC TCCGGCAGGT TACGCCGCTC   
  
  
- GAGGCTGACC GACGAAGCGA TCAACTACTC TACTGGTTAC CCGACAACGG TGCACAATCA TGCCACACAC   
  
  
- CCTAGCCGTT CCATCGGCCG ATAAAACAAC TCCGAAACTC GGCCGCGGAC AAAGTCGGGC CGGGCCTTTG   
  
  
- ACCGGGCTGG ACCGGCCCGA GCCTCAAGCT CCGACTCCAC AACATAGCAG TGAAGATACT CCGAACGGGA   
  
  
- ATAGAGTTTA AACGAGTGAA GTGCCGATTA GTTCGGTAGA ACCTCCGTAA ACTCCCGGTA CTAATACAGG   
  
  
- TGCAGTAGCT GAAGCCGAAG TACTGGCCGA ACGTTACCGG CCGAAACTAA GTTCGGGACC GAAATTCCGG   
  
  
- GCCACCCGGT GGTAGAGAAT CCGAGTGACC CTAACCGGGC AGAAATCCTT CACCCGGGCT TAGCTATGCA   
  
  
- CTTTAGCCCA ACTCCGATCG GGTTAACCGG GCTAGGTACT TGTACTCCAA GCGTAAAGCC CGACATCGTT   
  
  
- GAAGAGCCGA GCTCCTGCAC TTCGGCAGTT ACGAGCCTTA CCTGGGATCT CTTCGGCAGC GATACTTAAG   
  
  
- GCAGTACGTA GAAGTAGCTG AAAATCCTAA TCTGTAGTTG AGTCAAGATT TGACCTAAGC TTTGGATTTG   
  
  
- GGCTTCTAGC ATTGAAATCA TCTTGTTCTC CGCTTGGTAT TGGTTGGACT TAAAGAGGGG GCTAAGTGAC   
  
  
- TTCGTGATAC AATGATGAGT TGATACAAGC TGAGTAATCT CTGAACAGTT CAGGTTGGCG ACTTTCAGGA   
  
  
- TCGGCTCTAC ATATATGTTT TCCTCTATAG GTTGGATCAC ACGATGCTTC CGAGTCGGGC CCAGGTCTCC   
  
  
- GTGCTCGGCG AGCGGTTTAC CGTTCGAGCC TACCCGGTTC GGCCCAAGTT CGGCGATCCG GACTCAGTTT   
  
  
- TACGGGAATC CGTTCGATTG TACAACCATT GTAACAAGAG GAGTGTTCCC ATACTGCACC TCCTCTCTCT   
  
  
- TCCCACAAAC TGTGACCCGA CCGTATTGGC GGGAGAGTAT CACTGAAGCT GAACCGTTCA CCCCGGGTTT   
  
  
- TACCTGTCAG GTTCACACTG AGTACTCTAC AGTAGTAGGA GTCGAAGAAG TAGAAT

+     ABRE3a

| Site Name | Organism | Position | Strand | Matrix score. | sequence | function |
| --- | --- | --- | --- | --- | --- | --- |
| ABRE3a | Zea mays | 2800 | + | 6 | TACGTG |  |

>HU03G02797.1   
+ +Up\_Stream \_Len000CATTTT CAATTAGAAA TTTCTTCAAT TATATACCAT CTCCAAAAAT AGTCCTCATA   
  
  
+ AGTTGGCAGG TGAATGATCC TAATGGAAAA TTACTTTTAA CTGTTGAATT TGCAATAGGG TACCGAATTT   
  
  
+ GTGAAGTTTA TTGCCGCACG TGCCTAGCTT GTGACTTCTC GATAATCAAA AATTAATTAG AGAGAGACAG   
  
  
+ CTAAAGTTTT GAGAATAATA CTGAAAAATT ACATTAAATT TTATTTATGT TTATTTTCAT ACGCTATGTT   
  
  
+ TAACTGCTAT ACGCTGTGTT TAACTGCATC TCCTTCTATA TTTGATAAAT TGGTAAAAAA AAATAATTCA   
  
  
+ AAATTAAAGA CTAGTAATAT TTTCACTTAT CTGATAGATT TATAAGAAAT ATTTAGATAG CTAAATATAT   
  
  
+ TCTCTCCTGA AAGAGGAGAC TGATAGTTGA ATTTCATAGG ATATAAACAT GTATGTACAT TGAACGAATC   
  
  
+ TTTTTTCTAT GGTGATCGTT ATAAAGTTAT TTATTACTGT AAACATTAAG TGATAATTAG GATATGTTCT   
  
  
+ ATTTCACGTT TTAGATAGAT AGATAGATGT AAGGAGTTGT AAGAAGCTGT AAGAATATGT AAGGAGCTGT   
  
  
+ AAGGAGATGT AAGGAGCTGT AAGAAGATGT AAGAGAAACT GTAAGAAGCT ACAAGGATAG ATGTAACGAG   
  
  
+ TTGTTGAATT GTTGAAGAGT TGTATAGTGT AATTTGTTTT ACGATGAACG TTTTGGATGA AAAATAATAA   
  
  
+ TATTAAAATA ATATGCTTAT TAAAAATAAA TAAATAATTA AGTTAATTAC ACAACAATAA AATATATTAA   
  
  
+ TCCCCATAAA TTTAGTGAAT AAAACTTCGT CCACTTGAGA TGATTTTTTA TATGAACACT AGTTTGATAG   
  
  
+ AATAAACAAA GTATAAAAAA AAATAAAGTT TTTTGACAAA ATTGAATACA ACTCTTCAAA TAAATTCCTA   
  
  
+ TTTTAAGTAA ATAATTTTAA ATATAAATAA ATTTATGGTC ATCTATAATA ATATATATTT TATGTCGAAA   
  
  
+ CTCAAATATA ATTTACATCG TTATATTATA TTTTTATGTT TATGTAAATG TTGTGTTCAT AAGATATTTA   
  
  
+ TAAATTTTGT ATATTGGTAG TATCATTAAA AAAAATATAC ATATTTATGT AACTAAAATA AATGAATGTT   
  
  
+ ATAGAAAATC TATAAGATGT AAGGAGATGT ATGAAGATGT AAGGAGATGT AAGAAGATAT AAGGAGATGT   
  
  
+ AGAAAATGTA AGAAGATGTG AGAAGCTGTA AGGAGATGTA AGTTATTCAC TAAATCCTAA AAAATGCGAA   
  
  
+ ATAGAACAGG GGCTTAATTG AGCACTCAAG ATGCATAATT TGTGACGGAC CAAATGGATA AATATAGACC   
  
  
+ TGGCAAATGG GTTATTCGAT TTAGGTACGA GTATGGTTAA GTTTGGGTTT GGTTATGTTT CAGGATAAGT   
  
  
+ GACATTTCGA CTCAATTTGG TTTTGGTTGT ATGAACTTCG GCTCGAGATT TTTGGTTTGG CCAATACACT   
  
  
+ TATCATGCAA AAATAAGTAA CTTTATAGAA AATTTTGGAT CGGATATGAT GCGGTTCGGA TCAATTCGGA   
  
  
+ TTTTGGTTCA CATTCTGGTA AACCTATTTC GGATGTCAAG TAGGATATGA GTCCAGATCG TTGGATTTCG   
  
  
+ GGTATCAGCT CAATTTTACC AGATCTAGAT AAATCATAAA TGTACGGGTT TATTTTTTCC AGCTTGAATG   
  
  
+ ATTTTCTTTA TTAAAAAAAG ATAGATTTTA GATAAAACAC AACATTTCAT TTTCATTTGT TCCCCCCTAT   
  
  
+ AAATTTCTGT GACCACCTTC ACTTTCTTGC CCCATTCCCC CCCCCCCCCC GCTCTCTCTC TCTCTCTCTC   
  
  
+ TCTTCCCTTT ACAAAGGCAC CATTCAAATT CAAACCACTC TTTTTAAAAC CCCAAAAATT ATTCTCAAAA   
  
  
+ TAATACCTCA TAATATAAAT GGGTTCGTAT CACCATATAT GGGCCTCATC TCTCATGGGA ACATACGAAT   
  
  
+ TCAATGGCTT GCCCGATAGT TATCGGGTCA TGCCATCGGA GCCCCGGCAA CTAATCAATG GTCAAGCCTC   
  
  
+ AGATTTGGCC TCTTTGAGCC ATTATAGTAA CACCTTTCTC TCTGAACTCC CCCCTCTACC CACTCTGGGA   
  
  
+ CCCGGATCCG ACCCGGGTCT ATCGTGTCCG GGTCCTGCTC TGAGTCCAAT GGAGTGGATT GGTGACGCGT   
  
  
+ GGACACACCA GATTGAACAT GATCTGCCAC GTCAGCAGAG TCAACGGCTG TTGACTGATG GTGACATGGG   
  
  
+ GGAGGAGGAT TCCGGTATAA GGTTGGTGCA TGCCTTGCTG ACGTGCGCAG AGGCCGTCCA ATGCGGCGAG   
  
  
+ CTCCGACTGG CTGCTTCGCT AGTTGATGAG ATGACCAATG GGCTGTTGCC ACGTGTTAGT ACGGTGTGTG   
  
  
+ GGATCGGCAA GGTAGCCGGC TATTTTGTTG AGGCTTTGAG CCGGCGCCTG TTTCAGCCCG GCCCGGAAAC   
  
  
+ TGGCCCGACC TGGCCGGGCT CGGAGTTCGA GGCTGAGGTG TTGTATCGTC ACTTCTATGA GGCTTGCCCT   
  
  
+ TATCTCAAAT TTGCTCACTT CACGGCTAAT CAAGCCATCT TGGAGGCATT TGAGGGCCAT GATTATGTCC   
  
  
+ ACGTCATCGA CTTCGGCTTC ATGACCGGCT TGCAATGGCC GGCTTTGATT CAAGCCCTGG CTTTAAGGCC   
  
  
+ CGGTGGGCCA CCATCTCTTA GGCTCACTGG GATTGGCCCG TCTTTAGGAA GTGGGCCCGA ATCGATACGT   
  
  
+ GAAATCGGGT TGAGGCTAGC CCAATTGGCC CGATCCATGA ACATGAGGTT CGCATTTCGG GCTGTAGCAA   
  
  
+ CTTCTCGGCT CGAGGACGTG AAGCCGTCAA TGCTCGGAAT GGACCCTAGA GAAGCCGTCG CTATGAATTC   
  
  
+ CGTCATGCAT CTTCATCGAC TTTTAGGATT AGACATCAAC TCAGTTCTAA ACTGGATTCG AAACCTAAAC   
  
  
+ CCGAAGATCG TAACTTTAGT AGAACAAGAG GCGAACCATA ACCAACCTGA ATTTCTCCCC CGATTCACTG   
  
  
+ AAGCACTATG TTACTACTCA ACTATGTTCG ACTCATTAGA GACTTGTCAA GTCCAACCGC TGAAAGTCCT   
  
  
+ AGCCGAGATG TATATACAAA AGGAGATATC CAACCTAGTG TGCTACGAAG GCTCAGCCCG GGTCCAGAGG   
  
  
+ CACGAGCCGC TCGCCAAATG GCAAGCTCGG ATGGGCCAAG CCGGGTTCAA GCCGCTAGGC CTGAGTCAAA   
  
  
+ ATGCCCTTAG GCAAGCTAAC ATGTTGGTAA CATTGTTCTC CTCACAAGGG TATGACGTGG AGGAGAGAGA   
  
  
+ AGGGTGTTTG ACACTGGGCT GGCATAACCG CCCTCTCATA GTGACTTCGA CTTGGCAAGT GGGGCCCAAA   
  
  
+ ATGGACAGTC CAAGTGTGAC TCATGAGATG TCATCATCCT CAGCTTCTTC ATCTTA  

- +Up\_Stream \_Len000GTAAAA GTTAATCTTT AAAGAAGTTA ATATATGGTA GAGGTTTTTA TCAGGAGTAT   
  
  
- TCAACCGTCC ACTTACTAGG ATTACCTTTT AATGAAAATT GACAACTTAA ACGTTATCCC ATGGCTTAAA   
  
  
- CACTTCAAAT AACGGCGTGC ACGGATCGAA CACTGAAGAG CTATTAGTTT TTAATTAATC TCTCTCTGTC   
  
  
- GATTTCAAAA CTCTTATTAT GACTTTTTAA TGTAATTTAA AATAAATACA AATAAAAGTA TGCGATACAA   
  
  
- ATTGACGATA TGCGACACAA ATTGACGTAG AGGAAGATAT AAACTATTTA ACCATTTTTT TTTATTAAGT   
  
  
- TTTAATTTCT GATCATTATA AAAGTGAATA GACTATCTAA ATATTCTTTA TAAATCTATC GATTTATATA   
  
  
- AGAGAGGACT TTCTCCTCTG ACTATCAACT TAAAGTATCC TATATTTGTA CATACATGTA ACTTGCTTAG   
  
  
- AAAAAAGATA CCACTAGCAA TATTTCAATA AATAATGACA TTTGTAATTC ACTATTAATC CTATACAAGA   
  
  
- TAAAGTGCAA AATCTATCTA TCTATCTACA TTCCTCAACA TTCTTCGACA TTCTTATACA TTCCTCGACA   
  
  
- TTCCTCTACA TTCCTCGACA TTCTTCTACA TTCTCTTTGA CATTCTTCGA TGTTCCTATC TACATTGCTC   
  
  
- AACAACTTAA CAACTTCTCA ACATATCACA TTAAACAAAA TGCTACTTGC AAAACCTACT TTTTATTATT   
  
  
- ATAATTTTAT TATACGAATA ATTTTTATTT ATTTATTAAT TCAATTAATG TGTTGTTATT TTATATAATT   
  
  
- AGGGGTATTT AAATCACTTA TTTTGAAGCA GGTGAACTCT ACTAAAAAAT ATACTTGTGA TCAAACTATC   
  
  
- TTATTTGTTT CATATTTTTT TTTATTTCAA AAAACTGTTT TAACTTATGT TGAGAAGTTT ATTTAAGGAT   
  
  
- AAAATTCATT TATTAAAATT TATATTTATT TAAATACCAG TAGATATTAT TATATATAAA ATACAGCTTT   
  
  
- GAGTTTATAT TAAATGTAGC AATATAATAT AAAAATACAA ATACATTTAC AACACAAGTA TTCTATAAAT   
  
  
- ATTTAAAACA TATAACCATC ATAGTAATTT TTTTTATATG TATAAATACA TTGATTTTAT TTACTTACAA   
  
  
- TATCTTTTAG ATATTCTACA TTCCTCTACA TACTTCTACA TTCCTCTACA TTCTTCTATA TTCCTCTACA   
  
  
- TCTTTTACAT TCTTCTACAC TCTTCGACAT TCCTCTACAT TCAATAAGTG ATTTAGGATT TTTTACGCTT   
  
  
- TATCTTGTCC CCGAATTAAC TCGTGAGTTC TACGTATTAA ACACTGCCTG GTTTACCTAT TTATATCTGG   
  
  
- ACCGTTTACC CAATAAGCTA AATCCATGCT CATACCAATT CAAACCCAAA CCAATACAAA GTCCTATTCA   
  
  
- CTGTAAAGCT GAGTTAAACC AAAACCAACA TACTTGAAGC CGAGCTCTAA AAACCAAACC GGTTATGTGA   
  
  
- ATAGTACGTT TTTATTCATT GAAATATCTT TTAAAACCTA GCCTATACTA CGCCAAGCCT AGTTAAGCCT   
  
  
- AAAACCAAGT GTAAGACCAT TTGGATAAAG CCTACAGTTC ATCCTATACT CAGGTCTAGC AACCTAAAGC   
  
  
- CCATAGTCGA GTTAAAATGG TCTAGATCTA TTTAGTATTT ACATGCCCAA ATAAAAAAGG TCGAACTTAC   
  
  
- TAAAAGAAAT AATTTTTTTC TATCTAAAAT CTATTTTGTG TTGTAAAGTA AAAGTAAACA AGGGGGGATA   
  
  
- TTTAAAGACA CTGGTGGAAG TGAAAGAACG GGGTAAGGGG GGGGGGGGGG CGAGAGAGAG AGAGAGAGAG   
  
  
- AGAAGGGAAA TGTTTCCGTG GTAAGTTTAA GTTTGGTGAG AAAAATTTTG GGGTTTTTAA TAAGAGTTTT   
  
  
- ATTATGGAGT ATTATATTTA CCCAAGCATA GTGGTATATA CCCGGAGTAG AGAGTACCCT TGTATGCTTA   
  
  
- AGTTACCGAA CGGGCTATCA ATAGCCCAGT ACGGTAGCCT CGGGGCCGTT GATTAGTTAC CAGTTCGGAG   
  
  
- TCTAAACCGG AGAAACTCGG TAATATCATT GTGGAAAGAG AGACTTGAGG GGGGAGATGG GTGAGACCCT   
  
  
- GGGCCTAGGC TGGGCCCAGA TAGCACAGGC CCAGGACGAG ACTCAGGTTA CCTCACCTAA CCACTGCGCA   
  
  
- CCTGTGTGGT CTAACTTGTA CTAGACGGTG CAGTCGTCTC AGTTGCCGAC AACTGACTAC CACTGTACCC   
  
  
- CCTCCTCCTA AGGCCATATT CCAACCACGT ACGGAACGAC TGCACGCGTC TCCGGCAGGT TACGCCGCTC   
  
  
- GAGGCTGACC GACGAAGCGA TCAACTACTC TACTGGTTAC CCGACAACGG TGCACAATCA TGCCACACAC   
  
  
- CCTAGCCGTT CCATCGGCCG ATAAAACAAC TCCGAAACTC GGCCGCGGAC AAAGTCGGGC CGGGCCTTTG   
  
  
- ACCGGGCTGG ACCGGCCCGA GCCTCAAGCT CCGACTCCAC AACATAGCAG TGAAGATACT CCGAACGGGA   
  
  
- ATAGAGTTTA AACGAGTGAA GTGCCGATTA GTTCGGTAGA ACCTCCGTAA ACTCCCGGTA CTAATACAGG   
  
  
- TGCAGTAGCT GAAGCCGAAG TACTGGCCGA ACGTTACCGG CCGAAACTAA GTTCGGGACC GAAATTCCGG   
  
  
- GCCACCCGGT GGTAGAGAAT CCGAGTGACC CTAACCGGGC AGAAATCCTT CACCCGGGCT TAGCTATGCA   
  
  
- CTTTAGCCCA ACTCCGATCG GGTTAACCGG GCTAGGTACT TGTACTCCAA GCGTAAAGCC CGACATCGTT   
  
  
- GAAGAGCCGA GCTCCTGCAC TTCGGCAGTT ACGAGCCTTA CCTGGGATCT CTTCGGCAGC GATACTTAAG   
  
  
- GCAGTACGTA GAAGTAGCTG AAAATCCTAA TCTGTAGTTG AGTCAAGATT TGACCTAAGC TTTGGATTTG   
  
  
- GGCTTCTAGC ATTGAAATCA TCTTGTTCTC CGCTTGGTAT TGGTTGGACT TAAAGAGGGG GCTAAGTGAC   
  
  
- TTCGTGATAC AATGATGAGT TGATACAAGC TGAGTAATCT CTGAACAGTT CAGGTTGGCG ACTTTCAGGA   
  
  
- TCGGCTCTAC ATATATGTTT TCCTCTATAG GTTGGATCAC ACGATGCTTC CGAGTCGGGC CCAGGTCTCC   
  
  
- GTGCTCGGCG AGCGGTTTAC CGTTCGAGCC TACCCGGTTC GGCCCAAGTT CGGCGATCCG GACTCAGTTT   
  
  
- TACGGGAATC CGTTCGATTG TACAACCATT GTAACAAGAG GAGTGTTCCC ATACTGCACC TCCTCTCTCT   
  
  
- TCCCACAAAC TGTGACCCGA CCGTATTGGC GGGAGAGTAT CACTGAAGCT GAACCGTTCA CCCCGGGTTT   
  
  
- TACCTGTCAG GTTCACACTG AGTACTCTAC AGTAGTAGGA GTCGAAGAAG TAGAAT

+     ABRE4

| Site Name | Organism | Position | Strand | Matrix score. | sequence | function |
| --- | --- | --- | --- | --- | --- | --- |
| ABRE4 | Zea mays | 2800 | - | 6 | CACGTA |  |

>HU03G02797.1   
+ +Up\_Stream \_Len000CATTTT CAATTAGAAA TTTCTTCAAT TATATACCAT CTCCAAAAAT AGTCCTCATA   
  
  
+ AGTTGGCAGG TGAATGATCC TAATGGAAAA TTACTTTTAA CTGTTGAATT TGCAATAGGG TACCGAATTT   
  
  
+ GTGAAGTTTA TTGCCGCACG TGCCTAGCTT GTGACTTCTC GATAATCAAA AATTAATTAG AGAGAGACAG   
  
  
+ CTAAAGTTTT GAGAATAATA CTGAAAAATT ACATTAAATT TTATTTATGT TTATTTTCAT ACGCTATGTT   
  
  
+ TAACTGCTAT ACGCTGTGTT TAACTGCATC TCCTTCTATA TTTGATAAAT TGGTAAAAAA AAATAATTCA   
  
  
+ AAATTAAAGA CTAGTAATAT TTTCACTTAT CTGATAGATT TATAAGAAAT ATTTAGATAG CTAAATATAT   
  
  
+ TCTCTCCTGA AAGAGGAGAC TGATAGTTGA ATTTCATAGG ATATAAACAT GTATGTACAT TGAACGAATC   
  
  
+ TTTTTTCTAT GGTGATCGTT ATAAAGTTAT TTATTACTGT AAACATTAAG TGATAATTAG GATATGTTCT   
  
  
+ ATTTCACGTT TTAGATAGAT AGATAGATGT AAGGAGTTGT AAGAAGCTGT AAGAATATGT AAGGAGCTGT   
  
  
+ AAGGAGATGT AAGGAGCTGT AAGAAGATGT AAGAGAAACT GTAAGAAGCT ACAAGGATAG ATGTAACGAG   
  
  
+ TTGTTGAATT GTTGAAGAGT TGTATAGTGT AATTTGTTTT ACGATGAACG TTTTGGATGA AAAATAATAA   
  
  
+ TATTAAAATA ATATGCTTAT TAAAAATAAA TAAATAATTA AGTTAATTAC ACAACAATAA AATATATTAA   
  
  
+ TCCCCATAAA TTTAGTGAAT AAAACTTCGT CCACTTGAGA TGATTTTTTA TATGAACACT AGTTTGATAG   
  
  
+ AATAAACAAA GTATAAAAAA AAATAAAGTT TTTTGACAAA ATTGAATACA ACTCTTCAAA TAAATTCCTA   
  
  
+ TTTTAAGTAA ATAATTTTAA ATATAAATAA ATTTATGGTC ATCTATAATA ATATATATTT TATGTCGAAA   
  
  
+ CTCAAATATA ATTTACATCG TTATATTATA TTTTTATGTT TATGTAAATG TTGTGTTCAT AAGATATTTA   
  
  
+ TAAATTTTGT ATATTGGTAG TATCATTAAA AAAAATATAC ATATTTATGT AACTAAAATA AATGAATGTT   
  
  
+ ATAGAAAATC TATAAGATGT AAGGAGATGT ATGAAGATGT AAGGAGATGT AAGAAGATAT AAGGAGATGT   
  
  
+ AGAAAATGTA AGAAGATGTG AGAAGCTGTA AGGAGATGTA AGTTATTCAC TAAATCCTAA AAAATGCGAA   
  
  
+ ATAGAACAGG GGCTTAATTG AGCACTCAAG ATGCATAATT TGTGACGGAC CAAATGGATA AATATAGACC   
  
  
+ TGGCAAATGG GTTATTCGAT TTAGGTACGA GTATGGTTAA GTTTGGGTTT GGTTATGTTT CAGGATAAGT   
  
  
+ GACATTTCGA CTCAATTTGG TTTTGGTTGT ATGAACTTCG GCTCGAGATT TTTGGTTTGG CCAATACACT   
  
  
+ TATCATGCAA AAATAAGTAA CTTTATAGAA AATTTTGGAT CGGATATGAT GCGGTTCGGA TCAATTCGGA   
  
  
+ TTTTGGTTCA CATTCTGGTA AACCTATTTC GGATGTCAAG TAGGATATGA GTCCAGATCG TTGGATTTCG   
  
  
+ GGTATCAGCT CAATTTTACC AGATCTAGAT AAATCATAAA TGTACGGGTT TATTTTTTCC AGCTTGAATG   
  
  
+ ATTTTCTTTA TTAAAAAAAG ATAGATTTTA GATAAAACAC AACATTTCAT TTTCATTTGT TCCCCCCTAT   
  
  
+ AAATTTCTGT GACCACCTTC ACTTTCTTGC CCCATTCCCC CCCCCCCCCC GCTCTCTCTC TCTCTCTCTC   
  
  
+ TCTTCCCTTT ACAAAGGCAC CATTCAAATT CAAACCACTC TTTTTAAAAC CCCAAAAATT ATTCTCAAAA   
  
  
+ TAATACCTCA TAATATAAAT GGGTTCGTAT CACCATATAT GGGCCTCATC TCTCATGGGA ACATACGAAT   
  
  
+ TCAATGGCTT GCCCGATAGT TATCGGGTCA TGCCATCGGA GCCCCGGCAA CTAATCAATG GTCAAGCCTC   
  
  
+ AGATTTGGCC TCTTTGAGCC ATTATAGTAA CACCTTTCTC TCTGAACTCC CCCCTCTACC CACTCTGGGA   
  
  
+ CCCGGATCCG ACCCGGGTCT ATCGTGTCCG GGTCCTGCTC TGAGTCCAAT GGAGTGGATT GGTGACGCGT   
  
  
+ GGACACACCA GATTGAACAT GATCTGCCAC GTCAGCAGAG TCAACGGCTG TTGACTGATG GTGACATGGG   
  
  
+ GGAGGAGGAT TCCGGTATAA GGTTGGTGCA TGCCTTGCTG ACGTGCGCAG AGGCCGTCCA ATGCGGCGAG   
  
  
+ CTCCGACTGG CTGCTTCGCT AGTTGATGAG ATGACCAATG GGCTGTTGCC ACGTGTTAGT ACGGTGTGTG   
  
  
+ GGATCGGCAA GGTAGCCGGC TATTTTGTTG AGGCTTTGAG CCGGCGCCTG TTTCAGCCCG GCCCGGAAAC   
  
  
+ TGGCCCGACC TGGCCGGGCT CGGAGTTCGA GGCTGAGGTG TTGTATCGTC ACTTCTATGA GGCTTGCCCT   
  
  
+ TATCTCAAAT TTGCTCACTT CACGGCTAAT CAAGCCATCT TGGAGGCATT TGAGGGCCAT GATTATGTCC   
  
  
+ ACGTCATCGA CTTCGGCTTC ATGACCGGCT TGCAATGGCC GGCTTTGATT CAAGCCCTGG CTTTAAGGCC   
  
  
+ CGGTGGGCCA CCATCTCTTA GGCTCACTGG GATTGGCCCG TCTTTAGGAA GTGGGCCCGA ATCGATACGT   
  
  
+ GAAATCGGGT TGAGGCTAGC CCAATTGGCC CGATCCATGA ACATGAGGTT CGCATTTCGG GCTGTAGCAA   
  
  
+ CTTCTCGGCT CGAGGACGTG AAGCCGTCAA TGCTCGGAAT GGACCCTAGA GAAGCCGTCG CTATGAATTC   
  
  
+ CGTCATGCAT CTTCATCGAC TTTTAGGATT AGACATCAAC TCAGTTCTAA ACTGGATTCG AAACCTAAAC   
  
  
+ CCGAAGATCG TAACTTTAGT AGAACAAGAG GCGAACCATA ACCAACCTGA ATTTCTCCCC CGATTCACTG   
  
  
+ AAGCACTATG TTACTACTCA ACTATGTTCG ACTCATTAGA GACTTGTCAA GTCCAACCGC TGAAAGTCCT   
  
  
+ AGCCGAGATG TATATACAAA AGGAGATATC CAACCTAGTG TGCTACGAAG GCTCAGCCCG GGTCCAGAGG   
  
  
+ CACGAGCCGC TCGCCAAATG GCAAGCTCGG ATGGGCCAAG CCGGGTTCAA GCCGCTAGGC CTGAGTCAAA   
  
  
+ ATGCCCTTAG GCAAGCTAAC ATGTTGGTAA CATTGTTCTC CTCACAAGGG TATGACGTGG AGGAGAGAGA   
  
  
+ AGGGTGTTTG ACACTGGGCT GGCATAACCG CCCTCTCATA GTGACTTCGA CTTGGCAAGT GGGGCCCAAA   
  
  
+ ATGGACAGTC CAAGTGTGAC TCATGAGATG TCATCATCCT CAGCTTCTTC ATCTTA  

- +Up\_Stream \_Len000GTAAAA GTTAATCTTT AAAGAAGTTA ATATATGGTA GAGGTTTTTA TCAGGAGTAT   
  
  
- TCAACCGTCC ACTTACTAGG ATTACCTTTT AATGAAAATT GACAACTTAA ACGTTATCCC ATGGCTTAAA   
  
  
- CACTTCAAAT AACGGCGTGC ACGGATCGAA CACTGAAGAG CTATTAGTTT TTAATTAATC TCTCTCTGTC   
  
  
- GATTTCAAAA CTCTTATTAT GACTTTTTAA TGTAATTTAA AATAAATACA AATAAAAGTA TGCGATACAA   
  
  
- ATTGACGATA TGCGACACAA ATTGACGTAG AGGAAGATAT AAACTATTTA ACCATTTTTT TTTATTAAGT   
  
  
- TTTAATTTCT GATCATTATA AAAGTGAATA GACTATCTAA ATATTCTTTA TAAATCTATC GATTTATATA   
  
  
- AGAGAGGACT TTCTCCTCTG ACTATCAACT TAAAGTATCC TATATTTGTA CATACATGTA ACTTGCTTAG   
  
  
- AAAAAAGATA CCACTAGCAA TATTTCAATA AATAATGACA TTTGTAATTC ACTATTAATC CTATACAAGA   
  
  
- TAAAGTGCAA AATCTATCTA TCTATCTACA TTCCTCAACA TTCTTCGACA TTCTTATACA TTCCTCGACA   
  
  
- TTCCTCTACA TTCCTCGACA TTCTTCTACA TTCTCTTTGA CATTCTTCGA TGTTCCTATC TACATTGCTC   
  
  
- AACAACTTAA CAACTTCTCA ACATATCACA TTAAACAAAA TGCTACTTGC AAAACCTACT TTTTATTATT   
  
  
- ATAATTTTAT TATACGAATA ATTTTTATTT ATTTATTAAT TCAATTAATG TGTTGTTATT TTATATAATT   
  
  
- AGGGGTATTT AAATCACTTA TTTTGAAGCA GGTGAACTCT ACTAAAAAAT ATACTTGTGA TCAAACTATC   
  
  
- TTATTTGTTT CATATTTTTT TTTATTTCAA AAAACTGTTT TAACTTATGT TGAGAAGTTT ATTTAAGGAT   
  
  
- AAAATTCATT TATTAAAATT TATATTTATT TAAATACCAG TAGATATTAT TATATATAAA ATACAGCTTT   
  
  
- GAGTTTATAT TAAATGTAGC AATATAATAT AAAAATACAA ATACATTTAC AACACAAGTA TTCTATAAAT   
  
  
- ATTTAAAACA TATAACCATC ATAGTAATTT TTTTTATATG TATAAATACA TTGATTTTAT TTACTTACAA   
  
  
- TATCTTTTAG ATATTCTACA TTCCTCTACA TACTTCTACA TTCCTCTACA TTCTTCTATA TTCCTCTACA   
  
  
- TCTTTTACAT TCTTCTACAC TCTTCGACAT TCCTCTACAT TCAATAAGTG ATTTAGGATT TTTTACGCTT   
  
  
- TATCTTGTCC CCGAATTAAC TCGTGAGTTC TACGTATTAA ACACTGCCTG GTTTACCTAT TTATATCTGG   
  
  
- ACCGTTTACC CAATAAGCTA AATCCATGCT CATACCAATT CAAACCCAAA CCAATACAAA GTCCTATTCA   
  
  
- CTGTAAAGCT GAGTTAAACC AAAACCAACA TACTTGAAGC CGAGCTCTAA AAACCAAACC GGTTATGTGA   
  
  
- ATAGTACGTT TTTATTCATT GAAATATCTT TTAAAACCTA GCCTATACTA CGCCAAGCCT AGTTAAGCCT   
  
  
- AAAACCAAGT GTAAGACCAT TTGGATAAAG CCTACAGTTC ATCCTATACT CAGGTCTAGC AACCTAAAGC   
  
  
- CCATAGTCGA GTTAAAATGG TCTAGATCTA TTTAGTATTT ACATGCCCAA ATAAAAAAGG TCGAACTTAC   
  
  
- TAAAAGAAAT AATTTTTTTC TATCTAAAAT CTATTTTGTG TTGTAAAGTA AAAGTAAACA AGGGGGGATA   
  
  
- TTTAAAGACA CTGGTGGAAG TGAAAGAACG GGGTAAGGGG GGGGGGGGGG CGAGAGAGAG AGAGAGAGAG   
  
  
- AGAAGGGAAA TGTTTCCGTG GTAAGTTTAA GTTTGGTGAG AAAAATTTTG GGGTTTTTAA TAAGAGTTTT   
  
  
- ATTATGGAGT ATTATATTTA CCCAAGCATA GTGGTATATA CCCGGAGTAG AGAGTACCCT TGTATGCTTA   
  
  
- AGTTACCGAA CGGGCTATCA ATAGCCCAGT ACGGTAGCCT CGGGGCCGTT GATTAGTTAC CAGTTCGGAG   
  
  
- TCTAAACCGG AGAAACTCGG TAATATCATT GTGGAAAGAG AGACTTGAGG GGGGAGATGG GTGAGACCCT   
  
  
- GGGCCTAGGC TGGGCCCAGA TAGCACAGGC CCAGGACGAG ACTCAGGTTA CCTCACCTAA CCACTGCGCA   
  
  
- CCTGTGTGGT CTAACTTGTA CTAGACGGTG CAGTCGTCTC AGTTGCCGAC AACTGACTAC CACTGTACCC   
  
  
- CCTCCTCCTA AGGCCATATT CCAACCACGT ACGGAACGAC TGCACGCGTC TCCGGCAGGT TACGCCGCTC   
  
  
- GAGGCTGACC GACGAAGCGA TCAACTACTC TACTGGTTAC CCGACAACGG TGCACAATCA TGCCACACAC   
  
  
- CCTAGCCGTT CCATCGGCCG ATAAAACAAC TCCGAAACTC GGCCGCGGAC AAAGTCGGGC CGGGCCTTTG   
  
  
- ACCGGGCTGG ACCGGCCCGA GCCTCAAGCT CCGACTCCAC AACATAGCAG TGAAGATACT CCGAACGGGA   
  
  
- ATAGAGTTTA AACGAGTGAA GTGCCGATTA GTTCGGTAGA ACCTCCGTAA ACTCCCGGTA CTAATACAGG   
  
  
- TGCAGTAGCT GAAGCCGAAG TACTGGCCGA ACGTTACCGG CCGAAACTAA GTTCGGGACC GAAATTCCGG   
  
  
- GCCACCCGGT GGTAGAGAAT CCGAGTGACC CTAACCGGGC AGAAATCCTT CACCCGGGCT TAGCTATGCA   
  
  
- CTTTAGCCCA ACTCCGATCG GGTTAACCGG GCTAGGTACT TGTACTCCAA GCGTAAAGCC CGACATCGTT   
  
  
- GAAGAGCCGA GCTCCTGCAC TTCGGCAGTT ACGAGCCTTA CCTGGGATCT CTTCGGCAGC GATACTTAAG   
  
  
- GCAGTACGTA GAAGTAGCTG AAAATCCTAA TCTGTAGTTG AGTCAAGATT TGACCTAAGC TTTGGATTTG   
  
  
- GGCTTCTAGC ATTGAAATCA TCTTGTTCTC CGCTTGGTAT TGGTTGGACT TAAAGAGGGG GCTAAGTGAC   
  
  
- TTCGTGATAC AATGATGAGT TGATACAAGC TGAGTAATCT CTGAACAGTT CAGGTTGGCG ACTTTCAGGA   
  
  
- TCGGCTCTAC ATATATGTTT TCCTCTATAG GTTGGATCAC ACGATGCTTC CGAGTCGGGC CCAGGTCTCC   
  
  
- GTGCTCGGCG AGCGGTTTAC CGTTCGAGCC TACCCGGTTC GGCCCAAGTT CGGCGATCCG GACTCAGTTT   
  
  
- TACGGGAATC CGTTCGATTG TACAACCATT GTAACAAGAG GAGTGTTCCC ATACTGCACC TCCTCTCTCT   
  
  
- TCCCACAAAC TGTGACCCGA CCGTATTGGC GGGAGAGTAT CACTGAAGCT GAACCGTTCA CCCCGGGTTT   
  
  
- TACCTGTCAG GTTCACACTG AGTACTCTAC AGTAGTAGGA GTCGAAGAAG TAGAAT

+     ARE

| Site Name | Organism | Position | Strand | Matrix score. | sequence | function |
| --- | --- | --- | --- | --- | --- | --- |
| ARE | Zea mays | 1492 | - | 6 | AAACCA | cis-acting regulatory element essential for the anaerobic induction |
| ARE | Zea mays | 1926 | + | 6 | AAACCA | cis-acting regulatory element essential for the anaerobic induction |
| ARE | Zea mays | 1527 | - | 6 | AAACCA | cis-acting regulatory element essential for the anaerobic induction |

>HU03G02797.1   
+ +Up\_Stream \_Len000CATTTT CAATTAGAAA TTTCTTCAAT TATATACCAT CTCCAAAAAT AGTCCTCATA   
  
  
+ AGTTGGCAGG TGAATGATCC TAATGGAAAA TTACTTTTAA CTGTTGAATT TGCAATAGGG TACCGAATTT   
  
  
+ GTGAAGTTTA TTGCCGCACG TGCCTAGCTT GTGACTTCTC GATAATCAAA AATTAATTAG AGAGAGACAG   
  
  
+ CTAAAGTTTT GAGAATAATA CTGAAAAATT ACATTAAATT TTATTTATGT TTATTTTCAT ACGCTATGTT   
  
  
+ TAACTGCTAT ACGCTGTGTT TAACTGCATC TCCTTCTATA TTTGATAAAT TGGTAAAAAA AAATAATTCA   
  
  
+ AAATTAAAGA CTAGTAATAT TTTCACTTAT CTGATAGATT TATAAGAAAT ATTTAGATAG CTAAATATAT   
  
  
+ TCTCTCCTGA AAGAGGAGAC TGATAGTTGA ATTTCATAGG ATATAAACAT GTATGTACAT TGAACGAATC   
  
  
+ TTTTTTCTAT GGTGATCGTT ATAAAGTTAT TTATTACTGT AAACATTAAG TGATAATTAG GATATGTTCT   
  
  
+ ATTTCACGTT TTAGATAGAT AGATAGATGT AAGGAGTTGT AAGAAGCTGT AAGAATATGT AAGGAGCTGT   
  
  
+ AAGGAGATGT AAGGAGCTGT AAGAAGATGT AAGAGAAACT GTAAGAAGCT ACAAGGATAG ATGTAACGAG   
  
  
+ TTGTTGAATT GTTGAAGAGT TGTATAGTGT AATTTGTTTT ACGATGAACG TTTTGGATGA AAAATAATAA   
  
  
+ TATTAAAATA ATATGCTTAT TAAAAATAAA TAAATAATTA AGTTAATTAC ACAACAATAA AATATATTAA   
  
  
+ TCCCCATAAA TTTAGTGAAT AAAACTTCGT CCACTTGAGA TGATTTTTTA TATGAACACT AGTTTGATAG   
  
  
+ AATAAACAAA GTATAAAAAA AAATAAAGTT TTTTGACAAA ATTGAATACA ACTCTTCAAA TAAATTCCTA   
  
  
+ TTTTAAGTAA ATAATTTTAA ATATAAATAA ATTTATGGTC ATCTATAATA ATATATATTT TATGTCGAAA   
  
  
+ CTCAAATATA ATTTACATCG TTATATTATA TTTTTATGTT TATGTAAATG TTGTGTTCAT AAGATATTTA   
  
  
+ TAAATTTTGT ATATTGGTAG TATCATTAAA AAAAATATAC ATATTTATGT AACTAAAATA AATGAATGTT   
  
  
+ ATAGAAAATC TATAAGATGT AAGGAGATGT ATGAAGATGT AAGGAGATGT AAGAAGATAT AAGGAGATGT   
  
  
+ AGAAAATGTA AGAAGATGTG AGAAGCTGTA AGGAGATGTA AGTTATTCAC TAAATCCTAA AAAATGCGAA   
  
  
+ ATAGAACAGG GGCTTAATTG AGCACTCAAG ATGCATAATT TGTGACGGAC CAAATGGATA AATATAGACC   
  
  
+ TGGCAAATGG GTTATTCGAT TTAGGTACGA GTATGGTTAA GTTTGGGTTT GGTTATGTTT CAGGATAAGT   
  
  
+ GACATTTCGA CTCAATTTGG TTTTGGTTGT ATGAACTTCG GCTCGAGATT TTTGGTTTGG CCAATACACT   
  
  
+ TATCATGCAA AAATAAGTAA CTTTATAGAA AATTTTGGAT CGGATATGAT GCGGTTCGGA TCAATTCGGA   
  
  
+ TTTTGGTTCA CATTCTGGTA AACCTATTTC GGATGTCAAG TAGGATATGA GTCCAGATCG TTGGATTTCG   
  
  
+ GGTATCAGCT CAATTTTACC AGATCTAGAT AAATCATAAA TGTACGGGTT TATTTTTTCC AGCTTGAATG   
  
  
+ ATTTTCTTTA TTAAAAAAAG ATAGATTTTA GATAAAACAC AACATTTCAT TTTCATTTGT TCCCCCCTAT   
  
  
+ AAATTTCTGT GACCACCTTC ACTTTCTTGC CCCATTCCCC CCCCCCCCCC GCTCTCTCTC TCTCTCTCTC   
  
  
+ TCTTCCCTTT ACAAAGGCAC CATTCAAATT CAAACCACTC TTTTTAAAAC CCCAAAAATT ATTCTCAAAA   
  
  
+ TAATACCTCA TAATATAAAT GGGTTCGTAT CACCATATAT GGGCCTCATC TCTCATGGGA ACATACGAAT   
  
  
+ TCAATGGCTT GCCCGATAGT TATCGGGTCA TGCCATCGGA GCCCCGGCAA CTAATCAATG GTCAAGCCTC   
  
  
+ AGATTTGGCC TCTTTGAGCC ATTATAGTAA CACCTTTCTC TCTGAACTCC CCCCTCTACC CACTCTGGGA   
  
  
+ CCCGGATCCG ACCCGGGTCT ATCGTGTCCG GGTCCTGCTC TGAGTCCAAT GGAGTGGATT GGTGACGCGT   
  
  
+ GGACACACCA GATTGAACAT GATCTGCCAC GTCAGCAGAG TCAACGGCTG TTGACTGATG GTGACATGGG   
  
  
+ GGAGGAGGAT TCCGGTATAA GGTTGGTGCA TGCCTTGCTG ACGTGCGCAG AGGCCGTCCA ATGCGGCGAG   
  
  
+ CTCCGACTGG CTGCTTCGCT AGTTGATGAG ATGACCAATG GGCTGTTGCC ACGTGTTAGT ACGGTGTGTG   
  
  
+ GGATCGGCAA GGTAGCCGGC TATTTTGTTG AGGCTTTGAG CCGGCGCCTG TTTCAGCCCG GCCCGGAAAC   
  
  
+ TGGCCCGACC TGGCCGGGCT CGGAGTTCGA GGCTGAGGTG TTGTATCGTC ACTTCTATGA GGCTTGCCCT   
  
  
+ TATCTCAAAT TTGCTCACTT CACGGCTAAT CAAGCCATCT TGGAGGCATT TGAGGGCCAT GATTATGTCC   
  
  
+ ACGTCATCGA CTTCGGCTTC ATGACCGGCT TGCAATGGCC GGCTTTGATT CAAGCCCTGG CTTTAAGGCC   
  
  
+ CGGTGGGCCA CCATCTCTTA GGCTCACTGG GATTGGCCCG TCTTTAGGAA GTGGGCCCGA ATCGATACGT   
  
  
+ GAAATCGGGT TGAGGCTAGC CCAATTGGCC CGATCCATGA ACATGAGGTT CGCATTTCGG GCTGTAGCAA   
  
  
+ CTTCTCGGCT CGAGGACGTG AAGCCGTCAA TGCTCGGAAT GGACCCTAGA GAAGCCGTCG CTATGAATTC   
  
  
+ CGTCATGCAT CTTCATCGAC TTTTAGGATT AGACATCAAC TCAGTTCTAA ACTGGATTCG AAACCTAAAC   
  
  
+ CCGAAGATCG TAACTTTAGT AGAACAAGAG GCGAACCATA ACCAACCTGA ATTTCTCCCC CGATTCACTG   
  
  
+ AAGCACTATG TTACTACTCA ACTATGTTCG ACTCATTAGA GACTTGTCAA GTCCAACCGC TGAAAGTCCT   
  
  
+ AGCCGAGATG TATATACAAA AGGAGATATC CAACCTAGTG TGCTACGAAG GCTCAGCCCG GGTCCAGAGG   
  
  
+ CACGAGCCGC TCGCCAAATG GCAAGCTCGG ATGGGCCAAG CCGGGTTCAA GCCGCTAGGC CTGAGTCAAA   
  
  
+ ATGCCCTTAG GCAAGCTAAC ATGTTGGTAA CATTGTTCTC CTCACAAGGG TATGACGTGG AGGAGAGAGA   
  
  
+ AGGGTGTTTG ACACTGGGCT GGCATAACCG CCCTCTCATA GTGACTTCGA CTTGGCAAGT GGGGCCCAAA   
  
  
+ ATGGACAGTC CAAGTGTGAC TCATGAGATG TCATCATCCT CAGCTTCTTC ATCTTA  

- +Up\_Stream \_Len000GTAAAA GTTAATCTTT AAAGAAGTTA ATATATGGTA GAGGTTTTTA TCAGGAGTAT   
  
  
- TCAACCGTCC ACTTACTAGG ATTACCTTTT AATGAAAATT GACAACTTAA ACGTTATCCC ATGGCTTAAA   
  
  
- CACTTCAAAT AACGGCGTGC ACGGATCGAA CACTGAAGAG CTATTAGTTT TTAATTAATC TCTCTCTGTC   
  
  
- GATTTCAAAA CTCTTATTAT GACTTTTTAA TGTAATTTAA AATAAATACA AATAAAAGTA TGCGATACAA   
  
  
- ATTGACGATA TGCGACACAA ATTGACGTAG AGGAAGATAT AAACTATTTA ACCATTTTTT TTTATTAAGT   
  
  
- TTTAATTTCT GATCATTATA AAAGTGAATA GACTATCTAA ATATTCTTTA TAAATCTATC GATTTATATA   
  
  
- AGAGAGGACT TTCTCCTCTG ACTATCAACT TAAAGTATCC TATATTTGTA CATACATGTA ACTTGCTTAG   
  
  
- AAAAAAGATA CCACTAGCAA TATTTCAATA AATAATGACA TTTGTAATTC ACTATTAATC CTATACAAGA   
  
  
- TAAAGTGCAA AATCTATCTA TCTATCTACA TTCCTCAACA TTCTTCGACA TTCTTATACA TTCCTCGACA   
  
  
- TTCCTCTACA TTCCTCGACA TTCTTCTACA TTCTCTTTGA CATTCTTCGA TGTTCCTATC TACATTGCTC   
  
  
- AACAACTTAA CAACTTCTCA ACATATCACA TTAAACAAAA TGCTACTTGC AAAACCTACT TTTTATTATT   
  
  
- ATAATTTTAT TATACGAATA ATTTTTATTT ATTTATTAAT TCAATTAATG TGTTGTTATT TTATATAATT   
  
  
- AGGGGTATTT AAATCACTTA TTTTGAAGCA GGTGAACTCT ACTAAAAAAT ATACTTGTGA TCAAACTATC   
  
  
- TTATTTGTTT CATATTTTTT TTTATTTCAA AAAACTGTTT TAACTTATGT TGAGAAGTTT ATTTAAGGAT   
  
  
- AAAATTCATT TATTAAAATT TATATTTATT TAAATACCAG TAGATATTAT TATATATAAA ATACAGCTTT   
  
  
- GAGTTTATAT TAAATGTAGC AATATAATAT AAAAATACAA ATACATTTAC AACACAAGTA TTCTATAAAT   
  
  
- ATTTAAAACA TATAACCATC ATAGTAATTT TTTTTATATG TATAAATACA TTGATTTTAT TTACTTACAA   
  
  
- TATCTTTTAG ATATTCTACA TTCCTCTACA TACTTCTACA TTCCTCTACA TTCTTCTATA TTCCTCTACA   
  
  
- TCTTTTACAT TCTTCTACAC TCTTCGACAT TCCTCTACAT TCAATAAGTG ATTTAGGATT TTTTACGCTT   
  
  
- TATCTTGTCC CCGAATTAAC TCGTGAGTTC TACGTATTAA ACACTGCCTG GTTTACCTAT TTATATCTGG   
  
  
- ACCGTTTACC CAATAAGCTA AATCCATGCT CATACCAATT CAAACCCAAA CCAATACAAA GTCCTATTCA   
  
  
- CTGTAAAGCT GAGTTAAACC AAAACCAACA TACTTGAAGC CGAGCTCTAA AAACCAAACC GGTTATGTGA   
  
  
- ATAGTACGTT TTTATTCATT GAAATATCTT TTAAAACCTA GCCTATACTA CGCCAAGCCT AGTTAAGCCT   
  
  
- AAAACCAAGT GTAAGACCAT TTGGATAAAG CCTACAGTTC ATCCTATACT CAGGTCTAGC AACCTAAAGC   
  
  
- CCATAGTCGA GTTAAAATGG TCTAGATCTA TTTAGTATTT ACATGCCCAA ATAAAAAAGG TCGAACTTAC   
  
  
- TAAAAGAAAT AATTTTTTTC TATCTAAAAT CTATTTTGTG TTGTAAAGTA AAAGTAAACA AGGGGGGATA   
  
  
- TTTAAAGACA CTGGTGGAAG TGAAAGAACG GGGTAAGGGG GGGGGGGGGG CGAGAGAGAG AGAGAGAGAG   
  
  
- AGAAGGGAAA TGTTTCCGTG GTAAGTTTAA GTTTGGTGAG AAAAATTTTG GGGTTTTTAA TAAGAGTTTT   
  
  
- ATTATGGAGT ATTATATTTA CCCAAGCATA GTGGTATATA CCCGGAGTAG AGAGTACCCT TGTATGCTTA   
  
  
- AGTTACCGAA CGGGCTATCA ATAGCCCAGT ACGGTAGCCT CGGGGCCGTT GATTAGTTAC CAGTTCGGAG   
  
  
- TCTAAACCGG AGAAACTCGG TAATATCATT GTGGAAAGAG AGACTTGAGG GGGGAGATGG GTGAGACCCT   
  
  
- GGGCCTAGGC TGGGCCCAGA TAGCACAGGC CCAGGACGAG ACTCAGGTTA CCTCACCTAA CCACTGCGCA   
  
  
- CCTGTGTGGT CTAACTTGTA CTAGACGGTG CAGTCGTCTC AGTTGCCGAC AACTGACTAC CACTGTACCC   
  
  
- CCTCCTCCTA AGGCCATATT CCAACCACGT ACGGAACGAC TGCACGCGTC TCCGGCAGGT TACGCCGCTC   
  
  
- GAGGCTGACC GACGAAGCGA TCAACTACTC TACTGGTTAC CCGACAACGG TGCACAATCA TGCCACACAC   
  
  
- CCTAGCCGTT CCATCGGCCG ATAAAACAAC TCCGAAACTC GGCCGCGGAC AAAGTCGGGC CGGGCCTTTG   
  
  
- ACCGGGCTGG ACCGGCCCGA GCCTCAAGCT CCGACTCCAC AACATAGCAG TGAAGATACT CCGAACGGGA   
  
  
- ATAGAGTTTA AACGAGTGAA GTGCCGATTA GTTCGGTAGA ACCTCCGTAA ACTCCCGGTA CTAATACAGG   
  
  
- TGCAGTAGCT GAAGCCGAAG TACTGGCCGA ACGTTACCGG CCGAAACTAA GTTCGGGACC GAAATTCCGG   
  
  
- GCCACCCGGT GGTAGAGAAT CCGAGTGACC CTAACCGGGC AGAAATCCTT CACCCGGGCT TAGCTATGCA   
  
  
- CTTTAGCCCA ACTCCGATCG GGTTAACCGG GCTAGGTACT TGTACTCCAA GCGTAAAGCC CGACATCGTT   
  
  
- GAAGAGCCGA GCTCCTGCAC TTCGGCAGTT ACGAGCCTTA CCTGGGATCT CTTCGGCAGC GATACTTAAG   
  
  
- GCAGTACGTA GAAGTAGCTG AAAATCCTAA TCTGTAGTTG AGTCAAGATT TGACCTAAGC TTTGGATTTG   
  
  
- GGCTTCTAGC ATTGAAATCA TCTTGTTCTC CGCTTGGTAT TGGTTGGACT TAAAGAGGGG GCTAAGTGAC   
  
  
- TTCGTGATAC AATGATGAGT TGATACAAGC TGAGTAATCT CTGAACAGTT CAGGTTGGCG ACTTTCAGGA   
  
  
- TCGGCTCTAC ATATATGTTT TCCTCTATAG GTTGGATCAC ACGATGCTTC CGAGTCGGGC CCAGGTCTCC   
  
  
- GTGCTCGGCG AGCGGTTTAC CGTTCGAGCC TACCCGGTTC GGCCCAAGTT CGGCGATCCG GACTCAGTTT   
  
  
- TACGGGAATC CGTTCGATTG TACAACCATT GTAACAAGAG GAGTGTTCCC ATACTGCACC TCCTCTCTCT   
  
  
- TCCCACAAAC TGTGACCCGA CCGTATTGGC GGGAGAGTAT CACTGAAGCT GAACCGTTCA CCCCGGGTTT   
  
  
- TACCTGTCAG GTTCACACTG AGTACTCTAC AGTAGTAGGA GTCGAAGAAG TAGAAT

+     AT1-motif

| Site Name | Organism | Position | Strand | Matrix score. | sequence | function |
| --- | --- | --- | --- | --- | --- | --- |
| AT1-motif | Solanum tuberosum | 339 | - | 13 | AATTATTTTTTATT | part of a light responsive module |

>HU03G02797.1   
+ +Up\_Stream \_Len000CATTTT CAATTAGAAA TTTCTTCAAT TATATACCAT CTCCAAAAAT AGTCCTCATA   
  
  
+ AGTTGGCAGG TGAATGATCC TAATGGAAAA TTACTTTTAA CTGTTGAATT TGCAATAGGG TACCGAATTT   
  
  
+ GTGAAGTTTA TTGCCGCACG TGCCTAGCTT GTGACTTCTC GATAATCAAA AATTAATTAG AGAGAGACAG   
  
  
+ CTAAAGTTTT GAGAATAATA CTGAAAAATT ACATTAAATT TTATTTATGT TTATTTTCAT ACGCTATGTT   
  
  
+ TAACTGCTAT ACGCTGTGTT TAACTGCATC TCCTTCTATA TTTGATAAAT TGGTAAAAAA AAATAATTCA   
  
  
+ AAATTAAAGA CTAGTAATAT TTTCACTTAT CTGATAGATT TATAAGAAAT ATTTAGATAG CTAAATATAT   
  
  
+ TCTCTCCTGA AAGAGGAGAC TGATAGTTGA ATTTCATAGG ATATAAACAT GTATGTACAT TGAACGAATC   
  
  
+ TTTTTTCTAT GGTGATCGTT ATAAAGTTAT TTATTACTGT AAACATTAAG TGATAATTAG GATATGTTCT   
  
  
+ ATTTCACGTT TTAGATAGAT AGATAGATGT AAGGAGTTGT AAGAAGCTGT AAGAATATGT AAGGAGCTGT   
  
  
+ AAGGAGATGT AAGGAGCTGT AAGAAGATGT AAGAGAAACT GTAAGAAGCT ACAAGGATAG ATGTAACGAG   
  
  
+ TTGTTGAATT GTTGAAGAGT TGTATAGTGT AATTTGTTTT ACGATGAACG TTTTGGATGA AAAATAATAA   
  
  
+ TATTAAAATA ATATGCTTAT TAAAAATAAA TAAATAATTA AGTTAATTAC ACAACAATAA AATATATTAA   
  
  
+ TCCCCATAAA TTTAGTGAAT AAAACTTCGT CCACTTGAGA TGATTTTTTA TATGAACACT AGTTTGATAG   
  
  
+ AATAAACAAA GTATAAAAAA AAATAAAGTT TTTTGACAAA ATTGAATACA ACTCTTCAAA TAAATTCCTA   
  
  
+ TTTTAAGTAA ATAATTTTAA ATATAAATAA ATTTATGGTC ATCTATAATA ATATATATTT TATGTCGAAA   
  
  
+ CTCAAATATA ATTTACATCG TTATATTATA TTTTTATGTT TATGTAAATG TTGTGTTCAT AAGATATTTA   
  
  
+ TAAATTTTGT ATATTGGTAG TATCATTAAA AAAAATATAC ATATTTATGT AACTAAAATA AATGAATGTT   
  
  
+ ATAGAAAATC TATAAGATGT AAGGAGATGT ATGAAGATGT AAGGAGATGT AAGAAGATAT AAGGAGATGT   
  
  
+ AGAAAATGTA AGAAGATGTG AGAAGCTGTA AGGAGATGTA AGTTATTCAC TAAATCCTAA AAAATGCGAA   
  
  
+ ATAGAACAGG GGCTTAATTG AGCACTCAAG ATGCATAATT TGTGACGGAC CAAATGGATA AATATAGACC   
  
  
+ TGGCAAATGG GTTATTCGAT TTAGGTACGA GTATGGTTAA GTTTGGGTTT GGTTATGTTT CAGGATAAGT   
  
  
+ GACATTTCGA CTCAATTTGG TTTTGGTTGT ATGAACTTCG GCTCGAGATT TTTGGTTTGG CCAATACACT   
  
  
+ TATCATGCAA AAATAAGTAA CTTTATAGAA AATTTTGGAT CGGATATGAT GCGGTTCGGA TCAATTCGGA   
  
  
+ TTTTGGTTCA CATTCTGGTA AACCTATTTC GGATGTCAAG TAGGATATGA GTCCAGATCG TTGGATTTCG   
  
  
+ GGTATCAGCT CAATTTTACC AGATCTAGAT AAATCATAAA TGTACGGGTT TATTTTTTCC AGCTTGAATG   
  
  
+ ATTTTCTTTA TTAAAAAAAG ATAGATTTTA GATAAAACAC AACATTTCAT TTTCATTTGT TCCCCCCTAT   
  
  
+ AAATTTCTGT GACCACCTTC ACTTTCTTGC CCCATTCCCC CCCCCCCCCC GCTCTCTCTC TCTCTCTCTC   
  
  
+ TCTTCCCTTT ACAAAGGCAC CATTCAAATT CAAACCACTC TTTTTAAAAC CCCAAAAATT ATTCTCAAAA   
  
  
+ TAATACCTCA TAATATAAAT GGGTTCGTAT CACCATATAT GGGCCTCATC TCTCATGGGA ACATACGAAT   
  
  
+ TCAATGGCTT GCCCGATAGT TATCGGGTCA TGCCATCGGA GCCCCGGCAA CTAATCAATG GTCAAGCCTC   
  
  
+ AGATTTGGCC TCTTTGAGCC ATTATAGTAA CACCTTTCTC TCTGAACTCC CCCCTCTACC CACTCTGGGA   
  
  
+ CCCGGATCCG ACCCGGGTCT ATCGTGTCCG GGTCCTGCTC TGAGTCCAAT GGAGTGGATT GGTGACGCGT   
  
  
+ GGACACACCA GATTGAACAT GATCTGCCAC GTCAGCAGAG TCAACGGCTG TTGACTGATG GTGACATGGG   
  
  
+ GGAGGAGGAT TCCGGTATAA GGTTGGTGCA TGCCTTGCTG ACGTGCGCAG AGGCCGTCCA ATGCGGCGAG   
  
  
+ CTCCGACTGG CTGCTTCGCT AGTTGATGAG ATGACCAATG GGCTGTTGCC ACGTGTTAGT ACGGTGTGTG   
  
  
+ GGATCGGCAA GGTAGCCGGC TATTTTGTTG AGGCTTTGAG CCGGCGCCTG TTTCAGCCCG GCCCGGAAAC   
  
  
+ TGGCCCGACC TGGCCGGGCT CGGAGTTCGA GGCTGAGGTG TTGTATCGTC ACTTCTATGA GGCTTGCCCT   
  
  
+ TATCTCAAAT TTGCTCACTT CACGGCTAAT CAAGCCATCT TGGAGGCATT TGAGGGCCAT GATTATGTCC   
  
  
+ ACGTCATCGA CTTCGGCTTC ATGACCGGCT TGCAATGGCC GGCTTTGATT CAAGCCCTGG CTTTAAGGCC   
  
  
+ CGGTGGGCCA CCATCTCTTA GGCTCACTGG GATTGGCCCG TCTTTAGGAA GTGGGCCCGA ATCGATACGT   
  
  
+ GAAATCGGGT TGAGGCTAGC CCAATTGGCC CGATCCATGA ACATGAGGTT CGCATTTCGG GCTGTAGCAA   
  
  
+ CTTCTCGGCT CGAGGACGTG AAGCCGTCAA TGCTCGGAAT GGACCCTAGA GAAGCCGTCG CTATGAATTC   
  
  
+ CGTCATGCAT CTTCATCGAC TTTTAGGATT AGACATCAAC TCAGTTCTAA ACTGGATTCG AAACCTAAAC   
  
  
+ CCGAAGATCG TAACTTTAGT AGAACAAGAG GCGAACCATA ACCAACCTGA ATTTCTCCCC CGATTCACTG   
  
  
+ AAGCACTATG TTACTACTCA ACTATGTTCG ACTCATTAGA GACTTGTCAA GTCCAACCGC TGAAAGTCCT   
  
  
+ AGCCGAGATG TATATACAAA AGGAGATATC CAACCTAGTG TGCTACGAAG GCTCAGCCCG GGTCCAGAGG   
  
  
+ CACGAGCCGC TCGCCAAATG GCAAGCTCGG ATGGGCCAAG CCGGGTTCAA GCCGCTAGGC CTGAGTCAAA   
  
  
+ ATGCCCTTAG GCAAGCTAAC ATGTTGGTAA CATTGTTCTC CTCACAAGGG TATGACGTGG AGGAGAGAGA   
  
  
+ AGGGTGTTTG ACACTGGGCT GGCATAACCG CCCTCTCATA GTGACTTCGA CTTGGCAAGT GGGGCCCAAA   
  
  
+ ATGGACAGTC CAAGTGTGAC TCATGAGATG TCATCATCCT CAGCTTCTTC ATCTTA  

- +Up\_Stream \_Len000GTAAAA GTTAATCTTT AAAGAAGTTA ATATATGGTA GAGGTTTTTA TCAGGAGTAT   
  
  
- TCAACCGTCC ACTTACTAGG ATTACCTTTT AATGAAAATT GACAACTTAA ACGTTATCCC ATGGCTTAAA   
  
  
- CACTTCAAAT AACGGCGTGC ACGGATCGAA CACTGAAGAG CTATTAGTTT TTAATTAATC TCTCTCTGTC   
  
  
- GATTTCAAAA CTCTTATTAT GACTTTTTAA TGTAATTTAA AATAAATACA AATAAAAGTA TGCGATACAA   
  
  
- ATTGACGATA TGCGACACAA ATTGACGTAG AGGAAGATAT AAACTATTTA ACCATTTTTT TTTATTAAGT   
  
  
- TTTAATTTCT GATCATTATA AAAGTGAATA GACTATCTAA ATATTCTTTA TAAATCTATC GATTTATATA   
  
  
- AGAGAGGACT TTCTCCTCTG ACTATCAACT TAAAGTATCC TATATTTGTA CATACATGTA ACTTGCTTAG   
  
  
- AAAAAAGATA CCACTAGCAA TATTTCAATA AATAATGACA TTTGTAATTC ACTATTAATC CTATACAAGA   
  
  
- TAAAGTGCAA AATCTATCTA TCTATCTACA TTCCTCAACA TTCTTCGACA TTCTTATACA TTCCTCGACA   
  
  
- TTCCTCTACA TTCCTCGACA TTCTTCTACA TTCTCTTTGA CATTCTTCGA TGTTCCTATC TACATTGCTC   
  
  
- AACAACTTAA CAACTTCTCA ACATATCACA TTAAACAAAA TGCTACTTGC AAAACCTACT TTTTATTATT   
  
  
- ATAATTTTAT TATACGAATA ATTTTTATTT ATTTATTAAT TCAATTAATG TGTTGTTATT TTATATAATT   
  
  
- AGGGGTATTT AAATCACTTA TTTTGAAGCA GGTGAACTCT ACTAAAAAAT ATACTTGTGA TCAAACTATC   
  
  
- TTATTTGTTT CATATTTTTT TTTATTTCAA AAAACTGTTT TAACTTATGT TGAGAAGTTT ATTTAAGGAT   
  
  
- AAAATTCATT TATTAAAATT TATATTTATT TAAATACCAG TAGATATTAT TATATATAAA ATACAGCTTT   
  
  
- GAGTTTATAT TAAATGTAGC AATATAATAT AAAAATACAA ATACATTTAC AACACAAGTA TTCTATAAAT   
  
  
- ATTTAAAACA TATAACCATC ATAGTAATTT TTTTTATATG TATAAATACA TTGATTTTAT TTACTTACAA   
  
  
- TATCTTTTAG ATATTCTACA TTCCTCTACA TACTTCTACA TTCCTCTACA TTCTTCTATA TTCCTCTACA   
  
  
- TCTTTTACAT TCTTCTACAC TCTTCGACAT TCCTCTACAT TCAATAAGTG ATTTAGGATT TTTTACGCTT   
  
  
- TATCTTGTCC CCGAATTAAC TCGTGAGTTC TACGTATTAA ACACTGCCTG GTTTACCTAT TTATATCTGG   
  
  
- ACCGTTTACC CAATAAGCTA AATCCATGCT CATACCAATT CAAACCCAAA CCAATACAAA GTCCTATTCA   
  
  
- CTGTAAAGCT GAGTTAAACC AAAACCAACA TACTTGAAGC CGAGCTCTAA AAACCAAACC GGTTATGTGA   
  
  
- ATAGTACGTT TTTATTCATT GAAATATCTT TTAAAACCTA GCCTATACTA CGCCAAGCCT AGTTAAGCCT   
  
  
- AAAACCAAGT GTAAGACCAT TTGGATAAAG CCTACAGTTC ATCCTATACT CAGGTCTAGC AACCTAAAGC   
  
  
- CCATAGTCGA GTTAAAATGG TCTAGATCTA TTTAGTATTT ACATGCCCAA ATAAAAAAGG TCGAACTTAC   
  
  
- TAAAAGAAAT AATTTTTTTC TATCTAAAAT CTATTTTGTG TTGTAAAGTA AAAGTAAACA AGGGGGGATA   
  
  
- TTTAAAGACA CTGGTGGAAG TGAAAGAACG GGGTAAGGGG GGGGGGGGGG CGAGAGAGAG AGAGAGAGAG   
  
  
- AGAAGGGAAA TGTTTCCGTG GTAAGTTTAA GTTTGGTGAG AAAAATTTTG GGGTTTTTAA TAAGAGTTTT   
  
  
- ATTATGGAGT ATTATATTTA CCCAAGCATA GTGGTATATA CCCGGAGTAG AGAGTACCCT TGTATGCTTA   
  
  
- AGTTACCGAA CGGGCTATCA ATAGCCCAGT ACGGTAGCCT CGGGGCCGTT GATTAGTTAC CAGTTCGGAG   
  
  
- TCTAAACCGG AGAAACTCGG TAATATCATT GTGGAAAGAG AGACTTGAGG GGGGAGATGG GTGAGACCCT   
  
  
- GGGCCTAGGC TGGGCCCAGA TAGCACAGGC CCAGGACGAG ACTCAGGTTA CCTCACCTAA CCACTGCGCA   
  
  
- CCTGTGTGGT CTAACTTGTA CTAGACGGTG CAGTCGTCTC AGTTGCCGAC AACTGACTAC CACTGTACCC   
  
  
- CCTCCTCCTA AGGCCATATT CCAACCACGT ACGGAACGAC TGCACGCGTC TCCGGCAGGT TACGCCGCTC   
  
  
- GAGGCTGACC GACGAAGCGA TCAACTACTC TACTGGTTAC CCGACAACGG TGCACAATCA TGCCACACAC   
  
  
- CCTAGCCGTT CCATCGGCCG ATAAAACAAC TCCGAAACTC GGCCGCGGAC AAAGTCGGGC CGGGCCTTTG   
  
  
- ACCGGGCTGG ACCGGCCCGA GCCTCAAGCT CCGACTCCAC AACATAGCAG TGAAGATACT CCGAACGGGA   
  
  
- ATAGAGTTTA AACGAGTGAA GTGCCGATTA GTTCGGTAGA ACCTCCGTAA ACTCCCGGTA CTAATACAGG   
  
  
- TGCAGTAGCT GAAGCCGAAG TACTGGCCGA ACGTTACCGG CCGAAACTAA GTTCGGGACC GAAATTCCGG   
  
  
- GCCACCCGGT GGTAGAGAAT CCGAGTGACC CTAACCGGGC AGAAATCCTT CACCCGGGCT TAGCTATGCA   
  
  
- CTTTAGCCCA ACTCCGATCG GGTTAACCGG GCTAGGTACT TGTACTCCAA GCGTAAAGCC CGACATCGTT   
  
  
- GAAGAGCCGA GCTCCTGCAC TTCGGCAGTT ACGAGCCTTA CCTGGGATCT CTTCGGCAGC GATACTTAAG   
  
  
- GCAGTACGTA GAAGTAGCTG AAAATCCTAA TCTGTAGTTG AGTCAAGATT TGACCTAAGC TTTGGATTTG   
  
  
- GGCTTCTAGC ATTGAAATCA TCTTGTTCTC CGCTTGGTAT TGGTTGGACT TAAAGAGGGG GCTAAGTGAC   
  
  
- TTCGTGATAC AATGATGAGT TGATACAAGC TGAGTAATCT CTGAACAGTT CAGGTTGGCG ACTTTCAGGA   
  
  
- TCGGCTCTAC ATATATGTTT TCCTCTATAG GTTGGATCAC ACGATGCTTC CGAGTCGGGC CCAGGTCTCC   
  
  
- GTGCTCGGCG AGCGGTTTAC CGTTCGAGCC TACCCGGTTC GGCCCAAGTT CGGCGATCCG GACTCAGTTT   
  
  
- TACGGGAATC CGTTCGATTG TACAACCATT GTAACAAGAG GAGTGTTCCC ATACTGCACC TCCTCTCTCT   
  
  
- TCCCACAAAC TGTGACCCGA CCGTATTGGC GGGAGAGTAT CACTGAAGCT GAACCGTTCA CCCCGGGTTT   
  
  
- TACCTGTCAG GTTCACACTG AGTACTCTAC AGTAGTAGGA GTCGAAGAAG TAGAAT

+     AT~TATA-box

| Site Name | Organism | Position | Strand | Matrix score. | sequence | function |
| --- | --- | --- | --- | --- | --- | --- |
| AT~TATA-box | Arabidopsis thaliana | 3165 | - | 6 | TATATA |  |
| AT~TATA-box | Arabidopsis thaliana | 45 | + | 6 | TATATA |  |
| AT~TATA-box | Arabidopsis thaliana | 1036 | + | 6 | TATATA |  |

>HU03G02797.1   
+ +Up\_Stream \_Len000CATTTT CAATTAGAAA TTTCTTCAAT TATATACCAT CTCCAAAAAT AGTCCTCATA   
  
  
+ AGTTGGCAGG TGAATGATCC TAATGGAAAA TTACTTTTAA CTGTTGAATT TGCAATAGGG TACCGAATTT   
  
  
+ GTGAAGTTTA TTGCCGCACG TGCCTAGCTT GTGACTTCTC GATAATCAAA AATTAATTAG AGAGAGACAG   
  
  
+ CTAAAGTTTT GAGAATAATA CTGAAAAATT ACATTAAATT TTATTTATGT TTATTTTCAT ACGCTATGTT   
  
  
+ TAACTGCTAT ACGCTGTGTT TAACTGCATC TCCTTCTATA TTTGATAAAT TGGTAAAAAA AAATAATTCA   
  
  
+ AAATTAAAGA CTAGTAATAT TTTCACTTAT CTGATAGATT TATAAGAAAT ATTTAGATAG CTAAATATAT   
  
  
+ TCTCTCCTGA AAGAGGAGAC TGATAGTTGA ATTTCATAGG ATATAAACAT GTATGTACAT TGAACGAATC   
  
  
+ TTTTTTCTAT GGTGATCGTT ATAAAGTTAT TTATTACTGT AAACATTAAG TGATAATTAG GATATGTTCT   
  
  
+ ATTTCACGTT TTAGATAGAT AGATAGATGT AAGGAGTTGT AAGAAGCTGT AAGAATATGT AAGGAGCTGT   
  
  
+ AAGGAGATGT AAGGAGCTGT AAGAAGATGT AAGAGAAACT GTAAGAAGCT ACAAGGATAG ATGTAACGAG   
  
  
+ TTGTTGAATT GTTGAAGAGT TGTATAGTGT AATTTGTTTT ACGATGAACG TTTTGGATGA AAAATAATAA   
  
  
+ TATTAAAATA ATATGCTTAT TAAAAATAAA TAAATAATTA AGTTAATTAC ACAACAATAA AATATATTAA   
  
  
+ TCCCCATAAA TTTAGTGAAT AAAACTTCGT CCACTTGAGA TGATTTTTTA TATGAACACT AGTTTGATAG   
  
  
+ AATAAACAAA GTATAAAAAA AAATAAAGTT TTTTGACAAA ATTGAATACA ACTCTTCAAA TAAATTCCTA   
  
  
+ TTTTAAGTAA ATAATTTTAA ATATAAATAA ATTTATGGTC ATCTATAATA ATATATATTT TATGTCGAAA   
  
  
+ CTCAAATATA ATTTACATCG TTATATTATA TTTTTATGTT TATGTAAATG TTGTGTTCAT AAGATATTTA   
  
  
+ TAAATTTTGT ATATTGGTAG TATCATTAAA AAAAATATAC ATATTTATGT AACTAAAATA AATGAATGTT   
  
  
+ ATAGAAAATC TATAAGATGT AAGGAGATGT ATGAAGATGT AAGGAGATGT AAGAAGATAT AAGGAGATGT   
  
  
+ AGAAAATGTA AGAAGATGTG AGAAGCTGTA AGGAGATGTA AGTTATTCAC TAAATCCTAA AAAATGCGAA   
  
  
+ ATAGAACAGG GGCTTAATTG AGCACTCAAG ATGCATAATT TGTGACGGAC CAAATGGATA AATATAGACC   
  
  
+ TGGCAAATGG GTTATTCGAT TTAGGTACGA GTATGGTTAA GTTTGGGTTT GGTTATGTTT CAGGATAAGT   
  
  
+ GACATTTCGA CTCAATTTGG TTTTGGTTGT ATGAACTTCG GCTCGAGATT TTTGGTTTGG CCAATACACT   
  
  
+ TATCATGCAA AAATAAGTAA CTTTATAGAA AATTTTGGAT CGGATATGAT GCGGTTCGGA TCAATTCGGA   
  
  
+ TTTTGGTTCA CATTCTGGTA AACCTATTTC GGATGTCAAG TAGGATATGA GTCCAGATCG TTGGATTTCG   
  
  
+ GGTATCAGCT CAATTTTACC AGATCTAGAT AAATCATAAA TGTACGGGTT TATTTTTTCC AGCTTGAATG   
  
  
+ ATTTTCTTTA TTAAAAAAAG ATAGATTTTA GATAAAACAC AACATTTCAT TTTCATTTGT TCCCCCCTAT   
  
  
+ AAATTTCTGT GACCACCTTC ACTTTCTTGC CCCATTCCCC CCCCCCCCCC GCTCTCTCTC TCTCTCTCTC   
  
  
+ TCTTCCCTTT ACAAAGGCAC CATTCAAATT CAAACCACTC TTTTTAAAAC CCCAAAAATT ATTCTCAAAA   
  
  
+ TAATACCTCA TAATATAAAT GGGTTCGTAT CACCATATAT GGGCCTCATC TCTCATGGGA ACATACGAAT   
  
  
+ TCAATGGCTT GCCCGATAGT TATCGGGTCA TGCCATCGGA GCCCCGGCAA CTAATCAATG GTCAAGCCTC   
  
  
+ AGATTTGGCC TCTTTGAGCC ATTATAGTAA CACCTTTCTC TCTGAACTCC CCCCTCTACC CACTCTGGGA   
  
  
+ CCCGGATCCG ACCCGGGTCT ATCGTGTCCG GGTCCTGCTC TGAGTCCAAT GGAGTGGATT GGTGACGCGT   
  
  
+ GGACACACCA GATTGAACAT GATCTGCCAC GTCAGCAGAG TCAACGGCTG TTGACTGATG GTGACATGGG   
  
  
+ GGAGGAGGAT TCCGGTATAA GGTTGGTGCA TGCCTTGCTG ACGTGCGCAG AGGCCGTCCA ATGCGGCGAG   
  
  
+ CTCCGACTGG CTGCTTCGCT AGTTGATGAG ATGACCAATG GGCTGTTGCC ACGTGTTAGT ACGGTGTGTG   
  
  
+ GGATCGGCAA GGTAGCCGGC TATTTTGTTG AGGCTTTGAG CCGGCGCCTG TTTCAGCCCG GCCCGGAAAC   
  
  
+ TGGCCCGACC TGGCCGGGCT CGGAGTTCGA GGCTGAGGTG TTGTATCGTC ACTTCTATGA GGCTTGCCCT   
  
  
+ TATCTCAAAT TTGCTCACTT CACGGCTAAT CAAGCCATCT TGGAGGCATT TGAGGGCCAT GATTATGTCC   
  
  
+ ACGTCATCGA CTTCGGCTTC ATGACCGGCT TGCAATGGCC GGCTTTGATT CAAGCCCTGG CTTTAAGGCC   
  
  
+ CGGTGGGCCA CCATCTCTTA GGCTCACTGG GATTGGCCCG TCTTTAGGAA GTGGGCCCGA ATCGATACGT   
  
  
+ GAAATCGGGT TGAGGCTAGC CCAATTGGCC CGATCCATGA ACATGAGGTT CGCATTTCGG GCTGTAGCAA   
  
  
+ CTTCTCGGCT CGAGGACGTG AAGCCGTCAA TGCTCGGAAT GGACCCTAGA GAAGCCGTCG CTATGAATTC   
  
  
+ CGTCATGCAT CTTCATCGAC TTTTAGGATT AGACATCAAC TCAGTTCTAA ACTGGATTCG AAACCTAAAC   
  
  
+ CCGAAGATCG TAACTTTAGT AGAACAAGAG GCGAACCATA ACCAACCTGA ATTTCTCCCC CGATTCACTG   
  
  
+ AAGCACTATG TTACTACTCA ACTATGTTCG ACTCATTAGA GACTTGTCAA GTCCAACCGC TGAAAGTCCT   
  
  
+ AGCCGAGATG TATATACAAA AGGAGATATC CAACCTAGTG TGCTACGAAG GCTCAGCCCG GGTCCAGAGG   
  
  
+ CACGAGCCGC TCGCCAAATG GCAAGCTCGG ATGGGCCAAG CCGGGTTCAA GCCGCTAGGC CTGAGTCAAA   
  
  
+ ATGCCCTTAG GCAAGCTAAC ATGTTGGTAA CATTGTTCTC CTCACAAGGG TATGACGTGG AGGAGAGAGA   
  
  
+ AGGGTGTTTG ACACTGGGCT GGCATAACCG CCCTCTCATA GTGACTTCGA CTTGGCAAGT GGGGCCCAAA   
  
  
+ ATGGACAGTC CAAGTGTGAC TCATGAGATG TCATCATCCT CAGCTTCTTC ATCTTA  

- +Up\_Stream \_Len000GTAAAA GTTAATCTTT AAAGAAGTTA ATATATGGTA GAGGTTTTTA TCAGGAGTAT   
  
  
- TCAACCGTCC ACTTACTAGG ATTACCTTTT AATGAAAATT GACAACTTAA ACGTTATCCC ATGGCTTAAA   
  
  
- CACTTCAAAT AACGGCGTGC ACGGATCGAA CACTGAAGAG CTATTAGTTT TTAATTAATC TCTCTCTGTC   
  
  
- GATTTCAAAA CTCTTATTAT GACTTTTTAA TGTAATTTAA AATAAATACA AATAAAAGTA TGCGATACAA   
  
  
- ATTGACGATA TGCGACACAA ATTGACGTAG AGGAAGATAT AAACTATTTA ACCATTTTTT TTTATTAAGT   
  
  
- TTTAATTTCT GATCATTATA AAAGTGAATA GACTATCTAA ATATTCTTTA TAAATCTATC GATTTATATA   
  
  
- AGAGAGGACT TTCTCCTCTG ACTATCAACT TAAAGTATCC TATATTTGTA CATACATGTA ACTTGCTTAG   
  
  
- AAAAAAGATA CCACTAGCAA TATTTCAATA AATAATGACA TTTGTAATTC ACTATTAATC CTATACAAGA   
  
  
- TAAAGTGCAA AATCTATCTA TCTATCTACA TTCCTCAACA TTCTTCGACA TTCTTATACA TTCCTCGACA   
  
  
- TTCCTCTACA TTCCTCGACA TTCTTCTACA TTCTCTTTGA CATTCTTCGA TGTTCCTATC TACATTGCTC   
  
  
- AACAACTTAA CAACTTCTCA ACATATCACA TTAAACAAAA TGCTACTTGC AAAACCTACT TTTTATTATT   
  
  
- ATAATTTTAT TATACGAATA ATTTTTATTT ATTTATTAAT TCAATTAATG TGTTGTTATT TTATATAATT   
  
  
- AGGGGTATTT AAATCACTTA TTTTGAAGCA GGTGAACTCT ACTAAAAAAT ATACTTGTGA TCAAACTATC   
  
  
- TTATTTGTTT CATATTTTTT TTTATTTCAA AAAACTGTTT TAACTTATGT TGAGAAGTTT ATTTAAGGAT   
  
  
- AAAATTCATT TATTAAAATT TATATTTATT TAAATACCAG TAGATATTAT TATATATAAA ATACAGCTTT   
  
  
- GAGTTTATAT TAAATGTAGC AATATAATAT AAAAATACAA ATACATTTAC AACACAAGTA TTCTATAAAT   
  
  
- ATTTAAAACA TATAACCATC ATAGTAATTT TTTTTATATG TATAAATACA TTGATTTTAT TTACTTACAA   
  
  
- TATCTTTTAG ATATTCTACA TTCCTCTACA TACTTCTACA TTCCTCTACA TTCTTCTATA TTCCTCTACA   
  
  
- TCTTTTACAT TCTTCTACAC TCTTCGACAT TCCTCTACAT TCAATAAGTG ATTTAGGATT TTTTACGCTT   
  
  
- TATCTTGTCC CCGAATTAAC TCGTGAGTTC TACGTATTAA ACACTGCCTG GTTTACCTAT TTATATCTGG   
  
  
- ACCGTTTACC CAATAAGCTA AATCCATGCT CATACCAATT CAAACCCAAA CCAATACAAA GTCCTATTCA   
  
  
- CTGTAAAGCT GAGTTAAACC AAAACCAACA TACTTGAAGC CGAGCTCTAA AAACCAAACC GGTTATGTGA   
  
  
- ATAGTACGTT TTTATTCATT GAAATATCTT TTAAAACCTA GCCTATACTA CGCCAAGCCT AGTTAAGCCT   
  
  
- AAAACCAAGT GTAAGACCAT TTGGATAAAG CCTACAGTTC ATCCTATACT CAGGTCTAGC AACCTAAAGC   
  
  
- CCATAGTCGA GTTAAAATGG TCTAGATCTA TTTAGTATTT ACATGCCCAA ATAAAAAAGG TCGAACTTAC   
  
  
- TAAAAGAAAT AATTTTTTTC TATCTAAAAT CTATTTTGTG TTGTAAAGTA AAAGTAAACA AGGGGGGATA   
  
  
- TTTAAAGACA CTGGTGGAAG TGAAAGAACG GGGTAAGGGG GGGGGGGGGG CGAGAGAGAG AGAGAGAGAG   
  
  
- AGAAGGGAAA TGTTTCCGTG GTAAGTTTAA GTTTGGTGAG AAAAATTTTG GGGTTTTTAA TAAGAGTTTT   
  
  
- ATTATGGAGT ATTATATTTA CCCAAGCATA GTGGTATATA CCCGGAGTAG AGAGTACCCT TGTATGCTTA   
  
  
- AGTTACCGAA CGGGCTATCA ATAGCCCAGT ACGGTAGCCT CGGGGCCGTT GATTAGTTAC CAGTTCGGAG   
  
  
- TCTAAACCGG AGAAACTCGG TAATATCATT GTGGAAAGAG AGACTTGAGG GGGGAGATGG GTGAGACCCT   
  
  
- GGGCCTAGGC TGGGCCCAGA TAGCACAGGC CCAGGACGAG ACTCAGGTTA CCTCACCTAA CCACTGCGCA   
  
  
- CCTGTGTGGT CTAACTTGTA CTAGACGGTG CAGTCGTCTC AGTTGCCGAC AACTGACTAC CACTGTACCC   
  
  
- CCTCCTCCTA AGGCCATATT CCAACCACGT ACGGAACGAC TGCACGCGTC TCCGGCAGGT TACGCCGCTC   
  
  
- GAGGCTGACC GACGAAGCGA TCAACTACTC TACTGGTTAC CCGACAACGG TGCACAATCA TGCCACACAC   
  
  
- CCTAGCCGTT CCATCGGCCG ATAAAACAAC TCCGAAACTC GGCCGCGGAC AAAGTCGGGC CGGGCCTTTG   
  
  
- ACCGGGCTGG ACCGGCCCGA GCCTCAAGCT CCGACTCCAC AACATAGCAG TGAAGATACT CCGAACGGGA   
  
  
- ATAGAGTTTA AACGAGTGAA GTGCCGATTA GTTCGGTAGA ACCTCCGTAA ACTCCCGGTA CTAATACAGG   
  
  
- TGCAGTAGCT GAAGCCGAAG TACTGGCCGA ACGTTACCGG CCGAAACTAA GTTCGGGACC GAAATTCCGG   
  
  
- GCCACCCGGT GGTAGAGAAT CCGAGTGACC CTAACCGGGC AGAAATCCTT CACCCGGGCT TAGCTATGCA   
  
  
- CTTTAGCCCA ACTCCGATCG GGTTAACCGG GCTAGGTACT TGTACTCCAA GCGTAAAGCC CGACATCGTT   
  
  
- GAAGAGCCGA GCTCCTGCAC TTCGGCAGTT ACGAGCCTTA CCTGGGATCT CTTCGGCAGC GATACTTAAG   
  
  
- GCAGTACGTA GAAGTAGCTG AAAATCCTAA TCTGTAGTTG AGTCAAGATT TGACCTAAGC TTTGGATTTG   
  
  
- GGCTTCTAGC ATTGAAATCA TCTTGTTCTC CGCTTGGTAT TGGTTGGACT TAAAGAGGGG GCTAAGTGAC   
  
  
- TTCGTGATAC AATGATGAGT TGATACAAGC TGAGTAATCT CTGAACAGTT CAGGTTGGCG ACTTTCAGGA   
  
  
- TCGGCTCTAC ATATATGTTT TCCTCTATAG GTTGGATCAC ACGATGCTTC CGAGTCGGGC CCAGGTCTCC   
  
  
- GTGCTCGGCG AGCGGTTTAC CGTTCGAGCC TACCCGGTTC GGCCCAAGTT CGGCGATCCG GACTCAGTTT   
  
  
- TACGGGAATC CGTTCGATTG TACAACCATT GTAACAAGAG GAGTGTTCCC ATACTGCACC TCCTCTCTCT   
  
  
- TCCCACAAAC TGTGACCCGA CCGTATTGGC GGGAGAGTAT CACTGAAGCT GAACCGTTCA CCCCGGGTTT   
  
  
- TACCTGTCAG GTTCACACTG AGTACTCTAC AGTAGTAGGA GTCGAAGAAG TAGAAT

+     AuxRR-core

| Site Name | Organism | Position | Strand | Matrix score. | sequence | function |
| --- | --- | --- | --- | --- | --- | --- |
| AuxRR-core | Nicotiana tabacum | 2913 | - | 7 | GGTCCAT | cis-acting regulatory element involved in auxin responsiveness |

>HU03G02797.1   
+ +Up\_Stream \_Len000CATTTT CAATTAGAAA TTTCTTCAAT TATATACCAT CTCCAAAAAT AGTCCTCATA   
  
  
+ AGTTGGCAGG TGAATGATCC TAATGGAAAA TTACTTTTAA CTGTTGAATT TGCAATAGGG TACCGAATTT   
  
  
+ GTGAAGTTTA TTGCCGCACG TGCCTAGCTT GTGACTTCTC GATAATCAAA AATTAATTAG AGAGAGACAG   
  
  
+ CTAAAGTTTT GAGAATAATA CTGAAAAATT ACATTAAATT TTATTTATGT TTATTTTCAT ACGCTATGTT   
  
  
+ TAACTGCTAT ACGCTGTGTT TAACTGCATC TCCTTCTATA TTTGATAAAT TGGTAAAAAA AAATAATTCA   
  
  
+ AAATTAAAGA CTAGTAATAT TTTCACTTAT CTGATAGATT TATAAGAAAT ATTTAGATAG CTAAATATAT   
  
  
+ TCTCTCCTGA AAGAGGAGAC TGATAGTTGA ATTTCATAGG ATATAAACAT GTATGTACAT TGAACGAATC   
  
  
+ TTTTTTCTAT GGTGATCGTT ATAAAGTTAT TTATTACTGT AAACATTAAG TGATAATTAG GATATGTTCT   
  
  
+ ATTTCACGTT TTAGATAGAT AGATAGATGT AAGGAGTTGT AAGAAGCTGT AAGAATATGT AAGGAGCTGT   
  
  
+ AAGGAGATGT AAGGAGCTGT AAGAAGATGT AAGAGAAACT GTAAGAAGCT ACAAGGATAG ATGTAACGAG   
  
  
+ TTGTTGAATT GTTGAAGAGT TGTATAGTGT AATTTGTTTT ACGATGAACG TTTTGGATGA AAAATAATAA   
  
  
+ TATTAAAATA ATATGCTTAT TAAAAATAAA TAAATAATTA AGTTAATTAC ACAACAATAA AATATATTAA   
  
  
+ TCCCCATAAA TTTAGTGAAT AAAACTTCGT CCACTTGAGA TGATTTTTTA TATGAACACT AGTTTGATAG   
  
  
+ AATAAACAAA GTATAAAAAA AAATAAAGTT TTTTGACAAA ATTGAATACA ACTCTTCAAA TAAATTCCTA   
  
  
+ TTTTAAGTAA ATAATTTTAA ATATAAATAA ATTTATGGTC ATCTATAATA ATATATATTT TATGTCGAAA   
  
  
+ CTCAAATATA ATTTACATCG TTATATTATA TTTTTATGTT TATGTAAATG TTGTGTTCAT AAGATATTTA   
  
  
+ TAAATTTTGT ATATTGGTAG TATCATTAAA AAAAATATAC ATATTTATGT AACTAAAATA AATGAATGTT   
  
  
+ ATAGAAAATC TATAAGATGT AAGGAGATGT ATGAAGATGT AAGGAGATGT AAGAAGATAT AAGGAGATGT   
  
  
+ AGAAAATGTA AGAAGATGTG AGAAGCTGTA AGGAGATGTA AGTTATTCAC TAAATCCTAA AAAATGCGAA   
  
  
+ ATAGAACAGG GGCTTAATTG AGCACTCAAG ATGCATAATT TGTGACGGAC CAAATGGATA AATATAGACC   
  
  
+ TGGCAAATGG GTTATTCGAT TTAGGTACGA GTATGGTTAA GTTTGGGTTT GGTTATGTTT CAGGATAAGT   
  
  
+ GACATTTCGA CTCAATTTGG TTTTGGTTGT ATGAACTTCG GCTCGAGATT TTTGGTTTGG CCAATACACT   
  
  
+ TATCATGCAA AAATAAGTAA CTTTATAGAA AATTTTGGAT CGGATATGAT GCGGTTCGGA TCAATTCGGA   
  
  
+ TTTTGGTTCA CATTCTGGTA AACCTATTTC GGATGTCAAG TAGGATATGA GTCCAGATCG TTGGATTTCG   
  
  
+ GGTATCAGCT CAATTTTACC AGATCTAGAT AAATCATAAA TGTACGGGTT TATTTTTTCC AGCTTGAATG   
  
  
+ ATTTTCTTTA TTAAAAAAAG ATAGATTTTA GATAAAACAC AACATTTCAT TTTCATTTGT TCCCCCCTAT   
  
  
+ AAATTTCTGT GACCACCTTC ACTTTCTTGC CCCATTCCCC CCCCCCCCCC GCTCTCTCTC TCTCTCTCTC   
  
  
+ TCTTCCCTTT ACAAAGGCAC CATTCAAATT CAAACCACTC TTTTTAAAAC CCCAAAAATT ATTCTCAAAA   
  
  
+ TAATACCTCA TAATATAAAT GGGTTCGTAT CACCATATAT GGGCCTCATC TCTCATGGGA ACATACGAAT   
  
  
+ TCAATGGCTT GCCCGATAGT TATCGGGTCA TGCCATCGGA GCCCCGGCAA CTAATCAATG GTCAAGCCTC   
  
  
+ AGATTTGGCC TCTTTGAGCC ATTATAGTAA CACCTTTCTC TCTGAACTCC CCCCTCTACC CACTCTGGGA   
  
  
+ CCCGGATCCG ACCCGGGTCT ATCGTGTCCG GGTCCTGCTC TGAGTCCAAT GGAGTGGATT GGTGACGCGT   
  
  
+ GGACACACCA GATTGAACAT GATCTGCCAC GTCAGCAGAG TCAACGGCTG TTGACTGATG GTGACATGGG   
  
  
+ GGAGGAGGAT TCCGGTATAA GGTTGGTGCA TGCCTTGCTG ACGTGCGCAG AGGCCGTCCA ATGCGGCGAG   
  
  
+ CTCCGACTGG CTGCTTCGCT AGTTGATGAG ATGACCAATG GGCTGTTGCC ACGTGTTAGT ACGGTGTGTG   
  
  
+ GGATCGGCAA GGTAGCCGGC TATTTTGTTG AGGCTTTGAG CCGGCGCCTG TTTCAGCCCG GCCCGGAAAC   
  
  
+ TGGCCCGACC TGGCCGGGCT CGGAGTTCGA GGCTGAGGTG TTGTATCGTC ACTTCTATGA GGCTTGCCCT   
  
  
+ TATCTCAAAT TTGCTCACTT CACGGCTAAT CAAGCCATCT TGGAGGCATT TGAGGGCCAT GATTATGTCC   
  
  
+ ACGTCATCGA CTTCGGCTTC ATGACCGGCT TGCAATGGCC GGCTTTGATT CAAGCCCTGG CTTTAAGGCC   
  
  
+ CGGTGGGCCA CCATCTCTTA GGCTCACTGG GATTGGCCCG TCTTTAGGAA GTGGGCCCGA ATCGATACGT   
  
  
+ GAAATCGGGT TGAGGCTAGC CCAATTGGCC CGATCCATGA ACATGAGGTT CGCATTTCGG GCTGTAGCAA   
  
  
+ CTTCTCGGCT CGAGGACGTG AAGCCGTCAA TGCTCGGAAT GGACCCTAGA GAAGCCGTCG CTATGAATTC   
  
  
+ CGTCATGCAT CTTCATCGAC TTTTAGGATT AGACATCAAC TCAGTTCTAA ACTGGATTCG AAACCTAAAC   
  
  
+ CCGAAGATCG TAACTTTAGT AGAACAAGAG GCGAACCATA ACCAACCTGA ATTTCTCCCC CGATTCACTG   
  
  
+ AAGCACTATG TTACTACTCA ACTATGTTCG ACTCATTAGA GACTTGTCAA GTCCAACCGC TGAAAGTCCT   
  
  
+ AGCCGAGATG TATATACAAA AGGAGATATC CAACCTAGTG TGCTACGAAG GCTCAGCCCG GGTCCAGAGG   
  
  
+ CACGAGCCGC TCGCCAAATG GCAAGCTCGG ATGGGCCAAG CCGGGTTCAA GCCGCTAGGC CTGAGTCAAA   
  
  
+ ATGCCCTTAG GCAAGCTAAC ATGTTGGTAA CATTGTTCTC CTCACAAGGG TATGACGTGG AGGAGAGAGA   
  
  
+ AGGGTGTTTG ACACTGGGCT GGCATAACCG CCCTCTCATA GTGACTTCGA CTTGGCAAGT GGGGCCCAAA   
  
  
+ ATGGACAGTC CAAGTGTGAC TCATGAGATG TCATCATCCT CAGCTTCTTC ATCTTA  

- +Up\_Stream \_Len000GTAAAA GTTAATCTTT AAAGAAGTTA ATATATGGTA GAGGTTTTTA TCAGGAGTAT   
  
  
- TCAACCGTCC ACTTACTAGG ATTACCTTTT AATGAAAATT GACAACTTAA ACGTTATCCC ATGGCTTAAA   
  
  
- CACTTCAAAT AACGGCGTGC ACGGATCGAA CACTGAAGAG CTATTAGTTT TTAATTAATC TCTCTCTGTC   
  
  
- GATTTCAAAA CTCTTATTAT GACTTTTTAA TGTAATTTAA AATAAATACA AATAAAAGTA TGCGATACAA   
  
  
- ATTGACGATA TGCGACACAA ATTGACGTAG AGGAAGATAT AAACTATTTA ACCATTTTTT TTTATTAAGT   
  
  
- TTTAATTTCT GATCATTATA AAAGTGAATA GACTATCTAA ATATTCTTTA TAAATCTATC GATTTATATA   
  
  
- AGAGAGGACT TTCTCCTCTG ACTATCAACT TAAAGTATCC TATATTTGTA CATACATGTA ACTTGCTTAG   
  
  
- AAAAAAGATA CCACTAGCAA TATTTCAATA AATAATGACA TTTGTAATTC ACTATTAATC CTATACAAGA   
  
  
- TAAAGTGCAA AATCTATCTA TCTATCTACA TTCCTCAACA TTCTTCGACA TTCTTATACA TTCCTCGACA   
  
  
- TTCCTCTACA TTCCTCGACA TTCTTCTACA TTCTCTTTGA CATTCTTCGA TGTTCCTATC TACATTGCTC   
  
  
- AACAACTTAA CAACTTCTCA ACATATCACA TTAAACAAAA TGCTACTTGC AAAACCTACT TTTTATTATT   
  
  
- ATAATTTTAT TATACGAATA ATTTTTATTT ATTTATTAAT TCAATTAATG TGTTGTTATT TTATATAATT   
  
  
- AGGGGTATTT AAATCACTTA TTTTGAAGCA GGTGAACTCT ACTAAAAAAT ATACTTGTGA TCAAACTATC   
  
  
- TTATTTGTTT CATATTTTTT TTTATTTCAA AAAACTGTTT TAACTTATGT TGAGAAGTTT ATTTAAGGAT   
  
  
- AAAATTCATT TATTAAAATT TATATTTATT TAAATACCAG TAGATATTAT TATATATAAA ATACAGCTTT   
  
  
- GAGTTTATAT TAAATGTAGC AATATAATAT AAAAATACAA ATACATTTAC AACACAAGTA TTCTATAAAT   
  
  
- ATTTAAAACA TATAACCATC ATAGTAATTT TTTTTATATG TATAAATACA TTGATTTTAT TTACTTACAA   
  
  
- TATCTTTTAG ATATTCTACA TTCCTCTACA TACTTCTACA TTCCTCTACA TTCTTCTATA TTCCTCTACA   
  
  
- TCTTTTACAT TCTTCTACAC TCTTCGACAT TCCTCTACAT TCAATAAGTG ATTTAGGATT TTTTACGCTT   
  
  
- TATCTTGTCC CCGAATTAAC TCGTGAGTTC TACGTATTAA ACACTGCCTG GTTTACCTAT TTATATCTGG   
  
  
- ACCGTTTACC CAATAAGCTA AATCCATGCT CATACCAATT CAAACCCAAA CCAATACAAA GTCCTATTCA   
  
  
- CTGTAAAGCT GAGTTAAACC AAAACCAACA TACTTGAAGC CGAGCTCTAA AAACCAAACC GGTTATGTGA   
  
  
- ATAGTACGTT TTTATTCATT GAAATATCTT TTAAAACCTA GCCTATACTA CGCCAAGCCT AGTTAAGCCT   
  
  
- AAAACCAAGT GTAAGACCAT TTGGATAAAG CCTACAGTTC ATCCTATACT CAGGTCTAGC AACCTAAAGC   
  
  
- CCATAGTCGA GTTAAAATGG TCTAGATCTA TTTAGTATTT ACATGCCCAA ATAAAAAAGG TCGAACTTAC   
  
  
- TAAAAGAAAT AATTTTTTTC TATCTAAAAT CTATTTTGTG TTGTAAAGTA AAAGTAAACA AGGGGGGATA   
  
  
- TTTAAAGACA CTGGTGGAAG TGAAAGAACG GGGTAAGGGG GGGGGGGGGG CGAGAGAGAG AGAGAGAGAG   
  
  
- AGAAGGGAAA TGTTTCCGTG GTAAGTTTAA GTTTGGTGAG AAAAATTTTG GGGTTTTTAA TAAGAGTTTT   
  
  
- ATTATGGAGT ATTATATTTA CCCAAGCATA GTGGTATATA CCCGGAGTAG AGAGTACCCT TGTATGCTTA   
  
  
- AGTTACCGAA CGGGCTATCA ATAGCCCAGT ACGGTAGCCT CGGGGCCGTT GATTAGTTAC CAGTTCGGAG   
  
  
- TCTAAACCGG AGAAACTCGG TAATATCATT GTGGAAAGAG AGACTTGAGG GGGGAGATGG GTGAGACCCT   
  
  
- GGGCCTAGGC TGGGCCCAGA TAGCACAGGC CCAGGACGAG ACTCAGGTTA CCTCACCTAA CCACTGCGCA   
  
  
- CCTGTGTGGT CTAACTTGTA CTAGACGGTG CAGTCGTCTC AGTTGCCGAC AACTGACTAC CACTGTACCC   
  
  
- CCTCCTCCTA AGGCCATATT CCAACCACGT ACGGAACGAC TGCACGCGTC TCCGGCAGGT TACGCCGCTC   
  
  
- GAGGCTGACC GACGAAGCGA TCAACTACTC TACTGGTTAC CCGACAACGG TGCACAATCA TGCCACACAC   
  
  
- CCTAGCCGTT CCATCGGCCG ATAAAACAAC TCCGAAACTC GGCCGCGGAC AAAGTCGGGC CGGGCCTTTG   
  
  
- ACCGGGCTGG ACCGGCCCGA GCCTCAAGCT CCGACTCCAC AACATAGCAG TGAAGATACT CCGAACGGGA   
  
  
- ATAGAGTTTA AACGAGTGAA GTGCCGATTA GTTCGGTAGA ACCTCCGTAA ACTCCCGGTA CTAATACAGG   
  
  
- TGCAGTAGCT GAAGCCGAAG TACTGGCCGA ACGTTACCGG CCGAAACTAA GTTCGGGACC GAAATTCCGG   
  
  
- GCCACCCGGT GGTAGAGAAT CCGAGTGACC CTAACCGGGC AGAAATCCTT CACCCGGGCT TAGCTATGCA   
  
  
- CTTTAGCCCA ACTCCGATCG GGTTAACCGG GCTAGGTACT TGTACTCCAA GCGTAAAGCC CGACATCGTT   
  
  
- GAAGAGCCGA GCTCCTGCAC TTCGGCAGTT ACGAGCCTTA CCTGGGATCT CTTCGGCAGC GATACTTAAG   
  
  
- GCAGTACGTA GAAGTAGCTG AAAATCCTAA TCTGTAGTTG AGTCAAGATT TGACCTAAGC TTTGGATTTG   
  
  
- GGCTTCTAGC ATTGAAATCA TCTTGTTCTC CGCTTGGTAT TGGTTGGACT TAAAGAGGGG GCTAAGTGAC   
  
  
- TTCGTGATAC AATGATGAGT TGATACAAGC TGAGTAATCT CTGAACAGTT CAGGTTGGCG ACTTTCAGGA   
  
  
- TCGGCTCTAC ATATATGTTT TCCTCTATAG GTTGGATCAC ACGATGCTTC CGAGTCGGGC CCAGGTCTCC   
  
  
- GTGCTCGGCG AGCGGTTTAC CGTTCGAGCC TACCCGGTTC GGCCCAAGTT CGGCGATCCG GACTCAGTTT   
  
  
- TACGGGAATC CGTTCGATTG TACAACCATT GTAACAAGAG GAGTGTTCCC ATACTGCACC TCCTCTCTCT   
  
  
- TCCCACAAAC TGTGACCCGA CCGTATTGGC GGGAGAGTAT CACTGAAGCT GAACCGTTCA CCCCGGGTTT   
  
  
- TACCTGTCAG GTTCACACTG AGTACTCTAC AGTAGTAGGA GTCGAAGAAG TAGAAT

+     Box 4

| Site Name | Organism | Position | Strand | Matrix score. | sequence | function |
| --- | --- | --- | --- | --- | --- | --- |
| Box 4 | Petroselinum crispum | 196 | + | 6 | ATTAAT | part of a conserved DNA module involved in light responsiveness |
| Box 4 | Petroselinum crispum | 840 | + | 6 | ATTAAT | part of a conserved DNA module involved in light responsiveness |

>HU03G02797.1   
+ +Up\_Stream \_Len000CATTTT CAATTAGAAA TTTCTTCAAT TATATACCAT CTCCAAAAAT AGTCCTCATA   
  
  
+ AGTTGGCAGG TGAATGATCC TAATGGAAAA TTACTTTTAA CTGTTGAATT TGCAATAGGG TACCGAATTT   
  
  
+ GTGAAGTTTA TTGCCGCACG TGCCTAGCTT GTGACTTCTC GATAATCAAA AATTAATTAG AGAGAGACAG   
  
  
+ CTAAAGTTTT GAGAATAATA CTGAAAAATT ACATTAAATT TTATTTATGT TTATTTTCAT ACGCTATGTT   
  
  
+ TAACTGCTAT ACGCTGTGTT TAACTGCATC TCCTTCTATA TTTGATAAAT TGGTAAAAAA AAATAATTCA   
  
  
+ AAATTAAAGA CTAGTAATAT TTTCACTTAT CTGATAGATT TATAAGAAAT ATTTAGATAG CTAAATATAT   
  
  
+ TCTCTCCTGA AAGAGGAGAC TGATAGTTGA ATTTCATAGG ATATAAACAT GTATGTACAT TGAACGAATC   
  
  
+ TTTTTTCTAT GGTGATCGTT ATAAAGTTAT TTATTACTGT AAACATTAAG TGATAATTAG GATATGTTCT   
  
  
+ ATTTCACGTT TTAGATAGAT AGATAGATGT AAGGAGTTGT AAGAAGCTGT AAGAATATGT AAGGAGCTGT   
  
  
+ AAGGAGATGT AAGGAGCTGT AAGAAGATGT AAGAGAAACT GTAAGAAGCT ACAAGGATAG ATGTAACGAG   
  
  
+ TTGTTGAATT GTTGAAGAGT TGTATAGTGT AATTTGTTTT ACGATGAACG TTTTGGATGA AAAATAATAA   
  
  
+ TATTAAAATA ATATGCTTAT TAAAAATAAA TAAATAATTA AGTTAATTAC ACAACAATAA AATATATTAA   
  
  
+ TCCCCATAAA TTTAGTGAAT AAAACTTCGT CCACTTGAGA TGATTTTTTA TATGAACACT AGTTTGATAG   
  
  
+ AATAAACAAA GTATAAAAAA AAATAAAGTT TTTTGACAAA ATTGAATACA ACTCTTCAAA TAAATTCCTA   
  
  
+ TTTTAAGTAA ATAATTTTAA ATATAAATAA ATTTATGGTC ATCTATAATA ATATATATTT TATGTCGAAA   
  
  
+ CTCAAATATA ATTTACATCG TTATATTATA TTTTTATGTT TATGTAAATG TTGTGTTCAT AAGATATTTA   
  
  
+ TAAATTTTGT ATATTGGTAG TATCATTAAA AAAAATATAC ATATTTATGT AACTAAAATA AATGAATGTT   
  
  
+ ATAGAAAATC TATAAGATGT AAGGAGATGT ATGAAGATGT AAGGAGATGT AAGAAGATAT AAGGAGATGT   
  
  
+ AGAAAATGTA AGAAGATGTG AGAAGCTGTA AGGAGATGTA AGTTATTCAC TAAATCCTAA AAAATGCGAA   
  
  
+ ATAGAACAGG GGCTTAATTG AGCACTCAAG ATGCATAATT TGTGACGGAC CAAATGGATA AATATAGACC   
  
  
+ TGGCAAATGG GTTATTCGAT TTAGGTACGA GTATGGTTAA GTTTGGGTTT GGTTATGTTT CAGGATAAGT   
  
  
+ GACATTTCGA CTCAATTTGG TTTTGGTTGT ATGAACTTCG GCTCGAGATT TTTGGTTTGG CCAATACACT   
  
  
+ TATCATGCAA AAATAAGTAA CTTTATAGAA AATTTTGGAT CGGATATGAT GCGGTTCGGA TCAATTCGGA   
  
  
+ TTTTGGTTCA CATTCTGGTA AACCTATTTC GGATGTCAAG TAGGATATGA GTCCAGATCG TTGGATTTCG   
  
  
+ GGTATCAGCT CAATTTTACC AGATCTAGAT AAATCATAAA TGTACGGGTT TATTTTTTCC AGCTTGAATG   
  
  
+ ATTTTCTTTA TTAAAAAAAG ATAGATTTTA GATAAAACAC AACATTTCAT TTTCATTTGT TCCCCCCTAT   
  
  
+ AAATTTCTGT GACCACCTTC ACTTTCTTGC CCCATTCCCC CCCCCCCCCC GCTCTCTCTC TCTCTCTCTC   
  
  
+ TCTTCCCTTT ACAAAGGCAC CATTCAAATT CAAACCACTC TTTTTAAAAC CCCAAAAATT ATTCTCAAAA   
  
  
+ TAATACCTCA TAATATAAAT GGGTTCGTAT CACCATATAT GGGCCTCATC TCTCATGGGA ACATACGAAT   
  
  
+ TCAATGGCTT GCCCGATAGT TATCGGGTCA TGCCATCGGA GCCCCGGCAA CTAATCAATG GTCAAGCCTC   
  
  
+ AGATTTGGCC TCTTTGAGCC ATTATAGTAA CACCTTTCTC TCTGAACTCC CCCCTCTACC CACTCTGGGA   
  
  
+ CCCGGATCCG ACCCGGGTCT ATCGTGTCCG GGTCCTGCTC TGAGTCCAAT GGAGTGGATT GGTGACGCGT   
  
  
+ GGACACACCA GATTGAACAT GATCTGCCAC GTCAGCAGAG TCAACGGCTG TTGACTGATG GTGACATGGG   
  
  
+ GGAGGAGGAT TCCGGTATAA GGTTGGTGCA TGCCTTGCTG ACGTGCGCAG AGGCCGTCCA ATGCGGCGAG   
  
  
+ CTCCGACTGG CTGCTTCGCT AGTTGATGAG ATGACCAATG GGCTGTTGCC ACGTGTTAGT ACGGTGTGTG   
  
  
+ GGATCGGCAA GGTAGCCGGC TATTTTGTTG AGGCTTTGAG CCGGCGCCTG TTTCAGCCCG GCCCGGAAAC   
  
  
+ TGGCCCGACC TGGCCGGGCT CGGAGTTCGA GGCTGAGGTG TTGTATCGTC ACTTCTATGA GGCTTGCCCT   
  
  
+ TATCTCAAAT TTGCTCACTT CACGGCTAAT CAAGCCATCT TGGAGGCATT TGAGGGCCAT GATTATGTCC   
  
  
+ ACGTCATCGA CTTCGGCTTC ATGACCGGCT TGCAATGGCC GGCTTTGATT CAAGCCCTGG CTTTAAGGCC   
  
  
+ CGGTGGGCCA CCATCTCTTA GGCTCACTGG GATTGGCCCG TCTTTAGGAA GTGGGCCCGA ATCGATACGT   
  
  
+ GAAATCGGGT TGAGGCTAGC CCAATTGGCC CGATCCATGA ACATGAGGTT CGCATTTCGG GCTGTAGCAA   
  
  
+ CTTCTCGGCT CGAGGACGTG AAGCCGTCAA TGCTCGGAAT GGACCCTAGA GAAGCCGTCG CTATGAATTC   
  
  
+ CGTCATGCAT CTTCATCGAC TTTTAGGATT AGACATCAAC TCAGTTCTAA ACTGGATTCG AAACCTAAAC   
  
  
+ CCGAAGATCG TAACTTTAGT AGAACAAGAG GCGAACCATA ACCAACCTGA ATTTCTCCCC CGATTCACTG   
  
  
+ AAGCACTATG TTACTACTCA ACTATGTTCG ACTCATTAGA GACTTGTCAA GTCCAACCGC TGAAAGTCCT   
  
  
+ AGCCGAGATG TATATACAAA AGGAGATATC CAACCTAGTG TGCTACGAAG GCTCAGCCCG GGTCCAGAGG   
  
  
+ CACGAGCCGC TCGCCAAATG GCAAGCTCGG ATGGGCCAAG CCGGGTTCAA GCCGCTAGGC CTGAGTCAAA   
  
  
+ ATGCCCTTAG GCAAGCTAAC ATGTTGGTAA CATTGTTCTC CTCACAAGGG TATGACGTGG AGGAGAGAGA   
  
  
+ AGGGTGTTTG ACACTGGGCT GGCATAACCG CCCTCTCATA GTGACTTCGA CTTGGCAAGT GGGGCCCAAA   
  
  
+ ATGGACAGTC CAAGTGTGAC TCATGAGATG TCATCATCCT CAGCTTCTTC ATCTTA  

- +Up\_Stream \_Len000GTAAAA GTTAATCTTT AAAGAAGTTA ATATATGGTA GAGGTTTTTA TCAGGAGTAT   
  
  
- TCAACCGTCC ACTTACTAGG ATTACCTTTT AATGAAAATT GACAACTTAA ACGTTATCCC ATGGCTTAAA   
  
  
- CACTTCAAAT AACGGCGTGC ACGGATCGAA CACTGAAGAG CTATTAGTTT TTAATTAATC TCTCTCTGTC   
  
  
- GATTTCAAAA CTCTTATTAT GACTTTTTAA TGTAATTTAA AATAAATACA AATAAAAGTA TGCGATACAA   
  
  
- ATTGACGATA TGCGACACAA ATTGACGTAG AGGAAGATAT AAACTATTTA ACCATTTTTT TTTATTAAGT   
  
  
- TTTAATTTCT GATCATTATA AAAGTGAATA GACTATCTAA ATATTCTTTA TAAATCTATC GATTTATATA   
  
  
- AGAGAGGACT TTCTCCTCTG ACTATCAACT TAAAGTATCC TATATTTGTA CATACATGTA ACTTGCTTAG   
  
  
- AAAAAAGATA CCACTAGCAA TATTTCAATA AATAATGACA TTTGTAATTC ACTATTAATC CTATACAAGA   
  
  
- TAAAGTGCAA AATCTATCTA TCTATCTACA TTCCTCAACA TTCTTCGACA TTCTTATACA TTCCTCGACA   
  
  
- TTCCTCTACA TTCCTCGACA TTCTTCTACA TTCTCTTTGA CATTCTTCGA TGTTCCTATC TACATTGCTC   
  
  
- AACAACTTAA CAACTTCTCA ACATATCACA TTAAACAAAA TGCTACTTGC AAAACCTACT TTTTATTATT   
  
  
- ATAATTTTAT TATACGAATA ATTTTTATTT ATTTATTAAT TCAATTAATG TGTTGTTATT TTATATAATT   
  
  
- AGGGGTATTT AAATCACTTA TTTTGAAGCA GGTGAACTCT ACTAAAAAAT ATACTTGTGA TCAAACTATC   
  
  
- TTATTTGTTT CATATTTTTT TTTATTTCAA AAAACTGTTT TAACTTATGT TGAGAAGTTT ATTTAAGGAT   
  
  
- AAAATTCATT TATTAAAATT TATATTTATT TAAATACCAG TAGATATTAT TATATATAAA ATACAGCTTT   
  
  
- GAGTTTATAT TAAATGTAGC AATATAATAT AAAAATACAA ATACATTTAC AACACAAGTA TTCTATAAAT   
  
  
- ATTTAAAACA TATAACCATC ATAGTAATTT TTTTTATATG TATAAATACA TTGATTTTAT TTACTTACAA   
  
  
- TATCTTTTAG ATATTCTACA TTCCTCTACA TACTTCTACA TTCCTCTACA TTCTTCTATA TTCCTCTACA   
  
  
- TCTTTTACAT TCTTCTACAC TCTTCGACAT TCCTCTACAT TCAATAAGTG ATTTAGGATT TTTTACGCTT   
  
  
- TATCTTGTCC CCGAATTAAC TCGTGAGTTC TACGTATTAA ACACTGCCTG GTTTACCTAT TTATATCTGG   
  
  
- ACCGTTTACC CAATAAGCTA AATCCATGCT CATACCAATT CAAACCCAAA CCAATACAAA GTCCTATTCA   
  
  
- CTGTAAAGCT GAGTTAAACC AAAACCAACA TACTTGAAGC CGAGCTCTAA AAACCAAACC GGTTATGTGA   
  
  
- ATAGTACGTT TTTATTCATT GAAATATCTT TTAAAACCTA GCCTATACTA CGCCAAGCCT AGTTAAGCCT   
  
  
- AAAACCAAGT GTAAGACCAT TTGGATAAAG CCTACAGTTC ATCCTATACT CAGGTCTAGC AACCTAAAGC   
  
  
- CCATAGTCGA GTTAAAATGG TCTAGATCTA TTTAGTATTT ACATGCCCAA ATAAAAAAGG TCGAACTTAC   
  
  
- TAAAAGAAAT AATTTTTTTC TATCTAAAAT CTATTTTGTG TTGTAAAGTA AAAGTAAACA AGGGGGGATA   
  
  
- TTTAAAGACA CTGGTGGAAG TGAAAGAACG GGGTAAGGGG GGGGGGGGGG CGAGAGAGAG AGAGAGAGAG   
  
  
- AGAAGGGAAA TGTTTCCGTG GTAAGTTTAA GTTTGGTGAG AAAAATTTTG GGGTTTTTAA TAAGAGTTTT   
  
  
- ATTATGGAGT ATTATATTTA CCCAAGCATA GTGGTATATA CCCGGAGTAG AGAGTACCCT TGTATGCTTA   
  
  
- AGTTACCGAA CGGGCTATCA ATAGCCCAGT ACGGTAGCCT CGGGGCCGTT GATTAGTTAC CAGTTCGGAG   
  
  
- TCTAAACCGG AGAAACTCGG TAATATCATT GTGGAAAGAG AGACTTGAGG GGGGAGATGG GTGAGACCCT   
  
  
- GGGCCTAGGC TGGGCCCAGA TAGCACAGGC CCAGGACGAG ACTCAGGTTA CCTCACCTAA CCACTGCGCA   
  
  
- CCTGTGTGGT CTAACTTGTA CTAGACGGTG CAGTCGTCTC AGTTGCCGAC AACTGACTAC CACTGTACCC   
  
  
- CCTCCTCCTA AGGCCATATT CCAACCACGT ACGGAACGAC TGCACGCGTC TCCGGCAGGT TACGCCGCTC   
  
  
- GAGGCTGACC GACGAAGCGA TCAACTACTC TACTGGTTAC CCGACAACGG TGCACAATCA TGCCACACAC   
  
  
- CCTAGCCGTT CCATCGGCCG ATAAAACAAC TCCGAAACTC GGCCGCGGAC AAAGTCGGGC CGGGCCTTTG   
  
  
- ACCGGGCTGG ACCGGCCCGA GCCTCAAGCT CCGACTCCAC AACATAGCAG TGAAGATACT CCGAACGGGA   
  
  
- ATAGAGTTTA AACGAGTGAA GTGCCGATTA GTTCGGTAGA ACCTCCGTAA ACTCCCGGTA CTAATACAGG   
  
  
- TGCAGTAGCT GAAGCCGAAG TACTGGCCGA ACGTTACCGG CCGAAACTAA GTTCGGGACC GAAATTCCGG   
  
  
- GCCACCCGGT GGTAGAGAAT CCGAGTGACC CTAACCGGGC AGAAATCCTT CACCCGGGCT TAGCTATGCA   
  
  
- CTTTAGCCCA ACTCCGATCG GGTTAACCGG GCTAGGTACT TGTACTCCAA GCGTAAAGCC CGACATCGTT   
  
  
- GAAGAGCCGA GCTCCTGCAC TTCGGCAGTT ACGAGCCTTA CCTGGGATCT CTTCGGCAGC GATACTTAAG   
  
  
- GCAGTACGTA GAAGTAGCTG AAAATCCTAA TCTGTAGTTG AGTCAAGATT TGACCTAAGC TTTGGATTTG   
  
  
- GGCTTCTAGC ATTGAAATCA TCTTGTTCTC CGCTTGGTAT TGGTTGGACT TAAAGAGGGG GCTAAGTGAC   
  
  
- TTCGTGATAC AATGATGAGT TGATACAAGC TGAGTAATCT CTGAACAGTT CAGGTTGGCG ACTTTCAGGA   
  
  
- TCGGCTCTAC ATATATGTTT TCCTCTATAG GTTGGATCAC ACGATGCTTC CGAGTCGGGC CCAGGTCTCC   
  
  
- GTGCTCGGCG AGCGGTTTAC CGTTCGAGCC TACCCGGTTC GGCCCAAGTT CGGCGATCCG GACTCAGTTT   
  
  
- TACGGGAATC CGTTCGATTG TACAACCATT GTAACAAGAG GAGTGTTCCC ATACTGCACC TCCTCTCTCT   
  
  
- TCCCACAAAC TGTGACCCGA CCGTATTGGC GGGAGAGTAT CACTGAAGCT GAACCGTTCA CCCCGGGTTT   
  
  
- TACCTGTCAG GTTCACACTG AGTACTCTAC AGTAGTAGGA GTCGAAGAAG TAGAAT

+     CAAT-box

| Site Name | Organism | Position | Strand | Matrix score. | sequence | function |
| --- | --- | --- | --- | --- | --- | --- |
| CAAT-box | Nicotiana glutinosa | 712 | - | 4 | CAAT |  |
| CAAT-box | Nicotiana glutinosa | 127 | + | 4 | CAAT |  |
| CAAT-box | Nicotiana glutinosa | 3326 | - | 4 | CAAT |  |
| CAAT-box | Nicotiana glutinosa | 2256 | - | 4 | CAAT |  |
| CAAT-box | Arabidopsis thaliana | 2825 | + | 5 | CCAAT | common cis-acting element in promoter and enhancer regions |
| CAAT-box | Arabidopsis thaliana | 2766 | - | 5 | CCAAT | common cis-acting element in promoter and enhancer regions |
| CAAT-box | Nicotiana glutinosa | 25 | + | 4 | CAAT |  |
| CAAT-box | Nicotiana glutinosa | 1606 | + | 4 | CAAT |  |
| CAAT-box | Nicotiana glutinosa | 1536 | + | 4 | CAAT |  |
| CAAT-box | Arabidopsis thaliana | 2419 | + | 5 | CCAAT | common cis-acting element in promoter and enhancer regions |
| CAAT-box | Daucus carota | 1691 | + | 11 | AGCTCAATTTCA | common cis-acting element in promoter and enhancer regions |
| CAAT-box | Nicotiana glutinosa | 1695 | + | 4 | CAAT |  |
| CAAT-box | Nicotiana glutinosa | 2221 | + | 4 | CAAT |  |
| CAAT-box | Arabidopsis thaliana | 1535 | + | 5 | CCAAT | common cis-acting element in promoter and enhancer regions |
| CAAT-box | Nicotiana glutinosa | 829 | + | 4 | CAAT |  |
| CAAT-box | Nicotiana glutinosa | 41 | + | 4 | CAAT |  |
| CAAT-box | Nicotiana glutinosa | 2090 | + | 4 | CAAT |  |
| CAAT-box | Nicotiana glutinosa | 2036 | + | 4 | CAAT |  |
| CAAT-box | Pisum sativum | 971 | + | 5 | CAAAT | common cis-acting element in promoter and enhancer regions |
| CAAT-box | Pisum sativum | 736 | - | 5 | CAAAT | common cis-acting element in promoter and enhancer regions |
| CAAT-box | Arabidopsis thaliana | 333 | - | 5 | CCAAT | common cis-acting element in promoter and enhancer regions |
| CAAT-box | Nicotiana glutinosa | 2420 | + | 4 | CAAT |  |
| CAAT-box | Arabidopsis thaliana | 1137 | - | 5 | CCAAT | common cis-acting element in promoter and enhancer regions |
| CAAT-box | Nicotiana glutinosa | 2373 | + | 4 | CAAT |  |
| CAAT-box | Nicotiana glutinosa | 955 | - | 4 | CAAT |  |
| CAAT-box | Pisum sativum | 1408 | + | 5 | CAAAT | common cis-acting element in promoter and enhancer regions |
| CAAT-box | Nicotiana glutinosa | 2902 | + | 4 | CAAT |  |
| CAAT-box | Nicotiana glutinosa | 154 | - | 4 | CAAT |  |
| CAAT-box | Pisum sativum | 1809 | - | 5 | CAAAT | common cis-acting element in promoter and enhancer regions |
| CAAT-box | Pisum sativum | 1372 | - | 5 | CAAAT | common cis-acting element in promoter and enhancer regions |
| CAAT-box | Pisum sativum | 2603 | - | 5 | CAAAT | common cis-acting element in promoter and enhancer regions |
| CAAT-box | Nicotiana glutinosa | 483 | - | 4 | CAAT |  |
| CAAT-box | Arabidopsis thaliana | 2232 | - | 5 | CCAAT | common cis-acting element in promoter and enhancer regions |
| CAAT-box | Nicotiana glutinosa | 1487 | + | 4 | CAAT |  |
| CAAT-box | Pisum sativum | 3239 | + | 5 | CAAAT | common cis-acting element in promoter and enhancer regions |
| CAAT-box | Pisum sativum | 141 | - | 5 | CAAAT | common cis-acting element in promoter and enhancer regions |
| CAAT-box | Petunia hybrida | 76 | - | 7 | TGCCAAC | common cis-acting element in promoter and enhancer regions |
| CAAT-box | Pisum sativum | 1919 | + | 5 | CAAAT | common cis-acting element in promoter and enhancer regions |
| CAAT-box | Pisum sativum | 1385 | + | 5 | CAAAT | common cis-acting element in promoter and enhancer regions |
| CAAT-box | Nicotiana glutinosa | 2697 | + | 4 | CAAT |  |
| CAAT-box | Pisum sativum | 122 | - | 5 | CAAAT | common cis-acting element in promoter and enhancer regions |
| CAAT-box | Arabidopsis thaliana | 2372 | + | 5 | CCAAT | common cis-acting element in promoter and enhancer regions |
| CAAT-box | Pisum sativum | 1489 | - | 5 | CAAAT | common cis-acting element in promoter and enhancer regions |
| CAAT-box | Pisum sativum | 324 | - | 5 | CAAAT | common cis-acting element in promoter and enhancer regions |
| CAAT-box | Nicotiana glutinosa | 2826 | + | 4 | CAAT |  |
| CAAT-box | Pisum sativum | 1057 | + | 5 | CAAAT | common cis-acting element in promoter and enhancer regions |
| CAAT-box | Arabidopsis thaliana | 2828 | - | 5 | CCAAT | common cis-acting element in promoter and enhancer regions |
| CAAT-box | Nicotiana glutinosa | 1351 | - | 4 | CAAT |  |
| CAAT-box | Pisum sativum | 2107 | - | 5 | CAAAT | common cis-acting element in promoter and enhancer regions |
| CAAT-box | Arabidopsis thaliana | 2220 | + | 5 | CCAAT | common cis-acting element in promoter and enhancer regions |
| CAAT-box | Pisum sativum | 2642 | - | 5 | CAAAT | common cis-acting element in promoter and enhancer regions |
| CAAT-box | Pisum sativum | 2600 | + | 5 | CAAAT | common cis-acting element in promoter and enhancer regions |

>HU03G02797.1   
+ +Up\_Stream \_Len000CATTTT CAATTAGAAA TTTCTTCAAT TATATACCAT CTCCAAAAAT AGTCCTCATA   
  
  
+ AGTTGGCAGG TGAATGATCC TAATGGAAAA TTACTTTTAA CTGTTGAATT TGCAATAGGG TACCGAATTT   
  
  
+ GTGAAGTTTA TTGCCGCACG TGCCTAGCTT GTGACTTCTC GATAATCAAA AATTAATTAG AGAGAGACAG   
  
  
+ CTAAAGTTTT GAGAATAATA CTGAAAAATT ACATTAAATT TTATTTATGT TTATTTTCAT ACGCTATGTT   
  
  
+ TAACTGCTAT ACGCTGTGTT TAACTGCATC TCCTTCTATA TTTGATAAAT TGGTAAAAAA AAATAATTCA   
  
  
+ AAATTAAAGA CTAGTAATAT TTTCACTTAT CTGATAGATT TATAAGAAAT ATTTAGATAG CTAAATATAT   
  
  
+ TCTCTCCTGA AAGAGGAGAC TGATAGTTGA ATTTCATAGG ATATAAACAT GTATGTACAT TGAACGAATC   
  
  
+ TTTTTTCTAT GGTGATCGTT ATAAAGTTAT TTATTACTGT AAACATTAAG TGATAATTAG GATATGTTCT   
  
  
+ ATTTCACGTT TTAGATAGAT AGATAGATGT AAGGAGTTGT AAGAAGCTGT AAGAATATGT AAGGAGCTGT   
  
  
+ AAGGAGATGT AAGGAGCTGT AAGAAGATGT AAGAGAAACT GTAAGAAGCT ACAAGGATAG ATGTAACGAG   
  
  
+ TTGTTGAATT GTTGAAGAGT TGTATAGTGT AATTTGTTTT ACGATGAACG TTTTGGATGA AAAATAATAA   
  
  
+ TATTAAAATA ATATGCTTAT TAAAAATAAA TAAATAATTA AGTTAATTAC ACAACAATAA AATATATTAA   
  
  
+ TCCCCATAAA TTTAGTGAAT AAAACTTCGT CCACTTGAGA TGATTTTTTA TATGAACACT AGTTTGATAG   
  
  
+ AATAAACAAA GTATAAAAAA AAATAAAGTT TTTTGACAAA ATTGAATACA ACTCTTCAAA TAAATTCCTA   
  
  
+ TTTTAAGTAA ATAATTTTAA ATATAAATAA ATTTATGGTC ATCTATAATA ATATATATTT TATGTCGAAA   
  
  
+ CTCAAATATA ATTTACATCG TTATATTATA TTTTTATGTT TATGTAAATG TTGTGTTCAT AAGATATTTA   
  
  
+ TAAATTTTGT ATATTGGTAG TATCATTAAA AAAAATATAC ATATTTATGT AACTAAAATA AATGAATGTT   
  
  
+ ATAGAAAATC TATAAGATGT AAGGAGATGT ATGAAGATGT AAGGAGATGT AAGAAGATAT AAGGAGATGT   
  
  
+ AGAAAATGTA AGAAGATGTG AGAAGCTGTA AGGAGATGTA AGTTATTCAC TAAATCCTAA AAAATGCGAA   
  
  
+ ATAGAACAGG GGCTTAATTG AGCACTCAAG ATGCATAATT TGTGACGGAC CAAATGGATA AATATAGACC   
  
  
+ TGGCAAATGG GTTATTCGAT TTAGGTACGA GTATGGTTAA GTTTGGGTTT GGTTATGTTT CAGGATAAGT   
  
  
+ GACATTTCGA CTCAATTTGG TTTTGGTTGT ATGAACTTCG GCTCGAGATT TTTGGTTTGG CCAATACACT   
  
  
+ TATCATGCAA AAATAAGTAA CTTTATAGAA AATTTTGGAT CGGATATGAT GCGGTTCGGA TCAATTCGGA   
  
  
+ TTTTGGTTCA CATTCTGGTA AACCTATTTC GGATGTCAAG TAGGATATGA GTCCAGATCG TTGGATTTCG   
  
  
+ GGTATCAGCT CAATTTTACC AGATCTAGAT AAATCATAAA TGTACGGGTT TATTTTTTCC AGCTTGAATG   
  
  
+ ATTTTCTTTA TTAAAAAAAG ATAGATTTTA GATAAAACAC AACATTTCAT TTTCATTTGT TCCCCCCTAT   
  
  
+ AAATTTCTGT GACCACCTTC ACTTTCTTGC CCCATTCCCC CCCCCCCCCC GCTCTCTCTC TCTCTCTCTC   
  
  
+ TCTTCCCTTT ACAAAGGCAC CATTCAAATT CAAACCACTC TTTTTAAAAC CCCAAAAATT ATTCTCAAAA   
  
  
+ TAATACCTCA TAATATAAAT GGGTTCGTAT CACCATATAT GGGCCTCATC TCTCATGGGA ACATACGAAT   
  
  
+ TCAATGGCTT GCCCGATAGT TATCGGGTCA TGCCATCGGA GCCCCGGCAA CTAATCAATG GTCAAGCCTC   
  
  
+ AGATTTGGCC TCTTTGAGCC ATTATAGTAA CACCTTTCTC TCTGAACTCC CCCCTCTACC CACTCTGGGA   
  
  
+ CCCGGATCCG ACCCGGGTCT ATCGTGTCCG GGTCCTGCTC TGAGTCCAAT GGAGTGGATT GGTGACGCGT   
  
  
+ GGACACACCA GATTGAACAT GATCTGCCAC GTCAGCAGAG TCAACGGCTG TTGACTGATG GTGACATGGG   
  
  
+ GGAGGAGGAT TCCGGTATAA GGTTGGTGCA TGCCTTGCTG ACGTGCGCAG AGGCCGTCCA ATGCGGCGAG   
  
  
+ CTCCGACTGG CTGCTTCGCT AGTTGATGAG ATGACCAATG GGCTGTTGCC ACGTGTTAGT ACGGTGTGTG   
  
  
+ GGATCGGCAA GGTAGCCGGC TATTTTGTTG AGGCTTTGAG CCGGCGCCTG TTTCAGCCCG GCCCGGAAAC   
  
  
+ TGGCCCGACC TGGCCGGGCT CGGAGTTCGA GGCTGAGGTG TTGTATCGTC ACTTCTATGA GGCTTGCCCT   
  
  
+ TATCTCAAAT TTGCTCACTT CACGGCTAAT CAAGCCATCT TGGAGGCATT TGAGGGCCAT GATTATGTCC   
  
  
+ ACGTCATCGA CTTCGGCTTC ATGACCGGCT TGCAATGGCC GGCTTTGATT CAAGCCCTGG CTTTAAGGCC   
  
  
+ CGGTGGGCCA CCATCTCTTA GGCTCACTGG GATTGGCCCG TCTTTAGGAA GTGGGCCCGA ATCGATACGT   
  
  
+ GAAATCGGGT TGAGGCTAGC CCAATTGGCC CGATCCATGA ACATGAGGTT CGCATTTCGG GCTGTAGCAA   
  
  
+ CTTCTCGGCT CGAGGACGTG AAGCCGTCAA TGCTCGGAAT GGACCCTAGA GAAGCCGTCG CTATGAATTC   
  
  
+ CGTCATGCAT CTTCATCGAC TTTTAGGATT AGACATCAAC TCAGTTCTAA ACTGGATTCG AAACCTAAAC   
  
  
+ CCGAAGATCG TAACTTTAGT AGAACAAGAG GCGAACCATA ACCAACCTGA ATTTCTCCCC CGATTCACTG   
  
  
+ AAGCACTATG TTACTACTCA ACTATGTTCG ACTCATTAGA GACTTGTCAA GTCCAACCGC TGAAAGTCCT   
  
  
+ AGCCGAGATG TATATACAAA AGGAGATATC CAACCTAGTG TGCTACGAAG GCTCAGCCCG GGTCCAGAGG   
  
  
+ CACGAGCCGC TCGCCAAATG GCAAGCTCGG ATGGGCCAAG CCGGGTTCAA GCCGCTAGGC CTGAGTCAAA   
  
  
+ ATGCCCTTAG GCAAGCTAAC ATGTTGGTAA CATTGTTCTC CTCACAAGGG TATGACGTGG AGGAGAGAGA   
  
  
+ AGGGTGTTTG ACACTGGGCT GGCATAACCG CCCTCTCATA GTGACTTCGA CTTGGCAAGT GGGGCCCAAA   
  
  
+ ATGGACAGTC CAAGTGTGAC TCATGAGATG TCATCATCCT CAGCTTCTTC ATCTTA  

- +Up\_Stream \_Len000GTAAAA GTTAATCTTT AAAGAAGTTA ATATATGGTA GAGGTTTTTA TCAGGAGTAT   
  
  
- TCAACCGTCC ACTTACTAGG ATTACCTTTT AATGAAAATT GACAACTTAA ACGTTATCCC ATGGCTTAAA   
  
  
- CACTTCAAAT AACGGCGTGC ACGGATCGAA CACTGAAGAG CTATTAGTTT TTAATTAATC TCTCTCTGTC   
  
  
- GATTTCAAAA CTCTTATTAT GACTTTTTAA TGTAATTTAA AATAAATACA AATAAAAGTA TGCGATACAA   
  
  
- ATTGACGATA TGCGACACAA ATTGACGTAG AGGAAGATAT AAACTATTTA ACCATTTTTT TTTATTAAGT   
  
  
- TTTAATTTCT GATCATTATA AAAGTGAATA GACTATCTAA ATATTCTTTA TAAATCTATC GATTTATATA   
  
  
- AGAGAGGACT TTCTCCTCTG ACTATCAACT TAAAGTATCC TATATTTGTA CATACATGTA ACTTGCTTAG   
  
  
- AAAAAAGATA CCACTAGCAA TATTTCAATA AATAATGACA TTTGTAATTC ACTATTAATC CTATACAAGA   
  
  
- TAAAGTGCAA AATCTATCTA TCTATCTACA TTCCTCAACA TTCTTCGACA TTCTTATACA TTCCTCGACA   
  
  
- TTCCTCTACA TTCCTCGACA TTCTTCTACA TTCTCTTTGA CATTCTTCGA TGTTCCTATC TACATTGCTC   
  
  
- AACAACTTAA CAACTTCTCA ACATATCACA TTAAACAAAA TGCTACTTGC AAAACCTACT TTTTATTATT   
  
  
- ATAATTTTAT TATACGAATA ATTTTTATTT ATTTATTAAT TCAATTAATG TGTTGTTATT TTATATAATT   
  
  
- AGGGGTATTT AAATCACTTA TTTTGAAGCA GGTGAACTCT ACTAAAAAAT ATACTTGTGA TCAAACTATC   
  
  
- TTATTTGTTT CATATTTTTT TTTATTTCAA AAAACTGTTT TAACTTATGT TGAGAAGTTT ATTTAAGGAT   
  
  
- AAAATTCATT TATTAAAATT TATATTTATT TAAATACCAG TAGATATTAT TATATATAAA ATACAGCTTT   
  
  
- GAGTTTATAT TAAATGTAGC AATATAATAT AAAAATACAA ATACATTTAC AACACAAGTA TTCTATAAAT   
  
  
- ATTTAAAACA TATAACCATC ATAGTAATTT TTTTTATATG TATAAATACA TTGATTTTAT TTACTTACAA   
  
  
- TATCTTTTAG ATATTCTACA TTCCTCTACA TACTTCTACA TTCCTCTACA TTCTTCTATA TTCCTCTACA   
  
  
- TCTTTTACAT TCTTCTACAC TCTTCGACAT TCCTCTACAT TCAATAAGTG ATTTAGGATT TTTTACGCTT   
  
  
- TATCTTGTCC CCGAATTAAC TCGTGAGTTC TACGTATTAA ACACTGCCTG GTTTACCTAT TTATATCTGG   
  
  
- ACCGTTTACC CAATAAGCTA AATCCATGCT CATACCAATT CAAACCCAAA CCAATACAAA GTCCTATTCA   
  
  
- CTGTAAAGCT GAGTTAAACC AAAACCAACA TACTTGAAGC CGAGCTCTAA AAACCAAACC GGTTATGTGA   
  
  
- ATAGTACGTT TTTATTCATT GAAATATCTT TTAAAACCTA GCCTATACTA CGCCAAGCCT AGTTAAGCCT   
  
  
- AAAACCAAGT GTAAGACCAT TTGGATAAAG CCTACAGTTC ATCCTATACT CAGGTCTAGC AACCTAAAGC   
  
  
- CCATAGTCGA GTTAAAATGG TCTAGATCTA TTTAGTATTT ACATGCCCAA ATAAAAAAGG TCGAACTTAC   
  
  
- TAAAAGAAAT AATTTTTTTC TATCTAAAAT CTATTTTGTG TTGTAAAGTA AAAGTAAACA AGGGGGGATA   
  
  
- TTTAAAGACA CTGGTGGAAG TGAAAGAACG GGGTAAGGGG GGGGGGGGGG CGAGAGAGAG AGAGAGAGAG   
  
  
- AGAAGGGAAA TGTTTCCGTG GTAAGTTTAA GTTTGGTGAG AAAAATTTTG GGGTTTTTAA TAAGAGTTTT   
  
  
- ATTATGGAGT ATTATATTTA CCCAAGCATA GTGGTATATA CCCGGAGTAG AGAGTACCCT TGTATGCTTA   
  
  
- AGTTACCGAA CGGGCTATCA ATAGCCCAGT ACGGTAGCCT CGGGGCCGTT GATTAGTTAC CAGTTCGGAG   
  
  
- TCTAAACCGG AGAAACTCGG TAATATCATT GTGGAAAGAG AGACTTGAGG GGGGAGATGG GTGAGACCCT   
  
  
- GGGCCTAGGC TGGGCCCAGA TAGCACAGGC CCAGGACGAG ACTCAGGTTA CCTCACCTAA CCACTGCGCA   
  
  
- CCTGTGTGGT CTAACTTGTA CTAGACGGTG CAGTCGTCTC AGTTGCCGAC AACTGACTAC CACTGTACCC   
  
  
- CCTCCTCCTA AGGCCATATT CCAACCACGT ACGGAACGAC TGCACGCGTC TCCGGCAGGT TACGCCGCTC   
  
  
- GAGGCTGACC GACGAAGCGA TCAACTACTC TACTGGTTAC CCGACAACGG TGCACAATCA TGCCACACAC   
  
  
- CCTAGCCGTT CCATCGGCCG ATAAAACAAC TCCGAAACTC GGCCGCGGAC AAAGTCGGGC CGGGCCTTTG   
  
  
- ACCGGGCTGG ACCGGCCCGA GCCTCAAGCT CCGACTCCAC AACATAGCAG TGAAGATACT CCGAACGGGA   
  
  
- ATAGAGTTTA AACGAGTGAA GTGCCGATTA GTTCGGTAGA ACCTCCGTAA ACTCCCGGTA CTAATACAGG   
  
  
- TGCAGTAGCT GAAGCCGAAG TACTGGCCGA ACGTTACCGG CCGAAACTAA GTTCGGGACC GAAATTCCGG   
  
  
- GCCACCCGGT GGTAGAGAAT CCGAGTGACC CTAACCGGGC AGAAATCCTT CACCCGGGCT TAGCTATGCA   
  
  
- CTTTAGCCCA ACTCCGATCG GGTTAACCGG GCTAGGTACT TGTACTCCAA GCGTAAAGCC CGACATCGTT   
  
  
- GAAGAGCCGA GCTCCTGCAC TTCGGCAGTT ACGAGCCTTA CCTGGGATCT CTTCGGCAGC GATACTTAAG   
  
  
- GCAGTACGTA GAAGTAGCTG AAAATCCTAA TCTGTAGTTG AGTCAAGATT TGACCTAAGC TTTGGATTTG   
  
  
- GGCTTCTAGC ATTGAAATCA TCTTGTTCTC CGCTTGGTAT TGGTTGGACT TAAAGAGGGG GCTAAGTGAC   
  
  
- TTCGTGATAC AATGATGAGT TGATACAAGC TGAGTAATCT CTGAACAGTT CAGGTTGGCG ACTTTCAGGA   
  
  
- TCGGCTCTAC ATATATGTTT TCCTCTATAG GTTGGATCAC ACGATGCTTC CGAGTCGGGC CCAGGTCTCC   
  
  
- GTGCTCGGCG AGCGGTTTAC CGTTCGAGCC TACCCGGTTC GGCCCAAGTT CGGCGATCCG GACTCAGTTT   
  
  
- TACGGGAATC CGTTCGATTG TACAACCATT GTAACAAGAG GAGTGTTCCC ATACTGCACC TCCTCTCTCT   
  
  
- TCCCACAAAC TGTGACCCGA CCGTATTGGC GGGAGAGTAT CACTGAAGCT GAACCGTTCA CCCCGGGTTT   
  
  
- TACCTGTCAG GTTCACACTG AGTACTCTAC AGTAGTAGGA GTCGAAGAAG TAGAAT

+     CCAAT-box

| Site Name | Organism | Position | Strand | Matrix score. | sequence | function |
| --- | --- | --- | --- | --- | --- | --- |
| CCAAT-box | Hordeum vulgare | 2286 | + | 6 | CAACGG | MYBHv1 binding site |

>HU03G02797.1   
+ +Up\_Stream \_Len000CATTTT CAATTAGAAA TTTCTTCAAT TATATACCAT CTCCAAAAAT AGTCCTCATA   
  
  
+ AGTTGGCAGG TGAATGATCC TAATGGAAAA TTACTTTTAA CTGTTGAATT TGCAATAGGG TACCGAATTT   
  
  
+ GTGAAGTTTA TTGCCGCACG TGCCTAGCTT GTGACTTCTC GATAATCAAA AATTAATTAG AGAGAGACAG   
  
  
+ CTAAAGTTTT GAGAATAATA CTGAAAAATT ACATTAAATT TTATTTATGT TTATTTTCAT ACGCTATGTT   
  
  
+ TAACTGCTAT ACGCTGTGTT TAACTGCATC TCCTTCTATA TTTGATAAAT TGGTAAAAAA AAATAATTCA   
  
  
+ AAATTAAAGA CTAGTAATAT TTTCACTTAT CTGATAGATT TATAAGAAAT ATTTAGATAG CTAAATATAT   
  
  
+ TCTCTCCTGA AAGAGGAGAC TGATAGTTGA ATTTCATAGG ATATAAACAT GTATGTACAT TGAACGAATC   
  
  
+ TTTTTTCTAT GGTGATCGTT ATAAAGTTAT TTATTACTGT AAACATTAAG TGATAATTAG GATATGTTCT   
  
  
+ ATTTCACGTT TTAGATAGAT AGATAGATGT AAGGAGTTGT AAGAAGCTGT AAGAATATGT AAGGAGCTGT   
  
  
+ AAGGAGATGT AAGGAGCTGT AAGAAGATGT AAGAGAAACT GTAAGAAGCT ACAAGGATAG ATGTAACGAG   
  
  
+ TTGTTGAATT GTTGAAGAGT TGTATAGTGT AATTTGTTTT ACGATGAACG TTTTGGATGA AAAATAATAA   
  
  
+ TATTAAAATA ATATGCTTAT TAAAAATAAA TAAATAATTA AGTTAATTAC ACAACAATAA AATATATTAA   
  
  
+ TCCCCATAAA TTTAGTGAAT AAAACTTCGT CCACTTGAGA TGATTTTTTA TATGAACACT AGTTTGATAG   
  
  
+ AATAAACAAA GTATAAAAAA AAATAAAGTT TTTTGACAAA ATTGAATACA ACTCTTCAAA TAAATTCCTA   
  
  
+ TTTTAAGTAA ATAATTTTAA ATATAAATAA ATTTATGGTC ATCTATAATA ATATATATTT TATGTCGAAA   
  
  
+ CTCAAATATA ATTTACATCG TTATATTATA TTTTTATGTT TATGTAAATG TTGTGTTCAT AAGATATTTA   
  
  
+ TAAATTTTGT ATATTGGTAG TATCATTAAA AAAAATATAC ATATTTATGT AACTAAAATA AATGAATGTT   
  
  
+ ATAGAAAATC TATAAGATGT AAGGAGATGT ATGAAGATGT AAGGAGATGT AAGAAGATAT AAGGAGATGT   
  
  
+ AGAAAATGTA AGAAGATGTG AGAAGCTGTA AGGAGATGTA AGTTATTCAC TAAATCCTAA AAAATGCGAA   
  
  
+ ATAGAACAGG GGCTTAATTG AGCACTCAAG ATGCATAATT TGTGACGGAC CAAATGGATA AATATAGACC   
  
  
+ TGGCAAATGG GTTATTCGAT TTAGGTACGA GTATGGTTAA GTTTGGGTTT GGTTATGTTT CAGGATAAGT   
  
  
+ GACATTTCGA CTCAATTTGG TTTTGGTTGT ATGAACTTCG GCTCGAGATT TTTGGTTTGG CCAATACACT   
  
  
+ TATCATGCAA AAATAAGTAA CTTTATAGAA AATTTTGGAT CGGATATGAT GCGGTTCGGA TCAATTCGGA   
  
  
+ TTTTGGTTCA CATTCTGGTA AACCTATTTC GGATGTCAAG TAGGATATGA GTCCAGATCG TTGGATTTCG   
  
  
+ GGTATCAGCT CAATTTTACC AGATCTAGAT AAATCATAAA TGTACGGGTT TATTTTTTCC AGCTTGAATG   
  
  
+ ATTTTCTTTA TTAAAAAAAG ATAGATTTTA GATAAAACAC AACATTTCAT TTTCATTTGT TCCCCCCTAT   
  
  
+ AAATTTCTGT GACCACCTTC ACTTTCTTGC CCCATTCCCC CCCCCCCCCC GCTCTCTCTC TCTCTCTCTC   
  
  
+ TCTTCCCTTT ACAAAGGCAC CATTCAAATT CAAACCACTC TTTTTAAAAC CCCAAAAATT ATTCTCAAAA   
  
  
+ TAATACCTCA TAATATAAAT GGGTTCGTAT CACCATATAT GGGCCTCATC TCTCATGGGA ACATACGAAT   
  
  
+ TCAATGGCTT GCCCGATAGT TATCGGGTCA TGCCATCGGA GCCCCGGCAA CTAATCAATG GTCAAGCCTC   
  
  
+ AGATTTGGCC TCTTTGAGCC ATTATAGTAA CACCTTTCTC TCTGAACTCC CCCCTCTACC CACTCTGGGA   
  
  
+ CCCGGATCCG ACCCGGGTCT ATCGTGTCCG GGTCCTGCTC TGAGTCCAAT GGAGTGGATT GGTGACGCGT   
  
  
+ GGACACACCA GATTGAACAT GATCTGCCAC GTCAGCAGAG TCAACGGCTG TTGACTGATG GTGACATGGG   
  
  
+ GGAGGAGGAT TCCGGTATAA GGTTGGTGCA TGCCTTGCTG ACGTGCGCAG AGGCCGTCCA ATGCGGCGAG   
  
  
+ CTCCGACTGG CTGCTTCGCT AGTTGATGAG ATGACCAATG GGCTGTTGCC ACGTGTTAGT ACGGTGTGTG   
  
  
+ GGATCGGCAA GGTAGCCGGC TATTTTGTTG AGGCTTTGAG CCGGCGCCTG TTTCAGCCCG GCCCGGAAAC   
  
  
+ TGGCCCGACC TGGCCGGGCT CGGAGTTCGA GGCTGAGGTG TTGTATCGTC ACTTCTATGA GGCTTGCCCT   
  
  
+ TATCTCAAAT TTGCTCACTT CACGGCTAAT CAAGCCATCT TGGAGGCATT TGAGGGCCAT GATTATGTCC   
  
  
+ ACGTCATCGA CTTCGGCTTC ATGACCGGCT TGCAATGGCC GGCTTTGATT CAAGCCCTGG CTTTAAGGCC   
  
  
+ CGGTGGGCCA CCATCTCTTA GGCTCACTGG GATTGGCCCG TCTTTAGGAA GTGGGCCCGA ATCGATACGT   
  
  
+ GAAATCGGGT TGAGGCTAGC CCAATTGGCC CGATCCATGA ACATGAGGTT CGCATTTCGG GCTGTAGCAA   
  
  
+ CTTCTCGGCT CGAGGACGTG AAGCCGTCAA TGCTCGGAAT GGACCCTAGA GAAGCCGTCG CTATGAATTC   
  
  
+ CGTCATGCAT CTTCATCGAC TTTTAGGATT AGACATCAAC TCAGTTCTAA ACTGGATTCG AAACCTAAAC   
  
  
+ CCGAAGATCG TAACTTTAGT AGAACAAGAG GCGAACCATA ACCAACCTGA ATTTCTCCCC CGATTCACTG   
  
  
+ AAGCACTATG TTACTACTCA ACTATGTTCG ACTCATTAGA GACTTGTCAA GTCCAACCGC TGAAAGTCCT   
  
  
+ AGCCGAGATG TATATACAAA AGGAGATATC CAACCTAGTG TGCTACGAAG GCTCAGCCCG GGTCCAGAGG   
  
  
+ CACGAGCCGC TCGCCAAATG GCAAGCTCGG ATGGGCCAAG CCGGGTTCAA GCCGCTAGGC CTGAGTCAAA   
  
  
+ ATGCCCTTAG GCAAGCTAAC ATGTTGGTAA CATTGTTCTC CTCACAAGGG TATGACGTGG AGGAGAGAGA   
  
  
+ AGGGTGTTTG ACACTGGGCT GGCATAACCG CCCTCTCATA GTGACTTCGA CTTGGCAAGT GGGGCCCAAA   
  
  
+ ATGGACAGTC CAAGTGTGAC TCATGAGATG TCATCATCCT CAGCTTCTTC ATCTTA  

- +Up\_Stream \_Len000GTAAAA GTTAATCTTT AAAGAAGTTA ATATATGGTA GAGGTTTTTA TCAGGAGTAT   
  
  
- TCAACCGTCC ACTTACTAGG ATTACCTTTT AATGAAAATT GACAACTTAA ACGTTATCCC ATGGCTTAAA   
  
  
- CACTTCAAAT AACGGCGTGC ACGGATCGAA CACTGAAGAG CTATTAGTTT TTAATTAATC TCTCTCTGTC   
  
  
- GATTTCAAAA CTCTTATTAT GACTTTTTAA TGTAATTTAA AATAAATACA AATAAAAGTA TGCGATACAA   
  
  
- ATTGACGATA TGCGACACAA ATTGACGTAG AGGAAGATAT AAACTATTTA ACCATTTTTT TTTATTAAGT   
  
  
- TTTAATTTCT GATCATTATA AAAGTGAATA GACTATCTAA ATATTCTTTA TAAATCTATC GATTTATATA   
  
  
- AGAGAGGACT TTCTCCTCTG ACTATCAACT TAAAGTATCC TATATTTGTA CATACATGTA ACTTGCTTAG   
  
  
- AAAAAAGATA CCACTAGCAA TATTTCAATA AATAATGACA TTTGTAATTC ACTATTAATC CTATACAAGA   
  
  
- TAAAGTGCAA AATCTATCTA TCTATCTACA TTCCTCAACA TTCTTCGACA TTCTTATACA TTCCTCGACA   
  
  
- TTCCTCTACA TTCCTCGACA TTCTTCTACA TTCTCTTTGA CATTCTTCGA TGTTCCTATC TACATTGCTC   
  
  
- AACAACTTAA CAACTTCTCA ACATATCACA TTAAACAAAA TGCTACTTGC AAAACCTACT TTTTATTATT   
  
  
- ATAATTTTAT TATACGAATA ATTTTTATTT ATTTATTAAT TCAATTAATG TGTTGTTATT TTATATAATT   
  
  
- AGGGGTATTT AAATCACTTA TTTTGAAGCA GGTGAACTCT ACTAAAAAAT ATACTTGTGA TCAAACTATC   
  
  
- TTATTTGTTT CATATTTTTT TTTATTTCAA AAAACTGTTT TAACTTATGT TGAGAAGTTT ATTTAAGGAT   
  
  
- AAAATTCATT TATTAAAATT TATATTTATT TAAATACCAG TAGATATTAT TATATATAAA ATACAGCTTT   
  
  
- GAGTTTATAT TAAATGTAGC AATATAATAT AAAAATACAA ATACATTTAC AACACAAGTA TTCTATAAAT   
  
  
- ATTTAAAACA TATAACCATC ATAGTAATTT TTTTTATATG TATAAATACA TTGATTTTAT TTACTTACAA   
  
  
- TATCTTTTAG ATATTCTACA TTCCTCTACA TACTTCTACA TTCCTCTACA TTCTTCTATA TTCCTCTACA   
  
  
- TCTTTTACAT TCTTCTACAC TCTTCGACAT TCCTCTACAT TCAATAAGTG ATTTAGGATT TTTTACGCTT   
  
  
- TATCTTGTCC CCGAATTAAC TCGTGAGTTC TACGTATTAA ACACTGCCTG GTTTACCTAT TTATATCTGG   
  
  
- ACCGTTTACC CAATAAGCTA AATCCATGCT CATACCAATT CAAACCCAAA CCAATACAAA GTCCTATTCA   
  
  
- CTGTAAAGCT GAGTTAAACC AAAACCAACA TACTTGAAGC CGAGCTCTAA AAACCAAACC GGTTATGTGA   
  
  
- ATAGTACGTT TTTATTCATT GAAATATCTT TTAAAACCTA GCCTATACTA CGCCAAGCCT AGTTAAGCCT   
  
  
- AAAACCAAGT GTAAGACCAT TTGGATAAAG CCTACAGTTC ATCCTATACT CAGGTCTAGC AACCTAAAGC   
  
  
- CCATAGTCGA GTTAAAATGG TCTAGATCTA TTTAGTATTT ACATGCCCAA ATAAAAAAGG TCGAACTTAC   
  
  
- TAAAAGAAAT AATTTTTTTC TATCTAAAAT CTATTTTGTG TTGTAAAGTA AAAGTAAACA AGGGGGGATA   
  
  
- TTTAAAGACA CTGGTGGAAG TGAAAGAACG GGGTAAGGGG GGGGGGGGGG CGAGAGAGAG AGAGAGAGAG   
  
  
- AGAAGGGAAA TGTTTCCGTG GTAAGTTTAA GTTTGGTGAG AAAAATTTTG GGGTTTTTAA TAAGAGTTTT   
  
  
- ATTATGGAGT ATTATATTTA CCCAAGCATA GTGGTATATA CCCGGAGTAG AGAGTACCCT TGTATGCTTA   
  
  
- AGTTACCGAA CGGGCTATCA ATAGCCCAGT ACGGTAGCCT CGGGGCCGTT GATTAGTTAC CAGTTCGGAG   
  
  
- TCTAAACCGG AGAAACTCGG TAATATCATT GTGGAAAGAG AGACTTGAGG GGGGAGATGG GTGAGACCCT   
  
  
- GGGCCTAGGC TGGGCCCAGA TAGCACAGGC CCAGGACGAG ACTCAGGTTA CCTCACCTAA CCACTGCGCA   
  
  
- CCTGTGTGGT CTAACTTGTA CTAGACGGTG CAGTCGTCTC AGTTGCCGAC AACTGACTAC CACTGTACCC   
  
  
- CCTCCTCCTA AGGCCATATT CCAACCACGT ACGGAACGAC TGCACGCGTC TCCGGCAGGT TACGCCGCTC   
  
  
- GAGGCTGACC GACGAAGCGA TCAACTACTC TACTGGTTAC CCGACAACGG TGCACAATCA TGCCACACAC   
  
  
- CCTAGCCGTT CCATCGGCCG ATAAAACAAC TCCGAAACTC GGCCGCGGAC AAAGTCGGGC CGGGCCTTTG   
  
  
- ACCGGGCTGG ACCGGCCCGA GCCTCAAGCT CCGACTCCAC AACATAGCAG TGAAGATACT CCGAACGGGA   
  
  
- ATAGAGTTTA AACGAGTGAA GTGCCGATTA GTTCGGTAGA ACCTCCGTAA ACTCCCGGTA CTAATACAGG   
  
  
- TGCAGTAGCT GAAGCCGAAG TACTGGCCGA ACGTTACCGG CCGAAACTAA GTTCGGGACC GAAATTCCGG   
  
  
- GCCACCCGGT GGTAGAGAAT CCGAGTGACC CTAACCGGGC AGAAATCCTT CACCCGGGCT TAGCTATGCA   
  
  
- CTTTAGCCCA ACTCCGATCG GGTTAACCGG GCTAGGTACT TGTACTCCAA GCGTAAAGCC CGACATCGTT   
  
  
- GAAGAGCCGA GCTCCTGCAC TTCGGCAGTT ACGAGCCTTA CCTGGGATCT CTTCGGCAGC GATACTTAAG   
  
  
- GCAGTACGTA GAAGTAGCTG AAAATCCTAA TCTGTAGTTG AGTCAAGATT TGACCTAAGC TTTGGATTTG   
  
  
- GGCTTCTAGC ATTGAAATCA TCTTGTTCTC CGCTTGGTAT TGGTTGGACT TAAAGAGGGG GCTAAGTGAC   
  
  
- TTCGTGATAC AATGATGAGT TGATACAAGC TGAGTAATCT CTGAACAGTT CAGGTTGGCG ACTTTCAGGA   
  
  
- TCGGCTCTAC ATATATGTTT TCCTCTATAG GTTGGATCAC ACGATGCTTC CGAGTCGGGC CCAGGTCTCC   
  
  
- GTGCTCGGCG AGCGGTTTAC CGTTCGAGCC TACCCGGTTC GGCCCAAGTT CGGCGATCCG GACTCAGTTT   
  
  
- TACGGGAATC CGTTCGATTG TACAACCATT GTAACAAGAG GAGTGTTCCC ATACTGCACC TCCTCTCTCT   
  
  
- TCCCACAAAC TGTGACCCGA CCGTATTGGC GGGAGAGTAT CACTGAAGCT GAACCGTTCA CCCCGGGTTT   
  
  
- TACCTGTCAG GTTCACACTG AGTACTCTAC AGTAGTAGGA GTCGAAGAAG TAGAAT

+     CCGTCC motif

| Site Name | Organism | Position | Strand | Matrix score. | sequence | function |
| --- | --- | --- | --- | --- | --- | --- |
| CCGTCC motif | Nicotiana tabacum | 2368 | + | 6 | CCGTCC |  |

>HU03G02797.1   
+ +Up\_Stream \_Len000CATTTT CAATTAGAAA TTTCTTCAAT TATATACCAT CTCCAAAAAT AGTCCTCATA   
  
  
+ AGTTGGCAGG TGAATGATCC TAATGGAAAA TTACTTTTAA CTGTTGAATT TGCAATAGGG TACCGAATTT   
  
  
+ GTGAAGTTTA TTGCCGCACG TGCCTAGCTT GTGACTTCTC GATAATCAAA AATTAATTAG AGAGAGACAG   
  
  
+ CTAAAGTTTT GAGAATAATA CTGAAAAATT ACATTAAATT TTATTTATGT TTATTTTCAT ACGCTATGTT   
  
  
+ TAACTGCTAT ACGCTGTGTT TAACTGCATC TCCTTCTATA TTTGATAAAT TGGTAAAAAA AAATAATTCA   
  
  
+ AAATTAAAGA CTAGTAATAT TTTCACTTAT CTGATAGATT TATAAGAAAT ATTTAGATAG CTAAATATAT   
  
  
+ TCTCTCCTGA AAGAGGAGAC TGATAGTTGA ATTTCATAGG ATATAAACAT GTATGTACAT TGAACGAATC   
  
  
+ TTTTTTCTAT GGTGATCGTT ATAAAGTTAT TTATTACTGT AAACATTAAG TGATAATTAG GATATGTTCT   
  
  
+ ATTTCACGTT TTAGATAGAT AGATAGATGT AAGGAGTTGT AAGAAGCTGT AAGAATATGT AAGGAGCTGT   
  
  
+ AAGGAGATGT AAGGAGCTGT AAGAAGATGT AAGAGAAACT GTAAGAAGCT ACAAGGATAG ATGTAACGAG   
  
  
+ TTGTTGAATT GTTGAAGAGT TGTATAGTGT AATTTGTTTT ACGATGAACG TTTTGGATGA AAAATAATAA   
  
  
+ TATTAAAATA ATATGCTTAT TAAAAATAAA TAAATAATTA AGTTAATTAC ACAACAATAA AATATATTAA   
  
  
+ TCCCCATAAA TTTAGTGAAT AAAACTTCGT CCACTTGAGA TGATTTTTTA TATGAACACT AGTTTGATAG   
  
  
+ AATAAACAAA GTATAAAAAA AAATAAAGTT TTTTGACAAA ATTGAATACA ACTCTTCAAA TAAATTCCTA   
  
  
+ TTTTAAGTAA ATAATTTTAA ATATAAATAA ATTTATGGTC ATCTATAATA ATATATATTT TATGTCGAAA   
  
  
+ CTCAAATATA ATTTACATCG TTATATTATA TTTTTATGTT TATGTAAATG TTGTGTTCAT AAGATATTTA   
  
  
+ TAAATTTTGT ATATTGGTAG TATCATTAAA AAAAATATAC ATATTTATGT AACTAAAATA AATGAATGTT   
  
  
+ ATAGAAAATC TATAAGATGT AAGGAGATGT ATGAAGATGT AAGGAGATGT AAGAAGATAT AAGGAGATGT   
  
  
+ AGAAAATGTA AGAAGATGTG AGAAGCTGTA AGGAGATGTA AGTTATTCAC TAAATCCTAA AAAATGCGAA   
  
  
+ ATAGAACAGG GGCTTAATTG AGCACTCAAG ATGCATAATT TGTGACGGAC CAAATGGATA AATATAGACC   
  
  
+ TGGCAAATGG GTTATTCGAT TTAGGTACGA GTATGGTTAA GTTTGGGTTT GGTTATGTTT CAGGATAAGT   
  
  
+ GACATTTCGA CTCAATTTGG TTTTGGTTGT ATGAACTTCG GCTCGAGATT TTTGGTTTGG CCAATACACT   
  
  
+ TATCATGCAA AAATAAGTAA CTTTATAGAA AATTTTGGAT CGGATATGAT GCGGTTCGGA TCAATTCGGA   
  
  
+ TTTTGGTTCA CATTCTGGTA AACCTATTTC GGATGTCAAG TAGGATATGA GTCCAGATCG TTGGATTTCG   
  
  
+ GGTATCAGCT CAATTTTACC AGATCTAGAT AAATCATAAA TGTACGGGTT TATTTTTTCC AGCTTGAATG   
  
  
+ ATTTTCTTTA TTAAAAAAAG ATAGATTTTA GATAAAACAC AACATTTCAT TTTCATTTGT TCCCCCCTAT   
  
  
+ AAATTTCTGT GACCACCTTC ACTTTCTTGC CCCATTCCCC CCCCCCCCCC GCTCTCTCTC TCTCTCTCTC   
  
  
+ TCTTCCCTTT ACAAAGGCAC CATTCAAATT CAAACCACTC TTTTTAAAAC CCCAAAAATT ATTCTCAAAA   
  
  
+ TAATACCTCA TAATATAAAT GGGTTCGTAT CACCATATAT GGGCCTCATC TCTCATGGGA ACATACGAAT   
  
  
+ TCAATGGCTT GCCCGATAGT TATCGGGTCA TGCCATCGGA GCCCCGGCAA CTAATCAATG GTCAAGCCTC   
  
  
+ AGATTTGGCC TCTTTGAGCC ATTATAGTAA CACCTTTCTC TCTGAACTCC CCCCTCTACC CACTCTGGGA   
  
  
+ CCCGGATCCG ACCCGGGTCT ATCGTGTCCG GGTCCTGCTC TGAGTCCAAT GGAGTGGATT GGTGACGCGT   
  
  
+ GGACACACCA GATTGAACAT GATCTGCCAC GTCAGCAGAG TCAACGGCTG TTGACTGATG GTGACATGGG   
  
  
+ GGAGGAGGAT TCCGGTATAA GGTTGGTGCA TGCCTTGCTG ACGTGCGCAG AGGCCGTCCA ATGCGGCGAG   
  
  
+ CTCCGACTGG CTGCTTCGCT AGTTGATGAG ATGACCAATG GGCTGTTGCC ACGTGTTAGT ACGGTGTGTG   
  
  
+ GGATCGGCAA GGTAGCCGGC TATTTTGTTG AGGCTTTGAG CCGGCGCCTG TTTCAGCCCG GCCCGGAAAC   
  
  
+ TGGCCCGACC TGGCCGGGCT CGGAGTTCGA GGCTGAGGTG TTGTATCGTC ACTTCTATGA GGCTTGCCCT   
  
  
+ TATCTCAAAT TTGCTCACTT CACGGCTAAT CAAGCCATCT TGGAGGCATT TGAGGGCCAT GATTATGTCC   
  
  
+ ACGTCATCGA CTTCGGCTTC ATGACCGGCT TGCAATGGCC GGCTTTGATT CAAGCCCTGG CTTTAAGGCC   
  
  
+ CGGTGGGCCA CCATCTCTTA GGCTCACTGG GATTGGCCCG TCTTTAGGAA GTGGGCCCGA ATCGATACGT   
  
  
+ GAAATCGGGT TGAGGCTAGC CCAATTGGCC CGATCCATGA ACATGAGGTT CGCATTTCGG GCTGTAGCAA   
  
  
+ CTTCTCGGCT CGAGGACGTG AAGCCGTCAA TGCTCGGAAT GGACCCTAGA GAAGCCGTCG CTATGAATTC   
  
  
+ CGTCATGCAT CTTCATCGAC TTTTAGGATT AGACATCAAC TCAGTTCTAA ACTGGATTCG AAACCTAAAC   
  
  
+ CCGAAGATCG TAACTTTAGT AGAACAAGAG GCGAACCATA ACCAACCTGA ATTTCTCCCC CGATTCACTG   
  
  
+ AAGCACTATG TTACTACTCA ACTATGTTCG ACTCATTAGA GACTTGTCAA GTCCAACCGC TGAAAGTCCT   
  
  
+ AGCCGAGATG TATATACAAA AGGAGATATC CAACCTAGTG TGCTACGAAG GCTCAGCCCG GGTCCAGAGG   
  
  
+ CACGAGCCGC TCGCCAAATG GCAAGCTCGG ATGGGCCAAG CCGGGTTCAA GCCGCTAGGC CTGAGTCAAA   
  
  
+ ATGCCCTTAG GCAAGCTAAC ATGTTGGTAA CATTGTTCTC CTCACAAGGG TATGACGTGG AGGAGAGAGA   
  
  
+ AGGGTGTTTG ACACTGGGCT GGCATAACCG CCCTCTCATA GTGACTTCGA CTTGGCAAGT GGGGCCCAAA   
  
  
+ ATGGACAGTC CAAGTGTGAC TCATGAGATG TCATCATCCT CAGCTTCTTC ATCTTA  

- +Up\_Stream \_Len000GTAAAA GTTAATCTTT AAAGAAGTTA ATATATGGTA GAGGTTTTTA TCAGGAGTAT   
  
  
- TCAACCGTCC ACTTACTAGG ATTACCTTTT AATGAAAATT GACAACTTAA ACGTTATCCC ATGGCTTAAA   
  
  
- CACTTCAAAT AACGGCGTGC ACGGATCGAA CACTGAAGAG CTATTAGTTT TTAATTAATC TCTCTCTGTC   
  
  
- GATTTCAAAA CTCTTATTAT GACTTTTTAA TGTAATTTAA AATAAATACA AATAAAAGTA TGCGATACAA   
  
  
- ATTGACGATA TGCGACACAA ATTGACGTAG AGGAAGATAT AAACTATTTA ACCATTTTTT TTTATTAAGT   
  
  
- TTTAATTTCT GATCATTATA AAAGTGAATA GACTATCTAA ATATTCTTTA TAAATCTATC GATTTATATA   
  
  
- AGAGAGGACT TTCTCCTCTG ACTATCAACT TAAAGTATCC TATATTTGTA CATACATGTA ACTTGCTTAG   
  
  
- AAAAAAGATA CCACTAGCAA TATTTCAATA AATAATGACA TTTGTAATTC ACTATTAATC CTATACAAGA   
  
  
- TAAAGTGCAA AATCTATCTA TCTATCTACA TTCCTCAACA TTCTTCGACA TTCTTATACA TTCCTCGACA   
  
  
- TTCCTCTACA TTCCTCGACA TTCTTCTACA TTCTCTTTGA CATTCTTCGA TGTTCCTATC TACATTGCTC   
  
  
- AACAACTTAA CAACTTCTCA ACATATCACA TTAAACAAAA TGCTACTTGC AAAACCTACT TTTTATTATT   
  
  
- ATAATTTTAT TATACGAATA ATTTTTATTT ATTTATTAAT TCAATTAATG TGTTGTTATT TTATATAATT   
  
  
- AGGGGTATTT AAATCACTTA TTTTGAAGCA GGTGAACTCT ACTAAAAAAT ATACTTGTGA TCAAACTATC   
  
  
- TTATTTGTTT CATATTTTTT TTTATTTCAA AAAACTGTTT TAACTTATGT TGAGAAGTTT ATTTAAGGAT   
  
  
- AAAATTCATT TATTAAAATT TATATTTATT TAAATACCAG TAGATATTAT TATATATAAA ATACAGCTTT   
  
  
- GAGTTTATAT TAAATGTAGC AATATAATAT AAAAATACAA ATACATTTAC AACACAAGTA TTCTATAAAT   
  
  
- ATTTAAAACA TATAACCATC ATAGTAATTT TTTTTATATG TATAAATACA TTGATTTTAT TTACTTACAA   
  
  
- TATCTTTTAG ATATTCTACA TTCCTCTACA TACTTCTACA TTCCTCTACA TTCTTCTATA TTCCTCTACA   
  
  
- TCTTTTACAT TCTTCTACAC TCTTCGACAT TCCTCTACAT TCAATAAGTG ATTTAGGATT TTTTACGCTT   
  
  
- TATCTTGTCC CCGAATTAAC TCGTGAGTTC TACGTATTAA ACACTGCCTG GTTTACCTAT TTATATCTGG   
  
  
- ACCGTTTACC CAATAAGCTA AATCCATGCT CATACCAATT CAAACCCAAA CCAATACAAA GTCCTATTCA   
  
  
- CTGTAAAGCT GAGTTAAACC AAAACCAACA TACTTGAAGC CGAGCTCTAA AAACCAAACC GGTTATGTGA   
  
  
- ATAGTACGTT TTTATTCATT GAAATATCTT TTAAAACCTA GCCTATACTA CGCCAAGCCT AGTTAAGCCT   
  
  
- AAAACCAAGT GTAAGACCAT TTGGATAAAG CCTACAGTTC ATCCTATACT CAGGTCTAGC AACCTAAAGC   
  
  
- CCATAGTCGA GTTAAAATGG TCTAGATCTA TTTAGTATTT ACATGCCCAA ATAAAAAAGG TCGAACTTAC   
  
  
- TAAAAGAAAT AATTTTTTTC TATCTAAAAT CTATTTTGTG TTGTAAAGTA AAAGTAAACA AGGGGGGATA   
  
  
- TTTAAAGACA CTGGTGGAAG TGAAAGAACG GGGTAAGGGG GGGGGGGGGG CGAGAGAGAG AGAGAGAGAG   
  
  
- AGAAGGGAAA TGTTTCCGTG GTAAGTTTAA GTTTGGTGAG AAAAATTTTG GGGTTTTTAA TAAGAGTTTT   
  
  
- ATTATGGAGT ATTATATTTA CCCAAGCATA GTGGTATATA CCCGGAGTAG AGAGTACCCT TGTATGCTTA   
  
  
- AGTTACCGAA CGGGCTATCA ATAGCCCAGT ACGGTAGCCT CGGGGCCGTT GATTAGTTAC CAGTTCGGAG   
  
  
- TCTAAACCGG AGAAACTCGG TAATATCATT GTGGAAAGAG AGACTTGAGG GGGGAGATGG GTGAGACCCT   
  
  
- GGGCCTAGGC TGGGCCCAGA TAGCACAGGC CCAGGACGAG ACTCAGGTTA CCTCACCTAA CCACTGCGCA   
  
  
- CCTGTGTGGT CTAACTTGTA CTAGACGGTG CAGTCGTCTC AGTTGCCGAC AACTGACTAC CACTGTACCC   
  
  
- CCTCCTCCTA AGGCCATATT CCAACCACGT ACGGAACGAC TGCACGCGTC TCCGGCAGGT TACGCCGCTC   
  
  
- GAGGCTGACC GACGAAGCGA TCAACTACTC TACTGGTTAC CCGACAACGG TGCACAATCA TGCCACACAC   
  
  
- CCTAGCCGTT CCATCGGCCG ATAAAACAAC TCCGAAACTC GGCCGCGGAC AAAGTCGGGC CGGGCCTTTG   
  
  
- ACCGGGCTGG ACCGGCCCGA GCCTCAAGCT CCGACTCCAC AACATAGCAG TGAAGATACT CCGAACGGGA   
  
  
- ATAGAGTTTA AACGAGTGAA GTGCCGATTA GTTCGGTAGA ACCTCCGTAA ACTCCCGGTA CTAATACAGG   
  
  
- TGCAGTAGCT GAAGCCGAAG TACTGGCCGA ACGTTACCGG CCGAAACTAA GTTCGGGACC GAAATTCCGG   
  
  
- GCCACCCGGT GGTAGAGAAT CCGAGTGACC CTAACCGGGC AGAAATCCTT CACCCGGGCT TAGCTATGCA   
  
  
- CTTTAGCCCA ACTCCGATCG GGTTAACCGG GCTAGGTACT TGTACTCCAA GCGTAAAGCC CGACATCGTT   
  
  
- GAAGAGCCGA GCTCCTGCAC TTCGGCAGTT ACGAGCCTTA CCTGGGATCT CTTCGGCAGC GATACTTAAG   
  
  
- GCAGTACGTA GAAGTAGCTG AAAATCCTAA TCTGTAGTTG AGTCAAGATT TGACCTAAGC TTTGGATTTG   
  
  
- GGCTTCTAGC ATTGAAATCA TCTTGTTCTC CGCTTGGTAT TGGTTGGACT TAAAGAGGGG GCTAAGTGAC   
  
  
- TTCGTGATAC AATGATGAGT TGATACAAGC TGAGTAATCT CTGAACAGTT CAGGTTGGCG ACTTTCAGGA   
  
  
- TCGGCTCTAC ATATATGTTT TCCTCTATAG GTTGGATCAC ACGATGCTTC CGAGTCGGGC CCAGGTCTCC   
  
  
- GTGCTCGGCG AGCGGTTTAC CGTTCGAGCC TACCCGGTTC GGCCCAAGTT CGGCGATCCG GACTCAGTTT   
  
  
- TACGGGAATC CGTTCGATTG TACAACCATT GTAACAAGAG GAGTGTTCCC ATACTGCACC TCCTCTCTCT   
  
  
- TCCCACAAAC TGTGACCCGA CCGTATTGGC GGGAGAGTAT CACTGAAGCT GAACCGTTCA CCCCGGGTTT   
  
  
- TACCTGTCAG GTTCACACTG AGTACTCTAC AGTAGTAGGA GTCGAAGAAG TAGAAT

+     CCGTCC-box

| Site Name | Organism | Position | Strand | Matrix score. | sequence | function |
| --- | --- | --- | --- | --- | --- | --- |
| CCGTCC-box | Petroselinum hortense | 2368 | + | 6 | CCGTCC |  |

>HU03G02797.1   
+ +Up\_Stream \_Len000CATTTT CAATTAGAAA TTTCTTCAAT TATATACCAT CTCCAAAAAT AGTCCTCATA   
  
  
+ AGTTGGCAGG TGAATGATCC TAATGGAAAA TTACTTTTAA CTGTTGAATT TGCAATAGGG TACCGAATTT   
  
  
+ GTGAAGTTTA TTGCCGCACG TGCCTAGCTT GTGACTTCTC GATAATCAAA AATTAATTAG AGAGAGACAG   
  
  
+ CTAAAGTTTT GAGAATAATA CTGAAAAATT ACATTAAATT TTATTTATGT TTATTTTCAT ACGCTATGTT   
  
  
+ TAACTGCTAT ACGCTGTGTT TAACTGCATC TCCTTCTATA TTTGATAAAT TGGTAAAAAA AAATAATTCA   
  
  
+ AAATTAAAGA CTAGTAATAT TTTCACTTAT CTGATAGATT TATAAGAAAT ATTTAGATAG CTAAATATAT   
  
  
+ TCTCTCCTGA AAGAGGAGAC TGATAGTTGA ATTTCATAGG ATATAAACAT GTATGTACAT TGAACGAATC   
  
  
+ TTTTTTCTAT GGTGATCGTT ATAAAGTTAT TTATTACTGT AAACATTAAG TGATAATTAG GATATGTTCT   
  
  
+ ATTTCACGTT TTAGATAGAT AGATAGATGT AAGGAGTTGT AAGAAGCTGT AAGAATATGT AAGGAGCTGT   
  
  
+ AAGGAGATGT AAGGAGCTGT AAGAAGATGT AAGAGAAACT GTAAGAAGCT ACAAGGATAG ATGTAACGAG   
  
  
+ TTGTTGAATT GTTGAAGAGT TGTATAGTGT AATTTGTTTT ACGATGAACG TTTTGGATGA AAAATAATAA   
  
  
+ TATTAAAATA ATATGCTTAT TAAAAATAAA TAAATAATTA AGTTAATTAC ACAACAATAA AATATATTAA   
  
  
+ TCCCCATAAA TTTAGTGAAT AAAACTTCGT CCACTTGAGA TGATTTTTTA TATGAACACT AGTTTGATAG   
  
  
+ AATAAACAAA GTATAAAAAA AAATAAAGTT TTTTGACAAA ATTGAATACA ACTCTTCAAA TAAATTCCTA   
  
  
+ TTTTAAGTAA ATAATTTTAA ATATAAATAA ATTTATGGTC ATCTATAATA ATATATATTT TATGTCGAAA   
  
  
+ CTCAAATATA ATTTACATCG TTATATTATA TTTTTATGTT TATGTAAATG TTGTGTTCAT AAGATATTTA   
  
  
+ TAAATTTTGT ATATTGGTAG TATCATTAAA AAAAATATAC ATATTTATGT AACTAAAATA AATGAATGTT   
  
  
+ ATAGAAAATC TATAAGATGT AAGGAGATGT ATGAAGATGT AAGGAGATGT AAGAAGATAT AAGGAGATGT   
  
  
+ AGAAAATGTA AGAAGATGTG AGAAGCTGTA AGGAGATGTA AGTTATTCAC TAAATCCTAA AAAATGCGAA   
  
  
+ ATAGAACAGG GGCTTAATTG AGCACTCAAG ATGCATAATT TGTGACGGAC CAAATGGATA AATATAGACC   
  
  
+ TGGCAAATGG GTTATTCGAT TTAGGTACGA GTATGGTTAA GTTTGGGTTT GGTTATGTTT CAGGATAAGT   
  
  
+ GACATTTCGA CTCAATTTGG TTTTGGTTGT ATGAACTTCG GCTCGAGATT TTTGGTTTGG CCAATACACT   
  
  
+ TATCATGCAA AAATAAGTAA CTTTATAGAA AATTTTGGAT CGGATATGAT GCGGTTCGGA TCAATTCGGA   
  
  
+ TTTTGGTTCA CATTCTGGTA AACCTATTTC GGATGTCAAG TAGGATATGA GTCCAGATCG TTGGATTTCG   
  
  
+ GGTATCAGCT CAATTTTACC AGATCTAGAT AAATCATAAA TGTACGGGTT TATTTTTTCC AGCTTGAATG   
  
  
+ ATTTTCTTTA TTAAAAAAAG ATAGATTTTA GATAAAACAC AACATTTCAT TTTCATTTGT TCCCCCCTAT   
  
  
+ AAATTTCTGT GACCACCTTC ACTTTCTTGC CCCATTCCCC CCCCCCCCCC GCTCTCTCTC TCTCTCTCTC   
  
  
+ TCTTCCCTTT ACAAAGGCAC CATTCAAATT CAAACCACTC TTTTTAAAAC CCCAAAAATT ATTCTCAAAA   
  
  
+ TAATACCTCA TAATATAAAT GGGTTCGTAT CACCATATAT GGGCCTCATC TCTCATGGGA ACATACGAAT   
  
  
+ TCAATGGCTT GCCCGATAGT TATCGGGTCA TGCCATCGGA GCCCCGGCAA CTAATCAATG GTCAAGCCTC   
  
  
+ AGATTTGGCC TCTTTGAGCC ATTATAGTAA CACCTTTCTC TCTGAACTCC CCCCTCTACC CACTCTGGGA   
  
  
+ CCCGGATCCG ACCCGGGTCT ATCGTGTCCG GGTCCTGCTC TGAGTCCAAT GGAGTGGATT GGTGACGCGT   
  
  
+ GGACACACCA GATTGAACAT GATCTGCCAC GTCAGCAGAG TCAACGGCTG TTGACTGATG GTGACATGGG   
  
  
+ GGAGGAGGAT TCCGGTATAA GGTTGGTGCA TGCCTTGCTG ACGTGCGCAG AGGCCGTCCA ATGCGGCGAG   
  
  
+ CTCCGACTGG CTGCTTCGCT AGTTGATGAG ATGACCAATG GGCTGTTGCC ACGTGTTAGT ACGGTGTGTG   
  
  
+ GGATCGGCAA GGTAGCCGGC TATTTTGTTG AGGCTTTGAG CCGGCGCCTG TTTCAGCCCG GCCCGGAAAC   
  
  
+ TGGCCCGACC TGGCCGGGCT CGGAGTTCGA GGCTGAGGTG TTGTATCGTC ACTTCTATGA GGCTTGCCCT   
  
  
+ TATCTCAAAT TTGCTCACTT CACGGCTAAT CAAGCCATCT TGGAGGCATT TGAGGGCCAT GATTATGTCC   
  
  
+ ACGTCATCGA CTTCGGCTTC ATGACCGGCT TGCAATGGCC GGCTTTGATT CAAGCCCTGG CTTTAAGGCC   
  
  
+ CGGTGGGCCA CCATCTCTTA GGCTCACTGG GATTGGCCCG TCTTTAGGAA GTGGGCCCGA ATCGATACGT   
  
  
+ GAAATCGGGT TGAGGCTAGC CCAATTGGCC CGATCCATGA ACATGAGGTT CGCATTTCGG GCTGTAGCAA   
  
  
+ CTTCTCGGCT CGAGGACGTG AAGCCGTCAA TGCTCGGAAT GGACCCTAGA GAAGCCGTCG CTATGAATTC   
  
  
+ CGTCATGCAT CTTCATCGAC TTTTAGGATT AGACATCAAC TCAGTTCTAA ACTGGATTCG AAACCTAAAC   
  
  
+ CCGAAGATCG TAACTTTAGT AGAACAAGAG GCGAACCATA ACCAACCTGA ATTTCTCCCC CGATTCACTG   
  
  
+ AAGCACTATG TTACTACTCA ACTATGTTCG ACTCATTAGA GACTTGTCAA GTCCAACCGC TGAAAGTCCT   
  
  
+ AGCCGAGATG TATATACAAA AGGAGATATC CAACCTAGTG TGCTACGAAG GCTCAGCCCG GGTCCAGAGG   
  
  
+ CACGAGCCGC TCGCCAAATG GCAAGCTCGG ATGGGCCAAG CCGGGTTCAA GCCGCTAGGC CTGAGTCAAA   
  
  
+ ATGCCCTTAG GCAAGCTAAC ATGTTGGTAA CATTGTTCTC CTCACAAGGG TATGACGTGG AGGAGAGAGA   
  
  
+ AGGGTGTTTG ACACTGGGCT GGCATAACCG CCCTCTCATA GTGACTTCGA CTTGGCAAGT GGGGCCCAAA   
  
  
+ ATGGACAGTC CAAGTGTGAC TCATGAGATG TCATCATCCT CAGCTTCTTC ATCTTA  

- +Up\_Stream \_Len000GTAAAA GTTAATCTTT AAAGAAGTTA ATATATGGTA GAGGTTTTTA TCAGGAGTAT   
  
  
- TCAACCGTCC ACTTACTAGG ATTACCTTTT AATGAAAATT GACAACTTAA ACGTTATCCC ATGGCTTAAA   
  
  
- CACTTCAAAT AACGGCGTGC ACGGATCGAA CACTGAAGAG CTATTAGTTT TTAATTAATC TCTCTCTGTC   
  
  
- GATTTCAAAA CTCTTATTAT GACTTTTTAA TGTAATTTAA AATAAATACA AATAAAAGTA TGCGATACAA   
  
  
- ATTGACGATA TGCGACACAA ATTGACGTAG AGGAAGATAT AAACTATTTA ACCATTTTTT TTTATTAAGT   
  
  
- TTTAATTTCT GATCATTATA AAAGTGAATA GACTATCTAA ATATTCTTTA TAAATCTATC GATTTATATA   
  
  
- AGAGAGGACT TTCTCCTCTG ACTATCAACT TAAAGTATCC TATATTTGTA CATACATGTA ACTTGCTTAG   
  
  
- AAAAAAGATA CCACTAGCAA TATTTCAATA AATAATGACA TTTGTAATTC ACTATTAATC CTATACAAGA   
  
  
- TAAAGTGCAA AATCTATCTA TCTATCTACA TTCCTCAACA TTCTTCGACA TTCTTATACA TTCCTCGACA   
  
  
- TTCCTCTACA TTCCTCGACA TTCTTCTACA TTCTCTTTGA CATTCTTCGA TGTTCCTATC TACATTGCTC   
  
  
- AACAACTTAA CAACTTCTCA ACATATCACA TTAAACAAAA TGCTACTTGC AAAACCTACT TTTTATTATT   
  
  
- ATAATTTTAT TATACGAATA ATTTTTATTT ATTTATTAAT TCAATTAATG TGTTGTTATT TTATATAATT   
  
  
- AGGGGTATTT AAATCACTTA TTTTGAAGCA GGTGAACTCT ACTAAAAAAT ATACTTGTGA TCAAACTATC   
  
  
- TTATTTGTTT CATATTTTTT TTTATTTCAA AAAACTGTTT TAACTTATGT TGAGAAGTTT ATTTAAGGAT   
  
  
- AAAATTCATT TATTAAAATT TATATTTATT TAAATACCAG TAGATATTAT TATATATAAA ATACAGCTTT   
  
  
- GAGTTTATAT TAAATGTAGC AATATAATAT AAAAATACAA ATACATTTAC AACACAAGTA TTCTATAAAT   
  
  
- ATTTAAAACA TATAACCATC ATAGTAATTT TTTTTATATG TATAAATACA TTGATTTTAT TTACTTACAA   
  
  
- TATCTTTTAG ATATTCTACA TTCCTCTACA TACTTCTACA TTCCTCTACA TTCTTCTATA TTCCTCTACA   
  
  
- TCTTTTACAT TCTTCTACAC TCTTCGACAT TCCTCTACAT TCAATAAGTG ATTTAGGATT TTTTACGCTT   
  
  
- TATCTTGTCC CCGAATTAAC TCGTGAGTTC TACGTATTAA ACACTGCCTG GTTTACCTAT TTATATCTGG   
  
  
- ACCGTTTACC CAATAAGCTA AATCCATGCT CATACCAATT CAAACCCAAA CCAATACAAA GTCCTATTCA   
  
  
- CTGTAAAGCT GAGTTAAACC AAAACCAACA TACTTGAAGC CGAGCTCTAA AAACCAAACC GGTTATGTGA   
  
  
- ATAGTACGTT TTTATTCATT GAAATATCTT TTAAAACCTA GCCTATACTA CGCCAAGCCT AGTTAAGCCT   
  
  
- AAAACCAAGT GTAAGACCAT TTGGATAAAG CCTACAGTTC ATCCTATACT CAGGTCTAGC AACCTAAAGC   
  
  
- CCATAGTCGA GTTAAAATGG TCTAGATCTA TTTAGTATTT ACATGCCCAA ATAAAAAAGG TCGAACTTAC   
  
  
- TAAAAGAAAT AATTTTTTTC TATCTAAAAT CTATTTTGTG TTGTAAAGTA AAAGTAAACA AGGGGGGATA   
  
  
- TTTAAAGACA CTGGTGGAAG TGAAAGAACG GGGTAAGGGG GGGGGGGGGG CGAGAGAGAG AGAGAGAGAG   
  
  
- AGAAGGGAAA TGTTTCCGTG GTAAGTTTAA GTTTGGTGAG AAAAATTTTG GGGTTTTTAA TAAGAGTTTT   
  
  
- ATTATGGAGT ATTATATTTA CCCAAGCATA GTGGTATATA CCCGGAGTAG AGAGTACCCT TGTATGCTTA   
  
  
- AGTTACCGAA CGGGCTATCA ATAGCCCAGT ACGGTAGCCT CGGGGCCGTT GATTAGTTAC CAGTTCGGAG   
  
  
- TCTAAACCGG AGAAACTCGG TAATATCATT GTGGAAAGAG AGACTTGAGG GGGGAGATGG GTGAGACCCT   
  
  
- GGGCCTAGGC TGGGCCCAGA TAGCACAGGC CCAGGACGAG ACTCAGGTTA CCTCACCTAA CCACTGCGCA   
  
  
- CCTGTGTGGT CTAACTTGTA CTAGACGGTG CAGTCGTCTC AGTTGCCGAC AACTGACTAC CACTGTACCC   
  
  
- CCTCCTCCTA AGGCCATATT CCAACCACGT ACGGAACGAC TGCACGCGTC TCCGGCAGGT TACGCCGCTC   
  
  
- GAGGCTGACC GACGAAGCGA TCAACTACTC TACTGGTTAC CCGACAACGG TGCACAATCA TGCCACACAC   
  
  
- CCTAGCCGTT CCATCGGCCG ATAAAACAAC TCCGAAACTC GGCCGCGGAC AAAGTCGGGC CGGGCCTTTG   
  
  
- ACCGGGCTGG ACCGGCCCGA GCCTCAAGCT CCGACTCCAC AACATAGCAG TGAAGATACT CCGAACGGGA   
  
  
- ATAGAGTTTA AACGAGTGAA GTGCCGATTA GTTCGGTAGA ACCTCCGTAA ACTCCCGGTA CTAATACAGG   
  
  
- TGCAGTAGCT GAAGCCGAAG TACTGGCCGA ACGTTACCGG CCGAAACTAA GTTCGGGACC GAAATTCCGG   
  
  
- GCCACCCGGT GGTAGAGAAT CCGAGTGACC CTAACCGGGC AGAAATCCTT CACCCGGGCT TAGCTATGCA   
  
  
- CTTTAGCCCA ACTCCGATCG GGTTAACCGG GCTAGGTACT TGTACTCCAA GCGTAAAGCC CGACATCGTT   
  
  
- GAAGAGCCGA GCTCCTGCAC TTCGGCAGTT ACGAGCCTTA CCTGGGATCT CTTCGGCAGC GATACTTAAG   
  
  
- GCAGTACGTA GAAGTAGCTG AAAATCCTAA TCTGTAGTTG AGTCAAGATT TGACCTAAGC TTTGGATTTG   
  
  
- GGCTTCTAGC ATTGAAATCA TCTTGTTCTC CGCTTGGTAT TGGTTGGACT TAAAGAGGGG GCTAAGTGAC   
  
  
- TTCGTGATAC AATGATGAGT TGATACAAGC TGAGTAATCT CTGAACAGTT CAGGTTGGCG ACTTTCAGGA   
  
  
- TCGGCTCTAC ATATATGTTT TCCTCTATAG GTTGGATCAC ACGATGCTTC CGAGTCGGGC CCAGGTCTCC   
  
  
- GTGCTCGGCG AGCGGTTTAC CGTTCGAGCC TACCCGGTTC GGCCCAAGTT CGGCGATCCG GACTCAGTTT   
  
  
- TACGGGAATC CGTTCGATTG TACAACCATT GTAACAAGAG GAGTGTTCCC ATACTGCACC TCCTCTCTCT   
  
  
- TCCCACAAAC TGTGACCCGA CCGTATTGGC GGGAGAGTAT CACTGAAGCT GAACCGTTCA CCCCGGGTTT   
  
  
- TACCTGTCAG GTTCACACTG AGTACTCTAC AGTAGTAGGA GTCGAAGAAG TAGAAT

+     CGTCA-motif

| Site Name | Organism | Position | Strand | Matrix score. | sequence | function |
| --- | --- | --- | --- | --- | --- | --- |
| CGTCA-motif | Hordeum vulgare | 3347 | - | 5 | CGTCA | cis-acting regulatory element involved in the MeJA-responsiveness |
| CGTCA-motif | Hordeum vulgare | 2945 | + | 5 | CGTCA | cis-acting regulatory element involved in the MeJA-responsiveness |
| CGTCA-motif | Hordeum vulgare | 2666 | + | 5 | CGTCA | cis-acting regulatory element involved in the MeJA-responsiveness |
| CGTCA-motif | Hordeum vulgare | 2353 | - | 5 | CGTCA | cis-acting regulatory element involved in the MeJA-responsiveness |
| CGTCA-motif | Hordeum vulgare | 1377 | - | 5 | CGTCA | cis-acting regulatory element involved in the MeJA-responsiveness |
| CGTCA-motif | Hordeum vulgare | 2899 | + | 5 | CGTCA | cis-acting regulatory element involved in the MeJA-responsiveness |
| CGTCA-motif | Hordeum vulgare | 2571 | + | 5 | CGTCA | cis-acting regulatory element involved in the MeJA-responsiveness |
| CGTCA-motif | Hordeum vulgare | 2274 | + | 5 | CGTCA | cis-acting regulatory element involved in the MeJA-responsiveness |
| CGTCA-motif | Hordeum vulgare | 2237 | - | 5 | CGTCA | cis-acting regulatory element involved in the MeJA-responsiveness |

>HU03G02797.1   
+ +Up\_Stream \_Len000CATTTT CAATTAGAAA TTTCTTCAAT TATATACCAT CTCCAAAAAT AGTCCTCATA   
  
  
+ AGTTGGCAGG TGAATGATCC TAATGGAAAA TTACTTTTAA CTGTTGAATT TGCAATAGGG TACCGAATTT   
  
  
+ GTGAAGTTTA TTGCCGCACG TGCCTAGCTT GTGACTTCTC GATAATCAAA AATTAATTAG AGAGAGACAG   
  
  
+ CTAAAGTTTT GAGAATAATA CTGAAAAATT ACATTAAATT TTATTTATGT TTATTTTCAT ACGCTATGTT   
  
  
+ TAACTGCTAT ACGCTGTGTT TAACTGCATC TCCTTCTATA TTTGATAAAT TGGTAAAAAA AAATAATTCA   
  
  
+ AAATTAAAGA CTAGTAATAT TTTCACTTAT CTGATAGATT TATAAGAAAT ATTTAGATAG CTAAATATAT   
  
  
+ TCTCTCCTGA AAGAGGAGAC TGATAGTTGA ATTTCATAGG ATATAAACAT GTATGTACAT TGAACGAATC   
  
  
+ TTTTTTCTAT GGTGATCGTT ATAAAGTTAT TTATTACTGT AAACATTAAG TGATAATTAG GATATGTTCT   
  
  
+ ATTTCACGTT TTAGATAGAT AGATAGATGT AAGGAGTTGT AAGAAGCTGT AAGAATATGT AAGGAGCTGT   
  
  
+ AAGGAGATGT AAGGAGCTGT AAGAAGATGT AAGAGAAACT GTAAGAAGCT ACAAGGATAG ATGTAACGAG   
  
  
+ TTGTTGAATT GTTGAAGAGT TGTATAGTGT AATTTGTTTT ACGATGAACG TTTTGGATGA AAAATAATAA   
  
  
+ TATTAAAATA ATATGCTTAT TAAAAATAAA TAAATAATTA AGTTAATTAC ACAACAATAA AATATATTAA   
  
  
+ TCCCCATAAA TTTAGTGAAT AAAACTTCGT CCACTTGAGA TGATTTTTTA TATGAACACT AGTTTGATAG   
  
  
+ AATAAACAAA GTATAAAAAA AAATAAAGTT TTTTGACAAA ATTGAATACA ACTCTTCAAA TAAATTCCTA   
  
  
+ TTTTAAGTAA ATAATTTTAA ATATAAATAA ATTTATGGTC ATCTATAATA ATATATATTT TATGTCGAAA   
  
  
+ CTCAAATATA ATTTACATCG TTATATTATA TTTTTATGTT TATGTAAATG TTGTGTTCAT AAGATATTTA   
  
  
+ TAAATTTTGT ATATTGGTAG TATCATTAAA AAAAATATAC ATATTTATGT AACTAAAATA AATGAATGTT   
  
  
+ ATAGAAAATC TATAAGATGT AAGGAGATGT ATGAAGATGT AAGGAGATGT AAGAAGATAT AAGGAGATGT   
  
  
+ AGAAAATGTA AGAAGATGTG AGAAGCTGTA AGGAGATGTA AGTTATTCAC TAAATCCTAA AAAATGCGAA   
  
  
+ ATAGAACAGG GGCTTAATTG AGCACTCAAG ATGCATAATT TGTGACGGAC CAAATGGATA AATATAGACC   
  
  
+ TGGCAAATGG GTTATTCGAT TTAGGTACGA GTATGGTTAA GTTTGGGTTT GGTTATGTTT CAGGATAAGT   
  
  
+ GACATTTCGA CTCAATTTGG TTTTGGTTGT ATGAACTTCG GCTCGAGATT TTTGGTTTGG CCAATACACT   
  
  
+ TATCATGCAA AAATAAGTAA CTTTATAGAA AATTTTGGAT CGGATATGAT GCGGTTCGGA TCAATTCGGA   
  
  
+ TTTTGGTTCA CATTCTGGTA AACCTATTTC GGATGTCAAG TAGGATATGA GTCCAGATCG TTGGATTTCG   
  
  
+ GGTATCAGCT CAATTTTACC AGATCTAGAT AAATCATAAA TGTACGGGTT TATTTTTTCC AGCTTGAATG   
  
  
+ ATTTTCTTTA TTAAAAAAAG ATAGATTTTA GATAAAACAC AACATTTCAT TTTCATTTGT TCCCCCCTAT   
  
  
+ AAATTTCTGT GACCACCTTC ACTTTCTTGC CCCATTCCCC CCCCCCCCCC GCTCTCTCTC TCTCTCTCTC   
  
  
+ TCTTCCCTTT ACAAAGGCAC CATTCAAATT CAAACCACTC TTTTTAAAAC CCCAAAAATT ATTCTCAAAA   
  
  
+ TAATACCTCA TAATATAAAT GGGTTCGTAT CACCATATAT GGGCCTCATC TCTCATGGGA ACATACGAAT   
  
  
+ TCAATGGCTT GCCCGATAGT TATCGGGTCA TGCCATCGGA GCCCCGGCAA CTAATCAATG GTCAAGCCTC   
  
  
+ AGATTTGGCC TCTTTGAGCC ATTATAGTAA CACCTTTCTC TCTGAACTCC CCCCTCTACC CACTCTGGGA   
  
  
+ CCCGGATCCG ACCCGGGTCT ATCGTGTCCG GGTCCTGCTC TGAGTCCAAT GGAGTGGATT GGTGACGCGT   
  
  
+ GGACACACCA GATTGAACAT GATCTGCCAC GTCAGCAGAG TCAACGGCTG TTGACTGATG GTGACATGGG   
  
  
+ GGAGGAGGAT TCCGGTATAA GGTTGGTGCA TGCCTTGCTG ACGTGCGCAG AGGCCGTCCA ATGCGGCGAG   
  
  
+ CTCCGACTGG CTGCTTCGCT AGTTGATGAG ATGACCAATG GGCTGTTGCC ACGTGTTAGT ACGGTGTGTG   
  
  
+ GGATCGGCAA GGTAGCCGGC TATTTTGTTG AGGCTTTGAG CCGGCGCCTG TTTCAGCCCG GCCCGGAAAC   
  
  
+ TGGCCCGACC TGGCCGGGCT CGGAGTTCGA GGCTGAGGTG TTGTATCGTC ACTTCTATGA GGCTTGCCCT   
  
  
+ TATCTCAAAT TTGCTCACTT CACGGCTAAT CAAGCCATCT TGGAGGCATT TGAGGGCCAT GATTATGTCC   
  
  
+ ACGTCATCGA CTTCGGCTTC ATGACCGGCT TGCAATGGCC GGCTTTGATT CAAGCCCTGG CTTTAAGGCC   
  
  
+ CGGTGGGCCA CCATCTCTTA GGCTCACTGG GATTGGCCCG TCTTTAGGAA GTGGGCCCGA ATCGATACGT   
  
  
+ GAAATCGGGT TGAGGCTAGC CCAATTGGCC CGATCCATGA ACATGAGGTT CGCATTTCGG GCTGTAGCAA   
  
  
+ CTTCTCGGCT CGAGGACGTG AAGCCGTCAA TGCTCGGAAT GGACCCTAGA GAAGCCGTCG CTATGAATTC   
  
  
+ CGTCATGCAT CTTCATCGAC TTTTAGGATT AGACATCAAC TCAGTTCTAA ACTGGATTCG AAACCTAAAC   
  
  
+ CCGAAGATCG TAACTTTAGT AGAACAAGAG GCGAACCATA ACCAACCTGA ATTTCTCCCC CGATTCACTG   
  
  
+ AAGCACTATG TTACTACTCA ACTATGTTCG ACTCATTAGA GACTTGTCAA GTCCAACCGC TGAAAGTCCT   
  
  
+ AGCCGAGATG TATATACAAA AGGAGATATC CAACCTAGTG TGCTACGAAG GCTCAGCCCG GGTCCAGAGG   
  
  
+ CACGAGCCGC TCGCCAAATG GCAAGCTCGG ATGGGCCAAG CCGGGTTCAA GCCGCTAGGC CTGAGTCAAA   
  
  
+ ATGCCCTTAG GCAAGCTAAC ATGTTGGTAA CATTGTTCTC CTCACAAGGG TATGACGTGG AGGAGAGAGA   
  
  
+ AGGGTGTTTG ACACTGGGCT GGCATAACCG CCCTCTCATA GTGACTTCGA CTTGGCAAGT GGGGCCCAAA   
  
  
+ ATGGACAGTC CAAGTGTGAC TCATGAGATG TCATCATCCT CAGCTTCTTC ATCTTA  

- +Up\_Stream \_Len000GTAAAA GTTAATCTTT AAAGAAGTTA ATATATGGTA GAGGTTTTTA TCAGGAGTAT   
  
  
- TCAACCGTCC ACTTACTAGG ATTACCTTTT AATGAAAATT GACAACTTAA ACGTTATCCC ATGGCTTAAA   
  
  
- CACTTCAAAT AACGGCGTGC ACGGATCGAA CACTGAAGAG CTATTAGTTT TTAATTAATC TCTCTCTGTC   
  
  
- GATTTCAAAA CTCTTATTAT GACTTTTTAA TGTAATTTAA AATAAATACA AATAAAAGTA TGCGATACAA   
  
  
- ATTGACGATA TGCGACACAA ATTGACGTAG AGGAAGATAT AAACTATTTA ACCATTTTTT TTTATTAAGT   
  
  
- TTTAATTTCT GATCATTATA AAAGTGAATA GACTATCTAA ATATTCTTTA TAAATCTATC GATTTATATA   
  
  
- AGAGAGGACT TTCTCCTCTG ACTATCAACT TAAAGTATCC TATATTTGTA CATACATGTA ACTTGCTTAG   
  
  
- AAAAAAGATA CCACTAGCAA TATTTCAATA AATAATGACA TTTGTAATTC ACTATTAATC CTATACAAGA   
  
  
- TAAAGTGCAA AATCTATCTA TCTATCTACA TTCCTCAACA TTCTTCGACA TTCTTATACA TTCCTCGACA   
  
  
- TTCCTCTACA TTCCTCGACA TTCTTCTACA TTCTCTTTGA CATTCTTCGA TGTTCCTATC TACATTGCTC   
  
  
- AACAACTTAA CAACTTCTCA ACATATCACA TTAAACAAAA TGCTACTTGC AAAACCTACT TTTTATTATT   
  
  
- ATAATTTTAT TATACGAATA ATTTTTATTT ATTTATTAAT TCAATTAATG TGTTGTTATT TTATATAATT   
  
  
- AGGGGTATTT AAATCACTTA TTTTGAAGCA GGTGAACTCT ACTAAAAAAT ATACTTGTGA TCAAACTATC   
  
  
- TTATTTGTTT CATATTTTTT TTTATTTCAA AAAACTGTTT TAACTTATGT TGAGAAGTTT ATTTAAGGAT   
  
  
- AAAATTCATT TATTAAAATT TATATTTATT TAAATACCAG TAGATATTAT TATATATAAA ATACAGCTTT   
  
  
- GAGTTTATAT TAAATGTAGC AATATAATAT AAAAATACAA ATACATTTAC AACACAAGTA TTCTATAAAT   
  
  
- ATTTAAAACA TATAACCATC ATAGTAATTT TTTTTATATG TATAAATACA TTGATTTTAT TTACTTACAA   
  
  
- TATCTTTTAG ATATTCTACA TTCCTCTACA TACTTCTACA TTCCTCTACA TTCTTCTATA TTCCTCTACA   
  
  
- TCTTTTACAT TCTTCTACAC TCTTCGACAT TCCTCTACAT TCAATAAGTG ATTTAGGATT TTTTACGCTT   
  
  
- TATCTTGTCC CCGAATTAAC TCGTGAGTTC TACGTATTAA ACACTGCCTG GTTTACCTAT TTATATCTGG   
  
  
- ACCGTTTACC CAATAAGCTA AATCCATGCT CATACCAATT CAAACCCAAA CCAATACAAA GTCCTATTCA   
  
  
- CTGTAAAGCT GAGTTAAACC AAAACCAACA TACTTGAAGC CGAGCTCTAA AAACCAAACC GGTTATGTGA   
  
  
- ATAGTACGTT TTTATTCATT GAAATATCTT TTAAAACCTA GCCTATACTA CGCCAAGCCT AGTTAAGCCT   
  
  
- AAAACCAAGT GTAAGACCAT TTGGATAAAG CCTACAGTTC ATCCTATACT CAGGTCTAGC AACCTAAAGC   
  
  
- CCATAGTCGA GTTAAAATGG TCTAGATCTA TTTAGTATTT ACATGCCCAA ATAAAAAAGG TCGAACTTAC   
  
  
- TAAAAGAAAT AATTTTTTTC TATCTAAAAT CTATTTTGTG TTGTAAAGTA AAAGTAAACA AGGGGGGATA   
  
  
- TTTAAAGACA CTGGTGGAAG TGAAAGAACG GGGTAAGGGG GGGGGGGGGG CGAGAGAGAG AGAGAGAGAG   
  
  
- AGAAGGGAAA TGTTTCCGTG GTAAGTTTAA GTTTGGTGAG AAAAATTTTG GGGTTTTTAA TAAGAGTTTT   
  
  
- ATTATGGAGT ATTATATTTA CCCAAGCATA GTGGTATATA CCCGGAGTAG AGAGTACCCT TGTATGCTTA   
  
  
- AGTTACCGAA CGGGCTATCA ATAGCCCAGT ACGGTAGCCT CGGGGCCGTT GATTAGTTAC CAGTTCGGAG   
  
  
- TCTAAACCGG AGAAACTCGG TAATATCATT GTGGAAAGAG AGACTTGAGG GGGGAGATGG GTGAGACCCT   
  
  
- GGGCCTAGGC TGGGCCCAGA TAGCACAGGC CCAGGACGAG ACTCAGGTTA CCTCACCTAA CCACTGCGCA   
  
  
- CCTGTGTGGT CTAACTTGTA CTAGACGGTG CAGTCGTCTC AGTTGCCGAC AACTGACTAC CACTGTACCC   
  
  
- CCTCCTCCTA AGGCCATATT CCAACCACGT ACGGAACGAC TGCACGCGTC TCCGGCAGGT TACGCCGCTC   
  
  
- GAGGCTGACC GACGAAGCGA TCAACTACTC TACTGGTTAC CCGACAACGG TGCACAATCA TGCCACACAC   
  
  
- CCTAGCCGTT CCATCGGCCG ATAAAACAAC TCCGAAACTC GGCCGCGGAC AAAGTCGGGC CGGGCCTTTG   
  
  
- ACCGGGCTGG ACCGGCCCGA GCCTCAAGCT CCGACTCCAC AACATAGCAG TGAAGATACT CCGAACGGGA   
  
  
- ATAGAGTTTA AACGAGTGAA GTGCCGATTA GTTCGGTAGA ACCTCCGTAA ACTCCCGGTA CTAATACAGG   
  
  
- TGCAGTAGCT GAAGCCGAAG TACTGGCCGA ACGTTACCGG CCGAAACTAA GTTCGGGACC GAAATTCCGG   
  
  
- GCCACCCGGT GGTAGAGAAT CCGAGTGACC CTAACCGGGC AGAAATCCTT CACCCGGGCT TAGCTATGCA   
  
  
- CTTTAGCCCA ACTCCGATCG GGTTAACCGG GCTAGGTACT TGTACTCCAA GCGTAAAGCC CGACATCGTT   
  
  
- GAAGAGCCGA GCTCCTGCAC TTCGGCAGTT ACGAGCCTTA CCTGGGATCT CTTCGGCAGC GATACTTAAG   
  
  
- GCAGTACGTA GAAGTAGCTG AAAATCCTAA TCTGTAGTTG AGTCAAGATT TGACCTAAGC TTTGGATTTG   
  
  
- GGCTTCTAGC ATTGAAATCA TCTTGTTCTC CGCTTGGTAT TGGTTGGACT TAAAGAGGGG GCTAAGTGAC   
  
  
- TTCGTGATAC AATGATGAGT TGATACAAGC TGAGTAATCT CTGAACAGTT CAGGTTGGCG ACTTTCAGGA   
  
  
- TCGGCTCTAC ATATATGTTT TCCTCTATAG GTTGGATCAC ACGATGCTTC CGAGTCGGGC CCAGGTCTCC   
  
  
- GTGCTCGGCG AGCGGTTTAC CGTTCGAGCC TACCCGGTTC GGCCCAAGTT CGGCGATCCG GACTCAGTTT   
  
  
- TACGGGAATC CGTTCGATTG TACAACCATT GTAACAAGAG GAGTGTTCCC ATACTGCACC TCCTCTCTCT   
  
  
- TCCCACAAAC TGTGACCCGA CCGTATTGGC GGGAGAGTAT CACTGAAGCT GAACCGTTCA CCCCGGGTTT   
  
  
- TACCTGTCAG GTTCACACTG AGTACTCTAC AGTAGTAGGA GTCGAAGAAG TAGAAT

+     ERE

| Site Name | Organism | Position | Strand | Matrix score. | sequence | function |
| --- | --- | --- | --- | --- | --- | --- |
| ERE | Nicotiana glutinos | 455 | + | 8 | ATTTCATA |  |
| ERE | Nicotiana glutinos | 998 | + | 8 | ATTTTAAA |  |

>HU03G02797.1   
+ +Up\_Stream \_Len000CATTTT CAATTAGAAA TTTCTTCAAT TATATACCAT CTCCAAAAAT AGTCCTCATA   
  
  
+ AGTTGGCAGG TGAATGATCC TAATGGAAAA TTACTTTTAA CTGTTGAATT TGCAATAGGG TACCGAATTT   
  
  
+ GTGAAGTTTA TTGCCGCACG TGCCTAGCTT GTGACTTCTC GATAATCAAA AATTAATTAG AGAGAGACAG   
  
  
+ CTAAAGTTTT GAGAATAATA CTGAAAAATT ACATTAAATT TTATTTATGT TTATTTTCAT ACGCTATGTT   
  
  
+ TAACTGCTAT ACGCTGTGTT TAACTGCATC TCCTTCTATA TTTGATAAAT TGGTAAAAAA AAATAATTCA   
  
  
+ AAATTAAAGA CTAGTAATAT TTTCACTTAT CTGATAGATT TATAAGAAAT ATTTAGATAG CTAAATATAT   
  
  
+ TCTCTCCTGA AAGAGGAGAC TGATAGTTGA ATTTCATAGG ATATAAACAT GTATGTACAT TGAACGAATC   
  
  
+ TTTTTTCTAT GGTGATCGTT ATAAAGTTAT TTATTACTGT AAACATTAAG TGATAATTAG GATATGTTCT   
  
  
+ ATTTCACGTT TTAGATAGAT AGATAGATGT AAGGAGTTGT AAGAAGCTGT AAGAATATGT AAGGAGCTGT   
  
  
+ AAGGAGATGT AAGGAGCTGT AAGAAGATGT AAGAGAAACT GTAAGAAGCT ACAAGGATAG ATGTAACGAG   
  
  
+ TTGTTGAATT GTTGAAGAGT TGTATAGTGT AATTTGTTTT ACGATGAACG TTTTGGATGA AAAATAATAA   
  
  
+ TATTAAAATA ATATGCTTAT TAAAAATAAA TAAATAATTA AGTTAATTAC ACAACAATAA AATATATTAA   
  
  
+ TCCCCATAAA TTTAGTGAAT AAAACTTCGT CCACTTGAGA TGATTTTTTA TATGAACACT AGTTTGATAG   
  
  
+ AATAAACAAA GTATAAAAAA AAATAAAGTT TTTTGACAAA ATTGAATACA ACTCTTCAAA TAAATTCCTA   
  
  
+ TTTTAAGTAA ATAATTTTAA ATATAAATAA ATTTATGGTC ATCTATAATA ATATATATTT TATGTCGAAA   
  
  
+ CTCAAATATA ATTTACATCG TTATATTATA TTTTTATGTT TATGTAAATG TTGTGTTCAT AAGATATTTA   
  
  
+ TAAATTTTGT ATATTGGTAG TATCATTAAA AAAAATATAC ATATTTATGT AACTAAAATA AATGAATGTT   
  
  
+ ATAGAAAATC TATAAGATGT AAGGAGATGT ATGAAGATGT AAGGAGATGT AAGAAGATAT AAGGAGATGT   
  
  
+ AGAAAATGTA AGAAGATGTG AGAAGCTGTA AGGAGATGTA AGTTATTCAC TAAATCCTAA AAAATGCGAA   
  
  
+ ATAGAACAGG GGCTTAATTG AGCACTCAAG ATGCATAATT TGTGACGGAC CAAATGGATA AATATAGACC   
  
  
+ TGGCAAATGG GTTATTCGAT TTAGGTACGA GTATGGTTAA GTTTGGGTTT GGTTATGTTT CAGGATAAGT   
  
  
+ GACATTTCGA CTCAATTTGG TTTTGGTTGT ATGAACTTCG GCTCGAGATT TTTGGTTTGG CCAATACACT   
  
  
+ TATCATGCAA AAATAAGTAA CTTTATAGAA AATTTTGGAT CGGATATGAT GCGGTTCGGA TCAATTCGGA   
  
  
+ TTTTGGTTCA CATTCTGGTA AACCTATTTC GGATGTCAAG TAGGATATGA GTCCAGATCG TTGGATTTCG   
  
  
+ GGTATCAGCT CAATTTTACC AGATCTAGAT AAATCATAAA TGTACGGGTT TATTTTTTCC AGCTTGAATG   
  
  
+ ATTTTCTTTA TTAAAAAAAG ATAGATTTTA GATAAAACAC AACATTTCAT TTTCATTTGT TCCCCCCTAT   
  
  
+ AAATTTCTGT GACCACCTTC ACTTTCTTGC CCCATTCCCC CCCCCCCCCC GCTCTCTCTC TCTCTCTCTC   
  
  
+ TCTTCCCTTT ACAAAGGCAC CATTCAAATT CAAACCACTC TTTTTAAAAC CCCAAAAATT ATTCTCAAAA   
  
  
+ TAATACCTCA TAATATAAAT GGGTTCGTAT CACCATATAT GGGCCTCATC TCTCATGGGA ACATACGAAT   
  
  
+ TCAATGGCTT GCCCGATAGT TATCGGGTCA TGCCATCGGA GCCCCGGCAA CTAATCAATG GTCAAGCCTC   
  
  
+ AGATTTGGCC TCTTTGAGCC ATTATAGTAA CACCTTTCTC TCTGAACTCC CCCCTCTACC CACTCTGGGA   
  
  
+ CCCGGATCCG ACCCGGGTCT ATCGTGTCCG GGTCCTGCTC TGAGTCCAAT GGAGTGGATT GGTGACGCGT   
  
  
+ GGACACACCA GATTGAACAT GATCTGCCAC GTCAGCAGAG TCAACGGCTG TTGACTGATG GTGACATGGG   
  
  
+ GGAGGAGGAT TCCGGTATAA GGTTGGTGCA TGCCTTGCTG ACGTGCGCAG AGGCCGTCCA ATGCGGCGAG   
  
  
+ CTCCGACTGG CTGCTTCGCT AGTTGATGAG ATGACCAATG GGCTGTTGCC ACGTGTTAGT ACGGTGTGTG   
  
  
+ GGATCGGCAA GGTAGCCGGC TATTTTGTTG AGGCTTTGAG CCGGCGCCTG TTTCAGCCCG GCCCGGAAAC   
  
  
+ TGGCCCGACC TGGCCGGGCT CGGAGTTCGA GGCTGAGGTG TTGTATCGTC ACTTCTATGA GGCTTGCCCT   
  
  
+ TATCTCAAAT TTGCTCACTT CACGGCTAAT CAAGCCATCT TGGAGGCATT TGAGGGCCAT GATTATGTCC   
  
  
+ ACGTCATCGA CTTCGGCTTC ATGACCGGCT TGCAATGGCC GGCTTTGATT CAAGCCCTGG CTTTAAGGCC   
  
  
+ CGGTGGGCCA CCATCTCTTA GGCTCACTGG GATTGGCCCG TCTTTAGGAA GTGGGCCCGA ATCGATACGT   
  
  
+ GAAATCGGGT TGAGGCTAGC CCAATTGGCC CGATCCATGA ACATGAGGTT CGCATTTCGG GCTGTAGCAA   
  
  
+ CTTCTCGGCT CGAGGACGTG AAGCCGTCAA TGCTCGGAAT GGACCCTAGA GAAGCCGTCG CTATGAATTC   
  
  
+ CGTCATGCAT CTTCATCGAC TTTTAGGATT AGACATCAAC TCAGTTCTAA ACTGGATTCG AAACCTAAAC   
  
  
+ CCGAAGATCG TAACTTTAGT AGAACAAGAG GCGAACCATA ACCAACCTGA ATTTCTCCCC CGATTCACTG   
  
  
+ AAGCACTATG TTACTACTCA ACTATGTTCG ACTCATTAGA GACTTGTCAA GTCCAACCGC TGAAAGTCCT   
  
  
+ AGCCGAGATG TATATACAAA AGGAGATATC CAACCTAGTG TGCTACGAAG GCTCAGCCCG GGTCCAGAGG   
  
  
+ CACGAGCCGC TCGCCAAATG GCAAGCTCGG ATGGGCCAAG CCGGGTTCAA GCCGCTAGGC CTGAGTCAAA   
  
  
+ ATGCCCTTAG GCAAGCTAAC ATGTTGGTAA CATTGTTCTC CTCACAAGGG TATGACGTGG AGGAGAGAGA   
  
  
+ AGGGTGTTTG ACACTGGGCT GGCATAACCG CCCTCTCATA GTGACTTCGA CTTGGCAAGT GGGGCCCAAA   
  
  
+ ATGGACAGTC CAAGTGTGAC TCATGAGATG TCATCATCCT CAGCTTCTTC ATCTTA  

- +Up\_Stream \_Len000GTAAAA GTTAATCTTT AAAGAAGTTA ATATATGGTA GAGGTTTTTA TCAGGAGTAT   
  
  
- TCAACCGTCC ACTTACTAGG ATTACCTTTT AATGAAAATT GACAACTTAA ACGTTATCCC ATGGCTTAAA   
  
  
- CACTTCAAAT AACGGCGTGC ACGGATCGAA CACTGAAGAG CTATTAGTTT TTAATTAATC TCTCTCTGTC   
  
  
- GATTTCAAAA CTCTTATTAT GACTTTTTAA TGTAATTTAA AATAAATACA AATAAAAGTA TGCGATACAA   
  
  
- ATTGACGATA TGCGACACAA ATTGACGTAG AGGAAGATAT AAACTATTTA ACCATTTTTT TTTATTAAGT   
  
  
- TTTAATTTCT GATCATTATA AAAGTGAATA GACTATCTAA ATATTCTTTA TAAATCTATC GATTTATATA   
  
  
- AGAGAGGACT TTCTCCTCTG ACTATCAACT TAAAGTATCC TATATTTGTA CATACATGTA ACTTGCTTAG   
  
  
- AAAAAAGATA CCACTAGCAA TATTTCAATA AATAATGACA TTTGTAATTC ACTATTAATC CTATACAAGA   
  
  
- TAAAGTGCAA AATCTATCTA TCTATCTACA TTCCTCAACA TTCTTCGACA TTCTTATACA TTCCTCGACA   
  
  
- TTCCTCTACA TTCCTCGACA TTCTTCTACA TTCTCTTTGA CATTCTTCGA TGTTCCTATC TACATTGCTC   
  
  
- AACAACTTAA CAACTTCTCA ACATATCACA TTAAACAAAA TGCTACTTGC AAAACCTACT TTTTATTATT   
  
  
- ATAATTTTAT TATACGAATA ATTTTTATTT ATTTATTAAT TCAATTAATG TGTTGTTATT TTATATAATT   
  
  
- AGGGGTATTT AAATCACTTA TTTTGAAGCA GGTGAACTCT ACTAAAAAAT ATACTTGTGA TCAAACTATC   
  
  
- TTATTTGTTT CATATTTTTT TTTATTTCAA AAAACTGTTT TAACTTATGT TGAGAAGTTT ATTTAAGGAT   
  
  
- AAAATTCATT TATTAAAATT TATATTTATT TAAATACCAG TAGATATTAT TATATATAAA ATACAGCTTT   
  
  
- GAGTTTATAT TAAATGTAGC AATATAATAT AAAAATACAA ATACATTTAC AACACAAGTA TTCTATAAAT   
  
  
- ATTTAAAACA TATAACCATC ATAGTAATTT TTTTTATATG TATAAATACA TTGATTTTAT TTACTTACAA   
  
  
- TATCTTTTAG ATATTCTACA TTCCTCTACA TACTTCTACA TTCCTCTACA TTCTTCTATA TTCCTCTACA   
  
  
- TCTTTTACAT TCTTCTACAC TCTTCGACAT TCCTCTACAT TCAATAAGTG ATTTAGGATT TTTTACGCTT   
  
  
- TATCTTGTCC CCGAATTAAC TCGTGAGTTC TACGTATTAA ACACTGCCTG GTTTACCTAT TTATATCTGG   
  
  
- ACCGTTTACC CAATAAGCTA AATCCATGCT CATACCAATT CAAACCCAAA CCAATACAAA GTCCTATTCA   
  
  
- CTGTAAAGCT GAGTTAAACC AAAACCAACA TACTTGAAGC CGAGCTCTAA AAACCAAACC GGTTATGTGA   
  
  
- ATAGTACGTT TTTATTCATT GAAATATCTT TTAAAACCTA GCCTATACTA CGCCAAGCCT AGTTAAGCCT   
  
  
- AAAACCAAGT GTAAGACCAT TTGGATAAAG CCTACAGTTC ATCCTATACT CAGGTCTAGC AACCTAAAGC   
  
  
- CCATAGTCGA GTTAAAATGG TCTAGATCTA TTTAGTATTT ACATGCCCAA ATAAAAAAGG TCGAACTTAC   
  
  
- TAAAAGAAAT AATTTTTTTC TATCTAAAAT CTATTTTGTG TTGTAAAGTA AAAGTAAACA AGGGGGGATA   
  
  
- TTTAAAGACA CTGGTGGAAG TGAAAGAACG GGGTAAGGGG GGGGGGGGGG CGAGAGAGAG AGAGAGAGAG   
  
  
- AGAAGGGAAA TGTTTCCGTG GTAAGTTTAA GTTTGGTGAG AAAAATTTTG GGGTTTTTAA TAAGAGTTTT   
  
  
- ATTATGGAGT ATTATATTTA CCCAAGCATA GTGGTATATA CCCGGAGTAG AGAGTACCCT TGTATGCTTA   
  
  
- AGTTACCGAA CGGGCTATCA ATAGCCCAGT ACGGTAGCCT CGGGGCCGTT GATTAGTTAC CAGTTCGGAG   
  
  
- TCTAAACCGG AGAAACTCGG TAATATCATT GTGGAAAGAG AGACTTGAGG GGGGAGATGG GTGAGACCCT   
  
  
- GGGCCTAGGC TGGGCCCAGA TAGCACAGGC CCAGGACGAG ACTCAGGTTA CCTCACCTAA CCACTGCGCA   
  
  
- CCTGTGTGGT CTAACTTGTA CTAGACGGTG CAGTCGTCTC AGTTGCCGAC AACTGACTAC CACTGTACCC   
  
  
- CCTCCTCCTA AGGCCATATT CCAACCACGT ACGGAACGAC TGCACGCGTC TCCGGCAGGT TACGCCGCTC   
  
  
- GAGGCTGACC GACGAAGCGA TCAACTACTC TACTGGTTAC CCGACAACGG TGCACAATCA TGCCACACAC   
  
  
- CCTAGCCGTT CCATCGGCCG ATAAAACAAC TCCGAAACTC GGCCGCGGAC AAAGTCGGGC CGGGCCTTTG   
  
  
- ACCGGGCTGG ACCGGCCCGA GCCTCAAGCT CCGACTCCAC AACATAGCAG TGAAGATACT CCGAACGGGA   
  
  
- ATAGAGTTTA AACGAGTGAA GTGCCGATTA GTTCGGTAGA ACCTCCGTAA ACTCCCGGTA CTAATACAGG   
  
  
- TGCAGTAGCT GAAGCCGAAG TACTGGCCGA ACGTTACCGG CCGAAACTAA GTTCGGGACC GAAATTCCGG   
  
  
- GCCACCCGGT GGTAGAGAAT CCGAGTGACC CTAACCGGGC AGAAATCCTT CACCCGGGCT TAGCTATGCA   
  
  
- CTTTAGCCCA ACTCCGATCG GGTTAACCGG GCTAGGTACT TGTACTCCAA GCGTAAAGCC CGACATCGTT   
  
  
- GAAGAGCCGA GCTCCTGCAC TTCGGCAGTT ACGAGCCTTA CCTGGGATCT CTTCGGCAGC GATACTTAAG   
  
  
- GCAGTACGTA GAAGTAGCTG AAAATCCTAA TCTGTAGTTG AGTCAAGATT TGACCTAAGC TTTGGATTTG   
  
  
- GGCTTCTAGC ATTGAAATCA TCTTGTTCTC CGCTTGGTAT TGGTTGGACT TAAAGAGGGG GCTAAGTGAC   
  
  
- TTCGTGATAC AATGATGAGT TGATACAAGC TGAGTAATCT CTGAACAGTT CAGGTTGGCG ACTTTCAGGA   
  
  
- TCGGCTCTAC ATATATGTTT TCCTCTATAG GTTGGATCAC ACGATGCTTC CGAGTCGGGC CCAGGTCTCC   
  
  
- GTGCTCGGCG AGCGGTTTAC CGTTCGAGCC TACCCGGTTC GGCCCAAGTT CGGCGATCCG GACTCAGTTT   
  
  
- TACGGGAATC CGTTCGATTG TACAACCATT GTAACAAGAG GAGTGTTCCC ATACTGCACC TCCTCTCTCT   
  
  
- TCCCACAAAC TGTGACCCGA CCGTATTGGC GGGAGAGTAT CACTGAAGCT GAACCGTTCA CCCCGGGTTT   
  
  
- TACCTGTCAG GTTCACACTG AGTACTCTAC AGTAGTAGGA GTCGAAGAAG TAGAAT

+     G-Box

| Site Name | Organism | Position | Strand | Matrix score. | sequence | function |
| --- | --- | --- | --- | --- | --- | --- |
| G-Box | Pisum sativum | 161 | + | 6 | CACGTG | cis-acting regulatory element involved in light responsiveness |
| G-Box | Pisum sativum | 2434 | - | 6 | CACGTG | cis-acting regulatory element involved in light responsiveness |
| G-Box | Pisum sativum | 569 | + | 6 | CACGTT | cis-acting regulatory element involved in light responsiveness |

>HU03G02797.1   
+ +Up\_Stream \_Len000CATTTT CAATTAGAAA TTTCTTCAAT TATATACCAT CTCCAAAAAT AGTCCTCATA   
  
  
+ AGTTGGCAGG TGAATGATCC TAATGGAAAA TTACTTTTAA CTGTTGAATT TGCAATAGGG TACCGAATTT   
  
  
+ GTGAAGTTTA TTGCCGCACG TGCCTAGCTT GTGACTTCTC GATAATCAAA AATTAATTAG AGAGAGACAG   
  
  
+ CTAAAGTTTT GAGAATAATA CTGAAAAATT ACATTAAATT TTATTTATGT TTATTTTCAT ACGCTATGTT   
  
  
+ TAACTGCTAT ACGCTGTGTT TAACTGCATC TCCTTCTATA TTTGATAAAT TGGTAAAAAA AAATAATTCA   
  
  
+ AAATTAAAGA CTAGTAATAT TTTCACTTAT CTGATAGATT TATAAGAAAT ATTTAGATAG CTAAATATAT   
  
  
+ TCTCTCCTGA AAGAGGAGAC TGATAGTTGA ATTTCATAGG ATATAAACAT GTATGTACAT TGAACGAATC   
  
  
+ TTTTTTCTAT GGTGATCGTT ATAAAGTTAT TTATTACTGT AAACATTAAG TGATAATTAG GATATGTTCT   
  
  
+ ATTTCACGTT TTAGATAGAT AGATAGATGT AAGGAGTTGT AAGAAGCTGT AAGAATATGT AAGGAGCTGT   
  
  
+ AAGGAGATGT AAGGAGCTGT AAGAAGATGT AAGAGAAACT GTAAGAAGCT ACAAGGATAG ATGTAACGAG   
  
  
+ TTGTTGAATT GTTGAAGAGT TGTATAGTGT AATTTGTTTT ACGATGAACG TTTTGGATGA AAAATAATAA   
  
  
+ TATTAAAATA ATATGCTTAT TAAAAATAAA TAAATAATTA AGTTAATTAC ACAACAATAA AATATATTAA   
  
  
+ TCCCCATAAA TTTAGTGAAT AAAACTTCGT CCACTTGAGA TGATTTTTTA TATGAACACT AGTTTGATAG   
  
  
+ AATAAACAAA GTATAAAAAA AAATAAAGTT TTTTGACAAA ATTGAATACA ACTCTTCAAA TAAATTCCTA   
  
  
+ TTTTAAGTAA ATAATTTTAA ATATAAATAA ATTTATGGTC ATCTATAATA ATATATATTT TATGTCGAAA   
  
  
+ CTCAAATATA ATTTACATCG TTATATTATA TTTTTATGTT TATGTAAATG TTGTGTTCAT AAGATATTTA   
  
  
+ TAAATTTTGT ATATTGGTAG TATCATTAAA AAAAATATAC ATATTTATGT AACTAAAATA AATGAATGTT   
  
  
+ ATAGAAAATC TATAAGATGT AAGGAGATGT ATGAAGATGT AAGGAGATGT AAGAAGATAT AAGGAGATGT   
  
  
+ AGAAAATGTA AGAAGATGTG AGAAGCTGTA AGGAGATGTA AGTTATTCAC TAAATCCTAA AAAATGCGAA   
  
  
+ ATAGAACAGG GGCTTAATTG AGCACTCAAG ATGCATAATT TGTGACGGAC CAAATGGATA AATATAGACC   
  
  
+ TGGCAAATGG GTTATTCGAT TTAGGTACGA GTATGGTTAA GTTTGGGTTT GGTTATGTTT CAGGATAAGT   
  
  
+ GACATTTCGA CTCAATTTGG TTTTGGTTGT ATGAACTTCG GCTCGAGATT TTTGGTTTGG CCAATACACT   
  
  
+ TATCATGCAA AAATAAGTAA CTTTATAGAA AATTTTGGAT CGGATATGAT GCGGTTCGGA TCAATTCGGA   
  
  
+ TTTTGGTTCA CATTCTGGTA AACCTATTTC GGATGTCAAG TAGGATATGA GTCCAGATCG TTGGATTTCG   
  
  
+ GGTATCAGCT CAATTTTACC AGATCTAGAT AAATCATAAA TGTACGGGTT TATTTTTTCC AGCTTGAATG   
  
  
+ ATTTTCTTTA TTAAAAAAAG ATAGATTTTA GATAAAACAC AACATTTCAT TTTCATTTGT TCCCCCCTAT   
  
  
+ AAATTTCTGT GACCACCTTC ACTTTCTTGC CCCATTCCCC CCCCCCCCCC GCTCTCTCTC TCTCTCTCTC   
  
  
+ TCTTCCCTTT ACAAAGGCAC CATTCAAATT CAAACCACTC TTTTTAAAAC CCCAAAAATT ATTCTCAAAA   
  
  
+ TAATACCTCA TAATATAAAT GGGTTCGTAT CACCATATAT GGGCCTCATC TCTCATGGGA ACATACGAAT   
  
  
+ TCAATGGCTT GCCCGATAGT TATCGGGTCA TGCCATCGGA GCCCCGGCAA CTAATCAATG GTCAAGCCTC   
  
  
+ AGATTTGGCC TCTTTGAGCC ATTATAGTAA CACCTTTCTC TCTGAACTCC CCCCTCTACC CACTCTGGGA   
  
  
+ CCCGGATCCG ACCCGGGTCT ATCGTGTCCG GGTCCTGCTC TGAGTCCAAT GGAGTGGATT GGTGACGCGT   
  
  
+ GGACACACCA GATTGAACAT GATCTGCCAC GTCAGCAGAG TCAACGGCTG TTGACTGATG GTGACATGGG   
  
  
+ GGAGGAGGAT TCCGGTATAA GGTTGGTGCA TGCCTTGCTG ACGTGCGCAG AGGCCGTCCA ATGCGGCGAG   
  
  
+ CTCCGACTGG CTGCTTCGCT AGTTGATGAG ATGACCAATG GGCTGTTGCC ACGTGTTAGT ACGGTGTGTG   
  
  
+ GGATCGGCAA GGTAGCCGGC TATTTTGTTG AGGCTTTGAG CCGGCGCCTG TTTCAGCCCG GCCCGGAAAC   
  
  
+ TGGCCCGACC TGGCCGGGCT CGGAGTTCGA GGCTGAGGTG TTGTATCGTC ACTTCTATGA GGCTTGCCCT   
  
  
+ TATCTCAAAT TTGCTCACTT CACGGCTAAT CAAGCCATCT TGGAGGCATT TGAGGGCCAT GATTATGTCC   
  
  
+ ACGTCATCGA CTTCGGCTTC ATGACCGGCT TGCAATGGCC GGCTTTGATT CAAGCCCTGG CTTTAAGGCC   
  
  
+ CGGTGGGCCA CCATCTCTTA GGCTCACTGG GATTGGCCCG TCTTTAGGAA GTGGGCCCGA ATCGATACGT   
  
  
+ GAAATCGGGT TGAGGCTAGC CCAATTGGCC CGATCCATGA ACATGAGGTT CGCATTTCGG GCTGTAGCAA   
  
  
+ CTTCTCGGCT CGAGGACGTG AAGCCGTCAA TGCTCGGAAT GGACCCTAGA GAAGCCGTCG CTATGAATTC   
  
  
+ CGTCATGCAT CTTCATCGAC TTTTAGGATT AGACATCAAC TCAGTTCTAA ACTGGATTCG AAACCTAAAC   
  
  
+ CCGAAGATCG TAACTTTAGT AGAACAAGAG GCGAACCATA ACCAACCTGA ATTTCTCCCC CGATTCACTG   
  
  
+ AAGCACTATG TTACTACTCA ACTATGTTCG ACTCATTAGA GACTTGTCAA GTCCAACCGC TGAAAGTCCT   
  
  
+ AGCCGAGATG TATATACAAA AGGAGATATC CAACCTAGTG TGCTACGAAG GCTCAGCCCG GGTCCAGAGG   
  
  
+ CACGAGCCGC TCGCCAAATG GCAAGCTCGG ATGGGCCAAG CCGGGTTCAA GCCGCTAGGC CTGAGTCAAA   
  
  
+ ATGCCCTTAG GCAAGCTAAC ATGTTGGTAA CATTGTTCTC CTCACAAGGG TATGACGTGG AGGAGAGAGA   
  
  
+ AGGGTGTTTG ACACTGGGCT GGCATAACCG CCCTCTCATA GTGACTTCGA CTTGGCAAGT GGGGCCCAAA   
  
  
+ ATGGACAGTC CAAGTGTGAC TCATGAGATG TCATCATCCT CAGCTTCTTC ATCTTA  

- +Up\_Stream \_Len000GTAAAA GTTAATCTTT AAAGAAGTTA ATATATGGTA GAGGTTTTTA TCAGGAGTAT   
  
  
- TCAACCGTCC ACTTACTAGG ATTACCTTTT AATGAAAATT GACAACTTAA ACGTTATCCC ATGGCTTAAA   
  
  
- CACTTCAAAT AACGGCGTGC ACGGATCGAA CACTGAAGAG CTATTAGTTT TTAATTAATC TCTCTCTGTC   
  
  
- GATTTCAAAA CTCTTATTAT GACTTTTTAA TGTAATTTAA AATAAATACA AATAAAAGTA TGCGATACAA   
  
  
- ATTGACGATA TGCGACACAA ATTGACGTAG AGGAAGATAT AAACTATTTA ACCATTTTTT TTTATTAAGT   
  
  
- TTTAATTTCT GATCATTATA AAAGTGAATA GACTATCTAA ATATTCTTTA TAAATCTATC GATTTATATA   
  
  
- AGAGAGGACT TTCTCCTCTG ACTATCAACT TAAAGTATCC TATATTTGTA CATACATGTA ACTTGCTTAG   
  
  
- AAAAAAGATA CCACTAGCAA TATTTCAATA AATAATGACA TTTGTAATTC ACTATTAATC CTATACAAGA   
  
  
- TAAAGTGCAA AATCTATCTA TCTATCTACA TTCCTCAACA TTCTTCGACA TTCTTATACA TTCCTCGACA   
  
  
- TTCCTCTACA TTCCTCGACA TTCTTCTACA TTCTCTTTGA CATTCTTCGA TGTTCCTATC TACATTGCTC   
  
  
- AACAACTTAA CAACTTCTCA ACATATCACA TTAAACAAAA TGCTACTTGC AAAACCTACT TTTTATTATT   
  
  
- ATAATTTTAT TATACGAATA ATTTTTATTT ATTTATTAAT TCAATTAATG TGTTGTTATT TTATATAATT   
  
  
- AGGGGTATTT AAATCACTTA TTTTGAAGCA GGTGAACTCT ACTAAAAAAT ATACTTGTGA TCAAACTATC   
  
  
- TTATTTGTTT CATATTTTTT TTTATTTCAA AAAACTGTTT TAACTTATGT TGAGAAGTTT ATTTAAGGAT   
  
  
- AAAATTCATT TATTAAAATT TATATTTATT TAAATACCAG TAGATATTAT TATATATAAA ATACAGCTTT   
  
  
- GAGTTTATAT TAAATGTAGC AATATAATAT AAAAATACAA ATACATTTAC AACACAAGTA TTCTATAAAT   
  
  
- ATTTAAAACA TATAACCATC ATAGTAATTT TTTTTATATG TATAAATACA TTGATTTTAT TTACTTACAA   
  
  
- TATCTTTTAG ATATTCTACA TTCCTCTACA TACTTCTACA TTCCTCTACA TTCTTCTATA TTCCTCTACA   
  
  
- TCTTTTACAT TCTTCTACAC TCTTCGACAT TCCTCTACAT TCAATAAGTG ATTTAGGATT TTTTACGCTT   
  
  
- TATCTTGTCC CCGAATTAAC TCGTGAGTTC TACGTATTAA ACACTGCCTG GTTTACCTAT TTATATCTGG   
  
  
- ACCGTTTACC CAATAAGCTA AATCCATGCT CATACCAATT CAAACCCAAA CCAATACAAA GTCCTATTCA   
  
  
- CTGTAAAGCT GAGTTAAACC AAAACCAACA TACTTGAAGC CGAGCTCTAA AAACCAAACC GGTTATGTGA   
  
  
- ATAGTACGTT TTTATTCATT GAAATATCTT TTAAAACCTA GCCTATACTA CGCCAAGCCT AGTTAAGCCT   
  
  
- AAAACCAAGT GTAAGACCAT TTGGATAAAG CCTACAGTTC ATCCTATACT CAGGTCTAGC AACCTAAAGC   
  
  
- CCATAGTCGA GTTAAAATGG TCTAGATCTA TTTAGTATTT ACATGCCCAA ATAAAAAAGG TCGAACTTAC   
  
  
- TAAAAGAAAT AATTTTTTTC TATCTAAAAT CTATTTTGTG TTGTAAAGTA AAAGTAAACA AGGGGGGATA   
  
  
- TTTAAAGACA CTGGTGGAAG TGAAAGAACG GGGTAAGGGG GGGGGGGGGG CGAGAGAGAG AGAGAGAGAG   
  
  
- AGAAGGGAAA TGTTTCCGTG GTAAGTTTAA GTTTGGTGAG AAAAATTTTG GGGTTTTTAA TAAGAGTTTT   
  
  
- ATTATGGAGT ATTATATTTA CCCAAGCATA GTGGTATATA CCCGGAGTAG AGAGTACCCT TGTATGCTTA   
  
  
- AGTTACCGAA CGGGCTATCA ATAGCCCAGT ACGGTAGCCT CGGGGCCGTT GATTAGTTAC CAGTTCGGAG   
  
  
- TCTAAACCGG AGAAACTCGG TAATATCATT GTGGAAAGAG AGACTTGAGG GGGGAGATGG GTGAGACCCT   
  
  
- GGGCCTAGGC TGGGCCCAGA TAGCACAGGC CCAGGACGAG ACTCAGGTTA CCTCACCTAA CCACTGCGCA   
  
  
- CCTGTGTGGT CTAACTTGTA CTAGACGGTG CAGTCGTCTC AGTTGCCGAC AACTGACTAC CACTGTACCC   
  
  
- CCTCCTCCTA AGGCCATATT CCAACCACGT ACGGAACGAC TGCACGCGTC TCCGGCAGGT TACGCCGCTC   
  
  
- GAGGCTGACC GACGAAGCGA TCAACTACTC TACTGGTTAC CCGACAACGG TGCACAATCA TGCCACACAC   
  
  
- CCTAGCCGTT CCATCGGCCG ATAAAACAAC TCCGAAACTC GGCCGCGGAC AAAGTCGGGC CGGGCCTTTG   
  
  
- ACCGGGCTGG ACCGGCCCGA GCCTCAAGCT CCGACTCCAC AACATAGCAG TGAAGATACT CCGAACGGGA   
  
  
- ATAGAGTTTA AACGAGTGAA GTGCCGATTA GTTCGGTAGA ACCTCCGTAA ACTCCCGGTA CTAATACAGG   
  
  
- TGCAGTAGCT GAAGCCGAAG TACTGGCCGA ACGTTACCGG CCGAAACTAA GTTCGGGACC GAAATTCCGG   
  
  
- GCCACCCGGT GGTAGAGAAT CCGAGTGACC CTAACCGGGC AGAAATCCTT CACCCGGGCT TAGCTATGCA   
  
  
- CTTTAGCCCA ACTCCGATCG GGTTAACCGG GCTAGGTACT TGTACTCCAA GCGTAAAGCC CGACATCGTT   
  
  
- GAAGAGCCGA GCTCCTGCAC TTCGGCAGTT ACGAGCCTTA CCTGGGATCT CTTCGGCAGC GATACTTAAG   
  
  
- GCAGTACGTA GAAGTAGCTG AAAATCCTAA TCTGTAGTTG AGTCAAGATT TGACCTAAGC TTTGGATTTG   
  
  
- GGCTTCTAGC ATTGAAATCA TCTTGTTCTC CGCTTGGTAT TGGTTGGACT TAAAGAGGGG GCTAAGTGAC   
  
  
- TTCGTGATAC AATGATGAGT TGATACAAGC TGAGTAATCT CTGAACAGTT CAGGTTGGCG ACTTTCAGGA   
  
  
- TCGGCTCTAC ATATATGTTT TCCTCTATAG GTTGGATCAC ACGATGCTTC CGAGTCGGGC CCAGGTCTCC   
  
  
- GTGCTCGGCG AGCGGTTTAC CGTTCGAGCC TACCCGGTTC GGCCCAAGTT CGGCGATCCG GACTCAGTTT   
  
  
- TACGGGAATC CGTTCGATTG TACAACCATT GTAACAAGAG GAGTGTTCCC ATACTGCACC TCCTCTCTCT   
  
  
- TCCCACAAAC TGTGACCCGA CCGTATTGGC GGGAGAGTAT CACTGAAGCT GAACCGTTCA CCCCGGGTTT   
  
  
- TACCTGTCAG GTTCACACTG AGTACTCTAC AGTAGTAGGA GTCGAAGAAG TAGAAT

+     G-box

| Site Name | Organism | Position | Strand | Matrix score. | sequence | function |
| --- | --- | --- | --- | --- | --- | --- |
| G-box | Brassica oleracea | 2433 | - | 9 | TAACACGTAG | cis-acting regulatory element involved in light responsiveness |
| G-box | Brassica napus | 2432 | - | 9 | ACACGTGGC | cis-acting regulatory element involved in light responsiveness |
| G-box | Zea mays | 3348 | - | 6 | CACGTC | cis-acting regulatory element involved in light responsiveness |
| G-box | Arabidopsis thaliana | 2800 | + | 6 | TACGTG | cis-acting regulatory element involved in light responsiveness |
| G-box | Zea mays | 2664 | + | 6 | CACGTC | cis-acting regulatory element involved in light responsiveness |
| G-box | Arabidopsis thaliana | 2434 | - | 6 | CACGTG | cis-acting regulatory element involved in light responsiveness |
| G-box | Zea mays | 2889 | - | 6 | CACGTC | cis-acting regulatory element involved in light responsiveness |
| G-box | Arabidopsis thaliana | 161 | + | 6 | CACGTG | cis-acting regulatory element involved in light responsiveness |
| G-box | Lycopersicon esculentum | 2431 | - | 11 | tgACACGTGGCA | cis-acting regulatory element involved in light responsiveness |
| G-box | Zea mays | 2272 | + | 6 | CACGTC | cis-acting regulatory element involved in light responsiveness |
| G-box | Zea mays | 2354 | - | 6 | CACGTC | cis-acting regulatory element involved in light responsiveness |
| G-box | Lycopersicon esculentum | 2429 | - | 10.5 | ACACGTG(G/t)CACC | cis-acting regulatory element involved in light responsiveness |
| G-box | Nicotiana plumbaginifolia | 2269 | - | 10 | CAGACGTGGCA | cis-acting regulatory element involved in light responsiveness |

>HU03G02797.1   
+ +Up\_Stream \_Len000CATTTT CAATTAGAAA TTTCTTCAAT TATATACCAT CTCCAAAAAT AGTCCTCATA   
  
  
+ AGTTGGCAGG TGAATGATCC TAATGGAAAA TTACTTTTAA CTGTTGAATT TGCAATAGGG TACCGAATTT   
  
  
+ GTGAAGTTTA TTGCCGCACG TGCCTAGCTT GTGACTTCTC GATAATCAAA AATTAATTAG AGAGAGACAG   
  
  
+ CTAAAGTTTT GAGAATAATA CTGAAAAATT ACATTAAATT TTATTTATGT TTATTTTCAT ACGCTATGTT   
  
  
+ TAACTGCTAT ACGCTGTGTT TAACTGCATC TCCTTCTATA TTTGATAAAT TGGTAAAAAA AAATAATTCA   
  
  
+ AAATTAAAGA CTAGTAATAT TTTCACTTAT CTGATAGATT TATAAGAAAT ATTTAGATAG CTAAATATAT   
  
  
+ TCTCTCCTGA AAGAGGAGAC TGATAGTTGA ATTTCATAGG ATATAAACAT GTATGTACAT TGAACGAATC   
  
  
+ TTTTTTCTAT GGTGATCGTT ATAAAGTTAT TTATTACTGT AAACATTAAG TGATAATTAG GATATGTTCT   
  
  
+ ATTTCACGTT TTAGATAGAT AGATAGATGT AAGGAGTTGT AAGAAGCTGT AAGAATATGT AAGGAGCTGT   
  
  
+ AAGGAGATGT AAGGAGCTGT AAGAAGATGT AAGAGAAACT GTAAGAAGCT ACAAGGATAG ATGTAACGAG   
  
  
+ TTGTTGAATT GTTGAAGAGT TGTATAGTGT AATTTGTTTT ACGATGAACG TTTTGGATGA AAAATAATAA   
  
  
+ TATTAAAATA ATATGCTTAT TAAAAATAAA TAAATAATTA AGTTAATTAC ACAACAATAA AATATATTAA   
  
  
+ TCCCCATAAA TTTAGTGAAT AAAACTTCGT CCACTTGAGA TGATTTTTTA TATGAACACT AGTTTGATAG   
  
  
+ AATAAACAAA GTATAAAAAA AAATAAAGTT TTTTGACAAA ATTGAATACA ACTCTTCAAA TAAATTCCTA   
  
  
+ TTTTAAGTAA ATAATTTTAA ATATAAATAA ATTTATGGTC ATCTATAATA ATATATATTT TATGTCGAAA   
  
  
+ CTCAAATATA ATTTACATCG TTATATTATA TTTTTATGTT TATGTAAATG TTGTGTTCAT AAGATATTTA   
  
  
+ TAAATTTTGT ATATTGGTAG TATCATTAAA AAAAATATAC ATATTTATGT AACTAAAATA AATGAATGTT   
  
  
+ ATAGAAAATC TATAAGATGT AAGGAGATGT ATGAAGATGT AAGGAGATGT AAGAAGATAT AAGGAGATGT   
  
  
+ AGAAAATGTA AGAAGATGTG AGAAGCTGTA AGGAGATGTA AGTTATTCAC TAAATCCTAA AAAATGCGAA   
  
  
+ ATAGAACAGG GGCTTAATTG AGCACTCAAG ATGCATAATT TGTGACGGAC CAAATGGATA AATATAGACC   
  
  
+ TGGCAAATGG GTTATTCGAT TTAGGTACGA GTATGGTTAA GTTTGGGTTT GGTTATGTTT CAGGATAAGT   
  
  
+ GACATTTCGA CTCAATTTGG TTTTGGTTGT ATGAACTTCG GCTCGAGATT TTTGGTTTGG CCAATACACT   
  
  
+ TATCATGCAA AAATAAGTAA CTTTATAGAA AATTTTGGAT CGGATATGAT GCGGTTCGGA TCAATTCGGA   
  
  
+ TTTTGGTTCA CATTCTGGTA AACCTATTTC GGATGTCAAG TAGGATATGA GTCCAGATCG TTGGATTTCG   
  
  
+ GGTATCAGCT CAATTTTACC AGATCTAGAT AAATCATAAA TGTACGGGTT TATTTTTTCC AGCTTGAATG   
  
  
+ ATTTTCTTTA TTAAAAAAAG ATAGATTTTA GATAAAACAC AACATTTCAT TTTCATTTGT TCCCCCCTAT   
  
  
+ AAATTTCTGT GACCACCTTC ACTTTCTTGC CCCATTCCCC CCCCCCCCCC GCTCTCTCTC TCTCTCTCTC   
  
  
+ TCTTCCCTTT ACAAAGGCAC CATTCAAATT CAAACCACTC TTTTTAAAAC CCCAAAAATT ATTCTCAAAA   
  
  
+ TAATACCTCA TAATATAAAT GGGTTCGTAT CACCATATAT GGGCCTCATC TCTCATGGGA ACATACGAAT   
  
  
+ TCAATGGCTT GCCCGATAGT TATCGGGTCA TGCCATCGGA GCCCCGGCAA CTAATCAATG GTCAAGCCTC   
  
  
+ AGATTTGGCC TCTTTGAGCC ATTATAGTAA CACCTTTCTC TCTGAACTCC CCCCTCTACC CACTCTGGGA   
  
  
+ CCCGGATCCG ACCCGGGTCT ATCGTGTCCG GGTCCTGCTC TGAGTCCAAT GGAGTGGATT GGTGACGCGT   
  
  
+ GGACACACCA GATTGAACAT GATCTGCCAC GTCAGCAGAG TCAACGGCTG TTGACTGATG GTGACATGGG   
  
  
+ GGAGGAGGAT TCCGGTATAA GGTTGGTGCA TGCCTTGCTG ACGTGCGCAG AGGCCGTCCA ATGCGGCGAG   
  
  
+ CTCCGACTGG CTGCTTCGCT AGTTGATGAG ATGACCAATG GGCTGTTGCC ACGTGTTAGT ACGGTGTGTG   
  
  
+ GGATCGGCAA GGTAGCCGGC TATTTTGTTG AGGCTTTGAG CCGGCGCCTG TTTCAGCCCG GCCCGGAAAC   
  
  
+ TGGCCCGACC TGGCCGGGCT CGGAGTTCGA GGCTGAGGTG TTGTATCGTC ACTTCTATGA GGCTTGCCCT   
  
  
+ TATCTCAAAT TTGCTCACTT CACGGCTAAT CAAGCCATCT TGGAGGCATT TGAGGGCCAT GATTATGTCC   
  
  
+ ACGTCATCGA CTTCGGCTTC ATGACCGGCT TGCAATGGCC GGCTTTGATT CAAGCCCTGG CTTTAAGGCC   
  
  
+ CGGTGGGCCA CCATCTCTTA GGCTCACTGG GATTGGCCCG TCTTTAGGAA GTGGGCCCGA ATCGATACGT   
  
  
+ GAAATCGGGT TGAGGCTAGC CCAATTGGCC CGATCCATGA ACATGAGGTT CGCATTTCGG GCTGTAGCAA   
  
  
+ CTTCTCGGCT CGAGGACGTG AAGCCGTCAA TGCTCGGAAT GGACCCTAGA GAAGCCGTCG CTATGAATTC   
  
  
+ CGTCATGCAT CTTCATCGAC TTTTAGGATT AGACATCAAC TCAGTTCTAA ACTGGATTCG AAACCTAAAC   
  
  
+ CCGAAGATCG TAACTTTAGT AGAACAAGAG GCGAACCATA ACCAACCTGA ATTTCTCCCC CGATTCACTG   
  
  
+ AAGCACTATG TTACTACTCA ACTATGTTCG ACTCATTAGA GACTTGTCAA GTCCAACCGC TGAAAGTCCT   
  
  
+ AGCCGAGATG TATATACAAA AGGAGATATC CAACCTAGTG TGCTACGAAG GCTCAGCCCG GGTCCAGAGG   
  
  
+ CACGAGCCGC TCGCCAAATG GCAAGCTCGG ATGGGCCAAG CCGGGTTCAA GCCGCTAGGC CTGAGTCAAA   
  
  
+ ATGCCCTTAG GCAAGCTAAC ATGTTGGTAA CATTGTTCTC CTCACAAGGG TATGACGTGG AGGAGAGAGA   
  
  
+ AGGGTGTTTG ACACTGGGCT GGCATAACCG CCCTCTCATA GTGACTTCGA CTTGGCAAGT GGGGCCCAAA   
  
  
+ ATGGACAGTC CAAGTGTGAC TCATGAGATG TCATCATCCT CAGCTTCTTC ATCTTA  

- +Up\_Stream \_Len000GTAAAA GTTAATCTTT AAAGAAGTTA ATATATGGTA GAGGTTTTTA TCAGGAGTAT   
  
  
- TCAACCGTCC ACTTACTAGG ATTACCTTTT AATGAAAATT GACAACTTAA ACGTTATCCC ATGGCTTAAA   
  
  
- CACTTCAAAT AACGGCGTGC ACGGATCGAA CACTGAAGAG CTATTAGTTT TTAATTAATC TCTCTCTGTC   
  
  
- GATTTCAAAA CTCTTATTAT GACTTTTTAA TGTAATTTAA AATAAATACA AATAAAAGTA TGCGATACAA   
  
  
- ATTGACGATA TGCGACACAA ATTGACGTAG AGGAAGATAT AAACTATTTA ACCATTTTTT TTTATTAAGT   
  
  
- TTTAATTTCT GATCATTATA AAAGTGAATA GACTATCTAA ATATTCTTTA TAAATCTATC GATTTATATA   
  
  
- AGAGAGGACT TTCTCCTCTG ACTATCAACT TAAAGTATCC TATATTTGTA CATACATGTA ACTTGCTTAG   
  
  
- AAAAAAGATA CCACTAGCAA TATTTCAATA AATAATGACA TTTGTAATTC ACTATTAATC CTATACAAGA   
  
  
- TAAAGTGCAA AATCTATCTA TCTATCTACA TTCCTCAACA TTCTTCGACA TTCTTATACA TTCCTCGACA   
  
  
- TTCCTCTACA TTCCTCGACA TTCTTCTACA TTCTCTTTGA CATTCTTCGA TGTTCCTATC TACATTGCTC   
  
  
- AACAACTTAA CAACTTCTCA ACATATCACA TTAAACAAAA TGCTACTTGC AAAACCTACT TTTTATTATT   
  
  
- ATAATTTTAT TATACGAATA ATTTTTATTT ATTTATTAAT TCAATTAATG TGTTGTTATT TTATATAATT   
  
  
- AGGGGTATTT AAATCACTTA TTTTGAAGCA GGTGAACTCT ACTAAAAAAT ATACTTGTGA TCAAACTATC   
  
  
- TTATTTGTTT CATATTTTTT TTTATTTCAA AAAACTGTTT TAACTTATGT TGAGAAGTTT ATTTAAGGAT   
  
  
- AAAATTCATT TATTAAAATT TATATTTATT TAAATACCAG TAGATATTAT TATATATAAA ATACAGCTTT   
  
  
- GAGTTTATAT TAAATGTAGC AATATAATAT AAAAATACAA ATACATTTAC AACACAAGTA TTCTATAAAT   
  
  
- ATTTAAAACA TATAACCATC ATAGTAATTT TTTTTATATG TATAAATACA TTGATTTTAT TTACTTACAA   
  
  
- TATCTTTTAG ATATTCTACA TTCCTCTACA TACTTCTACA TTCCTCTACA TTCTTCTATA TTCCTCTACA   
  
  
- TCTTTTACAT TCTTCTACAC TCTTCGACAT TCCTCTACAT TCAATAAGTG ATTTAGGATT TTTTACGCTT   
  
  
- TATCTTGTCC CCGAATTAAC TCGTGAGTTC TACGTATTAA ACACTGCCTG GTTTACCTAT TTATATCTGG   
  
  
- ACCGTTTACC CAATAAGCTA AATCCATGCT CATACCAATT CAAACCCAAA CCAATACAAA GTCCTATTCA   
  
  
- CTGTAAAGCT GAGTTAAACC AAAACCAACA TACTTGAAGC CGAGCTCTAA AAACCAAACC GGTTATGTGA   
  
  
- ATAGTACGTT TTTATTCATT GAAATATCTT TTAAAACCTA GCCTATACTA CGCCAAGCCT AGTTAAGCCT   
  
  
- AAAACCAAGT GTAAGACCAT TTGGATAAAG CCTACAGTTC ATCCTATACT CAGGTCTAGC AACCTAAAGC   
  
  
- CCATAGTCGA GTTAAAATGG TCTAGATCTA TTTAGTATTT ACATGCCCAA ATAAAAAAGG TCGAACTTAC   
  
  
- TAAAAGAAAT AATTTTTTTC TATCTAAAAT CTATTTTGTG TTGTAAAGTA AAAGTAAACA AGGGGGGATA   
  
  
- TTTAAAGACA CTGGTGGAAG TGAAAGAACG GGGTAAGGGG GGGGGGGGGG CGAGAGAGAG AGAGAGAGAG   
  
  
- AGAAGGGAAA TGTTTCCGTG GTAAGTTTAA GTTTGGTGAG AAAAATTTTG GGGTTTTTAA TAAGAGTTTT   
  
  
- ATTATGGAGT ATTATATTTA CCCAAGCATA GTGGTATATA CCCGGAGTAG AGAGTACCCT TGTATGCTTA   
  
  
- AGTTACCGAA CGGGCTATCA ATAGCCCAGT ACGGTAGCCT CGGGGCCGTT GATTAGTTAC CAGTTCGGAG   
  
  
- TCTAAACCGG AGAAACTCGG TAATATCATT GTGGAAAGAG AGACTTGAGG GGGGAGATGG GTGAGACCCT   
  
  
- GGGCCTAGGC TGGGCCCAGA TAGCACAGGC CCAGGACGAG ACTCAGGTTA CCTCACCTAA CCACTGCGCA   
  
  
- CCTGTGTGGT CTAACTTGTA CTAGACGGTG CAGTCGTCTC AGTTGCCGAC AACTGACTAC CACTGTACCC   
  
  
- CCTCCTCCTA AGGCCATATT CCAACCACGT ACGGAACGAC TGCACGCGTC TCCGGCAGGT TACGCCGCTC   
  
  
- GAGGCTGACC GACGAAGCGA TCAACTACTC TACTGGTTAC CCGACAACGG TGCACAATCA TGCCACACAC   
  
  
- CCTAGCCGTT CCATCGGCCG ATAAAACAAC TCCGAAACTC GGCCGCGGAC AAAGTCGGGC CGGGCCTTTG   
  
  
- ACCGGGCTGG ACCGGCCCGA GCCTCAAGCT CCGACTCCAC AACATAGCAG TGAAGATACT CCGAACGGGA   
  
  
- ATAGAGTTTA AACGAGTGAA GTGCCGATTA GTTCGGTAGA ACCTCCGTAA ACTCCCGGTA CTAATACAGG   
  
  
- TGCAGTAGCT GAAGCCGAAG TACTGGCCGA ACGTTACCGG CCGAAACTAA GTTCGGGACC GAAATTCCGG   
  
  
- GCCACCCGGT GGTAGAGAAT CCGAGTGACC CTAACCGGGC AGAAATCCTT CACCCGGGCT TAGCTATGCA   
  
  
- CTTTAGCCCA ACTCCGATCG GGTTAACCGG GCTAGGTACT TGTACTCCAA GCGTAAAGCC CGACATCGTT   
  
  
- GAAGAGCCGA GCTCCTGCAC TTCGGCAGTT ACGAGCCTTA CCTGGGATCT CTTCGGCAGC GATACTTAAG   
  
  
- GCAGTACGTA GAAGTAGCTG AAAATCCTAA TCTGTAGTTG AGTCAAGATT TGACCTAAGC TTTGGATTTG   
  
  
- GGCTTCTAGC ATTGAAATCA TCTTGTTCTC CGCTTGGTAT TGGTTGGACT TAAAGAGGGG GCTAAGTGAC   
  
  
- TTCGTGATAC AATGATGAGT TGATACAAGC TGAGTAATCT CTGAACAGTT CAGGTTGGCG ACTTTCAGGA   
  
  
- TCGGCTCTAC ATATATGTTT TCCTCTATAG GTTGGATCAC ACGATGCTTC CGAGTCGGGC CCAGGTCTCC   
  
  
- GTGCTCGGCG AGCGGTTTAC CGTTCGAGCC TACCCGGTTC GGCCCAAGTT CGGCGATCCG GACTCAGTTT   
  
  
- TACGGGAATC CGTTCGATTG TACAACCATT GTAACAAGAG GAGTGTTCCC ATACTGCACC TCCTCTCTCT   
  
  
- TCCCACAAAC TGTGACCCGA CCGTATTGGC GGGAGAGTAT CACTGAAGCT GAACCGTTCA CCCCGGGTTT   
  
  
- TACCTGTCAG GTTCACACTG AGTACTCTAC AGTAGTAGGA GTCGAAGAAG TAGAAT

+     GC-motif

| Site Name | Organism | Position | Strand | Matrix score. | sequence | function |
| --- | --- | --- | --- | --- | --- | --- |
| GC-motif | Zea mays | 1870 | + | 6 | CCCCCG | enhancer-like element involved in anoxic specific inducibility |
| GC-motif | Zea mays | 3071 | + | 6 | CCCCCG | enhancer-like element involved in anoxic specific inducibility |

>HU03G02797.1   
+ +Up\_Stream \_Len000CATTTT CAATTAGAAA TTTCTTCAAT TATATACCAT CTCCAAAAAT AGTCCTCATA   
  
  
+ AGTTGGCAGG TGAATGATCC TAATGGAAAA TTACTTTTAA CTGTTGAATT TGCAATAGGG TACCGAATTT   
  
  
+ GTGAAGTTTA TTGCCGCACG TGCCTAGCTT GTGACTTCTC GATAATCAAA AATTAATTAG AGAGAGACAG   
  
  
+ CTAAAGTTTT GAGAATAATA CTGAAAAATT ACATTAAATT TTATTTATGT TTATTTTCAT ACGCTATGTT   
  
  
+ TAACTGCTAT ACGCTGTGTT TAACTGCATC TCCTTCTATA TTTGATAAAT TGGTAAAAAA AAATAATTCA   
  
  
+ AAATTAAAGA CTAGTAATAT TTTCACTTAT CTGATAGATT TATAAGAAAT ATTTAGATAG CTAAATATAT   
  
  
+ TCTCTCCTGA AAGAGGAGAC TGATAGTTGA ATTTCATAGG ATATAAACAT GTATGTACAT TGAACGAATC   
  
  
+ TTTTTTCTAT GGTGATCGTT ATAAAGTTAT TTATTACTGT AAACATTAAG TGATAATTAG GATATGTTCT   
  
  
+ ATTTCACGTT TTAGATAGAT AGATAGATGT AAGGAGTTGT AAGAAGCTGT AAGAATATGT AAGGAGCTGT   
  
  
+ AAGGAGATGT AAGGAGCTGT AAGAAGATGT AAGAGAAACT GTAAGAAGCT ACAAGGATAG ATGTAACGAG   
  
  
+ TTGTTGAATT GTTGAAGAGT TGTATAGTGT AATTTGTTTT ACGATGAACG TTTTGGATGA AAAATAATAA   
  
  
+ TATTAAAATA ATATGCTTAT TAAAAATAAA TAAATAATTA AGTTAATTAC ACAACAATAA AATATATTAA   
  
  
+ TCCCCATAAA TTTAGTGAAT AAAACTTCGT CCACTTGAGA TGATTTTTTA TATGAACACT AGTTTGATAG   
  
  
+ AATAAACAAA GTATAAAAAA AAATAAAGTT TTTTGACAAA ATTGAATACA ACTCTTCAAA TAAATTCCTA   
  
  
+ TTTTAAGTAA ATAATTTTAA ATATAAATAA ATTTATGGTC ATCTATAATA ATATATATTT TATGTCGAAA   
  
  
+ CTCAAATATA ATTTACATCG TTATATTATA TTTTTATGTT TATGTAAATG TTGTGTTCAT AAGATATTTA   
  
  
+ TAAATTTTGT ATATTGGTAG TATCATTAAA AAAAATATAC ATATTTATGT AACTAAAATA AATGAATGTT   
  
  
+ ATAGAAAATC TATAAGATGT AAGGAGATGT ATGAAGATGT AAGGAGATGT AAGAAGATAT AAGGAGATGT   
  
  
+ AGAAAATGTA AGAAGATGTG AGAAGCTGTA AGGAGATGTA AGTTATTCAC TAAATCCTAA AAAATGCGAA   
  
  
+ ATAGAACAGG GGCTTAATTG AGCACTCAAG ATGCATAATT TGTGACGGAC CAAATGGATA AATATAGACC   
  
  
+ TGGCAAATGG GTTATTCGAT TTAGGTACGA GTATGGTTAA GTTTGGGTTT GGTTATGTTT CAGGATAAGT   
  
  
+ GACATTTCGA CTCAATTTGG TTTTGGTTGT ATGAACTTCG GCTCGAGATT TTTGGTTTGG CCAATACACT   
  
  
+ TATCATGCAA AAATAAGTAA CTTTATAGAA AATTTTGGAT CGGATATGAT GCGGTTCGGA TCAATTCGGA   
  
  
+ TTTTGGTTCA CATTCTGGTA AACCTATTTC GGATGTCAAG TAGGATATGA GTCCAGATCG TTGGATTTCG   
  
  
+ GGTATCAGCT CAATTTTACC AGATCTAGAT AAATCATAAA TGTACGGGTT TATTTTTTCC AGCTTGAATG   
  
  
+ ATTTTCTTTA TTAAAAAAAG ATAGATTTTA GATAAAACAC AACATTTCAT TTTCATTTGT TCCCCCCTAT   
  
  
+ AAATTTCTGT GACCACCTTC ACTTTCTTGC CCCATTCCCC CCCCCCCCCC GCTCTCTCTC TCTCTCTCTC   
  
  
+ TCTTCCCTTT ACAAAGGCAC CATTCAAATT CAAACCACTC TTTTTAAAAC CCCAAAAATT ATTCTCAAAA   
  
  
+ TAATACCTCA TAATATAAAT GGGTTCGTAT CACCATATAT GGGCCTCATC TCTCATGGGA ACATACGAAT   
  
  
+ TCAATGGCTT GCCCGATAGT TATCGGGTCA TGCCATCGGA GCCCCGGCAA CTAATCAATG GTCAAGCCTC   
  
  
+ AGATTTGGCC TCTTTGAGCC ATTATAGTAA CACCTTTCTC TCTGAACTCC CCCCTCTACC CACTCTGGGA   
  
  
+ CCCGGATCCG ACCCGGGTCT ATCGTGTCCG GGTCCTGCTC TGAGTCCAAT GGAGTGGATT GGTGACGCGT   
  
  
+ GGACACACCA GATTGAACAT GATCTGCCAC GTCAGCAGAG TCAACGGCTG TTGACTGATG GTGACATGGG   
  
  
+ GGAGGAGGAT TCCGGTATAA GGTTGGTGCA TGCCTTGCTG ACGTGCGCAG AGGCCGTCCA ATGCGGCGAG   
  
  
+ CTCCGACTGG CTGCTTCGCT AGTTGATGAG ATGACCAATG GGCTGTTGCC ACGTGTTAGT ACGGTGTGTG   
  
  
+ GGATCGGCAA GGTAGCCGGC TATTTTGTTG AGGCTTTGAG CCGGCGCCTG TTTCAGCCCG GCCCGGAAAC   
  
  
+ TGGCCCGACC TGGCCGGGCT CGGAGTTCGA GGCTGAGGTG TTGTATCGTC ACTTCTATGA GGCTTGCCCT   
  
  
+ TATCTCAAAT TTGCTCACTT CACGGCTAAT CAAGCCATCT TGGAGGCATT TGAGGGCCAT GATTATGTCC   
  
  
+ ACGTCATCGA CTTCGGCTTC ATGACCGGCT TGCAATGGCC GGCTTTGATT CAAGCCCTGG CTTTAAGGCC   
  
  
+ CGGTGGGCCA CCATCTCTTA GGCTCACTGG GATTGGCCCG TCTTTAGGAA GTGGGCCCGA ATCGATACGT   
  
  
+ GAAATCGGGT TGAGGCTAGC CCAATTGGCC CGATCCATGA ACATGAGGTT CGCATTTCGG GCTGTAGCAA   
  
  
+ CTTCTCGGCT CGAGGACGTG AAGCCGTCAA TGCTCGGAAT GGACCCTAGA GAAGCCGTCG CTATGAATTC   
  
  
+ CGTCATGCAT CTTCATCGAC TTTTAGGATT AGACATCAAC TCAGTTCTAA ACTGGATTCG AAACCTAAAC   
  
  
+ CCGAAGATCG TAACTTTAGT AGAACAAGAG GCGAACCATA ACCAACCTGA ATTTCTCCCC CGATTCACTG   
  
  
+ AAGCACTATG TTACTACTCA ACTATGTTCG ACTCATTAGA GACTTGTCAA GTCCAACCGC TGAAAGTCCT   
  
  
+ AGCCGAGATG TATATACAAA AGGAGATATC CAACCTAGTG TGCTACGAAG GCTCAGCCCG GGTCCAGAGG   
  
  
+ CACGAGCCGC TCGCCAAATG GCAAGCTCGG ATGGGCCAAG CCGGGTTCAA GCCGCTAGGC CTGAGTCAAA   
  
  
+ ATGCCCTTAG GCAAGCTAAC ATGTTGGTAA CATTGTTCTC CTCACAAGGG TATGACGTGG AGGAGAGAGA   
  
  
+ AGGGTGTTTG ACACTGGGCT GGCATAACCG CCCTCTCATA GTGACTTCGA CTTGGCAAGT GGGGCCCAAA   
  
  
+ ATGGACAGTC CAAGTGTGAC TCATGAGATG TCATCATCCT CAGCTTCTTC ATCTTA  

- +Up\_Stream \_Len000GTAAAA GTTAATCTTT AAAGAAGTTA ATATATGGTA GAGGTTTTTA TCAGGAGTAT   
  
  
- TCAACCGTCC ACTTACTAGG ATTACCTTTT AATGAAAATT GACAACTTAA ACGTTATCCC ATGGCTTAAA   
  
  
- CACTTCAAAT AACGGCGTGC ACGGATCGAA CACTGAAGAG CTATTAGTTT TTAATTAATC TCTCTCTGTC   
  
  
- GATTTCAAAA CTCTTATTAT GACTTTTTAA TGTAATTTAA AATAAATACA AATAAAAGTA TGCGATACAA   
  
  
- ATTGACGATA TGCGACACAA ATTGACGTAG AGGAAGATAT AAACTATTTA ACCATTTTTT TTTATTAAGT   
  
  
- TTTAATTTCT GATCATTATA AAAGTGAATA GACTATCTAA ATATTCTTTA TAAATCTATC GATTTATATA   
  
  
- AGAGAGGACT TTCTCCTCTG ACTATCAACT TAAAGTATCC TATATTTGTA CATACATGTA ACTTGCTTAG   
  
  
- AAAAAAGATA CCACTAGCAA TATTTCAATA AATAATGACA TTTGTAATTC ACTATTAATC CTATACAAGA   
  
  
- TAAAGTGCAA AATCTATCTA TCTATCTACA TTCCTCAACA TTCTTCGACA TTCTTATACA TTCCTCGACA   
  
  
- TTCCTCTACA TTCCTCGACA TTCTTCTACA TTCTCTTTGA CATTCTTCGA TGTTCCTATC TACATTGCTC   
  
  
- AACAACTTAA CAACTTCTCA ACATATCACA TTAAACAAAA TGCTACTTGC AAAACCTACT TTTTATTATT   
  
  
- ATAATTTTAT TATACGAATA ATTTTTATTT ATTTATTAAT TCAATTAATG TGTTGTTATT TTATATAATT   
  
  
- AGGGGTATTT AAATCACTTA TTTTGAAGCA GGTGAACTCT ACTAAAAAAT ATACTTGTGA TCAAACTATC   
  
  
- TTATTTGTTT CATATTTTTT TTTATTTCAA AAAACTGTTT TAACTTATGT TGAGAAGTTT ATTTAAGGAT   
  
  
- AAAATTCATT TATTAAAATT TATATTTATT TAAATACCAG TAGATATTAT TATATATAAA ATACAGCTTT   
  
  
- GAGTTTATAT TAAATGTAGC AATATAATAT AAAAATACAA ATACATTTAC AACACAAGTA TTCTATAAAT   
  
  
- ATTTAAAACA TATAACCATC ATAGTAATTT TTTTTATATG TATAAATACA TTGATTTTAT TTACTTACAA   
  
  
- TATCTTTTAG ATATTCTACA TTCCTCTACA TACTTCTACA TTCCTCTACA TTCTTCTATA TTCCTCTACA   
  
  
- TCTTTTACAT TCTTCTACAC TCTTCGACAT TCCTCTACAT TCAATAAGTG ATTTAGGATT TTTTACGCTT   
  
  
- TATCTTGTCC CCGAATTAAC TCGTGAGTTC TACGTATTAA ACACTGCCTG GTTTACCTAT TTATATCTGG   
  
  
- ACCGTTTACC CAATAAGCTA AATCCATGCT CATACCAATT CAAACCCAAA CCAATACAAA GTCCTATTCA   
  
  
- CTGTAAAGCT GAGTTAAACC AAAACCAACA TACTTGAAGC CGAGCTCTAA AAACCAAACC GGTTATGTGA   
  
  
- ATAGTACGTT TTTATTCATT GAAATATCTT TTAAAACCTA GCCTATACTA CGCCAAGCCT AGTTAAGCCT   
  
  
- AAAACCAAGT GTAAGACCAT TTGGATAAAG CCTACAGTTC ATCCTATACT CAGGTCTAGC AACCTAAAGC   
  
  
- CCATAGTCGA GTTAAAATGG TCTAGATCTA TTTAGTATTT ACATGCCCAA ATAAAAAAGG TCGAACTTAC   
  
  
- TAAAAGAAAT AATTTTTTTC TATCTAAAAT CTATTTTGTG TTGTAAAGTA AAAGTAAACA AGGGGGGATA   
  
  
- TTTAAAGACA CTGGTGGAAG TGAAAGAACG GGGTAAGGGG GGGGGGGGGG CGAGAGAGAG AGAGAGAGAG   
  
  
- AGAAGGGAAA TGTTTCCGTG GTAAGTTTAA GTTTGGTGAG AAAAATTTTG GGGTTTTTAA TAAGAGTTTT   
  
  
- ATTATGGAGT ATTATATTTA CCCAAGCATA GTGGTATATA CCCGGAGTAG AGAGTACCCT TGTATGCTTA   
  
  
- AGTTACCGAA CGGGCTATCA ATAGCCCAGT ACGGTAGCCT CGGGGCCGTT GATTAGTTAC CAGTTCGGAG   
  
  
- TCTAAACCGG AGAAACTCGG TAATATCATT GTGGAAAGAG AGACTTGAGG GGGGAGATGG GTGAGACCCT   
  
  
- GGGCCTAGGC TGGGCCCAGA TAGCACAGGC CCAGGACGAG ACTCAGGTTA CCTCACCTAA CCACTGCGCA   
  
  
- CCTGTGTGGT CTAACTTGTA CTAGACGGTG CAGTCGTCTC AGTTGCCGAC AACTGACTAC CACTGTACCC   
  
  
- CCTCCTCCTA AGGCCATATT CCAACCACGT ACGGAACGAC TGCACGCGTC TCCGGCAGGT TACGCCGCTC   
  
  
- GAGGCTGACC GACGAAGCGA TCAACTACTC TACTGGTTAC CCGACAACGG TGCACAATCA TGCCACACAC   
  
  
- CCTAGCCGTT CCATCGGCCG ATAAAACAAC TCCGAAACTC GGCCGCGGAC AAAGTCGGGC CGGGCCTTTG   
  
  
- ACCGGGCTGG ACCGGCCCGA GCCTCAAGCT CCGACTCCAC AACATAGCAG TGAAGATACT CCGAACGGGA   
  
  
- ATAGAGTTTA AACGAGTGAA GTGCCGATTA GTTCGGTAGA ACCTCCGTAA ACTCCCGGTA CTAATACAGG   
  
  
- TGCAGTAGCT GAAGCCGAAG TACTGGCCGA ACGTTACCGG CCGAAACTAA GTTCGGGACC GAAATTCCGG   
  
  
- GCCACCCGGT GGTAGAGAAT CCGAGTGACC CTAACCGGGC AGAAATCCTT CACCCGGGCT TAGCTATGCA   
  
  
- CTTTAGCCCA ACTCCGATCG GGTTAACCGG GCTAGGTACT TGTACTCCAA GCGTAAAGCC CGACATCGTT   
  
  
- GAAGAGCCGA GCTCCTGCAC TTCGGCAGTT ACGAGCCTTA CCTGGGATCT CTTCGGCAGC GATACTTAAG   
  
  
- GCAGTACGTA GAAGTAGCTG AAAATCCTAA TCTGTAGTTG AGTCAAGATT TGACCTAAGC TTTGGATTTG   
  
  
- GGCTTCTAGC ATTGAAATCA TCTTGTTCTC CGCTTGGTAT TGGTTGGACT TAAAGAGGGG GCTAAGTGAC   
  
  
- TTCGTGATAC AATGATGAGT TGATACAAGC TGAGTAATCT CTGAACAGTT CAGGTTGGCG ACTTTCAGGA   
  
  
- TCGGCTCTAC ATATATGTTT TCCTCTATAG GTTGGATCAC ACGATGCTTC CGAGTCGGGC CCAGGTCTCC   
  
  
- GTGCTCGGCG AGCGGTTTAC CGTTCGAGCC TACCCGGTTC GGCCCAAGTT CGGCGATCCG GACTCAGTTT   
  
  
- TACGGGAATC CGTTCGATTG TACAACCATT GTAACAAGAG GAGTGTTCCC ATACTGCACC TCCTCTCTCT   
  
  
- TCCCACAAAC TGTGACCCGA CCGTATTGGC GGGAGAGTAT CACTGAAGCT GAACCGTTCA CCCCGGGTTT   
  
  
- TACCTGTCAG GTTCACACTG AGTACTCTAC AGTAGTAGGA GTCGAAGAAG TAGAAT

+     GCN4\_motif

| Site Name | Organism | Position | Strand | Matrix score. | sequence | function |
| --- | --- | --- | --- | --- | --- | --- |
| GCN4\_motif | Oryza sativa | 3286 | + | 7 | TGAGTCA | cis-regulatory element involved in endosperm expression |
| GCN4\_motif | Oryza sativa | 3451 | - | 7 | TGAGTCA | cis-regulatory element involved in endosperm expression |

>HU03G02797.1   
+ +Up\_Stream \_Len000CATTTT CAATTAGAAA TTTCTTCAAT TATATACCAT CTCCAAAAAT AGTCCTCATA   
  
  
+ AGTTGGCAGG TGAATGATCC TAATGGAAAA TTACTTTTAA CTGTTGAATT TGCAATAGGG TACCGAATTT   
  
  
+ GTGAAGTTTA TTGCCGCACG TGCCTAGCTT GTGACTTCTC GATAATCAAA AATTAATTAG AGAGAGACAG   
  
  
+ CTAAAGTTTT GAGAATAATA CTGAAAAATT ACATTAAATT TTATTTATGT TTATTTTCAT ACGCTATGTT   
  
  
+ TAACTGCTAT ACGCTGTGTT TAACTGCATC TCCTTCTATA TTTGATAAAT TGGTAAAAAA AAATAATTCA   
  
  
+ AAATTAAAGA CTAGTAATAT TTTCACTTAT CTGATAGATT TATAAGAAAT ATTTAGATAG CTAAATATAT   
  
  
+ TCTCTCCTGA AAGAGGAGAC TGATAGTTGA ATTTCATAGG ATATAAACAT GTATGTACAT TGAACGAATC   
  
  
+ TTTTTTCTAT GGTGATCGTT ATAAAGTTAT TTATTACTGT AAACATTAAG TGATAATTAG GATATGTTCT   
  
  
+ ATTTCACGTT TTAGATAGAT AGATAGATGT AAGGAGTTGT AAGAAGCTGT AAGAATATGT AAGGAGCTGT   
  
  
+ AAGGAGATGT AAGGAGCTGT AAGAAGATGT AAGAGAAACT GTAAGAAGCT ACAAGGATAG ATGTAACGAG   
  
  
+ TTGTTGAATT GTTGAAGAGT TGTATAGTGT AATTTGTTTT ACGATGAACG TTTTGGATGA AAAATAATAA   
  
  
+ TATTAAAATA ATATGCTTAT TAAAAATAAA TAAATAATTA AGTTAATTAC ACAACAATAA AATATATTAA   
  
  
+ TCCCCATAAA TTTAGTGAAT AAAACTTCGT CCACTTGAGA TGATTTTTTA TATGAACACT AGTTTGATAG   
  
  
+ AATAAACAAA GTATAAAAAA AAATAAAGTT TTTTGACAAA ATTGAATACA ACTCTTCAAA TAAATTCCTA   
  
  
+ TTTTAAGTAA ATAATTTTAA ATATAAATAA ATTTATGGTC ATCTATAATA ATATATATTT TATGTCGAAA   
  
  
+ CTCAAATATA ATTTACATCG TTATATTATA TTTTTATGTT TATGTAAATG TTGTGTTCAT AAGATATTTA   
  
  
+ TAAATTTTGT ATATTGGTAG TATCATTAAA AAAAATATAC ATATTTATGT AACTAAAATA AATGAATGTT   
  
  
+ ATAGAAAATC TATAAGATGT AAGGAGATGT ATGAAGATGT AAGGAGATGT AAGAAGATAT AAGGAGATGT   
  
  
+ AGAAAATGTA AGAAGATGTG AGAAGCTGTA AGGAGATGTA AGTTATTCAC TAAATCCTAA AAAATGCGAA   
  
  
+ ATAGAACAGG GGCTTAATTG AGCACTCAAG ATGCATAATT TGTGACGGAC CAAATGGATA AATATAGACC   
  
  
+ TGGCAAATGG GTTATTCGAT TTAGGTACGA GTATGGTTAA GTTTGGGTTT GGTTATGTTT CAGGATAAGT   
  
  
+ GACATTTCGA CTCAATTTGG TTTTGGTTGT ATGAACTTCG GCTCGAGATT TTTGGTTTGG CCAATACACT   
  
  
+ TATCATGCAA AAATAAGTAA CTTTATAGAA AATTTTGGAT CGGATATGAT GCGGTTCGGA TCAATTCGGA   
  
  
+ TTTTGGTTCA CATTCTGGTA AACCTATTTC GGATGTCAAG TAGGATATGA GTCCAGATCG TTGGATTTCG   
  
  
+ GGTATCAGCT CAATTTTACC AGATCTAGAT AAATCATAAA TGTACGGGTT TATTTTTTCC AGCTTGAATG   
  
  
+ ATTTTCTTTA TTAAAAAAAG ATAGATTTTA GATAAAACAC AACATTTCAT TTTCATTTGT TCCCCCCTAT   
  
  
+ AAATTTCTGT GACCACCTTC ACTTTCTTGC CCCATTCCCC CCCCCCCCCC GCTCTCTCTC TCTCTCTCTC   
  
  
+ TCTTCCCTTT ACAAAGGCAC CATTCAAATT CAAACCACTC TTTTTAAAAC CCCAAAAATT ATTCTCAAAA   
  
  
+ TAATACCTCA TAATATAAAT GGGTTCGTAT CACCATATAT GGGCCTCATC TCTCATGGGA ACATACGAAT   
  
  
+ TCAATGGCTT GCCCGATAGT TATCGGGTCA TGCCATCGGA GCCCCGGCAA CTAATCAATG GTCAAGCCTC   
  
  
+ AGATTTGGCC TCTTTGAGCC ATTATAGTAA CACCTTTCTC TCTGAACTCC CCCCTCTACC CACTCTGGGA   
  
  
+ CCCGGATCCG ACCCGGGTCT ATCGTGTCCG GGTCCTGCTC TGAGTCCAAT GGAGTGGATT GGTGACGCGT   
  
  
+ GGACACACCA GATTGAACAT GATCTGCCAC GTCAGCAGAG TCAACGGCTG TTGACTGATG GTGACATGGG   
  
  
+ GGAGGAGGAT TCCGGTATAA GGTTGGTGCA TGCCTTGCTG ACGTGCGCAG AGGCCGTCCA ATGCGGCGAG   
  
  
+ CTCCGACTGG CTGCTTCGCT AGTTGATGAG ATGACCAATG GGCTGTTGCC ACGTGTTAGT ACGGTGTGTG   
  
  
+ GGATCGGCAA GGTAGCCGGC TATTTTGTTG AGGCTTTGAG CCGGCGCCTG TTTCAGCCCG GCCCGGAAAC   
  
  
+ TGGCCCGACC TGGCCGGGCT CGGAGTTCGA GGCTGAGGTG TTGTATCGTC ACTTCTATGA GGCTTGCCCT   
  
  
+ TATCTCAAAT TTGCTCACTT CACGGCTAAT CAAGCCATCT TGGAGGCATT TGAGGGCCAT GATTATGTCC   
  
  
+ ACGTCATCGA CTTCGGCTTC ATGACCGGCT TGCAATGGCC GGCTTTGATT CAAGCCCTGG CTTTAAGGCC   
  
  
+ CGGTGGGCCA CCATCTCTTA GGCTCACTGG GATTGGCCCG TCTTTAGGAA GTGGGCCCGA ATCGATACGT   
  
  
+ GAAATCGGGT TGAGGCTAGC CCAATTGGCC CGATCCATGA ACATGAGGTT CGCATTTCGG GCTGTAGCAA   
  
  
+ CTTCTCGGCT CGAGGACGTG AAGCCGTCAA TGCTCGGAAT GGACCCTAGA GAAGCCGTCG CTATGAATTC   
  
  
+ CGTCATGCAT CTTCATCGAC TTTTAGGATT AGACATCAAC TCAGTTCTAA ACTGGATTCG AAACCTAAAC   
  
  
+ CCGAAGATCG TAACTTTAGT AGAACAAGAG GCGAACCATA ACCAACCTGA ATTTCTCCCC CGATTCACTG   
  
  
+ AAGCACTATG TTACTACTCA ACTATGTTCG ACTCATTAGA GACTTGTCAA GTCCAACCGC TGAAAGTCCT   
  
  
+ AGCCGAGATG TATATACAAA AGGAGATATC CAACCTAGTG TGCTACGAAG GCTCAGCCCG GGTCCAGAGG   
  
  
+ CACGAGCCGC TCGCCAAATG GCAAGCTCGG ATGGGCCAAG CCGGGTTCAA GCCGCTAGGC CTGAGTCAAA   
  
  
+ ATGCCCTTAG GCAAGCTAAC ATGTTGGTAA CATTGTTCTC CTCACAAGGG TATGACGTGG AGGAGAGAGA   
  
  
+ AGGGTGTTTG ACACTGGGCT GGCATAACCG CCCTCTCATA GTGACTTCGA CTTGGCAAGT GGGGCCCAAA   
  
  
+ ATGGACAGTC CAAGTGTGAC TCATGAGATG TCATCATCCT CAGCTTCTTC ATCTTA  

- +Up\_Stream \_Len000GTAAAA GTTAATCTTT AAAGAAGTTA ATATATGGTA GAGGTTTTTA TCAGGAGTAT   
  
  
- TCAACCGTCC ACTTACTAGG ATTACCTTTT AATGAAAATT GACAACTTAA ACGTTATCCC ATGGCTTAAA   
  
  
- CACTTCAAAT AACGGCGTGC ACGGATCGAA CACTGAAGAG CTATTAGTTT TTAATTAATC TCTCTCTGTC   
  
  
- GATTTCAAAA CTCTTATTAT GACTTTTTAA TGTAATTTAA AATAAATACA AATAAAAGTA TGCGATACAA   
  
  
- ATTGACGATA TGCGACACAA ATTGACGTAG AGGAAGATAT AAACTATTTA ACCATTTTTT TTTATTAAGT   
  
  
- TTTAATTTCT GATCATTATA AAAGTGAATA GACTATCTAA ATATTCTTTA TAAATCTATC GATTTATATA   
  
  
- AGAGAGGACT TTCTCCTCTG ACTATCAACT TAAAGTATCC TATATTTGTA CATACATGTA ACTTGCTTAG   
  
  
- AAAAAAGATA CCACTAGCAA TATTTCAATA AATAATGACA TTTGTAATTC ACTATTAATC CTATACAAGA   
  
  
- TAAAGTGCAA AATCTATCTA TCTATCTACA TTCCTCAACA TTCTTCGACA TTCTTATACA TTCCTCGACA   
  
  
- TTCCTCTACA TTCCTCGACA TTCTTCTACA TTCTCTTTGA CATTCTTCGA TGTTCCTATC TACATTGCTC   
  
  
- AACAACTTAA CAACTTCTCA ACATATCACA TTAAACAAAA TGCTACTTGC AAAACCTACT TTTTATTATT   
  
  
- ATAATTTTAT TATACGAATA ATTTTTATTT ATTTATTAAT TCAATTAATG TGTTGTTATT TTATATAATT   
  
  
- AGGGGTATTT AAATCACTTA TTTTGAAGCA GGTGAACTCT ACTAAAAAAT ATACTTGTGA TCAAACTATC   
  
  
- TTATTTGTTT CATATTTTTT TTTATTTCAA AAAACTGTTT TAACTTATGT TGAGAAGTTT ATTTAAGGAT   
  
  
- AAAATTCATT TATTAAAATT TATATTTATT TAAATACCAG TAGATATTAT TATATATAAA ATACAGCTTT   
  
  
- GAGTTTATAT TAAATGTAGC AATATAATAT AAAAATACAA ATACATTTAC AACACAAGTA TTCTATAAAT   
  
  
- ATTTAAAACA TATAACCATC ATAGTAATTT TTTTTATATG TATAAATACA TTGATTTTAT TTACTTACAA   
  
  
- TATCTTTTAG ATATTCTACA TTCCTCTACA TACTTCTACA TTCCTCTACA TTCTTCTATA TTCCTCTACA   
  
  
- TCTTTTACAT TCTTCTACAC TCTTCGACAT TCCTCTACAT TCAATAAGTG ATTTAGGATT TTTTACGCTT   
  
  
- TATCTTGTCC CCGAATTAAC TCGTGAGTTC TACGTATTAA ACACTGCCTG GTTTACCTAT TTATATCTGG   
  
  
- ACCGTTTACC CAATAAGCTA AATCCATGCT CATACCAATT CAAACCCAAA CCAATACAAA GTCCTATTCA   
  
  
- CTGTAAAGCT GAGTTAAACC AAAACCAACA TACTTGAAGC CGAGCTCTAA AAACCAAACC GGTTATGTGA   
  
  
- ATAGTACGTT TTTATTCATT GAAATATCTT TTAAAACCTA GCCTATACTA CGCCAAGCCT AGTTAAGCCT   
  
  
- AAAACCAAGT GTAAGACCAT TTGGATAAAG CCTACAGTTC ATCCTATACT CAGGTCTAGC AACCTAAAGC   
  
  
- CCATAGTCGA GTTAAAATGG TCTAGATCTA TTTAGTATTT ACATGCCCAA ATAAAAAAGG TCGAACTTAC   
  
  
- TAAAAGAAAT AATTTTTTTC TATCTAAAAT CTATTTTGTG TTGTAAAGTA AAAGTAAACA AGGGGGGATA   
  
  
- TTTAAAGACA CTGGTGGAAG TGAAAGAACG GGGTAAGGGG GGGGGGGGGG CGAGAGAGAG AGAGAGAGAG   
  
  
- AGAAGGGAAA TGTTTCCGTG GTAAGTTTAA GTTTGGTGAG AAAAATTTTG GGGTTTTTAA TAAGAGTTTT   
  
  
- ATTATGGAGT ATTATATTTA CCCAAGCATA GTGGTATATA CCCGGAGTAG AGAGTACCCT TGTATGCTTA   
  
  
- AGTTACCGAA CGGGCTATCA ATAGCCCAGT ACGGTAGCCT CGGGGCCGTT GATTAGTTAC CAGTTCGGAG   
  
  
- TCTAAACCGG AGAAACTCGG TAATATCATT GTGGAAAGAG AGACTTGAGG GGGGAGATGG GTGAGACCCT   
  
  
- GGGCCTAGGC TGGGCCCAGA TAGCACAGGC CCAGGACGAG ACTCAGGTTA CCTCACCTAA CCACTGCGCA   
  
  
- CCTGTGTGGT CTAACTTGTA CTAGACGGTG CAGTCGTCTC AGTTGCCGAC AACTGACTAC CACTGTACCC   
  
  
- CCTCCTCCTA AGGCCATATT CCAACCACGT ACGGAACGAC TGCACGCGTC TCCGGCAGGT TACGCCGCTC   
  
  
- GAGGCTGACC GACGAAGCGA TCAACTACTC TACTGGTTAC CCGACAACGG TGCACAATCA TGCCACACAC   
  
  
- CCTAGCCGTT CCATCGGCCG ATAAAACAAC TCCGAAACTC GGCCGCGGAC AAAGTCGGGC CGGGCCTTTG   
  
  
- ACCGGGCTGG ACCGGCCCGA GCCTCAAGCT CCGACTCCAC AACATAGCAG TGAAGATACT CCGAACGGGA   
  
  
- ATAGAGTTTA AACGAGTGAA GTGCCGATTA GTTCGGTAGA ACCTCCGTAA ACTCCCGGTA CTAATACAGG   
  
  
- TGCAGTAGCT GAAGCCGAAG TACTGGCCGA ACGTTACCGG CCGAAACTAA GTTCGGGACC GAAATTCCGG   
  
  
- GCCACCCGGT GGTAGAGAAT CCGAGTGACC CTAACCGGGC AGAAATCCTT CACCCGGGCT TAGCTATGCA   
  
  
- CTTTAGCCCA ACTCCGATCG GGTTAACCGG GCTAGGTACT TGTACTCCAA GCGTAAAGCC CGACATCGTT   
  
  
- GAAGAGCCGA GCTCCTGCAC TTCGGCAGTT ACGAGCCTTA CCTGGGATCT CTTCGGCAGC GATACTTAAG   
  
  
- GCAGTACGTA GAAGTAGCTG AAAATCCTAA TCTGTAGTTG AGTCAAGATT TGACCTAAGC TTTGGATTTG   
  
  
- GGCTTCTAGC ATTGAAATCA TCTTGTTCTC CGCTTGGTAT TGGTTGGACT TAAAGAGGGG GCTAAGTGAC   
  
  
- TTCGTGATAC AATGATGAGT TGATACAAGC TGAGTAATCT CTGAACAGTT CAGGTTGGCG ACTTTCAGGA   
  
  
- TCGGCTCTAC ATATATGTTT TCCTCTATAG GTTGGATCAC ACGATGCTTC CGAGTCGGGC CCAGGTCTCC   
  
  
- GTGCTCGGCG AGCGGTTTAC CGTTCGAGCC TACCCGGTTC GGCCCAAGTT CGGCGATCCG GACTCAGTTT   
  
  
- TACGGGAATC CGTTCGATTG TACAACCATT GTAACAAGAG GAGTGTTCCC ATACTGCACC TCCTCTCTCT   
  
  
- TCCCACAAAC TGTGACCCGA CCGTATTGGC GGGAGAGTAT CACTGAAGCT GAACCGTTCA CCCCGGGTTT   
  
  
- TACCTGTCAG GTTCACACTG AGTACTCTAC AGTAGTAGGA GTCGAAGAAG TAGAAT

+     GT1-motif

| Site Name | Organism | Position | Strand | Matrix score. | sequence | function |
| --- | --- | --- | --- | --- | --- | --- |
| GT1-motif | Arabidopsis thaliana | 1439 | + | 6 | GGTTAA | light responsive element |

>HU03G02797.1   
+ +Up\_Stream \_Len000CATTTT CAATTAGAAA TTTCTTCAAT TATATACCAT CTCCAAAAAT AGTCCTCATA   
  
  
+ AGTTGGCAGG TGAATGATCC TAATGGAAAA TTACTTTTAA CTGTTGAATT TGCAATAGGG TACCGAATTT   
  
  
+ GTGAAGTTTA TTGCCGCACG TGCCTAGCTT GTGACTTCTC GATAATCAAA AATTAATTAG AGAGAGACAG   
  
  
+ CTAAAGTTTT GAGAATAATA CTGAAAAATT ACATTAAATT TTATTTATGT TTATTTTCAT ACGCTATGTT   
  
  
+ TAACTGCTAT ACGCTGTGTT TAACTGCATC TCCTTCTATA TTTGATAAAT TGGTAAAAAA AAATAATTCA   
  
  
+ AAATTAAAGA CTAGTAATAT TTTCACTTAT CTGATAGATT TATAAGAAAT ATTTAGATAG CTAAATATAT   
  
  
+ TCTCTCCTGA AAGAGGAGAC TGATAGTTGA ATTTCATAGG ATATAAACAT GTATGTACAT TGAACGAATC   
  
  
+ TTTTTTCTAT GGTGATCGTT ATAAAGTTAT TTATTACTGT AAACATTAAG TGATAATTAG GATATGTTCT   
  
  
+ ATTTCACGTT TTAGATAGAT AGATAGATGT AAGGAGTTGT AAGAAGCTGT AAGAATATGT AAGGAGCTGT   
  
  
+ AAGGAGATGT AAGGAGCTGT AAGAAGATGT AAGAGAAACT GTAAGAAGCT ACAAGGATAG ATGTAACGAG   
  
  
+ TTGTTGAATT GTTGAAGAGT TGTATAGTGT AATTTGTTTT ACGATGAACG TTTTGGATGA AAAATAATAA   
  
  
+ TATTAAAATA ATATGCTTAT TAAAAATAAA TAAATAATTA AGTTAATTAC ACAACAATAA AATATATTAA   
  
  
+ TCCCCATAAA TTTAGTGAAT AAAACTTCGT CCACTTGAGA TGATTTTTTA TATGAACACT AGTTTGATAG   
  
  
+ AATAAACAAA GTATAAAAAA AAATAAAGTT TTTTGACAAA ATTGAATACA ACTCTTCAAA TAAATTCCTA   
  
  
+ TTTTAAGTAA ATAATTTTAA ATATAAATAA ATTTATGGTC ATCTATAATA ATATATATTT TATGTCGAAA   
  
  
+ CTCAAATATA ATTTACATCG TTATATTATA TTTTTATGTT TATGTAAATG TTGTGTTCAT AAGATATTTA   
  
  
+ TAAATTTTGT ATATTGGTAG TATCATTAAA AAAAATATAC ATATTTATGT AACTAAAATA AATGAATGTT   
  
  
+ ATAGAAAATC TATAAGATGT AAGGAGATGT ATGAAGATGT AAGGAGATGT AAGAAGATAT AAGGAGATGT   
  
  
+ AGAAAATGTA AGAAGATGTG AGAAGCTGTA AGGAGATGTA AGTTATTCAC TAAATCCTAA AAAATGCGAA   
  
  
+ ATAGAACAGG GGCTTAATTG AGCACTCAAG ATGCATAATT TGTGACGGAC CAAATGGATA AATATAGACC   
  
  
+ TGGCAAATGG GTTATTCGAT TTAGGTACGA GTATGGTTAA GTTTGGGTTT GGTTATGTTT CAGGATAAGT   
  
  
+ GACATTTCGA CTCAATTTGG TTTTGGTTGT ATGAACTTCG GCTCGAGATT TTTGGTTTGG CCAATACACT   
  
  
+ TATCATGCAA AAATAAGTAA CTTTATAGAA AATTTTGGAT CGGATATGAT GCGGTTCGGA TCAATTCGGA   
  
  
+ TTTTGGTTCA CATTCTGGTA AACCTATTTC GGATGTCAAG TAGGATATGA GTCCAGATCG TTGGATTTCG   
  
  
+ GGTATCAGCT CAATTTTACC AGATCTAGAT AAATCATAAA TGTACGGGTT TATTTTTTCC AGCTTGAATG   
  
  
+ ATTTTCTTTA TTAAAAAAAG ATAGATTTTA GATAAAACAC AACATTTCAT TTTCATTTGT TCCCCCCTAT   
  
  
+ AAATTTCTGT GACCACCTTC ACTTTCTTGC CCCATTCCCC CCCCCCCCCC GCTCTCTCTC TCTCTCTCTC   
  
  
+ TCTTCCCTTT ACAAAGGCAC CATTCAAATT CAAACCACTC TTTTTAAAAC CCCAAAAATT ATTCTCAAAA   
  
  
+ TAATACCTCA TAATATAAAT GGGTTCGTAT CACCATATAT GGGCCTCATC TCTCATGGGA ACATACGAAT   
  
  
+ TCAATGGCTT GCCCGATAGT TATCGGGTCA TGCCATCGGA GCCCCGGCAA CTAATCAATG GTCAAGCCTC   
  
  
+ AGATTTGGCC TCTTTGAGCC ATTATAGTAA CACCTTTCTC TCTGAACTCC CCCCTCTACC CACTCTGGGA   
  
  
+ CCCGGATCCG ACCCGGGTCT ATCGTGTCCG GGTCCTGCTC TGAGTCCAAT GGAGTGGATT GGTGACGCGT   
  
  
+ GGACACACCA GATTGAACAT GATCTGCCAC GTCAGCAGAG TCAACGGCTG TTGACTGATG GTGACATGGG   
  
  
+ GGAGGAGGAT TCCGGTATAA GGTTGGTGCA TGCCTTGCTG ACGTGCGCAG AGGCCGTCCA ATGCGGCGAG   
  
  
+ CTCCGACTGG CTGCTTCGCT AGTTGATGAG ATGACCAATG GGCTGTTGCC ACGTGTTAGT ACGGTGTGTG   
  
  
+ GGATCGGCAA GGTAGCCGGC TATTTTGTTG AGGCTTTGAG CCGGCGCCTG TTTCAGCCCG GCCCGGAAAC   
  
  
+ TGGCCCGACC TGGCCGGGCT CGGAGTTCGA GGCTGAGGTG TTGTATCGTC ACTTCTATGA GGCTTGCCCT   
  
  
+ TATCTCAAAT TTGCTCACTT CACGGCTAAT CAAGCCATCT TGGAGGCATT TGAGGGCCAT GATTATGTCC   
  
  
+ ACGTCATCGA CTTCGGCTTC ATGACCGGCT TGCAATGGCC GGCTTTGATT CAAGCCCTGG CTTTAAGGCC   
  
  
+ CGGTGGGCCA CCATCTCTTA GGCTCACTGG GATTGGCCCG TCTTTAGGAA GTGGGCCCGA ATCGATACGT   
  
  
+ GAAATCGGGT TGAGGCTAGC CCAATTGGCC CGATCCATGA ACATGAGGTT CGCATTTCGG GCTGTAGCAA   
  
  
+ CTTCTCGGCT CGAGGACGTG AAGCCGTCAA TGCTCGGAAT GGACCCTAGA GAAGCCGTCG CTATGAATTC   
  
  
+ CGTCATGCAT CTTCATCGAC TTTTAGGATT AGACATCAAC TCAGTTCTAA ACTGGATTCG AAACCTAAAC   
  
  
+ CCGAAGATCG TAACTTTAGT AGAACAAGAG GCGAACCATA ACCAACCTGA ATTTCTCCCC CGATTCACTG   
  
  
+ AAGCACTATG TTACTACTCA ACTATGTTCG ACTCATTAGA GACTTGTCAA GTCCAACCGC TGAAAGTCCT   
  
  
+ AGCCGAGATG TATATACAAA AGGAGATATC CAACCTAGTG TGCTACGAAG GCTCAGCCCG GGTCCAGAGG   
  
  
+ CACGAGCCGC TCGCCAAATG GCAAGCTCGG ATGGGCCAAG CCGGGTTCAA GCCGCTAGGC CTGAGTCAAA   
  
  
+ ATGCCCTTAG GCAAGCTAAC ATGTTGGTAA CATTGTTCTC CTCACAAGGG TATGACGTGG AGGAGAGAGA   
  
  
+ AGGGTGTTTG ACACTGGGCT GGCATAACCG CCCTCTCATA GTGACTTCGA CTTGGCAAGT GGGGCCCAAA   
  
  
+ ATGGACAGTC CAAGTGTGAC TCATGAGATG TCATCATCCT CAGCTTCTTC ATCTTA  

- +Up\_Stream \_Len000GTAAAA GTTAATCTTT AAAGAAGTTA ATATATGGTA GAGGTTTTTA TCAGGAGTAT   
  
  
- TCAACCGTCC ACTTACTAGG ATTACCTTTT AATGAAAATT GACAACTTAA ACGTTATCCC ATGGCTTAAA   
  
  
- CACTTCAAAT AACGGCGTGC ACGGATCGAA CACTGAAGAG CTATTAGTTT TTAATTAATC TCTCTCTGTC   
  
  
- GATTTCAAAA CTCTTATTAT GACTTTTTAA TGTAATTTAA AATAAATACA AATAAAAGTA TGCGATACAA   
  
  
- ATTGACGATA TGCGACACAA ATTGACGTAG AGGAAGATAT AAACTATTTA ACCATTTTTT TTTATTAAGT   
  
  
- TTTAATTTCT GATCATTATA AAAGTGAATA GACTATCTAA ATATTCTTTA TAAATCTATC GATTTATATA   
  
  
- AGAGAGGACT TTCTCCTCTG ACTATCAACT TAAAGTATCC TATATTTGTA CATACATGTA ACTTGCTTAG   
  
  
- AAAAAAGATA CCACTAGCAA TATTTCAATA AATAATGACA TTTGTAATTC ACTATTAATC CTATACAAGA   
  
  
- TAAAGTGCAA AATCTATCTA TCTATCTACA TTCCTCAACA TTCTTCGACA TTCTTATACA TTCCTCGACA   
  
  
- TTCCTCTACA TTCCTCGACA TTCTTCTACA TTCTCTTTGA CATTCTTCGA TGTTCCTATC TACATTGCTC   
  
  
- AACAACTTAA CAACTTCTCA ACATATCACA TTAAACAAAA TGCTACTTGC AAAACCTACT TTTTATTATT   
  
  
- ATAATTTTAT TATACGAATA ATTTTTATTT ATTTATTAAT TCAATTAATG TGTTGTTATT TTATATAATT   
  
  
- AGGGGTATTT AAATCACTTA TTTTGAAGCA GGTGAACTCT ACTAAAAAAT ATACTTGTGA TCAAACTATC   
  
  
- TTATTTGTTT CATATTTTTT TTTATTTCAA AAAACTGTTT TAACTTATGT TGAGAAGTTT ATTTAAGGAT   
  
  
- AAAATTCATT TATTAAAATT TATATTTATT TAAATACCAG TAGATATTAT TATATATAAA ATACAGCTTT   
  
  
- GAGTTTATAT TAAATGTAGC AATATAATAT AAAAATACAA ATACATTTAC AACACAAGTA TTCTATAAAT   
  
  
- ATTTAAAACA TATAACCATC ATAGTAATTT TTTTTATATG TATAAATACA TTGATTTTAT TTACTTACAA   
  
  
- TATCTTTTAG ATATTCTACA TTCCTCTACA TACTTCTACA TTCCTCTACA TTCTTCTATA TTCCTCTACA   
  
  
- TCTTTTACAT TCTTCTACAC TCTTCGACAT TCCTCTACAT TCAATAAGTG ATTTAGGATT TTTTACGCTT   
  
  
- TATCTTGTCC CCGAATTAAC TCGTGAGTTC TACGTATTAA ACACTGCCTG GTTTACCTAT TTATATCTGG   
  
  
- ACCGTTTACC CAATAAGCTA AATCCATGCT CATACCAATT CAAACCCAAA CCAATACAAA GTCCTATTCA   
  
  
- CTGTAAAGCT GAGTTAAACC AAAACCAACA TACTTGAAGC CGAGCTCTAA AAACCAAACC GGTTATGTGA   
  
  
- ATAGTACGTT TTTATTCATT GAAATATCTT TTAAAACCTA GCCTATACTA CGCCAAGCCT AGTTAAGCCT   
  
  
- AAAACCAAGT GTAAGACCAT TTGGATAAAG CCTACAGTTC ATCCTATACT CAGGTCTAGC AACCTAAAGC   
  
  
- CCATAGTCGA GTTAAAATGG TCTAGATCTA TTTAGTATTT ACATGCCCAA ATAAAAAAGG TCGAACTTAC   
  
  
- TAAAAGAAAT AATTTTTTTC TATCTAAAAT CTATTTTGTG TTGTAAAGTA AAAGTAAACA AGGGGGGATA   
  
  
- TTTAAAGACA CTGGTGGAAG TGAAAGAACG GGGTAAGGGG GGGGGGGGGG CGAGAGAGAG AGAGAGAGAG   
  
  
- AGAAGGGAAA TGTTTCCGTG GTAAGTTTAA GTTTGGTGAG AAAAATTTTG GGGTTTTTAA TAAGAGTTTT   
  
  
- ATTATGGAGT ATTATATTTA CCCAAGCATA GTGGTATATA CCCGGAGTAG AGAGTACCCT TGTATGCTTA   
  
  
- AGTTACCGAA CGGGCTATCA ATAGCCCAGT ACGGTAGCCT CGGGGCCGTT GATTAGTTAC CAGTTCGGAG   
  
  
- TCTAAACCGG AGAAACTCGG TAATATCATT GTGGAAAGAG AGACTTGAGG GGGGAGATGG GTGAGACCCT   
  
  
- GGGCCTAGGC TGGGCCCAGA TAGCACAGGC CCAGGACGAG ACTCAGGTTA CCTCACCTAA CCACTGCGCA   
  
  
- CCTGTGTGGT CTAACTTGTA CTAGACGGTG CAGTCGTCTC AGTTGCCGAC AACTGACTAC CACTGTACCC   
  
  
- CCTCCTCCTA AGGCCATATT CCAACCACGT ACGGAACGAC TGCACGCGTC TCCGGCAGGT TACGCCGCTC   
  
  
- GAGGCTGACC GACGAAGCGA TCAACTACTC TACTGGTTAC CCGACAACGG TGCACAATCA TGCCACACAC   
  
  
- CCTAGCCGTT CCATCGGCCG ATAAAACAAC TCCGAAACTC GGCCGCGGAC AAAGTCGGGC CGGGCCTTTG   
  
  
- ACCGGGCTGG ACCGGCCCGA GCCTCAAGCT CCGACTCCAC AACATAGCAG TGAAGATACT CCGAACGGGA   
  
  
- ATAGAGTTTA AACGAGTGAA GTGCCGATTA GTTCGGTAGA ACCTCCGTAA ACTCCCGGTA CTAATACAGG   
  
  
- TGCAGTAGCT GAAGCCGAAG TACTGGCCGA ACGTTACCGG CCGAAACTAA GTTCGGGACC GAAATTCCGG   
  
  
- GCCACCCGGT GGTAGAGAAT CCGAGTGACC CTAACCGGGC AGAAATCCTT CACCCGGGCT TAGCTATGCA   
  
  
- CTTTAGCCCA ACTCCGATCG GGTTAACCGG GCTAGGTACT TGTACTCCAA GCGTAAAGCC CGACATCGTT   
  
  
- GAAGAGCCGA GCTCCTGCAC TTCGGCAGTT ACGAGCCTTA CCTGGGATCT CTTCGGCAGC GATACTTAAG   
  
  
- GCAGTACGTA GAAGTAGCTG AAAATCCTAA TCTGTAGTTG AGTCAAGATT TGACCTAAGC TTTGGATTTG   
  
  
- GGCTTCTAGC ATTGAAATCA TCTTGTTCTC CGCTTGGTAT TGGTTGGACT TAAAGAGGGG GCTAAGTGAC   
  
  
- TTCGTGATAC AATGATGAGT TGATACAAGC TGAGTAATCT CTGAACAGTT CAGGTTGGCG ACTTTCAGGA   
  
  
- TCGGCTCTAC ATATATGTTT TCCTCTATAG GTTGGATCAC ACGATGCTTC CGAGTCGGGC CCAGGTCTCC   
  
  
- GTGCTCGGCG AGCGGTTTAC CGTTCGAGCC TACCCGGTTC GGCCCAAGTT CGGCGATCCG GACTCAGTTT   
  
  
- TACGGGAATC CGTTCGATTG TACAACCATT GTAACAAGAG GAGTGTTCCC ATACTGCACC TCCTCTCTCT   
  
  
- TCCCACAAAC TGTGACCCGA CCGTATTGGC GGGAGAGTAT CACTGAAGCT GAACCGTTCA CCCCGGGTTT   
  
  
- TACCTGTCAG GTTCACACTG AGTACTCTAC AGTAGTAGGA GTCGAAGAAG TAGAAT

+     Gap-box

| Site Name | Organism | Position | Strand | Matrix score. | sequence | function |
| --- | --- | --- | --- | --- | --- | --- |
| Gap-box | Arabidopsis thaliana | 1804 | - | 9.5 | CAAATGAA(A/G)A | part of a light responsive element |

>HU03G02797.1   
+ +Up\_Stream \_Len000CATTTT CAATTAGAAA TTTCTTCAAT TATATACCAT CTCCAAAAAT AGTCCTCATA   
  
  
+ AGTTGGCAGG TGAATGATCC TAATGGAAAA TTACTTTTAA CTGTTGAATT TGCAATAGGG TACCGAATTT   
  
  
+ GTGAAGTTTA TTGCCGCACG TGCCTAGCTT GTGACTTCTC GATAATCAAA AATTAATTAG AGAGAGACAG   
  
  
+ CTAAAGTTTT GAGAATAATA CTGAAAAATT ACATTAAATT TTATTTATGT TTATTTTCAT ACGCTATGTT   
  
  
+ TAACTGCTAT ACGCTGTGTT TAACTGCATC TCCTTCTATA TTTGATAAAT TGGTAAAAAA AAATAATTCA   
  
  
+ AAATTAAAGA CTAGTAATAT TTTCACTTAT CTGATAGATT TATAAGAAAT ATTTAGATAG CTAAATATAT   
  
  
+ TCTCTCCTGA AAGAGGAGAC TGATAGTTGA ATTTCATAGG ATATAAACAT GTATGTACAT TGAACGAATC   
  
  
+ TTTTTTCTAT GGTGATCGTT ATAAAGTTAT TTATTACTGT AAACATTAAG TGATAATTAG GATATGTTCT   
  
  
+ ATTTCACGTT TTAGATAGAT AGATAGATGT AAGGAGTTGT AAGAAGCTGT AAGAATATGT AAGGAGCTGT   
  
  
+ AAGGAGATGT AAGGAGCTGT AAGAAGATGT AAGAGAAACT GTAAGAAGCT ACAAGGATAG ATGTAACGAG   
  
  
+ TTGTTGAATT GTTGAAGAGT TGTATAGTGT AATTTGTTTT ACGATGAACG TTTTGGATGA AAAATAATAA   
  
  
+ TATTAAAATA ATATGCTTAT TAAAAATAAA TAAATAATTA AGTTAATTAC ACAACAATAA AATATATTAA   
  
  
+ TCCCCATAAA TTTAGTGAAT AAAACTTCGT CCACTTGAGA TGATTTTTTA TATGAACACT AGTTTGATAG   
  
  
+ AATAAACAAA GTATAAAAAA AAATAAAGTT TTTTGACAAA ATTGAATACA ACTCTTCAAA TAAATTCCTA   
  
  
+ TTTTAAGTAA ATAATTTTAA ATATAAATAA ATTTATGGTC ATCTATAATA ATATATATTT TATGTCGAAA   
  
  
+ CTCAAATATA ATTTACATCG TTATATTATA TTTTTATGTT TATGTAAATG TTGTGTTCAT AAGATATTTA   
  
  
+ TAAATTTTGT ATATTGGTAG TATCATTAAA AAAAATATAC ATATTTATGT AACTAAAATA AATGAATGTT   
  
  
+ ATAGAAAATC TATAAGATGT AAGGAGATGT ATGAAGATGT AAGGAGATGT AAGAAGATAT AAGGAGATGT   
  
  
+ AGAAAATGTA AGAAGATGTG AGAAGCTGTA AGGAGATGTA AGTTATTCAC TAAATCCTAA AAAATGCGAA   
  
  
+ ATAGAACAGG GGCTTAATTG AGCACTCAAG ATGCATAATT TGTGACGGAC CAAATGGATA AATATAGACC   
  
  
+ TGGCAAATGG GTTATTCGAT TTAGGTACGA GTATGGTTAA GTTTGGGTTT GGTTATGTTT CAGGATAAGT   
  
  
+ GACATTTCGA CTCAATTTGG TTTTGGTTGT ATGAACTTCG GCTCGAGATT TTTGGTTTGG CCAATACACT   
  
  
+ TATCATGCAA AAATAAGTAA CTTTATAGAA AATTTTGGAT CGGATATGAT GCGGTTCGGA TCAATTCGGA   
  
  
+ TTTTGGTTCA CATTCTGGTA AACCTATTTC GGATGTCAAG TAGGATATGA GTCCAGATCG TTGGATTTCG   
  
  
+ GGTATCAGCT CAATTTTACC AGATCTAGAT AAATCATAAA TGTACGGGTT TATTTTTTCC AGCTTGAATG   
  
  
+ ATTTTCTTTA TTAAAAAAAG ATAGATTTTA GATAAAACAC AACATTTCAT TTTCATTTGT TCCCCCCTAT   
  
  
+ AAATTTCTGT GACCACCTTC ACTTTCTTGC CCCATTCCCC CCCCCCCCCC GCTCTCTCTC TCTCTCTCTC   
  
  
+ TCTTCCCTTT ACAAAGGCAC CATTCAAATT CAAACCACTC TTTTTAAAAC CCCAAAAATT ATTCTCAAAA   
  
  
+ TAATACCTCA TAATATAAAT GGGTTCGTAT CACCATATAT GGGCCTCATC TCTCATGGGA ACATACGAAT   
  
  
+ TCAATGGCTT GCCCGATAGT TATCGGGTCA TGCCATCGGA GCCCCGGCAA CTAATCAATG GTCAAGCCTC   
  
  
+ AGATTTGGCC TCTTTGAGCC ATTATAGTAA CACCTTTCTC TCTGAACTCC CCCCTCTACC CACTCTGGGA   
  
  
+ CCCGGATCCG ACCCGGGTCT ATCGTGTCCG GGTCCTGCTC TGAGTCCAAT GGAGTGGATT GGTGACGCGT   
  
  
+ GGACACACCA GATTGAACAT GATCTGCCAC GTCAGCAGAG TCAACGGCTG TTGACTGATG GTGACATGGG   
  
  
+ GGAGGAGGAT TCCGGTATAA GGTTGGTGCA TGCCTTGCTG ACGTGCGCAG AGGCCGTCCA ATGCGGCGAG   
  
  
+ CTCCGACTGG CTGCTTCGCT AGTTGATGAG ATGACCAATG GGCTGTTGCC ACGTGTTAGT ACGGTGTGTG   
  
  
+ GGATCGGCAA GGTAGCCGGC TATTTTGTTG AGGCTTTGAG CCGGCGCCTG TTTCAGCCCG GCCCGGAAAC   
  
  
+ TGGCCCGACC TGGCCGGGCT CGGAGTTCGA GGCTGAGGTG TTGTATCGTC ACTTCTATGA GGCTTGCCCT   
  
  
+ TATCTCAAAT TTGCTCACTT CACGGCTAAT CAAGCCATCT TGGAGGCATT TGAGGGCCAT GATTATGTCC   
  
  
+ ACGTCATCGA CTTCGGCTTC ATGACCGGCT TGCAATGGCC GGCTTTGATT CAAGCCCTGG CTTTAAGGCC   
  
  
+ CGGTGGGCCA CCATCTCTTA GGCTCACTGG GATTGGCCCG TCTTTAGGAA GTGGGCCCGA ATCGATACGT   
  
  
+ GAAATCGGGT TGAGGCTAGC CCAATTGGCC CGATCCATGA ACATGAGGTT CGCATTTCGG GCTGTAGCAA   
  
  
+ CTTCTCGGCT CGAGGACGTG AAGCCGTCAA TGCTCGGAAT GGACCCTAGA GAAGCCGTCG CTATGAATTC   
  
  
+ CGTCATGCAT CTTCATCGAC TTTTAGGATT AGACATCAAC TCAGTTCTAA ACTGGATTCG AAACCTAAAC   
  
  
+ CCGAAGATCG TAACTTTAGT AGAACAAGAG GCGAACCATA ACCAACCTGA ATTTCTCCCC CGATTCACTG   
  
  
+ AAGCACTATG TTACTACTCA ACTATGTTCG ACTCATTAGA GACTTGTCAA GTCCAACCGC TGAAAGTCCT   
  
  
+ AGCCGAGATG TATATACAAA AGGAGATATC CAACCTAGTG TGCTACGAAG GCTCAGCCCG GGTCCAGAGG   
  
  
+ CACGAGCCGC TCGCCAAATG GCAAGCTCGG ATGGGCCAAG CCGGGTTCAA GCCGCTAGGC CTGAGTCAAA   
  
  
+ ATGCCCTTAG GCAAGCTAAC ATGTTGGTAA CATTGTTCTC CTCACAAGGG TATGACGTGG AGGAGAGAGA   
  
  
+ AGGGTGTTTG ACACTGGGCT GGCATAACCG CCCTCTCATA GTGACTTCGA CTTGGCAAGT GGGGCCCAAA   
  
  
+ ATGGACAGTC CAAGTGTGAC TCATGAGATG TCATCATCCT CAGCTTCTTC ATCTTA  

- +Up\_Stream \_Len000GTAAAA GTTAATCTTT AAAGAAGTTA ATATATGGTA GAGGTTTTTA TCAGGAGTAT   
  
  
- TCAACCGTCC ACTTACTAGG ATTACCTTTT AATGAAAATT GACAACTTAA ACGTTATCCC ATGGCTTAAA   
  
  
- CACTTCAAAT AACGGCGTGC ACGGATCGAA CACTGAAGAG CTATTAGTTT TTAATTAATC TCTCTCTGTC   
  
  
- GATTTCAAAA CTCTTATTAT GACTTTTTAA TGTAATTTAA AATAAATACA AATAAAAGTA TGCGATACAA   
  
  
- ATTGACGATA TGCGACACAA ATTGACGTAG AGGAAGATAT AAACTATTTA ACCATTTTTT TTTATTAAGT   
  
  
- TTTAATTTCT GATCATTATA AAAGTGAATA GACTATCTAA ATATTCTTTA TAAATCTATC GATTTATATA   
  
  
- AGAGAGGACT TTCTCCTCTG ACTATCAACT TAAAGTATCC TATATTTGTA CATACATGTA ACTTGCTTAG   
  
  
- AAAAAAGATA CCACTAGCAA TATTTCAATA AATAATGACA TTTGTAATTC ACTATTAATC CTATACAAGA   
  
  
- TAAAGTGCAA AATCTATCTA TCTATCTACA TTCCTCAACA TTCTTCGACA TTCTTATACA TTCCTCGACA   
  
  
- TTCCTCTACA TTCCTCGACA TTCTTCTACA TTCTCTTTGA CATTCTTCGA TGTTCCTATC TACATTGCTC   
  
  
- AACAACTTAA CAACTTCTCA ACATATCACA TTAAACAAAA TGCTACTTGC AAAACCTACT TTTTATTATT   
  
  
- ATAATTTTAT TATACGAATA ATTTTTATTT ATTTATTAAT TCAATTAATG TGTTGTTATT TTATATAATT   
  
  
- AGGGGTATTT AAATCACTTA TTTTGAAGCA GGTGAACTCT ACTAAAAAAT ATACTTGTGA TCAAACTATC   
  
  
- TTATTTGTTT CATATTTTTT TTTATTTCAA AAAACTGTTT TAACTTATGT TGAGAAGTTT ATTTAAGGAT   
  
  
- AAAATTCATT TATTAAAATT TATATTTATT TAAATACCAG TAGATATTAT TATATATAAA ATACAGCTTT   
  
  
- GAGTTTATAT TAAATGTAGC AATATAATAT AAAAATACAA ATACATTTAC AACACAAGTA TTCTATAAAT   
  
  
- ATTTAAAACA TATAACCATC ATAGTAATTT TTTTTATATG TATAAATACA TTGATTTTAT TTACTTACAA   
  
  
- TATCTTTTAG ATATTCTACA TTCCTCTACA TACTTCTACA TTCCTCTACA TTCTTCTATA TTCCTCTACA   
  
  
- TCTTTTACAT TCTTCTACAC TCTTCGACAT TCCTCTACAT TCAATAAGTG ATTTAGGATT TTTTACGCTT   
  
  
- TATCTTGTCC CCGAATTAAC TCGTGAGTTC TACGTATTAA ACACTGCCTG GTTTACCTAT TTATATCTGG   
  
  
- ACCGTTTACC CAATAAGCTA AATCCATGCT CATACCAATT CAAACCCAAA CCAATACAAA GTCCTATTCA   
  
  
- CTGTAAAGCT GAGTTAAACC AAAACCAACA TACTTGAAGC CGAGCTCTAA AAACCAAACC GGTTATGTGA   
  
  
- ATAGTACGTT TTTATTCATT GAAATATCTT TTAAAACCTA GCCTATACTA CGCCAAGCCT AGTTAAGCCT   
  
  
- AAAACCAAGT GTAAGACCAT TTGGATAAAG CCTACAGTTC ATCCTATACT CAGGTCTAGC AACCTAAAGC   
  
  
- CCATAGTCGA GTTAAAATGG TCTAGATCTA TTTAGTATTT ACATGCCCAA ATAAAAAAGG TCGAACTTAC   
  
  
- TAAAAGAAAT AATTTTTTTC TATCTAAAAT CTATTTTGTG TTGTAAAGTA AAAGTAAACA AGGGGGGATA   
  
  
- TTTAAAGACA CTGGTGGAAG TGAAAGAACG GGGTAAGGGG GGGGGGGGGG CGAGAGAGAG AGAGAGAGAG   
  
  
- AGAAGGGAAA TGTTTCCGTG GTAAGTTTAA GTTTGGTGAG AAAAATTTTG GGGTTTTTAA TAAGAGTTTT   
  
  
- ATTATGGAGT ATTATATTTA CCCAAGCATA GTGGTATATA CCCGGAGTAG AGAGTACCCT TGTATGCTTA   
  
  
- AGTTACCGAA CGGGCTATCA ATAGCCCAGT ACGGTAGCCT CGGGGCCGTT GATTAGTTAC CAGTTCGGAG   
  
  
- TCTAAACCGG AGAAACTCGG TAATATCATT GTGGAAAGAG AGACTTGAGG GGGGAGATGG GTGAGACCCT   
  
  
- GGGCCTAGGC TGGGCCCAGA TAGCACAGGC CCAGGACGAG ACTCAGGTTA CCTCACCTAA CCACTGCGCA   
  
  
- CCTGTGTGGT CTAACTTGTA CTAGACGGTG CAGTCGTCTC AGTTGCCGAC AACTGACTAC CACTGTACCC   
  
  
- CCTCCTCCTA AGGCCATATT CCAACCACGT ACGGAACGAC TGCACGCGTC TCCGGCAGGT TACGCCGCTC   
  
  
- GAGGCTGACC GACGAAGCGA TCAACTACTC TACTGGTTAC CCGACAACGG TGCACAATCA TGCCACACAC   
  
  
- CCTAGCCGTT CCATCGGCCG ATAAAACAAC TCCGAAACTC GGCCGCGGAC AAAGTCGGGC CGGGCCTTTG   
  
  
- ACCGGGCTGG ACCGGCCCGA GCCTCAAGCT CCGACTCCAC AACATAGCAG TGAAGATACT CCGAACGGGA   
  
  
- ATAGAGTTTA AACGAGTGAA GTGCCGATTA GTTCGGTAGA ACCTCCGTAA ACTCCCGGTA CTAATACAGG   
  
  
- TGCAGTAGCT GAAGCCGAAG TACTGGCCGA ACGTTACCGG CCGAAACTAA GTTCGGGACC GAAATTCCGG   
  
  
- GCCACCCGGT GGTAGAGAAT CCGAGTGACC CTAACCGGGC AGAAATCCTT CACCCGGGCT TAGCTATGCA   
  
  
- CTTTAGCCCA ACTCCGATCG GGTTAACCGG GCTAGGTACT TGTACTCCAA GCGTAAAGCC CGACATCGTT   
  
  
- GAAGAGCCGA GCTCCTGCAC TTCGGCAGTT ACGAGCCTTA CCTGGGATCT CTTCGGCAGC GATACTTAAG   
  
  
- GCAGTACGTA GAAGTAGCTG AAAATCCTAA TCTGTAGTTG AGTCAAGATT TGACCTAAGC TTTGGATTTG   
  
  
- GGCTTCTAGC ATTGAAATCA TCTTGTTCTC CGCTTGGTAT TGGTTGGACT TAAAGAGGGG GCTAAGTGAC   
  
  
- TTCGTGATAC AATGATGAGT TGATACAAGC TGAGTAATCT CTGAACAGTT CAGGTTGGCG ACTTTCAGGA   
  
  
- TCGGCTCTAC ATATATGTTT TCCTCTATAG GTTGGATCAC ACGATGCTTC CGAGTCGGGC CCAGGTCTCC   
  
  
- GTGCTCGGCG AGCGGTTTAC CGTTCGAGCC TACCCGGTTC GGCCCAAGTT CGGCGATCCG GACTCAGTTT   
  
  
- TACGGGAATC CGTTCGATTG TACAACCATT GTAACAAGAG GAGTGTTCCC ATACTGCACC TCCTCTCTCT   
  
  
- TCCCACAAAC TGTGACCCGA CCGTATTGGC GGGAGAGTAT CACTGAAGCT GAACCGTTCA CCCCGGGTTT   
  
  
- TACCTGTCAG GTTCACACTG AGTACTCTAC AGTAGTAGGA GTCGAAGAAG TAGAAT

+     I-box

| Site Name | Organism | Position | Strand | Matrix score. | sequence | function |
| --- | --- | --- | --- | --- | --- | --- |
| I-box | Triticum aestivum | 2592 | - | 8 | AGATAAGG | part of a light responsive element |

>HU03G02797.1   
+ +Up\_Stream \_Len000CATTTT CAATTAGAAA TTTCTTCAAT TATATACCAT CTCCAAAAAT AGTCCTCATA   
  
  
+ AGTTGGCAGG TGAATGATCC TAATGGAAAA TTACTTTTAA CTGTTGAATT TGCAATAGGG TACCGAATTT   
  
  
+ GTGAAGTTTA TTGCCGCACG TGCCTAGCTT GTGACTTCTC GATAATCAAA AATTAATTAG AGAGAGACAG   
  
  
+ CTAAAGTTTT GAGAATAATA CTGAAAAATT ACATTAAATT TTATTTATGT TTATTTTCAT ACGCTATGTT   
  
  
+ TAACTGCTAT ACGCTGTGTT TAACTGCATC TCCTTCTATA TTTGATAAAT TGGTAAAAAA AAATAATTCA   
  
  
+ AAATTAAAGA CTAGTAATAT TTTCACTTAT CTGATAGATT TATAAGAAAT ATTTAGATAG CTAAATATAT   
  
  
+ TCTCTCCTGA AAGAGGAGAC TGATAGTTGA ATTTCATAGG ATATAAACAT GTATGTACAT TGAACGAATC   
  
  
+ TTTTTTCTAT GGTGATCGTT ATAAAGTTAT TTATTACTGT AAACATTAAG TGATAATTAG GATATGTTCT   
  
  
+ ATTTCACGTT TTAGATAGAT AGATAGATGT AAGGAGTTGT AAGAAGCTGT AAGAATATGT AAGGAGCTGT   
  
  
+ AAGGAGATGT AAGGAGCTGT AAGAAGATGT AAGAGAAACT GTAAGAAGCT ACAAGGATAG ATGTAACGAG   
  
  
+ TTGTTGAATT GTTGAAGAGT TGTATAGTGT AATTTGTTTT ACGATGAACG TTTTGGATGA AAAATAATAA   
  
  
+ TATTAAAATA ATATGCTTAT TAAAAATAAA TAAATAATTA AGTTAATTAC ACAACAATAA AATATATTAA   
  
  
+ TCCCCATAAA TTTAGTGAAT AAAACTTCGT CCACTTGAGA TGATTTTTTA TATGAACACT AGTTTGATAG   
  
  
+ AATAAACAAA GTATAAAAAA AAATAAAGTT TTTTGACAAA ATTGAATACA ACTCTTCAAA TAAATTCCTA   
  
  
+ TTTTAAGTAA ATAATTTTAA ATATAAATAA ATTTATGGTC ATCTATAATA ATATATATTT TATGTCGAAA   
  
  
+ CTCAAATATA ATTTACATCG TTATATTATA TTTTTATGTT TATGTAAATG TTGTGTTCAT AAGATATTTA   
  
  
+ TAAATTTTGT ATATTGGTAG TATCATTAAA AAAAATATAC ATATTTATGT AACTAAAATA AATGAATGTT   
  
  
+ ATAGAAAATC TATAAGATGT AAGGAGATGT ATGAAGATGT AAGGAGATGT AAGAAGATAT AAGGAGATGT   
  
  
+ AGAAAATGTA AGAAGATGTG AGAAGCTGTA AGGAGATGTA AGTTATTCAC TAAATCCTAA AAAATGCGAA   
  
  
+ ATAGAACAGG GGCTTAATTG AGCACTCAAG ATGCATAATT TGTGACGGAC CAAATGGATA AATATAGACC   
  
  
+ TGGCAAATGG GTTATTCGAT TTAGGTACGA GTATGGTTAA GTTTGGGTTT GGTTATGTTT CAGGATAAGT   
  
  
+ GACATTTCGA CTCAATTTGG TTTTGGTTGT ATGAACTTCG GCTCGAGATT TTTGGTTTGG CCAATACACT   
  
  
+ TATCATGCAA AAATAAGTAA CTTTATAGAA AATTTTGGAT CGGATATGAT GCGGTTCGGA TCAATTCGGA   
  
  
+ TTTTGGTTCA CATTCTGGTA AACCTATTTC GGATGTCAAG TAGGATATGA GTCCAGATCG TTGGATTTCG   
  
  
+ GGTATCAGCT CAATTTTACC AGATCTAGAT AAATCATAAA TGTACGGGTT TATTTTTTCC AGCTTGAATG   
  
  
+ ATTTTCTTTA TTAAAAAAAG ATAGATTTTA GATAAAACAC AACATTTCAT TTTCATTTGT TCCCCCCTAT   
  
  
+ AAATTTCTGT GACCACCTTC ACTTTCTTGC CCCATTCCCC CCCCCCCCCC GCTCTCTCTC TCTCTCTCTC   
  
  
+ TCTTCCCTTT ACAAAGGCAC CATTCAAATT CAAACCACTC TTTTTAAAAC CCCAAAAATT ATTCTCAAAA   
  
  
+ TAATACCTCA TAATATAAAT GGGTTCGTAT CACCATATAT GGGCCTCATC TCTCATGGGA ACATACGAAT   
  
  
+ TCAATGGCTT GCCCGATAGT TATCGGGTCA TGCCATCGGA GCCCCGGCAA CTAATCAATG GTCAAGCCTC   
  
  
+ AGATTTGGCC TCTTTGAGCC ATTATAGTAA CACCTTTCTC TCTGAACTCC CCCCTCTACC CACTCTGGGA   
  
  
+ CCCGGATCCG ACCCGGGTCT ATCGTGTCCG GGTCCTGCTC TGAGTCCAAT GGAGTGGATT GGTGACGCGT   
  
  
+ GGACACACCA GATTGAACAT GATCTGCCAC GTCAGCAGAG TCAACGGCTG TTGACTGATG GTGACATGGG   
  
  
+ GGAGGAGGAT TCCGGTATAA GGTTGGTGCA TGCCTTGCTG ACGTGCGCAG AGGCCGTCCA ATGCGGCGAG   
  
  
+ CTCCGACTGG CTGCTTCGCT AGTTGATGAG ATGACCAATG GGCTGTTGCC ACGTGTTAGT ACGGTGTGTG   
  
  
+ GGATCGGCAA GGTAGCCGGC TATTTTGTTG AGGCTTTGAG CCGGCGCCTG TTTCAGCCCG GCCCGGAAAC   
  
  
+ TGGCCCGACC TGGCCGGGCT CGGAGTTCGA GGCTGAGGTG TTGTATCGTC ACTTCTATGA GGCTTGCCCT   
  
  
+ TATCTCAAAT TTGCTCACTT CACGGCTAAT CAAGCCATCT TGGAGGCATT TGAGGGCCAT GATTATGTCC   
  
  
+ ACGTCATCGA CTTCGGCTTC ATGACCGGCT TGCAATGGCC GGCTTTGATT CAAGCCCTGG CTTTAAGGCC   
  
  
+ CGGTGGGCCA CCATCTCTTA GGCTCACTGG GATTGGCCCG TCTTTAGGAA GTGGGCCCGA ATCGATACGT   
  
  
+ GAAATCGGGT TGAGGCTAGC CCAATTGGCC CGATCCATGA ACATGAGGTT CGCATTTCGG GCTGTAGCAA   
  
  
+ CTTCTCGGCT CGAGGACGTG AAGCCGTCAA TGCTCGGAAT GGACCCTAGA GAAGCCGTCG CTATGAATTC   
  
  
+ CGTCATGCAT CTTCATCGAC TTTTAGGATT AGACATCAAC TCAGTTCTAA ACTGGATTCG AAACCTAAAC   
  
  
+ CCGAAGATCG TAACTTTAGT AGAACAAGAG GCGAACCATA ACCAACCTGA ATTTCTCCCC CGATTCACTG   
  
  
+ AAGCACTATG TTACTACTCA ACTATGTTCG ACTCATTAGA GACTTGTCAA GTCCAACCGC TGAAAGTCCT   
  
  
+ AGCCGAGATG TATATACAAA AGGAGATATC CAACCTAGTG TGCTACGAAG GCTCAGCCCG GGTCCAGAGG   
  
  
+ CACGAGCCGC TCGCCAAATG GCAAGCTCGG ATGGGCCAAG CCGGGTTCAA GCCGCTAGGC CTGAGTCAAA   
  
  
+ ATGCCCTTAG GCAAGCTAAC ATGTTGGTAA CATTGTTCTC CTCACAAGGG TATGACGTGG AGGAGAGAGA   
  
  
+ AGGGTGTTTG ACACTGGGCT GGCATAACCG CCCTCTCATA GTGACTTCGA CTTGGCAAGT GGGGCCCAAA   
  
  
+ ATGGACAGTC CAAGTGTGAC TCATGAGATG TCATCATCCT CAGCTTCTTC ATCTTA  

- +Up\_Stream \_Len000GTAAAA GTTAATCTTT AAAGAAGTTA ATATATGGTA GAGGTTTTTA TCAGGAGTAT   
  
  
- TCAACCGTCC ACTTACTAGG ATTACCTTTT AATGAAAATT GACAACTTAA ACGTTATCCC ATGGCTTAAA   
  
  
- CACTTCAAAT AACGGCGTGC ACGGATCGAA CACTGAAGAG CTATTAGTTT TTAATTAATC TCTCTCTGTC   
  
  
- GATTTCAAAA CTCTTATTAT GACTTTTTAA TGTAATTTAA AATAAATACA AATAAAAGTA TGCGATACAA   
  
  
- ATTGACGATA TGCGACACAA ATTGACGTAG AGGAAGATAT AAACTATTTA ACCATTTTTT TTTATTAAGT   
  
  
- TTTAATTTCT GATCATTATA AAAGTGAATA GACTATCTAA ATATTCTTTA TAAATCTATC GATTTATATA   
  
  
- AGAGAGGACT TTCTCCTCTG ACTATCAACT TAAAGTATCC TATATTTGTA CATACATGTA ACTTGCTTAG   
  
  
- AAAAAAGATA CCACTAGCAA TATTTCAATA AATAATGACA TTTGTAATTC ACTATTAATC CTATACAAGA   
  
  
- TAAAGTGCAA AATCTATCTA TCTATCTACA TTCCTCAACA TTCTTCGACA TTCTTATACA TTCCTCGACA   
  
  
- TTCCTCTACA TTCCTCGACA TTCTTCTACA TTCTCTTTGA CATTCTTCGA TGTTCCTATC TACATTGCTC   
  
  
- AACAACTTAA CAACTTCTCA ACATATCACA TTAAACAAAA TGCTACTTGC AAAACCTACT TTTTATTATT   
  
  
- ATAATTTTAT TATACGAATA ATTTTTATTT ATTTATTAAT TCAATTAATG TGTTGTTATT TTATATAATT   
  
  
- AGGGGTATTT AAATCACTTA TTTTGAAGCA GGTGAACTCT ACTAAAAAAT ATACTTGTGA TCAAACTATC   
  
  
- TTATTTGTTT CATATTTTTT TTTATTTCAA AAAACTGTTT TAACTTATGT TGAGAAGTTT ATTTAAGGAT   
  
  
- AAAATTCATT TATTAAAATT TATATTTATT TAAATACCAG TAGATATTAT TATATATAAA ATACAGCTTT   
  
  
- GAGTTTATAT TAAATGTAGC AATATAATAT AAAAATACAA ATACATTTAC AACACAAGTA TTCTATAAAT   
  
  
- ATTTAAAACA TATAACCATC ATAGTAATTT TTTTTATATG TATAAATACA TTGATTTTAT TTACTTACAA   
  
  
- TATCTTTTAG ATATTCTACA TTCCTCTACA TACTTCTACA TTCCTCTACA TTCTTCTATA TTCCTCTACA   
  
  
- TCTTTTACAT TCTTCTACAC TCTTCGACAT TCCTCTACAT TCAATAAGTG ATTTAGGATT TTTTACGCTT   
  
  
- TATCTTGTCC CCGAATTAAC TCGTGAGTTC TACGTATTAA ACACTGCCTG GTTTACCTAT TTATATCTGG   
  
  
- ACCGTTTACC CAATAAGCTA AATCCATGCT CATACCAATT CAAACCCAAA CCAATACAAA GTCCTATTCA   
  
  
- CTGTAAAGCT GAGTTAAACC AAAACCAACA TACTTGAAGC CGAGCTCTAA AAACCAAACC GGTTATGTGA   
  
  
- ATAGTACGTT TTTATTCATT GAAATATCTT TTAAAACCTA GCCTATACTA CGCCAAGCCT AGTTAAGCCT   
  
  
- AAAACCAAGT GTAAGACCAT TTGGATAAAG CCTACAGTTC ATCCTATACT CAGGTCTAGC AACCTAAAGC   
  
  
- CCATAGTCGA GTTAAAATGG TCTAGATCTA TTTAGTATTT ACATGCCCAA ATAAAAAAGG TCGAACTTAC   
  
  
- TAAAAGAAAT AATTTTTTTC TATCTAAAAT CTATTTTGTG TTGTAAAGTA AAAGTAAACA AGGGGGGATA   
  
  
- TTTAAAGACA CTGGTGGAAG TGAAAGAACG GGGTAAGGGG GGGGGGGGGG CGAGAGAGAG AGAGAGAGAG   
  
  
- AGAAGGGAAA TGTTTCCGTG GTAAGTTTAA GTTTGGTGAG AAAAATTTTG GGGTTTTTAA TAAGAGTTTT   
  
  
- ATTATGGAGT ATTATATTTA CCCAAGCATA GTGGTATATA CCCGGAGTAG AGAGTACCCT TGTATGCTTA   
  
  
- AGTTACCGAA CGGGCTATCA ATAGCCCAGT ACGGTAGCCT CGGGGCCGTT GATTAGTTAC CAGTTCGGAG   
  
  
- TCTAAACCGG AGAAACTCGG TAATATCATT GTGGAAAGAG AGACTTGAGG GGGGAGATGG GTGAGACCCT   
  
  
- GGGCCTAGGC TGGGCCCAGA TAGCACAGGC CCAGGACGAG ACTCAGGTTA CCTCACCTAA CCACTGCGCA   
  
  
- CCTGTGTGGT CTAACTTGTA CTAGACGGTG CAGTCGTCTC AGTTGCCGAC AACTGACTAC CACTGTACCC   
  
  
- CCTCCTCCTA AGGCCATATT CCAACCACGT ACGGAACGAC TGCACGCGTC TCCGGCAGGT TACGCCGCTC   
  
  
- GAGGCTGACC GACGAAGCGA TCAACTACTC TACTGGTTAC CCGACAACGG TGCACAATCA TGCCACACAC   
  
  
- CCTAGCCGTT CCATCGGCCG ATAAAACAAC TCCGAAACTC GGCCGCGGAC AAAGTCGGGC CGGGCCTTTG   
  
  
- ACCGGGCTGG ACCGGCCCGA GCCTCAAGCT CCGACTCCAC AACATAGCAG TGAAGATACT CCGAACGGGA   
  
  
- ATAGAGTTTA AACGAGTGAA GTGCCGATTA GTTCGGTAGA ACCTCCGTAA ACTCCCGGTA CTAATACAGG   
  
  
- TGCAGTAGCT GAAGCCGAAG TACTGGCCGA ACGTTACCGG CCGAAACTAA GTTCGGGACC GAAATTCCGG   
  
  
- GCCACCCGGT GGTAGAGAAT CCGAGTGACC CTAACCGGGC AGAAATCCTT CACCCGGGCT TAGCTATGCA   
  
  
- CTTTAGCCCA ACTCCGATCG GGTTAACCGG GCTAGGTACT TGTACTCCAA GCGTAAAGCC CGACATCGTT   
  
  
- GAAGAGCCGA GCTCCTGCAC TTCGGCAGTT ACGAGCCTTA CCTGGGATCT CTTCGGCAGC GATACTTAAG   
  
  
- GCAGTACGTA GAAGTAGCTG AAAATCCTAA TCTGTAGTTG AGTCAAGATT TGACCTAAGC TTTGGATTTG   
  
  
- GGCTTCTAGC ATTGAAATCA TCTTGTTCTC CGCTTGGTAT TGGTTGGACT TAAAGAGGGG GCTAAGTGAC   
  
  
- TTCGTGATAC AATGATGAGT TGATACAAGC TGAGTAATCT CTGAACAGTT CAGGTTGGCG ACTTTCAGGA   
  
  
- TCGGCTCTAC ATATATGTTT TCCTCTATAG GTTGGATCAC ACGATGCTTC CGAGTCGGGC CCAGGTCTCC   
  
  
- GTGCTCGGCG AGCGGTTTAC CGTTCGAGCC TACCCGGTTC GGCCCAAGTT CGGCGATCCG GACTCAGTTT   
  
  
- TACGGGAATC CGTTCGATTG TACAACCATT GTAACAAGAG GAGTGTTCCC ATACTGCACC TCCTCTCTCT   
  
  
- TCCCACAAAC TGTGACCCGA CCGTATTGGC GGGAGAGTAT CACTGAAGCT GAACCGTTCA CCCCGGGTTT   
  
  
- TACCTGTCAG GTTCACACTG AGTACTCTAC AGTAGTAGGA GTCGAAGAAG TAGAAT

+     LTR

| Site Name | Organism | Position | Strand | Matrix score. | sequence | function |
| --- | --- | --- | --- | --- | --- | --- |
| LTR | Hordeum vulgare | 2859 | - | 6 | CCGAAA | cis-acting element involved in low-temperature responsiveness |
| LTR | Hordeum vulgare | 1680 | - | 6 | CCGAAA | cis-acting element involved in low-temperature responsiveness |
| LTR | Hordeum vulgare | 1641 | - | 6 | CCGAAA | cis-acting element involved in low-temperature responsiveness |

>HU03G02797.1   
+ +Up\_Stream \_Len000CATTTT CAATTAGAAA TTTCTTCAAT TATATACCAT CTCCAAAAAT AGTCCTCATA   
  
  
+ AGTTGGCAGG TGAATGATCC TAATGGAAAA TTACTTTTAA CTGTTGAATT TGCAATAGGG TACCGAATTT   
  
  
+ GTGAAGTTTA TTGCCGCACG TGCCTAGCTT GTGACTTCTC GATAATCAAA AATTAATTAG AGAGAGACAG   
  
  
+ CTAAAGTTTT GAGAATAATA CTGAAAAATT ACATTAAATT TTATTTATGT TTATTTTCAT ACGCTATGTT   
  
  
+ TAACTGCTAT ACGCTGTGTT TAACTGCATC TCCTTCTATA TTTGATAAAT TGGTAAAAAA AAATAATTCA   
  
  
+ AAATTAAAGA CTAGTAATAT TTTCACTTAT CTGATAGATT TATAAGAAAT ATTTAGATAG CTAAATATAT   
  
  
+ TCTCTCCTGA AAGAGGAGAC TGATAGTTGA ATTTCATAGG ATATAAACAT GTATGTACAT TGAACGAATC   
  
  
+ TTTTTTCTAT GGTGATCGTT ATAAAGTTAT TTATTACTGT AAACATTAAG TGATAATTAG GATATGTTCT   
  
  
+ ATTTCACGTT TTAGATAGAT AGATAGATGT AAGGAGTTGT AAGAAGCTGT AAGAATATGT AAGGAGCTGT   
  
  
+ AAGGAGATGT AAGGAGCTGT AAGAAGATGT AAGAGAAACT GTAAGAAGCT ACAAGGATAG ATGTAACGAG   
  
  
+ TTGTTGAATT GTTGAAGAGT TGTATAGTGT AATTTGTTTT ACGATGAACG TTTTGGATGA AAAATAATAA   
  
  
+ TATTAAAATA ATATGCTTAT TAAAAATAAA TAAATAATTA AGTTAATTAC ACAACAATAA AATATATTAA   
  
  
+ TCCCCATAAA TTTAGTGAAT AAAACTTCGT CCACTTGAGA TGATTTTTTA TATGAACACT AGTTTGATAG   
  
  
+ AATAAACAAA GTATAAAAAA AAATAAAGTT TTTTGACAAA ATTGAATACA ACTCTTCAAA TAAATTCCTA   
  
  
+ TTTTAAGTAA ATAATTTTAA ATATAAATAA ATTTATGGTC ATCTATAATA ATATATATTT TATGTCGAAA   
  
  
+ CTCAAATATA ATTTACATCG TTATATTATA TTTTTATGTT TATGTAAATG TTGTGTTCAT AAGATATTTA   
  
  
+ TAAATTTTGT ATATTGGTAG TATCATTAAA AAAAATATAC ATATTTATGT AACTAAAATA AATGAATGTT   
  
  
+ ATAGAAAATC TATAAGATGT AAGGAGATGT ATGAAGATGT AAGGAGATGT AAGAAGATAT AAGGAGATGT   
  
  
+ AGAAAATGTA AGAAGATGTG AGAAGCTGTA AGGAGATGTA AGTTATTCAC TAAATCCTAA AAAATGCGAA   
  
  
+ ATAGAACAGG GGCTTAATTG AGCACTCAAG ATGCATAATT TGTGACGGAC CAAATGGATA AATATAGACC   
  
  
+ TGGCAAATGG GTTATTCGAT TTAGGTACGA GTATGGTTAA GTTTGGGTTT GGTTATGTTT CAGGATAAGT   
  
  
+ GACATTTCGA CTCAATTTGG TTTTGGTTGT ATGAACTTCG GCTCGAGATT TTTGGTTTGG CCAATACACT   
  
  
+ TATCATGCAA AAATAAGTAA CTTTATAGAA AATTTTGGAT CGGATATGAT GCGGTTCGGA TCAATTCGGA   
  
  
+ TTTTGGTTCA CATTCTGGTA AACCTATTTC GGATGTCAAG TAGGATATGA GTCCAGATCG TTGGATTTCG   
  
  
+ GGTATCAGCT CAATTTTACC AGATCTAGAT AAATCATAAA TGTACGGGTT TATTTTTTCC AGCTTGAATG   
  
  
+ ATTTTCTTTA TTAAAAAAAG ATAGATTTTA GATAAAACAC AACATTTCAT TTTCATTTGT TCCCCCCTAT   
  
  
+ AAATTTCTGT GACCACCTTC ACTTTCTTGC CCCATTCCCC CCCCCCCCCC GCTCTCTCTC TCTCTCTCTC   
  
  
+ TCTTCCCTTT ACAAAGGCAC CATTCAAATT CAAACCACTC TTTTTAAAAC CCCAAAAATT ATTCTCAAAA   
  
  
+ TAATACCTCA TAATATAAAT GGGTTCGTAT CACCATATAT GGGCCTCATC TCTCATGGGA ACATACGAAT   
  
  
+ TCAATGGCTT GCCCGATAGT TATCGGGTCA TGCCATCGGA GCCCCGGCAA CTAATCAATG GTCAAGCCTC   
  
  
+ AGATTTGGCC TCTTTGAGCC ATTATAGTAA CACCTTTCTC TCTGAACTCC CCCCTCTACC CACTCTGGGA   
  
  
+ CCCGGATCCG ACCCGGGTCT ATCGTGTCCG GGTCCTGCTC TGAGTCCAAT GGAGTGGATT GGTGACGCGT   
  
  
+ GGACACACCA GATTGAACAT GATCTGCCAC GTCAGCAGAG TCAACGGCTG TTGACTGATG GTGACATGGG   
  
  
+ GGAGGAGGAT TCCGGTATAA GGTTGGTGCA TGCCTTGCTG ACGTGCGCAG AGGCCGTCCA ATGCGGCGAG   
  
  
+ CTCCGACTGG CTGCTTCGCT AGTTGATGAG ATGACCAATG GGCTGTTGCC ACGTGTTAGT ACGGTGTGTG   
  
  
+ GGATCGGCAA GGTAGCCGGC TATTTTGTTG AGGCTTTGAG CCGGCGCCTG TTTCAGCCCG GCCCGGAAAC   
  
  
+ TGGCCCGACC TGGCCGGGCT CGGAGTTCGA GGCTGAGGTG TTGTATCGTC ACTTCTATGA GGCTTGCCCT   
  
  
+ TATCTCAAAT TTGCTCACTT CACGGCTAAT CAAGCCATCT TGGAGGCATT TGAGGGCCAT GATTATGTCC   
  
  
+ ACGTCATCGA CTTCGGCTTC ATGACCGGCT TGCAATGGCC GGCTTTGATT CAAGCCCTGG CTTTAAGGCC   
  
  
+ CGGTGGGCCA CCATCTCTTA GGCTCACTGG GATTGGCCCG TCTTTAGGAA GTGGGCCCGA ATCGATACGT   
  
  
+ GAAATCGGGT TGAGGCTAGC CCAATTGGCC CGATCCATGA ACATGAGGTT CGCATTTCGG GCTGTAGCAA   
  
  
+ CTTCTCGGCT CGAGGACGTG AAGCCGTCAA TGCTCGGAAT GGACCCTAGA GAAGCCGTCG CTATGAATTC   
  
  
+ CGTCATGCAT CTTCATCGAC TTTTAGGATT AGACATCAAC TCAGTTCTAA ACTGGATTCG AAACCTAAAC   
  
  
+ CCGAAGATCG TAACTTTAGT AGAACAAGAG GCGAACCATA ACCAACCTGA ATTTCTCCCC CGATTCACTG   
  
  
+ AAGCACTATG TTACTACTCA ACTATGTTCG ACTCATTAGA GACTTGTCAA GTCCAACCGC TGAAAGTCCT   
  
  
+ AGCCGAGATG TATATACAAA AGGAGATATC CAACCTAGTG TGCTACGAAG GCTCAGCCCG GGTCCAGAGG   
  
  
+ CACGAGCCGC TCGCCAAATG GCAAGCTCGG ATGGGCCAAG CCGGGTTCAA GCCGCTAGGC CTGAGTCAAA   
  
  
+ ATGCCCTTAG GCAAGCTAAC ATGTTGGTAA CATTGTTCTC CTCACAAGGG TATGACGTGG AGGAGAGAGA   
  
  
+ AGGGTGTTTG ACACTGGGCT GGCATAACCG CCCTCTCATA GTGACTTCGA CTTGGCAAGT GGGGCCCAAA   
  
  
+ ATGGACAGTC CAAGTGTGAC TCATGAGATG TCATCATCCT CAGCTTCTTC ATCTTA  

- +Up\_Stream \_Len000GTAAAA GTTAATCTTT AAAGAAGTTA ATATATGGTA GAGGTTTTTA TCAGGAGTAT   
  
  
- TCAACCGTCC ACTTACTAGG ATTACCTTTT AATGAAAATT GACAACTTAA ACGTTATCCC ATGGCTTAAA   
  
  
- CACTTCAAAT AACGGCGTGC ACGGATCGAA CACTGAAGAG CTATTAGTTT TTAATTAATC TCTCTCTGTC   
  
  
- GATTTCAAAA CTCTTATTAT GACTTTTTAA TGTAATTTAA AATAAATACA AATAAAAGTA TGCGATACAA   
  
  
- ATTGACGATA TGCGACACAA ATTGACGTAG AGGAAGATAT AAACTATTTA ACCATTTTTT TTTATTAAGT   
  
  
- TTTAATTTCT GATCATTATA AAAGTGAATA GACTATCTAA ATATTCTTTA TAAATCTATC GATTTATATA   
  
  
- AGAGAGGACT TTCTCCTCTG ACTATCAACT TAAAGTATCC TATATTTGTA CATACATGTA ACTTGCTTAG   
  
  
- AAAAAAGATA CCACTAGCAA TATTTCAATA AATAATGACA TTTGTAATTC ACTATTAATC CTATACAAGA   
  
  
- TAAAGTGCAA AATCTATCTA TCTATCTACA TTCCTCAACA TTCTTCGACA TTCTTATACA TTCCTCGACA   
  
  
- TTCCTCTACA TTCCTCGACA TTCTTCTACA TTCTCTTTGA CATTCTTCGA TGTTCCTATC TACATTGCTC   
  
  
- AACAACTTAA CAACTTCTCA ACATATCACA TTAAACAAAA TGCTACTTGC AAAACCTACT TTTTATTATT   
  
  
- ATAATTTTAT TATACGAATA ATTTTTATTT ATTTATTAAT TCAATTAATG TGTTGTTATT TTATATAATT   
  
  
- AGGGGTATTT AAATCACTTA TTTTGAAGCA GGTGAACTCT ACTAAAAAAT ATACTTGTGA TCAAACTATC   
  
  
- TTATTTGTTT CATATTTTTT TTTATTTCAA AAAACTGTTT TAACTTATGT TGAGAAGTTT ATTTAAGGAT   
  
  
- AAAATTCATT TATTAAAATT TATATTTATT TAAATACCAG TAGATATTAT TATATATAAA ATACAGCTTT   
  
  
- GAGTTTATAT TAAATGTAGC AATATAATAT AAAAATACAA ATACATTTAC AACACAAGTA TTCTATAAAT   
  
  
- ATTTAAAACA TATAACCATC ATAGTAATTT TTTTTATATG TATAAATACA TTGATTTTAT TTACTTACAA   
  
  
- TATCTTTTAG ATATTCTACA TTCCTCTACA TACTTCTACA TTCCTCTACA TTCTTCTATA TTCCTCTACA   
  
  
- TCTTTTACAT TCTTCTACAC TCTTCGACAT TCCTCTACAT TCAATAAGTG ATTTAGGATT TTTTACGCTT   
  
  
- TATCTTGTCC CCGAATTAAC TCGTGAGTTC TACGTATTAA ACACTGCCTG GTTTACCTAT TTATATCTGG   
  
  
- ACCGTTTACC CAATAAGCTA AATCCATGCT CATACCAATT CAAACCCAAA CCAATACAAA GTCCTATTCA   
  
  
- CTGTAAAGCT GAGTTAAACC AAAACCAACA TACTTGAAGC CGAGCTCTAA AAACCAAACC GGTTATGTGA   
  
  
- ATAGTACGTT TTTATTCATT GAAATATCTT TTAAAACCTA GCCTATACTA CGCCAAGCCT AGTTAAGCCT   
  
  
- AAAACCAAGT GTAAGACCAT TTGGATAAAG CCTACAGTTC ATCCTATACT CAGGTCTAGC AACCTAAAGC   
  
  
- CCATAGTCGA GTTAAAATGG TCTAGATCTA TTTAGTATTT ACATGCCCAA ATAAAAAAGG TCGAACTTAC   
  
  
- TAAAAGAAAT AATTTTTTTC TATCTAAAAT CTATTTTGTG TTGTAAAGTA AAAGTAAACA AGGGGGGATA   
  
  
- TTTAAAGACA CTGGTGGAAG TGAAAGAACG GGGTAAGGGG GGGGGGGGGG CGAGAGAGAG AGAGAGAGAG   
  
  
- AGAAGGGAAA TGTTTCCGTG GTAAGTTTAA GTTTGGTGAG AAAAATTTTG GGGTTTTTAA TAAGAGTTTT   
  
  
- ATTATGGAGT ATTATATTTA CCCAAGCATA GTGGTATATA CCCGGAGTAG AGAGTACCCT TGTATGCTTA   
  
  
- AGTTACCGAA CGGGCTATCA ATAGCCCAGT ACGGTAGCCT CGGGGCCGTT GATTAGTTAC CAGTTCGGAG   
  
  
- TCTAAACCGG AGAAACTCGG TAATATCATT GTGGAAAGAG AGACTTGAGG GGGGAGATGG GTGAGACCCT   
  
  
- GGGCCTAGGC TGGGCCCAGA TAGCACAGGC CCAGGACGAG ACTCAGGTTA CCTCACCTAA CCACTGCGCA   
  
  
- CCTGTGTGGT CTAACTTGTA CTAGACGGTG CAGTCGTCTC AGTTGCCGAC AACTGACTAC CACTGTACCC   
  
  
- CCTCCTCCTA AGGCCATATT CCAACCACGT ACGGAACGAC TGCACGCGTC TCCGGCAGGT TACGCCGCTC   
  
  
- GAGGCTGACC GACGAAGCGA TCAACTACTC TACTGGTTAC CCGACAACGG TGCACAATCA TGCCACACAC   
  
  
- CCTAGCCGTT CCATCGGCCG ATAAAACAAC TCCGAAACTC GGCCGCGGAC AAAGTCGGGC CGGGCCTTTG   
  
  
- ACCGGGCTGG ACCGGCCCGA GCCTCAAGCT CCGACTCCAC AACATAGCAG TGAAGATACT CCGAACGGGA   
  
  
- ATAGAGTTTA AACGAGTGAA GTGCCGATTA GTTCGGTAGA ACCTCCGTAA ACTCCCGGTA CTAATACAGG   
  
  
- TGCAGTAGCT GAAGCCGAAG TACTGGCCGA ACGTTACCGG CCGAAACTAA GTTCGGGACC GAAATTCCGG   
  
  
- GCCACCCGGT GGTAGAGAAT CCGAGTGACC CTAACCGGGC AGAAATCCTT CACCCGGGCT TAGCTATGCA   
  
  
- CTTTAGCCCA ACTCCGATCG GGTTAACCGG GCTAGGTACT TGTACTCCAA GCGTAAAGCC CGACATCGTT   
  
  
- GAAGAGCCGA GCTCCTGCAC TTCGGCAGTT ACGAGCCTTA CCTGGGATCT CTTCGGCAGC GATACTTAAG   
  
  
- GCAGTACGTA GAAGTAGCTG AAAATCCTAA TCTGTAGTTG AGTCAAGATT TGACCTAAGC TTTGGATTTG   
  
  
- GGCTTCTAGC ATTGAAATCA TCTTGTTCTC CGCTTGGTAT TGGTTGGACT TAAAGAGGGG GCTAAGTGAC   
  
  
- TTCGTGATAC AATGATGAGT TGATACAAGC TGAGTAATCT CTGAACAGTT CAGGTTGGCG ACTTTCAGGA   
  
  
- TCGGCTCTAC ATATATGTTT TCCTCTATAG GTTGGATCAC ACGATGCTTC CGAGTCGGGC CCAGGTCTCC   
  
  
- GTGCTCGGCG AGCGGTTTAC CGTTCGAGCC TACCCGGTTC GGCCCAAGTT CGGCGATCCG GACTCAGTTT   
  
  
- TACGGGAATC CGTTCGATTG TACAACCATT GTAACAAGAG GAGTGTTCCC ATACTGCACC TCCTCTCTCT   
  
  
- TCCCACAAAC TGTGACCCGA CCGTATTGGC GGGAGAGTAT CACTGAAGCT GAACCGTTCA CCCCGGGTTT   
  
  
- TACCTGTCAG GTTCACACTG AGTACTCTAC AGTAGTAGGA GTCGAAGAAG TAGAAT

+     MRE

| Site Name | Organism | Position | Strand | Matrix score. | sequence | function |
| --- | --- | --- | --- | --- | --- | --- |
| MRE | Petroselinum crispum | 3006 | + | 7 | AACCTAA | MYB binding site involved in light responsiveness |

>HU03G02797.1   
+ +Up\_Stream \_Len000CATTTT CAATTAGAAA TTTCTTCAAT TATATACCAT CTCCAAAAAT AGTCCTCATA   
  
  
+ AGTTGGCAGG TGAATGATCC TAATGGAAAA TTACTTTTAA CTGTTGAATT TGCAATAGGG TACCGAATTT   
  
  
+ GTGAAGTTTA TTGCCGCACG TGCCTAGCTT GTGACTTCTC GATAATCAAA AATTAATTAG AGAGAGACAG   
  
  
+ CTAAAGTTTT GAGAATAATA CTGAAAAATT ACATTAAATT TTATTTATGT TTATTTTCAT ACGCTATGTT   
  
  
+ TAACTGCTAT ACGCTGTGTT TAACTGCATC TCCTTCTATA TTTGATAAAT TGGTAAAAAA AAATAATTCA   
  
  
+ AAATTAAAGA CTAGTAATAT TTTCACTTAT CTGATAGATT TATAAGAAAT ATTTAGATAG CTAAATATAT   
  
  
+ TCTCTCCTGA AAGAGGAGAC TGATAGTTGA ATTTCATAGG ATATAAACAT GTATGTACAT TGAACGAATC   
  
  
+ TTTTTTCTAT GGTGATCGTT ATAAAGTTAT TTATTACTGT AAACATTAAG TGATAATTAG GATATGTTCT   
  
  
+ ATTTCACGTT TTAGATAGAT AGATAGATGT AAGGAGTTGT AAGAAGCTGT AAGAATATGT AAGGAGCTGT   
  
  
+ AAGGAGATGT AAGGAGCTGT AAGAAGATGT AAGAGAAACT GTAAGAAGCT ACAAGGATAG ATGTAACGAG   
  
  
+ TTGTTGAATT GTTGAAGAGT TGTATAGTGT AATTTGTTTT ACGATGAACG TTTTGGATGA AAAATAATAA   
  
  
+ TATTAAAATA ATATGCTTAT TAAAAATAAA TAAATAATTA AGTTAATTAC ACAACAATAA AATATATTAA   
  
  
+ TCCCCATAAA TTTAGTGAAT AAAACTTCGT CCACTTGAGA TGATTTTTTA TATGAACACT AGTTTGATAG   
  
  
+ AATAAACAAA GTATAAAAAA AAATAAAGTT TTTTGACAAA ATTGAATACA ACTCTTCAAA TAAATTCCTA   
  
  
+ TTTTAAGTAA ATAATTTTAA ATATAAATAA ATTTATGGTC ATCTATAATA ATATATATTT TATGTCGAAA   
  
  
+ CTCAAATATA ATTTACATCG TTATATTATA TTTTTATGTT TATGTAAATG TTGTGTTCAT AAGATATTTA   
  
  
+ TAAATTTTGT ATATTGGTAG TATCATTAAA AAAAATATAC ATATTTATGT AACTAAAATA AATGAATGTT   
  
  
+ ATAGAAAATC TATAAGATGT AAGGAGATGT ATGAAGATGT AAGGAGATGT AAGAAGATAT AAGGAGATGT   
  
  
+ AGAAAATGTA AGAAGATGTG AGAAGCTGTA AGGAGATGTA AGTTATTCAC TAAATCCTAA AAAATGCGAA   
  
  
+ ATAGAACAGG GGCTTAATTG AGCACTCAAG ATGCATAATT TGTGACGGAC CAAATGGATA AATATAGACC   
  
  
+ TGGCAAATGG GTTATTCGAT TTAGGTACGA GTATGGTTAA GTTTGGGTTT GGTTATGTTT CAGGATAAGT   
  
  
+ GACATTTCGA CTCAATTTGG TTTTGGTTGT ATGAACTTCG GCTCGAGATT TTTGGTTTGG CCAATACACT   
  
  
+ TATCATGCAA AAATAAGTAA CTTTATAGAA AATTTTGGAT CGGATATGAT GCGGTTCGGA TCAATTCGGA   
  
  
+ TTTTGGTTCA CATTCTGGTA AACCTATTTC GGATGTCAAG TAGGATATGA GTCCAGATCG TTGGATTTCG   
  
  
+ GGTATCAGCT CAATTTTACC AGATCTAGAT AAATCATAAA TGTACGGGTT TATTTTTTCC AGCTTGAATG   
  
  
+ ATTTTCTTTA TTAAAAAAAG ATAGATTTTA GATAAAACAC AACATTTCAT TTTCATTTGT TCCCCCCTAT   
  
  
+ AAATTTCTGT GACCACCTTC ACTTTCTTGC CCCATTCCCC CCCCCCCCCC GCTCTCTCTC TCTCTCTCTC   
  
  
+ TCTTCCCTTT ACAAAGGCAC CATTCAAATT CAAACCACTC TTTTTAAAAC CCCAAAAATT ATTCTCAAAA   
  
  
+ TAATACCTCA TAATATAAAT GGGTTCGTAT CACCATATAT GGGCCTCATC TCTCATGGGA ACATACGAAT   
  
  
+ TCAATGGCTT GCCCGATAGT TATCGGGTCA TGCCATCGGA GCCCCGGCAA CTAATCAATG GTCAAGCCTC   
  
  
+ AGATTTGGCC TCTTTGAGCC ATTATAGTAA CACCTTTCTC TCTGAACTCC CCCCTCTACC CACTCTGGGA   
  
  
+ CCCGGATCCG ACCCGGGTCT ATCGTGTCCG GGTCCTGCTC TGAGTCCAAT GGAGTGGATT GGTGACGCGT   
  
  
+ GGACACACCA GATTGAACAT GATCTGCCAC GTCAGCAGAG TCAACGGCTG TTGACTGATG GTGACATGGG   
  
  
+ GGAGGAGGAT TCCGGTATAA GGTTGGTGCA TGCCTTGCTG ACGTGCGCAG AGGCCGTCCA ATGCGGCGAG   
  
  
+ CTCCGACTGG CTGCTTCGCT AGTTGATGAG ATGACCAATG GGCTGTTGCC ACGTGTTAGT ACGGTGTGTG   
  
  
+ GGATCGGCAA GGTAGCCGGC TATTTTGTTG AGGCTTTGAG CCGGCGCCTG TTTCAGCCCG GCCCGGAAAC   
  
  
+ TGGCCCGACC TGGCCGGGCT CGGAGTTCGA GGCTGAGGTG TTGTATCGTC ACTTCTATGA GGCTTGCCCT   
  
  
+ TATCTCAAAT TTGCTCACTT CACGGCTAAT CAAGCCATCT TGGAGGCATT TGAGGGCCAT GATTATGTCC   
  
  
+ ACGTCATCGA CTTCGGCTTC ATGACCGGCT TGCAATGGCC GGCTTTGATT CAAGCCCTGG CTTTAAGGCC   
  
  
+ CGGTGGGCCA CCATCTCTTA GGCTCACTGG GATTGGCCCG TCTTTAGGAA GTGGGCCCGA ATCGATACGT   
  
  
+ GAAATCGGGT TGAGGCTAGC CCAATTGGCC CGATCCATGA ACATGAGGTT CGCATTTCGG GCTGTAGCAA   
  
  
+ CTTCTCGGCT CGAGGACGTG AAGCCGTCAA TGCTCGGAAT GGACCCTAGA GAAGCCGTCG CTATGAATTC   
  
  
+ CGTCATGCAT CTTCATCGAC TTTTAGGATT AGACATCAAC TCAGTTCTAA ACTGGATTCG AAACCTAAAC   
  
  
+ CCGAAGATCG TAACTTTAGT AGAACAAGAG GCGAACCATA ACCAACCTGA ATTTCTCCCC CGATTCACTG   
  
  
+ AAGCACTATG TTACTACTCA ACTATGTTCG ACTCATTAGA GACTTGTCAA GTCCAACCGC TGAAAGTCCT   
  
  
+ AGCCGAGATG TATATACAAA AGGAGATATC CAACCTAGTG TGCTACGAAG GCTCAGCCCG GGTCCAGAGG   
  
  
+ CACGAGCCGC TCGCCAAATG GCAAGCTCGG ATGGGCCAAG CCGGGTTCAA GCCGCTAGGC CTGAGTCAAA   
  
  
+ ATGCCCTTAG GCAAGCTAAC ATGTTGGTAA CATTGTTCTC CTCACAAGGG TATGACGTGG AGGAGAGAGA   
  
  
+ AGGGTGTTTG ACACTGGGCT GGCATAACCG CCCTCTCATA GTGACTTCGA CTTGGCAAGT GGGGCCCAAA   
  
  
+ ATGGACAGTC CAAGTGTGAC TCATGAGATG TCATCATCCT CAGCTTCTTC ATCTTA  

- +Up\_Stream \_Len000GTAAAA GTTAATCTTT AAAGAAGTTA ATATATGGTA GAGGTTTTTA TCAGGAGTAT   
  
  
- TCAACCGTCC ACTTACTAGG ATTACCTTTT AATGAAAATT GACAACTTAA ACGTTATCCC ATGGCTTAAA   
  
  
- CACTTCAAAT AACGGCGTGC ACGGATCGAA CACTGAAGAG CTATTAGTTT TTAATTAATC TCTCTCTGTC   
  
  
- GATTTCAAAA CTCTTATTAT GACTTTTTAA TGTAATTTAA AATAAATACA AATAAAAGTA TGCGATACAA   
  
  
- ATTGACGATA TGCGACACAA ATTGACGTAG AGGAAGATAT AAACTATTTA ACCATTTTTT TTTATTAAGT   
  
  
- TTTAATTTCT GATCATTATA AAAGTGAATA GACTATCTAA ATATTCTTTA TAAATCTATC GATTTATATA   
  
  
- AGAGAGGACT TTCTCCTCTG ACTATCAACT TAAAGTATCC TATATTTGTA CATACATGTA ACTTGCTTAG   
  
  
- AAAAAAGATA CCACTAGCAA TATTTCAATA AATAATGACA TTTGTAATTC ACTATTAATC CTATACAAGA   
  
  
- TAAAGTGCAA AATCTATCTA TCTATCTACA TTCCTCAACA TTCTTCGACA TTCTTATACA TTCCTCGACA   
  
  
- TTCCTCTACA TTCCTCGACA TTCTTCTACA TTCTCTTTGA CATTCTTCGA TGTTCCTATC TACATTGCTC   
  
  
- AACAACTTAA CAACTTCTCA ACATATCACA TTAAACAAAA TGCTACTTGC AAAACCTACT TTTTATTATT   
  
  
- ATAATTTTAT TATACGAATA ATTTTTATTT ATTTATTAAT TCAATTAATG TGTTGTTATT TTATATAATT   
  
  
- AGGGGTATTT AAATCACTTA TTTTGAAGCA GGTGAACTCT ACTAAAAAAT ATACTTGTGA TCAAACTATC   
  
  
- TTATTTGTTT CATATTTTTT TTTATTTCAA AAAACTGTTT TAACTTATGT TGAGAAGTTT ATTTAAGGAT   
  
  
- AAAATTCATT TATTAAAATT TATATTTATT TAAATACCAG TAGATATTAT TATATATAAA ATACAGCTTT   
  
  
- GAGTTTATAT TAAATGTAGC AATATAATAT AAAAATACAA ATACATTTAC AACACAAGTA TTCTATAAAT   
  
  
- ATTTAAAACA TATAACCATC ATAGTAATTT TTTTTATATG TATAAATACA TTGATTTTAT TTACTTACAA   
  
  
- TATCTTTTAG ATATTCTACA TTCCTCTACA TACTTCTACA TTCCTCTACA TTCTTCTATA TTCCTCTACA   
  
  
- TCTTTTACAT TCTTCTACAC TCTTCGACAT TCCTCTACAT TCAATAAGTG ATTTAGGATT TTTTACGCTT   
  
  
- TATCTTGTCC CCGAATTAAC TCGTGAGTTC TACGTATTAA ACACTGCCTG GTTTACCTAT TTATATCTGG   
  
  
- ACCGTTTACC CAATAAGCTA AATCCATGCT CATACCAATT CAAACCCAAA CCAATACAAA GTCCTATTCA   
  
  
- CTGTAAAGCT GAGTTAAACC AAAACCAACA TACTTGAAGC CGAGCTCTAA AAACCAAACC GGTTATGTGA   
  
  
- ATAGTACGTT TTTATTCATT GAAATATCTT TTAAAACCTA GCCTATACTA CGCCAAGCCT AGTTAAGCCT   
  
  
- AAAACCAAGT GTAAGACCAT TTGGATAAAG CCTACAGTTC ATCCTATACT CAGGTCTAGC AACCTAAAGC   
  
  
- CCATAGTCGA GTTAAAATGG TCTAGATCTA TTTAGTATTT ACATGCCCAA ATAAAAAAGG TCGAACTTAC   
  
  
- TAAAAGAAAT AATTTTTTTC TATCTAAAAT CTATTTTGTG TTGTAAAGTA AAAGTAAACA AGGGGGGATA   
  
  
- TTTAAAGACA CTGGTGGAAG TGAAAGAACG GGGTAAGGGG GGGGGGGGGG CGAGAGAGAG AGAGAGAGAG   
  
  
- AGAAGGGAAA TGTTTCCGTG GTAAGTTTAA GTTTGGTGAG AAAAATTTTG GGGTTTTTAA TAAGAGTTTT   
  
  
- ATTATGGAGT ATTATATTTA CCCAAGCATA GTGGTATATA CCCGGAGTAG AGAGTACCCT TGTATGCTTA   
  
  
- AGTTACCGAA CGGGCTATCA ATAGCCCAGT ACGGTAGCCT CGGGGCCGTT GATTAGTTAC CAGTTCGGAG   
  
  
- TCTAAACCGG AGAAACTCGG TAATATCATT GTGGAAAGAG AGACTTGAGG GGGGAGATGG GTGAGACCCT   
  
  
- GGGCCTAGGC TGGGCCCAGA TAGCACAGGC CCAGGACGAG ACTCAGGTTA CCTCACCTAA CCACTGCGCA   
  
  
- CCTGTGTGGT CTAACTTGTA CTAGACGGTG CAGTCGTCTC AGTTGCCGAC AACTGACTAC CACTGTACCC   
  
  
- CCTCCTCCTA AGGCCATATT CCAACCACGT ACGGAACGAC TGCACGCGTC TCCGGCAGGT TACGCCGCTC   
  
  
- GAGGCTGACC GACGAAGCGA TCAACTACTC TACTGGTTAC CCGACAACGG TGCACAATCA TGCCACACAC   
  
  
- CCTAGCCGTT CCATCGGCCG ATAAAACAAC TCCGAAACTC GGCCGCGGAC AAAGTCGGGC CGGGCCTTTG   
  
  
- ACCGGGCTGG ACCGGCCCGA GCCTCAAGCT CCGACTCCAC AACATAGCAG TGAAGATACT CCGAACGGGA   
  
  
- ATAGAGTTTA AACGAGTGAA GTGCCGATTA GTTCGGTAGA ACCTCCGTAA ACTCCCGGTA CTAATACAGG   
  
  
- TGCAGTAGCT GAAGCCGAAG TACTGGCCGA ACGTTACCGG CCGAAACTAA GTTCGGGACC GAAATTCCGG   
  
  
- GCCACCCGGT GGTAGAGAAT CCGAGTGACC CTAACCGGGC AGAAATCCTT CACCCGGGCT TAGCTATGCA   
  
  
- CTTTAGCCCA ACTCCGATCG GGTTAACCGG GCTAGGTACT TGTACTCCAA GCGTAAAGCC CGACATCGTT   
  
  
- GAAGAGCCGA GCTCCTGCAC TTCGGCAGTT ACGAGCCTTA CCTGGGATCT CTTCGGCAGC GATACTTAAG   
  
  
- GCAGTACGTA GAAGTAGCTG AAAATCCTAA TCTGTAGTTG AGTCAAGATT TGACCTAAGC TTTGGATTTG   
  
  
- GGCTTCTAGC ATTGAAATCA TCTTGTTCTC CGCTTGGTAT TGGTTGGACT TAAAGAGGGG GCTAAGTGAC   
  
  
- TTCGTGATAC AATGATGAGT TGATACAAGC TGAGTAATCT CTGAACAGTT CAGGTTGGCG ACTTTCAGGA   
  
  
- TCGGCTCTAC ATATATGTTT TCCTCTATAG GTTGGATCAC ACGATGCTTC CGAGTCGGGC CCAGGTCTCC   
  
  
- GTGCTCGGCG AGCGGTTTAC CGTTCGAGCC TACCCGGTTC GGCCCAAGTT CGGCGATCCG GACTCAGTTT   
  
  
- TACGGGAATC CGTTCGATTG TACAACCATT GTAACAAGAG GAGTGTTCCC ATACTGCACC TCCTCTCTCT   
  
  
- TCCCACAAAC TGTGACCCGA CCGTATTGGC GGGAGAGTAT CACTGAAGCT GAACCGTTCA CCCCGGGTTT   
  
  
- TACCTGTCAG GTTCACACTG AGTACTCTAC AGTAGTAGGA GTCGAAGAAG TAGAAT

+     MYB

| Site Name | Organism | Position | Strand | Matrix score. | sequence | function |
| --- | --- | --- | --- | --- | --- | --- |
| MYB | Arabidopsis thaliana | 1454 | - | 6 | TAACCA |  |
| MYB | Arabidopsis thaliana | 2292 | - | 6 | CAACAG |  |
| MYB | Arabidopsis thaliana | 1438 | - | 6 | TAACCA |  |
| MYB | Arabidopsis thaliana | 3053 | + | 6 | TAACCA |  |
| MYB | Arabidopsis thaliana | 115 | - | 6 | CAACAG |  |
| MYB | Arabidopsis thaliana | 2427 | - | 6 | CAACAG |  |
| MYB | Arabidopsis thaliana | 1498 | - | 6 | CAACCA |  |

>HU03G02797.1   
+ +Up\_Stream \_Len000CATTTT CAATTAGAAA TTTCTTCAAT TATATACCAT CTCCAAAAAT AGTCCTCATA   
  
  
+ AGTTGGCAGG TGAATGATCC TAATGGAAAA TTACTTTTAA CTGTTGAATT TGCAATAGGG TACCGAATTT   
  
  
+ GTGAAGTTTA TTGCCGCACG TGCCTAGCTT GTGACTTCTC GATAATCAAA AATTAATTAG AGAGAGACAG   
  
  
+ CTAAAGTTTT GAGAATAATA CTGAAAAATT ACATTAAATT TTATTTATGT TTATTTTCAT ACGCTATGTT   
  
  
+ TAACTGCTAT ACGCTGTGTT TAACTGCATC TCCTTCTATA TTTGATAAAT TGGTAAAAAA AAATAATTCA   
  
  
+ AAATTAAAGA CTAGTAATAT TTTCACTTAT CTGATAGATT TATAAGAAAT ATTTAGATAG CTAAATATAT   
  
  
+ TCTCTCCTGA AAGAGGAGAC TGATAGTTGA ATTTCATAGG ATATAAACAT GTATGTACAT TGAACGAATC   
  
  
+ TTTTTTCTAT GGTGATCGTT ATAAAGTTAT TTATTACTGT AAACATTAAG TGATAATTAG GATATGTTCT   
  
  
+ ATTTCACGTT TTAGATAGAT AGATAGATGT AAGGAGTTGT AAGAAGCTGT AAGAATATGT AAGGAGCTGT   
  
  
+ AAGGAGATGT AAGGAGCTGT AAGAAGATGT AAGAGAAACT GTAAGAAGCT ACAAGGATAG ATGTAACGAG   
  
  
+ TTGTTGAATT GTTGAAGAGT TGTATAGTGT AATTTGTTTT ACGATGAACG TTTTGGATGA AAAATAATAA   
  
  
+ TATTAAAATA ATATGCTTAT TAAAAATAAA TAAATAATTA AGTTAATTAC ACAACAATAA AATATATTAA   
  
  
+ TCCCCATAAA TTTAGTGAAT AAAACTTCGT CCACTTGAGA TGATTTTTTA TATGAACACT AGTTTGATAG   
  
  
+ AATAAACAAA GTATAAAAAA AAATAAAGTT TTTTGACAAA ATTGAATACA ACTCTTCAAA TAAATTCCTA   
  
  
+ TTTTAAGTAA ATAATTTTAA ATATAAATAA ATTTATGGTC ATCTATAATA ATATATATTT TATGTCGAAA   
  
  
+ CTCAAATATA ATTTACATCG TTATATTATA TTTTTATGTT TATGTAAATG TTGTGTTCAT AAGATATTTA   
  
  
+ TAAATTTTGT ATATTGGTAG TATCATTAAA AAAAATATAC ATATTTATGT AACTAAAATA AATGAATGTT   
  
  
+ ATAGAAAATC TATAAGATGT AAGGAGATGT ATGAAGATGT AAGGAGATGT AAGAAGATAT AAGGAGATGT   
  
  
+ AGAAAATGTA AGAAGATGTG AGAAGCTGTA AGGAGATGTA AGTTATTCAC TAAATCCTAA AAAATGCGAA   
  
  
+ ATAGAACAGG GGCTTAATTG AGCACTCAAG ATGCATAATT TGTGACGGAC CAAATGGATA AATATAGACC   
  
  
+ TGGCAAATGG GTTATTCGAT TTAGGTACGA GTATGGTTAA GTTTGGGTTT GGTTATGTTT CAGGATAAGT   
  
  
+ GACATTTCGA CTCAATTTGG TTTTGGTTGT ATGAACTTCG GCTCGAGATT TTTGGTTTGG CCAATACACT   
  
  
+ TATCATGCAA AAATAAGTAA CTTTATAGAA AATTTTGGAT CGGATATGAT GCGGTTCGGA TCAATTCGGA   
  
  
+ TTTTGGTTCA CATTCTGGTA AACCTATTTC GGATGTCAAG TAGGATATGA GTCCAGATCG TTGGATTTCG   
  
  
+ GGTATCAGCT CAATTTTACC AGATCTAGAT AAATCATAAA TGTACGGGTT TATTTTTTCC AGCTTGAATG   
  
  
+ ATTTTCTTTA TTAAAAAAAG ATAGATTTTA GATAAAACAC AACATTTCAT TTTCATTTGT TCCCCCCTAT   
  
  
+ AAATTTCTGT GACCACCTTC ACTTTCTTGC CCCATTCCCC CCCCCCCCCC GCTCTCTCTC TCTCTCTCTC   
  
  
+ TCTTCCCTTT ACAAAGGCAC CATTCAAATT CAAACCACTC TTTTTAAAAC CCCAAAAATT ATTCTCAAAA   
  
  
+ TAATACCTCA TAATATAAAT GGGTTCGTAT CACCATATAT GGGCCTCATC TCTCATGGGA ACATACGAAT   
  
  
+ TCAATGGCTT GCCCGATAGT TATCGGGTCA TGCCATCGGA GCCCCGGCAA CTAATCAATG GTCAAGCCTC   
  
  
+ AGATTTGGCC TCTTTGAGCC ATTATAGTAA CACCTTTCTC TCTGAACTCC CCCCTCTACC CACTCTGGGA   
  
  
+ CCCGGATCCG ACCCGGGTCT ATCGTGTCCG GGTCCTGCTC TGAGTCCAAT GGAGTGGATT GGTGACGCGT   
  
  
+ GGACACACCA GATTGAACAT GATCTGCCAC GTCAGCAGAG TCAACGGCTG TTGACTGATG GTGACATGGG   
  
  
+ GGAGGAGGAT TCCGGTATAA GGTTGGTGCA TGCCTTGCTG ACGTGCGCAG AGGCCGTCCA ATGCGGCGAG   
  
  
+ CTCCGACTGG CTGCTTCGCT AGTTGATGAG ATGACCAATG GGCTGTTGCC ACGTGTTAGT ACGGTGTGTG   
  
  
+ GGATCGGCAA GGTAGCCGGC TATTTTGTTG AGGCTTTGAG CCGGCGCCTG TTTCAGCCCG GCCCGGAAAC   
  
  
+ TGGCCCGACC TGGCCGGGCT CGGAGTTCGA GGCTGAGGTG TTGTATCGTC ACTTCTATGA GGCTTGCCCT   
  
  
+ TATCTCAAAT TTGCTCACTT CACGGCTAAT CAAGCCATCT TGGAGGCATT TGAGGGCCAT GATTATGTCC   
  
  
+ ACGTCATCGA CTTCGGCTTC ATGACCGGCT TGCAATGGCC GGCTTTGATT CAAGCCCTGG CTTTAAGGCC   
  
  
+ CGGTGGGCCA CCATCTCTTA GGCTCACTGG GATTGGCCCG TCTTTAGGAA GTGGGCCCGA ATCGATACGT   
  
  
+ GAAATCGGGT TGAGGCTAGC CCAATTGGCC CGATCCATGA ACATGAGGTT CGCATTTCGG GCTGTAGCAA   
  
  
+ CTTCTCGGCT CGAGGACGTG AAGCCGTCAA TGCTCGGAAT GGACCCTAGA GAAGCCGTCG CTATGAATTC   
  
  
+ CGTCATGCAT CTTCATCGAC TTTTAGGATT AGACATCAAC TCAGTTCTAA ACTGGATTCG AAACCTAAAC   
  
  
+ CCGAAGATCG TAACTTTAGT AGAACAAGAG GCGAACCATA ACCAACCTGA ATTTCTCCCC CGATTCACTG   
  
  
+ AAGCACTATG TTACTACTCA ACTATGTTCG ACTCATTAGA GACTTGTCAA GTCCAACCGC TGAAAGTCCT   
  
  
+ AGCCGAGATG TATATACAAA AGGAGATATC CAACCTAGTG TGCTACGAAG GCTCAGCCCG GGTCCAGAGG   
  
  
+ CACGAGCCGC TCGCCAAATG GCAAGCTCGG ATGGGCCAAG CCGGGTTCAA GCCGCTAGGC CTGAGTCAAA   
  
  
+ ATGCCCTTAG GCAAGCTAAC ATGTTGGTAA CATTGTTCTC CTCACAAGGG TATGACGTGG AGGAGAGAGA   
  
  
+ AGGGTGTTTG ACACTGGGCT GGCATAACCG CCCTCTCATA GTGACTTCGA CTTGGCAAGT GGGGCCCAAA   
  
  
+ ATGGACAGTC CAAGTGTGAC TCATGAGATG TCATCATCCT CAGCTTCTTC ATCTTA  

- +Up\_Stream \_Len000GTAAAA GTTAATCTTT AAAGAAGTTA ATATATGGTA GAGGTTTTTA TCAGGAGTAT   
  
  
- TCAACCGTCC ACTTACTAGG ATTACCTTTT AATGAAAATT GACAACTTAA ACGTTATCCC ATGGCTTAAA   
  
  
- CACTTCAAAT AACGGCGTGC ACGGATCGAA CACTGAAGAG CTATTAGTTT TTAATTAATC TCTCTCTGTC   
  
  
- GATTTCAAAA CTCTTATTAT GACTTTTTAA TGTAATTTAA AATAAATACA AATAAAAGTA TGCGATACAA   
  
  
- ATTGACGATA TGCGACACAA ATTGACGTAG AGGAAGATAT AAACTATTTA ACCATTTTTT TTTATTAAGT   
  
  
- TTTAATTTCT GATCATTATA AAAGTGAATA GACTATCTAA ATATTCTTTA TAAATCTATC GATTTATATA   
  
  
- AGAGAGGACT TTCTCCTCTG ACTATCAACT TAAAGTATCC TATATTTGTA CATACATGTA ACTTGCTTAG   
  
  
- AAAAAAGATA CCACTAGCAA TATTTCAATA AATAATGACA TTTGTAATTC ACTATTAATC CTATACAAGA   
  
  
- TAAAGTGCAA AATCTATCTA TCTATCTACA TTCCTCAACA TTCTTCGACA TTCTTATACA TTCCTCGACA   
  
  
- TTCCTCTACA TTCCTCGACA TTCTTCTACA TTCTCTTTGA CATTCTTCGA TGTTCCTATC TACATTGCTC   
  
  
- AACAACTTAA CAACTTCTCA ACATATCACA TTAAACAAAA TGCTACTTGC AAAACCTACT TTTTATTATT   
  
  
- ATAATTTTAT TATACGAATA ATTTTTATTT ATTTATTAAT TCAATTAATG TGTTGTTATT TTATATAATT   
  
  
- AGGGGTATTT AAATCACTTA TTTTGAAGCA GGTGAACTCT ACTAAAAAAT ATACTTGTGA TCAAACTATC   
  
  
- TTATTTGTTT CATATTTTTT TTTATTTCAA AAAACTGTTT TAACTTATGT TGAGAAGTTT ATTTAAGGAT   
  
  
- AAAATTCATT TATTAAAATT TATATTTATT TAAATACCAG TAGATATTAT TATATATAAA ATACAGCTTT   
  
  
- GAGTTTATAT TAAATGTAGC AATATAATAT AAAAATACAA ATACATTTAC AACACAAGTA TTCTATAAAT   
  
  
- ATTTAAAACA TATAACCATC ATAGTAATTT TTTTTATATG TATAAATACA TTGATTTTAT TTACTTACAA   
  
  
- TATCTTTTAG ATATTCTACA TTCCTCTACA TACTTCTACA TTCCTCTACA TTCTTCTATA TTCCTCTACA   
  
  
- TCTTTTACAT TCTTCTACAC TCTTCGACAT TCCTCTACAT TCAATAAGTG ATTTAGGATT TTTTACGCTT   
  
  
- TATCTTGTCC CCGAATTAAC TCGTGAGTTC TACGTATTAA ACACTGCCTG GTTTACCTAT TTATATCTGG   
  
  
- ACCGTTTACC CAATAAGCTA AATCCATGCT CATACCAATT CAAACCCAAA CCAATACAAA GTCCTATTCA   
  
  
- CTGTAAAGCT GAGTTAAACC AAAACCAACA TACTTGAAGC CGAGCTCTAA AAACCAAACC GGTTATGTGA   
  
  
- ATAGTACGTT TTTATTCATT GAAATATCTT TTAAAACCTA GCCTATACTA CGCCAAGCCT AGTTAAGCCT   
  
  
- AAAACCAAGT GTAAGACCAT TTGGATAAAG CCTACAGTTC ATCCTATACT CAGGTCTAGC AACCTAAAGC   
  
  
- CCATAGTCGA GTTAAAATGG TCTAGATCTA TTTAGTATTT ACATGCCCAA ATAAAAAAGG TCGAACTTAC   
  
  
- TAAAAGAAAT AATTTTTTTC TATCTAAAAT CTATTTTGTG TTGTAAAGTA AAAGTAAACA AGGGGGGATA   
  
  
- TTTAAAGACA CTGGTGGAAG TGAAAGAACG GGGTAAGGGG GGGGGGGGGG CGAGAGAGAG AGAGAGAGAG   
  
  
- AGAAGGGAAA TGTTTCCGTG GTAAGTTTAA GTTTGGTGAG AAAAATTTTG GGGTTTTTAA TAAGAGTTTT   
  
  
- ATTATGGAGT ATTATATTTA CCCAAGCATA GTGGTATATA CCCGGAGTAG AGAGTACCCT TGTATGCTTA   
  
  
- AGTTACCGAA CGGGCTATCA ATAGCCCAGT ACGGTAGCCT CGGGGCCGTT GATTAGTTAC CAGTTCGGAG   
  
  
- TCTAAACCGG AGAAACTCGG TAATATCATT GTGGAAAGAG AGACTTGAGG GGGGAGATGG GTGAGACCCT   
  
  
- GGGCCTAGGC TGGGCCCAGA TAGCACAGGC CCAGGACGAG ACTCAGGTTA CCTCACCTAA CCACTGCGCA   
  
  
- CCTGTGTGGT CTAACTTGTA CTAGACGGTG CAGTCGTCTC AGTTGCCGAC AACTGACTAC CACTGTACCC   
  
  
- CCTCCTCCTA AGGCCATATT CCAACCACGT ACGGAACGAC TGCACGCGTC TCCGGCAGGT TACGCCGCTC   
  
  
- GAGGCTGACC GACGAAGCGA TCAACTACTC TACTGGTTAC CCGACAACGG TGCACAATCA TGCCACACAC   
  
  
- CCTAGCCGTT CCATCGGCCG ATAAAACAAC TCCGAAACTC GGCCGCGGAC AAAGTCGGGC CGGGCCTTTG   
  
  
- ACCGGGCTGG ACCGGCCCGA GCCTCAAGCT CCGACTCCAC AACATAGCAG TGAAGATACT CCGAACGGGA   
  
  
- ATAGAGTTTA AACGAGTGAA GTGCCGATTA GTTCGGTAGA ACCTCCGTAA ACTCCCGGTA CTAATACAGG   
  
  
- TGCAGTAGCT GAAGCCGAAG TACTGGCCGA ACGTTACCGG CCGAAACTAA GTTCGGGACC GAAATTCCGG   
  
  
- GCCACCCGGT GGTAGAGAAT CCGAGTGACC CTAACCGGGC AGAAATCCTT CACCCGGGCT TAGCTATGCA   
  
  
- CTTTAGCCCA ACTCCGATCG GGTTAACCGG GCTAGGTACT TGTACTCCAA GCGTAAAGCC CGACATCGTT   
  
  
- GAAGAGCCGA GCTCCTGCAC TTCGGCAGTT ACGAGCCTTA CCTGGGATCT CTTCGGCAGC GATACTTAAG   
  
  
- GCAGTACGTA GAAGTAGCTG AAAATCCTAA TCTGTAGTTG AGTCAAGATT TGACCTAAGC TTTGGATTTG   
  
  
- GGCTTCTAGC ATTGAAATCA TCTTGTTCTC CGCTTGGTAT TGGTTGGACT TAAAGAGGGG GCTAAGTGAC   
  
  
- TTCGTGATAC AATGATGAGT TGATACAAGC TGAGTAATCT CTGAACAGTT CAGGTTGGCG ACTTTCAGGA   
  
  
- TCGGCTCTAC ATATATGTTT TCCTCTATAG GTTGGATCAC ACGATGCTTC CGAGTCGGGC CCAGGTCTCC   
  
  
- GTGCTCGGCG AGCGGTTTAC CGTTCGAGCC TACCCGGTTC GGCCCAAGTT CGGCGATCCG GACTCAGTTT   
  
  
- TACGGGAATC CGTTCGATTG TACAACCATT GTAACAAGAG GAGTGTTCCC ATACTGCACC TCCTCTCTCT   
  
  
- TCCCACAAAC TGTGACCCGA CCGTATTGGC GGGAGAGTAT CACTGAAGCT GAACCGTTCA CCCCGGGTTT   
  
  
- TACCTGTCAG GTTCACACTG AGTACTCTAC AGTAGTAGGA GTCGAAGAAG TAGAAT

+     MYB recognition site

| Site Name | Organism | Position | Strand | Matrix score. | sequence | function |
| --- | --- | --- | --- | --- | --- | --- |
| MYB recognition site | Arabidopsis thaliana | 2286 | - | 6 | CCGTTG |  |

>HU03G02797.1   
+ +Up\_Stream \_Len000CATTTT CAATTAGAAA TTTCTTCAAT TATATACCAT CTCCAAAAAT AGTCCTCATA   
  
  
+ AGTTGGCAGG TGAATGATCC TAATGGAAAA TTACTTTTAA CTGTTGAATT TGCAATAGGG TACCGAATTT   
  
  
+ GTGAAGTTTA TTGCCGCACG TGCCTAGCTT GTGACTTCTC GATAATCAAA AATTAATTAG AGAGAGACAG   
  
  
+ CTAAAGTTTT GAGAATAATA CTGAAAAATT ACATTAAATT TTATTTATGT TTATTTTCAT ACGCTATGTT   
  
  
+ TAACTGCTAT ACGCTGTGTT TAACTGCATC TCCTTCTATA TTTGATAAAT TGGTAAAAAA AAATAATTCA   
  
  
+ AAATTAAAGA CTAGTAATAT TTTCACTTAT CTGATAGATT TATAAGAAAT ATTTAGATAG CTAAATATAT   
  
  
+ TCTCTCCTGA AAGAGGAGAC TGATAGTTGA ATTTCATAGG ATATAAACAT GTATGTACAT TGAACGAATC   
  
  
+ TTTTTTCTAT GGTGATCGTT ATAAAGTTAT TTATTACTGT AAACATTAAG TGATAATTAG GATATGTTCT   
  
  
+ ATTTCACGTT TTAGATAGAT AGATAGATGT AAGGAGTTGT AAGAAGCTGT AAGAATATGT AAGGAGCTGT   
  
  
+ AAGGAGATGT AAGGAGCTGT AAGAAGATGT AAGAGAAACT GTAAGAAGCT ACAAGGATAG ATGTAACGAG   
  
  
+ TTGTTGAATT GTTGAAGAGT TGTATAGTGT AATTTGTTTT ACGATGAACG TTTTGGATGA AAAATAATAA   
  
  
+ TATTAAAATA ATATGCTTAT TAAAAATAAA TAAATAATTA AGTTAATTAC ACAACAATAA AATATATTAA   
  
  
+ TCCCCATAAA TTTAGTGAAT AAAACTTCGT CCACTTGAGA TGATTTTTTA TATGAACACT AGTTTGATAG   
  
  
+ AATAAACAAA GTATAAAAAA AAATAAAGTT TTTTGACAAA ATTGAATACA ACTCTTCAAA TAAATTCCTA   
  
  
+ TTTTAAGTAA ATAATTTTAA ATATAAATAA ATTTATGGTC ATCTATAATA ATATATATTT TATGTCGAAA   
  
  
+ CTCAAATATA ATTTACATCG TTATATTATA TTTTTATGTT TATGTAAATG TTGTGTTCAT AAGATATTTA   
  
  
+ TAAATTTTGT ATATTGGTAG TATCATTAAA AAAAATATAC ATATTTATGT AACTAAAATA AATGAATGTT   
  
  
+ ATAGAAAATC TATAAGATGT AAGGAGATGT ATGAAGATGT AAGGAGATGT AAGAAGATAT AAGGAGATGT   
  
  
+ AGAAAATGTA AGAAGATGTG AGAAGCTGTA AGGAGATGTA AGTTATTCAC TAAATCCTAA AAAATGCGAA   
  
  
+ ATAGAACAGG GGCTTAATTG AGCACTCAAG ATGCATAATT TGTGACGGAC CAAATGGATA AATATAGACC   
  
  
+ TGGCAAATGG GTTATTCGAT TTAGGTACGA GTATGGTTAA GTTTGGGTTT GGTTATGTTT CAGGATAAGT   
  
  
+ GACATTTCGA CTCAATTTGG TTTTGGTTGT ATGAACTTCG GCTCGAGATT TTTGGTTTGG CCAATACACT   
  
  
+ TATCATGCAA AAATAAGTAA CTTTATAGAA AATTTTGGAT CGGATATGAT GCGGTTCGGA TCAATTCGGA   
  
  
+ TTTTGGTTCA CATTCTGGTA AACCTATTTC GGATGTCAAG TAGGATATGA GTCCAGATCG TTGGATTTCG   
  
  
+ GGTATCAGCT CAATTTTACC AGATCTAGAT AAATCATAAA TGTACGGGTT TATTTTTTCC AGCTTGAATG   
  
  
+ ATTTTCTTTA TTAAAAAAAG ATAGATTTTA GATAAAACAC AACATTTCAT TTTCATTTGT TCCCCCCTAT   
  
  
+ AAATTTCTGT GACCACCTTC ACTTTCTTGC CCCATTCCCC CCCCCCCCCC GCTCTCTCTC TCTCTCTCTC   
  
  
+ TCTTCCCTTT ACAAAGGCAC CATTCAAATT CAAACCACTC TTTTTAAAAC CCCAAAAATT ATTCTCAAAA   
  
  
+ TAATACCTCA TAATATAAAT GGGTTCGTAT CACCATATAT GGGCCTCATC TCTCATGGGA ACATACGAAT   
  
  
+ TCAATGGCTT GCCCGATAGT TATCGGGTCA TGCCATCGGA GCCCCGGCAA CTAATCAATG GTCAAGCCTC   
  
  
+ AGATTTGGCC TCTTTGAGCC ATTATAGTAA CACCTTTCTC TCTGAACTCC CCCCTCTACC CACTCTGGGA   
  
  
+ CCCGGATCCG ACCCGGGTCT ATCGTGTCCG GGTCCTGCTC TGAGTCCAAT GGAGTGGATT GGTGACGCGT   
  
  
+ GGACACACCA GATTGAACAT GATCTGCCAC GTCAGCAGAG TCAACGGCTG TTGACTGATG GTGACATGGG   
  
  
+ GGAGGAGGAT TCCGGTATAA GGTTGGTGCA TGCCTTGCTG ACGTGCGCAG AGGCCGTCCA ATGCGGCGAG   
  
  
+ CTCCGACTGG CTGCTTCGCT AGTTGATGAG ATGACCAATG GGCTGTTGCC ACGTGTTAGT ACGGTGTGTG   
  
  
+ GGATCGGCAA GGTAGCCGGC TATTTTGTTG AGGCTTTGAG CCGGCGCCTG TTTCAGCCCG GCCCGGAAAC   
  
  
+ TGGCCCGACC TGGCCGGGCT CGGAGTTCGA GGCTGAGGTG TTGTATCGTC ACTTCTATGA GGCTTGCCCT   
  
  
+ TATCTCAAAT TTGCTCACTT CACGGCTAAT CAAGCCATCT TGGAGGCATT TGAGGGCCAT GATTATGTCC   
  
  
+ ACGTCATCGA CTTCGGCTTC ATGACCGGCT TGCAATGGCC GGCTTTGATT CAAGCCCTGG CTTTAAGGCC   
  
  
+ CGGTGGGCCA CCATCTCTTA GGCTCACTGG GATTGGCCCG TCTTTAGGAA GTGGGCCCGA ATCGATACGT   
  
  
+ GAAATCGGGT TGAGGCTAGC CCAATTGGCC CGATCCATGA ACATGAGGTT CGCATTTCGG GCTGTAGCAA   
  
  
+ CTTCTCGGCT CGAGGACGTG AAGCCGTCAA TGCTCGGAAT GGACCCTAGA GAAGCCGTCG CTATGAATTC   
  
  
+ CGTCATGCAT CTTCATCGAC TTTTAGGATT AGACATCAAC TCAGTTCTAA ACTGGATTCG AAACCTAAAC   
  
  
+ CCGAAGATCG TAACTTTAGT AGAACAAGAG GCGAACCATA ACCAACCTGA ATTTCTCCCC CGATTCACTG   
  
  
+ AAGCACTATG TTACTACTCA ACTATGTTCG ACTCATTAGA GACTTGTCAA GTCCAACCGC TGAAAGTCCT   
  
  
+ AGCCGAGATG TATATACAAA AGGAGATATC CAACCTAGTG TGCTACGAAG GCTCAGCCCG GGTCCAGAGG   
  
  
+ CACGAGCCGC TCGCCAAATG GCAAGCTCGG ATGGGCCAAG CCGGGTTCAA GCCGCTAGGC CTGAGTCAAA   
  
  
+ ATGCCCTTAG GCAAGCTAAC ATGTTGGTAA CATTGTTCTC CTCACAAGGG TATGACGTGG AGGAGAGAGA   
  
  
+ AGGGTGTTTG ACACTGGGCT GGCATAACCG CCCTCTCATA GTGACTTCGA CTTGGCAAGT GGGGCCCAAA   
  
  
+ ATGGACAGTC CAAGTGTGAC TCATGAGATG TCATCATCCT CAGCTTCTTC ATCTTA  

- +Up\_Stream \_Len000GTAAAA GTTAATCTTT AAAGAAGTTA ATATATGGTA GAGGTTTTTA TCAGGAGTAT   
  
  
- TCAACCGTCC ACTTACTAGG ATTACCTTTT AATGAAAATT GACAACTTAA ACGTTATCCC ATGGCTTAAA   
  
  
- CACTTCAAAT AACGGCGTGC ACGGATCGAA CACTGAAGAG CTATTAGTTT TTAATTAATC TCTCTCTGTC   
  
  
- GATTTCAAAA CTCTTATTAT GACTTTTTAA TGTAATTTAA AATAAATACA AATAAAAGTA TGCGATACAA   
  
  
- ATTGACGATA TGCGACACAA ATTGACGTAG AGGAAGATAT AAACTATTTA ACCATTTTTT TTTATTAAGT   
  
  
- TTTAATTTCT GATCATTATA AAAGTGAATA GACTATCTAA ATATTCTTTA TAAATCTATC GATTTATATA   
  
  
- AGAGAGGACT TTCTCCTCTG ACTATCAACT TAAAGTATCC TATATTTGTA CATACATGTA ACTTGCTTAG   
  
  
- AAAAAAGATA CCACTAGCAA TATTTCAATA AATAATGACA TTTGTAATTC ACTATTAATC CTATACAAGA   
  
  
- TAAAGTGCAA AATCTATCTA TCTATCTACA TTCCTCAACA TTCTTCGACA TTCTTATACA TTCCTCGACA   
  
  
- TTCCTCTACA TTCCTCGACA TTCTTCTACA TTCTCTTTGA CATTCTTCGA TGTTCCTATC TACATTGCTC   
  
  
- AACAACTTAA CAACTTCTCA ACATATCACA TTAAACAAAA TGCTACTTGC AAAACCTACT TTTTATTATT   
  
  
- ATAATTTTAT TATACGAATA ATTTTTATTT ATTTATTAAT TCAATTAATG TGTTGTTATT TTATATAATT   
  
  
- AGGGGTATTT AAATCACTTA TTTTGAAGCA GGTGAACTCT ACTAAAAAAT ATACTTGTGA TCAAACTATC   
  
  
- TTATTTGTTT CATATTTTTT TTTATTTCAA AAAACTGTTT TAACTTATGT TGAGAAGTTT ATTTAAGGAT   
  
  
- AAAATTCATT TATTAAAATT TATATTTATT TAAATACCAG TAGATATTAT TATATATAAA ATACAGCTTT   
  
  
- GAGTTTATAT TAAATGTAGC AATATAATAT AAAAATACAA ATACATTTAC AACACAAGTA TTCTATAAAT   
  
  
- ATTTAAAACA TATAACCATC ATAGTAATTT TTTTTATATG TATAAATACA TTGATTTTAT TTACTTACAA   
  
  
- TATCTTTTAG ATATTCTACA TTCCTCTACA TACTTCTACA TTCCTCTACA TTCTTCTATA TTCCTCTACA   
  
  
- TCTTTTACAT TCTTCTACAC TCTTCGACAT TCCTCTACAT TCAATAAGTG ATTTAGGATT TTTTACGCTT   
  
  
- TATCTTGTCC CCGAATTAAC TCGTGAGTTC TACGTATTAA ACACTGCCTG GTTTACCTAT TTATATCTGG   
  
  
- ACCGTTTACC CAATAAGCTA AATCCATGCT CATACCAATT CAAACCCAAA CCAATACAAA GTCCTATTCA   
  
  
- CTGTAAAGCT GAGTTAAACC AAAACCAACA TACTTGAAGC CGAGCTCTAA AAACCAAACC GGTTATGTGA   
  
  
- ATAGTACGTT TTTATTCATT GAAATATCTT TTAAAACCTA GCCTATACTA CGCCAAGCCT AGTTAAGCCT   
  
  
- AAAACCAAGT GTAAGACCAT TTGGATAAAG CCTACAGTTC ATCCTATACT CAGGTCTAGC AACCTAAAGC   
  
  
- CCATAGTCGA GTTAAAATGG TCTAGATCTA TTTAGTATTT ACATGCCCAA ATAAAAAAGG TCGAACTTAC   
  
  
- TAAAAGAAAT AATTTTTTTC TATCTAAAAT CTATTTTGTG TTGTAAAGTA AAAGTAAACA AGGGGGGATA   
  
  
- TTTAAAGACA CTGGTGGAAG TGAAAGAACG GGGTAAGGGG GGGGGGGGGG CGAGAGAGAG AGAGAGAGAG   
  
  
- AGAAGGGAAA TGTTTCCGTG GTAAGTTTAA GTTTGGTGAG AAAAATTTTG GGGTTTTTAA TAAGAGTTTT   
  
  
- ATTATGGAGT ATTATATTTA CCCAAGCATA GTGGTATATA CCCGGAGTAG AGAGTACCCT TGTATGCTTA   
  
  
- AGTTACCGAA CGGGCTATCA ATAGCCCAGT ACGGTAGCCT CGGGGCCGTT GATTAGTTAC CAGTTCGGAG   
  
  
- TCTAAACCGG AGAAACTCGG TAATATCATT GTGGAAAGAG AGACTTGAGG GGGGAGATGG GTGAGACCCT   
  
  
- GGGCCTAGGC TGGGCCCAGA TAGCACAGGC CCAGGACGAG ACTCAGGTTA CCTCACCTAA CCACTGCGCA   
  
  
- CCTGTGTGGT CTAACTTGTA CTAGACGGTG CAGTCGTCTC AGTTGCCGAC AACTGACTAC CACTGTACCC   
  
  
- CCTCCTCCTA AGGCCATATT CCAACCACGT ACGGAACGAC TGCACGCGTC TCCGGCAGGT TACGCCGCTC   
  
  
- GAGGCTGACC GACGAAGCGA TCAACTACTC TACTGGTTAC CCGACAACGG TGCACAATCA TGCCACACAC   
  
  
- CCTAGCCGTT CCATCGGCCG ATAAAACAAC TCCGAAACTC GGCCGCGGAC AAAGTCGGGC CGGGCCTTTG   
  
  
- ACCGGGCTGG ACCGGCCCGA GCCTCAAGCT CCGACTCCAC AACATAGCAG TGAAGATACT CCGAACGGGA   
  
  
- ATAGAGTTTA AACGAGTGAA GTGCCGATTA GTTCGGTAGA ACCTCCGTAA ACTCCCGGTA CTAATACAGG   
  
  
- TGCAGTAGCT GAAGCCGAAG TACTGGCCGA ACGTTACCGG CCGAAACTAA GTTCGGGACC GAAATTCCGG   
  
  
- GCCACCCGGT GGTAGAGAAT CCGAGTGACC CTAACCGGGC AGAAATCCTT CACCCGGGCT TAGCTATGCA   
  
  
- CTTTAGCCCA ACTCCGATCG GGTTAACCGG GCTAGGTACT TGTACTCCAA GCGTAAAGCC CGACATCGTT   
  
  
- GAAGAGCCGA GCTCCTGCAC TTCGGCAGTT ACGAGCCTTA CCTGGGATCT CTTCGGCAGC GATACTTAAG   
  
  
- GCAGTACGTA GAAGTAGCTG AAAATCCTAA TCTGTAGTTG AGTCAAGATT TGACCTAAGC TTTGGATTTG   
  
  
- GGCTTCTAGC ATTGAAATCA TCTTGTTCTC CGCTTGGTAT TGGTTGGACT TAAAGAGGGG GCTAAGTGAC   
  
  
- TTCGTGATAC AATGATGAGT TGATACAAGC TGAGTAATCT CTGAACAGTT CAGGTTGGCG ACTTTCAGGA   
  
  
- TCGGCTCTAC ATATATGTTT TCCTCTATAG GTTGGATCAC ACGATGCTTC CGAGTCGGGC CCAGGTCTCC   
  
  
- GTGCTCGGCG AGCGGTTTAC CGTTCGAGCC TACCCGGTTC GGCCCAAGTT CGGCGATCCG GACTCAGTTT   
  
  
- TACGGGAATC CGTTCGATTG TACAACCATT GTAACAAGAG GAGTGTTCCC ATACTGCACC TCCTCTCTCT   
  
  
- TCCCACAAAC TGTGACCCGA CCGTATTGGC GGGAGAGTAT CACTGAAGCT GAACCGTTCA CCCCGGGTTT   
  
  
- TACCTGTCAG GTTCACACTG AGTACTCTAC AGTAGTAGGA GTCGAAGAAG TAGAAT

+     MYB-like sequence

| Site Name | Organism | Position | Strand | Matrix score. | sequence | function |
| --- | --- | --- | --- | --- | --- | --- |
| MYB-like sequence | Arabidopsis thaliana | 3053 | + | 6 | TAACCA |  |
| MYB-like sequence | Arabidopsis thaliana | 1454 | - | 6 | TAACCA |  |
| MYB-like sequence | Arabidopsis thaliana | 1438 | - | 6 | TAACCA |  |

>HU03G02797.1   
+ +Up\_Stream \_Len000CATTTT CAATTAGAAA TTTCTTCAAT TATATACCAT CTCCAAAAAT AGTCCTCATA   
  
  
+ AGTTGGCAGG TGAATGATCC TAATGGAAAA TTACTTTTAA CTGTTGAATT TGCAATAGGG TACCGAATTT   
  
  
+ GTGAAGTTTA TTGCCGCACG TGCCTAGCTT GTGACTTCTC GATAATCAAA AATTAATTAG AGAGAGACAG   
  
  
+ CTAAAGTTTT GAGAATAATA CTGAAAAATT ACATTAAATT TTATTTATGT TTATTTTCAT ACGCTATGTT   
  
  
+ TAACTGCTAT ACGCTGTGTT TAACTGCATC TCCTTCTATA TTTGATAAAT TGGTAAAAAA AAATAATTCA   
  
  
+ AAATTAAAGA CTAGTAATAT TTTCACTTAT CTGATAGATT TATAAGAAAT ATTTAGATAG CTAAATATAT   
  
  
+ TCTCTCCTGA AAGAGGAGAC TGATAGTTGA ATTTCATAGG ATATAAACAT GTATGTACAT TGAACGAATC   
  
  
+ TTTTTTCTAT GGTGATCGTT ATAAAGTTAT TTATTACTGT AAACATTAAG TGATAATTAG GATATGTTCT   
  
  
+ ATTTCACGTT TTAGATAGAT AGATAGATGT AAGGAGTTGT AAGAAGCTGT AAGAATATGT AAGGAGCTGT   
  
  
+ AAGGAGATGT AAGGAGCTGT AAGAAGATGT AAGAGAAACT GTAAGAAGCT ACAAGGATAG ATGTAACGAG   
  
  
+ TTGTTGAATT GTTGAAGAGT TGTATAGTGT AATTTGTTTT ACGATGAACG TTTTGGATGA AAAATAATAA   
  
  
+ TATTAAAATA ATATGCTTAT TAAAAATAAA TAAATAATTA AGTTAATTAC ACAACAATAA AATATATTAA   
  
  
+ TCCCCATAAA TTTAGTGAAT AAAACTTCGT CCACTTGAGA TGATTTTTTA TATGAACACT AGTTTGATAG   
  
  
+ AATAAACAAA GTATAAAAAA AAATAAAGTT TTTTGACAAA ATTGAATACA ACTCTTCAAA TAAATTCCTA   
  
  
+ TTTTAAGTAA ATAATTTTAA ATATAAATAA ATTTATGGTC ATCTATAATA ATATATATTT TATGTCGAAA   
  
  
+ CTCAAATATA ATTTACATCG TTATATTATA TTTTTATGTT TATGTAAATG TTGTGTTCAT AAGATATTTA   
  
  
+ TAAATTTTGT ATATTGGTAG TATCATTAAA AAAAATATAC ATATTTATGT AACTAAAATA AATGAATGTT   
  
  
+ ATAGAAAATC TATAAGATGT AAGGAGATGT ATGAAGATGT AAGGAGATGT AAGAAGATAT AAGGAGATGT   
  
  
+ AGAAAATGTA AGAAGATGTG AGAAGCTGTA AGGAGATGTA AGTTATTCAC TAAATCCTAA AAAATGCGAA   
  
  
+ ATAGAACAGG GGCTTAATTG AGCACTCAAG ATGCATAATT TGTGACGGAC CAAATGGATA AATATAGACC   
  
  
+ TGGCAAATGG GTTATTCGAT TTAGGTACGA GTATGGTTAA GTTTGGGTTT GGTTATGTTT CAGGATAAGT   
  
  
+ GACATTTCGA CTCAATTTGG TTTTGGTTGT ATGAACTTCG GCTCGAGATT TTTGGTTTGG CCAATACACT   
  
  
+ TATCATGCAA AAATAAGTAA CTTTATAGAA AATTTTGGAT CGGATATGAT GCGGTTCGGA TCAATTCGGA   
  
  
+ TTTTGGTTCA CATTCTGGTA AACCTATTTC GGATGTCAAG TAGGATATGA GTCCAGATCG TTGGATTTCG   
  
  
+ GGTATCAGCT CAATTTTACC AGATCTAGAT AAATCATAAA TGTACGGGTT TATTTTTTCC AGCTTGAATG   
  
  
+ ATTTTCTTTA TTAAAAAAAG ATAGATTTTA GATAAAACAC AACATTTCAT TTTCATTTGT TCCCCCCTAT   
  
  
+ AAATTTCTGT GACCACCTTC ACTTTCTTGC CCCATTCCCC CCCCCCCCCC GCTCTCTCTC TCTCTCTCTC   
  
  
+ TCTTCCCTTT ACAAAGGCAC CATTCAAATT CAAACCACTC TTTTTAAAAC CCCAAAAATT ATTCTCAAAA   
  
  
+ TAATACCTCA TAATATAAAT GGGTTCGTAT CACCATATAT GGGCCTCATC TCTCATGGGA ACATACGAAT   
  
  
+ TCAATGGCTT GCCCGATAGT TATCGGGTCA TGCCATCGGA GCCCCGGCAA CTAATCAATG GTCAAGCCTC   
  
  
+ AGATTTGGCC TCTTTGAGCC ATTATAGTAA CACCTTTCTC TCTGAACTCC CCCCTCTACC CACTCTGGGA   
  
  
+ CCCGGATCCG ACCCGGGTCT ATCGTGTCCG GGTCCTGCTC TGAGTCCAAT GGAGTGGATT GGTGACGCGT   
  
  
+ GGACACACCA GATTGAACAT GATCTGCCAC GTCAGCAGAG TCAACGGCTG TTGACTGATG GTGACATGGG   
  
  
+ GGAGGAGGAT TCCGGTATAA GGTTGGTGCA TGCCTTGCTG ACGTGCGCAG AGGCCGTCCA ATGCGGCGAG   
  
  
+ CTCCGACTGG CTGCTTCGCT AGTTGATGAG ATGACCAATG GGCTGTTGCC ACGTGTTAGT ACGGTGTGTG   
  
  
+ GGATCGGCAA GGTAGCCGGC TATTTTGTTG AGGCTTTGAG CCGGCGCCTG TTTCAGCCCG GCCCGGAAAC   
  
  
+ TGGCCCGACC TGGCCGGGCT CGGAGTTCGA GGCTGAGGTG TTGTATCGTC ACTTCTATGA GGCTTGCCCT   
  
  
+ TATCTCAAAT TTGCTCACTT CACGGCTAAT CAAGCCATCT TGGAGGCATT TGAGGGCCAT GATTATGTCC   
  
  
+ ACGTCATCGA CTTCGGCTTC ATGACCGGCT TGCAATGGCC GGCTTTGATT CAAGCCCTGG CTTTAAGGCC   
  
  
+ CGGTGGGCCA CCATCTCTTA GGCTCACTGG GATTGGCCCG TCTTTAGGAA GTGGGCCCGA ATCGATACGT   
  
  
+ GAAATCGGGT TGAGGCTAGC CCAATTGGCC CGATCCATGA ACATGAGGTT CGCATTTCGG GCTGTAGCAA   
  
  
+ CTTCTCGGCT CGAGGACGTG AAGCCGTCAA TGCTCGGAAT GGACCCTAGA GAAGCCGTCG CTATGAATTC   
  
  
+ CGTCATGCAT CTTCATCGAC TTTTAGGATT AGACATCAAC TCAGTTCTAA ACTGGATTCG AAACCTAAAC   
  
  
+ CCGAAGATCG TAACTTTAGT AGAACAAGAG GCGAACCATA ACCAACCTGA ATTTCTCCCC CGATTCACTG   
  
  
+ AAGCACTATG TTACTACTCA ACTATGTTCG ACTCATTAGA GACTTGTCAA GTCCAACCGC TGAAAGTCCT   
  
  
+ AGCCGAGATG TATATACAAA AGGAGATATC CAACCTAGTG TGCTACGAAG GCTCAGCCCG GGTCCAGAGG   
  
  
+ CACGAGCCGC TCGCCAAATG GCAAGCTCGG ATGGGCCAAG CCGGGTTCAA GCCGCTAGGC CTGAGTCAAA   
  
  
+ ATGCCCTTAG GCAAGCTAAC ATGTTGGTAA CATTGTTCTC CTCACAAGGG TATGACGTGG AGGAGAGAGA   
  
  
+ AGGGTGTTTG ACACTGGGCT GGCATAACCG CCCTCTCATA GTGACTTCGA CTTGGCAAGT GGGGCCCAAA   
  
  
+ ATGGACAGTC CAAGTGTGAC TCATGAGATG TCATCATCCT CAGCTTCTTC ATCTTA  

- +Up\_Stream \_Len000GTAAAA GTTAATCTTT AAAGAAGTTA ATATATGGTA GAGGTTTTTA TCAGGAGTAT   
  
  
- TCAACCGTCC ACTTACTAGG ATTACCTTTT AATGAAAATT GACAACTTAA ACGTTATCCC ATGGCTTAAA   
  
  
- CACTTCAAAT AACGGCGTGC ACGGATCGAA CACTGAAGAG CTATTAGTTT TTAATTAATC TCTCTCTGTC   
  
  
- GATTTCAAAA CTCTTATTAT GACTTTTTAA TGTAATTTAA AATAAATACA AATAAAAGTA TGCGATACAA   
  
  
- ATTGACGATA TGCGACACAA ATTGACGTAG AGGAAGATAT AAACTATTTA ACCATTTTTT TTTATTAAGT   
  
  
- TTTAATTTCT GATCATTATA AAAGTGAATA GACTATCTAA ATATTCTTTA TAAATCTATC GATTTATATA   
  
  
- AGAGAGGACT TTCTCCTCTG ACTATCAACT TAAAGTATCC TATATTTGTA CATACATGTA ACTTGCTTAG   
  
  
- AAAAAAGATA CCACTAGCAA TATTTCAATA AATAATGACA TTTGTAATTC ACTATTAATC CTATACAAGA   
  
  
- TAAAGTGCAA AATCTATCTA TCTATCTACA TTCCTCAACA TTCTTCGACA TTCTTATACA TTCCTCGACA   
  
  
- TTCCTCTACA TTCCTCGACA TTCTTCTACA TTCTCTTTGA CATTCTTCGA TGTTCCTATC TACATTGCTC   
  
  
- AACAACTTAA CAACTTCTCA ACATATCACA TTAAACAAAA TGCTACTTGC AAAACCTACT TTTTATTATT   
  
  
- ATAATTTTAT TATACGAATA ATTTTTATTT ATTTATTAAT TCAATTAATG TGTTGTTATT TTATATAATT   
  
  
- AGGGGTATTT AAATCACTTA TTTTGAAGCA GGTGAACTCT ACTAAAAAAT ATACTTGTGA TCAAACTATC   
  
  
- TTATTTGTTT CATATTTTTT TTTATTTCAA AAAACTGTTT TAACTTATGT TGAGAAGTTT ATTTAAGGAT   
  
  
- AAAATTCATT TATTAAAATT TATATTTATT TAAATACCAG TAGATATTAT TATATATAAA ATACAGCTTT   
  
  
- GAGTTTATAT TAAATGTAGC AATATAATAT AAAAATACAA ATACATTTAC AACACAAGTA TTCTATAAAT   
  
  
- ATTTAAAACA TATAACCATC ATAGTAATTT TTTTTATATG TATAAATACA TTGATTTTAT TTACTTACAA   
  
  
- TATCTTTTAG ATATTCTACA TTCCTCTACA TACTTCTACA TTCCTCTACA TTCTTCTATA TTCCTCTACA   
  
  
- TCTTTTACAT TCTTCTACAC TCTTCGACAT TCCTCTACAT TCAATAAGTG ATTTAGGATT TTTTACGCTT   
  
  
- TATCTTGTCC CCGAATTAAC TCGTGAGTTC TACGTATTAA ACACTGCCTG GTTTACCTAT TTATATCTGG   
  
  
- ACCGTTTACC CAATAAGCTA AATCCATGCT CATACCAATT CAAACCCAAA CCAATACAAA GTCCTATTCA   
  
  
- CTGTAAAGCT GAGTTAAACC AAAACCAACA TACTTGAAGC CGAGCTCTAA AAACCAAACC GGTTATGTGA   
  
  
- ATAGTACGTT TTTATTCATT GAAATATCTT TTAAAACCTA GCCTATACTA CGCCAAGCCT AGTTAAGCCT   
  
  
- AAAACCAAGT GTAAGACCAT TTGGATAAAG CCTACAGTTC ATCCTATACT CAGGTCTAGC AACCTAAAGC   
  
  
- CCATAGTCGA GTTAAAATGG TCTAGATCTA TTTAGTATTT ACATGCCCAA ATAAAAAAGG TCGAACTTAC   
  
  
- TAAAAGAAAT AATTTTTTTC TATCTAAAAT CTATTTTGTG TTGTAAAGTA AAAGTAAACA AGGGGGGATA   
  
  
- TTTAAAGACA CTGGTGGAAG TGAAAGAACG GGGTAAGGGG GGGGGGGGGG CGAGAGAGAG AGAGAGAGAG   
  
  
- AGAAGGGAAA TGTTTCCGTG GTAAGTTTAA GTTTGGTGAG AAAAATTTTG GGGTTTTTAA TAAGAGTTTT   
  
  
- ATTATGGAGT ATTATATTTA CCCAAGCATA GTGGTATATA CCCGGAGTAG AGAGTACCCT TGTATGCTTA   
  
  
- AGTTACCGAA CGGGCTATCA ATAGCCCAGT ACGGTAGCCT CGGGGCCGTT GATTAGTTAC CAGTTCGGAG   
  
  
- TCTAAACCGG AGAAACTCGG TAATATCATT GTGGAAAGAG AGACTTGAGG GGGGAGATGG GTGAGACCCT   
  
  
- GGGCCTAGGC TGGGCCCAGA TAGCACAGGC CCAGGACGAG ACTCAGGTTA CCTCACCTAA CCACTGCGCA   
  
  
- CCTGTGTGGT CTAACTTGTA CTAGACGGTG CAGTCGTCTC AGTTGCCGAC AACTGACTAC CACTGTACCC   
  
  
- CCTCCTCCTA AGGCCATATT CCAACCACGT ACGGAACGAC TGCACGCGTC TCCGGCAGGT TACGCCGCTC   
  
  
- GAGGCTGACC GACGAAGCGA TCAACTACTC TACTGGTTAC CCGACAACGG TGCACAATCA TGCCACACAC   
  
  
- CCTAGCCGTT CCATCGGCCG ATAAAACAAC TCCGAAACTC GGCCGCGGAC AAAGTCGGGC CGGGCCTTTG   
  
  
- ACCGGGCTGG ACCGGCCCGA GCCTCAAGCT CCGACTCCAC AACATAGCAG TGAAGATACT CCGAACGGGA   
  
  
- ATAGAGTTTA AACGAGTGAA GTGCCGATTA GTTCGGTAGA ACCTCCGTAA ACTCCCGGTA CTAATACAGG   
  
  
- TGCAGTAGCT GAAGCCGAAG TACTGGCCGA ACGTTACCGG CCGAAACTAA GTTCGGGACC GAAATTCCGG   
  
  
- GCCACCCGGT GGTAGAGAAT CCGAGTGACC CTAACCGGGC AGAAATCCTT CACCCGGGCT TAGCTATGCA   
  
  
- CTTTAGCCCA ACTCCGATCG GGTTAACCGG GCTAGGTACT TGTACTCCAA GCGTAAAGCC CGACATCGTT   
  
  
- GAAGAGCCGA GCTCCTGCAC TTCGGCAGTT ACGAGCCTTA CCTGGGATCT CTTCGGCAGC GATACTTAAG   
  
  
- GCAGTACGTA GAAGTAGCTG AAAATCCTAA TCTGTAGTTG AGTCAAGATT TGACCTAAGC TTTGGATTTG   
  
  
- GGCTTCTAGC ATTGAAATCA TCTTGTTCTC CGCTTGGTAT TGGTTGGACT TAAAGAGGGG GCTAAGTGAC   
  
  
- TTCGTGATAC AATGATGAGT TGATACAAGC TGAGTAATCT CTGAACAGTT CAGGTTGGCG ACTTTCAGGA   
  
  
- TCGGCTCTAC ATATATGTTT TCCTCTATAG GTTGGATCAC ACGATGCTTC CGAGTCGGGC CCAGGTCTCC   
  
  
- GTGCTCGGCG AGCGGTTTAC CGTTCGAGCC TACCCGGTTC GGCCCAAGTT CGGCGATCCG GACTCAGTTT   
  
  
- TACGGGAATC CGTTCGATTG TACAACCATT GTAACAAGAG GAGTGTTCCC ATACTGCACC TCCTCTCTCT   
  
  
- TCCCACAAAC TGTGACCCGA CCGTATTGGC GGGAGAGTAT CACTGAAGCT GAACCGTTCA CCCCGGGTTT   
  
  
- TACCTGTCAG GTTCACACTG AGTACTCTAC AGTAGTAGGA GTCGAAGAAG TAGAAT

+     MYC

| Site Name | Organism | Position | Strand | Matrix score. | sequence | function |
| --- | --- | --- | --- | --- | --- | --- |
| MYC | Arabidopsis thaliana | 3239 | - | 6 | CATTTG |  |
| MYC | Arabidopsis thaliana | 1385 | - | 6 | CATTTG |  |
| MYC | Arabidopsis thaliana | 1808 | + | 6 | CATTTG |  |
| MYC | Arabidopsis thaliana | 2826 | - | 6 | CAATTG |  |
| MYC | Arabidopsis thaliana | 1408 | - | 6 | CATTTG |  |
| MYC | Arabidopsis thaliana | 2641 | + | 6 | CATTTG |  |

>HU03G02797.1   
+ +Up\_Stream \_Len000CATTTT CAATTAGAAA TTTCTTCAAT TATATACCAT CTCCAAAAAT AGTCCTCATA   
  
  
+ AGTTGGCAGG TGAATGATCC TAATGGAAAA TTACTTTTAA CTGTTGAATT TGCAATAGGG TACCGAATTT   
  
  
+ GTGAAGTTTA TTGCCGCACG TGCCTAGCTT GTGACTTCTC GATAATCAAA AATTAATTAG AGAGAGACAG   
  
  
+ CTAAAGTTTT GAGAATAATA CTGAAAAATT ACATTAAATT TTATTTATGT TTATTTTCAT ACGCTATGTT   
  
  
+ TAACTGCTAT ACGCTGTGTT TAACTGCATC TCCTTCTATA TTTGATAAAT TGGTAAAAAA AAATAATTCA   
  
  
+ AAATTAAAGA CTAGTAATAT TTTCACTTAT CTGATAGATT TATAAGAAAT ATTTAGATAG CTAAATATAT   
  
  
+ TCTCTCCTGA AAGAGGAGAC TGATAGTTGA ATTTCATAGG ATATAAACAT GTATGTACAT TGAACGAATC   
  
  
+ TTTTTTCTAT GGTGATCGTT ATAAAGTTAT TTATTACTGT AAACATTAAG TGATAATTAG GATATGTTCT   
  
  
+ ATTTCACGTT TTAGATAGAT AGATAGATGT AAGGAGTTGT AAGAAGCTGT AAGAATATGT AAGGAGCTGT   
  
  
+ AAGGAGATGT AAGGAGCTGT AAGAAGATGT AAGAGAAACT GTAAGAAGCT ACAAGGATAG ATGTAACGAG   
  
  
+ TTGTTGAATT GTTGAAGAGT TGTATAGTGT AATTTGTTTT ACGATGAACG TTTTGGATGA AAAATAATAA   
  
  
+ TATTAAAATA ATATGCTTAT TAAAAATAAA TAAATAATTA AGTTAATTAC ACAACAATAA AATATATTAA   
  
  
+ TCCCCATAAA TTTAGTGAAT AAAACTTCGT CCACTTGAGA TGATTTTTTA TATGAACACT AGTTTGATAG   
  
  
+ AATAAACAAA GTATAAAAAA AAATAAAGTT TTTTGACAAA ATTGAATACA ACTCTTCAAA TAAATTCCTA   
  
  
+ TTTTAAGTAA ATAATTTTAA ATATAAATAA ATTTATGGTC ATCTATAATA ATATATATTT TATGTCGAAA   
  
  
+ CTCAAATATA ATTTACATCG TTATATTATA TTTTTATGTT TATGTAAATG TTGTGTTCAT AAGATATTTA   
  
  
+ TAAATTTTGT ATATTGGTAG TATCATTAAA AAAAATATAC ATATTTATGT AACTAAAATA AATGAATGTT   
  
  
+ ATAGAAAATC TATAAGATGT AAGGAGATGT ATGAAGATGT AAGGAGATGT AAGAAGATAT AAGGAGATGT   
  
  
+ AGAAAATGTA AGAAGATGTG AGAAGCTGTA AGGAGATGTA AGTTATTCAC TAAATCCTAA AAAATGCGAA   
  
  
+ ATAGAACAGG GGCTTAATTG AGCACTCAAG ATGCATAATT TGTGACGGAC CAAATGGATA AATATAGACC   
  
  
+ TGGCAAATGG GTTATTCGAT TTAGGTACGA GTATGGTTAA GTTTGGGTTT GGTTATGTTT CAGGATAAGT   
  
  
+ GACATTTCGA CTCAATTTGG TTTTGGTTGT ATGAACTTCG GCTCGAGATT TTTGGTTTGG CCAATACACT   
  
  
+ TATCATGCAA AAATAAGTAA CTTTATAGAA AATTTTGGAT CGGATATGAT GCGGTTCGGA TCAATTCGGA   
  
  
+ TTTTGGTTCA CATTCTGGTA AACCTATTTC GGATGTCAAG TAGGATATGA GTCCAGATCG TTGGATTTCG   
  
  
+ GGTATCAGCT CAATTTTACC AGATCTAGAT AAATCATAAA TGTACGGGTT TATTTTTTCC AGCTTGAATG   
  
  
+ ATTTTCTTTA TTAAAAAAAG ATAGATTTTA GATAAAACAC AACATTTCAT TTTCATTTGT TCCCCCCTAT   
  
  
+ AAATTTCTGT GACCACCTTC ACTTTCTTGC CCCATTCCCC CCCCCCCCCC GCTCTCTCTC TCTCTCTCTC   
  
  
+ TCTTCCCTTT ACAAAGGCAC CATTCAAATT CAAACCACTC TTTTTAAAAC CCCAAAAATT ATTCTCAAAA   
  
  
+ TAATACCTCA TAATATAAAT GGGTTCGTAT CACCATATAT GGGCCTCATC TCTCATGGGA ACATACGAAT   
  
  
+ TCAATGGCTT GCCCGATAGT TATCGGGTCA TGCCATCGGA GCCCCGGCAA CTAATCAATG GTCAAGCCTC   
  
  
+ AGATTTGGCC TCTTTGAGCC ATTATAGTAA CACCTTTCTC TCTGAACTCC CCCCTCTACC CACTCTGGGA   
  
  
+ CCCGGATCCG ACCCGGGTCT ATCGTGTCCG GGTCCTGCTC TGAGTCCAAT GGAGTGGATT GGTGACGCGT   
  
  
+ GGACACACCA GATTGAACAT GATCTGCCAC GTCAGCAGAG TCAACGGCTG TTGACTGATG GTGACATGGG   
  
  
+ GGAGGAGGAT TCCGGTATAA GGTTGGTGCA TGCCTTGCTG ACGTGCGCAG AGGCCGTCCA ATGCGGCGAG   
  
  
+ CTCCGACTGG CTGCTTCGCT AGTTGATGAG ATGACCAATG GGCTGTTGCC ACGTGTTAGT ACGGTGTGTG   
  
  
+ GGATCGGCAA GGTAGCCGGC TATTTTGTTG AGGCTTTGAG CCGGCGCCTG TTTCAGCCCG GCCCGGAAAC   
  
  
+ TGGCCCGACC TGGCCGGGCT CGGAGTTCGA GGCTGAGGTG TTGTATCGTC ACTTCTATGA GGCTTGCCCT   
  
  
+ TATCTCAAAT TTGCTCACTT CACGGCTAAT CAAGCCATCT TGGAGGCATT TGAGGGCCAT GATTATGTCC   
  
  
+ ACGTCATCGA CTTCGGCTTC ATGACCGGCT TGCAATGGCC GGCTTTGATT CAAGCCCTGG CTTTAAGGCC   
  
  
+ CGGTGGGCCA CCATCTCTTA GGCTCACTGG GATTGGCCCG TCTTTAGGAA GTGGGCCCGA ATCGATACGT   
  
  
+ GAAATCGGGT TGAGGCTAGC CCAATTGGCC CGATCCATGA ACATGAGGTT CGCATTTCGG GCTGTAGCAA   
  
  
+ CTTCTCGGCT CGAGGACGTG AAGCCGTCAA TGCTCGGAAT GGACCCTAGA GAAGCCGTCG CTATGAATTC   
  
  
+ CGTCATGCAT CTTCATCGAC TTTTAGGATT AGACATCAAC TCAGTTCTAA ACTGGATTCG AAACCTAAAC   
  
  
+ CCGAAGATCG TAACTTTAGT AGAACAAGAG GCGAACCATA ACCAACCTGA ATTTCTCCCC CGATTCACTG   
  
  
+ AAGCACTATG TTACTACTCA ACTATGTTCG ACTCATTAGA GACTTGTCAA GTCCAACCGC TGAAAGTCCT   
  
  
+ AGCCGAGATG TATATACAAA AGGAGATATC CAACCTAGTG TGCTACGAAG GCTCAGCCCG GGTCCAGAGG   
  
  
+ CACGAGCCGC TCGCCAAATG GCAAGCTCGG ATGGGCCAAG CCGGGTTCAA GCCGCTAGGC CTGAGTCAAA   
  
  
+ ATGCCCTTAG GCAAGCTAAC ATGTTGGTAA CATTGTTCTC CTCACAAGGG TATGACGTGG AGGAGAGAGA   
  
  
+ AGGGTGTTTG ACACTGGGCT GGCATAACCG CCCTCTCATA GTGACTTCGA CTTGGCAAGT GGGGCCCAAA   
  
  
+ ATGGACAGTC CAAGTGTGAC TCATGAGATG TCATCATCCT CAGCTTCTTC ATCTTA  

- +Up\_Stream \_Len000GTAAAA GTTAATCTTT AAAGAAGTTA ATATATGGTA GAGGTTTTTA TCAGGAGTAT   
  
  
- TCAACCGTCC ACTTACTAGG ATTACCTTTT AATGAAAATT GACAACTTAA ACGTTATCCC ATGGCTTAAA   
  
  
- CACTTCAAAT AACGGCGTGC ACGGATCGAA CACTGAAGAG CTATTAGTTT TTAATTAATC TCTCTCTGTC   
  
  
- GATTTCAAAA CTCTTATTAT GACTTTTTAA TGTAATTTAA AATAAATACA AATAAAAGTA TGCGATACAA   
  
  
- ATTGACGATA TGCGACACAA ATTGACGTAG AGGAAGATAT AAACTATTTA ACCATTTTTT TTTATTAAGT   
  
  
- TTTAATTTCT GATCATTATA AAAGTGAATA GACTATCTAA ATATTCTTTA TAAATCTATC GATTTATATA   
  
  
- AGAGAGGACT TTCTCCTCTG ACTATCAACT TAAAGTATCC TATATTTGTA CATACATGTA ACTTGCTTAG   
  
  
- AAAAAAGATA CCACTAGCAA TATTTCAATA AATAATGACA TTTGTAATTC ACTATTAATC CTATACAAGA   
  
  
- TAAAGTGCAA AATCTATCTA TCTATCTACA TTCCTCAACA TTCTTCGACA TTCTTATACA TTCCTCGACA   
  
  
- TTCCTCTACA TTCCTCGACA TTCTTCTACA TTCTCTTTGA CATTCTTCGA TGTTCCTATC TACATTGCTC   
  
  
- AACAACTTAA CAACTTCTCA ACATATCACA TTAAACAAAA TGCTACTTGC AAAACCTACT TTTTATTATT   
  
  
- ATAATTTTAT TATACGAATA ATTTTTATTT ATTTATTAAT TCAATTAATG TGTTGTTATT TTATATAATT   
  
  
- AGGGGTATTT AAATCACTTA TTTTGAAGCA GGTGAACTCT ACTAAAAAAT ATACTTGTGA TCAAACTATC   
  
  
- TTATTTGTTT CATATTTTTT TTTATTTCAA AAAACTGTTT TAACTTATGT TGAGAAGTTT ATTTAAGGAT   
  
  
- AAAATTCATT TATTAAAATT TATATTTATT TAAATACCAG TAGATATTAT TATATATAAA ATACAGCTTT   
  
  
- GAGTTTATAT TAAATGTAGC AATATAATAT AAAAATACAA ATACATTTAC AACACAAGTA TTCTATAAAT   
  
  
- ATTTAAAACA TATAACCATC ATAGTAATTT TTTTTATATG TATAAATACA TTGATTTTAT TTACTTACAA   
  
  
- TATCTTTTAG ATATTCTACA TTCCTCTACA TACTTCTACA TTCCTCTACA TTCTTCTATA TTCCTCTACA   
  
  
- TCTTTTACAT TCTTCTACAC TCTTCGACAT TCCTCTACAT TCAATAAGTG ATTTAGGATT TTTTACGCTT   
  
  
- TATCTTGTCC CCGAATTAAC TCGTGAGTTC TACGTATTAA ACACTGCCTG GTTTACCTAT TTATATCTGG   
  
  
- ACCGTTTACC CAATAAGCTA AATCCATGCT CATACCAATT CAAACCCAAA CCAATACAAA GTCCTATTCA   
  
  
- CTGTAAAGCT GAGTTAAACC AAAACCAACA TACTTGAAGC CGAGCTCTAA AAACCAAACC GGTTATGTGA   
  
  
- ATAGTACGTT TTTATTCATT GAAATATCTT TTAAAACCTA GCCTATACTA CGCCAAGCCT AGTTAAGCCT   
  
  
- AAAACCAAGT GTAAGACCAT TTGGATAAAG CCTACAGTTC ATCCTATACT CAGGTCTAGC AACCTAAAGC   
  
  
- CCATAGTCGA GTTAAAATGG TCTAGATCTA TTTAGTATTT ACATGCCCAA ATAAAAAAGG TCGAACTTAC   
  
  
- TAAAAGAAAT AATTTTTTTC TATCTAAAAT CTATTTTGTG TTGTAAAGTA AAAGTAAACA AGGGGGGATA   
  
  
- TTTAAAGACA CTGGTGGAAG TGAAAGAACG GGGTAAGGGG GGGGGGGGGG CGAGAGAGAG AGAGAGAGAG   
  
  
- AGAAGGGAAA TGTTTCCGTG GTAAGTTTAA GTTTGGTGAG AAAAATTTTG GGGTTTTTAA TAAGAGTTTT   
  
  
- ATTATGGAGT ATTATATTTA CCCAAGCATA GTGGTATATA CCCGGAGTAG AGAGTACCCT TGTATGCTTA   
  
  
- AGTTACCGAA CGGGCTATCA ATAGCCCAGT ACGGTAGCCT CGGGGCCGTT GATTAGTTAC CAGTTCGGAG   
  
  
- TCTAAACCGG AGAAACTCGG TAATATCATT GTGGAAAGAG AGACTTGAGG GGGGAGATGG GTGAGACCCT   
  
  
- GGGCCTAGGC TGGGCCCAGA TAGCACAGGC CCAGGACGAG ACTCAGGTTA CCTCACCTAA CCACTGCGCA   
  
  
- CCTGTGTGGT CTAACTTGTA CTAGACGGTG CAGTCGTCTC AGTTGCCGAC AACTGACTAC CACTGTACCC   
  
  
- CCTCCTCCTA AGGCCATATT CCAACCACGT ACGGAACGAC TGCACGCGTC TCCGGCAGGT TACGCCGCTC   
  
  
- GAGGCTGACC GACGAAGCGA TCAACTACTC TACTGGTTAC CCGACAACGG TGCACAATCA TGCCACACAC   
  
  
- CCTAGCCGTT CCATCGGCCG ATAAAACAAC TCCGAAACTC GGCCGCGGAC AAAGTCGGGC CGGGCCTTTG   
  
  
- ACCGGGCTGG ACCGGCCCGA GCCTCAAGCT CCGACTCCAC AACATAGCAG TGAAGATACT CCGAACGGGA   
  
  
- ATAGAGTTTA AACGAGTGAA GTGCCGATTA GTTCGGTAGA ACCTCCGTAA ACTCCCGGTA CTAATACAGG   
  
  
- TGCAGTAGCT GAAGCCGAAG TACTGGCCGA ACGTTACCGG CCGAAACTAA GTTCGGGACC GAAATTCCGG   
  
  
- GCCACCCGGT GGTAGAGAAT CCGAGTGACC CTAACCGGGC AGAAATCCTT CACCCGGGCT TAGCTATGCA   
  
  
- CTTTAGCCCA ACTCCGATCG GGTTAACCGG GCTAGGTACT TGTACTCCAA GCGTAAAGCC CGACATCGTT   
  
  
- GAAGAGCCGA GCTCCTGCAC TTCGGCAGTT ACGAGCCTTA CCTGGGATCT CTTCGGCAGC GATACTTAAG   
  
  
- GCAGTACGTA GAAGTAGCTG AAAATCCTAA TCTGTAGTTG AGTCAAGATT TGACCTAAGC TTTGGATTTG   
  
  
- GGCTTCTAGC ATTGAAATCA TCTTGTTCTC CGCTTGGTAT TGGTTGGACT TAAAGAGGGG GCTAAGTGAC   
  
  
- TTCGTGATAC AATGATGAGT TGATACAAGC TGAGTAATCT CTGAACAGTT CAGGTTGGCG ACTTTCAGGA   
  
  
- TCGGCTCTAC ATATATGTTT TCCTCTATAG GTTGGATCAC ACGATGCTTC CGAGTCGGGC CCAGGTCTCC   
  
  
- GTGCTCGGCG AGCGGTTTAC CGTTCGAGCC TACCCGGTTC GGCCCAAGTT CGGCGATCCG GACTCAGTTT   
  
  
- TACGGGAATC CGTTCGATTG TACAACCATT GTAACAAGAG GAGTGTTCCC ATACTGCACC TCCTCTCTCT   
  
  
- TCCCACAAAC TGTGACCCGA CCGTATTGGC GGGAGAGTAT CACTGAAGCT GAACCGTTCA CCCCGGGTTT   
  
  
- TACCTGTCAG GTTCACACTG AGTACTCTAC AGTAGTAGGA GTCGAAGAAG TAGAAT

+     Myb

| Site Name | Organism | Position | Strand | Matrix score. | sequence | function |
| --- | --- | --- | --- | --- | --- | --- |
| Myb | Arabidopsis thaliana | 112 | + | 6 | TAACTG |  |
| Myb | Arabidopsis thaliana | 285 | + | 6 | TAACTG |  |
| Myb | Arabidopsis thaliana | 305 | + | 6 | TAACTG |  |

>HU03G02797.1   
+ +Up\_Stream \_Len000CATTTT CAATTAGAAA TTTCTTCAAT TATATACCAT CTCCAAAAAT AGTCCTCATA   
  
  
+ AGTTGGCAGG TGAATGATCC TAATGGAAAA TTACTTTTAA CTGTTGAATT TGCAATAGGG TACCGAATTT   
  
  
+ GTGAAGTTTA TTGCCGCACG TGCCTAGCTT GTGACTTCTC GATAATCAAA AATTAATTAG AGAGAGACAG   
  
  
+ CTAAAGTTTT GAGAATAATA CTGAAAAATT ACATTAAATT TTATTTATGT TTATTTTCAT ACGCTATGTT   
  
  
+ TAACTGCTAT ACGCTGTGTT TAACTGCATC TCCTTCTATA TTTGATAAAT TGGTAAAAAA AAATAATTCA   
  
  
+ AAATTAAAGA CTAGTAATAT TTTCACTTAT CTGATAGATT TATAAGAAAT ATTTAGATAG CTAAATATAT   
  
  
+ TCTCTCCTGA AAGAGGAGAC TGATAGTTGA ATTTCATAGG ATATAAACAT GTATGTACAT TGAACGAATC   
  
  
+ TTTTTTCTAT GGTGATCGTT ATAAAGTTAT TTATTACTGT AAACATTAAG TGATAATTAG GATATGTTCT   
  
  
+ ATTTCACGTT TTAGATAGAT AGATAGATGT AAGGAGTTGT AAGAAGCTGT AAGAATATGT AAGGAGCTGT   
  
  
+ AAGGAGATGT AAGGAGCTGT AAGAAGATGT AAGAGAAACT GTAAGAAGCT ACAAGGATAG ATGTAACGAG   
  
  
+ TTGTTGAATT GTTGAAGAGT TGTATAGTGT AATTTGTTTT ACGATGAACG TTTTGGATGA AAAATAATAA   
  
  
+ TATTAAAATA ATATGCTTAT TAAAAATAAA TAAATAATTA AGTTAATTAC ACAACAATAA AATATATTAA   
  
  
+ TCCCCATAAA TTTAGTGAAT AAAACTTCGT CCACTTGAGA TGATTTTTTA TATGAACACT AGTTTGATAG   
  
  
+ AATAAACAAA GTATAAAAAA AAATAAAGTT TTTTGACAAA ATTGAATACA ACTCTTCAAA TAAATTCCTA   
  
  
+ TTTTAAGTAA ATAATTTTAA ATATAAATAA ATTTATGGTC ATCTATAATA ATATATATTT TATGTCGAAA   
  
  
+ CTCAAATATA ATTTACATCG TTATATTATA TTTTTATGTT TATGTAAATG TTGTGTTCAT AAGATATTTA   
  
  
+ TAAATTTTGT ATATTGGTAG TATCATTAAA AAAAATATAC ATATTTATGT AACTAAAATA AATGAATGTT   
  
  
+ ATAGAAAATC TATAAGATGT AAGGAGATGT ATGAAGATGT AAGGAGATGT AAGAAGATAT AAGGAGATGT   
  
  
+ AGAAAATGTA AGAAGATGTG AGAAGCTGTA AGGAGATGTA AGTTATTCAC TAAATCCTAA AAAATGCGAA   
  
  
+ ATAGAACAGG GGCTTAATTG AGCACTCAAG ATGCATAATT TGTGACGGAC CAAATGGATA AATATAGACC   
  
  
+ TGGCAAATGG GTTATTCGAT TTAGGTACGA GTATGGTTAA GTTTGGGTTT GGTTATGTTT CAGGATAAGT   
  
  
+ GACATTTCGA CTCAATTTGG TTTTGGTTGT ATGAACTTCG GCTCGAGATT TTTGGTTTGG CCAATACACT   
  
  
+ TATCATGCAA AAATAAGTAA CTTTATAGAA AATTTTGGAT CGGATATGAT GCGGTTCGGA TCAATTCGGA   
  
  
+ TTTTGGTTCA CATTCTGGTA AACCTATTTC GGATGTCAAG TAGGATATGA GTCCAGATCG TTGGATTTCG   
  
  
+ GGTATCAGCT CAATTTTACC AGATCTAGAT AAATCATAAA TGTACGGGTT TATTTTTTCC AGCTTGAATG   
  
  
+ ATTTTCTTTA TTAAAAAAAG ATAGATTTTA GATAAAACAC AACATTTCAT TTTCATTTGT TCCCCCCTAT   
  
  
+ AAATTTCTGT GACCACCTTC ACTTTCTTGC CCCATTCCCC CCCCCCCCCC GCTCTCTCTC TCTCTCTCTC   
  
  
+ TCTTCCCTTT ACAAAGGCAC CATTCAAATT CAAACCACTC TTTTTAAAAC CCCAAAAATT ATTCTCAAAA   
  
  
+ TAATACCTCA TAATATAAAT GGGTTCGTAT CACCATATAT GGGCCTCATC TCTCATGGGA ACATACGAAT   
  
  
+ TCAATGGCTT GCCCGATAGT TATCGGGTCA TGCCATCGGA GCCCCGGCAA CTAATCAATG GTCAAGCCTC   
  
  
+ AGATTTGGCC TCTTTGAGCC ATTATAGTAA CACCTTTCTC TCTGAACTCC CCCCTCTACC CACTCTGGGA   
  
  
+ CCCGGATCCG ACCCGGGTCT ATCGTGTCCG GGTCCTGCTC TGAGTCCAAT GGAGTGGATT GGTGACGCGT   
  
  
+ GGACACACCA GATTGAACAT GATCTGCCAC GTCAGCAGAG TCAACGGCTG TTGACTGATG GTGACATGGG   
  
  
+ GGAGGAGGAT TCCGGTATAA GGTTGGTGCA TGCCTTGCTG ACGTGCGCAG AGGCCGTCCA ATGCGGCGAG   
  
  
+ CTCCGACTGG CTGCTTCGCT AGTTGATGAG ATGACCAATG GGCTGTTGCC ACGTGTTAGT ACGGTGTGTG   
  
  
+ GGATCGGCAA GGTAGCCGGC TATTTTGTTG AGGCTTTGAG CCGGCGCCTG TTTCAGCCCG GCCCGGAAAC   
  
  
+ TGGCCCGACC TGGCCGGGCT CGGAGTTCGA GGCTGAGGTG TTGTATCGTC ACTTCTATGA GGCTTGCCCT   
  
  
+ TATCTCAAAT TTGCTCACTT CACGGCTAAT CAAGCCATCT TGGAGGCATT TGAGGGCCAT GATTATGTCC   
  
  
+ ACGTCATCGA CTTCGGCTTC ATGACCGGCT TGCAATGGCC GGCTTTGATT CAAGCCCTGG CTTTAAGGCC   
  
  
+ CGGTGGGCCA CCATCTCTTA GGCTCACTGG GATTGGCCCG TCTTTAGGAA GTGGGCCCGA ATCGATACGT   
  
  
+ GAAATCGGGT TGAGGCTAGC CCAATTGGCC CGATCCATGA ACATGAGGTT CGCATTTCGG GCTGTAGCAA   
  
  
+ CTTCTCGGCT CGAGGACGTG AAGCCGTCAA TGCTCGGAAT GGACCCTAGA GAAGCCGTCG CTATGAATTC   
  
  
+ CGTCATGCAT CTTCATCGAC TTTTAGGATT AGACATCAAC TCAGTTCTAA ACTGGATTCG AAACCTAAAC   
  
  
+ CCGAAGATCG TAACTTTAGT AGAACAAGAG GCGAACCATA ACCAACCTGA ATTTCTCCCC CGATTCACTG   
  
  
+ AAGCACTATG TTACTACTCA ACTATGTTCG ACTCATTAGA GACTTGTCAA GTCCAACCGC TGAAAGTCCT   
  
  
+ AGCCGAGATG TATATACAAA AGGAGATATC CAACCTAGTG TGCTACGAAG GCTCAGCCCG GGTCCAGAGG   
  
  
+ CACGAGCCGC TCGCCAAATG GCAAGCTCGG ATGGGCCAAG CCGGGTTCAA GCCGCTAGGC CTGAGTCAAA   
  
  
+ ATGCCCTTAG GCAAGCTAAC ATGTTGGTAA CATTGTTCTC CTCACAAGGG TATGACGTGG AGGAGAGAGA   
  
  
+ AGGGTGTTTG ACACTGGGCT GGCATAACCG CCCTCTCATA GTGACTTCGA CTTGGCAAGT GGGGCCCAAA   
  
  
+ ATGGACAGTC CAAGTGTGAC TCATGAGATG TCATCATCCT CAGCTTCTTC ATCTTA  

- +Up\_Stream \_Len000GTAAAA GTTAATCTTT AAAGAAGTTA ATATATGGTA GAGGTTTTTA TCAGGAGTAT   
  
  
- TCAACCGTCC ACTTACTAGG ATTACCTTTT AATGAAAATT GACAACTTAA ACGTTATCCC ATGGCTTAAA   
  
  
- CACTTCAAAT AACGGCGTGC ACGGATCGAA CACTGAAGAG CTATTAGTTT TTAATTAATC TCTCTCTGTC   
  
  
- GATTTCAAAA CTCTTATTAT GACTTTTTAA TGTAATTTAA AATAAATACA AATAAAAGTA TGCGATACAA   
  
  
- ATTGACGATA TGCGACACAA ATTGACGTAG AGGAAGATAT AAACTATTTA ACCATTTTTT TTTATTAAGT   
  
  
- TTTAATTTCT GATCATTATA AAAGTGAATA GACTATCTAA ATATTCTTTA TAAATCTATC GATTTATATA   
  
  
- AGAGAGGACT TTCTCCTCTG ACTATCAACT TAAAGTATCC TATATTTGTA CATACATGTA ACTTGCTTAG   
  
  
- AAAAAAGATA CCACTAGCAA TATTTCAATA AATAATGACA TTTGTAATTC ACTATTAATC CTATACAAGA   
  
  
- TAAAGTGCAA AATCTATCTA TCTATCTACA TTCCTCAACA TTCTTCGACA TTCTTATACA TTCCTCGACA   
  
  
- TTCCTCTACA TTCCTCGACA TTCTTCTACA TTCTCTTTGA CATTCTTCGA TGTTCCTATC TACATTGCTC   
  
  
- AACAACTTAA CAACTTCTCA ACATATCACA TTAAACAAAA TGCTACTTGC AAAACCTACT TTTTATTATT   
  
  
- ATAATTTTAT TATACGAATA ATTTTTATTT ATTTATTAAT TCAATTAATG TGTTGTTATT TTATATAATT   
  
  
- AGGGGTATTT AAATCACTTA TTTTGAAGCA GGTGAACTCT ACTAAAAAAT ATACTTGTGA TCAAACTATC   
  
  
- TTATTTGTTT CATATTTTTT TTTATTTCAA AAAACTGTTT TAACTTATGT TGAGAAGTTT ATTTAAGGAT   
  
  
- AAAATTCATT TATTAAAATT TATATTTATT TAAATACCAG TAGATATTAT TATATATAAA ATACAGCTTT   
  
  
- GAGTTTATAT TAAATGTAGC AATATAATAT AAAAATACAA ATACATTTAC AACACAAGTA TTCTATAAAT   
  
  
- ATTTAAAACA TATAACCATC ATAGTAATTT TTTTTATATG TATAAATACA TTGATTTTAT TTACTTACAA   
  
  
- TATCTTTTAG ATATTCTACA TTCCTCTACA TACTTCTACA TTCCTCTACA TTCTTCTATA TTCCTCTACA   
  
  
- TCTTTTACAT TCTTCTACAC TCTTCGACAT TCCTCTACAT TCAATAAGTG ATTTAGGATT TTTTACGCTT   
  
  
- TATCTTGTCC CCGAATTAAC TCGTGAGTTC TACGTATTAA ACACTGCCTG GTTTACCTAT TTATATCTGG   
  
  
- ACCGTTTACC CAATAAGCTA AATCCATGCT CATACCAATT CAAACCCAAA CCAATACAAA GTCCTATTCA   
  
  
- CTGTAAAGCT GAGTTAAACC AAAACCAACA TACTTGAAGC CGAGCTCTAA AAACCAAACC GGTTATGTGA   
  
  
- ATAGTACGTT TTTATTCATT GAAATATCTT TTAAAACCTA GCCTATACTA CGCCAAGCCT AGTTAAGCCT   
  
  
- AAAACCAAGT GTAAGACCAT TTGGATAAAG CCTACAGTTC ATCCTATACT CAGGTCTAGC AACCTAAAGC   
  
  
- CCATAGTCGA GTTAAAATGG TCTAGATCTA TTTAGTATTT ACATGCCCAA ATAAAAAAGG TCGAACTTAC   
  
  
- TAAAAGAAAT AATTTTTTTC TATCTAAAAT CTATTTTGTG TTGTAAAGTA AAAGTAAACA AGGGGGGATA   
  
  
- TTTAAAGACA CTGGTGGAAG TGAAAGAACG GGGTAAGGGG GGGGGGGGGG CGAGAGAGAG AGAGAGAGAG   
  
  
- AGAAGGGAAA TGTTTCCGTG GTAAGTTTAA GTTTGGTGAG AAAAATTTTG GGGTTTTTAA TAAGAGTTTT   
  
  
- ATTATGGAGT ATTATATTTA CCCAAGCATA GTGGTATATA CCCGGAGTAG AGAGTACCCT TGTATGCTTA   
  
  
- AGTTACCGAA CGGGCTATCA ATAGCCCAGT ACGGTAGCCT CGGGGCCGTT GATTAGTTAC CAGTTCGGAG   
  
  
- TCTAAACCGG AGAAACTCGG TAATATCATT GTGGAAAGAG AGACTTGAGG GGGGAGATGG GTGAGACCCT   
  
  
- GGGCCTAGGC TGGGCCCAGA TAGCACAGGC CCAGGACGAG ACTCAGGTTA CCTCACCTAA CCACTGCGCA   
  
  
- CCTGTGTGGT CTAACTTGTA CTAGACGGTG CAGTCGTCTC AGTTGCCGAC AACTGACTAC CACTGTACCC   
  
  
- CCTCCTCCTA AGGCCATATT CCAACCACGT ACGGAACGAC TGCACGCGTC TCCGGCAGGT TACGCCGCTC   
  
  
- GAGGCTGACC GACGAAGCGA TCAACTACTC TACTGGTTAC CCGACAACGG TGCACAATCA TGCCACACAC   
  
  
- CCTAGCCGTT CCATCGGCCG ATAAAACAAC TCCGAAACTC GGCCGCGGAC AAAGTCGGGC CGGGCCTTTG   
  
  
- ACCGGGCTGG ACCGGCCCGA GCCTCAAGCT CCGACTCCAC AACATAGCAG TGAAGATACT CCGAACGGGA   
  
  
- ATAGAGTTTA AACGAGTGAA GTGCCGATTA GTTCGGTAGA ACCTCCGTAA ACTCCCGGTA CTAATACAGG   
  
  
- TGCAGTAGCT GAAGCCGAAG TACTGGCCGA ACGTTACCGG CCGAAACTAA GTTCGGGACC GAAATTCCGG   
  
  
- GCCACCCGGT GGTAGAGAAT CCGAGTGACC CTAACCGGGC AGAAATCCTT CACCCGGGCT TAGCTATGCA   
  
  
- CTTTAGCCCA ACTCCGATCG GGTTAACCGG GCTAGGTACT TGTACTCCAA GCGTAAAGCC CGACATCGTT   
  
  
- GAAGAGCCGA GCTCCTGCAC TTCGGCAGTT ACGAGCCTTA CCTGGGATCT CTTCGGCAGC GATACTTAAG   
  
  
- GCAGTACGTA GAAGTAGCTG AAAATCCTAA TCTGTAGTTG AGTCAAGATT TGACCTAAGC TTTGGATTTG   
  
  
- GGCTTCTAGC ATTGAAATCA TCTTGTTCTC CGCTTGGTAT TGGTTGGACT TAAAGAGGGG GCTAAGTGAC   
  
  
- TTCGTGATAC AATGATGAGT TGATACAAGC TGAGTAATCT CTGAACAGTT CAGGTTGGCG ACTTTCAGGA   
  
  
- TCGGCTCTAC ATATATGTTT TCCTCTATAG GTTGGATCAC ACGATGCTTC CGAGTCGGGC CCAGGTCTCC   
  
  
- GTGCTCGGCG AGCGGTTTAC CGTTCGAGCC TACCCGGTTC GGCCCAAGTT CGGCGATCCG GACTCAGTTT   
  
  
- TACGGGAATC CGTTCGATTG TACAACCATT GTAACAAGAG GAGTGTTCCC ATACTGCACC TCCTCTCTCT   
  
  
- TCCCACAAAC TGTGACCCGA CCGTATTGGC GGGAGAGTAT CACTGAAGCT GAACCGTTCA CCCCGGGTTT   
  
  
- TACCTGTCAG GTTCACACTG AGTACTCTAC AGTAGTAGGA GTCGAAGAAG TAGAAT

+     Myb-binding site

| Site Name | Organism | Position | Strand | Matrix score. | sequence | function |
| --- | --- | --- | --- | --- | --- | --- |
| Myb-binding site | Nicotiana tabacum | 115 | - | 6 | CAACAG |  |
| Myb-binding site | Nicotiana tabacum | 2427 | - | 6 | CAACAG |  |
| Myb-binding site | Nicotiana tabacum | 2292 | - | 6 | CAACAG |  |

>HU03G02797.1   
+ +Up\_Stream \_Len000CATTTT CAATTAGAAA TTTCTTCAAT TATATACCAT CTCCAAAAAT AGTCCTCATA   
  
  
+ AGTTGGCAGG TGAATGATCC TAATGGAAAA TTACTTTTAA CTGTTGAATT TGCAATAGGG TACCGAATTT   
  
  
+ GTGAAGTTTA TTGCCGCACG TGCCTAGCTT GTGACTTCTC GATAATCAAA AATTAATTAG AGAGAGACAG   
  
  
+ CTAAAGTTTT GAGAATAATA CTGAAAAATT ACATTAAATT TTATTTATGT TTATTTTCAT ACGCTATGTT   
  
  
+ TAACTGCTAT ACGCTGTGTT TAACTGCATC TCCTTCTATA TTTGATAAAT TGGTAAAAAA AAATAATTCA   
  
  
+ AAATTAAAGA CTAGTAATAT TTTCACTTAT CTGATAGATT TATAAGAAAT ATTTAGATAG CTAAATATAT   
  
  
+ TCTCTCCTGA AAGAGGAGAC TGATAGTTGA ATTTCATAGG ATATAAACAT GTATGTACAT TGAACGAATC   
  
  
+ TTTTTTCTAT GGTGATCGTT ATAAAGTTAT TTATTACTGT AAACATTAAG TGATAATTAG GATATGTTCT   
  
  
+ ATTTCACGTT TTAGATAGAT AGATAGATGT AAGGAGTTGT AAGAAGCTGT AAGAATATGT AAGGAGCTGT   
  
  
+ AAGGAGATGT AAGGAGCTGT AAGAAGATGT AAGAGAAACT GTAAGAAGCT ACAAGGATAG ATGTAACGAG   
  
  
+ TTGTTGAATT GTTGAAGAGT TGTATAGTGT AATTTGTTTT ACGATGAACG TTTTGGATGA AAAATAATAA   
  
  
+ TATTAAAATA ATATGCTTAT TAAAAATAAA TAAATAATTA AGTTAATTAC ACAACAATAA AATATATTAA   
  
  
+ TCCCCATAAA TTTAGTGAAT AAAACTTCGT CCACTTGAGA TGATTTTTTA TATGAACACT AGTTTGATAG   
  
  
+ AATAAACAAA GTATAAAAAA AAATAAAGTT TTTTGACAAA ATTGAATACA ACTCTTCAAA TAAATTCCTA   
  
  
+ TTTTAAGTAA ATAATTTTAA ATATAAATAA ATTTATGGTC ATCTATAATA ATATATATTT TATGTCGAAA   
  
  
+ CTCAAATATA ATTTACATCG TTATATTATA TTTTTATGTT TATGTAAATG TTGTGTTCAT AAGATATTTA   
  
  
+ TAAATTTTGT ATATTGGTAG TATCATTAAA AAAAATATAC ATATTTATGT AACTAAAATA AATGAATGTT   
  
  
+ ATAGAAAATC TATAAGATGT AAGGAGATGT ATGAAGATGT AAGGAGATGT AAGAAGATAT AAGGAGATGT   
  
  
+ AGAAAATGTA AGAAGATGTG AGAAGCTGTA AGGAGATGTA AGTTATTCAC TAAATCCTAA AAAATGCGAA   
  
  
+ ATAGAACAGG GGCTTAATTG AGCACTCAAG ATGCATAATT TGTGACGGAC CAAATGGATA AATATAGACC   
  
  
+ TGGCAAATGG GTTATTCGAT TTAGGTACGA GTATGGTTAA GTTTGGGTTT GGTTATGTTT CAGGATAAGT   
  
  
+ GACATTTCGA CTCAATTTGG TTTTGGTTGT ATGAACTTCG GCTCGAGATT TTTGGTTTGG CCAATACACT   
  
  
+ TATCATGCAA AAATAAGTAA CTTTATAGAA AATTTTGGAT CGGATATGAT GCGGTTCGGA TCAATTCGGA   
  
  
+ TTTTGGTTCA CATTCTGGTA AACCTATTTC GGATGTCAAG TAGGATATGA GTCCAGATCG TTGGATTTCG   
  
  
+ GGTATCAGCT CAATTTTACC AGATCTAGAT AAATCATAAA TGTACGGGTT TATTTTTTCC AGCTTGAATG   
  
  
+ ATTTTCTTTA TTAAAAAAAG ATAGATTTTA GATAAAACAC AACATTTCAT TTTCATTTGT TCCCCCCTAT   
  
  
+ AAATTTCTGT GACCACCTTC ACTTTCTTGC CCCATTCCCC CCCCCCCCCC GCTCTCTCTC TCTCTCTCTC   
  
  
+ TCTTCCCTTT ACAAAGGCAC CATTCAAATT CAAACCACTC TTTTTAAAAC CCCAAAAATT ATTCTCAAAA   
  
  
+ TAATACCTCA TAATATAAAT GGGTTCGTAT CACCATATAT GGGCCTCATC TCTCATGGGA ACATACGAAT   
  
  
+ TCAATGGCTT GCCCGATAGT TATCGGGTCA TGCCATCGGA GCCCCGGCAA CTAATCAATG GTCAAGCCTC   
  
  
+ AGATTTGGCC TCTTTGAGCC ATTATAGTAA CACCTTTCTC TCTGAACTCC CCCCTCTACC CACTCTGGGA   
  
  
+ CCCGGATCCG ACCCGGGTCT ATCGTGTCCG GGTCCTGCTC TGAGTCCAAT GGAGTGGATT GGTGACGCGT   
  
  
+ GGACACACCA GATTGAACAT GATCTGCCAC GTCAGCAGAG TCAACGGCTG TTGACTGATG GTGACATGGG   
  
  
+ GGAGGAGGAT TCCGGTATAA GGTTGGTGCA TGCCTTGCTG ACGTGCGCAG AGGCCGTCCA ATGCGGCGAG   
  
  
+ CTCCGACTGG CTGCTTCGCT AGTTGATGAG ATGACCAATG GGCTGTTGCC ACGTGTTAGT ACGGTGTGTG   
  
  
+ GGATCGGCAA GGTAGCCGGC TATTTTGTTG AGGCTTTGAG CCGGCGCCTG TTTCAGCCCG GCCCGGAAAC   
  
  
+ TGGCCCGACC TGGCCGGGCT CGGAGTTCGA GGCTGAGGTG TTGTATCGTC ACTTCTATGA GGCTTGCCCT   
  
  
+ TATCTCAAAT TTGCTCACTT CACGGCTAAT CAAGCCATCT TGGAGGCATT TGAGGGCCAT GATTATGTCC   
  
  
+ ACGTCATCGA CTTCGGCTTC ATGACCGGCT TGCAATGGCC GGCTTTGATT CAAGCCCTGG CTTTAAGGCC   
  
  
+ CGGTGGGCCA CCATCTCTTA GGCTCACTGG GATTGGCCCG TCTTTAGGAA GTGGGCCCGA ATCGATACGT   
  
  
+ GAAATCGGGT TGAGGCTAGC CCAATTGGCC CGATCCATGA ACATGAGGTT CGCATTTCGG GCTGTAGCAA   
  
  
+ CTTCTCGGCT CGAGGACGTG AAGCCGTCAA TGCTCGGAAT GGACCCTAGA GAAGCCGTCG CTATGAATTC   
  
  
+ CGTCATGCAT CTTCATCGAC TTTTAGGATT AGACATCAAC TCAGTTCTAA ACTGGATTCG AAACCTAAAC   
  
  
+ CCGAAGATCG TAACTTTAGT AGAACAAGAG GCGAACCATA ACCAACCTGA ATTTCTCCCC CGATTCACTG   
  
  
+ AAGCACTATG TTACTACTCA ACTATGTTCG ACTCATTAGA GACTTGTCAA GTCCAACCGC TGAAAGTCCT   
  
  
+ AGCCGAGATG TATATACAAA AGGAGATATC CAACCTAGTG TGCTACGAAG GCTCAGCCCG GGTCCAGAGG   
  
  
+ CACGAGCCGC TCGCCAAATG GCAAGCTCGG ATGGGCCAAG CCGGGTTCAA GCCGCTAGGC CTGAGTCAAA   
  
  
+ ATGCCCTTAG GCAAGCTAAC ATGTTGGTAA CATTGTTCTC CTCACAAGGG TATGACGTGG AGGAGAGAGA   
  
  
+ AGGGTGTTTG ACACTGGGCT GGCATAACCG CCCTCTCATA GTGACTTCGA CTTGGCAAGT GGGGCCCAAA   
  
  
+ ATGGACAGTC CAAGTGTGAC TCATGAGATG TCATCATCCT CAGCTTCTTC ATCTTA  

- +Up\_Stream \_Len000GTAAAA GTTAATCTTT AAAGAAGTTA ATATATGGTA GAGGTTTTTA TCAGGAGTAT   
  
  
- TCAACCGTCC ACTTACTAGG ATTACCTTTT AATGAAAATT GACAACTTAA ACGTTATCCC ATGGCTTAAA   
  
  
- CACTTCAAAT AACGGCGTGC ACGGATCGAA CACTGAAGAG CTATTAGTTT TTAATTAATC TCTCTCTGTC   
  
  
- GATTTCAAAA CTCTTATTAT GACTTTTTAA TGTAATTTAA AATAAATACA AATAAAAGTA TGCGATACAA   
  
  
- ATTGACGATA TGCGACACAA ATTGACGTAG AGGAAGATAT AAACTATTTA ACCATTTTTT TTTATTAAGT   
  
  
- TTTAATTTCT GATCATTATA AAAGTGAATA GACTATCTAA ATATTCTTTA TAAATCTATC GATTTATATA   
  
  
- AGAGAGGACT TTCTCCTCTG ACTATCAACT TAAAGTATCC TATATTTGTA CATACATGTA ACTTGCTTAG   
  
  
- AAAAAAGATA CCACTAGCAA TATTTCAATA AATAATGACA TTTGTAATTC ACTATTAATC CTATACAAGA   
  
  
- TAAAGTGCAA AATCTATCTA TCTATCTACA TTCCTCAACA TTCTTCGACA TTCTTATACA TTCCTCGACA   
  
  
- TTCCTCTACA TTCCTCGACA TTCTTCTACA TTCTCTTTGA CATTCTTCGA TGTTCCTATC TACATTGCTC   
  
  
- AACAACTTAA CAACTTCTCA ACATATCACA TTAAACAAAA TGCTACTTGC AAAACCTACT TTTTATTATT   
  
  
- ATAATTTTAT TATACGAATA ATTTTTATTT ATTTATTAAT TCAATTAATG TGTTGTTATT TTATATAATT   
  
  
- AGGGGTATTT AAATCACTTA TTTTGAAGCA GGTGAACTCT ACTAAAAAAT ATACTTGTGA TCAAACTATC   
  
  
- TTATTTGTTT CATATTTTTT TTTATTTCAA AAAACTGTTT TAACTTATGT TGAGAAGTTT ATTTAAGGAT   
  
  
- AAAATTCATT TATTAAAATT TATATTTATT TAAATACCAG TAGATATTAT TATATATAAA ATACAGCTTT   
  
  
- GAGTTTATAT TAAATGTAGC AATATAATAT AAAAATACAA ATACATTTAC AACACAAGTA TTCTATAAAT   
  
  
- ATTTAAAACA TATAACCATC ATAGTAATTT TTTTTATATG TATAAATACA TTGATTTTAT TTACTTACAA   
  
  
- TATCTTTTAG ATATTCTACA TTCCTCTACA TACTTCTACA TTCCTCTACA TTCTTCTATA TTCCTCTACA   
  
  
- TCTTTTACAT TCTTCTACAC TCTTCGACAT TCCTCTACAT TCAATAAGTG ATTTAGGATT TTTTACGCTT   
  
  
- TATCTTGTCC CCGAATTAAC TCGTGAGTTC TACGTATTAA ACACTGCCTG GTTTACCTAT TTATATCTGG   
  
  
- ACCGTTTACC CAATAAGCTA AATCCATGCT CATACCAATT CAAACCCAAA CCAATACAAA GTCCTATTCA   
  
  
- CTGTAAAGCT GAGTTAAACC AAAACCAACA TACTTGAAGC CGAGCTCTAA AAACCAAACC GGTTATGTGA   
  
  
- ATAGTACGTT TTTATTCATT GAAATATCTT TTAAAACCTA GCCTATACTA CGCCAAGCCT AGTTAAGCCT   
  
  
- AAAACCAAGT GTAAGACCAT TTGGATAAAG CCTACAGTTC ATCCTATACT CAGGTCTAGC AACCTAAAGC   
  
  
- CCATAGTCGA GTTAAAATGG TCTAGATCTA TTTAGTATTT ACATGCCCAA ATAAAAAAGG TCGAACTTAC   
  
  
- TAAAAGAAAT AATTTTTTTC TATCTAAAAT CTATTTTGTG TTGTAAAGTA AAAGTAAACA AGGGGGGATA   
  
  
- TTTAAAGACA CTGGTGGAAG TGAAAGAACG GGGTAAGGGG GGGGGGGGGG CGAGAGAGAG AGAGAGAGAG   
  
  
- AGAAGGGAAA TGTTTCCGTG GTAAGTTTAA GTTTGGTGAG AAAAATTTTG GGGTTTTTAA TAAGAGTTTT   
  
  
- ATTATGGAGT ATTATATTTA CCCAAGCATA GTGGTATATA CCCGGAGTAG AGAGTACCCT TGTATGCTTA   
  
  
- AGTTACCGAA CGGGCTATCA ATAGCCCAGT ACGGTAGCCT CGGGGCCGTT GATTAGTTAC CAGTTCGGAG   
  
  
- TCTAAACCGG AGAAACTCGG TAATATCATT GTGGAAAGAG AGACTTGAGG GGGGAGATGG GTGAGACCCT   
  
  
- GGGCCTAGGC TGGGCCCAGA TAGCACAGGC CCAGGACGAG ACTCAGGTTA CCTCACCTAA CCACTGCGCA   
  
  
- CCTGTGTGGT CTAACTTGTA CTAGACGGTG CAGTCGTCTC AGTTGCCGAC AACTGACTAC CACTGTACCC   
  
  
- CCTCCTCCTA AGGCCATATT CCAACCACGT ACGGAACGAC TGCACGCGTC TCCGGCAGGT TACGCCGCTC   
  
  
- GAGGCTGACC GACGAAGCGA TCAACTACTC TACTGGTTAC CCGACAACGG TGCACAATCA TGCCACACAC   
  
  
- CCTAGCCGTT CCATCGGCCG ATAAAACAAC TCCGAAACTC GGCCGCGGAC AAAGTCGGGC CGGGCCTTTG   
  
  
- ACCGGGCTGG ACCGGCCCGA GCCTCAAGCT CCGACTCCAC AACATAGCAG TGAAGATACT CCGAACGGGA   
  
  
- ATAGAGTTTA AACGAGTGAA GTGCCGATTA GTTCGGTAGA ACCTCCGTAA ACTCCCGGTA CTAATACAGG   
  
  
- TGCAGTAGCT GAAGCCGAAG TACTGGCCGA ACGTTACCGG CCGAAACTAA GTTCGGGACC GAAATTCCGG   
  
  
- GCCACCCGGT GGTAGAGAAT CCGAGTGACC CTAACCGGGC AGAAATCCTT CACCCGGGCT TAGCTATGCA   
  
  
- CTTTAGCCCA ACTCCGATCG GGTTAACCGG GCTAGGTACT TGTACTCCAA GCGTAAAGCC CGACATCGTT   
  
  
- GAAGAGCCGA GCTCCTGCAC TTCGGCAGTT ACGAGCCTTA CCTGGGATCT CTTCGGCAGC GATACTTAAG   
  
  
- GCAGTACGTA GAAGTAGCTG AAAATCCTAA TCTGTAGTTG AGTCAAGATT TGACCTAAGC TTTGGATTTG   
  
  
- GGCTTCTAGC ATTGAAATCA TCTTGTTCTC CGCTTGGTAT TGGTTGGACT TAAAGAGGGG GCTAAGTGAC   
  
  
- TTCGTGATAC AATGATGAGT TGATACAAGC TGAGTAATCT CTGAACAGTT CAGGTTGGCG ACTTTCAGGA   
  
  
- TCGGCTCTAC ATATATGTTT TCCTCTATAG GTTGGATCAC ACGATGCTTC CGAGTCGGGC CCAGGTCTCC   
  
  
- GTGCTCGGCG AGCGGTTTAC CGTTCGAGCC TACCCGGTTC GGCCCAAGTT CGGCGATCCG GACTCAGTTT   
  
  
- TACGGGAATC CGTTCGATTG TACAACCATT GTAACAAGAG GAGTGTTCCC ATACTGCACC TCCTCTCTCT   
  
  
- TCCCACAAAC TGTGACCCGA CCGTATTGGC GGGAGAGTAT CACTGAAGCT GAACCGTTCA CCCCGGGTTT   
  
  
- TACCTGTCAG GTTCACACTG AGTACTCTAC AGTAGTAGGA GTCGAAGAAG TAGAAT

+     Myc

| Site Name | Organism | Position | Strand | Matrix score. | sequence | function |
| --- | --- | --- | --- | --- | --- | --- |
| Myc | Arabidopsis thaliana | 2748 | + | 7 | TCTCTTA |  |
| Myc | Arabidopsis thaliana | 664 | - | 7 | TCTCTTA |  |

>HU03G02797.1   
+ +Up\_Stream \_Len000CATTTT CAATTAGAAA TTTCTTCAAT TATATACCAT CTCCAAAAAT AGTCCTCATA   
  
  
+ AGTTGGCAGG TGAATGATCC TAATGGAAAA TTACTTTTAA CTGTTGAATT TGCAATAGGG TACCGAATTT   
  
  
+ GTGAAGTTTA TTGCCGCACG TGCCTAGCTT GTGACTTCTC GATAATCAAA AATTAATTAG AGAGAGACAG   
  
  
+ CTAAAGTTTT GAGAATAATA CTGAAAAATT ACATTAAATT TTATTTATGT TTATTTTCAT ACGCTATGTT   
  
  
+ TAACTGCTAT ACGCTGTGTT TAACTGCATC TCCTTCTATA TTTGATAAAT TGGTAAAAAA AAATAATTCA   
  
  
+ AAATTAAAGA CTAGTAATAT TTTCACTTAT CTGATAGATT TATAAGAAAT ATTTAGATAG CTAAATATAT   
  
  
+ TCTCTCCTGA AAGAGGAGAC TGATAGTTGA ATTTCATAGG ATATAAACAT GTATGTACAT TGAACGAATC   
  
  
+ TTTTTTCTAT GGTGATCGTT ATAAAGTTAT TTATTACTGT AAACATTAAG TGATAATTAG GATATGTTCT   
  
  
+ ATTTCACGTT TTAGATAGAT AGATAGATGT AAGGAGTTGT AAGAAGCTGT AAGAATATGT AAGGAGCTGT   
  
  
+ AAGGAGATGT AAGGAGCTGT AAGAAGATGT AAGAGAAACT GTAAGAAGCT ACAAGGATAG ATGTAACGAG   
  
  
+ TTGTTGAATT GTTGAAGAGT TGTATAGTGT AATTTGTTTT ACGATGAACG TTTTGGATGA AAAATAATAA   
  
  
+ TATTAAAATA ATATGCTTAT TAAAAATAAA TAAATAATTA AGTTAATTAC ACAACAATAA AATATATTAA   
  
  
+ TCCCCATAAA TTTAGTGAAT AAAACTTCGT CCACTTGAGA TGATTTTTTA TATGAACACT AGTTTGATAG   
  
  
+ AATAAACAAA GTATAAAAAA AAATAAAGTT TTTTGACAAA ATTGAATACA ACTCTTCAAA TAAATTCCTA   
  
  
+ TTTTAAGTAA ATAATTTTAA ATATAAATAA ATTTATGGTC ATCTATAATA ATATATATTT TATGTCGAAA   
  
  
+ CTCAAATATA ATTTACATCG TTATATTATA TTTTTATGTT TATGTAAATG TTGTGTTCAT AAGATATTTA   
  
  
+ TAAATTTTGT ATATTGGTAG TATCATTAAA AAAAATATAC ATATTTATGT AACTAAAATA AATGAATGTT   
  
  
+ ATAGAAAATC TATAAGATGT AAGGAGATGT ATGAAGATGT AAGGAGATGT AAGAAGATAT AAGGAGATGT   
  
  
+ AGAAAATGTA AGAAGATGTG AGAAGCTGTA AGGAGATGTA AGTTATTCAC TAAATCCTAA AAAATGCGAA   
  
  
+ ATAGAACAGG GGCTTAATTG AGCACTCAAG ATGCATAATT TGTGACGGAC CAAATGGATA AATATAGACC   
  
  
+ TGGCAAATGG GTTATTCGAT TTAGGTACGA GTATGGTTAA GTTTGGGTTT GGTTATGTTT CAGGATAAGT   
  
  
+ GACATTTCGA CTCAATTTGG TTTTGGTTGT ATGAACTTCG GCTCGAGATT TTTGGTTTGG CCAATACACT   
  
  
+ TATCATGCAA AAATAAGTAA CTTTATAGAA AATTTTGGAT CGGATATGAT GCGGTTCGGA TCAATTCGGA   
  
  
+ TTTTGGTTCA CATTCTGGTA AACCTATTTC GGATGTCAAG TAGGATATGA GTCCAGATCG TTGGATTTCG   
  
  
+ GGTATCAGCT CAATTTTACC AGATCTAGAT AAATCATAAA TGTACGGGTT TATTTTTTCC AGCTTGAATG   
  
  
+ ATTTTCTTTA TTAAAAAAAG ATAGATTTTA GATAAAACAC AACATTTCAT TTTCATTTGT TCCCCCCTAT   
  
  
+ AAATTTCTGT GACCACCTTC ACTTTCTTGC CCCATTCCCC CCCCCCCCCC GCTCTCTCTC TCTCTCTCTC   
  
  
+ TCTTCCCTTT ACAAAGGCAC CATTCAAATT CAAACCACTC TTTTTAAAAC CCCAAAAATT ATTCTCAAAA   
  
  
+ TAATACCTCA TAATATAAAT GGGTTCGTAT CACCATATAT GGGCCTCATC TCTCATGGGA ACATACGAAT   
  
  
+ TCAATGGCTT GCCCGATAGT TATCGGGTCA TGCCATCGGA GCCCCGGCAA CTAATCAATG GTCAAGCCTC   
  
  
+ AGATTTGGCC TCTTTGAGCC ATTATAGTAA CACCTTTCTC TCTGAACTCC CCCCTCTACC CACTCTGGGA   
  
  
+ CCCGGATCCG ACCCGGGTCT ATCGTGTCCG GGTCCTGCTC TGAGTCCAAT GGAGTGGATT GGTGACGCGT   
  
  
+ GGACACACCA GATTGAACAT GATCTGCCAC GTCAGCAGAG TCAACGGCTG TTGACTGATG GTGACATGGG   
  
  
+ GGAGGAGGAT TCCGGTATAA GGTTGGTGCA TGCCTTGCTG ACGTGCGCAG AGGCCGTCCA ATGCGGCGAG   
  
  
+ CTCCGACTGG CTGCTTCGCT AGTTGATGAG ATGACCAATG GGCTGTTGCC ACGTGTTAGT ACGGTGTGTG   
  
  
+ GGATCGGCAA GGTAGCCGGC TATTTTGTTG AGGCTTTGAG CCGGCGCCTG TTTCAGCCCG GCCCGGAAAC   
  
  
+ TGGCCCGACC TGGCCGGGCT CGGAGTTCGA GGCTGAGGTG TTGTATCGTC ACTTCTATGA GGCTTGCCCT   
  
  
+ TATCTCAAAT TTGCTCACTT CACGGCTAAT CAAGCCATCT TGGAGGCATT TGAGGGCCAT GATTATGTCC   
  
  
+ ACGTCATCGA CTTCGGCTTC ATGACCGGCT TGCAATGGCC GGCTTTGATT CAAGCCCTGG CTTTAAGGCC   
  
  
+ CGGTGGGCCA CCATCTCTTA GGCTCACTGG GATTGGCCCG TCTTTAGGAA GTGGGCCCGA ATCGATACGT   
  
  
+ GAAATCGGGT TGAGGCTAGC CCAATTGGCC CGATCCATGA ACATGAGGTT CGCATTTCGG GCTGTAGCAA   
  
  
+ CTTCTCGGCT CGAGGACGTG AAGCCGTCAA TGCTCGGAAT GGACCCTAGA GAAGCCGTCG CTATGAATTC   
  
  
+ CGTCATGCAT CTTCATCGAC TTTTAGGATT AGACATCAAC TCAGTTCTAA ACTGGATTCG AAACCTAAAC   
  
  
+ CCGAAGATCG TAACTTTAGT AGAACAAGAG GCGAACCATA ACCAACCTGA ATTTCTCCCC CGATTCACTG   
  
  
+ AAGCACTATG TTACTACTCA ACTATGTTCG ACTCATTAGA GACTTGTCAA GTCCAACCGC TGAAAGTCCT   
  
  
+ AGCCGAGATG TATATACAAA AGGAGATATC CAACCTAGTG TGCTACGAAG GCTCAGCCCG GGTCCAGAGG   
  
  
+ CACGAGCCGC TCGCCAAATG GCAAGCTCGG ATGGGCCAAG CCGGGTTCAA GCCGCTAGGC CTGAGTCAAA   
  
  
+ ATGCCCTTAG GCAAGCTAAC ATGTTGGTAA CATTGTTCTC CTCACAAGGG TATGACGTGG AGGAGAGAGA   
  
  
+ AGGGTGTTTG ACACTGGGCT GGCATAACCG CCCTCTCATA GTGACTTCGA CTTGGCAAGT GGGGCCCAAA   
  
  
+ ATGGACAGTC CAAGTGTGAC TCATGAGATG TCATCATCCT CAGCTTCTTC ATCTTA  

- +Up\_Stream \_Len000GTAAAA GTTAATCTTT AAAGAAGTTA ATATATGGTA GAGGTTTTTA TCAGGAGTAT   
  
  
- TCAACCGTCC ACTTACTAGG ATTACCTTTT AATGAAAATT GACAACTTAA ACGTTATCCC ATGGCTTAAA   
  
  
- CACTTCAAAT AACGGCGTGC ACGGATCGAA CACTGAAGAG CTATTAGTTT TTAATTAATC TCTCTCTGTC   
  
  
- GATTTCAAAA CTCTTATTAT GACTTTTTAA TGTAATTTAA AATAAATACA AATAAAAGTA TGCGATACAA   
  
  
- ATTGACGATA TGCGACACAA ATTGACGTAG AGGAAGATAT AAACTATTTA ACCATTTTTT TTTATTAAGT   
  
  
- TTTAATTTCT GATCATTATA AAAGTGAATA GACTATCTAA ATATTCTTTA TAAATCTATC GATTTATATA   
  
  
- AGAGAGGACT TTCTCCTCTG ACTATCAACT TAAAGTATCC TATATTTGTA CATACATGTA ACTTGCTTAG   
  
  
- AAAAAAGATA CCACTAGCAA TATTTCAATA AATAATGACA TTTGTAATTC ACTATTAATC CTATACAAGA   
  
  
- TAAAGTGCAA AATCTATCTA TCTATCTACA TTCCTCAACA TTCTTCGACA TTCTTATACA TTCCTCGACA   
  
  
- TTCCTCTACA TTCCTCGACA TTCTTCTACA TTCTCTTTGA CATTCTTCGA TGTTCCTATC TACATTGCTC   
  
  
- AACAACTTAA CAACTTCTCA ACATATCACA TTAAACAAAA TGCTACTTGC AAAACCTACT TTTTATTATT   
  
  
- ATAATTTTAT TATACGAATA ATTTTTATTT ATTTATTAAT TCAATTAATG TGTTGTTATT TTATATAATT   
  
  
- AGGGGTATTT AAATCACTTA TTTTGAAGCA GGTGAACTCT ACTAAAAAAT ATACTTGTGA TCAAACTATC   
  
  
- TTATTTGTTT CATATTTTTT TTTATTTCAA AAAACTGTTT TAACTTATGT TGAGAAGTTT ATTTAAGGAT   
  
  
- AAAATTCATT TATTAAAATT TATATTTATT TAAATACCAG TAGATATTAT TATATATAAA ATACAGCTTT   
  
  
- GAGTTTATAT TAAATGTAGC AATATAATAT AAAAATACAA ATACATTTAC AACACAAGTA TTCTATAAAT   
  
  
- ATTTAAAACA TATAACCATC ATAGTAATTT TTTTTATATG TATAAATACA TTGATTTTAT TTACTTACAA   
  
  
- TATCTTTTAG ATATTCTACA TTCCTCTACA TACTTCTACA TTCCTCTACA TTCTTCTATA TTCCTCTACA   
  
  
- TCTTTTACAT TCTTCTACAC TCTTCGACAT TCCTCTACAT TCAATAAGTG ATTTAGGATT TTTTACGCTT   
  
  
- TATCTTGTCC CCGAATTAAC TCGTGAGTTC TACGTATTAA ACACTGCCTG GTTTACCTAT TTATATCTGG   
  
  
- ACCGTTTACC CAATAAGCTA AATCCATGCT CATACCAATT CAAACCCAAA CCAATACAAA GTCCTATTCA   
  
  
- CTGTAAAGCT GAGTTAAACC AAAACCAACA TACTTGAAGC CGAGCTCTAA AAACCAAACC GGTTATGTGA   
  
  
- ATAGTACGTT TTTATTCATT GAAATATCTT TTAAAACCTA GCCTATACTA CGCCAAGCCT AGTTAAGCCT   
  
  
- AAAACCAAGT GTAAGACCAT TTGGATAAAG CCTACAGTTC ATCCTATACT CAGGTCTAGC AACCTAAAGC   
  
  
- CCATAGTCGA GTTAAAATGG TCTAGATCTA TTTAGTATTT ACATGCCCAA ATAAAAAAGG TCGAACTTAC   
  
  
- TAAAAGAAAT AATTTTTTTC TATCTAAAAT CTATTTTGTG TTGTAAAGTA AAAGTAAACA AGGGGGGATA   
  
  
- TTTAAAGACA CTGGTGGAAG TGAAAGAACG GGGTAAGGGG GGGGGGGGGG CGAGAGAGAG AGAGAGAGAG   
  
  
- AGAAGGGAAA TGTTTCCGTG GTAAGTTTAA GTTTGGTGAG AAAAATTTTG GGGTTTTTAA TAAGAGTTTT   
  
  
- ATTATGGAGT ATTATATTTA CCCAAGCATA GTGGTATATA CCCGGAGTAG AGAGTACCCT TGTATGCTTA   
  
  
- AGTTACCGAA CGGGCTATCA ATAGCCCAGT ACGGTAGCCT CGGGGCCGTT GATTAGTTAC CAGTTCGGAG   
  
  
- TCTAAACCGG AGAAACTCGG TAATATCATT GTGGAAAGAG AGACTTGAGG GGGGAGATGG GTGAGACCCT   
  
  
- GGGCCTAGGC TGGGCCCAGA TAGCACAGGC CCAGGACGAG ACTCAGGTTA CCTCACCTAA CCACTGCGCA   
  
  
- CCTGTGTGGT CTAACTTGTA CTAGACGGTG CAGTCGTCTC AGTTGCCGAC AACTGACTAC CACTGTACCC   
  
  
- CCTCCTCCTA AGGCCATATT CCAACCACGT ACGGAACGAC TGCACGCGTC TCCGGCAGGT TACGCCGCTC   
  
  
- GAGGCTGACC GACGAAGCGA TCAACTACTC TACTGGTTAC CCGACAACGG TGCACAATCA TGCCACACAC   
  
  
- CCTAGCCGTT CCATCGGCCG ATAAAACAAC TCCGAAACTC GGCCGCGGAC AAAGTCGGGC CGGGCCTTTG   
  
  
- ACCGGGCTGG ACCGGCCCGA GCCTCAAGCT CCGACTCCAC AACATAGCAG TGAAGATACT CCGAACGGGA   
  
  
- ATAGAGTTTA AACGAGTGAA GTGCCGATTA GTTCGGTAGA ACCTCCGTAA ACTCCCGGTA CTAATACAGG   
  
  
- TGCAGTAGCT GAAGCCGAAG TACTGGCCGA ACGTTACCGG CCGAAACTAA GTTCGGGACC GAAATTCCGG   
  
  
- GCCACCCGGT GGTAGAGAAT CCGAGTGACC CTAACCGGGC AGAAATCCTT CACCCGGGCT TAGCTATGCA   
  
  
- CTTTAGCCCA ACTCCGATCG GGTTAACCGG GCTAGGTACT TGTACTCCAA GCGTAAAGCC CGACATCGTT   
  
  
- GAAGAGCCGA GCTCCTGCAC TTCGGCAGTT ACGAGCCTTA CCTGGGATCT CTTCGGCAGC GATACTTAAG   
  
  
- GCAGTACGTA GAAGTAGCTG AAAATCCTAA TCTGTAGTTG AGTCAAGATT TGACCTAAGC TTTGGATTTG   
  
  
- GGCTTCTAGC ATTGAAATCA TCTTGTTCTC CGCTTGGTAT TGGTTGGACT TAAAGAGGGG GCTAAGTGAC   
  
  
- TTCGTGATAC AATGATGAGT TGATACAAGC TGAGTAATCT CTGAACAGTT CAGGTTGGCG ACTTTCAGGA   
  
  
- TCGGCTCTAC ATATATGTTT TCCTCTATAG GTTGGATCAC ACGATGCTTC CGAGTCGGGC CCAGGTCTCC   
  
  
- GTGCTCGGCG AGCGGTTTAC CGTTCGAGCC TACCCGGTTC GGCCCAAGTT CGGCGATCCG GACTCAGTTT   
  
  
- TACGGGAATC CGTTCGATTG TACAACCATT GTAACAAGAG GAGTGTTCCC ATACTGCACC TCCTCTCTCT   
  
  
- TCCCACAAAC TGTGACCCGA CCGTATTGGC GGGAGAGTAT CACTGAAGCT GAACCGTTCA CCCCGGGTTT   
  
  
- TACCTGTCAG GTTCACACTG AGTACTCTAC AGTAGTAGGA GTCGAAGAAG TAGAAT

+     O2-site

| Site Name | Organism | Position | Strand | Matrix score. | sequence | function |
| --- | --- | --- | --- | --- | --- | --- |
| O2-site | Zea mays | 2304 | + | 9 | GATGACATGG | cis-acting regulatory element involved in zein metabolism regulation |
| O2-site | Zea mays | 2663 | - | 9 | GATGATGTGG | cis-acting regulatory element involved in zein metabolism regulation |
| O2-site | Zea mays | 2409 | + | 8 | GATGA(C/T)(A/G)TG(A/G) | cis-acting regulatory element involved in zein metabolism regulation |

>HU03G02797.1   
+ +Up\_Stream \_Len000CATTTT CAATTAGAAA TTTCTTCAAT TATATACCAT CTCCAAAAAT AGTCCTCATA   
  
  
+ AGTTGGCAGG TGAATGATCC TAATGGAAAA TTACTTTTAA CTGTTGAATT TGCAATAGGG TACCGAATTT   
  
  
+ GTGAAGTTTA TTGCCGCACG TGCCTAGCTT GTGACTTCTC GATAATCAAA AATTAATTAG AGAGAGACAG   
  
  
+ CTAAAGTTTT GAGAATAATA CTGAAAAATT ACATTAAATT TTATTTATGT TTATTTTCAT ACGCTATGTT   
  
  
+ TAACTGCTAT ACGCTGTGTT TAACTGCATC TCCTTCTATA TTTGATAAAT TGGTAAAAAA AAATAATTCA   
  
  
+ AAATTAAAGA CTAGTAATAT TTTCACTTAT CTGATAGATT TATAAGAAAT ATTTAGATAG CTAAATATAT   
  
  
+ TCTCTCCTGA AAGAGGAGAC TGATAGTTGA ATTTCATAGG ATATAAACAT GTATGTACAT TGAACGAATC   
  
  
+ TTTTTTCTAT GGTGATCGTT ATAAAGTTAT TTATTACTGT AAACATTAAG TGATAATTAG GATATGTTCT   
  
  
+ ATTTCACGTT TTAGATAGAT AGATAGATGT AAGGAGTTGT AAGAAGCTGT AAGAATATGT AAGGAGCTGT   
  
  
+ AAGGAGATGT AAGGAGCTGT AAGAAGATGT AAGAGAAACT GTAAGAAGCT ACAAGGATAG ATGTAACGAG   
  
  
+ TTGTTGAATT GTTGAAGAGT TGTATAGTGT AATTTGTTTT ACGATGAACG TTTTGGATGA AAAATAATAA   
  
  
+ TATTAAAATA ATATGCTTAT TAAAAATAAA TAAATAATTA AGTTAATTAC ACAACAATAA AATATATTAA   
  
  
+ TCCCCATAAA TTTAGTGAAT AAAACTTCGT CCACTTGAGA TGATTTTTTA TATGAACACT AGTTTGATAG   
  
  
+ AATAAACAAA GTATAAAAAA AAATAAAGTT TTTTGACAAA ATTGAATACA ACTCTTCAAA TAAATTCCTA   
  
  
+ TTTTAAGTAA ATAATTTTAA ATATAAATAA ATTTATGGTC ATCTATAATA ATATATATTT TATGTCGAAA   
  
  
+ CTCAAATATA ATTTACATCG TTATATTATA TTTTTATGTT TATGTAAATG TTGTGTTCAT AAGATATTTA   
  
  
+ TAAATTTTGT ATATTGGTAG TATCATTAAA AAAAATATAC ATATTTATGT AACTAAAATA AATGAATGTT   
  
  
+ ATAGAAAATC TATAAGATGT AAGGAGATGT ATGAAGATGT AAGGAGATGT AAGAAGATAT AAGGAGATGT   
  
  
+ AGAAAATGTA AGAAGATGTG AGAAGCTGTA AGGAGATGTA AGTTATTCAC TAAATCCTAA AAAATGCGAA   
  
  
+ ATAGAACAGG GGCTTAATTG AGCACTCAAG ATGCATAATT TGTGACGGAC CAAATGGATA AATATAGACC   
  
  
+ TGGCAAATGG GTTATTCGAT TTAGGTACGA GTATGGTTAA GTTTGGGTTT GGTTATGTTT CAGGATAAGT   
  
  
+ GACATTTCGA CTCAATTTGG TTTTGGTTGT ATGAACTTCG GCTCGAGATT TTTGGTTTGG CCAATACACT   
  
  
+ TATCATGCAA AAATAAGTAA CTTTATAGAA AATTTTGGAT CGGATATGAT GCGGTTCGGA TCAATTCGGA   
  
  
+ TTTTGGTTCA CATTCTGGTA AACCTATTTC GGATGTCAAG TAGGATATGA GTCCAGATCG TTGGATTTCG   
  
  
+ GGTATCAGCT CAATTTTACC AGATCTAGAT AAATCATAAA TGTACGGGTT TATTTTTTCC AGCTTGAATG   
  
  
+ ATTTTCTTTA TTAAAAAAAG ATAGATTTTA GATAAAACAC AACATTTCAT TTTCATTTGT TCCCCCCTAT   
  
  
+ AAATTTCTGT GACCACCTTC ACTTTCTTGC CCCATTCCCC CCCCCCCCCC GCTCTCTCTC TCTCTCTCTC   
  
  
+ TCTTCCCTTT ACAAAGGCAC CATTCAAATT CAAACCACTC TTTTTAAAAC CCCAAAAATT ATTCTCAAAA   
  
  
+ TAATACCTCA TAATATAAAT GGGTTCGTAT CACCATATAT GGGCCTCATC TCTCATGGGA ACATACGAAT   
  
  
+ TCAATGGCTT GCCCGATAGT TATCGGGTCA TGCCATCGGA GCCCCGGCAA CTAATCAATG GTCAAGCCTC   
  
  
+ AGATTTGGCC TCTTTGAGCC ATTATAGTAA CACCTTTCTC TCTGAACTCC CCCCTCTACC CACTCTGGGA   
  
  
+ CCCGGATCCG ACCCGGGTCT ATCGTGTCCG GGTCCTGCTC TGAGTCCAAT GGAGTGGATT GGTGACGCGT   
  
  
+ GGACACACCA GATTGAACAT GATCTGCCAC GTCAGCAGAG TCAACGGCTG TTGACTGATG GTGACATGGG   
  
  
+ GGAGGAGGAT TCCGGTATAA GGTTGGTGCA TGCCTTGCTG ACGTGCGCAG AGGCCGTCCA ATGCGGCGAG   
  
  
+ CTCCGACTGG CTGCTTCGCT AGTTGATGAG ATGACCAATG GGCTGTTGCC ACGTGTTAGT ACGGTGTGTG   
  
  
+ GGATCGGCAA GGTAGCCGGC TATTTTGTTG AGGCTTTGAG CCGGCGCCTG TTTCAGCCCG GCCCGGAAAC   
  
  
+ TGGCCCGACC TGGCCGGGCT CGGAGTTCGA GGCTGAGGTG TTGTATCGTC ACTTCTATGA GGCTTGCCCT   
  
  
+ TATCTCAAAT TTGCTCACTT CACGGCTAAT CAAGCCATCT TGGAGGCATT TGAGGGCCAT GATTATGTCC   
  
  
+ ACGTCATCGA CTTCGGCTTC ATGACCGGCT TGCAATGGCC GGCTTTGATT CAAGCCCTGG CTTTAAGGCC   
  
  
+ CGGTGGGCCA CCATCTCTTA GGCTCACTGG GATTGGCCCG TCTTTAGGAA GTGGGCCCGA ATCGATACGT   
  
  
+ GAAATCGGGT TGAGGCTAGC CCAATTGGCC CGATCCATGA ACATGAGGTT CGCATTTCGG GCTGTAGCAA   
  
  
+ CTTCTCGGCT CGAGGACGTG AAGCCGTCAA TGCTCGGAAT GGACCCTAGA GAAGCCGTCG CTATGAATTC   
  
  
+ CGTCATGCAT CTTCATCGAC TTTTAGGATT AGACATCAAC TCAGTTCTAA ACTGGATTCG AAACCTAAAC   
  
  
+ CCGAAGATCG TAACTTTAGT AGAACAAGAG GCGAACCATA ACCAACCTGA ATTTCTCCCC CGATTCACTG   
  
  
+ AAGCACTATG TTACTACTCA ACTATGTTCG ACTCATTAGA GACTTGTCAA GTCCAACCGC TGAAAGTCCT   
  
  
+ AGCCGAGATG TATATACAAA AGGAGATATC CAACCTAGTG TGCTACGAAG GCTCAGCCCG GGTCCAGAGG   
  
  
+ CACGAGCCGC TCGCCAAATG GCAAGCTCGG ATGGGCCAAG CCGGGTTCAA GCCGCTAGGC CTGAGTCAAA   
  
  
+ ATGCCCTTAG GCAAGCTAAC ATGTTGGTAA CATTGTTCTC CTCACAAGGG TATGACGTGG AGGAGAGAGA   
  
  
+ AGGGTGTTTG ACACTGGGCT GGCATAACCG CCCTCTCATA GTGACTTCGA CTTGGCAAGT GGGGCCCAAA   
  
  
+ ATGGACAGTC CAAGTGTGAC TCATGAGATG TCATCATCCT CAGCTTCTTC ATCTTA  

- +Up\_Stream \_Len000GTAAAA GTTAATCTTT AAAGAAGTTA ATATATGGTA GAGGTTTTTA TCAGGAGTAT   
  
  
- TCAACCGTCC ACTTACTAGG ATTACCTTTT AATGAAAATT GACAACTTAA ACGTTATCCC ATGGCTTAAA   
  
  
- CACTTCAAAT AACGGCGTGC ACGGATCGAA CACTGAAGAG CTATTAGTTT TTAATTAATC TCTCTCTGTC   
  
  
- GATTTCAAAA CTCTTATTAT GACTTTTTAA TGTAATTTAA AATAAATACA AATAAAAGTA TGCGATACAA   
  
  
- ATTGACGATA TGCGACACAA ATTGACGTAG AGGAAGATAT AAACTATTTA ACCATTTTTT TTTATTAAGT   
  
  
- TTTAATTTCT GATCATTATA AAAGTGAATA GACTATCTAA ATATTCTTTA TAAATCTATC GATTTATATA   
  
  
- AGAGAGGACT TTCTCCTCTG ACTATCAACT TAAAGTATCC TATATTTGTA CATACATGTA ACTTGCTTAG   
  
  
- AAAAAAGATA CCACTAGCAA TATTTCAATA AATAATGACA TTTGTAATTC ACTATTAATC CTATACAAGA   
  
  
- TAAAGTGCAA AATCTATCTA TCTATCTACA TTCCTCAACA TTCTTCGACA TTCTTATACA TTCCTCGACA   
  
  
- TTCCTCTACA TTCCTCGACA TTCTTCTACA TTCTCTTTGA CATTCTTCGA TGTTCCTATC TACATTGCTC   
  
  
- AACAACTTAA CAACTTCTCA ACATATCACA TTAAACAAAA TGCTACTTGC AAAACCTACT TTTTATTATT   
  
  
- ATAATTTTAT TATACGAATA ATTTTTATTT ATTTATTAAT TCAATTAATG TGTTGTTATT TTATATAATT   
  
  
- AGGGGTATTT AAATCACTTA TTTTGAAGCA GGTGAACTCT ACTAAAAAAT ATACTTGTGA TCAAACTATC   
  
  
- TTATTTGTTT CATATTTTTT TTTATTTCAA AAAACTGTTT TAACTTATGT TGAGAAGTTT ATTTAAGGAT   
  
  
- AAAATTCATT TATTAAAATT TATATTTATT TAAATACCAG TAGATATTAT TATATATAAA ATACAGCTTT   
  
  
- GAGTTTATAT TAAATGTAGC AATATAATAT AAAAATACAA ATACATTTAC AACACAAGTA TTCTATAAAT   
  
  
- ATTTAAAACA TATAACCATC ATAGTAATTT TTTTTATATG TATAAATACA TTGATTTTAT TTACTTACAA   
  
  
- TATCTTTTAG ATATTCTACA TTCCTCTACA TACTTCTACA TTCCTCTACA TTCTTCTATA TTCCTCTACA   
  
  
- TCTTTTACAT TCTTCTACAC TCTTCGACAT TCCTCTACAT TCAATAAGTG ATTTAGGATT TTTTACGCTT   
  
  
- TATCTTGTCC CCGAATTAAC TCGTGAGTTC TACGTATTAA ACACTGCCTG GTTTACCTAT TTATATCTGG   
  
  
- ACCGTTTACC CAATAAGCTA AATCCATGCT CATACCAATT CAAACCCAAA CCAATACAAA GTCCTATTCA   
  
  
- CTGTAAAGCT GAGTTAAACC AAAACCAACA TACTTGAAGC CGAGCTCTAA AAACCAAACC GGTTATGTGA   
  
  
- ATAGTACGTT TTTATTCATT GAAATATCTT TTAAAACCTA GCCTATACTA CGCCAAGCCT AGTTAAGCCT   
  
  
- AAAACCAAGT GTAAGACCAT TTGGATAAAG CCTACAGTTC ATCCTATACT CAGGTCTAGC AACCTAAAGC   
  
  
- CCATAGTCGA GTTAAAATGG TCTAGATCTA TTTAGTATTT ACATGCCCAA ATAAAAAAGG TCGAACTTAC   
  
  
- TAAAAGAAAT AATTTTTTTC TATCTAAAAT CTATTTTGTG TTGTAAAGTA AAAGTAAACA AGGGGGGATA   
  
  
- TTTAAAGACA CTGGTGGAAG TGAAAGAACG GGGTAAGGGG GGGGGGGGGG CGAGAGAGAG AGAGAGAGAG   
  
  
- AGAAGGGAAA TGTTTCCGTG GTAAGTTTAA GTTTGGTGAG AAAAATTTTG GGGTTTTTAA TAAGAGTTTT   
  
  
- ATTATGGAGT ATTATATTTA CCCAAGCATA GTGGTATATA CCCGGAGTAG AGAGTACCCT TGTATGCTTA   
  
  
- AGTTACCGAA CGGGCTATCA ATAGCCCAGT ACGGTAGCCT CGGGGCCGTT GATTAGTTAC CAGTTCGGAG   
  
  
- TCTAAACCGG AGAAACTCGG TAATATCATT GTGGAAAGAG AGACTTGAGG GGGGAGATGG GTGAGACCCT   
  
  
- GGGCCTAGGC TGGGCCCAGA TAGCACAGGC CCAGGACGAG ACTCAGGTTA CCTCACCTAA CCACTGCGCA   
  
  
- CCTGTGTGGT CTAACTTGTA CTAGACGGTG CAGTCGTCTC AGTTGCCGAC AACTGACTAC CACTGTACCC   
  
  
- CCTCCTCCTA AGGCCATATT CCAACCACGT ACGGAACGAC TGCACGCGTC TCCGGCAGGT TACGCCGCTC   
  
  
- GAGGCTGACC GACGAAGCGA TCAACTACTC TACTGGTTAC CCGACAACGG TGCACAATCA TGCCACACAC   
  
  
- CCTAGCCGTT CCATCGGCCG ATAAAACAAC TCCGAAACTC GGCCGCGGAC AAAGTCGGGC CGGGCCTTTG   
  
  
- ACCGGGCTGG ACCGGCCCGA GCCTCAAGCT CCGACTCCAC AACATAGCAG TGAAGATACT CCGAACGGGA   
  
  
- ATAGAGTTTA AACGAGTGAA GTGCCGATTA GTTCGGTAGA ACCTCCGTAA ACTCCCGGTA CTAATACAGG   
  
  
- TGCAGTAGCT GAAGCCGAAG TACTGGCCGA ACGTTACCGG CCGAAACTAA GTTCGGGACC GAAATTCCGG   
  
  
- GCCACCCGGT GGTAGAGAAT CCGAGTGACC CTAACCGGGC AGAAATCCTT CACCCGGGCT TAGCTATGCA   
  
  
- CTTTAGCCCA ACTCCGATCG GGTTAACCGG GCTAGGTACT TGTACTCCAA GCGTAAAGCC CGACATCGTT   
  
  
- GAAGAGCCGA GCTCCTGCAC TTCGGCAGTT ACGAGCCTTA CCTGGGATCT CTTCGGCAGC GATACTTAAG   
  
  
- GCAGTACGTA GAAGTAGCTG AAAATCCTAA TCTGTAGTTG AGTCAAGATT TGACCTAAGC TTTGGATTTG   
  
  
- GGCTTCTAGC ATTGAAATCA TCTTGTTCTC CGCTTGGTAT TGGTTGGACT TAAAGAGGGG GCTAAGTGAC   
  
  
- TTCGTGATAC AATGATGAGT TGATACAAGC TGAGTAATCT CTGAACAGTT CAGGTTGGCG ACTTTCAGGA   
  
  
- TCGGCTCTAC ATATATGTTT TCCTCTATAG GTTGGATCAC ACGATGCTTC CGAGTCGGGC CCAGGTCTCC   
  
  
- GTGCTCGGCG AGCGGTTTAC CGTTCGAGCC TACCCGGTTC GGCCCAAGTT CGGCGATCCG GACTCAGTTT   
  
  
- TACGGGAATC CGTTCGATTG TACAACCATT GTAACAAGAG GAGTGTTCCC ATACTGCACC TCCTCTCTCT   
  
  
- TCCCACAAAC TGTGACCCGA CCGTATTGGC GGGAGAGTAT CACTGAAGCT GAACCGTTCA CCCCGGGTTT   
  
  
- TACCTGTCAG GTTCACACTG AGTACTCTAC AGTAGTAGGA GTCGAAGAAG TAGAAT

+     P-box

| Site Name | Organism | Position | Strand | Matrix score. | sequence | function |
| --- | --- | --- | --- | --- | --- | --- |
| P-box | Oryza sativa | 3171 | - | 7 | CCTTTTG | gibberellin-responsive element |

>HU03G02797.1   
+ +Up\_Stream \_Len000CATTTT CAATTAGAAA TTTCTTCAAT TATATACCAT CTCCAAAAAT AGTCCTCATA   
  
  
+ AGTTGGCAGG TGAATGATCC TAATGGAAAA TTACTTTTAA CTGTTGAATT TGCAATAGGG TACCGAATTT   
  
  
+ GTGAAGTTTA TTGCCGCACG TGCCTAGCTT GTGACTTCTC GATAATCAAA AATTAATTAG AGAGAGACAG   
  
  
+ CTAAAGTTTT GAGAATAATA CTGAAAAATT ACATTAAATT TTATTTATGT TTATTTTCAT ACGCTATGTT   
  
  
+ TAACTGCTAT ACGCTGTGTT TAACTGCATC TCCTTCTATA TTTGATAAAT TGGTAAAAAA AAATAATTCA   
  
  
+ AAATTAAAGA CTAGTAATAT TTTCACTTAT CTGATAGATT TATAAGAAAT ATTTAGATAG CTAAATATAT   
  
  
+ TCTCTCCTGA AAGAGGAGAC TGATAGTTGA ATTTCATAGG ATATAAACAT GTATGTACAT TGAACGAATC   
  
  
+ TTTTTTCTAT GGTGATCGTT ATAAAGTTAT TTATTACTGT AAACATTAAG TGATAATTAG GATATGTTCT   
  
  
+ ATTTCACGTT TTAGATAGAT AGATAGATGT AAGGAGTTGT AAGAAGCTGT AAGAATATGT AAGGAGCTGT   
  
  
+ AAGGAGATGT AAGGAGCTGT AAGAAGATGT AAGAGAAACT GTAAGAAGCT ACAAGGATAG ATGTAACGAG   
  
  
+ TTGTTGAATT GTTGAAGAGT TGTATAGTGT AATTTGTTTT ACGATGAACG TTTTGGATGA AAAATAATAA   
  
  
+ TATTAAAATA ATATGCTTAT TAAAAATAAA TAAATAATTA AGTTAATTAC ACAACAATAA AATATATTAA   
  
  
+ TCCCCATAAA TTTAGTGAAT AAAACTTCGT CCACTTGAGA TGATTTTTTA TATGAACACT AGTTTGATAG   
  
  
+ AATAAACAAA GTATAAAAAA AAATAAAGTT TTTTGACAAA ATTGAATACA ACTCTTCAAA TAAATTCCTA   
  
  
+ TTTTAAGTAA ATAATTTTAA ATATAAATAA ATTTATGGTC ATCTATAATA ATATATATTT TATGTCGAAA   
  
  
+ CTCAAATATA ATTTACATCG TTATATTATA TTTTTATGTT TATGTAAATG TTGTGTTCAT AAGATATTTA   
  
  
+ TAAATTTTGT ATATTGGTAG TATCATTAAA AAAAATATAC ATATTTATGT AACTAAAATA AATGAATGTT   
  
  
+ ATAGAAAATC TATAAGATGT AAGGAGATGT ATGAAGATGT AAGGAGATGT AAGAAGATAT AAGGAGATGT   
  
  
+ AGAAAATGTA AGAAGATGTG AGAAGCTGTA AGGAGATGTA AGTTATTCAC TAAATCCTAA AAAATGCGAA   
  
  
+ ATAGAACAGG GGCTTAATTG AGCACTCAAG ATGCATAATT TGTGACGGAC CAAATGGATA AATATAGACC   
  
  
+ TGGCAAATGG GTTATTCGAT TTAGGTACGA GTATGGTTAA GTTTGGGTTT GGTTATGTTT CAGGATAAGT   
  
  
+ GACATTTCGA CTCAATTTGG TTTTGGTTGT ATGAACTTCG GCTCGAGATT TTTGGTTTGG CCAATACACT   
  
  
+ TATCATGCAA AAATAAGTAA CTTTATAGAA AATTTTGGAT CGGATATGAT GCGGTTCGGA TCAATTCGGA   
  
  
+ TTTTGGTTCA CATTCTGGTA AACCTATTTC GGATGTCAAG TAGGATATGA GTCCAGATCG TTGGATTTCG   
  
  
+ GGTATCAGCT CAATTTTACC AGATCTAGAT AAATCATAAA TGTACGGGTT TATTTTTTCC AGCTTGAATG   
  
  
+ ATTTTCTTTA TTAAAAAAAG ATAGATTTTA GATAAAACAC AACATTTCAT TTTCATTTGT TCCCCCCTAT   
  
  
+ AAATTTCTGT GACCACCTTC ACTTTCTTGC CCCATTCCCC CCCCCCCCCC GCTCTCTCTC TCTCTCTCTC   
  
  
+ TCTTCCCTTT ACAAAGGCAC CATTCAAATT CAAACCACTC TTTTTAAAAC CCCAAAAATT ATTCTCAAAA   
  
  
+ TAATACCTCA TAATATAAAT GGGTTCGTAT CACCATATAT GGGCCTCATC TCTCATGGGA ACATACGAAT   
  
  
+ TCAATGGCTT GCCCGATAGT TATCGGGTCA TGCCATCGGA GCCCCGGCAA CTAATCAATG GTCAAGCCTC   
  
  
+ AGATTTGGCC TCTTTGAGCC ATTATAGTAA CACCTTTCTC TCTGAACTCC CCCCTCTACC CACTCTGGGA   
  
  
+ CCCGGATCCG ACCCGGGTCT ATCGTGTCCG GGTCCTGCTC TGAGTCCAAT GGAGTGGATT GGTGACGCGT   
  
  
+ GGACACACCA GATTGAACAT GATCTGCCAC GTCAGCAGAG TCAACGGCTG TTGACTGATG GTGACATGGG   
  
  
+ GGAGGAGGAT TCCGGTATAA GGTTGGTGCA TGCCTTGCTG ACGTGCGCAG AGGCCGTCCA ATGCGGCGAG   
  
  
+ CTCCGACTGG CTGCTTCGCT AGTTGATGAG ATGACCAATG GGCTGTTGCC ACGTGTTAGT ACGGTGTGTG   
  
  
+ GGATCGGCAA GGTAGCCGGC TATTTTGTTG AGGCTTTGAG CCGGCGCCTG TTTCAGCCCG GCCCGGAAAC   
  
  
+ TGGCCCGACC TGGCCGGGCT CGGAGTTCGA GGCTGAGGTG TTGTATCGTC ACTTCTATGA GGCTTGCCCT   
  
  
+ TATCTCAAAT TTGCTCACTT CACGGCTAAT CAAGCCATCT TGGAGGCATT TGAGGGCCAT GATTATGTCC   
  
  
+ ACGTCATCGA CTTCGGCTTC ATGACCGGCT TGCAATGGCC GGCTTTGATT CAAGCCCTGG CTTTAAGGCC   
  
  
+ CGGTGGGCCA CCATCTCTTA GGCTCACTGG GATTGGCCCG TCTTTAGGAA GTGGGCCCGA ATCGATACGT   
  
  
+ GAAATCGGGT TGAGGCTAGC CCAATTGGCC CGATCCATGA ACATGAGGTT CGCATTTCGG GCTGTAGCAA   
  
  
+ CTTCTCGGCT CGAGGACGTG AAGCCGTCAA TGCTCGGAAT GGACCCTAGA GAAGCCGTCG CTATGAATTC   
  
  
+ CGTCATGCAT CTTCATCGAC TTTTAGGATT AGACATCAAC TCAGTTCTAA ACTGGATTCG AAACCTAAAC   
  
  
+ CCGAAGATCG TAACTTTAGT AGAACAAGAG GCGAACCATA ACCAACCTGA ATTTCTCCCC CGATTCACTG   
  
  
+ AAGCACTATG TTACTACTCA ACTATGTTCG ACTCATTAGA GACTTGTCAA GTCCAACCGC TGAAAGTCCT   
  
  
+ AGCCGAGATG TATATACAAA AGGAGATATC CAACCTAGTG TGCTACGAAG GCTCAGCCCG GGTCCAGAGG   
  
  
+ CACGAGCCGC TCGCCAAATG GCAAGCTCGG ATGGGCCAAG CCGGGTTCAA GCCGCTAGGC CTGAGTCAAA   
  
  
+ ATGCCCTTAG GCAAGCTAAC ATGTTGGTAA CATTGTTCTC CTCACAAGGG TATGACGTGG AGGAGAGAGA   
  
  
+ AGGGTGTTTG ACACTGGGCT GGCATAACCG CCCTCTCATA GTGACTTCGA CTTGGCAAGT GGGGCCCAAA   
  
  
+ ATGGACAGTC CAAGTGTGAC TCATGAGATG TCATCATCCT CAGCTTCTTC ATCTTA  

- +Up\_Stream \_Len000GTAAAA GTTAATCTTT AAAGAAGTTA ATATATGGTA GAGGTTTTTA TCAGGAGTAT   
  
  
- TCAACCGTCC ACTTACTAGG ATTACCTTTT AATGAAAATT GACAACTTAA ACGTTATCCC ATGGCTTAAA   
  
  
- CACTTCAAAT AACGGCGTGC ACGGATCGAA CACTGAAGAG CTATTAGTTT TTAATTAATC TCTCTCTGTC   
  
  
- GATTTCAAAA CTCTTATTAT GACTTTTTAA TGTAATTTAA AATAAATACA AATAAAAGTA TGCGATACAA   
  
  
- ATTGACGATA TGCGACACAA ATTGACGTAG AGGAAGATAT AAACTATTTA ACCATTTTTT TTTATTAAGT   
  
  
- TTTAATTTCT GATCATTATA AAAGTGAATA GACTATCTAA ATATTCTTTA TAAATCTATC GATTTATATA   
  
  
- AGAGAGGACT TTCTCCTCTG ACTATCAACT TAAAGTATCC TATATTTGTA CATACATGTA ACTTGCTTAG   
  
  
- AAAAAAGATA CCACTAGCAA TATTTCAATA AATAATGACA TTTGTAATTC ACTATTAATC CTATACAAGA   
  
  
- TAAAGTGCAA AATCTATCTA TCTATCTACA TTCCTCAACA TTCTTCGACA TTCTTATACA TTCCTCGACA   
  
  
- TTCCTCTACA TTCCTCGACA TTCTTCTACA TTCTCTTTGA CATTCTTCGA TGTTCCTATC TACATTGCTC   
  
  
- AACAACTTAA CAACTTCTCA ACATATCACA TTAAACAAAA TGCTACTTGC AAAACCTACT TTTTATTATT   
  
  
- ATAATTTTAT TATACGAATA ATTTTTATTT ATTTATTAAT TCAATTAATG TGTTGTTATT TTATATAATT   
  
  
- AGGGGTATTT AAATCACTTA TTTTGAAGCA GGTGAACTCT ACTAAAAAAT ATACTTGTGA TCAAACTATC   
  
  
- TTATTTGTTT CATATTTTTT TTTATTTCAA AAAACTGTTT TAACTTATGT TGAGAAGTTT ATTTAAGGAT   
  
  
- AAAATTCATT TATTAAAATT TATATTTATT TAAATACCAG TAGATATTAT TATATATAAA ATACAGCTTT   
  
  
- GAGTTTATAT TAAATGTAGC AATATAATAT AAAAATACAA ATACATTTAC AACACAAGTA TTCTATAAAT   
  
  
- ATTTAAAACA TATAACCATC ATAGTAATTT TTTTTATATG TATAAATACA TTGATTTTAT TTACTTACAA   
  
  
- TATCTTTTAG ATATTCTACA TTCCTCTACA TACTTCTACA TTCCTCTACA TTCTTCTATA TTCCTCTACA   
  
  
- TCTTTTACAT TCTTCTACAC TCTTCGACAT TCCTCTACAT TCAATAAGTG ATTTAGGATT TTTTACGCTT   
  
  
- TATCTTGTCC CCGAATTAAC TCGTGAGTTC TACGTATTAA ACACTGCCTG GTTTACCTAT TTATATCTGG   
  
  
- ACCGTTTACC CAATAAGCTA AATCCATGCT CATACCAATT CAAACCCAAA CCAATACAAA GTCCTATTCA   
  
  
- CTGTAAAGCT GAGTTAAACC AAAACCAACA TACTTGAAGC CGAGCTCTAA AAACCAAACC GGTTATGTGA   
  
  
- ATAGTACGTT TTTATTCATT GAAATATCTT TTAAAACCTA GCCTATACTA CGCCAAGCCT AGTTAAGCCT   
  
  
- AAAACCAAGT GTAAGACCAT TTGGATAAAG CCTACAGTTC ATCCTATACT CAGGTCTAGC AACCTAAAGC   
  
  
- CCATAGTCGA GTTAAAATGG TCTAGATCTA TTTAGTATTT ACATGCCCAA ATAAAAAAGG TCGAACTTAC   
  
  
- TAAAAGAAAT AATTTTTTTC TATCTAAAAT CTATTTTGTG TTGTAAAGTA AAAGTAAACA AGGGGGGATA   
  
  
- TTTAAAGACA CTGGTGGAAG TGAAAGAACG GGGTAAGGGG GGGGGGGGGG CGAGAGAGAG AGAGAGAGAG   
  
  
- AGAAGGGAAA TGTTTCCGTG GTAAGTTTAA GTTTGGTGAG AAAAATTTTG GGGTTTTTAA TAAGAGTTTT   
  
  
- ATTATGGAGT ATTATATTTA CCCAAGCATA GTGGTATATA CCCGGAGTAG AGAGTACCCT TGTATGCTTA   
  
  
- AGTTACCGAA CGGGCTATCA ATAGCCCAGT ACGGTAGCCT CGGGGCCGTT GATTAGTTAC CAGTTCGGAG   
  
  
- TCTAAACCGG AGAAACTCGG TAATATCATT GTGGAAAGAG AGACTTGAGG GGGGAGATGG GTGAGACCCT   
  
  
- GGGCCTAGGC TGGGCCCAGA TAGCACAGGC CCAGGACGAG ACTCAGGTTA CCTCACCTAA CCACTGCGCA   
  
  
- CCTGTGTGGT CTAACTTGTA CTAGACGGTG CAGTCGTCTC AGTTGCCGAC AACTGACTAC CACTGTACCC   
  
  
- CCTCCTCCTA AGGCCATATT CCAACCACGT ACGGAACGAC TGCACGCGTC TCCGGCAGGT TACGCCGCTC   
  
  
- GAGGCTGACC GACGAAGCGA TCAACTACTC TACTGGTTAC CCGACAACGG TGCACAATCA TGCCACACAC   
  
  
- CCTAGCCGTT CCATCGGCCG ATAAAACAAC TCCGAAACTC GGCCGCGGAC AAAGTCGGGC CGGGCCTTTG   
  
  
- ACCGGGCTGG ACCGGCCCGA GCCTCAAGCT CCGACTCCAC AACATAGCAG TGAAGATACT CCGAACGGGA   
  
  
- ATAGAGTTTA AACGAGTGAA GTGCCGATTA GTTCGGTAGA ACCTCCGTAA ACTCCCGGTA CTAATACAGG   
  
  
- TGCAGTAGCT GAAGCCGAAG TACTGGCCGA ACGTTACCGG CCGAAACTAA GTTCGGGACC GAAATTCCGG   
  
  
- GCCACCCGGT GGTAGAGAAT CCGAGTGACC CTAACCGGGC AGAAATCCTT CACCCGGGCT TAGCTATGCA   
  
  
- CTTTAGCCCA ACTCCGATCG GGTTAACCGG GCTAGGTACT TGTACTCCAA GCGTAAAGCC CGACATCGTT   
  
  
- GAAGAGCCGA GCTCCTGCAC TTCGGCAGTT ACGAGCCTTA CCTGGGATCT CTTCGGCAGC GATACTTAAG   
  
  
- GCAGTACGTA GAAGTAGCTG AAAATCCTAA TCTGTAGTTG AGTCAAGATT TGACCTAAGC TTTGGATTTG   
  
  
- GGCTTCTAGC ATTGAAATCA TCTTGTTCTC CGCTTGGTAT TGGTTGGACT TAAAGAGGGG GCTAAGTGAC   
  
  
- TTCGTGATAC AATGATGAGT TGATACAAGC TGAGTAATCT CTGAACAGTT CAGGTTGGCG ACTTTCAGGA   
  
  
- TCGGCTCTAC ATATATGTTT TCCTCTATAG GTTGGATCAC ACGATGCTTC CGAGTCGGGC CCAGGTCTCC   
  
  
- GTGCTCGGCG AGCGGTTTAC CGTTCGAGCC TACCCGGTTC GGCCCAAGTT CGGCGATCCG GACTCAGTTT   
  
  
- TACGGGAATC CGTTCGATTG TACAACCATT GTAACAAGAG GAGTGTTCCC ATACTGCACC TCCTCTCTCT   
  
  
- TCCCACAAAC TGTGACCCGA CCGTATTGGC GGGAGAGTAT CACTGAAGCT GAACCGTTCA CCCCGGGTTT   
  
  
- TACCTGTCAG GTTCACACTG AGTACTCTAC AGTAGTAGGA GTCGAAGAAG TAGAAT

+     STRE

| Site Name | Organism | Position | Strand | Matrix score. | sequence | function |
| --- | --- | --- | --- | --- | --- | --- |
| STRE | Arabidopsis thaliana | 2155 | - | 5 | AGGGG |  |
| STRE | Arabidopsis thaliana | 1342 | + | 5 | AGGGG |  |
| STRE | Arabidopsis thaliana | 1818 | - | 5 | AGGGG |  |

>HU03G02797.1   
+ +Up\_Stream \_Len000CATTTT CAATTAGAAA TTTCTTCAAT TATATACCAT CTCCAAAAAT AGTCCTCATA   
  
  
+ AGTTGGCAGG TGAATGATCC TAATGGAAAA TTACTTTTAA CTGTTGAATT TGCAATAGGG TACCGAATTT   
  
  
+ GTGAAGTTTA TTGCCGCACG TGCCTAGCTT GTGACTTCTC GATAATCAAA AATTAATTAG AGAGAGACAG   
  
  
+ CTAAAGTTTT GAGAATAATA CTGAAAAATT ACATTAAATT TTATTTATGT TTATTTTCAT ACGCTATGTT   
  
  
+ TAACTGCTAT ACGCTGTGTT TAACTGCATC TCCTTCTATA TTTGATAAAT TGGTAAAAAA AAATAATTCA   
  
  
+ AAATTAAAGA CTAGTAATAT TTTCACTTAT CTGATAGATT TATAAGAAAT ATTTAGATAG CTAAATATAT   
  
  
+ TCTCTCCTGA AAGAGGAGAC TGATAGTTGA ATTTCATAGG ATATAAACAT GTATGTACAT TGAACGAATC   
  
  
+ TTTTTTCTAT GGTGATCGTT ATAAAGTTAT TTATTACTGT AAACATTAAG TGATAATTAG GATATGTTCT   
  
  
+ ATTTCACGTT TTAGATAGAT AGATAGATGT AAGGAGTTGT AAGAAGCTGT AAGAATATGT AAGGAGCTGT   
  
  
+ AAGGAGATGT AAGGAGCTGT AAGAAGATGT AAGAGAAACT GTAAGAAGCT ACAAGGATAG ATGTAACGAG   
  
  
+ TTGTTGAATT GTTGAAGAGT TGTATAGTGT AATTTGTTTT ACGATGAACG TTTTGGATGA AAAATAATAA   
  
  
+ TATTAAAATA ATATGCTTAT TAAAAATAAA TAAATAATTA AGTTAATTAC ACAACAATAA AATATATTAA   
  
  
+ TCCCCATAAA TTTAGTGAAT AAAACTTCGT CCACTTGAGA TGATTTTTTA TATGAACACT AGTTTGATAG   
  
  
+ AATAAACAAA GTATAAAAAA AAATAAAGTT TTTTGACAAA ATTGAATACA ACTCTTCAAA TAAATTCCTA   
  
  
+ TTTTAAGTAA ATAATTTTAA ATATAAATAA ATTTATGGTC ATCTATAATA ATATATATTT TATGTCGAAA   
  
  
+ CTCAAATATA ATTTACATCG TTATATTATA TTTTTATGTT TATGTAAATG TTGTGTTCAT AAGATATTTA   
  
  
+ TAAATTTTGT ATATTGGTAG TATCATTAAA AAAAATATAC ATATTTATGT AACTAAAATA AATGAATGTT   
  
  
+ ATAGAAAATC TATAAGATGT AAGGAGATGT ATGAAGATGT AAGGAGATGT AAGAAGATAT AAGGAGATGT   
  
  
+ AGAAAATGTA AGAAGATGTG AGAAGCTGTA AGGAGATGTA AGTTATTCAC TAAATCCTAA AAAATGCGAA   
  
  
+ ATAGAACAGG GGCTTAATTG AGCACTCAAG ATGCATAATT TGTGACGGAC CAAATGGATA AATATAGACC   
  
  
+ TGGCAAATGG GTTATTCGAT TTAGGTACGA GTATGGTTAA GTTTGGGTTT GGTTATGTTT CAGGATAAGT   
  
  
+ GACATTTCGA CTCAATTTGG TTTTGGTTGT ATGAACTTCG GCTCGAGATT TTTGGTTTGG CCAATACACT   
  
  
+ TATCATGCAA AAATAAGTAA CTTTATAGAA AATTTTGGAT CGGATATGAT GCGGTTCGGA TCAATTCGGA   
  
  
+ TTTTGGTTCA CATTCTGGTA AACCTATTTC GGATGTCAAG TAGGATATGA GTCCAGATCG TTGGATTTCG   
  
  
+ GGTATCAGCT CAATTTTACC AGATCTAGAT AAATCATAAA TGTACGGGTT TATTTTTTCC AGCTTGAATG   
  
  
+ ATTTTCTTTA TTAAAAAAAG ATAGATTTTA GATAAAACAC AACATTTCAT TTTCATTTGT TCCCCCCTAT   
  
  
+ AAATTTCTGT GACCACCTTC ACTTTCTTGC CCCATTCCCC CCCCCCCCCC GCTCTCTCTC TCTCTCTCTC   
  
  
+ TCTTCCCTTT ACAAAGGCAC CATTCAAATT CAAACCACTC TTTTTAAAAC CCCAAAAATT ATTCTCAAAA   
  
  
+ TAATACCTCA TAATATAAAT GGGTTCGTAT CACCATATAT GGGCCTCATC TCTCATGGGA ACATACGAAT   
  
  
+ TCAATGGCTT GCCCGATAGT TATCGGGTCA TGCCATCGGA GCCCCGGCAA CTAATCAATG GTCAAGCCTC   
  
  
+ AGATTTGGCC TCTTTGAGCC ATTATAGTAA CACCTTTCTC TCTGAACTCC CCCCTCTACC CACTCTGGGA   
  
  
+ CCCGGATCCG ACCCGGGTCT ATCGTGTCCG GGTCCTGCTC TGAGTCCAAT GGAGTGGATT GGTGACGCGT   
  
  
+ GGACACACCA GATTGAACAT GATCTGCCAC GTCAGCAGAG TCAACGGCTG TTGACTGATG GTGACATGGG   
  
  
+ GGAGGAGGAT TCCGGTATAA GGTTGGTGCA TGCCTTGCTG ACGTGCGCAG AGGCCGTCCA ATGCGGCGAG   
  
  
+ CTCCGACTGG CTGCTTCGCT AGTTGATGAG ATGACCAATG GGCTGTTGCC ACGTGTTAGT ACGGTGTGTG   
  
  
+ GGATCGGCAA GGTAGCCGGC TATTTTGTTG AGGCTTTGAG CCGGCGCCTG TTTCAGCCCG GCCCGGAAAC   
  
  
+ TGGCCCGACC TGGCCGGGCT CGGAGTTCGA GGCTGAGGTG TTGTATCGTC ACTTCTATGA GGCTTGCCCT   
  
  
+ TATCTCAAAT TTGCTCACTT CACGGCTAAT CAAGCCATCT TGGAGGCATT TGAGGGCCAT GATTATGTCC   
  
  
+ ACGTCATCGA CTTCGGCTTC ATGACCGGCT TGCAATGGCC GGCTTTGATT CAAGCCCTGG CTTTAAGGCC   
  
  
+ CGGTGGGCCA CCATCTCTTA GGCTCACTGG GATTGGCCCG TCTTTAGGAA GTGGGCCCGA ATCGATACGT   
  
  
+ GAAATCGGGT TGAGGCTAGC CCAATTGGCC CGATCCATGA ACATGAGGTT CGCATTTCGG GCTGTAGCAA   
  
  
+ CTTCTCGGCT CGAGGACGTG AAGCCGTCAA TGCTCGGAAT GGACCCTAGA GAAGCCGTCG CTATGAATTC   
  
  
+ CGTCATGCAT CTTCATCGAC TTTTAGGATT AGACATCAAC TCAGTTCTAA ACTGGATTCG AAACCTAAAC   
  
  
+ CCGAAGATCG TAACTTTAGT AGAACAAGAG GCGAACCATA ACCAACCTGA ATTTCTCCCC CGATTCACTG   
  
  
+ AAGCACTATG TTACTACTCA ACTATGTTCG ACTCATTAGA GACTTGTCAA GTCCAACCGC TGAAAGTCCT   
  
  
+ AGCCGAGATG TATATACAAA AGGAGATATC CAACCTAGTG TGCTACGAAG GCTCAGCCCG GGTCCAGAGG   
  
  
+ CACGAGCCGC TCGCCAAATG GCAAGCTCGG ATGGGCCAAG CCGGGTTCAA GCCGCTAGGC CTGAGTCAAA   
  
  
+ ATGCCCTTAG GCAAGCTAAC ATGTTGGTAA CATTGTTCTC CTCACAAGGG TATGACGTGG AGGAGAGAGA   
  
  
+ AGGGTGTTTG ACACTGGGCT GGCATAACCG CCCTCTCATA GTGACTTCGA CTTGGCAAGT GGGGCCCAAA   
  
  
+ ATGGACAGTC CAAGTGTGAC TCATGAGATG TCATCATCCT CAGCTTCTTC ATCTTA  

- +Up\_Stream \_Len000GTAAAA GTTAATCTTT AAAGAAGTTA ATATATGGTA GAGGTTTTTA TCAGGAGTAT   
  
  
- TCAACCGTCC ACTTACTAGG ATTACCTTTT AATGAAAATT GACAACTTAA ACGTTATCCC ATGGCTTAAA   
  
  
- CACTTCAAAT AACGGCGTGC ACGGATCGAA CACTGAAGAG CTATTAGTTT TTAATTAATC TCTCTCTGTC   
  
  
- GATTTCAAAA CTCTTATTAT GACTTTTTAA TGTAATTTAA AATAAATACA AATAAAAGTA TGCGATACAA   
  
  
- ATTGACGATA TGCGACACAA ATTGACGTAG AGGAAGATAT AAACTATTTA ACCATTTTTT TTTATTAAGT   
  
  
- TTTAATTTCT GATCATTATA AAAGTGAATA GACTATCTAA ATATTCTTTA TAAATCTATC GATTTATATA   
  
  
- AGAGAGGACT TTCTCCTCTG ACTATCAACT TAAAGTATCC TATATTTGTA CATACATGTA ACTTGCTTAG   
  
  
- AAAAAAGATA CCACTAGCAA TATTTCAATA AATAATGACA TTTGTAATTC ACTATTAATC CTATACAAGA   
  
  
- TAAAGTGCAA AATCTATCTA TCTATCTACA TTCCTCAACA TTCTTCGACA TTCTTATACA TTCCTCGACA   
  
  
- TTCCTCTACA TTCCTCGACA TTCTTCTACA TTCTCTTTGA CATTCTTCGA TGTTCCTATC TACATTGCTC   
  
  
- AACAACTTAA CAACTTCTCA ACATATCACA TTAAACAAAA TGCTACTTGC AAAACCTACT TTTTATTATT   
  
  
- ATAATTTTAT TATACGAATA ATTTTTATTT ATTTATTAAT TCAATTAATG TGTTGTTATT TTATATAATT   
  
  
- AGGGGTATTT AAATCACTTA TTTTGAAGCA GGTGAACTCT ACTAAAAAAT ATACTTGTGA TCAAACTATC   
  
  
- TTATTTGTTT CATATTTTTT TTTATTTCAA AAAACTGTTT TAACTTATGT TGAGAAGTTT ATTTAAGGAT   
  
  
- AAAATTCATT TATTAAAATT TATATTTATT TAAATACCAG TAGATATTAT TATATATAAA ATACAGCTTT   
  
  
- GAGTTTATAT TAAATGTAGC AATATAATAT AAAAATACAA ATACATTTAC AACACAAGTA TTCTATAAAT   
  
  
- ATTTAAAACA TATAACCATC ATAGTAATTT TTTTTATATG TATAAATACA TTGATTTTAT TTACTTACAA   
  
  
- TATCTTTTAG ATATTCTACA TTCCTCTACA TACTTCTACA TTCCTCTACA TTCTTCTATA TTCCTCTACA   
  
  
- TCTTTTACAT TCTTCTACAC TCTTCGACAT TCCTCTACAT TCAATAAGTG ATTTAGGATT TTTTACGCTT   
  
  
- TATCTTGTCC CCGAATTAAC TCGTGAGTTC TACGTATTAA ACACTGCCTG GTTTACCTAT TTATATCTGG   
  
  
- ACCGTTTACC CAATAAGCTA AATCCATGCT CATACCAATT CAAACCCAAA CCAATACAAA GTCCTATTCA   
  
  
- CTGTAAAGCT GAGTTAAACC AAAACCAACA TACTTGAAGC CGAGCTCTAA AAACCAAACC GGTTATGTGA   
  
  
- ATAGTACGTT TTTATTCATT GAAATATCTT TTAAAACCTA GCCTATACTA CGCCAAGCCT AGTTAAGCCT   
  
  
- AAAACCAAGT GTAAGACCAT TTGGATAAAG CCTACAGTTC ATCCTATACT CAGGTCTAGC AACCTAAAGC   
  
  
- CCATAGTCGA GTTAAAATGG TCTAGATCTA TTTAGTATTT ACATGCCCAA ATAAAAAAGG TCGAACTTAC   
  
  
- TAAAAGAAAT AATTTTTTTC TATCTAAAAT CTATTTTGTG TTGTAAAGTA AAAGTAAACA AGGGGGGATA   
  
  
- TTTAAAGACA CTGGTGGAAG TGAAAGAACG GGGTAAGGGG GGGGGGGGGG CGAGAGAGAG AGAGAGAGAG   
  
  
- AGAAGGGAAA TGTTTCCGTG GTAAGTTTAA GTTTGGTGAG AAAAATTTTG GGGTTTTTAA TAAGAGTTTT   
  
  
- ATTATGGAGT ATTATATTTA CCCAAGCATA GTGGTATATA CCCGGAGTAG AGAGTACCCT TGTATGCTTA   
  
  
- AGTTACCGAA CGGGCTATCA ATAGCCCAGT ACGGTAGCCT CGGGGCCGTT GATTAGTTAC CAGTTCGGAG   
  
  
- TCTAAACCGG AGAAACTCGG TAATATCATT GTGGAAAGAG AGACTTGAGG GGGGAGATGG GTGAGACCCT   
  
  
- GGGCCTAGGC TGGGCCCAGA TAGCACAGGC CCAGGACGAG ACTCAGGTTA CCTCACCTAA CCACTGCGCA   
  
  
- CCTGTGTGGT CTAACTTGTA CTAGACGGTG CAGTCGTCTC AGTTGCCGAC AACTGACTAC CACTGTACCC   
  
  
- CCTCCTCCTA AGGCCATATT CCAACCACGT ACGGAACGAC TGCACGCGTC TCCGGCAGGT TACGCCGCTC   
  
  
- GAGGCTGACC GACGAAGCGA TCAACTACTC TACTGGTTAC CCGACAACGG TGCACAATCA TGCCACACAC   
  
  
- CCTAGCCGTT CCATCGGCCG ATAAAACAAC TCCGAAACTC GGCCGCGGAC AAAGTCGGGC CGGGCCTTTG   
  
  
- ACCGGGCTGG ACCGGCCCGA GCCTCAAGCT CCGACTCCAC AACATAGCAG TGAAGATACT CCGAACGGGA   
  
  
- ATAGAGTTTA AACGAGTGAA GTGCCGATTA GTTCGGTAGA ACCTCCGTAA ACTCCCGGTA CTAATACAGG   
  
  
- TGCAGTAGCT GAAGCCGAAG TACTGGCCGA ACGTTACCGG CCGAAACTAA GTTCGGGACC GAAATTCCGG   
  
  
- GCCACCCGGT GGTAGAGAAT CCGAGTGACC CTAACCGGGC AGAAATCCTT CACCCGGGCT TAGCTATGCA   
  
  
- CTTTAGCCCA ACTCCGATCG GGTTAACCGG GCTAGGTACT TGTACTCCAA GCGTAAAGCC CGACATCGTT   
  
  
- GAAGAGCCGA GCTCCTGCAC TTCGGCAGTT ACGAGCCTTA CCTGGGATCT CTTCGGCAGC GATACTTAAG   
  
  
- GCAGTACGTA GAAGTAGCTG AAAATCCTAA TCTGTAGTTG AGTCAAGATT TGACCTAAGC TTTGGATTTG   
  
  
- GGCTTCTAGC ATTGAAATCA TCTTGTTCTC CGCTTGGTAT TGGTTGGACT TAAAGAGGGG GCTAAGTGAC   
  
  
- TTCGTGATAC AATGATGAGT TGATACAAGC TGAGTAATCT CTGAACAGTT CAGGTTGGCG ACTTTCAGGA   
  
  
- TCGGCTCTAC ATATATGTTT TCCTCTATAG GTTGGATCAC ACGATGCTTC CGAGTCGGGC CCAGGTCTCC   
  
  
- GTGCTCGGCG AGCGGTTTAC CGTTCGAGCC TACCCGGTTC GGCCCAAGTT CGGCGATCCG GACTCAGTTT   
  
  
- TACGGGAATC CGTTCGATTG TACAACCATT GTAACAAGAG GAGTGTTCCC ATACTGCACC TCCTCTCTCT   
  
  
- TCCCACAAAC TGTGACCCGA CCGTATTGGC GGGAGAGTAT CACTGAAGCT GAACCGTTCA CCCCGGGTTT   
  
  
- TACCTGTCAG GTTCACACTG AGTACTCTAC AGTAGTAGGA GTCGAAGAAG TAGAAT

+     Sp1

| Site Name | Organism | Position | Strand | Matrix score. | sequence | function |
| --- | --- | --- | --- | --- | --- | --- |
| Sp1 | Oryza sativa | 3392 | - | 6 | GGGCGG | light responsive element |

>HU03G02797.1   
+ +Up\_Stream \_Len000CATTTT CAATTAGAAA TTTCTTCAAT TATATACCAT CTCCAAAAAT AGTCCTCATA   
  
  
+ AGTTGGCAGG TGAATGATCC TAATGGAAAA TTACTTTTAA CTGTTGAATT TGCAATAGGG TACCGAATTT   
  
  
+ GTGAAGTTTA TTGCCGCACG TGCCTAGCTT GTGACTTCTC GATAATCAAA AATTAATTAG AGAGAGACAG   
  
  
+ CTAAAGTTTT GAGAATAATA CTGAAAAATT ACATTAAATT TTATTTATGT TTATTTTCAT ACGCTATGTT   
  
  
+ TAACTGCTAT ACGCTGTGTT TAACTGCATC TCCTTCTATA TTTGATAAAT TGGTAAAAAA AAATAATTCA   
  
  
+ AAATTAAAGA CTAGTAATAT TTTCACTTAT CTGATAGATT TATAAGAAAT ATTTAGATAG CTAAATATAT   
  
  
+ TCTCTCCTGA AAGAGGAGAC TGATAGTTGA ATTTCATAGG ATATAAACAT GTATGTACAT TGAACGAATC   
  
  
+ TTTTTTCTAT GGTGATCGTT ATAAAGTTAT TTATTACTGT AAACATTAAG TGATAATTAG GATATGTTCT   
  
  
+ ATTTCACGTT TTAGATAGAT AGATAGATGT AAGGAGTTGT AAGAAGCTGT AAGAATATGT AAGGAGCTGT   
  
  
+ AAGGAGATGT AAGGAGCTGT AAGAAGATGT AAGAGAAACT GTAAGAAGCT ACAAGGATAG ATGTAACGAG   
  
  
+ TTGTTGAATT GTTGAAGAGT TGTATAGTGT AATTTGTTTT ACGATGAACG TTTTGGATGA AAAATAATAA   
  
  
+ TATTAAAATA ATATGCTTAT TAAAAATAAA TAAATAATTA AGTTAATTAC ACAACAATAA AATATATTAA   
  
  
+ TCCCCATAAA TTTAGTGAAT AAAACTTCGT CCACTTGAGA TGATTTTTTA TATGAACACT AGTTTGATAG   
  
  
+ AATAAACAAA GTATAAAAAA AAATAAAGTT TTTTGACAAA ATTGAATACA ACTCTTCAAA TAAATTCCTA   
  
  
+ TTTTAAGTAA ATAATTTTAA ATATAAATAA ATTTATGGTC ATCTATAATA ATATATATTT TATGTCGAAA   
  
  
+ CTCAAATATA ATTTACATCG TTATATTATA TTTTTATGTT TATGTAAATG TTGTGTTCAT AAGATATTTA   
  
  
+ TAAATTTTGT ATATTGGTAG TATCATTAAA AAAAATATAC ATATTTATGT AACTAAAATA AATGAATGTT   
  
  
+ ATAGAAAATC TATAAGATGT AAGGAGATGT ATGAAGATGT AAGGAGATGT AAGAAGATAT AAGGAGATGT   
  
  
+ AGAAAATGTA AGAAGATGTG AGAAGCTGTA AGGAGATGTA AGTTATTCAC TAAATCCTAA AAAATGCGAA   
  
  
+ ATAGAACAGG GGCTTAATTG AGCACTCAAG ATGCATAATT TGTGACGGAC CAAATGGATA AATATAGACC   
  
  
+ TGGCAAATGG GTTATTCGAT TTAGGTACGA GTATGGTTAA GTTTGGGTTT GGTTATGTTT CAGGATAAGT   
  
  
+ GACATTTCGA CTCAATTTGG TTTTGGTTGT ATGAACTTCG GCTCGAGATT TTTGGTTTGG CCAATACACT   
  
  
+ TATCATGCAA AAATAAGTAA CTTTATAGAA AATTTTGGAT CGGATATGAT GCGGTTCGGA TCAATTCGGA   
  
  
+ TTTTGGTTCA CATTCTGGTA AACCTATTTC GGATGTCAAG TAGGATATGA GTCCAGATCG TTGGATTTCG   
  
  
+ GGTATCAGCT CAATTTTACC AGATCTAGAT AAATCATAAA TGTACGGGTT TATTTTTTCC AGCTTGAATG   
  
  
+ ATTTTCTTTA TTAAAAAAAG ATAGATTTTA GATAAAACAC AACATTTCAT TTTCATTTGT TCCCCCCTAT   
  
  
+ AAATTTCTGT GACCACCTTC ACTTTCTTGC CCCATTCCCC CCCCCCCCCC GCTCTCTCTC TCTCTCTCTC   
  
  
+ TCTTCCCTTT ACAAAGGCAC CATTCAAATT CAAACCACTC TTTTTAAAAC CCCAAAAATT ATTCTCAAAA   
  
  
+ TAATACCTCA TAATATAAAT GGGTTCGTAT CACCATATAT GGGCCTCATC TCTCATGGGA ACATACGAAT   
  
  
+ TCAATGGCTT GCCCGATAGT TATCGGGTCA TGCCATCGGA GCCCCGGCAA CTAATCAATG GTCAAGCCTC   
  
  
+ AGATTTGGCC TCTTTGAGCC ATTATAGTAA CACCTTTCTC TCTGAACTCC CCCCTCTACC CACTCTGGGA   
  
  
+ CCCGGATCCG ACCCGGGTCT ATCGTGTCCG GGTCCTGCTC TGAGTCCAAT GGAGTGGATT GGTGACGCGT   
  
  
+ GGACACACCA GATTGAACAT GATCTGCCAC GTCAGCAGAG TCAACGGCTG TTGACTGATG GTGACATGGG   
  
  
+ GGAGGAGGAT TCCGGTATAA GGTTGGTGCA TGCCTTGCTG ACGTGCGCAG AGGCCGTCCA ATGCGGCGAG   
  
  
+ CTCCGACTGG CTGCTTCGCT AGTTGATGAG ATGACCAATG GGCTGTTGCC ACGTGTTAGT ACGGTGTGTG   
  
  
+ GGATCGGCAA GGTAGCCGGC TATTTTGTTG AGGCTTTGAG CCGGCGCCTG TTTCAGCCCG GCCCGGAAAC   
  
  
+ TGGCCCGACC TGGCCGGGCT CGGAGTTCGA GGCTGAGGTG TTGTATCGTC ACTTCTATGA GGCTTGCCCT   
  
  
+ TATCTCAAAT TTGCTCACTT CACGGCTAAT CAAGCCATCT TGGAGGCATT TGAGGGCCAT GATTATGTCC   
  
  
+ ACGTCATCGA CTTCGGCTTC ATGACCGGCT TGCAATGGCC GGCTTTGATT CAAGCCCTGG CTTTAAGGCC   
  
  
+ CGGTGGGCCA CCATCTCTTA GGCTCACTGG GATTGGCCCG TCTTTAGGAA GTGGGCCCGA ATCGATACGT   
  
  
+ GAAATCGGGT TGAGGCTAGC CCAATTGGCC CGATCCATGA ACATGAGGTT CGCATTTCGG GCTGTAGCAA   
  
  
+ CTTCTCGGCT CGAGGACGTG AAGCCGTCAA TGCTCGGAAT GGACCCTAGA GAAGCCGTCG CTATGAATTC   
  
  
+ CGTCATGCAT CTTCATCGAC TTTTAGGATT AGACATCAAC TCAGTTCTAA ACTGGATTCG AAACCTAAAC   
  
  
+ CCGAAGATCG TAACTTTAGT AGAACAAGAG GCGAACCATA ACCAACCTGA ATTTCTCCCC CGATTCACTG   
  
  
+ AAGCACTATG TTACTACTCA ACTATGTTCG ACTCATTAGA GACTTGTCAA GTCCAACCGC TGAAAGTCCT   
  
  
+ AGCCGAGATG TATATACAAA AGGAGATATC CAACCTAGTG TGCTACGAAG GCTCAGCCCG GGTCCAGAGG   
  
  
+ CACGAGCCGC TCGCCAAATG GCAAGCTCGG ATGGGCCAAG CCGGGTTCAA GCCGCTAGGC CTGAGTCAAA   
  
  
+ ATGCCCTTAG GCAAGCTAAC ATGTTGGTAA CATTGTTCTC CTCACAAGGG TATGACGTGG AGGAGAGAGA   
  
  
+ AGGGTGTTTG ACACTGGGCT GGCATAACCG CCCTCTCATA GTGACTTCGA CTTGGCAAGT GGGGCCCAAA   
  
  
+ ATGGACAGTC CAAGTGTGAC TCATGAGATG TCATCATCCT CAGCTTCTTC ATCTTA  

- +Up\_Stream \_Len000GTAAAA GTTAATCTTT AAAGAAGTTA ATATATGGTA GAGGTTTTTA TCAGGAGTAT   
  
  
- TCAACCGTCC ACTTACTAGG ATTACCTTTT AATGAAAATT GACAACTTAA ACGTTATCCC ATGGCTTAAA   
  
  
- CACTTCAAAT AACGGCGTGC ACGGATCGAA CACTGAAGAG CTATTAGTTT TTAATTAATC TCTCTCTGTC   
  
  
- GATTTCAAAA CTCTTATTAT GACTTTTTAA TGTAATTTAA AATAAATACA AATAAAAGTA TGCGATACAA   
  
  
- ATTGACGATA TGCGACACAA ATTGACGTAG AGGAAGATAT AAACTATTTA ACCATTTTTT TTTATTAAGT   
  
  
- TTTAATTTCT GATCATTATA AAAGTGAATA GACTATCTAA ATATTCTTTA TAAATCTATC GATTTATATA   
  
  
- AGAGAGGACT TTCTCCTCTG ACTATCAACT TAAAGTATCC TATATTTGTA CATACATGTA ACTTGCTTAG   
  
  
- AAAAAAGATA CCACTAGCAA TATTTCAATA AATAATGACA TTTGTAATTC ACTATTAATC CTATACAAGA   
  
  
- TAAAGTGCAA AATCTATCTA TCTATCTACA TTCCTCAACA TTCTTCGACA TTCTTATACA TTCCTCGACA   
  
  
- TTCCTCTACA TTCCTCGACA TTCTTCTACA TTCTCTTTGA CATTCTTCGA TGTTCCTATC TACATTGCTC   
  
  
- AACAACTTAA CAACTTCTCA ACATATCACA TTAAACAAAA TGCTACTTGC AAAACCTACT TTTTATTATT   
  
  
- ATAATTTTAT TATACGAATA ATTTTTATTT ATTTATTAAT TCAATTAATG TGTTGTTATT TTATATAATT   
  
  
- AGGGGTATTT AAATCACTTA TTTTGAAGCA GGTGAACTCT ACTAAAAAAT ATACTTGTGA TCAAACTATC   
  
  
- TTATTTGTTT CATATTTTTT TTTATTTCAA AAAACTGTTT TAACTTATGT TGAGAAGTTT ATTTAAGGAT   
  
  
- AAAATTCATT TATTAAAATT TATATTTATT TAAATACCAG TAGATATTAT TATATATAAA ATACAGCTTT   
  
  
- GAGTTTATAT TAAATGTAGC AATATAATAT AAAAATACAA ATACATTTAC AACACAAGTA TTCTATAAAT   
  
  
- ATTTAAAACA TATAACCATC ATAGTAATTT TTTTTATATG TATAAATACA TTGATTTTAT TTACTTACAA   
  
  
- TATCTTTTAG ATATTCTACA TTCCTCTACA TACTTCTACA TTCCTCTACA TTCTTCTATA TTCCTCTACA   
  
  
- TCTTTTACAT TCTTCTACAC TCTTCGACAT TCCTCTACAT TCAATAAGTG ATTTAGGATT TTTTACGCTT   
  
  
- TATCTTGTCC CCGAATTAAC TCGTGAGTTC TACGTATTAA ACACTGCCTG GTTTACCTAT TTATATCTGG   
  
  
- ACCGTTTACC CAATAAGCTA AATCCATGCT CATACCAATT CAAACCCAAA CCAATACAAA GTCCTATTCA   
  
  
- CTGTAAAGCT GAGTTAAACC AAAACCAACA TACTTGAAGC CGAGCTCTAA AAACCAAACC GGTTATGTGA   
  
  
- ATAGTACGTT TTTATTCATT GAAATATCTT TTAAAACCTA GCCTATACTA CGCCAAGCCT AGTTAAGCCT   
  
  
- AAAACCAAGT GTAAGACCAT TTGGATAAAG CCTACAGTTC ATCCTATACT CAGGTCTAGC AACCTAAAGC   
  
  
- CCATAGTCGA GTTAAAATGG TCTAGATCTA TTTAGTATTT ACATGCCCAA ATAAAAAAGG TCGAACTTAC   
  
  
- TAAAAGAAAT AATTTTTTTC TATCTAAAAT CTATTTTGTG TTGTAAAGTA AAAGTAAACA AGGGGGGATA   
  
  
- TTTAAAGACA CTGGTGGAAG TGAAAGAACG GGGTAAGGGG GGGGGGGGGG CGAGAGAGAG AGAGAGAGAG   
  
  
- AGAAGGGAAA TGTTTCCGTG GTAAGTTTAA GTTTGGTGAG AAAAATTTTG GGGTTTTTAA TAAGAGTTTT   
  
  
- ATTATGGAGT ATTATATTTA CCCAAGCATA GTGGTATATA CCCGGAGTAG AGAGTACCCT TGTATGCTTA   
  
  
- AGTTACCGAA CGGGCTATCA ATAGCCCAGT ACGGTAGCCT CGGGGCCGTT GATTAGTTAC CAGTTCGGAG   
  
  
- TCTAAACCGG AGAAACTCGG TAATATCATT GTGGAAAGAG AGACTTGAGG GGGGAGATGG GTGAGACCCT   
  
  
- GGGCCTAGGC TGGGCCCAGA TAGCACAGGC CCAGGACGAG ACTCAGGTTA CCTCACCTAA CCACTGCGCA   
  
  
- CCTGTGTGGT CTAACTTGTA CTAGACGGTG CAGTCGTCTC AGTTGCCGAC AACTGACTAC CACTGTACCC   
  
  
- CCTCCTCCTA AGGCCATATT CCAACCACGT ACGGAACGAC TGCACGCGTC TCCGGCAGGT TACGCCGCTC   
  
  
- GAGGCTGACC GACGAAGCGA TCAACTACTC TACTGGTTAC CCGACAACGG TGCACAATCA TGCCACACAC   
  
  
- CCTAGCCGTT CCATCGGCCG ATAAAACAAC TCCGAAACTC GGCCGCGGAC AAAGTCGGGC CGGGCCTTTG   
  
  
- ACCGGGCTGG ACCGGCCCGA GCCTCAAGCT CCGACTCCAC AACATAGCAG TGAAGATACT CCGAACGGGA   
  
  
- ATAGAGTTTA AACGAGTGAA GTGCCGATTA GTTCGGTAGA ACCTCCGTAA ACTCCCGGTA CTAATACAGG   
  
  
- TGCAGTAGCT GAAGCCGAAG TACTGGCCGA ACGTTACCGG CCGAAACTAA GTTCGGGACC GAAATTCCGG   
  
  
- GCCACCCGGT GGTAGAGAAT CCGAGTGACC CTAACCGGGC AGAAATCCTT CACCCGGGCT TAGCTATGCA   
  
  
- CTTTAGCCCA ACTCCGATCG GGTTAACCGG GCTAGGTACT TGTACTCCAA GCGTAAAGCC CGACATCGTT   
  
  
- GAAGAGCCGA GCTCCTGCAC TTCGGCAGTT ACGAGCCTTA CCTGGGATCT CTTCGGCAGC GATACTTAAG   
  
  
- GCAGTACGTA GAAGTAGCTG AAAATCCTAA TCTGTAGTTG AGTCAAGATT TGACCTAAGC TTTGGATTTG   
  
  
- GGCTTCTAGC ATTGAAATCA TCTTGTTCTC CGCTTGGTAT TGGTTGGACT TAAAGAGGGG GCTAAGTGAC   
  
  
- TTCGTGATAC AATGATGAGT TGATACAAGC TGAGTAATCT CTGAACAGTT CAGGTTGGCG ACTTTCAGGA   
  
  
- TCGGCTCTAC ATATATGTTT TCCTCTATAG GTTGGATCAC ACGATGCTTC CGAGTCGGGC CCAGGTCTCC   
  
  
- GTGCTCGGCG AGCGGTTTAC CGTTCGAGCC TACCCGGTTC GGCCCAAGTT CGGCGATCCG GACTCAGTTT   
  
  
- TACGGGAATC CGTTCGATTG TACAACCATT GTAACAAGAG GAGTGTTCCC ATACTGCACC TCCTCTCTCT   
  
  
- TCCCACAAAC TGTGACCCGA CCGTATTGGC GGGAGAGTAT CACTGAAGCT GAACCGTTCA CCCCGGGTTT   
  
  
- TACCTGTCAG GTTCACACTG AGTACTCTAC AGTAGTAGGA GTCGAAGAAG TAGAAT

+     TATA-box

| Site Name | Organism | Position | Strand | Matrix score. | sequence | function |
| --- | --- | --- | --- | --- | --- | --- |
| TATA-box | Arabidopsis thaliana | 3167 | - | 4 | TATA | core promoter element around -30 of transcription start |
| TATA-box | Brassica napus | 1079 | + | 6 | ATTATA | core promoter element around -30 of transcription start |
| TATA-box | Arabidopsis thaliana | 2000 | - | 4 | TATA | core promoter element around -30 of transcription start |
| TATA-box | Helianthus annuus | 3163 | - | 6 | TATACA | core promoter element around -30 of transcription start |
| TATA-box | Arabidopsis thaliana | 837 | + | 4 | TATA | core promoter element around -30 of transcription start |
| TATA-box | Oryza sativa | 3169 | + | 7 | TACAAAA | core promoter element around -30 of transcription start |
| TATA-box | Arabidopsis thaliana | 3161 | - | 9 | taTATAAAtc | core promoter element around -30 of transcription start |
| TATA-box | Arabidopsis thaliana | 3165 | - | 6 | TATATA | core promoter element around -30 of transcription start |
| TATA-box | Arabidopsis thaliana | 1397 | + | 4 | TATA | core promoter element around -30 of transcription start |
| TATA-box | Arabidopsis thaliana | 2330 | - | 4 | TATA | core promoter element around -30 of transcription start |
| TATA-box | Arabidopsis thaliana | 2127 | - | 4 | TATA | core promoter element around -30 of transcription start |
| TATA-box | Helianthus annuus | 1132 | - | 6 | TATACA | core promoter element around -30 of transcription start |
| TATA-box | Brassica oleracea | 1977 | + | 6 | ATATAA | core promoter element around -30 of transcription start |
| TATA-box | Arabidopsis thaliana | 1568 | + | 4 | TATA | core promoter element around -30 of transcription start |
| TATA-box | Helianthus annuus | 1566 | - | 6 | TATAAA | core promoter element around -30 of transcription start |
| TATA-box | Brassica oleracea | 1251 | + | 6 | ATATAA | core promoter element around -30 of transcription start |
| TATA-box | Arabidopsis thaliana | 1194 | + | 4 | TATA | core promoter element around -30 of transcription start |
| TATA-box | Arabidopsis thaliana | 1076 | + | 4 | TATA | core promoter element around -30 of transcription start |
| TATA-box | Arabidopsis thaliana | 1134 | + | 4 | TATA | core promoter element around -30 of transcription start |
| TATA-box | Arabidopsis thaliana | 1061 | + | 4 | TATA | core promoter element around -30 of transcription start |
| TATA-box | Arabidopsis thaliana | 1006 | + | 4 | TATA | core promoter element around -30 of transcription start |
| TATA-box | Brassica oleracea | 1005 | + | 6 | ATATAA | core promoter element around -30 of transcription start |
| TATA-box | Brassica napus | 836 | + | 6 | ATATAT | core promoter element around -30 of transcription start |
| TATA-box | Helianthus annuus | 725 | - | 6 | TATACA | core promoter element around -30 of transcription start |
| TATA-box | Brassica napus | 2125 | + | 6 | ATTATA | core promoter element around -30 of transcription start |
| TATA-box | Oryza sativa | 1129 | - | 7 | TACAAAA | core promoter element around -30 of transcription start |
| TATA-box | Arabidopsis thaliana | 1978 | - | 4 | TATA | core promoter element around -30 of transcription start |
| TATA-box | Arabidopsis thaliana | 1123 | + | 4 | TATA | core promoter element around -30 of transcription start |
| TATA-box | Helianthus annuus | 1121 | - | 6 | TATAAA | core promoter element around -30 of transcription start |
| TATA-box | Brassica juncea | 1120 | - | 7 | TATAAAT | core promoter element around -30 of transcription start |
| TATA-box | Arabidopsis thaliana | 1080 | - | 5 | TATAA | core promoter element around -30 of transcription start |
| TATA-box | Arabidopsis thaliana | 727 | + | 4 | TATA | core promoter element around -30 of transcription start |
| TATA-box | Arabidopsis thaliana | 514 | + | 4 | TATA | core promoter element around -30 of transcription start |
| TATA-box | Brassica napus | 1999 | - | 6 | ATATAT | core promoter element around -30 of transcription start |
| TATA-box | Oryza sativa | 1093 | - | 8 | TACATAAA | core promoter element around -30 of transcription start |
| TATA-box | Brassica oleracea | 465 | + | 6 | ATATAA | core promoter element around -30 of transcription start |
| TATA-box | Arabidopsis thaliana | 321 | + | 4 | TATA | core promoter element around -30 of transcription start |
| TATA-box | Arabidopsis thaliana | 45 | + | 6 | TATATA | core promoter element around -30 of transcription start |
| TATA-box | Brassica napus | 419 | + | 6 | ATATAT | core promoter element around -30 of transcription start |
| TATA-box | Arabidopsis thaliana | 1710 | + | 9 | taTATAAAtc | core promoter element around -30 of transcription start |
| TATA-box | Arabidopsis thaliana | 513 | - | 5 | TATAA | core promoter element around -30 of transcription start |
| TATA-box | Arabidopsis thaliana | 1252 | + | 4 | TATA | core promoter element around -30 of transcription start |
| TATA-box | Arabidopsis thaliana | 1075 | - | 5 | TATAA | core promoter element around -30 of transcription start |
| TATA-box | Arabidopsis thaliana | 893 | + | 4 | TATA | core promoter element around -30 of transcription start |
| TATA-box | Arabidopsis thaliana | 2126 | - | 5 | TATAA | core promoter element around -30 of transcription start |
| TATA-box | Daucus carota | 1119 | - | 8 | TATAAATA | core promoter element around -30 of transcription start |
| TATA-box | Arabidopsis thaliana | 1122 | - | 5 | TATAA | core promoter element around -30 of transcription start |
| TATA-box | Arabidopsis thaliana | 1822 | - | 4 | TATA | core promoter element around -30 of transcription start |
| TATA-box | Arabidopsis thaliana | 394 | - | 5 | TATAA | core promoter element around -30 of transcription start |
| TATA-box | Brassica oleracea | 1060 | + | 6 | ATATAA | core promoter element around -30 of transcription start |
| TATA-box | Arabidopsis thaliana | 1160 | + | 4 | TATA | core promoter element around -30 of transcription start |
| TATA-box | Arabidopsis thaliana | 292 | + | 4 | TATA | core promoter element around -30 of transcription start |
| TATA-box | Arabidopsis thaliana | 1567 | - | 5 | TATAA | core promoter element around -30 of transcription start |
| TATA-box | Arabidopsis thaliana | 420 | + | 4 | TATA | core promoter element around -30 of transcription start |
| TATA-box | Arabidopsis thaliana | 44 | - | 7 | TATATAA | core promoter element around -30 of transcription start |
| TATA-box | Arabidopsis thaliana | 47 | + | 4 | TATA | core promoter element around -30 of transcription start |
| TATA-box | Arabidopsis thaliana | 1081 | + | 4 | TATA | core promoter element around -30 of transcription start |
| TATA-box | Arabidopsis thaliana | 1000 | - | 8 | TATTTAAA | core promoter element around -30 of transcription start |
| TATA-box | Brassica napus | 1037 | + | 6 | ATATAT | core promoter element around -30 of transcription start |
| TATA-box | Brassica napus | 1035 | + | 6 | ATATAT | core promoter element around -30 of transcription start |
| TATA-box | Brassica napus | 43 | + | 6 | ATTATA | core promoter element around -30 of transcription start |
| TATA-box | Avena sativa | 1003 | - | 12 | TATATTTATATTT | core promoter element around -30 of transcription start |
| TATA-box | Arabidopsis thaliana | 1205 | + | 4 | TATA | core promoter element around -30 of transcription start |
| TATA-box | Arabidopsis thaliana | 395 | + | 4 | TATA | core promoter element around -30 of transcription start |
| TATA-box | Oryza sativa | 1168 | - | 8 | TACATAAA | core promoter element around -30 of transcription start |
| TATA-box | Helianthus annuus | 393 | - | 6 | TATAAA | core promoter element around -30 of transcription start |
| TATA-box | Arabidopsis thaliana | 1193 | - | 5 | TATAA | core promoter element around -30 of transcription start |
| TATA-box | Brassica juncea | 392 | - | 7 | TATAAAT | core promoter element around -30 of transcription start |
| TATA-box | Helianthus annuus | 891 | - | 6 | TATAAA | core promoter element around -30 of transcription start |
| TATA-box | Arabidopsis thaliana | 889 | - | 9 | ccTATAAAaa | core promoter element around -30 of transcription start |
| TATA-box | Arabidopsis thaliana | 466 | + | 4 | TATA | core promoter element around -30 of transcription start |
| TATA-box | Arabidopsis thaliana | 1038 | + | 4 | TATA | core promoter element around -30 of transcription start |
| TATA-box | Arabidopsis thaliana | 1028 | + | 4 | TATA | core promoter element around -30 of transcription start |
| TATA-box | Arabidopsis thaliana | 1036 | + | 6 | TATATA | core promoter element around -30 of transcription start |
| TATA-box | Arabidopsis thaliana | 926 | + | 4 | TATA | core promoter element around -30 of transcription start |
| TATA-box | Pisum sativum | 890 | - | 7 | TATAAAA | core promoter element around -30 of transcription start |
| TATA-box | Arabidopsis thaliana | 892 | - | 5 | TATAA | core promoter element around -30 of transcription start |

>HU03G02797.1   
+ +Up\_Stream \_Len000CATTTT CAATTAGAAA TTTCTTCAAT TATATACCAT CTCCAAAAAT AGTCCTCATA   
  
  
+ AGTTGGCAGG TGAATGATCC TAATGGAAAA TTACTTTTAA CTGTTGAATT TGCAATAGGG TACCGAATTT   
  
  
+ GTGAAGTTTA TTGCCGCACG TGCCTAGCTT GTGACTTCTC GATAATCAAA AATTAATTAG AGAGAGACAG   
  
  
+ CTAAAGTTTT GAGAATAATA CTGAAAAATT ACATTAAATT TTATTTATGT TTATTTTCAT ACGCTATGTT   
  
  
+ TAACTGCTAT ACGCTGTGTT TAACTGCATC TCCTTCTATA TTTGATAAAT TGGTAAAAAA AAATAATTCA   
  
  
+ AAATTAAAGA CTAGTAATAT TTTCACTTAT CTGATAGATT TATAAGAAAT ATTTAGATAG CTAAATATAT   
  
  
+ TCTCTCCTGA AAGAGGAGAC TGATAGTTGA ATTTCATAGG ATATAAACAT GTATGTACAT TGAACGAATC   
  
  
+ TTTTTTCTAT GGTGATCGTT ATAAAGTTAT TTATTACTGT AAACATTAAG TGATAATTAG GATATGTTCT   
  
  
+ ATTTCACGTT TTAGATAGAT AGATAGATGT AAGGAGTTGT AAGAAGCTGT AAGAATATGT AAGGAGCTGT   
  
  
+ AAGGAGATGT AAGGAGCTGT AAGAAGATGT AAGAGAAACT GTAAGAAGCT ACAAGGATAG ATGTAACGAG   
  
  
+ TTGTTGAATT GTTGAAGAGT TGTATAGTGT AATTTGTTTT ACGATGAACG TTTTGGATGA AAAATAATAA   
  
  
+ TATTAAAATA ATATGCTTAT TAAAAATAAA TAAATAATTA AGTTAATTAC ACAACAATAA AATATATTAA   
  
  
+ TCCCCATAAA TTTAGTGAAT AAAACTTCGT CCACTTGAGA TGATTTTTTA TATGAACACT AGTTTGATAG   
  
  
+ AATAAACAAA GTATAAAAAA AAATAAAGTT TTTTGACAAA ATTGAATACA ACTCTTCAAA TAAATTCCTA   
  
  
+ TTTTAAGTAA ATAATTTTAA ATATAAATAA ATTTATGGTC ATCTATAATA ATATATATTT TATGTCGAAA   
  
  
+ CTCAAATATA ATTTACATCG TTATATTATA TTTTTATGTT TATGTAAATG TTGTGTTCAT AAGATATTTA   
  
  
+ TAAATTTTGT ATATTGGTAG TATCATTAAA AAAAATATAC ATATTTATGT AACTAAAATA AATGAATGTT   
  
  
+ ATAGAAAATC TATAAGATGT AAGGAGATGT ATGAAGATGT AAGGAGATGT AAGAAGATAT AAGGAGATGT   
  
  
+ AGAAAATGTA AGAAGATGTG AGAAGCTGTA AGGAGATGTA AGTTATTCAC TAAATCCTAA AAAATGCGAA   
  
  
+ ATAGAACAGG GGCTTAATTG AGCACTCAAG ATGCATAATT TGTGACGGAC CAAATGGATA AATATAGACC   
  
  
+ TGGCAAATGG GTTATTCGAT TTAGGTACGA GTATGGTTAA GTTTGGGTTT GGTTATGTTT CAGGATAAGT   
  
  
+ GACATTTCGA CTCAATTTGG TTTTGGTTGT ATGAACTTCG GCTCGAGATT TTTGGTTTGG CCAATACACT   
  
  
+ TATCATGCAA AAATAAGTAA CTTTATAGAA AATTTTGGAT CGGATATGAT GCGGTTCGGA TCAATTCGGA   
  
  
+ TTTTGGTTCA CATTCTGGTA AACCTATTTC GGATGTCAAG TAGGATATGA GTCCAGATCG TTGGATTTCG   
  
  
+ GGTATCAGCT CAATTTTACC AGATCTAGAT AAATCATAAA TGTACGGGTT TATTTTTTCC AGCTTGAATG   
  
  
+ ATTTTCTTTA TTAAAAAAAG ATAGATTTTA GATAAAACAC AACATTTCAT TTTCATTTGT TCCCCCCTAT   
  
  
+ AAATTTCTGT GACCACCTTC ACTTTCTTGC CCCATTCCCC CCCCCCCCCC GCTCTCTCTC TCTCTCTCTC   
  
  
+ TCTTCCCTTT ACAAAGGCAC CATTCAAATT CAAACCACTC TTTTTAAAAC CCCAAAAATT ATTCTCAAAA   
  
  
+ TAATACCTCA TAATATAAAT GGGTTCGTAT CACCATATAT GGGCCTCATC TCTCATGGGA ACATACGAAT   
  
  
+ TCAATGGCTT GCCCGATAGT TATCGGGTCA TGCCATCGGA GCCCCGGCAA CTAATCAATG GTCAAGCCTC   
  
  
+ AGATTTGGCC TCTTTGAGCC ATTATAGTAA CACCTTTCTC TCTGAACTCC CCCCTCTACC CACTCTGGGA   
  
  
+ CCCGGATCCG ACCCGGGTCT ATCGTGTCCG GGTCCTGCTC TGAGTCCAAT GGAGTGGATT GGTGACGCGT   
  
  
+ GGACACACCA GATTGAACAT GATCTGCCAC GTCAGCAGAG TCAACGGCTG TTGACTGATG GTGACATGGG   
  
  
+ GGAGGAGGAT TCCGGTATAA GGTTGGTGCA TGCCTTGCTG ACGTGCGCAG AGGCCGTCCA ATGCGGCGAG   
  
  
+ CTCCGACTGG CTGCTTCGCT AGTTGATGAG ATGACCAATG GGCTGTTGCC ACGTGTTAGT ACGGTGTGTG   
  
  
+ GGATCGGCAA GGTAGCCGGC TATTTTGTTG AGGCTTTGAG CCGGCGCCTG TTTCAGCCCG GCCCGGAAAC   
  
  
+ TGGCCCGACC TGGCCGGGCT CGGAGTTCGA GGCTGAGGTG TTGTATCGTC ACTTCTATGA GGCTTGCCCT   
  
  
+ TATCTCAAAT TTGCTCACTT CACGGCTAAT CAAGCCATCT TGGAGGCATT TGAGGGCCAT GATTATGTCC   
  
  
+ ACGTCATCGA CTTCGGCTTC ATGACCGGCT TGCAATGGCC GGCTTTGATT CAAGCCCTGG CTTTAAGGCC   
  
  
+ CGGTGGGCCA CCATCTCTTA GGCTCACTGG GATTGGCCCG TCTTTAGGAA GTGGGCCCGA ATCGATACGT   
  
  
+ GAAATCGGGT TGAGGCTAGC CCAATTGGCC CGATCCATGA ACATGAGGTT CGCATTTCGG GCTGTAGCAA   
  
  
+ CTTCTCGGCT CGAGGACGTG AAGCCGTCAA TGCTCGGAAT GGACCCTAGA GAAGCCGTCG CTATGAATTC   
  
  
+ CGTCATGCAT CTTCATCGAC TTTTAGGATT AGACATCAAC TCAGTTCTAA ACTGGATTCG AAACCTAAAC   
  
  
+ CCGAAGATCG TAACTTTAGT AGAACAAGAG GCGAACCATA ACCAACCTGA ATTTCTCCCC CGATTCACTG   
  
  
+ AAGCACTATG TTACTACTCA ACTATGTTCG ACTCATTAGA GACTTGTCAA GTCCAACCGC TGAAAGTCCT   
  
  
+ AGCCGAGATG TATATACAAA AGGAGATATC CAACCTAGTG TGCTACGAAG GCTCAGCCCG GGTCCAGAGG   
  
  
+ CACGAGCCGC TCGCCAAATG GCAAGCTCGG ATGGGCCAAG CCGGGTTCAA GCCGCTAGGC CTGAGTCAAA   
  
  
+ ATGCCCTTAG GCAAGCTAAC ATGTTGGTAA CATTGTTCTC CTCACAAGGG TATGACGTGG AGGAGAGAGA   
  
  
+ AGGGTGTTTG ACACTGGGCT GGCATAACCG CCCTCTCATA GTGACTTCGA CTTGGCAAGT GGGGCCCAAA   
  
  
+ ATGGACAGTC CAAGTGTGAC TCATGAGATG TCATCATCCT CAGCTTCTTC ATCTTA  

- +Up\_Stream \_Len000GTAAAA GTTAATCTTT AAAGAAGTTA ATATATGGTA GAGGTTTTTA TCAGGAGTAT   
  
  
- TCAACCGTCC ACTTACTAGG ATTACCTTTT AATGAAAATT GACAACTTAA ACGTTATCCC ATGGCTTAAA   
  
  
- CACTTCAAAT AACGGCGTGC ACGGATCGAA CACTGAAGAG CTATTAGTTT TTAATTAATC TCTCTCTGTC   
  
  
- GATTTCAAAA CTCTTATTAT GACTTTTTAA TGTAATTTAA AATAAATACA AATAAAAGTA TGCGATACAA   
  
  
- ATTGACGATA TGCGACACAA ATTGACGTAG AGGAAGATAT AAACTATTTA ACCATTTTTT TTTATTAAGT   
  
  
- TTTAATTTCT GATCATTATA AAAGTGAATA GACTATCTAA ATATTCTTTA TAAATCTATC GATTTATATA   
  
  
- AGAGAGGACT TTCTCCTCTG ACTATCAACT TAAAGTATCC TATATTTGTA CATACATGTA ACTTGCTTAG   
  
  
- AAAAAAGATA CCACTAGCAA TATTTCAATA AATAATGACA TTTGTAATTC ACTATTAATC CTATACAAGA   
  
  
- TAAAGTGCAA AATCTATCTA TCTATCTACA TTCCTCAACA TTCTTCGACA TTCTTATACA TTCCTCGACA   
  
  
- TTCCTCTACA TTCCTCGACA TTCTTCTACA TTCTCTTTGA CATTCTTCGA TGTTCCTATC TACATTGCTC   
  
  
- AACAACTTAA CAACTTCTCA ACATATCACA TTAAACAAAA TGCTACTTGC AAAACCTACT TTTTATTATT   
  
  
- ATAATTTTAT TATACGAATA ATTTTTATTT ATTTATTAAT TCAATTAATG TGTTGTTATT TTATATAATT   
  
  
- AGGGGTATTT AAATCACTTA TTTTGAAGCA GGTGAACTCT ACTAAAAAAT ATACTTGTGA TCAAACTATC   
  
  
- TTATTTGTTT CATATTTTTT TTTATTTCAA AAAACTGTTT TAACTTATGT TGAGAAGTTT ATTTAAGGAT   
  
  
- AAAATTCATT TATTAAAATT TATATTTATT TAAATACCAG TAGATATTAT TATATATAAA ATACAGCTTT   
  
  
- GAGTTTATAT TAAATGTAGC AATATAATAT AAAAATACAA ATACATTTAC AACACAAGTA TTCTATAAAT   
  
  
- ATTTAAAACA TATAACCATC ATAGTAATTT TTTTTATATG TATAAATACA TTGATTTTAT TTACTTACAA   
  
  
- TATCTTTTAG ATATTCTACA TTCCTCTACA TACTTCTACA TTCCTCTACA TTCTTCTATA TTCCTCTACA   
  
  
- TCTTTTACAT TCTTCTACAC TCTTCGACAT TCCTCTACAT TCAATAAGTG ATTTAGGATT TTTTACGCTT   
  
  
- TATCTTGTCC CCGAATTAAC TCGTGAGTTC TACGTATTAA ACACTGCCTG GTTTACCTAT TTATATCTGG   
  
  
- ACCGTTTACC CAATAAGCTA AATCCATGCT CATACCAATT CAAACCCAAA CCAATACAAA GTCCTATTCA   
  
  
- CTGTAAAGCT GAGTTAAACC AAAACCAACA TACTTGAAGC CGAGCTCTAA AAACCAAACC GGTTATGTGA   
  
  
- ATAGTACGTT TTTATTCATT GAAATATCTT TTAAAACCTA GCCTATACTA CGCCAAGCCT AGTTAAGCCT   
  
  
- AAAACCAAGT GTAAGACCAT TTGGATAAAG CCTACAGTTC ATCCTATACT CAGGTCTAGC AACCTAAAGC   
  
  
- CCATAGTCGA GTTAAAATGG TCTAGATCTA TTTAGTATTT ACATGCCCAA ATAAAAAAGG TCGAACTTAC   
  
  
- TAAAAGAAAT AATTTTTTTC TATCTAAAAT CTATTTTGTG TTGTAAAGTA AAAGTAAACA AGGGGGGATA   
  
  
- TTTAAAGACA CTGGTGGAAG TGAAAGAACG GGGTAAGGGG GGGGGGGGGG CGAGAGAGAG AGAGAGAGAG   
  
  
- AGAAGGGAAA TGTTTCCGTG GTAAGTTTAA GTTTGGTGAG AAAAATTTTG GGGTTTTTAA TAAGAGTTTT   
  
  
- ATTATGGAGT ATTATATTTA CCCAAGCATA GTGGTATATA CCCGGAGTAG AGAGTACCCT TGTATGCTTA   
  
  
- AGTTACCGAA CGGGCTATCA ATAGCCCAGT ACGGTAGCCT CGGGGCCGTT GATTAGTTAC CAGTTCGGAG   
  
  
- TCTAAACCGG AGAAACTCGG TAATATCATT GTGGAAAGAG AGACTTGAGG GGGGAGATGG GTGAGACCCT   
  
  
- GGGCCTAGGC TGGGCCCAGA TAGCACAGGC CCAGGACGAG ACTCAGGTTA CCTCACCTAA CCACTGCGCA   
  
  
- CCTGTGTGGT CTAACTTGTA CTAGACGGTG CAGTCGTCTC AGTTGCCGAC AACTGACTAC CACTGTACCC   
  
  
- CCTCCTCCTA AGGCCATATT CCAACCACGT ACGGAACGAC TGCACGCGTC TCCGGCAGGT TACGCCGCTC   
  
  
- GAGGCTGACC GACGAAGCGA TCAACTACTC TACTGGTTAC CCGACAACGG TGCACAATCA TGCCACACAC   
  
  
- CCTAGCCGTT CCATCGGCCG ATAAAACAAC TCCGAAACTC GGCCGCGGAC AAAGTCGGGC CGGGCCTTTG   
  
  
- ACCGGGCTGG ACCGGCCCGA GCCTCAAGCT CCGACTCCAC AACATAGCAG TGAAGATACT CCGAACGGGA   
  
  
- ATAGAGTTTA AACGAGTGAA GTGCCGATTA GTTCGGTAGA ACCTCCGTAA ACTCCCGGTA CTAATACAGG   
  
  
- TGCAGTAGCT GAAGCCGAAG TACTGGCCGA ACGTTACCGG CCGAAACTAA GTTCGGGACC GAAATTCCGG   
  
  
- GCCACCCGGT GGTAGAGAAT CCGAGTGACC CTAACCGGGC AGAAATCCTT CACCCGGGCT TAGCTATGCA   
  
  
- CTTTAGCCCA ACTCCGATCG GGTTAACCGG GCTAGGTACT TGTACTCCAA GCGTAAAGCC CGACATCGTT   
  
  
- GAAGAGCCGA GCTCCTGCAC TTCGGCAGTT ACGAGCCTTA CCTGGGATCT CTTCGGCAGC GATACTTAAG   
  
  
- GCAGTACGTA GAAGTAGCTG AAAATCCTAA TCTGTAGTTG AGTCAAGATT TGACCTAAGC TTTGGATTTG   
  
  
- GGCTTCTAGC ATTGAAATCA TCTTGTTCTC CGCTTGGTAT TGGTTGGACT TAAAGAGGGG GCTAAGTGAC   
  
  
- TTCGTGATAC AATGATGAGT TGATACAAGC TGAGTAATCT CTGAACAGTT CAGGTTGGCG ACTTTCAGGA   
  
  
- TCGGCTCTAC ATATATGTTT TCCTCTATAG GTTGGATCAC ACGATGCTTC CGAGTCGGGC CCAGGTCTCC   
  
  
- GTGCTCGGCG AGCGGTTTAC CGTTCGAGCC TACCCGGTTC GGCCCAAGTT CGGCGATCCG GACTCAGTTT   
  
  
- TACGGGAATC CGTTCGATTG TACAACCATT GTAACAAGAG GAGTGTTCCC ATACTGCACC TCCTCTCTCT   
  
  
- TCCCACAAAC TGTGACCCGA CCGTATTGGC GGGAGAGTAT CACTGAAGCT GAACCGTTCA CCCCGGGTTT   
  
  
- TACCTGTCAG GTTCACACTG AGTACTCTAC AGTAGTAGGA GTCGAAGAAG TAGAAT

+     TCA

| Site Name | Organism | Position | Strand | Matrix score. | sequence | function |
| --- | --- | --- | --- | --- | --- | --- |
| TCA | Pisum sativum | 1225 | - | 9 | TCATCTTCAT |  |
| TCA | Pisum sativum | 2841 | - | 9 | TCATCTTCAT |  |
| TCA | Pisum sativum | 1801 | + | 9 | TCATCTTCAT |  |
| TCA | Pisum sativum | 2951 | + | 9 | TCATCTTCAT |  |

>HU03G02797.1   
+ +Up\_Stream \_Len000CATTTT CAATTAGAAA TTTCTTCAAT TATATACCAT CTCCAAAAAT AGTCCTCATA   
  
  
+ AGTTGGCAGG TGAATGATCC TAATGGAAAA TTACTTTTAA CTGTTGAATT TGCAATAGGG TACCGAATTT   
  
  
+ GTGAAGTTTA TTGCCGCACG TGCCTAGCTT GTGACTTCTC GATAATCAAA AATTAATTAG AGAGAGACAG   
  
  
+ CTAAAGTTTT GAGAATAATA CTGAAAAATT ACATTAAATT TTATTTATGT TTATTTTCAT ACGCTATGTT   
  
  
+ TAACTGCTAT ACGCTGTGTT TAACTGCATC TCCTTCTATA TTTGATAAAT TGGTAAAAAA AAATAATTCA   
  
  
+ AAATTAAAGA CTAGTAATAT TTTCACTTAT CTGATAGATT TATAAGAAAT ATTTAGATAG CTAAATATAT   
  
  
+ TCTCTCCTGA AAGAGGAGAC TGATAGTTGA ATTTCATAGG ATATAAACAT GTATGTACAT TGAACGAATC   
  
  
+ TTTTTTCTAT GGTGATCGTT ATAAAGTTAT TTATTACTGT AAACATTAAG TGATAATTAG GATATGTTCT   
  
  
+ ATTTCACGTT TTAGATAGAT AGATAGATGT AAGGAGTTGT AAGAAGCTGT AAGAATATGT AAGGAGCTGT   
  
  
+ AAGGAGATGT AAGGAGCTGT AAGAAGATGT AAGAGAAACT GTAAGAAGCT ACAAGGATAG ATGTAACGAG   
  
  
+ TTGTTGAATT GTTGAAGAGT TGTATAGTGT AATTTGTTTT ACGATGAACG TTTTGGATGA AAAATAATAA   
  
  
+ TATTAAAATA ATATGCTTAT TAAAAATAAA TAAATAATTA AGTTAATTAC ACAACAATAA AATATATTAA   
  
  
+ TCCCCATAAA TTTAGTGAAT AAAACTTCGT CCACTTGAGA TGATTTTTTA TATGAACACT AGTTTGATAG   
  
  
+ AATAAACAAA GTATAAAAAA AAATAAAGTT TTTTGACAAA ATTGAATACA ACTCTTCAAA TAAATTCCTA   
  
  
+ TTTTAAGTAA ATAATTTTAA ATATAAATAA ATTTATGGTC ATCTATAATA ATATATATTT TATGTCGAAA   
  
  
+ CTCAAATATA ATTTACATCG TTATATTATA TTTTTATGTT TATGTAAATG TTGTGTTCAT AAGATATTTA   
  
  
+ TAAATTTTGT ATATTGGTAG TATCATTAAA AAAAATATAC ATATTTATGT AACTAAAATA AATGAATGTT   
  
  
+ ATAGAAAATC TATAAGATGT AAGGAGATGT ATGAAGATGT AAGGAGATGT AAGAAGATAT AAGGAGATGT   
  
  
+ AGAAAATGTA AGAAGATGTG AGAAGCTGTA AGGAGATGTA AGTTATTCAC TAAATCCTAA AAAATGCGAA   
  
  
+ ATAGAACAGG GGCTTAATTG AGCACTCAAG ATGCATAATT TGTGACGGAC CAAATGGATA AATATAGACC   
  
  
+ TGGCAAATGG GTTATTCGAT TTAGGTACGA GTATGGTTAA GTTTGGGTTT GGTTATGTTT CAGGATAAGT   
  
  
+ GACATTTCGA CTCAATTTGG TTTTGGTTGT ATGAACTTCG GCTCGAGATT TTTGGTTTGG CCAATACACT   
  
  
+ TATCATGCAA AAATAAGTAA CTTTATAGAA AATTTTGGAT CGGATATGAT GCGGTTCGGA TCAATTCGGA   
  
  
+ TTTTGGTTCA CATTCTGGTA AACCTATTTC GGATGTCAAG TAGGATATGA GTCCAGATCG TTGGATTTCG   
  
  
+ GGTATCAGCT CAATTTTACC AGATCTAGAT AAATCATAAA TGTACGGGTT TATTTTTTCC AGCTTGAATG   
  
  
+ ATTTTCTTTA TTAAAAAAAG ATAGATTTTA GATAAAACAC AACATTTCAT TTTCATTTGT TCCCCCCTAT   
  
  
+ AAATTTCTGT GACCACCTTC ACTTTCTTGC CCCATTCCCC CCCCCCCCCC GCTCTCTCTC TCTCTCTCTC   
  
  
+ TCTTCCCTTT ACAAAGGCAC CATTCAAATT CAAACCACTC TTTTTAAAAC CCCAAAAATT ATTCTCAAAA   
  
  
+ TAATACCTCA TAATATAAAT GGGTTCGTAT CACCATATAT GGGCCTCATC TCTCATGGGA ACATACGAAT   
  
  
+ TCAATGGCTT GCCCGATAGT TATCGGGTCA TGCCATCGGA GCCCCGGCAA CTAATCAATG GTCAAGCCTC   
  
  
+ AGATTTGGCC TCTTTGAGCC ATTATAGTAA CACCTTTCTC TCTGAACTCC CCCCTCTACC CACTCTGGGA   
  
  
+ CCCGGATCCG ACCCGGGTCT ATCGTGTCCG GGTCCTGCTC TGAGTCCAAT GGAGTGGATT GGTGACGCGT   
  
  
+ GGACACACCA GATTGAACAT GATCTGCCAC GTCAGCAGAG TCAACGGCTG TTGACTGATG GTGACATGGG   
  
  
+ GGAGGAGGAT TCCGGTATAA GGTTGGTGCA TGCCTTGCTG ACGTGCGCAG AGGCCGTCCA ATGCGGCGAG   
  
  
+ CTCCGACTGG CTGCTTCGCT AGTTGATGAG ATGACCAATG GGCTGTTGCC ACGTGTTAGT ACGGTGTGTG   
  
  
+ GGATCGGCAA GGTAGCCGGC TATTTTGTTG AGGCTTTGAG CCGGCGCCTG TTTCAGCCCG GCCCGGAAAC   
  
  
+ TGGCCCGACC TGGCCGGGCT CGGAGTTCGA GGCTGAGGTG TTGTATCGTC ACTTCTATGA GGCTTGCCCT   
  
  
+ TATCTCAAAT TTGCTCACTT CACGGCTAAT CAAGCCATCT TGGAGGCATT TGAGGGCCAT GATTATGTCC   
  
  
+ ACGTCATCGA CTTCGGCTTC ATGACCGGCT TGCAATGGCC GGCTTTGATT CAAGCCCTGG CTTTAAGGCC   
  
  
+ CGGTGGGCCA CCATCTCTTA GGCTCACTGG GATTGGCCCG TCTTTAGGAA GTGGGCCCGA ATCGATACGT   
  
  
+ GAAATCGGGT TGAGGCTAGC CCAATTGGCC CGATCCATGA ACATGAGGTT CGCATTTCGG GCTGTAGCAA   
  
  
+ CTTCTCGGCT CGAGGACGTG AAGCCGTCAA TGCTCGGAAT GGACCCTAGA GAAGCCGTCG CTATGAATTC   
  
  
+ CGTCATGCAT CTTCATCGAC TTTTAGGATT AGACATCAAC TCAGTTCTAA ACTGGATTCG AAACCTAAAC   
  
  
+ CCGAAGATCG TAACTTTAGT AGAACAAGAG GCGAACCATA ACCAACCTGA ATTTCTCCCC CGATTCACTG   
  
  
+ AAGCACTATG TTACTACTCA ACTATGTTCG ACTCATTAGA GACTTGTCAA GTCCAACCGC TGAAAGTCCT   
  
  
+ AGCCGAGATG TATATACAAA AGGAGATATC CAACCTAGTG TGCTACGAAG GCTCAGCCCG GGTCCAGAGG   
  
  
+ CACGAGCCGC TCGCCAAATG GCAAGCTCGG ATGGGCCAAG CCGGGTTCAA GCCGCTAGGC CTGAGTCAAA   
  
  
+ ATGCCCTTAG GCAAGCTAAC ATGTTGGTAA CATTGTTCTC CTCACAAGGG TATGACGTGG AGGAGAGAGA   
  
  
+ AGGGTGTTTG ACACTGGGCT GGCATAACCG CCCTCTCATA GTGACTTCGA CTTGGCAAGT GGGGCCCAAA   
  
  
+ ATGGACAGTC CAAGTGTGAC TCATGAGATG TCATCATCCT CAGCTTCTTC ATCTTA  

- +Up\_Stream \_Len000GTAAAA GTTAATCTTT AAAGAAGTTA ATATATGGTA GAGGTTTTTA TCAGGAGTAT   
  
  
- TCAACCGTCC ACTTACTAGG ATTACCTTTT AATGAAAATT GACAACTTAA ACGTTATCCC ATGGCTTAAA   
  
  
- CACTTCAAAT AACGGCGTGC ACGGATCGAA CACTGAAGAG CTATTAGTTT TTAATTAATC TCTCTCTGTC   
  
  
- GATTTCAAAA CTCTTATTAT GACTTTTTAA TGTAATTTAA AATAAATACA AATAAAAGTA TGCGATACAA   
  
  
- ATTGACGATA TGCGACACAA ATTGACGTAG AGGAAGATAT AAACTATTTA ACCATTTTTT TTTATTAAGT   
  
  
- TTTAATTTCT GATCATTATA AAAGTGAATA GACTATCTAA ATATTCTTTA TAAATCTATC GATTTATATA   
  
  
- AGAGAGGACT TTCTCCTCTG ACTATCAACT TAAAGTATCC TATATTTGTA CATACATGTA ACTTGCTTAG   
  
  
- AAAAAAGATA CCACTAGCAA TATTTCAATA AATAATGACA TTTGTAATTC ACTATTAATC CTATACAAGA   
  
  
- TAAAGTGCAA AATCTATCTA TCTATCTACA TTCCTCAACA TTCTTCGACA TTCTTATACA TTCCTCGACA   
  
  
- TTCCTCTACA TTCCTCGACA TTCTTCTACA TTCTCTTTGA CATTCTTCGA TGTTCCTATC TACATTGCTC   
  
  
- AACAACTTAA CAACTTCTCA ACATATCACA TTAAACAAAA TGCTACTTGC AAAACCTACT TTTTATTATT   
  
  
- ATAATTTTAT TATACGAATA ATTTTTATTT ATTTATTAAT TCAATTAATG TGTTGTTATT TTATATAATT   
  
  
- AGGGGTATTT AAATCACTTA TTTTGAAGCA GGTGAACTCT ACTAAAAAAT ATACTTGTGA TCAAACTATC   
  
  
- TTATTTGTTT CATATTTTTT TTTATTTCAA AAAACTGTTT TAACTTATGT TGAGAAGTTT ATTTAAGGAT   
  
  
- AAAATTCATT TATTAAAATT TATATTTATT TAAATACCAG TAGATATTAT TATATATAAA ATACAGCTTT   
  
  
- GAGTTTATAT TAAATGTAGC AATATAATAT AAAAATACAA ATACATTTAC AACACAAGTA TTCTATAAAT   
  
  
- ATTTAAAACA TATAACCATC ATAGTAATTT TTTTTATATG TATAAATACA TTGATTTTAT TTACTTACAA   
  
  
- TATCTTTTAG ATATTCTACA TTCCTCTACA TACTTCTACA TTCCTCTACA TTCTTCTATA TTCCTCTACA   
  
  
- TCTTTTACAT TCTTCTACAC TCTTCGACAT TCCTCTACAT TCAATAAGTG ATTTAGGATT TTTTACGCTT   
  
  
- TATCTTGTCC CCGAATTAAC TCGTGAGTTC TACGTATTAA ACACTGCCTG GTTTACCTAT TTATATCTGG   
  
  
- ACCGTTTACC CAATAAGCTA AATCCATGCT CATACCAATT CAAACCCAAA CCAATACAAA GTCCTATTCA   
  
  
- CTGTAAAGCT GAGTTAAACC AAAACCAACA TACTTGAAGC CGAGCTCTAA AAACCAAACC GGTTATGTGA   
  
  
- ATAGTACGTT TTTATTCATT GAAATATCTT TTAAAACCTA GCCTATACTA CGCCAAGCCT AGTTAAGCCT   
  
  
- AAAACCAAGT GTAAGACCAT TTGGATAAAG CCTACAGTTC ATCCTATACT CAGGTCTAGC AACCTAAAGC   
  
  
- CCATAGTCGA GTTAAAATGG TCTAGATCTA TTTAGTATTT ACATGCCCAA ATAAAAAAGG TCGAACTTAC   
  
  
- TAAAAGAAAT AATTTTTTTC TATCTAAAAT CTATTTTGTG TTGTAAAGTA AAAGTAAACA AGGGGGGATA   
  
  
- TTTAAAGACA CTGGTGGAAG TGAAAGAACG GGGTAAGGGG GGGGGGGGGG CGAGAGAGAG AGAGAGAGAG   
  
  
- AGAAGGGAAA TGTTTCCGTG GTAAGTTTAA GTTTGGTGAG AAAAATTTTG GGGTTTTTAA TAAGAGTTTT   
  
  
- ATTATGGAGT ATTATATTTA CCCAAGCATA GTGGTATATA CCCGGAGTAG AGAGTACCCT TGTATGCTTA   
  
  
- AGTTACCGAA CGGGCTATCA ATAGCCCAGT ACGGTAGCCT CGGGGCCGTT GATTAGTTAC CAGTTCGGAG   
  
  
- TCTAAACCGG AGAAACTCGG TAATATCATT GTGGAAAGAG AGACTTGAGG GGGGAGATGG GTGAGACCCT   
  
  
- GGGCCTAGGC TGGGCCCAGA TAGCACAGGC CCAGGACGAG ACTCAGGTTA CCTCACCTAA CCACTGCGCA   
  
  
- CCTGTGTGGT CTAACTTGTA CTAGACGGTG CAGTCGTCTC AGTTGCCGAC AACTGACTAC CACTGTACCC   
  
  
- CCTCCTCCTA AGGCCATATT CCAACCACGT ACGGAACGAC TGCACGCGTC TCCGGCAGGT TACGCCGCTC   
  
  
- GAGGCTGACC GACGAAGCGA TCAACTACTC TACTGGTTAC CCGACAACGG TGCACAATCA TGCCACACAC   
  
  
- CCTAGCCGTT CCATCGGCCG ATAAAACAAC TCCGAAACTC GGCCGCGGAC AAAGTCGGGC CGGGCCTTTG   
  
  
- ACCGGGCTGG ACCGGCCCGA GCCTCAAGCT CCGACTCCAC AACATAGCAG TGAAGATACT CCGAACGGGA   
  
  
- ATAGAGTTTA AACGAGTGAA GTGCCGATTA GTTCGGTAGA ACCTCCGTAA ACTCCCGGTA CTAATACAGG   
  
  
- TGCAGTAGCT GAAGCCGAAG TACTGGCCGA ACGTTACCGG CCGAAACTAA GTTCGGGACC GAAATTCCGG   
  
  
- GCCACCCGGT GGTAGAGAAT CCGAGTGACC CTAACCGGGC AGAAATCCTT CACCCGGGCT TAGCTATGCA   
  
  
- CTTTAGCCCA ACTCCGATCG GGTTAACCGG GCTAGGTACT TGTACTCCAA GCGTAAAGCC CGACATCGTT   
  
  
- GAAGAGCCGA GCTCCTGCAC TTCGGCAGTT ACGAGCCTTA CCTGGGATCT CTTCGGCAGC GATACTTAAG   
  
  
- GCAGTACGTA GAAGTAGCTG AAAATCCTAA TCTGTAGTTG AGTCAAGATT TGACCTAAGC TTTGGATTTG   
  
  
- GGCTTCTAGC ATTGAAATCA TCTTGTTCTC CGCTTGGTAT TGGTTGGACT TAAAGAGGGG GCTAAGTGAC   
  
  
- TTCGTGATAC AATGATGAGT TGATACAAGC TGAGTAATCT CTGAACAGTT CAGGTTGGCG ACTTTCAGGA   
  
  
- TCGGCTCTAC ATATATGTTT TCCTCTATAG GTTGGATCAC ACGATGCTTC CGAGTCGGGC CCAGGTCTCC   
  
  
- GTGCTCGGCG AGCGGTTTAC CGTTCGAGCC TACCCGGTTC GGCCCAAGTT CGGCGATCCG GACTCAGTTT   
  
  
- TACGGGAATC CGTTCGATTG TACAACCATT GTAACAAGAG GAGTGTTCCC ATACTGCACC TCCTCTCTCT   
  
  
- TCCCACAAAC TGTGACCCGA CCGTATTGGC GGGAGAGTAT CACTGAAGCT GAACCGTTCA CCCCGGGTTT   
  
  
- TACCTGTCAG GTTCACACTG AGTACTCTAC AGTAGTAGGA GTCGAAGAAG TAGAAT

+     TCA-element

| Site Name | Organism | Position | Strand | Matrix score. | sequence | function |
| --- | --- | --- | --- | --- | --- | --- |
| TCA-element | Nicotiana tabacum | 1769 | - | 9 | CCATCTTTTT | cis-acting element involved in salicylic acid responsiveness |

>HU03G02797.1   
+ +Up\_Stream \_Len000CATTTT CAATTAGAAA TTTCTTCAAT TATATACCAT CTCCAAAAAT AGTCCTCATA   
  
  
+ AGTTGGCAGG TGAATGATCC TAATGGAAAA TTACTTTTAA CTGTTGAATT TGCAATAGGG TACCGAATTT   
  
  
+ GTGAAGTTTA TTGCCGCACG TGCCTAGCTT GTGACTTCTC GATAATCAAA AATTAATTAG AGAGAGACAG   
  
  
+ CTAAAGTTTT GAGAATAATA CTGAAAAATT ACATTAAATT TTATTTATGT TTATTTTCAT ACGCTATGTT   
  
  
+ TAACTGCTAT ACGCTGTGTT TAACTGCATC TCCTTCTATA TTTGATAAAT TGGTAAAAAA AAATAATTCA   
  
  
+ AAATTAAAGA CTAGTAATAT TTTCACTTAT CTGATAGATT TATAAGAAAT ATTTAGATAG CTAAATATAT   
  
  
+ TCTCTCCTGA AAGAGGAGAC TGATAGTTGA ATTTCATAGG ATATAAACAT GTATGTACAT TGAACGAATC   
  
  
+ TTTTTTCTAT GGTGATCGTT ATAAAGTTAT TTATTACTGT AAACATTAAG TGATAATTAG GATATGTTCT   
  
  
+ ATTTCACGTT TTAGATAGAT AGATAGATGT AAGGAGTTGT AAGAAGCTGT AAGAATATGT AAGGAGCTGT   
  
  
+ AAGGAGATGT AAGGAGCTGT AAGAAGATGT AAGAGAAACT GTAAGAAGCT ACAAGGATAG ATGTAACGAG   
  
  
+ TTGTTGAATT GTTGAAGAGT TGTATAGTGT AATTTGTTTT ACGATGAACG TTTTGGATGA AAAATAATAA   
  
  
+ TATTAAAATA ATATGCTTAT TAAAAATAAA TAAATAATTA AGTTAATTAC ACAACAATAA AATATATTAA   
  
  
+ TCCCCATAAA TTTAGTGAAT AAAACTTCGT CCACTTGAGA TGATTTTTTA TATGAACACT AGTTTGATAG   
  
  
+ AATAAACAAA GTATAAAAAA AAATAAAGTT TTTTGACAAA ATTGAATACA ACTCTTCAAA TAAATTCCTA   
  
  
+ TTTTAAGTAA ATAATTTTAA ATATAAATAA ATTTATGGTC ATCTATAATA ATATATATTT TATGTCGAAA   
  
  
+ CTCAAATATA ATTTACATCG TTATATTATA TTTTTATGTT TATGTAAATG TTGTGTTCAT AAGATATTTA   
  
  
+ TAAATTTTGT ATATTGGTAG TATCATTAAA AAAAATATAC ATATTTATGT AACTAAAATA AATGAATGTT   
  
  
+ ATAGAAAATC TATAAGATGT AAGGAGATGT ATGAAGATGT AAGGAGATGT AAGAAGATAT AAGGAGATGT   
  
  
+ AGAAAATGTA AGAAGATGTG AGAAGCTGTA AGGAGATGTA AGTTATTCAC TAAATCCTAA AAAATGCGAA   
  
  
+ ATAGAACAGG GGCTTAATTG AGCACTCAAG ATGCATAATT TGTGACGGAC CAAATGGATA AATATAGACC   
  
  
+ TGGCAAATGG GTTATTCGAT TTAGGTACGA GTATGGTTAA GTTTGGGTTT GGTTATGTTT CAGGATAAGT   
  
  
+ GACATTTCGA CTCAATTTGG TTTTGGTTGT ATGAACTTCG GCTCGAGATT TTTGGTTTGG CCAATACACT   
  
  
+ TATCATGCAA AAATAAGTAA CTTTATAGAA AATTTTGGAT CGGATATGAT GCGGTTCGGA TCAATTCGGA   
  
  
+ TTTTGGTTCA CATTCTGGTA AACCTATTTC GGATGTCAAG TAGGATATGA GTCCAGATCG TTGGATTTCG   
  
  
+ GGTATCAGCT CAATTTTACC AGATCTAGAT AAATCATAAA TGTACGGGTT TATTTTTTCC AGCTTGAATG   
  
  
+ ATTTTCTTTA TTAAAAAAAG ATAGATTTTA GATAAAACAC AACATTTCAT TTTCATTTGT TCCCCCCTAT   
  
  
+ AAATTTCTGT GACCACCTTC ACTTTCTTGC CCCATTCCCC CCCCCCCCCC GCTCTCTCTC TCTCTCTCTC   
  
  
+ TCTTCCCTTT ACAAAGGCAC CATTCAAATT CAAACCACTC TTTTTAAAAC CCCAAAAATT ATTCTCAAAA   
  
  
+ TAATACCTCA TAATATAAAT GGGTTCGTAT CACCATATAT GGGCCTCATC TCTCATGGGA ACATACGAAT   
  
  
+ TCAATGGCTT GCCCGATAGT TATCGGGTCA TGCCATCGGA GCCCCGGCAA CTAATCAATG GTCAAGCCTC   
  
  
+ AGATTTGGCC TCTTTGAGCC ATTATAGTAA CACCTTTCTC TCTGAACTCC CCCCTCTACC CACTCTGGGA   
  
  
+ CCCGGATCCG ACCCGGGTCT ATCGTGTCCG GGTCCTGCTC TGAGTCCAAT GGAGTGGATT GGTGACGCGT   
  
  
+ GGACACACCA GATTGAACAT GATCTGCCAC GTCAGCAGAG TCAACGGCTG TTGACTGATG GTGACATGGG   
  
  
+ GGAGGAGGAT TCCGGTATAA GGTTGGTGCA TGCCTTGCTG ACGTGCGCAG AGGCCGTCCA ATGCGGCGAG   
  
  
+ CTCCGACTGG CTGCTTCGCT AGTTGATGAG ATGACCAATG GGCTGTTGCC ACGTGTTAGT ACGGTGTGTG   
  
  
+ GGATCGGCAA GGTAGCCGGC TATTTTGTTG AGGCTTTGAG CCGGCGCCTG TTTCAGCCCG GCCCGGAAAC   
  
  
+ TGGCCCGACC TGGCCGGGCT CGGAGTTCGA GGCTGAGGTG TTGTATCGTC ACTTCTATGA GGCTTGCCCT   
  
  
+ TATCTCAAAT TTGCTCACTT CACGGCTAAT CAAGCCATCT TGGAGGCATT TGAGGGCCAT GATTATGTCC   
  
  
+ ACGTCATCGA CTTCGGCTTC ATGACCGGCT TGCAATGGCC GGCTTTGATT CAAGCCCTGG CTTTAAGGCC   
  
  
+ CGGTGGGCCA CCATCTCTTA GGCTCACTGG GATTGGCCCG TCTTTAGGAA GTGGGCCCGA ATCGATACGT   
  
  
+ GAAATCGGGT TGAGGCTAGC CCAATTGGCC CGATCCATGA ACATGAGGTT CGCATTTCGG GCTGTAGCAA   
  
  
+ CTTCTCGGCT CGAGGACGTG AAGCCGTCAA TGCTCGGAAT GGACCCTAGA GAAGCCGTCG CTATGAATTC   
  
  
+ CGTCATGCAT CTTCATCGAC TTTTAGGATT AGACATCAAC TCAGTTCTAA ACTGGATTCG AAACCTAAAC   
  
  
+ CCGAAGATCG TAACTTTAGT AGAACAAGAG GCGAACCATA ACCAACCTGA ATTTCTCCCC CGATTCACTG   
  
  
+ AAGCACTATG TTACTACTCA ACTATGTTCG ACTCATTAGA GACTTGTCAA GTCCAACCGC TGAAAGTCCT   
  
  
+ AGCCGAGATG TATATACAAA AGGAGATATC CAACCTAGTG TGCTACGAAG GCTCAGCCCG GGTCCAGAGG   
  
  
+ CACGAGCCGC TCGCCAAATG GCAAGCTCGG ATGGGCCAAG CCGGGTTCAA GCCGCTAGGC CTGAGTCAAA   
  
  
+ ATGCCCTTAG GCAAGCTAAC ATGTTGGTAA CATTGTTCTC CTCACAAGGG TATGACGTGG AGGAGAGAGA   
  
  
+ AGGGTGTTTG ACACTGGGCT GGCATAACCG CCCTCTCATA GTGACTTCGA CTTGGCAAGT GGGGCCCAAA   
  
  
+ ATGGACAGTC CAAGTGTGAC TCATGAGATG TCATCATCCT CAGCTTCTTC ATCTTA  

- +Up\_Stream \_Len000GTAAAA GTTAATCTTT AAAGAAGTTA ATATATGGTA GAGGTTTTTA TCAGGAGTAT   
  
  
- TCAACCGTCC ACTTACTAGG ATTACCTTTT AATGAAAATT GACAACTTAA ACGTTATCCC ATGGCTTAAA   
  
  
- CACTTCAAAT AACGGCGTGC ACGGATCGAA CACTGAAGAG CTATTAGTTT TTAATTAATC TCTCTCTGTC   
  
  
- GATTTCAAAA CTCTTATTAT GACTTTTTAA TGTAATTTAA AATAAATACA AATAAAAGTA TGCGATACAA   
  
  
- ATTGACGATA TGCGACACAA ATTGACGTAG AGGAAGATAT AAACTATTTA ACCATTTTTT TTTATTAAGT   
  
  
- TTTAATTTCT GATCATTATA AAAGTGAATA GACTATCTAA ATATTCTTTA TAAATCTATC GATTTATATA   
  
  
- AGAGAGGACT TTCTCCTCTG ACTATCAACT TAAAGTATCC TATATTTGTA CATACATGTA ACTTGCTTAG   
  
  
- AAAAAAGATA CCACTAGCAA TATTTCAATA AATAATGACA TTTGTAATTC ACTATTAATC CTATACAAGA   
  
  
- TAAAGTGCAA AATCTATCTA TCTATCTACA TTCCTCAACA TTCTTCGACA TTCTTATACA TTCCTCGACA   
  
  
- TTCCTCTACA TTCCTCGACA TTCTTCTACA TTCTCTTTGA CATTCTTCGA TGTTCCTATC TACATTGCTC   
  
  
- AACAACTTAA CAACTTCTCA ACATATCACA TTAAACAAAA TGCTACTTGC AAAACCTACT TTTTATTATT   
  
  
- ATAATTTTAT TATACGAATA ATTTTTATTT ATTTATTAAT TCAATTAATG TGTTGTTATT TTATATAATT   
  
  
- AGGGGTATTT AAATCACTTA TTTTGAAGCA GGTGAACTCT ACTAAAAAAT ATACTTGTGA TCAAACTATC   
  
  
- TTATTTGTTT CATATTTTTT TTTATTTCAA AAAACTGTTT TAACTTATGT TGAGAAGTTT ATTTAAGGAT   
  
  
- AAAATTCATT TATTAAAATT TATATTTATT TAAATACCAG TAGATATTAT TATATATAAA ATACAGCTTT   
  
  
- GAGTTTATAT TAAATGTAGC AATATAATAT AAAAATACAA ATACATTTAC AACACAAGTA TTCTATAAAT   
  
  
- ATTTAAAACA TATAACCATC ATAGTAATTT TTTTTATATG TATAAATACA TTGATTTTAT TTACTTACAA   
  
  
- TATCTTTTAG ATATTCTACA TTCCTCTACA TACTTCTACA TTCCTCTACA TTCTTCTATA TTCCTCTACA   
  
  
- TCTTTTACAT TCTTCTACAC TCTTCGACAT TCCTCTACAT TCAATAAGTG ATTTAGGATT TTTTACGCTT   
  
  
- TATCTTGTCC CCGAATTAAC TCGTGAGTTC TACGTATTAA ACACTGCCTG GTTTACCTAT TTATATCTGG   
  
  
- ACCGTTTACC CAATAAGCTA AATCCATGCT CATACCAATT CAAACCCAAA CCAATACAAA GTCCTATTCA   
  
  
- CTGTAAAGCT GAGTTAAACC AAAACCAACA TACTTGAAGC CGAGCTCTAA AAACCAAACC GGTTATGTGA   
  
  
- ATAGTACGTT TTTATTCATT GAAATATCTT TTAAAACCTA GCCTATACTA CGCCAAGCCT AGTTAAGCCT   
  
  
- AAAACCAAGT GTAAGACCAT TTGGATAAAG CCTACAGTTC ATCCTATACT CAGGTCTAGC AACCTAAAGC   
  
  
- CCATAGTCGA GTTAAAATGG TCTAGATCTA TTTAGTATTT ACATGCCCAA ATAAAAAAGG TCGAACTTAC   
  
  
- TAAAAGAAAT AATTTTTTTC TATCTAAAAT CTATTTTGTG TTGTAAAGTA AAAGTAAACA AGGGGGGATA   
  
  
- TTTAAAGACA CTGGTGGAAG TGAAAGAACG GGGTAAGGGG GGGGGGGGGG CGAGAGAGAG AGAGAGAGAG   
  
  
- AGAAGGGAAA TGTTTCCGTG GTAAGTTTAA GTTTGGTGAG AAAAATTTTG GGGTTTTTAA TAAGAGTTTT   
  
  
- ATTATGGAGT ATTATATTTA CCCAAGCATA GTGGTATATA CCCGGAGTAG AGAGTACCCT TGTATGCTTA   
  
  
- AGTTACCGAA CGGGCTATCA ATAGCCCAGT ACGGTAGCCT CGGGGCCGTT GATTAGTTAC CAGTTCGGAG   
  
  
- TCTAAACCGG AGAAACTCGG TAATATCATT GTGGAAAGAG AGACTTGAGG GGGGAGATGG GTGAGACCCT   
  
  
- GGGCCTAGGC TGGGCCCAGA TAGCACAGGC CCAGGACGAG ACTCAGGTTA CCTCACCTAA CCACTGCGCA   
  
  
- CCTGTGTGGT CTAACTTGTA CTAGACGGTG CAGTCGTCTC AGTTGCCGAC AACTGACTAC CACTGTACCC   
  
  
- CCTCCTCCTA AGGCCATATT CCAACCACGT ACGGAACGAC TGCACGCGTC TCCGGCAGGT TACGCCGCTC   
  
  
- GAGGCTGACC GACGAAGCGA TCAACTACTC TACTGGTTAC CCGACAACGG TGCACAATCA TGCCACACAC   
  
  
- CCTAGCCGTT CCATCGGCCG ATAAAACAAC TCCGAAACTC GGCCGCGGAC AAAGTCGGGC CGGGCCTTTG   
  
  
- ACCGGGCTGG ACCGGCCCGA GCCTCAAGCT CCGACTCCAC AACATAGCAG TGAAGATACT CCGAACGGGA   
  
  
- ATAGAGTTTA AACGAGTGAA GTGCCGATTA GTTCGGTAGA ACCTCCGTAA ACTCCCGGTA CTAATACAGG   
  
  
- TGCAGTAGCT GAAGCCGAAG TACTGGCCGA ACGTTACCGG CCGAAACTAA GTTCGGGACC GAAATTCCGG   
  
  
- GCCACCCGGT GGTAGAGAAT CCGAGTGACC CTAACCGGGC AGAAATCCTT CACCCGGGCT TAGCTATGCA   
  
  
- CTTTAGCCCA ACTCCGATCG GGTTAACCGG GCTAGGTACT TGTACTCCAA GCGTAAAGCC CGACATCGTT   
  
  
- GAAGAGCCGA GCTCCTGCAC TTCGGCAGTT ACGAGCCTTA CCTGGGATCT CTTCGGCAGC GATACTTAAG   
  
  
- GCAGTACGTA GAAGTAGCTG AAAATCCTAA TCTGTAGTTG AGTCAAGATT TGACCTAAGC TTTGGATTTG   
  
  
- GGCTTCTAGC ATTGAAATCA TCTTGTTCTC CGCTTGGTAT TGGTTGGACT TAAAGAGGGG GCTAAGTGAC   
  
  
- TTCGTGATAC AATGATGAGT TGATACAAGC TGAGTAATCT CTGAACAGTT CAGGTTGGCG ACTTTCAGGA   
  
  
- TCGGCTCTAC ATATATGTTT TCCTCTATAG GTTGGATCAC ACGATGCTTC CGAGTCGGGC CCAGGTCTCC   
  
  
- GTGCTCGGCG AGCGGTTTAC CGTTCGAGCC TACCCGGTTC GGCCCAAGTT CGGCGATCCG GACTCAGTTT   
  
  
- TACGGGAATC CGTTCGATTG TACAACCATT GTAACAAGAG GAGTGTTCCC ATACTGCACC TCCTCTCTCT   
  
  
- TCCCACAAAC TGTGACCCGA CCGTATTGGC GGGAGAGTAT CACTGAAGCT GAACCGTTCA CCCCGGGTTT   
  
  
- TACCTGTCAG GTTCACACTG AGTACTCTAC AGTAGTAGGA GTCGAAGAAG TAGAAT

+     TCT-motif

| Site Name | Organism | Position | Strand | Matrix score. | sequence | function |
| --- | --- | --- | --- | --- | --- | --- |
| TCT-motif | Arabidopsis thaliana | 1272 | - | 6 | TCTTAC | part of a light responsive element |
| TCT-motif | Arabidopsis thaliana | 653 | - | 6 | TCTTAC | part of a light responsive element |
| TCT-motif | Arabidopsis thaliana | 663 | - | 6 | TCTTAC | part of a light responsive element |
| TCT-motif | Arabidopsis thaliana | 1243 | - | 6 | TCTTAC | part of a light responsive element |
| TCT-motif | Arabidopsis thaliana | 675 | - | 6 | TCTTAC | part of a light responsive element |
| TCT-motif | Arabidopsis thaliana | 613 | - | 6 | TCTTAC | part of a light responsive element |
| TCT-motif | Arabidopsis thaliana | 603 | - | 6 | TCTTAC | part of a light responsive element |

>HU03G02797.1   
+ +Up\_Stream \_Len000CATTTT CAATTAGAAA TTTCTTCAAT TATATACCAT CTCCAAAAAT AGTCCTCATA   
  
  
+ AGTTGGCAGG TGAATGATCC TAATGGAAAA TTACTTTTAA CTGTTGAATT TGCAATAGGG TACCGAATTT   
  
  
+ GTGAAGTTTA TTGCCGCACG TGCCTAGCTT GTGACTTCTC GATAATCAAA AATTAATTAG AGAGAGACAG   
  
  
+ CTAAAGTTTT GAGAATAATA CTGAAAAATT ACATTAAATT TTATTTATGT TTATTTTCAT ACGCTATGTT   
  
  
+ TAACTGCTAT ACGCTGTGTT TAACTGCATC TCCTTCTATA TTTGATAAAT TGGTAAAAAA AAATAATTCA   
  
  
+ AAATTAAAGA CTAGTAATAT TTTCACTTAT CTGATAGATT TATAAGAAAT ATTTAGATAG CTAAATATAT   
  
  
+ TCTCTCCTGA AAGAGGAGAC TGATAGTTGA ATTTCATAGG ATATAAACAT GTATGTACAT TGAACGAATC   
  
  
+ TTTTTTCTAT GGTGATCGTT ATAAAGTTAT TTATTACTGT AAACATTAAG TGATAATTAG GATATGTTCT   
  
  
+ ATTTCACGTT TTAGATAGAT AGATAGATGT AAGGAGTTGT AAGAAGCTGT AAGAATATGT AAGGAGCTGT   
  
  
+ AAGGAGATGT AAGGAGCTGT AAGAAGATGT AAGAGAAACT GTAAGAAGCT ACAAGGATAG ATGTAACGAG   
  
  
+ TTGTTGAATT GTTGAAGAGT TGTATAGTGT AATTTGTTTT ACGATGAACG TTTTGGATGA AAAATAATAA   
  
  
+ TATTAAAATA ATATGCTTAT TAAAAATAAA TAAATAATTA AGTTAATTAC ACAACAATAA AATATATTAA   
  
  
+ TCCCCATAAA TTTAGTGAAT AAAACTTCGT CCACTTGAGA TGATTTTTTA TATGAACACT AGTTTGATAG   
  
  
+ AATAAACAAA GTATAAAAAA AAATAAAGTT TTTTGACAAA ATTGAATACA ACTCTTCAAA TAAATTCCTA   
  
  
+ TTTTAAGTAA ATAATTTTAA ATATAAATAA ATTTATGGTC ATCTATAATA ATATATATTT TATGTCGAAA   
  
  
+ CTCAAATATA ATTTACATCG TTATATTATA TTTTTATGTT TATGTAAATG TTGTGTTCAT AAGATATTTA   
  
  
+ TAAATTTTGT ATATTGGTAG TATCATTAAA AAAAATATAC ATATTTATGT AACTAAAATA AATGAATGTT   
  
  
+ ATAGAAAATC TATAAGATGT AAGGAGATGT ATGAAGATGT AAGGAGATGT AAGAAGATAT AAGGAGATGT   
  
  
+ AGAAAATGTA AGAAGATGTG AGAAGCTGTA AGGAGATGTA AGTTATTCAC TAAATCCTAA AAAATGCGAA   
  
  
+ ATAGAACAGG GGCTTAATTG AGCACTCAAG ATGCATAATT TGTGACGGAC CAAATGGATA AATATAGACC   
  
  
+ TGGCAAATGG GTTATTCGAT TTAGGTACGA GTATGGTTAA GTTTGGGTTT GGTTATGTTT CAGGATAAGT   
  
  
+ GACATTTCGA CTCAATTTGG TTTTGGTTGT ATGAACTTCG GCTCGAGATT TTTGGTTTGG CCAATACACT   
  
  
+ TATCATGCAA AAATAAGTAA CTTTATAGAA AATTTTGGAT CGGATATGAT GCGGTTCGGA TCAATTCGGA   
  
  
+ TTTTGGTTCA CATTCTGGTA AACCTATTTC GGATGTCAAG TAGGATATGA GTCCAGATCG TTGGATTTCG   
  
  
+ GGTATCAGCT CAATTTTACC AGATCTAGAT AAATCATAAA TGTACGGGTT TATTTTTTCC AGCTTGAATG   
  
  
+ ATTTTCTTTA TTAAAAAAAG ATAGATTTTA GATAAAACAC AACATTTCAT TTTCATTTGT TCCCCCCTAT   
  
  
+ AAATTTCTGT GACCACCTTC ACTTTCTTGC CCCATTCCCC CCCCCCCCCC GCTCTCTCTC TCTCTCTCTC   
  
  
+ TCTTCCCTTT ACAAAGGCAC CATTCAAATT CAAACCACTC TTTTTAAAAC CCCAAAAATT ATTCTCAAAA   
  
  
+ TAATACCTCA TAATATAAAT GGGTTCGTAT CACCATATAT GGGCCTCATC TCTCATGGGA ACATACGAAT   
  
  
+ TCAATGGCTT GCCCGATAGT TATCGGGTCA TGCCATCGGA GCCCCGGCAA CTAATCAATG GTCAAGCCTC   
  
  
+ AGATTTGGCC TCTTTGAGCC ATTATAGTAA CACCTTTCTC TCTGAACTCC CCCCTCTACC CACTCTGGGA   
  
  
+ CCCGGATCCG ACCCGGGTCT ATCGTGTCCG GGTCCTGCTC TGAGTCCAAT GGAGTGGATT GGTGACGCGT   
  
  
+ GGACACACCA GATTGAACAT GATCTGCCAC GTCAGCAGAG TCAACGGCTG TTGACTGATG GTGACATGGG   
  
  
+ GGAGGAGGAT TCCGGTATAA GGTTGGTGCA TGCCTTGCTG ACGTGCGCAG AGGCCGTCCA ATGCGGCGAG   
  
  
+ CTCCGACTGG CTGCTTCGCT AGTTGATGAG ATGACCAATG GGCTGTTGCC ACGTGTTAGT ACGGTGTGTG   
  
  
+ GGATCGGCAA GGTAGCCGGC TATTTTGTTG AGGCTTTGAG CCGGCGCCTG TTTCAGCCCG GCCCGGAAAC   
  
  
+ TGGCCCGACC TGGCCGGGCT CGGAGTTCGA GGCTGAGGTG TTGTATCGTC ACTTCTATGA GGCTTGCCCT   
  
  
+ TATCTCAAAT TTGCTCACTT CACGGCTAAT CAAGCCATCT TGGAGGCATT TGAGGGCCAT GATTATGTCC   
  
  
+ ACGTCATCGA CTTCGGCTTC ATGACCGGCT TGCAATGGCC GGCTTTGATT CAAGCCCTGG CTTTAAGGCC   
  
  
+ CGGTGGGCCA CCATCTCTTA GGCTCACTGG GATTGGCCCG TCTTTAGGAA GTGGGCCCGA ATCGATACGT   
  
  
+ GAAATCGGGT TGAGGCTAGC CCAATTGGCC CGATCCATGA ACATGAGGTT CGCATTTCGG GCTGTAGCAA   
  
  
+ CTTCTCGGCT CGAGGACGTG AAGCCGTCAA TGCTCGGAAT GGACCCTAGA GAAGCCGTCG CTATGAATTC   
  
  
+ CGTCATGCAT CTTCATCGAC TTTTAGGATT AGACATCAAC TCAGTTCTAA ACTGGATTCG AAACCTAAAC   
  
  
+ CCGAAGATCG TAACTTTAGT AGAACAAGAG GCGAACCATA ACCAACCTGA ATTTCTCCCC CGATTCACTG   
  
  
+ AAGCACTATG TTACTACTCA ACTATGTTCG ACTCATTAGA GACTTGTCAA GTCCAACCGC TGAAAGTCCT   
  
  
+ AGCCGAGATG TATATACAAA AGGAGATATC CAACCTAGTG TGCTACGAAG GCTCAGCCCG GGTCCAGAGG   
  
  
+ CACGAGCCGC TCGCCAAATG GCAAGCTCGG ATGGGCCAAG CCGGGTTCAA GCCGCTAGGC CTGAGTCAAA   
  
  
+ ATGCCCTTAG GCAAGCTAAC ATGTTGGTAA CATTGTTCTC CTCACAAGGG TATGACGTGG AGGAGAGAGA   
  
  
+ AGGGTGTTTG ACACTGGGCT GGCATAACCG CCCTCTCATA GTGACTTCGA CTTGGCAAGT GGGGCCCAAA   
  
  
+ ATGGACAGTC CAAGTGTGAC TCATGAGATG TCATCATCCT CAGCTTCTTC ATCTTA  

- +Up\_Stream \_Len000GTAAAA GTTAATCTTT AAAGAAGTTA ATATATGGTA GAGGTTTTTA TCAGGAGTAT   
  
  
- TCAACCGTCC ACTTACTAGG ATTACCTTTT AATGAAAATT GACAACTTAA ACGTTATCCC ATGGCTTAAA   
  
  
- CACTTCAAAT AACGGCGTGC ACGGATCGAA CACTGAAGAG CTATTAGTTT TTAATTAATC TCTCTCTGTC   
  
  
- GATTTCAAAA CTCTTATTAT GACTTTTTAA TGTAATTTAA AATAAATACA AATAAAAGTA TGCGATACAA   
  
  
- ATTGACGATA TGCGACACAA ATTGACGTAG AGGAAGATAT AAACTATTTA ACCATTTTTT TTTATTAAGT   
  
  
- TTTAATTTCT GATCATTATA AAAGTGAATA GACTATCTAA ATATTCTTTA TAAATCTATC GATTTATATA   
  
  
- AGAGAGGACT TTCTCCTCTG ACTATCAACT TAAAGTATCC TATATTTGTA CATACATGTA ACTTGCTTAG   
  
  
- AAAAAAGATA CCACTAGCAA TATTTCAATA AATAATGACA TTTGTAATTC ACTATTAATC CTATACAAGA   
  
  
- TAAAGTGCAA AATCTATCTA TCTATCTACA TTCCTCAACA TTCTTCGACA TTCTTATACA TTCCTCGACA   
  
  
- TTCCTCTACA TTCCTCGACA TTCTTCTACA TTCTCTTTGA CATTCTTCGA TGTTCCTATC TACATTGCTC   
  
  
- AACAACTTAA CAACTTCTCA ACATATCACA TTAAACAAAA TGCTACTTGC AAAACCTACT TTTTATTATT   
  
  
- ATAATTTTAT TATACGAATA ATTTTTATTT ATTTATTAAT TCAATTAATG TGTTGTTATT TTATATAATT   
  
  
- AGGGGTATTT AAATCACTTA TTTTGAAGCA GGTGAACTCT ACTAAAAAAT ATACTTGTGA TCAAACTATC   
  
  
- TTATTTGTTT CATATTTTTT TTTATTTCAA AAAACTGTTT TAACTTATGT TGAGAAGTTT ATTTAAGGAT   
  
  
- AAAATTCATT TATTAAAATT TATATTTATT TAAATACCAG TAGATATTAT TATATATAAA ATACAGCTTT   
  
  
- GAGTTTATAT TAAATGTAGC AATATAATAT AAAAATACAA ATACATTTAC AACACAAGTA TTCTATAAAT   
  
  
- ATTTAAAACA TATAACCATC ATAGTAATTT TTTTTATATG TATAAATACA TTGATTTTAT TTACTTACAA   
  
  
- TATCTTTTAG ATATTCTACA TTCCTCTACA TACTTCTACA TTCCTCTACA TTCTTCTATA TTCCTCTACA   
  
  
- TCTTTTACAT TCTTCTACAC TCTTCGACAT TCCTCTACAT TCAATAAGTG ATTTAGGATT TTTTACGCTT   
  
  
- TATCTTGTCC CCGAATTAAC TCGTGAGTTC TACGTATTAA ACACTGCCTG GTTTACCTAT TTATATCTGG   
  
  
- ACCGTTTACC CAATAAGCTA AATCCATGCT CATACCAATT CAAACCCAAA CCAATACAAA GTCCTATTCA   
  
  
- CTGTAAAGCT GAGTTAAACC AAAACCAACA TACTTGAAGC CGAGCTCTAA AAACCAAACC GGTTATGTGA   
  
  
- ATAGTACGTT TTTATTCATT GAAATATCTT TTAAAACCTA GCCTATACTA CGCCAAGCCT AGTTAAGCCT   
  
  
- AAAACCAAGT GTAAGACCAT TTGGATAAAG CCTACAGTTC ATCCTATACT CAGGTCTAGC AACCTAAAGC   
  
  
- CCATAGTCGA GTTAAAATGG TCTAGATCTA TTTAGTATTT ACATGCCCAA ATAAAAAAGG TCGAACTTAC   
  
  
- TAAAAGAAAT AATTTTTTTC TATCTAAAAT CTATTTTGTG TTGTAAAGTA AAAGTAAACA AGGGGGGATA   
  
  
- TTTAAAGACA CTGGTGGAAG TGAAAGAACG GGGTAAGGGG GGGGGGGGGG CGAGAGAGAG AGAGAGAGAG   
  
  
- AGAAGGGAAA TGTTTCCGTG GTAAGTTTAA GTTTGGTGAG AAAAATTTTG GGGTTTTTAA TAAGAGTTTT
[truncated: 95,259 more chars]
