# Supplementary material for: Genome-Wide Identification and Expression Pattern of the GRAS Gene Family in Pitaya (Selenicereus undatus L.)
Source: Biology (Basel). 2022 Dec 21;12(1):11. doi: 10.3390/biology12010011 (PMC9854919; doi:10.3390/biology12010011)
Supplement: Supplementary file 1 [file biology-12-00011-s001.zip › Supplementary file S5/HU04G00047.1_plantcare.html]

Content-Type: text/html; charset=ISO-8859-1


PlantCARE


Webmaster Firefox specific output  
To save the result:
click on the frame with the right mouse button and save the source code as a text file with extension .html  
REFERENCE:PlantCARE: a database of plant cis-acting regulatory elements and a portal to tools for in silico analysis of promoter sequences.  
Lescot, M., Déhais, P., Moreau, Y., De Moor, B., Rouzé ,P.,and Rombauts, S.  
Nucleic Acids Res., Database issue(2002), 30(1):325-327.   


---

>HU04G00047.1   
+ +Up\_Stream \_Len000TCTTAA CATTAGTTGC TTGGTTCAAC CCATCAGTGA GAATATCTGA TTTCAAAAGT   
  
  
+ ATAGATTTAT ATAAACATAT GGACTACAGG GAAGGGAGTC GTTTCCTCAA AGTCTTAGAT TTACACTCAA   
  
  
+ AAGTTTGAGC GATACCAATA ATGTGATTCC AAACATGAGC CAAATATTAA GTATCCTATA CTAAGATCAA   
  
  
+ GAAGAGAATG AACCAAAAGA CTGTTAATTT TTCAATTAAG CTAAACTTAT TAACCTTTTT TTGGAATAGA   
  
  
+ TTTTTAAATT TTATCTTCAA TATAAGTTTT TCCAAGGCGA GAGACATATA GCAATTTTAA TTAAAGTTAA   
  
  
+ AGTAGTATAA CATTTTTTTT AATGGTTATA GGCTTATAGC ACCTTTTCTA GGTGAAAAGA GATATCTTGG   
  
  
+ CCTGATTTCC AGTAAAGTTT TTTTTTTAAA TATTGTCAAT TATTTTTGCA AATGACATTT GGTAATTTGG   
  
  
+ GGGATAATCT GATGTGTTAA AAGATACAAT GAAAATATCA GAAAATAGGA TGCCTCAAAT TTCACAAGAA   
  
  
+ AGAAAAAATC TTTTTGTTGT TAGTATTTCT ACACATCCAC AATTTTTACT CATGGTAAAA AAAAAAGGGC   
  
  
+ CAAATATCTC ACCAATATAA CCAAACTATA TGTTCCATTA TTTAGTAACG GTGATAAATA TGTAATGGGT   
  
  
+ CAAGTTACTA ATAAAATAGC CTACAATACT TGGATATCTA CATAAAATTG ATATAGAATA TATATTGCAA   
  
  
+ TACTTAAATC AAAATTAAAT GAAATATTTA CTTTTTTATT CTATTACAAA TAGCTTCTTA TAGAATAGCA   
  
  
+ ACAATTTTTA ATGTAAGTTT CATTCTTACC AGATTTGAAG AGGTCCGGAT ATAGAAAATC TTATTCGTAT   
  
  
+ AAGTACTCGC ATGAGGGCGG ATAAATTCTA AAATATCAAC CTCAACTCGC TTCTACTATT CCATCTAAGT   
  
  
+ TCAATTTATC ATATAAAAAA ATTCAATCCA CTTTAATGTA CTCAATTCAC TTCAACCTAT TTTAATCAAT   
  
  
+ TTACCCATGA AAAGAACAAG GTCCTAAGTA GATGTTTTCT ATTGATTGAT TTAAACAATT TTCACCTACC   
  
  
+ CAATTATCCC CAAAACTGAA AGAGGGGGCA AAGGGGGGTG GGTAATGCTG TGTAAAATAG GGGGAAAGGG   
  
  
+ CAAGAAAACT GTGTACAATG GTGGAGCAGG GGTATACCAT TTGGCCTTGA GTTGAGGGGG CACAAAAGCC   
  
  
+ CAGAGAGAGA GAGGGGTAAT GCCCACCAGA AATAAGCAGG AAGCGAGGGA AAAACACTAG AGAGAAACAG   
  
  
+ AGAAAGAGAG AGAGAGAGAG AGAGAAGGGG AAATTTACTA GAGAATGAGA GTGAAGAAGG GAACTTTATT   
  
  
+ TTAACTTTTT TATGATGCAC GCATCCATGG CAGAACAGGG AAGGGAAAGA AGGGTACAGA GTACTGCTGT   
  
  
+ CCCCAAAAAA TTAGTGTAGA AAACAGGAGA CACTCAACTA CAACAAGGGT AAGGAAGAAG AACAAGCAAG   
  
  
+ ATATCCCACC CAAAGAAAAA AAAAAAGAAG AAGAAGAAAA TTTTTCAGAG AGGAAAGAAA AGCAATCACA   
  
  
+ TGAATTAGAA GCCAACCCAA GCCTTCTTCA AAGAGAAGCA GCATAACATA GCATAGCTGG GGAGAGGATA   
  
  
+ GGAGAGAGAA ACTCAAGAAA AGGGGTACCC GGCTTAAATG TTGATGCAGA AAGGTATAGA TATGGCCACA   
  
  
+ CTACTACTAC CCAGGAGTTT ATCATTGGAG ATGGATTTCT CTTTCTGCCT CATCTTCTCC TCCATCCACC   
  
  
+ AGCAAAACTG GGTTTCCTTT CAGCTGGGTC TTTTCTAGAT CTAGAAACTA TGGTACCCAA ATCTCCCATT   
  
  
+ CCTCCATTTT TATTCTCAAA ATCTATCTTC TTAGACTTGT GGGTTTGATT CATTTCTTTT GGGTTTTTGA   
  
  
+ AAAGAATTTA ATTTGATTGT ATTGTGTTGA TTGGGGAATT TGTGGTGGTG TTAGATGCGA GCTATGCCCT   
  
  
+ ACAATTTGCA AGGCAAGGGT GTGGTAGAGG TTTCAAGCAT TTGTATTCCA CAAATCTCTT CCCCTGCTTC   
  
  
+ AAAGTGGAAG ACCAACAACA ACAACTTGGA CAACAAATTT CAGCAACAAC AAGAAGAAAT TGAGCAATTG   
  
  
+ CAGAGTAGTG GTGGCACTTT TCTCTCTCCT GATAACACCA ATACTAATAT TAGTACTACT CCGAGAGCGA   
  
  
+ CAAGTTCTGT TGATAGCGAA CACACATCTA CTCTGGATAC TACGGGCCCT CCCACTTCAA CATCTTCCTC   
  
  
+ CTTCAAGAGC ACCTCCACCG CCAACACAGC CGGTGTGGCG GACCCCGCAT GTACTCCCAA AGAGGATTGG   
  
  
+ GATGTCTCCG GCGGCGCTGC TGGCGGTGAC GGCGGTGGTG GGGGGCTTGG ATTGGAGGAG TGGGACAGCA   
  
  
+ TGTTCCCAAA TGGGGAGGGG GCTTTGCTCC CTTGGATCAT GGGTGAAGCT GATGACATGG GTATGGGTTT   
  
  
+ GAAGCATCTT TTGCAATCGG GTAACCCGGT TGACTATGAG GGCAATGCTG GTCTAGGGGT TGTCGATCAG   
  
  
+ GGTTCTGGAT TTGAGACTCT CTCTCCTCCT CCACCGCAGC CGGCGGCATG TGAATCTAAT GGTGGTGGTG   
  
  
+ TTGCTAATTT GGGGTTTCCA GGGAATAATG GTAAGATTTC TTCAATTTCA CATAATTGTT CATCTGGGAT   
  
  
+ TTTGAATGGT AAGGTGAACA ATAATGGGTT GAATCCCAAT TGTAACCCTC AAGCTCAAGG CAGTCTACTT   
  
  
+ GGTTTAATCC AAGGAGCTAC AGGTGTACAC GCACATCCTG ACGTTGGAGA CGAGAAACCC CAGATTTTGA   
  
  
+ ATCCACATTT GGTGATGAAC CCTCAGCAAG CTCAGAGCAT TGCAAACCCT AGCTTTTTGA TGCACTCATT   
  
  
+ AGGTTACTAT CAGCTGGAGC AACATCTATT TCAACCTCAG GCAAAACGCC TGAACACGGG TGCTGTTCTG   
  
  
+ GATCCTAACC TTGTTCAGCT TGCAAAGAAC CCATTTGCTG ATCAGGGTCA TGAGTTATTG TTGAGGAAGC   
  
  
+ AGCACCAACA GCTTGGTTTG CAACCATTGC CATTGGGTTT GGGTCCTCAG TTGGTCCCTC CGCAGAAGCC   
  
  
+ TGTGATGGGT TCAAAGCAGG GGAACCCCCA GCATTACCCG TTGCATGTTC ATCAGCAGCT GCAATTGCAA   
  
  
+ GAGCAGGCTG TCAAAGATCA GCTCTTCAAG GCGGCAGACC TTATTCAAAC TGGAAGTTTC TCACTCGCGC   
  
  
+ AAGAGATATT GGCGCGGCTC AATCACCAGC TCTCCCTCCC TGCAAAGCCC CTCATTAGGG CGGCTTTGTA   
  
  
+ TGTGAAGGAG GCCCTTCAAA TGCTCCTCCT AATGAGCAAC CCAGTTGCGG CTCCACCGTC CAAGATCCTC   
  
  
+ ACCCCTTATG ATGTTGTTCA CAAGATGAGC GCGTATAAGG TCTTCTCTGA GGTCTGCCCA ATCACTCAAT   
  
  
+ TTGTGAATTT CACTTGTACA CAGGCCATTC TCGAGGCTCT TGATGATTCT GATGCTATTC ATGTTGTCGA   
  
  
+ CTTTGATATT GGTTGCGGTG CTCAATGGGC ATCATTGATT CAGGAGCTGC CATTGAGGAA AAGGGGAGCT   
  
  
+ CCCTCTCTGA AAATTACAGC CATAGCTCCC ATGTTGACTG GCAGTCACTT TGAAATTAGC CTAGTATGTG   
  
  
+ AAAACCTTGT GCAATTTGCC AACGATATTG GTGTTGCTTG TGAGCTCCAA GTTGTCAACT TTGATTTGTT   
  
  
+ TGATCCATCT TCGCCATCAA TGCCGAACAT TAGTACTGCT GAGGATGAGT CAATTGCTGT TAGTATCCCC   
  
  
+ ATCTGGGCAT CTTCAATTAG GCCATCTGTT CTTCCTTCCA TCCTCCGATT CATTAAGCAA AAATCCCCCA   
  
  
+ AAATTGTGGT CTCGTTTGAT AGAGGATTCG ATCGTTTTGA TGTCCCTTTC CCCCAACATC TGTTGCATAC   
  
  
+ CCTAGAATCC TGCAGTAATT TATTGGACTC GCTTGATGGT CTCAATGTTG CATCGGACAT TGTGAGCAAG   
  
  
+ GTCGAGAAGT TCTTTGTTCA ACCTAGGATC GAAAATGCTG TGTTGGGTCG GGTCCATGCC CCTGACAAGA   
  
  
+ TGCCCCATTG GAAAAATCTC TTTGCTTCAG CCGGCTTCTT GCCCTTGCAA TTCAGTAATT TCACAGAAAC   
  
  
+ CCAGGCCGAT TATGTGGTGA AGAGAACCCC AACAAGAGGA TTTCACGTGG AGAAGCGCCA GGCATCACTC   
  
  
+ ATTCTGAGTT GGCAGAGGCA GGAGCTTGTG GCAGCTTCGG CATGGAAGTG TTG  

- +Up\_Stream \_Len000AGAATT GTAATCAACG AACCAAGTTG GGTAGTCACT CTTATAGACT AAAGTTTTCA   
  
  
- TATCTAAATA TATTTGTATA CCTGATGTCC CTTCCCTCAG CAAAGGAGTT TCAGAATCTA AATGTGAGTT   
  
  
- TTCAAACTCG CTATGGTTAT TACACTAAGG TTTGTACTCG GTTTATAATT CATAGGATAT GATTCTAGTT   
  
  
- CTTCTCTTAC TTGGTTTTCT GACAATTAAA AAGTTAATTC GATTTGAATA ATTGGAAAAA AACCTTATCT   
  
  
- AAAAATTTAA AATAGAAGTT ATATTCAAAA AGGTTCCGCT CTCTGTATAT CGTTAAAATT AATTTCAATT   
  
  
- TCATCATATT GTAAAAAAAA TTACCAATAT CCGAATATCG TGGAAAAGAT CCACTTTTCT CTATAGAACC   
  
  
- GGACTAAAGG TCATTTCAAA AAAAAAATTT ATAACAGTTA ATAAAAACGT TTACTGTAAA CCATTAAACC   
  
  
- CCCTATTAGA CTACACAATT TTCTATGTTA CTTTTATAGT CTTTTATCCT ACGGAGTTTA AAGTGTTCTT   
  
  
- TCTTTTTTAG AAAAACAACA ATCATAAAGA TGTGTAGGTG TTAAAAATGA GTACCATTTT TTTTTTCCCG   
  
  
- GTTTATAGAG TGGTTATATT GGTTTGATAT ACAAGGTAAT AAATCATTGC CACTATTTAT ACATTACCCA   
  
  
- GTTCAATGAT TATTTTATCG GATGTTATGA ACCTATAGAT GTATTTTAAC TATATCTTAT ATATAACGTT   
  
  
- ATGAATTTAG TTTTAATTTA CTTTATAAAT GAAAAAATAA GATAATGTTT ATCGAAGAAT ATCTTATCGT   
  
  
- TGTTAAAAAT TACATTCAAA GTAAGAATGG TCTAAACTTC TCCAGGCCTA TATCTTTTAG AATAAGCATA   
  
  
- TTCATGAGCG TACTCCCGCC TATTTAAGAT TTTATAGTTG GAGTTGAGCG AAGATGATAA GGTAGATTCA   
  
  
- AGTTAAATAG TATATTTTTT TAAGTTAGGT GAAATTACAT GAGTTAAGTG AAGTTGGATA AAATTAGTTA   
  
  
- AATGGGTACT TTTCTTGTTC CAGGATTCAT CTACAAAAGA TAACTAACTA AATTTGTTAA AAGTGGATGG   
  
  
- GTTAATAGGG GTTTTGACTT TCTCCCCCGT TTCCCCCCAC CCATTACGAC ACATTTTATC CCCCTTTCCC   
  
  
- GTTCTTTTGA CACATGTTAC CACCTCGTCC CCATATGGTA AACCGGAACT CAACTCCCCC GTGTTTTCGG   
  
  
- GTCTCTCTCT CTCCCCATTA CGGGTGGTCT TTATTCGTCC TTCGCTCCCT TTTTGTGATC TCTCTTTGTC   
  
  
- TCTTTCTCTC TCTCTCTCTC TCTCTTCCCC TTTAAATGAT CTCTTACTCT CACTTCTTCC CTTGAAATAA   
  
  
- AATTGAAAAA ATACTACGTG CGTAGGTACC GTCTTGTCCC TTCCCTTTCT TCCCATGTCT CATGACGACA   
  
  
- GGGGTTTTTT AATCACATCT TTTGTCCTCT GTGAGTTGAT GTTGTTCCCA TTCCTTCTTC TTGTTCGTTC   
  
  
- TATAGGGTGG GTTTCTTTTT TTTTTTCTTC TTCTTCTTTT AAAAAGTCTC TCCTTTCTTT TCGTTAGTGT   
  
  
- ACTTAATCTT CGGTTGGGTT CGGAAGAAGT TTCTCTTCGT CGTATTGTAT CGTATCGACC CCTCTCCTAT   
  
  
- CCTCTCTCTT TGAGTTCTTT TCCCCATGGG CCGAATTTAC AACTACGTCT TTCCATATCT ATACCGGTGT   
  
  
- GATGATGATG GGTCCTCAAA TAGTAACCTC TACCTAAAGA GAAAGACGGA GTAGAAGAGG AGGTAGGTGG   
  
  
- TCGTTTTGAC CCAAAGGAAA GTCGACCCAG AAAAGATCTA GATCTTTGAT ACCATGGGTT TAGAGGGTAA   
  
  
- GGAGGTAAAA ATAAGAGTTT TAGATAGAAG AATCTGAACA CCCAAACTAA GTAAAGAAAA CCCAAAAACT   
  
  
- TTTCTTAAAT TAAACTAACA TAACACAACT AACCCCTTAA ACACCACCAC AATCTACGCT CGATACGGGA   
  
  
- TGTTAAACGT TCCGTTCCCA CACCATCTCC AAAGTTCGTA AACATAAGGT GTTTAGAGAA GGGGACGAAG   
  
  
- TTTCACCTTC TGGTTGTTGT TGTTGAACCT GTTGTTTAAA GTCGTTGTTG TTCTTCTTTA ACTCGTTAAC   
  
  
- GTCTCATCAC CACCGTGAAA AGAGAGAGGA CTATTGTGGT TATGATTATA ATCATGATGA GGCTCTCGCT   
  
  
- GTTCAAGACA ACTATCGCTT GTGTGTAGAT GAGACCTATG ATGCCCGGGA GGGTGAAGTT GTAGAAGGAG   
  
  
- GAAGTTCTCG TGGAGGTGGC GGTTGTGTCG GCCACACCGC CTGGGGCGTA CATGAGGGTT TCTCCTAACC   
  
  
- CTACAGAGGC CGCCGCGACG ACCGCCACTG CCGCCACCAC CCCCCGAACC TAACCTCCTC ACCCTGTCGT   
  
  
- ACAAGGGTTT ACCCCTCCCC CGAAACGAGG GAACCTAGTA CCCACTTCGA CTACTGTACC CATACCCAAA   
  
  
- CTTCGTAGAA AACGTTAGCC CATTGGGCCA ACTGATACTC CCGTTACGAC CAGATCCCCA ACAGCTAGTC   
  
  
- CCAAGACCTA AACTCTGAGA GAGAGGAGGA GGTGGCGTCG GCCGCCGTAC ACTTAGATTA CCACCACCAC   
  
  
- AACGATTAAA CCCCAAAGGT CCCTTATTAC CATTCTAAAG AAGTTAAAGT GTATTAACAA GTAGACCCTA   
  
  
- AAACTTACCA TTCCACTTGT TATTACCCAA CTTAGGGTTA ACATTGGGAG TTCGAGTTCC GTCAGATGAA   
  
  
- CCAAATTAGG TTCCTCGATG TCCACATGTG CGTGTAGGAC TGCAACCTCT GCTCTTTGGG GTCTAAAACT   
  
  
- TAGGTGTAAA CCACTACTTG GGAGTCGTTC GAGTCTCGTA ACGTTTGGGA TCGAAAAACT ACGTGAGTAA   
  
  
- TCCAATGATA GTCGACCTCG TTGTAGATAA AGTTGGAGTC CGTTTTGCGG ACTTGTGCCC ACGACAAGAC   
  
  
- CTAGGATTGG AACAAGTCGA ACGTTTCTTG GGTAAACGAC TAGTCCCAGT ACTCAATAAC AACTCCTTCG   
  
  
- TCGTGGTTGT CGAACCAAAC GTTGGTAACG GTAACCCAAA CCCAGGAGTC AACCAGGGAG GCGTCTTCGG   
  
  
- ACACTACCCA AGTTTCGTCC CCTTGGGGGT CGTAATGGGC AACGTACAAG TAGTCGTCGA CGTTAACGTT   
  
  
- CTCGTCCGAC AGTTTCTAGT CGAGAAGTTC CGCCGTCTGG AATAAGTTTG ACCTTCAAAG AGTGAGCGCG   
  
  
- TTCTCTATAA CCGCGCCGAG TTAGTGGTCG AGAGGGAGGG ACGTTTCGGG GAGTAATCCC GCCGAAACAT   
  
  
- ACACTTCCTC CGGGAAGTTT ACGAGGAGGA TTACTCGTTG GGTCAACGCC GAGGTGGCAG GTTCTAGGAG   
  
  
- TGGGGAATAC TACAACAAGT GTTCTACTCG CGCATATTCC AGAAGAGACT CCAGACGGGT TAGTGAGTTA   
  
  
- AACACTTAAA GTGAACATGT GTCCGGTAAG AGCTCCGAGA ACTACTAAGA CTACGATAAG TACAACAGCT   
  
  
- GAAACTATAA CCAACGCCAC GAGTTACCCG TAGTAACTAA GTCCTCGACG GTAACTCCTT TTCCCCTCGA   
  
  
- GGGAGAGACT TTTAATGTCG GTATCGAGGG TACAACTGAC CGTCAGTGAA ACTTTAATCG GATCATACAC   
  
  
- TTTTGGAACA CGTTAAACGG TTGCTATAAC CACAACGAAC ACTCGAGGTT CAACAGTTGA AACTAAACAA   
  
  
- ACTAGGTAGA AGCGGTAGTT ACGGCTTGTA ATCATGACGA CTCCTACTCA GTTAACGACA ATCATAGGGG   
  
  
- TAGACCCGTA GAAGTTAATC CGGTAGACAA GAAGGAAGGT AGGAGGCTAA GTAATTCGTT TTTAGGGGGT   
  
  
- TTTAACACCA GAGCAAACTA TCTCCTAAGC TAGCAAAACT ACAGGGAAAG GGGGTTGTAG ACAACGTATG   
  
  
- GGATCTTAGG ACGTCATTAA ATAACCTGAG CGAACTACCA GAGTTACAAC GTAGCCTGTA ACACTCGTTC   
  
  
- CAGCTCTTCA AGAAACAAGT TGGATCCTAG CTTTTACGAC ACAACCCAGC CCAGGTACGG GGACTGTTCT   
  
  
- ACGGGGTAAC CTTTTTAGAG AAACGAAGTC GGCCGAAGAA CGGGAACGTT AAGTCATTAA AGTGTCTTTG   
  
  
- GGTCCGGCTA ATACACCACT TCTCTTGGGG TTGTTCTCCT AAAGTGCACC TCTTCGCGGT CCGTAGTGAG   
  
  
- TAAGACTCAA CCGTCTCCGT CCTCGAACAC CGTCGAAGCC GTACCTTCAC AAC

  
  
Motifs Found  

+   

| Site Name | Organism | Position | Strand | Matrix score. | sequence | function |
| --- | --- | --- | --- | --- | --- | --- |
|  | organism | 4286 | - | 4 | motif\_sequence | short\_function |
|  | organism | 2090 | + | 4 | motif\_sequence | short\_function |
|  | organism | 1020 | + | 4 | motif\_sequence | short\_function |
|  | organism | 3880 | + | 4 | motif\_sequence | short\_function |
|  | organism | 1781 | - | 4 | motif\_sequence | short\_function |
|  | organism | 3863 | + | 4 | motif\_sequence | short\_function |
|  | organism | 3964 | + | 4 | motif\_sequence | short\_function |
|  | organism | 1675 | - | 4 | motif\_sequence | short\_function |
|  | organism | 1685 | - | 4 | motif\_sequence | short\_function |
|  | organism | 215 | - | 4 | motif\_sequence | short\_function |
|  | organism | 3199 | + | 4 | motif\_sequence | short\_function |
|  | organism | 3324 | + | 4 | motif\_sequence | short\_function |
|  | organism | 2363 | + | 4 | motif\_sequence | short\_function |
|  | organism | 2270 | + | 4 | motif\_sequence | short\_function |
|  | organism | 3007 | + | 4 | motif\_sequence | short\_function |
|  | organism | 3809 | - | 4 | motif\_sequence | short\_function |
|  | organism | 1471 | + | 4 | motif\_sequence | short\_function |
|  | organism | 2198 | + | 4 | motif\_sequence | short\_function |
|  | organism | 2306 | + | 4 | motif\_sequence | short\_function |
|  | organism | 4049 | - | 4 | motif\_sequence | short\_function |
|  | organism | 1458 | - | 4 | motif\_sequence | short\_function |
|  | organism | 2454 | + | 4 | motif\_sequence | short\_function |
|  | organism | 1806 | + | 4 | motif\_sequence | short\_function |
|  | organism | 3791 | + | 4 | motif\_sequence | short\_function |
|  | organism | 3246 | + | 4 | motif\_sequence | short\_function |
|  | organism | 2615 | + | 4 | motif\_sequence | short\_function |
|  | organism | 2174 | - | 4 | motif\_sequence | short\_function |
|  | organism | 4223 | - | 4 | motif\_sequence | short\_function |
|  | organism | 2447 | - | 4 | motif\_sequence | short\_function |
|  | organism | 2835 | - | 4 | motif\_sequence | short\_function |
|  | organism | 1437 | - | 4 | motif\_sequence | short\_function |
|  | organism | 297 | + | 4 | motif\_sequence | short\_function |
|  | organism | 740 | + | 4 | motif\_sequence | short\_function |
|  | organism | 1082 | - | 4 | motif\_sequence | short\_function |
|  | organism | 2058 | - | 4 | motif\_sequence | short\_function |
|  | organism | 664 | + | 4 | motif\_sequence | short\_function |
|  | organism | 1885 | + | 4 | motif\_sequence | short\_function |
|  | organism | 1919 | + | 4 | motif\_sequence | short\_function |
|  | organism | 881 | - | 4 | motif\_sequence | short\_function |

>HU04G00047.1   
+ +Up\_Stream \_Len000TCTTAA CATTAGTTGC TTGGTTCAAC CCATCAGTGA GAATATCTGA TTTCAAAAGT   
  
  
+ ATAGATTTAT ATAAACATAT GGACTACAGG GAAGGGAGTC GTTTCCTCAA AGTCTTAGAT TTACACTCAA   
  
  
+ AAGTTTGAGC GATACCAATA ATGTGATTCC AAACATGAGC CAAATATTAA GTATCCTATA CTAAGATCAA   
  
  
+ GAAGAGAATG AACCAAAAGA CTGTTAATTT TTCAATTAAG CTAAACTTAT TAACCTTTTT TTGGAATAGA   
  
  
+ TTTTTAAATT TTATCTTCAA TATAAGTTTT TCCAAGGCGA GAGACATATA GCAATTTTAA TTAAAGTTAA   
  
  
+ AGTAGTATAA CATTTTTTTT AATGGTTATA GGCTTATAGC ACCTTTTCTA GGTGAAAAGA GATATCTTGG   
  
  
+ CCTGATTTCC AGTAAAGTTT TTTTTTTAAA TATTGTCAAT TATTTTTGCA AATGACATTT GGTAATTTGG   
  
  
+ GGGATAATCT GATGTGTTAA AAGATACAAT GAAAATATCA GAAAATAGGA TGCCTCAAAT TTCACAAGAA   
  
  
+ AGAAAAAATC TTTTTGTTGT TAGTATTTCT ACACATCCAC AATTTTTACT CATGGTAAAA AAAAAAGGGC   
  
  
+ CAAATATCTC ACCAATATAA CCAAACTATA TGTTCCATTA TTTAGTAACG GTGATAAATA TGTAATGGGT   
  
  
+ CAAGTTACTA ATAAAATAGC CTACAATACT TGGATATCTA CATAAAATTG ATATAGAATA TATATTGCAA   
  
  
+ TACTTAAATC AAAATTAAAT GAAATATTTA CTTTTTTATT CTATTACAAA TAGCTTCTTA TAGAATAGCA   
  
  
+ ACAATTTTTA ATGTAAGTTT CATTCTTACC AGATTTGAAG AGGTCCGGAT ATAGAAAATC TTATTCGTAT   
  
  
+ AAGTACTCGC ATGAGGGCGG ATAAATTCTA AAATATCAAC CTCAACTCGC TTCTACTATT CCATCTAAGT   
  
  
+ TCAATTTATC ATATAAAAAA ATTCAATCCA CTTTAATGTA CTCAATTCAC TTCAACCTAT TTTAATCAAT   
  
  
+ TTACCCATGA AAAGAACAAG GTCCTAAGTA GATGTTTTCT ATTGATTGAT TTAAACAATT TTCACCTACC   
  
  
+ CAATTATCCC CAAAACTGAA AGAGGGGGCA AAGGGGGGTG GGTAATGCTG TGTAAAATAG GGGGAAAGGG   
  
  
+ CAAGAAAACT GTGTACAATG GTGGAGCAGG GGTATACCAT TTGGCCTTGA GTTGAGGGGG CACAAAAGCC   
  
  
+ CAGAGAGAGA GAGGGGTAAT GCCCACCAGA AATAAGCAGG AAGCGAGGGA AAAACACTAG AGAGAAACAG   
  
  
+ AGAAAGAGAG AGAGAGAGAG AGAGAAGGGG AAATTTACTA GAGAATGAGA GTGAAGAAGG GAACTTTATT   
  
  
+ TTAACTTTTT TATGATGCAC GCATCCATGG CAGAACAGGG AAGGGAAAGA AGGGTACAGA GTACTGCTGT   
  
  
+ CCCCAAAAAA TTAGTGTAGA AAACAGGAGA CACTCAACTA CAACAAGGGT AAGGAAGAAG AACAAGCAAG   
  
  
+ ATATCCCACC CAAAGAAAAA AAAAAAGAAG AAGAAGAAAA TTTTTCAGAG AGGAAAGAAA AGCAATCACA   
  
  
+ TGAATTAGAA GCCAACCCAA GCCTTCTTCA AAGAGAAGCA GCATAACATA GCATAGCTGG GGAGAGGATA   
  
  
+ GGAGAGAGAA ACTCAAGAAA AGGGGTACCC GGCTTAAATG TTGATGCAGA AAGGTATAGA TATGGCCACA   
  
  
+ CTACTACTAC CCAGGAGTTT ATCATTGGAG ATGGATTTCT CTTTCTGCCT CATCTTCTCC TCCATCCACC   
  
  
+ AGCAAAACTG GGTTTCCTTT CAGCTGGGTC TTTTCTAGAT CTAGAAACTA TGGTACCCAA ATCTCCCATT   
  
  
+ CCTCCATTTT TATTCTCAAA ATCTATCTTC TTAGACTTGT GGGTTTGATT CATTTCTTTT GGGTTTTTGA   
  
  
+ AAAGAATTTA ATTTGATTGT ATTGTGTTGA TTGGGGAATT TGTGGTGGTG TTAGATGCGA GCTATGCCCT   
  
  
+ ACAATTTGCA AGGCAAGGGT GTGGTAGAGG TTTCAAGCAT TTGTATTCCA CAAATCTCTT CCCCTGCTTC   
  
  
+ AAAGTGGAAG ACCAACAACA ACAACTTGGA CAACAAATTT CAGCAACAAC AAGAAGAAAT TGAGCAATTG   
  
  
+ CAGAGTAGTG GTGGCACTTT TCTCTCTCCT GATAACACCA ATACTAATAT TAGTACTACT CCGAGAGCGA   
  
  
+ CAAGTTCTGT TGATAGCGAA CACACATCTA CTCTGGATAC TACGGGCCCT CCCACTTCAA CATCTTCCTC   
  
  
+ CTTCAAGAGC ACCTCCACCG CCAACACAGC CGGTGTGGCG GACCCCGCAT GTACTCCCAA AGAGGATTGG   
  
  
+ GATGTCTCCG GCGGCGCTGC TGGCGGTGAC GGCGGTGGTG GGGGGCTTGG ATTGGAGGAG TGGGACAGCA   
  
  
+ TGTTCCCAAA TGGGGAGGGG GCTTTGCTCC CTTGGATCAT GGGTGAAGCT GATGACATGG GTATGGGTTT   
  
  
+ GAAGCATCTT TTGCAATCGG GTAACCCGGT TGACTATGAG GGCAATGCTG GTCTAGGGGT TGTCGATCAG   
  
  
+ GGTTCTGGAT TTGAGACTCT CTCTCCTCCT CCACCGCAGC CGGCGGCATG TGAATCTAAT GGTGGTGGTG   
  
  
+ TTGCTAATTT GGGGTTTCCA GGGAATAATG GTAAGATTTC TTCAATTTCA CATAATTGTT CATCTGGGAT   
  
  
+ TTTGAATGGT AAGGTGAACA ATAATGGGTT GAATCCCAAT TGTAACCCTC AAGCTCAAGG CAGTCTACTT   
  
  
+ GGTTTAATCC AAGGAGCTAC AGGTGTACAC GCACATCCTG ACGTTGGAGA CGAGAAACCC CAGATTTTGA   
  
  
+ ATCCACATTT GGTGATGAAC CCTCAGCAAG CTCAGAGCAT TGCAAACCCT AGCTTTTTGA TGCACTCATT   
  
  
+ AGGTTACTAT CAGCTGGAGC AACATCTATT TCAACCTCAG GCAAAACGCC TGAACACGGG TGCTGTTCTG   
  
  
+ GATCCTAACC TTGTTCAGCT TGCAAAGAAC CCATTTGCTG ATCAGGGTCA TGAGTTATTG TTGAGGAAGC   
  
  
+ AGCACCAACA GCTTGGTTTG CAACCATTGC CATTGGGTTT GGGTCCTCAG TTGGTCCCTC CGCAGAAGCC   
  
  
+ TGTGATGGGT TCAAAGCAGG GGAACCCCCA GCATTACCCG TTGCATGTTC ATCAGCAGCT GCAATTGCAA   
  
  
+ GAGCAGGCTG TCAAAGATCA GCTCTTCAAG GCGGCAGACC TTATTCAAAC TGGAAGTTTC TCACTCGCGC   
  
  
+ AAGAGATATT GGCGCGGCTC AATCACCAGC TCTCCCTCCC TGCAAAGCCC CTCATTAGGG CGGCTTTGTA   
  
  
+ TGTGAAGGAG GCCCTTCAAA TGCTCCTCCT AATGAGCAAC CCAGTTGCGG CTCCACCGTC CAAGATCCTC   
  
  
+ ACCCCTTATG ATGTTGTTCA CAAGATGAGC GCGTATAAGG TCTTCTCTGA GGTCTGCCCA ATCACTCAAT   
  
  
+ TTGTGAATTT CACTTGTACA CAGGCCATTC TCGAGGCTCT TGATGATTCT GATGCTATTC ATGTTGTCGA   
  
  
+ CTTTGATATT GGTTGCGGTG CTCAATGGGC ATCATTGATT CAGGAGCTGC CATTGAGGAA AAGGGGAGCT   
  
  
+ CCCTCTCTGA AAATTACAGC CATAGCTCCC ATGTTGACTG GCAGTCACTT TGAAATTAGC CTAGTATGTG   
  
  
+ AAAACCTTGT GCAATTTGCC AACGATATTG GTGTTGCTTG TGAGCTCCAA GTTGTCAACT TTGATTTGTT   
  
  
+ TGATCCATCT TCGCCATCAA TGCCGAACAT TAGTACTGCT GAGGATGAGT CAATTGCTGT TAGTATCCCC   
  
  
+ ATCTGGGCAT CTTCAATTAG GCCATCTGTT CTTCCTTCCA TCCTCCGATT CATTAAGCAA AAATCCCCCA   
  
  
+ AAATTGTGGT CTCGTTTGAT AGAGGATTCG ATCGTTTTGA TGTCCCTTTC CCCCAACATC TGTTGCATAC   
  
  
+ CCTAGAATCC TGCAGTAATT TATTGGACTC GCTTGATGGT CTCAATGTTG CATCGGACAT TGTGAGCAAG   
  
  
+ GTCGAGAAGT TCTTTGTTCA ACCTAGGATC GAAAATGCTG TGTTGGGTCG GGTCCATGCC CCTGACAAGA   
  
  
+ TGCCCCATTG GAAAAATCTC TTTGCTTCAG CCGGCTTCTT GCCCTTGCAA TTCAGTAATT TCACAGAAAC   
  
  
+ CCAGGCCGAT TATGTGGTGA AGAGAACCCC AACAAGAGGA TTTCACGTGG AGAAGCGCCA GGCATCACTC   
  
  
+ ATTCTGAGTT GGCAGAGGCA GGAGCTTGTG GCAGCTTCGG CATGGAAGTG TTG  

- +Up\_Stream \_Len000AGAATT GTAATCAACG AACCAAGTTG GGTAGTCACT CTTATAGACT AAAGTTTTCA   
  
  
- TATCTAAATA TATTTGTATA CCTGATGTCC CTTCCCTCAG CAAAGGAGTT TCAGAATCTA AATGTGAGTT   
  
  
- TTCAAACTCG CTATGGTTAT TACACTAAGG TTTGTACTCG GTTTATAATT CATAGGATAT GATTCTAGTT   
  
  
- CTTCTCTTAC TTGGTTTTCT GACAATTAAA AAGTTAATTC GATTTGAATA ATTGGAAAAA AACCTTATCT   
  
  
- AAAAATTTAA AATAGAAGTT ATATTCAAAA AGGTTCCGCT CTCTGTATAT CGTTAAAATT AATTTCAATT   
  
  
- TCATCATATT GTAAAAAAAA TTACCAATAT CCGAATATCG TGGAAAAGAT CCACTTTTCT CTATAGAACC   
  
  
- GGACTAAAGG TCATTTCAAA AAAAAAATTT ATAACAGTTA ATAAAAACGT TTACTGTAAA CCATTAAACC   
  
  
- CCCTATTAGA CTACACAATT TTCTATGTTA CTTTTATAGT CTTTTATCCT ACGGAGTTTA AAGTGTTCTT   
  
  
- TCTTTTTTAG AAAAACAACA ATCATAAAGA TGTGTAGGTG TTAAAAATGA GTACCATTTT TTTTTTCCCG   
  
  
- GTTTATAGAG TGGTTATATT GGTTTGATAT ACAAGGTAAT AAATCATTGC CACTATTTAT ACATTACCCA   
  
  
- GTTCAATGAT TATTTTATCG GATGTTATGA ACCTATAGAT GTATTTTAAC TATATCTTAT ATATAACGTT   
  
  
- ATGAATTTAG TTTTAATTTA CTTTATAAAT GAAAAAATAA GATAATGTTT ATCGAAGAAT ATCTTATCGT   
  
  
- TGTTAAAAAT TACATTCAAA GTAAGAATGG TCTAAACTTC TCCAGGCCTA TATCTTTTAG AATAAGCATA   
  
  
- TTCATGAGCG TACTCCCGCC TATTTAAGAT TTTATAGTTG GAGTTGAGCG AAGATGATAA GGTAGATTCA   
  
  
- AGTTAAATAG TATATTTTTT TAAGTTAGGT GAAATTACAT GAGTTAAGTG AAGTTGGATA AAATTAGTTA   
  
  
- AATGGGTACT TTTCTTGTTC CAGGATTCAT CTACAAAAGA TAACTAACTA AATTTGTTAA AAGTGGATGG   
  
  
- GTTAATAGGG GTTTTGACTT TCTCCCCCGT TTCCCCCCAC CCATTACGAC ACATTTTATC CCCCTTTCCC   
  
  
- GTTCTTTTGA CACATGTTAC CACCTCGTCC CCATATGGTA AACCGGAACT CAACTCCCCC GTGTTTTCGG   
  
  
- GTCTCTCTCT CTCCCCATTA CGGGTGGTCT TTATTCGTCC TTCGCTCCCT TTTTGTGATC TCTCTTTGTC   
  
  
- TCTTTCTCTC TCTCTCTCTC TCTCTTCCCC TTTAAATGAT CTCTTACTCT CACTTCTTCC CTTGAAATAA   
  
  
- AATTGAAAAA ATACTACGTG CGTAGGTACC GTCTTGTCCC TTCCCTTTCT TCCCATGTCT CATGACGACA   
  
  
- GGGGTTTTTT AATCACATCT TTTGTCCTCT GTGAGTTGAT GTTGTTCCCA TTCCTTCTTC TTGTTCGTTC   
  
  
- TATAGGGTGG GTTTCTTTTT TTTTTTCTTC TTCTTCTTTT AAAAAGTCTC TCCTTTCTTT TCGTTAGTGT   
  
  
- ACTTAATCTT CGGTTGGGTT CGGAAGAAGT TTCTCTTCGT CGTATTGTAT CGTATCGACC CCTCTCCTAT   
  
  
- CCTCTCTCTT TGAGTTCTTT TCCCCATGGG CCGAATTTAC AACTACGTCT TTCCATATCT ATACCGGTGT   
  
  
- GATGATGATG GGTCCTCAAA TAGTAACCTC TACCTAAAGA GAAAGACGGA GTAGAAGAGG AGGTAGGTGG   
  
  
- TCGTTTTGAC CCAAAGGAAA GTCGACCCAG AAAAGATCTA GATCTTTGAT ACCATGGGTT TAGAGGGTAA   
  
  
- GGAGGTAAAA ATAAGAGTTT TAGATAGAAG AATCTGAACA CCCAAACTAA GTAAAGAAAA CCCAAAAACT   
  
  
- TTTCTTAAAT TAAACTAACA TAACACAACT AACCCCTTAA ACACCACCAC AATCTACGCT CGATACGGGA   
  
  
- TGTTAAACGT TCCGTTCCCA CACCATCTCC AAAGTTCGTA AACATAAGGT GTTTAGAGAA GGGGACGAAG   
  
  
- TTTCACCTTC TGGTTGTTGT TGTTGAACCT GTTGTTTAAA GTCGTTGTTG TTCTTCTTTA ACTCGTTAAC   
  
  
- GTCTCATCAC CACCGTGAAA AGAGAGAGGA CTATTGTGGT TATGATTATA ATCATGATGA GGCTCTCGCT   
  
  
- GTTCAAGACA ACTATCGCTT GTGTGTAGAT GAGACCTATG ATGCCCGGGA GGGTGAAGTT GTAGAAGGAG   
  
  
- GAAGTTCTCG TGGAGGTGGC GGTTGTGTCG GCCACACCGC CTGGGGCGTA CATGAGGGTT TCTCCTAACC   
  
  
- CTACAGAGGC CGCCGCGACG ACCGCCACTG CCGCCACCAC CCCCCGAACC TAACCTCCTC ACCCTGTCGT   
  
  
- ACAAGGGTTT ACCCCTCCCC CGAAACGAGG GAACCTAGTA CCCACTTCGA CTACTGTACC CATACCCAAA   
  
  
- CTTCGTAGAA AACGTTAGCC CATTGGGCCA ACTGATACTC CCGTTACGAC CAGATCCCCA ACAGCTAGTC   
  
  
- CCAAGACCTA AACTCTGAGA GAGAGGAGGA GGTGGCGTCG GCCGCCGTAC ACTTAGATTA CCACCACCAC   
  
  
- AACGATTAAA CCCCAAAGGT CCCTTATTAC CATTCTAAAG AAGTTAAAGT GTATTAACAA GTAGACCCTA   
  
  
- AAACTTACCA TTCCACTTGT TATTACCCAA CTTAGGGTTA ACATTGGGAG TTCGAGTTCC GTCAGATGAA   
  
  
- CCAAATTAGG TTCCTCGATG TCCACATGTG CGTGTAGGAC TGCAACCTCT GCTCTTTGGG GTCTAAAACT   
  
  
- TAGGTGTAAA CCACTACTTG GGAGTCGTTC GAGTCTCGTA ACGTTTGGGA TCGAAAAACT ACGTGAGTAA   
  
  
- TCCAATGATA GTCGACCTCG TTGTAGATAA AGTTGGAGTC CGTTTTGCGG ACTTGTGCCC ACGACAAGAC   
  
  
- CTAGGATTGG AACAAGTCGA ACGTTTCTTG GGTAAACGAC TAGTCCCAGT ACTCAATAAC AACTCCTTCG   
  
  
- TCGTGGTTGT CGAACCAAAC GTTGGTAACG GTAACCCAAA CCCAGGAGTC AACCAGGGAG GCGTCTTCGG   
  
  
- ACACTACCCA AGTTTCGTCC CCTTGGGGGT CGTAATGGGC AACGTACAAG TAGTCGTCGA CGTTAACGTT   
  
  
- CTCGTCCGAC AGTTTCTAGT CGAGAAGTTC CGCCGTCTGG AATAAGTTTG ACCTTCAAAG AGTGAGCGCG   
  
  
- TTCTCTATAA CCGCGCCGAG TTAGTGGTCG AGAGGGAGGG ACGTTTCGGG GAGTAATCCC GCCGAAACAT   
  
  
- ACACTTCCTC CGGGAAGTTT ACGAGGAGGA TTACTCGTTG GGTCAACGCC GAGGTGGCAG GTTCTAGGAG   
  
  
- TGGGGAATAC TACAACAAGT GTTCTACTCG CGCATATTCC AGAAGAGACT CCAGACGGGT TAGTGAGTTA   
  
  
- AACACTTAAA GTGAACATGT GTCCGGTAAG AGCTCCGAGA ACTACTAAGA CTACGATAAG TACAACAGCT   
  
  
- GAAACTATAA CCAACGCCAC GAGTTACCCG TAGTAACTAA GTCCTCGACG GTAACTCCTT TTCCCCTCGA   
  
  
- GGGAGAGACT TTTAATGTCG GTATCGAGGG TACAACTGAC CGTCAGTGAA ACTTTAATCG GATCATACAC   
  
  
- TTTTGGAACA CGTTAAACGG TTGCTATAAC CACAACGAAC ACTCGAGGTT CAACAGTTGA AACTAAACAA   
  
  
- ACTAGGTAGA AGCGGTAGTT ACGGCTTGTA ATCATGACGA CTCCTACTCA GTTAACGACA ATCATAGGGG   
  
  
- TAGACCCGTA GAAGTTAATC CGGTAGACAA GAAGGAAGGT AGGAGGCTAA GTAATTCGTT TTTAGGGGGT   
  
  
- TTTAACACCA GAGCAAACTA TCTCCTAAGC TAGCAAAACT ACAGGGAAAG GGGGTTGTAG ACAACGTATG   
  
  
- GGATCTTAGG ACGTCATTAA ATAACCTGAG CGAACTACCA GAGTTACAAC GTAGCCTGTA ACACTCGTTC   
  
  
- CAGCTCTTCA AGAAACAAGT TGGATCCTAG CTTTTACGAC ACAACCCAGC CCAGGTACGG GGACTGTTCT   
  
  
- ACGGGGTAAC CTTTTTAGAG AAACGAAGTC GGCCGAAGAA CGGGAACGTT AAGTCATTAA AGTGTCTTTG   
  
  
- GGTCCGGCTA ATACACCACT TCTCTTGGGG TTGTTCTCCT AAAGTGCACC TCTTCGCGGT CCGTAGTGAG   
  
  
- TAAGACTCAA CCGTCTCCGT CCTCGAACAC CGTCGAAGCC GTACCTTCAC AAC

+     3-AF1 binding site

| Site Name | Organism | Position | Strand | Matrix score. | sequence | function |
| --- | --- | --- | --- | --- | --- | --- |
| 3-AF1 binding site | Solanum tuberosum | 1589 | + | 10 | TAAGAGAGGAA | light responsive element |

>HU04G00047.1   
+ +Up\_Stream \_Len000TCTTAA CATTAGTTGC TTGGTTCAAC CCATCAGTGA GAATATCTGA TTTCAAAAGT   
  
  
+ ATAGATTTAT ATAAACATAT GGACTACAGG GAAGGGAGTC GTTTCCTCAA AGTCTTAGAT TTACACTCAA   
  
  
+ AAGTTTGAGC GATACCAATA ATGTGATTCC AAACATGAGC CAAATATTAA GTATCCTATA CTAAGATCAA   
  
  
+ GAAGAGAATG AACCAAAAGA CTGTTAATTT TTCAATTAAG CTAAACTTAT TAACCTTTTT TTGGAATAGA   
  
  
+ TTTTTAAATT TTATCTTCAA TATAAGTTTT TCCAAGGCGA GAGACATATA GCAATTTTAA TTAAAGTTAA   
  
  
+ AGTAGTATAA CATTTTTTTT AATGGTTATA GGCTTATAGC ACCTTTTCTA GGTGAAAAGA GATATCTTGG   
  
  
+ CCTGATTTCC AGTAAAGTTT TTTTTTTAAA TATTGTCAAT TATTTTTGCA AATGACATTT GGTAATTTGG   
  
  
+ GGGATAATCT GATGTGTTAA AAGATACAAT GAAAATATCA GAAAATAGGA TGCCTCAAAT TTCACAAGAA   
  
  
+ AGAAAAAATC TTTTTGTTGT TAGTATTTCT ACACATCCAC AATTTTTACT CATGGTAAAA AAAAAAGGGC   
  
  
+ CAAATATCTC ACCAATATAA CCAAACTATA TGTTCCATTA TTTAGTAACG GTGATAAATA TGTAATGGGT   
  
  
+ CAAGTTACTA ATAAAATAGC CTACAATACT TGGATATCTA CATAAAATTG ATATAGAATA TATATTGCAA   
  
  
+ TACTTAAATC AAAATTAAAT GAAATATTTA CTTTTTTATT CTATTACAAA TAGCTTCTTA TAGAATAGCA   
  
  
+ ACAATTTTTA ATGTAAGTTT CATTCTTACC AGATTTGAAG AGGTCCGGAT ATAGAAAATC TTATTCGTAT   
  
  
+ AAGTACTCGC ATGAGGGCGG ATAAATTCTA AAATATCAAC CTCAACTCGC TTCTACTATT CCATCTAAGT   
  
  
+ TCAATTTATC ATATAAAAAA ATTCAATCCA CTTTAATGTA CTCAATTCAC TTCAACCTAT TTTAATCAAT   
  
  
+ TTACCCATGA AAAGAACAAG GTCCTAAGTA GATGTTTTCT ATTGATTGAT TTAAACAATT TTCACCTACC   
  
  
+ CAATTATCCC CAAAACTGAA AGAGGGGGCA AAGGGGGGTG GGTAATGCTG TGTAAAATAG GGGGAAAGGG   
  
  
+ CAAGAAAACT GTGTACAATG GTGGAGCAGG GGTATACCAT TTGGCCTTGA GTTGAGGGGG CACAAAAGCC   
  
  
+ CAGAGAGAGA GAGGGGTAAT GCCCACCAGA AATAAGCAGG AAGCGAGGGA AAAACACTAG AGAGAAACAG   
  
  
+ AGAAAGAGAG AGAGAGAGAG AGAGAAGGGG AAATTTACTA GAGAATGAGA GTGAAGAAGG GAACTTTATT   
  
  
+ TTAACTTTTT TATGATGCAC GCATCCATGG CAGAACAGGG AAGGGAAAGA AGGGTACAGA GTACTGCTGT   
  
  
+ CCCCAAAAAA TTAGTGTAGA AAACAGGAGA CACTCAACTA CAACAAGGGT AAGGAAGAAG AACAAGCAAG   
  
  
+ ATATCCCACC CAAAGAAAAA AAAAAAGAAG AAGAAGAAAA TTTTTCAGAG AGGAAAGAAA AGCAATCACA   
  
  
+ TGAATTAGAA GCCAACCCAA GCCTTCTTCA AAGAGAAGCA GCATAACATA GCATAGCTGG GGAGAGGATA   
  
  
+ GGAGAGAGAA ACTCAAGAAA AGGGGTACCC GGCTTAAATG TTGATGCAGA AAGGTATAGA TATGGCCACA   
  
  
+ CTACTACTAC CCAGGAGTTT ATCATTGGAG ATGGATTTCT CTTTCTGCCT CATCTTCTCC TCCATCCACC   
  
  
+ AGCAAAACTG GGTTTCCTTT CAGCTGGGTC TTTTCTAGAT CTAGAAACTA TGGTACCCAA ATCTCCCATT   
  
  
+ CCTCCATTTT TATTCTCAAA ATCTATCTTC TTAGACTTGT GGGTTTGATT CATTTCTTTT GGGTTTTTGA   
  
  
+ AAAGAATTTA ATTTGATTGT ATTGTGTTGA TTGGGGAATT TGTGGTGGTG TTAGATGCGA GCTATGCCCT   
  
  
+ ACAATTTGCA AGGCAAGGGT GTGGTAGAGG TTTCAAGCAT TTGTATTCCA CAAATCTCTT CCCCTGCTTC   
  
  
+ AAAGTGGAAG ACCAACAACA ACAACTTGGA CAACAAATTT CAGCAACAAC AAGAAGAAAT TGAGCAATTG   
  
  
+ CAGAGTAGTG GTGGCACTTT TCTCTCTCCT GATAACACCA ATACTAATAT TAGTACTACT CCGAGAGCGA   
  
  
+ CAAGTTCTGT TGATAGCGAA CACACATCTA CTCTGGATAC TACGGGCCCT CCCACTTCAA CATCTTCCTC   
  
  
+ CTTCAAGAGC ACCTCCACCG CCAACACAGC CGGTGTGGCG GACCCCGCAT GTACTCCCAA AGAGGATTGG   
  
  
+ GATGTCTCCG GCGGCGCTGC TGGCGGTGAC GGCGGTGGTG GGGGGCTTGG ATTGGAGGAG TGGGACAGCA   
  
  
+ TGTTCCCAAA TGGGGAGGGG GCTTTGCTCC CTTGGATCAT GGGTGAAGCT GATGACATGG GTATGGGTTT   
  
  
+ GAAGCATCTT TTGCAATCGG GTAACCCGGT TGACTATGAG GGCAATGCTG GTCTAGGGGT TGTCGATCAG   
  
  
+ GGTTCTGGAT TTGAGACTCT CTCTCCTCCT CCACCGCAGC CGGCGGCATG TGAATCTAAT GGTGGTGGTG   
  
  
+ TTGCTAATTT GGGGTTTCCA GGGAATAATG GTAAGATTTC TTCAATTTCA CATAATTGTT CATCTGGGAT   
  
  
+ TTTGAATGGT AAGGTGAACA ATAATGGGTT GAATCCCAAT TGTAACCCTC AAGCTCAAGG CAGTCTACTT   
  
  
+ GGTTTAATCC AAGGAGCTAC AGGTGTACAC GCACATCCTG ACGTTGGAGA CGAGAAACCC CAGATTTTGA   
  
  
+ ATCCACATTT GGTGATGAAC CCTCAGCAAG CTCAGAGCAT TGCAAACCCT AGCTTTTTGA TGCACTCATT   
  
  
+ AGGTTACTAT CAGCTGGAGC AACATCTATT TCAACCTCAG GCAAAACGCC TGAACACGGG TGCTGTTCTG   
  
  
+ GATCCTAACC TTGTTCAGCT TGCAAAGAAC CCATTTGCTG ATCAGGGTCA TGAGTTATTG TTGAGGAAGC   
  
  
+ AGCACCAACA GCTTGGTTTG CAACCATTGC CATTGGGTTT GGGTCCTCAG TTGGTCCCTC CGCAGAAGCC   
  
  
+ TGTGATGGGT TCAAAGCAGG GGAACCCCCA GCATTACCCG TTGCATGTTC ATCAGCAGCT GCAATTGCAA   
  
  
+ GAGCAGGCTG TCAAAGATCA GCTCTTCAAG GCGGCAGACC TTATTCAAAC TGGAAGTTTC TCACTCGCGC   
  
  
+ AAGAGATATT GGCGCGGCTC AATCACCAGC TCTCCCTCCC TGCAAAGCCC CTCATTAGGG CGGCTTTGTA   
  
  
+ TGTGAAGGAG GCCCTTCAAA TGCTCCTCCT AATGAGCAAC CCAGTTGCGG CTCCACCGTC CAAGATCCTC   
  
  
+ ACCCCTTATG ATGTTGTTCA CAAGATGAGC GCGTATAAGG TCTTCTCTGA GGTCTGCCCA ATCACTCAAT   
  
  
+ TTGTGAATTT CACTTGTACA CAGGCCATTC TCGAGGCTCT TGATGATTCT GATGCTATTC ATGTTGTCGA   
  
  
+ CTTTGATATT GGTTGCGGTG CTCAATGGGC ATCATTGATT CAGGAGCTGC CATTGAGGAA AAGGGGAGCT   
  
  
+ CCCTCTCTGA AAATTACAGC CATAGCTCCC ATGTTGACTG GCAGTCACTT TGAAATTAGC CTAGTATGTG   
  
  
+ AAAACCTTGT GCAATTTGCC AACGATATTG GTGTTGCTTG TGAGCTCCAA GTTGTCAACT TTGATTTGTT   
  
  
+ TGATCCATCT TCGCCATCAA TGCCGAACAT TAGTACTGCT GAGGATGAGT CAATTGCTGT TAGTATCCCC   
  
  
+ ATCTGGGCAT CTTCAATTAG GCCATCTGTT CTTCCTTCCA TCCTCCGATT CATTAAGCAA AAATCCCCCA   
  
  
+ AAATTGTGGT CTCGTTTGAT AGAGGATTCG ATCGTTTTGA TGTCCCTTTC CCCCAACATC TGTTGCATAC   
  
  
+ CCTAGAATCC TGCAGTAATT TATTGGACTC GCTTGATGGT CTCAATGTTG CATCGGACAT TGTGAGCAAG   
  
  
+ GTCGAGAAGT TCTTTGTTCA ACCTAGGATC GAAAATGCTG TGTTGGGTCG GGTCCATGCC CCTGACAAGA   
  
  
+ TGCCCCATTG GAAAAATCTC TTTGCTTCAG CCGGCTTCTT GCCCTTGCAA TTCAGTAATT TCACAGAAAC   
  
  
+ CCAGGCCGAT TATGTGGTGA AGAGAACCCC AACAAGAGGA TTTCACGTGG AGAAGCGCCA GGCATCACTC   
  
  
+ ATTCTGAGTT GGCAGAGGCA GGAGCTTGTG GCAGCTTCGG CATGGAAGTG TTG  

- +Up\_Stream \_Len000AGAATT GTAATCAACG AACCAAGTTG GGTAGTCACT CTTATAGACT AAAGTTTTCA   
  
  
- TATCTAAATA TATTTGTATA CCTGATGTCC CTTCCCTCAG CAAAGGAGTT TCAGAATCTA AATGTGAGTT   
  
  
- TTCAAACTCG CTATGGTTAT TACACTAAGG TTTGTACTCG GTTTATAATT CATAGGATAT GATTCTAGTT   
  
  
- CTTCTCTTAC TTGGTTTTCT GACAATTAAA AAGTTAATTC GATTTGAATA ATTGGAAAAA AACCTTATCT   
  
  
- AAAAATTTAA AATAGAAGTT ATATTCAAAA AGGTTCCGCT CTCTGTATAT CGTTAAAATT AATTTCAATT   
  
  
- TCATCATATT GTAAAAAAAA TTACCAATAT CCGAATATCG TGGAAAAGAT CCACTTTTCT CTATAGAACC   
  
  
- GGACTAAAGG TCATTTCAAA AAAAAAATTT ATAACAGTTA ATAAAAACGT TTACTGTAAA CCATTAAACC   
  
  
- CCCTATTAGA CTACACAATT TTCTATGTTA CTTTTATAGT CTTTTATCCT ACGGAGTTTA AAGTGTTCTT   
  
  
- TCTTTTTTAG AAAAACAACA ATCATAAAGA TGTGTAGGTG TTAAAAATGA GTACCATTTT TTTTTTCCCG   
  
  
- GTTTATAGAG TGGTTATATT GGTTTGATAT ACAAGGTAAT AAATCATTGC CACTATTTAT ACATTACCCA   
  
  
- GTTCAATGAT TATTTTATCG GATGTTATGA ACCTATAGAT GTATTTTAAC TATATCTTAT ATATAACGTT   
  
  
- ATGAATTTAG TTTTAATTTA CTTTATAAAT GAAAAAATAA GATAATGTTT ATCGAAGAAT ATCTTATCGT   
  
  
- TGTTAAAAAT TACATTCAAA GTAAGAATGG TCTAAACTTC TCCAGGCCTA TATCTTTTAG AATAAGCATA   
  
  
- TTCATGAGCG TACTCCCGCC TATTTAAGAT TTTATAGTTG GAGTTGAGCG AAGATGATAA GGTAGATTCA   
  
  
- AGTTAAATAG TATATTTTTT TAAGTTAGGT GAAATTACAT GAGTTAAGTG AAGTTGGATA AAATTAGTTA   
  
  
- AATGGGTACT TTTCTTGTTC CAGGATTCAT CTACAAAAGA TAACTAACTA AATTTGTTAA AAGTGGATGG   
  
  
- GTTAATAGGG GTTTTGACTT TCTCCCCCGT TTCCCCCCAC CCATTACGAC ACATTTTATC CCCCTTTCCC   
  
  
- GTTCTTTTGA CACATGTTAC CACCTCGTCC CCATATGGTA AACCGGAACT CAACTCCCCC GTGTTTTCGG   
  
  
- GTCTCTCTCT CTCCCCATTA CGGGTGGTCT TTATTCGTCC TTCGCTCCCT TTTTGTGATC TCTCTTTGTC   
  
  
- TCTTTCTCTC TCTCTCTCTC TCTCTTCCCC TTTAAATGAT CTCTTACTCT CACTTCTTCC CTTGAAATAA   
  
  
- AATTGAAAAA ATACTACGTG CGTAGGTACC GTCTTGTCCC TTCCCTTTCT TCCCATGTCT CATGACGACA   
  
  
- GGGGTTTTTT AATCACATCT TTTGTCCTCT GTGAGTTGAT GTTGTTCCCA TTCCTTCTTC TTGTTCGTTC   
  
  
- TATAGGGTGG GTTTCTTTTT TTTTTTCTTC TTCTTCTTTT AAAAAGTCTC TCCTTTCTTT TCGTTAGTGT   
  
  
- ACTTAATCTT CGGTTGGGTT CGGAAGAAGT TTCTCTTCGT CGTATTGTAT CGTATCGACC CCTCTCCTAT   
  
  
- CCTCTCTCTT TGAGTTCTTT TCCCCATGGG CCGAATTTAC AACTACGTCT TTCCATATCT ATACCGGTGT   
  
  
- GATGATGATG GGTCCTCAAA TAGTAACCTC TACCTAAAGA GAAAGACGGA GTAGAAGAGG AGGTAGGTGG   
  
  
- TCGTTTTGAC CCAAAGGAAA GTCGACCCAG AAAAGATCTA GATCTTTGAT ACCATGGGTT TAGAGGGTAA   
  
  
- GGAGGTAAAA ATAAGAGTTT TAGATAGAAG AATCTGAACA CCCAAACTAA GTAAAGAAAA CCCAAAAACT   
  
  
- TTTCTTAAAT TAAACTAACA TAACACAACT AACCCCTTAA ACACCACCAC AATCTACGCT CGATACGGGA   
  
  
- TGTTAAACGT TCCGTTCCCA CACCATCTCC AAAGTTCGTA AACATAAGGT GTTTAGAGAA GGGGACGAAG   
  
  
- TTTCACCTTC TGGTTGTTGT TGTTGAACCT GTTGTTTAAA GTCGTTGTTG TTCTTCTTTA ACTCGTTAAC   
  
  
- GTCTCATCAC CACCGTGAAA AGAGAGAGGA CTATTGTGGT TATGATTATA ATCATGATGA GGCTCTCGCT   
  
  
- GTTCAAGACA ACTATCGCTT GTGTGTAGAT GAGACCTATG ATGCCCGGGA GGGTGAAGTT GTAGAAGGAG   
  
  
- GAAGTTCTCG TGGAGGTGGC GGTTGTGTCG GCCACACCGC CTGGGGCGTA CATGAGGGTT TCTCCTAACC   
  
  
- CTACAGAGGC CGCCGCGACG ACCGCCACTG CCGCCACCAC CCCCCGAACC TAACCTCCTC ACCCTGTCGT   
  
  
- ACAAGGGTTT ACCCCTCCCC CGAAACGAGG GAACCTAGTA CCCACTTCGA CTACTGTACC CATACCCAAA   
  
  
- CTTCGTAGAA AACGTTAGCC CATTGGGCCA ACTGATACTC CCGTTACGAC CAGATCCCCA ACAGCTAGTC   
  
  
- CCAAGACCTA AACTCTGAGA GAGAGGAGGA GGTGGCGTCG GCCGCCGTAC ACTTAGATTA CCACCACCAC   
  
  
- AACGATTAAA CCCCAAAGGT CCCTTATTAC CATTCTAAAG AAGTTAAAGT GTATTAACAA GTAGACCCTA   
  
  
- AAACTTACCA TTCCACTTGT TATTACCCAA CTTAGGGTTA ACATTGGGAG TTCGAGTTCC GTCAGATGAA   
  
  
- CCAAATTAGG TTCCTCGATG TCCACATGTG CGTGTAGGAC TGCAACCTCT GCTCTTTGGG GTCTAAAACT   
  
  
- TAGGTGTAAA CCACTACTTG GGAGTCGTTC GAGTCTCGTA ACGTTTGGGA TCGAAAAACT ACGTGAGTAA   
  
  
- TCCAATGATA GTCGACCTCG TTGTAGATAA AGTTGGAGTC CGTTTTGCGG ACTTGTGCCC ACGACAAGAC   
  
  
- CTAGGATTGG AACAAGTCGA ACGTTTCTTG GGTAAACGAC TAGTCCCAGT ACTCAATAAC AACTCCTTCG   
  
  
- TCGTGGTTGT CGAACCAAAC GTTGGTAACG GTAACCCAAA CCCAGGAGTC AACCAGGGAG GCGTCTTCGG   
  
  
- ACACTACCCA AGTTTCGTCC CCTTGGGGGT CGTAATGGGC AACGTACAAG TAGTCGTCGA CGTTAACGTT   
  
  
- CTCGTCCGAC AGTTTCTAGT CGAGAAGTTC CGCCGTCTGG AATAAGTTTG ACCTTCAAAG AGTGAGCGCG   
  
  
- TTCTCTATAA CCGCGCCGAG TTAGTGGTCG AGAGGGAGGG ACGTTTCGGG GAGTAATCCC GCCGAAACAT   
  
  
- ACACTTCCTC CGGGAAGTTT ACGAGGAGGA TTACTCGTTG GGTCAACGCC GAGGTGGCAG GTTCTAGGAG   
  
  
- TGGGGAATAC TACAACAAGT GTTCTACTCG CGCATATTCC AGAAGAGACT CCAGACGGGT TAGTGAGTTA   
  
  
- AACACTTAAA GTGAACATGT GTCCGGTAAG AGCTCCGAGA ACTACTAAGA CTACGATAAG TACAACAGCT   
  
  
- GAAACTATAA CCAACGCCAC GAGTTACCCG TAGTAACTAA GTCCTCGACG GTAACTCCTT TTCCCCTCGA   
  
  
- GGGAGAGACT TTTAATGTCG GTATCGAGGG TACAACTGAC CGTCAGTGAA ACTTTAATCG GATCATACAC   
  
  
- TTTTGGAACA CGTTAAACGG TTGCTATAAC CACAACGAAC ACTCGAGGTT CAACAGTTGA AACTAAACAA   
  
  
- ACTAGGTAGA AGCGGTAGTT ACGGCTTGTA ATCATGACGA CTCCTACTCA GTTAACGACA ATCATAGGGG   
  
  
- TAGACCCGTA GAAGTTAATC CGGTAGACAA GAAGGAAGGT AGGAGGCTAA GTAATTCGTT TTTAGGGGGT   
  
  
- TTTAACACCA GAGCAAACTA TCTCCTAAGC TAGCAAAACT ACAGGGAAAG GGGGTTGTAG ACAACGTATG   
  
  
- GGATCTTAGG ACGTCATTAA ATAACCTGAG CGAACTACCA GAGTTACAAC GTAGCCTGTA ACACTCGTTC   
  
  
- CAGCTCTTCA AGAAACAAGT TGGATCCTAG CTTTTACGAC ACAACCCAGC CCAGGTACGG GGACTGTTCT   
  
  
- ACGGGGTAAC CTTTTTAGAG AAACGAAGTC GGCCGAAGAA CGGGAACGTT AAGTCATTAA AGTGTCTTTG   
  
  
- GGTCCGGCTA ATACACCACT TCTCTTGGGG TTGTTCTCCT AAAGTGCACC TCTTCGCGGT CCGTAGTGAG   
  
  
- TAAGACTCAA CCGTCTCCGT CCTCGAACAC CGTCGAAGCC GTACCTTCAC AAC

+     A-box

| Site Name | Organism | Position | Strand | Matrix score. | sequence | function |
| --- | --- | --- | --- | --- | --- | --- |
| A-box | Petroselinum crispum | 3420 | + | 6 | CCGTCC | cis-acting regulatory element |

>HU04G00047.1   
+ +Up\_Stream \_Len000TCTTAA CATTAGTTGC TTGGTTCAAC CCATCAGTGA GAATATCTGA TTTCAAAAGT   
  
  
+ ATAGATTTAT ATAAACATAT GGACTACAGG GAAGGGAGTC GTTTCCTCAA AGTCTTAGAT TTACACTCAA   
  
  
+ AAGTTTGAGC GATACCAATA ATGTGATTCC AAACATGAGC CAAATATTAA GTATCCTATA CTAAGATCAA   
  
  
+ GAAGAGAATG AACCAAAAGA CTGTTAATTT TTCAATTAAG CTAAACTTAT TAACCTTTTT TTGGAATAGA   
  
  
+ TTTTTAAATT TTATCTTCAA TATAAGTTTT TCCAAGGCGA GAGACATATA GCAATTTTAA TTAAAGTTAA   
  
  
+ AGTAGTATAA CATTTTTTTT AATGGTTATA GGCTTATAGC ACCTTTTCTA GGTGAAAAGA GATATCTTGG   
  
  
+ CCTGATTTCC AGTAAAGTTT TTTTTTTAAA TATTGTCAAT TATTTTTGCA AATGACATTT GGTAATTTGG   
  
  
+ GGGATAATCT GATGTGTTAA AAGATACAAT GAAAATATCA GAAAATAGGA TGCCTCAAAT TTCACAAGAA   
  
  
+ AGAAAAAATC TTTTTGTTGT TAGTATTTCT ACACATCCAC AATTTTTACT CATGGTAAAA AAAAAAGGGC   
  
  
+ CAAATATCTC ACCAATATAA CCAAACTATA TGTTCCATTA TTTAGTAACG GTGATAAATA TGTAATGGGT   
  
  
+ CAAGTTACTA ATAAAATAGC CTACAATACT TGGATATCTA CATAAAATTG ATATAGAATA TATATTGCAA   
  
  
+ TACTTAAATC AAAATTAAAT GAAATATTTA CTTTTTTATT CTATTACAAA TAGCTTCTTA TAGAATAGCA   
  
  
+ ACAATTTTTA ATGTAAGTTT CATTCTTACC AGATTTGAAG AGGTCCGGAT ATAGAAAATC TTATTCGTAT   
  
  
+ AAGTACTCGC ATGAGGGCGG ATAAATTCTA AAATATCAAC CTCAACTCGC TTCTACTATT CCATCTAAGT   
  
  
+ TCAATTTATC ATATAAAAAA ATTCAATCCA CTTTAATGTA CTCAATTCAC TTCAACCTAT TTTAATCAAT   
  
  
+ TTACCCATGA AAAGAACAAG GTCCTAAGTA GATGTTTTCT ATTGATTGAT TTAAACAATT TTCACCTACC   
  
  
+ CAATTATCCC CAAAACTGAA AGAGGGGGCA AAGGGGGGTG GGTAATGCTG TGTAAAATAG GGGGAAAGGG   
  
  
+ CAAGAAAACT GTGTACAATG GTGGAGCAGG GGTATACCAT TTGGCCTTGA GTTGAGGGGG CACAAAAGCC   
  
  
+ CAGAGAGAGA GAGGGGTAAT GCCCACCAGA AATAAGCAGG AAGCGAGGGA AAAACACTAG AGAGAAACAG   
  
  
+ AGAAAGAGAG AGAGAGAGAG AGAGAAGGGG AAATTTACTA GAGAATGAGA GTGAAGAAGG GAACTTTATT   
  
  
+ TTAACTTTTT TATGATGCAC GCATCCATGG CAGAACAGGG AAGGGAAAGA AGGGTACAGA GTACTGCTGT   
  
  
+ CCCCAAAAAA TTAGTGTAGA AAACAGGAGA CACTCAACTA CAACAAGGGT AAGGAAGAAG AACAAGCAAG   
  
  
+ ATATCCCACC CAAAGAAAAA AAAAAAGAAG AAGAAGAAAA TTTTTCAGAG AGGAAAGAAA AGCAATCACA   
  
  
+ TGAATTAGAA GCCAACCCAA GCCTTCTTCA AAGAGAAGCA GCATAACATA GCATAGCTGG GGAGAGGATA   
  
  
+ GGAGAGAGAA ACTCAAGAAA AGGGGTACCC GGCTTAAATG TTGATGCAGA AAGGTATAGA TATGGCCACA   
  
  
+ CTACTACTAC CCAGGAGTTT ATCATTGGAG ATGGATTTCT CTTTCTGCCT CATCTTCTCC TCCATCCACC   
  
  
+ AGCAAAACTG GGTTTCCTTT CAGCTGGGTC TTTTCTAGAT CTAGAAACTA TGGTACCCAA ATCTCCCATT   
  
  
+ CCTCCATTTT TATTCTCAAA ATCTATCTTC TTAGACTTGT GGGTTTGATT CATTTCTTTT GGGTTTTTGA   
  
  
+ AAAGAATTTA ATTTGATTGT ATTGTGTTGA TTGGGGAATT TGTGGTGGTG TTAGATGCGA GCTATGCCCT   
  
  
+ ACAATTTGCA AGGCAAGGGT GTGGTAGAGG TTTCAAGCAT TTGTATTCCA CAAATCTCTT CCCCTGCTTC   
  
  
+ AAAGTGGAAG ACCAACAACA ACAACTTGGA CAACAAATTT CAGCAACAAC AAGAAGAAAT TGAGCAATTG   
  
  
+ CAGAGTAGTG GTGGCACTTT TCTCTCTCCT GATAACACCA ATACTAATAT TAGTACTACT CCGAGAGCGA   
  
  
+ CAAGTTCTGT TGATAGCGAA CACACATCTA CTCTGGATAC TACGGGCCCT CCCACTTCAA CATCTTCCTC   
  
  
+ CTTCAAGAGC ACCTCCACCG CCAACACAGC CGGTGTGGCG GACCCCGCAT GTACTCCCAA AGAGGATTGG   
  
  
+ GATGTCTCCG GCGGCGCTGC TGGCGGTGAC GGCGGTGGTG GGGGGCTTGG ATTGGAGGAG TGGGACAGCA   
  
  
+ TGTTCCCAAA TGGGGAGGGG GCTTTGCTCC CTTGGATCAT GGGTGAAGCT GATGACATGG GTATGGGTTT   
  
  
+ GAAGCATCTT TTGCAATCGG GTAACCCGGT TGACTATGAG GGCAATGCTG GTCTAGGGGT TGTCGATCAG   
  
  
+ GGTTCTGGAT TTGAGACTCT CTCTCCTCCT CCACCGCAGC CGGCGGCATG TGAATCTAAT GGTGGTGGTG   
  
  
+ TTGCTAATTT GGGGTTTCCA GGGAATAATG GTAAGATTTC TTCAATTTCA CATAATTGTT CATCTGGGAT   
  
  
+ TTTGAATGGT AAGGTGAACA ATAATGGGTT GAATCCCAAT TGTAACCCTC AAGCTCAAGG CAGTCTACTT   
  
  
+ GGTTTAATCC AAGGAGCTAC AGGTGTACAC GCACATCCTG ACGTTGGAGA CGAGAAACCC CAGATTTTGA   
  
  
+ ATCCACATTT GGTGATGAAC CCTCAGCAAG CTCAGAGCAT TGCAAACCCT AGCTTTTTGA TGCACTCATT   
  
  
+ AGGTTACTAT CAGCTGGAGC AACATCTATT TCAACCTCAG GCAAAACGCC TGAACACGGG TGCTGTTCTG   
  
  
+ GATCCTAACC TTGTTCAGCT TGCAAAGAAC CCATTTGCTG ATCAGGGTCA TGAGTTATTG TTGAGGAAGC   
  
  
+ AGCACCAACA GCTTGGTTTG CAACCATTGC CATTGGGTTT GGGTCCTCAG TTGGTCCCTC CGCAGAAGCC   
  
  
+ TGTGATGGGT TCAAAGCAGG GGAACCCCCA GCATTACCCG TTGCATGTTC ATCAGCAGCT GCAATTGCAA   
  
  
+ GAGCAGGCTG TCAAAGATCA GCTCTTCAAG GCGGCAGACC TTATTCAAAC TGGAAGTTTC TCACTCGCGC   
  
  
+ AAGAGATATT GGCGCGGCTC AATCACCAGC TCTCCCTCCC TGCAAAGCCC CTCATTAGGG CGGCTTTGTA   
  
  
+ TGTGAAGGAG GCCCTTCAAA TGCTCCTCCT AATGAGCAAC CCAGTTGCGG CTCCACCGTC CAAGATCCTC   
  
  
+ ACCCCTTATG ATGTTGTTCA CAAGATGAGC GCGTATAAGG TCTTCTCTGA GGTCTGCCCA ATCACTCAAT   
  
  
+ TTGTGAATTT CACTTGTACA CAGGCCATTC TCGAGGCTCT TGATGATTCT GATGCTATTC ATGTTGTCGA   
  
  
+ CTTTGATATT GGTTGCGGTG CTCAATGGGC ATCATTGATT CAGGAGCTGC CATTGAGGAA AAGGGGAGCT   
  
  
+ CCCTCTCTGA AAATTACAGC CATAGCTCCC ATGTTGACTG GCAGTCACTT TGAAATTAGC CTAGTATGTG   
  
  
+ AAAACCTTGT GCAATTTGCC AACGATATTG GTGTTGCTTG TGAGCTCCAA GTTGTCAACT TTGATTTGTT   
  
  
+ TGATCCATCT TCGCCATCAA TGCCGAACAT TAGTACTGCT GAGGATGAGT CAATTGCTGT TAGTATCCCC   
  
  
+ ATCTGGGCAT CTTCAATTAG GCCATCTGTT CTTCCTTCCA TCCTCCGATT CATTAAGCAA AAATCCCCCA   
  
  
+ AAATTGTGGT CTCGTTTGAT AGAGGATTCG ATCGTTTTGA TGTCCCTTTC CCCCAACATC TGTTGCATAC   
  
  
+ CCTAGAATCC TGCAGTAATT TATTGGACTC GCTTGATGGT CTCAATGTTG CATCGGACAT TGTGAGCAAG   
  
  
+ GTCGAGAAGT TCTTTGTTCA ACCTAGGATC GAAAATGCTG TGTTGGGTCG GGTCCATGCC CCTGACAAGA   
  
  
+ TGCCCCATTG GAAAAATCTC TTTGCTTCAG CCGGCTTCTT GCCCTTGCAA TTCAGTAATT TCACAGAAAC   
  
  
+ CCAGGCCGAT TATGTGGTGA AGAGAACCCC AACAAGAGGA TTTCACGTGG AGAAGCGCCA GGCATCACTC   
  
  
+ ATTCTGAGTT GGCAGAGGCA GGAGCTTGTG GCAGCTTCGG CATGGAAGTG TTG  

- +Up\_Stream \_Len000AGAATT GTAATCAACG AACCAAGTTG GGTAGTCACT CTTATAGACT AAAGTTTTCA   
  
  
- TATCTAAATA TATTTGTATA CCTGATGTCC CTTCCCTCAG CAAAGGAGTT TCAGAATCTA AATGTGAGTT   
  
  
- TTCAAACTCG CTATGGTTAT TACACTAAGG TTTGTACTCG GTTTATAATT CATAGGATAT GATTCTAGTT   
  
  
- CTTCTCTTAC TTGGTTTTCT GACAATTAAA AAGTTAATTC GATTTGAATA ATTGGAAAAA AACCTTATCT   
  
  
- AAAAATTTAA AATAGAAGTT ATATTCAAAA AGGTTCCGCT CTCTGTATAT CGTTAAAATT AATTTCAATT   
  
  
- TCATCATATT GTAAAAAAAA TTACCAATAT CCGAATATCG TGGAAAAGAT CCACTTTTCT CTATAGAACC   
  
  
- GGACTAAAGG TCATTTCAAA AAAAAAATTT ATAACAGTTA ATAAAAACGT TTACTGTAAA CCATTAAACC   
  
  
- CCCTATTAGA CTACACAATT TTCTATGTTA CTTTTATAGT CTTTTATCCT ACGGAGTTTA AAGTGTTCTT   
  
  
- TCTTTTTTAG AAAAACAACA ATCATAAAGA TGTGTAGGTG TTAAAAATGA GTACCATTTT TTTTTTCCCG   
  
  
- GTTTATAGAG TGGTTATATT GGTTTGATAT ACAAGGTAAT AAATCATTGC CACTATTTAT ACATTACCCA   
  
  
- GTTCAATGAT TATTTTATCG GATGTTATGA ACCTATAGAT GTATTTTAAC TATATCTTAT ATATAACGTT   
  
  
- ATGAATTTAG TTTTAATTTA CTTTATAAAT GAAAAAATAA GATAATGTTT ATCGAAGAAT ATCTTATCGT   
  
  
- TGTTAAAAAT TACATTCAAA GTAAGAATGG TCTAAACTTC TCCAGGCCTA TATCTTTTAG AATAAGCATA   
  
  
- TTCATGAGCG TACTCCCGCC TATTTAAGAT TTTATAGTTG GAGTTGAGCG AAGATGATAA GGTAGATTCA   
  
  
- AGTTAAATAG TATATTTTTT TAAGTTAGGT GAAATTACAT GAGTTAAGTG AAGTTGGATA AAATTAGTTA   
  
  
- AATGGGTACT TTTCTTGTTC CAGGATTCAT CTACAAAAGA TAACTAACTA AATTTGTTAA AAGTGGATGG   
  
  
- GTTAATAGGG GTTTTGACTT TCTCCCCCGT TTCCCCCCAC CCATTACGAC ACATTTTATC CCCCTTTCCC   
  
  
- GTTCTTTTGA CACATGTTAC CACCTCGTCC CCATATGGTA AACCGGAACT CAACTCCCCC GTGTTTTCGG   
  
  
- GTCTCTCTCT CTCCCCATTA CGGGTGGTCT TTATTCGTCC TTCGCTCCCT TTTTGTGATC TCTCTTTGTC   
  
  
- TCTTTCTCTC TCTCTCTCTC TCTCTTCCCC TTTAAATGAT CTCTTACTCT CACTTCTTCC CTTGAAATAA   
  
  
- AATTGAAAAA ATACTACGTG CGTAGGTACC GTCTTGTCCC TTCCCTTTCT TCCCATGTCT CATGACGACA   
  
  
- GGGGTTTTTT AATCACATCT TTTGTCCTCT GTGAGTTGAT GTTGTTCCCA TTCCTTCTTC TTGTTCGTTC   
  
  
- TATAGGGTGG GTTTCTTTTT TTTTTTCTTC TTCTTCTTTT AAAAAGTCTC TCCTTTCTTT TCGTTAGTGT   
  
  
- ACTTAATCTT CGGTTGGGTT CGGAAGAAGT TTCTCTTCGT CGTATTGTAT CGTATCGACC CCTCTCCTAT   
  
  
- CCTCTCTCTT TGAGTTCTTT TCCCCATGGG CCGAATTTAC AACTACGTCT TTCCATATCT ATACCGGTGT   
  
  
- GATGATGATG GGTCCTCAAA TAGTAACCTC TACCTAAAGA GAAAGACGGA GTAGAAGAGG AGGTAGGTGG   
  
  
- TCGTTTTGAC CCAAAGGAAA GTCGACCCAG AAAAGATCTA GATCTTTGAT ACCATGGGTT TAGAGGGTAA   
  
  
- GGAGGTAAAA ATAAGAGTTT TAGATAGAAG AATCTGAACA CCCAAACTAA GTAAAGAAAA CCCAAAAACT   
  
  
- TTTCTTAAAT TAAACTAACA TAACACAACT AACCCCTTAA ACACCACCAC AATCTACGCT CGATACGGGA   
  
  
- TGTTAAACGT TCCGTTCCCA CACCATCTCC AAAGTTCGTA AACATAAGGT GTTTAGAGAA GGGGACGAAG   
  
  
- TTTCACCTTC TGGTTGTTGT TGTTGAACCT GTTGTTTAAA GTCGTTGTTG TTCTTCTTTA ACTCGTTAAC   
  
  
- GTCTCATCAC CACCGTGAAA AGAGAGAGGA CTATTGTGGT TATGATTATA ATCATGATGA GGCTCTCGCT   
  
  
- GTTCAAGACA ACTATCGCTT GTGTGTAGAT GAGACCTATG ATGCCCGGGA GGGTGAAGTT GTAGAAGGAG   
  
  
- GAAGTTCTCG TGGAGGTGGC GGTTGTGTCG GCCACACCGC CTGGGGCGTA CATGAGGGTT TCTCCTAACC   
  
  
- CTACAGAGGC CGCCGCGACG ACCGCCACTG CCGCCACCAC CCCCCGAACC TAACCTCCTC ACCCTGTCGT   
  
  
- ACAAGGGTTT ACCCCTCCCC CGAAACGAGG GAACCTAGTA CCCACTTCGA CTACTGTACC CATACCCAAA   
  
  
- CTTCGTAGAA AACGTTAGCC CATTGGGCCA ACTGATACTC CCGTTACGAC CAGATCCCCA ACAGCTAGTC   
  
  
- CCAAGACCTA AACTCTGAGA GAGAGGAGGA GGTGGCGTCG GCCGCCGTAC ACTTAGATTA CCACCACCAC   
  
  
- AACGATTAAA CCCCAAAGGT CCCTTATTAC CATTCTAAAG AAGTTAAAGT GTATTAACAA GTAGACCCTA   
  
  
- AAACTTACCA TTCCACTTGT TATTACCCAA CTTAGGGTTA ACATTGGGAG TTCGAGTTCC GTCAGATGAA   
  
  
- CCAAATTAGG TTCCTCGATG TCCACATGTG CGTGTAGGAC TGCAACCTCT GCTCTTTGGG GTCTAAAACT   
  
  
- TAGGTGTAAA CCACTACTTG GGAGTCGTTC GAGTCTCGTA ACGTTTGGGA TCGAAAAACT ACGTGAGTAA   
  
  
- TCCAATGATA GTCGACCTCG TTGTAGATAA AGTTGGAGTC CGTTTTGCGG ACTTGTGCCC ACGACAAGAC   
  
  
- CTAGGATTGG AACAAGTCGA ACGTTTCTTG GGTAAACGAC TAGTCCCAGT ACTCAATAAC AACTCCTTCG   
  
  
- TCGTGGTTGT CGAACCAAAC GTTGGTAACG GTAACCCAAA CCCAGGAGTC AACCAGGGAG GCGTCTTCGG   
  
  
- ACACTACCCA AGTTTCGTCC CCTTGGGGGT CGTAATGGGC AACGTACAAG TAGTCGTCGA CGTTAACGTT   
  
  
- CTCGTCCGAC AGTTTCTAGT CGAGAAGTTC CGCCGTCTGG AATAAGTTTG ACCTTCAAAG AGTGAGCGCG   
  
  
- TTCTCTATAA CCGCGCCGAG TTAGTGGTCG AGAGGGAGGG ACGTTTCGGG GAGTAATCCC GCCGAAACAT   
  
  
- ACACTTCCTC CGGGAAGTTT ACGAGGAGGA TTACTCGTTG GGTCAACGCC GAGGTGGCAG GTTCTAGGAG   
  
  
- TGGGGAATAC TACAACAAGT GTTCTACTCG CGCATATTCC AGAAGAGACT CCAGACGGGT TAGTGAGTTA   
  
  
- AACACTTAAA GTGAACATGT GTCCGGTAAG AGCTCCGAGA ACTACTAAGA CTACGATAAG TACAACAGCT   
  
  
- GAAACTATAA CCAACGCCAC GAGTTACCCG TAGTAACTAA GTCCTCGACG GTAACTCCTT TTCCCCTCGA   
  
  
- GGGAGAGACT TTTAATGTCG GTATCGAGGG TACAACTGAC CGTCAGTGAA ACTTTAATCG GATCATACAC   
  
  
- TTTTGGAACA CGTTAAACGG TTGCTATAAC CACAACGAAC ACTCGAGGTT CAACAGTTGA AACTAAACAA   
  
  
- ACTAGGTAGA AGCGGTAGTT ACGGCTTGTA ATCATGACGA CTCCTACTCA GTTAACGACA ATCATAGGGG   
  
  
- TAGACCCGTA GAAGTTAATC CGGTAGACAA GAAGGAAGGT AGGAGGCTAA GTAATTCGTT TTTAGGGGGT   
  
  
- TTTAACACCA GAGCAAACTA TCTCCTAAGC TAGCAAAACT ACAGGGAAAG GGGGTTGTAG ACAACGTATG   
  
  
- GGATCTTAGG ACGTCATTAA ATAACCTGAG CGAACTACCA GAGTTACAAC GTAGCCTGTA ACACTCGTTC   
  
  
- CAGCTCTTCA AGAAACAAGT TGGATCCTAG CTTTTACGAC ACAACCCAGC CCAGGTACGG GGACTGTTCT   
  
  
- ACGGGGTAAC CTTTTTAGAG AAACGAAGTC GGCCGAAGAA CGGGAACGTT AAGTCATTAA AGTGTCTTTG   
  
  
- GGTCCGGCTA ATACACCACT TCTCTTGGGG TTGTTCTCCT AAAGTGCACC TCTTCGCGGT CCGTAGTGAG   
  
  
- TAAGACTCAA CCGTCTCCGT CCTCGAACAC CGTCGAAGCC GTACCTTCAC AAC

+     AAGAA-motif

| Site Name | Organism | Position | Strand | Matrix score. | sequence | function |
| --- | --- | --- | --- | --- | --- | --- |
| AAGAA-motif | Avena sativa | 618 | + | 9 | gGTAAAGAAA |  |
| AAGAA-motif | Avena sativa | 562 | + | 7 | GAAAGAA |  |
| AAGAA-motif | Avena sativa | 1597 | + | 7 | GAAAGAA |  |
| AAGAA-motif | Avena sativa | 1449 | + | 7 | GAAAGAA |  |

>HU04G00047.1   
+ +Up\_Stream \_Len000TCTTAA CATTAGTTGC TTGGTTCAAC CCATCAGTGA GAATATCTGA TTTCAAAAGT   
  
  
+ ATAGATTTAT ATAAACATAT GGACTACAGG GAAGGGAGTC GTTTCCTCAA AGTCTTAGAT TTACACTCAA   
  
  
+ AAGTTTGAGC GATACCAATA ATGTGATTCC AAACATGAGC CAAATATTAA GTATCCTATA CTAAGATCAA   
  
  
+ GAAGAGAATG AACCAAAAGA CTGTTAATTT TTCAATTAAG CTAAACTTAT TAACCTTTTT TTGGAATAGA   
  
  
+ TTTTTAAATT TTATCTTCAA TATAAGTTTT TCCAAGGCGA GAGACATATA GCAATTTTAA TTAAAGTTAA   
  
  
+ AGTAGTATAA CATTTTTTTT AATGGTTATA GGCTTATAGC ACCTTTTCTA GGTGAAAAGA GATATCTTGG   
  
  
+ CCTGATTTCC AGTAAAGTTT TTTTTTTAAA TATTGTCAAT TATTTTTGCA AATGACATTT GGTAATTTGG   
  
  
+ GGGATAATCT GATGTGTTAA AAGATACAAT GAAAATATCA GAAAATAGGA TGCCTCAAAT TTCACAAGAA   
  
  
+ AGAAAAAATC TTTTTGTTGT TAGTATTTCT ACACATCCAC AATTTTTACT CATGGTAAAA AAAAAAGGGC   
  
  
+ CAAATATCTC ACCAATATAA CCAAACTATA TGTTCCATTA TTTAGTAACG GTGATAAATA TGTAATGGGT   
  
  
+ CAAGTTACTA ATAAAATAGC CTACAATACT TGGATATCTA CATAAAATTG ATATAGAATA TATATTGCAA   
  
  
+ TACTTAAATC AAAATTAAAT GAAATATTTA CTTTTTTATT CTATTACAAA TAGCTTCTTA TAGAATAGCA   
  
  
+ ACAATTTTTA ATGTAAGTTT CATTCTTACC AGATTTGAAG AGGTCCGGAT ATAGAAAATC TTATTCGTAT   
  
  
+ AAGTACTCGC ATGAGGGCGG ATAAATTCTA AAATATCAAC CTCAACTCGC TTCTACTATT CCATCTAAGT   
  
  
+ TCAATTTATC ATATAAAAAA ATTCAATCCA CTTTAATGTA CTCAATTCAC TTCAACCTAT TTTAATCAAT   
  
  
+ TTACCCATGA AAAGAACAAG GTCCTAAGTA GATGTTTTCT ATTGATTGAT TTAAACAATT TTCACCTACC   
  
  
+ CAATTATCCC CAAAACTGAA AGAGGGGGCA AAGGGGGGTG GGTAATGCTG TGTAAAATAG GGGGAAAGGG   
  
  
+ CAAGAAAACT GTGTACAATG GTGGAGCAGG GGTATACCAT TTGGCCTTGA GTTGAGGGGG CACAAAAGCC   
  
  
+ CAGAGAGAGA GAGGGGTAAT GCCCACCAGA AATAAGCAGG AAGCGAGGGA AAAACACTAG AGAGAAACAG   
  
  
+ AGAAAGAGAG AGAGAGAGAG AGAGAAGGGG AAATTTACTA GAGAATGAGA GTGAAGAAGG GAACTTTATT   
  
  
+ TTAACTTTTT TATGATGCAC GCATCCATGG CAGAACAGGG AAGGGAAAGA AGGGTACAGA GTACTGCTGT   
  
  
+ CCCCAAAAAA TTAGTGTAGA AAACAGGAGA CACTCAACTA CAACAAGGGT AAGGAAGAAG AACAAGCAAG   
  
  
+ ATATCCCACC CAAAGAAAAA AAAAAAGAAG AAGAAGAAAA TTTTTCAGAG AGGAAAGAAA AGCAATCACA   
  
  
+ TGAATTAGAA GCCAACCCAA GCCTTCTTCA AAGAGAAGCA GCATAACATA GCATAGCTGG GGAGAGGATA   
  
  
+ GGAGAGAGAA ACTCAAGAAA AGGGGTACCC GGCTTAAATG TTGATGCAGA AAGGTATAGA TATGGCCACA   
  
  
+ CTACTACTAC CCAGGAGTTT ATCATTGGAG ATGGATTTCT CTTTCTGCCT CATCTTCTCC TCCATCCACC   
  
  
+ AGCAAAACTG GGTTTCCTTT CAGCTGGGTC TTTTCTAGAT CTAGAAACTA TGGTACCCAA ATCTCCCATT   
  
  
+ CCTCCATTTT TATTCTCAAA ATCTATCTTC TTAGACTTGT GGGTTTGATT CATTTCTTTT GGGTTTTTGA   
  
  
+ AAAGAATTTA ATTTGATTGT ATTGTGTTGA TTGGGGAATT TGTGGTGGTG TTAGATGCGA GCTATGCCCT   
  
  
+ ACAATTTGCA AGGCAAGGGT GTGGTAGAGG TTTCAAGCAT TTGTATTCCA CAAATCTCTT CCCCTGCTTC   
  
  
+ AAAGTGGAAG ACCAACAACA ACAACTTGGA CAACAAATTT CAGCAACAAC AAGAAGAAAT TGAGCAATTG   
  
  
+ CAGAGTAGTG GTGGCACTTT TCTCTCTCCT GATAACACCA ATACTAATAT TAGTACTACT CCGAGAGCGA   
  
  
+ CAAGTTCTGT TGATAGCGAA CACACATCTA CTCTGGATAC TACGGGCCCT CCCACTTCAA CATCTTCCTC   
  
  
+ CTTCAAGAGC ACCTCCACCG CCAACACAGC CGGTGTGGCG GACCCCGCAT GTACTCCCAA AGAGGATTGG   
  
  
+ GATGTCTCCG GCGGCGCTGC TGGCGGTGAC GGCGGTGGTG GGGGGCTTGG ATTGGAGGAG TGGGACAGCA   
  
  
+ TGTTCCCAAA TGGGGAGGGG GCTTTGCTCC CTTGGATCAT GGGTGAAGCT GATGACATGG GTATGGGTTT   
  
  
+ GAAGCATCTT TTGCAATCGG GTAACCCGGT TGACTATGAG GGCAATGCTG GTCTAGGGGT TGTCGATCAG   
  
  
+ GGTTCTGGAT TTGAGACTCT CTCTCCTCCT CCACCGCAGC CGGCGGCATG TGAATCTAAT GGTGGTGGTG   
  
  
+ TTGCTAATTT GGGGTTTCCA GGGAATAATG GTAAGATTTC TTCAATTTCA CATAATTGTT CATCTGGGAT   
  
  
+ TTTGAATGGT AAGGTGAACA ATAATGGGTT GAATCCCAAT TGTAACCCTC AAGCTCAAGG CAGTCTACTT   
  
  
+ GGTTTAATCC AAGGAGCTAC AGGTGTACAC GCACATCCTG ACGTTGGAGA CGAGAAACCC CAGATTTTGA   
  
  
+ ATCCACATTT GGTGATGAAC CCTCAGCAAG CTCAGAGCAT TGCAAACCCT AGCTTTTTGA TGCACTCATT   
  
  
+ AGGTTACTAT CAGCTGGAGC AACATCTATT TCAACCTCAG GCAAAACGCC TGAACACGGG TGCTGTTCTG   
  
  
+ GATCCTAACC TTGTTCAGCT TGCAAAGAAC CCATTTGCTG ATCAGGGTCA TGAGTTATTG TTGAGGAAGC   
  
  
+ AGCACCAACA GCTTGGTTTG CAACCATTGC CATTGGGTTT GGGTCCTCAG TTGGTCCCTC CGCAGAAGCC   
  
  
+ TGTGATGGGT TCAAAGCAGG GGAACCCCCA GCATTACCCG TTGCATGTTC ATCAGCAGCT GCAATTGCAA   
  
  
+ GAGCAGGCTG TCAAAGATCA GCTCTTCAAG GCGGCAGACC TTATTCAAAC TGGAAGTTTC TCACTCGCGC   
  
  
+ AAGAGATATT GGCGCGGCTC AATCACCAGC TCTCCCTCCC TGCAAAGCCC CTCATTAGGG CGGCTTTGTA   
  
  
+ TGTGAAGGAG GCCCTTCAAA TGCTCCTCCT AATGAGCAAC CCAGTTGCGG CTCCACCGTC CAAGATCCTC   
  
  
+ ACCCCTTATG ATGTTGTTCA CAAGATGAGC GCGTATAAGG TCTTCTCTGA GGTCTGCCCA ATCACTCAAT   
  
  
+ TTGTGAATTT CACTTGTACA CAGGCCATTC TCGAGGCTCT TGATGATTCT GATGCTATTC ATGTTGTCGA   
  
  
+ CTTTGATATT GGTTGCGGTG CTCAATGGGC ATCATTGATT CAGGAGCTGC CATTGAGGAA AAGGGGAGCT   
  
  
+ CCCTCTCTGA AAATTACAGC CATAGCTCCC ATGTTGACTG GCAGTCACTT TGAAATTAGC CTAGTATGTG   
  
  
+ AAAACCTTGT GCAATTTGCC AACGATATTG GTGTTGCTTG TGAGCTCCAA GTTGTCAACT TTGATTTGTT   
  
  
+ TGATCCATCT TCGCCATCAA TGCCGAACAT TAGTACTGCT GAGGATGAGT CAATTGCTGT TAGTATCCCC   
  
  
+ ATCTGGGCAT CTTCAATTAG GCCATCTGTT CTTCCTTCCA TCCTCCGATT CATTAAGCAA AAATCCCCCA   
  
  
+ AAATTGTGGT CTCGTTTGAT AGAGGATTCG ATCGTTTTGA TGTCCCTTTC CCCCAACATC TGTTGCATAC   
  
  
+ CCTAGAATCC TGCAGTAATT TATTGGACTC GCTTGATGGT CTCAATGTTG CATCGGACAT TGTGAGCAAG   
  
  
+ GTCGAGAAGT TCTTTGTTCA ACCTAGGATC GAAAATGCTG TGTTGGGTCG GGTCCATGCC CCTGACAAGA   
  
  
+ TGCCCCATTG GAAAAATCTC TTTGCTTCAG CCGGCTTCTT GCCCTTGCAA TTCAGTAATT TCACAGAAAC   
  
  
+ CCAGGCCGAT TATGTGGTGA AGAGAACCCC AACAAGAGGA TTTCACGTGG AGAAGCGCCA GGCATCACTC   
  
  
+ ATTCTGAGTT GGCAGAGGCA GGAGCTTGTG GCAGCTTCGG CATGGAAGTG TTG  

- +Up\_Stream \_Len000AGAATT GTAATCAACG AACCAAGTTG GGTAGTCACT CTTATAGACT AAAGTTTTCA   
  
  
- TATCTAAATA TATTTGTATA CCTGATGTCC CTTCCCTCAG CAAAGGAGTT TCAGAATCTA AATGTGAGTT   
  
  
- TTCAAACTCG CTATGGTTAT TACACTAAGG TTTGTACTCG GTTTATAATT CATAGGATAT GATTCTAGTT   
  
  
- CTTCTCTTAC TTGGTTTTCT GACAATTAAA AAGTTAATTC GATTTGAATA ATTGGAAAAA AACCTTATCT   
  
  
- AAAAATTTAA AATAGAAGTT ATATTCAAAA AGGTTCCGCT CTCTGTATAT CGTTAAAATT AATTTCAATT   
  
  
- TCATCATATT GTAAAAAAAA TTACCAATAT CCGAATATCG TGGAAAAGAT CCACTTTTCT CTATAGAACC   
  
  
- GGACTAAAGG TCATTTCAAA AAAAAAATTT ATAACAGTTA ATAAAAACGT TTACTGTAAA CCATTAAACC   
  
  
- CCCTATTAGA CTACACAATT TTCTATGTTA CTTTTATAGT CTTTTATCCT ACGGAGTTTA AAGTGTTCTT   
  
  
- TCTTTTTTAG AAAAACAACA ATCATAAAGA TGTGTAGGTG TTAAAAATGA GTACCATTTT TTTTTTCCCG   
  
  
- GTTTATAGAG TGGTTATATT GGTTTGATAT ACAAGGTAAT AAATCATTGC CACTATTTAT ACATTACCCA   
  
  
- GTTCAATGAT TATTTTATCG GATGTTATGA ACCTATAGAT GTATTTTAAC TATATCTTAT ATATAACGTT   
  
  
- ATGAATTTAG TTTTAATTTA CTTTATAAAT GAAAAAATAA GATAATGTTT ATCGAAGAAT ATCTTATCGT   
  
  
- TGTTAAAAAT TACATTCAAA GTAAGAATGG TCTAAACTTC TCCAGGCCTA TATCTTTTAG AATAAGCATA   
  
  
- TTCATGAGCG TACTCCCGCC TATTTAAGAT TTTATAGTTG GAGTTGAGCG AAGATGATAA GGTAGATTCA   
  
  
- AGTTAAATAG TATATTTTTT TAAGTTAGGT GAAATTACAT GAGTTAAGTG AAGTTGGATA AAATTAGTTA   
  
  
- AATGGGTACT TTTCTTGTTC CAGGATTCAT CTACAAAAGA TAACTAACTA AATTTGTTAA AAGTGGATGG   
  
  
- GTTAATAGGG GTTTTGACTT TCTCCCCCGT TTCCCCCCAC CCATTACGAC ACATTTTATC CCCCTTTCCC   
  
  
- GTTCTTTTGA CACATGTTAC CACCTCGTCC CCATATGGTA AACCGGAACT CAACTCCCCC GTGTTTTCGG   
  
  
- GTCTCTCTCT CTCCCCATTA CGGGTGGTCT TTATTCGTCC TTCGCTCCCT TTTTGTGATC TCTCTTTGTC   
  
  
- TCTTTCTCTC TCTCTCTCTC TCTCTTCCCC TTTAAATGAT CTCTTACTCT CACTTCTTCC CTTGAAATAA   
  
  
- AATTGAAAAA ATACTACGTG CGTAGGTACC GTCTTGTCCC TTCCCTTTCT TCCCATGTCT CATGACGACA   
  
  
- GGGGTTTTTT AATCACATCT TTTGTCCTCT GTGAGTTGAT GTTGTTCCCA TTCCTTCTTC TTGTTCGTTC   
  
  
- TATAGGGTGG GTTTCTTTTT TTTTTTCTTC TTCTTCTTTT AAAAAGTCTC TCCTTTCTTT TCGTTAGTGT   
  
  
- ACTTAATCTT CGGTTGGGTT CGGAAGAAGT TTCTCTTCGT CGTATTGTAT CGTATCGACC CCTCTCCTAT   
  
  
- CCTCTCTCTT TGAGTTCTTT TCCCCATGGG CCGAATTTAC AACTACGTCT TTCCATATCT ATACCGGTGT   
  
  
- GATGATGATG GGTCCTCAAA TAGTAACCTC TACCTAAAGA GAAAGACGGA GTAGAAGAGG AGGTAGGTGG   
  
  
- TCGTTTTGAC CCAAAGGAAA GTCGACCCAG AAAAGATCTA GATCTTTGAT ACCATGGGTT TAGAGGGTAA   
  
  
- GGAGGTAAAA ATAAGAGTTT TAGATAGAAG AATCTGAACA CCCAAACTAA GTAAAGAAAA CCCAAAAACT   
  
  
- TTTCTTAAAT TAAACTAACA TAACACAACT AACCCCTTAA ACACCACCAC AATCTACGCT CGATACGGGA   
  
  
- TGTTAAACGT TCCGTTCCCA CACCATCTCC AAAGTTCGTA AACATAAGGT GTTTAGAGAA GGGGACGAAG   
  
  
- TTTCACCTTC TGGTTGTTGT TGTTGAACCT GTTGTTTAAA GTCGTTGTTG TTCTTCTTTA ACTCGTTAAC   
  
  
- GTCTCATCAC CACCGTGAAA AGAGAGAGGA CTATTGTGGT TATGATTATA ATCATGATGA GGCTCTCGCT   
  
  
- GTTCAAGACA ACTATCGCTT GTGTGTAGAT GAGACCTATG ATGCCCGGGA GGGTGAAGTT GTAGAAGGAG   
  
  
- GAAGTTCTCG TGGAGGTGGC GGTTGTGTCG GCCACACCGC CTGGGGCGTA CATGAGGGTT TCTCCTAACC   
  
  
- CTACAGAGGC CGCCGCGACG ACCGCCACTG CCGCCACCAC CCCCCGAACC TAACCTCCTC ACCCTGTCGT   
  
  
- ACAAGGGTTT ACCCCTCCCC CGAAACGAGG GAACCTAGTA CCCACTTCGA CTACTGTACC CATACCCAAA   
  
  
- CTTCGTAGAA AACGTTAGCC CATTGGGCCA ACTGATACTC CCGTTACGAC CAGATCCCCA ACAGCTAGTC   
  
  
- CCAAGACCTA AACTCTGAGA GAGAGGAGGA GGTGGCGTCG GCCGCCGTAC ACTTAGATTA CCACCACCAC   
  
  
- AACGATTAAA CCCCAAAGGT CCCTTATTAC CATTCTAAAG AAGTTAAAGT GTATTAACAA GTAGACCCTA   
  
  
- AAACTTACCA TTCCACTTGT TATTACCCAA CTTAGGGTTA ACATTGGGAG TTCGAGTTCC GTCAGATGAA   
  
  
- CCAAATTAGG TTCCTCGATG TCCACATGTG CGTGTAGGAC TGCAACCTCT GCTCTTTGGG GTCTAAAACT   
  
  
- TAGGTGTAAA CCACTACTTG GGAGTCGTTC GAGTCTCGTA ACGTTTGGGA TCGAAAAACT ACGTGAGTAA   
  
  
- TCCAATGATA GTCGACCTCG TTGTAGATAA AGTTGGAGTC CGTTTTGCGG ACTTGTGCCC ACGACAAGAC   
  
  
- CTAGGATTGG AACAAGTCGA ACGTTTCTTG GGTAAACGAC TAGTCCCAGT ACTCAATAAC AACTCCTTCG   
  
  
- TCGTGGTTGT CGAACCAAAC GTTGGTAACG GTAACCCAAA CCCAGGAGTC AACCAGGGAG GCGTCTTCGG   
  
  
- ACACTACCCA AGTTTCGTCC CCTTGGGGGT CGTAATGGGC AACGTACAAG TAGTCGTCGA CGTTAACGTT   
  
  
- CTCGTCCGAC AGTTTCTAGT CGAGAAGTTC CGCCGTCTGG AATAAGTTTG ACCTTCAAAG AGTGAGCGCG   
  
  
- TTCTCTATAA CCGCGCCGAG TTAGTGGTCG AGAGGGAGGG ACGTTTCGGG GAGTAATCCC GCCGAAACAT   
  
  
- ACACTTCCTC CGGGAAGTTT ACGAGGAGGA TTACTCGTTG GGTCAACGCC GAGGTGGCAG GTTCTAGGAG   
  
  
- TGGGGAATAC TACAACAAGT GTTCTACTCG CGCATATTCC AGAAGAGACT CCAGACGGGT TAGTGAGTTA   
  
  
- AACACTTAAA GTGAACATGT GTCCGGTAAG AGCTCCGAGA ACTACTAAGA CTACGATAAG TACAACAGCT   
  
  
- GAAACTATAA CCAACGCCAC GAGTTACCCG TAGTAACTAA GTCCTCGACG GTAACTCCTT TTCCCCTCGA   
  
  
- GGGAGAGACT TTTAATGTCG GTATCGAGGG TACAACTGAC CGTCAGTGAA ACTTTAATCG GATCATACAC   
  
  
- TTTTGGAACA CGTTAAACGG TTGCTATAAC CACAACGAAC ACTCGAGGTT CAACAGTTGA AACTAAACAA   
  
  
- ACTAGGTAGA AGCGGTAGTT ACGGCTTGTA ATCATGACGA CTCCTACTCA GTTAACGACA ATCATAGGGG   
  
  
- TAGACCCGTA GAAGTTAATC CGGTAGACAA GAAGGAAGGT AGGAGGCTAA GTAATTCGTT TTTAGGGGGT   
  
  
- TTTAACACCA GAGCAAACTA TCTCCTAAGC TAGCAAAACT ACAGGGAAAG GGGGTTGTAG ACAACGTATG   
  
  
- GGATCTTAGG ACGTCATTAA ATAACCTGAG CGAACTACCA GAGTTACAAC GTAGCCTGTA ACACTCGTTC   
  
  
- CAGCTCTTCA AGAAACAAGT TGGATCCTAG CTTTTACGAC ACAACCCAGC CCAGGTACGG GGACTGTTCT   
  
  
- ACGGGGTAAC CTTTTTAGAG AAACGAAGTC GGCCGAAGAA CGGGAACGTT AAGTCATTAA AGTGTCTTTG   
  
  
- GGTCCGGCTA ATACACCACT TCTCTTGGGG TTGTTCTCCT AAAGTGCACC TCTTCGCGGT CCGTAGTGAG   
  
  
- TAAGACTCAA CCGTCTCCGT CCTCGAACAC CGTCGAAGCC GTACCTTCAC AAC

+     ABRE

| Site Name | Organism | Position | Strand | Matrix score. | sequence | function |
| --- | --- | --- | --- | --- | --- | --- |
| ABRE | Arabidopsis thaliana | 2547 | + | 7 | AACCCGG | cis-acting element involved in the abscisic acid responsiveness |
| ABRE | Arabidopsis thaliana | 4249 | + | 5 | ACGTG | cis-acting element involved in the abscisic acid responsiveness |
| ABRE | Arabidopsis thaliana | 4248 | - | 6 | CACGTG | cis-acting element involved in the abscisic acid responsiveness |

>HU04G00047.1   
+ +Up\_Stream \_Len000TCTTAA CATTAGTTGC TTGGTTCAAC CCATCAGTGA GAATATCTGA TTTCAAAAGT   
  
  
+ ATAGATTTAT ATAAACATAT GGACTACAGG GAAGGGAGTC GTTTCCTCAA AGTCTTAGAT TTACACTCAA   
  
  
+ AAGTTTGAGC GATACCAATA ATGTGATTCC AAACATGAGC CAAATATTAA GTATCCTATA CTAAGATCAA   
  
  
+ GAAGAGAATG AACCAAAAGA CTGTTAATTT TTCAATTAAG CTAAACTTAT TAACCTTTTT TTGGAATAGA   
  
  
+ TTTTTAAATT TTATCTTCAA TATAAGTTTT TCCAAGGCGA GAGACATATA GCAATTTTAA TTAAAGTTAA   
  
  
+ AGTAGTATAA CATTTTTTTT AATGGTTATA GGCTTATAGC ACCTTTTCTA GGTGAAAAGA GATATCTTGG   
  
  
+ CCTGATTTCC AGTAAAGTTT TTTTTTTAAA TATTGTCAAT TATTTTTGCA AATGACATTT GGTAATTTGG   
  
  
+ GGGATAATCT GATGTGTTAA AAGATACAAT GAAAATATCA GAAAATAGGA TGCCTCAAAT TTCACAAGAA   
  
  
+ AGAAAAAATC TTTTTGTTGT TAGTATTTCT ACACATCCAC AATTTTTACT CATGGTAAAA AAAAAAGGGC   
  
  
+ CAAATATCTC ACCAATATAA CCAAACTATA TGTTCCATTA TTTAGTAACG GTGATAAATA TGTAATGGGT   
  
  
+ CAAGTTACTA ATAAAATAGC CTACAATACT TGGATATCTA CATAAAATTG ATATAGAATA TATATTGCAA   
  
  
+ TACTTAAATC AAAATTAAAT GAAATATTTA CTTTTTTATT CTATTACAAA TAGCTTCTTA TAGAATAGCA   
  
  
+ ACAATTTTTA ATGTAAGTTT CATTCTTACC AGATTTGAAG AGGTCCGGAT ATAGAAAATC TTATTCGTAT   
  
  
+ AAGTACTCGC ATGAGGGCGG ATAAATTCTA AAATATCAAC CTCAACTCGC TTCTACTATT CCATCTAAGT   
  
  
+ TCAATTTATC ATATAAAAAA ATTCAATCCA CTTTAATGTA CTCAATTCAC TTCAACCTAT TTTAATCAAT   
  
  
+ TTACCCATGA AAAGAACAAG GTCCTAAGTA GATGTTTTCT ATTGATTGAT TTAAACAATT TTCACCTACC   
  
  
+ CAATTATCCC CAAAACTGAA AGAGGGGGCA AAGGGGGGTG GGTAATGCTG TGTAAAATAG GGGGAAAGGG   
  
  
+ CAAGAAAACT GTGTACAATG GTGGAGCAGG GGTATACCAT TTGGCCTTGA GTTGAGGGGG CACAAAAGCC   
  
  
+ CAGAGAGAGA GAGGGGTAAT GCCCACCAGA AATAAGCAGG AAGCGAGGGA AAAACACTAG AGAGAAACAG   
  
  
+ AGAAAGAGAG AGAGAGAGAG AGAGAAGGGG AAATTTACTA GAGAATGAGA GTGAAGAAGG GAACTTTATT   
  
  
+ TTAACTTTTT TATGATGCAC GCATCCATGG CAGAACAGGG AAGGGAAAGA AGGGTACAGA GTACTGCTGT   
  
  
+ CCCCAAAAAA TTAGTGTAGA AAACAGGAGA CACTCAACTA CAACAAGGGT AAGGAAGAAG AACAAGCAAG   
  
  
+ ATATCCCACC CAAAGAAAAA AAAAAAGAAG AAGAAGAAAA TTTTTCAGAG AGGAAAGAAA AGCAATCACA   
  
  
+ TGAATTAGAA GCCAACCCAA GCCTTCTTCA AAGAGAAGCA GCATAACATA GCATAGCTGG GGAGAGGATA   
  
  
+ GGAGAGAGAA ACTCAAGAAA AGGGGTACCC GGCTTAAATG TTGATGCAGA AAGGTATAGA TATGGCCACA   
  
  
+ CTACTACTAC CCAGGAGTTT ATCATTGGAG ATGGATTTCT CTTTCTGCCT CATCTTCTCC TCCATCCACC   
  
  
+ AGCAAAACTG GGTTTCCTTT CAGCTGGGTC TTTTCTAGAT CTAGAAACTA TGGTACCCAA ATCTCCCATT   
  
  
+ CCTCCATTTT TATTCTCAAA ATCTATCTTC TTAGACTTGT GGGTTTGATT CATTTCTTTT GGGTTTTTGA   
  
  
+ AAAGAATTTA ATTTGATTGT ATTGTGTTGA TTGGGGAATT TGTGGTGGTG TTAGATGCGA GCTATGCCCT   
  
  
+ ACAATTTGCA AGGCAAGGGT GTGGTAGAGG TTTCAAGCAT TTGTATTCCA CAAATCTCTT CCCCTGCTTC   
  
  
+ AAAGTGGAAG ACCAACAACA ACAACTTGGA CAACAAATTT CAGCAACAAC AAGAAGAAAT TGAGCAATTG   
  
  
+ CAGAGTAGTG GTGGCACTTT TCTCTCTCCT GATAACACCA ATACTAATAT TAGTACTACT CCGAGAGCGA   
  
  
+ CAAGTTCTGT TGATAGCGAA CACACATCTA CTCTGGATAC TACGGGCCCT CCCACTTCAA CATCTTCCTC   
  
  
+ CTTCAAGAGC ACCTCCACCG CCAACACAGC CGGTGTGGCG GACCCCGCAT GTACTCCCAA AGAGGATTGG   
  
  
+ GATGTCTCCG GCGGCGCTGC TGGCGGTGAC GGCGGTGGTG GGGGGCTTGG ATTGGAGGAG TGGGACAGCA   
  
  
+ TGTTCCCAAA TGGGGAGGGG GCTTTGCTCC CTTGGATCAT GGGTGAAGCT GATGACATGG GTATGGGTTT   
  
  
+ GAAGCATCTT TTGCAATCGG GTAACCCGGT TGACTATGAG GGCAATGCTG GTCTAGGGGT TGTCGATCAG   
  
  
+ GGTTCTGGAT TTGAGACTCT CTCTCCTCCT CCACCGCAGC CGGCGGCATG TGAATCTAAT GGTGGTGGTG   
  
  
+ TTGCTAATTT GGGGTTTCCA GGGAATAATG GTAAGATTTC TTCAATTTCA CATAATTGTT CATCTGGGAT   
  
  
+ TTTGAATGGT AAGGTGAACA ATAATGGGTT GAATCCCAAT TGTAACCCTC AAGCTCAAGG CAGTCTACTT   
  
  
+ GGTTTAATCC AAGGAGCTAC AGGTGTACAC GCACATCCTG ACGTTGGAGA CGAGAAACCC CAGATTTTGA   
  
  
+ ATCCACATTT GGTGATGAAC CCTCAGCAAG CTCAGAGCAT TGCAAACCCT AGCTTTTTGA TGCACTCATT   
  
  
+ AGGTTACTAT CAGCTGGAGC AACATCTATT TCAACCTCAG GCAAAACGCC TGAACACGGG TGCTGTTCTG   
  
  
+ GATCCTAACC TTGTTCAGCT TGCAAAGAAC CCATTTGCTG ATCAGGGTCA TGAGTTATTG TTGAGGAAGC   
  
  
+ AGCACCAACA GCTTGGTTTG CAACCATTGC CATTGGGTTT GGGTCCTCAG TTGGTCCCTC CGCAGAAGCC   
  
  
+ TGTGATGGGT TCAAAGCAGG GGAACCCCCA GCATTACCCG TTGCATGTTC ATCAGCAGCT GCAATTGCAA   
  
  
+ GAGCAGGCTG TCAAAGATCA GCTCTTCAAG GCGGCAGACC TTATTCAAAC TGGAAGTTTC TCACTCGCGC   
  
  
+ AAGAGATATT GGCGCGGCTC AATCACCAGC TCTCCCTCCC TGCAAAGCCC CTCATTAGGG CGGCTTTGTA   
  
  
+ TGTGAAGGAG GCCCTTCAAA TGCTCCTCCT AATGAGCAAC CCAGTTGCGG CTCCACCGTC CAAGATCCTC   
  
  
+ ACCCCTTATG ATGTTGTTCA CAAGATGAGC GCGTATAAGG TCTTCTCTGA GGTCTGCCCA ATCACTCAAT   
  
  
+ TTGTGAATTT CACTTGTACA CAGGCCATTC TCGAGGCTCT TGATGATTCT GATGCTATTC ATGTTGTCGA   
  
  
+ CTTTGATATT GGTTGCGGTG CTCAATGGGC ATCATTGATT CAGGAGCTGC CATTGAGGAA AAGGGGAGCT   
  
  
+ CCCTCTCTGA AAATTACAGC CATAGCTCCC ATGTTGACTG GCAGTCACTT TGAAATTAGC CTAGTATGTG   
  
  
+ AAAACCTTGT GCAATTTGCC AACGATATTG GTGTTGCTTG TGAGCTCCAA GTTGTCAACT TTGATTTGTT   
  
  
+ TGATCCATCT TCGCCATCAA TGCCGAACAT TAGTACTGCT GAGGATGAGT CAATTGCTGT TAGTATCCCC   
  
  
+ ATCTGGGCAT CTTCAATTAG GCCATCTGTT CTTCCTTCCA TCCTCCGATT CATTAAGCAA AAATCCCCCA   
  
  
+ AAATTGTGGT CTCGTTTGAT AGAGGATTCG ATCGTTTTGA TGTCCCTTTC CCCCAACATC TGTTGCATAC   
  
  
+ CCTAGAATCC TGCAGTAATT TATTGGACTC GCTTGATGGT CTCAATGTTG CATCGGACAT TGTGAGCAAG   
  
  
+ GTCGAGAAGT TCTTTGTTCA ACCTAGGATC GAAAATGCTG TGTTGGGTCG GGTCCATGCC CCTGACAAGA   
  
  
+ TGCCCCATTG GAAAAATCTC TTTGCTTCAG CCGGCTTCTT GCCCTTGCAA TTCAGTAATT TCACAGAAAC   
  
  
+ CCAGGCCGAT TATGTGGTGA AGAGAACCCC AACAAGAGGA TTTCACGTGG AGAAGCGCCA GGCATCACTC   
  
  
+ ATTCTGAGTT GGCAGAGGCA GGAGCTTGTG GCAGCTTCGG CATGGAAGTG TTG  

- +Up\_Stream \_Len000AGAATT GTAATCAACG AACCAAGTTG GGTAGTCACT CTTATAGACT AAAGTTTTCA   
  
  
- TATCTAAATA TATTTGTATA CCTGATGTCC CTTCCCTCAG CAAAGGAGTT TCAGAATCTA AATGTGAGTT   
  
  
- TTCAAACTCG CTATGGTTAT TACACTAAGG TTTGTACTCG GTTTATAATT CATAGGATAT GATTCTAGTT   
  
  
- CTTCTCTTAC TTGGTTTTCT GACAATTAAA AAGTTAATTC GATTTGAATA ATTGGAAAAA AACCTTATCT   
  
  
- AAAAATTTAA AATAGAAGTT ATATTCAAAA AGGTTCCGCT CTCTGTATAT CGTTAAAATT AATTTCAATT   
  
  
- TCATCATATT GTAAAAAAAA TTACCAATAT CCGAATATCG TGGAAAAGAT CCACTTTTCT CTATAGAACC   
  
  
- GGACTAAAGG TCATTTCAAA AAAAAAATTT ATAACAGTTA ATAAAAACGT TTACTGTAAA CCATTAAACC   
  
  
- CCCTATTAGA CTACACAATT TTCTATGTTA CTTTTATAGT CTTTTATCCT ACGGAGTTTA AAGTGTTCTT   
  
  
- TCTTTTTTAG AAAAACAACA ATCATAAAGA TGTGTAGGTG TTAAAAATGA GTACCATTTT TTTTTTCCCG   
  
  
- GTTTATAGAG TGGTTATATT GGTTTGATAT ACAAGGTAAT AAATCATTGC CACTATTTAT ACATTACCCA   
  
  
- GTTCAATGAT TATTTTATCG GATGTTATGA ACCTATAGAT GTATTTTAAC TATATCTTAT ATATAACGTT   
  
  
- ATGAATTTAG TTTTAATTTA CTTTATAAAT GAAAAAATAA GATAATGTTT ATCGAAGAAT ATCTTATCGT   
  
  
- TGTTAAAAAT TACATTCAAA GTAAGAATGG TCTAAACTTC TCCAGGCCTA TATCTTTTAG AATAAGCATA   
  
  
- TTCATGAGCG TACTCCCGCC TATTTAAGAT TTTATAGTTG GAGTTGAGCG AAGATGATAA GGTAGATTCA   
  
  
- AGTTAAATAG TATATTTTTT TAAGTTAGGT GAAATTACAT GAGTTAAGTG AAGTTGGATA AAATTAGTTA   
  
  
- AATGGGTACT TTTCTTGTTC CAGGATTCAT CTACAAAAGA TAACTAACTA AATTTGTTAA AAGTGGATGG   
  
  
- GTTAATAGGG GTTTTGACTT TCTCCCCCGT TTCCCCCCAC CCATTACGAC ACATTTTATC CCCCTTTCCC   
  
  
- GTTCTTTTGA CACATGTTAC CACCTCGTCC CCATATGGTA AACCGGAACT CAACTCCCCC GTGTTTTCGG   
  
  
- GTCTCTCTCT CTCCCCATTA CGGGTGGTCT TTATTCGTCC TTCGCTCCCT TTTTGTGATC TCTCTTTGTC   
  
  
- TCTTTCTCTC TCTCTCTCTC TCTCTTCCCC TTTAAATGAT CTCTTACTCT CACTTCTTCC CTTGAAATAA   
  
  
- AATTGAAAAA ATACTACGTG CGTAGGTACC GTCTTGTCCC TTCCCTTTCT TCCCATGTCT CATGACGACA   
  
  
- GGGGTTTTTT AATCACATCT TTTGTCCTCT GTGAGTTGAT GTTGTTCCCA TTCCTTCTTC TTGTTCGTTC   
  
  
- TATAGGGTGG GTTTCTTTTT TTTTTTCTTC TTCTTCTTTT AAAAAGTCTC TCCTTTCTTT TCGTTAGTGT   
  
  
- ACTTAATCTT CGGTTGGGTT CGGAAGAAGT TTCTCTTCGT CGTATTGTAT CGTATCGACC CCTCTCCTAT   
  
  
- CCTCTCTCTT TGAGTTCTTT TCCCCATGGG CCGAATTTAC AACTACGTCT TTCCATATCT ATACCGGTGT   
  
  
- GATGATGATG GGTCCTCAAA TAGTAACCTC TACCTAAAGA GAAAGACGGA GTAGAAGAGG AGGTAGGTGG   
  
  
- TCGTTTTGAC CCAAAGGAAA GTCGACCCAG AAAAGATCTA GATCTTTGAT ACCATGGGTT TAGAGGGTAA   
  
  
- GGAGGTAAAA ATAAGAGTTT TAGATAGAAG AATCTGAACA CCCAAACTAA GTAAAGAAAA CCCAAAAACT   
  
  
- TTTCTTAAAT TAAACTAACA TAACACAACT AACCCCTTAA ACACCACCAC AATCTACGCT CGATACGGGA   
  
  
- TGTTAAACGT TCCGTTCCCA CACCATCTCC AAAGTTCGTA AACATAAGGT GTTTAGAGAA GGGGACGAAG   
  
  
- TTTCACCTTC TGGTTGTTGT TGTTGAACCT GTTGTTTAAA GTCGTTGTTG TTCTTCTTTA ACTCGTTAAC   
  
  
- GTCTCATCAC CACCGTGAAA AGAGAGAGGA CTATTGTGGT TATGATTATA ATCATGATGA GGCTCTCGCT   
  
  
- GTTCAAGACA ACTATCGCTT GTGTGTAGAT GAGACCTATG ATGCCCGGGA GGGTGAAGTT GTAGAAGGAG   
  
  
- GAAGTTCTCG TGGAGGTGGC GGTTGTGTCG GCCACACCGC CTGGGGCGTA CATGAGGGTT TCTCCTAACC   
  
  
- CTACAGAGGC CGCCGCGACG ACCGCCACTG CCGCCACCAC CCCCCGAACC TAACCTCCTC ACCCTGTCGT   
  
  
- ACAAGGGTTT ACCCCTCCCC CGAAACGAGG GAACCTAGTA CCCACTTCGA CTACTGTACC CATACCCAAA   
  
  
- CTTCGTAGAA AACGTTAGCC CATTGGGCCA ACTGATACTC CCGTTACGAC CAGATCCCCA ACAGCTAGTC   
  
  
- CCAAGACCTA AACTCTGAGA GAGAGGAGGA GGTGGCGTCG GCCGCCGTAC ACTTAGATTA CCACCACCAC   
  
  
- AACGATTAAA CCCCAAAGGT CCCTTATTAC CATTCTAAAG AAGTTAAAGT GTATTAACAA GTAGACCCTA   
  
  
- AAACTTACCA TTCCACTTGT TATTACCCAA CTTAGGGTTA ACATTGGGAG TTCGAGTTCC GTCAGATGAA   
  
  
- CCAAATTAGG TTCCTCGATG TCCACATGTG CGTGTAGGAC TGCAACCTCT GCTCTTTGGG GTCTAAAACT   
  
  
- TAGGTGTAAA CCACTACTTG GGAGTCGTTC GAGTCTCGTA ACGTTTGGGA TCGAAAAACT ACGTGAGTAA   
  
  
- TCCAATGATA GTCGACCTCG TTGTAGATAA AGTTGGAGTC CGTTTTGCGG ACTTGTGCCC ACGACAAGAC   
  
  
- CTAGGATTGG AACAAGTCGA ACGTTTCTTG GGTAAACGAC TAGTCCCAGT ACTCAATAAC AACTCCTTCG   
  
  
- TCGTGGTTGT CGAACCAAAC GTTGGTAACG GTAACCCAAA CCCAGGAGTC AACCAGGGAG GCGTCTTCGG   
  
  
- ACACTACCCA AGTTTCGTCC CCTTGGGGGT CGTAATGGGC AACGTACAAG TAGTCGTCGA CGTTAACGTT   
  
  
- CTCGTCCGAC AGTTTCTAGT CGAGAAGTTC CGCCGTCTGG AATAAGTTTG ACCTTCAAAG AGTGAGCGCG   
  
  
- TTCTCTATAA CCGCGCCGAG TTAGTGGTCG AGAGGGAGGG ACGTTTCGGG GAGTAATCCC GCCGAAACAT   
  
  
- ACACTTCCTC CGGGAAGTTT ACGAGGAGGA TTACTCGTTG GGTCAACGCC GAGGTGGCAG GTTCTAGGAG   
  
  
- TGGGGAATAC TACAACAAGT GTTCTACTCG CGCATATTCC AGAAGAGACT CCAGACGGGT TAGTGAGTTA   
  
  
- AACACTTAAA GTGAACATGT GTCCGGTAAG AGCTCCGAGA ACTACTAAGA CTACGATAAG TACAACAGCT   
  
  
- GAAACTATAA CCAACGCCAC GAGTTACCCG TAGTAACTAA GTCCTCGACG GTAACTCCTT TTCCCCTCGA   
  
  
- GGGAGAGACT TTTAATGTCG GTATCGAGGG TACAACTGAC CGTCAGTGAA ACTTTAATCG GATCATACAC   
  
  
- TTTTGGAACA CGTTAAACGG TTGCTATAAC CACAACGAAC ACTCGAGGTT CAACAGTTGA AACTAAACAA   
  
  
- ACTAGGTAGA AGCGGTAGTT ACGGCTTGTA ATCATGACGA CTCCTACTCA GTTAACGACA ATCATAGGGG   
  
  
- TAGACCCGTA GAAGTTAATC CGGTAGACAA GAAGGAAGGT AGGAGGCTAA GTAATTCGTT TTTAGGGGGT   
  
  
- TTTAACACCA GAGCAAACTA TCTCCTAAGC TAGCAAAACT ACAGGGAAAG GGGGTTGTAG ACAACGTATG   
  
  
- GGATCTTAGG ACGTCATTAA ATAACCTGAG CGAACTACCA GAGTTACAAC GTAGCCTGTA ACACTCGTTC   
  
  
- CAGCTCTTCA AGAAACAAGT TGGATCCTAG CTTTTACGAC ACAACCCAGC CCAGGTACGG GGACTGTTCT   
  
  
- ACGGGGTAAC CTTTTTAGAG AAACGAAGTC GGCCGAAGAA CGGGAACGTT AAGTCATTAA AGTGTCTTTG   
  
  
- GGTCCGGCTA ATACACCACT TCTCTTGGGG TTGTTCTCCT AAAGTGCACC TCTTCGCGGT CCGTAGTGAG   
  
  
- TAAGACTCAA CCGTCTCCGT CCTCGAACAC CGTCGAAGCC GTACCTTCAC AAC

+     ACA-motif

| Site Name | Organism | Position | Strand | Matrix score. | sequence | function |
| --- | --- | --- | --- | --- | --- | --- |
| ACA-motif | Pisum sativum | 3656 | + | 12 | AATTACAGCCATT | part of gapA in (gapA-CMA1) involved with light responsiveness |

>HU04G00047.1   
+ +Up\_Stream \_Len000TCTTAA CATTAGTTGC TTGGTTCAAC CCATCAGTGA GAATATCTGA TTTCAAAAGT   
  
  
+ ATAGATTTAT ATAAACATAT GGACTACAGG GAAGGGAGTC GTTTCCTCAA AGTCTTAGAT TTACACTCAA   
  
  
+ AAGTTTGAGC GATACCAATA ATGTGATTCC AAACATGAGC CAAATATTAA GTATCCTATA CTAAGATCAA   
  
  
+ GAAGAGAATG AACCAAAAGA CTGTTAATTT TTCAATTAAG CTAAACTTAT TAACCTTTTT TTGGAATAGA   
  
  
+ TTTTTAAATT TTATCTTCAA TATAAGTTTT TCCAAGGCGA GAGACATATA GCAATTTTAA TTAAAGTTAA   
  
  
+ AGTAGTATAA CATTTTTTTT AATGGTTATA GGCTTATAGC ACCTTTTCTA GGTGAAAAGA GATATCTTGG   
  
  
+ CCTGATTTCC AGTAAAGTTT TTTTTTTAAA TATTGTCAAT TATTTTTGCA AATGACATTT GGTAATTTGG   
  
  
+ GGGATAATCT GATGTGTTAA AAGATACAAT GAAAATATCA GAAAATAGGA TGCCTCAAAT TTCACAAGAA   
  
  
+ AGAAAAAATC TTTTTGTTGT TAGTATTTCT ACACATCCAC AATTTTTACT CATGGTAAAA AAAAAAGGGC   
  
  
+ CAAATATCTC ACCAATATAA CCAAACTATA TGTTCCATTA TTTAGTAACG GTGATAAATA TGTAATGGGT   
  
  
+ CAAGTTACTA ATAAAATAGC CTACAATACT TGGATATCTA CATAAAATTG ATATAGAATA TATATTGCAA   
  
  
+ TACTTAAATC AAAATTAAAT GAAATATTTA CTTTTTTATT CTATTACAAA TAGCTTCTTA TAGAATAGCA   
  
  
+ ACAATTTTTA ATGTAAGTTT CATTCTTACC AGATTTGAAG AGGTCCGGAT ATAGAAAATC TTATTCGTAT   
  
  
+ AAGTACTCGC ATGAGGGCGG ATAAATTCTA AAATATCAAC CTCAACTCGC TTCTACTATT CCATCTAAGT   
  
  
+ TCAATTTATC ATATAAAAAA ATTCAATCCA CTTTAATGTA CTCAATTCAC TTCAACCTAT TTTAATCAAT   
  
  
+ TTACCCATGA AAAGAACAAG GTCCTAAGTA GATGTTTTCT ATTGATTGAT TTAAACAATT TTCACCTACC   
  
  
+ CAATTATCCC CAAAACTGAA AGAGGGGGCA AAGGGGGGTG GGTAATGCTG TGTAAAATAG GGGGAAAGGG   
  
  
+ CAAGAAAACT GTGTACAATG GTGGAGCAGG GGTATACCAT TTGGCCTTGA GTTGAGGGGG CACAAAAGCC   
  
  
+ CAGAGAGAGA GAGGGGTAAT GCCCACCAGA AATAAGCAGG AAGCGAGGGA AAAACACTAG AGAGAAACAG   
  
  
+ AGAAAGAGAG AGAGAGAGAG AGAGAAGGGG AAATTTACTA GAGAATGAGA GTGAAGAAGG GAACTTTATT   
  
  
+ TTAACTTTTT TATGATGCAC GCATCCATGG CAGAACAGGG AAGGGAAAGA AGGGTACAGA GTACTGCTGT   
  
  
+ CCCCAAAAAA TTAGTGTAGA AAACAGGAGA CACTCAACTA CAACAAGGGT AAGGAAGAAG AACAAGCAAG   
  
  
+ ATATCCCACC CAAAGAAAAA AAAAAAGAAG AAGAAGAAAA TTTTTCAGAG AGGAAAGAAA AGCAATCACA   
  
  
+ TGAATTAGAA GCCAACCCAA GCCTTCTTCA AAGAGAAGCA GCATAACATA GCATAGCTGG GGAGAGGATA   
  
  
+ GGAGAGAGAA ACTCAAGAAA AGGGGTACCC GGCTTAAATG TTGATGCAGA AAGGTATAGA TATGGCCACA   
  
  
+ CTACTACTAC CCAGGAGTTT ATCATTGGAG ATGGATTTCT CTTTCTGCCT CATCTTCTCC TCCATCCACC   
  
  
+ AGCAAAACTG GGTTTCCTTT CAGCTGGGTC TTTTCTAGAT CTAGAAACTA TGGTACCCAA ATCTCCCATT   
  
  
+ CCTCCATTTT TATTCTCAAA ATCTATCTTC TTAGACTTGT GGGTTTGATT CATTTCTTTT GGGTTTTTGA   
  
  
+ AAAGAATTTA ATTTGATTGT ATTGTGTTGA TTGGGGAATT TGTGGTGGTG TTAGATGCGA GCTATGCCCT   
  
  
+ ACAATTTGCA AGGCAAGGGT GTGGTAGAGG TTTCAAGCAT TTGTATTCCA CAAATCTCTT CCCCTGCTTC   
  
  
+ AAAGTGGAAG ACCAACAACA ACAACTTGGA CAACAAATTT CAGCAACAAC AAGAAGAAAT TGAGCAATTG   
  
  
+ CAGAGTAGTG GTGGCACTTT TCTCTCTCCT GATAACACCA ATACTAATAT TAGTACTACT CCGAGAGCGA   
  
  
+ CAAGTTCTGT TGATAGCGAA CACACATCTA CTCTGGATAC TACGGGCCCT CCCACTTCAA CATCTTCCTC   
  
  
+ CTTCAAGAGC ACCTCCACCG CCAACACAGC CGGTGTGGCG GACCCCGCAT GTACTCCCAA AGAGGATTGG   
  
  
+ GATGTCTCCG GCGGCGCTGC TGGCGGTGAC GGCGGTGGTG GGGGGCTTGG ATTGGAGGAG TGGGACAGCA   
  
  
+ TGTTCCCAAA TGGGGAGGGG GCTTTGCTCC CTTGGATCAT GGGTGAAGCT GATGACATGG GTATGGGTTT   
  
  
+ GAAGCATCTT TTGCAATCGG GTAACCCGGT TGACTATGAG GGCAATGCTG GTCTAGGGGT TGTCGATCAG   
  
  
+ GGTTCTGGAT TTGAGACTCT CTCTCCTCCT CCACCGCAGC CGGCGGCATG TGAATCTAAT GGTGGTGGTG   
  
  
+ TTGCTAATTT GGGGTTTCCA GGGAATAATG GTAAGATTTC TTCAATTTCA CATAATTGTT CATCTGGGAT   
  
  
+ TTTGAATGGT AAGGTGAACA ATAATGGGTT GAATCCCAAT TGTAACCCTC AAGCTCAAGG CAGTCTACTT   
  
  
+ GGTTTAATCC AAGGAGCTAC AGGTGTACAC GCACATCCTG ACGTTGGAGA CGAGAAACCC CAGATTTTGA   
  
  
+ ATCCACATTT GGTGATGAAC CCTCAGCAAG CTCAGAGCAT TGCAAACCCT AGCTTTTTGA TGCACTCATT   
  
  
+ AGGTTACTAT CAGCTGGAGC AACATCTATT TCAACCTCAG GCAAAACGCC TGAACACGGG TGCTGTTCTG   
  
  
+ GATCCTAACC TTGTTCAGCT TGCAAAGAAC CCATTTGCTG ATCAGGGTCA TGAGTTATTG TTGAGGAAGC   
  
  
+ AGCACCAACA GCTTGGTTTG CAACCATTGC CATTGGGTTT GGGTCCTCAG TTGGTCCCTC CGCAGAAGCC   
  
  
+ TGTGATGGGT TCAAAGCAGG GGAACCCCCA GCATTACCCG TTGCATGTTC ATCAGCAGCT GCAATTGCAA   
  
  
+ GAGCAGGCTG TCAAAGATCA GCTCTTCAAG GCGGCAGACC TTATTCAAAC TGGAAGTTTC TCACTCGCGC   
  
  
+ AAGAGATATT GGCGCGGCTC AATCACCAGC TCTCCCTCCC TGCAAAGCCC CTCATTAGGG CGGCTTTGTA   
  
  
+ TGTGAAGGAG GCCCTTCAAA TGCTCCTCCT AATGAGCAAC CCAGTTGCGG CTCCACCGTC CAAGATCCTC   
  
  
+ ACCCCTTATG ATGTTGTTCA CAAGATGAGC GCGTATAAGG TCTTCTCTGA GGTCTGCCCA ATCACTCAAT   
  
  
+ TTGTGAATTT CACTTGTACA CAGGCCATTC TCGAGGCTCT TGATGATTCT GATGCTATTC ATGTTGTCGA   
  
  
+ CTTTGATATT GGTTGCGGTG CTCAATGGGC ATCATTGATT CAGGAGCTGC CATTGAGGAA AAGGGGAGCT   
  
  
+ CCCTCTCTGA AAATTACAGC CATAGCTCCC ATGTTGACTG GCAGTCACTT TGAAATTAGC CTAGTATGTG   
  
  
+ AAAACCTTGT GCAATTTGCC AACGATATTG GTGTTGCTTG TGAGCTCCAA GTTGTCAACT TTGATTTGTT   
  
  
+ TGATCCATCT TCGCCATCAA TGCCGAACAT TAGTACTGCT GAGGATGAGT CAATTGCTGT TAGTATCCCC   
  
  
+ ATCTGGGCAT CTTCAATTAG GCCATCTGTT CTTCCTTCCA TCCTCCGATT CATTAAGCAA AAATCCCCCA   
  
  
+ AAATTGTGGT CTCGTTTGAT AGAGGATTCG ATCGTTTTGA TGTCCCTTTC CCCCAACATC TGTTGCATAC   
  
  
+ CCTAGAATCC TGCAGTAATT TATTGGACTC GCTTGATGGT CTCAATGTTG CATCGGACAT TGTGAGCAAG   
  
  
+ GTCGAGAAGT TCTTTGTTCA ACCTAGGATC GAAAATGCTG TGTTGGGTCG GGTCCATGCC CCTGACAAGA   
  
  
+ TGCCCCATTG GAAAAATCTC TTTGCTTCAG CCGGCTTCTT GCCCTTGCAA TTCAGTAATT TCACAGAAAC   
  
  
+ CCAGGCCGAT TATGTGGTGA AGAGAACCCC AACAAGAGGA TTTCACGTGG AGAAGCGCCA GGCATCACTC   
  
  
+ ATTCTGAGTT GGCAGAGGCA GGAGCTTGTG GCAGCTTCGG CATGGAAGTG TTG  

- +Up\_Stream \_Len000AGAATT GTAATCAACG AACCAAGTTG GGTAGTCACT CTTATAGACT AAAGTTTTCA   
  
  
- TATCTAAATA TATTTGTATA CCTGATGTCC CTTCCCTCAG CAAAGGAGTT TCAGAATCTA AATGTGAGTT   
  
  
- TTCAAACTCG CTATGGTTAT TACACTAAGG TTTGTACTCG GTTTATAATT CATAGGATAT GATTCTAGTT   
  
  
- CTTCTCTTAC TTGGTTTTCT GACAATTAAA AAGTTAATTC GATTTGAATA ATTGGAAAAA AACCTTATCT   
  
  
- AAAAATTTAA AATAGAAGTT ATATTCAAAA AGGTTCCGCT CTCTGTATAT CGTTAAAATT AATTTCAATT   
  
  
- TCATCATATT GTAAAAAAAA TTACCAATAT CCGAATATCG TGGAAAAGAT CCACTTTTCT CTATAGAACC   
  
  
- GGACTAAAGG TCATTTCAAA AAAAAAATTT ATAACAGTTA ATAAAAACGT TTACTGTAAA CCATTAAACC   
  
  
- CCCTATTAGA CTACACAATT TTCTATGTTA CTTTTATAGT CTTTTATCCT ACGGAGTTTA AAGTGTTCTT   
  
  
- TCTTTTTTAG AAAAACAACA ATCATAAAGA TGTGTAGGTG TTAAAAATGA GTACCATTTT TTTTTTCCCG   
  
  
- GTTTATAGAG TGGTTATATT GGTTTGATAT ACAAGGTAAT AAATCATTGC CACTATTTAT ACATTACCCA   
  
  
- GTTCAATGAT TATTTTATCG GATGTTATGA ACCTATAGAT GTATTTTAAC TATATCTTAT ATATAACGTT   
  
  
- ATGAATTTAG TTTTAATTTA CTTTATAAAT GAAAAAATAA GATAATGTTT ATCGAAGAAT ATCTTATCGT   
  
  
- TGTTAAAAAT TACATTCAAA GTAAGAATGG TCTAAACTTC TCCAGGCCTA TATCTTTTAG AATAAGCATA   
  
  
- TTCATGAGCG TACTCCCGCC TATTTAAGAT TTTATAGTTG GAGTTGAGCG AAGATGATAA GGTAGATTCA   
  
  
- AGTTAAATAG TATATTTTTT TAAGTTAGGT GAAATTACAT GAGTTAAGTG AAGTTGGATA AAATTAGTTA   
  
  
- AATGGGTACT TTTCTTGTTC CAGGATTCAT CTACAAAAGA TAACTAACTA AATTTGTTAA AAGTGGATGG   
  
  
- GTTAATAGGG GTTTTGACTT TCTCCCCCGT TTCCCCCCAC CCATTACGAC ACATTTTATC CCCCTTTCCC   
  
  
- GTTCTTTTGA CACATGTTAC CACCTCGTCC CCATATGGTA AACCGGAACT CAACTCCCCC GTGTTTTCGG   
  
  
- GTCTCTCTCT CTCCCCATTA CGGGTGGTCT TTATTCGTCC TTCGCTCCCT TTTTGTGATC TCTCTTTGTC   
  
  
- TCTTTCTCTC TCTCTCTCTC TCTCTTCCCC TTTAAATGAT CTCTTACTCT CACTTCTTCC CTTGAAATAA   
  
  
- AATTGAAAAA ATACTACGTG CGTAGGTACC GTCTTGTCCC TTCCCTTTCT TCCCATGTCT CATGACGACA   
  
  
- GGGGTTTTTT AATCACATCT TTTGTCCTCT GTGAGTTGAT GTTGTTCCCA TTCCTTCTTC TTGTTCGTTC   
  
  
- TATAGGGTGG GTTTCTTTTT TTTTTTCTTC TTCTTCTTTT AAAAAGTCTC TCCTTTCTTT TCGTTAGTGT   
  
  
- ACTTAATCTT CGGTTGGGTT CGGAAGAAGT TTCTCTTCGT CGTATTGTAT CGTATCGACC CCTCTCCTAT   
  
  
- CCTCTCTCTT TGAGTTCTTT TCCCCATGGG CCGAATTTAC AACTACGTCT TTCCATATCT ATACCGGTGT   
  
  
- GATGATGATG GGTCCTCAAA TAGTAACCTC TACCTAAAGA GAAAGACGGA GTAGAAGAGG AGGTAGGTGG   
  
  
- TCGTTTTGAC CCAAAGGAAA GTCGACCCAG AAAAGATCTA GATCTTTGAT ACCATGGGTT TAGAGGGTAA   
  
  
- GGAGGTAAAA ATAAGAGTTT TAGATAGAAG AATCTGAACA CCCAAACTAA GTAAAGAAAA CCCAAAAACT   
  
  
- TTTCTTAAAT TAAACTAACA TAACACAACT AACCCCTTAA ACACCACCAC AATCTACGCT CGATACGGGA   
  
  
- TGTTAAACGT TCCGTTCCCA CACCATCTCC AAAGTTCGTA AACATAAGGT GTTTAGAGAA GGGGACGAAG   
  
  
- TTTCACCTTC TGGTTGTTGT TGTTGAACCT GTTGTTTAAA GTCGTTGTTG TTCTTCTTTA ACTCGTTAAC   
  
  
- GTCTCATCAC CACCGTGAAA AGAGAGAGGA CTATTGTGGT TATGATTATA ATCATGATGA GGCTCTCGCT   
  
  
- GTTCAAGACA ACTATCGCTT GTGTGTAGAT GAGACCTATG ATGCCCGGGA GGGTGAAGTT GTAGAAGGAG   
  
  
- GAAGTTCTCG TGGAGGTGGC GGTTGTGTCG GCCACACCGC CTGGGGCGTA CATGAGGGTT TCTCCTAACC   
  
  
- CTACAGAGGC CGCCGCGACG ACCGCCACTG CCGCCACCAC CCCCCGAACC TAACCTCCTC ACCCTGTCGT   
  
  
- ACAAGGGTTT ACCCCTCCCC CGAAACGAGG GAACCTAGTA CCCACTTCGA CTACTGTACC CATACCCAAA   
  
  
- CTTCGTAGAA AACGTTAGCC CATTGGGCCA ACTGATACTC CCGTTACGAC CAGATCCCCA ACAGCTAGTC   
  
  
- CCAAGACCTA AACTCTGAGA GAGAGGAGGA GGTGGCGTCG GCCGCCGTAC ACTTAGATTA CCACCACCAC   
  
  
- AACGATTAAA CCCCAAAGGT CCCTTATTAC CATTCTAAAG AAGTTAAAGT GTATTAACAA GTAGACCCTA   
  
  
- AAACTTACCA TTCCACTTGT TATTACCCAA CTTAGGGTTA ACATTGGGAG TTCGAGTTCC GTCAGATGAA   
  
  
- CCAAATTAGG TTCCTCGATG TCCACATGTG CGTGTAGGAC TGCAACCTCT GCTCTTTGGG GTCTAAAACT   
  
  
- TAGGTGTAAA CCACTACTTG GGAGTCGTTC GAGTCTCGTA ACGTTTGGGA TCGAAAAACT ACGTGAGTAA   
  
  
- TCCAATGATA GTCGACCTCG TTGTAGATAA AGTTGGAGTC CGTTTTGCGG ACTTGTGCCC ACGACAAGAC   
  
  
- CTAGGATTGG AACAAGTCGA ACGTTTCTTG GGTAAACGAC TAGTCCCAGT ACTCAATAAC AACTCCTTCG   
  
  
- TCGTGGTTGT CGAACCAAAC GTTGGTAACG GTAACCCAAA CCCAGGAGTC AACCAGGGAG GCGTCTTCGG   
  
  
- ACACTACCCA AGTTTCGTCC CCTTGGGGGT CGTAATGGGC AACGTACAAG TAGTCGTCGA CGTTAACGTT   
  
  
- CTCGTCCGAC AGTTTCTAGT CGAGAAGTTC CGCCGTCTGG AATAAGTTTG ACCTTCAAAG AGTGAGCGCG   
  
  
- TTCTCTATAA CCGCGCCGAG TTAGTGGTCG AGAGGGAGGG ACGTTTCGGG GAGTAATCCC GCCGAAACAT   
  
  
- ACACTTCCTC CGGGAAGTTT ACGAGGAGGA TTACTCGTTG GGTCAACGCC GAGGTGGCAG GTTCTAGGAG   
  
  
- TGGGGAATAC TACAACAAGT GTTCTACTCG CGCATATTCC AGAAGAGACT CCAGACGGGT TAGTGAGTTA   
  
  
- AACACTTAAA GTGAACATGT GTCCGGTAAG AGCTCCGAGA ACTACTAAGA CTACGATAAG TACAACAGCT   
  
  
- GAAACTATAA CCAACGCCAC GAGTTACCCG TAGTAACTAA GTCCTCGACG GTAACTCCTT TTCCCCTCGA   
  
  
- GGGAGAGACT TTTAATGTCG GTATCGAGGG TACAACTGAC CGTCAGTGAA ACTTTAATCG GATCATACAC   
  
  
- TTTTGGAACA CGTTAAACGG TTGCTATAAC CACAACGAAC ACTCGAGGTT CAACAGTTGA AACTAAACAA   
  
  
- ACTAGGTAGA AGCGGTAGTT ACGGCTTGTA ATCATGACGA CTCCTACTCA GTTAACGACA ATCATAGGGG   
  
  
- TAGACCCGTA GAAGTTAATC CGGTAGACAA GAAGGAAGGT AGGAGGCTAA GTAATTCGTT TTTAGGGGGT   
  
  
- TTTAACACCA GAGCAAACTA TCTCCTAAGC TAGCAAAACT ACAGGGAAAG GGGGTTGTAG ACAACGTATG   
  
  
- GGATCTTAGG ACGTCATTAA ATAACCTGAG CGAACTACCA GAGTTACAAC GTAGCCTGTA ACACTCGTTC   
  
  
- CAGCTCTTCA AGAAACAAGT TGGATCCTAG CTTTTACGAC ACAACCCAGC CCAGGTACGG GGACTGTTCT   
  
  
- ACGGGGTAAC CTTTTTAGAG AAACGAAGTC GGCCGAAGAA CGGGAACGTT AAGTCATTAA AGTGTCTTTG   
  
  
- GGTCCGGCTA ATACACCACT TCTCTTGGGG TTGTTCTCCT AAAGTGCACC TCTTCGCGGT CCGTAGTGAG   
  
  
- TAAGACTCAA CCGTCTCCGT CCTCGAACAC CGTCGAAGCC GTACCTTCAC AAC

+     ACTCATCCT sequence

| Site Name | Organism | Position | Strand | Matrix score. | sequence | function |
| --- | --- | --- | --- | --- | --- | --- |
| ACTCATCCT sequence | Arabidopsis thaliana | 3826 | - | 9 | ACTCATCCT |  |

>HU04G00047.1   
+ +Up\_Stream \_Len000TCTTAA CATTAGTTGC TTGGTTCAAC CCATCAGTGA GAATATCTGA TTTCAAAAGT   
  
  
+ ATAGATTTAT ATAAACATAT GGACTACAGG GAAGGGAGTC GTTTCCTCAA AGTCTTAGAT TTACACTCAA   
  
  
+ AAGTTTGAGC GATACCAATA ATGTGATTCC AAACATGAGC CAAATATTAA GTATCCTATA CTAAGATCAA   
  
  
+ GAAGAGAATG AACCAAAAGA CTGTTAATTT TTCAATTAAG CTAAACTTAT TAACCTTTTT TTGGAATAGA   
  
  
+ TTTTTAAATT TTATCTTCAA TATAAGTTTT TCCAAGGCGA GAGACATATA GCAATTTTAA TTAAAGTTAA   
  
  
+ AGTAGTATAA CATTTTTTTT AATGGTTATA GGCTTATAGC ACCTTTTCTA GGTGAAAAGA GATATCTTGG   
  
  
+ CCTGATTTCC AGTAAAGTTT TTTTTTTAAA TATTGTCAAT TATTTTTGCA AATGACATTT GGTAATTTGG   
  
  
+ GGGATAATCT GATGTGTTAA AAGATACAAT GAAAATATCA GAAAATAGGA TGCCTCAAAT TTCACAAGAA   
  
  
+ AGAAAAAATC TTTTTGTTGT TAGTATTTCT ACACATCCAC AATTTTTACT CATGGTAAAA AAAAAAGGGC   
  
  
+ CAAATATCTC ACCAATATAA CCAAACTATA TGTTCCATTA TTTAGTAACG GTGATAAATA TGTAATGGGT   
  
  
+ CAAGTTACTA ATAAAATAGC CTACAATACT TGGATATCTA CATAAAATTG ATATAGAATA TATATTGCAA   
  
  
+ TACTTAAATC AAAATTAAAT GAAATATTTA CTTTTTTATT CTATTACAAA TAGCTTCTTA TAGAATAGCA   
  
  
+ ACAATTTTTA ATGTAAGTTT CATTCTTACC AGATTTGAAG AGGTCCGGAT ATAGAAAATC TTATTCGTAT   
  
  
+ AAGTACTCGC ATGAGGGCGG ATAAATTCTA AAATATCAAC CTCAACTCGC TTCTACTATT CCATCTAAGT   
  
  
+ TCAATTTATC ATATAAAAAA ATTCAATCCA CTTTAATGTA CTCAATTCAC TTCAACCTAT TTTAATCAAT   
  
  
+ TTACCCATGA AAAGAACAAG GTCCTAAGTA GATGTTTTCT ATTGATTGAT TTAAACAATT TTCACCTACC   
  
  
+ CAATTATCCC CAAAACTGAA AGAGGGGGCA AAGGGGGGTG GGTAATGCTG TGTAAAATAG GGGGAAAGGG   
  
  
+ CAAGAAAACT GTGTACAATG GTGGAGCAGG GGTATACCAT TTGGCCTTGA GTTGAGGGGG CACAAAAGCC   
  
  
+ CAGAGAGAGA GAGGGGTAAT GCCCACCAGA AATAAGCAGG AAGCGAGGGA AAAACACTAG AGAGAAACAG   
  
  
+ AGAAAGAGAG AGAGAGAGAG AGAGAAGGGG AAATTTACTA GAGAATGAGA GTGAAGAAGG GAACTTTATT   
  
  
+ TTAACTTTTT TATGATGCAC GCATCCATGG CAGAACAGGG AAGGGAAAGA AGGGTACAGA GTACTGCTGT   
  
  
+ CCCCAAAAAA TTAGTGTAGA AAACAGGAGA CACTCAACTA CAACAAGGGT AAGGAAGAAG AACAAGCAAG   
  
  
+ ATATCCCACC CAAAGAAAAA AAAAAAGAAG AAGAAGAAAA TTTTTCAGAG AGGAAAGAAA AGCAATCACA   
  
  
+ TGAATTAGAA GCCAACCCAA GCCTTCTTCA AAGAGAAGCA GCATAACATA GCATAGCTGG GGAGAGGATA   
  
  
+ GGAGAGAGAA ACTCAAGAAA AGGGGTACCC GGCTTAAATG TTGATGCAGA AAGGTATAGA TATGGCCACA   
  
  
+ CTACTACTAC CCAGGAGTTT ATCATTGGAG ATGGATTTCT CTTTCTGCCT CATCTTCTCC TCCATCCACC   
  
  
+ AGCAAAACTG GGTTTCCTTT CAGCTGGGTC TTTTCTAGAT CTAGAAACTA TGGTACCCAA ATCTCCCATT   
  
  
+ CCTCCATTTT TATTCTCAAA ATCTATCTTC TTAGACTTGT GGGTTTGATT CATTTCTTTT GGGTTTTTGA   
  
  
+ AAAGAATTTA ATTTGATTGT ATTGTGTTGA TTGGGGAATT TGTGGTGGTG TTAGATGCGA GCTATGCCCT   
  
  
+ ACAATTTGCA AGGCAAGGGT GTGGTAGAGG TTTCAAGCAT TTGTATTCCA CAAATCTCTT CCCCTGCTTC   
  
  
+ AAAGTGGAAG ACCAACAACA ACAACTTGGA CAACAAATTT CAGCAACAAC AAGAAGAAAT TGAGCAATTG   
  
  
+ CAGAGTAGTG GTGGCACTTT TCTCTCTCCT GATAACACCA ATACTAATAT TAGTACTACT CCGAGAGCGA   
  
  
+ CAAGTTCTGT TGATAGCGAA CACACATCTA CTCTGGATAC TACGGGCCCT CCCACTTCAA CATCTTCCTC   
  
  
+ CTTCAAGAGC ACCTCCACCG CCAACACAGC CGGTGTGGCG GACCCCGCAT GTACTCCCAA AGAGGATTGG   
  
  
+ GATGTCTCCG GCGGCGCTGC TGGCGGTGAC GGCGGTGGTG GGGGGCTTGG ATTGGAGGAG TGGGACAGCA   
  
  
+ TGTTCCCAAA TGGGGAGGGG GCTTTGCTCC CTTGGATCAT GGGTGAAGCT GATGACATGG GTATGGGTTT   
  
  
+ GAAGCATCTT TTGCAATCGG GTAACCCGGT TGACTATGAG GGCAATGCTG GTCTAGGGGT TGTCGATCAG   
  
  
+ GGTTCTGGAT TTGAGACTCT CTCTCCTCCT CCACCGCAGC CGGCGGCATG TGAATCTAAT GGTGGTGGTG   
  
  
+ TTGCTAATTT GGGGTTTCCA GGGAATAATG GTAAGATTTC TTCAATTTCA CATAATTGTT CATCTGGGAT   
  
  
+ TTTGAATGGT AAGGTGAACA ATAATGGGTT GAATCCCAAT TGTAACCCTC AAGCTCAAGG CAGTCTACTT   
  
  
+ GGTTTAATCC AAGGAGCTAC AGGTGTACAC GCACATCCTG ACGTTGGAGA CGAGAAACCC CAGATTTTGA   
  
  
+ ATCCACATTT GGTGATGAAC CCTCAGCAAG CTCAGAGCAT TGCAAACCCT AGCTTTTTGA TGCACTCATT   
  
  
+ AGGTTACTAT CAGCTGGAGC AACATCTATT TCAACCTCAG GCAAAACGCC TGAACACGGG TGCTGTTCTG   
  
  
+ GATCCTAACC TTGTTCAGCT TGCAAAGAAC CCATTTGCTG ATCAGGGTCA TGAGTTATTG TTGAGGAAGC   
  
  
+ AGCACCAACA GCTTGGTTTG CAACCATTGC CATTGGGTTT GGGTCCTCAG TTGGTCCCTC CGCAGAAGCC   
  
  
+ TGTGATGGGT TCAAAGCAGG GGAACCCCCA GCATTACCCG TTGCATGTTC ATCAGCAGCT GCAATTGCAA   
  
  
+ GAGCAGGCTG TCAAAGATCA GCTCTTCAAG GCGGCAGACC TTATTCAAAC TGGAAGTTTC TCACTCGCGC   
  
  
+ AAGAGATATT GGCGCGGCTC AATCACCAGC TCTCCCTCCC TGCAAAGCCC CTCATTAGGG CGGCTTTGTA   
  
  
+ TGTGAAGGAG GCCCTTCAAA TGCTCCTCCT AATGAGCAAC CCAGTTGCGG CTCCACCGTC CAAGATCCTC   
  
  
+ ACCCCTTATG ATGTTGTTCA CAAGATGAGC GCGTATAAGG TCTTCTCTGA GGTCTGCCCA ATCACTCAAT   
  
  
+ TTGTGAATTT CACTTGTACA CAGGCCATTC TCGAGGCTCT TGATGATTCT GATGCTATTC ATGTTGTCGA   
  
  
+ CTTTGATATT GGTTGCGGTG CTCAATGGGC ATCATTGATT CAGGAGCTGC CATTGAGGAA AAGGGGAGCT   
  
  
+ CCCTCTCTGA AAATTACAGC CATAGCTCCC ATGTTGACTG GCAGTCACTT TGAAATTAGC CTAGTATGTG   
  
  
+ AAAACCTTGT GCAATTTGCC AACGATATTG GTGTTGCTTG TGAGCTCCAA GTTGTCAACT TTGATTTGTT   
  
  
+ TGATCCATCT TCGCCATCAA TGCCGAACAT TAGTACTGCT GAGGATGAGT CAATTGCTGT TAGTATCCCC   
  
  
+ ATCTGGGCAT CTTCAATTAG GCCATCTGTT CTTCCTTCCA TCCTCCGATT CATTAAGCAA AAATCCCCCA   
  
  
+ AAATTGTGGT CTCGTTTGAT AGAGGATTCG ATCGTTTTGA TGTCCCTTTC CCCCAACATC TGTTGCATAC   
  
  
+ CCTAGAATCC TGCAGTAATT TATTGGACTC GCTTGATGGT CTCAATGTTG CATCGGACAT TGTGAGCAAG   
  
  
+ GTCGAGAAGT TCTTTGTTCA ACCTAGGATC GAAAATGCTG TGTTGGGTCG GGTCCATGCC CCTGACAAGA   
  
  
+ TGCCCCATTG GAAAAATCTC TTTGCTTCAG CCGGCTTCTT GCCCTTGCAA TTCAGTAATT TCACAGAAAC   
  
  
+ CCAGGCCGAT TATGTGGTGA AGAGAACCCC AACAAGAGGA TTTCACGTGG AGAAGCGCCA GGCATCACTC   
  
  
+ ATTCTGAGTT GGCAGAGGCA GGAGCTTGTG GCAGCTTCGG CATGGAAGTG TTG  

- +Up\_Stream \_Len000AGAATT GTAATCAACG AACCAAGTTG GGTAGTCACT CTTATAGACT AAAGTTTTCA   
  
  
- TATCTAAATA TATTTGTATA CCTGATGTCC CTTCCCTCAG CAAAGGAGTT TCAGAATCTA AATGTGAGTT   
  
  
- TTCAAACTCG CTATGGTTAT TACACTAAGG TTTGTACTCG GTTTATAATT CATAGGATAT GATTCTAGTT   
  
  
- CTTCTCTTAC TTGGTTTTCT GACAATTAAA AAGTTAATTC GATTTGAATA ATTGGAAAAA AACCTTATCT   
  
  
- AAAAATTTAA AATAGAAGTT ATATTCAAAA AGGTTCCGCT CTCTGTATAT CGTTAAAATT AATTTCAATT   
  
  
- TCATCATATT GTAAAAAAAA TTACCAATAT CCGAATATCG TGGAAAAGAT CCACTTTTCT CTATAGAACC   
  
  
- GGACTAAAGG TCATTTCAAA AAAAAAATTT ATAACAGTTA ATAAAAACGT TTACTGTAAA CCATTAAACC   
  
  
- CCCTATTAGA CTACACAATT TTCTATGTTA CTTTTATAGT CTTTTATCCT ACGGAGTTTA AAGTGTTCTT   
  
  
- TCTTTTTTAG AAAAACAACA ATCATAAAGA TGTGTAGGTG TTAAAAATGA GTACCATTTT TTTTTTCCCG   
  
  
- GTTTATAGAG TGGTTATATT GGTTTGATAT ACAAGGTAAT AAATCATTGC CACTATTTAT ACATTACCCA   
  
  
- GTTCAATGAT TATTTTATCG GATGTTATGA ACCTATAGAT GTATTTTAAC TATATCTTAT ATATAACGTT   
  
  
- ATGAATTTAG TTTTAATTTA CTTTATAAAT GAAAAAATAA GATAATGTTT ATCGAAGAAT ATCTTATCGT   
  
  
- TGTTAAAAAT TACATTCAAA GTAAGAATGG TCTAAACTTC TCCAGGCCTA TATCTTTTAG AATAAGCATA   
  
  
- TTCATGAGCG TACTCCCGCC TATTTAAGAT TTTATAGTTG GAGTTGAGCG AAGATGATAA GGTAGATTCA   
  
  
- AGTTAAATAG TATATTTTTT TAAGTTAGGT GAAATTACAT GAGTTAAGTG AAGTTGGATA AAATTAGTTA   
  
  
- AATGGGTACT TTTCTTGTTC CAGGATTCAT CTACAAAAGA TAACTAACTA AATTTGTTAA AAGTGGATGG   
  
  
- GTTAATAGGG GTTTTGACTT TCTCCCCCGT TTCCCCCCAC CCATTACGAC ACATTTTATC CCCCTTTCCC   
  
  
- GTTCTTTTGA CACATGTTAC CACCTCGTCC CCATATGGTA AACCGGAACT CAACTCCCCC GTGTTTTCGG   
  
  
- GTCTCTCTCT CTCCCCATTA CGGGTGGTCT TTATTCGTCC TTCGCTCCCT TTTTGTGATC TCTCTTTGTC   
  
  
- TCTTTCTCTC TCTCTCTCTC TCTCTTCCCC TTTAAATGAT CTCTTACTCT CACTTCTTCC CTTGAAATAA   
  
  
- AATTGAAAAA ATACTACGTG CGTAGGTACC GTCTTGTCCC TTCCCTTTCT TCCCATGTCT CATGACGACA   
  
  
- GGGGTTTTTT AATCACATCT TTTGTCCTCT GTGAGTTGAT GTTGTTCCCA TTCCTTCTTC TTGTTCGTTC   
  
  
- TATAGGGTGG GTTTCTTTTT TTTTTTCTTC TTCTTCTTTT AAAAAGTCTC TCCTTTCTTT TCGTTAGTGT   
  
  
- ACTTAATCTT CGGTTGGGTT CGGAAGAAGT TTCTCTTCGT CGTATTGTAT CGTATCGACC CCTCTCCTAT   
  
  
- CCTCTCTCTT TGAGTTCTTT TCCCCATGGG CCGAATTTAC AACTACGTCT TTCCATATCT ATACCGGTGT   
  
  
- GATGATGATG GGTCCTCAAA TAGTAACCTC TACCTAAAGA GAAAGACGGA GTAGAAGAGG AGGTAGGTGG   
  
  
- TCGTTTTGAC CCAAAGGAAA GTCGACCCAG AAAAGATCTA GATCTTTGAT ACCATGGGTT TAGAGGGTAA   
  
  
- GGAGGTAAAA ATAAGAGTTT TAGATAGAAG AATCTGAACA CCCAAACTAA GTAAAGAAAA CCCAAAAACT   
  
  
- TTTCTTAAAT TAAACTAACA TAACACAACT AACCCCTTAA ACACCACCAC AATCTACGCT CGATACGGGA   
  
  
- TGTTAAACGT TCCGTTCCCA CACCATCTCC AAAGTTCGTA AACATAAGGT GTTTAGAGAA GGGGACGAAG   
  
  
- TTTCACCTTC TGGTTGTTGT TGTTGAACCT GTTGTTTAAA GTCGTTGTTG TTCTTCTTTA ACTCGTTAAC   
  
  
- GTCTCATCAC CACCGTGAAA AGAGAGAGGA CTATTGTGGT TATGATTATA ATCATGATGA GGCTCTCGCT   
  
  
- GTTCAAGACA ACTATCGCTT GTGTGTAGAT GAGACCTATG ATGCCCGGGA GGGTGAAGTT GTAGAAGGAG   
  
  
- GAAGTTCTCG TGGAGGTGGC GGTTGTGTCG GCCACACCGC CTGGGGCGTA CATGAGGGTT TCTCCTAACC   
  
  
- CTACAGAGGC CGCCGCGACG ACCGCCACTG CCGCCACCAC CCCCCGAACC TAACCTCCTC ACCCTGTCGT   
  
  
- ACAAGGGTTT ACCCCTCCCC CGAAACGAGG GAACCTAGTA CCCACTTCGA CTACTGTACC CATACCCAAA   
  
  
- CTTCGTAGAA AACGTTAGCC CATTGGGCCA ACTGATACTC CCGTTACGAC CAGATCCCCA ACAGCTAGTC   
  
  
- CCAAGACCTA AACTCTGAGA GAGAGGAGGA GGTGGCGTCG GCCGCCGTAC ACTTAGATTA CCACCACCAC   
  
  
- AACGATTAAA CCCCAAAGGT CCCTTATTAC CATTCTAAAG AAGTTAAAGT GTATTAACAA GTAGACCCTA   
  
  
- AAACTTACCA TTCCACTTGT TATTACCCAA CTTAGGGTTA ACATTGGGAG TTCGAGTTCC GTCAGATGAA   
  
  
- CCAAATTAGG TTCCTCGATG TCCACATGTG CGTGTAGGAC TGCAACCTCT GCTCTTTGGG GTCTAAAACT   
  
  
- TAGGTGTAAA CCACTACTTG GGAGTCGTTC GAGTCTCGTA ACGTTTGGGA TCGAAAAACT ACGTGAGTAA   
  
  
- TCCAATGATA GTCGACCTCG TTGTAGATAA AGTTGGAGTC CGTTTTGCGG ACTTGTGCCC ACGACAAGAC   
  
  
- CTAGGATTGG AACAAGTCGA ACGTTTCTTG GGTAAACGAC TAGTCCCAGT ACTCAATAAC AACTCCTTCG   
  
  
- TCGTGGTTGT CGAACCAAAC GTTGGTAACG GTAACCCAAA CCCAGGAGTC AACCAGGGAG GCGTCTTCGG   
  
  
- ACACTACCCA AGTTTCGTCC CCTTGGGGGT CGTAATGGGC AACGTACAAG TAGTCGTCGA CGTTAACGTT   
  
  
- CTCGTCCGAC AGTTTCTAGT CGAGAAGTTC CGCCGTCTGG AATAAGTTTG ACCTTCAAAG AGTGAGCGCG   
  
  
- TTCTCTATAA CCGCGCCGAG TTAGTGGTCG AGAGGGAGGG ACGTTTCGGG GAGTAATCCC GCCGAAACAT   
  
  
- ACACTTCCTC CGGGAAGTTT ACGAGGAGGA TTACTCGTTG GGTCAACGCC GAGGTGGCAG GTTCTAGGAG   
  
  
- TGGGGAATAC TACAACAAGT GTTCTACTCG CGCATATTCC AGAAGAGACT CCAGACGGGT TAGTGAGTTA   
  
  
- AACACTTAAA GTGAACATGT GTCCGGTAAG AGCTCCGAGA ACTACTAAGA CTACGATAAG TACAACAGCT   
  
  
- GAAACTATAA CCAACGCCAC GAGTTACCCG TAGTAACTAA GTCCTCGACG GTAACTCCTT TTCCCCTCGA   
  
  
- GGGAGAGACT TTTAATGTCG GTATCGAGGG TACAACTGAC CGTCAGTGAA ACTTTAATCG GATCATACAC   
  
  
- TTTTGGAACA CGTTAAACGG TTGCTATAAC CACAACGAAC ACTCGAGGTT CAACAGTTGA AACTAAACAA   
  
  
- ACTAGGTAGA AGCGGTAGTT ACGGCTTGTA ATCATGACGA CTCCTACTCA GTTAACGACA ATCATAGGGG   
  
  
- TAGACCCGTA GAAGTTAATC CGGTAGACAA GAAGGAAGGT AGGAGGCTAA GTAATTCGTT TTTAGGGGGT   
  
  
- TTTAACACCA GAGCAAACTA TCTCCTAAGC TAGCAAAACT ACAGGGAAAG GGGGTTGTAG ACAACGTATG   
  
  
- GGATCTTAGG ACGTCATTAA ATAACCTGAG CGAACTACCA GAGTTACAAC GTAGCCTGTA ACACTCGTTC   
  
  
- CAGCTCTTCA AGAAACAAGT TGGATCCTAG CTTTTACGAC ACAACCCAGC CCAGGTACGG GGACTGTTCT   
  
  
- ACGGGGTAAC CTTTTTAGAG AAACGAAGTC GGCCGAAGAA CGGGAACGTT AAGTCATTAA AGTGTCTTTG   
  
  
- GGTCCGGCTA ATACACCACT TCTCTTGGGG TTGTTCTCCT AAAGTGCACC TCTTCGCGGT CCGTAGTGAG   
  
  
- TAAGACTCAA CCGTCTCCGT CCTCGAACAC CGTCGAAGCC GTACCTTCAC AAC

+     AE-box

| Site Name | Organism | Position | Strand | Matrix score. | sequence | function |
| --- | --- | --- | --- | --- | --- | --- |
| AE-box | Arabidopsis thaliana | 3278 | - | 8 | AGAAACTT | part of a module for light response |

>HU04G00047.1   
+ +Up\_Stream \_Len000TCTTAA CATTAGTTGC TTGGTTCAAC CCATCAGTGA GAATATCTGA TTTCAAAAGT   
  
  
+ ATAGATTTAT ATAAACATAT GGACTACAGG GAAGGGAGTC GTTTCCTCAA AGTCTTAGAT TTACACTCAA   
  
  
+ AAGTTTGAGC GATACCAATA ATGTGATTCC AAACATGAGC CAAATATTAA GTATCCTATA CTAAGATCAA   
  
  
+ GAAGAGAATG AACCAAAAGA CTGTTAATTT TTCAATTAAG CTAAACTTAT TAACCTTTTT TTGGAATAGA   
  
  
+ TTTTTAAATT TTATCTTCAA TATAAGTTTT TCCAAGGCGA GAGACATATA GCAATTTTAA TTAAAGTTAA   
  
  
+ AGTAGTATAA CATTTTTTTT AATGGTTATA GGCTTATAGC ACCTTTTCTA GGTGAAAAGA GATATCTTGG   
  
  
+ CCTGATTTCC AGTAAAGTTT TTTTTTTAAA TATTGTCAAT TATTTTTGCA AATGACATTT GGTAATTTGG   
  
  
+ GGGATAATCT GATGTGTTAA AAGATACAAT GAAAATATCA GAAAATAGGA TGCCTCAAAT TTCACAAGAA   
  
  
+ AGAAAAAATC TTTTTGTTGT TAGTATTTCT ACACATCCAC AATTTTTACT CATGGTAAAA AAAAAAGGGC   
  
  
+ CAAATATCTC ACCAATATAA CCAAACTATA TGTTCCATTA TTTAGTAACG GTGATAAATA TGTAATGGGT   
  
  
+ CAAGTTACTA ATAAAATAGC CTACAATACT TGGATATCTA CATAAAATTG ATATAGAATA TATATTGCAA   
  
  
+ TACTTAAATC AAAATTAAAT GAAATATTTA CTTTTTTATT CTATTACAAA TAGCTTCTTA TAGAATAGCA   
  
  
+ ACAATTTTTA ATGTAAGTTT CATTCTTACC AGATTTGAAG AGGTCCGGAT ATAGAAAATC TTATTCGTAT   
  
  
+ AAGTACTCGC ATGAGGGCGG ATAAATTCTA AAATATCAAC CTCAACTCGC TTCTACTATT CCATCTAAGT   
  
  
+ TCAATTTATC ATATAAAAAA ATTCAATCCA CTTTAATGTA CTCAATTCAC TTCAACCTAT TTTAATCAAT   
  
  
+ TTACCCATGA AAAGAACAAG GTCCTAAGTA GATGTTTTCT ATTGATTGAT TTAAACAATT TTCACCTACC   
  
  
+ CAATTATCCC CAAAACTGAA AGAGGGGGCA AAGGGGGGTG GGTAATGCTG TGTAAAATAG GGGGAAAGGG   
  
  
+ CAAGAAAACT GTGTACAATG GTGGAGCAGG GGTATACCAT TTGGCCTTGA GTTGAGGGGG CACAAAAGCC   
  
  
+ CAGAGAGAGA GAGGGGTAAT GCCCACCAGA AATAAGCAGG AAGCGAGGGA AAAACACTAG AGAGAAACAG   
  
  
+ AGAAAGAGAG AGAGAGAGAG AGAGAAGGGG AAATTTACTA GAGAATGAGA GTGAAGAAGG GAACTTTATT   
  
  
+ TTAACTTTTT TATGATGCAC GCATCCATGG CAGAACAGGG AAGGGAAAGA AGGGTACAGA GTACTGCTGT   
  
  
+ CCCCAAAAAA TTAGTGTAGA AAACAGGAGA CACTCAACTA CAACAAGGGT AAGGAAGAAG AACAAGCAAG   
  
  
+ ATATCCCACC CAAAGAAAAA AAAAAAGAAG AAGAAGAAAA TTTTTCAGAG AGGAAAGAAA AGCAATCACA   
  
  
+ TGAATTAGAA GCCAACCCAA GCCTTCTTCA AAGAGAAGCA GCATAACATA GCATAGCTGG GGAGAGGATA   
  
  
+ GGAGAGAGAA ACTCAAGAAA AGGGGTACCC GGCTTAAATG TTGATGCAGA AAGGTATAGA TATGGCCACA   
  
  
+ CTACTACTAC CCAGGAGTTT ATCATTGGAG ATGGATTTCT CTTTCTGCCT CATCTTCTCC TCCATCCACC   
  
  
+ AGCAAAACTG GGTTTCCTTT CAGCTGGGTC TTTTCTAGAT CTAGAAACTA TGGTACCCAA ATCTCCCATT   
  
  
+ CCTCCATTTT TATTCTCAAA ATCTATCTTC TTAGACTTGT GGGTTTGATT CATTTCTTTT GGGTTTTTGA   
  
  
+ AAAGAATTTA ATTTGATTGT ATTGTGTTGA TTGGGGAATT TGTGGTGGTG TTAGATGCGA GCTATGCCCT   
  
  
+ ACAATTTGCA AGGCAAGGGT GTGGTAGAGG TTTCAAGCAT TTGTATTCCA CAAATCTCTT CCCCTGCTTC   
  
  
+ AAAGTGGAAG ACCAACAACA ACAACTTGGA CAACAAATTT CAGCAACAAC AAGAAGAAAT TGAGCAATTG   
  
  
+ CAGAGTAGTG GTGGCACTTT TCTCTCTCCT GATAACACCA ATACTAATAT TAGTACTACT CCGAGAGCGA   
  
  
+ CAAGTTCTGT TGATAGCGAA CACACATCTA CTCTGGATAC TACGGGCCCT CCCACTTCAA CATCTTCCTC   
  
  
+ CTTCAAGAGC ACCTCCACCG CCAACACAGC CGGTGTGGCG GACCCCGCAT GTACTCCCAA AGAGGATTGG   
  
  
+ GATGTCTCCG GCGGCGCTGC TGGCGGTGAC GGCGGTGGTG GGGGGCTTGG ATTGGAGGAG TGGGACAGCA   
  
  
+ TGTTCCCAAA TGGGGAGGGG GCTTTGCTCC CTTGGATCAT GGGTGAAGCT GATGACATGG GTATGGGTTT   
  
  
+ GAAGCATCTT TTGCAATCGG GTAACCCGGT TGACTATGAG GGCAATGCTG GTCTAGGGGT TGTCGATCAG   
  
  
+ GGTTCTGGAT TTGAGACTCT CTCTCCTCCT CCACCGCAGC CGGCGGCATG TGAATCTAAT GGTGGTGGTG   
  
  
+ TTGCTAATTT GGGGTTTCCA GGGAATAATG GTAAGATTTC TTCAATTTCA CATAATTGTT CATCTGGGAT   
  
  
+ TTTGAATGGT AAGGTGAACA ATAATGGGTT GAATCCCAAT TGTAACCCTC AAGCTCAAGG CAGTCTACTT   
  
  
+ GGTTTAATCC AAGGAGCTAC AGGTGTACAC GCACATCCTG ACGTTGGAGA CGAGAAACCC CAGATTTTGA   
  
  
+ ATCCACATTT GGTGATGAAC CCTCAGCAAG CTCAGAGCAT TGCAAACCCT AGCTTTTTGA TGCACTCATT   
  
  
+ AGGTTACTAT CAGCTGGAGC AACATCTATT TCAACCTCAG GCAAAACGCC TGAACACGGG TGCTGTTCTG   
  
  
+ GATCCTAACC TTGTTCAGCT TGCAAAGAAC CCATTTGCTG ATCAGGGTCA TGAGTTATTG TTGAGGAAGC   
  
  
+ AGCACCAACA GCTTGGTTTG CAACCATTGC CATTGGGTTT GGGTCCTCAG TTGGTCCCTC CGCAGAAGCC   
  
  
+ TGTGATGGGT TCAAAGCAGG GGAACCCCCA GCATTACCCG TTGCATGTTC ATCAGCAGCT GCAATTGCAA   
  
  
+ GAGCAGGCTG TCAAAGATCA GCTCTTCAAG GCGGCAGACC TTATTCAAAC TGGAAGTTTC TCACTCGCGC   
  
  
+ AAGAGATATT GGCGCGGCTC AATCACCAGC TCTCCCTCCC TGCAAAGCCC CTCATTAGGG CGGCTTTGTA   
  
  
+ TGTGAAGGAG GCCCTTCAAA TGCTCCTCCT AATGAGCAAC CCAGTTGCGG CTCCACCGTC CAAGATCCTC   
  
  
+ ACCCCTTATG ATGTTGTTCA CAAGATGAGC GCGTATAAGG TCTTCTCTGA GGTCTGCCCA ATCACTCAAT   
  
  
+ TTGTGAATTT CACTTGTACA CAGGCCATTC TCGAGGCTCT TGATGATTCT GATGCTATTC ATGTTGTCGA   
  
  
+ CTTTGATATT GGTTGCGGTG CTCAATGGGC ATCATTGATT CAGGAGCTGC CATTGAGGAA AAGGGGAGCT   
  
  
+ CCCTCTCTGA AAATTACAGC CATAGCTCCC ATGTTGACTG GCAGTCACTT TGAAATTAGC CTAGTATGTG   
  
  
+ AAAACCTTGT GCAATTTGCC AACGATATTG GTGTTGCTTG TGAGCTCCAA GTTGTCAACT TTGATTTGTT   
  
  
+ TGATCCATCT TCGCCATCAA TGCCGAACAT TAGTACTGCT GAGGATGAGT CAATTGCTGT TAGTATCCCC   
  
  
+ ATCTGGGCAT CTTCAATTAG GCCATCTGTT CTTCCTTCCA TCCTCCGATT CATTAAGCAA AAATCCCCCA   
  
  
+ AAATTGTGGT CTCGTTTGAT AGAGGATTCG ATCGTTTTGA TGTCCCTTTC CCCCAACATC TGTTGCATAC   
  
  
+ CCTAGAATCC TGCAGTAATT TATTGGACTC GCTTGATGGT CTCAATGTTG CATCGGACAT TGTGAGCAAG   
  
  
+ GTCGAGAAGT TCTTTGTTCA ACCTAGGATC GAAAATGCTG TGTTGGGTCG GGTCCATGCC CCTGACAAGA   
  
  
+ TGCCCCATTG GAAAAATCTC TTTGCTTCAG CCGGCTTCTT GCCCTTGCAA TTCAGTAATT TCACAGAAAC   
  
  
+ CCAGGCCGAT TATGTGGTGA AGAGAACCCC AACAAGAGGA TTTCACGTGG AGAAGCGCCA GGCATCACTC   
  
  
+ ATTCTGAGTT GGCAGAGGCA GGAGCTTGTG GCAGCTTCGG CATGGAAGTG TTG  

- +Up\_Stream \_Len000AGAATT GTAATCAACG AACCAAGTTG GGTAGTCACT CTTATAGACT AAAGTTTTCA   
  
  
- TATCTAAATA TATTTGTATA CCTGATGTCC CTTCCCTCAG CAAAGGAGTT TCAGAATCTA AATGTGAGTT   
  
  
- TTCAAACTCG CTATGGTTAT TACACTAAGG TTTGTACTCG GTTTATAATT CATAGGATAT GATTCTAGTT   
  
  
- CTTCTCTTAC TTGGTTTTCT GACAATTAAA AAGTTAATTC GATTTGAATA ATTGGAAAAA AACCTTATCT   
  
  
- AAAAATTTAA AATAGAAGTT ATATTCAAAA AGGTTCCGCT CTCTGTATAT CGTTAAAATT AATTTCAATT   
  
  
- TCATCATATT GTAAAAAAAA TTACCAATAT CCGAATATCG TGGAAAAGAT CCACTTTTCT CTATAGAACC   
  
  
- GGACTAAAGG TCATTTCAAA AAAAAAATTT ATAACAGTTA ATAAAAACGT TTACTGTAAA CCATTAAACC   
  
  
- CCCTATTAGA CTACACAATT TTCTATGTTA CTTTTATAGT CTTTTATCCT ACGGAGTTTA AAGTGTTCTT   
  
  
- TCTTTTTTAG AAAAACAACA ATCATAAAGA TGTGTAGGTG TTAAAAATGA GTACCATTTT TTTTTTCCCG   
  
  
- GTTTATAGAG TGGTTATATT GGTTTGATAT ACAAGGTAAT AAATCATTGC CACTATTTAT ACATTACCCA   
  
  
- GTTCAATGAT TATTTTATCG GATGTTATGA ACCTATAGAT GTATTTTAAC TATATCTTAT ATATAACGTT   
  
  
- ATGAATTTAG TTTTAATTTA CTTTATAAAT GAAAAAATAA GATAATGTTT ATCGAAGAAT ATCTTATCGT   
  
  
- TGTTAAAAAT TACATTCAAA GTAAGAATGG TCTAAACTTC TCCAGGCCTA TATCTTTTAG AATAAGCATA   
  
  
- TTCATGAGCG TACTCCCGCC TATTTAAGAT TTTATAGTTG GAGTTGAGCG AAGATGATAA GGTAGATTCA   
  
  
- AGTTAAATAG TATATTTTTT TAAGTTAGGT GAAATTACAT GAGTTAAGTG AAGTTGGATA AAATTAGTTA   
  
  
- AATGGGTACT TTTCTTGTTC CAGGATTCAT CTACAAAAGA TAACTAACTA AATTTGTTAA AAGTGGATGG   
  
  
- GTTAATAGGG GTTTTGACTT TCTCCCCCGT TTCCCCCCAC CCATTACGAC ACATTTTATC CCCCTTTCCC   
  
  
- GTTCTTTTGA CACATGTTAC CACCTCGTCC CCATATGGTA AACCGGAACT CAACTCCCCC GTGTTTTCGG   
  
  
- GTCTCTCTCT CTCCCCATTA CGGGTGGTCT TTATTCGTCC TTCGCTCCCT TTTTGTGATC TCTCTTTGTC   
  
  
- TCTTTCTCTC TCTCTCTCTC TCTCTTCCCC TTTAAATGAT CTCTTACTCT CACTTCTTCC CTTGAAATAA   
  
  
- AATTGAAAAA ATACTACGTG CGTAGGTACC GTCTTGTCCC TTCCCTTTCT TCCCATGTCT CATGACGACA   
  
  
- GGGGTTTTTT AATCACATCT TTTGTCCTCT GTGAGTTGAT GTTGTTCCCA TTCCTTCTTC TTGTTCGTTC   
  
  
- TATAGGGTGG GTTTCTTTTT TTTTTTCTTC TTCTTCTTTT AAAAAGTCTC TCCTTTCTTT TCGTTAGTGT   
  
  
- ACTTAATCTT CGGTTGGGTT CGGAAGAAGT TTCTCTTCGT CGTATTGTAT CGTATCGACC CCTCTCCTAT   
  
  
- CCTCTCTCTT TGAGTTCTTT TCCCCATGGG CCGAATTTAC AACTACGTCT TTCCATATCT ATACCGGTGT   
  
  
- GATGATGATG GGTCCTCAAA TAGTAACCTC TACCTAAAGA GAAAGACGGA GTAGAAGAGG AGGTAGGTGG   
  
  
- TCGTTTTGAC CCAAAGGAAA GTCGACCCAG AAAAGATCTA GATCTTTGAT ACCATGGGTT TAGAGGGTAA   
  
  
- GGAGGTAAAA ATAAGAGTTT TAGATAGAAG AATCTGAACA CCCAAACTAA GTAAAGAAAA CCCAAAAACT   
  
  
- TTTCTTAAAT TAAACTAACA TAACACAACT AACCCCTTAA ACACCACCAC AATCTACGCT CGATACGGGA   
  
  
- TGTTAAACGT TCCGTTCCCA CACCATCTCC AAAGTTCGTA AACATAAGGT GTTTAGAGAA GGGGACGAAG   
  
  
- TTTCACCTTC TGGTTGTTGT TGTTGAACCT GTTGTTTAAA GTCGTTGTTG TTCTTCTTTA ACTCGTTAAC   
  
  
- GTCTCATCAC CACCGTGAAA AGAGAGAGGA CTATTGTGGT TATGATTATA ATCATGATGA GGCTCTCGCT   
  
  
- GTTCAAGACA ACTATCGCTT GTGTGTAGAT GAGACCTATG ATGCCCGGGA GGGTGAAGTT GTAGAAGGAG   
  
  
- GAAGTTCTCG TGGAGGTGGC GGTTGTGTCG GCCACACCGC CTGGGGCGTA CATGAGGGTT TCTCCTAACC   
  
  
- CTACAGAGGC CGCCGCGACG ACCGCCACTG CCGCCACCAC CCCCCGAACC TAACCTCCTC ACCCTGTCGT   
  
  
- ACAAGGGTTT ACCCCTCCCC CGAAACGAGG GAACCTAGTA CCCACTTCGA CTACTGTACC CATACCCAAA   
  
  
- CTTCGTAGAA AACGTTAGCC CATTGGGCCA ACTGATACTC CCGTTACGAC CAGATCCCCA ACAGCTAGTC   
  
  
- CCAAGACCTA AACTCTGAGA GAGAGGAGGA GGTGGCGTCG GCCGCCGTAC ACTTAGATTA CCACCACCAC   
  
  
- AACGATTAAA CCCCAAAGGT CCCTTATTAC CATTCTAAAG AAGTTAAAGT GTATTAACAA GTAGACCCTA   
  
  
- AAACTTACCA TTCCACTTGT TATTACCCAA CTTAGGGTTA ACATTGGGAG TTCGAGTTCC GTCAGATGAA   
  
  
- CCAAATTAGG TTCCTCGATG TCCACATGTG CGTGTAGGAC TGCAACCTCT GCTCTTTGGG GTCTAAAACT   
  
  
- TAGGTGTAAA CCACTACTTG GGAGTCGTTC GAGTCTCGTA ACGTTTGGGA TCGAAAAACT ACGTGAGTAA   
  
  
- TCCAATGATA GTCGACCTCG TTGTAGATAA AGTTGGAGTC CGTTTTGCGG ACTTGTGCCC ACGACAAGAC   
  
  
- CTAGGATTGG AACAAGTCGA ACGTTTCTTG GGTAAACGAC TAGTCCCAGT ACTCAATAAC AACTCCTTCG   
  
  
- TCGTGGTTGT CGAACCAAAC GTTGGTAACG GTAACCCAAA CCCAGGAGTC AACCAGGGAG GCGTCTTCGG   
  
  
- ACACTACCCA AGTTTCGTCC CCTTGGGGGT CGTAATGGGC AACGTACAAG TAGTCGTCGA CGTTAACGTT   
  
  
- CTCGTCCGAC AGTTTCTAGT CGAGAAGTTC CGCCGTCTGG AATAAGTTTG ACCTTCAAAG AGTGAGCGCG   
  
  
- TTCTCTATAA CCGCGCCGAG TTAGTGGTCG AGAGGGAGGG ACGTTTCGGG GAGTAATCCC GCCGAAACAT   
  
  
- ACACTTCCTC CGGGAAGTTT ACGAGGAGGA TTACTCGTTG GGTCAACGCC GAGGTGGCAG GTTCTAGGAG   
  
  
- TGGGGAATAC TACAACAAGT GTTCTACTCG CGCATATTCC AGAAGAGACT CCAGACGGGT TAGTGAGTTA   
  
  
- AACACTTAAA GTGAACATGT GTCCGGTAAG AGCTCCGAGA ACTACTAAGA CTACGATAAG TACAACAGCT   
  
  
- GAAACTATAA CCAACGCCAC GAGTTACCCG TAGTAACTAA GTCCTCGACG GTAACTCCTT TTCCCCTCGA   
  
  
- GGGAGAGACT TTTAATGTCG GTATCGAGGG TACAACTGAC CGTCAGTGAA ACTTTAATCG GATCATACAC   
  
  
- TTTTGGAACA CGTTAAACGG TTGCTATAAC CACAACGAAC ACTCGAGGTT CAACAGTTGA AACTAAACAA   
  
  
- ACTAGGTAGA AGCGGTAGTT ACGGCTTGTA ATCATGACGA CTCCTACTCA GTTAACGACA ATCATAGGGG   
  
  
- TAGACCCGTA GAAGTTAATC CGGTAGACAA GAAGGAAGGT AGGAGGCTAA GTAATTCGTT TTTAGGGGGT   
  
  
- TTTAACACCA GAGCAAACTA TCTCCTAAGC TAGCAAAACT ACAGGGAAAG GGGGTTGTAG ACAACGTATG   
  
  
- GGATCTTAGG ACGTCATTAA ATAACCTGAG CGAACTACCA GAGTTACAAC GTAGCCTGTA ACACTCGTTC   
  
  
- CAGCTCTTCA AGAAACAAGT TGGATCCTAG CTTTTACGAC ACAACCCAGC CCAGGTACGG GGACTGTTCT   
  
  
- ACGGGGTAAC CTTTTTAGAG AAACGAAGTC GGCCGAAGAA CGGGAACGTT AAGTCATTAA AGTGTCTTTG   
  
  
- GGTCCGGCTA ATACACCACT TCTCTTGGGG TTGTTCTCCT AAAGTGCACC TCTTCGCGGT CCGTAGTGAG   
  
  
- TAAGACTCAA CCGTCTCCGT CCTCGAACAC CGTCGAAGCC GTACCTTCAC AAC

+     ARE

| Site Name | Organism | Position | Strand | Matrix score. | sequence | function |
| --- | --- | --- | --- | --- | --- | --- |
| ARE | Zea mays | 2804 | - | 6 | AAACCA | cis-acting regulatory element essential for the anaerobic induction |
| ARE | Zea mays | 3098 | - | 6 | AAACCA | cis-acting regulatory element essential for the anaerobic induction |

>HU04G00047.1   
+ +Up\_Stream \_Len000TCTTAA CATTAGTTGC TTGGTTCAAC CCATCAGTGA GAATATCTGA TTTCAAAAGT   
  
  
+ ATAGATTTAT ATAAACATAT GGACTACAGG GAAGGGAGTC GTTTCCTCAA AGTCTTAGAT TTACACTCAA   
  
  
+ AAGTTTGAGC GATACCAATA ATGTGATTCC AAACATGAGC CAAATATTAA GTATCCTATA CTAAGATCAA   
  
  
+ GAAGAGAATG AACCAAAAGA CTGTTAATTT TTCAATTAAG CTAAACTTAT TAACCTTTTT TTGGAATAGA   
  
  
+ TTTTTAAATT TTATCTTCAA TATAAGTTTT TCCAAGGCGA GAGACATATA GCAATTTTAA TTAAAGTTAA   
  
  
+ AGTAGTATAA CATTTTTTTT AATGGTTATA GGCTTATAGC ACCTTTTCTA GGTGAAAAGA GATATCTTGG   
  
  
+ CCTGATTTCC AGTAAAGTTT TTTTTTTAAA TATTGTCAAT TATTTTTGCA AATGACATTT GGTAATTTGG   
  
  
+ GGGATAATCT GATGTGTTAA AAGATACAAT GAAAATATCA GAAAATAGGA TGCCTCAAAT TTCACAAGAA   
  
  
+ AGAAAAAATC TTTTTGTTGT TAGTATTTCT ACACATCCAC AATTTTTACT CATGGTAAAA AAAAAAGGGC   
  
  
+ CAAATATCTC ACCAATATAA CCAAACTATA TGTTCCATTA TTTAGTAACG GTGATAAATA TGTAATGGGT   
  
  
+ CAAGTTACTA ATAAAATAGC CTACAATACT TGGATATCTA CATAAAATTG ATATAGAATA TATATTGCAA   
  
  
+ TACTTAAATC AAAATTAAAT GAAATATTTA CTTTTTTATT CTATTACAAA TAGCTTCTTA TAGAATAGCA   
  
  
+ ACAATTTTTA ATGTAAGTTT CATTCTTACC AGATTTGAAG AGGTCCGGAT ATAGAAAATC TTATTCGTAT   
  
  
+ AAGTACTCGC ATGAGGGCGG ATAAATTCTA AAATATCAAC CTCAACTCGC TTCTACTATT CCATCTAAGT   
  
  
+ TCAATTTATC ATATAAAAAA ATTCAATCCA CTTTAATGTA CTCAATTCAC TTCAACCTAT TTTAATCAAT   
  
  
+ TTACCCATGA AAAGAACAAG GTCCTAAGTA GATGTTTTCT ATTGATTGAT TTAAACAATT TTCACCTACC   
  
  
+ CAATTATCCC CAAAACTGAA AGAGGGGGCA AAGGGGGGTG GGTAATGCTG TGTAAAATAG GGGGAAAGGG   
  
  
+ CAAGAAAACT GTGTACAATG GTGGAGCAGG GGTATACCAT TTGGCCTTGA GTTGAGGGGG CACAAAAGCC   
  
  
+ CAGAGAGAGA GAGGGGTAAT GCCCACCAGA AATAAGCAGG AAGCGAGGGA AAAACACTAG AGAGAAACAG   
  
  
+ AGAAAGAGAG AGAGAGAGAG AGAGAAGGGG AAATTTACTA GAGAATGAGA GTGAAGAAGG GAACTTTATT   
  
  
+ TTAACTTTTT TATGATGCAC GCATCCATGG CAGAACAGGG AAGGGAAAGA AGGGTACAGA GTACTGCTGT   
  
  
+ CCCCAAAAAA TTAGTGTAGA AAACAGGAGA CACTCAACTA CAACAAGGGT AAGGAAGAAG AACAAGCAAG   
  
  
+ ATATCCCACC CAAAGAAAAA AAAAAAGAAG AAGAAGAAAA TTTTTCAGAG AGGAAAGAAA AGCAATCACA   
  
  
+ TGAATTAGAA GCCAACCCAA GCCTTCTTCA AAGAGAAGCA GCATAACATA GCATAGCTGG GGAGAGGATA   
  
  
+ GGAGAGAGAA ACTCAAGAAA AGGGGTACCC GGCTTAAATG TTGATGCAGA AAGGTATAGA TATGGCCACA   
  
  
+ CTACTACTAC CCAGGAGTTT ATCATTGGAG ATGGATTTCT CTTTCTGCCT CATCTTCTCC TCCATCCACC   
  
  
+ AGCAAAACTG GGTTTCCTTT CAGCTGGGTC TTTTCTAGAT CTAGAAACTA TGGTACCCAA ATCTCCCATT   
  
  
+ CCTCCATTTT TATTCTCAAA ATCTATCTTC TTAGACTTGT GGGTTTGATT CATTTCTTTT GGGTTTTTGA   
  
  
+ AAAGAATTTA ATTTGATTGT ATTGTGTTGA TTGGGGAATT TGTGGTGGTG TTAGATGCGA GCTATGCCCT   
  
  
+ ACAATTTGCA AGGCAAGGGT GTGGTAGAGG TTTCAAGCAT TTGTATTCCA CAAATCTCTT CCCCTGCTTC   
  
  
+ AAAGTGGAAG ACCAACAACA ACAACTTGGA CAACAAATTT CAGCAACAAC AAGAAGAAAT TGAGCAATTG   
  
  
+ CAGAGTAGTG GTGGCACTTT TCTCTCTCCT GATAACACCA ATACTAATAT TAGTACTACT CCGAGAGCGA   
  
  
+ CAAGTTCTGT TGATAGCGAA CACACATCTA CTCTGGATAC TACGGGCCCT CCCACTTCAA CATCTTCCTC   
  
  
+ CTTCAAGAGC ACCTCCACCG CCAACACAGC CGGTGTGGCG GACCCCGCAT GTACTCCCAA AGAGGATTGG   
  
  
+ GATGTCTCCG GCGGCGCTGC TGGCGGTGAC GGCGGTGGTG GGGGGCTTGG ATTGGAGGAG TGGGACAGCA   
  
  
+ TGTTCCCAAA TGGGGAGGGG GCTTTGCTCC CTTGGATCAT GGGTGAAGCT GATGACATGG GTATGGGTTT   
  
  
+ GAAGCATCTT TTGCAATCGG GTAACCCGGT TGACTATGAG GGCAATGCTG GTCTAGGGGT TGTCGATCAG   
  
  
+ GGTTCTGGAT TTGAGACTCT CTCTCCTCCT CCACCGCAGC CGGCGGCATG TGAATCTAAT GGTGGTGGTG   
  
  
+ TTGCTAATTT GGGGTTTCCA GGGAATAATG GTAAGATTTC TTCAATTTCA CATAATTGTT CATCTGGGAT   
  
  
+ TTTGAATGGT AAGGTGAACA ATAATGGGTT GAATCCCAAT TGTAACCCTC AAGCTCAAGG CAGTCTACTT   
  
  
+ GGTTTAATCC AAGGAGCTAC AGGTGTACAC GCACATCCTG ACGTTGGAGA CGAGAAACCC CAGATTTTGA   
  
  
+ ATCCACATTT GGTGATGAAC CCTCAGCAAG CTCAGAGCAT TGCAAACCCT AGCTTTTTGA TGCACTCATT   
  
  
+ AGGTTACTAT CAGCTGGAGC AACATCTATT TCAACCTCAG GCAAAACGCC TGAACACGGG TGCTGTTCTG   
  
  
+ GATCCTAACC TTGTTCAGCT TGCAAAGAAC CCATTTGCTG ATCAGGGTCA TGAGTTATTG TTGAGGAAGC   
  
  
+ AGCACCAACA GCTTGGTTTG CAACCATTGC CATTGGGTTT GGGTCCTCAG TTGGTCCCTC CGCAGAAGCC   
  
  
+ TGTGATGGGT TCAAAGCAGG GGAACCCCCA GCATTACCCG TTGCATGTTC ATCAGCAGCT GCAATTGCAA   
  
  
+ GAGCAGGCTG TCAAAGATCA GCTCTTCAAG GCGGCAGACC TTATTCAAAC TGGAAGTTTC TCACTCGCGC   
  
  
+ AAGAGATATT GGCGCGGCTC AATCACCAGC TCTCCCTCCC TGCAAAGCCC CTCATTAGGG CGGCTTTGTA   
  
  
+ TGTGAAGGAG GCCCTTCAAA TGCTCCTCCT AATGAGCAAC CCAGTTGCGG CTCCACCGTC CAAGATCCTC   
  
  
+ ACCCCTTATG ATGTTGTTCA CAAGATGAGC GCGTATAAGG TCTTCTCTGA GGTCTGCCCA ATCACTCAAT   
  
  
+ TTGTGAATTT CACTTGTACA CAGGCCATTC TCGAGGCTCT TGATGATTCT GATGCTATTC ATGTTGTCGA   
  
  
+ CTTTGATATT GGTTGCGGTG CTCAATGGGC ATCATTGATT CAGGAGCTGC CATTGAGGAA AAGGGGAGCT   
  
  
+ CCCTCTCTGA AAATTACAGC CATAGCTCCC ATGTTGACTG GCAGTCACTT TGAAATTAGC CTAGTATGTG   
  
  
+ AAAACCTTGT GCAATTTGCC AACGATATTG GTGTTGCTTG TGAGCTCCAA GTTGTCAACT TTGATTTGTT   
  
  
+ TGATCCATCT TCGCCATCAA TGCCGAACAT TAGTACTGCT GAGGATGAGT CAATTGCTGT TAGTATCCCC   
  
  
+ ATCTGGGCAT CTTCAATTAG GCCATCTGTT CTTCCTTCCA TCCTCCGATT CATTAAGCAA AAATCCCCCA   
  
  
+ AAATTGTGGT CTCGTTTGAT AGAGGATTCG ATCGTTTTGA TGTCCCTTTC CCCCAACATC TGTTGCATAC   
  
  
+ CCTAGAATCC TGCAGTAATT TATTGGACTC GCTTGATGGT CTCAATGTTG CATCGGACAT TGTGAGCAAG   
  
  
+ GTCGAGAAGT TCTTTGTTCA ACCTAGGATC GAAAATGCTG TGTTGGGTCG GGTCCATGCC CCTGACAAGA   
  
  
+ TGCCCCATTG GAAAAATCTC TTTGCTTCAG CCGGCTTCTT GCCCTTGCAA TTCAGTAATT TCACAGAAAC   
  
  
+ CCAGGCCGAT TATGTGGTGA AGAGAACCCC AACAAGAGGA TTTCACGTGG AGAAGCGCCA GGCATCACTC   
  
  
+ ATTCTGAGTT GGCAGAGGCA GGAGCTTGTG GCAGCTTCGG CATGGAAGTG TTG  

- +Up\_Stream \_Len000AGAATT GTAATCAACG AACCAAGTTG GGTAGTCACT CTTATAGACT AAAGTTTTCA   
  
  
- TATCTAAATA TATTTGTATA CCTGATGTCC CTTCCCTCAG CAAAGGAGTT TCAGAATCTA AATGTGAGTT   
  
  
- TTCAAACTCG CTATGGTTAT TACACTAAGG TTTGTACTCG GTTTATAATT CATAGGATAT GATTCTAGTT   
  
  
- CTTCTCTTAC TTGGTTTTCT GACAATTAAA AAGTTAATTC GATTTGAATA ATTGGAAAAA AACCTTATCT   
  
  
- AAAAATTTAA AATAGAAGTT ATATTCAAAA AGGTTCCGCT CTCTGTATAT CGTTAAAATT AATTTCAATT   
  
  
- TCATCATATT GTAAAAAAAA TTACCAATAT CCGAATATCG TGGAAAAGAT CCACTTTTCT CTATAGAACC   
  
  
- GGACTAAAGG TCATTTCAAA AAAAAAATTT ATAACAGTTA ATAAAAACGT TTACTGTAAA CCATTAAACC   
  
  
- CCCTATTAGA CTACACAATT TTCTATGTTA CTTTTATAGT CTTTTATCCT ACGGAGTTTA AAGTGTTCTT   
  
  
- TCTTTTTTAG AAAAACAACA ATCATAAAGA TGTGTAGGTG TTAAAAATGA GTACCATTTT TTTTTTCCCG   
  
  
- GTTTATAGAG TGGTTATATT GGTTTGATAT ACAAGGTAAT AAATCATTGC CACTATTTAT ACATTACCCA   
  
  
- GTTCAATGAT TATTTTATCG GATGTTATGA ACCTATAGAT GTATTTTAAC TATATCTTAT ATATAACGTT   
  
  
- ATGAATTTAG TTTTAATTTA CTTTATAAAT GAAAAAATAA GATAATGTTT ATCGAAGAAT ATCTTATCGT   
  
  
- TGTTAAAAAT TACATTCAAA GTAAGAATGG TCTAAACTTC TCCAGGCCTA TATCTTTTAG AATAAGCATA   
  
  
- TTCATGAGCG TACTCCCGCC TATTTAAGAT TTTATAGTTG GAGTTGAGCG AAGATGATAA GGTAGATTCA   
  
  
- AGTTAAATAG TATATTTTTT TAAGTTAGGT GAAATTACAT GAGTTAAGTG AAGTTGGATA AAATTAGTTA   
  
  
- AATGGGTACT TTTCTTGTTC CAGGATTCAT CTACAAAAGA TAACTAACTA AATTTGTTAA AAGTGGATGG   
  
  
- GTTAATAGGG GTTTTGACTT TCTCCCCCGT TTCCCCCCAC CCATTACGAC ACATTTTATC CCCCTTTCCC   
  
  
- GTTCTTTTGA CACATGTTAC CACCTCGTCC CCATATGGTA AACCGGAACT CAACTCCCCC GTGTTTTCGG   
  
  
- GTCTCTCTCT CTCCCCATTA CGGGTGGTCT TTATTCGTCC TTCGCTCCCT TTTTGTGATC TCTCTTTGTC   
  
  
- TCTTTCTCTC TCTCTCTCTC TCTCTTCCCC TTTAAATGAT CTCTTACTCT CACTTCTTCC CTTGAAATAA   
  
  
- AATTGAAAAA ATACTACGTG CGTAGGTACC GTCTTGTCCC TTCCCTTTCT TCCCATGTCT CATGACGACA   
  
  
- GGGGTTTTTT AATCACATCT TTTGTCCTCT GTGAGTTGAT GTTGTTCCCA TTCCTTCTTC TTGTTCGTTC   
  
  
- TATAGGGTGG GTTTCTTTTT TTTTTTCTTC TTCTTCTTTT AAAAAGTCTC TCCTTTCTTT TCGTTAGTGT   
  
  
- ACTTAATCTT CGGTTGGGTT CGGAAGAAGT TTCTCTTCGT CGTATTGTAT CGTATCGACC CCTCTCCTAT   
  
  
- CCTCTCTCTT TGAGTTCTTT TCCCCATGGG CCGAATTTAC AACTACGTCT TTCCATATCT ATACCGGTGT   
  
  
- GATGATGATG GGTCCTCAAA TAGTAACCTC TACCTAAAGA GAAAGACGGA GTAGAAGAGG AGGTAGGTGG   
  
  
- TCGTTTTGAC CCAAAGGAAA GTCGACCCAG AAAAGATCTA GATCTTTGAT ACCATGGGTT TAGAGGGTAA   
  
  
- GGAGGTAAAA ATAAGAGTTT TAGATAGAAG AATCTGAACA CCCAAACTAA GTAAAGAAAA CCCAAAAACT   
  
  
- TTTCTTAAAT TAAACTAACA TAACACAACT AACCCCTTAA ACACCACCAC AATCTACGCT CGATACGGGA   
  
  
- TGTTAAACGT TCCGTTCCCA CACCATCTCC AAAGTTCGTA AACATAAGGT GTTTAGAGAA GGGGACGAAG   
  
  
- TTTCACCTTC TGGTTGTTGT TGTTGAACCT GTTGTTTAAA GTCGTTGTTG TTCTTCTTTA ACTCGTTAAC   
  
  
- GTCTCATCAC CACCGTGAAA AGAGAGAGGA CTATTGTGGT TATGATTATA ATCATGATGA GGCTCTCGCT   
  
  
- GTTCAAGACA ACTATCGCTT GTGTGTAGAT GAGACCTATG ATGCCCGGGA GGGTGAAGTT GTAGAAGGAG   
  
  
- GAAGTTCTCG TGGAGGTGGC GGTTGTGTCG GCCACACCGC CTGGGGCGTA CATGAGGGTT TCTCCTAACC   
  
  
- CTACAGAGGC CGCCGCGACG ACCGCCACTG CCGCCACCAC CCCCCGAACC TAACCTCCTC ACCCTGTCGT   
  
  
- ACAAGGGTTT ACCCCTCCCC CGAAACGAGG GAACCTAGTA CCCACTTCGA CTACTGTACC CATACCCAAA   
  
  
- CTTCGTAGAA AACGTTAGCC CATTGGGCCA ACTGATACTC CCGTTACGAC CAGATCCCCA ACAGCTAGTC   
  
  
- CCAAGACCTA AACTCTGAGA GAGAGGAGGA GGTGGCGTCG GCCGCCGTAC ACTTAGATTA CCACCACCAC   
  
  
- AACGATTAAA CCCCAAAGGT CCCTTATTAC CATTCTAAAG AAGTTAAAGT GTATTAACAA GTAGACCCTA   
  
  
- AAACTTACCA TTCCACTTGT TATTACCCAA CTTAGGGTTA ACATTGGGAG TTCGAGTTCC GTCAGATGAA   
  
  
- CCAAATTAGG TTCCTCGATG TCCACATGTG CGTGTAGGAC TGCAACCTCT GCTCTTTGGG GTCTAAAACT   
  
  
- TAGGTGTAAA CCACTACTTG GGAGTCGTTC GAGTCTCGTA ACGTTTGGGA TCGAAAAACT ACGTGAGTAA   
  
  
- TCCAATGATA GTCGACCTCG TTGTAGATAA AGTTGGAGTC CGTTTTGCGG ACTTGTGCCC ACGACAAGAC   
  
  
- CTAGGATTGG AACAAGTCGA ACGTTTCTTG GGTAAACGAC TAGTCCCAGT ACTCAATAAC AACTCCTTCG   
  
  
- TCGTGGTTGT CGAACCAAAC GTTGGTAACG GTAACCCAAA CCCAGGAGTC AACCAGGGAG GCGTCTTCGG   
  
  
- ACACTACCCA AGTTTCGTCC CCTTGGGGGT CGTAATGGGC AACGTACAAG TAGTCGTCGA CGTTAACGTT   
  
  
- CTCGTCCGAC AGTTTCTAGT CGAGAAGTTC CGCCGTCTGG AATAAGTTTG ACCTTCAAAG AGTGAGCGCG   
  
  
- TTCTCTATAA CCGCGCCGAG TTAGTGGTCG AGAGGGAGGG ACGTTTCGGG GAGTAATCCC GCCGAAACAT   
  
  
- ACACTTCCTC CGGGAAGTTT ACGAGGAGGA TTACTCGTTG GGTCAACGCC GAGGTGGCAG GTTCTAGGAG   
  
  
- TGGGGAATAC TACAACAAGT GTTCTACTCG CGCATATTCC AGAAGAGACT CCAGACGGGT TAGTGAGTTA   
  
  
- AACACTTAAA GTGAACATGT GTCCGGTAAG AGCTCCGAGA ACTACTAAGA CTACGATAAG TACAACAGCT   
  
  
- GAAACTATAA CCAACGCCAC GAGTTACCCG TAGTAACTAA GTCCTCGACG GTAACTCCTT TTCCCCTCGA   
  
  
- GGGAGAGACT TTTAATGTCG GTATCGAGGG TACAACTGAC CGTCAGTGAA ACTTTAATCG GATCATACAC   
  
  
- TTTTGGAACA CGTTAAACGG TTGCTATAAC CACAACGAAC ACTCGAGGTT CAACAGTTGA AACTAAACAA   
  
  
- ACTAGGTAGA AGCGGTAGTT ACGGCTTGTA ATCATGACGA CTCCTACTCA GTTAACGACA ATCATAGGGG   
  
  
- TAGACCCGTA GAAGTTAATC CGGTAGACAA GAAGGAAGGT AGGAGGCTAA GTAATTCGTT TTTAGGGGGT   
  
  
- TTTAACACCA GAGCAAACTA TCTCCTAAGC TAGCAAAACT ACAGGGAAAG GGGGTTGTAG ACAACGTATG   
  
  
- GGATCTTAGG ACGTCATTAA ATAACCTGAG CGAACTACCA GAGTTACAAC GTAGCCTGTA ACACTCGTTC   
  
  
- CAGCTCTTCA AGAAACAAGT TGGATCCTAG CTTTTACGAC ACAACCCAGC CCAGGTACGG GGACTGTTCT   
  
  
- ACGGGGTAAC CTTTTTAGAG AAACGAAGTC GGCCGAAGAA CGGGAACGTT AAGTCATTAA AGTGTCTTTG   
  
  
- GGTCCGGCTA ATACACCACT TCTCTTGGGG TTGTTCTCCT AAAGTGCACC TCTTCGCGGT CCGTAGTGAG   
  
  
- TAAGACTCAA CCGTCTCCGT CCTCGAACAC CGTCGAAGCC GTACCTTCAC AAC

+     ATCT-motif

| Site Name | Organism | Position | Strand | Matrix score. | sequence | function |
| --- | --- | --- | --- | --- | --- | --- |
| ATCT-motif | Pisum sativum | 277 | - | 9 | AATCTAATCC | part of a conserved DNA module involved in light responsiveness |

>HU04G00047.1   
+ +Up\_Stream \_Len000TCTTAA CATTAGTTGC TTGGTTCAAC CCATCAGTGA GAATATCTGA TTTCAAAAGT   
  
  
+ ATAGATTTAT ATAAACATAT GGACTACAGG GAAGGGAGTC GTTTCCTCAA AGTCTTAGAT TTACACTCAA   
  
  
+ AAGTTTGAGC GATACCAATA ATGTGATTCC AAACATGAGC CAAATATTAA GTATCCTATA CTAAGATCAA   
  
  
+ GAAGAGAATG AACCAAAAGA CTGTTAATTT TTCAATTAAG CTAAACTTAT TAACCTTTTT TTGGAATAGA   
  
  
+ TTTTTAAATT TTATCTTCAA TATAAGTTTT TCCAAGGCGA GAGACATATA GCAATTTTAA TTAAAGTTAA   
  
  
+ AGTAGTATAA CATTTTTTTT AATGGTTATA GGCTTATAGC ACCTTTTCTA GGTGAAAAGA GATATCTTGG   
  
  
+ CCTGATTTCC AGTAAAGTTT TTTTTTTAAA TATTGTCAAT TATTTTTGCA AATGACATTT GGTAATTTGG   
  
  
+ GGGATAATCT GATGTGTTAA AAGATACAAT GAAAATATCA GAAAATAGGA TGCCTCAAAT TTCACAAGAA   
  
  
+ AGAAAAAATC TTTTTGTTGT TAGTATTTCT ACACATCCAC AATTTTTACT CATGGTAAAA AAAAAAGGGC   
  
  
+ CAAATATCTC ACCAATATAA CCAAACTATA TGTTCCATTA TTTAGTAACG GTGATAAATA TGTAATGGGT   
  
  
+ CAAGTTACTA ATAAAATAGC CTACAATACT TGGATATCTA CATAAAATTG ATATAGAATA TATATTGCAA   
  
  
+ TACTTAAATC AAAATTAAAT GAAATATTTA CTTTTTTATT CTATTACAAA TAGCTTCTTA TAGAATAGCA   
  
  
+ ACAATTTTTA ATGTAAGTTT CATTCTTACC AGATTTGAAG AGGTCCGGAT ATAGAAAATC TTATTCGTAT   
  
  
+ AAGTACTCGC ATGAGGGCGG ATAAATTCTA AAATATCAAC CTCAACTCGC TTCTACTATT CCATCTAAGT   
  
  
+ TCAATTTATC ATATAAAAAA ATTCAATCCA CTTTAATGTA CTCAATTCAC TTCAACCTAT TTTAATCAAT   
  
  
+ TTACCCATGA AAAGAACAAG GTCCTAAGTA GATGTTTTCT ATTGATTGAT TTAAACAATT TTCACCTACC   
  
  
+ CAATTATCCC CAAAACTGAA AGAGGGGGCA AAGGGGGGTG GGTAATGCTG TGTAAAATAG GGGGAAAGGG   
  
  
+ CAAGAAAACT GTGTACAATG GTGGAGCAGG GGTATACCAT TTGGCCTTGA GTTGAGGGGG CACAAAAGCC   
  
  
+ CAGAGAGAGA GAGGGGTAAT GCCCACCAGA AATAAGCAGG AAGCGAGGGA AAAACACTAG AGAGAAACAG   
  
  
+ AGAAAGAGAG AGAGAGAGAG AGAGAAGGGG AAATTTACTA GAGAATGAGA GTGAAGAAGG GAACTTTATT   
  
  
+ TTAACTTTTT TATGATGCAC GCATCCATGG CAGAACAGGG AAGGGAAAGA AGGGTACAGA GTACTGCTGT   
  
  
+ CCCCAAAAAA TTAGTGTAGA AAACAGGAGA CACTCAACTA CAACAAGGGT AAGGAAGAAG AACAAGCAAG   
  
  
+ ATATCCCACC CAAAGAAAAA AAAAAAGAAG AAGAAGAAAA TTTTTCAGAG AGGAAAGAAA AGCAATCACA   
  
  
+ TGAATTAGAA GCCAACCCAA GCCTTCTTCA AAGAGAAGCA GCATAACATA GCATAGCTGG GGAGAGGATA   
  
  
+ GGAGAGAGAA ACTCAAGAAA AGGGGTACCC GGCTTAAATG TTGATGCAGA AAGGTATAGA TATGGCCACA   
  
  
+ CTACTACTAC CCAGGAGTTT ATCATTGGAG ATGGATTTCT CTTTCTGCCT CATCTTCTCC TCCATCCACC   
  
  
+ AGCAAAACTG GGTTTCCTTT CAGCTGGGTC TTTTCTAGAT CTAGAAACTA TGGTACCCAA ATCTCCCATT   
  
  
+ CCTCCATTTT TATTCTCAAA ATCTATCTTC TTAGACTTGT GGGTTTGATT CATTTCTTTT GGGTTTTTGA   
  
  
+ AAAGAATTTA ATTTGATTGT ATTGTGTTGA TTGGGGAATT TGTGGTGGTG TTAGATGCGA GCTATGCCCT   
  
  
+ ACAATTTGCA AGGCAAGGGT GTGGTAGAGG TTTCAAGCAT TTGTATTCCA CAAATCTCTT CCCCTGCTTC   
  
  
+ AAAGTGGAAG ACCAACAACA ACAACTTGGA CAACAAATTT CAGCAACAAC AAGAAGAAAT TGAGCAATTG   
  
  
+ CAGAGTAGTG GTGGCACTTT TCTCTCTCCT GATAACACCA ATACTAATAT TAGTACTACT CCGAGAGCGA   
  
  
+ CAAGTTCTGT TGATAGCGAA CACACATCTA CTCTGGATAC TACGGGCCCT CCCACTTCAA CATCTTCCTC   
  
  
+ CTTCAAGAGC ACCTCCACCG CCAACACAGC CGGTGTGGCG GACCCCGCAT GTACTCCCAA AGAGGATTGG   
  
  
+ GATGTCTCCG GCGGCGCTGC TGGCGGTGAC GGCGGTGGTG GGGGGCTTGG ATTGGAGGAG TGGGACAGCA   
  
  
+ TGTTCCCAAA TGGGGAGGGG GCTTTGCTCC CTTGGATCAT GGGTGAAGCT GATGACATGG GTATGGGTTT   
  
  
+ GAAGCATCTT TTGCAATCGG GTAACCCGGT TGACTATGAG GGCAATGCTG GTCTAGGGGT TGTCGATCAG   
  
  
+ GGTTCTGGAT TTGAGACTCT CTCTCCTCCT CCACCGCAGC CGGCGGCATG TGAATCTAAT GGTGGTGGTG   
  
  
+ TTGCTAATTT GGGGTTTCCA GGGAATAATG GTAAGATTTC TTCAATTTCA CATAATTGTT CATCTGGGAT   
  
  
+ TTTGAATGGT AAGGTGAACA ATAATGGGTT GAATCCCAAT TGTAACCCTC AAGCTCAAGG CAGTCTACTT   
  
  
+ GGTTTAATCC AAGGAGCTAC AGGTGTACAC GCACATCCTG ACGTTGGAGA CGAGAAACCC CAGATTTTGA   
  
  
+ ATCCACATTT GGTGATGAAC CCTCAGCAAG CTCAGAGCAT TGCAAACCCT AGCTTTTTGA TGCACTCATT   
  
  
+ AGGTTACTAT CAGCTGGAGC AACATCTATT TCAACCTCAG GCAAAACGCC TGAACACGGG TGCTGTTCTG   
  
  
+ GATCCTAACC TTGTTCAGCT TGCAAAGAAC CCATTTGCTG ATCAGGGTCA TGAGTTATTG TTGAGGAAGC   
  
  
+ AGCACCAACA GCTTGGTTTG CAACCATTGC CATTGGGTTT GGGTCCTCAG TTGGTCCCTC CGCAGAAGCC   
  
  
+ TGTGATGGGT TCAAAGCAGG GGAACCCCCA GCATTACCCG TTGCATGTTC ATCAGCAGCT GCAATTGCAA   
  
  
+ GAGCAGGCTG TCAAAGATCA GCTCTTCAAG GCGGCAGACC TTATTCAAAC TGGAAGTTTC TCACTCGCGC   
  
  
+ AAGAGATATT GGCGCGGCTC AATCACCAGC TCTCCCTCCC TGCAAAGCCC CTCATTAGGG CGGCTTTGTA   
  
  
+ TGTGAAGGAG GCCCTTCAAA TGCTCCTCCT AATGAGCAAC CCAGTTGCGG CTCCACCGTC CAAGATCCTC   
  
  
+ ACCCCTTATG ATGTTGTTCA CAAGATGAGC GCGTATAAGG TCTTCTCTGA GGTCTGCCCA ATCACTCAAT   
  
  
+ TTGTGAATTT CACTTGTACA CAGGCCATTC TCGAGGCTCT TGATGATTCT GATGCTATTC ATGTTGTCGA   
  
  
+ CTTTGATATT GGTTGCGGTG CTCAATGGGC ATCATTGATT CAGGAGCTGC CATTGAGGAA AAGGGGAGCT   
  
  
+ CCCTCTCTGA AAATTACAGC CATAGCTCCC ATGTTGACTG GCAGTCACTT TGAAATTAGC CTAGTATGTG   
  
  
+ AAAACCTTGT GCAATTTGCC AACGATATTG GTGTTGCTTG TGAGCTCCAA GTTGTCAACT TTGATTTGTT   
  
  
+ TGATCCATCT TCGCCATCAA TGCCGAACAT TAGTACTGCT GAGGATGAGT CAATTGCTGT TAGTATCCCC   
  
  
+ ATCTGGGCAT CTTCAATTAG GCCATCTGTT CTTCCTTCCA TCCTCCGATT CATTAAGCAA AAATCCCCCA   
  
  
+ AAATTGTGGT CTCGTTTGAT AGAGGATTCG ATCGTTTTGA TGTCCCTTTC CCCCAACATC TGTTGCATAC   
  
  
+ CCTAGAATCC TGCAGTAATT TATTGGACTC GCTTGATGGT CTCAATGTTG CATCGGACAT TGTGAGCAAG   
  
  
+ GTCGAGAAGT TCTTTGTTCA ACCTAGGATC GAAAATGCTG TGTTGGGTCG GGTCCATGCC CCTGACAAGA   
  
  
+ TGCCCCATTG GAAAAATCTC TTTGCTTCAG CCGGCTTCTT GCCCTTGCAA TTCAGTAATT TCACAGAAAC   
  
  
+ CCAGGCCGAT TATGTGGTGA AGAGAACCCC AACAAGAGGA TTTCACGTGG AGAAGCGCCA GGCATCACTC   
  
  
+ ATTCTGAGTT GGCAGAGGCA GGAGCTTGTG GCAGCTTCGG CATGGAAGTG TTG  

- +Up\_Stream \_Len000AGAATT GTAATCAACG AACCAAGTTG GGTAGTCACT CTTATAGACT AAAGTTTTCA   
  
  
- TATCTAAATA TATTTGTATA CCTGATGTCC CTTCCCTCAG CAAAGGAGTT TCAGAATCTA AATGTGAGTT   
  
  
- TTCAAACTCG CTATGGTTAT TACACTAAGG TTTGTACTCG GTTTATAATT CATAGGATAT GATTCTAGTT   
  
  
- CTTCTCTTAC TTGGTTTTCT GACAATTAAA AAGTTAATTC GATTTGAATA ATTGGAAAAA AACCTTATCT   
  
  
- AAAAATTTAA AATAGAAGTT ATATTCAAAA AGGTTCCGCT CTCTGTATAT CGTTAAAATT AATTTCAATT   
  
  
- TCATCATATT GTAAAAAAAA TTACCAATAT CCGAATATCG TGGAAAAGAT CCACTTTTCT CTATAGAACC   
  
  
- GGACTAAAGG TCATTTCAAA AAAAAAATTT ATAACAGTTA ATAAAAACGT TTACTGTAAA CCATTAAACC   
  
  
- CCCTATTAGA CTACACAATT TTCTATGTTA CTTTTATAGT CTTTTATCCT ACGGAGTTTA AAGTGTTCTT   
  
  
- TCTTTTTTAG AAAAACAACA ATCATAAAGA TGTGTAGGTG TTAAAAATGA GTACCATTTT TTTTTTCCCG   
  
  
- GTTTATAGAG TGGTTATATT GGTTTGATAT ACAAGGTAAT AAATCATTGC CACTATTTAT ACATTACCCA   
  
  
- GTTCAATGAT TATTTTATCG GATGTTATGA ACCTATAGAT GTATTTTAAC TATATCTTAT ATATAACGTT   
  
  
- ATGAATTTAG TTTTAATTTA CTTTATAAAT GAAAAAATAA GATAATGTTT ATCGAAGAAT ATCTTATCGT   
  
  
- TGTTAAAAAT TACATTCAAA GTAAGAATGG TCTAAACTTC TCCAGGCCTA TATCTTTTAG AATAAGCATA   
  
  
- TTCATGAGCG TACTCCCGCC TATTTAAGAT TTTATAGTTG GAGTTGAGCG AAGATGATAA GGTAGATTCA   
  
  
- AGTTAAATAG TATATTTTTT TAAGTTAGGT GAAATTACAT GAGTTAAGTG AAGTTGGATA AAATTAGTTA   
  
  
- AATGGGTACT TTTCTTGTTC CAGGATTCAT CTACAAAAGA TAACTAACTA AATTTGTTAA AAGTGGATGG   
  
  
- GTTAATAGGG GTTTTGACTT TCTCCCCCGT TTCCCCCCAC CCATTACGAC ACATTTTATC CCCCTTTCCC   
  
  
- GTTCTTTTGA CACATGTTAC CACCTCGTCC CCATATGGTA AACCGGAACT CAACTCCCCC GTGTTTTCGG   
  
  
- GTCTCTCTCT CTCCCCATTA CGGGTGGTCT TTATTCGTCC TTCGCTCCCT TTTTGTGATC TCTCTTTGTC   
  
  
- TCTTTCTCTC TCTCTCTCTC TCTCTTCCCC TTTAAATGAT CTCTTACTCT CACTTCTTCC CTTGAAATAA   
  
  
- AATTGAAAAA ATACTACGTG CGTAGGTACC GTCTTGTCCC TTCCCTTTCT TCCCATGTCT CATGACGACA   
  
  
- GGGGTTTTTT AATCACATCT TTTGTCCTCT GTGAGTTGAT GTTGTTCCCA TTCCTTCTTC TTGTTCGTTC   
  
  
- TATAGGGTGG GTTTCTTTTT TTTTTTCTTC TTCTTCTTTT AAAAAGTCTC TCCTTTCTTT TCGTTAGTGT   
  
  
- ACTTAATCTT CGGTTGGGTT CGGAAGAAGT TTCTCTTCGT CGTATTGTAT CGTATCGACC CCTCTCCTAT   
  
  
- CCTCTCTCTT TGAGTTCTTT TCCCCATGGG CCGAATTTAC AACTACGTCT TTCCATATCT ATACCGGTGT   
  
  
- GATGATGATG GGTCCTCAAA TAGTAACCTC TACCTAAAGA GAAAGACGGA GTAGAAGAGG AGGTAGGTGG   
  
  
- TCGTTTTGAC CCAAAGGAAA GTCGACCCAG AAAAGATCTA GATCTTTGAT ACCATGGGTT TAGAGGGTAA   
  
  
- GGAGGTAAAA ATAAGAGTTT TAGATAGAAG AATCTGAACA CCCAAACTAA GTAAAGAAAA CCCAAAAACT   
  
  
- TTTCTTAAAT TAAACTAACA TAACACAACT AACCCCTTAA ACACCACCAC AATCTACGCT CGATACGGGA   
  
  
- TGTTAAACGT TCCGTTCCCA CACCATCTCC AAAGTTCGTA AACATAAGGT GTTTAGAGAA GGGGACGAAG   
  
  
- TTTCACCTTC TGGTTGTTGT TGTTGAACCT GTTGTTTAAA GTCGTTGTTG TTCTTCTTTA ACTCGTTAAC   
  
  
- GTCTCATCAC CACCGTGAAA AGAGAGAGGA CTATTGTGGT TATGATTATA ATCATGATGA GGCTCTCGCT   
  
  
- GTTCAAGACA ACTATCGCTT GTGTGTAGAT GAGACCTATG ATGCCCGGGA GGGTGAAGTT GTAGAAGGAG   
  
  
- GAAGTTCTCG TGGAGGTGGC GGTTGTGTCG GCCACACCGC CTGGGGCGTA CATGAGGGTT TCTCCTAACC   
  
  
- CTACAGAGGC CGCCGCGACG ACCGCCACTG CCGCCACCAC CCCCCGAACC TAACCTCCTC ACCCTGTCGT   
  
  
- ACAAGGGTTT ACCCCTCCCC CGAAACGAGG GAACCTAGTA CCCACTTCGA CTACTGTACC CATACCCAAA   
  
  
- CTTCGTAGAA AACGTTAGCC CATTGGGCCA ACTGATACTC CCGTTACGAC CAGATCCCCA ACAGCTAGTC   
  
  
- CCAAGACCTA AACTCTGAGA GAGAGGAGGA GGTGGCGTCG GCCGCCGTAC ACTTAGATTA CCACCACCAC   
  
  
- AACGATTAAA CCCCAAAGGT CCCTTATTAC CATTCTAAAG AAGTTAAAGT GTATTAACAA GTAGACCCTA   
  
  
- AAACTTACCA TTCCACTTGT TATTACCCAA CTTAGGGTTA ACATTGGGAG TTCGAGTTCC GTCAGATGAA   
  
  
- CCAAATTAGG TTCCTCGATG TCCACATGTG CGTGTAGGAC TGCAACCTCT GCTCTTTGGG GTCTAAAACT   
  
  
- TAGGTGTAAA CCACTACTTG GGAGTCGTTC GAGTCTCGTA ACGTTTGGGA TCGAAAAACT ACGTGAGTAA   
  
  
- TCCAATGATA GTCGACCTCG TTGTAGATAA AGTTGGAGTC CGTTTTGCGG ACTTGTGCCC ACGACAAGAC   
  
  
- CTAGGATTGG AACAAGTCGA ACGTTTCTTG GGTAAACGAC TAGTCCCAGT ACTCAATAAC AACTCCTTCG   
  
  
- TCGTGGTTGT CGAACCAAAC GTTGGTAACG GTAACCCAAA CCCAGGAGTC AACCAGGGAG GCGTCTTCGG   
  
  
- ACACTACCCA AGTTTCGTCC CCTTGGGGGT CGTAATGGGC AACGTACAAG TAGTCGTCGA CGTTAACGTT   
  
  
- CTCGTCCGAC AGTTTCTAGT CGAGAAGTTC CGCCGTCTGG AATAAGTTTG ACCTTCAAAG AGTGAGCGCG   
  
  
- TTCTCTATAA CCGCGCCGAG TTAGTGGTCG AGAGGGAGGG ACGTTTCGGG GAGTAATCCC GCCGAAACAT   
  
  
- ACACTTCCTC CGGGAAGTTT ACGAGGAGGA TTACTCGTTG GGTCAACGCC GAGGTGGCAG GTTCTAGGAG   
  
  
- TGGGGAATAC TACAACAAGT GTTCTACTCG CGCATATTCC AGAAGAGACT CCAGACGGGT TAGTGAGTTA   
  
  
- AACACTTAAA GTGAACATGT GTCCGGTAAG AGCTCCGAGA ACTACTAAGA CTACGATAAG TACAACAGCT   
  
  
- GAAACTATAA CCAACGCCAC GAGTTACCCG TAGTAACTAA GTCCTCGACG GTAACTCCTT TTCCCCTCGA   
  
  
- GGGAGAGACT TTTAATGTCG GTATCGAGGG TACAACTGAC CGTCAGTGAA ACTTTAATCG GATCATACAC   
  
  
- TTTTGGAACA CGTTAAACGG TTGCTATAAC CACAACGAAC ACTCGAGGTT CAACAGTTGA AACTAAACAA   
  
  
- ACTAGGTAGA AGCGGTAGTT ACGGCTTGTA ATCATGACGA CTCCTACTCA GTTAACGACA ATCATAGGGG   
  
  
- TAGACCCGTA GAAGTTAATC CGGTAGACAA GAAGGAAGGT AGGAGGCTAA GTAATTCGTT TTTAGGGGGT   
  
  
- TTTAACACCA GAGCAAACTA TCTCCTAAGC TAGCAAAACT ACAGGGAAAG GGGGTTGTAG ACAACGTATG   
  
  
- GGATCTTAGG ACGTCATTAA ATAACCTGAG CGAACTACCA GAGTTACAAC GTAGCCTGTA ACACTCGTTC   
  
  
- CAGCTCTTCA AGAAACAAGT TGGATCCTAG CTTTTACGAC ACAACCCAGC CCAGGTACGG GGACTGTTCT   
  
  
- ACGGGGTAAC CTTTTTAGAG AAACGAAGTC GGCCGAAGAA CGGGAACGTT AAGTCATTAA AGTGTCTTTG   
  
  
- GGTCCGGCTA ATACACCACT TCTCTTGGGG TTGTTCTCCT AAAGTGCACC TCTTCGCGGT CCGTAGTGAG   
  
  
- TAAGACTCAA CCGTCTCCGT CCTCGAACAC CGTCGAAGCC GTACCTTCAC AAC

+     AT~TATA-box

| Site Name | Organism | Position | Strand | Matrix score. | sequence | function |
| --- | --- | --- | --- | --- | --- | --- |
| AT~TATA-box | Arabidopsis thaliana | 763 | + | 6 | TATATA |  |
| AT~TATA-box | Arabidopsis thaliana | 82 | + | 6 | TATATA |  |
| AT~TATA-box | Arabidopsis thaliana | 80 | - | 8 | TATATAAA |  |

>HU04G00047.1   
+ +Up\_Stream \_Len000TCTTAA CATTAGTTGC TTGGTTCAAC CCATCAGTGA GAATATCTGA TTTCAAAAGT   
  
  
+ ATAGATTTAT ATAAACATAT GGACTACAGG GAAGGGAGTC GTTTCCTCAA AGTCTTAGAT TTACACTCAA   
  
  
+ AAGTTTGAGC GATACCAATA ATGTGATTCC AAACATGAGC CAAATATTAA GTATCCTATA CTAAGATCAA   
  
  
+ GAAGAGAATG AACCAAAAGA CTGTTAATTT TTCAATTAAG CTAAACTTAT TAACCTTTTT TTGGAATAGA   
  
  
+ TTTTTAAATT TTATCTTCAA TATAAGTTTT TCCAAGGCGA GAGACATATA GCAATTTTAA TTAAAGTTAA   
  
  
+ AGTAGTATAA CATTTTTTTT AATGGTTATA GGCTTATAGC ACCTTTTCTA GGTGAAAAGA GATATCTTGG   
  
  
+ CCTGATTTCC AGTAAAGTTT TTTTTTTAAA TATTGTCAAT TATTTTTGCA AATGACATTT GGTAATTTGG   
  
  
+ GGGATAATCT GATGTGTTAA AAGATACAAT GAAAATATCA GAAAATAGGA TGCCTCAAAT TTCACAAGAA   
  
  
+ AGAAAAAATC TTTTTGTTGT TAGTATTTCT ACACATCCAC AATTTTTACT CATGGTAAAA AAAAAAGGGC   
  
  
+ CAAATATCTC ACCAATATAA CCAAACTATA TGTTCCATTA TTTAGTAACG GTGATAAATA TGTAATGGGT   
  
  
+ CAAGTTACTA ATAAAATAGC CTACAATACT TGGATATCTA CATAAAATTG ATATAGAATA TATATTGCAA   
  
  
+ TACTTAAATC AAAATTAAAT GAAATATTTA CTTTTTTATT CTATTACAAA TAGCTTCTTA TAGAATAGCA   
  
  
+ ACAATTTTTA ATGTAAGTTT CATTCTTACC AGATTTGAAG AGGTCCGGAT ATAGAAAATC TTATTCGTAT   
  
  
+ AAGTACTCGC ATGAGGGCGG ATAAATTCTA AAATATCAAC CTCAACTCGC TTCTACTATT CCATCTAAGT   
  
  
+ TCAATTTATC ATATAAAAAA ATTCAATCCA CTTTAATGTA CTCAATTCAC TTCAACCTAT TTTAATCAAT   
  
  
+ TTACCCATGA AAAGAACAAG GTCCTAAGTA GATGTTTTCT ATTGATTGAT TTAAACAATT TTCACCTACC   
  
  
+ CAATTATCCC CAAAACTGAA AGAGGGGGCA AAGGGGGGTG GGTAATGCTG TGTAAAATAG GGGGAAAGGG   
  
  
+ CAAGAAAACT GTGTACAATG GTGGAGCAGG GGTATACCAT TTGGCCTTGA GTTGAGGGGG CACAAAAGCC   
  
  
+ CAGAGAGAGA GAGGGGTAAT GCCCACCAGA AATAAGCAGG AAGCGAGGGA AAAACACTAG AGAGAAACAG   
  
  
+ AGAAAGAGAG AGAGAGAGAG AGAGAAGGGG AAATTTACTA GAGAATGAGA GTGAAGAAGG GAACTTTATT   
  
  
+ TTAACTTTTT TATGATGCAC GCATCCATGG CAGAACAGGG AAGGGAAAGA AGGGTACAGA GTACTGCTGT   
  
  
+ CCCCAAAAAA TTAGTGTAGA AAACAGGAGA CACTCAACTA CAACAAGGGT AAGGAAGAAG AACAAGCAAG   
  
  
+ ATATCCCACC CAAAGAAAAA AAAAAAGAAG AAGAAGAAAA TTTTTCAGAG AGGAAAGAAA AGCAATCACA   
  
  
+ TGAATTAGAA GCCAACCCAA GCCTTCTTCA AAGAGAAGCA GCATAACATA GCATAGCTGG GGAGAGGATA   
  
  
+ GGAGAGAGAA ACTCAAGAAA AGGGGTACCC GGCTTAAATG TTGATGCAGA AAGGTATAGA TATGGCCACA   
  
  
+ CTACTACTAC CCAGGAGTTT ATCATTGGAG ATGGATTTCT CTTTCTGCCT CATCTTCTCC TCCATCCACC   
  
  
+ AGCAAAACTG GGTTTCCTTT CAGCTGGGTC TTTTCTAGAT CTAGAAACTA TGGTACCCAA ATCTCCCATT   
  
  
+ CCTCCATTTT TATTCTCAAA ATCTATCTTC TTAGACTTGT GGGTTTGATT CATTTCTTTT GGGTTTTTGA   
  
  
+ AAAGAATTTA ATTTGATTGT ATTGTGTTGA TTGGGGAATT TGTGGTGGTG TTAGATGCGA GCTATGCCCT   
  
  
+ ACAATTTGCA AGGCAAGGGT GTGGTAGAGG TTTCAAGCAT TTGTATTCCA CAAATCTCTT CCCCTGCTTC   
  
  
+ AAAGTGGAAG ACCAACAACA ACAACTTGGA CAACAAATTT CAGCAACAAC AAGAAGAAAT TGAGCAATTG   
  
  
+ CAGAGTAGTG GTGGCACTTT TCTCTCTCCT GATAACACCA ATACTAATAT TAGTACTACT CCGAGAGCGA   
  
  
+ CAAGTTCTGT TGATAGCGAA CACACATCTA CTCTGGATAC TACGGGCCCT CCCACTTCAA CATCTTCCTC   
  
  
+ CTTCAAGAGC ACCTCCACCG CCAACACAGC CGGTGTGGCG GACCCCGCAT GTACTCCCAA AGAGGATTGG   
  
  
+ GATGTCTCCG GCGGCGCTGC TGGCGGTGAC GGCGGTGGTG GGGGGCTTGG ATTGGAGGAG TGGGACAGCA   
  
  
+ TGTTCCCAAA TGGGGAGGGG GCTTTGCTCC CTTGGATCAT GGGTGAAGCT GATGACATGG GTATGGGTTT   
  
  
+ GAAGCATCTT TTGCAATCGG GTAACCCGGT TGACTATGAG GGCAATGCTG GTCTAGGGGT TGTCGATCAG   
  
  
+ GGTTCTGGAT TTGAGACTCT CTCTCCTCCT CCACCGCAGC CGGCGGCATG TGAATCTAAT GGTGGTGGTG   
  
  
+ TTGCTAATTT GGGGTTTCCA GGGAATAATG GTAAGATTTC TTCAATTTCA CATAATTGTT CATCTGGGAT   
  
  
+ TTTGAATGGT AAGGTGAACA ATAATGGGTT GAATCCCAAT TGTAACCCTC AAGCTCAAGG CAGTCTACTT   
  
  
+ GGTTTAATCC AAGGAGCTAC AGGTGTACAC GCACATCCTG ACGTTGGAGA CGAGAAACCC CAGATTTTGA   
  
  
+ ATCCACATTT GGTGATGAAC CCTCAGCAAG CTCAGAGCAT TGCAAACCCT AGCTTTTTGA TGCACTCATT   
  
  
+ AGGTTACTAT CAGCTGGAGC AACATCTATT TCAACCTCAG GCAAAACGCC TGAACACGGG TGCTGTTCTG   
  
  
+ GATCCTAACC TTGTTCAGCT TGCAAAGAAC CCATTTGCTG ATCAGGGTCA TGAGTTATTG TTGAGGAAGC   
  
  
+ AGCACCAACA GCTTGGTTTG CAACCATTGC CATTGGGTTT GGGTCCTCAG TTGGTCCCTC CGCAGAAGCC   
  
  
+ TGTGATGGGT TCAAAGCAGG GGAACCCCCA GCATTACCCG TTGCATGTTC ATCAGCAGCT GCAATTGCAA   
  
  
+ GAGCAGGCTG TCAAAGATCA GCTCTTCAAG GCGGCAGACC TTATTCAAAC TGGAAGTTTC TCACTCGCGC   
  
  
+ AAGAGATATT GGCGCGGCTC AATCACCAGC TCTCCCTCCC TGCAAAGCCC CTCATTAGGG CGGCTTTGTA   
  
  
+ TGTGAAGGAG GCCCTTCAAA TGCTCCTCCT AATGAGCAAC CCAGTTGCGG CTCCACCGTC CAAGATCCTC   
  
  
+ ACCCCTTATG ATGTTGTTCA CAAGATGAGC GCGTATAAGG TCTTCTCTGA GGTCTGCCCA ATCACTCAAT   
  
  
+ TTGTGAATTT CACTTGTACA CAGGCCATTC TCGAGGCTCT TGATGATTCT GATGCTATTC ATGTTGTCGA   
  
  
+ CTTTGATATT GGTTGCGGTG CTCAATGGGC ATCATTGATT CAGGAGCTGC CATTGAGGAA AAGGGGAGCT   
  
  
+ CCCTCTCTGA AAATTACAGC CATAGCTCCC ATGTTGACTG GCAGTCACTT TGAAATTAGC CTAGTATGTG   
  
  
+ AAAACCTTGT GCAATTTGCC AACGATATTG GTGTTGCTTG TGAGCTCCAA GTTGTCAACT TTGATTTGTT   
  
  
+ TGATCCATCT TCGCCATCAA TGCCGAACAT TAGTACTGCT GAGGATGAGT CAATTGCTGT TAGTATCCCC   
  
  
+ ATCTGGGCAT CTTCAATTAG GCCATCTGTT CTTCCTTCCA TCCTCCGATT CATTAAGCAA AAATCCCCCA   
  
  
+ AAATTGTGGT CTCGTTTGAT AGAGGATTCG ATCGTTTTGA TGTCCCTTTC CCCCAACATC TGTTGCATAC   
  
  
+ CCTAGAATCC TGCAGTAATT TATTGGACTC GCTTGATGGT CTCAATGTTG CATCGGACAT TGTGAGCAAG   
  
  
+ GTCGAGAAGT TCTTTGTTCA ACCTAGGATC GAAAATGCTG TGTTGGGTCG GGTCCATGCC CCTGACAAGA   
  
  
+ TGCCCCATTG GAAAAATCTC TTTGCTTCAG CCGGCTTCTT GCCCTTGCAA TTCAGTAATT TCACAGAAAC   
  
  
+ CCAGGCCGAT TATGTGGTGA AGAGAACCCC AACAAGAGGA TTTCACGTGG AGAAGCGCCA GGCATCACTC   
  
  
+ ATTCTGAGTT GGCAGAGGCA GGAGCTTGTG GCAGCTTCGG CATGGAAGTG TTG  

- +Up\_Stream \_Len000AGAATT GTAATCAACG AACCAAGTTG GGTAGTCACT CTTATAGACT AAAGTTTTCA   
  
  
- TATCTAAATA TATTTGTATA CCTGATGTCC CTTCCCTCAG CAAAGGAGTT TCAGAATCTA AATGTGAGTT   
  
  
- TTCAAACTCG CTATGGTTAT TACACTAAGG TTTGTACTCG GTTTATAATT CATAGGATAT GATTCTAGTT   
  
  
- CTTCTCTTAC TTGGTTTTCT GACAATTAAA AAGTTAATTC GATTTGAATA ATTGGAAAAA AACCTTATCT   
  
  
- AAAAATTTAA AATAGAAGTT ATATTCAAAA AGGTTCCGCT CTCTGTATAT CGTTAAAATT AATTTCAATT   
  
  
- TCATCATATT GTAAAAAAAA TTACCAATAT CCGAATATCG TGGAAAAGAT CCACTTTTCT CTATAGAACC   
  
  
- GGACTAAAGG TCATTTCAAA AAAAAAATTT ATAACAGTTA ATAAAAACGT TTACTGTAAA CCATTAAACC   
  
  
- CCCTATTAGA CTACACAATT TTCTATGTTA CTTTTATAGT CTTTTATCCT ACGGAGTTTA AAGTGTTCTT   
  
  
- TCTTTTTTAG AAAAACAACA ATCATAAAGA TGTGTAGGTG TTAAAAATGA GTACCATTTT TTTTTTCCCG   
  
  
- GTTTATAGAG TGGTTATATT GGTTTGATAT ACAAGGTAAT AAATCATTGC CACTATTTAT ACATTACCCA   
  
  
- GTTCAATGAT TATTTTATCG GATGTTATGA ACCTATAGAT GTATTTTAAC TATATCTTAT ATATAACGTT   
  
  
- ATGAATTTAG TTTTAATTTA CTTTATAAAT GAAAAAATAA GATAATGTTT ATCGAAGAAT ATCTTATCGT   
  
  
- TGTTAAAAAT TACATTCAAA GTAAGAATGG TCTAAACTTC TCCAGGCCTA TATCTTTTAG AATAAGCATA   
  
  
- TTCATGAGCG TACTCCCGCC TATTTAAGAT TTTATAGTTG GAGTTGAGCG AAGATGATAA GGTAGATTCA   
  
  
- AGTTAAATAG TATATTTTTT TAAGTTAGGT GAAATTACAT GAGTTAAGTG AAGTTGGATA AAATTAGTTA   
  
  
- AATGGGTACT TTTCTTGTTC CAGGATTCAT CTACAAAAGA TAACTAACTA AATTTGTTAA AAGTGGATGG   
  
  
- GTTAATAGGG GTTTTGACTT TCTCCCCCGT TTCCCCCCAC CCATTACGAC ACATTTTATC CCCCTTTCCC   
  
  
- GTTCTTTTGA CACATGTTAC CACCTCGTCC CCATATGGTA AACCGGAACT CAACTCCCCC GTGTTTTCGG   
  
  
- GTCTCTCTCT CTCCCCATTA CGGGTGGTCT TTATTCGTCC TTCGCTCCCT TTTTGTGATC TCTCTTTGTC   
  
  
- TCTTTCTCTC TCTCTCTCTC TCTCTTCCCC TTTAAATGAT CTCTTACTCT CACTTCTTCC CTTGAAATAA   
  
  
- AATTGAAAAA ATACTACGTG CGTAGGTACC GTCTTGTCCC TTCCCTTTCT TCCCATGTCT CATGACGACA   
  
  
- GGGGTTTTTT AATCACATCT TTTGTCCTCT GTGAGTTGAT GTTGTTCCCA TTCCTTCTTC TTGTTCGTTC   
  
  
- TATAGGGTGG GTTTCTTTTT TTTTTTCTTC TTCTTCTTTT AAAAAGTCTC TCCTTTCTTT TCGTTAGTGT   
  
  
- ACTTAATCTT CGGTTGGGTT CGGAAGAAGT TTCTCTTCGT CGTATTGTAT CGTATCGACC CCTCTCCTAT   
  
  
- CCTCTCTCTT TGAGTTCTTT TCCCCATGGG CCGAATTTAC AACTACGTCT TTCCATATCT ATACCGGTGT   
  
  
- GATGATGATG GGTCCTCAAA TAGTAACCTC TACCTAAAGA GAAAGACGGA GTAGAAGAGG AGGTAGGTGG   
  
  
- TCGTTTTGAC CCAAAGGAAA GTCGACCCAG AAAAGATCTA GATCTTTGAT ACCATGGGTT TAGAGGGTAA   
  
  
- GGAGGTAAAA ATAAGAGTTT TAGATAGAAG AATCTGAACA CCCAAACTAA GTAAAGAAAA CCCAAAAACT   
  
  
- TTTCTTAAAT TAAACTAACA TAACACAACT AACCCCTTAA ACACCACCAC AATCTACGCT CGATACGGGA   
  
  
- TGTTAAACGT TCCGTTCCCA CACCATCTCC AAAGTTCGTA AACATAAGGT GTTTAGAGAA GGGGACGAAG   
  
  
- TTTCACCTTC TGGTTGTTGT TGTTGAACCT GTTGTTTAAA GTCGTTGTTG TTCTTCTTTA ACTCGTTAAC   
  
  
- GTCTCATCAC CACCGTGAAA AGAGAGAGGA CTATTGTGGT TATGATTATA ATCATGATGA GGCTCTCGCT   
  
  
- GTTCAAGACA ACTATCGCTT GTGTGTAGAT GAGACCTATG ATGCCCGGGA GGGTGAAGTT GTAGAAGGAG   
  
  
- GAAGTTCTCG TGGAGGTGGC GGTTGTGTCG GCCACACCGC CTGGGGCGTA CATGAGGGTT TCTCCTAACC   
  
  
- CTACAGAGGC CGCCGCGACG ACCGCCACTG CCGCCACCAC CCCCCGAACC TAACCTCCTC ACCCTGTCGT   
  
  
- ACAAGGGTTT ACCCCTCCCC CGAAACGAGG GAACCTAGTA CCCACTTCGA CTACTGTACC CATACCCAAA   
  
  
- CTTCGTAGAA AACGTTAGCC CATTGGGCCA ACTGATACTC CCGTTACGAC CAGATCCCCA ACAGCTAGTC   
  
  
- CCAAGACCTA AACTCTGAGA GAGAGGAGGA GGTGGCGTCG GCCGCCGTAC ACTTAGATTA CCACCACCAC   
  
  
- AACGATTAAA CCCCAAAGGT CCCTTATTAC CATTCTAAAG AAGTTAAAGT GTATTAACAA GTAGACCCTA   
  
  
- AAACTTACCA TTCCACTTGT TATTACCCAA CTTAGGGTTA ACATTGGGAG TTCGAGTTCC GTCAGATGAA   
  
  
- CCAAATTAGG TTCCTCGATG TCCACATGTG CGTGTAGGAC TGCAACCTCT GCTCTTTGGG GTCTAAAACT   
  
  
- TAGGTGTAAA CCACTACTTG GGAGTCGTTC GAGTCTCGTA ACGTTTGGGA TCGAAAAACT ACGTGAGTAA   
  
  
- TCCAATGATA GTCGACCTCG TTGTAGATAA AGTTGGAGTC CGTTTTGCGG ACTTGTGCCC ACGACAAGAC   
  
  
- CTAGGATTGG AACAAGTCGA ACGTTTCTTG GGTAAACGAC TAGTCCCAGT ACTCAATAAC AACTCCTTCG   
  
  
- TCGTGGTTGT CGAACCAAAC GTTGGTAACG GTAACCCAAA CCCAGGAGTC AACCAGGGAG GCGTCTTCGG   
  
  
- ACACTACCCA AGTTTCGTCC CCTTGGGGGT CGTAATGGGC AACGTACAAG TAGTCGTCGA CGTTAACGTT   
  
  
- CTCGTCCGAC AGTTTCTAGT CGAGAAGTTC CGCCGTCTGG AATAAGTTTG ACCTTCAAAG AGTGAGCGCG   
  
  
- TTCTCTATAA CCGCGCCGAG TTAGTGGTCG AGAGGGAGGG ACGTTTCGGG GAGTAATCCC GCCGAAACAT   
  
  
- ACACTTCCTC CGGGAAGTTT ACGAGGAGGA TTACTCGTTG GGTCAACGCC GAGGTGGCAG GTTCTAGGAG   
  
  
- TGGGGAATAC TACAACAAGT GTTCTACTCG CGCATATTCC AGAAGAGACT CCAGACGGGT TAGTGAGTTA   
  
  
- AACACTTAAA GTGAACATGT GTCCGGTAAG AGCTCCGAGA ACTACTAAGA CTACGATAAG TACAACAGCT   
  
  
- GAAACTATAA CCAACGCCAC GAGTTACCCG TAGTAACTAA GTCCTCGACG GTAACTCCTT TTCCCCTCGA   
  
  
- GGGAGAGACT TTTAATGTCG GTATCGAGGG TACAACTGAC CGTCAGTGAA ACTTTAATCG GATCATACAC   
  
  
- TTTTGGAACA CGTTAAACGG TTGCTATAAC CACAACGAAC ACTCGAGGTT CAACAGTTGA AACTAAACAA   
  
  
- ACTAGGTAGA AGCGGTAGTT ACGGCTTGTA ATCATGACGA CTCCTACTCA GTTAACGACA ATCATAGGGG   
  
  
- TAGACCCGTA GAAGTTAATC CGGTAGACAA GAAGGAAGGT AGGAGGCTAA GTAATTCGTT TTTAGGGGGT   
  
  
- TTTAACACCA GAGCAAACTA TCTCCTAAGC TAGCAAAACT ACAGGGAAAG GGGGTTGTAG ACAACGTATG   
  
  
- GGATCTTAGG ACGTCATTAA ATAACCTGAG CGAACTACCA GAGTTACAAC GTAGCCTGTA ACACTCGTTC   
  
  
- CAGCTCTTCA AGAAACAAGT TGGATCCTAG CTTTTACGAC ACAACCCAGC CCAGGTACGG GGACTGTTCT   
  
  
- ACGGGGTAAC CTTTTTAGAG AAACGAAGTC GGCCGAAGAA CGGGAACGTT AAGTCATTAA AGTGTCTTTG   
  
  
- GGTCCGGCTA ATACACCACT TCTCTTGGGG TTGTTCTCCT AAAGTGCACC TCTTCGCGGT CCGTAGTGAG   
  
  
- TAAGACTCAA CCGTCTCCGT CCTCGAACAC CGTCGAAGCC GTACCTTCAC AAC

+     AuxRR-core

| Site Name | Organism | Position | Strand | Matrix score. | sequence | function |
| --- | --- | --- | --- | --- | --- | --- |
| AuxRR-core | Nicotiana tabacum | 4115 | + | 7 | GGTCCAT | cis-acting regulatory element involved in auxin responsiveness |

>HU04G00047.1   
+ +Up\_Stream \_Len000TCTTAA CATTAGTTGC TTGGTTCAAC CCATCAGTGA GAATATCTGA TTTCAAAAGT   
  
  
+ ATAGATTTAT ATAAACATAT GGACTACAGG GAAGGGAGTC GTTTCCTCAA AGTCTTAGAT TTACACTCAA   
  
  
+ AAGTTTGAGC GATACCAATA ATGTGATTCC AAACATGAGC CAAATATTAA GTATCCTATA CTAAGATCAA   
  
  
+ GAAGAGAATG AACCAAAAGA CTGTTAATTT TTCAATTAAG CTAAACTTAT TAACCTTTTT TTGGAATAGA   
  
  
+ TTTTTAAATT TTATCTTCAA TATAAGTTTT TCCAAGGCGA GAGACATATA GCAATTTTAA TTAAAGTTAA   
  
  
+ AGTAGTATAA CATTTTTTTT AATGGTTATA GGCTTATAGC ACCTTTTCTA GGTGAAAAGA GATATCTTGG   
  
  
+ CCTGATTTCC AGTAAAGTTT TTTTTTTAAA TATTGTCAAT TATTTTTGCA AATGACATTT GGTAATTTGG   
  
  
+ GGGATAATCT GATGTGTTAA AAGATACAAT GAAAATATCA GAAAATAGGA TGCCTCAAAT TTCACAAGAA   
  
  
+ AGAAAAAATC TTTTTGTTGT TAGTATTTCT ACACATCCAC AATTTTTACT CATGGTAAAA AAAAAAGGGC   
  
  
+ CAAATATCTC ACCAATATAA CCAAACTATA TGTTCCATTA TTTAGTAACG GTGATAAATA TGTAATGGGT   
  
  
+ CAAGTTACTA ATAAAATAGC CTACAATACT TGGATATCTA CATAAAATTG ATATAGAATA TATATTGCAA   
  
  
+ TACTTAAATC AAAATTAAAT GAAATATTTA CTTTTTTATT CTATTACAAA TAGCTTCTTA TAGAATAGCA   
  
  
+ ACAATTTTTA ATGTAAGTTT CATTCTTACC AGATTTGAAG AGGTCCGGAT ATAGAAAATC TTATTCGTAT   
  
  
+ AAGTACTCGC ATGAGGGCGG ATAAATTCTA AAATATCAAC CTCAACTCGC TTCTACTATT CCATCTAAGT   
  
  
+ TCAATTTATC ATATAAAAAA ATTCAATCCA CTTTAATGTA CTCAATTCAC TTCAACCTAT TTTAATCAAT   
  
  
+ TTACCCATGA AAAGAACAAG GTCCTAAGTA GATGTTTTCT ATTGATTGAT TTAAACAATT TTCACCTACC   
  
  
+ CAATTATCCC CAAAACTGAA AGAGGGGGCA AAGGGGGGTG GGTAATGCTG TGTAAAATAG GGGGAAAGGG   
  
  
+ CAAGAAAACT GTGTACAATG GTGGAGCAGG GGTATACCAT TTGGCCTTGA GTTGAGGGGG CACAAAAGCC   
  
  
+ CAGAGAGAGA GAGGGGTAAT GCCCACCAGA AATAAGCAGG AAGCGAGGGA AAAACACTAG AGAGAAACAG   
  
  
+ AGAAAGAGAG AGAGAGAGAG AGAGAAGGGG AAATTTACTA GAGAATGAGA GTGAAGAAGG GAACTTTATT   
  
  
+ TTAACTTTTT TATGATGCAC GCATCCATGG CAGAACAGGG AAGGGAAAGA AGGGTACAGA GTACTGCTGT   
  
  
+ CCCCAAAAAA TTAGTGTAGA AAACAGGAGA CACTCAACTA CAACAAGGGT AAGGAAGAAG AACAAGCAAG   
  
  
+ ATATCCCACC CAAAGAAAAA AAAAAAGAAG AAGAAGAAAA TTTTTCAGAG AGGAAAGAAA AGCAATCACA   
  
  
+ TGAATTAGAA GCCAACCCAA GCCTTCTTCA AAGAGAAGCA GCATAACATA GCATAGCTGG GGAGAGGATA   
  
  
+ GGAGAGAGAA ACTCAAGAAA AGGGGTACCC GGCTTAAATG TTGATGCAGA AAGGTATAGA TATGGCCACA   
  
  
+ CTACTACTAC CCAGGAGTTT ATCATTGGAG ATGGATTTCT CTTTCTGCCT CATCTTCTCC TCCATCCACC   
  
  
+ AGCAAAACTG GGTTTCCTTT CAGCTGGGTC TTTTCTAGAT CTAGAAACTA TGGTACCCAA ATCTCCCATT   
  
  
+ CCTCCATTTT TATTCTCAAA ATCTATCTTC TTAGACTTGT GGGTTTGATT CATTTCTTTT GGGTTTTTGA   
  
  
+ AAAGAATTTA ATTTGATTGT ATTGTGTTGA TTGGGGAATT TGTGGTGGTG TTAGATGCGA GCTATGCCCT   
  
  
+ ACAATTTGCA AGGCAAGGGT GTGGTAGAGG TTTCAAGCAT TTGTATTCCA CAAATCTCTT CCCCTGCTTC   
  
  
+ AAAGTGGAAG ACCAACAACA ACAACTTGGA CAACAAATTT CAGCAACAAC AAGAAGAAAT TGAGCAATTG   
  
  
+ CAGAGTAGTG GTGGCACTTT TCTCTCTCCT GATAACACCA ATACTAATAT TAGTACTACT CCGAGAGCGA   
  
  
+ CAAGTTCTGT TGATAGCGAA CACACATCTA CTCTGGATAC TACGGGCCCT CCCACTTCAA CATCTTCCTC   
  
  
+ CTTCAAGAGC ACCTCCACCG CCAACACAGC CGGTGTGGCG GACCCCGCAT GTACTCCCAA AGAGGATTGG   
  
  
+ GATGTCTCCG GCGGCGCTGC TGGCGGTGAC GGCGGTGGTG GGGGGCTTGG ATTGGAGGAG TGGGACAGCA   
  
  
+ TGTTCCCAAA TGGGGAGGGG GCTTTGCTCC CTTGGATCAT GGGTGAAGCT GATGACATGG GTATGGGTTT   
  
  
+ GAAGCATCTT TTGCAATCGG GTAACCCGGT TGACTATGAG GGCAATGCTG GTCTAGGGGT TGTCGATCAG   
  
  
+ GGTTCTGGAT TTGAGACTCT CTCTCCTCCT CCACCGCAGC CGGCGGCATG TGAATCTAAT GGTGGTGGTG   
  
  
+ TTGCTAATTT GGGGTTTCCA GGGAATAATG GTAAGATTTC TTCAATTTCA CATAATTGTT CATCTGGGAT   
  
  
+ TTTGAATGGT AAGGTGAACA ATAATGGGTT GAATCCCAAT TGTAACCCTC AAGCTCAAGG CAGTCTACTT   
  
  
+ GGTTTAATCC AAGGAGCTAC AGGTGTACAC GCACATCCTG ACGTTGGAGA CGAGAAACCC CAGATTTTGA   
  
  
+ ATCCACATTT GGTGATGAAC CCTCAGCAAG CTCAGAGCAT TGCAAACCCT AGCTTTTTGA TGCACTCATT   
  
  
+ AGGTTACTAT CAGCTGGAGC AACATCTATT TCAACCTCAG GCAAAACGCC TGAACACGGG TGCTGTTCTG   
  
  
+ GATCCTAACC TTGTTCAGCT TGCAAAGAAC CCATTTGCTG ATCAGGGTCA TGAGTTATTG TTGAGGAAGC   
  
  
+ AGCACCAACA GCTTGGTTTG CAACCATTGC CATTGGGTTT GGGTCCTCAG TTGGTCCCTC CGCAGAAGCC   
  
  
+ TGTGATGGGT TCAAAGCAGG GGAACCCCCA GCATTACCCG TTGCATGTTC ATCAGCAGCT GCAATTGCAA   
  
  
+ GAGCAGGCTG TCAAAGATCA GCTCTTCAAG GCGGCAGACC TTATTCAAAC TGGAAGTTTC TCACTCGCGC   
  
  
+ AAGAGATATT GGCGCGGCTC AATCACCAGC TCTCCCTCCC TGCAAAGCCC CTCATTAGGG CGGCTTTGTA   
  
  
+ TGTGAAGGAG GCCCTTCAAA TGCTCCTCCT AATGAGCAAC CCAGTTGCGG CTCCACCGTC CAAGATCCTC   
  
  
+ ACCCCTTATG ATGTTGTTCA CAAGATGAGC GCGTATAAGG TCTTCTCTGA GGTCTGCCCA ATCACTCAAT   
  
  
+ TTGTGAATTT CACTTGTACA CAGGCCATTC TCGAGGCTCT TGATGATTCT GATGCTATTC ATGTTGTCGA   
  
  
+ CTTTGATATT GGTTGCGGTG CTCAATGGGC ATCATTGATT CAGGAGCTGC CATTGAGGAA AAGGGGAGCT   
  
  
+ CCCTCTCTGA AAATTACAGC CATAGCTCCC ATGTTGACTG GCAGTCACTT TGAAATTAGC CTAGTATGTG   
  
  
+ AAAACCTTGT GCAATTTGCC AACGATATTG GTGTTGCTTG TGAGCTCCAA GTTGTCAACT TTGATTTGTT   
  
  
+ TGATCCATCT TCGCCATCAA TGCCGAACAT TAGTACTGCT GAGGATGAGT CAATTGCTGT TAGTATCCCC   
  
  
+ ATCTGGGCAT CTTCAATTAG GCCATCTGTT CTTCCTTCCA TCCTCCGATT CATTAAGCAA AAATCCCCCA   
  
  
+ AAATTGTGGT CTCGTTTGAT AGAGGATTCG ATCGTTTTGA TGTCCCTTTC CCCCAACATC TGTTGCATAC   
  
  
+ CCTAGAATCC TGCAGTAATT TATTGGACTC GCTTGATGGT CTCAATGTTG CATCGGACAT TGTGAGCAAG   
  
  
+ GTCGAGAAGT TCTTTGTTCA ACCTAGGATC GAAAATGCTG TGTTGGGTCG GGTCCATGCC CCTGACAAGA   
  
  
+ TGCCCCATTG GAAAAATCTC TTTGCTTCAG CCGGCTTCTT GCCCTTGCAA TTCAGTAATT TCACAGAAAC   
  
  
+ CCAGGCCGAT TATGTGGTGA AGAGAACCCC AACAAGAGGA TTTCACGTGG AGAAGCGCCA GGCATCACTC   
  
  
+ ATTCTGAGTT GGCAGAGGCA GGAGCTTGTG GCAGCTTCGG CATGGAAGTG TTG  

- +Up\_Stream \_Len000AGAATT GTAATCAACG AACCAAGTTG GGTAGTCACT CTTATAGACT AAAGTTTTCA   
  
  
- TATCTAAATA TATTTGTATA CCTGATGTCC CTTCCCTCAG CAAAGGAGTT TCAGAATCTA AATGTGAGTT   
  
  
- TTCAAACTCG CTATGGTTAT TACACTAAGG TTTGTACTCG GTTTATAATT CATAGGATAT GATTCTAGTT   
  
  
- CTTCTCTTAC TTGGTTTTCT GACAATTAAA AAGTTAATTC GATTTGAATA ATTGGAAAAA AACCTTATCT   
  
  
- AAAAATTTAA AATAGAAGTT ATATTCAAAA AGGTTCCGCT CTCTGTATAT CGTTAAAATT AATTTCAATT   
  
  
- TCATCATATT GTAAAAAAAA TTACCAATAT CCGAATATCG TGGAAAAGAT CCACTTTTCT CTATAGAACC   
  
  
- GGACTAAAGG TCATTTCAAA AAAAAAATTT ATAACAGTTA ATAAAAACGT TTACTGTAAA CCATTAAACC   
  
  
- CCCTATTAGA CTACACAATT TTCTATGTTA CTTTTATAGT CTTTTATCCT ACGGAGTTTA AAGTGTTCTT   
  
  
- TCTTTTTTAG AAAAACAACA ATCATAAAGA TGTGTAGGTG TTAAAAATGA GTACCATTTT TTTTTTCCCG   
  
  
- GTTTATAGAG TGGTTATATT GGTTTGATAT ACAAGGTAAT AAATCATTGC CACTATTTAT ACATTACCCA   
  
  
- GTTCAATGAT TATTTTATCG GATGTTATGA ACCTATAGAT GTATTTTAAC TATATCTTAT ATATAACGTT   
  
  
- ATGAATTTAG TTTTAATTTA CTTTATAAAT GAAAAAATAA GATAATGTTT ATCGAAGAAT ATCTTATCGT   
  
  
- TGTTAAAAAT TACATTCAAA GTAAGAATGG TCTAAACTTC TCCAGGCCTA TATCTTTTAG AATAAGCATA   
  
  
- TTCATGAGCG TACTCCCGCC TATTTAAGAT TTTATAGTTG GAGTTGAGCG AAGATGATAA GGTAGATTCA   
  
  
- AGTTAAATAG TATATTTTTT TAAGTTAGGT GAAATTACAT GAGTTAAGTG AAGTTGGATA AAATTAGTTA   
  
  
- AATGGGTACT TTTCTTGTTC CAGGATTCAT CTACAAAAGA TAACTAACTA AATTTGTTAA AAGTGGATGG   
  
  
- GTTAATAGGG GTTTTGACTT TCTCCCCCGT TTCCCCCCAC CCATTACGAC ACATTTTATC CCCCTTTCCC   
  
  
- GTTCTTTTGA CACATGTTAC CACCTCGTCC CCATATGGTA AACCGGAACT CAACTCCCCC GTGTTTTCGG   
  
  
- GTCTCTCTCT CTCCCCATTA CGGGTGGTCT TTATTCGTCC TTCGCTCCCT TTTTGTGATC TCTCTTTGTC   
  
  
- TCTTTCTCTC TCTCTCTCTC TCTCTTCCCC TTTAAATGAT CTCTTACTCT CACTTCTTCC CTTGAAATAA   
  
  
- AATTGAAAAA ATACTACGTG CGTAGGTACC GTCTTGTCCC TTCCCTTTCT TCCCATGTCT CATGACGACA   
  
  
- GGGGTTTTTT AATCACATCT TTTGTCCTCT GTGAGTTGAT GTTGTTCCCA TTCCTTCTTC TTGTTCGTTC   
  
  
- TATAGGGTGG GTTTCTTTTT TTTTTTCTTC TTCTTCTTTT AAAAAGTCTC TCCTTTCTTT TCGTTAGTGT   
  
  
- ACTTAATCTT CGGTTGGGTT CGGAAGAAGT TTCTCTTCGT CGTATTGTAT CGTATCGACC CCTCTCCTAT   
  
  
- CCTCTCTCTT TGAGTTCTTT TCCCCATGGG CCGAATTTAC AACTACGTCT TTCCATATCT ATACCGGTGT   
  
  
- GATGATGATG GGTCCTCAAA TAGTAACCTC TACCTAAAGA GAAAGACGGA GTAGAAGAGG AGGTAGGTGG   
  
  
- TCGTTTTGAC CCAAAGGAAA GTCGACCCAG AAAAGATCTA GATCTTTGAT ACCATGGGTT TAGAGGGTAA   
  
  
- GGAGGTAAAA ATAAGAGTTT TAGATAGAAG AATCTGAACA CCCAAACTAA GTAAAGAAAA CCCAAAAACT   
  
  
- TTTCTTAAAT TAAACTAACA TAACACAACT AACCCCTTAA ACACCACCAC AATCTACGCT CGATACGGGA   
  
  
- TGTTAAACGT TCCGTTCCCA CACCATCTCC AAAGTTCGTA AACATAAGGT GTTTAGAGAA GGGGACGAAG   
  
  
- TTTCACCTTC TGGTTGTTGT TGTTGAACCT GTTGTTTAAA GTCGTTGTTG TTCTTCTTTA ACTCGTTAAC   
  
  
- GTCTCATCAC CACCGTGAAA AGAGAGAGGA CTATTGTGGT TATGATTATA ATCATGATGA GGCTCTCGCT   
  
  
- GTTCAAGACA ACTATCGCTT GTGTGTAGAT GAGACCTATG ATGCCCGGGA GGGTGAAGTT GTAGAAGGAG   
  
  
- GAAGTTCTCG TGGAGGTGGC GGTTGTGTCG GCCACACCGC CTGGGGCGTA CATGAGGGTT TCTCCTAACC   
  
  
- CTACAGAGGC CGCCGCGACG ACCGCCACTG CCGCCACCAC CCCCCGAACC TAACCTCCTC ACCCTGTCGT   
  
  
- ACAAGGGTTT ACCCCTCCCC CGAAACGAGG GAACCTAGTA CCCACTTCGA CTACTGTACC CATACCCAAA   
  
  
- CTTCGTAGAA AACGTTAGCC CATTGGGCCA ACTGATACTC CCGTTACGAC CAGATCCCCA ACAGCTAGTC   
  
  
- CCAAGACCTA AACTCTGAGA GAGAGGAGGA GGTGGCGTCG GCCGCCGTAC ACTTAGATTA CCACCACCAC   
  
  
- AACGATTAAA CCCCAAAGGT CCCTTATTAC CATTCTAAAG AAGTTAAAGT GTATTAACAA GTAGACCCTA   
  
  
- AAACTTACCA TTCCACTTGT TATTACCCAA CTTAGGGTTA ACATTGGGAG TTCGAGTTCC GTCAGATGAA   
  
  
- CCAAATTAGG TTCCTCGATG TCCACATGTG CGTGTAGGAC TGCAACCTCT GCTCTTTGGG GTCTAAAACT   
  
  
- TAGGTGTAAA CCACTACTTG GGAGTCGTTC GAGTCTCGTA ACGTTTGGGA TCGAAAAACT ACGTGAGTAA   
  
  
- TCCAATGATA GTCGACCTCG TTGTAGATAA AGTTGGAGTC CGTTTTGCGG ACTTGTGCCC ACGACAAGAC   
  
  
- CTAGGATTGG AACAAGTCGA ACGTTTCTTG GGTAAACGAC TAGTCCCAGT ACTCAATAAC AACTCCTTCG   
  
  
- TCGTGGTTGT CGAACCAAAC GTTGGTAACG GTAACCCAAA CCCAGGAGTC AACCAGGGAG GCGTCTTCGG   
  
  
- ACACTACCCA AGTTTCGTCC CCTTGGGGGT CGTAATGGGC AACGTACAAG TAGTCGTCGA CGTTAACGTT   
  
  
- CTCGTCCGAC AGTTTCTAGT CGAGAAGTTC CGCCGTCTGG AATAAGTTTG ACCTTCAAAG AGTGAGCGCG   
  
  
- TTCTCTATAA CCGCGCCGAG TTAGTGGTCG AGAGGGAGGG ACGTTTCGGG GAGTAATCCC GCCGAAACAT   
  
  
- ACACTTCCTC CGGGAAGTTT ACGAGGAGGA TTACTCGTTG GGTCAACGCC GAGGTGGCAG GTTCTAGGAG   
  
  
- TGGGGAATAC TACAACAAGT GTTCTACTCG CGCATATTCC AGAAGAGACT CCAGACGGGT TAGTGAGTTA   
  
  
- AACACTTAAA GTGAACATGT GTCCGGTAAG AGCTCCGAGA ACTACTAAGA CTACGATAAG TACAACAGCT   
  
  
- GAAACTATAA CCAACGCCAC GAGTTACCCG TAGTAACTAA GTCCTCGACG GTAACTCCTT TTCCCCTCGA   
  
  
- GGGAGAGACT TTTAATGTCG GTATCGAGGG TACAACTGAC CGTCAGTGAA ACTTTAATCG GATCATACAC   
  
  
- TTTTGGAACA CGTTAAACGG TTGCTATAAC CACAACGAAC ACTCGAGGTT CAACAGTTGA AACTAAACAA   
  
  
- ACTAGGTAGA AGCGGTAGTT ACGGCTTGTA ATCATGACGA CTCCTACTCA GTTAACGACA ATCATAGGGG   
  
  
- TAGACCCGTA GAAGTTAATC CGGTAGACAA GAAGGAAGGT AGGAGGCTAA GTAATTCGTT TTTAGGGGGT   
  
  
- TTTAACACCA GAGCAAACTA TCTCCTAAGC TAGCAAAACT ACAGGGAAAG GGGGTTGTAG ACAACGTATG   
  
  
- GGATCTTAGG ACGTCATTAA ATAACCTGAG CGAACTACCA GAGTTACAAC GTAGCCTGTA ACACTCGTTC   
  
  
- CAGCTCTTCA AGAAACAAGT TGGATCCTAG CTTTTACGAC ACAACCCAGC CCAGGTACGG GGACTGTTCT   
  
  
- ACGGGGTAAC CTTTTTAGAG AAACGAAGTC GGCCGAAGAA CGGGAACGTT AAGTCATTAA AGTGTCTTTG   
  
  
- GGTCCGGCTA ATACACCACT TCTCTTGGGG TTGTTCTCCT AAAGTGCACC TCTTCGCGGT CCGTAGTGAG   
  
  
- TAAGACTCAA CCGTCTCCGT CCTCGAACAC CGTCGAAGCC GTACCTTCAC AAC

+     CAAT-box

| Site Name | Organism | Position | Strand | Matrix score. | sequence | function |
| --- | --- | --- | --- | --- | --- | --- |
| CAAT-box | Nicotiana glutinosa | 3868 | + | 4 | CAAT |  |
| CAAT-box | Nicotiana glutinosa | 4037 | + | 4 | CAAT |  |
| CAAT-box | Nicotiana glutinosa | 3597 | + | 4 | CAAT |  |
| CAAT-box | Arabidopsis thaliana | 3582 | - | 5 | CCAAT | common cis-acting element in promoter and enhancer regions |
| CAAT-box | Arabidopsis thaliana | 4016 | - | 5 | CCAAT | common cis-acting element in promoter and enhancer regions |
| CAAT-box | Nicotiana glutinosa | 3501 | + | 4 | CAAT |  |
| CAAT-box | Arabidopsis thaliana | 3492 | + | 5 | CCAAT | common cis-acting element in promoter and enhancer regions |
| CAAT-box | Pisum sativum | 3381 | + | 5 | CAAAT | common cis-acting element in promoter and enhancer regions |
| CAAT-box | Nicotiana glutinosa | 3314 | + | 4 | CAAT |  |
| CAAT-box | Pisum sativum | 3778 | - | 5 | CAAAT | common cis-acting element in promoter and enhancer regions |
| CAAT-box | Arabidopsis thaliana | 3116 | - | 5 | CCAAT | common cis-acting element in promoter and enhancer regions |
| CAAT-box | Petunia hybrida | 4282 | - | 7 | TGCCAAC | common cis-acting element in promoter and enhancer regions |
| CAAT-box | Nicotiana glutinosa | 3835 | + | 4 | CAAT |  |
| CAAT-box | Nicotiana glutinosa | 3802 | + | 4 | CAAT |  |
| CAAT-box | Nicotiana glutinosa | 4182 | + | 4 | CAAT |  |
| CAAT-box | Arabidopsis thaliana | 3302 | - | 5 | CCAAT | common cis-acting element in promoter and enhancer regions |
| CAAT-box | Nicotiana glutinosa | 3216 | + | 4 | CAAT |  |
| CAAT-box | Nicotiana glutinosa | 2169 | + | 4 | CAAT |  |
| CAAT-box | Pisum sativum | 1975 | - | 5 | CAAAT | common cis-acting element in promoter and enhancer regions |
| CAAT-box | Pisum sativum | 1882 | + | 5 | CAAAT | common cis-acting element in promoter and enhancer regions |
| CAAT-box | Nicotiana glutinosa | 1125 | + | 4 | CAAT |  |
| CAAT-box | Nicotiana glutinosa | 3608 | - | 4 | CAAT |  |
| CAAT-box | Nicotiana glutinosa | 3218 | - | 4 | CAAT |  |
| CAAT-box | Nicotiana glutinosa | 3110 | - | 4 | CAAT |  |
| CAAT-box | Pisum sativum | 2038 | - | 5 | CAAAT | common cis-acting element in promoter and enhancer regions |
| CAAT-box | Nicotiana glutinosa | 2163 | - | 4 | CAAT |  |
| CAAT-box | Pisum sativum | 821 | + | 5 | CAAAT | common cis-acting element in promoter and enhancer regions |
| CAAT-box | Pisum sativum | 2085 | + | 5 | CAAAT | common cis-acting element in promoter and enhancer regions |
| CAAT-box | Arabidopsis thaliana | 2212 | + | 5 | CCAAT | common cis-acting element in promoter and enhancer regions |
| CAAT-box | Nicotiana glutinosa | 1980 | - | 4 | CAAT |  |
| CAAT-box | Nicotiana glutinosa | 3626 | - | 4 | CAAT |  |
| CAAT-box | Nicotiana glutinosa | 3493 | + | 4 | CAAT |  |
| CAAT-box | Pisum sativum | 3728 | - | 5 | CAAAT | common cis-acting element in promoter and enhancer regions |
| CAAT-box | Pisum sativum | 2002 | - | 5 | CAAAT | common cis-acting element in promoter and enhancer regions |
| CAAT-box | Pisum sativum | 3503 | - | 5 | CAAAT | common cis-acting element in promoter and enhancer regions |
| CAAT-box | Nicotiana glutinosa | 2913 | - | 4 | CAAT |  |
| CAAT-box | Pisum sativum | 2881 | - | 5 | CAAAT | common cis-acting element in promoter and enhancer regions |
| CAAT-box | Nicotiana glutinosa | 2773 | - | 4 | CAAT |  |
| CAAT-box | Pisum sativum | 2138 | + | 5 | CAAAT | common cis-acting element in promoter and enhancer regions |
| CAAT-box | Nicotiana glutinosa | 1027 | + | 4 | CAAT |  |
| CAAT-box | Pisum sativum | 2073 | - | 5 | CAAAT | common cis-acting element in promoter and enhancer regions |
| CAAT-box | Nicotiana glutinosa | 2036 | + | 4 | CAAT |  |
| CAAT-box | Nicotiana glutinosa | 1985 | - | 4 | CAAT |  |
| CAAT-box | Arabidopsis thaliana | 1994 | - | 5 | CCAAT | common cis-acting element in promoter and enhancer regions |
| CAAT-box | Nicotiana glutinosa | 3927 | - | 4 | CAAT |  |
| CAAT-box | Nicotiana glutinosa | 2753 | + | 4 | CAAT |  |
| CAAT-box | Nicotiana glutinosa | 1095 | - | 4 | CAAT |  |
| CAAT-box | Nicotiana glutinosa | 1607 | + | 4 | CAAT |  |
| CAAT-box | Pisum sativum | 1233 | - | 5 | CAAAT | common cis-acting element in promoter and enhancer regions |
| CAAT-box | Nicotiana glutinosa | 1210 | + | 4 | CAAT |  |
| CAAT-box | Nicotiana glutinosa | 1051 | + | 4 | CAAT |  |
| CAAT-box | Nicotiana glutinosa | 2171 | - | 4 | CAAT |  |
| CAAT-box | Nicotiana glutinosa | 2771 | + | 4 | CAAT |  |
| CAAT-box | Nicotiana glutinosa | 2707 | + | 4 | CAAT |  |
| CAAT-box | Arabidopsis thaliana | 1778 | - | 5 | CCAAT | common cis-acting element in promoter and enhancer regions |
| CAAT-box | Nicotiana glutinosa | 1110 | + | 4 | CAAT |  |
| CAAT-box | Arabidopsis thaliana | 3741 | - | 5 | CCAAT | common cis-acting element in promoter and enhancer regions |
| CAAT-box | Nicotiana glutinosa | 3726 | + | 4 | CAAT |  |
| CAAT-box | Nicotiana glutinosa | 1099 | - | 4 | CAAT |  |
| CAAT-box | Arabidopsis thaliana | 2770 | + | 5 | CCAAT | common cis-acting element in promoter and enhancer regions |
| CAAT-box | Arabidopsis thaliana | 1124 | + | 5 | CCAAT | common cis-acting element in promoter and enhancer regions |
| CAAT-box | Nicotiana glutinosa | 2719 | - | 4 | CAAT |  |
| CAAT-box | Nicotiana glutinosa | 1008 | + | 4 | CAAT |  |
| CAAT-box | Pisum sativum | 2671 | - | 5 | CAAAT | common cis-acting element in promoter and enhancer regions |
| CAAT-box | Nicotiana glutinosa | 768 | - | 4 | CAAT |  |
| CAAT-box | Nicotiana glutinosa | 4053 | - | 4 | CAAT |  |
| CAAT-box | Arabidopsis thaliana | 4141 | - | 5 | CCAAT | common cis-acting element in promoter and enhancer regions |
| CAAT-box | Nicotiana glutinosa | 2567 | + | 4 | CAAT |  |
| CAAT-box | Arabidopsis thaliana | 2435 | - | 5 | CCAAT | common cis-acting element in promoter and enhancer regions |
| CAAT-box | Pisum sativum | 635 | + | 5 | CAAAT | common cis-acting element in promoter and enhancer regions |
| CAAT-box | Petunia hybrida | 3731 | + | 7 | TGCCAAC | common cis-acting element in promoter and enhancer regions |
| CAAT-box | Pisum sativum | 877 | - | 5 | CAAAT | common cis-acting element in promoter and enhancer regions |
| CAAT-box | Pisum sativum | 3047 | - | 5 | CAAAT | common cis-acting element in promoter and enhancer regions |
| CAAT-box | Pisum sativum | 2603 | - | 5 | CAAAT | common cis-acting element in promoter and enhancer regions |
| CAAT-box | Nicotiana glutinosa | 2538 | + | 4 | CAAT |  |
| CAAT-box | Pisum sativum | 550 | + | 5 | CAAAT | common cis-acting element in promoter and enhancer regions |
| CAAT-box | Nicotiana glutinosa | 456 | - | 4 | CAAT |  |
| CAAT-box | Nicotiana glutinosa | 3837 | - | 4 | CAAT |  |
| CAAT-box | Nicotiana glutinosa | 604 | + | 4 | CAAT |  |
| CAAT-box | Nicotiana glutinosa | 247 | + | 4 | CAAT |  |
| CAAT-box | Pisum sativum | 481 | - | 5 | CAAAT | common cis-acting element in promoter and enhancer regions |
| CAAT-box | Pisum sativum | 185 | + | 5 | CAAAT | common cis-acting element in promoter and enhancer regions |
| CAAT-box | Pisum sativum | 473 | + | 5 | CAAAT | common cis-acting element in promoter and enhancer regions |
| CAAT-box | Nicotiana glutinosa | 3071 | - | 4 | CAAT |  |
| CAAT-box | Nicotiana glutinosa | 647 | + | 4 | CAAT |  |
| CAAT-box | Pisum sativum | 2461 | + | 5 | CAAAT | common cis-acting element in promoter and enhancer regions |
| CAAT-box | Nicotiana glutinosa | 2213 | + | 4 | CAAT |  |
| CAAT-box | Arabidopsis thaliana | 2380 | - | 5 | CCAAT | common cis-acting element in promoter and enhancer regions |
| CAAT-box | Nicotiana glutinosa | 302 | + | 4 | CAAT |  |
| CAAT-box | Arabidopsis thaliana | 159 | + | 5 | CCAAT | common cis-acting element in promoter and enhancer regions |
| CAAT-box | Nicotiana glutinosa | 751 | - | 4 | CAAT |  |
| CAAT-box | Pisum sativum | 489 | - | 5 | CAAAT | common cis-acting element in promoter and enhancer regions |
| CAAT-box | Nicotiana glutinosa | 986 | + | 4 | CAAT |  |
| CAAT-box | Nicotiana glutinosa | 521 | + | 4 | CAAT |  |
| CAAT-box | Arabidopsis thaliana | 646 | + | 5 | CCAAT | common cis-acting element in promoter and enhancer regions |
| CAAT-box | Nicotiana glutinosa | 336 | + | 4 | CAAT |  |
| CAAT-box | Nicotiana glutinosa | 846 | + | 4 | CAAT |  |
| CAAT-box | Nicotiana glutinosa | 160 | + | 4 | CAAT |  |
| CAAT-box | Nicotiana glutinosa | 728 | + | 4 | CAAT |  |
| CAAT-box | Nicotiana glutinosa | 461 | + | 4 | CAAT |  |
| CAAT-box | Nicotiana glutinosa | 772 | + | 4 | CAAT |  |

>HU04G00047.1   
+ +Up\_Stream \_Len000TCTTAA CATTAGTTGC TTGGTTCAAC CCATCAGTGA GAATATCTGA TTTCAAAAGT   
  
  
+ ATAGATTTAT ATAAACATAT GGACTACAGG GAAGGGAGTC GTTTCCTCAA AGTCTTAGAT TTACACTCAA   
  
  
+ AAGTTTGAGC GATACCAATA ATGTGATTCC AAACATGAGC CAAATATTAA GTATCCTATA CTAAGATCAA   
  
  
+ GAAGAGAATG AACCAAAAGA CTGTTAATTT TTCAATTAAG CTAAACTTAT TAACCTTTTT TTGGAATAGA   
  
  
+ TTTTTAAATT TTATCTTCAA TATAAGTTTT TCCAAGGCGA GAGACATATA GCAATTTTAA TTAAAGTTAA   
  
  
+ AGTAGTATAA CATTTTTTTT AATGGTTATA GGCTTATAGC ACCTTTTCTA GGTGAAAAGA GATATCTTGG   
  
  
+ CCTGATTTCC AGTAAAGTTT TTTTTTTAAA TATTGTCAAT TATTTTTGCA AATGACATTT GGTAATTTGG   
  
  
+ GGGATAATCT GATGTGTTAA AAGATACAAT GAAAATATCA GAAAATAGGA TGCCTCAAAT TTCACAAGAA   
  
  
+ AGAAAAAATC TTTTTGTTGT TAGTATTTCT ACACATCCAC AATTTTTACT CATGGTAAAA AAAAAAGGGC   
  
  
+ CAAATATCTC ACCAATATAA CCAAACTATA TGTTCCATTA TTTAGTAACG GTGATAAATA TGTAATGGGT   
  
  
+ CAAGTTACTA ATAAAATAGC CTACAATACT TGGATATCTA CATAAAATTG ATATAGAATA TATATTGCAA   
  
  
+ TACTTAAATC AAAATTAAAT GAAATATTTA CTTTTTTATT CTATTACAAA TAGCTTCTTA TAGAATAGCA   
  
  
+ ACAATTTTTA ATGTAAGTTT CATTCTTACC AGATTTGAAG AGGTCCGGAT ATAGAAAATC TTATTCGTAT   
  
  
+ AAGTACTCGC ATGAGGGCGG ATAAATTCTA AAATATCAAC CTCAACTCGC TTCTACTATT CCATCTAAGT   
  
  
+ TCAATTTATC ATATAAAAAA ATTCAATCCA CTTTAATGTA CTCAATTCAC TTCAACCTAT TTTAATCAAT   
  
  
+ TTACCCATGA AAAGAACAAG GTCCTAAGTA GATGTTTTCT ATTGATTGAT TTAAACAATT TTCACCTACC   
  
  
+ CAATTATCCC CAAAACTGAA AGAGGGGGCA AAGGGGGGTG GGTAATGCTG TGTAAAATAG GGGGAAAGGG   
  
  
+ CAAGAAAACT GTGTACAATG GTGGAGCAGG GGTATACCAT TTGGCCTTGA GTTGAGGGGG CACAAAAGCC   
  
  
+ CAGAGAGAGA GAGGGGTAAT GCCCACCAGA AATAAGCAGG AAGCGAGGGA AAAACACTAG AGAGAAACAG   
  
  
+ AGAAAGAGAG AGAGAGAGAG AGAGAAGGGG AAATTTACTA GAGAATGAGA GTGAAGAAGG GAACTTTATT   
  
  
+ TTAACTTTTT TATGATGCAC GCATCCATGG CAGAACAGGG AAGGGAAAGA AGGGTACAGA GTACTGCTGT   
  
  
+ CCCCAAAAAA TTAGTGTAGA AAACAGGAGA CACTCAACTA CAACAAGGGT AAGGAAGAAG AACAAGCAAG   
  
  
+ ATATCCCACC CAAAGAAAAA AAAAAAGAAG AAGAAGAAAA TTTTTCAGAG AGGAAAGAAA AGCAATCACA   
  
  
+ TGAATTAGAA GCCAACCCAA GCCTTCTTCA AAGAGAAGCA GCATAACATA GCATAGCTGG GGAGAGGATA   
  
  
+ GGAGAGAGAA ACTCAAGAAA AGGGGTACCC GGCTTAAATG TTGATGCAGA AAGGTATAGA TATGGCCACA   
  
  
+ CTACTACTAC CCAGGAGTTT ATCATTGGAG ATGGATTTCT CTTTCTGCCT CATCTTCTCC TCCATCCACC   
  
  
+ AGCAAAACTG GGTTTCCTTT CAGCTGGGTC TTTTCTAGAT CTAGAAACTA TGGTACCCAA ATCTCCCATT   
  
  
+ CCTCCATTTT TATTCTCAAA ATCTATCTTC TTAGACTTGT GGGTTTGATT CATTTCTTTT GGGTTTTTGA   
  
  
+ AAAGAATTTA ATTTGATTGT ATTGTGTTGA TTGGGGAATT TGTGGTGGTG TTAGATGCGA GCTATGCCCT   
  
  
+ ACAATTTGCA AGGCAAGGGT GTGGTAGAGG TTTCAAGCAT TTGTATTCCA CAAATCTCTT CCCCTGCTTC   
  
  
+ AAAGTGGAAG ACCAACAACA ACAACTTGGA CAACAAATTT CAGCAACAAC AAGAAGAAAT TGAGCAATTG   
  
  
+ CAGAGTAGTG GTGGCACTTT TCTCTCTCCT GATAACACCA ATACTAATAT TAGTACTACT CCGAGAGCGA   
  
  
+ CAAGTTCTGT TGATAGCGAA CACACATCTA CTCTGGATAC TACGGGCCCT CCCACTTCAA CATCTTCCTC   
  
  
+ CTTCAAGAGC ACCTCCACCG CCAACACAGC CGGTGTGGCG GACCCCGCAT GTACTCCCAA AGAGGATTGG   
  
  
+ GATGTCTCCG GCGGCGCTGC TGGCGGTGAC GGCGGTGGTG GGGGGCTTGG ATTGGAGGAG TGGGACAGCA   
  
  
+ TGTTCCCAAA TGGGGAGGGG GCTTTGCTCC CTTGGATCAT GGGTGAAGCT GATGACATGG GTATGGGTTT   
  
  
+ GAAGCATCTT TTGCAATCGG GTAACCCGGT TGACTATGAG GGCAATGCTG GTCTAGGGGT TGTCGATCAG   
  
  
+ GGTTCTGGAT TTGAGACTCT CTCTCCTCCT CCACCGCAGC CGGCGGCATG TGAATCTAAT GGTGGTGGTG   
  
  
+ TTGCTAATTT GGGGTTTCCA GGGAATAATG GTAAGATTTC TTCAATTTCA CATAATTGTT CATCTGGGAT   
  
  
+ TTTGAATGGT AAGGTGAACA ATAATGGGTT GAATCCCAAT TGTAACCCTC AAGCTCAAGG CAGTCTACTT   
  
  
+ GGTTTAATCC AAGGAGCTAC AGGTGTACAC GCACATCCTG ACGTTGGAGA CGAGAAACCC CAGATTTTGA   
  
  
+ ATCCACATTT GGTGATGAAC CCTCAGCAAG CTCAGAGCAT TGCAAACCCT AGCTTTTTGA TGCACTCATT   
  
  
+ AGGTTACTAT CAGCTGGAGC AACATCTATT TCAACCTCAG GCAAAACGCC TGAACACGGG TGCTGTTCTG   
  
  
+ GATCCTAACC TTGTTCAGCT TGCAAAGAAC CCATTTGCTG ATCAGGGTCA TGAGTTATTG TTGAGGAAGC   
  
  
+ AGCACCAACA GCTTGGTTTG CAACCATTGC CATTGGGTTT GGGTCCTCAG TTGGTCCCTC CGCAGAAGCC   
  
  
+ TGTGATGGGT TCAAAGCAGG GGAACCCCCA GCATTACCCG TTGCATGTTC ATCAGCAGCT GCAATTGCAA   
  
  
+ GAGCAGGCTG TCAAAGATCA GCTCTTCAAG GCGGCAGACC TTATTCAAAC TGGAAGTTTC TCACTCGCGC   
  
  
+ AAGAGATATT GGCGCGGCTC AATCACCAGC TCTCCCTCCC TGCAAAGCCC CTCATTAGGG CGGCTTTGTA   
  
  
+ TGTGAAGGAG GCCCTTCAAA TGCTCCTCCT AATGAGCAAC CCAGTTGCGG CTCCACCGTC CAAGATCCTC   
  
  
+ ACCCCTTATG ATGTTGTTCA CAAGATGAGC GCGTATAAGG TCTTCTCTGA GGTCTGCCCA ATCACTCAAT   
  
  
+ TTGTGAATTT CACTTGTACA CAGGCCATTC TCGAGGCTCT TGATGATTCT GATGCTATTC ATGTTGTCGA   
  
  
+ CTTTGATATT GGTTGCGGTG CTCAATGGGC ATCATTGATT CAGGAGCTGC CATTGAGGAA AAGGGGAGCT   
  
  
+ CCCTCTCTGA AAATTACAGC CATAGCTCCC ATGTTGACTG GCAGTCACTT TGAAATTAGC CTAGTATGTG   
  
  
+ AAAACCTTGT GCAATTTGCC AACGATATTG GTGTTGCTTG TGAGCTCCAA GTTGTCAACT TTGATTTGTT   
  
  
+ TGATCCATCT TCGCCATCAA TGCCGAACAT TAGTACTGCT GAGGATGAGT CAATTGCTGT TAGTATCCCC   
  
  
+ ATCTGGGCAT CTTCAATTAG GCCATCTGTT CTTCCTTCCA TCCTCCGATT CATTAAGCAA AAATCCCCCA   
  
  
+ AAATTGTGGT CTCGTTTGAT AGAGGATTCG ATCGTTTTGA TGTCCCTTTC CCCCAACATC TGTTGCATAC   
  
  
+ CCTAGAATCC TGCAGTAATT TATTGGACTC GCTTGATGGT CTCAATGTTG CATCGGACAT TGTGAGCAAG   
  
  
+ GTCGAGAAGT TCTTTGTTCA ACCTAGGATC GAAAATGCTG TGTTGGGTCG GGTCCATGCC CCTGACAAGA   
  
  
+ TGCCCCATTG GAAAAATCTC TTTGCTTCAG CCGGCTTCTT GCCCTTGCAA TTCAGTAATT TCACAGAAAC   
  
  
+ CCAGGCCGAT TATGTGGTGA AGAGAACCCC AACAAGAGGA TTTCACGTGG AGAAGCGCCA GGCATCACTC   
  
  
+ ATTCTGAGTT GGCAGAGGCA GGAGCTTGTG GCAGCTTCGG CATGGAAGTG TTG  

- +Up\_Stream \_Len000AGAATT GTAATCAACG AACCAAGTTG GGTAGTCACT CTTATAGACT AAAGTTTTCA   
  
  
- TATCTAAATA TATTTGTATA CCTGATGTCC CTTCCCTCAG CAAAGGAGTT TCAGAATCTA AATGTGAGTT   
  
  
- TTCAAACTCG CTATGGTTAT TACACTAAGG TTTGTACTCG GTTTATAATT CATAGGATAT GATTCTAGTT   
  
  
- CTTCTCTTAC TTGGTTTTCT GACAATTAAA AAGTTAATTC GATTTGAATA ATTGGAAAAA AACCTTATCT   
  
  
- AAAAATTTAA AATAGAAGTT ATATTCAAAA AGGTTCCGCT CTCTGTATAT CGTTAAAATT AATTTCAATT   
  
  
- TCATCATATT GTAAAAAAAA TTACCAATAT CCGAATATCG TGGAAAAGAT CCACTTTTCT CTATAGAACC   
  
  
- GGACTAAAGG TCATTTCAAA AAAAAAATTT ATAACAGTTA ATAAAAACGT TTACTGTAAA CCATTAAACC   
  
  
- CCCTATTAGA CTACACAATT TTCTATGTTA CTTTTATAGT CTTTTATCCT ACGGAGTTTA AAGTGTTCTT   
  
  
- TCTTTTTTAG AAAAACAACA ATCATAAAGA TGTGTAGGTG TTAAAAATGA GTACCATTTT TTTTTTCCCG   
  
  
- GTTTATAGAG TGGTTATATT GGTTTGATAT ACAAGGTAAT AAATCATTGC CACTATTTAT ACATTACCCA   
  
  
- GTTCAATGAT TATTTTATCG GATGTTATGA ACCTATAGAT GTATTTTAAC TATATCTTAT ATATAACGTT   
  
  
- ATGAATTTAG TTTTAATTTA CTTTATAAAT GAAAAAATAA GATAATGTTT ATCGAAGAAT ATCTTATCGT   
  
  
- TGTTAAAAAT TACATTCAAA GTAAGAATGG TCTAAACTTC TCCAGGCCTA TATCTTTTAG AATAAGCATA   
  
  
- TTCATGAGCG TACTCCCGCC TATTTAAGAT TTTATAGTTG GAGTTGAGCG AAGATGATAA GGTAGATTCA   
  
  
- AGTTAAATAG TATATTTTTT TAAGTTAGGT GAAATTACAT GAGTTAAGTG AAGTTGGATA AAATTAGTTA   
  
  
- AATGGGTACT TTTCTTGTTC CAGGATTCAT CTACAAAAGA TAACTAACTA AATTTGTTAA AAGTGGATGG   
  
  
- GTTAATAGGG GTTTTGACTT TCTCCCCCGT TTCCCCCCAC CCATTACGAC ACATTTTATC CCCCTTTCCC   
  
  
- GTTCTTTTGA CACATGTTAC CACCTCGTCC CCATATGGTA AACCGGAACT CAACTCCCCC GTGTTTTCGG   
  
  
- GTCTCTCTCT CTCCCCATTA CGGGTGGTCT TTATTCGTCC TTCGCTCCCT TTTTGTGATC TCTCTTTGTC   
  
  
- TCTTTCTCTC TCTCTCTCTC TCTCTTCCCC TTTAAATGAT CTCTTACTCT CACTTCTTCC CTTGAAATAA   
  
  
- AATTGAAAAA ATACTACGTG CGTAGGTACC GTCTTGTCCC TTCCCTTTCT TCCCATGTCT CATGACGACA   
  
  
- GGGGTTTTTT AATCACATCT TTTGTCCTCT GTGAGTTGAT GTTGTTCCCA TTCCTTCTTC TTGTTCGTTC   
  
  
- TATAGGGTGG GTTTCTTTTT TTTTTTCTTC TTCTTCTTTT AAAAAGTCTC TCCTTTCTTT TCGTTAGTGT   
  
  
- ACTTAATCTT CGGTTGGGTT CGGAAGAAGT TTCTCTTCGT CGTATTGTAT CGTATCGACC CCTCTCCTAT   
  
  
- CCTCTCTCTT TGAGTTCTTT TCCCCATGGG CCGAATTTAC AACTACGTCT TTCCATATCT ATACCGGTGT   
  
  
- GATGATGATG GGTCCTCAAA TAGTAACCTC TACCTAAAGA GAAAGACGGA GTAGAAGAGG AGGTAGGTGG   
  
  
- TCGTTTTGAC CCAAAGGAAA GTCGACCCAG AAAAGATCTA GATCTTTGAT ACCATGGGTT TAGAGGGTAA   
  
  
- GGAGGTAAAA ATAAGAGTTT TAGATAGAAG AATCTGAACA CCCAAACTAA GTAAAGAAAA CCCAAAAACT   
  
  
- TTTCTTAAAT TAAACTAACA TAACACAACT AACCCCTTAA ACACCACCAC AATCTACGCT CGATACGGGA   
  
  
- TGTTAAACGT TCCGTTCCCA CACCATCTCC AAAGTTCGTA AACATAAGGT GTTTAGAGAA GGGGACGAAG   
  
  
- TTTCACCTTC TGGTTGTTGT TGTTGAACCT GTTGTTTAAA GTCGTTGTTG TTCTTCTTTA ACTCGTTAAC   
  
  
- GTCTCATCAC CACCGTGAAA AGAGAGAGGA CTATTGTGGT TATGATTATA ATCATGATGA GGCTCTCGCT   
  
  
- GTTCAAGACA ACTATCGCTT GTGTGTAGAT GAGACCTATG ATGCCCGGGA GGGTGAAGTT GTAGAAGGAG   
  
  
- GAAGTTCTCG TGGAGGTGGC GGTTGTGTCG GCCACACCGC CTGGGGCGTA CATGAGGGTT TCTCCTAACC   
  
  
- CTACAGAGGC CGCCGCGACG ACCGCCACTG CCGCCACCAC CCCCCGAACC TAACCTCCTC ACCCTGTCGT   
  
  
- ACAAGGGTTT ACCCCTCCCC CGAAACGAGG GAACCTAGTA CCCACTTCGA CTACTGTACC CATACCCAAA   
  
  
- CTTCGTAGAA AACGTTAGCC CATTGGGCCA ACTGATACTC CCGTTACGAC CAGATCCCCA ACAGCTAGTC   
  
  
- CCAAGACCTA AACTCTGAGA GAGAGGAGGA GGTGGCGTCG GCCGCCGTAC ACTTAGATTA CCACCACCAC   
  
  
- AACGATTAAA CCCCAAAGGT CCCTTATTAC CATTCTAAAG AAGTTAAAGT GTATTAACAA GTAGACCCTA   
  
  
- AAACTTACCA TTCCACTTGT TATTACCCAA CTTAGGGTTA ACATTGGGAG TTCGAGTTCC GTCAGATGAA   
  
  
- CCAAATTAGG TTCCTCGATG TCCACATGTG CGTGTAGGAC TGCAACCTCT GCTCTTTGGG GTCTAAAACT   
  
  
- TAGGTGTAAA CCACTACTTG GGAGTCGTTC GAGTCTCGTA ACGTTTGGGA TCGAAAAACT ACGTGAGTAA   
  
  
- TCCAATGATA GTCGACCTCG TTGTAGATAA AGTTGGAGTC CGTTTTGCGG ACTTGTGCCC ACGACAAGAC   
  
  
- CTAGGATTGG AACAAGTCGA ACGTTTCTTG GGTAAACGAC TAGTCCCAGT ACTCAATAAC AACTCCTTCG   
  
  
- TCGTGGTTGT CGAACCAAAC GTTGGTAACG GTAACCCAAA CCCAGGAGTC AACCAGGGAG GCGTCTTCGG   
  
  
- ACACTACCCA AGTTTCGTCC CCTTGGGGGT CGTAATGGGC AACGTACAAG TAGTCGTCGA CGTTAACGTT   
  
  
- CTCGTCCGAC AGTTTCTAGT CGAGAAGTTC CGCCGTCTGG AATAAGTTTG ACCTTCAAAG AGTGAGCGCG   
  
  
- TTCTCTATAA CCGCGCCGAG TTAGTGGTCG AGAGGGAGGG ACGTTTCGGG GAGTAATCCC GCCGAAACAT   
  
  
- ACACTTCCTC CGGGAAGTTT ACGAGGAGGA TTACTCGTTG GGTCAACGCC GAGGTGGCAG GTTCTAGGAG   
  
  
- TGGGGAATAC TACAACAAGT GTTCTACTCG CGCATATTCC AGAAGAGACT CCAGACGGGT TAGTGAGTTA   
  
  
- AACACTTAAA GTGAACATGT GTCCGGTAAG AGCTCCGAGA ACTACTAAGA CTACGATAAG TACAACAGCT   
  
  
- GAAACTATAA CCAACGCCAC GAGTTACCCG TAGTAACTAA GTCCTCGACG GTAACTCCTT TTCCCCTCGA   
  
  
- GGGAGAGACT TTTAATGTCG GTATCGAGGG TACAACTGAC CGTCAGTGAA ACTTTAATCG GATCATACAC   
  
  
- TTTTGGAACA CGTTAAACGG TTGCTATAAC CACAACGAAC ACTCGAGGTT CAACAGTTGA AACTAAACAA   
  
  
- ACTAGGTAGA AGCGGTAGTT ACGGCTTGTA ATCATGACGA CTCCTACTCA GTTAACGACA ATCATAGGGG   
  
  
- TAGACCCGTA GAAGTTAATC CGGTAGACAA GAAGGAAGGT AGGAGGCTAA GTAATTCGTT TTTAGGGGGT   
  
  
- TTTAACACCA GAGCAAACTA TCTCCTAAGC TAGCAAAACT ACAGGGAAAG GGGGTTGTAG ACAACGTATG   
  
  
- GGATCTTAGG ACGTCATTAA ATAACCTGAG CGAACTACCA GAGTTACAAC GTAGCCTGTA ACACTCGTTC   
  
  
- CAGCTCTTCA AGAAACAAGT TGGATCCTAG CTTTTACGAC ACAACCCAGC CCAGGTACGG GGACTGTTCT   
  
  
- ACGGGGTAAC CTTTTTAGAG AAACGAAGTC GGCCGAAGAA CGGGAACGTT AAGTCATTAA AGTGTCTTTG   
  
  
- GGTCCGGCTA ATACACCACT TCTCTTGGGG TTGTTCTCCT AAAGTGCACC TCTTCGCGGT CCGTAGTGAG   
  
  
- TAAGACTCAA CCGTCTCCGT CCTCGAACAC CGTCGAAGCC GTACCTTCAC AAC

+     CCAAT-box

| Site Name | Organism | Position | Strand | Matrix score. | sequence | function |
| --- | --- | --- | --- | --- | --- | --- |
| CCAAT-box | Hordeum vulgare | 3192 | - | 6 | CAACGG | MYBHv1 binding site |

>HU04G00047.1   
+ +Up\_Stream \_Len000TCTTAA CATTAGTTGC TTGGTTCAAC CCATCAGTGA GAATATCTGA TTTCAAAAGT   
  
  
+ ATAGATTTAT ATAAACATAT GGACTACAGG GAAGGGAGTC GTTTCCTCAA AGTCTTAGAT TTACACTCAA   
  
  
+ AAGTTTGAGC GATACCAATA ATGTGATTCC AAACATGAGC CAAATATTAA GTATCCTATA CTAAGATCAA   
  
  
+ GAAGAGAATG AACCAAAAGA CTGTTAATTT TTCAATTAAG CTAAACTTAT TAACCTTTTT TTGGAATAGA   
  
  
+ TTTTTAAATT TTATCTTCAA TATAAGTTTT TCCAAGGCGA GAGACATATA GCAATTTTAA TTAAAGTTAA   
  
  
+ AGTAGTATAA CATTTTTTTT AATGGTTATA GGCTTATAGC ACCTTTTCTA GGTGAAAAGA GATATCTTGG   
  
  
+ CCTGATTTCC AGTAAAGTTT TTTTTTTAAA TATTGTCAAT TATTTTTGCA AATGACATTT GGTAATTTGG   
  
  
+ GGGATAATCT GATGTGTTAA AAGATACAAT GAAAATATCA GAAAATAGGA TGCCTCAAAT TTCACAAGAA   
  
  
+ AGAAAAAATC TTTTTGTTGT TAGTATTTCT ACACATCCAC AATTTTTACT CATGGTAAAA AAAAAAGGGC   
  
  
+ CAAATATCTC ACCAATATAA CCAAACTATA TGTTCCATTA TTTAGTAACG GTGATAAATA TGTAATGGGT   
  
  
+ CAAGTTACTA ATAAAATAGC CTACAATACT TGGATATCTA CATAAAATTG ATATAGAATA TATATTGCAA   
  
  
+ TACTTAAATC AAAATTAAAT GAAATATTTA CTTTTTTATT CTATTACAAA TAGCTTCTTA TAGAATAGCA   
  
  
+ ACAATTTTTA ATGTAAGTTT CATTCTTACC AGATTTGAAG AGGTCCGGAT ATAGAAAATC TTATTCGTAT   
  
  
+ AAGTACTCGC ATGAGGGCGG ATAAATTCTA AAATATCAAC CTCAACTCGC TTCTACTATT CCATCTAAGT   
  
  
+ TCAATTTATC ATATAAAAAA ATTCAATCCA CTTTAATGTA CTCAATTCAC TTCAACCTAT TTTAATCAAT   
  
  
+ TTACCCATGA AAAGAACAAG GTCCTAAGTA GATGTTTTCT ATTGATTGAT TTAAACAATT TTCACCTACC   
  
  
+ CAATTATCCC CAAAACTGAA AGAGGGGGCA AAGGGGGGTG GGTAATGCTG TGTAAAATAG GGGGAAAGGG   
  
  
+ CAAGAAAACT GTGTACAATG GTGGAGCAGG GGTATACCAT TTGGCCTTGA GTTGAGGGGG CACAAAAGCC   
  
  
+ CAGAGAGAGA GAGGGGTAAT GCCCACCAGA AATAAGCAGG AAGCGAGGGA AAAACACTAG AGAGAAACAG   
  
  
+ AGAAAGAGAG AGAGAGAGAG AGAGAAGGGG AAATTTACTA GAGAATGAGA GTGAAGAAGG GAACTTTATT   
  
  
+ TTAACTTTTT TATGATGCAC GCATCCATGG CAGAACAGGG AAGGGAAAGA AGGGTACAGA GTACTGCTGT   
  
  
+ CCCCAAAAAA TTAGTGTAGA AAACAGGAGA CACTCAACTA CAACAAGGGT AAGGAAGAAG AACAAGCAAG   
  
  
+ ATATCCCACC CAAAGAAAAA AAAAAAGAAG AAGAAGAAAA TTTTTCAGAG AGGAAAGAAA AGCAATCACA   
  
  
+ TGAATTAGAA GCCAACCCAA GCCTTCTTCA AAGAGAAGCA GCATAACATA GCATAGCTGG GGAGAGGATA   
  
  
+ GGAGAGAGAA ACTCAAGAAA AGGGGTACCC GGCTTAAATG TTGATGCAGA AAGGTATAGA TATGGCCACA   
  
  
+ CTACTACTAC CCAGGAGTTT ATCATTGGAG ATGGATTTCT CTTTCTGCCT CATCTTCTCC TCCATCCACC   
  
  
+ AGCAAAACTG GGTTTCCTTT CAGCTGGGTC TTTTCTAGAT CTAGAAACTA TGGTACCCAA ATCTCCCATT   
  
  
+ CCTCCATTTT TATTCTCAAA ATCTATCTTC TTAGACTTGT GGGTTTGATT CATTTCTTTT GGGTTTTTGA   
  
  
+ AAAGAATTTA ATTTGATTGT ATTGTGTTGA TTGGGGAATT TGTGGTGGTG TTAGATGCGA GCTATGCCCT   
  
  
+ ACAATTTGCA AGGCAAGGGT GTGGTAGAGG TTTCAAGCAT TTGTATTCCA CAAATCTCTT CCCCTGCTTC   
  
  
+ AAAGTGGAAG ACCAACAACA ACAACTTGGA CAACAAATTT CAGCAACAAC AAGAAGAAAT TGAGCAATTG   
  
  
+ CAGAGTAGTG GTGGCACTTT TCTCTCTCCT GATAACACCA ATACTAATAT TAGTACTACT CCGAGAGCGA   
  
  
+ CAAGTTCTGT TGATAGCGAA CACACATCTA CTCTGGATAC TACGGGCCCT CCCACTTCAA CATCTTCCTC   
  
  
+ CTTCAAGAGC ACCTCCACCG CCAACACAGC CGGTGTGGCG GACCCCGCAT GTACTCCCAA AGAGGATTGG   
  
  
+ GATGTCTCCG GCGGCGCTGC TGGCGGTGAC GGCGGTGGTG GGGGGCTTGG ATTGGAGGAG TGGGACAGCA   
  
  
+ TGTTCCCAAA TGGGGAGGGG GCTTTGCTCC CTTGGATCAT GGGTGAAGCT GATGACATGG GTATGGGTTT   
  
  
+ GAAGCATCTT TTGCAATCGG GTAACCCGGT TGACTATGAG GGCAATGCTG GTCTAGGGGT TGTCGATCAG   
  
  
+ GGTTCTGGAT TTGAGACTCT CTCTCCTCCT CCACCGCAGC CGGCGGCATG TGAATCTAAT GGTGGTGGTG   
  
  
+ TTGCTAATTT GGGGTTTCCA GGGAATAATG GTAAGATTTC TTCAATTTCA CATAATTGTT CATCTGGGAT   
  
  
+ TTTGAATGGT AAGGTGAACA ATAATGGGTT GAATCCCAAT TGTAACCCTC AAGCTCAAGG CAGTCTACTT   
  
  
+ GGTTTAATCC AAGGAGCTAC AGGTGTACAC GCACATCCTG ACGTTGGAGA CGAGAAACCC CAGATTTTGA   
  
  
+ ATCCACATTT GGTGATGAAC CCTCAGCAAG CTCAGAGCAT TGCAAACCCT AGCTTTTTGA TGCACTCATT   
  
  
+ AGGTTACTAT CAGCTGGAGC AACATCTATT TCAACCTCAG GCAAAACGCC TGAACACGGG TGCTGTTCTG   
  
  
+ GATCCTAACC TTGTTCAGCT TGCAAAGAAC CCATTTGCTG ATCAGGGTCA TGAGTTATTG TTGAGGAAGC   
  
  
+ AGCACCAACA GCTTGGTTTG CAACCATTGC CATTGGGTTT GGGTCCTCAG TTGGTCCCTC CGCAGAAGCC   
  
  
+ TGTGATGGGT TCAAAGCAGG GGAACCCCCA GCATTACCCG TTGCATGTTC ATCAGCAGCT GCAATTGCAA   
  
  
+ GAGCAGGCTG TCAAAGATCA GCTCTTCAAG GCGGCAGACC TTATTCAAAC TGGAAGTTTC TCACTCGCGC   
  
  
+ AAGAGATATT GGCGCGGCTC AATCACCAGC TCTCCCTCCC TGCAAAGCCC CTCATTAGGG CGGCTTTGTA   
  
  
+ TGTGAAGGAG GCCCTTCAAA TGCTCCTCCT AATGAGCAAC CCAGTTGCGG CTCCACCGTC CAAGATCCTC   
  
  
+ ACCCCTTATG ATGTTGTTCA CAAGATGAGC GCGTATAAGG TCTTCTCTGA GGTCTGCCCA ATCACTCAAT   
  
  
+ TTGTGAATTT CACTTGTACA CAGGCCATTC TCGAGGCTCT TGATGATTCT GATGCTATTC ATGTTGTCGA   
  
  
+ CTTTGATATT GGTTGCGGTG CTCAATGGGC ATCATTGATT CAGGAGCTGC CATTGAGGAA AAGGGGAGCT   
  
  
+ CCCTCTCTGA AAATTACAGC CATAGCTCCC ATGTTGACTG GCAGTCACTT TGAAATTAGC CTAGTATGTG   
  
  
+ AAAACCTTGT GCAATTTGCC AACGATATTG GTGTTGCTTG TGAGCTCCAA GTTGTCAACT TTGATTTGTT   
  
  
+ TGATCCATCT TCGCCATCAA TGCCGAACAT TAGTACTGCT GAGGATGAGT CAATTGCTGT TAGTATCCCC   
  
  
+ ATCTGGGCAT CTTCAATTAG GCCATCTGTT CTTCCTTCCA TCCTCCGATT CATTAAGCAA AAATCCCCCA   
  
  
+ AAATTGTGGT CTCGTTTGAT AGAGGATTCG ATCGTTTTGA TGTCCCTTTC CCCCAACATC TGTTGCATAC   
  
  
+ CCTAGAATCC TGCAGTAATT TATTGGACTC GCTTGATGGT CTCAATGTTG CATCGGACAT TGTGAGCAAG   
  
  
+ GTCGAGAAGT TCTTTGTTCA ACCTAGGATC GAAAATGCTG TGTTGGGTCG GGTCCATGCC CCTGACAAGA   
  
  
+ TGCCCCATTG GAAAAATCTC TTTGCTTCAG CCGGCTTCTT GCCCTTGCAA TTCAGTAATT TCACAGAAAC   
  
  
+ CCAGGCCGAT TATGTGGTGA AGAGAACCCC AACAAGAGGA TTTCACGTGG AGAAGCGCCA GGCATCACTC   
  
  
+ ATTCTGAGTT GGCAGAGGCA GGAGCTTGTG GCAGCTTCGG CATGGAAGTG TTG  

- +Up\_Stream \_Len000AGAATT GTAATCAACG AACCAAGTTG GGTAGTCACT CTTATAGACT AAAGTTTTCA   
  
  
- TATCTAAATA TATTTGTATA CCTGATGTCC CTTCCCTCAG CAAAGGAGTT TCAGAATCTA AATGTGAGTT   
  
  
- TTCAAACTCG CTATGGTTAT TACACTAAGG TTTGTACTCG GTTTATAATT CATAGGATAT GATTCTAGTT   
  
  
- CTTCTCTTAC TTGGTTTTCT GACAATTAAA AAGTTAATTC GATTTGAATA ATTGGAAAAA AACCTTATCT   
  
  
- AAAAATTTAA AATAGAAGTT ATATTCAAAA AGGTTCCGCT CTCTGTATAT CGTTAAAATT AATTTCAATT   
  
  
- TCATCATATT GTAAAAAAAA TTACCAATAT CCGAATATCG TGGAAAAGAT CCACTTTTCT CTATAGAACC   
  
  
- GGACTAAAGG TCATTTCAAA AAAAAAATTT ATAACAGTTA ATAAAAACGT TTACTGTAAA CCATTAAACC   
  
  
- CCCTATTAGA CTACACAATT TTCTATGTTA CTTTTATAGT CTTTTATCCT ACGGAGTTTA AAGTGTTCTT   
  
  
- TCTTTTTTAG AAAAACAACA ATCATAAAGA TGTGTAGGTG TTAAAAATGA GTACCATTTT TTTTTTCCCG   
  
  
- GTTTATAGAG TGGTTATATT GGTTTGATAT ACAAGGTAAT AAATCATTGC CACTATTTAT ACATTACCCA   
  
  
- GTTCAATGAT TATTTTATCG GATGTTATGA ACCTATAGAT GTATTTTAAC TATATCTTAT ATATAACGTT   
  
  
- ATGAATTTAG TTTTAATTTA CTTTATAAAT GAAAAAATAA GATAATGTTT ATCGAAGAAT ATCTTATCGT   
  
  
- TGTTAAAAAT TACATTCAAA GTAAGAATGG TCTAAACTTC TCCAGGCCTA TATCTTTTAG AATAAGCATA   
  
  
- TTCATGAGCG TACTCCCGCC TATTTAAGAT TTTATAGTTG GAGTTGAGCG AAGATGATAA GGTAGATTCA   
  
  
- AGTTAAATAG TATATTTTTT TAAGTTAGGT GAAATTACAT GAGTTAAGTG AAGTTGGATA AAATTAGTTA   
  
  
- AATGGGTACT TTTCTTGTTC CAGGATTCAT CTACAAAAGA TAACTAACTA AATTTGTTAA AAGTGGATGG   
  
  
- GTTAATAGGG GTTTTGACTT TCTCCCCCGT TTCCCCCCAC CCATTACGAC ACATTTTATC CCCCTTTCCC   
  
  
- GTTCTTTTGA CACATGTTAC CACCTCGTCC CCATATGGTA AACCGGAACT CAACTCCCCC GTGTTTTCGG   
  
  
- GTCTCTCTCT CTCCCCATTA CGGGTGGTCT TTATTCGTCC TTCGCTCCCT TTTTGTGATC TCTCTTTGTC   
  
  
- TCTTTCTCTC TCTCTCTCTC TCTCTTCCCC TTTAAATGAT CTCTTACTCT CACTTCTTCC CTTGAAATAA   
  
  
- AATTGAAAAA ATACTACGTG CGTAGGTACC GTCTTGTCCC TTCCCTTTCT TCCCATGTCT CATGACGACA   
  
  
- GGGGTTTTTT AATCACATCT TTTGTCCTCT GTGAGTTGAT GTTGTTCCCA TTCCTTCTTC TTGTTCGTTC   
  
  
- TATAGGGTGG GTTTCTTTTT TTTTTTCTTC TTCTTCTTTT AAAAAGTCTC TCCTTTCTTT TCGTTAGTGT   
  
  
- ACTTAATCTT CGGTTGGGTT CGGAAGAAGT TTCTCTTCGT CGTATTGTAT CGTATCGACC CCTCTCCTAT   
  
  
- CCTCTCTCTT TGAGTTCTTT TCCCCATGGG CCGAATTTAC AACTACGTCT TTCCATATCT ATACCGGTGT   
  
  
- GATGATGATG GGTCCTCAAA TAGTAACCTC TACCTAAAGA GAAAGACGGA GTAGAAGAGG AGGTAGGTGG   
  
  
- TCGTTTTGAC CCAAAGGAAA GTCGACCCAG AAAAGATCTA GATCTTTGAT ACCATGGGTT TAGAGGGTAA   
  
  
- GGAGGTAAAA ATAAGAGTTT TAGATAGAAG AATCTGAACA CCCAAACTAA GTAAAGAAAA CCCAAAAACT   
  
  
- TTTCTTAAAT TAAACTAACA TAACACAACT AACCCCTTAA ACACCACCAC AATCTACGCT CGATACGGGA   
  
  
- TGTTAAACGT TCCGTTCCCA CACCATCTCC AAAGTTCGTA AACATAAGGT GTTTAGAGAA GGGGACGAAG   
  
  
- TTTCACCTTC TGGTTGTTGT TGTTGAACCT GTTGTTTAAA GTCGTTGTTG TTCTTCTTTA ACTCGTTAAC   
  
  
- GTCTCATCAC CACCGTGAAA AGAGAGAGGA CTATTGTGGT TATGATTATA ATCATGATGA GGCTCTCGCT   
  
  
- GTTCAAGACA ACTATCGCTT GTGTGTAGAT GAGACCTATG ATGCCCGGGA GGGTGAAGTT GTAGAAGGAG   
  
  
- GAAGTTCTCG TGGAGGTGGC GGTTGTGTCG GCCACACCGC CTGGGGCGTA CATGAGGGTT TCTCCTAACC   
  
  
- CTACAGAGGC CGCCGCGACG ACCGCCACTG CCGCCACCAC CCCCCGAACC TAACCTCCTC ACCCTGTCGT   
  
  
- ACAAGGGTTT ACCCCTCCCC CGAAACGAGG GAACCTAGTA CCCACTTCGA CTACTGTACC CATACCCAAA   
  
  
- CTTCGTAGAA AACGTTAGCC CATTGGGCCA ACTGATACTC CCGTTACGAC CAGATCCCCA ACAGCTAGTC   
  
  
- CCAAGACCTA AACTCTGAGA GAGAGGAGGA GGTGGCGTCG GCCGCCGTAC ACTTAGATTA CCACCACCAC   
  
  
- AACGATTAAA CCCCAAAGGT CCCTTATTAC CATTCTAAAG AAGTTAAAGT GTATTAACAA GTAGACCCTA   
  
  
- AAACTTACCA TTCCACTTGT TATTACCCAA CTTAGGGTTA ACATTGGGAG TTCGAGTTCC GTCAGATGAA   
  
  
- CCAAATTAGG TTCCTCGATG TCCACATGTG CGTGTAGGAC TGCAACCTCT GCTCTTTGGG GTCTAAAACT   
  
  
- TAGGTGTAAA CCACTACTTG GGAGTCGTTC GAGTCTCGTA ACGTTTGGGA TCGAAAAACT ACGTGAGTAA   
  
  
- TCCAATGATA GTCGACCTCG TTGTAGATAA AGTTGGAGTC CGTTTTGCGG ACTTGTGCCC ACGACAAGAC   
  
  
- CTAGGATTGG AACAAGTCGA ACGTTTCTTG GGTAAACGAC TAGTCCCAGT ACTCAATAAC AACTCCTTCG   
  
  
- TCGTGGTTGT CGAACCAAAC GTTGGTAACG GTAACCCAAA CCCAGGAGTC AACCAGGGAG GCGTCTTCGG   
  
  
- ACACTACCCA AGTTTCGTCC CCTTGGGGGT CGTAATGGGC AACGTACAAG TAGTCGTCGA CGTTAACGTT   
  
  
- CTCGTCCGAC AGTTTCTAGT CGAGAAGTTC CGCCGTCTGG AATAAGTTTG ACCTTCAAAG AGTGAGCGCG   
  
  
- TTCTCTATAA CCGCGCCGAG TTAGTGGTCG AGAGGGAGGG ACGTTTCGGG GAGTAATCCC GCCGAAACAT   
  
  
- ACACTTCCTC CGGGAAGTTT ACGAGGAGGA TTACTCGTTG GGTCAACGCC GAGGTGGCAG GTTCTAGGAG   
  
  
- TGGGGAATAC TACAACAAGT GTTCTACTCG CGCATATTCC AGAAGAGACT CCAGACGGGT TAGTGAGTTA   
  
  
- AACACTTAAA GTGAACATGT GTCCGGTAAG AGCTCCGAGA ACTACTAAGA CTACGATAAG TACAACAGCT   
  
  
- GAAACTATAA CCAACGCCAC GAGTTACCCG TAGTAACTAA GTCCTCGACG GTAACTCCTT TTCCCCTCGA   
  
  
- GGGAGAGACT TTTAATGTCG GTATCGAGGG TACAACTGAC CGTCAGTGAA ACTTTAATCG GATCATACAC   
  
  
- TTTTGGAACA CGTTAAACGG TTGCTATAAC CACAACGAAC ACTCGAGGTT CAACAGTTGA AACTAAACAA   
  
  
- ACTAGGTAGA AGCGGTAGTT ACGGCTTGTA ATCATGACGA CTCCTACTCA GTTAACGACA ATCATAGGGG   
  
  
- TAGACCCGTA GAAGTTAATC CGGTAGACAA GAAGGAAGGT AGGAGGCTAA GTAATTCGTT TTTAGGGGGT   
  
  
- TTTAACACCA GAGCAAACTA TCTCCTAAGC TAGCAAAACT ACAGGGAAAG GGGGTTGTAG ACAACGTATG   
  
  
- GGATCTTAGG ACGTCATTAA ATAACCTGAG CGAACTACCA GAGTTACAAC GTAGCCTGTA ACACTCGTTC   
  
  
- CAGCTCTTCA AGAAACAAGT TGGATCCTAG CTTTTACGAC ACAACCCAGC CCAGGTACGG GGACTGTTCT   
  
  
- ACGGGGTAAC CTTTTTAGAG AAACGAAGTC GGCCGAAGAA CGGGAACGTT AAGTCATTAA AGTGTCTTTG   
  
  
- GGTCCGGCTA ATACACCACT TCTCTTGGGG TTGTTCTCCT AAAGTGCACC TCTTCGCGGT CCGTAGTGAG   
  
  
- TAAGACTCAA CCGTCTCCGT CCTCGAACAC CGTCGAAGCC GTACCTTCAC AAC

+     CCGTCC motif

| Site Name | Organism | Position | Strand | Matrix score. | sequence | function |
| --- | --- | --- | --- | --- | --- | --- |
| CCGTCC motif | Nicotiana tabacum | 3420 | + | 6 | CCGTCC |  |

>HU04G00047.1   
+ +Up\_Stream \_Len000TCTTAA CATTAGTTGC TTGGTTCAAC CCATCAGTGA GAATATCTGA TTTCAAAAGT   
  
  
+ ATAGATTTAT ATAAACATAT GGACTACAGG GAAGGGAGTC GTTTCCTCAA AGTCTTAGAT TTACACTCAA   
  
  
+ AAGTTTGAGC GATACCAATA ATGTGATTCC AAACATGAGC CAAATATTAA GTATCCTATA CTAAGATCAA   
  
  
+ GAAGAGAATG AACCAAAAGA CTGTTAATTT TTCAATTAAG CTAAACTTAT TAACCTTTTT TTGGAATAGA   
  
  
+ TTTTTAAATT TTATCTTCAA TATAAGTTTT TCCAAGGCGA GAGACATATA GCAATTTTAA TTAAAGTTAA   
  
  
+ AGTAGTATAA CATTTTTTTT AATGGTTATA GGCTTATAGC ACCTTTTCTA GGTGAAAAGA GATATCTTGG   
  
  
+ CCTGATTTCC AGTAAAGTTT TTTTTTTAAA TATTGTCAAT TATTTTTGCA AATGACATTT GGTAATTTGG   
  
  
+ GGGATAATCT GATGTGTTAA AAGATACAAT GAAAATATCA GAAAATAGGA TGCCTCAAAT TTCACAAGAA   
  
  
+ AGAAAAAATC TTTTTGTTGT TAGTATTTCT ACACATCCAC AATTTTTACT CATGGTAAAA AAAAAAGGGC   
  
  
+ CAAATATCTC ACCAATATAA CCAAACTATA TGTTCCATTA TTTAGTAACG GTGATAAATA TGTAATGGGT   
  
  
+ CAAGTTACTA ATAAAATAGC CTACAATACT TGGATATCTA CATAAAATTG ATATAGAATA TATATTGCAA   
  
  
+ TACTTAAATC AAAATTAAAT GAAATATTTA CTTTTTTATT CTATTACAAA TAGCTTCTTA TAGAATAGCA   
  
  
+ ACAATTTTTA ATGTAAGTTT CATTCTTACC AGATTTGAAG AGGTCCGGAT ATAGAAAATC TTATTCGTAT   
  
  
+ AAGTACTCGC ATGAGGGCGG ATAAATTCTA AAATATCAAC CTCAACTCGC TTCTACTATT CCATCTAAGT   
  
  
+ TCAATTTATC ATATAAAAAA ATTCAATCCA CTTTAATGTA CTCAATTCAC TTCAACCTAT TTTAATCAAT   
  
  
+ TTACCCATGA AAAGAACAAG GTCCTAAGTA GATGTTTTCT ATTGATTGAT TTAAACAATT TTCACCTACC   
  
  
+ CAATTATCCC CAAAACTGAA AGAGGGGGCA AAGGGGGGTG GGTAATGCTG TGTAAAATAG GGGGAAAGGG   
  
  
+ CAAGAAAACT GTGTACAATG GTGGAGCAGG GGTATACCAT TTGGCCTTGA GTTGAGGGGG CACAAAAGCC   
  
  
+ CAGAGAGAGA GAGGGGTAAT GCCCACCAGA AATAAGCAGG AAGCGAGGGA AAAACACTAG AGAGAAACAG   
  
  
+ AGAAAGAGAG AGAGAGAGAG AGAGAAGGGG AAATTTACTA GAGAATGAGA GTGAAGAAGG GAACTTTATT   
  
  
+ TTAACTTTTT TATGATGCAC GCATCCATGG CAGAACAGGG AAGGGAAAGA AGGGTACAGA GTACTGCTGT   
  
  
+ CCCCAAAAAA TTAGTGTAGA AAACAGGAGA CACTCAACTA CAACAAGGGT AAGGAAGAAG AACAAGCAAG   
  
  
+ ATATCCCACC CAAAGAAAAA AAAAAAGAAG AAGAAGAAAA TTTTTCAGAG AGGAAAGAAA AGCAATCACA   
  
  
+ TGAATTAGAA GCCAACCCAA GCCTTCTTCA AAGAGAAGCA GCATAACATA GCATAGCTGG GGAGAGGATA   
  
  
+ GGAGAGAGAA ACTCAAGAAA AGGGGTACCC GGCTTAAATG TTGATGCAGA AAGGTATAGA TATGGCCACA   
  
  
+ CTACTACTAC CCAGGAGTTT ATCATTGGAG ATGGATTTCT CTTTCTGCCT CATCTTCTCC TCCATCCACC   
  
  
+ AGCAAAACTG GGTTTCCTTT CAGCTGGGTC TTTTCTAGAT CTAGAAACTA TGGTACCCAA ATCTCCCATT   
  
  
+ CCTCCATTTT TATTCTCAAA ATCTATCTTC TTAGACTTGT GGGTTTGATT CATTTCTTTT GGGTTTTTGA   
  
  
+ AAAGAATTTA ATTTGATTGT ATTGTGTTGA TTGGGGAATT TGTGGTGGTG TTAGATGCGA GCTATGCCCT   
  
  
+ ACAATTTGCA AGGCAAGGGT GTGGTAGAGG TTTCAAGCAT TTGTATTCCA CAAATCTCTT CCCCTGCTTC   
  
  
+ AAAGTGGAAG ACCAACAACA ACAACTTGGA CAACAAATTT CAGCAACAAC AAGAAGAAAT TGAGCAATTG   
  
  
+ CAGAGTAGTG GTGGCACTTT TCTCTCTCCT GATAACACCA ATACTAATAT TAGTACTACT CCGAGAGCGA   
  
  
+ CAAGTTCTGT TGATAGCGAA CACACATCTA CTCTGGATAC TACGGGCCCT CCCACTTCAA CATCTTCCTC   
  
  
+ CTTCAAGAGC ACCTCCACCG CCAACACAGC CGGTGTGGCG GACCCCGCAT GTACTCCCAA AGAGGATTGG   
  
  
+ GATGTCTCCG GCGGCGCTGC TGGCGGTGAC GGCGGTGGTG GGGGGCTTGG ATTGGAGGAG TGGGACAGCA   
  
  
+ TGTTCCCAAA TGGGGAGGGG GCTTTGCTCC CTTGGATCAT GGGTGAAGCT GATGACATGG GTATGGGTTT   
  
  
+ GAAGCATCTT TTGCAATCGG GTAACCCGGT TGACTATGAG GGCAATGCTG GTCTAGGGGT TGTCGATCAG   
  
  
+ GGTTCTGGAT TTGAGACTCT CTCTCCTCCT CCACCGCAGC CGGCGGCATG TGAATCTAAT GGTGGTGGTG   
  
  
+ TTGCTAATTT GGGGTTTCCA GGGAATAATG GTAAGATTTC TTCAATTTCA CATAATTGTT CATCTGGGAT   
  
  
+ TTTGAATGGT AAGGTGAACA ATAATGGGTT GAATCCCAAT TGTAACCCTC AAGCTCAAGG CAGTCTACTT   
  
  
+ GGTTTAATCC AAGGAGCTAC AGGTGTACAC GCACATCCTG ACGTTGGAGA CGAGAAACCC CAGATTTTGA   
  
  
+ ATCCACATTT GGTGATGAAC CCTCAGCAAG CTCAGAGCAT TGCAAACCCT AGCTTTTTGA TGCACTCATT   
  
  
+ AGGTTACTAT CAGCTGGAGC AACATCTATT TCAACCTCAG GCAAAACGCC TGAACACGGG TGCTGTTCTG   
  
  
+ GATCCTAACC TTGTTCAGCT TGCAAAGAAC CCATTTGCTG ATCAGGGTCA TGAGTTATTG TTGAGGAAGC   
  
  
+ AGCACCAACA GCTTGGTTTG CAACCATTGC CATTGGGTTT GGGTCCTCAG TTGGTCCCTC CGCAGAAGCC   
  
  
+ TGTGATGGGT TCAAAGCAGG GGAACCCCCA GCATTACCCG TTGCATGTTC ATCAGCAGCT GCAATTGCAA   
  
  
+ GAGCAGGCTG TCAAAGATCA GCTCTTCAAG GCGGCAGACC TTATTCAAAC TGGAAGTTTC TCACTCGCGC   
  
  
+ AAGAGATATT GGCGCGGCTC AATCACCAGC TCTCCCTCCC TGCAAAGCCC CTCATTAGGG CGGCTTTGTA   
  
  
+ TGTGAAGGAG GCCCTTCAAA TGCTCCTCCT AATGAGCAAC CCAGTTGCGG CTCCACCGTC CAAGATCCTC   
  
  
+ ACCCCTTATG ATGTTGTTCA CAAGATGAGC GCGTATAAGG TCTTCTCTGA GGTCTGCCCA ATCACTCAAT   
  
  
+ TTGTGAATTT CACTTGTACA CAGGCCATTC TCGAGGCTCT TGATGATTCT GATGCTATTC ATGTTGTCGA   
  
  
+ CTTTGATATT GGTTGCGGTG CTCAATGGGC ATCATTGATT CAGGAGCTGC CATTGAGGAA AAGGGGAGCT   
  
  
+ CCCTCTCTGA AAATTACAGC CATAGCTCCC ATGTTGACTG GCAGTCACTT TGAAATTAGC CTAGTATGTG   
  
  
+ AAAACCTTGT GCAATTTGCC AACGATATTG GTGTTGCTTG TGAGCTCCAA GTTGTCAACT TTGATTTGTT   
  
  
+ TGATCCATCT TCGCCATCAA TGCCGAACAT TAGTACTGCT GAGGATGAGT CAATTGCTGT TAGTATCCCC   
  
  
+ ATCTGGGCAT CTTCAATTAG GCCATCTGTT CTTCCTTCCA TCCTCCGATT CATTAAGCAA AAATCCCCCA   
  
  
+ AAATTGTGGT CTCGTTTGAT AGAGGATTCG ATCGTTTTGA TGTCCCTTTC CCCCAACATC TGTTGCATAC   
  
  
+ CCTAGAATCC TGCAGTAATT TATTGGACTC GCTTGATGGT CTCAATGTTG CATCGGACAT TGTGAGCAAG   
  
  
+ GTCGAGAAGT TCTTTGTTCA ACCTAGGATC GAAAATGCTG TGTTGGGTCG GGTCCATGCC CCTGACAAGA   
  
  
+ TGCCCCATTG GAAAAATCTC TTTGCTTCAG CCGGCTTCTT GCCCTTGCAA TTCAGTAATT TCACAGAAAC   
  
  
+ CCAGGCCGAT TATGTGGTGA AGAGAACCCC AACAAGAGGA TTTCACGTGG AGAAGCGCCA GGCATCACTC   
  
  
+ ATTCTGAGTT GGCAGAGGCA GGAGCTTGTG GCAGCTTCGG CATGGAAGTG TTG  

- +Up\_Stream \_Len000AGAATT GTAATCAACG AACCAAGTTG GGTAGTCACT CTTATAGACT AAAGTTTTCA   
  
  
- TATCTAAATA TATTTGTATA CCTGATGTCC CTTCCCTCAG CAAAGGAGTT TCAGAATCTA AATGTGAGTT   
  
  
- TTCAAACTCG CTATGGTTAT TACACTAAGG TTTGTACTCG GTTTATAATT CATAGGATAT GATTCTAGTT   
  
  
- CTTCTCTTAC TTGGTTTTCT GACAATTAAA AAGTTAATTC GATTTGAATA ATTGGAAAAA AACCTTATCT   
  
  
- AAAAATTTAA AATAGAAGTT ATATTCAAAA AGGTTCCGCT CTCTGTATAT CGTTAAAATT AATTTCAATT   
  
  
- TCATCATATT GTAAAAAAAA TTACCAATAT CCGAATATCG TGGAAAAGAT CCACTTTTCT CTATAGAACC   
  
  
- GGACTAAAGG TCATTTCAAA AAAAAAATTT ATAACAGTTA ATAAAAACGT TTACTGTAAA CCATTAAACC   
  
  
- CCCTATTAGA CTACACAATT TTCTATGTTA CTTTTATAGT CTTTTATCCT ACGGAGTTTA AAGTGTTCTT   
  
  
- TCTTTTTTAG AAAAACAACA ATCATAAAGA TGTGTAGGTG TTAAAAATGA GTACCATTTT TTTTTTCCCG   
  
  
- GTTTATAGAG TGGTTATATT GGTTTGATAT ACAAGGTAAT AAATCATTGC CACTATTTAT ACATTACCCA   
  
  
- GTTCAATGAT TATTTTATCG GATGTTATGA ACCTATAGAT GTATTTTAAC TATATCTTAT ATATAACGTT   
  
  
- ATGAATTTAG TTTTAATTTA CTTTATAAAT GAAAAAATAA GATAATGTTT ATCGAAGAAT ATCTTATCGT   
  
  
- TGTTAAAAAT TACATTCAAA GTAAGAATGG TCTAAACTTC TCCAGGCCTA TATCTTTTAG AATAAGCATA   
  
  
- TTCATGAGCG TACTCCCGCC TATTTAAGAT TTTATAGTTG GAGTTGAGCG AAGATGATAA GGTAGATTCA   
  
  
- AGTTAAATAG TATATTTTTT TAAGTTAGGT GAAATTACAT GAGTTAAGTG AAGTTGGATA AAATTAGTTA   
  
  
- AATGGGTACT TTTCTTGTTC CAGGATTCAT CTACAAAAGA TAACTAACTA AATTTGTTAA AAGTGGATGG   
  
  
- GTTAATAGGG GTTTTGACTT TCTCCCCCGT TTCCCCCCAC CCATTACGAC ACATTTTATC CCCCTTTCCC   
  
  
- GTTCTTTTGA CACATGTTAC CACCTCGTCC CCATATGGTA AACCGGAACT CAACTCCCCC GTGTTTTCGG   
  
  
- GTCTCTCTCT CTCCCCATTA CGGGTGGTCT TTATTCGTCC TTCGCTCCCT TTTTGTGATC TCTCTTTGTC   
  
  
- TCTTTCTCTC TCTCTCTCTC TCTCTTCCCC TTTAAATGAT CTCTTACTCT CACTTCTTCC CTTGAAATAA   
  
  
- AATTGAAAAA ATACTACGTG CGTAGGTACC GTCTTGTCCC TTCCCTTTCT TCCCATGTCT CATGACGACA   
  
  
- GGGGTTTTTT AATCACATCT TTTGTCCTCT GTGAGTTGAT GTTGTTCCCA TTCCTTCTTC TTGTTCGTTC   
  
  
- TATAGGGTGG GTTTCTTTTT TTTTTTCTTC TTCTTCTTTT AAAAAGTCTC TCCTTTCTTT TCGTTAGTGT   
  
  
- ACTTAATCTT CGGTTGGGTT CGGAAGAAGT TTCTCTTCGT CGTATTGTAT CGTATCGACC CCTCTCCTAT   
  
  
- CCTCTCTCTT TGAGTTCTTT TCCCCATGGG CCGAATTTAC AACTACGTCT TTCCATATCT ATACCGGTGT   
  
  
- GATGATGATG GGTCCTCAAA TAGTAACCTC TACCTAAAGA GAAAGACGGA GTAGAAGAGG AGGTAGGTGG   
  
  
- TCGTTTTGAC CCAAAGGAAA GTCGACCCAG AAAAGATCTA GATCTTTGAT ACCATGGGTT TAGAGGGTAA   
  
  
- GGAGGTAAAA ATAAGAGTTT TAGATAGAAG AATCTGAACA CCCAAACTAA GTAAAGAAAA CCCAAAAACT   
  
  
- TTTCTTAAAT TAAACTAACA TAACACAACT AACCCCTTAA ACACCACCAC AATCTACGCT CGATACGGGA   
  
  
- TGTTAAACGT TCCGTTCCCA CACCATCTCC AAAGTTCGTA AACATAAGGT GTTTAGAGAA GGGGACGAAG   
  
  
- TTTCACCTTC TGGTTGTTGT TGTTGAACCT GTTGTTTAAA GTCGTTGTTG TTCTTCTTTA ACTCGTTAAC   
  
  
- GTCTCATCAC CACCGTGAAA AGAGAGAGGA CTATTGTGGT TATGATTATA ATCATGATGA GGCTCTCGCT   
  
  
- GTTCAAGACA ACTATCGCTT GTGTGTAGAT GAGACCTATG ATGCCCGGGA GGGTGAAGTT GTAGAAGGAG   
  
  
- GAAGTTCTCG TGGAGGTGGC GGTTGTGTCG GCCACACCGC CTGGGGCGTA CATGAGGGTT TCTCCTAACC   
  
  
- CTACAGAGGC CGCCGCGACG ACCGCCACTG CCGCCACCAC CCCCCGAACC TAACCTCCTC ACCCTGTCGT   
  
  
- ACAAGGGTTT ACCCCTCCCC CGAAACGAGG GAACCTAGTA CCCACTTCGA CTACTGTACC CATACCCAAA   
  
  
- CTTCGTAGAA AACGTTAGCC CATTGGGCCA ACTGATACTC CCGTTACGAC CAGATCCCCA ACAGCTAGTC   
  
  
- CCAAGACCTA AACTCTGAGA GAGAGGAGGA GGTGGCGTCG GCCGCCGTAC ACTTAGATTA CCACCACCAC   
  
  
- AACGATTAAA CCCCAAAGGT CCCTTATTAC CATTCTAAAG AAGTTAAAGT GTATTAACAA GTAGACCCTA   
  
  
- AAACTTACCA TTCCACTTGT TATTACCCAA CTTAGGGTTA ACATTGGGAG TTCGAGTTCC GTCAGATGAA   
  
  
- CCAAATTAGG TTCCTCGATG TCCACATGTG CGTGTAGGAC TGCAACCTCT GCTCTTTGGG GTCTAAAACT   
  
  
- TAGGTGTAAA CCACTACTTG GGAGTCGTTC GAGTCTCGTA ACGTTTGGGA TCGAAAAACT ACGTGAGTAA   
  
  
- TCCAATGATA GTCGACCTCG TTGTAGATAA AGTTGGAGTC CGTTTTGCGG ACTTGTGCCC ACGACAAGAC   
  
  
- CTAGGATTGG AACAAGTCGA ACGTTTCTTG GGTAAACGAC TAGTCCCAGT ACTCAATAAC AACTCCTTCG   
  
  
- TCGTGGTTGT CGAACCAAAC GTTGGTAACG GTAACCCAAA CCCAGGAGTC AACCAGGGAG GCGTCTTCGG   
  
  
- ACACTACCCA AGTTTCGTCC CCTTGGGGGT CGTAATGGGC AACGTACAAG TAGTCGTCGA CGTTAACGTT   
  
  
- CTCGTCCGAC AGTTTCTAGT CGAGAAGTTC CGCCGTCTGG AATAAGTTTG ACCTTCAAAG AGTGAGCGCG   
  
  
- TTCTCTATAA CCGCGCCGAG TTAGTGGTCG AGAGGGAGGG ACGTTTCGGG GAGTAATCCC GCCGAAACAT   
  
  
- ACACTTCCTC CGGGAAGTTT ACGAGGAGGA TTACTCGTTG GGTCAACGCC GAGGTGGCAG GTTCTAGGAG   
  
  
- TGGGGAATAC TACAACAAGT GTTCTACTCG CGCATATTCC AGAAGAGACT CCAGACGGGT TAGTGAGTTA   
  
  
- AACACTTAAA GTGAACATGT GTCCGGTAAG AGCTCCGAGA ACTACTAAGA CTACGATAAG TACAACAGCT   
  
  
- GAAACTATAA CCAACGCCAC GAGTTACCCG TAGTAACTAA GTCCTCGACG GTAACTCCTT TTCCCCTCGA   
  
  
- GGGAGAGACT TTTAATGTCG GTATCGAGGG TACAACTGAC CGTCAGTGAA ACTTTAATCG GATCATACAC   
  
  
- TTTTGGAACA CGTTAAACGG TTGCTATAAC CACAACGAAC ACTCGAGGTT CAACAGTTGA AACTAAACAA   
  
  
- ACTAGGTAGA AGCGGTAGTT ACGGCTTGTA ATCATGACGA CTCCTACTCA GTTAACGACA ATCATAGGGG   
  
  
- TAGACCCGTA GAAGTTAATC CGGTAGACAA GAAGGAAGGT AGGAGGCTAA GTAATTCGTT TTTAGGGGGT   
  
  
- TTTAACACCA GAGCAAACTA TCTCCTAAGC TAGCAAAACT ACAGGGAAAG GGGGTTGTAG ACAACGTATG   
  
  
- GGATCTTAGG ACGTCATTAA ATAACCTGAG CGAACTACCA GAGTTACAAC GTAGCCTGTA ACACTCGTTC   
  
  
- CAGCTCTTCA AGAAACAAGT TGGATCCTAG CTTTTACGAC ACAACCCAGC CCAGGTACGG GGACTGTTCT   
  
  
- ACGGGGTAAC CTTTTTAGAG AAACGAAGTC GGCCGAAGAA CGGGAACGTT AAGTCATTAA AGTGTCTTTG   
  
  
- GGTCCGGCTA ATACACCACT TCTCTTGGGG TTGTTCTCCT AAAGTGCACC TCTTCGCGGT CCGTAGTGAG   
  
  
- TAAGACTCAA CCGTCTCCGT CCTCGAACAC CGTCGAAGCC GTACCTTCAC AAC

+     CCGTCC-box

| Site Name | Organism | Position | Strand | Matrix score. | sequence | function |
| --- | --- | --- | --- | --- | --- | --- |
| CCGTCC-box | Petroselinum hortense | 3420 | + | 6 | CCGTCC |  |

>HU04G00047.1   
+ +Up\_Stream \_Len000TCTTAA CATTAGTTGC TTGGTTCAAC CCATCAGTGA GAATATCTGA TTTCAAAAGT   
  
  
+ ATAGATTTAT ATAAACATAT GGACTACAGG GAAGGGAGTC GTTTCCTCAA AGTCTTAGAT TTACACTCAA   
  
  
+ AAGTTTGAGC GATACCAATA ATGTGATTCC AAACATGAGC CAAATATTAA GTATCCTATA CTAAGATCAA   
  
  
+ GAAGAGAATG AACCAAAAGA CTGTTAATTT TTCAATTAAG CTAAACTTAT TAACCTTTTT TTGGAATAGA   
  
  
+ TTTTTAAATT TTATCTTCAA TATAAGTTTT TCCAAGGCGA GAGACATATA GCAATTTTAA TTAAAGTTAA   
  
  
+ AGTAGTATAA CATTTTTTTT AATGGTTATA GGCTTATAGC ACCTTTTCTA GGTGAAAAGA GATATCTTGG   
  
  
+ CCTGATTTCC AGTAAAGTTT TTTTTTTAAA TATTGTCAAT TATTTTTGCA AATGACATTT GGTAATTTGG   
  
  
+ GGGATAATCT GATGTGTTAA AAGATACAAT GAAAATATCA GAAAATAGGA TGCCTCAAAT TTCACAAGAA   
  
  
+ AGAAAAAATC TTTTTGTTGT TAGTATTTCT ACACATCCAC AATTTTTACT CATGGTAAAA AAAAAAGGGC   
  
  
+ CAAATATCTC ACCAATATAA CCAAACTATA TGTTCCATTA TTTAGTAACG GTGATAAATA TGTAATGGGT   
  
  
+ CAAGTTACTA ATAAAATAGC CTACAATACT TGGATATCTA CATAAAATTG ATATAGAATA TATATTGCAA   
  
  
+ TACTTAAATC AAAATTAAAT GAAATATTTA CTTTTTTATT CTATTACAAA TAGCTTCTTA TAGAATAGCA   
  
  
+ ACAATTTTTA ATGTAAGTTT CATTCTTACC AGATTTGAAG AGGTCCGGAT ATAGAAAATC TTATTCGTAT   
  
  
+ AAGTACTCGC ATGAGGGCGG ATAAATTCTA AAATATCAAC CTCAACTCGC TTCTACTATT CCATCTAAGT   
  
  
+ TCAATTTATC ATATAAAAAA ATTCAATCCA CTTTAATGTA CTCAATTCAC TTCAACCTAT TTTAATCAAT   
  
  
+ TTACCCATGA AAAGAACAAG GTCCTAAGTA GATGTTTTCT ATTGATTGAT TTAAACAATT TTCACCTACC   
  
  
+ CAATTATCCC CAAAACTGAA AGAGGGGGCA AAGGGGGGTG GGTAATGCTG TGTAAAATAG GGGGAAAGGG   
  
  
+ CAAGAAAACT GTGTACAATG GTGGAGCAGG GGTATACCAT TTGGCCTTGA GTTGAGGGGG CACAAAAGCC   
  
  
+ CAGAGAGAGA GAGGGGTAAT GCCCACCAGA AATAAGCAGG AAGCGAGGGA AAAACACTAG AGAGAAACAG   
  
  
+ AGAAAGAGAG AGAGAGAGAG AGAGAAGGGG AAATTTACTA GAGAATGAGA GTGAAGAAGG GAACTTTATT   
  
  
+ TTAACTTTTT TATGATGCAC GCATCCATGG CAGAACAGGG AAGGGAAAGA AGGGTACAGA GTACTGCTGT   
  
  
+ CCCCAAAAAA TTAGTGTAGA AAACAGGAGA CACTCAACTA CAACAAGGGT AAGGAAGAAG AACAAGCAAG   
  
  
+ ATATCCCACC CAAAGAAAAA AAAAAAGAAG AAGAAGAAAA TTTTTCAGAG AGGAAAGAAA AGCAATCACA   
  
  
+ TGAATTAGAA GCCAACCCAA GCCTTCTTCA AAGAGAAGCA GCATAACATA GCATAGCTGG GGAGAGGATA   
  
  
+ GGAGAGAGAA ACTCAAGAAA AGGGGTACCC GGCTTAAATG TTGATGCAGA AAGGTATAGA TATGGCCACA   
  
  
+ CTACTACTAC CCAGGAGTTT ATCATTGGAG ATGGATTTCT CTTTCTGCCT CATCTTCTCC TCCATCCACC   
  
  
+ AGCAAAACTG GGTTTCCTTT CAGCTGGGTC TTTTCTAGAT CTAGAAACTA TGGTACCCAA ATCTCCCATT   
  
  
+ CCTCCATTTT TATTCTCAAA ATCTATCTTC TTAGACTTGT GGGTTTGATT CATTTCTTTT GGGTTTTTGA   
  
  
+ AAAGAATTTA ATTTGATTGT ATTGTGTTGA TTGGGGAATT TGTGGTGGTG TTAGATGCGA GCTATGCCCT   
  
  
+ ACAATTTGCA AGGCAAGGGT GTGGTAGAGG TTTCAAGCAT TTGTATTCCA CAAATCTCTT CCCCTGCTTC   
  
  
+ AAAGTGGAAG ACCAACAACA ACAACTTGGA CAACAAATTT CAGCAACAAC AAGAAGAAAT TGAGCAATTG   
  
  
+ CAGAGTAGTG GTGGCACTTT TCTCTCTCCT GATAACACCA ATACTAATAT TAGTACTACT CCGAGAGCGA   
  
  
+ CAAGTTCTGT TGATAGCGAA CACACATCTA CTCTGGATAC TACGGGCCCT CCCACTTCAA CATCTTCCTC   
  
  
+ CTTCAAGAGC ACCTCCACCG CCAACACAGC CGGTGTGGCG GACCCCGCAT GTACTCCCAA AGAGGATTGG   
  
  
+ GATGTCTCCG GCGGCGCTGC TGGCGGTGAC GGCGGTGGTG GGGGGCTTGG ATTGGAGGAG TGGGACAGCA   
  
  
+ TGTTCCCAAA TGGGGAGGGG GCTTTGCTCC CTTGGATCAT GGGTGAAGCT GATGACATGG GTATGGGTTT   
  
  
+ GAAGCATCTT TTGCAATCGG GTAACCCGGT TGACTATGAG GGCAATGCTG GTCTAGGGGT TGTCGATCAG   
  
  
+ GGTTCTGGAT TTGAGACTCT CTCTCCTCCT CCACCGCAGC CGGCGGCATG TGAATCTAAT GGTGGTGGTG   
  
  
+ TTGCTAATTT GGGGTTTCCA GGGAATAATG GTAAGATTTC TTCAATTTCA CATAATTGTT CATCTGGGAT   
  
  
+ TTTGAATGGT AAGGTGAACA ATAATGGGTT GAATCCCAAT TGTAACCCTC AAGCTCAAGG CAGTCTACTT   
  
  
+ GGTTTAATCC AAGGAGCTAC AGGTGTACAC GCACATCCTG ACGTTGGAGA CGAGAAACCC CAGATTTTGA   
  
  
+ ATCCACATTT GGTGATGAAC CCTCAGCAAG CTCAGAGCAT TGCAAACCCT AGCTTTTTGA TGCACTCATT   
  
  
+ AGGTTACTAT CAGCTGGAGC AACATCTATT TCAACCTCAG GCAAAACGCC TGAACACGGG TGCTGTTCTG   
  
  
+ GATCCTAACC TTGTTCAGCT TGCAAAGAAC CCATTTGCTG ATCAGGGTCA TGAGTTATTG TTGAGGAAGC   
  
  
+ AGCACCAACA GCTTGGTTTG CAACCATTGC CATTGGGTTT GGGTCCTCAG TTGGTCCCTC CGCAGAAGCC   
  
  
+ TGTGATGGGT TCAAAGCAGG GGAACCCCCA GCATTACCCG TTGCATGTTC ATCAGCAGCT GCAATTGCAA   
  
  
+ GAGCAGGCTG TCAAAGATCA GCTCTTCAAG GCGGCAGACC TTATTCAAAC TGGAAGTTTC TCACTCGCGC   
  
  
+ AAGAGATATT GGCGCGGCTC AATCACCAGC TCTCCCTCCC TGCAAAGCCC CTCATTAGGG CGGCTTTGTA   
  
  
+ TGTGAAGGAG GCCCTTCAAA TGCTCCTCCT AATGAGCAAC CCAGTTGCGG CTCCACCGTC CAAGATCCTC   
  
  
+ ACCCCTTATG ATGTTGTTCA CAAGATGAGC GCGTATAAGG TCTTCTCTGA GGTCTGCCCA ATCACTCAAT   
  
  
+ TTGTGAATTT CACTTGTACA CAGGCCATTC TCGAGGCTCT TGATGATTCT GATGCTATTC ATGTTGTCGA   
  
  
+ CTTTGATATT GGTTGCGGTG CTCAATGGGC ATCATTGATT CAGGAGCTGC CATTGAGGAA AAGGGGAGCT   
  
  
+ CCCTCTCTGA AAATTACAGC CATAGCTCCC ATGTTGACTG GCAGTCACTT TGAAATTAGC CTAGTATGTG   
  
  
+ AAAACCTTGT GCAATTTGCC AACGATATTG GTGTTGCTTG TGAGCTCCAA GTTGTCAACT TTGATTTGTT   
  
  
+ TGATCCATCT TCGCCATCAA TGCCGAACAT TAGTACTGCT GAGGATGAGT CAATTGCTGT TAGTATCCCC   
  
  
+ ATCTGGGCAT CTTCAATTAG GCCATCTGTT CTTCCTTCCA TCCTCCGATT CATTAAGCAA AAATCCCCCA   
  
  
+ AAATTGTGGT CTCGTTTGAT AGAGGATTCG ATCGTTTTGA TGTCCCTTTC CCCCAACATC TGTTGCATAC   
  
  
+ CCTAGAATCC TGCAGTAATT TATTGGACTC GCTTGATGGT CTCAATGTTG CATCGGACAT TGTGAGCAAG   
  
  
+ GTCGAGAAGT TCTTTGTTCA ACCTAGGATC GAAAATGCTG TGTTGGGTCG GGTCCATGCC CCTGACAAGA   
  
  
+ TGCCCCATTG GAAAAATCTC TTTGCTTCAG CCGGCTTCTT GCCCTTGCAA TTCAGTAATT TCACAGAAAC   
  
  
+ CCAGGCCGAT TATGTGGTGA AGAGAACCCC AACAAGAGGA TTTCACGTGG AGAAGCGCCA GGCATCACTC   
  
  
+ ATTCTGAGTT GGCAGAGGCA GGAGCTTGTG GCAGCTTCGG CATGGAAGTG TTG  

- +Up\_Stream \_Len000AGAATT GTAATCAACG AACCAAGTTG GGTAGTCACT CTTATAGACT AAAGTTTTCA   
  
  
- TATCTAAATA TATTTGTATA CCTGATGTCC CTTCCCTCAG CAAAGGAGTT TCAGAATCTA AATGTGAGTT   
  
  
- TTCAAACTCG CTATGGTTAT TACACTAAGG TTTGTACTCG GTTTATAATT CATAGGATAT GATTCTAGTT   
  
  
- CTTCTCTTAC TTGGTTTTCT GACAATTAAA AAGTTAATTC GATTTGAATA ATTGGAAAAA AACCTTATCT   
  
  
- AAAAATTTAA AATAGAAGTT ATATTCAAAA AGGTTCCGCT CTCTGTATAT CGTTAAAATT AATTTCAATT   
  
  
- TCATCATATT GTAAAAAAAA TTACCAATAT CCGAATATCG TGGAAAAGAT CCACTTTTCT CTATAGAACC   
  
  
- GGACTAAAGG TCATTTCAAA AAAAAAATTT ATAACAGTTA ATAAAAACGT TTACTGTAAA CCATTAAACC   
  
  
- CCCTATTAGA CTACACAATT TTCTATGTTA CTTTTATAGT CTTTTATCCT ACGGAGTTTA AAGTGTTCTT   
  
  
- TCTTTTTTAG AAAAACAACA ATCATAAAGA TGTGTAGGTG TTAAAAATGA GTACCATTTT TTTTTTCCCG   
  
  
- GTTTATAGAG TGGTTATATT GGTTTGATAT ACAAGGTAAT AAATCATTGC CACTATTTAT ACATTACCCA   
  
  
- GTTCAATGAT TATTTTATCG GATGTTATGA ACCTATAGAT GTATTTTAAC TATATCTTAT ATATAACGTT   
  
  
- ATGAATTTAG TTTTAATTTA CTTTATAAAT GAAAAAATAA GATAATGTTT ATCGAAGAAT ATCTTATCGT   
  
  
- TGTTAAAAAT TACATTCAAA GTAAGAATGG TCTAAACTTC TCCAGGCCTA TATCTTTTAG AATAAGCATA   
  
  
- TTCATGAGCG TACTCCCGCC TATTTAAGAT TTTATAGTTG GAGTTGAGCG AAGATGATAA GGTAGATTCA   
  
  
- AGTTAAATAG TATATTTTTT TAAGTTAGGT GAAATTACAT GAGTTAAGTG AAGTTGGATA AAATTAGTTA   
  
  
- AATGGGTACT TTTCTTGTTC CAGGATTCAT CTACAAAAGA TAACTAACTA AATTTGTTAA AAGTGGATGG   
  
  
- GTTAATAGGG GTTTTGACTT TCTCCCCCGT TTCCCCCCAC CCATTACGAC ACATTTTATC CCCCTTTCCC   
  
  
- GTTCTTTTGA CACATGTTAC CACCTCGTCC CCATATGGTA AACCGGAACT CAACTCCCCC GTGTTTTCGG   
  
  
- GTCTCTCTCT CTCCCCATTA CGGGTGGTCT TTATTCGTCC TTCGCTCCCT TTTTGTGATC TCTCTTTGTC   
  
  
- TCTTTCTCTC TCTCTCTCTC TCTCTTCCCC TTTAAATGAT CTCTTACTCT CACTTCTTCC CTTGAAATAA   
  
  
- AATTGAAAAA ATACTACGTG CGTAGGTACC GTCTTGTCCC TTCCCTTTCT TCCCATGTCT CATGACGACA   
  
  
- GGGGTTTTTT AATCACATCT TTTGTCCTCT GTGAGTTGAT GTTGTTCCCA TTCCTTCTTC TTGTTCGTTC   
  
  
- TATAGGGTGG GTTTCTTTTT TTTTTTCTTC TTCTTCTTTT AAAAAGTCTC TCCTTTCTTT TCGTTAGTGT   
  
  
- ACTTAATCTT CGGTTGGGTT CGGAAGAAGT TTCTCTTCGT CGTATTGTAT CGTATCGACC CCTCTCCTAT   
  
  
- CCTCTCTCTT TGAGTTCTTT TCCCCATGGG CCGAATTTAC AACTACGTCT TTCCATATCT ATACCGGTGT   
  
  
- GATGATGATG GGTCCTCAAA TAGTAACCTC TACCTAAAGA GAAAGACGGA GTAGAAGAGG AGGTAGGTGG   
  
  
- TCGTTTTGAC CCAAAGGAAA GTCGACCCAG AAAAGATCTA GATCTTTGAT ACCATGGGTT TAGAGGGTAA   
  
  
- GGAGGTAAAA ATAAGAGTTT TAGATAGAAG AATCTGAACA CCCAAACTAA GTAAAGAAAA CCCAAAAACT   
  
  
- TTTCTTAAAT TAAACTAACA TAACACAACT AACCCCTTAA ACACCACCAC AATCTACGCT CGATACGGGA   
  
  
- TGTTAAACGT TCCGTTCCCA CACCATCTCC AAAGTTCGTA AACATAAGGT GTTTAGAGAA GGGGACGAAG   
  
  
- TTTCACCTTC TGGTTGTTGT TGTTGAACCT GTTGTTTAAA GTCGTTGTTG TTCTTCTTTA ACTCGTTAAC   
  
  
- GTCTCATCAC CACCGTGAAA AGAGAGAGGA CTATTGTGGT TATGATTATA ATCATGATGA GGCTCTCGCT   
  
  
- GTTCAAGACA ACTATCGCTT GTGTGTAGAT GAGACCTATG ATGCCCGGGA GGGTGAAGTT GTAGAAGGAG   
  
  
- GAAGTTCTCG TGGAGGTGGC GGTTGTGTCG GCCACACCGC CTGGGGCGTA CATGAGGGTT TCTCCTAACC   
  
  
- CTACAGAGGC CGCCGCGACG ACCGCCACTG CCGCCACCAC CCCCCGAACC TAACCTCCTC ACCCTGTCGT   
  
  
- ACAAGGGTTT ACCCCTCCCC CGAAACGAGG GAACCTAGTA CCCACTTCGA CTACTGTACC CATACCCAAA   
  
  
- CTTCGTAGAA AACGTTAGCC CATTGGGCCA ACTGATACTC CCGTTACGAC CAGATCCCCA ACAGCTAGTC   
  
  
- CCAAGACCTA AACTCTGAGA GAGAGGAGGA GGTGGCGTCG GCCGCCGTAC ACTTAGATTA CCACCACCAC   
  
  
- AACGATTAAA CCCCAAAGGT CCCTTATTAC CATTCTAAAG AAGTTAAAGT GTATTAACAA GTAGACCCTA   
  
  
- AAACTTACCA TTCCACTTGT TATTACCCAA CTTAGGGTTA ACATTGGGAG TTCGAGTTCC GTCAGATGAA   
  
  
- CCAAATTAGG TTCCTCGATG TCCACATGTG CGTGTAGGAC TGCAACCTCT GCTCTTTGGG GTCTAAAACT   
  
  
- TAGGTGTAAA CCACTACTTG GGAGTCGTTC GAGTCTCGTA ACGTTTGGGA TCGAAAAACT ACGTGAGTAA   
  
  
- TCCAATGATA GTCGACCTCG TTGTAGATAA AGTTGGAGTC CGTTTTGCGG ACTTGTGCCC ACGACAAGAC   
  
  
- CTAGGATTGG AACAAGTCGA ACGTTTCTTG GGTAAACGAC TAGTCCCAGT ACTCAATAAC AACTCCTTCG   
  
  
- TCGTGGTTGT CGAACCAAAC GTTGGTAACG GTAACCCAAA CCCAGGAGTC AACCAGGGAG GCGTCTTCGG   
  
  
- ACACTACCCA AGTTTCGTCC CCTTGGGGGT CGTAATGGGC AACGTACAAG TAGTCGTCGA CGTTAACGTT   
  
  
- CTCGTCCGAC AGTTTCTAGT CGAGAAGTTC CGCCGTCTGG AATAAGTTTG ACCTTCAAAG AGTGAGCGCG   
  
  
- TTCTCTATAA CCGCGCCGAG TTAGTGGTCG AGAGGGAGGG ACGTTTCGGG GAGTAATCCC GCCGAAACAT   
  
  
- ACACTTCCTC CGGGAAGTTT ACGAGGAGGA TTACTCGTTG GGTCAACGCC GAGGTGGCAG GTTCTAGGAG   
  
  
- TGGGGAATAC TACAACAAGT GTTCTACTCG CGCATATTCC AGAAGAGACT CCAGACGGGT TAGTGAGTTA   
  
  
- AACACTTAAA GTGAACATGT GTCCGGTAAG AGCTCCGAGA ACTACTAAGA CTACGATAAG TACAACAGCT   
  
  
- GAAACTATAA CCAACGCCAC GAGTTACCCG TAGTAACTAA GTCCTCGACG GTAACTCCTT TTCCCCTCGA   
  
  
- GGGAGAGACT TTTAATGTCG GTATCGAGGG TACAACTGAC CGTCAGTGAA ACTTTAATCG GATCATACAC   
  
  
- TTTTGGAACA CGTTAAACGG TTGCTATAAC CACAACGAAC ACTCGAGGTT CAACAGTTGA AACTAAACAA   
  
  
- ACTAGGTAGA AGCGGTAGTT ACGGCTTGTA ATCATGACGA CTCCTACTCA GTTAACGACA ATCATAGGGG   
  
  
- TAGACCCGTA GAAGTTAATC CGGTAGACAA GAAGGAAGGT AGGAGGCTAA GTAATTCGTT TTTAGGGGGT   
  
  
- TTTAACACCA GAGCAAACTA TCTCCTAAGC TAGCAAAACT ACAGGGAAAG GGGGTTGTAG ACAACGTATG   
  
  
- GGATCTTAGG ACGTCATTAA ATAACCTGAG CGAACTACCA GAGTTACAAC GTAGCCTGTA ACACTCGTTC   
  
  
- CAGCTCTTCA AGAAACAAGT TGGATCCTAG CTTTTACGAC ACAACCCAGC CCAGGTACGG GGACTGTTCT   
  
  
- ACGGGGTAAC CTTTTTAGAG AAACGAAGTC GGCCGAAGAA CGGGAACGTT AAGTCATTAA AGTGTCTTTG   
  
  
- GGTCCGGCTA ATACACCACT TCTCTTGGGG TTGTTCTCCT AAAGTGCACC TCTTCGCGGT CCGTAGTGAG   
  
  
- TAAGACTCAA CCGTCTCCGT CCTCGAACAC CGTCGAAGCC GTACCTTCAC AAC

+     CGTCA-motif

| Site Name | Organism | Position | Strand | Matrix score. | sequence | function |
| --- | --- | --- | --- | --- | --- | --- |
| CGTCA-motif | Hordeum vulgare | 2843 | - | 5 | CGTCA | cis-acting regulatory element involved in the MeJA-responsiveness |
| CGTCA-motif | Hordeum vulgare | 2411 | - | 5 | CGTCA | cis-acting regulatory element involved in the MeJA-responsiveness |

>HU04G00047.1   
+ +Up\_Stream \_Len000TCTTAA CATTAGTTGC TTGGTTCAAC CCATCAGTGA GAATATCTGA TTTCAAAAGT   
  
  
+ ATAGATTTAT ATAAACATAT GGACTACAGG GAAGGGAGTC GTTTCCTCAA AGTCTTAGAT TTACACTCAA   
  
  
+ AAGTTTGAGC GATACCAATA ATGTGATTCC AAACATGAGC CAAATATTAA GTATCCTATA CTAAGATCAA   
  
  
+ GAAGAGAATG AACCAAAAGA CTGTTAATTT TTCAATTAAG CTAAACTTAT TAACCTTTTT TTGGAATAGA   
  
  
+ TTTTTAAATT TTATCTTCAA TATAAGTTTT TCCAAGGCGA GAGACATATA GCAATTTTAA TTAAAGTTAA   
  
  
+ AGTAGTATAA CATTTTTTTT AATGGTTATA GGCTTATAGC ACCTTTTCTA GGTGAAAAGA GATATCTTGG   
  
  
+ CCTGATTTCC AGTAAAGTTT TTTTTTTAAA TATTGTCAAT TATTTTTGCA AATGACATTT GGTAATTTGG   
  
  
+ GGGATAATCT GATGTGTTAA AAGATACAAT GAAAATATCA GAAAATAGGA TGCCTCAAAT TTCACAAGAA   
  
  
+ AGAAAAAATC TTTTTGTTGT TAGTATTTCT ACACATCCAC AATTTTTACT CATGGTAAAA AAAAAAGGGC   
  
  
+ CAAATATCTC ACCAATATAA CCAAACTATA TGTTCCATTA TTTAGTAACG GTGATAAATA TGTAATGGGT   
  
  
+ CAAGTTACTA ATAAAATAGC CTACAATACT TGGATATCTA CATAAAATTG ATATAGAATA TATATTGCAA   
  
  
+ TACTTAAATC AAAATTAAAT GAAATATTTA CTTTTTTATT CTATTACAAA TAGCTTCTTA TAGAATAGCA   
  
  
+ ACAATTTTTA ATGTAAGTTT CATTCTTACC AGATTTGAAG AGGTCCGGAT ATAGAAAATC TTATTCGTAT   
  
  
+ AAGTACTCGC ATGAGGGCGG ATAAATTCTA AAATATCAAC CTCAACTCGC TTCTACTATT CCATCTAAGT   
  
  
+ TCAATTTATC ATATAAAAAA ATTCAATCCA CTTTAATGTA CTCAATTCAC TTCAACCTAT TTTAATCAAT   
  
  
+ TTACCCATGA AAAGAACAAG GTCCTAAGTA GATGTTTTCT ATTGATTGAT TTAAACAATT TTCACCTACC   
  
  
+ CAATTATCCC CAAAACTGAA AGAGGGGGCA AAGGGGGGTG GGTAATGCTG TGTAAAATAG GGGGAAAGGG   
  
  
+ CAAGAAAACT GTGTACAATG GTGGAGCAGG GGTATACCAT TTGGCCTTGA GTTGAGGGGG CACAAAAGCC   
  
  
+ CAGAGAGAGA GAGGGGTAAT GCCCACCAGA AATAAGCAGG AAGCGAGGGA AAAACACTAG AGAGAAACAG   
  
  
+ AGAAAGAGAG AGAGAGAGAG AGAGAAGGGG AAATTTACTA GAGAATGAGA GTGAAGAAGG GAACTTTATT   
  
  
+ TTAACTTTTT TATGATGCAC GCATCCATGG CAGAACAGGG AAGGGAAAGA AGGGTACAGA GTACTGCTGT   
  
  
+ CCCCAAAAAA TTAGTGTAGA AAACAGGAGA CACTCAACTA CAACAAGGGT AAGGAAGAAG AACAAGCAAG   
  
  
+ ATATCCCACC CAAAGAAAAA AAAAAAGAAG AAGAAGAAAA TTTTTCAGAG AGGAAAGAAA AGCAATCACA   
  
  
+ TGAATTAGAA GCCAACCCAA GCCTTCTTCA AAGAGAAGCA GCATAACATA GCATAGCTGG GGAGAGGATA   
  
  
+ GGAGAGAGAA ACTCAAGAAA AGGGGTACCC GGCTTAAATG TTGATGCAGA AAGGTATAGA TATGGCCACA   
  
  
+ CTACTACTAC CCAGGAGTTT ATCATTGGAG ATGGATTTCT CTTTCTGCCT CATCTTCTCC TCCATCCACC   
  
  
+ AGCAAAACTG GGTTTCCTTT CAGCTGGGTC TTTTCTAGAT CTAGAAACTA TGGTACCCAA ATCTCCCATT   
  
  
+ CCTCCATTTT TATTCTCAAA ATCTATCTTC TTAGACTTGT GGGTTTGATT CATTTCTTTT GGGTTTTTGA   
  
  
+ AAAGAATTTA ATTTGATTGT ATTGTGTTGA TTGGGGAATT TGTGGTGGTG TTAGATGCGA GCTATGCCCT   
  
  
+ ACAATTTGCA AGGCAAGGGT GTGGTAGAGG TTTCAAGCAT TTGTATTCCA CAAATCTCTT CCCCTGCTTC   
  
  
+ AAAGTGGAAG ACCAACAACA ACAACTTGGA CAACAAATTT CAGCAACAAC AAGAAGAAAT TGAGCAATTG   
  
  
+ CAGAGTAGTG GTGGCACTTT TCTCTCTCCT GATAACACCA ATACTAATAT TAGTACTACT CCGAGAGCGA   
  
  
+ CAAGTTCTGT TGATAGCGAA CACACATCTA CTCTGGATAC TACGGGCCCT CCCACTTCAA CATCTTCCTC   
  
  
+ CTTCAAGAGC ACCTCCACCG CCAACACAGC CGGTGTGGCG GACCCCGCAT GTACTCCCAA AGAGGATTGG   
  
  
+ GATGTCTCCG GCGGCGCTGC TGGCGGTGAC GGCGGTGGTG GGGGGCTTGG ATTGGAGGAG TGGGACAGCA   
  
  
+ TGTTCCCAAA TGGGGAGGGG GCTTTGCTCC CTTGGATCAT GGGTGAAGCT GATGACATGG GTATGGGTTT   
  
  
+ GAAGCATCTT TTGCAATCGG GTAACCCGGT TGACTATGAG GGCAATGCTG GTCTAGGGGT TGTCGATCAG   
  
  
+ GGTTCTGGAT TTGAGACTCT CTCTCCTCCT CCACCGCAGC CGGCGGCATG TGAATCTAAT GGTGGTGGTG   
  
  
+ TTGCTAATTT GGGGTTTCCA GGGAATAATG GTAAGATTTC TTCAATTTCA CATAATTGTT CATCTGGGAT   
  
  
+ TTTGAATGGT AAGGTGAACA ATAATGGGTT GAATCCCAAT TGTAACCCTC AAGCTCAAGG CAGTCTACTT   
  
  
+ GGTTTAATCC AAGGAGCTAC AGGTGTACAC GCACATCCTG ACGTTGGAGA CGAGAAACCC CAGATTTTGA   
  
  
+ ATCCACATTT GGTGATGAAC CCTCAGCAAG CTCAGAGCAT TGCAAACCCT AGCTTTTTGA TGCACTCATT   
  
  
+ AGGTTACTAT CAGCTGGAGC AACATCTATT TCAACCTCAG GCAAAACGCC TGAACACGGG TGCTGTTCTG   
  
  
+ GATCCTAACC TTGTTCAGCT TGCAAAGAAC CCATTTGCTG ATCAGGGTCA TGAGTTATTG TTGAGGAAGC   
  
  
+ AGCACCAACA GCTTGGTTTG CAACCATTGC CATTGGGTTT GGGTCCTCAG TTGGTCCCTC CGCAGAAGCC   
  
  
+ TGTGATGGGT TCAAAGCAGG GGAACCCCCA GCATTACCCG TTGCATGTTC ATCAGCAGCT GCAATTGCAA   
  
  
+ GAGCAGGCTG TCAAAGATCA GCTCTTCAAG GCGGCAGACC TTATTCAAAC TGGAAGTTTC TCACTCGCGC   
  
  
+ AAGAGATATT GGCGCGGCTC AATCACCAGC TCTCCCTCCC TGCAAAGCCC CTCATTAGGG CGGCTTTGTA   
  
  
+ TGTGAAGGAG GCCCTTCAAA TGCTCCTCCT AATGAGCAAC CCAGTTGCGG CTCCACCGTC CAAGATCCTC   
  
  
+ ACCCCTTATG ATGTTGTTCA CAAGATGAGC GCGTATAAGG TCTTCTCTGA GGTCTGCCCA ATCACTCAAT   
  
  
+ TTGTGAATTT CACTTGTACA CAGGCCATTC TCGAGGCTCT TGATGATTCT GATGCTATTC ATGTTGTCGA   
  
  
+ CTTTGATATT GGTTGCGGTG CTCAATGGGC ATCATTGATT CAGGAGCTGC CATTGAGGAA AAGGGGAGCT   
  
  
+ CCCTCTCTGA AAATTACAGC CATAGCTCCC ATGTTGACTG GCAGTCACTT TGAAATTAGC CTAGTATGTG   
  
  
+ AAAACCTTGT GCAATTTGCC AACGATATTG GTGTTGCTTG TGAGCTCCAA GTTGTCAACT TTGATTTGTT   
  
  
+ TGATCCATCT TCGCCATCAA TGCCGAACAT TAGTACTGCT GAGGATGAGT CAATTGCTGT TAGTATCCCC   
  
  
+ ATCTGGGCAT CTTCAATTAG GCCATCTGTT CTTCCTTCCA TCCTCCGATT CATTAAGCAA AAATCCCCCA   
  
  
+ AAATTGTGGT CTCGTTTGAT AGAGGATTCG ATCGTTTTGA TGTCCCTTTC CCCCAACATC TGTTGCATAC   
  
  
+ CCTAGAATCC TGCAGTAATT TATTGGACTC GCTTGATGGT CTCAATGTTG CATCGGACAT TGTGAGCAAG   
  
  
+ GTCGAGAAGT TCTTTGTTCA ACCTAGGATC GAAAATGCTG TGTTGGGTCG GGTCCATGCC CCTGACAAGA   
  
  
+ TGCCCCATTG GAAAAATCTC TTTGCTTCAG CCGGCTTCTT GCCCTTGCAA TTCAGTAATT TCACAGAAAC   
  
  
+ CCAGGCCGAT TATGTGGTGA AGAGAACCCC AACAAGAGGA TTTCACGTGG AGAAGCGCCA GGCATCACTC   
  
  
+ ATTCTGAGTT GGCAGAGGCA GGAGCTTGTG GCAGCTTCGG CATGGAAGTG TTG  

- +Up\_Stream \_Len000AGAATT GTAATCAACG AACCAAGTTG GGTAGTCACT CTTATAGACT AAAGTTTTCA   
  
  
- TATCTAAATA TATTTGTATA CCTGATGTCC CTTCCCTCAG CAAAGGAGTT TCAGAATCTA AATGTGAGTT   
  
  
- TTCAAACTCG CTATGGTTAT TACACTAAGG TTTGTACTCG GTTTATAATT CATAGGATAT GATTCTAGTT   
  
  
- CTTCTCTTAC TTGGTTTTCT GACAATTAAA AAGTTAATTC GATTTGAATA ATTGGAAAAA AACCTTATCT   
  
  
- AAAAATTTAA AATAGAAGTT ATATTCAAAA AGGTTCCGCT CTCTGTATAT CGTTAAAATT AATTTCAATT   
  
  
- TCATCATATT GTAAAAAAAA TTACCAATAT CCGAATATCG TGGAAAAGAT CCACTTTTCT CTATAGAACC   
  
  
- GGACTAAAGG TCATTTCAAA AAAAAAATTT ATAACAGTTA ATAAAAACGT TTACTGTAAA CCATTAAACC   
  
  
- CCCTATTAGA CTACACAATT TTCTATGTTA CTTTTATAGT CTTTTATCCT ACGGAGTTTA AAGTGTTCTT   
  
  
- TCTTTTTTAG AAAAACAACA ATCATAAAGA TGTGTAGGTG TTAAAAATGA GTACCATTTT TTTTTTCCCG   
  
  
- GTTTATAGAG TGGTTATATT GGTTTGATAT ACAAGGTAAT AAATCATTGC CACTATTTAT ACATTACCCA   
  
  
- GTTCAATGAT TATTTTATCG GATGTTATGA ACCTATAGAT GTATTTTAAC TATATCTTAT ATATAACGTT   
  
  
- ATGAATTTAG TTTTAATTTA CTTTATAAAT GAAAAAATAA GATAATGTTT ATCGAAGAAT ATCTTATCGT   
  
  
- TGTTAAAAAT TACATTCAAA GTAAGAATGG TCTAAACTTC TCCAGGCCTA TATCTTTTAG AATAAGCATA   
  
  
- TTCATGAGCG TACTCCCGCC TATTTAAGAT TTTATAGTTG GAGTTGAGCG AAGATGATAA GGTAGATTCA   
  
  
- AGTTAAATAG TATATTTTTT TAAGTTAGGT GAAATTACAT GAGTTAAGTG AAGTTGGATA AAATTAGTTA   
  
  
- AATGGGTACT TTTCTTGTTC CAGGATTCAT CTACAAAAGA TAACTAACTA AATTTGTTAA AAGTGGATGG   
  
  
- GTTAATAGGG GTTTTGACTT TCTCCCCCGT TTCCCCCCAC CCATTACGAC ACATTTTATC CCCCTTTCCC   
  
  
- GTTCTTTTGA CACATGTTAC CACCTCGTCC CCATATGGTA AACCGGAACT CAACTCCCCC GTGTTTTCGG   
  
  
- GTCTCTCTCT CTCCCCATTA CGGGTGGTCT TTATTCGTCC TTCGCTCCCT TTTTGTGATC TCTCTTTGTC   
  
  
- TCTTTCTCTC TCTCTCTCTC TCTCTTCCCC TTTAAATGAT CTCTTACTCT CACTTCTTCC CTTGAAATAA   
  
  
- AATTGAAAAA ATACTACGTG CGTAGGTACC GTCTTGTCCC TTCCCTTTCT TCCCATGTCT CATGACGACA   
  
  
- GGGGTTTTTT AATCACATCT TTTGTCCTCT GTGAGTTGAT GTTGTTCCCA TTCCTTCTTC TTGTTCGTTC   
  
  
- TATAGGGTGG GTTTCTTTTT TTTTTTCTTC TTCTTCTTTT AAAAAGTCTC TCCTTTCTTT TCGTTAGTGT   
  
  
- ACTTAATCTT CGGTTGGGTT CGGAAGAAGT TTCTCTTCGT CGTATTGTAT CGTATCGACC CCTCTCCTAT   
  
  
- CCTCTCTCTT TGAGTTCTTT TCCCCATGGG CCGAATTTAC AACTACGTCT TTCCATATCT ATACCGGTGT   
  
  
- GATGATGATG GGTCCTCAAA TAGTAACCTC TACCTAAAGA GAAAGACGGA GTAGAAGAGG AGGTAGGTGG   
  
  
- TCGTTTTGAC CCAAAGGAAA GTCGACCCAG AAAAGATCTA GATCTTTGAT ACCATGGGTT TAGAGGGTAA   
  
  
- GGAGGTAAAA ATAAGAGTTT TAGATAGAAG AATCTGAACA CCCAAACTAA GTAAAGAAAA CCCAAAAACT   
  
  
- TTTCTTAAAT TAAACTAACA TAACACAACT AACCCCTTAA ACACCACCAC AATCTACGCT CGATACGGGA   
  
  
- TGTTAAACGT TCCGTTCCCA CACCATCTCC AAAGTTCGTA AACATAAGGT GTTTAGAGAA GGGGACGAAG   
  
  
- TTTCACCTTC TGGTTGTTGT TGTTGAACCT GTTGTTTAAA GTCGTTGTTG TTCTTCTTTA ACTCGTTAAC   
  
  
- GTCTCATCAC CACCGTGAAA AGAGAGAGGA CTATTGTGGT TATGATTATA ATCATGATGA GGCTCTCGCT   
  
  
- GTTCAAGACA ACTATCGCTT GTGTGTAGAT GAGACCTATG ATGCCCGGGA GGGTGAAGTT GTAGAAGGAG   
  
  
- GAAGTTCTCG TGGAGGTGGC GGTTGTGTCG GCCACACCGC CTGGGGCGTA CATGAGGGTT TCTCCTAACC   
  
  
- CTACAGAGGC CGCCGCGACG ACCGCCACTG CCGCCACCAC CCCCCGAACC TAACCTCCTC ACCCTGTCGT   
  
  
- ACAAGGGTTT ACCCCTCCCC CGAAACGAGG GAACCTAGTA CCCACTTCGA CTACTGTACC CATACCCAAA   
  
  
- CTTCGTAGAA AACGTTAGCC CATTGGGCCA ACTGATACTC CCGTTACGAC CAGATCCCCA ACAGCTAGTC   
  
  
- CCAAGACCTA AACTCTGAGA GAGAGGAGGA GGTGGCGTCG GCCGCCGTAC ACTTAGATTA CCACCACCAC   
  
  
- AACGATTAAA CCCCAAAGGT CCCTTATTAC CATTCTAAAG AAGTTAAAGT GTATTAACAA GTAGACCCTA   
  
  
- AAACTTACCA TTCCACTTGT TATTACCCAA CTTAGGGTTA ACATTGGGAG TTCGAGTTCC GTCAGATGAA   
  
  
- CCAAATTAGG TTCCTCGATG TCCACATGTG CGTGTAGGAC TGCAACCTCT GCTCTTTGGG GTCTAAAACT   
  
  
- TAGGTGTAAA CCACTACTTG GGAGTCGTTC GAGTCTCGTA ACGTTTGGGA TCGAAAAACT ACGTGAGTAA   
  
  
- TCCAATGATA GTCGACCTCG TTGTAGATAA AGTTGGAGTC CGTTTTGCGG ACTTGTGCCC ACGACAAGAC   
  
  
- CTAGGATTGG AACAAGTCGA ACGTTTCTTG GGTAAACGAC TAGTCCCAGT ACTCAATAAC AACTCCTTCG   
  
  
- TCGTGGTTGT CGAACCAAAC GTTGGTAACG GTAACCCAAA CCCAGGAGTC AACCAGGGAG GCGTCTTCGG   
  
  
- ACACTACCCA AGTTTCGTCC CCTTGGGGGT CGTAATGGGC AACGTACAAG TAGTCGTCGA CGTTAACGTT   
  
  
- CTCGTCCGAC AGTTTCTAGT CGAGAAGTTC CGCCGTCTGG AATAAGTTTG ACCTTCAAAG AGTGAGCGCG   
  
  
- TTCTCTATAA CCGCGCCGAG TTAGTGGTCG AGAGGGAGGG ACGTTTCGGG GAGTAATCCC GCCGAAACAT   
  
  
- ACACTTCCTC CGGGAAGTTT ACGAGGAGGA TTACTCGTTG GGTCAACGCC GAGGTGGCAG GTTCTAGGAG   
  
  
- TGGGGAATAC TACAACAAGT GTTCTACTCG CGCATATTCC AGAAGAGACT CCAGACGGGT TAGTGAGTTA   
  
  
- AACACTTAAA GTGAACATGT GTCCGGTAAG AGCTCCGAGA ACTACTAAGA CTACGATAAG TACAACAGCT   
  
  
- GAAACTATAA CCAACGCCAC GAGTTACCCG TAGTAACTAA GTCCTCGACG GTAACTCCTT TTCCCCTCGA   
  
  
- GGGAGAGACT TTTAATGTCG GTATCGAGGG TACAACTGAC CGTCAGTGAA ACTTTAATCG GATCATACAC   
  
  
- TTTTGGAACA CGTTAAACGG TTGCTATAAC CACAACGAAC ACTCGAGGTT CAACAGTTGA AACTAAACAA   
  
  
- ACTAGGTAGA AGCGGTAGTT ACGGCTTGTA ATCATGACGA CTCCTACTCA GTTAACGACA ATCATAGGGG   
  
  
- TAGACCCGTA GAAGTTAATC CGGTAGACAA GAAGGAAGGT AGGAGGCTAA GTAATTCGTT TTTAGGGGGT   
  
  
- TTTAACACCA GAGCAAACTA TCTCCTAAGC TAGCAAAACT ACAGGGAAAG GGGGTTGTAG ACAACGTATG   
  
  
- GGATCTTAGG ACGTCATTAA ATAACCTGAG CGAACTACCA GAGTTACAAC GTAGCCTGTA ACACTCGTTC   
  
  
- CAGCTCTTCA AGAAACAAGT TGGATCCTAG CTTTTACGAC ACAACCCAGC CCAGGTACGG GGACTGTTCT   
  
  
- ACGGGGTAAC CTTTTTAGAG AAACGAAGTC GGCCGAAGAA CGGGAACGTT AAGTCATTAA AGTGTCTTTG   
  
  
- GGTCCGGCTA ATACACCACT TCTCTTGGGG TTGTTCTCCT AAAGTGCACC TCTTCGCGGT CCGTAGTGAG   
  
  
- TAAGACTCAA CCGTCTCCGT CCTCGAACAC CGTCGAAGCC GTACCTTCAC AAC

+     G-Box

| Site Name | Organism | Position | Strand | Matrix score. | sequence | function |
| --- | --- | --- | --- | --- | --- | --- |
| G-Box | Pisum sativum | 4245 | - | 9 | CACGTGAAA | cis-acting regulatory element involved in light responsiveness |
| G-Box | Pisum sativum | 4248 | - | 6 | CACGTG | cis-acting regulatory element involved in light responsiveness |

>HU04G00047.1   
+ +Up\_Stream \_Len000TCTTAA CATTAGTTGC TTGGTTCAAC CCATCAGTGA GAATATCTGA TTTCAAAAGT   
  
  
+ ATAGATTTAT ATAAACATAT GGACTACAGG GAAGGGAGTC GTTTCCTCAA AGTCTTAGAT TTACACTCAA   
  
  
+ AAGTTTGAGC GATACCAATA ATGTGATTCC AAACATGAGC CAAATATTAA GTATCCTATA CTAAGATCAA   
  
  
+ GAAGAGAATG AACCAAAAGA CTGTTAATTT TTCAATTAAG CTAAACTTAT TAACCTTTTT TTGGAATAGA   
  
  
+ TTTTTAAATT TTATCTTCAA TATAAGTTTT TCCAAGGCGA GAGACATATA GCAATTTTAA TTAAAGTTAA   
  
  
+ AGTAGTATAA CATTTTTTTT AATGGTTATA GGCTTATAGC ACCTTTTCTA GGTGAAAAGA GATATCTTGG   
  
  
+ CCTGATTTCC AGTAAAGTTT TTTTTTTAAA TATTGTCAAT TATTTTTGCA AATGACATTT GGTAATTTGG   
  
  
+ GGGATAATCT GATGTGTTAA AAGATACAAT GAAAATATCA GAAAATAGGA TGCCTCAAAT TTCACAAGAA   
  
  
+ AGAAAAAATC TTTTTGTTGT TAGTATTTCT ACACATCCAC AATTTTTACT CATGGTAAAA AAAAAAGGGC   
  
  
+ CAAATATCTC ACCAATATAA CCAAACTATA TGTTCCATTA TTTAGTAACG GTGATAAATA TGTAATGGGT   
  
  
+ CAAGTTACTA ATAAAATAGC CTACAATACT TGGATATCTA CATAAAATTG ATATAGAATA TATATTGCAA   
  
  
+ TACTTAAATC AAAATTAAAT GAAATATTTA CTTTTTTATT CTATTACAAA TAGCTTCTTA TAGAATAGCA   
  
  
+ ACAATTTTTA ATGTAAGTTT CATTCTTACC AGATTTGAAG AGGTCCGGAT ATAGAAAATC TTATTCGTAT   
  
  
+ AAGTACTCGC ATGAGGGCGG ATAAATTCTA AAATATCAAC CTCAACTCGC TTCTACTATT CCATCTAAGT   
  
  
+ TCAATTTATC ATATAAAAAA ATTCAATCCA CTTTAATGTA CTCAATTCAC TTCAACCTAT TTTAATCAAT   
  
  
+ TTACCCATGA AAAGAACAAG GTCCTAAGTA GATGTTTTCT ATTGATTGAT TTAAACAATT TTCACCTACC   
  
  
+ CAATTATCCC CAAAACTGAA AGAGGGGGCA AAGGGGGGTG GGTAATGCTG TGTAAAATAG GGGGAAAGGG   
  
  
+ CAAGAAAACT GTGTACAATG GTGGAGCAGG GGTATACCAT TTGGCCTTGA GTTGAGGGGG CACAAAAGCC   
  
  
+ CAGAGAGAGA GAGGGGTAAT GCCCACCAGA AATAAGCAGG AAGCGAGGGA AAAACACTAG AGAGAAACAG   
  
  
+ AGAAAGAGAG AGAGAGAGAG AGAGAAGGGG AAATTTACTA GAGAATGAGA GTGAAGAAGG GAACTTTATT   
  
  
+ TTAACTTTTT TATGATGCAC GCATCCATGG CAGAACAGGG AAGGGAAAGA AGGGTACAGA GTACTGCTGT   
  
  
+ CCCCAAAAAA TTAGTGTAGA AAACAGGAGA CACTCAACTA CAACAAGGGT AAGGAAGAAG AACAAGCAAG   
  
  
+ ATATCCCACC CAAAGAAAAA AAAAAAGAAG AAGAAGAAAA TTTTTCAGAG AGGAAAGAAA AGCAATCACA   
  
  
+ TGAATTAGAA GCCAACCCAA GCCTTCTTCA AAGAGAAGCA GCATAACATA GCATAGCTGG GGAGAGGATA   
  
  
+ GGAGAGAGAA ACTCAAGAAA AGGGGTACCC GGCTTAAATG TTGATGCAGA AAGGTATAGA TATGGCCACA   
  
  
+ CTACTACTAC CCAGGAGTTT ATCATTGGAG ATGGATTTCT CTTTCTGCCT CATCTTCTCC TCCATCCACC   
  
  
+ AGCAAAACTG GGTTTCCTTT CAGCTGGGTC TTTTCTAGAT CTAGAAACTA TGGTACCCAA ATCTCCCATT   
  
  
+ CCTCCATTTT TATTCTCAAA ATCTATCTTC TTAGACTTGT GGGTTTGATT CATTTCTTTT GGGTTTTTGA   
  
  
+ AAAGAATTTA ATTTGATTGT ATTGTGTTGA TTGGGGAATT TGTGGTGGTG TTAGATGCGA GCTATGCCCT   
  
  
+ ACAATTTGCA AGGCAAGGGT GTGGTAGAGG TTTCAAGCAT TTGTATTCCA CAAATCTCTT CCCCTGCTTC   
  
  
+ AAAGTGGAAG ACCAACAACA ACAACTTGGA CAACAAATTT CAGCAACAAC AAGAAGAAAT TGAGCAATTG   
  
  
+ CAGAGTAGTG GTGGCACTTT TCTCTCTCCT GATAACACCA ATACTAATAT TAGTACTACT CCGAGAGCGA   
  
  
+ CAAGTTCTGT TGATAGCGAA CACACATCTA CTCTGGATAC TACGGGCCCT CCCACTTCAA CATCTTCCTC   
  
  
+ CTTCAAGAGC ACCTCCACCG CCAACACAGC CGGTGTGGCG GACCCCGCAT GTACTCCCAA AGAGGATTGG   
  
  
+ GATGTCTCCG GCGGCGCTGC TGGCGGTGAC GGCGGTGGTG GGGGGCTTGG ATTGGAGGAG TGGGACAGCA   
  
  
+ TGTTCCCAAA TGGGGAGGGG GCTTTGCTCC CTTGGATCAT GGGTGAAGCT GATGACATGG GTATGGGTTT   
  
  
+ GAAGCATCTT TTGCAATCGG GTAACCCGGT TGACTATGAG GGCAATGCTG GTCTAGGGGT TGTCGATCAG   
  
  
+ GGTTCTGGAT TTGAGACTCT CTCTCCTCCT CCACCGCAGC CGGCGGCATG TGAATCTAAT GGTGGTGGTG   
  
  
+ TTGCTAATTT GGGGTTTCCA GGGAATAATG GTAAGATTTC TTCAATTTCA CATAATTGTT CATCTGGGAT   
  
  
+ TTTGAATGGT AAGGTGAACA ATAATGGGTT GAATCCCAAT TGTAACCCTC AAGCTCAAGG CAGTCTACTT   
  
  
+ GGTTTAATCC AAGGAGCTAC AGGTGTACAC GCACATCCTG ACGTTGGAGA CGAGAAACCC CAGATTTTGA   
  
  
+ ATCCACATTT GGTGATGAAC CCTCAGCAAG CTCAGAGCAT TGCAAACCCT AGCTTTTTGA TGCACTCATT   
  
  
+ AGGTTACTAT CAGCTGGAGC AACATCTATT TCAACCTCAG GCAAAACGCC TGAACACGGG TGCTGTTCTG   
  
  
+ GATCCTAACC TTGTTCAGCT TGCAAAGAAC CCATTTGCTG ATCAGGGTCA TGAGTTATTG TTGAGGAAGC   
  
  
+ AGCACCAACA GCTTGGTTTG CAACCATTGC CATTGGGTTT GGGTCCTCAG TTGGTCCCTC CGCAGAAGCC   
  
  
+ TGTGATGGGT TCAAAGCAGG GGAACCCCCA GCATTACCCG TTGCATGTTC ATCAGCAGCT GCAATTGCAA   
  
  
+ GAGCAGGCTG TCAAAGATCA GCTCTTCAAG GCGGCAGACC TTATTCAAAC TGGAAGTTTC TCACTCGCGC   
  
  
+ AAGAGATATT GGCGCGGCTC AATCACCAGC TCTCCCTCCC TGCAAAGCCC CTCATTAGGG CGGCTTTGTA   
  
  
+ TGTGAAGGAG GCCCTTCAAA TGCTCCTCCT AATGAGCAAC CCAGTTGCGG CTCCACCGTC CAAGATCCTC   
  
  
+ ACCCCTTATG ATGTTGTTCA CAAGATGAGC GCGTATAAGG TCTTCTCTGA GGTCTGCCCA ATCACTCAAT   
  
  
+ TTGTGAATTT CACTTGTACA CAGGCCATTC TCGAGGCTCT TGATGATTCT GATGCTATTC ATGTTGTCGA   
  
  
+ CTTTGATATT GGTTGCGGTG CTCAATGGGC ATCATTGATT CAGGAGCTGC CATTGAGGAA AAGGGGAGCT   
  
  
+ CCCTCTCTGA AAATTACAGC CATAGCTCCC ATGTTGACTG GCAGTCACTT TGAAATTAGC CTAGTATGTG   
  
  
+ AAAACCTTGT GCAATTTGCC AACGATATTG GTGTTGCTTG TGAGCTCCAA GTTGTCAACT TTGATTTGTT   
  
  
+ TGATCCATCT TCGCCATCAA TGCCGAACAT TAGTACTGCT GAGGATGAGT CAATTGCTGT TAGTATCCCC   
  
  
+ ATCTGGGCAT CTTCAATTAG GCCATCTGTT CTTCCTTCCA TCCTCCGATT CATTAAGCAA AAATCCCCCA   
  
  
+ AAATTGTGGT CTCGTTTGAT AGAGGATTCG ATCGTTTTGA TGTCCCTTTC CCCCAACATC TGTTGCATAC   
  
  
+ CCTAGAATCC TGCAGTAATT TATTGGACTC GCTTGATGGT CTCAATGTTG CATCGGACAT TGTGAGCAAG   
  
  
+ GTCGAGAAGT TCTTTGTTCA ACCTAGGATC GAAAATGCTG TGTTGGGTCG GGTCCATGCC CCTGACAAGA   
  
  
+ TGCCCCATTG GAAAAATCTC TTTGCTTCAG CCGGCTTCTT GCCCTTGCAA TTCAGTAATT TCACAGAAAC   
  
  
+ CCAGGCCGAT TATGTGGTGA AGAGAACCCC AACAAGAGGA TTTCACGTGG AGAAGCGCCA GGCATCACTC   
  
  
+ ATTCTGAGTT GGCAGAGGCA GGAGCTTGTG GCAGCTTCGG CATGGAAGTG TTG  

- +Up\_Stream \_Len000AGAATT GTAATCAACG AACCAAGTTG GGTAGTCACT CTTATAGACT AAAGTTTTCA   
  
  
- TATCTAAATA TATTTGTATA CCTGATGTCC CTTCCCTCAG CAAAGGAGTT TCAGAATCTA AATGTGAGTT   
  
  
- TTCAAACTCG CTATGGTTAT TACACTAAGG TTTGTACTCG GTTTATAATT CATAGGATAT GATTCTAGTT   
  
  
- CTTCTCTTAC TTGGTTTTCT GACAATTAAA AAGTTAATTC GATTTGAATA ATTGGAAAAA AACCTTATCT   
  
  
- AAAAATTTAA AATAGAAGTT ATATTCAAAA AGGTTCCGCT CTCTGTATAT CGTTAAAATT AATTTCAATT   
  
  
- TCATCATATT GTAAAAAAAA TTACCAATAT CCGAATATCG TGGAAAAGAT CCACTTTTCT CTATAGAACC   
  
  
- GGACTAAAGG TCATTTCAAA AAAAAAATTT ATAACAGTTA ATAAAAACGT TTACTGTAAA CCATTAAACC   
  
  
- CCCTATTAGA CTACACAATT TTCTATGTTA CTTTTATAGT CTTTTATCCT ACGGAGTTTA AAGTGTTCTT   
  
  
- TCTTTTTTAG AAAAACAACA ATCATAAAGA TGTGTAGGTG TTAAAAATGA GTACCATTTT TTTTTTCCCG   
  
  
- GTTTATAGAG TGGTTATATT GGTTTGATAT ACAAGGTAAT AAATCATTGC CACTATTTAT ACATTACCCA   
  
  
- GTTCAATGAT TATTTTATCG GATGTTATGA ACCTATAGAT GTATTTTAAC TATATCTTAT ATATAACGTT   
  
  
- ATGAATTTAG TTTTAATTTA CTTTATAAAT GAAAAAATAA GATAATGTTT ATCGAAGAAT ATCTTATCGT   
  
  
- TGTTAAAAAT TACATTCAAA GTAAGAATGG TCTAAACTTC TCCAGGCCTA TATCTTTTAG AATAAGCATA   
  
  
- TTCATGAGCG TACTCCCGCC TATTTAAGAT TTTATAGTTG GAGTTGAGCG AAGATGATAA GGTAGATTCA   
  
  
- AGTTAAATAG TATATTTTTT TAAGTTAGGT GAAATTACAT GAGTTAAGTG AAGTTGGATA AAATTAGTTA   
  
  
- AATGGGTACT TTTCTTGTTC CAGGATTCAT CTACAAAAGA TAACTAACTA AATTTGTTAA AAGTGGATGG   
  
  
- GTTAATAGGG GTTTTGACTT TCTCCCCCGT TTCCCCCCAC CCATTACGAC ACATTTTATC CCCCTTTCCC   
  
  
- GTTCTTTTGA CACATGTTAC CACCTCGTCC CCATATGGTA AACCGGAACT CAACTCCCCC GTGTTTTCGG   
  
  
- GTCTCTCTCT CTCCCCATTA CGGGTGGTCT TTATTCGTCC TTCGCTCCCT TTTTGTGATC TCTCTTTGTC   
  
  
- TCTTTCTCTC TCTCTCTCTC TCTCTTCCCC TTTAAATGAT CTCTTACTCT CACTTCTTCC CTTGAAATAA   
  
  
- AATTGAAAAA ATACTACGTG CGTAGGTACC GTCTTGTCCC TTCCCTTTCT TCCCATGTCT CATGACGACA   
  
  
- GGGGTTTTTT AATCACATCT TTTGTCCTCT GTGAGTTGAT GTTGTTCCCA TTCCTTCTTC TTGTTCGTTC   
  
  
- TATAGGGTGG GTTTCTTTTT TTTTTTCTTC TTCTTCTTTT AAAAAGTCTC TCCTTTCTTT TCGTTAGTGT   
  
  
- ACTTAATCTT CGGTTGGGTT CGGAAGAAGT TTCTCTTCGT CGTATTGTAT CGTATCGACC CCTCTCCTAT   
  
  
- CCTCTCTCTT TGAGTTCTTT TCCCCATGGG CCGAATTTAC AACTACGTCT TTCCATATCT ATACCGGTGT   
  
  
- GATGATGATG GGTCCTCAAA TAGTAACCTC TACCTAAAGA GAAAGACGGA GTAGAAGAGG AGGTAGGTGG   
  
  
- TCGTTTTGAC CCAAAGGAAA GTCGACCCAG AAAAGATCTA GATCTTTGAT ACCATGGGTT TAGAGGGTAA   
  
  
- GGAGGTAAAA ATAAGAGTTT TAGATAGAAG AATCTGAACA CCCAAACTAA GTAAAGAAAA CCCAAAAACT   
  
  
- TTTCTTAAAT TAAACTAACA TAACACAACT AACCCCTTAA ACACCACCAC AATCTACGCT CGATACGGGA   
  
  
- TGTTAAACGT TCCGTTCCCA CACCATCTCC AAAGTTCGTA AACATAAGGT GTTTAGAGAA GGGGACGAAG   
  
  
- TTTCACCTTC TGGTTGTTGT TGTTGAACCT GTTGTTTAAA GTCGTTGTTG TTCTTCTTTA ACTCGTTAAC   
  
  
- GTCTCATCAC CACCGTGAAA AGAGAGAGGA CTATTGTGGT TATGATTATA ATCATGATGA GGCTCTCGCT   
  
  
- GTTCAAGACA ACTATCGCTT GTGTGTAGAT GAGACCTATG ATGCCCGGGA GGGTGAAGTT GTAGAAGGAG   
  
  
- GAAGTTCTCG TGGAGGTGGC GGTTGTGTCG GCCACACCGC CTGGGGCGTA CATGAGGGTT TCTCCTAACC   
  
  
- CTACAGAGGC CGCCGCGACG ACCGCCACTG CCGCCACCAC CCCCCGAACC TAACCTCCTC ACCCTGTCGT   
  
  
- ACAAGGGTTT ACCCCTCCCC CGAAACGAGG GAACCTAGTA CCCACTTCGA CTACTGTACC CATACCCAAA   
  
  
- CTTCGTAGAA AACGTTAGCC CATTGGGCCA ACTGATACTC CCGTTACGAC CAGATCCCCA ACAGCTAGTC   
  
  
- CCAAGACCTA AACTCTGAGA GAGAGGAGGA GGTGGCGTCG GCCGCCGTAC ACTTAGATTA CCACCACCAC   
  
  
- AACGATTAAA CCCCAAAGGT CCCTTATTAC CATTCTAAAG AAGTTAAAGT GTATTAACAA GTAGACCCTA   
  
  
- AAACTTACCA TTCCACTTGT TATTACCCAA CTTAGGGTTA ACATTGGGAG TTCGAGTTCC GTCAGATGAA   
  
  
- CCAAATTAGG TTCCTCGATG TCCACATGTG CGTGTAGGAC TGCAACCTCT GCTCTTTGGG GTCTAAAACT   
  
  
- TAGGTGTAAA CCACTACTTG GGAGTCGTTC GAGTCTCGTA ACGTTTGGGA TCGAAAAACT ACGTGAGTAA   
  
  
- TCCAATGATA GTCGACCTCG TTGTAGATAA AGTTGGAGTC CGTTTTGCGG ACTTGTGCCC ACGACAAGAC   
  
  
- CTAGGATTGG AACAAGTCGA ACGTTTCTTG GGTAAACGAC TAGTCCCAGT ACTCAATAAC AACTCCTTCG   
  
  
- TCGTGGTTGT CGAACCAAAC GTTGGTAACG GTAACCCAAA CCCAGGAGTC AACCAGGGAG GCGTCTTCGG   
  
  
- ACACTACCCA AGTTTCGTCC CCTTGGGGGT CGTAATGGGC AACGTACAAG TAGTCGTCGA CGTTAACGTT   
  
  
- CTCGTCCGAC AGTTTCTAGT CGAGAAGTTC CGCCGTCTGG AATAAGTTTG ACCTTCAAAG AGTGAGCGCG   
  
  
- TTCTCTATAA CCGCGCCGAG TTAGTGGTCG AGAGGGAGGG ACGTTTCGGG GAGTAATCCC GCCGAAACAT   
  
  
- ACACTTCCTC CGGGAAGTTT ACGAGGAGGA TTACTCGTTG GGTCAACGCC GAGGTGGCAG GTTCTAGGAG   
  
  
- TGGGGAATAC TACAACAAGT GTTCTACTCG CGCATATTCC AGAAGAGACT CCAGACGGGT TAGTGAGTTA   
  
  
- AACACTTAAA GTGAACATGT GTCCGGTAAG AGCTCCGAGA ACTACTAAGA CTACGATAAG TACAACAGCT   
  
  
- GAAACTATAA CCAACGCCAC GAGTTACCCG TAGTAACTAA GTCCTCGACG GTAACTCCTT TTCCCCTCGA   
  
  
- GGGAGAGACT TTTAATGTCG GTATCGAGGG TACAACTGAC CGTCAGTGAA ACTTTAATCG GATCATACAC   
  
  
- TTTTGGAACA CGTTAAACGG TTGCTATAAC CACAACGAAC ACTCGAGGTT CAACAGTTGA AACTAAACAA   
  
  
- ACTAGGTAGA AGCGGTAGTT ACGGCTTGTA ATCATGACGA CTCCTACTCA GTTAACGACA ATCATAGGGG   
  
  
- TAGACCCGTA GAAGTTAATC CGGTAGACAA GAAGGAAGGT AGGAGGCTAA GTAATTCGTT TTTAGGGGGT   
  
  
- TTTAACACCA GAGCAAACTA TCTCCTAAGC TAGCAAAACT ACAGGGAAAG GGGGTTGTAG ACAACGTATG   
  
  
- GGATCTTAGG ACGTCATTAA ATAACCTGAG CGAACTACCA GAGTTACAAC GTAGCCTGTA ACACTCGTTC   
  
  
- CAGCTCTTCA AGAAACAAGT TGGATCCTAG CTTTTACGAC ACAACCCAGC CCAGGTACGG GGACTGTTCT   
  
  
- ACGGGGTAAC CTTTTTAGAG AAACGAAGTC GGCCGAAGAA CGGGAACGTT AAGTCATTAA AGTGTCTTTG   
  
  
- GGTCCGGCTA ATACACCACT TCTCTTGGGG TTGTTCTCCT AAAGTGCACC TCTTCGCGGT CCGTAGTGAG   
  
  
- TAAGACTCAA CCGTCTCCGT CCTCGAACAC CGTCGAAGCC GTACCTTCAC AAC

+     G-box

| Site Name | Organism | Position | Strand | Matrix score. | sequence | function |
| --- | --- | --- | --- | --- | --- | --- |
| G-box | Arabidopsis thaliana | 4248 | - | 6 | CACGTG | cis-acting regulatory element involved in light responsiveness |

>HU04G00047.1   
+ +Up\_Stream \_Len000TCTTAA CATTAGTTGC TTGGTTCAAC CCATCAGTGA GAATATCTGA TTTCAAAAGT   
  
  
+ ATAGATTTAT ATAAACATAT GGACTACAGG GAAGGGAGTC GTTTCCTCAA AGTCTTAGAT TTACACTCAA   
  
  
+ AAGTTTGAGC GATACCAATA ATGTGATTCC AAACATGAGC CAAATATTAA GTATCCTATA CTAAGATCAA   
  
  
+ GAAGAGAATG AACCAAAAGA CTGTTAATTT TTCAATTAAG CTAAACTTAT TAACCTTTTT TTGGAATAGA   
  
  
+ TTTTTAAATT TTATCTTCAA TATAAGTTTT TCCAAGGCGA GAGACATATA GCAATTTTAA TTAAAGTTAA   
  
  
+ AGTAGTATAA CATTTTTTTT AATGGTTATA GGCTTATAGC ACCTTTTCTA GGTGAAAAGA GATATCTTGG   
  
  
+ CCTGATTTCC AGTAAAGTTT TTTTTTTAAA TATTGTCAAT TATTTTTGCA AATGACATTT GGTAATTTGG   
  
  
+ GGGATAATCT GATGTGTTAA AAGATACAAT GAAAATATCA GAAAATAGGA TGCCTCAAAT TTCACAAGAA   
  
  
+ AGAAAAAATC TTTTTGTTGT TAGTATTTCT ACACATCCAC AATTTTTACT CATGGTAAAA AAAAAAGGGC   
  
  
+ CAAATATCTC ACCAATATAA CCAAACTATA TGTTCCATTA TTTAGTAACG GTGATAAATA TGTAATGGGT   
  
  
+ CAAGTTACTA ATAAAATAGC CTACAATACT TGGATATCTA CATAAAATTG ATATAGAATA TATATTGCAA   
  
  
+ TACTTAAATC AAAATTAAAT GAAATATTTA CTTTTTTATT CTATTACAAA TAGCTTCTTA TAGAATAGCA   
  
  
+ ACAATTTTTA ATGTAAGTTT CATTCTTACC AGATTTGAAG AGGTCCGGAT ATAGAAAATC TTATTCGTAT   
  
  
+ AAGTACTCGC ATGAGGGCGG ATAAATTCTA AAATATCAAC CTCAACTCGC TTCTACTATT CCATCTAAGT   
  
  
+ TCAATTTATC ATATAAAAAA ATTCAATCCA CTTTAATGTA CTCAATTCAC TTCAACCTAT TTTAATCAAT   
  
  
+ TTACCCATGA AAAGAACAAG GTCCTAAGTA GATGTTTTCT ATTGATTGAT TTAAACAATT TTCACCTACC   
  
  
+ CAATTATCCC CAAAACTGAA AGAGGGGGCA AAGGGGGGTG GGTAATGCTG TGTAAAATAG GGGGAAAGGG   
  
  
+ CAAGAAAACT GTGTACAATG GTGGAGCAGG GGTATACCAT TTGGCCTTGA GTTGAGGGGG CACAAAAGCC   
  
  
+ CAGAGAGAGA GAGGGGTAAT GCCCACCAGA AATAAGCAGG AAGCGAGGGA AAAACACTAG AGAGAAACAG   
  
  
+ AGAAAGAGAG AGAGAGAGAG AGAGAAGGGG AAATTTACTA GAGAATGAGA GTGAAGAAGG GAACTTTATT   
  
  
+ TTAACTTTTT TATGATGCAC GCATCCATGG CAGAACAGGG AAGGGAAAGA AGGGTACAGA GTACTGCTGT   
  
  
+ CCCCAAAAAA TTAGTGTAGA AAACAGGAGA CACTCAACTA CAACAAGGGT AAGGAAGAAG AACAAGCAAG   
  
  
+ ATATCCCACC CAAAGAAAAA AAAAAAGAAG AAGAAGAAAA TTTTTCAGAG AGGAAAGAAA AGCAATCACA   
  
  
+ TGAATTAGAA GCCAACCCAA GCCTTCTTCA AAGAGAAGCA GCATAACATA GCATAGCTGG GGAGAGGATA   
  
  
+ GGAGAGAGAA ACTCAAGAAA AGGGGTACCC GGCTTAAATG TTGATGCAGA AAGGTATAGA TATGGCCACA   
  
  
+ CTACTACTAC CCAGGAGTTT ATCATTGGAG ATGGATTTCT CTTTCTGCCT CATCTTCTCC TCCATCCACC   
  
  
+ AGCAAAACTG GGTTTCCTTT CAGCTGGGTC TTTTCTAGAT CTAGAAACTA TGGTACCCAA ATCTCCCATT   
  
  
+ CCTCCATTTT TATTCTCAAA ATCTATCTTC TTAGACTTGT GGGTTTGATT CATTTCTTTT GGGTTTTTGA   
  
  
+ AAAGAATTTA ATTTGATTGT ATTGTGTTGA TTGGGGAATT TGTGGTGGTG TTAGATGCGA GCTATGCCCT   
  
  
+ ACAATTTGCA AGGCAAGGGT GTGGTAGAGG TTTCAAGCAT TTGTATTCCA CAAATCTCTT CCCCTGCTTC   
  
  
+ AAAGTGGAAG ACCAACAACA ACAACTTGGA CAACAAATTT CAGCAACAAC AAGAAGAAAT TGAGCAATTG   
  
  
+ CAGAGTAGTG GTGGCACTTT TCTCTCTCCT GATAACACCA ATACTAATAT TAGTACTACT CCGAGAGCGA   
  
  
+ CAAGTTCTGT TGATAGCGAA CACACATCTA CTCTGGATAC TACGGGCCCT CCCACTTCAA CATCTTCCTC   
  
  
+ CTTCAAGAGC ACCTCCACCG CCAACACAGC CGGTGTGGCG GACCCCGCAT GTACTCCCAA AGAGGATTGG   
  
  
+ GATGTCTCCG GCGGCGCTGC TGGCGGTGAC GGCGGTGGTG GGGGGCTTGG ATTGGAGGAG TGGGACAGCA   
  
  
+ TGTTCCCAAA TGGGGAGGGG GCTTTGCTCC CTTGGATCAT GGGTGAAGCT GATGACATGG GTATGGGTTT   
  
  
+ GAAGCATCTT TTGCAATCGG GTAACCCGGT TGACTATGAG GGCAATGCTG GTCTAGGGGT TGTCGATCAG   
  
  
+ GGTTCTGGAT TTGAGACTCT CTCTCCTCCT CCACCGCAGC CGGCGGCATG TGAATCTAAT GGTGGTGGTG   
  
  
+ TTGCTAATTT GGGGTTTCCA GGGAATAATG GTAAGATTTC TTCAATTTCA CATAATTGTT CATCTGGGAT   
  
  
+ TTTGAATGGT AAGGTGAACA ATAATGGGTT GAATCCCAAT TGTAACCCTC AAGCTCAAGG CAGTCTACTT   
  
  
+ GGTTTAATCC AAGGAGCTAC AGGTGTACAC GCACATCCTG ACGTTGGAGA CGAGAAACCC CAGATTTTGA   
  
  
+ ATCCACATTT GGTGATGAAC CCTCAGCAAG CTCAGAGCAT TGCAAACCCT AGCTTTTTGA TGCACTCATT   
  
  
+ AGGTTACTAT CAGCTGGAGC AACATCTATT TCAACCTCAG GCAAAACGCC TGAACACGGG TGCTGTTCTG   
  
  
+ GATCCTAACC TTGTTCAGCT TGCAAAGAAC CCATTTGCTG ATCAGGGTCA TGAGTTATTG TTGAGGAAGC   
  
  
+ AGCACCAACA GCTTGGTTTG CAACCATTGC CATTGGGTTT GGGTCCTCAG TTGGTCCCTC CGCAGAAGCC   
  
  
+ TGTGATGGGT TCAAAGCAGG GGAACCCCCA GCATTACCCG TTGCATGTTC ATCAGCAGCT GCAATTGCAA   
  
  
+ GAGCAGGCTG TCAAAGATCA GCTCTTCAAG GCGGCAGACC TTATTCAAAC TGGAAGTTTC TCACTCGCGC   
  
  
+ AAGAGATATT GGCGCGGCTC AATCACCAGC TCTCCCTCCC TGCAAAGCCC CTCATTAGGG CGGCTTTGTA   
  
  
+ TGTGAAGGAG GCCCTTCAAA TGCTCCTCCT AATGAGCAAC CCAGTTGCGG CTCCACCGTC CAAGATCCTC   
  
  
+ ACCCCTTATG ATGTTGTTCA CAAGATGAGC GCGTATAAGG TCTTCTCTGA GGTCTGCCCA ATCACTCAAT   
  
  
+ TTGTGAATTT CACTTGTACA CAGGCCATTC TCGAGGCTCT TGATGATTCT GATGCTATTC ATGTTGTCGA   
  
  
+ CTTTGATATT GGTTGCGGTG CTCAATGGGC ATCATTGATT CAGGAGCTGC CATTGAGGAA AAGGGGAGCT   
  
  
+ CCCTCTCTGA AAATTACAGC CATAGCTCCC ATGTTGACTG GCAGTCACTT TGAAATTAGC CTAGTATGTG   
  
  
+ AAAACCTTGT GCAATTTGCC AACGATATTG GTGTTGCTTG TGAGCTCCAA GTTGTCAACT TTGATTTGTT   
  
  
+ TGATCCATCT TCGCCATCAA TGCCGAACAT TAGTACTGCT GAGGATGAGT CAATTGCTGT TAGTATCCCC   
  
  
+ ATCTGGGCAT CTTCAATTAG GCCATCTGTT CTTCCTTCCA TCCTCCGATT CATTAAGCAA AAATCCCCCA   
  
  
+ AAATTGTGGT CTCGTTTGAT AGAGGATTCG ATCGTTTTGA TGTCCCTTTC CCCCAACATC TGTTGCATAC   
  
  
+ CCTAGAATCC TGCAGTAATT TATTGGACTC GCTTGATGGT CTCAATGTTG CATCGGACAT TGTGAGCAAG   
  
  
+ GTCGAGAAGT TCTTTGTTCA ACCTAGGATC GAAAATGCTG TGTTGGGTCG GGTCCATGCC CCTGACAAGA   
  
  
+ TGCCCCATTG GAAAAATCTC TTTGCTTCAG CCGGCTTCTT GCCCTTGCAA TTCAGTAATT TCACAGAAAC   
  
  
+ CCAGGCCGAT TATGTGGTGA AGAGAACCCC AACAAGAGGA TTTCACGTGG AGAAGCGCCA GGCATCACTC   
  
  
+ ATTCTGAGTT GGCAGAGGCA GGAGCTTGTG GCAGCTTCGG CATGGAAGTG TTG  

- +Up\_Stream \_Len000AGAATT GTAATCAACG AACCAAGTTG GGTAGTCACT CTTATAGACT AAAGTTTTCA   
  
  
- TATCTAAATA TATTTGTATA CCTGATGTCC CTTCCCTCAG CAAAGGAGTT TCAGAATCTA AATGTGAGTT   
  
  
- TTCAAACTCG CTATGGTTAT TACACTAAGG TTTGTACTCG GTTTATAATT CATAGGATAT GATTCTAGTT   
  
  
- CTTCTCTTAC TTGGTTTTCT GACAATTAAA AAGTTAATTC GATTTGAATA ATTGGAAAAA AACCTTATCT   
  
  
- AAAAATTTAA AATAGAAGTT ATATTCAAAA AGGTTCCGCT CTCTGTATAT CGTTAAAATT AATTTCAATT   
  
  
- TCATCATATT GTAAAAAAAA TTACCAATAT CCGAATATCG TGGAAAAGAT CCACTTTTCT CTATAGAACC   
  
  
- GGACTAAAGG TCATTTCAAA AAAAAAATTT ATAACAGTTA ATAAAAACGT TTACTGTAAA CCATTAAACC   
  
  
- CCCTATTAGA CTACACAATT TTCTATGTTA CTTTTATAGT CTTTTATCCT ACGGAGTTTA AAGTGTTCTT   
  
  
- TCTTTTTTAG AAAAACAACA ATCATAAAGA TGTGTAGGTG TTAAAAATGA GTACCATTTT TTTTTTCCCG   
  
  
- GTTTATAGAG TGGTTATATT GGTTTGATAT ACAAGGTAAT AAATCATTGC CACTATTTAT ACATTACCCA   
  
  
- GTTCAATGAT TATTTTATCG GATGTTATGA ACCTATAGAT GTATTTTAAC TATATCTTAT ATATAACGTT   
  
  
- ATGAATTTAG TTTTAATTTA CTTTATAAAT GAAAAAATAA GATAATGTTT ATCGAAGAAT ATCTTATCGT   
  
  
- TGTTAAAAAT TACATTCAAA GTAAGAATGG TCTAAACTTC TCCAGGCCTA TATCTTTTAG AATAAGCATA   
  
  
- TTCATGAGCG TACTCCCGCC TATTTAAGAT TTTATAGTTG GAGTTGAGCG AAGATGATAA GGTAGATTCA   
  
  
- AGTTAAATAG TATATTTTTT TAAGTTAGGT GAAATTACAT GAGTTAAGTG AAGTTGGATA AAATTAGTTA   
  
  
- AATGGGTACT TTTCTTGTTC CAGGATTCAT CTACAAAAGA TAACTAACTA AATTTGTTAA AAGTGGATGG   
  
  
- GTTAATAGGG GTTTTGACTT TCTCCCCCGT TTCCCCCCAC CCATTACGAC ACATTTTATC CCCCTTTCCC   
  
  
- GTTCTTTTGA CACATGTTAC CACCTCGTCC CCATATGGTA AACCGGAACT CAACTCCCCC GTGTTTTCGG   
  
  
- GTCTCTCTCT CTCCCCATTA CGGGTGGTCT TTATTCGTCC TTCGCTCCCT TTTTGTGATC TCTCTTTGTC   
  
  
- TCTTTCTCTC TCTCTCTCTC TCTCTTCCCC TTTAAATGAT CTCTTACTCT CACTTCTTCC CTTGAAATAA   
  
  
- AATTGAAAAA ATACTACGTG CGTAGGTACC GTCTTGTCCC TTCCCTTTCT TCCCATGTCT CATGACGACA   
  
  
- GGGGTTTTTT AATCACATCT TTTGTCCTCT GTGAGTTGAT GTTGTTCCCA TTCCTTCTTC TTGTTCGTTC   
  
  
- TATAGGGTGG GTTTCTTTTT TTTTTTCTTC TTCTTCTTTT AAAAAGTCTC TCCTTTCTTT TCGTTAGTGT   
  
  
- ACTTAATCTT CGGTTGGGTT CGGAAGAAGT TTCTCTTCGT CGTATTGTAT CGTATCGACC CCTCTCCTAT   
  
  
- CCTCTCTCTT TGAGTTCTTT TCCCCATGGG CCGAATTTAC AACTACGTCT TTCCATATCT ATACCGGTGT   
  
  
- GATGATGATG GGTCCTCAAA TAGTAACCTC TACCTAAAGA GAAAGACGGA GTAGAAGAGG AGGTAGGTGG   
  
  
- TCGTTTTGAC CCAAAGGAAA GTCGACCCAG AAAAGATCTA GATCTTTGAT ACCATGGGTT TAGAGGGTAA   
  
  
- GGAGGTAAAA ATAAGAGTTT TAGATAGAAG AATCTGAACA CCCAAACTAA GTAAAGAAAA CCCAAAAACT   
  
  
- TTTCTTAAAT TAAACTAACA TAACACAACT AACCCCTTAA ACACCACCAC AATCTACGCT CGATACGGGA   
  
  
- TGTTAAACGT TCCGTTCCCA CACCATCTCC AAAGTTCGTA AACATAAGGT GTTTAGAGAA GGGGACGAAG   
  
  
- TTTCACCTTC TGGTTGTTGT TGTTGAACCT GTTGTTTAAA GTCGTTGTTG TTCTTCTTTA ACTCGTTAAC   
  
  
- GTCTCATCAC CACCGTGAAA AGAGAGAGGA CTATTGTGGT TATGATTATA ATCATGATGA GGCTCTCGCT   
  
  
- GTTCAAGACA ACTATCGCTT GTGTGTAGAT GAGACCTATG ATGCCCGGGA GGGTGAAGTT GTAGAAGGAG   
  
  
- GAAGTTCTCG TGGAGGTGGC GGTTGTGTCG GCCACACCGC CTGGGGCGTA CATGAGGGTT TCTCCTAACC   
  
  
- CTACAGAGGC CGCCGCGACG ACCGCCACTG CCGCCACCAC CCCCCGAACC TAACCTCCTC ACCCTGTCGT   
  
  
- ACAAGGGTTT ACCCCTCCCC CGAAACGAGG GAACCTAGTA CCCACTTCGA CTACTGTACC CATACCCAAA   
  
  
- CTTCGTAGAA AACGTTAGCC CATTGGGCCA ACTGATACTC CCGTTACGAC CAGATCCCCA ACAGCTAGTC   
  
  
- CCAAGACCTA AACTCTGAGA GAGAGGAGGA GGTGGCGTCG GCCGCCGTAC ACTTAGATTA CCACCACCAC   
  
  
- AACGATTAAA CCCCAAAGGT CCCTTATTAC CATTCTAAAG AAGTTAAAGT GTATTAACAA GTAGACCCTA   
  
  
- AAACTTACCA TTCCACTTGT TATTACCCAA CTTAGGGTTA ACATTGGGAG TTCGAGTTCC GTCAGATGAA   
  
  
- CCAAATTAGG TTCCTCGATG TCCACATGTG CGTGTAGGAC TGCAACCTCT GCTCTTTGGG GTCTAAAACT   
  
  
- TAGGTGTAAA CCACTACTTG GGAGTCGTTC GAGTCTCGTA ACGTTTGGGA TCGAAAAACT ACGTGAGTAA   
  
  
- TCCAATGATA GTCGACCTCG TTGTAGATAA AGTTGGAGTC CGTTTTGCGG ACTTGTGCCC ACGACAAGAC   
  
  
- CTAGGATTGG AACAAGTCGA ACGTTTCTTG GGTAAACGAC TAGTCCCAGT ACTCAATAAC AACTCCTTCG   
  
  
- TCGTGGTTGT CGAACCAAAC GTTGGTAACG GTAACCCAAA CCCAGGAGTC AACCAGGGAG GCGTCTTCGG   
  
  
- ACACTACCCA AGTTTCGTCC CCTTGGGGGT CGTAATGGGC AACGTACAAG TAGTCGTCGA CGTTAACGTT   
  
  
- CTCGTCCGAC AGTTTCTAGT CGAGAAGTTC CGCCGTCTGG AATAAGTTTG ACCTTCAAAG AGTGAGCGCG   
  
  
- TTCTCTATAA CCGCGCCGAG TTAGTGGTCG AGAGGGAGGG ACGTTTCGGG GAGTAATCCC GCCGAAACAT   
  
  
- ACACTTCCTC CGGGAAGTTT ACGAGGAGGA TTACTCGTTG GGTCAACGCC GAGGTGGCAG GTTCTAGGAG   
  
  
- TGGGGAATAC TACAACAAGT GTTCTACTCG CGCATATTCC AGAAGAGACT CCAGACGGGT TAGTGAGTTA   
  
  
- AACACTTAAA GTGAACATGT GTCCGGTAAG AGCTCCGAGA ACTACTAAGA CTACGATAAG TACAACAGCT   
  
  
- GAAACTATAA CCAACGCCAC GAGTTACCCG TAGTAACTAA GTCCTCGACG GTAACTCCTT TTCCCCTCGA   
  
  
- GGGAGAGACT TTTAATGTCG GTATCGAGGG TACAACTGAC CGTCAGTGAA ACTTTAATCG GATCATACAC   
  
  
- TTTTGGAACA CGTTAAACGG TTGCTATAAC CACAACGAAC ACTCGAGGTT CAACAGTTGA AACTAAACAA   
  
  
- ACTAGGTAGA AGCGGTAGTT ACGGCTTGTA ATCATGACGA CTCCTACTCA GTTAACGACA ATCATAGGGG   
  
  
- TAGACCCGTA GAAGTTAATC CGGTAGACAA GAAGGAAGGT AGGAGGCTAA GTAATTCGTT TTTAGGGGGT   
  
  
- TTTAACACCA GAGCAAACTA TCTCCTAAGC TAGCAAAACT ACAGGGAAAG GGGGTTGTAG ACAACGTATG   
  
  
- GGATCTTAGG ACGTCATTAA ATAACCTGAG CGAACTACCA GAGTTACAAC GTAGCCTGTA ACACTCGTTC   
  
  
- CAGCTCTTCA AGAAACAAGT TGGATCCTAG CTTTTACGAC ACAACCCAGC CCAGGTACGG GGACTGTTCT   
  
  
- ACGGGGTAAC CTTTTTAGAG AAACGAAGTC GGCCGAAGAA CGGGAACGTT AAGTCATTAA AGTGTCTTTG   
  
  
- GGTCCGGCTA ATACACCACT TCTCTTGGGG TTGTTCTCCT AAAGTGCACC TCTTCGCGGT CCGTAGTGAG   
  
  
- TAAGACTCAA CCGTCTCCGT CCTCGAACAC CGTCGAAGCC GTACCTTCAC AAC

+     GARE-motif

| Site Name | Organism | Position | Strand | Matrix score. | sequence | function |
| --- | --- | --- | --- | --- | --- | --- |
| GARE-motif | Brassica oleracea | 3983 | + | 7 | TCTGTTG | gibberellin-responsive element |
| GARE-motif | Brassica oleracea | 2250 | + | 7 | TCTGTTG | gibberellin-responsive element |

>HU04G00047.1   
+ +Up\_Stream \_Len000TCTTAA CATTAGTTGC TTGGTTCAAC CCATCAGTGA GAATATCTGA TTTCAAAAGT   
  
  
+ ATAGATTTAT ATAAACATAT GGACTACAGG GAAGGGAGTC GTTTCCTCAA AGTCTTAGAT TTACACTCAA   
  
  
+ AAGTTTGAGC GATACCAATA ATGTGATTCC AAACATGAGC CAAATATTAA GTATCCTATA CTAAGATCAA   
  
  
+ GAAGAGAATG AACCAAAAGA CTGTTAATTT TTCAATTAAG CTAAACTTAT TAACCTTTTT TTGGAATAGA   
  
  
+ TTTTTAAATT TTATCTTCAA TATAAGTTTT TCCAAGGCGA GAGACATATA GCAATTTTAA TTAAAGTTAA   
  
  
+ AGTAGTATAA CATTTTTTTT AATGGTTATA GGCTTATAGC ACCTTTTCTA GGTGAAAAGA GATATCTTGG   
  
  
+ CCTGATTTCC AGTAAAGTTT TTTTTTTAAA TATTGTCAAT TATTTTTGCA AATGACATTT GGTAATTTGG   
  
  
+ GGGATAATCT GATGTGTTAA AAGATACAAT GAAAATATCA GAAAATAGGA TGCCTCAAAT TTCACAAGAA   
  
  
+ AGAAAAAATC TTTTTGTTGT TAGTATTTCT ACACATCCAC AATTTTTACT CATGGTAAAA AAAAAAGGGC   
  
  
+ CAAATATCTC ACCAATATAA CCAAACTATA TGTTCCATTA TTTAGTAACG GTGATAAATA TGTAATGGGT   
  
  
+ CAAGTTACTA ATAAAATAGC CTACAATACT TGGATATCTA CATAAAATTG ATATAGAATA TATATTGCAA   
  
  
+ TACTTAAATC AAAATTAAAT GAAATATTTA CTTTTTTATT CTATTACAAA TAGCTTCTTA TAGAATAGCA   
  
  
+ ACAATTTTTA ATGTAAGTTT CATTCTTACC AGATTTGAAG AGGTCCGGAT ATAGAAAATC TTATTCGTAT   
  
  
+ AAGTACTCGC ATGAGGGCGG ATAAATTCTA AAATATCAAC CTCAACTCGC TTCTACTATT CCATCTAAGT   
  
  
+ TCAATTTATC ATATAAAAAA ATTCAATCCA CTTTAATGTA CTCAATTCAC TTCAACCTAT TTTAATCAAT   
  
  
+ TTACCCATGA AAAGAACAAG GTCCTAAGTA GATGTTTTCT ATTGATTGAT TTAAACAATT TTCACCTACC   
  
  
+ CAATTATCCC CAAAACTGAA AGAGGGGGCA AAGGGGGGTG GGTAATGCTG TGTAAAATAG GGGGAAAGGG   
  
  
+ CAAGAAAACT GTGTACAATG GTGGAGCAGG GGTATACCAT TTGGCCTTGA GTTGAGGGGG CACAAAAGCC   
  
  
+ CAGAGAGAGA GAGGGGTAAT GCCCACCAGA AATAAGCAGG AAGCGAGGGA AAAACACTAG AGAGAAACAG   
  
  
+ AGAAAGAGAG AGAGAGAGAG AGAGAAGGGG AAATTTACTA GAGAATGAGA GTGAAGAAGG GAACTTTATT   
  
  
+ TTAACTTTTT TATGATGCAC GCATCCATGG CAGAACAGGG AAGGGAAAGA AGGGTACAGA GTACTGCTGT   
  
  
+ CCCCAAAAAA TTAGTGTAGA AAACAGGAGA CACTCAACTA CAACAAGGGT AAGGAAGAAG AACAAGCAAG   
  
  
+ ATATCCCACC CAAAGAAAAA AAAAAAGAAG AAGAAGAAAA TTTTTCAGAG AGGAAAGAAA AGCAATCACA   
  
  
+ TGAATTAGAA GCCAACCCAA GCCTTCTTCA AAGAGAAGCA GCATAACATA GCATAGCTGG GGAGAGGATA   
  
  
+ GGAGAGAGAA ACTCAAGAAA AGGGGTACCC GGCTTAAATG TTGATGCAGA AAGGTATAGA TATGGCCACA   
  
  
+ CTACTACTAC CCAGGAGTTT ATCATTGGAG ATGGATTTCT CTTTCTGCCT CATCTTCTCC TCCATCCACC   
  
  
+ AGCAAAACTG GGTTTCCTTT CAGCTGGGTC TTTTCTAGAT CTAGAAACTA TGGTACCCAA ATCTCCCATT   
  
  
+ CCTCCATTTT TATTCTCAAA ATCTATCTTC TTAGACTTGT GGGTTTGATT CATTTCTTTT GGGTTTTTGA   
  
  
+ AAAGAATTTA ATTTGATTGT ATTGTGTTGA TTGGGGAATT TGTGGTGGTG TTAGATGCGA GCTATGCCCT   
  
  
+ ACAATTTGCA AGGCAAGGGT GTGGTAGAGG TTTCAAGCAT TTGTATTCCA CAAATCTCTT CCCCTGCTTC   
  
  
+ AAAGTGGAAG ACCAACAACA ACAACTTGGA CAACAAATTT CAGCAACAAC AAGAAGAAAT TGAGCAATTG   
  
  
+ CAGAGTAGTG GTGGCACTTT TCTCTCTCCT GATAACACCA ATACTAATAT TAGTACTACT CCGAGAGCGA   
  
  
+ CAAGTTCTGT TGATAGCGAA CACACATCTA CTCTGGATAC TACGGGCCCT CCCACTTCAA CATCTTCCTC   
  
  
+ CTTCAAGAGC ACCTCCACCG CCAACACAGC CGGTGTGGCG GACCCCGCAT GTACTCCCAA AGAGGATTGG   
  
  
+ GATGTCTCCG GCGGCGCTGC TGGCGGTGAC GGCGGTGGTG GGGGGCTTGG ATTGGAGGAG TGGGACAGCA   
  
  
+ TGTTCCCAAA TGGGGAGGGG GCTTTGCTCC CTTGGATCAT GGGTGAAGCT GATGACATGG GTATGGGTTT   
  
  
+ GAAGCATCTT TTGCAATCGG GTAACCCGGT TGACTATGAG GGCAATGCTG GTCTAGGGGT TGTCGATCAG   
  
  
+ GGTTCTGGAT TTGAGACTCT CTCTCCTCCT CCACCGCAGC CGGCGGCATG TGAATCTAAT GGTGGTGGTG   
  
  
+ TTGCTAATTT GGGGTTTCCA GGGAATAATG GTAAGATTTC TTCAATTTCA CATAATTGTT CATCTGGGAT   
  
  
+ TTTGAATGGT AAGGTGAACA ATAATGGGTT GAATCCCAAT TGTAACCCTC AAGCTCAAGG CAGTCTACTT   
  
  
+ GGTTTAATCC AAGGAGCTAC AGGTGTACAC GCACATCCTG ACGTTGGAGA CGAGAAACCC CAGATTTTGA   
  
  
+ ATCCACATTT GGTGATGAAC CCTCAGCAAG CTCAGAGCAT TGCAAACCCT AGCTTTTTGA TGCACTCATT   
  
  
+ AGGTTACTAT CAGCTGGAGC AACATCTATT TCAACCTCAG GCAAAACGCC TGAACACGGG TGCTGTTCTG   
  
  
+ GATCCTAACC TTGTTCAGCT TGCAAAGAAC CCATTTGCTG ATCAGGGTCA TGAGTTATTG TTGAGGAAGC   
  
  
+ AGCACCAACA GCTTGGTTTG CAACCATTGC CATTGGGTTT GGGTCCTCAG TTGGTCCCTC CGCAGAAGCC   
  
  
+ TGTGATGGGT TCAAAGCAGG GGAACCCCCA GCATTACCCG TTGCATGTTC ATCAGCAGCT GCAATTGCAA   
  
  
+ GAGCAGGCTG TCAAAGATCA GCTCTTCAAG GCGGCAGACC TTATTCAAAC TGGAAGTTTC TCACTCGCGC   
  
  
+ AAGAGATATT GGCGCGGCTC AATCACCAGC TCTCCCTCCC TGCAAAGCCC CTCATTAGGG CGGCTTTGTA   
  
  
+ TGTGAAGGAG GCCCTTCAAA TGCTCCTCCT AATGAGCAAC CCAGTTGCGG CTCCACCGTC CAAGATCCTC   
  
  
+ ACCCCTTATG ATGTTGTTCA CAAGATGAGC GCGTATAAGG TCTTCTCTGA GGTCTGCCCA ATCACTCAAT   
  
  
+ TTGTGAATTT CACTTGTACA CAGGCCATTC TCGAGGCTCT TGATGATTCT GATGCTATTC ATGTTGTCGA   
  
  
+ CTTTGATATT GGTTGCGGTG CTCAATGGGC ATCATTGATT CAGGAGCTGC CATTGAGGAA AAGGGGAGCT   
  
  
+ CCCTCTCTGA AAATTACAGC CATAGCTCCC ATGTTGACTG GCAGTCACTT TGAAATTAGC CTAGTATGTG   
  
  
+ AAAACCTTGT GCAATTTGCC AACGATATTG GTGTTGCTTG TGAGCTCCAA GTTGTCAACT TTGATTTGTT   
  
  
+ TGATCCATCT TCGCCATCAA TGCCGAACAT TAGTACTGCT GAGGATGAGT CAATTGCTGT TAGTATCCCC   
  
  
+ ATCTGGGCAT CTTCAATTAG GCCATCTGTT CTTCCTTCCA TCCTCCGATT CATTAAGCAA AAATCCCCCA   
  
  
+ AAATTGTGGT CTCGTTTGAT AGAGGATTCG ATCGTTTTGA TGTCCCTTTC CCCCAACATC TGTTGCATAC   
  
  
+ CCTAGAATCC TGCAGTAATT TATTGGACTC GCTTGATGGT CTCAATGTTG CATCGGACAT TGTGAGCAAG   
  
  
+ GTCGAGAAGT TCTTTGTTCA ACCTAGGATC GAAAATGCTG TGTTGGGTCG GGTCCATGCC CCTGACAAGA   
  
  
+ TGCCCCATTG GAAAAATCTC TTTGCTTCAG CCGGCTTCTT GCCCTTGCAA TTCAGTAATT TCACAGAAAC   
  
  
+ CCAGGCCGAT TATGTGGTGA AGAGAACCCC AACAAGAGGA TTTCACGTGG AGAAGCGCCA GGCATCACTC   
  
  
+ ATTCTGAGTT GGCAGAGGCA GGAGCTTGTG GCAGCTTCGG CATGGAAGTG TTG  

- +Up\_Stream \_Len000AGAATT GTAATCAACG AACCAAGTTG GGTAGTCACT CTTATAGACT AAAGTTTTCA   
  
  
- TATCTAAATA TATTTGTATA CCTGATGTCC CTTCCCTCAG CAAAGGAGTT TCAGAATCTA AATGTGAGTT   
  
  
- TTCAAACTCG CTATGGTTAT TACACTAAGG TTTGTACTCG GTTTATAATT CATAGGATAT GATTCTAGTT   
  
  
- CTTCTCTTAC TTGGTTTTCT GACAATTAAA AAGTTAATTC GATTTGAATA ATTGGAAAAA AACCTTATCT   
  
  
- AAAAATTTAA AATAGAAGTT ATATTCAAAA AGGTTCCGCT CTCTGTATAT CGTTAAAATT AATTTCAATT   
  
  
- TCATCATATT GTAAAAAAAA TTACCAATAT CCGAATATCG TGGAAAAGAT CCACTTTTCT CTATAGAACC   
  
  
- GGACTAAAGG TCATTTCAAA AAAAAAATTT ATAACAGTTA ATAAAAACGT TTACTGTAAA CCATTAAACC   
  
  
- CCCTATTAGA CTACACAATT TTCTATGTTA CTTTTATAGT CTTTTATCCT ACGGAGTTTA AAGTGTTCTT   
  
  
- TCTTTTTTAG AAAAACAACA ATCATAAAGA TGTGTAGGTG TTAAAAATGA GTACCATTTT TTTTTTCCCG   
  
  
- GTTTATAGAG TGGTTATATT GGTTTGATAT ACAAGGTAAT AAATCATTGC CACTATTTAT ACATTACCCA   
  
  
- GTTCAATGAT TATTTTATCG GATGTTATGA ACCTATAGAT GTATTTTAAC TATATCTTAT ATATAACGTT   
  
  
- ATGAATTTAG TTTTAATTTA CTTTATAAAT GAAAAAATAA GATAATGTTT ATCGAAGAAT ATCTTATCGT   
  
  
- TGTTAAAAAT TACATTCAAA GTAAGAATGG TCTAAACTTC TCCAGGCCTA TATCTTTTAG AATAAGCATA   
  
  
- TTCATGAGCG TACTCCCGCC TATTTAAGAT TTTATAGTTG GAGTTGAGCG AAGATGATAA GGTAGATTCA   
  
  
- AGTTAAATAG TATATTTTTT TAAGTTAGGT GAAATTACAT GAGTTAAGTG AAGTTGGATA AAATTAGTTA   
  
  
- AATGGGTACT TTTCTTGTTC CAGGATTCAT CTACAAAAGA TAACTAACTA AATTTGTTAA AAGTGGATGG   
  
  
- GTTAATAGGG GTTTTGACTT TCTCCCCCGT TTCCCCCCAC CCATTACGAC ACATTTTATC CCCCTTTCCC   
  
  
- GTTCTTTTGA CACATGTTAC CACCTCGTCC CCATATGGTA AACCGGAACT CAACTCCCCC GTGTTTTCGG   
  
  
- GTCTCTCTCT CTCCCCATTA CGGGTGGTCT TTATTCGTCC TTCGCTCCCT TTTTGTGATC TCTCTTTGTC   
  
  
- TCTTTCTCTC TCTCTCTCTC TCTCTTCCCC TTTAAATGAT CTCTTACTCT CACTTCTTCC CTTGAAATAA   
  
  
- AATTGAAAAA ATACTACGTG CGTAGGTACC GTCTTGTCCC TTCCCTTTCT TCCCATGTCT CATGACGACA   
  
  
- GGGGTTTTTT AATCACATCT TTTGTCCTCT GTGAGTTGAT GTTGTTCCCA TTCCTTCTTC TTGTTCGTTC   
  
  
- TATAGGGTGG GTTTCTTTTT TTTTTTCTTC TTCTTCTTTT AAAAAGTCTC TCCTTTCTTT TCGTTAGTGT   
  
  
- ACTTAATCTT CGGTTGGGTT CGGAAGAAGT TTCTCTTCGT CGTATTGTAT CGTATCGACC CCTCTCCTAT   
  
  
- CCTCTCTCTT TGAGTTCTTT TCCCCATGGG CCGAATTTAC AACTACGTCT TTCCATATCT ATACCGGTGT   
  
  
- GATGATGATG GGTCCTCAAA TAGTAACCTC TACCTAAAGA GAAAGACGGA GTAGAAGAGG AGGTAGGTGG   
  
  
- TCGTTTTGAC CCAAAGGAAA GTCGACCCAG AAAAGATCTA GATCTTTGAT ACCATGGGTT TAGAGGGTAA   
  
  
- GGAGGTAAAA ATAAGAGTTT TAGATAGAAG AATCTGAACA CCCAAACTAA GTAAAGAAAA CCCAAAAACT   
  
  
- TTTCTTAAAT TAAACTAACA TAACACAACT AACCCCTTAA ACACCACCAC AATCTACGCT CGATACGGGA   
  
  
- TGTTAAACGT TCCGTTCCCA CACCATCTCC AAAGTTCGTA AACATAAGGT GTTTAGAGAA GGGGACGAAG   
  
  
- TTTCACCTTC TGGTTGTTGT TGTTGAACCT GTTGTTTAAA GTCGTTGTTG TTCTTCTTTA ACTCGTTAAC   
  
  
- GTCTCATCAC CACCGTGAAA AGAGAGAGGA CTATTGTGGT TATGATTATA ATCATGATGA GGCTCTCGCT   
  
  
- GTTCAAGACA ACTATCGCTT GTGTGTAGAT GAGACCTATG ATGCCCGGGA GGGTGAAGTT GTAGAAGGAG   
  
  
- GAAGTTCTCG TGGAGGTGGC GGTTGTGTCG GCCACACCGC CTGGGGCGTA CATGAGGGTT TCTCCTAACC   
  
  
- CTACAGAGGC CGCCGCGACG ACCGCCACTG CCGCCACCAC CCCCCGAACC TAACCTCCTC ACCCTGTCGT   
  
  
- ACAAGGGTTT ACCCCTCCCC CGAAACGAGG GAACCTAGTA CCCACTTCGA CTACTGTACC CATACCCAAA   
  
  
- CTTCGTAGAA AACGTTAGCC CATTGGGCCA ACTGATACTC CCGTTACGAC CAGATCCCCA ACAGCTAGTC   
  
  
- CCAAGACCTA AACTCTGAGA GAGAGGAGGA GGTGGCGTCG GCCGCCGTAC ACTTAGATTA CCACCACCAC   
  
  
- AACGATTAAA CCCCAAAGGT CCCTTATTAC CATTCTAAAG AAGTTAAAGT GTATTAACAA GTAGACCCTA   
  
  
- AAACTTACCA TTCCACTTGT TATTACCCAA CTTAGGGTTA ACATTGGGAG TTCGAGTTCC GTCAGATGAA   
  
  
- CCAAATTAGG TTCCTCGATG TCCACATGTG CGTGTAGGAC TGCAACCTCT GCTCTTTGGG GTCTAAAACT   
  
  
- TAGGTGTAAA CCACTACTTG GGAGTCGTTC GAGTCTCGTA ACGTTTGGGA TCGAAAAACT ACGTGAGTAA   
  
  
- TCCAATGATA GTCGACCTCG TTGTAGATAA AGTTGGAGTC CGTTTTGCGG ACTTGTGCCC ACGACAAGAC   
  
  
- CTAGGATTGG AACAAGTCGA ACGTTTCTTG GGTAAACGAC TAGTCCCAGT ACTCAATAAC AACTCCTTCG   
  
  
- TCGTGGTTGT CGAACCAAAC GTTGGTAACG GTAACCCAAA CCCAGGAGTC AACCAGGGAG GCGTCTTCGG   
  
  
- ACACTACCCA AGTTTCGTCC CCTTGGGGGT CGTAATGGGC AACGTACAAG TAGTCGTCGA CGTTAACGTT   
  
  
- CTCGTCCGAC AGTTTCTAGT CGAGAAGTTC CGCCGTCTGG AATAAGTTTG ACCTTCAAAG AGTGAGCGCG   
  
  
- TTCTCTATAA CCGCGCCGAG TTAGTGGTCG AGAGGGAGGG ACGTTTCGGG GAGTAATCCC GCCGAAACAT   
  
  
- ACACTTCCTC CGGGAAGTTT ACGAGGAGGA TTACTCGTTG GGTCAACGCC GAGGTGGCAG GTTCTAGGAG   
  
  
- TGGGGAATAC TACAACAAGT GTTCTACTCG CGCATATTCC AGAAGAGACT CCAGACGGGT TAGTGAGTTA   
  
  
- AACACTTAAA GTGAACATGT GTCCGGTAAG AGCTCCGAGA ACTACTAAGA CTACGATAAG TACAACAGCT   
  
  
- GAAACTATAA CCAACGCCAC GAGTTACCCG TAGTAACTAA GTCCTCGACG GTAACTCCTT TTCCCCTCGA   
  
  
- GGGAGAGACT TTTAATGTCG GTATCGAGGG TACAACTGAC CGTCAGTGAA ACTTTAATCG GATCATACAC   
  
  
- TTTTGGAACA CGTTAAACGG TTGCTATAAC CACAACGAAC ACTCGAGGTT CAACAGTTGA AACTAAACAA   
  
  
- ACTAGGTAGA AGCGGTAGTT ACGGCTTGTA ATCATGACGA CTCCTACTCA GTTAACGACA ATCATAGGGG   
  
  
- TAGACCCGTA GAAGTTAATC CGGTAGACAA GAAGGAAGGT AGGAGGCTAA GTAATTCGTT TTTAGGGGGT   
  
  
- TTTAACACCA GAGCAAACTA TCTCCTAAGC TAGCAAAACT ACAGGGAAAG GGGGTTGTAG ACAACGTATG   
  
  
- GGATCTTAGG ACGTCATTAA ATAACCTGAG CGAACTACCA GAGTTACAAC GTAGCCTGTA ACACTCGTTC   
  
  
- CAGCTCTTCA AGAAACAAGT TGGATCCTAG CTTTTACGAC ACAACCCAGC CCAGGTACGG GGACTGTTCT   
  
  
- ACGGGGTAAC CTTTTTAGAG AAACGAAGTC GGCCGAAGAA CGGGAACGTT AAGTCATTAA AGTGTCTTTG   
  
  
- GGTCCGGCTA ATACACCACT TCTCTTGGGG TTGTTCTCCT AAAGTGCACC TCTTCGCGGT CCGTAGTGAG   
  
  
- TAAGACTCAA CCGTCTCCGT CCTCGAACAC CGTCGAAGCC GTACCTTCAC AAC

+     GATA-motif

| Site Name | Organism | Position | Strand | Matrix score. | sequence | function |
| --- | --- | --- | --- | --- | --- | --- |
| GATA-motif | Arabidopsis thaliana | 291 | - | 10 | AAGATAAGATT | part of a light responsive element |
| GATA-motif | Solanum tuberosum | 1519 | + | 9 | AAGGATAAGG | part of a light responsive element |
| GATA-motif | Arabidopsis thaliana | 1681 | + | 7 | GATAGGA | part of a light responsive element |

>HU04G00047.1   
+ +Up\_Stream \_Len000TCTTAA CATTAGTTGC TTGGTTCAAC CCATCAGTGA GAATATCTGA TTTCAAAAGT   
  
  
+ ATAGATTTAT ATAAACATAT GGACTACAGG GAAGGGAGTC GTTTCCTCAA AGTCTTAGAT TTACACTCAA   
  
  
+ AAGTTTGAGC GATACCAATA ATGTGATTCC AAACATGAGC CAAATATTAA GTATCCTATA CTAAGATCAA   
  
  
+ GAAGAGAATG AACCAAAAGA CTGTTAATTT TTCAATTAAG CTAAACTTAT TAACCTTTTT TTGGAATAGA   
  
  
+ TTTTTAAATT TTATCTTCAA TATAAGTTTT TCCAAGGCGA GAGACATATA GCAATTTTAA TTAAAGTTAA   
  
  
+ AGTAGTATAA CATTTTTTTT AATGGTTATA GGCTTATAGC ACCTTTTCTA GGTGAAAAGA GATATCTTGG   
  
  
+ CCTGATTTCC AGTAAAGTTT TTTTTTTAAA TATTGTCAAT TATTTTTGCA AATGACATTT GGTAATTTGG   
  
  
+ GGGATAATCT GATGTGTTAA AAGATACAAT GAAAATATCA GAAAATAGGA TGCCTCAAAT TTCACAAGAA   
  
  
+ AGAAAAAATC TTTTTGTTGT TAGTATTTCT ACACATCCAC AATTTTTACT CATGGTAAAA AAAAAAGGGC   
  
  
+ CAAATATCTC ACCAATATAA CCAAACTATA TGTTCCATTA TTTAGTAACG GTGATAAATA TGTAATGGGT   
  
  
+ CAAGTTACTA ATAAAATAGC CTACAATACT TGGATATCTA CATAAAATTG ATATAGAATA TATATTGCAA   
  
  
+ TACTTAAATC AAAATTAAAT GAAATATTTA CTTTTTTATT CTATTACAAA TAGCTTCTTA TAGAATAGCA   
  
  
+ ACAATTTTTA ATGTAAGTTT CATTCTTACC AGATTTGAAG AGGTCCGGAT ATAGAAAATC TTATTCGTAT   
  
  
+ AAGTACTCGC ATGAGGGCGG ATAAATTCTA AAATATCAAC CTCAACTCGC TTCTACTATT CCATCTAAGT   
  
  
+ TCAATTTATC ATATAAAAAA ATTCAATCCA CTTTAATGTA CTCAATTCAC TTCAACCTAT TTTAATCAAT   
  
  
+ TTACCCATGA AAAGAACAAG GTCCTAAGTA GATGTTTTCT ATTGATTGAT TTAAACAATT TTCACCTACC   
  
  
+ CAATTATCCC CAAAACTGAA AGAGGGGGCA AAGGGGGGTG GGTAATGCTG TGTAAAATAG GGGGAAAGGG   
  
  
+ CAAGAAAACT GTGTACAATG GTGGAGCAGG GGTATACCAT TTGGCCTTGA GTTGAGGGGG CACAAAAGCC   
  
  
+ CAGAGAGAGA GAGGGGTAAT GCCCACCAGA AATAAGCAGG AAGCGAGGGA AAAACACTAG AGAGAAACAG   
  
  
+ AGAAAGAGAG AGAGAGAGAG AGAGAAGGGG AAATTTACTA GAGAATGAGA GTGAAGAAGG GAACTTTATT   
  
  
+ TTAACTTTTT TATGATGCAC GCATCCATGG CAGAACAGGG AAGGGAAAGA AGGGTACAGA GTACTGCTGT   
  
  
+ CCCCAAAAAA TTAGTGTAGA AAACAGGAGA CACTCAACTA CAACAAGGGT AAGGAAGAAG AACAAGCAAG   
  
  
+ ATATCCCACC CAAAGAAAAA AAAAAAGAAG AAGAAGAAAA TTTTTCAGAG AGGAAAGAAA AGCAATCACA   
  
  
+ TGAATTAGAA GCCAACCCAA GCCTTCTTCA AAGAGAAGCA GCATAACATA GCATAGCTGG GGAGAGGATA   
  
  
+ GGAGAGAGAA ACTCAAGAAA AGGGGTACCC GGCTTAAATG TTGATGCAGA AAGGTATAGA TATGGCCACA   
  
  
+ CTACTACTAC CCAGGAGTTT ATCATTGGAG ATGGATTTCT CTTTCTGCCT CATCTTCTCC TCCATCCACC   
  
  
+ AGCAAAACTG GGTTTCCTTT CAGCTGGGTC TTTTCTAGAT CTAGAAACTA TGGTACCCAA ATCTCCCATT   
  
  
+ CCTCCATTTT TATTCTCAAA ATCTATCTTC TTAGACTTGT GGGTTTGATT CATTTCTTTT GGGTTTTTGA   
  
  
+ AAAGAATTTA ATTTGATTGT ATTGTGTTGA TTGGGGAATT TGTGGTGGTG TTAGATGCGA GCTATGCCCT   
  
  
+ ACAATTTGCA AGGCAAGGGT GTGGTAGAGG TTTCAAGCAT TTGTATTCCA CAAATCTCTT CCCCTGCTTC   
  
  
+ AAAGTGGAAG ACCAACAACA ACAACTTGGA CAACAAATTT CAGCAACAAC AAGAAGAAAT TGAGCAATTG   
  
  
+ CAGAGTAGTG GTGGCACTTT TCTCTCTCCT GATAACACCA ATACTAATAT TAGTACTACT CCGAGAGCGA   
  
  
+ CAAGTTCTGT TGATAGCGAA CACACATCTA CTCTGGATAC TACGGGCCCT CCCACTTCAA CATCTTCCTC   
  
  
+ CTTCAAGAGC ACCTCCACCG CCAACACAGC CGGTGTGGCG GACCCCGCAT GTACTCCCAA AGAGGATTGG   
  
  
+ GATGTCTCCG GCGGCGCTGC TGGCGGTGAC GGCGGTGGTG GGGGGCTTGG ATTGGAGGAG TGGGACAGCA   
  
  
+ TGTTCCCAAA TGGGGAGGGG GCTTTGCTCC CTTGGATCAT GGGTGAAGCT GATGACATGG GTATGGGTTT   
  
  
+ GAAGCATCTT TTGCAATCGG GTAACCCGGT TGACTATGAG GGCAATGCTG GTCTAGGGGT TGTCGATCAG   
  
  
+ GGTTCTGGAT TTGAGACTCT CTCTCCTCCT CCACCGCAGC CGGCGGCATG TGAATCTAAT GGTGGTGGTG   
  
  
+ TTGCTAATTT GGGGTTTCCA GGGAATAATG GTAAGATTTC TTCAATTTCA CATAATTGTT CATCTGGGAT   
  
  
+ TTTGAATGGT AAGGTGAACA ATAATGGGTT GAATCCCAAT TGTAACCCTC AAGCTCAAGG CAGTCTACTT   
  
  
+ GGTTTAATCC AAGGAGCTAC AGGTGTACAC GCACATCCTG ACGTTGGAGA CGAGAAACCC CAGATTTTGA   
  
  
+ ATCCACATTT GGTGATGAAC CCTCAGCAAG CTCAGAGCAT TGCAAACCCT AGCTTTTTGA TGCACTCATT   
  
  
+ AGGTTACTAT CAGCTGGAGC AACATCTATT TCAACCTCAG GCAAAACGCC TGAACACGGG TGCTGTTCTG   
  
  
+ GATCCTAACC TTGTTCAGCT TGCAAAGAAC CCATTTGCTG ATCAGGGTCA TGAGTTATTG TTGAGGAAGC   
  
  
+ AGCACCAACA GCTTGGTTTG CAACCATTGC CATTGGGTTT GGGTCCTCAG TTGGTCCCTC CGCAGAAGCC   
  
  
+ TGTGATGGGT TCAAAGCAGG GGAACCCCCA GCATTACCCG TTGCATGTTC ATCAGCAGCT GCAATTGCAA   
  
  
+ GAGCAGGCTG TCAAAGATCA GCTCTTCAAG GCGGCAGACC TTATTCAAAC TGGAAGTTTC TCACTCGCGC   
  
  
+ AAGAGATATT GGCGCGGCTC AATCACCAGC TCTCCCTCCC TGCAAAGCCC CTCATTAGGG CGGCTTTGTA   
  
  
+ TGTGAAGGAG GCCCTTCAAA TGCTCCTCCT AATGAGCAAC CCAGTTGCGG CTCCACCGTC CAAGATCCTC   
  
  
+ ACCCCTTATG ATGTTGTTCA CAAGATGAGC GCGTATAAGG TCTTCTCTGA GGTCTGCCCA ATCACTCAAT   
  
  
+ TTGTGAATTT CACTTGTACA CAGGCCATTC TCGAGGCTCT TGATGATTCT GATGCTATTC ATGTTGTCGA   
  
  
+ CTTTGATATT GGTTGCGGTG CTCAATGGGC ATCATTGATT CAGGAGCTGC CATTGAGGAA AAGGGGAGCT   
  
  
+ CCCTCTCTGA AAATTACAGC CATAGCTCCC ATGTTGACTG GCAGTCACTT TGAAATTAGC CTAGTATGTG   
  
  
+ AAAACCTTGT GCAATTTGCC AACGATATTG GTGTTGCTTG TGAGCTCCAA GTTGTCAACT TTGATTTGTT   
  
  
+ TGATCCATCT TCGCCATCAA TGCCGAACAT TAGTACTGCT GAGGATGAGT CAATTGCTGT TAGTATCCCC   
  
  
+ ATCTGGGCAT CTTCAATTAG GCCATCTGTT CTTCCTTCCA TCCTCCGATT CATTAAGCAA AAATCCCCCA   
  
  
+ AAATTGTGGT CTCGTTTGAT AGAGGATTCG ATCGTTTTGA TGTCCCTTTC CCCCAACATC TGTTGCATAC   
  
  
+ CCTAGAATCC TGCAGTAATT TATTGGACTC GCTTGATGGT CTCAATGTTG CATCGGACAT TGTGAGCAAG   
  
  
+ GTCGAGAAGT TCTTTGTTCA ACCTAGGATC GAAAATGCTG TGTTGGGTCG GGTCCATGCC CCTGACAAGA   
  
  
+ TGCCCCATTG GAAAAATCTC TTTGCTTCAG CCGGCTTCTT GCCCTTGCAA TTCAGTAATT TCACAGAAAC   
  
  
+ CCAGGCCGAT TATGTGGTGA AGAGAACCCC AACAAGAGGA TTTCACGTGG AGAAGCGCCA GGCATCACTC   
  
  
+ ATTCTGAGTT GGCAGAGGCA GGAGCTTGTG GCAGCTTCGG CATGGAAGTG TTG  

- +Up\_Stream \_Len000AGAATT GTAATCAACG AACCAAGTTG GGTAGTCACT CTTATAGACT AAAGTTTTCA   
  
  
- TATCTAAATA TATTTGTATA CCTGATGTCC CTTCCCTCAG CAAAGGAGTT TCAGAATCTA AATGTGAGTT   
  
  
- TTCAAACTCG CTATGGTTAT TACACTAAGG TTTGTACTCG GTTTATAATT CATAGGATAT GATTCTAGTT   
  
  
- CTTCTCTTAC TTGGTTTTCT GACAATTAAA AAGTTAATTC GATTTGAATA ATTGGAAAAA AACCTTATCT   
  
  
- AAAAATTTAA AATAGAAGTT ATATTCAAAA AGGTTCCGCT CTCTGTATAT CGTTAAAATT AATTTCAATT   
  
  
- TCATCATATT GTAAAAAAAA TTACCAATAT CCGAATATCG TGGAAAAGAT CCACTTTTCT CTATAGAACC   
  
  
- GGACTAAAGG TCATTTCAAA AAAAAAATTT ATAACAGTTA ATAAAAACGT TTACTGTAAA CCATTAAACC   
  
  
- CCCTATTAGA CTACACAATT TTCTATGTTA CTTTTATAGT CTTTTATCCT ACGGAGTTTA AAGTGTTCTT   
  
  
- TCTTTTTTAG AAAAACAACA ATCATAAAGA TGTGTAGGTG TTAAAAATGA GTACCATTTT TTTTTTCCCG   
  
  
- GTTTATAGAG TGGTTATATT GGTTTGATAT ACAAGGTAAT AAATCATTGC CACTATTTAT ACATTACCCA   
  
  
- GTTCAATGAT TATTTTATCG GATGTTATGA ACCTATAGAT GTATTTTAAC TATATCTTAT ATATAACGTT   
  
  
- ATGAATTTAG TTTTAATTTA CTTTATAAAT GAAAAAATAA GATAATGTTT ATCGAAGAAT ATCTTATCGT   
  
  
- TGTTAAAAAT TACATTCAAA GTAAGAATGG TCTAAACTTC TCCAGGCCTA TATCTTTTAG AATAAGCATA   
  
  
- TTCATGAGCG TACTCCCGCC TATTTAAGAT TTTATAGTTG GAGTTGAGCG AAGATGATAA GGTAGATTCA   
  
  
- AGTTAAATAG TATATTTTTT TAAGTTAGGT GAAATTACAT GAGTTAAGTG AAGTTGGATA AAATTAGTTA   
  
  
- AATGGGTACT TTTCTTGTTC CAGGATTCAT CTACAAAAGA TAACTAACTA AATTTGTTAA AAGTGGATGG   
  
  
- GTTAATAGGG GTTTTGACTT TCTCCCCCGT TTCCCCCCAC CCATTACGAC ACATTTTATC CCCCTTTCCC   
  
  
- GTTCTTTTGA CACATGTTAC CACCTCGTCC CCATATGGTA AACCGGAACT CAACTCCCCC GTGTTTTCGG   
  
  
- GTCTCTCTCT CTCCCCATTA CGGGTGGTCT TTATTCGTCC TTCGCTCCCT TTTTGTGATC TCTCTTTGTC   
  
  
- TCTTTCTCTC TCTCTCTCTC TCTCTTCCCC TTTAAATGAT CTCTTACTCT CACTTCTTCC CTTGAAATAA   
  
  
- AATTGAAAAA ATACTACGTG CGTAGGTACC GTCTTGTCCC TTCCCTTTCT TCCCATGTCT CATGACGACA   
  
  
- GGGGTTTTTT AATCACATCT TTTGTCCTCT GTGAGTTGAT GTTGTTCCCA TTCCTTCTTC TTGTTCGTTC   
  
  
- TATAGGGTGG GTTTCTTTTT TTTTTTCTTC TTCTTCTTTT AAAAAGTCTC TCCTTTCTTT TCGTTAGTGT   
  
  
- ACTTAATCTT CGGTTGGGTT CGGAAGAAGT TTCTCTTCGT CGTATTGTAT CGTATCGACC CCTCTCCTAT   
  
  
- CCTCTCTCTT TGAGTTCTTT TCCCCATGGG CCGAATTTAC AACTACGTCT TTCCATATCT ATACCGGTGT   
  
  
- GATGATGATG GGTCCTCAAA TAGTAACCTC TACCTAAAGA GAAAGACGGA GTAGAAGAGG AGGTAGGTGG   
  
  
- TCGTTTTGAC CCAAAGGAAA GTCGACCCAG AAAAGATCTA GATCTTTGAT ACCATGGGTT TAGAGGGTAA   
  
  
- GGAGGTAAAA ATAAGAGTTT TAGATAGAAG AATCTGAACA CCCAAACTAA GTAAAGAAAA CCCAAAAACT   
  
  
- TTTCTTAAAT TAAACTAACA TAACACAACT AACCCCTTAA ACACCACCAC AATCTACGCT CGATACGGGA   
  
  
- TGTTAAACGT TCCGTTCCCA CACCATCTCC AAAGTTCGTA AACATAAGGT GTTTAGAGAA GGGGACGAAG   
  
  
- TTTCACCTTC TGGTTGTTGT TGTTGAACCT GTTGTTTAAA GTCGTTGTTG TTCTTCTTTA ACTCGTTAAC   
  
  
- GTCTCATCAC CACCGTGAAA AGAGAGAGGA CTATTGTGGT TATGATTATA ATCATGATGA GGCTCTCGCT   
  
  
- GTTCAAGACA ACTATCGCTT GTGTGTAGAT GAGACCTATG ATGCCCGGGA GGGTGAAGTT GTAGAAGGAG   
  
  
- GAAGTTCTCG TGGAGGTGGC GGTTGTGTCG GCCACACCGC CTGGGGCGTA CATGAGGGTT TCTCCTAACC   
  
  
- CTACAGAGGC CGCCGCGACG ACCGCCACTG CCGCCACCAC CCCCCGAACC TAACCTCCTC ACCCTGTCGT   
  
  
- ACAAGGGTTT ACCCCTCCCC CGAAACGAGG GAACCTAGTA CCCACTTCGA CTACTGTACC CATACCCAAA   
  
  
- CTTCGTAGAA AACGTTAGCC CATTGGGCCA ACTGATACTC CCGTTACGAC CAGATCCCCA ACAGCTAGTC   
  
  
- CCAAGACCTA AACTCTGAGA GAGAGGAGGA GGTGGCGTCG GCCGCCGTAC ACTTAGATTA CCACCACCAC   
  
  
- AACGATTAAA CCCCAAAGGT CCCTTATTAC CATTCTAAAG AAGTTAAAGT GTATTAACAA GTAGACCCTA   
  
  
- AAACTTACCA TTCCACTTGT TATTACCCAA CTTAGGGTTA ACATTGGGAG TTCGAGTTCC GTCAGATGAA   
  
  
- CCAAATTAGG TTCCTCGATG TCCACATGTG CGTGTAGGAC TGCAACCTCT GCTCTTTGGG GTCTAAAACT   
  
  
- TAGGTGTAAA CCACTACTTG GGAGTCGTTC GAGTCTCGTA ACGTTTGGGA TCGAAAAACT ACGTGAGTAA   
  
  
- TCCAATGATA GTCGACCTCG TTGTAGATAA AGTTGGAGTC CGTTTTGCGG ACTTGTGCCC ACGACAAGAC   
  
  
- CTAGGATTGG AACAAGTCGA ACGTTTCTTG GGTAAACGAC TAGTCCCAGT ACTCAATAAC AACTCCTTCG   
  
  
- TCGTGGTTGT CGAACCAAAC GTTGGTAACG GTAACCCAAA CCCAGGAGTC AACCAGGGAG GCGTCTTCGG   
  
  
- ACACTACCCA AGTTTCGTCC CCTTGGGGGT CGTAATGGGC AACGTACAAG TAGTCGTCGA CGTTAACGTT   
  
  
- CTCGTCCGAC AGTTTCTAGT CGAGAAGTTC CGCCGTCTGG AATAAGTTTG ACCTTCAAAG AGTGAGCGCG   
  
  
- TTCTCTATAA CCGCGCCGAG TTAGTGGTCG AGAGGGAGGG ACGTTTCGGG GAGTAATCCC GCCGAAACAT   
  
  
- ACACTTCCTC CGGGAAGTTT ACGAGGAGGA TTACTCGTTG GGTCAACGCC GAGGTGGCAG GTTCTAGGAG   
  
  
- TGGGGAATAC TACAACAAGT GTTCTACTCG CGCATATTCC AGAAGAGACT CCAGACGGGT TAGTGAGTTA   
  
  
- AACACTTAAA GTGAACATGT GTCCGGTAAG AGCTCCGAGA ACTACTAAGA CTACGATAAG TACAACAGCT   
  
  
- GAAACTATAA CCAACGCCAC GAGTTACCCG TAGTAACTAA GTCCTCGACG GTAACTCCTT TTCCCCTCGA   
  
  
- GGGAGAGACT TTTAATGTCG GTATCGAGGG TACAACTGAC CGTCAGTGAA ACTTTAATCG GATCATACAC   
  
  
- TTTTGGAACA CGTTAAACGG TTGCTATAAC CACAACGAAC ACTCGAGGTT CAACAGTTGA AACTAAACAA   
  
  
- ACTAGGTAGA AGCGGTAGTT ACGGCTTGTA ATCATGACGA CTCCTACTCA GTTAACGACA ATCATAGGGG   
  
  
- TAGACCCGTA GAAGTTAATC CGGTAGACAA GAAGGAAGGT AGGAGGCTAA GTAATTCGTT TTTAGGGGGT   
  
  
- TTTAACACCA GAGCAAACTA TCTCCTAAGC TAGCAAAACT ACAGGGAAAG GGGGTTGTAG ACAACGTATG   
  
  
- GGATCTTAGG ACGTCATTAA ATAACCTGAG CGAACTACCA GAGTTACAAC GTAGCCTGTA ACACTCGTTC   
  
  
- CAGCTCTTCA AGAAACAAGT TGGATCCTAG CTTTTACGAC ACAACCCAGC CCAGGTACGG GGACTGTTCT   
  
  
- ACGGGGTAAC CTTTTTAGAG AAACGAAGTC GGCCGAAGAA CGGGAACGTT AAGTCATTAA AGTGTCTTTG   
  
  
- GGTCCGGCTA ATACACCACT TCTCTTGGGG TTGTTCTCCT AAAGTGCACC TCTTCGCGGT CCGTAGTGAG   
  
  
- TAAGACTCAA CCGTCTCCGT CCTCGAACAC CGTCGAAGCC GTACCTTCAC AAC

+     GCN4\_motif

| Site Name | Organism | Position | Strand | Matrix score. | sequence | function |
| --- | --- | --- | --- | --- | --- | --- |
| GCN4\_motif | Oryza sativa | 3830 | + | 7 | TGAGTCA | cis-regulatory element involved in endosperm expression |

>HU04G00047.1   
+ +Up\_Stream \_Len000TCTTAA CATTAGTTGC TTGGTTCAAC CCATCAGTGA GAATATCTGA TTTCAAAAGT   
  
  
+ ATAGATTTAT ATAAACATAT GGACTACAGG GAAGGGAGTC GTTTCCTCAA AGTCTTAGAT TTACACTCAA   
  
  
+ AAGTTTGAGC GATACCAATA ATGTGATTCC AAACATGAGC CAAATATTAA GTATCCTATA CTAAGATCAA   
  
  
+ GAAGAGAATG AACCAAAAGA CTGTTAATTT TTCAATTAAG CTAAACTTAT TAACCTTTTT TTGGAATAGA   
  
  
+ TTTTTAAATT TTATCTTCAA TATAAGTTTT TCCAAGGCGA GAGACATATA GCAATTTTAA TTAAAGTTAA   
  
  
+ AGTAGTATAA CATTTTTTTT AATGGTTATA GGCTTATAGC ACCTTTTCTA GGTGAAAAGA GATATCTTGG   
  
  
+ CCTGATTTCC AGTAAAGTTT TTTTTTTAAA TATTGTCAAT TATTTTTGCA AATGACATTT GGTAATTTGG   
  
  
+ GGGATAATCT GATGTGTTAA AAGATACAAT GAAAATATCA GAAAATAGGA TGCCTCAAAT TTCACAAGAA   
  
  
+ AGAAAAAATC TTTTTGTTGT TAGTATTTCT ACACATCCAC AATTTTTACT CATGGTAAAA AAAAAAGGGC   
  
  
+ CAAATATCTC ACCAATATAA CCAAACTATA TGTTCCATTA TTTAGTAACG GTGATAAATA TGTAATGGGT   
  
  
+ CAAGTTACTA ATAAAATAGC CTACAATACT TGGATATCTA CATAAAATTG ATATAGAATA TATATTGCAA   
  
  
+ TACTTAAATC AAAATTAAAT GAAATATTTA CTTTTTTATT CTATTACAAA TAGCTTCTTA TAGAATAGCA   
  
  
+ ACAATTTTTA ATGTAAGTTT CATTCTTACC AGATTTGAAG AGGTCCGGAT ATAGAAAATC TTATTCGTAT   
  
  
+ AAGTACTCGC ATGAGGGCGG ATAAATTCTA AAATATCAAC CTCAACTCGC TTCTACTATT CCATCTAAGT   
  
  
+ TCAATTTATC ATATAAAAAA ATTCAATCCA CTTTAATGTA CTCAATTCAC TTCAACCTAT TTTAATCAAT   
  
  
+ TTACCCATGA AAAGAACAAG GTCCTAAGTA GATGTTTTCT ATTGATTGAT TTAAACAATT TTCACCTACC   
  
  
+ CAATTATCCC CAAAACTGAA AGAGGGGGCA AAGGGGGGTG GGTAATGCTG TGTAAAATAG GGGGAAAGGG   
  
  
+ CAAGAAAACT GTGTACAATG GTGGAGCAGG GGTATACCAT TTGGCCTTGA GTTGAGGGGG CACAAAAGCC   
  
  
+ CAGAGAGAGA GAGGGGTAAT GCCCACCAGA AATAAGCAGG AAGCGAGGGA AAAACACTAG AGAGAAACAG   
  
  
+ AGAAAGAGAG AGAGAGAGAG AGAGAAGGGG AAATTTACTA GAGAATGAGA GTGAAGAAGG GAACTTTATT   
  
  
+ TTAACTTTTT TATGATGCAC GCATCCATGG CAGAACAGGG AAGGGAAAGA AGGGTACAGA GTACTGCTGT   
  
  
+ CCCCAAAAAA TTAGTGTAGA AAACAGGAGA CACTCAACTA CAACAAGGGT AAGGAAGAAG AACAAGCAAG   
  
  
+ ATATCCCACC CAAAGAAAAA AAAAAAGAAG AAGAAGAAAA TTTTTCAGAG AGGAAAGAAA AGCAATCACA   
  
  
+ TGAATTAGAA GCCAACCCAA GCCTTCTTCA AAGAGAAGCA GCATAACATA GCATAGCTGG GGAGAGGATA   
  
  
+ GGAGAGAGAA ACTCAAGAAA AGGGGTACCC GGCTTAAATG TTGATGCAGA AAGGTATAGA TATGGCCACA   
  
  
+ CTACTACTAC CCAGGAGTTT ATCATTGGAG ATGGATTTCT CTTTCTGCCT CATCTTCTCC TCCATCCACC   
  
  
+ AGCAAAACTG GGTTTCCTTT CAGCTGGGTC TTTTCTAGAT CTAGAAACTA TGGTACCCAA ATCTCCCATT   
  
  
+ CCTCCATTTT TATTCTCAAA ATCTATCTTC TTAGACTTGT GGGTTTGATT CATTTCTTTT GGGTTTTTGA   
  
  
+ AAAGAATTTA ATTTGATTGT ATTGTGTTGA TTGGGGAATT TGTGGTGGTG TTAGATGCGA GCTATGCCCT   
  
  
+ ACAATTTGCA AGGCAAGGGT GTGGTAGAGG TTTCAAGCAT TTGTATTCCA CAAATCTCTT CCCCTGCTTC   
  
  
+ AAAGTGGAAG ACCAACAACA ACAACTTGGA CAACAAATTT CAGCAACAAC AAGAAGAAAT TGAGCAATTG   
  
  
+ CAGAGTAGTG GTGGCACTTT TCTCTCTCCT GATAACACCA ATACTAATAT TAGTACTACT CCGAGAGCGA   
  
  
+ CAAGTTCTGT TGATAGCGAA CACACATCTA CTCTGGATAC TACGGGCCCT CCCACTTCAA CATCTTCCTC   
  
  
+ CTTCAAGAGC ACCTCCACCG CCAACACAGC CGGTGTGGCG GACCCCGCAT GTACTCCCAA AGAGGATTGG   
  
  
+ GATGTCTCCG GCGGCGCTGC TGGCGGTGAC GGCGGTGGTG GGGGGCTTGG ATTGGAGGAG TGGGACAGCA   
  
  
+ TGTTCCCAAA TGGGGAGGGG GCTTTGCTCC CTTGGATCAT GGGTGAAGCT GATGACATGG GTATGGGTTT   
  
  
+ GAAGCATCTT TTGCAATCGG GTAACCCGGT TGACTATGAG GGCAATGCTG GTCTAGGGGT TGTCGATCAG   
  
  
+ GGTTCTGGAT TTGAGACTCT CTCTCCTCCT CCACCGCAGC CGGCGGCATG TGAATCTAAT GGTGGTGGTG   
  
  
+ TTGCTAATTT GGGGTTTCCA GGGAATAATG GTAAGATTTC TTCAATTTCA CATAATTGTT CATCTGGGAT   
  
  
+ TTTGAATGGT AAGGTGAACA ATAATGGGTT GAATCCCAAT TGTAACCCTC AAGCTCAAGG CAGTCTACTT   
  
  
+ GGTTTAATCC AAGGAGCTAC AGGTGTACAC GCACATCCTG ACGTTGGAGA CGAGAAACCC CAGATTTTGA   
  
  
+ ATCCACATTT GGTGATGAAC CCTCAGCAAG CTCAGAGCAT TGCAAACCCT AGCTTTTTGA TGCACTCATT   
  
  
+ AGGTTACTAT CAGCTGGAGC AACATCTATT TCAACCTCAG GCAAAACGCC TGAACACGGG TGCTGTTCTG   
  
  
+ GATCCTAACC TTGTTCAGCT TGCAAAGAAC CCATTTGCTG ATCAGGGTCA TGAGTTATTG TTGAGGAAGC   
  
  
+ AGCACCAACA GCTTGGTTTG CAACCATTGC CATTGGGTTT GGGTCCTCAG TTGGTCCCTC CGCAGAAGCC   
  
  
+ TGTGATGGGT TCAAAGCAGG GGAACCCCCA GCATTACCCG TTGCATGTTC ATCAGCAGCT GCAATTGCAA   
  
  
+ GAGCAGGCTG TCAAAGATCA GCTCTTCAAG GCGGCAGACC TTATTCAAAC TGGAAGTTTC TCACTCGCGC   
  
  
+ AAGAGATATT GGCGCGGCTC AATCACCAGC TCTCCCTCCC TGCAAAGCCC CTCATTAGGG CGGCTTTGTA   
  
  
+ TGTGAAGGAG GCCCTTCAAA TGCTCCTCCT AATGAGCAAC CCAGTTGCGG CTCCACCGTC CAAGATCCTC   
  
  
+ ACCCCTTATG ATGTTGTTCA CAAGATGAGC GCGTATAAGG TCTTCTCTGA GGTCTGCCCA ATCACTCAAT   
  
  
+ TTGTGAATTT CACTTGTACA CAGGCCATTC TCGAGGCTCT TGATGATTCT GATGCTATTC ATGTTGTCGA   
  
  
+ CTTTGATATT GGTTGCGGTG CTCAATGGGC ATCATTGATT CAGGAGCTGC CATTGAGGAA AAGGGGAGCT   
  
  
+ CCCTCTCTGA AAATTACAGC CATAGCTCCC ATGTTGACTG GCAGTCACTT TGAAATTAGC CTAGTATGTG   
  
  
+ AAAACCTTGT GCAATTTGCC AACGATATTG GTGTTGCTTG TGAGCTCCAA GTTGTCAACT TTGATTTGTT   
  
  
+ TGATCCATCT TCGCCATCAA TGCCGAACAT TAGTACTGCT GAGGATGAGT CAATTGCTGT TAGTATCCCC   
  
  
+ ATCTGGGCAT CTTCAATTAG GCCATCTGTT CTTCCTTCCA TCCTCCGATT CATTAAGCAA AAATCCCCCA   
  
  
+ AAATTGTGGT CTCGTTTGAT AGAGGATTCG ATCGTTTTGA TGTCCCTTTC CCCCAACATC TGTTGCATAC   
  
  
+ CCTAGAATCC TGCAGTAATT TATTGGACTC GCTTGATGGT CTCAATGTTG CATCGGACAT TGTGAGCAAG   
  
  
+ GTCGAGAAGT TCTTTGTTCA ACCTAGGATC GAAAATGCTG TGTTGGGTCG GGTCCATGCC CCTGACAAGA   
  
  
+ TGCCCCATTG GAAAAATCTC TTTGCTTCAG CCGGCTTCTT GCCCTTGCAA TTCAGTAATT TCACAGAAAC   
  
  
+ CCAGGCCGAT TATGTGGTGA AGAGAACCCC AACAAGAGGA TTTCACGTGG AGAAGCGCCA GGCATCACTC   
  
  
+ ATTCTGAGTT GGCAGAGGCA GGAGCTTGTG GCAGCTTCGG CATGGAAGTG TTG  

- +Up\_Stream \_Len000AGAATT GTAATCAACG AACCAAGTTG GGTAGTCACT CTTATAGACT AAAGTTTTCA   
  
  
- TATCTAAATA TATTTGTATA CCTGATGTCC CTTCCCTCAG CAAAGGAGTT TCAGAATCTA AATGTGAGTT   
  
  
- TTCAAACTCG CTATGGTTAT TACACTAAGG TTTGTACTCG GTTTATAATT CATAGGATAT GATTCTAGTT   
  
  
- CTTCTCTTAC TTGGTTTTCT GACAATTAAA AAGTTAATTC GATTTGAATA ATTGGAAAAA AACCTTATCT   
  
  
- AAAAATTTAA AATAGAAGTT ATATTCAAAA AGGTTCCGCT CTCTGTATAT CGTTAAAATT AATTTCAATT   
  
  
- TCATCATATT GTAAAAAAAA TTACCAATAT CCGAATATCG TGGAAAAGAT CCACTTTTCT CTATAGAACC   
  
  
- GGACTAAAGG TCATTTCAAA AAAAAAATTT ATAACAGTTA ATAAAAACGT TTACTGTAAA CCATTAAACC   
  
  
- CCCTATTAGA CTACACAATT TTCTATGTTA CTTTTATAGT CTTTTATCCT ACGGAGTTTA AAGTGTTCTT   
  
  
- TCTTTTTTAG AAAAACAACA ATCATAAAGA TGTGTAGGTG TTAAAAATGA GTACCATTTT TTTTTTCCCG   
  
  
- GTTTATAGAG TGGTTATATT GGTTTGATAT ACAAGGTAAT AAATCATTGC CACTATTTAT ACATTACCCA   
  
  
- GTTCAATGAT TATTTTATCG GATGTTATGA ACCTATAGAT GTATTTTAAC TATATCTTAT ATATAACGTT   
  
  
- ATGAATTTAG TTTTAATTTA CTTTATAAAT GAAAAAATAA GATAATGTTT ATCGAAGAAT ATCTTATCGT   
  
  
- TGTTAAAAAT TACATTCAAA GTAAGAATGG TCTAAACTTC TCCAGGCCTA TATCTTTTAG AATAAGCATA   
  
  
- TTCATGAGCG TACTCCCGCC TATTTAAGAT TTTATAGTTG GAGTTGAGCG AAGATGATAA GGTAGATTCA   
  
  
- AGTTAAATAG TATATTTTTT TAAGTTAGGT GAAATTACAT GAGTTAAGTG AAGTTGGATA AAATTAGTTA   
  
  
- AATGGGTACT TTTCTTGTTC CAGGATTCAT CTACAAAAGA TAACTAACTA AATTTGTTAA AAGTGGATGG   
  
  
- GTTAATAGGG GTTTTGACTT TCTCCCCCGT TTCCCCCCAC CCATTACGAC ACATTTTATC CCCCTTTCCC   
  
  
- GTTCTTTTGA CACATGTTAC CACCTCGTCC CCATATGGTA AACCGGAACT CAACTCCCCC GTGTTTTCGG   
  
  
- GTCTCTCTCT CTCCCCATTA CGGGTGGTCT TTATTCGTCC TTCGCTCCCT TTTTGTGATC TCTCTTTGTC   
  
  
- TCTTTCTCTC TCTCTCTCTC TCTCTTCCCC TTTAAATGAT CTCTTACTCT CACTTCTTCC CTTGAAATAA   
  
  
- AATTGAAAAA ATACTACGTG CGTAGGTACC GTCTTGTCCC TTCCCTTTCT TCCCATGTCT CATGACGACA   
  
  
- GGGGTTTTTT AATCACATCT TTTGTCCTCT GTGAGTTGAT GTTGTTCCCA TTCCTTCTTC TTGTTCGTTC   
  
  
- TATAGGGTGG GTTTCTTTTT TTTTTTCTTC TTCTTCTTTT AAAAAGTCTC TCCTTTCTTT TCGTTAGTGT   
  
  
- ACTTAATCTT CGGTTGGGTT CGGAAGAAGT TTCTCTTCGT CGTATTGTAT CGTATCGACC CCTCTCCTAT   
  
  
- CCTCTCTCTT TGAGTTCTTT TCCCCATGGG CCGAATTTAC AACTACGTCT TTCCATATCT ATACCGGTGT   
  
  
- GATGATGATG GGTCCTCAAA TAGTAACCTC TACCTAAAGA GAAAGACGGA GTAGAAGAGG AGGTAGGTGG   
  
  
- TCGTTTTGAC CCAAAGGAAA GTCGACCCAG AAAAGATCTA GATCTTTGAT ACCATGGGTT TAGAGGGTAA   
  
  
- GGAGGTAAAA ATAAGAGTTT TAGATAGAAG AATCTGAACA CCCAAACTAA GTAAAGAAAA CCCAAAAACT   
  
  
- TTTCTTAAAT TAAACTAACA TAACACAACT AACCCCTTAA ACACCACCAC AATCTACGCT CGATACGGGA   
  
  
- TGTTAAACGT TCCGTTCCCA CACCATCTCC AAAGTTCGTA AACATAAGGT GTTTAGAGAA GGGGACGAAG   
  
  
- TTTCACCTTC TGGTTGTTGT TGTTGAACCT GTTGTTTAAA GTCGTTGTTG TTCTTCTTTA ACTCGTTAAC   
  
  
- GTCTCATCAC CACCGTGAAA AGAGAGAGGA CTATTGTGGT TATGATTATA ATCATGATGA GGCTCTCGCT   
  
  
- GTTCAAGACA ACTATCGCTT GTGTGTAGAT GAGACCTATG ATGCCCGGGA GGGTGAAGTT GTAGAAGGAG   
  
  
- GAAGTTCTCG TGGAGGTGGC GGTTGTGTCG GCCACACCGC CTGGGGCGTA CATGAGGGTT TCTCCTAACC   
  
  
- CTACAGAGGC CGCCGCGACG ACCGCCACTG CCGCCACCAC CCCCCGAACC TAACCTCCTC ACCCTGTCGT   
  
  
- ACAAGGGTTT ACCCCTCCCC CGAAACGAGG GAACCTAGTA CCCACTTCGA CTACTGTACC CATACCCAAA   
  
  
- CTTCGTAGAA AACGTTAGCC CATTGGGCCA ACTGATACTC CCGTTACGAC CAGATCCCCA ACAGCTAGTC   
  
  
- CCAAGACCTA AACTCTGAGA GAGAGGAGGA GGTGGCGTCG GCCGCCGTAC ACTTAGATTA CCACCACCAC   
  
  
- AACGATTAAA CCCCAAAGGT CCCTTATTAC CATTCTAAAG AAGTTAAAGT GTATTAACAA GTAGACCCTA   
  
  
- AAACTTACCA TTCCACTTGT TATTACCCAA CTTAGGGTTA ACATTGGGAG TTCGAGTTCC GTCAGATGAA   
  
  
- CCAAATTAGG TTCCTCGATG TCCACATGTG CGTGTAGGAC TGCAACCTCT GCTCTTTGGG GTCTAAAACT   
  
  
- TAGGTGTAAA CCACTACTTG GGAGTCGTTC GAGTCTCGTA ACGTTTGGGA TCGAAAAACT ACGTGAGTAA   
  
  
- TCCAATGATA GTCGACCTCG TTGTAGATAA AGTTGGAGTC CGTTTTGCGG ACTTGTGCCC ACGACAAGAC   
  
  
- CTAGGATTGG AACAAGTCGA ACGTTTCTTG GGTAAACGAC TAGTCCCAGT ACTCAATAAC AACTCCTTCG   
  
  
- TCGTGGTTGT CGAACCAAAC GTTGGTAACG GTAACCCAAA CCCAGGAGTC AACCAGGGAG GCGTCTTCGG   
  
  
- ACACTACCCA AGTTTCGTCC CCTTGGGGGT CGTAATGGGC AACGTACAAG TAGTCGTCGA CGTTAACGTT   
  
  
- CTCGTCCGAC AGTTTCTAGT CGAGAAGTTC CGCCGTCTGG AATAAGTTTG ACCTTCAAAG AGTGAGCGCG   
  
  
- TTCTCTATAA CCGCGCCGAG TTAGTGGTCG AGAGGGAGGG ACGTTTCGGG GAGTAATCCC GCCGAAACAT   
  
  
- ACACTTCCTC CGGGAAGTTT ACGAGGAGGA TTACTCGTTG GGTCAACGCC GAGGTGGCAG GTTCTAGGAG   
  
  
- TGGGGAATAC TACAACAAGT GTTCTACTCG CGCATATTCC AGAAGAGACT CCAGACGGGT TAGTGAGTTA   
  
  
- AACACTTAAA GTGAACATGT GTCCGGTAAG AGCTCCGAGA ACTACTAAGA CTACGATAAG TACAACAGCT   
  
  
- GAAACTATAA CCAACGCCAC GAGTTACCCG TAGTAACTAA GTCCTCGACG GTAACTCCTT TTCCCCTCGA   
  
  
- GGGAGAGACT TTTAATGTCG GTATCGAGGG TACAACTGAC CGTCAGTGAA ACTTTAATCG GATCATACAC   
  
  
- TTTTGGAACA CGTTAAACGG TTGCTATAAC CACAACGAAC ACTCGAGGTT CAACAGTTGA AACTAAACAA   
  
  
- ACTAGGTAGA AGCGGTAGTT ACGGCTTGTA ATCATGACGA CTCCTACTCA GTTAACGACA ATCATAGGGG   
  
  
- TAGACCCGTA GAAGTTAATC CGGTAGACAA GAAGGAAGGT AGGAGGCTAA GTAATTCGTT TTTAGGGGGT   
  
  
- TTTAACACCA GAGCAAACTA TCTCCTAAGC TAGCAAAACT ACAGGGAAAG GGGGTTGTAG ACAACGTATG   
  
  
- GGATCTTAGG ACGTCATTAA ATAACCTGAG CGAACTACCA GAGTTACAAC GTAGCCTGTA ACACTCGTTC   
  
  
- CAGCTCTTCA AGAAACAAGT TGGATCCTAG CTTTTACGAC ACAACCCAGC CCAGGTACGG GGACTGTTCT   
  
  
- ACGGGGTAAC CTTTTTAGAG AAACGAAGTC GGCCGAAGAA CGGGAACGTT AAGTCATTAA AGTGTCTTTG   
  
  
- GGTCCGGCTA ATACACCACT TCTCTTGGGG TTGTTCTCCT AAAGTGCACC TCTTCGCGGT CCGTAGTGAG   
  
  
- TAAGACTCAA CCGTCTCCGT CCTCGAACAC CGTCGAAGCC GTACCTTCAC AAC

+     GT1-motif

| Site Name | Organism | Position | Strand | Matrix score. | sequence | function |
| --- | --- | --- | --- | --- | --- | --- |
| GT1-motif | Avena sativa | 263 | - | 7 | GGTTAAT | light responsive element |
| GT1-motif | Arabidopsis thaliana | 264 | - | 6 | GGTTAA | light responsive element |

>HU04G00047.1   
+ +Up\_Stream \_Len000TCTTAA CATTAGTTGC TTGGTTCAAC CCATCAGTGA GAATATCTGA TTTCAAAAGT   
  
  
+ ATAGATTTAT ATAAACATAT GGACTACAGG GAAGGGAGTC GTTTCCTCAA AGTCTTAGAT TTACACTCAA   
  
  
+ AAGTTTGAGC GATACCAATA ATGTGATTCC AAACATGAGC CAAATATTAA GTATCCTATA CTAAGATCAA   
  
  
+ GAAGAGAATG AACCAAAAGA CTGTTAATTT TTCAATTAAG CTAAACTTAT TAACCTTTTT TTGGAATAGA   
  
  
+ TTTTTAAATT TTATCTTCAA TATAAGTTTT TCCAAGGCGA GAGACATATA GCAATTTTAA TTAAAGTTAA   
  
  
+ AGTAGTATAA CATTTTTTTT AATGGTTATA GGCTTATAGC ACCTTTTCTA GGTGAAAAGA GATATCTTGG   
  
  
+ CCTGATTTCC AGTAAAGTTT TTTTTTTAAA TATTGTCAAT TATTTTTGCA AATGACATTT GGTAATTTGG   
  
  
+ GGGATAATCT GATGTGTTAA AAGATACAAT GAAAATATCA GAAAATAGGA TGCCTCAAAT TTCACAAGAA   
  
  
+ AGAAAAAATC TTTTTGTTGT TAGTATTTCT ACACATCCAC AATTTTTACT CATGGTAAAA AAAAAAGGGC   
  
  
+ CAAATATCTC ACCAATATAA CCAAACTATA TGTTCCATTA TTTAGTAACG GTGATAAATA TGTAATGGGT   
  
  
+ CAAGTTACTA ATAAAATAGC CTACAATACT TGGATATCTA CATAAAATTG ATATAGAATA TATATTGCAA   
  
  
+ TACTTAAATC AAAATTAAAT GAAATATTTA CTTTTTTATT CTATTACAAA TAGCTTCTTA TAGAATAGCA   
  
  
+ ACAATTTTTA ATGTAAGTTT CATTCTTACC AGATTTGAAG AGGTCCGGAT ATAGAAAATC TTATTCGTAT   
  
  
+ AAGTACTCGC ATGAGGGCGG ATAAATTCTA AAATATCAAC CTCAACTCGC TTCTACTATT CCATCTAAGT   
  
  
+ TCAATTTATC ATATAAAAAA ATTCAATCCA CTTTAATGTA CTCAATTCAC TTCAACCTAT TTTAATCAAT   
  
  
+ TTACCCATGA AAAGAACAAG GTCCTAAGTA GATGTTTTCT ATTGATTGAT TTAAACAATT TTCACCTACC   
  
  
+ CAATTATCCC CAAAACTGAA AGAGGGGGCA AAGGGGGGTG GGTAATGCTG TGTAAAATAG GGGGAAAGGG   
  
  
+ CAAGAAAACT GTGTACAATG GTGGAGCAGG GGTATACCAT TTGGCCTTGA GTTGAGGGGG CACAAAAGCC   
  
  
+ CAGAGAGAGA GAGGGGTAAT GCCCACCAGA AATAAGCAGG AAGCGAGGGA AAAACACTAG AGAGAAACAG   
  
  
+ AGAAAGAGAG AGAGAGAGAG AGAGAAGGGG AAATTTACTA GAGAATGAGA GTGAAGAAGG GAACTTTATT   
  
  
+ TTAACTTTTT TATGATGCAC GCATCCATGG CAGAACAGGG AAGGGAAAGA AGGGTACAGA GTACTGCTGT   
  
  
+ CCCCAAAAAA TTAGTGTAGA AAACAGGAGA CACTCAACTA CAACAAGGGT AAGGAAGAAG AACAAGCAAG   
  
  
+ ATATCCCACC CAAAGAAAAA AAAAAAGAAG AAGAAGAAAA TTTTTCAGAG AGGAAAGAAA AGCAATCACA   
  
  
+ TGAATTAGAA GCCAACCCAA GCCTTCTTCA AAGAGAAGCA GCATAACATA GCATAGCTGG GGAGAGGATA   
  
  
+ GGAGAGAGAA ACTCAAGAAA AGGGGTACCC GGCTTAAATG TTGATGCAGA AAGGTATAGA TATGGCCACA   
  
  
+ CTACTACTAC CCAGGAGTTT ATCATTGGAG ATGGATTTCT CTTTCTGCCT CATCTTCTCC TCCATCCACC   
  
  
+ AGCAAAACTG GGTTTCCTTT CAGCTGGGTC TTTTCTAGAT CTAGAAACTA TGGTACCCAA ATCTCCCATT   
  
  
+ CCTCCATTTT TATTCTCAAA ATCTATCTTC TTAGACTTGT GGGTTTGATT CATTTCTTTT GGGTTTTTGA   
  
  
+ AAAGAATTTA ATTTGATTGT ATTGTGTTGA TTGGGGAATT TGTGGTGGTG TTAGATGCGA GCTATGCCCT   
  
  
+ ACAATTTGCA AGGCAAGGGT GTGGTAGAGG TTTCAAGCAT TTGTATTCCA CAAATCTCTT CCCCTGCTTC   
  
  
+ AAAGTGGAAG ACCAACAACA ACAACTTGGA CAACAAATTT CAGCAACAAC AAGAAGAAAT TGAGCAATTG   
  
  
+ CAGAGTAGTG GTGGCACTTT TCTCTCTCCT GATAACACCA ATACTAATAT TAGTACTACT CCGAGAGCGA   
  
  
+ CAAGTTCTGT TGATAGCGAA CACACATCTA CTCTGGATAC TACGGGCCCT CCCACTTCAA CATCTTCCTC   
  
  
+ CTTCAAGAGC ACCTCCACCG CCAACACAGC CGGTGTGGCG GACCCCGCAT GTACTCCCAA AGAGGATTGG   
  
  
+ GATGTCTCCG GCGGCGCTGC TGGCGGTGAC GGCGGTGGTG GGGGGCTTGG ATTGGAGGAG TGGGACAGCA   
  
  
+ TGTTCCCAAA TGGGGAGGGG GCTTTGCTCC CTTGGATCAT GGGTGAAGCT GATGACATGG GTATGGGTTT   
  
  
+ GAAGCATCTT TTGCAATCGG GTAACCCGGT TGACTATGAG GGCAATGCTG GTCTAGGGGT TGTCGATCAG   
  
  
+ GGTTCTGGAT TTGAGACTCT CTCTCCTCCT CCACCGCAGC CGGCGGCATG TGAATCTAAT GGTGGTGGTG   
  
  
+ TTGCTAATTT GGGGTTTCCA GGGAATAATG GTAAGATTTC TTCAATTTCA CATAATTGTT CATCTGGGAT   
  
  
+ TTTGAATGGT AAGGTGAACA ATAATGGGTT GAATCCCAAT TGTAACCCTC AAGCTCAAGG CAGTCTACTT   
  
  
+ GGTTTAATCC AAGGAGCTAC AGGTGTACAC GCACATCCTG ACGTTGGAGA CGAGAAACCC CAGATTTTGA   
  
  
+ ATCCACATTT GGTGATGAAC CCTCAGCAAG CTCAGAGCAT TGCAAACCCT AGCTTTTTGA TGCACTCATT   
  
  
+ AGGTTACTAT CAGCTGGAGC AACATCTATT TCAACCTCAG GCAAAACGCC TGAACACGGG TGCTGTTCTG   
  
  
+ GATCCTAACC TTGTTCAGCT TGCAAAGAAC CCATTTGCTG ATCAGGGTCA TGAGTTATTG TTGAGGAAGC   
  
  
+ AGCACCAACA GCTTGGTTTG CAACCATTGC CATTGGGTTT GGGTCCTCAG TTGGTCCCTC CGCAGAAGCC   
  
  
+ TGTGATGGGT TCAAAGCAGG GGAACCCCCA GCATTACCCG TTGCATGTTC ATCAGCAGCT GCAATTGCAA   
  
  
+ GAGCAGGCTG TCAAAGATCA GCTCTTCAAG GCGGCAGACC TTATTCAAAC TGGAAGTTTC TCACTCGCGC   
  
  
+ AAGAGATATT GGCGCGGCTC AATCACCAGC TCTCCCTCCC TGCAAAGCCC CTCATTAGGG CGGCTTTGTA   
  
  
+ TGTGAAGGAG GCCCTTCAAA TGCTCCTCCT AATGAGCAAC CCAGTTGCGG CTCCACCGTC CAAGATCCTC   
  
  
+ ACCCCTTATG ATGTTGTTCA CAAGATGAGC GCGTATAAGG TCTTCTCTGA GGTCTGCCCA ATCACTCAAT   
  
  
+ TTGTGAATTT CACTTGTACA CAGGCCATTC TCGAGGCTCT TGATGATTCT GATGCTATTC ATGTTGTCGA   
  
  
+ CTTTGATATT GGTTGCGGTG CTCAATGGGC ATCATTGATT CAGGAGCTGC CATTGAGGAA AAGGGGAGCT   
  
  
+ CCCTCTCTGA AAATTACAGC CATAGCTCCC ATGTTGACTG GCAGTCACTT TGAAATTAGC CTAGTATGTG   
  
  
+ AAAACCTTGT GCAATTTGCC AACGATATTG GTGTTGCTTG TGAGCTCCAA GTTGTCAACT TTGATTTGTT   
  
  
+ TGATCCATCT TCGCCATCAA TGCCGAACAT TAGTACTGCT GAGGATGAGT CAATTGCTGT TAGTATCCCC   
  
  
+ ATCTGGGCAT CTTCAATTAG GCCATCTGTT CTTCCTTCCA TCCTCCGATT CATTAAGCAA AAATCCCCCA   
  
  
+ AAATTGTGGT CTCGTTTGAT AGAGGATTCG ATCGTTTTGA TGTCCCTTTC CCCCAACATC TGTTGCATAC   
  
  
+ CCTAGAATCC TGCAGTAATT TATTGGACTC GCTTGATGGT CTCAATGTTG CATCGGACAT TGTGAGCAAG   
  
  
+ GTCGAGAAGT TCTTTGTTCA ACCTAGGATC GAAAATGCTG TGTTGGGTCG GGTCCATGCC CCTGACAAGA   
  
  
+ TGCCCCATTG GAAAAATCTC TTTGCTTCAG CCGGCTTCTT GCCCTTGCAA TTCAGTAATT TCACAGAAAC   
  
  
+ CCAGGCCGAT TATGTGGTGA AGAGAACCCC AACAAGAGGA TTTCACGTGG AGAAGCGCCA GGCATCACTC   
  
  
+ ATTCTGAGTT GGCAGAGGCA GGAGCTTGTG GCAGCTTCGG CATGGAAGTG TTG  

- +Up\_Stream \_Len000AGAATT GTAATCAACG AACCAAGTTG GGTAGTCACT CTTATAGACT AAAGTTTTCA   
  
  
- TATCTAAATA TATTTGTATA CCTGATGTCC CTTCCCTCAG CAAAGGAGTT TCAGAATCTA AATGTGAGTT   
  
  
- TTCAAACTCG CTATGGTTAT TACACTAAGG TTTGTACTCG GTTTATAATT CATAGGATAT GATTCTAGTT   
  
  
- CTTCTCTTAC TTGGTTTTCT GACAATTAAA AAGTTAATTC GATTTGAATA ATTGGAAAAA AACCTTATCT   
  
  
- AAAAATTTAA AATAGAAGTT ATATTCAAAA AGGTTCCGCT CTCTGTATAT CGTTAAAATT AATTTCAATT   
  
  
- TCATCATATT GTAAAAAAAA TTACCAATAT CCGAATATCG TGGAAAAGAT CCACTTTTCT CTATAGAACC   
  
  
- GGACTAAAGG TCATTTCAAA AAAAAAATTT ATAACAGTTA ATAAAAACGT TTACTGTAAA CCATTAAACC   
  
  
- CCCTATTAGA CTACACAATT TTCTATGTTA CTTTTATAGT CTTTTATCCT ACGGAGTTTA AAGTGTTCTT   
  
  
- TCTTTTTTAG AAAAACAACA ATCATAAAGA TGTGTAGGTG TTAAAAATGA GTACCATTTT TTTTTTCCCG   
  
  
- GTTTATAGAG TGGTTATATT GGTTTGATAT ACAAGGTAAT AAATCATTGC CACTATTTAT ACATTACCCA   
  
  
- GTTCAATGAT TATTTTATCG GATGTTATGA ACCTATAGAT GTATTTTAAC TATATCTTAT ATATAACGTT   
  
  
- ATGAATTTAG TTTTAATTTA CTTTATAAAT GAAAAAATAA GATAATGTTT ATCGAAGAAT ATCTTATCGT   
  
  
- TGTTAAAAAT TACATTCAAA GTAAGAATGG TCTAAACTTC TCCAGGCCTA TATCTTTTAG AATAAGCATA   
  
  
- TTCATGAGCG TACTCCCGCC TATTTAAGAT TTTATAGTTG GAGTTGAGCG AAGATGATAA GGTAGATTCA   
  
  
- AGTTAAATAG TATATTTTTT TAAGTTAGGT GAAATTACAT GAGTTAAGTG AAGTTGGATA AAATTAGTTA   
  
  
- AATGGGTACT TTTCTTGTTC CAGGATTCAT CTACAAAAGA TAACTAACTA AATTTGTTAA AAGTGGATGG   
  
  
- GTTAATAGGG GTTTTGACTT TCTCCCCCGT TTCCCCCCAC CCATTACGAC ACATTTTATC CCCCTTTCCC   
  
  
- GTTCTTTTGA CACATGTTAC CACCTCGTCC CCATATGGTA AACCGGAACT CAACTCCCCC GTGTTTTCGG   
  
  
- GTCTCTCTCT CTCCCCATTA CGGGTGGTCT TTATTCGTCC TTCGCTCCCT TTTTGTGATC TCTCTTTGTC   
  
  
- TCTTTCTCTC TCTCTCTCTC TCTCTTCCCC TTTAAATGAT CTCTTACTCT CACTTCTTCC CTTGAAATAA   
  
  
- AATTGAAAAA ATACTACGTG CGTAGGTACC GTCTTGTCCC TTCCCTTTCT TCCCATGTCT CATGACGACA   
  
  
- GGGGTTTTTT AATCACATCT TTTGTCCTCT GTGAGTTGAT GTTGTTCCCA TTCCTTCTTC TTGTTCGTTC   
  
  
- TATAGGGTGG GTTTCTTTTT TTTTTTCTTC TTCTTCTTTT AAAAAGTCTC TCCTTTCTTT TCGTTAGTGT   
  
  
- ACTTAATCTT CGGTTGGGTT CGGAAGAAGT TTCTCTTCGT CGTATTGTAT CGTATCGACC CCTCTCCTAT   
  
  
- CCTCTCTCTT TGAGTTCTTT TCCCCATGGG CCGAATTTAC AACTACGTCT TTCCATATCT ATACCGGTGT   
  
  
- GATGATGATG GGTCCTCAAA TAGTAACCTC TACCTAAAGA GAAAGACGGA GTAGAAGAGG AGGTAGGTGG   
  
  
- TCGTTTTGAC CCAAAGGAAA GTCGACCCAG AAAAGATCTA GATCTTTGAT ACCATGGGTT TAGAGGGTAA   
  
  
- GGAGGTAAAA ATAAGAGTTT TAGATAGAAG AATCTGAACA CCCAAACTAA GTAAAGAAAA CCCAAAAACT   
  
  
- TTTCTTAAAT TAAACTAACA TAACACAACT AACCCCTTAA ACACCACCAC AATCTACGCT CGATACGGGA   
  
  
- TGTTAAACGT TCCGTTCCCA CACCATCTCC AAAGTTCGTA AACATAAGGT GTTTAGAGAA GGGGACGAAG   
  
  
- TTTCACCTTC TGGTTGTTGT TGTTGAACCT GTTGTTTAAA GTCGTTGTTG TTCTTCTTTA ACTCGTTAAC   
  
  
- GTCTCATCAC CACCGTGAAA AGAGAGAGGA CTATTGTGGT TATGATTATA ATCATGATGA GGCTCTCGCT   
  
  
- GTTCAAGACA ACTATCGCTT GTGTGTAGAT GAGACCTATG ATGCCCGGGA GGGTGAAGTT GTAGAAGGAG   
  
  
- GAAGTTCTCG TGGAGGTGGC GGTTGTGTCG GCCACACCGC CTGGGGCGTA CATGAGGGTT TCTCCTAACC   
  
  
- CTACAGAGGC CGCCGCGACG ACCGCCACTG CCGCCACCAC CCCCCGAACC TAACCTCCTC ACCCTGTCGT   
  
  
- ACAAGGGTTT ACCCCTCCCC CGAAACGAGG GAACCTAGTA CCCACTTCGA CTACTGTACC CATACCCAAA   
  
  
- CTTCGTAGAA AACGTTAGCC CATTGGGCCA ACTGATACTC CCGTTACGAC CAGATCCCCA ACAGCTAGTC   
  
  
- CCAAGACCTA AACTCTGAGA GAGAGGAGGA GGTGGCGTCG GCCGCCGTAC ACTTAGATTA CCACCACCAC   
  
  
- AACGATTAAA CCCCAAAGGT CCCTTATTAC CATTCTAAAG AAGTTAAAGT GTATTAACAA GTAGACCCTA   
  
  
- AAACTTACCA TTCCACTTGT TATTACCCAA CTTAGGGTTA ACATTGGGAG TTCGAGTTCC GTCAGATGAA   
  
  
- CCAAATTAGG TTCCTCGATG TCCACATGTG CGTGTAGGAC TGCAACCTCT GCTCTTTGGG GTCTAAAACT   
  
  
- TAGGTGTAAA CCACTACTTG GGAGTCGTTC GAGTCTCGTA ACGTTTGGGA TCGAAAAACT ACGTGAGTAA   
  
  
- TCCAATGATA GTCGACCTCG TTGTAGATAA AGTTGGAGTC CGTTTTGCGG ACTTGTGCCC ACGACAAGAC   
  
  
- CTAGGATTGG AACAAGTCGA ACGTTTCTTG GGTAAACGAC TAGTCCCAGT ACTCAATAAC AACTCCTTCG   
  
  
- TCGTGGTTGT CGAACCAAAC GTTGGTAACG GTAACCCAAA CCCAGGAGTC AACCAGGGAG GCGTCTTCGG   
  
  
- ACACTACCCA AGTTTCGTCC CCTTGGGGGT CGTAATGGGC AACGTACAAG TAGTCGTCGA CGTTAACGTT   
  
  
- CTCGTCCGAC AGTTTCTAGT CGAGAAGTTC CGCCGTCTGG AATAAGTTTG ACCTTCAAAG AGTGAGCGCG   
  
  
- TTCTCTATAA CCGCGCCGAG TTAGTGGTCG AGAGGGAGGG ACGTTTCGGG GAGTAATCCC GCCGAAACAT   
  
  
- ACACTTCCTC CGGGAAGTTT ACGAGGAGGA TTACTCGTTG GGTCAACGCC GAGGTGGCAG GTTCTAGGAG   
  
  
- TGGGGAATAC TACAACAAGT GTTCTACTCG CGCATATTCC AGAAGAGACT CCAGACGGGT TAGTGAGTTA   
  
  
- AACACTTAAA GTGAACATGT GTCCGGTAAG AGCTCCGAGA ACTACTAAGA CTACGATAAG TACAACAGCT   
  
  
- GAAACTATAA CCAACGCCAC GAGTTACCCG TAGTAACTAA GTCCTCGACG GTAACTCCTT TTCCCCTCGA   
  
  
- GGGAGAGACT TTTAATGTCG GTATCGAGGG TACAACTGAC CGTCAGTGAA ACTTTAATCG GATCATACAC   
  
  
- TTTTGGAACA CGTTAAACGG TTGCTATAAC CACAACGAAC ACTCGAGGTT CAACAGTTGA AACTAAACAA   
  
  
- ACTAGGTAGA AGCGGTAGTT ACGGCTTGTA ATCATGACGA CTCCTACTCA GTTAACGACA ATCATAGGGG   
  
  
- TAGACCCGTA GAAGTTAATC CGGTAGACAA GAAGGAAGGT AGGAGGCTAA GTAATTCGTT TTTAGGGGGT   
  
  
- TTTAACACCA GAGCAAACTA TCTCCTAAGC TAGCAAAACT ACAGGGAAAG GGGGTTGTAG ACAACGTATG   
  
  
- GGATCTTAGG ACGTCATTAA ATAACCTGAG CGAACTACCA GAGTTACAAC GTAGCCTGTA ACACTCGTTC   
  
  
- CAGCTCTTCA AGAAACAAGT TGGATCCTAG CTTTTACGAC ACAACCCAGC CCAGGTACGG GGACTGTTCT   
  
  
- ACGGGGTAAC CTTTTTAGAG AAACGAAGTC GGCCGAAGAA CGGGAACGTT AAGTCATTAA AGTGTCTTTG   
  
  
- GGTCCGGCTA ATACACCACT TCTCTTGGGG TTGTTCTCCT AAAGTGCACC TCTTCGCGGT CCGTAGTGAG   
  
  
- TAAGACTCAA CCGTCTCCGT CCTCGAACAC CGTCGAAGCC GTACCTTCAC AAC

+     I-box

| Site Name | Organism | Position | Strand | Matrix score. | sequence | function |
| --- | --- | --- | --- | --- | --- | --- |
| I-box | Flaveria trinervia | 1778 | - | 10 | cCATATCCAAT | part of a light responsive element |
| I-box | Larix laricina | 3467 | + | 9 | GTATAAGGCC | part of a light responsive element |

>HU04G00047.1   
+ +Up\_Stream \_Len000TCTTAA CATTAGTTGC TTGGTTCAAC CCATCAGTGA GAATATCTGA TTTCAAAAGT   
  
  
+ ATAGATTTAT ATAAACATAT GGACTACAGG GAAGGGAGTC GTTTCCTCAA AGTCTTAGAT TTACACTCAA   
  
  
+ AAGTTTGAGC GATACCAATA ATGTGATTCC AAACATGAGC CAAATATTAA GTATCCTATA CTAAGATCAA   
  
  
+ GAAGAGAATG AACCAAAAGA CTGTTAATTT TTCAATTAAG CTAAACTTAT TAACCTTTTT TTGGAATAGA   
  
  
+ TTTTTAAATT TTATCTTCAA TATAAGTTTT TCCAAGGCGA GAGACATATA GCAATTTTAA TTAAAGTTAA   
  
  
+ AGTAGTATAA CATTTTTTTT AATGGTTATA GGCTTATAGC ACCTTTTCTA GGTGAAAAGA GATATCTTGG   
  
  
+ CCTGATTTCC AGTAAAGTTT TTTTTTTAAA TATTGTCAAT TATTTTTGCA AATGACATTT GGTAATTTGG   
  
  
+ GGGATAATCT GATGTGTTAA AAGATACAAT GAAAATATCA GAAAATAGGA TGCCTCAAAT TTCACAAGAA   
  
  
+ AGAAAAAATC TTTTTGTTGT TAGTATTTCT ACACATCCAC AATTTTTACT CATGGTAAAA AAAAAAGGGC   
  
  
+ CAAATATCTC ACCAATATAA CCAAACTATA TGTTCCATTA TTTAGTAACG GTGATAAATA TGTAATGGGT   
  
  
+ CAAGTTACTA ATAAAATAGC CTACAATACT TGGATATCTA CATAAAATTG ATATAGAATA TATATTGCAA   
  
  
+ TACTTAAATC AAAATTAAAT GAAATATTTA CTTTTTTATT CTATTACAAA TAGCTTCTTA TAGAATAGCA   
  
  
+ ACAATTTTTA ATGTAAGTTT CATTCTTACC AGATTTGAAG AGGTCCGGAT ATAGAAAATC TTATTCGTAT   
  
  
+ AAGTACTCGC ATGAGGGCGG ATAAATTCTA AAATATCAAC CTCAACTCGC TTCTACTATT CCATCTAAGT   
  
  
+ TCAATTTATC ATATAAAAAA ATTCAATCCA CTTTAATGTA CTCAATTCAC TTCAACCTAT TTTAATCAAT   
  
  
+ TTACCCATGA AAAGAACAAG GTCCTAAGTA GATGTTTTCT ATTGATTGAT TTAAACAATT TTCACCTACC   
  
  
+ CAATTATCCC CAAAACTGAA AGAGGGGGCA AAGGGGGGTG GGTAATGCTG TGTAAAATAG GGGGAAAGGG   
  
  
+ CAAGAAAACT GTGTACAATG GTGGAGCAGG GGTATACCAT TTGGCCTTGA GTTGAGGGGG CACAAAAGCC   
  
  
+ CAGAGAGAGA GAGGGGTAAT GCCCACCAGA AATAAGCAGG AAGCGAGGGA AAAACACTAG AGAGAAACAG   
  
  
+ AGAAAGAGAG AGAGAGAGAG AGAGAAGGGG AAATTTACTA GAGAATGAGA GTGAAGAAGG GAACTTTATT   
  
  
+ TTAACTTTTT TATGATGCAC GCATCCATGG CAGAACAGGG AAGGGAAAGA AGGGTACAGA GTACTGCTGT   
  
  
+ CCCCAAAAAA TTAGTGTAGA AAACAGGAGA CACTCAACTA CAACAAGGGT AAGGAAGAAG AACAAGCAAG   
  
  
+ ATATCCCACC CAAAGAAAAA AAAAAAGAAG AAGAAGAAAA TTTTTCAGAG AGGAAAGAAA AGCAATCACA   
  
  
+ TGAATTAGAA GCCAACCCAA GCCTTCTTCA AAGAGAAGCA GCATAACATA GCATAGCTGG GGAGAGGATA   
  
  
+ GGAGAGAGAA ACTCAAGAAA AGGGGTACCC GGCTTAAATG TTGATGCAGA AAGGTATAGA TATGGCCACA   
  
  
+ CTACTACTAC CCAGGAGTTT ATCATTGGAG ATGGATTTCT CTTTCTGCCT CATCTTCTCC TCCATCCACC   
  
  
+ AGCAAAACTG GGTTTCCTTT CAGCTGGGTC TTTTCTAGAT CTAGAAACTA TGGTACCCAA ATCTCCCATT   
  
  
+ CCTCCATTTT TATTCTCAAA ATCTATCTTC TTAGACTTGT GGGTTTGATT CATTTCTTTT GGGTTTTTGA   
  
  
+ AAAGAATTTA ATTTGATTGT ATTGTGTTGA TTGGGGAATT TGTGGTGGTG TTAGATGCGA GCTATGCCCT   
  
  
+ ACAATTTGCA AGGCAAGGGT GTGGTAGAGG TTTCAAGCAT TTGTATTCCA CAAATCTCTT CCCCTGCTTC   
  
  
+ AAAGTGGAAG ACCAACAACA ACAACTTGGA CAACAAATTT CAGCAACAAC AAGAAGAAAT TGAGCAATTG   
  
  
+ CAGAGTAGTG GTGGCACTTT TCTCTCTCCT GATAACACCA ATACTAATAT TAGTACTACT CCGAGAGCGA   
  
  
+ CAAGTTCTGT TGATAGCGAA CACACATCTA CTCTGGATAC TACGGGCCCT CCCACTTCAA CATCTTCCTC   
  
  
+ CTTCAAGAGC ACCTCCACCG CCAACACAGC CGGTGTGGCG GACCCCGCAT GTACTCCCAA AGAGGATTGG   
  
  
+ GATGTCTCCG GCGGCGCTGC TGGCGGTGAC GGCGGTGGTG GGGGGCTTGG ATTGGAGGAG TGGGACAGCA   
  
  
+ TGTTCCCAAA TGGGGAGGGG GCTTTGCTCC CTTGGATCAT GGGTGAAGCT GATGACATGG GTATGGGTTT   
  
  
+ GAAGCATCTT TTGCAATCGG GTAACCCGGT TGACTATGAG GGCAATGCTG GTCTAGGGGT TGTCGATCAG   
  
  
+ GGTTCTGGAT TTGAGACTCT CTCTCCTCCT CCACCGCAGC CGGCGGCATG TGAATCTAAT GGTGGTGGTG   
  
  
+ TTGCTAATTT GGGGTTTCCA GGGAATAATG GTAAGATTTC TTCAATTTCA CATAATTGTT CATCTGGGAT   
  
  
+ TTTGAATGGT AAGGTGAACA ATAATGGGTT GAATCCCAAT TGTAACCCTC AAGCTCAAGG CAGTCTACTT   
  
  
+ GGTTTAATCC AAGGAGCTAC AGGTGTACAC GCACATCCTG ACGTTGGAGA CGAGAAACCC CAGATTTTGA   
  
  
+ ATCCACATTT GGTGATGAAC CCTCAGCAAG CTCAGAGCAT TGCAAACCCT AGCTTTTTGA TGCACTCATT   
  
  
+ AGGTTACTAT CAGCTGGAGC AACATCTATT TCAACCTCAG GCAAAACGCC TGAACACGGG TGCTGTTCTG   
  
  
+ GATCCTAACC TTGTTCAGCT TGCAAAGAAC CCATTTGCTG ATCAGGGTCA TGAGTTATTG TTGAGGAAGC   
  
  
+ AGCACCAACA GCTTGGTTTG CAACCATTGC CATTGGGTTT GGGTCCTCAG TTGGTCCCTC CGCAGAAGCC   
  
  
+ TGTGATGGGT TCAAAGCAGG GGAACCCCCA GCATTACCCG TTGCATGTTC ATCAGCAGCT GCAATTGCAA   
  
  
+ GAGCAGGCTG TCAAAGATCA GCTCTTCAAG GCGGCAGACC TTATTCAAAC TGGAAGTTTC TCACTCGCGC   
  
  
+ AAGAGATATT GGCGCGGCTC AATCACCAGC TCTCCCTCCC TGCAAAGCCC CTCATTAGGG CGGCTTTGTA   
  
  
+ TGTGAAGGAG GCCCTTCAAA TGCTCCTCCT AATGAGCAAC CCAGTTGCGG CTCCACCGTC CAAGATCCTC   
  
  
+ ACCCCTTATG ATGTTGTTCA CAAGATGAGC GCGTATAAGG TCTTCTCTGA GGTCTGCCCA ATCACTCAAT   
  
  
+ TTGTGAATTT CACTTGTACA CAGGCCATTC TCGAGGCTCT TGATGATTCT GATGCTATTC ATGTTGTCGA   
  
  
+ CTTTGATATT GGTTGCGGTG CTCAATGGGC ATCATTGATT CAGGAGCTGC CATTGAGGAA AAGGGGAGCT   
  
  
+ CCCTCTCTGA AAATTACAGC CATAGCTCCC ATGTTGACTG GCAGTCACTT TGAAATTAGC CTAGTATGTG   
  
  
+ AAAACCTTGT GCAATTTGCC AACGATATTG GTGTTGCTTG TGAGCTCCAA GTTGTCAACT TTGATTTGTT   
  
  
+ TGATCCATCT TCGCCATCAA TGCCGAACAT TAGTACTGCT GAGGATGAGT CAATTGCTGT TAGTATCCCC   
  
  
+ ATCTGGGCAT CTTCAATTAG GCCATCTGTT CTTCCTTCCA TCCTCCGATT CATTAAGCAA AAATCCCCCA   
  
  
+ AAATTGTGGT CTCGTTTGAT AGAGGATTCG ATCGTTTTGA TGTCCCTTTC CCCCAACATC TGTTGCATAC   
  
  
+ CCTAGAATCC TGCAGTAATT TATTGGACTC GCTTGATGGT CTCAATGTTG CATCGGACAT TGTGAGCAAG   
  
  
+ GTCGAGAAGT TCTTTGTTCA ACCTAGGATC GAAAATGCTG TGTTGGGTCG GGTCCATGCC CCTGACAAGA   
  
  
+ TGCCCCATTG GAAAAATCTC TTTGCTTCAG CCGGCTTCTT GCCCTTGCAA TTCAGTAATT TCACAGAAAC   
  
  
+ CCAGGCCGAT TATGTGGTGA AGAGAACCCC AACAAGAGGA TTTCACGTGG AGAAGCGCCA GGCATCACTC   
  
  
+ ATTCTGAGTT GGCAGAGGCA GGAGCTTGTG GCAGCTTCGG CATGGAAGTG TTG  

- +Up\_Stream \_Len000AGAATT GTAATCAACG AACCAAGTTG GGTAGTCACT CTTATAGACT AAAGTTTTCA   
  
  
- TATCTAAATA TATTTGTATA CCTGATGTCC CTTCCCTCAG CAAAGGAGTT TCAGAATCTA AATGTGAGTT   
  
  
- TTCAAACTCG CTATGGTTAT TACACTAAGG TTTGTACTCG GTTTATAATT CATAGGATAT GATTCTAGTT   
  
  
- CTTCTCTTAC TTGGTTTTCT GACAATTAAA AAGTTAATTC GATTTGAATA ATTGGAAAAA AACCTTATCT   
  
  
- AAAAATTTAA AATAGAAGTT ATATTCAAAA AGGTTCCGCT CTCTGTATAT CGTTAAAATT AATTTCAATT   
  
  
- TCATCATATT GTAAAAAAAA TTACCAATAT CCGAATATCG TGGAAAAGAT CCACTTTTCT CTATAGAACC   
  
  
- GGACTAAAGG TCATTTCAAA AAAAAAATTT ATAACAGTTA ATAAAAACGT TTACTGTAAA CCATTAAACC   
  
  
- CCCTATTAGA CTACACAATT TTCTATGTTA CTTTTATAGT CTTTTATCCT ACGGAGTTTA AAGTGTTCTT   
  
  
- TCTTTTTTAG AAAAACAACA ATCATAAAGA TGTGTAGGTG TTAAAAATGA GTACCATTTT TTTTTTCCCG   
  
  
- GTTTATAGAG TGGTTATATT GGTTTGATAT ACAAGGTAAT AAATCATTGC CACTATTTAT ACATTACCCA   
  
  
- GTTCAATGAT TATTTTATCG GATGTTATGA ACCTATAGAT GTATTTTAAC TATATCTTAT ATATAACGTT   
  
  
- ATGAATTTAG TTTTAATTTA CTTTATAAAT GAAAAAATAA GATAATGTTT ATCGAAGAAT ATCTTATCGT   
  
  
- TGTTAAAAAT TACATTCAAA GTAAGAATGG TCTAAACTTC TCCAGGCCTA TATCTTTTAG AATAAGCATA   
  
  
- TTCATGAGCG TACTCCCGCC TATTTAAGAT TTTATAGTTG GAGTTGAGCG AAGATGATAA GGTAGATTCA   
  
  
- AGTTAAATAG TATATTTTTT TAAGTTAGGT GAAATTACAT GAGTTAAGTG AAGTTGGATA AAATTAGTTA   
  
  
- AATGGGTACT TTTCTTGTTC CAGGATTCAT CTACAAAAGA TAACTAACTA AATTTGTTAA AAGTGGATGG   
  
  
- GTTAATAGGG GTTTTGACTT TCTCCCCCGT TTCCCCCCAC CCATTACGAC ACATTTTATC CCCCTTTCCC   
  
  
- GTTCTTTTGA CACATGTTAC CACCTCGTCC CCATATGGTA AACCGGAACT CAACTCCCCC GTGTTTTCGG   
  
  
- GTCTCTCTCT CTCCCCATTA CGGGTGGTCT TTATTCGTCC TTCGCTCCCT TTTTGTGATC TCTCTTTGTC   
  
  
- TCTTTCTCTC TCTCTCTCTC TCTCTTCCCC TTTAAATGAT CTCTTACTCT CACTTCTTCC CTTGAAATAA   
  
  
- AATTGAAAAA ATACTACGTG CGTAGGTACC GTCTTGTCCC TTCCCTTTCT TCCCATGTCT CATGACGACA   
  
  
- GGGGTTTTTT AATCACATCT TTTGTCCTCT GTGAGTTGAT GTTGTTCCCA TTCCTTCTTC TTGTTCGTTC   
  
  
- TATAGGGTGG GTTTCTTTTT TTTTTTCTTC TTCTTCTTTT AAAAAGTCTC TCCTTTCTTT TCGTTAGTGT   
  
  
- ACTTAATCTT CGGTTGGGTT CGGAAGAAGT TTCTCTTCGT CGTATTGTAT CGTATCGACC CCTCTCCTAT   
  
  
- CCTCTCTCTT TGAGTTCTTT TCCCCATGGG CCGAATTTAC AACTACGTCT TTCCATATCT ATACCGGTGT   
  
  
- GATGATGATG GGTCCTCAAA TAGTAACCTC TACCTAAAGA GAAAGACGGA GTAGAAGAGG AGGTAGGTGG   
  
  
- TCGTTTTGAC CCAAAGGAAA GTCGACCCAG AAAAGATCTA GATCTTTGAT ACCATGGGTT TAGAGGGTAA   
  
  
- GGAGGTAAAA ATAAGAGTTT TAGATAGAAG AATCTGAACA CCCAAACTAA GTAAAGAAAA CCCAAAAACT   
  
  
- TTTCTTAAAT TAAACTAACA TAACACAACT AACCCCTTAA ACACCACCAC AATCTACGCT CGATACGGGA   
  
  
- TGTTAAACGT TCCGTTCCCA CACCATCTCC AAAGTTCGTA AACATAAGGT GTTTAGAGAA GGGGACGAAG   
  
  
- TTTCACCTTC TGGTTGTTGT TGTTGAACCT GTTGTTTAAA GTCGTTGTTG TTCTTCTTTA ACTCGTTAAC   
  
  
- GTCTCATCAC CACCGTGAAA AGAGAGAGGA CTATTGTGGT TATGATTATA ATCATGATGA GGCTCTCGCT   
  
  
- GTTCAAGACA ACTATCGCTT GTGTGTAGAT GAGACCTATG ATGCCCGGGA GGGTGAAGTT GTAGAAGGAG   
  
  
- GAAGTTCTCG TGGAGGTGGC GGTTGTGTCG GCCACACCGC CTGGGGCGTA CATGAGGGTT TCTCCTAACC   
  
  
- CTACAGAGGC CGCCGCGACG ACCGCCACTG CCGCCACCAC CCCCCGAACC TAACCTCCTC ACCCTGTCGT   
  
  
- ACAAGGGTTT ACCCCTCCCC CGAAACGAGG GAACCTAGTA CCCACTTCGA CTACTGTACC CATACCCAAA   
  
  
- CTTCGTAGAA AACGTTAGCC CATTGGGCCA ACTGATACTC CCGTTACGAC CAGATCCCCA ACAGCTAGTC   
  
  
- CCAAGACCTA AACTCTGAGA GAGAGGAGGA GGTGGCGTCG GCCGCCGTAC ACTTAGATTA CCACCACCAC   
  
  
- AACGATTAAA CCCCAAAGGT CCCTTATTAC CATTCTAAAG AAGTTAAAGT GTATTAACAA GTAGACCCTA   
  
  
- AAACTTACCA TTCCACTTGT TATTACCCAA CTTAGGGTTA ACATTGGGAG TTCGAGTTCC GTCAGATGAA   
  
  
- CCAAATTAGG TTCCTCGATG TCCACATGTG CGTGTAGGAC TGCAACCTCT GCTCTTTGGG GTCTAAAACT   
  
  
- TAGGTGTAAA CCACTACTTG GGAGTCGTTC GAGTCTCGTA ACGTTTGGGA TCGAAAAACT ACGTGAGTAA   
  
  
- TCCAATGATA GTCGACCTCG TTGTAGATAA AGTTGGAGTC CGTTTTGCGG ACTTGTGCCC ACGACAAGAC   
  
  
- CTAGGATTGG AACAAGTCGA ACGTTTCTTG GGTAAACGAC TAGTCCCAGT ACTCAATAAC AACTCCTTCG   
  
  
- TCGTGGTTGT CGAACCAAAC GTTGGTAACG GTAACCCAAA CCCAGGAGTC AACCAGGGAG GCGTCTTCGG   
  
  
- ACACTACCCA AGTTTCGTCC CCTTGGGGGT CGTAATGGGC AACGTACAAG TAGTCGTCGA CGTTAACGTT   
  
  
- CTCGTCCGAC AGTTTCTAGT CGAGAAGTTC CGCCGTCTGG AATAAGTTTG ACCTTCAAAG AGTGAGCGCG   
  
  
- TTCTCTATAA CCGCGCCGAG TTAGTGGTCG AGAGGGAGGG ACGTTTCGGG GAGTAATCCC GCCGAAACAT   
  
  
- ACACTTCCTC CGGGAAGTTT ACGAGGAGGA TTACTCGTTG GGTCAACGCC GAGGTGGCAG GTTCTAGGAG   
  
  
- TGGGGAATAC TACAACAAGT GTTCTACTCG CGCATATTCC AGAAGAGACT CCAGACGGGT TAGTGAGTTA   
  
  
- AACACTTAAA GTGAACATGT GTCCGGTAAG AGCTCCGAGA ACTACTAAGA CTACGATAAG TACAACAGCT   
  
  
- GAAACTATAA CCAACGCCAC GAGTTACCCG TAGTAACTAA GTCCTCGACG GTAACTCCTT TTCCCCTCGA   
  
  
- GGGAGAGACT TTTAATGTCG GTATCGAGGG TACAACTGAC CGTCAGTGAA ACTTTAATCG GATCATACAC   
  
  
- TTTTGGAACA CGTTAAACGG TTGCTATAAC CACAACGAAC ACTCGAGGTT CAACAGTTGA AACTAAACAA   
  
  
- ACTAGGTAGA AGCGGTAGTT ACGGCTTGTA ATCATGACGA CTCCTACTCA GTTAACGACA ATCATAGGGG   
  
  
- TAGACCCGTA GAAGTTAATC CGGTAGACAA GAAGGAAGGT AGGAGGCTAA GTAATTCGTT TTTAGGGGGT   
  
  
- TTTAACACCA GAGCAAACTA TCTCCTAAGC TAGCAAAACT ACAGGGAAAG GGGGTTGTAG ACAACGTATG   
  
  
- GGATCTTAGG ACGTCATTAA ATAACCTGAG CGAACTACCA GAGTTACAAC GTAGCCTGTA ACACTCGTTC   
  
  
- CAGCTCTTCA AGAAACAAGT TGGATCCTAG CTTTTACGAC ACAACCCAGC CCAGGTACGG GGACTGTTCT   
  
  
- ACGGGGTAAC CTTTTTAGAG AAACGAAGTC GGCCGAAGAA CGGGAACGTT AAGTCATTAA AGTGTCTTTG   
  
  
- GGTCCGGCTA ATACACCACT TCTCTTGGGG TTGTTCTCCT AAAGTGCACC TCTTCGCGGT CCGTAGTGAG   
  
  
- TAAGACTCAA CCGTCTCCGT CCTCGAACAC CGTCGAAGCC GTACCTTCAC AAC

+     MBS

| Site Name | Organism | Position | Strand | Matrix score. | sequence | function |
| --- | --- | --- | --- | --- | --- | --- |
| MBS | Arabidopsis thaliana | 3132 | - | 6 | CAACTG | MYB binding site involved in drought-inducibility |
| MBS | Arabidopsis thaliana | 3406 | - | 6 | CAACTG | MYB binding site involved in drought-inducibility |

>HU04G00047.1   
+ +Up\_Stream \_Len000TCTTAA CATTAGTTGC TTGGTTCAAC CCATCAGTGA GAATATCTGA TTTCAAAAGT   
  
  
+ ATAGATTTAT ATAAACATAT GGACTACAGG GAAGGGAGTC GTTTCCTCAA AGTCTTAGAT TTACACTCAA   
  
  
+ AAGTTTGAGC GATACCAATA ATGTGATTCC AAACATGAGC CAAATATTAA GTATCCTATA CTAAGATCAA   
  
  
+ GAAGAGAATG AACCAAAAGA CTGTTAATTT TTCAATTAAG CTAAACTTAT TAACCTTTTT TTGGAATAGA   
  
  
+ TTTTTAAATT TTATCTTCAA TATAAGTTTT TCCAAGGCGA GAGACATATA GCAATTTTAA TTAAAGTTAA   
  
  
+ AGTAGTATAA CATTTTTTTT AATGGTTATA GGCTTATAGC ACCTTTTCTA GGTGAAAAGA GATATCTTGG   
  
  
+ CCTGATTTCC AGTAAAGTTT TTTTTTTAAA TATTGTCAAT TATTTTTGCA AATGACATTT GGTAATTTGG   
  
  
+ GGGATAATCT GATGTGTTAA AAGATACAAT GAAAATATCA GAAAATAGGA TGCCTCAAAT TTCACAAGAA   
  
  
+ AGAAAAAATC TTTTTGTTGT TAGTATTTCT ACACATCCAC AATTTTTACT CATGGTAAAA AAAAAAGGGC   
  
  
+ CAAATATCTC ACCAATATAA CCAAACTATA TGTTCCATTA TTTAGTAACG GTGATAAATA TGTAATGGGT   
  
  
+ CAAGTTACTA ATAAAATAGC CTACAATACT TGGATATCTA CATAAAATTG ATATAGAATA TATATTGCAA   
  
  
+ TACTTAAATC AAAATTAAAT GAAATATTTA CTTTTTTATT CTATTACAAA TAGCTTCTTA TAGAATAGCA   
  
  
+ ACAATTTTTA ATGTAAGTTT CATTCTTACC AGATTTGAAG AGGTCCGGAT ATAGAAAATC TTATTCGTAT   
  
  
+ AAGTACTCGC ATGAGGGCGG ATAAATTCTA AAATATCAAC CTCAACTCGC TTCTACTATT CCATCTAAGT   
  
  
+ TCAATTTATC ATATAAAAAA ATTCAATCCA CTTTAATGTA CTCAATTCAC TTCAACCTAT TTTAATCAAT   
  
  
+ TTACCCATGA AAAGAACAAG GTCCTAAGTA GATGTTTTCT ATTGATTGAT TTAAACAATT TTCACCTACC   
  
  
+ CAATTATCCC CAAAACTGAA AGAGGGGGCA AAGGGGGGTG GGTAATGCTG TGTAAAATAG GGGGAAAGGG   
  
  
+ CAAGAAAACT GTGTACAATG GTGGAGCAGG GGTATACCAT TTGGCCTTGA GTTGAGGGGG CACAAAAGCC   
  
  
+ CAGAGAGAGA GAGGGGTAAT GCCCACCAGA AATAAGCAGG AAGCGAGGGA AAAACACTAG AGAGAAACAG   
  
  
+ AGAAAGAGAG AGAGAGAGAG AGAGAAGGGG AAATTTACTA GAGAATGAGA GTGAAGAAGG GAACTTTATT   
  
  
+ TTAACTTTTT TATGATGCAC GCATCCATGG CAGAACAGGG AAGGGAAAGA AGGGTACAGA GTACTGCTGT   
  
  
+ CCCCAAAAAA TTAGTGTAGA AAACAGGAGA CACTCAACTA CAACAAGGGT AAGGAAGAAG AACAAGCAAG   
  
  
+ ATATCCCACC CAAAGAAAAA AAAAAAGAAG AAGAAGAAAA TTTTTCAGAG AGGAAAGAAA AGCAATCACA   
  
  
+ TGAATTAGAA GCCAACCCAA GCCTTCTTCA AAGAGAAGCA GCATAACATA GCATAGCTGG GGAGAGGATA   
  
  
+ GGAGAGAGAA ACTCAAGAAA AGGGGTACCC GGCTTAAATG TTGATGCAGA AAGGTATAGA TATGGCCACA   
  
  
+ CTACTACTAC CCAGGAGTTT ATCATTGGAG ATGGATTTCT CTTTCTGCCT CATCTTCTCC TCCATCCACC   
  
  
+ AGCAAAACTG GGTTTCCTTT CAGCTGGGTC TTTTCTAGAT CTAGAAACTA TGGTACCCAA ATCTCCCATT   
  
  
+ CCTCCATTTT TATTCTCAAA ATCTATCTTC TTAGACTTGT GGGTTTGATT CATTTCTTTT GGGTTTTTGA   
  
  
+ AAAGAATTTA ATTTGATTGT ATTGTGTTGA TTGGGGAATT TGTGGTGGTG TTAGATGCGA GCTATGCCCT   
  
  
+ ACAATTTGCA AGGCAAGGGT GTGGTAGAGG TTTCAAGCAT TTGTATTCCA CAAATCTCTT CCCCTGCTTC   
  
  
+ AAAGTGGAAG ACCAACAACA ACAACTTGGA CAACAAATTT CAGCAACAAC AAGAAGAAAT TGAGCAATTG   
  
  
+ CAGAGTAGTG GTGGCACTTT TCTCTCTCCT GATAACACCA ATACTAATAT TAGTACTACT CCGAGAGCGA   
  
  
+ CAAGTTCTGT TGATAGCGAA CACACATCTA CTCTGGATAC TACGGGCCCT CCCACTTCAA CATCTTCCTC   
  
  
+ CTTCAAGAGC ACCTCCACCG CCAACACAGC CGGTGTGGCG GACCCCGCAT GTACTCCCAA AGAGGATTGG   
  
  
+ GATGTCTCCG GCGGCGCTGC TGGCGGTGAC GGCGGTGGTG GGGGGCTTGG ATTGGAGGAG TGGGACAGCA   
  
  
+ TGTTCCCAAA TGGGGAGGGG GCTTTGCTCC CTTGGATCAT GGGTGAAGCT GATGACATGG GTATGGGTTT   
  
  
+ GAAGCATCTT TTGCAATCGG GTAACCCGGT TGACTATGAG GGCAATGCTG GTCTAGGGGT TGTCGATCAG   
  
  
+ GGTTCTGGAT TTGAGACTCT CTCTCCTCCT CCACCGCAGC CGGCGGCATG TGAATCTAAT GGTGGTGGTG   
  
  
+ TTGCTAATTT GGGGTTTCCA GGGAATAATG GTAAGATTTC TTCAATTTCA CATAATTGTT CATCTGGGAT   
  
  
+ TTTGAATGGT AAGGTGAACA ATAATGGGTT GAATCCCAAT TGTAACCCTC AAGCTCAAGG CAGTCTACTT   
  
  
+ GGTTTAATCC AAGGAGCTAC AGGTGTACAC GCACATCCTG ACGTTGGAGA CGAGAAACCC CAGATTTTGA   
  
  
+ ATCCACATTT GGTGATGAAC CCTCAGCAAG CTCAGAGCAT TGCAAACCCT AGCTTTTTGA TGCACTCATT   
  
  
+ AGGTTACTAT CAGCTGGAGC AACATCTATT TCAACCTCAG GCAAAACGCC TGAACACGGG TGCTGTTCTG   
  
  
+ GATCCTAACC TTGTTCAGCT TGCAAAGAAC CCATTTGCTG ATCAGGGTCA TGAGTTATTG TTGAGGAAGC   
  
  
+ AGCACCAACA GCTTGGTTTG CAACCATTGC CATTGGGTTT GGGTCCTCAG TTGGTCCCTC CGCAGAAGCC   
  
  
+ TGTGATGGGT TCAAAGCAGG GGAACCCCCA GCATTACCCG TTGCATGTTC ATCAGCAGCT GCAATTGCAA   
  
  
+ GAGCAGGCTG TCAAAGATCA GCTCTTCAAG GCGGCAGACC TTATTCAAAC TGGAAGTTTC TCACTCGCGC   
  
  
+ AAGAGATATT GGCGCGGCTC AATCACCAGC TCTCCCTCCC TGCAAAGCCC CTCATTAGGG CGGCTTTGTA   
  
  
+ TGTGAAGGAG GCCCTTCAAA TGCTCCTCCT AATGAGCAAC CCAGTTGCGG CTCCACCGTC CAAGATCCTC   
  
  
+ ACCCCTTATG ATGTTGTTCA CAAGATGAGC GCGTATAAGG TCTTCTCTGA GGTCTGCCCA ATCACTCAAT   
  
  
+ TTGTGAATTT CACTTGTACA CAGGCCATTC TCGAGGCTCT TGATGATTCT GATGCTATTC ATGTTGTCGA   
  
  
+ CTTTGATATT GGTTGCGGTG CTCAATGGGC ATCATTGATT CAGGAGCTGC CATTGAGGAA AAGGGGAGCT   
  
  
+ CCCTCTCTGA AAATTACAGC CATAGCTCCC ATGTTGACTG GCAGTCACTT TGAAATTAGC CTAGTATGTG   
  
  
+ AAAACCTTGT GCAATTTGCC AACGATATTG GTGTTGCTTG TGAGCTCCAA GTTGTCAACT TTGATTTGTT   
  
  
+ TGATCCATCT TCGCCATCAA TGCCGAACAT TAGTACTGCT GAGGATGAGT CAATTGCTGT TAGTATCCCC   
  
  
+ ATCTGGGCAT CTTCAATTAG GCCATCTGTT CTTCCTTCCA TCCTCCGATT CATTAAGCAA AAATCCCCCA   
  
  
+ AAATTGTGGT CTCGTTTGAT AGAGGATTCG ATCGTTTTGA TGTCCCTTTC CCCCAACATC TGTTGCATAC   
  
  
+ CCTAGAATCC TGCAGTAATT TATTGGACTC GCTTGATGGT CTCAATGTTG CATCGGACAT TGTGAGCAAG   
  
  
+ GTCGAGAAGT TCTTTGTTCA ACCTAGGATC GAAAATGCTG TGTTGGGTCG GGTCCATGCC CCTGACAAGA   
  
  
+ TGCCCCATTG GAAAAATCTC TTTGCTTCAG CCGGCTTCTT GCCCTTGCAA TTCAGTAATT TCACAGAAAC   
  
  
+ CCAGGCCGAT TATGTGGTGA AGAGAACCCC AACAAGAGGA TTTCACGTGG AGAAGCGCCA GGCATCACTC   
  
  
+ ATTCTGAGTT GGCAGAGGCA GGAGCTTGTG GCAGCTTCGG CATGGAAGTG TTG  

- +Up\_Stream \_Len000AGAATT GTAATCAACG AACCAAGTTG GGTAGTCACT CTTATAGACT AAAGTTTTCA   
  
  
- TATCTAAATA TATTTGTATA CCTGATGTCC CTTCCCTCAG CAAAGGAGTT TCAGAATCTA AATGTGAGTT   
  
  
- TTCAAACTCG CTATGGTTAT TACACTAAGG TTTGTACTCG GTTTATAATT CATAGGATAT GATTCTAGTT   
  
  
- CTTCTCTTAC TTGGTTTTCT GACAATTAAA AAGTTAATTC GATTTGAATA ATTGGAAAAA AACCTTATCT   
  
  
- AAAAATTTAA AATAGAAGTT ATATTCAAAA AGGTTCCGCT CTCTGTATAT CGTTAAAATT AATTTCAATT   
  
  
- TCATCATATT GTAAAAAAAA TTACCAATAT CCGAATATCG TGGAAAAGAT CCACTTTTCT CTATAGAACC   
  
  
- GGACTAAAGG TCATTTCAAA AAAAAAATTT ATAACAGTTA ATAAAAACGT TTACTGTAAA CCATTAAACC   
  
  
- CCCTATTAGA CTACACAATT TTCTATGTTA CTTTTATAGT CTTTTATCCT ACGGAGTTTA AAGTGTTCTT   
  
  
- TCTTTTTTAG AAAAACAACA ATCATAAAGA TGTGTAGGTG TTAAAAATGA GTACCATTTT TTTTTTCCCG   
  
  
- GTTTATAGAG TGGTTATATT GGTTTGATAT ACAAGGTAAT AAATCATTGC CACTATTTAT ACATTACCCA   
  
  
- GTTCAATGAT TATTTTATCG GATGTTATGA ACCTATAGAT GTATTTTAAC TATATCTTAT ATATAACGTT   
  
  
- ATGAATTTAG TTTTAATTTA CTTTATAAAT GAAAAAATAA GATAATGTTT ATCGAAGAAT ATCTTATCGT   
  
  
- TGTTAAAAAT TACATTCAAA GTAAGAATGG TCTAAACTTC TCCAGGCCTA TATCTTTTAG AATAAGCATA   
  
  
- TTCATGAGCG TACTCCCGCC TATTTAAGAT TTTATAGTTG GAGTTGAGCG AAGATGATAA GGTAGATTCA   
  
  
- AGTTAAATAG TATATTTTTT TAAGTTAGGT GAAATTACAT GAGTTAAGTG AAGTTGGATA AAATTAGTTA   
  
  
- AATGGGTACT TTTCTTGTTC CAGGATTCAT CTACAAAAGA TAACTAACTA AATTTGTTAA AAGTGGATGG   
  
  
- GTTAATAGGG GTTTTGACTT TCTCCCCCGT TTCCCCCCAC CCATTACGAC ACATTTTATC CCCCTTTCCC   
  
  
- GTTCTTTTGA CACATGTTAC CACCTCGTCC CCATATGGTA AACCGGAACT CAACTCCCCC GTGTTTTCGG   
  
  
- GTCTCTCTCT CTCCCCATTA CGGGTGGTCT TTATTCGTCC TTCGCTCCCT TTTTGTGATC TCTCTTTGTC   
  
  
- TCTTTCTCTC TCTCTCTCTC TCTCTTCCCC TTTAAATGAT CTCTTACTCT CACTTCTTCC CTTGAAATAA   
  
  
- AATTGAAAAA ATACTACGTG CGTAGGTACC GTCTTGTCCC TTCCCTTTCT TCCCATGTCT CATGACGACA   
  
  
- GGGGTTTTTT AATCACATCT TTTGTCCTCT GTGAGTTGAT GTTGTTCCCA TTCCTTCTTC TTGTTCGTTC   
  
  
- TATAGGGTGG GTTTCTTTTT TTTTTTCTTC TTCTTCTTTT AAAAAGTCTC TCCTTTCTTT TCGTTAGTGT   
  
  
- ACTTAATCTT CGGTTGGGTT CGGAAGAAGT TTCTCTTCGT CGTATTGTAT CGTATCGACC CCTCTCCTAT   
  
  
- CCTCTCTCTT TGAGTTCTTT TCCCCATGGG CCGAATTTAC AACTACGTCT TTCCATATCT ATACCGGTGT   
  
  
- GATGATGATG GGTCCTCAAA TAGTAACCTC TACCTAAAGA GAAAGACGGA GTAGAAGAGG AGGTAGGTGG   
  
  
- TCGTTTTGAC CCAAAGGAAA GTCGACCCAG AAAAGATCTA GATCTTTGAT ACCATGGGTT TAGAGGGTAA   
  
  
- GGAGGTAAAA ATAAGAGTTT TAGATAGAAG AATCTGAACA CCCAAACTAA GTAAAGAAAA CCCAAAAACT   
  
  
- TTTCTTAAAT TAAACTAACA TAACACAACT AACCCCTTAA ACACCACCAC AATCTACGCT CGATACGGGA   
  
  
- TGTTAAACGT TCCGTTCCCA CACCATCTCC AAAGTTCGTA AACATAAGGT GTTTAGAGAA GGGGACGAAG   
  
  
- TTTCACCTTC TGGTTGTTGT TGTTGAACCT GTTGTTTAAA GTCGTTGTTG TTCTTCTTTA ACTCGTTAAC   
  
  
- GTCTCATCAC CACCGTGAAA AGAGAGAGGA CTATTGTGGT TATGATTATA ATCATGATGA GGCTCTCGCT   
  
  
- GTTCAAGACA ACTATCGCTT GTGTGTAGAT GAGACCTATG ATGCCCGGGA GGGTGAAGTT GTAGAAGGAG   
  
  
- GAAGTTCTCG TGGAGGTGGC GGTTGTGTCG GCCACACCGC CTGGGGCGTA CATGAGGGTT TCTCCTAACC   
  
  
- CTACAGAGGC CGCCGCGACG ACCGCCACTG CCGCCACCAC CCCCCGAACC TAACCTCCTC ACCCTGTCGT   
  
  
- ACAAGGGTTT ACCCCTCCCC CGAAACGAGG GAACCTAGTA CCCACTTCGA CTACTGTACC CATACCCAAA   
  
  
- CTTCGTAGAA AACGTTAGCC CATTGGGCCA ACTGATACTC CCGTTACGAC CAGATCCCCA ACAGCTAGTC   
  
  
- CCAAGACCTA AACTCTGAGA GAGAGGAGGA GGTGGCGTCG GCCGCCGTAC ACTTAGATTA CCACCACCAC   
  
  
- AACGATTAAA CCCCAAAGGT CCCTTATTAC CATTCTAAAG AAGTTAAAGT GTATTAACAA GTAGACCCTA   
  
  
- AAACTTACCA TTCCACTTGT TATTACCCAA CTTAGGGTTA ACATTGGGAG TTCGAGTTCC GTCAGATGAA   
  
  
- CCAAATTAGG TTCCTCGATG TCCACATGTG CGTGTAGGAC TGCAACCTCT GCTCTTTGGG GTCTAAAACT   
  
  
- TAGGTGTAAA CCACTACTTG GGAGTCGTTC GAGTCTCGTA ACGTTTGGGA TCGAAAAACT ACGTGAGTAA   
  
  
- TCCAATGATA GTCGACCTCG TTGTAGATAA AGTTGGAGTC CGTTTTGCGG ACTTGTGCCC ACGACAAGAC   
  
  
- CTAGGATTGG AACAAGTCGA ACGTTTCTTG GGTAAACGAC TAGTCCCAGT ACTCAATAAC AACTCCTTCG   
  
  
- TCGTGGTTGT CGAACCAAAC GTTGGTAACG GTAACCCAAA CCCAGGAGTC AACCAGGGAG GCGTCTTCGG   
  
  
- ACACTACCCA AGTTTCGTCC CCTTGGGGGT CGTAATGGGC AACGTACAAG TAGTCGTCGA CGTTAACGTT   
  
  
- CTCGTCCGAC AGTTTCTAGT CGAGAAGTTC CGCCGTCTGG AATAAGTTTG ACCTTCAAAG AGTGAGCGCG   
  
  
- TTCTCTATAA CCGCGCCGAG TTAGTGGTCG AGAGGGAGGG ACGTTTCGGG GAGTAATCCC GCCGAAACAT   
  
  
- ACACTTCCTC CGGGAAGTTT ACGAGGAGGA TTACTCGTTG GGTCAACGCC GAGGTGGCAG GTTCTAGGAG   
  
  
- TGGGGAATAC TACAACAAGT GTTCTACTCG CGCATATTCC AGAAGAGACT CCAGACGGGT TAGTGAGTTA   
  
  
- AACACTTAAA GTGAACATGT GTCCGGTAAG AGCTCCGAGA ACTACTAAGA CTACGATAAG TACAACAGCT   
  
  
- GAAACTATAA CCAACGCCAC GAGTTACCCG TAGTAACTAA GTCCTCGACG GTAACTCCTT TTCCCCTCGA   
  
  
- GGGAGAGACT TTTAATGTCG GTATCGAGGG TACAACTGAC CGTCAGTGAA ACTTTAATCG GATCATACAC   
  
  
- TTTTGGAACA CGTTAAACGG TTGCTATAAC CACAACGAAC ACTCGAGGTT CAACAGTTGA AACTAAACAA   
  
  
- ACTAGGTAGA AGCGGTAGTT ACGGCTTGTA ATCATGACGA CTCCTACTCA GTTAACGACA ATCATAGGGG   
  
  
- TAGACCCGTA GAAGTTAATC CGGTAGACAA GAAGGAAGGT AGGAGGCTAA GTAATTCGTT TTTAGGGGGT   
  
  
- TTTAACACCA GAGCAAACTA TCTCCTAAGC TAGCAAAACT ACAGGGAAAG GGGGTTGTAG ACAACGTATG   
  
  
- GGATCTTAGG ACGTCATTAA ATAACCTGAG CGAACTACCA GAGTTACAAC GTAGCCTGTA ACACTCGTTC   
  
  
- CAGCTCTTCA AGAAACAAGT TGGATCCTAG CTTTTACGAC ACAACCCAGC CCAGGTACGG GGACTGTTCT   
  
  
- ACGGGGTAAC CTTTTTAGAG AAACGAAGTC GGCCGAAGAA CGGGAACGTT AAGTCATTAA AGTGTCTTTG   
  
  
- GGTCCGGCTA ATACACCACT TCTCTTGGGG TTGTTCTCCT AAAGTGCACC TCTTCGCGGT CCGTAGTGAG   
  
  
- TAAGACTCAA CCGTCTCCGT CCTCGAACAC CGTCGAAGCC GTACCTTCAC AAC

+     MBSI

| Site Name | Organism | Position | Strand | Matrix score. | sequence | function |
| --- | --- | --- | --- | --- | --- | --- |
| MBSI | Petunia hybrida | 265 | - | 10.5 | aaaAaaC(G/C)GTTA | MYB binding site involved in flavonoid biosynthetic genes regulation |

>HU04G00047.1   
+ +Up\_Stream \_Len000TCTTAA CATTAGTTGC TTGGTTCAAC CCATCAGTGA GAATATCTGA TTTCAAAAGT   
  
  
+ ATAGATTTAT ATAAACATAT GGACTACAGG GAAGGGAGTC GTTTCCTCAA AGTCTTAGAT TTACACTCAA   
  
  
+ AAGTTTGAGC GATACCAATA ATGTGATTCC AAACATGAGC CAAATATTAA GTATCCTATA CTAAGATCAA   
  
  
+ GAAGAGAATG AACCAAAAGA CTGTTAATTT TTCAATTAAG CTAAACTTAT TAACCTTTTT TTGGAATAGA   
  
  
+ TTTTTAAATT TTATCTTCAA TATAAGTTTT TCCAAGGCGA GAGACATATA GCAATTTTAA TTAAAGTTAA   
  
  
+ AGTAGTATAA CATTTTTTTT AATGGTTATA GGCTTATAGC ACCTTTTCTA GGTGAAAAGA GATATCTTGG   
  
  
+ CCTGATTTCC AGTAAAGTTT TTTTTTTAAA TATTGTCAAT TATTTTTGCA AATGACATTT GGTAATTTGG   
  
  
+ GGGATAATCT GATGTGTTAA AAGATACAAT GAAAATATCA GAAAATAGGA TGCCTCAAAT TTCACAAGAA   
  
  
+ AGAAAAAATC TTTTTGTTGT TAGTATTTCT ACACATCCAC AATTTTTACT CATGGTAAAA AAAAAAGGGC   
  
  
+ CAAATATCTC ACCAATATAA CCAAACTATA TGTTCCATTA TTTAGTAACG GTGATAAATA TGTAATGGGT   
  
  
+ CAAGTTACTA ATAAAATAGC CTACAATACT TGGATATCTA CATAAAATTG ATATAGAATA TATATTGCAA   
  
  
+ TACTTAAATC AAAATTAAAT GAAATATTTA CTTTTTTATT CTATTACAAA TAGCTTCTTA TAGAATAGCA   
  
  
+ ACAATTTTTA ATGTAAGTTT CATTCTTACC AGATTTGAAG AGGTCCGGAT ATAGAAAATC TTATTCGTAT   
  
  
+ AAGTACTCGC ATGAGGGCGG ATAAATTCTA AAATATCAAC CTCAACTCGC TTCTACTATT CCATCTAAGT   
  
  
+ TCAATTTATC ATATAAAAAA ATTCAATCCA CTTTAATGTA CTCAATTCAC TTCAACCTAT TTTAATCAAT   
  
  
+ TTACCCATGA AAAGAACAAG GTCCTAAGTA GATGTTTTCT ATTGATTGAT TTAAACAATT TTCACCTACC   
  
  
+ CAATTATCCC CAAAACTGAA AGAGGGGGCA AAGGGGGGTG GGTAATGCTG TGTAAAATAG GGGGAAAGGG   
  
  
+ CAAGAAAACT GTGTACAATG GTGGAGCAGG GGTATACCAT TTGGCCTTGA GTTGAGGGGG CACAAAAGCC   
  
  
+ CAGAGAGAGA GAGGGGTAAT GCCCACCAGA AATAAGCAGG AAGCGAGGGA AAAACACTAG AGAGAAACAG   
  
  
+ AGAAAGAGAG AGAGAGAGAG AGAGAAGGGG AAATTTACTA GAGAATGAGA GTGAAGAAGG GAACTTTATT   
  
  
+ TTAACTTTTT TATGATGCAC GCATCCATGG CAGAACAGGG AAGGGAAAGA AGGGTACAGA GTACTGCTGT   
  
  
+ CCCCAAAAAA TTAGTGTAGA AAACAGGAGA CACTCAACTA CAACAAGGGT AAGGAAGAAG AACAAGCAAG   
  
  
+ ATATCCCACC CAAAGAAAAA AAAAAAGAAG AAGAAGAAAA TTTTTCAGAG AGGAAAGAAA AGCAATCACA   
  
  
+ TGAATTAGAA GCCAACCCAA GCCTTCTTCA AAGAGAAGCA GCATAACATA GCATAGCTGG GGAGAGGATA   
  
  
+ GGAGAGAGAA ACTCAAGAAA AGGGGTACCC GGCTTAAATG TTGATGCAGA AAGGTATAGA TATGGCCACA   
  
  
+ CTACTACTAC CCAGGAGTTT ATCATTGGAG ATGGATTTCT CTTTCTGCCT CATCTTCTCC TCCATCCACC   
  
  
+ AGCAAAACTG GGTTTCCTTT CAGCTGGGTC TTTTCTAGAT CTAGAAACTA TGGTACCCAA ATCTCCCATT   
  
  
+ CCTCCATTTT TATTCTCAAA ATCTATCTTC TTAGACTTGT GGGTTTGATT CATTTCTTTT GGGTTTTTGA   
  
  
+ AAAGAATTTA ATTTGATTGT ATTGTGTTGA TTGGGGAATT TGTGGTGGTG TTAGATGCGA GCTATGCCCT   
  
  
+ ACAATTTGCA AGGCAAGGGT GTGGTAGAGG TTTCAAGCAT TTGTATTCCA CAAATCTCTT CCCCTGCTTC   
  
  
+ AAAGTGGAAG ACCAACAACA ACAACTTGGA CAACAAATTT CAGCAACAAC AAGAAGAAAT TGAGCAATTG   
  
  
+ CAGAGTAGTG GTGGCACTTT TCTCTCTCCT GATAACACCA ATACTAATAT TAGTACTACT CCGAGAGCGA   
  
  
+ CAAGTTCTGT TGATAGCGAA CACACATCTA CTCTGGATAC TACGGGCCCT CCCACTTCAA CATCTTCCTC   
  
  
+ CTTCAAGAGC ACCTCCACCG CCAACACAGC CGGTGTGGCG GACCCCGCAT GTACTCCCAA AGAGGATTGG   
  
  
+ GATGTCTCCG GCGGCGCTGC TGGCGGTGAC GGCGGTGGTG GGGGGCTTGG ATTGGAGGAG TGGGACAGCA   
  
  
+ TGTTCCCAAA TGGGGAGGGG GCTTTGCTCC CTTGGATCAT GGGTGAAGCT GATGACATGG GTATGGGTTT   
  
  
+ GAAGCATCTT TTGCAATCGG GTAACCCGGT TGACTATGAG GGCAATGCTG GTCTAGGGGT TGTCGATCAG   
  
  
+ GGTTCTGGAT TTGAGACTCT CTCTCCTCCT CCACCGCAGC CGGCGGCATG TGAATCTAAT GGTGGTGGTG   
  
  
+ TTGCTAATTT GGGGTTTCCA GGGAATAATG GTAAGATTTC TTCAATTTCA CATAATTGTT CATCTGGGAT   
  
  
+ TTTGAATGGT AAGGTGAACA ATAATGGGTT GAATCCCAAT TGTAACCCTC AAGCTCAAGG CAGTCTACTT   
  
  
+ GGTTTAATCC AAGGAGCTAC AGGTGTACAC GCACATCCTG ACGTTGGAGA CGAGAAACCC CAGATTTTGA   
  
  
+ ATCCACATTT GGTGATGAAC CCTCAGCAAG CTCAGAGCAT TGCAAACCCT AGCTTTTTGA TGCACTCATT   
  
  
+ AGGTTACTAT CAGCTGGAGC AACATCTATT TCAACCTCAG GCAAAACGCC TGAACACGGG TGCTGTTCTG   
  
  
+ GATCCTAACC TTGTTCAGCT TGCAAAGAAC CCATTTGCTG ATCAGGGTCA TGAGTTATTG TTGAGGAAGC   
  
  
+ AGCACCAACA GCTTGGTTTG CAACCATTGC CATTGGGTTT GGGTCCTCAG TTGGTCCCTC CGCAGAAGCC   
  
  
+ TGTGATGGGT TCAAAGCAGG GGAACCCCCA GCATTACCCG TTGCATGTTC ATCAGCAGCT GCAATTGCAA   
  
  
+ GAGCAGGCTG TCAAAGATCA GCTCTTCAAG GCGGCAGACC TTATTCAAAC TGGAAGTTTC TCACTCGCGC   
  
  
+ AAGAGATATT GGCGCGGCTC AATCACCAGC TCTCCCTCCC TGCAAAGCCC CTCATTAGGG CGGCTTTGTA   
  
  
+ TGTGAAGGAG GCCCTTCAAA TGCTCCTCCT AATGAGCAAC CCAGTTGCGG CTCCACCGTC CAAGATCCTC   
  
  
+ ACCCCTTATG ATGTTGTTCA CAAGATGAGC GCGTATAAGG TCTTCTCTGA GGTCTGCCCA ATCACTCAAT   
  
  
+ TTGTGAATTT CACTTGTACA CAGGCCATTC TCGAGGCTCT TGATGATTCT GATGCTATTC ATGTTGTCGA   
  
  
+ CTTTGATATT GGTTGCGGTG CTCAATGGGC ATCATTGATT CAGGAGCTGC CATTGAGGAA AAGGGGAGCT   
  
  
+ CCCTCTCTGA AAATTACAGC CATAGCTCCC ATGTTGACTG GCAGTCACTT TGAAATTAGC CTAGTATGTG   
  
  
+ AAAACCTTGT GCAATTTGCC AACGATATTG GTGTTGCTTG TGAGCTCCAA GTTGTCAACT TTGATTTGTT   
  
  
+ TGATCCATCT TCGCCATCAA TGCCGAACAT TAGTACTGCT GAGGATGAGT CAATTGCTGT TAGTATCCCC   
  
  
+ ATCTGGGCAT CTTCAATTAG GCCATCTGTT CTTCCTTCCA TCCTCCGATT CATTAAGCAA AAATCCCCCA   
  
  
+ AAATTGTGGT CTCGTTTGAT AGAGGATTCG ATCGTTTTGA TGTCCCTTTC CCCCAACATC TGTTGCATAC   
  
  
+ CCTAGAATCC TGCAGTAATT TATTGGACTC GCTTGATGGT CTCAATGTTG CATCGGACAT TGTGAGCAAG   
  
  
+ GTCGAGAAGT TCTTTGTTCA ACCTAGGATC GAAAATGCTG TGTTGGGTCG GGTCCATGCC CCTGACAAGA   
  
  
+ TGCCCCATTG GAAAAATCTC TTTGCTTCAG CCGGCTTCTT GCCCTTGCAA TTCAGTAATT TCACAGAAAC   
  
  
+ CCAGGCCGAT TATGTGGTGA AGAGAACCCC AACAAGAGGA TTTCACGTGG AGAAGCGCCA GGCATCACTC   
  
  
+ ATTCTGAGTT GGCAGAGGCA GGAGCTTGTG GCAGCTTCGG CATGGAAGTG TTG  

- +Up\_Stream \_Len000AGAATT GTAATCAACG AACCAAGTTG GGTAGTCACT CTTATAGACT AAAGTTTTCA   
  
  
- TATCTAAATA TATTTGTATA CCTGATGTCC CTTCCCTCAG CAAAGGAGTT TCAGAATCTA AATGTGAGTT   
  
  
- TTCAAACTCG CTATGGTTAT TACACTAAGG TTTGTACTCG GTTTATAATT CATAGGATAT GATTCTAGTT   
  
  
- CTTCTCTTAC TTGGTTTTCT GACAATTAAA AAGTTAATTC GATTTGAATA ATTGGAAAAA AACCTTATCT   
  
  
- AAAAATTTAA AATAGAAGTT ATATTCAAAA AGGTTCCGCT CTCTGTATAT CGTTAAAATT AATTTCAATT   
  
  
- TCATCATATT GTAAAAAAAA TTACCAATAT CCGAATATCG TGGAAAAGAT CCACTTTTCT CTATAGAACC   
  
  
- GGACTAAAGG TCATTTCAAA AAAAAAATTT ATAACAGTTA ATAAAAACGT TTACTGTAAA CCATTAAACC   
  
  
- CCCTATTAGA CTACACAATT TTCTATGTTA CTTTTATAGT CTTTTATCCT ACGGAGTTTA AAGTGTTCTT   
  
  
- TCTTTTTTAG AAAAACAACA ATCATAAAGA TGTGTAGGTG TTAAAAATGA GTACCATTTT TTTTTTCCCG   
  
  
- GTTTATAGAG TGGTTATATT GGTTTGATAT ACAAGGTAAT AAATCATTGC CACTATTTAT ACATTACCCA   
  
  
- GTTCAATGAT TATTTTATCG GATGTTATGA ACCTATAGAT GTATTTTAAC TATATCTTAT ATATAACGTT   
  
  
- ATGAATTTAG TTTTAATTTA CTTTATAAAT GAAAAAATAA GATAATGTTT ATCGAAGAAT ATCTTATCGT   
  
  
- TGTTAAAAAT TACATTCAAA GTAAGAATGG TCTAAACTTC TCCAGGCCTA TATCTTTTAG AATAAGCATA   
  
  
- TTCATGAGCG TACTCCCGCC TATTTAAGAT TTTATAGTTG GAGTTGAGCG AAGATGATAA GGTAGATTCA   
  
  
- AGTTAAATAG TATATTTTTT TAAGTTAGGT GAAATTACAT GAGTTAAGTG AAGTTGGATA AAATTAGTTA   
  
  
- AATGGGTACT TTTCTTGTTC CAGGATTCAT CTACAAAAGA TAACTAACTA AATTTGTTAA AAGTGGATGG   
  
  
- GTTAATAGGG GTTTTGACTT TCTCCCCCGT TTCCCCCCAC CCATTACGAC ACATTTTATC CCCCTTTCCC   
  
  
- GTTCTTTTGA CACATGTTAC CACCTCGTCC CCATATGGTA AACCGGAACT CAACTCCCCC GTGTTTTCGG   
  
  
- GTCTCTCTCT CTCCCCATTA CGGGTGGTCT TTATTCGTCC TTCGCTCCCT TTTTGTGATC TCTCTTTGTC   
  
  
- TCTTTCTCTC TCTCTCTCTC TCTCTTCCCC TTTAAATGAT CTCTTACTCT CACTTCTTCC CTTGAAATAA   
  
  
- AATTGAAAAA ATACTACGTG CGTAGGTACC GTCTTGTCCC TTCCCTTTCT TCCCATGTCT CATGACGACA   
  
  
- GGGGTTTTTT AATCACATCT TTTGTCCTCT GTGAGTTGAT GTTGTTCCCA TTCCTTCTTC TTGTTCGTTC   
  
  
- TATAGGGTGG GTTTCTTTTT TTTTTTCTTC TTCTTCTTTT AAAAAGTCTC TCCTTTCTTT TCGTTAGTGT   
  
  
- ACTTAATCTT CGGTTGGGTT CGGAAGAAGT TTCTCTTCGT CGTATTGTAT CGTATCGACC CCTCTCCTAT   
  
  
- CCTCTCTCTT TGAGTTCTTT TCCCCATGGG CCGAATTTAC AACTACGTCT TTCCATATCT ATACCGGTGT   
  
  
- GATGATGATG GGTCCTCAAA TAGTAACCTC TACCTAAAGA GAAAGACGGA GTAGAAGAGG AGGTAGGTGG   
  
  
- TCGTTTTGAC CCAAAGGAAA GTCGACCCAG AAAAGATCTA GATCTTTGAT ACCATGGGTT TAGAGGGTAA   
  
  
- GGAGGTAAAA ATAAGAGTTT TAGATAGAAG AATCTGAACA CCCAAACTAA GTAAAGAAAA CCCAAAAACT   
  
  
- TTTCTTAAAT TAAACTAACA TAACACAACT AACCCCTTAA ACACCACCAC AATCTACGCT CGATACGGGA   
  
  
- TGTTAAACGT TCCGTTCCCA CACCATCTCC AAAGTTCGTA AACATAAGGT GTTTAGAGAA GGGGACGAAG   
  
  
- TTTCACCTTC TGGTTGTTGT TGTTGAACCT GTTGTTTAAA GTCGTTGTTG TTCTTCTTTA ACTCGTTAAC   
  
  
- GTCTCATCAC CACCGTGAAA AGAGAGAGGA CTATTGTGGT TATGATTATA ATCATGATGA GGCTCTCGCT   
  
  
- GTTCAAGACA ACTATCGCTT GTGTGTAGAT GAGACCTATG ATGCCCGGGA GGGTGAAGTT GTAGAAGGAG   
  
  
- GAAGTTCTCG TGGAGGTGGC GGTTGTGTCG GCCACACCGC CTGGGGCGTA CATGAGGGTT TCTCCTAACC   
  
  
- CTACAGAGGC CGCCGCGACG ACCGCCACTG CCGCCACCAC CCCCCGAACC TAACCTCCTC ACCCTGTCGT   
  
  
- ACAAGGGTTT ACCCCTCCCC CGAAACGAGG GAACCTAGTA CCCACTTCGA CTACTGTACC CATACCCAAA   
  
  
- CTTCGTAGAA AACGTTAGCC CATTGGGCCA ACTGATACTC CCGTTACGAC CAGATCCCCA ACAGCTAGTC   
  
  
- CCAAGACCTA AACTCTGAGA GAGAGGAGGA GGTGGCGTCG GCCGCCGTAC ACTTAGATTA CCACCACCAC   
  
  
- AACGATTAAA CCCCAAAGGT CCCTTATTAC CATTCTAAAG AAGTTAAAGT GTATTAACAA GTAGACCCTA   
  
  
- AAACTTACCA TTCCACTTGT TATTACCCAA CTTAGGGTTA ACATTGGGAG TTCGAGTTCC GTCAGATGAA   
  
  
- CCAAATTAGG TTCCTCGATG TCCACATGTG CGTGTAGGAC TGCAACCTCT GCTCTTTGGG GTCTAAAACT   
  
  
- TAGGTGTAAA CCACTACTTG GGAGTCGTTC GAGTCTCGTA ACGTTTGGGA TCGAAAAACT ACGTGAGTAA   
  
  
- TCCAATGATA GTCGACCTCG TTGTAGATAA AGTTGGAGTC CGTTTTGCGG ACTTGTGCCC ACGACAAGAC   
  
  
- CTAGGATTGG AACAAGTCGA ACGTTTCTTG GGTAAACGAC TAGTCCCAGT ACTCAATAAC AACTCCTTCG   
  
  
- TCGTGGTTGT CGAACCAAAC GTTGGTAACG GTAACCCAAA CCCAGGAGTC AACCAGGGAG GCGTCTTCGG   
  
  
- ACACTACCCA AGTTTCGTCC CCTTGGGGGT CGTAATGGGC AACGTACAAG TAGTCGTCGA CGTTAACGTT   
  
  
- CTCGTCCGAC AGTTTCTAGT CGAGAAGTTC CGCCGTCTGG AATAAGTTTG ACCTTCAAAG AGTGAGCGCG   
  
  
- TTCTCTATAA CCGCGCCGAG TTAGTGGTCG AGAGGGAGGG ACGTTTCGGG GAGTAATCCC GCCGAAACAT   
  
  
- ACACTTCCTC CGGGAAGTTT ACGAGGAGGA TTACTCGTTG GGTCAACGCC GAGGTGGCAG GTTCTAGGAG   
  
  
- TGGGGAATAC TACAACAAGT GTTCTACTCG CGCATATTCC AGAAGAGACT CCAGACGGGT TAGTGAGTTA   
  
  
- AACACTTAAA GTGAACATGT GTCCGGTAAG AGCTCCGAGA ACTACTAAGA CTACGATAAG TACAACAGCT   
  
  
- GAAACTATAA CCAACGCCAC GAGTTACCCG TAGTAACTAA GTCCTCGACG GTAACTCCTT TTCCCCTCGA   
  
  
- GGGAGAGACT TTTAATGTCG GTATCGAGGG TACAACTGAC CGTCAGTGAA ACTTTAATCG GATCATACAC   
  
  
- TTTTGGAACA CGTTAAACGG TTGCTATAAC CACAACGAAC ACTCGAGGTT CAACAGTTGA AACTAAACAA   
  
  
- ACTAGGTAGA AGCGGTAGTT ACGGCTTGTA ATCATGACGA CTCCTACTCA GTTAACGACA ATCATAGGGG   
  
  
- TAGACCCGTA GAAGTTAATC CGGTAGACAA GAAGGAAGGT AGGAGGCTAA GTAATTCGTT TTTAGGGGGT   
  
  
- TTTAACACCA GAGCAAACTA TCTCCTAAGC TAGCAAAACT ACAGGGAAAG GGGGTTGTAG ACAACGTATG   
  
  
- GGATCTTAGG ACGTCATTAA ATAACCTGAG CGAACTACCA GAGTTACAAC GTAGCCTGTA ACACTCGTTC   
  
  
- CAGCTCTTCA AGAAACAAGT TGGATCCTAG CTTTTACGAC ACAACCCAGC CCAGGTACGG GGACTGTTCT   
  
  
- ACGGGGTAAC CTTTTTAGAG AAACGAAGTC GGCCGAAGAA CGGGAACGTT AAGTCATTAA AGTGTCTTTG   
  
  
- GGTCCGGCTA ATACACCACT TCTCTTGGGG TTGTTCTCCT AAAGTGCACC TCTTCGCGGT CCGTAGTGAG   
  
  
- TAAGACTCAA CCGTCTCCGT CCTCGAACAC CGTCGAAGCC GTACCTTCAC AAC

+     MRE

| Site Name | Organism | Position | Strand | Matrix score. | sequence | function |
| --- | --- | --- | --- | --- | --- | --- |
| MRE | Petroselinum crispum | 2943 | - | 7 | AACCTAA | MYB binding site involved in light responsiveness |

>HU04G00047.1   
+ +Up\_Stream \_Len000TCTTAA CATTAGTTGC TTGGTTCAAC CCATCAGTGA GAATATCTGA TTTCAAAAGT   
  
  
+ ATAGATTTAT ATAAACATAT GGACTACAGG GAAGGGAGTC GTTTCCTCAA AGTCTTAGAT TTACACTCAA   
  
  
+ AAGTTTGAGC GATACCAATA ATGTGATTCC AAACATGAGC CAAATATTAA GTATCCTATA CTAAGATCAA   
  
  
+ GAAGAGAATG AACCAAAAGA CTGTTAATTT TTCAATTAAG CTAAACTTAT TAACCTTTTT TTGGAATAGA   
  
  
+ TTTTTAAATT TTATCTTCAA TATAAGTTTT TCCAAGGCGA GAGACATATA GCAATTTTAA TTAAAGTTAA   
  
  
+ AGTAGTATAA CATTTTTTTT AATGGTTATA GGCTTATAGC ACCTTTTCTA GGTGAAAAGA GATATCTTGG   
  
  
+ CCTGATTTCC AGTAAAGTTT TTTTTTTAAA TATTGTCAAT TATTTTTGCA AATGACATTT GGTAATTTGG   
  
  
+ GGGATAATCT GATGTGTTAA AAGATACAAT GAAAATATCA GAAAATAGGA TGCCTCAAAT TTCACAAGAA   
  
  
+ AGAAAAAATC TTTTTGTTGT TAGTATTTCT ACACATCCAC AATTTTTACT CATGGTAAAA AAAAAAGGGC   
  
  
+ CAAATATCTC ACCAATATAA CCAAACTATA TGTTCCATTA TTTAGTAACG GTGATAAATA TGTAATGGGT   
  
  
+ CAAGTTACTA ATAAAATAGC CTACAATACT TGGATATCTA CATAAAATTG ATATAGAATA TATATTGCAA   
  
  
+ TACTTAAATC AAAATTAAAT GAAATATTTA CTTTTTTATT CTATTACAAA TAGCTTCTTA TAGAATAGCA   
  
  
+ ACAATTTTTA ATGTAAGTTT CATTCTTACC AGATTTGAAG AGGTCCGGAT ATAGAAAATC TTATTCGTAT   
  
  
+ AAGTACTCGC ATGAGGGCGG ATAAATTCTA AAATATCAAC CTCAACTCGC TTCTACTATT CCATCTAAGT   
  
  
+ TCAATTTATC ATATAAAAAA ATTCAATCCA CTTTAATGTA CTCAATTCAC TTCAACCTAT TTTAATCAAT   
  
  
+ TTACCCATGA AAAGAACAAG GTCCTAAGTA GATGTTTTCT ATTGATTGAT TTAAACAATT TTCACCTACC   
  
  
+ CAATTATCCC CAAAACTGAA AGAGGGGGCA AAGGGGGGTG GGTAATGCTG TGTAAAATAG GGGGAAAGGG   
  
  
+ CAAGAAAACT GTGTACAATG GTGGAGCAGG GGTATACCAT TTGGCCTTGA GTTGAGGGGG CACAAAAGCC   
  
  
+ CAGAGAGAGA GAGGGGTAAT GCCCACCAGA AATAAGCAGG AAGCGAGGGA AAAACACTAG AGAGAAACAG   
  
  
+ AGAAAGAGAG AGAGAGAGAG AGAGAAGGGG AAATTTACTA GAGAATGAGA GTGAAGAAGG GAACTTTATT   
  
  
+ TTAACTTTTT TATGATGCAC GCATCCATGG CAGAACAGGG AAGGGAAAGA AGGGTACAGA GTACTGCTGT   
  
  
+ CCCCAAAAAA TTAGTGTAGA AAACAGGAGA CACTCAACTA CAACAAGGGT AAGGAAGAAG AACAAGCAAG   
  
  
+ ATATCCCACC CAAAGAAAAA AAAAAAGAAG AAGAAGAAAA TTTTTCAGAG AGGAAAGAAA AGCAATCACA   
  
  
+ TGAATTAGAA GCCAACCCAA GCCTTCTTCA AAGAGAAGCA GCATAACATA GCATAGCTGG GGAGAGGATA   
  
  
+ GGAGAGAGAA ACTCAAGAAA AGGGGTACCC GGCTTAAATG TTGATGCAGA AAGGTATAGA TATGGCCACA   
  
  
+ CTACTACTAC CCAGGAGTTT ATCATTGGAG ATGGATTTCT CTTTCTGCCT CATCTTCTCC TCCATCCACC   
  
  
+ AGCAAAACTG GGTTTCCTTT CAGCTGGGTC TTTTCTAGAT CTAGAAACTA TGGTACCCAA ATCTCCCATT   
  
  
+ CCTCCATTTT TATTCTCAAA ATCTATCTTC TTAGACTTGT GGGTTTGATT CATTTCTTTT GGGTTTTTGA   
  
  
+ AAAGAATTTA ATTTGATTGT ATTGTGTTGA TTGGGGAATT TGTGGTGGTG TTAGATGCGA GCTATGCCCT   
  
  
+ ACAATTTGCA AGGCAAGGGT GTGGTAGAGG TTTCAAGCAT TTGTATTCCA CAAATCTCTT CCCCTGCTTC   
  
  
+ AAAGTGGAAG ACCAACAACA ACAACTTGGA CAACAAATTT CAGCAACAAC AAGAAGAAAT TGAGCAATTG   
  
  
+ CAGAGTAGTG GTGGCACTTT TCTCTCTCCT GATAACACCA ATACTAATAT TAGTACTACT CCGAGAGCGA   
  
  
+ CAAGTTCTGT TGATAGCGAA CACACATCTA CTCTGGATAC TACGGGCCCT CCCACTTCAA CATCTTCCTC   
  
  
+ CTTCAAGAGC ACCTCCACCG CCAACACAGC CGGTGTGGCG GACCCCGCAT GTACTCCCAA AGAGGATTGG   
  
  
+ GATGTCTCCG GCGGCGCTGC TGGCGGTGAC GGCGGTGGTG GGGGGCTTGG ATTGGAGGAG TGGGACAGCA   
  
  
+ TGTTCCCAAA TGGGGAGGGG GCTTTGCTCC CTTGGATCAT GGGTGAAGCT GATGACATGG GTATGGGTTT   
  
  
+ GAAGCATCTT TTGCAATCGG GTAACCCGGT TGACTATGAG GGCAATGCTG GTCTAGGGGT TGTCGATCAG   
  
  
+ GGTTCTGGAT TTGAGACTCT CTCTCCTCCT CCACCGCAGC CGGCGGCATG TGAATCTAAT GGTGGTGGTG   
  
  
+ TTGCTAATTT GGGGTTTCCA GGGAATAATG GTAAGATTTC TTCAATTTCA CATAATTGTT CATCTGGGAT   
  
  
+ TTTGAATGGT AAGGTGAACA ATAATGGGTT GAATCCCAAT TGTAACCCTC AAGCTCAAGG CAGTCTACTT   
  
  
+ GGTTTAATCC AAGGAGCTAC AGGTGTACAC GCACATCCTG ACGTTGGAGA CGAGAAACCC CAGATTTTGA   
  
  
+ ATCCACATTT GGTGATGAAC CCTCAGCAAG CTCAGAGCAT TGCAAACCCT AGCTTTTTGA TGCACTCATT   
  
  
+ AGGTTACTAT CAGCTGGAGC AACATCTATT TCAACCTCAG GCAAAACGCC TGAACACGGG TGCTGTTCTG   
  
  
+ GATCCTAACC TTGTTCAGCT TGCAAAGAAC CCATTTGCTG ATCAGGGTCA TGAGTTATTG TTGAGGAAGC   
  
  
+ AGCACCAACA GCTTGGTTTG CAACCATTGC CATTGGGTTT GGGTCCTCAG TTGGTCCCTC CGCAGAAGCC   
  
  
+ TGTGATGGGT TCAAAGCAGG GGAACCCCCA GCATTACCCG TTGCATGTTC ATCAGCAGCT GCAATTGCAA   
  
  
+ GAGCAGGCTG TCAAAGATCA GCTCTTCAAG GCGGCAGACC TTATTCAAAC TGGAAGTTTC TCACTCGCGC   
  
  
+ AAGAGATATT GGCGCGGCTC AATCACCAGC TCTCCCTCCC TGCAAAGCCC CTCATTAGGG CGGCTTTGTA   
  
  
+ TGTGAAGGAG GCCCTTCAAA TGCTCCTCCT AATGAGCAAC CCAGTTGCGG CTCCACCGTC CAAGATCCTC   
  
  
+ ACCCCTTATG ATGTTGTTCA CAAGATGAGC GCGTATAAGG TCTTCTCTGA GGTCTGCCCA ATCACTCAAT   
  
  
+ TTGTGAATTT CACTTGTACA CAGGCCATTC TCGAGGCTCT TGATGATTCT GATGCTATTC ATGTTGTCGA   
  
  
+ CTTTGATATT GGTTGCGGTG CTCAATGGGC ATCATTGATT CAGGAGCTGC CATTGAGGAA AAGGGGAGCT   
  
  
+ CCCTCTCTGA AAATTACAGC CATAGCTCCC ATGTTGACTG GCAGTCACTT TGAAATTAGC CTAGTATGTG   
  
  
+ AAAACCTTGT GCAATTTGCC AACGATATTG GTGTTGCTTG TGAGCTCCAA GTTGTCAACT TTGATTTGTT   
  
  
+ TGATCCATCT TCGCCATCAA TGCCGAACAT TAGTACTGCT GAGGATGAGT CAATTGCTGT TAGTATCCCC   
  
  
+ ATCTGGGCAT CTTCAATTAG GCCATCTGTT CTTCCTTCCA TCCTCCGATT CATTAAGCAA AAATCCCCCA   
  
  
+ AAATTGTGGT CTCGTTTGAT AGAGGATTCG ATCGTTTTGA TGTCCCTTTC CCCCAACATC TGTTGCATAC   
  
  
+ CCTAGAATCC TGCAGTAATT TATTGGACTC GCTTGATGGT CTCAATGTTG CATCGGACAT TGTGAGCAAG   
  
  
+ GTCGAGAAGT TCTTTGTTCA ACCTAGGATC GAAAATGCTG TGTTGGGTCG GGTCCATGCC CCTGACAAGA   
  
  
+ TGCCCCATTG GAAAAATCTC TTTGCTTCAG CCGGCTTCTT GCCCTTGCAA TTCAGTAATT TCACAGAAAC   
  
  
+ CCAGGCCGAT TATGTGGTGA AGAGAACCCC AACAAGAGGA TTTCACGTGG AGAAGCGCCA GGCATCACTC   
  
  
+ ATTCTGAGTT GGCAGAGGCA GGAGCTTGTG GCAGCTTCGG CATGGAAGTG TTG  

- +Up\_Stream \_Len000AGAATT GTAATCAACG AACCAAGTTG GGTAGTCACT CTTATAGACT AAAGTTTTCA   
  
  
- TATCTAAATA TATTTGTATA CCTGATGTCC CTTCCCTCAG CAAAGGAGTT TCAGAATCTA AATGTGAGTT   
  
  
- TTCAAACTCG CTATGGTTAT TACACTAAGG TTTGTACTCG GTTTATAATT CATAGGATAT GATTCTAGTT   
  
  
- CTTCTCTTAC TTGGTTTTCT GACAATTAAA AAGTTAATTC GATTTGAATA ATTGGAAAAA AACCTTATCT   
  
  
- AAAAATTTAA AATAGAAGTT ATATTCAAAA AGGTTCCGCT CTCTGTATAT CGTTAAAATT AATTTCAATT   
  
  
- TCATCATATT GTAAAAAAAA TTACCAATAT CCGAATATCG TGGAAAAGAT CCACTTTTCT CTATAGAACC   
  
  
- GGACTAAAGG TCATTTCAAA AAAAAAATTT ATAACAGTTA ATAAAAACGT TTACTGTAAA CCATTAAACC   
  
  
- CCCTATTAGA CTACACAATT TTCTATGTTA CTTTTATAGT CTTTTATCCT ACGGAGTTTA AAGTGTTCTT   
  
  
- TCTTTTTTAG AAAAACAACA ATCATAAAGA TGTGTAGGTG TTAAAAATGA GTACCATTTT TTTTTTCCCG   
  
  
- GTTTATAGAG TGGTTATATT GGTTTGATAT ACAAGGTAAT AAATCATTGC CACTATTTAT ACATTACCCA   
  
  
- GTTCAATGAT TATTTTATCG GATGTTATGA ACCTATAGAT GTATTTTAAC TATATCTTAT ATATAACGTT   
  
  
- ATGAATTTAG TTTTAATTTA CTTTATAAAT GAAAAAATAA GATAATGTTT ATCGAAGAAT ATCTTATCGT   
  
  
- TGTTAAAAAT TACATTCAAA GTAAGAATGG TCTAAACTTC TCCAGGCCTA TATCTTTTAG AATAAGCATA   
  
  
- TTCATGAGCG TACTCCCGCC TATTTAAGAT TTTATAGTTG GAGTTGAGCG AAGATGATAA GGTAGATTCA   
  
  
- AGTTAAATAG TATATTTTTT TAAGTTAGGT GAAATTACAT GAGTTAAGTG AAGTTGGATA AAATTAGTTA   
  
  
- AATGGGTACT TTTCTTGTTC CAGGATTCAT CTACAAAAGA TAACTAACTA AATTTGTTAA AAGTGGATGG   
  
  
- GTTAATAGGG GTTTTGACTT TCTCCCCCGT TTCCCCCCAC CCATTACGAC ACATTTTATC CCCCTTTCCC   
  
  
- GTTCTTTTGA CACATGTTAC CACCTCGTCC CCATATGGTA AACCGGAACT CAACTCCCCC GTGTTTTCGG   
  
  
- GTCTCTCTCT CTCCCCATTA CGGGTGGTCT TTATTCGTCC TTCGCTCCCT TTTTGTGATC TCTCTTTGTC   
  
  
- TCTTTCTCTC TCTCTCTCTC TCTCTTCCCC TTTAAATGAT CTCTTACTCT CACTTCTTCC CTTGAAATAA   
  
  
- AATTGAAAAA ATACTACGTG CGTAGGTACC GTCTTGTCCC TTCCCTTTCT TCCCATGTCT CATGACGACA   
  
  
- GGGGTTTTTT AATCACATCT TTTGTCCTCT GTGAGTTGAT GTTGTTCCCA TTCCTTCTTC TTGTTCGTTC   
  
  
- TATAGGGTGG GTTTCTTTTT TTTTTTCTTC TTCTTCTTTT AAAAAGTCTC TCCTTTCTTT TCGTTAGTGT   
  
  
- ACTTAATCTT CGGTTGGGTT CGGAAGAAGT TTCTCTTCGT CGTATTGTAT CGTATCGACC CCTCTCCTAT   
  
  
- CCTCTCTCTT TGAGTTCTTT TCCCCATGGG CCGAATTTAC AACTACGTCT TTCCATATCT ATACCGGTGT   
  
  
- GATGATGATG GGTCCTCAAA TAGTAACCTC TACCTAAAGA GAAAGACGGA GTAGAAGAGG AGGTAGGTGG   
  
  
- TCGTTTTGAC CCAAAGGAAA GTCGACCCAG AAAAGATCTA GATCTTTGAT ACCATGGGTT TAGAGGGTAA   
  
  
- GGAGGTAAAA ATAAGAGTTT TAGATAGAAG AATCTGAACA CCCAAACTAA GTAAAGAAAA CCCAAAAACT   
  
  
- TTTCTTAAAT TAAACTAACA TAACACAACT AACCCCTTAA ACACCACCAC AATCTACGCT CGATACGGGA   
  
  
- TGTTAAACGT TCCGTTCCCA CACCATCTCC AAAGTTCGTA AACATAAGGT GTTTAGAGAA GGGGACGAAG   
  
  
- TTTCACCTTC TGGTTGTTGT TGTTGAACCT GTTGTTTAAA GTCGTTGTTG TTCTTCTTTA ACTCGTTAAC   
  
  
- GTCTCATCAC CACCGTGAAA AGAGAGAGGA CTATTGTGGT TATGATTATA ATCATGATGA GGCTCTCGCT   
  
  
- GTTCAAGACA ACTATCGCTT GTGTGTAGAT GAGACCTATG ATGCCCGGGA GGGTGAAGTT GTAGAAGGAG   
  
  
- GAAGTTCTCG TGGAGGTGGC GGTTGTGTCG GCCACACCGC CTGGGGCGTA CATGAGGGTT TCTCCTAACC   
  
  
- CTACAGAGGC CGCCGCGACG ACCGCCACTG CCGCCACCAC CCCCCGAACC TAACCTCCTC ACCCTGTCGT   
  
  
- ACAAGGGTTT ACCCCTCCCC CGAAACGAGG GAACCTAGTA CCCACTTCGA CTACTGTACC CATACCCAAA   
  
  
- CTTCGTAGAA AACGTTAGCC CATTGGGCCA ACTGATACTC CCGTTACGAC CAGATCCCCA ACAGCTAGTC   
  
  
- CCAAGACCTA AACTCTGAGA GAGAGGAGGA GGTGGCGTCG GCCGCCGTAC ACTTAGATTA CCACCACCAC   
  
  
- AACGATTAAA CCCCAAAGGT CCCTTATTAC CATTCTAAAG AAGTTAAAGT GTATTAACAA GTAGACCCTA   
  
  
- AAACTTACCA TTCCACTTGT TATTACCCAA CTTAGGGTTA ACATTGGGAG TTCGAGTTCC GTCAGATGAA   
  
  
- CCAAATTAGG TTCCTCGATG TCCACATGTG CGTGTAGGAC TGCAACCTCT GCTCTTTGGG GTCTAAAACT   
  
  
- TAGGTGTAAA CCACTACTTG GGAGTCGTTC GAGTCTCGTA ACGTTTGGGA TCGAAAAACT ACGTGAGTAA   
  
  
- TCCAATGATA GTCGACCTCG TTGTAGATAA AGTTGGAGTC CGTTTTGCGG ACTTGTGCCC ACGACAAGAC   
  
  
- CTAGGATTGG AACAAGTCGA ACGTTTCTTG GGTAAACGAC TAGTCCCAGT ACTCAATAAC AACTCCTTCG   
  
  
- TCGTGGTTGT CGAACCAAAC GTTGGTAACG GTAACCCAAA CCCAGGAGTC AACCAGGGAG GCGTCTTCGG   
  
  
- ACACTACCCA AGTTTCGTCC CCTTGGGGGT CGTAATGGGC AACGTACAAG TAGTCGTCGA CGTTAACGTT   
  
  
- CTCGTCCGAC AGTTTCTAGT CGAGAAGTTC CGCCGTCTGG AATAAGTTTG ACCTTCAAAG AGTGAGCGCG   
  
  
- TTCTCTATAA CCGCGCCGAG TTAGTGGTCG AGAGGGAGGG ACGTTTCGGG GAGTAATCCC GCCGAAACAT   
  
  
- ACACTTCCTC CGGGAAGTTT ACGAGGAGGA TTACTCGTTG GGTCAACGCC GAGGTGGCAG GTTCTAGGAG   
  
  
- TGGGGAATAC TACAACAAGT GTTCTACTCG CGCATATTCC AGAAGAGACT CCAGACGGGT TAGTGAGTTA   
  
  
- AACACTTAAA GTGAACATGT GTCCGGTAAG AGCTCCGAGA ACTACTAAGA CTACGATAAG TACAACAGCT   
  
  
- GAAACTATAA CCAACGCCAC GAGTTACCCG TAGTAACTAA GTCCTCGACG GTAACTCCTT TTCCCCTCGA   
  
  
- GGGAGAGACT TTTAATGTCG GTATCGAGGG TACAACTGAC CGTCAGTGAA ACTTTAATCG GATCATACAC   
  
  
- TTTTGGAACA CGTTAAACGG TTGCTATAAC CACAACGAAC ACTCGAGGTT CAACAGTTGA AACTAAACAA   
  
  
- ACTAGGTAGA AGCGGTAGTT ACGGCTTGTA ATCATGACGA CTCCTACTCA GTTAACGACA ATCATAGGGG   
  
  
- TAGACCCGTA GAAGTTAATC CGGTAGACAA GAAGGAAGGT AGGAGGCTAA GTAATTCGTT TTTAGGGGGT   
  
  
- TTTAACACCA GAGCAAACTA TCTCCTAAGC TAGCAAAACT ACAGGGAAAG GGGGTTGTAG ACAACGTATG   
  
  
- GGATCTTAGG ACGTCATTAA ATAACCTGAG CGAACTACCA GAGTTACAAC GTAGCCTGTA ACACTCGTTC   
  
  
- CAGCTCTTCA AGAAACAAGT TGGATCCTAG CTTTTACGAC ACAACCCAGC CCAGGTACGG GGACTGTTCT   
  
  
- ACGGGGTAAC CTTTTTAGAG AAACGAAGTC GGCCGAAGAA CGGGAACGTT AAGTCATTAA AGTGTCTTTG   
  
  
- GGTCCGGCTA ATACACCACT TCTCTTGGGG TTGTTCTCCT AAAGTGCACC TCTTCGCGGT CCGTAGTGAG   
  
  
- TAAGACTCAA CCGTCTCCGT CCTCGAACAC CGTCGAAGCC GTACCTTCAC AAC

+     MYB

| Site Name | Organism | Position | Strand | Matrix score. | sequence | function |
| --- | --- | --- | --- | --- | --- | --- |
| MYB | Arabidopsis thaliana | 652 | + | 6 | TAACCA |  |
| MYB | Arabidopsis thaliana | 3090 | + | 6 | CAACAG |  |
| MYB | Arabidopsis thaliana | 3105 | + | 6 | CAACCA |  |
| MYB | Arabidopsis thaliana | 377 | - | 6 | TAACCA |  |
| MYB | Arabidopsis thaliana | 3984 | - | 6 | CAACAG |  |
| MYB | Arabidopsis thaliana | 3584 | - | 6 | CAACCA |  |
| MYB | Arabidopsis thaliana | 2251 | - | 6 | CAACAG |  |

>HU04G00047.1   
+ +Up\_Stream \_Len000TCTTAA CATTAGTTGC TTGGTTCAAC CCATCAGTGA GAATATCTGA TTTCAAAAGT   
  
  
+ ATAGATTTAT ATAAACATAT GGACTACAGG GAAGGGAGTC GTTTCCTCAA AGTCTTAGAT TTACACTCAA   
  
  
+ AAGTTTGAGC GATACCAATA ATGTGATTCC AAACATGAGC CAAATATTAA GTATCCTATA CTAAGATCAA   
  
  
+ GAAGAGAATG AACCAAAAGA CTGTTAATTT TTCAATTAAG CTAAACTTAT TAACCTTTTT TTGGAATAGA   
  
  
+ TTTTTAAATT TTATCTTCAA TATAAGTTTT TCCAAGGCGA GAGACATATA GCAATTTTAA TTAAAGTTAA   
  
  
+ AGTAGTATAA CATTTTTTTT AATGGTTATA GGCTTATAGC ACCTTTTCTA GGTGAAAAGA GATATCTTGG   
  
  
+ CCTGATTTCC AGTAAAGTTT TTTTTTTAAA TATTGTCAAT TATTTTTGCA AATGACATTT GGTAATTTGG   
  
  
+ GGGATAATCT GATGTGTTAA AAGATACAAT GAAAATATCA GAAAATAGGA TGCCTCAAAT TTCACAAGAA   
  
  
+ AGAAAAAATC TTTTTGTTGT TAGTATTTCT ACACATCCAC AATTTTTACT CATGGTAAAA AAAAAAGGGC   
  
  
+ CAAATATCTC ACCAATATAA CCAAACTATA TGTTCCATTA TTTAGTAACG GTGATAAATA TGTAATGGGT   
  
  
+ CAAGTTACTA ATAAAATAGC CTACAATACT TGGATATCTA CATAAAATTG ATATAGAATA TATATTGCAA   
  
  
+ TACTTAAATC AAAATTAAAT GAAATATTTA CTTTTTTATT CTATTACAAA TAGCTTCTTA TAGAATAGCA   
  
  
+ ACAATTTTTA ATGTAAGTTT CATTCTTACC AGATTTGAAG AGGTCCGGAT ATAGAAAATC TTATTCGTAT   
  
  
+ AAGTACTCGC ATGAGGGCGG ATAAATTCTA AAATATCAAC CTCAACTCGC TTCTACTATT CCATCTAAGT   
  
  
+ TCAATTTATC ATATAAAAAA ATTCAATCCA CTTTAATGTA CTCAATTCAC TTCAACCTAT TTTAATCAAT   
  
  
+ TTACCCATGA AAAGAACAAG GTCCTAAGTA GATGTTTTCT ATTGATTGAT TTAAACAATT TTCACCTACC   
  
  
+ CAATTATCCC CAAAACTGAA AGAGGGGGCA AAGGGGGGTG GGTAATGCTG TGTAAAATAG GGGGAAAGGG   
  
  
+ CAAGAAAACT GTGTACAATG GTGGAGCAGG GGTATACCAT TTGGCCTTGA GTTGAGGGGG CACAAAAGCC   
  
  
+ CAGAGAGAGA GAGGGGTAAT GCCCACCAGA AATAAGCAGG AAGCGAGGGA AAAACACTAG AGAGAAACAG   
  
  
+ AGAAAGAGAG AGAGAGAGAG AGAGAAGGGG AAATTTACTA GAGAATGAGA GTGAAGAAGG GAACTTTATT   
  
  
+ TTAACTTTTT TATGATGCAC GCATCCATGG CAGAACAGGG AAGGGAAAGA AGGGTACAGA GTACTGCTGT   
  
  
+ CCCCAAAAAA TTAGTGTAGA AAACAGGAGA CACTCAACTA CAACAAGGGT AAGGAAGAAG AACAAGCAAG   
  
  
+ ATATCCCACC CAAAGAAAAA AAAAAAGAAG AAGAAGAAAA TTTTTCAGAG AGGAAAGAAA AGCAATCACA   
  
  
+ TGAATTAGAA GCCAACCCAA GCCTTCTTCA AAGAGAAGCA GCATAACATA GCATAGCTGG GGAGAGGATA   
  
  
+ GGAGAGAGAA ACTCAAGAAA AGGGGTACCC GGCTTAAATG TTGATGCAGA AAGGTATAGA TATGGCCACA   
  
  
+ CTACTACTAC CCAGGAGTTT ATCATTGGAG ATGGATTTCT CTTTCTGCCT CATCTTCTCC TCCATCCACC   
  
  
+ AGCAAAACTG GGTTTCCTTT CAGCTGGGTC TTTTCTAGAT CTAGAAACTA TGGTACCCAA ATCTCCCATT   
  
  
+ CCTCCATTTT TATTCTCAAA ATCTATCTTC TTAGACTTGT GGGTTTGATT CATTTCTTTT GGGTTTTTGA   
  
  
+ AAAGAATTTA ATTTGATTGT ATTGTGTTGA TTGGGGAATT TGTGGTGGTG TTAGATGCGA GCTATGCCCT   
  
  
+ ACAATTTGCA AGGCAAGGGT GTGGTAGAGG TTTCAAGCAT TTGTATTCCA CAAATCTCTT CCCCTGCTTC   
  
  
+ AAAGTGGAAG ACCAACAACA ACAACTTGGA CAACAAATTT CAGCAACAAC AAGAAGAAAT TGAGCAATTG   
  
  
+ CAGAGTAGTG GTGGCACTTT TCTCTCTCCT GATAACACCA ATACTAATAT TAGTACTACT CCGAGAGCGA   
  
  
+ CAAGTTCTGT TGATAGCGAA CACACATCTA CTCTGGATAC TACGGGCCCT CCCACTTCAA CATCTTCCTC   
  
  
+ CTTCAAGAGC ACCTCCACCG CCAACACAGC CGGTGTGGCG GACCCCGCAT GTACTCCCAA AGAGGATTGG   
  
  
+ GATGTCTCCG GCGGCGCTGC TGGCGGTGAC GGCGGTGGTG GGGGGCTTGG ATTGGAGGAG TGGGACAGCA   
  
  
+ TGTTCCCAAA TGGGGAGGGG GCTTTGCTCC CTTGGATCAT GGGTGAAGCT GATGACATGG GTATGGGTTT   
  
  
+ GAAGCATCTT TTGCAATCGG GTAACCCGGT TGACTATGAG GGCAATGCTG GTCTAGGGGT TGTCGATCAG   
  
  
+ GGTTCTGGAT TTGAGACTCT CTCTCCTCCT CCACCGCAGC CGGCGGCATG TGAATCTAAT GGTGGTGGTG   
  
  
+ TTGCTAATTT GGGGTTTCCA GGGAATAATG GTAAGATTTC TTCAATTTCA CATAATTGTT CATCTGGGAT   
  
  
+ TTTGAATGGT AAGGTGAACA ATAATGGGTT GAATCCCAAT TGTAACCCTC AAGCTCAAGG CAGTCTACTT   
  
  
+ GGTTTAATCC AAGGAGCTAC AGGTGTACAC GCACATCCTG ACGTTGGAGA CGAGAAACCC CAGATTTTGA   
  
  
+ ATCCACATTT GGTGATGAAC CCTCAGCAAG CTCAGAGCAT TGCAAACCCT AGCTTTTTGA TGCACTCATT   
  
  
+ AGGTTACTAT CAGCTGGAGC AACATCTATT TCAACCTCAG GCAAAACGCC TGAACACGGG TGCTGTTCTG   
  
  
+ GATCCTAACC TTGTTCAGCT TGCAAAGAAC CCATTTGCTG ATCAGGGTCA TGAGTTATTG TTGAGGAAGC   
  
  
+ AGCACCAACA GCTTGGTTTG CAACCATTGC CATTGGGTTT GGGTCCTCAG TTGGTCCCTC CGCAGAAGCC   
  
  
+ TGTGATGGGT TCAAAGCAGG GGAACCCCCA GCATTACCCG TTGCATGTTC ATCAGCAGCT GCAATTGCAA   
  
  
+ GAGCAGGCTG TCAAAGATCA GCTCTTCAAG GCGGCAGACC TTATTCAAAC TGGAAGTTTC TCACTCGCGC   
  
  
+ AAGAGATATT GGCGCGGCTC AATCACCAGC TCTCCCTCCC TGCAAAGCCC CTCATTAGGG CGGCTTTGTA   
  
  
+ TGTGAAGGAG GCCCTTCAAA TGCTCCTCCT AATGAGCAAC CCAGTTGCGG CTCCACCGTC CAAGATCCTC   
  
  
+ ACCCCTTATG ATGTTGTTCA CAAGATGAGC GCGTATAAGG TCTTCTCTGA GGTCTGCCCA ATCACTCAAT   
  
  
+ TTGTGAATTT CACTTGTACA CAGGCCATTC TCGAGGCTCT TGATGATTCT GATGCTATTC ATGTTGTCGA   
  
  
+ CTTTGATATT GGTTGCGGTG CTCAATGGGC ATCATTGATT CAGGAGCTGC CATTGAGGAA AAGGGGAGCT   
  
  
+ CCCTCTCTGA AAATTACAGC CATAGCTCCC ATGTTGACTG GCAGTCACTT TGAAATTAGC CTAGTATGTG   
  
  
+ AAAACCTTGT GCAATTTGCC AACGATATTG GTGTTGCTTG TGAGCTCCAA GTTGTCAACT TTGATTTGTT   
  
  
+ TGATCCATCT TCGCCATCAA TGCCGAACAT TAGTACTGCT GAGGATGAGT CAATTGCTGT TAGTATCCCC   
  
  
+ ATCTGGGCAT CTTCAATTAG GCCATCTGTT CTTCCTTCCA TCCTCCGATT CATTAAGCAA AAATCCCCCA   
  
  
+ AAATTGTGGT CTCGTTTGAT AGAGGATTCG ATCGTTTTGA TGTCCCTTTC CCCCAACATC TGTTGCATAC   
  
  
+ CCTAGAATCC TGCAGTAATT TATTGGACTC GCTTGATGGT CTCAATGTTG CATCGGACAT TGTGAGCAAG   
  
  
+ GTCGAGAAGT TCTTTGTTCA ACCTAGGATC GAAAATGCTG TGTTGGGTCG GGTCCATGCC CCTGACAAGA   
  
  
+ TGCCCCATTG GAAAAATCTC TTTGCTTCAG CCGGCTTCTT GCCCTTGCAA TTCAGTAATT TCACAGAAAC   
  
  
+ CCAGGCCGAT TATGTGGTGA AGAGAACCCC AACAAGAGGA TTTCACGTGG AGAAGCGCCA GGCATCACTC   
  
  
+ ATTCTGAGTT GGCAGAGGCA GGAGCTTGTG GCAGCTTCGG CATGGAAGTG TTG  

- +Up\_Stream \_Len000AGAATT GTAATCAACG AACCAAGTTG GGTAGTCACT CTTATAGACT AAAGTTTTCA   
  
  
- TATCTAAATA TATTTGTATA CCTGATGTCC CTTCCCTCAG CAAAGGAGTT TCAGAATCTA AATGTGAGTT   
  
  
- TTCAAACTCG CTATGGTTAT TACACTAAGG TTTGTACTCG GTTTATAATT CATAGGATAT GATTCTAGTT   
  
  
- CTTCTCTTAC TTGGTTTTCT GACAATTAAA AAGTTAATTC GATTTGAATA ATTGGAAAAA AACCTTATCT   
  
  
- AAAAATTTAA AATAGAAGTT ATATTCAAAA AGGTTCCGCT CTCTGTATAT CGTTAAAATT AATTTCAATT   
  
  
- TCATCATATT GTAAAAAAAA TTACCAATAT CCGAATATCG TGGAAAAGAT CCACTTTTCT CTATAGAACC   
  
  
- GGACTAAAGG TCATTTCAAA AAAAAAATTT ATAACAGTTA ATAAAAACGT TTACTGTAAA CCATTAAACC   
  
  
- CCCTATTAGA CTACACAATT TTCTATGTTA CTTTTATAGT CTTTTATCCT ACGGAGTTTA AAGTGTTCTT   
  
  
- TCTTTTTTAG AAAAACAACA ATCATAAAGA TGTGTAGGTG TTAAAAATGA GTACCATTTT TTTTTTCCCG   
  
  
- GTTTATAGAG TGGTTATATT GGTTTGATAT ACAAGGTAAT AAATCATTGC CACTATTTAT ACATTACCCA   
  
  
- GTTCAATGAT TATTTTATCG GATGTTATGA ACCTATAGAT GTATTTTAAC TATATCTTAT ATATAACGTT   
  
  
- ATGAATTTAG TTTTAATTTA CTTTATAAAT GAAAAAATAA GATAATGTTT ATCGAAGAAT ATCTTATCGT   
  
  
- TGTTAAAAAT TACATTCAAA GTAAGAATGG TCTAAACTTC TCCAGGCCTA TATCTTTTAG AATAAGCATA   
  
  
- TTCATGAGCG TACTCCCGCC TATTTAAGAT TTTATAGTTG GAGTTGAGCG AAGATGATAA GGTAGATTCA   
  
  
- AGTTAAATAG TATATTTTTT TAAGTTAGGT GAAATTACAT GAGTTAAGTG AAGTTGGATA AAATTAGTTA   
  
  
- AATGGGTACT TTTCTTGTTC CAGGATTCAT CTACAAAAGA TAACTAACTA AATTTGTTAA AAGTGGATGG   
  
  
- GTTAATAGGG GTTTTGACTT TCTCCCCCGT TTCCCCCCAC CCATTACGAC ACATTTTATC CCCCTTTCCC   
  
  
- GTTCTTTTGA CACATGTTAC CACCTCGTCC CCATATGGTA AACCGGAACT CAACTCCCCC GTGTTTTCGG   
  
  
- GTCTCTCTCT CTCCCCATTA CGGGTGGTCT TTATTCGTCC TTCGCTCCCT TTTTGTGATC TCTCTTTGTC   
  
  
- TCTTTCTCTC TCTCTCTCTC TCTCTTCCCC TTTAAATGAT CTCTTACTCT CACTTCTTCC CTTGAAATAA   
  
  
- AATTGAAAAA ATACTACGTG CGTAGGTACC GTCTTGTCCC TTCCCTTTCT TCCCATGTCT CATGACGACA   
  
  
- GGGGTTTTTT AATCACATCT TTTGTCCTCT GTGAGTTGAT GTTGTTCCCA TTCCTTCTTC TTGTTCGTTC   
  
  
- TATAGGGTGG GTTTCTTTTT TTTTTTCTTC TTCTTCTTTT AAAAAGTCTC TCCTTTCTTT TCGTTAGTGT   
  
  
- ACTTAATCTT CGGTTGGGTT CGGAAGAAGT TTCTCTTCGT CGTATTGTAT CGTATCGACC CCTCTCCTAT   
  
  
- CCTCTCTCTT TGAGTTCTTT TCCCCATGGG CCGAATTTAC AACTACGTCT TTCCATATCT ATACCGGTGT   
  
  
- GATGATGATG GGTCCTCAAA TAGTAACCTC TACCTAAAGA GAAAGACGGA GTAGAAGAGG AGGTAGGTGG   
  
  
- TCGTTTTGAC CCAAAGGAAA GTCGACCCAG AAAAGATCTA GATCTTTGAT ACCATGGGTT TAGAGGGTAA   
  
  
- GGAGGTAAAA ATAAGAGTTT TAGATAGAAG AATCTGAACA CCCAAACTAA GTAAAGAAAA CCCAAAAACT   
  
  
- TTTCTTAAAT TAAACTAACA TAACACAACT AACCCCTTAA ACACCACCAC AATCTACGCT CGATACGGGA   
  
  
- TGTTAAACGT TCCGTTCCCA CACCATCTCC AAAGTTCGTA AACATAAGGT GTTTAGAGAA GGGGACGAAG   
  
  
- TTTCACCTTC TGGTTGTTGT TGTTGAACCT GTTGTTTAAA GTCGTTGTTG TTCTTCTTTA ACTCGTTAAC   
  
  
- GTCTCATCAC CACCGTGAAA AGAGAGAGGA CTATTGTGGT TATGATTATA ATCATGATGA GGCTCTCGCT   
  
  
- GTTCAAGACA ACTATCGCTT GTGTGTAGAT GAGACCTATG ATGCCCGGGA GGGTGAAGTT GTAGAAGGAG   
  
  
- GAAGTTCTCG TGGAGGTGGC GGTTGTGTCG GCCACACCGC CTGGGGCGTA CATGAGGGTT TCTCCTAACC   
  
  
- CTACAGAGGC CGCCGCGACG ACCGCCACTG CCGCCACCAC CCCCCGAACC TAACCTCCTC ACCCTGTCGT   
  
  
- ACAAGGGTTT ACCCCTCCCC CGAAACGAGG GAACCTAGTA CCCACTTCGA CTACTGTACC CATACCCAAA   
  
  
- CTTCGTAGAA AACGTTAGCC CATTGGGCCA ACTGATACTC CCGTTACGAC CAGATCCCCA ACAGCTAGTC   
  
  
- CCAAGACCTA AACTCTGAGA GAGAGGAGGA GGTGGCGTCG GCCGCCGTAC ACTTAGATTA CCACCACCAC   
  
  
- AACGATTAAA CCCCAAAGGT CCCTTATTAC CATTCTAAAG AAGTTAAAGT GTATTAACAA GTAGACCCTA   
  
  
- AAACTTACCA TTCCACTTGT TATTACCCAA CTTAGGGTTA ACATTGGGAG TTCGAGTTCC GTCAGATGAA   
  
  
- CCAAATTAGG TTCCTCGATG TCCACATGTG CGTGTAGGAC TGCAACCTCT GCTCTTTGGG GTCTAAAACT   
  
  
- TAGGTGTAAA CCACTACTTG GGAGTCGTTC GAGTCTCGTA ACGTTTGGGA TCGAAAAACT ACGTGAGTAA   
  
  
- TCCAATGATA GTCGACCTCG TTGTAGATAA AGTTGGAGTC CGTTTTGCGG ACTTGTGCCC ACGACAAGAC   
  
  
- CTAGGATTGG AACAAGTCGA ACGTTTCTTG GGTAAACGAC TAGTCCCAGT ACTCAATAAC AACTCCTTCG   
  
  
- TCGTGGTTGT CGAACCAAAC GTTGGTAACG GTAACCCAAA CCCAGGAGTC AACCAGGGAG GCGTCTTCGG   
  
  
- ACACTACCCA AGTTTCGTCC CCTTGGGGGT CGTAATGGGC AACGTACAAG TAGTCGTCGA CGTTAACGTT   
  
  
- CTCGTCCGAC AGTTTCTAGT CGAGAAGTTC CGCCGTCTGG AATAAGTTTG ACCTTCAAAG AGTGAGCGCG   
  
  
- TTCTCTATAA CCGCGCCGAG TTAGTGGTCG AGAGGGAGGG ACGTTTCGGG GAGTAATCCC GCCGAAACAT   
  
  
- ACACTTCCTC CGGGAAGTTT ACGAGGAGGA TTACTCGTTG GGTCAACGCC GAGGTGGCAG GTTCTAGGAG   
  
  
- TGGGGAATAC TACAACAAGT GTTCTACTCG CGCATATTCC AGAAGAGACT CCAGACGGGT TAGTGAGTTA   
  
  
- AACACTTAAA GTGAACATGT GTCCGGTAAG AGCTCCGAGA ACTACTAAGA CTACGATAAG TACAACAGCT   
  
  
- GAAACTATAA CCAACGCCAC GAGTTACCCG TAGTAACTAA GTCCTCGACG GTAACTCCTT TTCCCCTCGA   
  
  
- GGGAGAGACT TTTAATGTCG GTATCGAGGG TACAACTGAC CGTCAGTGAA ACTTTAATCG GATCATACAC   
  
  
- TTTTGGAACA CGTTAAACGG TTGCTATAAC CACAACGAAC ACTCGAGGTT CAACAGTTGA AACTAAACAA   
  
  
- ACTAGGTAGA AGCGGTAGTT ACGGCTTGTA ATCATGACGA CTCCTACTCA GTTAACGACA ATCATAGGGG   
  
  
- TAGACCCGTA GAAGTTAATC CGGTAGACAA GAAGGAAGGT AGGAGGCTAA GTAATTCGTT TTTAGGGGGT   
  
  
- TTTAACACCA GAGCAAACTA TCTCCTAAGC TAGCAAAACT ACAGGGAAAG GGGGTTGTAG ACAACGTATG   
  
  
- GGATCTTAGG ACGTCATTAA ATAACCTGAG CGAACTACCA GAGTTACAAC GTAGCCTGTA ACACTCGTTC   
  
  
- CAGCTCTTCA AGAAACAAGT TGGATCCTAG CTTTTACGAC ACAACCCAGC CCAGGTACGG GGACTGTTCT   
  
  
- ACGGGGTAAC CTTTTTAGAG AAACGAAGTC GGCCGAAGAA CGGGAACGTT AAGTCATTAA AGTGTCTTTG   
  
  
- GGTCCGGCTA ATACACCACT TCTCTTGGGG TTGTTCTCCT AAAGTGCACC TCTTCGCGGT CCGTAGTGAG   
  
  
- TAAGACTCAA CCGTCTCCGT CCTCGAACAC CGTCGAAGCC GTACCTTCAC AAC

+     MYB recognition site

| Site Name | Organism | Position | Strand | Matrix score. | sequence | function |
| --- | --- | --- | --- | --- | --- | --- |
| MYB recognition site | Arabidopsis thaliana | 3192 | + | 6 | CCGTTG |  |

>HU04G00047.1   
+ +Up\_Stream \_Len000TCTTAA CATTAGTTGC TTGGTTCAAC CCATCAGTGA GAATATCTGA TTTCAAAAGT   
  
  
+ ATAGATTTAT ATAAACATAT GGACTACAGG GAAGGGAGTC GTTTCCTCAA AGTCTTAGAT TTACACTCAA   
  
  
+ AAGTTTGAGC GATACCAATA ATGTGATTCC AAACATGAGC CAAATATTAA GTATCCTATA CTAAGATCAA   
  
  
+ GAAGAGAATG AACCAAAAGA CTGTTAATTT TTCAATTAAG CTAAACTTAT TAACCTTTTT TTGGAATAGA   
  
  
+ TTTTTAAATT TTATCTTCAA TATAAGTTTT TCCAAGGCGA GAGACATATA GCAATTTTAA TTAAAGTTAA   
  
  
+ AGTAGTATAA CATTTTTTTT AATGGTTATA GGCTTATAGC ACCTTTTCTA GGTGAAAAGA GATATCTTGG   
  
  
+ CCTGATTTCC AGTAAAGTTT TTTTTTTAAA TATTGTCAAT TATTTTTGCA AATGACATTT GGTAATTTGG   
  
  
+ GGGATAATCT GATGTGTTAA AAGATACAAT GAAAATATCA GAAAATAGGA TGCCTCAAAT TTCACAAGAA   
  
  
+ AGAAAAAATC TTTTTGTTGT TAGTATTTCT ACACATCCAC AATTTTTACT CATGGTAAAA AAAAAAGGGC   
  
  
+ CAAATATCTC ACCAATATAA CCAAACTATA TGTTCCATTA TTTAGTAACG GTGATAAATA TGTAATGGGT   
  
  
+ CAAGTTACTA ATAAAATAGC CTACAATACT TGGATATCTA CATAAAATTG ATATAGAATA TATATTGCAA   
  
  
+ TACTTAAATC AAAATTAAAT GAAATATTTA CTTTTTTATT CTATTACAAA TAGCTTCTTA TAGAATAGCA   
  
  
+ ACAATTTTTA ATGTAAGTTT CATTCTTACC AGATTTGAAG AGGTCCGGAT ATAGAAAATC TTATTCGTAT   
  
  
+ AAGTACTCGC ATGAGGGCGG ATAAATTCTA AAATATCAAC CTCAACTCGC TTCTACTATT CCATCTAAGT   
  
  
+ TCAATTTATC ATATAAAAAA ATTCAATCCA CTTTAATGTA CTCAATTCAC TTCAACCTAT TTTAATCAAT   
  
  
+ TTACCCATGA AAAGAACAAG GTCCTAAGTA GATGTTTTCT ATTGATTGAT TTAAACAATT TTCACCTACC   
  
  
+ CAATTATCCC CAAAACTGAA AGAGGGGGCA AAGGGGGGTG GGTAATGCTG TGTAAAATAG GGGGAAAGGG   
  
  
+ CAAGAAAACT GTGTACAATG GTGGAGCAGG GGTATACCAT TTGGCCTTGA GTTGAGGGGG CACAAAAGCC   
  
  
+ CAGAGAGAGA GAGGGGTAAT GCCCACCAGA AATAAGCAGG AAGCGAGGGA AAAACACTAG AGAGAAACAG   
  
  
+ AGAAAGAGAG AGAGAGAGAG AGAGAAGGGG AAATTTACTA GAGAATGAGA GTGAAGAAGG GAACTTTATT   
  
  
+ TTAACTTTTT TATGATGCAC GCATCCATGG CAGAACAGGG AAGGGAAAGA AGGGTACAGA GTACTGCTGT   
  
  
+ CCCCAAAAAA TTAGTGTAGA AAACAGGAGA CACTCAACTA CAACAAGGGT AAGGAAGAAG AACAAGCAAG   
  
  
+ ATATCCCACC CAAAGAAAAA AAAAAAGAAG AAGAAGAAAA TTTTTCAGAG AGGAAAGAAA AGCAATCACA   
  
  
+ TGAATTAGAA GCCAACCCAA GCCTTCTTCA AAGAGAAGCA GCATAACATA GCATAGCTGG GGAGAGGATA   
  
  
+ GGAGAGAGAA ACTCAAGAAA AGGGGTACCC GGCTTAAATG TTGATGCAGA AAGGTATAGA TATGGCCACA   
  
  
+ CTACTACTAC CCAGGAGTTT ATCATTGGAG ATGGATTTCT CTTTCTGCCT CATCTTCTCC TCCATCCACC   
  
  
+ AGCAAAACTG GGTTTCCTTT CAGCTGGGTC TTTTCTAGAT CTAGAAACTA TGGTACCCAA ATCTCCCATT   
  
  
+ CCTCCATTTT TATTCTCAAA ATCTATCTTC TTAGACTTGT GGGTTTGATT CATTTCTTTT GGGTTTTTGA   
  
  
+ AAAGAATTTA ATTTGATTGT ATTGTGTTGA TTGGGGAATT TGTGGTGGTG TTAGATGCGA GCTATGCCCT   
  
  
+ ACAATTTGCA AGGCAAGGGT GTGGTAGAGG TTTCAAGCAT TTGTATTCCA CAAATCTCTT CCCCTGCTTC   
  
  
+ AAAGTGGAAG ACCAACAACA ACAACTTGGA CAACAAATTT CAGCAACAAC AAGAAGAAAT TGAGCAATTG   
  
  
+ CAGAGTAGTG GTGGCACTTT TCTCTCTCCT GATAACACCA ATACTAATAT TAGTACTACT CCGAGAGCGA   
  
  
+ CAAGTTCTGT TGATAGCGAA CACACATCTA CTCTGGATAC TACGGGCCCT CCCACTTCAA CATCTTCCTC   
  
  
+ CTTCAAGAGC ACCTCCACCG CCAACACAGC CGGTGTGGCG GACCCCGCAT GTACTCCCAA AGAGGATTGG   
  
  
+ GATGTCTCCG GCGGCGCTGC TGGCGGTGAC GGCGGTGGTG GGGGGCTTGG ATTGGAGGAG TGGGACAGCA   
  
  
+ TGTTCCCAAA TGGGGAGGGG GCTTTGCTCC CTTGGATCAT GGGTGAAGCT GATGACATGG GTATGGGTTT   
  
  
+ GAAGCATCTT TTGCAATCGG GTAACCCGGT TGACTATGAG GGCAATGCTG GTCTAGGGGT TGTCGATCAG   
  
  
+ GGTTCTGGAT TTGAGACTCT CTCTCCTCCT CCACCGCAGC CGGCGGCATG TGAATCTAAT GGTGGTGGTG   
  
  
+ TTGCTAATTT GGGGTTTCCA GGGAATAATG GTAAGATTTC TTCAATTTCA CATAATTGTT CATCTGGGAT   
  
  
+ TTTGAATGGT AAGGTGAACA ATAATGGGTT GAATCCCAAT TGTAACCCTC AAGCTCAAGG CAGTCTACTT   
  
  
+ GGTTTAATCC AAGGAGCTAC AGGTGTACAC GCACATCCTG ACGTTGGAGA CGAGAAACCC CAGATTTTGA   
  
  
+ ATCCACATTT GGTGATGAAC CCTCAGCAAG CTCAGAGCAT TGCAAACCCT AGCTTTTTGA TGCACTCATT   
  
  
+ AGGTTACTAT CAGCTGGAGC AACATCTATT TCAACCTCAG GCAAAACGCC TGAACACGGG TGCTGTTCTG   
  
  
+ GATCCTAACC TTGTTCAGCT TGCAAAGAAC CCATTTGCTG ATCAGGGTCA TGAGTTATTG TTGAGGAAGC   
  
  
+ AGCACCAACA GCTTGGTTTG CAACCATTGC CATTGGGTTT GGGTCCTCAG TTGGTCCCTC CGCAGAAGCC   
  
  
+ TGTGATGGGT TCAAAGCAGG GGAACCCCCA GCATTACCCG TTGCATGTTC ATCAGCAGCT GCAATTGCAA   
  
  
+ GAGCAGGCTG TCAAAGATCA GCTCTTCAAG GCGGCAGACC TTATTCAAAC TGGAAGTTTC TCACTCGCGC   
  
  
+ AAGAGATATT GGCGCGGCTC AATCACCAGC TCTCCCTCCC TGCAAAGCCC CTCATTAGGG CGGCTTTGTA   
  
  
+ TGTGAAGGAG GCCCTTCAAA TGCTCCTCCT AATGAGCAAC CCAGTTGCGG CTCCACCGTC CAAGATCCTC   
  
  
+ ACCCCTTATG ATGTTGTTCA CAAGATGAGC GCGTATAAGG TCTTCTCTGA GGTCTGCCCA ATCACTCAAT   
  
  
+ TTGTGAATTT CACTTGTACA CAGGCCATTC TCGAGGCTCT TGATGATTCT GATGCTATTC ATGTTGTCGA   
  
  
+ CTTTGATATT GGTTGCGGTG CTCAATGGGC ATCATTGATT CAGGAGCTGC CATTGAGGAA AAGGGGAGCT   
  
  
+ CCCTCTCTGA AAATTACAGC CATAGCTCCC ATGTTGACTG GCAGTCACTT TGAAATTAGC CTAGTATGTG   
  
  
+ AAAACCTTGT GCAATTTGCC AACGATATTG GTGTTGCTTG TGAGCTCCAA GTTGTCAACT TTGATTTGTT   
  
  
+ TGATCCATCT TCGCCATCAA TGCCGAACAT TAGTACTGCT GAGGATGAGT CAATTGCTGT TAGTATCCCC   
  
  
+ ATCTGGGCAT CTTCAATTAG GCCATCTGTT CTTCCTTCCA TCCTCCGATT CATTAAGCAA AAATCCCCCA   
  
  
+ AAATTGTGGT CTCGTTTGAT AGAGGATTCG ATCGTTTTGA TGTCCCTTTC CCCCAACATC TGTTGCATAC   
  
  
+ CCTAGAATCC TGCAGTAATT TATTGGACTC GCTTGATGGT CTCAATGTTG CATCGGACAT TGTGAGCAAG   
  
  
+ GTCGAGAAGT TCTTTGTTCA ACCTAGGATC GAAAATGCTG TGTTGGGTCG GGTCCATGCC CCTGACAAGA   
  
  
+ TGCCCCATTG GAAAAATCTC TTTGCTTCAG CCGGCTTCTT GCCCTTGCAA TTCAGTAATT TCACAGAAAC   
  
  
+ CCAGGCCGAT TATGTGGTGA AGAGAACCCC AACAAGAGGA TTTCACGTGG AGAAGCGCCA GGCATCACTC   
  
  
+ ATTCTGAGTT GGCAGAGGCA GGAGCTTGTG GCAGCTTCGG CATGGAAGTG TTG  

- +Up\_Stream \_Len000AGAATT GTAATCAACG AACCAAGTTG GGTAGTCACT CTTATAGACT AAAGTTTTCA   
  
  
- TATCTAAATA TATTTGTATA CCTGATGTCC CTTCCCTCAG CAAAGGAGTT TCAGAATCTA AATGTGAGTT   
  
  
- TTCAAACTCG CTATGGTTAT TACACTAAGG TTTGTACTCG GTTTATAATT CATAGGATAT GATTCTAGTT   
  
  
- CTTCTCTTAC TTGGTTTTCT GACAATTAAA AAGTTAATTC GATTTGAATA ATTGGAAAAA AACCTTATCT   
  
  
- AAAAATTTAA AATAGAAGTT ATATTCAAAA AGGTTCCGCT CTCTGTATAT CGTTAAAATT AATTTCAATT   
  
  
- TCATCATATT GTAAAAAAAA TTACCAATAT CCGAATATCG TGGAAAAGAT CCACTTTTCT CTATAGAACC   
  
  
- GGACTAAAGG TCATTTCAAA AAAAAAATTT ATAACAGTTA ATAAAAACGT TTACTGTAAA CCATTAAACC   
  
  
- CCCTATTAGA CTACACAATT TTCTATGTTA CTTTTATAGT CTTTTATCCT ACGGAGTTTA AAGTGTTCTT   
  
  
- TCTTTTTTAG AAAAACAACA ATCATAAAGA TGTGTAGGTG TTAAAAATGA GTACCATTTT TTTTTTCCCG   
  
  
- GTTTATAGAG TGGTTATATT GGTTTGATAT ACAAGGTAAT AAATCATTGC CACTATTTAT ACATTACCCA   
  
  
- GTTCAATGAT TATTTTATCG GATGTTATGA ACCTATAGAT GTATTTTAAC TATATCTTAT ATATAACGTT   
  
  
- ATGAATTTAG TTTTAATTTA CTTTATAAAT GAAAAAATAA GATAATGTTT ATCGAAGAAT ATCTTATCGT   
  
  
- TGTTAAAAAT TACATTCAAA GTAAGAATGG TCTAAACTTC TCCAGGCCTA TATCTTTTAG AATAAGCATA   
  
  
- TTCATGAGCG TACTCCCGCC TATTTAAGAT TTTATAGTTG GAGTTGAGCG AAGATGATAA GGTAGATTCA   
  
  
- AGTTAAATAG TATATTTTTT TAAGTTAGGT GAAATTACAT GAGTTAAGTG AAGTTGGATA AAATTAGTTA   
  
  
- AATGGGTACT TTTCTTGTTC CAGGATTCAT CTACAAAAGA TAACTAACTA AATTTGTTAA AAGTGGATGG   
  
  
- GTTAATAGGG GTTTTGACTT TCTCCCCCGT TTCCCCCCAC CCATTACGAC ACATTTTATC CCCCTTTCCC   
  
  
- GTTCTTTTGA CACATGTTAC CACCTCGTCC CCATATGGTA AACCGGAACT CAACTCCCCC GTGTTTTCGG   
  
  
- GTCTCTCTCT CTCCCCATTA CGGGTGGTCT TTATTCGTCC TTCGCTCCCT TTTTGTGATC TCTCTTTGTC   
  
  
- TCTTTCTCTC TCTCTCTCTC TCTCTTCCCC TTTAAATGAT CTCTTACTCT CACTTCTTCC CTTGAAATAA   
  
  
- AATTGAAAAA ATACTACGTG CGTAGGTACC GTCTTGTCCC TTCCCTTTCT TCCCATGTCT CATGACGACA   
  
  
- GGGGTTTTTT AATCACATCT TTTGTCCTCT GTGAGTTGAT GTTGTTCCCA TTCCTTCTTC TTGTTCGTTC   
  
  
- TATAGGGTGG GTTTCTTTTT TTTTTTCTTC TTCTTCTTTT AAAAAGTCTC TCCTTTCTTT TCGTTAGTGT   
  
  
- ACTTAATCTT CGGTTGGGTT CGGAAGAAGT TTCTCTTCGT CGTATTGTAT CGTATCGACC CCTCTCCTAT   
  
  
- CCTCTCTCTT TGAGTTCTTT TCCCCATGGG CCGAATTTAC AACTACGTCT TTCCATATCT ATACCGGTGT   
  
  
- GATGATGATG GGTCCTCAAA TAGTAACCTC TACCTAAAGA GAAAGACGGA GTAGAAGAGG AGGTAGGTGG   
  
  
- TCGTTTTGAC CCAAAGGAAA GTCGACCCAG AAAAGATCTA GATCTTTGAT ACCATGGGTT TAGAGGGTAA   
  
  
- GGAGGTAAAA ATAAGAGTTT TAGATAGAAG AATCTGAACA CCCAAACTAA GTAAAGAAAA CCCAAAAACT   
  
  
- TTTCTTAAAT TAAACTAACA TAACACAACT AACCCCTTAA ACACCACCAC AATCTACGCT CGATACGGGA   
  
  
- TGTTAAACGT TCCGTTCCCA CACCATCTCC AAAGTTCGTA AACATAAGGT GTTTAGAGAA GGGGACGAAG   
  
  
- TTTCACCTTC TGGTTGTTGT TGTTGAACCT GTTGTTTAAA GTCGTTGTTG TTCTTCTTTA ACTCGTTAAC   
  
  
- GTCTCATCAC CACCGTGAAA AGAGAGAGGA CTATTGTGGT TATGATTATA ATCATGATGA GGCTCTCGCT   
  
  
- GTTCAAGACA ACTATCGCTT GTGTGTAGAT GAGACCTATG ATGCCCGGGA GGGTGAAGTT GTAGAAGGAG   
  
  
- GAAGTTCTCG TGGAGGTGGC GGTTGTGTCG GCCACACCGC CTGGGGCGTA CATGAGGGTT TCTCCTAACC   
  
  
- CTACAGAGGC CGCCGCGACG ACCGCCACTG CCGCCACCAC CCCCCGAACC TAACCTCCTC ACCCTGTCGT   
  
  
- ACAAGGGTTT ACCCCTCCCC CGAAACGAGG GAACCTAGTA CCCACTTCGA CTACTGTACC CATACCCAAA   
  
  
- CTTCGTAGAA AACGTTAGCC CATTGGGCCA ACTGATACTC CCGTTACGAC CAGATCCCCA ACAGCTAGTC   
  
  
- CCAAGACCTA AACTCTGAGA GAGAGGAGGA GGTGGCGTCG GCCGCCGTAC ACTTAGATTA CCACCACCAC   
  
  
- AACGATTAAA CCCCAAAGGT CCCTTATTAC CATTCTAAAG AAGTTAAAGT GTATTAACAA GTAGACCCTA   
  
  
- AAACTTACCA TTCCACTTGT TATTACCCAA CTTAGGGTTA ACATTGGGAG TTCGAGTTCC GTCAGATGAA   
  
  
- CCAAATTAGG TTCCTCGATG TCCACATGTG CGTGTAGGAC TGCAACCTCT GCTCTTTGGG GTCTAAAACT   
  
  
- TAGGTGTAAA CCACTACTTG GGAGTCGTTC GAGTCTCGTA ACGTTTGGGA TCGAAAAACT ACGTGAGTAA   
  
  
- TCCAATGATA GTCGACCTCG TTGTAGATAA AGTTGGAGTC CGTTTTGCGG ACTTGTGCCC ACGACAAGAC   
  
  
- CTAGGATTGG AACAAGTCGA ACGTTTCTTG GGTAAACGAC TAGTCCCAGT ACTCAATAAC AACTCCTTCG   
  
  
- TCGTGGTTGT CGAACCAAAC GTTGGTAACG GTAACCCAAA CCCAGGAGTC AACCAGGGAG GCGTCTTCGG   
  
  
- ACACTACCCA AGTTTCGTCC CCTTGGGGGT CGTAATGGGC AACGTACAAG TAGTCGTCGA CGTTAACGTT   
  
  
- CTCGTCCGAC AGTTTCTAGT CGAGAAGTTC CGCCGTCTGG AATAAGTTTG ACCTTCAAAG AGTGAGCGCG   
  
  
- TTCTCTATAA CCGCGCCGAG TTAGTGGTCG AGAGGGAGGG ACGTTTCGGG GAGTAATCCC GCCGAAACAT   
  
  
- ACACTTCCTC CGGGAAGTTT ACGAGGAGGA TTACTCGTTG GGTCAACGCC GAGGTGGCAG GTTCTAGGAG   
  
  
- TGGGGAATAC TACAACAAGT GTTCTACTCG CGCATATTCC AGAAGAGACT CCAGACGGGT TAGTGAGTTA   
  
  
- AACACTTAAA GTGAACATGT GTCCGGTAAG AGCTCCGAGA ACTACTAAGA CTACGATAAG TACAACAGCT   
  
  
- GAAACTATAA CCAACGCCAC GAGTTACCCG TAGTAACTAA GTCCTCGACG GTAACTCCTT TTCCCCTCGA   
  
  
- GGGAGAGACT TTTAATGTCG GTATCGAGGG TACAACTGAC CGTCAGTGAA ACTTTAATCG GATCATACAC   
  
  
- TTTTGGAACA CGTTAAACGG TTGCTATAAC CACAACGAAC ACTCGAGGTT CAACAGTTGA AACTAAACAA   
  
  
- ACTAGGTAGA AGCGGTAGTT ACGGCTTGTA ATCATGACGA CTCCTACTCA GTTAACGACA ATCATAGGGG   
  
  
- TAGACCCGTA GAAGTTAATC CGGTAGACAA GAAGGAAGGT AGGAGGCTAA GTAATTCGTT TTTAGGGGGT   
  
  
- TTTAACACCA GAGCAAACTA TCTCCTAAGC TAGCAAAACT ACAGGGAAAG GGGGTTGTAG ACAACGTATG   
  
  
- GGATCTTAGG ACGTCATTAA ATAACCTGAG CGAACTACCA GAGTTACAAC GTAGCCTGTA ACACTCGTTC   
  
  
- CAGCTCTTCA AGAAACAAGT TGGATCCTAG CTTTTACGAC ACAACCCAGC CCAGGTACGG GGACTGTTCT   
  
  
- ACGGGGTAAC CTTTTTAGAG AAACGAAGTC GGCCGAAGAA CGGGAACGTT AAGTCATTAA AGTGTCTTTG   
  
  
- GGTCCGGCTA ATACACCACT TCTCTTGGGG TTGTTCTCCT AAAGTGCACC TCTTCGCGGT CCGTAGTGAG   
  
  
- TAAGACTCAA CCGTCTCCGT CCTCGAACAC CGTCGAAGCC GTACCTTCAC AAC

+     MYB-like sequence

| Site Name | Organism | Position | Strand | Matrix score. | sequence | function |
| --- | --- | --- | --- | --- | --- | --- |
| MYB-like sequence | Arabidopsis thaliana | 377 | - | 6 | TAACCA |  |
| MYB-like sequence | Arabidopsis thaliana | 652 | + | 6 | TAACCA |  |

>HU04G00047.1   
+ +Up\_Stream \_Len000TCTTAA CATTAGTTGC TTGGTTCAAC CCATCAGTGA GAATATCTGA TTTCAAAAGT   
  
  
+ ATAGATTTAT ATAAACATAT GGACTACAGG GAAGGGAGTC GTTTCCTCAA AGTCTTAGAT TTACACTCAA   
  
  
+ AAGTTTGAGC GATACCAATA ATGTGATTCC AAACATGAGC CAAATATTAA GTATCCTATA CTAAGATCAA   
  
  
+ GAAGAGAATG AACCAAAAGA CTGTTAATTT TTCAATTAAG CTAAACTTAT TAACCTTTTT TTGGAATAGA   
  
  
+ TTTTTAAATT TTATCTTCAA TATAAGTTTT TCCAAGGCGA GAGACATATA GCAATTTTAA TTAAAGTTAA   
  
  
+ AGTAGTATAA CATTTTTTTT AATGGTTATA GGCTTATAGC ACCTTTTCTA GGTGAAAAGA GATATCTTGG   
  
  
+ CCTGATTTCC AGTAAAGTTT TTTTTTTAAA TATTGTCAAT TATTTTTGCA AATGACATTT GGTAATTTGG   
  
  
+ GGGATAATCT GATGTGTTAA AAGATACAAT GAAAATATCA GAAAATAGGA TGCCTCAAAT TTCACAAGAA   
  
  
+ AGAAAAAATC TTTTTGTTGT TAGTATTTCT ACACATCCAC AATTTTTACT CATGGTAAAA AAAAAAGGGC   
  
  
+ CAAATATCTC ACCAATATAA CCAAACTATA TGTTCCATTA TTTAGTAACG GTGATAAATA TGTAATGGGT   
  
  
+ CAAGTTACTA ATAAAATAGC CTACAATACT TGGATATCTA CATAAAATTG ATATAGAATA TATATTGCAA   
  
  
+ TACTTAAATC AAAATTAAAT GAAATATTTA CTTTTTTATT CTATTACAAA TAGCTTCTTA TAGAATAGCA   
  
  
+ ACAATTTTTA ATGTAAGTTT CATTCTTACC AGATTTGAAG AGGTCCGGAT ATAGAAAATC TTATTCGTAT   
  
  
+ AAGTACTCGC ATGAGGGCGG ATAAATTCTA AAATATCAAC CTCAACTCGC TTCTACTATT CCATCTAAGT   
  
  
+ TCAATTTATC ATATAAAAAA ATTCAATCCA CTTTAATGTA CTCAATTCAC TTCAACCTAT TTTAATCAAT   
  
  
+ TTACCCATGA AAAGAACAAG GTCCTAAGTA GATGTTTTCT ATTGATTGAT TTAAACAATT TTCACCTACC   
  
  
+ CAATTATCCC CAAAACTGAA AGAGGGGGCA AAGGGGGGTG GGTAATGCTG TGTAAAATAG GGGGAAAGGG   
  
  
+ CAAGAAAACT GTGTACAATG GTGGAGCAGG GGTATACCAT TTGGCCTTGA GTTGAGGGGG CACAAAAGCC   
  
  
+ CAGAGAGAGA GAGGGGTAAT GCCCACCAGA AATAAGCAGG AAGCGAGGGA AAAACACTAG AGAGAAACAG   
  
  
+ AGAAAGAGAG AGAGAGAGAG AGAGAAGGGG AAATTTACTA GAGAATGAGA GTGAAGAAGG GAACTTTATT   
  
  
+ TTAACTTTTT TATGATGCAC GCATCCATGG CAGAACAGGG AAGGGAAAGA AGGGTACAGA GTACTGCTGT   
  
  
+ CCCCAAAAAA TTAGTGTAGA AAACAGGAGA CACTCAACTA CAACAAGGGT AAGGAAGAAG AACAAGCAAG   
  
  
+ ATATCCCACC CAAAGAAAAA AAAAAAGAAG AAGAAGAAAA TTTTTCAGAG AGGAAAGAAA AGCAATCACA   
  
  
+ TGAATTAGAA GCCAACCCAA GCCTTCTTCA AAGAGAAGCA GCATAACATA GCATAGCTGG GGAGAGGATA   
  
  
+ GGAGAGAGAA ACTCAAGAAA AGGGGTACCC GGCTTAAATG TTGATGCAGA AAGGTATAGA TATGGCCACA   
  
  
+ CTACTACTAC CCAGGAGTTT ATCATTGGAG ATGGATTTCT CTTTCTGCCT CATCTTCTCC TCCATCCACC   
  
  
+ AGCAAAACTG GGTTTCCTTT CAGCTGGGTC TTTTCTAGAT CTAGAAACTA TGGTACCCAA ATCTCCCATT   
  
  
+ CCTCCATTTT TATTCTCAAA ATCTATCTTC TTAGACTTGT GGGTTTGATT CATTTCTTTT GGGTTTTTGA   
  
  
+ AAAGAATTTA ATTTGATTGT ATTGTGTTGA TTGGGGAATT TGTGGTGGTG TTAGATGCGA GCTATGCCCT   
  
  
+ ACAATTTGCA AGGCAAGGGT GTGGTAGAGG TTTCAAGCAT TTGTATTCCA CAAATCTCTT CCCCTGCTTC   
  
  
+ AAAGTGGAAG ACCAACAACA ACAACTTGGA CAACAAATTT CAGCAACAAC AAGAAGAAAT TGAGCAATTG   
  
  
+ CAGAGTAGTG GTGGCACTTT TCTCTCTCCT GATAACACCA ATACTAATAT TAGTACTACT CCGAGAGCGA   
  
  
+ CAAGTTCTGT TGATAGCGAA CACACATCTA CTCTGGATAC TACGGGCCCT CCCACTTCAA CATCTTCCTC   
  
  
+ CTTCAAGAGC ACCTCCACCG CCAACACAGC CGGTGTGGCG GACCCCGCAT GTACTCCCAA AGAGGATTGG   
  
  
+ GATGTCTCCG GCGGCGCTGC TGGCGGTGAC GGCGGTGGTG GGGGGCTTGG ATTGGAGGAG TGGGACAGCA   
  
  
+ TGTTCCCAAA TGGGGAGGGG GCTTTGCTCC CTTGGATCAT GGGTGAAGCT GATGACATGG GTATGGGTTT   
  
  
+ GAAGCATCTT TTGCAATCGG GTAACCCGGT TGACTATGAG GGCAATGCTG GTCTAGGGGT TGTCGATCAG   
  
  
+ GGTTCTGGAT TTGAGACTCT CTCTCCTCCT CCACCGCAGC CGGCGGCATG TGAATCTAAT GGTGGTGGTG   
  
  
+ TTGCTAATTT GGGGTTTCCA GGGAATAATG GTAAGATTTC TTCAATTTCA CATAATTGTT CATCTGGGAT   
  
  
+ TTTGAATGGT AAGGTGAACA ATAATGGGTT GAATCCCAAT TGTAACCCTC AAGCTCAAGG CAGTCTACTT   
  
  
+ GGTTTAATCC AAGGAGCTAC AGGTGTACAC GCACATCCTG ACGTTGGAGA CGAGAAACCC CAGATTTTGA   
  
  
+ ATCCACATTT GGTGATGAAC CCTCAGCAAG CTCAGAGCAT TGCAAACCCT AGCTTTTTGA TGCACTCATT   
  
  
+ AGGTTACTAT CAGCTGGAGC AACATCTATT TCAACCTCAG GCAAAACGCC TGAACACGGG TGCTGTTCTG   
  
  
+ GATCCTAACC TTGTTCAGCT TGCAAAGAAC CCATTTGCTG ATCAGGGTCA TGAGTTATTG TTGAGGAAGC   
  
  
+ AGCACCAACA GCTTGGTTTG CAACCATTGC CATTGGGTTT GGGTCCTCAG TTGGTCCCTC CGCAGAAGCC   
  
  
+ TGTGATGGGT TCAAAGCAGG GGAACCCCCA GCATTACCCG TTGCATGTTC ATCAGCAGCT GCAATTGCAA   
  
  
+ GAGCAGGCTG TCAAAGATCA GCTCTTCAAG GCGGCAGACC TTATTCAAAC TGGAAGTTTC TCACTCGCGC   
  
  
+ AAGAGATATT GGCGCGGCTC AATCACCAGC TCTCCCTCCC TGCAAAGCCC CTCATTAGGG CGGCTTTGTA   
  
  
+ TGTGAAGGAG GCCCTTCAAA TGCTCCTCCT AATGAGCAAC CCAGTTGCGG CTCCACCGTC CAAGATCCTC   
  
  
+ ACCCCTTATG ATGTTGTTCA CAAGATGAGC GCGTATAAGG TCTTCTCTGA GGTCTGCCCA ATCACTCAAT   
  
  
+ TTGTGAATTT CACTTGTACA CAGGCCATTC TCGAGGCTCT TGATGATTCT GATGCTATTC ATGTTGTCGA   
  
  
+ CTTTGATATT GGTTGCGGTG CTCAATGGGC ATCATTGATT CAGGAGCTGC CATTGAGGAA AAGGGGAGCT   
  
  
+ CCCTCTCTGA AAATTACAGC CATAGCTCCC ATGTTGACTG GCAGTCACTT TGAAATTAGC CTAGTATGTG   
  
  
+ AAAACCTTGT GCAATTTGCC AACGATATTG GTGTTGCTTG TGAGCTCCAA GTTGTCAACT TTGATTTGTT   
  
  
+ TGATCCATCT TCGCCATCAA TGCCGAACAT TAGTACTGCT GAGGATGAGT CAATTGCTGT TAGTATCCCC   
  
  
+ ATCTGGGCAT CTTCAATTAG GCCATCTGTT CTTCCTTCCA TCCTCCGATT CATTAAGCAA AAATCCCCCA   
  
  
+ AAATTGTGGT CTCGTTTGAT AGAGGATTCG ATCGTTTTGA TGTCCCTTTC CCCCAACATC TGTTGCATAC   
  
  
+ CCTAGAATCC TGCAGTAATT TATTGGACTC GCTTGATGGT CTCAATGTTG CATCGGACAT TGTGAGCAAG   
  
  
+ GTCGAGAAGT TCTTTGTTCA ACCTAGGATC GAAAATGCTG TGTTGGGTCG GGTCCATGCC CCTGACAAGA   
  
  
+ TGCCCCATTG GAAAAATCTC TTTGCTTCAG CCGGCTTCTT GCCCTTGCAA TTCAGTAATT TCACAGAAAC   
  
  
+ CCAGGCCGAT TATGTGGTGA AGAGAACCCC AACAAGAGGA TTTCACGTGG AGAAGCGCCA GGCATCACTC   
  
  
+ ATTCTGAGTT GGCAGAGGCA GGAGCTTGTG GCAGCTTCGG CATGGAAGTG TTG  

- +Up\_Stream \_Len000AGAATT GTAATCAACG AACCAAGTTG GGTAGTCACT CTTATAGACT AAAGTTTTCA   
  
  
- TATCTAAATA TATTTGTATA CCTGATGTCC CTTCCCTCAG CAAAGGAGTT TCAGAATCTA AATGTGAGTT   
  
  
- TTCAAACTCG CTATGGTTAT TACACTAAGG TTTGTACTCG GTTTATAATT CATAGGATAT GATTCTAGTT   
  
  
- CTTCTCTTAC TTGGTTTTCT GACAATTAAA AAGTTAATTC GATTTGAATA ATTGGAAAAA AACCTTATCT   
  
  
- AAAAATTTAA AATAGAAGTT ATATTCAAAA AGGTTCCGCT CTCTGTATAT CGTTAAAATT AATTTCAATT   
  
  
- TCATCATATT GTAAAAAAAA TTACCAATAT CCGAATATCG TGGAAAAGAT CCACTTTTCT CTATAGAACC   
  
  
- GGACTAAAGG TCATTTCAAA AAAAAAATTT ATAACAGTTA ATAAAAACGT TTACTGTAAA CCATTAAACC   
  
  
- CCCTATTAGA CTACACAATT TTCTATGTTA CTTTTATAGT CTTTTATCCT ACGGAGTTTA AAGTGTTCTT   
  
  
- TCTTTTTTAG AAAAACAACA ATCATAAAGA TGTGTAGGTG TTAAAAATGA GTACCATTTT TTTTTTCCCG   
  
  
- GTTTATAGAG TGGTTATATT GGTTTGATAT ACAAGGTAAT AAATCATTGC CACTATTTAT ACATTACCCA   
  
  
- GTTCAATGAT TATTTTATCG GATGTTATGA ACCTATAGAT GTATTTTAAC TATATCTTAT ATATAACGTT   
  
  
- ATGAATTTAG TTTTAATTTA CTTTATAAAT GAAAAAATAA GATAATGTTT ATCGAAGAAT ATCTTATCGT   
  
  
- TGTTAAAAAT TACATTCAAA GTAAGAATGG TCTAAACTTC TCCAGGCCTA TATCTTTTAG AATAAGCATA   
  
  
- TTCATGAGCG TACTCCCGCC TATTTAAGAT TTTATAGTTG GAGTTGAGCG AAGATGATAA GGTAGATTCA   
  
  
- AGTTAAATAG TATATTTTTT TAAGTTAGGT GAAATTACAT GAGTTAAGTG AAGTTGGATA AAATTAGTTA   
  
  
- AATGGGTACT TTTCTTGTTC CAGGATTCAT CTACAAAAGA TAACTAACTA AATTTGTTAA AAGTGGATGG   
  
  
- GTTAATAGGG GTTTTGACTT TCTCCCCCGT TTCCCCCCAC CCATTACGAC ACATTTTATC CCCCTTTCCC   
  
  
- GTTCTTTTGA CACATGTTAC CACCTCGTCC CCATATGGTA AACCGGAACT CAACTCCCCC GTGTTTTCGG   
  
  
- GTCTCTCTCT CTCCCCATTA CGGGTGGTCT TTATTCGTCC TTCGCTCCCT TTTTGTGATC TCTCTTTGTC   
  
  
- TCTTTCTCTC TCTCTCTCTC TCTCTTCCCC TTTAAATGAT CTCTTACTCT CACTTCTTCC CTTGAAATAA   
  
  
- AATTGAAAAA ATACTACGTG CGTAGGTACC GTCTTGTCCC TTCCCTTTCT TCCCATGTCT CATGACGACA   
  
  
- GGGGTTTTTT AATCACATCT TTTGTCCTCT GTGAGTTGAT GTTGTTCCCA TTCCTTCTTC TTGTTCGTTC   
  
  
- TATAGGGTGG GTTTCTTTTT TTTTTTCTTC TTCTTCTTTT AAAAAGTCTC TCCTTTCTTT TCGTTAGTGT   
  
  
- ACTTAATCTT CGGTTGGGTT CGGAAGAAGT TTCTCTTCGT CGTATTGTAT CGTATCGACC CCTCTCCTAT   
  
  
- CCTCTCTCTT TGAGTTCTTT TCCCCATGGG CCGAATTTAC AACTACGTCT TTCCATATCT ATACCGGTGT   
  
  
- GATGATGATG GGTCCTCAAA TAGTAACCTC TACCTAAAGA GAAAGACGGA GTAGAAGAGG AGGTAGGTGG   
  
  
- TCGTTTTGAC CCAAAGGAAA GTCGACCCAG AAAAGATCTA GATCTTTGAT ACCATGGGTT TAGAGGGTAA   
  
  
- GGAGGTAAAA ATAAGAGTTT TAGATAGAAG AATCTGAACA CCCAAACTAA GTAAAGAAAA CCCAAAAACT   
  
  
- TTTCTTAAAT TAAACTAACA TAACACAACT AACCCCTTAA ACACCACCAC AATCTACGCT CGATACGGGA   
  
  
- TGTTAAACGT TCCGTTCCCA CACCATCTCC AAAGTTCGTA AACATAAGGT GTTTAGAGAA GGGGACGAAG   
  
  
- TTTCACCTTC TGGTTGTTGT TGTTGAACCT GTTGTTTAAA GTCGTTGTTG TTCTTCTTTA ACTCGTTAAC   
  
  
- GTCTCATCAC CACCGTGAAA AGAGAGAGGA CTATTGTGGT TATGATTATA ATCATGATGA GGCTCTCGCT   
  
  
- GTTCAAGACA ACTATCGCTT GTGTGTAGAT GAGACCTATG ATGCCCGGGA GGGTGAAGTT GTAGAAGGAG   
  
  
- GAAGTTCTCG TGGAGGTGGC GGTTGTGTCG GCCACACCGC CTGGGGCGTA CATGAGGGTT TCTCCTAACC   
  
  
- CTACAGAGGC CGCCGCGACG ACCGCCACTG CCGCCACCAC CCCCCGAACC TAACCTCCTC ACCCTGTCGT   
  
  
- ACAAGGGTTT ACCCCTCCCC CGAAACGAGG GAACCTAGTA CCCACTTCGA CTACTGTACC CATACCCAAA   
  
  
- CTTCGTAGAA AACGTTAGCC CATTGGGCCA ACTGATACTC CCGTTACGAC CAGATCCCCA ACAGCTAGTC   
  
  
- CCAAGACCTA AACTCTGAGA GAGAGGAGGA GGTGGCGTCG GCCGCCGTAC ACTTAGATTA CCACCACCAC   
  
  
- AACGATTAAA CCCCAAAGGT CCCTTATTAC CATTCTAAAG AAGTTAAAGT GTATTAACAA GTAGACCCTA   
  
  
- AAACTTACCA TTCCACTTGT TATTACCCAA CTTAGGGTTA ACATTGGGAG TTCGAGTTCC GTCAGATGAA   
  
  
- CCAAATTAGG TTCCTCGATG TCCACATGTG CGTGTAGGAC TGCAACCTCT GCTCTTTGGG GTCTAAAACT   
  
  
- TAGGTGTAAA CCACTACTTG GGAGTCGTTC GAGTCTCGTA ACGTTTGGGA TCGAAAAACT ACGTGAGTAA   
  
  
- TCCAATGATA GTCGACCTCG TTGTAGATAA AGTTGGAGTC CGTTTTGCGG ACTTGTGCCC ACGACAAGAC   
  
  
- CTAGGATTGG AACAAGTCGA ACGTTTCTTG GGTAAACGAC TAGTCCCAGT ACTCAATAAC AACTCCTTCG   
  
  
- TCGTGGTTGT CGAACCAAAC GTTGGTAACG GTAACCCAAA CCCAGGAGTC AACCAGGGAG GCGTCTTCGG   
  
  
- ACACTACCCA AGTTTCGTCC CCTTGGGGGT CGTAATGGGC AACGTACAAG TAGTCGTCGA CGTTAACGTT   
  
  
- CTCGTCCGAC AGTTTCTAGT CGAGAAGTTC CGCCGTCTGG AATAAGTTTG ACCTTCAAAG AGTGAGCGCG   
  
  
- TTCTCTATAA CCGCGCCGAG TTAGTGGTCG AGAGGGAGGG ACGTTTCGGG GAGTAATCCC GCCGAAACAT   
  
  
- ACACTTCCTC CGGGAAGTTT ACGAGGAGGA TTACTCGTTG GGTCAACGCC GAGGTGGCAG GTTCTAGGAG   
  
  
- TGGGGAATAC TACAACAAGT GTTCTACTCG CGCATATTCC AGAAGAGACT CCAGACGGGT TAGTGAGTTA   
  
  
- AACACTTAAA GTGAACATGT GTCCGGTAAG AGCTCCGAGA ACTACTAAGA CTACGATAAG TACAACAGCT   
  
  
- GAAACTATAA CCAACGCCAC GAGTTACCCG TAGTAACTAA GTCCTCGACG GTAACTCCTT TTCCCCTCGA   
  
  
- GGGAGAGACT TTTAATGTCG GTATCGAGGG TACAACTGAC CGTCAGTGAA ACTTTAATCG GATCATACAC   
  
  
- TTTTGGAACA CGTTAAACGG TTGCTATAAC CACAACGAAC ACTCGAGGTT CAACAGTTGA AACTAAACAA   
  
  
- ACTAGGTAGA AGCGGTAGTT ACGGCTTGTA ATCATGACGA CTCCTACTCA GTTAACGACA ATCATAGGGG   
  
  
- TAGACCCGTA GAAGTTAATC CGGTAGACAA GAAGGAAGGT AGGAGGCTAA GTAATTCGTT TTTAGGGGGT   
  
  
- TTTAACACCA GAGCAAACTA TCTCCTAAGC TAGCAAAACT ACAGGGAAAG GGGGTTGTAG ACAACGTATG   
  
  
- GGATCTTAGG ACGTCATTAA ATAACCTGAG CGAACTACCA GAGTTACAAC GTAGCCTGTA ACACTCGTTC   
  
  
- CAGCTCTTCA AGAAACAAGT TGGATCCTAG CTTTTACGAC ACAACCCAGC CCAGGTACGG GGACTGTTCT   
  
  
- ACGGGGTAAC CTTTTTAGAG AAACGAAGTC GGCCGAAGAA CGGGAACGTT AAGTCATTAA AGTGTCTTTG   
  
  
- GGTCCGGCTA ATACACCACT TCTCTTGGGG TTGTTCTCCT AAAGTGCACC TCTTCGCGGT CCGTAGTGAG   
  
  
- TAAGACTCAA CCGTCTCCGT CCTCGAACAC CGTCGAAGCC GTACCTTCAC AAC

+     MYC

| Site Name | Organism | Position | Strand | Matrix score. | sequence | function |
| --- | --- | --- | --- | --- | --- | --- |
| MYC | Arabidopsis thaliana | 3835 | - | 6 | CAATTG |  |
| MYC | Arabidopsis thaliana | 2072 | + | 6 | CATTTG |  |
| MYC | Arabidopsis thaliana | 2169 | - | 6 | CAATTG |  |
| MYC | Arabidopsis thaliana | 3216 | - | 6 | CAATTG |  |
| MYC | Arabidopsis thaliana | 2461 | - | 6 | CATTTG |  |
| MYC | Arabidopsis thaliana | 2641 | + | 6 | CATGTG |  |
| MYC | Arabidopsis thaliana | 2771 | - | 6 | CAATTG |  |
| MYC | Arabidopsis thaliana | 2880 | + | 6 | CATTTG |  |
| MYC | Arabidopsis thaliana | 3046 | + | 6 | CATTTG |  |
| MYC | Arabidopsis thaliana | 3381 | - | 6 | CATTTG |  |
| MYC | Arabidopsis thaliana | 473 | - | 6 | CATTTG |  |
| MYC | Arabidopsis thaliana | 1232 | + | 6 | CATTTG |  |
| MYC | Arabidopsis thaliana | 480 | + | 6 | CATTTG |  |
| MYC | Arabidopsis thaliana | 1611 | - | 6 | CATGTG |  |

>HU04G00047.1   
+ +Up\_Stream \_Len000TCTTAA CATTAGTTGC TTGGTTCAAC CCATCAGTGA GAATATCTGA TTTCAAAAGT   
  
  
+ ATAGATTTAT ATAAACATAT GGACTACAGG GAAGGGAGTC GTTTCCTCAA AGTCTTAGAT TTACACTCAA   
  
  
+ AAGTTTGAGC GATACCAATA ATGTGATTCC AAACATGAGC CAAATATTAA GTATCCTATA CTAAGATCAA   
  
  
+ GAAGAGAATG AACCAAAAGA CTGTTAATTT TTCAATTAAG CTAAACTTAT TAACCTTTTT TTGGAATAGA   
  
  
+ TTTTTAAATT TTATCTTCAA TATAAGTTTT TCCAAGGCGA GAGACATATA GCAATTTTAA TTAAAGTTAA   
  
  
+ AGTAGTATAA CATTTTTTTT AATGGTTATA GGCTTATAGC ACCTTTTCTA GGTGAAAAGA GATATCTTGG   
  
  
+ CCTGATTTCC AGTAAAGTTT TTTTTTTAAA TATTGTCAAT TATTTTTGCA AATGACATTT GGTAATTTGG   
  
  
+ GGGATAATCT GATGTGTTAA AAGATACAAT GAAAATATCA GAAAATAGGA TGCCTCAAAT TTCACAAGAA   
  
  
+ AGAAAAAATC TTTTTGTTGT TAGTATTTCT ACACATCCAC AATTTTTACT CATGGTAAAA AAAAAAGGGC   
  
  
+ CAAATATCTC ACCAATATAA CCAAACTATA TGTTCCATTA TTTAGTAACG GTGATAAATA TGTAATGGGT   
  
  
+ CAAGTTACTA ATAAAATAGC CTACAATACT TGGATATCTA CATAAAATTG ATATAGAATA TATATTGCAA   
  
  
+ TACTTAAATC AAAATTAAAT GAAATATTTA CTTTTTTATT CTATTACAAA TAGCTTCTTA TAGAATAGCA   
  
  
+ ACAATTTTTA ATGTAAGTTT CATTCTTACC AGATTTGAAG AGGTCCGGAT ATAGAAAATC TTATTCGTAT   
  
  
+ AAGTACTCGC ATGAGGGCGG ATAAATTCTA AAATATCAAC CTCAACTCGC TTCTACTATT CCATCTAAGT   
  
  
+ TCAATTTATC ATATAAAAAA ATTCAATCCA CTTTAATGTA CTCAATTCAC TTCAACCTAT TTTAATCAAT   
  
  
+ TTACCCATGA AAAGAACAAG GTCCTAAGTA GATGTTTTCT ATTGATTGAT TTAAACAATT TTCACCTACC   
  
  
+ CAATTATCCC CAAAACTGAA AGAGGGGGCA AAGGGGGGTG GGTAATGCTG TGTAAAATAG GGGGAAAGGG   
  
  
+ CAAGAAAACT GTGTACAATG GTGGAGCAGG GGTATACCAT TTGGCCTTGA GTTGAGGGGG CACAAAAGCC   
  
  
+ CAGAGAGAGA GAGGGGTAAT GCCCACCAGA AATAAGCAGG AAGCGAGGGA AAAACACTAG AGAGAAACAG   
  
  
+ AGAAAGAGAG AGAGAGAGAG AGAGAAGGGG AAATTTACTA GAGAATGAGA GTGAAGAAGG GAACTTTATT   
  
  
+ TTAACTTTTT TATGATGCAC GCATCCATGG CAGAACAGGG AAGGGAAAGA AGGGTACAGA GTACTGCTGT   
  
  
+ CCCCAAAAAA TTAGTGTAGA AAACAGGAGA CACTCAACTA CAACAAGGGT AAGGAAGAAG AACAAGCAAG   
  
  
+ ATATCCCACC CAAAGAAAAA AAAAAAGAAG AAGAAGAAAA TTTTTCAGAG AGGAAAGAAA AGCAATCACA   
  
  
+ TGAATTAGAA GCCAACCCAA GCCTTCTTCA AAGAGAAGCA GCATAACATA GCATAGCTGG GGAGAGGATA   
  
  
+ GGAGAGAGAA ACTCAAGAAA AGGGGTACCC GGCTTAAATG TTGATGCAGA AAGGTATAGA TATGGCCACA   
  
  
+ CTACTACTAC CCAGGAGTTT ATCATTGGAG ATGGATTTCT CTTTCTGCCT CATCTTCTCC TCCATCCACC   
  
  
+ AGCAAAACTG GGTTTCCTTT CAGCTGGGTC TTTTCTAGAT CTAGAAACTA TGGTACCCAA ATCTCCCATT   
  
  
+ CCTCCATTTT TATTCTCAAA ATCTATCTTC TTAGACTTGT GGGTTTGATT CATTTCTTTT GGGTTTTTGA   
  
  
+ AAAGAATTTA ATTTGATTGT ATTGTGTTGA TTGGGGAATT TGTGGTGGTG TTAGATGCGA GCTATGCCCT   
  
  
+ ACAATTTGCA AGGCAAGGGT GTGGTAGAGG TTTCAAGCAT TTGTATTCCA CAAATCTCTT CCCCTGCTTC   
  
  
+ AAAGTGGAAG ACCAACAACA ACAACTTGGA CAACAAATTT CAGCAACAAC AAGAAGAAAT TGAGCAATTG   
  
  
+ CAGAGTAGTG GTGGCACTTT TCTCTCTCCT GATAACACCA ATACTAATAT TAGTACTACT CCGAGAGCGA   
  
  
+ CAAGTTCTGT TGATAGCGAA CACACATCTA CTCTGGATAC TACGGGCCCT CCCACTTCAA CATCTTCCTC   
  
  
+ CTTCAAGAGC ACCTCCACCG CCAACACAGC CGGTGTGGCG GACCCCGCAT GTACTCCCAA AGAGGATTGG   
  
  
+ GATGTCTCCG GCGGCGCTGC TGGCGGTGAC GGCGGTGGTG GGGGGCTTGG ATTGGAGGAG TGGGACAGCA   
  
  
+ TGTTCCCAAA TGGGGAGGGG GCTTTGCTCC CTTGGATCAT GGGTGAAGCT GATGACATGG GTATGGGTTT   
  
  
+ GAAGCATCTT TTGCAATCGG GTAACCCGGT TGACTATGAG GGCAATGCTG GTCTAGGGGT TGTCGATCAG   
  
  
+ GGTTCTGGAT TTGAGACTCT CTCTCCTCCT CCACCGCAGC CGGCGGCATG TGAATCTAAT GGTGGTGGTG   
  
  
+ TTGCTAATTT GGGGTTTCCA GGGAATAATG GTAAGATTTC TTCAATTTCA CATAATTGTT CATCTGGGAT   
  
  
+ TTTGAATGGT AAGGTGAACA ATAATGGGTT GAATCCCAAT TGTAACCCTC AAGCTCAAGG CAGTCTACTT   
  
  
+ GGTTTAATCC AAGGAGCTAC AGGTGTACAC GCACATCCTG ACGTTGGAGA CGAGAAACCC CAGATTTTGA   
  
  
+ ATCCACATTT GGTGATGAAC CCTCAGCAAG CTCAGAGCAT TGCAAACCCT AGCTTTTTGA TGCACTCATT   
  
  
+ AGGTTACTAT CAGCTGGAGC AACATCTATT TCAACCTCAG GCAAAACGCC TGAACACGGG TGCTGTTCTG   
  
  
+ GATCCTAACC TTGTTCAGCT TGCAAAGAAC CCATTTGCTG ATCAGGGTCA TGAGTTATTG TTGAGGAAGC   
  
  
+ AGCACCAACA GCTTGGTTTG CAACCATTGC CATTGGGTTT GGGTCCTCAG TTGGTCCCTC CGCAGAAGCC   
  
  
+ TGTGATGGGT TCAAAGCAGG GGAACCCCCA GCATTACCCG TTGCATGTTC ATCAGCAGCT GCAATTGCAA   
  
  
+ GAGCAGGCTG TCAAAGATCA GCTCTTCAAG GCGGCAGACC TTATTCAAAC TGGAAGTTTC TCACTCGCGC   
  
  
+ AAGAGATATT GGCGCGGCTC AATCACCAGC TCTCCCTCCC TGCAAAGCCC CTCATTAGGG CGGCTTTGTA   
  
  
+ TGTGAAGGAG GCCCTTCAAA TGCTCCTCCT AATGAGCAAC CCAGTTGCGG CTCCACCGTC CAAGATCCTC   
  
  
+ ACCCCTTATG ATGTTGTTCA CAAGATGAGC GCGTATAAGG TCTTCTCTGA GGTCTGCCCA ATCACTCAAT   
  
  
+ TTGTGAATTT CACTTGTACA CAGGCCATTC TCGAGGCTCT TGATGATTCT GATGCTATTC ATGTTGTCGA   
  
  
+ CTTTGATATT GGTTGCGGTG CTCAATGGGC ATCATTGATT CAGGAGCTGC CATTGAGGAA AAGGGGAGCT   
  
  
+ CCCTCTCTGA AAATTACAGC CATAGCTCCC ATGTTGACTG GCAGTCACTT TGAAATTAGC CTAGTATGTG   
  
  
+ AAAACCTTGT GCAATTTGCC AACGATATTG GTGTTGCTTG TGAGCTCCAA GTTGTCAACT TTGATTTGTT   
  
  
+ TGATCCATCT TCGCCATCAA TGCCGAACAT TAGTACTGCT GAGGATGAGT CAATTGCTGT TAGTATCCCC   
  
  
+ ATCTGGGCAT CTTCAATTAG GCCATCTGTT CTTCCTTCCA TCCTCCGATT CATTAAGCAA AAATCCCCCA   
  
  
+ AAATTGTGGT CTCGTTTGAT AGAGGATTCG ATCGTTTTGA TGTCCCTTTC CCCCAACATC TGTTGCATAC   
  
  
+ CCTAGAATCC TGCAGTAATT TATTGGACTC GCTTGATGGT CTCAATGTTG CATCGGACAT TGTGAGCAAG   
  
  
+ GTCGAGAAGT TCTTTGTTCA ACCTAGGATC GAAAATGCTG TGTTGGGTCG GGTCCATGCC CCTGACAAGA   
  
  
+ TGCCCCATTG GAAAAATCTC TTTGCTTCAG CCGGCTTCTT GCCCTTGCAA TTCAGTAATT TCACAGAAAC   
  
  
+ CCAGGCCGAT TATGTGGTGA AGAGAACCCC AACAAGAGGA TTTCACGTGG AGAAGCGCCA GGCATCACTC   
  
  
+ ATTCTGAGTT GGCAGAGGCA GGAGCTTGTG GCAGCTTCGG CATGGAAGTG TTG  

- +Up\_Stream \_Len000AGAATT GTAATCAACG AACCAAGTTG GGTAGTCACT CTTATAGACT AAAGTTTTCA   
  
  
- TATCTAAATA TATTTGTATA CCTGATGTCC CTTCCCTCAG CAAAGGAGTT TCAGAATCTA AATGTGAGTT   
  
  
- TTCAAACTCG CTATGGTTAT TACACTAAGG TTTGTACTCG GTTTATAATT CATAGGATAT GATTCTAGTT   
  
  
- CTTCTCTTAC TTGGTTTTCT GACAATTAAA AAGTTAATTC GATTTGAATA ATTGGAAAAA AACCTTATCT   
  
  
- AAAAATTTAA AATAGAAGTT ATATTCAAAA AGGTTCCGCT CTCTGTATAT CGTTAAAATT AATTTCAATT   
  
  
- TCATCATATT GTAAAAAAAA TTACCAATAT CCGAATATCG TGGAAAAGAT CCACTTTTCT CTATAGAACC   
  
  
- GGACTAAAGG TCATTTCAAA AAAAAAATTT ATAACAGTTA ATAAAAACGT TTACTGTAAA CCATTAAACC   
  
  
- CCCTATTAGA CTACACAATT TTCTATGTTA CTTTTATAGT CTTTTATCCT ACGGAGTTTA AAGTGTTCTT   
  
  
- TCTTTTTTAG AAAAACAACA ATCATAAAGA TGTGTAGGTG TTAAAAATGA GTACCATTTT TTTTTTCCCG   
  
  
- GTTTATAGAG TGGTTATATT GGTTTGATAT ACAAGGTAAT AAATCATTGC CACTATTTAT ACATTACCCA   
  
  
- GTTCAATGAT TATTTTATCG GATGTTATGA ACCTATAGAT GTATTTTAAC TATATCTTAT ATATAACGTT   
  
  
- ATGAATTTAG TTTTAATTTA CTTTATAAAT GAAAAAATAA GATAATGTTT ATCGAAGAAT ATCTTATCGT   
  
  
- TGTTAAAAAT TACATTCAAA GTAAGAATGG TCTAAACTTC TCCAGGCCTA TATCTTTTAG AATAAGCATA   
  
  
- TTCATGAGCG TACTCCCGCC TATTTAAGAT TTTATAGTTG GAGTTGAGCG AAGATGATAA GGTAGATTCA   
  
  
- AGTTAAATAG TATATTTTTT TAAGTTAGGT GAAATTACAT GAGTTAAGTG AAGTTGGATA AAATTAGTTA   
  
  
- AATGGGTACT TTTCTTGTTC CAGGATTCAT CTACAAAAGA TAACTAACTA AATTTGTTAA AAGTGGATGG   
  
  
- GTTAATAGGG GTTTTGACTT TCTCCCCCGT TTCCCCCCAC CCATTACGAC ACATTTTATC CCCCTTTCCC   
  
  
- GTTCTTTTGA CACATGTTAC CACCTCGTCC CCATATGGTA AACCGGAACT CAACTCCCCC GTGTTTTCGG   
  
  
- GTCTCTCTCT CTCCCCATTA CGGGTGGTCT TTATTCGTCC TTCGCTCCCT TTTTGTGATC TCTCTTTGTC   
  
  
- TCTTTCTCTC TCTCTCTCTC TCTCTTCCCC TTTAAATGAT CTCTTACTCT CACTTCTTCC CTTGAAATAA   
  
  
- AATTGAAAAA ATACTACGTG CGTAGGTACC GTCTTGTCCC TTCCCTTTCT TCCCATGTCT CATGACGACA   
  
  
- GGGGTTTTTT AATCACATCT TTTGTCCTCT GTGAGTTGAT GTTGTTCCCA TTCCTTCTTC TTGTTCGTTC   
  
  
- TATAGGGTGG GTTTCTTTTT TTTTTTCTTC TTCTTCTTTT AAAAAGTCTC TCCTTTCTTT TCGTTAGTGT   
  
  
- ACTTAATCTT CGGTTGGGTT CGGAAGAAGT TTCTCTTCGT CGTATTGTAT CGTATCGACC CCTCTCCTAT   
  
  
- CCTCTCTCTT TGAGTTCTTT TCCCCATGGG CCGAATTTAC AACTACGTCT TTCCATATCT ATACCGGTGT   
  
  
- GATGATGATG GGTCCTCAAA TAGTAACCTC TACCTAAAGA GAAAGACGGA GTAGAAGAGG AGGTAGGTGG   
  
  
- TCGTTTTGAC CCAAAGGAAA GTCGACCCAG AAAAGATCTA GATCTTTGAT ACCATGGGTT TAGAGGGTAA   
  
  
- GGAGGTAAAA ATAAGAGTTT TAGATAGAAG AATCTGAACA CCCAAACTAA GTAAAGAAAA CCCAAAAACT   
  
  
- TTTCTTAAAT TAAACTAACA TAACACAACT AACCCCTTAA ACACCACCAC AATCTACGCT CGATACGGGA   
  
  
- TGTTAAACGT TCCGTTCCCA CACCATCTCC AAAGTTCGTA AACATAAGGT GTTTAGAGAA GGGGACGAAG   
  
  
- TTTCACCTTC TGGTTGTTGT TGTTGAACCT GTTGTTTAAA GTCGTTGTTG TTCTTCTTTA ACTCGTTAAC   
  
  
- GTCTCATCAC CACCGTGAAA AGAGAGAGGA CTATTGTGGT TATGATTATA ATCATGATGA GGCTCTCGCT   
  
  
- GTTCAAGACA ACTATCGCTT GTGTGTAGAT GAGACCTATG ATGCCCGGGA GGGTGAAGTT GTAGAAGGAG   
  
  
- GAAGTTCTCG TGGAGGTGGC GGTTGTGTCG GCCACACCGC CTGGGGCGTA CATGAGGGTT TCTCCTAACC   
  
  
- CTACAGAGGC CGCCGCGACG ACCGCCACTG CCGCCACCAC CCCCCGAACC TAACCTCCTC ACCCTGTCGT   
  
  
- ACAAGGGTTT ACCCCTCCCC CGAAACGAGG GAACCTAGTA CCCACTTCGA CTACTGTACC CATACCCAAA   
  
  
- CTTCGTAGAA AACGTTAGCC CATTGGGCCA ACTGATACTC CCGTTACGAC CAGATCCCCA ACAGCTAGTC   
  
  
- CCAAGACCTA AACTCTGAGA GAGAGGAGGA GGTGGCGTCG GCCGCCGTAC ACTTAGATTA CCACCACCAC   
  
  
- AACGATTAAA CCCCAAAGGT CCCTTATTAC CATTCTAAAG AAGTTAAAGT GTATTAACAA GTAGACCCTA   
  
  
- AAACTTACCA TTCCACTTGT TATTACCCAA CTTAGGGTTA ACATTGGGAG TTCGAGTTCC GTCAGATGAA   
  
  
- CCAAATTAGG TTCCTCGATG TCCACATGTG CGTGTAGGAC TGCAACCTCT GCTCTTTGGG GTCTAAAACT   
  
  
- TAGGTGTAAA CCACTACTTG GGAGTCGTTC GAGTCTCGTA ACGTTTGGGA TCGAAAAACT ACGTGAGTAA   
  
  
- TCCAATGATA GTCGACCTCG TTGTAGATAA AGTTGGAGTC CGTTTTGCGG ACTTGTGCCC ACGACAAGAC   
  
  
- CTAGGATTGG AACAAGTCGA ACGTTTCTTG GGTAAACGAC TAGTCCCAGT ACTCAATAAC AACTCCTTCG   
  
  
- TCGTGGTTGT CGAACCAAAC GTTGGTAACG GTAACCCAAA CCCAGGAGTC AACCAGGGAG GCGTCTTCGG   
  
  
- ACACTACCCA AGTTTCGTCC CCTTGGGGGT CGTAATGGGC AACGTACAAG TAGTCGTCGA CGTTAACGTT   
  
  
- CTCGTCCGAC AGTTTCTAGT CGAGAAGTTC CGCCGTCTGG AATAAGTTTG ACCTTCAAAG AGTGAGCGCG   
  
  
- TTCTCTATAA CCGCGCCGAG TTAGTGGTCG AGAGGGAGGG ACGTTTCGGG GAGTAATCCC GCCGAAACAT   
  
  
- ACACTTCCTC CGGGAAGTTT ACGAGGAGGA TTACTCGTTG GGTCAACGCC GAGGTGGCAG GTTCTAGGAG   
  
  
- TGGGGAATAC TACAACAAGT GTTCTACTCG CGCATATTCC AGAAGAGACT CCAGACGGGT TAGTGAGTTA   
  
  
- AACACTTAAA GTGAACATGT GTCCGGTAAG AGCTCCGAGA ACTACTAAGA CTACGATAAG TACAACAGCT   
  
  
- GAAACTATAA CCAACGCCAC GAGTTACCCG TAGTAACTAA GTCCTCGACG GTAACTCCTT TTCCCCTCGA   
  
  
- GGGAGAGACT TTTAATGTCG GTATCGAGGG TACAACTGAC CGTCAGTGAA ACTTTAATCG GATCATACAC   
  
  
- TTTTGGAACA CGTTAAACGG TTGCTATAAC CACAACGAAC ACTCGAGGTT CAACAGTTGA AACTAAACAA   
  
  
- ACTAGGTAGA AGCGGTAGTT ACGGCTTGTA ATCATGACGA CTCCTACTCA GTTAACGACA ATCATAGGGG   
  
  
- TAGACCCGTA GAAGTTAATC CGGTAGACAA GAAGGAAGGT AGGAGGCTAA GTAATTCGTT TTTAGGGGGT   
  
  
- TTTAACACCA GAGCAAACTA TCTCCTAAGC TAGCAAAACT ACAGGGAAAG GGGGTTGTAG ACAACGTATG   
  
  
- GGATCTTAGG ACGTCATTAA ATAACCTGAG CGAACTACCA GAGTTACAAC GTAGCCTGTA ACACTCGTTC   
  
  
- CAGCTCTTCA AGAAACAAGT TGGATCCTAG CTTTTACGAC ACAACCCAGC CCAGGTACGG GGACTGTTCT   
  
  
- ACGGGGTAAC CTTTTTAGAG AAACGAAGTC GGCCGAAGAA CGGGAACGTT AAGTCATTAA AGTGTCTTTG   
  
  
- GGTCCGGCTA ATACACCACT TCTCTTGGGG TTGTTCTCCT AAAGTGCACC TCTTCGCGGT CCGTAGTGAG   
  
  
- TAAGACTCAA CCGTCTCCGT CCTCGAACAC CGTCGAAGCC GTACCTTCAC AAC

+     Myb

| Site Name | Organism | Position | Strand | Matrix score. | sequence | function |
| --- | --- | --- | --- | --- | --- | --- |
| Myb | Arabidopsis thaliana | 3132 | - | 6 | CAACTG |  |
| Myb | Arabidopsis thaliana | 3406 | - | 6 | CAACTG |  |

>HU04G00047.1   
+ +Up\_Stream \_Len000TCTTAA CATTAGTTGC TTGGTTCAAC CCATCAGTGA GAATATCTGA TTTCAAAAGT   
  
  
+ ATAGATTTAT ATAAACATAT GGACTACAGG GAAGGGAGTC GTTTCCTCAA AGTCTTAGAT TTACACTCAA   
  
  
+ AAGTTTGAGC GATACCAATA ATGTGATTCC AAACATGAGC CAAATATTAA GTATCCTATA CTAAGATCAA   
  
  
+ GAAGAGAATG AACCAAAAGA CTGTTAATTT TTCAATTAAG CTAAACTTAT TAACCTTTTT TTGGAATAGA   
  
  
+ TTTTTAAATT TTATCTTCAA TATAAGTTTT TCCAAGGCGA GAGACATATA GCAATTTTAA TTAAAGTTAA   
  
  
+ AGTAGTATAA CATTTTTTTT AATGGTTATA GGCTTATAGC ACCTTTTCTA GGTGAAAAGA GATATCTTGG   
  
  
+ CCTGATTTCC AGTAAAGTTT TTTTTTTAAA TATTGTCAAT TATTTTTGCA AATGACATTT GGTAATTTGG   
  
  
+ GGGATAATCT GATGTGTTAA AAGATACAAT GAAAATATCA GAAAATAGGA TGCCTCAAAT TTCACAAGAA   
  
  
+ AGAAAAAATC TTTTTGTTGT TAGTATTTCT ACACATCCAC AATTTTTACT CATGGTAAAA AAAAAAGGGC   
  
  
+ CAAATATCTC ACCAATATAA CCAAACTATA TGTTCCATTA TTTAGTAACG GTGATAAATA TGTAATGGGT   
  
  
+ CAAGTTACTA ATAAAATAGC CTACAATACT TGGATATCTA CATAAAATTG ATATAGAATA TATATTGCAA   
  
  
+ TACTTAAATC AAAATTAAAT GAAATATTTA CTTTTTTATT CTATTACAAA TAGCTTCTTA TAGAATAGCA   
  
  
+ ACAATTTTTA ATGTAAGTTT CATTCTTACC AGATTTGAAG AGGTCCGGAT ATAGAAAATC TTATTCGTAT   
  
  
+ AAGTACTCGC ATGAGGGCGG ATAAATTCTA AAATATCAAC CTCAACTCGC TTCTACTATT CCATCTAAGT   
  
  
+ TCAATTTATC ATATAAAAAA ATTCAATCCA CTTTAATGTA CTCAATTCAC TTCAACCTAT TTTAATCAAT   
  
  
+ TTACCCATGA AAAGAACAAG GTCCTAAGTA GATGTTTTCT ATTGATTGAT TTAAACAATT TTCACCTACC   
  
  
+ CAATTATCCC CAAAACTGAA AGAGGGGGCA AAGGGGGGTG GGTAATGCTG TGTAAAATAG GGGGAAAGGG   
  
  
+ CAAGAAAACT GTGTACAATG GTGGAGCAGG GGTATACCAT TTGGCCTTGA GTTGAGGGGG CACAAAAGCC   
  
  
+ CAGAGAGAGA GAGGGGTAAT GCCCACCAGA AATAAGCAGG AAGCGAGGGA AAAACACTAG AGAGAAACAG   
  
  
+ AGAAAGAGAG AGAGAGAGAG AGAGAAGGGG AAATTTACTA GAGAATGAGA GTGAAGAAGG GAACTTTATT   
  
  
+ TTAACTTTTT TATGATGCAC GCATCCATGG CAGAACAGGG AAGGGAAAGA AGGGTACAGA GTACTGCTGT   
  
  
+ CCCCAAAAAA TTAGTGTAGA AAACAGGAGA CACTCAACTA CAACAAGGGT AAGGAAGAAG AACAAGCAAG   
  
  
+ ATATCCCACC CAAAGAAAAA AAAAAAGAAG AAGAAGAAAA TTTTTCAGAG AGGAAAGAAA AGCAATCACA   
  
  
+ TGAATTAGAA GCCAACCCAA GCCTTCTTCA AAGAGAAGCA GCATAACATA GCATAGCTGG GGAGAGGATA   
  
  
+ GGAGAGAGAA ACTCAAGAAA AGGGGTACCC GGCTTAAATG TTGATGCAGA AAGGTATAGA TATGGCCACA   
  
  
+ CTACTACTAC CCAGGAGTTT ATCATTGGAG ATGGATTTCT CTTTCTGCCT CATCTTCTCC TCCATCCACC   
  
  
+ AGCAAAACTG GGTTTCCTTT CAGCTGGGTC TTTTCTAGAT CTAGAAACTA TGGTACCCAA ATCTCCCATT   
  
  
+ CCTCCATTTT TATTCTCAAA ATCTATCTTC TTAGACTTGT GGGTTTGATT CATTTCTTTT GGGTTTTTGA   
  
  
+ AAAGAATTTA ATTTGATTGT ATTGTGTTGA TTGGGGAATT TGTGGTGGTG TTAGATGCGA GCTATGCCCT   
  
  
+ ACAATTTGCA AGGCAAGGGT GTGGTAGAGG TTTCAAGCAT TTGTATTCCA CAAATCTCTT CCCCTGCTTC   
  
  
+ AAAGTGGAAG ACCAACAACA ACAACTTGGA CAACAAATTT CAGCAACAAC AAGAAGAAAT TGAGCAATTG   
  
  
+ CAGAGTAGTG GTGGCACTTT TCTCTCTCCT GATAACACCA ATACTAATAT TAGTACTACT CCGAGAGCGA   
  
  
+ CAAGTTCTGT TGATAGCGAA CACACATCTA CTCTGGATAC TACGGGCCCT CCCACTTCAA CATCTTCCTC   
  
  
+ CTTCAAGAGC ACCTCCACCG CCAACACAGC CGGTGTGGCG GACCCCGCAT GTACTCCCAA AGAGGATTGG   
  
  
+ GATGTCTCCG GCGGCGCTGC TGGCGGTGAC GGCGGTGGTG GGGGGCTTGG ATTGGAGGAG TGGGACAGCA   
  
  
+ TGTTCCCAAA TGGGGAGGGG GCTTTGCTCC CTTGGATCAT GGGTGAAGCT GATGACATGG GTATGGGTTT   
  
  
+ GAAGCATCTT TTGCAATCGG GTAACCCGGT TGACTATGAG GGCAATGCTG GTCTAGGGGT TGTCGATCAG   
  
  
+ GGTTCTGGAT TTGAGACTCT CTCTCCTCCT CCACCGCAGC CGGCGGCATG TGAATCTAAT GGTGGTGGTG   
  
  
+ TTGCTAATTT GGGGTTTCCA GGGAATAATG GTAAGATTTC TTCAATTTCA CATAATTGTT CATCTGGGAT   
  
  
+ TTTGAATGGT AAGGTGAACA ATAATGGGTT GAATCCCAAT TGTAACCCTC AAGCTCAAGG CAGTCTACTT   
  
  
+ GGTTTAATCC AAGGAGCTAC AGGTGTACAC GCACATCCTG ACGTTGGAGA CGAGAAACCC CAGATTTTGA   
  
  
+ ATCCACATTT GGTGATGAAC CCTCAGCAAG CTCAGAGCAT TGCAAACCCT AGCTTTTTGA TGCACTCATT   
  
  
+ AGGTTACTAT CAGCTGGAGC AACATCTATT TCAACCTCAG GCAAAACGCC TGAACACGGG TGCTGTTCTG   
  
  
+ GATCCTAACC TTGTTCAGCT TGCAAAGAAC CCATTTGCTG ATCAGGGTCA TGAGTTATTG TTGAGGAAGC   
  
  
+ AGCACCAACA GCTTGGTTTG CAACCATTGC CATTGGGTTT GGGTCCTCAG TTGGTCCCTC CGCAGAAGCC   
  
  
+ TGTGATGGGT TCAAAGCAGG GGAACCCCCA GCATTACCCG TTGCATGTTC ATCAGCAGCT GCAATTGCAA   
  
  
+ GAGCAGGCTG TCAAAGATCA GCTCTTCAAG GCGGCAGACC TTATTCAAAC TGGAAGTTTC TCACTCGCGC   
  
  
+ AAGAGATATT GGCGCGGCTC AATCACCAGC TCTCCCTCCC TGCAAAGCCC CTCATTAGGG CGGCTTTGTA   
  
  
+ TGTGAAGGAG GCCCTTCAAA TGCTCCTCCT AATGAGCAAC CCAGTTGCGG CTCCACCGTC CAAGATCCTC   
  
  
+ ACCCCTTATG ATGTTGTTCA CAAGATGAGC GCGTATAAGG TCTTCTCTGA GGTCTGCCCA ATCACTCAAT   
  
  
+ TTGTGAATTT CACTTGTACA CAGGCCATTC TCGAGGCTCT TGATGATTCT GATGCTATTC ATGTTGTCGA   
  
  
+ CTTTGATATT GGTTGCGGTG CTCAATGGGC ATCATTGATT CAGGAGCTGC CATTGAGGAA AAGGGGAGCT   
  
  
+ CCCTCTCTGA AAATTACAGC CATAGCTCCC ATGTTGACTG GCAGTCACTT TGAAATTAGC CTAGTATGTG   
  
  
+ AAAACCTTGT GCAATTTGCC AACGATATTG GTGTTGCTTG TGAGCTCCAA GTTGTCAACT TTGATTTGTT   
  
  
+ TGATCCATCT TCGCCATCAA TGCCGAACAT TAGTACTGCT GAGGATGAGT CAATTGCTGT TAGTATCCCC   
  
  
+ ATCTGGGCAT CTTCAATTAG GCCATCTGTT CTTCCTTCCA TCCTCCGATT CATTAAGCAA AAATCCCCCA   
  
  
+ AAATTGTGGT CTCGTTTGAT AGAGGATTCG ATCGTTTTGA TGTCCCTTTC CCCCAACATC TGTTGCATAC   
  
  
+ CCTAGAATCC TGCAGTAATT TATTGGACTC GCTTGATGGT CTCAATGTTG CATCGGACAT TGTGAGCAAG   
  
  
+ GTCGAGAAGT TCTTTGTTCA ACCTAGGATC GAAAATGCTG TGTTGGGTCG GGTCCATGCC CCTGACAAGA   
  
  
+ TGCCCCATTG GAAAAATCTC TTTGCTTCAG CCGGCTTCTT GCCCTTGCAA TTCAGTAATT TCACAGAAAC   
  
  
+ CCAGGCCGAT TATGTGGTGA AGAGAACCCC AACAAGAGGA TTTCACGTGG AGAAGCGCCA GGCATCACTC   
  
  
+ ATTCTGAGTT GGCAGAGGCA GGAGCTTGTG GCAGCTTCGG CATGGAAGTG TTG  

- +Up\_Stream \_Len000AGAATT GTAATCAACG AACCAAGTTG GGTAGTCACT CTTATAGACT AAAGTTTTCA   
  
  
- TATCTAAATA TATTTGTATA CCTGATGTCC CTTCCCTCAG CAAAGGAGTT TCAGAATCTA AATGTGAGTT   
  
  
- TTCAAACTCG CTATGGTTAT TACACTAAGG TTTGTACTCG GTTTATAATT CATAGGATAT GATTCTAGTT   
  
  
- CTTCTCTTAC TTGGTTTTCT GACAATTAAA AAGTTAATTC GATTTGAATA ATTGGAAAAA AACCTTATCT   
  
  
- AAAAATTTAA AATAGAAGTT ATATTCAAAA AGGTTCCGCT CTCTGTATAT CGTTAAAATT AATTTCAATT   
  
  
- TCATCATATT GTAAAAAAAA TTACCAATAT CCGAATATCG TGGAAAAGAT CCACTTTTCT CTATAGAACC   
  
  
- GGACTAAAGG TCATTTCAAA AAAAAAATTT ATAACAGTTA ATAAAAACGT TTACTGTAAA CCATTAAACC   
  
  
- CCCTATTAGA CTACACAATT TTCTATGTTA CTTTTATAGT CTTTTATCCT ACGGAGTTTA AAGTGTTCTT   
  
  
- TCTTTTTTAG AAAAACAACA ATCATAAAGA TGTGTAGGTG TTAAAAATGA GTACCATTTT TTTTTTCCCG   
  
  
- GTTTATAGAG TGGTTATATT GGTTTGATAT ACAAGGTAAT AAATCATTGC CACTATTTAT ACATTACCCA   
  
  
- GTTCAATGAT TATTTTATCG GATGTTATGA ACCTATAGAT GTATTTTAAC TATATCTTAT ATATAACGTT   
  
  
- ATGAATTTAG TTTTAATTTA CTTTATAAAT GAAAAAATAA GATAATGTTT ATCGAAGAAT ATCTTATCGT   
  
  
- TGTTAAAAAT TACATTCAAA GTAAGAATGG TCTAAACTTC TCCAGGCCTA TATCTTTTAG AATAAGCATA   
  
  
- TTCATGAGCG TACTCCCGCC TATTTAAGAT TTTATAGTTG GAGTTGAGCG AAGATGATAA GGTAGATTCA   
  
  
- AGTTAAATAG TATATTTTTT TAAGTTAGGT GAAATTACAT GAGTTAAGTG AAGTTGGATA AAATTAGTTA   
  
  
- AATGGGTACT TTTCTTGTTC CAGGATTCAT CTACAAAAGA TAACTAACTA AATTTGTTAA AAGTGGATGG   
  
  
- GTTAATAGGG GTTTTGACTT TCTCCCCCGT TTCCCCCCAC CCATTACGAC ACATTTTATC CCCCTTTCCC   
  
  
- GTTCTTTTGA CACATGTTAC CACCTCGTCC CCATATGGTA AACCGGAACT CAACTCCCCC GTGTTTTCGG   
  
  
- GTCTCTCTCT CTCCCCATTA CGGGTGGTCT TTATTCGTCC TTCGCTCCCT TTTTGTGATC TCTCTTTGTC   
  
  
- TCTTTCTCTC TCTCTCTCTC TCTCTTCCCC TTTAAATGAT CTCTTACTCT CACTTCTTCC CTTGAAATAA   
  
  
- AATTGAAAAA ATACTACGTG CGTAGGTACC GTCTTGTCCC TTCCCTTTCT TCCCATGTCT CATGACGACA   
  
  
- GGGGTTTTTT AATCACATCT TTTGTCCTCT GTGAGTTGAT GTTGTTCCCA TTCCTTCTTC TTGTTCGTTC   
  
  
- TATAGGGTGG GTTTCTTTTT TTTTTTCTTC TTCTTCTTTT AAAAAGTCTC TCCTTTCTTT TCGTTAGTGT   
  
  
- ACTTAATCTT CGGTTGGGTT CGGAAGAAGT TTCTCTTCGT CGTATTGTAT CGTATCGACC CCTCTCCTAT   
  
  
- CCTCTCTCTT TGAGTTCTTT TCCCCATGGG CCGAATTTAC AACTACGTCT TTCCATATCT ATACCGGTGT   
  
  
- GATGATGATG GGTCCTCAAA TAGTAACCTC TACCTAAAGA GAAAGACGGA GTAGAAGAGG AGGTAGGTGG   
  
  
- TCGTTTTGAC CCAAAGGAAA GTCGACCCAG AAAAGATCTA GATCTTTGAT ACCATGGGTT TAGAGGGTAA   
  
  
- GGAGGTAAAA ATAAGAGTTT TAGATAGAAG AATCTGAACA CCCAAACTAA GTAAAGAAAA CCCAAAAACT   
  
  
- TTTCTTAAAT TAAACTAACA TAACACAACT AACCCCTTAA ACACCACCAC AATCTACGCT CGATACGGGA   
  
  
- TGTTAAACGT TCCGTTCCCA CACCATCTCC AAAGTTCGTA AACATAAGGT GTTTAGAGAA GGGGACGAAG   
  
  
- TTTCACCTTC TGGTTGTTGT TGTTGAACCT GTTGTTTAAA GTCGTTGTTG TTCTTCTTTA ACTCGTTAAC   
  
  
- GTCTCATCAC CACCGTGAAA AGAGAGAGGA CTATTGTGGT TATGATTATA ATCATGATGA GGCTCTCGCT   
  
  
- GTTCAAGACA ACTATCGCTT GTGTGTAGAT GAGACCTATG ATGCCCGGGA GGGTGAAGTT GTAGAAGGAG   
  
  
- GAAGTTCTCG TGGAGGTGGC GGTTGTGTCG GCCACACCGC CTGGGGCGTA CATGAGGGTT TCTCCTAACC   
  
  
- CTACAGAGGC CGCCGCGACG ACCGCCACTG CCGCCACCAC CCCCCGAACC TAACCTCCTC ACCCTGTCGT   
  
  
- ACAAGGGTTT ACCCCTCCCC CGAAACGAGG GAACCTAGTA CCCACTTCGA CTACTGTACC CATACCCAAA   
  
  
- CTTCGTAGAA AACGTTAGCC CATTGGGCCA ACTGATACTC CCGTTACGAC CAGATCCCCA ACAGCTAGTC   
  
  
- CCAAGACCTA AACTCTGAGA GAGAGGAGGA GGTGGCGTCG GCCGCCGTAC ACTTAGATTA CCACCACCAC   
  
  
- AACGATTAAA CCCCAAAGGT CCCTTATTAC CATTCTAAAG AAGTTAAAGT GTATTAACAA GTAGACCCTA   
  
  
- AAACTTACCA TTCCACTTGT TATTACCCAA CTTAGGGTTA ACATTGGGAG TTCGAGTTCC GTCAGATGAA   
  
  
- CCAAATTAGG TTCCTCGATG TCCACATGTG CGTGTAGGAC TGCAACCTCT GCTCTTTGGG GTCTAAAACT   
  
  
- TAGGTGTAAA CCACTACTTG GGAGTCGTTC GAGTCTCGTA ACGTTTGGGA TCGAAAAACT ACGTGAGTAA   
  
  
- TCCAATGATA GTCGACCTCG TTGTAGATAA AGTTGGAGTC CGTTTTGCGG ACTTGTGCCC ACGACAAGAC   
  
  
- CTAGGATTGG AACAAGTCGA ACGTTTCTTG GGTAAACGAC TAGTCCCAGT ACTCAATAAC AACTCCTTCG   
  
  
- TCGTGGTTGT CGAACCAAAC GTTGGTAACG GTAACCCAAA CCCAGGAGTC AACCAGGGAG GCGTCTTCGG   
  
  
- ACACTACCCA AGTTTCGTCC CCTTGGGGGT CGTAATGGGC AACGTACAAG TAGTCGTCGA CGTTAACGTT   
  
  
- CTCGTCCGAC AGTTTCTAGT CGAGAAGTTC CGCCGTCTGG AATAAGTTTG ACCTTCAAAG AGTGAGCGCG   
  
  
- TTCTCTATAA CCGCGCCGAG TTAGTGGTCG AGAGGGAGGG ACGTTTCGGG GAGTAATCCC GCCGAAACAT   
  
  
- ACACTTCCTC CGGGAAGTTT ACGAGGAGGA TTACTCGTTG GGTCAACGCC GAGGTGGCAG GTTCTAGGAG   
  
  
- TGGGGAATAC TACAACAAGT GTTCTACTCG CGCATATTCC AGAAGAGACT CCAGACGGGT TAGTGAGTTA   
  
  
- AACACTTAAA GTGAACATGT GTCCGGTAAG AGCTCCGAGA ACTACTAAGA CTACGATAAG TACAACAGCT   
  
  
- GAAACTATAA CCAACGCCAC GAGTTACCCG TAGTAACTAA GTCCTCGACG GTAACTCCTT TTCCCCTCGA   
  
  
- GGGAGAGACT TTTAATGTCG GTATCGAGGG TACAACTGAC CGTCAGTGAA ACTTTAATCG GATCATACAC   
  
  
- TTTTGGAACA CGTTAAACGG TTGCTATAAC CACAACGAAC ACTCGAGGTT CAACAGTTGA AACTAAACAA   
  
  
- ACTAGGTAGA AGCGGTAGTT ACGGCTTGTA ATCATGACGA CTCCTACTCA GTTAACGACA ATCATAGGGG   
  
  
- TAGACCCGTA GAAGTTAATC CGGTAGACAA GAAGGAAGGT AGGAGGCTAA GTAATTCGTT TTTAGGGGGT   
  
  
- TTTAACACCA GAGCAAACTA TCTCCTAAGC TAGCAAAACT ACAGGGAAAG GGGGTTGTAG ACAACGTATG   
  
  
- GGATCTTAGG ACGTCATTAA ATAACCTGAG CGAACTACCA GAGTTACAAC GTAGCCTGTA ACACTCGTTC   
  
  
- CAGCTCTTCA AGAAACAAGT TGGATCCTAG CTTTTACGAC ACAACCCAGC CCAGGTACGG GGACTGTTCT   
  
  
- ACGGGGTAAC CTTTTTAGAG AAACGAAGTC GGCCGAAGAA CGGGAACGTT AAGTCATTAA AGTGTCTTTG   
  
  
- GGTCCGGCTA ATACACCACT TCTCTTGGGG TTGTTCTCCT AAAGTGCACC TCTTCGCGGT CCGTAGTGAG   
  
  
- TAAGACTCAA CCGTCTCCGT CCTCGAACAC CGTCGAAGCC GTACCTTCAC AAC

+     Myb-binding site

| Site Name | Organism | Position | Strand | Matrix score. | sequence | function |
| --- | --- | --- | --- | --- | --- | --- |
| Myb-binding site | Nicotiana tabacum | 3090 | + | 6 | CAACAG |  |
| Myb-binding site | Nicotiana tabacum | 3984 | - | 6 | CAACAG |  |
| Myb-binding site | Nicotiana tabacum | 2251 | - | 6 | CAACAG |  |

>HU04G00047.1   
+ +Up\_Stream \_Len000TCTTAA CATTAGTTGC TTGGTTCAAC CCATCAGTGA GAATATCTGA TTTCAAAAGT   
  
  
+ ATAGATTTAT ATAAACATAT GGACTACAGG GAAGGGAGTC GTTTCCTCAA AGTCTTAGAT TTACACTCAA   
  
  
+ AAGTTTGAGC GATACCAATA ATGTGATTCC AAACATGAGC CAAATATTAA GTATCCTATA CTAAGATCAA   
  
  
+ GAAGAGAATG AACCAAAAGA CTGTTAATTT TTCAATTAAG CTAAACTTAT TAACCTTTTT TTGGAATAGA   
  
  
+ TTTTTAAATT TTATCTTCAA TATAAGTTTT TCCAAGGCGA GAGACATATA GCAATTTTAA TTAAAGTTAA   
  
  
+ AGTAGTATAA CATTTTTTTT AATGGTTATA GGCTTATAGC ACCTTTTCTA GGTGAAAAGA GATATCTTGG   
  
  
+ CCTGATTTCC AGTAAAGTTT TTTTTTTAAA TATTGTCAAT TATTTTTGCA AATGACATTT GGTAATTTGG   
  
  
+ GGGATAATCT GATGTGTTAA AAGATACAAT GAAAATATCA GAAAATAGGA TGCCTCAAAT TTCACAAGAA   
  
  
+ AGAAAAAATC TTTTTGTTGT TAGTATTTCT ACACATCCAC AATTTTTACT CATGGTAAAA AAAAAAGGGC   
  
  
+ CAAATATCTC ACCAATATAA CCAAACTATA TGTTCCATTA TTTAGTAACG GTGATAAATA TGTAATGGGT   
  
  
+ CAAGTTACTA ATAAAATAGC CTACAATACT TGGATATCTA CATAAAATTG ATATAGAATA TATATTGCAA   
  
  
+ TACTTAAATC AAAATTAAAT GAAATATTTA CTTTTTTATT CTATTACAAA TAGCTTCTTA TAGAATAGCA   
  
  
+ ACAATTTTTA ATGTAAGTTT CATTCTTACC AGATTTGAAG AGGTCCGGAT ATAGAAAATC TTATTCGTAT   
  
  
+ AAGTACTCGC ATGAGGGCGG ATAAATTCTA AAATATCAAC CTCAACTCGC TTCTACTATT CCATCTAAGT   
  
  
+ TCAATTTATC ATATAAAAAA ATTCAATCCA CTTTAATGTA CTCAATTCAC TTCAACCTAT TTTAATCAAT   
  
  
+ TTACCCATGA AAAGAACAAG GTCCTAAGTA GATGTTTTCT ATTGATTGAT TTAAACAATT TTCACCTACC   
  
  
+ CAATTATCCC CAAAACTGAA AGAGGGGGCA AAGGGGGGTG GGTAATGCTG TGTAAAATAG GGGGAAAGGG   
  
  
+ CAAGAAAACT GTGTACAATG GTGGAGCAGG GGTATACCAT TTGGCCTTGA GTTGAGGGGG CACAAAAGCC   
  
  
+ CAGAGAGAGA GAGGGGTAAT GCCCACCAGA AATAAGCAGG AAGCGAGGGA AAAACACTAG AGAGAAACAG   
  
  
+ AGAAAGAGAG AGAGAGAGAG AGAGAAGGGG AAATTTACTA GAGAATGAGA GTGAAGAAGG GAACTTTATT   
  
  
+ TTAACTTTTT TATGATGCAC GCATCCATGG CAGAACAGGG AAGGGAAAGA AGGGTACAGA GTACTGCTGT   
  
  
+ CCCCAAAAAA TTAGTGTAGA AAACAGGAGA CACTCAACTA CAACAAGGGT AAGGAAGAAG AACAAGCAAG   
  
  
+ ATATCCCACC CAAAGAAAAA AAAAAAGAAG AAGAAGAAAA TTTTTCAGAG AGGAAAGAAA AGCAATCACA   
  
  
+ TGAATTAGAA GCCAACCCAA GCCTTCTTCA AAGAGAAGCA GCATAACATA GCATAGCTGG GGAGAGGATA   
  
  
+ GGAGAGAGAA ACTCAAGAAA AGGGGTACCC GGCTTAAATG TTGATGCAGA AAGGTATAGA TATGGCCACA   
  
  
+ CTACTACTAC CCAGGAGTTT ATCATTGGAG ATGGATTTCT CTTTCTGCCT CATCTTCTCC TCCATCCACC   
  
  
+ AGCAAAACTG GGTTTCCTTT CAGCTGGGTC TTTTCTAGAT CTAGAAACTA TGGTACCCAA ATCTCCCATT   
  
  
+ CCTCCATTTT TATTCTCAAA ATCTATCTTC TTAGACTTGT GGGTTTGATT CATTTCTTTT GGGTTTTTGA   
  
  
+ AAAGAATTTA ATTTGATTGT ATTGTGTTGA TTGGGGAATT TGTGGTGGTG TTAGATGCGA GCTATGCCCT   
  
  
+ ACAATTTGCA AGGCAAGGGT GTGGTAGAGG TTTCAAGCAT TTGTATTCCA CAAATCTCTT CCCCTGCTTC   
  
  
+ AAAGTGGAAG ACCAACAACA ACAACTTGGA CAACAAATTT CAGCAACAAC AAGAAGAAAT TGAGCAATTG   
  
  
+ CAGAGTAGTG GTGGCACTTT TCTCTCTCCT GATAACACCA ATACTAATAT TAGTACTACT CCGAGAGCGA   
  
  
+ CAAGTTCTGT TGATAGCGAA CACACATCTA CTCTGGATAC TACGGGCCCT CCCACTTCAA CATCTTCCTC   
  
  
+ CTTCAAGAGC ACCTCCACCG CCAACACAGC CGGTGTGGCG GACCCCGCAT GTACTCCCAA AGAGGATTGG   
  
  
+ GATGTCTCCG GCGGCGCTGC TGGCGGTGAC GGCGGTGGTG GGGGGCTTGG ATTGGAGGAG TGGGACAGCA   
  
  
+ TGTTCCCAAA TGGGGAGGGG GCTTTGCTCC CTTGGATCAT GGGTGAAGCT GATGACATGG GTATGGGTTT   
  
  
+ GAAGCATCTT TTGCAATCGG GTAACCCGGT TGACTATGAG GGCAATGCTG GTCTAGGGGT TGTCGATCAG   
  
  
+ GGTTCTGGAT TTGAGACTCT CTCTCCTCCT CCACCGCAGC CGGCGGCATG TGAATCTAAT GGTGGTGGTG   
  
  
+ TTGCTAATTT GGGGTTTCCA GGGAATAATG GTAAGATTTC TTCAATTTCA CATAATTGTT CATCTGGGAT   
  
  
+ TTTGAATGGT AAGGTGAACA ATAATGGGTT GAATCCCAAT TGTAACCCTC AAGCTCAAGG CAGTCTACTT   
  
  
+ GGTTTAATCC AAGGAGCTAC AGGTGTACAC GCACATCCTG ACGTTGGAGA CGAGAAACCC CAGATTTTGA   
  
  
+ ATCCACATTT GGTGATGAAC CCTCAGCAAG CTCAGAGCAT TGCAAACCCT AGCTTTTTGA TGCACTCATT   
  
  
+ AGGTTACTAT CAGCTGGAGC AACATCTATT TCAACCTCAG GCAAAACGCC TGAACACGGG TGCTGTTCTG   
  
  
+ GATCCTAACC TTGTTCAGCT TGCAAAGAAC CCATTTGCTG ATCAGGGTCA TGAGTTATTG TTGAGGAAGC   
  
  
+ AGCACCAACA GCTTGGTTTG CAACCATTGC CATTGGGTTT GGGTCCTCAG TTGGTCCCTC CGCAGAAGCC   
  
  
+ TGTGATGGGT TCAAAGCAGG GGAACCCCCA GCATTACCCG TTGCATGTTC ATCAGCAGCT GCAATTGCAA   
  
  
+ GAGCAGGCTG TCAAAGATCA GCTCTTCAAG GCGGCAGACC TTATTCAAAC TGGAAGTTTC TCACTCGCGC   
  
  
+ AAGAGATATT GGCGCGGCTC AATCACCAGC TCTCCCTCCC TGCAAAGCCC CTCATTAGGG CGGCTTTGTA   
  
  
+ TGTGAAGGAG GCCCTTCAAA TGCTCCTCCT AATGAGCAAC CCAGTTGCGG CTCCACCGTC CAAGATCCTC   
  
  
+ ACCCCTTATG ATGTTGTTCA CAAGATGAGC GCGTATAAGG TCTTCTCTGA GGTCTGCCCA ATCACTCAAT   
  
  
+ TTGTGAATTT CACTTGTACA CAGGCCATTC TCGAGGCTCT TGATGATTCT GATGCTATTC ATGTTGTCGA   
  
  
+ CTTTGATATT GGTTGCGGTG CTCAATGGGC ATCATTGATT CAGGAGCTGC CATTGAGGAA AAGGGGAGCT   
  
  
+ CCCTCTCTGA AAATTACAGC CATAGCTCCC ATGTTGACTG GCAGTCACTT TGAAATTAGC CTAGTATGTG   
  
  
+ AAAACCTTGT GCAATTTGCC AACGATATTG GTGTTGCTTG TGAGCTCCAA GTTGTCAACT TTGATTTGTT   
  
  
+ TGATCCATCT TCGCCATCAA TGCCGAACAT TAGTACTGCT GAGGATGAGT CAATTGCTGT TAGTATCCCC   
  
  
+ ATCTGGGCAT CTTCAATTAG GCCATCTGTT CTTCCTTCCA TCCTCCGATT CATTAAGCAA AAATCCCCCA   
  
  
+ AAATTGTGGT CTCGTTTGAT AGAGGATTCG ATCGTTTTGA TGTCCCTTTC CCCCAACATC TGTTGCATAC   
  
  
+ CCTAGAATCC TGCAGTAATT TATTGGACTC GCTTGATGGT CTCAATGTTG CATCGGACAT TGTGAGCAAG   
  
  
+ GTCGAGAAGT TCTTTGTTCA ACCTAGGATC GAAAATGCTG TGTTGGGTCG GGTCCATGCC CCTGACAAGA   
  
  
+ TGCCCCATTG GAAAAATCTC TTTGCTTCAG CCGGCTTCTT GCCCTTGCAA TTCAGTAATT TCACAGAAAC   
  
  
+ CCAGGCCGAT TATGTGGTGA AGAGAACCCC AACAAGAGGA TTTCACGTGG AGAAGCGCCA GGCATCACTC   
  
  
+ ATTCTGAGTT GGCAGAGGCA GGAGCTTGTG GCAGCTTCGG CATGGAAGTG TTG  

- +Up\_Stream \_Len000AGAATT GTAATCAACG AACCAAGTTG GGTAGTCACT CTTATAGACT AAAGTTTTCA   
  
  
- TATCTAAATA TATTTGTATA CCTGATGTCC CTTCCCTCAG CAAAGGAGTT TCAGAATCTA AATGTGAGTT   
  
  
- TTCAAACTCG CTATGGTTAT TACACTAAGG TTTGTACTCG GTTTATAATT CATAGGATAT GATTCTAGTT   
  
  
- CTTCTCTTAC TTGGTTTTCT GACAATTAAA AAGTTAATTC GATTTGAATA ATTGGAAAAA AACCTTATCT   
  
  
- AAAAATTTAA AATAGAAGTT ATATTCAAAA AGGTTCCGCT CTCTGTATAT CGTTAAAATT AATTTCAATT   
  
  
- TCATCATATT GTAAAAAAAA TTACCAATAT CCGAATATCG TGGAAAAGAT CCACTTTTCT CTATAGAACC   
  
  
- GGACTAAAGG TCATTTCAAA AAAAAAATTT ATAACAGTTA ATAAAAACGT TTACTGTAAA CCATTAAACC   
  
  
- CCCTATTAGA CTACACAATT TTCTATGTTA CTTTTATAGT CTTTTATCCT ACGGAGTTTA AAGTGTTCTT   
  
  
- TCTTTTTTAG AAAAACAACA ATCATAAAGA TGTGTAGGTG TTAAAAATGA GTACCATTTT TTTTTTCCCG   
  
  
- GTTTATAGAG TGGTTATATT GGTTTGATAT ACAAGGTAAT AAATCATTGC CACTATTTAT ACATTACCCA   
  
  
- GTTCAATGAT TATTTTATCG GATGTTATGA ACCTATAGAT GTATTTTAAC TATATCTTAT ATATAACGTT   
  
  
- ATGAATTTAG TTTTAATTTA CTTTATAAAT GAAAAAATAA GATAATGTTT ATCGAAGAAT ATCTTATCGT   
  
  
- TGTTAAAAAT TACATTCAAA GTAAGAATGG TCTAAACTTC TCCAGGCCTA TATCTTTTAG AATAAGCATA   
  
  
- TTCATGAGCG TACTCCCGCC TATTTAAGAT TTTATAGTTG GAGTTGAGCG AAGATGATAA GGTAGATTCA   
  
  
- AGTTAAATAG TATATTTTTT TAAGTTAGGT GAAATTACAT GAGTTAAGTG AAGTTGGATA AAATTAGTTA   
  
  
- AATGGGTACT TTTCTTGTTC CAGGATTCAT CTACAAAAGA TAACTAACTA AATTTGTTAA AAGTGGATGG   
  
  
- GTTAATAGGG GTTTTGACTT TCTCCCCCGT TTCCCCCCAC CCATTACGAC ACATTTTATC CCCCTTTCCC   
  
  
- GTTCTTTTGA CACATGTTAC CACCTCGTCC CCATATGGTA AACCGGAACT CAACTCCCCC GTGTTTTCGG   
  
  
- GTCTCTCTCT CTCCCCATTA CGGGTGGTCT TTATTCGTCC TTCGCTCCCT TTTTGTGATC TCTCTTTGTC   
  
  
- TCTTTCTCTC TCTCTCTCTC TCTCTTCCCC TTTAAATGAT CTCTTACTCT CACTTCTTCC CTTGAAATAA   
  
  
- AATTGAAAAA ATACTACGTG CGTAGGTACC GTCTTGTCCC TTCCCTTTCT TCCCATGTCT CATGACGACA   
  
  
- GGGGTTTTTT AATCACATCT TTTGTCCTCT GTGAGTTGAT GTTGTTCCCA TTCCTTCTTC TTGTTCGTTC   
  
  
- TATAGGGTGG GTTTCTTTTT TTTTTTCTTC TTCTTCTTTT AAAAAGTCTC TCCTTTCTTT TCGTTAGTGT   
  
  
- ACTTAATCTT CGGTTGGGTT CGGAAGAAGT TTCTCTTCGT CGTATTGTAT CGTATCGACC CCTCTCCTAT   
  
  
- CCTCTCTCTT TGAGTTCTTT TCCCCATGGG CCGAATTTAC AACTACGTCT TTCCATATCT ATACCGGTGT   
  
  
- GATGATGATG GGTCCTCAAA TAGTAACCTC TACCTAAAGA GAAAGACGGA GTAGAAGAGG AGGTAGGTGG   
  
  
- TCGTTTTGAC CCAAAGGAAA GTCGACCCAG AAAAGATCTA GATCTTTGAT ACCATGGGTT TAGAGGGTAA   
  
  
- GGAGGTAAAA ATAAGAGTTT TAGATAGAAG AATCTGAACA CCCAAACTAA GTAAAGAAAA CCCAAAAACT   
  
  
- TTTCTTAAAT TAAACTAACA TAACACAACT AACCCCTTAA ACACCACCAC AATCTACGCT CGATACGGGA   
  
  
- TGTTAAACGT TCCGTTCCCA CACCATCTCC AAAGTTCGTA AACATAAGGT GTTTAGAGAA GGGGACGAAG   
  
  
- TTTCACCTTC TGGTTGTTGT TGTTGAACCT GTTGTTTAAA GTCGTTGTTG TTCTTCTTTA ACTCGTTAAC   
  
  
- GTCTCATCAC CACCGTGAAA AGAGAGAGGA CTATTGTGGT TATGATTATA ATCATGATGA GGCTCTCGCT   
  
  
- GTTCAAGACA ACTATCGCTT GTGTGTAGAT GAGACCTATG ATGCCCGGGA GGGTGAAGTT GTAGAAGGAG   
  
  
- GAAGTTCTCG TGGAGGTGGC GGTTGTGTCG GCCACACCGC CTGGGGCGTA CATGAGGGTT TCTCCTAACC   
  
  
- CTACAGAGGC CGCCGCGACG ACCGCCACTG CCGCCACCAC CCCCCGAACC TAACCTCCTC ACCCTGTCGT   
  
  
- ACAAGGGTTT ACCCCTCCCC CGAAACGAGG GAACCTAGTA CCCACTTCGA CTACTGTACC CATACCCAAA   
  
  
- CTTCGTAGAA AACGTTAGCC CATTGGGCCA ACTGATACTC CCGTTACGAC CAGATCCCCA ACAGCTAGTC   
  
  
- CCAAGACCTA AACTCTGAGA GAGAGGAGGA GGTGGCGTCG GCCGCCGTAC ACTTAGATTA CCACCACCAC   
  
  
- AACGATTAAA CCCCAAAGGT CCCTTATTAC CATTCTAAAG AAGTTAAAGT GTATTAACAA GTAGACCCTA   
  
  
- AAACTTACCA TTCCACTTGT TATTACCCAA CTTAGGGTTA ACATTGGGAG TTCGAGTTCC GTCAGATGAA   
  
  
- CCAAATTAGG TTCCTCGATG TCCACATGTG CGTGTAGGAC TGCAACCTCT GCTCTTTGGG GTCTAAAACT   
  
  
- TAGGTGTAAA CCACTACTTG GGAGTCGTTC GAGTCTCGTA ACGTTTGGGA TCGAAAAACT ACGTGAGTAA   
  
  
- TCCAATGATA GTCGACCTCG TTGTAGATAA AGTTGGAGTC CGTTTTGCGG ACTTGTGCCC ACGACAAGAC   
  
  
- CTAGGATTGG AACAAGTCGA ACGTTTCTTG GGTAAACGAC TAGTCCCAGT ACTCAATAAC AACTCCTTCG   
  
  
- TCGTGGTTGT CGAACCAAAC GTTGGTAACG GTAACCCAAA CCCAGGAGTC AACCAGGGAG GCGTCTTCGG   
  
  
- ACACTACCCA AGTTTCGTCC CCTTGGGGGT CGTAATGGGC AACGTACAAG TAGTCGTCGA CGTTAACGTT   
  
  
- CTCGTCCGAC AGTTTCTAGT CGAGAAGTTC CGCCGTCTGG AATAAGTTTG ACCTTCAAAG AGTGAGCGCG   
  
  
- TTCTCTATAA CCGCGCCGAG TTAGTGGTCG AGAGGGAGGG ACGTTTCGGG GAGTAATCCC GCCGAAACAT   
  
  
- ACACTTCCTC CGGGAAGTTT ACGAGGAGGA TTACTCGTTG GGTCAACGCC GAGGTGGCAG GTTCTAGGAG   
  
  
- TGGGGAATAC TACAACAAGT GTTCTACTCG CGCATATTCC AGAAGAGACT CCAGACGGGT TAGTGAGTTA   
  
  
- AACACTTAAA GTGAACATGT GTCCGGTAAG AGCTCCGAGA ACTACTAAGA CTACGATAAG TACAACAGCT   
  
  
- GAAACTATAA CCAACGCCAC GAGTTACCCG TAGTAACTAA GTCCTCGACG GTAACTCCTT TTCCCCTCGA   
  
  
- GGGAGAGACT TTTAATGTCG GTATCGAGGG TACAACTGAC CGTCAGTGAA ACTTTAATCG GATCATACAC   
  
  
- TTTTGGAACA CGTTAAACGG TTGCTATAAC CACAACGAAC ACTCGAGGTT CAACAGTTGA AACTAAACAA   
  
  
- ACTAGGTAGA AGCGGTAGTT ACGGCTTGTA ATCATGACGA CTCCTACTCA GTTAACGACA ATCATAGGGG   
  
  
- TAGACCCGTA GAAGTTAATC CGGTAGACAA GAAGGAAGGT AGGAGGCTAA GTAATTCGTT TTTAGGGGGT   
  
  
- TTTAACACCA GAGCAAACTA TCTCCTAAGC TAGCAAAACT ACAGGGAAAG GGGGTTGTAG ACAACGTATG   
  
  
- GGATCTTAGG ACGTCATTAA ATAACCTGAG CGAACTACCA GAGTTACAAC GTAGCCTGTA ACACTCGTTC   
  
  
- CAGCTCTTCA AGAAACAAGT TGGATCCTAG CTTTTACGAC ACAACCCAGC CCAGGTACGG GGACTGTTCT   
  
  
- ACGGGGTAAC CTTTTTAGAG AAACGAAGTC GGCCGAAGAA CGGGAACGTT AAGTCATTAA AGTGTCTTTG   
  
  
- GGTCCGGCTA ATACACCACT TCTCTTGGGG TTGTTCTCCT AAAGTGCACC TCTTCGCGGT CCGTAGTGAG   
  
  
- TAAGACTCAA CCGTCTCCGT CCTCGAACAC CGTCGAAGCC GTACCTTCAC AAC

+     O2-site

| Site Name | Organism | Position | Strand | Matrix score. | sequence | function |
| --- | --- | --- | --- | --- | --- | --- |
| O2-site | Zea mays | 2505 | + | 10 | GATGACATGG | cis-acting regulatory element involved in zein metabolism regulation |
| O2-site | Zea mays | 1031 | - | 9 | GTTGACGTGA | cis-acting regulatory element involved in zein metabolism regulation |
| O2-site | Zea mays | 4212 | + | 9 | GATGATGTGG | cis-acting regulatory element involved in zein metabolism regulation |

>HU04G00047.1   
+ +Up\_Stream \_Len000TCTTAA CATTAGTTGC TTGGTTCAAC CCATCAGTGA GAATATCTGA TTTCAAAAGT   
  
  
+ ATAGATTTAT ATAAACATAT GGACTACAGG GAAGGGAGTC GTTTCCTCAA AGTCTTAGAT TTACACTCAA   
  
  
+ AAGTTTGAGC GATACCAATA ATGTGATTCC AAACATGAGC CAAATATTAA GTATCCTATA CTAAGATCAA   
  
  
+ GAAGAGAATG AACCAAAAGA CTGTTAATTT TTCAATTAAG CTAAACTTAT TAACCTTTTT TTGGAATAGA   
  
  
+ TTTTTAAATT TTATCTTCAA TATAAGTTTT TCCAAGGCGA GAGACATATA GCAATTTTAA TTAAAGTTAA   
  
  
+ AGTAGTATAA CATTTTTTTT AATGGTTATA GGCTTATAGC ACCTTTTCTA GGTGAAAAGA GATATCTTGG   
  
  
+ CCTGATTTCC AGTAAAGTTT TTTTTTTAAA TATTGTCAAT TATTTTTGCA AATGACATTT GGTAATTTGG   
  
  
+ GGGATAATCT GATGTGTTAA AAGATACAAT GAAAATATCA GAAAATAGGA TGCCTCAAAT TTCACAAGAA   
  
  
+ AGAAAAAATC TTTTTGTTGT TAGTATTTCT ACACATCCAC AATTTTTACT CATGGTAAAA AAAAAAGGGC   
  
  
+ CAAATATCTC ACCAATATAA CCAAACTATA TGTTCCATTA TTTAGTAACG GTGATAAATA TGTAATGGGT   
  
  
+ CAAGTTACTA ATAAAATAGC CTACAATACT TGGATATCTA CATAAAATTG ATATAGAATA TATATTGCAA   
  
  
+ TACTTAAATC AAAATTAAAT GAAATATTTA CTTTTTTATT CTATTACAAA TAGCTTCTTA TAGAATAGCA   
  
  
+ ACAATTTTTA ATGTAAGTTT CATTCTTACC AGATTTGAAG AGGTCCGGAT ATAGAAAATC TTATTCGTAT   
  
  
+ AAGTACTCGC ATGAGGGCGG ATAAATTCTA AAATATCAAC CTCAACTCGC TTCTACTATT CCATCTAAGT   
  
  
+ TCAATTTATC ATATAAAAAA ATTCAATCCA CTTTAATGTA CTCAATTCAC TTCAACCTAT TTTAATCAAT   
  
  
+ TTACCCATGA AAAGAACAAG GTCCTAAGTA GATGTTTTCT ATTGATTGAT TTAAACAATT TTCACCTACC   
  
  
+ CAATTATCCC CAAAACTGAA AGAGGGGGCA AAGGGGGGTG GGTAATGCTG TGTAAAATAG GGGGAAAGGG   
  
  
+ CAAGAAAACT GTGTACAATG GTGGAGCAGG GGTATACCAT TTGGCCTTGA GTTGAGGGGG CACAAAAGCC   
  
  
+ CAGAGAGAGA GAGGGGTAAT GCCCACCAGA AATAAGCAGG AAGCGAGGGA AAAACACTAG AGAGAAACAG   
  
  
+ AGAAAGAGAG AGAGAGAGAG AGAGAAGGGG AAATTTACTA GAGAATGAGA GTGAAGAAGG GAACTTTATT   
  
  
+ TTAACTTTTT TATGATGCAC GCATCCATGG CAGAACAGGG AAGGGAAAGA AGGGTACAGA GTACTGCTGT   
  
  
+ CCCCAAAAAA TTAGTGTAGA AAACAGGAGA CACTCAACTA CAACAAGGGT AAGGAAGAAG AACAAGCAAG   
  
  
+ ATATCCCACC CAAAGAAAAA AAAAAAGAAG AAGAAGAAAA TTTTTCAGAG AGGAAAGAAA AGCAATCACA   
  
  
+ TGAATTAGAA GCCAACCCAA GCCTTCTTCA AAGAGAAGCA GCATAACATA GCATAGCTGG GGAGAGGATA   
  
  
+ GGAGAGAGAA ACTCAAGAAA AGGGGTACCC GGCTTAAATG TTGATGCAGA AAGGTATAGA TATGGCCACA   
  
  
+ CTACTACTAC CCAGGAGTTT ATCATTGGAG ATGGATTTCT CTTTCTGCCT CATCTTCTCC TCCATCCACC   
  
  
+ AGCAAAACTG GGTTTCCTTT CAGCTGGGTC TTTTCTAGAT CTAGAAACTA TGGTACCCAA ATCTCCCATT   
  
  
+ CCTCCATTTT TATTCTCAAA ATCTATCTTC TTAGACTTGT GGGTTTGATT CATTTCTTTT GGGTTTTTGA   
  
  
+ AAAGAATTTA ATTTGATTGT ATTGTGTTGA TTGGGGAATT TGTGGTGGTG TTAGATGCGA GCTATGCCCT   
  
  
+ ACAATTTGCA AGGCAAGGGT GTGGTAGAGG TTTCAAGCAT TTGTATTCCA CAAATCTCTT CCCCTGCTTC   
  
  
+ AAAGTGGAAG ACCAACAACA ACAACTTGGA CAACAAATTT CAGCAACAAC AAGAAGAAAT TGAGCAATTG   
  
  
+ CAGAGTAGTG GTGGCACTTT TCTCTCTCCT GATAACACCA ATACTAATAT TAGTACTACT CCGAGAGCGA   
  
  
+ CAAGTTCTGT TGATAGCGAA CACACATCTA CTCTGGATAC TACGGGCCCT CCCACTTCAA CATCTTCCTC   
  
  
+ CTTCAAGAGC ACCTCCACCG CCAACACAGC CGGTGTGGCG GACCCCGCAT GTACTCCCAA AGAGGATTGG   
  
  
+ GATGTCTCCG GCGGCGCTGC TGGCGGTGAC GGCGGTGGTG GGGGGCTTGG ATTGGAGGAG TGGGACAGCA   
  
  
+ TGTTCCCAAA TGGGGAGGGG GCTTTGCTCC CTTGGATCAT GGGTGAAGCT GATGACATGG GTATGGGTTT   
  
  
+ GAAGCATCTT TTGCAATCGG GTAACCCGGT TGACTATGAG GGCAATGCTG GTCTAGGGGT TGTCGATCAG   
  
  
+ GGTTCTGGAT TTGAGACTCT CTCTCCTCCT CCACCGCAGC CGGCGGCATG TGAATCTAAT GGTGGTGGTG   
  
  
+ TTGCTAATTT GGGGTTTCCA GGGAATAATG GTAAGATTTC TTCAATTTCA CATAATTGTT CATCTGGGAT   
  
  
+ TTTGAATGGT AAGGTGAACA ATAATGGGTT GAATCCCAAT TGTAACCCTC AAGCTCAAGG CAGTCTACTT   
  
  
+ GGTTTAATCC AAGGAGCTAC AGGTGTACAC GCACATCCTG ACGTTGGAGA CGAGAAACCC CAGATTTTGA   
  
  
+ ATCCACATTT GGTGATGAAC CCTCAGCAAG CTCAGAGCAT TGCAAACCCT AGCTTTTTGA TGCACTCATT   
  
  
+ AGGTTACTAT CAGCTGGAGC AACATCTATT TCAACCTCAG GCAAAACGCC TGAACACGGG TGCTGTTCTG   
  
  
+ GATCCTAACC TTGTTCAGCT TGCAAAGAAC CCATTTGCTG ATCAGGGTCA TGAGTTATTG TTGAGGAAGC   
  
  
+ AGCACCAACA GCTTGGTTTG CAACCATTGC CATTGGGTTT GGGTCCTCAG TTGGTCCCTC CGCAGAAGCC   
  
  
+ TGTGATGGGT TCAAAGCAGG GGAACCCCCA GCATTACCCG TTGCATGTTC ATCAGCAGCT GCAATTGCAA   
  
  
+ GAGCAGGCTG TCAAAGATCA GCTCTTCAAG GCGGCAGACC TTATTCAAAC TGGAAGTTTC TCACTCGCGC   
  
  
+ AAGAGATATT GGCGCGGCTC AATCACCAGC TCTCCCTCCC TGCAAAGCCC CTCATTAGGG CGGCTTTGTA   
  
  
+ TGTGAAGGAG GCCCTTCAAA TGCTCCTCCT AATGAGCAAC CCAGTTGCGG CTCCACCGTC CAAGATCCTC   
  
  
+ ACCCCTTATG ATGTTGTTCA CAAGATGAGC GCGTATAAGG TCTTCTCTGA GGTCTGCCCA ATCACTCAAT   
  
  
+ TTGTGAATTT CACTTGTACA CAGGCCATTC TCGAGGCTCT TGATGATTCT GATGCTATTC ATGTTGTCGA   
  
  
+ CTTTGATATT GGTTGCGGTG CTCAATGGGC ATCATTGATT CAGGAGCTGC CATTGAGGAA AAGGGGAGCT   
  
  
+ CCCTCTCTGA AAATTACAGC CATAGCTCCC ATGTTGACTG GCAGTCACTT TGAAATTAGC CTAGTATGTG   
  
  
+ AAAACCTTGT GCAATTTGCC AACGATATTG GTGTTGCTTG TGAGCTCCAA GTTGTCAACT TTGATTTGTT   
  
  
+ TGATCCATCT TCGCCATCAA TGCCGAACAT TAGTACTGCT GAGGATGAGT CAATTGCTGT TAGTATCCCC   
  
  
+ ATCTGGGCAT CTTCAATTAG GCCATCTGTT CTTCCTTCCA TCCTCCGATT CATTAAGCAA AAATCCCCCA   
  
  
+ AAATTGTGGT CTCGTTTGAT AGAGGATTCG ATCGTTTTGA TGTCCCTTTC CCCCAACATC TGTTGCATAC   
  
  
+ CCTAGAATCC TGCAGTAATT TATTGGACTC GCTTGATGGT CTCAATGTTG CATCGGACAT TGTGAGCAAG   
  
  
+ GTCGAGAAGT TCTTTGTTCA ACCTAGGATC GAAAATGCTG TGTTGGGTCG GGTCCATGCC CCTGACAAGA   
  
  
+ TGCCCCATTG GAAAAATCTC TTTGCTTCAG CCGGCTTCTT GCCCTTGCAA TTCAGTAATT TCACAGAAAC   
  
  
+ CCAGGCCGAT TATGTGGTGA AGAGAACCCC AACAAGAGGA TTTCACGTGG AGAAGCGCCA GGCATCACTC   
  
  
+ ATTCTGAGTT GGCAGAGGCA GGAGCTTGTG GCAGCTTCGG CATGGAAGTG TTG  

- +Up\_Stream \_Len000AGAATT GTAATCAACG AACCAAGTTG GGTAGTCACT CTTATAGACT AAAGTTTTCA   
  
  
- TATCTAAATA TATTTGTATA CCTGATGTCC CTTCCCTCAG CAAAGGAGTT TCAGAATCTA AATGTGAGTT   
  
  
- TTCAAACTCG CTATGGTTAT TACACTAAGG TTTGTACTCG GTTTATAATT CATAGGATAT GATTCTAGTT   
  
  
- CTTCTCTTAC TTGGTTTTCT GACAATTAAA AAGTTAATTC GATTTGAATA ATTGGAAAAA AACCTTATCT   
  
  
- AAAAATTTAA AATAGAAGTT ATATTCAAAA AGGTTCCGCT CTCTGTATAT CGTTAAAATT AATTTCAATT   
  
  
- TCATCATATT GTAAAAAAAA TTACCAATAT CCGAATATCG TGGAAAAGAT CCACTTTTCT CTATAGAACC   
  
  
- GGACTAAAGG TCATTTCAAA AAAAAAATTT ATAACAGTTA ATAAAAACGT TTACTGTAAA CCATTAAACC   
  
  
- CCCTATTAGA CTACACAATT TTCTATGTTA CTTTTATAGT CTTTTATCCT ACGGAGTTTA AAGTGTTCTT   
  
  
- TCTTTTTTAG AAAAACAACA ATCATAAAGA TGTGTAGGTG TTAAAAATGA GTACCATTTT TTTTTTCCCG   
  
  
- GTTTATAGAG TGGTTATATT GGTTTGATAT ACAAGGTAAT AAATCATTGC CACTATTTAT ACATTACCCA   
  
  
- GTTCAATGAT TATTTTATCG GATGTTATGA ACCTATAGAT GTATTTTAAC TATATCTTAT ATATAACGTT   
  
  
- ATGAATTTAG TTTTAATTTA CTTTATAAAT GAAAAAATAA GATAATGTTT ATCGAAGAAT ATCTTATCGT   
  
  
- TGTTAAAAAT TACATTCAAA GTAAGAATGG TCTAAACTTC TCCAGGCCTA TATCTTTTAG AATAAGCATA   
  
  
- TTCATGAGCG TACTCCCGCC TATTTAAGAT TTTATAGTTG GAGTTGAGCG AAGATGATAA GGTAGATTCA   
  
  
- AGTTAAATAG TATATTTTTT TAAGTTAGGT GAAATTACAT GAGTTAAGTG AAGTTGGATA AAATTAGTTA   
  
  
- AATGGGTACT TTTCTTGTTC CAGGATTCAT CTACAAAAGA TAACTAACTA AATTTGTTAA AAGTGGATGG   
  
  
- GTTAATAGGG GTTTTGACTT TCTCCCCCGT TTCCCCCCAC CCATTACGAC ACATTTTATC CCCCTTTCCC   
  
  
- GTTCTTTTGA CACATGTTAC CACCTCGTCC CCATATGGTA AACCGGAACT CAACTCCCCC GTGTTTTCGG   
  
  
- GTCTCTCTCT CTCCCCATTA CGGGTGGTCT TTATTCGTCC TTCGCTCCCT TTTTGTGATC TCTCTTTGTC
[truncated: 190,012 more chars]
