# Supplementary material for: Genome-Wide Identification and Expression Pattern of the GRAS Gene Family in Pitaya (Selenicereus undatus L.)
Source: Biology (Basel). 2022 Dec 21;12(1):11. doi: 10.3390/biology12010011 (PMC9854919; doi:10.3390/biology12010011)
Supplement: Supplementary file 1 [file biology-12-00011-s001.zip › Supplementary file S5/HU04G00048.1_plantcare.html]

Content-Type: text/html; charset=ISO-8859-1


PlantCARE


Webmaster Firefox specific output  
To save the result:
click on the frame with the right mouse button and save the source code as a text file with extension .html  
REFERENCE:PlantCARE: a database of plant cis-acting regulatory elements and a portal to tools for in silico analysis of promoter sequences.  
Lescot, M., Déhais, P., Moreau, Y., De Moor, B., Rouzé ,P.,and Rombauts, S.  
Nucleic Acids Res., Database issue(2002), 30(1):325-327.   


---

>HU04G00048.1   
+ -Up\_Stream \_Len000TTTGTA TTTAAATTTT ACAATATTTA TAAAAATTTT GTATTGATTA ATTGTGCTAA   
  
  
+ CTAACACTAA TAATTGGGGG AGAAAAGAAG GGAAAATAAA AAGAAATTAA GAAGAAGAAA AGAAAACACC   
  
  
+ TTTGAAAAGG CAAAAAACTA AAATAAAACA ATAATGAACA GAAGGAGAGA AGGATGGATA AACAACAAAT   
  
  
+ GTCGCTTTTA GGACAGCAAT AGTCTATGTG ATTGGCCTTA GTTCCTGTTG GAGGATTGCT AACAAAAGCA   
  
  
+ ACAGCAAAAA ATAATACTAA TTATTACATA TTTTTTAACT CTTAAAGCTA ATCAATCATC CTCCATTAAT   
  
  
+ TGATTAATTA TTTGCCAAAA ACACAAATCA ACTGGGGCAA CTATAAGAGA TCTACCTGCT TTAAGATATA   
  
  
+ TAAACTCAAT TGTTTCATTT TATTTTATTT TAGATTTTTT GGCCTTTGGT CTCTATTGAG CTAAAATCTC   
  
  
+ GTTATTTTCG GATCATGAAA AATACAACTC GATCTTTTGA TATAAGTCAT CTTTATTTGT AAATTCTGAA   
  
  
+ CTTAAAATCT TACTTGAAGA TCAGGAATAG AAGCACTCGA TTTTTGATAA ATCCTATGTG TTCTTCTTAA   
  
  
+ AATTCATAAT CTAATTCATA TTTTAGATGT ACCCGTTGCC TACTTTGGCA AGTTTAGATT ATGATTTTTA   
  
  
+ TTTTTTTTAA TTACTTGTAT TTTTCTTTTG GATTGATAGT CAATCTACAT CATCTTTGAT CAATTGCGAC   
  
  
+ AGTTTCATTT TCCCAACAAA AATTTCGAGT GTATTCTTTT TAATTTATCA TAAGTATTTA TTGTCAACAT   
  
  
+ TCAACATGCA TCAACAATCA TTAAACACTT TTCGTCACTC CTCAATACTG GTCAGCACTT AAGATTTTGT   
  
  
+ CTTCTAAACT TATCGTAAGT AACTTATTGT TGATTAATTG ATGCTGACCA AATGATACTG AGTGCTAAAA   
  
  
+ TTATAATTAA TAAATACAAT TAAGAGTTGA TCAATGTCGA TGATTCAAAT CAATGTGATT GGTGCTGAAT   
  
  
+ AGTAATTATA AGCACCTATA AAGACTTGAA AAGAACAAAC ATTTAATTAG ATTTTATAAT TATAATTTCC   
  
  
+ TAATATTTAG CATTCATCAG CAAATTCAGC ACACACCAAC ATTAGTCAAC ACCTATCAAC ACTTATAAGT   
  
  
+ ACCAATTAAT AATAATTACC TATAATAGAT TAAAAGTTAG GCCCTTAATG TTTCCCACAT TATTAGATAT   
  
  
+ ATCAAGGGAG GGGGAAAAAA ATAATACTCC CCCATCTTAA AAAATTTATC CCTTTTTTTC TTTTTAATGT   
  
  
+ TCCAAAACAA AAATATGCTT CTAAATTATG ATTGCAATAT GAATGTGTAC GGTTTCAAAC CCAATTTTCT   
  
  
+ CCAACAATTC TTCTACTATA TTGTAATTAT ATTATACTAC AATTTGTATC TCATCTATGA TCTACCCCCC   
  
  
+ AAAAACCAAA TACTCATATA AAGTTTTATT TTCTTTCCAT CCCACTCTAA AAAAAAAAAA TGTTGGGAAA   
  
  
+ AAGTTGATTT ATTTAATTTG AAATGGTACG GTGAATATAG ATTTGTAGAG GGGCAGTTGA TAGAAAAAGG   
  
  
+ TGAAGACAAA GGAAGGGCAC ATTTGCCTTT TCATAGAAGG AAGAGACCAA GGTACTGGGG ACTGGGGGAA   
  
  
+ GGGCTCAAGA GAAGAGAGAG ACTGAGAAGA GTGCTGCTAC TCCTTCCTAC CTACACACAA AATTGCTGCG   
  
  
+ GGGGGTTCAA GTTCAACTTG AACGAGGCAA ATACCCTCAT TGAACCCACC AAATCGAAAT CTTCAATGTA   
  
  
+ CACCCCCATT ATACACGCAT TCAGAGACAT AGATGTCGAG GGGGAGATGA ATTAGCAAGA AGATGTCGAG   
  
  
+ CTAAAATGCA TTTCGAGGTT ACTAGAGCCA GAAATGTGGG CACATCCCAG AAGATCTCAG TAGAGAGAGT   
  
  
+ AACTTCAGAA AAGTTTCAAT TTTTATTCGA AATTTGCCTC TTGTTTGTGT AGATATGATG GGAACTTCGC   
  
  
+ CTTACAATCT GCAATGTGAG GGTTTGGTTG AAGGAGTTGC AGTCAATCCT CAATTTTATG AATCAACCCC   
  
  
+ AAAGTGGAAG AAGGGCATCA AAGAAGAGTC AATTGAGAGC GAACCCACTT CGGTTTTGGA CAACCCTAGC   
  
  
+ CCTCCAAATT CTGCATCTAC TCTCTCTTCC TGCTTCAATG GTGGAAGTAG TGGGCTTGGG ATGGACGATT   
  
  
+ TGGAGGGTTT GTCTTTTGGA GAAGGGTCCC TTCTCCCTTG GTTCATGGGT GAAATTGAAG ACCCTAGTTT   
  
  
+ GAGTTTCAAG CACCTCCTTC AACCTTCAAA TCCCTTGGAG TTTGGTGGCA ATGATGGGCT TGAAGCAGCT   
  
  
+ ATTCAGAGTG CTGGTATGGG TAATTTTAAT TTGGTTGAAA GTTTTAATTC TGATTTGAGG TCTTGTAATT   
  
  
+ CTGGGTCTAT TTGCAATGGG GGGAATGATT CTGTCAATGA AAAGGGATGT GTTCTTAATT ATGCTTCAGA   
  
  
+ GCAGGGTCAT GTGGTTGCTG TATCTCCAGT AGAACAGTTT AGGATTAGTG ATGTGAAACA TGAGGTGTTG   
  
  
+ AACCCAAAAT TTGGCTTTTG TGAGCAAGGC TCTACAATCA GTCAGAACTC TGGTTATGTG CATATACTGG   
  
  
+ GTTATAACCA GCTAGAATCT CCCCTTGAGC CTCCGGCAAA GCGCCACAAT GCTGGGAATG CTCTTTACAC   
  
  
+ TAGTGTTGTT CCAGTTCCAA AGAGTTCGTT TTTAGGTGAG GATCATGAAA TTCTACTCAG AAATCAAGAG   
  
  
+ CAGCAGTTTC TGATGCAACA ACAGTGGTTA ATGGGTTTAG CCCCTCAGTT TACACCCCAG CACCCGCCAA   
  
  
+ AGCCATTAGA CGACCGAAAG CAACCAAACT CGCAAACTTT GGTTCTGCGC GATCAGTTAG TTAAGATTGC   
  
  
+ GGAGATGTTC CAAACCGGTA ACTTTTCGCT TGCCCAAGTG ATATTGGCCC GGCTCAATCA GCAATTGTCT   
  
  
+ CTTTCAGGGA ACCCCTTTGT AAGAGCTGCT ACTTTCCATG TCAAGGAGGA ACTTGAAAGG CTCCTGACTA   
  
  
+ TCAATAGCTT CTCTGCGGCT CCACCCCAAC CTAAAAGCCT TACTCTGTCA GATGTTGTGG ATAGGATGAA   
  
  
+ TGCATACAAG CTTTTTTCAG AGGCATCCCC AGTTATGCAA TTTACCGATT TTACTTGTAC CCAGGCTTTG   
  
  
+ CTCGAAGCTC TTGATGATGC TGATTATATC CACATACTAG ATTTTGACAT TGGTTGTGGT TCCCAATGGG   
  
  
+ CATCCTTTAT TCGAGAGCTT CCTCTCAGGA AAAGGGGCAC TCCATTTTTG AAAATTACAG CCTTTGCCTC   
  
  
+ TTCTTTAACC CATAATCCAT TTGAACTAGC CCTTGTATGT GATAACATTC TGGAATTTGC AAAAGAGTTC   
  
  
+ GGTGTCTCTG TCGACCTTCA GGTCATGAAT TTGGACTTGT TTGACCCAAC TACTTGCACA ATACCCAATT   
  
  
+ TTCAATCCAC TGAGAACGCA GCAGTTGCTG TCAACTTCCC TATATGGGCG TGCTCACACT GCCCGTTTGT   
  
  
+ ACTCAGTCAA CTCCTGTCCT TCATCAAGCA ATGTTCCCCA AAAATCATGA CGAGCTTTGA TAGAGGTTGT   
  
  
+ GATCATTTTG AGTTGTCTTT TCCATCGCAC ATCATCCGTG TATTGGACTC ATGCTCTAAT ATGTTGGAGT   
  
  
+ CCCTCCATGG TTCAAGCGTG ACATTAGACA CCTCAAAGAA GATTGAAAAG TTCTTGATCA AGCCTATAAT   
  
  
+ TGAGAGGGCA ATATTGGGGC GAATTCATGC ACCAGACAAG ATGCAGGCAA ACAAGGCACA CCGGTGGAAG   
  
  
+ AATCTCTTCA CTTCTGCTGG GTTCTTCCCT TTGCCATTTA GTAGTTTCAC TGAATCTCAG GCCAATCTAG   
  
  
+ TGGCTCAACG AACCCCAGTG AGGGGTTTTC AGGTGGAAAA GCAGCAAGCA TCACTTGTGC TCAGGTGGAT   
  
  
+ GTCTCATGAG CTCGTGGCAG CTTCAGCTTG GAGGTGCTA  

- -Up\_Stream \_Len000AAACAT AAATTTAAAA TGTTATAAAT ATTTTTAAAA CATAACTAAT TAACACGATT   
  
  
- GATTGTGATT ATTAACCCCC TCTTTTCTTC CCTTTTATTT TTCTTTAATT CTTCTTCTTT TCTTTTGTGG   
  
  
- AAACTTTTCC GTTTTTTGAT TTTATTTTGT TATTACTTGT CTTCCTCTCT TCCTACCTAT TTGTTGTTTA   
  
  
- CAGCGAAAAT CCTGTCGTTA TCAGATACAC TAACCGGAAT CAAGGACAAC CTCCTAACGA TTGTTTTCGT   
  
  
- TGTCGTTTTT TATTATGATT AATAATGTAT AAAAAATTGA GAATTTCGAT TAGTTAGTAG GAGGTAATTA   
  
  
- ACTAATTAAT AAACGGTTTT TGTGTTTAGT TGACCCCGTT GATATTCTCT AGATGGACGA AATTCTATAT   
  
  
- ATTTGAGTTA ACAAAGTAAA ATAAAATAAA ATCTAAAAAA CCGGAAACCA GAGATAACTC GATTTTAGAG   
  
  
- CAATAAAAGC CTAGTACTTT TTATGTTGAG CTAGAAAACT ATATTCAGTA GAAATAAACA TTTAAGACTT   
  
  
- GAATTTTAGA ATGAACTTCT AGTCCTTATC TTCGTGAGCT AAAAACTATT TAGGATACAC AAGAAGAATT   
  
  
- TTAAGTATTA GATTAAGTAT AAAATCTACA TGGGCAACGG ATGAAACCGT TCAAATCTAA TACTAAAAAT   
  
  
- AAAAAAAATT AATGAACATA AAAAGAAAAC CTAACTATCA GTTAGATGTA GTAGAAACTA GTTAACGCTG   
  
  
- TCAAAGTAAA AGGGTTGTTT TTAAAGCTCA CATAAGAAAA ATTAAATAGT ATTCATAAAT AACAGTTGTA   
  
  
- AGTTGTACGT AGTTGTTAGT AATTTGTGAA AAGCAGTGAG GAGTTATGAC CAGTCGTGAA TTCTAAAACA   
  
  
- GAAGATTTGA ATAGCATTCA TTGAATAACA ACTAATTAAC TACGACTGGT TTACTATGAC TCACGATTTT   
  
  
- AATATTAATT ATTTATGTTA ATTCTCAACT AGTTACAGCT ACTAAGTTTA GTTACACTAA CCACGACTTA   
  
  
- TCATTAATAT TCGTGGATAT TTCTGAACTT TTCTTGTTTG TAAATTAATC TAAAATATTA ATATTAAAGG   
  
  
- ATTATAAATC GTAAGTAGTC GTTTAAGTCG TGTGTGGTTG TAATCAGTTG TGGATAGTTG TGAATATTCA   
  
  
- TGGTTAATTA TTATTAATGG ATATTATCTA ATTTTCAATC CGGGAATTAC AAAGGGTGTA ATAATCTATA   
  
  
- TAGTTCCCTC CCCCTTTTTT TATTATGAGG GGGTAGAATT TTTTAAATAG GGAAAAAAAG AAAAATTACA   
  
  
- AGGTTTTGTT TTTATACGAA GATTTAATAC TAACGTTATA CTTACACATG CCAAAGTTTG GGTTAAAAGA   
  
  
- GGTTGTTAAG AAGATGATAT AACATTAATA TAATATGATG TTAAACATAG AGTAGATACT AGATGGGGGG   
  
  
- TTTTTGGTTT ATGAGTATAT TTCAAAATAA AAGAAAGGTA GGGTGAGATT TTTTTTTTTT ACAACCCTTT   
  
  
- TTCAACTAAA TAAATTAAAC TTTACCATGC CACTTATATC TAAACATCTC CCCGTCAACT ATCTTTTTCC   
  
  
- ACTTCTGTTT CCTTCCCGTG TAAACGGAAA AGTATCTTCC TTCTCTGGTT CCATGACCCC TGACCCCCTT   
  
  
- CCCGAGTTCT CTTCTCTCTC TGACTCTTCT CACGACGATG AGGAAGGATG GATGTGTGTT TTAACGACGC   
  
  
- CCCCCAAGTT CAAGTTGAAC TTGCTCCGTT TATGGGAGTA ACTTGGGTGG TTTAGCTTTA GAAGTTACAT   
  
  
- GTGGGGGTAA TATGTGCGTA AGTCTCTGTA TCTACAGCTC CCCCTCTACT TAATCGTTCT TCTACAGCTC   
  
  
- GATTTTACGT AAAGCTCCAA TGATCTCGGT CTTTACACCC GTGTAGGGTC TTCTAGAGTC ATCTCTCTCA   
  
  
- TTGAAGTCTT TTCAAAGTTA AAAATAAGCT TTAAACGGAG AACAAACACA TCTATACTAC CCTTGAAGCG   
  
  
- GAATGTTAGA CGTTACACTC CCAAACCAAC TTCCTCAACG TCAGTTAGGA GTTAAAATAC TTAGTTGGGG   
  
  
- TTTCACCTTC TTCCCGTAGT TTCTTCTCAG TTAACTCTCG CTTGGGTGAA GCCAAAACCT GTTGGGATCG   
  
  
- GGAGGTTTAA GACGTAGATG AGAGAGAAGG ACGAAGTTAC CACCTTCATC ACCCGAACCC TACCTGCTAA   
  
  
- ACCTCCCAAA CAGAAAACCT CTTCCCAGGG AAGAGGGAAC CAAGTACCCA CTTTAACTTC TGGGATCAAA   
  
  
- CTCAAAGTTC GTGGAGGAAG TTGGAAGTTT AGGGAACCTC AAACCACCGT TACTACCCGA ACTTCGTCGA   
  
  
- TAAGTCTCAC GACCATACCC ATTAAAATTA AACCAACTTT CAAAATTAAG ACTAAACTCC AGAACATTAA   
  
  
- GACCCAGATA AACGTTACCC CCCTTACTAA GACAGTTACT TTTCCCTACA CAAGAATTAA TACGAAGTCT   
  
  
- CGTCCCAGTA CACCAACGAC ATAGAGGTCA TCTTGTCAAA TCCTAATCAC TACACTTTGT ACTCCACAAC   
  
  
- TTGGGTTTTA AACCGAAAAC ACTCGTTCCG AGATGTTAGT CAGTCTTGAG ACCAATACAC GTATATGACC   
  
  
- CAATATTGGT CGATCTTAGA GGGGAACTCG GAGGCCGTTT CGCGGTGTTA CGACCCTTAC GAGAAATGTG   
  
  
- ATCACAACAA GGTCAAGGTT TCTCAAGCAA AAATCCACTC CTAGTACTTT AAGATGAGTC TTTAGTTCTC   
  
  
- GTCGTCAAAG ACTACGTTGT TGTCACCAAT TACCCAAATC GGGGAGTCAA ATGTGGGGTC GTGGGCGGTT   
  
  
- TCGGTAATCT GCTGGCTTTC GTTGGTTTGA GCGTTTGAAA CCAAGACGCG CTAGTCAATC AATTCTAACG   
  
  
- CCTCTACAAG GTTTGGCCAT TGAAAAGCGA ACGGGTTCAC TATAACCGGG CCGAGTTAGT CGTTAACAGA   
  
  
- GAAAGTCCCT TGGGGAAACA TTCTCGACGA TGAAAGGTAC AGTTCCTCCT TGAACTTTCC GAGGACTGAT   
  
  
- AGTTATCGAA GAGACGCCGA GGTGGGGTTG GATTTTCGGA ATGAGACAGT CTACAACACC TATCCTACTT   
  
  
- ACGTATGTTC GAAAAAAGTC TCCGTAGGGG TCAATACGTT AAATGGCTAA AATGAACATG GGTCCGAAAC   
  
  
- GAGCTTCGAG AACTACTACG ACTAATATAG GTGTATGATC TAAAACTGTA ACCAACACCA AGGGTTACCC   
  
  
- GTAGGAAATA AGCTCTCGAA GGAGAGTCCT TTTCCCCGTG AGGTAAAAAC TTTTAATGTC GGAAACGGAG   
  
  
- AAGAAATTGG GTATTAGGTA AACTTGATCG GGAACATACA CTATTGTAAG ACCTTAAACG TTTTCTCAAG   
  
  
- CCACAGAGAC AGCTGGAAGT CCAGTACTTA AACCTGAACA AACTGGGTTG ATGAACGTGT TATGGGTTAA   
  
  
- AAGTTAGGTG ACTCTTGCGT CGTCAACGAC AGTTGAAGGG ATATACCCGC ACGAGTGTGA CGGGCAAACA   
  
  
- TGAGTCAGTT GAGGACAGGA AGTAGTTCGT TACAAGGGGT TTTTAGTACT GCTCGAAACT ATCTCCAACA   
  
  
- CTAGTAAAAC TCAACAGAAA AGGTAGCGTG TAGTAGGCAC ATAACCTGAG TACGAGATTA TACAACCTCA   
  
  
- GGGAGGTACC AAGTTCGCAC TGTAATCTGT GGAGTTTCTT CTAACTTTTC AAGAACTAGT TCGGATATTA   
  
  
- ACTCTCCCGT TATAACCCCG CTTAAGTACG TGGTCTGTTC TACGTCCGTT TGTTCCGTGT GGCCACCTTC   
  
  
- TTAGAGAAGT GAAGACGACC CAAGAAGGGA AACGGTAAAT CATCAAAGTG ACTTAGAGTC CGGTTAGATC   
  
  
- ACCGAGTTGC TTGGGGTCAC TCCCCAAAAG TCCACCTTTT CGTCGTTCGT AGTGAACACG AGTCCACCTA   
  
  
- CAGAGTACTC GAGCACCGTC GAAGTCGAAC CTCCACGAT

  
  
Motifs Found  

+   

| Site Name | Organism | Position | Strand | Matrix score. | sequence | function |
| --- | --- | --- | --- | --- | --- | --- |
|  | organism | 1695 | - | 4 | motif\_sequence | short\_function |
|  | organism | 1867 | - | 4 | motif\_sequence | short\_function |
|  | organism | 3095 | + | 4 | motif\_sequence | short\_function |
|  | organism | 225 | - | 4 | motif\_sequence | short\_function |
|  | organism | 404 | + | 4 | motif\_sequence | short\_function |
|  | organism | 3858 | + | 4 | motif\_sequence | short\_function |
|  | organism | 3752 | - | 4 | motif\_sequence | short\_function |
|  | organism | 3671 | - | 4 | motif\_sequence | short\_function |
|  | organism | 3588 | + | 4 | motif\_sequence | short\_function |
|  | organism | 2624 | + | 4 | motif\_sequence | short\_function |
|  | organism | 1934 | - | 4 | motif\_sequence | short\_function |
|  | organism | 747 | + | 4 | motif\_sequence | short\_function |
|  | organism | 1464 | + | 4 | motif\_sequence | short\_function |
|  | organism | 180 | - | 4 | motif\_sequence | short\_function |
|  | organism | 1944 | - | 4 | motif\_sequence | short\_function |
|  | organism | 1813 | + | 4 | motif\_sequence | short\_function |
|  | organism | 580 | - | 4 | motif\_sequence | short\_function |
|  | organism | 661 | + | 4 | motif\_sequence | short\_function |
|  | organism | 1820 | + | 4 | motif\_sequence | short\_function |
|  | organism | 3605 | + | 4 | motif\_sequence | short\_function |
|  | organism | 1331 | + | 4 | motif\_sequence | short\_function |
|  | organism | 1631 | - | 4 | motif\_sequence | short\_function |
|  | organism | 3362 | + | 4 | motif\_sequence | short\_function |
|  | organism | 2949 | + | 4 | motif\_sequence | short\_function |
|  | organism | 2945 | - | 4 | motif\_sequence | short\_function |
|  | organism | 2681 | + | 4 | motif\_sequence | short\_function |
|  | organism | 2650 | + | 4 | motif\_sequence | short\_function |
|  | organism | 2198 | + | 4 | motif\_sequence | short\_function |
|  | organism | 2013 | - | 4 | motif\_sequence | short\_function |
|  | organism | 188 | - | 4 | motif\_sequence | short\_function |
|  | organism | 1883 | - | 4 | motif\_sequence | short\_function |
|  | organism | 2546 | + | 4 | motif\_sequence | short\_function |
|  | organism | 1589 | - | 4 | motif\_sequence | short\_function |
|  | organism | 1654 | - | 4 | motif\_sequence | short\_function |
|  | organism | 1710 | - | 4 | motif\_sequence | short\_function |
|  | organism | 2189 | + | 4 | motif\_sequence | short\_function |
|  | organism | 2556 | - | 4 | motif\_sequence | short\_function |
|  | organism | 1954 | - | 4 | motif\_sequence | short\_function |
|  | organism | 2127 | - | 4 | motif\_sequence | short\_function |
|  | organism | 2041 | + | 4 | motif\_sequence | short\_function |

>HU04G00048.1   
+ -Up\_Stream \_Len000TTTGTA TTTAAATTTT ACAATATTTA TAAAAATTTT GTATTGATTA ATTGTGCTAA   
  
  
+ CTAACACTAA TAATTGGGGG AGAAAAGAAG GGAAAATAAA AAGAAATTAA GAAGAAGAAA AGAAAACACC   
  
  
+ TTTGAAAAGG CAAAAAACTA AAATAAAACA ATAATGAACA GAAGGAGAGA AGGATGGATA AACAACAAAT   
  
  
+ GTCGCTTTTA GGACAGCAAT AGTCTATGTG ATTGGCCTTA GTTCCTGTTG GAGGATTGCT AACAAAAGCA   
  
  
+ ACAGCAAAAA ATAATACTAA TTATTACATA TTTTTTAACT CTTAAAGCTA ATCAATCATC CTCCATTAAT   
  
  
+ TGATTAATTA TTTGCCAAAA ACACAAATCA ACTGGGGCAA CTATAAGAGA TCTACCTGCT TTAAGATATA   
  
  
+ TAAACTCAAT TGTTTCATTT TATTTTATTT TAGATTTTTT GGCCTTTGGT CTCTATTGAG CTAAAATCTC   
  
  
+ GTTATTTTCG GATCATGAAA AATACAACTC GATCTTTTGA TATAAGTCAT CTTTATTTGT AAATTCTGAA   
  
  
+ CTTAAAATCT TACTTGAAGA TCAGGAATAG AAGCACTCGA TTTTTGATAA ATCCTATGTG TTCTTCTTAA   
  
  
+ AATTCATAAT CTAATTCATA TTTTAGATGT ACCCGTTGCC TACTTTGGCA AGTTTAGATT ATGATTTTTA   
  
  
+ TTTTTTTTAA TTACTTGTAT TTTTCTTTTG GATTGATAGT CAATCTACAT CATCTTTGAT CAATTGCGAC   
  
  
+ AGTTTCATTT TCCCAACAAA AATTTCGAGT GTATTCTTTT TAATTTATCA TAAGTATTTA TTGTCAACAT   
  
  
+ TCAACATGCA TCAACAATCA TTAAACACTT TTCGTCACTC CTCAATACTG GTCAGCACTT AAGATTTTGT   
  
  
+ CTTCTAAACT TATCGTAAGT AACTTATTGT TGATTAATTG ATGCTGACCA AATGATACTG AGTGCTAAAA   
  
  
+ TTATAATTAA TAAATACAAT TAAGAGTTGA TCAATGTCGA TGATTCAAAT CAATGTGATT GGTGCTGAAT   
  
  
+ AGTAATTATA AGCACCTATA AAGACTTGAA AAGAACAAAC ATTTAATTAG ATTTTATAAT TATAATTTCC   
  
  
+ TAATATTTAG CATTCATCAG CAAATTCAGC ACACACCAAC ATTAGTCAAC ACCTATCAAC ACTTATAAGT   
  
  
+ ACCAATTAAT AATAATTACC TATAATAGAT TAAAAGTTAG GCCCTTAATG TTTCCCACAT TATTAGATAT   
  
  
+ ATCAAGGGAG GGGGAAAAAA ATAATACTCC CCCATCTTAA AAAATTTATC CCTTTTTTTC TTTTTAATGT   
  
  
+ TCCAAAACAA AAATATGCTT CTAAATTATG ATTGCAATAT GAATGTGTAC GGTTTCAAAC CCAATTTTCT   
  
  
+ CCAACAATTC TTCTACTATA TTGTAATTAT ATTATACTAC AATTTGTATC TCATCTATGA TCTACCCCCC   
  
  
+ AAAAACCAAA TACTCATATA AAGTTTTATT TTCTTTCCAT CCCACTCTAA AAAAAAAAAA TGTTGGGAAA   
  
  
+ AAGTTGATTT ATTTAATTTG AAATGGTACG GTGAATATAG ATTTGTAGAG GGGCAGTTGA TAGAAAAAGG   
  
  
+ TGAAGACAAA GGAAGGGCAC ATTTGCCTTT TCATAGAAGG AAGAGACCAA GGTACTGGGG ACTGGGGGAA   
  
  
+ GGGCTCAAGA GAAGAGAGAG ACTGAGAAGA GTGCTGCTAC TCCTTCCTAC CTACACACAA AATTGCTGCG   
  
  
+ GGGGGTTCAA GTTCAACTTG AACGAGGCAA ATACCCTCAT TGAACCCACC AAATCGAAAT CTTCAATGTA   
  
  
+ CACCCCCATT ATACACGCAT TCAGAGACAT AGATGTCGAG GGGGAGATGA ATTAGCAAGA AGATGTCGAG   
  
  
+ CTAAAATGCA TTTCGAGGTT ACTAGAGCCA GAAATGTGGG CACATCCCAG AAGATCTCAG TAGAGAGAGT   
  
  
+ AACTTCAGAA AAGTTTCAAT TTTTATTCGA AATTTGCCTC TTGTTTGTGT AGATATGATG GGAACTTCGC   
  
  
+ CTTACAATCT GCAATGTGAG GGTTTGGTTG AAGGAGTTGC AGTCAATCCT CAATTTTATG AATCAACCCC   
  
  
+ AAAGTGGAAG AAGGGCATCA AAGAAGAGTC AATTGAGAGC GAACCCACTT CGGTTTTGGA CAACCCTAGC   
  
  
+ CCTCCAAATT CTGCATCTAC TCTCTCTTCC TGCTTCAATG GTGGAAGTAG TGGGCTTGGG ATGGACGATT   
  
  
+ TGGAGGGTTT GTCTTTTGGA GAAGGGTCCC TTCTCCCTTG GTTCATGGGT GAAATTGAAG ACCCTAGTTT   
  
  
+ GAGTTTCAAG CACCTCCTTC AACCTTCAAA TCCCTTGGAG TTTGGTGGCA ATGATGGGCT TGAAGCAGCT   
  
  
+ ATTCAGAGTG CTGGTATGGG TAATTTTAAT TTGGTTGAAA GTTTTAATTC TGATTTGAGG TCTTGTAATT   
  
  
+ CTGGGTCTAT TTGCAATGGG GGGAATGATT CTGTCAATGA AAAGGGATGT GTTCTTAATT ATGCTTCAGA   
  
  
+ GCAGGGTCAT GTGGTTGCTG TATCTCCAGT AGAACAGTTT AGGATTAGTG ATGTGAAACA TGAGGTGTTG   
  
  
+ AACCCAAAAT TTGGCTTTTG TGAGCAAGGC TCTACAATCA GTCAGAACTC TGGTTATGTG CATATACTGG   
  
  
+ GTTATAACCA GCTAGAATCT CCCCTTGAGC CTCCGGCAAA GCGCCACAAT GCTGGGAATG CTCTTTACAC   
  
  
+ TAGTGTTGTT CCAGTTCCAA AGAGTTCGTT TTTAGGTGAG GATCATGAAA TTCTACTCAG AAATCAAGAG   
  
  
+ CAGCAGTTTC TGATGCAACA ACAGTGGTTA ATGGGTTTAG CCCCTCAGTT TACACCCCAG CACCCGCCAA   
  
  
+ AGCCATTAGA CGACCGAAAG CAACCAAACT CGCAAACTTT GGTTCTGCGC GATCAGTTAG TTAAGATTGC   
  
  
+ GGAGATGTTC CAAACCGGTA ACTTTTCGCT TGCCCAAGTG ATATTGGCCC GGCTCAATCA GCAATTGTCT   
  
  
+ CTTTCAGGGA ACCCCTTTGT AAGAGCTGCT ACTTTCCATG TCAAGGAGGA ACTTGAAAGG CTCCTGACTA   
  
  
+ TCAATAGCTT CTCTGCGGCT CCACCCCAAC CTAAAAGCCT TACTCTGTCA GATGTTGTGG ATAGGATGAA   
  
  
+ TGCATACAAG CTTTTTTCAG AGGCATCCCC AGTTATGCAA TTTACCGATT TTACTTGTAC CCAGGCTTTG   
  
  
+ CTCGAAGCTC TTGATGATGC TGATTATATC CACATACTAG ATTTTGACAT TGGTTGTGGT TCCCAATGGG   
  
  
+ CATCCTTTAT TCGAGAGCTT CCTCTCAGGA AAAGGGGCAC TCCATTTTTG AAAATTACAG CCTTTGCCTC   
  
  
+ TTCTTTAACC CATAATCCAT TTGAACTAGC CCTTGTATGT GATAACATTC TGGAATTTGC AAAAGAGTTC   
  
  
+ GGTGTCTCTG TCGACCTTCA GGTCATGAAT TTGGACTTGT TTGACCCAAC TACTTGCACA ATACCCAATT   
  
  
+ TTCAATCCAC TGAGAACGCA GCAGTTGCTG TCAACTTCCC TATATGGGCG TGCTCACACT GCCCGTTTGT   
  
  
+ ACTCAGTCAA CTCCTGTCCT TCATCAAGCA ATGTTCCCCA AAAATCATGA CGAGCTTTGA TAGAGGTTGT   
  
  
+ GATCATTTTG AGTTGTCTTT TCCATCGCAC ATCATCCGTG TATTGGACTC ATGCTCTAAT ATGTTGGAGT   
  
  
+ CCCTCCATGG TTCAAGCGTG ACATTAGACA CCTCAAAGAA GATTGAAAAG TTCTTGATCA AGCCTATAAT   
  
  
+ TGAGAGGGCA ATATTGGGGC GAATTCATGC ACCAGACAAG ATGCAGGCAA ACAAGGCACA CCGGTGGAAG   
  
  
+ AATCTCTTCA CTTCTGCTGG GTTCTTCCCT TTGCCATTTA GTAGTTTCAC TGAATCTCAG GCCAATCTAG   
  
  
+ TGGCTCAACG AACCCCAGTG AGGGGTTTTC AGGTGGAAAA GCAGCAAGCA TCACTTGTGC TCAGGTGGAT   
  
  
+ GTCTCATGAG CTCGTGGCAG CTTCAGCTTG GAGGTGCTA  

- -Up\_Stream \_Len000AAACAT AAATTTAAAA TGTTATAAAT ATTTTTAAAA CATAACTAAT TAACACGATT   
  
  
- GATTGTGATT ATTAACCCCC TCTTTTCTTC CCTTTTATTT TTCTTTAATT CTTCTTCTTT TCTTTTGTGG   
  
  
- AAACTTTTCC GTTTTTTGAT TTTATTTTGT TATTACTTGT CTTCCTCTCT TCCTACCTAT TTGTTGTTTA   
  
  
- CAGCGAAAAT CCTGTCGTTA TCAGATACAC TAACCGGAAT CAAGGACAAC CTCCTAACGA TTGTTTTCGT   
  
  
- TGTCGTTTTT TATTATGATT AATAATGTAT AAAAAATTGA GAATTTCGAT TAGTTAGTAG GAGGTAATTA   
  
  
- ACTAATTAAT AAACGGTTTT TGTGTTTAGT TGACCCCGTT GATATTCTCT AGATGGACGA AATTCTATAT   
  
  
- ATTTGAGTTA ACAAAGTAAA ATAAAATAAA ATCTAAAAAA CCGGAAACCA GAGATAACTC GATTTTAGAG   
  
  
- CAATAAAAGC CTAGTACTTT TTATGTTGAG CTAGAAAACT ATATTCAGTA GAAATAAACA TTTAAGACTT   
  
  
- GAATTTTAGA ATGAACTTCT AGTCCTTATC TTCGTGAGCT AAAAACTATT TAGGATACAC AAGAAGAATT   
  
  
- TTAAGTATTA GATTAAGTAT AAAATCTACA TGGGCAACGG ATGAAACCGT TCAAATCTAA TACTAAAAAT   
  
  
- AAAAAAAATT AATGAACATA AAAAGAAAAC CTAACTATCA GTTAGATGTA GTAGAAACTA GTTAACGCTG   
  
  
- TCAAAGTAAA AGGGTTGTTT TTAAAGCTCA CATAAGAAAA ATTAAATAGT ATTCATAAAT AACAGTTGTA   
  
  
- AGTTGTACGT AGTTGTTAGT AATTTGTGAA AAGCAGTGAG GAGTTATGAC CAGTCGTGAA TTCTAAAACA   
  
  
- GAAGATTTGA ATAGCATTCA TTGAATAACA ACTAATTAAC TACGACTGGT TTACTATGAC TCACGATTTT   
  
  
- AATATTAATT ATTTATGTTA ATTCTCAACT AGTTACAGCT ACTAAGTTTA GTTACACTAA CCACGACTTA   
  
  
- TCATTAATAT TCGTGGATAT TTCTGAACTT TTCTTGTTTG TAAATTAATC TAAAATATTA ATATTAAAGG   
  
  
- ATTATAAATC GTAAGTAGTC GTTTAAGTCG TGTGTGGTTG TAATCAGTTG TGGATAGTTG TGAATATTCA   
  
  
- TGGTTAATTA TTATTAATGG ATATTATCTA ATTTTCAATC CGGGAATTAC AAAGGGTGTA ATAATCTATA   
  
  
- TAGTTCCCTC CCCCTTTTTT TATTATGAGG GGGTAGAATT TTTTAAATAG GGAAAAAAAG AAAAATTACA   
  
  
- AGGTTTTGTT TTTATACGAA GATTTAATAC TAACGTTATA CTTACACATG CCAAAGTTTG GGTTAAAAGA   
  
  
- GGTTGTTAAG AAGATGATAT AACATTAATA TAATATGATG TTAAACATAG AGTAGATACT AGATGGGGGG   
  
  
- TTTTTGGTTT ATGAGTATAT TTCAAAATAA AAGAAAGGTA GGGTGAGATT TTTTTTTTTT ACAACCCTTT   
  
  
- TTCAACTAAA TAAATTAAAC TTTACCATGC CACTTATATC TAAACATCTC CCCGTCAACT ATCTTTTTCC   
  
  
- ACTTCTGTTT CCTTCCCGTG TAAACGGAAA AGTATCTTCC TTCTCTGGTT CCATGACCCC TGACCCCCTT   
  
  
- CCCGAGTTCT CTTCTCTCTC TGACTCTTCT CACGACGATG AGGAAGGATG GATGTGTGTT TTAACGACGC   
  
  
- CCCCCAAGTT CAAGTTGAAC TTGCTCCGTT TATGGGAGTA ACTTGGGTGG TTTAGCTTTA GAAGTTACAT   
  
  
- GTGGGGGTAA TATGTGCGTA AGTCTCTGTA TCTACAGCTC CCCCTCTACT TAATCGTTCT TCTACAGCTC   
  
  
- GATTTTACGT AAAGCTCCAA TGATCTCGGT CTTTACACCC GTGTAGGGTC TTCTAGAGTC ATCTCTCTCA   
  
  
- TTGAAGTCTT TTCAAAGTTA AAAATAAGCT TTAAACGGAG AACAAACACA TCTATACTAC CCTTGAAGCG   
  
  
- GAATGTTAGA CGTTACACTC CCAAACCAAC TTCCTCAACG TCAGTTAGGA GTTAAAATAC TTAGTTGGGG   
  
  
- TTTCACCTTC TTCCCGTAGT TTCTTCTCAG TTAACTCTCG CTTGGGTGAA GCCAAAACCT GTTGGGATCG   
  
  
- GGAGGTTTAA GACGTAGATG AGAGAGAAGG ACGAAGTTAC CACCTTCATC ACCCGAACCC TACCTGCTAA   
  
  
- ACCTCCCAAA CAGAAAACCT CTTCCCAGGG AAGAGGGAAC CAAGTACCCA CTTTAACTTC TGGGATCAAA   
  
  
- CTCAAAGTTC GTGGAGGAAG TTGGAAGTTT AGGGAACCTC AAACCACCGT TACTACCCGA ACTTCGTCGA   
  
  
- TAAGTCTCAC GACCATACCC ATTAAAATTA AACCAACTTT CAAAATTAAG ACTAAACTCC AGAACATTAA   
  
  
- GACCCAGATA AACGTTACCC CCCTTACTAA GACAGTTACT TTTCCCTACA CAAGAATTAA TACGAAGTCT   
  
  
- CGTCCCAGTA CACCAACGAC ATAGAGGTCA TCTTGTCAAA TCCTAATCAC TACACTTTGT ACTCCACAAC   
  
  
- TTGGGTTTTA AACCGAAAAC ACTCGTTCCG AGATGTTAGT CAGTCTTGAG ACCAATACAC GTATATGACC   
  
  
- CAATATTGGT CGATCTTAGA GGGGAACTCG GAGGCCGTTT CGCGGTGTTA CGACCCTTAC GAGAAATGTG   
  
  
- ATCACAACAA GGTCAAGGTT TCTCAAGCAA AAATCCACTC CTAGTACTTT AAGATGAGTC TTTAGTTCTC   
  
  
- GTCGTCAAAG ACTACGTTGT TGTCACCAAT TACCCAAATC GGGGAGTCAA ATGTGGGGTC GTGGGCGGTT   
  
  
- TCGGTAATCT GCTGGCTTTC GTTGGTTTGA GCGTTTGAAA CCAAGACGCG CTAGTCAATC AATTCTAACG   
  
  
- CCTCTACAAG GTTTGGCCAT TGAAAAGCGA ACGGGTTCAC TATAACCGGG CCGAGTTAGT CGTTAACAGA   
  
  
- GAAAGTCCCT TGGGGAAACA TTCTCGACGA TGAAAGGTAC AGTTCCTCCT TGAACTTTCC GAGGACTGAT   
  
  
- AGTTATCGAA GAGACGCCGA GGTGGGGTTG GATTTTCGGA ATGAGACAGT CTACAACACC TATCCTACTT   
  
  
- ACGTATGTTC GAAAAAAGTC TCCGTAGGGG TCAATACGTT AAATGGCTAA AATGAACATG GGTCCGAAAC   
  
  
- GAGCTTCGAG AACTACTACG ACTAATATAG GTGTATGATC TAAAACTGTA ACCAACACCA AGGGTTACCC   
  
  
- GTAGGAAATA AGCTCTCGAA GGAGAGTCCT TTTCCCCGTG AGGTAAAAAC TTTTAATGTC GGAAACGGAG   
  
  
- AAGAAATTGG GTATTAGGTA AACTTGATCG GGAACATACA CTATTGTAAG ACCTTAAACG TTTTCTCAAG   
  
  
- CCACAGAGAC AGCTGGAAGT CCAGTACTTA AACCTGAACA AACTGGGTTG ATGAACGTGT TATGGGTTAA   
  
  
- AAGTTAGGTG ACTCTTGCGT CGTCAACGAC AGTTGAAGGG ATATACCCGC ACGAGTGTGA CGGGCAAACA   
  
  
- TGAGTCAGTT GAGGACAGGA AGTAGTTCGT TACAAGGGGT TTTTAGTACT GCTCGAAACT ATCTCCAACA   
  
  
- CTAGTAAAAC TCAACAGAAA AGGTAGCGTG TAGTAGGCAC ATAACCTGAG TACGAGATTA TACAACCTCA   
  
  
- GGGAGGTACC AAGTTCGCAC TGTAATCTGT GGAGTTTCTT CTAACTTTTC AAGAACTAGT TCGGATATTA   
  
  
- ACTCTCCCGT TATAACCCCG CTTAAGTACG TGGTCTGTTC TACGTCCGTT TGTTCCGTGT GGCCACCTTC   
  
  
- TTAGAGAAGT GAAGACGACC CAAGAAGGGA AACGGTAAAT CATCAAAGTG ACTTAGAGTC CGGTTAGATC   
  
  
- ACCGAGTTGC TTGGGGTCAC TCCCCAAAAG TCCACCTTTT CGTCGTTCGT AGTGAACACG AGTCCACCTA   
  
  
- CAGAGTACTC GAGCACCGTC GAAGTCGAAC CTCCACGAT

+     AAGAA-motif

| Site Name | Organism | Position | Strand | Matrix score. | sequence | function |
| --- | --- | --- | --- | --- | --- | --- |
| AAGAA-motif | Avena sativa | 1505 | - | 7 | GAAAGAA |  |

>HU04G00048.1   
+ -Up\_Stream \_Len000TTTGTA TTTAAATTTT ACAATATTTA TAAAAATTTT GTATTGATTA ATTGTGCTAA   
  
  
+ CTAACACTAA TAATTGGGGG AGAAAAGAAG GGAAAATAAA AAGAAATTAA GAAGAAGAAA AGAAAACACC   
  
  
+ TTTGAAAAGG CAAAAAACTA AAATAAAACA ATAATGAACA GAAGGAGAGA AGGATGGATA AACAACAAAT   
  
  
+ GTCGCTTTTA GGACAGCAAT AGTCTATGTG ATTGGCCTTA GTTCCTGTTG GAGGATTGCT AACAAAAGCA   
  
  
+ ACAGCAAAAA ATAATACTAA TTATTACATA TTTTTTAACT CTTAAAGCTA ATCAATCATC CTCCATTAAT   
  
  
+ TGATTAATTA TTTGCCAAAA ACACAAATCA ACTGGGGCAA CTATAAGAGA TCTACCTGCT TTAAGATATA   
  
  
+ TAAACTCAAT TGTTTCATTT TATTTTATTT TAGATTTTTT GGCCTTTGGT CTCTATTGAG CTAAAATCTC   
  
  
+ GTTATTTTCG GATCATGAAA AATACAACTC GATCTTTTGA TATAAGTCAT CTTTATTTGT AAATTCTGAA   
  
  
+ CTTAAAATCT TACTTGAAGA TCAGGAATAG AAGCACTCGA TTTTTGATAA ATCCTATGTG TTCTTCTTAA   
  
  
+ AATTCATAAT CTAATTCATA TTTTAGATGT ACCCGTTGCC TACTTTGGCA AGTTTAGATT ATGATTTTTA   
  
  
+ TTTTTTTTAA TTACTTGTAT TTTTCTTTTG GATTGATAGT CAATCTACAT CATCTTTGAT CAATTGCGAC   
  
  
+ AGTTTCATTT TCCCAACAAA AATTTCGAGT GTATTCTTTT TAATTTATCA TAAGTATTTA TTGTCAACAT   
  
  
+ TCAACATGCA TCAACAATCA TTAAACACTT TTCGTCACTC CTCAATACTG GTCAGCACTT AAGATTTTGT   
  
  
+ CTTCTAAACT TATCGTAAGT AACTTATTGT TGATTAATTG ATGCTGACCA AATGATACTG AGTGCTAAAA   
  
  
+ TTATAATTAA TAAATACAAT TAAGAGTTGA TCAATGTCGA TGATTCAAAT CAATGTGATT GGTGCTGAAT   
  
  
+ AGTAATTATA AGCACCTATA AAGACTTGAA AAGAACAAAC ATTTAATTAG ATTTTATAAT TATAATTTCC   
  
  
+ TAATATTTAG CATTCATCAG CAAATTCAGC ACACACCAAC ATTAGTCAAC ACCTATCAAC ACTTATAAGT   
  
  
+ ACCAATTAAT AATAATTACC TATAATAGAT TAAAAGTTAG GCCCTTAATG TTTCCCACAT TATTAGATAT   
  
  
+ ATCAAGGGAG GGGGAAAAAA ATAATACTCC CCCATCTTAA AAAATTTATC CCTTTTTTTC TTTTTAATGT   
  
  
+ TCCAAAACAA AAATATGCTT CTAAATTATG ATTGCAATAT GAATGTGTAC GGTTTCAAAC CCAATTTTCT   
  
  
+ CCAACAATTC TTCTACTATA TTGTAATTAT ATTATACTAC AATTTGTATC TCATCTATGA TCTACCCCCC   
  
  
+ AAAAACCAAA TACTCATATA AAGTTTTATT TTCTTTCCAT CCCACTCTAA AAAAAAAAAA TGTTGGGAAA   
  
  
+ AAGTTGATTT ATTTAATTTG AAATGGTACG GTGAATATAG ATTTGTAGAG GGGCAGTTGA TAGAAAAAGG   
  
  
+ TGAAGACAAA GGAAGGGCAC ATTTGCCTTT TCATAGAAGG AAGAGACCAA GGTACTGGGG ACTGGGGGAA   
  
  
+ GGGCTCAAGA GAAGAGAGAG ACTGAGAAGA GTGCTGCTAC TCCTTCCTAC CTACACACAA AATTGCTGCG   
  
  
+ GGGGGTTCAA GTTCAACTTG AACGAGGCAA ATACCCTCAT TGAACCCACC AAATCGAAAT CTTCAATGTA   
  
  
+ CACCCCCATT ATACACGCAT TCAGAGACAT AGATGTCGAG GGGGAGATGA ATTAGCAAGA AGATGTCGAG   
  
  
+ CTAAAATGCA TTTCGAGGTT ACTAGAGCCA GAAATGTGGG CACATCCCAG AAGATCTCAG TAGAGAGAGT   
  
  
+ AACTTCAGAA AAGTTTCAAT TTTTATTCGA AATTTGCCTC TTGTTTGTGT AGATATGATG GGAACTTCGC   
  
  
+ CTTACAATCT GCAATGTGAG GGTTTGGTTG AAGGAGTTGC AGTCAATCCT CAATTTTATG AATCAACCCC   
  
  
+ AAAGTGGAAG AAGGGCATCA AAGAAGAGTC AATTGAGAGC GAACCCACTT CGGTTTTGGA CAACCCTAGC   
  
  
+ CCTCCAAATT CTGCATCTAC TCTCTCTTCC TGCTTCAATG GTGGAAGTAG TGGGCTTGGG ATGGACGATT   
  
  
+ TGGAGGGTTT GTCTTTTGGA GAAGGGTCCC TTCTCCCTTG GTTCATGGGT GAAATTGAAG ACCCTAGTTT   
  
  
+ GAGTTTCAAG CACCTCCTTC AACCTTCAAA TCCCTTGGAG TTTGGTGGCA ATGATGGGCT TGAAGCAGCT   
  
  
+ ATTCAGAGTG CTGGTATGGG TAATTTTAAT TTGGTTGAAA GTTTTAATTC TGATTTGAGG TCTTGTAATT   
  
  
+ CTGGGTCTAT TTGCAATGGG GGGAATGATT CTGTCAATGA AAAGGGATGT GTTCTTAATT ATGCTTCAGA   
  
  
+ GCAGGGTCAT GTGGTTGCTG TATCTCCAGT AGAACAGTTT AGGATTAGTG ATGTGAAACA TGAGGTGTTG   
  
  
+ AACCCAAAAT TTGGCTTTTG TGAGCAAGGC TCTACAATCA GTCAGAACTC TGGTTATGTG CATATACTGG   
  
  
+ GTTATAACCA GCTAGAATCT CCCCTTGAGC CTCCGGCAAA GCGCCACAAT GCTGGGAATG CTCTTTACAC   
  
  
+ TAGTGTTGTT CCAGTTCCAA AGAGTTCGTT TTTAGGTGAG GATCATGAAA TTCTACTCAG AAATCAAGAG   
  
  
+ CAGCAGTTTC TGATGCAACA ACAGTGGTTA ATGGGTTTAG CCCCTCAGTT TACACCCCAG CACCCGCCAA   
  
  
+ AGCCATTAGA CGACCGAAAG CAACCAAACT CGCAAACTTT GGTTCTGCGC GATCAGTTAG TTAAGATTGC   
  
  
+ GGAGATGTTC CAAACCGGTA ACTTTTCGCT TGCCCAAGTG ATATTGGCCC GGCTCAATCA GCAATTGTCT   
  
  
+ CTTTCAGGGA ACCCCTTTGT AAGAGCTGCT ACTTTCCATG TCAAGGAGGA ACTTGAAAGG CTCCTGACTA   
  
  
+ TCAATAGCTT CTCTGCGGCT CCACCCCAAC CTAAAAGCCT TACTCTGTCA GATGTTGTGG ATAGGATGAA   
  
  
+ TGCATACAAG CTTTTTTCAG AGGCATCCCC AGTTATGCAA TTTACCGATT TTACTTGTAC CCAGGCTTTG   
  
  
+ CTCGAAGCTC TTGATGATGC TGATTATATC CACATACTAG ATTTTGACAT TGGTTGTGGT TCCCAATGGG   
  
  
+ CATCCTTTAT TCGAGAGCTT CCTCTCAGGA AAAGGGGCAC TCCATTTTTG AAAATTACAG CCTTTGCCTC   
  
  
+ TTCTTTAACC CATAATCCAT TTGAACTAGC CCTTGTATGT GATAACATTC TGGAATTTGC AAAAGAGTTC   
  
  
+ GGTGTCTCTG TCGACCTTCA GGTCATGAAT TTGGACTTGT TTGACCCAAC TACTTGCACA ATACCCAATT   
  
  
+ TTCAATCCAC TGAGAACGCA GCAGTTGCTG TCAACTTCCC TATATGGGCG TGCTCACACT GCCCGTTTGT   
  
  
+ ACTCAGTCAA CTCCTGTCCT TCATCAAGCA ATGTTCCCCA AAAATCATGA CGAGCTTTGA TAGAGGTTGT   
  
  
+ GATCATTTTG AGTTGTCTTT TCCATCGCAC ATCATCCGTG TATTGGACTC ATGCTCTAAT ATGTTGGAGT   
  
  
+ CCCTCCATGG TTCAAGCGTG ACATTAGACA CCTCAAAGAA GATTGAAAAG TTCTTGATCA AGCCTATAAT   
  
  
+ TGAGAGGGCA ATATTGGGGC GAATTCATGC ACCAGACAAG ATGCAGGCAA ACAAGGCACA CCGGTGGAAG   
  
  
+ AATCTCTTCA CTTCTGCTGG GTTCTTCCCT TTGCCATTTA GTAGTTTCAC TGAATCTCAG GCCAATCTAG   
  
  
+ TGGCTCAACG AACCCCAGTG AGGGGTTTTC AGGTGGAAAA GCAGCAAGCA TCACTTGTGC TCAGGTGGAT   
  
  
+ GTCTCATGAG CTCGTGGCAG CTTCAGCTTG GAGGTGCTA  

- -Up\_Stream \_Len000AAACAT AAATTTAAAA TGTTATAAAT ATTTTTAAAA CATAACTAAT TAACACGATT   
  
  
- GATTGTGATT ATTAACCCCC TCTTTTCTTC CCTTTTATTT TTCTTTAATT CTTCTTCTTT TCTTTTGTGG   
  
  
- AAACTTTTCC GTTTTTTGAT TTTATTTTGT TATTACTTGT CTTCCTCTCT TCCTACCTAT TTGTTGTTTA   
  
  
- CAGCGAAAAT CCTGTCGTTA TCAGATACAC TAACCGGAAT CAAGGACAAC CTCCTAACGA TTGTTTTCGT   
  
  
- TGTCGTTTTT TATTATGATT AATAATGTAT AAAAAATTGA GAATTTCGAT TAGTTAGTAG GAGGTAATTA   
  
  
- ACTAATTAAT AAACGGTTTT TGTGTTTAGT TGACCCCGTT GATATTCTCT AGATGGACGA AATTCTATAT   
  
  
- ATTTGAGTTA ACAAAGTAAA ATAAAATAAA ATCTAAAAAA CCGGAAACCA GAGATAACTC GATTTTAGAG   
  
  
- CAATAAAAGC CTAGTACTTT TTATGTTGAG CTAGAAAACT ATATTCAGTA GAAATAAACA TTTAAGACTT   
  
  
- GAATTTTAGA ATGAACTTCT AGTCCTTATC TTCGTGAGCT AAAAACTATT TAGGATACAC AAGAAGAATT   
  
  
- TTAAGTATTA GATTAAGTAT AAAATCTACA TGGGCAACGG ATGAAACCGT TCAAATCTAA TACTAAAAAT   
  
  
- AAAAAAAATT AATGAACATA AAAAGAAAAC CTAACTATCA GTTAGATGTA GTAGAAACTA GTTAACGCTG   
  
  
- TCAAAGTAAA AGGGTTGTTT TTAAAGCTCA CATAAGAAAA ATTAAATAGT ATTCATAAAT AACAGTTGTA   
  
  
- AGTTGTACGT AGTTGTTAGT AATTTGTGAA AAGCAGTGAG GAGTTATGAC CAGTCGTGAA TTCTAAAACA   
  
  
- GAAGATTTGA ATAGCATTCA TTGAATAACA ACTAATTAAC TACGACTGGT TTACTATGAC TCACGATTTT   
  
  
- AATATTAATT ATTTATGTTA ATTCTCAACT AGTTACAGCT ACTAAGTTTA GTTACACTAA CCACGACTTA   
  
  
- TCATTAATAT TCGTGGATAT TTCTGAACTT TTCTTGTTTG TAAATTAATC TAAAATATTA ATATTAAAGG   
  
  
- ATTATAAATC GTAAGTAGTC GTTTAAGTCG TGTGTGGTTG TAATCAGTTG TGGATAGTTG TGAATATTCA   
  
  
- TGGTTAATTA TTATTAATGG ATATTATCTA ATTTTCAATC CGGGAATTAC AAAGGGTGTA ATAATCTATA   
  
  
- TAGTTCCCTC CCCCTTTTTT TATTATGAGG GGGTAGAATT TTTTAAATAG GGAAAAAAAG AAAAATTACA   
  
  
- AGGTTTTGTT TTTATACGAA GATTTAATAC TAACGTTATA CTTACACATG CCAAAGTTTG GGTTAAAAGA   
  
  
- GGTTGTTAAG AAGATGATAT AACATTAATA TAATATGATG TTAAACATAG AGTAGATACT AGATGGGGGG   
  
  
- TTTTTGGTTT ATGAGTATAT TTCAAAATAA AAGAAAGGTA GGGTGAGATT TTTTTTTTTT ACAACCCTTT   
  
  
- TTCAACTAAA TAAATTAAAC TTTACCATGC CACTTATATC TAAACATCTC CCCGTCAACT ATCTTTTTCC   
  
  
- ACTTCTGTTT CCTTCCCGTG TAAACGGAAA AGTATCTTCC TTCTCTGGTT CCATGACCCC TGACCCCCTT   
  
  
- CCCGAGTTCT CTTCTCTCTC TGACTCTTCT CACGACGATG AGGAAGGATG GATGTGTGTT TTAACGACGC   
  
  
- CCCCCAAGTT CAAGTTGAAC TTGCTCCGTT TATGGGAGTA ACTTGGGTGG TTTAGCTTTA GAAGTTACAT   
  
  
- GTGGGGGTAA TATGTGCGTA AGTCTCTGTA TCTACAGCTC CCCCTCTACT TAATCGTTCT TCTACAGCTC   
  
  
- GATTTTACGT AAAGCTCCAA TGATCTCGGT CTTTACACCC GTGTAGGGTC TTCTAGAGTC ATCTCTCTCA   
  
  
- TTGAAGTCTT TTCAAAGTTA AAAATAAGCT TTAAACGGAG AACAAACACA TCTATACTAC CCTTGAAGCG   
  
  
- GAATGTTAGA CGTTACACTC CCAAACCAAC TTCCTCAACG TCAGTTAGGA GTTAAAATAC TTAGTTGGGG   
  
  
- TTTCACCTTC TTCCCGTAGT TTCTTCTCAG TTAACTCTCG CTTGGGTGAA GCCAAAACCT GTTGGGATCG   
  
  
- GGAGGTTTAA GACGTAGATG AGAGAGAAGG ACGAAGTTAC CACCTTCATC ACCCGAACCC TACCTGCTAA   
  
  
- ACCTCCCAAA CAGAAAACCT CTTCCCAGGG AAGAGGGAAC CAAGTACCCA CTTTAACTTC TGGGATCAAA   
  
  
- CTCAAAGTTC GTGGAGGAAG TTGGAAGTTT AGGGAACCTC AAACCACCGT TACTACCCGA ACTTCGTCGA   
  
  
- TAAGTCTCAC GACCATACCC ATTAAAATTA AACCAACTTT CAAAATTAAG ACTAAACTCC AGAACATTAA   
  
  
- GACCCAGATA AACGTTACCC CCCTTACTAA GACAGTTACT TTTCCCTACA CAAGAATTAA TACGAAGTCT   
  
  
- CGTCCCAGTA CACCAACGAC ATAGAGGTCA TCTTGTCAAA TCCTAATCAC TACACTTTGT ACTCCACAAC   
  
  
- TTGGGTTTTA AACCGAAAAC ACTCGTTCCG AGATGTTAGT CAGTCTTGAG ACCAATACAC GTATATGACC   
  
  
- CAATATTGGT CGATCTTAGA GGGGAACTCG GAGGCCGTTT CGCGGTGTTA CGACCCTTAC GAGAAATGTG   
  
  
- ATCACAACAA GGTCAAGGTT TCTCAAGCAA AAATCCACTC CTAGTACTTT AAGATGAGTC TTTAGTTCTC   
  
  
- GTCGTCAAAG ACTACGTTGT TGTCACCAAT TACCCAAATC GGGGAGTCAA ATGTGGGGTC GTGGGCGGTT   
  
  
- TCGGTAATCT GCTGGCTTTC GTTGGTTTGA GCGTTTGAAA CCAAGACGCG CTAGTCAATC AATTCTAACG   
  
  
- CCTCTACAAG GTTTGGCCAT TGAAAAGCGA ACGGGTTCAC TATAACCGGG CCGAGTTAGT CGTTAACAGA   
  
  
- GAAAGTCCCT TGGGGAAACA TTCTCGACGA TGAAAGGTAC AGTTCCTCCT TGAACTTTCC GAGGACTGAT   
  
  
- AGTTATCGAA GAGACGCCGA GGTGGGGTTG GATTTTCGGA ATGAGACAGT CTACAACACC TATCCTACTT   
  
  
- ACGTATGTTC GAAAAAAGTC TCCGTAGGGG TCAATACGTT AAATGGCTAA AATGAACATG GGTCCGAAAC   
  
  
- GAGCTTCGAG AACTACTACG ACTAATATAG GTGTATGATC TAAAACTGTA ACCAACACCA AGGGTTACCC   
  
  
- GTAGGAAATA AGCTCTCGAA GGAGAGTCCT TTTCCCCGTG AGGTAAAAAC TTTTAATGTC GGAAACGGAG   
  
  
- AAGAAATTGG GTATTAGGTA AACTTGATCG GGAACATACA CTATTGTAAG ACCTTAAACG TTTTCTCAAG   
  
  
- CCACAGAGAC AGCTGGAAGT CCAGTACTTA AACCTGAACA AACTGGGTTG ATGAACGTGT TATGGGTTAA   
  
  
- AAGTTAGGTG ACTCTTGCGT CGTCAACGAC AGTTGAAGGG ATATACCCGC ACGAGTGTGA CGGGCAAACA   
  
  
- TGAGTCAGTT GAGGACAGGA AGTAGTTCGT TACAAGGGGT TTTTAGTACT GCTCGAAACT ATCTCCAACA   
  
  
- CTAGTAAAAC TCAACAGAAA AGGTAGCGTG TAGTAGGCAC ATAACCTGAG TACGAGATTA TACAACCTCA   
  
  
- GGGAGGTACC AAGTTCGCAC TGTAATCTGT GGAGTTTCTT CTAACTTTTC AAGAACTAGT TCGGATATTA   
  
  
- ACTCTCCCGT TATAACCCCG CTTAAGTACG TGGTCTGTTC TACGTCCGTT TGTTCCGTGT GGCCACCTTC   
  
  
- TTAGAGAAGT GAAGACGACC CAAGAAGGGA AACGGTAAAT CATCAAAGTG ACTTAGAGTC CGGTTAGATC   
  
  
- ACCGAGTTGC TTGGGGTCAC TCCCCAAAAG TCCACCTTTT CGTCGTTCGT AGTGAACACG AGTCCACCTA   
  
  
- CAGAGTACTC GAGCACCGTC GAAGTCGAAC CTCCACGAT

+     ACA-motif

| Site Name | Organism | Position | Strand | Matrix score. | sequence | function |
| --- | --- | --- | --- | --- | --- | --- |
| ACA-motif | Pisum sativum | 3347 | + | 12 | AATTACAGCCATT | part of gapA in (gapA-CMA1) involved with light responsiveness |

>HU04G00048.1   
+ -Up\_Stream \_Len000TTTGTA TTTAAATTTT ACAATATTTA TAAAAATTTT GTATTGATTA ATTGTGCTAA   
  
  
+ CTAACACTAA TAATTGGGGG AGAAAAGAAG GGAAAATAAA AAGAAATTAA GAAGAAGAAA AGAAAACACC   
  
  
+ TTTGAAAAGG CAAAAAACTA AAATAAAACA ATAATGAACA GAAGGAGAGA AGGATGGATA AACAACAAAT   
  
  
+ GTCGCTTTTA GGACAGCAAT AGTCTATGTG ATTGGCCTTA GTTCCTGTTG GAGGATTGCT AACAAAAGCA   
  
  
+ ACAGCAAAAA ATAATACTAA TTATTACATA TTTTTTAACT CTTAAAGCTA ATCAATCATC CTCCATTAAT   
  
  
+ TGATTAATTA TTTGCCAAAA ACACAAATCA ACTGGGGCAA CTATAAGAGA TCTACCTGCT TTAAGATATA   
  
  
+ TAAACTCAAT TGTTTCATTT TATTTTATTT TAGATTTTTT GGCCTTTGGT CTCTATTGAG CTAAAATCTC   
  
  
+ GTTATTTTCG GATCATGAAA AATACAACTC GATCTTTTGA TATAAGTCAT CTTTATTTGT AAATTCTGAA   
  
  
+ CTTAAAATCT TACTTGAAGA TCAGGAATAG AAGCACTCGA TTTTTGATAA ATCCTATGTG TTCTTCTTAA   
  
  
+ AATTCATAAT CTAATTCATA TTTTAGATGT ACCCGTTGCC TACTTTGGCA AGTTTAGATT ATGATTTTTA   
  
  
+ TTTTTTTTAA TTACTTGTAT TTTTCTTTTG GATTGATAGT CAATCTACAT CATCTTTGAT CAATTGCGAC   
  
  
+ AGTTTCATTT TCCCAACAAA AATTTCGAGT GTATTCTTTT TAATTTATCA TAAGTATTTA TTGTCAACAT   
  
  
+ TCAACATGCA TCAACAATCA TTAAACACTT TTCGTCACTC CTCAATACTG GTCAGCACTT AAGATTTTGT   
  
  
+ CTTCTAAACT TATCGTAAGT AACTTATTGT TGATTAATTG ATGCTGACCA AATGATACTG AGTGCTAAAA   
  
  
+ TTATAATTAA TAAATACAAT TAAGAGTTGA TCAATGTCGA TGATTCAAAT CAATGTGATT GGTGCTGAAT   
  
  
+ AGTAATTATA AGCACCTATA AAGACTTGAA AAGAACAAAC ATTTAATTAG ATTTTATAAT TATAATTTCC   
  
  
+ TAATATTTAG CATTCATCAG CAAATTCAGC ACACACCAAC ATTAGTCAAC ACCTATCAAC ACTTATAAGT   
  
  
+ ACCAATTAAT AATAATTACC TATAATAGAT TAAAAGTTAG GCCCTTAATG TTTCCCACAT TATTAGATAT   
  
  
+ ATCAAGGGAG GGGGAAAAAA ATAATACTCC CCCATCTTAA AAAATTTATC CCTTTTTTTC TTTTTAATGT   
  
  
+ TCCAAAACAA AAATATGCTT CTAAATTATG ATTGCAATAT GAATGTGTAC GGTTTCAAAC CCAATTTTCT   
  
  
+ CCAACAATTC TTCTACTATA TTGTAATTAT ATTATACTAC AATTTGTATC TCATCTATGA TCTACCCCCC   
  
  
+ AAAAACCAAA TACTCATATA AAGTTTTATT TTCTTTCCAT CCCACTCTAA AAAAAAAAAA TGTTGGGAAA   
  
  
+ AAGTTGATTT ATTTAATTTG AAATGGTACG GTGAATATAG ATTTGTAGAG GGGCAGTTGA TAGAAAAAGG   
  
  
+ TGAAGACAAA GGAAGGGCAC ATTTGCCTTT TCATAGAAGG AAGAGACCAA GGTACTGGGG ACTGGGGGAA   
  
  
+ GGGCTCAAGA GAAGAGAGAG ACTGAGAAGA GTGCTGCTAC TCCTTCCTAC CTACACACAA AATTGCTGCG   
  
  
+ GGGGGTTCAA GTTCAACTTG AACGAGGCAA ATACCCTCAT TGAACCCACC AAATCGAAAT CTTCAATGTA   
  
  
+ CACCCCCATT ATACACGCAT TCAGAGACAT AGATGTCGAG GGGGAGATGA ATTAGCAAGA AGATGTCGAG   
  
  
+ CTAAAATGCA TTTCGAGGTT ACTAGAGCCA GAAATGTGGG CACATCCCAG AAGATCTCAG TAGAGAGAGT   
  
  
+ AACTTCAGAA AAGTTTCAAT TTTTATTCGA AATTTGCCTC TTGTTTGTGT AGATATGATG GGAACTTCGC   
  
  
+ CTTACAATCT GCAATGTGAG GGTTTGGTTG AAGGAGTTGC AGTCAATCCT CAATTTTATG AATCAACCCC   
  
  
+ AAAGTGGAAG AAGGGCATCA AAGAAGAGTC AATTGAGAGC GAACCCACTT CGGTTTTGGA CAACCCTAGC   
  
  
+ CCTCCAAATT CTGCATCTAC TCTCTCTTCC TGCTTCAATG GTGGAAGTAG TGGGCTTGGG ATGGACGATT   
  
  
+ TGGAGGGTTT GTCTTTTGGA GAAGGGTCCC TTCTCCCTTG GTTCATGGGT GAAATTGAAG ACCCTAGTTT   
  
  
+ GAGTTTCAAG CACCTCCTTC AACCTTCAAA TCCCTTGGAG TTTGGTGGCA ATGATGGGCT TGAAGCAGCT   
  
  
+ ATTCAGAGTG CTGGTATGGG TAATTTTAAT TTGGTTGAAA GTTTTAATTC TGATTTGAGG TCTTGTAATT   
  
  
+ CTGGGTCTAT TTGCAATGGG GGGAATGATT CTGTCAATGA AAAGGGATGT GTTCTTAATT ATGCTTCAGA   
  
  
+ GCAGGGTCAT GTGGTTGCTG TATCTCCAGT AGAACAGTTT AGGATTAGTG ATGTGAAACA TGAGGTGTTG   
  
  
+ AACCCAAAAT TTGGCTTTTG TGAGCAAGGC TCTACAATCA GTCAGAACTC TGGTTATGTG CATATACTGG   
  
  
+ GTTATAACCA GCTAGAATCT CCCCTTGAGC CTCCGGCAAA GCGCCACAAT GCTGGGAATG CTCTTTACAC   
  
  
+ TAGTGTTGTT CCAGTTCCAA AGAGTTCGTT TTTAGGTGAG GATCATGAAA TTCTACTCAG AAATCAAGAG   
  
  
+ CAGCAGTTTC TGATGCAACA ACAGTGGTTA ATGGGTTTAG CCCCTCAGTT TACACCCCAG CACCCGCCAA   
  
  
+ AGCCATTAGA CGACCGAAAG CAACCAAACT CGCAAACTTT GGTTCTGCGC GATCAGTTAG TTAAGATTGC   
  
  
+ GGAGATGTTC CAAACCGGTA ACTTTTCGCT TGCCCAAGTG ATATTGGCCC GGCTCAATCA GCAATTGTCT   
  
  
+ CTTTCAGGGA ACCCCTTTGT AAGAGCTGCT ACTTTCCATG TCAAGGAGGA ACTTGAAAGG CTCCTGACTA   
  
  
+ TCAATAGCTT CTCTGCGGCT CCACCCCAAC CTAAAAGCCT TACTCTGTCA GATGTTGTGG ATAGGATGAA   
  
  
+ TGCATACAAG CTTTTTTCAG AGGCATCCCC AGTTATGCAA TTTACCGATT TTACTTGTAC CCAGGCTTTG   
  
  
+ CTCGAAGCTC TTGATGATGC TGATTATATC CACATACTAG ATTTTGACAT TGGTTGTGGT TCCCAATGGG   
  
  
+ CATCCTTTAT TCGAGAGCTT CCTCTCAGGA AAAGGGGCAC TCCATTTTTG AAAATTACAG CCTTTGCCTC   
  
  
+ TTCTTTAACC CATAATCCAT TTGAACTAGC CCTTGTATGT GATAACATTC TGGAATTTGC AAAAGAGTTC   
  
  
+ GGTGTCTCTG TCGACCTTCA GGTCATGAAT TTGGACTTGT TTGACCCAAC TACTTGCACA ATACCCAATT   
  
  
+ TTCAATCCAC TGAGAACGCA GCAGTTGCTG TCAACTTCCC TATATGGGCG TGCTCACACT GCCCGTTTGT   
  
  
+ ACTCAGTCAA CTCCTGTCCT TCATCAAGCA ATGTTCCCCA AAAATCATGA CGAGCTTTGA TAGAGGTTGT   
  
  
+ GATCATTTTG AGTTGTCTTT TCCATCGCAC ATCATCCGTG TATTGGACTC ATGCTCTAAT ATGTTGGAGT   
  
  
+ CCCTCCATGG TTCAAGCGTG ACATTAGACA CCTCAAAGAA GATTGAAAAG TTCTTGATCA AGCCTATAAT   
  
  
+ TGAGAGGGCA ATATTGGGGC GAATTCATGC ACCAGACAAG ATGCAGGCAA ACAAGGCACA CCGGTGGAAG   
  
  
+ AATCTCTTCA CTTCTGCTGG GTTCTTCCCT TTGCCATTTA GTAGTTTCAC TGAATCTCAG GCCAATCTAG   
  
  
+ TGGCTCAACG AACCCCAGTG AGGGGTTTTC AGGTGGAAAA GCAGCAAGCA TCACTTGTGC TCAGGTGGAT   
  
  
+ GTCTCATGAG CTCGTGGCAG CTTCAGCTTG GAGGTGCTA  

- -Up\_Stream \_Len000AAACAT AAATTTAAAA TGTTATAAAT ATTTTTAAAA CATAACTAAT TAACACGATT   
  
  
- GATTGTGATT ATTAACCCCC TCTTTTCTTC CCTTTTATTT TTCTTTAATT CTTCTTCTTT TCTTTTGTGG   
  
  
- AAACTTTTCC GTTTTTTGAT TTTATTTTGT TATTACTTGT CTTCCTCTCT TCCTACCTAT TTGTTGTTTA   
  
  
- CAGCGAAAAT CCTGTCGTTA TCAGATACAC TAACCGGAAT CAAGGACAAC CTCCTAACGA TTGTTTTCGT   
  
  
- TGTCGTTTTT TATTATGATT AATAATGTAT AAAAAATTGA GAATTTCGAT TAGTTAGTAG GAGGTAATTA   
  
  
- ACTAATTAAT AAACGGTTTT TGTGTTTAGT TGACCCCGTT GATATTCTCT AGATGGACGA AATTCTATAT   
  
  
- ATTTGAGTTA ACAAAGTAAA ATAAAATAAA ATCTAAAAAA CCGGAAACCA GAGATAACTC GATTTTAGAG   
  
  
- CAATAAAAGC CTAGTACTTT TTATGTTGAG CTAGAAAACT ATATTCAGTA GAAATAAACA TTTAAGACTT   
  
  
- GAATTTTAGA ATGAACTTCT AGTCCTTATC TTCGTGAGCT AAAAACTATT TAGGATACAC AAGAAGAATT   
  
  
- TTAAGTATTA GATTAAGTAT AAAATCTACA TGGGCAACGG ATGAAACCGT TCAAATCTAA TACTAAAAAT   
  
  
- AAAAAAAATT AATGAACATA AAAAGAAAAC CTAACTATCA GTTAGATGTA GTAGAAACTA GTTAACGCTG   
  
  
- TCAAAGTAAA AGGGTTGTTT TTAAAGCTCA CATAAGAAAA ATTAAATAGT ATTCATAAAT AACAGTTGTA   
  
  
- AGTTGTACGT AGTTGTTAGT AATTTGTGAA AAGCAGTGAG GAGTTATGAC CAGTCGTGAA TTCTAAAACA   
  
  
- GAAGATTTGA ATAGCATTCA TTGAATAACA ACTAATTAAC TACGACTGGT TTACTATGAC TCACGATTTT   
  
  
- AATATTAATT ATTTATGTTA ATTCTCAACT AGTTACAGCT ACTAAGTTTA GTTACACTAA CCACGACTTA   
  
  
- TCATTAATAT TCGTGGATAT TTCTGAACTT TTCTTGTTTG TAAATTAATC TAAAATATTA ATATTAAAGG   
  
  
- ATTATAAATC GTAAGTAGTC GTTTAAGTCG TGTGTGGTTG TAATCAGTTG TGGATAGTTG TGAATATTCA   
  
  
- TGGTTAATTA TTATTAATGG ATATTATCTA ATTTTCAATC CGGGAATTAC AAAGGGTGTA ATAATCTATA   
  
  
- TAGTTCCCTC CCCCTTTTTT TATTATGAGG GGGTAGAATT TTTTAAATAG GGAAAAAAAG AAAAATTACA   
  
  
- AGGTTTTGTT TTTATACGAA GATTTAATAC TAACGTTATA CTTACACATG CCAAAGTTTG GGTTAAAAGA   
  
  
- GGTTGTTAAG AAGATGATAT AACATTAATA TAATATGATG TTAAACATAG AGTAGATACT AGATGGGGGG   
  
  
- TTTTTGGTTT ATGAGTATAT TTCAAAATAA AAGAAAGGTA GGGTGAGATT TTTTTTTTTT ACAACCCTTT   
  
  
- TTCAACTAAA TAAATTAAAC TTTACCATGC CACTTATATC TAAACATCTC CCCGTCAACT ATCTTTTTCC   
  
  
- ACTTCTGTTT CCTTCCCGTG TAAACGGAAA AGTATCTTCC TTCTCTGGTT CCATGACCCC TGACCCCCTT   
  
  
- CCCGAGTTCT CTTCTCTCTC TGACTCTTCT CACGACGATG AGGAAGGATG GATGTGTGTT TTAACGACGC   
  
  
- CCCCCAAGTT CAAGTTGAAC TTGCTCCGTT TATGGGAGTA ACTTGGGTGG TTTAGCTTTA GAAGTTACAT   
  
  
- GTGGGGGTAA TATGTGCGTA AGTCTCTGTA TCTACAGCTC CCCCTCTACT TAATCGTTCT TCTACAGCTC   
  
  
- GATTTTACGT AAAGCTCCAA TGATCTCGGT CTTTACACCC GTGTAGGGTC TTCTAGAGTC ATCTCTCTCA   
  
  
- TTGAAGTCTT TTCAAAGTTA AAAATAAGCT TTAAACGGAG AACAAACACA TCTATACTAC CCTTGAAGCG   
  
  
- GAATGTTAGA CGTTACACTC CCAAACCAAC TTCCTCAACG TCAGTTAGGA GTTAAAATAC TTAGTTGGGG   
  
  
- TTTCACCTTC TTCCCGTAGT TTCTTCTCAG TTAACTCTCG CTTGGGTGAA GCCAAAACCT GTTGGGATCG   
  
  
- GGAGGTTTAA GACGTAGATG AGAGAGAAGG ACGAAGTTAC CACCTTCATC ACCCGAACCC TACCTGCTAA   
  
  
- ACCTCCCAAA CAGAAAACCT CTTCCCAGGG AAGAGGGAAC CAAGTACCCA CTTTAACTTC TGGGATCAAA   
  
  
- CTCAAAGTTC GTGGAGGAAG TTGGAAGTTT AGGGAACCTC AAACCACCGT TACTACCCGA ACTTCGTCGA   
  
  
- TAAGTCTCAC GACCATACCC ATTAAAATTA AACCAACTTT CAAAATTAAG ACTAAACTCC AGAACATTAA   
  
  
- GACCCAGATA AACGTTACCC CCCTTACTAA GACAGTTACT TTTCCCTACA CAAGAATTAA TACGAAGTCT   
  
  
- CGTCCCAGTA CACCAACGAC ATAGAGGTCA TCTTGTCAAA TCCTAATCAC TACACTTTGT ACTCCACAAC   
  
  
- TTGGGTTTTA AACCGAAAAC ACTCGTTCCG AGATGTTAGT CAGTCTTGAG ACCAATACAC GTATATGACC   
  
  
- CAATATTGGT CGATCTTAGA GGGGAACTCG GAGGCCGTTT CGCGGTGTTA CGACCCTTAC GAGAAATGTG   
  
  
- ATCACAACAA GGTCAAGGTT TCTCAAGCAA AAATCCACTC CTAGTACTTT AAGATGAGTC TTTAGTTCTC   
  
  
- GTCGTCAAAG ACTACGTTGT TGTCACCAAT TACCCAAATC GGGGAGTCAA ATGTGGGGTC GTGGGCGGTT   
  
  
- TCGGTAATCT GCTGGCTTTC GTTGGTTTGA GCGTTTGAAA CCAAGACGCG CTAGTCAATC AATTCTAACG   
  
  
- CCTCTACAAG GTTTGGCCAT TGAAAAGCGA ACGGGTTCAC TATAACCGGG CCGAGTTAGT CGTTAACAGA   
  
  
- GAAAGTCCCT TGGGGAAACA TTCTCGACGA TGAAAGGTAC AGTTCCTCCT TGAACTTTCC GAGGACTGAT   
  
  
- AGTTATCGAA GAGACGCCGA GGTGGGGTTG GATTTTCGGA ATGAGACAGT CTACAACACC TATCCTACTT   
  
  
- ACGTATGTTC GAAAAAAGTC TCCGTAGGGG TCAATACGTT AAATGGCTAA AATGAACATG GGTCCGAAAC   
  
  
- GAGCTTCGAG AACTACTACG ACTAATATAG GTGTATGATC TAAAACTGTA ACCAACACCA AGGGTTACCC   
  
  
- GTAGGAAATA AGCTCTCGAA GGAGAGTCCT TTTCCCCGTG AGGTAAAAAC TTTTAATGTC GGAAACGGAG   
  
  
- AAGAAATTGG GTATTAGGTA AACTTGATCG GGAACATACA CTATTGTAAG ACCTTAAACG TTTTCTCAAG   
  
  
- CCACAGAGAC AGCTGGAAGT CCAGTACTTA AACCTGAACA AACTGGGTTG ATGAACGTGT TATGGGTTAA   
  
  
- AAGTTAGGTG ACTCTTGCGT CGTCAACGAC AGTTGAAGGG ATATACCCGC ACGAGTGTGA CGGGCAAACA   
  
  
- TGAGTCAGTT GAGGACAGGA AGTAGTTCGT TACAAGGGGT TTTTAGTACT GCTCGAAACT ATCTCCAACA   
  
  
- CTAGTAAAAC TCAACAGAAA AGGTAGCGTG TAGTAGGCAC ATAACCTGAG TACGAGATTA TACAACCTCA   
  
  
- GGGAGGTACC AAGTTCGCAC TGTAATCTGT GGAGTTTCTT CTAACTTTTC AAGAACTAGT TCGGATATTA   
  
  
- ACTCTCCCGT TATAACCCCG CTTAAGTACG TGGTCTGTTC TACGTCCGTT TGTTCCGTGT GGCCACCTTC   
  
  
- TTAGAGAAGT GAAGACGACC CAAGAAGGGA AACGGTAAAT CATCAAAGTG ACTTAGAGTC CGGTTAGATC   
  
  
- ACCGAGTTGC TTGGGGTCAC TCCCCAAAAG TCCACCTTTT CGTCGTTCGT AGTGAACACG AGTCCACCTA   
  
  
- CAGAGTACTC GAGCACCGTC GAAGTCGAAC CTCCACGAT

+     ARE

| Site Name | Organism | Position | Strand | Matrix score. | sequence | function |
| --- | --- | --- | --- | --- | --- | --- |
| ARE | Zea mays | 1477 | + | 6 | AAACCA | cis-acting regulatory element essential for the anaerobic induction |

>HU04G00048.1   
+ -Up\_Stream \_Len000TTTGTA TTTAAATTTT ACAATATTTA TAAAAATTTT GTATTGATTA ATTGTGCTAA   
  
  
+ CTAACACTAA TAATTGGGGG AGAAAAGAAG GGAAAATAAA AAGAAATTAA GAAGAAGAAA AGAAAACACC   
  
  
+ TTTGAAAAGG CAAAAAACTA AAATAAAACA ATAATGAACA GAAGGAGAGA AGGATGGATA AACAACAAAT   
  
  
+ GTCGCTTTTA GGACAGCAAT AGTCTATGTG ATTGGCCTTA GTTCCTGTTG GAGGATTGCT AACAAAAGCA   
  
  
+ ACAGCAAAAA ATAATACTAA TTATTACATA TTTTTTAACT CTTAAAGCTA ATCAATCATC CTCCATTAAT   
  
  
+ TGATTAATTA TTTGCCAAAA ACACAAATCA ACTGGGGCAA CTATAAGAGA TCTACCTGCT TTAAGATATA   
  
  
+ TAAACTCAAT TGTTTCATTT TATTTTATTT TAGATTTTTT GGCCTTTGGT CTCTATTGAG CTAAAATCTC   
  
  
+ GTTATTTTCG GATCATGAAA AATACAACTC GATCTTTTGA TATAAGTCAT CTTTATTTGT AAATTCTGAA   
  
  
+ CTTAAAATCT TACTTGAAGA TCAGGAATAG AAGCACTCGA TTTTTGATAA ATCCTATGTG TTCTTCTTAA   
  
  
+ AATTCATAAT CTAATTCATA TTTTAGATGT ACCCGTTGCC TACTTTGGCA AGTTTAGATT ATGATTTTTA   
  
  
+ TTTTTTTTAA TTACTTGTAT TTTTCTTTTG GATTGATAGT CAATCTACAT CATCTTTGAT CAATTGCGAC   
  
  
+ AGTTTCATTT TCCCAACAAA AATTTCGAGT GTATTCTTTT TAATTTATCA TAAGTATTTA TTGTCAACAT   
  
  
+ TCAACATGCA TCAACAATCA TTAAACACTT TTCGTCACTC CTCAATACTG GTCAGCACTT AAGATTTTGT   
  
  
+ CTTCTAAACT TATCGTAAGT AACTTATTGT TGATTAATTG ATGCTGACCA AATGATACTG AGTGCTAAAA   
  
  
+ TTATAATTAA TAAATACAAT TAAGAGTTGA TCAATGTCGA TGATTCAAAT CAATGTGATT GGTGCTGAAT   
  
  
+ AGTAATTATA AGCACCTATA AAGACTTGAA AAGAACAAAC ATTTAATTAG ATTTTATAAT TATAATTTCC   
  
  
+ TAATATTTAG CATTCATCAG CAAATTCAGC ACACACCAAC ATTAGTCAAC ACCTATCAAC ACTTATAAGT   
  
  
+ ACCAATTAAT AATAATTACC TATAATAGAT TAAAAGTTAG GCCCTTAATG TTTCCCACAT TATTAGATAT   
  
  
+ ATCAAGGGAG GGGGAAAAAA ATAATACTCC CCCATCTTAA AAAATTTATC CCTTTTTTTC TTTTTAATGT   
  
  
+ TCCAAAACAA AAATATGCTT CTAAATTATG ATTGCAATAT GAATGTGTAC GGTTTCAAAC CCAATTTTCT   
  
  
+ CCAACAATTC TTCTACTATA TTGTAATTAT ATTATACTAC AATTTGTATC TCATCTATGA TCTACCCCCC   
  
  
+ AAAAACCAAA TACTCATATA AAGTTTTATT TTCTTTCCAT CCCACTCTAA AAAAAAAAAA TGTTGGGAAA   
  
  
+ AAGTTGATTT ATTTAATTTG AAATGGTACG GTGAATATAG ATTTGTAGAG GGGCAGTTGA TAGAAAAAGG   
  
  
+ TGAAGACAAA GGAAGGGCAC ATTTGCCTTT TCATAGAAGG AAGAGACCAA GGTACTGGGG ACTGGGGGAA   
  
  
+ GGGCTCAAGA GAAGAGAGAG ACTGAGAAGA GTGCTGCTAC TCCTTCCTAC CTACACACAA AATTGCTGCG   
  
  
+ GGGGGTTCAA GTTCAACTTG AACGAGGCAA ATACCCTCAT TGAACCCACC AAATCGAAAT CTTCAATGTA   
  
  
+ CACCCCCATT ATACACGCAT TCAGAGACAT AGATGTCGAG GGGGAGATGA ATTAGCAAGA AGATGTCGAG   
  
  
+ CTAAAATGCA TTTCGAGGTT ACTAGAGCCA GAAATGTGGG CACATCCCAG AAGATCTCAG TAGAGAGAGT   
  
  
+ AACTTCAGAA AAGTTTCAAT TTTTATTCGA AATTTGCCTC TTGTTTGTGT AGATATGATG GGAACTTCGC   
  
  
+ CTTACAATCT GCAATGTGAG GGTTTGGTTG AAGGAGTTGC AGTCAATCCT CAATTTTATG AATCAACCCC   
  
  
+ AAAGTGGAAG AAGGGCATCA AAGAAGAGTC AATTGAGAGC GAACCCACTT CGGTTTTGGA CAACCCTAGC   
  
  
+ CCTCCAAATT CTGCATCTAC TCTCTCTTCC TGCTTCAATG GTGGAAGTAG TGGGCTTGGG ATGGACGATT   
  
  
+ TGGAGGGTTT GTCTTTTGGA GAAGGGTCCC TTCTCCCTTG GTTCATGGGT GAAATTGAAG ACCCTAGTTT   
  
  
+ GAGTTTCAAG CACCTCCTTC AACCTTCAAA TCCCTTGGAG TTTGGTGGCA ATGATGGGCT TGAAGCAGCT   
  
  
+ ATTCAGAGTG CTGGTATGGG TAATTTTAAT TTGGTTGAAA GTTTTAATTC TGATTTGAGG TCTTGTAATT   
  
  
+ CTGGGTCTAT TTGCAATGGG GGGAATGATT CTGTCAATGA AAAGGGATGT GTTCTTAATT ATGCTTCAGA   
  
  
+ GCAGGGTCAT GTGGTTGCTG TATCTCCAGT AGAACAGTTT AGGATTAGTG ATGTGAAACA TGAGGTGTTG   
  
  
+ AACCCAAAAT TTGGCTTTTG TGAGCAAGGC TCTACAATCA GTCAGAACTC TGGTTATGTG CATATACTGG   
  
  
+ GTTATAACCA GCTAGAATCT CCCCTTGAGC CTCCGGCAAA GCGCCACAAT GCTGGGAATG CTCTTTACAC   
  
  
+ TAGTGTTGTT CCAGTTCCAA AGAGTTCGTT TTTAGGTGAG GATCATGAAA TTCTACTCAG AAATCAAGAG   
  
  
+ CAGCAGTTTC TGATGCAACA ACAGTGGTTA ATGGGTTTAG CCCCTCAGTT TACACCCCAG CACCCGCCAA   
  
  
+ AGCCATTAGA CGACCGAAAG CAACCAAACT CGCAAACTTT GGTTCTGCGC GATCAGTTAG TTAAGATTGC   
  
  
+ GGAGATGTTC CAAACCGGTA ACTTTTCGCT TGCCCAAGTG ATATTGGCCC GGCTCAATCA GCAATTGTCT   
  
  
+ CTTTCAGGGA ACCCCTTTGT AAGAGCTGCT ACTTTCCATG TCAAGGAGGA ACTTGAAAGG CTCCTGACTA   
  
  
+ TCAATAGCTT CTCTGCGGCT CCACCCCAAC CTAAAAGCCT TACTCTGTCA GATGTTGTGG ATAGGATGAA   
  
  
+ TGCATACAAG CTTTTTTCAG AGGCATCCCC AGTTATGCAA TTTACCGATT TTACTTGTAC CCAGGCTTTG   
  
  
+ CTCGAAGCTC TTGATGATGC TGATTATATC CACATACTAG ATTTTGACAT TGGTTGTGGT TCCCAATGGG   
  
  
+ CATCCTTTAT TCGAGAGCTT CCTCTCAGGA AAAGGGGCAC TCCATTTTTG AAAATTACAG CCTTTGCCTC   
  
  
+ TTCTTTAACC CATAATCCAT TTGAACTAGC CCTTGTATGT GATAACATTC TGGAATTTGC AAAAGAGTTC   
  
  
+ GGTGTCTCTG TCGACCTTCA GGTCATGAAT TTGGACTTGT TTGACCCAAC TACTTGCACA ATACCCAATT   
  
  
+ TTCAATCCAC TGAGAACGCA GCAGTTGCTG TCAACTTCCC TATATGGGCG TGCTCACACT GCCCGTTTGT   
  
  
+ ACTCAGTCAA CTCCTGTCCT TCATCAAGCA ATGTTCCCCA AAAATCATGA CGAGCTTTGA TAGAGGTTGT   
  
  
+ GATCATTTTG AGTTGTCTTT TCCATCGCAC ATCATCCGTG TATTGGACTC ATGCTCTAAT ATGTTGGAGT   
  
  
+ CCCTCCATGG TTCAAGCGTG ACATTAGACA CCTCAAAGAA GATTGAAAAG TTCTTGATCA AGCCTATAAT   
  
  
+ TGAGAGGGCA ATATTGGGGC GAATTCATGC ACCAGACAAG ATGCAGGCAA ACAAGGCACA CCGGTGGAAG   
  
  
+ AATCTCTTCA CTTCTGCTGG GTTCTTCCCT TTGCCATTTA GTAGTTTCAC TGAATCTCAG GCCAATCTAG   
  
  
+ TGGCTCAACG AACCCCAGTG AGGGGTTTTC AGGTGGAAAA GCAGCAAGCA TCACTTGTGC TCAGGTGGAT   
  
  
+ GTCTCATGAG CTCGTGGCAG CTTCAGCTTG GAGGTGCTA  

- -Up\_Stream \_Len000AAACAT AAATTTAAAA TGTTATAAAT ATTTTTAAAA CATAACTAAT TAACACGATT   
  
  
- GATTGTGATT ATTAACCCCC TCTTTTCTTC CCTTTTATTT TTCTTTAATT CTTCTTCTTT TCTTTTGTGG   
  
  
- AAACTTTTCC GTTTTTTGAT TTTATTTTGT TATTACTTGT CTTCCTCTCT TCCTACCTAT TTGTTGTTTA   
  
  
- CAGCGAAAAT CCTGTCGTTA TCAGATACAC TAACCGGAAT CAAGGACAAC CTCCTAACGA TTGTTTTCGT   
  
  
- TGTCGTTTTT TATTATGATT AATAATGTAT AAAAAATTGA GAATTTCGAT TAGTTAGTAG GAGGTAATTA   
  
  
- ACTAATTAAT AAACGGTTTT TGTGTTTAGT TGACCCCGTT GATATTCTCT AGATGGACGA AATTCTATAT   
  
  
- ATTTGAGTTA ACAAAGTAAA ATAAAATAAA ATCTAAAAAA CCGGAAACCA GAGATAACTC GATTTTAGAG   
  
  
- CAATAAAAGC CTAGTACTTT TTATGTTGAG CTAGAAAACT ATATTCAGTA GAAATAAACA TTTAAGACTT   
  
  
- GAATTTTAGA ATGAACTTCT AGTCCTTATC TTCGTGAGCT AAAAACTATT TAGGATACAC AAGAAGAATT   
  
  
- TTAAGTATTA GATTAAGTAT AAAATCTACA TGGGCAACGG ATGAAACCGT TCAAATCTAA TACTAAAAAT   
  
  
- AAAAAAAATT AATGAACATA AAAAGAAAAC CTAACTATCA GTTAGATGTA GTAGAAACTA GTTAACGCTG   
  
  
- TCAAAGTAAA AGGGTTGTTT TTAAAGCTCA CATAAGAAAA ATTAAATAGT ATTCATAAAT AACAGTTGTA   
  
  
- AGTTGTACGT AGTTGTTAGT AATTTGTGAA AAGCAGTGAG GAGTTATGAC CAGTCGTGAA TTCTAAAACA   
  
  
- GAAGATTTGA ATAGCATTCA TTGAATAACA ACTAATTAAC TACGACTGGT TTACTATGAC TCACGATTTT   
  
  
- AATATTAATT ATTTATGTTA ATTCTCAACT AGTTACAGCT ACTAAGTTTA GTTACACTAA CCACGACTTA   
  
  
- TCATTAATAT TCGTGGATAT TTCTGAACTT TTCTTGTTTG TAAATTAATC TAAAATATTA ATATTAAAGG   
  
  
- ATTATAAATC GTAAGTAGTC GTTTAAGTCG TGTGTGGTTG TAATCAGTTG TGGATAGTTG TGAATATTCA   
  
  
- TGGTTAATTA TTATTAATGG ATATTATCTA ATTTTCAATC CGGGAATTAC AAAGGGTGTA ATAATCTATA   
  
  
- TAGTTCCCTC CCCCTTTTTT TATTATGAGG GGGTAGAATT TTTTAAATAG GGAAAAAAAG AAAAATTACA   
  
  
- AGGTTTTGTT TTTATACGAA GATTTAATAC TAACGTTATA CTTACACATG CCAAAGTTTG GGTTAAAAGA   
  
  
- GGTTGTTAAG AAGATGATAT AACATTAATA TAATATGATG TTAAACATAG AGTAGATACT AGATGGGGGG   
  
  
- TTTTTGGTTT ATGAGTATAT TTCAAAATAA AAGAAAGGTA GGGTGAGATT TTTTTTTTTT ACAACCCTTT   
  
  
- TTCAACTAAA TAAATTAAAC TTTACCATGC CACTTATATC TAAACATCTC CCCGTCAACT ATCTTTTTCC   
  
  
- ACTTCTGTTT CCTTCCCGTG TAAACGGAAA AGTATCTTCC TTCTCTGGTT CCATGACCCC TGACCCCCTT   
  
  
- CCCGAGTTCT CTTCTCTCTC TGACTCTTCT CACGACGATG AGGAAGGATG GATGTGTGTT TTAACGACGC   
  
  
- CCCCCAAGTT CAAGTTGAAC TTGCTCCGTT TATGGGAGTA ACTTGGGTGG TTTAGCTTTA GAAGTTACAT   
  
  
- GTGGGGGTAA TATGTGCGTA AGTCTCTGTA TCTACAGCTC CCCCTCTACT TAATCGTTCT TCTACAGCTC   
  
  
- GATTTTACGT AAAGCTCCAA TGATCTCGGT CTTTACACCC GTGTAGGGTC TTCTAGAGTC ATCTCTCTCA   
  
  
- TTGAAGTCTT TTCAAAGTTA AAAATAAGCT TTAAACGGAG AACAAACACA TCTATACTAC CCTTGAAGCG   
  
  
- GAATGTTAGA CGTTACACTC CCAAACCAAC TTCCTCAACG TCAGTTAGGA GTTAAAATAC TTAGTTGGGG   
  
  
- TTTCACCTTC TTCCCGTAGT TTCTTCTCAG TTAACTCTCG CTTGGGTGAA GCCAAAACCT GTTGGGATCG   
  
  
- GGAGGTTTAA GACGTAGATG AGAGAGAAGG ACGAAGTTAC CACCTTCATC ACCCGAACCC TACCTGCTAA   
  
  
- ACCTCCCAAA CAGAAAACCT CTTCCCAGGG AAGAGGGAAC CAAGTACCCA CTTTAACTTC TGGGATCAAA   
  
  
- CTCAAAGTTC GTGGAGGAAG TTGGAAGTTT AGGGAACCTC AAACCACCGT TACTACCCGA ACTTCGTCGA   
  
  
- TAAGTCTCAC GACCATACCC ATTAAAATTA AACCAACTTT CAAAATTAAG ACTAAACTCC AGAACATTAA   
  
  
- GACCCAGATA AACGTTACCC CCCTTACTAA GACAGTTACT TTTCCCTACA CAAGAATTAA TACGAAGTCT   
  
  
- CGTCCCAGTA CACCAACGAC ATAGAGGTCA TCTTGTCAAA TCCTAATCAC TACACTTTGT ACTCCACAAC   
  
  
- TTGGGTTTTA AACCGAAAAC ACTCGTTCCG AGATGTTAGT CAGTCTTGAG ACCAATACAC GTATATGACC   
  
  
- CAATATTGGT CGATCTTAGA GGGGAACTCG GAGGCCGTTT CGCGGTGTTA CGACCCTTAC GAGAAATGTG   
  
  
- ATCACAACAA GGTCAAGGTT TCTCAAGCAA AAATCCACTC CTAGTACTTT AAGATGAGTC TTTAGTTCTC   
  
  
- GTCGTCAAAG ACTACGTTGT TGTCACCAAT TACCCAAATC GGGGAGTCAA ATGTGGGGTC GTGGGCGGTT   
  
  
- TCGGTAATCT GCTGGCTTTC GTTGGTTTGA GCGTTTGAAA CCAAGACGCG CTAGTCAATC AATTCTAACG   
  
  
- CCTCTACAAG GTTTGGCCAT TGAAAAGCGA ACGGGTTCAC TATAACCGGG CCGAGTTAGT CGTTAACAGA   
  
  
- GAAAGTCCCT TGGGGAAACA TTCTCGACGA TGAAAGGTAC AGTTCCTCCT TGAACTTTCC GAGGACTGAT   
  
  
- AGTTATCGAA GAGACGCCGA GGTGGGGTTG GATTTTCGGA ATGAGACAGT CTACAACACC TATCCTACTT   
  
  
- ACGTATGTTC GAAAAAAGTC TCCGTAGGGG TCAATACGTT AAATGGCTAA AATGAACATG GGTCCGAAAC   
  
  
- GAGCTTCGAG AACTACTACG ACTAATATAG GTGTATGATC TAAAACTGTA ACCAACACCA AGGGTTACCC   
  
  
- GTAGGAAATA AGCTCTCGAA GGAGAGTCCT TTTCCCCGTG AGGTAAAAAC TTTTAATGTC GGAAACGGAG   
  
  
- AAGAAATTGG GTATTAGGTA AACTTGATCG GGAACATACA CTATTGTAAG ACCTTAAACG TTTTCTCAAG   
  
  
- CCACAGAGAC AGCTGGAAGT CCAGTACTTA AACCTGAACA AACTGGGTTG ATGAACGTGT TATGGGTTAA   
  
  
- AAGTTAGGTG ACTCTTGCGT CGTCAACGAC AGTTGAAGGG ATATACCCGC ACGAGTGTGA CGGGCAAACA   
  
  
- TGAGTCAGTT GAGGACAGGA AGTAGTTCGT TACAAGGGGT TTTTAGTACT GCTCGAAACT ATCTCCAACA   
  
  
- CTAGTAAAAC TCAACAGAAA AGGTAGCGTG TAGTAGGCAC ATAACCTGAG TACGAGATTA TACAACCTCA   
  
  
- GGGAGGTACC AAGTTCGCAC TGTAATCTGT GGAGTTTCTT CTAACTTTTC AAGAACTAGT TCGGATATTA   
  
  
- ACTCTCCCGT TATAACCCCG CTTAAGTACG TGGTCTGTTC TACGTCCGTT TGTTCCGTGT GGCCACCTTC   
  
  
- TTAGAGAAGT GAAGACGACC CAAGAAGGGA AACGGTAAAT CATCAAAGTG ACTTAGAGTC CGGTTAGATC   
  
  
- ACCGAGTTGC TTGGGGTCAC TCCCCAAAAG TCCACCTTTT CGTCGTTCGT AGTGAACACG AGTCCACCTA   
  
  
- CAGAGTACTC GAGCACCGTC GAAGTCGAAC CTCCACGAT

+     ATCT-motif

| Site Name | Organism | Position | Strand | Matrix score. | sequence | function |
| --- | --- | --- | --- | --- | --- | --- |
| ATCT-motif | Pisum sativum | 642 | + | 9 | AATCTAATCC | part of a conserved DNA module involved in light responsiveness |

>HU04G00048.1   
+ -Up\_Stream \_Len000TTTGTA TTTAAATTTT ACAATATTTA TAAAAATTTT GTATTGATTA ATTGTGCTAA   
  
  
+ CTAACACTAA TAATTGGGGG AGAAAAGAAG GGAAAATAAA AAGAAATTAA GAAGAAGAAA AGAAAACACC   
  
  
+ TTTGAAAAGG CAAAAAACTA AAATAAAACA ATAATGAACA GAAGGAGAGA AGGATGGATA AACAACAAAT   
  
  
+ GTCGCTTTTA GGACAGCAAT AGTCTATGTG ATTGGCCTTA GTTCCTGTTG GAGGATTGCT AACAAAAGCA   
  
  
+ ACAGCAAAAA ATAATACTAA TTATTACATA TTTTTTAACT CTTAAAGCTA ATCAATCATC CTCCATTAAT   
  
  
+ TGATTAATTA TTTGCCAAAA ACACAAATCA ACTGGGGCAA CTATAAGAGA TCTACCTGCT TTAAGATATA   
  
  
+ TAAACTCAAT TGTTTCATTT TATTTTATTT TAGATTTTTT GGCCTTTGGT CTCTATTGAG CTAAAATCTC   
  
  
+ GTTATTTTCG GATCATGAAA AATACAACTC GATCTTTTGA TATAAGTCAT CTTTATTTGT AAATTCTGAA   
  
  
+ CTTAAAATCT TACTTGAAGA TCAGGAATAG AAGCACTCGA TTTTTGATAA ATCCTATGTG TTCTTCTTAA   
  
  
+ AATTCATAAT CTAATTCATA TTTTAGATGT ACCCGTTGCC TACTTTGGCA AGTTTAGATT ATGATTTTTA   
  
  
+ TTTTTTTTAA TTACTTGTAT TTTTCTTTTG GATTGATAGT CAATCTACAT CATCTTTGAT CAATTGCGAC   
  
  
+ AGTTTCATTT TCCCAACAAA AATTTCGAGT GTATTCTTTT TAATTTATCA TAAGTATTTA TTGTCAACAT   
  
  
+ TCAACATGCA TCAACAATCA TTAAACACTT TTCGTCACTC CTCAATACTG GTCAGCACTT AAGATTTTGT   
  
  
+ CTTCTAAACT TATCGTAAGT AACTTATTGT TGATTAATTG ATGCTGACCA AATGATACTG AGTGCTAAAA   
  
  
+ TTATAATTAA TAAATACAAT TAAGAGTTGA TCAATGTCGA TGATTCAAAT CAATGTGATT GGTGCTGAAT   
  
  
+ AGTAATTATA AGCACCTATA AAGACTTGAA AAGAACAAAC ATTTAATTAG ATTTTATAAT TATAATTTCC   
  
  
+ TAATATTTAG CATTCATCAG CAAATTCAGC ACACACCAAC ATTAGTCAAC ACCTATCAAC ACTTATAAGT   
  
  
+ ACCAATTAAT AATAATTACC TATAATAGAT TAAAAGTTAG GCCCTTAATG TTTCCCACAT TATTAGATAT   
  
  
+ ATCAAGGGAG GGGGAAAAAA ATAATACTCC CCCATCTTAA AAAATTTATC CCTTTTTTTC TTTTTAATGT   
  
  
+ TCCAAAACAA AAATATGCTT CTAAATTATG ATTGCAATAT GAATGTGTAC GGTTTCAAAC CCAATTTTCT   
  
  
+ CCAACAATTC TTCTACTATA TTGTAATTAT ATTATACTAC AATTTGTATC TCATCTATGA TCTACCCCCC   
  
  
+ AAAAACCAAA TACTCATATA AAGTTTTATT TTCTTTCCAT CCCACTCTAA AAAAAAAAAA TGTTGGGAAA   
  
  
+ AAGTTGATTT ATTTAATTTG AAATGGTACG GTGAATATAG ATTTGTAGAG GGGCAGTTGA TAGAAAAAGG   
  
  
+ TGAAGACAAA GGAAGGGCAC ATTTGCCTTT TCATAGAAGG AAGAGACCAA GGTACTGGGG ACTGGGGGAA   
  
  
+ GGGCTCAAGA GAAGAGAGAG ACTGAGAAGA GTGCTGCTAC TCCTTCCTAC CTACACACAA AATTGCTGCG   
  
  
+ GGGGGTTCAA GTTCAACTTG AACGAGGCAA ATACCCTCAT TGAACCCACC AAATCGAAAT CTTCAATGTA   
  
  
+ CACCCCCATT ATACACGCAT TCAGAGACAT AGATGTCGAG GGGGAGATGA ATTAGCAAGA AGATGTCGAG   
  
  
+ CTAAAATGCA TTTCGAGGTT ACTAGAGCCA GAAATGTGGG CACATCCCAG AAGATCTCAG TAGAGAGAGT   
  
  
+ AACTTCAGAA AAGTTTCAAT TTTTATTCGA AATTTGCCTC TTGTTTGTGT AGATATGATG GGAACTTCGC   
  
  
+ CTTACAATCT GCAATGTGAG GGTTTGGTTG AAGGAGTTGC AGTCAATCCT CAATTTTATG AATCAACCCC   
  
  
+ AAAGTGGAAG AAGGGCATCA AAGAAGAGTC AATTGAGAGC GAACCCACTT CGGTTTTGGA CAACCCTAGC   
  
  
+ CCTCCAAATT CTGCATCTAC TCTCTCTTCC TGCTTCAATG GTGGAAGTAG TGGGCTTGGG ATGGACGATT   
  
  
+ TGGAGGGTTT GTCTTTTGGA GAAGGGTCCC TTCTCCCTTG GTTCATGGGT GAAATTGAAG ACCCTAGTTT   
  
  
+ GAGTTTCAAG CACCTCCTTC AACCTTCAAA TCCCTTGGAG TTTGGTGGCA ATGATGGGCT TGAAGCAGCT   
  
  
+ ATTCAGAGTG CTGGTATGGG TAATTTTAAT TTGGTTGAAA GTTTTAATTC TGATTTGAGG TCTTGTAATT   
  
  
+ CTGGGTCTAT TTGCAATGGG GGGAATGATT CTGTCAATGA AAAGGGATGT GTTCTTAATT ATGCTTCAGA   
  
  
+ GCAGGGTCAT GTGGTTGCTG TATCTCCAGT AGAACAGTTT AGGATTAGTG ATGTGAAACA TGAGGTGTTG   
  
  
+ AACCCAAAAT TTGGCTTTTG TGAGCAAGGC TCTACAATCA GTCAGAACTC TGGTTATGTG CATATACTGG   
  
  
+ GTTATAACCA GCTAGAATCT CCCCTTGAGC CTCCGGCAAA GCGCCACAAT GCTGGGAATG CTCTTTACAC   
  
  
+ TAGTGTTGTT CCAGTTCCAA AGAGTTCGTT TTTAGGTGAG GATCATGAAA TTCTACTCAG AAATCAAGAG   
  
  
+ CAGCAGTTTC TGATGCAACA ACAGTGGTTA ATGGGTTTAG CCCCTCAGTT TACACCCCAG CACCCGCCAA   
  
  
+ AGCCATTAGA CGACCGAAAG CAACCAAACT CGCAAACTTT GGTTCTGCGC GATCAGTTAG TTAAGATTGC   
  
  
+ GGAGATGTTC CAAACCGGTA ACTTTTCGCT TGCCCAAGTG ATATTGGCCC GGCTCAATCA GCAATTGTCT   
  
  
+ CTTTCAGGGA ACCCCTTTGT AAGAGCTGCT ACTTTCCATG TCAAGGAGGA ACTTGAAAGG CTCCTGACTA   
  
  
+ TCAATAGCTT CTCTGCGGCT CCACCCCAAC CTAAAAGCCT TACTCTGTCA GATGTTGTGG ATAGGATGAA   
  
  
+ TGCATACAAG CTTTTTTCAG AGGCATCCCC AGTTATGCAA TTTACCGATT TTACTTGTAC CCAGGCTTTG   
  
  
+ CTCGAAGCTC TTGATGATGC TGATTATATC CACATACTAG ATTTTGACAT TGGTTGTGGT TCCCAATGGG   
  
  
+ CATCCTTTAT TCGAGAGCTT CCTCTCAGGA AAAGGGGCAC TCCATTTTTG AAAATTACAG CCTTTGCCTC   
  
  
+ TTCTTTAACC CATAATCCAT TTGAACTAGC CCTTGTATGT GATAACATTC TGGAATTTGC AAAAGAGTTC   
  
  
+ GGTGTCTCTG TCGACCTTCA GGTCATGAAT TTGGACTTGT TTGACCCAAC TACTTGCACA ATACCCAATT   
  
  
+ TTCAATCCAC TGAGAACGCA GCAGTTGCTG TCAACTTCCC TATATGGGCG TGCTCACACT GCCCGTTTGT   
  
  
+ ACTCAGTCAA CTCCTGTCCT TCATCAAGCA ATGTTCCCCA AAAATCATGA CGAGCTTTGA TAGAGGTTGT   
  
  
+ GATCATTTTG AGTTGTCTTT TCCATCGCAC ATCATCCGTG TATTGGACTC ATGCTCTAAT ATGTTGGAGT   
  
  
+ CCCTCCATGG TTCAAGCGTG ACATTAGACA CCTCAAAGAA GATTGAAAAG TTCTTGATCA AGCCTATAAT   
  
  
+ TGAGAGGGCA ATATTGGGGC GAATTCATGC ACCAGACAAG ATGCAGGCAA ACAAGGCACA CCGGTGGAAG   
  
  
+ AATCTCTTCA CTTCTGCTGG GTTCTTCCCT TTGCCATTTA GTAGTTTCAC TGAATCTCAG GCCAATCTAG   
  
  
+ TGGCTCAACG AACCCCAGTG AGGGGTTTTC AGGTGGAAAA GCAGCAAGCA TCACTTGTGC TCAGGTGGAT   
  
  
+ GTCTCATGAG CTCGTGGCAG CTTCAGCTTG GAGGTGCTA  

- -Up\_Stream \_Len000AAACAT AAATTTAAAA TGTTATAAAT ATTTTTAAAA CATAACTAAT TAACACGATT   
  
  
- GATTGTGATT ATTAACCCCC TCTTTTCTTC CCTTTTATTT TTCTTTAATT CTTCTTCTTT TCTTTTGTGG   
  
  
- AAACTTTTCC GTTTTTTGAT TTTATTTTGT TATTACTTGT CTTCCTCTCT TCCTACCTAT TTGTTGTTTA   
  
  
- CAGCGAAAAT CCTGTCGTTA TCAGATACAC TAACCGGAAT CAAGGACAAC CTCCTAACGA TTGTTTTCGT   
  
  
- TGTCGTTTTT TATTATGATT AATAATGTAT AAAAAATTGA GAATTTCGAT TAGTTAGTAG GAGGTAATTA   
  
  
- ACTAATTAAT AAACGGTTTT TGTGTTTAGT TGACCCCGTT GATATTCTCT AGATGGACGA AATTCTATAT   
  
  
- ATTTGAGTTA ACAAAGTAAA ATAAAATAAA ATCTAAAAAA CCGGAAACCA GAGATAACTC GATTTTAGAG   
  
  
- CAATAAAAGC CTAGTACTTT TTATGTTGAG CTAGAAAACT ATATTCAGTA GAAATAAACA TTTAAGACTT   
  
  
- GAATTTTAGA ATGAACTTCT AGTCCTTATC TTCGTGAGCT AAAAACTATT TAGGATACAC AAGAAGAATT   
  
  
- TTAAGTATTA GATTAAGTAT AAAATCTACA TGGGCAACGG ATGAAACCGT TCAAATCTAA TACTAAAAAT   
  
  
- AAAAAAAATT AATGAACATA AAAAGAAAAC CTAACTATCA GTTAGATGTA GTAGAAACTA GTTAACGCTG   
  
  
- TCAAAGTAAA AGGGTTGTTT TTAAAGCTCA CATAAGAAAA ATTAAATAGT ATTCATAAAT AACAGTTGTA   
  
  
- AGTTGTACGT AGTTGTTAGT AATTTGTGAA AAGCAGTGAG GAGTTATGAC CAGTCGTGAA TTCTAAAACA   
  
  
- GAAGATTTGA ATAGCATTCA TTGAATAACA ACTAATTAAC TACGACTGGT TTACTATGAC TCACGATTTT   
  
  
- AATATTAATT ATTTATGTTA ATTCTCAACT AGTTACAGCT ACTAAGTTTA GTTACACTAA CCACGACTTA   
  
  
- TCATTAATAT TCGTGGATAT TTCTGAACTT TTCTTGTTTG TAAATTAATC TAAAATATTA ATATTAAAGG   
  
  
- ATTATAAATC GTAAGTAGTC GTTTAAGTCG TGTGTGGTTG TAATCAGTTG TGGATAGTTG TGAATATTCA   
  
  
- TGGTTAATTA TTATTAATGG ATATTATCTA ATTTTCAATC CGGGAATTAC AAAGGGTGTA ATAATCTATA   
  
  
- TAGTTCCCTC CCCCTTTTTT TATTATGAGG GGGTAGAATT TTTTAAATAG GGAAAAAAAG AAAAATTACA   
  
  
- AGGTTTTGTT TTTATACGAA GATTTAATAC TAACGTTATA CTTACACATG CCAAAGTTTG GGTTAAAAGA   
  
  
- GGTTGTTAAG AAGATGATAT AACATTAATA TAATATGATG TTAAACATAG AGTAGATACT AGATGGGGGG   
  
  
- TTTTTGGTTT ATGAGTATAT TTCAAAATAA AAGAAAGGTA GGGTGAGATT TTTTTTTTTT ACAACCCTTT   
  
  
- TTCAACTAAA TAAATTAAAC TTTACCATGC CACTTATATC TAAACATCTC CCCGTCAACT ATCTTTTTCC   
  
  
- ACTTCTGTTT CCTTCCCGTG TAAACGGAAA AGTATCTTCC TTCTCTGGTT CCATGACCCC TGACCCCCTT   
  
  
- CCCGAGTTCT CTTCTCTCTC TGACTCTTCT CACGACGATG AGGAAGGATG GATGTGTGTT TTAACGACGC   
  
  
- CCCCCAAGTT CAAGTTGAAC TTGCTCCGTT TATGGGAGTA ACTTGGGTGG TTTAGCTTTA GAAGTTACAT   
  
  
- GTGGGGGTAA TATGTGCGTA AGTCTCTGTA TCTACAGCTC CCCCTCTACT TAATCGTTCT TCTACAGCTC   
  
  
- GATTTTACGT AAAGCTCCAA TGATCTCGGT CTTTACACCC GTGTAGGGTC TTCTAGAGTC ATCTCTCTCA   
  
  
- TTGAAGTCTT TTCAAAGTTA AAAATAAGCT TTAAACGGAG AACAAACACA TCTATACTAC CCTTGAAGCG   
  
  
- GAATGTTAGA CGTTACACTC CCAAACCAAC TTCCTCAACG TCAGTTAGGA GTTAAAATAC TTAGTTGGGG   
  
  
- TTTCACCTTC TTCCCGTAGT TTCTTCTCAG TTAACTCTCG CTTGGGTGAA GCCAAAACCT GTTGGGATCG   
  
  
- GGAGGTTTAA GACGTAGATG AGAGAGAAGG ACGAAGTTAC CACCTTCATC ACCCGAACCC TACCTGCTAA   
  
  
- ACCTCCCAAA CAGAAAACCT CTTCCCAGGG AAGAGGGAAC CAAGTACCCA CTTTAACTTC TGGGATCAAA   
  
  
- CTCAAAGTTC GTGGAGGAAG TTGGAAGTTT AGGGAACCTC AAACCACCGT TACTACCCGA ACTTCGTCGA   
  
  
- TAAGTCTCAC GACCATACCC ATTAAAATTA AACCAACTTT CAAAATTAAG ACTAAACTCC AGAACATTAA   
  
  
- GACCCAGATA AACGTTACCC CCCTTACTAA GACAGTTACT TTTCCCTACA CAAGAATTAA TACGAAGTCT   
  
  
- CGTCCCAGTA CACCAACGAC ATAGAGGTCA TCTTGTCAAA TCCTAATCAC TACACTTTGT ACTCCACAAC   
  
  
- TTGGGTTTTA AACCGAAAAC ACTCGTTCCG AGATGTTAGT CAGTCTTGAG ACCAATACAC GTATATGACC   
  
  
- CAATATTGGT CGATCTTAGA GGGGAACTCG GAGGCCGTTT CGCGGTGTTA CGACCCTTAC GAGAAATGTG   
  
  
- ATCACAACAA GGTCAAGGTT TCTCAAGCAA AAATCCACTC CTAGTACTTT AAGATGAGTC TTTAGTTCTC   
  
  
- GTCGTCAAAG ACTACGTTGT TGTCACCAAT TACCCAAATC GGGGAGTCAA ATGTGGGGTC GTGGGCGGTT   
  
  
- TCGGTAATCT GCTGGCTTTC GTTGGTTTGA GCGTTTGAAA CCAAGACGCG CTAGTCAATC AATTCTAACG   
  
  
- CCTCTACAAG GTTTGGCCAT TGAAAAGCGA ACGGGTTCAC TATAACCGGG CCGAGTTAGT CGTTAACAGA   
  
  
- GAAAGTCCCT TGGGGAAACA TTCTCGACGA TGAAAGGTAC AGTTCCTCCT TGAACTTTCC GAGGACTGAT   
  
  
- AGTTATCGAA GAGACGCCGA GGTGGGGTTG GATTTTCGGA ATGAGACAGT CTACAACACC TATCCTACTT   
  
  
- ACGTATGTTC GAAAAAAGTC TCCGTAGGGG TCAATACGTT AAATGGCTAA AATGAACATG GGTCCGAAAC   
  
  
- GAGCTTCGAG AACTACTACG ACTAATATAG GTGTATGATC TAAAACTGTA ACCAACACCA AGGGTTACCC   
  
  
- GTAGGAAATA AGCTCTCGAA GGAGAGTCCT TTTCCCCGTG AGGTAAAAAC TTTTAATGTC GGAAACGGAG   
  
  
- AAGAAATTGG GTATTAGGTA AACTTGATCG GGAACATACA CTATTGTAAG ACCTTAAACG TTTTCTCAAG   
  
  
- CCACAGAGAC AGCTGGAAGT CCAGTACTTA AACCTGAACA AACTGGGTTG ATGAACGTGT TATGGGTTAA   
  
  
- AAGTTAGGTG ACTCTTGCGT CGTCAACGAC AGTTGAAGGG ATATACCCGC ACGAGTGTGA CGGGCAAACA   
  
  
- TGAGTCAGTT GAGGACAGGA AGTAGTTCGT TACAAGGGGT TTTTAGTACT GCTCGAAACT ATCTCCAACA   
  
  
- CTAGTAAAAC TCAACAGAAA AGGTAGCGTG TAGTAGGCAC ATAACCTGAG TACGAGATTA TACAACCTCA   
  
  
- GGGAGGTACC AAGTTCGCAC TGTAATCTGT GGAGTTTCTT CTAACTTTTC AAGAACTAGT TCGGATATTA   
  
  
- ACTCTCCCGT TATAACCCCG CTTAAGTACG TGGTCTGTTC TACGTCCGTT TGTTCCGTGT GGCCACCTTC   
  
  
- TTAGAGAAGT GAAGACGACC CAAGAAGGGA AACGGTAAAT CATCAAAGTG ACTTAGAGTC CGGTTAGATC   
  
  
- ACCGAGTTGC TTGGGGTCAC TCCCCAAAAG TCCACCTTTT CGTCGTTCGT AGTGAACACG AGTCCACCTA   
  
  
- CAGAGTACTC GAGCACCGTC GAAGTCGAAC CTCCACGAT

+     AT~TATA-box

| Site Name | Organism | Position | Strand | Matrix score. | sequence | function |
| --- | --- | --- | --- | --- | --- | --- |
| AT~TATA-box | Arabidopsis thaliana | 421 | + | 6 | TATATA |  |

>HU04G00048.1   
+ -Up\_Stream \_Len000TTTGTA TTTAAATTTT ACAATATTTA TAAAAATTTT GTATTGATTA ATTGTGCTAA   
  
  
+ CTAACACTAA TAATTGGGGG AGAAAAGAAG GGAAAATAAA AAGAAATTAA GAAGAAGAAA AGAAAACACC   
  
  
+ TTTGAAAAGG CAAAAAACTA AAATAAAACA ATAATGAACA GAAGGAGAGA AGGATGGATA AACAACAAAT   
  
  
+ GTCGCTTTTA GGACAGCAAT AGTCTATGTG ATTGGCCTTA GTTCCTGTTG GAGGATTGCT AACAAAAGCA   
  
  
+ ACAGCAAAAA ATAATACTAA TTATTACATA TTTTTTAACT CTTAAAGCTA ATCAATCATC CTCCATTAAT   
  
  
+ TGATTAATTA TTTGCCAAAA ACACAAATCA ACTGGGGCAA CTATAAGAGA TCTACCTGCT TTAAGATATA   
  
  
+ TAAACTCAAT TGTTTCATTT TATTTTATTT TAGATTTTTT GGCCTTTGGT CTCTATTGAG CTAAAATCTC   
  
  
+ GTTATTTTCG GATCATGAAA AATACAACTC GATCTTTTGA TATAAGTCAT CTTTATTTGT AAATTCTGAA   
  
  
+ CTTAAAATCT TACTTGAAGA TCAGGAATAG AAGCACTCGA TTTTTGATAA ATCCTATGTG TTCTTCTTAA   
  
  
+ AATTCATAAT CTAATTCATA TTTTAGATGT ACCCGTTGCC TACTTTGGCA AGTTTAGATT ATGATTTTTA   
  
  
+ TTTTTTTTAA TTACTTGTAT TTTTCTTTTG GATTGATAGT CAATCTACAT CATCTTTGAT CAATTGCGAC   
  
  
+ AGTTTCATTT TCCCAACAAA AATTTCGAGT GTATTCTTTT TAATTTATCA TAAGTATTTA TTGTCAACAT   
  
  
+ TCAACATGCA TCAACAATCA TTAAACACTT TTCGTCACTC CTCAATACTG GTCAGCACTT AAGATTTTGT   
  
  
+ CTTCTAAACT TATCGTAAGT AACTTATTGT TGATTAATTG ATGCTGACCA AATGATACTG AGTGCTAAAA   
  
  
+ TTATAATTAA TAAATACAAT TAAGAGTTGA TCAATGTCGA TGATTCAAAT CAATGTGATT GGTGCTGAAT   
  
  
+ AGTAATTATA AGCACCTATA AAGACTTGAA AAGAACAAAC ATTTAATTAG ATTTTATAAT TATAATTTCC   
  
  
+ TAATATTTAG CATTCATCAG CAAATTCAGC ACACACCAAC ATTAGTCAAC ACCTATCAAC ACTTATAAGT   
  
  
+ ACCAATTAAT AATAATTACC TATAATAGAT TAAAAGTTAG GCCCTTAATG TTTCCCACAT TATTAGATAT   
  
  
+ ATCAAGGGAG GGGGAAAAAA ATAATACTCC CCCATCTTAA AAAATTTATC CCTTTTTTTC TTTTTAATGT   
  
  
+ TCCAAAACAA AAATATGCTT CTAAATTATG ATTGCAATAT GAATGTGTAC GGTTTCAAAC CCAATTTTCT   
  
  
+ CCAACAATTC TTCTACTATA TTGTAATTAT ATTATACTAC AATTTGTATC TCATCTATGA TCTACCCCCC   
  
  
+ AAAAACCAAA TACTCATATA AAGTTTTATT TTCTTTCCAT CCCACTCTAA AAAAAAAAAA TGTTGGGAAA   
  
  
+ AAGTTGATTT ATTTAATTTG AAATGGTACG GTGAATATAG ATTTGTAGAG GGGCAGTTGA TAGAAAAAGG   
  
  
+ TGAAGACAAA GGAAGGGCAC ATTTGCCTTT TCATAGAAGG AAGAGACCAA GGTACTGGGG ACTGGGGGAA   
  
  
+ GGGCTCAAGA GAAGAGAGAG ACTGAGAAGA GTGCTGCTAC TCCTTCCTAC CTACACACAA AATTGCTGCG   
  
  
+ GGGGGTTCAA GTTCAACTTG AACGAGGCAA ATACCCTCAT TGAACCCACC AAATCGAAAT CTTCAATGTA   
  
  
+ CACCCCCATT ATACACGCAT TCAGAGACAT AGATGTCGAG GGGGAGATGA ATTAGCAAGA AGATGTCGAG   
  
  
+ CTAAAATGCA TTTCGAGGTT ACTAGAGCCA GAAATGTGGG CACATCCCAG AAGATCTCAG TAGAGAGAGT   
  
  
+ AACTTCAGAA AAGTTTCAAT TTTTATTCGA AATTTGCCTC TTGTTTGTGT AGATATGATG GGAACTTCGC   
  
  
+ CTTACAATCT GCAATGTGAG GGTTTGGTTG AAGGAGTTGC AGTCAATCCT CAATTTTATG AATCAACCCC   
  
  
+ AAAGTGGAAG AAGGGCATCA AAGAAGAGTC AATTGAGAGC GAACCCACTT CGGTTTTGGA CAACCCTAGC   
  
  
+ CCTCCAAATT CTGCATCTAC TCTCTCTTCC TGCTTCAATG GTGGAAGTAG TGGGCTTGGG ATGGACGATT   
  
  
+ TGGAGGGTTT GTCTTTTGGA GAAGGGTCCC TTCTCCCTTG GTTCATGGGT GAAATTGAAG ACCCTAGTTT   
  
  
+ GAGTTTCAAG CACCTCCTTC AACCTTCAAA TCCCTTGGAG TTTGGTGGCA ATGATGGGCT TGAAGCAGCT   
  
  
+ ATTCAGAGTG CTGGTATGGG TAATTTTAAT TTGGTTGAAA GTTTTAATTC TGATTTGAGG TCTTGTAATT   
  
  
+ CTGGGTCTAT TTGCAATGGG GGGAATGATT CTGTCAATGA AAAGGGATGT GTTCTTAATT ATGCTTCAGA   
  
  
+ GCAGGGTCAT GTGGTTGCTG TATCTCCAGT AGAACAGTTT AGGATTAGTG ATGTGAAACA TGAGGTGTTG   
  
  
+ AACCCAAAAT TTGGCTTTTG TGAGCAAGGC TCTACAATCA GTCAGAACTC TGGTTATGTG CATATACTGG   
  
  
+ GTTATAACCA GCTAGAATCT CCCCTTGAGC CTCCGGCAAA GCGCCACAAT GCTGGGAATG CTCTTTACAC   
  
  
+ TAGTGTTGTT CCAGTTCCAA AGAGTTCGTT TTTAGGTGAG GATCATGAAA TTCTACTCAG AAATCAAGAG   
  
  
+ CAGCAGTTTC TGATGCAACA ACAGTGGTTA ATGGGTTTAG CCCCTCAGTT TACACCCCAG CACCCGCCAA   
  
  
+ AGCCATTAGA CGACCGAAAG CAACCAAACT CGCAAACTTT GGTTCTGCGC GATCAGTTAG TTAAGATTGC   
  
  
+ GGAGATGTTC CAAACCGGTA ACTTTTCGCT TGCCCAAGTG ATATTGGCCC GGCTCAATCA GCAATTGTCT   
  
  
+ CTTTCAGGGA ACCCCTTTGT AAGAGCTGCT ACTTTCCATG TCAAGGAGGA ACTTGAAAGG CTCCTGACTA   
  
  
+ TCAATAGCTT CTCTGCGGCT CCACCCCAAC CTAAAAGCCT TACTCTGTCA GATGTTGTGG ATAGGATGAA   
  
  
+ TGCATACAAG CTTTTTTCAG AGGCATCCCC AGTTATGCAA TTTACCGATT TTACTTGTAC CCAGGCTTTG   
  
  
+ CTCGAAGCTC TTGATGATGC TGATTATATC CACATACTAG ATTTTGACAT TGGTTGTGGT TCCCAATGGG   
  
  
+ CATCCTTTAT TCGAGAGCTT CCTCTCAGGA AAAGGGGCAC TCCATTTTTG AAAATTACAG CCTTTGCCTC   
  
  
+ TTCTTTAACC CATAATCCAT TTGAACTAGC CCTTGTATGT GATAACATTC TGGAATTTGC AAAAGAGTTC   
  
  
+ GGTGTCTCTG TCGACCTTCA GGTCATGAAT TTGGACTTGT TTGACCCAAC TACTTGCACA ATACCCAATT   
  
  
+ TTCAATCCAC TGAGAACGCA GCAGTTGCTG TCAACTTCCC TATATGGGCG TGCTCACACT GCCCGTTTGT   
  
  
+ ACTCAGTCAA CTCCTGTCCT TCATCAAGCA ATGTTCCCCA AAAATCATGA CGAGCTTTGA TAGAGGTTGT   
  
  
+ GATCATTTTG AGTTGTCTTT TCCATCGCAC ATCATCCGTG TATTGGACTC ATGCTCTAAT ATGTTGGAGT   
  
  
+ CCCTCCATGG TTCAAGCGTG ACATTAGACA CCTCAAAGAA GATTGAAAAG TTCTTGATCA AGCCTATAAT   
  
  
+ TGAGAGGGCA ATATTGGGGC GAATTCATGC ACCAGACAAG ATGCAGGCAA ACAAGGCACA CCGGTGGAAG   
  
  
+ AATCTCTTCA CTTCTGCTGG GTTCTTCCCT TTGCCATTTA GTAGTTTCAC TGAATCTCAG GCCAATCTAG   
  
  
+ TGGCTCAACG AACCCCAGTG AGGGGTTTTC AGGTGGAAAA GCAGCAAGCA TCACTTGTGC TCAGGTGGAT   
  
  
+ GTCTCATGAG CTCGTGGCAG CTTCAGCTTG GAGGTGCTA  

- -Up\_Stream \_Len000AAACAT AAATTTAAAA TGTTATAAAT ATTTTTAAAA CATAACTAAT TAACACGATT   
  
  
- GATTGTGATT ATTAACCCCC TCTTTTCTTC CCTTTTATTT TTCTTTAATT CTTCTTCTTT TCTTTTGTGG   
  
  
- AAACTTTTCC GTTTTTTGAT TTTATTTTGT TATTACTTGT CTTCCTCTCT TCCTACCTAT TTGTTGTTTA   
  
  
- CAGCGAAAAT CCTGTCGTTA TCAGATACAC TAACCGGAAT CAAGGACAAC CTCCTAACGA TTGTTTTCGT   
  
  
- TGTCGTTTTT TATTATGATT AATAATGTAT AAAAAATTGA GAATTTCGAT TAGTTAGTAG GAGGTAATTA   
  
  
- ACTAATTAAT AAACGGTTTT TGTGTTTAGT TGACCCCGTT GATATTCTCT AGATGGACGA AATTCTATAT   
  
  
- ATTTGAGTTA ACAAAGTAAA ATAAAATAAA ATCTAAAAAA CCGGAAACCA GAGATAACTC GATTTTAGAG   
  
  
- CAATAAAAGC CTAGTACTTT TTATGTTGAG CTAGAAAACT ATATTCAGTA GAAATAAACA TTTAAGACTT   
  
  
- GAATTTTAGA ATGAACTTCT AGTCCTTATC TTCGTGAGCT AAAAACTATT TAGGATACAC AAGAAGAATT   
  
  
- TTAAGTATTA GATTAAGTAT AAAATCTACA TGGGCAACGG ATGAAACCGT TCAAATCTAA TACTAAAAAT   
  
  
- AAAAAAAATT AATGAACATA AAAAGAAAAC CTAACTATCA GTTAGATGTA GTAGAAACTA GTTAACGCTG   
  
  
- TCAAAGTAAA AGGGTTGTTT TTAAAGCTCA CATAAGAAAA ATTAAATAGT ATTCATAAAT AACAGTTGTA   
  
  
- AGTTGTACGT AGTTGTTAGT AATTTGTGAA AAGCAGTGAG GAGTTATGAC CAGTCGTGAA TTCTAAAACA   
  
  
- GAAGATTTGA ATAGCATTCA TTGAATAACA ACTAATTAAC TACGACTGGT TTACTATGAC TCACGATTTT   
  
  
- AATATTAATT ATTTATGTTA ATTCTCAACT AGTTACAGCT ACTAAGTTTA GTTACACTAA CCACGACTTA   
  
  
- TCATTAATAT TCGTGGATAT TTCTGAACTT TTCTTGTTTG TAAATTAATC TAAAATATTA ATATTAAAGG   
  
  
- ATTATAAATC GTAAGTAGTC GTTTAAGTCG TGTGTGGTTG TAATCAGTTG TGGATAGTTG TGAATATTCA   
  
  
- TGGTTAATTA TTATTAATGG ATATTATCTA ATTTTCAATC CGGGAATTAC AAAGGGTGTA ATAATCTATA   
  
  
- TAGTTCCCTC CCCCTTTTTT TATTATGAGG GGGTAGAATT TTTTAAATAG GGAAAAAAAG AAAAATTACA   
  
  
- AGGTTTTGTT TTTATACGAA GATTTAATAC TAACGTTATA CTTACACATG CCAAAGTTTG GGTTAAAAGA   
  
  
- GGTTGTTAAG AAGATGATAT AACATTAATA TAATATGATG TTAAACATAG AGTAGATACT AGATGGGGGG   
  
  
- TTTTTGGTTT ATGAGTATAT TTCAAAATAA AAGAAAGGTA GGGTGAGATT TTTTTTTTTT ACAACCCTTT   
  
  
- TTCAACTAAA TAAATTAAAC TTTACCATGC CACTTATATC TAAACATCTC CCCGTCAACT ATCTTTTTCC   
  
  
- ACTTCTGTTT CCTTCCCGTG TAAACGGAAA AGTATCTTCC TTCTCTGGTT CCATGACCCC TGACCCCCTT   
  
  
- CCCGAGTTCT CTTCTCTCTC TGACTCTTCT CACGACGATG AGGAAGGATG GATGTGTGTT TTAACGACGC   
  
  
- CCCCCAAGTT CAAGTTGAAC TTGCTCCGTT TATGGGAGTA ACTTGGGTGG TTTAGCTTTA GAAGTTACAT   
  
  
- GTGGGGGTAA TATGTGCGTA AGTCTCTGTA TCTACAGCTC CCCCTCTACT TAATCGTTCT TCTACAGCTC   
  
  
- GATTTTACGT AAAGCTCCAA TGATCTCGGT CTTTACACCC GTGTAGGGTC TTCTAGAGTC ATCTCTCTCA   
  
  
- TTGAAGTCTT TTCAAAGTTA AAAATAAGCT TTAAACGGAG AACAAACACA TCTATACTAC CCTTGAAGCG   
  
  
- GAATGTTAGA CGTTACACTC CCAAACCAAC TTCCTCAACG TCAGTTAGGA GTTAAAATAC TTAGTTGGGG   
  
  
- TTTCACCTTC TTCCCGTAGT TTCTTCTCAG TTAACTCTCG CTTGGGTGAA GCCAAAACCT GTTGGGATCG   
  
  
- GGAGGTTTAA GACGTAGATG AGAGAGAAGG ACGAAGTTAC CACCTTCATC ACCCGAACCC TACCTGCTAA   
  
  
- ACCTCCCAAA CAGAAAACCT CTTCCCAGGG AAGAGGGAAC CAAGTACCCA CTTTAACTTC TGGGATCAAA   
  
  
- CTCAAAGTTC GTGGAGGAAG TTGGAAGTTT AGGGAACCTC AAACCACCGT TACTACCCGA ACTTCGTCGA   
  
  
- TAAGTCTCAC GACCATACCC ATTAAAATTA AACCAACTTT CAAAATTAAG ACTAAACTCC AGAACATTAA   
  
  
- GACCCAGATA AACGTTACCC CCCTTACTAA GACAGTTACT TTTCCCTACA CAAGAATTAA TACGAAGTCT   
  
  
- CGTCCCAGTA CACCAACGAC ATAGAGGTCA TCTTGTCAAA TCCTAATCAC TACACTTTGT ACTCCACAAC   
  
  
- TTGGGTTTTA AACCGAAAAC ACTCGTTCCG AGATGTTAGT CAGTCTTGAG ACCAATACAC GTATATGACC   
  
  
- CAATATTGGT CGATCTTAGA GGGGAACTCG GAGGCCGTTT CGCGGTGTTA CGACCCTTAC GAGAAATGTG   
  
  
- ATCACAACAA GGTCAAGGTT TCTCAAGCAA AAATCCACTC CTAGTACTTT AAGATGAGTC TTTAGTTCTC   
  
  
- GTCGTCAAAG ACTACGTTGT TGTCACCAAT TACCCAAATC GGGGAGTCAA ATGTGGGGTC GTGGGCGGTT   
  
  
- TCGGTAATCT GCTGGCTTTC GTTGGTTTGA GCGTTTGAAA CCAAGACGCG CTAGTCAATC AATTCTAACG   
  
  
- CCTCTACAAG GTTTGGCCAT TGAAAAGCGA ACGGGTTCAC TATAACCGGG CCGAGTTAGT CGTTAACAGA   
  
  
- GAAAGTCCCT TGGGGAAACA TTCTCGACGA TGAAAGGTAC AGTTCCTCCT TGAACTTTCC GAGGACTGAT   
  
  
- AGTTATCGAA GAGACGCCGA GGTGGGGTTG GATTTTCGGA ATGAGACAGT CTACAACACC TATCCTACTT   
  
  
- ACGTATGTTC GAAAAAAGTC TCCGTAGGGG TCAATACGTT AAATGGCTAA AATGAACATG GGTCCGAAAC   
  
  
- GAGCTTCGAG AACTACTACG ACTAATATAG GTGTATGATC TAAAACTGTA ACCAACACCA AGGGTTACCC   
  
  
- GTAGGAAATA AGCTCTCGAA GGAGAGTCCT TTTCCCCGTG AGGTAAAAAC TTTTAATGTC GGAAACGGAG   
  
  
- AAGAAATTGG GTATTAGGTA AACTTGATCG GGAACATACA CTATTGTAAG ACCTTAAACG TTTTCTCAAG   
  
  
- CCACAGAGAC AGCTGGAAGT CCAGTACTTA AACCTGAACA AACTGGGTTG ATGAACGTGT TATGGGTTAA   
  
  
- AAGTTAGGTG ACTCTTGCGT CGTCAACGAC AGTTGAAGGG ATATACCCGC ACGAGTGTGA CGGGCAAACA   
  
  
- TGAGTCAGTT GAGGACAGGA AGTAGTTCGT TACAAGGGGT TTTTAGTACT GCTCGAAACT ATCTCCAACA   
  
  
- CTAGTAAAAC TCAACAGAAA AGGTAGCGTG TAGTAGGCAC ATAACCTGAG TACGAGATTA TACAACCTCA   
  
  
- GGGAGGTACC AAGTTCGCAC TGTAATCTGT GGAGTTTCTT CTAACTTTTC AAGAACTAGT TCGGATATTA   
  
  
- ACTCTCCCGT TATAACCCCG CTTAAGTACG TGGTCTGTTC TACGTCCGTT TGTTCCGTGT GGCCACCTTC   
  
  
- TTAGAGAAGT GAAGACGACC CAAGAAGGGA AACGGTAAAT CATCAAAGTG ACTTAGAGTC CGGTTAGATC   
  
  
- ACCGAGTTGC TTGGGGTCAC TCCCCAAAAG TCCACCTTTT CGTCGTTCGT AGTGAACACG AGTCCACCTA   
  
  
- CAGAGTACTC GAGCACCGTC GAAGTCGAAC CTCCACGAT

+     Box 4

| Site Name | Organism | Position | Strand | Matrix score. | sequence | function |
| --- | --- | --- | --- | --- | --- | --- |
| Box 4 | Petroselinum crispum | 990 | + | 6 | ATTAAT | part of a conserved DNA module involved in light responsiveness |
| Box 4 | Petroselinum crispum | 1199 | + | 6 | ATTAAT | part of a conserved DNA module involved in light responsiveness |
| Box 4 | Petroselinum crispum | 947 | + | 6 | ATTAAT | part of a conserved DNA module involved in light responsiveness |
| Box 4 | Petroselinum crispum | 349 | + | 6 | ATTAAT | part of a conserved DNA module involved in light responsiveness |
| Box 4 | Petroselinum crispum | 61 | + | 6 | ATTAAT | part of a conserved DNA module involved in light responsiveness |
| Box 4 | Petroselinum crispum | 357 | + | 6 | ATTAAT | part of a conserved DNA module involved in light responsiveness |

>HU04G00048.1   
+ -Up\_Stream \_Len000TTTGTA TTTAAATTTT ACAATATTTA TAAAAATTTT GTATTGATTA ATTGTGCTAA   
  
  
+ CTAACACTAA TAATTGGGGG AGAAAAGAAG GGAAAATAAA AAGAAATTAA GAAGAAGAAA AGAAAACACC   
  
  
+ TTTGAAAAGG CAAAAAACTA AAATAAAACA ATAATGAACA GAAGGAGAGA AGGATGGATA AACAACAAAT   
  
  
+ GTCGCTTTTA GGACAGCAAT AGTCTATGTG ATTGGCCTTA GTTCCTGTTG GAGGATTGCT AACAAAAGCA   
  
  
+ ACAGCAAAAA ATAATACTAA TTATTACATA TTTTTTAACT CTTAAAGCTA ATCAATCATC CTCCATTAAT   
  
  
+ TGATTAATTA TTTGCCAAAA ACACAAATCA ACTGGGGCAA CTATAAGAGA TCTACCTGCT TTAAGATATA   
  
  
+ TAAACTCAAT TGTTTCATTT TATTTTATTT TAGATTTTTT GGCCTTTGGT CTCTATTGAG CTAAAATCTC   
  
  
+ GTTATTTTCG GATCATGAAA AATACAACTC GATCTTTTGA TATAAGTCAT CTTTATTTGT AAATTCTGAA   
  
  
+ CTTAAAATCT TACTTGAAGA TCAGGAATAG AAGCACTCGA TTTTTGATAA ATCCTATGTG TTCTTCTTAA   
  
  
+ AATTCATAAT CTAATTCATA TTTTAGATGT ACCCGTTGCC TACTTTGGCA AGTTTAGATT ATGATTTTTA   
  
  
+ TTTTTTTTAA TTACTTGTAT TTTTCTTTTG GATTGATAGT CAATCTACAT CATCTTTGAT CAATTGCGAC   
  
  
+ AGTTTCATTT TCCCAACAAA AATTTCGAGT GTATTCTTTT TAATTTATCA TAAGTATTTA TTGTCAACAT   
  
  
+ TCAACATGCA TCAACAATCA TTAAACACTT TTCGTCACTC CTCAATACTG GTCAGCACTT AAGATTTTGT   
  
  
+ CTTCTAAACT TATCGTAAGT AACTTATTGT TGATTAATTG ATGCTGACCA AATGATACTG AGTGCTAAAA   
  
  
+ TTATAATTAA TAAATACAAT TAAGAGTTGA TCAATGTCGA TGATTCAAAT CAATGTGATT GGTGCTGAAT   
  
  
+ AGTAATTATA AGCACCTATA AAGACTTGAA AAGAACAAAC ATTTAATTAG ATTTTATAAT TATAATTTCC   
  
  
+ TAATATTTAG CATTCATCAG CAAATTCAGC ACACACCAAC ATTAGTCAAC ACCTATCAAC ACTTATAAGT   
  
  
+ ACCAATTAAT AATAATTACC TATAATAGAT TAAAAGTTAG GCCCTTAATG TTTCCCACAT TATTAGATAT   
  
  
+ ATCAAGGGAG GGGGAAAAAA ATAATACTCC CCCATCTTAA AAAATTTATC CCTTTTTTTC TTTTTAATGT   
  
  
+ TCCAAAACAA AAATATGCTT CTAAATTATG ATTGCAATAT GAATGTGTAC GGTTTCAAAC CCAATTTTCT   
  
  
+ CCAACAATTC TTCTACTATA TTGTAATTAT ATTATACTAC AATTTGTATC TCATCTATGA TCTACCCCCC   
  
  
+ AAAAACCAAA TACTCATATA AAGTTTTATT TTCTTTCCAT CCCACTCTAA AAAAAAAAAA TGTTGGGAAA   
  
  
+ AAGTTGATTT ATTTAATTTG AAATGGTACG GTGAATATAG ATTTGTAGAG GGGCAGTTGA TAGAAAAAGG   
  
  
+ TGAAGACAAA GGAAGGGCAC ATTTGCCTTT TCATAGAAGG AAGAGACCAA GGTACTGGGG ACTGGGGGAA   
  
  
+ GGGCTCAAGA GAAGAGAGAG ACTGAGAAGA GTGCTGCTAC TCCTTCCTAC CTACACACAA AATTGCTGCG   
  
  
+ GGGGGTTCAA GTTCAACTTG AACGAGGCAA ATACCCTCAT TGAACCCACC AAATCGAAAT CTTCAATGTA   
  
  
+ CACCCCCATT ATACACGCAT TCAGAGACAT AGATGTCGAG GGGGAGATGA ATTAGCAAGA AGATGTCGAG   
  
  
+ CTAAAATGCA TTTCGAGGTT ACTAGAGCCA GAAATGTGGG CACATCCCAG AAGATCTCAG TAGAGAGAGT   
  
  
+ AACTTCAGAA AAGTTTCAAT TTTTATTCGA AATTTGCCTC TTGTTTGTGT AGATATGATG GGAACTTCGC   
  
  
+ CTTACAATCT GCAATGTGAG GGTTTGGTTG AAGGAGTTGC AGTCAATCCT CAATTTTATG AATCAACCCC   
  
  
+ AAAGTGGAAG AAGGGCATCA AAGAAGAGTC AATTGAGAGC GAACCCACTT CGGTTTTGGA CAACCCTAGC   
  
  
+ CCTCCAAATT CTGCATCTAC TCTCTCTTCC TGCTTCAATG GTGGAAGTAG TGGGCTTGGG ATGGACGATT   
  
  
+ TGGAGGGTTT GTCTTTTGGA GAAGGGTCCC TTCTCCCTTG GTTCATGGGT GAAATTGAAG ACCCTAGTTT   
  
  
+ GAGTTTCAAG CACCTCCTTC AACCTTCAAA TCCCTTGGAG TTTGGTGGCA ATGATGGGCT TGAAGCAGCT   
  
  
+ ATTCAGAGTG CTGGTATGGG TAATTTTAAT TTGGTTGAAA GTTTTAATTC TGATTTGAGG TCTTGTAATT   
  
  
+ CTGGGTCTAT TTGCAATGGG GGGAATGATT CTGTCAATGA AAAGGGATGT GTTCTTAATT ATGCTTCAGA   
  
  
+ GCAGGGTCAT GTGGTTGCTG TATCTCCAGT AGAACAGTTT AGGATTAGTG ATGTGAAACA TGAGGTGTTG   
  
  
+ AACCCAAAAT TTGGCTTTTG TGAGCAAGGC TCTACAATCA GTCAGAACTC TGGTTATGTG CATATACTGG   
  
  
+ GTTATAACCA GCTAGAATCT CCCCTTGAGC CTCCGGCAAA GCGCCACAAT GCTGGGAATG CTCTTTACAC   
  
  
+ TAGTGTTGTT CCAGTTCCAA AGAGTTCGTT TTTAGGTGAG GATCATGAAA TTCTACTCAG AAATCAAGAG   
  
  
+ CAGCAGTTTC TGATGCAACA ACAGTGGTTA ATGGGTTTAG CCCCTCAGTT TACACCCCAG CACCCGCCAA   
  
  
+ AGCCATTAGA CGACCGAAAG CAACCAAACT CGCAAACTTT GGTTCTGCGC GATCAGTTAG TTAAGATTGC   
  
  
+ GGAGATGTTC CAAACCGGTA ACTTTTCGCT TGCCCAAGTG ATATTGGCCC GGCTCAATCA GCAATTGTCT   
  
  
+ CTTTCAGGGA ACCCCTTTGT AAGAGCTGCT ACTTTCCATG TCAAGGAGGA ACTTGAAAGG CTCCTGACTA   
  
  
+ TCAATAGCTT CTCTGCGGCT CCACCCCAAC CTAAAAGCCT TACTCTGTCA GATGTTGTGG ATAGGATGAA   
  
  
+ TGCATACAAG CTTTTTTCAG AGGCATCCCC AGTTATGCAA TTTACCGATT TTACTTGTAC CCAGGCTTTG   
  
  
+ CTCGAAGCTC TTGATGATGC TGATTATATC CACATACTAG ATTTTGACAT TGGTTGTGGT TCCCAATGGG   
  
  
+ CATCCTTTAT TCGAGAGCTT CCTCTCAGGA AAAGGGGCAC TCCATTTTTG AAAATTACAG CCTTTGCCTC   
  
  
+ TTCTTTAACC CATAATCCAT TTGAACTAGC CCTTGTATGT GATAACATTC TGGAATTTGC AAAAGAGTTC   
  
  
+ GGTGTCTCTG TCGACCTTCA GGTCATGAAT TTGGACTTGT TTGACCCAAC TACTTGCACA ATACCCAATT   
  
  
+ TTCAATCCAC TGAGAACGCA GCAGTTGCTG TCAACTTCCC TATATGGGCG TGCTCACACT GCCCGTTTGT   
  
  
+ ACTCAGTCAA CTCCTGTCCT TCATCAAGCA ATGTTCCCCA AAAATCATGA CGAGCTTTGA TAGAGGTTGT   
  
  
+ GATCATTTTG AGTTGTCTTT TCCATCGCAC ATCATCCGTG TATTGGACTC ATGCTCTAAT ATGTTGGAGT   
  
  
+ CCCTCCATGG TTCAAGCGTG ACATTAGACA CCTCAAAGAA GATTGAAAAG TTCTTGATCA AGCCTATAAT   
  
  
+ TGAGAGGGCA ATATTGGGGC GAATTCATGC ACCAGACAAG ATGCAGGCAA ACAAGGCACA CCGGTGGAAG   
  
  
+ AATCTCTTCA CTTCTGCTGG GTTCTTCCCT TTGCCATTTA GTAGTTTCAC TGAATCTCAG GCCAATCTAG   
  
  
+ TGGCTCAACG AACCCCAGTG AGGGGTTTTC AGGTGGAAAA GCAGCAAGCA TCACTTGTGC TCAGGTGGAT   
  
  
+ GTCTCATGAG CTCGTGGCAG CTTCAGCTTG GAGGTGCTA  

- -Up\_Stream \_Len000AAACAT AAATTTAAAA TGTTATAAAT ATTTTTAAAA CATAACTAAT TAACACGATT   
  
  
- GATTGTGATT ATTAACCCCC TCTTTTCTTC CCTTTTATTT TTCTTTAATT CTTCTTCTTT TCTTTTGTGG   
  
  
- AAACTTTTCC GTTTTTTGAT TTTATTTTGT TATTACTTGT CTTCCTCTCT TCCTACCTAT TTGTTGTTTA   
  
  
- CAGCGAAAAT CCTGTCGTTA TCAGATACAC TAACCGGAAT CAAGGACAAC CTCCTAACGA TTGTTTTCGT   
  
  
- TGTCGTTTTT TATTATGATT AATAATGTAT AAAAAATTGA GAATTTCGAT TAGTTAGTAG GAGGTAATTA   
  
  
- ACTAATTAAT AAACGGTTTT TGTGTTTAGT TGACCCCGTT GATATTCTCT AGATGGACGA AATTCTATAT   
  
  
- ATTTGAGTTA ACAAAGTAAA ATAAAATAAA ATCTAAAAAA CCGGAAACCA GAGATAACTC GATTTTAGAG   
  
  
- CAATAAAAGC CTAGTACTTT TTATGTTGAG CTAGAAAACT ATATTCAGTA GAAATAAACA TTTAAGACTT   
  
  
- GAATTTTAGA ATGAACTTCT AGTCCTTATC TTCGTGAGCT AAAAACTATT TAGGATACAC AAGAAGAATT   
  
  
- TTAAGTATTA GATTAAGTAT AAAATCTACA TGGGCAACGG ATGAAACCGT TCAAATCTAA TACTAAAAAT   
  
  
- AAAAAAAATT AATGAACATA AAAAGAAAAC CTAACTATCA GTTAGATGTA GTAGAAACTA GTTAACGCTG   
  
  
- TCAAAGTAAA AGGGTTGTTT TTAAAGCTCA CATAAGAAAA ATTAAATAGT ATTCATAAAT AACAGTTGTA   
  
  
- AGTTGTACGT AGTTGTTAGT AATTTGTGAA AAGCAGTGAG GAGTTATGAC CAGTCGTGAA TTCTAAAACA   
  
  
- GAAGATTTGA ATAGCATTCA TTGAATAACA ACTAATTAAC TACGACTGGT TTACTATGAC TCACGATTTT   
  
  
- AATATTAATT ATTTATGTTA ATTCTCAACT AGTTACAGCT ACTAAGTTTA GTTACACTAA CCACGACTTA   
  
  
- TCATTAATAT TCGTGGATAT TTCTGAACTT TTCTTGTTTG TAAATTAATC TAAAATATTA ATATTAAAGG   
  
  
- ATTATAAATC GTAAGTAGTC GTTTAAGTCG TGTGTGGTTG TAATCAGTTG TGGATAGTTG TGAATATTCA   
  
  
- TGGTTAATTA TTATTAATGG ATATTATCTA ATTTTCAATC CGGGAATTAC AAAGGGTGTA ATAATCTATA   
  
  
- TAGTTCCCTC CCCCTTTTTT TATTATGAGG GGGTAGAATT TTTTAAATAG GGAAAAAAAG AAAAATTACA   
  
  
- AGGTTTTGTT TTTATACGAA GATTTAATAC TAACGTTATA CTTACACATG CCAAAGTTTG GGTTAAAAGA   
  
  
- GGTTGTTAAG AAGATGATAT AACATTAATA TAATATGATG TTAAACATAG AGTAGATACT AGATGGGGGG   
  
  
- TTTTTGGTTT ATGAGTATAT TTCAAAATAA AAGAAAGGTA GGGTGAGATT TTTTTTTTTT ACAACCCTTT   
  
  
- TTCAACTAAA TAAATTAAAC TTTACCATGC CACTTATATC TAAACATCTC CCCGTCAACT ATCTTTTTCC   
  
  
- ACTTCTGTTT CCTTCCCGTG TAAACGGAAA AGTATCTTCC TTCTCTGGTT CCATGACCCC TGACCCCCTT   
  
  
- CCCGAGTTCT CTTCTCTCTC TGACTCTTCT CACGACGATG AGGAAGGATG GATGTGTGTT TTAACGACGC   
  
  
- CCCCCAAGTT CAAGTTGAAC TTGCTCCGTT TATGGGAGTA ACTTGGGTGG TTTAGCTTTA GAAGTTACAT   
  
  
- GTGGGGGTAA TATGTGCGTA AGTCTCTGTA TCTACAGCTC CCCCTCTACT TAATCGTTCT TCTACAGCTC   
  
  
- GATTTTACGT AAAGCTCCAA TGATCTCGGT CTTTACACCC GTGTAGGGTC TTCTAGAGTC ATCTCTCTCA   
  
  
- TTGAAGTCTT TTCAAAGTTA AAAATAAGCT TTAAACGGAG AACAAACACA TCTATACTAC CCTTGAAGCG   
  
  
- GAATGTTAGA CGTTACACTC CCAAACCAAC TTCCTCAACG TCAGTTAGGA GTTAAAATAC TTAGTTGGGG   
  
  
- TTTCACCTTC TTCCCGTAGT TTCTTCTCAG TTAACTCTCG CTTGGGTGAA GCCAAAACCT GTTGGGATCG   
  
  
- GGAGGTTTAA GACGTAGATG AGAGAGAAGG ACGAAGTTAC CACCTTCATC ACCCGAACCC TACCTGCTAA   
  
  
- ACCTCCCAAA CAGAAAACCT CTTCCCAGGG AAGAGGGAAC CAAGTACCCA CTTTAACTTC TGGGATCAAA   
  
  
- CTCAAAGTTC GTGGAGGAAG TTGGAAGTTT AGGGAACCTC AAACCACCGT TACTACCCGA ACTTCGTCGA   
  
  
- TAAGTCTCAC GACCATACCC ATTAAAATTA AACCAACTTT CAAAATTAAG ACTAAACTCC AGAACATTAA   
  
  
- GACCCAGATA AACGTTACCC CCCTTACTAA GACAGTTACT TTTCCCTACA CAAGAATTAA TACGAAGTCT   
  
  
- CGTCCCAGTA CACCAACGAC ATAGAGGTCA TCTTGTCAAA TCCTAATCAC TACACTTTGT ACTCCACAAC   
  
  
- TTGGGTTTTA AACCGAAAAC ACTCGTTCCG AGATGTTAGT CAGTCTTGAG ACCAATACAC GTATATGACC   
  
  
- CAATATTGGT CGATCTTAGA GGGGAACTCG GAGGCCGTTT CGCGGTGTTA CGACCCTTAC GAGAAATGTG   
  
  
- ATCACAACAA GGTCAAGGTT TCTCAAGCAA AAATCCACTC CTAGTACTTT AAGATGAGTC TTTAGTTCTC   
  
  
- GTCGTCAAAG ACTACGTTGT TGTCACCAAT TACCCAAATC GGGGAGTCAA ATGTGGGGTC GTGGGCGGTT   
  
  
- TCGGTAATCT GCTGGCTTTC GTTGGTTTGA GCGTTTGAAA CCAAGACGCG CTAGTCAATC AATTCTAACG   
  
  
- CCTCTACAAG GTTTGGCCAT TGAAAAGCGA ACGGGTTCAC TATAACCGGG CCGAGTTAGT CGTTAACAGA   
  
  
- GAAAGTCCCT TGGGGAAACA TTCTCGACGA TGAAAGGTAC AGTTCCTCCT TGAACTTTCC GAGGACTGAT   
  
  
- AGTTATCGAA GAGACGCCGA GGTGGGGTTG GATTTTCGGA ATGAGACAGT CTACAACACC TATCCTACTT   
  
  
- ACGTATGTTC GAAAAAAGTC TCCGTAGGGG TCAATACGTT AAATGGCTAA AATGAACATG GGTCCGAAAC   
  
  
- GAGCTTCGAG AACTACTACG ACTAATATAG GTGTATGATC TAAAACTGTA ACCAACACCA AGGGTTACCC   
  
  
- GTAGGAAATA AGCTCTCGAA GGAGAGTCCT TTTCCCCGTG AGGTAAAAAC TTTTAATGTC GGAAACGGAG   
  
  
- AAGAAATTGG GTATTAGGTA AACTTGATCG GGAACATACA CTATTGTAAG ACCTTAAACG TTTTCTCAAG   
  
  
- CCACAGAGAC AGCTGGAAGT CCAGTACTTA AACCTGAACA AACTGGGTTG ATGAACGTGT TATGGGTTAA   
  
  
- AAGTTAGGTG ACTCTTGCGT CGTCAACGAC AGTTGAAGGG ATATACCCGC ACGAGTGTGA CGGGCAAACA   
  
  
- TGAGTCAGTT GAGGACAGGA AGTAGTTCGT TACAAGGGGT TTTTAGTACT GCTCGAAACT ATCTCCAACA   
  
  
- CTAGTAAAAC TCAACAGAAA AGGTAGCGTG TAGTAGGCAC ATAACCTGAG TACGAGATTA TACAACCTCA   
  
  
- GGGAGGTACC AAGTTCGCAC TGTAATCTGT GGAGTTTCTT CTAACTTTTC AAGAACTAGT TCGGATATTA   
  
  
- ACTCTCCCGT TATAACCCCG CTTAAGTACG TGGTCTGTTC TACGTCCGTT TGTTCCGTGT GGCCACCTTC   
  
  
- TTAGAGAAGT GAAGACGACC CAAGAAGGGA AACGGTAAAT CATCAAAGTG ACTTAGAGTC CGGTTAGATC   
  
  
- ACCGAGTTGC TTGGGGTCAC TCCCCAAAAG TCCACCTTTT CGTCGTTCGT AGTGAACACG AGTCCACCTA   
  
  
- CAGAGTACTC GAGCACCGTC GAAGTCGAAC CTCCACGAT

+     CAAT-box

| Site Name | Organism | Position | Strand | Matrix score. | sequence | function |
| --- | --- | --- | --- | --- | --- | --- |
| CAAT-box | Nicotiana glutinosa | 2629 | + | 4 | CAAT |  |
| CAAT-box | Pisum sativum | 2603 | - | 5 | CAAAT | common cis-acting element in promoter and enhancer regions |
| CAAT-box | Nicotiana glutinosa | 2134 | + | 4 | CAAT |  |
| CAAT-box | Nicotiana glutinosa | 3917 | + | 4 | CAAT |  |
| CAAT-box | Arabidopsis thaliana | 3916 | + | 5 | CCAAT | common cis-acting element in promoter and enhancer regions |
| CAAT-box | Arabidopsis thaliana | 3797 | - | 5 | CCAAT | common cis-acting element in promoter and enhancer regions |
| CAAT-box | Nicotiana glutinosa | 3783 | - | 4 | CAAT |  |
| CAAT-box | Nicotiana glutinosa | 3756 | - | 4 | CAAT |  |
| CAAT-box | Nicotiana glutinosa | 3507 | + | 4 | CAAT |  |
| CAAT-box | Arabidopsis thaliana | 3686 | - | 5 | CCAAT | common cis-acting element in promoter and enhancer regions |
| CAAT-box | Nicotiana glutinosa | 2363 | + | 4 | CAAT |  |
| CAAT-box | Nicotiana glutinosa | 2711 | + | 4 | CAAT |  |
| CAAT-box | Nicotiana glutinosa | 2999 | + | 4 | CAAT |  |
| CAAT-box | Pisum sativum | 2413 | - | 5 | CAAAT | common cis-acting element in promoter and enhancer regions |
| CAAT-box | Arabidopsis thaliana | 2987 | - | 5 | CCAAT | common cis-acting element in promoter and enhancer regions |
| CAAT-box | Nicotiana glutinosa | 2085 | + | 4 | CAAT |  |
| CAAT-box | Nicotiana glutinosa | 3603 | + | 4 | CAAT |  |
| CAAT-box | Pisum sativum | 2437 | - | 5 | CAAAT | common cis-acting element in promoter and enhancer regions |
| CAAT-box | Nicotiana glutinosa | 2039 | + | 4 | CAAT |  |
| CAAT-box | Nicotiana glutinosa | 2468 | + | 4 | CAAT |  |
| CAAT-box | Nicotiana glutinosa | 2940 | - | 4 | CAAT |  |
| CAAT-box | Nicotiana glutinosa | 3793 | + | 4 | CAAT |  |
| CAAT-box | Nicotiana glutinosa | 3500 | + | 4 | CAAT |  |
| CAAT-box | Nicotiana glutinosa | 2298 | - | 4 | CAAT |  |
| CAAT-box | Arabidopsis thaliana | 3499 | + | 5 | CCAAT | common cis-acting element in promoter and enhancer regions |
| CAAT-box | Nicotiana glutinosa | 3006 | + | 4 | CAAT |  |
| CAAT-box | Nicotiana glutinosa | 3008 | - | 4 | CAAT |  |
| CAAT-box | Nicotiana glutinosa | 3493 | + | 4 | CAAT |  |
| CAAT-box | Pisum sativum | 3463 | - | 5 | CAAAT | common cis-acting element in promoter and enhancer regions |
| CAAT-box | Nicotiana glutinosa | 2489 | + | 4 | CAAT |  |
| CAAT-box | Nicotiana glutinosa | 3086 | + | 4 | CAAT |  |
| CAAT-box | Arabidopsis thaliana | 3287 | + | 5 | CCAAT | common cis-acting element in promoter and enhancer regions |
| CAAT-box | Nicotiana glutinosa | 3192 | + | 4 | CAAT |  |
| CAAT-box | Arabidopsis thaliana | 3273 | - | 5 | CCAAT | common cis-acting element in promoter and enhancer regions |
| CAAT-box | Pisum sativum | 2341 | + | 5 | CAAAT | common cis-acting element in promoter and enhancer regions |
| CAAT-box | Pisum sativum | 2463 | - | 5 | CAAAT | common cis-acting element in promoter and enhancer regions |
| CAAT-box | Arabidopsis thaliana | 3498 | + | 8 | CCCAATTT | common cis-acting element in promoter and enhancer regions |
| CAAT-box | Pisum sativum | 2242 | - | 5 | CAAAT | common cis-acting element in promoter and enhancer regions |
| CAAT-box | Nicotiana glutinosa | 2210 | + | 4 | CAAT |  |
| CAAT-box | Pisum sativum | 3419 | - | 5 | CAAAT | common cis-acting element in promoter and enhancer regions |
| CAAT-box | Pisum sativum | 3383 | - | 5 | CAAAT | common cis-acting element in promoter and enhancer regions |
| CAAT-box | Pisum sativum | 2179 | + | 5 | CAAAT | common cis-acting element in promoter and enhancer regions |
| CAAT-box | Nicotiana glutinosa | 3288 | + | 4 | CAAT |  |
| CAAT-box | Nicotiana glutinosa | 2136 | - | 4 | CAAT |  |
| CAAT-box | Nicotiana glutinosa | 2078 | + | 4 | CAAT |  |
| CAAT-box | Nicotiana glutinosa | 2046 | + | 4 | CAAT |  |
| CAAT-box | Pisum sativum | 1996 | - | 5 | CAAAT | common cis-acting element in promoter and enhancer regions |
| CAAT-box | Nicotiana glutinosa | 1981 | + | 4 | CAAT |  |
| CAAT-box | Nicotiana glutinosa | 1818 | + | 4 | CAAT |  |
| CAAT-box | Pisum sativum | 1782 | + | 5 | CAAAT | common cis-acting element in promoter and enhancer regions |
| CAAT-box | Pisum sativum | 1585 | - | 5 | CAAAT | common cis-acting element in promoter and enhancer regions |
| CAAT-box | Nicotiana glutinosa | 1369 | + | 4 | CAAT |  |
| CAAT-box | Nicotiana glutinosa | 1001 | + | 4 | CAAT |  |
| CAAT-box | Pisum sativum | 963 | + | 5 | CAAAT | common cis-acting element in promoter and enhancer regions |
| CAAT-box | Pisum sativum | 1145 | + | 5 | CAAAT | common cis-acting element in promoter and enhancer regions |
| CAAT-box | Pisum sativum | 1481 | + | 5 | CAAAT | common cis-acting element in promoter and enhancer regions |
| CAAT-box | Arabidopsis thaliana | 1395 | + | 5 | CCAAT | common cis-acting element in promoter and enhancer regions |
| CAAT-box | Pisum sativum | 1635 | - | 5 | CAAAT | common cis-acting element in promoter and enhancer regions |
| CAAT-box | Nicotiana glutinosa | 1424 | - | 4 | CAAT |  |
| CAAT-box | Nicotiana glutinosa | 859 | + | 4 | CAAT |  |
| CAAT-box | Pisum sativum | 1804 | + | 5 | CAAAT | common cis-acting element in promoter and enhancer regions |
| CAAT-box | Nicotiana glutinosa | 1409 | + | 4 | CAAT |  |
| CAAT-box | Nicotiana glutinosa | 1396 | + | 4 | CAAT |  |
| CAAT-box | Nicotiana glutinosa | 951 | - | 4 | CAAT |  |
| CAAT-box | Nicotiana glutinosa | 940 | - | 4 | CAAT |  |
| CAAT-box | Nicotiana glutinosa | 1016 | + | 4 | CAAT |  |
| CAAT-box | Nicotiana glutinosa | 767 | - | 4 | CAAT |  |
| CAAT-box | Pisum sativum | 364 | - | 5 | CAAAT | common cis-acting element in promoter and enhancer regions |
| CAAT-box | Nicotiana glutinosa | 353 | - | 4 | CAAT |  |
| CAAT-box | Nicotiana glutinosa | 433 | - | 4 | CAAT |  |
| CAAT-box | Pisum sativum | 1446 | - | 5 | CAAAT | common cis-acting element in promoter and enhancer regions |
| CAAT-box | Arabidopsis thaliana | 1394 | + | 8 | CCCAATTT | common cis-acting element in promoter and enhancer regions |
| CAAT-box | Arabidopsis thaliana | 1196 | + | 5 | CCAAT | common cis-acting element in promoter and enhancer regions |
| CAAT-box | Nicotiana glutinosa | 1035 | + | 4 | CAAT |  |
| CAAT-box | Nicotiana glutinosa | 1197 | + | 4 | CAAT |  |
| CAAT-box | Pisum sativum | 1030 | + | 5 | CAAAT | common cis-acting element in promoter and enhancer regions |
| CAAT-box | Nicotiana glutinosa | 1365 | - | 4 | CAAT |  |
| CAAT-box | Arabidopsis thaliana | 1042 | - | 5 | CCAAT | common cis-acting element in promoter and enhancer regions |
| CAAT-box | Nicotiana glutinosa | 1444 | + | 4 | CAAT |  |
| CAAT-box | Nicotiana glutinosa | 1793 | - | 4 | CAAT |  |
| CAAT-box | Nicotiana glutinosa | 337 | + | 4 | CAAT |  |
| CAAT-box | Pisum sativum | 1560 | - | 5 | CAAAT | common cis-acting element in promoter and enhancer regions |
| CAAT-box | Pisum sativum | 210 | + | 5 | CAAAT | common cis-acting element in promoter and enhancer regions |
| CAAT-box | Arabidopsis thaliana | 87 | - | 5 | CCAAT | common cis-acting element in promoter and enhancer regions |
| CAAT-box | Nicotiana glutinosa | 736 | - | 4 | CAAT |  |
| CAAT-box | Pisum sativum | 549 | - | 5 | CAAAT | common cis-acting element in promoter and enhancer regions |
| CAAT-box | Nicotiana glutinosa | 1746 | - | 4 | CAAT |  |
| CAAT-box | Nicotiana glutinosa | 834 | - | 4 | CAAT |  |
| CAAT-box | Nicotiana glutinosa | 479 | - | 4 | CAAT |  |
| CAAT-box | Nicotiana glutinosa | 765 | + | 4 | CAAT |  |
| CAAT-box | Nicotiana glutinosa | 431 | + | 4 | CAAT |  |
| CAAT-box | Nicotiana glutinosa | 745 | + | 4 | CAAT |  |
| CAAT-box | Pisum sativum | 378 | + | 5 | CAAAT | common cis-acting element in promoter and enhancer regions |
| CAAT-box | Nicotiana glutinosa | 269 | - | 4 | CAAT |  |
| CAAT-box | Nicotiana glutinosa | 887 | + | 4 | CAAT |  |
| CAAT-box | Arabidopsis thaliana | 245 | - | 5 | CCAAT | common cis-acting element in promoter and enhancer regions |
| CAAT-box | Nicotiana glutinosa | 173 | + | 4 | CAAT |  |
| CAAT-box | Nicotiana glutinosa | 231 | + | 4 | CAAT |  |
| CAAT-box | Nicotiana glutinosa | 65 | - | 4 | CAAT |  |
| CAAT-box | Nicotiana glutinosa | 57 | - | 4 | CAAT |  |
| CAAT-box | Nicotiana glutinosa | 36 | + | 4 | CAAT |  |

>HU04G00048.1   
+ -Up\_Stream \_Len000TTTGTA TTTAAATTTT ACAATATTTA TAAAAATTTT GTATTGATTA ATTGTGCTAA   
  
  
+ CTAACACTAA TAATTGGGGG AGAAAAGAAG GGAAAATAAA AAGAAATTAA GAAGAAGAAA AGAAAACACC   
  
  
+ TTTGAAAAGG CAAAAAACTA AAATAAAACA ATAATGAACA GAAGGAGAGA AGGATGGATA AACAACAAAT   
  
  
+ GTCGCTTTTA GGACAGCAAT AGTCTATGTG ATTGGCCTTA GTTCCTGTTG GAGGATTGCT AACAAAAGCA   
  
  
+ ACAGCAAAAA ATAATACTAA TTATTACATA TTTTTTAACT CTTAAAGCTA ATCAATCATC CTCCATTAAT   
  
  
+ TGATTAATTA TTTGCCAAAA ACACAAATCA ACTGGGGCAA CTATAAGAGA TCTACCTGCT TTAAGATATA   
  
  
+ TAAACTCAAT TGTTTCATTT TATTTTATTT TAGATTTTTT GGCCTTTGGT CTCTATTGAG CTAAAATCTC   
  
  
+ GTTATTTTCG GATCATGAAA AATACAACTC GATCTTTTGA TATAAGTCAT CTTTATTTGT AAATTCTGAA   
  
  
+ CTTAAAATCT TACTTGAAGA TCAGGAATAG AAGCACTCGA TTTTTGATAA ATCCTATGTG TTCTTCTTAA   
  
  
+ AATTCATAAT CTAATTCATA TTTTAGATGT ACCCGTTGCC TACTTTGGCA AGTTTAGATT ATGATTTTTA   
  
  
+ TTTTTTTTAA TTACTTGTAT TTTTCTTTTG GATTGATAGT CAATCTACAT CATCTTTGAT CAATTGCGAC   
  
  
+ AGTTTCATTT TCCCAACAAA AATTTCGAGT GTATTCTTTT TAATTTATCA TAAGTATTTA TTGTCAACAT   
  
  
+ TCAACATGCA TCAACAATCA TTAAACACTT TTCGTCACTC CTCAATACTG GTCAGCACTT AAGATTTTGT   
  
  
+ CTTCTAAACT TATCGTAAGT AACTTATTGT TGATTAATTG ATGCTGACCA AATGATACTG AGTGCTAAAA   
  
  
+ TTATAATTAA TAAATACAAT TAAGAGTTGA TCAATGTCGA TGATTCAAAT CAATGTGATT GGTGCTGAAT   
  
  
+ AGTAATTATA AGCACCTATA AAGACTTGAA AAGAACAAAC ATTTAATTAG ATTTTATAAT TATAATTTCC   
  
  
+ TAATATTTAG CATTCATCAG CAAATTCAGC ACACACCAAC ATTAGTCAAC ACCTATCAAC ACTTATAAGT   
  
  
+ ACCAATTAAT AATAATTACC TATAATAGAT TAAAAGTTAG GCCCTTAATG TTTCCCACAT TATTAGATAT   
  
  
+ ATCAAGGGAG GGGGAAAAAA ATAATACTCC CCCATCTTAA AAAATTTATC CCTTTTTTTC TTTTTAATGT   
  
  
+ TCCAAAACAA AAATATGCTT CTAAATTATG ATTGCAATAT GAATGTGTAC GGTTTCAAAC CCAATTTTCT   
  
  
+ CCAACAATTC TTCTACTATA TTGTAATTAT ATTATACTAC AATTTGTATC TCATCTATGA TCTACCCCCC   
  
  
+ AAAAACCAAA TACTCATATA AAGTTTTATT TTCTTTCCAT CCCACTCTAA AAAAAAAAAA TGTTGGGAAA   
  
  
+ AAGTTGATTT ATTTAATTTG AAATGGTACG GTGAATATAG ATTTGTAGAG GGGCAGTTGA TAGAAAAAGG   
  
  
+ TGAAGACAAA GGAAGGGCAC ATTTGCCTTT TCATAGAAGG AAGAGACCAA GGTACTGGGG ACTGGGGGAA   
  
  
+ GGGCTCAAGA GAAGAGAGAG ACTGAGAAGA GTGCTGCTAC TCCTTCCTAC CTACACACAA AATTGCTGCG   
  
  
+ GGGGGTTCAA GTTCAACTTG AACGAGGCAA ATACCCTCAT TGAACCCACC AAATCGAAAT CTTCAATGTA   
  
  
+ CACCCCCATT ATACACGCAT TCAGAGACAT AGATGTCGAG GGGGAGATGA ATTAGCAAGA AGATGTCGAG   
  
  
+ CTAAAATGCA TTTCGAGGTT ACTAGAGCCA GAAATGTGGG CACATCCCAG AAGATCTCAG TAGAGAGAGT   
  
  
+ AACTTCAGAA AAGTTTCAAT TTTTATTCGA AATTTGCCTC TTGTTTGTGT AGATATGATG GGAACTTCGC   
  
  
+ CTTACAATCT GCAATGTGAG GGTTTGGTTG AAGGAGTTGC AGTCAATCCT CAATTTTATG AATCAACCCC   
  
  
+ AAAGTGGAAG AAGGGCATCA AAGAAGAGTC AATTGAGAGC GAACCCACTT CGGTTTTGGA CAACCCTAGC   
  
  
+ CCTCCAAATT CTGCATCTAC TCTCTCTTCC TGCTTCAATG GTGGAAGTAG TGGGCTTGGG ATGGACGATT   
  
  
+ TGGAGGGTTT GTCTTTTGGA GAAGGGTCCC TTCTCCCTTG GTTCATGGGT GAAATTGAAG ACCCTAGTTT   
  
  
+ GAGTTTCAAG CACCTCCTTC AACCTTCAAA TCCCTTGGAG TTTGGTGGCA ATGATGGGCT TGAAGCAGCT   
  
  
+ ATTCAGAGTG CTGGTATGGG TAATTTTAAT TTGGTTGAAA GTTTTAATTC TGATTTGAGG TCTTGTAATT   
  
  
+ CTGGGTCTAT TTGCAATGGG GGGAATGATT CTGTCAATGA AAAGGGATGT GTTCTTAATT ATGCTTCAGA   
  
  
+ GCAGGGTCAT GTGGTTGCTG TATCTCCAGT AGAACAGTTT AGGATTAGTG ATGTGAAACA TGAGGTGTTG   
  
  
+ AACCCAAAAT TTGGCTTTTG TGAGCAAGGC TCTACAATCA GTCAGAACTC TGGTTATGTG CATATACTGG   
  
  
+ GTTATAACCA GCTAGAATCT CCCCTTGAGC CTCCGGCAAA GCGCCACAAT GCTGGGAATG CTCTTTACAC   
  
  
+ TAGTGTTGTT CCAGTTCCAA AGAGTTCGTT TTTAGGTGAG GATCATGAAA TTCTACTCAG AAATCAAGAG   
  
  
+ CAGCAGTTTC TGATGCAACA ACAGTGGTTA ATGGGTTTAG CCCCTCAGTT TACACCCCAG CACCCGCCAA   
  
  
+ AGCCATTAGA CGACCGAAAG CAACCAAACT CGCAAACTTT GGTTCTGCGC GATCAGTTAG TTAAGATTGC   
  
  
+ GGAGATGTTC CAAACCGGTA ACTTTTCGCT TGCCCAAGTG ATATTGGCCC GGCTCAATCA GCAATTGTCT   
  
  
+ CTTTCAGGGA ACCCCTTTGT AAGAGCTGCT ACTTTCCATG TCAAGGAGGA ACTTGAAAGG CTCCTGACTA   
  
  
+ TCAATAGCTT CTCTGCGGCT CCACCCCAAC CTAAAAGCCT TACTCTGTCA GATGTTGTGG ATAGGATGAA   
  
  
+ TGCATACAAG CTTTTTTCAG AGGCATCCCC AGTTATGCAA TTTACCGATT TTACTTGTAC CCAGGCTTTG   
  
  
+ CTCGAAGCTC TTGATGATGC TGATTATATC CACATACTAG ATTTTGACAT TGGTTGTGGT TCCCAATGGG   
  
  
+ CATCCTTTAT TCGAGAGCTT CCTCTCAGGA AAAGGGGCAC TCCATTTTTG AAAATTACAG CCTTTGCCTC   
  
  
+ TTCTTTAACC CATAATCCAT TTGAACTAGC CCTTGTATGT GATAACATTC TGGAATTTGC AAAAGAGTTC   
  
  
+ GGTGTCTCTG TCGACCTTCA GGTCATGAAT TTGGACTTGT TTGACCCAAC TACTTGCACA ATACCCAATT   
  
  
+ TTCAATCCAC TGAGAACGCA GCAGTTGCTG TCAACTTCCC TATATGGGCG TGCTCACACT GCCCGTTTGT   
  
  
+ ACTCAGTCAA CTCCTGTCCT TCATCAAGCA ATGTTCCCCA AAAATCATGA CGAGCTTTGA TAGAGGTTGT   
  
  
+ GATCATTTTG AGTTGTCTTT TCCATCGCAC ATCATCCGTG TATTGGACTC ATGCTCTAAT ATGTTGGAGT   
  
  
+ CCCTCCATGG TTCAAGCGTG ACATTAGACA CCTCAAAGAA GATTGAAAAG TTCTTGATCA AGCCTATAAT   
  
  
+ TGAGAGGGCA ATATTGGGGC GAATTCATGC ACCAGACAAG ATGCAGGCAA ACAAGGCACA CCGGTGGAAG   
  
  
+ AATCTCTTCA CTTCTGCTGG GTTCTTCCCT TTGCCATTTA GTAGTTTCAC TGAATCTCAG GCCAATCTAG   
  
  
+ TGGCTCAACG AACCCCAGTG AGGGGTTTTC AGGTGGAAAA GCAGCAAGCA TCACTTGTGC TCAGGTGGAT   
  
  
+ GTCTCATGAG CTCGTGGCAG CTTCAGCTTG GAGGTGCTA  

- -Up\_Stream \_Len000AAACAT AAATTTAAAA TGTTATAAAT ATTTTTAAAA CATAACTAAT TAACACGATT   
  
  
- GATTGTGATT ATTAACCCCC TCTTTTCTTC CCTTTTATTT TTCTTTAATT CTTCTTCTTT TCTTTTGTGG   
  
  
- AAACTTTTCC GTTTTTTGAT TTTATTTTGT TATTACTTGT CTTCCTCTCT TCCTACCTAT TTGTTGTTTA   
  
  
- CAGCGAAAAT CCTGTCGTTA TCAGATACAC TAACCGGAAT CAAGGACAAC CTCCTAACGA TTGTTTTCGT   
  
  
- TGTCGTTTTT TATTATGATT AATAATGTAT AAAAAATTGA GAATTTCGAT TAGTTAGTAG GAGGTAATTA   
  
  
- ACTAATTAAT AAACGGTTTT TGTGTTTAGT TGACCCCGTT GATATTCTCT AGATGGACGA AATTCTATAT   
  
  
- ATTTGAGTTA ACAAAGTAAA ATAAAATAAA ATCTAAAAAA CCGGAAACCA GAGATAACTC GATTTTAGAG   
  
  
- CAATAAAAGC CTAGTACTTT TTATGTTGAG CTAGAAAACT ATATTCAGTA GAAATAAACA TTTAAGACTT   
  
  
- GAATTTTAGA ATGAACTTCT AGTCCTTATC TTCGTGAGCT AAAAACTATT TAGGATACAC AAGAAGAATT   
  
  
- TTAAGTATTA GATTAAGTAT AAAATCTACA TGGGCAACGG ATGAAACCGT TCAAATCTAA TACTAAAAAT   
  
  
- AAAAAAAATT AATGAACATA AAAAGAAAAC CTAACTATCA GTTAGATGTA GTAGAAACTA GTTAACGCTG   
  
  
- TCAAAGTAAA AGGGTTGTTT TTAAAGCTCA CATAAGAAAA ATTAAATAGT ATTCATAAAT AACAGTTGTA   
  
  
- AGTTGTACGT AGTTGTTAGT AATTTGTGAA AAGCAGTGAG GAGTTATGAC CAGTCGTGAA TTCTAAAACA   
  
  
- GAAGATTTGA ATAGCATTCA TTGAATAACA ACTAATTAAC TACGACTGGT TTACTATGAC TCACGATTTT   
  
  
- AATATTAATT ATTTATGTTA ATTCTCAACT AGTTACAGCT ACTAAGTTTA GTTACACTAA CCACGACTTA   
  
  
- TCATTAATAT TCGTGGATAT TTCTGAACTT TTCTTGTTTG TAAATTAATC TAAAATATTA ATATTAAAGG   
  
  
- ATTATAAATC GTAAGTAGTC GTTTAAGTCG TGTGTGGTTG TAATCAGTTG TGGATAGTTG TGAATATTCA   
  
  
- TGGTTAATTA TTATTAATGG ATATTATCTA ATTTTCAATC CGGGAATTAC AAAGGGTGTA ATAATCTATA   
  
  
- TAGTTCCCTC CCCCTTTTTT TATTATGAGG GGGTAGAATT TTTTAAATAG GGAAAAAAAG AAAAATTACA   
  
  
- AGGTTTTGTT TTTATACGAA GATTTAATAC TAACGTTATA CTTACACATG CCAAAGTTTG GGTTAAAAGA   
  
  
- GGTTGTTAAG AAGATGATAT AACATTAATA TAATATGATG TTAAACATAG AGTAGATACT AGATGGGGGG   
  
  
- TTTTTGGTTT ATGAGTATAT TTCAAAATAA AAGAAAGGTA GGGTGAGATT TTTTTTTTTT ACAACCCTTT   
  
  
- TTCAACTAAA TAAATTAAAC TTTACCATGC CACTTATATC TAAACATCTC CCCGTCAACT ATCTTTTTCC   
  
  
- ACTTCTGTTT CCTTCCCGTG TAAACGGAAA AGTATCTTCC TTCTCTGGTT CCATGACCCC TGACCCCCTT   
  
  
- CCCGAGTTCT CTTCTCTCTC TGACTCTTCT CACGACGATG AGGAAGGATG GATGTGTGTT TTAACGACGC   
  
  
- CCCCCAAGTT CAAGTTGAAC TTGCTCCGTT TATGGGAGTA ACTTGGGTGG TTTAGCTTTA GAAGTTACAT   
  
  
- GTGGGGGTAA TATGTGCGTA AGTCTCTGTA TCTACAGCTC CCCCTCTACT TAATCGTTCT TCTACAGCTC   
  
  
- GATTTTACGT AAAGCTCCAA TGATCTCGGT CTTTACACCC GTGTAGGGTC TTCTAGAGTC ATCTCTCTCA   
  
  
- TTGAAGTCTT TTCAAAGTTA AAAATAAGCT TTAAACGGAG AACAAACACA TCTATACTAC CCTTGAAGCG   
  
  
- GAATGTTAGA CGTTACACTC CCAAACCAAC TTCCTCAACG TCAGTTAGGA GTTAAAATAC TTAGTTGGGG   
  
  
- TTTCACCTTC TTCCCGTAGT TTCTTCTCAG TTAACTCTCG CTTGGGTGAA GCCAAAACCT GTTGGGATCG   
  
  
- GGAGGTTTAA GACGTAGATG AGAGAGAAGG ACGAAGTTAC CACCTTCATC ACCCGAACCC TACCTGCTAA   
  
  
- ACCTCCCAAA CAGAAAACCT CTTCCCAGGG AAGAGGGAAC CAAGTACCCA CTTTAACTTC TGGGATCAAA   
  
  
- CTCAAAGTTC GTGGAGGAAG TTGGAAGTTT AGGGAACCTC AAACCACCGT TACTACCCGA ACTTCGTCGA   
  
  
- TAAGTCTCAC GACCATACCC ATTAAAATTA AACCAACTTT CAAAATTAAG ACTAAACTCC AGAACATTAA   
  
  
- GACCCAGATA AACGTTACCC CCCTTACTAA GACAGTTACT TTTCCCTACA CAAGAATTAA TACGAAGTCT   
  
  
- CGTCCCAGTA CACCAACGAC ATAGAGGTCA TCTTGTCAAA TCCTAATCAC TACACTTTGT ACTCCACAAC   
  
  
- TTGGGTTTTA AACCGAAAAC ACTCGTTCCG AGATGTTAGT CAGTCTTGAG ACCAATACAC GTATATGACC   
  
  
- CAATATTGGT CGATCTTAGA GGGGAACTCG GAGGCCGTTT CGCGGTGTTA CGACCCTTAC GAGAAATGTG   
  
  
- ATCACAACAA GGTCAAGGTT TCTCAAGCAA AAATCCACTC CTAGTACTTT AAGATGAGTC TTTAGTTCTC   
  
  
- GTCGTCAAAG ACTACGTTGT TGTCACCAAT TACCCAAATC GGGGAGTCAA ATGTGGGGTC GTGGGCGGTT   
  
  
- TCGGTAATCT GCTGGCTTTC GTTGGTTTGA GCGTTTGAAA CCAAGACGCG CTAGTCAATC AATTCTAACG   
  
  
- CCTCTACAAG GTTTGGCCAT TGAAAAGCGA ACGGGTTCAC TATAACCGGG CCGAGTTAGT CGTTAACAGA   
  
  
- GAAAGTCCCT TGGGGAAACA TTCTCGACGA TGAAAGGTAC AGTTCCTCCT TGAACTTTCC GAGGACTGAT   
  
  
- AGTTATCGAA GAGACGCCGA GGTGGGGTTG GATTTTCGGA ATGAGACAGT CTACAACACC TATCCTACTT   
  
  
- ACGTATGTTC GAAAAAAGTC TCCGTAGGGG TCAATACGTT AAATGGCTAA AATGAACATG GGTCCGAAAC   
  
  
- GAGCTTCGAG AACTACTACG ACTAATATAG GTGTATGATC TAAAACTGTA ACCAACACCA AGGGTTACCC   
  
  
- GTAGGAAATA AGCTCTCGAA GGAGAGTCCT TTTCCCCGTG AGGTAAAAAC TTTTAATGTC GGAAACGGAG   
  
  
- AAGAAATTGG GTATTAGGTA AACTTGATCG GGAACATACA CTATTGTAAG ACCTTAAACG TTTTCTCAAG   
  
  
- CCACAGAGAC AGCTGGAAGT CCAGTACTTA AACCTGAACA AACTGGGTTG ATGAACGTGT TATGGGTTAA   
  
  
- AAGTTAGGTG ACTCTTGCGT CGTCAACGAC AGTTGAAGGG ATATACCCGC ACGAGTGTGA CGGGCAAACA   
  
  
- TGAGTCAGTT GAGGACAGGA AGTAGTTCGT TACAAGGGGT TTTTAGTACT GCTCGAAACT ATCTCCAACA   
  
  
- CTAGTAAAAC TCAACAGAAA AGGTAGCGTG TAGTAGGCAC ATAACCTGAG TACGAGATTA TACAACCTCA   
  
  
- GGGAGGTACC AAGTTCGCAC TGTAATCTGT GGAGTTTCTT CTAACTTTTC AAGAACTAGT TCGGATATTA   
  
  
- ACTCTCCCGT TATAACCCCG CTTAAGTACG TGGTCTGTTC TACGTCCGTT TGTTCCGTGT GGCCACCTTC   
  
  
- TTAGAGAAGT GAAGACGACC CAAGAAGGGA AACGGTAAAT CATCAAAGTG ACTTAGAGTC CGGTTAGATC   
  
  
- ACCGAGTTGC TTGGGGTCAC TCCCCAAAAG TCCACCTTTT CGTCGTTCGT AGTGAACACG AGTCCACCTA   
  
  
- CAGAGTACTC GAGCACCGTC GAAGTCGAAC CTCCACGAT

+     CAT-box

| Site Name | Organism | Position | Strand | Matrix score. | sequence | function |
| --- | --- | --- | --- | --- | --- | --- |
| CAT-box | Arabidopsis thaliana | 3923 | - | 6 | GCCACT | cis-acting regulatory element related to meristem expression |

>HU04G00048.1   
+ -Up\_Stream \_Len000TTTGTA TTTAAATTTT ACAATATTTA TAAAAATTTT GTATTGATTA ATTGTGCTAA   
  
  
+ CTAACACTAA TAATTGGGGG AGAAAAGAAG GGAAAATAAA AAGAAATTAA GAAGAAGAAA AGAAAACACC   
  
  
+ TTTGAAAAGG CAAAAAACTA AAATAAAACA ATAATGAACA GAAGGAGAGA AGGATGGATA AACAACAAAT   
  
  
+ GTCGCTTTTA GGACAGCAAT AGTCTATGTG ATTGGCCTTA GTTCCTGTTG GAGGATTGCT AACAAAAGCA   
  
  
+ ACAGCAAAAA ATAATACTAA TTATTACATA TTTTTTAACT CTTAAAGCTA ATCAATCATC CTCCATTAAT   
  
  
+ TGATTAATTA TTTGCCAAAA ACACAAATCA ACTGGGGCAA CTATAAGAGA TCTACCTGCT TTAAGATATA   
  
  
+ TAAACTCAAT TGTTTCATTT TATTTTATTT TAGATTTTTT GGCCTTTGGT CTCTATTGAG CTAAAATCTC   
  
  
+ GTTATTTTCG GATCATGAAA AATACAACTC GATCTTTTGA TATAAGTCAT CTTTATTTGT AAATTCTGAA   
  
  
+ CTTAAAATCT TACTTGAAGA TCAGGAATAG AAGCACTCGA TTTTTGATAA ATCCTATGTG TTCTTCTTAA   
  
  
+ AATTCATAAT CTAATTCATA TTTTAGATGT ACCCGTTGCC TACTTTGGCA AGTTTAGATT ATGATTTTTA   
  
  
+ TTTTTTTTAA TTACTTGTAT TTTTCTTTTG GATTGATAGT CAATCTACAT CATCTTTGAT CAATTGCGAC   
  
  
+ AGTTTCATTT TCCCAACAAA AATTTCGAGT GTATTCTTTT TAATTTATCA TAAGTATTTA TTGTCAACAT   
  
  
+ TCAACATGCA TCAACAATCA TTAAACACTT TTCGTCACTC CTCAATACTG GTCAGCACTT AAGATTTTGT   
  
  
+ CTTCTAAACT TATCGTAAGT AACTTATTGT TGATTAATTG ATGCTGACCA AATGATACTG AGTGCTAAAA   
  
  
+ TTATAATTAA TAAATACAAT TAAGAGTTGA TCAATGTCGA TGATTCAAAT CAATGTGATT GGTGCTGAAT   
  
  
+ AGTAATTATA AGCACCTATA AAGACTTGAA AAGAACAAAC ATTTAATTAG ATTTTATAAT TATAATTTCC   
  
  
+ TAATATTTAG CATTCATCAG CAAATTCAGC ACACACCAAC ATTAGTCAAC ACCTATCAAC ACTTATAAGT   
  
  
+ ACCAATTAAT AATAATTACC TATAATAGAT TAAAAGTTAG GCCCTTAATG TTTCCCACAT TATTAGATAT   
  
  
+ ATCAAGGGAG GGGGAAAAAA ATAATACTCC CCCATCTTAA AAAATTTATC CCTTTTTTTC TTTTTAATGT   
  
  
+ TCCAAAACAA AAATATGCTT CTAAATTATG ATTGCAATAT GAATGTGTAC GGTTTCAAAC CCAATTTTCT   
  
  
+ CCAACAATTC TTCTACTATA TTGTAATTAT ATTATACTAC AATTTGTATC TCATCTATGA TCTACCCCCC   
  
  
+ AAAAACCAAA TACTCATATA AAGTTTTATT TTCTTTCCAT CCCACTCTAA AAAAAAAAAA TGTTGGGAAA   
  
  
+ AAGTTGATTT ATTTAATTTG AAATGGTACG GTGAATATAG ATTTGTAGAG GGGCAGTTGA TAGAAAAAGG   
  
  
+ TGAAGACAAA GGAAGGGCAC ATTTGCCTTT TCATAGAAGG AAGAGACCAA GGTACTGGGG ACTGGGGGAA   
  
  
+ GGGCTCAAGA GAAGAGAGAG ACTGAGAAGA GTGCTGCTAC TCCTTCCTAC CTACACACAA AATTGCTGCG   
  
  
+ GGGGGTTCAA GTTCAACTTG AACGAGGCAA ATACCCTCAT TGAACCCACC AAATCGAAAT CTTCAATGTA   
  
  
+ CACCCCCATT ATACACGCAT TCAGAGACAT AGATGTCGAG GGGGAGATGA ATTAGCAAGA AGATGTCGAG   
  
  
+ CTAAAATGCA TTTCGAGGTT ACTAGAGCCA GAAATGTGGG CACATCCCAG AAGATCTCAG TAGAGAGAGT   
  
  
+ AACTTCAGAA AAGTTTCAAT TTTTATTCGA AATTTGCCTC TTGTTTGTGT AGATATGATG GGAACTTCGC   
  
  
+ CTTACAATCT GCAATGTGAG GGTTTGGTTG AAGGAGTTGC AGTCAATCCT CAATTTTATG AATCAACCCC   
  
  
+ AAAGTGGAAG AAGGGCATCA AAGAAGAGTC AATTGAGAGC GAACCCACTT CGGTTTTGGA CAACCCTAGC   
  
  
+ CCTCCAAATT CTGCATCTAC TCTCTCTTCC TGCTTCAATG GTGGAAGTAG TGGGCTTGGG ATGGACGATT   
  
  
+ TGGAGGGTTT GTCTTTTGGA GAAGGGTCCC TTCTCCCTTG GTTCATGGGT GAAATTGAAG ACCCTAGTTT   
  
  
+ GAGTTTCAAG CACCTCCTTC AACCTTCAAA TCCCTTGGAG TTTGGTGGCA ATGATGGGCT TGAAGCAGCT   
  
  
+ ATTCAGAGTG CTGGTATGGG TAATTTTAAT TTGGTTGAAA GTTTTAATTC TGATTTGAGG TCTTGTAATT   
  
  
+ CTGGGTCTAT TTGCAATGGG GGGAATGATT CTGTCAATGA AAAGGGATGT GTTCTTAATT ATGCTTCAGA   
  
  
+ GCAGGGTCAT GTGGTTGCTG TATCTCCAGT AGAACAGTTT AGGATTAGTG ATGTGAAACA TGAGGTGTTG   
  
  
+ AACCCAAAAT TTGGCTTTTG TGAGCAAGGC TCTACAATCA GTCAGAACTC TGGTTATGTG CATATACTGG   
  
  
+ GTTATAACCA GCTAGAATCT CCCCTTGAGC CTCCGGCAAA GCGCCACAAT GCTGGGAATG CTCTTTACAC   
  
  
+ TAGTGTTGTT CCAGTTCCAA AGAGTTCGTT TTTAGGTGAG GATCATGAAA TTCTACTCAG AAATCAAGAG   
  
  
+ CAGCAGTTTC TGATGCAACA ACAGTGGTTA ATGGGTTTAG CCCCTCAGTT TACACCCCAG CACCCGCCAA   
  
  
+ AGCCATTAGA CGACCGAAAG CAACCAAACT CGCAAACTTT GGTTCTGCGC GATCAGTTAG TTAAGATTGC   
  
  
+ GGAGATGTTC CAAACCGGTA ACTTTTCGCT TGCCCAAGTG ATATTGGCCC GGCTCAATCA GCAATTGTCT   
  
  
+ CTTTCAGGGA ACCCCTTTGT AAGAGCTGCT ACTTTCCATG TCAAGGAGGA ACTTGAAAGG CTCCTGACTA   
  
  
+ TCAATAGCTT CTCTGCGGCT CCACCCCAAC CTAAAAGCCT TACTCTGTCA GATGTTGTGG ATAGGATGAA   
  
  
+ TGCATACAAG CTTTTTTCAG AGGCATCCCC AGTTATGCAA TTTACCGATT TTACTTGTAC CCAGGCTTTG   
  
  
+ CTCGAAGCTC TTGATGATGC TGATTATATC CACATACTAG ATTTTGACAT TGGTTGTGGT TCCCAATGGG   
  
  
+ CATCCTTTAT TCGAGAGCTT CCTCTCAGGA AAAGGGGCAC TCCATTTTTG AAAATTACAG CCTTTGCCTC   
  
  
+ TTCTTTAACC CATAATCCAT TTGAACTAGC CCTTGTATGT GATAACATTC TGGAATTTGC AAAAGAGTTC   
  
  
+ GGTGTCTCTG TCGACCTTCA GGTCATGAAT TTGGACTTGT TTGACCCAAC TACTTGCACA ATACCCAATT   
  
  
+ TTCAATCCAC TGAGAACGCA GCAGTTGCTG TCAACTTCCC TATATGGGCG TGCTCACACT GCCCGTTTGT   
  
  
+ ACTCAGTCAA CTCCTGTCCT TCATCAAGCA ATGTTCCCCA AAAATCATGA CGAGCTTTGA TAGAGGTTGT   
  
  
+ GATCATTTTG AGTTGTCTTT TCCATCGCAC ATCATCCGTG TATTGGACTC ATGCTCTAAT ATGTTGGAGT   
  
  
+ CCCTCCATGG TTCAAGCGTG ACATTAGACA CCTCAAAGAA GATTGAAAAG TTCTTGATCA AGCCTATAAT   
  
  
+ TGAGAGGGCA ATATTGGGGC GAATTCATGC ACCAGACAAG ATGCAGGCAA ACAAGGCACA CCGGTGGAAG   
  
  
+ AATCTCTTCA CTTCTGCTGG GTTCTTCCCT TTGCCATTTA GTAGTTTCAC TGAATCTCAG GCCAATCTAG   
  
  
+ TGGCTCAACG AACCCCAGTG AGGGGTTTTC AGGTGGAAAA GCAGCAAGCA TCACTTGTGC TCAGGTGGAT   
  
  
+ GTCTCATGAG CTCGTGGCAG CTTCAGCTTG GAGGTGCTA  

- -Up\_Stream \_Len000AAACAT AAATTTAAAA TGTTATAAAT ATTTTTAAAA CATAACTAAT TAACACGATT   
  
  
- GATTGTGATT ATTAACCCCC TCTTTTCTTC CCTTTTATTT TTCTTTAATT CTTCTTCTTT TCTTTTGTGG   
  
  
- AAACTTTTCC GTTTTTTGAT TTTATTTTGT TATTACTTGT CTTCCTCTCT TCCTACCTAT TTGTTGTTTA   
  
  
- CAGCGAAAAT CCTGTCGTTA TCAGATACAC TAACCGGAAT CAAGGACAAC CTCCTAACGA TTGTTTTCGT   
  
  
- TGTCGTTTTT TATTATGATT AATAATGTAT AAAAAATTGA GAATTTCGAT TAGTTAGTAG GAGGTAATTA   
  
  
- ACTAATTAAT AAACGGTTTT TGTGTTTAGT TGACCCCGTT GATATTCTCT AGATGGACGA AATTCTATAT   
  
  
- ATTTGAGTTA ACAAAGTAAA ATAAAATAAA ATCTAAAAAA CCGGAAACCA GAGATAACTC GATTTTAGAG   
  
  
- CAATAAAAGC CTAGTACTTT TTATGTTGAG CTAGAAAACT ATATTCAGTA GAAATAAACA TTTAAGACTT   
  
  
- GAATTTTAGA ATGAACTTCT AGTCCTTATC TTCGTGAGCT AAAAACTATT TAGGATACAC AAGAAGAATT   
  
  
- TTAAGTATTA GATTAAGTAT AAAATCTACA TGGGCAACGG ATGAAACCGT TCAAATCTAA TACTAAAAAT   
  
  
- AAAAAAAATT AATGAACATA AAAAGAAAAC CTAACTATCA GTTAGATGTA GTAGAAACTA GTTAACGCTG   
  
  
- TCAAAGTAAA AGGGTTGTTT TTAAAGCTCA CATAAGAAAA ATTAAATAGT ATTCATAAAT AACAGTTGTA   
  
  
- AGTTGTACGT AGTTGTTAGT AATTTGTGAA AAGCAGTGAG GAGTTATGAC CAGTCGTGAA TTCTAAAACA   
  
  
- GAAGATTTGA ATAGCATTCA TTGAATAACA ACTAATTAAC TACGACTGGT TTACTATGAC TCACGATTTT   
  
  
- AATATTAATT ATTTATGTTA ATTCTCAACT AGTTACAGCT ACTAAGTTTA GTTACACTAA CCACGACTTA   
  
  
- TCATTAATAT TCGTGGATAT TTCTGAACTT TTCTTGTTTG TAAATTAATC TAAAATATTA ATATTAAAGG   
  
  
- ATTATAAATC GTAAGTAGTC GTTTAAGTCG TGTGTGGTTG TAATCAGTTG TGGATAGTTG TGAATATTCA   
  
  
- TGGTTAATTA TTATTAATGG ATATTATCTA ATTTTCAATC CGGGAATTAC AAAGGGTGTA ATAATCTATA   
  
  
- TAGTTCCCTC CCCCTTTTTT TATTATGAGG GGGTAGAATT TTTTAAATAG GGAAAAAAAG AAAAATTACA   
  
  
- AGGTTTTGTT TTTATACGAA GATTTAATAC TAACGTTATA CTTACACATG CCAAAGTTTG GGTTAAAAGA   
  
  
- GGTTGTTAAG AAGATGATAT AACATTAATA TAATATGATG TTAAACATAG AGTAGATACT AGATGGGGGG   
  
  
- TTTTTGGTTT ATGAGTATAT TTCAAAATAA AAGAAAGGTA GGGTGAGATT TTTTTTTTTT ACAACCCTTT   
  
  
- TTCAACTAAA TAAATTAAAC TTTACCATGC CACTTATATC TAAACATCTC CCCGTCAACT ATCTTTTTCC   
  
  
- ACTTCTGTTT CCTTCCCGTG TAAACGGAAA AGTATCTTCC TTCTCTGGTT CCATGACCCC TGACCCCCTT   
  
  
- CCCGAGTTCT CTTCTCTCTC TGACTCTTCT CACGACGATG AGGAAGGATG GATGTGTGTT TTAACGACGC   
  
  
- CCCCCAAGTT CAAGTTGAAC TTGCTCCGTT TATGGGAGTA ACTTGGGTGG TTTAGCTTTA GAAGTTACAT   
  
  
- GTGGGGGTAA TATGTGCGTA AGTCTCTGTA TCTACAGCTC CCCCTCTACT TAATCGTTCT TCTACAGCTC   
  
  
- GATTTTACGT AAAGCTCCAA TGATCTCGGT CTTTACACCC GTGTAGGGTC TTCTAGAGTC ATCTCTCTCA   
  
  
- TTGAAGTCTT TTCAAAGTTA AAAATAAGCT TTAAACGGAG AACAAACACA TCTATACTAC CCTTGAAGCG   
  
  
- GAATGTTAGA CGTTACACTC CCAAACCAAC TTCCTCAACG TCAGTTAGGA GTTAAAATAC TTAGTTGGGG   
  
  
- TTTCACCTTC TTCCCGTAGT TTCTTCTCAG TTAACTCTCG CTTGGGTGAA GCCAAAACCT GTTGGGATCG   
  
  
- GGAGGTTTAA GACGTAGATG AGAGAGAAGG ACGAAGTTAC CACCTTCATC ACCCGAACCC TACCTGCTAA   
  
  
- ACCTCCCAAA CAGAAAACCT CTTCCCAGGG AAGAGGGAAC CAAGTACCCA CTTTAACTTC TGGGATCAAA   
  
  
- CTCAAAGTTC GTGGAGGAAG TTGGAAGTTT AGGGAACCTC AAACCACCGT TACTACCCGA ACTTCGTCGA   
  
  
- TAAGTCTCAC GACCATACCC ATTAAAATTA AACCAACTTT CAAAATTAAG ACTAAACTCC AGAACATTAA   
  
  
- GACCCAGATA AACGTTACCC CCCTTACTAA GACAGTTACT TTTCCCTACA CAAGAATTAA TACGAAGTCT   
  
  
- CGTCCCAGTA CACCAACGAC ATAGAGGTCA TCTTGTCAAA TCCTAATCAC TACACTTTGT ACTCCACAAC   
  
  
- TTGGGTTTTA AACCGAAAAC ACTCGTTCCG AGATGTTAGT CAGTCTTGAG ACCAATACAC GTATATGACC   
  
  
- CAATATTGGT CGATCTTAGA GGGGAACTCG GAGGCCGTTT CGCGGTGTTA CGACCCTTAC GAGAAATGTG   
  
  
- ATCACAACAA GGTCAAGGTT TCTCAAGCAA AAATCCACTC CTAGTACTTT AAGATGAGTC TTTAGTTCTC   
  
  
- GTCGTCAAAG ACTACGTTGT TGTCACCAAT TACCCAAATC GGGGAGTCAA ATGTGGGGTC GTGGGCGGTT   
  
  
- TCGGTAATCT GCTGGCTTTC GTTGGTTTGA GCGTTTGAAA CCAAGACGCG CTAGTCAATC AATTCTAACG   
  
  
- CCTCTACAAG GTTTGGCCAT TGAAAAGCGA ACGGGTTCAC TATAACCGGG CCGAGTTAGT CGTTAACAGA   
  
  
- GAAAGTCCCT TGGGGAAACA TTCTCGACGA TGAAAGGTAC AGTTCCTCCT TGAACTTTCC GAGGACTGAT   
  
  
- AGTTATCGAA GAGACGCCGA GGTGGGGTTG GATTTTCGGA ATGAGACAGT CTACAACACC TATCCTACTT   
  
  
- ACGTATGTTC GAAAAAAGTC TCCGTAGGGG TCAATACGTT AAATGGCTAA AATGAACATG GGTCCGAAAC   
  
  
- GAGCTTCGAG AACTACTACG ACTAATATAG GTGTATGATC TAAAACTGTA ACCAACACCA AGGGTTACCC   
  
  
- GTAGGAAATA AGCTCTCGAA GGAGAGTCCT TTTCCCCGTG AGGTAAAAAC TTTTAATGTC GGAAACGGAG   
  
  
- AAGAAATTGG GTATTAGGTA AACTTGATCG GGAACATACA CTATTGTAAG ACCTTAAACG TTTTCTCAAG   
  
  
- CCACAGAGAC AGCTGGAAGT CCAGTACTTA AACCTGAACA AACTGGGTTG ATGAACGTGT TATGGGTTAA   
  
  
- AAGTTAGGTG ACTCTTGCGT CGTCAACGAC AGTTGAAGGG ATATACCCGC ACGAGTGTGA CGGGCAAACA   
  
  
- TGAGTCAGTT GAGGACAGGA AGTAGTTCGT TACAAGGGGT TTTTAGTACT GCTCGAAACT ATCTCCAACA   
  
  
- CTAGTAAAAC TCAACAGAAA AGGTAGCGTG TAGTAGGCAC ATAACCTGAG TACGAGATTA TACAACCTCA   
  
  
- GGGAGGTACC AAGTTCGCAC TGTAATCTGT GGAGTTTCTT CTAACTTTTC AAGAACTAGT TCGGATATTA   
  
  
- ACTCTCCCGT TATAACCCCG CTTAAGTACG TGGTCTGTTC TACGTCCGTT TGTTCCGTGT GGCCACCTTC   
  
  
- TTAGAGAAGT GAAGACGACC CAAGAAGGGA AACGGTAAAT CATCAAAGTG ACTTAGAGTC CGGTTAGATC   
  
  
- ACCGAGTTGC TTGGGGTCAC TCCCCAAAAG TCCACCTTTT CGTCGTTCGT AGTGAACACG AGTCCACCTA   
  
  
- CAGAGTACTC GAGCACCGTC GAAGTCGAAC CTCCACGAT

+     CCAAT-box

| Site Name | Organism | Position | Strand | Matrix score. | sequence | function |
| --- | --- | --- | --- | --- | --- | --- |
| CCAAT-box | Hordeum vulgare | 667 | - | 6 | CAACGG | MYBHv1 binding site |

>HU04G00048.1   
+ -Up\_Stream \_Len000TTTGTA TTTAAATTTT ACAATATTTA TAAAAATTTT GTATTGATTA ATTGTGCTAA   
  
  
+ CTAACACTAA TAATTGGGGG AGAAAAGAAG GGAAAATAAA AAGAAATTAA GAAGAAGAAA AGAAAACACC   
  
  
+ TTTGAAAAGG CAAAAAACTA AAATAAAACA ATAATGAACA GAAGGAGAGA AGGATGGATA AACAACAAAT   
  
  
+ GTCGCTTTTA GGACAGCAAT AGTCTATGTG ATTGGCCTTA GTTCCTGTTG GAGGATTGCT AACAAAAGCA   
  
  
+ ACAGCAAAAA ATAATACTAA TTATTACATA TTTTTTAACT CTTAAAGCTA ATCAATCATC CTCCATTAAT   
  
  
+ TGATTAATTA TTTGCCAAAA ACACAAATCA ACTGGGGCAA CTATAAGAGA TCTACCTGCT TTAAGATATA   
  
  
+ TAAACTCAAT TGTTTCATTT TATTTTATTT TAGATTTTTT GGCCTTTGGT CTCTATTGAG CTAAAATCTC   
  
  
+ GTTATTTTCG GATCATGAAA AATACAACTC GATCTTTTGA TATAAGTCAT CTTTATTTGT AAATTCTGAA   
  
  
+ CTTAAAATCT TACTTGAAGA TCAGGAATAG AAGCACTCGA TTTTTGATAA ATCCTATGTG TTCTTCTTAA   
  
  
+ AATTCATAAT CTAATTCATA TTTTAGATGT ACCCGTTGCC TACTTTGGCA AGTTTAGATT ATGATTTTTA   
  
  
+ TTTTTTTTAA TTACTTGTAT TTTTCTTTTG GATTGATAGT CAATCTACAT CATCTTTGAT CAATTGCGAC   
  
  
+ AGTTTCATTT TCCCAACAAA AATTTCGAGT GTATTCTTTT TAATTTATCA TAAGTATTTA TTGTCAACAT   
  
  
+ TCAACATGCA TCAACAATCA TTAAACACTT TTCGTCACTC CTCAATACTG GTCAGCACTT AAGATTTTGT   
  
  
+ CTTCTAAACT TATCGTAAGT AACTTATTGT TGATTAATTG ATGCTGACCA AATGATACTG AGTGCTAAAA   
  
  
+ TTATAATTAA TAAATACAAT TAAGAGTTGA TCAATGTCGA TGATTCAAAT CAATGTGATT GGTGCTGAAT   
  
  
+ AGTAATTATA AGCACCTATA AAGACTTGAA AAGAACAAAC ATTTAATTAG ATTTTATAAT TATAATTTCC   
  
  
+ TAATATTTAG CATTCATCAG CAAATTCAGC ACACACCAAC ATTAGTCAAC ACCTATCAAC ACTTATAAGT   
  
  
+ ACCAATTAAT AATAATTACC TATAATAGAT TAAAAGTTAG GCCCTTAATG TTTCCCACAT TATTAGATAT   
  
  
+ ATCAAGGGAG GGGGAAAAAA ATAATACTCC CCCATCTTAA AAAATTTATC CCTTTTTTTC TTTTTAATGT   
  
  
+ TCCAAAACAA AAATATGCTT CTAAATTATG ATTGCAATAT GAATGTGTAC GGTTTCAAAC CCAATTTTCT   
  
  
+ CCAACAATTC TTCTACTATA TTGTAATTAT ATTATACTAC AATTTGTATC TCATCTATGA TCTACCCCCC   
  
  
+ AAAAACCAAA TACTCATATA AAGTTTTATT TTCTTTCCAT CCCACTCTAA AAAAAAAAAA TGTTGGGAAA   
  
  
+ AAGTTGATTT ATTTAATTTG AAATGGTACG GTGAATATAG ATTTGTAGAG GGGCAGTTGA TAGAAAAAGG   
  
  
+ TGAAGACAAA GGAAGGGCAC ATTTGCCTTT TCATAGAAGG AAGAGACCAA GGTACTGGGG ACTGGGGGAA   
  
  
+ GGGCTCAAGA GAAGAGAGAG ACTGAGAAGA GTGCTGCTAC TCCTTCCTAC CTACACACAA AATTGCTGCG   
  
  
+ GGGGGTTCAA GTTCAACTTG AACGAGGCAA ATACCCTCAT TGAACCCACC AAATCGAAAT CTTCAATGTA   
  
  
+ CACCCCCATT ATACACGCAT TCAGAGACAT AGATGTCGAG GGGGAGATGA ATTAGCAAGA AGATGTCGAG   
  
  
+ CTAAAATGCA TTTCGAGGTT ACTAGAGCCA GAAATGTGGG CACATCCCAG AAGATCTCAG TAGAGAGAGT   
  
  
+ AACTTCAGAA AAGTTTCAAT TTTTATTCGA AATTTGCCTC TTGTTTGTGT AGATATGATG GGAACTTCGC   
  
  
+ CTTACAATCT GCAATGTGAG GGTTTGGTTG AAGGAGTTGC AGTCAATCCT CAATTTTATG AATCAACCCC   
  
  
+ AAAGTGGAAG AAGGGCATCA AAGAAGAGTC AATTGAGAGC GAACCCACTT CGGTTTTGGA CAACCCTAGC   
  
  
+ CCTCCAAATT CTGCATCTAC TCTCTCTTCC TGCTTCAATG GTGGAAGTAG TGGGCTTGGG ATGGACGATT   
  
  
+ TGGAGGGTTT GTCTTTTGGA GAAGGGTCCC TTCTCCCTTG GTTCATGGGT GAAATTGAAG ACCCTAGTTT   
  
  
+ GAGTTTCAAG CACCTCCTTC AACCTTCAAA TCCCTTGGAG TTTGGTGGCA ATGATGGGCT TGAAGCAGCT   
  
  
+ ATTCAGAGTG CTGGTATGGG TAATTTTAAT TTGGTTGAAA GTTTTAATTC TGATTTGAGG TCTTGTAATT   
  
  
+ CTGGGTCTAT TTGCAATGGG GGGAATGATT CTGTCAATGA AAAGGGATGT GTTCTTAATT ATGCTTCAGA   
  
  
+ GCAGGGTCAT GTGGTTGCTG TATCTCCAGT AGAACAGTTT AGGATTAGTG ATGTGAAACA TGAGGTGTTG   
  
  
+ AACCCAAAAT TTGGCTTTTG TGAGCAAGGC TCTACAATCA GTCAGAACTC TGGTTATGTG CATATACTGG   
  
  
+ GTTATAACCA GCTAGAATCT CCCCTTGAGC CTCCGGCAAA GCGCCACAAT GCTGGGAATG CTCTTTACAC   
  
  
+ TAGTGTTGTT CCAGTTCCAA AGAGTTCGTT TTTAGGTGAG GATCATGAAA TTCTACTCAG AAATCAAGAG   
  
  
+ CAGCAGTTTC TGATGCAACA ACAGTGGTTA ATGGGTTTAG CCCCTCAGTT TACACCCCAG CACCCGCCAA   
  
  
+ AGCCATTAGA CGACCGAAAG CAACCAAACT CGCAAACTTT GGTTCTGCGC GATCAGTTAG TTAAGATTGC   
  
  
+ GGAGATGTTC CAAACCGGTA ACTTTTCGCT TGCCCAAGTG ATATTGGCCC GGCTCAATCA GCAATTGTCT   
  
  
+ CTTTCAGGGA ACCCCTTTGT AAGAGCTGCT ACTTTCCATG TCAAGGAGGA ACTTGAAAGG CTCCTGACTA   
  
  
+ TCAATAGCTT CTCTGCGGCT CCACCCCAAC CTAAAAGCCT TACTCTGTCA GATGTTGTGG ATAGGATGAA   
  
  
+ TGCATACAAG CTTTTTTCAG AGGCATCCCC AGTTATGCAA TTTACCGATT TTACTTGTAC CCAGGCTTTG   
  
  
+ CTCGAAGCTC TTGATGATGC TGATTATATC CACATACTAG ATTTTGACAT TGGTTGTGGT TCCCAATGGG   
  
  
+ CATCCTTTAT TCGAGAGCTT CCTCTCAGGA AAAGGGGCAC TCCATTTTTG AAAATTACAG CCTTTGCCTC   
  
  
+ TTCTTTAACC CATAATCCAT TTGAACTAGC CCTTGTATGT GATAACATTC TGGAATTTGC AAAAGAGTTC   
  
  
+ GGTGTCTCTG TCGACCTTCA GGTCATGAAT TTGGACTTGT TTGACCCAAC TACTTGCACA ATACCCAATT   
  
  
+ TTCAATCCAC TGAGAACGCA GCAGTTGCTG TCAACTTCCC TATATGGGCG TGCTCACACT GCCCGTTTGT   
  
  
+ ACTCAGTCAA CTCCTGTCCT TCATCAAGCA ATGTTCCCCA AAAATCATGA CGAGCTTTGA TAGAGGTTGT   
  
  
+ GATCATTTTG AGTTGTCTTT TCCATCGCAC ATCATCCGTG TATTGGACTC ATGCTCTAAT ATGTTGGAGT   
  
  
+ CCCTCCATGG TTCAAGCGTG ACATTAGACA CCTCAAAGAA GATTGAAAAG TTCTTGATCA AGCCTATAAT   
  
  
+ TGAGAGGGCA ATATTGGGGC GAATTCATGC ACCAGACAAG ATGCAGGCAA ACAAGGCACA CCGGTGGAAG   
  
  
+ AATCTCTTCA CTTCTGCTGG GTTCTTCCCT TTGCCATTTA GTAGTTTCAC TGAATCTCAG GCCAATCTAG   
  
  
+ TGGCTCAACG AACCCCAGTG AGGGGTTTTC AGGTGGAAAA GCAGCAAGCA TCACTTGTGC TCAGGTGGAT   
  
  
+ GTCTCATGAG CTCGTGGCAG CTTCAGCTTG GAGGTGCTA  

- -Up\_Stream \_Len000AAACAT AAATTTAAAA TGTTATAAAT ATTTTTAAAA CATAACTAAT TAACACGATT   
  
  
- GATTGTGATT ATTAACCCCC TCTTTTCTTC CCTTTTATTT TTCTTTAATT CTTCTTCTTT TCTTTTGTGG   
  
  
- AAACTTTTCC GTTTTTTGAT TTTATTTTGT TATTACTTGT CTTCCTCTCT TCCTACCTAT TTGTTGTTTA   
  
  
- CAGCGAAAAT CCTGTCGTTA TCAGATACAC TAACCGGAAT CAAGGACAAC CTCCTAACGA TTGTTTTCGT   
  
  
- TGTCGTTTTT TATTATGATT AATAATGTAT AAAAAATTGA GAATTTCGAT TAGTTAGTAG GAGGTAATTA   
  
  
- ACTAATTAAT AAACGGTTTT TGTGTTTAGT TGACCCCGTT GATATTCTCT AGATGGACGA AATTCTATAT   
  
  
- ATTTGAGTTA ACAAAGTAAA ATAAAATAAA ATCTAAAAAA CCGGAAACCA GAGATAACTC GATTTTAGAG   
  
  
- CAATAAAAGC CTAGTACTTT TTATGTTGAG CTAGAAAACT ATATTCAGTA GAAATAAACA TTTAAGACTT   
  
  
- GAATTTTAGA ATGAACTTCT AGTCCTTATC TTCGTGAGCT AAAAACTATT TAGGATACAC AAGAAGAATT   
  
  
- TTAAGTATTA GATTAAGTAT AAAATCTACA TGGGCAACGG ATGAAACCGT TCAAATCTAA TACTAAAAAT   
  
  
- AAAAAAAATT AATGAACATA AAAAGAAAAC CTAACTATCA GTTAGATGTA GTAGAAACTA GTTAACGCTG   
  
  
- TCAAAGTAAA AGGGTTGTTT TTAAAGCTCA CATAAGAAAA ATTAAATAGT ATTCATAAAT AACAGTTGTA   
  
  
- AGTTGTACGT AGTTGTTAGT AATTTGTGAA AAGCAGTGAG GAGTTATGAC CAGTCGTGAA TTCTAAAACA   
  
  
- GAAGATTTGA ATAGCATTCA TTGAATAACA ACTAATTAAC TACGACTGGT TTACTATGAC TCACGATTTT   
  
  
- AATATTAATT ATTTATGTTA ATTCTCAACT AGTTACAGCT ACTAAGTTTA GTTACACTAA CCACGACTTA   
  
  
- TCATTAATAT TCGTGGATAT TTCTGAACTT TTCTTGTTTG TAAATTAATC TAAAATATTA ATATTAAAGG   
  
  
- ATTATAAATC GTAAGTAGTC GTTTAAGTCG TGTGTGGTTG TAATCAGTTG TGGATAGTTG TGAATATTCA   
  
  
- TGGTTAATTA TTATTAATGG ATATTATCTA ATTTTCAATC CGGGAATTAC AAAGGGTGTA ATAATCTATA   
  
  
- TAGTTCCCTC CCCCTTTTTT TATTATGAGG GGGTAGAATT TTTTAAATAG GGAAAAAAAG AAAAATTACA   
  
  
- AGGTTTTGTT TTTATACGAA GATTTAATAC TAACGTTATA CTTACACATG CCAAAGTTTG GGTTAAAAGA   
  
  
- GGTTGTTAAG AAGATGATAT AACATTAATA TAATATGATG TTAAACATAG AGTAGATACT AGATGGGGGG   
  
  
- TTTTTGGTTT ATGAGTATAT TTCAAAATAA AAGAAAGGTA GGGTGAGATT TTTTTTTTTT ACAACCCTTT   
  
  
- TTCAACTAAA TAAATTAAAC TTTACCATGC CACTTATATC TAAACATCTC CCCGTCAACT ATCTTTTTCC   
  
  
- ACTTCTGTTT CCTTCCCGTG TAAACGGAAA AGTATCTTCC TTCTCTGGTT CCATGACCCC TGACCCCCTT   
  
  
- CCCGAGTTCT CTTCTCTCTC TGACTCTTCT CACGACGATG AGGAAGGATG GATGTGTGTT TTAACGACGC   
  
  
- CCCCCAAGTT CAAGTTGAAC TTGCTCCGTT TATGGGAGTA ACTTGGGTGG TTTAGCTTTA GAAGTTACAT   
  
  
- GTGGGGGTAA TATGTGCGTA AGTCTCTGTA TCTACAGCTC CCCCTCTACT TAATCGTTCT TCTACAGCTC   
  
  
- GATTTTACGT AAAGCTCCAA TGATCTCGGT CTTTACACCC GTGTAGGGTC TTCTAGAGTC ATCTCTCTCA   
  
  
- TTGAAGTCTT TTCAAAGTTA AAAATAAGCT TTAAACGGAG AACAAACACA TCTATACTAC CCTTGAAGCG   
  
  
- GAATGTTAGA CGTTACACTC CCAAACCAAC TTCCTCAACG TCAGTTAGGA GTTAAAATAC TTAGTTGGGG   
  
  
- TTTCACCTTC TTCCCGTAGT TTCTTCTCAG TTAACTCTCG CTTGGGTGAA GCCAAAACCT GTTGGGATCG   
  
  
- GGAGGTTTAA GACGTAGATG AGAGAGAAGG ACGAAGTTAC CACCTTCATC ACCCGAACCC TACCTGCTAA   
  
  
- ACCTCCCAAA CAGAAAACCT CTTCCCAGGG AAGAGGGAAC CAAGTACCCA CTTTAACTTC TGGGATCAAA   
  
  
- CTCAAAGTTC GTGGAGGAAG TTGGAAGTTT AGGGAACCTC AAACCACCGT TACTACCCGA ACTTCGTCGA   
  
  
- TAAGTCTCAC GACCATACCC ATTAAAATTA AACCAACTTT CAAAATTAAG ACTAAACTCC AGAACATTAA   
  
  
- GACCCAGATA AACGTTACCC CCCTTACTAA GACAGTTACT TTTCCCTACA CAAGAATTAA TACGAAGTCT   
  
  
- CGTCCCAGTA CACCAACGAC ATAGAGGTCA TCTTGTCAAA TCCTAATCAC TACACTTTGT ACTCCACAAC   
  
  
- TTGGGTTTTA AACCGAAAAC ACTCGTTCCG AGATGTTAGT CAGTCTTGAG ACCAATACAC GTATATGACC   
  
  
- CAATATTGGT CGATCTTAGA GGGGAACTCG GAGGCCGTTT CGCGGTGTTA CGACCCTTAC GAGAAATGTG   
  
  
- ATCACAACAA GGTCAAGGTT TCTCAAGCAA AAATCCACTC CTAGTACTTT AAGATGAGTC TTTAGTTCTC   
  
  
- GTCGTCAAAG ACTACGTTGT TGTCACCAAT TACCCAAATC GGGGAGTCAA ATGTGGGGTC GTGGGCGGTT   
  
  
- TCGGTAATCT GCTGGCTTTC GTTGGTTTGA GCGTTTGAAA CCAAGACGCG CTAGTCAATC AATTCTAACG   
  
  
- CCTCTACAAG GTTTGGCCAT TGAAAAGCGA ACGGGTTCAC TATAACCGGG CCGAGTTAGT CGTTAACAGA   
  
  
- GAAAGTCCCT TGGGGAAACA TTCTCGACGA TGAAAGGTAC AGTTCCTCCT TGAACTTTCC GAGGACTGAT   
  
  
- AGTTATCGAA GAGACGCCGA GGTGGGGTTG GATTTTCGGA ATGAGACAGT CTACAACACC TATCCTACTT   
  
  
- ACGTATGTTC GAAAAAAGTC TCCGTAGGGG TCAATACGTT AAATGGCTAA AATGAACATG GGTCCGAAAC   
  
  
- GAGCTTCGAG AACTACTACG ACTAATATAG GTGTATGATC TAAAACTGTA ACCAACACCA AGGGTTACCC   
  
  
- GTAGGAAATA AGCTCTCGAA GGAGAGTCCT TTTCCCCGTG AGGTAAAAAC TTTTAATGTC GGAAACGGAG   
  
  
- AAGAAATTGG GTATTAGGTA AACTTGATCG GGAACATACA CTATTGTAAG ACCTTAAACG TTTTCTCAAG   
  
  
- CCACAGAGAC AGCTGGAAGT CCAGTACTTA AACCTGAACA AACTGGGTTG ATGAACGTGT TATGGGTTAA   
  
  
- AAGTTAGGTG ACTCTTGCGT CGTCAACGAC AGTTGAAGGG ATATACCCGC ACGAGTGTGA CGGGCAAACA   
  
  
- TGAGTCAGTT GAGGACAGGA AGTAGTTCGT TACAAGGGGT TTTTAGTACT GCTCGAAACT ATCTCCAACA   
  
  
- CTAGTAAAAC TCAACAGAAA AGGTAGCGTG TAGTAGGCAC ATAACCTGAG TACGAGATTA TACAACCTCA   
  
  
- GGGAGGTACC AAGTTCGCAC TGTAATCTGT GGAGTTTCTT CTAACTTTTC AAGAACTAGT TCGGATATTA   
  
  
- ACTCTCCCGT TATAACCCCG CTTAAGTACG TGGTCTGTTC TACGTCCGTT TGTTCCGTGT GGCCACCTTC   
  
  
- TTAGAGAAGT GAAGACGACC CAAGAAGGGA AACGGTAAAT CATCAAAGTG ACTTAGAGTC CGGTTAGATC   
  
  
- ACCGAGTTGC TTGGGGTCAC TCCCCAAAAG TCCACCTTTT CGTCGTTCGT AGTGAACACG AGTCCACCTA   
  
  
- CAGAGTACTC GAGCACCGTC GAAGTCGAAC CTCCACGAT

+     CGTCA-motif

| Site Name | Organism | Position | Strand | Matrix score. | sequence | function |
| --- | --- | --- | --- | --- | --- | --- |
| CGTCA-motif | Hordeum vulgare | 3622 | - | 5 | CGTCA | cis-acting regulatory element involved in the MeJA-responsiveness |
| CGTCA-motif | Hordeum vulgare | 877 | + | 5 | CGTCA | cis-acting regulatory element involved in the MeJA-responsiveness |

>HU04G00048.1   
+ -Up\_Stream \_Len000TTTGTA TTTAAATTTT ACAATATTTA TAAAAATTTT GTATTGATTA ATTGTGCTAA   
  
  
+ CTAACACTAA TAATTGGGGG AGAAAAGAAG GGAAAATAAA AAGAAATTAA GAAGAAGAAA AGAAAACACC   
  
  
+ TTTGAAAAGG CAAAAAACTA AAATAAAACA ATAATGAACA GAAGGAGAGA AGGATGGATA AACAACAAAT   
  
  
+ GTCGCTTTTA GGACAGCAAT AGTCTATGTG ATTGGCCTTA GTTCCTGTTG GAGGATTGCT AACAAAAGCA   
  
  
+ ACAGCAAAAA ATAATACTAA TTATTACATA TTTTTTAACT CTTAAAGCTA ATCAATCATC CTCCATTAAT   
  
  
+ TGATTAATTA TTTGCCAAAA ACACAAATCA ACTGGGGCAA CTATAAGAGA TCTACCTGCT TTAAGATATA   
  
  
+ TAAACTCAAT TGTTTCATTT TATTTTATTT TAGATTTTTT GGCCTTTGGT CTCTATTGAG CTAAAATCTC   
  
  
+ GTTATTTTCG GATCATGAAA AATACAACTC GATCTTTTGA TATAAGTCAT CTTTATTTGT AAATTCTGAA   
  
  
+ CTTAAAATCT TACTTGAAGA TCAGGAATAG AAGCACTCGA TTTTTGATAA ATCCTATGTG TTCTTCTTAA   
  
  
+ AATTCATAAT CTAATTCATA TTTTAGATGT ACCCGTTGCC TACTTTGGCA AGTTTAGATT ATGATTTTTA   
  
  
+ TTTTTTTTAA TTACTTGTAT TTTTCTTTTG GATTGATAGT CAATCTACAT CATCTTTGAT CAATTGCGAC   
  
  
+ AGTTTCATTT TCCCAACAAA AATTTCGAGT GTATTCTTTT TAATTTATCA TAAGTATTTA TTGTCAACAT   
  
  
+ TCAACATGCA TCAACAATCA TTAAACACTT TTCGTCACTC CTCAATACTG GTCAGCACTT AAGATTTTGT   
  
  
+ CTTCTAAACT TATCGTAAGT AACTTATTGT TGATTAATTG ATGCTGACCA AATGATACTG AGTGCTAAAA   
  
  
+ TTATAATTAA TAAATACAAT TAAGAGTTGA TCAATGTCGA TGATTCAAAT CAATGTGATT GGTGCTGAAT   
  
  
+ AGTAATTATA AGCACCTATA AAGACTTGAA AAGAACAAAC ATTTAATTAG ATTTTATAAT TATAATTTCC   
  
  
+ TAATATTTAG CATTCATCAG CAAATTCAGC ACACACCAAC ATTAGTCAAC ACCTATCAAC ACTTATAAGT   
  
  
+ ACCAATTAAT AATAATTACC TATAATAGAT TAAAAGTTAG GCCCTTAATG TTTCCCACAT TATTAGATAT   
  
  
+ ATCAAGGGAG GGGGAAAAAA ATAATACTCC CCCATCTTAA AAAATTTATC CCTTTTTTTC TTTTTAATGT   
  
  
+ TCCAAAACAA AAATATGCTT CTAAATTATG ATTGCAATAT GAATGTGTAC GGTTTCAAAC CCAATTTTCT   
  
  
+ CCAACAATTC TTCTACTATA TTGTAATTAT ATTATACTAC AATTTGTATC TCATCTATGA TCTACCCCCC   
  
  
+ AAAAACCAAA TACTCATATA AAGTTTTATT TTCTTTCCAT CCCACTCTAA AAAAAAAAAA TGTTGGGAAA   
  
  
+ AAGTTGATTT ATTTAATTTG AAATGGTACG GTGAATATAG ATTTGTAGAG GGGCAGTTGA TAGAAAAAGG   
  
  
+ TGAAGACAAA GGAAGGGCAC ATTTGCCTTT TCATAGAAGG AAGAGACCAA GGTACTGGGG ACTGGGGGAA   
  
  
+ GGGCTCAAGA GAAGAGAGAG ACTGAGAAGA GTGCTGCTAC TCCTTCCTAC CTACACACAA AATTGCTGCG   
  
  
+ GGGGGTTCAA GTTCAACTTG AACGAGGCAA ATACCCTCAT TGAACCCACC AAATCGAAAT CTTCAATGTA   
  
  
+ CACCCCCATT ATACACGCAT TCAGAGACAT AGATGTCGAG GGGGAGATGA ATTAGCAAGA AGATGTCGAG   
  
  
+ CTAAAATGCA TTTCGAGGTT ACTAGAGCCA GAAATGTGGG CACATCCCAG AAGATCTCAG TAGAGAGAGT   
  
  
+ AACTTCAGAA AAGTTTCAAT TTTTATTCGA AATTTGCCTC TTGTTTGTGT AGATATGATG GGAACTTCGC   
  
  
+ CTTACAATCT GCAATGTGAG GGTTTGGTTG AAGGAGTTGC AGTCAATCCT CAATTTTATG AATCAACCCC   
  
  
+ AAAGTGGAAG AAGGGCATCA AAGAAGAGTC AATTGAGAGC GAACCCACTT CGGTTTTGGA CAACCCTAGC   
  
  
+ CCTCCAAATT CTGCATCTAC TCTCTCTTCC TGCTTCAATG GTGGAAGTAG TGGGCTTGGG ATGGACGATT   
  
  
+ TGGAGGGTTT GTCTTTTGGA GAAGGGTCCC TTCTCCCTTG GTTCATGGGT GAAATTGAAG ACCCTAGTTT   
  
  
+ GAGTTTCAAG CACCTCCTTC AACCTTCAAA TCCCTTGGAG TTTGGTGGCA ATGATGGGCT TGAAGCAGCT   
  
  
+ ATTCAGAGTG CTGGTATGGG TAATTTTAAT TTGGTTGAAA GTTTTAATTC TGATTTGAGG TCTTGTAATT   
  
  
+ CTGGGTCTAT TTGCAATGGG GGGAATGATT CTGTCAATGA AAAGGGATGT GTTCTTAATT ATGCTTCAGA   
  
  
+ GCAGGGTCAT GTGGTTGCTG TATCTCCAGT AGAACAGTTT AGGATTAGTG ATGTGAAACA TGAGGTGTTG   
  
  
+ AACCCAAAAT TTGGCTTTTG TGAGCAAGGC TCTACAATCA GTCAGAACTC TGGTTATGTG CATATACTGG   
  
  
+ GTTATAACCA GCTAGAATCT CCCCTTGAGC CTCCGGCAAA GCGCCACAAT GCTGGGAATG CTCTTTACAC   
  
  
+ TAGTGTTGTT CCAGTTCCAA AGAGTTCGTT TTTAGGTGAG GATCATGAAA TTCTACTCAG AAATCAAGAG   
  
  
+ CAGCAGTTTC TGATGCAACA ACAGTGGTTA ATGGGTTTAG CCCCTCAGTT TACACCCCAG CACCCGCCAA   
  
  
+ AGCCATTAGA CGACCGAAAG CAACCAAACT CGCAAACTTT GGTTCTGCGC GATCAGTTAG TTAAGATTGC   
  
  
+ GGAGATGTTC CAAACCGGTA ACTTTTCGCT TGCCCAAGTG ATATTGGCCC GGCTCAATCA GCAATTGTCT   
  
  
+ CTTTCAGGGA ACCCCTTTGT AAGAGCTGCT ACTTTCCATG TCAAGGAGGA ACTTGAAAGG CTCCTGACTA   
  
  
+ TCAATAGCTT CTCTGCGGCT CCACCCCAAC CTAAAAGCCT TACTCTGTCA GATGTTGTGG ATAGGATGAA   
  
  
+ TGCATACAAG CTTTTTTCAG AGGCATCCCC AGTTATGCAA TTTACCGATT TTACTTGTAC CCAGGCTTTG   
  
  
+ CTCGAAGCTC TTGATGATGC TGATTATATC CACATACTAG ATTTTGACAT TGGTTGTGGT TCCCAATGGG   
  
  
+ CATCCTTTAT TCGAGAGCTT CCTCTCAGGA AAAGGGGCAC TCCATTTTTG AAAATTACAG CCTTTGCCTC   
  
  
+ TTCTTTAACC CATAATCCAT TTGAACTAGC CCTTGTATGT GATAACATTC TGGAATTTGC AAAAGAGTTC   
  
  
+ GGTGTCTCTG TCGACCTTCA GGTCATGAAT TTGGACTTGT TTGACCCAAC TACTTGCACA ATACCCAATT   
  
  
+ TTCAATCCAC TGAGAACGCA GCAGTTGCTG TCAACTTCCC TATATGGGCG TGCTCACACT GCCCGTTTGT   
  
  
+ ACTCAGTCAA CTCCTGTCCT TCATCAAGCA ATGTTCCCCA AAAATCATGA CGAGCTTTGA TAGAGGTTGT   
  
  
+ GATCATTTTG AGTTGTCTTT TCCATCGCAC ATCATCCGTG TATTGGACTC ATGCTCTAAT ATGTTGGAGT   
  
  
+ CCCTCCATGG TTCAAGCGTG ACATTAGACA CCTCAAAGAA GATTGAAAAG TTCTTGATCA AGCCTATAAT   
  
  
+ TGAGAGGGCA ATATTGGGGC GAATTCATGC ACCAGACAAG ATGCAGGCAA ACAAGGCACA CCGGTGGAAG   
  
  
+ AATCTCTTCA CTTCTGCTGG GTTCTTCCCT TTGCCATTTA GTAGTTTCAC TGAATCTCAG GCCAATCTAG   
  
  
+ TGGCTCAACG AACCCCAGTG AGGGGTTTTC AGGTGGAAAA GCAGCAAGCA TCACTTGTGC TCAGGTGGAT   
  
  
+ GTCTCATGAG CTCGTGGCAG CTTCAGCTTG GAGGTGCTA  

- -Up\_Stream \_Len000AAACAT AAATTTAAAA TGTTATAAAT ATTTTTAAAA CATAACTAAT TAACACGATT   
  
  
- GATTGTGATT ATTAACCCCC TCTTTTCTTC CCTTTTATTT TTCTTTAATT CTTCTTCTTT TCTTTTGTGG   
  
  
- AAACTTTTCC GTTTTTTGAT TTTATTTTGT TATTACTTGT CTTCCTCTCT TCCTACCTAT TTGTTGTTTA   
  
  
- CAGCGAAAAT CCTGTCGTTA TCAGATACAC TAACCGGAAT CAAGGACAAC CTCCTAACGA TTGTTTTCGT   
  
  
- TGTCGTTTTT TATTATGATT AATAATGTAT AAAAAATTGA GAATTTCGAT TAGTTAGTAG GAGGTAATTA   
  
  
- ACTAATTAAT AAACGGTTTT TGTGTTTAGT TGACCCCGTT GATATTCTCT AGATGGACGA AATTCTATAT   
  
  
- ATTTGAGTTA ACAAAGTAAA ATAAAATAAA ATCTAAAAAA CCGGAAACCA GAGATAACTC GATTTTAGAG   
  
  
- CAATAAAAGC CTAGTACTTT TTATGTTGAG CTAGAAAACT ATATTCAGTA GAAATAAACA TTTAAGACTT   
  
  
- GAATTTTAGA ATGAACTTCT AGTCCTTATC TTCGTGAGCT AAAAACTATT TAGGATACAC AAGAAGAATT   
  
  
- TTAAGTATTA GATTAAGTAT AAAATCTACA TGGGCAACGG ATGAAACCGT TCAAATCTAA TACTAAAAAT   
  
  
- AAAAAAAATT AATGAACATA AAAAGAAAAC CTAACTATCA GTTAGATGTA GTAGAAACTA GTTAACGCTG   
  
  
- TCAAAGTAAA AGGGTTGTTT TTAAAGCTCA CATAAGAAAA ATTAAATAGT ATTCATAAAT AACAGTTGTA   
  
  
- AGTTGTACGT AGTTGTTAGT AATTTGTGAA AAGCAGTGAG GAGTTATGAC CAGTCGTGAA TTCTAAAACA   
  
  
- GAAGATTTGA ATAGCATTCA TTGAATAACA ACTAATTAAC TACGACTGGT TTACTATGAC TCACGATTTT   
  
  
- AATATTAATT ATTTATGTTA ATTCTCAACT AGTTACAGCT ACTAAGTTTA GTTACACTAA CCACGACTTA   
  
  
- TCATTAATAT TCGTGGATAT TTCTGAACTT TTCTTGTTTG TAAATTAATC TAAAATATTA ATATTAAAGG   
  
  
- ATTATAAATC GTAAGTAGTC GTTTAAGTCG TGTGTGGTTG TAATCAGTTG TGGATAGTTG TGAATATTCA   
  
  
- TGGTTAATTA TTATTAATGG ATATTATCTA ATTTTCAATC CGGGAATTAC AAAGGGTGTA ATAATCTATA   
  
  
- TAGTTCCCTC CCCCTTTTTT TATTATGAGG GGGTAGAATT TTTTAAATAG GGAAAAAAAG AAAAATTACA   
  
  
- AGGTTTTGTT TTTATACGAA GATTTAATAC TAACGTTATA CTTACACATG CCAAAGTTTG GGTTAAAAGA   
  
  
- GGTTGTTAAG AAGATGATAT AACATTAATA TAATATGATG TTAAACATAG AGTAGATACT AGATGGGGGG   
  
  
- TTTTTGGTTT ATGAGTATAT TTCAAAATAA AAGAAAGGTA GGGTGAGATT TTTTTTTTTT ACAACCCTTT   
  
  
- TTCAACTAAA TAAATTAAAC TTTACCATGC CACTTATATC TAAACATCTC CCCGTCAACT ATCTTTTTCC   
  
  
- ACTTCTGTTT CCTTCCCGTG TAAACGGAAA AGTATCTTCC TTCTCTGGTT CCATGACCCC TGACCCCCTT   
  
  
- CCCGAGTTCT CTTCTCTCTC TGACTCTTCT CACGACGATG AGGAAGGATG GATGTGTGTT TTAACGACGC   
  
  
- CCCCCAAGTT CAAGTTGAAC TTGCTCCGTT TATGGGAGTA ACTTGGGTGG TTTAGCTTTA GAAGTTACAT   
  
  
- GTGGGGGTAA TATGTGCGTA AGTCTCTGTA TCTACAGCTC CCCCTCTACT TAATCGTTCT TCTACAGCTC   
  
  
- GATTTTACGT AAAGCTCCAA TGATCTCGGT CTTTACACCC GTGTAGGGTC TTCTAGAGTC ATCTCTCTCA   
  
  
- TTGAAGTCTT TTCAAAGTTA AAAATAAGCT TTAAACGGAG AACAAACACA TCTATACTAC CCTTGAAGCG   
  
  
- GAATGTTAGA CGTTACACTC CCAAACCAAC TTCCTCAACG TCAGTTAGGA GTTAAAATAC TTAGTTGGGG   
  
  
- TTTCACCTTC TTCCCGTAGT TTCTTCTCAG TTAACTCTCG CTTGGGTGAA GCCAAAACCT GTTGGGATCG   
  
  
- GGAGGTTTAA GACGTAGATG AGAGAGAAGG ACGAAGTTAC CACCTTCATC ACCCGAACCC TACCTGCTAA   
  
  
- ACCTCCCAAA CAGAAAACCT CTTCCCAGGG AAGAGGGAAC CAAGTACCCA CTTTAACTTC TGGGATCAAA   
  
  
- CTCAAAGTTC GTGGAGGAAG TTGGAAGTTT AGGGAACCTC AAACCACCGT TACTACCCGA ACTTCGTCGA   
  
  
- TAAGTCTCAC GACCATACCC ATTAAAATTA AACCAACTTT CAAAATTAAG ACTAAACTCC AGAACATTAA   
  
  
- GACCCAGATA AACGTTACCC CCCTTACTAA GACAGTTACT TTTCCCTACA CAAGAATTAA TACGAAGTCT   
  
  
- CGTCCCAGTA CACCAACGAC ATAGAGGTCA TCTTGTCAAA TCCTAATCAC TACACTTTGT ACTCCACAAC   
  
  
- TTGGGTTTTA AACCGAAAAC ACTCGTTCCG AGATGTTAGT CAGTCTTGAG ACCAATACAC GTATATGACC   
  
  
- CAATATTGGT CGATCTTAGA GGGGAACTCG GAGGCCGTTT CGCGGTGTTA CGACCCTTAC GAGAAATGTG   
  
  
- ATCACAACAA GGTCAAGGTT TCTCAAGCAA AAATCCACTC CTAGTACTTT AAGATGAGTC TTTAGTTCTC   
  
  
- GTCGTCAAAG ACTACGTTGT TGTCACCAAT TACCCAAATC GGGGAGTCAA ATGTGGGGTC GTGGGCGGTT   
  
  
- TCGGTAATCT GCTGGCTTTC GTTGGTTTGA GCGTTTGAAA CCAAGACGCG CTAGTCAATC AATTCTAACG   
  
  
- CCTCTACAAG GTTTGGCCAT TGAAAAGCGA ACGGGTTCAC TATAACCGGG CCGAGTTAGT CGTTAACAGA   
  
  
- GAAAGTCCCT TGGGGAAACA TTCTCGACGA TGAAAGGTAC AGTTCCTCCT TGAACTTTCC GAGGACTGAT   
  
  
- AGTTATCGAA GAGACGCCGA GGTGGGGTTG GATTTTCGGA ATGAGACAGT CTACAACACC TATCCTACTT   
  
  
- ACGTATGTTC GAAAAAAGTC TCCGTAGGGG TCAATACGTT AAATGGCTAA AATGAACATG GGTCCGAAAC   
  
  
- GAGCTTCGAG AACTACTACG ACTAATATAG GTGTATGATC TAAAACTGTA ACCAACACCA AGGGTTACCC   
  
  
- GTAGGAAATA AGCTCTCGAA GGAGAGTCCT TTTCCCCGTG AGGTAAAAAC TTTTAATGTC GGAAACGGAG   
  
  
- AAGAAATTGG GTATTAGGTA AACTTGATCG GGAACATACA CTATTGTAAG ACCTTAAACG TTTTCTCAAG   
  
  
- CCACAGAGAC AGCTGGAAGT CCAGTACTTA AACCTGAACA AACTGGGTTG ATGAACGTGT TATGGGTTAA   
  
  
- AAGTTAGGTG ACTCTTGCGT CGTCAACGAC AGTTGAAGGG ATATACCCGC ACGAGTGTGA CGGGCAAACA   
  
  
- TGAGTCAGTT GAGGACAGGA AGTAGTTCGT TACAAGGGGT TTTTAGTACT GCTCGAAACT ATCTCCAACA   
  
  
- CTAGTAAAAC TCAACAGAAA AGGTAGCGTG TAGTAGGCAC ATAACCTGAG TACGAGATTA TACAACCTCA   
  
  
- GGGAGGTACC AAGTTCGCAC TGTAATCTGT GGAGTTTCTT CTAACTTTTC AAGAACTAGT TCGGATATTA   
  
  
- ACTCTCCCGT TATAACCCCG CTTAAGTACG TGGTCTGTTC TACGTCCGTT TGTTCCGTGT GGCCACCTTC   
  
  
- TTAGAGAAGT GAAGACGACC CAAGAAGGGA AACGGTAAAT CATCAAAGTG ACTTAGAGTC CGGTTAGATC   
  
  
- ACCGAGTTGC TTGGGGTCAC TCCCCAAAAG TCCACCTTTT CGTCGTTCGT AGTGAACACG AGTCCACCTA   
  
  
- CAGAGTACTC GAGCACCGTC GAAGTCGAAC CTCCACGAT

+     GATA-motif

| Site Name | Organism | Position | Strand | Matrix score. | sequence | function |
| --- | --- | --- | --- | --- | --- | --- |
| GATA-motif | Arabidopsis thaliana | 3144 | + | 7 | GATAGGA | part of a light responsive element |

>HU04G00048.1   
+ -Up\_Stream \_Len000TTTGTA TTTAAATTTT ACAATATTTA TAAAAATTTT GTATTGATTA ATTGTGCTAA   
  
  
+ CTAACACTAA TAATTGGGGG AGAAAAGAAG GGAAAATAAA AAGAAATTAA GAAGAAGAAA AGAAAACACC   
  
  
+ TTTGAAAAGG CAAAAAACTA AAATAAAACA ATAATGAACA GAAGGAGAGA AGGATGGATA AACAACAAAT   
  
  
+ GTCGCTTTTA GGACAGCAAT AGTCTATGTG ATTGGCCTTA GTTCCTGTTG GAGGATTGCT AACAAAAGCA   
  
  
+ ACAGCAAAAA ATAATACTAA TTATTACATA TTTTTTAACT CTTAAAGCTA ATCAATCATC CTCCATTAAT   
  
  
+ TGATTAATTA TTTGCCAAAA ACACAAATCA ACTGGGGCAA CTATAAGAGA TCTACCTGCT TTAAGATATA   
  
  
+ TAAACTCAAT TGTTTCATTT TATTTTATTT TAGATTTTTT GGCCTTTGGT CTCTATTGAG CTAAAATCTC   
  
  
+ GTTATTTTCG GATCATGAAA AATACAACTC GATCTTTTGA TATAAGTCAT CTTTATTTGT AAATTCTGAA   
  
  
+ CTTAAAATCT TACTTGAAGA TCAGGAATAG AAGCACTCGA TTTTTGATAA ATCCTATGTG TTCTTCTTAA   
  
  
+ AATTCATAAT CTAATTCATA TTTTAGATGT ACCCGTTGCC TACTTTGGCA AGTTTAGATT ATGATTTTTA   
  
  
+ TTTTTTTTAA TTACTTGTAT TTTTCTTTTG GATTGATAGT CAATCTACAT CATCTTTGAT CAATTGCGAC   
  
  
+ AGTTTCATTT TCCCAACAAA AATTTCGAGT GTATTCTTTT TAATTTATCA TAAGTATTTA TTGTCAACAT   
  
  
+ TCAACATGCA TCAACAATCA TTAAACACTT TTCGTCACTC CTCAATACTG GTCAGCACTT AAGATTTTGT   
  
  
+ CTTCTAAACT TATCGTAAGT AACTTATTGT TGATTAATTG ATGCTGACCA AATGATACTG AGTGCTAAAA   
  
  
+ TTATAATTAA TAAATACAAT TAAGAGTTGA TCAATGTCGA TGATTCAAAT CAATGTGATT GGTGCTGAAT   
  
  
+ AGTAATTATA AGCACCTATA AAGACTTGAA AAGAACAAAC ATTTAATTAG ATTTTATAAT TATAATTTCC   
  
  
+ TAATATTTAG CATTCATCAG CAAATTCAGC ACACACCAAC ATTAGTCAAC ACCTATCAAC ACTTATAAGT   
  
  
+ ACCAATTAAT AATAATTACC TATAATAGAT TAAAAGTTAG GCCCTTAATG TTTCCCACAT TATTAGATAT   
  
  
+ ATCAAGGGAG GGGGAAAAAA ATAATACTCC CCCATCTTAA AAAATTTATC CCTTTTTTTC TTTTTAATGT   
  
  
+ TCCAAAACAA AAATATGCTT CTAAATTATG ATTGCAATAT GAATGTGTAC GGTTTCAAAC CCAATTTTCT   
  
  
+ CCAACAATTC TTCTACTATA TTGTAATTAT ATTATACTAC AATTTGTATC TCATCTATGA TCTACCCCCC   
  
  
+ AAAAACCAAA TACTCATATA AAGTTTTATT TTCTTTCCAT CCCACTCTAA AAAAAAAAAA TGTTGGGAAA   
  
  
+ AAGTTGATTT ATTTAATTTG AAATGGTACG GTGAATATAG ATTTGTAGAG GGGCAGTTGA TAGAAAAAGG   
  
  
+ TGAAGACAAA GGAAGGGCAC ATTTGCCTTT TCATAGAAGG AAGAGACCAA GGTACTGGGG ACTGGGGGAA   
  
  
+ GGGCTCAAGA GAAGAGAGAG ACTGAGAAGA GTGCTGCTAC TCCTTCCTAC CTACACACAA AATTGCTGCG   
  
  
+ GGGGGTTCAA GTTCAACTTG AACGAGGCAA ATACCCTCAT TGAACCCACC AAATCGAAAT CTTCAATGTA   
  
  
+ CACCCCCATT ATACACGCAT TCAGAGACAT AGATGTCGAG GGGGAGATGA ATTAGCAAGA AGATGTCGAG   
  
  
+ CTAAAATGCA TTTCGAGGTT ACTAGAGCCA GAAATGTGGG CACATCCCAG AAGATCTCAG TAGAGAGAGT   
  
  
+ AACTTCAGAA AAGTTTCAAT TTTTATTCGA AATTTGCCTC TTGTTTGTGT AGATATGATG GGAACTTCGC   
  
  
+ CTTACAATCT GCAATGTGAG GGTTTGGTTG AAGGAGTTGC AGTCAATCCT CAATTTTATG AATCAACCCC   
  
  
+ AAAGTGGAAG AAGGGCATCA AAGAAGAGTC AATTGAGAGC GAACCCACTT CGGTTTTGGA CAACCCTAGC   
  
  
+ CCTCCAAATT CTGCATCTAC TCTCTCTTCC TGCTTCAATG GTGGAAGTAG TGGGCTTGGG ATGGACGATT   
  
  
+ TGGAGGGTTT GTCTTTTGGA GAAGGGTCCC TTCTCCCTTG GTTCATGGGT GAAATTGAAG ACCCTAGTTT   
  
  
+ GAGTTTCAAG CACCTCCTTC AACCTTCAAA TCCCTTGGAG TTTGGTGGCA ATGATGGGCT TGAAGCAGCT   
  
  
+ ATTCAGAGTG CTGGTATGGG TAATTTTAAT TTGGTTGAAA GTTTTAATTC TGATTTGAGG TCTTGTAATT   
  
  
+ CTGGGTCTAT TTGCAATGGG GGGAATGATT CTGTCAATGA AAAGGGATGT GTTCTTAATT ATGCTTCAGA   
  
  
+ GCAGGGTCAT GTGGTTGCTG TATCTCCAGT AGAACAGTTT AGGATTAGTG ATGTGAAACA TGAGGTGTTG   
  
  
+ AACCCAAAAT TTGGCTTTTG TGAGCAAGGC TCTACAATCA GTCAGAACTC TGGTTATGTG CATATACTGG   
  
  
+ GTTATAACCA GCTAGAATCT CCCCTTGAGC CTCCGGCAAA GCGCCACAAT GCTGGGAATG CTCTTTACAC   
  
  
+ TAGTGTTGTT CCAGTTCCAA AGAGTTCGTT TTTAGGTGAG GATCATGAAA TTCTACTCAG AAATCAAGAG   
  
  
+ CAGCAGTTTC TGATGCAACA ACAGTGGTTA ATGGGTTTAG CCCCTCAGTT TACACCCCAG CACCCGCCAA   
  
  
+ AGCCATTAGA CGACCGAAAG CAACCAAACT CGCAAACTTT GGTTCTGCGC GATCAGTTAG TTAAGATTGC   
  
  
+ GGAGATGTTC CAAACCGGTA ACTTTTCGCT TGCCCAAGTG ATATTGGCCC GGCTCAATCA GCAATTGTCT   
  
  
+ CTTTCAGGGA ACCCCTTTGT AAGAGCTGCT ACTTTCCATG TCAAGGAGGA ACTTGAAAGG CTCCTGACTA   
  
  
+ TCAATAGCTT CTCTGCGGCT CCACCCCAAC CTAAAAGCCT TACTCTGTCA GATGTTGTGG ATAGGATGAA   
  
  
+ TGCATACAAG CTTTTTTCAG AGGCATCCCC AGTTATGCAA TTTACCGATT TTACTTGTAC CCAGGCTTTG   
  
  
+ CTCGAAGCTC TTGATGATGC TGATTATATC CACATACTAG ATTTTGACAT TGGTTGTGGT TCCCAATGGG   
  
  
+ CATCCTTTAT TCGAGAGCTT CCTCTCAGGA AAAGGGGCAC TCCATTTTTG AAAATTACAG CCTTTGCCTC   
  
  
+ TTCTTTAACC CATAATCCAT TTGAACTAGC CCTTGTATGT GATAACATTC TGGAATTTGC AAAAGAGTTC   
  
  
+ GGTGTCTCTG TCGACCTTCA GGTCATGAAT TTGGACTTGT TTGACCCAAC TACTTGCACA ATACCCAATT   
  
  
+ TTCAATCCAC TGAGAACGCA GCAGTTGCTG TCAACTTCCC TATATGGGCG TGCTCACACT GCCCGTTTGT   
  
  
+ ACTCAGTCAA CTCCTGTCCT TCATCAAGCA ATGTTCCCCA AAAATCATGA CGAGCTTTGA TAGAGGTTGT   
  
  
+ GATCATTTTG AGTTGTCTTT TCCATCGCAC ATCATCCGTG TATTGGACTC ATGCTCTAAT ATGTTGGAGT   
  
  
+ CCCTCCATGG TTCAAGCGTG ACATTAGACA CCTCAAAGAA GATTGAAAAG TTCTTGATCA AGCCTATAAT   
  
  
+ TGAGAGGGCA ATATTGGGGC GAATTCATGC ACCAGACAAG ATGCAGGCAA ACAAGGCACA CCGGTGGAAG   
  
  
+ AATCTCTTCA CTTCTGCTGG GTTCTTCCCT TTGCCATTTA GTAGTTTCAC TGAATCTCAG GCCAATCTAG   
  
  
+ TGGCTCAACG AACCCCAGTG AGGGGTTTTC AGGTGGAAAA GCAGCAAGCA TCACTTGTGC TCAGGTGGAT   
  
  
+ GTCTCATGAG CTCGTGGCAG CTTCAGCTTG GAGGTGCTA  

- -Up\_Stream \_Len000AAACAT AAATTTAAAA TGTTATAAAT ATTTTTAAAA CATAACTAAT TAACACGATT   
  
  
- GATTGTGATT ATTAACCCCC TCTTTTCTTC CCTTTTATTT TTCTTTAATT CTTCTTCTTT TCTTTTGTGG   
  
  
- AAACTTTTCC GTTTTTTGAT TTTATTTTGT TATTACTTGT CTTCCTCTCT TCCTACCTAT TTGTTGTTTA   
  
  
- CAGCGAAAAT CCTGTCGTTA TCAGATACAC TAACCGGAAT CAAGGACAAC CTCCTAACGA TTGTTTTCGT   
  
  
- TGTCGTTTTT TATTATGATT AATAATGTAT AAAAAATTGA GAATTTCGAT TAGTTAGTAG GAGGTAATTA   
  
  
- ACTAATTAAT AAACGGTTTT TGTGTTTAGT TGACCCCGTT GATATTCTCT AGATGGACGA AATTCTATAT   
  
  
- ATTTGAGTTA ACAAAGTAAA ATAAAATAAA ATCTAAAAAA CCGGAAACCA GAGATAACTC GATTTTAGAG   
  
  
- CAATAAAAGC CTAGTACTTT TTATGTTGAG CTAGAAAACT ATATTCAGTA GAAATAAACA TTTAAGACTT   
  
  
- GAATTTTAGA ATGAACTTCT AGTCCTTATC TTCGTGAGCT AAAAACTATT TAGGATACAC AAGAAGAATT   
  
  
- TTAAGTATTA GATTAAGTAT AAAATCTACA TGGGCAACGG ATGAAACCGT TCAAATCTAA TACTAAAAAT   
  
  
- AAAAAAAATT AATGAACATA AAAAGAAAAC CTAACTATCA GTTAGATGTA GTAGAAACTA GTTAACGCTG   
  
  
- TCAAAGTAAA AGGGTTGTTT TTAAAGCTCA CATAAGAAAA ATTAAATAGT ATTCATAAAT AACAGTTGTA   
  
  
- AGTTGTACGT AGTTGTTAGT AATTTGTGAA AAGCAGTGAG GAGTTATGAC CAGTCGTGAA TTCTAAAACA   
  
  
- GAAGATTTGA ATAGCATTCA TTGAATAACA ACTAATTAAC TACGACTGGT TTACTATGAC TCACGATTTT   
  
  
- AATATTAATT ATTTATGTTA ATTCTCAACT AGTTACAGCT ACTAAGTTTA GTTACACTAA CCACGACTTA   
  
  
- TCATTAATAT TCGTGGATAT TTCTGAACTT TTCTTGTTTG TAAATTAATC TAAAATATTA ATATTAAAGG   
  
  
- ATTATAAATC GTAAGTAGTC GTTTAAGTCG TGTGTGGTTG TAATCAGTTG TGGATAGTTG TGAATATTCA   
  
  
- TGGTTAATTA TTATTAATGG ATATTATCTA ATTTTCAATC CGGGAATTAC AAAGGGTGTA ATAATCTATA   
  
  
- TAGTTCCCTC CCCCTTTTTT TATTATGAGG GGGTAGAATT TTTTAAATAG GGAAAAAAAG AAAAATTACA   
  
  
- AGGTTTTGTT TTTATACGAA GATTTAATAC TAACGTTATA CTTACACATG CCAAAGTTTG GGTTAAAAGA   
  
  
- GGTTGTTAAG AAGATGATAT AACATTAATA TAATATGATG TTAAACATAG AGTAGATACT AGATGGGGGG   
  
  
- TTTTTGGTTT ATGAGTATAT TTCAAAATAA AAGAAAGGTA GGGTGAGATT TTTTTTTTTT ACAACCCTTT   
  
  
- TTCAACTAAA TAAATTAAAC TTTACCATGC CACTTATATC TAAACATCTC CCCGTCAACT ATCTTTTTCC   
  
  
- ACTTCTGTTT CCTTCCCGTG TAAACGGAAA AGTATCTTCC TTCTCTGGTT CCATGACCCC TGACCCCCTT   
  
  
- CCCGAGTTCT CTTCTCTCTC TGACTCTTCT CACGACGATG AGGAAGGATG GATGTGTGTT TTAACGACGC   
  
  
- CCCCCAAGTT CAAGTTGAAC TTGCTCCGTT TATGGGAGTA ACTTGGGTGG TTTAGCTTTA GAAGTTACAT   
  
  
- GTGGGGGTAA TATGTGCGTA AGTCTCTGTA TCTACAGCTC CCCCTCTACT TAATCGTTCT TCTACAGCTC   
  
  
- GATTTTACGT AAAGCTCCAA TGATCTCGGT CTTTACACCC GTGTAGGGTC TTCTAGAGTC ATCTCTCTCA   
  
  
- TTGAAGTCTT TTCAAAGTTA AAAATAAGCT TTAAACGGAG AACAAACACA TCTATACTAC CCTTGAAGCG   
  
  
- GAATGTTAGA CGTTACACTC CCAAACCAAC TTCCTCAACG TCAGTTAGGA GTTAAAATAC TTAGTTGGGG   
  
  
- TTTCACCTTC TTCCCGTAGT TTCTTCTCAG TTAACTCTCG CTTGGGTGAA GCCAAAACCT GTTGGGATCG   
  
  
- GGAGGTTTAA GACGTAGATG AGAGAGAAGG ACGAAGTTAC CACCTTCATC ACCCGAACCC TACCTGCTAA   
  
  
- ACCTCCCAAA CAGAAAACCT CTTCCCAGGG AAGAGGGAAC CAAGTACCCA CTTTAACTTC TGGGATCAAA   
  
  
- CTCAAAGTTC GTGGAGGAAG TTGGAAGTTT AGGGAACCTC AAACCACCGT TACTACCCGA ACTTCGTCGA   
  
  
- TAAGTCTCAC GACCATACCC ATTAAAATTA AACCAACTTT CAAAATTAAG ACTAAACTCC AGAACATTAA   
  
  
- GACCCAGATA AACGTTACCC CCCTTACTAA GACAGTTACT TTTCCCTACA CAAGAATTAA TACGAAGTCT   
  
  
- CGTCCCAGTA CACCAACGAC ATAGAGGTCA TCTTGTCAAA TCCTAATCAC TACACTTTGT ACTCCACAAC   
  
  
- TTGGGTTTTA AACCGAAAAC ACTCGTTCCG AGATGTTAGT CAGTCTTGAG ACCAATACAC GTATATGACC   
  
  
- CAATATTGGT CGATCTTAGA GGGGAACTCG GAGGCCGTTT CGCGGTGTTA CGACCCTTAC GAGAAATGTG   
  
  
- ATCACAACAA GGTCAAGGTT TCTCAAGCAA AAATCCACTC CTAGTACTTT AAGATGAGTC TTTAGTTCTC   
  
  
- GTCGTCAAAG ACTACGTTGT TGTCACCAAT TACCCAAATC GGGGAGTCAA ATGTGGGGTC GTGGGCGGTT   
  
  
- TCGGTAATCT GCTGGCTTTC GTTGGTTTGA GCGTTTGAAA CCAAGACGCG CTAGTCAATC AATTCTAACG   
  
  
- CCTCTACAAG GTTTGGCCAT TGAAAAGCGA ACGGGTTCAC TATAACCGGG CCGAGTTAGT CGTTAACAGA   
  
  
- GAAAGTCCCT TGGGGAAACA TTCTCGACGA TGAAAGGTAC AGTTCCTCCT TGAACTTTCC GAGGACTGAT   
  
  
- AGTTATCGAA GAGACGCCGA GGTGGGGTTG GATTTTCGGA ATGAGACAGT CTACAACACC TATCCTACTT   
  
  
- ACGTATGTTC GAAAAAAGTC TCCGTAGGGG TCAATACGTT AAATGGCTAA AATGAACATG GGTCCGAAAC   
  
  
- GAGCTTCGAG AACTACTACG ACTAATATAG GTGTATGATC TAAAACTGTA ACCAACACCA AGGGTTACCC   
  
  
- GTAGGAAATA AGCTCTCGAA GGAGAGTCCT TTTCCCCGTG AGGTAAAAAC TTTTAATGTC GGAAACGGAG   
  
  
- AAGAAATTGG GTATTAGGTA AACTTGATCG GGAACATACA CTATTGTAAG ACCTTAAACG TTTTCTCAAG   
  
  
- CCACAGAGAC AGCTGGAAGT CCAGTACTTA AACCTGAACA AACTGGGTTG ATGAACGTGT TATGGGTTAA   
  
  
- AAGTTAGGTG ACTCTTGCGT CGTCAACGAC AGTTGAAGGG ATATACCCGC ACGAGTGTGA CGGGCAAACA   
  
  
- TGAGTCAGTT GAGGACAGGA AGTAGTTCGT TACAAGGGGT TTTTAGTACT GCTCGAAACT ATCTCCAACA   
  
  
- CTAGTAAAAC TCAACAGAAA AGGTAGCGTG TAGTAGGCAC ATAACCTGAG TACGAGATTA TACAACCTCA   
  
  
- GGGAGGTACC AAGTTCGCAC TGTAATCTGT GGAGTTTCTT CTAACTTTTC AAGAACTAGT TCGGATATTA   
  
  
- ACTCTCCCGT TATAACCCCG CTTAAGTACG TGGTCTGTTC TACGTCCGTT TGTTCCGTGT GGCCACCTTC   
  
  
- TTAGAGAAGT GAAGACGACC CAAGAAGGGA AACGGTAAAT CATCAAAGTG ACTTAGAGTC CGGTTAGATC   
  
  
- ACCGAGTTGC TTGGGGTCAC TCCCCAAAAG TCCACCTTTT CGTCGTTCGT AGTGAACACG AGTCCACCTA   
  
  
- CAGAGTACTC GAGCACCGTC GAAGTCGAAC CTCCACGAT

+     GC-motif

| Site Name | Organism | Position | Strand | Matrix score. | sequence | function |
| --- | --- | --- | --- | --- | --- | --- |
| GC-motif | Zea mays | 1753 | - | 6 | CCCCCG | enhancer-like element involved in anoxic specific inducibility |

>HU04G00048.1   
+ -Up\_Stream \_Len000TTTGTA TTTAAATTTT ACAATATTTA TAAAAATTTT GTATTGATTA ATTGTGCTAA   
  
  
+ CTAACACTAA TAATTGGGGG AGAAAAGAAG GGAAAATAAA AAGAAATTAA GAAGAAGAAA AGAAAACACC   
  
  
+ TTTGAAAAGG CAAAAAACTA AAATAAAACA ATAATGAACA GAAGGAGAGA AGGATGGATA AACAACAAAT   
  
  
+ GTCGCTTTTA GGACAGCAAT AGTCTATGTG ATTGGCCTTA GTTCCTGTTG GAGGATTGCT AACAAAAGCA   
  
  
+ ACAGCAAAAA ATAATACTAA TTATTACATA TTTTTTAACT CTTAAAGCTA ATCAATCATC CTCCATTAAT   
  
  
+ TGATTAATTA TTTGCCAAAA ACACAAATCA ACTGGGGCAA CTATAAGAGA TCTACCTGCT TTAAGATATA   
  
  
+ TAAACTCAAT TGTTTCATTT TATTTTATTT TAGATTTTTT GGCCTTTGGT CTCTATTGAG CTAAAATCTC   
  
  
+ GTTATTTTCG GATCATGAAA AATACAACTC GATCTTTTGA TATAAGTCAT CTTTATTTGT AAATTCTGAA   
  
  
+ CTTAAAATCT TACTTGAAGA TCAGGAATAG AAGCACTCGA TTTTTGATAA ATCCTATGTG TTCTTCTTAA   
  
  
+ AATTCATAAT CTAATTCATA TTTTAGATGT ACCCGTTGCC TACTTTGGCA AGTTTAGATT ATGATTTTTA   
  
  
+ TTTTTTTTAA TTACTTGTAT TTTTCTTTTG GATTGATAGT CAATCTACAT CATCTTTGAT CAATTGCGAC   
  
  
+ AGTTTCATTT TCCCAACAAA AATTTCGAGT GTATTCTTTT TAATTTATCA TAAGTATTTA TTGTCAACAT   
  
  
+ TCAACATGCA TCAACAATCA TTAAACACTT TTCGTCACTC CTCAATACTG GTCAGCACTT AAGATTTTGT   
  
  
+ CTTCTAAACT TATCGTAAGT AACTTATTGT TGATTAATTG ATGCTGACCA AATGATACTG AGTGCTAAAA   
  
  
+ TTATAATTAA TAAATACAAT TAAGAGTTGA TCAATGTCGA TGATTCAAAT CAATGTGATT GGTGCTGAAT   
  
  
+ AGTAATTATA AGCACCTATA AAGACTTGAA AAGAACAAAC ATTTAATTAG ATTTTATAAT TATAATTTCC   
  
  
+ TAATATTTAG CATTCATCAG CAAATTCAGC ACACACCAAC ATTAGTCAAC ACCTATCAAC ACTTATAAGT   
  
  
+ ACCAATTAAT AATAATTACC TATAATAGAT TAAAAGTTAG GCCCTTAATG TTTCCCACAT TATTAGATAT   
  
  
+ ATCAAGGGAG GGGGAAAAAA ATAATACTCC CCCATCTTAA AAAATTTATC CCTTTTTTTC TTTTTAATGT   
  
  
+ TCCAAAACAA AAATATGCTT CTAAATTATG ATTGCAATAT GAATGTGTAC GGTTTCAAAC CCAATTTTCT   
  
  
+ CCAACAATTC TTCTACTATA TTGTAATTAT ATTATACTAC AATTTGTATC TCATCTATGA TCTACCCCCC   
  
  
+ AAAAACCAAA TACTCATATA AAGTTTTATT TTCTTTCCAT CCCACTCTAA AAAAAAAAAA TGTTGGGAAA   
  
  
+ AAGTTGATTT ATTTAATTTG AAATGGTACG GTGAATATAG ATTTGTAGAG GGGCAGTTGA TAGAAAAAGG   
  
  
+ TGAAGACAAA GGAAGGGCAC ATTTGCCTTT TCATAGAAGG AAGAGACCAA GGTACTGGGG ACTGGGGGAA   
  
  
+ GGGCTCAAGA GAAGAGAGAG ACTGAGAAGA GTGCTGCTAC TCCTTCCTAC CTACACACAA AATTGCTGCG   
  
  
+ GGGGGTTCAA GTTCAACTTG AACGAGGCAA ATACCCTCAT TGAACCCACC AAATCGAAAT CTTCAATGTA   
  
  
+ CACCCCCATT ATACACGCAT TCAGAGACAT AGATGTCGAG GGGGAGATGA ATTAGCAAGA AGATGTCGAG   
  
  
+ CTAAAATGCA TTTCGAGGTT ACTAGAGCCA GAAATGTGGG CACATCCCAG AAGATCTCAG TAGAGAGAGT   
  
  
+ AACTTCAGAA AAGTTTCAAT TTTTATTCGA AATTTGCCTC TTGTTTGTGT AGATATGATG GGAACTTCGC   
  
  
+ CTTACAATCT GCAATGTGAG GGTTTGGTTG AAGGAGTTGC AGTCAATCCT CAATTTTATG AATCAACCCC   
  
  
+ AAAGTGGAAG AAGGGCATCA AAGAAGAGTC AATTGAGAGC GAACCCACTT CGGTTTTGGA CAACCCTAGC   
  
  
+ CCTCCAAATT CTGCATCTAC TCTCTCTTCC TGCTTCAATG GTGGAAGTAG TGGGCTTGGG ATGGACGATT   
  
  
+ TGGAGGGTTT GTCTTTTGGA GAAGGGTCCC TTCTCCCTTG GTTCATGGGT GAAATTGAAG ACCCTAGTTT   
  
  
+ GAGTTTCAAG CACCTCCTTC AACCTTCAAA TCCCTTGGAG TTTGGTGGCA ATGATGGGCT TGAAGCAGCT   
  
  
+ ATTCAGAGTG CTGGTATGGG TAATTTTAAT TTGGTTGAAA GTTTTAATTC TGATTTGAGG TCTTGTAATT   
  
  
+ CTGGGTCTAT TTGCAATGGG GGGAATGATT CTGTCAATGA AAAGGGATGT GTTCTTAATT ATGCTTCAGA   
  
  
+ GCAGGGTCAT GTGGTTGCTG TATCTCCAGT AGAACAGTTT AGGATTAGTG ATGTGAAACA TGAGGTGTTG   
  
  
+ AACCCAAAAT TTGGCTTTTG TGAGCAAGGC TCTACAATCA GTCAGAACTC TGGTTATGTG CATATACTGG   
  
  
+ GTTATAACCA GCTAGAATCT CCCCTTGAGC CTCCGGCAAA GCGCCACAAT GCTGGGAATG CTCTTTACAC   
  
  
+ TAGTGTTGTT CCAGTTCCAA AGAGTTCGTT TTTAGGTGAG GATCATGAAA TTCTACTCAG AAATCAAGAG   
  
  
+ CAGCAGTTTC TGATGCAACA ACAGTGGTTA ATGGGTTTAG CCCCTCAGTT TACACCCCAG CACCCGCCAA   
  
  
+ AGCCATTAGA CGACCGAAAG CAACCAAACT CGCAAACTTT GGTTCTGCGC GATCAGTTAG TTAAGATTGC   
  
  
+ GGAGATGTTC CAAACCGGTA ACTTTTCGCT TGCCCAAGTG ATATTGGCCC GGCTCAATCA GCAATTGTCT   
  
  
+ CTTTCAGGGA ACCCCTTTGT AAGAGCTGCT ACTTTCCATG TCAAGGAGGA ACTTGAAAGG CTCCTGACTA   
  
  
+ TCAATAGCTT CTCTGCGGCT CCACCCCAAC CTAAAAGCCT TACTCTGTCA GATGTTGTGG ATAGGATGAA   
  
  
+ TGCATACAAG CTTTTTTCAG AGGCATCCCC AGTTATGCAA TTTACCGATT TTACTTGTAC CCAGGCTTTG   
  
  
+ CTCGAAGCTC TTGATGATGC TGATTATATC CACATACTAG ATTTTGACAT TGGTTGTGGT TCCCAATGGG   
  
  
+ CATCCTTTAT TCGAGAGCTT CCTCTCAGGA AAAGGGGCAC TCCATTTTTG AAAATTACAG CCTTTGCCTC   
  
  
+ TTCTTTAACC CATAATCCAT TTGAACTAGC CCTTGTATGT GATAACATTC TGGAATTTGC AAAAGAGTTC   
  
  
+ GGTGTCTCTG TCGACCTTCA GGTCATGAAT TTGGACTTGT TTGACCCAAC TACTTGCACA ATACCCAATT   
  
  
+ TTCAATCCAC TGAGAACGCA GCAGTTGCTG TCAACTTCCC TATATGGGCG TGCTCACACT GCCCGTTTGT   
  
  
+ ACTCAGTCAA CTCCTGTCCT TCATCAAGCA ATGTTCCCCA AAAATCATGA CGAGCTTTGA TAGAGGTTGT   
  
  
+ GATCATTTTG AGTTGTCTTT TCCATCGCAC ATCATCCGTG TATTGGACTC ATGCTCTAAT ATGTTGGAGT   
  
  
+ CCCTCCATGG TTCAAGCGTG ACATTAGACA CCTCAAAGAA GATTGAAAAG TTCTTGATCA AGCCTATAAT   
  
  
+ TGAGAGGGCA ATATTGGGGC GAATTCATGC ACCAGACAAG ATGCAGGCAA ACAAGGCACA CCGGTGGAAG   
  
  
+ AATCTCTTCA CTTCTGCTGG GTTCTTCCCT TTGCCATTTA GTAGTTTCAC TGAATCTCAG GCCAATCTAG   
  
  
+ TGGCTCAACG AACCCCAGTG AGGGGTTTTC AGGTGGAAAA GCAGCAAGCA TCACTTGTGC TCAGGTGGAT   
  
  
+ GTCTCATGAG CTCGTGGCAG CTTCAGCTTG GAGGTGCTA  

- -Up\_Stream \_Len000AAACAT AAATTTAAAA TGTTATAAAT ATTTTTAAAA CATAACTAAT TAACACGATT   
  
  
- GATTGTGATT ATTAACCCCC TCTTTTCTTC CCTTTTATTT TTCTTTAATT CTTCTTCTTT TCTTTTGTGG   
  
  
- AAACTTTTCC GTTTTTTGAT TTTATTTTGT TATTACTTGT CTTCCTCTCT TCCTACCTAT TTGTTGTTTA   
  
  
- CAGCGAAAAT CCTGTCGTTA TCAGATACAC TAACCGGAAT CAAGGACAAC CTCCTAACGA TTGTTTTCGT   
  
  
- TGTCGTTTTT TATTATGATT AATAATGTAT AAAAAATTGA GAATTTCGAT TAGTTAGTAG GAGGTAATTA   
  
  
- ACTAATTAAT AAACGGTTTT TGTGTTTAGT TGACCCCGTT GATATTCTCT AGATGGACGA AATTCTATAT   
  
  
- ATTTGAGTTA ACAAAGTAAA ATAAAATAAA ATCTAAAAAA CCGGAAACCA GAGATAACTC GATTTTAGAG   
  
  
- CAATAAAAGC CTAGTACTTT TTATGTTGAG CTAGAAAACT ATATTCAGTA GAAATAAACA TTTAAGACTT   
  
  
- GAATTTTAGA ATGAACTTCT AGTCCTTATC TTCGTGAGCT AAAAACTATT TAGGATACAC AAGAAGAATT   
  
  
- TTAAGTATTA GATTAAGTAT AAAATCTACA TGGGCAACGG ATGAAACCGT TCAAATCTAA TACTAAAAAT   
  
  
- AAAAAAAATT AATGAACATA AAAAGAAAAC CTAACTATCA GTTAGATGTA GTAGAAACTA GTTAACGCTG   
  
  
- TCAAAGTAAA AGGGTTGTTT TTAAAGCTCA CATAAGAAAA ATTAAATAGT ATTCATAAAT AACAGTTGTA   
  
  
- AGTTGTACGT AGTTGTTAGT AATTTGTGAA AAGCAGTGAG GAGTTATGAC CAGTCGTGAA TTCTAAAACA   
  
  
- GAAGATTTGA ATAGCATTCA TTGAATAACA ACTAATTAAC TACGACTGGT TTACTATGAC TCACGATTTT   
  
  
- AATATTAATT ATTTATGTTA ATTCTCAACT AGTTACAGCT ACTAAGTTTA GTTACACTAA CCACGACTTA   
  
  
- TCATTAATAT TCGTGGATAT TTCTGAACTT TTCTTGTTTG TAAATTAATC TAAAATATTA ATATTAAAGG   
  
  
- ATTATAAATC GTAAGTAGTC GTTTAAGTCG TGTGTGGTTG TAATCAGTTG TGGATAGTTG TGAATATTCA   
  
  
- TGGTTAATTA TTATTAATGG ATATTATCTA ATTTTCAATC CGGGAATTAC AAAGGGTGTA ATAATCTATA   
  
  
- TAGTTCCCTC CCCCTTTTTT TATTATGAGG GGGTAGAATT TTTTAAATAG GGAAAAAAAG AAAAATTACA   
  
  
- AGGTTTTGTT TTTATACGAA GATTTAATAC TAACGTTATA CTTACACATG CCAAAGTTTG GGTTAAAAGA   
  
  
- GGTTGTTAAG AAGATGATAT AACATTAATA TAATATGATG TTAAACATAG AGTAGATACT AGATGGGGGG   
  
  
- TTTTTGGTTT ATGAGTATAT TTCAAAATAA AAGAAAGGTA GGGTGAGATT TTTTTTTTTT ACAACCCTTT   
  
  
- TTCAACTAAA TAAATTAAAC TTTACCATGC CACTTATATC TAAACATCTC CCCGTCAACT ATCTTTTTCC   
  
  
- ACTTCTGTTT CCTTCCCGTG TAAACGGAAA AGTATCTTCC TTCTCTGGTT CCATGACCCC TGACCCCCTT   
  
  
- CCCGAGTTCT CTTCTCTCTC TGACTCTTCT CACGACGATG AGGAAGGATG GATGTGTGTT TTAACGACGC   
  
  
- CCCCCAAGTT CAAGTTGAAC TTGCTCCGTT TATGGGAGTA ACTTGGGTGG TTTAGCTTTA GAAGTTACAT   
  
  
- GTGGGGGTAA TATGTGCGTA AGTCTCTGTA TCTACAGCTC CCCCTCTACT TAATCGTTCT TCTACAGCTC   
  
  
- GATTTTACGT AAAGCTCCAA TGATCTCGGT CTTTACACCC GTGTAGGGTC TTCTAGAGTC ATCTCTCTCA   
  
  
- TTGAAGTCTT TTCAAAGTTA AAAATAAGCT TTAAACGGAG AACAAACACA TCTATACTAC CCTTGAAGCG   
  
  
- GAATGTTAGA CGTTACACTC CCAAACCAAC TTCCTCAACG TCAGTTAGGA GTTAAAATAC TTAGTTGGGG   
  
  
- TTTCACCTTC TTCCCGTAGT TTCTTCTCAG TTAACTCTCG CTTGGGTGAA GCCAAAACCT GTTGGGATCG   
  
  
- GGAGGTTTAA GACGTAGATG AGAGAGAAGG ACGAAGTTAC CACCTTCATC ACCCGAACCC TACCTGCTAA   
  
  
- ACCTCCCAAA CAGAAAACCT CTTCCCAGGG AAGAGGGAAC CAAGTACCCA CTTTAACTTC TGGGATCAAA   
  
  
- CTCAAAGTTC GTGGAGGAAG TTGGAAGTTT AGGGAACCTC AAACCACCGT TACTACCCGA ACTTCGTCGA   
  
  
- TAAGTCTCAC GACCATACCC ATTAAAATTA AACCAACTTT CAAAATTAAG ACTAAACTCC AGAACATTAA   
  
  
- GACCCAGATA AACGTTACCC CCCTTACTAA GACAGTTACT TTTCCCTACA CAAGAATTAA TACGAAGTCT   
  
  
- CGTCCCAGTA CACCAACGAC ATAGAGGTCA TCTTGTCAAA TCCTAATCAC TACACTTTGT ACTCCACAAC   
  
  
- TTGGGTTTTA AACCGAAAAC ACTCGTTCCG AGATGTTAGT CAGTCTTGAG ACCAATACAC GTATATGACC   
  
  
- CAATATTGGT CGATCTTAGA GGGGAACTCG GAGGCCGTTT CGCGGTGTTA CGACCCTTAC GAGAAATGTG   
  
  
- ATCACAACAA GGTCAAGGTT TCTCAAGCAA AAATCCACTC CTAGTACTTT AAGATGAGTC TTTAGTTCTC   
  
  
- GTCGTCAAAG ACTACGTTGT TGTCACCAAT TACCCAAATC GGGGAGTCAA ATGTGGGGTC GTGGGCGGTT   
  
  
- TCGGTAATCT GCTGGCTTTC GTTGGTTTGA GCGTTTGAAA CCAAGACGCG CTAGTCAATC AATTCTAACG   
  
  
- CCTCTACAAG GTTTGGCCAT TGAAAAGCGA ACGGGTTCAC TATAACCGGG CCGAGTTAGT CGTTAACAGA   
  
  
- GAAAGTCCCT TGGGGAAACA TTCTCGACGA TGAAAGGTAC AGTTCCTCCT TGAACTTTCC GAGGACTGAT   
  
  
- AGTTATCGAA GAGACGCCGA GGTGGGGTTG GATTTTCGGA ATGAGACAGT CTACAACACC TATCCTACTT   
  
  
- ACGTATGTTC GAAAAAAGTC TCCGTAGGGG TCAATACGTT AAATGGCTAA AATGAACATG GGTCCGAAAC   
  
  
- GAGCTTCGAG AACTACTACG ACTAATATAG GTGTATGATC TAAAACTGTA ACCAACACCA AGGGTTACCC   
  
  
- GTAGGAAATA AGCTCTCGAA GGAGAGTCCT TTTCCCCGTG AGGTAAAAAC TTTTAATGTC GGAAACGGAG   
  
  
- AAGAAATTGG GTATTAGGTA AACTTGATCG GGAACATACA CTATTGTAAG ACCTTAAACG TTTTCTCAAG   
  
  
- CCACAGAGAC AGCTGGAAGT CCAGTACTTA AACCTGAACA AACTGGGTTG ATGAACGTGT TATGGGTTAA   
  
  
- AAGTTAGGTG ACTCTTGCGT CGTCAACGAC AGTTGAAGGG ATATACCCGC ACGAGTGTGA CGGGCAAACA   
  
  
- TGAGTCAGTT GAGGACAGGA AGTAGTTCGT TACAAGGGGT TTTTAGTACT GCTCGAAACT ATCTCCAACA   
  
  
- CTAGTAAAAC TCAACAGAAA AGGTAGCGTG TAGTAGGCAC ATAACCTGAG TACGAGATTA TACAACCTCA   
  
  
- GGGAGGTACC AAGTTCGCAC TGTAATCTGT GGAGTTTCTT CTAACTTTTC AAGAACTAGT TCGGATATTA   
  
  
- ACTCTCCCGT TATAACCCCG CTTAAGTACG TGGTCTGTTC TACGTCCGTT TGTTCCGTGT GGCCACCTTC   
  
  
- TTAGAGAAGT GAAGACGACC CAAGAAGGGA AACGGTAAAT CATCAAAGTG ACTTAGAGTC CGGTTAGATC   
  
  
- ACCGAGTTGC TTGGGGTCAC TCCCCAAAAG TCCACCTTTT CGTCGTTCGT AGTGAACACG AGTCCACCTA   
  
  
- CAGAGTACTC GAGCACCGTC GAAGTCGAAC CTCCACGAT

+     GT1-motif

| Site Name | Organism | Position | Strand | Matrix score. | sequence | function |
| --- | --- | --- | --- | --- | --- | --- |
| GT1-motif | Arabidopsis thaliana | 3369 | - | 6 | GGTTAA | light responsive element |
| GT1-motif | Avena sativa | 2830 | + | 7 | GGTTAAT | light responsive element |

>HU04G00048.1   
+ -Up\_Stream \_Len000TTTGTA TTTAAATTTT ACAATATTTA TAAAAATTTT GTATTGATTA ATTGTGCTAA   
  
  
+ CTAACACTAA TAATTGGGGG AGAAAAGAAG GGAAAATAAA AAGAAATTAA GAAGAAGAAA AGAAAACACC   
  
  
+ TTTGAAAAGG CAAAAAACTA AAATAAAACA ATAATGAACA GAAGGAGAGA AGGATGGATA AACAACAAAT   
  
  
+ GTCGCTTTTA GGACAGCAAT AGTCTATGTG ATTGGCCTTA GTTCCTGTTG GAGGATTGCT AACAAAAGCA   
  
  
+ ACAGCAAAAA ATAATACTAA TTATTACATA TTTTTTAACT CTTAAAGCTA ATCAATCATC CTCCATTAAT   
  
  
+ TGATTAATTA TTTGCCAAAA ACACAAATCA ACTGGGGCAA CTATAAGAGA TCTACCTGCT TTAAGATATA   
  
  
+ TAAACTCAAT TGTTTCATTT TATTTTATTT TAGATTTTTT GGCCTTTGGT CTCTATTGAG CTAAAATCTC   
  
  
+ GTTATTTTCG GATCATGAAA AATACAACTC GATCTTTTGA TATAAGTCAT CTTTATTTGT AAATTCTGAA   
  
  
+ CTTAAAATCT TACTTGAAGA TCAGGAATAG AAGCACTCGA TTTTTGATAA ATCCTATGTG TTCTTCTTAA   
  
  
+ AATTCATAAT CTAATTCATA TTTTAGATGT ACCCGTTGCC TACTTTGGCA AGTTTAGATT ATGATTTTTA   
  
  
+ TTTTTTTTAA TTACTTGTAT TTTTCTTTTG GATTGATAGT CAATCTACAT CATCTTTGAT CAATTGCGAC   
  
  
+ AGTTTCATTT TCCCAACAAA AATTTCGAGT GTATTCTTTT TAATTTATCA TAAGTATTTA TTGTCAACAT   
  
  
+ TCAACATGCA TCAACAATCA TTAAACACTT TTCGTCACTC CTCAATACTG GTCAGCACTT AAGATTTTGT   
  
  
+ CTTCTAAACT TATCGTAAGT AACTTATTGT TGATTAATTG ATGCTGACCA AATGATACTG AGTGCTAAAA   
  
  
+ TTATAATTAA TAAATACAAT TAAGAGTTGA TCAATGTCGA TGATTCAAAT CAATGTGATT GGTGCTGAAT   
  
  
+ AGTAATTATA AGCACCTATA AAGACTTGAA AAGAACAAAC ATTTAATTAG ATTTTATAAT TATAATTTCC   
  
  
+ TAATATTTAG CATTCATCAG CAAATTCAGC ACACACCAAC ATTAGTCAAC ACCTATCAAC ACTTATAAGT   
  
  
+ ACCAATTAAT AATAATTACC TATAATAGAT TAAAAGTTAG GCCCTTAATG TTTCCCACAT TATTAGATAT   
  
  
+ ATCAAGGGAG GGGGAAAAAA ATAATACTCC CCCATCTTAA AAAATTTATC CCTTTTTTTC TTTTTAATGT   
  
  
+ TCCAAAACAA AAATATGCTT CTAAATTATG ATTGCAATAT GAATGTGTAC GGTTTCAAAC CCAATTTTCT   
  
  
+ CCAACAATTC TTCTACTATA TTGTAATTAT ATTATACTAC AATTTGTATC TCATCTATGA TCTACCCCCC   
  
  
+ AAAAACCAAA TACTCATATA AAGTTTTATT TTCTTTCCAT CCCACTCTAA AAAAAAAAAA TGTTGGGAAA   
  
  
+ AAGTTGATTT ATTTAATTTG AAATGGTACG GTGAATATAG ATTTGTAGAG GGGCAGTTGA TAGAAAAAGG   
  
  
+ TGAAGACAAA GGAAGGGCAC ATTTGCCTTT TCATAGAAGG AAGAGACCAA GGTACTGGGG ACTGGGGGAA   
  
  
+ GGGCTCAAGA GAAGAGAGAG ACTGAGAAGA GTGCTGCTAC TCCTTCCTAC CTACACACAA AATTGCTGCG   
  
  
+ GGGGGTTCAA GTTCAACTTG AACGAGGCAA ATACCCTCAT TGAACCCACC AAATCGAAAT CTTCAATGTA   
  
  
+ CACCCCCATT ATACACGCAT TCAGAGACAT AGATGTCGAG GGGGAGATGA ATTAGCAAGA AGATGTCGAG   
  
  
+ CTAAAATGCA TTTCGAGGTT ACTAGAGCCA GAAATGTGGG CACATCCCAG AAGATCTCAG TAGAGAGAGT   
  
  
+ AACTTCAGAA AAGTTTCAAT TTTTATTCGA AATTTGCCTC TTGTTTGTGT AGATATGATG GGAACTTCGC   
  
  
+ CTTACAATCT GCAATGTGAG GGTTTGGTTG AAGGAGTTGC AGTCAATCCT CAATTTTATG AATCAACCCC   
  
  
+ AAAGTGGAAG AAGGGCATCA AAGAAGAGTC AATTGAGAGC GAACCCACTT CGGTTTTGGA CAACCCTAGC   
  
  
+ CCTCCAAATT CTGCATCTAC TCTCTCTTCC TGCTTCAATG GTGGAAGTAG TGGGCTTGGG ATGGACGATT   
  
  
+ TGGAGGGTTT GTCTTTTGGA GAAGGGTCCC TTCTCCCTTG GTTCATGGGT GAAATTGAAG ACCCTAGTTT   
  
  
+ GAGTTTCAAG CACCTCCTTC AACCTTCAAA TCCCTTGGAG TTTGGTGGCA ATGATGGGCT TGAAGCAGCT   
  
  
+ ATTCAGAGTG CTGGTATGGG TAATTTTAAT TTGGTTGAAA GTTTTAATTC TGATTTGAGG TCTTGTAATT   
  
  
+ CTGGGTCTAT TTGCAATGGG GGGAATGATT CTGTCAATGA AAAGGGATGT GTTCTTAATT ATGCTTCAGA   
  
  
+ GCAGGGTCAT GTGGTTGCTG TATCTCCAGT AGAACAGTTT AGGATTAGTG ATGTGAAACA TGAGGTGTTG   
  
  
+ AACCCAAAAT TTGGCTTTTG TGAGCAAGGC TCTACAATCA GTCAGAACTC TGGTTATGTG CATATACTGG   
  
  
+ GTTATAACCA GCTAGAATCT CCCCTTGAGC CTCCGGCAAA GCGCCACAAT GCTGGGAATG CTCTTTACAC   
  
  
+ TAGTGTTGTT CCAGTTCCAA AGAGTTCGTT TTTAGGTGAG GATCATGAAA TTCTACTCAG AAATCAAGAG   
  
  
+ CAGCAGTTTC TGATGCAACA ACAGTGGTTA ATGGGTTTAG CCCCTCAGTT TACACCCCAG CACCCGCCAA   
  
  
+ AGCCATTAGA CGACCGAAAG CAACCAAACT CGCAAACTTT GGTTCTGCGC GATCAGTTAG TTAAGATTGC   
  
  
+ GGAGATGTTC CAAACCGGTA ACTTTTCGCT TGCCCAAGTG ATATTGGCCC GGCTCAATCA GCAATTGTCT   
  
  
+ CTTTCAGGGA ACCCCTTTGT AAGAGCTGCT ACTTTCCATG TCAAGGAGGA ACTTGAAAGG CTCCTGACTA   
  
  
+ TCAATAGCTT CTCTGCGGCT CCACCCCAAC CTAAAAGCCT TACTCTGTCA GATGTTGTGG ATAGGATGAA   
  
  
+ TGCATACAAG CTTTTTTCAG AGGCATCCCC AGTTATGCAA TTTACCGATT TTACTTGTAC CCAGGCTTTG   
  
  
+ CTCGAAGCTC TTGATGATGC TGATTATATC CACATACTAG ATTTTGACAT TGGTTGTGGT TCCCAATGGG   
  
  
+ CATCCTTTAT TCGAGAGCTT CCTCTCAGGA AAAGGGGCAC TCCATTTTTG AAAATTACAG CCTTTGCCTC   
  
  
+ TTCTTTAACC CATAATCCAT TTGAACTAGC CCTTGTATGT GATAACATTC TGGAATTTGC AAAAGAGTTC   
  
  
+ GGTGTCTCTG TCGACCTTCA GGTCATGAAT TTGGACTTGT TTGACCCAAC TACTTGCACA ATACCCAATT   
  
  
+ TTCAATCCAC TGAGAACGCA GCAGTTGCTG TCAACTTCCC TATATGGGCG TGCTCACACT GCCCGTTTGT   
  
  
+ ACTCAGTCAA CTCCTGTCCT TCATCAAGCA ATGTTCCCCA AAAATCATGA CGAGCTTTGA TAGAGGTTGT   
  
  
+ GATCATTTTG AGTTGTCTTT TCCATCGCAC ATCATCCGTG TATTGGACTC ATGCTCTAAT ATGTTGGAGT   
  
  
+ CCCTCCATGG TTCAAGCGTG ACATTAGACA CCTCAAAGAA GATTGAAAAG TTCTTGATCA AGCCTATAAT   
  
  
+ TGAGAGGGCA ATATTGGGGC GAATTCATGC ACCAGACAAG ATGCAGGCAA ACAAGGCACA CCGGTGGAAG   
  
  
+ AATCTCTTCA CTTCTGCTGG GTTCTTCCCT TTGCCATTTA GTAGTTTCAC TGAATCTCAG GCCAATCTAG   
  
  
+ TGGCTCAACG AACCCCAGTG AGGGGTTTTC AGGTGGAAAA GCAGCAAGCA TCACTTGTGC TCAGGTGGAT   
  
  
+ GTCTCATGAG CTCGTGGCAG CTTCAGCTTG GAGGTGCTA  

- -Up\_Stream \_Len000AAACAT AAATTTAAAA TGTTATAAAT ATTTTTAAAA CATAACTAAT TAACACGATT   
  
  
- GATTGTGATT ATTAACCCCC TCTTTTCTTC CCTTTTATTT TTCTTTAATT CTTCTTCTTT TCTTTTGTGG   
  
  
- AAACTTTTCC GTTTTTTGAT TTTATTTTGT TATTACTTGT CTTCCTCTCT TCCTACCTAT TTGTTGTTTA   
  
  
- CAGCGAAAAT CCTGTCGTTA TCAGATACAC TAACCGGAAT CAAGGACAAC CTCCTAACGA TTGTTTTCGT   
  
  
- TGTCGTTTTT TATTATGATT AATAATGTAT AAAAAATTGA GAATTTCGAT TAGTTAGTAG GAGGTAATTA   
  
  
- ACTAATTAAT AAACGGTTTT TGTGTTTAGT TGACCCCGTT GATATTCTCT AGATGGACGA AATTCTATAT   
  
  
- ATTTGAGTTA ACAAAGTAAA ATAAAATAAA ATCTAAAAAA CCGGAAACCA GAGATAACTC GATTTTAGAG   
  
  
- CAATAAAAGC CTAGTACTTT TTATGTTGAG CTAGAAAACT ATATTCAGTA GAAATAAACA TTTAAGACTT   
  
  
- GAATTTTAGA ATGAACTTCT AGTCCTTATC TTCGTGAGCT AAAAACTATT TAGGATACAC AAGAAGAATT   
  
  
- TTAAGTATTA GATTAAGTAT AAAATCTACA TGGGCAACGG ATGAAACCGT TCAAATCTAA TACTAAAAAT   
  
  
- AAAAAAAATT AATGAACATA AAAAGAAAAC CTAACTATCA GTTAGATGTA GTAGAAACTA GTTAACGCTG   
  
  
- TCAAAGTAAA AGGGTTGTTT TTAAAGCTCA CATAAGAAAA ATTAAATAGT ATTCATAAAT AACAGTTGTA   
  
  
- AGTTGTACGT AGTTGTTAGT AATTTGTGAA AAGCAGTGAG GAGTTATGAC CAGTCGTGAA TTCTAAAACA   
  
  
- GAAGATTTGA ATAGCATTCA TTGAATAACA ACTAATTAAC TACGACTGGT TTACTATGAC TCACGATTTT   
  
  
- AATATTAATT ATTTATGTTA ATTCTCAACT AGTTACAGCT ACTAAGTTTA GTTACACTAA CCACGACTTA   
  
  
- TCATTAATAT TCGTGGATAT TTCTGAACTT TTCTTGTTTG TAAATTAATC TAAAATATTA ATATTAAAGG   
  
  
- ATTATAAATC GTAAGTAGTC GTTTAAGTCG TGTGTGGTTG TAATCAGTTG TGGATAGTTG TGAATATTCA   
  
  
- TGGTTAATTA TTATTAATGG ATATTATCTA ATTTTCAATC CGGGAATTAC AAAGGGTGTA ATAATCTATA   
  
  
- TAGTTCCCTC CCCCTTTTTT TATTATGAGG GGGTAGAATT TTTTAAATAG GGAAAAAAAG AAAAATTACA   
  
  
- AGGTTTTGTT TTTATACGAA GATTTAATAC TAACGTTATA CTTACACATG CCAAAGTTTG GGTTAAAAGA   
  
  
- GGTTGTTAAG AAGATGATAT AACATTAATA TAATATGATG TTAAACATAG AGTAGATACT AGATGGGGGG   
  
  
- TTTTTGGTTT ATGAGTATAT TTCAAAATAA AAGAAAGGTA GGGTGAGATT TTTTTTTTTT ACAACCCTTT   
  
  
- TTCAACTAAA TAAATTAAAC TTTACCATGC CACTTATATC TAAACATCTC CCCGTCAACT ATCTTTTTCC   
  
  
- ACTTCTGTTT CCTTCCCGTG TAAACGGAAA AGTATCTTCC TTCTCTGGTT CCATGACCCC TGACCCCCTT   
  
  
- CCCGAGTTCT CTTCTCTCTC TGACTCTTCT CACGACGATG AGGAAGGATG GATGTGTGTT TTAACGACGC   
  
  
- CCCCCAAGTT CAAGTTGAAC TTGCTCCGTT TATGGGAGTA ACTTGGGTGG TTTAGCTTTA GAAGTTACAT   
  
  
- GTGGGGGTAA TATGTGCGTA AGTCTCTGTA TCTACAGCTC CCCCTCTACT TAATCGTTCT TCTACAGCTC   
  
  
- GATTTTACGT AAAGCTCCAA TGATCTCGGT CTTTACACCC GTGTAGGGTC TTCTAGAGTC ATCTCTCTCA   
  
  
- TTGAAGTCTT TTCAAAGTTA AAAATAAGCT TTAAACGGAG AACAAACACA TCTATACTAC CCTTGAAGCG   
  
  
- GAATGTTAGA CGTTACACTC CCAAACCAAC TTCCTCAACG TCAGTTAGGA GTTAAAATAC TTAGTTGGGG   
  
  
- TTTCACCTTC TTCCCGTAGT TTCTTCTCAG TTAACTCTCG CTTGGGTGAA GCCAAAACCT GTTGGGATCG   
  
  
- GGAGGTTTAA GACGTAGATG AGAGAGAAGG ACGAAGTTAC CACCTTCATC ACCCGAACCC TACCTGCTAA   
  
  
- ACCTCCCAAA CAGAAAACCT CTTCCCAGGG AAGAGGGAAC CAAGTACCCA CTTTAACTTC TGGGATCAAA   
  
  
- CTCAAAGTTC GTGGAGGAAG TTGGAAGTTT AGGGAACCTC AAACCACCGT TACTACCCGA ACTTCGTCGA   
  
  
- TAAGTCTCAC GACCATACCC ATTAAAATTA AACCAACTTT CAAAATTAAG ACTAAACTCC AGAACATTAA   
  
  
- GACCCAGATA AACGTTACCC CCCTTACTAA GACAGTTACT TTTCCCTACA CAAGAATTAA TACGAAGTCT   
  
  
- CGTCCCAGTA CACCAACGAC ATAGAGGTCA TCTTGTCAAA TCCTAATCAC TACACTTTGT ACTCCACAAC   
  
  
- TTGGGTTTTA AACCGAAAAC ACTCGTTCCG AGATGTTAGT CAGTCTTGAG ACCAATACAC GTATATGACC   
  
  
- CAATATTGGT CGATCTTAGA GGGGAACTCG GAGGCCGTTT CGCGGTGTTA CGACCCTTAC GAGAAATGTG   
  
  
- ATCACAACAA GGTCAAGGTT TCTCAAGCAA AAATCCACTC CTAGTACTTT AAGATGAGTC TTTAGTTCTC   
  
  
- GTCGTCAAAG ACTACGTTGT TGTCACCAAT TACCCAAATC GGGGAGTCAA ATGTGGGGTC GTGGGCGGTT   
  
  
- TCGGTAATCT GCTGGCTTTC GTTGGTTTGA GCGTTTGAAA CCAAGACGCG CTAGTCAATC AATTCTAACG   
  
  
- CCTCTACAAG GTTTGGCCAT TGAAAAGCGA ACGGGTTCAC TATAACCGGG CCGAGTTAGT CGTTAACAGA   
  
  
- GAAAGTCCCT TGGGGAAACA TTCTCGACGA TGAAAGGTAC AGTTCCTCCT TGAACTTTCC GAGGACTGAT   
  
  
- AGTTATCGAA GAGACGCCGA GGTGGGGTTG GATTTTCGGA ATGAGACAGT CTACAACACC TATCCTACTT   
  
  
- ACGTATGTTC GAAAAAAGTC TCCGTAGGGG TCAATACGTT AAATGGCTAA AATGAACATG GGTCCGAAAC   
  
  
- GAGCTTCGAG AACTACTACG ACTAATATAG GTGTATGATC TAAAACTGTA ACCAACACCA AGGGTTACCC   
  
  
- GTAGGAAATA AGCTCTCGAA GGAGAGTCCT TTTCCCCGTG AGGTAAAAAC TTTTAATGTC GGAAACGGAG   
  
  
- AAGAAATTGG GTATTAGGTA AACTTGATCG GGAACATACA CTATTGTAAG ACCTTAAACG TTTTCTCAAG   
  
  
- CCACAGAGAC AGCTGGAAGT CCAGTACTTA AACCTGAACA AACTGGGTTG ATGAACGTGT TATGGGTTAA   
  
  
- AAGTTAGGTG ACTCTTGCGT CGTCAACGAC AGTTGAAGGG ATATACCCGC ACGAGTGTGA CGGGCAAACA   
  
  
- TGAGTCAGTT GAGGACAGGA AGTAGTTCGT TACAAGGGGT TTTTAGTACT GCTCGAAACT ATCTCCAACA   
  
  
- CTAGTAAAAC TCAACAGAAA AGGTAGCGTG TAGTAGGCAC ATAACCTGAG TACGAGATTA TACAACCTCA   
  
  
- GGGAGGTACC AAGTTCGCAC TGTAATCTGT GGAGTTTCTT CTAACTTTTC AAGAACTAGT TCGGATATTA   
  
  
- ACTCTCCCGT TATAACCCCG CTTAAGTACG TGGTCTGTTC TACGTCCGTT TGTTCCGTGT GGCCACCTTC   
  
  
- TTAGAGAAGT GAAGACGACC CAAGAAGGGA AACGGTAAAT CATCAAAGTG ACTTAGAGTC CGGTTAGATC   
  
  
- ACCGAGTTGC TTGGGGTCAC TCCCCAAAAG TCCACCTTTT CGTCGTTCGT AGTGAACACG AGTCCACCTA   
  
  
- CAGAGTACTC GAGCACCGTC GAAGTCGAAC CTCCACGAT

+     LTR

| Site Name | Organism | Position | Strand | Matrix score. | sequence | function |
| --- | --- | --- | --- | --- | --- | --- |
| LTR | Hordeum vulgare | 500 | - | 6 | CCGAAA | cis-acting element involved in low-temperature responsiveness |
| LTR | Hordeum vulgare | 2888 | + | 6 | CCGAAA | cis-acting element involved in low-temperature responsiveness |

>HU04G00048.1   
+ -Up\_Stream \_Len000TTTGTA TTTAAATTTT ACAATATTTA TAAAAATTTT GTATTGATTA ATTGTGCTAA   
  
  
+ CTAACACTAA TAATTGGGGG AGAAAAGAAG GGAAAATAAA AAGAAATTAA GAAGAAGAAA AGAAAACACC   
  
  
+ TTTGAAAAGG CAAAAAACTA AAATAAAACA ATAATGAACA GAAGGAGAGA AGGATGGATA AACAACAAAT   
  
  
+ GTCGCTTTTA GGACAGCAAT AGTCTATGTG ATTGGCCTTA GTTCCTGTTG GAGGATTGCT AACAAAAGCA   
  
  
+ ACAGCAAAAA ATAATACTAA TTATTACATA TTTTTTAACT CTTAAAGCTA ATCAATCATC CTCCATTAAT   
  
  
+ TGATTAATTA TTTGCCAAAA ACACAAATCA ACTGGGGCAA CTATAAGAGA TCTACCTGCT TTAAGATATA   
  
  
+ TAAACTCAAT TGTTTCATTT TATTTTATTT TAGATTTTTT GGCCTTTGGT CTCTATTGAG CTAAAATCTC   
  
  
+ GTTATTTTCG GATCATGAAA AATACAACTC GATCTTTTGA TATAAGTCAT CTTTATTTGT AAATTCTGAA   
  
  
+ CTTAAAATCT TACTTGAAGA TCAGGAATAG AAGCACTCGA TTTTTGATAA ATCCTATGTG TTCTTCTTAA   
  
  
+ AATTCATAAT CTAATTCATA TTTTAGATGT ACCCGTTGCC TACTTTGGCA AGTTTAGATT ATGATTTTTA   
  
  
+ TTTTTTTTAA TTACTTGTAT TTTTCTTTTG GATTGATAGT CAATCTACAT CATCTTTGAT CAATTGCGAC   
  
  
+ AGTTTCATTT TCCCAACAAA AATTTCGAGT GTATTCTTTT TAATTTATCA TAAGTATTTA TTGTCAACAT   
  
  
+ TCAACATGCA TCAACAATCA TTAAACACTT TTCGTCACTC CTCAATACTG GTCAGCACTT AAGATTTTGT   
  
  
+ CTTCTAAACT TATCGTAAGT AACTTATTGT TGATTAATTG ATGCTGACCA AATGATACTG AGTGCTAAAA   
  
  
+ TTATAATTAA TAAATACAAT TAAGAGTTGA TCAATGTCGA TGATTCAAAT CAATGTGATT GGTGCTGAAT   
  
  
+ AGTAATTATA AGCACCTATA AAGACTTGAA AAGAACAAAC ATTTAATTAG ATTTTATAAT TATAATTTCC   
  
  
+ TAATATTTAG CATTCATCAG CAAATTCAGC ACACACCAAC ATTAGTCAAC ACCTATCAAC ACTTATAAGT   
  
  
+ ACCAATTAAT AATAATTACC TATAATAGAT TAAAAGTTAG GCCCTTAATG TTTCCCACAT TATTAGATAT   
  
  
+ ATCAAGGGAG GGGGAAAAAA ATAATACTCC CCCATCTTAA AAAATTTATC CCTTTTTTTC TTTTTAATGT   
  
  
+ TCCAAAACAA AAATATGCTT CTAAATTATG ATTGCAATAT GAATGTGTAC GGTTTCAAAC CCAATTTTCT   
  
  
+ CCAACAATTC TTCTACTATA TTGTAATTAT ATTATACTAC AATTTGTATC TCATCTATGA TCTACCCCCC   
  
  
+ AAAAACCAAA TACTCATATA AAGTTTTATT TTCTTTCCAT CCCACTCTAA AAAAAAAAAA TGTTGGGAAA   
  
  
+ AAGTTGATTT ATTTAATTTG AAATGGTACG GTGAATATAG ATTTGTAGAG GGGCAGTTGA TAGAAAAAGG   
  
  
+ TGAAGACAAA GGAAGGGCAC ATTTGCCTTT TCATAGAAGG AAGAGACCAA GGTACTGGGG ACTGGGGGAA   
  
  
+ GGGCTCAAGA GAAGAGAGAG ACTGAGAAGA GTGCTGCTAC TCCTTCCTAC CTACACACAA AATTGCTGCG   
  
  
+ GGGGGTTCAA GTTCAACTTG AACGAGGCAA ATACCCTCAT TGAACCCACC AAATCGAAAT CTTCAATGTA   
  
  
+ CACCCCCATT ATACACGCAT TCAGAGACAT AGATGTCGAG GGGGAGATGA ATTAGCAAGA AGATGTCGAG   
  
  
+ CTAAAATGCA TTTCGAGGTT ACTAGAGCCA GAAATGTGGG CACATCCCAG AAGATCTCAG TAGAGAGAGT   
  
  
+ AACTTCAGAA AAGTTTCAAT TTTTATTCGA AATTTGCCTC TTGTTTGTGT AGATATGATG GGAACTTCGC   
  
  
+ CTTACAATCT GCAATGTGAG GGTTTGGTTG AAGGAGTTGC AGTCAATCCT CAATTTTATG AATCAACCCC   
  
  
+ AAAGTGGAAG AAGGGCATCA AAGAAGAGTC AATTGAGAGC GAACCCACTT CGGTTTTGGA CAACCCTAGC   
  
  
+ CCTCCAAATT CTGCATCTAC TCTCTCTTCC TGCTTCAATG GTGGAAGTAG TGGGCTTGGG ATGGACGATT   
  
  
+ TGGAGGGTTT GTCTTTTGGA GAAGGGTCCC TTCTCCCTTG GTTCATGGGT GAAATTGAAG ACCCTAGTTT   
  
  
+ GAGTTTCAAG CACCTCCTTC AACCTTCAAA TCCCTTGGAG TTTGGTGGCA ATGATGGGCT TGAAGCAGCT   
  
  
+ ATTCAGAGTG CTGGTATGGG TAATTTTAAT TTGGTTGAAA GTTTTAATTC TGATTTGAGG TCTTGTAATT   
  
  
+ CTGGGTCTAT TTGCAATGGG GGGAATGATT CTGTCAATGA AAAGGGATGT GTTCTTAATT ATGCTTCAGA   
  
  
+ GCAGGGTCAT GTGGTTGCTG TATCTCCAGT AGAACAGTTT AGGATTAGTG ATGTGAAACA TGAGGTGTTG   
  
  
+ AACCCAAAAT TTGGCTTTTG TGAGCAAGGC TCTACAATCA GTCAGAACTC TGGTTATGTG CATATACTGG   
  
  
+ GTTATAACCA GCTAGAATCT CCCCTTGAGC CTCCGGCAAA GCGCCACAAT GCTGGGAATG CTCTTTACAC   
  
  
+ TAGTGTTGTT CCAGTTCCAA AGAGTTCGTT TTTAGGTGAG GATCATGAAA TTCTACTCAG AAATCAAGAG   
  
  
+ CAGCAGTTTC TGATGCAACA ACAGTGGTTA ATGGGTTTAG CCCCTCAGTT TACACCCCAG CACCCGCCAA   
  
  
+ AGCCATTAGA CGACCGAAAG CAACCAAACT CGCAAACTTT GGTTCTGCGC GATCAGTTAG TTAAGATTGC   
  
  
+ GGAGATGTTC CAAACCGGTA ACTTTTCGCT TGCCCAAGTG ATATTGGCCC GGCTCAATCA GCAATTGTCT   
  
  
+ CTTTCAGGGA ACCCCTTTGT AAGAGCTGCT ACTTTCCATG TCAAGGAGGA ACTTGAAAGG CTCCTGACTA   
  
  
+ TCAATAGCTT CTCTGCGGCT CCACCCCAAC CTAAAAGCCT TACTCTGTCA GATGTTGTGG ATAGGATGAA   
  
  
+ TGCATACAAG CTTTTTTCAG AGGCATCCCC AGTTATGCAA TTTACCGATT TTACTTGTAC CCAGGCTTTG   
  
  
+ CTCGAAGCTC TTGATGATGC TGATTATATC CACATACTAG ATTTTGACAT TGGTTGTGGT TCCCAATGGG   
  
  
+ CATCCTTTAT TCGAGAGCTT CCTCTCAGGA AAAGGGGCAC TCCATTTTTG AAAATTACAG CCTTTGCCTC   
  
  
+ TTCTTTAACC CATAATCCAT TTGAACTAGC CCTTGTATGT GATAACATTC TGGAATTTGC AAAAGAGTTC   
  
  
+ GGTGTCTCTG TCGACCTTCA GGTCATGAAT TTGGACTTGT TTGACCCAAC TACTTGCACA ATACCCAATT   
  
  
+ TTCAATCCAC TGAGAACGCA GCAGTTGCTG TCAACTTCCC TATATGGGCG TGCTCACACT GCCCGTTTGT   
  
  
+ ACTCAGTCAA CTCCTGTCCT TCATCAAGCA ATGTTCCCCA AAAATCATGA CGAGCTTTGA TAGAGGTTGT   
  
  
+ GATCATTTTG AGTTGTCTTT TCCATCGCAC ATCATCCGTG TATTGGACTC ATGCTCTAAT ATGTTGGAGT   
  
  
+ CCCTCCATGG TTCAAGCGTG ACATTAGACA CCTCAAAGAA GATTGAAAAG TTCTTGATCA AGCCTATAAT   
  
  
+ TGAGAGGGCA ATATTGGGGC GAATTCATGC ACCAGACAAG ATGCAGGCAA ACAAGGCACA CCGGTGGAAG   
  
  
+ AATCTCTTCA CTTCTGCTGG GTTCTTCCCT TTGCCATTTA GTAGTTTCAC TGAATCTCAG GCCAATCTAG   
  
  
+ TGGCTCAACG AACCCCAGTG AGGGGTTTTC AGGTGGAAAA GCAGCAAGCA TCACTTGTGC TCAGGTGGAT   
  
  
+ GTCTCATGAG CTCGTGGCAG CTTCAGCTTG GAGGTGCTA  

- -Up\_Stream \_Len000AAACAT AAATTTAAAA TGTTATAAAT ATTTTTAAAA CATAACTAAT TAACACGATT   
  
  
- GATTGTGATT ATTAACCCCC TCTTTTCTTC CCTTTTATTT TTCTTTAATT CTTCTTCTTT TCTTTTGTGG   
  
  
- AAACTTTTCC GTTTTTTGAT TTTATTTTGT TATTACTTGT CTTCCTCTCT TCCTACCTAT TTGTTGTTTA   
  
  
- CAGCGAAAAT CCTGTCGTTA TCAGATACAC TAACCGGAAT CAAGGACAAC CTCCTAACGA TTGTTTTCGT   
  
  
- TGTCGTTTTT TATTATGATT AATAATGTAT AAAAAATTGA GAATTTCGAT TAGTTAGTAG GAGGTAATTA   
  
  
- ACTAATTAAT AAACGGTTTT TGTGTTTAGT TGACCCCGTT GATATTCTCT AGATGGACGA AATTCTATAT   
  
  
- ATTTGAGTTA ACAAAGTAAA ATAAAATAAA ATCTAAAAAA CCGGAAACCA GAGATAACTC GATTTTAGAG   
  
  
- CAATAAAAGC CTAGTACTTT TTATGTTGAG CTAGAAAACT ATATTCAGTA GAAATAAACA TTTAAGACTT   
  
  
- GAATTTTAGA ATGAACTTCT AGTCCTTATC TTCGTGAGCT AAAAACTATT TAGGATACAC AAGAAGAATT   
  
  
- TTAAGTATTA GATTAAGTAT AAAATCTACA TGGGCAACGG ATGAAACCGT TCAAATCTAA TACTAAAAAT   
  
  
- AAAAAAAATT AATGAACATA AAAAGAAAAC CTAACTATCA GTTAGATGTA GTAGAAACTA GTTAACGCTG   
  
  
- TCAAAGTAAA AGGGTTGTTT TTAAAGCTCA CATAAGAAAA ATTAAATAGT ATTCATAAAT AACAGTTGTA   
  
  
- AGTTGTACGT AGTTGTTAGT AATTTGTGAA AAGCAGTGAG GAGTTATGAC CAGTCGTGAA TTCTAAAACA   
  
  
- GAAGATTTGA ATAGCATTCA TTGAATAACA ACTAATTAAC TACGACTGGT TTACTATGAC TCACGATTTT   
  
  
- AATATTAATT ATTTATGTTA ATTCTCAACT AGTTACAGCT ACTAAGTTTA GTTACACTAA CCACGACTTA   
  
  
- TCATTAATAT TCGTGGATAT TTCTGAACTT TTCTTGTTTG TAAATTAATC TAAAATATTA ATATTAAAGG   
  
  
- ATTATAAATC GTAAGTAGTC GTTTAAGTCG TGTGTGGTTG TAATCAGTTG TGGATAGTTG TGAATATTCA   
  
  
- TGGTTAATTA TTATTAATGG ATATTATCTA ATTTTCAATC CGGGAATTAC AAAGGGTGTA ATAATCTATA   
  
  
- TAGTTCCCTC CCCCTTTTTT TATTATGAGG GGGTAGAATT TTTTAAATAG GGAAAAAAAG AAAAATTACA   
  
  
- AGGTTTTGTT TTTATACGAA GATTTAATAC TAACGTTATA CTTACACATG CCAAAGTTTG GGTTAAAAGA   
  
  
- GGTTGTTAAG AAGATGATAT AACATTAATA TAATATGATG TTAAACATAG AGTAGATACT AGATGGGGGG   
  
  
- TTTTTGGTTT ATGAGTATAT TTCAAAATAA AAGAAAGGTA GGGTGAGATT TTTTTTTTTT ACAACCCTTT   
  
  
- TTCAACTAAA TAAATTAAAC TTTACCATGC CACTTATATC TAAACATCTC CCCGTCAACT ATCTTTTTCC   
  
  
- ACTTCTGTTT CCTTCCCGTG TAAACGGAAA AGTATCTTCC TTCTCTGGTT CCATGACCCC TGACCCCCTT   
  
  
- CCCGAGTTCT CTTCTCTCTC TGACTCTTCT CACGACGATG AGGAAGGATG GATGTGTGTT TTAACGACGC   
  
  
- CCCCCAAGTT CAAGTTGAAC TTGCTCCGTT TATGGGAGTA ACTTGGGTGG TTTAGCTTTA GAAGTTACAT   
  
  
- GTGGGGGTAA TATGTGCGTA AGTCTCTGTA TCTACAGCTC CCCCTCTACT TAATCGTTCT TCTACAGCTC   
  
  
- GATTTTACGT AAAGCTCCAA TGATCTCGGT CTTTACACCC GTGTAGGGTC TTCTAGAGTC ATCTCTCTCA   
  
  
- TTGAAGTCTT TTCAAAGTTA AAAATAAGCT TTAAACGGAG AACAAACACA TCTATACTAC CCTTGAAGCG   
  
  
- GAATGTTAGA CGTTACACTC CCAAACCAAC TTCCTCAACG TCAGTTAGGA GTTAAAATAC TTAGTTGGGG   
  
  
- TTTCACCTTC TTCCCGTAGT TTCTTCTCAG TTAACTCTCG CTTGGGTGAA GCCAAAACCT GTTGGGATCG   
  
  
- GGAGGTTTAA GACGTAGATG AGAGAGAAGG ACGAAGTTAC CACCTTCATC ACCCGAACCC TACCTGCTAA   
  
  
- ACCTCCCAAA CAGAAAACCT CTTCCCAGGG AAGAGGGAAC CAAGTACCCA CTTTAACTTC TGGGATCAAA   
  
  
- CTCAAAGTTC GTGGAGGAAG TTGGAAGTTT AGGGAACCTC AAACCACCGT TACTACCCGA ACTTCGTCGA   
  
  
- TAAGTCTCAC GACCATACCC ATTAAAATTA AACCAACTTT CAAAATTAAG ACTAAACTCC AGAACATTAA   
  
  
- GACCCAGATA AACGTTACCC CCCTTACTAA GACAGTTACT TTTCCCTACA CAAGAATTAA TACGAAGTCT   
  
  
- CGTCCCAGTA CACCAACGAC ATAGAGGTCA TCTTGTCAAA TCCTAATCAC TACACTTTGT ACTCCACAAC   
  
  
- TTGGGTTTTA AACCGAAAAC ACTCGTTCCG AGATGTTAGT CAGTCTTGAG ACCAATACAC GTATATGACC   
  
  
- CAATATTGGT CGATCTTAGA GGGGAACTCG GAGGCCGTTT CGCGGTGTTA CGACCCTTAC GAGAAATGTG   
  
  
- ATCACAACAA GGTCAAGGTT TCTCAAGCAA AAATCCACTC CTAGTACTTT AAGATGAGTC TTTAGTTCTC   
  
  
- GTCGTCAAAG ACTACGTTGT TGTCACCAAT TACCCAAATC GGGGAGTCAA ATGTGGGGTC GTGGGCGGTT   
  
  
- TCGGTAATCT GCTGGCTTTC GTTGGTTTGA GCGTTTGAAA CCAAGACGCG CTAGTCAATC AATTCTAACG   
  
  
- CCTCTACAAG GTTTGGCCAT TGAAAAGCGA ACGGGTTCAC TATAACCGGG CCGAGTTAGT CGTTAACAGA   
  
  
- GAAAGTCCCT TGGGGAAACA TTCTCGACGA TGAAAGGTAC AGTTCCTCCT TGAACTTTCC GAGGACTGAT   
  
  
- AGTTATCGAA GAGACGCCGA GGTGGGGTTG GATTTTCGGA ATGAGACAGT CTACAACACC TATCCTACTT   
  
  
- ACGTATGTTC GAAAAAAGTC TCCGTAGGGG TCAATACGTT AAATGGCTAA AATGAACATG GGTCCGAAAC   
  
  
- GAGCTTCGAG AACTACTACG ACTAATATAG GTGTATGATC TAAAACTGTA ACCAACACCA AGGGTTACCC   
  
  
- GTAGGAAATA AGCTCTCGAA GGAGAGTCCT TTTCCCCGTG AGGTAAAAAC TTTTAATGTC GGAAACGGAG   
  
  
- AAGAAATTGG GTATTAGGTA AACTTGATCG GGAACATACA CTATTGTAAG ACCTTAAACG TTTTCTCAAG   
  
  
- CCACAGAGAC AGCTGGAAGT CCAGTACTTA AACCTGAACA AACTGGGTTG ATGAACGTGT TATGGGTTAA   
  
  
- AAGTTAGGTG ACTCTTGCGT CGTCAACGAC AGTTGAAGGG ATATACCCGC ACGAGTGTGA CGGGCAAACA   
  
  
- TGAGTCAGTT GAGGACAGGA AGTAGTTCGT TACAAGGGGT TTTTAGTACT GCTCGAAACT ATCTCCAACA   
  
  
- CTAGTAAAAC TCAACAGAAA AGGTAGCGTG TAGTAGGCAC ATAACCTGAG TACGAGATTA TACAACCTCA   
  
  
- GGGAGGTACC AAGTTCGCAC TGTAATCTGT GGAGTTTCTT CTAACTTTTC AAGAACTAGT TCGGATATTA   
  
  
- ACTCTCCCGT TATAACCCCG CTTAAGTACG TGGTCTGTTC TACGTCCGTT TGTTCCGTGT GGCCACCTTC   
  
  
- TTAGAGAAGT GAAGACGACC CAAGAAGGGA AACGGTAAAT CATCAAAGTG ACTTAGAGTC CGGTTAGATC   
  
  
- ACCGAGTTGC TTGGGGTCAC TCCCCAAAAG TCCACCTTTT CGTCGTTCGT AGTGAACACG AGTCCACCTA   
  
  
- CAGAGTACTC GAGCACCGTC GAAGTCGAAC CTCCACGAT

+     MBS

| Site Name | Organism | Position | Strand | Matrix score. | sequence | function |
| --- | --- | --- | --- | --- | --- | --- |
| MBS | Arabidopsis thaliana | 3526 | - | 6 | CAACTG | MYB binding site involved in drought-inducibility |
| MBS | Arabidopsis thaliana | 383 | + | 6 | CAACTG | MYB binding site involved in drought-inducibility |
| MBS | Arabidopsis thaliana | 1598 | - | 6 | CAACTG | MYB binding site involved in drought-inducibility |

>HU04G00048.1   
+ -Up\_Stream \_Len000TTTGTA TTTAAATTTT ACAATATTTA TAAAAATTTT GTATTGATTA ATTGTGCTAA   
  
  
+ CTAACACTAA TAATTGGGGG AGAAAAGAAG GGAAAATAAA AAGAAATTAA GAAGAAGAAA AGAAAACACC   
  
  
+ TTTGAAAAGG CAAAAAACTA AAATAAAACA ATAATGAACA GAAGGAGAGA AGGATGGATA AACAACAAAT   
  
  
+ GTCGCTTTTA GGACAGCAAT AGTCTATGTG ATTGGCCTTA GTTCCTGTTG GAGGATTGCT AACAAAAGCA   
  
  
+ ACAGCAAAAA ATAATACTAA TTATTACATA TTTTTTAACT CTTAAAGCTA ATCAATCATC CTCCATTAAT   
  
  
+ TGATTAATTA TTTGCCAAAA ACACAAATCA ACTGGGGCAA CTATAAGAGA TCTACCTGCT TTAAGATATA   
  
  
+ TAAACTCAAT TGTTTCATTT TATTTTATTT TAGATTTTTT GGCCTTTGGT CTCTATTGAG CTAAAATCTC   
  
  
+ GTTATTTTCG GATCATGAAA AATACAACTC GATCTTTTGA TATAAGTCAT CTTTATTTGT AAATTCTGAA   
  
  
+ CTTAAAATCT TACTTGAAGA TCAGGAATAG AAGCACTCGA TTTTTGATAA ATCCTATGTG TTCTTCTTAA   
  
  
+ AATTCATAAT CTAATTCATA TTTTAGATGT ACCCGTTGCC TACTTTGGCA AGTTTAGATT ATGATTTTTA   
  
  
+ TTTTTTTTAA TTACTTGTAT TTTTCTTTTG GATTGATAGT CAATCTACAT CATCTTTGAT CAATTGCGAC   
  
  
+ AGTTTCATTT TCCCAACAAA AATTTCGAGT GTATTCTTTT TAATTTATCA TAAGTATTTA TTGTCAACAT   
  
  
+ TCAACATGCA TCAACAATCA TTAAACACTT TTCGTCACTC CTCAATACTG GTCAGCACTT AAGATTTTGT   
  
  
+ CTTCTAAACT TATCGTAAGT AACTTATTGT TGATTAATTG ATGCTGACCA AATGATACTG AGTGCTAAAA   
  
  
+ TTATAATTAA TAAATACAAT TAAGAGTTGA TCAATGTCGA TGATTCAAAT CAATGTGATT GGTGCTGAAT   
  
  
+ AGTAATTATA AGCACCTATA AAGACTTGAA AAGAACAAAC ATTTAATTAG ATTTTATAAT TATAATTTCC   
  
  
+ TAATATTTAG CATTCATCAG CAAATTCAGC ACACACCAAC ATTAGTCAAC ACCTATCAAC ACTTATAAGT   
  
  
+ ACCAATTAAT AATAATTACC TATAATAGAT TAAAAGTTAG GCCCTTAATG TTTCCCACAT TATTAGATAT   
  
  
+ ATCAAGGGAG GGGGAAAAAA ATAATACTCC CCCATCTTAA AAAATTTATC CCTTTTTTTC TTTTTAATGT   
  
  
+ TCCAAAACAA AAATATGCTT CTAAATTATG ATTGCAATAT GAATGTGTAC GGTTTCAAAC CCAATTTTCT   
  
  
+ CCAACAATTC TTCTACTATA TTGTAATTAT ATTATACTAC AATTTGTATC TCATCTATGA TCTACCCCCC   
  
  
+ AAAAACCAAA TACTCATATA AAGTTTTATT TTCTTTCCAT CCCACTCTAA AAAAAAAAAA TGTTGGGAAA   
  
  
+ AAGTTGATTT ATTTAATTTG AAATGGTACG GTGAATATAG ATTTGTAGAG GGGCAGTTGA TAGAAAAAGG   
  
  
+ TGAAGACAAA GGAAGGGCAC ATTTGCCTTT TCATAGAAGG AAGAGACCAA GGTACTGGGG ACTGGGGGAA   
  
  
+ GGGCTCAAGA GAAGAGAGAG ACTGAGAAGA GTGCTGCTAC TCCTTCCTAC CTACACACAA AATTGCTGCG   
  
  
+ GGGGGTTCAA GTTCAACTTG AACGAGGCAA ATACCCTCAT TGAACCCACC AAATCGAAAT CTTCAATGTA   
  
  
+ CACCCCCATT ATACACGCAT TCAGAGACAT AGATGTCGAG GGGGAGATGA ATTAGCAAGA AGATGTCGAG   
  
  
+ CTAAAATGCA TTTCGAGGTT ACTAGAGCCA GAAATGTGGG CACATCCCAG AAGATCTCAG TAGAGAGAGT   
  
  
+ AACTTCAGAA AAGTTTCAAT TTTTATTCGA AATTTGCCTC TTGTTTGTGT AGATATGATG GGAACTTCGC   
  
  
+ CTTACAATCT GCAATGTGAG GGTTTGGTTG AAGGAGTTGC AGTCAATCCT CAATTTTATG AATCAACCCC   
  
  
+ AAAGTGGAAG AAGGGCATCA AAGAAGAGTC AATTGAGAGC GAACCCACTT CGGTTTTGGA CAACCCTAGC   
  
  
+ CCTCCAAATT CTGCATCTAC TCTCTCTTCC TGCTTCAATG GTGGAAGTAG TGGGCTTGGG ATGGACGATT   
  
  
+ TGGAGGGTTT GTCTTTTGGA GAAGGGTCCC TTCTCCCTTG GTTCATGGGT GAAATTGAAG ACCCTAGTTT   
  
  
+ GAGTTTCAAG CACCTCCTTC AACCTTCAAA TCCCTTGGAG TTTGGTGGCA ATGATGGGCT TGAAGCAGCT   
  
  
+ ATTCAGAGTG CTGGTATGGG TAATTTTAAT TTGGTTGAAA GTTTTAATTC TGATTTGAGG TCTTGTAATT   
  
  
+ CTGGGTCTAT TTGCAATGGG GGGAATGATT CTGTCAATGA AAAGGGATGT GTTCTTAATT ATGCTTCAGA   
  
  
+ GCAGGGTCAT GTGGTTGCTG TATCTCCAGT AGAACAGTTT AGGATTAGTG ATGTGAAACA TGAGGTGTTG   
  
  
+ AACCCAAAAT TTGGCTTTTG TGAGCAAGGC TCTACAATCA GTCAGAACTC TGGTTATGTG CATATACTGG   
  
  
+ GTTATAACCA GCTAGAATCT CCCCTTGAGC CTCCGGCAAA GCGCCACAAT GCTGGGAATG CTCTTTACAC   
  
  
+ TAGTGTTGTT CCAGTTCCAA AGAGTTCGTT TTTAGGTGAG GATCATGAAA TTCTACTCAG AAATCAAGAG   
  
  
+ CAGCAGTTTC TGATGCAACA ACAGTGGTTA ATGGGTTTAG CCCCTCAGTT TACACCCCAG CACCCGCCAA   
  
  
+ AGCCATTAGA CGACCGAAAG CAACCAAACT CGCAAACTTT GGTTCTGCGC GATCAGTTAG TTAAGATTGC   
  
  
+ GGAGATGTTC CAAACCGGTA ACTTTTCGCT TGCCCAAGTG ATATTGGCCC GGCTCAATCA GCAATTGTCT   
  
  
+ CTTTCAGGGA ACCCCTTTGT AAGAGCTGCT ACTTTCCATG TCAAGGAGGA ACTTGAAAGG CTCCTGACTA   
  
  
+ TCAATAGCTT CTCTGCGGCT CCACCCCAAC CTAAAAGCCT TACTCTGTCA GATGTTGTGG ATAGGATGAA   
  
  
+ TGCATACAAG CTTTTTTCAG AGGCATCCCC AGTTATGCAA TTTACCGATT TTACTTGTAC CCAGGCTTTG   
  
  
+ CTCGAAGCTC TTGATGATGC TGATTATATC CACATACTAG ATTTTGACAT TGGTTGTGGT TCCCAATGGG   
  
  
+ CATCCTTTAT TCGAGAGCTT CCTCTCAGGA AAAGGGGCAC TCCATTTTTG AAAATTACAG CCTTTGCCTC   
  
  
+ TTCTTTAACC CATAATCCAT TTGAACTAGC CCTTGTATGT GATAACATTC TGGAATTTGC AAAAGAGTTC   
  
  
+ GGTGTCTCTG TCGACCTTCA GGTCATGAAT TTGGACTTGT TTGACCCAAC TACTTGCACA ATACCCAATT   
  
  
+ TTCAATCCAC TGAGAACGCA GCAGTTGCTG TCAACTTCCC TATATGGGCG TGCTCACACT GCCCGTTTGT   
  
  
+ ACTCAGTCAA CTCCTGTCCT TCATCAAGCA ATGTTCCCCA AAAATCATGA CGAGCTTTGA TAGAGGTTGT   
  
  
+ GATCATTTTG AGTTGTCTTT TCCATCGCAC ATCATCCGTG TATTGGACTC ATGCTCTAAT ATGTTGGAGT   
  
  
+ CCCTCCATGG TTCAAGCGTG ACATTAGACA CCTCAAAGAA GATTGAAAAG TTCTTGATCA AGCCTATAAT   
  
  
+ TGAGAGGGCA ATATTGGGGC GAATTCATGC ACCAGACAAG ATGCAGGCAA ACAAGGCACA CCGGTGGAAG   
  
  
+ AATCTCTTCA CTTCTGCTGG GTTCTTCCCT TTGCCATTTA GTAGTTTCAC TGAATCTCAG GCCAATCTAG   
  
  
+ TGGCTCAACG AACCCCAGTG AGGGGTTTTC AGGTGGAAAA GCAGCAAGCA TCACTTGTGC TCAGGTGGAT   
  
  
+ GTCTCATGAG CTCGTGGCAG CTTCAGCTTG GAGGTGCTA  

- -Up\_Stream \_Len000AAACAT AAATTTAAAA TGTTATAAAT ATTTTTAAAA CATAACTAAT TAACACGATT   
  
  
- GATTGTGATT ATTAACCCCC TCTTTTCTTC CCTTTTATTT TTCTTTAATT CTTCTTCTTT TCTTTTGTGG   
  
  
- AAACTTTTCC GTTTTTTGAT TTTATTTTGT TATTACTTGT CTTCCTCTCT TCCTACCTAT TTGTTGTTTA   
  
  
- CAGCGAAAAT CCTGTCGTTA TCAGATACAC TAACCGGAAT CAAGGACAAC CTCCTAACGA TTGTTTTCGT   
  
  
- TGTCGTTTTT TATTATGATT AATAATGTAT AAAAAATTGA GAATTTCGAT TAGTTAGTAG GAGGTAATTA   
  
  
- ACTAATTAAT AAACGGTTTT TGTGTTTAGT TGACCCCGTT GATATTCTCT AGATGGACGA AATTCTATAT   
  
  
- ATTTGAGTTA ACAAAGTAAA ATAAAATAAA ATCTAAAAAA CCGGAAACCA GAGATAACTC GATTTTAGAG   
  
  
- CAATAAAAGC CTAGTACTTT TTATGTTGAG CTAGAAAACT ATATTCAGTA GAAATAAACA TTTAAGACTT   
  
  
- GAATTTTAGA ATGAACTTCT AGTCCTTATC TTCGTGAGCT AAAAACTATT TAGGATACAC AAGAAGAATT   
  
  
- TTAAGTATTA GATTAAGTAT AAAATCTACA TGGGCAACGG ATGAAACCGT TCAAATCTAA TACTAAAAAT   
  
  
- AAAAAAAATT AATGAACATA AAAAGAAAAC CTAACTATCA GTTAGATGTA GTAGAAACTA GTTAACGCTG   
  
  
- TCAAAGTAAA AGGGTTGTTT TTAAAGCTCA CATAAGAAAA ATTAAATAGT ATTCATAAAT AACAGTTGTA   
  
  
- AGTTGTACGT AGTTGTTAGT AATTTGTGAA AAGCAGTGAG GAGTTATGAC CAGTCGTGAA TTCTAAAACA   
  
  
- GAAGATTTGA ATAGCATTCA TTGAATAACA ACTAATTAAC TACGACTGGT TTACTATGAC TCACGATTTT   
  
  
- AATATTAATT ATTTATGTTA ATTCTCAACT AGTTACAGCT ACTAAGTTTA GTTACACTAA CCACGACTTA   
  
  
- TCATTAATAT TCGTGGATAT TTCTGAACTT TTCTTGTTTG TAAATTAATC TAAAATATTA ATATTAAAGG   
  
  
- ATTATAAATC GTAAGTAGTC GTTTAAGTCG TGTGTGGTTG TAATCAGTTG TGGATAGTTG TGAATATTCA   
  
  
- TGGTTAATTA TTATTAATGG ATATTATCTA ATTTTCAATC CGGGAATTAC AAAGGGTGTA ATAATCTATA   
  
  
- TAGTTCCCTC CCCCTTTTTT TATTATGAGG GGGTAGAATT TTTTAAATAG GGAAAAAAAG AAAAATTACA   
  
  
- AGGTTTTGTT TTTATACGAA GATTTAATAC TAACGTTATA CTTACACATG CCAAAGTTTG GGTTAAAAGA   
  
  
- GGTTGTTAAG AAGATGATAT AACATTAATA TAATATGATG TTAAACATAG AGTAGATACT AGATGGGGGG   
  
  
- TTTTTGGTTT ATGAGTATAT TTCAAAATAA AAGAAAGGTA GGGTGAGATT TTTTTTTTTT ACAACCCTTT   
  
  
- TTCAACTAAA TAAATTAAAC TTTACCATGC CACTTATATC TAAACATCTC CCCGTCAACT ATCTTTTTCC   
  
  
- ACTTCTGTTT CCTTCCCGTG TAAACGGAAA AGTATCTTCC TTCTCTGGTT CCATGACCCC TGACCCCCTT   
  
  
- CCCGAGTTCT CTTCTCTCTC TGACTCTTCT CACGACGATG AGGAAGGATG GATGTGTGTT TTAACGACGC   
  
  
- CCCCCAAGTT CAAGTTGAAC TTGCTCCGTT TATGGGAGTA ACTTGGGTGG TTTAGCTTTA GAAGTTACAT   
  
  
- GTGGGGGTAA TATGTGCGTA AGTCTCTGTA TCTACAGCTC CCCCTCTACT TAATCGTTCT TCTACAGCTC   
  
  
- GATTTTACGT AAAGCTCCAA TGATCTCGGT CTTTACACCC GTGTAGGGTC TTCTAGAGTC ATCTCTCTCA   
  
  
- TTGAAGTCTT TTCAAAGTTA AAAATAAGCT TTAAACGGAG AACAAACACA TCTATACTAC CCTTGAAGCG   
  
  
- GAATGTTAGA CGTTACACTC CCAAACCAAC TTCCTCAACG TCAGTTAGGA GTTAAAATAC TTAGTTGGGG   
  
  
- TTTCACCTTC TTCCCGTAGT TTCTTCTCAG TTAACTCTCG CTTGGGTGAA GCCAAAACCT GTTGGGATCG   
  
  
- GGAGGTTTAA GACGTAGATG AGAGAGAAGG ACGAAGTTAC CACCTTCATC ACCCGAACCC TACCTGCTAA   
  
  
- ACCTCCCAAA CAGAAAACCT CTTCCCAGGG AAGAGGGAAC CAAGTACCCA CTTTAACTTC TGGGATCAAA   
  
  
- CTCAAAGTTC GTGGAGGAAG TTGGAAGTTT AGGGAACCTC AAACCACCGT TACTACCCGA ACTTCGTCGA   
  
  
- TAAGTCTCAC GACCATACCC ATTAAAATTA AACCAACTTT CAAAATTAAG ACTAAACTCC AGAACATTAA   
  
  
- GACCCAGATA AACGTTACCC CCCTTACTAA GACAGTTACT TTTCCCTACA CAAGAATTAA TACGAAGTCT   
  
  
- CGTCCCAGTA CACCAACGAC ATAGAGGTCA TCTTGTCAAA TCCTAATCAC TACACTTTGT ACTCCACAAC   
  
  
- TTGGGTTTTA AACCGAAAAC ACTCGTTCCG AGATGTTAGT CAGTCTTGAG ACCAATACAC GTATATGACC   
  
  
- CAATATTGGT CGATCTTAGA GGGGAACTCG GAGGCCGTTT CGCGGTGTTA CGACCCTTAC GAGAAATGTG   
  
  
- ATCACAACAA GGTCAAGGTT TCTCAAGCAA AAATCCACTC CTAGTACTTT AAGATGAGTC TTTAGTTCTC   
  
  
- GTCGTCAAAG ACTACGTTGT TGTCACCAAT TACCCAAATC GGGGAGTCAA ATGTGGGGTC GTGGGCGGTT   
  
  
- TCGGTAATCT GCTGGCTTTC GTTGGTTTGA GCGTTTGAAA CCAAGACGCG CTAGTCAATC AATTCTAACG   
  
  
- CCTCTACAAG GTTTGGCCAT TGAAAAGCGA ACGGGTTCAC TATAACCGGG CCGAGTTAGT CGTTAACAGA   
  
  
- GAAAGTCCCT TGGGGAAACA TTCTCGACGA TGAAAGGTAC AGTTCCTCCT TGAACTTTCC GAGGACTGAT   
  
  
- AGTTATCGAA GAGACGCCGA GGTGGGGTTG GATTTTCGGA ATGAGACAGT CTACAACACC TATCCTACTT   
  
  
- ACGTATGTTC GAAAAAAGTC TCCGTAGGGG TCAATACGTT AAATGGCTAA AATGAACATG GGTCCGAAAC   
  
  
- GAGCTTCGAG AACTACTACG ACTAATATAG GTGTATGATC TAAAACTGTA ACCAACACCA AGGGTTACCC   
  
  
- GTAGGAAATA AGCTCTCGAA GGAGAGTCCT TTTCCCCGTG AGGTAAAAAC TTTTAATGTC GGAAACGGAG   
  
  
- AAGAAATTGG GTATTAGGTA AACTTGATCG GGAACATACA CTATTGTAAG ACCTTAAACG TTTTCTCAAG   
  
  
- CCACAGAGAC AGCTGGAAGT CCAGTACTTA AACCTGAACA AACTGGGTTG ATGAACGTGT TATGGGTTAA   
  
  
- AAGTTAGGTG ACTCTTGCGT CGTCAACGAC AGTTGAAGGG ATATACCCGC ACGAGTGTGA CGGGCAAACA   
  
  
- TGAGTCAGTT GAGGACAGGA AGTAGTTCGT TACAAGGGGT TTTTAGTACT GCTCGAAACT ATCTCCAACA   
  
  
- CTAGTAAAAC TCAACAGAAA AGGTAGCGTG TAGTAGGCAC ATAACCTGAG TACGAGATTA TACAACCTCA   
  
  
- GGGAGGTACC AAGTTCGCAC TGTAATCTGT GGAGTTTCTT CTAACTTTTC AAGAACTAGT TCGGATATTA   
  
  
- ACTCTCCCGT TATAACCCCG CTTAAGTACG TGGTCTGTTC TACGTCCGTT TGTTCCGTGT GGCCACCTTC   
  
  
- TTAGAGAAGT GAAGACGACC CAAGAAGGGA AACGGTAAAT CATCAAAGTG ACTTAGAGTC CGGTTAGATC   
  
  
- ACCGAGTTGC TTGGGGTCAC TCCCCAAAAG TCCACCTTTT CGTCGTTCGT AGTGAACACG AGTCCACCTA   
  
  
- CAGAGTACTC GAGCACCGTC GAAGTCGAAC CTCCACGAT

+     MRE

| Site Name | Organism | Position | Strand | Matrix score. | sequence | function |
| --- | --- | --- | --- | --- | --- | --- |
| MRE | Petroselinum crispum | 3112 | + | 7 | AACCTAA | MYB binding site involved in light responsiveness |

>HU04G00048.1   
+ -Up\_Stream \_Len000TTTGTA TTTAAATTTT ACAATATTTA TAAAAATTTT GTATTGATTA ATTGTGCTAA   
  
  
+ CTAACACTAA TAATTGGGGG AGAAAAGAAG GGAAAATAAA AAGAAATTAA GAAGAAGAAA AGAAAACACC   
  
  
+ TTTGAAAAGG CAAAAAACTA AAATAAAACA ATAATGAACA GAAGGAGAGA AGGATGGATA AACAACAAAT   
  
  
+ GTCGCTTTTA GGACAGCAAT AGTCTATGTG ATTGGCCTTA GTTCCTGTTG GAGGATTGCT AACAAAAGCA   
  
  
+ ACAGCAAAAA ATAATACTAA TTATTACATA TTTTTTAACT CTTAAAGCTA ATCAATCATC CTCCATTAAT   
  
  
+ TGATTAATTA TTTGCCAAAA ACACAAATCA ACTGGGGCAA CTATAAGAGA TCTACCTGCT TTAAGATATA   
  
  
+ TAAACTCAAT TGTTTCATTT TATTTTATTT TAGATTTTTT GGCCTTTGGT CTCTATTGAG CTAAAATCTC   
  
  
+ GTTATTTTCG GATCATGAAA AATACAACTC GATCTTTTGA TATAAGTCAT CTTTATTTGT AAATTCTGAA   
  
  
+ CTTAAAATCT TACTTGAAGA TCAGGAATAG AAGCACTCGA TTTTTGATAA ATCCTATGTG TTCTTCTTAA   
  
  
+ AATTCATAAT CTAATTCATA TTTTAGATGT ACCCGTTGCC TACTTTGGCA AGTTTAGATT ATGATTTTTA   
  
  
+ TTTTTTTTAA TTACTTGTAT TTTTCTTTTG GATTGATAGT CAATCTACAT CATCTTTGAT CAATTGCGAC   
  
  
+ AGTTTCATTT TCCCAACAAA AATTTCGAGT GTATTCTTTT TAATTTATCA TAAGTATTTA TTGTCAACAT   
  
  
+ TCAACATGCA TCAACAATCA TTAAACACTT TTCGTCACTC CTCAATACTG GTCAGCACTT AAGATTTTGT   
  
  
+ CTTCTAAACT TATCGTAAGT AACTTATTGT TGATTAATTG ATGCTGACCA AATGATACTG AGTGCTAAAA   
  
  
+ TTATAATTAA TAAATACAAT TAAGAGTTGA TCAATGTCGA TGATTCAAAT CAATGTGATT GGTGCTGAAT   
  
  
+ AGTAATTATA AGCACCTATA AAGACTTGAA AAGAACAAAC ATTTAATTAG ATTTTATAAT TATAATTTCC   
  
  
+ TAATATTTAG CATTCATCAG CAAATTCAGC ACACACCAAC ATTAGTCAAC ACCTATCAAC ACTTATAAGT   
  
  
+ ACCAATTAAT AATAATTACC TATAATAGAT TAAAAGTTAG GCCCTTAATG TTTCCCACAT TATTAGATAT   
  
  
+ ATCAAGGGAG GGGGAAAAAA ATAATACTCC CCCATCTTAA AAAATTTATC CCTTTTTTTC TTTTTAATGT   
  
  
+ TCCAAAACAA AAATATGCTT CTAAATTATG ATTGCAATAT GAATGTGTAC GGTTTCAAAC CCAATTTTCT   
  
  
+ CCAACAATTC TTCTACTATA TTGTAATTAT ATTATACTAC AATTTGTATC TCATCTATGA TCTACCCCCC   
  
  
+ AAAAACCAAA TACTCATATA AAGTTTTATT TTCTTTCCAT CCCACTCTAA AAAAAAAAAA TGTTGGGAAA   
  
  
+ AAGTTGATTT ATTTAATTTG AAATGGTACG GTGAATATAG ATTTGTAGAG GGGCAGTTGA TAGAAAAAGG   
  
  
+ TGAAGACAAA GGAAGGGCAC ATTTGCCTTT TCATAGAAGG AAGAGACCAA GGTACTGGGG ACTGGGGGAA   
  
  
+ GGGCTCAAGA GAAGAGAGAG ACTGAGAAGA GTGCTGCTAC TCCTTCCTAC CTACACACAA AATTGCTGCG   
  
  
+ GGGGGTTCAA GTTCAACTTG AACGAGGCAA ATACCCTCAT TGAACCCACC AAATCGAAAT CTTCAATGTA   
  
  
+ CACCCCCATT ATACACGCAT TCAGAGACAT AGATGTCGAG GGGGAGATGA ATTAGCAAGA AGATGTCGAG   
  
  
+ CTAAAATGCA TTTCGAGGTT ACTAGAGCCA GAAATGTGGG CACATCCCAG AAGATCTCAG TAGAGAGAGT   
  
  
+ AACTTCAGAA AAGTTTCAAT TTTTATTCGA AATTTGCCTC TTGTTTGTGT AGATATGATG GGAACTTCGC   
  
  
+ CTTACAATCT GCAATGTGAG GGTTTGGTTG AAGGAGTTGC AGTCAATCCT CAATTTTATG AATCAACCCC   
  
  
+ AAAGTGGAAG AAGGGCATCA AAGAAGAGTC AATTGAGAGC GAACCCACTT CGGTTTTGGA CAACCCTAGC   
  
  
+ CCTCCAAATT CTGCATCTAC TCTCTCTTCC TGCTTCAATG GTGGAAGTAG TGGGCTTGGG ATGGACGATT   
  
  
+ TGGAGGGTTT GTCTTTTGGA GAAGGGTCCC TTCTCCCTTG GTTCATGGGT GAAATTGAAG ACCCTAGTTT   
  
  
+ GAGTTTCAAG CACCTCCTTC AACCTTCAAA TCCCTTGGAG TTTGGTGGCA ATGATGGGCT TGAAGCAGCT   
  
  
+ ATTCAGAGTG CTGGTATGGG TAATTTTAAT TTGGTTGAAA GTTTTAATTC TGATTTGAGG TCTTGTAATT   
  
  
+ CTGGGTCTAT TTGCAATGGG GGGAATGATT CTGTCAATGA AAAGGGATGT GTTCTTAATT ATGCTTCAGA   
  
  
+ GCAGGGTCAT GTGGTTGCTG TATCTCCAGT AGAACAGTTT AGGATTAGTG ATGTGAAACA TGAGGTGTTG   
  
  
+ AACCCAAAAT TTGGCTTTTG TGAGCAAGGC TCTACAATCA GTCAGAACTC TGGTTATGTG CATATACTGG   
  
  
+ GTTATAACCA GCTAGAATCT CCCCTTGAGC CTCCGGCAAA GCGCCACAAT GCTGGGAATG CTCTTTACAC   
  
  
+ TAGTGTTGTT CCAGTTCCAA AGAGTTCGTT TTTAGGTGAG GATCATGAAA TTCTACTCAG AAATCAAGAG   
  
  
+ CAGCAGTTTC TGATGCAACA ACAGTGGTTA ATGGGTTTAG CCCCTCAGTT TACACCCCAG CACCCGCCAA   
  
  
+ AGCCATTAGA CGACCGAAAG CAACCAAACT CGCAAACTTT GGTTCTGCGC GATCAGTTAG TTAAGATTGC   
  
  
+ GGAGATGTTC CAAACCGGTA ACTTTTCGCT TGCCCAAGTG ATATTGGCCC GGCTCAATCA GCAATTGTCT   
  
  
+ CTTTCAGGGA ACCCCTTTGT AAGAGCTGCT ACTTTCCATG TCAAGGAGGA ACTTGAAAGG CTCCTGACTA   
  
  
+ TCAATAGCTT CTCTGCGGCT CCACCCCAAC CTAAAAGCCT TACTCTGTCA GATGTTGTGG ATAGGATGAA   
  
  
+ TGCATACAAG CTTTTTTCAG AGGCATCCCC AGTTATGCAA TTTACCGATT TTACTTGTAC CCAGGCTTTG   
  
  
+ CTCGAAGCTC TTGATGATGC TGATTATATC CACATACTAG ATTTTGACAT TGGTTGTGGT TCCCAATGGG   
  
  
+ CATCCTTTAT TCGAGAGCTT CCTCTCAGGA AAAGGGGCAC TCCATTTTTG AAAATTACAG CCTTTGCCTC   
  
  
+ TTCTTTAACC CATAATCCAT TTGAACTAGC CCTTGTATGT GATAACATTC TGGAATTTGC AAAAGAGTTC   
  
  
+ GGTGTCTCTG TCGACCTTCA GGTCATGAAT TTGGACTTGT TTGACCCAAC TACTTGCACA ATACCCAATT   
  
  
+ TTCAATCCAC TGAGAACGCA GCAGTTGCTG TCAACTTCCC TATATGGGCG TGCTCACACT GCCCGTTTGT   
  
  
+ ACTCAGTCAA CTCCTGTCCT TCATCAAGCA ATGTTCCCCA AAAATCATGA CGAGCTTTGA TAGAGGTTGT   
  
  
+ GATCATTTTG AGTTGTCTTT TCCATCGCAC ATCATCCGTG TATTGGACTC ATGCTCTAAT ATGTTGGAGT   
  
  
+ CCCTCCATGG TTCAAGCGTG ACATTAGACA CCTCAAAGAA GATTGAAAAG TTCTTGATCA AGCCTATAAT   
  
  
+ TGAGAGGGCA ATATTGGGGC GAATTCATGC ACCAGACAAG ATGCAGGCAA ACAAGGCACA CCGGTGGAAG   
  
  
+ AATCTCTTCA CTTCTGCTGG GTTCTTCCCT TTGCCATTTA GTAGTTTCAC TGAATCTCAG GCCAATCTAG   
  
  
+ TGGCTCAACG AACCCCAGTG AGGGGTTTTC AGGTGGAAAA GCAGCAAGCA TCACTTGTGC TCAGGTGGAT   
  
  
+ GTCTCATGAG CTCGTGGCAG CTTCAGCTTG GAGGTGCTA  

- -Up\_Stream \_Len000AAACAT AAATTTAAAA TGTTATAAAT ATTTTTAAAA CATAACTAAT TAACACGATT   
  
  
- GATTGTGATT ATTAACCCCC TCTTTTCTTC CCTTTTATTT TTCTTTAATT CTTCTTCTTT TCTTTTGTGG   
  
  
- AAACTTTTCC GTTTTTTGAT TTTATTTTGT TATTACTTGT CTTCCTCTCT TCCTACCTAT TTGTTGTTTA   
  
  
- CAGCGAAAAT CCTGTCGTTA TCAGATACAC TAACCGGAAT CAAGGACAAC CTCCTAACGA TTGTTTTCGT   
  
  
- TGTCGTTTTT TATTATGATT AATAATGTAT AAAAAATTGA GAATTTCGAT TAGTTAGTAG GAGGTAATTA   
  
  
- ACTAATTAAT AAACGGTTTT TGTGTTTAGT TGACCCCGTT GATATTCTCT AGATGGACGA AATTCTATAT   
  
  
- ATTTGAGTTA ACAAAGTAAA ATAAAATAAA ATCTAAAAAA CCGGAAACCA GAGATAACTC GATTTTAGAG   
  
  
- CAATAAAAGC CTAGTACTTT TTATGTTGAG CTAGAAAACT ATATTCAGTA GAAATAAACA TTTAAGACTT   
  
  
- GAATTTTAGA ATGAACTTCT AGTCCTTATC TTCGTGAGCT AAAAACTATT TAGGATACAC AAGAAGAATT   
  
  
- TTAAGTATTA GATTAAGTAT AAAATCTACA TGGGCAACGG ATGAAACCGT TCAAATCTAA TACTAAAAAT   
  
  
- AAAAAAAATT AATGAACATA AAAAGAAAAC CTAACTATCA GTTAGATGTA GTAGAAACTA GTTAACGCTG   
  
  
- TCAAAGTAAA AGGGTTGTTT TTAAAGCTCA CATAAGAAAA ATTAAATAGT ATTCATAAAT AACAGTTGTA   
  
  
- AGTTGTACGT AGTTGTTAGT AATTTGTGAA AAGCAGTGAG GAGTTATGAC CAGTCGTGAA TTCTAAAACA   
  
  
- GAAGATTTGA ATAGCATTCA TTGAATAACA ACTAATTAAC TACGACTGGT TTACTATGAC TCACGATTTT   
  
  
- AATATTAATT ATTTATGTTA ATTCTCAACT AGTTACAGCT ACTAAGTTTA GTTACACTAA CCACGACTTA   
  
  
- TCATTAATAT TCGTGGATAT TTCTGAACTT TTCTTGTTTG TAAATTAATC TAAAATATTA ATATTAAAGG   
  
  
- ATTATAAATC GTAAGTAGTC GTTTAAGTCG TGTGTGGTTG TAATCAGTTG TGGATAGTTG TGAATATTCA   
  
  
- TGGTTAATTA TTATTAATGG ATATTATCTA ATTTTCAATC CGGGAATTAC AAAGGGTGTA ATAATCTATA   
  
  
- TAGTTCCCTC CCCCTTTTTT TATTATGAGG GGGTAGAATT TTTTAAATAG GGAAAAAAAG AAAAATTACA   
  
  
- AGGTTTTGTT TTTATACGAA GATTTAATAC TAACGTTATA CTTACACATG CCAAAGTTTG GGTTAAAAGA   
  
  
- GGTTGTTAAG AAGATGATAT AACATTAATA TAATATGATG TTAAACATAG AGTAGATACT AGATGGGGGG   
  
  
- TTTTTGGTTT ATGAGTATAT TTCAAAATAA AAGAAAGGTA GGGTGAGATT TTTTTTTTTT ACAACCCTTT   
  
  
- TTCAACTAAA TAAATTAAAC TTTACCATGC CACTTATATC TAAACATCTC CCCGTCAACT ATCTTTTTCC   
  
  
- ACTTCTGTTT CCTTCCCGTG TAAACGGAAA AGTATCTTCC TTCTCTGGTT CCATGACCCC TGACCCCCTT   
  
  
- CCCGAGTTCT CTTCTCTCTC TGACTCTTCT CACGACGATG AGGAAGGATG GATGTGTGTT TTAACGACGC   
  
  
- CCCCCAAGTT CAAGTTGAAC TTGCTCCGTT TATGGGAGTA ACTTGGGTGG TTTAGCTTTA GAAGTTACAT   
  
  
- GTGGGGGTAA TATGTGCGTA AGTCTCTGTA TCTACAGCTC CCCCTCTACT TAATCGTTCT TCTACAGCTC   
  
  
- GATTTTACGT AAAGCTCCAA TGATCTCGGT CTTTACACCC GTGTAGGGTC TTCTAGAGTC ATCTCTCTCA   
  
  
- TTGAAGTCTT TTCAAAGTTA AAAATAAGCT TTAAACGGAG AACAAACACA TCTATACTAC CCTTGAAGCG   
  
  
- GAATGTTAGA CGTTACACTC CCAAACCAAC TTCCTCAACG TCAGTTAGGA GTTAAAATAC TTAGTTGGGG   
  
  
- TTTCACCTTC TTCCCGTAGT TTCTTCTCAG TTAACTCTCG CTTGGGTGAA GCCAAAACCT GTTGGGATCG   
  
  
- GGAGGTTTAA GACGTAGATG AGAGAGAAGG ACGAAGTTAC CACCTTCATC ACCCGAACCC TACCTGCTAA   
  
  
- ACCTCCCAAA CAGAAAACCT CTTCCCAGGG AAGAGGGAAC CAAGTACCCA CTTTAACTTC TGGGATCAAA   
  
  
- CTCAAAGTTC GTGGAGGAAG TTGGAAGTTT AGGGAACCTC AAACCACCGT TACTACCCGA ACTTCGTCGA   
  
  
- TAAGTCTCAC GACCATACCC ATTAAAATTA AACCAACTTT CAAAATTAAG ACTAAACTCC AGAACATTAA   
  
  
- GACCCAGATA AACGTTACCC CCCTTACTAA GACAGTTACT TTTCCCTACA CAAGAATTAA TACGAAGTCT   
  
  
- CGTCCCAGTA CACCAACGAC ATAGAGGTCA TCTTGTCAAA TCCTAATCAC TACACTTTGT ACTCCACAAC   
  
  
- TTGGGTTTTA AACCGAAAAC ACTCGTTCCG AGATGTTAGT CAGTCTTGAG ACCAATACAC GTATATGACC   
  
  
- CAATATTGGT CGATCTTAGA GGGGAACTCG GAGGCCGTTT CGCGGTGTTA CGACCCTTAC GAGAAATGTG   
  
  
- ATCACAACAA GGTCAAGGTT TCTCAAGCAA AAATCCACTC CTAGTACTTT AAGATGAGTC TTTAGTTCTC   
  
  
- GTCGTCAAAG ACTACGTTGT TGTCACCAAT TACCCAAATC GGGGAGTCAA ATGTGGGGTC GTGGGCGGTT   
  
  
- TCGGTAATCT GCTGGCTTTC GTTGGTTTGA GCGTTTGAAA CCAAGACGCG CTAGTCAATC AATTCTAACG   
  
  
- CCTCTACAAG GTTTGGCCAT TGAAAAGCGA ACGGGTTCAC TATAACCGGG CCGAGTTAGT CGTTAACAGA   
  
  
- GAAAGTCCCT TGGGGAAACA TTCTCGACGA TGAAAGGTAC AGTTCCTCCT TGAACTTTCC GAGGACTGAT   
  
  
- AGTTATCGAA GAGACGCCGA GGTGGGGTTG GATTTTCGGA ATGAGACAGT CTACAACACC TATCCTACTT   
  
  
- ACGTATGTTC GAAAAAAGTC TCCGTAGGGG TCAATACGTT AAATGGCTAA AATGAACATG GGTCCGAAAC   
  
  
- GAGCTTCGAG AACTACTACG ACTAATATAG GTGTATGATC TAAAACTGTA ACCAACACCA AGGGTTACCC   
  
  
- GTAGGAAATA AGCTCTCGAA GGAGAGTCCT TTTCCCCGTG AGGTAAAAAC TTTTAATGTC GGAAACGGAG   
  
  
- AAGAAATTGG GTATTAGGTA AACTTGATCG GGAACATACA CTATTGTAAG ACCTTAAACG TTTTCTCAAG   
  
  
- CCACAGAGAC AGCTGGAAGT CCAGTACTTA AACCTGAACA AACTGGGTTG ATGAACGTGT TATGGGTTAA   
  
  
- AAGTTAGGTG ACTCTTGCGT CGTCAACGAC AGTTGAAGGG ATATACCCGC ACGAGTGTGA CGGGCAAACA   
  
  
- TGAGTCAGTT GAGGACAGGA AGTAGTTCGT TACAAGGGGT TTTTAGTACT GCTCGAAACT ATCTCCAACA   
  
  
- CTAGTAAAAC TCAACAGAAA AGGTAGCGTG TAGTAGGCAC ATAACCTGAG TACGAGATTA TACAACCTCA   
  
  
- GGGAGGTACC AAGTTCGCAC TGTAATCTGT GGAGTTTCTT CTAACTTTTC AAGAACTAGT TCGGATATTA   
  
  
- ACTCTCCCGT TATAACCCCG CTTAAGTACG TGGTCTGTTC TACGTCCGTT TGTTCCGTGT GGCCACCTTC   
  
  
- TTAGAGAAGT GAAGACGACC CAAGAAGGGA AACGGTAAAT CATCAAAGTG ACTTAGAGTC CGGTTAGATC   
  
  
- ACCGAGTTGC TTGGGGTCAC TCCCCAAAAG TCCACCTTTT CGTCGTTCGT AGTGAACACG AGTCCACCTA   
  
  
- CAGAGTACTC GAGCACCGTC GAAGTCGAAC CTCCACGAT

+     MYB

| Site Name | Organism | Position | Strand | Matrix score. | sequence | function |
| --- | --- | --- | --- | --- | --- | --- |
| MYB | Arabidopsis thaliana | 2895 | + | 6 | CAACCA |  |
| MYB | Arabidopsis thaliana | 2829 | - | 6 | TAACCA |  |
| MYB | Arabidopsis thaliana | 2669 | + | 6 | TAACCA |  |
| MYB | Arabidopsis thaliana | 2536 | - | 6 | CAACCA |  |
| MYB | Arabidopsis thaliana | 2645 | - | 6 | TAACCA |  |
| MYB | Arabidopsis thaliana | 259 | - | 6 | CAACAG |  |
| MYB | Arabidopsis thaliana | 3275 | - | 6 | CAACCA |  |
| MYB | Arabidopsis thaliana | 2416 | - | 6 | CAACCA |  |
| MYB | Arabidopsis thaliana | 2823 | + | 6 | CAACAG |  |
| MYB | Arabidopsis thaliana | 283 | + | 6 | CAACAG |  |
| MYB | Arabidopsis thaliana | 2059 | - | 6 | CAACCA |  |

>HU04G00048.1   
+ -Up\_Stream \_Len000TTTGTA TTTAAATTTT ACAATATTTA TAAAAATTTT GTATTGATTA ATTGTGCTAA   
  
  
+ CTAACACTAA TAATTGGGGG AGAAAAGAAG GGAAAATAAA AAGAAATTAA GAAGAAGAAA AGAAAACACC   
  
  
+ TTTGAAAAGG CAAAAAACTA AAATAAAACA ATAATGAACA GAAGGAGAGA AGGATGGATA AACAACAAAT   
  
  
+ GTCGCTTTTA GGACAGCAAT AGTCTATGTG ATTGGCCTTA GTTCCTGTTG GAGGATTGCT AACAAAAGCA   
  
  
+ ACAGCAAAAA ATAATACTAA TTATTACATA TTTTTTAACT CTTAAAGCTA ATCAATCATC CTCCATTAAT   
  
  
+ TGATTAATTA TTTGCCAAAA ACACAAATCA ACTGGGGCAA CTATAAGAGA TCTACCTGCT TTAAGATATA   
  
  
+ TAAACTCAAT TGTTTCATTT TATTTTATTT TAGATTTTTT GGCCTTTGGT CTCTATTGAG CTAAAATCTC   
  
  
+ GTTATTTTCG GATCATGAAA AATACAACTC GATCTTTTGA TATAAGTCAT CTTTATTTGT AAATTCTGAA   
  
  
+ CTTAAAATCT TACTTGAAGA TCAGGAATAG AAGCACTCGA TTTTTGATAA ATCCTATGTG TTCTTCTTAA   
  
  
+ AATTCATAAT CTAATTCATA TTTTAGATGT ACCCGTTGCC TACTTTGGCA AGTTTAGATT ATGATTTTTA   
  
  
+ TTTTTTTTAA TTACTTGTAT TTTTCTTTTG GATTGATAGT CAATCTACAT CATCTTTGAT CAATTGCGAC   
  
  
+ AGTTTCATTT TCCCAACAAA AATTTCGAGT GTATTCTTTT TAATTTATCA TAAGTATTTA TTGTCAACAT   
  
  
+ TCAACATGCA TCAACAATCA TTAAACACTT TTCGTCACTC CTCAATACTG GTCAGCACTT AAGATTTTGT   
  
  
+ CTTCTAAACT TATCGTAAGT AACTTATTGT TGATTAATTG ATGCTGACCA AATGATACTG AGTGCTAAAA   
  
  
+ TTATAATTAA TAAATACAAT TAAGAGTTGA TCAATGTCGA TGATTCAAAT CAATGTGATT GGTGCTGAAT   
  
  
+ AGTAATTATA AGCACCTATA AAGACTTGAA AAGAACAAAC ATTTAATTAG ATTTTATAAT TATAATTTCC   
  
  
+ TAATATTTAG CATTCATCAG CAAATTCAGC ACACACCAAC ATTAGTCAAC ACCTATCAAC ACTTATAAGT   
  
  
+ ACCAATTAAT AATAATTACC TATAATAGAT TAAAAGTTAG GCCCTTAATG TTTCCCACAT TATTAGATAT   
  
  
+ ATCAAGGGAG GGGGAAAAAA ATAATACTCC CCCATCTTAA AAAATTTATC CCTTTTTTTC TTTTTAATGT   
  
  
+ TCCAAAACAA AAATATGCTT CTAAATTATG ATTGCAATAT GAATGTGTAC GGTTTCAAAC CCAATTTTCT   
  
  
+ CCAACAATTC TTCTACTATA TTGTAATTAT ATTATACTAC AATTTGTATC TCATCTATGA TCTACCCCCC   
  
  
+ AAAAACCAAA TACTCATATA AAGTTTTATT TTCTTTCCAT CCCACTCTAA AAAAAAAAAA TGTTGGGAAA   
  
  
+ AAGTTGATTT ATTTAATTTG AAATGGTACG GTGAATATAG ATTTGTAGAG GGGCAGTTGA TAGAAAAAGG   
  
  
+ TGAAGACAAA GGAAGGGCAC ATTTGCCTTT TCATAGAAGG AAGAGACCAA GGTACTGGGG ACTGGGGGAA   
  
  
+ GGGCTCAAGA GAAGAGAGAG ACTGAGAAGA GTGCTGCTAC TCCTTCCTAC CTACACACAA AATTGCTGCG   
  
  
+ GGGGGTTCAA GTTCAACTTG AACGAGGCAA ATACCCTCAT TGAACCCACC AAATCGAAAT CTTCAATGTA   
  
  
+ CACCCCCATT ATACACGCAT TCAGAGACAT AGATGTCGAG GGGGAGATGA ATTAGCAAGA AGATGTCGAG   
  
  
+ CTAAAATGCA TTTCGAGGTT ACTAGAGCCA GAAATGTGGG CACATCCCAG AAGATCTCAG TAGAGAGAGT   
  
  
+ AACTTCAGAA AAGTTTCAAT TTTTATTCGA AATTTGCCTC TTGTTTGTGT AGATATGATG GGAACTTCGC   
  
  
+ CTTACAATCT GCAATGTGAG GGTTTGGTTG AAGGAGTTGC AGTCAATCCT CAATTTTATG AATCAACCCC   
  
  
+ AAAGTGGAAG AAGGGCATCA AAGAAGAGTC AATTGAGAGC GAACCCACTT CGGTTTTGGA CAACCCTAGC   
  
  
+ CCTCCAAATT CTGCATCTAC TCTCTCTTCC TGCTTCAATG GTGGAAGTAG TGGGCTTGGG ATGGACGATT   
  
  
+ TGGAGGGTTT GTCTTTTGGA GAAGGGTCCC TTCTCCCTTG GTTCATGGGT GAAATTGAAG ACCCTAGTTT   
  
  
+ GAGTTTCAAG CACCTCCTTC AACCTTCAAA TCCCTTGGAG TTTGGTGGCA ATGATGGGCT TGAAGCAGCT   
  
  
+ ATTCAGAGTG CTGGTATGGG TAATTTTAAT TTGGTTGAAA GTTTTAATTC TGATTTGAGG TCTTGTAATT   
  
  
+ CTGGGTCTAT TTGCAATGGG GGGAATGATT CTGTCAATGA AAAGGGATGT GTTCTTAATT ATGCTTCAGA   
  
  
+ GCAGGGTCAT GTGGTTGCTG TATCTCCAGT AGAACAGTTT AGGATTAGTG ATGTGAAACA TGAGGTGTTG   
  
  
+ AACCCAAAAT TTGGCTTTTG TGAGCAAGGC TCTACAATCA GTCAGAACTC TGGTTATGTG CATATACTGG   
  
  
+ GTTATAACCA GCTAGAATCT CCCCTTGAGC CTCCGGCAAA GCGCCACAAT GCTGGGAATG CTCTTTACAC   
  
  
+ TAGTGTTGTT CCAGTTCCAA AGAGTTCGTT TTTAGGTGAG GATCATGAAA TTCTACTCAG AAATCAAGAG   
  
  
+ CAGCAGTTTC TGATGCAACA ACAGTGGTTA ATGGGTTTAG CCCCTCAGTT TACACCCCAG CACCCGCCAA   
  
  
+ AGCCATTAGA CGACCGAAAG CAACCAAACT CGCAAACTTT GGTTCTGCGC GATCAGTTAG TTAAGATTGC   
  
  
+ GGAGATGTTC CAAACCGGTA ACTTTTCGCT TGCCCAAGTG ATATTGGCCC GGCTCAATCA GCAATTGTCT   
  
  
+ CTTTCAGGGA ACCCCTTTGT AAGAGCTGCT ACTTTCCATG TCAAGGAGGA ACTTGAAAGG CTCCTGACTA   
  
  
+ TCAATAGCTT CTCTGCGGCT CCACCCCAAC CTAAAAGCCT TACTCTGTCA GATGTTGTGG ATAGGATGAA   
  
  
+ TGCATACAAG CTTTTTTCAG AGGCATCCCC AGTTATGCAA TTTACCGATT TTACTTGTAC CCAGGCTTTG   
  
  
+ CTCGAAGCTC TTGATGATGC TGATTATATC CACATACTAG ATTTTGACAT TGGTTGTGGT TCCCAATGGG   
  
  
+ CATCCTTTAT TCGAGAGCTT CCTCTCAGGA AAAGGGGCAC TCCATTTTTG AAAATTACAG CCTTTGCCTC   
  
  
+ TTCTTTAACC CATAATCCAT TTGAACTAGC CCTTGTATGT GATAACATTC TGGAATTTGC AAAAGAGTTC   
  
  
+ GGTGTCTCTG TCGACCTTCA GGTCATGAAT TTGGACTTGT TTGACCCAAC TACTTGCACA ATACCCAATT   
  
  
+ TTCAATCCAC TGAGAACGCA GCAGTTGCTG TCAACTTCCC TATATGGGCG TGCTCACACT GCCCGTTTGT   
  
  
+ ACTCAGTCAA CTCCTGTCCT TCATCAAGCA ATGTTCCCCA AAAATCATGA CGAGCTTTGA TAGAGGTTGT   
  
  
+ GATCATTTTG AGTTGTCTTT TCCATCGCAC ATCATCCGTG TATTGGACTC ATGCTCTAAT ATGTTGGAGT   
  
  
+ CCCTCCATGG TTCAAGCGTG ACATTAGACA CCTCAAAGAA GATTGAAAAG TTCTTGATCA AGCCTATAAT   
  
  
+ TGAGAGGGCA ATATTGGGGC GAATTCATGC ACCAGACAAG ATGCAGGCAA ACAAGGCACA CCGGTGGAAG   
  
  
+ AATCTCTTCA CTTCTGCTGG GTTCTTCCCT TTGCCATTTA GTAGTTTCAC TGAATCTCAG GCCAATCTAG   
  
  
+ TGGCTCAACG AACCCCAGTG AGGGGTTTTC AGGTGGAAAA GCAGCAAGCA TCACTTGTGC TCAGGTGGAT   
  
  
+ GTCTCATGAG CTCGTGGCAG CTTCAGCTTG GAGGTGCTA  

- -Up\_Stream \_Len000AAACAT AAATTTAAAA TGTTATAAAT ATTTTTAAAA CATAACTAAT TAACACGATT   
  
  
- GATTGTGATT ATTAACCCCC TCTTTTCTTC CCTTTTATTT TTCTTTAATT CTTCTTCTTT TCTTTTGTGG   
  
  
- AAACTTTTCC GTTTTTTGAT TTTATTTTGT TATTACTTGT CTTCCTCTCT TCCTACCTAT TTGTTGTTTA   
  
  
- CAGCGAAAAT CCTGTCGTTA TCAGATACAC TAACCGGAAT CAAGGACAAC CTCCTAACGA TTGTTTTCGT   
  
  
- TGTCGTTTTT TATTATGATT AATAATGTAT AAAAAATTGA GAATTTCGAT TAGTTAGTAG GAGGTAATTA   
  
  
- ACTAATTAAT AAACGGTTTT TGTGTTTAGT TGACCCCGTT GATATTCTCT AGATGGACGA AATTCTATAT   
  
  
- ATTTGAGTTA ACAAAGTAAA ATAAAATAAA ATCTAAAAAA CCGGAAACCA GAGATAACTC GATTTTAGAG   
  
  
- CAATAAAAGC CTAGTACTTT TTATGTTGAG CTAGAAAACT ATATTCAGTA GAAATAAACA TTTAAGACTT   
  
  
- GAATTTTAGA ATGAACTTCT AGTCCTTATC TTCGTGAGCT AAAAACTATT TAGGATACAC AAGAAGAATT   
  
  
- TTAAGTATTA GATTAAGTAT AAAATCTACA TGGGCAACGG ATGAAACCGT TCAAATCTAA TACTAAAAAT   
  
  
- AAAAAAAATT AATGAACATA AAAAGAAAAC CTAACTATCA GTTAGATGTA GTAGAAACTA GTTAACGCTG   
  
  
- TCAAAGTAAA AGGGTTGTTT TTAAAGCTCA CATAAGAAAA ATTAAATAGT ATTCATAAAT AACAGTTGTA   
  
  
- AGTTGTACGT AGTTGTTAGT AATTTGTGAA AAGCAGTGAG GAGTTATGAC CAGTCGTGAA TTCTAAAACA   
  
  
- GAAGATTTGA ATAGCATTCA TTGAATAACA ACTAATTAAC TACGACTGGT TTACTATGAC TCACGATTTT   
  
  
- AATATTAATT ATTTATGTTA ATTCTCAACT AGTTACAGCT ACTAAGTTTA GTTACACTAA CCACGACTTA   
  
  
- TCATTAATAT TCGTGGATAT TTCTGAACTT TTCTTGTTTG TAAATTAATC TAAAATATTA ATATTAAAGG   
  
  
- ATTATAAATC GTAAGTAGTC GTTTAAGTCG TGTGTGGTTG TAATCAGTTG TGGATAGTTG TGAATATTCA   
  
  
- TGGTTAATTA TTATTAATGG ATATTATCTA ATTTTCAATC CGGGAATTAC AAAGGGTGTA ATAATCTATA   
  
  
- TAGTTCCCTC CCCCTTTTTT TATTATGAGG GGGTAGAATT TTTTAAATAG GGAAAAAAAG AAAAATTACA   
  
  
- AGGTTTTGTT TTTATACGAA GATTTAATAC TAACGTTATA CTTACACATG CCAAAGTTTG GGTTAAAAGA   
  
  
- GGTTGTTAAG AAGATGATAT AACATTAATA TAATATGATG TTAAACATAG AGTAGATACT AGATGGGGGG   
  
  
- TTTTTGGTTT ATGAGTATAT TTCAAAATAA AAGAAAGGTA GGGTGAGATT TTTTTTTTTT ACAACCCTTT   
  
  
- TTCAACTAAA TAAATTAAAC TTTACCATGC CACTTATATC TAAACATCTC CCCGTCAACT ATCTTTTTCC   
  
  
- ACTTCTGTTT CCTTCCCGTG TAAACGGAAA AGTATCTTCC TTCTCTGGTT CCATGACCCC TGACCCCCTT   
  
  
- CCCGAGTTCT CTTCTCTCTC TGACTCTTCT CACGACGATG AGGAAGGATG GATGTGTGTT TTAACGACGC   
  
  
- CCCCCAAGTT CAAGTTGAAC TTGCTCCGTT TATGGGAGTA ACTTGGGTGG TTTAGCTTTA GAAGTTACAT   
  
  
- GTGGGGGTAA TATGTGCGTA AGTCTCTGTA TCTACAGCTC CCCCTCTACT TAATCGTTCT TCTACAGCTC   
  
  
- GATTTTACGT AAAGCTCCAA TGATCTCGGT CTTTACACCC GTGTAGGGTC TTCTAGAGTC ATCTCTCTCA   
  
  
- TTGAAGTCTT TTCAAAGTTA AAAATAAGCT TTAAACGGAG AACAAACACA TCTATACTAC CCTTGAAGCG   
  
  
- GAATGTTAGA CGTTACACTC CCAAACCAAC TTCCTCAACG TCAGTTAGGA GTTAAAATAC TTAGTTGGGG   
  
  
- TTTCACCTTC TTCCCGTAGT TTCTTCTCAG TTAACTCTCG CTTGGGTGAA GCCAAAACCT GTTGGGATCG   
  
  
- GGAGGTTTAA GACGTAGATG AGAGAGAAGG ACGAAGTTAC CACCTTCATC ACCCGAACCC TACCTGCTAA   
  
  
- ACCTCCCAAA CAGAAAACCT CTTCCCAGGG AAGAGGGAAC CAAGTACCCA CTTTAACTTC TGGGATCAAA   
  
  
- CTCAAAGTTC GTGGAGGAAG TTGGAAGTTT AGGGAACCTC AAACCACCGT TACTACCCGA ACTTCGTCGA   
  
  
- TAAGTCTCAC GACCATACCC ATTAAAATTA AACCAACTTT CAAAATTAAG ACTAAACTCC AGAACATTAA   
  
  
- GACCCAGATA AACGTTACCC CCCTTACTAA GACAGTTACT TTTCCCTACA CAAGAATTAA TACGAAGTCT   
  
  
- CGTCCCAGTA CACCAACGAC ATAGAGGTCA TCTTGTCAAA TCCTAATCAC TACACTTTGT ACTCCACAAC   
  
  
- TTGGGTTTTA AACCGAAAAC ACTCGTTCCG AGATGTTAGT CAGTCTTGAG ACCAATACAC GTATATGACC   
  
  
- CAATATTGGT CGATCTTAGA GGGGAACTCG GAGGCCGTTT CGCGGTGTTA CGACCCTTAC GAGAAATGTG   
  
  
- ATCACAACAA GGTCAAGGTT TCTCAAGCAA AAATCCACTC CTAGTACTTT AAGATGAGTC TTTAGTTCTC   
  
  
- GTCGTCAAAG ACTACGTTGT TGTCACCAAT TACCCAAATC GGGGAGTCAA ATGTGGGGTC GTGGGCGGTT   
  
  
- TCGGTAATCT GCTGGCTTTC GTTGGTTTGA GCGTTTGAAA CCAAGACGCG CTAGTCAATC AATTCTAACG   
  
  
- CCTCTACAAG GTTTGGCCAT TGAAAAGCGA ACGGGTTCAC TATAACCGGG CCGAGTTAGT CGTTAACAGA   
  
  
- GAAAGTCCCT TGGGGAAACA TTCTCGACGA TGAAAGGTAC AGTTCCTCCT TGAACTTTCC GAGGACTGAT   
  
  
- AGTTATCGAA GAGACGCCGA GGTGGGGTTG GATTTTCGGA ATGAGACAGT CTACAACACC TATCCTACTT   
  
  
- ACGTATGTTC GAAAAAAGTC TCCGTAGGGG TCAATACGTT AAATGGCTAA AATGAACATG GGTCCGAAAC   
  
  
- GAGCTTCGAG AACTACTACG ACTAATATAG GTGTATGATC TAAAACTGTA ACCAACACCA AGGGTTACCC   
  
  
- GTAGGAAATA AGCTCTCGAA GGAGAGTCCT TTTCCCCGTG AGGTAAAAAC TTTTAATGTC GGAAACGGAG   
  
  
- AAGAAATTGG GTATTAGGTA AACTTGATCG GGAACATACA CTATTGTAAG ACCTTAAACG TTTTCTCAAG   
  
  
- CCACAGAGAC AGCTGGAAGT CCAGTACTTA AACCTGAACA AACTGGGTTG ATGAACGTGT TATGGGTTAA   
  
  
- AAGTTAGGTG ACTCTTGCGT CGTCAACGAC AGTTGAAGGG ATATACCCGC ACGAGTGTGA CGGGCAAACA   
  
  
- TGAGTCAGTT GAGGACAGGA AGTAGTTCGT TACAAGGGGT TTTTAGTACT GCTCGAAACT ATCTCCAACA   
  
  
- CTAGTAAAAC TCAACAGAAA AGGTAGCGTG TAGTAGGCAC ATAACCTGAG TACGAGATTA TACAACCTCA   
  
  
- GGGAGGTACC AAGTTCGCAC TGTAATCTGT GGAGTTTCTT CTAACTTTTC AAGAACTAGT TCGGATATTA   
  
  
- ACTCTCCCGT TATAACCCCG CTTAAGTACG TGGTCTGTTC TACGTCCGTT TGTTCCGTGT GGCCACCTTC   
  
  
- TTAGAGAAGT GAAGACGACC CAAGAAGGGA AACGGTAAAT CATCAAAGTG ACTTAGAGTC CGGTTAGATC   
  
  
- ACCGAGTTGC TTGGGGTCAC TCCCCAAAAG TCCACCTTTT CGTCGTTCGT AGTGAACACG AGTCCACCTA   
  
  
- CAGAGTACTC GAGCACCGTC GAAGTCGAAC CTCCACGAT

+     MYB recognition site

| Site Name | Organism | Position | Strand | Matrix score. | sequence | function |
| --- | --- | --- | --- | --- | --- | --- |
| MYB recognition site | Arabidopsis thaliana | 667 | + | 6 | CCGTTG |  |

>HU04G00048.1   
+ -Up\_Stream \_Len000TTTGTA TTTAAATTTT ACAATATTTA TAAAAATTTT GTATTGATTA ATTGTGCTAA   
  
  
+ CTAACACTAA TAATTGGGGG AGAAAAGAAG GGAAAATAAA AAGAAATTAA GAAGAAGAAA AGAAAACACC   
  
  
+ TTTGAAAAGG CAAAAAACTA AAATAAAACA ATAATGAACA GAAGGAGAGA AGGATGGATA AACAACAAAT   
  
  
+ GTCGCTTTTA GGACAGCAAT AGTCTATGTG ATTGGCCTTA GTTCCTGTTG GAGGATTGCT AACAAAAGCA   
  
  
+ ACAGCAAAAA ATAATACTAA TTATTACATA TTTTTTAACT CTTAAAGCTA ATCAATCATC CTCCATTAAT   
  
  
+ TGATTAATTA TTTGCCAAAA ACACAAATCA ACTGGGGCAA CTATAAGAGA TCTACCTGCT TTAAGATATA   
  
  
+ TAAACTCAAT TGTTTCATTT TATTTTATTT TAGATTTTTT GGCCTTTGGT CTCTATTGAG CTAAAATCTC   
  
  
+ GTTATTTTCG GATCATGAAA AATACAACTC GATCTTTTGA TATAAGTCAT CTTTATTTGT AAATTCTGAA   
  
  
+ CTTAAAATCT TACTTGAAGA TCAGGAATAG AAGCACTCGA TTTTTGATAA ATCCTATGTG TTCTTCTTAA   
  
  
+ AATTCATAAT CTAATTCATA TTTTAGATGT ACCCGTTGCC TACTTTGGCA AGTTTAGATT ATGATTTTTA   
  
  
+ TTTTTTTTAA TTACTTGTAT TTTTCTTTTG GATTGATAGT CAATCTACAT CATCTTTGAT CAATTGCGAC   
  
  
+ AGTTTCATTT TCCCAACAAA AATTTCGAGT GTATTCTTTT TAATTTATCA TAAGTATTTA TTGTCAACAT   
  
  
+ TCAACATGCA TCAACAATCA TTAAACACTT TTCGTCACTC CTCAATACTG GTCAGCACTT AAGATTTTGT   
  
  
+ CTTCTAAACT TATCGTAAGT AACTTATTGT TGATTAATTG ATGCTGACCA AATGATACTG AGTGCTAAAA   
  
  
+ TTATAATTAA TAAATACAAT TAAGAGTTGA TCAATGTCGA TGATTCAAAT CAATGTGATT GGTGCTGAAT   
  
  
+ AGTAATTATA AGCACCTATA AAGACTTGAA AAGAACAAAC ATTTAATTAG ATTTTATAAT TATAATTTCC   
  
  
+ TAATATTTAG CATTCATCAG CAAATTCAGC ACACACCAAC ATTAGTCAAC ACCTATCAAC ACTTATAAGT   
  
  
+ ACCAATTAAT AATAATTACC TATAATAGAT TAAAAGTTAG GCCCTTAATG TTTCCCACAT TATTAGATAT   
  
  
+ ATCAAGGGAG GGGGAAAAAA ATAATACTCC CCCATCTTAA AAAATTTATC CCTTTTTTTC TTTTTAATGT   
  
  
+ TCCAAAACAA AAATATGCTT CTAAATTATG ATTGCAATAT GAATGTGTAC GGTTTCAAAC CCAATTTTCT   
  
  
+ CCAACAATTC TTCTACTATA TTGTAATTAT ATTATACTAC AATTTGTATC TCATCTATGA TCTACCCCCC   
  
  
+ AAAAACCAAA TACTCATATA AAGTTTTATT TTCTTTCCAT CCCACTCTAA AAAAAAAAAA TGTTGGGAAA   
  
  
+ AAGTTGATTT ATTTAATTTG AAATGGTACG GTGAATATAG ATTTGTAGAG GGGCAGTTGA TAGAAAAAGG   
  
  
+ TGAAGACAAA GGAAGGGCAC ATTTGCCTTT TCATAGAAGG AAGAGACCAA GGTACTGGGG ACTGGGGGAA   
  
  
+ GGGCTCAAGA GAAGAGAGAG ACTGAGAAGA GTGCTGCTAC TCCTTCCTAC CTACACACAA AATTGCTGCG   
  
  
+ GGGGGTTCAA GTTCAACTTG AACGAGGCAA ATACCCTCAT TGAACCCACC AAATCGAAAT CTTCAATGTA   
  
  
+ CACCCCCATT ATACACGCAT TCAGAGACAT AGATGTCGAG GGGGAGATGA ATTAGCAAGA AGATGTCGAG   
  
  
+ CTAAAATGCA TTTCGAGGTT ACTAGAGCCA GAAATGTGGG CACATCCCAG AAGATCTCAG TAGAGAGAGT   
  
  
+ AACTTCAGAA AAGTTTCAAT TTTTATTCGA AATTTGCCTC TTGTTTGTGT AGATATGATG GGAACTTCGC   
  
  
+ CTTACAATCT GCAATGTGAG GGTTTGGTTG AAGGAGTTGC AGTCAATCCT CAATTTTATG AATCAACCCC   
  
  
+ AAAGTGGAAG AAGGGCATCA AAGAAGAGTC AATTGAGAGC GAACCCACTT CGGTTTTGGA CAACCCTAGC   
  
  
+ CCTCCAAATT CTGCATCTAC TCTCTCTTCC TGCTTCAATG GTGGAAGTAG TGGGCTTGGG ATGGACGATT   
  
  
+ TGGAGGGTTT GTCTTTTGGA GAAGGGTCCC TTCTCCCTTG GTTCATGGGT GAAATTGAAG ACCCTAGTTT   
  
  
+ GAGTTTCAAG CACCTCCTTC AACCTTCAAA TCCCTTGGAG TTTGGTGGCA ATGATGGGCT TGAAGCAGCT   
  
  
+ ATTCAGAGTG CTGGTATGGG TAATTTTAAT TTGGTTGAAA GTTTTAATTC TGATTTGAGG TCTTGTAATT   
  
  
+ CTGGGTCTAT TTGCAATGGG GGGAATGATT CTGTCAATGA AAAGGGATGT GTTCTTAATT ATGCTTCAGA   
  
  
+ GCAGGGTCAT GTGGTTGCTG TATCTCCAGT AGAACAGTTT AGGATTAGTG ATGTGAAACA TGAGGTGTTG   
  
  
+ AACCCAAAAT TTGGCTTTTG TGAGCAAGGC TCTACAATCA GTCAGAACTC TGGTTATGTG CATATACTGG   
  
  
+ GTTATAACCA GCTAGAATCT CCCCTTGAGC CTCCGGCAAA GCGCCACAAT GCTGGGAATG CTCTTTACAC   
  
  
+ TAGTGTTGTT CCAGTTCCAA AGAGTTCGTT TTTAGGTGAG GATCATGAAA TTCTACTCAG AAATCAAGAG   
  
  
+ CAGCAGTTTC TGATGCAACA ACAGTGGTTA ATGGGTTTAG CCCCTCAGTT TACACCCCAG CACCCGCCAA   
  
  
+ AGCCATTAGA CGACCGAAAG CAACCAAACT CGCAAACTTT GGTTCTGCGC GATCAGTTAG TTAAGATTGC   
  
  
+ GGAGATGTTC CAAACCGGTA ACTTTTCGCT TGCCCAAGTG ATATTGGCCC GGCTCAATCA GCAATTGTCT   
  
  
+ CTTTCAGGGA ACCCCTTTGT AAGAGCTGCT ACTTTCCATG TCAAGGAGGA ACTTGAAAGG CTCCTGACTA   
  
  
+ TCAATAGCTT CTCTGCGGCT CCACCCCAAC CTAAAAGCCT TACTCTGTCA GATGTTGTGG ATAGGATGAA   
  
  
+ TGCATACAAG CTTTTTTCAG AGGCATCCCC AGTTATGCAA TTTACCGATT TTACTTGTAC CCAGGCTTTG   
  
  
+ CTCGAAGCTC TTGATGATGC TGATTATATC CACATACTAG ATTTTGACAT TGGTTGTGGT TCCCAATGGG   
  
  
+ CATCCTTTAT TCGAGAGCTT CCTCTCAGGA AAAGGGGCAC TCCATTTTTG AAAATTACAG CCTTTGCCTC   
  
  
+ TTCTTTAACC CATAATCCAT TTGAACTAGC CCTTGTATGT GATAACATTC TGGAATTTGC AAAAGAGTTC   
  
  
+ GGTGTCTCTG TCGACCTTCA GGTCATGAAT TTGGACTTGT TTGACCCAAC TACTTGCACA ATACCCAATT   
  
  
+ TTCAATCCAC TGAGAACGCA GCAGTTGCTG TCAACTTCCC TATATGGGCG TGCTCACACT GCCCGTTTGT   
  
  
+ ACTCAGTCAA CTCCTGTCCT TCATCAAGCA ATGTTCCCCA AAAATCATGA CGAGCTTTGA TAGAGGTTGT   
  
  
+ GATCATTTTG AGTTGTCTTT TCCATCGCAC ATCATCCGTG TATTGGACTC ATGCTCTAAT ATGTTGGAGT   
  
  
+ CCCTCCATGG TTCAAGCGTG ACATTAGACA CCTCAAAGAA GATTGAAAAG TTCTTGATCA AGCCTATAAT   
  
  
+ TGAGAGGGCA ATATTGGGGC GAATTCATGC ACCAGACAAG ATGCAGGCAA ACAAGGCACA CCGGTGGAAG   
  
  
+ AATCTCTTCA CTTCTGCTGG GTTCTTCCCT TTGCCATTTA GTAGTTTCAC TGAATCTCAG GCCAATCTAG   
  
  
+ TGGCTCAACG AACCCCAGTG AGGGGTTTTC AGGTGGAAAA GCAGCAAGCA TCACTTGTGC TCAGGTGGAT   
  
  
+ GTCTCATGAG CTCGTGGCAG CTTCAGCTTG GAGGTGCTA  

- -Up\_Stream \_Len000AAACAT AAATTTAAAA TGTTATAAAT ATTTTTAAAA CATAACTAAT TAACACGATT   
  
  
- GATTGTGATT ATTAACCCCC TCTTTTCTTC CCTTTTATTT TTCTTTAATT CTTCTTCTTT TCTTTTGTGG   
  
  
- AAACTTTTCC GTTTTTTGAT TTTATTTTGT TATTACTTGT CTTCCTCTCT TCCTACCTAT TTGTTGTTTA   
  
  
- CAGCGAAAAT CCTGTCGTTA TCAGATACAC TAACCGGAAT CAAGGACAAC CTCCTAACGA TTGTTTTCGT   
  
  
- TGTCGTTTTT TATTATGATT AATAATGTAT AAAAAATTGA GAATTTCGAT TAGTTAGTAG GAGGTAATTA   
  
  
- ACTAATTAAT AAACGGTTTT TGTGTTTAGT TGACCCCGTT GATATTCTCT AGATGGACGA AATTCTATAT   
  
  
- ATTTGAGTTA ACAAAGTAAA ATAAAATAAA ATCTAAAAAA CCGGAAACCA GAGATAACTC GATTTTAGAG   
  
  
- CAATAAAAGC CTAGTACTTT TTATGTTGAG CTAGAAAACT ATATTCAGTA GAAATAAACA TTTAAGACTT   
  
  
- GAATTTTAGA ATGAACTTCT AGTCCTTATC TTCGTGAGCT AAAAACTATT TAGGATACAC AAGAAGAATT   
  
  
- TTAAGTATTA GATTAAGTAT AAAATCTACA TGGGCAACGG ATGAAACCGT TCAAATCTAA TACTAAAAAT   
  
  
- AAAAAAAATT AATGAACATA AAAAGAAAAC CTAACTATCA GTTAGATGTA GTAGAAACTA GTTAACGCTG   
  
  
- TCAAAGTAAA AGGGTTGTTT TTAAAGCTCA CATAAGAAAA ATTAAATAGT ATTCATAAAT AACAGTTGTA   
  
  
- AGTTGTACGT AGTTGTTAGT AATTTGTGAA AAGCAGTGAG GAGTTATGAC CAGTCGTGAA TTCTAAAACA   
  
  
- GAAGATTTGA ATAGCATTCA TTGAATAACA ACTAATTAAC TACGACTGGT TTACTATGAC TCACGATTTT   
  
  
- AATATTAATT ATTTATGTTA ATTCTCAACT AGTTACAGCT ACTAAGTTTA GTTACACTAA CCACGACTTA   
  
  
- TCATTAATAT TCGTGGATAT TTCTGAACTT TTCTTGTTTG TAAATTAATC TAAAATATTA ATATTAAAGG   
  
  
- ATTATAAATC GTAAGTAGTC GTTTAAGTCG TGTGTGGTTG TAATCAGTTG TGGATAGTTG TGAATATTCA   
  
  
- TGGTTAATTA TTATTAATGG ATATTATCTA ATTTTCAATC CGGGAATTAC AAAGGGTGTA ATAATCTATA   
  
  
- TAGTTCCCTC CCCCTTTTTT TATTATGAGG GGGTAGAATT TTTTAAATAG GGAAAAAAAG AAAAATTACA   
  
  
- AGGTTTTGTT TTTATACGAA GATTTAATAC TAACGTTATA CTTACACATG CCAAAGTTTG GGTTAAAAGA   
  
  
- GGTTGTTAAG AAGATGATAT AACATTAATA TAATATGATG TTAAACATAG AGTAGATACT AGATGGGGGG   
  
  
- TTTTTGGTTT ATGAGTATAT TTCAAAATAA AAGAAAGGTA GGGTGAGATT TTTTTTTTTT ACAACCCTTT   
  
  
- TTCAACTAAA TAAATTAAAC TTTACCATGC CACTTATATC TAAACATCTC CCCGTCAACT ATCTTTTTCC   
  
  
- ACTTCTGTTT CCTTCCCGTG TAAACGGAAA AGTATCTTCC TTCTCTGGTT CCATGACCCC TGACCCCCTT   
  
  
- CCCGAGTTCT CTTCTCTCTC TGACTCTTCT CACGACGATG AGGAAGGATG GATGTGTGTT TTAACGACGC   
  
  
- CCCCCAAGTT CAAGTTGAAC TTGCTCCGTT TATGGGAGTA ACTTGGGTGG TTTAGCTTTA GAAGTTACAT   
  
  
- GTGGGGGTAA TATGTGCGTA AGTCTCTGTA TCTACAGCTC CCCCTCTACT TAATCGTTCT TCTACAGCTC   
  
  
- GATTTTACGT AAAGCTCCAA TGATCTCGGT CTTTACACCC GTGTAGGGTC TTCTAGAGTC ATCTCTCTCA   
  
  
- TTGAAGTCTT TTCAAAGTTA AAAATAAGCT TTAAACGGAG AACAAACACA TCTATACTAC CCTTGAAGCG   
  
  
- GAATGTTAGA CGTTACACTC CCAAACCAAC TTCCTCAACG TCAGTTAGGA GTTAAAATAC TTAGTTGGGG   
  
  
- TTTCACCTTC TTCCCGTAGT TTCTTCTCAG TTAACTCTCG CTTGGGTGAA GCCAAAACCT GTTGGGATCG   
  
  
- GGAGGTTTAA GACGTAGATG AGAGAGAAGG ACGAAGTTAC CACCTTCATC ACCCGAACCC TACCTGCTAA   
  
  
- ACCTCCCAAA CAGAAAACCT CTTCCCAGGG AAGAGGGAAC CAAGTACCCA CTTTAACTTC TGGGATCAAA   
  
  
- CTCAAAGTTC GTGGAGGAAG TTGGAAGTTT AGGGAACCTC AAACCACCGT TACTACCCGA ACTTCGTCGA   
  
  
- TAAGTCTCAC GACCATACCC ATTAAAATTA AACCAACTTT CAAAATTAAG ACTAAACTCC AGAACATTAA   
  
  
- GACCCAGATA AACGTTACCC CCCTTACTAA GACAGTTACT TTTCCCTACA CAAGAATTAA TACGAAGTCT   
  
  
- CGTCCCAGTA CACCAACGAC ATAGAGGTCA TCTTGTCAAA TCCTAATCAC TACACTTTGT ACTCCACAAC   
  
  
- TTGGGTTTTA AACCGAAAAC ACTCGTTCCG AGATGTTAGT CAGTCTTGAG ACCAATACAC GTATATGACC   
  
  
- CAATATTGGT CGATCTTAGA GGGGAACTCG GAGGCCGTTT CGCGGTGTTA CGACCCTTAC GAGAAATGTG   
  
  
- ATCACAACAA GGTCAAGGTT TCTCAAGCAA AAATCCACTC CTAGTACTTT AAGATGAGTC TTTAGTTCTC   
  
  
- GTCGTCAAAG ACTACGTTGT TGTCACCAAT TACCCAAATC GGGGAGTCAA ATGTGGGGTC GTGGGCGGTT   
  
  
- TCGGTAATCT GCTGGCTTTC GTTGGTTTGA GCGTTTGAAA CCAAGACGCG CTAGTCAATC AATTCTAACG   
  
  
- CCTCTACAAG GTTTGGCCAT TGAAAAGCGA ACGGGTTCAC TATAACCGGG CCGAGTTAGT CGTTAACAGA   
  
  
- GAAAGTCCCT TGGGGAAACA TTCTCGACGA TGAAAGGTAC AGTTCCTCCT TGAACTTTCC GAGGACTGAT   
  
  
- AGTTATCGAA GAGACGCCGA GGTGGGGTTG GATTTTCGGA ATGAGACAGT CTACAACACC TATCCTACTT   
  
  
- ACGTATGTTC GAAAAAAGTC TCCGTAGGGG TCAATACGTT AAATGGCTAA AATGAACATG GGTCCGAAAC   
  
  
- GAGCTTCGAG AACTACTACG ACTAATATAG GTGTATGATC TAAAACTGTA ACCAACACCA AGGGTTACCC   
  
  
- GTAGGAAATA AGCTCTCGAA GGAGAGTCCT TTTCCCCGTG AGGTAAAAAC TTTTAATGTC GGAAACGGAG   
  
  
- AAGAAATTGG GTATTAGGTA AACTTGATCG GGAACATACA CTATTGTAAG ACCTTAAACG TTTTCTCAAG   
  
  
- CCACAGAGAC AGCTGGAAGT CCAGTACTTA AACCTGAACA AACTGGGTTG ATGAACGTGT TATGGGTTAA   
  
  
- AAGTTAGGTG ACTCTTGCGT CGTCAACGAC AGTTGAAGGG ATATACCCGC ACGAGTGTGA CGGGCAAACA   
  
  
- TGAGTCAGTT GAGGACAGGA AGTAGTTCGT TACAAGGGGT TTTTAGTACT GCTCGAAACT ATCTCCAACA   
  
  
- CTAGTAAAAC TCAACAGAAA AGGTAGCGTG TAGTAGGCAC ATAACCTGAG TACGAGATTA TACAACCTCA   
  
  
- GGGAGGTACC AAGTTCGCAC TGTAATCTGT GGAGTTTCTT CTAACTTTTC AAGAACTAGT TCGGATATTA   
  
  
- ACTCTCCCGT TATAACCCCG CTTAAGTACG TGGTCTGTTC TACGTCCGTT TGTTCCGTGT GGCCACCTTC   
  
  
- TTAGAGAAGT GAAGACGACC CAAGAAGGGA AACGGTAAAT CATCAAAGTG ACTTAGAGTC CGGTTAGATC   
  
  
- ACCGAGTTGC TTGGGGTCAC TCCCCAAAAG TCCACCTTTT CGTCGTTCGT AGTGAACACG AGTCCACCTA   
  
  
- CAGAGTACTC GAGCACCGTC GAAGTCGAAC CTCCACGAT

+     MYB-like sequence

| Site Name | Organism | Position | Strand | Matrix score. | sequence | function |
| --- | --- | --- | --- | --- | --- | --- |
| MYB-like sequence | Arabidopsis thaliana | 2829 | - | 6 | TAACCA |  |
| MYB-like sequence | Arabidopsis thaliana | 2645 | - | 6 | TAACCA |  |
| MYB-like sequence | Arabidopsis thaliana | 2669 | + | 6 | TAACCA |  |

>HU04G00048.1   
+ -Up\_Stream \_Len000TTTGTA TTTAAATTTT ACAATATTTA TAAAAATTTT GTATTGATTA ATTGTGCTAA   
  
  
+ CTAACACTAA TAATTGGGGG AGAAAAGAAG GGAAAATAAA AAGAAATTAA GAAGAAGAAA AGAAAACACC   
  
  
+ TTTGAAAAGG CAAAAAACTA AAATAAAACA ATAATGAACA GAAGGAGAGA AGGATGGATA AACAACAAAT   
  
  
+ GTCGCTTTTA GGACAGCAAT AGTCTATGTG ATTGGCCTTA GTTCCTGTTG GAGGATTGCT AACAAAAGCA   
  
  
+ ACAGCAAAAA ATAATACTAA TTATTACATA TTTTTTAACT CTTAAAGCTA ATCAATCATC CTCCATTAAT   
  
  
+ TGATTAATTA TTTGCCAAAA ACACAAATCA ACTGGGGCAA CTATAAGAGA TCTACCTGCT TTAAGATATA   
  
  
+ TAAACTCAAT TGTTTCATTT TATTTTATTT TAGATTTTTT GGCCTTTGGT CTCTATTGAG CTAAAATCTC   
  
  
+ GTTATTTTCG GATCATGAAA AATACAACTC GATCTTTTGA TATAAGTCAT CTTTATTTGT AAATTCTGAA   
  
  
+ CTTAAAATCT TACTTGAAGA TCAGGAATAG AAGCACTCGA TTTTTGATAA ATCCTATGTG TTCTTCTTAA   
  
  
+ AATTCATAAT CTAATTCATA TTTTAGATGT ACCCGTTGCC TACTTTGGCA AGTTTAGATT ATGATTTTTA   
  
  
+ TTTTTTTTAA TTACTTGTAT TTTTCTTTTG GATTGATAGT CAATCTACAT CATCTTTGAT CAATTGCGAC   
  
  
+ AGTTTCATTT TCCCAACAAA AATTTCGAGT GTATTCTTTT TAATTTATCA TAAGTATTTA TTGTCAACAT   
  
  
+ TCAACATGCA TCAACAATCA TTAAACACTT TTCGTCACTC CTCAATACTG GTCAGCACTT AAGATTTTGT   
  
  
+ CTTCTAAACT TATCGTAAGT AACTTATTGT TGATTAATTG ATGCTGACCA AATGATACTG AGTGCTAAAA   
  
  
+ TTATAATTAA TAAATACAAT TAAGAGTTGA TCAATGTCGA TGATTCAAAT CAATGTGATT GGTGCTGAAT   
  
  
+ AGTAATTATA AGCACCTATA AAGACTTGAA AAGAACAAAC ATTTAATTAG ATTTTATAAT TATAATTTCC   
  
  
+ TAATATTTAG CATTCATCAG CAAATTCAGC ACACACCAAC ATTAGTCAAC ACCTATCAAC ACTTATAAGT   
  
  
+ ACCAATTAAT AATAATTACC TATAATAGAT TAAAAGTTAG GCCCTTAATG TTTCCCACAT TATTAGATAT   
  
  
+ ATCAAGGGAG GGGGAAAAAA ATAATACTCC CCCATCTTAA AAAATTTATC CCTTTTTTTC TTTTTAATGT   
  
  
+ TCCAAAACAA AAATATGCTT CTAAATTATG ATTGCAATAT GAATGTGTAC GGTTTCAAAC CCAATTTTCT   
  
  
+ CCAACAATTC TTCTACTATA TTGTAATTAT ATTATACTAC AATTTGTATC TCATCTATGA TCTACCCCCC   
  
  
+ AAAAACCAAA TACTCATATA AAGTTTTATT TTCTTTCCAT CCCACTCTAA AAAAAAAAAA TGTTGGGAAA   
  
  
+ AAGTTGATTT ATTTAATTTG AAATGGTACG GTGAATATAG ATTTGTAGAG GGGCAGTTGA TAGAAAAAGG   
  
  
+ TGAAGACAAA GGAAGGGCAC ATTTGCCTTT TCATAGAAGG AAGAGACCAA GGTACTGGGG ACTGGGGGAA   
  
  
+ GGGCTCAAGA GAAGAGAGAG ACTGAGAAGA GTGCTGCTAC TCCTTCCTAC CTACACACAA AATTGCTGCG   
  
  
+ GGGGGTTCAA GTTCAACTTG AACGAGGCAA ATACCCTCAT TGAACCCACC AAATCGAAAT CTTCAATGTA   
  
  
+ CACCCCCATT ATACACGCAT TCAGAGACAT AGATGTCGAG GGGGAGATGA ATTAGCAAGA AGATGTCGAG   
  
  
+ CTAAAATGCA TTTCGAGGTT ACTAGAGCCA GAAATGTGGG CACATCCCAG AAGATCTCAG TAGAGAGAGT   
  
  
+ AACTTCAGAA AAGTTTCAAT TTTTATTCGA AATTTGCCTC TTGTTTGTGT AGATATGATG GGAACTTCGC   
  
  
+ CTTACAATCT GCAATGTGAG GGTTTGGTTG AAGGAGTTGC AGTCAATCCT CAATTTTATG AATCAACCCC   
  
  
+ AAAGTGGAAG AAGGGCATCA AAGAAGAGTC AATTGAGAGC GAACCCACTT CGGTTTTGGA CAACCCTAGC   
  
  
+ CCTCCAAATT CTGCATCTAC TCTCTCTTCC TGCTTCAATG GTGGAAGTAG TGGGCTTGGG ATGGACGATT   
  
  
+ TGGAGGGTTT GTCTTTTGGA GAAGGGTCCC TTCTCCCTTG GTTCATGGGT GAAATTGAAG ACCCTAGTTT   
  
  
+ GAGTTTCAAG CACCTCCTTC AACCTTCAAA TCCCTTGGAG TTTGGTGGCA ATGATGGGCT TGAAGCAGCT   
  
  
+ ATTCAGAGTG CTGGTATGGG TAATTTTAAT TTGGTTGAAA GTTTTAATTC TGATTTGAGG TCTTGTAATT   
  
  
+ CTGGGTCTAT TTGCAATGGG GGGAATGATT CTGTCAATGA AAAGGGATGT GTTCTTAATT ATGCTTCAGA   
  
  
+ GCAGGGTCAT GTGGTTGCTG TATCTCCAGT AGAACAGTTT AGGATTAGTG ATGTGAAACA TGAGGTGTTG   
  
  
+ AACCCAAAAT TTGGCTTTTG TGAGCAAGGC TCTACAATCA GTCAGAACTC TGGTTATGTG CATATACTGG   
  
  
+ GTTATAACCA GCTAGAATCT CCCCTTGAGC CTCCGGCAAA GCGCCACAAT GCTGGGAATG CTCTTTACAC   
  
  
+ TAGTGTTGTT CCAGTTCCAA AGAGTTCGTT TTTAGGTGAG GATCATGAAA TTCTACTCAG AAATCAAGAG   
  
  
+ CAGCAGTTTC TGATGCAACA ACAGTGGTTA ATGGGTTTAG CCCCTCAGTT TACACCCCAG CACCCGCCAA   
  
  
+ AGCCATTAGA CGACCGAAAG CAACCAAACT CGCAAACTTT GGTTCTGCGC GATCAGTTAG TTAAGATTGC   
  
  
+ GGAGATGTTC CAAACCGGTA ACTTTTCGCT TGCCCAAGTG ATATTGGCCC GGCTCAATCA GCAATTGTCT   
  
  
+ CTTTCAGGGA ACCCCTTTGT AAGAGCTGCT ACTTTCCATG TCAAGGAGGA ACTTGAAAGG CTCCTGACTA   
  
  
+ TCAATAGCTT CTCTGCGGCT CCACCCCAAC CTAAAAGCCT TACTCTGTCA GATGTTGTGG ATAGGATGAA   
  
  
+ TGCATACAAG CTTTTTTCAG AGGCATCCCC AGTTATGCAA TTTACCGATT TTACTTGTAC CCAGGCTTTG   
  
  
+ CTCGAAGCTC TTGATGATGC TGATTATATC CACATACTAG ATTTTGACAT TGGTTGTGGT TCCCAATGGG   
  
  
+ CATCCTTTAT TCGAGAGCTT CCTCTCAGGA AAAGGGGCAC TCCATTTTTG AAAATTACAG CCTTTGCCTC   
  
  
+ TTCTTTAACC CATAATCCAT TTGAACTAGC CCTTGTATGT GATAACATTC TGGAATTTGC AAAAGAGTTC   
  
  
+ GGTGTCTCTG TCGACCTTCA GGTCATGAAT TTGGACTTGT TTGACCCAAC TACTTGCACA ATACCCAATT   
  
  
+ TTCAATCCAC TGAGAACGCA GCAGTTGCTG TCAACTTCCC TATATGGGCG TGCTCACACT GCCCGTTTGT   
  
  
+ ACTCAGTCAA CTCCTGTCCT TCATCAAGCA ATGTTCCCCA AAAATCATGA CGAGCTTTGA TAGAGGTTGT   
  
  
+ GATCATTTTG AGTTGTCTTT TCCATCGCAC ATCATCCGTG TATTGGACTC ATGCTCTAAT ATGTTGGAGT   
  
  
+ CCCTCCATGG TTCAAGCGTG ACATTAGACA CCTCAAAGAA GATTGAAAAG TTCTTGATCA AGCCTATAAT   
  
  
+ TGAGAGGGCA ATATTGGGGC GAATTCATGC ACCAGACAAG ATGCAGGCAA ACAAGGCACA CCGGTGGAAG   
  
  
+ AATCTCTTCA CTTCTGCTGG GTTCTTCCCT TTGCCATTTA GTAGTTTCAC TGAATCTCAG GCCAATCTAG   
  
  
+ TGGCTCAACG AACCCCAGTG AGGGGTTTTC AGGTGGAAAA GCAGCAAGCA TCACTTGTGC TCAGGTGGAT   
  
  
+ GTCTCATGAG CTCGTGGCAG CTTCAGCTTG GAGGTGCTA  

- -Up\_Stream \_Len000AAACAT AAATTTAAAA TGTTATAAAT ATTTTTAAAA CATAACTAAT TAACACGATT   
  
  
- GATTGTGATT ATTAACCCCC TCTTTTCTTC CCTTTTATTT TTCTTTAATT CTTCTTCTTT TCTTTTGTGG   
  
  
- AAACTTTTCC GTTTTTTGAT TTTATTTTGT TATTACTTGT CTTCCTCTCT TCCTACCTAT TTGTTGTTTA   
  
  
- CAGCGAAAAT CCTGTCGTTA TCAGATACAC TAACCGGAAT CAAGGACAAC CTCCTAACGA TTGTTTTCGT   
  
  
- TGTCGTTTTT TATTATGATT AATAATGTAT AAAAAATTGA GAATTTCGAT TAGTTAGTAG GAGGTAATTA   
  
  
- ACTAATTAAT AAACGGTTTT TGTGTTTAGT TGACCCCGTT GATATTCTCT AGATGGACGA AATTCTATAT   
  
  
- ATTTGAGTTA ACAAAGTAAA ATAAAATAAA ATCTAAAAAA CCGGAAACCA GAGATAACTC GATTTTAGAG   
  
  
- CAATAAAAGC CTAGTACTTT TTATGTTGAG CTAGAAAACT ATATTCAGTA GAAATAAACA TTTAAGACTT   
  
  
- GAATTTTAGA ATGAACTTCT AGTCCTTATC TTCGTGAGCT AAAAACTATT TAGGATACAC AAGAAGAATT   
  
  
- TTAAGTATTA GATTAAGTAT AAAATCTACA TGGGCAACGG ATGAAACCGT TCAAATCTAA TACTAAAAAT   
  
  
- AAAAAAAATT AATGAACATA AAAAGAAAAC CTAACTATCA GTTAGATGTA GTAGAAACTA GTTAACGCTG   
  
  
- TCAAAGTAAA AGGGTTGTTT TTAAAGCTCA CATAAGAAAA ATTAAATAGT ATTCATAAAT AACAGTTGTA   
  
  
- AGTTGTACGT AGTTGTTAGT AATTTGTGAA AAGCAGTGAG GAGTTATGAC CAGTCGTGAA TTCTAAAACA   
  
  
- GAAGATTTGA ATAGCATTCA TTGAATAACA ACTAATTAAC TACGACTGGT TTACTATGAC TCACGATTTT   
  
  
- AATATTAATT ATTTATGTTA ATTCTCAACT AGTTACAGCT ACTAAGTTTA GTTACACTAA CCACGACTTA   
  
  
- TCATTAATAT TCGTGGATAT TTCTGAACTT TTCTTGTTTG TAAATTAATC TAAAATATTA ATATTAAAGG   
  
  
- ATTATAAATC GTAAGTAGTC GTTTAAGTCG TGTGTGGTTG TAATCAGTTG TGGATAGTTG TGAATATTCA   
  
  
- TGGTTAATTA TTATTAATGG ATATTATCTA ATTTTCAATC CGGGAATTAC AAAGGGTGTA ATAATCTATA   
  
  
- TAGTTCCCTC CCCCTTTTTT TATTATGAGG GGGTAGAATT TTTTAAATAG GGAAAAAAAG AAAAATTACA   
  
  
- AGGTTTTGTT TTTATACGAA GATTTAATAC TAACGTTATA CTTACACATG CCAAAGTTTG GGTTAAAAGA   
  
  
- GGTTGTTAAG AAGATGATAT AACATTAATA TAATATGATG TTAAACATAG AGTAGATACT AGATGGGGGG   
  
  
- TTTTTGGTTT ATGAGTATAT TTCAAAATAA AAGAAAGGTA GGGTGAGATT TTTTTTTTTT ACAACCCTTT   
  
  
- TTCAACTAAA TAAATTAAAC TTTACCATGC CACTTATATC TAAACATCTC CCCGTCAACT ATCTTTTTCC   
  
  
- ACTTCTGTTT CCTTCCCGTG TAAACGGAAA AGTATCTTCC TTCTCTGGTT CCATGACCCC TGACCCCCTT   
  
  
- CCCGAGTTCT CTTCTCTCTC TGACTCTTCT CACGACGATG AGGAAGGATG GATGTGTGTT TTAACGACGC   
  
  
- CCCCCAAGTT CAAGTTGAAC TTGCTCCGTT TATGGGAGTA ACTTGGGTGG TTTAGCTTTA GAAGTTACAT   
  
  
- GTGGGGGTAA TATGTGCGTA AGTCTCTGTA TCTACAGCTC CCCCTCTACT TAATCGTTCT TCTACAGCTC   
  
  
- GATTTTACGT AAAGCTCCAA TGATCTCGGT CTTTACACCC GTGTAGGGTC TTCTAGAGTC ATCTCTCTCA   
  
  
- TTGAAGTCTT TTCAAAGTTA AAAATAAGCT TTAAACGGAG AACAAACACA TCTATACTAC CCTTGAAGCG   
  
  
- GAATGTTAGA CGTTACACTC CCAAACCAAC TTCCTCAACG TCAGTTAGGA GTTAAAATAC TTAGTTGGGG   
  
  
- TTTCACCTTC TTCCCGTAGT TTCTTCTCAG TTAACTCTCG CTTGGGTGAA GCCAAAACCT GTTGGGATCG   
  
  
- GGAGGTTTAA GACGTAGATG AGAGAGAAGG ACGAAGTTAC CACCTTCATC ACCCGAACCC TACCTGCTAA   
  
  
- ACCTCCCAAA CAGAAAACCT CTTCCCAGGG AAGAGGGAAC CAAGTACCCA CTTTAACTTC TGGGATCAAA   
  
  
- CTCAAAGTTC GTGGAGGAAG TTGGAAGTTT AGGGAACCTC AAACCACCGT TACTACCCGA ACTTCGTCGA   
  
  
- TAAGTCTCAC GACCATACCC ATTAAAATTA AACCAACTTT CAAAATTAAG ACTAAACTCC AGAACATTAA   
  
  
- GACCCAGATA AACGTTACCC CCCTTACTAA GACAGTTACT TTTCCCTACA CAAGAATTAA TACGAAGTCT   
  
  
- CGTCCCAGTA CACCAACGAC ATAGAGGTCA TCTTGTCAAA TCCTAATCAC TACACTTTGT ACTCCACAAC   
  
  
- TTGGGTTTTA AACCGAAAAC ACTCGTTCCG AGATGTTAGT CAGTCTTGAG ACCAATACAC GTATATGACC   
  
  
- CAATATTGGT CGATCTTAGA GGGGAACTCG GAGGCCGTTT CGCGGTGTTA CGACCCTTAC GAGAAATGTG   
  
  
- ATCACAACAA GGTCAAGGTT TCTCAAGCAA AAATCCACTC CTAGTACTTT AAGATGAGTC TTTAGTTCTC   
  
  
- GTCGTCAAAG ACTACGTTGT TGTCACCAAT TACCCAAATC GGGGAGTCAA ATGTGGGGTC GTGGGCGGTT   
  
  
- TCGGTAATCT GCTGGCTTTC GTTGGTTTGA GCGTTTGAAA CCAAGACGCG CTAGTCAATC AATTCTAACG   
  
  
- CCTCTACAAG GTTTGGCCAT TGAAAAGCGA ACGGGTTCAC TATAACCGGG CCGAGTTAGT CGTTAACAGA   
  
  
- GAAAGTCCCT TGGGGAAACA TTCTCGACGA TGAAAGGTAC AGTTCCTCCT TGAACTTTCC GAGGACTGAT   
  
  
- AGTTATCGAA GAGACGCCGA GGTGGGGTTG GATTTTCGGA ATGAGACAGT CTACAACACC TATCCTACTT   
  
  
- ACGTATGTTC GAAAAAAGTC TCCGTAGGGG TCAATACGTT AAATGGCTAA AATGAACATG GGTCCGAAAC   
  
  
- GAGCTTCGAG AACTACTACG ACTAATATAG GTGTATGATC TAAAACTGTA ACCAACACCA AGGGTTACCC   
  
  
- GTAGGAAATA AGCTCTCGAA GGAGAGTCCT TTTCCCCGTG AGGTAAAAAC TTTTAATGTC GGAAACGGAG   
  
  
- AAGAAATTGG GTATTAGGTA AACTTGATCG GGAACATACA CTATTGTAAG ACCTTAAACG TTTTCTCAAG   
  
  
- CCACAGAGAC AGCTGGAAGT CCAGTACTTA AACCTGAACA AACTGGGTTG ATGAACGTGT TATGGGTTAA   
  
  
- AAGTTAGGTG ACTCTTGCGT CGTCAACGAC AGTTGAAGGG ATATACCCGC ACGAGTGTGA CGGGCAAACA   
  
  
- TGAGTCAGTT GAGGACAGGA AGTAGTTCGT TACAAGGGGT TTTTAGTACT GCTCGAAACT ATCTCCAACA   
  
  
- CTAGTAAAAC TCAACAGAAA AGGTAGCGTG TAGTAGGCAC ATAACCTGAG TACGAGATTA TACAACCTCA   
  
  
- GGGAGGTACC AAGTTCGCAC TGTAATCTGT GGAGTTTCTT CTAACTTTTC AAGAACTAGT TCGGATATTA   
  
  
- ACTCTCCCGT TATAACCCCG CTTAAGTACG TGGTCTGTTC TACGTCCGTT TGTTCCGTGT GGCCACCTTC   
  
  
- TTAGAGAAGT GAAGACGACC CAAGAAGGGA AACGGTAAAT CATCAAAGTG ACTTAGAGTC CGGTTAGATC   
  
  
- ACCGAGTTGC TTGGGGTCAC TCCCCAAAAG TCCACCTTTT CGTCGTTCGT AGTGAACACG AGTCCACCTA   
  
  
- CAGAGTACTC GAGCACCGTC GAAGTCGAAC CTCCACGAT

+     MYC

| Site Name | Organism | Position | Strand | Matrix score. | sequence | function |
| --- | --- | --- | --- | --- | --- | --- |
| MYC | Arabidopsis thaliana | 3382 | + | 6 | CATTTG |  |
| MYC | Arabidopsis thaliana | 2532 | + | 6 | CATGTG |  |
| MYC | Arabidopsis thaliana | 2134 | - | 6 | CAATTG |  |
| MYC | Arabidopsis thaliana | 1634 | + | 6 | CATTTG |  |
| MYC | Arabidopsis thaliana | 765 | + | 6 | CAATTG |  |
| MYC | Arabidopsis thaliana | 963 | - | 6 | CATTTG |  |
| MYC | Arabidopsis thaliana | 210 | - | 6 | CATTTG |  |
| MYC | Arabidopsis thaliana | 431 | + | 6 | CAATTG |  |
| MYC | Arabidopsis thaliana | 3006 | - | 6 | CAATTG |  |

>HU04G00048.1   
+ -Up\_Stream \_Len000TTTGTA TTTAAATTTT ACAATATTTA TAAAAATTTT GTATTGATTA ATTGTGCTAA   
  
  
+ CTAACACTAA TAATTGGGGG AGAAAAGAAG GGAAAATAAA AAGAAATTAA GAAGAAGAAA AGAAAACACC   
  
  
+ TTTGAAAAGG CAAAAAACTA AAATAAAACA ATAATGAACA GAAGGAGAGA AGGATGGATA AACAACAAAT   
  
  
+ GTCGCTTTTA GGACAGCAAT AGTCTATGTG ATTGGCCTTA GTTCCTGTTG GAGGATTGCT AACAAAAGCA   
  
  
+ ACAGCAAAAA ATAATACTAA TTATTACATA TTTTTTAACT CTTAAAGCTA ATCAATCATC CTCCATTAAT   
  
  
+ TGATTAATTA TTTGCCAAAA ACACAAATCA ACTGGGGCAA CTATAAGAGA TCTACCTGCT TTAAGATATA   
  
  
+ TAAACTCAAT TGTTTCATTT TATTTTATTT TAGATTTTTT GGCCTTTGGT CTCTATTGAG CTAAAATCTC   
  
  
+ GTTATTTTCG GATCATGAAA AATACAACTC GATCTTTTGA TATAAGTCAT CTTTATTTGT AAATTCTGAA   
  
  
+ CTTAAAATCT TACTTGAAGA TCAGGAATAG AAGCACTCGA TTTTTGATAA ATCCTATGTG TTCTTCTTAA   
  
  
+ AATTCATAAT CTAATTCATA TTTTAGATGT ACCCGTTGCC TACTTTGGCA AGTTTAGATT ATGATTTTTA   
  
  
+ TTTTTTTTAA TTACTTGTAT TTTTCTTTTG GATTGATAGT CAATCTACAT CATCTTTGAT CAATTGCGAC   
  
  
+ AGTTTCATTT TCCCAACAAA AATTTCGAGT GTATTCTTTT TAATTTATCA TAAGTATTTA TTGTCAACAT   
  
  
+ TCAACATGCA TCAACAATCA TTAAACACTT TTCGTCACTC CTCAATACTG GTCAGCACTT AAGATTTTGT   
  
  
+ CTTCTAAACT TATCGTAAGT AACTTATTGT TGATTAATTG ATGCTGACCA AATGATACTG AGTGCTAAAA   
  
  
+ TTATAATTAA TAAATACAAT TAAGAGTTGA TCAATGTCGA TGATTCAAAT CAATGTGATT GGTGCTGAAT   
  
  
+ AGTAATTATA AGCACCTATA AAGACTTGAA AAGAACAAAC ATTTAATTAG ATTTTATAAT TATAATTTCC   
  
  
+ TAATATTTAG CATTCATCAG CAAATTCAGC ACACACCAAC ATTAGTCAAC ACCTATCAAC ACTTATAAGT   
  
  
+ ACCAATTAAT AATAATTACC TATAATAGAT TAAAAGTTAG GCCCTTAATG TTTCCCACAT TATTAGATAT   
  
  
+ ATCAAGGGAG GGGGAAAAAA ATAATACTCC CCCATCTTAA AAAATTTATC CCTTTTTTTC TTTTTAATGT   
  
  
+ TCCAAAACAA AAATATGCTT CTAAATTATG ATTGCAATAT GAATGTGTAC GGTTTCAAAC CCAATTTTCT   
  
  
+ CCAACAATTC TTCTACTATA TTGTAATTAT ATTATACTAC AATTTGTATC TCATCTATGA TCTACCCCCC   
  
  
+ AAAAACCAAA TACTCATATA AAGTTTTATT TTCTTTCCAT CCCACTCTAA AAAAAAAAAA TGTTGGGAAA   
  
  
+ AAGTTGATTT ATTTAATTTG AAATGGTACG GTGAATATAG ATTTGTAGAG GGGCAGTTGA TAGAAAAAGG   
  
  
+ TGAAGACAAA GGAAGGGCAC ATTTGCCTTT TCATAGAAGG AAGAGACCAA GGTACTGGGG ACTGGGGGAA   
  
  
+ GGGCTCAAGA GAAGAGAGAG ACTGAGAAGA GTGCTGCTAC TCCTTCCTAC CTACACACAA AATTGCTGCG   
  
  
+ GGGGGTTCAA GTTCAACTTG AACGAGGCAA ATACCCTCAT TGAACCCACC AAATCGAAAT CTTCAATGTA   
  
  
+ CACCCCCATT ATACACGCAT TCAGAGACAT AGATGTCGAG GGGGAGATGA ATTAGCAAGA AGATGTCGAG   
  
  
+ CTAAAATGCA TTTCGAGGTT ACTAGAGCCA GAAATGTGGG CACATCCCAG AAGATCTCAG TAGAGAGAGT   
  
  
+ AACTTCAGAA AAGTTTCAAT TTTTATTCGA AATTTGCCTC TTGTTTGTGT AGATATGATG GGAACTTCGC   
  
  
+ CTTACAATCT GCAATGTGAG GGTTTGGTTG AAGGAGTTGC AGTCAATCCT CAATTTTATG AATCAACCCC   
  
  
+ AAAGTGGAAG AAGGGCATCA AAGAAGAGTC AATTGAGAGC GAACCCACTT CGGTTTTGGA CAACCCTAGC   
  
  
+ CCTCCAAATT CTGCATCTAC TCTCTCTTCC TGCTTCAATG GTGGAAGTAG TGGGCTTGGG ATGGACGATT   
  
  
+ TGGAGGGTTT GTCTTTTGGA GAAGGGTCCC TTCTCCCTTG GTTCATGGGT GAAATTGAAG ACCCTAGTTT   
  
  
+ GAGTTTCAAG CACCTCCTTC AACCTTCAAA TCCCTTGGAG TTTGGTGGCA ATGATGGGCT TGAAGCAGCT   
  
  
+ ATTCAGAGTG CTGGTATGGG TAATTTTAAT TTGGTTGAAA GTTTTAATTC TGATTTGAGG TCTTGTAATT   
  
  
+ CTGGGTCTAT TTGCAATGGG GGGAATGATT CTGTCAATGA AAAGGGATGT GTTCTTAATT ATGCTTCAGA   
  
  
+ GCAGGGTCAT GTGGTTGCTG TATCTCCAGT AGAACAGTTT AGGATTAGTG ATGTGAAACA TGAGGTGTTG   
  
  
+ AACCCAAAAT TTGGCTTTTG TGAGCAAGGC TCTACAATCA GTCAGAACTC TGGTTATGTG CATATACTGG   
  
  
+ GTTATAACCA GCTAGAATCT CCCCTTGAGC CTCCGGCAAA GCGCCACAAT GCTGGGAATG CTCTTTACAC   
  
  
+ TAGTGTTGTT CCAGTTCCAA AGAGTTCGTT TTTAGGTGAG GATCATGAAA TTCTACTCAG AAATCAAGAG   
  
  
+ CAGCAGTTTC TGATGCAACA ACAGTGGTTA ATGGGTTTAG CCCCTCAGTT TACACCCCAG CACCCGCCAA   
  
  
+ AGCCATTAGA CGACCGAAAG CAACCAAACT CGCAAACTTT GGTTCTGCGC GATCAGTTAG TTAAGATTGC   
  
  
+ GGAGATGTTC CAAACCGGTA ACTTTTCGCT TGCCCAAGTG ATATTGGCCC GGCTCAATCA GCAATTGTCT   
  
  
+ CTTTCAGGGA ACCCCTTTGT AAGAGCTGCT ACTTTCCATG TCAAGGAGGA ACTTGAAAGG CTCCTGACTA   
  
  
+ TCAATAGCTT CTCTGCGGCT CCACCCCAAC CTAAAAGCCT TACTCTGTCA GATGTTGTGG ATAGGATGAA   
  
  
+ TGCATACAAG CTTTTTTCAG AGGCATCCCC AGTTATGCAA TTTACCGATT TTACTTGTAC CCAGGCTTTG   
  
  
+ CTCGAAGCTC TTGATGATGC TGATTATATC CACATACTAG ATTTTGACAT TGGTTGTGGT TCCCAATGGG   
  
  
+ CATCCTTTAT TCGAGAGCTT CCTCTCAGGA AAAGGGGCAC TCCATTTTTG AAAATTACAG CCTTTGCCTC   
  
  
+ TTCTTTAACC CATAATCCAT TTGAACTAGC CCTTGTATGT GATAACATTC TGGAATTTGC AAAAGAGTTC   
  
  
+ GGTGTCTCTG TCGACCTTCA GGTCATGAAT TTGGACTTGT TTGACCCAAC TACTTGCACA ATACCCAATT   
  
  
+ TTCAATCCAC TGAGAACGCA GCAGTTGCTG TCAACTTCCC TATATGGGCG TGCTCACACT GCCCGTTTGT   
  
  
+ ACTCAGTCAA CTCCTGTCCT TCATCAAGCA ATGTTCCCCA AAAATCATGA CGAGCTTTGA TAGAGGTTGT   
  
  
+ GATCATTTTG AGTTGTCTTT TCCATCGCAC ATCATCCGTG TATTGGACTC ATGCTCTAAT ATGTTGGAGT   
  
  
+ CCCTCCATGG TTCAAGCGTG ACATTAGACA CCTCAAAGAA GATTGAAAAG TTCTTGATCA AGCCTATAAT   
  
  
+ TGAGAGGGCA ATATTGGGGC GAATTCATGC ACCAGACAAG ATGCAGGCAA ACAAGGCACA CCGGTGGAAG   
  
  
+ AATCTCTTCA CTTCTGCTGG GTTCTTCCCT TTGCCATTTA GTAGTTTCAC TGAATCTCAG GCCAATCTAG   
  
  
+ TGGCTCAACG AACCCCAGTG AGGGGTTTTC AGGTGGAAAA GCAGCAAGCA TCACTTGTGC TCAGGTGGAT   
  
  
+ GTCTCATGAG CTCGTGGCAG CTTCAGCTTG GAGGTGCTA  

- -Up\_Stream \_Len000AAACAT AAATTTAAAA TGTTATAAAT ATTTTTAAAA CATAACTAAT TAACACGATT   
  
  
- GATTGTGATT ATTAACCCCC TCTTTTCTTC CCTTTTATTT TTCTTTAATT CTTCTTCTTT TCTTTTGTGG   
  
  
- AAACTTTTCC GTTTTTTGAT TTTATTTTGT TATTACTTGT CTTCCTCTCT TCCTACCTAT TTGTTGTTTA   
  
  
- CAGCGAAAAT CCTGTCGTTA TCAGATACAC TAACCGGAAT CAAGGACAAC CTCCTAACGA TTGTTTTCGT   
  
  
- TGTCGTTTTT TATTATGATT AATAATGTAT AAAAAATTGA GAATTTCGAT TAGTTAGTAG GAGGTAATTA   
  
  
- ACTAATTAAT AAACGGTTTT TGTGTTTAGT TGACCCCGTT GATATTCTCT AGATGGACGA AATTCTATAT   
  
  
- ATTTGAGTTA ACAAAGTAAA ATAAAATAAA ATCTAAAAAA CCGGAAACCA GAGATAACTC GATTTTAGAG   
  
  
- CAATAAAAGC CTAGTACTTT TTATGTTGAG CTAGAAAACT ATATTCAGTA GAAATAAACA TTTAAGACTT   
  
  
- GAATTTTAGA ATGAACTTCT AGTCCTTATC TTCGTGAGCT AAAAACTATT TAGGATACAC AAGAAGAATT   
  
  
- TTAAGTATTA GATTAAGTAT AAAATCTACA TGGGCAACGG ATGAAACCGT TCAAATCTAA TACTAAAAAT   
  
  
- AAAAAAAATT AATGAACATA AAAAGAAAAC CTAACTATCA GTTAGATGTA GTAGAAACTA GTTAACGCTG   
  
  
- TCAAAGTAAA AGGGTTGTTT TTAAAGCTCA CATAAGAAAA ATTAAATAGT ATTCATAAAT AACAGTTGTA   
  
  
- AGTTGTACGT AGTTGTTAGT AATTTGTGAA AAGCAGTGAG GAGTTATGAC CAGTCGTGAA TTCTAAAACA   
  
  
- GAAGATTTGA ATAGCATTCA TTGAATAACA ACTAATTAAC TACGACTGGT TTACTATGAC TCACGATTTT   
  
  
- AATATTAATT ATTTATGTTA ATTCTCAACT AGTTACAGCT ACTAAGTTTA GTTACACTAA CCACGACTTA   
  
  
- TCATTAATAT TCGTGGATAT TTCTGAACTT TTCTTGTTTG TAAATTAATC TAAAATATTA ATATTAAAGG   
  
  
- ATTATAAATC GTAAGTAGTC GTTTAAGTCG TGTGTGGTTG TAATCAGTTG TGGATAGTTG TGAATATTCA   
  
  
- TGGTTAATTA TTATTAATGG ATATTATCTA ATTTTCAATC CGGGAATTAC AAAGGGTGTA ATAATCTATA   
  
  
- TAGTTCCCTC CCCCTTTTTT TATTATGAGG GGGTAGAATT TTTTAAATAG GGAAAAAAAG AAAAATTACA   
  
  
- AGGTTTTGTT TTTATACGAA GATTTAATAC TAACGTTATA CTTACACATG CCAAAGTTTG GGTTAAAAGA   
  
  
- GGTTGTTAAG AAGATGATAT AACATTAATA TAATATGATG TTAAACATAG AGTAGATACT AGATGGGGGG   
  
  
- TTTTTGGTTT ATGAGTATAT TTCAAAATAA AAGAAAGGTA GGGTGAGATT TTTTTTTTTT ACAACCCTTT   
  
  
- TTCAACTAAA TAAATTAAAC TTTACCATGC CACTTATATC TAAACATCTC CCCGTCAACT ATCTTTTTCC   
  
  
- ACTTCTGTTT CCTTCCCGTG TAAACGGAAA AGTATCTTCC TTCTCTGGTT CCATGACCCC TGACCCCCTT   
  
  
- CCCGAGTTCT CTTCTCTCTC TGACTCTTCT CACGACGATG AGGAAGGATG GATGTGTGTT TTAACGACGC   
  
  
- CCCCCAAGTT CAAGTTGAAC TTGCTCCGTT TATGGGAGTA ACTTGGGTGG TTTAGCTTTA GAAGTTACAT   
  
  
- GTGGGGGTAA TATGTGCGTA AGTCTCTGTA TCTACAGCTC CCCCTCTACT TAATCGTTCT TCTACAGCTC   
  
  
- GATTTTACGT AAAGCTCCAA TGATCTCGGT CTTTACACCC GTGTAGGGTC TTCTAGAGTC ATCTCTCTCA   
  
  
- TTGAAGTCTT TTCAAAGTTA AAAATAAGCT TTAAACGGAG AACAAACACA TCTATACTAC CCTTGAAGCG   
  
  
- GAATGTTAGA CGTTACACTC CCAAACCAAC TTCCTCAACG TCAGTTAGGA GTTAAAATAC TTAGTTGGGG   
  
  
- TTTCACCTTC TTCCCGTAGT TTCTTCTCAG TTAACTCTCG CTTGGGTGAA GCCAAAACCT GTTGGGATCG   
  
  
- GGAGGTTTAA GACGTAGATG AGAGAGAAGG ACGAAGTTAC CACCTTCATC ACCCGAACCC TACCTGCTAA   
  
  
- ACCTCCCAAA CAGAAAACCT CTTCCCAGGG AAGAGGGAAC CAAGTACCCA CTTTAACTTC TGGGATCAAA   
  
  
- CTCAAAGTTC GTGGAGGAAG TTGGAAGTTT AGGGAACCTC AAACCACCGT TACTACCCGA ACTTCGTCGA   
  
  
- TAAGTCTCAC GACCATACCC ATTAAAATTA AACCAACTTT CAAAATTAAG ACTAAACTCC AGAACATTAA   
  
  
- GACCCAGATA AACGTTACCC CCCTTACTAA GACAGTTACT TTTCCCTACA CAAGAATTAA TACGAAGTCT   
  
  
- CGTCCCAGTA CACCAACGAC ATAGAGGTCA TCTTGTCAAA TCCTAATCAC TACACTTTGT ACTCCACAAC   
  
  
- TTGGGTTTTA AACCGAAAAC ACTCGTTCCG AGATGTTAGT CAGTCTTGAG ACCAATACAC GTATATGACC   
  
  
- CAATATTGGT CGATCTTAGA GGGGAACTCG GAGGCCGTTT CGCGGTGTTA CGACCCTTAC GAGAAATGTG   
  
  
- ATCACAACAA GGTCAAGGTT TCTCAAGCAA AAATCCACTC CTAGTACTTT AAGATGAGTC TTTAGTTCTC   
  
  
- GTCGTCAAAG ACTACGTTGT TGTCACCAAT TACCCAAATC GGGGAGTCAA ATGTGGGGTC GTGGGCGGTT   
  
  
- TCGGTAATCT GCTGGCTTTC GTTGGTTTGA GCGTTTGAAA CCAAGACGCG CTAGTCAATC AATTCTAACG   
  
  
- CCTCTACAAG GTTTGGCCAT TGAAAAGCGA ACGGGTTCAC TATAACCGGG CCGAGTTAGT CGTTAACAGA   
  
  
- GAAAGTCCCT TGGGGAAACA TTCTCGACGA TGAAAGGTAC AGTTCCTCCT TGAACTTTCC GAGGACTGAT   
  
  
- AGTTATCGAA GAGACGCCGA GGTGGGGTTG GATTTTCGGA ATGAGACAGT CTACAACACC TATCCTACTT   
  
  
- ACGTATGTTC GAAAAAAGTC TCCGTAGGGG TCAATACGTT AAATGGCTAA AATGAACATG GGTCCGAAAC   
  
  
- GAGCTTCGAG AACTACTACG ACTAATATAG GTGTATGATC TAAAACTGTA ACCAACACCA AGGGTTACCC   
  
  
- GTAGGAAATA AGCTCTCGAA GGAGAGTCCT TTTCCCCGTG AGGTAAAAAC TTTTAATGTC GGAAACGGAG   
  
  
- AAGAAATTGG GTATTAGGTA AACTTGATCG GGAACATACA CTATTGTAAG ACCTTAAACG TTTTCTCAAG   
  
  
- CCACAGAGAC AGCTGGAAGT CCAGTACTTA AACCTGAACA AACTGGGTTG ATGAACGTGT TATGGGTTAA   
  
  
- AAGTTAGGTG ACTCTTGCGT CGTCAACGAC AGTTGAAGGG ATATACCCGC ACGAGTGTGA CGGGCAAACA   
  
  
- TGAGTCAGTT GAGGACAGGA AGTAGTTCGT TACAAGGGGT TTTTAGTACT GCTCGAAACT ATCTCCAACA   
  
  
- CTAGTAAAAC TCAACAGAAA AGGTAGCGTG TAGTAGGCAC ATAACCTGAG TACGAGATTA TACAACCTCA   
  
  
- GGGAGGTACC AAGTTCGCAC TGTAATCTGT GGAGTTTCTT CTAACTTTTC AAGAACTAGT TCGGATATTA   
  
  
- ACTCTCCCGT TATAACCCCG CTTAAGTACG TGGTCTGTTC TACGTCCGTT TGTTCCGTGT GGCCACCTTC   
  
  
- TTAGAGAAGT GAAGACGACC CAAGAAGGGA AACGGTAAAT CATCAAAGTG ACTTAGAGTC CGGTTAGATC   
  
  
- ACCGAGTTGC TTGGGGTCAC TCCCCAAAAG TCCACCTTTT CGTCGTTCGT AGTGAACACG AGTCCACCTA   
  
  
- CAGAGTACTC GAGCACCGTC GAAGTCGAAC CTCCACGAT

+     Myb

| Site Name | Organism | Position | Strand | Matrix score. | sequence | function |
| --- | --- | --- | --- | --- | --- | --- |
| Myb | Arabidopsis thaliana | 3526 | - | 6 | CAACTG |  |
| Myb | Arabidopsis thaliana | 3184 | - | 6 | TAACTG |  |
| Myb | Arabidopsis thaliana | 2928 | - | 6 | TAACTG |  |
| Myb | Arabidopsis thaliana | 1598 | - | 6 | CAACTG |  |
| Myb | Arabidopsis thaliana | 383 | + | 6 | CAACTG |  |

>HU04G00048.1   
+ -Up\_Stream \_Len000TTTGTA TTTAAATTTT ACAATATTTA TAAAAATTTT GTATTGATTA ATTGTGCTAA   
  
  
+ CTAACACTAA TAATTGGGGG AGAAAAGAAG GGAAAATAAA AAGAAATTAA GAAGAAGAAA AGAAAACACC   
  
  
+ TTTGAAAAGG CAAAAAACTA AAATAAAACA ATAATGAACA GAAGGAGAGA AGGATGGATA AACAACAAAT   
  
  
+ GTCGCTTTTA GGACAGCAAT AGTCTATGTG ATTGGCCTTA GTTCCTGTTG GAGGATTGCT AACAAAAGCA   
  
  
+ ACAGCAAAAA ATAATACTAA TTATTACATA TTTTTTAACT CTTAAAGCTA ATCAATCATC CTCCATTAAT   
  
  
+ TGATTAATTA TTTGCCAAAA ACACAAATCA ACTGGGGCAA CTATAAGAGA TCTACCTGCT TTAAGATATA   
  
  
+ TAAACTCAAT TGTTTCATTT TATTTTATTT TAGATTTTTT GGCCTTTGGT CTCTATTGAG CTAAAATCTC   
  
  
+ GTTATTTTCG GATCATGAAA AATACAACTC GATCTTTTGA TATAAGTCAT CTTTATTTGT AAATTCTGAA   
  
  
+ CTTAAAATCT TACTTGAAGA TCAGGAATAG AAGCACTCGA TTTTTGATAA ATCCTATGTG TTCTTCTTAA   
  
  
+ AATTCATAAT CTAATTCATA TTTTAGATGT ACCCGTTGCC TACTTTGGCA AGTTTAGATT ATGATTTTTA   
  
  
+ TTTTTTTTAA TTACTTGTAT TTTTCTTTTG GATTGATAGT CAATCTACAT CATCTTTGAT CAATTGCGAC   
  
  
+ AGTTTCATTT TCCCAACAAA AATTTCGAGT GTATTCTTTT TAATTTATCA TAAGTATTTA TTGTCAACAT   
  
  
+ TCAACATGCA TCAACAATCA TTAAACACTT TTCGTCACTC CTCAATACTG GTCAGCACTT AAGATTTTGT   
  
  
+ CTTCTAAACT TATCGTAAGT AACTTATTGT TGATTAATTG ATGCTGACCA AATGATACTG AGTGCTAAAA   
  
  
+ TTATAATTAA TAAATACAAT TAAGAGTTGA TCAATGTCGA TGATTCAAAT CAATGTGATT GGTGCTGAAT   
  
  
+ AGTAATTATA AGCACCTATA AAGACTTGAA AAGAACAAAC ATTTAATTAG ATTTTATAAT TATAATTTCC   
  
  
+ TAATATTTAG CATTCATCAG CAAATTCAGC ACACACCAAC ATTAGTCAAC ACCTATCAAC ACTTATAAGT   
  
  
+ ACCAATTAAT AATAATTACC TATAATAGAT TAAAAGTTAG GCCCTTAATG TTTCCCACAT TATTAGATAT   
  
  
+ ATCAAGGGAG GGGGAAAAAA ATAATACTCC CCCATCTTAA AAAATTTATC CCTTTTTTTC TTTTTAATGT   
  
  
+ TCCAAAACAA AAATATGCTT CTAAATTATG ATTGCAATAT GAATGTGTAC GGTTTCAAAC CCAATTTTCT   
  
  
+ CCAACAATTC TTCTACTATA TTGTAATTAT ATTATACTAC AATTTGTATC TCATCTATGA TCTACCCCCC   
  
  
+ AAAAACCAAA TACTCATATA AAGTTTTATT TTCTTTCCAT CCCACTCTAA AAAAAAAAAA TGTTGGGAAA   
  
  
+ AAGTTGATTT ATTTAATTTG AAATGGTACG GTGAATATAG ATTTGTAGAG GGGCAGTTGA TAGAAAAAGG   
  
  
+ TGAAGACAAA GGAAGGGCAC ATTTGCCTTT TCATAGAAGG AAGAGACCAA GGTACTGGGG ACTGGGGGAA   
  
  
+ GGGCTCAAGA GAAGAGAGAG ACTGAGAAGA GTGCTGCTAC TCCTTCCTAC CTACACACAA AATTGCTGCG   
  
  
+ GGGGGTTCAA GTTCAACTTG AACGAGGCAA ATACCCTCAT TGAACCCACC AAATCGAAAT CTTCAATGTA   
  
  
+ CACCCCCATT ATACACGCAT TCAGAGACAT AGATGTCGAG GGGGAGATGA ATTAGCAAGA AGATGTCGAG   
  
  
+ CTAAAATGCA TTTCGAGGTT ACTAGAGCCA GAAATGTGGG CACATCCCAG AAGATCTCAG TAGAGAGAGT   
  
  
+ AACTTCAGAA AAGTTTCAAT TTTTATTCGA AATTTGCCTC TTGTTTGTGT AGATATGATG GGAACTTCGC   
  
  
+ CTTACAATCT GCAATGTGAG GGTTTGGTTG AAGGAGTTGC AGTCAATCCT CAATTTTATG AATCAACCCC   
  
  
+ AAAGTGGAAG AAGGGCATCA AAGAAGAGTC AATTGAGAGC GAACCCACTT CGGTTTTGGA CAACCCTAGC   
  
  
+ CCTCCAAATT CTGCATCTAC TCTCTCTTCC TGCTTCAATG GTGGAAGTAG TGGGCTTGGG ATGGACGATT   
  
  
+ TGGAGGGTTT GTCTTTTGGA GAAGGGTCCC TTCTCCCTTG GTTCATGGGT GAAATTGAAG ACCCTAGTTT   
  
  
+ GAGTTTCAAG CACCTCCTTC AACCTTCAAA TCCCTTGGAG TTTGGTGGCA ATGATGGGCT TGAAGCAGCT   
  
  
+ ATTCAGAGTG CTGGTATGGG TAATTTTAAT TTGGTTGAAA GTTTTAATTC TGATTTGAGG TCTTGTAATT   
  
  
+ CTGGGTCTAT TTGCAATGGG GGGAATGATT CTGTCAATGA AAAGGGATGT GTTCTTAATT ATGCTTCAGA   
  
  
+ GCAGGGTCAT GTGGTTGCTG TATCTCCAGT AGAACAGTTT AGGATTAGTG ATGTGAAACA TGAGGTGTTG   
  
  
+ AACCCAAAAT TTGGCTTTTG TGAGCAAGGC TCTACAATCA GTCAGAACTC TGGTTATGTG CATATACTGG   
  
  
+ GTTATAACCA GCTAGAATCT CCCCTTGAGC CTCCGGCAAA GCGCCACAAT GCTGGGAATG CTCTTTACAC   
  
  
+ TAGTGTTGTT CCAGTTCCAA AGAGTTCGTT TTTAGGTGAG GATCATGAAA TTCTACTCAG AAATCAAGAG   
  
  
+ CAGCAGTTTC TGATGCAACA ACAGTGGTTA ATGGGTTTAG CCCCTCAGTT TACACCCCAG CACCCGCCAA   
  
  
+ AGCCATTAGA CGACCGAAAG CAACCAAACT CGCAAACTTT GGTTCTGCGC GATCAGTTAG TTAAGATTGC   
  
  
+ GGAGATGTTC CAAACCGGTA ACTTTTCGCT TGCCCAAGTG ATATTGGCCC GGCTCAATCA GCAATTGTCT   
  
  
+ CTTTCAGGGA ACCCCTTTGT AAGAGCTGCT ACTTTCCATG TCAAGGAGGA ACTTGAAAGG CTCCTGACTA   
  
  
+ TCAATAGCTT CTCTGCGGCT CCACCCCAAC CTAAAAGCCT TACTCTGTCA GATGTTGTGG ATAGGATGAA   
  
  
+ TGCATACAAG CTTTTTTCAG AGGCATCCCC AGTTATGCAA TTTACCGATT TTACTTGTAC CCAGGCTTTG   
  
  
+ CTCGAAGCTC TTGATGATGC TGATTATATC CACATACTAG ATTTTGACAT TGGTTGTGGT TCCCAATGGG   
  
  
+ CATCCTTTAT TCGAGAGCTT CCTCTCAGGA AAAGGGGCAC TCCATTTTTG AAAATTACAG CCTTTGCCTC   
  
  
+ TTCTTTAACC CATAATCCAT TTGAACTAGC CCTTGTATGT GATAACATTC TGGAATTTGC AAAAGAGTTC   
  
  
+ GGTGTCTCTG TCGACCTTCA GGTCATGAAT TTGGACTTGT TTGACCCAAC TACTTGCACA ATACCCAATT   
  
  
+ TTCAATCCAC TGAGAACGCA GCAGTTGCTG TCAACTTCCC TATATGGGCG TGCTCACACT GCCCGTTTGT   
  
  
+ ACTCAGTCAA CTCCTGTCCT TCATCAAGCA ATGTTCCCCA AAAATCATGA CGAGCTTTGA TAGAGGTTGT   
  
  
+ GATCATTTTG AGTTGTCTTT TCCATCGCAC ATCATCCGTG TATTGGACTC ATGCTCTAAT ATGTTGGAGT   
  
  
+ CCCTCCATGG TTCAAGCGTG ACATTAGACA CCTCAAAGAA GATTGAAAAG TTCTTGATCA AGCCTATAAT   
  
  
+ TGAGAGGGCA ATATTGGGGC GAATTCATGC ACCAGACAAG ATGCAGGCAA ACAAGGCACA CCGGTGGAAG   
  
  
+ AATCTCTTCA CTTCTGCTGG GTTCTTCCCT TTGCCATTTA GTAGTTTCAC TGAATCTCAG GCCAATCTAG   
  
  
+ TGGCTCAACG AACCCCAGTG AGGGGTTTTC AGGTGGAAAA GCAGCAAGCA TCACTTGTGC TCAGGTGGAT   
  
  
+ GTCTCATGAG CTCGTGGCAG CTTCAGCTTG GAGGTGCTA  

- -Up\_Stream \_Len000AAACAT AAATTTAAAA TGTTATAAAT ATTTTTAAAA CATAACTAAT TAACACGATT   
  
  
- GATTGTGATT ATTAACCCCC TCTTTTCTTC CCTTTTATTT TTCTTTAATT CTTCTTCTTT TCTTTTGTGG   
  
  
- AAACTTTTCC GTTTTTTGAT TTTATTTTGT TATTACTTGT CTTCCTCTCT TCCTACCTAT TTGTTGTTTA   
  
  
- CAGCGAAAAT CCTGTCGTTA TCAGATACAC TAACCGGAAT CAAGGACAAC CTCCTAACGA TTGTTTTCGT   
  
  
- TGTCGTTTTT TATTATGATT AATAATGTAT AAAAAATTGA GAATTTCGAT TAGTTAGTAG GAGGTAATTA   
  
  
- ACTAATTAAT AAACGGTTTT TGTGTTTAGT TGACCCCGTT GATATTCTCT AGATGGACGA AATTCTATAT   
  
  
- ATTTGAGTTA ACAAAGTAAA ATAAAATAAA ATCTAAAAAA CCGGAAACCA GAGATAACTC GATTTTAGAG   
  
  
- CAATAAAAGC CTAGTACTTT TTATGTTGAG CTAGAAAACT ATATTCAGTA GAAATAAACA TTTAAGACTT   
  
  
- GAATTTTAGA ATGAACTTCT AGTCCTTATC TTCGTGAGCT AAAAACTATT TAGGATACAC AAGAAGAATT   
  
  
- TTAAGTATTA GATTAAGTAT AAAATCTACA TGGGCAACGG ATGAAACCGT TCAAATCTAA TACTAAAAAT   
  
  
- AAAAAAAATT AATGAACATA AAAAGAAAAC CTAACTATCA GTTAGATGTA GTAGAAACTA GTTAACGCTG   
  
  
- TCAAAGTAAA AGGGTTGTTT TTAAAGCTCA CATAAGAAAA ATTAAATAGT ATTCATAAAT AACAGTTGTA   
  
  
- AGTTGTACGT AGTTGTTAGT AATTTGTGAA AAGCAGTGAG GAGTTATGAC CAGTCGTGAA TTCTAAAACA   
  
  
- GAAGATTTGA ATAGCATTCA TTGAATAACA ACTAATTAAC TACGACTGGT TTACTATGAC TCACGATTTT   
  
  
- AATATTAATT ATTTATGTTA ATTCTCAACT AGTTACAGCT ACTAAGTTTA GTTACACTAA CCACGACTTA   
  
  
- TCATTAATAT TCGTGGATAT TTCTGAACTT TTCTTGTTTG TAAATTAATC TAAAATATTA ATATTAAAGG   
  
  
- ATTATAAATC GTAAGTAGTC GTTTAAGTCG TGTGTGGTTG TAATCAGTTG TGGATAGTTG TGAATATTCA   
  
  
- TGGTTAATTA TTATTAATGG ATATTATCTA ATTTTCAATC CGGGAATTAC AAAGGGTGTA ATAATCTATA   
  
  
- TAGTTCCCTC CCCCTTTTTT TATTATGAGG GGGTAGAATT TTTTAAATAG GGAAAAAAAG AAAAATTACA   
  
  
- AGGTTTTGTT TTTATACGAA GATTTAATAC TAACGTTATA CTTACACATG CCAAAGTTTG GGTTAAAAGA   
  
  
- GGTTGTTAAG AAGATGATAT AACATTAATA TAATATGATG TTAAACATAG AGTAGATACT AGATGGGGGG   
  
  
- TTTTTGGTTT ATGAGTATAT TTCAAAATAA AAGAAAGGTA GGGTGAGATT TTTTTTTTTT ACAACCCTTT   
  
  
- TTCAACTAAA TAAATTAAAC TTTACCATGC CACTTATATC TAAACATCTC CCCGTCAACT ATCTTTTTCC   
  
  
- ACTTCTGTTT CCTTCCCGTG TAAACGGAAA AGTATCTTCC TTCTCTGGTT CCATGACCCC TGACCCCCTT   
  
  
- CCCGAGTTCT CTTCTCTCTC TGACTCTTCT CACGACGATG AGGAAGGATG GATGTGTGTT TTAACGACGC   
  
  
- CCCCCAAGTT CAAGTTGAAC TTGCTCCGTT TATGGGAGTA ACTTGGGTGG TTTAGCTTTA GAAGTTACAT   
  
  
- GTGGGGGTAA TATGTGCGTA AGTCTCTGTA TCTACAGCTC CCCCTCTACT TAATCGTTCT TCTACAGCTC   
  
  
- GATTTTACGT AAAGCTCCAA TGATCTCGGT CTTTACACCC GTGTAGGGTC TTCTAGAGTC ATCTCTCTCA   
  
  
- TTGAAGTCTT TTCAAAGTTA AAAATAAGCT TTAAACGGAG AACAAACACA TCTATACTAC CCTTGAAGCG   
  
  
- GAATGTTAGA CGTTACACTC CCAAACCAAC TTCCTCAACG TCAGTTAGGA GTTAAAATAC TTAGTTGGGG   
  
  
- TTTCACCTTC TTCCCGTAGT TTCTTCTCAG TTAACTCTCG CTTGGGTGAA GCCAAAACCT GTTGGGATCG   
  
  
- GGAGGTTTAA GACGTAGATG AGAGAGAAGG ACGAAGTTAC CACCTTCATC ACCCGAACCC TACCTGCTAA   
  
  
- ACCTCCCAAA CAGAAAACCT CTTCCCAGGG AAGAGGGAAC CAAGTACCCA CTTTAACTTC TGGGATCAAA   
  
  
- CTCAAAGTTC GTGGAGGAAG TTGGAAGTTT AGGGAACCTC AAACCACCGT TACTACCCGA ACTTCGTCGA   
  
  
- TAAGTCTCAC GACCATACCC ATTAAAATTA AACCAACTTT CAAAATTAAG ACTAAACTCC AGAACATTAA   
  
  
- GACCCAGATA AACGTTACCC CCCTTACTAA GACAGTTACT TTTCCCTACA CAAGAATTAA TACGAAGTCT   
  
  
- CGTCCCAGTA CACCAACGAC ATAGAGGTCA TCTTGTCAAA TCCTAATCAC TACACTTTGT ACTCCACAAC   
  
  
- TTGGGTTTTA AACCGAAAAC ACTCGTTCCG AGATGTTAGT CAGTCTTGAG ACCAATACAC GTATATGACC   
  
  
- CAATATTGGT CGATCTTAGA GGGGAACTCG GAGGCCGTTT CGCGGTGTTA CGACCCTTAC GAGAAATGTG   
  
  
- ATCACAACAA GGTCAAGGTT TCTCAAGCAA AAATCCACTC CTAGTACTTT AAGATGAGTC TTTAGTTCTC   
  
  
- GTCGTCAAAG ACTACGTTGT TGTCACCAAT TACCCAAATC GGGGAGTCAA ATGTGGGGTC GTGGGCGGTT   
  
  
- TCGGTAATCT GCTGGCTTTC GTTGGTTTGA GCGTTTGAAA CCAAGACGCG CTAGTCAATC AATTCTAACG   
  
  
- CCTCTACAAG GTTTGGCCAT TGAAAAGCGA ACGGGTTCAC TATAACCGGG CCGAGTTAGT CGTTAACAGA   
  
  
- GAAAGTCCCT TGGGGAAACA TTCTCGACGA TGAAAGGTAC AGTTCCTCCT TGAACTTTCC GAGGACTGAT   
  
  
- AGTTATCGAA GAGACGCCGA GGTGGGGTTG GATTTTCGGA ATGAGACAGT CTACAACACC TATCCTACTT   
  
  
- ACGTATGTTC GAAAAAAGTC TCCGTAGGGG TCAATACGTT AAATGGCTAA AATGAACATG GGTCCGAAAC   
  
  
- GAGCTTCGAG AACTACTACG ACTAATATAG GTGTATGATC TAAAACTGTA ACCAACACCA AGGGTTACCC   
  
  
- GTAGGAAATA AGCTCTCGAA GGAGAGTCCT TTTCCCCGTG AGGTAAAAAC TTTTAATGTC GGAAACGGAG   
  
  
- AAGAAATTGG GTATTAGGTA AACTTGATCG GGAACATACA CTATTGTAAG ACCTTAAACG TTTTCTCAAG   
  
  
- CCACAGAGAC AGCTGGAAGT CCAGTACTTA AACCTGAACA AACTGGGTTG ATGAACGTGT TATGGGTTAA   
  
  
- AAGTTAGGTG ACTCTTGCGT CGTCAACGAC AGTTGAAGGG ATATACCCGC ACGAGTGTGA CGGGCAAACA   
  
  
- TGAGTCAGTT GAGGACAGGA AGTAGTTCGT TACAAGGGGT TTTTAGTACT GCTCGAAACT ATCTCCAACA   
  
  
- CTAGTAAAAC TCAACAGAAA AGGTAGCGTG TAGTAGGCAC ATAACCTGAG TACGAGATTA TACAACCTCA   
  
  
- GGGAGGTACC AAGTTCGCAC TGTAATCTGT GGAGTTTCTT CTAACTTTTC AAGAACTAGT TCGGATATTA   
  
  
- ACTCTCCCGT TATAACCCCG CTTAAGTACG TGGTCTGTTC TACGTCCGTT TGTTCCGTGT GGCCACCTTC   
  
  
- TTAGAGAAGT GAAGACGACC CAAGAAGGGA AACGGTAAAT CATCAAAGTG ACTTAGAGTC CGGTTAGATC   
  
  
- ACCGAGTTGC TTGGGGTCAC TCCCCAAAAG TCCACCTTTT CGTCGTTCGT AGTGAACACG AGTCCACCTA   
  
  
- CAGAGTACTC GAGCACCGTC GAAGTCGAAC CTCCACGAT

+     Myb-binding site

| Site Name | Organism | Position | Strand | Matrix score. | sequence | function |
| --- | --- | --- | --- | --- | --- | --- |
| Myb-binding site | Nicotiana tabacum | 259 | - | 6 | CAACAG |  |
| Myb-binding site | Nicotiana tabacum | 283 | + | 6 | CAACAG |  |
| Myb-binding site | Nicotiana tabacum | 2823 | + | 6 | CAACAG |  |

>HU04G00048.1   
+ -Up\_Stream \_Len000TTTGTA TTTAAATTTT ACAATATTTA TAAAAATTTT GTATTGATTA ATTGTGCTAA   
  
  
+ CTAACACTAA TAATTGGGGG AGAAAAGAAG GGAAAATAAA AAGAAATTAA GAAGAAGAAA AGAAAACACC   
  
  
+ TTTGAAAAGG CAAAAAACTA AAATAAAACA ATAATGAACA GAAGGAGAGA AGGATGGATA AACAACAAAT   
  
  
+ GTCGCTTTTA GGACAGCAAT AGTCTATGTG ATTGGCCTTA GTTCCTGTTG GAGGATTGCT AACAAAAGCA   
  
  
+ ACAGCAAAAA ATAATACTAA TTATTACATA TTTTTTAACT CTTAAAGCTA ATCAATCATC CTCCATTAAT   
  
  
+ TGATTAATTA TTTGCCAAAA ACACAAATCA ACTGGGGCAA CTATAAGAGA TCTACCTGCT TTAAGATATA   
  
  
+ TAAACTCAAT TGTTTCATTT TATTTTATTT TAGATTTTTT GGCCTTTGGT CTCTATTGAG CTAAAATCTC   
  
  
+ GTTATTTTCG GATCATGAAA AATACAACTC GATCTTTTGA TATAAGTCAT CTTTATTTGT AAATTCTGAA   
  
  
+ CTTAAAATCT TACTTGAAGA TCAGGAATAG AAGCACTCGA TTTTTGATAA ATCCTATGTG TTCTTCTTAA   
  
  
+ AATTCATAAT CTAATTCATA TTTTAGATGT ACCCGTTGCC TACTTTGGCA AGTTTAGATT ATGATTTTTA   
  
  
+ TTTTTTTTAA TTACTTGTAT TTTTCTTTTG GATTGATAGT CAATCTACAT CATCTTTGAT CAATTGCGAC   
  
  
+ AGTTTCATTT TCCCAACAAA AATTTCGAGT GTATTCTTTT TAATTTATCA TAAGTATTTA TTGTCAACAT   
  
  
+ TCAACATGCA TCAACAATCA TTAAACACTT TTCGTCACTC CTCAATACTG GTCAGCACTT AAGATTTTGT   
  
  
+ CTTCTAAACT TATCGTAAGT AACTTATTGT TGATTAATTG ATGCTGACCA AATGATACTG AGTGCTAAAA   
  
  
+ TTATAATTAA TAAATACAAT TAAGAGTTGA TCAATGTCGA TGATTCAAAT CAATGTGATT GGTGCTGAAT   
  
  
+ AGTAATTATA AGCACCTATA AAGACTTGAA AAGAACAAAC ATTTAATTAG ATTTTATAAT TATAATTTCC   
  
  
+ TAATATTTAG CATTCATCAG CAAATTCAGC ACACACCAAC ATTAGTCAAC ACCTATCAAC ACTTATAAGT   
  
  
+ ACCAATTAAT AATAATTACC TATAATAGAT TAAAAGTTAG GCCCTTAATG TTTCCCACAT TATTAGATAT   
  
  
+ ATCAAGGGAG GGGGAAAAAA ATAATACTCC CCCATCTTAA AAAATTTATC CCTTTTTTTC TTTTTAATGT   
  
  
+ TCCAAAACAA AAATATGCTT CTAAATTATG ATTGCAATAT GAATGTGTAC GGTTTCAAAC CCAATTTTCT   
  
  
+ CCAACAATTC TTCTACTATA TTGTAATTAT ATTATACTAC AATTTGTATC TCATCTATGA TCTACCCCCC   
  
  
+ AAAAACCAAA TACTCATATA AAGTTTTATT TTCTTTCCAT CCCACTCTAA AAAAAAAAAA TGTTGGGAAA   
  
  
+ AAGTTGATTT ATTTAATTTG AAATGGTACG GTGAATATAG ATTTGTAGAG GGGCAGTTGA TAGAAAAAGG   
  
  
+ TGAAGACAAA GGAAGGGCAC ATTTGCCTTT TCATAGAAGG AAGAGACCAA GGTACTGGGG ACTGGGGGAA   
  
  
+ GGGCTCAAGA GAAGAGAGAG ACTGAGAAGA GTGCTGCTAC TCCTTCCTAC CTACACACAA AATTGCTGCG   
  
  
+ GGGGGTTCAA GTTCAACTTG AACGAGGCAA ATACCCTCAT TGAACCCACC AAATCGAAAT CTTCAATGTA   
  
  
+ CACCCCCATT ATACACGCAT TCAGAGACAT AGATGTCGAG GGGGAGATGA ATTAGCAAGA AGATGTCGAG   
  
  
+ CTAAAATGCA TTTCGAGGTT ACTAGAGCCA GAAATGTGGG CACATCCCAG AAGATCTCAG TAGAGAGAGT   
  
  
+ AACTTCAGAA AAGTTTCAAT TTTTATTCGA AATTTGCCTC TTGTTTGTGT AGATATGATG GGAACTTCGC   
  
  
+ CTTACAATCT GCAATGTGAG GGTTTGGTTG AAGGAGTTGC AGTCAATCCT CAATTTTATG AATCAACCCC   
  
  
+ AAAGTGGAAG AAGGGCATCA AAGAAGAGTC AATTGAGAGC GAACCCACTT CGGTTTTGGA CAACCCTAGC   
  
  
+ CCTCCAAATT CTGCATCTAC TCTCTCTTCC TGCTTCAATG GTGGAAGTAG TGGGCTTGGG ATGGACGATT   
  
  
+ TGGAGGGTTT GTCTTTTGGA GAAGGGTCCC TTCTCCCTTG GTTCATGGGT GAAATTGAAG ACCCTAGTTT   
  
  
+ GAGTTTCAAG CACCTCCTTC AACCTTCAAA TCCCTTGGAG TTTGGTGGCA ATGATGGGCT TGAAGCAGCT   
  
  
+ ATTCAGAGTG CTGGTATGGG TAATTTTAAT TTGGTTGAAA GTTTTAATTC TGATTTGAGG TCTTGTAATT   
  
  
+ CTGGGTCTAT TTGCAATGGG GGGAATGATT CTGTCAATGA AAAGGGATGT GTTCTTAATT ATGCTTCAGA   
  
  
+ GCAGGGTCAT GTGGTTGCTG TATCTCCAGT AGAACAGTTT AGGATTAGTG ATGTGAAACA TGAGGTGTTG   
  
  
+ AACCCAAAAT TTGGCTTTTG TGAGCAAGGC TCTACAATCA GTCAGAACTC TGGTTATGTG CATATACTGG   
  
  
+ GTTATAACCA GCTAGAATCT CCCCTTGAGC CTCCGGCAAA GCGCCACAAT GCTGGGAATG CTCTTTACAC   
  
  
+ TAGTGTTGTT CCAGTTCCAA AGAGTTCGTT TTTAGGTGAG GATCATGAAA TTCTACTCAG AAATCAAGAG   
  
  
+ CAGCAGTTTC TGATGCAACA ACAGTGGTTA ATGGGTTTAG CCCCTCAGTT TACACCCCAG CACCCGCCAA   
  
  
+ AGCCATTAGA CGACCGAAAG CAACCAAACT CGCAAACTTT GGTTCTGCGC GATCAGTTAG TTAAGATTGC   
  
  
+ GGAGATGTTC CAAACCGGTA ACTTTTCGCT TGCCCAAGTG ATATTGGCCC GGCTCAATCA GCAATTGTCT   
  
  
+ CTTTCAGGGA ACCCCTTTGT AAGAGCTGCT ACTTTCCATG TCAAGGAGGA ACTTGAAAGG CTCCTGACTA   
  
  
+ TCAATAGCTT CTCTGCGGCT CCACCCCAAC CTAAAAGCCT TACTCTGTCA GATGTTGTGG ATAGGATGAA   
  
  
+ TGCATACAAG CTTTTTTCAG AGGCATCCCC AGTTATGCAA TTTACCGATT TTACTTGTAC CCAGGCTTTG   
  
  
+ CTCGAAGCTC TTGATGATGC TGATTATATC CACATACTAG ATTTTGACAT TGGTTGTGGT TCCCAATGGG   
  
  
+ CATCCTTTAT TCGAGAGCTT CCTCTCAGGA AAAGGGGCAC TCCATTTTTG AAAATTACAG CCTTTGCCTC   
  
  
+ TTCTTTAACC CATAATCCAT TTGAACTAGC CCTTGTATGT GATAACATTC TGGAATTTGC AAAAGAGTTC   
  
  
+ GGTGTCTCTG TCGACCTTCA GGTCATGAAT TTGGACTTGT TTGACCCAAC TACTTGCACA ATACCCAATT   
  
  
+ TTCAATCCAC TGAGAACGCA GCAGTTGCTG TCAACTTCCC TATATGGGCG TGCTCACACT GCCCGTTTGT   
  
  
+ ACTCAGTCAA CTCCTGTCCT TCATCAAGCA ATGTTCCCCA AAAATCATGA CGAGCTTTGA TAGAGGTTGT   
  
  
+ GATCATTTTG AGTTGTCTTT TCCATCGCAC ATCATCCGTG TATTGGACTC ATGCTCTAAT ATGTTGGAGT   
  
  
+ CCCTCCATGG TTCAAGCGTG ACATTAGACA CCTCAAAGAA GATTGAAAAG TTCTTGATCA AGCCTATAAT   
  
  
+ TGAGAGGGCA ATATTGGGGC GAATTCATGC ACCAGACAAG ATGCAGGCAA ACAAGGCACA CCGGTGGAAG   
  
  
+ AATCTCTTCA CTTCTGCTGG GTTCTTCCCT TTGCCATTTA GTAGTTTCAC TGAATCTCAG GCCAATCTAG   
  
  
+ TGGCTCAACG AACCCCAGTG AGGGGTTTTC AGGTGGAAAA GCAGCAAGCA TCACTTGTGC TCAGGTGGAT   
  
  
+ GTCTCATGAG CTCGTGGCAG CTTCAGCTTG GAGGTGCTA  

- -Up\_Stream \_Len000AAACAT AAATTTAAAA TGTTATAAAT ATTTTTAAAA CATAACTAAT TAACACGATT   
  
  
- GATTGTGATT ATTAACCCCC TCTTTTCTTC CCTTTTATTT TTCTTTAATT CTTCTTCTTT TCTTTTGTGG   
  
  
- AAACTTTTCC GTTTTTTGAT TTTATTTTGT TATTACTTGT CTTCCTCTCT TCCTACCTAT TTGTTGTTTA   
  
  
- CAGCGAAAAT CCTGTCGTTA TCAGATACAC TAACCGGAAT CAAGGACAAC CTCCTAACGA TTGTTTTCGT   
  
  
- TGTCGTTTTT TATTATGATT AATAATGTAT AAAAAATTGA GAATTTCGAT TAGTTAGTAG GAGGTAATTA   
  
  
- ACTAATTAAT AAACGGTTTT TGTGTTTAGT TGACCCCGTT GATATTCTCT AGATGGACGA AATTCTATAT   
  
  
- ATTTGAGTTA ACAAAGTAAA ATAAAATAAA ATCTAAAAAA CCGGAAACCA GAGATAACTC GATTTTAGAG   
  
  
- CAATAAAAGC CTAGTACTTT TTATGTTGAG CTAGAAAACT ATATTCAGTA GAAATAAACA TTTAAGACTT   
  
  
- GAATTTTAGA ATGAACTTCT AGTCCTTATC TTCGTGAGCT AAAAACTATT TAGGATACAC AAGAAGAATT   
  
  
- TTAAGTATTA GATTAAGTAT AAAATCTACA TGGGCAACGG ATGAAACCGT TCAAATCTAA TACTAAAAAT   
  
  
- AAAAAAAATT AATGAACATA AAAAGAAAAC CTAACTATCA GTTAGATGTA GTAGAAACTA GTTAACGCTG   
  
  
- TCAAAGTAAA AGGGTTGTTT TTAAAGCTCA CATAAGAAAA ATTAAATAGT ATTCATAAAT AACAGTTGTA   
  
  
- AGTTGTACGT AGTTGTTAGT AATTTGTGAA AAGCAGTGAG GAGTTATGAC CAGTCGTGAA TTCTAAAACA   
  
  
- GAAGATTTGA ATAGCATTCA TTGAATAACA ACTAATTAAC TACGACTGGT TTACTATGAC TCACGATTTT   
  
  
- AATATTAATT ATTTATGTTA ATTCTCAACT AGTTACAGCT ACTAAGTTTA GTTACACTAA CCACGACTTA   
  
  
- TCATTAATAT TCGTGGATAT TTCTGAACTT TTCTTGTTTG TAAATTAATC TAAAATATTA ATATTAAAGG   
  
  
- ATTATAAATC GTAAGTAGTC GTTTAAGTCG TGTGTGGTTG TAATCAGTTG TGGATAGTTG TGAATATTCA   
  
  
- TGGTTAATTA TTATTAATGG ATATTATCTA ATTTTCAATC CGGGAATTAC AAAGGGTGTA ATAATCTATA   
  
  
- TAGTTCCCTC CCCCTTTTTT TATTATGAGG GGGTAGAATT TTTTAAATAG GGAAAAAAAG AAAAATTACA   
  
  
- AGGTTTTGTT TTTATACGAA GATTTAATAC TAACGTTATA CTTACACATG CCAAAGTTTG GGTTAAAAGA   
  
  
- GGTTGTTAAG AAGATGATAT AACATTAATA TAATATGATG TTAAACATAG AGTAGATACT AGATGGGGGG   
  
  
- TTTTTGGTTT ATGAGTATAT TTCAAAATAA AAGAAAGGTA GGGTGAGATT TTTTTTTTTT ACAACCCTTT   
  
  
- TTCAACTAAA TAAATTAAAC TTTACCATGC CACTTATATC TAAACATCTC CCCGTCAACT ATCTTTTTCC   
  
  
- ACTTCTGTTT CCTTCCCGTG TAAACGGAAA AGTATCTTCC TTCTCTGGTT CCATGACCCC TGACCCCCTT   
  
  
- CCCGAGTTCT CTTCTCTCTC TGACTCTTCT CACGACGATG AGGAAGGATG GATGTGTGTT TTAACGACGC   
  
  
- CCCCCAAGTT CAAGTTGAAC TTGCTCCGTT TATGGGAGTA ACTTGGGTGG TTTAGCTTTA GAAGTTACAT   
  
  
- GTGGGGGTAA TATGTGCGTA AGTCTCTGTA TCTACAGCTC CCCCTCTACT TAATCGTTCT TCTACAGCTC   
  
  
- GATTTTACGT AAAGCTCCAA TGATCTCGGT CTTTACACCC GTGTAGGGTC TTCTAGAGTC ATCTCTCTCA   
  
  
- TTGAAGTCTT TTCAAAGTTA AAAATAAGCT TTAAACGGAG AACAAACACA TCTATACTAC CCTTGAAGCG   
  
  
- GAATGTTAGA CGTTACACTC CCAAACCAAC TTCCTCAACG TCAGTTAGGA GTTAAAATAC TTAGTTGGGG   
  
  
- TTTCACCTTC TTCCCGTAGT TTCTTCTCAG TTAACTCTCG CTTGGGTGAA GCCAAAACCT GTTGGGATCG   
  
  
- GGAGGTTTAA GACGTAGATG AGAGAGAAGG ACGAAGTTAC CACCTTCATC ACCCGAACCC TACCTGCTAA   
  
  
- ACCTCCCAAA CAGAAAACCT CTTCCCAGGG AAGAGGGAAC CAAGTACCCA CTTTAACTTC TGGGATCAAA   
  
  
- CTCAAAGTTC GTGGAGGAAG TTGGAAGTTT AGGGAACCTC AAACCACCGT TACTACCCGA ACTTCGTCGA   
  
  
- TAAGTCTCAC GACCATACCC ATTAAAATTA AACCAACTTT CAAAATTAAG ACTAAACTCC AGAACATTAA   
  
  
- GACCCAGATA AACGTTACCC CCCTTACTAA GACAGTTACT TTTCCCTACA CAAGAATTAA TACGAAGTCT   
  
  
- CGTCCCAGTA CACCAACGAC ATAGAGGTCA TCTTGTCAAA TCCTAATCAC TACACTTTGT ACTCCACAAC   
  
  
- TTGGGTTTTA AACCGAAAAC ACTCGTTCCG AGATGTTAGT CAGTCTTGAG ACCAATACAC GTATATGACC   
  
  
- CAATATTGGT CGATCTTAGA GGGGAACTCG GAGGCCGTTT CGCGGTGTTA CGACCCTTAC GAGAAATGTG   
  
  
- ATCACAACAA GGTCAAGGTT TCTCAAGCAA AAATCCACTC CTAGTACTTT AAGATGAGTC TTTAGTTCTC   
  
  
- GTCGTCAAAG ACTACGTTGT TGTCACCAAT TACCCAAATC GGGGAGTCAA ATGTGGGGTC GTGGGCGGTT   
  
  
- TCGGTAATCT GCTGGCTTTC GTTGGTTTGA GCGTTTGAAA CCAAGACGCG CTAGTCAATC AATTCTAACG   
  
  
- CCTCTACAAG GTTTGGCCAT TGAAAAGCGA ACGGGTTCAC TATAACCGGG CCGAGTTAGT CGTTAACAGA   
  
  
- GAAAGTCCCT TGGGGAAACA TTCTCGACGA TGAAAGGTAC AGTTCCTCCT TGAACTTTCC GAGGACTGAT   
  
  
- AGTTATCGAA GAGACGCCGA GGTGGGGTTG GATTTTCGGA ATGAGACAGT CTACAACACC TATCCTACTT   
  
  
- ACGTATGTTC GAAAAAAGTC TCCGTAGGGG TCAATACGTT AAATGGCTAA AATGAACATG GGTCCGAAAC   
  
  
- GAGCTTCGAG AACTACTACG ACTAATATAG GTGTATGATC TAAAACTGTA ACCAACACCA AGGGTTACCC   
  
  
- GTAGGAAATA AGCTCTCGAA GGAGAGTCCT TTTCCCCGTG AGGTAAAAAC TTTTAATGTC GGAAACGGAG   
  
  
- AAGAAATTGG GTATTAGGTA AACTTGATCG GGAACATACA CTATTGTAAG ACCTTAAACG TTTTCTCAAG   
  
  
- CCACAGAGAC AGCTGGAAGT CCAGTACTTA AACCTGAACA AACTGGGTTG ATGAACGTGT TATGGGTTAA   
  
  
- AAGTTAGGTG ACTCTTGCGT CGTCAACGAC AGTTGAAGGG ATATACCCGC ACGAGTGTGA CGGGCAAACA   
  
  
- TGAGTCAGTT GAGGACAGGA AGTAGTTCGT TACAAGGGGT TTTTAGTACT GCTCGAAACT ATCTCCAACA   
  
  
- CTAGTAAAAC TCAACAGAAA AGGTAGCGTG TAGTAGGCAC ATAACCTGAG TACGAGATTA TACAACCTCA   
  
  
- GGGAGGTACC AAGTTCGCAC TGTAATCTGT GGAGTTTCTT CTAACTTTTC AAGAACTAGT TCGGATATTA   
  
  
- ACTCTCCCGT TATAACCCCG CTTAAGTACG TGGTCTGTTC TACGTCCGTT TGTTCCGTGT GGCCACCTTC   
  
  
- TTAGAGAAGT GAAGACGACC CAAGAAGGGA AACGGTAAAT CATCAAAGTG ACTTAGAGTC CGGTTAGATC   
  
  
- ACCGAGTTGC TTGGGGTCAC TCCCCAAAAG TCCACCTTTT CGTCGTTCGT AGTGAACACG AGTCCACCTA   
  
  
- CAGAGTACTC GAGCACCGTC GAAGTCGAAC CTCCACGAT

+     Myc

| Site Name | Organism | Position | Strand | Matrix score. | sequence | function |
| --- | --- | --- | --- | --- | --- | --- |
| Myc | Arabidopsis thaliana | 398 | - | 7 | TCTCTTA |  |

>HU04G00048.1   
+ -Up\_Stream \_Len000TTTGTA TTTAAATTTT ACAATATTTA TAAAAATTTT GTATTGATTA ATTGTGCTAA   
  
  
+ CTAACACTAA TAATTGGGGG AGAAAAGAAG GGAAAATAAA AAGAAATTAA GAAGAAGAAA AGAAAACACC   
  
  
+ TTTGAAAAGG CAAAAAACTA AAATAAAACA ATAATGAACA GAAGGAGAGA AGGATGGATA AACAACAAAT   
  
  
+ GTCGCTTTTA GGACAGCAAT AGTCTATGTG ATTGGCCTTA GTTCCTGTTG GAGGATTGCT AACAAAAGCA   
  
  
+ ACAGCAAAAA ATAATACTAA TTATTACATA TTTTTTAACT CTTAAAGCTA ATCAATCATC CTCCATTAAT   
  
  
+ TGATTAATTA TTTGCCAAAA ACACAAATCA ACTGGGGCAA CTATAAGAGA TCTACCTGCT TTAAGATATA   
  
  
+ TAAACTCAAT TGTTTCATTT TATTTTATTT TAGATTTTTT GGCCTTTGGT CTCTATTGAG CTAAAATCTC   
  
  
+ GTTATTTTCG GATCATGAAA AATACAACTC GATCTTTTGA TATAAGTCAT CTTTATTTGT AAATTCTGAA   
  
  
+ CTTAAAATCT TACTTGAAGA TCAGGAATAG AAGCACTCGA TTTTTGATAA ATCCTATGTG TTCTTCTTAA   
  
  
+ AATTCATAAT CTAATTCATA TTTTAGATGT ACCCGTTGCC TACTTTGGCA AGTTTAGATT ATGATTTTTA   
  
  
+ TTTTTTTTAA TTACTTGTAT TTTTCTTTTG GATTGATAGT CAATCTACAT CATCTTTGAT CAATTGCGAC   
  
  
+ AGTTTCATTT TCCCAACAAA AATTTCGAGT GTATTCTTTT TAATTTATCA TAAGTATTTA TTGTCAACAT   
  
  
+ TCAACATGCA TCAACAATCA TTAAACACTT TTCGTCACTC CTCAATACTG GTCAGCACTT AAGATTTTGT   
  
  
+ CTTCTAAACT TATCGTAAGT AACTTATTGT TGATTAATTG ATGCTGACCA AATGATACTG AGTGCTAAAA   
  
  
+ TTATAATTAA TAAATACAAT TAAGAGTTGA TCAATGTCGA TGATTCAAAT CAATGTGATT GGTGCTGAAT   
  
  
+ AGTAATTATA AGCACCTATA AAGACTTGAA AAGAACAAAC ATTTAATTAG ATTTTATAAT TATAATTTCC   
  
  
+ TAATATTTAG CATTCATCAG CAAATTCAGC ACACACCAAC ATTAGTCAAC ACCTATCAAC ACTTATAAGT   
  
  
+ ACCAATTAAT AATAATTACC TATAATAGAT TAAAAGTTAG GCCCTTAATG TTTCCCACAT TATTAGATAT   
  
  
+ ATCAAGGGAG GGGGAAAAAA ATAATACTCC CCCATCTTAA AAAATTTATC CCTTTTTTTC TTTTTAATGT   
  
  
+ TCCAAAACAA AAATATGCTT CTAAATTATG ATTGCAATAT GAATGTGTAC GGTTTCAAAC CCAATTTTCT   
  
  
+ CCAACAATTC TTCTACTATA TTGTAATTAT ATTATACTAC AATTTGTATC TCATCTATGA TCTACCCCCC   
  
  
+ AAAAACCAAA TACTCATATA AAGTTTTATT TTCTTTCCAT CCCACTCTAA AAAAAAAAAA TGTTGGGAAA   
  
  
+ AAGTTGATTT ATTTAATTTG AAATGGTACG GTGAATATAG ATTTGTAGAG GGGCAGTTGA TAGAAAAAGG   
  
  
+ TGAAGACAAA GGAAGGGCAC ATTTGCCTTT TCATAGAAGG AAGAGACCAA GGTACTGGGG ACTGGGGGAA   
  
  
+ GGGCTCAAGA GAAGAGAGAG ACTGAGAAGA GTGCTGCTAC TCCTTCCTAC CTACACACAA AATTGCTGCG   
  
  
+ GGGGGTTCAA GTTCAACTTG AACGAGGCAA ATACCCTCAT TGAACCCACC AAATCGAAAT CTTCAATGTA   
  
  
+ CACCCCCATT ATACACGCAT TCAGAGACAT AGATGTCGAG GGGGAGATGA ATTAGCAAGA AGATGTCGAG   
  
  
+ CTAAAATGCA TTTCGAGGTT ACTAGAGCCA GAAATGTGGG CACATCCCAG AAGATCTCAG TAGAGAGAGT   
  
  
+ AACTTCAGAA AAGTTTCAAT TTTTATTCGA AATTTGCCTC TTGTTTGTGT AGATATGATG GGAACTTCGC   
  
  
+ CTTACAATCT GCAATGTGAG GGTTTGGTTG AAGGAGTTGC AGTCAATCCT CAATTTTATG AATCAACCCC   
  
  
+ AAAGTGGAAG AAGGGCATCA AAGAAGAGTC AATTGAGAGC GAACCCACTT CGGTTTTGGA CAACCCTAGC   
  
  
+ CCTCCAAATT CTGCATCTAC TCTCTCTTCC TGCTTCAATG GTGGAAGTAG TGGGCTTGGG ATGGACGATT   
  
  
+ TGGAGGGTTT GTCTTTTGGA GAAGGGTCCC TTCTCCCTTG GTTCATGGGT GAAATTGAAG ACCCTAGTTT   
  
  
+ GAGTTTCAAG CACCTCCTTC AACCTTCAAA TCCCTTGGAG TTTGGTGGCA ATGATGGGCT TGAAGCAGCT   
  
  
+ ATTCAGAGTG CTGGTATGGG TAATTTTAAT TTGGTTGAAA GTTTTAATTC TGATTTGAGG TCTTGTAATT   
  
  
+ CTGGGTCTAT TTGCAATGGG GGGAATGATT CTGTCAATGA AAAGGGATGT GTTCTTAATT ATGCTTCAGA   
  
  
+ GCAGGGTCAT GTGGTTGCTG TATCTCCAGT AGAACAGTTT AGGATTAGTG ATGTGAAACA TGAGGTGTTG   
  
  
+ AACCCAAAAT TTGGCTTTTG TGAGCAAGGC TCTACAATCA GTCAGAACTC TGGTTATGTG CATATACTGG   
  
  
+ GTTATAACCA GCTAGAATCT CCCCTTGAGC CTCCGGCAAA GCGCCACAAT GCTGGGAATG CTCTTTACAC   
  
  
+ TAGTGTTGTT CCAGTTCCAA AGAGTTCGTT TTTAGGTGAG GATCATGAAA TTCTACTCAG AAATCAAGAG   
  
  
+ CAGCAGTTTC TGATGCAACA ACAGTGGTTA ATGGGTTTAG CCCCTCAGTT TACACCCCAG CACCCGCCAA   
  
  
+ AGCCATTAGA CGACCGAAAG CAACCAAACT CGCAAACTTT GGTTCTGCGC GATCAGTTAG TTAAGATTGC   
  
  
+ GGAGATGTTC CAAACCGGTA ACTTTTCGCT TGCCCAAGTG ATATTGGCCC GGCTCAATCA GCAATTGTCT   
  
  
+ CTTTCAGGGA ACCCCTTTGT AAGAGCTGCT ACTTTCCATG TCAAGGAGGA ACTTGAAAGG CTCCTGACTA   
  
  
+ TCAATAGCTT CTCTGCGGCT CCACCCCAAC CTAAAAGCCT TACTCTGTCA GATGTTGTGG ATAGGATGAA   
  
  
+ TGCATACAAG CTTTTTTCAG AGGCATCCCC AGTTATGCAA TTTACCGATT TTACTTGTAC CCAGGCTTTG   
  
  
+ CTCGAAGCTC TTGATGATGC TGATTATATC CACATACTAG ATTTTGACAT TGGTTGTGGT TCCCAATGGG   
  
  
+ CATCCTTTAT TCGAGAGCTT CCTCTCAGGA AAAGGGGCAC TCCATTTTTG AAAATTACAG CCTTTGCCTC   
  
  
+ TTCTTTAACC CATAATCCAT TTGAACTAGC CCTTGTATGT GATAACATTC TGGAATTTGC AAAAGAGTTC   
  
  
+ GGTGTCTCTG TCGACCTTCA GGTCATGAAT TTGGACTTGT TTGACCCAAC TACTTGCACA ATACCCAATT   
  
  
+ TTCAATCCAC TGAGAACGCA GCAGTTGCTG TCAACTTCCC TATATGGGCG TGCTCACACT GCCCGTTTGT   
  
  
+ ACTCAGTCAA CTCCTGTCCT TCATCAAGCA ATGTTCCCCA AAAATCATGA CGAGCTTTGA TAGAGGTTGT   
  
  
+ GATCATTTTG AGTTGTCTTT TCCATCGCAC ATCATCCGTG TATTGGACTC ATGCTCTAAT ATGTTGGAGT   
  
  
+ CCCTCCATGG TTCAAGCGTG ACATTAGACA CCTCAAAGAA GATTGAAAAG TTCTTGATCA AGCCTATAAT   
  
  
+ TGAGAGGGCA ATATTGGGGC GAATTCATGC ACCAGACAAG ATGCAGGCAA ACAAGGCACA CCGGTGGAAG   
  
  
+ AATCTCTTCA CTTCTGCTGG GTTCTTCCCT TTGCCATTTA GTAGTTTCAC TGAATCTCAG GCCAATCTAG   
  
  
+ TGGCTCAACG AACCCCAGTG AGGGGTTTTC AGGTGGAAAA GCAGCAAGCA TCACTTGTGC TCAGGTGGAT   
  
  
+ GTCTCATGAG CTCGTGGCAG CTTCAGCTTG GAGGTGCTA  

- -Up\_Stream \_Len000AAACAT AAATTTAAAA TGTTATAAAT ATTTTTAAAA CATAACTAAT TAACACGATT   
  
  
- GATTGTGATT ATTAACCCCC TCTTTTCTTC CCTTTTATTT TTCTTTAATT CTTCTTCTTT TCTTTTGTGG   
  
  
- AAACTTTTCC GTTTTTTGAT TTTATTTTGT TATTACTTGT CTTCCTCTCT TCCTACCTAT TTGTTGTTTA   
  
  
- CAGCGAAAAT CCTGTCGTTA TCAGATACAC TAACCGGAAT CAAGGACAAC CTCCTAACGA TTGTTTTCGT   
  
  
- TGTCGTTTTT TATTATGATT AATAATGTAT AAAAAATTGA GAATTTCGAT TAGTTAGTAG GAGGTAATTA   
  
  
- ACTAATTAAT AAACGGTTTT TGTGTTTAGT TGACCCCGTT GATATTCTCT AGATGGACGA AATTCTATAT   
  
  
- ATTTGAGTTA ACAAAGTAAA ATAAAATAAA ATCTAAAAAA CCGGAAACCA GAGATAACTC GATTTTAGAG   
  
  
- CAATAAAAGC CTAGTACTTT TTATGTTGAG CTAGAAAACT ATATTCAGTA GAAATAAACA TTTAAGACTT   
  
  
- GAATTTTAGA ATGAACTTCT AGTCCTTATC TTCGTGAGCT AAAAACTATT TAGGATACAC AAGAAGAATT   
  
  
- TTAAGTATTA GATTAAGTAT AAAATCTACA TGGGCAACGG ATGAAACCGT TCAAATCTAA TACTAAAAAT   
  
  
- AAAAAAAATT AATGAACATA AAAAGAAAAC CTAACTATCA GTTAGATGTA GTAGAAACTA GTTAACGCTG   
  
  
- TCAAAGTAAA AGGGTTGTTT TTAAAGCTCA CATAAGAAAA ATTAAATAGT ATTCATAAAT AACAGTTGTA   
  
  
- AGTTGTACGT AGTTGTTAGT AATTTGTGAA AAGCAGTGAG GAGTTATGAC CAGTCGTGAA TTCTAAAACA   
  
  
- GAAGATTTGA ATAGCATTCA TTGAATAACA ACTAATTAAC TACGACTGGT TTACTATGAC TCACGATTTT   
  
  
- AATATTAATT ATTTATGTTA ATTCTCAACT AGTTACAGCT ACTAAGTTTA GTTACACTAA CCACGACTTA   
  
  
- TCATTAATAT TCGTGGATAT TTCTGAACTT TTCTTGTTTG TAAATTAATC TAAAATATTA ATATTAAAGG   
  
  
- ATTATAAATC GTAAGTAGTC GTTTAAGTCG TGTGTGGTTG TAATCAGTTG TGGATAGTTG TGAATATTCA   
  
  
- TGGTTAATTA TTATTAATGG ATATTATCTA ATTTTCAATC CGGGAATTAC AAAGGGTGTA ATAATCTATA   
  
  
- TAGTTCCCTC CCCCTTTTTT TATTATGAGG GGGTAGAATT TTTTAAATAG GGAAAAAAAG AAAAATTACA   
  
  
- AGGTTTTGTT TTTATACGAA GATTTAATAC TAACGTTATA CTTACACATG CCAAAGTTTG GGTTAAAAGA   
  
  
- GGTTGTTAAG AAGATGATAT AACATTAATA TAATATGATG TTAAACATAG AGTAGATACT AGATGGGGGG   
  
  
- TTTTTGGTTT ATGAGTATAT TTCAAAATAA AAGAAAGGTA GGGTGAGATT TTTTTTTTTT ACAACCCTTT   
  
  
- TTCAACTAAA TAAATTAAAC TTTACCATGC CACTTATATC TAAACATCTC CCCGTCAACT ATCTTTTTCC   
  
  
- ACTTCTGTTT CCTTCCCGTG TAAACGGAAA AGTATCTTCC TTCTCTGGTT CCATGACCCC TGACCCCCTT   
  
  
- CCCGAGTTCT CTTCTCTCTC TGACTCTTCT CACGACGATG AGGAAGGATG GATGTGTGTT TTAACGACGC   
  
  
- CCCCCAAGTT CAAGTTGAAC TTGCTCCGTT TATGGGAGTA ACTTGGGTGG TTTAGCTTTA GAAGTTACAT   
  
  
- GTGGGGGTAA TATGTGCGTA AGTCTCTGTA TCTACAGCTC CCCCTCTACT TAATCGTTCT TCTACAGCTC   
  
  
- GATTTTACGT AAAGCTCCAA TGATCTCGGT CTTTACACCC GTGTAGGGTC TTCTAGAGTC ATCTCTCTCA   
  
  
- TTGAAGTCTT TTCAAAGTTA AAAATAAGCT TTAAACGGAG AACAAACACA TCTATACTAC CCTTGAAGCG   
  
  
- GAATGTTAGA CGTTACACTC CCAAACCAAC TTCCTCAACG TCAGTTAGGA GTTAAAATAC TTAGTTGGGG   
  
  
- TTTCACCTTC TTCCCGTAGT TTCTTCTCAG TTAACTCTCG CTTGGGTGAA GCCAAAACCT GTTGGGATCG   
  
  
- GGAGGTTTAA GACGTAGATG AGAGAGAAGG ACGAAGTTAC CACCTTCATC ACCCGAACCC TACCTGCTAA   
  
  
- ACCTCCCAAA CAGAAAACCT CTTCCCAGGG AAGAGGGAAC CAAGTACCCA CTTTAACTTC TGGGATCAAA   
  
  
- CTCAAAGTTC GTGGAGGAAG TTGGAAGTTT AGGGAACCTC AAACCACCGT TACTACCCGA ACTTCGTCGA   
  
  
- TAAGTCTCAC GACCATACCC ATTAAAATTA AACCAACTTT CAAAATTAAG ACTAAACTCC AGAACATTAA   
  
  
- GACCCAGATA AACGTTACCC CCCTTACTAA GACAGTTACT TTTCCCTACA CAAGAATTAA TACGAAGTCT   
  
  
- CGTCCCAGTA CACCAACGAC ATAGAGGTCA TCTTGTCAAA TCCTAATCAC TACACTTTGT ACTCCACAAC   
  
  
- TTGGGTTTTA AACCGAAAAC ACTCGTTCCG AGATGTTAGT CAGTCTTGAG ACCAATACAC GTATATGACC   
  
  
- CAATATTGGT CGATCTTAGA GGGGAACTCG GAGGCCGTTT CGCGGTGTTA CGACCCTTAC GAGAAATGTG   
  
  
- ATCACAACAA GGTCAAGGTT TCTCAAGCAA AAATCCACTC CTAGTACTTT AAGATGAGTC TTTAGTTCTC   
  
  
- GTCGTCAAAG ACTACGTTGT TGTCACCAAT TACCCAAATC GGGGAGTCAA ATGTGGGGTC GTGGGCGGTT   
  
  
- TCGGTAATCT GCTGGCTTTC GTTGGTTTGA GCGTTTGAAA CCAAGACGCG CTAGTCAATC AATTCTAACG   
  
  
- CCTCTACAAG GTTTGGCCAT TGAAAAGCGA ACGGGTTCAC TATAACCGGG CCGAGTTAGT CGTTAACAGA   
  
  
- GAAAGTCCCT TGGGGAAACA TTCTCGACGA TGAAAGGTAC AGTTCCTCCT TGAACTTTCC GAGGACTGAT   
  
  
- AGTTATCGAA GAGACGCCGA GGTGGGGTTG GATTTTCGGA ATGAGACAGT CTACAACACC TATCCTACTT   
  
  
- ACGTATGTTC GAAAAAAGTC TCCGTAGGGG TCAATACGTT AAATGGCTAA AATGAACATG GGTCCGAAAC   
  
  
- GAGCTTCGAG AACTACTACG ACTAATATAG GTGTATGATC TAAAACTGTA ACCAACACCA AGGGTTACCC   
  
  
- GTAGGAAATA AGCTCTCGAA GGAGAGTCCT TTTCCCCGTG AGGTAAAAAC TTTTAATGTC GGAAACGGAG   
  
  
- AAGAAATTGG GTATTAGGTA AACTTGATCG GGAACATACA CTATTGTAAG ACCTTAAACG TTTTCTCAAG   
  
  
- CCACAGAGAC AGCTGGAAGT CCAGTACTTA AACCTGAACA AACTGGGTTG ATGAACGTGT TATGGGTTAA   
  
  
- AAGTTAGGTG ACTCTTGCGT CGTCAACGAC AGTTGAAGGG ATATACCCGC ACGAGTGTGA CGGGCAAACA   
  
  
- TGAGTCAGTT GAGGACAGGA AGTAGTTCGT TACAAGGGGT TTTTAGTACT GCTCGAAACT ATCTCCAACA   
  
  
- CTAGTAAAAC TCAACAGAAA AGGTAGCGTG TAGTAGGCAC ATAACCTGAG TACGAGATTA TACAACCTCA   
  
  
- GGGAGGTACC AAGTTCGCAC TGTAATCTGT GGAGTTTCTT CTAACTTTTC AAGAACTAGT TCGGATATTA   
  
  
- ACTCTCCCGT TATAACCCCG CTTAAGTACG TGGTCTGTTC TACGTCCGTT TGTTCCGTGT GGCCACCTTC   
  
  
- TTAGAGAAGT GAAGACGACC CAAGAAGGGA AACGGTAAAT CATCAAAGTG ACTTAGAGTC CGGTTAGATC   
  
  
- ACCGAGTTGC TTGGGGTCAC TCCCCAAAAG TCCACCTTTT CGTCGTTCGT AGTGAACACG AGTCCACCTA   
  
  
- CAGAGTACTC GAGCACCGTC GAAGTCGAAC CTCCACGAT

+     O2-site

| Site Name | Organism | Position | Strand | Matrix score. | sequence | function |
| --- | --- | --- | --- | --- | --- | --- |
| O2-site | Zea mays | 749 | - | 9 | GATGATGTGG | cis-acting regulatory element involved in zein metabolism regulation |
| O2-site | Zea mays | 3671 | - | 9 | GATGATGTGG | cis-acting regulatory element involved in zein metabolism regulation |
| O2-site | Zea mays | 3135 | + | 9 | GATGATGTGG | cis-acting regulatory element involved in zein metabolism regulation |

>HU04G00048.1   
+ -Up\_Stream \_Len000TTTGTA TTTAAATTTT ACAATATTTA TAAAAATTTT GTATTGATTA ATTGTGCTAA   
  
  
+ CTAACACTAA TAATTGGGGG AGAAAAGAAG GGAAAATAAA AAGAAATTAA GAAGAAGAAA AGAAAACACC   
  
  
+ TTTGAAAAGG CAAAAAACTA AAATAAAACA ATAATGAACA GAAGGAGAGA AGGATGGATA AACAACAAAT   
  
  
+ GTCGCTTTTA GGACAGCAAT AGTCTATGTG ATTGGCCTTA GTTCCTGTTG GAGGATTGCT AACAAAAGCA   
  
  
+ ACAGCAAAAA ATAATACTAA TTATTACATA TTTTTTAACT CTTAAAGCTA ATCAATCATC CTCCATTAAT   
  
  
+ TGATTAATTA TTTGCCAAAA ACACAAATCA ACTGGGGCAA CTATAAGAGA TCTACCTGCT TTAAGATATA   
  
  
+ TAAACTCAAT TGTTTCATTT TATTTTATTT TAGATTTTTT GGCCTTTGGT CTCTATTGAG CTAAAATCTC   
  
  
+ GTTATTTTCG GATCATGAAA AATACAACTC GATCTTTTGA TATAAGTCAT CTTTATTTGT AAATTCTGAA   
  
  
+ CTTAAAATCT TACTTGAAGA TCAGGAATAG AAGCACTCGA TTTTTGATAA ATCCTATGTG TTCTTCTTAA   
  
  
+ AATTCATAAT CTAATTCATA TTTTAGATGT ACCCGTTGCC TACTTTGGCA AGTTTAGATT ATGATTTTTA   
  
  
+ TTTTTTTTAA TTACTTGTAT TTTTCTTTTG GATTGATAGT CAATCTACAT CATCTTTGAT CAATTGCGAC   
  
  
+ AGTTTCATTT TCCCAACAAA AATTTCGAGT GTATTCTTTT TAATTTATCA TAAGTATTTA TTGTCAACAT   
  
  
+ TCAACATGCA TCAACAATCA TTAAACACTT TTCGTCACTC CTCAATACTG GTCAGCACTT AAGATTTTGT   
  
  
+ CTTCTAAACT TATCGTAAGT AACTTATTGT TGATTAATTG ATGCTGACCA AATGATACTG AGTGCTAAAA   
  
  
+ TTATAATTAA TAAATACAAT TAAGAGTTGA TCAATGTCGA TGATTCAAAT CAATGTGATT GGTGCTGAAT   
  
  
+ AGTAATTATA AGCACCTATA AAGACTTGAA AAGAACAAAC ATTTAATTAG ATTTTATAAT TATAATTTCC   
  
  
+ TAATATTTAG CATTCATCAG CAAATTCAGC ACACACCAAC ATTAGTCAAC ACCTATCAAC ACTTATAAGT   
  
  
+ ACCAATTAAT AATAATTACC TATAATAGAT TAAAAGTTAG GCCCTTAATG TTTCCCACAT TATTAGATAT   
  
  
+ ATCAAGGGAG GGGGAAAAAA ATAATACTCC CCCATCTTAA AAAATTTATC CCTTTTTTTC TTTTTAATGT   
  
  
+ TCCAAAACAA AAATATGCTT CTAAATTATG ATTGCAATAT GAATGTGTAC GGTTTCAAAC CCAATTTTCT   
  
  
+ CCAACAATTC TTCTACTATA TTGTAATTAT ATTATACTAC AATTTGTATC TCATCTATGA TCTACCCCCC   
  
  
+ AAAAACCAAA TACTCATATA AAGTTTTATT TTCTTTCCAT CCCACTCTAA AAAAAAAAAA TGTTGGGAAA   
  
  
+ AAGTTGATTT ATTTAATTTG AAATGGTACG GTGAATATAG ATTTGTAGAG GGGCAGTTGA TAGAAAAAGG   
  
  
+ TGAAGACAAA GGAAGGGCAC ATTTGCCTTT TCATAGAAGG AAGAGACCAA GGTACTGGGG ACTGGGGGAA   
  
  
+ GGGCTCAAGA GAAGAGAGAG ACTGAGAAGA GTGCTGCTAC TCCTTCCTAC CTACACACAA AATTGCTGCG   
  
  
+ GGGGGTTCAA GTTCAACTTG AACGAGGCAA ATACCCTCAT TGAACCCACC AAATCGAAAT CTTCAATGTA   
  
  
+ CACCCCCATT ATACACGCAT TCAGAGACAT AGATGTCGAG GGGGAGATGA ATTAGCAAGA AGATGTCGAG   
  
  
+ CTAAAATGCA TTTCGAGGTT ACTAGAGCCA GAAATGTGGG CACATCCCAG AAGATCTCAG TAGAGAGAGT   
  
  
+ AACTTCAGAA AAGTTTCAAT TTTTATTCGA AATTTGCCTC TTGTTTGTGT AGATATGATG GGAACTTCGC   
  
  
+ CTTACAATCT GCAATGTGAG GGTTTGGTTG AAGGAGTTGC AGTCAATCCT CAATTTTATG AATCAACCCC   
  
  
+ AAAGTGGAAG AAGGGCATCA AAGAAGAGTC AATTGAGAGC GAACCCACTT CGGTTTTGGA CAACCCTAGC   
  
  
+ CCTCCAAATT CTGCATCTAC TCTCTCTTCC TGCTTCAATG GTGGAAGTAG TGGGCTTGGG ATGGACGATT   
  
  
+ TGGAGGGTTT GTCTTTTGGA GAAGGGTCCC TTCTCCCTTG GTTCATGGGT GAAATTGAAG ACCCTAGTTT   
  
  
+ GAGTTTCAAG CACCTCCTTC AACCTTCAAA TCCCTTGGAG TTTGGTGGCA ATGATGGGCT TGAAGCAGCT   
  
  
+ ATTCAGAGTG CTGGTATGGG TAATTTTAAT TTGGTTGAAA GTTTTAATTC TGATTTGAGG TCTTGTAATT   
  
  
+ CTGGGTCTAT TTGCAATGGG GGGAATGATT CTGTCAATGA AAAGGGATGT GTTCTTAATT ATGCTTCAGA   
  
  
+ GCAGGGTCAT GTGGTTGCTG TATCTCCAGT AGAACAGTTT AGGATTAGTG ATGTGAAACA TGAGGTGTTG   
  
  
+ AACCCAAAAT TTGGCTTTTG TGAGCAAGGC TCTACAATCA GTCAGAACTC TGGTTATGTG CATATACTGG   
  
  
+ GTTATAACCA GCTAGAATCT CCCCTTGAGC CTCCGGCAAA GCGCCACAAT GCTGGGAATG CTCTTTACAC   
  
  
+ TAGTGTTGTT CCAGTTCCAA AGAGTTCGTT TTTAGGTGAG GATCATGAAA TTCTACTCAG AAATCAAGAG   
  
  
+ CAGCAGTTTC TGATGCAACA ACAGTGGTTA ATGGGTTTAG CCCCTCAGTT TACACCCCAG CACCCGCCAA   
  
  
+ AGCCATTAGA CGACCGAAAG CAACCAAACT CGCAAACTTT GGTTCTGCGC GATCAGTTAG TTAAGATTGC   
  
  
+ GGAGATGTTC CAAACCGGTA ACTTTTCGCT TGCCCAAGTG ATATTGGCCC GGCTCAATCA GCAATTGTCT   
  
  
+ CTTTCAGGGA ACCCCTTTGT AAGAGCTGCT ACTTTCCATG TCAAGGAGGA ACTTGAAAGG CTCCTGACTA   
  
  
+ TCAATAGCTT CTCTGCGGCT CCACCCCAAC CTAAAAGCCT TACTCTGTCA GATGTTGTGG ATAGGATGAA   
  
  
+ TGCATACAAG CTTTTTTCAG AGGCATCCCC AGTTATGCAA TTTACCGATT TTACTTGTAC CCAGGCTTTG   
  
  
+ CTCGAAGCTC TTGATGATGC TGATTATATC CACATACTAG ATTTTGACAT TGGTTGTGGT TCCCAATGGG   
  
  
+ CATCCTTTAT TCGAGAGCTT CCTCTCAGGA AAAGGGGCAC TCCATTTTTG AAAATTACAG CCTTTGCCTC   
  
  
+ TTCTTTAACC CATAATCCAT TTGAACTAGC CCTTGTATGT GATAACATTC TGGAATTTGC AAAAGAGTTC   
  
  
+ GGTGTCTCTG TCGACCTTCA GGTCATGAAT TTGGACTTGT TTGACCCAAC TACTTGCACA ATACCCAATT   
  
  
+ TTCAATCCAC TGAGAACGCA GCAGTTGCTG TCAACTTCCC TATATGGGCG TGCTCACACT GCCCGTTTGT   
  
  
+ ACTCAGTCAA CTCCTGTCCT TCATCAAGCA ATGTTCCCCA AAAATCATGA CGAGCTTTGA TAGAGGTTGT   
  
  
+ GATCATTTTG AGTTGTCTTT TCCATCGCAC ATCATCCGTG TATTGGACTC ATGCTCTAAT ATGTTGGAGT   
  
  
+ CCCTCCATGG TTCAAGCGTG ACATTAGACA CCTCAAAGAA GATTGAAAAG TTCTTGATCA AGCCTATAAT   
  
  
+ TGAGAGGGCA ATATTGGGGC GAATTCATGC ACCAGACAAG ATGCAGGCAA ACAAGGCACA CCGGTGGAAG   
  
  
+ AATCTCTTCA CTTCTGCTGG GTTCTTCCCT TTGCCATTTA GTAGTTTCAC TGAATCTCAG GCCAATCTAG   
  
  
+ TGGCTCAACG AACCCCAGTG AGGGGTTTTC AGGTGGAAAA GCAGCAAGCA TCACTTGTGC TCAGGTGGAT   
  
  
+ GTCTCATGAG CTCGTGGCAG CTTCAGCTTG GAGGTGCTA  

- -Up\_Stream \_Len000AAACAT AAATTTAAAA TGTTATAAAT ATTTTTAAAA CATAACTAAT TAACACGATT   
  
  
- GATTGTGATT ATTAACCCCC TCTTTTCTTC CCTTTTATTT TTCTTTAATT CTTCTTCTTT TCTTTTGTGG   
  
  
- AAACTTTTCC GTTTTTTGAT TTTATTTTGT TATTACTTGT CTTCCTCTCT TCCTACCTAT TTGTTGTTTA   
  
  
- CAGCGAAAAT CCTGTCGTTA TCAGATACAC TAACCGGAAT CAAGGACAAC CTCCTAACGA TTGTTTTCGT   
  
  
- TGTCGTTTTT TATTATGATT AATAATGTAT AAAAAATTGA GAATTTCGAT TAGTTAGTAG GAGGTAATTA   
  
  
- ACTAATTAAT AAACGGTTTT TGTGTTTAGT TGACCCCGTT GATATTCTCT AGATGGACGA AATTCTATAT   
  
  
- ATTTGAGTTA ACAAAGTAAA ATAAAATAAA ATCTAAAAAA CCGGAAACCA GAGATAACTC GATTTTAGAG   
  
  
- CAATAAAAGC CTAGTACTTT TTATGTTGAG CTAGAAAACT ATATTCAGTA GAAATAAACA TTTAAGACTT   
  
  
- GAATTTTAGA ATGAACTTCT AGTCCTTATC TTCGTGAGCT AAAAACTATT TAGGATACAC AAGAAGAATT   
  
  
- TTAAGTATTA GATTAAGTAT AAAATCTACA TGGGCAACGG ATGAAACCGT TCAAATCTAA TACTAAAAAT   
  
  
- AAAAAAAATT AATGAACATA AAAAGAAAAC CTAACTATCA GTTAGATGTA GTAGAAACTA GTTAACGCTG   
  
  
- TCAAAGTAAA AGGGTTGTTT TTAAAGCTCA CATAAGAAAA ATTAAATAGT ATTCATAAAT AACAGTTGTA   
  
  
- AGTTGTACGT AGTTGTTAGT AATTTGTGAA AAGCAGTGAG GAGTTATGAC CAGTCGTGAA TTCTAAAACA   
  
  
- GAAGATTTGA ATAGCATTCA TTGAATAACA ACTAATTAAC TACGACTGGT TTACTATGAC TCACGATTTT   
  
  
- AATATTAATT ATTTATGTTA ATTCTCAACT AGTTACAGCT ACTAAGTTTA GTTACACTAA CCACGACTTA   
  
  
- TCATTAATAT TCGTGGATAT TTCTGAACTT TTCTTGTTTG TAAATTAATC TAAAATATTA ATATTAAAGG   
  
  
- ATTATAAATC GTAAGTAGTC GTTTAAGTCG TGTGTGGTTG TAATCAGTTG TGGATAGTTG TGAATATTCA   
  
  
- TGGTTAATTA TTATTAATGG ATATTATCTA ATTTTCAATC CGGGAATTAC AAAGGGTGTA ATAATCTATA   
  
  
- TAGTTCCCTC CCCCTTTTTT TATTATGAGG GGGTAGAATT TTTTAAATAG GGAAAAAAAG AAAAATTACA   
  
  
- AGGTTTTGTT TTTATACGAA GATTTAATAC TAACGTTATA CTTACACATG CCAAAGTTTG GGTTAAAAGA   
  
  
- GGTTGTTAAG AAGATGATAT AACATTAATA TAATATGATG TTAAACATAG AGTAGATACT AGATGGGGGG   
  
  
- TTTTTGGTTT ATGAGTATAT TTCAAAATAA AAGAAAGGTA GGGTGAGATT TTTTTTTTTT ACAACCCTTT   
  
  
- TTCAACTAAA TAAATTAAAC TTTACCATGC CACTTATATC TAAACATCTC CCCGTCAACT ATCTTTTTCC   
  
  
- ACTTCTGTTT CCTTCCCGTG TAAACGGAAA AGTATCTTCC TTCTCTGGTT CCATGACCCC TGACCCCCTT   
  
  
- CCCGAGTTCT CTTCTCTCTC TGACTCTTCT CACGACGATG AGGAAGGATG GATGTGTGTT TTAACGACGC   
  
  
- CCCCCAAGTT CAAGTTGAAC TTGCTCCGTT TATGGGAGTA ACTTGGGTGG TTTAGCTTTA GAAGTTACAT   
  
  
- GTGGGGGTAA TATGTGCGTA AGTCTCTGTA TCTACAGCTC CCCCTCTACT TAATCGTTCT TCTACAGCTC   
  
  
- GATTTTACGT AAAGCTCCAA TGATCTCGGT CTTTACACCC GTGTAGGGTC TTCTAGAGTC ATCTCTCTCA   
  
  
- TTGAAGTCTT TTCAAAGTTA AAAATAAGCT TTAAACGGAG AACAAACACA TCTATACTAC CCTTGAAGCG   
  
  
- GAATGTTAGA CGTTACACTC CCAAACCAAC TTCCTCAACG TCAGTTAGGA GTTAAAATAC TTAGTTGGGG   
  
  
- TTTCACCTTC TTCCCGTAGT TTCTTCTCAG TTAACTCTCG CTTGGGTGAA GCCAAAACCT GTTGGGATCG   
  
  
- GGAGGTTTAA GACGTAGATG AGAGAGAAGG ACGAAGTTAC CACCTTCATC ACCCGAACCC TACCTGCTAA   
  
  
- ACCTCCCAAA CAGAAAACCT CTTCCCAGGG AAGAGGGAAC CAAGTACCCA CTTTAACTTC TGGGATCAAA   
  
  
- CTCAAAGTTC GTGGAGGAAG TTGGAAGTTT AGGGAACCTC AAACCACCGT TACTACCCGA ACTTCGTCGA   
  
  
- TAAGTCTCAC GACCATACCC ATTAAAATTA AACCAACTTT CAAAATTAAG ACTAAACTCC AGAACATTAA   
  
  
- GACCCAGATA AACGTTACCC CCCTTACTAA GACAGTTACT TTTCCCTACA CAAGAATTAA TACGAAGTCT   
  
  
- CGTCCCAGTA CACCAACGAC ATAGAGGTCA TCTTGTCAAA TCCTAATCAC TACACTTTGT ACTCCACAAC   
  
  
- TTGGGTTTTA AACCGAAAAC ACTCGTTCCG AGATGTTAGT CAGTCTTGAG ACCAATACAC GTATATGACC   
  
  
- CAATATTGGT CGATCTTAGA GGGGAACTCG GAGGCCGTTT CGCGGTGTTA CGACCCTTAC GAGAAATGTG   
  
  
- ATCACAACAA GGTCAAGGTT TCTCAAGCAA AAATCCACTC CTAGTACTTT AAGATGAGTC TTTAGTTCTC   
  
  
- GTCGTCAAAG ACTACGTTGT TGTCACCAAT TACCCAAATC GGGGAGTCAA ATGTGGGGTC GTGGGCGGTT   
  
  
- TCGGTAATCT GCTGGCTTTC GTTGGTTTGA GCGTTTGAAA CCAAGACGCG CTAGTCAATC AATTCTAACG   
  
  
- CCTCTACAAG GTTTGGCCAT TGAAAAGCGA ACGGGTTCAC TATAACCGGG CCGAGTTAGT CGTTAACAGA   
  
  
- GAAAGTCCCT TGGGGAAACA TTCTCGACGA TGAAAGGTAC AGTTCCTCCT TGAACTTTCC GAGGACTGAT   
  
  
- AGTTATCGAA GAGACGCCGA GGTGGGGTTG GATTTTCGGA ATGAGACAGT CTACAACACC TATCCTACTT   
  
  
- ACGTATGTTC GAAAAAAGTC TCCGTAGGGG TCAATACGTT AAATGGCTAA AATGAACATG GGTCCGAAAC   
  
  
- GAGCTTCGAG AACTACTACG ACTAATATAG GTGTATGATC TAAAACTGTA ACCAACACCA AGGGTTACCC   
  
  
- GTAGGAAATA AGCTCTCGAA GGAGAGTCCT TTTCCCCGTG AGGTAAAAAC TTTTAATGTC GGAAACGGAG   
  
  
- AAGAAATTGG GTATTAGGTA AACTTGATCG GGAACATACA CTATTGTAAG ACCTTAAACG TTTTCTCAAG   
  
  
- CCACAGAGAC AGCTGGAAGT CCAGTACTTA AACCTGAACA AACTGGGTTG ATGAACGTGT TATGGGTTAA   
  
  
- AAGTTAGGTG ACTCTTGCGT CGTCAACGAC AGTTGAAGGG ATATACCCGC ACGAGTGTGA CGGGCAAACA   
  
  
- TGAGTCAGTT GAGGACAGGA AGTAGTTCGT TACAAGGGGT TTTTAGTACT GCTCGAAACT ATCTCCAACA   
  
  
- CTAGTAAAAC TCAACAGAAA AGGTAGCGTG TAGTAGGCAC ATAACCTGAG TACGAGATTA TACAACCTCA   
  
  
- GGGAGGTACC AAGTTCGCAC TGTAATCTGT GGAGTTTCTT CTAACTTTTC AAGAACTAGT TCGGATATTA   
  
  
- ACTCTCCCGT TATAACCCCG CTTAAGTACG TGGTCTGTTC TACGTCCGTT TGTTCCGTGT GGCCACCTTC   
  
  
- TTAGAGAAGT GAAGACGACC CAAGAAGGGA AACGGTAAAT CATCAAAGTG ACTTAGAGTC CGGTTAGATC   
  
  
- ACCGAGTTGC TTGGGGTCAC TCCCCAAAAG TCCACCTTTT CGTCGTTCGT AGTGAACACG AGTCCACCTA   
  
  
- CAGAGTACTC GAGCACCGTC GAAGTCGAAC CTCCACGAT

+     STRE

| Site Name | Organism | Position | Strand | Matrix score. | sequence | function |
| --- | --- | --- | --- | --- | --- | --- |
| STRE | Arabidopsis thaliana | 3327 | + | 5 | AGGGG |  |
| STRE | Arabidopsis thaliana | 3945 | + | 5 | AGGGG |  |
| STRE | Arabidopsis thaliana | 3026 | - | 5 | AGGGG |  |
| STRE | Arabidopsis thaliana | 2845 | - | 5 | AGGGG |  |
| STRE | Arabidopsis thaliana | 2685 | - | 5 | AGGGG |  |
| STRE | Arabidopsis thaliana | 1863 | + | 5 | AGGGG |  |
| STRE | Arabidopsis thaliana | 1593 | + | 5 | AGGGG |  |
| STRE | Arabidopsis thaliana | 1273 | + | 5 | AGGGG |  |

>HU04G00048.1   
+ -Up\_Stream \_Len000TTTGTA TTTAAATTTT ACAATATTTA TAAAAATTTT GTATTGATTA ATTGTGCTAA   
  
  
+ CTAACACTAA TAATTGGGGG AGAAAAGAAG GGAAAATAAA AAGAAATTAA GAAGAAGAAA AGAAAACACC   
  
  
+ TTTGAAAAGG CAAAAAACTA AAATAAAACA ATAATGAACA GAAGGAGAGA AGGATGGATA AACAACAAAT   
  
  
+ GTCGCTTTTA GGACAGCAAT AGTCTATGTG ATTGGCCTTA GTTCCTGTTG GAGGATTGCT AACAAAAGCA   
  
  
+ ACAGCAAAAA ATAATACTAA TTATTACATA TTTTTTAACT CTTAAAGCTA ATCAATCATC CTCCATTAAT   
  
  
+ TGATTAATTA TTTGCCAAAA ACACAAATCA ACTGGGGCAA CTATAAGAGA TCTACCTGCT TTAAGATATA   
  
  
+ TAAACTCAAT TGTTTCATTT TATTTTATTT TAGATTTTTT GGCCTTTGGT CTCTATTGAG CTAAAATCTC   
  
  
+ GTTATTTTCG GATCATGAAA AATACAACTC GATCTTTTGA TATAAGTCAT CTTTATTTGT AAATTCTGAA   
  
  
+ CTTAAAATCT TACTTGAAGA TCAGGAATAG AAGCACTCGA TTTTTGATAA ATCCTATGTG TTCTTCTTAA   
  
  
+ AATTCATAAT CTAATTCATA TTTTAGATGT ACCCGTTGCC TACTTTGGCA AGTTTAGATT ATGATTTTTA   
  
  
+ TTTTTTTTAA TTACTTGTAT TTTTCTTTTG GATTGATAGT CAATCTACAT CATCTTTGAT CAATTGCGAC   
  
  
+ AGTTTCATTT TCCCAACAAA AATTTCGAGT GTATTCTTTT TAATTTATCA TAAGTATTTA TTGTCAACAT   
  
  
+ TCAACATGCA TCAACAATCA TTAAACACTT TTCGTCACTC CTCAATACTG GTCAGCACTT AAGATTTTGT   
  
  
+ CTTCTAAACT TATCGTAAGT AACTTATTGT TGATTAATTG ATGCTGACCA AATGATACTG AGTGCTAAAA   
  
  
+ TTATAATTAA TAAATACAAT TAAGAGTTGA TCAATGTCGA TGATTCAAAT CAATGTGATT GGTGCTGAAT   
  
  
+ AGTAATTATA AGCACCTATA AAGACTTGAA AAGAACAAAC ATTTAATTAG ATTTTATAAT TATAATTTCC   
  
  
+ TAATATTTAG CATTCATCAG CAAATTCAGC ACACACCAAC ATTAGTCAAC ACCTATCAAC ACTTATAAGT   
  
  
+ ACCAATTAAT AATAATTACC TATAATAGAT TAAAAGTTAG GCCCTTAATG TTTCCCACAT TATTAGATAT   
  
  
+ ATCAAGGGAG GGGGAAAAAA ATAATACTCC CCCATCTTAA AAAATTTATC CCTTTTTTTC TTTTTAATGT   
  
  
+ TCCAAAACAA AAATATGCTT CTAAATTATG ATTGCAATAT GAATGTGTAC GGTTTCAAAC CCAATTTTCT   
  
  
+ CCAACAATTC TTCTACTATA TTGTAATTAT ATTATACTAC AATTTGTATC TCATCTATGA TCTACCCCCC   
  
  
+ AAAAACCAAA TACTCATATA AAGTTTTATT TTCTTTCCAT CCCACTCTAA AAAAAAAAAA TGTTGGGAAA   
  
  
+ AAGTTGATTT ATTTAATTTG AAATGGTACG GTGAATATAG ATTTGTAGAG GGGCAGTTGA TAGAAAAAGG   
  
  
+ TGAAGACAAA GGAAGGGCAC ATTTGCCTTT TCATAGAAGG AAGAGACCAA GGTACTGGGG ACTGGGGGAA   
  
  
+ GGGCTCAAGA GAAGAGAGAG ACTGAGAAGA GTGCTGCTAC TCCTTCCTAC CTACACACAA AATTGCTGCG   
  
  
+ GGGGGTTCAA GTTCAACTTG AACGAGGCAA ATACCCTCAT TGAACCCACC AAATCGAAAT CTTCAATGTA   
  
  
+ CACCCCCATT ATACACGCAT TCAGAGACAT AGATGTCGAG GGGGAGATGA ATTAGCAAGA AGATGTCGAG   
  
  
+ CTAAAATGCA TTTCGAGGTT ACTAGAGCCA GAAATGTGGG CACATCCCAG AAGATCTCAG TAGAGAGAGT   
  
  
+ AACTTCAGAA AAGTTTCAAT TTTTATTCGA AATTTGCCTC TTGTTTGTGT AGATATGATG GGAACTTCGC   
  
  
+ CTTACAATCT GCAATGTGAG GGTTTGGTTG AAGGAGTTGC AGTCAATCCT CAATTTTATG AATCAACCCC   
  
  
+ AAAGTGGAAG AAGGGCATCA AAGAAGAGTC AATTGAGAGC GAACCCACTT CGGTTTTGGA CAACCCTAGC   
  
  
+ CCTCCAAATT CTGCATCTAC TCTCTCTTCC TGCTTCAATG GTGGAAGTAG TGGGCTTGGG ATGGACGATT   
  
  
+ TGGAGGGTTT GTCTTTTGGA GAAGGGTCCC TTCTCCCTTG GTTCATGGGT GAAATTGAAG ACCCTAGTTT   
  
  
+ GAGTTTCAAG CACCTCCTTC AACCTTCAAA TCCCTTGGAG TTTGGTGGCA ATGATGGGCT TGAAGCAGCT   
  
  
+ ATTCAGAGTG CTGGTATGGG TAATTTTAAT TTGGTTGAAA GTTTTAATTC TGATTTGAGG TCTTGTAATT   
  
  
+ CTGGGTCTAT TTGCAATGGG GGGAATGATT CTGTCAATGA AAAGGGATGT GTTCTTAATT ATGCTTCAGA   
  
  
+ GCAGGGTCAT GTGGTTGCTG TATCTCCAGT AGAACAGTTT AGGATTAGTG ATGTGAAACA TGAGGTGTTG   
  
  
+ AACCCAAAAT TTGGCTTTTG TGAGCAAGGC TCTACAATCA GTCAGAACTC TGGTTATGTG CATATACTGG   
  
  
+ GTTATAACCA GCTAGAATCT CCCCTTGAGC CTCCGGCAAA GCGCCACAAT GCTGGGAATG CTCTTTACAC   
  
  
+ TAGTGTTGTT CCAGTTCCAA AGAGTTCGTT TTTAGGTGAG GATCATGAAA TTCTACTCAG AAATCAAGAG   
  
  
+ CAGCAGTTTC TGATGCAACA ACAGTGGTTA ATGGGTTTAG CCCCTCAGTT TACACCCCAG CACCCGCCAA   
  
  
+ AGCCATTAGA CGACCGAAAG CAACCAAACT CGCAAACTTT GGTTCTGCGC GATCAGTTAG TTAAGATTGC   
  
  
+ GGAGATGTTC CAAACCGGTA ACTTTTCGCT TGCCCAAGTG ATATTGGCCC GGCTCAATCA GCAATTGTCT   
  
  
+ CTTTCAGGGA ACCCCTTTGT AAGAGCTGCT ACTTTCCATG TCAAGGAGGA ACTTGAAAGG CTCCTGACTA   
  
  
+ TCAATAGCTT CTCTGCGGCT CCACCCCAAC CTAAAAGCCT TACTCTGTCA GATGTTGTGG ATAGGATGAA   
  
  
+ TGCATACAAG CTTTTTTCAG AGGCATCCCC AGTTATGCAA TTTACCGATT TTACTTGTAC CCAGGCTTTG   
  
  
+ CTCGAAGCTC TTGATGATGC TGATTATATC CACATACTAG ATTTTGACAT TGGTTGTGGT TCCCAATGGG   
  
  
+ CATCCTTTAT TCGAGAGCTT CCTCTCAGGA AAAGGGGCAC TCCATTTTTG AAAATTACAG CCTTTGCCTC   
  
  
+ TTCTTTAACC CATAATCCAT TTGAACTAGC CCTTGTATGT GATAACATTC TGGAATTTGC AAAAGAGTTC   
  
  
+ GGTGTCTCTG TCGACCTTCA GGTCATGAAT TTGGACTTGT TTGACCCAAC TACTTGCACA ATACCCAATT   
  
  
+ TTCAATCCAC TGAGAACGCA GCAGTTGCTG TCAACTTCCC TATATGGGCG TGCTCACACT GCCCGTTTGT   
  
  
+ ACTCAGTCAA CTCCTGTCCT TCATCAAGCA ATGTTCCCCA AAAATCATGA CGAGCTTTGA TAGAGGTTGT   
  
  
+ GATCATTTTG AGTTGTCTTT TCCATCGCAC ATCATCCGTG TATTGGACTC ATGCTCTAAT ATGTTGGAGT   
  
  
+ CCCTCCATGG TTCAAGCGTG ACATTAGACA CCTCAAAGAA GATTGAAAAG TTCTTGATCA AGCCTATAAT   
  
  
+ TGAGAGGGCA ATATTGGGGC GAATTCATGC ACCAGACAAG ATGCAGGCAA ACAAGGCACA CCGGTGGAAG   
  
  
+ AATCTCTTCA CTTCTGCTGG GTTCTTCCCT TTGCCATTTA GTAGTTTCAC TGAATCTCAG GCCAATCTAG   
  
  
+ TGGCTCAACG AACCCCAGTG AGGGGTTTTC AGGTGGAAAA GCAGCAAGCA TCACTTGTGC TCAGGTGGAT   
  
  
+ GTCTCATGAG CTCGTGGCAG CTTCAGCTTG GAGGTGCTA  

- -Up\_Stream \_Len000AAACAT AAATTTAAAA TGTTATAAAT ATTTTTAAAA CATAACTAAT TAACACGATT   
  
  
- GATTGTGATT ATTAACCCCC TCTTTTCTTC CCTTTTATTT TTCTTTAATT CTTCTTCTTT TCTTTTGTGG   
  
  
- AAACTTTTCC GTTTTTTGAT TTTATTTTGT TATTACTTGT CTTCCTCTCT TCCTACCTAT TTGTTGTTTA   
  
  
- CAGCGAAAAT CCTGTCGTTA TCAGATACAC TAACCGGAAT CAAGGACAAC CTCCTAACGA TTGTTTTCGT   
  
  
- TGTCGTTTTT TATTATGATT AATAATGTAT AAAAAATTGA GAATTTCGAT TAGTTAGTAG GAGGTAATTA   
  
  
- ACTAATTAAT AAACGGTTTT TGTGTTTAGT TGACCCCGTT GATATTCTCT AGATGGACGA AATTCTATAT   
  
  
- ATTTGAGTTA ACAAAGTAAA ATAAAATAAA ATCTAAAAAA CCGGAAACCA GAGATAACTC GATTTTAGAG   
  
  
- CAATAAAAGC CTAGTACTTT TTATGTTGAG CTAGAAAACT ATATTCAGTA GAAATAAACA TTTAAGACTT   
  
  
- GAATTTTAGA ATGAACTTCT AGTCCTTATC TTCGTGAGCT AAAAACTATT TAGGATACAC AAGAAGAATT   
  
  
- TTAAGTATTA GATTAAGTAT AAAATCTACA TGGGCAACGG ATGAAACCGT TCAAATCTAA TACTAAAAAT   
  
  
- AAAAAAAATT AATGAACATA AAAAGAAAAC CTAACTATCA GTTAGATGTA GTAGAAACTA GTTAACGCTG   
  
  
- TCAAAGTAAA AGGGTTGTTT TTAAAGCTCA CATAAGAAAA ATTAAATAGT ATTCATAAAT AACAGTTGTA   
  
  
- AGTTGTACGT AGTTGTTAGT AATTTGTGAA AAGCAGTGAG GAGTTATGAC CAGTCGTGAA TTCTAAAACA   
  
  
- GAAGATTTGA ATAGCATTCA TTGAATAACA ACTAATTAAC TACGACTGGT TTACTATGAC TCACGATTTT   
  
  
- AATATTAATT ATTTATGTTA ATTCTCAACT AGTTACAGCT ACTAAGTTTA GTTACACTAA CCACGACTTA   
  
  
- TCATTAATAT TCGTGGATAT TTCTGAACTT TTCTTGTTTG TAAATTAATC TAAAATATTA ATATTAAAGG   
  
  
- ATTATAAATC GTAAGTAGTC GTTTAAGTCG TGTGTGGTTG TAATCAGTTG TGGATAGTTG TGAATATTCA   
  
  
- TGGTTAATTA TTATTAATGG ATATTATCTA ATTTTCAATC CGGGAATTAC AAAGGGTGTA ATAATCTATA   
  
  
- TAGTTCCCTC CCCCTTTTTT TATTATGAGG GGGTAGAATT TTTTAAATAG GGAAAAAAAG AAAAATTACA   
  
  
- AGGTTTTGTT TTTATACGAA GATTTAATAC TAACGTTATA CTTACACATG CCAAAGTTTG GGTTAAAAGA   
  
  
- GGTTGTTAAG AAGATGATAT AACATTAATA TAATATGATG TTAAACATAG AGTAGATACT AGATGGGGGG   
  
  
- TTTTTGGTTT ATGAGTATAT TTCAAAATAA AAGAAAGGTA GGGTGAGATT TTTTTTTTTT ACAACCCTTT   
  
  
- TTCAACTAAA TAAATTAAAC TTTACCATGC CACTTATATC TAAACATCTC CCCGTCAACT ATCTTTTTCC   
  
  
- ACTTCTGTTT CCTTCCCGTG TAAACGGAAA AGTATCTTCC TTCTCTGGTT CCATGACCCC TGACCCCCTT   
  
  
- CCCGAGTTCT CTTCTCTCTC TGACTCTTCT CACGACGATG AGGAAGGATG GATGTGTGTT TTAACGACGC   
  
  
- CCCCCAAGTT CAAGTTGAAC TTGCTCCGTT TATGGGAGTA ACTTGGGTGG TTTAGCTTTA GAAGTTACAT   
  
  
- GTGGGGGTAA TATGTGCGTA AGTCTCTGTA TCTACAGCTC CCCCTCTACT TAATCGTTCT TCTACAGCTC   
  
  
- GATTTTACGT AAAGCTCCAA TGATCTCGGT CTTTACACCC GTGTAGGGTC TTCTAGAGTC ATCTCTCTCA   
  
  
- TTGAAGTCTT TTCAAAGTTA AAAATAAGCT TTAAACGGAG AACAAACACA TCTATACTAC CCTTGAAGCG   
  
  
- GAATGTTAGA CGTTACACTC CCAAACCAAC TTCCTCAACG TCAGTTAGGA GTTAAAATAC TTAGTTGGGG   
  
  
- TTTCACCTTC TTCCCGTAGT TTCTTCTCAG TTAACTCTCG CTTGGGTGAA GCCAAAACCT GTTGGGATCG   
  
  
- GGAGGTTTAA GACGTAGATG AGAGAGAAGG ACGAAGTTAC CACCTTCATC ACCCGAACCC TACCTGCTAA   
  
  
- ACCTCCCAAA CAGAAAACCT CTTCCCAGGG AAGAGGGAAC CAAGTACCCA CTTTAACTTC TGGGATCAAA   
  
  
- CTCAAAGTTC GTGGAGGAAG TTGGAAGTTT AGGGAACCTC AAACCACCGT TACTACCCGA ACTTCGTCGA   
  
  
- TAAGTCTCAC GACCATACCC ATTAAAATTA AACCAACTTT CAAAATTAAG ACTAAACTCC AGAACATTAA   
  
  
- GACCCAGATA AACGTTACCC CCCTTACTAA GACAGTTACT TTTCCCTACA CAAGAATTAA TACGAAGTCT   
  
  
- CGTCCCAGTA CACCAACGAC ATAGAGGTCA TCTTGTCAAA TCCTAATCAC TACACTTTGT ACTCCACAAC   
  
  
- TTGGGTTTTA AACCGAAAAC ACTCGTTCCG AGATGTTAGT CAGTCTTGAG ACCAATACAC GTATATGACC   
  
  
- CAATATTGGT CGATCTTAGA GGGGAACTCG GAGGCCGTTT CGCGGTGTTA CGACCCTTAC GAGAAATGTG   
  
  
- ATCACAACAA GGTCAAGGTT TCTCAAGCAA AAATCCACTC CTAGTACTTT AAGATGAGTC TTTAGTTCTC   
  
  
- GTCGTCAAAG ACTACGTTGT TGTCACCAAT TACCCAAATC GGGGAGTCAA ATGTGGGGTC GTGGGCGGTT   
  
  
- TCGGTAATCT GCTGGCTTTC GTTGGTTTGA GCGTTTGAAA CCAAGACGCG CTAGTCAATC AATTCTAACG   
  
  
- CCTCTACAAG GTTTGGCCAT TGAAAAGCGA ACGGGTTCAC TATAACCGGG CCGAGTTAGT CGTTAACAGA   
  
  
- GAAAGTCCCT TGGGGAAACA TTCTCGACGA TGAAAGGTAC AGTTCCTCCT TGAACTTTCC GAGGACTGAT   
  
  
- AGTTATCGAA GAGACGCCGA GGTGGGGTTG GATTTTCGGA ATGAGACAGT CTACAACACC TATCCTACTT   
  
  
- ACGTATGTTC GAAAAAAGTC TCCGTAGGGG TCAATACGTT AAATGGCTAA AATGAACATG GGTCCGAAAC   
  
  
- GAGCTTCGAG AACTACTACG ACTAATATAG GTGTATGATC TAAAACTGTA ACCAACACCA AGGGTTACCC   
  
  
- GTAGGAAATA AGCTCTCGAA GGAGAGTCCT TTTCCCCGTG AGGTAAAAAC TTTTAATGTC GGAAACGGAG   
  
  
- AAGAAATTGG GTATTAGGTA AACTTGATCG GGAACATACA CTATTGTAAG ACCTTAAACG TTTTCTCAAG   
  
  
- CCACAGAGAC AGCTGGAAGT CCAGTACTTA AACCTGAACA AACTGGGTTG ATGAACGTGT TATGGGTTAA   
  
  
- AAGTTAGGTG ACTCTTGCGT CGTCAACGAC AGTTGAAGGG ATATACCCGC ACGAGTGTGA CGGGCAAACA   
  
  
- TGAGTCAGTT GAGGACAGGA AGTAGTTCGT TACAAGGGGT TTTTAGTACT GCTCGAAACT ATCTCCAACA   
  
  
- CTAGTAAAAC TCAACAGAAA AGGTAGCGTG TAGTAGGCAC ATAACCTGAG TACGAGATTA TACAACCTCA   
  
  
- GGGAGGTACC AAGTTCGCAC TGTAATCTGT GGAGTTTCTT CTAACTTTTC AAGAACTAGT TCGGATATTA   
  
  
- ACTCTCCCGT TATAACCCCG CTTAAGTACG TGGTCTGTTC TACGTCCGTT TGTTCCGTGT GGCCACCTTC   
  
  
- TTAGAGAAGT GAAGACGACC CAAGAAGGGA AACGGTAAAT CATCAAAGTG ACTTAGAGTC CGGTTAGATC   
  
  
- ACCGAGTTGC TTGGGGTCAC TCCCCAAAAG TCCACCTTTT CGTCGTTCGT AGTGAACACG AGTCCACCTA   
  
  
- CAGAGTACTC GAGCACCGTC GAAGTCGAAC CTCCACGAT

+     TATA

| Site Name | Organism | Position | Strand | Matrix score. | sequence | function |
| --- | --- | --- | --- | --- | --- | --- |
| TATA | Arabidopsis thaliana | 1105 | - | 8 | TATAAAAT |  |

>HU04G00048.1   
+ -Up\_Stream \_Len000TTTGTA TTTAAATTTT ACAATATTTA TAAAAATTTT GTATTGATTA ATTGTGCTAA   
  
  
+ CTAACACTAA TAATTGGGGG AGAAAAGAAG GGAAAATAAA AAGAAATTAA GAAGAAGAAA AGAAAACACC   
  
  
+ TTTGAAAAGG CAAAAAACTA AAATAAAACA ATAATGAACA GAAGGAGAGA AGGATGGATA AACAACAAAT   
  
  
+ GTCGCTTTTA GGACAGCAAT AGTCTATGTG ATTGGCCTTA GTTCCTGTTG GAGGATTGCT AACAAAAGCA   
  
  
+ ACAGCAAAAA ATAATACTAA TTATTACATA TTTTTTAACT CTTAAAGCTA ATCAATCATC CTCCATTAAT   
  
  
+ TGATTAATTA TTTGCCAAAA ACACAAATCA ACTGGGGCAA CTATAAGAGA TCTACCTGCT TTAAGATATA   
  
  
+ TAAACTCAAT TGTTTCATTT TATTTTATTT TAGATTTTTT GGCCTTTGGT CTCTATTGAG CTAAAATCTC   
  
  
+ GTTATTTTCG GATCATGAAA AATACAACTC GATCTTTTGA TATAAGTCAT CTTTATTTGT AAATTCTGAA   
  
  
+ CTTAAAATCT TACTTGAAGA TCAGGAATAG AAGCACTCGA TTTTTGATAA ATCCTATGTG TTCTTCTTAA   
  
  
+ AATTCATAAT CTAATTCATA TTTTAGATGT ACCCGTTGCC TACTTTGGCA AGTTTAGATT ATGATTTTTA   
  
  
+ TTTTTTTTAA TTACTTGTAT TTTTCTTTTG GATTGATAGT CAATCTACAT CATCTTTGAT CAATTGCGAC   
  
  
+ AGTTTCATTT TCCCAACAAA AATTTCGAGT GTATTCTTTT TAATTTATCA TAAGTATTTA TTGTCAACAT   
  
  
+ TCAACATGCA TCAACAATCA TTAAACACTT TTCGTCACTC CTCAATACTG GTCAGCACTT AAGATTTTGT   
  
  
+ CTTCTAAACT TATCGTAAGT AACTTATTGT TGATTAATTG ATGCTGACCA AATGATACTG AGTGCTAAAA   
  
  
+ TTATAATTAA TAAATACAAT TAAGAGTTGA TCAATGTCGA TGATTCAAAT CAATGTGATT GGTGCTGAAT   
  
  
+ AGTAATTATA AGCACCTATA AAGACTTGAA AAGAACAAAC ATTTAATTAG ATTTTATAAT TATAATTTCC   
  
  
+ TAATATTTAG CATTCATCAG CAAATTCAGC ACACACCAAC ATTAGTCAAC ACCTATCAAC ACTTATAAGT   
  
  
+ ACCAATTAAT AATAATTACC TATAATAGAT TAAAAGTTAG GCCCTTAATG TTTCCCACAT TATTAGATAT   
  
  
+ ATCAAGGGAG GGGGAAAAAA ATAATACTCC CCCATCTTAA AAAATTTATC CCTTTTTTTC TTTTTAATGT   
  
  
+ TCCAAAACAA AAATATGCTT CTAAATTATG ATTGCAATAT GAATGTGTAC GGTTTCAAAC CCAATTTTCT   
  
  
+ CCAACAATTC TTCTACTATA TTGTAATTAT ATTATACTAC AATTTGTATC TCATCTATGA TCTACCCCCC   
  
  
+ AAAAACCAAA TACTCATATA AAGTTTTATT TTCTTTCCAT CCCACTCTAA AAAAAAAAAA TGTTGGGAAA   
  
  
+ AAGTTGATTT ATTTAATTTG AAATGGTACG GTGAATATAG ATTTGTAGAG GGGCAGTTGA TAGAAAAAGG   
  
  
+ TGAAGACAAA GGAAGGGCAC ATTTGCCTTT TCATAGAAGG AAGAGACCAA GGTACTGGGG ACTGGGGGAA   
  
  
+ GGGCTCAAGA GAAGAGAGAG ACTGAGAAGA GTGCTGCTAC TCCTTCCTAC CTACACACAA AATTGCTGCG   
  
  
+ GGGGGTTCAA GTTCAACTTG AACGAGGCAA ATACCCTCAT TGAACCCACC AAATCGAAAT CTTCAATGTA   
  
  
+ CACCCCCATT ATACACGCAT TCAGAGACAT AGATGTCGAG GGGGAGATGA ATTAGCAAGA AGATGTCGAG   
  
  
+ CTAAAATGCA TTTCGAGGTT ACTAGAGCCA GAAATGTGGG CACATCCCAG AAGATCTCAG TAGAGAGAGT   
  
  
+ AACTTCAGAA AAGTTTCAAT TTTTATTCGA AATTTGCCTC TTGTTTGTGT AGATATGATG GGAACTTCGC   
  
  
+ CTTACAATCT GCAATGTGAG GGTTTGGTTG AAGGAGTTGC AGTCAATCCT CAATTTTATG AATCAACCCC   
  
  
+ AAAGTGGAAG AAGGGCATCA AAGAAGAGTC AATTGAGAGC GAACCCACTT CGGTTTTGGA CAACCCTAGC   
  
  
+ CCTCCAAATT CTGCATCTAC TCTCTCTTCC TGCTTCAATG GTGGAAGTAG TGGGCTTGGG ATGGACGATT   
  
  
+ TGGAGGGTTT GTCTTTTGGA GAAGGGTCCC TTCTCCCTTG GTTCATGGGT GAAATTGAAG ACCCTAGTTT   
  
  
+ GAGTTTCAAG CACCTCCTTC AACCTTCAAA TCCCTTGGAG TTTGGTGGCA ATGATGGGCT TGAAGCAGCT   
  
  
+ ATTCAGAGTG CTGGTATGGG TAATTTTAAT TTGGTTGAAA GTTTTAATTC TGATTTGAGG TCTTGTAATT   
  
  
+ CTGGGTCTAT TTGCAATGGG GGGAATGATT CTGTCAATGA AAAGGGATGT GTTCTTAATT ATGCTTCAGA   
  
  
+ GCAGGGTCAT GTGGTTGCTG TATCTCCAGT AGAACAGTTT AGGATTAGTG ATGTGAAACA TGAGGTGTTG   
  
  
+ AACCCAAAAT TTGGCTTTTG TGAGCAAGGC TCTACAATCA GTCAGAACTC TGGTTATGTG CATATACTGG   
  
  
+ GTTATAACCA GCTAGAATCT CCCCTTGAGC CTCCGGCAAA GCGCCACAAT GCTGGGAATG CTCTTTACAC   
  
  
+ TAGTGTTGTT CCAGTTCCAA AGAGTTCGTT TTTAGGTGAG GATCATGAAA TTCTACTCAG AAATCAAGAG   
  
  
+ CAGCAGTTTC TGATGCAACA ACAGTGGTTA ATGGGTTTAG CCCCTCAGTT TACACCCCAG CACCCGCCAA   
  
  
+ AGCCATTAGA CGACCGAAAG CAACCAAACT CGCAAACTTT GGTTCTGCGC GATCAGTTAG TTAAGATTGC   
  
  
+ GGAGATGTTC CAAACCGGTA ACTTTTCGCT TGCCCAAGTG ATATTGGCCC GGCTCAATCA GCAATTGTCT   
  
  
+ CTTTCAGGGA ACCCCTTTGT AAGAGCTGCT ACTTTCCATG TCAAGGAGGA ACTTGAAAGG CTCCTGACTA   
  
  
+ TCAATAGCTT CTCTGCGGCT CCACCCCAAC CTAAAAGCCT TACTCTGTCA GATGTTGTGG ATAGGATGAA   
  
  
+ TGCATACAAG CTTTTTTCAG AGGCATCCCC AGTTATGCAA TTTACCGATT TTACTTGTAC CCAGGCTTTG   
  
  
+ CTCGAAGCTC TTGATGATGC TGATTATATC CACATACTAG ATTTTGACAT TGGTTGTGGT TCCCAATGGG   
  
  
+ CATCCTTTAT TCGAGAGCTT CCTCTCAGGA AAAGGGGCAC TCCATTTTTG AAAATTACAG CCTTTGCCTC   
  
  
+ TTCTTTAACC CATAATCCAT TTGAACTAGC CCTTGTATGT GATAACATTC TGGAATTTGC AAAAGAGTTC   
  
  
+ GGTGTCTCTG TCGACCTTCA GGTCATGAAT TTGGACTTGT TTGACCCAAC TACTTGCACA ATACCCAATT   
  
  
+ TTCAATCCAC TGAGAACGCA GCAGTTGCTG TCAACTTCCC TATATGGGCG TGCTCACACT GCCCGTTTGT   
  
  
+ ACTCAGTCAA CTCCTGTCCT TCATCAAGCA ATGTTCCCCA AAAATCATGA CGAGCTTTGA TAGAGGTTGT   
  
  
+ GATCATTTTG AGTTGTCTTT TCCATCGCAC ATCATCCGTG TATTGGACTC ATGCTCTAAT ATGTTGGAGT   
  
  
+ CCCTCCATGG TTCAAGCGTG ACATTAGACA CCTCAAAGAA GATTGAAAAG TTCTTGATCA AGCCTATAAT   
  
  
+ TGAGAGGGCA ATATTGGGGC GAATTCATGC ACCAGACAAG ATGCAGGCAA ACAAGGCACA CCGGTGGAAG   
  
  
+ AATCTCTTCA CTTCTGCTGG GTTCTTCCCT TTGCCATTTA GTAGTTTCAC TGAATCTCAG GCCAATCTAG   
  
  
+ TGGCTCAACG AACCCCAGTG AGGGGTTTTC AGGTGGAAAA GCAGCAAGCA TCACTTGTGC TCAGGTGGAT   
  
  
+ GTCTCATGAG CTCGTGGCAG CTTCAGCTTG GAGGTGCTA  

- -Up\_Stream \_Len000AAACAT AAATTTAAAA TGTTATAAAT ATTTTTAAAA CATAACTAAT TAACACGATT   
  
  
- GATTGTGATT ATTAACCCCC TCTTTTCTTC CCTTTTATTT TTCTTTAATT CTTCTTCTTT TCTTTTGTGG   
  
  
- AAACTTTTCC GTTTTTTGAT TTTATTTTGT TATTACTTGT CTTCCTCTCT TCCTACCTAT TTGTTGTTTA   
  
  
- CAGCGAAAAT CCTGTCGTTA TCAGATACAC TAACCGGAAT CAAGGACAAC CTCCTAACGA TTGTTTTCGT   
  
  
- TGTCGTTTTT TATTATGATT AATAATGTAT AAAAAATTGA GAATTTCGAT TAGTTAGTAG GAGGTAATTA   
  
  
- ACTAATTAAT AAACGGTTTT TGTGTTTAGT TGACCCCGTT GATATTCTCT AGATGGACGA AATTCTATAT   
  
  
- ATTTGAGTTA ACAAAGTAAA ATAAAATAAA ATCTAAAAAA CCGGAAACCA GAGATAACTC GATTTTAGAG   
  
  
- CAATAAAAGC CTAGTACTTT TTATGTTGAG CTAGAAAACT ATATTCAGTA GAAATAAACA TTTAAGACTT   
  
  
- GAATTTTAGA ATGAACTTCT AGTCCTTATC TTCGTGAGCT AAAAACTATT TAGGATACAC AAGAAGAATT   
  
  
- TTAAGTATTA GATTAAGTAT AAAATCTACA TGGGCAACGG ATGAAACCGT TCAAATCTAA TACTAAAAAT   
  
  
- AAAAAAAATT AATGAACATA AAAAGAAAAC CTAACTATCA GTTAGATGTA GTAGAAACTA GTTAACGCTG   
  
  
- TCAAAGTAAA AGGGTTGTTT TTAAAGCTCA CATAAGAAAA ATTAAATAGT ATTCATAAAT AACAGTTGTA   
  
  
- AGTTGTACGT AGTTGTTAGT AATTTGTGAA AAGCAGTGAG GAGTTATGAC CAGTCGTGAA TTCTAAAACA   
  
  
- GAAGATTTGA ATAGCATTCA TTGAATAACA ACTAATTAAC TACGACTGGT TTACTATGAC TCACGATTTT   
  
  
- AATATTAATT ATTTATGTTA ATTCTCAACT AGTTACAGCT ACTAAGTTTA GTTACACTAA CCACGACTTA   
  
  
- TCATTAATAT TCGTGGATAT TTCTGAACTT TTCTTGTTTG TAAATTAATC TAAAATATTA ATATTAAAGG   
  
  
- ATTATAAATC GTAAGTAGTC GTTTAAGTCG TGTGTGGTTG TAATCAGTTG TGGATAGTTG TGAATATTCA   
  
  
- TGGTTAATTA TTATTAATGG ATATTATCTA ATTTTCAATC CGGGAATTAC AAAGGGTGTA ATAATCTATA   
  
  
- TAGTTCCCTC CCCCTTTTTT TATTATGAGG GGGTAGAATT TTTTAAATAG GGAAAAAAAG AAAAATTACA   
  
  
- AGGTTTTGTT TTTATACGAA GATTTAATAC TAACGTTATA CTTACACATG CCAAAGTTTG GGTTAAAAGA   
  
  
- GGTTGTTAAG AAGATGATAT AACATTAATA TAATATGATG TTAAACATAG AGTAGATACT AGATGGGGGG   
  
  
- TTTTTGGTTT ATGAGTATAT TTCAAAATAA AAGAAAGGTA GGGTGAGATT TTTTTTTTTT ACAACCCTTT   
  
  
- TTCAACTAAA TAAATTAAAC TTTACCATGC CACTTATATC TAAACATCTC CCCGTCAACT ATCTTTTTCC   
  
  
- ACTTCTGTTT CCTTCCCGTG TAAACGGAAA AGTATCTTCC TTCTCTGGTT CCATGACCCC TGACCCCCTT   
  
  
- CCCGAGTTCT CTTCTCTCTC TGACTCTTCT CACGACGATG AGGAAGGATG GATGTGTGTT TTAACGACGC   
  
  
- CCCCCAAGTT CAAGTTGAAC TTGCTCCGTT TATGGGAGTA ACTTGGGTGG TTTAGCTTTA GAAGTTACAT   
  
  
- GTGGGGGTAA TATGTGCGTA AGTCTCTGTA TCTACAGCTC CCCCTCTACT TAATCGTTCT TCTACAGCTC   
  
  
- GATTTTACGT AAAGCTCCAA TGATCTCGGT CTTTACACCC GTGTAGGGTC TTCTAGAGTC ATCTCTCTCA   
  
  
- TTGAAGTCTT TTCAAAGTTA AAAATAAGCT TTAAACGGAG AACAAACACA TCTATACTAC CCTTGAAGCG   
  
  
- GAATGTTAGA CGTTACACTC CCAAACCAAC TTCCTCAACG TCAGTTAGGA GTTAAAATAC TTAGTTGGGG   
  
  
- TTTCACCTTC TTCCCGTAGT TTCTTCTCAG TTAACTCTCG CTTGGGTGAA GCCAAAACCT GTTGGGATCG   
  
  
- GGAGGTTTAA GACGTAGATG AGAGAGAAGG ACGAAGTTAC CACCTTCATC ACCCGAACCC TACCTGCTAA   
  
  
- ACCTCCCAAA CAGAAAACCT CTTCCCAGGG AAGAGGGAAC CAAGTACCCA CTTTAACTTC TGGGATCAAA   
  
  
- CTCAAAGTTC GTGGAGGAAG TTGGAAGTTT AGGGAACCTC AAACCACCGT TACTACCCGA ACTTCGTCGA   
  
  
- TAAGTCTCAC GACCATACCC ATTAAAATTA AACCAACTTT CAAAATTAAG ACTAAACTCC AGAACATTAA   
  
  
- GACCCAGATA AACGTTACCC CCCTTACTAA GACAGTTACT TTTCCCTACA CAAGAATTAA TACGAAGTCT   
  
  
- CGTCCCAGTA CACCAACGAC ATAGAGGTCA TCTTGTCAAA TCCTAATCAC TACACTTTGT ACTCCACAAC   
  
  
- TTGGGTTTTA AACCGAAAAC ACTCGTTCCG AGATGTTAGT CAGTCTTGAG ACCAATACAC GTATATGACC   
  
  
- CAATATTGGT CGATCTTAGA GGGGAACTCG GAGGCCGTTT CGCGGTGTTA CGACCCTTAC GAGAAATGTG   
  
  
- ATCACAACAA GGTCAAGGTT TCTCAAGCAA AAATCCACTC CTAGTACTTT AAGATGAGTC TTTAGTTCTC   
  
  
- GTCGTCAAAG ACTACGTTGT TGTCACCAAT TACCCAAATC GGGGAGTCAA ATGTGGGGTC GTGGGCGGTT   
  
  
- TCGGTAATCT GCTGGCTTTC GTTGGTTTGA GCGTTTGAAA CCAAGACGCG CTAGTCAATC AATTCTAACG   
  
  
- CCTCTACAAG GTTTGGCCAT TGAAAAGCGA ACGGGTTCAC TATAACCGGG CCGAGTTAGT CGTTAACAGA   
  
  
- GAAAGTCCCT TGGGGAAACA TTCTCGACGA TGAAAGGTAC AGTTCCTCCT TGAACTTTCC GAGGACTGAT   
  
  
- AGTTATCGAA GAGACGCCGA GGTGGGGTTG GATTTTCGGA ATGAGACAGT CTACAACACC TATCCTACTT   
  
  
- ACGTATGTTC GAAAAAAGTC TCCGTAGGGG TCAATACGTT AAATGGCTAA AATGAACATG GGTCCGAAAC   
  
  
- GAGCTTCGAG AACTACTACG ACTAATATAG GTGTATGATC TAAAACTGTA ACCAACACCA AGGGTTACCC   
  
  
- GTAGGAAATA AGCTCTCGAA GGAGAGTCCT TTTCCCCGTG AGGTAAAAAC TTTTAATGTC GGAAACGGAG   
  
  
- AAGAAATTGG GTATTAGGTA AACTTGATCG GGAACATACA CTATTGTAAG ACCTTAAACG TTTTCTCAAG   
  
  
- CCACAGAGAC AGCTGGAAGT CCAGTACTTA AACCTGAACA AACTGGGTTG ATGAACGTGT TATGGGTTAA   
  
  
- AAGTTAGGTG ACTCTTGCGT CGTCAACGAC AGTTGAAGGG ATATACCCGC ACGAGTGTGA CGGGCAAACA   
  
  
- TGAGTCAGTT GAGGACAGGA AGTAGTTCGT TACAAGGGGT TTTTAGTACT GCTCGAAACT ATCTCCAACA   
  
  
- CTAGTAAAAC TCAACAGAAA AGGTAGCGTG TAGTAGGCAC ATAACCTGAG TACGAGATTA TACAACCTCA   
  
  
- GGGAGGTACC AAGTTCGCAC TGTAATCTGT GGAGTTTCTT CTAACTTTTC AAGAACTAGT TCGGATATTA   
  
  
- ACTCTCCCGT TATAACCCCG CTTAAGTACG TGGTCTGTTC TACGTCCGTT TGTTCCGTGT GGCCACCTTC   
  
  
- TTAGAGAAGT GAAGACGACC CAAGAAGGGA AACGGTAAAT CATCAAAGTG ACTTAGAGTC CGGTTAGATC   
  
  
- ACCGAGTTGC TTGGGGTCAC TCCCCAAAAG TCCACCTTTT CGTCGTTCGT AGTGAACACG AGTCCACCTA   
  
  
- CAGAGTACTC GAGCACCGTC GAAGTCGAAC CTCCACGAT

+     TATA-box

| Site Name | Organism | Position | Strand | Matrix score. | sequence | function |
| --- | --- | --- | --- | --- | --- | --- |
| TATA-box | Arabidopsis thaliana | 1109 | + | 4 | TATA | core promoter element around -30 of transcription start |
| TATA-box | Helianthus annuus | 1107 | - | 6 | TATAAA | core promoter element around -30 of transcription start |
| TATA-box | Arabidopsis thaliana | 396 | + | 4 | TATA | core promoter element around -30 of transcription start |
| TATA-box | Arabidopsis thaliana | 2666 | - | 5 | TATAA | core promoter element around -30 of transcription start |
| TATA-box | Brassica napus | 1832 | + | 6 | ATTATA | core promoter element around -30 of transcription start |
| TATA-box | Arabidopsis thaliana | 1437 | + | 4 | TATA | core promoter element around -30 of transcription start |
| TATA-box | Arabidopsis thaliana | 1432 | + | 4 | TATA | core promoter element around -30 of transcription start |
| TATA-box | Brassica oleracea | 1490 | + | 6 | ATATAA | core promoter element around -30 of transcription start |
| TATA-box | Arabidopsis thaliana | 1436 | - | 5 | TATAA | core promoter element around -30 of transcription start |
| TATA-box | Brassica napus | 1435 | + | 6 | ATTATA | core promoter element around -30 of transcription start |
| TATA-box | Arabidopsis thaliana | 1431 | - | 5 | TATAA | core promoter element around -30 of transcription start |
| TATA-box | Arabidopsis thaliana | 1262 | + | 4 | TATA | core promoter element around -30 of transcription start |
| TATA-box | Arabidopsis thaliana | 1108 | - | 5 | TATAA | core promoter element around -30 of transcription start |
| TATA-box | Arabidopsis thaliana | 1215 | + | 4 | TATA | core promoter element around -30 of transcription start |
| TATA-box | Arabidopsis thaliana | 1188 | + | 4 | TATA | core promoter element around -30 of transcription start |
| TATA-box | Arabidopsis thaliana | 1114 | - | 5 | TATAA | core promoter element around -30 of transcription start |
| TATA-box | Brassica napus | 1059 | + | 6 | ATTATA | core promoter element around -30 of transcription start |
| TATA-box | Arabidopsis thaliana | 423 | + | 4 | TATA | core promoter element around -30 of transcription start |
| TATA-box | Arabidopsis thaliana | 1061 | + | 4 | TATA | core promoter element around -30 of transcription start |
| TATA-box | Daucus carota | 39 | - | 8 | TATAAATA | core promoter element around -30 of transcription start |
| TATA-box | Arabidopsis thaliana | 986 | + | 4 | TATA | core promoter element around -30 of transcription start |
| TATA-box | Brassica napus | 1261 | + | 6 | ATATAT | core promoter element around -30 of transcription start |
| TATA-box | Brassica oleracea | 422 | + | 6 | ATATAA | core promoter element around -30 of transcription start |
| TATA-box | Arabidopsis thaliana | 1115 | + | 4 | TATA | core promoter element around -30 of transcription start |
| TATA-box | Brassica napus | 1430 | + | 6 | ATTATA | core promoter element around -30 of transcription start |
| TATA-box | Arabidopsis thaliana | 1421 | + | 4 | TATA | core promoter element around -30 of transcription start |
| TATA-box | Arabidopsis thaliana | 985 | - | 5 | TATAA | core promoter element around -30 of transcription start |
| TATA-box | Brassica napus | 984 | + | 6 | ATTATA | core promoter element around -30 of transcription start |
| TATA-box | Arabidopsis thaliana | 421 | + | 6 | TATATA | core promoter element around -30 of transcription start |
| TATA-box | Arabidopsis thaliana | 1187 | - | 5 | TATAA | core promoter element around -30 of transcription start |
| TATA-box | Arabidopsis thaliana | 1071 | + | 4 | TATA | core promoter element around -30 of transcription start |
| TATA-box | Brassica napus | 420 | + | 6 | ATATAT | core promoter element around -30 of transcription start |
| TATA-box | Brassica napus | 1113 | + | 6 | ATTATA | core promoter element around -30 of transcription start |
| TATA-box | Arabidopsis thaliana | 3545 | - | 4 | TATA | core promoter element around -30 of transcription start |
| TATA-box | Brassica napus | 3247 | + | 6 | ATTATA | core promoter element around -30 of transcription start |
| TATA-box | Arabidopsis thaliana | 3779 | - | 4 | TATA | core promoter element around -30 of transcription start |
| TATA-box | Arabidopsis thaliana | 535 | + | 4 | TATA | core promoter element around -30 of transcription start |
| TATA-box | Brassica juncea | 40 | - | 7 | TATAAAT | core promoter element around -30 of transcription start |
| TATA-box | Arabidopsis thaliana | 2667 | - | 4 | TATA | core promoter element around -30 of transcription start |
| TATA-box | Arabidopsis thaliana | 3249 | - | 4 | TATA | core promoter element around -30 of transcription start |
| TATA-box | Arabidopsis thaliana | 3248 | - | 5 | TATAA | core promoter element around -30 of transcription start |
| TATA-box | Arabidopsis thaliana | 2657 | - | 4 | TATA | core promoter element around -30 of transcription start |
| TATA-box | Arabidopsis thaliana | 1833 | - | 5 | TATAA | core promoter element around -30 of transcription start |
| TATA-box | Arabidopsis thaliana | 1580 | + | 4 | TATA | core promoter element around -30 of transcription start |
| TATA-box | Arabidopsis thaliana | 1060 | - | 5 | TATAA | core promoter element around -30 of transcription start |
| TATA-box | Arabidopsis thaliana | 23 | + | 8 | TATTTAAA | core promoter element around -30 of transcription start |
| TATA-box | Pisum sativum | 1106 | - | 7 | TATAAAA | core promoter element around -30 of transcription start |
| TATA-box | Arabidopsis thaliana | 1834 | + | 4 | TATA | core promoter element around -30 of transcription start |
| TATA-box | Arabidopsis thaliana | 1491 | + | 4 | TATA | core promoter element around -30 of transcription start |
| TATA-box | Arabidopsis thaliana | 1550 | - | 9 | taTATAAAtc | core promoter element around -30 of transcription start |
| TATA-box | Brassica oleracea | 534 | + | 6 | ATATAA | core promoter element around -30 of transcription start |
| TATA-box | Arabidopsis thaliana | 1069 | + | 9 | ccTATAAAaa | core promoter element around -30 of transcription start |
| TATA-box | Oryza sativa | 51 | - | 7 | TACAAAA | core promoter element around -30 of transcription start |
| TATA-box | Arabidopsis thaliana | 43 | + | 4 | TATA | core promoter element around -30 of transcription start |
| TATA-box | Helianthus annuus | 41 | - | 6 | TATAAA | core promoter element around -30 of transcription start |
| TATA-box | Arabidopsis thaliana | 42 | - | 5 | TATAA | core promoter element around -30 of transcription start |

>HU04G00048.1   
+ -Up\_Stream \_Len000TTTGTA TTTAAATTTT ACAATATTTA TAAAAATTTT GTATTGATTA ATTGTGCTAA   
  
  
+ CTAACACTAA TAATTGGGGG AGAAAAGAAG GGAAAATAAA AAGAAATTAA GAAGAAGAAA AGAAAACACC   
  
  
+ TTTGAAAAGG CAAAAAACTA AAATAAAACA ATAATGAACA GAAGGAGAGA AGGATGGATA AACAACAAAT   
  
  
+ GTCGCTTTTA GGACAGCAAT AGTCTATGTG ATTGGCCTTA GTTCCTGTTG GAGGATTGCT AACAAAAGCA   
  
  
+ ACAGCAAAAA ATAATACTAA TTATTACATA TTTTTTAACT CTTAAAGCTA ATCAATCATC CTCCATTAAT   
  
  
+ TGATTAATTA TTTGCCAAAA ACACAAATCA ACTGGGGCAA CTATAAGAGA TCTACCTGCT TTAAGATATA   
  
  
+ TAAACTCAAT TGTTTCATTT TATTTTATTT TAGATTTTTT GGCCTTTGGT CTCTATTGAG CTAAAATCTC   
  
  
+ GTTATTTTCG GATCATGAAA AATACAACTC GATCTTTTGA TATAAGTCAT CTTTATTTGT AAATTCTGAA   
  
  
+ CTTAAAATCT TACTTGAAGA TCAGGAATAG AAGCACTCGA TTTTTGATAA ATCCTATGTG TTCTTCTTAA   
  
  
+ AATTCATAAT CTAATTCATA TTTTAGATGT ACCCGTTGCC TACTTTGGCA AGTTTAGATT ATGATTTTTA   
  
  
+ TTTTTTTTAA TTACTTGTAT TTTTCTTTTG GATTGATAGT CAATCTACAT CATCTTTGAT CAATTGCGAC   
  
  
+ AGTTTCATTT TCCCAACAAA AATTTCGAGT GTATTCTTTT TAATTTATCA TAAGTATTTA TTGTCAACAT   
  
  
+ TCAACATGCA TCAACAATCA TTAAACACTT TTCGTCACTC CTCAATACTG GTCAGCACTT AAGATTTTGT   
  
  
+ CTTCTAAACT TATCGTAAGT AACTTATTGT TGATTAATTG ATGCTGACCA AATGATACTG AGTGCTAAAA   
  
  
+ TTATAATTAA TAAATACAAT TAAGAGTTGA TCAATGTCGA TGATTCAAAT CAATGTGATT GGTGCTGAAT   
  
  
+ AGTAATTATA AGCACCTATA AAGACTTGAA AAGAACAAAC ATTTAATTAG ATTTTATAAT TATAATTTCC   
  
  
+ TAATATTTAG CATTCATCAG CAAATTCAGC ACACACCAAC ATTAGTCAAC ACCTATCAAC ACTTATAAGT   
  
  
+ ACCAATTAAT AATAATTACC TATAATAGAT TAAAAGTTAG GCCCTTAATG TTTCCCACAT TATTAGATAT   
  
  
+ ATCAAGGGAG GGGGAAAAAA ATAATACTCC CCCATCTTAA AAAATTTATC CCTTTTTTTC TTTTTAATGT   
  
  
+ TCCAAAACAA AAATATGCTT CTAAATTATG ATTGCAATAT GAATGTGTAC GGTTTCAAAC CCAATTTTCT   
  
  
+ CCAACAATTC TTCTACTATA TTGTAATTAT ATTATACTAC AATTTGTATC TCATCTATGA TCTACCCCCC   
  
  
+ AAAAACCAAA TACTCATATA AAGTTTTATT TTCTTTCCAT CCCACTCTAA AAAAAAAAAA TGTTGGGAAA   
  
  
+ AAGTTGATTT ATTTAATTTG AAATGGTACG GTGAATATAG ATTTGTAGAG GGGCAGTTGA TAGAAAAAGG   
  
  
+ TGAAGACAAA GGAAGGGCAC ATTTGCCTTT TCATAGAAGG AAGAGACCAA GGTACTGGGG ACTGGGGGAA   
  
  
+ GGGCTCAAGA GAAGAGAGAG ACTGAGAAGA GTGCTGCTAC TCCTTCCTAC CTACACACAA AATTGCTGCG   
  
  
+ GGGGGTTCAA GTTCAACTTG AACGAGGCAA ATACCCTCAT TGAACCCACC AAATCGAAAT CTTCAATGTA   
  
  
+ CACCCCCATT ATACACGCAT TCAGAGACAT AGATGTCGAG GGGGAGATGA ATTAGCAAGA AGATGTCGAG   
  
  
+ CTAAAATGCA TTTCGAGGTT ACTAGAGCCA GAAATGTGGG CACATCCCAG AAGATCTCAG TAGAGAGAGT   
  
  
+ AACTTCAGAA AAGTTTCAAT TTTTATTCGA AATTTGCCTC TTGTTTGTGT AGATATGATG GGAACTTCGC   
  
  
+ CTTACAATCT GCAATGTGAG GGTTTGGTTG AAGGAGTTGC AGTCAATCCT CAATTTTATG AATCAACCCC   
  
  
+ AAAGTGGAAG AAGGGCATCA AAGAAGAGTC AATTGAGAGC GAACCCACTT CGGTTTTGGA CAACCCTAGC   
  
  
+ CCTCCAAATT CTGCATCTAC TCTCTCTTCC TGCTTCAATG GTGGAAGTAG TGGGCTTGGG ATGGACGATT   
  
  
+ TGGAGGGTTT GTCTTTTGGA GAAGGGTCCC TTCTCCCTTG GTTCATGGGT GAAATTGAAG ACCCTAGTTT   
  
  
+ GAGTTTCAAG CACCTCCTTC AACCTTCAAA TCCCTTGGAG TTTGGTGGCA ATGATGGGCT TGAAGCAGCT   
  
  
+ ATTCAGAGTG CTGGTATGGG TAATTTTAAT TTGGTTGAAA GTTTTAATTC TGATTTGAGG TCTTGTAATT   
  
  
+ CTGGGTCTAT TTGCAATGGG GGGAATGATT CTGTCAATGA AAAGGGATGT GTTCTTAATT ATGCTTCAGA   
  
  
+ GCAGGGTCAT GTGGTTGCTG TATCTCCAGT AGAACAGTTT AGGATTAGTG ATGTGAAACA TGAGGTGTTG   
  
  
+ AACCCAAAAT TTGGCTTTTG TGAGCAAGGC TCTACAATCA GTCAGAACTC TGGTTATGTG CATATACTGG   
  
  
+ GTTATAACCA GCTAGAATCT CCCCTTGAGC CTCCGGCAAA GCGCCACAAT GCTGGGAATG CTCTTTACAC   
  
  
+ TAGTGTTGTT CCAGTTCCAA AGAGTTCGTT TTTAGGTGAG GATCATGAAA TTCTACTCAG AAATCAAGAG   
  
  
+ CAGCAGTTTC TGATGCAACA ACAGTGGTTA ATGGGTTTAG CCCCTCAGTT TACACCCCAG CACCCGCCAA   
  
  
+ AGCCATTAGA CGACCGAAAG CAACCAAACT CGCAAACTTT GGTTCTGCGC GATCAGTTAG TTAAGATTGC   
  
  
+ GGAGATGTTC CAAACCGGTA ACTTTTCGCT TGCCCAAGTG ATATTGGCCC GGCTCAATCA GCAATTGTCT   
  
  
+ CTTTCAGGGA ACCCCTTTGT AAGAGCTGCT ACTTTCCATG TCAAGGAGGA ACTTGAAAGG CTCCTGACTA   
  
  
+ TCAATAGCTT CTCTGCGGCT CCACCCCAAC CTAAAAGCCT TACTCTGTCA GATGTTGTGG ATAGGATGAA   
  
  
+ TGCATACAAG CTTTTTTCAG AGGCATCCCC AGTTATGCAA TTTACCGATT TTACTTGTAC CCAGGCTTTG   
  
  
+ CTCGAAGCTC TTGATGATGC TGATTATATC CACATACTAG ATTTTGACAT TGGTTGTGGT TCCCAATGGG   
  
  
+ CATCCTTTAT TCGAGAGCTT CCTCTCAGGA AAAGGGGCAC TCCATTTTTG AAAATTACAG CCTTTGCCTC   
  
  
+ TTCTTTAACC CATAATCCAT TTGAACTAGC CCTTGTATGT GATAACATTC TGGAATTTGC AAAAGAGTTC   
  
  
+ GGTGTCTCTG TCGACCTTCA GGTCATGAAT TTGGACTTGT TTGACCCAAC TACTTGCACA ATACCCAATT   
  
  
+ TTCAATCCAC TGAGAACGCA GCAGTTGCTG TCAACTTCCC TATATGGGCG TGCTCACACT GCCCGTTTGT   
  
  
+ ACTCAGTCAA CTCCTGTCCT TCATCAAGCA ATGTTCCCCA AAAATCATGA CGAGCTTTGA TAGAGGTTGT   
  
  
+ GATCATTTTG AGTTGTCTTT TCCATCGCAC ATCATCCGTG TATTGGACTC ATGCTCTAAT ATGTTGGAGT   
  
  
+ CCCTCCATGG TTCAAGCGTG ACATTAGACA CCTCAAAGAA GATTGAAAAG TTCTTGATCA AGCCTATAAT   
  
  
+ TGAGAGGGCA ATATTGGGGC GAATTCATGC ACCAGACAAG ATGCAGGCAA ACAAGGCACA CCGGTGGAAG   
  
  
+ AATCTCTTCA CTTCTGCTGG GTTCTTCCCT TTGCCATTTA GTAGTTTCAC TGAATCTCAG GCCAATCTAG   
  
  
+ TGGCTCAACG AACCCCAGTG AGGGGTTTTC AGGTGGAAAA GCAGCAAGCA TCACTTGTGC TCAGGTGGAT   
  
  
+ GTCTCATGAG CTCGTGGCAG CTTCAGCTTG GAGGTGCTA  

- -Up\_Stream \_Len000AAACAT AAATTTAAAA TGTTATAAAT ATTTTTAAAA CATAACTAAT TAACACGATT   
  
  
- GATTGTGATT ATTAACCCCC TCTTTTCTTC CCTTTTATTT TTCTTTAATT CTTCTTCTTT TCTTTTGTGG   
  
  
- AAACTTTTCC GTTTTTTGAT TTTATTTTGT TATTACTTGT CTTCCTCTCT TCCTACCTAT TTGTTGTTTA   
  
  
- CAGCGAAAAT CCTGTCGTTA TCAGATACAC TAACCGGAAT CAAGGACAAC CTCCTAACGA TTGTTTTCGT   
  
  
- TGTCGTTTTT TATTATGATT AATAATGTAT AAAAAATTGA GAATTTCGAT TAGTTAGTAG GAGGTAATTA   
  
  
- ACTAATTAAT AAACGGTTTT TGTGTTTAGT TGACCCCGTT GATATTCTCT AGATGGACGA AATTCTATAT   
  
  
- ATTTGAGTTA ACAAAGTAAA ATAAAATAAA ATCTAAAAAA CCGGAAACCA GAGATAACTC GATTTTAGAG   
  
  
- CAATAAAAGC CTAGTACTTT TTATGTTGAG CTAGAAAACT ATATTCAGTA GAAATAAACA TTTAAGACTT   
  
  
- GAATTTTAGA ATGAACTTCT AGTCCTTATC TTCGTGAGCT AAAAACTATT TAGGATACAC AAGAAGAATT   
  
  
- TTAAGTATTA GATTAAGTAT AAAATCTACA TGGGCAACGG ATGAAACCGT TCAAATCTAA TACTAAAAAT   
  
  
- AAAAAAAATT AATGAACATA AAAAGAAAAC CTAACTATCA GTTAGATGTA GTAGAAACTA GTTAACGCTG   
  
  
- TCAAAGTAAA AGGGTTGTTT TTAAAGCTCA CATAAGAAAA ATTAAATAGT ATTCATAAAT AACAGTTGTA   
  
  
- AGTTGTACGT AGTTGTTAGT AATTTGTGAA AAGCAGTGAG GAGTTATGAC CAGTCGTGAA TTCTAAAACA   
  
  
- GAAGATTTGA ATAGCATTCA TTGAATAACA ACTAATTAAC TACGACTGGT TTACTATGAC TCACGATTTT   
  
  
- AATATTAATT ATTTATGTTA ATTCTCAACT AGTTACAGCT ACTAAGTTTA GTTACACTAA CCACGACTTA   
  
  
- TCATTAATAT TCGTGGATAT TTCTGAACTT TTCTTGTTTG TAAATTAATC TAAAATATTA ATATTAAAGG   
  
  
- ATTATAAATC GTAAGTAGTC GTTTAAGTCG TGTGTGGTTG TAATCAGTTG TGGATAGTTG TGAATATTCA   
  
  
- TGGTTAATTA TTATTAATGG ATATTATCTA ATTTTCAATC CGGGAATTAC AAAGGGTGTA ATAATCTATA   
  
  
- TAGTTCCCTC CCCCTTTTTT TATTATGAGG GGGTAGAATT TTTTAAATAG GGAAAAAAAG AAAAATTACA   
  
  
- AGGTTTTGTT TTTATACGAA GATTTAATAC TAACGTTATA CTTACACATG CCAAAGTTTG GGTTAAAAGA   
  
  
- GGTTGTTAAG AAGATGATAT AACATTAATA TAATATGATG TTAAACATAG AGTAGATACT AGATGGGGGG   
  
  
- TTTTTGGTTT ATGAGTATAT TTCAAAATAA AAGAAAGGTA GGGTGAGATT TTTTTTTTTT ACAACCCTTT   
  
  
- TTCAACTAAA TAAATTAAAC TTTACCATGC CACTTATATC TAAACATCTC CCCGTCAACT ATCTTTTTCC   
  
  
- ACTTCTGTTT CCTTCCCGTG TAAACGGAAA AGTATCTTCC TTCTCTGGTT CCATGACCCC TGACCCCCTT   
  
  
- CCCGAGTTCT CTTCTCTCTC TGACTCTTCT CACGACGATG AGGAAGGATG GATGTGTGTT TTAACGACGC   
  
  
- CCCCCAAGTT CAAGTTGAAC TTGCTCCGTT TATGGGAGTA ACTTGGGTGG TTTAGCTTTA GAAGTTACAT   
  
  
- GTGGGGGTAA TATGTGCGTA AGTCTCTGTA TCTACAGCTC CCCCTCTACT TAATCGTTCT TCTACAGCTC   
  
  
- GATTTTACGT AAAGCTCCAA TGATCTCGGT CTTTACACCC GTGTAGGGTC TTCTAGAGTC ATCTCTCTCA   
  
  
- TTGAAGTCTT TTCAAAGTTA AAAATAAGCT TTAAACGGAG AACAAACACA TCTATACTAC CCTTGAAGCG   
  
  
- GAATGTTAGA CGTTACACTC CCAAACCAAC TTCCTCAACG TCAGTTAGGA GTTAAAATAC TTAGTTGGGG   
  
  
- TTTCACCTTC TTCCCGTAGT TTCTTCTCAG TTAACTCTCG CTTGGGTGAA GCCAAAACCT GTTGGGATCG   
  
  
- GGAGGTTTAA GACGTAGATG AGAGAGAAGG ACGAAGTTAC CACCTTCATC ACCCGAACCC TACCTGCTAA   
  
  
- ACCTCCCAAA CAGAAAACCT CTTCCCAGGG AAGAGGGAAC CAAGTACCCA CTTTAACTTC TGGGATCAAA   
  
  
- CTCAAAGTTC GTGGAGGAAG TTGGAAGTTT AGGGAACCTC AAACCACCGT TACTACCCGA ACTTCGTCGA   
  
  
- TAAGTCTCAC GACCATACCC ATTAAAATTA AACCAACTTT CAAAATTAAG ACTAAACTCC AGAACATTAA   
  
  
- GACCCAGATA AACGTTACCC CCCTTACTAA GACAGTTACT TTTCCCTACA CAAGAATTAA TACGAAGTCT   
  
  
- CGTCCCAGTA CACCAACGAC ATAGAGGTCA TCTTGTCAAA TCCTAATCAC TACACTTTGT ACTCCACAAC   
  
  
- TTGGGTTTTA AACCGAAAAC ACTCGTTCCG AGATGTTAGT CAGTCTTGAG ACCAATACAC GTATATGACC   
  
  
- CAATATTGGT CGATCTTAGA GGGGAACTCG GAGGCCGTTT CGCGGTGTTA CGACCCTTAC GAGAAATGTG   
  
  
- ATCACAACAA GGTCAAGGTT TCTCAAGCAA AAATCCACTC CTAGTACTTT AAGATGAGTC TTTAGTTCTC   
  
  
- GTCGTCAAAG ACTACGTTGT TGTCACCAAT TACCCAAATC GGGGAGTCAA ATGTGGGGTC GTGGGCGGTT   
  
  
- TCGGTAATCT GCTGGCTTTC GTTGGTTTGA GCGTTTGAAA CCAAGACGCG CTAGTCAATC AATTCTAACG   
  
  
- CCTCTACAAG GTTTGGCCAT TGAAAAGCGA ACGGGTTCAC TATAACCGGG CCGAGTTAGT CGTTAACAGA   
  
  
- GAAAGTCCCT TGGGGAAACA TTCTCGACGA TGAAAGGTAC AGTTCCTCCT TGAACTTTCC GAGGACTGAT   
  
  
- AGTTATCGAA GAGACGCCGA GGTGGGGTTG GATTTTCGGA ATGAGACAGT CTACAACACC TATCCTACTT   
  
  
- ACGTATGTTC GAAAAAAGTC TCCGTAGGGG TCAATACGTT AAATGGCTAA AATGAACATG GGTCCGAAAC   
  
  
- GAGCTTCGAG AACTACTACG ACTAATATAG GTGTATGATC TAAAACTGTA ACCAACACCA AGGGTTACCC   
  
  
- GTAGGAAATA AGCTCTCGAA GGAGAGTCCT TTTCCCCGTG AGGTAAAAAC TTTTAATGTC GGAAACGGAG   
  
  
- AAGAAATTGG GTATTAGGTA AACTTGATCG GGAACATACA CTATTGTAAG ACCTTAAACG TTTTCTCAAG   
  
  
- CCACAGAGAC AGCTGGAAGT CCAGTACTTA AACCTGAACA AACTGGGTTG ATGAACGTGT TATGGGTTAA   
  
  
- AAGTTAGGTG ACTCTTGCGT CGTCAACGAC AGTTGAAGGG ATATACCCGC ACGAGTGTGA CGGGCAAACA   
  
  
- TGAGTCAGTT GAGGACAGGA AGTAGTTCGT TACAAGGGGT TTTTAGTACT GCTCGAAACT ATCTCCAACA   
  
  
- CTAGTAAAAC TCAACAGAAA AGGTAGCGTG TAGTAGGCAC ATAACCTGAG TACGAGATTA TACAACCTCA   
  
  
- GGGAGGTACC AAGTTCGCAC TGTAATCTGT GGAGTTTCTT CTAACTTTTC AAGAACTAGT TCGGATATTA   
  
  
- ACTCTCCCGT TATAACCCCG CTTAAGTACG TGGTCTGTTC TACGTCCGTT TGTTCCGTGT GGCCACCTTC   
  
  
- TTAGAGAAGT GAAGACGACC CAAGAAGGGA AACGGTAAAT CATCAAAGTG ACTTAGAGTC CGGTTAGATC   
  
  
- ACCGAGTTGC TTGGGGTCAC TCCCCAAAAG TCCACCTTTT CGTCGTTCGT AGTGAACACG AGTCCACCTA   
  
  
- CAGAGTACTC GAGCACCGTC GAAGTCGAAC CTCCACGAT

+     TCA

| Site Name | Organism | Position | Strand | Matrix score. | sequence | function |
| --- | --- | --- | --- | --- | --- | --- |
| TCA | Pisum sativum | 541 | + | 9 | TCATCTTCAT |  |

>HU04G00048.1   
+ -Up\_Stream \_Len000TTTGTA TTTAAATTTT ACAATATTTA TAAAAATTTT GTATTGATTA ATTGTGCTAA   
  
  
+ CTAACACTAA TAATTGGGGG AGAAAAGAAG GGAAAATAAA AAGAAATTAA GAAGAAGAAA AGAAAACACC   
  
  
+ TTTGAAAAGG CAAAAAACTA AAATAAAACA ATAATGAACA GAAGGAGAGA AGGATGGATA AACAACAAAT   
  
  
+ GTCGCTTTTA GGACAGCAAT AGTCTATGTG ATTGGCCTTA GTTCCTGTTG GAGGATTGCT AACAAAAGCA   
  
  
+ ACAGCAAAAA ATAATACTAA TTATTACATA TTTTTTAACT CTTAAAGCTA ATCAATCATC CTCCATTAAT   
  
  
+ TGATTAATTA TTTGCCAAAA ACACAAATCA ACTGGGGCAA CTATAAGAGA TCTACCTGCT TTAAGATATA   
  
  
+ TAAACTCAAT TGTTTCATTT TATTTTATTT TAGATTTTTT GGCCTTTGGT CTCTATTGAG CTAAAATCTC   
  
  
+ GTTATTTTCG GATCATGAAA AATACAACTC GATCTTTTGA TATAAGTCAT CTTTATTTGT AAATTCTGAA   
  
  
+ CTTAAAATCT TACTTGAAGA TCAGGAATAG AAGCACTCGA TTTTTGATAA ATCCTATGTG TTCTTCTTAA   
  
  
+ AATTCATAAT CTAATTCATA TTTTAGATGT ACCCGTTGCC TACTTTGGCA AGTTTAGATT ATGATTTTTA   
  
  
+ TTTTTTTTAA TTACTTGTAT TTTTCTTTTG GATTGATAGT CAATCTACAT CATCTTTGAT CAATTGCGAC   
  
  
+ AGTTTCATTT TCCCAACAAA AATTTCGAGT GTATTCTTTT TAATTTATCA TAAGTATTTA TTGTCAACAT   
  
  
+ TCAACATGCA TCAACAATCA TTAAACACTT TTCGTCACTC CTCAATACTG GTCAGCACTT AAGATTTTGT   
  
  
+ CTTCTAAACT TATCGTAAGT AACTTATTGT TGATTAATTG ATGCTGACCA AATGATACTG AGTGCTAAAA   
  
  
+ TTATAATTAA TAAATACAAT TAAGAGTTGA TCAATGTCGA TGATTCAAAT CAATGTGATT GGTGCTGAAT   
  
  
+ AGTAATTATA AGCACCTATA AAGACTTGAA AAGAACAAAC ATTTAATTAG ATTTTATAAT TATAATTTCC   
  
  
+ TAATATTTAG CATTCATCAG CAAATTCAGC ACACACCAAC ATTAGTCAAC ACCTATCAAC ACTTATAAGT   
  
  
+ ACCAATTAAT AATAATTACC TATAATAGAT TAAAAGTTAG GCCCTTAATG TTTCCCACAT TATTAGATAT   
  
  
+ ATCAAGGGAG GGGGAAAAAA ATAATACTCC CCCATCTTAA AAAATTTATC CCTTTTTTTC TTTTTAATGT   
  
  
+ TCCAAAACAA AAATATGCTT CTAAATTATG ATTGCAATAT GAATGTGTAC GGTTTCAAAC CCAATTTTCT   
  
  
+ CCAACAATTC TTCTACTATA TTGTAATTAT ATTATACTAC AATTTGTATC TCATCTATGA TCTACCCCCC   
  
  
+ AAAAACCAAA TACTCATATA AAGTTTTATT TTCTTTCCAT CCCACTCTAA AAAAAAAAAA TGTTGGGAAA   
  
  
+ AAGTTGATTT ATTTAATTTG AAATGGTACG GTGAATATAG ATTTGTAGAG GGGCAGTTGA TAGAAAAAGG   
  
  
+ TGAAGACAAA GGAAGGGCAC ATTTGCCTTT TCATAGAAGG AAGAGACCAA GGTACTGGGG ACTGGGGGAA   
  
  
+ GGGCTCAAGA GAAGAGAGAG ACTGAGAAGA GTGCTGCTAC TCCTTCCTAC CTACACACAA AATTGCTGCG   
  
  
+ GGGGGTTCAA GTTCAACTTG AACGAGGCAA ATACCCTCAT TGAACCCACC AAATCGAAAT CTTCAATGTA   
  
  
+ CACCCCCATT ATACACGCAT TCAGAGACAT AGATGTCGAG GGGGAGATGA ATTAGCAAGA AGATGTCGAG   
  
  
+ CTAAAATGCA TTTCGAGGTT ACTAGAGCCA GAAATGTGGG CACATCCCAG AAGATCTCAG TAGAGAGAGT   
  
  
+ AACTTCAGAA AAGTTTCAAT TTTTATTCGA AATTTGCCTC TTGTTTGTGT AGATATGATG GGAACTTCGC   
  
  
+ CTTACAATCT GCAATGTGAG GGTTTGGTTG AAGGAGTTGC AGTCAATCCT CAATTTTATG AATCAACCCC   
  
  
+ AAAGTGGAAG AAGGGCATCA AAGAAGAGTC AATTGAGAGC GAACCCACTT CGGTTTTGGA CAACCCTAGC   
  
  
+ CCTCCAAATT CTGCATCTAC TCTCTCTTCC TGCTTCAATG GTGGAAGTAG TGGGCTTGGG ATGGACGATT   
  
  
+ TGGAGGGTTT GTCTTTTGGA GAAGGGTCCC TTCTCCCTTG GTTCATGGGT GAAATTGAAG ACCCTAGTTT   
  
  
+ GAGTTTCAAG CACCTCCTTC AACCTTCAAA TCCCTTGGAG TTTGGTGGCA ATGATGGGCT TGAAGCAGCT   
  
  
+ ATTCAGAGTG CTGGTATGGG TAATTTTAAT TTGGTTGAAA GTTTTAATTC TGATTTGAGG TCTTGTAATT   
  
  
+ CTGGGTCTAT TTGCAATGGG GGGAATGATT CTGTCAATGA AAAGGGATGT GTTCTTAATT ATGCTTCAGA   
  
  
+ GCAGGGTCAT GTGGTTGCTG TATCTCCAGT AGAACAGTTT AGGATTAGTG ATGTGAAACA TGAGGTGTTG   
  
  
+ AACCCAAAAT TTGGCTTTTG TGAGCAAGGC TCTACAATCA GTCAGAACTC TGGTTATGTG CATATACTGG   
  
  
+ GTTATAACCA GCTAGAATCT CCCCTTGAGC CTCCGGCAAA GCGCCACAAT GCTGGGAATG CTCTTTACAC   
  
  
+ TAGTGTTGTT CCAGTTCCAA AGAGTTCGTT TTTAGGTGAG GATCATGAAA TTCTACTCAG AAATCAAGAG   
  
  
+ CAGCAGTTTC TGATGCAACA ACAGTGGTTA ATGGGTTTAG CCCCTCAGTT TACACCCCAG CACCCGCCAA   
  
  
+ AGCCATTAGA CGACCGAAAG CAACCAAACT CGCAAACTTT GGTTCTGCGC GATCAGTTAG TTAAGATTGC   
  
  
+ GGAGATGTTC CAAACCGGTA ACTTTTCGCT TGCCCAAGTG ATATTGGCCC GGCTCAATCA GCAATTGTCT   
  
  
+ CTTTCAGGGA ACCCCTTTGT AAGAGCTGCT ACTTTCCATG TCAAGGAGGA ACTTGAAAGG CTCCTGACTA   
  
  
+ TCAATAGCTT CTCTGCGGCT CCACCCCAAC CTAAAAGCCT TACTCTGTCA GATGTTGTGG ATAGGATGAA   
  
  
+ TGCATACAAG CTTTTTTCAG AGGCATCCCC AGTTATGCAA TTTACCGATT TTACTTGTAC CCAGGCTTTG   
  
  
+ CTCGAAGCTC TTGATGATGC TGATTATATC CACATACTAG ATTTTGACAT TGGTTGTGGT TCCCAATGGG   
  
  
+ CATCCTTTAT TCGAGAGCTT CCTCTCAGGA AAAGGGGCAC TCCATTTTTG AAAATTACAG CCTTTGCCTC   
  
  
+ TTCTTTAACC CATAATCCAT TTGAACTAGC CCTTGTATGT GATAACATTC TGGAATTTGC AAAAGAGTTC   
  
  
+ GGTGTCTCTG TCGACCTTCA GGTCATGAAT TTGGACTTGT TTGACCCAAC TACTTGCACA ATACCCAATT   
  
  
+ TTCAATCCAC TGAGAACGCA GCAGTTGCTG TCAACTTCCC TATATGGGCG TGCTCACACT GCCCGTTTGT   
  
  
+ ACTCAGTCAA CTCCTGTCCT TCATCAAGCA ATGTTCCCCA AAAATCATGA CGAGCTTTGA TAGAGGTTGT   
  
  
+ GATCATTTTG AGTTGTCTTT TCCATCGCAC ATCATCCGTG TATTGGACTC ATGCTCTAAT ATGTTGGAGT   
  
  
+ CCCTCCATGG TTCAAGCGTG ACATTAGACA CCTCAAAGAA GATTGAAAAG TTCTTGATCA AGCCTATAAT   
  
  
+ TGAGAGGGCA ATATTGGGGC GAATTCATGC ACCAGACAAG ATGCAGGCAA ACAAGGCACA CCGGTGGAAG   
  
  
+ AATCTCTTCA CTTCTGCTGG GTTCTTCCCT TTGCCATTTA GTAGTTTCAC TGAATCTCAG GCCAATCTAG   
  
  
+ TGGCTCAACG AACCCCAGTG AGGGGTTTTC AGGTGGAAAA GCAGCAAGCA TCACTTGTGC TCAGGTGGAT   
  
  
+ GTCTCATGAG CTCGTGGCAG CTTCAGCTTG GAGGTGCTA  

- -Up\_Stream \_Len000AAACAT AAATTTAAAA TGTTATAAAT ATTTTTAAAA CATAACTAAT TAACACGATT   
  
  
- GATTGTGATT ATTAACCCCC TCTTTTCTTC CCTTTTATTT TTCTTTAATT CTTCTTCTTT TCTTTTGTGG   
  
  
- AAACTTTTCC GTTTTTTGAT TTTATTTTGT TATTACTTGT CTTCCTCTCT TCCTACCTAT TTGTTGTTTA   
  
  
- CAGCGAAAAT CCTGTCGTTA TCAGATACAC TAACCGGAAT CAAGGACAAC CTCCTAACGA TTGTTTTCGT   
  
  
- TGTCGTTTTT TATTATGATT AATAATGTAT AAAAAATTGA GAATTTCGAT TAGTTAGTAG GAGGTAATTA   
  
  
- ACTAATTAAT AAACGGTTTT TGTGTTTAGT TGACCCCGTT GATATTCTCT AGATGGACGA AATTCTATAT   
  
  
- ATTTGAGTTA ACAAAGTAAA ATAAAATAAA ATCTAAAAAA CCGGAAACCA GAGATAACTC GATTTTAGAG   
  
  
- CAATAAAAGC CTAGTACTTT TTATGTTGAG CTAGAAAACT ATATTCAGTA GAAATAAACA TTTAAGACTT   
  
  
- GAATTTTAGA ATGAACTTCT AGTCCTTATC TTCGTGAGCT AAAAACTATT TAGGATACAC AAGAAGAATT   
  
  
- TTAAGTATTA GATTAAGTAT AAAATCTACA TGGGCAACGG ATGAAACCGT TCAAATCTAA TACTAAAAAT   
  
  
- AAAAAAAATT AATGAACATA AAAAGAAAAC CTAACTATCA GTTAGATGTA GTAGAAACTA GTTAACGCTG   
  
  
- TCAAAGTAAA AGGGTTGTTT TTAAAGCTCA CATAAGAAAA ATTAAATAGT ATTCATAAAT AACAGTTGTA   
  
  
- AGTTGTACGT AGTTGTTAGT AATTTGTGAA AAGCAGTGAG GAGTTATGAC CAGTCGTGAA TTCTAAAACA   
  
  
- GAAGATTTGA ATAGCATTCA TTGAATAACA ACTAATTAAC TACGACTGGT TTACTATGAC TCACGATTTT   
  
  
- AATATTAATT ATTTATGTTA ATTCTCAACT AGTTACAGCT ACTAAGTTTA GTTACACTAA CCACGACTTA   
  
  
- TCATTAATAT TCGTGGATAT TTCTGAACTT TTCTTGTTTG TAAATTAATC TAAAATATTA ATATTAAAGG   
  
  
- ATTATAAATC GTAAGTAGTC GTTTAAGTCG TGTGTGGTTG TAATCAGTTG TGGATAGTTG TGAATATTCA   
  
  
- TGGTTAATTA TTATTAATGG ATATTATCTA ATTTTCAATC CGGGAATTAC AAAGGGTGTA ATAATCTATA   
  
  
- TAGTTCCCTC CCCCTTTTTT TATTATGAGG GGGTAGAATT TTTTAAATAG GGAAAAAAAG AAAAATTACA   
  
  
- AGGTTTTGTT TTTATACGAA GATTTAATAC TAACGTTATA CTTACACATG CCAAAGTTTG GGTTAAAAGA   
  
  
- GGTTGTTAAG AAGATGATAT AACATTAATA TAATATGATG TTAAACATAG AGTAGATACT AGATGGGGGG   
  
  
- TTTTTGGTTT ATGAGTATAT TTCAAAATAA AAGAAAGGTA GGGTGAGATT TTTTTTTTTT ACAACCCTTT   
  
  
- TTCAACTAAA TAAATTAAAC TTTACCATGC CACTTATATC TAAACATCTC CCCGTCAACT ATCTTTTTCC   
  
  
- ACTTCTGTTT CCTTCCCGTG TAAACGGAAA AGTATCTTCC TTCTCTGGTT CCATGACCCC TGACCCCCTT   
  
  
- CCCGAGTTCT CTTCTCTCTC TGACTCTTCT CACGACGATG AGGAAGGATG GATGTGTGTT TTAACGACGC   
  
  
- CCCCCAAGTT CAAGTTGAAC TTGCTCCGTT TATGGGAGTA ACTTGGGTGG TTTAGCTTTA GAAGTTACAT   
  
  
- GTGGGGGTAA TATGTGCGTA AGTCTCTGTA TCTACAGCTC CCCCTCTACT TAATCGTTCT TCTACAGCTC   
  
  
- GATTTTACGT AAAGCTCCAA TGATCTCGGT CTTTACACCC GTGTAGGGTC TTCTAGAGTC ATCTCTCTCA   
  
  
- TTGAAGTCTT TTCAAAGTTA AAAATAAGCT TTAAACGGAG AACAAACACA TCTATACTAC CCTTGAAGCG   
  
  
- GAATGTTAGA CGTTACACTC CCAAACCAAC TTCCTCAACG TCAGTTAGGA GTTAAAATAC TTAGTTGGGG   
  
  
- TTTCACCTTC TTCCCGTAGT TTCTTCTCAG TTAACTCTCG CTTGGGTGAA GCCAAAACCT GTTGGGATCG   
  
  
- GGAGGTTTAA GACGTAGATG AGAGAGAAGG ACGAAGTTAC CACCTTCATC ACCCGAACCC TACCTGCTAA   
  
  
- ACCTCCCAAA CAGAAAACCT CTTCCCAGGG AAGAGGGAAC CAAGTACCCA CTTTAACTTC TGGGATCAAA   
  
  
- CTCAAAGTTC GTGGAGGAAG TTGGAAGTTT AGGGAACCTC AAACCACCGT TACTACCCGA ACTTCGTCGA   
  
  
- TAAGTCTCAC GACCATACCC ATTAAAATTA AACCAACTTT CAAAATTAAG ACTAAACTCC AGAACATTAA   
  
  
- GACCCAGATA AACGTTACCC CCCTTACTAA GACAGTTACT TTTCCCTACA CAAGAATTAA TACGAAGTCT   
  
  
- CGTCCCAGTA CACCAACGAC ATAGAGGTCA TCTTGTCAAA TCCTAATCAC TACACTTTGT ACTCCACAAC   
  
  
- TTGGGTTTTA AACCGAAAAC ACTCGTTCCG AGATGTTAGT CAGTCTTGAG ACCAATACAC GTATATGACC   
  
  
- CAATATTGGT CGATCTTAGA GGGGAACTCG GAGGCCGTTT CGCGGTGTTA CGACCCTTAC GAGAAATGTG   
  
  
- ATCACAACAA GGTCAAGGTT TCTCAAGCAA AAATCCACTC CTAGTACTTT AAGATGAGTC TTTAGTTCTC   
  
  
- GTCGTCAAAG ACTACGTTGT TGTCACCAAT TACCCAAATC GGGGAGTCAA ATGTGGGGTC GTGGGCGGTT   
  
  
- TCGGTAATCT GCTGGCTTTC GTTGGTTTGA GCGTTTGAAA CCAAGACGCG CTAGTCAATC AATTCTAACG   
  
  
- CCTCTACAAG GTTTGGCCAT TGAAAAGCGA ACGGGTTCAC TATAACCGGG CCGAGTTAGT CGTTAACAGA   
  
  
- GAAAGTCCCT TGGGGAAACA TTCTCGACGA TGAAAGGTAC AGTTCCTCCT TGAACTTTCC GAGGACTGAT   
  
  
- AGTTATCGAA GAGACGCCGA GGTGGGGTTG GATTTTCGGA ATGAGACAGT CTACAACACC TATCCTACTT   
  
  
- ACGTATGTTC GAAAAAAGTC TCCGTAGGGG TCAATACGTT AAATGGCTAA AATGAACATG GGTCCGAAAC   
  
  
- GAGCTTCGAG AACTACTACG ACTAATATAG GTGTATGATC TAAAACTGTA ACCAACACCA AGGGTTACCC   
  
  
- GTAGGAAATA AGCTCTCGAA GGAGAGTCCT TTTCCCCGTG AGGTAAAAAC TTTTAATGTC GGAAACGGAG   
  
  
- AAGAAATTGG GTATTAGGTA AACTTGATCG GGAACATACA CTATTGTAAG ACCTTAAACG TTTTCTCAAG   
  
  
- CCACAGAGAC AGCTGGAAGT CCAGTACTTA AACCTGAACA AACTGGGTTG ATGAACGTGT TATGGGTTAA   
  
  
- AAGTTAGGTG ACTCTTGCGT CGTCAACGAC AGTTGAAGGG ATATACCCGC ACGAGTGTGA CGGGCAAACA   
  
  
- TGAGTCAGTT GAGGACAGGA AGTAGTTCGT TACAAGGGGT TTTTAGTACT GCTCGAAACT ATCTCCAACA   
  
  
- CTAGTAAAAC TCAACAGAAA AGGTAGCGTG TAGTAGGCAC ATAACCTGAG TACGAGATTA TACAACCTCA   
  
  
- GGGAGGTACC AAGTTCGCAC TGTAATCTGT GGAGTTTCTT CTAACTTTTC AAGAACTAGT TCGGATATTA   
  
  
- ACTCTCCCGT TATAACCCCG CTTAAGTACG TGGTCTGTTC TACGTCCGTT TGTTCCGTGT GGCCACCTTC   
  
  
- TTAGAGAAGT GAAGACGACC CAAGAAGGGA AACGGTAAAT CATCAAAGTG ACTTAGAGTC CGGTTAGATC   
  
  
- ACCGAGTTGC TTGGGGTCAC TCCCCAAAAG TCCACCTTTT CGTCGTTCGT AGTGAACACG AGTCCACCTA   
  
  
- CAGAGTACTC GAGCACCGTC GAAGTCGAAC CTCCACGAT

+     TCCC-motif

| Site Name | Organism | Position | Strand | Matrix score. | sequence | function |
| --- | --- | --- | --- | --- | --- | --- |
| TCCC-motif | Spinacia oleracea | 2276 | + | 7 | TCTCCCT | part of a light responsive element |

>HU04G00048.1   
+ -Up\_Stream \_Len000TTTGTA TTTAAATTTT ACAATATTTA TAAAAATTTT GTATTGATTA ATTGTGCTAA   
  
  
+ CTAACACTAA TAATTGGGGG AGAAAAGAAG GGAAAATAAA AAGAAATTAA GAAGAAGAAA AGAAAACACC   
  
  
+ TTTGAAAAGG CAAAAAACTA AAATAAAACA ATAATGAACA GAAGGAGAGA AGGATGGATA AACAACAAAT   
  
  
+ GTCGCTTTTA GGACAGCAAT AGTCTATGTG ATTGGCCTTA GTTCCTGTTG GAGGATTGCT AACAAAAGCA   
  
  
+ ACAGCAAAAA ATAATACTAA TTATTACATA TTTTTTAACT CTTAAAGCTA ATCAATCATC CTCCATTAAT   
  
  
+ TGATTAATTA TTTGCCAAAA ACACAAATCA ACTGGGGCAA CTATAAGAGA TCTACCTGCT TTAAGATATA   
  
  
+ TAAACTCAAT TGTTTCATTT TATTTTATTT TAGATTTTTT GGCCTTTGGT CTCTATTGAG CTAAAATCTC   
  
  
+ GTTATTTTCG GATCATGAAA AATACAACTC GATCTTTTGA TATAAGTCAT CTTTATTTGT AAATTCTGAA   
  
  
+ CTTAAAATCT TACTTGAAGA TCAGGAATAG AAGCACTCGA TTTTTGATAA ATCCTATGTG TTCTTCTTAA   
  
  
+ AATTCATAAT CTAATTCATA TTTTAGATGT ACCCGTTGCC TACTTTGGCA AGTTTAGATT ATGATTTTTA   
  
  
+ TTTTTTTTAA TTACTTGTAT TTTTCTTTTG GATTGATAGT CAATCTACAT CATCTTTGAT CAATTGCGAC   
  
  
+ AGTTTCATTT TCCCAACAAA AATTTCGAGT GTATTCTTTT TAATTTATCA TAAGTATTTA TTGTCAACAT   
  
  
+ TCAACATGCA TCAACAATCA TTAAACACTT TTCGTCACTC CTCAATACTG GTCAGCACTT AAGATTTTGT   
  
  
+ CTTCTAAACT TATCGTAAGT AACTTATTGT TGATTAATTG ATGCTGACCA AATGATACTG AGTGCTAAAA   
  
  
+ TTATAATTAA TAAATACAAT TAAGAGTTGA TCAATGTCGA TGATTCAAAT CAATGTGATT GGTGCTGAAT   
  
  
+ AGTAATTATA AGCACCTATA AAGACTTGAA AAGAACAAAC ATTTAATTAG ATTTTATAAT TATAATTTCC   
  
  
+ TAATATTTAG CATTCATCAG CAAATTCAGC ACACACCAAC ATTAGTCAAC ACCTATCAAC ACTTATAAGT   
  
  
+ ACCAATTAAT AATAATTACC TATAATAGAT TAAAAGTTAG GCCCTTAATG TTTCCCACAT TATTAGATAT   
  
  
+ ATCAAGGGAG GGGGAAAAAA ATAATACTCC CCCATCTTAA AAAATTTATC CCTTTTTTTC TTTTTAATGT   
  
  
+ TCCAAAACAA AAATATGCTT CTAAATTATG ATTGCAATAT GAATGTGTAC GGTTTCAAAC CCAATTTTCT   
  
  
+ CCAACAATTC TTCTACTATA TTGTAATTAT ATTATACTAC AATTTGTATC TCATCTATGA TCTACCCCCC   
  
  
+ AAAAACCAAA TACTCATATA AAGTTTTATT TTCTTTCCAT CCCACTCTAA AAAAAAAAAA TGTTGGGAAA   
  
  
+ AAGTTGATTT ATTTAATTTG AAATGGTACG GTGAATATAG ATTTGTAGAG GGGCAGTTGA TAGAAAAAGG   
  
  
+ TGAAGACAAA GGAAGGGCAC ATTTGCCTTT TCATAGAAGG AAGAGACCAA GGTACTGGGG ACTGGGGGAA   
  
  
+ GGGCTCAAGA GAAGAGAGAG ACTGAGAAGA GTGCTGCTAC TCCTTCCTAC CTACACACAA AATTGCTGCG   
  
  
+ GGGGGTTCAA GTTCAACTTG AACGAGGCAA ATACCCTCAT TGAACCCACC AAATCGAAAT CTTCAATGTA   
  
  
+ CACCCCCATT ATACACGCAT TCAGAGACAT AGATGTCGAG GGGGAGATGA ATTAGCAAGA AGATGTCGAG   
  
  
+ CTAAAATGCA TTTCGAGGTT ACTAGAGCCA GAAATGTGGG CACATCCCAG AAGATCTCAG TAGAGAGAGT   
  
  
+ AACTTCAGAA AAGTTTCAAT TTTTATTCGA AATTTGCCTC TTGTTTGTGT AGATATGATG GGAACTTCGC   
  
  
+ CTTACAATCT GCAATGTGAG GGTTTGGTTG AAGGAGTTGC AGTCAATCCT CAATTTTATG AATCAACCCC   
  
  
+ AAAGTGGAAG AAGGGCATCA AAGAAGAGTC AATTGAGAGC GAACCCACTT CGGTTTTGGA CAACCCTAGC   
  
  
+ CCTCCAAATT CTGCATCTAC TCTCTCTTCC TGCTTCAATG GTGGAAGTAG TGGGCTTGGG ATGGACGATT   
  
  
+ TGGAGGGTTT GTCTTTTGGA GAAGGGTCCC TTCTCCCTTG GTTCATGGGT GAAATTGAAG ACCCTAGTTT   
  
  
+ GAGTTTCAAG CACCTCCTTC AACCTTCAAA TCCCTTGGAG TTTGGTGGCA ATGATGGGCT TGAAGCAGCT   
  
  
+ ATTCAGAGTG CTGGTATGGG TAATTTTAAT TTGGTTGAAA GTTTTAATTC TGATTTGAGG TCTTGTAATT   
  
  
+ CTGGGTCTAT TTGCAATGGG GGGAATGATT CTGTCAATGA AAAGGGATGT GTTCTTAATT ATGCTTCAGA   
  
  
+ GCAGGGTCAT GTGGTTGCTG TATCTCCAGT AGAACAGTTT AGGATTAGTG ATGTGAAACA TGAGGTGTTG   
  
  
+ AACCCAAAAT TTGGCTTTTG TGAGCAAGGC TCTACAATCA GTCAGAACTC TGGTTATGTG CATATACTGG   
  
  
+ GTTATAACCA GCTAGAATCT CCCCTTGAGC CTCCGGCAAA GCGCCACAAT GCTGGGAATG CTCTTTACAC   
  
  
+ TAGTGTTGTT CCAGTTCCAA AGAGTTCGTT TTTAGGTGAG GATCATGAAA TTCTACTCAG AAATCAAGAG   
  
  
+ CAGCAGTTTC TGATGCAACA ACAGTGGTTA ATGGGTTTAG CCCCTCAGTT TACACCCCAG CACCCGCCAA   
  
  
+ AGCCATTAGA CGACCGAAAG CAACCAAACT CGCAAACTTT GGTTCTGCGC GATCAGTTAG TTAAGATTGC   
  
  
+ GGAGATGTTC CAAACCGGTA ACTTTTCGCT TGCCCAAGTG ATATTGGCCC GGCTCAATCA GCAATTGTCT   
  
  
+ CTTTCAGGGA ACCCCTTTGT AAGAGCTGCT ACTTTCCATG TCAAGGAGGA ACTTGAAAGG CTCCTGACTA   
  
  
+ TCAATAGCTT CTCTGCGGCT CCACCCCAAC CTAAAAGCCT TACTCTGTCA GATGTTGTGG ATAGGATGAA   
  
  
+ TGCATACAAG CTTTTTTCAG AGGCATCCCC AGTTATGCAA TTTACCGATT TTACTTGTAC CCAGGCTTTG   
  
  
+ CTCGAAGCTC TTGATGATGC TGATTATATC CACATACTAG ATTTTGACAT TGGTTGTGGT TCCCAATGGG   
  
  
+ CATCCTTTAT TCGAGAGCTT CCTCTCAGGA AAAGGGGCAC TCCATTTTTG AAAATTACAG CCTTTGCCTC   
  
  
+ TTCTTTAACC CATAATCCAT TTGAACTAGC CCTTGTATGT GATAACATTC TGGAATTTGC AAAAGAGTTC   
  
  
+ GGTGTCTCTG TCGACCTTCA GGTCATGAAT TTGGACTTGT TTGACCCAAC TACTTGCACA ATACCCAATT   
  
  
+ TTCAATCCAC TGAGAACGCA GCAGTTGCTG TCAACTTCCC TATATGGGCG TGCTCACACT GCCCGTTTGT   
  
  
+ ACTCAGTCAA CTCCTGTCCT TCATCAAGCA ATGTTCCCCA AAAATCATGA CGAGCTTTGA TAGAGGTTGT   
  
  
+ GATCATTTTG AGTTGTCTTT TCCATCGCAC ATCATCCGTG TATTGGACTC ATGCTCTAAT ATGTTGGAGT   
  
  
+ CCCTCCATGG TTCAAGCGTG ACATTAGACA CCTCAAAGAA GATTGAAAAG TTCTTGATCA AGCCTATAAT   
  
  
+ TGAGAGGGCA ATATTGGGGC GAATTCATGC ACCAGACAAG ATGCAGGCAA ACAAGGCACA CCGGTGGAAG   
  
  
+ AATCTCTTCA CTTCTGCTGG GTTCTTCCCT TTGCCATTTA GTAGTTTCAC TGAATCTCAG GCCAATCTAG   
  
  
+ TGGCTCAACG AACCCCAGTG AGGGGTTTTC AGGTGGAAAA GCAGCAAGCA TCACTTGTGC TCAGGTGGAT   
  
  
+ GTCTCATGAG CTCGTGGCAG CTTCAGCTTG GAGGTGCTA  

- -Up\_Stream \_Len000AAACAT AAATTTAAAA TGTTATAAAT ATTTTTAAAA CATAACTAAT TAACACGATT   
  
  
- GATTGTGATT ATTAACCCCC TCTTTTCTTC CCTTTTATTT TTCTTTAATT CTTCTTCTTT TCTTTTGTGG   
  
  
- AAACTTTTCC GTTTTTTGAT TTTATTTTGT TATTACTTGT CTTCCTCTCT TCCTACCTAT TTGTTGTTTA   
  
  
- CAGCGAAAAT CCTGTCGTTA TCAGATACAC TAACCGGAAT CAAGGACAAC CTCCTAACGA TTGTTTTCGT   
  
  
- TGTCGTTTTT TATTATGATT AATAATGTAT AAAAAATTGA GAATTTCGAT TAGTTAGTAG GAGGTAATTA   
  
  
- ACTAATTAAT AAACGGTTTT TGTGTTTAGT TGACCCCGTT GATATTCTCT AGATGGACGA AATTCTATAT   
  
  
- ATTTGAGTTA ACAAAGTAAA ATAAAATAAA ATCTAAAAAA CCGGAAACCA GAGATAACTC GATTTTAGAG   
  
  
- CAATAAAAGC CTAGTACTTT TTATGTTGAG CTAGAAAACT ATATTCAGTA GAAATAAACA TTTAAGACTT   
  
  
- GAATTTTAGA ATGAACTTCT AGTCCTTATC TTCGTGAGCT AAAAACTATT TAGGATACAC AAGAAGAATT   
  
  
- TTAAGTATTA GATTAAGTAT AAAATCTACA TGGGCAACGG ATGAAACCGT TCAAATCTAA TACTAAAAAT   
  
  
- AAAAAAAATT AATGAACATA AAAAGAAAAC CTAACTATCA GTTAGATGTA GTAGAAACTA GTTAACGCTG   
  
  
- TCAAAGTAAA AGGGTTGTTT TTAAAGCTCA CATAAGAAAA ATTAAATAGT ATTCATAAAT AACAGTTGTA   
  
  
- AGTTGTACGT AGTTGTTAGT AATTTGTGAA AAGCAGTGAG GAGTTATGAC CAGTCGTGAA TTCTAAAACA   
  
  
- GAAGATTTGA ATAGCATTCA TTGAATAACA ACTAATTAAC TACGACTGGT TTACTATGAC TCACGATTTT   
  
  
- AATATTAATT ATTTATGTTA ATTCTCAACT AGTTACAGCT ACTAAGTTTA GTTACACTAA CCACGACTTA   
  
  
- TCATTAATAT TCGTGGATAT TTCTGAACTT TTCTTGTTTG TAAATTAATC TAAAATATTA ATATTAAAGG   
  
  
- ATTATAAATC GTAAGTAGTC GTTTAAGTCG TGTGTGGTTG TAATCAGTTG TGGATAGTTG TGAATATTCA   
  
  
- TGGTTAATTA TTATTAATGG ATATTATCTA ATTTTCAATC CGGGAATTAC AAAGGGTGTA ATAATCTATA   
  
  
- TAGTTCCCTC CCCCTTTTTT TATTATGAGG GGGTAGAATT TTTTAAATAG GGAAAAAAAG AAAAATTACA   
  
  
- AGGTTTTGTT TTTATACGAA GATTTAATAC TAACGTTATA CTTACACATG CCAAAGTTTG GGTTAAAAGA   
  
  
- GGTTGTTAAG AAGATGATAT AACATTAATA TAATATGATG TTAAACATAG AGTAGATACT AGATGGGGGG   
  
  
- TTTTTGGTTT ATGAGTATAT TTCAAAATAA AAGAAAGGTA GGGTGAGATT TTTTTTTTTT ACAACCCTTT   
  
  
- TTCAACTAAA TAAATTAAAC TTTACCATGC CACTTATATC TAAACATCTC CCCGTCAACT ATCTTTTTCC   
  
  
- ACTTCTGTTT CCTTCCCGTG TAAACGGAAA AGTATCTTCC TTCTCTGGTT CCATGACCCC TGACCCCCTT   
  
  
- CCCGAGTTCT CTTCTCTCTC TGACTCTTCT CACGACGATG AGGAAGGATG GATGTGTGTT TTAACGACGC   
  
  
- CCCCCAAGTT CAAGTTGAAC TTGCTCCGTT TATGGGAGTA ACTTGGGTGG TTTAGCTTTA GAAGTTACAT   
  
  
- GTGGGGGTAA TATGTGCGTA AGTCTCTGTA TCTACAGCTC CCCCTCTACT TAATCGTTCT TCTACAGCTC   
  
  
- GATTTTACGT AAAGCTCCAA TGATCTCGGT CTTTACACCC GTGTAGGGTC TTCTAGAGTC ATCTCTCTCA   
  
  
- TTGAAGTCTT TTCAAAGTTA AAAATAAGCT TTAAACGGAG AACAAACACA TCTATACTAC CCTTGAAGCG   
  
  
- GAATGTTAGA CGTTACACTC CCAAACCAAC TTCCTCAACG TCAGTTAGGA GTTAAAATAC TTAGTTGGGG   
  
  
- TTTCACCTTC TTCCCGTAGT TTCTTCTCAG TTAACTCTCG CTTGGGTGAA GCCAAAACCT GTTGGGATCG   
  
  
- GGAGGTTTAA GACGTAGATG AGAGAGAAGG ACGAAGTTAC CACCTTCATC ACCCGAACCC TACCTGCTAA   
  
  
- ACCTCCCAAA CAGAAAACCT CTTCCCAGGG AAGAGGGAAC CAAGTACCCA CTTTAACTTC TGGGATCAAA   
  
  
- CTCAAAGTTC GTGGAGGAAG TTGGAAGTTT AGGGAACCTC AAACCACCGT TACTACCCGA ACTTCGTCGA   
  
  
- TAAGTCTCAC GACCATACCC ATTAAAATTA AACCAACTTT CAAAATTAAG ACTAAACTCC AGAACATTAA   
  
  
- GACCCAGATA AACGTTACCC CCCTTACTAA GACAGTTACT TTTCCCTACA CAAGAATTAA TACGAAGTCT   
  
  
- CGTCCCAGTA CACCAACGAC ATAGAGGTCA TCTTGTCAAA TCCTAATCAC TACACTTTGT ACTCCACAAC   
  
  
- TTGGGTTTTA AACCGAAAAC ACTCGTTCCG AGATGTTAGT CAGTCTTGAG ACCAATACAC GTATATGACC   
  
  
- CAATATTGGT CGATCTTAGA GGGGAACTCG GAGGCCGTTT CGCGGTGTTA CGACCCTTAC GAGAAATGTG   
  
  
- ATCACAACAA GGTCAAGGTT TCTCAAGCAA AAATCCACTC CTAGTACTTT AAGATGAGTC TTTAGTTCTC   
  
  
- GTCGTCAAAG ACTACGTTGT TGTCACCAAT TACCCAAATC GGGGAGTCAA ATGTGGGGTC GTGGGCGGTT   
  
  
- TCGGTAATCT GCTGGCTTTC GTTGGTTTGA GCGTTTGAAA CCAAGACGCG CTAGTCAATC AATTCTAACG   
  
  
- CCTCTACAAG GTTTGGCCAT TGAAAAGCGA ACGGGTTCAC TATAACCGGG CCGAGTTAGT CGTTAACAGA   
  
  
- GAAAGTCCCT TGGGGAAACA TTCTCGACGA TGAAAGGTAC AGTTCCTCCT TGAACTTTCC GAGGACTGAT   
  
  
- AGTTATCGAA GAGACGCCGA GGTGGGGTTG GATTTTCGGA ATGAGACAGT CTACAACACC TATCCTACTT   
  
  
- ACGTATGTTC GAAAAAAGTC TCCGTAGGGG TCAATACGTT AAATGGCTAA AATGAACATG GGTCCGAAAC   
  
  
- GAGCTTCGAG AACTACTACG ACTAATATAG GTGTATGATC TAAAACTGTA ACCAACACCA AGGGTTACCC   
  
  
- GTAGGAAATA AGCTCTCGAA GGAGAGTCCT TTTCCCCGTG AGGTAAAAAC TTTTAATGTC GGAAACGGAG   
  
  
- AAGAAATTGG GTATTAGGTA AACTTGATCG GGAACATACA CTATTGTAAG ACCTTAAACG TTTTCTCAAG   
  
  
- CCACAGAGAC AGCTGGAAGT CCAGTACTTA AACCTGAACA AACTGGGTTG ATGAACGTGT TATGGGTTAA   
  
  
- AAGTTAGGTG ACTCTTGCGT CGTCAACGAC AGTTGAAGGG ATATACCCGC ACGAGTGTGA CGGGCAAACA   
  
  
- TGAGTCAGTT GAGGACAGGA AGTAGTTCGT TACAAGGGGT TTTTAGTACT GCTCGAAACT ATCTCCAACA   
  
  
- CTAGTAAAAC TCAACAGAAA AGGTAGCGTG TAGTAGGCAC ATAACCTGAG TACGAGATTA TACAACCTCA   
  
  
- GGGAGGTACC AAGTTCGCAC TGTAATCTGT GGAGTTTCTT CTAACTTTTC AAGAACTAGT TCGGATATTA   
  
  
- ACTCTCCCGT TATAACCCCG CTTAAGTACG TGGTCTGTTC TACGTCCGTT TGTTCCGTGT GGCCACCTTC   
  
  
- TTAGAGAAGT GAAGACGACC CAAGAAGGGA AACGGTAAAT CATCAAAGTG ACTTAGAGTC CGGTTAGATC   
  
  
- ACCGAGTTGC TTGGGGTCAC TCCCCAAAAG TCCACCTTTT CGTCGTTCGT AGTGAACACG AGTCCACCTA   
  
  
- CAGAGTACTC GAGCACCGTC GAAGTCGAAC CTCCACGAT

+     TCT-motif

| Site Name | Organism | Position | Strand | Matrix score. | sequence | function |
| --- | --- | --- | --- | --- | --- | --- |
| TCT-motif | Arabidopsis thaliana | 572 | + | 6 | TCTTAC | part of a light responsive element |
| TCT-motif | Arabidopsis thaliana | 3033 | - | 6 | TCTTAC | part of a light responsive element |

>HU04G00048.1   
+ -Up\_Stream \_Len000TTTGTA TTTAAATTTT ACAATATTTA TAAAAATTTT GTATTGATTA ATTGTGCTAA   
  
  
+ CTAACACTAA TAATTGGGGG AGAAAAGAAG GGAAAATAAA AAGAAATTAA GAAGAAGAAA AGAAAACACC   
  
  
+ TTTGAAAAGG CAAAAAACTA AAATAAAACA ATAATGAACA GAAGGAGAGA AGGATGGATA AACAACAAAT   
  
  
+ GTCGCTTTTA GGACAGCAAT AGTCTATGTG ATTGGCCTTA GTTCCTGTTG GAGGATTGCT AACAAAAGCA   
  
  
+ ACAGCAAAAA ATAATACTAA TTATTACATA TTTTTTAACT CTTAAAGCTA ATCAATCATC CTCCATTAAT   
  
  
+ TGATTAATTA TTTGCCAAAA ACACAAATCA ACTGGGGCAA CTATAAGAGA TCTACCTGCT TTAAGATATA   
  
  
+ TAAACTCAAT TGTTTCATTT TATTTTATTT TAGATTTTTT GGCCTTTGGT CTCTATTGAG CTAAAATCTC   
  
  
+ GTTATTTTCG GATCATGAAA AATACAACTC GATCTTTTGA TATAAGTCAT CTTTATTTGT AAATTCTGAA   
  
  
+ CTTAAAATCT TACTTGAAGA TCAGGAATAG AAGCACTCGA TTTTTGATAA ATCCTATGTG TTCTTCTTAA   
  
  
+ AATTCATAAT CTAATTCATA TTTTAGATGT ACCCGTTGCC TACTTTGGCA AGTTTAGATT ATGATTTTTA   
  
  
+ TTTTTTTTAA TTACTTGTAT TTTTCTTTTG GATTGATAGT CAATCTACAT CATCTTTGAT CAATTGCGAC   
  
  
+ AGTTTCATTT TCCCAACAAA AATTTCGAGT GTATTCTTTT TAATTTATCA TAAGTATTTA TTGTCAACAT   
  
  
+ TCAACATGCA TCAACAATCA TTAAACACTT TTCGTCACTC CTCAATACTG GTCAGCACTT AAGATTTTGT   
  
  
+ CTTCTAAACT TATCGTAAGT AACTTATTGT TGATTAATTG ATGCTGACCA AATGATACTG AGTGCTAAAA   
  
  
+ TTATAATTAA TAAATACAAT TAAGAGTTGA TCAATGTCGA TGATTCAAAT CAATGTGATT GGTGCTGAAT   
  
  
+ AGTAATTATA AGCACCTATA AAGACTTGAA AAGAACAAAC ATTTAATTAG ATTTTATAAT TATAATTTCC   
  
  
+ TAATATTTAG CATTCATCAG CAAATTCAGC ACACACCAAC ATTAGTCAAC ACCTATCAAC ACTTATAAGT   
  
  
+ ACCAATTAAT AATAATTACC TATAATAGAT TAAAAGTTAG GCCCTTAATG TTTCCCACAT TATTAGATAT   
  
  
+ ATCAAGGGAG GGGGAAAAAA ATAATACTCC CCCATCTTAA AAAATTTATC CCTTTTTTTC TTTTTAATGT   
  
  
+ TCCAAAACAA AAATATGCTT CTAAATTATG ATTGCAATAT GAATGTGTAC GGTTTCAAAC CCAATTTTCT   
  
  
+ CCAACAATTC TTCTACTATA TTGTAATTAT ATTATACTAC AATTTGTATC TCATCTATGA TCTACCCCCC   
  
  
+ AAAAACCAAA TACTCATATA AAGTTTTATT TTCTTTCCAT CCCACTCTAA AAAAAAAAAA TGTTGGGAAA   
  
  
+ AAGTTGATTT ATTTAATTTG AAATGGTACG GTGAATATAG ATTTGTAGAG GGGCAGTTGA TAGAAAAAGG   
  
  
+ TGAAGACAAA GGAAGGGCAC ATTTGCCTTT TCATAGAAGG AAGAGACCAA GGTACTGGGG ACTGGGGGAA   
  
  
+ GGGCTCAAGA GAAGAGAGAG ACTGAGAAGA GTGCTGCTAC TCCTTCCTAC CTACACACAA AATTGCTGCG   
  
  
+ GGGGGTTCAA GTTCAACTTG AACGAGGCAA ATACCCTCAT TGAACCCACC AAATCGAAAT CTTCAATGTA   
  
  
+ CACCCCCATT ATACACGCAT TCAGAGACAT AGATGTCGAG GGGGAGATGA ATTAGCAAGA AGATGTCGAG   
  
  
+ CTAAAATGCA TTTCGAGGTT ACTAGAGCCA GAAATGTGGG CACATCCCAG AAGATCTCAG TAGAGAGAGT   
  
  
+ AACTTCAGAA AAGTTTCAAT TTTTATTCGA AATTTGCCTC TTGTTTGTGT AGATATGATG GGAACTTCGC   
  
  
+ CTTACAATCT GCAATGTGAG GGTTTGGTTG AAGGAGTTGC AGTCAATCCT CAATTTTATG AATCAACCCC   
  
  
+ AAAGTGGAAG AAGGGCATCA AAGAAGAGTC AATTGAGAGC GAACCCACTT CGGTTTTGGA CAACCCTAGC   
  
  
+ CCTCCAAATT CTGCATCTAC TCTCTCTTCC TGCTTCAATG GTGGAAGTAG TGGGCTTGGG ATGGACGATT   
  
  
+ TGGAGGGTTT GTCTTTTGGA GAAGGGTCCC TTCTCCCTTG GTTCATGGGT GAAATTGAAG ACCCTAGTTT   
  
  
+ GAGTTTCAAG CACCTCCTTC AACCTTCAAA TCCCTTGGAG TTTGGTGGCA ATGATGGGCT TGAAGCAGCT   
  
  
+ ATTCAGAGTG CTGGTATGGG TAATTTTAAT TTGGTTGAAA GTTTTAATTC TGATTTGAGG TCTTGTAATT   
  
  
+ CTGGGTCTAT TTGCAATGGG GGGAATGATT CTGTCAATGA AAAGGGATGT GTTCTTAATT ATGCTTCAGA   
  
  
+ GCAGGGTCAT GTGGTTGCTG TATCTCCAGT AGAACAGTTT AGGATTAGTG ATGTGAAACA TGAGGTGTTG   
  
  
+ AACCCAAAAT TTGGCTTTTG TGAGCAAGGC TCTACAATCA GTCAGAACTC TGGTTATGTG CATATACTGG   
  
  
+ GTTATAACCA GCTAGAATCT CCCCTTGAGC CTCCGGCAAA GCGCCACAAT GCTGGGAATG CTCTTTACAC   
  
  
+ TAGTGTTGTT CCAGTTCCAA AGAGTTCGTT TTTAGGTGAG GATCATGAAA TTCTACTCAG AAATCAAGAG   
  
  
+ CAGCAGTTTC TGATGCAACA ACAGTGGTTA ATGGGTTTAG CCCCTCAGTT TACACCCCAG CACCCGCCAA   
  
  
+ AGCCATTAGA CGACCGAAAG CAACCAAACT CGCAAACTTT GGTTCTGCGC GATCAGTTAG TTAAGATTGC   
  
  
+ GGAGATGTTC CAAACCGGTA ACTTTTCGCT TGCCCAAGTG ATATTGGCCC GGCTCAATCA GCAATTGTCT   
  
  
+ CTTTCAGGGA ACCCCTTTGT AAGAGCTGCT ACTTTCCATG TCAAGGAGGA ACTTGAAAGG CTCCTGACTA   
  
  
+ TCAATAGCTT CTCTGCGGCT CCACCCCAAC CTAAAAGCCT TACTCTGTCA GATGTTGTGG ATAGGATGAA   
  
  
+ TGCATACAAG CTTTTTTCAG AGGCATCCCC AGTTATGCAA TTTACCGATT TTACTTGTAC CCAGGCTTTG   
  
  
+ CTCGAAGCTC TTGATGATGC TGATTATATC CACATACTAG ATTTTGACAT TGGTTGTGGT TCCCAATGGG   
  
  
+ CATCCTTTAT TCGAGAGCTT CCTCTCAGGA AAAGGGGCAC TCCATTTTTG AAAATTACAG CCTTTGCCTC   
  
  
+ TTCTTTAACC CATAATCCAT TTGAACTAGC CCTTGTATGT GATAACATTC TGGAATTTGC AAAAGAGTTC   
  
  
+ GGTGTCTCTG TCGACCTTCA GGTCATGAAT TTGGACTTGT TTGACCCAAC TACTTGCACA ATACCCAATT   
  
  
+ TTCAATCCAC TGAGAACGCA GCAGTTGCTG TCAACTTCCC TATATGGGCG TGCTCACACT GCCCGTTTGT   
  
  
+ ACTCAGTCAA CTCCTGTCCT TCATCAAGCA ATGTTCCCCA AAAATCATGA CGAGCTTTGA TAGAGGTTGT   
  
  
+ GATCATTTTG AGTTGTCTTT TCCATCGCAC ATCATCCGTG TATTGGACTC ATGCTCTAAT ATGTTGGAGT   
  
  
+ CCCTCCATGG TTCAAGCGTG ACATTAGACA CCTCAAAGAA GATTGAAAAG TTCTTGATCA AGCCTATAAT   
  
  
+ TGAGAGGGCA ATATTGGGGC GAATTCATGC ACCAGACAAG ATGCAGGCAA ACAAGGCACA CCGGTGGAAG   
  
  
+ AATCTCTTCA CTTCTGCTGG GTTCTTCCCT TTGCCATTTA GTAGTTTCAC TGAATCTCAG GCCAATCTAG   
  
  
+ TGGCTCAACG AACCCCAGTG AGGGGTTTTC AGGTGGAAAA GCAGCAAGCA TCACTTGTGC TCAGGTGGAT   
  
  
+ GTCTCATGAG CTCGTGGCAG CTTCAGCTTG GAGGTGCTA  

- -Up\_Stream \_Len000AAACAT AAATTTAAAA TGTTATAAAT ATTTTTAAAA CATAACTAAT TAACACGATT   
  
  
- GATTGTGATT ATTAACCCCC TCTTTTCTTC CCTTTTATTT TTCTTTAATT CTTCTTCTTT TCTTTTGTGG   
  
  
- AAACTTTTCC GTTTTTTGAT TTTATTTTGT TATTACTTGT CTTCCTCTCT TCCTACCTAT TTGTTGTTTA   
  
  
- CAGCGAAAAT CCTGTCGTTA TCAGATACAC TAACCGGAAT CAAGGACAAC CTCCTAACGA TTGTTTTCGT   
  
  
- TGTCGTTTTT TATTATGATT AATAATGTAT AAAAAATTGA GAATTTCGAT TAGTTAGTAG GAGGTAATTA   
  
  
- ACTAATTAAT AAACGGTTTT TGTGTTTAGT TGACCCCGTT GATATTCTCT AGATGGACGA AATTCTATAT   
  
  
- ATTTGAGTTA ACAAAGTAAA ATAAAATAAA ATCTAAAAAA CCGGAAACCA GAGATAACTC GATTTTAGAG   
  
  
- CAATAAAAGC CTAGTACTTT TTATGTTGAG CTAGAAAACT ATATTCAGTA GAAATAAACA TTTAAGACTT   
  
  
- GAATTTTAGA ATGAACTTCT AGTCCTTATC TTCGTGAGCT AAAAACTATT TAGGATACAC AAGAAGAATT   
  
  
- TTAAGTATTA GATTAAGTAT AAAATCTACA TGGGCAACGG ATGAAACCGT TCAAATCTAA TACTAAAAAT   
  
  
- AAAAAAAATT AATGAACATA AAAAGAAAAC CTAACTATCA GTTAGATGTA GTAGAAACTA GTTAACGCTG   
  
  
- TCAAAGTAAA AGGGTTGTTT TTAAAGCTCA CATAAGAAAA ATTAAATAGT ATTCATAAAT AACAGTTGTA   
  
  
- AGTTGTACGT AGTTGTTAGT AATTTGTGAA AAGCAGTGAG GAGTTATGAC CAGTCGTGAA TTCTAAAACA   
  
  
- GAAGATTTGA ATAGCATTCA TTGAATAACA ACTAATTAAC TACGACTGGT TTACTATGAC TCACGATTTT   
  
  
- AATATTAATT ATTTATGTTA ATTCTCAACT AGTTACAGCT ACTAAGTTTA GTTACACTAA CCACGACTTA   
  
  
- TCATTAATAT TCGTGGATAT TTCTGAACTT TTCTTGTTTG TAAATTAATC TAAAATATTA ATATTAAAGG   
  
  
- ATTATAAATC GTAAGTAGTC GTTTAAGTCG TGTGTGGTTG TAATCAGTTG TGGATAGTTG TGAATATTCA   
  
  
- TGGTTAATTA TTATTAATGG ATATTATCTA ATTTTCAATC CGGGAATTAC AAAGGGTGTA ATAATCTATA   
  
  
- TAGTTCCCTC CCCCTTTTTT TATTATGAGG GGGTAGAATT TTTTAAATAG GGAAAAAAAG AAAAATTACA   
  
  
- AGGTTTTGTT TTTATACGAA GATTTAATAC TAACGTTATA CTTACACATG CCAAAGTTTG GGTTAAAAGA   
  
  
- GGTTGTTAAG AAGATGATAT AACATTAATA TAATATGATG TTAAACATAG AGTAGATACT AGATGGGGGG   
  
  
- TTTTTGGTTT ATGAGTATAT TTCAAAATAA AAGAAAGGTA GGGTGAGATT TTTTTTTTTT ACAACCCTTT   
  
  
- TTCAACTAAA TAAATTAAAC TTTACCATGC CACTTATATC TAAACATCTC CCCGTCAACT ATCTTTTTCC   
  
  
- ACTTCTGTTT CCTTCCCGTG TAAACGGAAA AGTATCTTCC TTCTCTGGTT CCATGACCCC TGACCCCCTT   
  
  
- CCCGAGTTCT CTTCTCTCTC TGACTCTTCT CACGACGATG AGGAAGGATG GATGTGTGTT TTAACGACGC   
  
  
- CCCCCAAGTT CAAGTTGAAC TTGCTCCGTT TATGGGAGTA ACTTGGGTGG TTTAGCTTTA GAAGTTACAT   
  
  
- GTGGGGGTAA TATGTGCGTA AGTCTCTGTA TCTACAGCTC CCCCTCTACT TAATCGTTCT TCTACAGCTC   
  
  
- GATTTTACGT AAAGCTCCAA TGATCTCGGT CTTTACACCC GTGTAGGGTC TTCTAGAGTC ATCTCTCTCA   
  
  
- TTGAAGTCTT TTCAAAGTTA AAAATAAGCT TTAAACGGAG AACAAACACA TCTATACTAC CCTTGAAGCG   
  
  
- GAATGTTAGA CGTTACACTC CCAAACCAAC TTCCTCAACG TCAGTTAGGA GTTAAAATAC TTAGTTGGGG   
  
  
- TTTCACCTTC TTCCCGTAGT TTCTTCTCAG TTAACTCTCG CTTGGGTGAA GCCAAAACCT GTTGGGATCG   
  
  
- GGAGGTTTAA GACGTAGATG AGAGAGAAGG ACGAAGTTAC CACCTTCATC ACCCGAACCC TACCTGCTAA   
  
  
- ACCTCCCAAA CAGAAAACCT CTTCCCAGGG AAGAGGGAAC CAAGTACCCA CTTTAACTTC TGGGATCAAA   
  
  
- CTCAAAGTTC GTGGAGGAAG TTGGAAGTTT AGGGAACCTC AAACCACCGT TACTACCCGA ACTTCGTCGA   
  
  
- TAAGTCTCAC GACCATACCC ATTAAAATTA AACCAACTTT CAAAATTAAG ACTAAACTCC AGAACATTAA   
  
  
- GACCCAGATA AACGTTACCC CCCTTACTAA GACAGTTACT TTTCCCTACA CAAGAATTAA TACGAAGTCT   
  
  
- CGTCCCAGTA CACCAACGAC ATAGAGGTCA TCTTGTCAAA TCCTAATCAC TACACTTTGT ACTCCACAAC   
  
  
- TTGGGTTTTA AACCGAAAAC ACTCGTTCCG AGATGTTAGT CAGTCTTGAG ACCAATACAC GTATATGACC   
  
  
- CAATATTGGT CGATCTTAGA GGGGAACTCG GAGGCCGTTT CGCGGTGTTA CGACCCTTAC GAGAAATGTG   
  
  
- ATCACAACAA GGTCAAGGTT TCTCAAGCAA AAATCCACTC CTAGTACTTT AAGATGAGTC TTTAGTTCTC   
  
  
- GTCGTCAAAG ACTACGTTGT TGTCACCAAT TACCCAAATC GGGGAGTCAA ATGTGGGGTC GTGGGCGGTT   
  
  
- TCGGTAATCT GCTGGCTTTC GTTGGTTTGA GCGTTTGAAA CCAAGACGCG CTAGTCAATC AATTCTAACG   
  
  
- CCTCTACAAG GTTTGGCCAT TGAAAAGCGA ACGGGTTCAC TATAACCGGG CCGAGTTAGT CGTTAACAGA   
  
  
- GAAAGTCCCT TGGGGAAACA TTCTCGACGA TGAAAGGTAC AGTTCCTCCT TGAACTTTCC GAGGACTGAT   
  
  
- AGTTATCGAA GAGACGCCGA GGTGGGGTTG GATTTTCGGA ATGAGACAGT CTACAACACC TATCCTACTT   
  
  
- ACGTATGTTC GAAAAAAGTC TCCGTAGGGG TCAATACGTT AAATGGCTAA AATGAACATG GGTCCGAAAC   
  
  
- GAGCTTCGAG AACTACTACG ACTAATATAG GTGTATGATC TAAAACTGTA ACCAACACCA AGGGTTACCC   
  
  
- GTAGGAAATA AGCTCTCGAA GGAGAGTCCT TTTCCCCGTG AGGTAAAAAC TTTTAATGTC GGAAACGGAG   
  
  
- AAGAAATTGG GTATTAGGTA AACTTGATCG GGAACATACA CTATTGTAAG ACCTTAAACG TTTTCTCAAG   
  
  
- CCACAGAGAC AGCTGGAAGT CCAGTACTTA AACCTGAACA AACTGGGTTG ATGAACGTGT TATGGGTTAA   
  
  
- AAGTTAGGTG ACTCTTGCGT CGTCAACGAC AGTTGAAGGG ATATACCCGC ACGAGTGTGA CGGGCAAACA   
  
  
- TGAGTCAGTT GAGGACAGGA AGTAGTTCGT TACAAGGGGT TTTTAGTACT GCTCGAAACT ATCTCCAACA   
  
  
- CTAGTAAAAC TCAACAGAAA AGGTAGCGTG TAGTAGGCAC ATAACCTGAG TACGAGATTA TACAACCTCA   
  
  
- GGGAGGTACC AAGTTCGCAC TGTAATCTGT GGAGTTTCTT CTAACTTTTC AAGAACTAGT TCGGATATTA   
  
  
- ACTCTCCCGT TATAACCCCG CTTAAGTACG TGGTCTGTTC TACGTCCGTT TGTTCCGTGT GGCCACCTTC   
  
  
- TTAGAGAAGT GAAGACGACC CAAGAAGGGA AACGGTAAAT CATCAAAGTG ACTTAGAGTC CGGTTAGATC   
  
  
- ACCGAGTTGC TTGGGGTCAC TCCCCAAAAG TCCACCTTTT CGTCGTTCGT AGTGAACACG AGTCCACCTA   
  
  
- CAGAGTACTC GAGCACCGTC GAAGTCGAAC CTCCACGAT

+     TGACG-motif

| Site Name | Organism | Position | Strand | Matrix score. | sequence | function |
| --- | --- | --- | --- | --- | --- | --- |
| TGACG-motif | Hordeum vulgare | 3622 | + | 5 | TGACG | cis-acting regulatory element involved in the MeJA-responsiveness |
| TGACG-motif | Hordeum vulgare | 877 | - | 5 | TGACG | cis-acting regulatory element involved in the MeJA-responsiveness |

>HU04G00048.1   
+ -Up\_Stream \_Len000TTTGTA TTTAAATTTT ACAATATTTA TAAAAATTTT GTATTGATTA ATTGTGCTAA   
  
  
+ CTAACACTAA TAATTGGGGG AGAAAAGAAG GGAAAATAAA AAGAAATTAA GAAGAAGAAA AGAAAACACC   
  
  
+ TTTGAAAAGG CAAAAAACTA AAATAAAACA ATAATGAACA GAAGGAGAGA AGGATGGATA AACAACAAAT   
  
  
+ GTCGCTTTTA GGACAGCAAT AGTCTATGTG ATTGGCCTTA GTTCCTGTTG GAGGATTGCT AACAAAAGCA   
  
  
+ ACAGCAAAAA ATAATACTAA TTATTACATA TTTTTTAACT CTTAAAGCTA ATCAATCATC CTCCATTAAT   
  
  
+ TGATTAATTA TTTGCCAAAA ACACAAATCA ACTGGGGCAA CTATAAGAGA TCTACCTGCT TTAAGATATA   
  
  
+ TAAACTCAAT TGTTTCATTT TATTTTATTT TAGATTTTTT GGCCTTTGGT CTCTATTGAG CTAAAATCTC   
  
  
+ GTTATTTTCG GATCATGAAA AATACAACTC GATCTTTTGA TATAAGTCAT CTTTATTTGT AAATTCTGAA   
  
  
+ CTTAAAATCT TACTTGAAGA TCAGGAATAG AAGCACTCGA TTTTTGATAA ATCCTATGTG TTCTTCTTAA   
  
  
+ AATTCATAAT CTAATTCATA TTTTAGATGT ACCCGTTGCC TACTTTGGCA AGTTTAGATT ATGATTTTTA   
  
  
+ TTTTTTTTAA TTACTTGTAT TTTTCTTTTG GATTGATAGT CAATCTACAT CATCTTTGAT CAATTGCGAC   
  
  
+ AGTTTCATTT TCCCAACAAA AATTTCGAGT GTATTCTTTT TAATTTATCA TAAGTATTTA TTGTCAACAT   
  
  
+ TCAACATGCA TCAACAATCA TTAAACACTT TTCGTCACTC CTCAATACTG GTCAGCACTT AAGATTTTGT   
  
  
+ CTTCTAAACT TATCGTAAGT AACTTATTGT TGATTAATTG ATGCTGACCA AATGATACTG AGTGCTAAAA   
  
  
+ TTATAATTAA TAAATACAAT TAAGAGTTGA TCAATGTCGA TGATTCAAAT CAATGTGATT GGTGCTGAAT   
  
  
+ AGTAATTATA AGCACCTATA AAGACTTGAA AAGAACAAAC ATTTAATTAG ATTTTATAAT TATAATTTCC   
  
  
+ TAATATTTAG CATTCATCAG CAAATTCAGC ACACACCAAC ATTAGTCAAC ACCTATCAAC ACTTATAAGT   
  
  
+ ACCAATTAAT AATAATTACC TATAATAGAT TAAAAGTTAG GCCCTTAATG TTTCCCACAT TATTAGATAT   
  
  
+ ATCAAGGGAG GGGGAAAAAA ATAATACTCC CCCATCTTAA AAAATTTATC CCTTTTTTTC TTTTTAATGT   
  
  
+ TCCAAAACAA AAATATGCTT CTAAATTATG ATTGCAATAT GAATGTGTAC GGTTTCAAAC CCAATTTTCT   
  
  
+ CCAACAATTC TTCTACTATA TTGTAATTAT ATTATACTAC AATTTGTATC TCATCTATGA TCTACCCCCC   
  
  
+ AAAAACCAAA TACTCATATA AAGTTTTATT TTCTTTCCAT CCCACTCTAA AAAAAAAAAA TGTTGGGAAA   
  
  
+ AAGTTGATTT ATTTAATTTG AAATGGTACG GTGAATATAG ATTTGTAGAG GGGCAGTTGA TAGAAAAAGG   
  
  
+ TGAAGACAAA GGAAGGGCAC ATTTGCCTTT TCATAGAAGG AAGAGACCAA GGTACTGGGG ACTGGGGGAA   
  
  
+ GGGCTCAAGA GAAGAGAGAG ACTGAGAAGA GTGCTGCTAC TCCTTCCTAC CTACACACAA AATTGCTGCG   
  
  
+ GGGGGTTCAA GTTCAACTTG AACGAGGCAA ATACCCTCAT TGAACCCACC AAATCGAAAT CTTCAATGTA   
  
  
+ CACCCCCATT ATACACGCAT TCAGAGACAT AGATGTCGAG GGGGAGATGA ATTAGCAAGA AGATGTCGAG   
  
  
+ CTAAAATGCA TTTCGAGGTT ACTAGAGCCA GAAATGTGGG CACATCCCAG AAGATCTCAG TAGAGAGAGT   
  
  
+ AACTTCAGAA AAGTTTCAAT TTTTATTCGA AATTTGCCTC TTGTTTGTGT AGATATGATG GGAACTTCGC   
  
  
+ CTTACAATCT GCAATGTGAG GGTTTGGTTG AAGGAGTTGC AGTCAATCCT CAATTTTATG AATCAACCCC   
  
  
+ AAAGTGGAAG AAGGGCATCA AAGAAGAGTC AATTGAGAGC GAACCCACTT CGGTTTTGGA CAACCCTAGC   
  
  
+ CCTCCAAATT CTGCATCTAC TCTCTCTTCC TGCTTCAATG GTGGAAGTAG TGGGCTTGGG ATGGACGATT   
  
  
+ TGGAGGGTTT GTCTTTTGGA GAAGGGTCCC TTCTCCCTTG GTTCATGGGT GAAATTGAAG ACCCTAGTTT   
  
  
+ GAGTTTCAAG CACCTCCTTC AACCTTCAAA TCCCTTGGAG TTTGGTGGCA ATGATGGGCT TGAAGCAGCT   
  
  
+ ATTCAGAGTG CTGGTATGGG TAATTTTAAT TTGGTTGAAA GTTTTAATTC TGATTTGAGG TCTTGTAATT   
  
  
+ CTGGGTCTAT TTGCAATGGG GGGAATGATT CTGTCAATGA AAAGGGATGT GTTCTTAATT ATGCTTCAGA   
  
  
+ GCAGGGTCAT GTGGTTGCTG TATCTCCAGT AGAACAGTTT AGGATTAGTG ATGTGAAACA TGAGGTGTTG   
  
  
+ AACCCAAAAT TTGGCTTTTG TGAGCAAGGC TCTACAATCA GTCAGAACTC TGGTTATGTG CATATACTGG   
  
  
+ GTTATAACCA GCTAGAATCT CCCCTTGAGC CTCCGGCAAA GCGCCACAAT GCTGGGAATG CTCTTTACAC   
  
  
+ TAGTGTTGTT CCAGTTCCAA AGAGTTCGTT TTTAGGTGAG GATCATGAAA TTCTACTCAG AAATCAAGAG   
  
  
+ CAGCAGTTTC TGATGCAACA ACAGTGGTTA ATGGGTTTAG CCCCTCAGTT TACACCCCAG CACCCGCCAA   
  
  
+ AGCCATTAGA CGACCGAAAG CAACCAAACT CGCAAACTTT GGTTCTGCGC GATCAGTTAG TTAAGATTGC   
  
  
+ GGAGATGTTC CAAACCGGTA ACTTTTCGCT TGCCCAAGTG ATATTGGCCC GGCTCAATCA GCAATTGTCT   
  
  
+ CTTTCAGGGA ACCCCTTTGT AAGAGCTGCT ACTTTCCATG TCAAGGAGGA ACTTGAAAGG CTCCTGACTA   
  
  
+ TCAATAGCTT CTCTGCGGCT CCACCCCAAC CTAAAAGCCT TACTCTGTCA GATGTTGTGG ATAGGATGAA   
  
  
+ TGCATACAAG CTTTTTTCAG AGGCATCCCC AGTTATGCAA TTTACCGATT TTACTTGTAC CCAGGCTTTG   
  
  
+ CTCGAAGCTC TTGATGATGC TGATTATATC CACATACTAG ATTTTGACAT TGGTTGTGGT TCCCAATGGG   
  
  
+ CATCCTTTAT TCGAGAGCTT CCTCTCAGGA AAAGGGGCAC TCCATTTTTG AAAATTACAG CCTTTGCCTC   
  
  
+ TTCTTTAACC CATAATCCAT TTGAACTAGC CCTTGTATGT GATAACATTC TGGAATTTGC AAAAGAGTTC   
  
  
+ GGTGTCTCTG TCGACCTTCA GGTCATGAAT TTGGACTTGT TTGACCCAAC TACTTGCACA ATACCCAATT   
  
  
+ TTCAATCCAC TGAGAACGCA GCAGTTGCTG TCAACTTCCC TATATGGGCG TGCTCACACT GCCCGTTTGT   
  
  
+ ACTCAGTCAA CTCCTGTCCT TCATCAAGCA ATGTTCCCCA AAAATCATGA CGAGCTTTGA TAGAGGTTGT   
  
  
+ GATCATTTTG AGTTGTCTTT TCCATCGCAC ATCATCCGTG TATTGGACTC ATGCTCTAAT ATGTTGGAGT   
  
  
+ CCCTCCATGG TTCAAGCGTG ACATTAGACA CCTCAAAGAA GATTGAAAAG TTCTTGATCA AGCCTATAAT   
  
  
+ TGAGAGGGCA ATATTGGGGC GAATTCATGC ACCAGACAAG ATGCAGGCAA ACAAGGCACA CCGGTGGAAG   
  
  
+ AATCTCTTCA CTTCTGCTGG GTTCTTCCCT TTGCCATTTA GTAGTTTCAC TGAATCTCAG GCCAATCTAG   
  
  
+ TGGCTCAACG AACCCCAGTG AGGGGTTTTC AGGTGGAAAA GCAGCAAGCA TCACTTGTGC TCAGGTGGAT   
  
  
+ GTCTCATGAG CTCGTGGCAG CTTCAGCTTG GAGGTGCTA  

- -Up\_Stream \_Len000AAACAT AAATTTAAAA TGTTATAAAT ATTTTTAAAA CATAACTAAT TAACACGATT   
  
  
- GATTGTGATT ATTAACCCCC TCTTTTCTTC CCTTTTATTT TTCTTTAATT CTTCTTCTTT TCTTTTGTGG   
  
  
- AAACTTTTCC GTTTTTTGAT TTTATTTTGT TATTACTTGT CTTCCTCTCT TCCTACCTAT TTGTTGTTTA   
  
  
- CAGCGAAAAT CCTGTCGTTA TCAGATACAC TAACCGGAAT CAAGGACAAC CTCCTAACGA TTGTTTTCGT   
  
  
- TGTCGTTTTT TATTATGATT AATAATGTAT AAAAAATTGA GAATTTCGAT TAGTTAGTAG GAGGTAATTA   
  
  
- ACTAATTAAT AAACGGTTTT TGTGTTTAGT TGACCCCGTT GATATTCTCT AGATGGACGA AATTCTATAT   
  
  
- ATTTGAGTTA ACAAAGTAAA ATAAAATAAA ATCTAAAAAA CCGGAAACCA GAGATAACTC GATTTTAGAG   
  
  
- CAATAAAAGC CTAGTACTTT TTATGTTGAG CTAGAAAACT ATATTCAGTA GAAATAAACA TTTAAGACTT   
  
  
- GAATTTTAGA ATGAACTTCT AGTCCTTATC TTCGTGAGCT AAAAACTATT TAGGATACAC AAGAAGAATT   
  
  
- TTAAGTATTA GATTAAGTAT AAAATCTACA TGGGCAACGG ATGAAACCGT TCAAATCTAA TACTAAAAAT   
  
  
- AAAAAAAATT AATGAACATA AAAAGAAAAC CTAACTATCA GTTAGATGTA GTAGAAACTA GTTAACGCTG   
  
  
- TCAAAGTAAA AGGGTTGTTT TTAAAGCTCA CATAAGAAAA ATTAAATAGT ATTCATAAAT AACAGTTGTA   
  
  
- AGTTGTACGT AGTTGTTAGT AATTTGTGAA AAGCAGTGAG GAGTTATGAC CAGTCGTGAA TTCTAAAACA   
  
  
- GAAGATTTGA ATAGCATTCA TTGAATAACA ACTAATTAAC TACGACTGGT TTACTATGAC TCACGATTTT   
  
  
- AATATTAATT ATTTATGTTA ATTCTCAACT AGTTACAGCT ACTAAGTTTA GTTACACTAA CCACGACTTA   
  
  
- TCATTAATAT TCGTGGATAT TTCTGAACTT TTCTTGTTTG TAAATTAATC TAAAATATTA ATATTAAAGG   
  
  
- ATTATAAATC GTAAGTAGTC GTTTAAGTCG TGTGTGGTTG TAATCAGTTG TGGATAGTTG TGAATATTCA   
  
  
- TGGTTAATTA TTATTAATGG ATATTATCTA ATTTTCAATC CGGGAATTAC AAAGGGTGTA ATAATCTATA   
  
  
- TAGTTCCCTC CCCCTTTTTT TATTATGAGG GGGTAGAATT TTTTAAATAG GGAAAAAAAG AAAAATTACA   
  
  
- AGGTTTTGTT TTTATACGAA GATTTAATAC TAACGTTATA CTTACACATG CCAAAGTTTG GGTTAAAAGA   
  
  
- GGTTGTTAAG AAGATGATAT AACATTAATA TAATATGATG TTAAACATAG AGTAGATACT AGATGGGGGG   
  
  
- TTTTTGGTTT ATGAGTATAT TTCAAAATAA AAGAAAGGTA GGGTGAGATT TTTTTTTTTT ACAACCCTTT   
  
  
- TTCAACTAAA TAAATTAAAC TTTACCATGC CACTTATATC TAAACATCTC CCCGTCAACT ATCTTTTTCC   
  
  
- ACTTCTGTTT CCTTCCCGTG TAAACGGAAA AGTATCTTCC TTCTCTGGTT CCATGACCCC TGACCCCCTT   
  
  
- CCCGAGTTCT CTTCTCTCTC TGACTCTTCT CACGACGATG AGGAAGGATG GATGTGTGTT TTAACGACGC   
  
  
- CCCCCAAGTT CAAGTTGAAC TTGCTCCGTT TATGGGAGTA ACTTGGGTGG TTTAGCTTTA GAAGTTACAT   
  
  
- GTGGGGGTAA TATGTGCGTA AGTCTCTGTA TCTACAGCTC CCCCTCTACT TAATCGTTCT TCTACAGCTC   
  
  
- GATTTTACGT AAAGCTCCAA TGATCTCGGT CTTTACACCC GTGTAGGGTC TTCTAGAGTC ATCTCTCTCA   
  
  
- TTGAAGTCTT TTCAAAGTTA AAAATAAGCT TTAAACGGAG AACAAACACA TCTATACTAC CCTTGAAGCG   
  
  
- GAATGTTAGA CGTTACACTC CCAAACCAAC TTCCTCAACG TCAGTTAGGA GTTAAAATAC TTAGTTGGGG   
  
  
- TTTCACCTTC TTCCCGTAGT TTCTTCTCAG TTAACTCTCG CTTGGGTGAA GCCAAAACCT GTTGGGATCG   
  
  
- GGAGGTTTAA GACGTAGATG AGAGAGAAGG ACGAAGTTAC CACCTTCATC ACCCGAACCC TACCTGCTAA   
  
  
- ACCTCCCAAA CAGAAAACCT CTTCCCAGGG AAGAGGGAAC CAAGTACCCA CTTTAACTTC TGGGATCAAA   
  
  
- CTCAAAGTTC GTGGAGGAAG TTGGAAGTTT AGGGAACCTC AAACCACCGT TACTACCCGA ACTTCGTCGA   
  
  
- TAAGTCTCAC GACCATACCC ATTAAAATTA AACCAACTTT CAAAATTAAG ACTAAACTCC AGAACATTAA   
  
  
- GACCCAGATA AACGTTACCC CCCTTACTAA GACAGTTACT TTTCCCTACA CAAGAATTAA TACGAAGTCT   
  
  
- CGTCCCAGTA CACCAACGAC ATAGAGGTCA TCTTGTCAAA TCCTAATCAC TACACTTTGT ACTCCACAAC   
  
  
- TTGGGTTTTA AACCGAAAAC ACTCGTTCCG AGATGTTAGT CAGTCTTGAG ACCAATACAC GTATATGACC   
  
  
- CAATATTGGT CGATCTTAGA GGGGAACTCG GAGGCCGTTT CGCGGTGTTA CGACCCTTAC GAGAAATGTG   
  
  
- ATCACAACAA GGTCAAGGTT TCTCAAGCAA AAATCCACTC CTAGTACTTT AAGATGAGTC TTTAGTTCTC   
  
  
- GTCGTCAAAG ACTACGTTGT TGTCACCAAT TACCCAAATC GGGGAGTCAA ATGTGGGGTC GTGGGCGGTT   
  
  
- TCGGTAATCT GCTGGCTTTC GTTGGTTTGA GCGTTTGAAA CCAAGACGCG CTAGTCAATC AATTCTAACG   
  
  
- CCTCTACAAG GTTTGGCCAT TGAAAAGCGA ACGGGTTCAC TATAACCGGG CCGAGTTAGT CGTTAACAGA   
  
  
- GAAAGTCCCT TGGGGAAACA TTCTCGACGA TGAAAGGTAC AGTTCCTCCT TGAACTTTCC GAGGACTGAT   
  
  
- AGTTATCGAA GAGACGCCGA GGTGGGGTTG GATTTTCGGA ATGAGACAGT CTACAACACC TATCCTACTT   
  
  
- ACGTATGTTC GAAAAAAGTC TCCGTAGGGG TCAATACGTT AAATGGCTAA AATGAACATG GGTCCGAAAC   
  
  
- GAGCTTCGAG AACTACTACG ACTAATATAG GTGTATGATC TAAAACTGTA ACCAACACCA AGGGTTACCC   
  
  
- GTAGGAAATA AGCTCTCGAA GGAGAGTCCT TTTCCCCGTG AGGTAAAAAC TTTTAATGTC GGAAACGGAG   
  
  
- AAGAAATTGG GTATTAGGTA AACTTGATCG GGAACATACA CTATTGTAAG ACCTTAAACG TTTTCTCAAG   
  
  
- CCACAGAGAC AGCTGGAAGT CCAGTACTTA AACCTGAACA AACTGGGTTG ATGAACGTGT TATGGGTTAA   
  
  
- AAGTTAGGTG ACTCTTGCGT CGTCAACGAC AGTTGAAGGG ATATACCCGC ACGAGTGTGA CGGGCAAACA   
  
  
- TGAGTCAGTT GAGGACAGGA AGTAGTTCGT TACAAGGGGT TTTTAGTACT GCTCGAAACT ATCTCCAACA   
  
  
- CTAGTAAAAC TCAACAGAAA AGGTAGCGTG TAGTAGGCAC ATAACCTGAG TACGAGATTA TACAACCTCA   
  
  
- GGGAGGTACC AAGTTCGCAC TGTAATCTGT GGAGTTTCTT CTAACTTTTC AAGAACTAGT TCGGATATTA   
  
  
- ACTCTCCCGT TATAACCCCG CTTAAGTACG TGGTCTGTTC TACGTCCGTT TGTTCCGTGT GGCCACCTTC   
  
  
- TTAGAGAAGT GAAGACGACC CAAGAAGGGA AACGGTAAAT CATCAAAGTG ACTTAGAGTC CGGTTAGATC   
  
  
- ACCGAGTTGC TTGGGGTCAC TCCCCAAAAG TCCACCTTTT CGTCGTTCGT AGTGAACACG AGTCCACCTA   
  
  
- CAGAGTACTC GAGCACCGTC GAAGTCGAAC CTCCACGAT

+     Unnamed\_\_1

| Site Name | Organism | Position | Strand | Matrix score. | sequence | function |
| --- | --- | --- | --- | --- | --- | --- |
| Unnamed\_\_1 | Zea mays | 4007 | + | 5 | CGTGG |  |

>HU04G00048.1   
+ -Up\_Stream \_Len000TTTGTA TTTAAATTTT ACAATATTTA TAAAAATTTT GTATTGATTA ATTGTGCTAA   
  
  
+ CTAACACTAA TAATTGGGGG AGAAAAGAAG GGAAAATAAA AAGAAATTAA GAAGAAGAAA AGAAAACACC   
  
  
+ TTTGAAAAGG CAAAAAACTA AAATAAAACA ATAATGAACA GAAGGAGAGA AGGATGGATA AACAACAAAT   
  
  
+ GTCGCTTTTA GGACAGCAAT AGTCTATGTG ATTGGCCTTA GTTCCTGTTG GAGGATTGCT AACAAAAGCA   
  
  
+ ACAGCAAAAA ATAATACTAA TTATTACATA TTTTTTAACT CTTAAAGCTA ATCAATCATC CTCCATTAAT   
  
  
+ TGATTAATTA TTTGCCAAAA ACACAAATCA ACTGGGGCAA CTATAAGAGA TCTACCTGCT TTAAGATATA   
  
  
+ TAAACTCAAT TGTTTCATTT TATTTTATTT TAGATTTTTT GGCCTTTGGT CTCTATTGAG CTAAAATCTC   
  
  
+ GTTATTTTCG GATCATGAAA AATACAACTC GATCTTTTGA TATAAGTCAT CTTTATTTGT AAATTCTGAA   
  
  
+ CTTAAAATCT TACTTGAAGA TCAGGAATAG AAGCACTCGA TTTTTGATAA ATCCTATGTG TTCTTCTTAA   
  
  
+ AATTCATAAT CTAATTCATA TTTTAGATGT ACCCGTTGCC TACTTTGGCA AGTTTAGATT ATGATTTTTA   
  
  
+ TTTTTTTTAA TTACTTGTAT TTTTCTTTTG GATTGATAGT CAATCTACAT CATCTTTGAT CAATTGCGAC   
  
  
+ AGTTTCATTT TCCCAACAAA AATTTCGAGT GTATTCTTTT TAATTTATCA TAAGTATTTA TTGTCAACAT   
  
  
+ TCAACATGCA TCAACAATCA TTAAACACTT TTCGTCACTC CTCAATACTG GTCAGCACTT AAGATTTTGT   
  
  
+ CTTCTAAACT TATCGTAAGT AACTTATTGT TGATTAATTG ATGCTGACCA AATGATACTG AGTGCTAAAA   
  
  
+ TTATAATTAA TAAATACAAT TAAGAGTTGA TCAATGTCGA TGATTCAAAT CAATGTGATT GGTGCTGAAT   
  
  
+ AGTAATTATA AGCACCTATA AAGACTTGAA AAGAACAAAC ATTTAATTAG ATTTTATAAT TATAATTTCC   
  
  
+ TAATATTTAG CATTCATCAG CAAATTCAGC ACACACCAAC ATTAGTCAAC ACCTATCAAC ACTTATAAGT   
  
  
+ ACCAATTAAT AATAATTACC TATAATAGAT TAAAAGTTAG GCCCTTAATG TTTCCCACAT TATTAGATAT   
  
  
+ ATCAAGGGAG GGGGAAAAAA ATAATACTCC CCCATCTTAA AAAATTTATC CCTTTTTTTC TTTTTAATGT   
  
  
+ TCCAAAACAA AAATATGCTT CTAAATTATG ATTGCAATAT GAATGTGTAC GGTTTCAAAC CCAATTTTCT   
  
  
+ CCAACAATTC TTCTACTATA TTGTAATTAT ATTATACTAC AATTTGTATC TCATCTATGA TCTACCCCCC   
  
  
+ AAAAACCAAA TACTCATATA AAGTTTTATT TTCTTTCCAT CCCACTCTAA AAAAAAAAAA TGTTGGGAAA   
  
  
+ AAGTTGATTT ATTTAATTTG AAATGGTACG GTGAATATAG ATTTGTAGAG GGGCAGTTGA TAGAAAAAGG   
  
  
+ TGAAGACAAA GGAAGGGCAC ATTTGCCTTT TCATAGAAGG AAGAGACCAA GGTACTGGGG ACTGGGGGAA   
  
  
+ GGGCTCAAGA GAAGAGAGAG ACTGAGAAGA GTGCTGCTAC TCCTTCCTAC CTACACACAA AATTGCTGCG   
  
  
+ GGGGGTTCAA GTTCAACTTG AACGAGGCAA ATACCCTCAT TGAACCCACC AAATCGAAAT CTTCAATGTA   
  
  
+ CACCCCCATT ATACACGCAT TCAGAGACAT AGATGTCGAG GGGGAGATGA ATTAGCAAGA AGATGTCGAG   
  
  
+ CTAAAATGCA TTTCGAGGTT ACTAGAGCCA GAAATGTGGG CACATCCCAG AAGATCTCAG TAGAGAGAGT   
  
  
+ AACTTCAGAA AAGTTTCAAT TTTTATTCGA AATTTGCCTC TTGTTTGTGT AGATATGATG GGAACTTCGC   
  
  
+ CTTACAATCT GCAATGTGAG GGTTTGGTTG AAGGAGTTGC AGTCAATCCT CAATTTTATG AATCAACCCC   
  
  
+ AAAGTGGAAG AAGGGCATCA AAGAAGAGTC AATTGAGAGC GAACCCACTT CGGTTTTGGA CAACCCTAGC   
  
  
+ CCTCCAAATT CTGCATCTAC TCTCTCTTCC TGCTTCAATG GTGGAAGTAG TGGGCTTGGG ATGGACGATT   
  
  
+ TGGAGGGTTT GTCTTTTGGA GAAGGGTCCC TTCTCCCTTG GTTCATGGGT GAAATTGAAG ACCCTAGTTT   
  
  
+ GAGTTTCAAG CACCTCCTTC AACCTTCAAA TCCCTTGGAG TTTGGTGGCA ATGATGGGCT TGAAGCAGCT   
  
  
+ ATTCAGAGTG CTGGTATGGG TAATTTTAAT TTGGTTGAAA GTTTTAATTC TGATTTGAGG TCTTGTAATT   
  
  
+ CTGGGTCTAT TTGCAATGGG GGGAATGATT CTGTCAATGA AAAGGGATGT GTTCTTAATT ATGCTTCAGA   
  
  
+ GCAGGGTCAT GTGGTTGCTG TATCTCCAGT AGAACAGTTT AGGATTAGTG ATGTGAAACA TGAGGTGTTG   
  
  
+ AACCCAAAAT TTGGCTTTTG TGAGCAAGGC TCTACAATCA GTCAGAACTC TGGTTATGTG CATATACTGG   
  
  
+ GTTATAACCA GCTAGAATCT CCCCTTGAGC CTCCGGCAAA GCGCCACAAT GCTGGGAATG CTCTTTACAC   
  
  
+ TAGTGTTGTT CCAGTTCCAA AGAGTTCGTT TTTAGGTGAG GATCATGAAA TTCTACTCAG AAATCAAGAG   
  
  
+ CAGCAGTTTC TGATGCAACA ACAGTGGTTA ATGGGTTTAG CCCCTCAGTT TACACCCCAG CACCCGCCAA   
  
  
+ AGCCATTAGA CGACCGAAAG CAACCAAACT CGCAAACTTT GGTTCTGCGC GATCAGTTAG TTAAGATTGC   
  
  
+ GGAGATGTTC CAAACCGGTA ACTTTTCGCT TGCCCAAGTG ATATTGGCCC GGCTCAATCA GCAATTGTCT   
  
  
+ CTTTCAGGGA ACCCCTTTGT AAGAGCTGCT ACTTTCCATG TCAAGGAGGA ACTTGAAAGG CTCCTGACTA   
  
  
+ TCAATAGCTT CTCTGCGGCT CCACCCCAAC CTAAAAGCCT TACTCTGTCA GATGTTGTGG ATAGGATGAA   
  
  
+ TGCATACAAG CTTTTTTCAG AGGCATCCCC AGTTATGCAA TTTACCGATT TTACTTGTAC CCAGGCTTTG   
  
  
+ CTCGAAGCTC TTGATGATGC TGATTATATC CACATACTAG ATTTTGACAT TGGTTGTGGT TCCCAATGGG   
  
  
+ CATCCTTTAT TCGAGAGCTT CCTCTCAGGA AAAGGGGCAC TCCATTTTTG AAAATTACAG CCTTTGCCTC   
  
  
+ TTCTTTAACC CATAATCCAT TTGAACTAGC CCTTGTATGT GATAACATTC TGGAATTTGC AAAAGAGTTC   
  
  
+ GGTGTCTCTG TCGACCTTCA GGTCATGAAT TTGGACTTGT TTGACCCAAC TACTTGCACA ATACCCAATT   
  
  
+ TTCAATCCAC TGAGAACGCA GCAGTTGCTG TCAACTTCCC TATATGGGCG TGCTCACACT GCCCGTTTGT   
  
  
+ ACTCAGTCAA CTCCTGTCCT TCATCAAGCA ATGTTCCCCA AAAATCATGA CGAGCTTTGA TAGAGGTTGT   
  
  
+ GATCATTTTG AGTTGTCTTT TCCATCGCAC ATCATCCGTG TATTGGACTC ATGCTCTAAT ATGTTGGAGT   
  
  
+ CCCTCCATGG TTCAAGCGTG ACATTAGACA CCTCAAAGAA GATTGAAAAG TTCTTGATCA AGCCTATAAT   
  
  
+ TGAGAGGGCA ATATTGGGGC GAATTCATGC ACCAGACAAG ATGCAGGCAA ACAAGGCACA CCGGTGGAAG   
  
  
+ AATCTCTTCA CTTCTGCTGG GTTCTTCCCT TTGCCATTTA GTAGTTTCAC TGAATCTCAG GCCAATCTAG   
  
  
+ TGGCTCAACG AACCCCAGTG AGGGGTTTTC AGGTGGAAAA GCAGCAAGCA TCACTTGTGC TCAGGTGGAT   
  
  
+ GTCTCATGAG CTCGTGGCAG CTTCAGCTTG GAGGTGCTA  

- -Up\_Stream \_Len000AAACAT AAATTTAAAA TGTTATAAAT ATTTTTAAAA CATAACTAAT TAACACGATT   
  
  
- GATTGTGATT ATTAACCCCC TCTTTTCTTC CCTTTTATTT TTCTTTAATT CTTCTTCTTT TCTTTTGTGG   
  
  
- AAACTTTTCC GTTTTTTGAT TTTATTTTGT TATTACTTGT CTTCCTCTCT TCCTACCTAT TTGTTGTTTA   
  
  
- CAGCGAAAAT CCTGTCGTTA TCAGATACAC TAACCGGAAT CAAGGACAAC CTCCTAACGA TTGTTTTCGT   
  
  
- TGTCGTTTTT TATTATGATT AATAATGTAT AAAAAATTGA GAATTTCGAT TAGTTAGTAG GAGGTAATTA   
  
  
- ACTAATTAAT AAACGGTTTT TGTGTTTAGT TGACCCCGTT GATATTCTCT AGATGGACGA AATTCTATAT   
  
  
- ATTTGAGTTA ACAAAGTAAA ATAAAATAAA ATCTAAAAAA CCGGAAACCA GAGATAACTC GATTTTAGAG   
  
  
- CAATAAAAGC CTAGTACTTT TTATGTTGAG CTAGAAAACT ATATTCAGTA GAAATAAACA TTTAAGACTT   
  
  
- GAATTTTAGA ATGAACTTCT AGTCCTTATC TTCGTGAGCT AAAAACTATT TAGGATACAC AAGAAGAATT   
  
  
- TTAAGTATTA GATTAAGTAT AAAATCTACA TGGGCAACGG ATGAAACCGT TCAAATCTAA TACTAAAAAT   
  
  
- AAAAAAAATT AATGAACATA AAAAGAAAAC CTAACTATCA GTTAGATGTA GTAGAAACTA GTTAACGCTG   
  
  
- TCAAAGTAAA AGGGTTGTTT TTAAAGCTCA CATAAGAAAA ATTAAATAGT ATTCATAAAT AACAGTTGTA   
  
  
- AGTTGTACGT AGTTGTTAGT AATTTGTGAA AAGCAGTGAG GAGTTATGAC CAGTCGTGAA TTCTAAAACA   
  
  
- GAAGATTTGA ATAGCATTCA TTGAATAACA ACTAATTAAC TACGACTGGT TTACTATGAC TCACGATTTT   
  
  
- AATATTAATT ATTTATGTTA ATTCTCAACT AGTTACAGCT ACTAAGTTTA GTTACACTAA CCACGACTTA   
  
  
- TCATTAATAT TCGTGGATAT TTCTGAACTT TTCTTGTTTG TAAATTAATC TAAAATATTA ATATTAAAGG   
  
  
- ATTATAAATC GTAAGTAGTC GTTTAAGTCG TGTGTGGTTG TAATCAGTTG TGGATAGTTG TGAATATTCA   
  
  
- TGGTTAATTA TTATTAATGG ATATTATCTA ATTTTCAATC CGGGAATTAC AAAGGGTGTA ATAATCTATA   
  
  
- TAGTTCCCTC CCCCTTTTTT TATTATGAGG GGGTAGAATT TTTTAAATAG GGAAAAAAAG AAAAATTACA   
  
  
- AGGTTTTGTT TTTATACGAA GATTTAATAC TAACGTTATA CTTACACATG CCAAAGTTTG GGTTAAAAGA   
  
  
- GGTTGTTAAG AAGATGATAT AACATTAATA TAATATGATG TTAAACATAG AGTAGATACT AGATGGGGGG   
  
  
- TTTTTGGTTT ATGAGTATAT TTCAAAATAA AAGAAAGGTA GGGTGAGATT TTTTTTTTTT ACAACCCTTT   
  
  
- TTCAACTAAA TAAATTAAAC TTTACCATGC CACTTATATC TAAACATCTC CCCGTCAACT ATCTTTTTCC   
  
  
- ACTTCTGTTT CCTTCCCGTG TAAACGGAAA AGTATCTTCC TTCTCTGGTT CCATGACCCC TGACCCCCTT   
  
  
- CCCGAGTTCT CTTCTCTCTC TGACTCTTCT CACGACGATG AGGAAGGATG GATGTGTGTT TTAACGACGC   
  
  
- CCCCCAAGTT CAAGTTGAAC TTGCTCCGTT TATGGGAGTA ACTTGGGTGG TTTAGCTTTA GAAGTTACAT   
  
  
- GTGGGGGTAA TATGTGCGTA AGTCTCTGTA TCTACAGCTC CCCCTCTACT TAATCGTTCT TCTACAGCTC   
  
  
- GATTTTACGT AAAGCTCCAA TGATCTCGGT CTTTACACCC GTGTAGGGTC TTCTAGAGTC ATCTCTCTCA   
  
  
- TTGAAGTCTT TTCAAAGTTA AAAATAAGCT TTAAACGGAG AACAAACACA TCTATACTAC CCTTGAAGCG   
  
  
- GAATGTTAGA CGTTACACTC CCAAACCAAC TTCCTCAACG TCAGTTAGGA GTTAAAATAC TTAGTTGGGG   
  
  
- TTTCACCTTC TTCCCGTAGT TTCTTCTCAG TTAACTCTCG CTTGGGTGAA GCCAAAACCT GTTGGGATCG   
  
  
- GGAGGTTTAA GACGTAGATG AGAGAGAAGG ACGAAGTTAC CACCTTCATC ACCCGAACCC TACCTGCTAA   
  
  
- ACCTCCCAAA CAGAAAACCT CTTCCCAGGG AAGAGGGAAC CAAGTACCCA CTTTAACTTC TGGGATCAAA   
  
  
- CTCAAAGTTC GTGGAGGAAG TTGGAAGTTT AGGGAACCTC AAACCACCGT TACTACCCGA ACTTCGTCGA   
  
  
- TAAGTCTCAC GACCATACCC ATTAAAATTA AACCAACTTT CAAAATTAAG ACTAAACTCC AGAACATTAA   
  
  
- GACCCAGATA AACGTTACCC CCCTTACTAA GACAGTTACT TTTCCCTACA CAAGAATTAA TACGAAGTCT   
  
  
- CGTCCCAGTA CACCAACGAC ATAGAGGTCA TCTTGTCAAA TCCTAATCAC TACACTTTGT ACTCCACAAC   
  
  
- TTGGGTTTTA AACCGAAAAC ACTCGTTCCG AGATGTTAGT CAGTCTTGAG ACCAATACAC GTATATGACC   
  
  
- CAATATTGGT CGATCTTAGA GGGGAACTCG GAGGCCGTTT CGCGGTGTTA CGACCCTTAC GAGAAATGTG   
  
  
- ATCACAACAA GGTCAAGGTT TCTCAAGCAA AAATCCACTC CTAGTACTTT AAGATGAGTC TTTAGTTCTC   
  
  
- GTCGTCAAAG ACTACGTTGT TGTCACCAAT TACCCAAATC GGGGAGTCAA ATGTGGGGTC GTGGGCGGTT   
  
  
- TCGGTAATCT GCTGGCTTTC GTTGGTTTGA GCGTTTGAAA CCAAGACGCG CTAGTCAATC AATTCTAACG   
  
  
- CCTCTACAAG GTTTGGCCAT TGAAAAGCGA ACGGGTTCAC TATAACCGGG CCGAGTTAGT CGTTAACAGA   
  
  
- GAAAGTCCCT TGGGGAAACA TTCTCGACGA TGAAAGGTAC AGTTCCTCCT TGAACTTTCC GAGGACTGAT   
  
  
- AGTTATCGAA GAGACGCCGA GGTGGGGTTG GATTTTCGGA ATGAGACAGT CTACAACACC TATCCTACTT   
  
  
- ACGTATGTTC GAAAAAAGTC TCCGTAGGGG TCAATACGTT AAATGGCTAA AATGAACATG GGTCCGAAAC   
  
  
- GAGCTTCGAG AACTACTACG ACTAATATAG GTGTATGATC TAAAACTGTA ACCAACACCA AGGGTTACCC   
  
  
- GTAGGAAATA AGCTCTCGAA GGAGAGTCCT TTTCCCCGTG AGGTAAAAAC TTTTAATGTC GGAAACGGAG   
  
  
- AAGAAATTGG GTATTAGGTA AACTTGATCG GGAACATACA CTATTGTAAG ACCTTAAACG TTTTCTCAAG   
  
  
- CCACAGAGAC AGCTGGAAGT CCAGTACTTA AACCTGAACA AACTGGGTTG ATGAACGTGT TATGGGTTAA   
  
  
- AAGTTAGGTG ACTCTTGCGT CGTCAACGAC AGTTGAAGGG ATATACCCGC ACGAGTGTGA CGGGCAAACA   
  
  
- TGAGTCAGTT GAGGACAGGA AGTAGTTCGT TACAAGGGGT TTTTAGTACT GCTCGAAACT ATCTCCAACA   
  
  
- CTAGTAAAAC TCAACAGAAA AGGTAGCGTG TAGTAGGCAC ATAACCTGAG TACGAGATTA TACAACCTCA   
  
  
- GGGAGGTACC AAGTTCGCAC TGTAATCTGT GGAGTTTCTT CTAACTTTTC AAGAACTAGT TCGGATATTA   
  
  
- ACTCTCCCGT TATAACCCCG CTTAAGTACG TGGTCTGTTC TACGTCCGTT TGTTCCGTGT GGCCACCTTC   
  
  
- TTAGAGAAGT GAAGACGACC CAAGAAGGGA AACGGTAAAT CATCAAAGTG ACTTAGAGTC CGGTTAGATC   
  
  
- ACCGAGTTGC TTGGGGTCAC TCCCCAAAAG TCCACCTTTT CGTCGTTCGT AGTGAACACG AGTCCACCTA   
  
  
- CAGAGTACTC GAGCACCGTC GAAGTCGAAC CTCCACGAT

+     Unnamed\_\_4

| Site Name | Organism | Position | Strand | Matrix score. | sequence | function |
| --- | --- | --- | --- | --- | --- | --- |
| Unnamed\_\_4 | Petroselinum hortense | 4024 | - | 4 | CTCC |  |
| Unnamed\_\_4 | Petroselinum hortense | 3075 | + | 4 | CTCC |  |
| Unnamed\_\_4 | Petroselinum hortense | 2246 | - | 4 | CTCC |  |
| Unnamed\_\_4 | Petroselinum hortense | 1403 | + | 4 | CTCC |  |
| Unnamed\_\_4 | Petroselinum hortense | 3585 | + | 4 | CTCC |  |
| Unnamed\_\_4 | Petroselinum hortense | 3334 | + | 4 | CTCC |  |
| Unnamed\_\_4 | Petroselinum hortense | 2328 | + | 4 | CTCC |  |
| Unnamed\_\_4 | Petroselinum hortense | 2067 | - | 4 | CTCC |  |
| Unnamed\_\_4 | Petroselinum hortense | 2176 | + | 4 | CTCC |  |
| Unnamed\_\_4 | Petroselinum hortense | 3710 | - | 4 | CTCC |  |
| Unnamed\_\_4 | Petroselinum hortense | 3059 | - | 4 | CTCC |  |
| Unnamed\_\_4 | Petroselinum hortense | 1291 | + | 4 | CTCC |  |
| Unnamed\_\_4 | Petroselinum hortense | 2695 | + | 4 | CTCC |  |
| Unnamed\_\_4 | Petroselinum hortense | 1867 | - | 4 | CTCC |  |
| Unnamed\_\_4 | Petroselinum hortense | 3717 | + | 4 | CTCC |  |
| Unnamed\_\_4 | Petroselinum hortense | 3103 | + | 4 | CTCC |  |
| Unnamed\_\_4 | Petroselinum hortense | 2683 | + | 4 | CTCC |  |
| Unnamed\_\_4 | Petroselinum hortense | 2548 | + | 4 | CTCC |  |
| Unnamed\_\_4 | Petroselinum hortense | 2351 | - | 4 | CTCC |  |
| Unnamed\_\_4 | Petroselinum hortense | 1724 | + | 4 | CTCC |  |
| Unnamed\_\_4 | Petroselinum hortense | 2945 | - | 4 | CTCC |  |
| Unnamed\_\_4 | Petroselinum hortense | 1271 | - | 4 | CTCC |  |
| Unnamed\_\_4 | Petroselinum hortense | 345 | + | 4 | CTCC |  |
| Unnamed\_\_4 | Petroselinum hortense | 2262 | - | 4 | CTCC |  |
| Unnamed\_\_4 | Petroselinum hortense | 882 | + | 4 | CTCC |  |
| Unnamed\_\_4 | Petroselinum hortense | 264 | - | 4 | CTCC |  |
| Unnamed\_\_4 | Petroselinum hortense | 2277 | + | 4 | CTCC |  |
| Unnamed\_\_4 | Petroselinum hortense | 93 | - | 4 | CTCC |  |
| Unnamed\_\_4 | Petroselinum hortense | 188 | - | 4 | CTCC |  |

>HU04G00048.1   
+ -Up\_Stream \_Len000TTTGTA TTTAAATTTT ACAATATTTA TAAAAATTTT GTATTGATTA ATTGTGCTAA   
  
  
+ CTAACACTAA TAATTGGGGG AGAAAAGAAG GGAAAATAAA AAGAAATTAA GAAGAAGAAA AGAAAACACC   
  
  
+ TTTGAAAAGG CAAAAAACTA AAATAAAACA ATAATGAACA GAAGGAGAGA AGGATGGATA AACAACAAAT   
  
  
+ GTCGCTTTTA GGACAGCAAT AGTCTATGTG ATTGGCCTTA GTTCCTGTTG GAGGATTGCT AACAAAAGCA   
  
  
+ ACAGCAAAAA ATAATACTAA TTATTACATA TTTTTTAACT CTTAAAGCTA ATCAATCATC CTCCATTAAT   
  
  
+ TGATTAATTA TTTGCCAAAA ACACAAATCA ACTGGGGCAA CTATAAGAGA TCTACCTGCT TTAAGATATA   
  
  
+ TAAACTCAAT TGTTTCATTT TATTTTATTT TAGATTTTTT GGCCTTTGGT CTCTATTGAG CTAAAATCTC   
  
  
+ GTTATTTTCG GATCATGAAA AATACAACTC GATCTTTTGA TATAAGTCAT CTTTATTTGT AAATTCTGAA   
  
  
+ CTTAAAATCT TACTTGAAGA TCAGGAATAG AAGCACTCGA TTTTTGATAA ATCCTATGTG TTCTTCTTAA   
  
  
+ AATTCATAAT CTAATTCATA TTTTAGATGT ACCCGTTGCC TACTTTGGCA AGTTTAGATT ATGATTTTTA   
  
  
+ TTTTTTTTAA TTACTTGTAT TTTTCTTTTG GATTGATAGT CAATCTACAT CATCTTTGAT CAATTGCGAC   
  
  
+ AGTTTCATTT TCCCAACAAA AATTTCGAGT GTATTCTTTT TAATTTATCA TAAGTATTTA TTGTCAACAT   
  
  
+ TCAACATGCA TCAACAATCA TTAAACACTT TTCGTCACTC CTCAATACTG GTCAGCACTT AAGATTTTGT   
  
  
+ CTTCTAAACT TATCGTAAGT AACTTATTGT TGATTAATTG ATGCTGACCA AATGATACTG AGTGCTAAAA   
  
  
+ TTATAATTAA TAAATACAAT TAAGAGTTGA TCAATGTCGA TGATTCAAAT CAATGTGATT GGTGCTGAAT   
  
  
+ AGTAATTATA AGCACCTATA AAGACTTGAA AAGAACAAAC ATTTAATTAG ATTTTATAAT TATAATTTCC   
  
  
+ TAATATTTAG CATTCATCAG CAAATTCAGC ACACACCAAC ATTAGTCAAC ACCTATCAAC ACTTATAAGT   
  
  
+ ACCAATTAAT AATAATTACC TATAATAGAT TAAAAGTTAG GCCCTTAATG TTTCCCACAT TATTAGATAT   
  
  
+ ATCAAGGGAG GGGGAAAAAA ATAATACTCC CCCATCTTAA AAAATTTATC CCTTTTTTTC TTTTTAATGT   
  
  
+ TCCAAAACAA AAATATGCTT CTAAATTATG ATTGCAATAT GAATGTGTAC GGTTTCAAAC CCAATTTTCT   
  
  
+ CCAACAATTC TTCTACTATA TTGTAATTAT ATTATACTAC AATTTGTATC TCATCTATGA TCTACCCCCC   
  
  
+ AAAAACCAAA TACTCATATA AAGTTTTATT TTCTTTCCAT CCCACTCTAA AAAAAAAAAA TGTTGGGAAA   
  
  
+ AAGTTGATTT ATTTAATTTG AAATGGTACG GTGAATATAG ATTTGTAGAG GGGCAGTTGA TAGAAAAAGG   
  
  
+ TGAAGACAAA GGAAGGGCAC ATTTGCCTTT TCATAGAAGG AAGAGACCAA GGTACTGGGG ACTGGGGGAA   
  
  
+ GGGCTCAAGA GAAGAGAGAG ACTGAGAAGA GTGCTGCTAC TCCTTCCTAC CTACACACAA AATTGCTGCG   
  
  
+ GGGGGTTCAA GTTCAACTTG AACGAGGCAA ATACCCTCAT TGAACCCACC AAATCGAAAT CTTCAATGTA   
  
  
+ CACCCCCATT ATACACGCAT TCAGAGACAT AGATGTCGAG GGGGAGATGA ATTAGCAAGA AGATGTCGAG   
  
  
+ CTAAAATGCA TTTCGAGGTT ACTAGAGCCA GAAATGTGGG CACATCCCAG AAGATCTCAG TAGAGAGAGT   
  
  
+ AACTTCAGAA AAGTTTCAAT TTTTATTCGA AATTTGCCTC TTGTTTGTGT AGATATGATG GGAACTTCGC   
  
  
+ CTTACAATCT GCAATGTGAG GGTTTGGTTG AAGGAGTTGC AGTCAATCCT CAATTTTATG AATCAACCCC   
  
  
+ AAAGTGGAAG AAGGGCATCA AAGAAGAGTC AATTGAGAGC GAACCCACTT CGGTTTTGGA CAACCCTAGC   
  
  
+ CCTCCAAATT CTGCATCTAC TCTCTCTTCC TGCTTCAATG GTGGAAGTAG TGGGCTTGGG ATGGACGATT   
  
  
+ TGGAGGGTTT GTCTTTTGGA GAAGGGTCCC TTCTCCCTTG GTTCATGGGT GAAATTGAAG ACCCTAGTTT   
  
  
+ GAGTTTCAAG CACCTCCTTC AACCTTCAAA TCCCTTGGAG TTTGGTGGCA ATGATGGGCT TGAAGCAGCT   
  
  
+ ATTCAGAGTG CTGGTATGGG TAATTTTAAT TTGGTTGAAA GTTTTAATTC TGATTTGAGG TCTTGTAATT   
  
  
+ CTGGGTCTAT TTGCAATGGG GGGAATGATT CTGTCAATGA AAAGGGATGT GTTCTTAATT ATGCTTCAGA   
  
  
+ GCAGGGTCAT GTGGTTGCTG TATCTCCAGT AGAACAGTTT AGGATTAGTG ATGTGAAACA TGAGGTGTTG   
  
  
+ AACCCAAAAT TTGGCTTTTG TGAGCAAGGC TCTACAATCA GTCAGAACTC TGGTTATGTG CATATACTGG   
  
  
+ GTTATAACCA GCTAGAATCT CCCCTTGAGC CTCCGGCAAA GCGCCACAAT GCTGGGAATG CTCTTTACAC   
  
  
+ TAGTGTTGTT CCAGTTCCAA AGAGTTCGTT TTTAGGTGAG GATCATGAAA TTCTACTCAG AAATCAAGAG   
  
  
+ CAGCAGTTTC TGATGCAACA ACAGTGGTTA ATGGGTTTAG CCCCTCAGTT TACACCCCAG CACCCGCCAA   
  
  
+ AGCCATTAGA CGACCGAAAG CAACCAAACT CGCAAACTTT GGTTCTGCGC GATCAGTTAG TTAAGATTGC   
  
  
+ GGAGATGTTC CAAACCGGTA ACTTTTCGCT TGCCCAAGTG ATATTGGCCC GGCTCAATCA GCAATTGTCT   
  
  
+ CTTTCAGGGA ACCCCTTTGT AAGAGCTGCT ACTTTCCATG TCAAGGAGGA ACTTGAAAGG CTCCTGACTA   
  
  
+ TCAATAGCTT CTCTGCGGCT CCACCCCAAC CTAAAAGCCT TACTCTGTCA GATGTTGTGG ATAGGATGAA   
  
  
+ TGCATACAAG CTTTTTTCAG AGGCATCCCC AGTTATGCAA TTTACCGATT TTACTTGTAC CCAGGCTTTG   
  
  
+ CTCGAAGCTC TTGATGATGC TGATTATATC CACATACTAG ATTTTGACAT TGGTTGTGGT TCCCAATGGG   
  
  
+ CATCCTTTAT TCGAGAGCTT CCTCTCAGGA AAAGGGGCAC TCCATTTTTG AAAATTACAG CCTTTGCCTC   
  
  
+ TTCTTTAACC CATAATCCAT TTGAACTAGC CCTTGTATGT GATAACATTC TGGAATTTGC AAAAGAGTTC   
  
  
+ GGTGTCTCTG TCGACCTTCA GGTCATGAAT TTGGACTTGT TTGACCCAAC TACTTGCACA ATACCCAATT   
  
  
+ TTCAATCCAC TGAGAACGCA GCAGTTGCTG TCAACTTCCC TATATGGGCG TGCTCACACT GCCCGTTTGT   
  
  
+ ACTCAGTCAA CTCCTGTCCT TCATCAAGCA ATGTTCCCCA AAAATCATGA CGAGCTTTGA TAGAGGTTGT   
  
  
+ GATCATTTTG AGTTGTCTTT TCCATCGCAC ATCATCCGTG TATTGGACTC ATGCTCTAAT ATGTTGGAGT   
  
  
+ CCCTCCATGG TTCAAGCGTG ACATTAGACA CCTCAAAGAA GATTGAAAAG TTCTTGATCA AGCCTATAAT   
  
  
+ TGAGAGGGCA ATATTGGGGC GAATTCATGC ACCAGACAAG ATGCAGGCAA ACAAGGCACA CCGGTGGAAG   
  
  
+ AATCTCTTCA CTTCTGCTGG GTTCTTCCCT TTGCCATTTA GTAGTTTCAC TGAATCTCAG GCCAATCTAG   
  
  
+ TGGCTCAACG AACCCCAGTG AGGGGTTTTC AGGTGGAAAA GCAGCAAGCA TCACTTGTGC TCAGGTGGAT   
  
  
+ GTCTCATGAG CTCGTGGCAG CTTCAGCTTG GAGGTGCTA  

- -Up\_Stream \_Len000AAACAT AAATTTAAAA TGTTATAAAT ATTTTTAAAA CATAACTAAT TAACACGATT   
  
  
- GATTGTGATT ATTAACCCCC TCTTTTCTTC CCTTTTATTT TTCTTTAATT CTTCTTCTTT TCTTTTGTGG   
  
  
- AAACTTTTCC GTTTTTTGAT TTTATTTTGT TATTACTTGT CTTCCTCTCT TCCTACCTAT TTGTTGTTTA   
  
  
- CAGCGAAAAT CCTGTCGTTA TCAGATACAC TAACCGGAAT CAAGGACAAC CTCCTAACGA TTGTTTTCGT   
  
  
- TGTCGTTTTT TATTATGATT AATAATGTAT AAAAAATTGA GAATTTCGAT TAGTTAGTAG GAGGTAATTA   
  
  
- ACTAATTAAT AAACGGTTTT TGTGTTTAGT TGACCCCGTT GATATTCTCT AGATGGACGA AATTCTATAT   
  
  
- ATTTGAGTTA ACAAAGTAAA ATAAAATAAA ATCTAAAAAA CCGGAAACCA GAGATAACTC GATTTTAGAG   
  
  
- CAATAAAAGC CTAGTACTTT TTATGTTGAG CTAGAAAACT ATATTCAGTA GAAATAAACA TTTAAGACTT   
  
  
- GAATTTTAGA ATGAACTTCT AGTCCTTATC TTCGTGAGCT AAAAACTATT TAGGATACAC AAGAAGAATT   
  
  
- TTAAGTATTA GATTAAGTAT AAAATCTACA TGGGCAACGG ATGAAACCGT TCAAATCTAA TACTAAAAAT   
  
  
- AAAAAAAATT AATGAACATA AAAAGAAAAC CTAACTATCA GTTAGATGTA GTAGAAACTA GTTAACGCTG   
  
  
- TCAAAGTAAA AGGGTTGTTT TTAAAGCTCA CATAAGAAAA ATTAAATAGT ATTCATAAAT AACAGTTGTA   
  
  
- AGTTGTACGT AGTTGTTAGT AATTTGTGAA AAGCAGTGAG GAGTTATGAC CAGTCGTGAA TTCTAAAACA   
  
  
- GAAGATTTGA ATAGCATTCA TTGAATAACA ACTAATTAAC TACGACTGGT TTACTATGAC TCACGATTTT   
  
  
- AATATTAATT ATTTATGTTA ATTCTCAACT AGTTACAGCT ACTAAGTTTA GTTACACTAA CCACGACTTA   
  
  
- TCATTAATAT TCGTGGATAT TTCTGAACTT TTCTTGTTTG TAAATTAATC TAAAATATTA ATATTAAAGG   
  
  
- ATTATAAATC GTAAGTAGTC GTTTAAGTCG TGTGTGGTTG TAATCAGTTG TGGATAGTTG TGAATATTCA   
  
  
- TGGTTAATTA TTATTAATGG ATATTATCTA ATTTTCAATC CGGGAATTAC AAAGGGTGTA ATAATCTATA   
  
  
- TAGTTCCCTC CCCCTTTTTT TATTATGAGG GGGTAGAATT TTTTAAATAG GGAAAAAAAG AAAAATTACA   
  
  
- AGGTTTTGTT TTTATACGAA GATTTAATAC TAACGTTATA CTTACACATG CCAAAGTTTG GGTTAAAAGA   
  
  
- GGTTGTTAAG AAGATGATAT AACATTAATA TAATATGATG TTAAACATAG AGTAGATACT AGATGGGGGG   
  
  
- TTTTTGGTTT ATGAGTATAT TTCAAAATAA AAGAAAGGTA GGGTGAGATT TTTTTTTTTT ACAACCCTTT   
  
  
- TTCAACTAAA TAAATTAAAC TTTACCATGC CACTTATATC TAAACATCTC CCCGTCAACT ATCTTTTTCC   
  
  
- ACTTCTGTTT CCTTCCCGTG TAAACGGAAA AGTATCTTCC TTCTCTGGTT CCATGACCCC TGACCCCCTT   
  
  
- CCCGAGTTCT CTTCTCTCTC TGACTCTTCT CACGACGATG AGGAAGGATG GATGTGTGTT TTAACGACGC   
  
  
- CCCCCAAGTT CAAGTTGAAC TTGCTCCGTT TATGGGAGTA ACTTGGGTGG TTTAGCTTTA GAAGTTACAT   
  
  
- GTGGGGGTAA TATGTGCGTA AGTCTCTGTA TCTACAGCTC CCCCTCTACT TAATCGTTCT TCTACAGCTC   
  
  
- GATTTTACGT AAAGCTCCAA TGATCTCGGT CTTTACACCC GTGTAGGGTC TTCTAGAGTC ATCTCTCTCA   
  
  
- TTGAAGTCTT TTCAAAGTTA AAAATAAGCT TTAAACGGAG AACAAACACA TCTATACTAC CCTTGAAGCG   
  
  
- GAATGTTAGA CGTTACACTC CCAAACCAAC TTCCTCAACG TCAGTTAGGA GTTAAAATAC TTAGTTGGGG   
  
  
- TTTCACCTTC TTCCCGTAGT TTCTTCTCAG TTAACTCTCG CTTGGGTGAA GCCAAAACCT GTTGGGATCG   
  
  
- GGAGGTTTAA GACGTAGATG AGAGAGAAGG ACGAAGTTAC CACCTTCATC ACCCGAACCC TACCTGCTAA   
  
  
- ACCTCCCAAA CAGAAAACCT CTTCCCAGGG AAGAGGGAAC CAAGTACCCA CTTTAACTTC TGGGATCAAA   
  
  
- CTCAAAGTTC GTGGAGGAAG TTGGAAGTTT AGGGAACCTC AAACCACCGT TACTACCCGA ACTTCGTCGA   
  
  
- TAAGTCTCAC GACCATACCC ATTAAAATTA AACCAACTTT CAAAATTAAG ACTAAACTCC AGAACATTAA   
  
  
- GACCCAGATA AACGTTACCC CCCTTACTAA GACAGTTACT TTTCCCTACA CAAGAATTAA TACGAAGTCT   
  
  
- CGTCCCAGTA CACCAACGAC ATAGAGGTCA TCTTGTCAAA TCCTAATCAC TACACTTTGT ACTCCACAAC   
  
  
- TTGGGTTTTA AACCGAAAAC ACTCGTTCCG AGATGTTAGT CAGTCTTGAG ACCAATACAC GTATATGACC   
  
  
- CAATATTGGT CGATCTTAGA GGGGAACTCG GAGGCCGTTT CGCGGTGTTA CGACCCTTAC GAGAAATGTG   
  
  
- ATCACAACAA GGTCAAGGTT TCTCAAGCAA AAATCCACTC CTAGTACTTT AAGATGAGTC TTTAGTTCTC   
  
  
- GTCGTCAAAG ACTACGTTGT TGTCACCAAT TACCCAAATC GGGGAGTCAA ATGTGGGGTC GTGGGCGGTT   
  
  
- TCGGTAATCT GCTGGCTTTC GTTGGTTTGA GCGTTTGAAA CCAAGACGCG CTAGTCAATC AATTCTAACG   
  
  
- CCTCTACAAG GTTTGGCCAT TGAAAAGCGA ACGGGTTCAC TATAACCGGG CCGAGTTAGT CGTTAACAGA   
  
  
- GAAAGTCCCT TGGGGAAACA TTCTCGACGA TGAAAGGTAC AGTTCCTCCT TGAACTTTCC GAGGACTGAT   
  
  
- AGTTATCGAA GAGACGCCGA GGTGGGGTTG GATTTTCGGA ATGAGACAGT CTACAACACC TATCCTACTT   
  
  
- ACGTATGTTC GAAAAAAGTC TCCGTAGGGG TCAATACGTT AAATGGCTAA AATGAACATG GGTCCGAAAC   
  
  
- GAGCTTCGAG AACTACTACG ACTAATATAG GTGTATGATC TAAAACTGTA ACCAACACCA AGGGTTACCC   
  
  
- GTAGGAAATA AGCTCTCGAA GGAGAGTCCT TTTCCCCGTG AGGTAAAAAC TTTTAATGTC GGAAACGGAG   
  
  
- AAGAAATTGG GTATTAGGTA AACTTGATCG GGAACATACA CTATTGTAAG ACCTTAAACG TTTTCTCAAG   
  
  
- CCACAGAGAC AGCTGGAAGT CCAGTACTTA AACCTGAACA AACTGGGTTG ATGAACGTGT TATGGGTTAA   
  
  
- AAGTTAGGTG ACTCTTGCGT CGTCAACGAC AGTTGAAGGG ATATACCCGC ACGAGTGTGA CGGGCAAACA   
  
  
- TGAGTCAGTT GAGGACAGGA AGTAGTTCGT TACAAGGGGT TTTTAGTACT GCTCGAAACT ATCTCCAACA   
  
  
- CTAGTAAAAC TCAACAGAAA AGGTAGCGTG TAGTAGGCAC ATAACCTGAG TACGAGATTA TACAACCTCA   
  
  
- GGGAGGTACC AAGTTCGCAC TGTAATCTGT GGAGTTTCTT CTAACTTTTC AAGAACTAGT TCGGATATTA   
  
  
- ACTCTCCCGT TATAACCCCG CTTAAGTACG TGGTCTGTTC TACGTCCGTT TGTTCCGTGT GGCCACCTTC   
  
  
- TTAGAGAAGT GAAGACGACC CAAGAAGGGA AACGGTAAAT CATCAAAGTG ACTTAGAGTC CGGTTAGATC   
  
  
- ACCGAGTTGC TTGGGGTCAC TCCCCAAAAG TCCACCTTTT CGTCGTTCGT AGTGAACACG AGTCCACCTA   
  
  
- CAGAGTACTC GAGCACCGTC GAAGTCGAAC CTCCACGAT

+     W box

| Site Name | Organism | Position | Strand | Matrix score. | sequence | function |
| --- | --- | --- | --- | --- | --- | --- |
| W box | Arabidopsis thaliana | 3475 | + | 6 | TTGACC |  |

>HU04G00048.1   
+ -Up\_Stream \_Len000TTTGTA TTTAAATTTT ACAATATTTA TAAAAATTTT GTATTGATTA ATTGTGCTAA   
  
  
+ CTAACACTAA TAATTGGGGG AGAAAAGAAG GGAAAATAAA AAGAAATTAA GAAGAAGAAA AGAAAACACC   
  
  
+ TTTGAAAAGG CAAAAAACTA AAATAAAACA ATAATGAACA GAAGGAGAGA AGGATGGATA AACAACAAAT   
  
  
+ GTCGCTTTTA GGACAGCAAT AGTCTATGTG ATTGGCCTTA GTTCCTGTTG GAGGATTGCT AACAAAAGCA   
  
  
+ ACAGCAAAAA ATAATACTAA TTATTACATA TTTTTTAACT CTTAAAGCTA ATCAATCATC CTCCATTAAT   
  
  
+ TGATTAATTA TTTGCCAAAA ACACAAATCA ACTGGGGCAA CTATAAGAGA TCTACCTGCT TTAAGATATA   
  
  
+ TAAACTCAAT TGTTTCATTT TATTTTATTT TAGATTTTTT GGCCTTTGGT CTCTATTGAG CTAAAATCTC   
  
  
+ GTTATTTTCG GATCATGAAA AATACAACTC GATCTTTTGA TATAAGTCAT CTTTATTTGT AAATTCTGAA   
  
  
+ CTTAAAATCT TACTTGAAGA TCAGGAATAG AAGCACTCGA TTTTTGATAA ATCCTATGTG TTCTTCTTAA   
  
  
+ AATTCATAAT CTAATTCATA TTTTAGATGT ACCCGTTGCC TACTTTGGCA AGTTTAGATT ATGATTTTTA   
  
  
+ TTTTTTTTAA TTACTTGTAT TTTTCTTTTG GATTGATAGT CAATCTACAT CATCTTTGAT CAATTGCGAC   
  
  
+ AGTTTCATTT TCCCAACAAA AATTTCGAGT GTATTCTTTT TAATTTATCA TAAGTATTTA TTGTCAACAT   
  
  
+ TCAACATGCA TCAACAATCA TTAAACACTT TTCGTCACTC CTCAATACTG GTCAGCACTT AAGATTTTGT   
  
  
+ CTTCTAAACT TATCGTAAGT AACTTATTGT TGATTAATTG ATGCTGACCA AATGATACTG AGTGCTAAAA   
  
  
+ TTATAATTAA TAAATACAAT TAAGAGTTGA TCAATGTCGA TGATTCAAAT CAATGTGATT GGTGCTGAAT   
  
  
+ AGTAATTATA AGCACCTATA AAGACTTGAA AAGAACAAAC ATTTAATTAG ATTTTATAAT TATAATTTCC   
  
  
+ TAATATTTAG CATTCATCAG CAAATTCAGC ACACACCAAC ATTAGTCAAC ACCTATCAAC ACTTATAAGT   
  
  
+ ACCAATTAAT AATAATTACC TATAATAGAT TAAAAGTTAG GCCCTTAATG TTTCCCACAT TATTAGATAT   
  
  
+ ATCAAGGGAG GGGGAAAAAA ATAATACTCC CCCATCTTAA AAAATTTATC CCTTTTTTTC TTTTTAATGT   
  
  
+ TCCAAAACAA AAATATGCTT CTAAATTATG ATTGCAATAT GAATGTGTAC GGTTTCAAAC CCAATTTTCT   
  
  
+ CCAACAATTC TTCTACTATA TTGTAATTAT ATTATACTAC AATTTGTATC TCATCTATGA TCTACCCCCC   
  
  
+ AAAAACCAAA TACTCATATA AAGTTTTATT TTCTTTCCAT CCCACTCTAA AAAAAAAAAA TGTTGGGAAA   
  
  
+ AAGTTGATTT ATTTAATTTG AAATGGTACG GTGAATATAG ATTTGTAGAG GGGCAGTTGA TAGAAAAAGG   
  
  
+ TGAAGACAAA GGAAGGGCAC ATTTGCCTTT TCATAGAAGG AAGAGACCAA GGTACTGGGG ACTGGGGGAA   
  
  
+ GGGCTCAAGA GAAGAGAGAG ACTGAGAAGA GTGCTGCTAC TCCTTCCTAC CTACACACAA AATTGCTGCG   
  
  
+ GGGGGTTCAA GTTCAACTTG AACGAGGCAA ATACCCTCAT TGAACCCACC AAATCGAAAT CTTCAATGTA   
  
  
+ CACCCCCATT ATACACGCAT TCAGAGACAT AGATGTCGAG GGGGAGATGA ATTAGCAAGA AGATGTCGAG   
  
  
+ CTAAAATGCA TTTCGAGGTT ACTAGAGCCA GAAATGTGGG CACATCCCAG AAGATCTCAG TAGAGAGAGT   
  
  
+ AACTTCAGAA AAGTTTCAAT TTTTATTCGA AATTTGCCTC TTGTTTGTGT AGATATGATG GGAACTTCGC   
  
  
+ CTTACAATCT GCAATGTGAG GGTTTGGTTG AAGGAGTTGC AGTCAATCCT CAATTTTATG AATCAACCCC   
  
  
+ AAAGTGGAAG AAGGGCATCA AAGAAGAGTC AATTGAGAGC GAACCCACTT CGGTTTTGGA CAACCCTAGC   
  
  
+ CCTCCAAATT CTGCATCTAC TCTCTCTTCC TGCTTCAATG GTGGAAGTAG TGGGCTTGGG ATGGACGATT   
  
  
+ TGGAGGGTTT GTCTTTTGGA GAAGGGTCCC TTCTCCCTTG GTTCATGGGT GAAATTGAAG ACCCTAGTTT   
  
  
+ GAGTTTCAAG CACCTCCTTC AACCTTCAAA TCCCTTGGAG TTTGGTGGCA ATGATGGGCT TGAAGCAGCT   
  
  
+ ATTCAGAGTG CTGGTATGGG TAATTTTAAT TTGGTTGAAA GTTTTAATTC TGATTTGAGG TCTTGTAATT   
  
  
+ CTGGGTCTAT TTGCAATGGG GGGAATGATT CTGTCAATGA AAAGGGATGT GTTCTTAATT ATGCTTCAGA   
  
  
+ GCAGGGTCAT GTGGTTGCTG TATCTCCAGT AGAACAGTTT AGGATTAGTG ATGTGAAACA TGAGGTGTTG   
  
  
+ AACCCAAAAT TTGGCTTTTG TGAGCAAGGC TCTACAATCA GTCAGAACTC TGGTTATGTG CATATACTGG   
  
  
+ GTTATAACCA GCTAGAATCT CCCCTTGAGC CTCCGGCAAA GCGCCACAAT GCTGGGAATG CTCTTTACAC   
  
  
+ TAGTGTTGTT CCAGTTCCAA AGAGTTCGTT TTTAGGTGAG GATCATGAAA TTCTACTCAG AAATCAAGAG   
  
  
+ CAGCAGTTTC TGATGCAACA ACAGTGGTTA ATGGGTTTAG CCCCTCAGTT TACACCCCAG CACCCGCCAA   
  
  
+ AGCCATTAGA CGACCGAAAG CAACCAAACT CGCAAACTTT GGTTCTGCGC GATCAGTTAG TTAAGATTGC   
  
  
+ GGAGATGTTC CAAACCGGTA ACTTTTCGCT TGCCCAAGTG ATATTGGCCC GGCTCAATCA GCAATTGTCT   
  
  
+ CTTTCAGGGA ACCCCTTTGT AAGAGCTGCT ACTTTCCATG TCAAGGAGGA ACTTGAAAGG CTCCTGACTA   
  
  
+ TCAATAGCTT CTCTGCGGCT CCACCCCAAC CTAAAAGCCT TACTCTGTCA GATGTTGTGG ATAGGATGAA   
  
  
+ TGCATACAAG CTTTTTTCAG AGGCATCCCC AGTTATGCAA TTTACCGATT TTACTTGTAC CCAGGCTTTG   
  
  
+ CTCGAAGCTC TTGATGATGC TGATTATATC CACATACTAG ATTTTGACAT TGGTTGTGGT TCCCAATGGG   
  
  
+ CATCCTTTAT TCGAGAGCTT CCTCTCAGGA AAAGGGGCAC TCCATTTTTG AAAATTACAG CCTTTGCCTC   
  
  
+ TTCTTTAACC CATAATCCAT TTGAACTAGC CCTTGTATGT GATAACATTC TGGAATTTGC AAAAGAGTTC   
  
  
+ GGTGTCTCTG TCGACCTTCA GGTCATGAAT TTGGACTTGT TTGACCCAAC TACTTGCACA ATACCCAATT   
  
  
+ TTCAATCCAC TGAGAACGCA GCAGTTGCTG TCAACTTCCC TATATGGGCG TGCTCACACT GCCCGTTTGT   
  
  
+ ACTCAGTCAA CTCCTGTCCT TCATCAAGCA ATGTTCCCCA AAAATCATGA CGAGCTTTGA TAGAGGTTGT   
  
  
+ GATCATTTTG AGTTGTCTTT TCCATCGCAC ATCATCCGTG TATTGGACTC ATGCTCTAAT ATGTTGGAGT   
  
  
+ CCCTCCATGG TTCAAGCGTG ACATTAGACA CCTCAAAGAA GATTGAAAAG TTCTTGATCA AGCCTATAAT   
  
  
+ TGAGAGGGCA ATATTGGGGC GAATTCATGC ACCAGACAAG ATGCAGGCAA ACAAGGCACA CCGGTGGAAG   
  
  
+ AATCTCTTCA CTTCTGCTGG GTTCTTCCCT TTGCCATTTA GTAGTTTCAC TGAATCTCAG GCCAATCTAG   
  
  
+ TGGCTCAACG AACCCCAGTG AGGGGTTTTC AGGTGGAAAA GCAGCAAGCA TCACTTGTGC TCAGGTGGAT   
  
  
+ GTCTCATGAG CTCGTGGCAG CTTCAGCTTG GAGGTGCTA  

- -Up\_Stream \_Len000AAACAT AAATTTAAAA TGTTATAAAT ATTTTTAAAA CATAACTAAT TAACACGATT   
  
  
- GATTGTGATT ATTAACCCCC TCTTTTCTTC CCTTTTATTT TTCTTTAATT CTTCTTCTTT TCTTTTGTGG   
  
  
- AAACTTTTCC GTTTTTTGAT TTTATTTTGT TATTACTTGT CTTCCTCTCT TCCTACCTAT TTGTTGTTTA   
  
  
- CAGCGAAAAT CCTGTCGTTA TCAGATACAC TAACCGGAAT CAAGGACAAC CTCCTAACGA TTGTTTTCGT   
  
  
- TGTCGTTTTT TATTATGATT AATAATGTAT AAAAAATTGA GAATTTCGAT TAGTTAGTAG GAGGTAATTA   
  
  
- ACTAATTAAT AAACGGTTTT TGTGTTTAGT TGACCCCGTT GATATTCTCT AGATGGACGA AATTCTATAT   
  
  
- ATTTGAGTTA ACAAAGTAAA ATAAAATAAA ATCTAAAAAA CCGGAAACCA GAGATAACTC GATTTTAGAG   
  
  
- CAATAAAAGC CTAGTACTTT TTATGTTGAG CTAGAAAACT ATATTCAGTA GAAATAAACA TTTAAGACTT   
  
  
- GAATTTTAGA ATGAACTTCT AGTCCTTATC TTCGTGAGCT AAAAACTATT TAGGATACAC AAGAAGAATT   
  
  
- TTAAGTATTA GATTAAGTAT AAAATCTACA TGGGCAACGG ATGAAACCGT TCAAATCTAA TACTAAAAAT   
  
  
- AAAAAAAATT AATGAACATA AAAAGAAAAC CTAACTATCA GTTAGATGTA GTAGAAACTA GTTAACGCTG   
  
  
- TCAAAGTAAA AGGGTTGTTT TTAAAGCTCA CATAAGAAAA ATTAAATAGT ATTCATAAAT AACAGTTGTA   
  
  
- AGTTGTACGT AGTTGTTAGT AATTTGTGAA AAGCAGTGAG GAGTTATGAC CAGTCGTGAA TTCTAAAACA   
  
  
- GAAGATTTGA ATAGCATTCA TTGAATAACA ACTAATTAAC TACGACTGGT TTACTATGAC TCACGATTTT   
  
  
- AATATTAATT ATTTATGTTA ATTCTCAACT AGTTACAGCT ACTAAGTTTA GTTACACTAA CCACGACTTA   
  
  
- TCATTAATAT TCGTGGATAT TTCTGAACTT TTCTTGTTTG TAAATTAATC TAAAATATTA ATATTAAAGG   
  
  
- ATTATAAATC GTAAGTAGTC GTTTAAGTCG TGTGTGGTTG TAATCAGTTG TGGATAGTTG TGAATATTCA   
  
  
- TGGTTAATTA TTATTAATGG ATATTATCTA ATTTTCAATC CGGGAATTAC AAAGGGTGTA ATAATCTATA   
  
  
- TAGTTCCCTC CCCCTTTTTT TATTATGAGG GGGTAGAATT TTTTAAATAG GGAAAAAAAG AAAAATTACA   
  
  
- AGGTTTTGTT TTTATACGAA GATTTAATAC TAACGTTATA CTTACACATG CCAAAGTTTG GGTTAAAAGA   
  
  
- GGTTGTTAAG AAGATGATAT AACATTAATA TAATATGATG TTAAACATAG AGTAGATACT AGATGGGGGG   
  
  
- TTTTTGGTTT ATGAGTATAT TTCAAAATAA AAGAAAGGTA GGGTGAGATT TTTTTTTTTT ACAACCCTTT   
  
  
- TTCAACTAAA TAAATTAAAC TTTACCATGC CACTTATATC TAAACATCTC CCCGTCAACT ATCTTTTTCC   
  
  
- ACTTCTGTTT CCTTCCCGTG TAAACGGAAA AGTATCTTCC TTCTCTGGTT CCATGACCCC TGACCCCCTT   
  
  
- CCCGAGTTCT CTTCTCTCTC TGACTCTTCT CACGACGATG AGGAAGGATG GATGTGTGTT TTAACGACGC   
  
  
- CCCCCAAGTT CAAGTTGAAC TTGCTCCGTT TATGGGAGTA ACTTGGGTGG TTTAGCTTTA GAAGTTACAT   
  
  
- GTGGGGGTAA TATGTGCGTA AGTCTCTGTA TCTACAGCTC CCCCTCTACT TAATCGTTCT TCTACAGCTC   
  
  
- GATTTTACGT AAAGCTCCAA TGATCTCGGT CTTTACACCC GTGTAGGGTC TTCTAGAGTC ATCTCTCTCA   
  
  
- TTGAAGTCTT TTCAAAGTTA AAAATAAGCT TTAAACGGAG AACAAACACA TCTATACTAC CCTTGAAGCG   
  
  
- GAATGTTAGA CGTTACACTC CCAAACCAAC TTCCTCAACG TCAGTTAGGA GTTAAAATAC TTAGTTGGGG   
  
  
- TTTCACCTTC TTCCCGTAGT TTCTTCTCAG TTAACTCTCG CTTGGGTGAA GCCAAAACCT GTTGGGATCG   
  
  
- GGAGGTTTAA GACGTAGATG AGAGAGAAGG ACGAAGTTAC CACCTTCATC ACCCGAACCC TACCTGCTAA   
  
  
- ACCTCCCAAA CAGAAAACCT CTTCCCAGGG AAGAGGGAAC CAAGTACCCA CTTTAACTTC TGGGATCAAA   
  
  
- CTCAAAGTTC GTGGAGGAAG TTGGAAGTTT AGGGAACCTC AAACCACCGT TACTACCCGA ACTTCGTCGA   
  
  
- TAAGTCTCAC GACCATACCC ATTAAAATTA AACCAACTTT CAAAATTAAG ACTAAACTCC AGAACATTAA   
  
  
- GACCCAGATA AACGTTACCC CCCTTACTAA GACAGTTACT TTTCCCTACA CAAGAATTAA TACGAAGTCT   
  
  
- CGTCCCAGTA CACCAACGAC ATAGAGGTCA TCTTGTCAAA TCCTAATCAC TACACTTTGT ACTCCACAAC   
  
  
- TTGGGTTTTA AACCGAAAAC ACTCGTTCCG AGATGTTAGT CAGTCTTGAG ACCAATACAC GTATATGACC   
  
  
- CAATATTGGT CGATCTTAGA GGGGAACTCG GAGGCCGTTT CGCGGTGTTA CGACCCTTAC GAGAAATGTG   
  
  
- ATCACAACAA GGTCAAGGTT TCTCAAGCAA AAATCCACTC CTAGTACTTT AAGATGAGTC TTTAGTTCTC   
  
  
- GTCGTCAAAG ACTACGTTGT TGTCACCAAT TACCCAAATC GGGGAGTCAA ATGTGGGGTC GTGGGCGGTT   
  
  
- TCGGTAATCT GCTGGCTTTC GTTGGTTTGA GCGTTTGAAA CCAAGACGCG CTAGTCAATC AATTCTAACG   
  
  
- CCTCTACAAG GTTTGGCCAT TGAAAAGCGA ACGGGTTCAC TATAACCGGG CCGAGTTAGT CGTTAACAGA   
  
  
- GAAAGTCCCT TGGGGAAACA TTCTCGACGA TGAAAGGTAC AGTTCCTCCT TGAACTTTCC GAGGACTGAT   
  
  
- AGTTATCGAA GAGACGCCGA GGTGGGGTTG GATTTTCGGA ATGAGACAGT CTACAACACC TATCCTACTT   
  
  
- ACGTATGTTC GAAAAAAGTC TCCGTAGGGG TCAATACGTT AAATGGCTAA AATGAACATG GGTCCGAAAC   
  
  
- GAGCTTCGAG AACTACTACG ACTAATATAG GTGTATGATC TAAAACTGTA ACCAACACCA AGGGTTACCC   
  
  
- GTAGGAAATA AGCTCTCGAA GGAGAGTCCT TTTCCCCGTG AGGTAAAAAC TTTTAATGTC GGAAACGGAG   
  
  
- AAGAAATTGG GTATTAGGTA AACTTGATCG GGAACATACA CTATTGTAAG ACCTTAAACG TTTTCTCAAG   
  
  
- CCACAGAGAC AGCTGGAAGT CCAGTACTTA AACCTGAACA AACTGGGTTG ATGAACGTGT TATGGGTTAA   
  
  
- AAGTTAGGTG ACTCTTGCGT CGTCAACGAC AGTTGAAGGG ATATACCCGC ACGAGTGTGA CGGGCAAACA   
  
  
- TGAGTCAGTT GAGGACAGGA AGTAGTTCGT TACAAGGGGT TTTTAGTACT GCTCGAAACT ATCTCCAACA   
  
  
- CTAGTAAAAC TCAACAGAAA AGGTAGCGTG TAGTAGGCAC ATAACCTGAG TACGAGATTA TACAACCTCA   
  
  
- GGGAGGTACC AAGTTCGCAC TGTAATCTGT GGAGTTTCTT CTAACTTTTC AAGAACTAGT TCGGATATTA   
  
  
- ACTCTCCCGT TATAACCCCG CTTAAGTACG TGGTCTGTTC TACGTCCGTT TGTTCCGTGT GGCCACCTTC   
  
  
- TTAGAGAAGT GAAGACGACC CAAGAAGGGA AACGGTAAAT CATCAAAGTG ACTTAGAGTC CGGTTAGATC   
  
  
- ACCGAGTTGC TTGGGGTCAC TCCCCAAAAG TCCACCTTTT CGTCGTTCGT AGTGAACACG AGTCCACCTA   
  
  
- CAGAGTACTC GAGCACCGTC GAAGTCGAAC CTCCACGAT

+     WRE3

| Site Name | Organism | Position | Strand | Matrix score. | sequence | function |
| --- | --- | --- | --- | --- | --- | --- |
| WRE3 | Pisum sativum | 3987 | - | 6 | CCACCT |  |
| WRE3 | Pisum sativum | 3955 | - | 6 | CCACCT |  |

>HU04G00048.1   
+ -Up\_Stream \_Len000TTTGTA TTTAAATTTT ACAATATTTA TAAAAATTTT GTATTGATTA ATTGTGCTAA   
  
  
+ CTAACACTAA TAATTGGGGG AGAAAAGAAG GGAAAATAAA AAGAAATTAA GAAGAAGAAA AGAAAACACC   
  
  
+ TTTGAAAAGG CAAAAAACTA AAATAAAACA ATAATGAACA GAAGGAGAGA AGGATGGATA AACAACAAAT   
  
  
+ GTCGCTTTTA GGACAGCAAT AGTCTATGTG ATTGGCCTTA GTTCCTGTTG GAGGATTGCT AACAAAAGCA   
  
  
+ ACAGCAAAAA ATAATACTAA TTATTACATA TTTTTTAACT CTTAAAGCTA ATCAATCATC CTCCATTAAT   
  
  
+ TGATTAATTA TTTGCCAAAA ACACAAATCA ACTGGGGCAA CTATAAGAGA TCTACCTGCT TTAAGATATA   
  
  
+ TAAACTCAAT TGTTTCATTT TATTTTATTT TAGATTTTTT GGCCTTTGGT CTCTATTGAG CTAAAATCTC   
  
  
+ GTTATTTTCG GATCATGAAA AATACAACTC GATCTTTTGA TATAAGTCAT CTTTATTTGT AAATTCTGAA   
  
  
+ CTTAAAATCT TACTTGAAGA TCAGGAATAG AAGCACTCGA TTTTTGATAA ATCCTATGTG TTCTTCTTAA   
  
  
+ AATTCATAAT CTAATTCATA TTTTAGATGT ACCCGTTGCC TACTTTGGCA AGTTTAGATT ATGATTTTTA   
  
  
+ TTTTTTTTAA TTACTTGTAT TTTTCTTTTG GATTGATAGT CAATCTACAT CATCTTTGAT CAATTGCGAC   
  
  
+ AGTTTCATTT TCCCAACAAA AATTTCGAGT GTATTCTTTT TAATTTATCA TAAGTATTTA TTGTCAACAT   
  
  
+ TCAACATGCA TCAACAATCA TTAAACACTT TTCGTCACTC CTCAATACTG GTCAGCACTT AAGATTTTGT   
  
  
+ CTTCTAAACT TATCGTAAGT AACTTATTGT TGATTAATTG ATGCTGACCA AATGATACTG AGTGCTAAAA   
  
  
+ TTATAATTAA TAAATACAAT TAAGAGTTGA TCAATGTCGA TGATTCAAAT CAATGTGATT GGTGCTGAAT   
  
  
+ AGTAATTATA AGCACCTATA AAGACTTGAA AAGAACAAAC ATTTAATTAG ATTTTATAAT TATAATTTCC   
  
  
+ TAATATTTAG CATTCATCAG CAAATTCAGC ACACACCAAC ATTAGTCAAC ACCTATCAAC ACTTATAAGT   
  
  
+ ACCAATTAAT AATAATTACC TATAATAGAT TAAAAGTTAG GCCCTTAATG TTTCCCACAT TATTAGATAT   
  
  
+ ATCAAGGGAG GGGGAAAAAA ATAATACTCC CCCATCTTAA AAAATTTATC CCTTTTTTTC TTTTTAATGT   
  
  
+ TCCAAAACAA AAATATGCTT CTAAATTATG ATTGCAATAT GAATGTGTAC GGTTTCAAAC CCAATTTTCT   
  
  
+ CCAACAATTC TTCTACTATA TTGTAATTAT ATTATACTAC AATTTGTATC TCATCTATGA TCTACCCCCC   
  
  
+ AAAAACCAAA TACTCATATA AAGTTTTATT TTCTTTCCAT CCCACTCTAA AAAAAAAAAA TGTTGGGAAA   
  
  
+ AAGTTGATTT ATTTAATTTG AAATGGTACG GTGAATATAG ATTTGTAGAG GGGCAGTTGA TAGAAAAAGG   
  
  
+ TGAAGACAAA GGAAGGGCAC ATTTGCCTTT TCATAGAAGG AAGAGACCAA GGTACTGGGG ACTGGGGGAA   
  
  
+ GGGCTCAAGA GAAGAGAGAG ACTGAGAAGA GTGCTGCTAC TCCTTCCTAC CTACACACAA AATTGCTGCG   
  
  
+ GGGGGTTCAA GTTCAACTTG AACGAGGCAA ATACCCTCAT TGAACCCACC AAATCGAAAT CTTCAATGTA   
  
  
+ CACCCCCATT ATACACGCAT TCAGAGACAT AGATGTCGAG GGGGAGATGA ATTAGCAAGA AGATGTCGAG   
  
  
+ CTAAAATGCA TTTCGAGGTT ACTAGAGCCA GAAATGTGGG CACATCCCAG AAGATCTCAG TAGAGAGAGT   
  
  
+ AACTTCAGAA AAGTTTCAAT TTTTATTCGA AATTTGCCTC TTGTTTGTGT AGATATGATG GGAACTTCGC   
  
  
+ CTTACAATCT GCAATGTGAG GGTTTGGTTG AAGGAGTTGC AGTCAATCCT CAATTTTATG AATCAACCCC   
  
  
+ AAAGTGGAAG AAGGGCATCA AAGAAGAGTC AATTGAGAGC GAACCCACTT CGGTTTTGGA CAACCCTAGC   
  
  
+ CCTCCAAATT CTGCATCTAC TCTCTCTTCC TGCTTCAATG GTGGAAGTAG TGGGCTTGGG ATGGACGATT   
  
  
+ TGGAGGGTTT GTCTTTTGGA GAAGGGTCCC TTCTCCCTTG GTTCATGGGT GAAATTGAAG ACCCTAGTTT   
  
  
+ GAGTTTCAAG CACCTCCTTC AACCTTCAAA TCCCTTGGAG TTTGGTGGCA ATGATGGGCT TGAAGCAGCT   
  
  
+ ATTCAGAGTG CTGGTATGGG TAATTTTAAT TTGGTTGAAA GTTTTAATTC TGATTTGAGG TCTTGTAATT
[truncated: 27,887 more chars]
