# Supplementary material for: Genome-Wide Identification and Expression Pattern of the GRAS Gene Family in Pitaya (Selenicereus undatus L.)
Source: Biology (Basel). 2022 Dec 21;12(1):11. doi: 10.3390/biology12010011 (PMC9854919; doi:10.3390/biology12010011)
Supplement: Supplementary file 1 [file biology-12-00011-s001.zip › Supplementary file S5/HU04G00148.1_plantcare.html]

Content-Type: text/html; charset=ISO-8859-1


PlantCARE


Webmaster Firefox specific output  
To save the result:
click on the frame with the right mouse button and save the source code as a text file with extension .html  
REFERENCE:PlantCARE: a database of plant cis-acting regulatory elements and a portal to tools for in silico analysis of promoter sequences.  
Lescot, M., Déhais, P., Moreau, Y., De Moor, B., Rouzé ,P.,and Rombauts, S.  
Nucleic Acids Res., Database issue(2002), 30(1):325-327.   


---

>HU04G00148.1   
+ -Up\_Stream \_Len000ATAATG GGTGATAACT TTTGAAGGAC AATATACAAA TCACTTATTT ATTAAAGTCA   
  
  
+ AGGGGACATT TCAACTATAA CATCATTGTT TATAAAGTAT TTGAAAATTG CAACTCTTGT AATAGATAAT   
  
  
+ AATGCTTTAT AGACTTCACC AATCACTAAT TTGTAGAATC AAAAAATTAA TTGAAAAGCA AACAAAAGGA   
  
  
+ AAAGGCACAT AAACCTAAAT TATGTAAACA AATGAGAACA TATATGCCAG CTTATCAACG TTAATTATAT   
  
  
+ TTGTTATCAC TCTTAATATC TGTATTCATA GTAAATGAAA ATAAGTATCG ATTTTTTATT AAACCTAAAA   
  
  
+ TCTTCTGTCA ATAATTTTTC TTCCGATAAA AAAAAAATTG GACCATATTA AATTAACATT AATAAATTCA   
  
  
+ TATAATTACT TTGAATGTCT GAAATATAAT GCTCTTCGGT ATATTAAGGT CTTATAAATT TCTCATTAAT   
  
  
+ TTGAAATCTC TTATAATATA TTGGGTCAAC AAGTGCTAGC TTGTTTTTTT TTCCTGGTTA ATTGCATTTA   
  
  
+ AAAAAATAAT TTTTAATTAC AATTGCAAGG GGAGGAATCA TTTACAAAAT ATCAAAAGTG GAAAAGGCAA   
  
  
+ AAAGAAACAA ATTATAAAAC AGAAAAAAGA TTAGAAAACG CAAACAAAGA CGAAATGAGG AACAGTAAGA   
  
  
+ ATCAGTAAAA AAATGTAGTG CAACGGTCAC ATACGGGAAG GCAAGCACAA CAATCATGAA AGAGACAGGC   
  
  
+ ACGTGCGTTC CTCGTGATCA GCAGCCTTCC CTACTTCCCC GCCAAGTGGA GTCCGTACTA ACTCCTGCCA   
  
  
+ TTCCCTCCCA ACGGTCATAT TCCGCCGACA TGCCTCAACG GTCAGATTTC CTGCCCCATC TGGACCGTCC   
  
  
+ GATCAAGATC CAACGGCCAT AAGGCGCACT CTCCAATACC CTAGTGCCCA TTTCTGGAAC AGCCCCAAAA   
  
  
+ ATCTCAGTAT TCGCCATCAT TTTTCAATAC TGGGGTTACA TAAACAGTCT AACAAGGAAG GACCCAACAC   
  
  
+ AGAGGAGAGA GAAAAAAGCT AGCTTTGATG GGGACAGCCA TGGATGGAGG TGAAGAGAGA GGAGGTTGGG   
  
  
+ ACCTTTTATT TTGCTCACTT TCCATTCTTA TATAAAAACA GCTCTAGTTT ATCTCTTATG GCTTTTTTGG   
  
  
+ GTTTGTTCAT AGAGAGGGGG GAATGTGATA TGATTTGATT TGATGTTAGA AATTCTCATT AGTTTTTTCT   
  
  
+ TTTTGTTTGC AATTTTGATG TGTTAGGGTT TGAGGATCAT CATTTTTGAG TAGGGAACTT TCTGGGTTTT   
  
  
+ GCTTGCTGAT TGATTGCCAA TGCTATGGAT TTCTTGGTGA GTCAATCTTC AATTGCTGAT CTGGGTTCGT   
  
  
+ TTGTTCTTCA GAGTTTCACC TGCCAATTTG ATCAAGTATG TCCCTTCACT TCTCCTGCTT CAATTCTCTC   
  
  
+ TCAAATTCAC GCATTTTTCC AATGAATTTC AAATAGGAGT CCTGGAATCC GATGATATTT TAGGTTTTTT   
  
  
+ CCTGACTTCA TTGCATTAGT TTGTTCATAT CTGTGACCAA TTCTTTCATT TGTTATATGA TCTGATTGTT   
  
  
+ TAGTTAGCTA TTCATTCATT GCATATACAT TGGTTTATTC ATTGCAGAGA CACTCACATA CACACACACA   
  
  
+ TACAGGGGAC ACTAGTATTT TTTCTCTCTA ACTCATCGGA TTCCCTTTGA TACTCTGTTT TTTGGGGGGT   
  
  
+ TGATTTGATT TGATTTGATT GACCTTAATT TTGAACTCAC ACACACTCGC ACATAGATAT ATTCTTTCTG   
  
  
+ TTTGTGACAT ATACAGCATC ATATAGAAAT ATATTGCACT GCATATTTGA GCTCAAGACA GGGATATCAT   
  
  
+ CACAAACTGA GTGTGAGGGA GGCAAAAAAC AGAGGAAAAG AAGGGAATTG GGAGTGTAAT CAAAACAAGA   
  
  
+ GAGAGATTGA GGAGAGAGGA AAAAACAAAA TTGGGAGAAG GTTAATAGAG AAGAATGTTG GCTGGGTGTT   
  
  
+ CATCCACATT GCTGTCACCA AGGTATAGAT TGAGGAGTGA AGCTACATCA CAGTTCCAAG CCTGCCATCA   
  
  
+ TCCAATGAGC ACACAGAGAA TTGATTTGCC CTGCACTTTC CCAAGAAAGG ATGTTTCGAA GCCACAATCG   
  
  
+ GTTCGCCCCG TTGGCCTATC TGTCGAGAAG CCGGTTGAAG TCAGGGCCCT GAAGCAGACC ATCCGAGTCC   
  
  
+ CACCATCGCC GGAGGGTAGA AGAGAGATTA AGGCAGATTT CTGGGGTGAT AGAAGAAAGA GCTTGAAGAG   
  
  
+ GAGACTAGCA GAACAGGGGA GCTCTTTTGA TGATGGTGGT GACGAATCTT GTGTTGGGAG AACCAAAAGG   
  
  
+ AAGAAGGGTA GCTTTGATTT TGGTGAATCT GATGAAATTT CTCCAAAAAT TGAGGACACA ATGAGTTTTG   
  
  
+ GACATCTGGG TAGTGGTGGA AATTTCTGGG TTCATCCTGG TTTTGGTGTT GTTAATGTTA ATACTAATTA   
  
  
+ CCCTCAAGTG CCATTTTCTC TCACTTGTTC AGGGGAGGAA GAAAGAGTAT GTTTTGTTCC TACTGATGTG   
  
  
+ ATCTCACCAG CCATCATGCC TCCATTGTCA CACAATCCTT GGGTTGAATC TGGTGTTACT GAGGTCACAG   
  
  
+ AATATGGTGG GGGTGACAAA GACGGCGAAC CTAGTCATGG GTTTGTGAGG GGTACAACAA CAACGTCCGG   
  
  
+ GTCAAGTTCA TCTTCTGAGA GTCATAGTTT TGGGCATAGG CTCAATGAGA GCTCATCTGA CCCTGAAATC   
  
  
+ AGAAATGGTT CCATGTTGCC TAACCCTGGT CATGGTCCTG GCACCAGTCA TGGTCCTGGA ACCGGTCTGG   
  
  
+ CTCACAACCA CAATGATCAA ACCGAGCAAC AAGGGTTCGA GCTCATTAGC TTTCTCATGG GTTGTGTCGA   
  
  
+ AGCGATCAGT TCAAGGAACA TTGCAGCCAT CAATCATTTC ATAGCTAAGC TGGGCGAGCA GGCTTCTCCA   
  
  
+ AGGGCTCGGT CTGCCATTAG CCGCCTCACA GCCTACTTCA CCGAAGCATT AGCCTTGAGG GTCACAAGAT   
  
  
+ TTTGGCCTCA TATCTTTCAC ATAAGTATCC CTCGAGAGTT TGATCGATTT GATGATGAAT CGGGGGCAGC   
  
  
+ TGCAATGAGG CTTTTGAATC AGATCAGCCC AATTCCAAAG TTCGTTCATT TTACAGCCAA TGAGATGTTA   
  
  
+ TTGAGGGCAT TTGAAGGGAA GGACAAGGTG CATATCATAG ACTTCGACAT CAAGCAAGGC TTACAATGGC   
  
  
+ CCGGGTTTTT CCAAAGCTTA GCCATGAGGG AAAACCCCCC GAGCCATGTC AGGATAACAG GCGTAGGGGA   
  
  
+ TTCGAAGCAA GAATTGGTTG AGACAGGAGA AAGACTAGCC GGGTTTGCTG GGGCATTCAA CCTCTCCTTC   
  
  
+ GAGTTCCACC CAGTTGTGGA CCGGTTGGAA GATGTTAGGC TATGGATGCT TCATGTTAAG GAAGGTGAAA   
  
  
+ GTGTCGCGGT AAATTGCATT TTGCAGCTCC ACAAGACACT CTATGACCCC CATGGTGCCA CATTCAGGGA   
  
  
+ CTTCATGGGT TTAATCCGAA GCACAAATCC CATAGCATTG GTTATGGCTG AGCAAGAAGC TGATCACAAC   
  
  
+ GAACCCACCT TAGAAGGCCG AGTATGCAAC TCACTTGGAT ACTATGCAGC CCTTTTCGAT GCCATAGATT   
  
  
+ GCAGCCTTCC TTTCGAGAGC TCAGCAAGGT TGAAGATCGA AGAGATGTTT GGCCGGGAAA TCAGGAACAT   
  
  
+ AATAGCCTGT GAAGGGGCAG ACAGGATCGA AAGACATGAG AAGTTCGAGA AATGGAAGAG AAGGATCGAG   
  
  
+ CAGGAAGGGT TCCGGTGCAT GCGGACTAGC GAAAGGGAGG TGATGCAGAG CCAAATGCTG CTTAAGATGT   
  
  
+ ACTCGAACGA GAGCTACAAC ATGCAAAGAC AAGGCAATGA AGAAGCAATA TCACTAGTAT GGTTAGATCA   
  
  
+ GCCACTCTAC ACTGTTTCTT TGTGGGCTCC AAGTGAATTT GCTGCAGGGA GTTCTTCCAG TTTTTCTCTG   
  
  
+ CCATCTTG  

- -Up\_Stream \_Len000TATTAC CCACTATTGA AAACTTCCTG TTATATGTTT AGTGAATAAA TAATTTCAGT   
  
  
- TCCCCTGTAA AGTTGATATT GTAGTAACAA ATATTTCATA AACTTTTAAC GTTGAGAACA TTATCTATTA   
  
  
- TTACGAAATA TCTGAAGTGG TTAGTGATTA AACATCTTAG TTTTTTAATT AACTTTTCGT TTGTTTTCCT   
  
  
- TTTCCGTGTA TTTGGATTTA ATACATTTGT TTACTCTTGT ATATACGGTC GAATAGTTGC AATTAATATA   
  
  
- AACAATAGTG AGAATTATAG ACATAAGTAT CATTTACTTT TATTCATAGC TAAAAAATAA TTTGGATTTT   
  
  
- AGAAGACAGT TATTAAAAAG AAGGCTATTT TTTTTTTAAC CTGGTATAAT TTAATTGTAA TTATTTAAGT   
  
  
- ATATTAATGA AACTTACAGA CTTTATATTA CGAGAAGCCA TATAATTCCA GAATATTTAA AGAGTAATTA   
  
  
- AACTTTAGAG AATATTATAT AACCCAGTTG TTCACGATCG AACAAAAAAA AAGGACCAAT TAACGTAAAT   
  
  
- TTTTTTATTA AAAATTAATG TTAACGTTCC CCTCCTTAGT AAATGTTTTA TAGTTTTCAC CTTTTCCGTT   
  
  
- TTTCTTTGTT TAATATTTTG TCTTTTTTCT AATCTTTTGC GTTTGTTTCT GCTTTACTCC TTGTCATTCT   
  
  
- TAGTCATTTT TTTACATCAC GTTGCCAGTG TATGCCCTTC CGTTCGTGTT GTTAGTACTT TCTCTGTCCG   
  
  
- TGCACGCAAG GAGCACTAGT CGTCGGAAGG GATGAAGGGG CGGTTCACCT CAGGCATGAT TGAGGACGGT   
  
  
- AAGGGAGGGT TGCCAGTATA AGGCGGCTGT ACGGAGTTGC CAGTCTAAAG GACGGGGTAG ACCTGGCAGG   
  
  
- CTAGTTCTAG GTTGCCGGTA TTCCGCGTGA GAGGTTATGG GATCACGGGT AAAGACCTTG TCGGGGTTTT   
  
  
- TAGAGTCATA AGCGGTAGTA AAAAGTTATG ACCCCAATGT ATTTGTCAGA TTGTTCCTTC CTGGGTTGTG   
  
  
- TCTCCTCTCT CTTTTTTCGA TCGAAACTAC CCCTGTCGGT ACCTACCTCC ACTTCTCTCT CCTCCAACCC   
  
  
- TGGAAAATAA AACGAGTGAA AGGTAAGAAT ATATTTTTGT CGAGATCAAA TAGAGAATAC CGAAAAAACC   
  
  
- CAAACAAGTA TCTCTCCCCC CTTACACTAT ACTAAACTAA ACTACAATCT TTAAGAGTAA TCAAAAAAGA   
  
  
- AAAACAAACG TTAAAACTAC ACAATCCCAA ACTCCTAGTA GTAAAAACTC ATCCCTTGAA AGACCCAAAA   
  
  
- CGAACGACTA ACTAACGGTT ACGATACCTA AAGAACCACT CAGTTAGAAG TTAACGACTA GACCCAAGCA   
  
  
- AACAAGAAGT CTCAAAGTGG ACGGTTAAAC TAGTTCATAC AGGGAAGTGA AGAGGACGAA GTTAAGAGAG   
  
  
- AGTTTAAGTG CGTAAAAAGG TTACTTAAAG TTTATCCTCA GGACCTTAGG CTACTATAAA ATCCAAAAAA   
  
  
- GGACTGAAGT AACGTAATCA AACAAGTATA GACACTGGTT AAGAAAGTAA ACAATATACT AGACTAACAA   
  
  
- ATCAATCGAT AAGTAAGTAA CGTATATGTA ACCAAATAAG TAACGTCTCT GTGAGTGTAT GTGTGTGTGT   
  
  
- ATGTCCCCTG TGATCATAAA AAAGAGAGAT TGAGTAGCCT AAGGGAAACT ATGAGACAAA AAACCCCCCA   
  
  
- ACTAAACTAA ACTAAACTAA CTGGAATTAA AACTTGAGTG TGTGTGAGCG TGTATCTATA TAAGAAAGAC   
  
  
- AAACACTGTA TATGTCGTAG TATATCTTTA TATAACGTGA CGTATAAACT CGAGTTCTGT CCCTATAGTA   
  
  
- GTGTTTGACT CACACTCCCT CCGTTTTTTG TCTCCTTTTC TTCCCTTAAC CCTCACATTA GTTTTGTTCT   
  
  
- CTCTCTAACT CCTCTCTCCT TTTTTGTTTT AACCCTCTTC CAATTATCTC TTCTTACAAC CGACCCACAA   
  
  
- GTAGGTGTAA CGACAGTGGT TCCATATCTA ACTCCTCACT TCGATGTAGT GTCAAGGTTC GGACGGTAGT   
  
  
- AGGTTACTCG TGTGTCTCTT AACTAAACGG GACGTGAAAG GGTTCTTTCC TACAAAGCTT CGGTGTTAGC   
  
  
- CAAGCGGGGC AACCGGATAG ACAGCTCTTC GGCCAACTTC AGTCCCGGGA CTTCGTCTGG TAGGCTCAGG   
  
  
- GTGGTAGCGG CCTCCCATCT TCTCTCTAAT TCCGTCTAAA GACCCCACTA TCTTCTTTCT CGAACTTCTC   
  
  
- CTCTGATCGT CTTGTCCCCT CGAGAAAACT ACTACCACCA CTGCTTAGAA CACAACCCTC TTGGTTTTCC   
  
  
- TTCTTCCCAT CGAAACTAAA ACCACTTAGA CTACTTTAAA GAGGTTTTTA ACTCCTGTGT TACTCAAAAC   
  
  
- CTGTAGACCC ATCACCACCT TTAAAGACCC AAGTAGGACC AAAACCACAA CAATTACAAT TATGATTAAT   
  
  
- GGGAGTTCAC GGTAAAAGAG AGTGAACAAG TCCCCTCCTT CTTTCTCATA CAAAACAAGG ATGACTACAC   
  
  
- TAGAGTGGTC GGTAGTACGG AGGTAACAGT GTGTTAGGAA CCCAACTTAG ACCACAATGA CTCCAGTGTC   
  
  
- TTATACCACC CCCACTGTTT CTGCCGCTTG GATCAGTACC CAAACACTCC CCATGTTGTT GTTGCAGGCC   
  
  
- CAGTTCAAGT AGAAGACTCT CAGTATCAAA ACCCGTATCC GAGTTACTCT CGAGTAGACT GGGACTTTAG   
  
  
- TCTTTACCAA GGTACAACGG ATTGGGACCA GTACCAGGAC CGTGGTCAGT ACCAGGACCT TGGCCAGACC   
  
  
- GAGTGTTGGT GTTACTAGTT TGGCTCGTTG TTCCCAAGCT CGAGTAATCG AAAGAGTACC CAACACAGCT   
  
  
- TCGCTAGTCA AGTTCCTTGT AACGTCGGTA GTTAGTAAAG TATCGATTCG ACCCGCTCGT CCGAAGAGGT   
  
  
- TCCCGAGCCA GACGGTAATC GGCGGAGTGT CGGATGAAGT GGCTTCGTAA TCGGAACTCC CAGTGTTCTA   
  
  
- AAACCGGAGT ATAGAAAGTG TATTCATAGG GAGCTCTCAA ACTAGCTAAA CTACTACTTA GCCCCCGTCG   
  
  
- ACGTTACTCC GAAAACTTAG TCTAGTCGGG TTAAGGTTTC AAGCAAGTAA AATGTCGGTT ACTCTACAAT   
  
  
- AACTCCCGTA AACTTCCCTT CCTGTTCCAC GTATAGTATC TGAAGCTGTA GTTCGTTCCG AATGTTACCG   
  
  
- GGCCCAAAAA GGTTTCGAAT CGGTACTCCC TTTTGGGGGG CTCGGTACAG TCCTATTGTC CGCATCCCCT   
  
  
- AAGCTTCGTT CTTAACCAAC TCTGTCCTCT TTCTGATCGG CCCAAACGAC CCCGTAAGTT GGAGAGGAAG   
  
  
- CTCAAGGTGG GTCAACACCT GGCCAACCTT CTACAATCCG ATACCTACGA AGTACAATTC CTTCCACTTT   
  
  
- CACAGCGCCA TTTAACGTAA AACGTCGAGG TGTTCTGTGA GATACTGGGG GTACCACGGT GTAAGTCCCT   
  
  
- GAAGTACCCA AATTAGGCTT CGTGTTTAGG GTATCGTAAC CAATACCGAC TCGTTCTTCG ACTAGTGTTG   
  
  
- CTTGGGTGGA ATCTTCCGGC TCATACGTTG AGTGAACCTA TGATACGTCG GGAAAAGCTA CGGTATCTAA   
  
  
- CGTCGGAAGG AAAGCTCTCG AGTCGTTCCA ACTTCTAGCT TCTCTACAAA CCGGCCCTTT AGTCCTTGTA   
  
  
- TTATCGGACA CTTCCCCGTC TGTCCTAGCT TTCTGTACTC TTCAAGCTCT TTACCTTCTC TTCCTAGCTC   
  
  
- GTCCTTCCCA AGGCCACGTA CGCCTGATCG CTTTCCCTCC ACTACGTCTC GGTTTACGAC GAATTCTACA   
  
  
- TGAGCTTGCT CTCGATGTTG TACGTTTCTG TTCCGTTACT TCTTCGTTAT AGTGATCATA CCAATCTAGT   
  
  
- CGGTGAGATG TGACAAAGAA ACACCCGAGG TTCACTTAAA CGACGTCCCT CAAGAAGGTC AAAAAGAGAC   
  
  
- GGTAGAAC

  
  
Motifs Found  

+   

| Site Name | Organism | Position | Strand | Matrix score. | sequence | function |
| --- | --- | --- | --- | --- | --- | --- |
|  | organism | 3921 | + | 4 | motif\_sequence | short\_function |
|  | organism | 354 | + | 4 | motif\_sequence | short\_function |
|  | organism | 1379 | + | 4 | motif\_sequence | short\_function |
|  | organism | 4060 | + | 4 | motif\_sequence | short\_function |
|  | organism | 1058 | - | 4 | motif\_sequence | short\_function |
|  | organism | 2277 | - | 4 | motif\_sequence | short\_function |
|  | organism | 971 | - | 4 | motif\_sequence | short\_function |
|  | organism | 943 | + | 4 | motif\_sequence | short\_function |
|  | organism | 1658 | - | 4 | motif\_sequence | short\_function |
|  | organism | 3779 | - | 4 | motif\_sequence | short\_function |
|  | organism | 3426 | + | 4 | motif\_sequence | short\_function |
|  | organism | 3746 | - | 4 | motif\_sequence | short\_function |
|  | organism | 1803 | - | 4 | motif\_sequence | short\_function |
|  | organism | 456 | + | 4 | motif\_sequence | short\_function |
|  | organism | 3839 | - | 4 | motif\_sequence | short\_function |
|  | organism | 694 | - | 4 | motif\_sequence | short\_function |
|  | organism | 1442 | + | 4 | motif\_sequence | short\_function |
|  | organism | 3999 | + | 4 | motif\_sequence | short\_function |
|  | organism | 1086 | - | 4 | motif\_sequence | short\_function |
|  | organism | 2960 | - | 4 | motif\_sequence | short\_function |
|  | organism | 1106 | - | 4 | motif\_sequence | short\_function |
|  | organism | 250 | - | 4 | motif\_sequence | short\_function |
|  | organism | 3462 | - | 4 | motif\_sequence | short\_function |
|  | organism | 2263 | - | 4 | motif\_sequence | short\_function |
|  | organism | 3753 | - | 4 | motif\_sequence | short\_function |
|  | organism | 219 | - | 4 | motif\_sequence | short\_function |
|  | organism | 2454 | - | 4 | motif\_sequence | short\_function |
|  | organism | 2744 | + | 4 | motif\_sequence | short\_function |
|  | organism | 1975 | - | 4 | motif\_sequence | short\_function |
|  | organism | 78 | - | 4 | motif\_sequence | short\_function |
|  | organism | 3899 | - | 4 | motif\_sequence | short\_function |
|  | organism | 2309 | - | 4 | motif\_sequence | short\_function |
|  | organism | 2325 | - | 4 | motif\_sequence | short\_function |

>HU04G00148.1   
+ -Up\_Stream \_Len000ATAATG GGTGATAACT TTTGAAGGAC AATATACAAA TCACTTATTT ATTAAAGTCA   
  
  
+ AGGGGACATT TCAACTATAA CATCATTGTT TATAAAGTAT TTGAAAATTG CAACTCTTGT AATAGATAAT   
  
  
+ AATGCTTTAT AGACTTCACC AATCACTAAT TTGTAGAATC AAAAAATTAA TTGAAAAGCA AACAAAAGGA   
  
  
+ AAAGGCACAT AAACCTAAAT TATGTAAACA AATGAGAACA TATATGCCAG CTTATCAACG TTAATTATAT   
  
  
+ TTGTTATCAC TCTTAATATC TGTATTCATA GTAAATGAAA ATAAGTATCG ATTTTTTATT AAACCTAAAA   
  
  
+ TCTTCTGTCA ATAATTTTTC TTCCGATAAA AAAAAAATTG GACCATATTA AATTAACATT AATAAATTCA   
  
  
+ TATAATTACT TTGAATGTCT GAAATATAAT GCTCTTCGGT ATATTAAGGT CTTATAAATT TCTCATTAAT   
  
  
+ TTGAAATCTC TTATAATATA TTGGGTCAAC AAGTGCTAGC TTGTTTTTTT TTCCTGGTTA ATTGCATTTA   
  
  
+ AAAAAATAAT TTTTAATTAC AATTGCAAGG GGAGGAATCA TTTACAAAAT ATCAAAAGTG GAAAAGGCAA   
  
  
+ AAAGAAACAA ATTATAAAAC AGAAAAAAGA TTAGAAAACG CAAACAAAGA CGAAATGAGG AACAGTAAGA   
  
  
+ ATCAGTAAAA AAATGTAGTG CAACGGTCAC ATACGGGAAG GCAAGCACAA CAATCATGAA AGAGACAGGC   
  
  
+ ACGTGCGTTC CTCGTGATCA GCAGCCTTCC CTACTTCCCC GCCAAGTGGA GTCCGTACTA ACTCCTGCCA   
  
  
+ TTCCCTCCCA ACGGTCATAT TCCGCCGACA TGCCTCAACG GTCAGATTTC CTGCCCCATC TGGACCGTCC   
  
  
+ GATCAAGATC CAACGGCCAT AAGGCGCACT CTCCAATACC CTAGTGCCCA TTTCTGGAAC AGCCCCAAAA   
  
  
+ ATCTCAGTAT TCGCCATCAT TTTTCAATAC TGGGGTTACA TAAACAGTCT AACAAGGAAG GACCCAACAC   
  
  
+ AGAGGAGAGA GAAAAAAGCT AGCTTTGATG GGGACAGCCA TGGATGGAGG TGAAGAGAGA GGAGGTTGGG   
  
  
+ ACCTTTTATT TTGCTCACTT TCCATTCTTA TATAAAAACA GCTCTAGTTT ATCTCTTATG GCTTTTTTGG   
  
  
+ GTTTGTTCAT AGAGAGGGGG GAATGTGATA TGATTTGATT TGATGTTAGA AATTCTCATT AGTTTTTTCT   
  
  
+ TTTTGTTTGC AATTTTGATG TGTTAGGGTT TGAGGATCAT CATTTTTGAG TAGGGAACTT TCTGGGTTTT   
  
  
+ GCTTGCTGAT TGATTGCCAA TGCTATGGAT TTCTTGGTGA GTCAATCTTC AATTGCTGAT CTGGGTTCGT   
  
  
+ TTGTTCTTCA GAGTTTCACC TGCCAATTTG ATCAAGTATG TCCCTTCACT TCTCCTGCTT CAATTCTCTC   
  
  
+ TCAAATTCAC GCATTTTTCC AATGAATTTC AAATAGGAGT CCTGGAATCC GATGATATTT TAGGTTTTTT   
  
  
+ CCTGACTTCA TTGCATTAGT TTGTTCATAT CTGTGACCAA TTCTTTCATT TGTTATATGA TCTGATTGTT   
  
  
+ TAGTTAGCTA TTCATTCATT GCATATACAT TGGTTTATTC ATTGCAGAGA CACTCACATA CACACACACA   
  
  
+ TACAGGGGAC ACTAGTATTT TTTCTCTCTA ACTCATCGGA TTCCCTTTGA TACTCTGTTT TTTGGGGGGT   
  
  
+ TGATTTGATT TGATTTGATT GACCTTAATT TTGAACTCAC ACACACTCGC ACATAGATAT ATTCTTTCTG   
  
  
+ TTTGTGACAT ATACAGCATC ATATAGAAAT ATATTGCACT GCATATTTGA GCTCAAGACA GGGATATCAT   
  
  
+ CACAAACTGA GTGTGAGGGA GGCAAAAAAC AGAGGAAAAG AAGGGAATTG GGAGTGTAAT CAAAACAAGA   
  
  
+ GAGAGATTGA GGAGAGAGGA AAAAACAAAA TTGGGAGAAG GTTAATAGAG AAGAATGTTG GCTGGGTGTT   
  
  
+ CATCCACATT GCTGTCACCA AGGTATAGAT TGAGGAGTGA AGCTACATCA CAGTTCCAAG CCTGCCATCA   
  
  
+ TCCAATGAGC ACACAGAGAA TTGATTTGCC CTGCACTTTC CCAAGAAAGG ATGTTTCGAA GCCACAATCG   
  
  
+ GTTCGCCCCG TTGGCCTATC TGTCGAGAAG CCGGTTGAAG TCAGGGCCCT GAAGCAGACC ATCCGAGTCC   
  
  
+ CACCATCGCC GGAGGGTAGA AGAGAGATTA AGGCAGATTT CTGGGGTGAT AGAAGAAAGA GCTTGAAGAG   
  
  
+ GAGACTAGCA GAACAGGGGA GCTCTTTTGA TGATGGTGGT GACGAATCTT GTGTTGGGAG AACCAAAAGG   
  
  
+ AAGAAGGGTA GCTTTGATTT TGGTGAATCT GATGAAATTT CTCCAAAAAT TGAGGACACA ATGAGTTTTG   
  
  
+ GACATCTGGG TAGTGGTGGA AATTTCTGGG TTCATCCTGG TTTTGGTGTT GTTAATGTTA ATACTAATTA   
  
  
+ CCCTCAAGTG CCATTTTCTC TCACTTGTTC AGGGGAGGAA GAAAGAGTAT GTTTTGTTCC TACTGATGTG   
  
  
+ ATCTCACCAG CCATCATGCC TCCATTGTCA CACAATCCTT GGGTTGAATC TGGTGTTACT GAGGTCACAG   
  
  
+ AATATGGTGG GGGTGACAAA GACGGCGAAC CTAGTCATGG GTTTGTGAGG GGTACAACAA CAACGTCCGG   
  
  
+ GTCAAGTTCA TCTTCTGAGA GTCATAGTTT TGGGCATAGG CTCAATGAGA GCTCATCTGA CCCTGAAATC   
  
  
+ AGAAATGGTT CCATGTTGCC TAACCCTGGT CATGGTCCTG GCACCAGTCA TGGTCCTGGA ACCGGTCTGG   
  
  
+ CTCACAACCA CAATGATCAA ACCGAGCAAC AAGGGTTCGA GCTCATTAGC TTTCTCATGG GTTGTGTCGA   
  
  
+ AGCGATCAGT TCAAGGAACA TTGCAGCCAT CAATCATTTC ATAGCTAAGC TGGGCGAGCA GGCTTCTCCA   
  
  
+ AGGGCTCGGT CTGCCATTAG CCGCCTCACA GCCTACTTCA CCGAAGCATT AGCCTTGAGG GTCACAAGAT   
  
  
+ TTTGGCCTCA TATCTTTCAC ATAAGTATCC CTCGAGAGTT TGATCGATTT GATGATGAAT CGGGGGCAGC   
  
  
+ TGCAATGAGG CTTTTGAATC AGATCAGCCC AATTCCAAAG TTCGTTCATT TTACAGCCAA TGAGATGTTA   
  
  
+ TTGAGGGCAT TTGAAGGGAA GGACAAGGTG CATATCATAG ACTTCGACAT CAAGCAAGGC TTACAATGGC   
  
  
+ CCGGGTTTTT CCAAAGCTTA GCCATGAGGG AAAACCCCCC GAGCCATGTC AGGATAACAG GCGTAGGGGA   
  
  
+ TTCGAAGCAA GAATTGGTTG AGACAGGAGA AAGACTAGCC GGGTTTGCTG GGGCATTCAA CCTCTCCTTC   
  
  
+ GAGTTCCACC CAGTTGTGGA CCGGTTGGAA GATGTTAGGC TATGGATGCT TCATGTTAAG GAAGGTGAAA   
  
  
+ GTGTCGCGGT AAATTGCATT TTGCAGCTCC ACAAGACACT CTATGACCCC CATGGTGCCA CATTCAGGGA   
  
  
+ CTTCATGGGT TTAATCCGAA GCACAAATCC CATAGCATTG GTTATGGCTG AGCAAGAAGC TGATCACAAC   
  
  
+ GAACCCACCT TAGAAGGCCG AGTATGCAAC TCACTTGGAT ACTATGCAGC CCTTTTCGAT GCCATAGATT   
  
  
+ GCAGCCTTCC TTTCGAGAGC TCAGCAAGGT TGAAGATCGA AGAGATGTTT GGCCGGGAAA TCAGGAACAT   
  
  
+ AATAGCCTGT GAAGGGGCAG ACAGGATCGA AAGACATGAG AAGTTCGAGA AATGGAAGAG AAGGATCGAG   
  
  
+ CAGGAAGGGT TCCGGTGCAT GCGGACTAGC GAAAGGGAGG TGATGCAGAG CCAAATGCTG CTTAAGATGT   
  
  
+ ACTCGAACGA GAGCTACAAC ATGCAAAGAC AAGGCAATGA AGAAGCAATA TCACTAGTAT GGTTAGATCA   
  
  
+ GCCACTCTAC ACTGTTTCTT TGTGGGCTCC AAGTGAATTT GCTGCAGGGA GTTCTTCCAG TTTTTCTCTG   
  
  
+ CCATCTTGA  

- -Up\_Stream \_Len

000TATTAC CCACTATTGA AAACTTCCTG TTATATGTTT AGTGAATAAA TAATTTCAGT   
  
  
- TCCCCTGTAA AGTTGATATT GTAGTAACAA ATATTTCATA AACTTTTAAC GTTGAGAACA TTATCTATTA   
  
  
- TTACGAAATA TCTGAAGTGG TTAGTGATTA AACATCTTAG TTTTTTAATT AACTTTTCGT TTGTTTTCCT   
  
  
- TTTCCGTGTA TTTGGATTTA ATACATTTGT TTACTCTTGT ATATACGGTC GAATAGTTGC AATTAATATA   
  
  
- AACAATAGTG AGAATTATAG ACATAAGTAT CATTTACTTT TATTCATAGC TAAAAAATAA TTTGGATTTT   
  
  
- AGAAGACAGT TATTAAAAAG AAGGCTATTT TTTTTTTAAC CTGGTATAAT TTAATTGTAA TTATTTAAGT   
  
  
- ATATTAATGA AACTTACAGA CTTTATATTA CGAGAAGCCA TATAATTCCA GAATATTTAA AGAGTAATTA   
  
  
- AACTTTAGAG AATATTATAT AACCCAGTTG TTCACGATCG AACAAAAAAA AAGGACCAAT TAACGTAAAT   
  
  
- TTTTTTATTA AAAATTAATG TTAACGTTCC CCTCCTTAGT AAATGTTTTA TAGTTTTCAC CTTTTCCGTT   
  
  
- TTTCTTTGTT TAATATTTTG TCTTTTTTCT AATCTTTTGC GTTTGTTTCT GCTTTACTCC TTGTCATTCT   
  
  
- TAGTCATTTT TTTACATCAC GTTGCCAGTG TATGCCCTTC CGTTCGTGTT GTTAGTACTT TCTCTGTCCG   
  
  
- TGCACGCAAG GAGCACTAGT CGTCGGAAGG GATGAAGGGG CGGTTCACCT CAGGCATGAT TGAGGACGGT   
  
  
- AAGGGAGGGT TGCCAGTATA AGGCGGCTGT ACGGAGTTGC CAGTCTAAAG GACGGGGTAG ACCTGGCAGG   
  
  
- CTAGTTCTAG GTTGCCGGTA TTCCGCGTGA GAGGTTATGG GATCACGGGT AAAGACCTTG TCGGGGTTTT   
  
  
- TAGAGTCATA AGCGGTAGTA AAAAGTTATG ACCCCAATGT ATTTGTCAGA TTGTTCCTTC CTGGGTTGTG   
  
  
- TCTCCTCTCT CTTTTTTCGA TCGAAACTAC CCCTGTCGGT ACCTACCTCC ACTTCTCTCT CCTCCAACCC   
  
  
- TGGAAAATAA AACGAGTGAA AGGTAAGAAT ATATTTTTGT CGAGATCAAA TAGAGAATAC CGAAAAAACC   
  
  
- CAAACAAGTA TCTCTCCCCC CTTACACTAT ACTAAACTAA ACTACAATCT TTAAGAGTAA TCAAAAAAGA   
  
  
- AAAACAAACG TTAAAACTAC ACAATCCCAA ACTCCTAGTA GTAAAAACTC ATCCCTTGAA AGACCCAAAA   
  
  
- CGAACGACTA ACTAACGGTT ACGATACCTA AAGAACCACT CAGTTAGAAG TTAACGACTA GACCCAAGCA   
  
  
- AACAAGAAGT CTCAAAGTGG ACGGTTAAAC TAGTTCATAC AGGGAAGTGA AGAGGACGAA GTTAAGAGAG   
  
  
- AGTTTAAGTG CGTAAAAAGG TTACTTAAAG TTTATCCTCA GGACCTTAGG CTACTATAAA ATCCAAAAAA   
  
  
- GGACTGAAGT AACGTAATCA AACAAGTATA GACACTGGTT AAGAAAGTAA ACAATATACT AGACTAACAA   
  
  
- ATCAATCGAT AAGTAAGTAA CGTATATGTA ACCAAATAAG TAACGTCTCT GTGAGTGTAT GTGTGTGTGT   
  
  
- ATGTCCCCTG TGATCATAAA AAAGAGAGAT TGAGTAGCCT AAGGGAAACT ATGAGACAAA AAACCCCCCA   
  
  
- ACTAAACTAA ACTAAACTAA CTGGAATTAA AACTTGAGTG TGTGTGAGCG TGTATCTATA TAAGAAAGAC   
  
  
- AAACACTGTA TATGTCGTAG TATATCTTTA TATAACGTGA CGTATAAACT CGAGTTCTGT CCCTATAGTA   
  
  
- GTGTTTGACT CACACTCCCT CCGTTTTTTG TCTCCTTTTC TTCCCTTAAC CCTCACATTA GTTTTGTTCT   
  
  
- CTCTCTAACT CCTCTCTCCT TTTTTGTTTT AACCCTCTTC CAATTATCTC TTCTTACAAC CGACCCACAA   
  
  
- GTAGGTGTAA CGACAGTGGT TCCATATCTA ACTCCTCACT TCGATGTAGT GTCAAGGTTC GGACGGTAGT   
  
  
- AGGTTACTCG TGTGTCTCTT AACTAAACGG GACGTGAAAG GGTTCTTTCC TACAAAGCTT CGGTGTTAGC   
  
  
- CAAGCGGGGC AACCGGATAG ACAGCTCTTC GGCCAACTTC AGTCCCGGGA CTTCGTCTGG TAGGCTCAGG   
  
  
- GTGGTAGCGG CCTCCCATCT TCTCTCTAAT TCCGTCTAAA GACCCCACTA TCTTCTTTCT CGAACTTCTC   
  
  
- CTCTGATCGT CTTGTCCCCT CGAGAAAACT ACTACCACCA CTGCTTAGAA CACAACCCTC TTGGTTTTCC   
  
  
- TTCTTCCCAT CGAAACTAAA ACCACTTAGA CTACTTTAAA GAGGTTTTTA ACTCCTGTGT TACTCAAAAC   
  
  
- CTGTAGACCC ATCACCACCT TTAAAGACCC AAGTAGGACC AAAACCACAA CAATTACAAT TATGATTAAT   
  
  
- GGGAGTTCAC GGTAAAAGAG AGTGAACAAG TCCCCTCCTT CTTTCTCATA CAAAACAAGG ATGACTACAC   
  
  
- TAGAGTGGTC GGTAGTACGG AGGTAACAGT GTGTTAGGAA CCCAACTTAG ACCACAATGA CTCCAGTGTC   
  
  
- TTATACCACC CCCACTGTTT CTGCCGCTTG GATCAGTACC CAAACACTCC CCATGTTGTT GTTGCAGGCC   
  
  
- CAGTTCAAGT AGAAGACTCT CAGTATCAAA ACCCGTATCC GAGTTACTCT CGAGTAGACT GGGACTTTAG   
  
  
- TCTTTACCAA GGTACAACGG ATTGGGACCA GTACCAGGAC CGTGGTCAGT ACCAGGACCT TGGCCAGACC   
  
  
- GAGTGTTGGT GTTACTAGTT TGGCTCGTTG TTCCCAAGCT CGAGTAATCG AAAGAGTACC CAACACAGCT   
  
  
- TCGCTAGTCA AGTTCCTTGT AACGTCGGTA GTTAGTAAAG TATCGATTCG ACCCGCTCGT CCGAAGAGGT   
  
  
- TCCCGAGCCA GACGGTAATC GGCGGAGTGT CGGATGAAGT GGCTTCGTAA TCGGAACTCC CAGTGTTCTA   
  
  
- AAACCGGAGT ATAGAAAGTG TATTCATAGG GAGCTCTCAA ACTAGCTAAA CTACTACTTA GCCCCCGTCG   
  
  
- ACGTTACTCC GAAAACTTAG TCTAGTCGGG TTAAGGTTTC AAGCAAGTAA AATGTCGGTT ACTCTACAAT   
  
  
- AACTCCCGTA AACTTCCCTT CCTGTTCCAC GTATAGTATC TGAAGCTGTA GTTCGTTCCG AATGTTACCG   
  
  
- GGCCCAAAAA GGTTTCGAAT CGGTACTCCC TTTTGGGGGG CTCGGTACAG TCCTATTGTC CGCATCCCCT   
  
  
- AAGCTTCGTT CTTAACCAAC TCTGTCCTCT TTCTGATCGG CCCAAACGAC CCCGTAAGTT GGAGAGGAAG   
  
  
- CTCAAGGTGG GTCAACACCT GGCCAACCTT CTACAATCCG ATACCTACGA AGTACAATTC CTTCCACTTT   
  
  
- CACAGCGCCA TTTAACGTAA AACGTCGAGG TGTTCTGTGA GATACTGGGG GTACCACGGT GTAAGTCCCT   
  
  
- GAAGTACCCA AATTAGGCTT CGTGTTTAGG GTATCGTAAC CAATACCGAC TCGTTCTTCG ACTAGTGTTG   
  
  
- CTTGGGTGGA ATCTTCCGGC TCATACGTTG AGTGAACCTA TGATACGTCG GGAAAAGCTA CGGTATCTAA   
  
  
- CGTCGGAAGG AAAGCTCTCG AGTCGTTCCA ACTTCTAGCT TCTCTACAAA CCGGCCCTTT AGTCCTTGTA   
  
  
- TTATCGGACA CTTCCCCGTC TGTCCTAGCT TTCTGTACTC TTCAAGCTCT TTACCTTCTC TTCCTAGCTC   
  
  
- GTCCTTCCCA AGGCCACGTA CGCCTGATCG CTTTCCCTCC ACTACGTCTC GGTTTACGAC GAATTCTACA   
  
  
- TGAGCTTGCT CTCGATGTTG TACGTTTCTG TTCCGTTACT TCTTCGTTAT AGTGATCATA CCAATCTAGT   
  
  
- CGGTGAGATG TGACAAAGAA ACACCCGAGG TTCACTTAAA CGACGTCCCT CAAGAAGGTC AAAAAGAGAC   
  
  
- GGTAGAAC

+     A-box

| Site Name | Organism | Position | Strand | Matrix score. | sequence | function |
| --- | --- | --- | --- | --- | --- | --- |
| A-box | Petroselinum crispum | 909 | + | 6 | CCGTCC | cis-acting regulatory element |

>HU04G00148.1   
+ -Up\_Stream \_Len000ATAATG GGTGATAACT TTTGAAGGAC AATATACAAA TCACTTATTT ATTAAAGTCA   
  
  
+ AGGGGACATT TCAACTATAA CATCATTGTT TATAAAGTAT TTGAAAATTG CAACTCTTGT AATAGATAAT   
  
  
+ AATGCTTTAT AGACTTCACC AATCACTAAT TTGTAGAATC AAAAAATTAA TTGAAAAGCA AACAAAAGGA   
  
  
+ AAAGGCACAT AAACCTAAAT TATGTAAACA AATGAGAACA TATATGCCAG CTTATCAACG TTAATTATAT   
  
  
+ TTGTTATCAC TCTTAATATC TGTATTCATA GTAAATGAAA ATAAGTATCG ATTTTTTATT AAACCTAAAA   
  
  
+ TCTTCTGTCA ATAATTTTTC TTCCGATAAA AAAAAAATTG GACCATATTA AATTAACATT AATAAATTCA   
  
  
+ TATAATTACT TTGAATGTCT GAAATATAAT GCTCTTCGGT ATATTAAGGT CTTATAAATT TCTCATTAAT   
  
  
+ TTGAAATCTC TTATAATATA TTGGGTCAAC AAGTGCTAGC TTGTTTTTTT TTCCTGGTTA ATTGCATTTA   
  
  
+ AAAAAATAAT TTTTAATTAC AATTGCAAGG GGAGGAATCA TTTACAAAAT ATCAAAAGTG GAAAAGGCAA   
  
  
+ AAAGAAACAA ATTATAAAAC AGAAAAAAGA TTAGAAAACG CAAACAAAGA CGAAATGAGG AACAGTAAGA   
  
  
+ ATCAGTAAAA AAATGTAGTG CAACGGTCAC ATACGGGAAG GCAAGCACAA CAATCATGAA AGAGACAGGC   
  
  
+ ACGTGCGTTC CTCGTGATCA GCAGCCTTCC CTACTTCCCC GCCAAGTGGA GTCCGTACTA ACTCCTGCCA   
  
  
+ TTCCCTCCCA ACGGTCATAT TCCGCCGACA TGCCTCAACG GTCAGATTTC CTGCCCCATC TGGACCGTCC   
  
  
+ GATCAAGATC CAACGGCCAT AAGGCGCACT CTCCAATACC CTAGTGCCCA TTTCTGGAAC AGCCCCAAAA   
  
  
+ ATCTCAGTAT TCGCCATCAT TTTTCAATAC TGGGGTTACA TAAACAGTCT AACAAGGAAG GACCCAACAC   
  
  
+ AGAGGAGAGA GAAAAAAGCT AGCTTTGATG GGGACAGCCA TGGATGGAGG TGAAGAGAGA GGAGGTTGGG   
  
  
+ ACCTTTTATT TTGCTCACTT TCCATTCTTA TATAAAAACA GCTCTAGTTT ATCTCTTATG GCTTTTTTGG   
  
  
+ GTTTGTTCAT AGAGAGGGGG GAATGTGATA TGATTTGATT TGATGTTAGA AATTCTCATT AGTTTTTTCT   
  
  
+ TTTTGTTTGC AATTTTGATG TGTTAGGGTT TGAGGATCAT CATTTTTGAG TAGGGAACTT TCTGGGTTTT   
  
  
+ GCTTGCTGAT TGATTGCCAA TGCTATGGAT TTCTTGGTGA GTCAATCTTC AATTGCTGAT CTGGGTTCGT   
  
  
+ TTGTTCTTCA GAGTTTCACC TGCCAATTTG ATCAAGTATG TCCCTTCACT TCTCCTGCTT CAATTCTCTC   
  
  
+ TCAAATTCAC GCATTTTTCC AATGAATTTC AAATAGGAGT CCTGGAATCC GATGATATTT TAGGTTTTTT   
  
  
+ CCTGACTTCA TTGCATTAGT TTGTTCATAT CTGTGACCAA TTCTTTCATT TGTTATATGA TCTGATTGTT   
  
  
+ TAGTTAGCTA TTCATTCATT GCATATACAT TGGTTTATTC ATTGCAGAGA CACTCACATA CACACACACA   
  
  
+ TACAGGGGAC ACTAGTATTT TTTCTCTCTA ACTCATCGGA TTCCCTTTGA TACTCTGTTT TTTGGGGGGT   
  
  
+ TGATTTGATT TGATTTGATT GACCTTAATT TTGAACTCAC ACACACTCGC ACATAGATAT ATTCTTTCTG   
  
  
+ TTTGTGACAT ATACAGCATC ATATAGAAAT ATATTGCACT GCATATTTGA GCTCAAGACA GGGATATCAT   
  
  
+ CACAAACTGA GTGTGAGGGA GGCAAAAAAC AGAGGAAAAG AAGGGAATTG GGAGTGTAAT CAAAACAAGA   
  
  
+ GAGAGATTGA GGAGAGAGGA AAAAACAAAA TTGGGAGAAG GTTAATAGAG AAGAATGTTG GCTGGGTGTT   
  
  
+ CATCCACATT GCTGTCACCA AGGTATAGAT TGAGGAGTGA AGCTACATCA CAGTTCCAAG CCTGCCATCA   
  
  
+ TCCAATGAGC ACACAGAGAA TTGATTTGCC CTGCACTTTC CCAAGAAAGG ATGTTTCGAA GCCACAATCG   
  
  
+ GTTCGCCCCG TTGGCCTATC TGTCGAGAAG CCGGTTGAAG TCAGGGCCCT GAAGCAGACC ATCCGAGTCC   
  
  
+ CACCATCGCC GGAGGGTAGA AGAGAGATTA AGGCAGATTT CTGGGGTGAT AGAAGAAAGA GCTTGAAGAG   
  
  
+ GAGACTAGCA GAACAGGGGA GCTCTTTTGA TGATGGTGGT GACGAATCTT GTGTTGGGAG AACCAAAAGG   
  
  
+ AAGAAGGGTA GCTTTGATTT TGGTGAATCT GATGAAATTT CTCCAAAAAT TGAGGACACA ATGAGTTTTG   
  
  
+ GACATCTGGG TAGTGGTGGA AATTTCTGGG TTCATCCTGG TTTTGGTGTT GTTAATGTTA ATACTAATTA   
  
  
+ CCCTCAAGTG CCATTTTCTC TCACTTGTTC AGGGGAGGAA GAAAGAGTAT GTTTTGTTCC TACTGATGTG   
  
  
+ ATCTCACCAG CCATCATGCC TCCATTGTCA CACAATCCTT GGGTTGAATC TGGTGTTACT GAGGTCACAG   
  
  
+ AATATGGTGG GGGTGACAAA GACGGCGAAC CTAGTCATGG GTTTGTGAGG GGTACAACAA CAACGTCCGG   
  
  
+ GTCAAGTTCA TCTTCTGAGA GTCATAGTTT TGGGCATAGG CTCAATGAGA GCTCATCTGA CCCTGAAATC   
  
  
+ AGAAATGGTT CCATGTTGCC TAACCCTGGT CATGGTCCTG GCACCAGTCA TGGTCCTGGA ACCGGTCTGG   
  
  
+ CTCACAACCA CAATGATCAA ACCGAGCAAC AAGGGTTCGA GCTCATTAGC TTTCTCATGG GTTGTGTCGA   
  
  
+ AGCGATCAGT TCAAGGAACA TTGCAGCCAT CAATCATTTC ATAGCTAAGC TGGGCGAGCA GGCTTCTCCA   
  
  
+ AGGGCTCGGT CTGCCATTAG CCGCCTCACA GCCTACTTCA CCGAAGCATT AGCCTTGAGG GTCACAAGAT   
  
  
+ TTTGGCCTCA TATCTTTCAC ATAAGTATCC CTCGAGAGTT TGATCGATTT GATGATGAAT CGGGGGCAGC   
  
  
+ TGCAATGAGG CTTTTGAATC AGATCAGCCC AATTCCAAAG TTCGTTCATT TTACAGCCAA TGAGATGTTA   
  
  
+ TTGAGGGCAT TTGAAGGGAA GGACAAGGTG CATATCATAG ACTTCGACAT CAAGCAAGGC TTACAATGGC   
  
  
+ CCGGGTTTTT CCAAAGCTTA GCCATGAGGG AAAACCCCCC GAGCCATGTC AGGATAACAG GCGTAGGGGA   
  
  
+ TTCGAAGCAA GAATTGGTTG AGACAGGAGA AAGACTAGCC GGGTTTGCTG GGGCATTCAA CCTCTCCTTC   
  
  
+ GAGTTCCACC CAGTTGTGGA CCGGTTGGAA GATGTTAGGC TATGGATGCT TCATGTTAAG GAAGGTGAAA   
  
  
+ GTGTCGCGGT AAATTGCATT TTGCAGCTCC ACAAGACACT CTATGACCCC CATGGTGCCA CATTCAGGGA   
  
  
+ CTTCATGGGT TTAATCCGAA GCACAAATCC CATAGCATTG GTTATGGCTG AGCAAGAAGC TGATCACAAC   
  
  
+ GAACCCACCT TAGAAGGCCG AGTATGCAAC TCACTTGGAT ACTATGCAGC CCTTTTCGAT GCCATAGATT   
  
  
+ GCAGCCTTCC TTTCGAGAGC TCAGCAAGGT TGAAGATCGA AGAGATGTTT GGCCGGGAAA TCAGGAACAT   
  
  
+ AATAGCCTGT GAAGGGGCAG ACAGGATCGA AAGACATGAG AAGTTCGAGA AATGGAAGAG AAGGATCGAG   
  
  
+ CAGGAAGGGT TCCGGTGCAT GCGGACTAGC GAAAGGGAGG TGATGCAGAG CCAAATGCTG CTTAAGATGT   
  
  
+ ACTCGAACGA GAGCTACAAC ATGCAAAGAC AAGGCAATGA AGAAGCAATA TCACTAGTAT GGTTAGATCA   
  
  
+ GCCACTCTAC ACTGTTTCTT TGTGGGCTCC AAGTGAATTT GCTGCAGGGA GTTCTTCCAG TTTTTCTCTG   
  
  
+ CCATCTTG  

- -Up\_Stream \_Len000TATTAC CCACTATTGA AAACTTCCTG TTATATGTTT AGTGAATAAA TAATTTCAGT   
  
  
- TCCCCTGTAA AGTTGATATT GTAGTAACAA ATATTTCATA AACTTTTAAC GTTGAGAACA TTATCTATTA   
  
  
- TTACGAAATA TCTGAAGTGG TTAGTGATTA AACATCTTAG TTTTTTAATT AACTTTTCGT TTGTTTTCCT   
  
  
- TTTCCGTGTA TTTGGATTTA ATACATTTGT TTACTCTTGT ATATACGGTC GAATAGTTGC AATTAATATA   
  
  
- AACAATAGTG AGAATTATAG ACATAAGTAT CATTTACTTT TATTCATAGC TAAAAAATAA TTTGGATTTT   
  
  
- AGAAGACAGT TATTAAAAAG AAGGCTATTT TTTTTTTAAC CTGGTATAAT TTAATTGTAA TTATTTAAGT   
  
  
- ATATTAATGA AACTTACAGA CTTTATATTA CGAGAAGCCA TATAATTCCA GAATATTTAA AGAGTAATTA   
  
  
- AACTTTAGAG AATATTATAT AACCCAGTTG TTCACGATCG AACAAAAAAA AAGGACCAAT TAACGTAAAT   
  
  
- TTTTTTATTA AAAATTAATG TTAACGTTCC CCTCCTTAGT AAATGTTTTA TAGTTTTCAC CTTTTCCGTT   
  
  
- TTTCTTTGTT TAATATTTTG TCTTTTTTCT AATCTTTTGC GTTTGTTTCT GCTTTACTCC TTGTCATTCT   
  
  
- TAGTCATTTT TTTACATCAC GTTGCCAGTG TATGCCCTTC CGTTCGTGTT GTTAGTACTT TCTCTGTCCG   
  
  
- TGCACGCAAG GAGCACTAGT CGTCGGAAGG GATGAAGGGG CGGTTCACCT CAGGCATGAT TGAGGACGGT   
  
  
- AAGGGAGGGT TGCCAGTATA AGGCGGCTGT ACGGAGTTGC CAGTCTAAAG GACGGGGTAG ACCTGGCAGG   
  
  
- CTAGTTCTAG GTTGCCGGTA TTCCGCGTGA GAGGTTATGG GATCACGGGT AAAGACCTTG TCGGGGTTTT   
  
  
- TAGAGTCATA AGCGGTAGTA AAAAGTTATG ACCCCAATGT ATTTGTCAGA TTGTTCCTTC CTGGGTTGTG   
  
  
- TCTCCTCTCT CTTTTTTCGA TCGAAACTAC CCCTGTCGGT ACCTACCTCC ACTTCTCTCT CCTCCAACCC   
  
  
- TGGAAAATAA AACGAGTGAA AGGTAAGAAT ATATTTTTGT CGAGATCAAA TAGAGAATAC CGAAAAAACC   
  
  
- CAAACAAGTA TCTCTCCCCC CTTACACTAT ACTAAACTAA ACTACAATCT TTAAGAGTAA TCAAAAAAGA   
  
  
- AAAACAAACG TTAAAACTAC ACAATCCCAA ACTCCTAGTA GTAAAAACTC ATCCCTTGAA AGACCCAAAA   
  
  
- CGAACGACTA ACTAACGGTT ACGATACCTA AAGAACCACT CAGTTAGAAG TTAACGACTA GACCCAAGCA   
  
  
- AACAAGAAGT CTCAAAGTGG ACGGTTAAAC TAGTTCATAC AGGGAAGTGA AGAGGACGAA GTTAAGAGAG   
  
  
- AGTTTAAGTG CGTAAAAAGG TTACTTAAAG TTTATCCTCA GGACCTTAGG CTACTATAAA ATCCAAAAAA   
  
  
- GGACTGAAGT AACGTAATCA AACAAGTATA GACACTGGTT AAGAAAGTAA ACAATATACT AGACTAACAA   
  
  
- ATCAATCGAT AAGTAAGTAA CGTATATGTA ACCAAATAAG TAACGTCTCT GTGAGTGTAT GTGTGTGTGT   
  
  
- ATGTCCCCTG TGATCATAAA AAAGAGAGAT TGAGTAGCCT AAGGGAAACT ATGAGACAAA AAACCCCCCA   
  
  
- ACTAAACTAA ACTAAACTAA CTGGAATTAA AACTTGAGTG TGTGTGAGCG TGTATCTATA TAAGAAAGAC   
  
  
- AAACACTGTA TATGTCGTAG TATATCTTTA TATAACGTGA CGTATAAACT CGAGTTCTGT CCCTATAGTA   
  
  
- GTGTTTGACT CACACTCCCT CCGTTTTTTG TCTCCTTTTC TTCCCTTAAC CCTCACATTA GTTTTGTTCT   
  
  
- CTCTCTAACT CCTCTCTCCT TTTTTGTTTT AACCCTCTTC CAATTATCTC TTCTTACAAC CGACCCACAA   
  
  
- GTAGGTGTAA CGACAGTGGT TCCATATCTA ACTCCTCACT TCGATGTAGT GTCAAGGTTC GGACGGTAGT   
  
  
- AGGTTACTCG TGTGTCTCTT AACTAAACGG GACGTGAAAG GGTTCTTTCC TACAAAGCTT CGGTGTTAGC   
  
  
- CAAGCGGGGC AACCGGATAG ACAGCTCTTC GGCCAACTTC AGTCCCGGGA CTTCGTCTGG TAGGCTCAGG   
  
  
- GTGGTAGCGG CCTCCCATCT TCTCTCTAAT TCCGTCTAAA GACCCCACTA TCTTCTTTCT CGAACTTCTC   
  
  
- CTCTGATCGT CTTGTCCCCT CGAGAAAACT ACTACCACCA CTGCTTAGAA CACAACCCTC TTGGTTTTCC   
  
  
- TTCTTCCCAT CGAAACTAAA ACCACTTAGA CTACTTTAAA GAGGTTTTTA ACTCCTGTGT TACTCAAAAC   
  
  
- CTGTAGACCC ATCACCACCT TTAAAGACCC AAGTAGGACC AAAACCACAA CAATTACAAT TATGATTAAT   
  
  
- GGGAGTTCAC GGTAAAAGAG AGTGAACAAG TCCCCTCCTT CTTTCTCATA CAAAACAAGG ATGACTACAC   
  
  
- TAGAGTGGTC GGTAGTACGG AGGTAACAGT GTGTTAGGAA CCCAACTTAG ACCACAATGA CTCCAGTGTC   
  
  
- TTATACCACC CCCACTGTTT CTGCCGCTTG GATCAGTACC CAAACACTCC CCATGTTGTT GTTGCAGGCC   
  
  
- CAGTTCAAGT AGAAGACTCT CAGTATCAAA ACCCGTATCC GAGTTACTCT CGAGTAGACT GGGACTTTAG   
  
  
- TCTTTACCAA GGTACAACGG ATTGGGACCA GTACCAGGAC CGTGGTCAGT ACCAGGACCT TGGCCAGACC   
  
  
- GAGTGTTGGT GTTACTAGTT TGGCTCGTTG TTCCCAAGCT CGAGTAATCG AAAGAGTACC CAACACAGCT   
  
  
- TCGCTAGTCA AGTTCCTTGT AACGTCGGTA GTTAGTAAAG TATCGATTCG ACCCGCTCGT CCGAAGAGGT   
  
  
- TCCCGAGCCA GACGGTAATC GGCGGAGTGT CGGATGAAGT GGCTTCGTAA TCGGAACTCC CAGTGTTCTA   
  
  
- AAACCGGAGT ATAGAAAGTG TATTCATAGG GAGCTCTCAA ACTAGCTAAA CTACTACTTA GCCCCCGTCG   
  
  
- ACGTTACTCC GAAAACTTAG TCTAGTCGGG TTAAGGTTTC AAGCAAGTAA AATGTCGGTT ACTCTACAAT   
  
  
- AACTCCCGTA AACTTCCCTT CCTGTTCCAC GTATAGTATC TGAAGCTGTA GTTCGTTCCG AATGTTACCG   
  
  
- GGCCCAAAAA GGTTTCGAAT CGGTACTCCC TTTTGGGGGG CTCGGTACAG TCCTATTGTC CGCATCCCCT   
  
  
- AAGCTTCGTT CTTAACCAAC TCTGTCCTCT TTCTGATCGG CCCAAACGAC CCCGTAAGTT GGAGAGGAAG   
  
  
- CTCAAGGTGG GTCAACACCT GGCCAACCTT CTACAATCCG ATACCTACGA AGTACAATTC CTTCCACTTT   
  
  
- CACAGCGCCA TTTAACGTAA AACGTCGAGG TGTTCTGTGA GATACTGGGG GTACCACGGT GTAAGTCCCT   
  
  
- GAAGTACCCA AATTAGGCTT CGTGTTTAGG GTATCGTAAC CAATACCGAC TCGTTCTTCG ACTAGTGTTG   
  
  
- CTTGGGTGGA ATCTTCCGGC TCATACGTTG AGTGAACCTA TGATACGTCG GGAAAAGCTA CGGTATCTAA   
  
  
- CGTCGGAAGG AAAGCTCTCG AGTCGTTCCA ACTTCTAGCT TCTCTACAAA CCGGCCCTTT AGTCCTTGTA   
  
  
- TTATCGGACA CTTCCCCGTC TGTCCTAGCT TTCTGTACTC TTCAAGCTCT TTACCTTCTC TTCCTAGCTC   
  
  
- GTCCTTCCCA AGGCCACGTA CGCCTGATCG CTTTCCCTCC ACTACGTCTC GGTTTACGAC GAATTCTACA   
  
  
- TGAGCTTGCT CTCGATGTTG TACGTTTCTG TTCCGTTACT TCTTCGTTAT AGTGATCATA CCAATCTAGT   
  
  
- CGGTGAGATG TGACAAAGAA ACACCCGAGG TTCACTTAAA CGACGTCCCT CAAGAAGGTC AAAAAGAGAC   
  
  
- GGTAGAAC

+     AAGAA-motif

| Site Name | Organism | Position | Strand | Matrix score. | sequence | function |
| --- | --- | --- | --- | --- | --- | --- |
| AAGAA-motif | Avena sativa | 1585 | - | 7 | GAAAGAA |  |
| AAGAA-motif | Avena sativa | 1816 | - | 7 | GAAAGAA |  |

>HU04G00148.1   
+ -Up\_Stream \_Len000ATAATG GGTGATAACT TTTGAAGGAC AATATACAAA TCACTTATTT ATTAAAGTCA   
  
  
+ AGGGGACATT TCAACTATAA CATCATTGTT TATAAAGTAT TTGAAAATTG CAACTCTTGT AATAGATAAT   
  
  
+ AATGCTTTAT AGACTTCACC AATCACTAAT TTGTAGAATC AAAAAATTAA TTGAAAAGCA AACAAAAGGA   
  
  
+ AAAGGCACAT AAACCTAAAT TATGTAAACA AATGAGAACA TATATGCCAG CTTATCAACG TTAATTATAT   
  
  
+ TTGTTATCAC TCTTAATATC TGTATTCATA GTAAATGAAA ATAAGTATCG ATTTTTTATT AAACCTAAAA   
  
  
+ TCTTCTGTCA ATAATTTTTC TTCCGATAAA AAAAAAATTG GACCATATTA AATTAACATT AATAAATTCA   
  
  
+ TATAATTACT TTGAATGTCT GAAATATAAT GCTCTTCGGT ATATTAAGGT CTTATAAATT TCTCATTAAT   
  
  
+ TTGAAATCTC TTATAATATA TTGGGTCAAC AAGTGCTAGC TTGTTTTTTT TTCCTGGTTA ATTGCATTTA   
  
  
+ AAAAAATAAT TTTTAATTAC AATTGCAAGG GGAGGAATCA TTTACAAAAT ATCAAAAGTG GAAAAGGCAA   
  
  
+ AAAGAAACAA ATTATAAAAC AGAAAAAAGA TTAGAAAACG CAAACAAAGA CGAAATGAGG AACAGTAAGA   
  
  
+ ATCAGTAAAA AAATGTAGTG CAACGGTCAC ATACGGGAAG GCAAGCACAA CAATCATGAA AGAGACAGGC   
  
  
+ ACGTGCGTTC CTCGTGATCA GCAGCCTTCC CTACTTCCCC GCCAAGTGGA GTCCGTACTA ACTCCTGCCA   
  
  
+ TTCCCTCCCA ACGGTCATAT TCCGCCGACA TGCCTCAACG GTCAGATTTC CTGCCCCATC TGGACCGTCC   
  
  
+ GATCAAGATC CAACGGCCAT AAGGCGCACT CTCCAATACC CTAGTGCCCA TTTCTGGAAC AGCCCCAAAA   
  
  
+ ATCTCAGTAT TCGCCATCAT TTTTCAATAC TGGGGTTACA TAAACAGTCT AACAAGGAAG GACCCAACAC   
  
  
+ AGAGGAGAGA GAAAAAAGCT AGCTTTGATG GGGACAGCCA TGGATGGAGG TGAAGAGAGA GGAGGTTGGG   
  
  
+ ACCTTTTATT TTGCTCACTT TCCATTCTTA TATAAAAACA GCTCTAGTTT ATCTCTTATG GCTTTTTTGG   
  
  
+ GTTTGTTCAT AGAGAGGGGG GAATGTGATA TGATTTGATT TGATGTTAGA AATTCTCATT AGTTTTTTCT   
  
  
+ TTTTGTTTGC AATTTTGATG TGTTAGGGTT TGAGGATCAT CATTTTTGAG TAGGGAACTT TCTGGGTTTT   
  
  
+ GCTTGCTGAT TGATTGCCAA TGCTATGGAT TTCTTGGTGA GTCAATCTTC AATTGCTGAT CTGGGTTCGT   
  
  
+ TTGTTCTTCA GAGTTTCACC TGCCAATTTG ATCAAGTATG TCCCTTCACT TCTCCTGCTT CAATTCTCTC   
  
  
+ TCAAATTCAC GCATTTTTCC AATGAATTTC AAATAGGAGT CCTGGAATCC GATGATATTT TAGGTTTTTT   
  
  
+ CCTGACTTCA TTGCATTAGT TTGTTCATAT CTGTGACCAA TTCTTTCATT TGTTATATGA TCTGATTGTT   
  
  
+ TAGTTAGCTA TTCATTCATT GCATATACAT TGGTTTATTC ATTGCAGAGA CACTCACATA CACACACACA   
  
  
+ TACAGGGGAC ACTAGTATTT TTTCTCTCTA ACTCATCGGA TTCCCTTTGA TACTCTGTTT TTTGGGGGGT   
  
  
+ TGATTTGATT TGATTTGATT GACCTTAATT TTGAACTCAC ACACACTCGC ACATAGATAT ATTCTTTCTG   
  
  
+ TTTGTGACAT ATACAGCATC ATATAGAAAT ATATTGCACT GCATATTTGA GCTCAAGACA GGGATATCAT   
  
  
+ CACAAACTGA GTGTGAGGGA GGCAAAAAAC AGAGGAAAAG AAGGGAATTG GGAGTGTAAT CAAAACAAGA   
  
  
+ GAGAGATTGA GGAGAGAGGA AAAAACAAAA TTGGGAGAAG GTTAATAGAG AAGAATGTTG GCTGGGTGTT   
  
  
+ CATCCACATT GCTGTCACCA AGGTATAGAT TGAGGAGTGA AGCTACATCA CAGTTCCAAG CCTGCCATCA   
  
  
+ TCCAATGAGC ACACAGAGAA TTGATTTGCC CTGCACTTTC CCAAGAAAGG ATGTTTCGAA GCCACAATCG   
  
  
+ GTTCGCCCCG TTGGCCTATC TGTCGAGAAG CCGGTTGAAG TCAGGGCCCT GAAGCAGACC ATCCGAGTCC   
  
  
+ CACCATCGCC GGAGGGTAGA AGAGAGATTA AGGCAGATTT CTGGGGTGAT AGAAGAAAGA GCTTGAAGAG   
  
  
+ GAGACTAGCA GAACAGGGGA GCTCTTTTGA TGATGGTGGT GACGAATCTT GTGTTGGGAG AACCAAAAGG   
  
  
+ AAGAAGGGTA GCTTTGATTT TGGTGAATCT GATGAAATTT CTCCAAAAAT TGAGGACACA ATGAGTTTTG   
  
  
+ GACATCTGGG TAGTGGTGGA AATTTCTGGG TTCATCCTGG TTTTGGTGTT GTTAATGTTA ATACTAATTA   
  
  
+ CCCTCAAGTG CCATTTTCTC TCACTTGTTC AGGGGAGGAA GAAAGAGTAT GTTTTGTTCC TACTGATGTG   
  
  
+ ATCTCACCAG CCATCATGCC TCCATTGTCA CACAATCCTT GGGTTGAATC TGGTGTTACT GAGGTCACAG   
  
  
+ AATATGGTGG GGGTGACAAA GACGGCGAAC CTAGTCATGG GTTTGTGAGG GGTACAACAA CAACGTCCGG   
  
  
+ GTCAAGTTCA TCTTCTGAGA GTCATAGTTT TGGGCATAGG CTCAATGAGA GCTCATCTGA CCCTGAAATC   
  
  
+ AGAAATGGTT CCATGTTGCC TAACCCTGGT CATGGTCCTG GCACCAGTCA TGGTCCTGGA ACCGGTCTGG   
  
  
+ CTCACAACCA CAATGATCAA ACCGAGCAAC AAGGGTTCGA GCTCATTAGC TTTCTCATGG GTTGTGTCGA   
  
  
+ AGCGATCAGT TCAAGGAACA TTGCAGCCAT CAATCATTTC ATAGCTAAGC TGGGCGAGCA GGCTTCTCCA   
  
  
+ AGGGCTCGGT CTGCCATTAG CCGCCTCACA GCCTACTTCA CCGAAGCATT AGCCTTGAGG GTCACAAGAT   
  
  
+ TTTGGCCTCA TATCTTTCAC ATAAGTATCC CTCGAGAGTT TGATCGATTT GATGATGAAT CGGGGGCAGC   
  
  
+ TGCAATGAGG CTTTTGAATC AGATCAGCCC AATTCCAAAG TTCGTTCATT TTACAGCCAA TGAGATGTTA   
  
  
+ TTGAGGGCAT TTGAAGGGAA GGACAAGGTG CATATCATAG ACTTCGACAT CAAGCAAGGC TTACAATGGC   
  
  
+ CCGGGTTTTT CCAAAGCTTA GCCATGAGGG AAAACCCCCC GAGCCATGTC AGGATAACAG GCGTAGGGGA   
  
  
+ TTCGAAGCAA GAATTGGTTG AGACAGGAGA AAGACTAGCC GGGTTTGCTG GGGCATTCAA CCTCTCCTTC   
  
  
+ GAGTTCCACC CAGTTGTGGA CCGGTTGGAA GATGTTAGGC TATGGATGCT TCATGTTAAG GAAGGTGAAA   
  
  
+ GTGTCGCGGT AAATTGCATT TTGCAGCTCC ACAAGACACT CTATGACCCC CATGGTGCCA CATTCAGGGA   
  
  
+ CTTCATGGGT TTAATCCGAA GCACAAATCC CATAGCATTG GTTATGGCTG AGCAAGAAGC TGATCACAAC   
  
  
+ GAACCCACCT TAGAAGGCCG AGTATGCAAC TCACTTGGAT ACTATGCAGC CCTTTTCGAT GCCATAGATT   
  
  
+ GCAGCCTTCC TTTCGAGAGC TCAGCAAGGT TGAAGATCGA AGAGATGTTT GGCCGGGAAA TCAGGAACAT   
  
  
+ AATAGCCTGT GAAGGGGCAG ACAGGATCGA AAGACATGAG AAGTTCGAGA AATGGAAGAG AAGGATCGAG   
  
  
+ CAGGAAGGGT TCCGGTGCAT GCGGACTAGC GAAAGGGAGG TGATGCAGAG CCAAATGCTG CTTAAGATGT   
  
  
+ ACTCGAACGA GAGCTACAAC ATGCAAAGAC AAGGCAATGA AGAAGCAATA TCACTAGTAT GGTTAGATCA   
  
  
+ GCCACTCTAC ACTGTTTCTT TGTGGGCTCC AAGTGAATTT GCTGCAGGGA GTTCTTCCAG TTTTTCTCTG   
  
  
+ CCATCTTG  

- -Up\_Stream \_Len000TATTAC CCACTATTGA AAACTTCCTG TTATATGTTT AGTGAATAAA TAATTTCAGT   
  
  
- TCCCCTGTAA AGTTGATATT GTAGTAACAA ATATTTCATA AACTTTTAAC GTTGAGAACA TTATCTATTA   
  
  
- TTACGAAATA TCTGAAGTGG TTAGTGATTA AACATCTTAG TTTTTTAATT AACTTTTCGT TTGTTTTCCT   
  
  
- TTTCCGTGTA TTTGGATTTA ATACATTTGT TTACTCTTGT ATATACGGTC GAATAGTTGC AATTAATATA   
  
  
- AACAATAGTG AGAATTATAG ACATAAGTAT CATTTACTTT TATTCATAGC TAAAAAATAA TTTGGATTTT   
  
  
- AGAAGACAGT TATTAAAAAG AAGGCTATTT TTTTTTTAAC CTGGTATAAT TTAATTGTAA TTATTTAAGT   
  
  
- ATATTAATGA AACTTACAGA CTTTATATTA CGAGAAGCCA TATAATTCCA GAATATTTAA AGAGTAATTA   
  
  
- AACTTTAGAG AATATTATAT AACCCAGTTG TTCACGATCG AACAAAAAAA AAGGACCAAT TAACGTAAAT   
  
  
- TTTTTTATTA AAAATTAATG TTAACGTTCC CCTCCTTAGT AAATGTTTTA TAGTTTTCAC CTTTTCCGTT   
  
  
- TTTCTTTGTT TAATATTTTG TCTTTTTTCT AATCTTTTGC GTTTGTTTCT GCTTTACTCC TTGTCATTCT   
  
  
- TAGTCATTTT TTTACATCAC GTTGCCAGTG TATGCCCTTC CGTTCGTGTT GTTAGTACTT TCTCTGTCCG   
  
  
- TGCACGCAAG GAGCACTAGT CGTCGGAAGG GATGAAGGGG CGGTTCACCT CAGGCATGAT TGAGGACGGT   
  
  
- AAGGGAGGGT TGCCAGTATA AGGCGGCTGT ACGGAGTTGC CAGTCTAAAG GACGGGGTAG ACCTGGCAGG   
  
  
- CTAGTTCTAG GTTGCCGGTA TTCCGCGTGA GAGGTTATGG GATCACGGGT AAAGACCTTG TCGGGGTTTT   
  
  
- TAGAGTCATA AGCGGTAGTA AAAAGTTATG ACCCCAATGT ATTTGTCAGA TTGTTCCTTC CTGGGTTGTG   
  
  
- TCTCCTCTCT CTTTTTTCGA TCGAAACTAC CCCTGTCGGT ACCTACCTCC ACTTCTCTCT CCTCCAACCC   
  
  
- TGGAAAATAA AACGAGTGAA AGGTAAGAAT ATATTTTTGT CGAGATCAAA TAGAGAATAC CGAAAAAACC   
  
  
- CAAACAAGTA TCTCTCCCCC CTTACACTAT ACTAAACTAA ACTACAATCT TTAAGAGTAA TCAAAAAAGA   
  
  
- AAAACAAACG TTAAAACTAC ACAATCCCAA ACTCCTAGTA GTAAAAACTC ATCCCTTGAA AGACCCAAAA   
  
  
- CGAACGACTA ACTAACGGTT ACGATACCTA AAGAACCACT CAGTTAGAAG TTAACGACTA GACCCAAGCA   
  
  
- AACAAGAAGT CTCAAAGTGG ACGGTTAAAC TAGTTCATAC AGGGAAGTGA AGAGGACGAA GTTAAGAGAG   
  
  
- AGTTTAAGTG CGTAAAAAGG TTACTTAAAG TTTATCCTCA GGACCTTAGG CTACTATAAA ATCCAAAAAA   
  
  
- GGACTGAAGT AACGTAATCA AACAAGTATA GACACTGGTT AAGAAAGTAA ACAATATACT AGACTAACAA   
  
  
- ATCAATCGAT AAGTAAGTAA CGTATATGTA ACCAAATAAG TAACGTCTCT GTGAGTGTAT GTGTGTGTGT   
  
  
- ATGTCCCCTG TGATCATAAA AAAGAGAGAT TGAGTAGCCT AAGGGAAACT ATGAGACAAA AAACCCCCCA   
  
  
- ACTAAACTAA ACTAAACTAA CTGGAATTAA AACTTGAGTG TGTGTGAGCG TGTATCTATA TAAGAAAGAC   
  
  
- AAACACTGTA TATGTCGTAG TATATCTTTA TATAACGTGA CGTATAAACT CGAGTTCTGT CCCTATAGTA   
  
  
- GTGTTTGACT CACACTCCCT CCGTTTTTTG TCTCCTTTTC TTCCCTTAAC CCTCACATTA GTTTTGTTCT   
  
  
- CTCTCTAACT CCTCTCTCCT TTTTTGTTTT AACCCTCTTC CAATTATCTC TTCTTACAAC CGACCCACAA   
  
  
- GTAGGTGTAA CGACAGTGGT TCCATATCTA ACTCCTCACT TCGATGTAGT GTCAAGGTTC GGACGGTAGT   
  
  
- AGGTTACTCG TGTGTCTCTT AACTAAACGG GACGTGAAAG GGTTCTTTCC TACAAAGCTT CGGTGTTAGC   
  
  
- CAAGCGGGGC AACCGGATAG ACAGCTCTTC GGCCAACTTC AGTCCCGGGA CTTCGTCTGG TAGGCTCAGG   
  
  
- GTGGTAGCGG CCTCCCATCT TCTCTCTAAT TCCGTCTAAA GACCCCACTA TCTTCTTTCT CGAACTTCTC   
  
  
- CTCTGATCGT CTTGTCCCCT CGAGAAAACT ACTACCACCA CTGCTTAGAA CACAACCCTC TTGGTTTTCC   
  
  
- TTCTTCCCAT CGAAACTAAA ACCACTTAGA CTACTTTAAA GAGGTTTTTA ACTCCTGTGT TACTCAAAAC   
  
  
- CTGTAGACCC ATCACCACCT TTAAAGACCC AAGTAGGACC AAAACCACAA CAATTACAAT TATGATTAAT   
  
  
- GGGAGTTCAC GGTAAAAGAG AGTGAACAAG TCCCCTCCTT CTTTCTCATA CAAAACAAGG ATGACTACAC   
  
  
- TAGAGTGGTC GGTAGTACGG AGGTAACAGT GTGTTAGGAA CCCAACTTAG ACCACAATGA CTCCAGTGTC   
  
  
- TTATACCACC CCCACTGTTT CTGCCGCTTG GATCAGTACC CAAACACTCC CCATGTTGTT GTTGCAGGCC   
  
  
- CAGTTCAAGT AGAAGACTCT CAGTATCAAA ACCCGTATCC GAGTTACTCT CGAGTAGACT GGGACTTTAG   
  
  
- TCTTTACCAA GGTACAACGG ATTGGGACCA GTACCAGGAC CGTGGTCAGT ACCAGGACCT TGGCCAGACC   
  
  
- GAGTGTTGGT GTTACTAGTT TGGCTCGTTG TTCCCAAGCT CGAGTAATCG AAAGAGTACC CAACACAGCT   
  
  
- TCGCTAGTCA AGTTCCTTGT AACGTCGGTA GTTAGTAAAG TATCGATTCG ACCCGCTCGT CCGAAGAGGT   
  
  
- TCCCGAGCCA GACGGTAATC GGCGGAGTGT CGGATGAAGT GGCTTCGTAA TCGGAACTCC CAGTGTTCTA   
  
  
- AAACCGGAGT ATAGAAAGTG TATTCATAGG GAGCTCTCAA ACTAGCTAAA CTACTACTTA GCCCCCGTCG   
  
  
- ACGTTACTCC GAAAACTTAG TCTAGTCGGG TTAAGGTTTC AAGCAAGTAA AATGTCGGTT ACTCTACAAT   
  
  
- AACTCCCGTA AACTTCCCTT CCTGTTCCAC GTATAGTATC TGAAGCTGTA GTTCGTTCCG AATGTTACCG   
  
  
- GGCCCAAAAA GGTTTCGAAT CGGTACTCCC TTTTGGGGGG CTCGGTACAG TCCTATTGTC CGCATCCCCT   
  
  
- AAGCTTCGTT CTTAACCAAC TCTGTCCTCT TTCTGATCGG CCCAAACGAC CCCGTAAGTT GGAGAGGAAG   
  
  
- CTCAAGGTGG GTCAACACCT GGCCAACCTT CTACAATCCG ATACCTACGA AGTACAATTC CTTCCACTTT   
  
  
- CACAGCGCCA TTTAACGTAA AACGTCGAGG TGTTCTGTGA GATACTGGGG GTACCACGGT GTAAGTCCCT   
  
  
- GAAGTACCCA AATTAGGCTT CGTGTTTAGG GTATCGTAAC CAATACCGAC TCGTTCTTCG ACTAGTGTTG   
  
  
- CTTGGGTGGA ATCTTCCGGC TCATACGTTG AGTGAACCTA TGATACGTCG GGAAAAGCTA CGGTATCTAA   
  
  
- CGTCGGAAGG AAAGCTCTCG AGTCGTTCCA ACTTCTAGCT TCTCTACAAA CCGGCCCTTT AGTCCTTGTA   
  
  
- TTATCGGACA CTTCCCCGTC TGTCCTAGCT TTCTGTACTC TTCAAGCTCT TTACCTTCTC TTCCTAGCTC   
  
  
- GTCCTTCCCA AGGCCACGTA CGCCTGATCG CTTTCCCTCC ACTACGTCTC GGTTTACGAC GAATTCTACA   
  
  
- TGAGCTTGCT CTCGATGTTG TACGTTTCTG TTCCGTTACT TCTTCGTTAT AGTGATCATA CCAATCTAGT   
  
  
- CGGTGAGATG TGACAAAGAA ACACCCGAGG TTCACTTAAA CGACGTCCCT CAAGAAGGTC AAAAAGAGAC   
  
  
- GGTAGAAC

+     ABRE

| Site Name | Organism | Position | Strand | Matrix score. | sequence | function |
| --- | --- | --- | --- | --- | --- | --- |
| ABRE | Arabidopsis thaliana | 775 | + | 5 | ACGTG | cis-acting element involved in the abscisic acid responsiveness |
| ABRE | Arabidopsis thaliana | 3403 | - | 7 | AACCCGG | cis-acting element involved in the abscisic acid responsiveness |
| ABRE | Hordeum vulgare | 772 | - | 9 | CGCACGTGTC | cis-acting element involved in the abscisic acid responsiveness |
| ABRE | Arabidopsis thaliana | 774 | + | 6 | CACGTG | cis-acting element involved in the abscisic acid responsiveness |
| ABRE | Arabidopsis thaliana | 3295 | - | 7 | AACCCGG | cis-acting element involved in the abscisic acid responsiveness |

>HU04G00148.1   
+ -Up\_Stream \_Len000ATAATG GGTGATAACT TTTGAAGGAC AATATACAAA TCACTTATTT ATTAAAGTCA   
  
  
+ AGGGGACATT TCAACTATAA CATCATTGTT TATAAAGTAT TTGAAAATTG CAACTCTTGT AATAGATAAT   
  
  
+ AATGCTTTAT AGACTTCACC AATCACTAAT TTGTAGAATC AAAAAATTAA TTGAAAAGCA AACAAAAGGA   
  
  
+ AAAGGCACAT AAACCTAAAT TATGTAAACA AATGAGAACA TATATGCCAG CTTATCAACG TTAATTATAT   
  
  
+ TTGTTATCAC TCTTAATATC TGTATTCATA GTAAATGAAA ATAAGTATCG ATTTTTTATT AAACCTAAAA   
  
  
+ TCTTCTGTCA ATAATTTTTC TTCCGATAAA AAAAAAATTG GACCATATTA AATTAACATT AATAAATTCA   
  
  
+ TATAATTACT TTGAATGTCT GAAATATAAT GCTCTTCGGT ATATTAAGGT CTTATAAATT TCTCATTAAT   
  
  
+ TTGAAATCTC TTATAATATA TTGGGTCAAC AAGTGCTAGC TTGTTTTTTT TTCCTGGTTA ATTGCATTTA   
  
  
+ AAAAAATAAT TTTTAATTAC AATTGCAAGG GGAGGAATCA TTTACAAAAT ATCAAAAGTG GAAAAGGCAA   
  
  
+ AAAGAAACAA ATTATAAAAC AGAAAAAAGA TTAGAAAACG CAAACAAAGA CGAAATGAGG AACAGTAAGA   
  
  
+ ATCAGTAAAA AAATGTAGTG CAACGGTCAC ATACGGGAAG GCAAGCACAA CAATCATGAA AGAGACAGGC   
  
  
+ ACGTGCGTTC CTCGTGATCA GCAGCCTTCC CTACTTCCCC GCCAAGTGGA GTCCGTACTA ACTCCTGCCA   
  
  
+ TTCCCTCCCA ACGGTCATAT TCCGCCGACA TGCCTCAACG GTCAGATTTC CTGCCCCATC TGGACCGTCC   
  
  
+ GATCAAGATC CAACGGCCAT AAGGCGCACT CTCCAATACC CTAGTGCCCA TTTCTGGAAC AGCCCCAAAA   
  
  
+ ATCTCAGTAT TCGCCATCAT TTTTCAATAC TGGGGTTACA TAAACAGTCT AACAAGGAAG GACCCAACAC   
  
  
+ AGAGGAGAGA GAAAAAAGCT AGCTTTGATG GGGACAGCCA TGGATGGAGG TGAAGAGAGA GGAGGTTGGG   
  
  
+ ACCTTTTATT TTGCTCACTT TCCATTCTTA TATAAAAACA GCTCTAGTTT ATCTCTTATG GCTTTTTTGG   
  
  
+ GTTTGTTCAT AGAGAGGGGG GAATGTGATA TGATTTGATT TGATGTTAGA AATTCTCATT AGTTTTTTCT   
  
  
+ TTTTGTTTGC AATTTTGATG TGTTAGGGTT TGAGGATCAT CATTTTTGAG TAGGGAACTT TCTGGGTTTT   
  
  
+ GCTTGCTGAT TGATTGCCAA TGCTATGGAT TTCTTGGTGA GTCAATCTTC AATTGCTGAT CTGGGTTCGT   
  
  
+ TTGTTCTTCA GAGTTTCACC TGCCAATTTG ATCAAGTATG TCCCTTCACT TCTCCTGCTT CAATTCTCTC   
  
  
+ TCAAATTCAC GCATTTTTCC AATGAATTTC AAATAGGAGT CCTGGAATCC GATGATATTT TAGGTTTTTT   
  
  
+ CCTGACTTCA TTGCATTAGT TTGTTCATAT CTGTGACCAA TTCTTTCATT TGTTATATGA TCTGATTGTT   
  
  
+ TAGTTAGCTA TTCATTCATT GCATATACAT TGGTTTATTC ATTGCAGAGA CACTCACATA CACACACACA   
  
  
+ TACAGGGGAC ACTAGTATTT TTTCTCTCTA ACTCATCGGA TTCCCTTTGA TACTCTGTTT TTTGGGGGGT   
  
  
+ TGATTTGATT TGATTTGATT GACCTTAATT TTGAACTCAC ACACACTCGC ACATAGATAT ATTCTTTCTG   
  
  
+ TTTGTGACAT ATACAGCATC ATATAGAAAT ATATTGCACT GCATATTTGA GCTCAAGACA GGGATATCAT   
  
  
+ CACAAACTGA GTGTGAGGGA GGCAAAAAAC AGAGGAAAAG AAGGGAATTG GGAGTGTAAT CAAAACAAGA   
  
  
+ GAGAGATTGA GGAGAGAGGA AAAAACAAAA TTGGGAGAAG GTTAATAGAG AAGAATGTTG GCTGGGTGTT   
  
  
+ CATCCACATT GCTGTCACCA AGGTATAGAT TGAGGAGTGA AGCTACATCA CAGTTCCAAG CCTGCCATCA   
  
  
+ TCCAATGAGC ACACAGAGAA TTGATTTGCC CTGCACTTTC CCAAGAAAGG ATGTTTCGAA GCCACAATCG   
  
  
+ GTTCGCCCCG TTGGCCTATC TGTCGAGAAG CCGGTTGAAG TCAGGGCCCT GAAGCAGACC ATCCGAGTCC   
  
  
+ CACCATCGCC GGAGGGTAGA AGAGAGATTA AGGCAGATTT CTGGGGTGAT AGAAGAAAGA GCTTGAAGAG   
  
  
+ GAGACTAGCA GAACAGGGGA GCTCTTTTGA TGATGGTGGT GACGAATCTT GTGTTGGGAG AACCAAAAGG   
  
  
+ AAGAAGGGTA GCTTTGATTT TGGTGAATCT GATGAAATTT CTCCAAAAAT TGAGGACACA ATGAGTTTTG   
  
  
+ GACATCTGGG TAGTGGTGGA AATTTCTGGG TTCATCCTGG TTTTGGTGTT GTTAATGTTA ATACTAATTA   
  
  
+ CCCTCAAGTG CCATTTTCTC TCACTTGTTC AGGGGAGGAA GAAAGAGTAT GTTTTGTTCC TACTGATGTG   
  
  
+ ATCTCACCAG CCATCATGCC TCCATTGTCA CACAATCCTT GGGTTGAATC TGGTGTTACT GAGGTCACAG   
  
  
+ AATATGGTGG GGGTGACAAA GACGGCGAAC CTAGTCATGG GTTTGTGAGG GGTACAACAA CAACGTCCGG   
  
  
+ GTCAAGTTCA TCTTCTGAGA GTCATAGTTT TGGGCATAGG CTCAATGAGA GCTCATCTGA CCCTGAAATC   
  
  
+ AGAAATGGTT CCATGTTGCC TAACCCTGGT CATGGTCCTG GCACCAGTCA TGGTCCTGGA ACCGGTCTGG   
  
  
+ CTCACAACCA CAATGATCAA ACCGAGCAAC AAGGGTTCGA GCTCATTAGC TTTCTCATGG GTTGTGTCGA   
  
  
+ AGCGATCAGT TCAAGGAACA TTGCAGCCAT CAATCATTTC ATAGCTAAGC TGGGCGAGCA GGCTTCTCCA   
  
  
+ AGGGCTCGGT CTGCCATTAG CCGCCTCACA GCCTACTTCA CCGAAGCATT AGCCTTGAGG GTCACAAGAT   
  
  
+ TTTGGCCTCA TATCTTTCAC ATAAGTATCC CTCGAGAGTT TGATCGATTT GATGATGAAT CGGGGGCAGC   
  
  
+ TGCAATGAGG CTTTTGAATC AGATCAGCCC AATTCCAAAG TTCGTTCATT TTACAGCCAA TGAGATGTTA   
  
  
+ TTGAGGGCAT TTGAAGGGAA GGACAAGGTG CATATCATAG ACTTCGACAT CAAGCAAGGC TTACAATGGC   
  
  
+ CCGGGTTTTT CCAAAGCTTA GCCATGAGGG AAAACCCCCC GAGCCATGTC AGGATAACAG GCGTAGGGGA   
  
  
+ TTCGAAGCAA GAATTGGTTG AGACAGGAGA AAGACTAGCC GGGTTTGCTG GGGCATTCAA CCTCTCCTTC   
  
  
+ GAGTTCCACC CAGTTGTGGA CCGGTTGGAA GATGTTAGGC TATGGATGCT TCATGTTAAG GAAGGTGAAA   
  
  
+ GTGTCGCGGT AAATTGCATT TTGCAGCTCC ACAAGACACT CTATGACCCC CATGGTGCCA CATTCAGGGA   
  
  
+ CTTCATGGGT TTAATCCGAA GCACAAATCC CATAGCATTG GTTATGGCTG AGCAAGAAGC TGATCACAAC   
  
  
+ GAACCCACCT TAGAAGGCCG AGTATGCAAC TCACTTGGAT ACTATGCAGC CCTTTTCGAT GCCATAGATT   
  
  
+ GCAGCCTTCC TTTCGAGAGC TCAGCAAGGT TGAAGATCGA AGAGATGTTT GGCCGGGAAA TCAGGAACAT   
  
  
+ AATAGCCTGT GAAGGGGCAG ACAGGATCGA AAGACATGAG AAGTTCGAGA AATGGAAGAG AAGGATCGAG   
  
  
+ CAGGAAGGGT TCCGGTGCAT GCGGACTAGC GAAAGGGAGG TGATGCAGAG CCAAATGCTG CTTAAGATGT   
  
  
+ ACTCGAACGA GAGCTACAAC ATGCAAAGAC AAGGCAATGA AGAAGCAATA TCACTAGTAT GGTTAGATCA   
  
  
+ GCCACTCTAC ACTGTTTCTT TGTGGGCTCC AAGTGAATTT GCTGCAGGGA GTTCTTCCAG TTTTTCTCTG   
  
  
+ CCATCTTG  

- -Up\_Stream \_Len000TATTAC CCACTATTGA AAACTTCCTG TTATATGTTT AGTGAATAAA TAATTTCAGT   
  
  
- TCCCCTGTAA AGTTGATATT GTAGTAACAA ATATTTCATA AACTTTTAAC GTTGAGAACA TTATCTATTA   
  
  
- TTACGAAATA TCTGAAGTGG TTAGTGATTA AACATCTTAG TTTTTTAATT AACTTTTCGT TTGTTTTCCT   
  
  
- TTTCCGTGTA TTTGGATTTA ATACATTTGT TTACTCTTGT ATATACGGTC GAATAGTTGC AATTAATATA   
  
  
- AACAATAGTG AGAATTATAG ACATAAGTAT CATTTACTTT TATTCATAGC TAAAAAATAA TTTGGATTTT   
  
  
- AGAAGACAGT TATTAAAAAG AAGGCTATTT TTTTTTTAAC CTGGTATAAT TTAATTGTAA TTATTTAAGT   
  
  
- ATATTAATGA AACTTACAGA CTTTATATTA CGAGAAGCCA TATAATTCCA GAATATTTAA AGAGTAATTA   
  
  
- AACTTTAGAG AATATTATAT AACCCAGTTG TTCACGATCG AACAAAAAAA AAGGACCAAT TAACGTAAAT   
  
  
- TTTTTTATTA AAAATTAATG TTAACGTTCC CCTCCTTAGT AAATGTTTTA TAGTTTTCAC CTTTTCCGTT   
  
  
- TTTCTTTGTT TAATATTTTG TCTTTTTTCT AATCTTTTGC GTTTGTTTCT GCTTTACTCC TTGTCATTCT   
  
  
- TAGTCATTTT TTTACATCAC GTTGCCAGTG TATGCCCTTC CGTTCGTGTT GTTAGTACTT TCTCTGTCCG   
  
  
- TGCACGCAAG GAGCACTAGT CGTCGGAAGG GATGAAGGGG CGGTTCACCT CAGGCATGAT TGAGGACGGT   
  
  
- AAGGGAGGGT TGCCAGTATA AGGCGGCTGT ACGGAGTTGC CAGTCTAAAG GACGGGGTAG ACCTGGCAGG   
  
  
- CTAGTTCTAG GTTGCCGGTA TTCCGCGTGA GAGGTTATGG GATCACGGGT AAAGACCTTG TCGGGGTTTT   
  
  
- TAGAGTCATA AGCGGTAGTA AAAAGTTATG ACCCCAATGT ATTTGTCAGA TTGTTCCTTC CTGGGTTGTG   
  
  
- TCTCCTCTCT CTTTTTTCGA TCGAAACTAC CCCTGTCGGT ACCTACCTCC ACTTCTCTCT CCTCCAACCC   
  
  
- TGGAAAATAA AACGAGTGAA AGGTAAGAAT ATATTTTTGT CGAGATCAAA TAGAGAATAC CGAAAAAACC   
  
  
- CAAACAAGTA TCTCTCCCCC CTTACACTAT ACTAAACTAA ACTACAATCT TTAAGAGTAA TCAAAAAAGA   
  
  
- AAAACAAACG TTAAAACTAC ACAATCCCAA ACTCCTAGTA GTAAAAACTC ATCCCTTGAA AGACCCAAAA   
  
  
- CGAACGACTA ACTAACGGTT ACGATACCTA AAGAACCACT CAGTTAGAAG TTAACGACTA GACCCAAGCA   
  
  
- AACAAGAAGT CTCAAAGTGG ACGGTTAAAC TAGTTCATAC AGGGAAGTGA AGAGGACGAA GTTAAGAGAG   
  
  
- AGTTTAAGTG CGTAAAAAGG TTACTTAAAG TTTATCCTCA GGACCTTAGG CTACTATAAA ATCCAAAAAA   
  
  
- GGACTGAAGT AACGTAATCA AACAAGTATA GACACTGGTT AAGAAAGTAA ACAATATACT AGACTAACAA   
  
  
- ATCAATCGAT AAGTAAGTAA CGTATATGTA ACCAAATAAG TAACGTCTCT GTGAGTGTAT GTGTGTGTGT   
  
  
- ATGTCCCCTG TGATCATAAA AAAGAGAGAT TGAGTAGCCT AAGGGAAACT ATGAGACAAA AAACCCCCCA   
  
  
- ACTAAACTAA ACTAAACTAA CTGGAATTAA AACTTGAGTG TGTGTGAGCG TGTATCTATA TAAGAAAGAC   
  
  
- AAACACTGTA TATGTCGTAG TATATCTTTA TATAACGTGA CGTATAAACT CGAGTTCTGT CCCTATAGTA   
  
  
- GTGTTTGACT CACACTCCCT CCGTTTTTTG TCTCCTTTTC TTCCCTTAAC CCTCACATTA GTTTTGTTCT   
  
  
- CTCTCTAACT CCTCTCTCCT TTTTTGTTTT AACCCTCTTC CAATTATCTC TTCTTACAAC CGACCCACAA   
  
  
- GTAGGTGTAA CGACAGTGGT TCCATATCTA ACTCCTCACT TCGATGTAGT GTCAAGGTTC GGACGGTAGT   
  
  
- AGGTTACTCG TGTGTCTCTT AACTAAACGG GACGTGAAAG GGTTCTTTCC TACAAAGCTT CGGTGTTAGC   
  
  
- CAAGCGGGGC AACCGGATAG ACAGCTCTTC GGCCAACTTC AGTCCCGGGA CTTCGTCTGG TAGGCTCAGG   
  
  
- GTGGTAGCGG CCTCCCATCT TCTCTCTAAT TCCGTCTAAA GACCCCACTA TCTTCTTTCT CGAACTTCTC   
  
  
- CTCTGATCGT CTTGTCCCCT CGAGAAAACT ACTACCACCA CTGCTTAGAA CACAACCCTC TTGGTTTTCC   
  
  
- TTCTTCCCAT CGAAACTAAA ACCACTTAGA CTACTTTAAA GAGGTTTTTA ACTCCTGTGT TACTCAAAAC   
  
  
- CTGTAGACCC ATCACCACCT TTAAAGACCC AAGTAGGACC AAAACCACAA CAATTACAAT TATGATTAAT   
  
  
- GGGAGTTCAC GGTAAAAGAG AGTGAACAAG TCCCCTCCTT CTTTCTCATA CAAAACAAGG ATGACTACAC   
  
  
- TAGAGTGGTC GGTAGTACGG AGGTAACAGT GTGTTAGGAA CCCAACTTAG ACCACAATGA CTCCAGTGTC   
  
  
- TTATACCACC CCCACTGTTT CTGCCGCTTG GATCAGTACC CAAACACTCC CCATGTTGTT GTTGCAGGCC   
  
  
- CAGTTCAAGT AGAAGACTCT CAGTATCAAA ACCCGTATCC GAGTTACTCT CGAGTAGACT GGGACTTTAG   
  
  
- TCTTTACCAA GGTACAACGG ATTGGGACCA GTACCAGGAC CGTGGTCAGT ACCAGGACCT TGGCCAGACC   
  
  
- GAGTGTTGGT GTTACTAGTT TGGCTCGTTG TTCCCAAGCT CGAGTAATCG AAAGAGTACC CAACACAGCT   
  
  
- TCGCTAGTCA AGTTCCTTGT AACGTCGGTA GTTAGTAAAG TATCGATTCG ACCCGCTCGT CCGAAGAGGT   
  
  
- TCCCGAGCCA GACGGTAATC GGCGGAGTGT CGGATGAAGT GGCTTCGTAA TCGGAACTCC CAGTGTTCTA   
  
  
- AAACCGGAGT ATAGAAAGTG TATTCATAGG GAGCTCTCAA ACTAGCTAAA CTACTACTTA GCCCCCGTCG   
  
  
- ACGTTACTCC GAAAACTTAG TCTAGTCGGG TTAAGGTTTC AAGCAAGTAA AATGTCGGTT ACTCTACAAT   
  
  
- AACTCCCGTA AACTTCCCTT CCTGTTCCAC GTATAGTATC TGAAGCTGTA GTTCGTTCCG AATGTTACCG   
  
  
- GGCCCAAAAA GGTTTCGAAT CGGTACTCCC TTTTGGGGGG CTCGGTACAG TCCTATTGTC CGCATCCCCT   
  
  
- AAGCTTCGTT CTTAACCAAC TCTGTCCTCT TTCTGATCGG CCCAAACGAC CCCGTAAGTT GGAGAGGAAG   
  
  
- CTCAAGGTGG GTCAACACCT GGCCAACCTT CTACAATCCG ATACCTACGA AGTACAATTC CTTCCACTTT   
  
  
- CACAGCGCCA TTTAACGTAA AACGTCGAGG TGTTCTGTGA GATACTGGGG GTACCACGGT GTAAGTCCCT   
  
  
- GAAGTACCCA AATTAGGCTT CGTGTTTAGG GTATCGTAAC CAATACCGAC TCGTTCTTCG ACTAGTGTTG   
  
  
- CTTGGGTGGA ATCTTCCGGC TCATACGTTG AGTGAACCTA TGATACGTCG GGAAAAGCTA CGGTATCTAA   
  
  
- CGTCGGAAGG AAAGCTCTCG AGTCGTTCCA ACTTCTAGCT TCTCTACAAA CCGGCCCTTT AGTCCTTGTA   
  
  
- TTATCGGACA CTTCCCCGTC TGTCCTAGCT TTCTGTACTC TTCAAGCTCT TTACCTTCTC TTCCTAGCTC   
  
  
- GTCCTTCCCA AGGCCACGTA CGCCTGATCG CTTTCCCTCC ACTACGTCTC GGTTTACGAC GAATTCTACA   
  
  
- TGAGCTTGCT CTCGATGTTG TACGTTTCTG TTCCGTTACT TCTTCGTTAT AGTGATCATA CCAATCTAGT   
  
  
- CGGTGAGATG TGACAAAGAA ACACCCGAGG TTCACTTAAA CGACGTCCCT CAAGAAGGTC AAAAAGAGAC   
  
  
- GGTAGAAC

+     AE-box

| Site Name | Organism | Position | Strand | Matrix score. | sequence | function |
| --- | --- | --- | --- | --- | --- | --- |
| AE-box | Arabidopsis thaliana | 637 | + | 8 | AGAAACAA | part of a module for light response |

>HU04G00148.1   
+ -Up\_Stream \_Len000ATAATG GGTGATAACT TTTGAAGGAC AATATACAAA TCACTTATTT ATTAAAGTCA   
  
  
+ AGGGGACATT TCAACTATAA CATCATTGTT TATAAAGTAT TTGAAAATTG CAACTCTTGT AATAGATAAT   
  
  
+ AATGCTTTAT AGACTTCACC AATCACTAAT TTGTAGAATC AAAAAATTAA TTGAAAAGCA AACAAAAGGA   
  
  
+ AAAGGCACAT AAACCTAAAT TATGTAAACA AATGAGAACA TATATGCCAG CTTATCAACG TTAATTATAT   
  
  
+ TTGTTATCAC TCTTAATATC TGTATTCATA GTAAATGAAA ATAAGTATCG ATTTTTTATT AAACCTAAAA   
  
  
+ TCTTCTGTCA ATAATTTTTC TTCCGATAAA AAAAAAATTG GACCATATTA AATTAACATT AATAAATTCA   
  
  
+ TATAATTACT TTGAATGTCT GAAATATAAT GCTCTTCGGT ATATTAAGGT CTTATAAATT TCTCATTAAT   
  
  
+ TTGAAATCTC TTATAATATA TTGGGTCAAC AAGTGCTAGC TTGTTTTTTT TTCCTGGTTA ATTGCATTTA   
  
  
+ AAAAAATAAT TTTTAATTAC AATTGCAAGG GGAGGAATCA TTTACAAAAT ATCAAAAGTG GAAAAGGCAA   
  
  
+ AAAGAAACAA ATTATAAAAC AGAAAAAAGA TTAGAAAACG CAAACAAAGA CGAAATGAGG AACAGTAAGA   
  
  
+ ATCAGTAAAA AAATGTAGTG CAACGGTCAC ATACGGGAAG GCAAGCACAA CAATCATGAA AGAGACAGGC   
  
  
+ ACGTGCGTTC CTCGTGATCA GCAGCCTTCC CTACTTCCCC GCCAAGTGGA GTCCGTACTA ACTCCTGCCA   
  
  
+ TTCCCTCCCA ACGGTCATAT TCCGCCGACA TGCCTCAACG GTCAGATTTC CTGCCCCATC TGGACCGTCC   
  
  
+ GATCAAGATC CAACGGCCAT AAGGCGCACT CTCCAATACC CTAGTGCCCA TTTCTGGAAC AGCCCCAAAA   
  
  
+ ATCTCAGTAT TCGCCATCAT TTTTCAATAC TGGGGTTACA TAAACAGTCT AACAAGGAAG GACCCAACAC   
  
  
+ AGAGGAGAGA GAAAAAAGCT AGCTTTGATG GGGACAGCCA TGGATGGAGG TGAAGAGAGA GGAGGTTGGG   
  
  
+ ACCTTTTATT TTGCTCACTT TCCATTCTTA TATAAAAACA GCTCTAGTTT ATCTCTTATG GCTTTTTTGG   
  
  
+ GTTTGTTCAT AGAGAGGGGG GAATGTGATA TGATTTGATT TGATGTTAGA AATTCTCATT AGTTTTTTCT   
  
  
+ TTTTGTTTGC AATTTTGATG TGTTAGGGTT TGAGGATCAT CATTTTTGAG TAGGGAACTT TCTGGGTTTT   
  
  
+ GCTTGCTGAT TGATTGCCAA TGCTATGGAT TTCTTGGTGA GTCAATCTTC AATTGCTGAT CTGGGTTCGT   
  
  
+ TTGTTCTTCA GAGTTTCACC TGCCAATTTG ATCAAGTATG TCCCTTCACT TCTCCTGCTT CAATTCTCTC   
  
  
+ TCAAATTCAC GCATTTTTCC AATGAATTTC AAATAGGAGT CCTGGAATCC GATGATATTT TAGGTTTTTT   
  
  
+ CCTGACTTCA TTGCATTAGT TTGTTCATAT CTGTGACCAA TTCTTTCATT TGTTATATGA TCTGATTGTT   
  
  
+ TAGTTAGCTA TTCATTCATT GCATATACAT TGGTTTATTC ATTGCAGAGA CACTCACATA CACACACACA   
  
  
+ TACAGGGGAC ACTAGTATTT TTTCTCTCTA ACTCATCGGA TTCCCTTTGA TACTCTGTTT TTTGGGGGGT   
  
  
+ TGATTTGATT TGATTTGATT GACCTTAATT TTGAACTCAC ACACACTCGC ACATAGATAT ATTCTTTCTG   
  
  
+ TTTGTGACAT ATACAGCATC ATATAGAAAT ATATTGCACT GCATATTTGA GCTCAAGACA GGGATATCAT   
  
  
+ CACAAACTGA GTGTGAGGGA GGCAAAAAAC AGAGGAAAAG AAGGGAATTG GGAGTGTAAT CAAAACAAGA   
  
  
+ GAGAGATTGA GGAGAGAGGA AAAAACAAAA TTGGGAGAAG GTTAATAGAG AAGAATGTTG GCTGGGTGTT   
  
  
+ CATCCACATT GCTGTCACCA AGGTATAGAT TGAGGAGTGA AGCTACATCA CAGTTCCAAG CCTGCCATCA   
  
  
+ TCCAATGAGC ACACAGAGAA TTGATTTGCC CTGCACTTTC CCAAGAAAGG ATGTTTCGAA GCCACAATCG   
  
  
+ GTTCGCCCCG TTGGCCTATC TGTCGAGAAG CCGGTTGAAG TCAGGGCCCT GAAGCAGACC ATCCGAGTCC   
  
  
+ CACCATCGCC GGAGGGTAGA AGAGAGATTA AGGCAGATTT CTGGGGTGAT AGAAGAAAGA GCTTGAAGAG   
  
  
+ GAGACTAGCA GAACAGGGGA GCTCTTTTGA TGATGGTGGT GACGAATCTT GTGTTGGGAG AACCAAAAGG   
  
  
+ AAGAAGGGTA GCTTTGATTT TGGTGAATCT GATGAAATTT CTCCAAAAAT TGAGGACACA ATGAGTTTTG   
  
  
+ GACATCTGGG TAGTGGTGGA AATTTCTGGG TTCATCCTGG TTTTGGTGTT GTTAATGTTA ATACTAATTA   
  
  
+ CCCTCAAGTG CCATTTTCTC TCACTTGTTC AGGGGAGGAA GAAAGAGTAT GTTTTGTTCC TACTGATGTG   
  
  
+ ATCTCACCAG CCATCATGCC TCCATTGTCA CACAATCCTT GGGTTGAATC TGGTGTTACT GAGGTCACAG   
  
  
+ AATATGGTGG GGGTGACAAA GACGGCGAAC CTAGTCATGG GTTTGTGAGG GGTACAACAA CAACGTCCGG   
  
  
+ GTCAAGTTCA TCTTCTGAGA GTCATAGTTT TGGGCATAGG CTCAATGAGA GCTCATCTGA CCCTGAAATC   
  
  
+ AGAAATGGTT CCATGTTGCC TAACCCTGGT CATGGTCCTG GCACCAGTCA TGGTCCTGGA ACCGGTCTGG   
  
  
+ CTCACAACCA CAATGATCAA ACCGAGCAAC AAGGGTTCGA GCTCATTAGC TTTCTCATGG GTTGTGTCGA   
  
  
+ AGCGATCAGT TCAAGGAACA TTGCAGCCAT CAATCATTTC ATAGCTAAGC TGGGCGAGCA GGCTTCTCCA   
  
  
+ AGGGCTCGGT CTGCCATTAG CCGCCTCACA GCCTACTTCA CCGAAGCATT AGCCTTGAGG GTCACAAGAT   
  
  
+ TTTGGCCTCA TATCTTTCAC ATAAGTATCC CTCGAGAGTT TGATCGATTT GATGATGAAT CGGGGGCAGC   
  
  
+ TGCAATGAGG CTTTTGAATC AGATCAGCCC AATTCCAAAG TTCGTTCATT TTACAGCCAA TGAGATGTTA   
  
  
+ TTGAGGGCAT TTGAAGGGAA GGACAAGGTG CATATCATAG ACTTCGACAT CAAGCAAGGC TTACAATGGC   
  
  
+ CCGGGTTTTT CCAAAGCTTA GCCATGAGGG AAAACCCCCC GAGCCATGTC AGGATAACAG GCGTAGGGGA   
  
  
+ TTCGAAGCAA GAATTGGTTG AGACAGGAGA AAGACTAGCC GGGTTTGCTG GGGCATTCAA CCTCTCCTTC   
  
  
+ GAGTTCCACC CAGTTGTGGA CCGGTTGGAA GATGTTAGGC TATGGATGCT TCATGTTAAG GAAGGTGAAA   
  
  
+ GTGTCGCGGT AAATTGCATT TTGCAGCTCC ACAAGACACT CTATGACCCC CATGGTGCCA CATTCAGGGA   
  
  
+ CTTCATGGGT TTAATCCGAA GCACAAATCC CATAGCATTG GTTATGGCTG AGCAAGAAGC TGATCACAAC   
  
  
+ GAACCCACCT TAGAAGGCCG AGTATGCAAC TCACTTGGAT ACTATGCAGC CCTTTTCGAT GCCATAGATT   
  
  
+ GCAGCCTTCC TTTCGAGAGC TCAGCAAGGT TGAAGATCGA AGAGATGTTT GGCCGGGAAA TCAGGAACAT   
  
  
+ AATAGCCTGT GAAGGGGCAG ACAGGATCGA AAGACATGAG AAGTTCGAGA AATGGAAGAG AAGGATCGAG   
  
  
+ CAGGAAGGGT TCCGGTGCAT GCGGACTAGC GAAAGGGAGG TGATGCAGAG CCAAATGCTG CTTAAGATGT   
  
  
+ ACTCGAACGA GAGCTACAAC ATGCAAAGAC AAGGCAATGA AGAAGCAATA TCACTAGTAT GGTTAGATCA   
  
  
+ GCCACTCTAC ACTGTTTCTT TGTGGGCTCC AAGTGAATTT GCTGCAGGGA GTTCTTCCAG TTTTTCTCTG   
  
  
+ CCATCTTG  

- -Up\_Stream \_Len000TATTAC CCACTATTGA AAACTTCCTG TTATATGTTT AGTGAATAAA TAATTTCAGT   
  
  
- TCCCCTGTAA AGTTGATATT GTAGTAACAA ATATTTCATA AACTTTTAAC GTTGAGAACA TTATCTATTA   
  
  
- TTACGAAATA TCTGAAGTGG TTAGTGATTA AACATCTTAG TTTTTTAATT AACTTTTCGT TTGTTTTCCT   
  
  
- TTTCCGTGTA TTTGGATTTA ATACATTTGT TTACTCTTGT ATATACGGTC GAATAGTTGC AATTAATATA   
  
  
- AACAATAGTG AGAATTATAG ACATAAGTAT CATTTACTTT TATTCATAGC TAAAAAATAA TTTGGATTTT   
  
  
- AGAAGACAGT TATTAAAAAG AAGGCTATTT TTTTTTTAAC CTGGTATAAT TTAATTGTAA TTATTTAAGT   
  
  
- ATATTAATGA AACTTACAGA CTTTATATTA CGAGAAGCCA TATAATTCCA GAATATTTAA AGAGTAATTA   
  
  
- AACTTTAGAG AATATTATAT AACCCAGTTG TTCACGATCG AACAAAAAAA AAGGACCAAT TAACGTAAAT   
  
  
- TTTTTTATTA AAAATTAATG TTAACGTTCC CCTCCTTAGT AAATGTTTTA TAGTTTTCAC CTTTTCCGTT   
  
  
- TTTCTTTGTT TAATATTTTG TCTTTTTTCT AATCTTTTGC GTTTGTTTCT GCTTTACTCC TTGTCATTCT   
  
  
- TAGTCATTTT TTTACATCAC GTTGCCAGTG TATGCCCTTC CGTTCGTGTT GTTAGTACTT TCTCTGTCCG   
  
  
- TGCACGCAAG GAGCACTAGT CGTCGGAAGG GATGAAGGGG CGGTTCACCT CAGGCATGAT TGAGGACGGT   
  
  
- AAGGGAGGGT TGCCAGTATA AGGCGGCTGT ACGGAGTTGC CAGTCTAAAG GACGGGGTAG ACCTGGCAGG   
  
  
- CTAGTTCTAG GTTGCCGGTA TTCCGCGTGA GAGGTTATGG GATCACGGGT AAAGACCTTG TCGGGGTTTT   
  
  
- TAGAGTCATA AGCGGTAGTA AAAAGTTATG ACCCCAATGT ATTTGTCAGA TTGTTCCTTC CTGGGTTGTG   
  
  
- TCTCCTCTCT CTTTTTTCGA TCGAAACTAC CCCTGTCGGT ACCTACCTCC ACTTCTCTCT CCTCCAACCC   
  
  
- TGGAAAATAA AACGAGTGAA AGGTAAGAAT ATATTTTTGT CGAGATCAAA TAGAGAATAC CGAAAAAACC   
  
  
- CAAACAAGTA TCTCTCCCCC CTTACACTAT ACTAAACTAA ACTACAATCT TTAAGAGTAA TCAAAAAAGA   
  
  
- AAAACAAACG TTAAAACTAC ACAATCCCAA ACTCCTAGTA GTAAAAACTC ATCCCTTGAA AGACCCAAAA   
  
  
- CGAACGACTA ACTAACGGTT ACGATACCTA AAGAACCACT CAGTTAGAAG TTAACGACTA GACCCAAGCA   
  
  
- AACAAGAAGT CTCAAAGTGG ACGGTTAAAC TAGTTCATAC AGGGAAGTGA AGAGGACGAA GTTAAGAGAG   
  
  
- AGTTTAAGTG CGTAAAAAGG TTACTTAAAG TTTATCCTCA GGACCTTAGG CTACTATAAA ATCCAAAAAA   
  
  
- GGACTGAAGT AACGTAATCA AACAAGTATA GACACTGGTT AAGAAAGTAA ACAATATACT AGACTAACAA   
  
  
- ATCAATCGAT AAGTAAGTAA CGTATATGTA ACCAAATAAG TAACGTCTCT GTGAGTGTAT GTGTGTGTGT   
  
  
- ATGTCCCCTG TGATCATAAA AAAGAGAGAT TGAGTAGCCT AAGGGAAACT ATGAGACAAA AAACCCCCCA   
  
  
- ACTAAACTAA ACTAAACTAA CTGGAATTAA AACTTGAGTG TGTGTGAGCG TGTATCTATA TAAGAAAGAC   
  
  
- AAACACTGTA TATGTCGTAG TATATCTTTA TATAACGTGA CGTATAAACT CGAGTTCTGT CCCTATAGTA   
  
  
- GTGTTTGACT CACACTCCCT CCGTTTTTTG TCTCCTTTTC TTCCCTTAAC CCTCACATTA GTTTTGTTCT   
  
  
- CTCTCTAACT CCTCTCTCCT TTTTTGTTTT AACCCTCTTC CAATTATCTC TTCTTACAAC CGACCCACAA   
  
  
- GTAGGTGTAA CGACAGTGGT TCCATATCTA ACTCCTCACT TCGATGTAGT GTCAAGGTTC GGACGGTAGT   
  
  
- AGGTTACTCG TGTGTCTCTT AACTAAACGG GACGTGAAAG GGTTCTTTCC TACAAAGCTT CGGTGTTAGC   
  
  
- CAAGCGGGGC AACCGGATAG ACAGCTCTTC GGCCAACTTC AGTCCCGGGA CTTCGTCTGG TAGGCTCAGG   
  
  
- GTGGTAGCGG CCTCCCATCT TCTCTCTAAT TCCGTCTAAA GACCCCACTA TCTTCTTTCT CGAACTTCTC   
  
  
- CTCTGATCGT CTTGTCCCCT CGAGAAAACT ACTACCACCA CTGCTTAGAA CACAACCCTC TTGGTTTTCC   
  
  
- TTCTTCCCAT CGAAACTAAA ACCACTTAGA CTACTTTAAA GAGGTTTTTA ACTCCTGTGT TACTCAAAAC   
  
  
- CTGTAGACCC ATCACCACCT TTAAAGACCC AAGTAGGACC AAAACCACAA CAATTACAAT TATGATTAAT   
  
  
- GGGAGTTCAC GGTAAAAGAG AGTGAACAAG TCCCCTCCTT CTTTCTCATA CAAAACAAGG ATGACTACAC   
  
  
- TAGAGTGGTC GGTAGTACGG AGGTAACAGT GTGTTAGGAA CCCAACTTAG ACCACAATGA CTCCAGTGTC   
  
  
- TTATACCACC CCCACTGTTT CTGCCGCTTG GATCAGTACC CAAACACTCC CCATGTTGTT GTTGCAGGCC   
  
  
- CAGTTCAAGT AGAAGACTCT CAGTATCAAA ACCCGTATCC GAGTTACTCT CGAGTAGACT GGGACTTTAG   
  
  
- TCTTTACCAA GGTACAACGG ATTGGGACCA GTACCAGGAC CGTGGTCAGT ACCAGGACCT TGGCCAGACC   
  
  
- GAGTGTTGGT GTTACTAGTT TGGCTCGTTG TTCCCAAGCT CGAGTAATCG AAAGAGTACC CAACACAGCT   
  
  
- TCGCTAGTCA AGTTCCTTGT AACGTCGGTA GTTAGTAAAG TATCGATTCG ACCCGCTCGT CCGAAGAGGT   
  
  
- TCCCGAGCCA GACGGTAATC GGCGGAGTGT CGGATGAAGT GGCTTCGTAA TCGGAACTCC CAGTGTTCTA   
  
  
- AAACCGGAGT ATAGAAAGTG TATTCATAGG GAGCTCTCAA ACTAGCTAAA CTACTACTTA GCCCCCGTCG   
  
  
- ACGTTACTCC GAAAACTTAG TCTAGTCGGG TTAAGGTTTC AAGCAAGTAA AATGTCGGTT ACTCTACAAT   
  
  
- AACTCCCGTA AACTTCCCTT CCTGTTCCAC GTATAGTATC TGAAGCTGTA GTTCGTTCCG AATGTTACCG   
  
  
- GGCCCAAAAA GGTTTCGAAT CGGTACTCCC TTTTGGGGGG CTCGGTACAG TCCTATTGTC CGCATCCCCT   
  
  
- AAGCTTCGTT CTTAACCAAC TCTGTCCTCT TTCTGATCGG CCCAAACGAC CCCGTAAGTT GGAGAGGAAG   
  
  
- CTCAAGGTGG GTCAACACCT GGCCAACCTT CTACAATCCG ATACCTACGA AGTACAATTC CTTCCACTTT   
  
  
- CACAGCGCCA TTTAACGTAA AACGTCGAGG TGTTCTGTGA GATACTGGGG GTACCACGGT GTAAGTCCCT   
  
  
- GAAGTACCCA AATTAGGCTT CGTGTTTAGG GTATCGTAAC CAATACCGAC TCGTTCTTCG ACTAGTGTTG   
  
  
- CTTGGGTGGA ATCTTCCGGC TCATACGTTG AGTGAACCTA TGATACGTCG GGAAAAGCTA CGGTATCTAA   
  
  
- CGTCGGAAGG AAAGCTCTCG AGTCGTTCCA ACTTCTAGCT TCTCTACAAA CCGGCCCTTT AGTCCTTGTA   
  
  
- TTATCGGACA CTTCCCCGTC TGTCCTAGCT TTCTGTACTC TTCAAGCTCT TTACCTTCTC TTCCTAGCTC   
  
  
- GTCCTTCCCA AGGCCACGTA CGCCTGATCG CTTTCCCTCC ACTACGTCTC GGTTTACGAC GAATTCTACA   
  
  
- TGAGCTTGCT CTCGATGTTG TACGTTTCTG TTCCGTTACT TCTTCGTTAT AGTGATCATA CCAATCTAGT   
  
  
- CGGTGAGATG TGACAAAGAA ACACCCGAGG TTCACTTAAA CGACGTCCCT CAAGAAGGTC AAAAAGAGAC   
  
  
- GGTAGAAC

+     AP-1

| Site Name | Organism | Position | Strand | Matrix score. | sequence | function |
| --- | --- | --- | --- | --- | --- | --- |
| AP-1 | Arabidopsis thaliana | 1712 | - | 8 | TGAGTTAG |  |

>HU04G00148.1   
+ -Up\_Stream \_Len000ATAATG GGTGATAACT TTTGAAGGAC AATATACAAA TCACTTATTT ATTAAAGTCA   
  
  
+ AGGGGACATT TCAACTATAA CATCATTGTT TATAAAGTAT TTGAAAATTG CAACTCTTGT AATAGATAAT   
  
  
+ AATGCTTTAT AGACTTCACC AATCACTAAT TTGTAGAATC AAAAAATTAA TTGAAAAGCA AACAAAAGGA   
  
  
+ AAAGGCACAT AAACCTAAAT TATGTAAACA AATGAGAACA TATATGCCAG CTTATCAACG TTAATTATAT   
  
  
+ TTGTTATCAC TCTTAATATC TGTATTCATA GTAAATGAAA ATAAGTATCG ATTTTTTATT AAACCTAAAA   
  
  
+ TCTTCTGTCA ATAATTTTTC TTCCGATAAA AAAAAAATTG GACCATATTA AATTAACATT AATAAATTCA   
  
  
+ TATAATTACT TTGAATGTCT GAAATATAAT GCTCTTCGGT ATATTAAGGT CTTATAAATT TCTCATTAAT   
  
  
+ TTGAAATCTC TTATAATATA TTGGGTCAAC AAGTGCTAGC TTGTTTTTTT TTCCTGGTTA ATTGCATTTA   
  
  
+ AAAAAATAAT TTTTAATTAC AATTGCAAGG GGAGGAATCA TTTACAAAAT ATCAAAAGTG GAAAAGGCAA   
  
  
+ AAAGAAACAA ATTATAAAAC AGAAAAAAGA TTAGAAAACG CAAACAAAGA CGAAATGAGG AACAGTAAGA   
  
  
+ ATCAGTAAAA AAATGTAGTG CAACGGTCAC ATACGGGAAG GCAAGCACAA CAATCATGAA AGAGACAGGC   
  
  
+ ACGTGCGTTC CTCGTGATCA GCAGCCTTCC CTACTTCCCC GCCAAGTGGA GTCCGTACTA ACTCCTGCCA   
  
  
+ TTCCCTCCCA ACGGTCATAT TCCGCCGACA TGCCTCAACG GTCAGATTTC CTGCCCCATC TGGACCGTCC   
  
  
+ GATCAAGATC CAACGGCCAT AAGGCGCACT CTCCAATACC CTAGTGCCCA TTTCTGGAAC AGCCCCAAAA   
  
  
+ ATCTCAGTAT TCGCCATCAT TTTTCAATAC TGGGGTTACA TAAACAGTCT AACAAGGAAG GACCCAACAC   
  
  
+ AGAGGAGAGA GAAAAAAGCT AGCTTTGATG GGGACAGCCA TGGATGGAGG TGAAGAGAGA GGAGGTTGGG   
  
  
+ ACCTTTTATT TTGCTCACTT TCCATTCTTA TATAAAAACA GCTCTAGTTT ATCTCTTATG GCTTTTTTGG   
  
  
+ GTTTGTTCAT AGAGAGGGGG GAATGTGATA TGATTTGATT TGATGTTAGA AATTCTCATT AGTTTTTTCT   
  
  
+ TTTTGTTTGC AATTTTGATG TGTTAGGGTT TGAGGATCAT CATTTTTGAG TAGGGAACTT TCTGGGTTTT   
  
  
+ GCTTGCTGAT TGATTGCCAA TGCTATGGAT TTCTTGGTGA GTCAATCTTC AATTGCTGAT CTGGGTTCGT   
  
  
+ TTGTTCTTCA GAGTTTCACC TGCCAATTTG ATCAAGTATG TCCCTTCACT TCTCCTGCTT CAATTCTCTC   
  
  
+ TCAAATTCAC GCATTTTTCC AATGAATTTC AAATAGGAGT CCTGGAATCC GATGATATTT TAGGTTTTTT   
  
  
+ CCTGACTTCA TTGCATTAGT TTGTTCATAT CTGTGACCAA TTCTTTCATT TGTTATATGA TCTGATTGTT   
  
  
+ TAGTTAGCTA TTCATTCATT GCATATACAT TGGTTTATTC ATTGCAGAGA CACTCACATA CACACACACA   
  
  
+ TACAGGGGAC ACTAGTATTT TTTCTCTCTA ACTCATCGGA TTCCCTTTGA TACTCTGTTT TTTGGGGGGT   
  
  
+ TGATTTGATT TGATTTGATT GACCTTAATT TTGAACTCAC ACACACTCGC ACATAGATAT ATTCTTTCTG   
  
  
+ TTTGTGACAT ATACAGCATC ATATAGAAAT ATATTGCACT GCATATTTGA GCTCAAGACA GGGATATCAT   
  
  
+ CACAAACTGA GTGTGAGGGA GGCAAAAAAC AGAGGAAAAG AAGGGAATTG GGAGTGTAAT CAAAACAAGA   
  
  
+ GAGAGATTGA GGAGAGAGGA AAAAACAAAA TTGGGAGAAG GTTAATAGAG AAGAATGTTG GCTGGGTGTT   
  
  
+ CATCCACATT GCTGTCACCA AGGTATAGAT TGAGGAGTGA AGCTACATCA CAGTTCCAAG CCTGCCATCA   
  
  
+ TCCAATGAGC ACACAGAGAA TTGATTTGCC CTGCACTTTC CCAAGAAAGG ATGTTTCGAA GCCACAATCG   
  
  
+ GTTCGCCCCG TTGGCCTATC TGTCGAGAAG CCGGTTGAAG TCAGGGCCCT GAAGCAGACC ATCCGAGTCC   
  
  
+ CACCATCGCC GGAGGGTAGA AGAGAGATTA AGGCAGATTT CTGGGGTGAT AGAAGAAAGA GCTTGAAGAG   
  
  
+ GAGACTAGCA GAACAGGGGA GCTCTTTTGA TGATGGTGGT GACGAATCTT GTGTTGGGAG AACCAAAAGG   
  
  
+ AAGAAGGGTA GCTTTGATTT TGGTGAATCT GATGAAATTT CTCCAAAAAT TGAGGACACA ATGAGTTTTG   
  
  
+ GACATCTGGG TAGTGGTGGA AATTTCTGGG TTCATCCTGG TTTTGGTGTT GTTAATGTTA ATACTAATTA   
  
  
+ CCCTCAAGTG CCATTTTCTC TCACTTGTTC AGGGGAGGAA GAAAGAGTAT GTTTTGTTCC TACTGATGTG   
  
  
+ ATCTCACCAG CCATCATGCC TCCATTGTCA CACAATCCTT GGGTTGAATC TGGTGTTACT GAGGTCACAG   
  
  
+ AATATGGTGG GGGTGACAAA GACGGCGAAC CTAGTCATGG GTTTGTGAGG GGTACAACAA CAACGTCCGG   
  
  
+ GTCAAGTTCA TCTTCTGAGA GTCATAGTTT TGGGCATAGG CTCAATGAGA GCTCATCTGA CCCTGAAATC   
  
  
+ AGAAATGGTT CCATGTTGCC TAACCCTGGT CATGGTCCTG GCACCAGTCA TGGTCCTGGA ACCGGTCTGG   
  
  
+ CTCACAACCA CAATGATCAA ACCGAGCAAC AAGGGTTCGA GCTCATTAGC TTTCTCATGG GTTGTGTCGA   
  
  
+ AGCGATCAGT TCAAGGAACA TTGCAGCCAT CAATCATTTC ATAGCTAAGC TGGGCGAGCA GGCTTCTCCA   
  
  
+ AGGGCTCGGT CTGCCATTAG CCGCCTCACA GCCTACTTCA CCGAAGCATT AGCCTTGAGG GTCACAAGAT   
  
  
+ TTTGGCCTCA TATCTTTCAC ATAAGTATCC CTCGAGAGTT TGATCGATTT GATGATGAAT CGGGGGCAGC   
  
  
+ TGCAATGAGG CTTTTGAATC AGATCAGCCC AATTCCAAAG TTCGTTCATT TTACAGCCAA TGAGATGTTA   
  
  
+ TTGAGGGCAT TTGAAGGGAA GGACAAGGTG CATATCATAG ACTTCGACAT CAAGCAAGGC TTACAATGGC   
  
  
+ CCGGGTTTTT CCAAAGCTTA GCCATGAGGG AAAACCCCCC GAGCCATGTC AGGATAACAG GCGTAGGGGA   
  
  
+ TTCGAAGCAA GAATTGGTTG AGACAGGAGA AAGACTAGCC GGGTTTGCTG GGGCATTCAA CCTCTCCTTC   
  
  
+ GAGTTCCACC CAGTTGTGGA CCGGTTGGAA GATGTTAGGC TATGGATGCT TCATGTTAAG GAAGGTGAAA   
  
  
+ GTGTCGCGGT AAATTGCATT TTGCAGCTCC ACAAGACACT CTATGACCCC CATGGTGCCA CATTCAGGGA   
  
  
+ CTTCATGGGT TTAATCCGAA GCACAAATCC CATAGCATTG GTTATGGCTG AGCAAGAAGC TGATCACAAC   
  
  
+ GAACCCACCT TAGAAGGCCG AGTATGCAAC TCACTTGGAT ACTATGCAGC CCTTTTCGAT GCCATAGATT   
  
  
+ GCAGCCTTCC TTTCGAGAGC TCAGCAAGGT TGAAGATCGA AGAGATGTTT GGCCGGGAAA TCAGGAACAT   
  
  
+ AATAGCCTGT GAAGGGGCAG ACAGGATCGA AAGACATGAG AAGTTCGAGA AATGGAAGAG AAGGATCGAG   
  
  
+ CAGGAAGGGT TCCGGTGCAT GCGGACTAGC GAAAGGGAGG TGATGCAGAG CCAAATGCTG CTTAAGATGT   
  
  
+ ACTCGAACGA GAGCTACAAC ATGCAAAGAC AAGGCAATGA AGAAGCAATA TCACTAGTAT GGTTAGATCA   
  
  
+ GCCACTCTAC ACTGTTTCTT TGTGGGCTCC AAGTGAATTT GCTGCAGGGA GTTCTTCCAG TTTTTCTCTG   
  
  
+ CCATCTTG  

- -Up\_Stream \_Len000TATTAC CCACTATTGA AAACTTCCTG TTATATGTTT AGTGAATAAA TAATTTCAGT   
  
  
- TCCCCTGTAA AGTTGATATT GTAGTAACAA ATATTTCATA AACTTTTAAC GTTGAGAACA TTATCTATTA   
  
  
- TTACGAAATA TCTGAAGTGG TTAGTGATTA AACATCTTAG TTTTTTAATT AACTTTTCGT TTGTTTTCCT   
  
  
- TTTCCGTGTA TTTGGATTTA ATACATTTGT TTACTCTTGT ATATACGGTC GAATAGTTGC AATTAATATA   
  
  
- AACAATAGTG AGAATTATAG ACATAAGTAT CATTTACTTT TATTCATAGC TAAAAAATAA TTTGGATTTT   
  
  
- AGAAGACAGT TATTAAAAAG AAGGCTATTT TTTTTTTAAC CTGGTATAAT TTAATTGTAA TTATTTAAGT   
  
  
- ATATTAATGA AACTTACAGA CTTTATATTA CGAGAAGCCA TATAATTCCA GAATATTTAA AGAGTAATTA   
  
  
- AACTTTAGAG AATATTATAT AACCCAGTTG TTCACGATCG AACAAAAAAA AAGGACCAAT TAACGTAAAT   
  
  
- TTTTTTATTA AAAATTAATG TTAACGTTCC CCTCCTTAGT AAATGTTTTA TAGTTTTCAC CTTTTCCGTT   
  
  
- TTTCTTTGTT TAATATTTTG TCTTTTTTCT AATCTTTTGC GTTTGTTTCT GCTTTACTCC TTGTCATTCT   
  
  
- TAGTCATTTT TTTACATCAC GTTGCCAGTG TATGCCCTTC CGTTCGTGTT GTTAGTACTT TCTCTGTCCG   
  
  
- TGCACGCAAG GAGCACTAGT CGTCGGAAGG GATGAAGGGG CGGTTCACCT CAGGCATGAT TGAGGACGGT   
  
  
- AAGGGAGGGT TGCCAGTATA AGGCGGCTGT ACGGAGTTGC CAGTCTAAAG GACGGGGTAG ACCTGGCAGG   
  
  
- CTAGTTCTAG GTTGCCGGTA TTCCGCGTGA GAGGTTATGG GATCACGGGT AAAGACCTTG TCGGGGTTTT   
  
  
- TAGAGTCATA AGCGGTAGTA AAAAGTTATG ACCCCAATGT ATTTGTCAGA TTGTTCCTTC CTGGGTTGTG   
  
  
- TCTCCTCTCT CTTTTTTCGA TCGAAACTAC CCCTGTCGGT ACCTACCTCC ACTTCTCTCT CCTCCAACCC   
  
  
- TGGAAAATAA AACGAGTGAA AGGTAAGAAT ATATTTTTGT CGAGATCAAA TAGAGAATAC CGAAAAAACC   
  
  
- CAAACAAGTA TCTCTCCCCC CTTACACTAT ACTAAACTAA ACTACAATCT TTAAGAGTAA TCAAAAAAGA   
  
  
- AAAACAAACG TTAAAACTAC ACAATCCCAA ACTCCTAGTA GTAAAAACTC ATCCCTTGAA AGACCCAAAA   
  
  
- CGAACGACTA ACTAACGGTT ACGATACCTA AAGAACCACT CAGTTAGAAG TTAACGACTA GACCCAAGCA   
  
  
- AACAAGAAGT CTCAAAGTGG ACGGTTAAAC TAGTTCATAC AGGGAAGTGA AGAGGACGAA GTTAAGAGAG   
  
  
- AGTTTAAGTG CGTAAAAAGG TTACTTAAAG TTTATCCTCA GGACCTTAGG CTACTATAAA ATCCAAAAAA   
  
  
- GGACTGAAGT AACGTAATCA AACAAGTATA GACACTGGTT AAGAAAGTAA ACAATATACT AGACTAACAA   
  
  
- ATCAATCGAT AAGTAAGTAA CGTATATGTA ACCAAATAAG TAACGTCTCT GTGAGTGTAT GTGTGTGTGT   
  
  
- ATGTCCCCTG TGATCATAAA AAAGAGAGAT TGAGTAGCCT AAGGGAAACT ATGAGACAAA AAACCCCCCA   
  
  
- ACTAAACTAA ACTAAACTAA CTGGAATTAA AACTTGAGTG TGTGTGAGCG TGTATCTATA TAAGAAAGAC   
  
  
- AAACACTGTA TATGTCGTAG TATATCTTTA TATAACGTGA CGTATAAACT CGAGTTCTGT CCCTATAGTA   
  
  
- GTGTTTGACT CACACTCCCT CCGTTTTTTG TCTCCTTTTC TTCCCTTAAC CCTCACATTA GTTTTGTTCT   
  
  
- CTCTCTAACT CCTCTCTCCT TTTTTGTTTT AACCCTCTTC CAATTATCTC TTCTTACAAC CGACCCACAA   
  
  
- GTAGGTGTAA CGACAGTGGT TCCATATCTA ACTCCTCACT TCGATGTAGT GTCAAGGTTC GGACGGTAGT   
  
  
- AGGTTACTCG TGTGTCTCTT AACTAAACGG GACGTGAAAG GGTTCTTTCC TACAAAGCTT CGGTGTTAGC   
  
  
- CAAGCGGGGC AACCGGATAG ACAGCTCTTC GGCCAACTTC AGTCCCGGGA CTTCGTCTGG TAGGCTCAGG   
  
  
- GTGGTAGCGG CCTCCCATCT TCTCTCTAAT TCCGTCTAAA GACCCCACTA TCTTCTTTCT CGAACTTCTC   
  
  
- CTCTGATCGT CTTGTCCCCT CGAGAAAACT ACTACCACCA CTGCTTAGAA CACAACCCTC TTGGTTTTCC   
  
  
- TTCTTCCCAT CGAAACTAAA ACCACTTAGA CTACTTTAAA GAGGTTTTTA ACTCCTGTGT TACTCAAAAC   
  
  
- CTGTAGACCC ATCACCACCT TTAAAGACCC AAGTAGGACC AAAACCACAA CAATTACAAT TATGATTAAT   
  
  
- GGGAGTTCAC GGTAAAAGAG AGTGAACAAG TCCCCTCCTT CTTTCTCATA CAAAACAAGG ATGACTACAC   
  
  
- TAGAGTGGTC GGTAGTACGG AGGTAACAGT GTGTTAGGAA CCCAACTTAG ACCACAATGA CTCCAGTGTC   
  
  
- TTATACCACC CCCACTGTTT CTGCCGCTTG GATCAGTACC CAAACACTCC CCATGTTGTT GTTGCAGGCC   
  
  
- CAGTTCAAGT AGAAGACTCT CAGTATCAAA ACCCGTATCC GAGTTACTCT CGAGTAGACT GGGACTTTAG   
  
  
- TCTTTACCAA GGTACAACGG ATTGGGACCA GTACCAGGAC CGTGGTCAGT ACCAGGACCT TGGCCAGACC   
  
  
- GAGTGTTGGT GTTACTAGTT TGGCTCGTTG TTCCCAAGCT CGAGTAATCG AAAGAGTACC CAACACAGCT   
  
  
- TCGCTAGTCA AGTTCCTTGT AACGTCGGTA GTTAGTAAAG TATCGATTCG ACCCGCTCGT CCGAAGAGGT   
  
  
- TCCCGAGCCA GACGGTAATC GGCGGAGTGT CGGATGAAGT GGCTTCGTAA TCGGAACTCC CAGTGTTCTA   
  
  
- AAACCGGAGT ATAGAAAGTG TATTCATAGG GAGCTCTCAA ACTAGCTAAA CTACTACTTA GCCCCCGTCG   
  
  
- ACGTTACTCC GAAAACTTAG TCTAGTCGGG TTAAGGTTTC AAGCAAGTAA AATGTCGGTT ACTCTACAAT   
  
  
- AACTCCCGTA AACTTCCCTT CCTGTTCCAC GTATAGTATC TGAAGCTGTA GTTCGTTCCG AATGTTACCG   
  
  
- GGCCCAAAAA GGTTTCGAAT CGGTACTCCC TTTTGGGGGG CTCGGTACAG TCCTATTGTC CGCATCCCCT   
  
  
- AAGCTTCGTT CTTAACCAAC TCTGTCCTCT TTCTGATCGG CCCAAACGAC CCCGTAAGTT GGAGAGGAAG   
  
  
- CTCAAGGTGG GTCAACACCT GGCCAACCTT CTACAATCCG ATACCTACGA AGTACAATTC CTTCCACTTT   
  
  
- CACAGCGCCA TTTAACGTAA AACGTCGAGG TGTTCTGTGA GATACTGGGG GTACCACGGT GTAAGTCCCT   
  
  
- GAAGTACCCA AATTAGGCTT CGTGTTTAGG GTATCGTAAC CAATACCGAC TCGTTCTTCG ACTAGTGTTG   
  
  
- CTTGGGTGGA ATCTTCCGGC TCATACGTTG AGTGAACCTA TGATACGTCG GGAAAAGCTA CGGTATCTAA   
  
  
- CGTCGGAAGG AAAGCTCTCG AGTCGTTCCA ACTTCTAGCT TCTCTACAAA CCGGCCCTTT AGTCCTTGTA   
  
  
- TTATCGGACA CTTCCCCGTC TGTCCTAGCT TTCTGTACTC TTCAAGCTCT TTACCTTCTC TTCCTAGCTC   
  
  
- GTCCTTCCCA AGGCCACGTA CGCCTGATCG CTTTCCCTCC ACTACGTCTC GGTTTACGAC GAATTCTACA   
  
  
- TGAGCTTGCT CTCGATGTTG TACGTTTCTG TTCCGTTACT TCTTCGTTAT AGTGATCATA CCAATCTAGT   
  
  
- CGGTGAGATG TGACAAAGAA ACACCCGAGG TTCACTTAAA CGACGTCCCT CAAGAAGGTC AAAAAGAGAC   
  
  
- GGTAGAAC

+     ARE

| Site Name | Organism | Position | Strand | Matrix score. | sequence | function |
| --- | --- | --- | --- | --- | --- | --- |
| ARE | Zea mays | 1645 | - | 6 | AAACCA | cis-acting regulatory element essential for the anaerobic induction |
| ARE | Zea mays | 2492 | - | 6 | AAACCA | cis-acting regulatory element essential for the anaerobic induction |

>HU04G00148.1   
+ -Up\_Stream \_Len000ATAATG GGTGATAACT TTTGAAGGAC AATATACAAA TCACTTATTT ATTAAAGTCA   
  
  
+ AGGGGACATT TCAACTATAA CATCATTGTT TATAAAGTAT TTGAAAATTG CAACTCTTGT AATAGATAAT   
  
  
+ AATGCTTTAT AGACTTCACC AATCACTAAT TTGTAGAATC AAAAAATTAA TTGAAAAGCA AACAAAAGGA   
  
  
+ AAAGGCACAT AAACCTAAAT TATGTAAACA AATGAGAACA TATATGCCAG CTTATCAACG TTAATTATAT   
  
  
+ TTGTTATCAC TCTTAATATC TGTATTCATA GTAAATGAAA ATAAGTATCG ATTTTTTATT AAACCTAAAA   
  
  
+ TCTTCTGTCA ATAATTTTTC TTCCGATAAA AAAAAAATTG GACCATATTA AATTAACATT AATAAATTCA   
  
  
+ TATAATTACT TTGAATGTCT GAAATATAAT GCTCTTCGGT ATATTAAGGT CTTATAAATT TCTCATTAAT   
  
  
+ TTGAAATCTC TTATAATATA TTGGGTCAAC AAGTGCTAGC TTGTTTTTTT TTCCTGGTTA ATTGCATTTA   
  
  
+ AAAAAATAAT TTTTAATTAC AATTGCAAGG GGAGGAATCA TTTACAAAAT ATCAAAAGTG GAAAAGGCAA   
  
  
+ AAAGAAACAA ATTATAAAAC AGAAAAAAGA TTAGAAAACG CAAACAAAGA CGAAATGAGG AACAGTAAGA   
  
  
+ ATCAGTAAAA AAATGTAGTG CAACGGTCAC ATACGGGAAG GCAAGCACAA CAATCATGAA AGAGACAGGC   
  
  
+ ACGTGCGTTC CTCGTGATCA GCAGCCTTCC CTACTTCCCC GCCAAGTGGA GTCCGTACTA ACTCCTGCCA   
  
  
+ TTCCCTCCCA ACGGTCATAT TCCGCCGACA TGCCTCAACG GTCAGATTTC CTGCCCCATC TGGACCGTCC   
  
  
+ GATCAAGATC CAACGGCCAT AAGGCGCACT CTCCAATACC CTAGTGCCCA TTTCTGGAAC AGCCCCAAAA   
  
  
+ ATCTCAGTAT TCGCCATCAT TTTTCAATAC TGGGGTTACA TAAACAGTCT AACAAGGAAG GACCCAACAC   
  
  
+ AGAGGAGAGA GAAAAAAGCT AGCTTTGATG GGGACAGCCA TGGATGGAGG TGAAGAGAGA GGAGGTTGGG   
  
  
+ ACCTTTTATT TTGCTCACTT TCCATTCTTA TATAAAAACA GCTCTAGTTT ATCTCTTATG GCTTTTTTGG   
  
  
+ GTTTGTTCAT AGAGAGGGGG GAATGTGATA TGATTTGATT TGATGTTAGA AATTCTCATT AGTTTTTTCT   
  
  
+ TTTTGTTTGC AATTTTGATG TGTTAGGGTT TGAGGATCAT CATTTTTGAG TAGGGAACTT TCTGGGTTTT   
  
  
+ GCTTGCTGAT TGATTGCCAA TGCTATGGAT TTCTTGGTGA GTCAATCTTC AATTGCTGAT CTGGGTTCGT   
  
  
+ TTGTTCTTCA GAGTTTCACC TGCCAATTTG ATCAAGTATG TCCCTTCACT TCTCCTGCTT CAATTCTCTC   
  
  
+ TCAAATTCAC GCATTTTTCC AATGAATTTC AAATAGGAGT CCTGGAATCC GATGATATTT TAGGTTTTTT   
  
  
+ CCTGACTTCA TTGCATTAGT TTGTTCATAT CTGTGACCAA TTCTTTCATT TGTTATATGA TCTGATTGTT   
  
  
+ TAGTTAGCTA TTCATTCATT GCATATACAT TGGTTTATTC ATTGCAGAGA CACTCACATA CACACACACA   
  
  
+ TACAGGGGAC ACTAGTATTT TTTCTCTCTA ACTCATCGGA TTCCCTTTGA TACTCTGTTT TTTGGGGGGT   
  
  
+ TGATTTGATT TGATTTGATT GACCTTAATT TTGAACTCAC ACACACTCGC ACATAGATAT ATTCTTTCTG   
  
  
+ TTTGTGACAT ATACAGCATC ATATAGAAAT ATATTGCACT GCATATTTGA GCTCAAGACA GGGATATCAT   
  
  
+ CACAAACTGA GTGTGAGGGA GGCAAAAAAC AGAGGAAAAG AAGGGAATTG GGAGTGTAAT CAAAACAAGA   
  
  
+ GAGAGATTGA GGAGAGAGGA AAAAACAAAA TTGGGAGAAG GTTAATAGAG AAGAATGTTG GCTGGGTGTT   
  
  
+ CATCCACATT GCTGTCACCA AGGTATAGAT TGAGGAGTGA AGCTACATCA CAGTTCCAAG CCTGCCATCA   
  
  
+ TCCAATGAGC ACACAGAGAA TTGATTTGCC CTGCACTTTC CCAAGAAAGG ATGTTTCGAA GCCACAATCG   
  
  
+ GTTCGCCCCG TTGGCCTATC TGTCGAGAAG CCGGTTGAAG TCAGGGCCCT GAAGCAGACC ATCCGAGTCC   
  
  
+ CACCATCGCC GGAGGGTAGA AGAGAGATTA AGGCAGATTT CTGGGGTGAT AGAAGAAAGA GCTTGAAGAG   
  
  
+ GAGACTAGCA GAACAGGGGA GCTCTTTTGA TGATGGTGGT GACGAATCTT GTGTTGGGAG AACCAAAAGG   
  
  
+ AAGAAGGGTA GCTTTGATTT TGGTGAATCT GATGAAATTT CTCCAAAAAT TGAGGACACA ATGAGTTTTG   
  
  
+ GACATCTGGG TAGTGGTGGA AATTTCTGGG TTCATCCTGG TTTTGGTGTT GTTAATGTTA ATACTAATTA   
  
  
+ CCCTCAAGTG CCATTTTCTC TCACTTGTTC AGGGGAGGAA GAAAGAGTAT GTTTTGTTCC TACTGATGTG   
  
  
+ ATCTCACCAG CCATCATGCC TCCATTGTCA CACAATCCTT GGGTTGAATC TGGTGTTACT GAGGTCACAG   
  
  
+ AATATGGTGG GGGTGACAAA GACGGCGAAC CTAGTCATGG GTTTGTGAGG GGTACAACAA CAACGTCCGG   
  
  
+ GTCAAGTTCA TCTTCTGAGA GTCATAGTTT TGGGCATAGG CTCAATGAGA GCTCATCTGA CCCTGAAATC   
  
  
+ AGAAATGGTT CCATGTTGCC TAACCCTGGT CATGGTCCTG GCACCAGTCA TGGTCCTGGA ACCGGTCTGG   
  
  
+ CTCACAACCA CAATGATCAA ACCGAGCAAC AAGGGTTCGA GCTCATTAGC TTTCTCATGG GTTGTGTCGA   
  
  
+ AGCGATCAGT TCAAGGAACA TTGCAGCCAT CAATCATTTC ATAGCTAAGC TGGGCGAGCA GGCTTCTCCA   
  
  
+ AGGGCTCGGT CTGCCATTAG CCGCCTCACA GCCTACTTCA CCGAAGCATT AGCCTTGAGG GTCACAAGAT   
  
  
+ TTTGGCCTCA TATCTTTCAC ATAAGTATCC CTCGAGAGTT TGATCGATTT GATGATGAAT CGGGGGCAGC   
  
  
+ TGCAATGAGG CTTTTGAATC AGATCAGCCC AATTCCAAAG TTCGTTCATT TTACAGCCAA TGAGATGTTA   
  
  
+ TTGAGGGCAT TTGAAGGGAA GGACAAGGTG CATATCATAG ACTTCGACAT CAAGCAAGGC TTACAATGGC   
  
  
+ CCGGGTTTTT CCAAAGCTTA GCCATGAGGG AAAACCCCCC GAGCCATGTC AGGATAACAG GCGTAGGGGA   
  
  
+ TTCGAAGCAA GAATTGGTTG AGACAGGAGA AAGACTAGCC GGGTTTGCTG GGGCATTCAA CCTCTCCTTC   
  
  
+ GAGTTCCACC CAGTTGTGGA CCGGTTGGAA GATGTTAGGC TATGGATGCT TCATGTTAAG GAAGGTGAAA   
  
  
+ GTGTCGCGGT AAATTGCATT TTGCAGCTCC ACAAGACACT CTATGACCCC CATGGTGCCA CATTCAGGGA   
  
  
+ CTTCATGGGT TTAATCCGAA GCACAAATCC CATAGCATTG GTTATGGCTG AGCAAGAAGC TGATCACAAC   
  
  
+ GAACCCACCT TAGAAGGCCG AGTATGCAAC TCACTTGGAT ACTATGCAGC CCTTTTCGAT GCCATAGATT   
  
  
+ GCAGCCTTCC TTTCGAGAGC TCAGCAAGGT TGAAGATCGA AGAGATGTTT GGCCGGGAAA TCAGGAACAT   
  
  
+ AATAGCCTGT GAAGGGGCAG ACAGGATCGA AAGACATGAG AAGTTCGAGA AATGGAAGAG AAGGATCGAG   
  
  
+ CAGGAAGGGT TCCGGTGCAT GCGGACTAGC GAAAGGGAGG TGATGCAGAG CCAAATGCTG CTTAAGATGT   
  
  
+ ACTCGAACGA GAGCTACAAC ATGCAAAGAC AAGGCAATGA AGAAGCAATA TCACTAGTAT GGTTAGATCA   
  
  
+ GCCACTCTAC ACTGTTTCTT TGTGGGCTCC AAGTGAATTT GCTGCAGGGA GTTCTTCCAG TTTTTCTCTG   
  
  
+ CCATCTTG  

- -Up\_Stream \_Len000TATTAC CCACTATTGA AAACTTCCTG TTATATGTTT AGTGAATAAA TAATTTCAGT   
  
  
- TCCCCTGTAA AGTTGATATT GTAGTAACAA ATATTTCATA AACTTTTAAC GTTGAGAACA TTATCTATTA   
  
  
- TTACGAAATA TCTGAAGTGG TTAGTGATTA AACATCTTAG TTTTTTAATT AACTTTTCGT TTGTTTTCCT   
  
  
- TTTCCGTGTA TTTGGATTTA ATACATTTGT TTACTCTTGT ATATACGGTC GAATAGTTGC AATTAATATA   
  
  
- AACAATAGTG AGAATTATAG ACATAAGTAT CATTTACTTT TATTCATAGC TAAAAAATAA TTTGGATTTT   
  
  
- AGAAGACAGT TATTAAAAAG AAGGCTATTT TTTTTTTAAC CTGGTATAAT TTAATTGTAA TTATTTAAGT   
  
  
- ATATTAATGA AACTTACAGA CTTTATATTA CGAGAAGCCA TATAATTCCA GAATATTTAA AGAGTAATTA   
  
  
- AACTTTAGAG AATATTATAT AACCCAGTTG TTCACGATCG AACAAAAAAA AAGGACCAAT TAACGTAAAT   
  
  
- TTTTTTATTA AAAATTAATG TTAACGTTCC CCTCCTTAGT AAATGTTTTA TAGTTTTCAC CTTTTCCGTT   
  
  
- TTTCTTTGTT TAATATTTTG TCTTTTTTCT AATCTTTTGC GTTTGTTTCT GCTTTACTCC TTGTCATTCT   
  
  
- TAGTCATTTT TTTACATCAC GTTGCCAGTG TATGCCCTTC CGTTCGTGTT GTTAGTACTT TCTCTGTCCG   
  
  
- TGCACGCAAG GAGCACTAGT CGTCGGAAGG GATGAAGGGG CGGTTCACCT CAGGCATGAT TGAGGACGGT   
  
  
- AAGGGAGGGT TGCCAGTATA AGGCGGCTGT ACGGAGTTGC CAGTCTAAAG GACGGGGTAG ACCTGGCAGG   
  
  
- CTAGTTCTAG GTTGCCGGTA TTCCGCGTGA GAGGTTATGG GATCACGGGT AAAGACCTTG TCGGGGTTTT   
  
  
- TAGAGTCATA AGCGGTAGTA AAAAGTTATG ACCCCAATGT ATTTGTCAGA TTGTTCCTTC CTGGGTTGTG   
  
  
- TCTCCTCTCT CTTTTTTCGA TCGAAACTAC CCCTGTCGGT ACCTACCTCC ACTTCTCTCT CCTCCAACCC   
  
  
- TGGAAAATAA AACGAGTGAA AGGTAAGAAT ATATTTTTGT CGAGATCAAA TAGAGAATAC CGAAAAAACC   
  
  
- CAAACAAGTA TCTCTCCCCC CTTACACTAT ACTAAACTAA ACTACAATCT TTAAGAGTAA TCAAAAAAGA   
  
  
- AAAACAAACG TTAAAACTAC ACAATCCCAA ACTCCTAGTA GTAAAAACTC ATCCCTTGAA AGACCCAAAA   
  
  
- CGAACGACTA ACTAACGGTT ACGATACCTA AAGAACCACT CAGTTAGAAG TTAACGACTA GACCCAAGCA   
  
  
- AACAAGAAGT CTCAAAGTGG ACGGTTAAAC TAGTTCATAC AGGGAAGTGA AGAGGACGAA GTTAAGAGAG   
  
  
- AGTTTAAGTG CGTAAAAAGG TTACTTAAAG TTTATCCTCA GGACCTTAGG CTACTATAAA ATCCAAAAAA   
  
  
- GGACTGAAGT AACGTAATCA AACAAGTATA GACACTGGTT AAGAAAGTAA ACAATATACT AGACTAACAA   
  
  
- ATCAATCGAT AAGTAAGTAA CGTATATGTA ACCAAATAAG TAACGTCTCT GTGAGTGTAT GTGTGTGTGT   
  
  
- ATGTCCCCTG TGATCATAAA AAAGAGAGAT TGAGTAGCCT AAGGGAAACT ATGAGACAAA AAACCCCCCA   
  
  
- ACTAAACTAA ACTAAACTAA CTGGAATTAA AACTTGAGTG TGTGTGAGCG TGTATCTATA TAAGAAAGAC   
  
  
- AAACACTGTA TATGTCGTAG TATATCTTTA TATAACGTGA CGTATAAACT CGAGTTCTGT CCCTATAGTA   
  
  
- GTGTTTGACT CACACTCCCT CCGTTTTTTG TCTCCTTTTC TTCCCTTAAC CCTCACATTA GTTTTGTTCT   
  
  
- CTCTCTAACT CCTCTCTCCT TTTTTGTTTT AACCCTCTTC CAATTATCTC TTCTTACAAC CGACCCACAA   
  
  
- GTAGGTGTAA CGACAGTGGT TCCATATCTA ACTCCTCACT TCGATGTAGT GTCAAGGTTC GGACGGTAGT   
  
  
- AGGTTACTCG TGTGTCTCTT AACTAAACGG GACGTGAAAG GGTTCTTTCC TACAAAGCTT CGGTGTTAGC   
  
  
- CAAGCGGGGC AACCGGATAG ACAGCTCTTC GGCCAACTTC AGTCCCGGGA CTTCGTCTGG TAGGCTCAGG   
  
  
- GTGGTAGCGG CCTCCCATCT TCTCTCTAAT TCCGTCTAAA GACCCCACTA TCTTCTTTCT CGAACTTCTC   
  
  
- CTCTGATCGT CTTGTCCCCT CGAGAAAACT ACTACCACCA CTGCTTAGAA CACAACCCTC TTGGTTTTCC   
  
  
- TTCTTCCCAT CGAAACTAAA ACCACTTAGA CTACTTTAAA GAGGTTTTTA ACTCCTGTGT TACTCAAAAC   
  
  
- CTGTAGACCC ATCACCACCT TTAAAGACCC AAGTAGGACC AAAACCACAA CAATTACAAT TATGATTAAT   
  
  
- GGGAGTTCAC GGTAAAAGAG AGTGAACAAG TCCCCTCCTT CTTTCTCATA CAAAACAAGG ATGACTACAC   
  
  
- TAGAGTGGTC GGTAGTACGG AGGTAACAGT GTGTTAGGAA CCCAACTTAG ACCACAATGA CTCCAGTGTC   
  
  
- TTATACCACC CCCACTGTTT CTGCCGCTTG GATCAGTACC CAAACACTCC CCATGTTGTT GTTGCAGGCC   
  
  
- CAGTTCAAGT AGAAGACTCT CAGTATCAAA ACCCGTATCC GAGTTACTCT CGAGTAGACT GGGACTTTAG   
  
  
- TCTTTACCAA GGTACAACGG ATTGGGACCA GTACCAGGAC CGTGGTCAGT ACCAGGACCT TGGCCAGACC   
  
  
- GAGTGTTGGT GTTACTAGTT TGGCTCGTTG TTCCCAAGCT CGAGTAATCG AAAGAGTACC CAACACAGCT   
  
  
- TCGCTAGTCA AGTTCCTTGT AACGTCGGTA GTTAGTAAAG TATCGATTCG ACCCGCTCGT CCGAAGAGGT   
  
  
- TCCCGAGCCA GACGGTAATC GGCGGAGTGT CGGATGAAGT GGCTTCGTAA TCGGAACTCC CAGTGTTCTA   
  
  
- AAACCGGAGT ATAGAAAGTG TATTCATAGG GAGCTCTCAA ACTAGCTAAA CTACTACTTA GCCCCCGTCG   
  
  
- ACGTTACTCC GAAAACTTAG TCTAGTCGGG TTAAGGTTTC AAGCAAGTAA AATGTCGGTT ACTCTACAAT   
  
  
- AACTCCCGTA AACTTCCCTT CCTGTTCCAC GTATAGTATC TGAAGCTGTA GTTCGTTCCG AATGTTACCG   
  
  
- GGCCCAAAAA GGTTTCGAAT CGGTACTCCC TTTTGGGGGG CTCGGTACAG TCCTATTGTC CGCATCCCCT   
  
  
- AAGCTTCGTT CTTAACCAAC TCTGTCCTCT TTCTGATCGG CCCAAACGAC CCCGTAAGTT GGAGAGGAAG   
  
  
- CTCAAGGTGG GTCAACACCT GGCCAACCTT CTACAATCCG ATACCTACGA AGTACAATTC CTTCCACTTT   
  
  
- CACAGCGCCA TTTAACGTAA AACGTCGAGG TGTTCTGTGA GATACTGGGG GTACCACGGT GTAAGTCCCT   
  
  
- GAAGTACCCA AATTAGGCTT CGTGTTTAGG GTATCGTAAC CAATACCGAC TCGTTCTTCG ACTAGTGTTG   
  
  
- CTTGGGTGGA ATCTTCCGGC TCATACGTTG AGTGAACCTA TGATACGTCG GGAAAAGCTA CGGTATCTAA   
  
  
- CGTCGGAAGG AAAGCTCTCG AGTCGTTCCA ACTTCTAGCT TCTCTACAAA CCGGCCCTTT AGTCCTTGTA   
  
  
- TTATCGGACA CTTCCCCGTC TGTCCTAGCT TTCTGTACTC TTCAAGCTCT TTACCTTCTC TTCCTAGCTC   
  
  
- GTCCTTCCCA AGGCCACGTA CGCCTGATCG CTTTCCCTCC ACTACGTCTC GGTTTACGAC GAATTCTACA   
  
  
- TGAGCTTGCT CTCGATGTTG TACGTTTCTG TTCCGTTACT TCTTCGTTAT AGTGATCATA CCAATCTAGT   
  
  
- CGGTGAGATG TGACAAAGAA ACACCCGAGG TTCACTTAAA CGACGTCCCT CAAGAAGGTC AAAAAGAGAC   
  
  
- GGTAGAAC

+     AT~TATA-box

| Site Name | Organism | Position | Strand | Matrix score. | sequence | function |
| --- | --- | --- | --- | --- | --- | --- |
| AT~TATA-box | Arabidopsis thaliana | 1153 | + | 6 | TATATA |  |

>HU04G00148.1   
+ -Up\_Stream \_Len000ATAATG GGTGATAACT TTTGAAGGAC AATATACAAA TCACTTATTT ATTAAAGTCA   
  
  
+ AGGGGACATT TCAACTATAA CATCATTGTT TATAAAGTAT TTGAAAATTG CAACTCTTGT AATAGATAAT   
  
  
+ AATGCTTTAT AGACTTCACC AATCACTAAT TTGTAGAATC AAAAAATTAA TTGAAAAGCA AACAAAAGGA   
  
  
+ AAAGGCACAT AAACCTAAAT TATGTAAACA AATGAGAACA TATATGCCAG CTTATCAACG TTAATTATAT   
  
  
+ TTGTTATCAC TCTTAATATC TGTATTCATA GTAAATGAAA ATAAGTATCG ATTTTTTATT AAACCTAAAA   
  
  
+ TCTTCTGTCA ATAATTTTTC TTCCGATAAA AAAAAAATTG GACCATATTA AATTAACATT AATAAATTCA   
  
  
+ TATAATTACT TTGAATGTCT GAAATATAAT GCTCTTCGGT ATATTAAGGT CTTATAAATT TCTCATTAAT   
  
  
+ TTGAAATCTC TTATAATATA TTGGGTCAAC AAGTGCTAGC TTGTTTTTTT TTCCTGGTTA ATTGCATTTA   
  
  
+ AAAAAATAAT TTTTAATTAC AATTGCAAGG GGAGGAATCA TTTACAAAAT ATCAAAAGTG GAAAAGGCAA   
  
  
+ AAAGAAACAA ATTATAAAAC AGAAAAAAGA TTAGAAAACG CAAACAAAGA CGAAATGAGG AACAGTAAGA   
  
  
+ ATCAGTAAAA AAATGTAGTG CAACGGTCAC ATACGGGAAG GCAAGCACAA CAATCATGAA AGAGACAGGC   
  
  
+ ACGTGCGTTC CTCGTGATCA GCAGCCTTCC CTACTTCCCC GCCAAGTGGA GTCCGTACTA ACTCCTGCCA   
  
  
+ TTCCCTCCCA ACGGTCATAT TCCGCCGACA TGCCTCAACG GTCAGATTTC CTGCCCCATC TGGACCGTCC   
  
  
+ GATCAAGATC CAACGGCCAT AAGGCGCACT CTCCAATACC CTAGTGCCCA TTTCTGGAAC AGCCCCAAAA   
  
  
+ ATCTCAGTAT TCGCCATCAT TTTTCAATAC TGGGGTTACA TAAACAGTCT AACAAGGAAG GACCCAACAC   
  
  
+ AGAGGAGAGA GAAAAAAGCT AGCTTTGATG GGGACAGCCA TGGATGGAGG TGAAGAGAGA GGAGGTTGGG   
  
  
+ ACCTTTTATT TTGCTCACTT TCCATTCTTA TATAAAAACA GCTCTAGTTT ATCTCTTATG GCTTTTTTGG   
  
  
+ GTTTGTTCAT AGAGAGGGGG GAATGTGATA TGATTTGATT TGATGTTAGA AATTCTCATT AGTTTTTTCT   
  
  
+ TTTTGTTTGC AATTTTGATG TGTTAGGGTT TGAGGATCAT CATTTTTGAG TAGGGAACTT TCTGGGTTTT   
  
  
+ GCTTGCTGAT TGATTGCCAA TGCTATGGAT TTCTTGGTGA GTCAATCTTC AATTGCTGAT CTGGGTTCGT   
  
  
+ TTGTTCTTCA GAGTTTCACC TGCCAATTTG ATCAAGTATG TCCCTTCACT TCTCCTGCTT CAATTCTCTC   
  
  
+ TCAAATTCAC GCATTTTTCC AATGAATTTC AAATAGGAGT CCTGGAATCC GATGATATTT TAGGTTTTTT   
  
  
+ CCTGACTTCA TTGCATTAGT TTGTTCATAT CTGTGACCAA TTCTTTCATT TGTTATATGA TCTGATTGTT   
  
  
+ TAGTTAGCTA TTCATTCATT GCATATACAT TGGTTTATTC ATTGCAGAGA CACTCACATA CACACACACA   
  
  
+ TACAGGGGAC ACTAGTATTT TTTCTCTCTA ACTCATCGGA TTCCCTTTGA TACTCTGTTT TTTGGGGGGT   
  
  
+ TGATTTGATT TGATTTGATT GACCTTAATT TTGAACTCAC ACACACTCGC ACATAGATAT ATTCTTTCTG   
  
  
+ TTTGTGACAT ATACAGCATC ATATAGAAAT ATATTGCACT GCATATTTGA GCTCAAGACA GGGATATCAT   
  
  
+ CACAAACTGA GTGTGAGGGA GGCAAAAAAC AGAGGAAAAG AAGGGAATTG GGAGTGTAAT CAAAACAAGA   
  
  
+ GAGAGATTGA GGAGAGAGGA AAAAACAAAA TTGGGAGAAG GTTAATAGAG AAGAATGTTG GCTGGGTGTT   
  
  
+ CATCCACATT GCTGTCACCA AGGTATAGAT TGAGGAGTGA AGCTACATCA CAGTTCCAAG CCTGCCATCA   
  
  
+ TCCAATGAGC ACACAGAGAA TTGATTTGCC CTGCACTTTC CCAAGAAAGG ATGTTTCGAA GCCACAATCG   
  
  
+ GTTCGCCCCG TTGGCCTATC TGTCGAGAAG CCGGTTGAAG TCAGGGCCCT GAAGCAGACC ATCCGAGTCC   
  
  
+ CACCATCGCC GGAGGGTAGA AGAGAGATTA AGGCAGATTT CTGGGGTGAT AGAAGAAAGA GCTTGAAGAG   
  
  
+ GAGACTAGCA GAACAGGGGA GCTCTTTTGA TGATGGTGGT GACGAATCTT GTGTTGGGAG AACCAAAAGG   
  
  
+ AAGAAGGGTA GCTTTGATTT TGGTGAATCT GATGAAATTT CTCCAAAAAT TGAGGACACA ATGAGTTTTG   
  
  
+ GACATCTGGG TAGTGGTGGA AATTTCTGGG TTCATCCTGG TTTTGGTGTT GTTAATGTTA ATACTAATTA   
  
  
+ CCCTCAAGTG CCATTTTCTC TCACTTGTTC AGGGGAGGAA GAAAGAGTAT GTTTTGTTCC TACTGATGTG   
  
  
+ ATCTCACCAG CCATCATGCC TCCATTGTCA CACAATCCTT GGGTTGAATC TGGTGTTACT GAGGTCACAG   
  
  
+ AATATGGTGG GGGTGACAAA GACGGCGAAC CTAGTCATGG GTTTGTGAGG GGTACAACAA CAACGTCCGG   
  
  
+ GTCAAGTTCA TCTTCTGAGA GTCATAGTTT TGGGCATAGG CTCAATGAGA GCTCATCTGA CCCTGAAATC   
  
  
+ AGAAATGGTT CCATGTTGCC TAACCCTGGT CATGGTCCTG GCACCAGTCA TGGTCCTGGA ACCGGTCTGG   
  
  
+ CTCACAACCA CAATGATCAA ACCGAGCAAC AAGGGTTCGA GCTCATTAGC TTTCTCATGG GTTGTGTCGA   
  
  
+ AGCGATCAGT TCAAGGAACA TTGCAGCCAT CAATCATTTC ATAGCTAAGC TGGGCGAGCA GGCTTCTCCA   
  
  
+ AGGGCTCGGT CTGCCATTAG CCGCCTCACA GCCTACTTCA CCGAAGCATT AGCCTTGAGG GTCACAAGAT   
  
  
+ TTTGGCCTCA TATCTTTCAC ATAAGTATCC CTCGAGAGTT TGATCGATTT GATGATGAAT CGGGGGCAGC   
  
  
+ TGCAATGAGG CTTTTGAATC AGATCAGCCC AATTCCAAAG TTCGTTCATT TTACAGCCAA TGAGATGTTA   
  
  
+ TTGAGGGCAT TTGAAGGGAA GGACAAGGTG CATATCATAG ACTTCGACAT CAAGCAAGGC TTACAATGGC   
  
  
+ CCGGGTTTTT CCAAAGCTTA GCCATGAGGG AAAACCCCCC GAGCCATGTC AGGATAACAG GCGTAGGGGA   
  
  
+ TTCGAAGCAA GAATTGGTTG AGACAGGAGA AAGACTAGCC GGGTTTGCTG GGGCATTCAA CCTCTCCTTC   
  
  
+ GAGTTCCACC CAGTTGTGGA CCGGTTGGAA GATGTTAGGC TATGGATGCT TCATGTTAAG GAAGGTGAAA   
  
  
+ GTGTCGCGGT AAATTGCATT TTGCAGCTCC ACAAGACACT CTATGACCCC CATGGTGCCA CATTCAGGGA   
  
  
+ CTTCATGGGT TTAATCCGAA GCACAAATCC CATAGCATTG GTTATGGCTG AGCAAGAAGC TGATCACAAC   
  
  
+ GAACCCACCT TAGAAGGCCG AGTATGCAAC TCACTTGGAT ACTATGCAGC CCTTTTCGAT GCCATAGATT   
  
  
+ GCAGCCTTCC TTTCGAGAGC TCAGCAAGGT TGAAGATCGA AGAGATGTTT GGCCGGGAAA TCAGGAACAT   
  
  
+ AATAGCCTGT GAAGGGGCAG ACAGGATCGA AAGACATGAG AAGTTCGAGA AATGGAAGAG AAGGATCGAG   
  
  
+ CAGGAAGGGT TCCGGTGCAT GCGGACTAGC GAAAGGGAGG TGATGCAGAG CCAAATGCTG CTTAAGATGT   
  
  
+ ACTCGAACGA GAGCTACAAC ATGCAAAGAC AAGGCAATGA AGAAGCAATA TCACTAGTAT GGTTAGATCA   
  
  
+ GCCACTCTAC ACTGTTTCTT TGTGGGCTCC AAGTGAATTT GCTGCAGGGA GTTCTTCCAG TTTTTCTCTG   
  
  
+ CCATCTTG  

- -Up\_Stream \_Len000TATTAC CCACTATTGA AAACTTCCTG TTATATGTTT AGTGAATAAA TAATTTCAGT   
  
  
- TCCCCTGTAA AGTTGATATT GTAGTAACAA ATATTTCATA AACTTTTAAC GTTGAGAACA TTATCTATTA   
  
  
- TTACGAAATA TCTGAAGTGG TTAGTGATTA AACATCTTAG TTTTTTAATT AACTTTTCGT TTGTTTTCCT   
  
  
- TTTCCGTGTA TTTGGATTTA ATACATTTGT TTACTCTTGT ATATACGGTC GAATAGTTGC AATTAATATA   
  
  
- AACAATAGTG AGAATTATAG ACATAAGTAT CATTTACTTT TATTCATAGC TAAAAAATAA TTTGGATTTT   
  
  
- AGAAGACAGT TATTAAAAAG AAGGCTATTT TTTTTTTAAC CTGGTATAAT TTAATTGTAA TTATTTAAGT   
  
  
- ATATTAATGA AACTTACAGA CTTTATATTA CGAGAAGCCA TATAATTCCA GAATATTTAA AGAGTAATTA   
  
  
- AACTTTAGAG AATATTATAT AACCCAGTTG TTCACGATCG AACAAAAAAA AAGGACCAAT TAACGTAAAT   
  
  
- TTTTTTATTA AAAATTAATG TTAACGTTCC CCTCCTTAGT AAATGTTTTA TAGTTTTCAC CTTTTCCGTT   
  
  
- TTTCTTTGTT TAATATTTTG TCTTTTTTCT AATCTTTTGC GTTTGTTTCT GCTTTACTCC TTGTCATTCT   
  
  
- TAGTCATTTT TTTACATCAC GTTGCCAGTG TATGCCCTTC CGTTCGTGTT GTTAGTACTT TCTCTGTCCG   
  
  
- TGCACGCAAG GAGCACTAGT CGTCGGAAGG GATGAAGGGG CGGTTCACCT CAGGCATGAT TGAGGACGGT   
  
  
- AAGGGAGGGT TGCCAGTATA AGGCGGCTGT ACGGAGTTGC CAGTCTAAAG GACGGGGTAG ACCTGGCAGG   
  
  
- CTAGTTCTAG GTTGCCGGTA TTCCGCGTGA GAGGTTATGG GATCACGGGT AAAGACCTTG TCGGGGTTTT   
  
  
- TAGAGTCATA AGCGGTAGTA AAAAGTTATG ACCCCAATGT ATTTGTCAGA TTGTTCCTTC CTGGGTTGTG   
  
  
- TCTCCTCTCT CTTTTTTCGA TCGAAACTAC CCCTGTCGGT ACCTACCTCC ACTTCTCTCT CCTCCAACCC   
  
  
- TGGAAAATAA AACGAGTGAA AGGTAAGAAT ATATTTTTGT CGAGATCAAA TAGAGAATAC CGAAAAAACC   
  
  
- CAAACAAGTA TCTCTCCCCC CTTACACTAT ACTAAACTAA ACTACAATCT TTAAGAGTAA TCAAAAAAGA   
  
  
- AAAACAAACG TTAAAACTAC ACAATCCCAA ACTCCTAGTA GTAAAAACTC ATCCCTTGAA AGACCCAAAA   
  
  
- CGAACGACTA ACTAACGGTT ACGATACCTA AAGAACCACT CAGTTAGAAG TTAACGACTA GACCCAAGCA   
  
  
- AACAAGAAGT CTCAAAGTGG ACGGTTAAAC TAGTTCATAC AGGGAAGTGA AGAGGACGAA GTTAAGAGAG   
  
  
- AGTTTAAGTG CGTAAAAAGG TTACTTAAAG TTTATCCTCA GGACCTTAGG CTACTATAAA ATCCAAAAAA   
  
  
- GGACTGAAGT AACGTAATCA AACAAGTATA GACACTGGTT AAGAAAGTAA ACAATATACT AGACTAACAA   
  
  
- ATCAATCGAT AAGTAAGTAA CGTATATGTA ACCAAATAAG TAACGTCTCT GTGAGTGTAT GTGTGTGTGT   
  
  
- ATGTCCCCTG TGATCATAAA AAAGAGAGAT TGAGTAGCCT AAGGGAAACT ATGAGACAAA AAACCCCCCA   
  
  
- ACTAAACTAA ACTAAACTAA CTGGAATTAA AACTTGAGTG TGTGTGAGCG TGTATCTATA TAAGAAAGAC   
  
  
- AAACACTGTA TATGTCGTAG TATATCTTTA TATAACGTGA CGTATAAACT CGAGTTCTGT CCCTATAGTA   
  
  
- GTGTTTGACT CACACTCCCT CCGTTTTTTG TCTCCTTTTC TTCCCTTAAC CCTCACATTA GTTTTGTTCT   
  
  
- CTCTCTAACT CCTCTCTCCT TTTTTGTTTT AACCCTCTTC CAATTATCTC TTCTTACAAC CGACCCACAA   
  
  
- GTAGGTGTAA CGACAGTGGT TCCATATCTA ACTCCTCACT TCGATGTAGT GTCAAGGTTC GGACGGTAGT   
  
  
- AGGTTACTCG TGTGTCTCTT AACTAAACGG GACGTGAAAG GGTTCTTTCC TACAAAGCTT CGGTGTTAGC   
  
  
- CAAGCGGGGC AACCGGATAG ACAGCTCTTC GGCCAACTTC AGTCCCGGGA CTTCGTCTGG TAGGCTCAGG   
  
  
- GTGGTAGCGG CCTCCCATCT TCTCTCTAAT TCCGTCTAAA GACCCCACTA TCTTCTTTCT CGAACTTCTC   
  
  
- CTCTGATCGT CTTGTCCCCT CGAGAAAACT ACTACCACCA CTGCTTAGAA CACAACCCTC TTGGTTTTCC   
  
  
- TTCTTCCCAT CGAAACTAAA ACCACTTAGA CTACTTTAAA GAGGTTTTTA ACTCCTGTGT TACTCAAAAC   
  
  
- CTGTAGACCC ATCACCACCT TTAAAGACCC AAGTAGGACC AAAACCACAA CAATTACAAT TATGATTAAT   
  
  
- GGGAGTTCAC GGTAAAAGAG AGTGAACAAG TCCCCTCCTT CTTTCTCATA CAAAACAAGG ATGACTACAC   
  
  
- TAGAGTGGTC GGTAGTACGG AGGTAACAGT GTGTTAGGAA CCCAACTTAG ACCACAATGA CTCCAGTGTC   
  
  
- TTATACCACC CCCACTGTTT CTGCCGCTTG GATCAGTACC CAAACACTCC CCATGTTGTT GTTGCAGGCC   
  
  
- CAGTTCAAGT AGAAGACTCT CAGTATCAAA ACCCGTATCC GAGTTACTCT CGAGTAGACT GGGACTTTAG   
  
  
- TCTTTACCAA GGTACAACGG ATTGGGACCA GTACCAGGAC CGTGGTCAGT ACCAGGACCT TGGCCAGACC   
  
  
- GAGTGTTGGT GTTACTAGTT TGGCTCGTTG TTCCCAAGCT CGAGTAATCG AAAGAGTACC CAACACAGCT   
  
  
- TCGCTAGTCA AGTTCCTTGT AACGTCGGTA GTTAGTAAAG TATCGATTCG ACCCGCTCGT CCGAAGAGGT   
  
  
- TCCCGAGCCA GACGGTAATC GGCGGAGTGT CGGATGAAGT GGCTTCGTAA TCGGAACTCC CAGTGTTCTA   
  
  
- AAACCGGAGT ATAGAAAGTG TATTCATAGG GAGCTCTCAA ACTAGCTAAA CTACTACTTA GCCCCCGTCG   
  
  
- ACGTTACTCC GAAAACTTAG TCTAGTCGGG TTAAGGTTTC AAGCAAGTAA AATGTCGGTT ACTCTACAAT   
  
  
- AACTCCCGTA AACTTCCCTT CCTGTTCCAC GTATAGTATC TGAAGCTGTA GTTCGTTCCG AATGTTACCG   
  
  
- GGCCCAAAAA GGTTTCGAAT CGGTACTCCC TTTTGGGGGG CTCGGTACAG TCCTATTGTC CGCATCCCCT   
  
  
- AAGCTTCGTT CTTAACCAAC TCTGTCCTCT TTCTGATCGG CCCAAACGAC CCCGTAAGTT GGAGAGGAAG   
  
  
- CTCAAGGTGG GTCAACACCT GGCCAACCTT CTACAATCCG ATACCTACGA AGTACAATTC CTTCCACTTT   
  
  
- CACAGCGCCA TTTAACGTAA AACGTCGAGG TGTTCTGTGA GATACTGGGG GTACCACGGT GTAAGTCCCT   
  
  
- GAAGTACCCA AATTAGGCTT CGTGTTTAGG GTATCGTAAC CAATACCGAC TCGTTCTTCG ACTAGTGTTG   
  
  
- CTTGGGTGGA ATCTTCCGGC TCATACGTTG AGTGAACCTA TGATACGTCG GGAAAAGCTA CGGTATCTAA   
  
  
- CGTCGGAAGG AAAGCTCTCG AGTCGTTCCA ACTTCTAGCT TCTCTACAAA CCGGCCCTTT AGTCCTTGTA   
  
  
- TTATCGGACA CTTCCCCGTC TGTCCTAGCT TTCTGTACTC TTCAAGCTCT TTACCTTCTC TTCCTAGCTC   
  
  
- GTCCTTCCCA AGGCCACGTA CGCCTGATCG CTTTCCCTCC ACTACGTCTC GGTTTACGAC GAATTCTACA   
  
  
- TGAGCTTGCT CTCGATGTTG TACGTTTCTG TTCCGTTACT TCTTCGTTAT AGTGATCATA CCAATCTAGT   
  
  
- CGGTGAGATG TGACAAAGAA ACACCCGAGG TTCACTTAAA CGACGTCCCT CAAGAAGGTC AAAAAGAGAC   
  
  
- GGTAGAAC

+     Box 4

| Site Name | Organism | Position | Strand | Matrix score. | sequence | function |
| --- | --- | --- | --- | --- | --- | --- |
| Box 4 | Petroselinum crispum | 412 | + | 6 | ATTAAT | part of a conserved DNA module involved in light responsiveness |
| Box 4 | Petroselinum crispum | 190 | + | 6 | ATTAAT | part of a conserved DNA module involved in light responsiveness |
| Box 4 | Petroselinum crispum | 489 | + | 6 | ATTAAT | part of a conserved DNA module involved in light responsiveness |

>HU04G00148.1   
+ -Up\_Stream \_Len000ATAATG GGTGATAACT TTTGAAGGAC AATATACAAA TCACTTATTT ATTAAAGTCA   
  
  
+ AGGGGACATT TCAACTATAA CATCATTGTT TATAAAGTAT TTGAAAATTG CAACTCTTGT AATAGATAAT   
  
  
+ AATGCTTTAT AGACTTCACC AATCACTAAT TTGTAGAATC AAAAAATTAA TTGAAAAGCA AACAAAAGGA   
  
  
+ AAAGGCACAT AAACCTAAAT TATGTAAACA AATGAGAACA TATATGCCAG CTTATCAACG TTAATTATAT   
  
  
+ TTGTTATCAC TCTTAATATC TGTATTCATA GTAAATGAAA ATAAGTATCG ATTTTTTATT AAACCTAAAA   
  
  
+ TCTTCTGTCA ATAATTTTTC TTCCGATAAA AAAAAAATTG GACCATATTA AATTAACATT AATAAATTCA   
  
  
+ TATAATTACT TTGAATGTCT GAAATATAAT GCTCTTCGGT ATATTAAGGT CTTATAAATT TCTCATTAAT   
  
  
+ TTGAAATCTC TTATAATATA TTGGGTCAAC AAGTGCTAGC TTGTTTTTTT TTCCTGGTTA ATTGCATTTA   
  
  
+ AAAAAATAAT TTTTAATTAC AATTGCAAGG GGAGGAATCA TTTACAAAAT ATCAAAAGTG GAAAAGGCAA   
  
  
+ AAAGAAACAA ATTATAAAAC AGAAAAAAGA TTAGAAAACG CAAACAAAGA CGAAATGAGG AACAGTAAGA   
  
  
+ ATCAGTAAAA AAATGTAGTG CAACGGTCAC ATACGGGAAG GCAAGCACAA CAATCATGAA AGAGACAGGC   
  
  
+ ACGTGCGTTC CTCGTGATCA GCAGCCTTCC CTACTTCCCC GCCAAGTGGA GTCCGTACTA ACTCCTGCCA   
  
  
+ TTCCCTCCCA ACGGTCATAT TCCGCCGACA TGCCTCAACG GTCAGATTTC CTGCCCCATC TGGACCGTCC   
  
  
+ GATCAAGATC CAACGGCCAT AAGGCGCACT CTCCAATACC CTAGTGCCCA TTTCTGGAAC AGCCCCAAAA   
  
  
+ ATCTCAGTAT TCGCCATCAT TTTTCAATAC TGGGGTTACA TAAACAGTCT AACAAGGAAG GACCCAACAC   
  
  
+ AGAGGAGAGA GAAAAAAGCT AGCTTTGATG GGGACAGCCA TGGATGGAGG TGAAGAGAGA GGAGGTTGGG   
  
  
+ ACCTTTTATT TTGCTCACTT TCCATTCTTA TATAAAAACA GCTCTAGTTT ATCTCTTATG GCTTTTTTGG   
  
  
+ GTTTGTTCAT AGAGAGGGGG GAATGTGATA TGATTTGATT TGATGTTAGA AATTCTCATT AGTTTTTTCT   
  
  
+ TTTTGTTTGC AATTTTGATG TGTTAGGGTT TGAGGATCAT CATTTTTGAG TAGGGAACTT TCTGGGTTTT   
  
  
+ GCTTGCTGAT TGATTGCCAA TGCTATGGAT TTCTTGGTGA GTCAATCTTC AATTGCTGAT CTGGGTTCGT   
  
  
+ TTGTTCTTCA GAGTTTCACC TGCCAATTTG ATCAAGTATG TCCCTTCACT TCTCCTGCTT CAATTCTCTC   
  
  
+ TCAAATTCAC GCATTTTTCC AATGAATTTC AAATAGGAGT CCTGGAATCC GATGATATTT TAGGTTTTTT   
  
  
+ CCTGACTTCA TTGCATTAGT TTGTTCATAT CTGTGACCAA TTCTTTCATT TGTTATATGA TCTGATTGTT   
  
  
+ TAGTTAGCTA TTCATTCATT GCATATACAT TGGTTTATTC ATTGCAGAGA CACTCACATA CACACACACA   
  
  
+ TACAGGGGAC ACTAGTATTT TTTCTCTCTA ACTCATCGGA TTCCCTTTGA TACTCTGTTT TTTGGGGGGT   
  
  
+ TGATTTGATT TGATTTGATT GACCTTAATT TTGAACTCAC ACACACTCGC ACATAGATAT ATTCTTTCTG   
  
  
+ TTTGTGACAT ATACAGCATC ATATAGAAAT ATATTGCACT GCATATTTGA GCTCAAGACA GGGATATCAT   
  
  
+ CACAAACTGA GTGTGAGGGA GGCAAAAAAC AGAGGAAAAG AAGGGAATTG GGAGTGTAAT CAAAACAAGA   
  
  
+ GAGAGATTGA GGAGAGAGGA AAAAACAAAA TTGGGAGAAG GTTAATAGAG AAGAATGTTG GCTGGGTGTT   
  
  
+ CATCCACATT GCTGTCACCA AGGTATAGAT TGAGGAGTGA AGCTACATCA CAGTTCCAAG CCTGCCATCA   
  
  
+ TCCAATGAGC ACACAGAGAA TTGATTTGCC CTGCACTTTC CCAAGAAAGG ATGTTTCGAA GCCACAATCG   
  
  
+ GTTCGCCCCG TTGGCCTATC TGTCGAGAAG CCGGTTGAAG TCAGGGCCCT GAAGCAGACC ATCCGAGTCC   
  
  
+ CACCATCGCC GGAGGGTAGA AGAGAGATTA AGGCAGATTT CTGGGGTGAT AGAAGAAAGA GCTTGAAGAG   
  
  
+ GAGACTAGCA GAACAGGGGA GCTCTTTTGA TGATGGTGGT GACGAATCTT GTGTTGGGAG AACCAAAAGG   
  
  
+ AAGAAGGGTA GCTTTGATTT TGGTGAATCT GATGAAATTT CTCCAAAAAT TGAGGACACA ATGAGTTTTG   
  
  
+ GACATCTGGG TAGTGGTGGA AATTTCTGGG TTCATCCTGG TTTTGGTGTT GTTAATGTTA ATACTAATTA   
  
  
+ CCCTCAAGTG CCATTTTCTC TCACTTGTTC AGGGGAGGAA GAAAGAGTAT GTTTTGTTCC TACTGATGTG   
  
  
+ ATCTCACCAG CCATCATGCC TCCATTGTCA CACAATCCTT GGGTTGAATC TGGTGTTACT GAGGTCACAG   
  
  
+ AATATGGTGG GGGTGACAAA GACGGCGAAC CTAGTCATGG GTTTGTGAGG GGTACAACAA CAACGTCCGG   
  
  
+ GTCAAGTTCA TCTTCTGAGA GTCATAGTTT TGGGCATAGG CTCAATGAGA GCTCATCTGA CCCTGAAATC   
  
  
+ AGAAATGGTT CCATGTTGCC TAACCCTGGT CATGGTCCTG GCACCAGTCA TGGTCCTGGA ACCGGTCTGG   
  
  
+ CTCACAACCA CAATGATCAA ACCGAGCAAC AAGGGTTCGA GCTCATTAGC TTTCTCATGG GTTGTGTCGA   
  
  
+ AGCGATCAGT TCAAGGAACA TTGCAGCCAT CAATCATTTC ATAGCTAAGC TGGGCGAGCA GGCTTCTCCA   
  
  
+ AGGGCTCGGT CTGCCATTAG CCGCCTCACA GCCTACTTCA CCGAAGCATT AGCCTTGAGG GTCACAAGAT   
  
  
+ TTTGGCCTCA TATCTTTCAC ATAAGTATCC CTCGAGAGTT TGATCGATTT GATGATGAAT CGGGGGCAGC   
  
  
+ TGCAATGAGG CTTTTGAATC AGATCAGCCC AATTCCAAAG TTCGTTCATT TTACAGCCAA TGAGATGTTA   
  
  
+ TTGAGGGCAT TTGAAGGGAA GGACAAGGTG CATATCATAG ACTTCGACAT CAAGCAAGGC TTACAATGGC   
  
  
+ CCGGGTTTTT CCAAAGCTTA GCCATGAGGG AAAACCCCCC GAGCCATGTC AGGATAACAG GCGTAGGGGA   
  
  
+ TTCGAAGCAA GAATTGGTTG AGACAGGAGA AAGACTAGCC GGGTTTGCTG GGGCATTCAA CCTCTCCTTC   
  
  
+ GAGTTCCACC CAGTTGTGGA CCGGTTGGAA GATGTTAGGC TATGGATGCT TCATGTTAAG GAAGGTGAAA   
  
  
+ GTGTCGCGGT AAATTGCATT TTGCAGCTCC ACAAGACACT CTATGACCCC CATGGTGCCA CATTCAGGGA   
  
  
+ CTTCATGGGT TTAATCCGAA GCACAAATCC CATAGCATTG GTTATGGCTG AGCAAGAAGC TGATCACAAC   
  
  
+ GAACCCACCT TAGAAGGCCG AGTATGCAAC TCACTTGGAT ACTATGCAGC CCTTTTCGAT GCCATAGATT   
  
  
+ GCAGCCTTCC TTTCGAGAGC TCAGCAAGGT TGAAGATCGA AGAGATGTTT GGCCGGGAAA TCAGGAACAT   
  
  
+ AATAGCCTGT GAAGGGGCAG ACAGGATCGA AAGACATGAG AAGTTCGAGA AATGGAAGAG AAGGATCGAG   
  
  
+ CAGGAAGGGT TCCGGTGCAT GCGGACTAGC GAAAGGGAGG TGATGCAGAG CCAAATGCTG CTTAAGATGT   
  
  
+ ACTCGAACGA GAGCTACAAC ATGCAAAGAC AAGGCAATGA AGAAGCAATA TCACTAGTAT GGTTAGATCA   
  
  
+ GCCACTCTAC ACTGTTTCTT TGTGGGCTCC AAGTGAATTT GCTGCAGGGA GTTCTTCCAG TTTTTCTCTG   
  
  
+ CCATCTTG  

- -Up\_Stream \_Len000TATTAC CCACTATTGA AAACTTCCTG TTATATGTTT AGTGAATAAA TAATTTCAGT   
  
  
- TCCCCTGTAA AGTTGATATT GTAGTAACAA ATATTTCATA AACTTTTAAC GTTGAGAACA TTATCTATTA   
  
  
- TTACGAAATA TCTGAAGTGG TTAGTGATTA AACATCTTAG TTTTTTAATT AACTTTTCGT TTGTTTTCCT   
  
  
- TTTCCGTGTA TTTGGATTTA ATACATTTGT TTACTCTTGT ATATACGGTC GAATAGTTGC AATTAATATA   
  
  
- AACAATAGTG AGAATTATAG ACATAAGTAT CATTTACTTT TATTCATAGC TAAAAAATAA TTTGGATTTT   
  
  
- AGAAGACAGT TATTAAAAAG AAGGCTATTT TTTTTTTAAC CTGGTATAAT TTAATTGTAA TTATTTAAGT   
  
  
- ATATTAATGA AACTTACAGA CTTTATATTA CGAGAAGCCA TATAATTCCA GAATATTTAA AGAGTAATTA   
  
  
- AACTTTAGAG AATATTATAT AACCCAGTTG TTCACGATCG AACAAAAAAA AAGGACCAAT TAACGTAAAT   
  
  
- TTTTTTATTA AAAATTAATG TTAACGTTCC CCTCCTTAGT AAATGTTTTA TAGTTTTCAC CTTTTCCGTT   
  
  
- TTTCTTTGTT TAATATTTTG TCTTTTTTCT AATCTTTTGC GTTTGTTTCT GCTTTACTCC TTGTCATTCT   
  
  
- TAGTCATTTT TTTACATCAC GTTGCCAGTG TATGCCCTTC CGTTCGTGTT GTTAGTACTT TCTCTGTCCG   
  
  
- TGCACGCAAG GAGCACTAGT CGTCGGAAGG GATGAAGGGG CGGTTCACCT CAGGCATGAT TGAGGACGGT   
  
  
- AAGGGAGGGT TGCCAGTATA AGGCGGCTGT ACGGAGTTGC CAGTCTAAAG GACGGGGTAG ACCTGGCAGG   
  
  
- CTAGTTCTAG GTTGCCGGTA TTCCGCGTGA GAGGTTATGG GATCACGGGT AAAGACCTTG TCGGGGTTTT   
  
  
- TAGAGTCATA AGCGGTAGTA AAAAGTTATG ACCCCAATGT ATTTGTCAGA TTGTTCCTTC CTGGGTTGTG   
  
  
- TCTCCTCTCT CTTTTTTCGA TCGAAACTAC CCCTGTCGGT ACCTACCTCC ACTTCTCTCT CCTCCAACCC   
  
  
- TGGAAAATAA AACGAGTGAA AGGTAAGAAT ATATTTTTGT CGAGATCAAA TAGAGAATAC CGAAAAAACC   
  
  
- CAAACAAGTA TCTCTCCCCC CTTACACTAT ACTAAACTAA ACTACAATCT TTAAGAGTAA TCAAAAAAGA   
  
  
- AAAACAAACG TTAAAACTAC ACAATCCCAA ACTCCTAGTA GTAAAAACTC ATCCCTTGAA AGACCCAAAA   
  
  
- CGAACGACTA ACTAACGGTT ACGATACCTA AAGAACCACT CAGTTAGAAG TTAACGACTA GACCCAAGCA   
  
  
- AACAAGAAGT CTCAAAGTGG ACGGTTAAAC TAGTTCATAC AGGGAAGTGA AGAGGACGAA GTTAAGAGAG   
  
  
- AGTTTAAGTG CGTAAAAAGG TTACTTAAAG TTTATCCTCA GGACCTTAGG CTACTATAAA ATCCAAAAAA   
  
  
- GGACTGAAGT AACGTAATCA AACAAGTATA GACACTGGTT AAGAAAGTAA ACAATATACT AGACTAACAA   
  
  
- ATCAATCGAT AAGTAAGTAA CGTATATGTA ACCAAATAAG TAACGTCTCT GTGAGTGTAT GTGTGTGTGT   
  
  
- ATGTCCCCTG TGATCATAAA AAAGAGAGAT TGAGTAGCCT AAGGGAAACT ATGAGACAAA AAACCCCCCA   
  
  
- ACTAAACTAA ACTAAACTAA CTGGAATTAA AACTTGAGTG TGTGTGAGCG TGTATCTATA TAAGAAAGAC   
  
  
- AAACACTGTA TATGTCGTAG TATATCTTTA TATAACGTGA CGTATAAACT CGAGTTCTGT CCCTATAGTA   
  
  
- GTGTTTGACT CACACTCCCT CCGTTTTTTG TCTCCTTTTC TTCCCTTAAC CCTCACATTA GTTTTGTTCT   
  
  
- CTCTCTAACT CCTCTCTCCT TTTTTGTTTT AACCCTCTTC CAATTATCTC TTCTTACAAC CGACCCACAA   
  
  
- GTAGGTGTAA CGACAGTGGT TCCATATCTA ACTCCTCACT TCGATGTAGT GTCAAGGTTC GGACGGTAGT   
  
  
- AGGTTACTCG TGTGTCTCTT AACTAAACGG GACGTGAAAG GGTTCTTTCC TACAAAGCTT CGGTGTTAGC   
  
  
- CAAGCGGGGC AACCGGATAG ACAGCTCTTC GGCCAACTTC AGTCCCGGGA CTTCGTCTGG TAGGCTCAGG   
  
  
- GTGGTAGCGG CCTCCCATCT TCTCTCTAAT TCCGTCTAAA GACCCCACTA TCTTCTTTCT CGAACTTCTC   
  
  
- CTCTGATCGT CTTGTCCCCT CGAGAAAACT ACTACCACCA CTGCTTAGAA CACAACCCTC TTGGTTTTCC   
  
  
- TTCTTCCCAT CGAAACTAAA ACCACTTAGA CTACTTTAAA GAGGTTTTTA ACTCCTGTGT TACTCAAAAC   
  
  
- CTGTAGACCC ATCACCACCT TTAAAGACCC AAGTAGGACC AAAACCACAA CAATTACAAT TATGATTAAT   
  
  
- GGGAGTTCAC GGTAAAAGAG AGTGAACAAG TCCCCTCCTT CTTTCTCATA CAAAACAAGG ATGACTACAC   
  
  
- TAGAGTGGTC GGTAGTACGG AGGTAACAGT GTGTTAGGAA CCCAACTTAG ACCACAATGA CTCCAGTGTC   
  
  
- TTATACCACC CCCACTGTTT CTGCCGCTTG GATCAGTACC CAAACACTCC CCATGTTGTT GTTGCAGGCC   
  
  
- CAGTTCAAGT AGAAGACTCT CAGTATCAAA ACCCGTATCC GAGTTACTCT CGAGTAGACT GGGACTTTAG   
  
  
- TCTTTACCAA GGTACAACGG ATTGGGACCA GTACCAGGAC CGTGGTCAGT ACCAGGACCT TGGCCAGACC   
  
  
- GAGTGTTGGT GTTACTAGTT TGGCTCGTTG TTCCCAAGCT CGAGTAATCG AAAGAGTACC CAACACAGCT   
  
  
- TCGCTAGTCA AGTTCCTTGT AACGTCGGTA GTTAGTAAAG TATCGATTCG ACCCGCTCGT CCGAAGAGGT   
  
  
- TCCCGAGCCA GACGGTAATC GGCGGAGTGT CGGATGAAGT GGCTTCGTAA TCGGAACTCC CAGTGTTCTA   
  
  
- AAACCGGAGT ATAGAAAGTG TATTCATAGG GAGCTCTCAA ACTAGCTAAA CTACTACTTA GCCCCCGTCG   
  
  
- ACGTTACTCC GAAAACTTAG TCTAGTCGGG TTAAGGTTTC AAGCAAGTAA AATGTCGGTT ACTCTACAAT   
  
  
- AACTCCCGTA AACTTCCCTT CCTGTTCCAC GTATAGTATC TGAAGCTGTA GTTCGTTCCG AATGTTACCG   
  
  
- GGCCCAAAAA GGTTTCGAAT CGGTACTCCC TTTTGGGGGG CTCGGTACAG TCCTATTGTC CGCATCCCCT   
  
  
- AAGCTTCGTT CTTAACCAAC TCTGTCCTCT TTCTGATCGG CCCAAACGAC CCCGTAAGTT GGAGAGGAAG   
  
  
- CTCAAGGTGG GTCAACACCT GGCCAACCTT CTACAATCCG ATACCTACGA AGTACAATTC CTTCCACTTT   
  
  
- CACAGCGCCA TTTAACGTAA AACGTCGAGG TGTTCTGTGA GATACTGGGG GTACCACGGT GTAAGTCCCT   
  
  
- GAAGTACCCA AATTAGGCTT CGTGTTTAGG GTATCGTAAC CAATACCGAC TCGTTCTTCG ACTAGTGTTG   
  
  
- CTTGGGTGGA ATCTTCCGGC TCATACGTTG AGTGAACCTA TGATACGTCG GGAAAAGCTA CGGTATCTAA   
  
  
- CGTCGGAAGG AAAGCTCTCG AGTCGTTCCA ACTTCTAGCT TCTCTACAAA CCGGCCCTTT AGTCCTTGTA   
  
  
- TTATCGGACA CTTCCCCGTC TGTCCTAGCT TTCTGTACTC TTCAAGCTCT TTACCTTCTC TTCCTAGCTC   
  
  
- GTCCTTCCCA AGGCCACGTA CGCCTGATCG CTTTCCCTCC ACTACGTCTC GGTTTACGAC GAATTCTACA   
  
  
- TGAGCTTGCT CTCGATGTTG TACGTTTCTG TTCCGTTACT TCTTCGTTAT AGTGATCATA CCAATCTAGT   
  
  
- CGGTGAGATG TGACAAAGAA ACACCCGAGG TTCACTTAAA CGACGTCCCT CAAGAAGGTC AAAAAGAGAC   
  
  
- GGTAGAAC

+     CAAT-box

| Site Name | Organism | Position | Strand | Matrix score. | sequence | function |
| --- | --- | --- | --- | --- | --- | --- |
| CAAT-box | Pisum sativum | 3906 | + | 5 | CAAAT | common cis-acting element in promoter and enhancer regions |
| CAAT-box | Nicotiana glutinosa | 2618 | - | 4 | CAAT |  |
| CAAT-box | Arabidopsis thaliana | 3611 | - | 5 | CCAAT | common cis-acting element in promoter and enhancer regions |
| CAAT-box | Nicotiana glutinosa | 3970 | + | 4 | CAAT |  |
| CAAT-box | Nicotiana glutinosa | 2443 | + | 4 | CAAT |  |
| CAAT-box | Nicotiana glutinosa | 3712 | - | 4 | CAAT |  |
| CAAT-box | Nicotiana glutinosa | 1970 | - | 4 | CAAT |  |
| CAAT-box | Pisum sativum | 283 | - | 5 | CAAAT | common cis-acting element in promoter and enhancer regions |
| CAAT-box | Pisum sativum | 243 | + | 5 | CAAAT | common cis-acting element in promoter and enhancer regions |
| CAAT-box | Nicotiana glutinosa | 1554 | - | 4 | CAAT |  |
| CAAT-box | Nicotiana glutinosa | 164 | + | 4 | CAAT |  |
| CAAT-box | Nicotiana glutinosa | 3959 | + | 4 | CAAT |  |
| CAAT-box | Arabidopsis thaliana | 3377 | - | 5 | CCAAT | common cis-acting element in promoter and enhancer regions |
| CAAT-box | Nicotiana glutinosa | 194 | - | 4 | CAAT |  |
| CAAT-box | Pisum sativum | 3131 | - | 5 | CAAAT | common cis-acting element in promoter and enhancer regions |
| CAAT-box | Nicotiana glutinosa | 3157 | + | 4 | CAAT |  |
| CAAT-box | Pisum sativum | 1869 | - | 5 | CAAAT | common cis-acting element in promoter and enhancer regions |
| CAAT-box | Nicotiana glutinosa | 1582 | + | 4 | CAAT |  |
| CAAT-box | Nicotiana glutinosa | 3288 | + | 4 | CAAT |  |
| CAAT-box | Pisum sativum | 3233 | - | 5 | CAAAT | common cis-acting element in promoter and enhancer regions |
| CAAT-box | Arabidopsis thaliana | 1493 | + | 5 | CCAAT | common cis-acting element in promoter and enhancer regions |
| CAAT-box | Pisum sativum | 1476 | + | 5 | CAAAT | common cis-acting element in promoter and enhancer regions |
| CAAT-box | Pisum sativum | 4031 | - | 5 | CAAAT | common cis-acting element in promoter and enhancer regions |
| CAAT-box | Nicotiana glutinosa | 1772 | - | 4 | CAAT |  |
| CAAT-box | Arabidopsis thaliana | 3211 | + | 5 | CCAAT | common cis-acting element in promoter and enhancer regions |
| CAAT-box | Pisum sativum | 1762 | - | 5 | CAAAT | common cis-acting element in promoter and enhancer regions |
| CAAT-box | Pisum sativum | 1757 | - | 5 | CAAAT | common cis-acting element in promoter and enhancer regions |
| CAAT-box | Nicotiana glutinosa | 1343 | - | 4 | CAAT |  |
| CAAT-box | Arabidopsis thaliana | 1427 | + | 5 | CCAAT | common cis-acting element in promoter and enhancer regions |
| CAAT-box | Nicotiana glutinosa | 1384 | + | 4 | CAAT |  |
| CAAT-box | Nicotiana glutinosa | 1377 | + | 4 | CAAT |  |
| CAAT-box | Pisum sativum | 51 | + | 5 | CAAAT | common cis-acting element in promoter and enhancer regions |
| CAAT-box | Nicotiana glutinosa | 3517 | - | 4 | CAAT |  |
| CAAT-box | Nicotiana glutinosa | 121 | - | 4 | CAAT |  |
| CAAT-box | Nicotiana glutinosa | 44 | + | 4 | CAAT |  |
| CAAT-box | Pisum sativum | 113 | - | 5 | CAAAT | common cis-acting element in promoter and enhancer regions |
| CAAT-box | Pisum sativum | 1227 | - | 5 | CAAAT | common cis-acting element in promoter and enhancer regions |
| CAAT-box | Arabidopsis thaliana | 1351 | + | 5 | CCAAT | common cis-acting element in promoter and enhancer regions |
| CAAT-box | Nicotiana glutinosa | 1009 | + | 4 | CAAT |  |
| CAAT-box | Nicotiana glutinosa | 2964 | - | 4 | CAAT |  |
| CAAT-box | Pisum sativum | 1504 | + | 5 | CAAAT | common cis-acting element in promoter and enhancer regions |
| CAAT-box | Arabidopsis thaliana | 514 | - | 5 | CCAAT | common cis-acting element in promoter and enhancer regions |
| CAAT-box | Nicotiana glutinosa | 555 | - | 4 | CAAT |  |
| CAAT-box | Arabidopsis thaliana | 391 | - | 5 | CCAAT | common cis-acting element in promoter and enhancer regions |
| CAAT-box | Nicotiana glutinosa | 2975 | + | 4 | CAAT |  |
| CAAT-box | Nicotiana glutinosa | 1352 | + | 4 | CAAT |  |
| CAAT-box | Arabidopsis thaliana | 163 | + | 5 | CCAAT | common cis-acting element in promoter and enhancer regions |
| CAAT-box | Nicotiana glutinosa | 1857 | - | 4 | CAAT |  |
| CAAT-box | Nicotiana glutinosa | 584 | + | 4 | CAAT |  |
| CAAT-box | Pisum sativum | 1767 | - | 5 | CAAAT | common cis-acting element in promoter and enhancer regions |
| CAAT-box | Arabidopsis thaliana | 1643 | - | 5 | CCAAT | common cis-acting element in promoter and enhancer regions |
| CAAT-box | Nicotiana glutinosa | 586 | - | 4 | CAAT |  |
| CAAT-box | Pisum sativum | 173 | - | 5 | CAAAT | common cis-acting element in promoter and enhancer regions |
| CAAT-box | Nicotiana glutinosa | 1609 | - | 4 | CAAT |  |
| CAAT-box | Pisum sativum | 1592 | - | 5 | CAAAT | common cis-acting element in promoter and enhancer regions |
| CAAT-box | Pisum sativum | 3598 | + | 5 | CAAAT | common cis-acting element in promoter and enhancer regions |
| CAAT-box | Nicotiana glutinosa | 99 | - | 4 | CAAT |  |
| CAAT-box | Nicotiana glutinosa | 2433 | - | 4 | CAAT |  |
| CAAT-box | Arabidopsis thaliana | 947 | + | 5 | CCAAT | common cis-acting element in promoter and enhancer regions |
| CAAT-box | Arabidopsis thaliana | 3183 | + | 5 | CCAAT | common cis-acting element in promoter and enhancer regions |
| CAAT-box | Pisum sativum | 642 | + | 5 | CAAAT | common cis-acting element in promoter and enhancer regions |
| CAAT-box | Arabidopsis thaliana | 1941 | - | 5 | CCAAT | common cis-acting element in promoter and enhancer regions |
| CAAT-box | Nicotiana glutinosa | 363 | + | 4 | CAAT |  |
| CAAT-box | Nicotiana glutinosa | 1428 | + | 4 | CAAT |  |
| CAAT-box | Nicotiana glutinosa | 2777 | + | 4 | CAAT |  |
| CAAT-box | Pisum sativum | 493 | - | 5 | CAAAT | common cis-acting element in promoter and enhancer regions |
| CAAT-box | Nicotiana glutinosa | 2063 | - | 4 | CAAT |  |
| CAAT-box | Nicotiana glutinosa | 755 | + | 4 | CAAT |  |
| CAAT-box | Nicotiana glutinosa | 948 | + | 4 | CAAT |  |
| CAAT-box | Nicotiana glutinosa | 1274 | + | 4 | CAAT |  |
| CAAT-box | Nicotiana glutinosa | 3224 | - | 4 | CAAT |  |
| CAAT-box | Nicotiana glutinosa | 2042 | - | 4 | CAAT |  |
| CAAT-box | Nicotiana glutinosa | 1465 | + | 4 | CAAT |  |
| CAAT-box | Nicotiana glutinosa | 1655 | - | 4 | CAAT |  |
| CAAT-box | Nicotiana glutinosa | 1632 | - | 4 | CAAT |  |
| CAAT-box | Nicotiana glutinosa | 2885 | + | 4 | CAAT |  |
| CAAT-box | Nicotiana glutinosa | 1386 | - | 4 | CAAT |  |
| CAAT-box | Nicotiana glutinosa | 1494 | + | 4 | CAAT |  |
| CAAT-box | Nicotiana glutinosa | 3184 | + | 4 | CAAT |  |
| CAAT-box | Pisum sativum | 1232 | - | 5 | CAAAT | common cis-acting element in promoter and enhancer regions |
| CAAT-box | Nicotiana glutinosa | 2124 | - | 4 | CAAT |  |
| CAAT-box | Nicotiana glutinosa | 2627 | + | 4 | CAAT |  |
| CAAT-box | Arabidopsis thaliana | 1581 | + | 5 | CCAAT | common cis-acting element in promoter and enhancer regions |
| CAAT-box | Nicotiana glutinosa | 3212 | + | 4 | CAAT |  |
| CAAT-box | Arabidopsis thaliana | 1992 | - | 8 | CCCAATTT | common cis-acting element in promoter and enhancer regions |
| CAAT-box | Pisum sativum | 2128 | - | 5 | CAAAT | common cis-acting element in promoter and enhancer regions |
| CAAT-box | Pisum sativum | 1430 | - | 5 | CAAAT | common cis-acting element in promoter and enhancer regions |
| CAAT-box | Nicotiana glutinosa | 1347 | - | 4 | CAAT |  |
| CAAT-box | Arabidopsis thaliana | 1994 | - | 5 | CCAAT | common cis-acting element in promoter and enhancer regions |
| CAAT-box | Nicotiana glutinosa | 2107 | + | 4 | CAAT |  |
| CAAT-box | Nicotiana glutinosa | 2169 | + | 4 | CAAT |  |
| CAAT-box | Arabidopsis thaliana | 2106 | + | 5 | CCAAT | common cis-acting element in promoter and enhancer regions |

>HU04G00148.1   
+ -Up\_Stream \_Len000ATAATG GGTGATAACT TTTGAAGGAC AATATACAAA TCACTTATTT ATTAAAGTCA   
  
  
+ AGGGGACATT TCAACTATAA CATCATTGTT TATAAAGTAT TTGAAAATTG CAACTCTTGT AATAGATAAT   
  
  
+ AATGCTTTAT AGACTTCACC AATCACTAAT TTGTAGAATC AAAAAATTAA TTGAAAAGCA AACAAAAGGA   
  
  
+ AAAGGCACAT AAACCTAAAT TATGTAAACA AATGAGAACA TATATGCCAG CTTATCAACG TTAATTATAT   
  
  
+ TTGTTATCAC TCTTAATATC TGTATTCATA GTAAATGAAA ATAAGTATCG ATTTTTTATT AAACCTAAAA   
  
  
+ TCTTCTGTCA ATAATTTTTC TTCCGATAAA AAAAAAATTG GACCATATTA AATTAACATT AATAAATTCA   
  
  
+ TATAATTACT TTGAATGTCT GAAATATAAT GCTCTTCGGT ATATTAAGGT CTTATAAATT TCTCATTAAT   
  
  
+ TTGAAATCTC TTATAATATA TTGGGTCAAC AAGTGCTAGC TTGTTTTTTT TTCCTGGTTA ATTGCATTTA   
  
  
+ AAAAAATAAT TTTTAATTAC AATTGCAAGG GGAGGAATCA TTTACAAAAT ATCAAAAGTG GAAAAGGCAA   
  
  
+ AAAGAAACAA ATTATAAAAC AGAAAAAAGA TTAGAAAACG CAAACAAAGA CGAAATGAGG AACAGTAAGA   
  
  
+ ATCAGTAAAA AAATGTAGTG CAACGGTCAC ATACGGGAAG GCAAGCACAA CAATCATGAA AGAGACAGGC   
  
  
+ ACGTGCGTTC CTCGTGATCA GCAGCCTTCC CTACTTCCCC GCCAAGTGGA GTCCGTACTA ACTCCTGCCA   
  
  
+ TTCCCTCCCA ACGGTCATAT TCCGCCGACA TGCCTCAACG GTCAGATTTC CTGCCCCATC TGGACCGTCC   
  
  
+ GATCAAGATC CAACGGCCAT AAGGCGCACT CTCCAATACC CTAGTGCCCA TTTCTGGAAC AGCCCCAAAA   
  
  
+ ATCTCAGTAT TCGCCATCAT TTTTCAATAC TGGGGTTACA TAAACAGTCT AACAAGGAAG GACCCAACAC   
  
  
+ AGAGGAGAGA GAAAAAAGCT AGCTTTGATG GGGACAGCCA TGGATGGAGG TGAAGAGAGA GGAGGTTGGG   
  
  
+ ACCTTTTATT TTGCTCACTT TCCATTCTTA TATAAAAACA GCTCTAGTTT ATCTCTTATG GCTTTTTTGG   
  
  
+ GTTTGTTCAT AGAGAGGGGG GAATGTGATA TGATTTGATT TGATGTTAGA AATTCTCATT AGTTTTTTCT   
  
  
+ TTTTGTTTGC AATTTTGATG TGTTAGGGTT TGAGGATCAT CATTTTTGAG TAGGGAACTT TCTGGGTTTT   
  
  
+ GCTTGCTGAT TGATTGCCAA TGCTATGGAT TTCTTGGTGA GTCAATCTTC AATTGCTGAT CTGGGTTCGT   
  
  
+ TTGTTCTTCA GAGTTTCACC TGCCAATTTG ATCAAGTATG TCCCTTCACT TCTCCTGCTT CAATTCTCTC   
  
  
+ TCAAATTCAC GCATTTTTCC AATGAATTTC AAATAGGAGT CCTGGAATCC GATGATATTT TAGGTTTTTT   
  
  
+ CCTGACTTCA TTGCATTAGT TTGTTCATAT CTGTGACCAA TTCTTTCATT TGTTATATGA TCTGATTGTT   
  
  
+ TAGTTAGCTA TTCATTCATT GCATATACAT TGGTTTATTC ATTGCAGAGA CACTCACATA CACACACACA   
  
  
+ TACAGGGGAC ACTAGTATTT TTTCTCTCTA ACTCATCGGA TTCCCTTTGA TACTCTGTTT TTTGGGGGGT   
  
  
+ TGATTTGATT TGATTTGATT GACCTTAATT TTGAACTCAC ACACACTCGC ACATAGATAT ATTCTTTCTG   
  
  
+ TTTGTGACAT ATACAGCATC ATATAGAAAT ATATTGCACT GCATATTTGA GCTCAAGACA GGGATATCAT   
  
  
+ CACAAACTGA GTGTGAGGGA GGCAAAAAAC AGAGGAAAAG AAGGGAATTG GGAGTGTAAT CAAAACAAGA   
  
  
+ GAGAGATTGA GGAGAGAGGA AAAAACAAAA TTGGGAGAAG GTTAATAGAG AAGAATGTTG GCTGGGTGTT   
  
  
+ CATCCACATT GCTGTCACCA AGGTATAGAT TGAGGAGTGA AGCTACATCA CAGTTCCAAG CCTGCCATCA   
  
  
+ TCCAATGAGC ACACAGAGAA TTGATTTGCC CTGCACTTTC CCAAGAAAGG ATGTTTCGAA GCCACAATCG   
  
  
+ GTTCGCCCCG TTGGCCTATC TGTCGAGAAG CCGGTTGAAG TCAGGGCCCT GAAGCAGACC ATCCGAGTCC   
  
  
+ CACCATCGCC GGAGGGTAGA AGAGAGATTA AGGCAGATTT CTGGGGTGAT AGAAGAAAGA GCTTGAAGAG   
  
  
+ GAGACTAGCA GAACAGGGGA GCTCTTTTGA TGATGGTGGT GACGAATCTT GTGTTGGGAG AACCAAAAGG   
  
  
+ AAGAAGGGTA GCTTTGATTT TGGTGAATCT GATGAAATTT CTCCAAAAAT TGAGGACACA ATGAGTTTTG   
  
  
+ GACATCTGGG TAGTGGTGGA AATTTCTGGG TTCATCCTGG TTTTGGTGTT GTTAATGTTA ATACTAATTA   
  
  
+ CCCTCAAGTG CCATTTTCTC TCACTTGTTC AGGGGAGGAA GAAAGAGTAT GTTTTGTTCC TACTGATGTG   
  
  
+ ATCTCACCAG CCATCATGCC TCCATTGTCA CACAATCCTT GGGTTGAATC TGGTGTTACT GAGGTCACAG   
  
  
+ AATATGGTGG GGGTGACAAA GACGGCGAAC CTAGTCATGG GTTTGTGAGG GGTACAACAA CAACGTCCGG   
  
  
+ GTCAAGTTCA TCTTCTGAGA GTCATAGTTT TGGGCATAGG CTCAATGAGA GCTCATCTGA CCCTGAAATC   
  
  
+ AGAAATGGTT CCATGTTGCC TAACCCTGGT CATGGTCCTG GCACCAGTCA TGGTCCTGGA ACCGGTCTGG   
  
  
+ CTCACAACCA CAATGATCAA ACCGAGCAAC AAGGGTTCGA GCTCATTAGC TTTCTCATGG GTTGTGTCGA   
  
  
+ AGCGATCAGT TCAAGGAACA TTGCAGCCAT CAATCATTTC ATAGCTAAGC TGGGCGAGCA GGCTTCTCCA   
  
  
+ AGGGCTCGGT CTGCCATTAG CCGCCTCACA GCCTACTTCA CCGAAGCATT AGCCTTGAGG GTCACAAGAT   
  
  
+ TTTGGCCTCA TATCTTTCAC ATAAGTATCC CTCGAGAGTT TGATCGATTT GATGATGAAT CGGGGGCAGC   
  
  
+ TGCAATGAGG CTTTTGAATC AGATCAGCCC AATTCCAAAG TTCGTTCATT TTACAGCCAA TGAGATGTTA   
  
  
+ TTGAGGGCAT TTGAAGGGAA GGACAAGGTG CATATCATAG ACTTCGACAT CAAGCAAGGC TTACAATGGC   
  
  
+ CCGGGTTTTT CCAAAGCTTA GCCATGAGGG AAAACCCCCC GAGCCATGTC AGGATAACAG GCGTAGGGGA   
  
  
+ TTCGAAGCAA GAATTGGTTG AGACAGGAGA AAGACTAGCC GGGTTTGCTG GGGCATTCAA CCTCTCCTTC   
  
  
+ GAGTTCCACC CAGTTGTGGA CCGGTTGGAA GATGTTAGGC TATGGATGCT TCATGTTAAG GAAGGTGAAA   
  
  
+ GTGTCGCGGT AAATTGCATT TTGCAGCTCC ACAAGACACT CTATGACCCC CATGGTGCCA CATTCAGGGA   
  
  
+ CTTCATGGGT TTAATCCGAA GCACAAATCC CATAGCATTG GTTATGGCTG AGCAAGAAGC TGATCACAAC   
  
  
+ GAACCCACCT TAGAAGGCCG AGTATGCAAC TCACTTGGAT ACTATGCAGC CCTTTTCGAT GCCATAGATT   
  
  
+ GCAGCCTTCC TTTCGAGAGC TCAGCAAGGT TGAAGATCGA AGAGATGTTT GGCCGGGAAA TCAGGAACAT   
  
  
+ AATAGCCTGT GAAGGGGCAG ACAGGATCGA AAGACATGAG AAGTTCGAGA AATGGAAGAG AAGGATCGAG   
  
  
+ CAGGAAGGGT TCCGGTGCAT GCGGACTAGC GAAAGGGAGG TGATGCAGAG CCAAATGCTG CTTAAGATGT   
  
  
+ ACTCGAACGA GAGCTACAAC ATGCAAAGAC AAGGCAATGA AGAAGCAATA TCACTAGTAT GGTTAGATCA   
  
  
+ GCCACTCTAC ACTGTTTCTT TGTGGGCTCC AAGTGAATTT GCTGCAGGGA GTTCTTCCAG TTTTTCTCTG   
  
  
+ CCATCTTG  

- -Up\_Stream \_Len000TATTAC CCACTATTGA AAACTTCCTG TTATATGTTT AGTGAATAAA TAATTTCAGT   
  
  
- TCCCCTGTAA AGTTGATATT GTAGTAACAA ATATTTCATA AACTTTTAAC GTTGAGAACA TTATCTATTA   
  
  
- TTACGAAATA TCTGAAGTGG TTAGTGATTA AACATCTTAG TTTTTTAATT AACTTTTCGT TTGTTTTCCT   
  
  
- TTTCCGTGTA TTTGGATTTA ATACATTTGT TTACTCTTGT ATATACGGTC GAATAGTTGC AATTAATATA   
  
  
- AACAATAGTG AGAATTATAG ACATAAGTAT CATTTACTTT TATTCATAGC TAAAAAATAA TTTGGATTTT   
  
  
- AGAAGACAGT TATTAAAAAG AAGGCTATTT TTTTTTTAAC CTGGTATAAT TTAATTGTAA TTATTTAAGT   
  
  
- ATATTAATGA AACTTACAGA CTTTATATTA CGAGAAGCCA TATAATTCCA GAATATTTAA AGAGTAATTA   
  
  
- AACTTTAGAG AATATTATAT AACCCAGTTG TTCACGATCG AACAAAAAAA AAGGACCAAT TAACGTAAAT   
  
  
- TTTTTTATTA AAAATTAATG TTAACGTTCC CCTCCTTAGT AAATGTTTTA TAGTTTTCAC CTTTTCCGTT   
  
  
- TTTCTTTGTT TAATATTTTG TCTTTTTTCT AATCTTTTGC GTTTGTTTCT GCTTTACTCC TTGTCATTCT   
  
  
- TAGTCATTTT TTTACATCAC GTTGCCAGTG TATGCCCTTC CGTTCGTGTT GTTAGTACTT TCTCTGTCCG   
  
  
- TGCACGCAAG GAGCACTAGT CGTCGGAAGG GATGAAGGGG CGGTTCACCT CAGGCATGAT TGAGGACGGT   
  
  
- AAGGGAGGGT TGCCAGTATA AGGCGGCTGT ACGGAGTTGC CAGTCTAAAG GACGGGGTAG ACCTGGCAGG   
  
  
- CTAGTTCTAG GTTGCCGGTA TTCCGCGTGA GAGGTTATGG GATCACGGGT AAAGACCTTG TCGGGGTTTT   
  
  
- TAGAGTCATA AGCGGTAGTA AAAAGTTATG ACCCCAATGT ATTTGTCAGA TTGTTCCTTC CTGGGTTGTG   
  
  
- TCTCCTCTCT CTTTTTTCGA TCGAAACTAC CCCTGTCGGT ACCTACCTCC ACTTCTCTCT CCTCCAACCC   
  
  
- TGGAAAATAA AACGAGTGAA AGGTAAGAAT ATATTTTTGT CGAGATCAAA TAGAGAATAC CGAAAAAACC   
  
  
- CAAACAAGTA TCTCTCCCCC CTTACACTAT ACTAAACTAA ACTACAATCT TTAAGAGTAA TCAAAAAAGA   
  
  
- AAAACAAACG TTAAAACTAC ACAATCCCAA ACTCCTAGTA GTAAAAACTC ATCCCTTGAA AGACCCAAAA   
  
  
- CGAACGACTA ACTAACGGTT ACGATACCTA AAGAACCACT CAGTTAGAAG TTAACGACTA GACCCAAGCA   
  
  
- AACAAGAAGT CTCAAAGTGG ACGGTTAAAC TAGTTCATAC AGGGAAGTGA AGAGGACGAA GTTAAGAGAG   
  
  
- AGTTTAAGTG CGTAAAAAGG TTACTTAAAG TTTATCCTCA GGACCTTAGG CTACTATAAA ATCCAAAAAA   
  
  
- GGACTGAAGT AACGTAATCA AACAAGTATA GACACTGGTT AAGAAAGTAA ACAATATACT AGACTAACAA   
  
  
- ATCAATCGAT AAGTAAGTAA CGTATATGTA ACCAAATAAG TAACGTCTCT GTGAGTGTAT GTGTGTGTGT   
  
  
- ATGTCCCCTG TGATCATAAA AAAGAGAGAT TGAGTAGCCT AAGGGAAACT ATGAGACAAA AAACCCCCCA   
  
  
- ACTAAACTAA ACTAAACTAA CTGGAATTAA AACTTGAGTG TGTGTGAGCG TGTATCTATA TAAGAAAGAC   
  
  
- AAACACTGTA TATGTCGTAG TATATCTTTA TATAACGTGA CGTATAAACT CGAGTTCTGT CCCTATAGTA   
  
  
- GTGTTTGACT CACACTCCCT CCGTTTTTTG TCTCCTTTTC TTCCCTTAAC CCTCACATTA GTTTTGTTCT   
  
  
- CTCTCTAACT CCTCTCTCCT TTTTTGTTTT AACCCTCTTC CAATTATCTC TTCTTACAAC CGACCCACAA   
  
  
- GTAGGTGTAA CGACAGTGGT TCCATATCTA ACTCCTCACT TCGATGTAGT GTCAAGGTTC GGACGGTAGT   
  
  
- AGGTTACTCG TGTGTCTCTT AACTAAACGG GACGTGAAAG GGTTCTTTCC TACAAAGCTT CGGTGTTAGC   
  
  
- CAAGCGGGGC AACCGGATAG ACAGCTCTTC GGCCAACTTC AGTCCCGGGA CTTCGTCTGG TAGGCTCAGG   
  
  
- GTGGTAGCGG CCTCCCATCT TCTCTCTAAT TCCGTCTAAA GACCCCACTA TCTTCTTTCT CGAACTTCTC   
  
  
- CTCTGATCGT CTTGTCCCCT CGAGAAAACT ACTACCACCA CTGCTTAGAA CACAACCCTC TTGGTTTTCC   
  
  
- TTCTTCCCAT CGAAACTAAA ACCACTTAGA CTACTTTAAA GAGGTTTTTA ACTCCTGTGT TACTCAAAAC   
  
  
- CTGTAGACCC ATCACCACCT TTAAAGACCC AAGTAGGACC AAAACCACAA CAATTACAAT TATGATTAAT   
  
  
- GGGAGTTCAC GGTAAAAGAG AGTGAACAAG TCCCCTCCTT CTTTCTCATA CAAAACAAGG ATGACTACAC   
  
  
- TAGAGTGGTC GGTAGTACGG AGGTAACAGT GTGTTAGGAA CCCAACTTAG ACCACAATGA CTCCAGTGTC   
  
  
- TTATACCACC CCCACTGTTT CTGCCGCTTG GATCAGTACC CAAACACTCC CCATGTTGTT GTTGCAGGCC   
  
  
- CAGTTCAAGT AGAAGACTCT CAGTATCAAA ACCCGTATCC GAGTTACTCT CGAGTAGACT GGGACTTTAG   
  
  
- TCTTTACCAA GGTACAACGG ATTGGGACCA GTACCAGGAC CGTGGTCAGT ACCAGGACCT TGGCCAGACC   
  
  
- GAGTGTTGGT GTTACTAGTT TGGCTCGTTG TTCCCAAGCT CGAGTAATCG AAAGAGTACC CAACACAGCT   
  
  
- TCGCTAGTCA AGTTCCTTGT AACGTCGGTA GTTAGTAAAG TATCGATTCG ACCCGCTCGT CCGAAGAGGT   
  
  
- TCCCGAGCCA GACGGTAATC GGCGGAGTGT CGGATGAAGT GGCTTCGTAA TCGGAACTCC CAGTGTTCTA   
  
  
- AAACCGGAGT ATAGAAAGTG TATTCATAGG GAGCTCTCAA ACTAGCTAAA CTACTACTTA GCCCCCGTCG   
  
  
- ACGTTACTCC GAAAACTTAG TCTAGTCGGG TTAAGGTTTC AAGCAAGTAA AATGTCGGTT ACTCTACAAT   
  
  
- AACTCCCGTA AACTTCCCTT CCTGTTCCAC GTATAGTATC TGAAGCTGTA GTTCGTTCCG AATGTTACCG   
  
  
- GGCCCAAAAA GGTTTCGAAT CGGTACTCCC TTTTGGGGGG CTCGGTACAG TCCTATTGTC CGCATCCCCT   
  
  
- AAGCTTCGTT CTTAACCAAC TCTGTCCTCT TTCTGATCGG CCCAAACGAC CCCGTAAGTT GGAGAGGAAG   
  
  
- CTCAAGGTGG GTCAACACCT GGCCAACCTT CTACAATCCG ATACCTACGA AGTACAATTC CTTCCACTTT   
  
  
- CACAGCGCCA TTTAACGTAA AACGTCGAGG TGTTCTGTGA GATACTGGGG GTACCACGGT GTAAGTCCCT   
  
  
- GAAGTACCCA AATTAGGCTT CGTGTTTAGG GTATCGTAAC CAATACCGAC TCGTTCTTCG ACTAGTGTTG   
  
  
- CTTGGGTGGA ATCTTCCGGC TCATACGTTG AGTGAACCTA TGATACGTCG GGAAAAGCTA CGGTATCTAA   
  
  
- CGTCGGAAGG AAAGCTCTCG AGTCGTTCCA ACTTCTAGCT TCTCTACAAA CCGGCCCTTT AGTCCTTGTA   
  
  
- TTATCGGACA CTTCCCCGTC TGTCCTAGCT TTCTGTACTC TTCAAGCTCT TTACCTTCTC TTCCTAGCTC   
  
  
- GTCCTTCCCA AGGCCACGTA CGCCTGATCG CTTTCCCTCC ACTACGTCTC GGTTTACGAC GAATTCTACA   
  
  
- TGAGCTTGCT CTCGATGTTG TACGTTTCTG TTCCGTTACT TCTTCGTTAT AGTGATCATA CCAATCTAGT   
  
  
- CGGTGAGATG TGACAAAGAA ACACCCGAGG TTCACTTAAA CGACGTCCCT CAAGAAGGTC AAAAAGAGAC   
  
  
- GGTAGAAC

+     CARE

| Site Name | Organism | Position | Strand | Matrix score. | sequence | function |
| --- | --- | --- | --- | --- | --- | --- |
| CARE | Oryza sativa | 3671 | + | 8 | CAACTCAC |  |

>HU04G00148.1   
+ -Up\_Stream \_Len000ATAATG GGTGATAACT TTTGAAGGAC AATATACAAA TCACTTATTT ATTAAAGTCA   
  
  
+ AGGGGACATT TCAACTATAA CATCATTGTT TATAAAGTAT TTGAAAATTG CAACTCTTGT AATAGATAAT   
  
  
+ AATGCTTTAT AGACTTCACC AATCACTAAT TTGTAGAATC AAAAAATTAA TTGAAAAGCA AACAAAAGGA   
  
  
+ AAAGGCACAT AAACCTAAAT TATGTAAACA AATGAGAACA TATATGCCAG CTTATCAACG TTAATTATAT   
  
  
+ TTGTTATCAC TCTTAATATC TGTATTCATA GTAAATGAAA ATAAGTATCG ATTTTTTATT AAACCTAAAA   
  
  
+ TCTTCTGTCA ATAATTTTTC TTCCGATAAA AAAAAAATTG GACCATATTA AATTAACATT AATAAATTCA   
  
  
+ TATAATTACT TTGAATGTCT GAAATATAAT GCTCTTCGGT ATATTAAGGT CTTATAAATT TCTCATTAAT   
  
  
+ TTGAAATCTC TTATAATATA TTGGGTCAAC AAGTGCTAGC TTGTTTTTTT TTCCTGGTTA ATTGCATTTA   
  
  
+ AAAAAATAAT TTTTAATTAC AATTGCAAGG GGAGGAATCA TTTACAAAAT ATCAAAAGTG GAAAAGGCAA   
  
  
+ AAAGAAACAA ATTATAAAAC AGAAAAAAGA TTAGAAAACG CAAACAAAGA CGAAATGAGG AACAGTAAGA   
  
  
+ ATCAGTAAAA AAATGTAGTG CAACGGTCAC ATACGGGAAG GCAAGCACAA CAATCATGAA AGAGACAGGC   
  
  
+ ACGTGCGTTC CTCGTGATCA GCAGCCTTCC CTACTTCCCC GCCAAGTGGA GTCCGTACTA ACTCCTGCCA   
  
  
+ TTCCCTCCCA ACGGTCATAT TCCGCCGACA TGCCTCAACG GTCAGATTTC CTGCCCCATC TGGACCGTCC   
  
  
+ GATCAAGATC CAACGGCCAT AAGGCGCACT CTCCAATACC CTAGTGCCCA TTTCTGGAAC AGCCCCAAAA   
  
  
+ ATCTCAGTAT TCGCCATCAT TTTTCAATAC TGGGGTTACA TAAACAGTCT AACAAGGAAG GACCCAACAC   
  
  
+ AGAGGAGAGA GAAAAAAGCT AGCTTTGATG GGGACAGCCA TGGATGGAGG TGAAGAGAGA GGAGGTTGGG   
  
  
+ ACCTTTTATT TTGCTCACTT TCCATTCTTA TATAAAAACA GCTCTAGTTT ATCTCTTATG GCTTTTTTGG   
  
  
+ GTTTGTTCAT AGAGAGGGGG GAATGTGATA TGATTTGATT TGATGTTAGA AATTCTCATT AGTTTTTTCT   
  
  
+ TTTTGTTTGC AATTTTGATG TGTTAGGGTT TGAGGATCAT CATTTTTGAG TAGGGAACTT TCTGGGTTTT   
  
  
+ GCTTGCTGAT TGATTGCCAA TGCTATGGAT TTCTTGGTGA GTCAATCTTC AATTGCTGAT CTGGGTTCGT   
  
  
+ TTGTTCTTCA GAGTTTCACC TGCCAATTTG ATCAAGTATG TCCCTTCACT TCTCCTGCTT CAATTCTCTC   
  
  
+ TCAAATTCAC GCATTTTTCC AATGAATTTC AAATAGGAGT CCTGGAATCC GATGATATTT TAGGTTTTTT   
  
  
+ CCTGACTTCA TTGCATTAGT TTGTTCATAT CTGTGACCAA TTCTTTCATT TGTTATATGA TCTGATTGTT   
  
  
+ TAGTTAGCTA TTCATTCATT GCATATACAT TGGTTTATTC ATTGCAGAGA CACTCACATA CACACACACA   
  
  
+ TACAGGGGAC ACTAGTATTT TTTCTCTCTA ACTCATCGGA TTCCCTTTGA TACTCTGTTT TTTGGGGGGT   
  
  
+ TGATTTGATT TGATTTGATT GACCTTAATT TTGAACTCAC ACACACTCGC ACATAGATAT ATTCTTTCTG   
  
  
+ TTTGTGACAT ATACAGCATC ATATAGAAAT ATATTGCACT GCATATTTGA GCTCAAGACA GGGATATCAT   
  
  
+ CACAAACTGA GTGTGAGGGA GGCAAAAAAC AGAGGAAAAG AAGGGAATTG GGAGTGTAAT CAAAACAAGA   
  
  
+ GAGAGATTGA GGAGAGAGGA AAAAACAAAA TTGGGAGAAG GTTAATAGAG AAGAATGTTG GCTGGGTGTT   
  
  
+ CATCCACATT GCTGTCACCA AGGTATAGAT TGAGGAGTGA AGCTACATCA CAGTTCCAAG CCTGCCATCA   
  
  
+ TCCAATGAGC ACACAGAGAA TTGATTTGCC CTGCACTTTC CCAAGAAAGG ATGTTTCGAA GCCACAATCG   
  
  
+ GTTCGCCCCG TTGGCCTATC TGTCGAGAAG CCGGTTGAAG TCAGGGCCCT GAAGCAGACC ATCCGAGTCC   
  
  
+ CACCATCGCC GGAGGGTAGA AGAGAGATTA AGGCAGATTT CTGGGGTGAT AGAAGAAAGA GCTTGAAGAG   
  
  
+ GAGACTAGCA GAACAGGGGA GCTCTTTTGA TGATGGTGGT GACGAATCTT GTGTTGGGAG AACCAAAAGG   
  
  
+ AAGAAGGGTA GCTTTGATTT TGGTGAATCT GATGAAATTT CTCCAAAAAT TGAGGACACA ATGAGTTTTG   
  
  
+ GACATCTGGG TAGTGGTGGA AATTTCTGGG TTCATCCTGG TTTTGGTGTT GTTAATGTTA ATACTAATTA   
  
  
+ CCCTCAAGTG CCATTTTCTC TCACTTGTTC AGGGGAGGAA GAAAGAGTAT GTTTTGTTCC TACTGATGTG   
  
  
+ ATCTCACCAG CCATCATGCC TCCATTGTCA CACAATCCTT GGGTTGAATC TGGTGTTACT GAGGTCACAG   
  
  
+ AATATGGTGG GGGTGACAAA GACGGCGAAC CTAGTCATGG GTTTGTGAGG GGTACAACAA CAACGTCCGG   
  
  
+ GTCAAGTTCA TCTTCTGAGA GTCATAGTTT TGGGCATAGG CTCAATGAGA GCTCATCTGA CCCTGAAATC   
  
  
+ AGAAATGGTT CCATGTTGCC TAACCCTGGT CATGGTCCTG GCACCAGTCA TGGTCCTGGA ACCGGTCTGG   
  
  
+ CTCACAACCA CAATGATCAA ACCGAGCAAC AAGGGTTCGA GCTCATTAGC TTTCTCATGG GTTGTGTCGA   
  
  
+ AGCGATCAGT TCAAGGAACA TTGCAGCCAT CAATCATTTC ATAGCTAAGC TGGGCGAGCA GGCTTCTCCA   
  
  
+ AGGGCTCGGT CTGCCATTAG CCGCCTCACA GCCTACTTCA CCGAAGCATT AGCCTTGAGG GTCACAAGAT   
  
  
+ TTTGGCCTCA TATCTTTCAC ATAAGTATCC CTCGAGAGTT TGATCGATTT GATGATGAAT CGGGGGCAGC   
  
  
+ TGCAATGAGG CTTTTGAATC AGATCAGCCC AATTCCAAAG TTCGTTCATT TTACAGCCAA TGAGATGTTA   
  
  
+ TTGAGGGCAT TTGAAGGGAA GGACAAGGTG CATATCATAG ACTTCGACAT CAAGCAAGGC TTACAATGGC   
  
  
+ CCGGGTTTTT CCAAAGCTTA GCCATGAGGG AAAACCCCCC GAGCCATGTC AGGATAACAG GCGTAGGGGA   
  
  
+ TTCGAAGCAA GAATTGGTTG AGACAGGAGA AAGACTAGCC GGGTTTGCTG GGGCATTCAA CCTCTCCTTC   
  
  
+ GAGTTCCACC CAGTTGTGGA CCGGTTGGAA GATGTTAGGC TATGGATGCT TCATGTTAAG GAAGGTGAAA   
  
  
+ GTGTCGCGGT AAATTGCATT TTGCAGCTCC ACAAGACACT CTATGACCCC CATGGTGCCA CATTCAGGGA   
  
  
+ CTTCATGGGT TTAATCCGAA GCACAAATCC CATAGCATTG GTTATGGCTG AGCAAGAAGC TGATCACAAC   
  
  
+ GAACCCACCT TAGAAGGCCG AGTATGCAAC TCACTTGGAT ACTATGCAGC CCTTTTCGAT GCCATAGATT   
  
  
+ GCAGCCTTCC TTTCGAGAGC TCAGCAAGGT TGAAGATCGA AGAGATGTTT GGCCGGGAAA TCAGGAACAT   
  
  
+ AATAGCCTGT GAAGGGGCAG ACAGGATCGA AAGACATGAG AAGTTCGAGA AATGGAAGAG AAGGATCGAG   
  
  
+ CAGGAAGGGT TCCGGTGCAT GCGGACTAGC GAAAGGGAGG TGATGCAGAG CCAAATGCTG CTTAAGATGT   
  
  
+ ACTCGAACGA GAGCTACAAC ATGCAAAGAC AAGGCAATGA AGAAGCAATA TCACTAGTAT GGTTAGATCA   
  
  
+ GCCACTCTAC ACTGTTTCTT TGTGGGCTCC AAGTGAATTT GCTGCAGGGA GTTCTTCCAG TTTTTCTCTG   
  
  
+ CCATCTTG  

- -Up\_Stream \_Len000TATTAC CCACTATTGA AAACTTCCTG TTATATGTTT AGTGAATAAA TAATTTCAGT   
  
  
- TCCCCTGTAA AGTTGATATT GTAGTAACAA ATATTTCATA AACTTTTAAC GTTGAGAACA TTATCTATTA   
  
  
- TTACGAAATA TCTGAAGTGG TTAGTGATTA AACATCTTAG TTTTTTAATT AACTTTTCGT TTGTTTTCCT   
  
  
- TTTCCGTGTA TTTGGATTTA ATACATTTGT TTACTCTTGT ATATACGGTC GAATAGTTGC AATTAATATA   
  
  
- AACAATAGTG AGAATTATAG ACATAAGTAT CATTTACTTT TATTCATAGC TAAAAAATAA TTTGGATTTT   
  
  
- AGAAGACAGT TATTAAAAAG AAGGCTATTT TTTTTTTAAC CTGGTATAAT TTAATTGTAA TTATTTAAGT   
  
  
- ATATTAATGA AACTTACAGA CTTTATATTA CGAGAAGCCA TATAATTCCA GAATATTTAA AGAGTAATTA   
  
  
- AACTTTAGAG AATATTATAT AACCCAGTTG TTCACGATCG AACAAAAAAA AAGGACCAAT TAACGTAAAT   
  
  
- TTTTTTATTA AAAATTAATG TTAACGTTCC CCTCCTTAGT AAATGTTTTA TAGTTTTCAC CTTTTCCGTT   
  
  
- TTTCTTTGTT TAATATTTTG TCTTTTTTCT AATCTTTTGC GTTTGTTTCT GCTTTACTCC TTGTCATTCT   
  
  
- TAGTCATTTT TTTACATCAC GTTGCCAGTG TATGCCCTTC CGTTCGTGTT GTTAGTACTT TCTCTGTCCG   
  
  
- TGCACGCAAG GAGCACTAGT CGTCGGAAGG GATGAAGGGG CGGTTCACCT CAGGCATGAT TGAGGACGGT   
  
  
- AAGGGAGGGT TGCCAGTATA AGGCGGCTGT ACGGAGTTGC CAGTCTAAAG GACGGGGTAG ACCTGGCAGG   
  
  
- CTAGTTCTAG GTTGCCGGTA TTCCGCGTGA GAGGTTATGG GATCACGGGT AAAGACCTTG TCGGGGTTTT   
  
  
- TAGAGTCATA AGCGGTAGTA AAAAGTTATG ACCCCAATGT ATTTGTCAGA TTGTTCCTTC CTGGGTTGTG   
  
  
- TCTCCTCTCT CTTTTTTCGA TCGAAACTAC CCCTGTCGGT ACCTACCTCC ACTTCTCTCT CCTCCAACCC   
  
  
- TGGAAAATAA AACGAGTGAA AGGTAAGAAT ATATTTTTGT CGAGATCAAA TAGAGAATAC CGAAAAAACC   
  
  
- CAAACAAGTA TCTCTCCCCC CTTACACTAT ACTAAACTAA ACTACAATCT TTAAGAGTAA TCAAAAAAGA   
  
  
- AAAACAAACG TTAAAACTAC ACAATCCCAA ACTCCTAGTA GTAAAAACTC ATCCCTTGAA AGACCCAAAA   
  
  
- CGAACGACTA ACTAACGGTT ACGATACCTA AAGAACCACT CAGTTAGAAG TTAACGACTA GACCCAAGCA   
  
  
- AACAAGAAGT CTCAAAGTGG ACGGTTAAAC TAGTTCATAC AGGGAAGTGA AGAGGACGAA GTTAAGAGAG   
  
  
- AGTTTAAGTG CGTAAAAAGG TTACTTAAAG TTTATCCTCA GGACCTTAGG CTACTATAAA ATCCAAAAAA   
  
  
- GGACTGAAGT AACGTAATCA AACAAGTATA GACACTGGTT AAGAAAGTAA ACAATATACT AGACTAACAA   
  
  
- ATCAATCGAT AAGTAAGTAA CGTATATGTA ACCAAATAAG TAACGTCTCT GTGAGTGTAT GTGTGTGTGT   
  
  
- ATGTCCCCTG TGATCATAAA AAAGAGAGAT TGAGTAGCCT AAGGGAAACT ATGAGACAAA AAACCCCCCA   
  
  
- ACTAAACTAA ACTAAACTAA CTGGAATTAA AACTTGAGTG TGTGTGAGCG TGTATCTATA TAAGAAAGAC   
  
  
- AAACACTGTA TATGTCGTAG TATATCTTTA TATAACGTGA CGTATAAACT CGAGTTCTGT CCCTATAGTA   
  
  
- GTGTTTGACT CACACTCCCT CCGTTTTTTG TCTCCTTTTC TTCCCTTAAC CCTCACATTA GTTTTGTTCT   
  
  
- CTCTCTAACT CCTCTCTCCT TTTTTGTTTT AACCCTCTTC CAATTATCTC TTCTTACAAC CGACCCACAA   
  
  
- GTAGGTGTAA CGACAGTGGT TCCATATCTA ACTCCTCACT TCGATGTAGT GTCAAGGTTC GGACGGTAGT   
  
  
- AGGTTACTCG TGTGTCTCTT AACTAAACGG GACGTGAAAG GGTTCTTTCC TACAAAGCTT CGGTGTTAGC   
  
  
- CAAGCGGGGC AACCGGATAG ACAGCTCTTC GGCCAACTTC AGTCCCGGGA CTTCGTCTGG TAGGCTCAGG   
  
  
- GTGGTAGCGG CCTCCCATCT TCTCTCTAAT TCCGTCTAAA GACCCCACTA TCTTCTTTCT CGAACTTCTC   
  
  
- CTCTGATCGT CTTGTCCCCT CGAGAAAACT ACTACCACCA CTGCTTAGAA CACAACCCTC TTGGTTTTCC   
  
  
- TTCTTCCCAT CGAAACTAAA ACCACTTAGA CTACTTTAAA GAGGTTTTTA ACTCCTGTGT TACTCAAAAC   
  
  
- CTGTAGACCC ATCACCACCT TTAAAGACCC AAGTAGGACC AAAACCACAA CAATTACAAT TATGATTAAT   
  
  
- GGGAGTTCAC GGTAAAAGAG AGTGAACAAG TCCCCTCCTT CTTTCTCATA CAAAACAAGG ATGACTACAC   
  
  
- TAGAGTGGTC GGTAGTACGG AGGTAACAGT GTGTTAGGAA CCCAACTTAG ACCACAATGA CTCCAGTGTC   
  
  
- TTATACCACC CCCACTGTTT CTGCCGCTTG GATCAGTACC CAAACACTCC CCATGTTGTT GTTGCAGGCC   
  
  
- CAGTTCAAGT AGAAGACTCT CAGTATCAAA ACCCGTATCC GAGTTACTCT CGAGTAGACT GGGACTTTAG   
  
  
- TCTTTACCAA GGTACAACGG ATTGGGACCA GTACCAGGAC CGTGGTCAGT ACCAGGACCT TGGCCAGACC   
  
  
- GAGTGTTGGT GTTACTAGTT TGGCTCGTTG TTCCCAAGCT CGAGTAATCG AAAGAGTACC CAACACAGCT   
  
  
- TCGCTAGTCA AGTTCCTTGT AACGTCGGTA GTTAGTAAAG TATCGATTCG ACCCGCTCGT CCGAAGAGGT   
  
  
- TCCCGAGCCA GACGGTAATC GGCGGAGTGT CGGATGAAGT GGCTTCGTAA TCGGAACTCC CAGTGTTCTA   
  
  
- AAACCGGAGT ATAGAAAGTG TATTCATAGG GAGCTCTCAA ACTAGCTAAA CTACTACTTA GCCCCCGTCG   
  
  
- ACGTTACTCC GAAAACTTAG TCTAGTCGGG TTAAGGTTTC AAGCAAGTAA AATGTCGGTT ACTCTACAAT   
  
  
- AACTCCCGTA AACTTCCCTT CCTGTTCCAC GTATAGTATC TGAAGCTGTA GTTCGTTCCG AATGTTACCG   
  
  
- GGCCCAAAAA GGTTTCGAAT CGGTACTCCC TTTTGGGGGG CTCGGTACAG TCCTATTGTC CGCATCCCCT   
  
  
- AAGCTTCGTT CTTAACCAAC TCTGTCCTCT TTCTGATCGG CCCAAACGAC CCCGTAAGTT GGAGAGGAAG   
  
  
- CTCAAGGTGG GTCAACACCT GGCCAACCTT CTACAATCCG ATACCTACGA AGTACAATTC CTTCCACTTT   
  
  
- CACAGCGCCA TTTAACGTAA AACGTCGAGG TGTTCTGTGA GATACTGGGG GTACCACGGT GTAAGTCCCT   
  
  
- GAAGTACCCA AATTAGGCTT CGTGTTTAGG GTATCGTAAC CAATACCGAC TCGTTCTTCG ACTAGTGTTG   
  
  
- CTTGGGTGGA ATCTTCCGGC TCATACGTTG AGTGAACCTA TGATACGTCG GGAAAAGCTA CGGTATCTAA   
  
  
- CGTCGGAAGG AAAGCTCTCG AGTCGTTCCA ACTTCTAGCT TCTCTACAAA CCGGCCCTTT AGTCCTTGTA   
  
  
- TTATCGGACA CTTCCCCGTC TGTCCTAGCT TTCTGTACTC TTCAAGCTCT TTACCTTCTC TTCCTAGCTC   
  
  
- GTCCTTCCCA AGGCCACGTA CGCCTGATCG CTTTCCCTCC ACTACGTCTC GGTTTACGAC GAATTCTACA   
  
  
- TGAGCTTGCT CTCGATGTTG TACGTTTCTG TTCCGTTACT TCTTCGTTAT AGTGATCATA CCAATCTAGT   
  
  
- CGGTGAGATG TGACAAAGAA ACACCCGAGG TTCACTTAAA CGACGTCCCT CAAGAAGGTC AAAAAGAGAC   
  
  
- GGTAGAAC

+     CAT-box

| Site Name | Organism | Position | Strand | Matrix score. | sequence | function |
| --- | --- | --- | --- | --- | --- | --- |
| CAT-box | Arabidopsis thaliana | 3995 | + | 6 | GCCACT | cis-acting regulatory element related to meristem expression |

>HU04G00148.1   
+ -Up\_Stream \_Len000ATAATG GGTGATAACT TTTGAAGGAC AATATACAAA TCACTTATTT ATTAAAGTCA   
  
  
+ AGGGGACATT TCAACTATAA CATCATTGTT TATAAAGTAT TTGAAAATTG CAACTCTTGT AATAGATAAT   
  
  
+ AATGCTTTAT AGACTTCACC AATCACTAAT TTGTAGAATC AAAAAATTAA TTGAAAAGCA AACAAAAGGA   
  
  
+ AAAGGCACAT AAACCTAAAT TATGTAAACA AATGAGAACA TATATGCCAG CTTATCAACG TTAATTATAT   
  
  
+ TTGTTATCAC TCTTAATATC TGTATTCATA GTAAATGAAA ATAAGTATCG ATTTTTTATT AAACCTAAAA   
  
  
+ TCTTCTGTCA ATAATTTTTC TTCCGATAAA AAAAAAATTG GACCATATTA AATTAACATT AATAAATTCA   
  
  
+ TATAATTACT TTGAATGTCT GAAATATAAT GCTCTTCGGT ATATTAAGGT CTTATAAATT TCTCATTAAT   
  
  
+ TTGAAATCTC TTATAATATA TTGGGTCAAC AAGTGCTAGC TTGTTTTTTT TTCCTGGTTA ATTGCATTTA   
  
  
+ AAAAAATAAT TTTTAATTAC AATTGCAAGG GGAGGAATCA TTTACAAAAT ATCAAAAGTG GAAAAGGCAA   
  
  
+ AAAGAAACAA ATTATAAAAC AGAAAAAAGA TTAGAAAACG CAAACAAAGA CGAAATGAGG AACAGTAAGA   
  
  
+ ATCAGTAAAA AAATGTAGTG CAACGGTCAC ATACGGGAAG GCAAGCACAA CAATCATGAA AGAGACAGGC   
  
  
+ ACGTGCGTTC CTCGTGATCA GCAGCCTTCC CTACTTCCCC GCCAAGTGGA GTCCGTACTA ACTCCTGCCA   
  
  
+ TTCCCTCCCA ACGGTCATAT TCCGCCGACA TGCCTCAACG GTCAGATTTC CTGCCCCATC TGGACCGTCC   
  
  
+ GATCAAGATC CAACGGCCAT AAGGCGCACT CTCCAATACC CTAGTGCCCA TTTCTGGAAC AGCCCCAAAA   
  
  
+ ATCTCAGTAT TCGCCATCAT TTTTCAATAC TGGGGTTACA TAAACAGTCT AACAAGGAAG GACCCAACAC   
  
  
+ AGAGGAGAGA GAAAAAAGCT AGCTTTGATG GGGACAGCCA TGGATGGAGG TGAAGAGAGA GGAGGTTGGG   
  
  
+ ACCTTTTATT TTGCTCACTT TCCATTCTTA TATAAAAACA GCTCTAGTTT ATCTCTTATG GCTTTTTTGG   
  
  
+ GTTTGTTCAT AGAGAGGGGG GAATGTGATA TGATTTGATT TGATGTTAGA AATTCTCATT AGTTTTTTCT   
  
  
+ TTTTGTTTGC AATTTTGATG TGTTAGGGTT TGAGGATCAT CATTTTTGAG TAGGGAACTT TCTGGGTTTT   
  
  
+ GCTTGCTGAT TGATTGCCAA TGCTATGGAT TTCTTGGTGA GTCAATCTTC AATTGCTGAT CTGGGTTCGT   
  
  
+ TTGTTCTTCA GAGTTTCACC TGCCAATTTG ATCAAGTATG TCCCTTCACT TCTCCTGCTT CAATTCTCTC   
  
  
+ TCAAATTCAC GCATTTTTCC AATGAATTTC AAATAGGAGT CCTGGAATCC GATGATATTT TAGGTTTTTT   
  
  
+ CCTGACTTCA TTGCATTAGT TTGTTCATAT CTGTGACCAA TTCTTTCATT TGTTATATGA TCTGATTGTT   
  
  
+ TAGTTAGCTA TTCATTCATT GCATATACAT TGGTTTATTC ATTGCAGAGA CACTCACATA CACACACACA   
  
  
+ TACAGGGGAC ACTAGTATTT TTTCTCTCTA ACTCATCGGA TTCCCTTTGA TACTCTGTTT TTTGGGGGGT   
  
  
+ TGATTTGATT TGATTTGATT GACCTTAATT TTGAACTCAC ACACACTCGC ACATAGATAT ATTCTTTCTG   
  
  
+ TTTGTGACAT ATACAGCATC ATATAGAAAT ATATTGCACT GCATATTTGA GCTCAAGACA GGGATATCAT   
  
  
+ CACAAACTGA GTGTGAGGGA GGCAAAAAAC AGAGGAAAAG AAGGGAATTG GGAGTGTAAT CAAAACAAGA   
  
  
+ GAGAGATTGA GGAGAGAGGA AAAAACAAAA TTGGGAGAAG GTTAATAGAG AAGAATGTTG GCTGGGTGTT   
  
  
+ CATCCACATT GCTGTCACCA AGGTATAGAT TGAGGAGTGA AGCTACATCA CAGTTCCAAG CCTGCCATCA   
  
  
+ TCCAATGAGC ACACAGAGAA TTGATTTGCC CTGCACTTTC CCAAGAAAGG ATGTTTCGAA GCCACAATCG   
  
  
+ GTTCGCCCCG TTGGCCTATC TGTCGAGAAG CCGGTTGAAG TCAGGGCCCT GAAGCAGACC ATCCGAGTCC   
  
  
+ CACCATCGCC GGAGGGTAGA AGAGAGATTA AGGCAGATTT CTGGGGTGAT AGAAGAAAGA GCTTGAAGAG   
  
  
+ GAGACTAGCA GAACAGGGGA GCTCTTTTGA TGATGGTGGT GACGAATCTT GTGTTGGGAG AACCAAAAGG   
  
  
+ AAGAAGGGTA GCTTTGATTT TGGTGAATCT GATGAAATTT CTCCAAAAAT TGAGGACACA ATGAGTTTTG   
  
  
+ GACATCTGGG TAGTGGTGGA AATTTCTGGG TTCATCCTGG TTTTGGTGTT GTTAATGTTA ATACTAATTA   
  
  
+ CCCTCAAGTG CCATTTTCTC TCACTTGTTC AGGGGAGGAA GAAAGAGTAT GTTTTGTTCC TACTGATGTG   
  
  
+ ATCTCACCAG CCATCATGCC TCCATTGTCA CACAATCCTT GGGTTGAATC TGGTGTTACT GAGGTCACAG   
  
  
+ AATATGGTGG GGGTGACAAA GACGGCGAAC CTAGTCATGG GTTTGTGAGG GGTACAACAA CAACGTCCGG   
  
  
+ GTCAAGTTCA TCTTCTGAGA GTCATAGTTT TGGGCATAGG CTCAATGAGA GCTCATCTGA CCCTGAAATC   
  
  
+ AGAAATGGTT CCATGTTGCC TAACCCTGGT CATGGTCCTG GCACCAGTCA TGGTCCTGGA ACCGGTCTGG   
  
  
+ CTCACAACCA CAATGATCAA ACCGAGCAAC AAGGGTTCGA GCTCATTAGC TTTCTCATGG GTTGTGTCGA   
  
  
+ AGCGATCAGT TCAAGGAACA TTGCAGCCAT CAATCATTTC ATAGCTAAGC TGGGCGAGCA GGCTTCTCCA   
  
  
+ AGGGCTCGGT CTGCCATTAG CCGCCTCACA GCCTACTTCA CCGAAGCATT AGCCTTGAGG GTCACAAGAT   
  
  
+ TTTGGCCTCA TATCTTTCAC ATAAGTATCC CTCGAGAGTT TGATCGATTT GATGATGAAT CGGGGGCAGC   
  
  
+ TGCAATGAGG CTTTTGAATC AGATCAGCCC AATTCCAAAG TTCGTTCATT TTACAGCCAA TGAGATGTTA   
  
  
+ TTGAGGGCAT TTGAAGGGAA GGACAAGGTG CATATCATAG ACTTCGACAT CAAGCAAGGC TTACAATGGC   
  
  
+ CCGGGTTTTT CCAAAGCTTA GCCATGAGGG AAAACCCCCC GAGCCATGTC AGGATAACAG GCGTAGGGGA   
  
  
+ TTCGAAGCAA GAATTGGTTG AGACAGGAGA AAGACTAGCC GGGTTTGCTG GGGCATTCAA CCTCTCCTTC   
  
  
+ GAGTTCCACC CAGTTGTGGA CCGGTTGGAA GATGTTAGGC TATGGATGCT TCATGTTAAG GAAGGTGAAA   
  
  
+ GTGTCGCGGT AAATTGCATT TTGCAGCTCC ACAAGACACT CTATGACCCC CATGGTGCCA CATTCAGGGA   
  
  
+ CTTCATGGGT TTAATCCGAA GCACAAATCC CATAGCATTG GTTATGGCTG AGCAAGAAGC TGATCACAAC   
  
  
+ GAACCCACCT TAGAAGGCCG AGTATGCAAC TCACTTGGAT ACTATGCAGC CCTTTTCGAT GCCATAGATT   
  
  
+ GCAGCCTTCC TTTCGAGAGC TCAGCAAGGT TGAAGATCGA AGAGATGTTT GGCCGGGAAA TCAGGAACAT   
  
  
+ AATAGCCTGT GAAGGGGCAG ACAGGATCGA AAGACATGAG AAGTTCGAGA AATGGAAGAG AAGGATCGAG   
  
  
+ CAGGAAGGGT TCCGGTGCAT GCGGACTAGC GAAAGGGAGG TGATGCAGAG CCAAATGCTG CTTAAGATGT   
  
  
+ ACTCGAACGA GAGCTACAAC ATGCAAAGAC AAGGCAATGA AGAAGCAATA TCACTAGTAT GGTTAGATCA   
  
  
+ GCCACTCTAC ACTGTTTCTT TGTGGGCTCC AAGTGAATTT GCTGCAGGGA GTTCTTCCAG TTTTTCTCTG   
  
  
+ CCATCTTG  

- -Up\_Stream \_Len000TATTAC CCACTATTGA AAACTTCCTG TTATATGTTT AGTGAATAAA TAATTTCAGT   
  
  
- TCCCCTGTAA AGTTGATATT GTAGTAACAA ATATTTCATA AACTTTTAAC GTTGAGAACA TTATCTATTA   
  
  
- TTACGAAATA TCTGAAGTGG TTAGTGATTA AACATCTTAG TTTTTTAATT AACTTTTCGT TTGTTTTCCT   
  
  
- TTTCCGTGTA TTTGGATTTA ATACATTTGT TTACTCTTGT ATATACGGTC GAATAGTTGC AATTAATATA   
  
  
- AACAATAGTG AGAATTATAG ACATAAGTAT CATTTACTTT TATTCATAGC TAAAAAATAA TTTGGATTTT   
  
  
- AGAAGACAGT TATTAAAAAG AAGGCTATTT TTTTTTTAAC CTGGTATAAT TTAATTGTAA TTATTTAAGT   
  
  
- ATATTAATGA AACTTACAGA CTTTATATTA CGAGAAGCCA TATAATTCCA GAATATTTAA AGAGTAATTA   
  
  
- AACTTTAGAG AATATTATAT AACCCAGTTG TTCACGATCG AACAAAAAAA AAGGACCAAT TAACGTAAAT   
  
  
- TTTTTTATTA AAAATTAATG TTAACGTTCC CCTCCTTAGT AAATGTTTTA TAGTTTTCAC CTTTTCCGTT   
  
  
- TTTCTTTGTT TAATATTTTG TCTTTTTTCT AATCTTTTGC GTTTGTTTCT GCTTTACTCC TTGTCATTCT   
  
  
- TAGTCATTTT TTTACATCAC GTTGCCAGTG TATGCCCTTC CGTTCGTGTT GTTAGTACTT TCTCTGTCCG   
  
  
- TGCACGCAAG GAGCACTAGT CGTCGGAAGG GATGAAGGGG CGGTTCACCT CAGGCATGAT TGAGGACGGT   
  
  
- AAGGGAGGGT TGCCAGTATA AGGCGGCTGT ACGGAGTTGC CAGTCTAAAG GACGGGGTAG ACCTGGCAGG   
  
  
- CTAGTTCTAG GTTGCCGGTA TTCCGCGTGA GAGGTTATGG GATCACGGGT AAAGACCTTG TCGGGGTTTT   
  
  
- TAGAGTCATA AGCGGTAGTA AAAAGTTATG ACCCCAATGT ATTTGTCAGA TTGTTCCTTC CTGGGTTGTG   
  
  
- TCTCCTCTCT CTTTTTTCGA TCGAAACTAC CCCTGTCGGT ACCTACCTCC ACTTCTCTCT CCTCCAACCC   
  
  
- TGGAAAATAA AACGAGTGAA AGGTAAGAAT ATATTTTTGT CGAGATCAAA TAGAGAATAC CGAAAAAACC   
  
  
- CAAACAAGTA TCTCTCCCCC CTTACACTAT ACTAAACTAA ACTACAATCT TTAAGAGTAA TCAAAAAAGA   
  
  
- AAAACAAACG TTAAAACTAC ACAATCCCAA ACTCCTAGTA GTAAAAACTC ATCCCTTGAA AGACCCAAAA   
  
  
- CGAACGACTA ACTAACGGTT ACGATACCTA AAGAACCACT CAGTTAGAAG TTAACGACTA GACCCAAGCA   
  
  
- AACAAGAAGT CTCAAAGTGG ACGGTTAAAC TAGTTCATAC AGGGAAGTGA AGAGGACGAA GTTAAGAGAG   
  
  
- AGTTTAAGTG CGTAAAAAGG TTACTTAAAG TTTATCCTCA GGACCTTAGG CTACTATAAA ATCCAAAAAA   
  
  
- GGACTGAAGT AACGTAATCA AACAAGTATA GACACTGGTT AAGAAAGTAA ACAATATACT AGACTAACAA   
  
  
- ATCAATCGAT AAGTAAGTAA CGTATATGTA ACCAAATAAG TAACGTCTCT GTGAGTGTAT GTGTGTGTGT   
  
  
- ATGTCCCCTG TGATCATAAA AAAGAGAGAT TGAGTAGCCT AAGGGAAACT ATGAGACAAA AAACCCCCCA   
  
  
- ACTAAACTAA ACTAAACTAA CTGGAATTAA AACTTGAGTG TGTGTGAGCG TGTATCTATA TAAGAAAGAC   
  
  
- AAACACTGTA TATGTCGTAG TATATCTTTA TATAACGTGA CGTATAAACT CGAGTTCTGT CCCTATAGTA   
  
  
- GTGTTTGACT CACACTCCCT CCGTTTTTTG TCTCCTTTTC TTCCCTTAAC CCTCACATTA GTTTTGTTCT   
  
  
- CTCTCTAACT CCTCTCTCCT TTTTTGTTTT AACCCTCTTC CAATTATCTC TTCTTACAAC CGACCCACAA   
  
  
- GTAGGTGTAA CGACAGTGGT TCCATATCTA ACTCCTCACT TCGATGTAGT GTCAAGGTTC GGACGGTAGT   
  
  
- AGGTTACTCG TGTGTCTCTT AACTAAACGG GACGTGAAAG GGTTCTTTCC TACAAAGCTT CGGTGTTAGC   
  
  
- CAAGCGGGGC AACCGGATAG ACAGCTCTTC GGCCAACTTC AGTCCCGGGA CTTCGTCTGG TAGGCTCAGG   
  
  
- GTGGTAGCGG CCTCCCATCT TCTCTCTAAT TCCGTCTAAA GACCCCACTA TCTTCTTTCT CGAACTTCTC   
  
  
- CTCTGATCGT CTTGTCCCCT CGAGAAAACT ACTACCACCA CTGCTTAGAA CACAACCCTC TTGGTTTTCC   
  
  
- TTCTTCCCAT CGAAACTAAA ACCACTTAGA CTACTTTAAA GAGGTTTTTA ACTCCTGTGT TACTCAAAAC   
  
  
- CTGTAGACCC ATCACCACCT TTAAAGACCC AAGTAGGACC AAAACCACAA CAATTACAAT TATGATTAAT   
  
  
- GGGAGTTCAC GGTAAAAGAG AGTGAACAAG TCCCCTCCTT CTTTCTCATA CAAAACAAGG ATGACTACAC   
  
  
- TAGAGTGGTC GGTAGTACGG AGGTAACAGT GTGTTAGGAA CCCAACTTAG ACCACAATGA CTCCAGTGTC   
  
  
- TTATACCACC CCCACTGTTT CTGCCGCTTG GATCAGTACC CAAACACTCC CCATGTTGTT GTTGCAGGCC   
  
  
- CAGTTCAAGT AGAAGACTCT CAGTATCAAA ACCCGTATCC GAGTTACTCT CGAGTAGACT GGGACTTTAG   
  
  
- TCTTTACCAA GGTACAACGG ATTGGGACCA GTACCAGGAC CGTGGTCAGT ACCAGGACCT TGGCCAGACC   
  
  
- GAGTGTTGGT GTTACTAGTT TGGCTCGTTG TTCCCAAGCT CGAGTAATCG AAAGAGTACC CAACACAGCT   
  
  
- TCGCTAGTCA AGTTCCTTGT AACGTCGGTA GTTAGTAAAG TATCGATTCG ACCCGCTCGT CCGAAGAGGT   
  
  
- TCCCGAGCCA GACGGTAATC GGCGGAGTGT CGGATGAAGT GGCTTCGTAA TCGGAACTCC CAGTGTTCTA   
  
  
- AAACCGGAGT ATAGAAAGTG TATTCATAGG GAGCTCTCAA ACTAGCTAAA CTACTACTTA GCCCCCGTCG   
  
  
- ACGTTACTCC GAAAACTTAG TCTAGTCGGG TTAAGGTTTC AAGCAAGTAA AATGTCGGTT ACTCTACAAT   
  
  
- AACTCCCGTA AACTTCCCTT CCTGTTCCAC GTATAGTATC TGAAGCTGTA GTTCGTTCCG AATGTTACCG   
  
  
- GGCCCAAAAA GGTTTCGAAT CGGTACTCCC TTTTGGGGGG CTCGGTACAG TCCTATTGTC CGCATCCCCT   
  
  
- AAGCTTCGTT CTTAACCAAC TCTGTCCTCT TTCTGATCGG CCCAAACGAC CCCGTAAGTT GGAGAGGAAG   
  
  
- CTCAAGGTGG GTCAACACCT GGCCAACCTT CTACAATCCG ATACCTACGA AGTACAATTC CTTCCACTTT   
  
  
- CACAGCGCCA TTTAACGTAA AACGTCGAGG TGTTCTGTGA GATACTGGGG GTACCACGGT GTAAGTCCCT   
  
  
- GAAGTACCCA AATTAGGCTT CGTGTTTAGG GTATCGTAAC CAATACCGAC TCGTTCTTCG ACTAGTGTTG   
  
  
- CTTGGGTGGA ATCTTCCGGC TCATACGTTG AGTGAACCTA TGATACGTCG GGAAAAGCTA CGGTATCTAA   
  
  
- CGTCGGAAGG AAAGCTCTCG AGTCGTTCCA ACTTCTAGCT TCTCTACAAA CCGGCCCTTT AGTCCTTGTA   
  
  
- TTATCGGACA CTTCCCCGTC TGTCCTAGCT TTCTGTACTC TTCAAGCTCT TTACCTTCTC TTCCTAGCTC   
  
  
- GTCCTTCCCA AGGCCACGTA CGCCTGATCG CTTTCCCTCC ACTACGTCTC GGTTTACGAC GAATTCTACA   
  
  
- TGAGCTTGCT CTCGATGTTG TACGTTTCTG TTCCGTTACT TCTTCGTTAT AGTGATCATA CCAATCTAGT   
  
  
- CGGTGAGATG TGACAAAGAA ACACCCGAGG TTCACTTAAA CGACGTCCCT CAAGAAGGTC AAAAAGAGAC   
  
  
- GGTAGAAC

+     CCAAT-box

| Site Name | Organism | Position | Strand | Matrix score. | sequence | function |
| --- | --- | --- | --- | --- | --- | --- |
| CCAAT-box | Hordeum vulgare | 853 | + | 6 | CAACGG | MYBHv1 binding site |
| CCAAT-box | Hordeum vulgare | 2182 | - | 6 | CAACGG | MYBHv1 binding site |
| CCAAT-box | Hordeum vulgare | 725 | + | 6 | CAACGG | MYBHv1 binding site |
| CCAAT-box | Hordeum vulgare | 925 | + | 6 | CAACGG | MYBHv1 binding site |
| CCAAT-box | Hordeum vulgare | 880 | + | 6 | CAACGG | MYBHv1 binding site |

>HU04G00148.1   
+ -Up\_Stream \_Len000ATAATG GGTGATAACT TTTGAAGGAC AATATACAAA TCACTTATTT ATTAAAGTCA   
  
  
+ AGGGGACATT TCAACTATAA CATCATTGTT TATAAAGTAT TTGAAAATTG CAACTCTTGT AATAGATAAT   
  
  
+ AATGCTTTAT AGACTTCACC AATCACTAAT TTGTAGAATC AAAAAATTAA TTGAAAAGCA AACAAAAGGA   
  
  
+ AAAGGCACAT AAACCTAAAT TATGTAAACA AATGAGAACA TATATGCCAG CTTATCAACG TTAATTATAT   
  
  
+ TTGTTATCAC TCTTAATATC TGTATTCATA GTAAATGAAA ATAAGTATCG ATTTTTTATT AAACCTAAAA   
  
  
+ TCTTCTGTCA ATAATTTTTC TTCCGATAAA AAAAAAATTG GACCATATTA AATTAACATT AATAAATTCA   
  
  
+ TATAATTACT TTGAATGTCT GAAATATAAT GCTCTTCGGT ATATTAAGGT CTTATAAATT TCTCATTAAT   
  
  
+ TTGAAATCTC TTATAATATA TTGGGTCAAC AAGTGCTAGC TTGTTTTTTT TTCCTGGTTA ATTGCATTTA   
  
  
+ AAAAAATAAT TTTTAATTAC AATTGCAAGG GGAGGAATCA TTTACAAAAT ATCAAAAGTG GAAAAGGCAA   
  
  
+ AAAGAAACAA ATTATAAAAC AGAAAAAAGA TTAGAAAACG CAAACAAAGA CGAAATGAGG AACAGTAAGA   
  
  
+ ATCAGTAAAA AAATGTAGTG CAACGGTCAC ATACGGGAAG GCAAGCACAA CAATCATGAA AGAGACAGGC   
  
  
+ ACGTGCGTTC CTCGTGATCA GCAGCCTTCC CTACTTCCCC GCCAAGTGGA GTCCGTACTA ACTCCTGCCA   
  
  
+ TTCCCTCCCA ACGGTCATAT TCCGCCGACA TGCCTCAACG GTCAGATTTC CTGCCCCATC TGGACCGTCC   
  
  
+ GATCAAGATC CAACGGCCAT AAGGCGCACT CTCCAATACC CTAGTGCCCA TTTCTGGAAC AGCCCCAAAA   
  
  
+ ATCTCAGTAT TCGCCATCAT TTTTCAATAC TGGGGTTACA TAAACAGTCT AACAAGGAAG GACCCAACAC   
  
  
+ AGAGGAGAGA GAAAAAAGCT AGCTTTGATG GGGACAGCCA TGGATGGAGG TGAAGAGAGA GGAGGTTGGG   
  
  
+ ACCTTTTATT TTGCTCACTT TCCATTCTTA TATAAAAACA GCTCTAGTTT ATCTCTTATG GCTTTTTTGG   
  
  
+ GTTTGTTCAT AGAGAGGGGG GAATGTGATA TGATTTGATT TGATGTTAGA AATTCTCATT AGTTTTTTCT   
  
  
+ TTTTGTTTGC AATTTTGATG TGTTAGGGTT TGAGGATCAT CATTTTTGAG TAGGGAACTT TCTGGGTTTT   
  
  
+ GCTTGCTGAT TGATTGCCAA TGCTATGGAT TTCTTGGTGA GTCAATCTTC AATTGCTGAT CTGGGTTCGT   
  
  
+ TTGTTCTTCA GAGTTTCACC TGCCAATTTG ATCAAGTATG TCCCTTCACT TCTCCTGCTT CAATTCTCTC   
  
  
+ TCAAATTCAC GCATTTTTCC AATGAATTTC AAATAGGAGT CCTGGAATCC GATGATATTT TAGGTTTTTT   
  
  
+ CCTGACTTCA TTGCATTAGT TTGTTCATAT CTGTGACCAA TTCTTTCATT TGTTATATGA TCTGATTGTT   
  
  
+ TAGTTAGCTA TTCATTCATT GCATATACAT TGGTTTATTC ATTGCAGAGA CACTCACATA CACACACACA   
  
  
+ TACAGGGGAC ACTAGTATTT TTTCTCTCTA ACTCATCGGA TTCCCTTTGA TACTCTGTTT TTTGGGGGGT   
  
  
+ TGATTTGATT TGATTTGATT GACCTTAATT TTGAACTCAC ACACACTCGC ACATAGATAT ATTCTTTCTG   
  
  
+ TTTGTGACAT ATACAGCATC ATATAGAAAT ATATTGCACT GCATATTTGA GCTCAAGACA GGGATATCAT   
  
  
+ CACAAACTGA GTGTGAGGGA GGCAAAAAAC AGAGGAAAAG AAGGGAATTG GGAGTGTAAT CAAAACAAGA   
  
  
+ GAGAGATTGA GGAGAGAGGA AAAAACAAAA TTGGGAGAAG GTTAATAGAG AAGAATGTTG GCTGGGTGTT   
  
  
+ CATCCACATT GCTGTCACCA AGGTATAGAT TGAGGAGTGA AGCTACATCA CAGTTCCAAG CCTGCCATCA   
  
  
+ TCCAATGAGC ACACAGAGAA TTGATTTGCC CTGCACTTTC CCAAGAAAGG ATGTTTCGAA GCCACAATCG   
  
  
+ GTTCGCCCCG TTGGCCTATC TGTCGAGAAG CCGGTTGAAG TCAGGGCCCT GAAGCAGACC ATCCGAGTCC   
  
  
+ CACCATCGCC GGAGGGTAGA AGAGAGATTA AGGCAGATTT CTGGGGTGAT AGAAGAAAGA GCTTGAAGAG   
  
  
+ GAGACTAGCA GAACAGGGGA GCTCTTTTGA TGATGGTGGT GACGAATCTT GTGTTGGGAG AACCAAAAGG   
  
  
+ AAGAAGGGTA GCTTTGATTT TGGTGAATCT GATGAAATTT CTCCAAAAAT TGAGGACACA ATGAGTTTTG   
  
  
+ GACATCTGGG TAGTGGTGGA AATTTCTGGG TTCATCCTGG TTTTGGTGTT GTTAATGTTA ATACTAATTA   
  
  
+ CCCTCAAGTG CCATTTTCTC TCACTTGTTC AGGGGAGGAA GAAAGAGTAT GTTTTGTTCC TACTGATGTG   
  
  
+ ATCTCACCAG CCATCATGCC TCCATTGTCA CACAATCCTT GGGTTGAATC TGGTGTTACT GAGGTCACAG   
  
  
+ AATATGGTGG GGGTGACAAA GACGGCGAAC CTAGTCATGG GTTTGTGAGG GGTACAACAA CAACGTCCGG   
  
  
+ GTCAAGTTCA TCTTCTGAGA GTCATAGTTT TGGGCATAGG CTCAATGAGA GCTCATCTGA CCCTGAAATC   
  
  
+ AGAAATGGTT CCATGTTGCC TAACCCTGGT CATGGTCCTG GCACCAGTCA TGGTCCTGGA ACCGGTCTGG   
  
  
+ CTCACAACCA CAATGATCAA ACCGAGCAAC AAGGGTTCGA GCTCATTAGC TTTCTCATGG GTTGTGTCGA   
  
  
+ AGCGATCAGT TCAAGGAACA TTGCAGCCAT CAATCATTTC ATAGCTAAGC TGGGCGAGCA GGCTTCTCCA   
  
  
+ AGGGCTCGGT CTGCCATTAG CCGCCTCACA GCCTACTTCA CCGAAGCATT AGCCTTGAGG GTCACAAGAT   
  
  
+ TTTGGCCTCA TATCTTTCAC ATAAGTATCC CTCGAGAGTT TGATCGATTT GATGATGAAT CGGGGGCAGC   
  
  
+ TGCAATGAGG CTTTTGAATC AGATCAGCCC AATTCCAAAG TTCGTTCATT TTACAGCCAA TGAGATGTTA   
  
  
+ TTGAGGGCAT TTGAAGGGAA GGACAAGGTG CATATCATAG ACTTCGACAT CAAGCAAGGC TTACAATGGC   
  
  
+ CCGGGTTTTT CCAAAGCTTA GCCATGAGGG AAAACCCCCC GAGCCATGTC AGGATAACAG GCGTAGGGGA   
  
  
+ TTCGAAGCAA GAATTGGTTG AGACAGGAGA AAGACTAGCC GGGTTTGCTG GGGCATTCAA CCTCTCCTTC   
  
  
+ GAGTTCCACC CAGTTGTGGA CCGGTTGGAA GATGTTAGGC TATGGATGCT TCATGTTAAG GAAGGTGAAA   
  
  
+ GTGTCGCGGT AAATTGCATT TTGCAGCTCC ACAAGACACT CTATGACCCC CATGGTGCCA CATTCAGGGA   
  
  
+ CTTCATGGGT TTAATCCGAA GCACAAATCC CATAGCATTG GTTATGGCTG AGCAAGAAGC TGATCACAAC   
  
  
+ GAACCCACCT TAGAAGGCCG AGTATGCAAC TCACTTGGAT ACTATGCAGC CCTTTTCGAT GCCATAGATT   
  
  
+ GCAGCCTTCC TTTCGAGAGC TCAGCAAGGT TGAAGATCGA AGAGATGTTT GGCCGGGAAA TCAGGAACAT   
  
  
+ AATAGCCTGT GAAGGGGCAG ACAGGATCGA AAGACATGAG AAGTTCGAGA AATGGAAGAG AAGGATCGAG   
  
  
+ CAGGAAGGGT TCCGGTGCAT GCGGACTAGC GAAAGGGAGG TGATGCAGAG CCAAATGCTG CTTAAGATGT   
  
  
+ ACTCGAACGA GAGCTACAAC ATGCAAAGAC AAGGCAATGA AGAAGCAATA TCACTAGTAT GGTTAGATCA   
  
  
+ GCCACTCTAC ACTGTTTCTT TGTGGGCTCC AAGTGAATTT GCTGCAGGGA GTTCTTCCAG TTTTTCTCTG   
  
  
+ CCATCTTG  

- -Up\_Stream \_Len000TATTAC CCACTATTGA AAACTTCCTG TTATATGTTT AGTGAATAAA TAATTTCAGT   
  
  
- TCCCCTGTAA AGTTGATATT GTAGTAACAA ATATTTCATA AACTTTTAAC GTTGAGAACA TTATCTATTA   
  
  
- TTACGAAATA TCTGAAGTGG TTAGTGATTA AACATCTTAG TTTTTTAATT AACTTTTCGT TTGTTTTCCT   
  
  
- TTTCCGTGTA TTTGGATTTA ATACATTTGT TTACTCTTGT ATATACGGTC GAATAGTTGC AATTAATATA   
  
  
- AACAATAGTG AGAATTATAG ACATAAGTAT CATTTACTTT TATTCATAGC TAAAAAATAA TTTGGATTTT   
  
  
- AGAAGACAGT TATTAAAAAG AAGGCTATTT TTTTTTTAAC CTGGTATAAT TTAATTGTAA TTATTTAAGT   
  
  
- ATATTAATGA AACTTACAGA CTTTATATTA CGAGAAGCCA TATAATTCCA GAATATTTAA AGAGTAATTA   
  
  
- AACTTTAGAG AATATTATAT AACCCAGTTG TTCACGATCG AACAAAAAAA AAGGACCAAT TAACGTAAAT   
  
  
- TTTTTTATTA AAAATTAATG TTAACGTTCC CCTCCTTAGT AAATGTTTTA TAGTTTTCAC CTTTTCCGTT   
  
  
- TTTCTTTGTT TAATATTTTG TCTTTTTTCT AATCTTTTGC GTTTGTTTCT GCTTTACTCC TTGTCATTCT   
  
  
- TAGTCATTTT TTTACATCAC GTTGCCAGTG TATGCCCTTC CGTTCGTGTT GTTAGTACTT TCTCTGTCCG   
  
  
- TGCACGCAAG GAGCACTAGT CGTCGGAAGG GATGAAGGGG CGGTTCACCT CAGGCATGAT TGAGGACGGT   
  
  
- AAGGGAGGGT TGCCAGTATA AGGCGGCTGT ACGGAGTTGC CAGTCTAAAG GACGGGGTAG ACCTGGCAGG   
  
  
- CTAGTTCTAG GTTGCCGGTA TTCCGCGTGA GAGGTTATGG GATCACGGGT AAAGACCTTG TCGGGGTTTT   
  
  
- TAGAGTCATA AGCGGTAGTA AAAAGTTATG ACCCCAATGT ATTTGTCAGA TTGTTCCTTC CTGGGTTGTG   
  
  
- TCTCCTCTCT CTTTTTTCGA TCGAAACTAC CCCTGTCGGT ACCTACCTCC ACTTCTCTCT CCTCCAACCC   
  
  
- TGGAAAATAA AACGAGTGAA AGGTAAGAAT ATATTTTTGT CGAGATCAAA TAGAGAATAC CGAAAAAACC   
  
  
- CAAACAAGTA TCTCTCCCCC CTTACACTAT ACTAAACTAA ACTACAATCT TTAAGAGTAA TCAAAAAAGA   
  
  
- AAAACAAACG TTAAAACTAC ACAATCCCAA ACTCCTAGTA GTAAAAACTC ATCCCTTGAA AGACCCAAAA   
  
  
- CGAACGACTA ACTAACGGTT ACGATACCTA AAGAACCACT CAGTTAGAAG TTAACGACTA GACCCAAGCA   
  
  
- AACAAGAAGT CTCAAAGTGG ACGGTTAAAC TAGTTCATAC AGGGAAGTGA AGAGGACGAA GTTAAGAGAG   
  
  
- AGTTTAAGTG CGTAAAAAGG TTACTTAAAG TTTATCCTCA GGACCTTAGG CTACTATAAA ATCCAAAAAA   
  
  
- GGACTGAAGT AACGTAATCA AACAAGTATA GACACTGGTT AAGAAAGTAA ACAATATACT AGACTAACAA   
  
  
- ATCAATCGAT AAGTAAGTAA CGTATATGTA ACCAAATAAG TAACGTCTCT GTGAGTGTAT GTGTGTGTGT   
  
  
- ATGTCCCCTG TGATCATAAA AAAGAGAGAT TGAGTAGCCT AAGGGAAACT ATGAGACAAA AAACCCCCCA   
  
  
- ACTAAACTAA ACTAAACTAA CTGGAATTAA AACTTGAGTG TGTGTGAGCG TGTATCTATA TAAGAAAGAC   
  
  
- AAACACTGTA TATGTCGTAG TATATCTTTA TATAACGTGA CGTATAAACT CGAGTTCTGT CCCTATAGTA   
  
  
- GTGTTTGACT CACACTCCCT CCGTTTTTTG TCTCCTTTTC TTCCCTTAAC CCTCACATTA GTTTTGTTCT   
  
  
- CTCTCTAACT CCTCTCTCCT TTTTTGTTTT AACCCTCTTC CAATTATCTC TTCTTACAAC CGACCCACAA   
  
  
- GTAGGTGTAA CGACAGTGGT TCCATATCTA ACTCCTCACT TCGATGTAGT GTCAAGGTTC GGACGGTAGT   
  
  
- AGGTTACTCG TGTGTCTCTT AACTAAACGG GACGTGAAAG GGTTCTTTCC TACAAAGCTT CGGTGTTAGC   
  
  
- CAAGCGGGGC AACCGGATAG ACAGCTCTTC GGCCAACTTC AGTCCCGGGA CTTCGTCTGG TAGGCTCAGG   
  
  
- GTGGTAGCGG CCTCCCATCT TCTCTCTAAT TCCGTCTAAA GACCCCACTA TCTTCTTTCT CGAACTTCTC   
  
  
- CTCTGATCGT CTTGTCCCCT CGAGAAAACT ACTACCACCA CTGCTTAGAA CACAACCCTC TTGGTTTTCC   
  
  
- TTCTTCCCAT CGAAACTAAA ACCACTTAGA CTACTTTAAA GAGGTTTTTA ACTCCTGTGT TACTCAAAAC   
  
  
- CTGTAGACCC ATCACCACCT TTAAAGACCC AAGTAGGACC AAAACCACAA CAATTACAAT TATGATTAAT   
  
  
- GGGAGTTCAC GGTAAAAGAG AGTGAACAAG TCCCCTCCTT CTTTCTCATA CAAAACAAGG ATGACTACAC   
  
  
- TAGAGTGGTC GGTAGTACGG AGGTAACAGT GTGTTAGGAA CCCAACTTAG ACCACAATGA CTCCAGTGTC   
  
  
- TTATACCACC CCCACTGTTT CTGCCGCTTG GATCAGTACC CAAACACTCC CCATGTTGTT GTTGCAGGCC   
  
  
- CAGTTCAAGT AGAAGACTCT CAGTATCAAA ACCCGTATCC GAGTTACTCT CGAGTAGACT GGGACTTTAG   
  
  
- TCTTTACCAA GGTACAACGG ATTGGGACCA GTACCAGGAC CGTGGTCAGT ACCAGGACCT TGGCCAGACC   
  
  
- GAGTGTTGGT GTTACTAGTT TGGCTCGTTG TTCCCAAGCT CGAGTAATCG AAAGAGTACC CAACACAGCT   
  
  
- TCGCTAGTCA AGTTCCTTGT AACGTCGGTA GTTAGTAAAG TATCGATTCG ACCCGCTCGT CCGAAGAGGT   
  
  
- TCCCGAGCCA GACGGTAATC GGCGGAGTGT CGGATGAAGT GGCTTCGTAA TCGGAACTCC CAGTGTTCTA   
  
  
- AAACCGGAGT ATAGAAAGTG TATTCATAGG GAGCTCTCAA ACTAGCTAAA CTACTACTTA GCCCCCGTCG   
  
  
- ACGTTACTCC GAAAACTTAG TCTAGTCGGG TTAAGGTTTC AAGCAAGTAA AATGTCGGTT ACTCTACAAT   
  
  
- AACTCCCGTA AACTTCCCTT CCTGTTCCAC GTATAGTATC TGAAGCTGTA GTTCGTTCCG AATGTTACCG   
  
  
- GGCCCAAAAA GGTTTCGAAT CGGTACTCCC TTTTGGGGGG CTCGGTACAG TCCTATTGTC CGCATCCCCT   
  
  
- AAGCTTCGTT CTTAACCAAC TCTGTCCTCT TTCTGATCGG CCCAAACGAC CCCGTAAGTT GGAGAGGAAG   
  
  
- CTCAAGGTGG GTCAACACCT GGCCAACCTT CTACAATCCG ATACCTACGA AGTACAATTC CTTCCACTTT   
  
  
- CACAGCGCCA TTTAACGTAA AACGTCGAGG TGTTCTGTGA GATACTGGGG GTACCACGGT GTAAGTCCCT   
  
  
- GAAGTACCCA AATTAGGCTT CGTGTTTAGG GTATCGTAAC CAATACCGAC TCGTTCTTCG ACTAGTGTTG   
  
  
- CTTGGGTGGA ATCTTCCGGC TCATACGTTG AGTGAACCTA TGATACGTCG GGAAAAGCTA CGGTATCTAA   
  
  
- CGTCGGAAGG AAAGCTCTCG AGTCGTTCCA ACTTCTAGCT TCTCTACAAA CCGGCCCTTT AGTCCTTGTA   
  
  
- TTATCGGACA CTTCCCCGTC TGTCCTAGCT TTCTGTACTC TTCAAGCTCT TTACCTTCTC TTCCTAGCTC   
  
  
- GTCCTTCCCA AGGCCACGTA CGCCTGATCG CTTTCCCTCC ACTACGTCTC GGTTTACGAC GAATTCTACA   
  
  
- TGAGCTTGCT CTCGATGTTG TACGTTTCTG TTCCGTTACT TCTTCGTTAT AGTGATCATA CCAATCTAGT   
  
  
- CGGTGAGATG TGACAAAGAA ACACCCGAGG TTCACTTAAA CGACGTCCCT CAAGAAGGTC AAAAAGAGAC   
  
  
- GGTAGAAC

+     CCGTCC motif

| Site Name | Organism | Position | Strand | Matrix score. | sequence | function |
| --- | --- | --- | --- | --- | --- | --- |
| CCGTCC motif | Nicotiana tabacum | 909 | + | 6 | CCGTCC |  |

>HU04G00148.1   
+ -Up\_Stream \_Len000ATAATG GGTGATAACT TTTGAAGGAC AATATACAAA TCACTTATTT ATTAAAGTCA   
  
  
+ AGGGGACATT TCAACTATAA CATCATTGTT TATAAAGTAT TTGAAAATTG CAACTCTTGT AATAGATAAT   
  
  
+ AATGCTTTAT AGACTTCACC AATCACTAAT TTGTAGAATC AAAAAATTAA TTGAAAAGCA AACAAAAGGA   
  
  
+ AAAGGCACAT AAACCTAAAT TATGTAAACA AATGAGAACA TATATGCCAG CTTATCAACG TTAATTATAT   
  
  
+ TTGTTATCAC TCTTAATATC TGTATTCATA GTAAATGAAA ATAAGTATCG ATTTTTTATT AAACCTAAAA   
  
  
+ TCTTCTGTCA ATAATTTTTC TTCCGATAAA AAAAAAATTG GACCATATTA AATTAACATT AATAAATTCA   
  
  
+ TATAATTACT TTGAATGTCT GAAATATAAT GCTCTTCGGT ATATTAAGGT CTTATAAATT TCTCATTAAT   
  
  
+ TTGAAATCTC TTATAATATA TTGGGTCAAC AAGTGCTAGC TTGTTTTTTT TTCCTGGTTA ATTGCATTTA   
  
  
+ AAAAAATAAT TTTTAATTAC AATTGCAAGG GGAGGAATCA TTTACAAAAT ATCAAAAGTG GAAAAGGCAA   
  
  
+ AAAGAAACAA ATTATAAAAC AGAAAAAAGA TTAGAAAACG CAAACAAAGA CGAAATGAGG AACAGTAAGA   
  
  
+ ATCAGTAAAA AAATGTAGTG CAACGGTCAC ATACGGGAAG GCAAGCACAA CAATCATGAA AGAGACAGGC   
  
  
+ ACGTGCGTTC CTCGTGATCA GCAGCCTTCC CTACTTCCCC GCCAAGTGGA GTCCGTACTA ACTCCTGCCA   
  
  
+ TTCCCTCCCA ACGGTCATAT TCCGCCGACA TGCCTCAACG GTCAGATTTC CTGCCCCATC TGGACCGTCC   
  
  
+ GATCAAGATC CAACGGCCAT AAGGCGCACT CTCCAATACC CTAGTGCCCA TTTCTGGAAC AGCCCCAAAA   
  
  
+ ATCTCAGTAT TCGCCATCAT TTTTCAATAC TGGGGTTACA TAAACAGTCT AACAAGGAAG GACCCAACAC   
  
  
+ AGAGGAGAGA GAAAAAAGCT AGCTTTGATG GGGACAGCCA TGGATGGAGG TGAAGAGAGA GGAGGTTGGG   
  
  
+ ACCTTTTATT TTGCTCACTT TCCATTCTTA TATAAAAACA GCTCTAGTTT ATCTCTTATG GCTTTTTTGG   
  
  
+ GTTTGTTCAT AGAGAGGGGG GAATGTGATA TGATTTGATT TGATGTTAGA AATTCTCATT AGTTTTTTCT   
  
  
+ TTTTGTTTGC AATTTTGATG TGTTAGGGTT TGAGGATCAT CATTTTTGAG TAGGGAACTT TCTGGGTTTT   
  
  
+ GCTTGCTGAT TGATTGCCAA TGCTATGGAT TTCTTGGTGA GTCAATCTTC AATTGCTGAT CTGGGTTCGT   
  
  
+ TTGTTCTTCA GAGTTTCACC TGCCAATTTG ATCAAGTATG TCCCTTCACT TCTCCTGCTT CAATTCTCTC   
  
  
+ TCAAATTCAC GCATTTTTCC AATGAATTTC AAATAGGAGT CCTGGAATCC GATGATATTT TAGGTTTTTT   
  
  
+ CCTGACTTCA TTGCATTAGT TTGTTCATAT CTGTGACCAA TTCTTTCATT TGTTATATGA TCTGATTGTT   
  
  
+ TAGTTAGCTA TTCATTCATT GCATATACAT TGGTTTATTC ATTGCAGAGA CACTCACATA CACACACACA   
  
  
+ TACAGGGGAC ACTAGTATTT TTTCTCTCTA ACTCATCGGA TTCCCTTTGA TACTCTGTTT TTTGGGGGGT   
  
  
+ TGATTTGATT TGATTTGATT GACCTTAATT TTGAACTCAC ACACACTCGC ACATAGATAT ATTCTTTCTG   
  
  
+ TTTGTGACAT ATACAGCATC ATATAGAAAT ATATTGCACT GCATATTTGA GCTCAAGACA GGGATATCAT   
  
  
+ CACAAACTGA GTGTGAGGGA GGCAAAAAAC AGAGGAAAAG AAGGGAATTG GGAGTGTAAT CAAAACAAGA   
  
  
+ GAGAGATTGA GGAGAGAGGA AAAAACAAAA TTGGGAGAAG GTTAATAGAG AAGAATGTTG GCTGGGTGTT   
  
  
+ CATCCACATT GCTGTCACCA AGGTATAGAT TGAGGAGTGA AGCTACATCA CAGTTCCAAG CCTGCCATCA   
  
  
+ TCCAATGAGC ACACAGAGAA TTGATTTGCC CTGCACTTTC CCAAGAAAGG ATGTTTCGAA GCCACAATCG   
  
  
+ GTTCGCCCCG TTGGCCTATC TGTCGAGAAG CCGGTTGAAG TCAGGGCCCT GAAGCAGACC ATCCGAGTCC   
  
  
+ CACCATCGCC GGAGGGTAGA AGAGAGATTA AGGCAGATTT CTGGGGTGAT AGAAGAAAGA GCTTGAAGAG   
  
  
+ GAGACTAGCA GAACAGGGGA GCTCTTTTGA TGATGGTGGT GACGAATCTT GTGTTGGGAG AACCAAAAGG   
  
  
+ AAGAAGGGTA GCTTTGATTT TGGTGAATCT GATGAAATTT CTCCAAAAAT TGAGGACACA ATGAGTTTTG   
  
  
+ GACATCTGGG TAGTGGTGGA AATTTCTGGG TTCATCCTGG TTTTGGTGTT GTTAATGTTA ATACTAATTA   
  
  
+ CCCTCAAGTG CCATTTTCTC TCACTTGTTC AGGGGAGGAA GAAAGAGTAT GTTTTGTTCC TACTGATGTG   
  
  
+ ATCTCACCAG CCATCATGCC TCCATTGTCA CACAATCCTT GGGTTGAATC TGGTGTTACT GAGGTCACAG   
  
  
+ AATATGGTGG GGGTGACAAA GACGGCGAAC CTAGTCATGG GTTTGTGAGG GGTACAACAA CAACGTCCGG   
  
  
+ GTCAAGTTCA TCTTCTGAGA GTCATAGTTT TGGGCATAGG CTCAATGAGA GCTCATCTGA CCCTGAAATC   
  
  
+ AGAAATGGTT CCATGTTGCC TAACCCTGGT CATGGTCCTG GCACCAGTCA TGGTCCTGGA ACCGGTCTGG   
  
  
+ CTCACAACCA CAATGATCAA ACCGAGCAAC AAGGGTTCGA GCTCATTAGC TTTCTCATGG GTTGTGTCGA   
  
  
+ AGCGATCAGT TCAAGGAACA TTGCAGCCAT CAATCATTTC ATAGCTAAGC TGGGCGAGCA GGCTTCTCCA   
  
  
+ AGGGCTCGGT CTGCCATTAG CCGCCTCACA GCCTACTTCA CCGAAGCATT AGCCTTGAGG GTCACAAGAT   
  
  
+ TTTGGCCTCA TATCTTTCAC ATAAGTATCC CTCGAGAGTT TGATCGATTT GATGATGAAT CGGGGGCAGC   
  
  
+ TGCAATGAGG CTTTTGAATC AGATCAGCCC AATTCCAAAG TTCGTTCATT TTACAGCCAA TGAGATGTTA   
  
  
+ TTGAGGGCAT TTGAAGGGAA GGACAAGGTG CATATCATAG ACTTCGACAT CAAGCAAGGC TTACAATGGC   
  
  
+ CCGGGTTTTT CCAAAGCTTA GCCATGAGGG AAAACCCCCC GAGCCATGTC AGGATAACAG GCGTAGGGGA   
  
  
+ TTCGAAGCAA GAATTGGTTG AGACAGGAGA AAGACTAGCC GGGTTTGCTG GGGCATTCAA CCTCTCCTTC   
  
  
+ GAGTTCCACC CAGTTGTGGA CCGGTTGGAA GATGTTAGGC TATGGATGCT TCATGTTAAG GAAGGTGAAA   
  
  
+ GTGTCGCGGT AAATTGCATT TTGCAGCTCC ACAAGACACT CTATGACCCC CATGGTGCCA CATTCAGGGA   
  
  
+ CTTCATGGGT TTAATCCGAA GCACAAATCC CATAGCATTG GTTATGGCTG AGCAAGAAGC TGATCACAAC   
  
  
+ GAACCCACCT TAGAAGGCCG AGTATGCAAC TCACTTGGAT ACTATGCAGC CCTTTTCGAT GCCATAGATT   
  
  
+ GCAGCCTTCC TTTCGAGAGC TCAGCAAGGT TGAAGATCGA AGAGATGTTT GGCCGGGAAA TCAGGAACAT   
  
  
+ AATAGCCTGT GAAGGGGCAG ACAGGATCGA AAGACATGAG AAGTTCGAGA AATGGAAGAG AAGGATCGAG   
  
  
+ CAGGAAGGGT TCCGGTGCAT GCGGACTAGC GAAAGGGAGG TGATGCAGAG CCAAATGCTG CTTAAGATGT   
  
  
+ ACTCGAACGA GAGCTACAAC ATGCAAAGAC AAGGCAATGA AGAAGCAATA TCACTAGTAT GGTTAGATCA   
  
  
+ GCCACTCTAC ACTGTTTCTT TGTGGGCTCC AAGTGAATTT GCTGCAGGGA GTTCTTCCAG TTTTTCTCTG   
  
  
+ CCATCTTG  

- -Up\_Stream \_Len000TATTAC CCACTATTGA AAACTTCCTG TTATATGTTT AGTGAATAAA TAATTTCAGT   
  
  
- TCCCCTGTAA AGTTGATATT GTAGTAACAA ATATTTCATA AACTTTTAAC GTTGAGAACA TTATCTATTA   
  
  
- TTACGAAATA TCTGAAGTGG TTAGTGATTA AACATCTTAG TTTTTTAATT AACTTTTCGT TTGTTTTCCT   
  
  
- TTTCCGTGTA TTTGGATTTA ATACATTTGT TTACTCTTGT ATATACGGTC GAATAGTTGC AATTAATATA   
  
  
- AACAATAGTG AGAATTATAG ACATAAGTAT CATTTACTTT TATTCATAGC TAAAAAATAA TTTGGATTTT   
  
  
- AGAAGACAGT TATTAAAAAG AAGGCTATTT TTTTTTTAAC CTGGTATAAT TTAATTGTAA TTATTTAAGT   
  
  
- ATATTAATGA AACTTACAGA CTTTATATTA CGAGAAGCCA TATAATTCCA GAATATTTAA AGAGTAATTA   
  
  
- AACTTTAGAG AATATTATAT AACCCAGTTG TTCACGATCG AACAAAAAAA AAGGACCAAT TAACGTAAAT   
  
  
- TTTTTTATTA AAAATTAATG TTAACGTTCC CCTCCTTAGT AAATGTTTTA TAGTTTTCAC CTTTTCCGTT   
  
  
- TTTCTTTGTT TAATATTTTG TCTTTTTTCT AATCTTTTGC GTTTGTTTCT GCTTTACTCC TTGTCATTCT   
  
  
- TAGTCATTTT TTTACATCAC GTTGCCAGTG TATGCCCTTC CGTTCGTGTT GTTAGTACTT TCTCTGTCCG   
  
  
- TGCACGCAAG GAGCACTAGT CGTCGGAAGG GATGAAGGGG CGGTTCACCT CAGGCATGAT TGAGGACGGT   
  
  
- AAGGGAGGGT TGCCAGTATA AGGCGGCTGT ACGGAGTTGC CAGTCTAAAG GACGGGGTAG ACCTGGCAGG   
  
  
- CTAGTTCTAG GTTGCCGGTA TTCCGCGTGA GAGGTTATGG GATCACGGGT AAAGACCTTG TCGGGGTTTT   
  
  
- TAGAGTCATA AGCGGTAGTA AAAAGTTATG ACCCCAATGT ATTTGTCAGA TTGTTCCTTC CTGGGTTGTG   
  
  
- TCTCCTCTCT CTTTTTTCGA TCGAAACTAC CCCTGTCGGT ACCTACCTCC ACTTCTCTCT CCTCCAACCC   
  
  
- TGGAAAATAA AACGAGTGAA AGGTAAGAAT ATATTTTTGT CGAGATCAAA TAGAGAATAC CGAAAAAACC   
  
  
- CAAACAAGTA TCTCTCCCCC CTTACACTAT ACTAAACTAA ACTACAATCT TTAAGAGTAA TCAAAAAAGA   
  
  
- AAAACAAACG TTAAAACTAC ACAATCCCAA ACTCCTAGTA GTAAAAACTC ATCCCTTGAA AGACCCAAAA   
  
  
- CGAACGACTA ACTAACGGTT ACGATACCTA AAGAACCACT CAGTTAGAAG TTAACGACTA GACCCAAGCA   
  
  
- AACAAGAAGT CTCAAAGTGG ACGGTTAAAC TAGTTCATAC AGGGAAGTGA AGAGGACGAA GTTAAGAGAG   
  
  
- AGTTTAAGTG CGTAAAAAGG TTACTTAAAG TTTATCCTCA GGACCTTAGG CTACTATAAA ATCCAAAAAA   
  
  
- GGACTGAAGT AACGTAATCA AACAAGTATA GACACTGGTT AAGAAAGTAA ACAATATACT AGACTAACAA   
  
  
- ATCAATCGAT AAGTAAGTAA CGTATATGTA ACCAAATAAG TAACGTCTCT GTGAGTGTAT GTGTGTGTGT   
  
  
- ATGTCCCCTG TGATCATAAA AAAGAGAGAT TGAGTAGCCT AAGGGAAACT ATGAGACAAA AAACCCCCCA   
  
  
- ACTAAACTAA ACTAAACTAA CTGGAATTAA AACTTGAGTG TGTGTGAGCG TGTATCTATA TAAGAAAGAC   
  
  
- AAACACTGTA TATGTCGTAG TATATCTTTA TATAACGTGA CGTATAAACT CGAGTTCTGT CCCTATAGTA   
  
  
- GTGTTTGACT CACACTCCCT CCGTTTTTTG TCTCCTTTTC TTCCCTTAAC CCTCACATTA GTTTTGTTCT   
  
  
- CTCTCTAACT CCTCTCTCCT TTTTTGTTTT AACCCTCTTC CAATTATCTC TTCTTACAAC CGACCCACAA   
  
  
- GTAGGTGTAA CGACAGTGGT TCCATATCTA ACTCCTCACT TCGATGTAGT GTCAAGGTTC GGACGGTAGT   
  
  
- AGGTTACTCG TGTGTCTCTT AACTAAACGG GACGTGAAAG GGTTCTTTCC TACAAAGCTT CGGTGTTAGC   
  
  
- CAAGCGGGGC AACCGGATAG ACAGCTCTTC GGCCAACTTC AGTCCCGGGA CTTCGTCTGG TAGGCTCAGG   
  
  
- GTGGTAGCGG CCTCCCATCT TCTCTCTAAT TCCGTCTAAA GACCCCACTA TCTTCTTTCT CGAACTTCTC   
  
  
- CTCTGATCGT CTTGTCCCCT CGAGAAAACT ACTACCACCA CTGCTTAGAA CACAACCCTC TTGGTTTTCC   
  
  
- TTCTTCCCAT CGAAACTAAA ACCACTTAGA CTACTTTAAA GAGGTTTTTA ACTCCTGTGT TACTCAAAAC   
  
  
- CTGTAGACCC ATCACCACCT TTAAAGACCC AAGTAGGACC AAAACCACAA CAATTACAAT TATGATTAAT   
  
  
- GGGAGTTCAC GGTAAAAGAG AGTGAACAAG TCCCCTCCTT CTTTCTCATA CAAAACAAGG ATGACTACAC   
  
  
- TAGAGTGGTC GGTAGTACGG AGGTAACAGT GTGTTAGGAA CCCAACTTAG ACCACAATGA CTCCAGTGTC   
  
  
- TTATACCACC CCCACTGTTT CTGCCGCTTG GATCAGTACC CAAACACTCC CCATGTTGTT GTTGCAGGCC   
  
  
- CAGTTCAAGT AGAAGACTCT CAGTATCAAA ACCCGTATCC GAGTTACTCT CGAGTAGACT GGGACTTTAG   
  
  
- TCTTTACCAA GGTACAACGG ATTGGGACCA GTACCAGGAC CGTGGTCAGT ACCAGGACCT TGGCCAGACC   
  
  
- GAGTGTTGGT GTTACTAGTT TGGCTCGTTG TTCCCAAGCT CGAGTAATCG AAAGAGTACC CAACACAGCT   
  
  
- TCGCTAGTCA AGTTCCTTGT AACGTCGGTA GTTAGTAAAG TATCGATTCG ACCCGCTCGT CCGAAGAGGT   
  
  
- TCCCGAGCCA GACGGTAATC GGCGGAGTGT CGGATGAAGT GGCTTCGTAA TCGGAACTCC CAGTGTTCTA   
  
  
- AAACCGGAGT ATAGAAAGTG TATTCATAGG GAGCTCTCAA ACTAGCTAAA CTACTACTTA GCCCCCGTCG   
  
  
- ACGTTACTCC GAAAACTTAG TCTAGTCGGG TTAAGGTTTC AAGCAAGTAA AATGTCGGTT ACTCTACAAT   
  
  
- AACTCCCGTA AACTTCCCTT CCTGTTCCAC GTATAGTATC TGAAGCTGTA GTTCGTTCCG AATGTTACCG   
  
  
- GGCCCAAAAA GGTTTCGAAT CGGTACTCCC TTTTGGGGGG CTCGGTACAG TCCTATTGTC CGCATCCCCT   
  
  
- AAGCTTCGTT CTTAACCAAC TCTGTCCTCT TTCTGATCGG CCCAAACGAC CCCGTAAGTT GGAGAGGAAG   
  
  
- CTCAAGGTGG GTCAACACCT GGCCAACCTT CTACAATCCG ATACCTACGA AGTACAATTC CTTCCACTTT   
  
  
- CACAGCGCCA TTTAACGTAA AACGTCGAGG TGTTCTGTGA GATACTGGGG GTACCACGGT GTAAGTCCCT   
  
  
- GAAGTACCCA AATTAGGCTT CGTGTTTAGG GTATCGTAAC CAATACCGAC TCGTTCTTCG ACTAGTGTTG   
  
  
- CTTGGGTGGA ATCTTCCGGC TCATACGTTG AGTGAACCTA TGATACGTCG GGAAAAGCTA CGGTATCTAA   
  
  
- CGTCGGAAGG AAAGCTCTCG AGTCGTTCCA ACTTCTAGCT TCTCTACAAA CCGGCCCTTT AGTCCTTGTA   
  
  
- TTATCGGACA CTTCCCCGTC TGTCCTAGCT TTCTGTACTC TTCAAGCTCT TTACCTTCTC TTCCTAGCTC   
  
  
- GTCCTTCCCA AGGCCACGTA CGCCTGATCG CTTTCCCTCC ACTACGTCTC GGTTTACGAC GAATTCTACA   
  
  
- TGAGCTTGCT CTCGATGTTG TACGTTTCTG TTCCGTTACT TCTTCGTTAT AGTGATCATA CCAATCTAGT   
  
  
- CGGTGAGATG TGACAAAGAA ACACCCGAGG TTCACTTAAA CGACGTCCCT CAAGAAGGTC AAAAAGAGAC   
  
  
- GGTAGAAC

+     CCGTCC-box

| Site Name | Organism | Position | Strand | Matrix score. | sequence | function |
| --- | --- | --- | --- | --- | --- | --- |
| CCGTCC-box | Petroselinum hortense | 909 | + | 6 | CCGTCC |  |

>HU04G00148.1   
+ -Up\_Stream \_Len000ATAATG GGTGATAACT TTTGAAGGAC AATATACAAA TCACTTATTT ATTAAAGTCA   
  
  
+ AGGGGACATT TCAACTATAA CATCATTGTT TATAAAGTAT TTGAAAATTG CAACTCTTGT AATAGATAAT   
  
  
+ AATGCTTTAT AGACTTCACC AATCACTAAT TTGTAGAATC AAAAAATTAA TTGAAAAGCA AACAAAAGGA   
  
  
+ AAAGGCACAT AAACCTAAAT TATGTAAACA AATGAGAACA TATATGCCAG CTTATCAACG TTAATTATAT   
  
  
+ TTGTTATCAC TCTTAATATC TGTATTCATA GTAAATGAAA ATAAGTATCG ATTTTTTATT AAACCTAAAA   
  
  
+ TCTTCTGTCA ATAATTTTTC TTCCGATAAA AAAAAAATTG GACCATATTA AATTAACATT AATAAATTCA   
  
  
+ TATAATTACT TTGAATGTCT GAAATATAAT GCTCTTCGGT ATATTAAGGT CTTATAAATT TCTCATTAAT   
  
  
+ TTGAAATCTC TTATAATATA TTGGGTCAAC AAGTGCTAGC TTGTTTTTTT TTCCTGGTTA ATTGCATTTA   
  
  
+ AAAAAATAAT TTTTAATTAC AATTGCAAGG GGAGGAATCA TTTACAAAAT ATCAAAAGTG GAAAAGGCAA   
  
  
+ AAAGAAACAA ATTATAAAAC AGAAAAAAGA TTAGAAAACG CAAACAAAGA CGAAATGAGG AACAGTAAGA   
  
  
+ ATCAGTAAAA AAATGTAGTG CAACGGTCAC ATACGGGAAG GCAAGCACAA CAATCATGAA AGAGACAGGC   
  
  
+ ACGTGCGTTC CTCGTGATCA GCAGCCTTCC CTACTTCCCC GCCAAGTGGA GTCCGTACTA ACTCCTGCCA   
  
  
+ TTCCCTCCCA ACGGTCATAT TCCGCCGACA TGCCTCAACG GTCAGATTTC CTGCCCCATC TGGACCGTCC   
  
  
+ GATCAAGATC CAACGGCCAT AAGGCGCACT CTCCAATACC CTAGTGCCCA TTTCTGGAAC AGCCCCAAAA   
  
  
+ ATCTCAGTAT TCGCCATCAT TTTTCAATAC TGGGGTTACA TAAACAGTCT AACAAGGAAG GACCCAACAC   
  
  
+ AGAGGAGAGA GAAAAAAGCT AGCTTTGATG GGGACAGCCA TGGATGGAGG TGAAGAGAGA GGAGGTTGGG   
  
  
+ ACCTTTTATT TTGCTCACTT TCCATTCTTA TATAAAAACA GCTCTAGTTT ATCTCTTATG GCTTTTTTGG   
  
  
+ GTTTGTTCAT AGAGAGGGGG GAATGTGATA TGATTTGATT TGATGTTAGA AATTCTCATT AGTTTTTTCT   
  
  
+ TTTTGTTTGC AATTTTGATG TGTTAGGGTT TGAGGATCAT CATTTTTGAG TAGGGAACTT TCTGGGTTTT   
  
  
+ GCTTGCTGAT TGATTGCCAA TGCTATGGAT TTCTTGGTGA GTCAATCTTC AATTGCTGAT CTGGGTTCGT   
  
  
+ TTGTTCTTCA GAGTTTCACC TGCCAATTTG ATCAAGTATG TCCCTTCACT TCTCCTGCTT CAATTCTCTC   
  
  
+ TCAAATTCAC GCATTTTTCC AATGAATTTC AAATAGGAGT CCTGGAATCC GATGATATTT TAGGTTTTTT   
  
  
+ CCTGACTTCA TTGCATTAGT TTGTTCATAT CTGTGACCAA TTCTTTCATT TGTTATATGA TCTGATTGTT   
  
  
+ TAGTTAGCTA TTCATTCATT GCATATACAT TGGTTTATTC ATTGCAGAGA CACTCACATA CACACACACA   
  
  
+ TACAGGGGAC ACTAGTATTT TTTCTCTCTA ACTCATCGGA TTCCCTTTGA TACTCTGTTT TTTGGGGGGT   
  
  
+ TGATTTGATT TGATTTGATT GACCTTAATT TTGAACTCAC ACACACTCGC ACATAGATAT ATTCTTTCTG   
  
  
+ TTTGTGACAT ATACAGCATC ATATAGAAAT ATATTGCACT GCATATTTGA GCTCAAGACA GGGATATCAT   
  
  
+ CACAAACTGA GTGTGAGGGA GGCAAAAAAC AGAGGAAAAG AAGGGAATTG GGAGTGTAAT CAAAACAAGA   
  
  
+ GAGAGATTGA GGAGAGAGGA AAAAACAAAA TTGGGAGAAG GTTAATAGAG AAGAATGTTG GCTGGGTGTT   
  
  
+ CATCCACATT GCTGTCACCA AGGTATAGAT TGAGGAGTGA AGCTACATCA CAGTTCCAAG CCTGCCATCA   
  
  
+ TCCAATGAGC ACACAGAGAA TTGATTTGCC CTGCACTTTC CCAAGAAAGG ATGTTTCGAA GCCACAATCG   
  
  
+ GTTCGCCCCG TTGGCCTATC TGTCGAGAAG CCGGTTGAAG TCAGGGCCCT GAAGCAGACC ATCCGAGTCC   
  
  
+ CACCATCGCC GGAGGGTAGA AGAGAGATTA AGGCAGATTT CTGGGGTGAT AGAAGAAAGA GCTTGAAGAG   
  
  
+ GAGACTAGCA GAACAGGGGA GCTCTTTTGA TGATGGTGGT GACGAATCTT GTGTTGGGAG AACCAAAAGG   
  
  
+ AAGAAGGGTA GCTTTGATTT TGGTGAATCT GATGAAATTT CTCCAAAAAT TGAGGACACA ATGAGTTTTG   
  
  
+ GACATCTGGG TAGTGGTGGA AATTTCTGGG TTCATCCTGG TTTTGGTGTT GTTAATGTTA ATACTAATTA   
  
  
+ CCCTCAAGTG CCATTTTCTC TCACTTGTTC AGGGGAGGAA GAAAGAGTAT GTTTTGTTCC TACTGATGTG   
  
  
+ ATCTCACCAG CCATCATGCC TCCATTGTCA CACAATCCTT GGGTTGAATC TGGTGTTACT GAGGTCACAG   
  
  
+ AATATGGTGG GGGTGACAAA GACGGCGAAC CTAGTCATGG GTTTGTGAGG GGTACAACAA CAACGTCCGG   
  
  
+ GTCAAGTTCA TCTTCTGAGA GTCATAGTTT TGGGCATAGG CTCAATGAGA GCTCATCTGA CCCTGAAATC   
  
  
+ AGAAATGGTT CCATGTTGCC TAACCCTGGT CATGGTCCTG GCACCAGTCA TGGTCCTGGA ACCGGTCTGG   
  
  
+ CTCACAACCA CAATGATCAA ACCGAGCAAC AAGGGTTCGA GCTCATTAGC TTTCTCATGG GTTGTGTCGA   
  
  
+ AGCGATCAGT TCAAGGAACA TTGCAGCCAT CAATCATTTC ATAGCTAAGC TGGGCGAGCA GGCTTCTCCA   
  
  
+ AGGGCTCGGT CTGCCATTAG CCGCCTCACA GCCTACTTCA CCGAAGCATT AGCCTTGAGG GTCACAAGAT   
  
  
+ TTTGGCCTCA TATCTTTCAC ATAAGTATCC CTCGAGAGTT TGATCGATTT GATGATGAAT CGGGGGCAGC   
  
  
+ TGCAATGAGG CTTTTGAATC AGATCAGCCC AATTCCAAAG TTCGTTCATT TTACAGCCAA TGAGATGTTA   
  
  
+ TTGAGGGCAT TTGAAGGGAA GGACAAGGTG CATATCATAG ACTTCGACAT CAAGCAAGGC TTACAATGGC   
  
  
+ CCGGGTTTTT CCAAAGCTTA GCCATGAGGG AAAACCCCCC GAGCCATGTC AGGATAACAG GCGTAGGGGA   
  
  
+ TTCGAAGCAA GAATTGGTTG AGACAGGAGA AAGACTAGCC GGGTTTGCTG GGGCATTCAA CCTCTCCTTC   
  
  
+ GAGTTCCACC CAGTTGTGGA CCGGTTGGAA GATGTTAGGC TATGGATGCT TCATGTTAAG GAAGGTGAAA   
  
  
+ GTGTCGCGGT AAATTGCATT TTGCAGCTCC ACAAGACACT CTATGACCCC CATGGTGCCA CATTCAGGGA   
  
  
+ CTTCATGGGT TTAATCCGAA GCACAAATCC CATAGCATTG GTTATGGCTG AGCAAGAAGC TGATCACAAC   
  
  
+ GAACCCACCT TAGAAGGCCG AGTATGCAAC TCACTTGGAT ACTATGCAGC CCTTTTCGAT GCCATAGATT   
  
  
+ GCAGCCTTCC TTTCGAGAGC TCAGCAAGGT TGAAGATCGA AGAGATGTTT GGCCGGGAAA TCAGGAACAT   
  
  
+ AATAGCCTGT GAAGGGGCAG ACAGGATCGA AAGACATGAG AAGTTCGAGA AATGGAAGAG AAGGATCGAG   
  
  
+ CAGGAAGGGT TCCGGTGCAT GCGGACTAGC GAAAGGGAGG TGATGCAGAG CCAAATGCTG CTTAAGATGT   
  
  
+ ACTCGAACGA GAGCTACAAC ATGCAAAGAC AAGGCAATGA AGAAGCAATA TCACTAGTAT GGTTAGATCA   
  
  
+ GCCACTCTAC ACTGTTTCTT TGTGGGCTCC AAGTGAATTT GCTGCAGGGA GTTCTTCCAG TTTTTCTCTG   
  
  
+ CCATCTTG  

- -Up\_Stream \_Len000TATTAC CCACTATTGA AAACTTCCTG TTATATGTTT AGTGAATAAA TAATTTCAGT   
  
  
- TCCCCTGTAA AGTTGATATT GTAGTAACAA ATATTTCATA AACTTTTAAC GTTGAGAACA TTATCTATTA   
  
  
- TTACGAAATA TCTGAAGTGG TTAGTGATTA AACATCTTAG TTTTTTAATT AACTTTTCGT TTGTTTTCCT   
  
  
- TTTCCGTGTA TTTGGATTTA ATACATTTGT TTACTCTTGT ATATACGGTC GAATAGTTGC AATTAATATA   
  
  
- AACAATAGTG AGAATTATAG ACATAAGTAT CATTTACTTT TATTCATAGC TAAAAAATAA TTTGGATTTT   
  
  
- AGAAGACAGT TATTAAAAAG AAGGCTATTT TTTTTTTAAC CTGGTATAAT TTAATTGTAA TTATTTAAGT   
  
  
- ATATTAATGA AACTTACAGA CTTTATATTA CGAGAAGCCA TATAATTCCA GAATATTTAA AGAGTAATTA   
  
  
- AACTTTAGAG AATATTATAT AACCCAGTTG TTCACGATCG AACAAAAAAA AAGGACCAAT TAACGTAAAT   
  
  
- TTTTTTATTA AAAATTAATG TTAACGTTCC CCTCCTTAGT AAATGTTTTA TAGTTTTCAC CTTTTCCGTT   
  
  
- TTTCTTTGTT TAATATTTTG TCTTTTTTCT AATCTTTTGC GTTTGTTTCT GCTTTACTCC TTGTCATTCT   
  
  
- TAGTCATTTT TTTACATCAC GTTGCCAGTG TATGCCCTTC CGTTCGTGTT GTTAGTACTT TCTCTGTCCG   
  
  
- TGCACGCAAG GAGCACTAGT CGTCGGAAGG GATGAAGGGG CGGTTCACCT CAGGCATGAT TGAGGACGGT   
  
  
- AAGGGAGGGT TGCCAGTATA AGGCGGCTGT ACGGAGTTGC CAGTCTAAAG GACGGGGTAG ACCTGGCAGG   
  
  
- CTAGTTCTAG GTTGCCGGTA TTCCGCGTGA GAGGTTATGG GATCACGGGT AAAGACCTTG TCGGGGTTTT   
  
  
- TAGAGTCATA AGCGGTAGTA AAAAGTTATG ACCCCAATGT ATTTGTCAGA TTGTTCCTTC CTGGGTTGTG   
  
  
- TCTCCTCTCT CTTTTTTCGA TCGAAACTAC CCCTGTCGGT ACCTACCTCC ACTTCTCTCT CCTCCAACCC   
  
  
- TGGAAAATAA AACGAGTGAA AGGTAAGAAT ATATTTTTGT CGAGATCAAA TAGAGAATAC CGAAAAAACC   
  
  
- CAAACAAGTA TCTCTCCCCC CTTACACTAT ACTAAACTAA ACTACAATCT TTAAGAGTAA TCAAAAAAGA   
  
  
- AAAACAAACG TTAAAACTAC ACAATCCCAA ACTCCTAGTA GTAAAAACTC ATCCCTTGAA AGACCCAAAA   
  
  
- CGAACGACTA ACTAACGGTT ACGATACCTA AAGAACCACT CAGTTAGAAG TTAACGACTA GACCCAAGCA   
  
  
- AACAAGAAGT CTCAAAGTGG ACGGTTAAAC TAGTTCATAC AGGGAAGTGA AGAGGACGAA GTTAAGAGAG   
  
  
- AGTTTAAGTG CGTAAAAAGG TTACTTAAAG TTTATCCTCA GGACCTTAGG CTACTATAAA ATCCAAAAAA   
  
  
- GGACTGAAGT AACGTAATCA AACAAGTATA GACACTGGTT AAGAAAGTAA ACAATATACT AGACTAACAA   
  
  
- ATCAATCGAT AAGTAAGTAA CGTATATGTA ACCAAATAAG TAACGTCTCT GTGAGTGTAT GTGTGTGTGT   
  
  
- ATGTCCCCTG TGATCATAAA AAAGAGAGAT TGAGTAGCCT AAGGGAAACT ATGAGACAAA AAACCCCCCA   
  
  
- ACTAAACTAA ACTAAACTAA CTGGAATTAA AACTTGAGTG TGTGTGAGCG TGTATCTATA TAAGAAAGAC   
  
  
- AAACACTGTA TATGTCGTAG TATATCTTTA TATAACGTGA CGTATAAACT CGAGTTCTGT CCCTATAGTA   
  
  
- GTGTTTGACT CACACTCCCT CCGTTTTTTG TCTCCTTTTC TTCCCTTAAC CCTCACATTA GTTTTGTTCT   
  
  
- CTCTCTAACT CCTCTCTCCT TTTTTGTTTT AACCCTCTTC CAATTATCTC TTCTTACAAC CGACCCACAA   
  
  
- GTAGGTGTAA CGACAGTGGT TCCATATCTA ACTCCTCACT TCGATGTAGT GTCAAGGTTC GGACGGTAGT   
  
  
- AGGTTACTCG TGTGTCTCTT AACTAAACGG GACGTGAAAG GGTTCTTTCC TACAAAGCTT CGGTGTTAGC   
  
  
- CAAGCGGGGC AACCGGATAG ACAGCTCTTC GGCCAACTTC AGTCCCGGGA CTTCGTCTGG TAGGCTCAGG   
  
  
- GTGGTAGCGG CCTCCCATCT TCTCTCTAAT TCCGTCTAAA GACCCCACTA TCTTCTTTCT CGAACTTCTC   
  
  
- CTCTGATCGT CTTGTCCCCT CGAGAAAACT ACTACCACCA CTGCTTAGAA CACAACCCTC TTGGTTTTCC   
  
  
- TTCTTCCCAT CGAAACTAAA ACCACTTAGA CTACTTTAAA GAGGTTTTTA ACTCCTGTGT TACTCAAAAC   
  
  
- CTGTAGACCC ATCACCACCT TTAAAGACCC AAGTAGGACC AAAACCACAA CAATTACAAT TATGATTAAT   
  
  
- GGGAGTTCAC GGTAAAAGAG AGTGAACAAG TCCCCTCCTT CTTTCTCATA CAAAACAAGG ATGACTACAC   
  
  
- TAGAGTGGTC GGTAGTACGG AGGTAACAGT GTGTTAGGAA CCCAACTTAG ACCACAATGA CTCCAGTGTC   
  
  
- TTATACCACC CCCACTGTTT CTGCCGCTTG GATCAGTACC CAAACACTCC CCATGTTGTT GTTGCAGGCC   
  
  
- CAGTTCAAGT AGAAGACTCT CAGTATCAAA ACCCGTATCC GAGTTACTCT CGAGTAGACT GGGACTTTAG   
  
  
- TCTTTACCAA GGTACAACGG ATTGGGACCA GTACCAGGAC CGTGGTCAGT ACCAGGACCT TGGCCAGACC   
  
  
- GAGTGTTGGT GTTACTAGTT TGGCTCGTTG TTCCCAAGCT CGAGTAATCG AAAGAGTACC CAACACAGCT   
  
  
- TCGCTAGTCA AGTTCCTTGT AACGTCGGTA GTTAGTAAAG TATCGATTCG ACCCGCTCGT CCGAAGAGGT   
  
  
- TCCCGAGCCA GACGGTAATC GGCGGAGTGT CGGATGAAGT GGCTTCGTAA TCGGAACTCC CAGTGTTCTA   
  
  
- AAACCGGAGT ATAGAAAGTG TATTCATAGG GAGCTCTCAA ACTAGCTAAA CTACTACTTA GCCCCCGTCG   
  
  
- ACGTTACTCC GAAAACTTAG TCTAGTCGGG TTAAGGTTTC AAGCAAGTAA AATGTCGGTT ACTCTACAAT   
  
  
- AACTCCCGTA AACTTCCCTT CCTGTTCCAC GTATAGTATC TGAAGCTGTA GTTCGTTCCG AATGTTACCG   
  
  
- GGCCCAAAAA GGTTTCGAAT CGGTACTCCC TTTTGGGGGG CTCGGTACAG TCCTATTGTC CGCATCCCCT   
  
  
- AAGCTTCGTT CTTAACCAAC TCTGTCCTCT TTCTGATCGG CCCAAACGAC CCCGTAAGTT GGAGAGGAAG   
  
  
- CTCAAGGTGG GTCAACACCT GGCCAACCTT CTACAATCCG ATACCTACGA AGTACAATTC CTTCCACTTT   
  
  
- CACAGCGCCA TTTAACGTAA AACGTCGAGG TGTTCTGTGA GATACTGGGG GTACCACGGT GTAAGTCCCT   
  
  
- GAAGTACCCA AATTAGGCTT CGTGTTTAGG GTATCGTAAC CAATACCGAC TCGTTCTTCG ACTAGTGTTG   
  
  
- CTTGGGTGGA ATCTTCCGGC TCATACGTTG AGTGAACCTA TGATACGTCG GGAAAAGCTA CGGTATCTAA   
  
  
- CGTCGGAAGG AAAGCTCTCG AGTCGTTCCA ACTTCTAGCT TCTCTACAAA CCGGCCCTTT AGTCCTTGTA   
  
  
- TTATCGGACA CTTCCCCGTC TGTCCTAGCT TTCTGTACTC TTCAAGCTCT TTACCTTCTC TTCCTAGCTC   
  
  
- GTCCTTCCCA AGGCCACGTA CGCCTGATCG CTTTCCCTCC ACTACGTCTC GGTTTACGAC GAATTCTACA   
  
  
- TGAGCTTGCT CTCGATGTTG TACGTTTCTG TTCCGTTACT TCTTCGTTAT AGTGATCATA CCAATCTAGT   
  
  
- CGGTGAGATG TGACAAAGAA ACACCCGAGG TTCACTTAAA CGACGTCCCT CAAGAAGGTC AAAAAGAGAC   
  
  
- GGTAGAAC

+     CGTCA-motif

| Site Name | Organism | Position | Strand | Matrix score. | sequence | function |
| --- | --- | --- | --- | --- | --- | --- |
| CGTCA-motif | Hordeum vulgare | 2354 | - | 5 | CGTCA | cis-acting regulatory element involved in the MeJA-responsiveness |

>HU04G00148.1   
+ -Up\_Stream \_Len000ATAATG GGTGATAACT TTTGAAGGAC AATATACAAA TCACTTATTT ATTAAAGTCA   
  
  
+ AGGGGACATT TCAACTATAA CATCATTGTT TATAAAGTAT TTGAAAATTG CAACTCTTGT AATAGATAAT   
  
  
+ AATGCTTTAT AGACTTCACC AATCACTAAT TTGTAGAATC AAAAAATTAA TTGAAAAGCA AACAAAAGGA   
  
  
+ AAAGGCACAT AAACCTAAAT TATGTAAACA AATGAGAACA TATATGCCAG CTTATCAACG TTAATTATAT   
  
  
+ TTGTTATCAC TCTTAATATC TGTATTCATA GTAAATGAAA ATAAGTATCG ATTTTTTATT AAACCTAAAA   
  
  
+ TCTTCTGTCA ATAATTTTTC TTCCGATAAA AAAAAAATTG GACCATATTA AATTAACATT AATAAATTCA   
  
  
+ TATAATTACT TTGAATGTCT GAAATATAAT GCTCTTCGGT ATATTAAGGT CTTATAAATT TCTCATTAAT   
  
  
+ TTGAAATCTC TTATAATATA TTGGGTCAAC AAGTGCTAGC TTGTTTTTTT TTCCTGGTTA ATTGCATTTA   
  
  
+ AAAAAATAAT TTTTAATTAC AATTGCAAGG GGAGGAATCA TTTACAAAAT ATCAAAAGTG GAAAAGGCAA   
  
  
+ AAAGAAACAA ATTATAAAAC AGAAAAAAGA TTAGAAAACG CAAACAAAGA CGAAATGAGG AACAGTAAGA   
  
  
+ ATCAGTAAAA AAATGTAGTG CAACGGTCAC ATACGGGAAG GCAAGCACAA CAATCATGAA AGAGACAGGC   
  
  
+ ACGTGCGTTC CTCGTGATCA GCAGCCTTCC CTACTTCCCC GCCAAGTGGA GTCCGTACTA ACTCCTGCCA   
  
  
+ TTCCCTCCCA ACGGTCATAT TCCGCCGACA TGCCTCAACG GTCAGATTTC CTGCCCCATC TGGACCGTCC   
  
  
+ GATCAAGATC CAACGGCCAT AAGGCGCACT CTCCAATACC CTAGTGCCCA TTTCTGGAAC AGCCCCAAAA   
  
  
+ ATCTCAGTAT TCGCCATCAT TTTTCAATAC TGGGGTTACA TAAACAGTCT AACAAGGAAG GACCCAACAC   
  
  
+ AGAGGAGAGA GAAAAAAGCT AGCTTTGATG GGGACAGCCA TGGATGGAGG TGAAGAGAGA GGAGGTTGGG   
  
  
+ ACCTTTTATT TTGCTCACTT TCCATTCTTA TATAAAAACA GCTCTAGTTT ATCTCTTATG GCTTTTTTGG   
  
  
+ GTTTGTTCAT AGAGAGGGGG GAATGTGATA TGATTTGATT TGATGTTAGA AATTCTCATT AGTTTTTTCT   
  
  
+ TTTTGTTTGC AATTTTGATG TGTTAGGGTT TGAGGATCAT CATTTTTGAG TAGGGAACTT TCTGGGTTTT   
  
  
+ GCTTGCTGAT TGATTGCCAA TGCTATGGAT TTCTTGGTGA GTCAATCTTC AATTGCTGAT CTGGGTTCGT   
  
  
+ TTGTTCTTCA GAGTTTCACC TGCCAATTTG ATCAAGTATG TCCCTTCACT TCTCCTGCTT CAATTCTCTC   
  
  
+ TCAAATTCAC GCATTTTTCC AATGAATTTC AAATAGGAGT CCTGGAATCC GATGATATTT TAGGTTTTTT   
  
  
+ CCTGACTTCA TTGCATTAGT TTGTTCATAT CTGTGACCAA TTCTTTCATT TGTTATATGA TCTGATTGTT   
  
  
+ TAGTTAGCTA TTCATTCATT GCATATACAT TGGTTTATTC ATTGCAGAGA CACTCACATA CACACACACA   
  
  
+ TACAGGGGAC ACTAGTATTT TTTCTCTCTA ACTCATCGGA TTCCCTTTGA TACTCTGTTT TTTGGGGGGT   
  
  
+ TGATTTGATT TGATTTGATT GACCTTAATT TTGAACTCAC ACACACTCGC ACATAGATAT ATTCTTTCTG   
  
  
+ TTTGTGACAT ATACAGCATC ATATAGAAAT ATATTGCACT GCATATTTGA GCTCAAGACA GGGATATCAT   
  
  
+ CACAAACTGA GTGTGAGGGA GGCAAAAAAC AGAGGAAAAG AAGGGAATTG GGAGTGTAAT CAAAACAAGA   
  
  
+ GAGAGATTGA GGAGAGAGGA AAAAACAAAA TTGGGAGAAG GTTAATAGAG AAGAATGTTG GCTGGGTGTT   
  
  
+ CATCCACATT GCTGTCACCA AGGTATAGAT TGAGGAGTGA AGCTACATCA CAGTTCCAAG CCTGCCATCA   
  
  
+ TCCAATGAGC ACACAGAGAA TTGATTTGCC CTGCACTTTC CCAAGAAAGG ATGTTTCGAA GCCACAATCG   
  
  
+ GTTCGCCCCG TTGGCCTATC TGTCGAGAAG CCGGTTGAAG TCAGGGCCCT GAAGCAGACC ATCCGAGTCC   
  
  
+ CACCATCGCC GGAGGGTAGA AGAGAGATTA AGGCAGATTT CTGGGGTGAT AGAAGAAAGA GCTTGAAGAG   
  
  
+ GAGACTAGCA GAACAGGGGA GCTCTTTTGA TGATGGTGGT GACGAATCTT GTGTTGGGAG AACCAAAAGG   
  
  
+ AAGAAGGGTA GCTTTGATTT TGGTGAATCT GATGAAATTT CTCCAAAAAT TGAGGACACA ATGAGTTTTG   
  
  
+ GACATCTGGG TAGTGGTGGA AATTTCTGGG TTCATCCTGG TTTTGGTGTT GTTAATGTTA ATACTAATTA   
  
  
+ CCCTCAAGTG CCATTTTCTC TCACTTGTTC AGGGGAGGAA GAAAGAGTAT GTTTTGTTCC TACTGATGTG   
  
  
+ ATCTCACCAG CCATCATGCC TCCATTGTCA CACAATCCTT GGGTTGAATC TGGTGTTACT GAGGTCACAG   
  
  
+ AATATGGTGG GGGTGACAAA GACGGCGAAC CTAGTCATGG GTTTGTGAGG GGTACAACAA CAACGTCCGG   
  
  
+ GTCAAGTTCA TCTTCTGAGA GTCATAGTTT TGGGCATAGG CTCAATGAGA GCTCATCTGA CCCTGAAATC   
  
  
+ AGAAATGGTT CCATGTTGCC TAACCCTGGT CATGGTCCTG GCACCAGTCA TGGTCCTGGA ACCGGTCTGG   
  
  
+ CTCACAACCA CAATGATCAA ACCGAGCAAC AAGGGTTCGA GCTCATTAGC TTTCTCATGG GTTGTGTCGA   
  
  
+ AGCGATCAGT TCAAGGAACA TTGCAGCCAT CAATCATTTC ATAGCTAAGC TGGGCGAGCA GGCTTCTCCA   
  
  
+ AGGGCTCGGT CTGCCATTAG CCGCCTCACA GCCTACTTCA CCGAAGCATT AGCCTTGAGG GTCACAAGAT   
  
  
+ TTTGGCCTCA TATCTTTCAC ATAAGTATCC CTCGAGAGTT TGATCGATTT GATGATGAAT CGGGGGCAGC   
  
  
+ TGCAATGAGG CTTTTGAATC AGATCAGCCC AATTCCAAAG TTCGTTCATT TTACAGCCAA TGAGATGTTA   
  
  
+ TTGAGGGCAT TTGAAGGGAA GGACAAGGTG CATATCATAG ACTTCGACAT CAAGCAAGGC TTACAATGGC   
  
  
+ CCGGGTTTTT CCAAAGCTTA GCCATGAGGG AAAACCCCCC GAGCCATGTC AGGATAACAG GCGTAGGGGA   
  
  
+ TTCGAAGCAA GAATTGGTTG AGACAGGAGA AAGACTAGCC GGGTTTGCTG GGGCATTCAA CCTCTCCTTC   
  
  
+ GAGTTCCACC CAGTTGTGGA CCGGTTGGAA GATGTTAGGC TATGGATGCT TCATGTTAAG GAAGGTGAAA   
  
  
+ GTGTCGCGGT AAATTGCATT TTGCAGCTCC ACAAGACACT CTATGACCCC CATGGTGCCA CATTCAGGGA   
  
  
+ CTTCATGGGT TTAATCCGAA GCACAAATCC CATAGCATTG GTTATGGCTG AGCAAGAAGC TGATCACAAC   
  
  
+ GAACCCACCT TAGAAGGCCG AGTATGCAAC TCACTTGGAT ACTATGCAGC CCTTTTCGAT GCCATAGATT   
  
  
+ GCAGCCTTCC TTTCGAGAGC TCAGCAAGGT TGAAGATCGA AGAGATGTTT GGCCGGGAAA TCAGGAACAT   
  
  
+ AATAGCCTGT GAAGGGGCAG ACAGGATCGA AAGACATGAG AAGTTCGAGA AATGGAAGAG AAGGATCGAG   
  
  
+ CAGGAAGGGT TCCGGTGCAT GCGGACTAGC GAAAGGGAGG TGATGCAGAG CCAAATGCTG CTTAAGATGT   
  
  
+ ACTCGAACGA GAGCTACAAC ATGCAAAGAC AAGGCAATGA AGAAGCAATA TCACTAGTAT GGTTAGATCA   
  
  
+ GCCACTCTAC ACTGTTTCTT TGTGGGCTCC AAGTGAATTT GCTGCAGGGA GTTCTTCCAG TTTTTCTCTG   
  
  
+ CCATCTTG  

- -Up\_Stream \_Len000TATTAC CCACTATTGA AAACTTCCTG TTATATGTTT AGTGAATAAA TAATTTCAGT   
  
  
- TCCCCTGTAA AGTTGATATT GTAGTAACAA ATATTTCATA AACTTTTAAC GTTGAGAACA TTATCTATTA   
  
  
- TTACGAAATA TCTGAAGTGG TTAGTGATTA AACATCTTAG TTTTTTAATT AACTTTTCGT TTGTTTTCCT   
  
  
- TTTCCGTGTA TTTGGATTTA ATACATTTGT TTACTCTTGT ATATACGGTC GAATAGTTGC AATTAATATA   
  
  
- AACAATAGTG AGAATTATAG ACATAAGTAT CATTTACTTT TATTCATAGC TAAAAAATAA TTTGGATTTT   
  
  
- AGAAGACAGT TATTAAAAAG AAGGCTATTT TTTTTTTAAC CTGGTATAAT TTAATTGTAA TTATTTAAGT   
  
  
- ATATTAATGA AACTTACAGA CTTTATATTA CGAGAAGCCA TATAATTCCA GAATATTTAA AGAGTAATTA   
  
  
- AACTTTAGAG AATATTATAT AACCCAGTTG TTCACGATCG AACAAAAAAA AAGGACCAAT TAACGTAAAT   
  
  
- TTTTTTATTA AAAATTAATG TTAACGTTCC CCTCCTTAGT AAATGTTTTA TAGTTTTCAC CTTTTCCGTT   
  
  
- TTTCTTTGTT TAATATTTTG TCTTTTTTCT AATCTTTTGC GTTTGTTTCT GCTTTACTCC TTGTCATTCT   
  
  
- TAGTCATTTT TTTACATCAC GTTGCCAGTG TATGCCCTTC CGTTCGTGTT GTTAGTACTT TCTCTGTCCG   
  
  
- TGCACGCAAG GAGCACTAGT CGTCGGAAGG GATGAAGGGG CGGTTCACCT CAGGCATGAT TGAGGACGGT   
  
  
- AAGGGAGGGT TGCCAGTATA AGGCGGCTGT ACGGAGTTGC CAGTCTAAAG GACGGGGTAG ACCTGGCAGG   
  
  
- CTAGTTCTAG GTTGCCGGTA TTCCGCGTGA GAGGTTATGG GATCACGGGT AAAGACCTTG TCGGGGTTTT   
  
  
- TAGAGTCATA AGCGGTAGTA AAAAGTTATG ACCCCAATGT ATTTGTCAGA TTGTTCCTTC CTGGGTTGTG   
  
  
- TCTCCTCTCT CTTTTTTCGA TCGAAACTAC CCCTGTCGGT ACCTACCTCC ACTTCTCTCT CCTCCAACCC   
  
  
- TGGAAAATAA AACGAGTGAA AGGTAAGAAT ATATTTTTGT CGAGATCAAA TAGAGAATAC CGAAAAAACC   
  
  
- CAAACAAGTA TCTCTCCCCC CTTACACTAT ACTAAACTAA ACTACAATCT TTAAGAGTAA TCAAAAAAGA   
  
  
- AAAACAAACG TTAAAACTAC ACAATCCCAA ACTCCTAGTA GTAAAAACTC ATCCCTTGAA AGACCCAAAA   
  
  
- CGAACGACTA ACTAACGGTT ACGATACCTA AAGAACCACT CAGTTAGAAG TTAACGACTA GACCCAAGCA   
  
  
- AACAAGAAGT CTCAAAGTGG ACGGTTAAAC TAGTTCATAC AGGGAAGTGA AGAGGACGAA GTTAAGAGAG   
  
  
- AGTTTAAGTG CGTAAAAAGG TTACTTAAAG TTTATCCTCA GGACCTTAGG CTACTATAAA ATCCAAAAAA   
  
  
- GGACTGAAGT AACGTAATCA AACAAGTATA GACACTGGTT AAGAAAGTAA ACAATATACT AGACTAACAA   
  
  
- ATCAATCGAT AAGTAAGTAA CGTATATGTA ACCAAATAAG TAACGTCTCT GTGAGTGTAT GTGTGTGTGT   
  
  
- ATGTCCCCTG TGATCATAAA AAAGAGAGAT TGAGTAGCCT AAGGGAAACT ATGAGACAAA AAACCCCCCA   
  
  
- ACTAAACTAA ACTAAACTAA CTGGAATTAA AACTTGAGTG TGTGTGAGCG TGTATCTATA TAAGAAAGAC   
  
  
- AAACACTGTA TATGTCGTAG TATATCTTTA TATAACGTGA CGTATAAACT CGAGTTCTGT CCCTATAGTA   
  
  
- GTGTTTGACT CACACTCCCT CCGTTTTTTG TCTCCTTTTC TTCCCTTAAC CCTCACATTA GTTTTGTTCT   
  
  
- CTCTCTAACT CCTCTCTCCT TTTTTGTTTT AACCCTCTTC CAATTATCTC TTCTTACAAC CGACCCACAA   
  
  
- GTAGGTGTAA CGACAGTGGT TCCATATCTA ACTCCTCACT TCGATGTAGT GTCAAGGTTC GGACGGTAGT   
  
  
- AGGTTACTCG TGTGTCTCTT AACTAAACGG GACGTGAAAG GGTTCTTTCC TACAAAGCTT CGGTGTTAGC   
  
  
- CAAGCGGGGC AACCGGATAG ACAGCTCTTC GGCCAACTTC AGTCCCGGGA CTTCGTCTGG TAGGCTCAGG   
  
  
- GTGGTAGCGG CCTCCCATCT TCTCTCTAAT TCCGTCTAAA GACCCCACTA TCTTCTTTCT CGAACTTCTC   
  
  
- CTCTGATCGT CTTGTCCCCT CGAGAAAACT ACTACCACCA CTGCTTAGAA CACAACCCTC TTGGTTTTCC   
  
  
- TTCTTCCCAT CGAAACTAAA ACCACTTAGA CTACTTTAAA GAGGTTTTTA ACTCCTGTGT TACTCAAAAC   
  
  
- CTGTAGACCC ATCACCACCT TTAAAGACCC AAGTAGGACC AAAACCACAA CAATTACAAT TATGATTAAT   
  
  
- GGGAGTTCAC GGTAAAAGAG AGTGAACAAG TCCCCTCCTT CTTTCTCATA CAAAACAAGG ATGACTACAC   
  
  
- TAGAGTGGTC GGTAGTACGG AGGTAACAGT GTGTTAGGAA CCCAACTTAG ACCACAATGA CTCCAGTGTC   
  
  
- TTATACCACC CCCACTGTTT CTGCCGCTTG GATCAGTACC CAAACACTCC CCATGTTGTT GTTGCAGGCC   
  
  
- CAGTTCAAGT AGAAGACTCT CAGTATCAAA ACCCGTATCC GAGTTACTCT CGAGTAGACT GGGACTTTAG   
  
  
- TCTTTACCAA GGTACAACGG ATTGGGACCA GTACCAGGAC CGTGGTCAGT ACCAGGACCT TGGCCAGACC   
  
  
- GAGTGTTGGT GTTACTAGTT TGGCTCGTTG TTCCCAAGCT CGAGTAATCG AAAGAGTACC CAACACAGCT   
  
  
- TCGCTAGTCA AGTTCCTTGT AACGTCGGTA GTTAGTAAAG TATCGATTCG ACCCGCTCGT CCGAAGAGGT   
  
  
- TCCCGAGCCA GACGGTAATC GGCGGAGTGT CGGATGAAGT GGCTTCGTAA TCGGAACTCC CAGTGTTCTA   
  
  
- AAACCGGAGT ATAGAAAGTG TATTCATAGG GAGCTCTCAA ACTAGCTAAA CTACTACTTA GCCCCCGTCG   
  
  
- ACGTTACTCC GAAAACTTAG TCTAGTCGGG TTAAGGTTTC AAGCAAGTAA AATGTCGGTT ACTCTACAAT   
  
  
- AACTCCCGTA AACTTCCCTT CCTGTTCCAC GTATAGTATC TGAAGCTGTA GTTCGTTCCG AATGTTACCG   
  
  
- GGCCCAAAAA GGTTTCGAAT CGGTACTCCC TTTTGGGGGG CTCGGTACAG TCCTATTGTC CGCATCCCCT   
  
  
- AAGCTTCGTT CTTAACCAAC TCTGTCCTCT TTCTGATCGG CCCAAACGAC CCCGTAAGTT GGAGAGGAAG   
  
  
- CTCAAGGTGG GTCAACACCT GGCCAACCTT CTACAATCCG ATACCTACGA AGTACAATTC CTTCCACTTT   
  
  
- CACAGCGCCA TTTAACGTAA AACGTCGAGG TGTTCTGTGA GATACTGGGG GTACCACGGT GTAAGTCCCT   
  
  
- GAAGTACCCA AATTAGGCTT CGTGTTTAGG GTATCGTAAC CAATACCGAC TCGTTCTTCG ACTAGTGTTG   
  
  
- CTTGGGTGGA ATCTTCCGGC TCATACGTTG AGTGAACCTA TGATACGTCG GGAAAAGCTA CGGTATCTAA   
  
  
- CGTCGGAAGG AAAGCTCTCG AGTCGTTCCA ACTTCTAGCT TCTCTACAAA CCGGCCCTTT AGTCCTTGTA   
  
  
- TTATCGGACA CTTCCCCGTC TGTCCTAGCT TTCTGTACTC TTCAAGCTCT TTACCTTCTC TTCCTAGCTC   
  
  
- GTCCTTCCCA AGGCCACGTA CGCCTGATCG CTTTCCCTCC ACTACGTCTC GGTTTACGAC GAATTCTACA   
  
  
- TGAGCTTGCT CTCGATGTTG TACGTTTCTG TTCCGTTACT TCTTCGTTAT AGTGATCATA CCAATCTAGT   
  
  
- CGGTGAGATG TGACAAAGAA ACACCCGAGG TTCACTTAAA CGACGTCCCT CAAGAAGGTC AAAAAGAGAC   
  
  
- GGTAGAAC

+     CTAG-motif

| Site Name | Organism | Position | Strand | Matrix score. | sequence | function |
| --- | --- | --- | --- | --- | --- | --- |
| CTAG-motif | Avena sativa | 2318 | + | 10 | ACTAGCAGAA |  |

>HU04G00148.1   
+ -Up\_Stream \_Len000ATAATG GGTGATAACT TTTGAAGGAC AATATACAAA TCACTTATTT ATTAAAGTCA   
  
  
+ AGGGGACATT TCAACTATAA CATCATTGTT TATAAAGTAT TTGAAAATTG CAACTCTTGT AATAGATAAT   
  
  
+ AATGCTTTAT AGACTTCACC AATCACTAAT TTGTAGAATC AAAAAATTAA TTGAAAAGCA AACAAAAGGA   
  
  
+ AAAGGCACAT AAACCTAAAT TATGTAAACA AATGAGAACA TATATGCCAG CTTATCAACG TTAATTATAT   
  
  
+ TTGTTATCAC TCTTAATATC TGTATTCATA GTAAATGAAA ATAAGTATCG ATTTTTTATT AAACCTAAAA   
  
  
+ TCTTCTGTCA ATAATTTTTC TTCCGATAAA AAAAAAATTG GACCATATTA AATTAACATT AATAAATTCA   
  
  
+ TATAATTACT TTGAATGTCT GAAATATAAT GCTCTTCGGT ATATTAAGGT CTTATAAATT TCTCATTAAT   
  
  
+ TTGAAATCTC TTATAATATA TTGGGTCAAC AAGTGCTAGC TTGTTTTTTT TTCCTGGTTA ATTGCATTTA   
  
  
+ AAAAAATAAT TTTTAATTAC AATTGCAAGG GGAGGAATCA TTTACAAAAT ATCAAAAGTG GAAAAGGCAA   
  
  
+ AAAGAAACAA ATTATAAAAC AGAAAAAAGA TTAGAAAACG CAAACAAAGA CGAAATGAGG AACAGTAAGA   
  
  
+ ATCAGTAAAA AAATGTAGTG CAACGGTCAC ATACGGGAAG GCAAGCACAA CAATCATGAA AGAGACAGGC   
  
  
+ ACGTGCGTTC CTCGTGATCA GCAGCCTTCC CTACTTCCCC GCCAAGTGGA GTCCGTACTA ACTCCTGCCA   
  
  
+ TTCCCTCCCA ACGGTCATAT TCCGCCGACA TGCCTCAACG GTCAGATTTC CTGCCCCATC TGGACCGTCC   
  
  
+ GATCAAGATC CAACGGCCAT AAGGCGCACT CTCCAATACC CTAGTGCCCA TTTCTGGAAC AGCCCCAAAA   
  
  
+ ATCTCAGTAT TCGCCATCAT TTTTCAATAC TGGGGTTACA TAAACAGTCT AACAAGGAAG GACCCAACAC   
  
  
+ AGAGGAGAGA GAAAAAAGCT AGCTTTGATG GGGACAGCCA TGGATGGAGG TGAAGAGAGA GGAGGTTGGG   
  
  
+ ACCTTTTATT TTGCTCACTT TCCATTCTTA TATAAAAACA GCTCTAGTTT ATCTCTTATG GCTTTTTTGG   
  
  
+ GTTTGTTCAT AGAGAGGGGG GAATGTGATA TGATTTGATT TGATGTTAGA AATTCTCATT AGTTTTTTCT   
  
  
+ TTTTGTTTGC AATTTTGATG TGTTAGGGTT TGAGGATCAT CATTTTTGAG TAGGGAACTT TCTGGGTTTT   
  
  
+ GCTTGCTGAT TGATTGCCAA TGCTATGGAT TTCTTGGTGA GTCAATCTTC AATTGCTGAT CTGGGTTCGT   
  
  
+ TTGTTCTTCA GAGTTTCACC TGCCAATTTG ATCAAGTATG TCCCTTCACT TCTCCTGCTT CAATTCTCTC   
  
  
+ TCAAATTCAC GCATTTTTCC AATGAATTTC AAATAGGAGT CCTGGAATCC GATGATATTT TAGGTTTTTT   
  
  
+ CCTGACTTCA TTGCATTAGT TTGTTCATAT CTGTGACCAA TTCTTTCATT TGTTATATGA TCTGATTGTT   
  
  
+ TAGTTAGCTA TTCATTCATT GCATATACAT TGGTTTATTC ATTGCAGAGA CACTCACATA CACACACACA   
  
  
+ TACAGGGGAC ACTAGTATTT TTTCTCTCTA ACTCATCGGA TTCCCTTTGA TACTCTGTTT TTTGGGGGGT   
  
  
+ TGATTTGATT TGATTTGATT GACCTTAATT TTGAACTCAC ACACACTCGC ACATAGATAT ATTCTTTCTG   
  
  
+ TTTGTGACAT ATACAGCATC ATATAGAAAT ATATTGCACT GCATATTTGA GCTCAAGACA GGGATATCAT   
  
  
+ CACAAACTGA GTGTGAGGGA GGCAAAAAAC AGAGGAAAAG AAGGGAATTG GGAGTGTAAT CAAAACAAGA   
  
  
+ GAGAGATTGA GGAGAGAGGA AAAAACAAAA TTGGGAGAAG GTTAATAGAG AAGAATGTTG GCTGGGTGTT   
  
  
+ CATCCACATT GCTGTCACCA AGGTATAGAT TGAGGAGTGA AGCTACATCA CAGTTCCAAG CCTGCCATCA   
  
  
+ TCCAATGAGC ACACAGAGAA TTGATTTGCC CTGCACTTTC CCAAGAAAGG ATGTTTCGAA GCCACAATCG   
  
  
+ GTTCGCCCCG TTGGCCTATC TGTCGAGAAG CCGGTTGAAG TCAGGGCCCT GAAGCAGACC ATCCGAGTCC   
  
  
+ CACCATCGCC GGAGGGTAGA AGAGAGATTA AGGCAGATTT CTGGGGTGAT AGAAGAAAGA GCTTGAAGAG   
  
  
+ GAGACTAGCA GAACAGGGGA GCTCTTTTGA TGATGGTGGT GACGAATCTT GTGTTGGGAG AACCAAAAGG   
  
  
+ AAGAAGGGTA GCTTTGATTT TGGTGAATCT GATGAAATTT CTCCAAAAAT TGAGGACACA ATGAGTTTTG   
  
  
+ GACATCTGGG TAGTGGTGGA AATTTCTGGG TTCATCCTGG TTTTGGTGTT GTTAATGTTA ATACTAATTA   
  
  
+ CCCTCAAGTG CCATTTTCTC TCACTTGTTC AGGGGAGGAA GAAAGAGTAT GTTTTGTTCC TACTGATGTG   
  
  
+ ATCTCACCAG CCATCATGCC TCCATTGTCA CACAATCCTT GGGTTGAATC TGGTGTTACT GAGGTCACAG   
  
  
+ AATATGGTGG GGGTGACAAA GACGGCGAAC CTAGTCATGG GTTTGTGAGG GGTACAACAA CAACGTCCGG   
  
  
+ GTCAAGTTCA TCTTCTGAGA GTCATAGTTT TGGGCATAGG CTCAATGAGA GCTCATCTGA CCCTGAAATC   
  
  
+ AGAAATGGTT CCATGTTGCC TAACCCTGGT CATGGTCCTG GCACCAGTCA TGGTCCTGGA ACCGGTCTGG   
  
  
+ CTCACAACCA CAATGATCAA ACCGAGCAAC AAGGGTTCGA GCTCATTAGC TTTCTCATGG GTTGTGTCGA   
  
  
+ AGCGATCAGT TCAAGGAACA TTGCAGCCAT CAATCATTTC ATAGCTAAGC TGGGCGAGCA GGCTTCTCCA   
  
  
+ AGGGCTCGGT CTGCCATTAG CCGCCTCACA GCCTACTTCA CCGAAGCATT AGCCTTGAGG GTCACAAGAT   
  
  
+ TTTGGCCTCA TATCTTTCAC ATAAGTATCC CTCGAGAGTT TGATCGATTT GATGATGAAT CGGGGGCAGC   
  
  
+ TGCAATGAGG CTTTTGAATC AGATCAGCCC AATTCCAAAG TTCGTTCATT TTACAGCCAA TGAGATGTTA   
  
  
+ TTGAGGGCAT TTGAAGGGAA GGACAAGGTG CATATCATAG ACTTCGACAT CAAGCAAGGC TTACAATGGC   
  
  
+ CCGGGTTTTT CCAAAGCTTA GCCATGAGGG AAAACCCCCC GAGCCATGTC AGGATAACAG GCGTAGGGGA   
  
  
+ TTCGAAGCAA GAATTGGTTG AGACAGGAGA AAGACTAGCC GGGTTTGCTG GGGCATTCAA CCTCTCCTTC   
  
  
+ GAGTTCCACC CAGTTGTGGA CCGGTTGGAA GATGTTAGGC TATGGATGCT TCATGTTAAG GAAGGTGAAA   
  
  
+ GTGTCGCGGT AAATTGCATT TTGCAGCTCC ACAAGACACT CTATGACCCC CATGGTGCCA CATTCAGGGA   
  
  
+ CTTCATGGGT TTAATCCGAA GCACAAATCC CATAGCATTG GTTATGGCTG AGCAAGAAGC TGATCACAAC   
  
  
+ GAACCCACCT TAGAAGGCCG AGTATGCAAC TCACTTGGAT ACTATGCAGC CCTTTTCGAT GCCATAGATT   
  
  
+ GCAGCCTTCC TTTCGAGAGC TCAGCAAGGT TGAAGATCGA AGAGATGTTT GGCCGGGAAA TCAGGAACAT   
  
  
+ AATAGCCTGT GAAGGGGCAG ACAGGATCGA AAGACATGAG AAGTTCGAGA AATGGAAGAG AAGGATCGAG   
  
  
+ CAGGAAGGGT TCCGGTGCAT GCGGACTAGC GAAAGGGAGG TGATGCAGAG CCAAATGCTG CTTAAGATGT   
  
  
+ ACTCGAACGA GAGCTACAAC ATGCAAAGAC AAGGCAATGA AGAAGCAATA TCACTAGTAT GGTTAGATCA   
  
  
+ GCCACTCTAC ACTGTTTCTT TGTGGGCTCC AAGTGAATTT GCTGCAGGGA GTTCTTCCAG TTTTTCTCTG   
  
  
+ CCATCTTG  

- -Up\_Stream \_Len000TATTAC CCACTATTGA AAACTTCCTG TTATATGTTT AGTGAATAAA TAATTTCAGT   
  
  
- TCCCCTGTAA AGTTGATATT GTAGTAACAA ATATTTCATA AACTTTTAAC GTTGAGAACA TTATCTATTA   
  
  
- TTACGAAATA TCTGAAGTGG TTAGTGATTA AACATCTTAG TTTTTTAATT AACTTTTCGT TTGTTTTCCT   
  
  
- TTTCCGTGTA TTTGGATTTA ATACATTTGT TTACTCTTGT ATATACGGTC GAATAGTTGC AATTAATATA   
  
  
- AACAATAGTG AGAATTATAG ACATAAGTAT CATTTACTTT TATTCATAGC TAAAAAATAA TTTGGATTTT   
  
  
- AGAAGACAGT TATTAAAAAG AAGGCTATTT TTTTTTTAAC CTGGTATAAT TTAATTGTAA TTATTTAAGT   
  
  
- ATATTAATGA AACTTACAGA CTTTATATTA CGAGAAGCCA TATAATTCCA GAATATTTAA AGAGTAATTA   
  
  
- AACTTTAGAG AATATTATAT AACCCAGTTG TTCACGATCG AACAAAAAAA AAGGACCAAT TAACGTAAAT   
  
  
- TTTTTTATTA AAAATTAATG TTAACGTTCC CCTCCTTAGT AAATGTTTTA TAGTTTTCAC CTTTTCCGTT   
  
  
- TTTCTTTGTT TAATATTTTG TCTTTTTTCT AATCTTTTGC GTTTGTTTCT GCTTTACTCC TTGTCATTCT   
  
  
- TAGTCATTTT TTTACATCAC GTTGCCAGTG TATGCCCTTC CGTTCGTGTT GTTAGTACTT TCTCTGTCCG   
  
  
- TGCACGCAAG GAGCACTAGT CGTCGGAAGG GATGAAGGGG CGGTTCACCT CAGGCATGAT TGAGGACGGT   
  
  
- AAGGGAGGGT TGCCAGTATA AGGCGGCTGT ACGGAGTTGC CAGTCTAAAG GACGGGGTAG ACCTGGCAGG   
  
  
- CTAGTTCTAG GTTGCCGGTA TTCCGCGTGA GAGGTTATGG GATCACGGGT AAAGACCTTG TCGGGGTTTT   
  
  
- TAGAGTCATA AGCGGTAGTA AAAAGTTATG ACCCCAATGT ATTTGTCAGA TTGTTCCTTC CTGGGTTGTG   
  
  
- TCTCCTCTCT CTTTTTTCGA TCGAAACTAC CCCTGTCGGT ACCTACCTCC ACTTCTCTCT CCTCCAACCC   
  
  
- TGGAAAATAA AACGAGTGAA AGGTAAGAAT ATATTTTTGT CGAGATCAAA TAGAGAATAC CGAAAAAACC   
  
  
- CAAACAAGTA TCTCTCCCCC CTTACACTAT ACTAAACTAA ACTACAATCT TTAAGAGTAA TCAAAAAAGA   
  
  
- AAAACAAACG TTAAAACTAC ACAATCCCAA ACTCCTAGTA GTAAAAACTC ATCCCTTGAA AGACCCAAAA   
  
  
- CGAACGACTA ACTAACGGTT ACGATACCTA AAGAACCACT CAGTTAGAAG TTAACGACTA GACCCAAGCA   
  
  
- AACAAGAAGT CTCAAAGTGG ACGGTTAAAC TAGTTCATAC AGGGAAGTGA AGAGGACGAA GTTAAGAGAG   
  
  
- AGTTTAAGTG CGTAAAAAGG TTACTTAAAG TTTATCCTCA GGACCTTAGG CTACTATAAA ATCCAAAAAA   
  
  
- GGACTGAAGT AACGTAATCA AACAAGTATA GACACTGGTT AAGAAAGTAA ACAATATACT AGACTAACAA   
  
  
- ATCAATCGAT AAGTAAGTAA CGTATATGTA ACCAAATAAG TAACGTCTCT GTGAGTGTAT GTGTGTGTGT   
  
  
- ATGTCCCCTG TGATCATAAA AAAGAGAGAT TGAGTAGCCT AAGGGAAACT ATGAGACAAA AAACCCCCCA   
  
  
- ACTAAACTAA ACTAAACTAA CTGGAATTAA AACTTGAGTG TGTGTGAGCG TGTATCTATA TAAGAAAGAC   
  
  
- AAACACTGTA TATGTCGTAG TATATCTTTA TATAACGTGA CGTATAAACT CGAGTTCTGT CCCTATAGTA   
  
  
- GTGTTTGACT CACACTCCCT CCGTTTTTTG TCTCCTTTTC TTCCCTTAAC CCTCACATTA GTTTTGTTCT   
  
  
- CTCTCTAACT CCTCTCTCCT TTTTTGTTTT AACCCTCTTC CAATTATCTC TTCTTACAAC CGACCCACAA   
  
  
- GTAGGTGTAA CGACAGTGGT TCCATATCTA ACTCCTCACT TCGATGTAGT GTCAAGGTTC GGACGGTAGT   
  
  
- AGGTTACTCG TGTGTCTCTT AACTAAACGG GACGTGAAAG GGTTCTTTCC TACAAAGCTT CGGTGTTAGC   
  
  
- CAAGCGGGGC AACCGGATAG ACAGCTCTTC GGCCAACTTC AGTCCCGGGA CTTCGTCTGG TAGGCTCAGG   
  
  
- GTGGTAGCGG CCTCCCATCT TCTCTCTAAT TCCGTCTAAA GACCCCACTA TCTTCTTTCT CGAACTTCTC   
  
  
- CTCTGATCGT CTTGTCCCCT CGAGAAAACT ACTACCACCA CTGCTTAGAA CACAACCCTC TTGGTTTTCC   
  
  
- TTCTTCCCAT CGAAACTAAA ACCACTTAGA CTACTTTAAA GAGGTTTTTA ACTCCTGTGT TACTCAAAAC   
  
  
- CTGTAGACCC ATCACCACCT TTAAAGACCC AAGTAGGACC AAAACCACAA CAATTACAAT TATGATTAAT   
  
  
- GGGAGTTCAC GGTAAAAGAG AGTGAACAAG TCCCCTCCTT CTTTCTCATA CAAAACAAGG ATGACTACAC   
  
  
- TAGAGTGGTC GGTAGTACGG AGGTAACAGT GTGTTAGGAA CCCAACTTAG ACCACAATGA CTCCAGTGTC   
  
  
- TTATACCACC CCCACTGTTT CTGCCGCTTG GATCAGTACC CAAACACTCC CCATGTTGTT GTTGCAGGCC   
  
  
- CAGTTCAAGT AGAAGACTCT CAGTATCAAA ACCCGTATCC GAGTTACTCT CGAGTAGACT GGGACTTTAG   
  
  
- TCTTTACCAA GGTACAACGG ATTGGGACCA GTACCAGGAC CGTGGTCAGT ACCAGGACCT TGGCCAGACC   
  
  
- GAGTGTTGGT GTTACTAGTT TGGCTCGTTG TTCCCAAGCT CGAGTAATCG AAAGAGTACC CAACACAGCT   
  
  
- TCGCTAGTCA AGTTCCTTGT AACGTCGGTA GTTAGTAAAG TATCGATTCG ACCCGCTCGT CCGAAGAGGT   
  
  
- TCCCGAGCCA GACGGTAATC GGCGGAGTGT CGGATGAAGT GGCTTCGTAA TCGGAACTCC CAGTGTTCTA   
  
  
- AAACCGGAGT ATAGAAAGTG TATTCATAGG GAGCTCTCAA ACTAGCTAAA CTACTACTTA GCCCCCGTCG   
  
  
- ACGTTACTCC GAAAACTTAG TCTAGTCGGG TTAAGGTTTC AAGCAAGTAA AATGTCGGTT ACTCTACAAT   
  
  
- AACTCCCGTA AACTTCCCTT CCTGTTCCAC GTATAGTATC TGAAGCTGTA GTTCGTTCCG AATGTTACCG   
  
  
- GGCCCAAAAA GGTTTCGAAT CGGTACTCCC TTTTGGGGGG CTCGGTACAG TCCTATTGTC CGCATCCCCT   
  
  
- AAGCTTCGTT CTTAACCAAC TCTGTCCTCT TTCTGATCGG CCCAAACGAC CCCGTAAGTT GGAGAGGAAG   
  
  
- CTCAAGGTGG GTCAACACCT GGCCAACCTT CTACAATCCG ATACCTACGA AGTACAATTC CTTCCACTTT   
  
  
- CACAGCGCCA TTTAACGTAA AACGTCGAGG TGTTCTGTGA GATACTGGGG GTACCACGGT GTAAGTCCCT   
  
  
- GAAGTACCCA AATTAGGCTT CGTGTTTAGG GTATCGTAAC CAATACCGAC TCGTTCTTCG ACTAGTGTTG   
  
  
- CTTGGGTGGA ATCTTCCGGC TCATACGTTG AGTGAACCTA TGATACGTCG GGAAAAGCTA CGGTATCTAA   
  
  
- CGTCGGAAGG AAAGCTCTCG AGTCGTTCCA ACTTCTAGCT TCTCTACAAA CCGGCCCTTT AGTCCTTGTA   
  
  
- TTATCGGACA CTTCCCCGTC TGTCCTAGCT TTCTGTACTC TTCAAGCTCT TTACCTTCTC TTCCTAGCTC   
  
  
- GTCCTTCCCA AGGCCACGTA CGCCTGATCG CTTTCCCTCC ACTACGTCTC GGTTTACGAC GAATTCTACA   
  
  
- TGAGCTTGCT CTCGATGTTG TACGTTTCTG TTCCGTTACT TCTTCGTTAT AGTGATCATA CCAATCTAGT   
  
  
- CGGTGAGATG TGACAAAGAA ACACCCGAGG TTCACTTAAA CGACGTCCCT CAAGAAGGTC AAAAAGAGAC   
  
  
- GGTAGAAC

+     DRE core

| Site Name | Organism | Position | Strand | Matrix score. | sequence | function |
| --- | --- | --- | --- | --- | --- | --- |
| DRE core | Arabidopsis thaliana | 868 | + | 6 | GCCGAC |  |

>HU04G00148.1   
+ -Up\_Stream \_Len000ATAATG GGTGATAACT TTTGAAGGAC AATATACAAA TCACTTATTT ATTAAAGTCA   
  
  
+ AGGGGACATT TCAACTATAA CATCATTGTT TATAAAGTAT TTGAAAATTG CAACTCTTGT AATAGATAAT   
  
  
+ AATGCTTTAT AGACTTCACC AATCACTAAT TTGTAGAATC AAAAAATTAA TTGAAAAGCA AACAAAAGGA   
  
  
+ AAAGGCACAT AAACCTAAAT TATGTAAACA AATGAGAACA TATATGCCAG CTTATCAACG TTAATTATAT   
  
  
+ TTGTTATCAC TCTTAATATC TGTATTCATA GTAAATGAAA ATAAGTATCG ATTTTTTATT AAACCTAAAA   
  
  
+ TCTTCTGTCA ATAATTTTTC TTCCGATAAA AAAAAAATTG GACCATATTA AATTAACATT AATAAATTCA   
  
  
+ TATAATTACT TTGAATGTCT GAAATATAAT GCTCTTCGGT ATATTAAGGT CTTATAAATT TCTCATTAAT   
  
  
+ TTGAAATCTC TTATAATATA TTGGGTCAAC AAGTGCTAGC TTGTTTTTTT TTCCTGGTTA ATTGCATTTA   
  
  
+ AAAAAATAAT TTTTAATTAC AATTGCAAGG GGAGGAATCA TTTACAAAAT ATCAAAAGTG GAAAAGGCAA   
  
  
+ AAAGAAACAA ATTATAAAAC AGAAAAAAGA TTAGAAAACG CAAACAAAGA CGAAATGAGG AACAGTAAGA   
  
  
+ ATCAGTAAAA AAATGTAGTG CAACGGTCAC ATACGGGAAG GCAAGCACAA CAATCATGAA AGAGACAGGC   
  
  
+ ACGTGCGTTC CTCGTGATCA GCAGCCTTCC CTACTTCCCC GCCAAGTGGA GTCCGTACTA ACTCCTGCCA   
  
  
+ TTCCCTCCCA ACGGTCATAT TCCGCCGACA TGCCTCAACG GTCAGATTTC CTGCCCCATC TGGACCGTCC   
  
  
+ GATCAAGATC CAACGGCCAT AAGGCGCACT CTCCAATACC CTAGTGCCCA TTTCTGGAAC AGCCCCAAAA   
  
  
+ ATCTCAGTAT TCGCCATCAT TTTTCAATAC TGGGGTTACA TAAACAGTCT AACAAGGAAG GACCCAACAC   
  
  
+ AGAGGAGAGA GAAAAAAGCT AGCTTTGATG GGGACAGCCA TGGATGGAGG TGAAGAGAGA GGAGGTTGGG   
  
  
+ ACCTTTTATT TTGCTCACTT TCCATTCTTA TATAAAAACA GCTCTAGTTT ATCTCTTATG GCTTTTTTGG   
  
  
+ GTTTGTTCAT AGAGAGGGGG GAATGTGATA TGATTTGATT TGATGTTAGA AATTCTCATT AGTTTTTTCT   
  
  
+ TTTTGTTTGC AATTTTGATG TGTTAGGGTT TGAGGATCAT CATTTTTGAG TAGGGAACTT TCTGGGTTTT   
  
  
+ GCTTGCTGAT TGATTGCCAA TGCTATGGAT TTCTTGGTGA GTCAATCTTC AATTGCTGAT CTGGGTTCGT   
  
  
+ TTGTTCTTCA GAGTTTCACC TGCCAATTTG ATCAAGTATG TCCCTTCACT TCTCCTGCTT CAATTCTCTC   
  
  
+ TCAAATTCAC GCATTTTTCC AATGAATTTC AAATAGGAGT CCTGGAATCC GATGATATTT TAGGTTTTTT   
  
  
+ CCTGACTTCA TTGCATTAGT TTGTTCATAT CTGTGACCAA TTCTTTCATT TGTTATATGA TCTGATTGTT   
  
  
+ TAGTTAGCTA TTCATTCATT GCATATACAT TGGTTTATTC ATTGCAGAGA CACTCACATA CACACACACA   
  
  
+ TACAGGGGAC ACTAGTATTT TTTCTCTCTA ACTCATCGGA TTCCCTTTGA TACTCTGTTT TTTGGGGGGT   
  
  
+ TGATTTGATT TGATTTGATT GACCTTAATT TTGAACTCAC ACACACTCGC ACATAGATAT ATTCTTTCTG   
  
  
+ TTTGTGACAT ATACAGCATC ATATAGAAAT ATATTGCACT GCATATTTGA GCTCAAGACA GGGATATCAT   
  
  
+ CACAAACTGA GTGTGAGGGA GGCAAAAAAC AGAGGAAAAG AAGGGAATTG GGAGTGTAAT CAAAACAAGA   
  
  
+ GAGAGATTGA GGAGAGAGGA AAAAACAAAA TTGGGAGAAG GTTAATAGAG AAGAATGTTG GCTGGGTGTT   
  
  
+ CATCCACATT GCTGTCACCA AGGTATAGAT TGAGGAGTGA AGCTACATCA CAGTTCCAAG CCTGCCATCA   
  
  
+ TCCAATGAGC ACACAGAGAA TTGATTTGCC CTGCACTTTC CCAAGAAAGG ATGTTTCGAA GCCACAATCG   
  
  
+ GTTCGCCCCG TTGGCCTATC TGTCGAGAAG CCGGTTGAAG TCAGGGCCCT GAAGCAGACC ATCCGAGTCC   
  
  
+ CACCATCGCC GGAGGGTAGA AGAGAGATTA AGGCAGATTT CTGGGGTGAT AGAAGAAAGA GCTTGAAGAG   
  
  
+ GAGACTAGCA GAACAGGGGA GCTCTTTTGA TGATGGTGGT GACGAATCTT GTGTTGGGAG AACCAAAAGG   
  
  
+ AAGAAGGGTA GCTTTGATTT TGGTGAATCT GATGAAATTT CTCCAAAAAT TGAGGACACA ATGAGTTTTG   
  
  
+ GACATCTGGG TAGTGGTGGA AATTTCTGGG TTCATCCTGG TTTTGGTGTT GTTAATGTTA ATACTAATTA   
  
  
+ CCCTCAAGTG CCATTTTCTC TCACTTGTTC AGGGGAGGAA GAAAGAGTAT GTTTTGTTCC TACTGATGTG   
  
  
+ ATCTCACCAG CCATCATGCC TCCATTGTCA CACAATCCTT GGGTTGAATC TGGTGTTACT GAGGTCACAG   
  
  
+ AATATGGTGG GGGTGACAAA GACGGCGAAC CTAGTCATGG GTTTGTGAGG GGTACAACAA CAACGTCCGG   
  
  
+ GTCAAGTTCA TCTTCTGAGA GTCATAGTTT TGGGCATAGG CTCAATGAGA GCTCATCTGA CCCTGAAATC   
  
  
+ AGAAATGGTT CCATGTTGCC TAACCCTGGT CATGGTCCTG GCACCAGTCA TGGTCCTGGA ACCGGTCTGG   
  
  
+ CTCACAACCA CAATGATCAA ACCGAGCAAC AAGGGTTCGA GCTCATTAGC TTTCTCATGG GTTGTGTCGA   
  
  
+ AGCGATCAGT TCAAGGAACA TTGCAGCCAT CAATCATTTC ATAGCTAAGC TGGGCGAGCA GGCTTCTCCA   
  
  
+ AGGGCTCGGT CTGCCATTAG CCGCCTCACA GCCTACTTCA CCGAAGCATT AGCCTTGAGG GTCACAAGAT   
  
  
+ TTTGGCCTCA TATCTTTCAC ATAAGTATCC CTCGAGAGTT TGATCGATTT GATGATGAAT CGGGGGCAGC   
  
  
+ TGCAATGAGG CTTTTGAATC AGATCAGCCC AATTCCAAAG TTCGTTCATT TTACAGCCAA TGAGATGTTA   
  
  
+ TTGAGGGCAT TTGAAGGGAA GGACAAGGTG CATATCATAG ACTTCGACAT CAAGCAAGGC TTACAATGGC   
  
  
+ CCGGGTTTTT CCAAAGCTTA GCCATGAGGG AAAACCCCCC GAGCCATGTC AGGATAACAG GCGTAGGGGA   
  
  
+ TTCGAAGCAA GAATTGGTTG AGACAGGAGA AAGACTAGCC GGGTTTGCTG GGGCATTCAA CCTCTCCTTC   
  
  
+ GAGTTCCACC CAGTTGTGGA CCGGTTGGAA GATGTTAGGC TATGGATGCT TCATGTTAAG GAAGGTGAAA   
  
  
+ GTGTCGCGGT AAATTGCATT TTGCAGCTCC ACAAGACACT CTATGACCCC CATGGTGCCA CATTCAGGGA   
  
  
+ CTTCATGGGT TTAATCCGAA GCACAAATCC CATAGCATTG GTTATGGCTG AGCAAGAAGC TGATCACAAC   
  
  
+ GAACCCACCT TAGAAGGCCG AGTATGCAAC TCACTTGGAT ACTATGCAGC CCTTTTCGAT GCCATAGATT   
  
  
+ GCAGCCTTCC TTTCGAGAGC TCAGCAAGGT TGAAGATCGA AGAGATGTTT GGCCGGGAAA TCAGGAACAT   
  
  
+ AATAGCCTGT GAAGGGGCAG ACAGGATCGA AAGACATGAG AAGTTCGAGA AATGGAAGAG AAGGATCGAG   
  
  
+ CAGGAAGGGT TCCGGTGCAT GCGGACTAGC GAAAGGGAGG TGATGCAGAG CCAAATGCTG CTTAAGATGT   
  
  
+ ACTCGAACGA GAGCTACAAC ATGCAAAGAC AAGGCAATGA AGAAGCAATA TCACTAGTAT GGTTAGATCA   
  
  
+ GCCACTCTAC ACTGTTTCTT TGTGGGCTCC AAGTGAATTT GCTGCAGGGA GTTCTTCCAG TTTTTCTCTG   
  
  
+ CCATCTTG  

- -Up\_Stream \_Len000TATTAC CCACTATTGA AAACTTCCTG TTATATGTTT AGTGAATAAA TAATTTCAGT   
  
  
- TCCCCTGTAA AGTTGATATT GTAGTAACAA ATATTTCATA AACTTTTAAC GTTGAGAACA TTATCTATTA   
  
  
- TTACGAAATA TCTGAAGTGG TTAGTGATTA AACATCTTAG TTTTTTAATT AACTTTTCGT TTGTTTTCCT   
  
  
- TTTCCGTGTA TTTGGATTTA ATACATTTGT TTACTCTTGT ATATACGGTC GAATAGTTGC AATTAATATA   
  
  
- AACAATAGTG AGAATTATAG ACATAAGTAT CATTTACTTT TATTCATAGC TAAAAAATAA TTTGGATTTT   
  
  
- AGAAGACAGT TATTAAAAAG AAGGCTATTT TTTTTTTAAC CTGGTATAAT TTAATTGTAA TTATTTAAGT   
  
  
- ATATTAATGA AACTTACAGA CTTTATATTA CGAGAAGCCA TATAATTCCA GAATATTTAA AGAGTAATTA   
  
  
- AACTTTAGAG AATATTATAT AACCCAGTTG TTCACGATCG AACAAAAAAA AAGGACCAAT TAACGTAAAT   
  
  
- TTTTTTATTA AAAATTAATG TTAACGTTCC CCTCCTTAGT AAATGTTTTA TAGTTTTCAC CTTTTCCGTT   
  
  
- TTTCTTTGTT TAATATTTTG TCTTTTTTCT AATCTTTTGC GTTTGTTTCT GCTTTACTCC TTGTCATTCT   
  
  
- TAGTCATTTT TTTACATCAC GTTGCCAGTG TATGCCCTTC CGTTCGTGTT GTTAGTACTT TCTCTGTCCG   
  
  
- TGCACGCAAG GAGCACTAGT CGTCGGAAGG GATGAAGGGG CGGTTCACCT CAGGCATGAT TGAGGACGGT   
  
  
- AAGGGAGGGT TGCCAGTATA AGGCGGCTGT ACGGAGTTGC CAGTCTAAAG GACGGGGTAG ACCTGGCAGG   
  
  
- CTAGTTCTAG GTTGCCGGTA TTCCGCGTGA GAGGTTATGG GATCACGGGT AAAGACCTTG TCGGGGTTTT   
  
  
- TAGAGTCATA AGCGGTAGTA AAAAGTTATG ACCCCAATGT ATTTGTCAGA TTGTTCCTTC CTGGGTTGTG   
  
  
- TCTCCTCTCT CTTTTTTCGA TCGAAACTAC CCCTGTCGGT ACCTACCTCC ACTTCTCTCT CCTCCAACCC   
  
  
- TGGAAAATAA AACGAGTGAA AGGTAAGAAT ATATTTTTGT CGAGATCAAA TAGAGAATAC CGAAAAAACC   
  
  
- CAAACAAGTA TCTCTCCCCC CTTACACTAT ACTAAACTAA ACTACAATCT TTAAGAGTAA TCAAAAAAGA   
  
  
- AAAACAAACG TTAAAACTAC ACAATCCCAA ACTCCTAGTA GTAAAAACTC ATCCCTTGAA AGACCCAAAA   
  
  
- CGAACGACTA ACTAACGGTT ACGATACCTA AAGAACCACT CAGTTAGAAG TTAACGACTA GACCCAAGCA   
  
  
- AACAAGAAGT CTCAAAGTGG ACGGTTAAAC TAGTTCATAC AGGGAAGTGA AGAGGACGAA GTTAAGAGAG   
  
  
- AGTTTAAGTG CGTAAAAAGG TTACTTAAAG TTTATCCTCA GGACCTTAGG CTACTATAAA ATCCAAAAAA   
  
  
- GGACTGAAGT AACGTAATCA AACAAGTATA GACACTGGTT AAGAAAGTAA ACAATATACT AGACTAACAA   
  
  
- ATCAATCGAT AAGTAAGTAA CGTATATGTA ACCAAATAAG TAACGTCTCT GTGAGTGTAT GTGTGTGTGT   
  
  
- ATGTCCCCTG TGATCATAAA AAAGAGAGAT TGAGTAGCCT AAGGGAAACT ATGAGACAAA AAACCCCCCA   
  
  
- ACTAAACTAA ACTAAACTAA CTGGAATTAA AACTTGAGTG TGTGTGAGCG TGTATCTATA TAAGAAAGAC   
  
  
- AAACACTGTA TATGTCGTAG TATATCTTTA TATAACGTGA CGTATAAACT CGAGTTCTGT CCCTATAGTA   
  
  
- GTGTTTGACT CACACTCCCT CCGTTTTTTG TCTCCTTTTC TTCCCTTAAC CCTCACATTA GTTTTGTTCT   
  
  
- CTCTCTAACT CCTCTCTCCT TTTTTGTTTT AACCCTCTTC CAATTATCTC TTCTTACAAC CGACCCACAA   
  
  
- GTAGGTGTAA CGACAGTGGT TCCATATCTA ACTCCTCACT TCGATGTAGT GTCAAGGTTC GGACGGTAGT   
  
  
- AGGTTACTCG TGTGTCTCTT AACTAAACGG GACGTGAAAG GGTTCTTTCC TACAAAGCTT CGGTGTTAGC   
  
  
- CAAGCGGGGC AACCGGATAG ACAGCTCTTC GGCCAACTTC AGTCCCGGGA CTTCGTCTGG TAGGCTCAGG   
  
  
- GTGGTAGCGG CCTCCCATCT TCTCTCTAAT TCCGTCTAAA GACCCCACTA TCTTCTTTCT CGAACTTCTC   
  
  
- CTCTGATCGT CTTGTCCCCT CGAGAAAACT ACTACCACCA CTGCTTAGAA CACAACCCTC TTGGTTTTCC   
  
  
- TTCTTCCCAT CGAAACTAAA ACCACTTAGA CTACTTTAAA GAGGTTTTTA ACTCCTGTGT TACTCAAAAC   
  
  
- CTGTAGACCC ATCACCACCT TTAAAGACCC AAGTAGGACC AAAACCACAA CAATTACAAT TATGATTAAT   
  
  
- GGGAGTTCAC GGTAAAAGAG AGTGAACAAG TCCCCTCCTT CTTTCTCATA CAAAACAAGG ATGACTACAC   
  
  
- TAGAGTGGTC GGTAGTACGG AGGTAACAGT GTGTTAGGAA CCCAACTTAG ACCACAATGA CTCCAGTGTC   
  
  
- TTATACCACC CCCACTGTTT CTGCCGCTTG GATCAGTACC CAAACACTCC CCATGTTGTT GTTGCAGGCC   
  
  
- CAGTTCAAGT AGAAGACTCT CAGTATCAAA ACCCGTATCC GAGTTACTCT CGAGTAGACT GGGACTTTAG   
  
  
- TCTTTACCAA GGTACAACGG ATTGGGACCA GTACCAGGAC CGTGGTCAGT ACCAGGACCT TGGCCAGACC   
  
  
- GAGTGTTGGT GTTACTAGTT TGGCTCGTTG TTCCCAAGCT CGAGTAATCG AAAGAGTACC CAACACAGCT   
  
  
- TCGCTAGTCA AGTTCCTTGT AACGTCGGTA GTTAGTAAAG TATCGATTCG ACCCGCTCGT CCGAAGAGGT   
  
  
- TCCCGAGCCA GACGGTAATC GGCGGAGTGT CGGATGAAGT GGCTTCGTAA TCGGAACTCC CAGTGTTCTA   
  
  
- AAACCGGAGT ATAGAAAGTG TATTCATAGG GAGCTCTCAA ACTAGCTAAA CTACTACTTA GCCCCCGTCG   
  
  
- ACGTTACTCC GAAAACTTAG TCTAGTCGGG TTAAGGTTTC AAGCAAGTAA AATGTCGGTT ACTCTACAAT   
  
  
- AACTCCCGTA AACTTCCCTT CCTGTTCCAC GTATAGTATC TGAAGCTGTA GTTCGTTCCG AATGTTACCG   
  
  
- GGCCCAAAAA GGTTTCGAAT CGGTACTCCC TTTTGGGGGG CTCGGTACAG TCCTATTGTC CGCATCCCCT   
  
  
- AAGCTTCGTT CTTAACCAAC TCTGTCCTCT TTCTGATCGG CCCAAACGAC CCCGTAAGTT GGAGAGGAAG   
  
  
- CTCAAGGTGG GTCAACACCT GGCCAACCTT CTACAATCCG ATACCTACGA AGTACAATTC CTTCCACTTT   
  
  
- CACAGCGCCA TTTAACGTAA AACGTCGAGG TGTTCTGTGA GATACTGGGG GTACCACGGT GTAAGTCCCT   
  
  
- GAAGTACCCA AATTAGGCTT CGTGTTTAGG GTATCGTAAC CAATACCGAC TCGTTCTTCG ACTAGTGTTG   
  
  
- CTTGGGTGGA ATCTTCCGGC TCATACGTTG AGTGAACCTA TGATACGTCG GGAAAAGCTA CGGTATCTAA   
  
  
- CGTCGGAAGG AAAGCTCTCG AGTCGTTCCA ACTTCTAGCT TCTCTACAAA CCGGCCCTTT AGTCCTTGTA   
  
  
- TTATCGGACA CTTCCCCGTC TGTCCTAGCT TTCTGTACTC TTCAAGCTCT TTACCTTCTC TTCCTAGCTC   
  
  
- GTCCTTCCCA AGGCCACGTA CGCCTGATCG CTTTCCCTCC ACTACGTCTC GGTTTACGAC GAATTCTACA   
  
  
- TGAGCTTGCT CTCGATGTTG TACGTTTCTG TTCCGTTACT TCTTCGTTAT AGTGATCATA CCAATCTAGT   
  
  
- CGGTGAGATG TGACAAAGAA ACACCCGAGG TTCACTTAAA CGACGTCCCT CAAGAAGGTC AAAAAGAGAC   
  
  
- GGTAGAAC

+     ERE

| Site Name | Organism | Position | Strand | Matrix score. | sequence | function |
| --- | --- | --- | --- | --- | --- | --- |
| ERE | Nicotiana glutinos | 2980 | + | 8 | ATTTCATA |  |

>HU04G00148.1   
+ -Up\_Stream \_Len000ATAATG GGTGATAACT TTTGAAGGAC AATATACAAA TCACTTATTT ATTAAAGTCA   
  
  
+ AGGGGACATT TCAACTATAA CATCATTGTT TATAAAGTAT TTGAAAATTG CAACTCTTGT AATAGATAAT   
  
  
+ AATGCTTTAT AGACTTCACC AATCACTAAT TTGTAGAATC AAAAAATTAA TTGAAAAGCA AACAAAAGGA   
  
  
+ AAAGGCACAT AAACCTAAAT TATGTAAACA AATGAGAACA TATATGCCAG CTTATCAACG TTAATTATAT   
  
  
+ TTGTTATCAC TCTTAATATC TGTATTCATA GTAAATGAAA ATAAGTATCG ATTTTTTATT AAACCTAAAA   
  
  
+ TCTTCTGTCA ATAATTTTTC TTCCGATAAA AAAAAAATTG GACCATATTA AATTAACATT AATAAATTCA   
  
  
+ TATAATTACT TTGAATGTCT GAAATATAAT GCTCTTCGGT ATATTAAGGT CTTATAAATT TCTCATTAAT   
  
  
+ TTGAAATCTC TTATAATATA TTGGGTCAAC AAGTGCTAGC TTGTTTTTTT TTCCTGGTTA ATTGCATTTA   
  
  
+ AAAAAATAAT TTTTAATTAC AATTGCAAGG GGAGGAATCA TTTACAAAAT ATCAAAAGTG GAAAAGGCAA   
  
  
+ AAAGAAACAA ATTATAAAAC AGAAAAAAGA TTAGAAAACG CAAACAAAGA CGAAATGAGG AACAGTAAGA   
  
  
+ ATCAGTAAAA AAATGTAGTG CAACGGTCAC ATACGGGAAG GCAAGCACAA CAATCATGAA AGAGACAGGC   
  
  
+ ACGTGCGTTC CTCGTGATCA GCAGCCTTCC CTACTTCCCC GCCAAGTGGA GTCCGTACTA ACTCCTGCCA   
  
  
+ TTCCCTCCCA ACGGTCATAT TCCGCCGACA TGCCTCAACG GTCAGATTTC CTGCCCCATC TGGACCGTCC   
  
  
+ GATCAAGATC CAACGGCCAT AAGGCGCACT CTCCAATACC CTAGTGCCCA TTTCTGGAAC AGCCCCAAAA   
  
  
+ ATCTCAGTAT TCGCCATCAT TTTTCAATAC TGGGGTTACA TAAACAGTCT AACAAGGAAG GACCCAACAC   
  
  
+ AGAGGAGAGA GAAAAAAGCT AGCTTTGATG GGGACAGCCA TGGATGGAGG TGAAGAGAGA GGAGGTTGGG   
  
  
+ ACCTTTTATT TTGCTCACTT TCCATTCTTA TATAAAAACA GCTCTAGTTT ATCTCTTATG GCTTTTTTGG   
  
  
+ GTTTGTTCAT AGAGAGGGGG GAATGTGATA TGATTTGATT TGATGTTAGA AATTCTCATT AGTTTTTTCT   
  
  
+ TTTTGTTTGC AATTTTGATG TGTTAGGGTT TGAGGATCAT CATTTTTGAG TAGGGAACTT TCTGGGTTTT   
  
  
+ GCTTGCTGAT TGATTGCCAA TGCTATGGAT TTCTTGGTGA GTCAATCTTC AATTGCTGAT CTGGGTTCGT   
  
  
+ TTGTTCTTCA GAGTTTCACC TGCCAATTTG ATCAAGTATG TCCCTTCACT TCTCCTGCTT CAATTCTCTC   
  
  
+ TCAAATTCAC GCATTTTTCC AATGAATTTC AAATAGGAGT CCTGGAATCC GATGATATTT TAGGTTTTTT   
  
  
+ CCTGACTTCA TTGCATTAGT TTGTTCATAT CTGTGACCAA TTCTTTCATT TGTTATATGA TCTGATTGTT   
  
  
+ TAGTTAGCTA TTCATTCATT GCATATACAT TGGTTTATTC ATTGCAGAGA CACTCACATA CACACACACA   
  
  
+ TACAGGGGAC ACTAGTATTT TTTCTCTCTA ACTCATCGGA TTCCCTTTGA TACTCTGTTT TTTGGGGGGT   
  
  
+ TGATTTGATT TGATTTGATT GACCTTAATT TTGAACTCAC ACACACTCGC ACATAGATAT ATTCTTTCTG   
  
  
+ TTTGTGACAT ATACAGCATC ATATAGAAAT ATATTGCACT GCATATTTGA GCTCAAGACA GGGATATCAT   
  
  
+ CACAAACTGA GTGTGAGGGA GGCAAAAAAC AGAGGAAAAG AAGGGAATTG GGAGTGTAAT CAAAACAAGA   
  
  
+ GAGAGATTGA GGAGAGAGGA AAAAACAAAA TTGGGAGAAG GTTAATAGAG AAGAATGTTG GCTGGGTGTT   
  
  
+ CATCCACATT GCTGTCACCA AGGTATAGAT TGAGGAGTGA AGCTACATCA CAGTTCCAAG CCTGCCATCA   
  
  
+ TCCAATGAGC ACACAGAGAA TTGATTTGCC CTGCACTTTC CCAAGAAAGG ATGTTTCGAA GCCACAATCG   
  
  
+ GTTCGCCCCG TTGGCCTATC TGTCGAGAAG CCGGTTGAAG TCAGGGCCCT GAAGCAGACC ATCCGAGTCC   
  
  
+ CACCATCGCC GGAGGGTAGA AGAGAGATTA AGGCAGATTT CTGGGGTGAT AGAAGAAAGA GCTTGAAGAG   
  
  
+ GAGACTAGCA GAACAGGGGA GCTCTTTTGA TGATGGTGGT GACGAATCTT GTGTTGGGAG AACCAAAAGG   
  
  
+ AAGAAGGGTA GCTTTGATTT TGGTGAATCT GATGAAATTT CTCCAAAAAT TGAGGACACA ATGAGTTTTG   
  
  
+ GACATCTGGG TAGTGGTGGA AATTTCTGGG TTCATCCTGG TTTTGGTGTT GTTAATGTTA ATACTAATTA   
  
  
+ CCCTCAAGTG CCATTTTCTC TCACTTGTTC AGGGGAGGAA GAAAGAGTAT GTTTTGTTCC TACTGATGTG   
  
  
+ ATCTCACCAG CCATCATGCC TCCATTGTCA CACAATCCTT GGGTTGAATC TGGTGTTACT GAGGTCACAG   
  
  
+ AATATGGTGG GGGTGACAAA GACGGCGAAC CTAGTCATGG GTTTGTGAGG GGTACAACAA CAACGTCCGG   
  
  
+ GTCAAGTTCA TCTTCTGAGA GTCATAGTTT TGGGCATAGG CTCAATGAGA GCTCATCTGA CCCTGAAATC   
  
  
+ AGAAATGGTT CCATGTTGCC TAACCCTGGT CATGGTCCTG GCACCAGTCA TGGTCCTGGA ACCGGTCTGG   
  
  
+ CTCACAACCA CAATGATCAA ACCGAGCAAC AAGGGTTCGA GCTCATTAGC TTTCTCATGG GTTGTGTCGA   
  
  
+ AGCGATCAGT TCAAGGAACA TTGCAGCCAT CAATCATTTC ATAGCTAAGC TGGGCGAGCA GGCTTCTCCA   
  
  
+ AGGGCTCGGT CTGCCATTAG CCGCCTCACA GCCTACTTCA CCGAAGCATT AGCCTTGAGG GTCACAAGAT   
  
  
+ TTTGGCCTCA TATCTTTCAC ATAAGTATCC CTCGAGAGTT TGATCGATTT GATGATGAAT CGGGGGCAGC   
  
  
+ TGCAATGAGG CTTTTGAATC AGATCAGCCC AATTCCAAAG TTCGTTCATT TTACAGCCAA TGAGATGTTA   
  
  
+ TTGAGGGCAT TTGAAGGGAA GGACAAGGTG CATATCATAG ACTTCGACAT CAAGCAAGGC TTACAATGGC   
  
  
+ CCGGGTTTTT CCAAAGCTTA GCCATGAGGG AAAACCCCCC GAGCCATGTC AGGATAACAG GCGTAGGGGA   
  
  
+ TTCGAAGCAA GAATTGGTTG AGACAGGAGA AAGACTAGCC GGGTTTGCTG GGGCATTCAA CCTCTCCTTC   
  
  
+ GAGTTCCACC CAGTTGTGGA CCGGTTGGAA GATGTTAGGC TATGGATGCT TCATGTTAAG GAAGGTGAAA   
  
  
+ GTGTCGCGGT AAATTGCATT TTGCAGCTCC ACAAGACACT CTATGACCCC CATGGTGCCA CATTCAGGGA   
  
  
+ CTTCATGGGT TTAATCCGAA GCACAAATCC CATAGCATTG GTTATGGCTG AGCAAGAAGC TGATCACAAC   
  
  
+ GAACCCACCT TAGAAGGCCG AGTATGCAAC TCACTTGGAT ACTATGCAGC CCTTTTCGAT GCCATAGATT   
  
  
+ GCAGCCTTCC TTTCGAGAGC TCAGCAAGGT TGAAGATCGA AGAGATGTTT GGCCGGGAAA TCAGGAACAT   
  
  
+ AATAGCCTGT GAAGGGGCAG ACAGGATCGA AAGACATGAG AAGTTCGAGA AATGGAAGAG AAGGATCGAG   
  
  
+ CAGGAAGGGT TCCGGTGCAT GCGGACTAGC GAAAGGGAGG TGATGCAGAG CCAAATGCTG CTTAAGATGT   
  
  
+ ACTCGAACGA GAGCTACAAC ATGCAAAGAC AAGGCAATGA AGAAGCAATA TCACTAGTAT GGTTAGATCA   
  
  
+ GCCACTCTAC ACTGTTTCTT TGTGGGCTCC AAGTGAATTT GCTGCAGGGA GTTCTTCCAG TTTTTCTCTG   
  
  
+ CCATCTTG  

- -Up\_Stream \_Len000TATTAC CCACTATTGA AAACTTCCTG TTATATGTTT AGTGAATAAA TAATTTCAGT   
  
  
- TCCCCTGTAA AGTTGATATT GTAGTAACAA ATATTTCATA AACTTTTAAC GTTGAGAACA TTATCTATTA   
  
  
- TTACGAAATA TCTGAAGTGG TTAGTGATTA AACATCTTAG TTTTTTAATT AACTTTTCGT TTGTTTTCCT   
  
  
- TTTCCGTGTA TTTGGATTTA ATACATTTGT TTACTCTTGT ATATACGGTC GAATAGTTGC AATTAATATA   
  
  
- AACAATAGTG AGAATTATAG ACATAAGTAT CATTTACTTT TATTCATAGC TAAAAAATAA TTTGGATTTT   
  
  
- AGAAGACAGT TATTAAAAAG AAGGCTATTT TTTTTTTAAC CTGGTATAAT TTAATTGTAA TTATTTAAGT   
  
  
- ATATTAATGA AACTTACAGA CTTTATATTA CGAGAAGCCA TATAATTCCA GAATATTTAA AGAGTAATTA   
  
  
- AACTTTAGAG AATATTATAT AACCCAGTTG TTCACGATCG AACAAAAAAA AAGGACCAAT TAACGTAAAT   
  
  
- TTTTTTATTA AAAATTAATG TTAACGTTCC CCTCCTTAGT AAATGTTTTA TAGTTTTCAC CTTTTCCGTT   
  
  
- TTTCTTTGTT TAATATTTTG TCTTTTTTCT AATCTTTTGC GTTTGTTTCT GCTTTACTCC TTGTCATTCT   
  
  
- TAGTCATTTT TTTACATCAC GTTGCCAGTG TATGCCCTTC CGTTCGTGTT GTTAGTACTT TCTCTGTCCG   
  
  
- TGCACGCAAG GAGCACTAGT CGTCGGAAGG GATGAAGGGG CGGTTCACCT CAGGCATGAT TGAGGACGGT   
  
  
- AAGGGAGGGT TGCCAGTATA AGGCGGCTGT ACGGAGTTGC CAGTCTAAAG GACGGGGTAG ACCTGGCAGG   
  
  
- CTAGTTCTAG GTTGCCGGTA TTCCGCGTGA GAGGTTATGG GATCACGGGT AAAGACCTTG TCGGGGTTTT   
  
  
- TAGAGTCATA AGCGGTAGTA AAAAGTTATG ACCCCAATGT ATTTGTCAGA TTGTTCCTTC CTGGGTTGTG   
  
  
- TCTCCTCTCT CTTTTTTCGA TCGAAACTAC CCCTGTCGGT ACCTACCTCC ACTTCTCTCT CCTCCAACCC   
  
  
- TGGAAAATAA AACGAGTGAA AGGTAAGAAT ATATTTTTGT CGAGATCAAA TAGAGAATAC CGAAAAAACC   
  
  
- CAAACAAGTA TCTCTCCCCC CTTACACTAT ACTAAACTAA ACTACAATCT TTAAGAGTAA TCAAAAAAGA   
  
  
- AAAACAAACG TTAAAACTAC ACAATCCCAA ACTCCTAGTA GTAAAAACTC ATCCCTTGAA AGACCCAAAA   
  
  
- CGAACGACTA ACTAACGGTT ACGATACCTA AAGAACCACT CAGTTAGAAG TTAACGACTA GACCCAAGCA   
  
  
- AACAAGAAGT CTCAAAGTGG ACGGTTAAAC TAGTTCATAC AGGGAAGTGA AGAGGACGAA GTTAAGAGAG   
  
  
- AGTTTAAGTG CGTAAAAAGG TTACTTAAAG TTTATCCTCA GGACCTTAGG CTACTATAAA ATCCAAAAAA   
  
  
- GGACTGAAGT AACGTAATCA AACAAGTATA GACACTGGTT AAGAAAGTAA ACAATATACT AGACTAACAA   
  
  
- ATCAATCGAT AAGTAAGTAA CGTATATGTA ACCAAATAAG TAACGTCTCT GTGAGTGTAT GTGTGTGTGT   
  
  
- ATGTCCCCTG TGATCATAAA AAAGAGAGAT TGAGTAGCCT AAGGGAAACT ATGAGACAAA AAACCCCCCA   
  
  
- ACTAAACTAA ACTAAACTAA CTGGAATTAA AACTTGAGTG TGTGTGAGCG TGTATCTATA TAAGAAAGAC   
  
  
- AAACACTGTA TATGTCGTAG TATATCTTTA TATAACGTGA CGTATAAACT CGAGTTCTGT CCCTATAGTA   
  
  
- GTGTTTGACT CACACTCCCT CCGTTTTTTG TCTCCTTTTC TTCCCTTAAC CCTCACATTA GTTTTGTTCT   
  
  
- CTCTCTAACT CCTCTCTCCT TTTTTGTTTT AACCCTCTTC CAATTATCTC TTCTTACAAC CGACCCACAA   
  
  
- GTAGGTGTAA CGACAGTGGT TCCATATCTA ACTCCTCACT TCGATGTAGT GTCAAGGTTC GGACGGTAGT   
  
  
- AGGTTACTCG TGTGTCTCTT AACTAAACGG GACGTGAAAG GGTTCTTTCC TACAAAGCTT CGGTGTTAGC   
  
  
- CAAGCGGGGC AACCGGATAG ACAGCTCTTC GGCCAACTTC AGTCCCGGGA CTTCGTCTGG TAGGCTCAGG   
  
  
- GTGGTAGCGG CCTCCCATCT TCTCTCTAAT TCCGTCTAAA GACCCCACTA TCTTCTTTCT CGAACTTCTC   
  
  
- CTCTGATCGT CTTGTCCCCT CGAGAAAACT ACTACCACCA CTGCTTAGAA CACAACCCTC TTGGTTTTCC   
  
  
- TTCTTCCCAT CGAAACTAAA ACCACTTAGA CTACTTTAAA GAGGTTTTTA ACTCCTGTGT TACTCAAAAC   
  
  
- CTGTAGACCC ATCACCACCT TTAAAGACCC AAGTAGGACC AAAACCACAA CAATTACAAT TATGATTAAT   
  
  
- GGGAGTTCAC GGTAAAAGAG AGTGAACAAG TCCCCTCCTT CTTTCTCATA CAAAACAAGG ATGACTACAC   
  
  
- TAGAGTGGTC GGTAGTACGG AGGTAACAGT GTGTTAGGAA CCCAACTTAG ACCACAATGA CTCCAGTGTC   
  
  
- TTATACCACC CCCACTGTTT CTGCCGCTTG GATCAGTACC CAAACACTCC CCATGTTGTT GTTGCAGGCC   
  
  
- CAGTTCAAGT AGAAGACTCT CAGTATCAAA ACCCGTATCC GAGTTACTCT CGAGTAGACT GGGACTTTAG   
  
  
- TCTTTACCAA GGTACAACGG ATTGGGACCA GTACCAGGAC CGTGGTCAGT ACCAGGACCT TGGCCAGACC   
  
  
- GAGTGTTGGT GTTACTAGTT TGGCTCGTTG TTCCCAAGCT CGAGTAATCG AAAGAGTACC CAACACAGCT   
  
  
- TCGCTAGTCA AGTTCCTTGT AACGTCGGTA GTTAGTAAAG TATCGATTCG ACCCGCTCGT CCGAAGAGGT   
  
  
- TCCCGAGCCA GACGGTAATC GGCGGAGTGT CGGATGAAGT GGCTTCGTAA TCGGAACTCC CAGTGTTCTA   
  
  
- AAACCGGAGT ATAGAAAGTG TATTCATAGG GAGCTCTCAA ACTAGCTAAA CTACTACTTA GCCCCCGTCG   
  
  
- ACGTTACTCC GAAAACTTAG TCTAGTCGGG TTAAGGTTTC AAGCAAGTAA AATGTCGGTT ACTCTACAAT   
  
  
- AACTCCCGTA AACTTCCCTT CCTGTTCCAC GTATAGTATC TGAAGCTGTA GTTCGTTCCG AATGTTACCG   
  
  
- GGCCCAAAAA GGTTTCGAAT CGGTACTCCC TTTTGGGGGG CTCGGTACAG TCCTATTGTC CGCATCCCCT   
  
  
- AAGCTTCGTT CTTAACCAAC TCTGTCCTCT TTCTGATCGG CCCAAACGAC CCCGTAAGTT GGAGAGGAAG   
  
  
- CTCAAGGTGG GTCAACACCT GGCCAACCTT CTACAATCCG ATACCTACGA AGTACAATTC CTTCCACTTT   
  
  
- CACAGCGCCA TTTAACGTAA AACGTCGAGG TGTTCTGTGA GATACTGGGG GTACCACGGT GTAAGTCCCT   
  
  
- GAAGTACCCA AATTAGGCTT CGTGTTTAGG GTATCGTAAC CAATACCGAC TCGTTCTTCG ACTAGTGTTG   
  
  
- CTTGGGTGGA ATCTTCCGGC TCATACGTTG AGTGAACCTA TGATACGTCG GGAAAAGCTA CGGTATCTAA   
  
  
- CGTCGGAAGG AAAGCTCTCG AGTCGTTCCA ACTTCTAGCT TCTCTACAAA CCGGCCCTTT AGTCCTTGTA   
  
  
- TTATCGGACA CTTCCCCGTC TGTCCTAGCT TTCTGTACTC TTCAAGCTCT TTACCTTCTC TTCCTAGCTC   
  
  
- GTCCTTCCCA AGGCCACGTA CGCCTGATCG CTTTCCCTCC ACTACGTCTC GGTTTACGAC GAATTCTACA   
  
  
- TGAGCTTGCT CTCGATGTTG TACGTTTCTG TTCCGTTACT TCTTCGTTAT AGTGATCATA CCAATCTAGT   
  
  
- CGGTGAGATG TGACAAAGAA ACACCCGAGG TTCACTTAAA CGACGTCCCT CAAGAAGGTC AAAAAGAGAC   
  
  
- GGTAGAAC

+     G-Box

| Site Name | Organism | Position | Strand | Matrix score. | sequence | function |
| --- | --- | --- | --- | --- | --- | --- |
| G-Box | Pisum sativum | 774 | + | 6 | CACGTG | cis-acting regulatory element involved in light responsiveness |

>HU04G00148.1   
+ -Up\_Stream \_Len000ATAATG GGTGATAACT TTTGAAGGAC AATATACAAA TCACTTATTT ATTAAAGTCA   
  
  
+ AGGGGACATT TCAACTATAA CATCATTGTT TATAAAGTAT TTGAAAATTG CAACTCTTGT AATAGATAAT   
  
  
+ AATGCTTTAT AGACTTCACC AATCACTAAT TTGTAGAATC AAAAAATTAA TTGAAAAGCA AACAAAAGGA   
  
  
+ AAAGGCACAT AAACCTAAAT TATGTAAACA AATGAGAACA TATATGCCAG CTTATCAACG TTAATTATAT   
  
  
+ TTGTTATCAC TCTTAATATC TGTATTCATA GTAAATGAAA ATAAGTATCG ATTTTTTATT AAACCTAAAA   
  
  
+ TCTTCTGTCA ATAATTTTTC TTCCGATAAA AAAAAAATTG GACCATATTA AATTAACATT AATAAATTCA   
  
  
+ TATAATTACT TTGAATGTCT GAAATATAAT GCTCTTCGGT ATATTAAGGT CTTATAAATT TCTCATTAAT   
  
  
+ TTGAAATCTC TTATAATATA TTGGGTCAAC AAGTGCTAGC TTGTTTTTTT TTCCTGGTTA ATTGCATTTA   
  
  
+ AAAAAATAAT TTTTAATTAC AATTGCAAGG GGAGGAATCA TTTACAAAAT ATCAAAAGTG GAAAAGGCAA   
  
  
+ AAAGAAACAA ATTATAAAAC AGAAAAAAGA TTAGAAAACG CAAACAAAGA CGAAATGAGG AACAGTAAGA   
  
  
+ ATCAGTAAAA AAATGTAGTG CAACGGTCAC ATACGGGAAG GCAAGCACAA CAATCATGAA AGAGACAGGC   
  
  
+ ACGTGCGTTC CTCGTGATCA GCAGCCTTCC CTACTTCCCC GCCAAGTGGA GTCCGTACTA ACTCCTGCCA   
  
  
+ TTCCCTCCCA ACGGTCATAT TCCGCCGACA TGCCTCAACG GTCAGATTTC CTGCCCCATC TGGACCGTCC   
  
  
+ GATCAAGATC CAACGGCCAT AAGGCGCACT CTCCAATACC CTAGTGCCCA TTTCTGGAAC AGCCCCAAAA   
  
  
+ ATCTCAGTAT TCGCCATCAT TTTTCAATAC TGGGGTTACA TAAACAGTCT AACAAGGAAG GACCCAACAC   
  
  
+ AGAGGAGAGA GAAAAAAGCT AGCTTTGATG GGGACAGCCA TGGATGGAGG TGAAGAGAGA GGAGGTTGGG   
  
  
+ ACCTTTTATT TTGCTCACTT TCCATTCTTA TATAAAAACA GCTCTAGTTT ATCTCTTATG GCTTTTTTGG   
  
  
+ GTTTGTTCAT AGAGAGGGGG GAATGTGATA TGATTTGATT TGATGTTAGA AATTCTCATT AGTTTTTTCT   
  
  
+ TTTTGTTTGC AATTTTGATG TGTTAGGGTT TGAGGATCAT CATTTTTGAG TAGGGAACTT TCTGGGTTTT   
  
  
+ GCTTGCTGAT TGATTGCCAA TGCTATGGAT TTCTTGGTGA GTCAATCTTC AATTGCTGAT CTGGGTTCGT   
  
  
+ TTGTTCTTCA GAGTTTCACC TGCCAATTTG ATCAAGTATG TCCCTTCACT TCTCCTGCTT CAATTCTCTC   
  
  
+ TCAAATTCAC GCATTTTTCC AATGAATTTC AAATAGGAGT CCTGGAATCC GATGATATTT TAGGTTTTTT   
  
  
+ CCTGACTTCA TTGCATTAGT TTGTTCATAT CTGTGACCAA TTCTTTCATT TGTTATATGA TCTGATTGTT   
  
  
+ TAGTTAGCTA TTCATTCATT GCATATACAT TGGTTTATTC ATTGCAGAGA CACTCACATA CACACACACA   
  
  
+ TACAGGGGAC ACTAGTATTT TTTCTCTCTA ACTCATCGGA TTCCCTTTGA TACTCTGTTT TTTGGGGGGT   
  
  
+ TGATTTGATT TGATTTGATT GACCTTAATT TTGAACTCAC ACACACTCGC ACATAGATAT ATTCTTTCTG   
  
  
+ TTTGTGACAT ATACAGCATC ATATAGAAAT ATATTGCACT GCATATTTGA GCTCAAGACA GGGATATCAT   
  
  
+ CACAAACTGA GTGTGAGGGA GGCAAAAAAC AGAGGAAAAG AAGGGAATTG GGAGTGTAAT CAAAACAAGA   
  
  
+ GAGAGATTGA GGAGAGAGGA AAAAACAAAA TTGGGAGAAG GTTAATAGAG AAGAATGTTG GCTGGGTGTT   
  
  
+ CATCCACATT GCTGTCACCA AGGTATAGAT TGAGGAGTGA AGCTACATCA CAGTTCCAAG CCTGCCATCA   
  
  
+ TCCAATGAGC ACACAGAGAA TTGATTTGCC CTGCACTTTC CCAAGAAAGG ATGTTTCGAA GCCACAATCG   
  
  
+ GTTCGCCCCG TTGGCCTATC TGTCGAGAAG CCGGTTGAAG TCAGGGCCCT GAAGCAGACC ATCCGAGTCC   
  
  
+ CACCATCGCC GGAGGGTAGA AGAGAGATTA AGGCAGATTT CTGGGGTGAT AGAAGAAAGA GCTTGAAGAG   
  
  
+ GAGACTAGCA GAACAGGGGA GCTCTTTTGA TGATGGTGGT GACGAATCTT GTGTTGGGAG AACCAAAAGG   
  
  
+ AAGAAGGGTA GCTTTGATTT TGGTGAATCT GATGAAATTT CTCCAAAAAT TGAGGACACA ATGAGTTTTG   
  
  
+ GACATCTGGG TAGTGGTGGA AATTTCTGGG TTCATCCTGG TTTTGGTGTT GTTAATGTTA ATACTAATTA   
  
  
+ CCCTCAAGTG CCATTTTCTC TCACTTGTTC AGGGGAGGAA GAAAGAGTAT GTTTTGTTCC TACTGATGTG   
  
  
+ ATCTCACCAG CCATCATGCC TCCATTGTCA CACAATCCTT GGGTTGAATC TGGTGTTACT GAGGTCACAG   
  
  
+ AATATGGTGG GGGTGACAAA GACGGCGAAC CTAGTCATGG GTTTGTGAGG GGTACAACAA CAACGTCCGG   
  
  
+ GTCAAGTTCA TCTTCTGAGA GTCATAGTTT TGGGCATAGG CTCAATGAGA GCTCATCTGA CCCTGAAATC   
  
  
+ AGAAATGGTT CCATGTTGCC TAACCCTGGT CATGGTCCTG GCACCAGTCA TGGTCCTGGA ACCGGTCTGG   
  
  
+ CTCACAACCA CAATGATCAA ACCGAGCAAC AAGGGTTCGA GCTCATTAGC TTTCTCATGG GTTGTGTCGA   
  
  
+ AGCGATCAGT TCAAGGAACA TTGCAGCCAT CAATCATTTC ATAGCTAAGC TGGGCGAGCA GGCTTCTCCA   
  
  
+ AGGGCTCGGT CTGCCATTAG CCGCCTCACA GCCTACTTCA CCGAAGCATT AGCCTTGAGG GTCACAAGAT   
  
  
+ TTTGGCCTCA TATCTTTCAC ATAAGTATCC CTCGAGAGTT TGATCGATTT GATGATGAAT CGGGGGCAGC   
  
  
+ TGCAATGAGG CTTTTGAATC AGATCAGCCC AATTCCAAAG TTCGTTCATT TTACAGCCAA TGAGATGTTA   
  
  
+ TTGAGGGCAT TTGAAGGGAA GGACAAGGTG CATATCATAG ACTTCGACAT CAAGCAAGGC TTACAATGGC   
  
  
+ CCGGGTTTTT CCAAAGCTTA GCCATGAGGG AAAACCCCCC GAGCCATGTC AGGATAACAG GCGTAGGGGA   
  
  
+ TTCGAAGCAA GAATTGGTTG AGACAGGAGA AAGACTAGCC GGGTTTGCTG GGGCATTCAA CCTCTCCTTC   
  
  
+ GAGTTCCACC CAGTTGTGGA CCGGTTGGAA GATGTTAGGC TATGGATGCT TCATGTTAAG GAAGGTGAAA   
  
  
+ GTGTCGCGGT AAATTGCATT TTGCAGCTCC ACAAGACACT CTATGACCCC CATGGTGCCA CATTCAGGGA   
  
  
+ CTTCATGGGT TTAATCCGAA GCACAAATCC CATAGCATTG GTTATGGCTG AGCAAGAAGC TGATCACAAC   
  
  
+ GAACCCACCT TAGAAGGCCG AGTATGCAAC TCACTTGGAT ACTATGCAGC CCTTTTCGAT GCCATAGATT   
  
  
+ GCAGCCTTCC TTTCGAGAGC TCAGCAAGGT TGAAGATCGA AGAGATGTTT GGCCGGGAAA TCAGGAACAT   
  
  
+ AATAGCCTGT GAAGGGGCAG ACAGGATCGA AAGACATGAG AAGTTCGAGA AATGGAAGAG AAGGATCGAG   
  
  
+ CAGGAAGGGT TCCGGTGCAT GCGGACTAGC GAAAGGGAGG TGATGCAGAG CCAAATGCTG CTTAAGATGT   
  
  
+ ACTCGAACGA GAGCTACAAC ATGCAAAGAC AAGGCAATGA AGAAGCAATA TCACTAGTAT GGTTAGATCA   
  
  
+ GCCACTCTAC ACTGTTTCTT TGTGGGCTCC AAGTGAATTT GCTGCAGGGA GTTCTTCCAG TTTTTCTCTG   
  
  
+ CCATCTTG  

- -Up\_Stream \_Len000TATTAC CCACTATTGA AAACTTCCTG TTATATGTTT AGTGAATAAA TAATTTCAGT   
  
  
- TCCCCTGTAA AGTTGATATT GTAGTAACAA ATATTTCATA AACTTTTAAC GTTGAGAACA TTATCTATTA   
  
  
- TTACGAAATA TCTGAAGTGG TTAGTGATTA AACATCTTAG TTTTTTAATT AACTTTTCGT TTGTTTTCCT   
  
  
- TTTCCGTGTA TTTGGATTTA ATACATTTGT TTACTCTTGT ATATACGGTC GAATAGTTGC AATTAATATA   
  
  
- AACAATAGTG AGAATTATAG ACATAAGTAT CATTTACTTT TATTCATAGC TAAAAAATAA TTTGGATTTT   
  
  
- AGAAGACAGT TATTAAAAAG AAGGCTATTT TTTTTTTAAC CTGGTATAAT TTAATTGTAA TTATTTAAGT   
  
  
- ATATTAATGA AACTTACAGA CTTTATATTA CGAGAAGCCA TATAATTCCA GAATATTTAA AGAGTAATTA   
  
  
- AACTTTAGAG AATATTATAT AACCCAGTTG TTCACGATCG AACAAAAAAA AAGGACCAAT TAACGTAAAT   
  
  
- TTTTTTATTA AAAATTAATG TTAACGTTCC CCTCCTTAGT AAATGTTTTA TAGTTTTCAC CTTTTCCGTT   
  
  
- TTTCTTTGTT TAATATTTTG TCTTTTTTCT AATCTTTTGC GTTTGTTTCT GCTTTACTCC TTGTCATTCT   
  
  
- TAGTCATTTT TTTACATCAC GTTGCCAGTG TATGCCCTTC CGTTCGTGTT GTTAGTACTT TCTCTGTCCG   
  
  
- TGCACGCAAG GAGCACTAGT CGTCGGAAGG GATGAAGGGG CGGTTCACCT CAGGCATGAT TGAGGACGGT   
  
  
- AAGGGAGGGT TGCCAGTATA AGGCGGCTGT ACGGAGTTGC CAGTCTAAAG GACGGGGTAG ACCTGGCAGG   
  
  
- CTAGTTCTAG GTTGCCGGTA TTCCGCGTGA GAGGTTATGG GATCACGGGT AAAGACCTTG TCGGGGTTTT   
  
  
- TAGAGTCATA AGCGGTAGTA AAAAGTTATG ACCCCAATGT ATTTGTCAGA TTGTTCCTTC CTGGGTTGTG   
  
  
- TCTCCTCTCT CTTTTTTCGA TCGAAACTAC CCCTGTCGGT ACCTACCTCC ACTTCTCTCT CCTCCAACCC   
  
  
- TGGAAAATAA AACGAGTGAA AGGTAAGAAT ATATTTTTGT CGAGATCAAA TAGAGAATAC CGAAAAAACC   
  
  
- CAAACAAGTA TCTCTCCCCC CTTACACTAT ACTAAACTAA ACTACAATCT TTAAGAGTAA TCAAAAAAGA   
  
  
- AAAACAAACG TTAAAACTAC ACAATCCCAA ACTCCTAGTA GTAAAAACTC ATCCCTTGAA AGACCCAAAA   
  
  
- CGAACGACTA ACTAACGGTT ACGATACCTA AAGAACCACT CAGTTAGAAG TTAACGACTA GACCCAAGCA   
  
  
- AACAAGAAGT CTCAAAGTGG ACGGTTAAAC TAGTTCATAC AGGGAAGTGA AGAGGACGAA GTTAAGAGAG   
  
  
- AGTTTAAGTG CGTAAAAAGG TTACTTAAAG TTTATCCTCA GGACCTTAGG CTACTATAAA ATCCAAAAAA   
  
  
- GGACTGAAGT AACGTAATCA AACAAGTATA GACACTGGTT AAGAAAGTAA ACAATATACT AGACTAACAA   
  
  
- ATCAATCGAT AAGTAAGTAA CGTATATGTA ACCAAATAAG TAACGTCTCT GTGAGTGTAT GTGTGTGTGT   
  
  
- ATGTCCCCTG TGATCATAAA AAAGAGAGAT TGAGTAGCCT AAGGGAAACT ATGAGACAAA AAACCCCCCA   
  
  
- ACTAAACTAA ACTAAACTAA CTGGAATTAA AACTTGAGTG TGTGTGAGCG TGTATCTATA TAAGAAAGAC   
  
  
- AAACACTGTA TATGTCGTAG TATATCTTTA TATAACGTGA CGTATAAACT CGAGTTCTGT CCCTATAGTA   
  
  
- GTGTTTGACT CACACTCCCT CCGTTTTTTG TCTCCTTTTC TTCCCTTAAC CCTCACATTA GTTTTGTTCT   
  
  
- CTCTCTAACT CCTCTCTCCT TTTTTGTTTT AACCCTCTTC CAATTATCTC TTCTTACAAC CGACCCACAA   
  
  
- GTAGGTGTAA CGACAGTGGT TCCATATCTA ACTCCTCACT TCGATGTAGT GTCAAGGTTC GGACGGTAGT   
  
  
- AGGTTACTCG TGTGTCTCTT AACTAAACGG GACGTGAAAG GGTTCTTTCC TACAAAGCTT CGGTGTTAGC   
  
  
- CAAGCGGGGC AACCGGATAG ACAGCTCTTC GGCCAACTTC AGTCCCGGGA CTTCGTCTGG TAGGCTCAGG   
  
  
- GTGGTAGCGG CCTCCCATCT TCTCTCTAAT TCCGTCTAAA GACCCCACTA TCTTCTTTCT CGAACTTCTC   
  
  
- CTCTGATCGT CTTGTCCCCT CGAGAAAACT ACTACCACCA CTGCTTAGAA CACAACCCTC TTGGTTTTCC   
  
  
- TTCTTCCCAT CGAAACTAAA ACCACTTAGA CTACTTTAAA GAGGTTTTTA ACTCCTGTGT TACTCAAAAC   
  
  
- CTGTAGACCC ATCACCACCT TTAAAGACCC AAGTAGGACC AAAACCACAA CAATTACAAT TATGATTAAT   
  
  
- GGGAGTTCAC GGTAAAAGAG AGTGAACAAG TCCCCTCCTT CTTTCTCATA CAAAACAAGG ATGACTACAC   
  
  
- TAGAGTGGTC GGTAGTACGG AGGTAACAGT GTGTTAGGAA CCCAACTTAG ACCACAATGA CTCCAGTGTC   
  
  
- TTATACCACC CCCACTGTTT CTGCCGCTTG GATCAGTACC CAAACACTCC CCATGTTGTT GTTGCAGGCC   
  
  
- CAGTTCAAGT AGAAGACTCT CAGTATCAAA ACCCGTATCC GAGTTACTCT CGAGTAGACT GGGACTTTAG   
  
  
- TCTTTACCAA GGTACAACGG ATTGGGACCA GTACCAGGAC CGTGGTCAGT ACCAGGACCT TGGCCAGACC   
  
  
- GAGTGTTGGT GTTACTAGTT TGGCTCGTTG TTCCCAAGCT CGAGTAATCG AAAGAGTACC CAACACAGCT   
  
  
- TCGCTAGTCA AGTTCCTTGT AACGTCGGTA GTTAGTAAAG TATCGATTCG ACCCGCTCGT CCGAAGAGGT   
  
  
- TCCCGAGCCA GACGGTAATC GGCGGAGTGT CGGATGAAGT GGCTTCGTAA TCGGAACTCC CAGTGTTCTA   
  
  
- AAACCGGAGT ATAGAAAGTG TATTCATAGG GAGCTCTCAA ACTAGCTAAA CTACTACTTA GCCCCCGTCG   
  
  
- ACGTTACTCC GAAAACTTAG TCTAGTCGGG TTAAGGTTTC AAGCAAGTAA AATGTCGGTT ACTCTACAAT   
  
  
- AACTCCCGTA AACTTCCCTT CCTGTTCCAC GTATAGTATC TGAAGCTGTA GTTCGTTCCG AATGTTACCG   
  
  
- GGCCCAAAAA GGTTTCGAAT CGGTACTCCC TTTTGGGGGG CTCGGTACAG TCCTATTGTC CGCATCCCCT   
  
  
- AAGCTTCGTT CTTAACCAAC TCTGTCCTCT TTCTGATCGG CCCAAACGAC CCCGTAAGTT GGAGAGGAAG   
  
  
- CTCAAGGTGG GTCAACACCT GGCCAACCTT CTACAATCCG ATACCTACGA AGTACAATTC CTTCCACTTT   
  
  
- CACAGCGCCA TTTAACGTAA AACGTCGAGG TGTTCTGTGA GATACTGGGG GTACCACGGT GTAAGTCCCT   
  
  
- GAAGTACCCA AATTAGGCTT CGTGTTTAGG GTATCGTAAC CAATACCGAC TCGTTCTTCG ACTAGTGTTG   
  
  
- CTTGGGTGGA ATCTTCCGGC TCATACGTTG AGTGAACCTA TGATACGTCG GGAAAAGCTA CGGTATCTAA   
  
  
- CGTCGGAAGG AAAGCTCTCG AGTCGTTCCA ACTTCTAGCT TCTCTACAAA CCGGCCCTTT AGTCCTTGTA   
  
  
- TTATCGGACA CTTCCCCGTC TGTCCTAGCT TTCTGTACTC TTCAAGCTCT TTACCTTCTC TTCCTAGCTC   
  
  
- GTCCTTCCCA AGGCCACGTA CGCCTGATCG CTTTCCCTCC ACTACGTCTC GGTTTACGAC GAATTCTACA   
  
  
- TGAGCTTGCT CTCGATGTTG TACGTTTCTG TTCCGTTACT TCTTCGTTAT AGTGATCATA CCAATCTAGT   
  
  
- CGGTGAGATG TGACAAAGAA ACACCCGAGG TTCACTTAAA CGACGTCCCT CAAGAAGGTC AAAAAGAGAC   
  
  
- GGTAGAAC

+     G-box

| Site Name | Organism | Position | Strand | Matrix score. | sequence | function |
| --- | --- | --- | --- | --- | --- | --- |
| G-box | Arabidopsis thaliana | 815 | + | 9 | GCCACGTGGA | cis-acting regulatory element involved in light responsiveness |
| G-box | Arabidopsis thaliana | 774 | + | 6 | CACGTG | cis-acting regulatory element involved in light responsiveness |

>HU04G00148.1   
+ -Up\_Stream \_Len000ATAATG GGTGATAACT TTTGAAGGAC AATATACAAA TCACTTATTT ATTAAAGTCA   
  
  
+ AGGGGACATT TCAACTATAA CATCATTGTT TATAAAGTAT TTGAAAATTG CAACTCTTGT AATAGATAAT   
  
  
+ AATGCTTTAT AGACTTCACC AATCACTAAT TTGTAGAATC AAAAAATTAA TTGAAAAGCA AACAAAAGGA   
  
  
+ AAAGGCACAT AAACCTAAAT TATGTAAACA AATGAGAACA TATATGCCAG CTTATCAACG TTAATTATAT   
  
  
+ TTGTTATCAC TCTTAATATC TGTATTCATA GTAAATGAAA ATAAGTATCG ATTTTTTATT AAACCTAAAA   
  
  
+ TCTTCTGTCA ATAATTTTTC TTCCGATAAA AAAAAAATTG GACCATATTA AATTAACATT AATAAATTCA   
  
  
+ TATAATTACT TTGAATGTCT GAAATATAAT GCTCTTCGGT ATATTAAGGT CTTATAAATT TCTCATTAAT   
  
  
+ TTGAAATCTC TTATAATATA TTGGGTCAAC AAGTGCTAGC TTGTTTTTTT TTCCTGGTTA ATTGCATTTA   
  
  
+ AAAAAATAAT TTTTAATTAC AATTGCAAGG GGAGGAATCA TTTACAAAAT ATCAAAAGTG GAAAAGGCAA   
  
  
+ AAAGAAACAA ATTATAAAAC AGAAAAAAGA TTAGAAAACG CAAACAAAGA CGAAATGAGG AACAGTAAGA   
  
  
+ ATCAGTAAAA AAATGTAGTG CAACGGTCAC ATACGGGAAG GCAAGCACAA CAATCATGAA AGAGACAGGC   
  
  
+ ACGTGCGTTC CTCGTGATCA GCAGCCTTCC CTACTTCCCC GCCAAGTGGA GTCCGTACTA ACTCCTGCCA   
  
  
+ TTCCCTCCCA ACGGTCATAT TCCGCCGACA TGCCTCAACG GTCAGATTTC CTGCCCCATC TGGACCGTCC   
  
  
+ GATCAAGATC CAACGGCCAT AAGGCGCACT CTCCAATACC CTAGTGCCCA TTTCTGGAAC AGCCCCAAAA   
  
  
+ ATCTCAGTAT TCGCCATCAT TTTTCAATAC TGGGGTTACA TAAACAGTCT AACAAGGAAG GACCCAACAC   
  
  
+ AGAGGAGAGA GAAAAAAGCT AGCTTTGATG GGGACAGCCA TGGATGGAGG TGAAGAGAGA GGAGGTTGGG   
  
  
+ ACCTTTTATT TTGCTCACTT TCCATTCTTA TATAAAAACA GCTCTAGTTT ATCTCTTATG GCTTTTTTGG   
  
  
+ GTTTGTTCAT AGAGAGGGGG GAATGTGATA TGATTTGATT TGATGTTAGA AATTCTCATT AGTTTTTTCT   
  
  
+ TTTTGTTTGC AATTTTGATG TGTTAGGGTT TGAGGATCAT CATTTTTGAG TAGGGAACTT TCTGGGTTTT   
  
  
+ GCTTGCTGAT TGATTGCCAA TGCTATGGAT TTCTTGGTGA GTCAATCTTC AATTGCTGAT CTGGGTTCGT   
  
  
+ TTGTTCTTCA GAGTTTCACC TGCCAATTTG ATCAAGTATG TCCCTTCACT TCTCCTGCTT CAATTCTCTC   
  
  
+ TCAAATTCAC GCATTTTTCC AATGAATTTC AAATAGGAGT CCTGGAATCC GATGATATTT TAGGTTTTTT   
  
  
+ CCTGACTTCA TTGCATTAGT TTGTTCATAT CTGTGACCAA TTCTTTCATT TGTTATATGA TCTGATTGTT   
  
  
+ TAGTTAGCTA TTCATTCATT GCATATACAT TGGTTTATTC ATTGCAGAGA CACTCACATA CACACACACA   
  
  
+ TACAGGGGAC ACTAGTATTT TTTCTCTCTA ACTCATCGGA TTCCCTTTGA TACTCTGTTT TTTGGGGGGT   
  
  
+ TGATTTGATT TGATTTGATT GACCTTAATT TTGAACTCAC ACACACTCGC ACATAGATAT ATTCTTTCTG   
  
  
+ TTTGTGACAT ATACAGCATC ATATAGAAAT ATATTGCACT GCATATTTGA GCTCAAGACA GGGATATCAT   
  
  
+ CACAAACTGA GTGTGAGGGA GGCAAAAAAC AGAGGAAAAG AAGGGAATTG GGAGTGTAAT CAAAACAAGA   
  
  
+ GAGAGATTGA GGAGAGAGGA AAAAACAAAA TTGGGAGAAG GTTAATAGAG AAGAATGTTG GCTGGGTGTT   
  
  
+ CATCCACATT GCTGTCACCA AGGTATAGAT TGAGGAGTGA AGCTACATCA CAGTTCCAAG CCTGCCATCA   
  
  
+ TCCAATGAGC ACACAGAGAA TTGATTTGCC CTGCACTTTC CCAAGAAAGG ATGTTTCGAA GCCACAATCG   
  
  
+ GTTCGCCCCG TTGGCCTATC TGTCGAGAAG CCGGTTGAAG TCAGGGCCCT GAAGCAGACC ATCCGAGTCC   
  
  
+ CACCATCGCC GGAGGGTAGA AGAGAGATTA AGGCAGATTT CTGGGGTGAT AGAAGAAAGA GCTTGAAGAG   
  
  
+ GAGACTAGCA GAACAGGGGA GCTCTTTTGA TGATGGTGGT GACGAATCTT GTGTTGGGAG AACCAAAAGG   
  
  
+ AAGAAGGGTA GCTTTGATTT TGGTGAATCT GATGAAATTT CTCCAAAAAT TGAGGACACA ATGAGTTTTG   
  
  
+ GACATCTGGG TAGTGGTGGA AATTTCTGGG TTCATCCTGG TTTTGGTGTT GTTAATGTTA ATACTAATTA   
  
  
+ CCCTCAAGTG CCATTTTCTC TCACTTGTTC AGGGGAGGAA GAAAGAGTAT GTTTTGTTCC TACTGATGTG   
  
  
+ ATCTCACCAG CCATCATGCC TCCATTGTCA CACAATCCTT GGGTTGAATC TGGTGTTACT GAGGTCACAG   
  
  
+ AATATGGTGG GGGTGACAAA GACGGCGAAC CTAGTCATGG GTTTGTGAGG GGTACAACAA CAACGTCCGG   
  
  
+ GTCAAGTTCA TCTTCTGAGA GTCATAGTTT TGGGCATAGG CTCAATGAGA GCTCATCTGA CCCTGAAATC   
  
  
+ AGAAATGGTT CCATGTTGCC TAACCCTGGT CATGGTCCTG GCACCAGTCA TGGTCCTGGA ACCGGTCTGG   
  
  
+ CTCACAACCA CAATGATCAA ACCGAGCAAC AAGGGTTCGA GCTCATTAGC TTTCTCATGG GTTGTGTCGA   
  
  
+ AGCGATCAGT TCAAGGAACA TTGCAGCCAT CAATCATTTC ATAGCTAAGC TGGGCGAGCA GGCTTCTCCA   
  
  
+ AGGGCTCGGT CTGCCATTAG CCGCCTCACA GCCTACTTCA CCGAAGCATT AGCCTTGAGG GTCACAAGAT   
  
  
+ TTTGGCCTCA TATCTTTCAC ATAAGTATCC CTCGAGAGTT TGATCGATTT GATGATGAAT CGGGGGCAGC   
  
  
+ TGCAATGAGG CTTTTGAATC AGATCAGCCC AATTCCAAAG TTCGTTCATT TTACAGCCAA TGAGATGTTA   
  
  
+ TTGAGGGCAT TTGAAGGGAA GGACAAGGTG CATATCATAG ACTTCGACAT CAAGCAAGGC TTACAATGGC   
  
  
+ CCGGGTTTTT CCAAAGCTTA GCCATGAGGG AAAACCCCCC GAGCCATGTC AGGATAACAG GCGTAGGGGA   
  
  
+ TTCGAAGCAA GAATTGGTTG AGACAGGAGA AAGACTAGCC GGGTTTGCTG GGGCATTCAA CCTCTCCTTC   
  
  
+ GAGTTCCACC CAGTTGTGGA CCGGTTGGAA GATGTTAGGC TATGGATGCT TCATGTTAAG GAAGGTGAAA   
  
  
+ GTGTCGCGGT AAATTGCATT TTGCAGCTCC ACAAGACACT CTATGACCCC CATGGTGCCA CATTCAGGGA   
  
  
+ CTTCATGGGT TTAATCCGAA GCACAAATCC CATAGCATTG GTTATGGCTG AGCAAGAAGC TGATCACAAC   
  
  
+ GAACCCACCT TAGAAGGCCG AGTATGCAAC TCACTTGGAT ACTATGCAGC CCTTTTCGAT GCCATAGATT   
  
  
+ GCAGCCTTCC TTTCGAGAGC TCAGCAAGGT TGAAGATCGA AGAGATGTTT GGCCGGGAAA TCAGGAACAT   
  
  
+ AATAGCCTGT GAAGGGGCAG ACAGGATCGA AAGACATGAG AAGTTCGAGA AATGGAAGAG AAGGATCGAG   
  
  
+ CAGGAAGGGT TCCGGTGCAT GCGGACTAGC GAAAGGGAGG TGATGCAGAG CCAAATGCTG CTTAAGATGT   
  
  
+ ACTCGAACGA GAGCTACAAC ATGCAAAGAC AAGGCAATGA AGAAGCAATA TCACTAGTAT GGTTAGATCA   
  
  
+ GCCACTCTAC ACTGTTTCTT TGTGGGCTCC AAGTGAATTT GCTGCAGGGA GTTCTTCCAG TTTTTCTCTG   
  
  
+ CCATCTTG  

- -Up\_Stream \_Len000TATTAC CCACTATTGA AAACTTCCTG TTATATGTTT AGTGAATAAA TAATTTCAGT   
  
  
- TCCCCTGTAA AGTTGATATT GTAGTAACAA ATATTTCATA AACTTTTAAC GTTGAGAACA TTATCTATTA   
  
  
- TTACGAAATA TCTGAAGTGG TTAGTGATTA AACATCTTAG TTTTTTAATT AACTTTTCGT TTGTTTTCCT   
  
  
- TTTCCGTGTA TTTGGATTTA ATACATTTGT TTACTCTTGT ATATACGGTC GAATAGTTGC AATTAATATA   
  
  
- AACAATAGTG AGAATTATAG ACATAAGTAT CATTTACTTT TATTCATAGC TAAAAAATAA TTTGGATTTT   
  
  
- AGAAGACAGT TATTAAAAAG AAGGCTATTT TTTTTTTAAC CTGGTATAAT TTAATTGTAA TTATTTAAGT   
  
  
- ATATTAATGA AACTTACAGA CTTTATATTA CGAGAAGCCA TATAATTCCA GAATATTTAA AGAGTAATTA   
  
  
- AACTTTAGAG AATATTATAT AACCCAGTTG TTCACGATCG AACAAAAAAA AAGGACCAAT TAACGTAAAT   
  
  
- TTTTTTATTA AAAATTAATG TTAACGTTCC CCTCCTTAGT AAATGTTTTA TAGTTTTCAC CTTTTCCGTT   
  
  
- TTTCTTTGTT TAATATTTTG TCTTTTTTCT AATCTTTTGC GTTTGTTTCT GCTTTACTCC TTGTCATTCT   
  
  
- TAGTCATTTT TTTACATCAC GTTGCCAGTG TATGCCCTTC CGTTCGTGTT GTTAGTACTT TCTCTGTCCG   
  
  
- TGCACGCAAG GAGCACTAGT CGTCGGAAGG GATGAAGGGG CGGTTCACCT CAGGCATGAT TGAGGACGGT   
  
  
- AAGGGAGGGT TGCCAGTATA AGGCGGCTGT ACGGAGTTGC CAGTCTAAAG GACGGGGTAG ACCTGGCAGG   
  
  
- CTAGTTCTAG GTTGCCGGTA TTCCGCGTGA GAGGTTATGG GATCACGGGT AAAGACCTTG TCGGGGTTTT   
  
  
- TAGAGTCATA AGCGGTAGTA AAAAGTTATG ACCCCAATGT ATTTGTCAGA TTGTTCCTTC CTGGGTTGTG   
  
  
- TCTCCTCTCT CTTTTTTCGA TCGAAACTAC CCCTGTCGGT ACCTACCTCC ACTTCTCTCT CCTCCAACCC   
  
  
- TGGAAAATAA AACGAGTGAA AGGTAAGAAT ATATTTTTGT CGAGATCAAA TAGAGAATAC CGAAAAAACC   
  
  
- CAAACAAGTA TCTCTCCCCC CTTACACTAT ACTAAACTAA ACTACAATCT TTAAGAGTAA TCAAAAAAGA   
  
  
- AAAACAAACG TTAAAACTAC ACAATCCCAA ACTCCTAGTA GTAAAAACTC ATCCCTTGAA AGACCCAAAA   
  
  
- CGAACGACTA ACTAACGGTT ACGATACCTA AAGAACCACT CAGTTAGAAG TTAACGACTA GACCCAAGCA   
  
  
- AACAAGAAGT CTCAAAGTGG ACGGTTAAAC TAGTTCATAC AGGGAAGTGA AGAGGACGAA GTTAAGAGAG   
  
  
- AGTTTAAGTG CGTAAAAAGG TTACTTAAAG TTTATCCTCA GGACCTTAGG CTACTATAAA ATCCAAAAAA   
  
  
- GGACTGAAGT AACGTAATCA AACAAGTATA GACACTGGTT AAGAAAGTAA ACAATATACT AGACTAACAA   
  
  
- ATCAATCGAT AAGTAAGTAA CGTATATGTA ACCAAATAAG TAACGTCTCT GTGAGTGTAT GTGTGTGTGT   
  
  
- ATGTCCCCTG TGATCATAAA AAAGAGAGAT TGAGTAGCCT AAGGGAAACT ATGAGACAAA AAACCCCCCA   
  
  
- ACTAAACTAA ACTAAACTAA CTGGAATTAA AACTTGAGTG TGTGTGAGCG TGTATCTATA TAAGAAAGAC   
  
  
- AAACACTGTA TATGTCGTAG TATATCTTTA TATAACGTGA CGTATAAACT CGAGTTCTGT CCCTATAGTA   
  
  
- GTGTTTGACT CACACTCCCT CCGTTTTTTG TCTCCTTTTC TTCCCTTAAC CCTCACATTA GTTTTGTTCT   
  
  
- CTCTCTAACT CCTCTCTCCT TTTTTGTTTT AACCCTCTTC CAATTATCTC TTCTTACAAC CGACCCACAA   
  
  
- GTAGGTGTAA CGACAGTGGT TCCATATCTA ACTCCTCACT TCGATGTAGT GTCAAGGTTC GGACGGTAGT   
  
  
- AGGTTACTCG TGTGTCTCTT AACTAAACGG GACGTGAAAG GGTTCTTTCC TACAAAGCTT CGGTGTTAGC   
  
  
- CAAGCGGGGC AACCGGATAG ACAGCTCTTC GGCCAACTTC AGTCCCGGGA CTTCGTCTGG TAGGCTCAGG   
  
  
- GTGGTAGCGG CCTCCCATCT TCTCTCTAAT TCCGTCTAAA GACCCCACTA TCTTCTTTCT CGAACTTCTC   
  
  
- CTCTGATCGT CTTGTCCCCT CGAGAAAACT ACTACCACCA CTGCTTAGAA CACAACCCTC TTGGTTTTCC   
  
  
- TTCTTCCCAT CGAAACTAAA ACCACTTAGA CTACTTTAAA GAGGTTTTTA ACTCCTGTGT TACTCAAAAC   
  
  
- CTGTAGACCC ATCACCACCT TTAAAGACCC AAGTAGGACC AAAACCACAA CAATTACAAT TATGATTAAT   
  
  
- GGGAGTTCAC GGTAAAAGAG AGTGAACAAG TCCCCTCCTT CTTTCTCATA CAAAACAAGG ATGACTACAC   
  
  
- TAGAGTGGTC GGTAGTACGG AGGTAACAGT GTGTTAGGAA CCCAACTTAG ACCACAATGA CTCCAGTGTC   
  
  
- TTATACCACC CCCACTGTTT CTGCCGCTTG GATCAGTACC CAAACACTCC CCATGTTGTT GTTGCAGGCC   
  
  
- CAGTTCAAGT AGAAGACTCT CAGTATCAAA ACCCGTATCC GAGTTACTCT CGAGTAGACT GGGACTTTAG   
  
  
- TCTTTACCAA GGTACAACGG ATTGGGACCA GTACCAGGAC CGTGGTCAGT ACCAGGACCT TGGCCAGACC   
  
  
- GAGTGTTGGT GTTACTAGTT TGGCTCGTTG TTCCCAAGCT CGAGTAATCG AAAGAGTACC CAACACAGCT   
  
  
- TCGCTAGTCA AGTTCCTTGT AACGTCGGTA GTTAGTAAAG TATCGATTCG ACCCGCTCGT CCGAAGAGGT   
  
  
- TCCCGAGCCA GACGGTAATC GGCGGAGTGT CGGATGAAGT GGCTTCGTAA TCGGAACTCC CAGTGTTCTA   
  
  
- AAACCGGAGT ATAGAAAGTG TATTCATAGG GAGCTCTCAA ACTAGCTAAA CTACTACTTA GCCCCCGTCG   
  
  
- ACGTTACTCC GAAAACTTAG TCTAGTCGGG TTAAGGTTTC AAGCAAGTAA AATGTCGGTT ACTCTACAAT   
  
  
- AACTCCCGTA AACTTCCCTT CCTGTTCCAC GTATAGTATC TGAAGCTGTA GTTCGTTCCG AATGTTACCG   
  
  
- GGCCCAAAAA GGTTTCGAAT CGGTACTCCC TTTTGGGGGG CTCGGTACAG TCCTATTGTC CGCATCCCCT   
  
  
- AAGCTTCGTT CTTAACCAAC TCTGTCCTCT TTCTGATCGG CCCAAACGAC CCCGTAAGTT GGAGAGGAAG   
  
  
- CTCAAGGTGG GTCAACACCT GGCCAACCTT CTACAATCCG ATACCTACGA AGTACAATTC CTTCCACTTT   
  
  
- CACAGCGCCA TTTAACGTAA AACGTCGAGG TGTTCTGTGA GATACTGGGG GTACCACGGT GTAAGTCCCT   
  
  
- GAAGTACCCA AATTAGGCTT CGTGTTTAGG GTATCGTAAC CAATACCGAC TCGTTCTTCG ACTAGTGTTG   
  
  
- CTTGGGTGGA ATCTTCCGGC TCATACGTTG AGTGAACCTA TGATACGTCG GGAAAAGCTA CGGTATCTAA   
  
  
- CGTCGGAAGG AAAGCTCTCG AGTCGTTCCA ACTTCTAGCT TCTCTACAAA CCGGCCCTTT AGTCCTTGTA   
  
  
- TTATCGGACA CTTCCCCGTC TGTCCTAGCT TTCTGTACTC TTCAAGCTCT TTACCTTCTC TTCCTAGCTC   
  
  
- GTCCTTCCCA AGGCCACGTA CGCCTGATCG CTTTCCCTCC ACTACGTCTC GGTTTACGAC GAATTCTACA   
  
  
- TGAGCTTGCT CTCGATGTTG TACGTTTCTG TTCCGTTACT TCTTCGTTAT AGTGATCATA CCAATCTAGT   
  
  
- CGGTGAGATG TGACAAAGAA ACACCCGAGG TTCACTTAAA CGACGTCCCT CAAGAAGGTC AAAAAGAGAC   
  
  
- GGTAGAAC

+     GA-motif

| Site Name | Organism | Position | Strand | Matrix score. | sequence | function |
| --- | --- | --- | --- | --- | --- | --- |
| GA-motif | Arabidopsis thaliana | 136 | + | 8 | ATAGATAA | part of a light responsive element |

>HU04G00148.1   
+ -Up\_Stream \_Len000ATAATG GGTGATAACT TTTGAAGGAC AATATACAAA TCACTTATTT ATTAAAGTCA   
  
  
+ AGGGGACATT TCAACTATAA CATCATTGTT TATAAAGTAT TTGAAAATTG CAACTCTTGT AATAGATAAT   
  
  
+ AATGCTTTAT AGACTTCACC AATCACTAAT TTGTAGAATC AAAAAATTAA TTGAAAAGCA AACAAAAGGA   
  
  
+ AAAGGCACAT AAACCTAAAT TATGTAAACA AATGAGAACA TATATGCCAG CTTATCAACG TTAATTATAT   
  
  
+ TTGTTATCAC TCTTAATATC TGTATTCATA GTAAATGAAA ATAAGTATCG ATTTTTTATT AAACCTAAAA   
  
  
+ TCTTCTGTCA ATAATTTTTC TTCCGATAAA AAAAAAATTG GACCATATTA AATTAACATT AATAAATTCA   
  
  
+ TATAATTACT TTGAATGTCT GAAATATAAT GCTCTTCGGT ATATTAAGGT CTTATAAATT TCTCATTAAT   
  
  
+ TTGAAATCTC TTATAATATA TTGGGTCAAC AAGTGCTAGC TTGTTTTTTT TTCCTGGTTA ATTGCATTTA   
  
  
+ AAAAAATAAT TTTTAATTAC AATTGCAAGG GGAGGAATCA TTTACAAAAT ATCAAAAGTG GAAAAGGCAA   
  
  
+ AAAGAAACAA ATTATAAAAC AGAAAAAAGA TTAGAAAACG CAAACAAAGA CGAAATGAGG AACAGTAAGA   
  
  
+ ATCAGTAAAA AAATGTAGTG CAACGGTCAC ATACGGGAAG GCAAGCACAA CAATCATGAA AGAGACAGGC   
  
  
+ ACGTGCGTTC CTCGTGATCA GCAGCCTTCC CTACTTCCCC GCCAAGTGGA GTCCGTACTA ACTCCTGCCA   
  
  
+ TTCCCTCCCA ACGGTCATAT TCCGCCGACA TGCCTCAACG GTCAGATTTC CTGCCCCATC TGGACCGTCC   
  
  
+ GATCAAGATC CAACGGCCAT AAGGCGCACT CTCCAATACC CTAGTGCCCA TTTCTGGAAC AGCCCCAAAA   
  
  
+ ATCTCAGTAT TCGCCATCAT TTTTCAATAC TGGGGTTACA TAAACAGTCT AACAAGGAAG GACCCAACAC   
  
  
+ AGAGGAGAGA GAAAAAAGCT AGCTTTGATG GGGACAGCCA TGGATGGAGG TGAAGAGAGA GGAGGTTGGG   
  
  
+ ACCTTTTATT TTGCTCACTT TCCATTCTTA TATAAAAACA GCTCTAGTTT ATCTCTTATG GCTTTTTTGG   
  
  
+ GTTTGTTCAT AGAGAGGGGG GAATGTGATA TGATTTGATT TGATGTTAGA AATTCTCATT AGTTTTTTCT   
  
  
+ TTTTGTTTGC AATTTTGATG TGTTAGGGTT TGAGGATCAT CATTTTTGAG TAGGGAACTT TCTGGGTTTT   
  
  
+ GCTTGCTGAT TGATTGCCAA TGCTATGGAT TTCTTGGTGA GTCAATCTTC AATTGCTGAT CTGGGTTCGT   
  
  
+ TTGTTCTTCA GAGTTTCACC TGCCAATTTG ATCAAGTATG TCCCTTCACT TCTCCTGCTT CAATTCTCTC   
  
  
+ TCAAATTCAC GCATTTTTCC AATGAATTTC AAATAGGAGT CCTGGAATCC GATGATATTT TAGGTTTTTT   
  
  
+ CCTGACTTCA TTGCATTAGT TTGTTCATAT CTGTGACCAA TTCTTTCATT TGTTATATGA TCTGATTGTT   
  
  
+ TAGTTAGCTA TTCATTCATT GCATATACAT TGGTTTATTC ATTGCAGAGA CACTCACATA CACACACACA   
  
  
+ TACAGGGGAC ACTAGTATTT TTTCTCTCTA ACTCATCGGA TTCCCTTTGA TACTCTGTTT TTTGGGGGGT   
  
  
+ TGATTTGATT TGATTTGATT GACCTTAATT TTGAACTCAC ACACACTCGC ACATAGATAT ATTCTTTCTG   
  
  
+ TTTGTGACAT ATACAGCATC ATATAGAAAT ATATTGCACT GCATATTTGA GCTCAAGACA GGGATATCAT   
  
  
+ CACAAACTGA GTGTGAGGGA GGCAAAAAAC AGAGGAAAAG AAGGGAATTG GGAGTGTAAT CAAAACAAGA   
  
  
+ GAGAGATTGA GGAGAGAGGA AAAAACAAAA TTGGGAGAAG GTTAATAGAG AAGAATGTTG GCTGGGTGTT   
  
  
+ CATCCACATT GCTGTCACCA AGGTATAGAT TGAGGAGTGA AGCTACATCA CAGTTCCAAG CCTGCCATCA   
  
  
+ TCCAATGAGC ACACAGAGAA TTGATTTGCC CTGCACTTTC CCAAGAAAGG ATGTTTCGAA GCCACAATCG   
  
  
+ GTTCGCCCCG TTGGCCTATC TGTCGAGAAG CCGGTTGAAG TCAGGGCCCT GAAGCAGACC ATCCGAGTCC   
  
  
+ CACCATCGCC GGAGGGTAGA AGAGAGATTA AGGCAGATTT CTGGGGTGAT AGAAGAAAGA GCTTGAAGAG   
  
  
+ GAGACTAGCA GAACAGGGGA GCTCTTTTGA TGATGGTGGT GACGAATCTT GTGTTGGGAG AACCAAAAGG   
  
  
+ AAGAAGGGTA GCTTTGATTT TGGTGAATCT GATGAAATTT CTCCAAAAAT TGAGGACACA ATGAGTTTTG   
  
  
+ GACATCTGGG TAGTGGTGGA AATTTCTGGG TTCATCCTGG TTTTGGTGTT GTTAATGTTA ATACTAATTA   
  
  
+ CCCTCAAGTG CCATTTTCTC TCACTTGTTC AGGGGAGGAA GAAAGAGTAT GTTTTGTTCC TACTGATGTG   
  
  
+ ATCTCACCAG CCATCATGCC TCCATTGTCA CACAATCCTT GGGTTGAATC TGGTGTTACT GAGGTCACAG   
  
  
+ AATATGGTGG GGGTGACAAA GACGGCGAAC CTAGTCATGG GTTTGTGAGG GGTACAACAA CAACGTCCGG   
  
  
+ GTCAAGTTCA TCTTCTGAGA GTCATAGTTT TGGGCATAGG CTCAATGAGA GCTCATCTGA CCCTGAAATC   
  
  
+ AGAAATGGTT CCATGTTGCC TAACCCTGGT CATGGTCCTG GCACCAGTCA TGGTCCTGGA ACCGGTCTGG   
  
  
+ CTCACAACCA CAATGATCAA ACCGAGCAAC AAGGGTTCGA GCTCATTAGC TTTCTCATGG GTTGTGTCGA   
  
  
+ AGCGATCAGT TCAAGGAACA TTGCAGCCAT CAATCATTTC ATAGCTAAGC TGGGCGAGCA GGCTTCTCCA   
  
  
+ AGGGCTCGGT CTGCCATTAG CCGCCTCACA GCCTACTTCA CCGAAGCATT AGCCTTGAGG GTCACAAGAT   
  
  
+ TTTGGCCTCA TATCTTTCAC ATAAGTATCC CTCGAGAGTT TGATCGATTT GATGATGAAT CGGGGGCAGC   
  
  
+ TGCAATGAGG CTTTTGAATC AGATCAGCCC AATTCCAAAG TTCGTTCATT TTACAGCCAA TGAGATGTTA   
  
  
+ TTGAGGGCAT TTGAAGGGAA GGACAAGGTG CATATCATAG ACTTCGACAT CAAGCAAGGC TTACAATGGC   
  
  
+ CCGGGTTTTT CCAAAGCTTA GCCATGAGGG AAAACCCCCC GAGCCATGTC AGGATAACAG GCGTAGGGGA   
  
  
+ TTCGAAGCAA GAATTGGTTG AGACAGGAGA AAGACTAGCC GGGTTTGCTG GGGCATTCAA CCTCTCCTTC   
  
  
+ GAGTTCCACC CAGTTGTGGA CCGGTTGGAA GATGTTAGGC TATGGATGCT TCATGTTAAG GAAGGTGAAA   
  
  
+ GTGTCGCGGT AAATTGCATT TTGCAGCTCC ACAAGACACT CTATGACCCC CATGGTGCCA CATTCAGGGA   
  
  
+ CTTCATGGGT TTAATCCGAA GCACAAATCC CATAGCATTG GTTATGGCTG AGCAAGAAGC TGATCACAAC   
  
  
+ GAACCCACCT TAGAAGGCCG AGTATGCAAC TCACTTGGAT ACTATGCAGC CCTTTTCGAT GCCATAGATT   
  
  
+ GCAGCCTTCC TTTCGAGAGC TCAGCAAGGT TGAAGATCGA AGAGATGTTT GGCCGGGAAA TCAGGAACAT   
  
  
+ AATAGCCTGT GAAGGGGCAG ACAGGATCGA AAGACATGAG AAGTTCGAGA AATGGAAGAG AAGGATCGAG   
  
  
+ CAGGAAGGGT TCCGGTGCAT GCGGACTAGC GAAAGGGAGG TGATGCAGAG CCAAATGCTG CTTAAGATGT   
  
  
+ ACTCGAACGA GAGCTACAAC ATGCAAAGAC AAGGCAATGA AGAAGCAATA TCACTAGTAT GGTTAGATCA   
  
  
+ GCCACTCTAC ACTGTTTCTT TGTGGGCTCC AAGTGAATTT GCTGCAGGGA GTTCTTCCAG TTTTTCTCTG   
  
  
+ CCATCTTG  

- -Up\_Stream \_Len000TATTAC CCACTATTGA AAACTTCCTG TTATATGTTT AGTGAATAAA TAATTTCAGT   
  
  
- TCCCCTGTAA AGTTGATATT GTAGTAACAA ATATTTCATA AACTTTTAAC GTTGAGAACA TTATCTATTA   
  
  
- TTACGAAATA TCTGAAGTGG TTAGTGATTA AACATCTTAG TTTTTTAATT AACTTTTCGT TTGTTTTCCT   
  
  
- TTTCCGTGTA TTTGGATTTA ATACATTTGT TTACTCTTGT ATATACGGTC GAATAGTTGC AATTAATATA   
  
  
- AACAATAGTG AGAATTATAG ACATAAGTAT CATTTACTTT TATTCATAGC TAAAAAATAA TTTGGATTTT   
  
  
- AGAAGACAGT TATTAAAAAG AAGGCTATTT TTTTTTTAAC CTGGTATAAT TTAATTGTAA TTATTTAAGT   
  
  
- ATATTAATGA AACTTACAGA CTTTATATTA CGAGAAGCCA TATAATTCCA GAATATTTAA AGAGTAATTA   
  
  
- AACTTTAGAG AATATTATAT AACCCAGTTG TTCACGATCG AACAAAAAAA AAGGACCAAT TAACGTAAAT   
  
  
- TTTTTTATTA AAAATTAATG TTAACGTTCC CCTCCTTAGT AAATGTTTTA TAGTTTTCAC CTTTTCCGTT   
  
  
- TTTCTTTGTT TAATATTTTG TCTTTTTTCT AATCTTTTGC GTTTGTTTCT GCTTTACTCC TTGTCATTCT   
  
  
- TAGTCATTTT TTTACATCAC GTTGCCAGTG TATGCCCTTC CGTTCGTGTT GTTAGTACTT TCTCTGTCCG   
  
  
- TGCACGCAAG GAGCACTAGT CGTCGGAAGG GATGAAGGGG CGGTTCACCT CAGGCATGAT TGAGGACGGT   
  
  
- AAGGGAGGGT TGCCAGTATA AGGCGGCTGT ACGGAGTTGC CAGTCTAAAG GACGGGGTAG ACCTGGCAGG   
  
  
- CTAGTTCTAG GTTGCCGGTA TTCCGCGTGA GAGGTTATGG GATCACGGGT AAAGACCTTG TCGGGGTTTT   
  
  
- TAGAGTCATA AGCGGTAGTA AAAAGTTATG ACCCCAATGT ATTTGTCAGA TTGTTCCTTC CTGGGTTGTG   
  
  
- TCTCCTCTCT CTTTTTTCGA TCGAAACTAC CCCTGTCGGT ACCTACCTCC ACTTCTCTCT CCTCCAACCC   
  
  
- TGGAAAATAA AACGAGTGAA AGGTAAGAAT ATATTTTTGT CGAGATCAAA TAGAGAATAC CGAAAAAACC   
  
  
- CAAACAAGTA TCTCTCCCCC CTTACACTAT ACTAAACTAA ACTACAATCT TTAAGAGTAA TCAAAAAAGA   
  
  
- AAAACAAACG TTAAAACTAC ACAATCCCAA ACTCCTAGTA GTAAAAACTC ATCCCTTGAA AGACCCAAAA   
  
  
- CGAACGACTA ACTAACGGTT ACGATACCTA AAGAACCACT CAGTTAGAAG TTAACGACTA GACCCAAGCA   
  
  
- AACAAGAAGT CTCAAAGTGG ACGGTTAAAC TAGTTCATAC AGGGAAGTGA AGAGGACGAA GTTAAGAGAG   
  
  
- AGTTTAAGTG CGTAAAAAGG TTACTTAAAG TTTATCCTCA GGACCTTAGG CTACTATAAA ATCCAAAAAA   
  
  
- GGACTGAAGT AACGTAATCA AACAAGTATA GACACTGGTT AAGAAAGTAA ACAATATACT AGACTAACAA   
  
  
- ATCAATCGAT AAGTAAGTAA CGTATATGTA ACCAAATAAG TAACGTCTCT GTGAGTGTAT GTGTGTGTGT   
  
  
- ATGTCCCCTG TGATCATAAA AAAGAGAGAT TGAGTAGCCT AAGGGAAACT ATGAGACAAA AAACCCCCCA   
  
  
- ACTAAACTAA ACTAAACTAA CTGGAATTAA AACTTGAGTG TGTGTGAGCG TGTATCTATA TAAGAAAGAC   
  
  
- AAACACTGTA TATGTCGTAG TATATCTTTA TATAACGTGA CGTATAAACT CGAGTTCTGT CCCTATAGTA   
  
  
- GTGTTTGACT CACACTCCCT CCGTTTTTTG TCTCCTTTTC TTCCCTTAAC CCTCACATTA GTTTTGTTCT   
  
  
- CTCTCTAACT CCTCTCTCCT TTTTTGTTTT AACCCTCTTC CAATTATCTC TTCTTACAAC CGACCCACAA   
  
  
- GTAGGTGTAA CGACAGTGGT TCCATATCTA ACTCCTCACT TCGATGTAGT GTCAAGGTTC GGACGGTAGT   
  
  
- AGGTTACTCG TGTGTCTCTT AACTAAACGG GACGTGAAAG GGTTCTTTCC TACAAAGCTT CGGTGTTAGC   
  
  
- CAAGCGGGGC AACCGGATAG ACAGCTCTTC GGCCAACTTC AGTCCCGGGA CTTCGTCTGG TAGGCTCAGG   
  
  
- GTGGTAGCGG CCTCCCATCT TCTCTCTAAT TCCGTCTAAA GACCCCACTA TCTTCTTTCT CGAACTTCTC   
  
  
- CTCTGATCGT CTTGTCCCCT CGAGAAAACT ACTACCACCA CTGCTTAGAA CACAACCCTC TTGGTTTTCC   
  
  
- TTCTTCCCAT CGAAACTAAA ACCACTTAGA CTACTTTAAA GAGGTTTTTA ACTCCTGTGT TACTCAAAAC   
  
  
- CTGTAGACCC ATCACCACCT TTAAAGACCC AAGTAGGACC AAAACCACAA CAATTACAAT TATGATTAAT   
  
  
- GGGAGTTCAC GGTAAAAGAG AGTGAACAAG TCCCCTCCTT CTTTCTCATA CAAAACAAGG ATGACTACAC   
  
  
- TAGAGTGGTC GGTAGTACGG AGGTAACAGT GTGTTAGGAA CCCAACTTAG ACCACAATGA CTCCAGTGTC   
  
  
- TTATACCACC CCCACTGTTT CTGCCGCTTG GATCAGTACC CAAACACTCC CCATGTTGTT GTTGCAGGCC   
  
  
- CAGTTCAAGT AGAAGACTCT CAGTATCAAA ACCCGTATCC GAGTTACTCT CGAGTAGACT GGGACTTTAG   
  
  
- TCTTTACCAA GGTACAACGG ATTGGGACCA GTACCAGGAC CGTGGTCAGT ACCAGGACCT TGGCCAGACC   
  
  
- GAGTGTTGGT GTTACTAGTT TGGCTCGTTG TTCCCAAGCT CGAGTAATCG AAAGAGTACC CAACACAGCT   
  
  
- TCGCTAGTCA AGTTCCTTGT AACGTCGGTA GTTAGTAAAG TATCGATTCG ACCCGCTCGT CCGAAGAGGT   
  
  
- TCCCGAGCCA GACGGTAATC GGCGGAGTGT CGGATGAAGT GGCTTCGTAA TCGGAACTCC CAGTGTTCTA   
  
  
- AAACCGGAGT ATAGAAAGTG TATTCATAGG GAGCTCTCAA ACTAGCTAAA CTACTACTTA GCCCCCGTCG   
  
  
- ACGTTACTCC GAAAACTTAG TCTAGTCGGG TTAAGGTTTC AAGCAAGTAA AATGTCGGTT ACTCTACAAT   
  
  
- AACTCCCGTA AACTTCCCTT CCTGTTCCAC GTATAGTATC TGAAGCTGTA GTTCGTTCCG AATGTTACCG   
  
  
- GGCCCAAAAA GGTTTCGAAT CGGTACTCCC TTTTGGGGGG CTCGGTACAG TCCTATTGTC CGCATCCCCT   
  
  
- AAGCTTCGTT CTTAACCAAC TCTGTCCTCT TTCTGATCGG CCCAAACGAC CCCGTAAGTT GGAGAGGAAG   
  
  
- CTCAAGGTGG GTCAACACCT GGCCAACCTT CTACAATCCG ATACCTACGA AGTACAATTC CTTCCACTTT   
  
  
- CACAGCGCCA TTTAACGTAA AACGTCGAGG TGTTCTGTGA GATACTGGGG GTACCACGGT GTAAGTCCCT   
  
  
- GAAGTACCCA AATTAGGCTT CGTGTTTAGG GTATCGTAAC CAATACCGAC TCGTTCTTCG ACTAGTGTTG   
  
  
- CTTGGGTGGA ATCTTCCGGC TCATACGTTG AGTGAACCTA TGATACGTCG GGAAAAGCTA CGGTATCTAA   
  
  
- CGTCGGAAGG AAAGCTCTCG AGTCGTTCCA ACTTCTAGCT TCTCTACAAA CCGGCCCTTT AGTCCTTGTA   
  
  
- TTATCGGACA CTTCCCCGTC TGTCCTAGCT TTCTGTACTC TTCAAGCTCT TTACCTTCTC TTCCTAGCTC   
  
  
- GTCCTTCCCA AGGCCACGTA CGCCTGATCG CTTTCCCTCC ACTACGTCTC GGTTTACGAC GAATTCTACA   
  
  
- TGAGCTTGCT CTCGATGTTG TACGTTTCTG TTCCGTTACT TCTTCGTTAT AGTGATCATA CCAATCTAGT   
  
  
- CGGTGAGATG TGACAAAGAA ACACCCGAGG TTCACTTAAA CGACGTCCCT CAAGAAGGTC AAAAAGAGAC   
  
  
- GGTAGAAC

+     GATA-motif

| Site Name | Organism | Position | Strand | Matrix score. | sequence | function |
| --- | --- | --- | --- | --- | --- | --- |
| GATA-motif | Solanum tuberosum | 210 | + | 9 | AAGGATAAGG | part of a light responsive element |
| GATA-motif | Solanum tuberosum | 3243 | + | 9 | AAGGATAAGG | part of a light responsive element |

>HU04G00148.1   
+ -Up\_Stream \_Len000ATAATG GGTGATAACT TTTGAAGGAC AATATACAAA TCACTTATTT ATTAAAGTCA   
  
  
+ AGGGGACATT TCAACTATAA CATCATTGTT TATAAAGTAT TTGAAAATTG CAACTCTTGT AATAGATAAT   
  
  
+ AATGCTTTAT AGACTTCACC AATCACTAAT TTGTAGAATC AAAAAATTAA TTGAAAAGCA AACAAAAGGA   
  
  
+ AAAGGCACAT AAACCTAAAT TATGTAAACA AATGAGAACA TATATGCCAG CTTATCAACG TTAATTATAT   
  
  
+ TTGTTATCAC TCTTAATATC TGTATTCATA GTAAATGAAA ATAAGTATCG ATTTTTTATT AAACCTAAAA   
  
  
+ TCTTCTGTCA ATAATTTTTC TTCCGATAAA AAAAAAATTG GACCATATTA AATTAACATT AATAAATTCA   
  
  
+ TATAATTACT TTGAATGTCT GAAATATAAT GCTCTTCGGT ATATTAAGGT CTTATAAATT TCTCATTAAT   
  
  
+ TTGAAATCTC TTATAATATA TTGGGTCAAC AAGTGCTAGC TTGTTTTTTT TTCCTGGTTA ATTGCATTTA   
  
  
+ AAAAAATAAT TTTTAATTAC AATTGCAAGG GGAGGAATCA TTTACAAAAT ATCAAAAGTG GAAAAGGCAA   
  
  
+ AAAGAAACAA ATTATAAAAC AGAAAAAAGA TTAGAAAACG CAAACAAAGA CGAAATGAGG AACAGTAAGA   
  
  
+ ATCAGTAAAA AAATGTAGTG CAACGGTCAC ATACGGGAAG GCAAGCACAA CAATCATGAA AGAGACAGGC   
  
  
+ ACGTGCGTTC CTCGTGATCA GCAGCCTTCC CTACTTCCCC GCCAAGTGGA GTCCGTACTA ACTCCTGCCA   
  
  
+ TTCCCTCCCA ACGGTCATAT TCCGCCGACA TGCCTCAACG GTCAGATTTC CTGCCCCATC TGGACCGTCC   
  
  
+ GATCAAGATC CAACGGCCAT AAGGCGCACT CTCCAATACC CTAGTGCCCA TTTCTGGAAC AGCCCCAAAA   
  
  
+ ATCTCAGTAT TCGCCATCAT TTTTCAATAC TGGGGTTACA TAAACAGTCT AACAAGGAAG GACCCAACAC   
  
  
+ AGAGGAGAGA GAAAAAAGCT AGCTTTGATG GGGACAGCCA TGGATGGAGG TGAAGAGAGA GGAGGTTGGG   
  
  
+ ACCTTTTATT TTGCTCACTT TCCATTCTTA TATAAAAACA GCTCTAGTTT ATCTCTTATG GCTTTTTTGG   
  
  
+ GTTTGTTCAT AGAGAGGGGG GAATGTGATA TGATTTGATT TGATGTTAGA AATTCTCATT AGTTTTTTCT   
  
  
+ TTTTGTTTGC AATTTTGATG TGTTAGGGTT TGAGGATCAT CATTTTTGAG TAGGGAACTT TCTGGGTTTT   
  
  
+ GCTTGCTGAT TGATTGCCAA TGCTATGGAT TTCTTGGTGA GTCAATCTTC AATTGCTGAT CTGGGTTCGT   
  
  
+ TTGTTCTTCA GAGTTTCACC TGCCAATTTG ATCAAGTATG TCCCTTCACT TCTCCTGCTT CAATTCTCTC   
  
  
+ TCAAATTCAC GCATTTTTCC AATGAATTTC AAATAGGAGT CCTGGAATCC GATGATATTT TAGGTTTTTT   
  
  
+ CCTGACTTCA TTGCATTAGT TTGTTCATAT CTGTGACCAA TTCTTTCATT TGTTATATGA TCTGATTGTT   
  
  
+ TAGTTAGCTA TTCATTCATT GCATATACAT TGGTTTATTC ATTGCAGAGA CACTCACATA CACACACACA   
  
  
+ TACAGGGGAC ACTAGTATTT TTTCTCTCTA ACTCATCGGA TTCCCTTTGA TACTCTGTTT TTTGGGGGGT   
  
  
+ TGATTTGATT TGATTTGATT GACCTTAATT TTGAACTCAC ACACACTCGC ACATAGATAT ATTCTTTCTG   
  
  
+ TTTGTGACAT ATACAGCATC ATATAGAAAT ATATTGCACT GCATATTTGA GCTCAAGACA GGGATATCAT   
  
  
+ CACAAACTGA GTGTGAGGGA GGCAAAAAAC AGAGGAAAAG AAGGGAATTG GGAGTGTAAT CAAAACAAGA   
  
  
+ GAGAGATTGA GGAGAGAGGA AAAAACAAAA TTGGGAGAAG GTTAATAGAG AAGAATGTTG GCTGGGTGTT   
  
  
+ CATCCACATT GCTGTCACCA AGGTATAGAT TGAGGAGTGA AGCTACATCA CAGTTCCAAG CCTGCCATCA   
  
  
+ TCCAATGAGC ACACAGAGAA TTGATTTGCC CTGCACTTTC CCAAGAAAGG ATGTTTCGAA GCCACAATCG   
  
  
+ GTTCGCCCCG TTGGCCTATC TGTCGAGAAG CCGGTTGAAG TCAGGGCCCT GAAGCAGACC ATCCGAGTCC   
  
  
+ CACCATCGCC GGAGGGTAGA AGAGAGATTA AGGCAGATTT CTGGGGTGAT AGAAGAAAGA GCTTGAAGAG   
  
  
+ GAGACTAGCA GAACAGGGGA GCTCTTTTGA TGATGGTGGT GACGAATCTT GTGTTGGGAG AACCAAAAGG   
  
  
+ AAGAAGGGTA GCTTTGATTT TGGTGAATCT GATGAAATTT CTCCAAAAAT TGAGGACACA ATGAGTTTTG   
  
  
+ GACATCTGGG TAGTGGTGGA AATTTCTGGG TTCATCCTGG TTTTGGTGTT GTTAATGTTA ATACTAATTA   
  
  
+ CCCTCAAGTG CCATTTTCTC TCACTTGTTC AGGGGAGGAA GAAAGAGTAT GTTTTGTTCC TACTGATGTG   
  
  
+ ATCTCACCAG CCATCATGCC TCCATTGTCA CACAATCCTT GGGTTGAATC TGGTGTTACT GAGGTCACAG   
  
  
+ AATATGGTGG GGGTGACAAA GACGGCGAAC CTAGTCATGG GTTTGTGAGG GGTACAACAA CAACGTCCGG   
  
  
+ GTCAAGTTCA TCTTCTGAGA GTCATAGTTT TGGGCATAGG CTCAATGAGA GCTCATCTGA CCCTGAAATC   
  
  
+ AGAAATGGTT CCATGTTGCC TAACCCTGGT CATGGTCCTG GCACCAGTCA TGGTCCTGGA ACCGGTCTGG   
  
  
+ CTCACAACCA CAATGATCAA ACCGAGCAAC AAGGGTTCGA GCTCATTAGC TTTCTCATGG GTTGTGTCGA   
  
  
+ AGCGATCAGT TCAAGGAACA TTGCAGCCAT CAATCATTTC ATAGCTAAGC TGGGCGAGCA GGCTTCTCCA   
  
  
+ AGGGCTCGGT CTGCCATTAG CCGCCTCACA GCCTACTTCA CCGAAGCATT AGCCTTGAGG GTCACAAGAT   
  
  
+ TTTGGCCTCA TATCTTTCAC ATAAGTATCC CTCGAGAGTT TGATCGATTT GATGATGAAT CGGGGGCAGC   
  
  
+ TGCAATGAGG CTTTTGAATC AGATCAGCCC AATTCCAAAG TTCGTTCATT TTACAGCCAA TGAGATGTTA   
  
  
+ TTGAGGGCAT TTGAAGGGAA GGACAAGGTG CATATCATAG ACTTCGACAT CAAGCAAGGC TTACAATGGC   
  
  
+ CCGGGTTTTT CCAAAGCTTA GCCATGAGGG AAAACCCCCC GAGCCATGTC AGGATAACAG GCGTAGGGGA   
  
  
+ TTCGAAGCAA GAATTGGTTG AGACAGGAGA AAGACTAGCC GGGTTTGCTG GGGCATTCAA CCTCTCCTTC   
  
  
+ GAGTTCCACC CAGTTGTGGA CCGGTTGGAA GATGTTAGGC TATGGATGCT TCATGTTAAG GAAGGTGAAA   
  
  
+ GTGTCGCGGT AAATTGCATT TTGCAGCTCC ACAAGACACT CTATGACCCC CATGGTGCCA CATTCAGGGA   
  
  
+ CTTCATGGGT TTAATCCGAA GCACAAATCC CATAGCATTG GTTATGGCTG AGCAAGAAGC TGATCACAAC   
  
  
+ GAACCCACCT TAGAAGGCCG AGTATGCAAC TCACTTGGAT ACTATGCAGC CCTTTTCGAT GCCATAGATT   
  
  
+ GCAGCCTTCC TTTCGAGAGC TCAGCAAGGT TGAAGATCGA AGAGATGTTT GGCCGGGAAA TCAGGAACAT   
  
  
+ AATAGCCTGT GAAGGGGCAG ACAGGATCGA AAGACATGAG AAGTTCGAGA AATGGAAGAG AAGGATCGAG   
  
  
+ CAGGAAGGGT TCCGGTGCAT GCGGACTAGC GAAAGGGAGG TGATGCAGAG CCAAATGCTG CTTAAGATGT   
  
  
+ ACTCGAACGA GAGCTACAAC ATGCAAAGAC AAGGCAATGA AGAAGCAATA TCACTAGTAT GGTTAGATCA   
  
  
+ GCCACTCTAC ACTGTTTCTT TGTGGGCTCC AAGTGAATTT GCTGCAGGGA GTTCTTCCAG TTTTTCTCTG   
  
  
+ CCATCTTG  

- -Up\_Stream \_Len000TATTAC CCACTATTGA AAACTTCCTG TTATATGTTT AGTGAATAAA TAATTTCAGT   
  
  
- TCCCCTGTAA AGTTGATATT GTAGTAACAA ATATTTCATA AACTTTTAAC GTTGAGAACA TTATCTATTA   
  
  
- TTACGAAATA TCTGAAGTGG TTAGTGATTA AACATCTTAG TTTTTTAATT AACTTTTCGT TTGTTTTCCT   
  
  
- TTTCCGTGTA TTTGGATTTA ATACATTTGT TTACTCTTGT ATATACGGTC GAATAGTTGC AATTAATATA   
  
  
- AACAATAGTG AGAATTATAG ACATAAGTAT CATTTACTTT TATTCATAGC TAAAAAATAA TTTGGATTTT   
  
  
- AGAAGACAGT TATTAAAAAG AAGGCTATTT TTTTTTTAAC CTGGTATAAT TTAATTGTAA TTATTTAAGT   
  
  
- ATATTAATGA AACTTACAGA CTTTATATTA CGAGAAGCCA TATAATTCCA GAATATTTAA AGAGTAATTA   
  
  
- AACTTTAGAG AATATTATAT AACCCAGTTG TTCACGATCG AACAAAAAAA AAGGACCAAT TAACGTAAAT   
  
  
- TTTTTTATTA AAAATTAATG TTAACGTTCC CCTCCTTAGT AAATGTTTTA TAGTTTTCAC CTTTTCCGTT   
  
  
- TTTCTTTGTT TAATATTTTG TCTTTTTTCT AATCTTTTGC GTTTGTTTCT GCTTTACTCC TTGTCATTCT   
  
  
- TAGTCATTTT TTTACATCAC GTTGCCAGTG TATGCCCTTC CGTTCGTGTT GTTAGTACTT TCTCTGTCCG   
  
  
- TGCACGCAAG GAGCACTAGT CGTCGGAAGG GATGAAGGGG CGGTTCACCT CAGGCATGAT TGAGGACGGT   
  
  
- AAGGGAGGGT TGCCAGTATA AGGCGGCTGT ACGGAGTTGC CAGTCTAAAG GACGGGGTAG ACCTGGCAGG   
  
  
- CTAGTTCTAG GTTGCCGGTA TTCCGCGTGA GAGGTTATGG GATCACGGGT AAAGACCTTG TCGGGGTTTT   
  
  
- TAGAGTCATA AGCGGTAGTA AAAAGTTATG ACCCCAATGT ATTTGTCAGA TTGTTCCTTC CTGGGTTGTG   
  
  
- TCTCCTCTCT CTTTTTTCGA TCGAAACTAC CCCTGTCGGT ACCTACCTCC ACTTCTCTCT CCTCCAACCC   
  
  
- TGGAAAATAA AACGAGTGAA AGGTAAGAAT ATATTTTTGT CGAGATCAAA TAGAGAATAC CGAAAAAACC   
  
  
- CAAACAAGTA TCTCTCCCCC CTTACACTAT ACTAAACTAA ACTACAATCT TTAAGAGTAA TCAAAAAAGA   
  
  
- AAAACAAACG TTAAAACTAC ACAATCCCAA ACTCCTAGTA GTAAAAACTC ATCCCTTGAA AGACCCAAAA   
  
  
- CGAACGACTA ACTAACGGTT ACGATACCTA AAGAACCACT CAGTTAGAAG TTAACGACTA GACCCAAGCA   
  
  
- AACAAGAAGT CTCAAAGTGG ACGGTTAAAC TAGTTCATAC AGGGAAGTGA AGAGGACGAA GTTAAGAGAG   
  
  
- AGTTTAAGTG CGTAAAAAGG TTACTTAAAG TTTATCCTCA GGACCTTAGG CTACTATAAA ATCCAAAAAA   
  
  
- GGACTGAAGT AACGTAATCA AACAAGTATA GACACTGGTT AAGAAAGTAA ACAATATACT AGACTAACAA   
  
  
- ATCAATCGAT AAGTAAGTAA CGTATATGTA ACCAAATAAG TAACGTCTCT GTGAGTGTAT GTGTGTGTGT   
  
  
- ATGTCCCCTG TGATCATAAA AAAGAGAGAT TGAGTAGCCT AAGGGAAACT ATGAGACAAA AAACCCCCCA   
  
  
- ACTAAACTAA ACTAAACTAA CTGGAATTAA AACTTGAGTG TGTGTGAGCG TGTATCTATA TAAGAAAGAC   
  
  
- AAACACTGTA TATGTCGTAG TATATCTTTA TATAACGTGA CGTATAAACT CGAGTTCTGT CCCTATAGTA   
  
  
- GTGTTTGACT CACACTCCCT CCGTTTTTTG TCTCCTTTTC TTCCCTTAAC CCTCACATTA GTTTTGTTCT   
  
  
- CTCTCTAACT CCTCTCTCCT TTTTTGTTTT AACCCTCTTC CAATTATCTC TTCTTACAAC CGACCCACAA   
  
  
- GTAGGTGTAA CGACAGTGGT TCCATATCTA ACTCCTCACT TCGATGTAGT GTCAAGGTTC GGACGGTAGT   
  
  
- AGGTTACTCG TGTGTCTCTT AACTAAACGG GACGTGAAAG GGTTCTTTCC TACAAAGCTT CGGTGTTAGC   
  
  
- CAAGCGGGGC AACCGGATAG ACAGCTCTTC GGCCAACTTC AGTCCCGGGA CTTCGTCTGG TAGGCTCAGG   
  
  
- GTGGTAGCGG CCTCCCATCT TCTCTCTAAT TCCGTCTAAA GACCCCACTA TCTTCTTTCT CGAACTTCTC   
  
  
- CTCTGATCGT CTTGTCCCCT CGAGAAAACT ACTACCACCA CTGCTTAGAA CACAACCCTC TTGGTTTTCC   
  
  
- TTCTTCCCAT CGAAACTAAA ACCACTTAGA CTACTTTAAA GAGGTTTTTA ACTCCTGTGT TACTCAAAAC   
  
  
- CTGTAGACCC ATCACCACCT TTAAAGACCC AAGTAGGACC AAAACCACAA CAATTACAAT TATGATTAAT   
  
  
- GGGAGTTCAC GGTAAAAGAG AGTGAACAAG TCCCCTCCTT CTTTCTCATA CAAAACAAGG ATGACTACAC   
  
  
- TAGAGTGGTC GGTAGTACGG AGGTAACAGT GTGTTAGGAA CCCAACTTAG ACCACAATGA CTCCAGTGTC   
  
  
- TTATACCACC CCCACTGTTT CTGCCGCTTG GATCAGTACC CAAACACTCC CCATGTTGTT GTTGCAGGCC   
  
  
- CAGTTCAAGT AGAAGACTCT CAGTATCAAA ACCCGTATCC GAGTTACTCT CGAGTAGACT GGGACTTTAG   
  
  
- TCTTTACCAA GGTACAACGG ATTGGGACCA GTACCAGGAC CGTGGTCAGT ACCAGGACCT TGGCCAGACC   
  
  
- GAGTGTTGGT GTTACTAGTT TGGCTCGTTG TTCCCAAGCT CGAGTAATCG AAAGAGTACC CAACACAGCT   
  
  
- TCGCTAGTCA AGTTCCTTGT AACGTCGGTA GTTAGTAAAG TATCGATTCG ACCCGCTCGT CCGAAGAGGT   
  
  
- TCCCGAGCCA GACGGTAATC GGCGGAGTGT CGGATGAAGT GGCTTCGTAA TCGGAACTCC CAGTGTTCTA   
  
  
- AAACCGGAGT ATAGAAAGTG TATTCATAGG GAGCTCTCAA ACTAGCTAAA CTACTACTTA GCCCCCGTCG   
  
  
- ACGTTACTCC GAAAACTTAG TCTAGTCGGG TTAAGGTTTC AAGCAAGTAA AATGTCGGTT ACTCTACAAT   
  
  
- AACTCCCGTA AACTTCCCTT CCTGTTCCAC GTATAGTATC TGAAGCTGTA GTTCGTTCCG AATGTTACCG   
  
  
- GGCCCAAAAA GGTTTCGAAT CGGTACTCCC TTTTGGGGGG CTCGGTACAG TCCTATTGTC CGCATCCCCT   
  
  
- AAGCTTCGTT CTTAACCAAC TCTGTCCTCT TTCTGATCGG CCCAAACGAC CCCGTAAGTT GGAGAGGAAG   
  
  
- CTCAAGGTGG GTCAACACCT GGCCAACCTT CTACAATCCG ATACCTACGA AGTACAATTC CTTCCACTTT   
  
  
- CACAGCGCCA TTTAACGTAA AACGTCGAGG TGTTCTGTGA GATACTGGGG GTACCACGGT GTAAGTCCCT   
  
  
- GAAGTACCCA AATTAGGCTT CGTGTTTAGG GTATCGTAAC CAATACCGAC TCGTTCTTCG ACTAGTGTTG   
  
  
- CTTGGGTGGA ATCTTCCGGC TCATACGTTG AGTGAACCTA TGATACGTCG GGAAAAGCTA CGGTATCTAA   
  
  
- CGTCGGAAGG AAAGCTCTCG AGTCGTTCCA ACTTCTAGCT TCTCTACAAA CCGGCCCTTT AGTCCTTGTA   
  
  
- TTATCGGACA CTTCCCCGTC TGTCCTAGCT TTCTGTACTC TTCAAGCTCT TTACCTTCTC TTCCTAGCTC   
  
  
- GTCCTTCCCA AGGCCACGTA CGCCTGATCG CTTTCCCTCC ACTACGTCTC GGTTTACGAC GAATTCTACA   
  
  
- TGAGCTTGCT CTCGATGTTG TACGTTTCTG TTCCGTTACT TCTTCGTTAT AGTGATCATA CCAATCTAGT   
  
  
- CGGTGAGATG TGACAAAGAA ACACCCGAGG TTCACTTAAA CGACGTCCCT CAAGAAGGTC AAAAAGAGAC   
  
  
- GGTAGAAC

+     GC-motif

| Site Name | Organism | Position | Strand | Matrix score. | sequence | function |
| --- | --- | --- | --- | --- | --- | --- |
| GC-motif | Zea mays | 3145 | - | 6 | CCCCCG | enhancer-like element involved in anoxic specific inducibility |
| GC-motif | Zea mays | 3330 | + | 6 | CCCCCG | enhancer-like element involved in anoxic specific inducibility |

>HU04G00148.1   
+ -Up\_Stream \_Len000ATAATG GGTGATAACT TTTGAAGGAC AATATACAAA TCACTTATTT ATTAAAGTCA   
  
  
+ AGGGGACATT TCAACTATAA CATCATTGTT TATAAAGTAT TTGAAAATTG CAACTCTTGT AATAGATAAT   
  
  
+ AATGCTTTAT AGACTTCACC AATCACTAAT TTGTAGAATC AAAAAATTAA TTGAAAAGCA AACAAAAGGA   
  
  
+ AAAGGCACAT AAACCTAAAT TATGTAAACA AATGAGAACA TATATGCCAG CTTATCAACG TTAATTATAT   
  
  
+ TTGTTATCAC TCTTAATATC TGTATTCATA GTAAATGAAA ATAAGTATCG ATTTTTTATT AAACCTAAAA   
  
  
+ TCTTCTGTCA ATAATTTTTC TTCCGATAAA AAAAAAATTG GACCATATTA AATTAACATT AATAAATTCA   
  
  
+ TATAATTACT TTGAATGTCT GAAATATAAT GCTCTTCGGT ATATTAAGGT CTTATAAATT TCTCATTAAT   
  
  
+ TTGAAATCTC TTATAATATA TTGGGTCAAC AAGTGCTAGC TTGTTTTTTT TTCCTGGTTA ATTGCATTTA   
  
  
+ AAAAAATAAT TTTTAATTAC AATTGCAAGG GGAGGAATCA TTTACAAAAT ATCAAAAGTG GAAAAGGCAA   
  
  
+ AAAGAAACAA ATTATAAAAC AGAAAAAAGA TTAGAAAACG CAAACAAAGA CGAAATGAGG AACAGTAAGA   
  
  
+ ATCAGTAAAA AAATGTAGTG CAACGGTCAC ATACGGGAAG GCAAGCACAA CAATCATGAA AGAGACAGGC   
  
  
+ ACGTGCGTTC CTCGTGATCA GCAGCCTTCC CTACTTCCCC GCCAAGTGGA GTCCGTACTA ACTCCTGCCA   
  
  
+ TTCCCTCCCA ACGGTCATAT TCCGCCGACA TGCCTCAACG GTCAGATTTC CTGCCCCATC TGGACCGTCC   
  
  
+ GATCAAGATC CAACGGCCAT AAGGCGCACT CTCCAATACC CTAGTGCCCA TTTCTGGAAC AGCCCCAAAA   
  
  
+ ATCTCAGTAT TCGCCATCAT TTTTCAATAC TGGGGTTACA TAAACAGTCT AACAAGGAAG GACCCAACAC   
  
  
+ AGAGGAGAGA GAAAAAAGCT AGCTTTGATG GGGACAGCCA TGGATGGAGG TGAAGAGAGA GGAGGTTGGG   
  
  
+ ACCTTTTATT TTGCTCACTT TCCATTCTTA TATAAAAACA GCTCTAGTTT ATCTCTTATG GCTTTTTTGG   
  
  
+ GTTTGTTCAT AGAGAGGGGG GAATGTGATA TGATTTGATT TGATGTTAGA AATTCTCATT AGTTTTTTCT   
  
  
+ TTTTGTTTGC AATTTTGATG TGTTAGGGTT TGAGGATCAT CATTTTTGAG TAGGGAACTT TCTGGGTTTT   
  
  
+ GCTTGCTGAT TGATTGCCAA TGCTATGGAT TTCTTGGTGA GTCAATCTTC AATTGCTGAT CTGGGTTCGT   
  
  
+ TTGTTCTTCA GAGTTTCACC TGCCAATTTG ATCAAGTATG TCCCTTCACT TCTCCTGCTT CAATTCTCTC   
  
  
+ TCAAATTCAC GCATTTTTCC AATGAATTTC AAATAGGAGT CCTGGAATCC GATGATATTT TAGGTTTTTT   
  
  
+ CCTGACTTCA TTGCATTAGT TTGTTCATAT CTGTGACCAA TTCTTTCATT TGTTATATGA TCTGATTGTT   
  
  
+ TAGTTAGCTA TTCATTCATT GCATATACAT TGGTTTATTC ATTGCAGAGA CACTCACATA CACACACACA   
  
  
+ TACAGGGGAC ACTAGTATTT TTTCTCTCTA ACTCATCGGA TTCCCTTTGA TACTCTGTTT TTTGGGGGGT   
  
  
+ TGATTTGATT TGATTTGATT GACCTTAATT TTGAACTCAC ACACACTCGC ACATAGATAT ATTCTTTCTG   
  
  
+ TTTGTGACAT ATACAGCATC ATATAGAAAT ATATTGCACT GCATATTTGA GCTCAAGACA GGGATATCAT   
  
  
+ CACAAACTGA GTGTGAGGGA GGCAAAAAAC AGAGGAAAAG AAGGGAATTG GGAGTGTAAT CAAAACAAGA   
  
  
+ GAGAGATTGA GGAGAGAGGA AAAAACAAAA TTGGGAGAAG GTTAATAGAG AAGAATGTTG GCTGGGTGTT   
  
  
+ CATCCACATT GCTGTCACCA AGGTATAGAT TGAGGAGTGA AGCTACATCA CAGTTCCAAG CCTGCCATCA   
  
  
+ TCCAATGAGC ACACAGAGAA TTGATTTGCC CTGCACTTTC CCAAGAAAGG ATGTTTCGAA GCCACAATCG   
  
  
+ GTTCGCCCCG TTGGCCTATC TGTCGAGAAG CCGGTTGAAG TCAGGGCCCT GAAGCAGACC ATCCGAGTCC   
  
  
+ CACCATCGCC GGAGGGTAGA AGAGAGATTA AGGCAGATTT CTGGGGTGAT AGAAGAAAGA GCTTGAAGAG   
  
  
+ GAGACTAGCA GAACAGGGGA GCTCTTTTGA TGATGGTGGT GACGAATCTT GTGTTGGGAG AACCAAAAGG   
  
  
+ AAGAAGGGTA GCTTTGATTT TGGTGAATCT GATGAAATTT CTCCAAAAAT TGAGGACACA ATGAGTTTTG   
  
  
+ GACATCTGGG TAGTGGTGGA AATTTCTGGG TTCATCCTGG TTTTGGTGTT GTTAATGTTA ATACTAATTA   
  
  
+ CCCTCAAGTG CCATTTTCTC TCACTTGTTC AGGGGAGGAA GAAAGAGTAT GTTTTGTTCC TACTGATGTG   
  
  
+ ATCTCACCAG CCATCATGCC TCCATTGTCA CACAATCCTT GGGTTGAATC TGGTGTTACT GAGGTCACAG   
  
  
+ AATATGGTGG GGGTGACAAA GACGGCGAAC CTAGTCATGG GTTTGTGAGG GGTACAACAA CAACGTCCGG   
  
  
+ GTCAAGTTCA TCTTCTGAGA GTCATAGTTT TGGGCATAGG CTCAATGAGA GCTCATCTGA CCCTGAAATC   
  
  
+ AGAAATGGTT CCATGTTGCC TAACCCTGGT CATGGTCCTG GCACCAGTCA TGGTCCTGGA ACCGGTCTGG   
  
  
+ CTCACAACCA CAATGATCAA ACCGAGCAAC AAGGGTTCGA GCTCATTAGC TTTCTCATGG GTTGTGTCGA   
  
  
+ AGCGATCAGT TCAAGGAACA TTGCAGCCAT CAATCATTTC ATAGCTAAGC TGGGCGAGCA GGCTTCTCCA   
  
  
+ AGGGCTCGGT CTGCCATTAG CCGCCTCACA GCCTACTTCA CCGAAGCATT AGCCTTGAGG GTCACAAGAT   
  
  
+ TTTGGCCTCA TATCTTTCAC ATAAGTATCC CTCGAGAGTT TGATCGATTT GATGATGAAT CGGGGGCAGC   
  
  
+ TGCAATGAGG CTTTTGAATC AGATCAGCCC AATTCCAAAG TTCGTTCATT TTACAGCCAA TGAGATGTTA   
  
  
+ TTGAGGGCAT TTGAAGGGAA GGACAAGGTG CATATCATAG ACTTCGACAT CAAGCAAGGC TTACAATGGC   
  
  
+ CCGGGTTTTT CCAAAGCTTA GCCATGAGGG AAAACCCCCC GAGCCATGTC AGGATAACAG GCGTAGGGGA   
  
  
+ TTCGAAGCAA GAATTGGTTG AGACAGGAGA AAGACTAGCC GGGTTTGCTG GGGCATTCAA CCTCTCCTTC   
  
  
+ GAGTTCCACC CAGTTGTGGA CCGGTTGGAA GATGTTAGGC TATGGATGCT TCATGTTAAG GAAGGTGAAA   
  
  
+ GTGTCGCGGT AAATTGCATT TTGCAGCTCC ACAAGACACT CTATGACCCC CATGGTGCCA CATTCAGGGA   
  
  
+ CTTCATGGGT TTAATCCGAA GCACAAATCC CATAGCATTG GTTATGGCTG AGCAAGAAGC TGATCACAAC   
  
  
+ GAACCCACCT TAGAAGGCCG AGTATGCAAC TCACTTGGAT ACTATGCAGC CCTTTTCGAT GCCATAGATT   
  
  
+ GCAGCCTTCC TTTCGAGAGC TCAGCAAGGT TGAAGATCGA AGAGATGTTT GGCCGGGAAA TCAGGAACAT   
  
  
+ AATAGCCTGT GAAGGGGCAG ACAGGATCGA AAGACATGAG AAGTTCGAGA AATGGAAGAG AAGGATCGAG   
  
  
+ CAGGAAGGGT TCCGGTGCAT GCGGACTAGC GAAAGGGAGG TGATGCAGAG CCAAATGCTG CTTAAGATGT   
  
  
+ ACTCGAACGA GAGCTACAAC ATGCAAAGAC AAGGCAATGA AGAAGCAATA TCACTAGTAT GGTTAGATCA   
  
  
+ GCCACTCTAC ACTGTTTCTT TGTGGGCTCC AAGTGAATTT GCTGCAGGGA GTTCTTCCAG TTTTTCTCTG   
  
  
+ CCATCTTG  

- -Up\_Stream \_Len000TATTAC CCACTATTGA AAACTTCCTG TTATATGTTT AGTGAATAAA TAATTTCAGT   
  
  
- TCCCCTGTAA AGTTGATATT GTAGTAACAA ATATTTCATA AACTTTTAAC GTTGAGAACA TTATCTATTA   
  
  
- TTACGAAATA TCTGAAGTGG TTAGTGATTA AACATCTTAG TTTTTTAATT AACTTTTCGT TTGTTTTCCT   
  
  
- TTTCCGTGTA TTTGGATTTA ATACATTTGT TTACTCTTGT ATATACGGTC GAATAGTTGC AATTAATATA   
  
  
- AACAATAGTG AGAATTATAG ACATAAGTAT CATTTACTTT TATTCATAGC TAAAAAATAA TTTGGATTTT   
  
  
- AGAAGACAGT TATTAAAAAG AAGGCTATTT TTTTTTTAAC CTGGTATAAT TTAATTGTAA TTATTTAAGT   
  
  
- ATATTAATGA AACTTACAGA CTTTATATTA CGAGAAGCCA TATAATTCCA GAATATTTAA AGAGTAATTA   
  
  
- AACTTTAGAG AATATTATAT AACCCAGTTG TTCACGATCG AACAAAAAAA AAGGACCAAT TAACGTAAAT   
  
  
- TTTTTTATTA AAAATTAATG TTAACGTTCC CCTCCTTAGT AAATGTTTTA TAGTTTTCAC CTTTTCCGTT   
  
  
- TTTCTTTGTT TAATATTTTG TCTTTTTTCT AATCTTTTGC GTTTGTTTCT GCTTTACTCC TTGTCATTCT   
  
  
- TAGTCATTTT TTTACATCAC GTTGCCAGTG TATGCCCTTC CGTTCGTGTT GTTAGTACTT TCTCTGTCCG   
  
  
- TGCACGCAAG GAGCACTAGT CGTCGGAAGG GATGAAGGGG CGGTTCACCT CAGGCATGAT TGAGGACGGT   
  
  
- AAGGGAGGGT TGCCAGTATA AGGCGGCTGT ACGGAGTTGC CAGTCTAAAG GACGGGGTAG ACCTGGCAGG   
  
  
- CTAGTTCTAG GTTGCCGGTA TTCCGCGTGA GAGGTTATGG GATCACGGGT AAAGACCTTG TCGGGGTTTT   
  
  
- TAGAGTCATA AGCGGTAGTA AAAAGTTATG ACCCCAATGT ATTTGTCAGA TTGTTCCTTC CTGGGTTGTG   
  
  
- TCTCCTCTCT CTTTTTTCGA TCGAAACTAC CCCTGTCGGT ACCTACCTCC ACTTCTCTCT CCTCCAACCC   
  
  
- TGGAAAATAA AACGAGTGAA AGGTAAGAAT ATATTTTTGT CGAGATCAAA TAGAGAATAC CGAAAAAACC   
  
  
- CAAACAAGTA TCTCTCCCCC CTTACACTAT ACTAAACTAA ACTACAATCT TTAAGAGTAA TCAAAAAAGA   
  
  
- AAAACAAACG TTAAAACTAC ACAATCCCAA ACTCCTAGTA GTAAAAACTC ATCCCTTGAA AGACCCAAAA   
  
  
- CGAACGACTA ACTAACGGTT ACGATACCTA AAGAACCACT CAGTTAGAAG TTAACGACTA GACCCAAGCA   
  
  
- AACAAGAAGT CTCAAAGTGG ACGGTTAAAC TAGTTCATAC AGGGAAGTGA AGAGGACGAA GTTAAGAGAG   
  
  
- AGTTTAAGTG CGTAAAAAGG TTACTTAAAG TTTATCCTCA GGACCTTAGG CTACTATAAA ATCCAAAAAA   
  
  
- GGACTGAAGT AACGTAATCA AACAAGTATA GACACTGGTT AAGAAAGTAA ACAATATACT AGACTAACAA   
  
  
- ATCAATCGAT AAGTAAGTAA CGTATATGTA ACCAAATAAG TAACGTCTCT GTGAGTGTAT GTGTGTGTGT   
  
  
- ATGTCCCCTG TGATCATAAA AAAGAGAGAT TGAGTAGCCT AAGGGAAACT ATGAGACAAA AAACCCCCCA   
  
  
- ACTAAACTAA ACTAAACTAA CTGGAATTAA AACTTGAGTG TGTGTGAGCG TGTATCTATA TAAGAAAGAC   
  
  
- AAACACTGTA TATGTCGTAG TATATCTTTA TATAACGTGA CGTATAAACT CGAGTTCTGT CCCTATAGTA   
  
  
- GTGTTTGACT CACACTCCCT CCGTTTTTTG TCTCCTTTTC TTCCCTTAAC CCTCACATTA GTTTTGTTCT   
  
  
- CTCTCTAACT CCTCTCTCCT TTTTTGTTTT AACCCTCTTC CAATTATCTC TTCTTACAAC CGACCCACAA   
  
  
- GTAGGTGTAA CGACAGTGGT TCCATATCTA ACTCCTCACT TCGATGTAGT GTCAAGGTTC GGACGGTAGT   
  
  
- AGGTTACTCG TGTGTCTCTT AACTAAACGG GACGTGAAAG GGTTCTTTCC TACAAAGCTT CGGTGTTAGC   
  
  
- CAAGCGGGGC AACCGGATAG ACAGCTCTTC GGCCAACTTC AGTCCCGGGA CTTCGTCTGG TAGGCTCAGG   
  
  
- GTGGTAGCGG CCTCCCATCT TCTCTCTAAT TCCGTCTAAA GACCCCACTA TCTTCTTTCT CGAACTTCTC   
  
  
- CTCTGATCGT CTTGTCCCCT CGAGAAAACT ACTACCACCA CTGCTTAGAA CACAACCCTC TTGGTTTTCC   
  
  
- TTCTTCCCAT CGAAACTAAA ACCACTTAGA CTACTTTAAA GAGGTTTTTA ACTCCTGTGT TACTCAAAAC   
  
  
- CTGTAGACCC ATCACCACCT TTAAAGACCC AAGTAGGACC AAAACCACAA CAATTACAAT TATGATTAAT   
  
  
- GGGAGTTCAC GGTAAAAGAG AGTGAACAAG TCCCCTCCTT CTTTCTCATA CAAAACAAGG ATGACTACAC   
  
  
- TAGAGTGGTC GGTAGTACGG AGGTAACAGT GTGTTAGGAA CCCAACTTAG ACCACAATGA CTCCAGTGTC   
  
  
- TTATACCACC CCCACTGTTT CTGCCGCTTG GATCAGTACC CAAACACTCC CCATGTTGTT GTTGCAGGCC   
  
  
- CAGTTCAAGT AGAAGACTCT CAGTATCAAA ACCCGTATCC GAGTTACTCT CGAGTAGACT GGGACTTTAG   
  
  
- TCTTTACCAA GGTACAACGG ATTGGGACCA GTACCAGGAC CGTGGTCAGT ACCAGGACCT TGGCCAGACC   
  
  
- GAGTGTTGGT GTTACTAGTT TGGCTCGTTG TTCCCAAGCT CGAGTAATCG AAAGAGTACC CAACACAGCT   
  
  
- TCGCTAGTCA AGTTCCTTGT AACGTCGGTA GTTAGTAAAG TATCGATTCG ACCCGCTCGT CCGAAGAGGT   
  
  
- TCCCGAGCCA GACGGTAATC GGCGGAGTGT CGGATGAAGT GGCTTCGTAA TCGGAACTCC CAGTGTTCTA   
  
  
- AAACCGGAGT ATAGAAAGTG TATTCATAGG GAGCTCTCAA ACTAGCTAAA CTACTACTTA GCCCCCGTCG   
  
  
- ACGTTACTCC GAAAACTTAG TCTAGTCGGG TTAAGGTTTC AAGCAAGTAA AATGTCGGTT ACTCTACAAT   
  
  
- AACTCCCGTA AACTTCCCTT CCTGTTCCAC GTATAGTATC TGAAGCTGTA GTTCGTTCCG AATGTTACCG   
  
  
- GGCCCAAAAA GGTTTCGAAT CGGTACTCCC TTTTGGGGGG CTCGGTACAG TCCTATTGTC CGCATCCCCT   
  
  
- AAGCTTCGTT CTTAACCAAC TCTGTCCTCT TTCTGATCGG CCCAAACGAC CCCGTAAGTT GGAGAGGAAG   
  
  
- CTCAAGGTGG GTCAACACCT GGCCAACCTT CTACAATCCG ATACCTACGA AGTACAATTC CTTCCACTTT   
  
  
- CACAGCGCCA TTTAACGTAA AACGTCGAGG TGTTCTGTGA GATACTGGGG GTACCACGGT GTAAGTCCCT   
  
  
- GAAGTACCCA AATTAGGCTT CGTGTTTAGG GTATCGTAAC CAATACCGAC TCGTTCTTCG ACTAGTGTTG   
  
  
- CTTGGGTGGA ATCTTCCGGC TCATACGTTG AGTGAACCTA TGATACGTCG GGAAAAGCTA CGGTATCTAA   
  
  
- CGTCGGAAGG AAAGCTCTCG AGTCGTTCCA ACTTCTAGCT TCTCTACAAA CCGGCCCTTT AGTCCTTGTA   
  
  
- TTATCGGACA CTTCCCCGTC TGTCCTAGCT TTCTGTACTC TTCAAGCTCT TTACCTTCTC TTCCTAGCTC   
  
  
- GTCCTTCCCA AGGCCACGTA CGCCTGATCG CTTTCCCTCC ACTACGTCTC GGTTTACGAC GAATTCTACA   
  
  
- TGAGCTTGCT CTCGATGTTG TACGTTTCTG TTCCGTTACT TCTTCGTTAT AGTGATCATA CCAATCTAGT   
  
  
- CGGTGAGATG TGACAAAGAA ACACCCGAGG TTCACTTAAA CGACGTCCCT CAAGAAGGTC AAAAAGAGAC   
  
  
- GGTAGAAC

+     GCN4\_motif

| Site Name | Organism | Position | Strand | Matrix score. | sequence | function |
| --- | --- | --- | --- | --- | --- | --- |
| GCN4\_motif | Oryza sativa | 1372 | + | 7 | TGAGTCA | cis-regulatory element involved in endosperm expression |

>HU04G00148.1   
+ -Up\_Stream \_Len000ATAATG GGTGATAACT TTTGAAGGAC AATATACAAA TCACTTATTT ATTAAAGTCA   
  
  
+ AGGGGACATT TCAACTATAA CATCATTGTT TATAAAGTAT TTGAAAATTG CAACTCTTGT AATAGATAAT   
  
  
+ AATGCTTTAT AGACTTCACC AATCACTAAT TTGTAGAATC AAAAAATTAA TTGAAAAGCA AACAAAAGGA   
  
  
+ AAAGGCACAT AAACCTAAAT TATGTAAACA AATGAGAACA TATATGCCAG CTTATCAACG TTAATTATAT   
  
  
+ TTGTTATCAC TCTTAATATC TGTATTCATA GTAAATGAAA ATAAGTATCG ATTTTTTATT AAACCTAAAA   
  
  
+ TCTTCTGTCA ATAATTTTTC TTCCGATAAA AAAAAAATTG GACCATATTA AATTAACATT AATAAATTCA   
  
  
+ TATAATTACT TTGAATGTCT GAAATATAAT GCTCTTCGGT ATATTAAGGT CTTATAAATT TCTCATTAAT   
  
  
+ TTGAAATCTC TTATAATATA TTGGGTCAAC AAGTGCTAGC TTGTTTTTTT TTCCTGGTTA ATTGCATTTA   
  
  
+ AAAAAATAAT TTTTAATTAC AATTGCAAGG GGAGGAATCA TTTACAAAAT ATCAAAAGTG GAAAAGGCAA   
  
  
+ AAAGAAACAA ATTATAAAAC AGAAAAAAGA TTAGAAAACG CAAACAAAGA CGAAATGAGG AACAGTAAGA   
  
  
+ ATCAGTAAAA AAATGTAGTG CAACGGTCAC ATACGGGAAG GCAAGCACAA CAATCATGAA AGAGACAGGC   
  
  
+ ACGTGCGTTC CTCGTGATCA GCAGCCTTCC CTACTTCCCC GCCAAGTGGA GTCCGTACTA ACTCCTGCCA   
  
  
+ TTCCCTCCCA ACGGTCATAT TCCGCCGACA TGCCTCAACG GTCAGATTTC CTGCCCCATC TGGACCGTCC   
  
  
+ GATCAAGATC CAACGGCCAT AAGGCGCACT CTCCAATACC CTAGTGCCCA TTTCTGGAAC AGCCCCAAAA   
  
  
+ ATCTCAGTAT TCGCCATCAT TTTTCAATAC TGGGGTTACA TAAACAGTCT AACAAGGAAG GACCCAACAC   
  
  
+ AGAGGAGAGA GAAAAAAGCT AGCTTTGATG GGGACAGCCA TGGATGGAGG TGAAGAGAGA GGAGGTTGGG   
  
  
+ ACCTTTTATT TTGCTCACTT TCCATTCTTA TATAAAAACA GCTCTAGTTT ATCTCTTATG GCTTTTTTGG   
  
  
+ GTTTGTTCAT AGAGAGGGGG GAATGTGATA TGATTTGATT TGATGTTAGA AATTCTCATT AGTTTTTTCT   
  
  
+ TTTTGTTTGC AATTTTGATG TGTTAGGGTT TGAGGATCAT CATTTTTGAG TAGGGAACTT TCTGGGTTTT   
  
  
+ GCTTGCTGAT TGATTGCCAA TGCTATGGAT TTCTTGGTGA GTCAATCTTC AATTGCTGAT CTGGGTTCGT   
  
  
+ TTGTTCTTCA GAGTTTCACC TGCCAATTTG ATCAAGTATG TCCCTTCACT TCTCCTGCTT CAATTCTCTC   
  
  
+ TCAAATTCAC GCATTTTTCC AATGAATTTC AAATAGGAGT CCTGGAATCC GATGATATTT TAGGTTTTTT   
  
  
+ CCTGACTTCA TTGCATTAGT TTGTTCATAT CTGTGACCAA TTCTTTCATT TGTTATATGA TCTGATTGTT   
  
  
+ TAGTTAGCTA TTCATTCATT GCATATACAT TGGTTTATTC ATTGCAGAGA CACTCACATA CACACACACA   
  
  
+ TACAGGGGAC ACTAGTATTT TTTCTCTCTA ACTCATCGGA TTCCCTTTGA TACTCTGTTT TTTGGGGGGT   
  
  
+ TGATTTGATT TGATTTGATT GACCTTAATT TTGAACTCAC ACACACTCGC ACATAGATAT ATTCTTTCTG   
  
  
+ TTTGTGACAT ATACAGCATC ATATAGAAAT ATATTGCACT GCATATTTGA GCTCAAGACA GGGATATCAT   
  
  
+ CACAAACTGA GTGTGAGGGA GGCAAAAAAC AGAGGAAAAG AAGGGAATTG GGAGTGTAAT CAAAACAAGA   
  
  
+ GAGAGATTGA GGAGAGAGGA AAAAACAAAA TTGGGAGAAG GTTAATAGAG AAGAATGTTG GCTGGGTGTT   
  
  
+ CATCCACATT GCTGTCACCA AGGTATAGAT TGAGGAGTGA AGCTACATCA CAGTTCCAAG CCTGCCATCA   
  
  
+ TCCAATGAGC ACACAGAGAA TTGATTTGCC CTGCACTTTC CCAAGAAAGG ATGTTTCGAA GCCACAATCG   
  
  
+ GTTCGCCCCG TTGGCCTATC TGTCGAGAAG CCGGTTGAAG TCAGGGCCCT GAAGCAGACC ATCCGAGTCC   
  
  
+ CACCATCGCC GGAGGGTAGA AGAGAGATTA AGGCAGATTT CTGGGGTGAT AGAAGAAAGA GCTTGAAGAG   
  
  
+ GAGACTAGCA GAACAGGGGA GCTCTTTTGA TGATGGTGGT GACGAATCTT GTGTTGGGAG AACCAAAAGG   
  
  
+ AAGAAGGGTA GCTTTGATTT TGGTGAATCT GATGAAATTT CTCCAAAAAT TGAGGACACA ATGAGTTTTG   
  
  
+ GACATCTGGG TAGTGGTGGA AATTTCTGGG TTCATCCTGG TTTTGGTGTT GTTAATGTTA ATACTAATTA   
  
  
+ CCCTCAAGTG CCATTTTCTC TCACTTGTTC AGGGGAGGAA GAAAGAGTAT GTTTTGTTCC TACTGATGTG   
  
  
+ ATCTCACCAG CCATCATGCC TCCATTGTCA CACAATCCTT GGGTTGAATC TGGTGTTACT GAGGTCACAG   
  
  
+ AATATGGTGG GGGTGACAAA GACGGCGAAC CTAGTCATGG GTTTGTGAGG GGTACAACAA CAACGTCCGG   
  
  
+ GTCAAGTTCA TCTTCTGAGA GTCATAGTTT TGGGCATAGG CTCAATGAGA GCTCATCTGA CCCTGAAATC   
  
  
+ AGAAATGGTT CCATGTTGCC TAACCCTGGT CATGGTCCTG GCACCAGTCA TGGTCCTGGA ACCGGTCTGG   
  
  
+ CTCACAACCA CAATGATCAA ACCGAGCAAC AAGGGTTCGA GCTCATTAGC TTTCTCATGG GTTGTGTCGA   
  
  
+ AGCGATCAGT TCAAGGAACA TTGCAGCCAT CAATCATTTC ATAGCTAAGC TGGGCGAGCA GGCTTCTCCA   
  
  
+ AGGGCTCGGT CTGCCATTAG CCGCCTCACA GCCTACTTCA CCGAAGCATT AGCCTTGAGG GTCACAAGAT   
  
  
+ TTTGGCCTCA TATCTTTCAC ATAAGTATCC CTCGAGAGTT TGATCGATTT GATGATGAAT CGGGGGCAGC   
  
  
+ TGCAATGAGG CTTTTGAATC AGATCAGCCC AATTCCAAAG TTCGTTCATT TTACAGCCAA TGAGATGTTA   
  
  
+ TTGAGGGCAT TTGAAGGGAA GGACAAGGTG CATATCATAG ACTTCGACAT CAAGCAAGGC TTACAATGGC   
  
  
+ CCGGGTTTTT CCAAAGCTTA GCCATGAGGG AAAACCCCCC GAGCCATGTC AGGATAACAG GCGTAGGGGA   
  
  
+ TTCGAAGCAA GAATTGGTTG AGACAGGAGA AAGACTAGCC GGGTTTGCTG GGGCATTCAA CCTCTCCTTC   
  
  
+ GAGTTCCACC CAGTTGTGGA CCGGTTGGAA GATGTTAGGC TATGGATGCT TCATGTTAAG GAAGGTGAAA   
  
  
+ GTGTCGCGGT AAATTGCATT TTGCAGCTCC ACAAGACACT CTATGACCCC CATGGTGCCA CATTCAGGGA   
  
  
+ CTTCATGGGT TTAATCCGAA GCACAAATCC CATAGCATTG GTTATGGCTG AGCAAGAAGC TGATCACAAC   
  
  
+ GAACCCACCT TAGAAGGCCG AGTATGCAAC TCACTTGGAT ACTATGCAGC CCTTTTCGAT GCCATAGATT   
  
  
+ GCAGCCTTCC TTTCGAGAGC TCAGCAAGGT TGAAGATCGA AGAGATGTTT GGCCGGGAAA TCAGGAACAT   
  
  
+ AATAGCCTGT GAAGGGGCAG ACAGGATCGA AAGACATGAG AAGTTCGAGA AATGGAAGAG AAGGATCGAG   
  
  
+ CAGGAAGGGT TCCGGTGCAT GCGGACTAGC GAAAGGGAGG TGATGCAGAG CCAAATGCTG CTTAAGATGT   
  
  
+ ACTCGAACGA GAGCTACAAC ATGCAAAGAC AAGGCAATGA AGAAGCAATA TCACTAGTAT GGTTAGATCA   
  
  
+ GCCACTCTAC ACTGTTTCTT TGTGGGCTCC AAGTGAATTT GCTGCAGGGA GTTCTTCCAG TTTTTCTCTG   
  
  
+ CCATCTTG  

- -Up\_Stream \_Len000TATTAC CCACTATTGA AAACTTCCTG TTATATGTTT AGTGAATAAA TAATTTCAGT   
  
  
- TCCCCTGTAA AGTTGATATT GTAGTAACAA ATATTTCATA AACTTTTAAC GTTGAGAACA TTATCTATTA   
  
  
- TTACGAAATA TCTGAAGTGG TTAGTGATTA AACATCTTAG TTTTTTAATT AACTTTTCGT TTGTTTTCCT   
  
  
- TTTCCGTGTA TTTGGATTTA ATACATTTGT TTACTCTTGT ATATACGGTC GAATAGTTGC AATTAATATA   
  
  
- AACAATAGTG AGAATTATAG ACATAAGTAT CATTTACTTT TATTCATAGC TAAAAAATAA TTTGGATTTT   
  
  
- AGAAGACAGT TATTAAAAAG AAGGCTATTT TTTTTTTAAC CTGGTATAAT TTAATTGTAA TTATTTAAGT   
  
  
- ATATTAATGA AACTTACAGA CTTTATATTA CGAGAAGCCA TATAATTCCA GAATATTTAA AGAGTAATTA   
  
  
- AACTTTAGAG AATATTATAT AACCCAGTTG TTCACGATCG AACAAAAAAA AAGGACCAAT TAACGTAAAT   
  
  
- TTTTTTATTA AAAATTAATG TTAACGTTCC CCTCCTTAGT AAATGTTTTA TAGTTTTCAC CTTTTCCGTT   
  
  
- TTTCTTTGTT TAATATTTTG TCTTTTTTCT AATCTTTTGC GTTTGTTTCT GCTTTACTCC TTGTCATTCT   
  
  
- TAGTCATTTT TTTACATCAC GTTGCCAGTG TATGCCCTTC CGTTCGTGTT GTTAGTACTT TCTCTGTCCG   
  
  
- TGCACGCAAG GAGCACTAGT CGTCGGAAGG GATGAAGGGG CGGTTCACCT CAGGCATGAT TGAGGACGGT   
  
  
- AAGGGAGGGT TGCCAGTATA AGGCGGCTGT ACGGAGTTGC CAGTCTAAAG GACGGGGTAG ACCTGGCAGG   
  
  
- CTAGTTCTAG GTTGCCGGTA TTCCGCGTGA GAGGTTATGG GATCACGGGT AAAGACCTTG TCGGGGTTTT   
  
  
- TAGAGTCATA AGCGGTAGTA AAAAGTTATG ACCCCAATGT ATTTGTCAGA TTGTTCCTTC CTGGGTTGTG   
  
  
- TCTCCTCTCT CTTTTTTCGA TCGAAACTAC CCCTGTCGGT ACCTACCTCC ACTTCTCTCT CCTCCAACCC   
  
  
- TGGAAAATAA AACGAGTGAA AGGTAAGAAT ATATTTTTGT CGAGATCAAA TAGAGAATAC CGAAAAAACC   
  
  
- CAAACAAGTA TCTCTCCCCC CTTACACTAT ACTAAACTAA ACTACAATCT TTAAGAGTAA TCAAAAAAGA   
  
  
- AAAACAAACG TTAAAACTAC ACAATCCCAA ACTCCTAGTA GTAAAAACTC ATCCCTTGAA AGACCCAAAA   
  
  
- CGAACGACTA ACTAACGGTT ACGATACCTA AAGAACCACT CAGTTAGAAG TTAACGACTA GACCCAAGCA   
  
  
- AACAAGAAGT CTCAAAGTGG ACGGTTAAAC TAGTTCATAC AGGGAAGTGA AGAGGACGAA GTTAAGAGAG   
  
  
- AGTTTAAGTG CGTAAAAAGG TTACTTAAAG TTTATCCTCA GGACCTTAGG CTACTATAAA ATCCAAAAAA   
  
  
- GGACTGAAGT AACGTAATCA AACAAGTATA GACACTGGTT AAGAAAGTAA ACAATATACT AGACTAACAA   
  
  
- ATCAATCGAT AAGTAAGTAA CGTATATGTA ACCAAATAAG TAACGTCTCT GTGAGTGTAT GTGTGTGTGT   
  
  
- ATGTCCCCTG TGATCATAAA AAAGAGAGAT TGAGTAGCCT AAGGGAAACT ATGAGACAAA AAACCCCCCA   
  
  
- ACTAAACTAA ACTAAACTAA CTGGAATTAA AACTTGAGTG TGTGTGAGCG TGTATCTATA TAAGAAAGAC   
  
  
- AAACACTGTA TATGTCGTAG TATATCTTTA TATAACGTGA CGTATAAACT CGAGTTCTGT CCCTATAGTA   
  
  
- GTGTTTGACT CACACTCCCT CCGTTTTTTG TCTCCTTTTC TTCCCTTAAC CCTCACATTA GTTTTGTTCT   
  
  
- CTCTCTAACT CCTCTCTCCT TTTTTGTTTT AACCCTCTTC CAATTATCTC TTCTTACAAC CGACCCACAA   
  
  
- GTAGGTGTAA CGACAGTGGT TCCATATCTA ACTCCTCACT TCGATGTAGT GTCAAGGTTC GGACGGTAGT   
  
  
- AGGTTACTCG TGTGTCTCTT AACTAAACGG GACGTGAAAG GGTTCTTTCC TACAAAGCTT CGGTGTTAGC   
  
  
- CAAGCGGGGC AACCGGATAG ACAGCTCTTC GGCCAACTTC AGTCCCGGGA CTTCGTCTGG TAGGCTCAGG   
  
  
- GTGGTAGCGG CCTCCCATCT TCTCTCTAAT TCCGTCTAAA GACCCCACTA TCTTCTTTCT CGAACTTCTC   
  
  
- CTCTGATCGT CTTGTCCCCT CGAGAAAACT ACTACCACCA CTGCTTAGAA CACAACCCTC TTGGTTTTCC   
  
  
- TTCTTCCCAT CGAAACTAAA ACCACTTAGA CTACTTTAAA GAGGTTTTTA ACTCCTGTGT TACTCAAAAC   
  
  
- CTGTAGACCC ATCACCACCT TTAAAGACCC AAGTAGGACC AAAACCACAA CAATTACAAT TATGATTAAT   
  
  
- GGGAGTTCAC GGTAAAAGAG AGTGAACAAG TCCCCTCCTT CTTTCTCATA CAAAACAAGG ATGACTACAC   
  
  
- TAGAGTGGTC GGTAGTACGG AGGTAACAGT GTGTTAGGAA CCCAACTTAG ACCACAATGA CTCCAGTGTC   
  
  
- TTATACCACC CCCACTGTTT CTGCCGCTTG GATCAGTACC CAAACACTCC CCATGTTGTT GTTGCAGGCC   
  
  
- CAGTTCAAGT AGAAGACTCT CAGTATCAAA ACCCGTATCC GAGTTACTCT CGAGTAGACT GGGACTTTAG   
  
  
- TCTTTACCAA GGTACAACGG ATTGGGACCA GTACCAGGAC CGTGGTCAGT ACCAGGACCT TGGCCAGACC   
  
  
- GAGTGTTGGT GTTACTAGTT TGGCTCGTTG TTCCCAAGCT CGAGTAATCG AAAGAGTACC CAACACAGCT   
  
  
- TCGCTAGTCA AGTTCCTTGT AACGTCGGTA GTTAGTAAAG TATCGATTCG ACCCGCTCGT CCGAAGAGGT   
  
  
- TCCCGAGCCA GACGGTAATC GGCGGAGTGT CGGATGAAGT GGCTTCGTAA TCGGAACTCC CAGTGTTCTA   
  
  
- AAACCGGAGT ATAGAAAGTG TATTCATAGG GAGCTCTCAA ACTAGCTAAA CTACTACTTA GCCCCCGTCG   
  
  
- ACGTTACTCC GAAAACTTAG TCTAGTCGGG TTAAGGTTTC AAGCAAGTAA AATGTCGGTT ACTCTACAAT   
  
  
- AACTCCCGTA AACTTCCCTT CCTGTTCCAC GTATAGTATC TGAAGCTGTA GTTCGTTCCG AATGTTACCG   
  
  
- GGCCCAAAAA GGTTTCGAAT CGGTACTCCC TTTTGGGGGG CTCGGTACAG TCCTATTGTC CGCATCCCCT   
  
  
- AAGCTTCGTT CTTAACCAAC TCTGTCCTCT TTCTGATCGG CCCAAACGAC CCCGTAAGTT GGAGAGGAAG   
  
  
- CTCAAGGTGG GTCAACACCT GGCCAACCTT CTACAATCCG ATACCTACGA AGTACAATTC CTTCCACTTT   
  
  
- CACAGCGCCA TTTAACGTAA AACGTCGAGG TGTTCTGTGA GATACTGGGG GTACCACGGT GTAAGTCCCT   
  
  
- GAAGTACCCA AATTAGGCTT CGTGTTTAGG GTATCGTAAC CAATACCGAC TCGTTCTTCG ACTAGTGTTG   
  
  
- CTTGGGTGGA ATCTTCCGGC TCATACGTTG AGTGAACCTA TGATACGTCG GGAAAAGCTA CGGTATCTAA   
  
  
- CGTCGGAAGG AAAGCTCTCG AGTCGTTCCA ACTTCTAGCT TCTCTACAAA CCGGCCCTTT AGTCCTTGTA   
  
  
- TTATCGGACA CTTCCCCGTC TGTCCTAGCT TTCTGTACTC TTCAAGCTCT TTACCTTCTC TTCCTAGCTC   
  
  
- GTCCTTCCCA AGGCCACGTA CGCCTGATCG CTTTCCCTCC ACTACGTCTC GGTTTACGAC GAATTCTACA   
  
  
- TGAGCTTGCT CTCGATGTTG TACGTTTCTG TTCCGTTACT TCTTCGTTAT AGTGATCATA CCAATCTAGT   
  
  
- CGGTGAGATG TGACAAAGAA ACACCCGAGG TTCACTTAAA CGACGTCCCT CAAGAAGGTC AAAAAGAGAC   
  
  
- GGTAGAAC

+     GT1-motif

| Site Name | Organism | Position | Strand | Matrix score. | sequence | function |
| --- | --- | --- | --- | --- | --- | --- |
| GT1-motif | Avena sativa | 550 | + | 7 | GGTTAAT | light responsive element |
| GT1-motif | Avena sativa | 2004 | + | 7 | GGTTAAT | light responsive element |

>HU04G00148.1   
+ -Up\_Stream \_Len000ATAATG GGTGATAACT TTTGAAGGAC AATATACAAA TCACTTATTT ATTAAAGTCA   
  
  
+ AGGGGACATT TCAACTATAA CATCATTGTT TATAAAGTAT TTGAAAATTG CAACTCTTGT AATAGATAAT   
  
  
+ AATGCTTTAT AGACTTCACC AATCACTAAT TTGTAGAATC AAAAAATTAA TTGAAAAGCA AACAAAAGGA   
  
  
+ AAAGGCACAT AAACCTAAAT TATGTAAACA AATGAGAACA TATATGCCAG CTTATCAACG TTAATTATAT   
  
  
+ TTGTTATCAC TCTTAATATC TGTATTCATA GTAAATGAAA ATAAGTATCG ATTTTTTATT AAACCTAAAA   
  
  
+ TCTTCTGTCA ATAATTTTTC TTCCGATAAA AAAAAAATTG GACCATATTA AATTAACATT AATAAATTCA   
  
  
+ TATAATTACT TTGAATGTCT GAAATATAAT GCTCTTCGGT ATATTAAGGT CTTATAAATT TCTCATTAAT   
  
  
+ TTGAAATCTC TTATAATATA TTGGGTCAAC AAGTGCTAGC TTGTTTTTTT TTCCTGGTTA ATTGCATTTA   
  
  
+ AAAAAATAAT TTTTAATTAC AATTGCAAGG GGAGGAATCA TTTACAAAAT ATCAAAAGTG GAAAAGGCAA   
  
  
+ AAAGAAACAA ATTATAAAAC AGAAAAAAGA TTAGAAAACG CAAACAAAGA CGAAATGAGG AACAGTAAGA   
  
  
+ ATCAGTAAAA AAATGTAGTG CAACGGTCAC ATACGGGAAG GCAAGCACAA CAATCATGAA AGAGACAGGC   
  
  
+ ACGTGCGTTC CTCGTGATCA GCAGCCTTCC CTACTTCCCC GCCAAGTGGA GTCCGTACTA ACTCCTGCCA   
  
  
+ TTCCCTCCCA ACGGTCATAT TCCGCCGACA TGCCTCAACG GTCAGATTTC CTGCCCCATC TGGACCGTCC   
  
  
+ GATCAAGATC CAACGGCCAT AAGGCGCACT CTCCAATACC CTAGTGCCCA TTTCTGGAAC AGCCCCAAAA   
  
  
+ ATCTCAGTAT TCGCCATCAT TTTTCAATAC TGGGGTTACA TAAACAGTCT AACAAGGAAG GACCCAACAC   
  
  
+ AGAGGAGAGA GAAAAAAGCT AGCTTTGATG GGGACAGCCA TGGATGGAGG TGAAGAGAGA GGAGGTTGGG   
  
  
+ ACCTTTTATT TTGCTCACTT TCCATTCTTA TATAAAAACA GCTCTAGTTT ATCTCTTATG GCTTTTTTGG   
  
  
+ GTTTGTTCAT AGAGAGGGGG GAATGTGATA TGATTTGATT TGATGTTAGA AATTCTCATT AGTTTTTTCT   
  
  
+ TTTTGTTTGC AATTTTGATG TGTTAGGGTT TGAGGATCAT CATTTTTGAG TAGGGAACTT TCTGGGTTTT   
  
  
+ GCTTGCTGAT TGATTGCCAA TGCTATGGAT TTCTTGGTGA GTCAATCTTC AATTGCTGAT CTGGGTTCGT   
  
  
+ TTGTTCTTCA GAGTTTCACC TGCCAATTTG ATCAAGTATG TCCCTTCACT TCTCCTGCTT CAATTCTCTC   
  
  
+ TCAAATTCAC GCATTTTTCC AATGAATTTC AAATAGGAGT CCTGGAATCC GATGATATTT TAGGTTTTTT   
  
  
+ CCTGACTTCA TTGCATTAGT TTGTTCATAT CTGTGACCAA TTCTTTCATT TGTTATATGA TCTGATTGTT   
  
  
+ TAGTTAGCTA TTCATTCATT GCATATACAT TGGTTTATTC ATTGCAGAGA CACTCACATA CACACACACA   
  
  
+ TACAGGGGAC ACTAGTATTT TTTCTCTCTA ACTCATCGGA TTCCCTTTGA TACTCTGTTT TTTGGGGGGT   
  
  
+ TGATTTGATT TGATTTGATT GACCTTAATT TTGAACTCAC ACACACTCGC ACATAGATAT ATTCTTTCTG   
  
  
+ TTTGTGACAT ATACAGCATC ATATAGAAAT ATATTGCACT GCATATTTGA GCTCAAGACA GGGATATCAT   
  
  
+ CACAAACTGA GTGTGAGGGA GGCAAAAAAC AGAGGAAAAG AAGGGAATTG GGAGTGTAAT CAAAACAAGA   
  
  
+ GAGAGATTGA GGAGAGAGGA AAAAACAAAA TTGGGAGAAG GTTAATAGAG AAGAATGTTG GCTGGGTGTT   
  
  
+ CATCCACATT GCTGTCACCA AGGTATAGAT TGAGGAGTGA AGCTACATCA CAGTTCCAAG CCTGCCATCA   
  
  
+ TCCAATGAGC ACACAGAGAA TTGATTTGCC CTGCACTTTC CCAAGAAAGG ATGTTTCGAA GCCACAATCG   
  
  
+ GTTCGCCCCG TTGGCCTATC TGTCGAGAAG CCGGTTGAAG TCAGGGCCCT GAAGCAGACC ATCCGAGTCC   
  
  
+ CACCATCGCC GGAGGGTAGA AGAGAGATTA AGGCAGATTT CTGGGGTGAT AGAAGAAAGA GCTTGAAGAG   
  
  
+ GAGACTAGCA GAACAGGGGA GCTCTTTTGA TGATGGTGGT GACGAATCTT GTGTTGGGAG AACCAAAAGG   
  
  
+ AAGAAGGGTA GCTTTGATTT TGGTGAATCT GATGAAATTT CTCCAAAAAT TGAGGACACA ATGAGTTTTG   
  
  
+ GACATCTGGG TAGTGGTGGA AATTTCTGGG TTCATCCTGG TTTTGGTGTT GTTAATGTTA ATACTAATTA   
  
  
+ CCCTCAAGTG CCATTTTCTC TCACTTGTTC AGGGGAGGAA GAAAGAGTAT GTTTTGTTCC TACTGATGTG   
  
  
+ ATCTCACCAG CCATCATGCC TCCATTGTCA CACAATCCTT GGGTTGAATC TGGTGTTACT GAGGTCACAG   
  
  
+ AATATGGTGG GGGTGACAAA GACGGCGAAC CTAGTCATGG GTTTGTGAGG GGTACAACAA CAACGTCCGG   
  
  
+ GTCAAGTTCA TCTTCTGAGA GTCATAGTTT TGGGCATAGG CTCAATGAGA GCTCATCTGA CCCTGAAATC   
  
  
+ AGAAATGGTT CCATGTTGCC TAACCCTGGT CATGGTCCTG GCACCAGTCA TGGTCCTGGA ACCGGTCTGG   
  
  
+ CTCACAACCA CAATGATCAA ACCGAGCAAC AAGGGTTCGA GCTCATTAGC TTTCTCATGG GTTGTGTCGA   
  
  
+ AGCGATCAGT TCAAGGAACA TTGCAGCCAT CAATCATTTC ATAGCTAAGC TGGGCGAGCA GGCTTCTCCA   
  
  
+ AGGGCTCGGT CTGCCATTAG CCGCCTCACA GCCTACTTCA CCGAAGCATT AGCCTTGAGG GTCACAAGAT   
  
  
+ TTTGGCCTCA TATCTTTCAC ATAAGTATCC CTCGAGAGTT TGATCGATTT GATGATGAAT CGGGGGCAGC   
  
  
+ TGCAATGAGG CTTTTGAATC AGATCAGCCC AATTCCAAAG TTCGTTCATT TTACAGCCAA TGAGATGTTA   
  
  
+ TTGAGGGCAT TTGAAGGGAA GGACAAGGTG CATATCATAG ACTTCGACAT CAAGCAAGGC TTACAATGGC   
  
  
+ CCGGGTTTTT CCAAAGCTTA GCCATGAGGG AAAACCCCCC GAGCCATGTC AGGATAACAG GCGTAGGGGA   
  
  
+ TTCGAAGCAA GAATTGGTTG AGACAGGAGA AAGACTAGCC GGGTTTGCTG GGGCATTCAA CCTCTCCTTC   
  
  
+ GAGTTCCACC CAGTTGTGGA CCGGTTGGAA GATGTTAGGC TATGGATGCT TCATGTTAAG GAAGGTGAAA   
  
  
+ GTGTCGCGGT AAATTGCATT TTGCAGCTCC ACAAGACACT CTATGACCCC CATGGTGCCA CATTCAGGGA   
  
  
+ CTTCATGGGT TTAATCCGAA GCACAAATCC CATAGCATTG GTTATGGCTG AGCAAGAAGC TGATCACAAC   
  
  
+ GAACCCACCT TAGAAGGCCG AGTATGCAAC TCACTTGGAT ACTATGCAGC CCTTTTCGAT GCCATAGATT   
  
  
+ GCAGCCTTCC TTTCGAGAGC TCAGCAAGGT TGAAGATCGA AGAGATGTTT GGCCGGGAAA TCAGGAACAT   
  
  
+ AATAGCCTGT GAAGGGGCAG ACAGGATCGA AAGACATGAG AAGTTCGAGA AATGGAAGAG AAGGATCGAG   
  
  
+ CAGGAAGGGT TCCGGTGCAT GCGGACTAGC GAAAGGGAGG TGATGCAGAG CCAAATGCTG CTTAAGATGT   
  
  
+ ACTCGAACGA GAGCTACAAC ATGCAAAGAC AAGGCAATGA AGAAGCAATA TCACTAGTAT GGTTAGATCA   
  
  
+ GCCACTCTAC ACTGTTTCTT TGTGGGCTCC AAGTGAATTT GCTGCAGGGA GTTCTTCCAG TTTTTCTCTG   
  
  
+ CCATCTTG  

- -Up\_Stream \_Len000TATTAC CCACTATTGA AAACTTCCTG TTATATGTTT AGTGAATAAA TAATTTCAGT   
  
  
- TCCCCTGTAA AGTTGATATT GTAGTAACAA ATATTTCATA AACTTTTAAC GTTGAGAACA TTATCTATTA   
  
  
- TTACGAAATA TCTGAAGTGG TTAGTGATTA AACATCTTAG TTTTTTAATT AACTTTTCGT TTGTTTTCCT   
  
  
- TTTCCGTGTA TTTGGATTTA ATACATTTGT TTACTCTTGT ATATACGGTC GAATAGTTGC AATTAATATA   
  
  
- AACAATAGTG AGAATTATAG ACATAAGTAT CATTTACTTT TATTCATAGC TAAAAAATAA TTTGGATTTT   
  
  
- AGAAGACAGT TATTAAAAAG AAGGCTATTT TTTTTTTAAC CTGGTATAAT TTAATTGTAA TTATTTAAGT   
  
  
- ATATTAATGA AACTTACAGA CTTTATATTA CGAGAAGCCA TATAATTCCA GAATATTTAA AGAGTAATTA   
  
  
- AACTTTAGAG AATATTATAT AACCCAGTTG TTCACGATCG AACAAAAAAA AAGGACCAAT TAACGTAAAT   
  
  
- TTTTTTATTA AAAATTAATG TTAACGTTCC CCTCCTTAGT AAATGTTTTA TAGTTTTCAC CTTTTCCGTT   
  
  
- TTTCTTTGTT TAATATTTTG TCTTTTTTCT AATCTTTTGC GTTTGTTTCT GCTTTACTCC TTGTCATTCT   
  
  
- TAGTCATTTT TTTACATCAC GTTGCCAGTG TATGCCCTTC CGTTCGTGTT GTTAGTACTT TCTCTGTCCG   
  
  
- TGCACGCAAG GAGCACTAGT CGTCGGAAGG GATGAAGGGG CGGTTCACCT CAGGCATGAT TGAGGACGGT   
  
  
- AAGGGAGGGT TGCCAGTATA AGGCGGCTGT ACGGAGTTGC CAGTCTAAAG GACGGGGTAG ACCTGGCAGG   
  
  
- CTAGTTCTAG GTTGCCGGTA TTCCGCGTGA GAGGTTATGG GATCACGGGT AAAGACCTTG TCGGGGTTTT   
  
  
- TAGAGTCATA AGCGGTAGTA AAAAGTTATG ACCCCAATGT ATTTGTCAGA TTGTTCCTTC CTGGGTTGTG   
  
  
- TCTCCTCTCT CTTTTTTCGA TCGAAACTAC CCCTGTCGGT ACCTACCTCC ACTTCTCTCT CCTCCAACCC   
  
  
- TGGAAAATAA AACGAGTGAA AGGTAAGAAT ATATTTTTGT CGAGATCAAA TAGAGAATAC CGAAAAAACC   
  
  
- CAAACAAGTA TCTCTCCCCC CTTACACTAT ACTAAACTAA ACTACAATCT TTAAGAGTAA TCAAAAAAGA   
  
  
- AAAACAAACG TTAAAACTAC ACAATCCCAA ACTCCTAGTA GTAAAAACTC ATCCCTTGAA AGACCCAAAA   
  
  
- CGAACGACTA ACTAACGGTT ACGATACCTA AAGAACCACT CAGTTAGAAG TTAACGACTA GACCCAAGCA   
  
  
- AACAAGAAGT CTCAAAGTGG ACGGTTAAAC TAGTTCATAC AGGGAAGTGA AGAGGACGAA GTTAAGAGAG   
  
  
- AGTTTAAGTG CGTAAAAAGG TTACTTAAAG TTTATCCTCA GGACCTTAGG CTACTATAAA ATCCAAAAAA   
  
  
- GGACTGAAGT AACGTAATCA AACAAGTATA GACACTGGTT AAGAAAGTAA ACAATATACT AGACTAACAA   
  
  
- ATCAATCGAT AAGTAAGTAA CGTATATGTA ACCAAATAAG TAACGTCTCT GTGAGTGTAT GTGTGTGTGT   
  
  
- ATGTCCCCTG TGATCATAAA AAAGAGAGAT TGAGTAGCCT AAGGGAAACT ATGAGACAAA AAACCCCCCA   
  
  
- ACTAAACTAA ACTAAACTAA CTGGAATTAA AACTTGAGTG TGTGTGAGCG TGTATCTATA TAAGAAAGAC   
  
  
- AAACACTGTA TATGTCGTAG TATATCTTTA TATAACGTGA CGTATAAACT CGAGTTCTGT CCCTATAGTA   
  
  
- GTGTTTGACT CACACTCCCT CCGTTTTTTG TCTCCTTTTC TTCCCTTAAC CCTCACATTA GTTTTGTTCT   
  
  
- CTCTCTAACT CCTCTCTCCT TTTTTGTTTT AACCCTCTTC CAATTATCTC TTCTTACAAC CGACCCACAA   
  
  
- GTAGGTGTAA CGACAGTGGT TCCATATCTA ACTCCTCACT TCGATGTAGT GTCAAGGTTC GGACGGTAGT   
  
  
- AGGTTACTCG TGTGTCTCTT AACTAAACGG GACGTGAAAG GGTTCTTTCC TACAAAGCTT CGGTGTTAGC   
  
  
- CAAGCGGGGC AACCGGATAG ACAGCTCTTC GGCCAACTTC AGTCCCGGGA CTTCGTCTGG TAGGCTCAGG   
  
  
- GTGGTAGCGG CCTCCCATCT TCTCTCTAAT TCCGTCTAAA GACCCCACTA TCTTCTTTCT CGAACTTCTC   
  
  
- CTCTGATCGT CTTGTCCCCT CGAGAAAACT ACTACCACCA CTGCTTAGAA CACAACCCTC TTGGTTTTCC   
  
  
- TTCTTCCCAT CGAAACTAAA ACCACTTAGA CTACTTTAAA GAGGTTTTTA ACTCCTGTGT TACTCAAAAC   
  
  
- CTGTAGACCC ATCACCACCT TTAAAGACCC AAGTAGGACC AAAACCACAA CAATTACAAT TATGATTAAT   
  
  
- GGGAGTTCAC GGTAAAAGAG AGTGAACAAG TCCCCTCCTT CTTTCTCATA CAAAACAAGG ATGACTACAC   
  
  
- TAGAGTGGTC GGTAGTACGG AGGTAACAGT GTGTTAGGAA CCCAACTTAG ACCACAATGA CTCCAGTGTC   
  
  
- TTATACCACC CCCACTGTTT CTGCCGCTTG GATCAGTACC CAAACACTCC CCATGTTGTT GTTGCAGGCC   
  
  
- CAGTTCAAGT AGAAGACTCT CAGTATCAAA ACCCGTATCC GAGTTACTCT CGAGTAGACT GGGACTTTAG   
  
  
- TCTTTACCAA GGTACAACGG ATTGGGACCA GTACCAGGAC CGTGGTCAGT ACCAGGACCT TGGCCAGACC   
  
  
- GAGTGTTGGT GTTACTAGTT TGGCTCGTTG TTCCCAAGCT CGAGTAATCG AAAGAGTACC CAACACAGCT   
  
  
- TCGCTAGTCA AGTTCCTTGT AACGTCGGTA GTTAGTAAAG TATCGATTCG ACCCGCTCGT CCGAAGAGGT   
  
  
- TCCCGAGCCA GACGGTAATC GGCGGAGTGT CGGATGAAGT GGCTTCGTAA TCGGAACTCC CAGTGTTCTA   
  
  
- AAACCGGAGT ATAGAAAGTG TATTCATAGG GAGCTCTCAA ACTAGCTAAA CTACTACTTA GCCCCCGTCG   
  
  
- ACGTTACTCC GAAAACTTAG TCTAGTCGGG TTAAGGTTTC AAGCAAGTAA AATGTCGGTT ACTCTACAAT   
  
  
- AACTCCCGTA AACTTCCCTT CCTGTTCCAC GTATAGTATC TGAAGCTGTA GTTCGTTCCG AATGTTACCG   
  
  
- GGCCCAAAAA GGTTTCGAAT CGGTACTCCC TTTTGGGGGG CTCGGTACAG TCCTATTGTC CGCATCCCCT   
  
  
- AAGCTTCGTT CTTAACCAAC TCTGTCCTCT TTCTGATCGG CCCAAACGAC CCCGTAAGTT GGAGAGGAAG   
  
  
- CTCAAGGTGG GTCAACACCT GGCCAACCTT CTACAATCCG ATACCTACGA AGTACAATTC CTTCCACTTT   
  
  
- CACAGCGCCA TTTAACGTAA AACGTCGAGG TGTTCTGTGA GATACTGGGG GTACCACGGT GTAAGTCCCT   
  
  
- GAAGTACCCA AATTAGGCTT CGTGTTTAGG GTATCGTAAC CAATACCGAC TCGTTCTTCG ACTAGTGTTG   
  
  
- CTTGGGTGGA ATCTTCCGGC TCATACGTTG AGTGAACCTA TGATACGTCG GGAAAAGCTA CGGTATCTAA   
  
  
- CGTCGGAAGG AAAGCTCTCG AGTCGTTCCA ACTTCTAGCT TCTCTACAAA CCGGCCCTTT AGTCCTTGTA   
  
  
- TTATCGGACA CTTCCCCGTC TGTCCTAGCT TTCTGTACTC TTCAAGCTCT TTACCTTCTC TTCCTAGCTC   
  
  
- GTCCTTCCCA AGGCCACGTA CGCCTGATCG CTTTCCCTCC ACTACGTCTC GGTTTACGAC GAATTCTACA   
  
  
- TGAGCTTGCT CTCGATGTTG TACGTTTCTG TTCCGTTACT TCTTCGTTAT AGTGATCATA CCAATCTAGT   
  
  
- CGGTGAGATG TGACAAAGAA ACACCCGAGG TTCACTTAAA CGACGTCCCT CAAGAAGGTC AAAAAGAGAC   
  
  
- GGTAGAAC

+     I-box

| Site Name | Organism | Position | Strand | Matrix score. | sequence | function |
| --- | --- | --- | --- | --- | --- | --- |
| I-box | Zea mays | 931 | + | 9 | cGATAAGGCG | part of a light responsive element |
| I-box | Flaveria trinervia | 3611 | - | 10 | cCATATCCAAT | part of a light responsive element |
| I-box | Zea mays | 3245 | + | 9 | gGATAAGGTG | part of a light responsive element |

>HU04G00148.1   
+ -Up\_Stream \_Len000ATAATG GGTGATAACT TTTGAAGGAC AATATACAAA TCACTTATTT ATTAAAGTCA   
  
  
+ AGGGGACATT TCAACTATAA CATCATTGTT TATAAAGTAT TTGAAAATTG CAACTCTTGT AATAGATAAT   
  
  
+ AATGCTTTAT AGACTTCACC AATCACTAAT TTGTAGAATC AAAAAATTAA TTGAAAAGCA AACAAAAGGA   
  
  
+ AAAGGCACAT AAACCTAAAT TATGTAAACA AATGAGAACA TATATGCCAG CTTATCAACG TTAATTATAT   
  
  
+ TTGTTATCAC TCTTAATATC TGTATTCATA GTAAATGAAA ATAAGTATCG ATTTTTTATT AAACCTAAAA   
  
  
+ TCTTCTGTCA ATAATTTTTC TTCCGATAAA AAAAAAATTG GACCATATTA AATTAACATT AATAAATTCA   
  
  
+ TATAATTACT TTGAATGTCT GAAATATAAT GCTCTTCGGT ATATTAAGGT CTTATAAATT TCTCATTAAT   
  
  
+ TTGAAATCTC TTATAATATA TTGGGTCAAC AAGTGCTAGC TTGTTTTTTT TTCCTGGTTA ATTGCATTTA   
  
  
+ AAAAAATAAT TTTTAATTAC AATTGCAAGG GGAGGAATCA TTTACAAAAT ATCAAAAGTG GAAAAGGCAA   
  
  
+ AAAGAAACAA ATTATAAAAC AGAAAAAAGA TTAGAAAACG CAAACAAAGA CGAAATGAGG AACAGTAAGA   
  
  
+ ATCAGTAAAA AAATGTAGTG CAACGGTCAC ATACGGGAAG GCAAGCACAA CAATCATGAA AGAGACAGGC   
  
  
+ ACGTGCGTTC CTCGTGATCA GCAGCCTTCC CTACTTCCCC GCCAAGTGGA GTCCGTACTA ACTCCTGCCA   
  
  
+ TTCCCTCCCA ACGGTCATAT TCCGCCGACA TGCCTCAACG GTCAGATTTC CTGCCCCATC TGGACCGTCC   
  
  
+ GATCAAGATC CAACGGCCAT AAGGCGCACT CTCCAATACC CTAGTGCCCA TTTCTGGAAC AGCCCCAAAA   
  
  
+ ATCTCAGTAT TCGCCATCAT TTTTCAATAC TGGGGTTACA TAAACAGTCT AACAAGGAAG GACCCAACAC   
  
  
+ AGAGGAGAGA GAAAAAAGCT AGCTTTGATG GGGACAGCCA TGGATGGAGG TGAAGAGAGA GGAGGTTGGG   
  
  
+ ACCTTTTATT TTGCTCACTT TCCATTCTTA TATAAAAACA GCTCTAGTTT ATCTCTTATG GCTTTTTTGG   
  
  
+ GTTTGTTCAT AGAGAGGGGG GAATGTGATA TGATTTGATT TGATGTTAGA AATTCTCATT AGTTTTTTCT   
  
  
+ TTTTGTTTGC AATTTTGATG TGTTAGGGTT TGAGGATCAT CATTTTTGAG TAGGGAACTT TCTGGGTTTT   
  
  
+ GCTTGCTGAT TGATTGCCAA TGCTATGGAT TTCTTGGTGA GTCAATCTTC AATTGCTGAT CTGGGTTCGT   
  
  
+ TTGTTCTTCA GAGTTTCACC TGCCAATTTG ATCAAGTATG TCCCTTCACT TCTCCTGCTT CAATTCTCTC   
  
  
+ TCAAATTCAC GCATTTTTCC AATGAATTTC AAATAGGAGT CCTGGAATCC GATGATATTT TAGGTTTTTT   
  
  
+ CCTGACTTCA TTGCATTAGT TTGTTCATAT CTGTGACCAA TTCTTTCATT TGTTATATGA TCTGATTGTT   
  
  
+ TAGTTAGCTA TTCATTCATT GCATATACAT TGGTTTATTC ATTGCAGAGA CACTCACATA CACACACACA   
  
  
+ TACAGGGGAC ACTAGTATTT TTTCTCTCTA ACTCATCGGA TTCCCTTTGA TACTCTGTTT TTTGGGGGGT   
  
  
+ TGATTTGATT TGATTTGATT GACCTTAATT TTGAACTCAC ACACACTCGC ACATAGATAT ATTCTTTCTG   
  
  
+ TTTGTGACAT ATACAGCATC ATATAGAAAT ATATTGCACT GCATATTTGA GCTCAAGACA GGGATATCAT   
  
  
+ CACAAACTGA GTGTGAGGGA GGCAAAAAAC AGAGGAAAAG AAGGGAATTG GGAGTGTAAT CAAAACAAGA   
  
  
+ GAGAGATTGA GGAGAGAGGA AAAAACAAAA TTGGGAGAAG GTTAATAGAG AAGAATGTTG GCTGGGTGTT   
  
  
+ CATCCACATT GCTGTCACCA AGGTATAGAT TGAGGAGTGA AGCTACATCA CAGTTCCAAG CCTGCCATCA   
  
  
+ TCCAATGAGC ACACAGAGAA TTGATTTGCC CTGCACTTTC CCAAGAAAGG ATGTTTCGAA GCCACAATCG   
  
  
+ GTTCGCCCCG TTGGCCTATC TGTCGAGAAG CCGGTTGAAG TCAGGGCCCT GAAGCAGACC ATCCGAGTCC   
  
  
+ CACCATCGCC GGAGGGTAGA AGAGAGATTA AGGCAGATTT CTGGGGTGAT AGAAGAAAGA GCTTGAAGAG   
  
  
+ GAGACTAGCA GAACAGGGGA GCTCTTTTGA TGATGGTGGT GACGAATCTT GTGTTGGGAG AACCAAAAGG   
  
  
+ AAGAAGGGTA GCTTTGATTT TGGTGAATCT GATGAAATTT CTCCAAAAAT TGAGGACACA ATGAGTTTTG   
  
  
+ GACATCTGGG TAGTGGTGGA AATTTCTGGG TTCATCCTGG TTTTGGTGTT GTTAATGTTA ATACTAATTA   
  
  
+ CCCTCAAGTG CCATTTTCTC TCACTTGTTC AGGGGAGGAA GAAAGAGTAT GTTTTGTTCC TACTGATGTG   
  
  
+ ATCTCACCAG CCATCATGCC TCCATTGTCA CACAATCCTT GGGTTGAATC TGGTGTTACT GAGGTCACAG   
  
  
+ AATATGGTGG GGGTGACAAA GACGGCGAAC CTAGTCATGG GTTTGTGAGG GGTACAACAA CAACGTCCGG   
  
  
+ GTCAAGTTCA TCTTCTGAGA GTCATAGTTT TGGGCATAGG CTCAATGAGA GCTCATCTGA CCCTGAAATC   
  
  
+ AGAAATGGTT CCATGTTGCC TAACCCTGGT CATGGTCCTG GCACCAGTCA TGGTCCTGGA ACCGGTCTGG   
  
  
+ CTCACAACCA CAATGATCAA ACCGAGCAAC AAGGGTTCGA GCTCATTAGC TTTCTCATGG GTTGTGTCGA   
  
  
+ AGCGATCAGT TCAAGGAACA TTGCAGCCAT CAATCATTTC ATAGCTAAGC TGGGCGAGCA GGCTTCTCCA   
  
  
+ AGGGCTCGGT CTGCCATTAG CCGCCTCACA GCCTACTTCA CCGAAGCATT AGCCTTGAGG GTCACAAGAT   
  
  
+ TTTGGCCTCA TATCTTTCAC ATAAGTATCC CTCGAGAGTT TGATCGATTT GATGATGAAT CGGGGGCAGC   
  
  
+ TGCAATGAGG CTTTTGAATC AGATCAGCCC AATTCCAAAG TTCGTTCATT TTACAGCCAA TGAGATGTTA   
  
  
+ TTGAGGGCAT TTGAAGGGAA GGACAAGGTG CATATCATAG ACTTCGACAT CAAGCAAGGC TTACAATGGC   
  
  
+ CCGGGTTTTT CCAAAGCTTA GCCATGAGGG AAAACCCCCC GAGCCATGTC AGGATAACAG GCGTAGGGGA   
  
  
+ TTCGAAGCAA GAATTGGTTG AGACAGGAGA AAGACTAGCC GGGTTTGCTG GGGCATTCAA CCTCTCCTTC   
  
  
+ GAGTTCCACC CAGTTGTGGA CCGGTTGGAA GATGTTAGGC TATGGATGCT TCATGTTAAG GAAGGTGAAA   
  
  
+ GTGTCGCGGT AAATTGCATT TTGCAGCTCC ACAAGACACT CTATGACCCC CATGGTGCCA CATTCAGGGA   
  
  
+ CTTCATGGGT TTAATCCGAA GCACAAATCC CATAGCATTG GTTATGGCTG AGCAAGAAGC TGATCACAAC   
  
  
+ GAACCCACCT TAGAAGGCCG AGTATGCAAC TCACTTGGAT ACTATGCAGC CCTTTTCGAT GCCATAGATT   
  
  
+ GCAGCCTTCC TTTCGAGAGC TCAGCAAGGT TGAAGATCGA AGAGATGTTT GGCCGGGAAA TCAGGAACAT   
  
  
+ AATAGCCTGT GAAGGGGCAG ACAGGATCGA AAGACATGAG AAGTTCGAGA AATGGAAGAG AAGGATCGAG   
  
  
+ CAGGAAGGGT TCCGGTGCAT GCGGACTAGC GAAAGGGAGG TGATGCAGAG CCAAATGCTG CTTAAGATGT   
  
  
+ ACTCGAACGA GAGCTACAAC ATGCAAAGAC AAGGCAATGA AGAAGCAATA TCACTAGTAT GGTTAGATCA   
  
  
+ GCCACTCTAC ACTGTTTCTT TGTGGGCTCC AAGTGAATTT GCTGCAGGGA GTTCTTCCAG TTTTTCTCTG   
  
  
+ CCATCTTG  

- -Up\_Stream \_Len000TATTAC CCACTATTGA AAACTTCCTG TTATATGTTT AGTGAATAAA TAATTTCAGT   
  
  
- TCCCCTGTAA AGTTGATATT GTAGTAACAA ATATTTCATA AACTTTTAAC GTTGAGAACA TTATCTATTA   
  
  
- TTACGAAATA TCTGAAGTGG TTAGTGATTA AACATCTTAG TTTTTTAATT AACTTTTCGT TTGTTTTCCT   
  
  
- TTTCCGTGTA TTTGGATTTA ATACATTTGT TTACTCTTGT ATATACGGTC GAATAGTTGC AATTAATATA   
  
  
- AACAATAGTG AGAATTATAG ACATAAGTAT CATTTACTTT TATTCATAGC TAAAAAATAA TTTGGATTTT   
  
  
- AGAAGACAGT TATTAAAAAG AAGGCTATTT TTTTTTTAAC CTGGTATAAT TTAATTGTAA TTATTTAAGT   
  
  
- ATATTAATGA AACTTACAGA CTTTATATTA CGAGAAGCCA TATAATTCCA GAATATTTAA AGAGTAATTA   
  
  
- AACTTTAGAG AATATTATAT AACCCAGTTG TTCACGATCG AACAAAAAAA AAGGACCAAT TAACGTAAAT   
  
  
- TTTTTTATTA AAAATTAATG TTAACGTTCC CCTCCTTAGT AAATGTTTTA TAGTTTTCAC CTTTTCCGTT   
  
  
- TTTCTTTGTT TAATATTTTG TCTTTTTTCT AATCTTTTGC GTTTGTTTCT GCTTTACTCC TTGTCATTCT   
  
  
- TAGTCATTTT TTTACATCAC GTTGCCAGTG TATGCCCTTC CGTTCGTGTT GTTAGTACTT TCTCTGTCCG   
  
  
- TGCACGCAAG GAGCACTAGT CGTCGGAAGG GATGAAGGGG CGGTTCACCT CAGGCATGAT TGAGGACGGT   
  
  
- AAGGGAGGGT TGCCAGTATA AGGCGGCTGT ACGGAGTTGC CAGTCTAAAG GACGGGGTAG ACCTGGCAGG   
  
  
- CTAGTTCTAG GTTGCCGGTA TTCCGCGTGA GAGGTTATGG GATCACGGGT AAAGACCTTG TCGGGGTTTT   
  
  
- TAGAGTCATA AGCGGTAGTA AAAAGTTATG ACCCCAATGT ATTTGTCAGA TTGTTCCTTC CTGGGTTGTG   
  
  
- TCTCCTCTCT CTTTTTTCGA TCGAAACTAC CCCTGTCGGT ACCTACCTCC ACTTCTCTCT CCTCCAACCC   
  
  
- TGGAAAATAA AACGAGTGAA AGGTAAGAAT ATATTTTTGT CGAGATCAAA TAGAGAATAC CGAAAAAACC   
  
  
- CAAACAAGTA TCTCTCCCCC CTTACACTAT ACTAAACTAA ACTACAATCT TTAAGAGTAA TCAAAAAAGA   
  
  
- AAAACAAACG TTAAAACTAC ACAATCCCAA ACTCCTAGTA GTAAAAACTC ATCCCTTGAA AGACCCAAAA   
  
  
- CGAACGACTA ACTAACGGTT ACGATACCTA AAGAACCACT CAGTTAGAAG TTAACGACTA GACCCAAGCA   
  
  
- AACAAGAAGT CTCAAAGTGG ACGGTTAAAC TAGTTCATAC AGGGAAGTGA AGAGGACGAA GTTAAGAGAG   
  
  
- AGTTTAAGTG CGTAAAAAGG TTACTTAAAG TTTATCCTCA GGACCTTAGG CTACTATAAA ATCCAAAAAA   
  
  
- GGACTGAAGT AACGTAATCA AACAAGTATA GACACTGGTT AAGAAAGTAA ACAATATACT AGACTAACAA   
  
  
- ATCAATCGAT AAGTAAGTAA CGTATATGTA ACCAAATAAG TAACGTCTCT GTGAGTGTAT GTGTGTGTGT   
  
  
- ATGTCCCCTG TGATCATAAA AAAGAGAGAT TGAGTAGCCT AAGGGAAACT ATGAGACAAA AAACCCCCCA   
  
  
- ACTAAACTAA ACTAAACTAA CTGGAATTAA AACTTGAGTG TGTGTGAGCG TGTATCTATA TAAGAAAGAC   
  
  
- AAACACTGTA TATGTCGTAG TATATCTTTA TATAACGTGA CGTATAAACT CGAGTTCTGT CCCTATAGTA   
  
  
- GTGTTTGACT CACACTCCCT CCGTTTTTTG TCTCCTTTTC TTCCCTTAAC CCTCACATTA GTTTTGTTCT   
  
  
- CTCTCTAACT CCTCTCTCCT TTTTTGTTTT AACCCTCTTC CAATTATCTC TTCTTACAAC CGACCCACAA   
  
  
- GTAGGTGTAA CGACAGTGGT TCCATATCTA ACTCCTCACT TCGATGTAGT GTCAAGGTTC GGACGGTAGT   
  
  
- AGGTTACTCG TGTGTCTCTT AACTAAACGG GACGTGAAAG GGTTCTTTCC TACAAAGCTT CGGTGTTAGC   
  
  
- CAAGCGGGGC AACCGGATAG ACAGCTCTTC GGCCAACTTC AGTCCCGGGA CTTCGTCTGG TAGGCTCAGG   
  
  
- GTGGTAGCGG CCTCCCATCT TCTCTCTAAT TCCGTCTAAA GACCCCACTA TCTTCTTTCT CGAACTTCTC   
  
  
- CTCTGATCGT CTTGTCCCCT CGAGAAAACT ACTACCACCA CTGCTTAGAA CACAACCCTC TTGGTTTTCC   
  
  
- TTCTTCCCAT CGAAACTAAA ACCACTTAGA CTACTTTAAA GAGGTTTTTA ACTCCTGTGT TACTCAAAAC   
  
  
- CTGTAGACCC ATCACCACCT TTAAAGACCC AAGTAGGACC AAAACCACAA CAATTACAAT TATGATTAAT   
  
  
- GGGAGTTCAC GGTAAAAGAG AGTGAACAAG TCCCCTCCTT CTTTCTCATA CAAAACAAGG ATGACTACAC   
  
  
- TAGAGTGGTC GGTAGTACGG AGGTAACAGT GTGTTAGGAA CCCAACTTAG ACCACAATGA CTCCAGTGTC   
  
  
- TTATACCACC CCCACTGTTT CTGCCGCTTG GATCAGTACC CAAACACTCC CCATGTTGTT GTTGCAGGCC   
  
  
- CAGTTCAAGT AGAAGACTCT CAGTATCAAA ACCCGTATCC GAGTTACTCT CGAGTAGACT GGGACTTTAG   
  
  
- TCTTTACCAA GGTACAACGG ATTGGGACCA GTACCAGGAC CGTGGTCAGT ACCAGGACCT TGGCCAGACC   
  
  
- GAGTGTTGGT GTTACTAGTT TGGCTCGTTG TTCCCAAGCT CGAGTAATCG AAAGAGTACC CAACACAGCT   
  
  
- TCGCTAGTCA AGTTCCTTGT AACGTCGGTA GTTAGTAAAG TATCGATTCG ACCCGCTCGT CCGAAGAGGT   
  
  
- TCCCGAGCCA GACGGTAATC GGCGGAGTGT CGGATGAAGT GGCTTCGTAA TCGGAACTCC CAGTGTTCTA   
  
  
- AAACCGGAGT ATAGAAAGTG TATTCATAGG GAGCTCTCAA ACTAGCTAAA CTACTACTTA GCCCCCGTCG   
  
  
- ACGTTACTCC GAAAACTTAG TCTAGTCGGG TTAAGGTTTC AAGCAAGTAA AATGTCGGTT ACTCTACAAT   
  
  
- AACTCCCGTA AACTTCCCTT CCTGTTCCAC GTATAGTATC TGAAGCTGTA GTTCGTTCCG AATGTTACCG   
  
  
- GGCCCAAAAA GGTTTCGAAT CGGTACTCCC TTTTGGGGGG CTCGGTACAG TCCTATTGTC CGCATCCCCT   
  
  
- AAGCTTCGTT CTTAACCAAC TCTGTCCTCT TTCTGATCGG CCCAAACGAC CCCGTAAGTT GGAGAGGAAG   
  
  
- CTCAAGGTGG GTCAACACCT GGCCAACCTT CTACAATCCG ATACCTACGA AGTACAATTC CTTCCACTTT   
  
  
- CACAGCGCCA TTTAACGTAA AACGTCGAGG TGTTCTGTGA GATACTGGGG GTACCACGGT GTAAGTCCCT   
  
  
- GAAGTACCCA AATTAGGCTT CGTGTTTAGG GTATCGTAAC CAATACCGAC TCGTTCTTCG ACTAGTGTTG   
  
  
- CTTGGGTGGA ATCTTCCGGC TCATACGTTG AGTGAACCTA TGATACGTCG GGAAAAGCTA CGGTATCTAA   
  
  
- CGTCGGAAGG AAAGCTCTCG AGTCGTTCCA ACTTCTAGCT TCTCTACAAA CCGGCCCTTT AGTCCTTGTA   
  
  
- TTATCGGACA CTTCCCCGTC TGTCCTAGCT TTCTGTACTC TTCAAGCTCT TTACCTTCTC TTCCTAGCTC   
  
  
- GTCCTTCCCA AGGCCACGTA CGCCTGATCG CTTTCCCTCC ACTACGTCTC GGTTTACGAC GAATTCTACA   
  
  
- TGAGCTTGCT CTCGATGTTG TACGTTTCTG TTCCGTTACT TCTTCGTTAT AGTGATCATA CCAATCTAGT   
  
  
- CGGTGAGATG TGACAAAGAA ACACCCGAGG TTCACTTAAA CGACGTCCCT CAAGAAGGTC AAAAAGAGAC   
  
  
- GGTAGAAC

+     MBS

| Site Name | Organism | Position | Strand | Matrix score. | sequence | function |
| --- | --- | --- | --- | --- | --- | --- |
| MBS | Arabidopsis thaliana | 3445 | - | 6 | CAACTG | MYB binding site involved in drought-inducibility |

>HU04G00148.1   
+ -Up\_Stream \_Len000ATAATG GGTGATAACT TTTGAAGGAC AATATACAAA TCACTTATTT ATTAAAGTCA   
  
  
+ AGGGGACATT TCAACTATAA CATCATTGTT TATAAAGTAT TTGAAAATTG CAACTCTTGT AATAGATAAT   
  
  
+ AATGCTTTAT AGACTTCACC AATCACTAAT TTGTAGAATC AAAAAATTAA TTGAAAAGCA AACAAAAGGA   
  
  
+ AAAGGCACAT AAACCTAAAT TATGTAAACA AATGAGAACA TATATGCCAG CTTATCAACG TTAATTATAT   
  
  
+ TTGTTATCAC TCTTAATATC TGTATTCATA GTAAATGAAA ATAAGTATCG ATTTTTTATT AAACCTAAAA   
  
  
+ TCTTCTGTCA ATAATTTTTC TTCCGATAAA AAAAAAATTG GACCATATTA AATTAACATT AATAAATTCA   
  
  
+ TATAATTACT TTGAATGTCT GAAATATAAT GCTCTTCGGT ATATTAAGGT CTTATAAATT TCTCATTAAT   
  
  
+ TTGAAATCTC TTATAATATA TTGGGTCAAC AAGTGCTAGC TTGTTTTTTT TTCCTGGTTA ATTGCATTTA   
  
  
+ AAAAAATAAT TTTTAATTAC AATTGCAAGG GGAGGAATCA TTTACAAAAT ATCAAAAGTG GAAAAGGCAA   
  
  
+ AAAGAAACAA ATTATAAAAC AGAAAAAAGA TTAGAAAACG CAAACAAAGA CGAAATGAGG AACAGTAAGA   
  
  
+ ATCAGTAAAA AAATGTAGTG CAACGGTCAC ATACGGGAAG GCAAGCACAA CAATCATGAA AGAGACAGGC   
  
  
+ ACGTGCGTTC CTCGTGATCA GCAGCCTTCC CTACTTCCCC GCCAAGTGGA GTCCGTACTA ACTCCTGCCA   
  
  
+ TTCCCTCCCA ACGGTCATAT TCCGCCGACA TGCCTCAACG GTCAGATTTC CTGCCCCATC TGGACCGTCC   
  
  
+ GATCAAGATC CAACGGCCAT AAGGCGCACT CTCCAATACC CTAGTGCCCA TTTCTGGAAC AGCCCCAAAA   
  
  
+ ATCTCAGTAT TCGCCATCAT TTTTCAATAC TGGGGTTACA TAAACAGTCT AACAAGGAAG GACCCAACAC   
  
  
+ AGAGGAGAGA GAAAAAAGCT AGCTTTGATG GGGACAGCCA TGGATGGAGG TGAAGAGAGA GGAGGTTGGG   
  
  
+ ACCTTTTATT TTGCTCACTT TCCATTCTTA TATAAAAACA GCTCTAGTTT ATCTCTTATG GCTTTTTTGG   
  
  
+ GTTTGTTCAT AGAGAGGGGG GAATGTGATA TGATTTGATT TGATGTTAGA AATTCTCATT AGTTTTTTCT   
  
  
+ TTTTGTTTGC AATTTTGATG TGTTAGGGTT TGAGGATCAT CATTTTTGAG TAGGGAACTT TCTGGGTTTT   
  
  
+ GCTTGCTGAT TGATTGCCAA TGCTATGGAT TTCTTGGTGA GTCAATCTTC AATTGCTGAT CTGGGTTCGT   
  
  
+ TTGTTCTTCA GAGTTTCACC TGCCAATTTG ATCAAGTATG TCCCTTCACT TCTCCTGCTT CAATTCTCTC   
  
  
+ TCAAATTCAC GCATTTTTCC AATGAATTTC AAATAGGAGT CCTGGAATCC GATGATATTT TAGGTTTTTT   
  
  
+ CCTGACTTCA TTGCATTAGT TTGTTCATAT CTGTGACCAA TTCTTTCATT TGTTATATGA TCTGATTGTT   
  
  
+ TAGTTAGCTA TTCATTCATT GCATATACAT TGGTTTATTC ATTGCAGAGA CACTCACATA CACACACACA   
  
  
+ TACAGGGGAC ACTAGTATTT TTTCTCTCTA ACTCATCGGA TTCCCTTTGA TACTCTGTTT TTTGGGGGGT   
  
  
+ TGATTTGATT TGATTTGATT GACCTTAATT TTGAACTCAC ACACACTCGC ACATAGATAT ATTCTTTCTG   
  
  
+ TTTGTGACAT ATACAGCATC ATATAGAAAT ATATTGCACT GCATATTTGA GCTCAAGACA GGGATATCAT   
  
  
+ CACAAACTGA GTGTGAGGGA GGCAAAAAAC AGAGGAAAAG AAGGGAATTG GGAGTGTAAT CAAAACAAGA   
  
  
+ GAGAGATTGA GGAGAGAGGA AAAAACAAAA TTGGGAGAAG GTTAATAGAG AAGAATGTTG GCTGGGTGTT   
  
  
+ CATCCACATT GCTGTCACCA AGGTATAGAT TGAGGAGTGA AGCTACATCA CAGTTCCAAG CCTGCCATCA   
  
  
+ TCCAATGAGC ACACAGAGAA TTGATTTGCC CTGCACTTTC CCAAGAAAGG ATGTTTCGAA GCCACAATCG   
  
  
+ GTTCGCCCCG TTGGCCTATC TGTCGAGAAG CCGGTTGAAG TCAGGGCCCT GAAGCAGACC ATCCGAGTCC   
  
  
+ CACCATCGCC GGAGGGTAGA AGAGAGATTA AGGCAGATTT CTGGGGTGAT AGAAGAAAGA GCTTGAAGAG   
  
  
+ GAGACTAGCA GAACAGGGGA GCTCTTTTGA TGATGGTGGT GACGAATCTT GTGTTGGGAG AACCAAAAGG   
  
  
+ AAGAAGGGTA GCTTTGATTT TGGTGAATCT GATGAAATTT CTCCAAAAAT TGAGGACACA ATGAGTTTTG   
  
  
+ GACATCTGGG TAGTGGTGGA AATTTCTGGG TTCATCCTGG TTTTGGTGTT GTTAATGTTA ATACTAATTA   
  
  
+ CCCTCAAGTG CCATTTTCTC TCACTTGTTC AGGGGAGGAA GAAAGAGTAT GTTTTGTTCC TACTGATGTG   
  
  
+ ATCTCACCAG CCATCATGCC TCCATTGTCA CACAATCCTT GGGTTGAATC TGGTGTTACT GAGGTCACAG   
  
  
+ AATATGGTGG GGGTGACAAA GACGGCGAAC CTAGTCATGG GTTTGTGAGG GGTACAACAA CAACGTCCGG   
  
  
+ GTCAAGTTCA TCTTCTGAGA GTCATAGTTT TGGGCATAGG CTCAATGAGA GCTCATCTGA CCCTGAAATC   
  
  
+ AGAAATGGTT CCATGTTGCC TAACCCTGGT CATGGTCCTG GCACCAGTCA TGGTCCTGGA ACCGGTCTGG   
  
  
+ CTCACAACCA CAATGATCAA ACCGAGCAAC AAGGGTTCGA GCTCATTAGC TTTCTCATGG GTTGTGTCGA   
  
  
+ AGCGATCAGT TCAAGGAACA TTGCAGCCAT CAATCATTTC ATAGCTAAGC TGGGCGAGCA GGCTTCTCCA   
  
  
+ AGGGCTCGGT CTGCCATTAG CCGCCTCACA GCCTACTTCA CCGAAGCATT AGCCTTGAGG GTCACAAGAT   
  
  
+ TTTGGCCTCA TATCTTTCAC ATAAGTATCC CTCGAGAGTT TGATCGATTT GATGATGAAT CGGGGGCAGC   
  
  
+ TGCAATGAGG CTTTTGAATC AGATCAGCCC AATTCCAAAG TTCGTTCATT TTACAGCCAA TGAGATGTTA   
  
  
+ TTGAGGGCAT TTGAAGGGAA GGACAAGGTG CATATCATAG ACTTCGACAT CAAGCAAGGC TTACAATGGC   
  
  
+ CCGGGTTTTT CCAAAGCTTA GCCATGAGGG AAAACCCCCC GAGCCATGTC AGGATAACAG GCGTAGGGGA   
  
  
+ TTCGAAGCAA GAATTGGTTG AGACAGGAGA AAGACTAGCC GGGTTTGCTG GGGCATTCAA CCTCTCCTTC   
  
  
+ GAGTTCCACC CAGTTGTGGA CCGGTTGGAA GATGTTAGGC TATGGATGCT TCATGTTAAG GAAGGTGAAA   
  
  
+ GTGTCGCGGT AAATTGCATT TTGCAGCTCC ACAAGACACT CTATGACCCC CATGGTGCCA CATTCAGGGA   
  
  
+ CTTCATGGGT TTAATCCGAA GCACAAATCC CATAGCATTG GTTATGGCTG AGCAAGAAGC TGATCACAAC   
  
  
+ GAACCCACCT TAGAAGGCCG AGTATGCAAC TCACTTGGAT ACTATGCAGC CCTTTTCGAT GCCATAGATT   
  
  
+ GCAGCCTTCC TTTCGAGAGC TCAGCAAGGT TGAAGATCGA AGAGATGTTT GGCCGGGAAA TCAGGAACAT   
  
  
+ AATAGCCTGT GAAGGGGCAG ACAGGATCGA AAGACATGAG AAGTTCGAGA AATGGAAGAG AAGGATCGAG   
  
  
+ CAGGAAGGGT TCCGGTGCAT GCGGACTAGC GAAAGGGAGG TGATGCAGAG CCAAATGCTG CTTAAGATGT   
  
  
+ ACTCGAACGA GAGCTACAAC ATGCAAAGAC AAGGCAATGA AGAAGCAATA TCACTAGTAT GGTTAGATCA   
  
  
+ GCCACTCTAC ACTGTTTCTT TGTGGGCTCC AAGTGAATTT GCTGCAGGGA GTTCTTCCAG TTTTTCTCTG   
  
  
+ CCATCTTG  

- -Up\_Stream \_Len000TATTAC CCACTATTGA AAACTTCCTG TTATATGTTT AGTGAATAAA TAATTTCAGT   
  
  
- TCCCCTGTAA AGTTGATATT GTAGTAACAA ATATTTCATA AACTTTTAAC GTTGAGAACA TTATCTATTA   
  
  
- TTACGAAATA TCTGAAGTGG TTAGTGATTA AACATCTTAG TTTTTTAATT AACTTTTCGT TTGTTTTCCT   
  
  
- TTTCCGTGTA TTTGGATTTA ATACATTTGT TTACTCTTGT ATATACGGTC GAATAGTTGC AATTAATATA   
  
  
- AACAATAGTG AGAATTATAG ACATAAGTAT CATTTACTTT TATTCATAGC TAAAAAATAA TTTGGATTTT   
  
  
- AGAAGACAGT TATTAAAAAG AAGGCTATTT TTTTTTTAAC CTGGTATAAT TTAATTGTAA TTATTTAAGT   
  
  
- ATATTAATGA AACTTACAGA CTTTATATTA CGAGAAGCCA TATAATTCCA GAATATTTAA AGAGTAATTA   
  
  
- AACTTTAGAG AATATTATAT AACCCAGTTG TTCACGATCG AACAAAAAAA AAGGACCAAT TAACGTAAAT   
  
  
- TTTTTTATTA AAAATTAATG TTAACGTTCC CCTCCTTAGT AAATGTTTTA TAGTTTTCAC CTTTTCCGTT   
  
  
- TTTCTTTGTT TAATATTTTG TCTTTTTTCT AATCTTTTGC GTTTGTTTCT GCTTTACTCC TTGTCATTCT   
  
  
- TAGTCATTTT TTTACATCAC GTTGCCAGTG TATGCCCTTC CGTTCGTGTT GTTAGTACTT TCTCTGTCCG   
  
  
- TGCACGCAAG GAGCACTAGT CGTCGGAAGG GATGAAGGGG CGGTTCACCT CAGGCATGAT TGAGGACGGT   
  
  
- AAGGGAGGGT TGCCAGTATA AGGCGGCTGT ACGGAGTTGC CAGTCTAAAG GACGGGGTAG ACCTGGCAGG   
  
  
- CTAGTTCTAG GTTGCCGGTA TTCCGCGTGA GAGGTTATGG GATCACGGGT AAAGACCTTG TCGGGGTTTT   
  
  
- TAGAGTCATA AGCGGTAGTA AAAAGTTATG ACCCCAATGT ATTTGTCAGA TTGTTCCTTC CTGGGTTGTG   
  
  
- TCTCCTCTCT CTTTTTTCGA TCGAAACTAC CCCTGTCGGT ACCTACCTCC ACTTCTCTCT CCTCCAACCC   
  
  
- TGGAAAATAA AACGAGTGAA AGGTAAGAAT ATATTTTTGT CGAGATCAAA TAGAGAATAC CGAAAAAACC   
  
  
- CAAACAAGTA TCTCTCCCCC CTTACACTAT ACTAAACTAA ACTACAATCT TTAAGAGTAA TCAAAAAAGA   
  
  
- AAAACAAACG TTAAAACTAC ACAATCCCAA ACTCCTAGTA GTAAAAACTC ATCCCTTGAA AGACCCAAAA   
  
  
- CGAACGACTA ACTAACGGTT ACGATACCTA AAGAACCACT CAGTTAGAAG TTAACGACTA GACCCAAGCA   
  
  
- AACAAGAAGT CTCAAAGTGG ACGGTTAAAC TAGTTCATAC AGGGAAGTGA AGAGGACGAA GTTAAGAGAG   
  
  
- AGTTTAAGTG CGTAAAAAGG TTACTTAAAG TTTATCCTCA GGACCTTAGG CTACTATAAA ATCCAAAAAA   
  
  
- GGACTGAAGT AACGTAATCA AACAAGTATA GACACTGGTT AAGAAAGTAA ACAATATACT AGACTAACAA   
  
  
- ATCAATCGAT AAGTAAGTAA CGTATATGTA ACCAAATAAG TAACGTCTCT GTGAGTGTAT GTGTGTGTGT   
  
  
- ATGTCCCCTG TGATCATAAA AAAGAGAGAT TGAGTAGCCT AAGGGAAACT ATGAGACAAA AAACCCCCCA   
  
  
- ACTAAACTAA ACTAAACTAA CTGGAATTAA AACTTGAGTG TGTGTGAGCG TGTATCTATA TAAGAAAGAC   
  
  
- AAACACTGTA TATGTCGTAG TATATCTTTA TATAACGTGA CGTATAAACT CGAGTTCTGT CCCTATAGTA   
  
  
- GTGTTTGACT CACACTCCCT CCGTTTTTTG TCTCCTTTTC TTCCCTTAAC CCTCACATTA GTTTTGTTCT   
  
  
- CTCTCTAACT CCTCTCTCCT TTTTTGTTTT AACCCTCTTC CAATTATCTC TTCTTACAAC CGACCCACAA   
  
  
- GTAGGTGTAA CGACAGTGGT TCCATATCTA ACTCCTCACT TCGATGTAGT GTCAAGGTTC GGACGGTAGT   
  
  
- AGGTTACTCG TGTGTCTCTT AACTAAACGG GACGTGAAAG GGTTCTTTCC TACAAAGCTT CGGTGTTAGC   
  
  
- CAAGCGGGGC AACCGGATAG ACAGCTCTTC GGCCAACTTC AGTCCCGGGA CTTCGTCTGG TAGGCTCAGG   
  
  
- GTGGTAGCGG CCTCCCATCT TCTCTCTAAT TCCGTCTAAA GACCCCACTA TCTTCTTTCT CGAACTTCTC   
  
  
- CTCTGATCGT CTTGTCCCCT CGAGAAAACT ACTACCACCA CTGCTTAGAA CACAACCCTC TTGGTTTTCC   
  
  
- TTCTTCCCAT CGAAACTAAA ACCACTTAGA CTACTTTAAA GAGGTTTTTA ACTCCTGTGT TACTCAAAAC   
  
  
- CTGTAGACCC ATCACCACCT TTAAAGACCC AAGTAGGACC AAAACCACAA CAATTACAAT TATGATTAAT   
  
  
- GGGAGTTCAC GGTAAAAGAG AGTGAACAAG TCCCCTCCTT CTTTCTCATA CAAAACAAGG ATGACTACAC   
  
  
- TAGAGTGGTC GGTAGTACGG AGGTAACAGT GTGTTAGGAA CCCAACTTAG ACCACAATGA CTCCAGTGTC   
  
  
- TTATACCACC CCCACTGTTT CTGCCGCTTG GATCAGTACC CAAACACTCC CCATGTTGTT GTTGCAGGCC   
  
  
- CAGTTCAAGT AGAAGACTCT CAGTATCAAA ACCCGTATCC GAGTTACTCT CGAGTAGACT GGGACTTTAG   
  
  
- TCTTTACCAA GGTACAACGG ATTGGGACCA GTACCAGGAC CGTGGTCAGT ACCAGGACCT TGGCCAGACC   
  
  
- GAGTGTTGGT GTTACTAGTT TGGCTCGTTG TTCCCAAGCT CGAGTAATCG AAAGAGTACC CAACACAGCT   
  
  
- TCGCTAGTCA AGTTCCTTGT AACGTCGGTA GTTAGTAAAG TATCGATTCG ACCCGCTCGT CCGAAGAGGT   
  
  
- TCCCGAGCCA GACGGTAATC GGCGGAGTGT CGGATGAAGT GGCTTCGTAA TCGGAACTCC CAGTGTTCTA   
  
  
- AAACCGGAGT ATAGAAAGTG TATTCATAGG GAGCTCTCAA ACTAGCTAAA CTACTACTTA GCCCCCGTCG   
  
  
- ACGTTACTCC GAAAACTTAG TCTAGTCGGG TTAAGGTTTC AAGCAAGTAA AATGTCGGTT ACTCTACAAT   
  
  
- AACTCCCGTA AACTTCCCTT CCTGTTCCAC GTATAGTATC TGAAGCTGTA GTTCGTTCCG AATGTTACCG   
  
  
- GGCCCAAAAA GGTTTCGAAT CGGTACTCCC TTTTGGGGGG CTCGGTACAG TCCTATTGTC CGCATCCCCT   
  
  
- AAGCTTCGTT CTTAACCAAC TCTGTCCTCT TTCTGATCGG CCCAAACGAC CCCGTAAGTT GGAGAGGAAG   
  
  
- CTCAAGGTGG GTCAACACCT GGCCAACCTT CTACAATCCG ATACCTACGA AGTACAATTC CTTCCACTTT   
  
  
- CACAGCGCCA TTTAACGTAA AACGTCGAGG TGTTCTGTGA GATACTGGGG GTACCACGGT GTAAGTCCCT   
  
  
- GAAGTACCCA AATTAGGCTT CGTGTTTAGG GTATCGTAAC CAATACCGAC TCGTTCTTCG ACTAGTGTTG   
  
  
- CTTGGGTGGA ATCTTCCGGC TCATACGTTG AGTGAACCTA TGATACGTCG GGAAAAGCTA CGGTATCTAA   
  
  
- CGTCGGAAGG AAAGCTCTCG AGTCGTTCCA ACTTCTAGCT TCTCTACAAA CCGGCCCTTT AGTCCTTGTA   
  
  
- TTATCGGACA CTTCCCCGTC TGTCCTAGCT TTCTGTACTC TTCAAGCTCT TTACCTTCTC TTCCTAGCTC   
  
  
- GTCCTTCCCA AGGCCACGTA CGCCTGATCG CTTTCCCTCC ACTACGTCTC GGTTTACGAC GAATTCTACA   
  
  
- TGAGCTTGCT CTCGATGTTG TACGTTTCTG TTCCGTTACT TCTTCGTTAT AGTGATCATA CCAATCTAGT   
  
  
- CGGTGAGATG TGACAAAGAA ACACCCGAGG TTCACTTAAA CGACGTCCCT CAAGAAGGTC AAAAAGAGAC   
  
  
- GGTAGAAC

+     MRE

| Site Name | Organism | Position | Strand | Matrix score. | sequence | function |
| --- | --- | --- | --- | --- | --- | --- |
| MRE | Petroselinum crispum | 1534 | - | 7 | AACCTAA | MYB binding site involved in light responsiveness |
| MRE | Petroselinum crispum | 346 | + | 7 | AACCTAA | MYB binding site involved in light responsiveness |
| MRE | Petroselinum crispum | 226 | + | 7 | AACCTAA | MYB binding site involved in light responsiveness |

>HU04G00148.1   
+ -Up\_Stream \_Len000ATAATG GGTGATAACT TTTGAAGGAC AATATACAAA TCACTTATTT ATTAAAGTCA   
  
  
+ AGGGGACATT TCAACTATAA CATCATTGTT TATAAAGTAT TTGAAAATTG CAACTCTTGT AATAGATAAT   
  
  
+ AATGCTTTAT AGACTTCACC AATCACTAAT TTGTAGAATC AAAAAATTAA TTGAAAAGCA AACAAAAGGA   
  
  
+ AAAGGCACAT AAACCTAAAT TATGTAAACA AATGAGAACA TATATGCCAG CTTATCAACG TTAATTATAT   
  
  
+ TTGTTATCAC TCTTAATATC TGTATTCATA GTAAATGAAA ATAAGTATCG ATTTTTTATT AAACCTAAAA   
  
  
+ TCTTCTGTCA ATAATTTTTC TTCCGATAAA AAAAAAATTG GACCATATTA AATTAACATT AATAAATTCA   
  
  
+ TATAATTACT TTGAATGTCT GAAATATAAT GCTCTTCGGT ATATTAAGGT CTTATAAATT TCTCATTAAT   
  
  
+ TTGAAATCTC TTATAATATA TTGGGTCAAC AAGTGCTAGC TTGTTTTTTT TTCCTGGTTA ATTGCATTTA   
  
  
+ AAAAAATAAT TTTTAATTAC AATTGCAAGG GGAGGAATCA TTTACAAAAT ATCAAAAGTG GAAAAGGCAA   
  
  
+ AAAGAAACAA ATTATAAAAC AGAAAAAAGA TTAGAAAACG CAAACAAAGA CGAAATGAGG AACAGTAAGA   
  
  
+ ATCAGTAAAA AAATGTAGTG CAACGGTCAC ATACGGGAAG GCAAGCACAA CAATCATGAA AGAGACAGGC   
  
  
+ ACGTGCGTTC CTCGTGATCA GCAGCCTTCC CTACTTCCCC GCCAAGTGGA GTCCGTACTA ACTCCTGCCA   
  
  
+ TTCCCTCCCA ACGGTCATAT TCCGCCGACA TGCCTCAACG GTCAGATTTC CTGCCCCATC TGGACCGTCC   
  
  
+ GATCAAGATC CAACGGCCAT AAGGCGCACT CTCCAATACC CTAGTGCCCA TTTCTGGAAC AGCCCCAAAA   
  
  
+ ATCTCAGTAT TCGCCATCAT TTTTCAATAC TGGGGTTACA TAAACAGTCT AACAAGGAAG GACCCAACAC   
  
  
+ AGAGGAGAGA GAAAAAAGCT AGCTTTGATG GGGACAGCCA TGGATGGAGG TGAAGAGAGA GGAGGTTGGG   
  
  
+ ACCTTTTATT TTGCTCACTT TCCATTCTTA TATAAAAACA GCTCTAGTTT ATCTCTTATG GCTTTTTTGG   
  
  
+ GTTTGTTCAT AGAGAGGGGG GAATGTGATA TGATTTGATT TGATGTTAGA AATTCTCATT AGTTTTTTCT   
  
  
+ TTTTGTTTGC AATTTTGATG TGTTAGGGTT TGAGGATCAT CATTTTTGAG TAGGGAACTT TCTGGGTTTT   
  
  
+ GCTTGCTGAT TGATTGCCAA TGCTATGGAT TTCTTGGTGA GTCAATCTTC AATTGCTGAT CTGGGTTCGT   
  
  
+ TTGTTCTTCA GAGTTTCACC TGCCAATTTG ATCAAGTATG TCCCTTCACT TCTCCTGCTT CAATTCTCTC   
  
  
+ TCAAATTCAC GCATTTTTCC AATGAATTTC AAATAGGAGT CCTGGAATCC GATGATATTT TAGGTTTTTT   
  
  
+ CCTGACTTCA TTGCATTAGT TTGTTCATAT CTGTGACCAA TTCTTTCATT TGTTATATGA TCTGATTGTT   
  
  
+ TAGTTAGCTA TTCATTCATT GCATATACAT TGGTTTATTC ATTGCAGAGA CACTCACATA CACACACACA   
  
  
+ TACAGGGGAC ACTAGTATTT TTTCTCTCTA ACTCATCGGA TTCCCTTTGA TACTCTGTTT TTTGGGGGGT   
  
  
+ TGATTTGATT TGATTTGATT GACCTTAATT TTGAACTCAC ACACACTCGC ACATAGATAT ATTCTTTCTG   
  
  
+ TTTGTGACAT ATACAGCATC ATATAGAAAT ATATTGCACT GCATATTTGA GCTCAAGACA GGGATATCAT   
  
  
+ CACAAACTGA GTGTGAGGGA GGCAAAAAAC AGAGGAAAAG AAGGGAATTG GGAGTGTAAT CAAAACAAGA   
  
  
+ GAGAGATTGA GGAGAGAGGA AAAAACAAAA TTGGGAGAAG GTTAATAGAG AAGAATGTTG GCTGGGTGTT   
  
  
+ CATCCACATT GCTGTCACCA AGGTATAGAT TGAGGAGTGA AGCTACATCA CAGTTCCAAG CCTGCCATCA   
  
  
+ TCCAATGAGC ACACAGAGAA TTGATTTGCC CTGCACTTTC CCAAGAAAGG ATGTTTCGAA GCCACAATCG   
  
  
+ GTTCGCCCCG TTGGCCTATC TGTCGAGAAG CCGGTTGAAG TCAGGGCCCT GAAGCAGACC ATCCGAGTCC   
  
  
+ CACCATCGCC GGAGGGTAGA AGAGAGATTA AGGCAGATTT CTGGGGTGAT AGAAGAAAGA GCTTGAAGAG   
  
  
+ GAGACTAGCA GAACAGGGGA GCTCTTTTGA TGATGGTGGT GACGAATCTT GTGTTGGGAG AACCAAAAGG   
  
  
+ AAGAAGGGTA GCTTTGATTT TGGTGAATCT GATGAAATTT CTCCAAAAAT TGAGGACACA ATGAGTTTTG   
  
  
+ GACATCTGGG TAGTGGTGGA AATTTCTGGG TTCATCCTGG TTTTGGTGTT GTTAATGTTA ATACTAATTA   
  
  
+ CCCTCAAGTG CCATTTTCTC TCACTTGTTC AGGGGAGGAA GAAAGAGTAT GTTTTGTTCC TACTGATGTG   
  
  
+ ATCTCACCAG CCATCATGCC TCCATTGTCA CACAATCCTT GGGTTGAATC TGGTGTTACT GAGGTCACAG   
  
  
+ AATATGGTGG GGGTGACAAA GACGGCGAAC CTAGTCATGG GTTTGTGAGG GGTACAACAA CAACGTCCGG   
  
  
+ GTCAAGTTCA TCTTCTGAGA GTCATAGTTT TGGGCATAGG CTCAATGAGA GCTCATCTGA CCCTGAAATC   
  
  
+ AGAAATGGTT CCATGTTGCC TAACCCTGGT CATGGTCCTG GCACCAGTCA TGGTCCTGGA ACCGGTCTGG   
  
  
+ CTCACAACCA CAATGATCAA ACCGAGCAAC AAGGGTTCGA GCTCATTAGC TTTCTCATGG GTTGTGTCGA   
  
  
+ AGCGATCAGT TCAAGGAACA TTGCAGCCAT CAATCATTTC ATAGCTAAGC TGGGCGAGCA GGCTTCTCCA   
  
  
+ AGGGCTCGGT CTGCCATTAG CCGCCTCACA GCCTACTTCA CCGAAGCATT AGCCTTGAGG GTCACAAGAT   
  
  
+ TTTGGCCTCA TATCTTTCAC ATAAGTATCC CTCGAGAGTT TGATCGATTT GATGATGAAT CGGGGGCAGC   
  
  
+ TGCAATGAGG CTTTTGAATC AGATCAGCCC AATTCCAAAG TTCGTTCATT TTACAGCCAA TGAGATGTTA   
  
  
+ TTGAGGGCAT TTGAAGGGAA GGACAAGGTG CATATCATAG ACTTCGACAT CAAGCAAGGC TTACAATGGC   
  
  
+ CCGGGTTTTT CCAAAGCTTA GCCATGAGGG AAAACCCCCC GAGCCATGTC AGGATAACAG GCGTAGGGGA   
  
  
+ TTCGAAGCAA GAATTGGTTG AGACAGGAGA AAGACTAGCC GGGTTTGCTG GGGCATTCAA CCTCTCCTTC   
  
  
+ GAGTTCCACC CAGTTGTGGA CCGGTTGGAA GATGTTAGGC TATGGATGCT TCATGTTAAG GAAGGTGAAA   
  
  
+ GTGTCGCGGT AAATTGCATT TTGCAGCTCC ACAAGACACT CTATGACCCC CATGGTGCCA CATTCAGGGA   
  
  
+ CTTCATGGGT TTAATCCGAA GCACAAATCC CATAGCATTG GTTATGGCTG AGCAAGAAGC TGATCACAAC   
  
  
+ GAACCCACCT TAGAAGGCCG AGTATGCAAC TCACTTGGAT ACTATGCAGC CCTTTTCGAT GCCATAGATT   
  
  
+ GCAGCCTTCC TTTCGAGAGC TCAGCAAGGT TGAAGATCGA AGAGATGTTT GGCCGGGAAA TCAGGAACAT   
  
  
+ AATAGCCTGT GAAGGGGCAG ACAGGATCGA AAGACATGAG AAGTTCGAGA AATGGAAGAG AAGGATCGAG   
  
  
+ CAGGAAGGGT TCCGGTGCAT GCGGACTAGC GAAAGGGAGG TGATGCAGAG CCAAATGCTG CTTAAGATGT   
  
  
+ ACTCGAACGA GAGCTACAAC ATGCAAAGAC AAGGCAATGA AGAAGCAATA TCACTAGTAT GGTTAGATCA   
  
  
+ GCCACTCTAC ACTGTTTCTT TGTGGGCTCC AAGTGAATTT GCTGCAGGGA GTTCTTCCAG TTTTTCTCTG   
  
  
+ CCATCTTG  

- -Up\_Stream \_Len000TATTAC CCACTATTGA AAACTTCCTG TTATATGTTT AGTGAATAAA TAATTTCAGT   
  
  
- TCCCCTGTAA AGTTGATATT GTAGTAACAA ATATTTCATA AACTTTTAAC GTTGAGAACA TTATCTATTA   
  
  
- TTACGAAATA TCTGAAGTGG TTAGTGATTA AACATCTTAG TTTTTTAATT AACTTTTCGT TTGTTTTCCT   
  
  
- TTTCCGTGTA TTTGGATTTA ATACATTTGT TTACTCTTGT ATATACGGTC GAATAGTTGC AATTAATATA   
  
  
- AACAATAGTG AGAATTATAG ACATAAGTAT CATTTACTTT TATTCATAGC TAAAAAATAA TTTGGATTTT   
  
  
- AGAAGACAGT TATTAAAAAG AAGGCTATTT TTTTTTTAAC CTGGTATAAT TTAATTGTAA TTATTTAAGT   
  
  
- ATATTAATGA AACTTACAGA CTTTATATTA CGAGAAGCCA TATAATTCCA GAATATTTAA AGAGTAATTA   
  
  
- AACTTTAGAG AATATTATAT AACCCAGTTG TTCACGATCG AACAAAAAAA AAGGACCAAT TAACGTAAAT   
  
  
- TTTTTTATTA AAAATTAATG TTAACGTTCC CCTCCTTAGT AAATGTTTTA TAGTTTTCAC CTTTTCCGTT   
  
  
- TTTCTTTGTT TAATATTTTG TCTTTTTTCT AATCTTTTGC GTTTGTTTCT GCTTTACTCC TTGTCATTCT   
  
  
- TAGTCATTTT TTTACATCAC GTTGCCAGTG TATGCCCTTC CGTTCGTGTT GTTAGTACTT TCTCTGTCCG   
  
  
- TGCACGCAAG GAGCACTAGT CGTCGGAAGG GATGAAGGGG CGGTTCACCT CAGGCATGAT TGAGGACGGT   
  
  
- AAGGGAGGGT TGCCAGTATA AGGCGGCTGT ACGGAGTTGC CAGTCTAAAG GACGGGGTAG ACCTGGCAGG   
  
  
- CTAGTTCTAG GTTGCCGGTA TTCCGCGTGA GAGGTTATGG GATCACGGGT AAAGACCTTG TCGGGGTTTT   
  
  
- TAGAGTCATA AGCGGTAGTA AAAAGTTATG ACCCCAATGT ATTTGTCAGA TTGTTCCTTC CTGGGTTGTG   
  
  
- TCTCCTCTCT CTTTTTTCGA TCGAAACTAC CCCTGTCGGT ACCTACCTCC ACTTCTCTCT CCTCCAACCC   
  
  
- TGGAAAATAA AACGAGTGAA AGGTAAGAAT ATATTTTTGT CGAGATCAAA TAGAGAATAC CGAAAAAACC   
  
  
- CAAACAAGTA TCTCTCCCCC CTTACACTAT ACTAAACTAA ACTACAATCT TTAAGAGTAA TCAAAAAAGA   
  
  
- AAAACAAACG TTAAAACTAC ACAATCCCAA ACTCCTAGTA GTAAAAACTC ATCCCTTGAA AGACCCAAAA   
  
  
- CGAACGACTA ACTAACGGTT ACGATACCTA AAGAACCACT CAGTTAGAAG TTAACGACTA GACCCAAGCA   
  
  
- AACAAGAAGT CTCAAAGTGG ACGGTTAAAC TAGTTCATAC AGGGAAGTGA AGAGGACGAA GTTAAGAGAG   
  
  
- AGTTTAAGTG CGTAAAAAGG TTACTTAAAG TTTATCCTCA GGACCTTAGG CTACTATAAA ATCCAAAAAA   
  
  
- GGACTGAAGT AACGTAATCA AACAAGTATA GACACTGGTT AAGAAAGTAA ACAATATACT AGACTAACAA   
  
  
- ATCAATCGAT AAGTAAGTAA CGTATATGTA ACCAAATAAG TAACGTCTCT GTGAGTGTAT GTGTGTGTGT   
  
  
- ATGTCCCCTG TGATCATAAA AAAGAGAGAT TGAGTAGCCT AAGGGAAACT ATGAGACAAA AAACCCCCCA   
  
  
- ACTAAACTAA ACTAAACTAA CTGGAATTAA AACTTGAGTG TGTGTGAGCG TGTATCTATA TAAGAAAGAC   
  
  
- AAACACTGTA TATGTCGTAG TATATCTTTA TATAACGTGA CGTATAAACT CGAGTTCTGT CCCTATAGTA   
  
  
- GTGTTTGACT CACACTCCCT CCGTTTTTTG TCTCCTTTTC TTCCCTTAAC CCTCACATTA GTTTTGTTCT   
  
  
- CTCTCTAACT CCTCTCTCCT TTTTTGTTTT AACCCTCTTC CAATTATCTC TTCTTACAAC CGACCCACAA   
  
  
- GTAGGTGTAA CGACAGTGGT TCCATATCTA ACTCCTCACT TCGATGTAGT GTCAAGGTTC GGACGGTAGT   
  
  
- AGGTTACTCG TGTGTCTCTT AACTAAACGG GACGTGAAAG GGTTCTTTCC TACAAAGCTT CGGTGTTAGC   
  
  
- CAAGCGGGGC AACCGGATAG ACAGCTCTTC GGCCAACTTC AGTCCCGGGA CTTCGTCTGG TAGGCTCAGG   
  
  
- GTGGTAGCGG CCTCCCATCT TCTCTCTAAT TCCGTCTAAA GACCCCACTA TCTTCTTTCT CGAACTTCTC   
  
  
- CTCTGATCGT CTTGTCCCCT CGAGAAAACT ACTACCACCA CTGCTTAGAA CACAACCCTC TTGGTTTTCC   
  
  
- TTCTTCCCAT CGAAACTAAA ACCACTTAGA CTACTTTAAA GAGGTTTTTA ACTCCTGTGT TACTCAAAAC   
  
  
- CTGTAGACCC ATCACCACCT TTAAAGACCC AAGTAGGACC AAAACCACAA CAATTACAAT TATGATTAAT   
  
  
- GGGAGTTCAC GGTAAAAGAG AGTGAACAAG TCCCCTCCTT CTTTCTCATA CAAAACAAGG ATGACTACAC   
  
  
- TAGAGTGGTC GGTAGTACGG AGGTAACAGT GTGTTAGGAA CCCAACTTAG ACCACAATGA CTCCAGTGTC   
  
  
- TTATACCACC CCCACTGTTT CTGCCGCTTG GATCAGTACC CAAACACTCC CCATGTTGTT GTTGCAGGCC   
  
  
- CAGTTCAAGT AGAAGACTCT CAGTATCAAA ACCCGTATCC GAGTTACTCT CGAGTAGACT GGGACTTTAG   
  
  
- TCTTTACCAA GGTACAACGG ATTGGGACCA GTACCAGGAC CGTGGTCAGT ACCAGGACCT TGGCCAGACC   
  
  
- GAGTGTTGGT GTTACTAGTT TGGCTCGTTG TTCCCAAGCT CGAGTAATCG AAAGAGTACC CAACACAGCT   
  
  
- TCGCTAGTCA AGTTCCTTGT AACGTCGGTA GTTAGTAAAG TATCGATTCG ACCCGCTCGT CCGAAGAGGT   
  
  
- TCCCGAGCCA GACGGTAATC GGCGGAGTGT CGGATGAAGT GGCTTCGTAA TCGGAACTCC CAGTGTTCTA   
  
  
- AAACCGGAGT ATAGAAAGTG TATTCATAGG GAGCTCTCAA ACTAGCTAAA CTACTACTTA GCCCCCGTCG   
  
  
- ACGTTACTCC GAAAACTTAG TCTAGTCGGG TTAAGGTTTC AAGCAAGTAA AATGTCGGTT ACTCTACAAT   
  
  
- AACTCCCGTA AACTTCCCTT CCTGTTCCAC GTATAGTATC TGAAGCTGTA GTTCGTTCCG AATGTTACCG   
  
  
- GGCCCAAAAA GGTTTCGAAT CGGTACTCCC TTTTGGGGGG CTCGGTACAG TCCTATTGTC CGCATCCCCT   
  
  
- AAGCTTCGTT CTTAACCAAC TCTGTCCTCT TTCTGATCGG CCCAAACGAC CCCGTAAGTT GGAGAGGAAG   
  
  
- CTCAAGGTGG GTCAACACCT GGCCAACCTT CTACAATCCG ATACCTACGA AGTACAATTC CTTCCACTTT   
  
  
- CACAGCGCCA TTTAACGTAA AACGTCGAGG TGTTCTGTGA GATACTGGGG GTACCACGGT GTAAGTCCCT   
  
  
- GAAGTACCCA AATTAGGCTT CGTGTTTAGG GTATCGTAAC CAATACCGAC TCGTTCTTCG ACTAGTGTTG   
  
  
- CTTGGGTGGA ATCTTCCGGC TCATACGTTG AGTGAACCTA TGATACGTCG GGAAAAGCTA CGGTATCTAA   
  
  
- CGTCGGAAGG AAAGCTCTCG AGTCGTTCCA ACTTCTAGCT TCTCTACAAA CCGGCCCTTT AGTCCTTGTA   
  
  
- TTATCGGACA CTTCCCCGTC TGTCCTAGCT TTCTGTACTC TTCAAGCTCT TTACCTTCTC TTCCTAGCTC   
  
  
- GTCCTTCCCA AGGCCACGTA CGCCTGATCG CTTTCCCTCC ACTACGTCTC GGTTTACGAC GAATTCTACA   
  
  
- TGAGCTTGCT CTCGATGTTG TACGTTTCTG TTCCGTTACT TCTTCGTTAT AGTGATCATA CCAATCTAGT   
  
  
- CGGTGAGATG TGACAAAGAA ACACCCGAGG TTCACTTAAA CGACGTCCCT CAAGAAGGTC AAAAAGAGAC   
  
  
- GGTAGAAC

+     MSA-like

| Site Name | Organism | Position | Strand | Matrix score. | sequence | function |
| --- | --- | --- | --- | --- | --- | --- |
| MSA-like | Catharanthus roseus | 851 | + | 9 | (T/C)C(T/C)AACGG(T/C)(T/C)A | cis-acting element involved in cell cycle regulation |
| MSA-like | Catharanthus roseus | 923 | + | 9 | (T/C)C(T/C)AACGG(T/C)(T/C)A | cis-acting element involved in cell cycle regulation |

>HU04G00148.1   
+ -Up\_Stream \_Len000ATAATG GGTGATAACT TTTGAAGGAC AATATACAAA TCACTTATTT ATTAAAGTCA   
  
  
+ AGGGGACATT TCAACTATAA CATCATTGTT TATAAAGTAT TTGAAAATTG CAACTCTTGT AATAGATAAT   
  
  
+ AATGCTTTAT AGACTTCACC AATCACTAAT TTGTAGAATC AAAAAATTAA TTGAAAAGCA AACAAAAGGA   
  
  
+ AAAGGCACAT AAACCTAAAT TATGTAAACA AATGAGAACA TATATGCCAG CTTATCAACG TTAATTATAT   
  
  
+ TTGTTATCAC TCTTAATATC TGTATTCATA GTAAATGAAA ATAAGTATCG ATTTTTTATT AAACCTAAAA   
  
  
+ TCTTCTGTCA ATAATTTTTC TTCCGATAAA AAAAAAATTG GACCATATTA AATTAACATT AATAAATTCA   
  
  
+ TATAATTACT TTGAATGTCT GAAATATAAT GCTCTTCGGT ATATTAAGGT CTTATAAATT TCTCATTAAT   
  
  
+ TTGAAATCTC TTATAATATA TTGGGTCAAC AAGTGCTAGC TTGTTTTTTT TTCCTGGTTA ATTGCATTTA   
  
  
+ AAAAAATAAT TTTTAATTAC AATTGCAAGG GGAGGAATCA TTTACAAAAT ATCAAAAGTG GAAAAGGCAA   
  
  
+ AAAGAAACAA ATTATAAAAC AGAAAAAAGA TTAGAAAACG CAAACAAAGA CGAAATGAGG AACAGTAAGA   
  
  
+ ATCAGTAAAA AAATGTAGTG CAACGGTCAC ATACGGGAAG GCAAGCACAA CAATCATGAA AGAGACAGGC   
  
  
+ ACGTGCGTTC CTCGTGATCA GCAGCCTTCC CTACTTCCCC GCCAAGTGGA GTCCGTACTA ACTCCTGCCA   
  
  
+ TTCCCTCCCA ACGGTCATAT TCCGCCGACA TGCCTCAACG GTCAGATTTC CTGCCCCATC TGGACCGTCC   
  
  
+ GATCAAGATC CAACGGCCAT AAGGCGCACT CTCCAATACC CTAGTGCCCA TTTCTGGAAC AGCCCCAAAA   
  
  
+ ATCTCAGTAT TCGCCATCAT TTTTCAATAC TGGGGTTACA TAAACAGTCT AACAAGGAAG GACCCAACAC   
  
  
+ AGAGGAGAGA GAAAAAAGCT AGCTTTGATG GGGACAGCCA TGGATGGAGG TGAAGAGAGA GGAGGTTGGG   
  
  
+ ACCTTTTATT TTGCTCACTT TCCATTCTTA TATAAAAACA GCTCTAGTTT ATCTCTTATG GCTTTTTTGG   
  
  
+ GTTTGTTCAT AGAGAGGGGG GAATGTGATA TGATTTGATT TGATGTTAGA AATTCTCATT AGTTTTTTCT   
  
  
+ TTTTGTTTGC AATTTTGATG TGTTAGGGTT TGAGGATCAT CATTTTTGAG TAGGGAACTT TCTGGGTTTT   
  
  
+ GCTTGCTGAT TGATTGCCAA TGCTATGGAT TTCTTGGTGA GTCAATCTTC AATTGCTGAT CTGGGTTCGT   
  
  
+ TTGTTCTTCA GAGTTTCACC TGCCAATTTG ATCAAGTATG TCCCTTCACT TCTCCTGCTT CAATTCTCTC   
  
  
+ TCAAATTCAC GCATTTTTCC AATGAATTTC AAATAGGAGT CCTGGAATCC GATGATATTT TAGGTTTTTT   
  
  
+ CCTGACTTCA TTGCATTAGT TTGTTCATAT CTGTGACCAA TTCTTTCATT TGTTATATGA TCTGATTGTT   
  
  
+ TAGTTAGCTA TTCATTCATT GCATATACAT TGGTTTATTC ATTGCAGAGA CACTCACATA CACACACACA   
  
  
+ TACAGGGGAC ACTAGTATTT TTTCTCTCTA ACTCATCGGA TTCCCTTTGA TACTCTGTTT TTTGGGGGGT   
  
  
+ TGATTTGATT TGATTTGATT GACCTTAATT TTGAACTCAC ACACACTCGC ACATAGATAT ATTCTTTCTG   
  
  
+ TTTGTGACAT ATACAGCATC ATATAGAAAT ATATTGCACT GCATATTTGA GCTCAAGACA GGGATATCAT   
  
  
+ CACAAACTGA GTGTGAGGGA GGCAAAAAAC AGAGGAAAAG AAGGGAATTG GGAGTGTAAT CAAAACAAGA   
  
  
+ GAGAGATTGA GGAGAGAGGA AAAAACAAAA TTGGGAGAAG GTTAATAGAG AAGAATGTTG GCTGGGTGTT   
  
  
+ CATCCACATT GCTGTCACCA AGGTATAGAT TGAGGAGTGA AGCTACATCA CAGTTCCAAG CCTGCCATCA   
  
  
+ TCCAATGAGC ACACAGAGAA TTGATTTGCC CTGCACTTTC CCAAGAAAGG ATGTTTCGAA GCCACAATCG   
  
  
+ GTTCGCCCCG TTGGCCTATC TGTCGAGAAG CCGGTTGAAG TCAGGGCCCT GAAGCAGACC ATCCGAGTCC   
  
  
+ CACCATCGCC GGAGGGTAGA AGAGAGATTA AGGCAGATTT CTGGGGTGAT AGAAGAAAGA GCTTGAAGAG   
  
  
+ GAGACTAGCA GAACAGGGGA GCTCTTTTGA TGATGGTGGT GACGAATCTT GTGTTGGGAG AACCAAAAGG   
  
  
+ AAGAAGGGTA GCTTTGATTT TGGTGAATCT GATGAAATTT CTCCAAAAAT TGAGGACACA ATGAGTTTTG   
  
  
+ GACATCTGGG TAGTGGTGGA AATTTCTGGG TTCATCCTGG TTTTGGTGTT GTTAATGTTA ATACTAATTA   
  
  
+ CCCTCAAGTG CCATTTTCTC TCACTTGTTC AGGGGAGGAA GAAAGAGTAT GTTTTGTTCC TACTGATGTG   
  
  
+ ATCTCACCAG CCATCATGCC TCCATTGTCA CACAATCCTT GGGTTGAATC TGGTGTTACT GAGGTCACAG   
  
  
+ AATATGGTGG GGGTGACAAA GACGGCGAAC CTAGTCATGG GTTTGTGAGG GGTACAACAA CAACGTCCGG   
  
  
+ GTCAAGTTCA TCTTCTGAGA GTCATAGTTT TGGGCATAGG CTCAATGAGA GCTCATCTGA CCCTGAAATC   
  
  
+ AGAAATGGTT CCATGTTGCC TAACCCTGGT CATGGTCCTG GCACCAGTCA TGGTCCTGGA ACCGGTCTGG   
  
  
+ CTCACAACCA CAATGATCAA ACCGAGCAAC AAGGGTTCGA GCTCATTAGC TTTCTCATGG GTTGTGTCGA   
  
  
+ AGCGATCAGT TCAAGGAACA TTGCAGCCAT CAATCATTTC ATAGCTAAGC TGGGCGAGCA GGCTTCTCCA   
  
  
+ AGGGCTCGGT CTGCCATTAG CCGCCTCACA GCCTACTTCA CCGAAGCATT AGCCTTGAGG GTCACAAGAT   
  
  
+ TTTGGCCTCA TATCTTTCAC ATAAGTATCC CTCGAGAGTT TGATCGATTT GATGATGAAT CGGGGGCAGC   
  
  
+ TGCAATGAGG CTTTTGAATC AGATCAGCCC AATTCCAAAG TTCGTTCATT TTACAGCCAA TGAGATGTTA   
  
  
+ TTGAGGGCAT TTGAAGGGAA GGACAAGGTG CATATCATAG ACTTCGACAT CAAGCAAGGC TTACAATGGC   
  
  
+ CCGGGTTTTT CCAAAGCTTA GCCATGAGGG AAAACCCCCC GAGCCATGTC AGGATAACAG GCGTAGGGGA   
  
  
+ TTCGAAGCAA GAATTGGTTG AGACAGGAGA AAGACTAGCC GGGTTTGCTG GGGCATTCAA CCTCTCCTTC   
  
  
+ GAGTTCCACC CAGTTGTGGA CCGGTTGGAA GATGTTAGGC TATGGATGCT TCATGTTAAG GAAGGTGAAA   
  
  
+ GTGTCGCGGT AAATTGCATT TTGCAGCTCC ACAAGACACT CTATGACCCC CATGGTGCCA CATTCAGGGA   
  
  
+ CTTCATGGGT TTAATCCGAA GCACAAATCC CATAGCATTG GTTATGGCTG AGCAAGAAGC TGATCACAAC   
  
  
+ GAACCCACCT TAGAAGGCCG AGTATGCAAC TCACTTGGAT ACTATGCAGC CCTTTTCGAT GCCATAGATT   
  
  
+ GCAGCCTTCC TTTCGAGAGC TCAGCAAGGT TGAAGATCGA AGAGATGTTT GGCCGGGAAA TCAGGAACAT   
  
  
+ AATAGCCTGT GAAGGGGCAG ACAGGATCGA AAGACATGAG AAGTTCGAGA AATGGAAGAG AAGGATCGAG   
  
  
+ CAGGAAGGGT TCCGGTGCAT GCGGACTAGC GAAAGGGAGG TGATGCAGAG CCAAATGCTG CTTAAGATGT   
  
  
+ ACTCGAACGA GAGCTACAAC ATGCAAAGAC AAGGCAATGA AGAAGCAATA TCACTAGTAT GGTTAGATCA   
  
  
+ GCCACTCTAC ACTGTTTCTT TGTGGGCTCC AAGTGAATTT GCTGCAGGGA GTTCTTCCAG TTTTTCTCTG   
  
  
+ CCATCTTG  

- -Up\_Stream \_Len000TATTAC CCACTATTGA AAACTTCCTG TTATATGTTT AGTGAATAAA TAATTTCAGT   
  
  
- TCCCCTGTAA AGTTGATATT GTAGTAACAA ATATTTCATA AACTTTTAAC GTTGAGAACA TTATCTATTA   
  
  
- TTACGAAATA TCTGAAGTGG TTAGTGATTA AACATCTTAG TTTTTTAATT AACTTTTCGT TTGTTTTCCT   
  
  
- TTTCCGTGTA TTTGGATTTA ATACATTTGT TTACTCTTGT ATATACGGTC GAATAGTTGC AATTAATATA   
  
  
- AACAATAGTG AGAATTATAG ACATAAGTAT CATTTACTTT TATTCATAGC TAAAAAATAA TTTGGATTTT   
  
  
- AGAAGACAGT TATTAAAAAG AAGGCTATTT TTTTTTTAAC CTGGTATAAT TTAATTGTAA TTATTTAAGT   
  
  
- ATATTAATGA AACTTACAGA CTTTATATTA CGAGAAGCCA TATAATTCCA GAATATTTAA AGAGTAATTA   
  
  
- AACTTTAGAG AATATTATAT AACCCAGTTG TTCACGATCG AACAAAAAAA AAGGACCAAT TAACGTAAAT   
  
  
- TTTTTTATTA AAAATTAATG TTAACGTTCC CCTCCTTAGT AAATGTTTTA TAGTTTTCAC CTTTTCCGTT   
  
  
- TTTCTTTGTT TAATATTTTG TCTTTTTTCT AATCTTTTGC GTTTGTTTCT GCTTTACTCC TTGTCATTCT   
  
  
- TAGTCATTTT TTTACATCAC GTTGCCAGTG TATGCCCTTC CGTTCGTGTT GTTAGTACTT TCTCTGTCCG   
  
  
- TGCACGCAAG GAGCACTAGT CGTCGGAAGG GATGAAGGGG CGGTTCACCT CAGGCATGAT TGAGGACGGT   
  
  
- AAGGGAGGGT TGCCAGTATA AGGCGGCTGT ACGGAGTTGC CAGTCTAAAG GACGGGGTAG ACCTGGCAGG   
  
  
- CTAGTTCTAG GTTGCCGGTA TTCCGCGTGA GAGGTTATGG GATCACGGGT AAAGACCTTG TCGGGGTTTT   
  
  
- TAGAGTCATA AGCGGTAGTA AAAAGTTATG ACCCCAATGT ATTTGTCAGA TTGTTCCTTC CTGGGTTGTG   
  
  
- TCTCCTCTCT CTTTTTTCGA TCGAAACTAC CCCTGTCGGT ACCTACCTCC ACTTCTCTCT CCTCCAACCC   
  
  
- TGGAAAATAA AACGAGTGAA AGGTAAGAAT ATATTTTTGT CGAGATCAAA TAGAGAATAC CGAAAAAACC   
  
  
- CAAACAAGTA TCTCTCCCCC CTTACACTAT ACTAAACTAA ACTACAATCT TTAAGAGTAA TCAAAAAAGA   
  
  
- AAAACAAACG TTAAAACTAC ACAATCCCAA ACTCCTAGTA GTAAAAACTC ATCCCTTGAA AGACCCAAAA   
  
  
- CGAACGACTA ACTAACGGTT ACGATACCTA AAGAACCACT CAGTTAGAAG TTAACGACTA GACCCAAGCA   
  
  
- AACAAGAAGT CTCAAAGTGG ACGGTTAAAC TAGTTCATAC AGGGAAGTGA AGAGGACGAA GTTAAGAGAG   
  
  
- AGTTTAAGTG CGTAAAAAGG TTACTTAAAG TTTATCCTCA GGACCTTAGG CTACTATAAA ATCCAAAAAA   
  
  
- GGACTGAAGT AACGTAATCA AACAAGTATA GACACTGGTT AAGAAAGTAA ACAATATACT AGACTAACAA   
  
  
- ATCAATCGAT AAGTAAGTAA CGTATATGTA ACCAAATAAG TAACGTCTCT GTGAGTGTAT GTGTGTGTGT   
  
  
- ATGTCCCCTG TGATCATAAA AAAGAGAGAT TGAGTAGCCT AAGGGAAACT ATGAGACAAA AAACCCCCCA   
  
  
- ACTAAACTAA ACTAAACTAA CTGGAATTAA AACTTGAGTG TGTGTGAGCG TGTATCTATA TAAGAAAGAC   
  
  
- AAACACTGTA TATGTCGTAG TATATCTTTA TATAACGTGA CGTATAAACT CGAGTTCTGT CCCTATAGTA   
  
  
- GTGTTTGACT CACACTCCCT CCGTTTTTTG TCTCCTTTTC TTCCCTTAAC CCTCACATTA GTTTTGTTCT   
  
  
- CTCTCTAACT CCTCTCTCCT TTTTTGTTTT AACCCTCTTC CAATTATCTC TTCTTACAAC CGACCCACAA   
  
  
- GTAGGTGTAA CGACAGTGGT TCCATATCTA ACTCCTCACT TCGATGTAGT GTCAAGGTTC GGACGGTAGT   
  
  
- AGGTTACTCG TGTGTCTCTT AACTAAACGG GACGTGAAAG GGTTCTTTCC TACAAAGCTT CGGTGTTAGC   
  
  
- CAAGCGGGGC AACCGGATAG ACAGCTCTTC GGCCAACTTC AGTCCCGGGA CTTCGTCTGG TAGGCTCAGG   
  
  
- GTGGTAGCGG CCTCCCATCT TCTCTCTAAT TCCGTCTAAA GACCCCACTA TCTTCTTTCT CGAACTTCTC   
  
  
- CTCTGATCGT CTTGTCCCCT CGAGAAAACT ACTACCACCA CTGCTTAGAA CACAACCCTC TTGGTTTTCC   
  
  
- TTCTTCCCAT CGAAACTAAA ACCACTTAGA CTACTTTAAA GAGGTTTTTA ACTCCTGTGT TACTCAAAAC   
  
  
- CTGTAGACCC ATCACCACCT TTAAAGACCC AAGTAGGACC AAAACCACAA CAATTACAAT TATGATTAAT   
  
  
- GGGAGTTCAC GGTAAAAGAG AGTGAACAAG TCCCCTCCTT CTTTCTCATA CAAAACAAGG ATGACTACAC   
  
  
- TAGAGTGGTC GGTAGTACGG AGGTAACAGT GTGTTAGGAA CCCAACTTAG ACCACAATGA CTCCAGTGTC   
  
  
- TTATACCACC CCCACTGTTT CTGCCGCTTG GATCAGTACC CAAACACTCC CCATGTTGTT GTTGCAGGCC   
  
  
- CAGTTCAAGT AGAAGACTCT CAGTATCAAA ACCCGTATCC GAGTTACTCT CGAGTAGACT GGGACTTTAG   
  
  
- TCTTTACCAA GGTACAACGG ATTGGGACCA GTACCAGGAC CGTGGTCAGT ACCAGGACCT TGGCCAGACC   
  
  
- GAGTGTTGGT GTTACTAGTT TGGCTCGTTG TTCCCAAGCT CGAGTAATCG AAAGAGTACC CAACACAGCT   
  
  
- TCGCTAGTCA AGTTCCTTGT AACGTCGGTA GTTAGTAAAG TATCGATTCG ACCCGCTCGT CCGAAGAGGT   
  
  
- TCCCGAGCCA GACGGTAATC GGCGGAGTGT CGGATGAAGT GGCTTCGTAA TCGGAACTCC CAGTGTTCTA   
  
  
- AAACCGGAGT ATAGAAAGTG TATTCATAGG GAGCTCTCAA ACTAGCTAAA CTACTACTTA GCCCCCGTCG   
  
  
- ACGTTACTCC GAAAACTTAG TCTAGTCGGG TTAAGGTTTC AAGCAAGTAA AATGTCGGTT ACTCTACAAT   
  
  
- AACTCCCGTA AACTTCCCTT CCTGTTCCAC GTATAGTATC TGAAGCTGTA GTTCGTTCCG AATGTTACCG   
  
  
- GGCCCAAAAA GGTTTCGAAT CGGTACTCCC TTTTGGGGGG CTCGGTACAG TCCTATTGTC CGCATCCCCT   
  
  
- AAGCTTCGTT CTTAACCAAC TCTGTCCTCT TTCTGATCGG CCCAAACGAC CCCGTAAGTT GGAGAGGAAG   
  
  
- CTCAAGGTGG GTCAACACCT GGCCAACCTT CTACAATCCG ATACCTACGA AGTACAATTC CTTCCACTTT   
  
  
- CACAGCGCCA TTTAACGTAA AACGTCGAGG TGTTCTGTGA GATACTGGGG GTACCACGGT GTAAGTCCCT   
  
  
- GAAGTACCCA AATTAGGCTT CGTGTTTAGG GTATCGTAAC CAATACCGAC TCGTTCTTCG ACTAGTGTTG   
  
  
- CTTGGGTGGA ATCTTCCGGC TCATACGTTG AGTGAACCTA TGATACGTCG GGAAAAGCTA CGGTATCTAA   
  
  
- CGTCGGAAGG AAAGCTCTCG AGTCGTTCCA ACTTCTAGCT TCTCTACAAA CCGGCCCTTT AGTCCTTGTA   
  
  
- TTATCGGACA CTTCCCCGTC TGTCCTAGCT TTCTGTACTC TTCAAGCTCT TTACCTTCTC TTCCTAGCTC   
  
  
- GTCCTTCCCA AGGCCACGTA CGCCTGATCG CTTTCCCTCC ACTACGTCTC GGTTTACGAC GAATTCTACA   
  
  
- TGAGCTTGCT CTCGATGTTG TACGTTTCTG TTCCGTTACT TCTTCGTTAT AGTGATCATA CCAATCTAGT   
  
  
- CGGTGAGATG TGACAAAGAA ACACCCGAGG TTCACTTAAA CGACGTCCCT CAAGAAGGTC AAAAAGAGAC   
  
  
- GGTAGAAC

+     MYB

| Site Name | Organism | Position | Strand | Matrix score. | sequence | function |
| --- | --- | --- | --- | --- | --- | --- |
| MYB | Arabidopsis thaliana | 2879 | + | 6 | CAACCA |  |
| MYB | Arabidopsis thaliana | 549 | - | 6 | TAACCA |  |
| MYB | Arabidopsis thaliana | 3984 | - | 6 | TAACCA |  |
| MYB | Arabidopsis thaliana | 3379 | - | 6 | CAACCA |  |
| MYB | Arabidopsis thaliana | 3613 | - | 6 | TAACCA |  |

>HU04G00148.1   
+ -Up\_Stream \_Len000ATAATG GGTGATAACT TTTGAAGGAC AATATACAAA TCACTTATTT ATTAAAGTCA   
  
  
+ AGGGGACATT TCAACTATAA CATCATTGTT TATAAAGTAT TTGAAAATTG CAACTCTTGT AATAGATAAT   
  
  
+ AATGCTTTAT AGACTTCACC AATCACTAAT TTGTAGAATC AAAAAATTAA TTGAAAAGCA AACAAAAGGA   
  
  
+ AAAGGCACAT AAACCTAAAT TATGTAAACA AATGAGAACA TATATGCCAG CTTATCAACG TTAATTATAT   
  
  
+ TTGTTATCAC TCTTAATATC TGTATTCATA GTAAATGAAA ATAAGTATCG ATTTTTTATT AAACCTAAAA   
  
  
+ TCTTCTGTCA ATAATTTTTC TTCCGATAAA AAAAAAATTG GACCATATTA AATTAACATT AATAAATTCA   
  
  
+ TATAATTACT TTGAATGTCT GAAATATAAT GCTCTTCGGT ATATTAAGGT CTTATAAATT TCTCATTAAT   
  
  
+ TTGAAATCTC TTATAATATA TTGGGTCAAC AAGTGCTAGC TTGTTTTTTT TTCCTGGTTA ATTGCATTTA   
  
  
+ AAAAAATAAT TTTTAATTAC AATTGCAAGG GGAGGAATCA TTTACAAAAT ATCAAAAGTG GAAAAGGCAA   
  
  
+ AAAGAAACAA ATTATAAAAC AGAAAAAAGA TTAGAAAACG CAAACAAAGA CGAAATGAGG AACAGTAAGA   
  
  
+ ATCAGTAAAA AAATGTAGTG CAACGGTCAC ATACGGGAAG GCAAGCACAA CAATCATGAA AGAGACAGGC   
  
  
+ ACGTGCGTTC CTCGTGATCA GCAGCCTTCC CTACTTCCCC GCCAAGTGGA GTCCGTACTA ACTCCTGCCA   
  
  
+ TTCCCTCCCA ACGGTCATAT TCCGCCGACA TGCCTCAACG GTCAGATTTC CTGCCCCATC TGGACCGTCC   
  
  
+ GATCAAGATC CAACGGCCAT AAGGCGCACT CTCCAATACC CTAGTGCCCA TTTCTGGAAC AGCCCCAAAA   
  
  
+ ATCTCAGTAT TCGCCATCAT TTTTCAATAC TGGGGTTACA TAAACAGTCT AACAAGGAAG GACCCAACAC   
  
  
+ AGAGGAGAGA GAAAAAAGCT AGCTTTGATG GGGACAGCCA TGGATGGAGG TGAAGAGAGA GGAGGTTGGG   
  
  
+ ACCTTTTATT TTGCTCACTT TCCATTCTTA TATAAAAACA GCTCTAGTTT ATCTCTTATG GCTTTTTTGG   
  
  
+ GTTTGTTCAT AGAGAGGGGG GAATGTGATA TGATTTGATT TGATGTTAGA AATTCTCATT AGTTTTTTCT   
  
  
+ TTTTGTTTGC AATTTTGATG TGTTAGGGTT TGAGGATCAT CATTTTTGAG TAGGGAACTT TCTGGGTTTT   
  
  
+ GCTTGCTGAT TGATTGCCAA TGCTATGGAT TTCTTGGTGA GTCAATCTTC AATTGCTGAT CTGGGTTCGT   
  
  
+ TTGTTCTTCA GAGTTTCACC TGCCAATTTG ATCAAGTATG TCCCTTCACT TCTCCTGCTT CAATTCTCTC   
  
  
+ TCAAATTCAC GCATTTTTCC AATGAATTTC AAATAGGAGT CCTGGAATCC GATGATATTT TAGGTTTTTT   
  
  
+ CCTGACTTCA TTGCATTAGT TTGTTCATAT CTGTGACCAA TTCTTTCATT TGTTATATGA TCTGATTGTT   
  
  
+ TAGTTAGCTA TTCATTCATT GCATATACAT TGGTTTATTC ATTGCAGAGA CACTCACATA CACACACACA   
  
  
+ TACAGGGGAC ACTAGTATTT TTTCTCTCTA ACTCATCGGA TTCCCTTTGA TACTCTGTTT TTTGGGGGGT   
  
  
+ TGATTTGATT TGATTTGATT GACCTTAATT TTGAACTCAC ACACACTCGC ACATAGATAT ATTCTTTCTG   
  
  
+ TTTGTGACAT ATACAGCATC ATATAGAAAT ATATTGCACT GCATATTTGA GCTCAAGACA GGGATATCAT   
  
  
+ CACAAACTGA GTGTGAGGGA GGCAAAAAAC AGAGGAAAAG AAGGGAATTG GGAGTGTAAT CAAAACAAGA   
  
  
+ GAGAGATTGA GGAGAGAGGA AAAAACAAAA TTGGGAGAAG GTTAATAGAG AAGAATGTTG GCTGGGTGTT   
  
  
+ CATCCACATT GCTGTCACCA AGGTATAGAT TGAGGAGTGA AGCTACATCA CAGTTCCAAG CCTGCCATCA   
  
  
+ TCCAATGAGC ACACAGAGAA TTGATTTGCC CTGCACTTTC CCAAGAAAGG ATGTTTCGAA GCCACAATCG   
  
  
+ GTTCGCCCCG TTGGCCTATC TGTCGAGAAG CCGGTTGAAG TCAGGGCCCT GAAGCAGACC ATCCGAGTCC   
  
  
+ CACCATCGCC GGAGGGTAGA AGAGAGATTA AGGCAGATTT CTGGGGTGAT AGAAGAAAGA GCTTGAAGAG   
  
  
+ GAGACTAGCA GAACAGGGGA GCTCTTTTGA TGATGGTGGT GACGAATCTT GTGTTGGGAG AACCAAAAGG   
  
  
+ AAGAAGGGTA GCTTTGATTT TGGTGAATCT GATGAAATTT CTCCAAAAAT TGAGGACACA ATGAGTTTTG   
  
  
+ GACATCTGGG TAGTGGTGGA AATTTCTGGG TTCATCCTGG TTTTGGTGTT GTTAATGTTA ATACTAATTA   
  
  
+ CCCTCAAGTG CCATTTTCTC TCACTTGTTC AGGGGAGGAA GAAAGAGTAT GTTTTGTTCC TACTGATGTG   
  
  
+ ATCTCACCAG CCATCATGCC TCCATTGTCA CACAATCCTT GGGTTGAATC TGGTGTTACT GAGGTCACAG   
  
  
+ AATATGGTGG GGGTGACAAA GACGGCGAAC CTAGTCATGG GTTTGTGAGG GGTACAACAA CAACGTCCGG   
  
  
+ GTCAAGTTCA TCTTCTGAGA GTCATAGTTT TGGGCATAGG CTCAATGAGA GCTCATCTGA CCCTGAAATC   
  
  
+ AGAAATGGTT CCATGTTGCC TAACCCTGGT CATGGTCCTG GCACCAGTCA TGGTCCTGGA ACCGGTCTGG   
  
  
+ CTCACAACCA CAATGATCAA ACCGAGCAAC AAGGGTTCGA GCTCATTAGC TTTCTCATGG GTTGTGTCGA   
  
  
+ AGCGATCAGT TCAAGGAACA TTGCAGCCAT CAATCATTTC ATAGCTAAGC TGGGCGAGCA GGCTTCTCCA   
  
  
+ AGGGCTCGGT CTGCCATTAG CCGCCTCACA GCCTACTTCA CCGAAGCATT AGCCTTGAGG GTCACAAGAT   
  
  
+ TTTGGCCTCA TATCTTTCAC ATAAGTATCC CTCGAGAGTT TGATCGATTT GATGATGAAT CGGGGGCAGC   
  
  
+ TGCAATGAGG CTTTTGAATC AGATCAGCCC AATTCCAAAG TTCGTTCATT TTACAGCCAA TGAGATGTTA   
  
  
+ TTGAGGGCAT TTGAAGGGAA GGACAAGGTG CATATCATAG ACTTCGACAT CAAGCAAGGC TTACAATGGC   
  
  
+ CCGGGTTTTT CCAAAGCTTA GCCATGAGGG AAAACCCCCC GAGCCATGTC AGGATAACAG GCGTAGGGGA   
  
  
+ TTCGAAGCAA GAATTGGTTG AGACAGGAGA AAGACTAGCC GGGTTTGCTG GGGCATTCAA CCTCTCCTTC   
  
  
+ GAGTTCCACC CAGTTGTGGA CCGGTTGGAA GATGTTAGGC TATGGATGCT TCATGTTAAG GAAGGTGAAA   
  
  
+ GTGTCGCGGT AAATTGCATT TTGCAGCTCC ACAAGACACT CTATGACCCC CATGGTGCCA CATTCAGGGA   
  
  
+ CTTCATGGGT TTAATCCGAA GCACAAATCC CATAGCATTG GTTATGGCTG AGCAAGAAGC TGATCACAAC   
  
  
+ GAACCCACCT TAGAAGGCCG AGTATGCAAC TCACTTGGAT ACTATGCAGC CCTTTTCGAT GCCATAGATT   
  
  
+ GCAGCCTTCC TTTCGAGAGC TCAGCAAGGT TGAAGATCGA AGAGATGTTT GGCCGGGAAA TCAGGAACAT   
  
  
+ AATAGCCTGT GAAGGGGCAG ACAGGATCGA AAGACATGAG AAGTTCGAGA AATGGAAGAG AAGGATCGAG   
  
  
+ CAGGAAGGGT TCCGGTGCAT GCGGACTAGC GAAAGGGAGG TGATGCAGAG CCAAATGCTG CTTAAGATGT   
  
  
+ ACTCGAACGA GAGCTACAAC ATGCAAAGAC AAGGCAATGA AGAAGCAATA TCACTAGTAT GGTTAGATCA   
  
  
+ GCCACTCTAC ACTGTTTCTT TGTGGGCTCC AAGTGAATTT GCTGCAGGGA GTTCTTCCAG TTTTTCTCTG   
  
  
+ CCATCTTG  

- -Up\_Stream \_Len000TATTAC CCACTATTGA AAACTTCCTG TTATATGTTT AGTGAATAAA TAATTTCAGT   
  
  
- TCCCCTGTAA AGTTGATATT GTAGTAACAA ATATTTCATA AACTTTTAAC GTTGAGAACA TTATCTATTA   
  
  
- TTACGAAATA TCTGAAGTGG TTAGTGATTA AACATCTTAG TTTTTTAATT AACTTTTCGT TTGTTTTCCT   
  
  
- TTTCCGTGTA TTTGGATTTA ATACATTTGT TTACTCTTGT ATATACGGTC GAATAGTTGC AATTAATATA   
  
  
- AACAATAGTG AGAATTATAG ACATAAGTAT CATTTACTTT TATTCATAGC TAAAAAATAA TTTGGATTTT   
  
  
- AGAAGACAGT TATTAAAAAG AAGGCTATTT TTTTTTTAAC CTGGTATAAT TTAATTGTAA TTATTTAAGT   
  
  
- ATATTAATGA AACTTACAGA CTTTATATTA CGAGAAGCCA TATAATTCCA GAATATTTAA AGAGTAATTA   
  
  
- AACTTTAGAG AATATTATAT AACCCAGTTG TTCACGATCG AACAAAAAAA AAGGACCAAT TAACGTAAAT   
  
  
- TTTTTTATTA AAAATTAATG TTAACGTTCC CCTCCTTAGT AAATGTTTTA TAGTTTTCAC CTTTTCCGTT   
  
  
- TTTCTTTGTT TAATATTTTG TCTTTTTTCT AATCTTTTGC GTTTGTTTCT GCTTTACTCC TTGTCATTCT   
  
  
- TAGTCATTTT TTTACATCAC GTTGCCAGTG TATGCCCTTC CGTTCGTGTT GTTAGTACTT TCTCTGTCCG   
  
  
- TGCACGCAAG GAGCACTAGT CGTCGGAAGG GATGAAGGGG CGGTTCACCT CAGGCATGAT TGAGGACGGT   
  
  
- AAGGGAGGGT TGCCAGTATA AGGCGGCTGT ACGGAGTTGC CAGTCTAAAG GACGGGGTAG ACCTGGCAGG   
  
  
- CTAGTTCTAG GTTGCCGGTA TTCCGCGTGA GAGGTTATGG GATCACGGGT AAAGACCTTG TCGGGGTTTT   
  
  
- TAGAGTCATA AGCGGTAGTA AAAAGTTATG ACCCCAATGT ATTTGTCAGA TTGTTCCTTC CTGGGTTGTG   
  
  
- TCTCCTCTCT CTTTTTTCGA TCGAAACTAC CCCTGTCGGT ACCTACCTCC ACTTCTCTCT CCTCCAACCC   
  
  
- TGGAAAATAA AACGAGTGAA AGGTAAGAAT ATATTTTTGT CGAGATCAAA TAGAGAATAC CGAAAAAACC   
  
  
- CAAACAAGTA TCTCTCCCCC CTTACACTAT ACTAAACTAA ACTACAATCT TTAAGAGTAA TCAAAAAAGA   
  
  
- AAAACAAACG TTAAAACTAC ACAATCCCAA ACTCCTAGTA GTAAAAACTC ATCCCTTGAA AGACCCAAAA   
  
  
- CGAACGACTA ACTAACGGTT ACGATACCTA AAGAACCACT CAGTTAGAAG TTAACGACTA GACCCAAGCA   
  
  
- AACAAGAAGT CTCAAAGTGG ACGGTTAAAC TAGTTCATAC AGGGAAGTGA AGAGGACGAA GTTAAGAGAG   
  
  
- AGTTTAAGTG CGTAAAAAGG TTACTTAAAG TTTATCCTCA GGACCTTAGG CTACTATAAA ATCCAAAAAA   
  
  
- GGACTGAAGT AACGTAATCA AACAAGTATA GACACTGGTT AAGAAAGTAA ACAATATACT AGACTAACAA   
  
  
- ATCAATCGAT AAGTAAGTAA CGTATATGTA ACCAAATAAG TAACGTCTCT GTGAGTGTAT GTGTGTGTGT   
  
  
- ATGTCCCCTG TGATCATAAA AAAGAGAGAT TGAGTAGCCT AAGGGAAACT ATGAGACAAA AAACCCCCCA   
  
  
- ACTAAACTAA ACTAAACTAA CTGGAATTAA AACTTGAGTG TGTGTGAGCG TGTATCTATA TAAGAAAGAC   
  
  
- AAACACTGTA TATGTCGTAG TATATCTTTA TATAACGTGA CGTATAAACT CGAGTTCTGT CCCTATAGTA   
  
  
- GTGTTTGACT CACACTCCCT CCGTTTTTTG TCTCCTTTTC TTCCCTTAAC CCTCACATTA GTTTTGTTCT   
  
  
- CTCTCTAACT CCTCTCTCCT TTTTTGTTTT AACCCTCTTC CAATTATCTC TTCTTACAAC CGACCCACAA   
  
  
- GTAGGTGTAA CGACAGTGGT TCCATATCTA ACTCCTCACT TCGATGTAGT GTCAAGGTTC GGACGGTAGT   
  
  
- AGGTTACTCG TGTGTCTCTT AACTAAACGG GACGTGAAAG GGTTCTTTCC TACAAAGCTT CGGTGTTAGC   
  
  
- CAAGCGGGGC AACCGGATAG ACAGCTCTTC GGCCAACTTC AGTCCCGGGA CTTCGTCTGG TAGGCTCAGG   
  
  
- GTGGTAGCGG CCTCCCATCT TCTCTCTAAT TCCGTCTAAA GACCCCACTA TCTTCTTTCT CGAACTTCTC   
  
  
- CTCTGATCGT CTTGTCCCCT CGAGAAAACT ACTACCACCA CTGCTTAGAA CACAACCCTC TTGGTTTTCC   
  
  
- TTCTTCCCAT CGAAACTAAA ACCACTTAGA CTACTTTAAA GAGGTTTTTA ACTCCTGTGT TACTCAAAAC   
  
  
- CTGTAGACCC ATCACCACCT TTAAAGACCC AAGTAGGACC AAAACCACAA CAATTACAAT TATGATTAAT   
  
  
- GGGAGTTCAC GGTAAAAGAG AGTGAACAAG TCCCCTCCTT CTTTCTCATA CAAAACAAGG ATGACTACAC   
  
  
- TAGAGTGGTC GGTAGTACGG AGGTAACAGT GTGTTAGGAA CCCAACTTAG ACCACAATGA CTCCAGTGTC   
  
  
- TTATACCACC CCCACTGTTT CTGCCGCTTG GATCAGTACC CAAACACTCC CCATGTTGTT GTTGCAGGCC   
  
  
- CAGTTCAAGT AGAAGACTCT CAGTATCAAA ACCCGTATCC GAGTTACTCT CGAGTAGACT GGGACTTTAG   
  
  
- TCTTTACCAA GGTACAACGG ATTGGGACCA GTACCAGGAC CGTGGTCAGT ACCAGGACCT TGGCCAGACC   
  
  
- GAGTGTTGGT GTTACTAGTT TGGCTCGTTG TTCCCAAGCT CGAGTAATCG AAAGAGTACC CAACACAGCT   
  
  
- TCGCTAGTCA AGTTCCTTGT AACGTCGGTA GTTAGTAAAG TATCGATTCG ACCCGCTCGT CCGAAGAGGT   
  
  
- TCCCGAGCCA GACGGTAATC GGCGGAGTGT CGGATGAAGT GGCTTCGTAA TCGGAACTCC CAGTGTTCTA   
  
  
- AAACCGGAGT ATAGAAAGTG TATTCATAGG GAGCTCTCAA ACTAGCTAAA CTACTACTTA GCCCCCGTCG   
  
  
- ACGTTACTCC GAAAACTTAG TCTAGTCGGG TTAAGGTTTC AAGCAAGTAA AATGTCGGTT ACTCTACAAT   
  
  
- AACTCCCGTA AACTTCCCTT CCTGTTCCAC GTATAGTATC TGAAGCTGTA GTTCGTTCCG AATGTTACCG   
  
  
- GGCCCAAAAA GGTTTCGAAT CGGTACTCCC TTTTGGGGGG CTCGGTACAG TCCTATTGTC CGCATCCCCT   
  
  
- AAGCTTCGTT CTTAACCAAC TCTGTCCTCT TTCTGATCGG CCCAAACGAC CCCGTAAGTT GGAGAGGAAG   
  
  
- CTCAAGGTGG GTCAACACCT GGCCAACCTT CTACAATCCG ATACCTACGA AGTACAATTC CTTCCACTTT   
  
  
- CACAGCGCCA TTTAACGTAA AACGTCGAGG TGTTCTGTGA GATACTGGGG GTACCACGGT GTAAGTCCCT   
  
  
- GAAGTACCCA AATTAGGCTT CGTGTTTAGG GTATCGTAAC CAATACCGAC TCGTTCTTCG ACTAGTGTTG   
  
  
- CTTGGGTGGA ATCTTCCGGC TCATACGTTG AGTGAACCTA TGATACGTCG GGAAAAGCTA CGGTATCTAA   
  
  
- CGTCGGAAGG AAAGCTCTCG AGTCGTTCCA ACTTCTAGCT TCTCTACAAA CCGGCCCTTT AGTCCTTGTA   
  
  
- TTATCGGACA CTTCCCCGTC TGTCCTAGCT TTCTGTACTC TTCAAGCTCT TTACCTTCTC TTCCTAGCTC   
  
  
- GTCCTTCCCA AGGCCACGTA CGCCTGATCG CTTTCCCTCC ACTACGTCTC GGTTTACGAC GAATTCTACA   
  
  
- TGAGCTTGCT CTCGATGTTG TACGTTTCTG TTCCGTTACT TCTTCGTTAT AGTGATCATA CCAATCTAGT   
  
  
- CGGTGAGATG TGACAAAGAA ACACCCGAGG TTCACTTAAA CGACGTCCCT CAAGAAGGTC AAAAAGAGAC   
  
  
- GGTAGAAC

+     MYB recognition site

| Site Name | Organism | Position | Strand | Matrix score. | sequence | function |
| --- | --- | --- | --- | --- | --- | --- |
| MYB recognition site | Arabidopsis thaliana | 853 | - | 6 | CCGTTG |  |
| MYB recognition site | Arabidopsis thaliana | 725 | - | 6 | CCGTTG |  |
| MYB recognition site | Arabidopsis thaliana | 925 | - | 6 | CCGTTG |  |
| MYB recognition site | Arabidopsis thaliana | 2182 | + | 6 | CCGTTG |  |
| MYB recognition site | Arabidopsis thaliana | 880 | - | 6 | CCGTTG |  |

>HU04G00148.1   
+ -Up\_Stream \_Len000ATAATG GGTGATAACT TTTGAAGGAC AATATACAAA TCACTTATTT ATTAAAGTCA   
  
  
+ AGGGGACATT TCAACTATAA CATCATTGTT TATAAAGTAT TTGAAAATTG CAACTCTTGT AATAGATAAT   
  
  
+ AATGCTTTAT AGACTTCACC AATCACTAAT TTGTAGAATC AAAAAATTAA TTGAAAAGCA AACAAAAGGA   
  
  
+ AAAGGCACAT AAACCTAAAT TATGTAAACA AATGAGAACA TATATGCCAG CTTATCAACG TTAATTATAT   
  
  
+ TTGTTATCAC TCTTAATATC TGTATTCATA GTAAATGAAA ATAAGTATCG ATTTTTTATT AAACCTAAAA   
  
  
+ TCTTCTGTCA ATAATTTTTC TTCCGATAAA AAAAAAATTG GACCATATTA AATTAACATT AATAAATTCA   
  
  
+ TATAATTACT TTGAATGTCT GAAATATAAT GCTCTTCGGT ATATTAAGGT CTTATAAATT TCTCATTAAT   
  
  
+ TTGAAATCTC TTATAATATA TTGGGTCAAC AAGTGCTAGC TTGTTTTTTT TTCCTGGTTA ATTGCATTTA   
  
  
+ AAAAAATAAT TTTTAATTAC AATTGCAAGG GGAGGAATCA TTTACAAAAT ATCAAAAGTG GAAAAGGCAA   
  
  
+ AAAGAAACAA ATTATAAAAC AGAAAAAAGA TTAGAAAACG CAAACAAAGA CGAAATGAGG AACAGTAAGA   
  
  
+ ATCAGTAAAA AAATGTAGTG CAACGGTCAC ATACGGGAAG GCAAGCACAA CAATCATGAA AGAGACAGGC   
  
  
+ ACGTGCGTTC CTCGTGATCA GCAGCCTTCC CTACTTCCCC GCCAAGTGGA GTCCGTACTA ACTCCTGCCA   
  
  
+ TTCCCTCCCA ACGGTCATAT TCCGCCGACA TGCCTCAACG GTCAGATTTC CTGCCCCATC TGGACCGTCC   
  
  
+ GATCAAGATC CAACGGCCAT AAGGCGCACT CTCCAATACC CTAGTGCCCA TTTCTGGAAC AGCCCCAAAA   
  
  
+ ATCTCAGTAT TCGCCATCAT TTTTCAATAC TGGGGTTACA TAAACAGTCT AACAAGGAAG GACCCAACAC   
  
  
+ AGAGGAGAGA GAAAAAAGCT AGCTTTGATG GGGACAGCCA TGGATGGAGG TGAAGAGAGA GGAGGTTGGG   
  
  
+ ACCTTTTATT TTGCTCACTT TCCATTCTTA TATAAAAACA GCTCTAGTTT ATCTCTTATG GCTTTTTTGG   
  
  
+ GTTTGTTCAT AGAGAGGGGG GAATGTGATA TGATTTGATT TGATGTTAGA AATTCTCATT AGTTTTTTCT   
  
  
+ TTTTGTTTGC AATTTTGATG TGTTAGGGTT TGAGGATCAT CATTTTTGAG TAGGGAACTT TCTGGGTTTT   
  
  
+ GCTTGCTGAT TGATTGCCAA TGCTATGGAT TTCTTGGTGA GTCAATCTTC AATTGCTGAT CTGGGTTCGT   
  
  
+ TTGTTCTTCA GAGTTTCACC TGCCAATTTG ATCAAGTATG TCCCTTCACT TCTCCTGCTT CAATTCTCTC   
  
  
+ TCAAATTCAC GCATTTTTCC AATGAATTTC AAATAGGAGT CCTGGAATCC GATGATATTT TAGGTTTTTT   
  
  
+ CCTGACTTCA TTGCATTAGT TTGTTCATAT CTGTGACCAA TTCTTTCATT TGTTATATGA TCTGATTGTT   
  
  
+ TAGTTAGCTA TTCATTCATT GCATATACAT TGGTTTATTC ATTGCAGAGA CACTCACATA CACACACACA   
  
  
+ TACAGGGGAC ACTAGTATTT TTTCTCTCTA ACTCATCGGA TTCCCTTTGA TACTCTGTTT TTTGGGGGGT   
  
  
+ TGATTTGATT TGATTTGATT GACCTTAATT TTGAACTCAC ACACACTCGC ACATAGATAT ATTCTTTCTG   
  
  
+ TTTGTGACAT ATACAGCATC ATATAGAAAT ATATTGCACT GCATATTTGA GCTCAAGACA GGGATATCAT   
  
  
+ CACAAACTGA GTGTGAGGGA GGCAAAAAAC AGAGGAAAAG AAGGGAATTG GGAGTGTAAT CAAAACAAGA   
  
  
+ GAGAGATTGA GGAGAGAGGA AAAAACAAAA TTGGGAGAAG GTTAATAGAG AAGAATGTTG GCTGGGTGTT   
  
  
+ CATCCACATT GCTGTCACCA AGGTATAGAT TGAGGAGTGA AGCTACATCA CAGTTCCAAG CCTGCCATCA   
  
  
+ TCCAATGAGC ACACAGAGAA TTGATTTGCC CTGCACTTTC CCAAGAAAGG ATGTTTCGAA GCCACAATCG   
  
  
+ GTTCGCCCCG TTGGCCTATC TGTCGAGAAG CCGGTTGAAG TCAGGGCCCT GAAGCAGACC ATCCGAGTCC   
  
  
+ CACCATCGCC GGAGGGTAGA AGAGAGATTA AGGCAGATTT CTGGGGTGAT AGAAGAAAGA GCTTGAAGAG   
  
  
+ GAGACTAGCA GAACAGGGGA GCTCTTTTGA TGATGGTGGT GACGAATCTT GTGTTGGGAG AACCAAAAGG   
  
  
+ AAGAAGGGTA GCTTTGATTT TGGTGAATCT GATGAAATTT CTCCAAAAAT TGAGGACACA ATGAGTTTTG   
  
  
+ GACATCTGGG TAGTGGTGGA AATTTCTGGG TTCATCCTGG TTTTGGTGTT GTTAATGTTA ATACTAATTA   
  
  
+ CCCTCAAGTG CCATTTTCTC TCACTTGTTC AGGGGAGGAA GAAAGAGTAT GTTTTGTTCC TACTGATGTG   
  
  
+ ATCTCACCAG CCATCATGCC TCCATTGTCA CACAATCCTT GGGTTGAATC TGGTGTTACT GAGGTCACAG   
  
  
+ AATATGGTGG GGGTGACAAA GACGGCGAAC CTAGTCATGG GTTTGTGAGG GGTACAACAA CAACGTCCGG   
  
  
+ GTCAAGTTCA TCTTCTGAGA GTCATAGTTT TGGGCATAGG CTCAATGAGA GCTCATCTGA CCCTGAAATC   
  
  
+ AGAAATGGTT CCATGTTGCC TAACCCTGGT CATGGTCCTG GCACCAGTCA TGGTCCTGGA ACCGGTCTGG   
  
  
+ CTCACAACCA CAATGATCAA ACCGAGCAAC AAGGGTTCGA GCTCATTAGC TTTCTCATGG GTTGTGTCGA   
  
  
+ AGCGATCAGT TCAAGGAACA TTGCAGCCAT CAATCATTTC ATAGCTAAGC TGGGCGAGCA GGCTTCTCCA   
  
  
+ AGGGCTCGGT CTGCCATTAG CCGCCTCACA GCCTACTTCA CCGAAGCATT AGCCTTGAGG GTCACAAGAT   
  
  
+ TTTGGCCTCA TATCTTTCAC ATAAGTATCC CTCGAGAGTT TGATCGATTT GATGATGAAT CGGGGGCAGC   
  
  
+ TGCAATGAGG CTTTTGAATC AGATCAGCCC AATTCCAAAG TTCGTTCATT TTACAGCCAA TGAGATGTTA   
  
  
+ TTGAGGGCAT TTGAAGGGAA GGACAAGGTG CATATCATAG ACTTCGACAT CAAGCAAGGC TTACAATGGC   
  
  
+ CCGGGTTTTT CCAAAGCTTA GCCATGAGGG AAAACCCCCC GAGCCATGTC AGGATAACAG GCGTAGGGGA   
  
  
+ TTCGAAGCAA GAATTGGTTG AGACAGGAGA AAGACTAGCC GGGTTTGCTG GGGCATTCAA CCTCTCCTTC   
  
  
+ GAGTTCCACC CAGTTGTGGA CCGGTTGGAA GATGTTAGGC TATGGATGCT TCATGTTAAG GAAGGTGAAA   
  
  
+ GTGTCGCGGT AAATTGCATT TTGCAGCTCC ACAAGACACT CTATGACCCC CATGGTGCCA CATTCAGGGA   
  
  
+ CTTCATGGGT TTAATCCGAA GCACAAATCC CATAGCATTG GTTATGGCTG AGCAAGAAGC TGATCACAAC   
  
  
+ GAACCCACCT TAGAAGGCCG AGTATGCAAC TCACTTGGAT ACTATGCAGC CCTTTTCGAT GCCATAGATT   
  
  
+ GCAGCCTTCC TTTCGAGAGC TCAGCAAGGT TGAAGATCGA AGAGATGTTT GGCCGGGAAA TCAGGAACAT   
  
  
+ AATAGCCTGT GAAGGGGCAG ACAGGATCGA AAGACATGAG AAGTTCGAGA AATGGAAGAG AAGGATCGAG   
  
  
+ CAGGAAGGGT TCCGGTGCAT GCGGACTAGC GAAAGGGAGG TGATGCAGAG CCAAATGCTG CTTAAGATGT   
  
  
+ ACTCGAACGA GAGCTACAAC ATGCAAAGAC AAGGCAATGA AGAAGCAATA TCACTAGTAT GGTTAGATCA   
  
  
+ GCCACTCTAC ACTGTTTCTT TGTGGGCTCC AAGTGAATTT GCTGCAGGGA GTTCTTCCAG TTTTTCTCTG   
  
  
+ CCATCTTG  

- -Up\_Stream \_Len000TATTAC CCACTATTGA AAACTTCCTG TTATATGTTT AGTGAATAAA TAATTTCAGT   
  
  
- TCCCCTGTAA AGTTGATATT GTAGTAACAA ATATTTCATA AACTTTTAAC GTTGAGAACA TTATCTATTA   
  
  
- TTACGAAATA TCTGAAGTGG TTAGTGATTA AACATCTTAG TTTTTTAATT AACTTTTCGT TTGTTTTCCT   
  
  
- TTTCCGTGTA TTTGGATTTA ATACATTTGT TTACTCTTGT ATATACGGTC GAATAGTTGC AATTAATATA   
  
  
- AACAATAGTG AGAATTATAG ACATAAGTAT CATTTACTTT TATTCATAGC TAAAAAATAA TTTGGATTTT   
  
  
- AGAAGACAGT TATTAAAAAG AAGGCTATTT TTTTTTTAAC CTGGTATAAT TTAATTGTAA TTATTTAAGT   
  
  
- ATATTAATGA AACTTACAGA CTTTATATTA CGAGAAGCCA TATAATTCCA GAATATTTAA AGAGTAATTA   
  
  
- AACTTTAGAG AATATTATAT AACCCAGTTG TTCACGATCG AACAAAAAAA AAGGACCAAT TAACGTAAAT   
  
  
- TTTTTTATTA AAAATTAATG TTAACGTTCC CCTCCTTAGT AAATGTTTTA TAGTTTTCAC CTTTTCCGTT   
  
  
- TTTCTTTGTT TAATATTTTG TCTTTTTTCT AATCTTTTGC GTTTGTTTCT GCTTTACTCC TTGTCATTCT   
  
  
- TAGTCATTTT TTTACATCAC GTTGCCAGTG TATGCCCTTC CGTTCGTGTT GTTAGTACTT TCTCTGTCCG   
  
  
- TGCACGCAAG GAGCACTAGT CGTCGGAAGG GATGAAGGGG CGGTTCACCT CAGGCATGAT TGAGGACGGT   
  
  
- AAGGGAGGGT TGCCAGTATA AGGCGGCTGT ACGGAGTTGC CAGTCTAAAG GACGGGGTAG ACCTGGCAGG   
  
  
- CTAGTTCTAG GTTGCCGGTA TTCCGCGTGA GAGGTTATGG GATCACGGGT AAAGACCTTG TCGGGGTTTT   
  
  
- TAGAGTCATA AGCGGTAGTA AAAAGTTATG ACCCCAATGT ATTTGTCAGA TTGTTCCTTC CTGGGTTGTG   
  
  
- TCTCCTCTCT CTTTTTTCGA TCGAAACTAC CCCTGTCGGT ACCTACCTCC ACTTCTCTCT CCTCCAACCC   
  
  
- TGGAAAATAA AACGAGTGAA AGGTAAGAAT ATATTTTTGT CGAGATCAAA TAGAGAATAC CGAAAAAACC   
  
  
- CAAACAAGTA TCTCTCCCCC CTTACACTAT ACTAAACTAA ACTACAATCT TTAAGAGTAA TCAAAAAAGA   
  
  
- AAAACAAACG TTAAAACTAC ACAATCCCAA ACTCCTAGTA GTAAAAACTC ATCCCTTGAA AGACCCAAAA   
  
  
- CGAACGACTA ACTAACGGTT ACGATACCTA AAGAACCACT CAGTTAGAAG TTAACGACTA GACCCAAGCA   
  
  
- AACAAGAAGT CTCAAAGTGG ACGGTTAAAC TAGTTCATAC AGGGAAGTGA AGAGGACGAA GTTAAGAGAG   
  
  
- AGTTTAAGTG CGTAAAAAGG TTACTTAAAG TTTATCCTCA GGACCTTAGG CTACTATAAA ATCCAAAAAA   
  
  
- GGACTGAAGT AACGTAATCA AACAAGTATA GACACTGGTT AAGAAAGTAA ACAATATACT AGACTAACAA   
  
  
- ATCAATCGAT AAGTAAGTAA CGTATATGTA ACCAAATAAG TAACGTCTCT GTGAGTGTAT GTGTGTGTGT   
  
  
- ATGTCCCCTG TGATCATAAA AAAGAGAGAT TGAGTAGCCT AAGGGAAACT ATGAGACAAA AAACCCCCCA   
  
  
- ACTAAACTAA ACTAAACTAA CTGGAATTAA AACTTGAGTG TGTGTGAGCG TGTATCTATA TAAGAAAGAC   
  
  
- AAACACTGTA TATGTCGTAG TATATCTTTA TATAACGTGA CGTATAAACT CGAGTTCTGT CCCTATAGTA   
  
  
- GTGTTTGACT CACACTCCCT CCGTTTTTTG TCTCCTTTTC TTCCCTTAAC CCTCACATTA GTTTTGTTCT   
  
  
- CTCTCTAACT CCTCTCTCCT TTTTTGTTTT AACCCTCTTC CAATTATCTC TTCTTACAAC CGACCCACAA   
  
  
- GTAGGTGTAA CGACAGTGGT TCCATATCTA ACTCCTCACT TCGATGTAGT GTCAAGGTTC GGACGGTAGT   
  
  
- AGGTTACTCG TGTGTCTCTT AACTAAACGG GACGTGAAAG GGTTCTTTCC TACAAAGCTT CGGTGTTAGC   
  
  
- CAAGCGGGGC AACCGGATAG ACAGCTCTTC GGCCAACTTC AGTCCCGGGA CTTCGTCTGG TAGGCTCAGG   
  
  
- GTGGTAGCGG CCTCCCATCT TCTCTCTAAT TCCGTCTAAA GACCCCACTA TCTTCTTTCT CGAACTTCTC   
  
  
- CTCTGATCGT CTTGTCCCCT CGAGAAAACT ACTACCACCA CTGCTTAGAA CACAACCCTC TTGGTTTTCC   
  
  
- TTCTTCCCAT CGAAACTAAA ACCACTTAGA CTACTTTAAA GAGGTTTTTA ACTCCTGTGT TACTCAAAAC   
  
  
- CTGTAGACCC ATCACCACCT TTAAAGACCC AAGTAGGACC AAAACCACAA CAATTACAAT TATGATTAAT   
  
  
- GGGAGTTCAC GGTAAAAGAG AGTGAACAAG TCCCCTCCTT CTTTCTCATA CAAAACAAGG ATGACTACAC   
  
  
- TAGAGTGGTC GGTAGTACGG AGGTAACAGT GTGTTAGGAA CCCAACTTAG ACCACAATGA CTCCAGTGTC   
  
  
- TTATACCACC CCCACTGTTT CTGCCGCTTG GATCAGTACC CAAACACTCC CCATGTTGTT GTTGCAGGCC   
  
  
- CAGTTCAAGT AGAAGACTCT CAGTATCAAA ACCCGTATCC GAGTTACTCT CGAGTAGACT GGGACTTTAG   
  
  
- TCTTTACCAA GGTACAACGG ATTGGGACCA GTACCAGGAC CGTGGTCAGT ACCAGGACCT TGGCCAGACC   
  
  
- GAGTGTTGGT GTTACTAGTT TGGCTCGTTG TTCCCAAGCT CGAGTAATCG AAAGAGTACC CAACACAGCT   
  
  
- TCGCTAGTCA AGTTCCTTGT AACGTCGGTA GTTAGTAAAG TATCGATTCG ACCCGCTCGT CCGAAGAGGT   
  
  
- TCCCGAGCCA GACGGTAATC GGCGGAGTGT CGGATGAAGT GGCTTCGTAA TCGGAACTCC CAGTGTTCTA   
  
  
- AAACCGGAGT ATAGAAAGTG TATTCATAGG GAGCTCTCAA ACTAGCTAAA CTACTACTTA GCCCCCGTCG   
  
  
- ACGTTACTCC GAAAACTTAG TCTAGTCGGG TTAAGGTTTC AAGCAAGTAA AATGTCGGTT ACTCTACAAT   
  
  
- AACTCCCGTA AACTTCCCTT CCTGTTCCAC GTATAGTATC TGAAGCTGTA GTTCGTTCCG AATGTTACCG   
  
  
- GGCCCAAAAA GGTTTCGAAT CGGTACTCCC TTTTGGGGGG CTCGGTACAG TCCTATTGTC CGCATCCCCT   
  
  
- AAGCTTCGTT CTTAACCAAC TCTGTCCTCT TTCTGATCGG CCCAAACGAC CCCGTAAGTT GGAGAGGAAG   
  
  
- CTCAAGGTGG GTCAACACCT GGCCAACCTT CTACAATCCG ATACCTACGA AGTACAATTC CTTCCACTTT   
  
  
- CACAGCGCCA TTTAACGTAA AACGTCGAGG TGTTCTGTGA GATACTGGGG GTACCACGGT GTAAGTCCCT   
  
  
- GAAGTACCCA AATTAGGCTT CGTGTTTAGG GTATCGTAAC CAATACCGAC TCGTTCTTCG ACTAGTGTTG   
  
  
- CTTGGGTGGA ATCTTCCGGC TCATACGTTG AGTGAACCTA TGATACGTCG GGAAAAGCTA CGGTATCTAA   
  
  
- CGTCGGAAGG AAAGCTCTCG AGTCGTTCCA ACTTCTAGCT TCTCTACAAA CCGGCCCTTT AGTCCTTGTA   
  
  
- TTATCGGACA CTTCCCCGTC TGTCCTAGCT TTCTGTACTC TTCAAGCTCT TTACCTTCTC TTCCTAGCTC   
  
  
- GTCCTTCCCA AGGCCACGTA CGCCTGATCG CTTTCCCTCC ACTACGTCTC GGTTTACGAC GAATTCTACA   
  
  
- TGAGCTTGCT CTCGATGTTG TACGTTTCTG TTCCGTTACT TCTTCGTTAT AGTGATCATA CCAATCTAGT   
  
  
- CGGTGAGATG TGACAAAGAA ACACCCGAGG TTCACTTAAA CGACGTCCCT CAAGAAGGTC AAAAAGAGAC   
  
  
- GGTAGAAC

+     MYB-like sequence

| Site Name | Organism | Position | Strand | Matrix score. | sequence | function |
| --- | --- | --- | --- | --- | --- | --- |
| MYB-like sequence | Arabidopsis thaliana | 3984 | - | 6 | TAACCA |  |
| MYB-like sequence | Arabidopsis thaliana | 3613 | - | 6 | TAACCA |  |
| MYB-like sequence | Arabidopsis thaliana | 549 | - | 6 | TAACCA |  |

>HU04G00148.1   
+ -Up\_Stream \_Len000ATAATG GGTGATAACT TTTGAAGGAC AATATACAAA TCACTTATTT ATTAAAGTCA   
  
  
+ AGGGGACATT TCAACTATAA CATCATTGTT TATAAAGTAT TTGAAAATTG CAACTCTTGT AATAGATAAT   
  
  
+ AATGCTTTAT AGACTTCACC AATCACTAAT TTGTAGAATC AAAAAATTAA TTGAAAAGCA AACAAAAGGA   
  
  
+ AAAGGCACAT AAACCTAAAT TATGTAAACA AATGAGAACA TATATGCCAG CTTATCAACG TTAATTATAT   
  
  
+ TTGTTATCAC TCTTAATATC TGTATTCATA GTAAATGAAA ATAAGTATCG ATTTTTTATT AAACCTAAAA   
  
  
+ TCTTCTGTCA ATAATTTTTC TTCCGATAAA AAAAAAATTG GACCATATTA AATTAACATT AATAAATTCA   
  
  
+ TATAATTACT TTGAATGTCT GAAATATAAT GCTCTTCGGT ATATTAAGGT CTTATAAATT TCTCATTAAT   
  
  
+ TTGAAATCTC TTATAATATA TTGGGTCAAC AAGTGCTAGC TTGTTTTTTT TTCCTGGTTA ATTGCATTTA   
  
  
+ AAAAAATAAT TTTTAATTAC AATTGCAAGG GGAGGAATCA TTTACAAAAT ATCAAAAGTG GAAAAGGCAA   
  
  
+ AAAGAAACAA ATTATAAAAC AGAAAAAAGA TTAGAAAACG CAAACAAAGA CGAAATGAGG AACAGTAAGA   
  
  
+ ATCAGTAAAA AAATGTAGTG CAACGGTCAC ATACGGGAAG GCAAGCACAA CAATCATGAA AGAGACAGGC   
  
  
+ ACGTGCGTTC CTCGTGATCA GCAGCCTTCC CTACTTCCCC GCCAAGTGGA GTCCGTACTA ACTCCTGCCA   
  
  
+ TTCCCTCCCA ACGGTCATAT TCCGCCGACA TGCCTCAACG GTCAGATTTC CTGCCCCATC TGGACCGTCC   
  
  
+ GATCAAGATC CAACGGCCAT AAGGCGCACT CTCCAATACC CTAGTGCCCA TTTCTGGAAC AGCCCCAAAA   
  
  
+ ATCTCAGTAT TCGCCATCAT TTTTCAATAC TGGGGTTACA TAAACAGTCT AACAAGGAAG GACCCAACAC   
  
  
+ AGAGGAGAGA GAAAAAAGCT AGCTTTGATG GGGACAGCCA TGGATGGAGG TGAAGAGAGA GGAGGTTGGG   
  
  
+ ACCTTTTATT TTGCTCACTT TCCATTCTTA TATAAAAACA GCTCTAGTTT ATCTCTTATG GCTTTTTTGG   
  
  
+ GTTTGTTCAT AGAGAGGGGG GAATGTGATA TGATTTGATT TGATGTTAGA AATTCTCATT AGTTTTTTCT   
  
  
+ TTTTGTTTGC AATTTTGATG TGTTAGGGTT TGAGGATCAT CATTTTTGAG TAGGGAACTT TCTGGGTTTT   
  
  
+ GCTTGCTGAT TGATTGCCAA TGCTATGGAT TTCTTGGTGA GTCAATCTTC AATTGCTGAT CTGGGTTCGT   
  
  
+ TTGTTCTTCA GAGTTTCACC TGCCAATTTG ATCAAGTATG TCCCTTCACT TCTCCTGCTT CAATTCTCTC   
  
  
+ TCAAATTCAC GCATTTTTCC AATGAATTTC AAATAGGAGT CCTGGAATCC GATGATATTT TAGGTTTTTT   
  
  
+ CCTGACTTCA TTGCATTAGT TTGTTCATAT CTGTGACCAA TTCTTTCATT TGTTATATGA TCTGATTGTT   
  
  
+ TAGTTAGCTA TTCATTCATT GCATATACAT TGGTTTATTC ATTGCAGAGA CACTCACATA CACACACACA   
  
  
+ TACAGGGGAC ACTAGTATTT TTTCTCTCTA ACTCATCGGA TTCCCTTTGA TACTCTGTTT TTTGGGGGGT   
  
  
+ TGATTTGATT TGATTTGATT GACCTTAATT TTGAACTCAC ACACACTCGC ACATAGATAT ATTCTTTCTG   
  
  
+ TTTGTGACAT ATACAGCATC ATATAGAAAT ATATTGCACT GCATATTTGA GCTCAAGACA GGGATATCAT   
  
  
+ CACAAACTGA GTGTGAGGGA GGCAAAAAAC AGAGGAAAAG AAGGGAATTG GGAGTGTAAT CAAAACAAGA   
  
  
+ GAGAGATTGA GGAGAGAGGA AAAAACAAAA TTGGGAGAAG GTTAATAGAG AAGAATGTTG GCTGGGTGTT   
  
  
+ CATCCACATT GCTGTCACCA AGGTATAGAT TGAGGAGTGA AGCTACATCA CAGTTCCAAG CCTGCCATCA   
  
  
+ TCCAATGAGC ACACAGAGAA TTGATTTGCC CTGCACTTTC CCAAGAAAGG ATGTTTCGAA GCCACAATCG   
  
  
+ GTTCGCCCCG TTGGCCTATC TGTCGAGAAG CCGGTTGAAG TCAGGGCCCT GAAGCAGACC ATCCGAGTCC   
  
  
+ CACCATCGCC GGAGGGTAGA AGAGAGATTA AGGCAGATTT CTGGGGTGAT AGAAGAAAGA GCTTGAAGAG   
  
  
+ GAGACTAGCA GAACAGGGGA GCTCTTTTGA TGATGGTGGT GACGAATCTT GTGTTGGGAG AACCAAAAGG   
  
  
+ AAGAAGGGTA GCTTTGATTT TGGTGAATCT GATGAAATTT CTCCAAAAAT TGAGGACACA ATGAGTTTTG   
  
  
+ GACATCTGGG TAGTGGTGGA AATTTCTGGG TTCATCCTGG TTTTGGTGTT GTTAATGTTA ATACTAATTA   
  
  
+ CCCTCAAGTG CCATTTTCTC TCACTTGTTC AGGGGAGGAA GAAAGAGTAT GTTTTGTTCC TACTGATGTG   
  
  
+ ATCTCACCAG CCATCATGCC TCCATTGTCA CACAATCCTT GGGTTGAATC TGGTGTTACT GAGGTCACAG   
  
  
+ AATATGGTGG GGGTGACAAA GACGGCGAAC CTAGTCATGG GTTTGTGAGG GGTACAACAA CAACGTCCGG   
  
  
+ GTCAAGTTCA TCTTCTGAGA GTCATAGTTT TGGGCATAGG CTCAATGAGA GCTCATCTGA CCCTGAAATC   
  
  
+ AGAAATGGTT CCATGTTGCC TAACCCTGGT CATGGTCCTG GCACCAGTCA TGGTCCTGGA ACCGGTCTGG   
  
  
+ CTCACAACCA CAATGATCAA ACCGAGCAAC AAGGGTTCGA GCTCATTAGC TTTCTCATGG GTTGTGTCGA   
  
  
+ AGCGATCAGT TCAAGGAACA TTGCAGCCAT CAATCATTTC ATAGCTAAGC TGGGCGAGCA GGCTTCTCCA   
  
  
+ AGGGCTCGGT CTGCCATTAG CCGCCTCACA GCCTACTTCA CCGAAGCATT AGCCTTGAGG GTCACAAGAT   
  
  
+ TTTGGCCTCA TATCTTTCAC ATAAGTATCC CTCGAGAGTT TGATCGATTT GATGATGAAT CGGGGGCAGC   
  
  
+ TGCAATGAGG CTTTTGAATC AGATCAGCCC AATTCCAAAG TTCGTTCATT TTACAGCCAA TGAGATGTTA   
  
  
+ TTGAGGGCAT TTGAAGGGAA GGACAAGGTG CATATCATAG ACTTCGACAT CAAGCAAGGC TTACAATGGC   
  
  
+ CCGGGTTTTT CCAAAGCTTA GCCATGAGGG AAAACCCCCC GAGCCATGTC AGGATAACAG GCGTAGGGGA   
  
  
+ TTCGAAGCAA GAATTGGTTG AGACAGGAGA AAGACTAGCC GGGTTTGCTG GGGCATTCAA CCTCTCCTTC   
  
  
+ GAGTTCCACC CAGTTGTGGA CCGGTTGGAA GATGTTAGGC TATGGATGCT TCATGTTAAG GAAGGTGAAA   
  
  
+ GTGTCGCGGT AAATTGCATT TTGCAGCTCC ACAAGACACT CTATGACCCC CATGGTGCCA CATTCAGGGA   
  
  
+ CTTCATGGGT TTAATCCGAA GCACAAATCC CATAGCATTG GTTATGGCTG AGCAAGAAGC TGATCACAAC   
  
  
+ GAACCCACCT TAGAAGGCCG AGTATGCAAC TCACTTGGAT ACTATGCAGC CCTTTTCGAT GCCATAGATT   
  
  
+ GCAGCCTTCC TTTCGAGAGC TCAGCAAGGT TGAAGATCGA AGAGATGTTT GGCCGGGAAA TCAGGAACAT   
  
  
+ AATAGCCTGT GAAGGGGCAG ACAGGATCGA AAGACATGAG AAGTTCGAGA AATGGAAGAG AAGGATCGAG   
  
  
+ CAGGAAGGGT TCCGGTGCAT GCGGACTAGC GAAAGGGAGG TGATGCAGAG CCAAATGCTG CTTAAGATGT   
  
  
+ ACTCGAACGA GAGCTACAAC ATGCAAAGAC AAGGCAATGA AGAAGCAATA TCACTAGTAT GGTTAGATCA   
  
  
+ GCCACTCTAC ACTGTTTCTT TGTGGGCTCC AAGTGAATTT GCTGCAGGGA GTTCTTCCAG TTTTTCTCTG   
  
  
+ CCATCTTG  

- -Up\_Stream \_Len000TATTAC CCACTATTGA AAACTTCCTG TTATATGTTT AGTGAATAAA TAATTTCAGT   
  
  
- TCCCCTGTAA AGTTGATATT GTAGTAACAA ATATTTCATA AACTTTTAAC GTTGAGAACA TTATCTATTA   
  
  
- TTACGAAATA TCTGAAGTGG TTAGTGATTA AACATCTTAG TTTTTTAATT AACTTTTCGT TTGTTTTCCT   
  
  
- TTTCCGTGTA TTTGGATTTA ATACATTTGT TTACTCTTGT ATATACGGTC GAATAGTTGC AATTAATATA   
  
  
- AACAATAGTG AGAATTATAG ACATAAGTAT CATTTACTTT TATTCATAGC TAAAAAATAA TTTGGATTTT   
  
  
- AGAAGACAGT TATTAAAAAG AAGGCTATTT TTTTTTTAAC CTGGTATAAT TTAATTGTAA TTATTTAAGT   
  
  
- ATATTAATGA AACTTACAGA CTTTATATTA CGAGAAGCCA TATAATTCCA GAATATTTAA AGAGTAATTA   
  
  
- AACTTTAGAG AATATTATAT AACCCAGTTG TTCACGATCG AACAAAAAAA AAGGACCAAT TAACGTAAAT   
  
  
- TTTTTTATTA AAAATTAATG TTAACGTTCC CCTCCTTAGT AAATGTTTTA TAGTTTTCAC CTTTTCCGTT   
  
  
- TTTCTTTGTT TAATATTTTG TCTTTTTTCT AATCTTTTGC GTTTGTTTCT GCTTTACTCC TTGTCATTCT   
  
  
- TAGTCATTTT TTTACATCAC GTTGCCAGTG TATGCCCTTC CGTTCGTGTT GTTAGTACTT TCTCTGTCCG   
  
  
- TGCACGCAAG GAGCACTAGT CGTCGGAAGG GATGAAGGGG CGGTTCACCT CAGGCATGAT TGAGGACGGT   
  
  
- AAGGGAGGGT TGCCAGTATA AGGCGGCTGT ACGGAGTTGC CAGTCTAAAG GACGGGGTAG ACCTGGCAGG   
  
  
- CTAGTTCTAG GTTGCCGGTA TTCCGCGTGA GAGGTTATGG GATCACGGGT AAAGACCTTG TCGGGGTTTT   
  
  
- TAGAGTCATA AGCGGTAGTA AAAAGTTATG ACCCCAATGT ATTTGTCAGA TTGTTCCTTC CTGGGTTGTG   
  
  
- TCTCCTCTCT CTTTTTTCGA TCGAAACTAC CCCTGTCGGT ACCTACCTCC ACTTCTCTCT CCTCCAACCC   
  
  
- TGGAAAATAA AACGAGTGAA AGGTAAGAAT ATATTTTTGT CGAGATCAAA TAGAGAATAC CGAAAAAACC   
  
  
- CAAACAAGTA TCTCTCCCCC CTTACACTAT ACTAAACTAA ACTACAATCT TTAAGAGTAA TCAAAAAAGA   
  
  
- AAAACAAACG TTAAAACTAC ACAATCCCAA ACTCCTAGTA GTAAAAACTC ATCCCTTGAA AGACCCAAAA   
  
  
- CGAACGACTA ACTAACGGTT ACGATACCTA AAGAACCACT CAGTTAGAAG TTAACGACTA GACCCAAGCA   
  
  
- AACAAGAAGT CTCAAAGTGG ACGGTTAAAC TAGTTCATAC AGGGAAGTGA AGAGGACGAA GTTAAGAGAG   
  
  
- AGTTTAAGTG CGTAAAAAGG TTACTTAAAG TTTATCCTCA GGACCTTAGG CTACTATAAA ATCCAAAAAA   
  
  
- GGACTGAAGT AACGTAATCA AACAAGTATA GACACTGGTT AAGAAAGTAA ACAATATACT AGACTAACAA   
  
  
- ATCAATCGAT AAGTAAGTAA CGTATATGTA ACCAAATAAG TAACGTCTCT GTGAGTGTAT GTGTGTGTGT   
  
  
- ATGTCCCCTG TGATCATAAA AAAGAGAGAT TGAGTAGCCT AAGGGAAACT ATGAGACAAA AAACCCCCCA   
  
  
- ACTAAACTAA ACTAAACTAA CTGGAATTAA AACTTGAGTG TGTGTGAGCG TGTATCTATA TAAGAAAGAC   
  
  
- AAACACTGTA TATGTCGTAG TATATCTTTA TATAACGTGA CGTATAAACT CGAGTTCTGT CCCTATAGTA   
  
  
- GTGTTTGACT CACACTCCCT CCGTTTTTTG TCTCCTTTTC TTCCCTTAAC CCTCACATTA GTTTTGTTCT   
  
  
- CTCTCTAACT CCTCTCTCCT TTTTTGTTTT AACCCTCTTC CAATTATCTC TTCTTACAAC CGACCCACAA   
  
  
- GTAGGTGTAA CGACAGTGGT TCCATATCTA ACTCCTCACT TCGATGTAGT GTCAAGGTTC GGACGGTAGT   
  
  
- AGGTTACTCG TGTGTCTCTT AACTAAACGG GACGTGAAAG GGTTCTTTCC TACAAAGCTT CGGTGTTAGC   
  
  
- CAAGCGGGGC AACCGGATAG ACAGCTCTTC GGCCAACTTC AGTCCCGGGA CTTCGTCTGG TAGGCTCAGG   
  
  
- GTGGTAGCGG CCTCCCATCT TCTCTCTAAT TCCGTCTAAA GACCCCACTA TCTTCTTTCT CGAACTTCTC   
  
  
- CTCTGATCGT CTTGTCCCCT CGAGAAAACT ACTACCACCA CTGCTTAGAA CACAACCCTC TTGGTTTTCC   
  
  
- TTCTTCCCAT CGAAACTAAA ACCACTTAGA CTACTTTAAA GAGGTTTTTA ACTCCTGTGT TACTCAAAAC   
  
  
- CTGTAGACCC ATCACCACCT TTAAAGACCC AAGTAGGACC AAAACCACAA CAATTACAAT TATGATTAAT   
  
  
- GGGAGTTCAC GGTAAAAGAG AGTGAACAAG TCCCCTCCTT CTTTCTCATA CAAAACAAGG ATGACTACAC   
  
  
- TAGAGTGGTC GGTAGTACGG AGGTAACAGT GTGTTAGGAA CCCAACTTAG ACCACAATGA CTCCAGTGTC   
  
  
- TTATACCACC CCCACTGTTT CTGCCGCTTG GATCAGTACC CAAACACTCC CCATGTTGTT GTTGCAGGCC   
  
  
- CAGTTCAAGT AGAAGACTCT CAGTATCAAA ACCCGTATCC GAGTTACTCT CGAGTAGACT GGGACTTTAG   
  
  
- TCTTTACCAA GGTACAACGG ATTGGGACCA GTACCAGGAC CGTGGTCAGT ACCAGGACCT TGGCCAGACC   
  
  
- GAGTGTTGGT GTTACTAGTT TGGCTCGTTG TTCCCAAGCT CGAGTAATCG AAAGAGTACC CAACACAGCT   
  
  
- TCGCTAGTCA AGTTCCTTGT AACGTCGGTA GTTAGTAAAG TATCGATTCG ACCCGCTCGT CCGAAGAGGT   
  
  
- TCCCGAGCCA GACGGTAATC GGCGGAGTGT CGGATGAAGT GGCTTCGTAA TCGGAACTCC CAGTGTTCTA   
  
  
- AAACCGGAGT ATAGAAAGTG TATTCATAGG GAGCTCTCAA ACTAGCTAAA CTACTACTTA GCCCCCGTCG   
  
  
- ACGTTACTCC GAAAACTTAG TCTAGTCGGG TTAAGGTTTC AAGCAAGTAA AATGTCGGTT ACTCTACAAT   
  
  
- AACTCCCGTA AACTTCCCTT CCTGTTCCAC GTATAGTATC TGAAGCTGTA GTTCGTTCCG AATGTTACCG   
  
  
- GGCCCAAAAA GGTTTCGAAT CGGTACTCCC TTTTGGGGGG CTCGGTACAG TCCTATTGTC CGCATCCCCT   
  
  
- AAGCTTCGTT CTTAACCAAC TCTGTCCTCT TTCTGATCGG CCCAAACGAC CCCGTAAGTT GGAGAGGAAG   
  
  
- CTCAAGGTGG GTCAACACCT GGCCAACCTT CTACAATCCG ATACCTACGA AGTACAATTC CTTCCACTTT   
  
  
- CACAGCGCCA TTTAACGTAA AACGTCGAGG TGTTCTGTGA GATACTGGGG GTACCACGGT GTAAGTCCCT   
  
  
- GAAGTACCCA AATTAGGCTT CGTGTTTAGG GTATCGTAAC CAATACCGAC TCGTTCTTCG ACTAGTGTTG   
  
  
- CTTGGGTGGA ATCTTCCGGC TCATACGTTG AGTGAACCTA TGATACGTCG GGAAAAGCTA CGGTATCTAA   
  
  
- CGTCGGAAGG AAAGCTCTCG AGTCGTTCCA ACTTCTAGCT TCTCTACAAA CCGGCCCTTT AGTCCTTGTA   
  
  
- TTATCGGACA CTTCCCCGTC TGTCCTAGCT TTCTGTACTC TTCAAGCTCT TTACCTTCTC TTCCTAGCTC   
  
  
- GTCCTTCCCA AGGCCACGTA CGCCTGATCG CTTTCCCTCC ACTACGTCTC GGTTTACGAC GAATTCTACA   
  
  
- TGAGCTTGCT CTCGATGTTG TACGTTTCTG TTCCGTTACT TCTTCGTTAT AGTGATCATA CCAATCTAGT   
  
  
- CGGTGAGATG TGACAAAGAA ACACCCGAGG TTCACTTAAA CGACGTCCCT CAAGAAGGTC AAAAAGAGAC   
  
  
- GGTAGAAC

+     MYC

| Site Name | Organism | Position | Strand | Matrix score. | sequence | function |
| --- | --- | --- | --- | --- | --- | --- |
| MYC | Arabidopsis thaliana | 3906 | - | 6 | CATTTG |  |
| MYC | Arabidopsis thaliana | 1384 | + | 6 | CAATTG |  |
| MYC | Arabidopsis thaliana | 584 | + | 6 | CAATTG |  |
| MYC | Arabidopsis thaliana | 3232 | + | 6 | CATTTG |  |
| MYC | Arabidopsis thaliana | 1591 | + | 6 | CATTTG |  |
| MYC | Arabidopsis thaliana | 243 | - | 6 | CATTTG |  |

>HU04G00148.1   
+ -Up\_Stream \_Len000ATAATG GGTGATAACT TTTGAAGGAC AATATACAAA TCACTTATTT ATTAAAGTCA   
  
  
+ AGGGGACATT TCAACTATAA CATCATTGTT TATAAAGTAT TTGAAAATTG CAACTCTTGT AATAGATAAT   
  
  
+ AATGCTTTAT AGACTTCACC AATCACTAAT TTGTAGAATC AAAAAATTAA TTGAAAAGCA AACAAAAGGA   
  
  
+ AAAGGCACAT AAACCTAAAT TATGTAAACA AATGAGAACA TATATGCCAG CTTATCAACG TTAATTATAT   
  
  
+ TTGTTATCAC TCTTAATATC TGTATTCATA GTAAATGAAA ATAAGTATCG ATTTTTTATT AAACCTAAAA   
  
  
+ TCTTCTGTCA ATAATTTTTC TTCCGATAAA AAAAAAATTG GACCATATTA AATTAACATT AATAAATTCA   
  
  
+ TATAATTACT TTGAATGTCT GAAATATAAT GCTCTTCGGT ATATTAAGGT CTTATAAATT TCTCATTAAT   
  
  
+ TTGAAATCTC TTATAATATA TTGGGTCAAC AAGTGCTAGC TTGTTTTTTT TTCCTGGTTA ATTGCATTTA   
  
  
+ AAAAAATAAT TTTTAATTAC AATTGCAAGG GGAGGAATCA TTTACAAAAT ATCAAAAGTG GAAAAGGCAA   
  
  
+ AAAGAAACAA ATTATAAAAC AGAAAAAAGA TTAGAAAACG CAAACAAAGA CGAAATGAGG AACAGTAAGA   
  
  
+ ATCAGTAAAA AAATGTAGTG CAACGGTCAC ATACGGGAAG GCAAGCACAA CAATCATGAA AGAGACAGGC   
  
  
+ ACGTGCGTTC CTCGTGATCA GCAGCCTTCC CTACTTCCCC GCCAAGTGGA GTCCGTACTA ACTCCTGCCA   
  
  
+ TTCCCTCCCA ACGGTCATAT TCCGCCGACA TGCCTCAACG GTCAGATTTC CTGCCCCATC TGGACCGTCC   
  
  
+ GATCAAGATC CAACGGCCAT AAGGCGCACT CTCCAATACC CTAGTGCCCA TTTCTGGAAC AGCCCCAAAA   
  
  
+ ATCTCAGTAT TCGCCATCAT TTTTCAATAC TGGGGTTACA TAAACAGTCT AACAAGGAAG GACCCAACAC   
  
  
+ AGAGGAGAGA GAAAAAAGCT AGCTTTGATG GGGACAGCCA TGGATGGAGG TGAAGAGAGA GGAGGTTGGG   
  
  
+ ACCTTTTATT TTGCTCACTT TCCATTCTTA TATAAAAACA GCTCTAGTTT ATCTCTTATG GCTTTTTTGG   
  
  
+ GTTTGTTCAT AGAGAGGGGG GAATGTGATA TGATTTGATT TGATGTTAGA AATTCTCATT AGTTTTTTCT   
  
  
+ TTTTGTTTGC AATTTTGATG TGTTAGGGTT TGAGGATCAT CATTTTTGAG TAGGGAACTT TCTGGGTTTT   
  
  
+ GCTTGCTGAT TGATTGCCAA TGCTATGGAT TTCTTGGTGA GTCAATCTTC AATTGCTGAT CTGGGTTCGT   
  
  
+ TTGTTCTTCA GAGTTTCACC TGCCAATTTG ATCAAGTATG TCCCTTCACT TCTCCTGCTT CAATTCTCTC   
  
  
+ TCAAATTCAC GCATTTTTCC AATGAATTTC AAATAGGAGT CCTGGAATCC GATGATATTT TAGGTTTTTT   
  
  
+ CCTGACTTCA TTGCATTAGT TTGTTCATAT CTGTGACCAA TTCTTTCATT TGTTATATGA TCTGATTGTT   
  
  
+ TAGTTAGCTA TTCATTCATT GCATATACAT TGGTTTATTC ATTGCAGAGA CACTCACATA CACACACACA   
  
  
+ TACAGGGGAC ACTAGTATTT TTTCTCTCTA ACTCATCGGA TTCCCTTTGA TACTCTGTTT TTTGGGGGGT   
  
  
+ TGATTTGATT TGATTTGATT GACCTTAATT TTGAACTCAC ACACACTCGC ACATAGATAT ATTCTTTCTG   
  
  
+ TTTGTGACAT ATACAGCATC ATATAGAAAT ATATTGCACT GCATATTTGA GCTCAAGACA GGGATATCAT   
  
  
+ CACAAACTGA GTGTGAGGGA GGCAAAAAAC AGAGGAAAAG AAGGGAATTG GGAGTGTAAT CAAAACAAGA   
  
  
+ GAGAGATTGA GGAGAGAGGA AAAAACAAAA TTGGGAGAAG GTTAATAGAG AAGAATGTTG GCTGGGTGTT   
  
  
+ CATCCACATT GCTGTCACCA AGGTATAGAT TGAGGAGTGA AGCTACATCA CAGTTCCAAG CCTGCCATCA   
  
  
+ TCCAATGAGC ACACAGAGAA TTGATTTGCC CTGCACTTTC CCAAGAAAGG ATGTTTCGAA GCCACAATCG   
  
  
+ GTTCGCCCCG TTGGCCTATC TGTCGAGAAG CCGGTTGAAG TCAGGGCCCT GAAGCAGACC ATCCGAGTCC   
  
  
+ CACCATCGCC GGAGGGTAGA AGAGAGATTA AGGCAGATTT CTGGGGTGAT AGAAGAAAGA GCTTGAAGAG   
  
  
+ GAGACTAGCA GAACAGGGGA GCTCTTTTGA TGATGGTGGT GACGAATCTT GTGTTGGGAG AACCAAAAGG   
  
  
+ AAGAAGGGTA GCTTTGATTT TGGTGAATCT GATGAAATTT CTCCAAAAAT TGAGGACACA ATGAGTTTTG   
  
  
+ GACATCTGGG TAGTGGTGGA AATTTCTGGG TTCATCCTGG TTTTGGTGTT GTTAATGTTA ATACTAATTA   
  
  
+ CCCTCAAGTG CCATTTTCTC TCACTTGTTC AGGGGAGGAA GAAAGAGTAT GTTTTGTTCC TACTGATGTG   
  
  
+ ATCTCACCAG CCATCATGCC TCCATTGTCA CACAATCCTT GGGTTGAATC TGGTGTTACT GAGGTCACAG   
  
  
+ AATATGGTGG GGGTGACAAA GACGGCGAAC CTAGTCATGG GTTTGTGAGG GGTACAACAA CAACGTCCGG   
  
  
+ GTCAAGTTCA TCTTCTGAGA GTCATAGTTT TGGGCATAGG CTCAATGAGA GCTCATCTGA CCCTGAAATC   
  
  
+ AGAAATGGTT CCATGTTGCC TAACCCTGGT CATGGTCCTG GCACCAGTCA TGGTCCTGGA ACCGGTCTGG   
  
  
+ CTCACAACCA CAATGATCAA ACCGAGCAAC AAGGGTTCGA GCTCATTAGC TTTCTCATGG GTTGTGTCGA   
  
  
+ AGCGATCAGT TCAAGGAACA TTGCAGCCAT CAATCATTTC ATAGCTAAGC TGGGCGAGCA GGCTTCTCCA   
  
  
+ AGGGCTCGGT CTGCCATTAG CCGCCTCACA GCCTACTTCA CCGAAGCATT AGCCTTGAGG GTCACAAGAT   
  
  
+ TTTGGCCTCA TATCTTTCAC ATAAGTATCC CTCGAGAGTT TGATCGATTT GATGATGAAT CGGGGGCAGC   
  
  
+ TGCAATGAGG CTTTTGAATC AGATCAGCCC AATTCCAAAG TTCGTTCATT TTACAGCCAA TGAGATGTTA   
  
  
+ TTGAGGGCAT TTGAAGGGAA GGACAAGGTG CATATCATAG ACTTCGACAT CAAGCAAGGC TTACAATGGC   
  
  
+ CCGGGTTTTT CCAAAGCTTA GCCATGAGGG AAAACCCCCC GAGCCATGTC AGGATAACAG GCGTAGGGGA   
  
  
+ TTCGAAGCAA GAATTGGTTG AGACAGGAGA AAGACTAGCC GGGTTTGCTG GGGCATTCAA CCTCTCCTTC   
  
  
+ GAGTTCCACC CAGTTGTGGA CCGGTTGGAA GATGTTAGGC TATGGATGCT TCATGTTAAG GAAGGTGAAA   
  
  
+ GTGTCGCGGT AAATTGCATT TTGCAGCTCC ACAAGACACT CTATGACCCC CATGGTGCCA CATTCAGGGA   
  
  
+ CTTCATGGGT TTAATCCGAA GCACAAATCC CATAGCATTG GTTATGGCTG AGCAAGAAGC TGATCACAAC   
  
  
+ GAACCCACCT TAGAAGGCCG AGTATGCAAC TCACTTGGAT ACTATGCAGC CCTTTTCGAT GCCATAGATT   
  
  
+ GCAGCCTTCC TTTCGAGAGC TCAGCAAGGT TGAAGATCGA AGAGATGTTT GGCCGGGAAA TCAGGAACAT   
  
  
+ AATAGCCTGT GAAGGGGCAG ACAGGATCGA AAGACATGAG AAGTTCGAGA AATGGAAGAG AAGGATCGAG   
  
  
+ CAGGAAGGGT TCCGGTGCAT GCGGACTAGC GAAAGGGAGG TGATGCAGAG CCAAATGCTG CTTAAGATGT   
  
  
+ ACTCGAACGA GAGCTACAAC ATGCAAAGAC AAGGCAATGA AGAAGCAATA TCACTAGTAT GGTTAGATCA   
  
  
+ GCCACTCTAC ACTGTTTCTT TGTGGGCTCC AAGTGAATTT GCTGCAGGGA GTTCTTCCAG TTTTTCTCTG   
  
  
+ CCATCTTG  

- -Up\_Stream \_Len000TATTAC CCACTATTGA AAACTTCCTG TTATATGTTT AGTGAATAAA TAATTTCAGT   
  
  
- TCCCCTGTAA AGTTGATATT GTAGTAACAA ATATTTCATA AACTTTTAAC GTTGAGAACA TTATCTATTA   
  
  
- TTACGAAATA TCTGAAGTGG TTAGTGATTA AACATCTTAG TTTTTTAATT AACTTTTCGT TTGTTTTCCT   
  
  
- TTTCCGTGTA TTTGGATTTA ATACATTTGT TTACTCTTGT ATATACGGTC GAATAGTTGC AATTAATATA   
  
  
- AACAATAGTG AGAATTATAG ACATAAGTAT CATTTACTTT TATTCATAGC TAAAAAATAA TTTGGATTTT   
  
  
- AGAAGACAGT TATTAAAAAG AAGGCTATTT TTTTTTTAAC CTGGTATAAT TTAATTGTAA TTATTTAAGT   
  
  
- ATATTAATGA AACTTACAGA CTTTATATTA CGAGAAGCCA TATAATTCCA GAATATTTAA AGAGTAATTA   
  
  
- AACTTTAGAG AATATTATAT AACCCAGTTG TTCACGATCG AACAAAAAAA AAGGACCAAT TAACGTAAAT   
  
  
- TTTTTTATTA AAAATTAATG TTAACGTTCC CCTCCTTAGT AAATGTTTTA TAGTTTTCAC CTTTTCCGTT   
  
  
- TTTCTTTGTT TAATATTTTG TCTTTTTTCT AATCTTTTGC GTTTGTTTCT GCTTTACTCC TTGTCATTCT   
  
  
- TAGTCATTTT TTTACATCAC GTTGCCAGTG TATGCCCTTC CGTTCGTGTT GTTAGTACTT TCTCTGTCCG   
  
  
- TGCACGCAAG GAGCACTAGT CGTCGGAAGG GATGAAGGGG CGGTTCACCT CAGGCATGAT TGAGGACGGT   
  
  
- AAGGGAGGGT TGCCAGTATA AGGCGGCTGT ACGGAGTTGC CAGTCTAAAG GACGGGGTAG ACCTGGCAGG   
  
  
- CTAGTTCTAG GTTGCCGGTA TTCCGCGTGA GAGGTTATGG GATCACGGGT AAAGACCTTG TCGGGGTTTT   
  
  
- TAGAGTCATA AGCGGTAGTA AAAAGTTATG ACCCCAATGT ATTTGTCAGA TTGTTCCTTC CTGGGTTGTG   
  
  
- TCTCCTCTCT CTTTTTTCGA TCGAAACTAC CCCTGTCGGT ACCTACCTCC ACTTCTCTCT CCTCCAACCC   
  
  
- TGGAAAATAA AACGAGTGAA AGGTAAGAAT ATATTTTTGT CGAGATCAAA TAGAGAATAC CGAAAAAACC   
  
  
- CAAACAAGTA TCTCTCCCCC CTTACACTAT ACTAAACTAA ACTACAATCT TTAAGAGTAA TCAAAAAAGA   
  
  
- AAAACAAACG TTAAAACTAC ACAATCCCAA ACTCCTAGTA GTAAAAACTC ATCCCTTGAA AGACCCAAAA   
  
  
- CGAACGACTA ACTAACGGTT ACGATACCTA AAGAACCACT CAGTTAGAAG TTAACGACTA GACCCAAGCA   
  
  
- AACAAGAAGT CTCAAAGTGG ACGGTTAAAC TAGTTCATAC AGGGAAGTGA AGAGGACGAA GTTAAGAGAG   
  
  
- AGTTTAAGTG CGTAAAAAGG TTACTTAAAG TTTATCCTCA GGACCTTAGG CTACTATAAA ATCCAAAAAA   
  
  
- GGACTGAAGT AACGTAATCA AACAAGTATA GACACTGGTT AAGAAAGTAA ACAATATACT AGACTAACAA   
  
  
- ATCAATCGAT AAGTAAGTAA CGTATATGTA ACCAAATAAG TAACGTCTCT GTGAGTGTAT GTGTGTGTGT   
  
  
- ATGTCCCCTG TGATCATAAA AAAGAGAGAT TGAGTAGCCT AAGGGAAACT ATGAGACAAA AAACCCCCCA   
  
  
- ACTAAACTAA ACTAAACTAA CTGGAATTAA AACTTGAGTG TGTGTGAGCG TGTATCTATA TAAGAAAGAC   
  
  
- AAACACTGTA TATGTCGTAG TATATCTTTA TATAACGTGA CGTATAAACT CGAGTTCTGT CCCTATAGTA   
  
  
- GTGTTTGACT CACACTCCCT CCGTTTTTTG TCTCCTTTTC TTCCCTTAAC CCTCACATTA GTTTTGTTCT   
  
  
- CTCTCTAACT CCTCTCTCCT TTTTTGTTTT AACCCTCTTC CAATTATCTC TTCTTACAAC CGACCCACAA   
  
  
- GTAGGTGTAA CGACAGTGGT TCCATATCTA ACTCCTCACT TCGATGTAGT GTCAAGGTTC GGACGGTAGT   
  
  
- AGGTTACTCG TGTGTCTCTT AACTAAACGG GACGTGAAAG GGTTCTTTCC TACAAAGCTT CGGTGTTAGC   
  
  
- CAAGCGGGGC AACCGGATAG ACAGCTCTTC GGCCAACTTC AGTCCCGGGA CTTCGTCTGG TAGGCTCAGG   
  
  
- GTGGTAGCGG CCTCCCATCT TCTCTCTAAT TCCGTCTAAA GACCCCACTA TCTTCTTTCT CGAACTTCTC   
  
  
- CTCTGATCGT CTTGTCCCCT CGAGAAAACT ACTACCACCA CTGCTTAGAA CACAACCCTC TTGGTTTTCC   
  
  
- TTCTTCCCAT CGAAACTAAA ACCACTTAGA CTACTTTAAA GAGGTTTTTA ACTCCTGTGT TACTCAAAAC   
  
  
- CTGTAGACCC ATCACCACCT TTAAAGACCC AAGTAGGACC AAAACCACAA CAATTACAAT TATGATTAAT   
  
  
- GGGAGTTCAC GGTAAAAGAG AGTGAACAAG TCCCCTCCTT CTTTCTCATA CAAAACAAGG ATGACTACAC   
  
  
- TAGAGTGGTC GGTAGTACGG AGGTAACAGT GTGTTAGGAA CCCAACTTAG ACCACAATGA CTCCAGTGTC   
  
  
- TTATACCACC CCCACTGTTT CTGCCGCTTG GATCAGTACC CAAACACTCC CCATGTTGTT GTTGCAGGCC   
  
  
- CAGTTCAAGT AGAAGACTCT CAGTATCAAA ACCCGTATCC GAGTTACTCT CGAGTAGACT GGGACTTTAG   
  
  
- TCTTTACCAA GGTACAACGG ATTGGGACCA GTACCAGGAC CGTGGTCAGT ACCAGGACCT TGGCCAGACC   
  
  
- GAGTGTTGGT GTTACTAGTT TGGCTCGTTG TTCCCAAGCT CGAGTAATCG AAAGAGTACC CAACACAGCT   
  
  
- TCGCTAGTCA AGTTCCTTGT AACGTCGGTA GTTAGTAAAG TATCGATTCG ACCCGCTCGT CCGAAGAGGT   
  
  
- TCCCGAGCCA GACGGTAATC GGCGGAGTGT CGGATGAAGT GGCTTCGTAA TCGGAACTCC CAGTGTTCTA   
  
  
- AAACCGGAGT ATAGAAAGTG TATTCATAGG GAGCTCTCAA ACTAGCTAAA CTACTACTTA GCCCCCGTCG   
  
  
- ACGTTACTCC GAAAACTTAG TCTAGTCGGG TTAAGGTTTC AAGCAAGTAA AATGTCGGTT ACTCTACAAT   
  
  
- AACTCCCGTA AACTTCCCTT CCTGTTCCAC GTATAGTATC TGAAGCTGTA GTTCGTTCCG AATGTTACCG   
  
  
- GGCCCAAAAA GGTTTCGAAT CGGTACTCCC TTTTGGGGGG CTCGGTACAG TCCTATTGTC CGCATCCCCT   
  
  
- AAGCTTCGTT CTTAACCAAC TCTGTCCTCT TTCTGATCGG CCCAAACGAC CCCGTAAGTT GGAGAGGAAG   
  
  
- CTCAAGGTGG GTCAACACCT GGCCAACCTT CTACAATCCG ATACCTACGA AGTACAATTC CTTCCACTTT   
  
  
- CACAGCGCCA TTTAACGTAA AACGTCGAGG TGTTCTGTGA GATACTGGGG GTACCACGGT GTAAGTCCCT   
  
  
- GAAGTACCCA AATTAGGCTT CGTGTTTAGG GTATCGTAAC CAATACCGAC TCGTTCTTCG ACTAGTGTTG   
  
  
- CTTGGGTGGA ATCTTCCGGC TCATACGTTG AGTGAACCTA TGATACGTCG GGAAAAGCTA CGGTATCTAA   
  
  
- CGTCGGAAGG AAAGCTCTCG AGTCGTTCCA ACTTCTAGCT TCTCTACAAA CCGGCCCTTT AGTCCTTGTA   
  
  
- TTATCGGACA CTTCCCCGTC TGTCCTAGCT TTCTGTACTC TTCAAGCTCT TTACCTTCTC TTCCTAGCTC   
  
  
- GTCCTTCCCA AGGCCACGTA CGCCTGATCG CTTTCCCTCC ACTACGTCTC GGTTTACGAC GAATTCTACA   
  
  
- TGAGCTTGCT CTCGATGTTG TACGTTTCTG TTCCGTTACT TCTTCGTTAT AGTGATCATA CCAATCTAGT   
  
  
- CGGTGAGATG TGACAAAGAA ACACCCGAGG TTCACTTAAA CGACGTCCCT CAAGAAGGTC AAAAAGAGAC   
  
  
- GGTAGAAC

+     Myb

| Site Name | Organism | Position | Strand | Matrix score. | sequence | function |
| --- | --- | --- | --- | --- | --- | --- |
| Myb | Arabidopsis thaliana | 3445 | - | 6 | CAACTG |  |

>HU04G00148.1   
+ -Up\_Stream \_Len000ATAATG GGTGATAACT TTTGAAGGAC AATATACAAA TCACTTATTT ATTAAAGTCA   
  
  
+ AGGGGACATT TCAACTATAA CATCATTGTT TATAAAGTAT TTGAAAATTG CAACTCTTGT AATAGATAAT   
  
  
+ AATGCTTTAT AGACTTCACC AATCACTAAT TTGTAGAATC AAAAAATTAA TTGAAAAGCA AACAAAAGGA   
  
  
+ AAAGGCACAT AAACCTAAAT TATGTAAACA AATGAGAACA TATATGCCAG CTTATCAACG TTAATTATAT   
  
  
+ TTGTTATCAC TCTTAATATC TGTATTCATA GTAAATGAAA ATAAGTATCG ATTTTTTATT AAACCTAAAA   
  
  
+ TCTTCTGTCA ATAATTTTTC TTCCGATAAA AAAAAAATTG GACCATATTA AATTAACATT AATAAATTCA   
  
  
+ TATAATTACT TTGAATGTCT GAAATATAAT GCTCTTCGGT ATATTAAGGT CTTATAAATT TCTCATTAAT   
  
  
+ TTGAAATCTC TTATAATATA TTGGGTCAAC AAGTGCTAGC TTGTTTTTTT TTCCTGGTTA ATTGCATTTA   
  
  
+ AAAAAATAAT TTTTAATTAC AATTGCAAGG GGAGGAATCA TTTACAAAAT ATCAAAAGTG GAAAAGGCAA   
  
  
+ AAAGAAACAA ATTATAAAAC AGAAAAAAGA TTAGAAAACG CAAACAAAGA CGAAATGAGG AACAGTAAGA   
  
  
+ ATCAGTAAAA AAATGTAGTG CAACGGTCAC ATACGGGAAG GCAAGCACAA CAATCATGAA AGAGACAGGC   
  
  
+ ACGTGCGTTC CTCGTGATCA GCAGCCTTCC CTACTTCCCC GCCAAGTGGA GTCCGTACTA ACTCCTGCCA   
  
  
+ TTCCCTCCCA ACGGTCATAT TCCGCCGACA TGCCTCAACG GTCAGATTTC CTGCCCCATC TGGACCGTCC   
  
  
+ GATCAAGATC CAACGGCCAT AAGGCGCACT CTCCAATACC CTAGTGCCCA TTTCTGGAAC AGCCCCAAAA   
  
  
+ ATCTCAGTAT TCGCCATCAT TTTTCAATAC TGGGGTTACA TAAACAGTCT AACAAGGAAG GACCCAACAC   
  
  
+ AGAGGAGAGA GAAAAAAGCT AGCTTTGATG GGGACAGCCA TGGATGGAGG TGAAGAGAGA GGAGGTTGGG   
  
  
+ ACCTTTTATT TTGCTCACTT TCCATTCTTA TATAAAAACA GCTCTAGTTT ATCTCTTATG GCTTTTTTGG   
  
  
+ GTTTGTTCAT AGAGAGGGGG GAATGTGATA TGATTTGATT TGATGTTAGA AATTCTCATT AGTTTTTTCT   
  
  
+ TTTTGTTTGC AATTTTGATG TGTTAGGGTT TGAGGATCAT CATTTTTGAG TAGGGAACTT TCTGGGTTTT   
  
  
+ GCTTGCTGAT TGATTGCCAA TGCTATGGAT TTCTTGGTGA GTCAATCTTC AATTGCTGAT CTGGGTTCGT   
  
  
+ TTGTTCTTCA GAGTTTCACC TGCCAATTTG ATCAAGTATG TCCCTTCACT TCTCCTGCTT CAATTCTCTC   
  
  
+ TCAAATTCAC GCATTTTTCC AATGAATTTC AAATAGGAGT CCTGGAATCC GATGATATTT TAGGTTTTTT   
  
  
+ CCTGACTTCA TTGCATTAGT TTGTTCATAT CTGTGACCAA TTCTTTCATT TGTTATATGA TCTGATTGTT   
  
  
+ TAGTTAGCTA TTCATTCATT GCATATACAT TGGTTTATTC ATTGCAGAGA CACTCACATA CACACACACA   
  
  
+ TACAGGGGAC ACTAGTATTT TTTCTCTCTA ACTCATCGGA TTCCCTTTGA TACTCTGTTT TTTGGGGGGT   
  
  
+ TGATTTGATT TGATTTGATT GACCTTAATT TTGAACTCAC ACACACTCGC ACATAGATAT ATTCTTTCTG   
  
  
+ TTTGTGACAT ATACAGCATC ATATAGAAAT ATATTGCACT GCATATTTGA GCTCAAGACA GGGATATCAT   
  
  
+ CACAAACTGA GTGTGAGGGA GGCAAAAAAC AGAGGAAAAG AAGGGAATTG GGAGTGTAAT CAAAACAAGA   
  
  
+ GAGAGATTGA GGAGAGAGGA AAAAACAAAA TTGGGAGAAG GTTAATAGAG AAGAATGTTG GCTGGGTGTT   
  
  
+ CATCCACATT GCTGTCACCA AGGTATAGAT TGAGGAGTGA AGCTACATCA CAGTTCCAAG CCTGCCATCA   
  
  
+ TCCAATGAGC ACACAGAGAA TTGATTTGCC CTGCACTTTC CCAAGAAAGG ATGTTTCGAA GCCACAATCG   
  
  
+ GTTCGCCCCG TTGGCCTATC TGTCGAGAAG CCGGTTGAAG TCAGGGCCCT GAAGCAGACC ATCCGAGTCC   
  
  
+ CACCATCGCC GGAGGGTAGA AGAGAGATTA AGGCAGATTT CTGGGGTGAT AGAAGAAAGA GCTTGAAGAG   
  
  
+ GAGACTAGCA GAACAGGGGA GCTCTTTTGA TGATGGTGGT GACGAATCTT GTGTTGGGAG AACCAAAAGG   
  
  
+ AAGAAGGGTA GCTTTGATTT TGGTGAATCT GATGAAATTT CTCCAAAAAT TGAGGACACA ATGAGTTTTG   
  
  
+ GACATCTGGG TAGTGGTGGA AATTTCTGGG TTCATCCTGG TTTTGGTGTT GTTAATGTTA ATACTAATTA   
  
  
+ CCCTCAAGTG CCATTTTCTC TCACTTGTTC AGGGGAGGAA GAAAGAGTAT GTTTTGTTCC TACTGATGTG   
  
  
+ ATCTCACCAG CCATCATGCC TCCATTGTCA CACAATCCTT GGGTTGAATC TGGTGTTACT GAGGTCACAG   
  
  
+ AATATGGTGG GGGTGACAAA GACGGCGAAC CTAGTCATGG GTTTGTGAGG GGTACAACAA CAACGTCCGG   
  
  
+ GTCAAGTTCA TCTTCTGAGA GTCATAGTTT TGGGCATAGG CTCAATGAGA GCTCATCTGA CCCTGAAATC   
  
  
+ AGAAATGGTT CCATGTTGCC TAACCCTGGT CATGGTCCTG GCACCAGTCA TGGTCCTGGA ACCGGTCTGG   
  
  
+ CTCACAACCA CAATGATCAA ACCGAGCAAC AAGGGTTCGA GCTCATTAGC TTTCTCATGG GTTGTGTCGA   
  
  
+ AGCGATCAGT TCAAGGAACA TTGCAGCCAT CAATCATTTC ATAGCTAAGC TGGGCGAGCA GGCTTCTCCA   
  
  
+ AGGGCTCGGT CTGCCATTAG CCGCCTCACA GCCTACTTCA CCGAAGCATT AGCCTTGAGG GTCACAAGAT   
  
  
+ TTTGGCCTCA TATCTTTCAC ATAAGTATCC CTCGAGAGTT TGATCGATTT GATGATGAAT CGGGGGCAGC   
  
  
+ TGCAATGAGG CTTTTGAATC AGATCAGCCC AATTCCAAAG TTCGTTCATT TTACAGCCAA TGAGATGTTA   
  
  
+ TTGAGGGCAT TTGAAGGGAA GGACAAGGTG CATATCATAG ACTTCGACAT CAAGCAAGGC TTACAATGGC   
  
  
+ CCGGGTTTTT CCAAAGCTTA GCCATGAGGG AAAACCCCCC GAGCCATGTC AGGATAACAG GCGTAGGGGA   
  
  
+ TTCGAAGCAA GAATTGGTTG AGACAGGAGA AAGACTAGCC GGGTTTGCTG GGGCATTCAA CCTCTCCTTC   
  
  
+ GAGTTCCACC CAGTTGTGGA CCGGTTGGAA GATGTTAGGC TATGGATGCT TCATGTTAAG GAAGGTGAAA   
  
  
+ GTGTCGCGGT AAATTGCATT TTGCAGCTCC ACAAGACACT CTATGACCCC CATGGTGCCA CATTCAGGGA   
  
  
+ CTTCATGGGT TTAATCCGAA GCACAAATCC CATAGCATTG GTTATGGCTG AGCAAGAAGC TGATCACAAC   
  
  
+ GAACCCACCT TAGAAGGCCG AGTATGCAAC TCACTTGGAT ACTATGCAGC CCTTTTCGAT GCCATAGATT   
  
  
+ GCAGCCTTCC TTTCGAGAGC TCAGCAAGGT TGAAGATCGA AGAGATGTTT GGCCGGGAAA TCAGGAACAT   
  
  
+ AATAGCCTGT GAAGGGGCAG ACAGGATCGA AAGACATGAG AAGTTCGAGA AATGGAAGAG AAGGATCGAG   
  
  
+ CAGGAAGGGT TCCGGTGCAT GCGGACTAGC GAAAGGGAGG TGATGCAGAG CCAAATGCTG CTTAAGATGT   
  
  
+ ACTCGAACGA GAGCTACAAC ATGCAAAGAC AAGGCAATGA AGAAGCAATA TCACTAGTAT GGTTAGATCA   
  
  
+ GCCACTCTAC ACTGTTTCTT TGTGGGCTCC AAGTGAATTT GCTGCAGGGA GTTCTTCCAG TTTTTCTCTG   
  
  
+ CCATCTTG  

- -Up\_Stream \_Len000TATTAC CCACTATTGA AAACTTCCTG TTATATGTTT AGTGAATAAA TAATTTCAGT   
  
  
- TCCCCTGTAA AGTTGATATT GTAGTAACAA ATATTTCATA AACTTTTAAC GTTGAGAACA TTATCTATTA   
  
  
- TTACGAAATA TCTGAAGTGG TTAGTGATTA AACATCTTAG TTTTTTAATT AACTTTTCGT TTGTTTTCCT   
  
  
- TTTCCGTGTA TTTGGATTTA ATACATTTGT TTACTCTTGT ATATACGGTC GAATAGTTGC AATTAATATA   
  
  
- AACAATAGTG AGAATTATAG ACATAAGTAT CATTTACTTT TATTCATAGC TAAAAAATAA TTTGGATTTT   
  
  
- AGAAGACAGT TATTAAAAAG AAGGCTATTT TTTTTTTAAC CTGGTATAAT TTAATTGTAA TTATTTAAGT   
  
  
- ATATTAATGA AACTTACAGA CTTTATATTA CGAGAAGCCA TATAATTCCA GAATATTTAA AGAGTAATTA   
  
  
- AACTTTAGAG AATATTATAT AACCCAGTTG TTCACGATCG AACAAAAAAA AAGGACCAAT TAACGTAAAT   
  
  
- TTTTTTATTA AAAATTAATG TTAACGTTCC CCTCCTTAGT AAATGTTTTA TAGTTTTCAC CTTTTCCGTT   
  
  
- TTTCTTTGTT TAATATTTTG TCTTTTTTCT AATCTTTTGC GTTTGTTTCT GCTTTACTCC TTGTCATTCT   
  
  
- TAGTCATTTT TTTACATCAC GTTGCCAGTG TATGCCCTTC CGTTCGTGTT GTTAGTACTT TCTCTGTCCG   
  
  
- TGCACGCAAG GAGCACTAGT CGTCGGAAGG GATGAAGGGG CGGTTCACCT CAGGCATGAT TGAGGACGGT   
  
  
- AAGGGAGGGT TGCCAGTATA AGGCGGCTGT ACGGAGTTGC CAGTCTAAAG GACGGGGTAG ACCTGGCAGG   
  
  
- CTAGTTCTAG GTTGCCGGTA TTCCGCGTGA GAGGTTATGG GATCACGGGT AAAGACCTTG TCGGGGTTTT   
  
  
- TAGAGTCATA AGCGGTAGTA AAAAGTTATG ACCCCAATGT ATTTGTCAGA TTGTTCCTTC CTGGGTTGTG   
  
  
- TCTCCTCTCT CTTTTTTCGA TCGAAACTAC CCCTGTCGGT ACCTACCTCC ACTTCTCTCT CCTCCAACCC   
  
  
- TGGAAAATAA AACGAGTGAA AGGTAAGAAT ATATTTTTGT CGAGATCAAA TAGAGAATAC CGAAAAAACC   
  
  
- CAAACAAGTA TCTCTCCCCC CTTACACTAT ACTAAACTAA ACTACAATCT TTAAGAGTAA TCAAAAAAGA   
  
  
- AAAACAAACG TTAAAACTAC ACAATCCCAA ACTCCTAGTA GTAAAAACTC ATCCCTTGAA AGACCCAAAA   
  
  
- CGAACGACTA ACTAACGGTT ACGATACCTA AAGAACCACT CAGTTAGAAG TTAACGACTA GACCCAAGCA   
  
  
- AACAAGAAGT CTCAAAGTGG ACGGTTAAAC TAGTTCATAC AGGGAAGTGA AGAGGACGAA GTTAAGAGAG   
  
  
- AGTTTAAGTG CGTAAAAAGG TTACTTAAAG TTTATCCTCA GGACCTTAGG CTACTATAAA ATCCAAAAAA   
  
  
- GGACTGAAGT AACGTAATCA AACAAGTATA GACACTGGTT AAGAAAGTAA ACAATATACT AGACTAACAA   
  
  
- ATCAATCGAT AAGTAAGTAA CGTATATGTA ACCAAATAAG TAACGTCTCT GTGAGTGTAT GTGTGTGTGT   
  
  
- ATGTCCCCTG TGATCATAAA AAAGAGAGAT TGAGTAGCCT AAGGGAAACT ATGAGACAAA AAACCCCCCA   
  
  
- ACTAAACTAA ACTAAACTAA CTGGAATTAA AACTTGAGTG TGTGTGAGCG TGTATCTATA TAAGAAAGAC   
  
  
- AAACACTGTA TATGTCGTAG TATATCTTTA TATAACGTGA CGTATAAACT CGAGTTCTGT CCCTATAGTA   
  
  
- GTGTTTGACT CACACTCCCT CCGTTTTTTG TCTCCTTTTC TTCCCTTAAC CCTCACATTA GTTTTGTTCT   
  
  
- CTCTCTAACT CCTCTCTCCT TTTTTGTTTT AACCCTCTTC CAATTATCTC TTCTTACAAC CGACCCACAA   
  
  
- GTAGGTGTAA CGACAGTGGT TCCATATCTA ACTCCTCACT TCGATGTAGT GTCAAGGTTC GGACGGTAGT   
  
  
- AGGTTACTCG TGTGTCTCTT AACTAAACGG GACGTGAAAG GGTTCTTTCC TACAAAGCTT CGGTGTTAGC   
  
  
- CAAGCGGGGC AACCGGATAG ACAGCTCTTC GGCCAACTTC AGTCCCGGGA CTTCGTCTGG TAGGCTCAGG   
  
  
- GTGGTAGCGG CCTCCCATCT TCTCTCTAAT TCCGTCTAAA GACCCCACTA TCTTCTTTCT CGAACTTCTC   
  
  
- CTCTGATCGT CTTGTCCCCT CGAGAAAACT ACTACCACCA CTGCTTAGAA CACAACCCTC TTGGTTTTCC   
  
  
- TTCTTCCCAT CGAAACTAAA ACCACTTAGA CTACTTTAAA GAGGTTTTTA ACTCCTGTGT TACTCAAAAC   
  
  
- CTGTAGACCC ATCACCACCT TTAAAGACCC AAGTAGGACC AAAACCACAA CAATTACAAT TATGATTAAT   
  
  
- GGGAGTTCAC GGTAAAAGAG AGTGAACAAG TCCCCTCCTT CTTTCTCATA CAAAACAAGG ATGACTACAC   
  
  
- TAGAGTGGTC GGTAGTACGG AGGTAACAGT GTGTTAGGAA CCCAACTTAG ACCACAATGA CTCCAGTGTC   
  
  
- TTATACCACC CCCACTGTTT CTGCCGCTTG GATCAGTACC CAAACACTCC CCATGTTGTT GTTGCAGGCC   
  
  
- CAGTTCAAGT AGAAGACTCT CAGTATCAAA ACCCGTATCC GAGTTACTCT CGAGTAGACT GGGACTTTAG   
  
  
- TCTTTACCAA GGTACAACGG ATTGGGACCA GTACCAGGAC CGTGGTCAGT ACCAGGACCT TGGCCAGACC   
  
  
- GAGTGTTGGT GTTACTAGTT TGGCTCGTTG TTCCCAAGCT CGAGTAATCG AAAGAGTACC CAACACAGCT   
  
  
- TCGCTAGTCA AGTTCCTTGT AACGTCGGTA GTTAGTAAAG TATCGATTCG ACCCGCTCGT CCGAAGAGGT   
  
  
- TCCCGAGCCA GACGGTAATC GGCGGAGTGT CGGATGAAGT GGCTTCGTAA TCGGAACTCC CAGTGTTCTA   
  
  
- AAACCGGAGT ATAGAAAGTG TATTCATAGG GAGCTCTCAA ACTAGCTAAA CTACTACTTA GCCCCCGTCG   
  
  
- ACGTTACTCC GAAAACTTAG TCTAGTCGGG TTAAGGTTTC AAGCAAGTAA AATGTCGGTT ACTCTACAAT   
  
  
- AACTCCCGTA AACTTCCCTT CCTGTTCCAC GTATAGTATC TGAAGCTGTA GTTCGTTCCG AATGTTACCG   
  
  
- GGCCCAAAAA GGTTTCGAAT CGGTACTCCC TTTTGGGGGG CTCGGTACAG TCCTATTGTC CGCATCCCCT   
  
  
- AAGCTTCGTT CTTAACCAAC TCTGTCCTCT TTCTGATCGG CCCAAACGAC CCCGTAAGTT GGAGAGGAAG   
  
  
- CTCAAGGTGG GTCAACACCT GGCCAACCTT CTACAATCCG ATACCTACGA AGTACAATTC CTTCCACTTT   
  
  
- CACAGCGCCA TTTAACGTAA AACGTCGAGG TGTTCTGTGA GATACTGGGG GTACCACGGT GTAAGTCCCT   
  
  
- GAAGTACCCA AATTAGGCTT CGTGTTTAGG GTATCGTAAC CAATACCGAC TCGTTCTTCG ACTAGTGTTG   
  
  
- CTTGGGTGGA ATCTTCCGGC TCATACGTTG AGTGAACCTA TGATACGTCG GGAAAAGCTA CGGTATCTAA   
  
  
- CGTCGGAAGG AAAGCTCTCG AGTCGTTCCA ACTTCTAGCT TCTCTACAAA CCGGCCCTTT AGTCCTTGTA   
  
  
- TTATCGGACA CTTCCCCGTC TGTCCTAGCT TTCTGTACTC TTCAAGCTCT TTACCTTCTC TTCCTAGCTC   
  
  
- GTCCTTCCCA AGGCCACGTA CGCCTGATCG CTTTCCCTCC ACTACGTCTC GGTTTACGAC GAATTCTACA   
  
  
- TGAGCTTGCT CTCGATGTTG TACGTTTCTG TTCCGTTACT TCTTCGTTAT AGTGATCATA CCAATCTAGT   
  
  
- CGGTGAGATG TGACAAAGAA ACACCCGAGG TTCACTTAAA CGACGTCCCT CAAGAAGGTC AAAAAGAGAC   
  
  
- GGTAGAAC

+     Myc

| Site Name | Organism | Position | Strand | Matrix score. | sequence | function |
| --- | --- | --- | --- | --- | --- | --- |
| Myc | Arabidopsis thaliana | 501 | + | 7 | TCTCTTA |  |
| Myc | Arabidopsis thaliana | 1176 | + | 7 | TCTCTTA |  |

>HU04G00148.1   
+ -Up\_Stream \_Len000ATAATG GGTGATAACT TTTGAAGGAC AATATACAAA TCACTTATTT ATTAAAGTCA   
  
  
+ AGGGGACATT TCAACTATAA CATCATTGTT TATAAAGTAT TTGAAAATTG CAACTCTTGT AATAGATAAT   
  
  
+ AATGCTTTAT AGACTTCACC AATCACTAAT TTGTAGAATC AAAAAATTAA TTGAAAAGCA AACAAAAGGA   
  
  
+ AAAGGCACAT AAACCTAAAT TATGTAAACA AATGAGAACA TATATGCCAG CTTATCAACG TTAATTATAT   
  
  
+ TTGTTATCAC TCTTAATATC TGTATTCATA GTAAATGAAA ATAAGTATCG ATTTTTTATT AAACCTAAAA   
  
  
+ TCTTCTGTCA ATAATTTTTC TTCCGATAAA AAAAAAATTG GACCATATTA AATTAACATT AATAAATTCA   
  
  
+ TATAATTACT TTGAATGTCT GAAATATAAT GCTCTTCGGT ATATTAAGGT CTTATAAATT TCTCATTAAT   
  
  
+ TTGAAATCTC TTATAATATA TTGGGTCAAC AAGTGCTAGC TTGTTTTTTT TTCCTGGTTA ATTGCATTTA   
  
  
+ AAAAAATAAT TTTTAATTAC AATTGCAAGG GGAGGAATCA TTTACAAAAT ATCAAAAGTG GAAAAGGCAA   
  
  
+ AAAGAAACAA ATTATAAAAC AGAAAAAAGA TTAGAAAACG CAAACAAAGA CGAAATGAGG AACAGTAAGA   
  
  
+ ATCAGTAAAA AAATGTAGTG CAACGGTCAC ATACGGGAAG GCAAGCACAA CAATCATGAA AGAGACAGGC   
  
  
+ ACGTGCGTTC CTCGTGATCA GCAGCCTTCC CTACTTCCCC GCCAAGTGGA GTCCGTACTA ACTCCTGCCA   
  
  
+ TTCCCTCCCA ACGGTCATAT TCCGCCGACA TGCCTCAACG GTCAGATTTC CTGCCCCATC TGGACCGTCC   
  
  
+ GATCAAGATC CAACGGCCAT AAGGCGCACT CTCCAATACC CTAGTGCCCA TTTCTGGAAC AGCCCCAAAA   
  
  
+ ATCTCAGTAT TCGCCATCAT TTTTCAATAC TGGGGTTACA TAAACAGTCT AACAAGGAAG GACCCAACAC   
  
  
+ AGAGGAGAGA GAAAAAAGCT AGCTTTGATG GGGACAGCCA TGGATGGAGG TGAAGAGAGA GGAGGTTGGG   
  
  
+ ACCTTTTATT TTGCTCACTT TCCATTCTTA TATAAAAACA GCTCTAGTTT ATCTCTTATG GCTTTTTTGG   
  
  
+ GTTTGTTCAT AGAGAGGGGG GAATGTGATA TGATTTGATT TGATGTTAGA AATTCTCATT AGTTTTTTCT   
  
  
+ TTTTGTTTGC AATTTTGATG TGTTAGGGTT TGAGGATCAT CATTTTTGAG TAGGGAACTT TCTGGGTTTT   
  
  
+ GCTTGCTGAT TGATTGCCAA TGCTATGGAT TTCTTGGTGA GTCAATCTTC AATTGCTGAT CTGGGTTCGT   
  
  
+ TTGTTCTTCA GAGTTTCACC TGCCAATTTG ATCAAGTATG TCCCTTCACT TCTCCTGCTT CAATTCTCTC   
  
  
+ TCAAATTCAC GCATTTTTCC AATGAATTTC AAATAGGAGT CCTGGAATCC GATGATATTT TAGGTTTTTT   
  
  
+ CCTGACTTCA TTGCATTAGT TTGTTCATAT CTGTGACCAA TTCTTTCATT TGTTATATGA TCTGATTGTT   
  
  
+ TAGTTAGCTA TTCATTCATT GCATATACAT TGGTTTATTC ATTGCAGAGA CACTCACATA CACACACACA   
  
  
+ TACAGGGGAC ACTAGTATTT TTTCTCTCTA ACTCATCGGA TTCCCTTTGA TACTCTGTTT TTTGGGGGGT   
  
  
+ TGATTTGATT TGATTTGATT GACCTTAATT TTGAACTCAC ACACACTCGC ACATAGATAT ATTCTTTCTG   
  
  
+ TTTGTGACAT ATACAGCATC ATATAGAAAT ATATTGCACT GCATATTTGA GCTCAAGACA GGGATATCAT   
  
  
+ CACAAACTGA GTGTGAGGGA GGCAAAAAAC AGAGGAAAAG AAGGGAATTG GGAGTGTAAT CAAAACAAGA   
  
  
+ GAGAGATTGA GGAGAGAGGA AAAAACAAAA TTGGGAGAAG GTTAATAGAG AAGAATGTTG GCTGGGTGTT   
  
  
+ CATCCACATT GCTGTCACCA AGGTATAGAT TGAGGAGTGA AGCTACATCA CAGTTCCAAG CCTGCCATCA   
  
  
+ TCCAATGAGC ACACAGAGAA TTGATTTGCC CTGCACTTTC CCAAGAAAGG ATGTTTCGAA GCCACAATCG   
  
  
+ GTTCGCCCCG TTGGCCTATC TGTCGAGAAG CCGGTTGAAG TCAGGGCCCT GAAGCAGACC ATCCGAGTCC   
  
  
+ CACCATCGCC GGAGGGTAGA AGAGAGATTA AGGCAGATTT CTGGGGTGAT AGAAGAAAGA GCTTGAAGAG   
  
  
+ GAGACTAGCA GAACAGGGGA GCTCTTTTGA TGATGGTGGT GACGAATCTT GTGTTGGGAG AACCAAAAGG   
  
  
+ AAGAAGGGTA GCTTTGATTT TGGTGAATCT GATGAAATTT CTCCAAAAAT TGAGGACACA ATGAGTTTTG   
  
  
+ GACATCTGGG TAGTGGTGGA AATTTCTGGG TTCATCCTGG TTTTGGTGTT GTTAATGTTA ATACTAATTA   
  
  
+ CCCTCAAGTG CCATTTTCTC TCACTTGTTC AGGGGAGGAA GAAAGAGTAT GTTTTGTTCC TACTGATGTG   
  
  
+ ATCTCACCAG CCATCATGCC TCCATTGTCA CACAATCCTT GGGTTGAATC TGGTGTTACT GAGGTCACAG   
  
  
+ AATATGGTGG GGGTGACAAA GACGGCGAAC CTAGTCATGG GTTTGTGAGG GGTACAACAA CAACGTCCGG   
  
  
+ GTCAAGTTCA TCTTCTGAGA GTCATAGTTT TGGGCATAGG CTCAATGAGA GCTCATCTGA CCCTGAAATC   
  
  
+ AGAAATGGTT CCATGTTGCC TAACCCTGGT CATGGTCCTG GCACCAGTCA TGGTCCTGGA ACCGGTCTGG   
  
  
+ CTCACAACCA CAATGATCAA ACCGAGCAAC AAGGGTTCGA GCTCATTAGC TTTCTCATGG GTTGTGTCGA   
  
  
+ AGCGATCAGT TCAAGGAACA TTGCAGCCAT CAATCATTTC ATAGCTAAGC TGGGCGAGCA GGCTTCTCCA   
  
  
+ AGGGCTCGGT CTGCCATTAG CCGCCTCACA GCCTACTTCA CCGAAGCATT AGCCTTGAGG GTCACAAGAT   
  
  
+ TTTGGCCTCA TATCTTTCAC ATAAGTATCC CTCGAGAGTT TGATCGATTT GATGATGAAT CGGGGGCAGC   
  
  
+ TGCAATGAGG CTTTTGAATC AGATCAGCCC AATTCCAAAG TTCGTTCATT TTACAGCCAA TGAGATGTTA   
  
  
+ TTGAGGGCAT TTGAAGGGAA GGACAAGGTG CATATCATAG ACTTCGACAT CAAGCAAGGC TTACAATGGC   
  
  
+ CCGGGTTTTT CCAAAGCTTA GCCATGAGGG AAAACCCCCC GAGCCATGTC AGGATAACAG GCGTAGGGGA   
  
  
+ TTCGAAGCAA GAATTGGTTG AGACAGGAGA AAGACTAGCC GGGTTTGCTG GGGCATTCAA CCTCTCCTTC   
  
  
+ GAGTTCCACC CAGTTGTGGA CCGGTTGGAA GATGTTAGGC TATGGATGCT TCATGTTAAG GAAGGTGAAA   
  
  
+ GTGTCGCGGT AAATTGCATT TTGCAGCTCC ACAAGACACT CTATGACCCC CATGGTGCCA CATTCAGGGA   
  
  
+ CTTCATGGGT TTAATCCGAA GCACAAATCC CATAGCATTG GTTATGGCTG AGCAAGAAGC TGATCACAAC   
  
  
+ GAACCCACCT TAGAAGGCCG AGTATGCAAC TCACTTGGAT ACTATGCAGC CCTTTTCGAT GCCATAGATT   
  
  
+ GCAGCCTTCC TTTCGAGAGC TCAGCAAGGT TGAAGATCGA AGAGATGTTT GGCCGGGAAA TCAGGAACAT   
  
  
+ AATAGCCTGT GAAGGGGCAG ACAGGATCGA AAGACATGAG AAGTTCGAGA AATGGAAGAG AAGGATCGAG   
  
  
+ CAGGAAGGGT TCCGGTGCAT GCGGACTAGC GAAAGGGAGG TGATGCAGAG CCAAATGCTG CTTAAGATGT   
  
  
+ ACTCGAACGA GAGCTACAAC ATGCAAAGAC AAGGCAATGA AGAAGCAATA TCACTAGTAT GGTTAGATCA   
  
  
+ GCCACTCTAC ACTGTTTCTT TGTGGGCTCC AAGTGAATTT GCTGCAGGGA GTTCTTCCAG TTTTTCTCTG   
  
  
+ CCATCTTG  

- -Up\_Stream \_Len000TATTAC CCACTATTGA AAACTTCCTG TTATATGTTT AGTGAATAAA TAATTTCAGT   
  
  
- TCCCCTGTAA AGTTGATATT GTAGTAACAA ATATTTCATA AACTTTTAAC GTTGAGAACA TTATCTATTA   
  
  
- TTACGAAATA TCTGAAGTGG TTAGTGATTA AACATCTTAG TTTTTTAATT AACTTTTCGT TTGTTTTCCT   
  
  
- TTTCCGTGTA TTTGGATTTA ATACATTTGT TTACTCTTGT ATATACGGTC GAATAGTTGC AATTAATATA   
  
  
- AACAATAGTG AGAATTATAG ACATAAGTAT CATTTACTTT TATTCATAGC TAAAAAATAA TTTGGATTTT   
  
  
- AGAAGACAGT TATTAAAAAG AAGGCTATTT TTTTTTTAAC CTGGTATAAT TTAATTGTAA TTATTTAAGT   
  
  
- ATATTAATGA AACTTACAGA CTTTATATTA CGAGAAGCCA TATAATTCCA GAATATTTAA AGAGTAATTA   
  
  
- AACTTTAGAG AATATTATAT AACCCAGTTG TTCACGATCG AACAAAAAAA AAGGACCAAT TAACGTAAAT   
  
  
- TTTTTTATTA AAAATTAATG TTAACGTTCC CCTCCTTAGT AAATGTTTTA TAGTTTTCAC CTTTTCCGTT   
  
  
- TTTCTTTGTT TAATATTTTG TCTTTTTTCT AATCTTTTGC GTTTGTTTCT GCTTTACTCC TTGTCATTCT   
  
  
- TAGTCATTTT TTTACATCAC GTTGCCAGTG TATGCCCTTC CGTTCGTGTT GTTAGTACTT TCTCTGTCCG   
  
  
- TGCACGCAAG GAGCACTAGT CGTCGGAAGG GATGAAGGGG CGGTTCACCT CAGGCATGAT TGAGGACGGT   
  
  
- AAGGGAGGGT TGCCAGTATA AGGCGGCTGT ACGGAGTTGC CAGTCTAAAG GACGGGGTAG ACCTGGCAGG   
  
  
- CTAGTTCTAG GTTGCCGGTA TTCCGCGTGA GAGGTTATGG GATCACGGGT AAAGACCTTG TCGGGGTTTT   
  
  
- TAGAGTCATA AGCGGTAGTA AAAAGTTATG ACCCCAATGT ATTTGTCAGA TTGTTCCTTC CTGGGTTGTG   
  
  
- TCTCCTCTCT CTTTTTTCGA TCGAAACTAC CCCTGTCGGT ACCTACCTCC ACTTCTCTCT CCTCCAACCC   
  
  
- TGGAAAATAA AACGAGTGAA AGGTAAGAAT ATATTTTTGT CGAGATCAAA TAGAGAATAC CGAAAAAACC   
  
  
- CAAACAAGTA TCTCTCCCCC CTTACACTAT ACTAAACTAA ACTACAATCT TTAAGAGTAA TCAAAAAAGA   
  
  
- AAAACAAACG TTAAAACTAC ACAATCCCAA ACTCCTAGTA GTAAAAACTC ATCCCTTGAA AGACCCAAAA   
  
  
- CGAACGACTA ACTAACGGTT ACGATACCTA AAGAACCACT CAGTTAGAAG TTAACGACTA GACCCAAGCA   
  
  
- AACAAGAAGT CTCAAAGTGG ACGGTTAAAC TAGTTCATAC AGGGAAGTGA AGAGGACGAA GTTAAGAGAG   
  
  
- AGTTTAAGTG CGTAAAAAGG TTACTTAAAG TTTATCCTCA GGACCTTAGG CTACTATAAA ATCCAAAAAA   
  
  
- GGACTGAAGT AACGTAATCA AACAAGTATA GACACTGGTT AAGAAAGTAA ACAATATACT AGACTAACAA   
  
  
- ATCAATCGAT AAGTAAGTAA CGTATATGTA ACCAAATAAG TAACGTCTCT GTGAGTGTAT GTGTGTGTGT   
  
  
- ATGTCCCCTG TGATCATAAA AAAGAGAGAT TGAGTAGCCT AAGGGAAACT ATGAGACAAA AAACCCCCCA   
  
  
- ACTAAACTAA ACTAAACTAA CTGGAATTAA AACTTGAGTG TGTGTGAGCG TGTATCTATA TAAGAAAGAC   
  
  
- AAACACTGTA TATGTCGTAG TATATCTTTA TATAACGTGA CGTATAAACT CGAGTTCTGT CCCTATAGTA   
  
  
- GTGTTTGACT CACACTCCCT CCGTTTTTTG TCTCCTTTTC TTCCCTTAAC CCTCACATTA GTTTTGTTCT   
  
  
- CTCTCTAACT CCTCTCTCCT TTTTTGTTTT AACCCTCTTC CAATTATCTC TTCTTACAAC CGACCCACAA   
  
  
- GTAGGTGTAA CGACAGTGGT TCCATATCTA ACTCCTCACT TCGATGTAGT GTCAAGGTTC GGACGGTAGT   
  
  
- AGGTTACTCG TGTGTCTCTT AACTAAACGG GACGTGAAAG GGTTCTTTCC TACAAAGCTT CGGTGTTAGC   
  
  
- CAAGCGGGGC AACCGGATAG ACAGCTCTTC GGCCAACTTC AGTCCCGGGA CTTCGTCTGG TAGGCTCAGG   
  
  
- GTGGTAGCGG CCTCCCATCT TCTCTCTAAT TCCGTCTAAA GACCCCACTA TCTTCTTTCT CGAACTTCTC   
  
  
- CTCTGATCGT CTTGTCCCCT CGAGAAAACT ACTACCACCA CTGCTTAGAA CACAACCCTC TTGGTTTTCC   
  
  
- TTCTTCCCAT CGAAACTAAA ACCACTTAGA CTACTTTAAA GAGGTTTTTA ACTCCTGTGT TACTCAAAAC   
  
  
- CTGTAGACCC ATCACCACCT TTAAAGACCC AAGTAGGACC AAAACCACAA CAATTACAAT TATGATTAAT   
  
  
- GGGAGTTCAC GGTAAAAGAG AGTGAACAAG TCCCCTCCTT CTTTCTCATA CAAAACAAGG ATGACTACAC   
  
  
- TAGAGTGGTC GGTAGTACGG AGGTAACAGT GTGTTAGGAA CCCAACTTAG ACCACAATGA CTCCAGTGTC   
  
  
- TTATACCACC CCCACTGTTT CTGCCGCTTG GATCAGTACC CAAACACTCC CCATGTTGTT GTTGCAGGCC   
  
  
- CAGTTCAAGT AGAAGACTCT CAGTATCAAA ACCCGTATCC GAGTTACTCT CGAGTAGACT GGGACTTTAG   
  
  
- TCTTTACCAA GGTACAACGG ATTGGGACCA GTACCAGGAC CGTGGTCAGT ACCAGGACCT TGGCCAGACC   
  
  
- GAGTGTTGGT GTTACTAGTT TGGCTCGTTG TTCCCAAGCT CGAGTAATCG AAAGAGTACC CAACACAGCT
[truncated: 124,455 more chars]
