# Supplementary material for: Genome-Wide Identification and Expression Pattern of the GRAS Gene Family in Pitaya (Selenicereus undatus L.)
Source: Biology (Basel). 2022 Dec 21;12(1):11. doi: 10.3390/biology12010011 (PMC9854919; doi:10.3390/biology12010011)
Supplement: Supplementary file 1 [file biology-12-00011-s001.zip › Supplementary file S5/HU05G00466.1_plantcare.html]

Content-Type: text/html; charset=ISO-8859-1


PlantCARE


Webmaster Firefox specific output  
To save the result:
click on the frame with the right mouse button and save the source code as a text file with extension .html  
REFERENCE:PlantCARE: a database of plant cis-acting regulatory elements and a portal to tools for in silico analysis of promoter sequences.  
Lescot, M., Déhais, P., Moreau, Y., De Moor, B., Rouzé ,P.,and Rombauts, S.  
Nucleic Acids Res., Database issue(2002), 30(1):325-327.   


---

>HU05G00466.1   
+ +Up\_Stream \_Len000ACATTG GGTGGGATTT AAATCCTCTA TTTTTTCTTA GGGGAAAAAT ACTTGGGAAA   
  
  
+ TTTCTCAATA ACTGAAAATC TAAATAATTC GTGACTTTGT TAGTAGGTTT AATGTGATTT ACTACCGTGA   
  
  
+ GTGTCACTTT TAGATGGTAC TTTAAGAAAG CATTATAAGT AAAACAATAT ATATCGTAGC ATCATACTAC   
  
  
+ TTGTGTAATA AATTAACAGC AACCTCAATT GTACTTATTT GATTTCGCAT TGTAATCAAT TTTCCCAAAT   
  
  
+ GATTTACTAT CTTGGTCTTA CTCAGTTGTA CTTCACACTC AATAGTTCAA TTATTTTTCA TTAGACTAAT   
  
  
+ GAGCGAGTCC CTTCTACAAG AGCCTACTAC TACTCTAATA ATGTAAGGAC TATTGGCTAA TGGGGAACTA   
  
  
+ AGCCTACGAC TTGTTAGGCA TAGGTGAATT TTCCTTACAA GAGAGTTTAA TTTACACTTG ATATTAACTC   
  
  
+ GAGAACGAGA AGCTGTCTTA ATAGATACCC ACATAGAAAA TGAAGCAAAT GCCCTTGATT ATTGGAGGGT   
  
  
+ TGGGCAAATA TATTCAGTAA CAATGTTGTT TGGCAAATAT CGTCTGAAAA TTGTTTTTGA CAAATATTGT   
  
  
+ TTGGAAGAAT TTTTCGAGAT TAAATATTAA AAATGTTAAA ATTTTAATCT AAATATCAAA CAAATATTGA   
  
  
+ AAAATGCTTT ATATTATCTA AATCTCTAAT TAAAATTTGT GTATTGTCCA CTAGAAAAAT GTATAGTGAT   
  
  
+ ATTACCGACA TATATGTGAT CTCTGGTGTA ATAGCCTACC CTAATGTCTA GGAAAATATT TAAAATCCCC   
  
  
+ AGTGGTCAAA AGGAAGTTAC GCACACTAGG GCAAGACACT TCATTAGGCT AGTTGGTGAA AGCTAAGTCA   
  
  
+ TGAAATTTAG ACCATGGCCA TGTCTACTTG TAGATCCAAC TACCAACAAT CCATTTTTAT TTTGGGGAAA   
  
  
+ GCTTGAAGGG TAATTTGGTA AATGTACACG GGAACCTTTT GCTATAAAAG AAGTGCTTCT TGAAAAAACA   
  
  
+ CCCTGTAGTA AAACAGGGCA AAAAAGCCAA CACTTCTGTT TCTGGCAGAG AAACAGTAGT GTACCACTTG   
  
  
+ GAGTTGGAGT AGACACGATA CACAAACCCA GTAGAGAGAG AAACAGGGGA GAGAAAGAGT ACACAAATTT   
  
  
+ GTGCAGGAGA AGGGTAAGGA AGAAGGAGAA GGAACCCCCT CCAAAAAAAA CCCATATAAA AAATAATAAA   
  
  
+ GAAAAAACAG GAGTGAGTTA GAGGGTACCC AAATCTCCAA TCTAAAACTA GAATCTAGGG TCTCTTTTCA   
  
  
+ TTTGCTTGAA AAATTTCTGT TCATGGGTTT TGTGTTTTTA GGGGGATTTG TTTGATTTTT GTAGTCTTTG   
  
  
+ TGGATTTCCT GTGTTATAGA TGCAAGCTAT GCTCTTCAAT TTGCAAGGAA CAGCATCTGG GGGGAGAGAA   
  
  
+ TTTGAGGAAT TTCAAGTACC CATTTCAATT TCAACAAGTA AAATCATCAT TGAAGGATAC TCAAACCCAA   
  
  
+ GCCCAGTTGA TAGTGAACCA ACTTCTACTC TGGATTCTAC TCCAAGTCCC AGCCCTCCCA CCCTCTCCTC   
  
  
+ CTCCTCCAAG AACTCCTACC AAAACGCTGC CGTTTTGGCT CACCCTCCTC AGCTTCACCA TGGATCTGAT   
  
  
+ AATGTGTAAG ACCCCAACCC TTTTGCTTTT ATTTTCCTCA TGCTCCATTC TCATGATTTG TACTTCTGTT   
  
  
+ ATGTAATTGG AGATGTGTCT AAATTTGGAA AAGTTCGTGG GTTTCGTGTT TGAGAATTTA GAATGACATT   
  
  
+ CTTTAATTAG TAATTTGAAG TTTGCATGCT TAATTTAGCT AATCACTGAT TAATTAAAAT AAATGAAGTG   
  
  
+ ATGTAGGTAT GGGCTGTTAC ACAATGTCAC CGGTGGTGGT GACAGCGGCG GGGCGGCGGC GGTTGGATTG   
  
  
+ GAAGAATGGG ACAGCGTGTT TCCAAATGGG GATGGAGCTT TACTCCCTTG GATCATGGGG GAAGGTGATG   
  
  
+ ATTTGGGTCT GAATTTGAAG CATCTCTTAC AATCTGGTTA CCCTGTTGAG TATGAAGGCA ATGCAGGACT   
  
  
+ TGGTGTTGTT GATCAGACAT CTCTTCTTGG AGGTGGTGAT TTGGGGTTTT CTGGTTCTGG GTCTGTGAAT   
  
  
+ AATGGCAAAA TTGGTTCCAT TTTGGGTAAT TGTTCATCTG GGGATTTGGA TTCTAAGGTT TCTAGTGACG   
  
  
+ GGTTGAATTC CAATTGTAGT TCACTGGGGA GTATGAGTAT CCTTGGTTCA ATCCATGGAC CTTTGCCTAA   
  
  
+ TACTGGTGGG TTTGTGTTCC CACAAACACA ACTATTTGAT CTTGGTGATG AGAAGCCTCA GATTTTGAAC   
  
  
+ CCACAATTGA TGACGATGAA TCCAAATCAG GCTCAGAGCA TGGGAAACCC TAGCTTTTTT GTGCCCTCCT   
  
  
+ TGGGTTATTG TCAGTTAGAG CAACATATGG TTCAACCACA GGCGAAACGC CATAGCCCTG GTGTAGTTTT   
  
  
+ GAACTCTGAT GTTATTGCCA AAACCCCGTT TCTCGATCAG GGTCATGAGT TTTTGTTGAG GAAACAACAC   
  
  
+ CATCAACAGA TTTTGCAGCA ACTTCCTATG GGTTTGGCTC ATCAGTCGGT CCCTCAGCAC CTCCAGCAAA   
  
  
+ AGGCGATGAA AGACCAGGTT GTTAAGGACC AGCTTTTGAA GGCAGCTGAC CTGATTCAAA CTGGGAATTT   
  
  
+ CTCACTTGCG CAAGAGATAT TGGCGCGGCT CAATCACCAG CTCTCCCTTC CTGCGAAGCC CCTCATTAGG   
  
  
+ GCAGCTTTGT ATGTCATGGA GGCCTTGCAA ATGCTCATCT TAATGAGCAA TCCTGTAGCA CCTCCACCGA   
  
  
+ TGAAGACGCT TTCCCCTATA GATGTTGTTC ATAAGATGAA TGCCTACAAG GCCTTCTCTG AGGTCTCTCC   
  
  
+ TATTACTCAG TTTACGAATT TCACTTGTGC CCAAGCCATT CTTGAAGCTC TCGATGATGC GGATTGCGTT   
  
  
+ CATGTCATTG ACTTTGATAT TGGTTGTGGG GCTCAATGGG CATCACTGAT TCAGGAGCTT CCATTGAGGA   
  
  
+ AAAAGGGAGC TCCATCCCTG AAAATTACAG CCATAGCTCC CCTGTCAATA AGCCACACTT TTGAACTCAA   
  
  
+ CCTAGCACGG GAAAACCTTG TGCAATTCGC CAATGATGTT GGTGTTGCTT TTGAGCTGCA AGTTGTGAAC   
  
  
+ TTGGATTTAT TTGATCCATC TTCATCTTCA ATGCCAAATG TTGGAACCTC TGGGGATGAG TTGATCGCTG   
  
  
+ TTAGTATACC CATTTGGGCA TGTTCATATC GGCCGTCTAT TCTCCCATCC ATCCTCCGGT TCATTAAGCA   
  
  
+ ACTAGCCCCC AAGATTGTCG TCTCGTTAGA TAGAGGGTCT GATCGTTGCG ACACTCTGTT CCCACAACAT   
  
  
+ CTTATCCATA TCTTAGAGTC CTGCACAAAT TTCTTGGAAT CGCTCGACGG TCTCAATGTA GCATCAGATA   
  
  
+ TTGTAAACAA GGTTGAGAAG TACTTCCTCC AGCCTAAGAT CGAAAACACC GTGTTGGGCC GCGTTCATGC   
  
  
+ CCCCAATAAG ATGCCACATT GGAAGACCCT CTTTGCTTCA GCCGGGTTAT TGCCCTTTCA GTTCAGTAAC   
  
  
+ TTCACAGAAA CACAAGCTGA TTATGTGGTG AAGCGAACCC CAGGGAGGGG ATTTCACATA GAAAAGCGCC   
  
  
+ ACGCATCTTT AGTGCTAAGT TGGCAAAGGC GGGAGCTTGT GACAGCCTCG GCATGGAGGT TTGAGAAGCT   
  
  
+ GTA  

- +Up\_Stream \_Len000TGTAAC CCACCCTAAA TTTAGGAGAT AAAAAAGAAT CCCCTTTTTA TGAACCCTTT   
  
  
- AAAGAGTTAT TGACTTTTAG ATTTATTAAG CACTGAAACA ATCATCCAAA TTACACTAAA TGATGGCACT   
  
  
- CACAGTGAAA ATCTACCATG AAATTCTTTC GTAATATTCA TTTTGTTATA TATAGCATCG TAGTATGATG   
  
  
- AACACATTAT TTAATTGTCG TTGGAGTTAA CATGAATAAA CTAAAGCGTA ACATTAGTTA AAAGGGTTTA   
  
  
- CTAAATGATA GAACCAGAAT GAGTCAACAT GAAGTGTGAG TTATCAAGTT AATAAAAAGT AATCTGATTA   
  
  
- CTCGCTCAGG GAAGATGTTC TCGGATGATG ATGAGATTAT TACATTCCTG ATAACCGATT ACCCCTTGAT   
  
  
- TCGGATGCTG AACAATCCGT ATCCACTTAA AAGGAATGTT CTCTCAAATT AAATGTGAAC TATAATTGAG   
  
  
- CTCTTGCTCT TCGACAGAAT TATCTATGGG TGTATCTTTT ACTTCGTTTA CGGGAACTAA TAACCTCCCA   
  
  
- ACCCGTTTAT ATAAGTCATT GTTACAACAA ACCGTTTATA GCAGACTTTT AACAAAAACT GTTTATAACA   
  
  
- AACCTTCTTA AAAAGCTCTA ATTTATAATT TTTACAATTT TAAAATTAGA TTTATAGTTT GTTTATAACT   
  
  
- TTTTACGAAA TATAATAGAT TTAGAGATTA ATTTTAAACA CATAACAGGT GATCTTTTTA CATATCACTA   
  
  
- TAATGGCTGT ATATACACTA GAGACCACAT TATCGGATGG GATTACAGAT CCTTTTATAA ATTTTAGGGG   
  
  
- TCACCAGTTT TCCTTCAATG CGTGTGATCC CGTTCTGTGA AGTAATCCGA TCAACCACTT TCGATTCAGT   
  
  
- ACTTTAAATC TGGTACCGGT ACAGATGAAC ATCTAGGTTG ATGGTTGTTA GGTAAAAATA AAACCCCTTT   
  
  
- CGAACTTCCC ATTAAACCAT TTACATGTGC CCTTGGAAAA CGATATTTTC TTCACGAAGA ACTTTTTTGT   
  
  
- GGGACATCAT TTTGTCCCGT TTTTTCGGTT GTGAAGACAA AGACCGTCTC TTTGTCATCA CATGGTGAAC   
  
  
- CTCAACCTCA TCTGTGCTAT GTGTTTGGGT CATCTCTCTC TTTGTCCCCT CTCTTTCTCA TGTGTTTAAA   
  
  
- CACGTCCTCT TCCCATTCCT TCTTCCTCTT CCTTGGGGGA GGTTTTTTTT GGGTATATTT TTTATTATTT   
  
  
- CTTTTTTGTC CTCACTCAAT CTCCCATGGG TTTAGAGGTT AGATTTTGAT CTTAGATCCC AGAGAAAAGT   
  
  
- AAACGAACTT TTTAAAGACA AGTACCCAAA ACACAAAAAT CCCCCTAAAC AAACTAAAAA CATCAGAAAC   
  
  
- ACCTAAAGGA CACAATATCT ACGTTCGATA CGAGAAGTTA AACGTTCCTT GTCGTAGACC CCCCTCTCTT   
  
  
- AAACTCCTTA AAGTTCATGG GTAAAGTTAA AGTTGTTCAT TTTAGTAGTA ACTTCCTATG AGTTTGGGTT   
  
  
- CGGGTCAACT ATCACTTGGT TGAAGATGAG ACCTAAGATG AGGTTCAGGG TCGGGAGGGT GGGAGAGGAG   
  
  
- GAGGAGGTTC TTGAGGATGG TTTTGCGACG GCAAAACCGA GTGGGAGGAG TCGAAGTGGT ACCTAGACTA   
  
  
- TTACACATTC TGGGGTTGGG AAAACGAAAA TAAAAGGAGT ACGAGGTAAG AGTACTAAAC ATGAAGACAA   
  
  
- TACATTAACC TCTACACAGA TTTAAACCTT TTCAAGCACC CAAAGCACAA ACTCTTAAAT CTTACTGTAA   
  
  
- GAAATTAATC ATTAAACTTC AAACGTACGA ATTAAATCGA TTAGTGACTA ATTAATTTTA TTTACTTCAC   
  
  
- TACATCCATA CCCGACAATG TGTTACAGTG GCCACCACCA CTGTCGCCGC CCCGCCGCCG CCAACCTAAC   
  
  
- CTTCTTACCC TGTCGCACAA AGGTTTACCC CTACCTCGAA ATGAGGGAAC CTAGTACCCC CTTCCACTAC   
  
  
- TAAACCCAGA CTTAAACTTC GTAGAGAATG TTAGACCAAT GGGACAACTC ATACTTCCGT TACGTCCTGA   
  
  
- ACCACAACAA CTAGTCTGTA GAGAAGAACC TCCACCACTA AACCCCAAAA GACCAAGACC CAGACACTTA   
  
  
- TTACCGTTTT AACCAAGGTA AAACCCATTA ACAAGTAGAC CCCTAAACCT AAGATTCCAA AGATCACTGC   
  
  
- CCAACTTAAG GTTAACATCA AGTGACCCCT CATACTCATA GGAACCAAGT TAGGTACCTG GAAACGGATT   
  
  
- ATGACCACCC AAACACAAGG GTGTTTGTGT TGATAAACTA GAACCACTAC TCTTCGGAGT CTAAAACTTG   
  
  
- GGTGTTAACT ACTGCTACTT AGGTTTAGTC CGAGTCTCGT ACCCTTTGGG ATCGAAAAAA CACGGGAGGA   
  
  
- ACCCAATAAC AGTCAATCTC GTTGTATACC AAGTTGGTGT CCGCTTTGCG GTATCGGGAC CACATCAAAA   
  
  
- CTTGAGACTA CAATAACGGT TTTGGGGCAA AGAGCTAGTC CCAGTACTCA AAAACAACTC CTTTGTTGTG   
  
  
- GTAGTTGTCT AAAACGTCGT TGAAGGATAC CCAAACCGAG TAGTCAGCCA GGGAGTCGTG GAGGTCGTTT   
  
  
- TCCGCTACTT TCTGGTCCAA CAATTCCTGG TCGAAAACTT CCGTCGACTG GACTAAGTTT GACCCTTAAA   
  
  
- GAGTGAACGC GTTCTCTATA ACCGCGCCGA GTTAGTGGTC GAGAGGGAAG GACGCTTCGG GGAGTAATCC   
  
  
- CGTCGAAACA TACAGTACCT CCGGAACGTT TACGAGTAGA ATTACTCGTT AGGACATCGT GGAGGTGGCT   
  
  
- ACTTCTGCGA AAGGGGATAT CTACAACAAG TATTCTACTT ACGGATGTTC CGGAAGAGAC TCCAGAGAGG   
  
  
- ATAATGAGTC AAATGCTTAA AGTGAACACG GGTTCGGTAA GAACTTCGAG AGCTACTACG CCTAACGCAA   
  
  
- GTACAGTAAC TGAAACTATA ACCAACACCC CGAGTTACCC GTAGTGACTA AGTCCTCGAA GGTAACTCCT   
  
  
- TTTTCCCTCG AGGTAGGGAC TTTTAATGTC GGTATCGAGG GGACAGTTAT TCGGTGTGAA AACTTGAGTT   
  
  
- GGATCGTGCC CTTTTGGAAC ACGTTAAGCG GTTACTACAA CCACAACGAA AACTCGACGT TCAACACTTG   
  
  
- AACCTAAATA AACTAGGTAG AAGTAGAAGT TACGGTTTAC AACCTTGGAG ACCCCTACTC AACTAGCGAC   
  
  
- AATCATATGG GTAAACCCGT ACAAGTATAG CCGGCAGATA AGAGGGTAGG TAGGAGGCCA AGTAATTCGT   
  
  
- TGATCGGGGG TTCTAACAGC AGAGCAATCT ATCTCCCAGA CTAGCAACGC TGTGAGACAA GGGTGTTGTA   
  
  
- GAATAGGTAT AGAATCTCAG GACGTGTTTA AAGAACCTTA GCGAGCTGCC AGAGTTACAT CGTAGTCTAT   
  
  
- AACATTTGTT CCAACTCTTC ATGAAGGAGG TCGGATTCTA GCTTTTGTGG CACAACCCGG CGCAAGTACG   
  
  
- GGGGTTATTC TACGGTGTAA CCTTCTGGGA GAAACGAAGT CGGCCCAATA ACGGGAAAGT CAAGTCATTG   
  
  
- AAGTGTCTTT GTGTTCGACT AATACACCAC TTCGCTTGGG GTCCCTCCCC TAAAGTGTAT CTTTTCGCGG   
  
  
- TGCGTAGAAA TCACGATTCA ACCGTTTCCG CCCTCGAACA CTGTCGGAGC CGTACCTCCA AACTCTTCGA   
  
  
- CAT

  
  
Motifs Found  

+   

| Site Name | Organism | Position | Strand | Matrix score. | sequence | function |
| --- | --- | --- | --- | --- | --- | --- |
|  | organism | 1297 | + | 4 | motif\_sequence | short\_function |
|  | organism | 2125 | + | 4 | motif\_sequence | short\_function |
|  | organism | 3314 | + | 4 | motif\_sequence | short\_function |
|  | organism | 3248 | + | 4 | motif\_sequence | short\_function |
|  | organism | 1172 | - | 4 | motif\_sequence | short\_function |
|  | organism | 1973 | - | 4 | motif\_sequence | short\_function |
|  | organism | 3242 | + | 4 | motif\_sequence | short\_function |
|  | organism | 944 | - | 4 | motif\_sequence | short\_function |
|  | organism | 3420 | + | 4 | motif\_sequence | short\_function |
|  | organism | 1351 | + | 4 | motif\_sequence | short\_function |
|  | organism | 1763 | - | 4 | motif\_sequence | short\_function |
|  | organism | 1436 | + | 4 | motif\_sequence | short\_function |
|  | organism | 1452 | - | 4 | motif\_sequence | short\_function |
|  | organism | 2939 | + | 4 | motif\_sequence | short\_function |
|  | organism | 1607 | + | 4 | motif\_sequence | short\_function |
|  | organism | 1467 | - | 4 | motif\_sequence | short\_function |
|  | organism | 1099 | - | 4 | motif\_sequence | short\_function |
|  | organism | 1155 | - | 4 | motif\_sequence | short\_function |
|  | organism | 2775 | + | 4 | motif\_sequence | short\_function |
|  | organism | 1006 | + | 4 | motif\_sequence | short\_function |

>HU05G00466.1   
+ +Up\_Stream \_Len000ACATTG GGTGGGATTT AAATCCTCTA TTTTTTCTTA GGGGAAAAAT ACTTGGGAAA   
  
  
+ TTTCTCAATA ACTGAAAATC TAAATAATTC GTGACTTTGT TAGTAGGTTT AATGTGATTT ACTACCGTGA   
  
  
+ GTGTCACTTT TAGATGGTAC TTTAAGAAAG CATTATAAGT AAAACAATAT ATATCGTAGC ATCATACTAC   
  
  
+ TTGTGTAATA AATTAACAGC AACCTCAATT GTACTTATTT GATTTCGCAT TGTAATCAAT TTTCCCAAAT   
  
  
+ GATTTACTAT CTTGGTCTTA CTCAGTTGTA CTTCACACTC AATAGTTCAA TTATTTTTCA TTAGACTAAT   
  
  
+ GAGCGAGTCC CTTCTACAAG AGCCTACTAC TACTCTAATA ATGTAAGGAC TATTGGCTAA TGGGGAACTA   
  
  
+ AGCCTACGAC TTGTTAGGCA TAGGTGAATT TTCCTTACAA GAGAGTTTAA TTTACACTTG ATATTAACTC   
  
  
+ GAGAACGAGA AGCTGTCTTA ATAGATACCC ACATAGAAAA TGAAGCAAAT GCCCTTGATT ATTGGAGGGT   
  
  
+ TGGGCAAATA TATTCAGTAA CAATGTTGTT TGGCAAATAT CGTCTGAAAA TTGTTTTTGA CAAATATTGT   
  
  
+ TTGGAAGAAT TTTTCGAGAT TAAATATTAA AAATGTTAAA ATTTTAATCT AAATATCAAA CAAATATTGA   
  
  
+ AAAATGCTTT ATATTATCTA AATCTCTAAT TAAAATTTGT GTATTGTCCA CTAGAAAAAT GTATAGTGAT   
  
  
+ ATTACCGACA TATATGTGAT CTCTGGTGTA ATAGCCTACC CTAATGTCTA GGAAAATATT TAAAATCCCC   
  
  
+ AGTGGTCAAA AGGAAGTTAC GCACACTAGG GCAAGACACT TCATTAGGCT AGTTGGTGAA AGCTAAGTCA   
  
  
+ TGAAATTTAG ACCATGGCCA TGTCTACTTG TAGATCCAAC TACCAACAAT CCATTTTTAT TTTGGGGAAA   
  
  
+ GCTTGAAGGG TAATTTGGTA AATGTACACG GGAACCTTTT GCTATAAAAG AAGTGCTTCT TGAAAAAACA   
  
  
+ CCCTGTAGTA AAACAGGGCA AAAAAGCCAA CACTTCTGTT TCTGGCAGAG AAACAGTAGT GTACCACTTG   
  
  
+ GAGTTGGAGT AGACACGATA CACAAACCCA GTAGAGAGAG AAACAGGGGA GAGAAAGAGT ACACAAATTT   
  
  
+ GTGCAGGAGA AGGGTAAGGA AGAAGGAGAA GGAACCCCCT CCAAAAAAAA CCCATATAAA AAATAATAAA   
  
  
+ GAAAAAACAG GAGTGAGTTA GAGGGTACCC AAATCTCCAA TCTAAAACTA GAATCTAGGG TCTCTTTTCA   
  
  
+ TTTGCTTGAA AAATTTCTGT TCATGGGTTT TGTGTTTTTA GGGGGATTTG TTTGATTTTT GTAGTCTTTG   
  
  
+ TGGATTTCCT GTGTTATAGA TGCAAGCTAT GCTCTTCAAT TTGCAAGGAA CAGCATCTGG GGGGAGAGAA   
  
  
+ TTTGAGGAAT TTCAAGTACC CATTTCAATT TCAACAAGTA AAATCATCAT TGAAGGATAC TCAAACCCAA   
  
  
+ GCCCAGTTGA TAGTGAACCA ACTTCTACTC TGGATTCTAC TCCAAGTCCC AGCCCTCCCA CCCTCTCCTC   
  
  
+ CTCCTCCAAG AACTCCTACC AAAACGCTGC CGTTTTGGCT CACCCTCCTC AGCTTCACCA TGGATCTGAT   
  
  
+ AATGTGTAAG ACCCCAACCC TTTTGCTTTT ATTTTCCTCA TGCTCCATTC TCATGATTTG TACTTCTGTT   
  
  
+ ATGTAATTGG AGATGTGTCT AAATTTGGAA AAGTTCGTGG GTTTCGTGTT TGAGAATTTA GAATGACATT   
  
  
+ CTTTAATTAG TAATTTGAAG TTTGCATGCT TAATTTAGCT AATCACTGAT TAATTAAAAT AAATGAAGTG   
  
  
+ ATGTAGGTAT GGGCTGTTAC ACAATGTCAC CGGTGGTGGT GACAGCGGCG GGGCGGCGGC GGTTGGATTG   
  
  
+ GAAGAATGGG ACAGCGTGTT TCCAAATGGG GATGGAGCTT TACTCCCTTG GATCATGGGG GAAGGTGATG   
  
  
+ ATTTGGGTCT GAATTTGAAG CATCTCTTAC AATCTGGTTA CCCTGTTGAG TATGAAGGCA ATGCAGGACT   
  
  
+ TGGTGTTGTT GATCAGACAT CTCTTCTTGG AGGTGGTGAT TTGGGGTTTT CTGGTTCTGG GTCTGTGAAT   
  
  
+ AATGGCAAAA TTGGTTCCAT TTTGGGTAAT TGTTCATCTG GGGATTTGGA TTCTAAGGTT TCTAGTGACG   
  
  
+ GGTTGAATTC CAATTGTAGT TCACTGGGGA GTATGAGTAT CCTTGGTTCA ATCCATGGAC CTTTGCCTAA   
  
  
+ TACTGGTGGG TTTGTGTTCC CACAAACACA ACTATTTGAT CTTGGTGATG AGAAGCCTCA GATTTTGAAC   
  
  
+ CCACAATTGA TGACGATGAA TCCAAATCAG GCTCAGAGCA TGGGAAACCC TAGCTTTTTT GTGCCCTCCT   
  
  
+ TGGGTTATTG TCAGTTAGAG CAACATATGG TTCAACCACA GGCGAAACGC CATAGCCCTG GTGTAGTTTT   
  
  
+ GAACTCTGAT GTTATTGCCA AAACCCCGTT TCTCGATCAG GGTCATGAGT TTTTGTTGAG GAAACAACAC   
  
  
+ CATCAACAGA TTTTGCAGCA ACTTCCTATG GGTTTGGCTC ATCAGTCGGT CCCTCAGCAC CTCCAGCAAA   
  
  
+ AGGCGATGAA AGACCAGGTT GTTAAGGACC AGCTTTTGAA GGCAGCTGAC CTGATTCAAA CTGGGAATTT   
  
  
+ CTCACTTGCG CAAGAGATAT TGGCGCGGCT CAATCACCAG CTCTCCCTTC CTGCGAAGCC CCTCATTAGG   
  
  
+ GCAGCTTTGT ATGTCATGGA GGCCTTGCAA ATGCTCATCT TAATGAGCAA TCCTGTAGCA CCTCCACCGA   
  
  
+ TGAAGACGCT TTCCCCTATA GATGTTGTTC ATAAGATGAA TGCCTACAAG GCCTTCTCTG AGGTCTCTCC   
  
  
+ TATTACTCAG TTTACGAATT TCACTTGTGC CCAAGCCATT CTTGAAGCTC TCGATGATGC GGATTGCGTT   
  
  
+ CATGTCATTG ACTTTGATAT TGGTTGTGGG GCTCAATGGG CATCACTGAT TCAGGAGCTT CCATTGAGGA   
  
  
+ AAAAGGGAGC TCCATCCCTG AAAATTACAG CCATAGCTCC CCTGTCAATA AGCCACACTT TTGAACTCAA   
  
  
+ CCTAGCACGG GAAAACCTTG TGCAATTCGC CAATGATGTT GGTGTTGCTT TTGAGCTGCA AGTTGTGAAC   
  
  
+ TTGGATTTAT TTGATCCATC TTCATCTTCA ATGCCAAATG TTGGAACCTC TGGGGATGAG TTGATCGCTG   
  
  
+ TTAGTATACC CATTTGGGCA TGTTCATATC GGCCGTCTAT TCTCCCATCC ATCCTCCGGT TCATTAAGCA   
  
  
+ ACTAGCCCCC AAGATTGTCG TCTCGTTAGA TAGAGGGTCT GATCGTTGCG ACACTCTGTT CCCACAACAT   
  
  
+ CTTATCCATA TCTTAGAGTC CTGCACAAAT TTCTTGGAAT CGCTCGACGG TCTCAATGTA GCATCAGATA   
  
  
+ TTGTAAACAA GGTTGAGAAG TACTTCCTCC AGCCTAAGAT CGAAAACACC GTGTTGGGCC GCGTTCATGC   
  
  
+ CCCCAATAAG ATGCCACATT GGAAGACCCT CTTTGCTTCA GCCGGGTTAT TGCCCTTTCA GTTCAGTAAC   
  
  
+ TTCACAGAAA CACAAGCTGA TTATGTGGTG AAGCGAACCC CAGGGAGGGG ATTTCACATA GAAAAGCGCC   
  
  
+ ACGCATCTTT AGTGCTAAGT TGGCAAAGGC GGGAGCTTGT GACAGCCTCG GCATGGAGGT TTGAGAAGCT   
  
  
+ GTA  

- +Up\_Stream \_Len000TGTAAC CCACCCTAAA TTTAGGAGAT AAAAAAGAAT CCCCTTTTTA TGAACCCTTT   
  
  
- AAAGAGTTAT TGACTTTTAG ATTTATTAAG CACTGAAACA ATCATCCAAA TTACACTAAA TGATGGCACT   
  
  
- CACAGTGAAA ATCTACCATG AAATTCTTTC GTAATATTCA TTTTGTTATA TATAGCATCG TAGTATGATG   
  
  
- AACACATTAT TTAATTGTCG TTGGAGTTAA CATGAATAAA CTAAAGCGTA ACATTAGTTA AAAGGGTTTA   
  
  
- CTAAATGATA GAACCAGAAT GAGTCAACAT GAAGTGTGAG TTATCAAGTT AATAAAAAGT AATCTGATTA   
  
  
- CTCGCTCAGG GAAGATGTTC TCGGATGATG ATGAGATTAT TACATTCCTG ATAACCGATT ACCCCTTGAT   
  
  
- TCGGATGCTG AACAATCCGT ATCCACTTAA AAGGAATGTT CTCTCAAATT AAATGTGAAC TATAATTGAG   
  
  
- CTCTTGCTCT TCGACAGAAT TATCTATGGG TGTATCTTTT ACTTCGTTTA CGGGAACTAA TAACCTCCCA   
  
  
- ACCCGTTTAT ATAAGTCATT GTTACAACAA ACCGTTTATA GCAGACTTTT AACAAAAACT GTTTATAACA   
  
  
- AACCTTCTTA AAAAGCTCTA ATTTATAATT TTTACAATTT TAAAATTAGA TTTATAGTTT GTTTATAACT   
  
  
- TTTTACGAAA TATAATAGAT TTAGAGATTA ATTTTAAACA CATAACAGGT GATCTTTTTA CATATCACTA   
  
  
- TAATGGCTGT ATATACACTA GAGACCACAT TATCGGATGG GATTACAGAT CCTTTTATAA ATTTTAGGGG   
  
  
- TCACCAGTTT TCCTTCAATG CGTGTGATCC CGTTCTGTGA AGTAATCCGA TCAACCACTT TCGATTCAGT   
  
  
- ACTTTAAATC TGGTACCGGT ACAGATGAAC ATCTAGGTTG ATGGTTGTTA GGTAAAAATA AAACCCCTTT   
  
  
- CGAACTTCCC ATTAAACCAT TTACATGTGC CCTTGGAAAA CGATATTTTC TTCACGAAGA ACTTTTTTGT   
  
  
- GGGACATCAT TTTGTCCCGT TTTTTCGGTT GTGAAGACAA AGACCGTCTC TTTGTCATCA CATGGTGAAC   
  
  
- CTCAACCTCA TCTGTGCTAT GTGTTTGGGT CATCTCTCTC TTTGTCCCCT CTCTTTCTCA TGTGTTTAAA   
  
  
- CACGTCCTCT TCCCATTCCT TCTTCCTCTT CCTTGGGGGA GGTTTTTTTT GGGTATATTT TTTATTATTT   
  
  
- CTTTTTTGTC CTCACTCAAT CTCCCATGGG TTTAGAGGTT AGATTTTGAT CTTAGATCCC AGAGAAAAGT   
  
  
- AAACGAACTT TTTAAAGACA AGTACCCAAA ACACAAAAAT CCCCCTAAAC AAACTAAAAA CATCAGAAAC   
  
  
- ACCTAAAGGA CACAATATCT ACGTTCGATA CGAGAAGTTA AACGTTCCTT GTCGTAGACC CCCCTCTCTT   
  
  
- AAACTCCTTA AAGTTCATGG GTAAAGTTAA AGTTGTTCAT TTTAGTAGTA ACTTCCTATG AGTTTGGGTT   
  
  
- CGGGTCAACT ATCACTTGGT TGAAGATGAG ACCTAAGATG AGGTTCAGGG TCGGGAGGGT GGGAGAGGAG   
  
  
- GAGGAGGTTC TTGAGGATGG TTTTGCGACG GCAAAACCGA GTGGGAGGAG TCGAAGTGGT ACCTAGACTA   
  
  
- TTACACATTC TGGGGTTGGG AAAACGAAAA TAAAAGGAGT ACGAGGTAAG AGTACTAAAC ATGAAGACAA   
  
  
- TACATTAACC TCTACACAGA TTTAAACCTT TTCAAGCACC CAAAGCACAA ACTCTTAAAT CTTACTGTAA   
  
  
- GAAATTAATC ATTAAACTTC AAACGTACGA ATTAAATCGA TTAGTGACTA ATTAATTTTA TTTACTTCAC   
  
  
- TACATCCATA CCCGACAATG TGTTACAGTG GCCACCACCA CTGTCGCCGC CCCGCCGCCG CCAACCTAAC   
  
  
- CTTCTTACCC TGTCGCACAA AGGTTTACCC CTACCTCGAA ATGAGGGAAC CTAGTACCCC CTTCCACTAC   
  
  
- TAAACCCAGA CTTAAACTTC GTAGAGAATG TTAGACCAAT GGGACAACTC ATACTTCCGT TACGTCCTGA   
  
  
- ACCACAACAA CTAGTCTGTA GAGAAGAACC TCCACCACTA AACCCCAAAA GACCAAGACC CAGACACTTA   
  
  
- TTACCGTTTT AACCAAGGTA AAACCCATTA ACAAGTAGAC CCCTAAACCT AAGATTCCAA AGATCACTGC   
  
  
- CCAACTTAAG GTTAACATCA AGTGACCCCT CATACTCATA GGAACCAAGT TAGGTACCTG GAAACGGATT   
  
  
- ATGACCACCC AAACACAAGG GTGTTTGTGT TGATAAACTA GAACCACTAC TCTTCGGAGT CTAAAACTTG   
  
  
- GGTGTTAACT ACTGCTACTT AGGTTTAGTC CGAGTCTCGT ACCCTTTGGG ATCGAAAAAA CACGGGAGGA   
  
  
- ACCCAATAAC AGTCAATCTC GTTGTATACC AAGTTGGTGT CCGCTTTGCG GTATCGGGAC CACATCAAAA   
  
  
- CTTGAGACTA CAATAACGGT TTTGGGGCAA AGAGCTAGTC CCAGTACTCA AAAACAACTC CTTTGTTGTG   
  
  
- GTAGTTGTCT AAAACGTCGT TGAAGGATAC CCAAACCGAG TAGTCAGCCA GGGAGTCGTG GAGGTCGTTT   
  
  
- TCCGCTACTT TCTGGTCCAA CAATTCCTGG TCGAAAACTT CCGTCGACTG GACTAAGTTT GACCCTTAAA   
  
  
- GAGTGAACGC GTTCTCTATA ACCGCGCCGA GTTAGTGGTC GAGAGGGAAG GACGCTTCGG GGAGTAATCC   
  
  
- CGTCGAAACA TACAGTACCT CCGGAACGTT TACGAGTAGA ATTACTCGTT AGGACATCGT GGAGGTGGCT   
  
  
- ACTTCTGCGA AAGGGGATAT CTACAACAAG TATTCTACTT ACGGATGTTC CGGAAGAGAC TCCAGAGAGG   
  
  
- ATAATGAGTC AAATGCTTAA AGTGAACACG GGTTCGGTAA GAACTTCGAG AGCTACTACG CCTAACGCAA   
  
  
- GTACAGTAAC TGAAACTATA ACCAACACCC CGAGTTACCC GTAGTGACTA AGTCCTCGAA GGTAACTCCT   
  
  
- TTTTCCCTCG AGGTAGGGAC TTTTAATGTC GGTATCGAGG GGACAGTTAT TCGGTGTGAA AACTTGAGTT   
  
  
- GGATCGTGCC CTTTTGGAAC ACGTTAAGCG GTTACTACAA CCACAACGAA AACTCGACGT TCAACACTTG   
  
  
- AACCTAAATA AACTAGGTAG AAGTAGAAGT TACGGTTTAC AACCTTGGAG ACCCCTACTC AACTAGCGAC   
  
  
- AATCATATGG GTAAACCCGT ACAAGTATAG CCGGCAGATA AGAGGGTAGG TAGGAGGCCA AGTAATTCGT   
  
  
- TGATCGGGGG TTCTAACAGC AGAGCAATCT ATCTCCCAGA CTAGCAACGC TGTGAGACAA GGGTGTTGTA   
  
  
- GAATAGGTAT AGAATCTCAG GACGTGTTTA AAGAACCTTA GCGAGCTGCC AGAGTTACAT CGTAGTCTAT   
  
  
- AACATTTGTT CCAACTCTTC ATGAAGGAGG TCGGATTCTA GCTTTTGTGG CACAACCCGG CGCAAGTACG   
  
  
- GGGGTTATTC TACGGTGTAA CCTTCTGGGA GAAACGAAGT CGGCCCAATA ACGGGAAAGT CAAGTCATTG   
  
  
- AAGTGTCTTT GTGTTCGACT AATACACCAC TTCGCTTGGG GTCCCTCCCC TAAAGTGTAT CTTTTCGCGG   
  
  
- TGCGTAGAAA TCACGATTCA ACCGTTTCCG CCCTCGAACA CTGTCGGAGC CGTACCTCCA AACTCTTCGA   
  
  
- CAT

+     3-AF3 binding site

| Site Name | Organism | Position | Strand | Matrix score. | sequence | function |
| --- | --- | --- | --- | --- | --- | --- |
| 3-AF3 binding site | Pisum sativum | 3389 | - | 10 | CACTATCTAAC | part of a conserved DNA module array (CMA3) |

>HU05G00466.1   
+ +Up\_Stream \_Len000ACATTG GGTGGGATTT AAATCCTCTA TTTTTTCTTA GGGGAAAAAT ACTTGGGAAA   
  
  
+ TTTCTCAATA ACTGAAAATC TAAATAATTC GTGACTTTGT TAGTAGGTTT AATGTGATTT ACTACCGTGA   
  
  
+ GTGTCACTTT TAGATGGTAC TTTAAGAAAG CATTATAAGT AAAACAATAT ATATCGTAGC ATCATACTAC   
  
  
+ TTGTGTAATA AATTAACAGC AACCTCAATT GTACTTATTT GATTTCGCAT TGTAATCAAT TTTCCCAAAT   
  
  
+ GATTTACTAT CTTGGTCTTA CTCAGTTGTA CTTCACACTC AATAGTTCAA TTATTTTTCA TTAGACTAAT   
  
  
+ GAGCGAGTCC CTTCTACAAG AGCCTACTAC TACTCTAATA ATGTAAGGAC TATTGGCTAA TGGGGAACTA   
  
  
+ AGCCTACGAC TTGTTAGGCA TAGGTGAATT TTCCTTACAA GAGAGTTTAA TTTACACTTG ATATTAACTC   
  
  
+ GAGAACGAGA AGCTGTCTTA ATAGATACCC ACATAGAAAA TGAAGCAAAT GCCCTTGATT ATTGGAGGGT   
  
  
+ TGGGCAAATA TATTCAGTAA CAATGTTGTT TGGCAAATAT CGTCTGAAAA TTGTTTTTGA CAAATATTGT   
  
  
+ TTGGAAGAAT TTTTCGAGAT TAAATATTAA AAATGTTAAA ATTTTAATCT AAATATCAAA CAAATATTGA   
  
  
+ AAAATGCTTT ATATTATCTA AATCTCTAAT TAAAATTTGT GTATTGTCCA CTAGAAAAAT GTATAGTGAT   
  
  
+ ATTACCGACA TATATGTGAT CTCTGGTGTA ATAGCCTACC CTAATGTCTA GGAAAATATT TAAAATCCCC   
  
  
+ AGTGGTCAAA AGGAAGTTAC GCACACTAGG GCAAGACACT TCATTAGGCT AGTTGGTGAA AGCTAAGTCA   
  
  
+ TGAAATTTAG ACCATGGCCA TGTCTACTTG TAGATCCAAC TACCAACAAT CCATTTTTAT TTTGGGGAAA   
  
  
+ GCTTGAAGGG TAATTTGGTA AATGTACACG GGAACCTTTT GCTATAAAAG AAGTGCTTCT TGAAAAAACA   
  
  
+ CCCTGTAGTA AAACAGGGCA AAAAAGCCAA CACTTCTGTT TCTGGCAGAG AAACAGTAGT GTACCACTTG   
  
  
+ GAGTTGGAGT AGACACGATA CACAAACCCA GTAGAGAGAG AAACAGGGGA GAGAAAGAGT ACACAAATTT   
  
  
+ GTGCAGGAGA AGGGTAAGGA AGAAGGAGAA GGAACCCCCT CCAAAAAAAA CCCATATAAA AAATAATAAA   
  
  
+ GAAAAAACAG GAGTGAGTTA GAGGGTACCC AAATCTCCAA TCTAAAACTA GAATCTAGGG TCTCTTTTCA   
  
  
+ TTTGCTTGAA AAATTTCTGT TCATGGGTTT TGTGTTTTTA GGGGGATTTG TTTGATTTTT GTAGTCTTTG   
  
  
+ TGGATTTCCT GTGTTATAGA TGCAAGCTAT GCTCTTCAAT TTGCAAGGAA CAGCATCTGG GGGGAGAGAA   
  
  
+ TTTGAGGAAT TTCAAGTACC CATTTCAATT TCAACAAGTA AAATCATCAT TGAAGGATAC TCAAACCCAA   
  
  
+ GCCCAGTTGA TAGTGAACCA ACTTCTACTC TGGATTCTAC TCCAAGTCCC AGCCCTCCCA CCCTCTCCTC   
  
  
+ CTCCTCCAAG AACTCCTACC AAAACGCTGC CGTTTTGGCT CACCCTCCTC AGCTTCACCA TGGATCTGAT   
  
  
+ AATGTGTAAG ACCCCAACCC TTTTGCTTTT ATTTTCCTCA TGCTCCATTC TCATGATTTG TACTTCTGTT   
  
  
+ ATGTAATTGG AGATGTGTCT AAATTTGGAA AAGTTCGTGG GTTTCGTGTT TGAGAATTTA GAATGACATT   
  
  
+ CTTTAATTAG TAATTTGAAG TTTGCATGCT TAATTTAGCT AATCACTGAT TAATTAAAAT AAATGAAGTG   
  
  
+ ATGTAGGTAT GGGCTGTTAC ACAATGTCAC CGGTGGTGGT GACAGCGGCG GGGCGGCGGC GGTTGGATTG   
  
  
+ GAAGAATGGG ACAGCGTGTT TCCAAATGGG GATGGAGCTT TACTCCCTTG GATCATGGGG GAAGGTGATG   
  
  
+ ATTTGGGTCT GAATTTGAAG CATCTCTTAC AATCTGGTTA CCCTGTTGAG TATGAAGGCA ATGCAGGACT   
  
  
+ TGGTGTTGTT GATCAGACAT CTCTTCTTGG AGGTGGTGAT TTGGGGTTTT CTGGTTCTGG GTCTGTGAAT   
  
  
+ AATGGCAAAA TTGGTTCCAT TTTGGGTAAT TGTTCATCTG GGGATTTGGA TTCTAAGGTT TCTAGTGACG   
  
  
+ GGTTGAATTC CAATTGTAGT TCACTGGGGA GTATGAGTAT CCTTGGTTCA ATCCATGGAC CTTTGCCTAA   
  
  
+ TACTGGTGGG TTTGTGTTCC CACAAACACA ACTATTTGAT CTTGGTGATG AGAAGCCTCA GATTTTGAAC   
  
  
+ CCACAATTGA TGACGATGAA TCCAAATCAG GCTCAGAGCA TGGGAAACCC TAGCTTTTTT GTGCCCTCCT   
  
  
+ TGGGTTATTG TCAGTTAGAG CAACATATGG TTCAACCACA GGCGAAACGC CATAGCCCTG GTGTAGTTTT   
  
  
+ GAACTCTGAT GTTATTGCCA AAACCCCGTT TCTCGATCAG GGTCATGAGT TTTTGTTGAG GAAACAACAC   
  
  
+ CATCAACAGA TTTTGCAGCA ACTTCCTATG GGTTTGGCTC ATCAGTCGGT CCCTCAGCAC CTCCAGCAAA   
  
  
+ AGGCGATGAA AGACCAGGTT GTTAAGGACC AGCTTTTGAA GGCAGCTGAC CTGATTCAAA CTGGGAATTT   
  
  
+ CTCACTTGCG CAAGAGATAT TGGCGCGGCT CAATCACCAG CTCTCCCTTC CTGCGAAGCC CCTCATTAGG   
  
  
+ GCAGCTTTGT ATGTCATGGA GGCCTTGCAA ATGCTCATCT TAATGAGCAA TCCTGTAGCA CCTCCACCGA   
  
  
+ TGAAGACGCT TTCCCCTATA GATGTTGTTC ATAAGATGAA TGCCTACAAG GCCTTCTCTG AGGTCTCTCC   
  
  
+ TATTACTCAG TTTACGAATT TCACTTGTGC CCAAGCCATT CTTGAAGCTC TCGATGATGC GGATTGCGTT   
  
  
+ CATGTCATTG ACTTTGATAT TGGTTGTGGG GCTCAATGGG CATCACTGAT TCAGGAGCTT CCATTGAGGA   
  
  
+ AAAAGGGAGC TCCATCCCTG AAAATTACAG CCATAGCTCC CCTGTCAATA AGCCACACTT TTGAACTCAA   
  
  
+ CCTAGCACGG GAAAACCTTG TGCAATTCGC CAATGATGTT GGTGTTGCTT TTGAGCTGCA AGTTGTGAAC   
  
  
+ TTGGATTTAT TTGATCCATC TTCATCTTCA ATGCCAAATG TTGGAACCTC TGGGGATGAG TTGATCGCTG   
  
  
+ TTAGTATACC CATTTGGGCA TGTTCATATC GGCCGTCTAT TCTCCCATCC ATCCTCCGGT TCATTAAGCA   
  
  
+ ACTAGCCCCC AAGATTGTCG TCTCGTTAGA TAGAGGGTCT GATCGTTGCG ACACTCTGTT CCCACAACAT   
  
  
+ CTTATCCATA TCTTAGAGTC CTGCACAAAT TTCTTGGAAT CGCTCGACGG TCTCAATGTA GCATCAGATA   
  
  
+ TTGTAAACAA GGTTGAGAAG TACTTCCTCC AGCCTAAGAT CGAAAACACC GTGTTGGGCC GCGTTCATGC   
  
  
+ CCCCAATAAG ATGCCACATT GGAAGACCCT CTTTGCTTCA GCCGGGTTAT TGCCCTTTCA GTTCAGTAAC   
  
  
+ TTCACAGAAA CACAAGCTGA TTATGTGGTG AAGCGAACCC CAGGGAGGGG ATTTCACATA GAAAAGCGCC   
  
  
+ ACGCATCTTT AGTGCTAAGT TGGCAAAGGC GGGAGCTTGT GACAGCCTCG GCATGGAGGT TTGAGAAGCT   
  
  
+ GTA  

- +Up\_Stream \_Len000TGTAAC CCACCCTAAA TTTAGGAGAT AAAAAAGAAT CCCCTTTTTA TGAACCCTTT   
  
  
- AAAGAGTTAT TGACTTTTAG ATTTATTAAG CACTGAAACA ATCATCCAAA TTACACTAAA TGATGGCACT   
  
  
- CACAGTGAAA ATCTACCATG AAATTCTTTC GTAATATTCA TTTTGTTATA TATAGCATCG TAGTATGATG   
  
  
- AACACATTAT TTAATTGTCG TTGGAGTTAA CATGAATAAA CTAAAGCGTA ACATTAGTTA AAAGGGTTTA   
  
  
- CTAAATGATA GAACCAGAAT GAGTCAACAT GAAGTGTGAG TTATCAAGTT AATAAAAAGT AATCTGATTA   
  
  
- CTCGCTCAGG GAAGATGTTC TCGGATGATG ATGAGATTAT TACATTCCTG ATAACCGATT ACCCCTTGAT   
  
  
- TCGGATGCTG AACAATCCGT ATCCACTTAA AAGGAATGTT CTCTCAAATT AAATGTGAAC TATAATTGAG   
  
  
- CTCTTGCTCT TCGACAGAAT TATCTATGGG TGTATCTTTT ACTTCGTTTA CGGGAACTAA TAACCTCCCA   
  
  
- ACCCGTTTAT ATAAGTCATT GTTACAACAA ACCGTTTATA GCAGACTTTT AACAAAAACT GTTTATAACA   
  
  
- AACCTTCTTA AAAAGCTCTA ATTTATAATT TTTACAATTT TAAAATTAGA TTTATAGTTT GTTTATAACT   
  
  
- TTTTACGAAA TATAATAGAT TTAGAGATTA ATTTTAAACA CATAACAGGT GATCTTTTTA CATATCACTA   
  
  
- TAATGGCTGT ATATACACTA GAGACCACAT TATCGGATGG GATTACAGAT CCTTTTATAA ATTTTAGGGG   
  
  
- TCACCAGTTT TCCTTCAATG CGTGTGATCC CGTTCTGTGA AGTAATCCGA TCAACCACTT TCGATTCAGT   
  
  
- ACTTTAAATC TGGTACCGGT ACAGATGAAC ATCTAGGTTG ATGGTTGTTA GGTAAAAATA AAACCCCTTT   
  
  
- CGAACTTCCC ATTAAACCAT TTACATGTGC CCTTGGAAAA CGATATTTTC TTCACGAAGA ACTTTTTTGT   
  
  
- GGGACATCAT TTTGTCCCGT TTTTTCGGTT GTGAAGACAA AGACCGTCTC TTTGTCATCA CATGGTGAAC   
  
  
- CTCAACCTCA TCTGTGCTAT GTGTTTGGGT CATCTCTCTC TTTGTCCCCT CTCTTTCTCA TGTGTTTAAA   
  
  
- CACGTCCTCT TCCCATTCCT TCTTCCTCTT CCTTGGGGGA GGTTTTTTTT GGGTATATTT TTTATTATTT   
  
  
- CTTTTTTGTC CTCACTCAAT CTCCCATGGG TTTAGAGGTT AGATTTTGAT CTTAGATCCC AGAGAAAAGT   
  
  
- AAACGAACTT TTTAAAGACA AGTACCCAAA ACACAAAAAT CCCCCTAAAC AAACTAAAAA CATCAGAAAC   
  
  
- ACCTAAAGGA CACAATATCT ACGTTCGATA CGAGAAGTTA AACGTTCCTT GTCGTAGACC CCCCTCTCTT   
  
  
- AAACTCCTTA AAGTTCATGG GTAAAGTTAA AGTTGTTCAT TTTAGTAGTA ACTTCCTATG AGTTTGGGTT   
  
  
- CGGGTCAACT ATCACTTGGT TGAAGATGAG ACCTAAGATG AGGTTCAGGG TCGGGAGGGT GGGAGAGGAG   
  
  
- GAGGAGGTTC TTGAGGATGG TTTTGCGACG GCAAAACCGA GTGGGAGGAG TCGAAGTGGT ACCTAGACTA   
  
  
- TTACACATTC TGGGGTTGGG AAAACGAAAA TAAAAGGAGT ACGAGGTAAG AGTACTAAAC ATGAAGACAA   
  
  
- TACATTAACC TCTACACAGA TTTAAACCTT TTCAAGCACC CAAAGCACAA ACTCTTAAAT CTTACTGTAA   
  
  
- GAAATTAATC ATTAAACTTC AAACGTACGA ATTAAATCGA TTAGTGACTA ATTAATTTTA TTTACTTCAC   
  
  
- TACATCCATA CCCGACAATG TGTTACAGTG GCCACCACCA CTGTCGCCGC CCCGCCGCCG CCAACCTAAC   
  
  
- CTTCTTACCC TGTCGCACAA AGGTTTACCC CTACCTCGAA ATGAGGGAAC CTAGTACCCC CTTCCACTAC   
  
  
- TAAACCCAGA CTTAAACTTC GTAGAGAATG TTAGACCAAT GGGACAACTC ATACTTCCGT TACGTCCTGA   
  
  
- ACCACAACAA CTAGTCTGTA GAGAAGAACC TCCACCACTA AACCCCAAAA GACCAAGACC CAGACACTTA   
  
  
- TTACCGTTTT AACCAAGGTA AAACCCATTA ACAAGTAGAC CCCTAAACCT AAGATTCCAA AGATCACTGC   
  
  
- CCAACTTAAG GTTAACATCA AGTGACCCCT CATACTCATA GGAACCAAGT TAGGTACCTG GAAACGGATT   
  
  
- ATGACCACCC AAACACAAGG GTGTTTGTGT TGATAAACTA GAACCACTAC TCTTCGGAGT CTAAAACTTG   
  
  
- GGTGTTAACT ACTGCTACTT AGGTTTAGTC CGAGTCTCGT ACCCTTTGGG ATCGAAAAAA CACGGGAGGA   
  
  
- ACCCAATAAC AGTCAATCTC GTTGTATACC AAGTTGGTGT CCGCTTTGCG GTATCGGGAC CACATCAAAA   
  
  
- CTTGAGACTA CAATAACGGT TTTGGGGCAA AGAGCTAGTC CCAGTACTCA AAAACAACTC CTTTGTTGTG   
  
  
- GTAGTTGTCT AAAACGTCGT TGAAGGATAC CCAAACCGAG TAGTCAGCCA GGGAGTCGTG GAGGTCGTTT   
  
  
- TCCGCTACTT TCTGGTCCAA CAATTCCTGG TCGAAAACTT CCGTCGACTG GACTAAGTTT GACCCTTAAA   
  
  
- GAGTGAACGC GTTCTCTATA ACCGCGCCGA GTTAGTGGTC GAGAGGGAAG GACGCTTCGG GGAGTAATCC   
  
  
- CGTCGAAACA TACAGTACCT CCGGAACGTT TACGAGTAGA ATTACTCGTT AGGACATCGT GGAGGTGGCT   
  
  
- ACTTCTGCGA AAGGGGATAT CTACAACAAG TATTCTACTT ACGGATGTTC CGGAAGAGAC TCCAGAGAGG   
  
  
- ATAATGAGTC AAATGCTTAA AGTGAACACG GGTTCGGTAA GAACTTCGAG AGCTACTACG CCTAACGCAA   
  
  
- GTACAGTAAC TGAAACTATA ACCAACACCC CGAGTTACCC GTAGTGACTA AGTCCTCGAA GGTAACTCCT   
  
  
- TTTTCCCTCG AGGTAGGGAC TTTTAATGTC GGTATCGAGG GGACAGTTAT TCGGTGTGAA AACTTGAGTT   
  
  
- GGATCGTGCC CTTTTGGAAC ACGTTAAGCG GTTACTACAA CCACAACGAA AACTCGACGT TCAACACTTG   
  
  
- AACCTAAATA AACTAGGTAG AAGTAGAAGT TACGGTTTAC AACCTTGGAG ACCCCTACTC AACTAGCGAC   
  
  
- AATCATATGG GTAAACCCGT ACAAGTATAG CCGGCAGATA AGAGGGTAGG TAGGAGGCCA AGTAATTCGT   
  
  
- TGATCGGGGG TTCTAACAGC AGAGCAATCT ATCTCCCAGA CTAGCAACGC TGTGAGACAA GGGTGTTGTA   
  
  
- GAATAGGTAT AGAATCTCAG GACGTGTTTA AAGAACCTTA GCGAGCTGCC AGAGTTACAT CGTAGTCTAT   
  
  
- AACATTTGTT CCAACTCTTC ATGAAGGAGG TCGGATTCTA GCTTTTGTGG CACAACCCGG CGCAAGTACG   
  
  
- GGGGTTATTC TACGGTGTAA CCTTCTGGGA GAAACGAAGT CGGCCCAATA ACGGGAAAGT CAAGTCATTG   
  
  
- AAGTGTCTTT GTGTTCGACT AATACACCAC TTCGCTTGGG GTCCCTCCCC TAAAGTGTAT CTTTTCGCGG   
  
  
- TGCGTAGAAA TCACGATTCA ACCGTTTCCG CCCTCGAACA CTGTCGGAGC CGTACCTCCA AACTCTTCGA   
  
  
- CAT

+     ABRE

| Site Name | Organism | Position | Strand | Matrix score. | sequence | function |
| --- | --- | --- | --- | --- | --- | --- |
| ABRE | Arabidopsis thaliana | 3616 | - | 7 | AACCCGG | cis-acting element involved in the abscisic acid responsiveness |

>HU05G00466.1   
+ +Up\_Stream \_Len000ACATTG GGTGGGATTT AAATCCTCTA TTTTTTCTTA GGGGAAAAAT ACTTGGGAAA   
  
  
+ TTTCTCAATA ACTGAAAATC TAAATAATTC GTGACTTTGT TAGTAGGTTT AATGTGATTT ACTACCGTGA   
  
  
+ GTGTCACTTT TAGATGGTAC TTTAAGAAAG CATTATAAGT AAAACAATAT ATATCGTAGC ATCATACTAC   
  
  
+ TTGTGTAATA AATTAACAGC AACCTCAATT GTACTTATTT GATTTCGCAT TGTAATCAAT TTTCCCAAAT   
  
  
+ GATTTACTAT CTTGGTCTTA CTCAGTTGTA CTTCACACTC AATAGTTCAA TTATTTTTCA TTAGACTAAT   
  
  
+ GAGCGAGTCC CTTCTACAAG AGCCTACTAC TACTCTAATA ATGTAAGGAC TATTGGCTAA TGGGGAACTA   
  
  
+ AGCCTACGAC TTGTTAGGCA TAGGTGAATT TTCCTTACAA GAGAGTTTAA TTTACACTTG ATATTAACTC   
  
  
+ GAGAACGAGA AGCTGTCTTA ATAGATACCC ACATAGAAAA TGAAGCAAAT GCCCTTGATT ATTGGAGGGT   
  
  
+ TGGGCAAATA TATTCAGTAA CAATGTTGTT TGGCAAATAT CGTCTGAAAA TTGTTTTTGA CAAATATTGT   
  
  
+ TTGGAAGAAT TTTTCGAGAT TAAATATTAA AAATGTTAAA ATTTTAATCT AAATATCAAA CAAATATTGA   
  
  
+ AAAATGCTTT ATATTATCTA AATCTCTAAT TAAAATTTGT GTATTGTCCA CTAGAAAAAT GTATAGTGAT   
  
  
+ ATTACCGACA TATATGTGAT CTCTGGTGTA ATAGCCTACC CTAATGTCTA GGAAAATATT TAAAATCCCC   
  
  
+ AGTGGTCAAA AGGAAGTTAC GCACACTAGG GCAAGACACT TCATTAGGCT AGTTGGTGAA AGCTAAGTCA   
  
  
+ TGAAATTTAG ACCATGGCCA TGTCTACTTG TAGATCCAAC TACCAACAAT CCATTTTTAT TTTGGGGAAA   
  
  
+ GCTTGAAGGG TAATTTGGTA AATGTACACG GGAACCTTTT GCTATAAAAG AAGTGCTTCT TGAAAAAACA   
  
  
+ CCCTGTAGTA AAACAGGGCA AAAAAGCCAA CACTTCTGTT TCTGGCAGAG AAACAGTAGT GTACCACTTG   
  
  
+ GAGTTGGAGT AGACACGATA CACAAACCCA GTAGAGAGAG AAACAGGGGA GAGAAAGAGT ACACAAATTT   
  
  
+ GTGCAGGAGA AGGGTAAGGA AGAAGGAGAA GGAACCCCCT CCAAAAAAAA CCCATATAAA AAATAATAAA   
  
  
+ GAAAAAACAG GAGTGAGTTA GAGGGTACCC AAATCTCCAA TCTAAAACTA GAATCTAGGG TCTCTTTTCA   
  
  
+ TTTGCTTGAA AAATTTCTGT TCATGGGTTT TGTGTTTTTA GGGGGATTTG TTTGATTTTT GTAGTCTTTG   
  
  
+ TGGATTTCCT GTGTTATAGA TGCAAGCTAT GCTCTTCAAT TTGCAAGGAA CAGCATCTGG GGGGAGAGAA   
  
  
+ TTTGAGGAAT TTCAAGTACC CATTTCAATT TCAACAAGTA AAATCATCAT TGAAGGATAC TCAAACCCAA   
  
  
+ GCCCAGTTGA TAGTGAACCA ACTTCTACTC TGGATTCTAC TCCAAGTCCC AGCCCTCCCA CCCTCTCCTC   
  
  
+ CTCCTCCAAG AACTCCTACC AAAACGCTGC CGTTTTGGCT CACCCTCCTC AGCTTCACCA TGGATCTGAT   
  
  
+ AATGTGTAAG ACCCCAACCC TTTTGCTTTT ATTTTCCTCA TGCTCCATTC TCATGATTTG TACTTCTGTT   
  
  
+ ATGTAATTGG AGATGTGTCT AAATTTGGAA AAGTTCGTGG GTTTCGTGTT TGAGAATTTA GAATGACATT   
  
  
+ CTTTAATTAG TAATTTGAAG TTTGCATGCT TAATTTAGCT AATCACTGAT TAATTAAAAT AAATGAAGTG   
  
  
+ ATGTAGGTAT GGGCTGTTAC ACAATGTCAC CGGTGGTGGT GACAGCGGCG GGGCGGCGGC GGTTGGATTG   
  
  
+ GAAGAATGGG ACAGCGTGTT TCCAAATGGG GATGGAGCTT TACTCCCTTG GATCATGGGG GAAGGTGATG   
  
  
+ ATTTGGGTCT GAATTTGAAG CATCTCTTAC AATCTGGTTA CCCTGTTGAG TATGAAGGCA ATGCAGGACT   
  
  
+ TGGTGTTGTT GATCAGACAT CTCTTCTTGG AGGTGGTGAT TTGGGGTTTT CTGGTTCTGG GTCTGTGAAT   
  
  
+ AATGGCAAAA TTGGTTCCAT TTTGGGTAAT TGTTCATCTG GGGATTTGGA TTCTAAGGTT TCTAGTGACG   
  
  
+ GGTTGAATTC CAATTGTAGT TCACTGGGGA GTATGAGTAT CCTTGGTTCA ATCCATGGAC CTTTGCCTAA   
  
  
+ TACTGGTGGG TTTGTGTTCC CACAAACACA ACTATTTGAT CTTGGTGATG AGAAGCCTCA GATTTTGAAC   
  
  
+ CCACAATTGA TGACGATGAA TCCAAATCAG GCTCAGAGCA TGGGAAACCC TAGCTTTTTT GTGCCCTCCT   
  
  
+ TGGGTTATTG TCAGTTAGAG CAACATATGG TTCAACCACA GGCGAAACGC CATAGCCCTG GTGTAGTTTT   
  
  
+ GAACTCTGAT GTTATTGCCA AAACCCCGTT TCTCGATCAG GGTCATGAGT TTTTGTTGAG GAAACAACAC   
  
  
+ CATCAACAGA TTTTGCAGCA ACTTCCTATG GGTTTGGCTC ATCAGTCGGT CCCTCAGCAC CTCCAGCAAA   
  
  
+ AGGCGATGAA AGACCAGGTT GTTAAGGACC AGCTTTTGAA GGCAGCTGAC CTGATTCAAA CTGGGAATTT   
  
  
+ CTCACTTGCG CAAGAGATAT TGGCGCGGCT CAATCACCAG CTCTCCCTTC CTGCGAAGCC CCTCATTAGG   
  
  
+ GCAGCTTTGT ATGTCATGGA GGCCTTGCAA ATGCTCATCT TAATGAGCAA TCCTGTAGCA CCTCCACCGA   
  
  
+ TGAAGACGCT TTCCCCTATA GATGTTGTTC ATAAGATGAA TGCCTACAAG GCCTTCTCTG AGGTCTCTCC   
  
  
+ TATTACTCAG TTTACGAATT TCACTTGTGC CCAAGCCATT CTTGAAGCTC TCGATGATGC GGATTGCGTT   
  
  
+ CATGTCATTG ACTTTGATAT TGGTTGTGGG GCTCAATGGG CATCACTGAT TCAGGAGCTT CCATTGAGGA   
  
  
+ AAAAGGGAGC TCCATCCCTG AAAATTACAG CCATAGCTCC CCTGTCAATA AGCCACACTT TTGAACTCAA   
  
  
+ CCTAGCACGG GAAAACCTTG TGCAATTCGC CAATGATGTT GGTGTTGCTT TTGAGCTGCA AGTTGTGAAC   
  
  
+ TTGGATTTAT TTGATCCATC TTCATCTTCA ATGCCAAATG TTGGAACCTC TGGGGATGAG TTGATCGCTG   
  
  
+ TTAGTATACC CATTTGGGCA TGTTCATATC GGCCGTCTAT TCTCCCATCC ATCCTCCGGT TCATTAAGCA   
  
  
+ ACTAGCCCCC AAGATTGTCG TCTCGTTAGA TAGAGGGTCT GATCGTTGCG ACACTCTGTT CCCACAACAT   
  
  
+ CTTATCCATA TCTTAGAGTC CTGCACAAAT TTCTTGGAAT CGCTCGACGG TCTCAATGTA GCATCAGATA   
  
  
+ TTGTAAACAA GGTTGAGAAG TACTTCCTCC AGCCTAAGAT CGAAAACACC GTGTTGGGCC GCGTTCATGC   
  
  
+ CCCCAATAAG ATGCCACATT GGAAGACCCT CTTTGCTTCA GCCGGGTTAT TGCCCTTTCA GTTCAGTAAC   
  
  
+ TTCACAGAAA CACAAGCTGA TTATGTGGTG AAGCGAACCC CAGGGAGGGG ATTTCACATA GAAAAGCGCC   
  
  
+ ACGCATCTTT AGTGCTAAGT TGGCAAAGGC GGGAGCTTGT GACAGCCTCG GCATGGAGGT TTGAGAAGCT   
  
  
+ GTA  

- +Up\_Stream \_Len000TGTAAC CCACCCTAAA TTTAGGAGAT AAAAAAGAAT CCCCTTTTTA TGAACCCTTT   
  
  
- AAAGAGTTAT TGACTTTTAG ATTTATTAAG CACTGAAACA ATCATCCAAA TTACACTAAA TGATGGCACT   
  
  
- CACAGTGAAA ATCTACCATG AAATTCTTTC GTAATATTCA TTTTGTTATA TATAGCATCG TAGTATGATG   
  
  
- AACACATTAT TTAATTGTCG TTGGAGTTAA CATGAATAAA CTAAAGCGTA ACATTAGTTA AAAGGGTTTA   
  
  
- CTAAATGATA GAACCAGAAT GAGTCAACAT GAAGTGTGAG TTATCAAGTT AATAAAAAGT AATCTGATTA   
  
  
- CTCGCTCAGG GAAGATGTTC TCGGATGATG ATGAGATTAT TACATTCCTG ATAACCGATT ACCCCTTGAT   
  
  
- TCGGATGCTG AACAATCCGT ATCCACTTAA AAGGAATGTT CTCTCAAATT AAATGTGAAC TATAATTGAG   
  
  
- CTCTTGCTCT TCGACAGAAT TATCTATGGG TGTATCTTTT ACTTCGTTTA CGGGAACTAA TAACCTCCCA   
  
  
- ACCCGTTTAT ATAAGTCATT GTTACAACAA ACCGTTTATA GCAGACTTTT AACAAAAACT GTTTATAACA   
  
  
- AACCTTCTTA AAAAGCTCTA ATTTATAATT TTTACAATTT TAAAATTAGA TTTATAGTTT GTTTATAACT   
  
  
- TTTTACGAAA TATAATAGAT TTAGAGATTA ATTTTAAACA CATAACAGGT GATCTTTTTA CATATCACTA   
  
  
- TAATGGCTGT ATATACACTA GAGACCACAT TATCGGATGG GATTACAGAT CCTTTTATAA ATTTTAGGGG   
  
  
- TCACCAGTTT TCCTTCAATG CGTGTGATCC CGTTCTGTGA AGTAATCCGA TCAACCACTT TCGATTCAGT   
  
  
- ACTTTAAATC TGGTACCGGT ACAGATGAAC ATCTAGGTTG ATGGTTGTTA GGTAAAAATA AAACCCCTTT   
  
  
- CGAACTTCCC ATTAAACCAT TTACATGTGC CCTTGGAAAA CGATATTTTC TTCACGAAGA ACTTTTTTGT   
  
  
- GGGACATCAT TTTGTCCCGT TTTTTCGGTT GTGAAGACAA AGACCGTCTC TTTGTCATCA CATGGTGAAC   
  
  
- CTCAACCTCA TCTGTGCTAT GTGTTTGGGT CATCTCTCTC TTTGTCCCCT CTCTTTCTCA TGTGTTTAAA   
  
  
- CACGTCCTCT TCCCATTCCT TCTTCCTCTT CCTTGGGGGA GGTTTTTTTT GGGTATATTT TTTATTATTT   
  
  
- CTTTTTTGTC CTCACTCAAT CTCCCATGGG TTTAGAGGTT AGATTTTGAT CTTAGATCCC AGAGAAAAGT   
  
  
- AAACGAACTT TTTAAAGACA AGTACCCAAA ACACAAAAAT CCCCCTAAAC AAACTAAAAA CATCAGAAAC   
  
  
- ACCTAAAGGA CACAATATCT ACGTTCGATA CGAGAAGTTA AACGTTCCTT GTCGTAGACC CCCCTCTCTT   
  
  
- AAACTCCTTA AAGTTCATGG GTAAAGTTAA AGTTGTTCAT TTTAGTAGTA ACTTCCTATG AGTTTGGGTT   
  
  
- CGGGTCAACT ATCACTTGGT TGAAGATGAG ACCTAAGATG AGGTTCAGGG TCGGGAGGGT GGGAGAGGAG   
  
  
- GAGGAGGTTC TTGAGGATGG TTTTGCGACG GCAAAACCGA GTGGGAGGAG TCGAAGTGGT ACCTAGACTA   
  
  
- TTACACATTC TGGGGTTGGG AAAACGAAAA TAAAAGGAGT ACGAGGTAAG AGTACTAAAC ATGAAGACAA   
  
  
- TACATTAACC TCTACACAGA TTTAAACCTT TTCAAGCACC CAAAGCACAA ACTCTTAAAT CTTACTGTAA   
  
  
- GAAATTAATC ATTAAACTTC AAACGTACGA ATTAAATCGA TTAGTGACTA ATTAATTTTA TTTACTTCAC   
  
  
- TACATCCATA CCCGACAATG TGTTACAGTG GCCACCACCA CTGTCGCCGC CCCGCCGCCG CCAACCTAAC   
  
  
- CTTCTTACCC TGTCGCACAA AGGTTTACCC CTACCTCGAA ATGAGGGAAC CTAGTACCCC CTTCCACTAC   
  
  
- TAAACCCAGA CTTAAACTTC GTAGAGAATG TTAGACCAAT GGGACAACTC ATACTTCCGT TACGTCCTGA   
  
  
- ACCACAACAA CTAGTCTGTA GAGAAGAACC TCCACCACTA AACCCCAAAA GACCAAGACC CAGACACTTA   
  
  
- TTACCGTTTT AACCAAGGTA AAACCCATTA ACAAGTAGAC CCCTAAACCT AAGATTCCAA AGATCACTGC   
  
  
- CCAACTTAAG GTTAACATCA AGTGACCCCT CATACTCATA GGAACCAAGT TAGGTACCTG GAAACGGATT   
  
  
- ATGACCACCC AAACACAAGG GTGTTTGTGT TGATAAACTA GAACCACTAC TCTTCGGAGT CTAAAACTTG   
  
  
- GGTGTTAACT ACTGCTACTT AGGTTTAGTC CGAGTCTCGT ACCCTTTGGG ATCGAAAAAA CACGGGAGGA   
  
  
- ACCCAATAAC AGTCAATCTC GTTGTATACC AAGTTGGTGT CCGCTTTGCG GTATCGGGAC CACATCAAAA   
  
  
- CTTGAGACTA CAATAACGGT TTTGGGGCAA AGAGCTAGTC CCAGTACTCA AAAACAACTC CTTTGTTGTG   
  
  
- GTAGTTGTCT AAAACGTCGT TGAAGGATAC CCAAACCGAG TAGTCAGCCA GGGAGTCGTG GAGGTCGTTT   
  
  
- TCCGCTACTT TCTGGTCCAA CAATTCCTGG TCGAAAACTT CCGTCGACTG GACTAAGTTT GACCCTTAAA   
  
  
- GAGTGAACGC GTTCTCTATA ACCGCGCCGA GTTAGTGGTC GAGAGGGAAG GACGCTTCGG GGAGTAATCC   
  
  
- CGTCGAAACA TACAGTACCT CCGGAACGTT TACGAGTAGA ATTACTCGTT AGGACATCGT GGAGGTGGCT   
  
  
- ACTTCTGCGA AAGGGGATAT CTACAACAAG TATTCTACTT ACGGATGTTC CGGAAGAGAC TCCAGAGAGG   
  
  
- ATAATGAGTC AAATGCTTAA AGTGAACACG GGTTCGGTAA GAACTTCGAG AGCTACTACG CCTAACGCAA   
  
  
- GTACAGTAAC TGAAACTATA ACCAACACCC CGAGTTACCC GTAGTGACTA AGTCCTCGAA GGTAACTCCT   
  
  
- TTTTCCCTCG AGGTAGGGAC TTTTAATGTC GGTATCGAGG GGACAGTTAT TCGGTGTGAA AACTTGAGTT   
  
  
- GGATCGTGCC CTTTTGGAAC ACGTTAAGCG GTTACTACAA CCACAACGAA AACTCGACGT TCAACACTTG   
  
  
- AACCTAAATA AACTAGGTAG AAGTAGAAGT TACGGTTTAC AACCTTGGAG ACCCCTACTC AACTAGCGAC   
  
  
- AATCATATGG GTAAACCCGT ACAAGTATAG CCGGCAGATA AGAGGGTAGG TAGGAGGCCA AGTAATTCGT   
  
  
- TGATCGGGGG TTCTAACAGC AGAGCAATCT ATCTCCCAGA CTAGCAACGC TGTGAGACAA GGGTGTTGTA   
  
  
- GAATAGGTAT AGAATCTCAG GACGTGTTTA AAGAACCTTA GCGAGCTGCC AGAGTTACAT CGTAGTCTAT   
  
  
- AACATTTGTT CCAACTCTTC ATGAAGGAGG TCGGATTCTA GCTTTTGTGG CACAACCCGG CGCAAGTACG   
  
  
- GGGGTTATTC TACGGTGTAA CCTTCTGGGA GAAACGAAGT CGGCCCAATA ACGGGAAAGT CAAGTCATTG   
  
  
- AAGTGTCTTT GTGTTCGACT AATACACCAC TTCGCTTGGG GTCCCTCCCC TAAAGTGTAT CTTTTCGCGG   
  
  
- TGCGTAGAAA TCACGATTCA ACCGTTTCCG CCCTCGAACA CTGTCGGAGC CGTACCTCCA AACTCTTCGA   
  
  
- CAT

+     ACA-motif

| Site Name | Organism | Position | Strand | Matrix score. | sequence | function |
| --- | --- | --- | --- | --- | --- | --- |
| ACA-motif | Pisum sativum | 3107 | + | 12 | AATTACAGCCATT | part of gapA in (gapA-CMA1) involved with light responsiveness |

>HU05G00466.1   
+ +Up\_Stream \_Len000ACATTG GGTGGGATTT AAATCCTCTA TTTTTTCTTA GGGGAAAAAT ACTTGGGAAA   
  
  
+ TTTCTCAATA ACTGAAAATC TAAATAATTC GTGACTTTGT TAGTAGGTTT AATGTGATTT ACTACCGTGA   
  
  
+ GTGTCACTTT TAGATGGTAC TTTAAGAAAG CATTATAAGT AAAACAATAT ATATCGTAGC ATCATACTAC   
  
  
+ TTGTGTAATA AATTAACAGC AACCTCAATT GTACTTATTT GATTTCGCAT TGTAATCAAT TTTCCCAAAT   
  
  
+ GATTTACTAT CTTGGTCTTA CTCAGTTGTA CTTCACACTC AATAGTTCAA TTATTTTTCA TTAGACTAAT   
  
  
+ GAGCGAGTCC CTTCTACAAG AGCCTACTAC TACTCTAATA ATGTAAGGAC TATTGGCTAA TGGGGAACTA   
  
  
+ AGCCTACGAC TTGTTAGGCA TAGGTGAATT TTCCTTACAA GAGAGTTTAA TTTACACTTG ATATTAACTC   
  
  
+ GAGAACGAGA AGCTGTCTTA ATAGATACCC ACATAGAAAA TGAAGCAAAT GCCCTTGATT ATTGGAGGGT   
  
  
+ TGGGCAAATA TATTCAGTAA CAATGTTGTT TGGCAAATAT CGTCTGAAAA TTGTTTTTGA CAAATATTGT   
  
  
+ TTGGAAGAAT TTTTCGAGAT TAAATATTAA AAATGTTAAA ATTTTAATCT AAATATCAAA CAAATATTGA   
  
  
+ AAAATGCTTT ATATTATCTA AATCTCTAAT TAAAATTTGT GTATTGTCCA CTAGAAAAAT GTATAGTGAT   
  
  
+ ATTACCGACA TATATGTGAT CTCTGGTGTA ATAGCCTACC CTAATGTCTA GGAAAATATT TAAAATCCCC   
  
  
+ AGTGGTCAAA AGGAAGTTAC GCACACTAGG GCAAGACACT TCATTAGGCT AGTTGGTGAA AGCTAAGTCA   
  
  
+ TGAAATTTAG ACCATGGCCA TGTCTACTTG TAGATCCAAC TACCAACAAT CCATTTTTAT TTTGGGGAAA   
  
  
+ GCTTGAAGGG TAATTTGGTA AATGTACACG GGAACCTTTT GCTATAAAAG AAGTGCTTCT TGAAAAAACA   
  
  
+ CCCTGTAGTA AAACAGGGCA AAAAAGCCAA CACTTCTGTT TCTGGCAGAG AAACAGTAGT GTACCACTTG   
  
  
+ GAGTTGGAGT AGACACGATA CACAAACCCA GTAGAGAGAG AAACAGGGGA GAGAAAGAGT ACACAAATTT   
  
  
+ GTGCAGGAGA AGGGTAAGGA AGAAGGAGAA GGAACCCCCT CCAAAAAAAA CCCATATAAA AAATAATAAA   
  
  
+ GAAAAAACAG GAGTGAGTTA GAGGGTACCC AAATCTCCAA TCTAAAACTA GAATCTAGGG TCTCTTTTCA   
  
  
+ TTTGCTTGAA AAATTTCTGT TCATGGGTTT TGTGTTTTTA GGGGGATTTG TTTGATTTTT GTAGTCTTTG   
  
  
+ TGGATTTCCT GTGTTATAGA TGCAAGCTAT GCTCTTCAAT TTGCAAGGAA CAGCATCTGG GGGGAGAGAA   
  
  
+ TTTGAGGAAT TTCAAGTACC CATTTCAATT TCAACAAGTA AAATCATCAT TGAAGGATAC TCAAACCCAA   
  
  
+ GCCCAGTTGA TAGTGAACCA ACTTCTACTC TGGATTCTAC TCCAAGTCCC AGCCCTCCCA CCCTCTCCTC   
  
  
+ CTCCTCCAAG AACTCCTACC AAAACGCTGC CGTTTTGGCT CACCCTCCTC AGCTTCACCA TGGATCTGAT   
  
  
+ AATGTGTAAG ACCCCAACCC TTTTGCTTTT ATTTTCCTCA TGCTCCATTC TCATGATTTG TACTTCTGTT   
  
  
+ ATGTAATTGG AGATGTGTCT AAATTTGGAA AAGTTCGTGG GTTTCGTGTT TGAGAATTTA GAATGACATT   
  
  
+ CTTTAATTAG TAATTTGAAG TTTGCATGCT TAATTTAGCT AATCACTGAT TAATTAAAAT AAATGAAGTG   
  
  
+ ATGTAGGTAT GGGCTGTTAC ACAATGTCAC CGGTGGTGGT GACAGCGGCG GGGCGGCGGC GGTTGGATTG   
  
  
+ GAAGAATGGG ACAGCGTGTT TCCAAATGGG GATGGAGCTT TACTCCCTTG GATCATGGGG GAAGGTGATG   
  
  
+ ATTTGGGTCT GAATTTGAAG CATCTCTTAC AATCTGGTTA CCCTGTTGAG TATGAAGGCA ATGCAGGACT   
  
  
+ TGGTGTTGTT GATCAGACAT CTCTTCTTGG AGGTGGTGAT TTGGGGTTTT CTGGTTCTGG GTCTGTGAAT   
  
  
+ AATGGCAAAA TTGGTTCCAT TTTGGGTAAT TGTTCATCTG GGGATTTGGA TTCTAAGGTT TCTAGTGACG   
  
  
+ GGTTGAATTC CAATTGTAGT TCACTGGGGA GTATGAGTAT CCTTGGTTCA ATCCATGGAC CTTTGCCTAA   
  
  
+ TACTGGTGGG TTTGTGTTCC CACAAACACA ACTATTTGAT CTTGGTGATG AGAAGCCTCA GATTTTGAAC   
  
  
+ CCACAATTGA TGACGATGAA TCCAAATCAG GCTCAGAGCA TGGGAAACCC TAGCTTTTTT GTGCCCTCCT   
  
  
+ TGGGTTATTG TCAGTTAGAG CAACATATGG TTCAACCACA GGCGAAACGC CATAGCCCTG GTGTAGTTTT   
  
  
+ GAACTCTGAT GTTATTGCCA AAACCCCGTT TCTCGATCAG GGTCATGAGT TTTTGTTGAG GAAACAACAC   
  
  
+ CATCAACAGA TTTTGCAGCA ACTTCCTATG GGTTTGGCTC ATCAGTCGGT CCCTCAGCAC CTCCAGCAAA   
  
  
+ AGGCGATGAA AGACCAGGTT GTTAAGGACC AGCTTTTGAA GGCAGCTGAC CTGATTCAAA CTGGGAATTT   
  
  
+ CTCACTTGCG CAAGAGATAT TGGCGCGGCT CAATCACCAG CTCTCCCTTC CTGCGAAGCC CCTCATTAGG   
  
  
+ GCAGCTTTGT ATGTCATGGA GGCCTTGCAA ATGCTCATCT TAATGAGCAA TCCTGTAGCA CCTCCACCGA   
  
  
+ TGAAGACGCT TTCCCCTATA GATGTTGTTC ATAAGATGAA TGCCTACAAG GCCTTCTCTG AGGTCTCTCC   
  
  
+ TATTACTCAG TTTACGAATT TCACTTGTGC CCAAGCCATT CTTGAAGCTC TCGATGATGC GGATTGCGTT   
  
  
+ CATGTCATTG ACTTTGATAT TGGTTGTGGG GCTCAATGGG CATCACTGAT TCAGGAGCTT CCATTGAGGA   
  
  
+ AAAAGGGAGC TCCATCCCTG AAAATTACAG CCATAGCTCC CCTGTCAATA AGCCACACTT TTGAACTCAA   
  
  
+ CCTAGCACGG GAAAACCTTG TGCAATTCGC CAATGATGTT GGTGTTGCTT TTGAGCTGCA AGTTGTGAAC   
  
  
+ TTGGATTTAT TTGATCCATC TTCATCTTCA ATGCCAAATG TTGGAACCTC TGGGGATGAG TTGATCGCTG   
  
  
+ TTAGTATACC CATTTGGGCA TGTTCATATC GGCCGTCTAT TCTCCCATCC ATCCTCCGGT TCATTAAGCA   
  
  
+ ACTAGCCCCC AAGATTGTCG TCTCGTTAGA TAGAGGGTCT GATCGTTGCG ACACTCTGTT CCCACAACAT   
  
  
+ CTTATCCATA TCTTAGAGTC CTGCACAAAT TTCTTGGAAT CGCTCGACGG TCTCAATGTA GCATCAGATA   
  
  
+ TTGTAAACAA GGTTGAGAAG TACTTCCTCC AGCCTAAGAT CGAAAACACC GTGTTGGGCC GCGTTCATGC   
  
  
+ CCCCAATAAG ATGCCACATT GGAAGACCCT CTTTGCTTCA GCCGGGTTAT TGCCCTTTCA GTTCAGTAAC   
  
  
+ TTCACAGAAA CACAAGCTGA TTATGTGGTG AAGCGAACCC CAGGGAGGGG ATTTCACATA GAAAAGCGCC   
  
  
+ ACGCATCTTT AGTGCTAAGT TGGCAAAGGC GGGAGCTTGT GACAGCCTCG GCATGGAGGT TTGAGAAGCT   
  
  
+ GTA  

- +Up\_Stream \_Len000TGTAAC CCACCCTAAA TTTAGGAGAT AAAAAAGAAT CCCCTTTTTA TGAACCCTTT   
  
  
- AAAGAGTTAT TGACTTTTAG ATTTATTAAG CACTGAAACA ATCATCCAAA TTACACTAAA TGATGGCACT   
  
  
- CACAGTGAAA ATCTACCATG AAATTCTTTC GTAATATTCA TTTTGTTATA TATAGCATCG TAGTATGATG   
  
  
- AACACATTAT TTAATTGTCG TTGGAGTTAA CATGAATAAA CTAAAGCGTA ACATTAGTTA AAAGGGTTTA   
  
  
- CTAAATGATA GAACCAGAAT GAGTCAACAT GAAGTGTGAG TTATCAAGTT AATAAAAAGT AATCTGATTA   
  
  
- CTCGCTCAGG GAAGATGTTC TCGGATGATG ATGAGATTAT TACATTCCTG ATAACCGATT ACCCCTTGAT   
  
  
- TCGGATGCTG AACAATCCGT ATCCACTTAA AAGGAATGTT CTCTCAAATT AAATGTGAAC TATAATTGAG   
  
  
- CTCTTGCTCT TCGACAGAAT TATCTATGGG TGTATCTTTT ACTTCGTTTA CGGGAACTAA TAACCTCCCA   
  
  
- ACCCGTTTAT ATAAGTCATT GTTACAACAA ACCGTTTATA GCAGACTTTT AACAAAAACT GTTTATAACA   
  
  
- AACCTTCTTA AAAAGCTCTA ATTTATAATT TTTACAATTT TAAAATTAGA TTTATAGTTT GTTTATAACT   
  
  
- TTTTACGAAA TATAATAGAT TTAGAGATTA ATTTTAAACA CATAACAGGT GATCTTTTTA CATATCACTA   
  
  
- TAATGGCTGT ATATACACTA GAGACCACAT TATCGGATGG GATTACAGAT CCTTTTATAA ATTTTAGGGG   
  
  
- TCACCAGTTT TCCTTCAATG CGTGTGATCC CGTTCTGTGA AGTAATCCGA TCAACCACTT TCGATTCAGT   
  
  
- ACTTTAAATC TGGTACCGGT ACAGATGAAC ATCTAGGTTG ATGGTTGTTA GGTAAAAATA AAACCCCTTT   
  
  
- CGAACTTCCC ATTAAACCAT TTACATGTGC CCTTGGAAAA CGATATTTTC TTCACGAAGA ACTTTTTTGT   
  
  
- GGGACATCAT TTTGTCCCGT TTTTTCGGTT GTGAAGACAA AGACCGTCTC TTTGTCATCA CATGGTGAAC   
  
  
- CTCAACCTCA TCTGTGCTAT GTGTTTGGGT CATCTCTCTC TTTGTCCCCT CTCTTTCTCA TGTGTTTAAA   
  
  
- CACGTCCTCT TCCCATTCCT TCTTCCTCTT CCTTGGGGGA GGTTTTTTTT GGGTATATTT TTTATTATTT   
  
  
- CTTTTTTGTC CTCACTCAAT CTCCCATGGG TTTAGAGGTT AGATTTTGAT CTTAGATCCC AGAGAAAAGT   
  
  
- AAACGAACTT TTTAAAGACA AGTACCCAAA ACACAAAAAT CCCCCTAAAC AAACTAAAAA CATCAGAAAC   
  
  
- ACCTAAAGGA CACAATATCT ACGTTCGATA CGAGAAGTTA AACGTTCCTT GTCGTAGACC CCCCTCTCTT   
  
  
- AAACTCCTTA AAGTTCATGG GTAAAGTTAA AGTTGTTCAT TTTAGTAGTA ACTTCCTATG AGTTTGGGTT   
  
  
- CGGGTCAACT ATCACTTGGT TGAAGATGAG ACCTAAGATG AGGTTCAGGG TCGGGAGGGT GGGAGAGGAG   
  
  
- GAGGAGGTTC TTGAGGATGG TTTTGCGACG GCAAAACCGA GTGGGAGGAG TCGAAGTGGT ACCTAGACTA   
  
  
- TTACACATTC TGGGGTTGGG AAAACGAAAA TAAAAGGAGT ACGAGGTAAG AGTACTAAAC ATGAAGACAA   
  
  
- TACATTAACC TCTACACAGA TTTAAACCTT TTCAAGCACC CAAAGCACAA ACTCTTAAAT CTTACTGTAA   
  
  
- GAAATTAATC ATTAAACTTC AAACGTACGA ATTAAATCGA TTAGTGACTA ATTAATTTTA TTTACTTCAC   
  
  
- TACATCCATA CCCGACAATG TGTTACAGTG GCCACCACCA CTGTCGCCGC CCCGCCGCCG CCAACCTAAC   
  
  
- CTTCTTACCC TGTCGCACAA AGGTTTACCC CTACCTCGAA ATGAGGGAAC CTAGTACCCC CTTCCACTAC   
  
  
- TAAACCCAGA CTTAAACTTC GTAGAGAATG TTAGACCAAT GGGACAACTC ATACTTCCGT TACGTCCTGA   
  
  
- ACCACAACAA CTAGTCTGTA GAGAAGAACC TCCACCACTA AACCCCAAAA GACCAAGACC CAGACACTTA   
  
  
- TTACCGTTTT AACCAAGGTA AAACCCATTA ACAAGTAGAC CCCTAAACCT AAGATTCCAA AGATCACTGC   
  
  
- CCAACTTAAG GTTAACATCA AGTGACCCCT CATACTCATA GGAACCAAGT TAGGTACCTG GAAACGGATT   
  
  
- ATGACCACCC AAACACAAGG GTGTTTGTGT TGATAAACTA GAACCACTAC TCTTCGGAGT CTAAAACTTG   
  
  
- GGTGTTAACT ACTGCTACTT AGGTTTAGTC CGAGTCTCGT ACCCTTTGGG ATCGAAAAAA CACGGGAGGA   
  
  
- ACCCAATAAC AGTCAATCTC GTTGTATACC AAGTTGGTGT CCGCTTTGCG GTATCGGGAC CACATCAAAA   
  
  
- CTTGAGACTA CAATAACGGT TTTGGGGCAA AGAGCTAGTC CCAGTACTCA AAAACAACTC CTTTGTTGTG   
  
  
- GTAGTTGTCT AAAACGTCGT TGAAGGATAC CCAAACCGAG TAGTCAGCCA GGGAGTCGTG GAGGTCGTTT   
  
  
- TCCGCTACTT TCTGGTCCAA CAATTCCTGG TCGAAAACTT CCGTCGACTG GACTAAGTTT GACCCTTAAA   
  
  
- GAGTGAACGC GTTCTCTATA ACCGCGCCGA GTTAGTGGTC GAGAGGGAAG GACGCTTCGG GGAGTAATCC   
  
  
- CGTCGAAACA TACAGTACCT CCGGAACGTT TACGAGTAGA ATTACTCGTT AGGACATCGT GGAGGTGGCT   
  
  
- ACTTCTGCGA AAGGGGATAT CTACAACAAG TATTCTACTT ACGGATGTTC CGGAAGAGAC TCCAGAGAGG   
  
  
- ATAATGAGTC AAATGCTTAA AGTGAACACG GGTTCGGTAA GAACTTCGAG AGCTACTACG CCTAACGCAA   
  
  
- GTACAGTAAC TGAAACTATA ACCAACACCC CGAGTTACCC GTAGTGACTA AGTCCTCGAA GGTAACTCCT   
  
  
- TTTTCCCTCG AGGTAGGGAC TTTTAATGTC GGTATCGAGG GGACAGTTAT TCGGTGTGAA AACTTGAGTT   
  
  
- GGATCGTGCC CTTTTGGAAC ACGTTAAGCG GTTACTACAA CCACAACGAA AACTCGACGT TCAACACTTG   
  
  
- AACCTAAATA AACTAGGTAG AAGTAGAAGT TACGGTTTAC AACCTTGGAG ACCCCTACTC AACTAGCGAC   
  
  
- AATCATATGG GTAAACCCGT ACAAGTATAG CCGGCAGATA AGAGGGTAGG TAGGAGGCCA AGTAATTCGT   
  
  
- TGATCGGGGG TTCTAACAGC AGAGCAATCT ATCTCCCAGA CTAGCAACGC TGTGAGACAA GGGTGTTGTA   
  
  
- GAATAGGTAT AGAATCTCAG GACGTGTTTA AAGAACCTTA GCGAGCTGCC AGAGTTACAT CGTAGTCTAT   
  
  
- AACATTTGTT CCAACTCTTC ATGAAGGAGG TCGGATTCTA GCTTTTGTGG CACAACCCGG CGCAAGTACG   
  
  
- GGGGTTATTC TACGGTGTAA CCTTCTGGGA GAAACGAAGT CGGCCCAATA ACGGGAAAGT CAAGTCATTG   
  
  
- AAGTGTCTTT GTGTTCGACT AATACACCAC TTCGCTTGGG GTCCCTCCCC TAAAGTGTAT CTTTTCGCGG   
  
  
- TGCGTAGAAA TCACGATTCA ACCGTTTCCG CCCTCGAACA CTGTCGGAGC CGTACCTCCA AACTCTTCGA   
  
  
- CAT

+     AP-1

| Site Name | Organism | Position | Strand | Matrix score. | sequence | function |
| --- | --- | --- | --- | --- | --- | --- |
| AP-1 | Arabidopsis thaliana | 1278 | + | 8 | TGAGTTAG |  |

>HU05G00466.1   
+ +Up\_Stream \_Len000ACATTG GGTGGGATTT AAATCCTCTA TTTTTTCTTA GGGGAAAAAT ACTTGGGAAA   
  
  
+ TTTCTCAATA ACTGAAAATC TAAATAATTC GTGACTTTGT TAGTAGGTTT AATGTGATTT ACTACCGTGA   
  
  
+ GTGTCACTTT TAGATGGTAC TTTAAGAAAG CATTATAAGT AAAACAATAT ATATCGTAGC ATCATACTAC   
  
  
+ TTGTGTAATA AATTAACAGC AACCTCAATT GTACTTATTT GATTTCGCAT TGTAATCAAT TTTCCCAAAT   
  
  
+ GATTTACTAT CTTGGTCTTA CTCAGTTGTA CTTCACACTC AATAGTTCAA TTATTTTTCA TTAGACTAAT   
  
  
+ GAGCGAGTCC CTTCTACAAG AGCCTACTAC TACTCTAATA ATGTAAGGAC TATTGGCTAA TGGGGAACTA   
  
  
+ AGCCTACGAC TTGTTAGGCA TAGGTGAATT TTCCTTACAA GAGAGTTTAA TTTACACTTG ATATTAACTC   
  
  
+ GAGAACGAGA AGCTGTCTTA ATAGATACCC ACATAGAAAA TGAAGCAAAT GCCCTTGATT ATTGGAGGGT   
  
  
+ TGGGCAAATA TATTCAGTAA CAATGTTGTT TGGCAAATAT CGTCTGAAAA TTGTTTTTGA CAAATATTGT   
  
  
+ TTGGAAGAAT TTTTCGAGAT TAAATATTAA AAATGTTAAA ATTTTAATCT AAATATCAAA CAAATATTGA   
  
  
+ AAAATGCTTT ATATTATCTA AATCTCTAAT TAAAATTTGT GTATTGTCCA CTAGAAAAAT GTATAGTGAT   
  
  
+ ATTACCGACA TATATGTGAT CTCTGGTGTA ATAGCCTACC CTAATGTCTA GGAAAATATT TAAAATCCCC   
  
  
+ AGTGGTCAAA AGGAAGTTAC GCACACTAGG GCAAGACACT TCATTAGGCT AGTTGGTGAA AGCTAAGTCA   
  
  
+ TGAAATTTAG ACCATGGCCA TGTCTACTTG TAGATCCAAC TACCAACAAT CCATTTTTAT TTTGGGGAAA   
  
  
+ GCTTGAAGGG TAATTTGGTA AATGTACACG GGAACCTTTT GCTATAAAAG AAGTGCTTCT TGAAAAAACA   
  
  
+ CCCTGTAGTA AAACAGGGCA AAAAAGCCAA CACTTCTGTT TCTGGCAGAG AAACAGTAGT GTACCACTTG   
  
  
+ GAGTTGGAGT AGACACGATA CACAAACCCA GTAGAGAGAG AAACAGGGGA GAGAAAGAGT ACACAAATTT   
  
  
+ GTGCAGGAGA AGGGTAAGGA AGAAGGAGAA GGAACCCCCT CCAAAAAAAA CCCATATAAA AAATAATAAA   
  
  
+ GAAAAAACAG GAGTGAGTTA GAGGGTACCC AAATCTCCAA TCTAAAACTA GAATCTAGGG TCTCTTTTCA   
  
  
+ TTTGCTTGAA AAATTTCTGT TCATGGGTTT TGTGTTTTTA GGGGGATTTG TTTGATTTTT GTAGTCTTTG   
  
  
+ TGGATTTCCT GTGTTATAGA TGCAAGCTAT GCTCTTCAAT TTGCAAGGAA CAGCATCTGG GGGGAGAGAA   
  
  
+ TTTGAGGAAT TTCAAGTACC CATTTCAATT TCAACAAGTA AAATCATCAT TGAAGGATAC TCAAACCCAA   
  
  
+ GCCCAGTTGA TAGTGAACCA ACTTCTACTC TGGATTCTAC TCCAAGTCCC AGCCCTCCCA CCCTCTCCTC   
  
  
+ CTCCTCCAAG AACTCCTACC AAAACGCTGC CGTTTTGGCT CACCCTCCTC AGCTTCACCA TGGATCTGAT   
  
  
+ AATGTGTAAG ACCCCAACCC TTTTGCTTTT ATTTTCCTCA TGCTCCATTC TCATGATTTG TACTTCTGTT   
  
  
+ ATGTAATTGG AGATGTGTCT AAATTTGGAA AAGTTCGTGG GTTTCGTGTT TGAGAATTTA GAATGACATT   
  
  
+ CTTTAATTAG TAATTTGAAG TTTGCATGCT TAATTTAGCT AATCACTGAT TAATTAAAAT AAATGAAGTG   
  
  
+ ATGTAGGTAT GGGCTGTTAC ACAATGTCAC CGGTGGTGGT GACAGCGGCG GGGCGGCGGC GGTTGGATTG   
  
  
+ GAAGAATGGG ACAGCGTGTT TCCAAATGGG GATGGAGCTT TACTCCCTTG GATCATGGGG GAAGGTGATG   
  
  
+ ATTTGGGTCT GAATTTGAAG CATCTCTTAC AATCTGGTTA CCCTGTTGAG TATGAAGGCA ATGCAGGACT   
  
  
+ TGGTGTTGTT GATCAGACAT CTCTTCTTGG AGGTGGTGAT TTGGGGTTTT CTGGTTCTGG GTCTGTGAAT   
  
  
+ AATGGCAAAA TTGGTTCCAT TTTGGGTAAT TGTTCATCTG GGGATTTGGA TTCTAAGGTT TCTAGTGACG   
  
  
+ GGTTGAATTC CAATTGTAGT TCACTGGGGA GTATGAGTAT CCTTGGTTCA ATCCATGGAC CTTTGCCTAA   
  
  
+ TACTGGTGGG TTTGTGTTCC CACAAACACA ACTATTTGAT CTTGGTGATG AGAAGCCTCA GATTTTGAAC   
  
  
+ CCACAATTGA TGACGATGAA TCCAAATCAG GCTCAGAGCA TGGGAAACCC TAGCTTTTTT GTGCCCTCCT   
  
  
+ TGGGTTATTG TCAGTTAGAG CAACATATGG TTCAACCACA GGCGAAACGC CATAGCCCTG GTGTAGTTTT   
  
  
+ GAACTCTGAT GTTATTGCCA AAACCCCGTT TCTCGATCAG GGTCATGAGT TTTTGTTGAG GAAACAACAC   
  
  
+ CATCAACAGA TTTTGCAGCA ACTTCCTATG GGTTTGGCTC ATCAGTCGGT CCCTCAGCAC CTCCAGCAAA   
  
  
+ AGGCGATGAA AGACCAGGTT GTTAAGGACC AGCTTTTGAA GGCAGCTGAC CTGATTCAAA CTGGGAATTT   
  
  
+ CTCACTTGCG CAAGAGATAT TGGCGCGGCT CAATCACCAG CTCTCCCTTC CTGCGAAGCC CCTCATTAGG   
  
  
+ GCAGCTTTGT ATGTCATGGA GGCCTTGCAA ATGCTCATCT TAATGAGCAA TCCTGTAGCA CCTCCACCGA   
  
  
+ TGAAGACGCT TTCCCCTATA GATGTTGTTC ATAAGATGAA TGCCTACAAG GCCTTCTCTG AGGTCTCTCC   
  
  
+ TATTACTCAG TTTACGAATT TCACTTGTGC CCAAGCCATT CTTGAAGCTC TCGATGATGC GGATTGCGTT   
  
  
+ CATGTCATTG ACTTTGATAT TGGTTGTGGG GCTCAATGGG CATCACTGAT TCAGGAGCTT CCATTGAGGA   
  
  
+ AAAAGGGAGC TCCATCCCTG AAAATTACAG CCATAGCTCC CCTGTCAATA AGCCACACTT TTGAACTCAA   
  
  
+ CCTAGCACGG GAAAACCTTG TGCAATTCGC CAATGATGTT GGTGTTGCTT TTGAGCTGCA AGTTGTGAAC   
  
  
+ TTGGATTTAT TTGATCCATC TTCATCTTCA ATGCCAAATG TTGGAACCTC TGGGGATGAG TTGATCGCTG   
  
  
+ TTAGTATACC CATTTGGGCA TGTTCATATC GGCCGTCTAT TCTCCCATCC ATCCTCCGGT TCATTAAGCA   
  
  
+ ACTAGCCCCC AAGATTGTCG TCTCGTTAGA TAGAGGGTCT GATCGTTGCG ACACTCTGTT CCCACAACAT   
  
  
+ CTTATCCATA TCTTAGAGTC CTGCACAAAT TTCTTGGAAT CGCTCGACGG TCTCAATGTA GCATCAGATA   
  
  
+ TTGTAAACAA GGTTGAGAAG TACTTCCTCC AGCCTAAGAT CGAAAACACC GTGTTGGGCC GCGTTCATGC   
  
  
+ CCCCAATAAG ATGCCACATT GGAAGACCCT CTTTGCTTCA GCCGGGTTAT TGCCCTTTCA GTTCAGTAAC   
  
  
+ TTCACAGAAA CACAAGCTGA TTATGTGGTG AAGCGAACCC CAGGGAGGGG ATTTCACATA GAAAAGCGCC   
  
  
+ ACGCATCTTT AGTGCTAAGT TGGCAAAGGC GGGAGCTTGT GACAGCCTCG GCATGGAGGT TTGAGAAGCT   
  
  
+ GTA  

- +Up\_Stream \_Len000TGTAAC CCACCCTAAA TTTAGGAGAT AAAAAAGAAT CCCCTTTTTA TGAACCCTTT   
  
  
- AAAGAGTTAT TGACTTTTAG ATTTATTAAG CACTGAAACA ATCATCCAAA TTACACTAAA TGATGGCACT   
  
  
- CACAGTGAAA ATCTACCATG AAATTCTTTC GTAATATTCA TTTTGTTATA TATAGCATCG TAGTATGATG   
  
  
- AACACATTAT TTAATTGTCG TTGGAGTTAA CATGAATAAA CTAAAGCGTA ACATTAGTTA AAAGGGTTTA   
  
  
- CTAAATGATA GAACCAGAAT GAGTCAACAT GAAGTGTGAG TTATCAAGTT AATAAAAAGT AATCTGATTA   
  
  
- CTCGCTCAGG GAAGATGTTC TCGGATGATG ATGAGATTAT TACATTCCTG ATAACCGATT ACCCCTTGAT   
  
  
- TCGGATGCTG AACAATCCGT ATCCACTTAA AAGGAATGTT CTCTCAAATT AAATGTGAAC TATAATTGAG   
  
  
- CTCTTGCTCT TCGACAGAAT TATCTATGGG TGTATCTTTT ACTTCGTTTA CGGGAACTAA TAACCTCCCA   
  
  
- ACCCGTTTAT ATAAGTCATT GTTACAACAA ACCGTTTATA GCAGACTTTT AACAAAAACT GTTTATAACA   
  
  
- AACCTTCTTA AAAAGCTCTA ATTTATAATT TTTACAATTT TAAAATTAGA TTTATAGTTT GTTTATAACT   
  
  
- TTTTACGAAA TATAATAGAT TTAGAGATTA ATTTTAAACA CATAACAGGT GATCTTTTTA CATATCACTA   
  
  
- TAATGGCTGT ATATACACTA GAGACCACAT TATCGGATGG GATTACAGAT CCTTTTATAA ATTTTAGGGG   
  
  
- TCACCAGTTT TCCTTCAATG CGTGTGATCC CGTTCTGTGA AGTAATCCGA TCAACCACTT TCGATTCAGT   
  
  
- ACTTTAAATC TGGTACCGGT ACAGATGAAC ATCTAGGTTG ATGGTTGTTA GGTAAAAATA AAACCCCTTT   
  
  
- CGAACTTCCC ATTAAACCAT TTACATGTGC CCTTGGAAAA CGATATTTTC TTCACGAAGA ACTTTTTTGT   
  
  
- GGGACATCAT TTTGTCCCGT TTTTTCGGTT GTGAAGACAA AGACCGTCTC TTTGTCATCA CATGGTGAAC   
  
  
- CTCAACCTCA TCTGTGCTAT GTGTTTGGGT CATCTCTCTC TTTGTCCCCT CTCTTTCTCA TGTGTTTAAA   
  
  
- CACGTCCTCT TCCCATTCCT TCTTCCTCTT CCTTGGGGGA GGTTTTTTTT GGGTATATTT TTTATTATTT   
  
  
- CTTTTTTGTC CTCACTCAAT CTCCCATGGG TTTAGAGGTT AGATTTTGAT CTTAGATCCC AGAGAAAAGT   
  
  
- AAACGAACTT TTTAAAGACA AGTACCCAAA ACACAAAAAT CCCCCTAAAC AAACTAAAAA CATCAGAAAC   
  
  
- ACCTAAAGGA CACAATATCT ACGTTCGATA CGAGAAGTTA AACGTTCCTT GTCGTAGACC CCCCTCTCTT   
  
  
- AAACTCCTTA AAGTTCATGG GTAAAGTTAA AGTTGTTCAT TTTAGTAGTA ACTTCCTATG AGTTTGGGTT   
  
  
- CGGGTCAACT ATCACTTGGT TGAAGATGAG ACCTAAGATG AGGTTCAGGG TCGGGAGGGT GGGAGAGGAG   
  
  
- GAGGAGGTTC TTGAGGATGG TTTTGCGACG GCAAAACCGA GTGGGAGGAG TCGAAGTGGT ACCTAGACTA   
  
  
- TTACACATTC TGGGGTTGGG AAAACGAAAA TAAAAGGAGT ACGAGGTAAG AGTACTAAAC ATGAAGACAA   
  
  
- TACATTAACC TCTACACAGA TTTAAACCTT TTCAAGCACC CAAAGCACAA ACTCTTAAAT CTTACTGTAA   
  
  
- GAAATTAATC ATTAAACTTC AAACGTACGA ATTAAATCGA TTAGTGACTA ATTAATTTTA TTTACTTCAC   
  
  
- TACATCCATA CCCGACAATG TGTTACAGTG GCCACCACCA CTGTCGCCGC CCCGCCGCCG CCAACCTAAC   
  
  
- CTTCTTACCC TGTCGCACAA AGGTTTACCC CTACCTCGAA ATGAGGGAAC CTAGTACCCC CTTCCACTAC   
  
  
- TAAACCCAGA CTTAAACTTC GTAGAGAATG TTAGACCAAT GGGACAACTC ATACTTCCGT TACGTCCTGA   
  
  
- ACCACAACAA CTAGTCTGTA GAGAAGAACC TCCACCACTA AACCCCAAAA GACCAAGACC CAGACACTTA   
  
  
- TTACCGTTTT AACCAAGGTA AAACCCATTA ACAAGTAGAC CCCTAAACCT AAGATTCCAA AGATCACTGC   
  
  
- CCAACTTAAG GTTAACATCA AGTGACCCCT CATACTCATA GGAACCAAGT TAGGTACCTG GAAACGGATT   
  
  
- ATGACCACCC AAACACAAGG GTGTTTGTGT TGATAAACTA GAACCACTAC TCTTCGGAGT CTAAAACTTG   
  
  
- GGTGTTAACT ACTGCTACTT AGGTTTAGTC CGAGTCTCGT ACCCTTTGGG ATCGAAAAAA CACGGGAGGA   
  
  
- ACCCAATAAC AGTCAATCTC GTTGTATACC AAGTTGGTGT CCGCTTTGCG GTATCGGGAC CACATCAAAA   
  
  
- CTTGAGACTA CAATAACGGT TTTGGGGCAA AGAGCTAGTC CCAGTACTCA AAAACAACTC CTTTGTTGTG   
  
  
- GTAGTTGTCT AAAACGTCGT TGAAGGATAC CCAAACCGAG TAGTCAGCCA GGGAGTCGTG GAGGTCGTTT   
  
  
- TCCGCTACTT TCTGGTCCAA CAATTCCTGG TCGAAAACTT CCGTCGACTG GACTAAGTTT GACCCTTAAA   
  
  
- GAGTGAACGC GTTCTCTATA ACCGCGCCGA GTTAGTGGTC GAGAGGGAAG GACGCTTCGG GGAGTAATCC   
  
  
- CGTCGAAACA TACAGTACCT CCGGAACGTT TACGAGTAGA ATTACTCGTT AGGACATCGT GGAGGTGGCT   
  
  
- ACTTCTGCGA AAGGGGATAT CTACAACAAG TATTCTACTT ACGGATGTTC CGGAAGAGAC TCCAGAGAGG   
  
  
- ATAATGAGTC AAATGCTTAA AGTGAACACG GGTTCGGTAA GAACTTCGAG AGCTACTACG CCTAACGCAA   
  
  
- GTACAGTAAC TGAAACTATA ACCAACACCC CGAGTTACCC GTAGTGACTA AGTCCTCGAA GGTAACTCCT   
  
  
- TTTTCCCTCG AGGTAGGGAC TTTTAATGTC GGTATCGAGG GGACAGTTAT TCGGTGTGAA AACTTGAGTT   
  
  
- GGATCGTGCC CTTTTGGAAC ACGTTAAGCG GTTACTACAA CCACAACGAA AACTCGACGT TCAACACTTG   
  
  
- AACCTAAATA AACTAGGTAG AAGTAGAAGT TACGGTTTAC AACCTTGGAG ACCCCTACTC AACTAGCGAC   
  
  
- AATCATATGG GTAAACCCGT ACAAGTATAG CCGGCAGATA AGAGGGTAGG TAGGAGGCCA AGTAATTCGT   
  
  
- TGATCGGGGG TTCTAACAGC AGAGCAATCT ATCTCCCAGA CTAGCAACGC TGTGAGACAA GGGTGTTGTA   
  
  
- GAATAGGTAT AGAATCTCAG GACGTGTTTA AAGAACCTTA GCGAGCTGCC AGAGTTACAT CGTAGTCTAT   
  
  
- AACATTTGTT CCAACTCTTC ATGAAGGAGG TCGGATTCTA GCTTTTGTGG CACAACCCGG CGCAAGTACG   
  
  
- GGGGTTATTC TACGGTGTAA CCTTCTGGGA GAAACGAAGT CGGCCCAATA ACGGGAAAGT CAAGTCATTG   
  
  
- AAGTGTCTTT GTGTTCGACT AATACACCAC TTCGCTTGGG GTCCCTCCCC TAAAGTGTAT CTTTTCGCGG   
  
  
- TGCGTAGAAA TCACGATTCA ACCGTTTCCG CCCTCGAACA CTGTCGGAGC CGTACCTCCA AACTCTTCGA   
  
  
- CAT

+     AT1-motif

| Site Name | Organism | Position | Strand | Matrix score. | sequence | function |
| --- | --- | --- | --- | --- | --- | --- |
| AT1-motif | Solanum tuberosum | 333 | + | 13 | AATTATTTTTTATT | part of a light responsive module |

>HU05G00466.1   
+ +Up\_Stream \_Len000ACATTG GGTGGGATTT AAATCCTCTA TTTTTTCTTA GGGGAAAAAT ACTTGGGAAA   
  
  
+ TTTCTCAATA ACTGAAAATC TAAATAATTC GTGACTTTGT TAGTAGGTTT AATGTGATTT ACTACCGTGA   
  
  
+ GTGTCACTTT TAGATGGTAC TTTAAGAAAG CATTATAAGT AAAACAATAT ATATCGTAGC ATCATACTAC   
  
  
+ TTGTGTAATA AATTAACAGC AACCTCAATT GTACTTATTT GATTTCGCAT TGTAATCAAT TTTCCCAAAT   
  
  
+ GATTTACTAT CTTGGTCTTA CTCAGTTGTA CTTCACACTC AATAGTTCAA TTATTTTTCA TTAGACTAAT   
  
  
+ GAGCGAGTCC CTTCTACAAG AGCCTACTAC TACTCTAATA ATGTAAGGAC TATTGGCTAA TGGGGAACTA   
  
  
+ AGCCTACGAC TTGTTAGGCA TAGGTGAATT TTCCTTACAA GAGAGTTTAA TTTACACTTG ATATTAACTC   
  
  
+ GAGAACGAGA AGCTGTCTTA ATAGATACCC ACATAGAAAA TGAAGCAAAT GCCCTTGATT ATTGGAGGGT   
  
  
+ TGGGCAAATA TATTCAGTAA CAATGTTGTT TGGCAAATAT CGTCTGAAAA TTGTTTTTGA CAAATATTGT   
  
  
+ TTGGAAGAAT TTTTCGAGAT TAAATATTAA AAATGTTAAA ATTTTAATCT AAATATCAAA CAAATATTGA   
  
  
+ AAAATGCTTT ATATTATCTA AATCTCTAAT TAAAATTTGT GTATTGTCCA CTAGAAAAAT GTATAGTGAT   
  
  
+ ATTACCGACA TATATGTGAT CTCTGGTGTA ATAGCCTACC CTAATGTCTA GGAAAATATT TAAAATCCCC   
  
  
+ AGTGGTCAAA AGGAAGTTAC GCACACTAGG GCAAGACACT TCATTAGGCT AGTTGGTGAA AGCTAAGTCA   
  
  
+ TGAAATTTAG ACCATGGCCA TGTCTACTTG TAGATCCAAC TACCAACAAT CCATTTTTAT TTTGGGGAAA   
  
  
+ GCTTGAAGGG TAATTTGGTA AATGTACACG GGAACCTTTT GCTATAAAAG AAGTGCTTCT TGAAAAAACA   
  
  
+ CCCTGTAGTA AAACAGGGCA AAAAAGCCAA CACTTCTGTT TCTGGCAGAG AAACAGTAGT GTACCACTTG   
  
  
+ GAGTTGGAGT AGACACGATA CACAAACCCA GTAGAGAGAG AAACAGGGGA GAGAAAGAGT ACACAAATTT   
  
  
+ GTGCAGGAGA AGGGTAAGGA AGAAGGAGAA GGAACCCCCT CCAAAAAAAA CCCATATAAA AAATAATAAA   
  
  
+ GAAAAAACAG GAGTGAGTTA GAGGGTACCC AAATCTCCAA TCTAAAACTA GAATCTAGGG TCTCTTTTCA   
  
  
+ TTTGCTTGAA AAATTTCTGT TCATGGGTTT TGTGTTTTTA GGGGGATTTG TTTGATTTTT GTAGTCTTTG   
  
  
+ TGGATTTCCT GTGTTATAGA TGCAAGCTAT GCTCTTCAAT TTGCAAGGAA CAGCATCTGG GGGGAGAGAA   
  
  
+ TTTGAGGAAT TTCAAGTACC CATTTCAATT TCAACAAGTA AAATCATCAT TGAAGGATAC TCAAACCCAA   
  
  
+ GCCCAGTTGA TAGTGAACCA ACTTCTACTC TGGATTCTAC TCCAAGTCCC AGCCCTCCCA CCCTCTCCTC   
  
  
+ CTCCTCCAAG AACTCCTACC AAAACGCTGC CGTTTTGGCT CACCCTCCTC AGCTTCACCA TGGATCTGAT   
  
  
+ AATGTGTAAG ACCCCAACCC TTTTGCTTTT ATTTTCCTCA TGCTCCATTC TCATGATTTG TACTTCTGTT   
  
  
+ ATGTAATTGG AGATGTGTCT AAATTTGGAA AAGTTCGTGG GTTTCGTGTT TGAGAATTTA GAATGACATT   
  
  
+ CTTTAATTAG TAATTTGAAG TTTGCATGCT TAATTTAGCT AATCACTGAT TAATTAAAAT AAATGAAGTG   
  
  
+ ATGTAGGTAT GGGCTGTTAC ACAATGTCAC CGGTGGTGGT GACAGCGGCG GGGCGGCGGC GGTTGGATTG   
  
  
+ GAAGAATGGG ACAGCGTGTT TCCAAATGGG GATGGAGCTT TACTCCCTTG GATCATGGGG GAAGGTGATG   
  
  
+ ATTTGGGTCT GAATTTGAAG CATCTCTTAC AATCTGGTTA CCCTGTTGAG TATGAAGGCA ATGCAGGACT   
  
  
+ TGGTGTTGTT GATCAGACAT CTCTTCTTGG AGGTGGTGAT TTGGGGTTTT CTGGTTCTGG GTCTGTGAAT   
  
  
+ AATGGCAAAA TTGGTTCCAT TTTGGGTAAT TGTTCATCTG GGGATTTGGA TTCTAAGGTT TCTAGTGACG   
  
  
+ GGTTGAATTC CAATTGTAGT TCACTGGGGA GTATGAGTAT CCTTGGTTCA ATCCATGGAC CTTTGCCTAA   
  
  
+ TACTGGTGGG TTTGTGTTCC CACAAACACA ACTATTTGAT CTTGGTGATG AGAAGCCTCA GATTTTGAAC   
  
  
+ CCACAATTGA TGACGATGAA TCCAAATCAG GCTCAGAGCA TGGGAAACCC TAGCTTTTTT GTGCCCTCCT   
  
  
+ TGGGTTATTG TCAGTTAGAG CAACATATGG TTCAACCACA GGCGAAACGC CATAGCCCTG GTGTAGTTTT   
  
  
+ GAACTCTGAT GTTATTGCCA AAACCCCGTT TCTCGATCAG GGTCATGAGT TTTTGTTGAG GAAACAACAC   
  
  
+ CATCAACAGA TTTTGCAGCA ACTTCCTATG GGTTTGGCTC ATCAGTCGGT CCCTCAGCAC CTCCAGCAAA   
  
  
+ AGGCGATGAA AGACCAGGTT GTTAAGGACC AGCTTTTGAA GGCAGCTGAC CTGATTCAAA CTGGGAATTT   
  
  
+ CTCACTTGCG CAAGAGATAT TGGCGCGGCT CAATCACCAG CTCTCCCTTC CTGCGAAGCC CCTCATTAGG   
  
  
+ GCAGCTTTGT ATGTCATGGA GGCCTTGCAA ATGCTCATCT TAATGAGCAA TCCTGTAGCA CCTCCACCGA   
  
  
+ TGAAGACGCT TTCCCCTATA GATGTTGTTC ATAAGATGAA TGCCTACAAG GCCTTCTCTG AGGTCTCTCC   
  
  
+ TATTACTCAG TTTACGAATT TCACTTGTGC CCAAGCCATT CTTGAAGCTC TCGATGATGC GGATTGCGTT   
  
  
+ CATGTCATTG ACTTTGATAT TGGTTGTGGG GCTCAATGGG CATCACTGAT TCAGGAGCTT CCATTGAGGA   
  
  
+ AAAAGGGAGC TCCATCCCTG AAAATTACAG CCATAGCTCC CCTGTCAATA AGCCACACTT TTGAACTCAA   
  
  
+ CCTAGCACGG GAAAACCTTG TGCAATTCGC CAATGATGTT GGTGTTGCTT TTGAGCTGCA AGTTGTGAAC   
  
  
+ TTGGATTTAT TTGATCCATC TTCATCTTCA ATGCCAAATG TTGGAACCTC TGGGGATGAG TTGATCGCTG   
  
  
+ TTAGTATACC CATTTGGGCA TGTTCATATC GGCCGTCTAT TCTCCCATCC ATCCTCCGGT TCATTAAGCA   
  
  
+ ACTAGCCCCC AAGATTGTCG TCTCGTTAGA TAGAGGGTCT GATCGTTGCG ACACTCTGTT CCCACAACAT   
  
  
+ CTTATCCATA TCTTAGAGTC CTGCACAAAT TTCTTGGAAT CGCTCGACGG TCTCAATGTA GCATCAGATA   
  
  
+ TTGTAAACAA GGTTGAGAAG TACTTCCTCC AGCCTAAGAT CGAAAACACC GTGTTGGGCC GCGTTCATGC   
  
  
+ CCCCAATAAG ATGCCACATT GGAAGACCCT CTTTGCTTCA GCCGGGTTAT TGCCCTTTCA GTTCAGTAAC   
  
  
+ TTCACAGAAA CACAAGCTGA TTATGTGGTG AAGCGAACCC CAGGGAGGGG ATTTCACATA GAAAAGCGCC   
  
  
+ ACGCATCTTT AGTGCTAAGT TGGCAAAGGC GGGAGCTTGT GACAGCCTCG GCATGGAGGT TTGAGAAGCT   
  
  
+ GTA  

- +Up\_Stream \_Len000TGTAAC CCACCCTAAA TTTAGGAGAT AAAAAAGAAT CCCCTTTTTA TGAACCCTTT   
  
  
- AAAGAGTTAT TGACTTTTAG ATTTATTAAG CACTGAAACA ATCATCCAAA TTACACTAAA TGATGGCACT   
  
  
- CACAGTGAAA ATCTACCATG AAATTCTTTC GTAATATTCA TTTTGTTATA TATAGCATCG TAGTATGATG   
  
  
- AACACATTAT TTAATTGTCG TTGGAGTTAA CATGAATAAA CTAAAGCGTA ACATTAGTTA AAAGGGTTTA   
  
  
- CTAAATGATA GAACCAGAAT GAGTCAACAT GAAGTGTGAG TTATCAAGTT AATAAAAAGT AATCTGATTA   
  
  
- CTCGCTCAGG GAAGATGTTC TCGGATGATG ATGAGATTAT TACATTCCTG ATAACCGATT ACCCCTTGAT   
  
  
- TCGGATGCTG AACAATCCGT ATCCACTTAA AAGGAATGTT CTCTCAAATT AAATGTGAAC TATAATTGAG   
  
  
- CTCTTGCTCT TCGACAGAAT TATCTATGGG TGTATCTTTT ACTTCGTTTA CGGGAACTAA TAACCTCCCA   
  
  
- ACCCGTTTAT ATAAGTCATT GTTACAACAA ACCGTTTATA GCAGACTTTT AACAAAAACT GTTTATAACA   
  
  
- AACCTTCTTA AAAAGCTCTA ATTTATAATT TTTACAATTT TAAAATTAGA TTTATAGTTT GTTTATAACT   
  
  
- TTTTACGAAA TATAATAGAT TTAGAGATTA ATTTTAAACA CATAACAGGT GATCTTTTTA CATATCACTA   
  
  
- TAATGGCTGT ATATACACTA GAGACCACAT TATCGGATGG GATTACAGAT CCTTTTATAA ATTTTAGGGG   
  
  
- TCACCAGTTT TCCTTCAATG CGTGTGATCC CGTTCTGTGA AGTAATCCGA TCAACCACTT TCGATTCAGT   
  
  
- ACTTTAAATC TGGTACCGGT ACAGATGAAC ATCTAGGTTG ATGGTTGTTA GGTAAAAATA AAACCCCTTT   
  
  
- CGAACTTCCC ATTAAACCAT TTACATGTGC CCTTGGAAAA CGATATTTTC TTCACGAAGA ACTTTTTTGT   
  
  
- GGGACATCAT TTTGTCCCGT TTTTTCGGTT GTGAAGACAA AGACCGTCTC TTTGTCATCA CATGGTGAAC   
  
  
- CTCAACCTCA TCTGTGCTAT GTGTTTGGGT CATCTCTCTC TTTGTCCCCT CTCTTTCTCA TGTGTTTAAA   
  
  
- CACGTCCTCT TCCCATTCCT TCTTCCTCTT CCTTGGGGGA GGTTTTTTTT GGGTATATTT TTTATTATTT   
  
  
- CTTTTTTGTC CTCACTCAAT CTCCCATGGG TTTAGAGGTT AGATTTTGAT CTTAGATCCC AGAGAAAAGT   
  
  
- AAACGAACTT TTTAAAGACA AGTACCCAAA ACACAAAAAT CCCCCTAAAC AAACTAAAAA CATCAGAAAC   
  
  
- ACCTAAAGGA CACAATATCT ACGTTCGATA CGAGAAGTTA AACGTTCCTT GTCGTAGACC CCCCTCTCTT   
  
  
- AAACTCCTTA AAGTTCATGG GTAAAGTTAA AGTTGTTCAT TTTAGTAGTA ACTTCCTATG AGTTTGGGTT   
  
  
- CGGGTCAACT ATCACTTGGT TGAAGATGAG ACCTAAGATG AGGTTCAGGG TCGGGAGGGT GGGAGAGGAG   
  
  
- GAGGAGGTTC TTGAGGATGG TTTTGCGACG GCAAAACCGA GTGGGAGGAG TCGAAGTGGT ACCTAGACTA   
  
  
- TTACACATTC TGGGGTTGGG AAAACGAAAA TAAAAGGAGT ACGAGGTAAG AGTACTAAAC ATGAAGACAA   
  
  
- TACATTAACC TCTACACAGA TTTAAACCTT TTCAAGCACC CAAAGCACAA ACTCTTAAAT CTTACTGTAA   
  
  
- GAAATTAATC ATTAAACTTC AAACGTACGA ATTAAATCGA TTAGTGACTA ATTAATTTTA TTTACTTCAC   
  
  
- TACATCCATA CCCGACAATG TGTTACAGTG GCCACCACCA CTGTCGCCGC CCCGCCGCCG CCAACCTAAC   
  
  
- CTTCTTACCC TGTCGCACAA AGGTTTACCC CTACCTCGAA ATGAGGGAAC CTAGTACCCC CTTCCACTAC   
  
  
- TAAACCCAGA CTTAAACTTC GTAGAGAATG TTAGACCAAT GGGACAACTC ATACTTCCGT TACGTCCTGA   
  
  
- ACCACAACAA CTAGTCTGTA GAGAAGAACC TCCACCACTA AACCCCAAAA GACCAAGACC CAGACACTTA   
  
  
- TTACCGTTTT AACCAAGGTA AAACCCATTA ACAAGTAGAC CCCTAAACCT AAGATTCCAA AGATCACTGC   
  
  
- CCAACTTAAG GTTAACATCA AGTGACCCCT CATACTCATA GGAACCAAGT TAGGTACCTG GAAACGGATT   
  
  
- ATGACCACCC AAACACAAGG GTGTTTGTGT TGATAAACTA GAACCACTAC TCTTCGGAGT CTAAAACTTG   
  
  
- GGTGTTAACT ACTGCTACTT AGGTTTAGTC CGAGTCTCGT ACCCTTTGGG ATCGAAAAAA CACGGGAGGA   
  
  
- ACCCAATAAC AGTCAATCTC GTTGTATACC AAGTTGGTGT CCGCTTTGCG GTATCGGGAC CACATCAAAA   
  
  
- CTTGAGACTA CAATAACGGT TTTGGGGCAA AGAGCTAGTC CCAGTACTCA AAAACAACTC CTTTGTTGTG   
  
  
- GTAGTTGTCT AAAACGTCGT TGAAGGATAC CCAAACCGAG TAGTCAGCCA GGGAGTCGTG GAGGTCGTTT   
  
  
- TCCGCTACTT TCTGGTCCAA CAATTCCTGG TCGAAAACTT CCGTCGACTG GACTAAGTTT GACCCTTAAA   
  
  
- GAGTGAACGC GTTCTCTATA ACCGCGCCGA GTTAGTGGTC GAGAGGGAAG GACGCTTCGG GGAGTAATCC   
  
  
- CGTCGAAACA TACAGTACCT CCGGAACGTT TACGAGTAGA ATTACTCGTT AGGACATCGT GGAGGTGGCT   
  
  
- ACTTCTGCGA AAGGGGATAT CTACAACAAG TATTCTACTT ACGGATGTTC CGGAAGAGAC TCCAGAGAGG   
  
  
- ATAATGAGTC AAATGCTTAA AGTGAACACG GGTTCGGTAA GAACTTCGAG AGCTACTACG CCTAACGCAA   
  
  
- GTACAGTAAC TGAAACTATA ACCAACACCC CGAGTTACCC GTAGTGACTA AGTCCTCGAA GGTAACTCCT   
  
  
- TTTTCCCTCG AGGTAGGGAC TTTTAATGTC GGTATCGAGG GGACAGTTAT TCGGTGTGAA AACTTGAGTT   
  
  
- GGATCGTGCC CTTTTGGAAC ACGTTAAGCG GTTACTACAA CCACAACGAA AACTCGACGT TCAACACTTG   
  
  
- AACCTAAATA AACTAGGTAG AAGTAGAAGT TACGGTTTAC AACCTTGGAG ACCCCTACTC AACTAGCGAC   
  
  
- AATCATATGG GTAAACCCGT ACAAGTATAG CCGGCAGATA AGAGGGTAGG TAGGAGGCCA AGTAATTCGT   
  
  
- TGATCGGGGG TTCTAACAGC AGAGCAATCT ATCTCCCAGA CTAGCAACGC TGTGAGACAA GGGTGTTGTA   
  
  
- GAATAGGTAT AGAATCTCAG GACGTGTTTA AAGAACCTTA GCGAGCTGCC AGAGTTACAT CGTAGTCTAT   
  
  
- AACATTTGTT CCAACTCTTC ATGAAGGAGG TCGGATTCTA GCTTTTGTGG CACAACCCGG CGCAAGTACG   
  
  
- GGGGTTATTC TACGGTGTAA CCTTCTGGGA GAAACGAAGT CGGCCCAATA ACGGGAAAGT CAAGTCATTG   
  
  
- AAGTGTCTTT GTGTTCGACT AATACACCAC TTCGCTTGGG GTCCCTCCCC TAAAGTGTAT CTTTTCGCGG   
  
  
- TGCGTAGAAA TCACGATTCA ACCGTTTCCG CCCTCGAACA CTGTCGGAGC CGTACCTCCA AACTCTTCGA   
  
  
- CAT

+     AT~TATA-box

| Site Name | Organism | Position | Strand | Matrix score. | sequence | function |
| --- | --- | --- | --- | --- | --- | --- |
| AT~TATA-box | Arabidopsis thaliana | 192 | + | 6 | TATATA |  |

>HU05G00466.1   
+ +Up\_Stream \_Len000ACATTG GGTGGGATTT AAATCCTCTA TTTTTTCTTA GGGGAAAAAT ACTTGGGAAA   
  
  
+ TTTCTCAATA ACTGAAAATC TAAATAATTC GTGACTTTGT TAGTAGGTTT AATGTGATTT ACTACCGTGA   
  
  
+ GTGTCACTTT TAGATGGTAC TTTAAGAAAG CATTATAAGT AAAACAATAT ATATCGTAGC ATCATACTAC   
  
  
+ TTGTGTAATA AATTAACAGC AACCTCAATT GTACTTATTT GATTTCGCAT TGTAATCAAT TTTCCCAAAT   
  
  
+ GATTTACTAT CTTGGTCTTA CTCAGTTGTA CTTCACACTC AATAGTTCAA TTATTTTTCA TTAGACTAAT   
  
  
+ GAGCGAGTCC CTTCTACAAG AGCCTACTAC TACTCTAATA ATGTAAGGAC TATTGGCTAA TGGGGAACTA   
  
  
+ AGCCTACGAC TTGTTAGGCA TAGGTGAATT TTCCTTACAA GAGAGTTTAA TTTACACTTG ATATTAACTC   
  
  
+ GAGAACGAGA AGCTGTCTTA ATAGATACCC ACATAGAAAA TGAAGCAAAT GCCCTTGATT ATTGGAGGGT   
  
  
+ TGGGCAAATA TATTCAGTAA CAATGTTGTT TGGCAAATAT CGTCTGAAAA TTGTTTTTGA CAAATATTGT   
  
  
+ TTGGAAGAAT TTTTCGAGAT TAAATATTAA AAATGTTAAA ATTTTAATCT AAATATCAAA CAAATATTGA   
  
  
+ AAAATGCTTT ATATTATCTA AATCTCTAAT TAAAATTTGT GTATTGTCCA CTAGAAAAAT GTATAGTGAT   
  
  
+ ATTACCGACA TATATGTGAT CTCTGGTGTA ATAGCCTACC CTAATGTCTA GGAAAATATT TAAAATCCCC   
  
  
+ AGTGGTCAAA AGGAAGTTAC GCACACTAGG GCAAGACACT TCATTAGGCT AGTTGGTGAA AGCTAAGTCA   
  
  
+ TGAAATTTAG ACCATGGCCA TGTCTACTTG TAGATCCAAC TACCAACAAT CCATTTTTAT TTTGGGGAAA   
  
  
+ GCTTGAAGGG TAATTTGGTA AATGTACACG GGAACCTTTT GCTATAAAAG AAGTGCTTCT TGAAAAAACA   
  
  
+ CCCTGTAGTA AAACAGGGCA AAAAAGCCAA CACTTCTGTT TCTGGCAGAG AAACAGTAGT GTACCACTTG   
  
  
+ GAGTTGGAGT AGACACGATA CACAAACCCA GTAGAGAGAG AAACAGGGGA GAGAAAGAGT ACACAAATTT   
  
  
+ GTGCAGGAGA AGGGTAAGGA AGAAGGAGAA GGAACCCCCT CCAAAAAAAA CCCATATAAA AAATAATAAA   
  
  
+ GAAAAAACAG GAGTGAGTTA GAGGGTACCC AAATCTCCAA TCTAAAACTA GAATCTAGGG TCTCTTTTCA   
  
  
+ TTTGCTTGAA AAATTTCTGT TCATGGGTTT TGTGTTTTTA GGGGGATTTG TTTGATTTTT GTAGTCTTTG   
  
  
+ TGGATTTCCT GTGTTATAGA TGCAAGCTAT GCTCTTCAAT TTGCAAGGAA CAGCATCTGG GGGGAGAGAA   
  
  
+ TTTGAGGAAT TTCAAGTACC CATTTCAATT TCAACAAGTA AAATCATCAT TGAAGGATAC TCAAACCCAA   
  
  
+ GCCCAGTTGA TAGTGAACCA ACTTCTACTC TGGATTCTAC TCCAAGTCCC AGCCCTCCCA CCCTCTCCTC   
  
  
+ CTCCTCCAAG AACTCCTACC AAAACGCTGC CGTTTTGGCT CACCCTCCTC AGCTTCACCA TGGATCTGAT   
  
  
+ AATGTGTAAG ACCCCAACCC TTTTGCTTTT ATTTTCCTCA TGCTCCATTC TCATGATTTG TACTTCTGTT   
  
  
+ ATGTAATTGG AGATGTGTCT AAATTTGGAA AAGTTCGTGG GTTTCGTGTT TGAGAATTTA GAATGACATT   
  
  
+ CTTTAATTAG TAATTTGAAG TTTGCATGCT TAATTTAGCT AATCACTGAT TAATTAAAAT AAATGAAGTG   
  
  
+ ATGTAGGTAT GGGCTGTTAC ACAATGTCAC CGGTGGTGGT GACAGCGGCG GGGCGGCGGC GGTTGGATTG   
  
  
+ GAAGAATGGG ACAGCGTGTT TCCAAATGGG GATGGAGCTT TACTCCCTTG GATCATGGGG GAAGGTGATG   
  
  
+ ATTTGGGTCT GAATTTGAAG CATCTCTTAC AATCTGGTTA CCCTGTTGAG TATGAAGGCA ATGCAGGACT   
  
  
+ TGGTGTTGTT GATCAGACAT CTCTTCTTGG AGGTGGTGAT TTGGGGTTTT CTGGTTCTGG GTCTGTGAAT   
  
  
+ AATGGCAAAA TTGGTTCCAT TTTGGGTAAT TGTTCATCTG GGGATTTGGA TTCTAAGGTT TCTAGTGACG   
  
  
+ GGTTGAATTC CAATTGTAGT TCACTGGGGA GTATGAGTAT CCTTGGTTCA ATCCATGGAC CTTTGCCTAA   
  
  
+ TACTGGTGGG TTTGTGTTCC CACAAACACA ACTATTTGAT CTTGGTGATG AGAAGCCTCA GATTTTGAAC   
  
  
+ CCACAATTGA TGACGATGAA TCCAAATCAG GCTCAGAGCA TGGGAAACCC TAGCTTTTTT GTGCCCTCCT   
  
  
+ TGGGTTATTG TCAGTTAGAG CAACATATGG TTCAACCACA GGCGAAACGC CATAGCCCTG GTGTAGTTTT   
  
  
+ GAACTCTGAT GTTATTGCCA AAACCCCGTT TCTCGATCAG GGTCATGAGT TTTTGTTGAG GAAACAACAC   
  
  
+ CATCAACAGA TTTTGCAGCA ACTTCCTATG GGTTTGGCTC ATCAGTCGGT CCCTCAGCAC CTCCAGCAAA   
  
  
+ AGGCGATGAA AGACCAGGTT GTTAAGGACC AGCTTTTGAA GGCAGCTGAC CTGATTCAAA CTGGGAATTT   
  
  
+ CTCACTTGCG CAAGAGATAT TGGCGCGGCT CAATCACCAG CTCTCCCTTC CTGCGAAGCC CCTCATTAGG   
  
  
+ GCAGCTTTGT ATGTCATGGA GGCCTTGCAA ATGCTCATCT TAATGAGCAA TCCTGTAGCA CCTCCACCGA   
  
  
+ TGAAGACGCT TTCCCCTATA GATGTTGTTC ATAAGATGAA TGCCTACAAG GCCTTCTCTG AGGTCTCTCC   
  
  
+ TATTACTCAG TTTACGAATT TCACTTGTGC CCAAGCCATT CTTGAAGCTC TCGATGATGC GGATTGCGTT   
  
  
+ CATGTCATTG ACTTTGATAT TGGTTGTGGG GCTCAATGGG CATCACTGAT TCAGGAGCTT CCATTGAGGA   
  
  
+ AAAAGGGAGC TCCATCCCTG AAAATTACAG CCATAGCTCC CCTGTCAATA AGCCACACTT TTGAACTCAA   
  
  
+ CCTAGCACGG GAAAACCTTG TGCAATTCGC CAATGATGTT GGTGTTGCTT TTGAGCTGCA AGTTGTGAAC   
  
  
+ TTGGATTTAT TTGATCCATC TTCATCTTCA ATGCCAAATG TTGGAACCTC TGGGGATGAG TTGATCGCTG   
  
  
+ TTAGTATACC CATTTGGGCA TGTTCATATC GGCCGTCTAT TCTCCCATCC ATCCTCCGGT TCATTAAGCA   
  
  
+ ACTAGCCCCC AAGATTGTCG TCTCGTTAGA TAGAGGGTCT GATCGTTGCG ACACTCTGTT CCCACAACAT   
  
  
+ CTTATCCATA TCTTAGAGTC CTGCACAAAT TTCTTGGAAT CGCTCGACGG TCTCAATGTA GCATCAGATA   
  
  
+ TTGTAAACAA GGTTGAGAAG TACTTCCTCC AGCCTAAGAT CGAAAACACC GTGTTGGGCC GCGTTCATGC   
  
  
+ CCCCAATAAG ATGCCACATT GGAAGACCCT CTTTGCTTCA GCCGGGTTAT TGCCCTTTCA GTTCAGTAAC   
  
  
+ TTCACAGAAA CACAAGCTGA TTATGTGGTG AAGCGAACCC CAGGGAGGGG ATTTCACATA GAAAAGCGCC   
  
  
+ ACGCATCTTT AGTGCTAAGT TGGCAAAGGC GGGAGCTTGT GACAGCCTCG GCATGGAGGT TTGAGAAGCT   
  
  
+ GTA  

- +Up\_Stream \_Len000TGTAAC CCACCCTAAA TTTAGGAGAT AAAAAAGAAT CCCCTTTTTA TGAACCCTTT   
  
  
- AAAGAGTTAT TGACTTTTAG ATTTATTAAG CACTGAAACA ATCATCCAAA TTACACTAAA TGATGGCACT   
  
  
- CACAGTGAAA ATCTACCATG AAATTCTTTC GTAATATTCA TTTTGTTATA TATAGCATCG TAGTATGATG   
  
  
- AACACATTAT TTAATTGTCG TTGGAGTTAA CATGAATAAA CTAAAGCGTA ACATTAGTTA AAAGGGTTTA   
  
  
- CTAAATGATA GAACCAGAAT GAGTCAACAT GAAGTGTGAG TTATCAAGTT AATAAAAAGT AATCTGATTA   
  
  
- CTCGCTCAGG GAAGATGTTC TCGGATGATG ATGAGATTAT TACATTCCTG ATAACCGATT ACCCCTTGAT   
  
  
- TCGGATGCTG AACAATCCGT ATCCACTTAA AAGGAATGTT CTCTCAAATT AAATGTGAAC TATAATTGAG   
  
  
- CTCTTGCTCT TCGACAGAAT TATCTATGGG TGTATCTTTT ACTTCGTTTA CGGGAACTAA TAACCTCCCA   
  
  
- ACCCGTTTAT ATAAGTCATT GTTACAACAA ACCGTTTATA GCAGACTTTT AACAAAAACT GTTTATAACA   
  
  
- AACCTTCTTA AAAAGCTCTA ATTTATAATT TTTACAATTT TAAAATTAGA TTTATAGTTT GTTTATAACT   
  
  
- TTTTACGAAA TATAATAGAT TTAGAGATTA ATTTTAAACA CATAACAGGT GATCTTTTTA CATATCACTA   
  
  
- TAATGGCTGT ATATACACTA GAGACCACAT TATCGGATGG GATTACAGAT CCTTTTATAA ATTTTAGGGG   
  
  
- TCACCAGTTT TCCTTCAATG CGTGTGATCC CGTTCTGTGA AGTAATCCGA TCAACCACTT TCGATTCAGT   
  
  
- ACTTTAAATC TGGTACCGGT ACAGATGAAC ATCTAGGTTG ATGGTTGTTA GGTAAAAATA AAACCCCTTT   
  
  
- CGAACTTCCC ATTAAACCAT TTACATGTGC CCTTGGAAAA CGATATTTTC TTCACGAAGA ACTTTTTTGT   
  
  
- GGGACATCAT TTTGTCCCGT TTTTTCGGTT GTGAAGACAA AGACCGTCTC TTTGTCATCA CATGGTGAAC   
  
  
- CTCAACCTCA TCTGTGCTAT GTGTTTGGGT CATCTCTCTC TTTGTCCCCT CTCTTTCTCA TGTGTTTAAA   
  
  
- CACGTCCTCT TCCCATTCCT TCTTCCTCTT CCTTGGGGGA GGTTTTTTTT GGGTATATTT TTTATTATTT   
  
  
- CTTTTTTGTC CTCACTCAAT CTCCCATGGG TTTAGAGGTT AGATTTTGAT CTTAGATCCC AGAGAAAAGT   
  
  
- AAACGAACTT TTTAAAGACA AGTACCCAAA ACACAAAAAT CCCCCTAAAC AAACTAAAAA CATCAGAAAC   
  
  
- ACCTAAAGGA CACAATATCT ACGTTCGATA CGAGAAGTTA AACGTTCCTT GTCGTAGACC CCCCTCTCTT   
  
  
- AAACTCCTTA AAGTTCATGG GTAAAGTTAA AGTTGTTCAT TTTAGTAGTA ACTTCCTATG AGTTTGGGTT   
  
  
- CGGGTCAACT ATCACTTGGT TGAAGATGAG ACCTAAGATG AGGTTCAGGG TCGGGAGGGT GGGAGAGGAG   
  
  
- GAGGAGGTTC TTGAGGATGG TTTTGCGACG GCAAAACCGA GTGGGAGGAG TCGAAGTGGT ACCTAGACTA   
  
  
- TTACACATTC TGGGGTTGGG AAAACGAAAA TAAAAGGAGT ACGAGGTAAG AGTACTAAAC ATGAAGACAA   
  
  
- TACATTAACC TCTACACAGA TTTAAACCTT TTCAAGCACC CAAAGCACAA ACTCTTAAAT CTTACTGTAA   
  
  
- GAAATTAATC ATTAAACTTC AAACGTACGA ATTAAATCGA TTAGTGACTA ATTAATTTTA TTTACTTCAC   
  
  
- TACATCCATA CCCGACAATG TGTTACAGTG GCCACCACCA CTGTCGCCGC CCCGCCGCCG CCAACCTAAC   
  
  
- CTTCTTACCC TGTCGCACAA AGGTTTACCC CTACCTCGAA ATGAGGGAAC CTAGTACCCC CTTCCACTAC   
  
  
- TAAACCCAGA CTTAAACTTC GTAGAGAATG TTAGACCAAT GGGACAACTC ATACTTCCGT TACGTCCTGA   
  
  
- ACCACAACAA CTAGTCTGTA GAGAAGAACC TCCACCACTA AACCCCAAAA GACCAAGACC CAGACACTTA   
  
  
- TTACCGTTTT AACCAAGGTA AAACCCATTA ACAAGTAGAC CCCTAAACCT AAGATTCCAA AGATCACTGC   
  
  
- CCAACTTAAG GTTAACATCA AGTGACCCCT CATACTCATA GGAACCAAGT TAGGTACCTG GAAACGGATT   
  
  
- ATGACCACCC AAACACAAGG GTGTTTGTGT TGATAAACTA GAACCACTAC TCTTCGGAGT CTAAAACTTG   
  
  
- GGTGTTAACT ACTGCTACTT AGGTTTAGTC CGAGTCTCGT ACCCTTTGGG ATCGAAAAAA CACGGGAGGA   
  
  
- ACCCAATAAC AGTCAATCTC GTTGTATACC AAGTTGGTGT CCGCTTTGCG GTATCGGGAC CACATCAAAA   
  
  
- CTTGAGACTA CAATAACGGT TTTGGGGCAA AGAGCTAGTC CCAGTACTCA AAAACAACTC CTTTGTTGTG   
  
  
- GTAGTTGTCT AAAACGTCGT TGAAGGATAC CCAAACCGAG TAGTCAGCCA GGGAGTCGTG GAGGTCGTTT   
  
  
- TCCGCTACTT TCTGGTCCAA CAATTCCTGG TCGAAAACTT CCGTCGACTG GACTAAGTTT GACCCTTAAA   
  
  
- GAGTGAACGC GTTCTCTATA ACCGCGCCGA GTTAGTGGTC GAGAGGGAAG GACGCTTCGG GGAGTAATCC   
  
  
- CGTCGAAACA TACAGTACCT CCGGAACGTT TACGAGTAGA ATTACTCGTT AGGACATCGT GGAGGTGGCT   
  
  
- ACTTCTGCGA AAGGGGATAT CTACAACAAG TATTCTACTT ACGGATGTTC CGGAAGAGAC TCCAGAGAGG   
  
  
- ATAATGAGTC AAATGCTTAA AGTGAACACG GGTTCGGTAA GAACTTCGAG AGCTACTACG CCTAACGCAA   
  
  
- GTACAGTAAC TGAAACTATA ACCAACACCC CGAGTTACCC GTAGTGACTA AGTCCTCGAA GGTAACTCCT   
  
  
- TTTTCCCTCG AGGTAGGGAC TTTTAATGTC GGTATCGAGG GGACAGTTAT TCGGTGTGAA AACTTGAGTT   
  
  
- GGATCGTGCC CTTTTGGAAC ACGTTAAGCG GTTACTACAA CCACAACGAA AACTCGACGT TCAACACTTG   
  
  
- AACCTAAATA AACTAGGTAG AAGTAGAAGT TACGGTTTAC AACCTTGGAG ACCCCTACTC AACTAGCGAC   
  
  
- AATCATATGG GTAAACCCGT ACAAGTATAG CCGGCAGATA AGAGGGTAGG TAGGAGGCCA AGTAATTCGT   
  
  
- TGATCGGGGG TTCTAACAGC AGAGCAATCT ATCTCCCAGA CTAGCAACGC TGTGAGACAA GGGTGTTGTA   
  
  
- GAATAGGTAT AGAATCTCAG GACGTGTTTA AAGAACCTTA GCGAGCTGCC AGAGTTACAT CGTAGTCTAT   
  
  
- AACATTTGTT CCAACTCTTC ATGAAGGAGG TCGGATTCTA GCTTTTGTGG CACAACCCGG CGCAAGTACG   
  
  
- GGGGTTATTC TACGGTGTAA CCTTCTGGGA GAAACGAAGT CGGCCCAATA ACGGGAAAGT CAAGTCATTG   
  
  
- AAGTGTCTTT GTGTTCGACT AATACACCAC TTCGCTTGGG GTCCCTCCCC TAAAGTGTAT CTTTTCGCGG   
  
  
- TGCGTAGAAA TCACGATTCA ACCGTTTCCG CCCTCGAACA CTGTCGGAGC CGTACCTCCA AACTCTTCGA   
  
  
- CAT

+     AuxRR-core

| Site Name | Organism | Position | Strand | Matrix score. | sequence | function |
| --- | --- | --- | --- | --- | --- | --- |
| AuxRR-core | Nicotiana tabacum | 2299 | - | 7 | GGTCCAT | cis-acting regulatory element involved in auxin responsiveness |

>HU05G00466.1   
+ +Up\_Stream \_Len000ACATTG GGTGGGATTT AAATCCTCTA TTTTTTCTTA GGGGAAAAAT ACTTGGGAAA   
  
  
+ TTTCTCAATA ACTGAAAATC TAAATAATTC GTGACTTTGT TAGTAGGTTT AATGTGATTT ACTACCGTGA   
  
  
+ GTGTCACTTT TAGATGGTAC TTTAAGAAAG CATTATAAGT AAAACAATAT ATATCGTAGC ATCATACTAC   
  
  
+ TTGTGTAATA AATTAACAGC AACCTCAATT GTACTTATTT GATTTCGCAT TGTAATCAAT TTTCCCAAAT   
  
  
+ GATTTACTAT CTTGGTCTTA CTCAGTTGTA CTTCACACTC AATAGTTCAA TTATTTTTCA TTAGACTAAT   
  
  
+ GAGCGAGTCC CTTCTACAAG AGCCTACTAC TACTCTAATA ATGTAAGGAC TATTGGCTAA TGGGGAACTA   
  
  
+ AGCCTACGAC TTGTTAGGCA TAGGTGAATT TTCCTTACAA GAGAGTTTAA TTTACACTTG ATATTAACTC   
  
  
+ GAGAACGAGA AGCTGTCTTA ATAGATACCC ACATAGAAAA TGAAGCAAAT GCCCTTGATT ATTGGAGGGT   
  
  
+ TGGGCAAATA TATTCAGTAA CAATGTTGTT TGGCAAATAT CGTCTGAAAA TTGTTTTTGA CAAATATTGT   
  
  
+ TTGGAAGAAT TTTTCGAGAT TAAATATTAA AAATGTTAAA ATTTTAATCT AAATATCAAA CAAATATTGA   
  
  
+ AAAATGCTTT ATATTATCTA AATCTCTAAT TAAAATTTGT GTATTGTCCA CTAGAAAAAT GTATAGTGAT   
  
  
+ ATTACCGACA TATATGTGAT CTCTGGTGTA ATAGCCTACC CTAATGTCTA GGAAAATATT TAAAATCCCC   
  
  
+ AGTGGTCAAA AGGAAGTTAC GCACACTAGG GCAAGACACT TCATTAGGCT AGTTGGTGAA AGCTAAGTCA   
  
  
+ TGAAATTTAG ACCATGGCCA TGTCTACTTG TAGATCCAAC TACCAACAAT CCATTTTTAT TTTGGGGAAA   
  
  
+ GCTTGAAGGG TAATTTGGTA AATGTACACG GGAACCTTTT GCTATAAAAG AAGTGCTTCT TGAAAAAACA   
  
  
+ CCCTGTAGTA AAACAGGGCA AAAAAGCCAA CACTTCTGTT TCTGGCAGAG AAACAGTAGT GTACCACTTG   
  
  
+ GAGTTGGAGT AGACACGATA CACAAACCCA GTAGAGAGAG AAACAGGGGA GAGAAAGAGT ACACAAATTT   
  
  
+ GTGCAGGAGA AGGGTAAGGA AGAAGGAGAA GGAACCCCCT CCAAAAAAAA CCCATATAAA AAATAATAAA   
  
  
+ GAAAAAACAG GAGTGAGTTA GAGGGTACCC AAATCTCCAA TCTAAAACTA GAATCTAGGG TCTCTTTTCA   
  
  
+ TTTGCTTGAA AAATTTCTGT TCATGGGTTT TGTGTTTTTA GGGGGATTTG TTTGATTTTT GTAGTCTTTG   
  
  
+ TGGATTTCCT GTGTTATAGA TGCAAGCTAT GCTCTTCAAT TTGCAAGGAA CAGCATCTGG GGGGAGAGAA   
  
  
+ TTTGAGGAAT TTCAAGTACC CATTTCAATT TCAACAAGTA AAATCATCAT TGAAGGATAC TCAAACCCAA   
  
  
+ GCCCAGTTGA TAGTGAACCA ACTTCTACTC TGGATTCTAC TCCAAGTCCC AGCCCTCCCA CCCTCTCCTC   
  
  
+ CTCCTCCAAG AACTCCTACC AAAACGCTGC CGTTTTGGCT CACCCTCCTC AGCTTCACCA TGGATCTGAT   
  
  
+ AATGTGTAAG ACCCCAACCC TTTTGCTTTT ATTTTCCTCA TGCTCCATTC TCATGATTTG TACTTCTGTT   
  
  
+ ATGTAATTGG AGATGTGTCT AAATTTGGAA AAGTTCGTGG GTTTCGTGTT TGAGAATTTA GAATGACATT   
  
  
+ CTTTAATTAG TAATTTGAAG TTTGCATGCT TAATTTAGCT AATCACTGAT TAATTAAAAT AAATGAAGTG   
  
  
+ ATGTAGGTAT GGGCTGTTAC ACAATGTCAC CGGTGGTGGT GACAGCGGCG GGGCGGCGGC GGTTGGATTG   
  
  
+ GAAGAATGGG ACAGCGTGTT TCCAAATGGG GATGGAGCTT TACTCCCTTG GATCATGGGG GAAGGTGATG   
  
  
+ ATTTGGGTCT GAATTTGAAG CATCTCTTAC AATCTGGTTA CCCTGTTGAG TATGAAGGCA ATGCAGGACT   
  
  
+ TGGTGTTGTT GATCAGACAT CTCTTCTTGG AGGTGGTGAT TTGGGGTTTT CTGGTTCTGG GTCTGTGAAT   
  
  
+ AATGGCAAAA TTGGTTCCAT TTTGGGTAAT TGTTCATCTG GGGATTTGGA TTCTAAGGTT TCTAGTGACG   
  
  
+ GGTTGAATTC CAATTGTAGT TCACTGGGGA GTATGAGTAT CCTTGGTTCA ATCCATGGAC CTTTGCCTAA   
  
  
+ TACTGGTGGG TTTGTGTTCC CACAAACACA ACTATTTGAT CTTGGTGATG AGAAGCCTCA GATTTTGAAC   
  
  
+ CCACAATTGA TGACGATGAA TCCAAATCAG GCTCAGAGCA TGGGAAACCC TAGCTTTTTT GTGCCCTCCT   
  
  
+ TGGGTTATTG TCAGTTAGAG CAACATATGG TTCAACCACA GGCGAAACGC CATAGCCCTG GTGTAGTTTT   
  
  
+ GAACTCTGAT GTTATTGCCA AAACCCCGTT TCTCGATCAG GGTCATGAGT TTTTGTTGAG GAAACAACAC   
  
  
+ CATCAACAGA TTTTGCAGCA ACTTCCTATG GGTTTGGCTC ATCAGTCGGT CCCTCAGCAC CTCCAGCAAA   
  
  
+ AGGCGATGAA AGACCAGGTT GTTAAGGACC AGCTTTTGAA GGCAGCTGAC CTGATTCAAA CTGGGAATTT   
  
  
+ CTCACTTGCG CAAGAGATAT TGGCGCGGCT CAATCACCAG CTCTCCCTTC CTGCGAAGCC CCTCATTAGG   
  
  
+ GCAGCTTTGT ATGTCATGGA GGCCTTGCAA ATGCTCATCT TAATGAGCAA TCCTGTAGCA CCTCCACCGA   
  
  
+ TGAAGACGCT TTCCCCTATA GATGTTGTTC ATAAGATGAA TGCCTACAAG GCCTTCTCTG AGGTCTCTCC   
  
  
+ TATTACTCAG TTTACGAATT TCACTTGTGC CCAAGCCATT CTTGAAGCTC TCGATGATGC GGATTGCGTT   
  
  
+ CATGTCATTG ACTTTGATAT TGGTTGTGGG GCTCAATGGG CATCACTGAT TCAGGAGCTT CCATTGAGGA   
  
  
+ AAAAGGGAGC TCCATCCCTG AAAATTACAG CCATAGCTCC CCTGTCAATA AGCCACACTT TTGAACTCAA   
  
  
+ CCTAGCACGG GAAAACCTTG TGCAATTCGC CAATGATGTT GGTGTTGCTT TTGAGCTGCA AGTTGTGAAC   
  
  
+ TTGGATTTAT TTGATCCATC TTCATCTTCA ATGCCAAATG TTGGAACCTC TGGGGATGAG TTGATCGCTG   
  
  
+ TTAGTATACC CATTTGGGCA TGTTCATATC GGCCGTCTAT TCTCCCATCC ATCCTCCGGT TCATTAAGCA   
  
  
+ ACTAGCCCCC AAGATTGTCG TCTCGTTAGA TAGAGGGTCT GATCGTTGCG ACACTCTGTT CCCACAACAT   
  
  
+ CTTATCCATA TCTTAGAGTC CTGCACAAAT TTCTTGGAAT CGCTCGACGG TCTCAATGTA GCATCAGATA   
  
  
+ TTGTAAACAA GGTTGAGAAG TACTTCCTCC AGCCTAAGAT CGAAAACACC GTGTTGGGCC GCGTTCATGC   
  
  
+ CCCCAATAAG ATGCCACATT GGAAGACCCT CTTTGCTTCA GCCGGGTTAT TGCCCTTTCA GTTCAGTAAC   
  
  
+ TTCACAGAAA CACAAGCTGA TTATGTGGTG AAGCGAACCC CAGGGAGGGG ATTTCACATA GAAAAGCGCC   
  
  
+ ACGCATCTTT AGTGCTAAGT TGGCAAAGGC GGGAGCTTGT GACAGCCTCG GCATGGAGGT TTGAGAAGCT   
  
  
+ GTA  

- +Up\_Stream \_Len000TGTAAC CCACCCTAAA TTTAGGAGAT AAAAAAGAAT CCCCTTTTTA TGAACCCTTT   
  
  
- AAAGAGTTAT TGACTTTTAG ATTTATTAAG CACTGAAACA ATCATCCAAA TTACACTAAA TGATGGCACT   
  
  
- CACAGTGAAA ATCTACCATG AAATTCTTTC GTAATATTCA TTTTGTTATA TATAGCATCG TAGTATGATG   
  
  
- AACACATTAT TTAATTGTCG TTGGAGTTAA CATGAATAAA CTAAAGCGTA ACATTAGTTA AAAGGGTTTA   
  
  
- CTAAATGATA GAACCAGAAT GAGTCAACAT GAAGTGTGAG TTATCAAGTT AATAAAAAGT AATCTGATTA   
  
  
- CTCGCTCAGG GAAGATGTTC TCGGATGATG ATGAGATTAT TACATTCCTG ATAACCGATT ACCCCTTGAT   
  
  
- TCGGATGCTG AACAATCCGT ATCCACTTAA AAGGAATGTT CTCTCAAATT AAATGTGAAC TATAATTGAG   
  
  
- CTCTTGCTCT TCGACAGAAT TATCTATGGG TGTATCTTTT ACTTCGTTTA CGGGAACTAA TAACCTCCCA   
  
  
- ACCCGTTTAT ATAAGTCATT GTTACAACAA ACCGTTTATA GCAGACTTTT AACAAAAACT GTTTATAACA   
  
  
- AACCTTCTTA AAAAGCTCTA ATTTATAATT TTTACAATTT TAAAATTAGA TTTATAGTTT GTTTATAACT   
  
  
- TTTTACGAAA TATAATAGAT TTAGAGATTA ATTTTAAACA CATAACAGGT GATCTTTTTA CATATCACTA   
  
  
- TAATGGCTGT ATATACACTA GAGACCACAT TATCGGATGG GATTACAGAT CCTTTTATAA ATTTTAGGGG   
  
  
- TCACCAGTTT TCCTTCAATG CGTGTGATCC CGTTCTGTGA AGTAATCCGA TCAACCACTT TCGATTCAGT   
  
  
- ACTTTAAATC TGGTACCGGT ACAGATGAAC ATCTAGGTTG ATGGTTGTTA GGTAAAAATA AAACCCCTTT   
  
  
- CGAACTTCCC ATTAAACCAT TTACATGTGC CCTTGGAAAA CGATATTTTC TTCACGAAGA ACTTTTTTGT   
  
  
- GGGACATCAT TTTGTCCCGT TTTTTCGGTT GTGAAGACAA AGACCGTCTC TTTGTCATCA CATGGTGAAC   
  
  
- CTCAACCTCA TCTGTGCTAT GTGTTTGGGT CATCTCTCTC TTTGTCCCCT CTCTTTCTCA TGTGTTTAAA   
  
  
- CACGTCCTCT TCCCATTCCT TCTTCCTCTT CCTTGGGGGA GGTTTTTTTT GGGTATATTT TTTATTATTT   
  
  
- CTTTTTTGTC CTCACTCAAT CTCCCATGGG TTTAGAGGTT AGATTTTGAT CTTAGATCCC AGAGAAAAGT   
  
  
- AAACGAACTT TTTAAAGACA AGTACCCAAA ACACAAAAAT CCCCCTAAAC AAACTAAAAA CATCAGAAAC   
  
  
- ACCTAAAGGA CACAATATCT ACGTTCGATA CGAGAAGTTA AACGTTCCTT GTCGTAGACC CCCCTCTCTT   
  
  
- AAACTCCTTA AAGTTCATGG GTAAAGTTAA AGTTGTTCAT TTTAGTAGTA ACTTCCTATG AGTTTGGGTT   
  
  
- CGGGTCAACT ATCACTTGGT TGAAGATGAG ACCTAAGATG AGGTTCAGGG TCGGGAGGGT GGGAGAGGAG   
  
  
- GAGGAGGTTC TTGAGGATGG TTTTGCGACG GCAAAACCGA GTGGGAGGAG TCGAAGTGGT ACCTAGACTA   
  
  
- TTACACATTC TGGGGTTGGG AAAACGAAAA TAAAAGGAGT ACGAGGTAAG AGTACTAAAC ATGAAGACAA   
  
  
- TACATTAACC TCTACACAGA TTTAAACCTT TTCAAGCACC CAAAGCACAA ACTCTTAAAT CTTACTGTAA   
  
  
- GAAATTAATC ATTAAACTTC AAACGTACGA ATTAAATCGA TTAGTGACTA ATTAATTTTA TTTACTTCAC   
  
  
- TACATCCATA CCCGACAATG TGTTACAGTG GCCACCACCA CTGTCGCCGC CCCGCCGCCG CCAACCTAAC   
  
  
- CTTCTTACCC TGTCGCACAA AGGTTTACCC CTACCTCGAA ATGAGGGAAC CTAGTACCCC CTTCCACTAC   
  
  
- TAAACCCAGA CTTAAACTTC GTAGAGAATG TTAGACCAAT GGGACAACTC ATACTTCCGT TACGTCCTGA   
  
  
- ACCACAACAA CTAGTCTGTA GAGAAGAACC TCCACCACTA AACCCCAAAA GACCAAGACC CAGACACTTA   
  
  
- TTACCGTTTT AACCAAGGTA AAACCCATTA ACAAGTAGAC CCCTAAACCT AAGATTCCAA AGATCACTGC   
  
  
- CCAACTTAAG GTTAACATCA AGTGACCCCT CATACTCATA GGAACCAAGT TAGGTACCTG GAAACGGATT   
  
  
- ATGACCACCC AAACACAAGG GTGTTTGTGT TGATAAACTA GAACCACTAC TCTTCGGAGT CTAAAACTTG   
  
  
- GGTGTTAACT ACTGCTACTT AGGTTTAGTC CGAGTCTCGT ACCCTTTGGG ATCGAAAAAA CACGGGAGGA   
  
  
- ACCCAATAAC AGTCAATCTC GTTGTATACC AAGTTGGTGT CCGCTTTGCG GTATCGGGAC CACATCAAAA   
  
  
- CTTGAGACTA CAATAACGGT TTTGGGGCAA AGAGCTAGTC CCAGTACTCA AAAACAACTC CTTTGTTGTG   
  
  
- GTAGTTGTCT AAAACGTCGT TGAAGGATAC CCAAACCGAG TAGTCAGCCA GGGAGTCGTG GAGGTCGTTT   
  
  
- TCCGCTACTT TCTGGTCCAA CAATTCCTGG TCGAAAACTT CCGTCGACTG GACTAAGTTT GACCCTTAAA   
  
  
- GAGTGAACGC GTTCTCTATA ACCGCGCCGA GTTAGTGGTC GAGAGGGAAG GACGCTTCGG GGAGTAATCC   
  
  
- CGTCGAAACA TACAGTACCT CCGGAACGTT TACGAGTAGA ATTACTCGTT AGGACATCGT GGAGGTGGCT   
  
  
- ACTTCTGCGA AAGGGGATAT CTACAACAAG TATTCTACTT ACGGATGTTC CGGAAGAGAC TCCAGAGAGG   
  
  
- ATAATGAGTC AAATGCTTAA AGTGAACACG GGTTCGGTAA GAACTTCGAG AGCTACTACG CCTAACGCAA   
  
  
- GTACAGTAAC TGAAACTATA ACCAACACCC CGAGTTACCC GTAGTGACTA AGTCCTCGAA GGTAACTCCT   
  
  
- TTTTCCCTCG AGGTAGGGAC TTTTAATGTC GGTATCGAGG GGACAGTTAT TCGGTGTGAA AACTTGAGTT   
  
  
- GGATCGTGCC CTTTTGGAAC ACGTTAAGCG GTTACTACAA CCACAACGAA AACTCGACGT TCAACACTTG   
  
  
- AACCTAAATA AACTAGGTAG AAGTAGAAGT TACGGTTTAC AACCTTGGAG ACCCCTACTC AACTAGCGAC   
  
  
- AATCATATGG GTAAACCCGT ACAAGTATAG CCGGCAGATA AGAGGGTAGG TAGGAGGCCA AGTAATTCGT   
  
  
- TGATCGGGGG TTCTAACAGC AGAGCAATCT ATCTCCCAGA CTAGCAACGC TGTGAGACAA GGGTGTTGTA   
  
  
- GAATAGGTAT AGAATCTCAG GACGTGTTTA AAGAACCTTA GCGAGCTGCC AGAGTTACAT CGTAGTCTAT   
  
  
- AACATTTGTT CCAACTCTTC ATGAAGGAGG TCGGATTCTA GCTTTTGTGG CACAACCCGG CGCAAGTACG   
  
  
- GGGGTTATTC TACGGTGTAA CCTTCTGGGA GAAACGAAGT CGGCCCAATA ACGGGAAAGT CAAGTCATTG   
  
  
- AAGTGTCTTT GTGTTCGACT AATACACCAC TTCGCTTGGG GTCCCTCCCC TAAAGTGTAT CTTTTCGCGG   
  
  
- TGCGTAGAAA TCACGATTCA ACCGTTTCCG CCCTCGAACA CTGTCGGAGC CGTACCTCCA AACTCTTCGA   
  
  
- CAT

+     Box 4

| Site Name | Organism | Position | Strand | Matrix score. | sequence | function |
| --- | --- | --- | --- | --- | --- | --- |
| Box 4 | Petroselinum crispum | 1873 | + | 6 | ATTAAT | part of a conserved DNA module involved in light responsiveness |

>HU05G00466.1   
+ +Up\_Stream \_Len000ACATTG GGTGGGATTT AAATCCTCTA TTTTTTCTTA GGGGAAAAAT ACTTGGGAAA   
  
  
+ TTTCTCAATA ACTGAAAATC TAAATAATTC GTGACTTTGT TAGTAGGTTT AATGTGATTT ACTACCGTGA   
  
  
+ GTGTCACTTT TAGATGGTAC TTTAAGAAAG CATTATAAGT AAAACAATAT ATATCGTAGC ATCATACTAC   
  
  
+ TTGTGTAATA AATTAACAGC AACCTCAATT GTACTTATTT GATTTCGCAT TGTAATCAAT TTTCCCAAAT   
  
  
+ GATTTACTAT CTTGGTCTTA CTCAGTTGTA CTTCACACTC AATAGTTCAA TTATTTTTCA TTAGACTAAT   
  
  
+ GAGCGAGTCC CTTCTACAAG AGCCTACTAC TACTCTAATA ATGTAAGGAC TATTGGCTAA TGGGGAACTA   
  
  
+ AGCCTACGAC TTGTTAGGCA TAGGTGAATT TTCCTTACAA GAGAGTTTAA TTTACACTTG ATATTAACTC   
  
  
+ GAGAACGAGA AGCTGTCTTA ATAGATACCC ACATAGAAAA TGAAGCAAAT GCCCTTGATT ATTGGAGGGT   
  
  
+ TGGGCAAATA TATTCAGTAA CAATGTTGTT TGGCAAATAT CGTCTGAAAA TTGTTTTTGA CAAATATTGT   
  
  
+ TTGGAAGAAT TTTTCGAGAT TAAATATTAA AAATGTTAAA ATTTTAATCT AAATATCAAA CAAATATTGA   
  
  
+ AAAATGCTTT ATATTATCTA AATCTCTAAT TAAAATTTGT GTATTGTCCA CTAGAAAAAT GTATAGTGAT   
  
  
+ ATTACCGACA TATATGTGAT CTCTGGTGTA ATAGCCTACC CTAATGTCTA GGAAAATATT TAAAATCCCC   
  
  
+ AGTGGTCAAA AGGAAGTTAC GCACACTAGG GCAAGACACT TCATTAGGCT AGTTGGTGAA AGCTAAGTCA   
  
  
+ TGAAATTTAG ACCATGGCCA TGTCTACTTG TAGATCCAAC TACCAACAAT CCATTTTTAT TTTGGGGAAA   
  
  
+ GCTTGAAGGG TAATTTGGTA AATGTACACG GGAACCTTTT GCTATAAAAG AAGTGCTTCT TGAAAAAACA   
  
  
+ CCCTGTAGTA AAACAGGGCA AAAAAGCCAA CACTTCTGTT TCTGGCAGAG AAACAGTAGT GTACCACTTG   
  
  
+ GAGTTGGAGT AGACACGATA CACAAACCCA GTAGAGAGAG AAACAGGGGA GAGAAAGAGT ACACAAATTT   
  
  
+ GTGCAGGAGA AGGGTAAGGA AGAAGGAGAA GGAACCCCCT CCAAAAAAAA CCCATATAAA AAATAATAAA   
  
  
+ GAAAAAACAG GAGTGAGTTA GAGGGTACCC AAATCTCCAA TCTAAAACTA GAATCTAGGG TCTCTTTTCA   
  
  
+ TTTGCTTGAA AAATTTCTGT TCATGGGTTT TGTGTTTTTA GGGGGATTTG TTTGATTTTT GTAGTCTTTG   
  
  
+ TGGATTTCCT GTGTTATAGA TGCAAGCTAT GCTCTTCAAT TTGCAAGGAA CAGCATCTGG GGGGAGAGAA   
  
  
+ TTTGAGGAAT TTCAAGTACC CATTTCAATT TCAACAAGTA AAATCATCAT TGAAGGATAC TCAAACCCAA   
  
  
+ GCCCAGTTGA TAGTGAACCA ACTTCTACTC TGGATTCTAC TCCAAGTCCC AGCCCTCCCA CCCTCTCCTC   
  
  
+ CTCCTCCAAG AACTCCTACC AAAACGCTGC CGTTTTGGCT CACCCTCCTC AGCTTCACCA TGGATCTGAT   
  
  
+ AATGTGTAAG ACCCCAACCC TTTTGCTTTT ATTTTCCTCA TGCTCCATTC TCATGATTTG TACTTCTGTT   
  
  
+ ATGTAATTGG AGATGTGTCT AAATTTGGAA AAGTTCGTGG GTTTCGTGTT TGAGAATTTA GAATGACATT   
  
  
+ CTTTAATTAG TAATTTGAAG TTTGCATGCT TAATTTAGCT AATCACTGAT TAATTAAAAT AAATGAAGTG   
  
  
+ ATGTAGGTAT GGGCTGTTAC ACAATGTCAC CGGTGGTGGT GACAGCGGCG GGGCGGCGGC GGTTGGATTG   
  
  
+ GAAGAATGGG ACAGCGTGTT TCCAAATGGG GATGGAGCTT TACTCCCTTG GATCATGGGG GAAGGTGATG   
  
  
+ ATTTGGGTCT GAATTTGAAG CATCTCTTAC AATCTGGTTA CCCTGTTGAG TATGAAGGCA ATGCAGGACT   
  
  
+ TGGTGTTGTT GATCAGACAT CTCTTCTTGG AGGTGGTGAT TTGGGGTTTT CTGGTTCTGG GTCTGTGAAT   
  
  
+ AATGGCAAAA TTGGTTCCAT TTTGGGTAAT TGTTCATCTG GGGATTTGGA TTCTAAGGTT TCTAGTGACG   
  
  
+ GGTTGAATTC CAATTGTAGT TCACTGGGGA GTATGAGTAT CCTTGGTTCA ATCCATGGAC CTTTGCCTAA   
  
  
+ TACTGGTGGG TTTGTGTTCC CACAAACACA ACTATTTGAT CTTGGTGATG AGAAGCCTCA GATTTTGAAC   
  
  
+ CCACAATTGA TGACGATGAA TCCAAATCAG GCTCAGAGCA TGGGAAACCC TAGCTTTTTT GTGCCCTCCT   
  
  
+ TGGGTTATTG TCAGTTAGAG CAACATATGG TTCAACCACA GGCGAAACGC CATAGCCCTG GTGTAGTTTT   
  
  
+ GAACTCTGAT GTTATTGCCA AAACCCCGTT TCTCGATCAG GGTCATGAGT TTTTGTTGAG GAAACAACAC   
  
  
+ CATCAACAGA TTTTGCAGCA ACTTCCTATG GGTTTGGCTC ATCAGTCGGT CCCTCAGCAC CTCCAGCAAA   
  
  
+ AGGCGATGAA AGACCAGGTT GTTAAGGACC AGCTTTTGAA GGCAGCTGAC CTGATTCAAA CTGGGAATTT   
  
  
+ CTCACTTGCG CAAGAGATAT TGGCGCGGCT CAATCACCAG CTCTCCCTTC CTGCGAAGCC CCTCATTAGG   
  
  
+ GCAGCTTTGT ATGTCATGGA GGCCTTGCAA ATGCTCATCT TAATGAGCAA TCCTGTAGCA CCTCCACCGA   
  
  
+ TGAAGACGCT TTCCCCTATA GATGTTGTTC ATAAGATGAA TGCCTACAAG GCCTTCTCTG AGGTCTCTCC   
  
  
+ TATTACTCAG TTTACGAATT TCACTTGTGC CCAAGCCATT CTTGAAGCTC TCGATGATGC GGATTGCGTT   
  
  
+ CATGTCATTG ACTTTGATAT TGGTTGTGGG GCTCAATGGG CATCACTGAT TCAGGAGCTT CCATTGAGGA   
  
  
+ AAAAGGGAGC TCCATCCCTG AAAATTACAG CCATAGCTCC CCTGTCAATA AGCCACACTT TTGAACTCAA   
  
  
+ CCTAGCACGG GAAAACCTTG TGCAATTCGC CAATGATGTT GGTGTTGCTT TTGAGCTGCA AGTTGTGAAC   
  
  
+ TTGGATTTAT TTGATCCATC TTCATCTTCA ATGCCAAATG TTGGAACCTC TGGGGATGAG TTGATCGCTG   
  
  
+ TTAGTATACC CATTTGGGCA TGTTCATATC GGCCGTCTAT TCTCCCATCC ATCCTCCGGT TCATTAAGCA   
  
  
+ ACTAGCCCCC AAGATTGTCG TCTCGTTAGA TAGAGGGTCT GATCGTTGCG ACACTCTGTT CCCACAACAT   
  
  
+ CTTATCCATA TCTTAGAGTC CTGCACAAAT TTCTTGGAAT CGCTCGACGG TCTCAATGTA GCATCAGATA   
  
  
+ TTGTAAACAA GGTTGAGAAG TACTTCCTCC AGCCTAAGAT CGAAAACACC GTGTTGGGCC GCGTTCATGC   
  
  
+ CCCCAATAAG ATGCCACATT GGAAGACCCT CTTTGCTTCA GCCGGGTTAT TGCCCTTTCA GTTCAGTAAC   
  
  
+ TTCACAGAAA CACAAGCTGA TTATGTGGTG AAGCGAACCC CAGGGAGGGG ATTTCACATA GAAAAGCGCC   
  
  
+ ACGCATCTTT AGTGCTAAGT TGGCAAAGGC GGGAGCTTGT GACAGCCTCG GCATGGAGGT TTGAGAAGCT   
  
  
+ GTA  

- +Up\_Stream \_Len000TGTAAC CCACCCTAAA TTTAGGAGAT AAAAAAGAAT CCCCTTTTTA TGAACCCTTT   
  
  
- AAAGAGTTAT TGACTTTTAG ATTTATTAAG CACTGAAACA ATCATCCAAA TTACACTAAA TGATGGCACT   
  
  
- CACAGTGAAA ATCTACCATG AAATTCTTTC GTAATATTCA TTTTGTTATA TATAGCATCG TAGTATGATG   
  
  
- AACACATTAT TTAATTGTCG TTGGAGTTAA CATGAATAAA CTAAAGCGTA ACATTAGTTA AAAGGGTTTA   
  
  
- CTAAATGATA GAACCAGAAT GAGTCAACAT GAAGTGTGAG TTATCAAGTT AATAAAAAGT AATCTGATTA   
  
  
- CTCGCTCAGG GAAGATGTTC TCGGATGATG ATGAGATTAT TACATTCCTG ATAACCGATT ACCCCTTGAT   
  
  
- TCGGATGCTG AACAATCCGT ATCCACTTAA AAGGAATGTT CTCTCAAATT AAATGTGAAC TATAATTGAG   
  
  
- CTCTTGCTCT TCGACAGAAT TATCTATGGG TGTATCTTTT ACTTCGTTTA CGGGAACTAA TAACCTCCCA   
  
  
- ACCCGTTTAT ATAAGTCATT GTTACAACAA ACCGTTTATA GCAGACTTTT AACAAAAACT GTTTATAACA   
  
  
- AACCTTCTTA AAAAGCTCTA ATTTATAATT TTTACAATTT TAAAATTAGA TTTATAGTTT GTTTATAACT   
  
  
- TTTTACGAAA TATAATAGAT TTAGAGATTA ATTTTAAACA CATAACAGGT GATCTTTTTA CATATCACTA   
  
  
- TAATGGCTGT ATATACACTA GAGACCACAT TATCGGATGG GATTACAGAT CCTTTTATAA ATTTTAGGGG   
  
  
- TCACCAGTTT TCCTTCAATG CGTGTGATCC CGTTCTGTGA AGTAATCCGA TCAACCACTT TCGATTCAGT   
  
  
- ACTTTAAATC TGGTACCGGT ACAGATGAAC ATCTAGGTTG ATGGTTGTTA GGTAAAAATA AAACCCCTTT   
  
  
- CGAACTTCCC ATTAAACCAT TTACATGTGC CCTTGGAAAA CGATATTTTC TTCACGAAGA ACTTTTTTGT   
  
  
- GGGACATCAT TTTGTCCCGT TTTTTCGGTT GTGAAGACAA AGACCGTCTC TTTGTCATCA CATGGTGAAC   
  
  
- CTCAACCTCA TCTGTGCTAT GTGTTTGGGT CATCTCTCTC TTTGTCCCCT CTCTTTCTCA TGTGTTTAAA   
  
  
- CACGTCCTCT TCCCATTCCT TCTTCCTCTT CCTTGGGGGA GGTTTTTTTT GGGTATATTT TTTATTATTT   
  
  
- CTTTTTTGTC CTCACTCAAT CTCCCATGGG TTTAGAGGTT AGATTTTGAT CTTAGATCCC AGAGAAAAGT   
  
  
- AAACGAACTT TTTAAAGACA AGTACCCAAA ACACAAAAAT CCCCCTAAAC AAACTAAAAA CATCAGAAAC   
  
  
- ACCTAAAGGA CACAATATCT ACGTTCGATA CGAGAAGTTA AACGTTCCTT GTCGTAGACC CCCCTCTCTT   
  
  
- AAACTCCTTA AAGTTCATGG GTAAAGTTAA AGTTGTTCAT TTTAGTAGTA ACTTCCTATG AGTTTGGGTT   
  
  
- CGGGTCAACT ATCACTTGGT TGAAGATGAG ACCTAAGATG AGGTTCAGGG TCGGGAGGGT GGGAGAGGAG   
  
  
- GAGGAGGTTC TTGAGGATGG TTTTGCGACG GCAAAACCGA GTGGGAGGAG TCGAAGTGGT ACCTAGACTA   
  
  
- TTACACATTC TGGGGTTGGG AAAACGAAAA TAAAAGGAGT ACGAGGTAAG AGTACTAAAC ATGAAGACAA   
  
  
- TACATTAACC TCTACACAGA TTTAAACCTT TTCAAGCACC CAAAGCACAA ACTCTTAAAT CTTACTGTAA   
  
  
- GAAATTAATC ATTAAACTTC AAACGTACGA ATTAAATCGA TTAGTGACTA ATTAATTTTA TTTACTTCAC   
  
  
- TACATCCATA CCCGACAATG TGTTACAGTG GCCACCACCA CTGTCGCCGC CCCGCCGCCG CCAACCTAAC   
  
  
- CTTCTTACCC TGTCGCACAA AGGTTTACCC CTACCTCGAA ATGAGGGAAC CTAGTACCCC CTTCCACTAC   
  
  
- TAAACCCAGA CTTAAACTTC GTAGAGAATG TTAGACCAAT GGGACAACTC ATACTTCCGT TACGTCCTGA   
  
  
- ACCACAACAA CTAGTCTGTA GAGAAGAACC TCCACCACTA AACCCCAAAA GACCAAGACC CAGACACTTA   
  
  
- TTACCGTTTT AACCAAGGTA AAACCCATTA ACAAGTAGAC CCCTAAACCT AAGATTCCAA AGATCACTGC   
  
  
- CCAACTTAAG GTTAACATCA AGTGACCCCT CATACTCATA GGAACCAAGT TAGGTACCTG GAAACGGATT   
  
  
- ATGACCACCC AAACACAAGG GTGTTTGTGT TGATAAACTA GAACCACTAC TCTTCGGAGT CTAAAACTTG   
  
  
- GGTGTTAACT ACTGCTACTT AGGTTTAGTC CGAGTCTCGT ACCCTTTGGG ATCGAAAAAA CACGGGAGGA   
  
  
- ACCCAATAAC AGTCAATCTC GTTGTATACC AAGTTGGTGT CCGCTTTGCG GTATCGGGAC CACATCAAAA   
  
  
- CTTGAGACTA CAATAACGGT TTTGGGGCAA AGAGCTAGTC CCAGTACTCA AAAACAACTC CTTTGTTGTG   
  
  
- GTAGTTGTCT AAAACGTCGT TGAAGGATAC CCAAACCGAG TAGTCAGCCA GGGAGTCGTG GAGGTCGTTT   
  
  
- TCCGCTACTT TCTGGTCCAA CAATTCCTGG TCGAAAACTT CCGTCGACTG GACTAAGTTT GACCCTTAAA   
  
  
- GAGTGAACGC GTTCTCTATA ACCGCGCCGA GTTAGTGGTC GAGAGGGAAG GACGCTTCGG GGAGTAATCC   
  
  
- CGTCGAAACA TACAGTACCT CCGGAACGTT TACGAGTAGA ATTACTCGTT AGGACATCGT GGAGGTGGCT   
  
  
- ACTTCTGCGA AAGGGGATAT CTACAACAAG TATTCTACTT ACGGATGTTC CGGAAGAGAC TCCAGAGAGG   
  
  
- ATAATGAGTC AAATGCTTAA AGTGAACACG GGTTCGGTAA GAACTTCGAG AGCTACTACG CCTAACGCAA   
  
  
- GTACAGTAAC TGAAACTATA ACCAACACCC CGAGTTACCC GTAGTGACTA AGTCCTCGAA GGTAACTCCT   
  
  
- TTTTCCCTCG AGGTAGGGAC TTTTAATGTC GGTATCGAGG GGACAGTTAT TCGGTGTGAA AACTTGAGTT   
  
  
- GGATCGTGCC CTTTTGGAAC ACGTTAAGCG GTTACTACAA CCACAACGAA AACTCGACGT TCAACACTTG   
  
  
- AACCTAAATA AACTAGGTAG AAGTAGAAGT TACGGTTTAC AACCTTGGAG ACCCCTACTC AACTAGCGAC   
  
  
- AATCATATGG GTAAACCCGT ACAAGTATAG CCGGCAGATA AGAGGGTAGG TAGGAGGCCA AGTAATTCGT   
  
  
- TGATCGGGGG TTCTAACAGC AGAGCAATCT ATCTCCCAGA CTAGCAACGC TGTGAGACAA GGGTGTTGTA   
  
  
- GAATAGGTAT AGAATCTCAG GACGTGTTTA AAGAACCTTA GCGAGCTGCC AGAGTTACAT CGTAGTCTAT   
  
  
- AACATTTGTT CCAACTCTTC ATGAAGGAGG TCGGATTCTA GCTTTTGTGG CACAACCCGG CGCAAGTACG   
  
  
- GGGGTTATTC TACGGTGTAA CCTTCTGGGA GAAACGAAGT CGGCCCAATA ACGGGAAAGT CAAGTCATTG   
  
  
- AAGTGTCTTT GTGTTCGACT AATACACCAC TTCGCTTGGG GTCCCTCCCC TAAAGTGTAT CTTTTCGCGG   
  
  
- TGCGTAGAAA TCACGATTCA ACCGTTTCCG CCCTCGAACA CTGTCGGAGC CGTACCTCCA AACTCTTCGA   
  
  
- CAT

+     CAAT-box

| Site Name | Organism | Position | Strand | Matrix score. | sequence | function |
| --- | --- | --- | --- | --- | --- | --- |
| CAAT-box | Nicotiana glutinosa | 2293 | + | 4 | CAAT |  |
| CAAT-box | Nicotiana glutinosa | 2257 | - | 4 | CAAT |  |
| CAAT-box | Nicotiana glutinosa | 2388 | + | 4 | CAAT |  |
| CAAT-box | Arabidopsis thaliana | 3592 | - | 5 | CCAAT | common cis-acting element in promoter and enhancer regions |
| CAAT-box | Nicotiana glutinosa | 3623 | - | 4 | CAAT |  |
| CAAT-box | Pisum sativum | 3460 | + | 5 | CAAAT | common cis-acting element in promoter and enhancer regions |
| CAAT-box | Nicotiana glutinosa | 3378 | - | 4 | CAAT |  |
| CAAT-box | Nicotiana glutinosa | 3185 | + | 4 | CAAT |  |
| CAAT-box | Arabidopsis thaliana | 1961 | - | 5 | CCAAT | common cis-acting element in promoter and enhancer regions |
| CAAT-box | Nicotiana glutinosa | 3177 | + | 4 | CAAT |  |
| CAAT-box | Pisum sativum | 2143 | - | 5 | CAAAT | common cis-acting element in promoter and enhancer regions |
| CAAT-box | Nicotiana glutinosa | 747 | - | 4 | CAAT |  |
| CAAT-box | Pisum sativum | 280 | + | 5 | CAAAT | common cis-acting element in promoter and enhancer regions |
| CAAT-box | Pisum sativum | 569 | + | 5 | CAAAT | common cis-acting element in promoter and enhancer regions |
| CAAT-box | Arabidopsis thaliana | 555 | - | 5 | CCAAT | common cis-acting element in promoter and enhancer regions |
| CAAT-box | Pisum sativum | 598 | + | 5 | CAAAT | common cis-acting element in promoter and enhancer regions |
| CAAT-box | Nicotiana glutinosa | 2538 | - | 4 | CAAT |  |
| CAAT-box | Pisum sativum | 1294 | + | 5 | CAAAT | common cis-acting element in promoter and enhancer regions |
| CAAT-box | Pisum sativum | 1443 | - | 5 | CAAAT | common cis-acting element in promoter and enhancer regions |
| CAAT-box | Pisum sativum | 1380 | - | 5 | CAAAT | common cis-acting element in promoter and enhancer regions |
| CAAT-box | Nicotiana glutinosa | 630 | - | 4 | CAAT |  |
| CAAT-box | Petunia hybrida | 3733 | - | 7 | TGCCAAC | common cis-acting element in promoter and enhancer regions |
| CAAT-box | Pisum sativum | 540 | + | 5 | CAAAT | common cis-acting element in promoter and enhancer regions |
| CAAT-box | Nicotiana glutinosa | 189 | + | 4 | CAAT |  |
| CAAT-box | Nicotiana glutinosa | 1916 | + | 4 | CAAT |  |
| CAAT-box | Nicotiana glutinosa | 3578 | + | 4 | CAAT |  |
| CAAT-box | Nicotiana glutinosa | 242 | - | 4 | CAAT |  |
| CAAT-box | Pisum sativum | 2047 | - | 5 | CAAAT | common cis-acting element in promoter and enhancer regions |
| CAAT-box | Nicotiana glutinosa | 961 | + | 4 | CAAT |  |
| CAAT-box | Arabidopsis thaliana | 3577 | + | 5 | CCAAT | common cis-acting element in promoter and enhancer regions |
| CAAT-box | Arabidopsis thaliana | 3033 | - | 5 | CCAAT | common cis-acting element in promoter and enhancer regions |
| CAAT-box | Arabidopsis thaliana | 1760 | - | 5 | CCAAT | common cis-acting element in promoter and enhancer regions |
| CAAT-box | Arabidopsis thaliana | 1301 | + | 5 | CCAAT | common cis-acting element in promoter and enhancer regions |
| CAAT-box | Nicotiana glutinosa | 2203 | - | 4 | CAAT |  |
| CAAT-box | Arabidopsis thaliana | 406 | - | 5 | CCAAT | common cis-acting element in promoter and enhancer regions |
| CAAT-box | Nicotiana glutinosa | 2765 | + | 4 | CAAT |  |
| CAAT-box | Pisum sativum | 3259 | + | 5 | CAAAT | common cis-acting element in promoter and enhancer regions |
| CAAT-box | Pisum sativum | 2832 | + | 5 | CAAAT | common cis-acting element in promoter and enhancer regions |
| CAAT-box | Nicotiana glutinosa | 585 | + | 4 | CAAT |  |
| CAAT-box | Pisum sativum | 625 | + | 5 | CAAAT | common cis-acting element in promoter and enhancer regions |
| CAAT-box | Nicotiana glutinosa | 324 | + | 4 | CAAT |  |
| CAAT-box | Nicotiana glutinosa | 3488 | + | 4 | CAAT |  |
| CAAT-box | Pisum sativum | 2035 | - | 5 | CAAAT | common cis-acting element in promoter and enhancer regions |
| CAAT-box | Nicotiana glutinosa | 2461 | - | 4 | CAAT |  |
| CAAT-box | Pisum sativum | 1777 | - | 5 | CAAAT | common cis-acting element in promoter and enhancer regions |
| CAAT-box | Nicotiana glutinosa | 240 | + | 4 | CAAT |  |
| CAAT-box | Nicotiana glutinosa | 3048 | + | 4 | CAAT |  |
| CAAT-box | Pisum sativum | 1987 | + | 5 | CAAAT | common cis-acting element in promoter and enhancer regions |
| CAAT-box | Nicotiana glutinosa | 2064 | + | 4 | CAAT |  |
| CAAT-box | Nicotiana glutinosa | 614 | - | 4 | CAAT |  |
| CAAT-box | Nicotiana glutinosa | 3504 | - | 4 | CAAT |  |
| CAAT-box | Pisum sativum | 251 | - | 5 | CAAAT | common cis-acting element in promoter and enhancer regions |
| CAAT-box | Nicotiana glutinosa | 1523 | - | 4 | CAAT |  |
| CAAT-box | Nicotiana glutinosa | 2255 | + | 4 | CAAT |  |
| CAAT-box | Arabidopsis thaliana | 2254 | + | 5 | CCAAT | common cis-acting element in promoter and enhancer regions |
| CAAT-box | Arabidopsis thaliana | 3184 | + | 5 | CCAAT | common cis-acting element in promoter and enhancer regions |
| CAAT-box | Pisum sativum | 739 | - | 5 | CAAAT | common cis-acting element in promoter and enhancer regions |
| CAAT-box | Pisum sativum | 1474 | - | 5 | CAAAT | common cis-acting element in promoter and enhancer regions |
| CAAT-box | Nicotiana glutinosa | 3130 | + | 4 | CAAT |  |
| CAAT-box | Nicotiana glutinosa | 332 | + | 4 | CAAT |  |
| CAAT-box | Pisum sativum | 1837 | - | 5 | CAAAT | common cis-acting element in promoter and enhancer regions |
| CAAT-box | Nicotiana glutinosa | 700 | - | 4 | CAAT |  |
| CAAT-box | Pisum sativum | 3306 | - | 5 | CAAAT | common cis-acting element in promoter and enhancer regions |
| CAAT-box | Pisum sativum | 1334 | - | 5 | CAAAT | common cis-acting element in promoter and enhancer regions |
| CAAT-box | Nicotiana glutinosa | 3253 | + | 4 | CAAT |  |
| CAAT-box | Pisum sativum | 3233 | - | 5 | CAAAT | common cis-acting element in promoter and enhancer regions |
| CAAT-box | Arabidopsis thaliana | 21 | - | 5 | CCAAT | common cis-acting element in promoter and enhancer regions |
| CAAT-box | Pisum sativum | 695 | + | 5 | CAAAT | common cis-acting element in promoter and enhancer regions |
| CAAT-box | Nicotiana glutinosa | 2852 | + | 4 | CAAT |  |
| CAAT-box | Arabidopsis thaliana | 2753 | - | 5 | CCAAT | common cis-acting element in promoter and enhancer regions |
| CAAT-box | Nicotiana glutinosa | 1441 | + | 4 | CAAT |  |
| CAAT-box | Nicotiana glutinosa | 80 | + | 4 | CAAT |  |
| CAAT-box | Nicotiana glutinosa | 1500 | + | 4 | CAAT |  |
| CAAT-box | Nicotiana glutinosa | 3021 | - | 4 | CAAT |  |
| CAAT-box | Nicotiana glutinosa | 1302 | + | 4 | CAAT |  |
| CAAT-box | Nicotiana glutinosa | 263 | - | 4 | CAAT |  |
| CAAT-box | Nicotiana glutinosa | 2390 | - | 4 | CAAT |  |
| CAAT-box | Nicotiana glutinosa | 271 | + | 4 | CAAT |  |
| CAAT-box | Pisum sativum | 1740 | - | 5 | CAAAT | common cis-acting element in promoter and enhancer regions |
| CAAT-box | Nicotiana glutinosa | 3077 | - | 4 | CAAT |  |
| CAAT-box | Pisum sativum | 997 | - | 5 | CAAAT | common cis-acting element in promoter and enhancer regions |
| CAAT-box | Pisum sativum | 1188 | + | 5 | CAAAT | common cis-acting element in promoter and enhancer regions |
| CAAT-box | Pisum sativum | 1191 | - | 5 | CAAAT | common cis-acting element in promoter and enhancer regions |
| CAAT-box | Arabidopsis thaliana | 2184 | - | 5 | CCAAT | common cis-acting element in promoter and enhancer regions |
| CAAT-box | Nicotiana glutinosa | 2093 | + | 4 | CAAT |  |
| CAAT-box | Pisum sativum | 2348 | - | 5 | CAAAT | common cis-acting element in promoter and enhancer regions |
| CAAT-box | Nicotiana glutinosa | 3007 | - | 4 | CAAT |  |
| CAAT-box | Pisum sativum | 2218 | - | 5 | CAAAT | common cis-acting element in promoter and enhancer regions |
| CAAT-box | Pisum sativum | 2407 | + | 5 | CAAAT | common cis-acting element in promoter and enhancer regions |

>HU05G00466.1   
+ +Up\_Stream \_Len000ACATTG GGTGGGATTT AAATCCTCTA TTTTTTCTTA GGGGAAAAAT ACTTGGGAAA   
  
  
+ TTTCTCAATA ACTGAAAATC TAAATAATTC GTGACTTTGT TAGTAGGTTT AATGTGATTT ACTACCGTGA   
  
  
+ GTGTCACTTT TAGATGGTAC TTTAAGAAAG CATTATAAGT AAAACAATAT ATATCGTAGC ATCATACTAC   
  
  
+ TTGTGTAATA AATTAACAGC AACCTCAATT GTACTTATTT GATTTCGCAT TGTAATCAAT TTTCCCAAAT   
  
  
+ GATTTACTAT CTTGGTCTTA CTCAGTTGTA CTTCACACTC AATAGTTCAA TTATTTTTCA TTAGACTAAT   
  
  
+ GAGCGAGTCC CTTCTACAAG AGCCTACTAC TACTCTAATA ATGTAAGGAC TATTGGCTAA TGGGGAACTA   
  
  
+ AGCCTACGAC TTGTTAGGCA TAGGTGAATT TTCCTTACAA GAGAGTTTAA TTTACACTTG ATATTAACTC   
  
  
+ GAGAACGAGA AGCTGTCTTA ATAGATACCC ACATAGAAAA TGAAGCAAAT GCCCTTGATT ATTGGAGGGT   
  
  
+ TGGGCAAATA TATTCAGTAA CAATGTTGTT TGGCAAATAT CGTCTGAAAA TTGTTTTTGA CAAATATTGT   
  
  
+ TTGGAAGAAT TTTTCGAGAT TAAATATTAA AAATGTTAAA ATTTTAATCT AAATATCAAA CAAATATTGA   
  
  
+ AAAATGCTTT ATATTATCTA AATCTCTAAT TAAAATTTGT GTATTGTCCA CTAGAAAAAT GTATAGTGAT   
  
  
+ ATTACCGACA TATATGTGAT CTCTGGTGTA ATAGCCTACC CTAATGTCTA GGAAAATATT TAAAATCCCC   
  
  
+ AGTGGTCAAA AGGAAGTTAC GCACACTAGG GCAAGACACT TCATTAGGCT AGTTGGTGAA AGCTAAGTCA   
  
  
+ TGAAATTTAG ACCATGGCCA TGTCTACTTG TAGATCCAAC TACCAACAAT CCATTTTTAT TTTGGGGAAA   
  
  
+ GCTTGAAGGG TAATTTGGTA AATGTACACG GGAACCTTTT GCTATAAAAG AAGTGCTTCT TGAAAAAACA   
  
  
+ CCCTGTAGTA AAACAGGGCA AAAAAGCCAA CACTTCTGTT TCTGGCAGAG AAACAGTAGT GTACCACTTG   
  
  
+ GAGTTGGAGT AGACACGATA CACAAACCCA GTAGAGAGAG AAACAGGGGA GAGAAAGAGT ACACAAATTT   
  
  
+ GTGCAGGAGA AGGGTAAGGA AGAAGGAGAA GGAACCCCCT CCAAAAAAAA CCCATATAAA AAATAATAAA   
  
  
+ GAAAAAACAG GAGTGAGTTA GAGGGTACCC AAATCTCCAA TCTAAAACTA GAATCTAGGG TCTCTTTTCA   
  
  
+ TTTGCTTGAA AAATTTCTGT TCATGGGTTT TGTGTTTTTA GGGGGATTTG TTTGATTTTT GTAGTCTTTG   
  
  
+ TGGATTTCCT GTGTTATAGA TGCAAGCTAT GCTCTTCAAT TTGCAAGGAA CAGCATCTGG GGGGAGAGAA   
  
  
+ TTTGAGGAAT TTCAAGTACC CATTTCAATT TCAACAAGTA AAATCATCAT TGAAGGATAC TCAAACCCAA   
  
  
+ GCCCAGTTGA TAGTGAACCA ACTTCTACTC TGGATTCTAC TCCAAGTCCC AGCCCTCCCA CCCTCTCCTC   
  
  
+ CTCCTCCAAG AACTCCTACC AAAACGCTGC CGTTTTGGCT CACCCTCCTC AGCTTCACCA TGGATCTGAT   
  
  
+ AATGTGTAAG ACCCCAACCC TTTTGCTTTT ATTTTCCTCA TGCTCCATTC TCATGATTTG TACTTCTGTT   
  
  
+ ATGTAATTGG AGATGTGTCT AAATTTGGAA AAGTTCGTGG GTTTCGTGTT TGAGAATTTA GAATGACATT   
  
  
+ CTTTAATTAG TAATTTGAAG TTTGCATGCT TAATTTAGCT AATCACTGAT TAATTAAAAT AAATGAAGTG   
  
  
+ ATGTAGGTAT GGGCTGTTAC ACAATGTCAC CGGTGGTGGT GACAGCGGCG GGGCGGCGGC GGTTGGATTG   
  
  
+ GAAGAATGGG ACAGCGTGTT TCCAAATGGG GATGGAGCTT TACTCCCTTG GATCATGGGG GAAGGTGATG   
  
  
+ ATTTGGGTCT GAATTTGAAG CATCTCTTAC AATCTGGTTA CCCTGTTGAG TATGAAGGCA ATGCAGGACT   
  
  
+ TGGTGTTGTT GATCAGACAT CTCTTCTTGG AGGTGGTGAT TTGGGGTTTT CTGGTTCTGG GTCTGTGAAT   
  
  
+ AATGGCAAAA TTGGTTCCAT TTTGGGTAAT TGTTCATCTG GGGATTTGGA TTCTAAGGTT TCTAGTGACG   
  
  
+ GGTTGAATTC CAATTGTAGT TCACTGGGGA GTATGAGTAT CCTTGGTTCA ATCCATGGAC CTTTGCCTAA   
  
  
+ TACTGGTGGG TTTGTGTTCC CACAAACACA ACTATTTGAT CTTGGTGATG AGAAGCCTCA GATTTTGAAC   
  
  
+ CCACAATTGA TGACGATGAA TCCAAATCAG GCTCAGAGCA TGGGAAACCC TAGCTTTTTT GTGCCCTCCT   
  
  
+ TGGGTTATTG TCAGTTAGAG CAACATATGG TTCAACCACA GGCGAAACGC CATAGCCCTG GTGTAGTTTT   
  
  
+ GAACTCTGAT GTTATTGCCA AAACCCCGTT TCTCGATCAG GGTCATGAGT TTTTGTTGAG GAAACAACAC   
  
  
+ CATCAACAGA TTTTGCAGCA ACTTCCTATG GGTTTGGCTC ATCAGTCGGT CCCTCAGCAC CTCCAGCAAA   
  
  
+ AGGCGATGAA AGACCAGGTT GTTAAGGACC AGCTTTTGAA GGCAGCTGAC CTGATTCAAA CTGGGAATTT   
  
  
+ CTCACTTGCG CAAGAGATAT TGGCGCGGCT CAATCACCAG CTCTCCCTTC CTGCGAAGCC CCTCATTAGG   
  
  
+ GCAGCTTTGT ATGTCATGGA GGCCTTGCAA ATGCTCATCT TAATGAGCAA TCCTGTAGCA CCTCCACCGA   
  
  
+ TGAAGACGCT TTCCCCTATA GATGTTGTTC ATAAGATGAA TGCCTACAAG GCCTTCTCTG AGGTCTCTCC   
  
  
+ TATTACTCAG TTTACGAATT TCACTTGTGC CCAAGCCATT CTTGAAGCTC TCGATGATGC GGATTGCGTT   
  
  
+ CATGTCATTG ACTTTGATAT TGGTTGTGGG GCTCAATGGG CATCACTGAT TCAGGAGCTT CCATTGAGGA   
  
  
+ AAAAGGGAGC TCCATCCCTG AAAATTACAG CCATAGCTCC CCTGTCAATA AGCCACACTT TTGAACTCAA   
  
  
+ CCTAGCACGG GAAAACCTTG TGCAATTCGC CAATGATGTT GGTGTTGCTT TTGAGCTGCA AGTTGTGAAC   
  
  
+ TTGGATTTAT TTGATCCATC TTCATCTTCA ATGCCAAATG TTGGAACCTC TGGGGATGAG TTGATCGCTG   
  
  
+ TTAGTATACC CATTTGGGCA TGTTCATATC GGCCGTCTAT TCTCCCATCC ATCCTCCGGT TCATTAAGCA   
  
  
+ ACTAGCCCCC AAGATTGTCG TCTCGTTAGA TAGAGGGTCT GATCGTTGCG ACACTCTGTT CCCACAACAT   
  
  
+ CTTATCCATA TCTTAGAGTC CTGCACAAAT TTCTTGGAAT CGCTCGACGG TCTCAATGTA GCATCAGATA   
  
  
+ TTGTAAACAA GGTTGAGAAG TACTTCCTCC AGCCTAAGAT CGAAAACACC GTGTTGGGCC GCGTTCATGC   
  
  
+ CCCCAATAAG ATGCCACATT GGAAGACCCT CTTTGCTTCA GCCGGGTTAT TGCCCTTTCA GTTCAGTAAC   
  
  
+ TTCACAGAAA CACAAGCTGA TTATGTGGTG AAGCGAACCC CAGGGAGGGG ATTTCACATA GAAAAGCGCC   
  
  
+ ACGCATCTTT AGTGCTAAGT TGGCAAAGGC GGGAGCTTGT GACAGCCTCG GCATGGAGGT TTGAGAAGCT   
  
  
+ GTA  

- +Up\_Stream \_Len000TGTAAC CCACCCTAAA TTTAGGAGAT AAAAAAGAAT CCCCTTTTTA TGAACCCTTT   
  
  
- AAAGAGTTAT TGACTTTTAG ATTTATTAAG CACTGAAACA ATCATCCAAA TTACACTAAA TGATGGCACT   
  
  
- CACAGTGAAA ATCTACCATG AAATTCTTTC GTAATATTCA TTTTGTTATA TATAGCATCG TAGTATGATG   
  
  
- AACACATTAT TTAATTGTCG TTGGAGTTAA CATGAATAAA CTAAAGCGTA ACATTAGTTA AAAGGGTTTA   
  
  
- CTAAATGATA GAACCAGAAT GAGTCAACAT GAAGTGTGAG TTATCAAGTT AATAAAAAGT AATCTGATTA   
  
  
- CTCGCTCAGG GAAGATGTTC TCGGATGATG ATGAGATTAT TACATTCCTG ATAACCGATT ACCCCTTGAT   
  
  
- TCGGATGCTG AACAATCCGT ATCCACTTAA AAGGAATGTT CTCTCAAATT AAATGTGAAC TATAATTGAG   
  
  
- CTCTTGCTCT TCGACAGAAT TATCTATGGG TGTATCTTTT ACTTCGTTTA CGGGAACTAA TAACCTCCCA   
  
  
- ACCCGTTTAT ATAAGTCATT GTTACAACAA ACCGTTTATA GCAGACTTTT AACAAAAACT GTTTATAACA   
  
  
- AACCTTCTTA AAAAGCTCTA ATTTATAATT TTTACAATTT TAAAATTAGA TTTATAGTTT GTTTATAACT   
  
  
- TTTTACGAAA TATAATAGAT TTAGAGATTA ATTTTAAACA CATAACAGGT GATCTTTTTA CATATCACTA   
  
  
- TAATGGCTGT ATATACACTA GAGACCACAT TATCGGATGG GATTACAGAT CCTTTTATAA ATTTTAGGGG   
  
  
- TCACCAGTTT TCCTTCAATG CGTGTGATCC CGTTCTGTGA AGTAATCCGA TCAACCACTT TCGATTCAGT   
  
  
- ACTTTAAATC TGGTACCGGT ACAGATGAAC ATCTAGGTTG ATGGTTGTTA GGTAAAAATA AAACCCCTTT   
  
  
- CGAACTTCCC ATTAAACCAT TTACATGTGC CCTTGGAAAA CGATATTTTC TTCACGAAGA ACTTTTTTGT   
  
  
- GGGACATCAT TTTGTCCCGT TTTTTCGGTT GTGAAGACAA AGACCGTCTC TTTGTCATCA CATGGTGAAC   
  
  
- CTCAACCTCA TCTGTGCTAT GTGTTTGGGT CATCTCTCTC TTTGTCCCCT CTCTTTCTCA TGTGTTTAAA   
  
  
- CACGTCCTCT TCCCATTCCT TCTTCCTCTT CCTTGGGGGA GGTTTTTTTT GGGTATATTT TTTATTATTT   
  
  
- CTTTTTTGTC CTCACTCAAT CTCCCATGGG TTTAGAGGTT AGATTTTGAT CTTAGATCCC AGAGAAAAGT   
  
  
- AAACGAACTT TTTAAAGACA AGTACCCAAA ACACAAAAAT CCCCCTAAAC AAACTAAAAA CATCAGAAAC   
  
  
- ACCTAAAGGA CACAATATCT ACGTTCGATA CGAGAAGTTA AACGTTCCTT GTCGTAGACC CCCCTCTCTT   
  
  
- AAACTCCTTA AAGTTCATGG GTAAAGTTAA AGTTGTTCAT TTTAGTAGTA ACTTCCTATG AGTTTGGGTT   
  
  
- CGGGTCAACT ATCACTTGGT TGAAGATGAG ACCTAAGATG AGGTTCAGGG TCGGGAGGGT GGGAGAGGAG   
  
  
- GAGGAGGTTC TTGAGGATGG TTTTGCGACG GCAAAACCGA GTGGGAGGAG TCGAAGTGGT ACCTAGACTA   
  
  
- TTACACATTC TGGGGTTGGG AAAACGAAAA TAAAAGGAGT ACGAGGTAAG AGTACTAAAC ATGAAGACAA   
  
  
- TACATTAACC TCTACACAGA TTTAAACCTT TTCAAGCACC CAAAGCACAA ACTCTTAAAT CTTACTGTAA   
  
  
- GAAATTAATC ATTAAACTTC AAACGTACGA ATTAAATCGA TTAGTGACTA ATTAATTTTA TTTACTTCAC   
  
  
- TACATCCATA CCCGACAATG TGTTACAGTG GCCACCACCA CTGTCGCCGC CCCGCCGCCG CCAACCTAAC   
  
  
- CTTCTTACCC TGTCGCACAA AGGTTTACCC CTACCTCGAA ATGAGGGAAC CTAGTACCCC CTTCCACTAC   
  
  
- TAAACCCAGA CTTAAACTTC GTAGAGAATG TTAGACCAAT GGGACAACTC ATACTTCCGT TACGTCCTGA   
  
  
- ACCACAACAA CTAGTCTGTA GAGAAGAACC TCCACCACTA AACCCCAAAA GACCAAGACC CAGACACTTA   
  
  
- TTACCGTTTT AACCAAGGTA AAACCCATTA ACAAGTAGAC CCCTAAACCT AAGATTCCAA AGATCACTGC   
  
  
- CCAACTTAAG GTTAACATCA AGTGACCCCT CATACTCATA GGAACCAAGT TAGGTACCTG GAAACGGATT   
  
  
- ATGACCACCC AAACACAAGG GTGTTTGTGT TGATAAACTA GAACCACTAC TCTTCGGAGT CTAAAACTTG   
  
  
- GGTGTTAACT ACTGCTACTT AGGTTTAGTC CGAGTCTCGT ACCCTTTGGG ATCGAAAAAA CACGGGAGGA   
  
  
- ACCCAATAAC AGTCAATCTC GTTGTATACC AAGTTGGTGT CCGCTTTGCG GTATCGGGAC CACATCAAAA   
  
  
- CTTGAGACTA CAATAACGGT TTTGGGGCAA AGAGCTAGTC CCAGTACTCA AAAACAACTC CTTTGTTGTG   
  
  
- GTAGTTGTCT AAAACGTCGT TGAAGGATAC CCAAACCGAG TAGTCAGCCA GGGAGTCGTG GAGGTCGTTT   
  
  
- TCCGCTACTT TCTGGTCCAA CAATTCCTGG TCGAAAACTT CCGTCGACTG GACTAAGTTT GACCCTTAAA   
  
  
- GAGTGAACGC GTTCTCTATA ACCGCGCCGA GTTAGTGGTC GAGAGGGAAG GACGCTTCGG GGAGTAATCC   
  
  
- CGTCGAAACA TACAGTACCT CCGGAACGTT TACGAGTAGA ATTACTCGTT AGGACATCGT GGAGGTGGCT   
  
  
- ACTTCTGCGA AAGGGGATAT CTACAACAAG TATTCTACTT ACGGATGTTC CGGAAGAGAC TCCAGAGAGG   
  
  
- ATAATGAGTC AAATGCTTAA AGTGAACACG GGTTCGGTAA GAACTTCGAG AGCTACTACG CCTAACGCAA   
  
  
- GTACAGTAAC TGAAACTATA ACCAACACCC CGAGTTACCC GTAGTGACTA AGTCCTCGAA GGTAACTCCT   
  
  
- TTTTCCCTCG AGGTAGGGAC TTTTAATGTC GGTATCGAGG GGACAGTTAT TCGGTGTGAA AACTTGAGTT   
  
  
- GGATCGTGCC CTTTTGGAAC ACGTTAAGCG GTTACTACAA CCACAACGAA AACTCGACGT TCAACACTTG   
  
  
- AACCTAAATA AACTAGGTAG AAGTAGAAGT TACGGTTTAC AACCTTGGAG ACCCCTACTC AACTAGCGAC   
  
  
- AATCATATGG GTAAACCCGT ACAAGTATAG CCGGCAGATA AGAGGGTAGG TAGGAGGCCA AGTAATTCGT   
  
  
- TGATCGGGGG TTCTAACAGC AGAGCAATCT ATCTCCCAGA CTAGCAACGC TGTGAGACAA GGGTGTTGTA   
  
  
- GAATAGGTAT AGAATCTCAG GACGTGTTTA AAGAACCTTA GCGAGCTGCC AGAGTTACAT CGTAGTCTAT   
  
  
- AACATTTGTT CCAACTCTTC ATGAAGGAGG TCGGATTCTA GCTTTTGTGG CACAACCCGG CGCAAGTACG   
  
  
- GGGGTTATTC TACGGTGTAA CCTTCTGGGA GAAACGAAGT CGGCCCAATA ACGGGAAAGT CAAGTCATTG   
  
  
- AAGTGTCTTT GTGTTCGACT AATACACCAC TTCGCTTGGG GTCCCTCCCC TAAAGTGTAT CTTTTCGCGG   
  
  
- TGCGTAGAAA TCACGATTCA ACCGTTTCCG CCCTCGAACA CTGTCGGAGC CGTACCTCCA AACTCTTCGA   
  
  
- CAT

+     CGTCA-motif

| Site Name | Organism | Position | Strand | Matrix score. | sequence | function |
| --- | --- | --- | --- | --- | --- | --- |
| CGTCA-motif | Hordeum vulgare | 2395 | - | 5 | CGTCA | cis-acting regulatory element involved in the MeJA-responsiveness |
| CGTCA-motif | Hordeum vulgare | 2240 | - | 5 | CGTCA | cis-acting regulatory element involved in the MeJA-responsiveness |

>HU05G00466.1   
+ +Up\_Stream \_Len000ACATTG GGTGGGATTT AAATCCTCTA TTTTTTCTTA GGGGAAAAAT ACTTGGGAAA   
  
  
+ TTTCTCAATA ACTGAAAATC TAAATAATTC GTGACTTTGT TAGTAGGTTT AATGTGATTT ACTACCGTGA   
  
  
+ GTGTCACTTT TAGATGGTAC TTTAAGAAAG CATTATAAGT AAAACAATAT ATATCGTAGC ATCATACTAC   
  
  
+ TTGTGTAATA AATTAACAGC AACCTCAATT GTACTTATTT GATTTCGCAT TGTAATCAAT TTTCCCAAAT   
  
  
+ GATTTACTAT CTTGGTCTTA CTCAGTTGTA CTTCACACTC AATAGTTCAA TTATTTTTCA TTAGACTAAT   
  
  
+ GAGCGAGTCC CTTCTACAAG AGCCTACTAC TACTCTAATA ATGTAAGGAC TATTGGCTAA TGGGGAACTA   
  
  
+ AGCCTACGAC TTGTTAGGCA TAGGTGAATT TTCCTTACAA GAGAGTTTAA TTTACACTTG ATATTAACTC   
  
  
+ GAGAACGAGA AGCTGTCTTA ATAGATACCC ACATAGAAAA TGAAGCAAAT GCCCTTGATT ATTGGAGGGT   
  
  
+ TGGGCAAATA TATTCAGTAA CAATGTTGTT TGGCAAATAT CGTCTGAAAA TTGTTTTTGA CAAATATTGT   
  
  
+ TTGGAAGAAT TTTTCGAGAT TAAATATTAA AAATGTTAAA ATTTTAATCT AAATATCAAA CAAATATTGA   
  
  
+ AAAATGCTTT ATATTATCTA AATCTCTAAT TAAAATTTGT GTATTGTCCA CTAGAAAAAT GTATAGTGAT   
  
  
+ ATTACCGACA TATATGTGAT CTCTGGTGTA ATAGCCTACC CTAATGTCTA GGAAAATATT TAAAATCCCC   
  
  
+ AGTGGTCAAA AGGAAGTTAC GCACACTAGG GCAAGACACT TCATTAGGCT AGTTGGTGAA AGCTAAGTCA   
  
  
+ TGAAATTTAG ACCATGGCCA TGTCTACTTG TAGATCCAAC TACCAACAAT CCATTTTTAT TTTGGGGAAA   
  
  
+ GCTTGAAGGG TAATTTGGTA AATGTACACG GGAACCTTTT GCTATAAAAG AAGTGCTTCT TGAAAAAACA   
  
  
+ CCCTGTAGTA AAACAGGGCA AAAAAGCCAA CACTTCTGTT TCTGGCAGAG AAACAGTAGT GTACCACTTG   
  
  
+ GAGTTGGAGT AGACACGATA CACAAACCCA GTAGAGAGAG AAACAGGGGA GAGAAAGAGT ACACAAATTT   
  
  
+ GTGCAGGAGA AGGGTAAGGA AGAAGGAGAA GGAACCCCCT CCAAAAAAAA CCCATATAAA AAATAATAAA   
  
  
+ GAAAAAACAG GAGTGAGTTA GAGGGTACCC AAATCTCCAA TCTAAAACTA GAATCTAGGG TCTCTTTTCA   
  
  
+ TTTGCTTGAA AAATTTCTGT TCATGGGTTT TGTGTTTTTA GGGGGATTTG TTTGATTTTT GTAGTCTTTG   
  
  
+ TGGATTTCCT GTGTTATAGA TGCAAGCTAT GCTCTTCAAT TTGCAAGGAA CAGCATCTGG GGGGAGAGAA   
  
  
+ TTTGAGGAAT TTCAAGTACC CATTTCAATT TCAACAAGTA AAATCATCAT TGAAGGATAC TCAAACCCAA   
  
  
+ GCCCAGTTGA TAGTGAACCA ACTTCTACTC TGGATTCTAC TCCAAGTCCC AGCCCTCCCA CCCTCTCCTC   
  
  
+ CTCCTCCAAG AACTCCTACC AAAACGCTGC CGTTTTGGCT CACCCTCCTC AGCTTCACCA TGGATCTGAT   
  
  
+ AATGTGTAAG ACCCCAACCC TTTTGCTTTT ATTTTCCTCA TGCTCCATTC TCATGATTTG TACTTCTGTT   
  
  
+ ATGTAATTGG AGATGTGTCT AAATTTGGAA AAGTTCGTGG GTTTCGTGTT TGAGAATTTA GAATGACATT   
  
  
+ CTTTAATTAG TAATTTGAAG TTTGCATGCT TAATTTAGCT AATCACTGAT TAATTAAAAT AAATGAAGTG   
  
  
+ ATGTAGGTAT GGGCTGTTAC ACAATGTCAC CGGTGGTGGT GACAGCGGCG GGGCGGCGGC GGTTGGATTG   
  
  
+ GAAGAATGGG ACAGCGTGTT TCCAAATGGG GATGGAGCTT TACTCCCTTG GATCATGGGG GAAGGTGATG   
  
  
+ ATTTGGGTCT GAATTTGAAG CATCTCTTAC AATCTGGTTA CCCTGTTGAG TATGAAGGCA ATGCAGGACT   
  
  
+ TGGTGTTGTT GATCAGACAT CTCTTCTTGG AGGTGGTGAT TTGGGGTTTT CTGGTTCTGG GTCTGTGAAT   
  
  
+ AATGGCAAAA TTGGTTCCAT TTTGGGTAAT TGTTCATCTG GGGATTTGGA TTCTAAGGTT TCTAGTGACG   
  
  
+ GGTTGAATTC CAATTGTAGT TCACTGGGGA GTATGAGTAT CCTTGGTTCA ATCCATGGAC CTTTGCCTAA   
  
  
+ TACTGGTGGG TTTGTGTTCC CACAAACACA ACTATTTGAT CTTGGTGATG AGAAGCCTCA GATTTTGAAC   
  
  
+ CCACAATTGA TGACGATGAA TCCAAATCAG GCTCAGAGCA TGGGAAACCC TAGCTTTTTT GTGCCCTCCT   
  
  
+ TGGGTTATTG TCAGTTAGAG CAACATATGG TTCAACCACA GGCGAAACGC CATAGCCCTG GTGTAGTTTT   
  
  
+ GAACTCTGAT GTTATTGCCA AAACCCCGTT TCTCGATCAG GGTCATGAGT TTTTGTTGAG GAAACAACAC   
  
  
+ CATCAACAGA TTTTGCAGCA ACTTCCTATG GGTTTGGCTC ATCAGTCGGT CCCTCAGCAC CTCCAGCAAA   
  
  
+ AGGCGATGAA AGACCAGGTT GTTAAGGACC AGCTTTTGAA GGCAGCTGAC CTGATTCAAA CTGGGAATTT   
  
  
+ CTCACTTGCG CAAGAGATAT TGGCGCGGCT CAATCACCAG CTCTCCCTTC CTGCGAAGCC CCTCATTAGG   
  
  
+ GCAGCTTTGT ATGTCATGGA GGCCTTGCAA ATGCTCATCT TAATGAGCAA TCCTGTAGCA CCTCCACCGA   
  
  
+ TGAAGACGCT TTCCCCTATA GATGTTGTTC ATAAGATGAA TGCCTACAAG GCCTTCTCTG AGGTCTCTCC   
  
  
+ TATTACTCAG TTTACGAATT TCACTTGTGC CCAAGCCATT CTTGAAGCTC TCGATGATGC GGATTGCGTT   
  
  
+ CATGTCATTG ACTTTGATAT TGGTTGTGGG GCTCAATGGG CATCACTGAT TCAGGAGCTT CCATTGAGGA   
  
  
+ AAAAGGGAGC TCCATCCCTG AAAATTACAG CCATAGCTCC CCTGTCAATA AGCCACACTT TTGAACTCAA   
  
  
+ CCTAGCACGG GAAAACCTTG TGCAATTCGC CAATGATGTT GGTGTTGCTT TTGAGCTGCA AGTTGTGAAC   
  
  
+ TTGGATTTAT TTGATCCATC TTCATCTTCA ATGCCAAATG TTGGAACCTC TGGGGATGAG TTGATCGCTG   
  
  
+ TTAGTATACC CATTTGGGCA TGTTCATATC GGCCGTCTAT TCTCCCATCC ATCCTCCGGT TCATTAAGCA   
  
  
+ ACTAGCCCCC AAGATTGTCG TCTCGTTAGA TAGAGGGTCT GATCGTTGCG ACACTCTGTT CCCACAACAT   
  
  
+ CTTATCCATA TCTTAGAGTC CTGCACAAAT TTCTTGGAAT CGCTCGACGG TCTCAATGTA GCATCAGATA   
  
  
+ TTGTAAACAA GGTTGAGAAG TACTTCCTCC AGCCTAAGAT CGAAAACACC GTGTTGGGCC GCGTTCATGC   
  
  
+ CCCCAATAAG ATGCCACATT GGAAGACCCT CTTTGCTTCA GCCGGGTTAT TGCCCTTTCA GTTCAGTAAC   
  
  
+ TTCACAGAAA CACAAGCTGA TTATGTGGTG AAGCGAACCC CAGGGAGGGG ATTTCACATA GAAAAGCGCC   
  
  
+ ACGCATCTTT AGTGCTAAGT TGGCAAAGGC GGGAGCTTGT GACAGCCTCG GCATGGAGGT TTGAGAAGCT   
  
  
+ GTA  

- +Up\_Stream \_Len000TGTAAC CCACCCTAAA TTTAGGAGAT AAAAAAGAAT CCCCTTTTTA TGAACCCTTT   
  
  
- AAAGAGTTAT TGACTTTTAG ATTTATTAAG CACTGAAACA ATCATCCAAA TTACACTAAA TGATGGCACT   
  
  
- CACAGTGAAA ATCTACCATG AAATTCTTTC GTAATATTCA TTTTGTTATA TATAGCATCG TAGTATGATG   
  
  
- AACACATTAT TTAATTGTCG TTGGAGTTAA CATGAATAAA CTAAAGCGTA ACATTAGTTA AAAGGGTTTA   
  
  
- CTAAATGATA GAACCAGAAT GAGTCAACAT GAAGTGTGAG TTATCAAGTT AATAAAAAGT AATCTGATTA   
  
  
- CTCGCTCAGG GAAGATGTTC TCGGATGATG ATGAGATTAT TACATTCCTG ATAACCGATT ACCCCTTGAT   
  
  
- TCGGATGCTG AACAATCCGT ATCCACTTAA AAGGAATGTT CTCTCAAATT AAATGTGAAC TATAATTGAG   
  
  
- CTCTTGCTCT TCGACAGAAT TATCTATGGG TGTATCTTTT ACTTCGTTTA CGGGAACTAA TAACCTCCCA   
  
  
- ACCCGTTTAT ATAAGTCATT GTTACAACAA ACCGTTTATA GCAGACTTTT AACAAAAACT GTTTATAACA   
  
  
- AACCTTCTTA AAAAGCTCTA ATTTATAATT TTTACAATTT TAAAATTAGA TTTATAGTTT GTTTATAACT   
  
  
- TTTTACGAAA TATAATAGAT TTAGAGATTA ATTTTAAACA CATAACAGGT GATCTTTTTA CATATCACTA   
  
  
- TAATGGCTGT ATATACACTA GAGACCACAT TATCGGATGG GATTACAGAT CCTTTTATAA ATTTTAGGGG   
  
  
- TCACCAGTTT TCCTTCAATG CGTGTGATCC CGTTCTGTGA AGTAATCCGA TCAACCACTT TCGATTCAGT   
  
  
- ACTTTAAATC TGGTACCGGT ACAGATGAAC ATCTAGGTTG ATGGTTGTTA GGTAAAAATA AAACCCCTTT   
  
  
- CGAACTTCCC ATTAAACCAT TTACATGTGC CCTTGGAAAA CGATATTTTC TTCACGAAGA ACTTTTTTGT   
  
  
- GGGACATCAT TTTGTCCCGT TTTTTCGGTT GTGAAGACAA AGACCGTCTC TTTGTCATCA CATGGTGAAC   
  
  
- CTCAACCTCA TCTGTGCTAT GTGTTTGGGT CATCTCTCTC TTTGTCCCCT CTCTTTCTCA TGTGTTTAAA   
  
  
- CACGTCCTCT TCCCATTCCT TCTTCCTCTT CCTTGGGGGA GGTTTTTTTT GGGTATATTT TTTATTATTT   
  
  
- CTTTTTTGTC CTCACTCAAT CTCCCATGGG TTTAGAGGTT AGATTTTGAT CTTAGATCCC AGAGAAAAGT   
  
  
- AAACGAACTT TTTAAAGACA AGTACCCAAA ACACAAAAAT CCCCCTAAAC AAACTAAAAA CATCAGAAAC   
  
  
- ACCTAAAGGA CACAATATCT ACGTTCGATA CGAGAAGTTA AACGTTCCTT GTCGTAGACC CCCCTCTCTT   
  
  
- AAACTCCTTA AAGTTCATGG GTAAAGTTAA AGTTGTTCAT TTTAGTAGTA ACTTCCTATG AGTTTGGGTT   
  
  
- CGGGTCAACT ATCACTTGGT TGAAGATGAG ACCTAAGATG AGGTTCAGGG TCGGGAGGGT GGGAGAGGAG   
  
  
- GAGGAGGTTC TTGAGGATGG TTTTGCGACG GCAAAACCGA GTGGGAGGAG TCGAAGTGGT ACCTAGACTA   
  
  
- TTACACATTC TGGGGTTGGG AAAACGAAAA TAAAAGGAGT ACGAGGTAAG AGTACTAAAC ATGAAGACAA   
  
  
- TACATTAACC TCTACACAGA TTTAAACCTT TTCAAGCACC CAAAGCACAA ACTCTTAAAT CTTACTGTAA   
  
  
- GAAATTAATC ATTAAACTTC AAACGTACGA ATTAAATCGA TTAGTGACTA ATTAATTTTA TTTACTTCAC   
  
  
- TACATCCATA CCCGACAATG TGTTACAGTG GCCACCACCA CTGTCGCCGC CCCGCCGCCG CCAACCTAAC   
  
  
- CTTCTTACCC TGTCGCACAA AGGTTTACCC CTACCTCGAA ATGAGGGAAC CTAGTACCCC CTTCCACTAC   
  
  
- TAAACCCAGA CTTAAACTTC GTAGAGAATG TTAGACCAAT GGGACAACTC ATACTTCCGT TACGTCCTGA   
  
  
- ACCACAACAA CTAGTCTGTA GAGAAGAACC TCCACCACTA AACCCCAAAA GACCAAGACC CAGACACTTA   
  
  
- TTACCGTTTT AACCAAGGTA AAACCCATTA ACAAGTAGAC CCCTAAACCT AAGATTCCAA AGATCACTGC   
  
  
- CCAACTTAAG GTTAACATCA AGTGACCCCT CATACTCATA GGAACCAAGT TAGGTACCTG GAAACGGATT   
  
  
- ATGACCACCC AAACACAAGG GTGTTTGTGT TGATAAACTA GAACCACTAC TCTTCGGAGT CTAAAACTTG   
  
  
- GGTGTTAACT ACTGCTACTT AGGTTTAGTC CGAGTCTCGT ACCCTTTGGG ATCGAAAAAA CACGGGAGGA   
  
  
- ACCCAATAAC AGTCAATCTC GTTGTATACC AAGTTGGTGT CCGCTTTGCG GTATCGGGAC CACATCAAAA   
  
  
- CTTGAGACTA CAATAACGGT TTTGGGGCAA AGAGCTAGTC CCAGTACTCA AAAACAACTC CTTTGTTGTG   
  
  
- GTAGTTGTCT AAAACGTCGT TGAAGGATAC CCAAACCGAG TAGTCAGCCA GGGAGTCGTG GAGGTCGTTT   
  
  
- TCCGCTACTT TCTGGTCCAA CAATTCCTGG TCGAAAACTT CCGTCGACTG GACTAAGTTT GACCCTTAAA   
  
  
- GAGTGAACGC GTTCTCTATA ACCGCGCCGA GTTAGTGGTC GAGAGGGAAG GACGCTTCGG GGAGTAATCC   
  
  
- CGTCGAAACA TACAGTACCT CCGGAACGTT TACGAGTAGA ATTACTCGTT AGGACATCGT GGAGGTGGCT   
  
  
- ACTTCTGCGA AAGGGGATAT CTACAACAAG TATTCTACTT ACGGATGTTC CGGAAGAGAC TCCAGAGAGG   
  
  
- ATAATGAGTC AAATGCTTAA AGTGAACACG GGTTCGGTAA GAACTTCGAG AGCTACTACG CCTAACGCAA   
  
  
- GTACAGTAAC TGAAACTATA ACCAACACCC CGAGTTACCC GTAGTGACTA AGTCCTCGAA GGTAACTCCT   
  
  
- TTTTCCCTCG AGGTAGGGAC TTTTAATGTC GGTATCGAGG GGACAGTTAT TCGGTGTGAA AACTTGAGTT   
  
  
- GGATCGTGCC CTTTTGGAAC ACGTTAAGCG GTTACTACAA CCACAACGAA AACTCGACGT TCAACACTTG   
  
  
- AACCTAAATA AACTAGGTAG AAGTAGAAGT TACGGTTTAC AACCTTGGAG ACCCCTACTC AACTAGCGAC   
  
  
- AATCATATGG GTAAACCCGT ACAAGTATAG CCGGCAGATA AGAGGGTAGG TAGGAGGCCA AGTAATTCGT   
  
  
- TGATCGGGGG TTCTAACAGC AGAGCAATCT ATCTCCCAGA CTAGCAACGC TGTGAGACAA GGGTGTTGTA   
  
  
- GAATAGGTAT AGAATCTCAG GACGTGTTTA AAGAACCTTA GCGAGCTGCC AGAGTTACAT CGTAGTCTAT   
  
  
- AACATTTGTT CCAACTCTTC ATGAAGGAGG TCGGATTCTA GCTTTTGTGG CACAACCCGG CGCAAGTACG   
  
  
- GGGGTTATTC TACGGTGTAA CCTTCTGGGA GAAACGAAGT CGGCCCAATA ACGGGAAAGT CAAGTCATTG   
  
  
- AAGTGTCTTT GTGTTCGACT AATACACCAC TTCGCTTGGG GTCCCTCCCC TAAAGTGTAT CTTTTCGCGG   
  
  
- TGCGTAGAAA TCACGATTCA ACCGTTTCCG CCCTCGAACA CTGTCGGAGC CGTACCTCCA AACTCTTCGA   
  
  
- CAT

+     DRE

| Site Name | Organism | Position | Strand | Matrix score. | sequence | function |
| --- | --- | --- | --- | --- | --- | --- |
| DRE | Arabidopsis thaliana | 777 | + | 9 | TACCGACAT | "cis-acting element involved in dehydration, low-temp, salt stresses |

>HU05G00466.1   
+ +Up\_Stream \_Len000ACATTG GGTGGGATTT AAATCCTCTA TTTTTTCTTA GGGGAAAAAT ACTTGGGAAA   
  
  
+ TTTCTCAATA ACTGAAAATC TAAATAATTC GTGACTTTGT TAGTAGGTTT AATGTGATTT ACTACCGTGA   
  
  
+ GTGTCACTTT TAGATGGTAC TTTAAGAAAG CATTATAAGT AAAACAATAT ATATCGTAGC ATCATACTAC   
  
  
+ TTGTGTAATA AATTAACAGC AACCTCAATT GTACTTATTT GATTTCGCAT TGTAATCAAT TTTCCCAAAT   
  
  
+ GATTTACTAT CTTGGTCTTA CTCAGTTGTA CTTCACACTC AATAGTTCAA TTATTTTTCA TTAGACTAAT   
  
  
+ GAGCGAGTCC CTTCTACAAG AGCCTACTAC TACTCTAATA ATGTAAGGAC TATTGGCTAA TGGGGAACTA   
  
  
+ AGCCTACGAC TTGTTAGGCA TAGGTGAATT TTCCTTACAA GAGAGTTTAA TTTACACTTG ATATTAACTC   
  
  
+ GAGAACGAGA AGCTGTCTTA ATAGATACCC ACATAGAAAA TGAAGCAAAT GCCCTTGATT ATTGGAGGGT   
  
  
+ TGGGCAAATA TATTCAGTAA CAATGTTGTT TGGCAAATAT CGTCTGAAAA TTGTTTTTGA CAAATATTGT   
  
  
+ TTGGAAGAAT TTTTCGAGAT TAAATATTAA AAATGTTAAA ATTTTAATCT AAATATCAAA CAAATATTGA   
  
  
+ AAAATGCTTT ATATTATCTA AATCTCTAAT TAAAATTTGT GTATTGTCCA CTAGAAAAAT GTATAGTGAT   
  
  
+ ATTACCGACA TATATGTGAT CTCTGGTGTA ATAGCCTACC CTAATGTCTA GGAAAATATT TAAAATCCCC   
  
  
+ AGTGGTCAAA AGGAAGTTAC GCACACTAGG GCAAGACACT TCATTAGGCT AGTTGGTGAA AGCTAAGTCA   
  
  
+ TGAAATTTAG ACCATGGCCA TGTCTACTTG TAGATCCAAC TACCAACAAT CCATTTTTAT TTTGGGGAAA   
  
  
+ GCTTGAAGGG TAATTTGGTA AATGTACACG GGAACCTTTT GCTATAAAAG AAGTGCTTCT TGAAAAAACA   
  
  
+ CCCTGTAGTA AAACAGGGCA AAAAAGCCAA CACTTCTGTT TCTGGCAGAG AAACAGTAGT GTACCACTTG   
  
  
+ GAGTTGGAGT AGACACGATA CACAAACCCA GTAGAGAGAG AAACAGGGGA GAGAAAGAGT ACACAAATTT   
  
  
+ GTGCAGGAGA AGGGTAAGGA AGAAGGAGAA GGAACCCCCT CCAAAAAAAA CCCATATAAA AAATAATAAA   
  
  
+ GAAAAAACAG GAGTGAGTTA GAGGGTACCC AAATCTCCAA TCTAAAACTA GAATCTAGGG TCTCTTTTCA   
  
  
+ TTTGCTTGAA AAATTTCTGT TCATGGGTTT TGTGTTTTTA GGGGGATTTG TTTGATTTTT GTAGTCTTTG   
  
  
+ TGGATTTCCT GTGTTATAGA TGCAAGCTAT GCTCTTCAAT TTGCAAGGAA CAGCATCTGG GGGGAGAGAA   
  
  
+ TTTGAGGAAT TTCAAGTACC CATTTCAATT TCAACAAGTA AAATCATCAT TGAAGGATAC TCAAACCCAA   
  
  
+ GCCCAGTTGA TAGTGAACCA ACTTCTACTC TGGATTCTAC TCCAAGTCCC AGCCCTCCCA CCCTCTCCTC   
  
  
+ CTCCTCCAAG AACTCCTACC AAAACGCTGC CGTTTTGGCT CACCCTCCTC AGCTTCACCA TGGATCTGAT   
  
  
+ AATGTGTAAG ACCCCAACCC TTTTGCTTTT ATTTTCCTCA TGCTCCATTC TCATGATTTG TACTTCTGTT   
  
  
+ ATGTAATTGG AGATGTGTCT AAATTTGGAA AAGTTCGTGG GTTTCGTGTT TGAGAATTTA GAATGACATT   
  
  
+ CTTTAATTAG TAATTTGAAG TTTGCATGCT TAATTTAGCT AATCACTGAT TAATTAAAAT AAATGAAGTG   
  
  
+ ATGTAGGTAT GGGCTGTTAC ACAATGTCAC CGGTGGTGGT GACAGCGGCG GGGCGGCGGC GGTTGGATTG   
  
  
+ GAAGAATGGG ACAGCGTGTT TCCAAATGGG GATGGAGCTT TACTCCCTTG GATCATGGGG GAAGGTGATG   
  
  
+ ATTTGGGTCT GAATTTGAAG CATCTCTTAC AATCTGGTTA CCCTGTTGAG TATGAAGGCA ATGCAGGACT   
  
  
+ TGGTGTTGTT GATCAGACAT CTCTTCTTGG AGGTGGTGAT TTGGGGTTTT CTGGTTCTGG GTCTGTGAAT   
  
  
+ AATGGCAAAA TTGGTTCCAT TTTGGGTAAT TGTTCATCTG GGGATTTGGA TTCTAAGGTT TCTAGTGACG   
  
  
+ GGTTGAATTC CAATTGTAGT TCACTGGGGA GTATGAGTAT CCTTGGTTCA ATCCATGGAC CTTTGCCTAA   
  
  
+ TACTGGTGGG TTTGTGTTCC CACAAACACA ACTATTTGAT CTTGGTGATG AGAAGCCTCA GATTTTGAAC   
  
  
+ CCACAATTGA TGACGATGAA TCCAAATCAG GCTCAGAGCA TGGGAAACCC TAGCTTTTTT GTGCCCTCCT   
  
  
+ TGGGTTATTG TCAGTTAGAG CAACATATGG TTCAACCACA GGCGAAACGC CATAGCCCTG GTGTAGTTTT   
  
  
+ GAACTCTGAT GTTATTGCCA AAACCCCGTT TCTCGATCAG GGTCATGAGT TTTTGTTGAG GAAACAACAC   
  
  
+ CATCAACAGA TTTTGCAGCA ACTTCCTATG GGTTTGGCTC ATCAGTCGGT CCCTCAGCAC CTCCAGCAAA   
  
  
+ AGGCGATGAA AGACCAGGTT GTTAAGGACC AGCTTTTGAA GGCAGCTGAC CTGATTCAAA CTGGGAATTT   
  
  
+ CTCACTTGCG CAAGAGATAT TGGCGCGGCT CAATCACCAG CTCTCCCTTC CTGCGAAGCC CCTCATTAGG   
  
  
+ GCAGCTTTGT ATGTCATGGA GGCCTTGCAA ATGCTCATCT TAATGAGCAA TCCTGTAGCA CCTCCACCGA   
  
  
+ TGAAGACGCT TTCCCCTATA GATGTTGTTC ATAAGATGAA TGCCTACAAG GCCTTCTCTG AGGTCTCTCC   
  
  
+ TATTACTCAG TTTACGAATT TCACTTGTGC CCAAGCCATT CTTGAAGCTC TCGATGATGC GGATTGCGTT   
  
  
+ CATGTCATTG ACTTTGATAT TGGTTGTGGG GCTCAATGGG CATCACTGAT TCAGGAGCTT CCATTGAGGA   
  
  
+ AAAAGGGAGC TCCATCCCTG AAAATTACAG CCATAGCTCC CCTGTCAATA AGCCACACTT TTGAACTCAA   
  
  
+ CCTAGCACGG GAAAACCTTG TGCAATTCGC CAATGATGTT GGTGTTGCTT TTGAGCTGCA AGTTGTGAAC   
  
  
+ TTGGATTTAT TTGATCCATC TTCATCTTCA ATGCCAAATG TTGGAACCTC TGGGGATGAG TTGATCGCTG   
  
  
+ TTAGTATACC CATTTGGGCA TGTTCATATC GGCCGTCTAT TCTCCCATCC ATCCTCCGGT TCATTAAGCA   
  
  
+ ACTAGCCCCC AAGATTGTCG TCTCGTTAGA TAGAGGGTCT GATCGTTGCG ACACTCTGTT CCCACAACAT   
  
  
+ CTTATCCATA TCTTAGAGTC CTGCACAAAT TTCTTGGAAT CGCTCGACGG TCTCAATGTA GCATCAGATA   
  
  
+ TTGTAAACAA GGTTGAGAAG TACTTCCTCC AGCCTAAGAT CGAAAACACC GTGTTGGGCC GCGTTCATGC   
  
  
+ CCCCAATAAG ATGCCACATT GGAAGACCCT CTTTGCTTCA GCCGGGTTAT TGCCCTTTCA GTTCAGTAAC   
  
  
+ TTCACAGAAA CACAAGCTGA TTATGTGGTG AAGCGAACCC CAGGGAGGGG ATTTCACATA GAAAAGCGCC   
  
  
+ ACGCATCTTT AGTGCTAAGT TGGCAAAGGC GGGAGCTTGT GACAGCCTCG GCATGGAGGT TTGAGAAGCT   
  
  
+ GTA  

- +Up\_Stream \_Len000TGTAAC CCACCCTAAA TTTAGGAGAT AAAAAAGAAT CCCCTTTTTA TGAACCCTTT   
  
  
- AAAGAGTTAT TGACTTTTAG ATTTATTAAG CACTGAAACA ATCATCCAAA TTACACTAAA TGATGGCACT   
  
  
- CACAGTGAAA ATCTACCATG AAATTCTTTC GTAATATTCA TTTTGTTATA TATAGCATCG TAGTATGATG   
  
  
- AACACATTAT TTAATTGTCG TTGGAGTTAA CATGAATAAA CTAAAGCGTA ACATTAGTTA AAAGGGTTTA   
  
  
- CTAAATGATA GAACCAGAAT GAGTCAACAT GAAGTGTGAG TTATCAAGTT AATAAAAAGT AATCTGATTA   
  
  
- CTCGCTCAGG GAAGATGTTC TCGGATGATG ATGAGATTAT TACATTCCTG ATAACCGATT ACCCCTTGAT   
  
  
- TCGGATGCTG AACAATCCGT ATCCACTTAA AAGGAATGTT CTCTCAAATT AAATGTGAAC TATAATTGAG   
  
  
- CTCTTGCTCT TCGACAGAAT TATCTATGGG TGTATCTTTT ACTTCGTTTA CGGGAACTAA TAACCTCCCA   
  
  
- ACCCGTTTAT ATAAGTCATT GTTACAACAA ACCGTTTATA GCAGACTTTT AACAAAAACT GTTTATAACA   
  
  
- AACCTTCTTA AAAAGCTCTA ATTTATAATT TTTACAATTT TAAAATTAGA TTTATAGTTT GTTTATAACT   
  
  
- TTTTACGAAA TATAATAGAT TTAGAGATTA ATTTTAAACA CATAACAGGT GATCTTTTTA CATATCACTA   
  
  
- TAATGGCTGT ATATACACTA GAGACCACAT TATCGGATGG GATTACAGAT CCTTTTATAA ATTTTAGGGG   
  
  
- TCACCAGTTT TCCTTCAATG CGTGTGATCC CGTTCTGTGA AGTAATCCGA TCAACCACTT TCGATTCAGT   
  
  
- ACTTTAAATC TGGTACCGGT ACAGATGAAC ATCTAGGTTG ATGGTTGTTA GGTAAAAATA AAACCCCTTT   
  
  
- CGAACTTCCC ATTAAACCAT TTACATGTGC CCTTGGAAAA CGATATTTTC TTCACGAAGA ACTTTTTTGT   
  
  
- GGGACATCAT TTTGTCCCGT TTTTTCGGTT GTGAAGACAA AGACCGTCTC TTTGTCATCA CATGGTGAAC   
  
  
- CTCAACCTCA TCTGTGCTAT GTGTTTGGGT CATCTCTCTC TTTGTCCCCT CTCTTTCTCA TGTGTTTAAA   
  
  
- CACGTCCTCT TCCCATTCCT TCTTCCTCTT CCTTGGGGGA GGTTTTTTTT GGGTATATTT TTTATTATTT   
  
  
- CTTTTTTGTC CTCACTCAAT CTCCCATGGG TTTAGAGGTT AGATTTTGAT CTTAGATCCC AGAGAAAAGT   
  
  
- AAACGAACTT TTTAAAGACA AGTACCCAAA ACACAAAAAT CCCCCTAAAC AAACTAAAAA CATCAGAAAC   
  
  
- ACCTAAAGGA CACAATATCT ACGTTCGATA CGAGAAGTTA AACGTTCCTT GTCGTAGACC CCCCTCTCTT   
  
  
- AAACTCCTTA AAGTTCATGG GTAAAGTTAA AGTTGTTCAT TTTAGTAGTA ACTTCCTATG AGTTTGGGTT   
  
  
- CGGGTCAACT ATCACTTGGT TGAAGATGAG ACCTAAGATG AGGTTCAGGG TCGGGAGGGT GGGAGAGGAG   
  
  
- GAGGAGGTTC TTGAGGATGG TTTTGCGACG GCAAAACCGA GTGGGAGGAG TCGAAGTGGT ACCTAGACTA   
  
  
- TTACACATTC TGGGGTTGGG AAAACGAAAA TAAAAGGAGT ACGAGGTAAG AGTACTAAAC ATGAAGACAA   
  
  
- TACATTAACC TCTACACAGA TTTAAACCTT TTCAAGCACC CAAAGCACAA ACTCTTAAAT CTTACTGTAA   
  
  
- GAAATTAATC ATTAAACTTC AAACGTACGA ATTAAATCGA TTAGTGACTA ATTAATTTTA TTTACTTCAC   
  
  
- TACATCCATA CCCGACAATG TGTTACAGTG GCCACCACCA CTGTCGCCGC CCCGCCGCCG CCAACCTAAC   
  
  
- CTTCTTACCC TGTCGCACAA AGGTTTACCC CTACCTCGAA ATGAGGGAAC CTAGTACCCC CTTCCACTAC   
  
  
- TAAACCCAGA CTTAAACTTC GTAGAGAATG TTAGACCAAT GGGACAACTC ATACTTCCGT TACGTCCTGA   
  
  
- ACCACAACAA CTAGTCTGTA GAGAAGAACC TCCACCACTA AACCCCAAAA GACCAAGACC CAGACACTTA   
  
  
- TTACCGTTTT AACCAAGGTA AAACCCATTA ACAAGTAGAC CCCTAAACCT AAGATTCCAA AGATCACTGC   
  
  
- CCAACTTAAG GTTAACATCA AGTGACCCCT CATACTCATA GGAACCAAGT TAGGTACCTG GAAACGGATT   
  
  
- ATGACCACCC AAACACAAGG GTGTTTGTGT TGATAAACTA GAACCACTAC TCTTCGGAGT CTAAAACTTG   
  
  
- GGTGTTAACT ACTGCTACTT AGGTTTAGTC CGAGTCTCGT ACCCTTTGGG ATCGAAAAAA CACGGGAGGA   
  
  
- ACCCAATAAC AGTCAATCTC GTTGTATACC AAGTTGGTGT CCGCTTTGCG GTATCGGGAC CACATCAAAA   
  
  
- CTTGAGACTA CAATAACGGT TTTGGGGCAA AGAGCTAGTC CCAGTACTCA AAAACAACTC CTTTGTTGTG   
  
  
- GTAGTTGTCT AAAACGTCGT TGAAGGATAC CCAAACCGAG TAGTCAGCCA GGGAGTCGTG GAGGTCGTTT   
  
  
- TCCGCTACTT TCTGGTCCAA CAATTCCTGG TCGAAAACTT CCGTCGACTG GACTAAGTTT GACCCTTAAA   
  
  
- GAGTGAACGC GTTCTCTATA ACCGCGCCGA GTTAGTGGTC GAGAGGGAAG GACGCTTCGG GGAGTAATCC   
  
  
- CGTCGAAACA TACAGTACCT CCGGAACGTT TACGAGTAGA ATTACTCGTT AGGACATCGT GGAGGTGGCT   
  
  
- ACTTCTGCGA AAGGGGATAT CTACAACAAG TATTCTACTT ACGGATGTTC CGGAAGAGAC TCCAGAGAGG   
  
  
- ATAATGAGTC AAATGCTTAA AGTGAACACG GGTTCGGTAA GAACTTCGAG AGCTACTACG CCTAACGCAA   
  
  
- GTACAGTAAC TGAAACTATA ACCAACACCC CGAGTTACCC GTAGTGACTA AGTCCTCGAA GGTAACTCCT   
  
  
- TTTTCCCTCG AGGTAGGGAC TTTTAATGTC GGTATCGAGG GGACAGTTAT TCGGTGTGAA AACTTGAGTT   
  
  
- GGATCGTGCC CTTTTGGAAC ACGTTAAGCG GTTACTACAA CCACAACGAA AACTCGACGT TCAACACTTG   
  
  
- AACCTAAATA AACTAGGTAG AAGTAGAAGT TACGGTTTAC AACCTTGGAG ACCCCTACTC AACTAGCGAC   
  
  
- AATCATATGG GTAAACCCGT ACAAGTATAG CCGGCAGATA AGAGGGTAGG TAGGAGGCCA AGTAATTCGT   
  
  
- TGATCGGGGG TTCTAACAGC AGAGCAATCT ATCTCCCAGA CTAGCAACGC TGTGAGACAA GGGTGTTGTA   
  
  
- GAATAGGTAT AGAATCTCAG GACGTGTTTA AAGAACCTTA GCGAGCTGCC AGAGTTACAT CGTAGTCTAT   
  
  
- AACATTTGTT CCAACTCTTC ATGAAGGAGG TCGGATTCTA GCTTTTGTGG CACAACCCGG CGCAAGTACG   
  
  
- GGGGTTATTC TACGGTGTAA CCTTCTGGGA GAAACGAAGT CGGCCCAATA ACGGGAAAGT CAAGTCATTG   
  
  
- AAGTGTCTTT GTGTTCGACT AATACACCAC TTCGCTTGGG GTCCCTCCCC TAAAGTGTAT CTTTTCGCGG   
  
  
- TGCGTAGAAA TCACGATTCA ACCGTTTCCG CCCTCGAACA CTGTCGGAGC CGTACCTCCA AACTCTTCGA   
  
  
- CAT

+     ERE

| Site Name | Organism | Position | Strand | Matrix score. | sequence | function |
| --- | --- | --- | --- | --- | --- | --- |
| ERE | Nicotiana glutinos | 833 | - | 8 | ATTTTAAA |  |

>HU05G00466.1   
+ +Up\_Stream \_Len000ACATTG GGTGGGATTT AAATCCTCTA TTTTTTCTTA GGGGAAAAAT ACTTGGGAAA   
  
  
+ TTTCTCAATA ACTGAAAATC TAAATAATTC GTGACTTTGT TAGTAGGTTT AATGTGATTT ACTACCGTGA   
  
  
+ GTGTCACTTT TAGATGGTAC TTTAAGAAAG CATTATAAGT AAAACAATAT ATATCGTAGC ATCATACTAC   
  
  
+ TTGTGTAATA AATTAACAGC AACCTCAATT GTACTTATTT GATTTCGCAT TGTAATCAAT TTTCCCAAAT   
  
  
+ GATTTACTAT CTTGGTCTTA CTCAGTTGTA CTTCACACTC AATAGTTCAA TTATTTTTCA TTAGACTAAT   
  
  
+ GAGCGAGTCC CTTCTACAAG AGCCTACTAC TACTCTAATA ATGTAAGGAC TATTGGCTAA TGGGGAACTA   
  
  
+ AGCCTACGAC TTGTTAGGCA TAGGTGAATT TTCCTTACAA GAGAGTTTAA TTTACACTTG ATATTAACTC   
  
  
+ GAGAACGAGA AGCTGTCTTA ATAGATACCC ACATAGAAAA TGAAGCAAAT GCCCTTGATT ATTGGAGGGT   
  
  
+ TGGGCAAATA TATTCAGTAA CAATGTTGTT TGGCAAATAT CGTCTGAAAA TTGTTTTTGA CAAATATTGT   
  
  
+ TTGGAAGAAT TTTTCGAGAT TAAATATTAA AAATGTTAAA ATTTTAATCT AAATATCAAA CAAATATTGA   
  
  
+ AAAATGCTTT ATATTATCTA AATCTCTAAT TAAAATTTGT GTATTGTCCA CTAGAAAAAT GTATAGTGAT   
  
  
+ ATTACCGACA TATATGTGAT CTCTGGTGTA ATAGCCTACC CTAATGTCTA GGAAAATATT TAAAATCCCC   
  
  
+ AGTGGTCAAA AGGAAGTTAC GCACACTAGG GCAAGACACT TCATTAGGCT AGTTGGTGAA AGCTAAGTCA   
  
  
+ TGAAATTTAG ACCATGGCCA TGTCTACTTG TAGATCCAAC TACCAACAAT CCATTTTTAT TTTGGGGAAA   
  
  
+ GCTTGAAGGG TAATTTGGTA AATGTACACG GGAACCTTTT GCTATAAAAG AAGTGCTTCT TGAAAAAACA   
  
  
+ CCCTGTAGTA AAACAGGGCA AAAAAGCCAA CACTTCTGTT TCTGGCAGAG AAACAGTAGT GTACCACTTG   
  
  
+ GAGTTGGAGT AGACACGATA CACAAACCCA GTAGAGAGAG AAACAGGGGA GAGAAAGAGT ACACAAATTT   
  
  
+ GTGCAGGAGA AGGGTAAGGA AGAAGGAGAA GGAACCCCCT CCAAAAAAAA CCCATATAAA AAATAATAAA   
  
  
+ GAAAAAACAG GAGTGAGTTA GAGGGTACCC AAATCTCCAA TCTAAAACTA GAATCTAGGG TCTCTTTTCA   
  
  
+ TTTGCTTGAA AAATTTCTGT TCATGGGTTT TGTGTTTTTA GGGGGATTTG TTTGATTTTT GTAGTCTTTG   
  
  
+ TGGATTTCCT GTGTTATAGA TGCAAGCTAT GCTCTTCAAT TTGCAAGGAA CAGCATCTGG GGGGAGAGAA   
  
  
+ TTTGAGGAAT TTCAAGTACC CATTTCAATT TCAACAAGTA AAATCATCAT TGAAGGATAC TCAAACCCAA   
  
  
+ GCCCAGTTGA TAGTGAACCA ACTTCTACTC TGGATTCTAC TCCAAGTCCC AGCCCTCCCA CCCTCTCCTC   
  
  
+ CTCCTCCAAG AACTCCTACC AAAACGCTGC CGTTTTGGCT CACCCTCCTC AGCTTCACCA TGGATCTGAT   
  
  
+ AATGTGTAAG ACCCCAACCC TTTTGCTTTT ATTTTCCTCA TGCTCCATTC TCATGATTTG TACTTCTGTT   
  
  
+ ATGTAATTGG AGATGTGTCT AAATTTGGAA AAGTTCGTGG GTTTCGTGTT TGAGAATTTA GAATGACATT   
  
  
+ CTTTAATTAG TAATTTGAAG TTTGCATGCT TAATTTAGCT AATCACTGAT TAATTAAAAT AAATGAAGTG   
  
  
+ ATGTAGGTAT GGGCTGTTAC ACAATGTCAC CGGTGGTGGT GACAGCGGCG GGGCGGCGGC GGTTGGATTG   
  
  
+ GAAGAATGGG ACAGCGTGTT TCCAAATGGG GATGGAGCTT TACTCCCTTG GATCATGGGG GAAGGTGATG   
  
  
+ ATTTGGGTCT GAATTTGAAG CATCTCTTAC AATCTGGTTA CCCTGTTGAG TATGAAGGCA ATGCAGGACT   
  
  
+ TGGTGTTGTT GATCAGACAT CTCTTCTTGG AGGTGGTGAT TTGGGGTTTT CTGGTTCTGG GTCTGTGAAT   
  
  
+ AATGGCAAAA TTGGTTCCAT TTTGGGTAAT TGTTCATCTG GGGATTTGGA TTCTAAGGTT TCTAGTGACG   
  
  
+ GGTTGAATTC CAATTGTAGT TCACTGGGGA GTATGAGTAT CCTTGGTTCA ATCCATGGAC CTTTGCCTAA   
  
  
+ TACTGGTGGG TTTGTGTTCC CACAAACACA ACTATTTGAT CTTGGTGATG AGAAGCCTCA GATTTTGAAC   
  
  
+ CCACAATTGA TGACGATGAA TCCAAATCAG GCTCAGAGCA TGGGAAACCC TAGCTTTTTT GTGCCCTCCT   
  
  
+ TGGGTTATTG TCAGTTAGAG CAACATATGG TTCAACCACA GGCGAAACGC CATAGCCCTG GTGTAGTTTT   
  
  
+ GAACTCTGAT GTTATTGCCA AAACCCCGTT TCTCGATCAG GGTCATGAGT TTTTGTTGAG GAAACAACAC   
  
  
+ CATCAACAGA TTTTGCAGCA ACTTCCTATG GGTTTGGCTC ATCAGTCGGT CCCTCAGCAC CTCCAGCAAA   
  
  
+ AGGCGATGAA AGACCAGGTT GTTAAGGACC AGCTTTTGAA GGCAGCTGAC CTGATTCAAA CTGGGAATTT   
  
  
+ CTCACTTGCG CAAGAGATAT TGGCGCGGCT CAATCACCAG CTCTCCCTTC CTGCGAAGCC CCTCATTAGG   
  
  
+ GCAGCTTTGT ATGTCATGGA GGCCTTGCAA ATGCTCATCT TAATGAGCAA TCCTGTAGCA CCTCCACCGA   
  
  
+ TGAAGACGCT TTCCCCTATA GATGTTGTTC ATAAGATGAA TGCCTACAAG GCCTTCTCTG AGGTCTCTCC   
  
  
+ TATTACTCAG TTTACGAATT TCACTTGTGC CCAAGCCATT CTTGAAGCTC TCGATGATGC GGATTGCGTT   
  
  
+ CATGTCATTG ACTTTGATAT TGGTTGTGGG GCTCAATGGG CATCACTGAT TCAGGAGCTT CCATTGAGGA   
  
  
+ AAAAGGGAGC TCCATCCCTG AAAATTACAG CCATAGCTCC CCTGTCAATA AGCCACACTT TTGAACTCAA   
  
  
+ CCTAGCACGG GAAAACCTTG TGCAATTCGC CAATGATGTT GGTGTTGCTT TTGAGCTGCA AGTTGTGAAC   
  
  
+ TTGGATTTAT TTGATCCATC TTCATCTTCA ATGCCAAATG TTGGAACCTC TGGGGATGAG TTGATCGCTG   
  
  
+ TTAGTATACC CATTTGGGCA TGTTCATATC GGCCGTCTAT TCTCCCATCC ATCCTCCGGT TCATTAAGCA   
  
  
+ ACTAGCCCCC AAGATTGTCG TCTCGTTAGA TAGAGGGTCT GATCGTTGCG ACACTCTGTT CCCACAACAT   
  
  
+ CTTATCCATA TCTTAGAGTC CTGCACAAAT TTCTTGGAAT CGCTCGACGG TCTCAATGTA GCATCAGATA   
  
  
+ TTGTAAACAA GGTTGAGAAG TACTTCCTCC AGCCTAAGAT CGAAAACACC GTGTTGGGCC GCGTTCATGC   
  
  
+ CCCCAATAAG ATGCCACATT GGAAGACCCT CTTTGCTTCA GCCGGGTTAT TGCCCTTTCA GTTCAGTAAC   
  
  
+ TTCACAGAAA CACAAGCTGA TTATGTGGTG AAGCGAACCC CAGGGAGGGG ATTTCACATA GAAAAGCGCC   
  
  
+ ACGCATCTTT AGTGCTAAGT TGGCAAAGGC GGGAGCTTGT GACAGCCTCG GCATGGAGGT TTGAGAAGCT   
  
  
+ GTA  

- +Up\_Stream \_Len000TGTAAC CCACCCTAAA TTTAGGAGAT AAAAAAGAAT CCCCTTTTTA TGAACCCTTT   
  
  
- AAAGAGTTAT TGACTTTTAG ATTTATTAAG CACTGAAACA ATCATCCAAA TTACACTAAA TGATGGCACT   
  
  
- CACAGTGAAA ATCTACCATG AAATTCTTTC GTAATATTCA TTTTGTTATA TATAGCATCG TAGTATGATG   
  
  
- AACACATTAT TTAATTGTCG TTGGAGTTAA CATGAATAAA CTAAAGCGTA ACATTAGTTA AAAGGGTTTA   
  
  
- CTAAATGATA GAACCAGAAT GAGTCAACAT GAAGTGTGAG TTATCAAGTT AATAAAAAGT AATCTGATTA   
  
  
- CTCGCTCAGG GAAGATGTTC TCGGATGATG ATGAGATTAT TACATTCCTG ATAACCGATT ACCCCTTGAT   
  
  
- TCGGATGCTG AACAATCCGT ATCCACTTAA AAGGAATGTT CTCTCAAATT AAATGTGAAC TATAATTGAG   
  
  
- CTCTTGCTCT TCGACAGAAT TATCTATGGG TGTATCTTTT ACTTCGTTTA CGGGAACTAA TAACCTCCCA   
  
  
- ACCCGTTTAT ATAAGTCATT GTTACAACAA ACCGTTTATA GCAGACTTTT AACAAAAACT GTTTATAACA   
  
  
- AACCTTCTTA AAAAGCTCTA ATTTATAATT TTTACAATTT TAAAATTAGA TTTATAGTTT GTTTATAACT   
  
  
- TTTTACGAAA TATAATAGAT TTAGAGATTA ATTTTAAACA CATAACAGGT GATCTTTTTA CATATCACTA   
  
  
- TAATGGCTGT ATATACACTA GAGACCACAT TATCGGATGG GATTACAGAT CCTTTTATAA ATTTTAGGGG   
  
  
- TCACCAGTTT TCCTTCAATG CGTGTGATCC CGTTCTGTGA AGTAATCCGA TCAACCACTT TCGATTCAGT   
  
  
- ACTTTAAATC TGGTACCGGT ACAGATGAAC ATCTAGGTTG ATGGTTGTTA GGTAAAAATA AAACCCCTTT   
  
  
- CGAACTTCCC ATTAAACCAT TTACATGTGC CCTTGGAAAA CGATATTTTC TTCACGAAGA ACTTTTTTGT   
  
  
- GGGACATCAT TTTGTCCCGT TTTTTCGGTT GTGAAGACAA AGACCGTCTC TTTGTCATCA CATGGTGAAC   
  
  
- CTCAACCTCA TCTGTGCTAT GTGTTTGGGT CATCTCTCTC TTTGTCCCCT CTCTTTCTCA TGTGTTTAAA   
  
  
- CACGTCCTCT TCCCATTCCT TCTTCCTCTT CCTTGGGGGA GGTTTTTTTT GGGTATATTT TTTATTATTT   
  
  
- CTTTTTTGTC CTCACTCAAT CTCCCATGGG TTTAGAGGTT AGATTTTGAT CTTAGATCCC AGAGAAAAGT   
  
  
- AAACGAACTT TTTAAAGACA AGTACCCAAA ACACAAAAAT CCCCCTAAAC AAACTAAAAA CATCAGAAAC   
  
  
- ACCTAAAGGA CACAATATCT ACGTTCGATA CGAGAAGTTA AACGTTCCTT GTCGTAGACC CCCCTCTCTT   
  
  
- AAACTCCTTA AAGTTCATGG GTAAAGTTAA AGTTGTTCAT TTTAGTAGTA ACTTCCTATG AGTTTGGGTT   
  
  
- CGGGTCAACT ATCACTTGGT TGAAGATGAG ACCTAAGATG AGGTTCAGGG TCGGGAGGGT GGGAGAGGAG   
  
  
- GAGGAGGTTC TTGAGGATGG TTTTGCGACG GCAAAACCGA GTGGGAGGAG TCGAAGTGGT ACCTAGACTA   
  
  
- TTACACATTC TGGGGTTGGG AAAACGAAAA TAAAAGGAGT ACGAGGTAAG AGTACTAAAC ATGAAGACAA   
  
  
- TACATTAACC TCTACACAGA TTTAAACCTT TTCAAGCACC CAAAGCACAA ACTCTTAAAT CTTACTGTAA   
  
  
- GAAATTAATC ATTAAACTTC AAACGTACGA ATTAAATCGA TTAGTGACTA ATTAATTTTA TTTACTTCAC   
  
  
- TACATCCATA CCCGACAATG TGTTACAGTG GCCACCACCA CTGTCGCCGC CCCGCCGCCG CCAACCTAAC   
  
  
- CTTCTTACCC TGTCGCACAA AGGTTTACCC CTACCTCGAA ATGAGGGAAC CTAGTACCCC CTTCCACTAC   
  
  
- TAAACCCAGA CTTAAACTTC GTAGAGAATG TTAGACCAAT GGGACAACTC ATACTTCCGT TACGTCCTGA   
  
  
- ACCACAACAA CTAGTCTGTA GAGAAGAACC TCCACCACTA AACCCCAAAA GACCAAGACC CAGACACTTA   
  
  
- TTACCGTTTT AACCAAGGTA AAACCCATTA ACAAGTAGAC CCCTAAACCT AAGATTCCAA AGATCACTGC   
  
  
- CCAACTTAAG GTTAACATCA AGTGACCCCT CATACTCATA GGAACCAAGT TAGGTACCTG GAAACGGATT   
  
  
- ATGACCACCC AAACACAAGG GTGTTTGTGT TGATAAACTA GAACCACTAC TCTTCGGAGT CTAAAACTTG   
  
  
- GGTGTTAACT ACTGCTACTT AGGTTTAGTC CGAGTCTCGT ACCCTTTGGG ATCGAAAAAA CACGGGAGGA   
  
  
- ACCCAATAAC AGTCAATCTC GTTGTATACC AAGTTGGTGT CCGCTTTGCG GTATCGGGAC CACATCAAAA   
  
  
- CTTGAGACTA CAATAACGGT TTTGGGGCAA AGAGCTAGTC CCAGTACTCA AAAACAACTC CTTTGTTGTG   
  
  
- GTAGTTGTCT AAAACGTCGT TGAAGGATAC CCAAACCGAG TAGTCAGCCA GGGAGTCGTG GAGGTCGTTT   
  
  
- TCCGCTACTT TCTGGTCCAA CAATTCCTGG TCGAAAACTT CCGTCGACTG GACTAAGTTT GACCCTTAAA   
  
  
- GAGTGAACGC GTTCTCTATA ACCGCGCCGA GTTAGTGGTC GAGAGGGAAG GACGCTTCGG GGAGTAATCC   
  
  
- CGTCGAAACA TACAGTACCT CCGGAACGTT TACGAGTAGA ATTACTCGTT AGGACATCGT GGAGGTGGCT   
  
  
- ACTTCTGCGA AAGGGGATAT CTACAACAAG TATTCTACTT ACGGATGTTC CGGAAGAGAC TCCAGAGAGG   
  
  
- ATAATGAGTC AAATGCTTAA AGTGAACACG GGTTCGGTAA GAACTTCGAG AGCTACTACG CCTAACGCAA   
  
  
- GTACAGTAAC TGAAACTATA ACCAACACCC CGAGTTACCC GTAGTGACTA AGTCCTCGAA GGTAACTCCT   
  
  
- TTTTCCCTCG AGGTAGGGAC TTTTAATGTC GGTATCGAGG GGACAGTTAT TCGGTGTGAA AACTTGAGTT   
  
  
- GGATCGTGCC CTTTTGGAAC ACGTTAAGCG GTTACTACAA CCACAACGAA AACTCGACGT TCAACACTTG   
  
  
- AACCTAAATA AACTAGGTAG AAGTAGAAGT TACGGTTTAC AACCTTGGAG ACCCCTACTC AACTAGCGAC   
  
  
- AATCATATGG GTAAACCCGT ACAAGTATAG CCGGCAGATA AGAGGGTAGG TAGGAGGCCA AGTAATTCGT   
  
  
- TGATCGGGGG TTCTAACAGC AGAGCAATCT ATCTCCCAGA CTAGCAACGC TGTGAGACAA GGGTGTTGTA   
  
  
- GAATAGGTAT AGAATCTCAG GACGTGTTTA AAGAACCTTA GCGAGCTGCC AGAGTTACAT CGTAGTCTAT   
  
  
- AACATTTGTT CCAACTCTTC ATGAAGGAGG TCGGATTCTA GCTTTTGTGG CACAACCCGG CGCAAGTACG   
  
  
- GGGGTTATTC TACGGTGTAA CCTTCTGGGA GAAACGAAGT CGGCCCAATA ACGGGAAAGT CAAGTCATTG   
  
  
- AAGTGTCTTT GTGTTCGACT AATACACCAC TTCGCTTGGG GTCCCTCCCC TAAAGTGTAT CTTTTCGCGG   
  
  
- TGCGTAGAAA TCACGATTCA ACCGTTTCCG CCCTCGAACA CTGTCGGAGC CGTACCTCCA AACTCTTCGA   
  
  
- CAT

+     GARE-motif

| Site Name | Organism | Position | Strand | Matrix score. | sequence | function |
| --- | --- | --- | --- | --- | --- | --- |
| GARE-motif | Brassica oleracea | 2598 | - | 7 | TCTGTTG | gibberellin-responsive element |

>HU05G00466.1   
+ +Up\_Stream \_Len000ACATTG GGTGGGATTT AAATCCTCTA TTTTTTCTTA GGGGAAAAAT ACTTGGGAAA   
  
  
+ TTTCTCAATA ACTGAAAATC TAAATAATTC GTGACTTTGT TAGTAGGTTT AATGTGATTT ACTACCGTGA   
  
  
+ GTGTCACTTT TAGATGGTAC TTTAAGAAAG CATTATAAGT AAAACAATAT ATATCGTAGC ATCATACTAC   
  
  
+ TTGTGTAATA AATTAACAGC AACCTCAATT GTACTTATTT GATTTCGCAT TGTAATCAAT TTTCCCAAAT   
  
  
+ GATTTACTAT CTTGGTCTTA CTCAGTTGTA CTTCACACTC AATAGTTCAA TTATTTTTCA TTAGACTAAT   
  
  
+ GAGCGAGTCC CTTCTACAAG AGCCTACTAC TACTCTAATA ATGTAAGGAC TATTGGCTAA TGGGGAACTA   
  
  
+ AGCCTACGAC TTGTTAGGCA TAGGTGAATT TTCCTTACAA GAGAGTTTAA TTTACACTTG ATATTAACTC   
  
  
+ GAGAACGAGA AGCTGTCTTA ATAGATACCC ACATAGAAAA TGAAGCAAAT GCCCTTGATT ATTGGAGGGT   
  
  
+ TGGGCAAATA TATTCAGTAA CAATGTTGTT TGGCAAATAT CGTCTGAAAA TTGTTTTTGA CAAATATTGT   
  
  
+ TTGGAAGAAT TTTTCGAGAT TAAATATTAA AAATGTTAAA ATTTTAATCT AAATATCAAA CAAATATTGA   
  
  
+ AAAATGCTTT ATATTATCTA AATCTCTAAT TAAAATTTGT GTATTGTCCA CTAGAAAAAT GTATAGTGAT   
  
  
+ ATTACCGACA TATATGTGAT CTCTGGTGTA ATAGCCTACC CTAATGTCTA GGAAAATATT TAAAATCCCC   
  
  
+ AGTGGTCAAA AGGAAGTTAC GCACACTAGG GCAAGACACT TCATTAGGCT AGTTGGTGAA AGCTAAGTCA   
  
  
+ TGAAATTTAG ACCATGGCCA TGTCTACTTG TAGATCCAAC TACCAACAAT CCATTTTTAT TTTGGGGAAA   
  
  
+ GCTTGAAGGG TAATTTGGTA AATGTACACG GGAACCTTTT GCTATAAAAG AAGTGCTTCT TGAAAAAACA   
  
  
+ CCCTGTAGTA AAACAGGGCA AAAAAGCCAA CACTTCTGTT TCTGGCAGAG AAACAGTAGT GTACCACTTG   
  
  
+ GAGTTGGAGT AGACACGATA CACAAACCCA GTAGAGAGAG AAACAGGGGA GAGAAAGAGT ACACAAATTT   
  
  
+ GTGCAGGAGA AGGGTAAGGA AGAAGGAGAA GGAACCCCCT CCAAAAAAAA CCCATATAAA AAATAATAAA   
  
  
+ GAAAAAACAG GAGTGAGTTA GAGGGTACCC AAATCTCCAA TCTAAAACTA GAATCTAGGG TCTCTTTTCA   
  
  
+ TTTGCTTGAA AAATTTCTGT TCATGGGTTT TGTGTTTTTA GGGGGATTTG TTTGATTTTT GTAGTCTTTG   
  
  
+ TGGATTTCCT GTGTTATAGA TGCAAGCTAT GCTCTTCAAT TTGCAAGGAA CAGCATCTGG GGGGAGAGAA   
  
  
+ TTTGAGGAAT TTCAAGTACC CATTTCAATT TCAACAAGTA AAATCATCAT TGAAGGATAC TCAAACCCAA   
  
  
+ GCCCAGTTGA TAGTGAACCA ACTTCTACTC TGGATTCTAC TCCAAGTCCC AGCCCTCCCA CCCTCTCCTC   
  
  
+ CTCCTCCAAG AACTCCTACC AAAACGCTGC CGTTTTGGCT CACCCTCCTC AGCTTCACCA TGGATCTGAT   
  
  
+ AATGTGTAAG ACCCCAACCC TTTTGCTTTT ATTTTCCTCA TGCTCCATTC TCATGATTTG TACTTCTGTT   
  
  
+ ATGTAATTGG AGATGTGTCT AAATTTGGAA AAGTTCGTGG GTTTCGTGTT TGAGAATTTA GAATGACATT   
  
  
+ CTTTAATTAG TAATTTGAAG TTTGCATGCT TAATTTAGCT AATCACTGAT TAATTAAAAT AAATGAAGTG   
  
  
+ ATGTAGGTAT GGGCTGTTAC ACAATGTCAC CGGTGGTGGT GACAGCGGCG GGGCGGCGGC GGTTGGATTG   
  
  
+ GAAGAATGGG ACAGCGTGTT TCCAAATGGG GATGGAGCTT TACTCCCTTG GATCATGGGG GAAGGTGATG   
  
  
+ ATTTGGGTCT GAATTTGAAG CATCTCTTAC AATCTGGTTA CCCTGTTGAG TATGAAGGCA ATGCAGGACT   
  
  
+ TGGTGTTGTT GATCAGACAT CTCTTCTTGG AGGTGGTGAT TTGGGGTTTT CTGGTTCTGG GTCTGTGAAT   
  
  
+ AATGGCAAAA TTGGTTCCAT TTTGGGTAAT TGTTCATCTG GGGATTTGGA TTCTAAGGTT TCTAGTGACG   
  
  
+ GGTTGAATTC CAATTGTAGT TCACTGGGGA GTATGAGTAT CCTTGGTTCA ATCCATGGAC CTTTGCCTAA   
  
  
+ TACTGGTGGG TTTGTGTTCC CACAAACACA ACTATTTGAT CTTGGTGATG AGAAGCCTCA GATTTTGAAC   
  
  
+ CCACAATTGA TGACGATGAA TCCAAATCAG GCTCAGAGCA TGGGAAACCC TAGCTTTTTT GTGCCCTCCT   
  
  
+ TGGGTTATTG TCAGTTAGAG CAACATATGG TTCAACCACA GGCGAAACGC CATAGCCCTG GTGTAGTTTT   
  
  
+ GAACTCTGAT GTTATTGCCA AAACCCCGTT TCTCGATCAG GGTCATGAGT TTTTGTTGAG GAAACAACAC   
  
  
+ CATCAACAGA TTTTGCAGCA ACTTCCTATG GGTTTGGCTC ATCAGTCGGT CCCTCAGCAC CTCCAGCAAA   
  
  
+ AGGCGATGAA AGACCAGGTT GTTAAGGACC AGCTTTTGAA GGCAGCTGAC CTGATTCAAA CTGGGAATTT   
  
  
+ CTCACTTGCG CAAGAGATAT TGGCGCGGCT CAATCACCAG CTCTCCCTTC CTGCGAAGCC CCTCATTAGG   
  
  
+ GCAGCTTTGT ATGTCATGGA GGCCTTGCAA ATGCTCATCT TAATGAGCAA TCCTGTAGCA CCTCCACCGA   
  
  
+ TGAAGACGCT TTCCCCTATA GATGTTGTTC ATAAGATGAA TGCCTACAAG GCCTTCTCTG AGGTCTCTCC   
  
  
+ TATTACTCAG TTTACGAATT TCACTTGTGC CCAAGCCATT CTTGAAGCTC TCGATGATGC GGATTGCGTT   
  
  
+ CATGTCATTG ACTTTGATAT TGGTTGTGGG GCTCAATGGG CATCACTGAT TCAGGAGCTT CCATTGAGGA   
  
  
+ AAAAGGGAGC TCCATCCCTG AAAATTACAG CCATAGCTCC CCTGTCAATA AGCCACACTT TTGAACTCAA   
  
  
+ CCTAGCACGG GAAAACCTTG TGCAATTCGC CAATGATGTT GGTGTTGCTT TTGAGCTGCA AGTTGTGAAC   
  
  
+ TTGGATTTAT TTGATCCATC TTCATCTTCA ATGCCAAATG TTGGAACCTC TGGGGATGAG TTGATCGCTG   
  
  
+ TTAGTATACC CATTTGGGCA TGTTCATATC GGCCGTCTAT TCTCCCATCC ATCCTCCGGT TCATTAAGCA   
  
  
+ ACTAGCCCCC AAGATTGTCG TCTCGTTAGA TAGAGGGTCT GATCGTTGCG ACACTCTGTT CCCACAACAT   
  
  
+ CTTATCCATA TCTTAGAGTC CTGCACAAAT TTCTTGGAAT CGCTCGACGG TCTCAATGTA GCATCAGATA   
  
  
+ TTGTAAACAA GGTTGAGAAG TACTTCCTCC AGCCTAAGAT CGAAAACACC GTGTTGGGCC GCGTTCATGC   
  
  
+ CCCCAATAAG ATGCCACATT GGAAGACCCT CTTTGCTTCA GCCGGGTTAT TGCCCTTTCA GTTCAGTAAC   
  
  
+ TTCACAGAAA CACAAGCTGA TTATGTGGTG AAGCGAACCC CAGGGAGGGG ATTTCACATA GAAAAGCGCC   
  
  
+ ACGCATCTTT AGTGCTAAGT TGGCAAAGGC GGGAGCTTGT GACAGCCTCG GCATGGAGGT TTGAGAAGCT   
  
  
+ GTA  

- +Up\_Stream \_Len000TGTAAC CCACCCTAAA TTTAGGAGAT AAAAAAGAAT CCCCTTTTTA TGAACCCTTT   
  
  
- AAAGAGTTAT TGACTTTTAG ATTTATTAAG CACTGAAACA ATCATCCAAA TTACACTAAA TGATGGCACT   
  
  
- CACAGTGAAA ATCTACCATG AAATTCTTTC GTAATATTCA TTTTGTTATA TATAGCATCG TAGTATGATG   
  
  
- AACACATTAT TTAATTGTCG TTGGAGTTAA CATGAATAAA CTAAAGCGTA ACATTAGTTA AAAGGGTTTA   
  
  
- CTAAATGATA GAACCAGAAT GAGTCAACAT GAAGTGTGAG TTATCAAGTT AATAAAAAGT AATCTGATTA   
  
  
- CTCGCTCAGG GAAGATGTTC TCGGATGATG ATGAGATTAT TACATTCCTG ATAACCGATT ACCCCTTGAT   
  
  
- TCGGATGCTG AACAATCCGT ATCCACTTAA AAGGAATGTT CTCTCAAATT AAATGTGAAC TATAATTGAG   
  
  
- CTCTTGCTCT TCGACAGAAT TATCTATGGG TGTATCTTTT ACTTCGTTTA CGGGAACTAA TAACCTCCCA   
  
  
- ACCCGTTTAT ATAAGTCATT GTTACAACAA ACCGTTTATA GCAGACTTTT AACAAAAACT GTTTATAACA   
  
  
- AACCTTCTTA AAAAGCTCTA ATTTATAATT TTTACAATTT TAAAATTAGA TTTATAGTTT GTTTATAACT   
  
  
- TTTTACGAAA TATAATAGAT TTAGAGATTA ATTTTAAACA CATAACAGGT GATCTTTTTA CATATCACTA   
  
  
- TAATGGCTGT ATATACACTA GAGACCACAT TATCGGATGG GATTACAGAT CCTTTTATAA ATTTTAGGGG   
  
  
- TCACCAGTTT TCCTTCAATG CGTGTGATCC CGTTCTGTGA AGTAATCCGA TCAACCACTT TCGATTCAGT   
  
  
- ACTTTAAATC TGGTACCGGT ACAGATGAAC ATCTAGGTTG ATGGTTGTTA GGTAAAAATA AAACCCCTTT   
  
  
- CGAACTTCCC ATTAAACCAT TTACATGTGC CCTTGGAAAA CGATATTTTC TTCACGAAGA ACTTTTTTGT   
  
  
- GGGACATCAT TTTGTCCCGT TTTTTCGGTT GTGAAGACAA AGACCGTCTC TTTGTCATCA CATGGTGAAC   
  
  
- CTCAACCTCA TCTGTGCTAT GTGTTTGGGT CATCTCTCTC TTTGTCCCCT CTCTTTCTCA TGTGTTTAAA   
  
  
- CACGTCCTCT TCCCATTCCT TCTTCCTCTT CCTTGGGGGA GGTTTTTTTT GGGTATATTT TTTATTATTT   
  
  
- CTTTTTTGTC CTCACTCAAT CTCCCATGGG TTTAGAGGTT AGATTTTGAT CTTAGATCCC AGAGAAAAGT   
  
  
- AAACGAACTT TTTAAAGACA AGTACCCAAA ACACAAAAAT CCCCCTAAAC AAACTAAAAA CATCAGAAAC   
  
  
- ACCTAAAGGA CACAATATCT ACGTTCGATA CGAGAAGTTA AACGTTCCTT GTCGTAGACC CCCCTCTCTT   
  
  
- AAACTCCTTA AAGTTCATGG GTAAAGTTAA AGTTGTTCAT TTTAGTAGTA ACTTCCTATG AGTTTGGGTT   
  
  
- CGGGTCAACT ATCACTTGGT TGAAGATGAG ACCTAAGATG AGGTTCAGGG TCGGGAGGGT GGGAGAGGAG   
  
  
- GAGGAGGTTC TTGAGGATGG TTTTGCGACG GCAAAACCGA GTGGGAGGAG TCGAAGTGGT ACCTAGACTA   
  
  
- TTACACATTC TGGGGTTGGG AAAACGAAAA TAAAAGGAGT ACGAGGTAAG AGTACTAAAC ATGAAGACAA   
  
  
- TACATTAACC TCTACACAGA TTTAAACCTT TTCAAGCACC CAAAGCACAA ACTCTTAAAT CTTACTGTAA   
  
  
- GAAATTAATC ATTAAACTTC AAACGTACGA ATTAAATCGA TTAGTGACTA ATTAATTTTA TTTACTTCAC   
  
  
- TACATCCATA CCCGACAATG TGTTACAGTG GCCACCACCA CTGTCGCCGC CCCGCCGCCG CCAACCTAAC   
  
  
- CTTCTTACCC TGTCGCACAA AGGTTTACCC CTACCTCGAA ATGAGGGAAC CTAGTACCCC CTTCCACTAC   
  
  
- TAAACCCAGA CTTAAACTTC GTAGAGAATG TTAGACCAAT GGGACAACTC ATACTTCCGT TACGTCCTGA   
  
  
- ACCACAACAA CTAGTCTGTA GAGAAGAACC TCCACCACTA AACCCCAAAA GACCAAGACC CAGACACTTA   
  
  
- TTACCGTTTT AACCAAGGTA AAACCCATTA ACAAGTAGAC CCCTAAACCT AAGATTCCAA AGATCACTGC   
  
  
- CCAACTTAAG GTTAACATCA AGTGACCCCT CATACTCATA GGAACCAAGT TAGGTACCTG GAAACGGATT   
  
  
- ATGACCACCC AAACACAAGG GTGTTTGTGT TGATAAACTA GAACCACTAC TCTTCGGAGT CTAAAACTTG   
  
  
- GGTGTTAACT ACTGCTACTT AGGTTTAGTC CGAGTCTCGT ACCCTTTGGG ATCGAAAAAA CACGGGAGGA   
  
  
- ACCCAATAAC AGTCAATCTC GTTGTATACC AAGTTGGTGT CCGCTTTGCG GTATCGGGAC CACATCAAAA   
  
  
- CTTGAGACTA CAATAACGGT TTTGGGGCAA AGAGCTAGTC CCAGTACTCA AAAACAACTC CTTTGTTGTG   
  
  
- GTAGTTGTCT AAAACGTCGT TGAAGGATAC CCAAACCGAG TAGTCAGCCA GGGAGTCGTG GAGGTCGTTT   
  
  
- TCCGCTACTT TCTGGTCCAA CAATTCCTGG TCGAAAACTT CCGTCGACTG GACTAAGTTT GACCCTTAAA   
  
  
- GAGTGAACGC GTTCTCTATA ACCGCGCCGA GTTAGTGGTC GAGAGGGAAG GACGCTTCGG GGAGTAATCC   
  
  
- CGTCGAAACA TACAGTACCT CCGGAACGTT TACGAGTAGA ATTACTCGTT AGGACATCGT GGAGGTGGCT   
  
  
- ACTTCTGCGA AAGGGGATAT CTACAACAAG TATTCTACTT ACGGATGTTC CGGAAGAGAC TCCAGAGAGG   
  
  
- ATAATGAGTC AAATGCTTAA AGTGAACACG GGTTCGGTAA GAACTTCGAG AGCTACTACG CCTAACGCAA   
  
  
- GTACAGTAAC TGAAACTATA ACCAACACCC CGAGTTACCC GTAGTGACTA AGTCCTCGAA GGTAACTCCT   
  
  
- TTTTCCCTCG AGGTAGGGAC TTTTAATGTC GGTATCGAGG GGACAGTTAT TCGGTGTGAA AACTTGAGTT   
  
  
- GGATCGTGCC CTTTTGGAAC ACGTTAAGCG GTTACTACAA CCACAACGAA AACTCGACGT TCAACACTTG   
  
  
- AACCTAAATA AACTAGGTAG AAGTAGAAGT TACGGTTTAC AACCTTGGAG ACCCCTACTC AACTAGCGAC   
  
  
- AATCATATGG GTAAACCCGT ACAAGTATAG CCGGCAGATA AGAGGGTAGG TAGGAGGCCA AGTAATTCGT   
  
  
- TGATCGGGGG TTCTAACAGC AGAGCAATCT ATCTCCCAGA CTAGCAACGC TGTGAGACAA GGGTGTTGTA   
  
  
- GAATAGGTAT AGAATCTCAG GACGTGTTTA AAGAACCTTA GCGAGCTGCC AGAGTTACAT CGTAGTCTAT   
  
  
- AACATTTGTT CCAACTCTTC ATGAAGGAGG TCGGATTCTA GCTTTTGTGG CACAACCCGG CGCAAGTACG   
  
  
- GGGGTTATTC TACGGTGTAA CCTTCTGGGA GAAACGAAGT CGGCCCAATA ACGGGAAAGT CAAGTCATTG   
  
  
- AAGTGTCTTT GTGTTCGACT AATACACCAC TTCGCTTGGG GTCCCTCCCC TAAAGTGTAT CTTTTCGCGG   
  
  
- TGCGTAGAAA TCACGATTCA ACCGTTTCCG CCCTCGAACA CTGTCGGAGC CGTACCTCCA AACTCTTCGA   
  
  
- CAT

+     GATA-motif

| Site Name | Organism | Position | Strand | Matrix score. | sequence | function |
| --- | --- | --- | --- | --- | --- | --- |
| GATA-motif | Solanum tuberosum | 1217 | + | 9 | AAGGATAAGG | part of a light responsive element |
| GATA-motif | Solanum tuberosum | 1204 | + | 9 | AAGGATAAGG | part of a light responsive element |

>HU05G00466.1   
+ +Up\_Stream \_Len000ACATTG GGTGGGATTT AAATCCTCTA TTTTTTCTTA GGGGAAAAAT ACTTGGGAAA   
  
  
+ TTTCTCAATA ACTGAAAATC TAAATAATTC GTGACTTTGT TAGTAGGTTT AATGTGATTT ACTACCGTGA   
  
  
+ GTGTCACTTT TAGATGGTAC TTTAAGAAAG CATTATAAGT AAAACAATAT ATATCGTAGC ATCATACTAC   
  
  
+ TTGTGTAATA AATTAACAGC AACCTCAATT GTACTTATTT GATTTCGCAT TGTAATCAAT TTTCCCAAAT   
  
  
+ GATTTACTAT CTTGGTCTTA CTCAGTTGTA CTTCACACTC AATAGTTCAA TTATTTTTCA TTAGACTAAT   
  
  
+ GAGCGAGTCC CTTCTACAAG AGCCTACTAC TACTCTAATA ATGTAAGGAC TATTGGCTAA TGGGGAACTA   
  
  
+ AGCCTACGAC TTGTTAGGCA TAGGTGAATT TTCCTTACAA GAGAGTTTAA TTTACACTTG ATATTAACTC   
  
  
+ GAGAACGAGA AGCTGTCTTA ATAGATACCC ACATAGAAAA TGAAGCAAAT GCCCTTGATT ATTGGAGGGT   
  
  
+ TGGGCAAATA TATTCAGTAA CAATGTTGTT TGGCAAATAT CGTCTGAAAA TTGTTTTTGA CAAATATTGT   
  
  
+ TTGGAAGAAT TTTTCGAGAT TAAATATTAA AAATGTTAAA ATTTTAATCT AAATATCAAA CAAATATTGA   
  
  
+ AAAATGCTTT ATATTATCTA AATCTCTAAT TAAAATTTGT GTATTGTCCA CTAGAAAAAT GTATAGTGAT   
  
  
+ ATTACCGACA TATATGTGAT CTCTGGTGTA ATAGCCTACC CTAATGTCTA GGAAAATATT TAAAATCCCC   
  
  
+ AGTGGTCAAA AGGAAGTTAC GCACACTAGG GCAAGACACT TCATTAGGCT AGTTGGTGAA AGCTAAGTCA   
  
  
+ TGAAATTTAG ACCATGGCCA TGTCTACTTG TAGATCCAAC TACCAACAAT CCATTTTTAT TTTGGGGAAA   
  
  
+ GCTTGAAGGG TAATTTGGTA AATGTACACG GGAACCTTTT GCTATAAAAG AAGTGCTTCT TGAAAAAACA   
  
  
+ CCCTGTAGTA AAACAGGGCA AAAAAGCCAA CACTTCTGTT TCTGGCAGAG AAACAGTAGT GTACCACTTG   
  
  
+ GAGTTGGAGT AGACACGATA CACAAACCCA GTAGAGAGAG AAACAGGGGA GAGAAAGAGT ACACAAATTT   
  
  
+ GTGCAGGAGA AGGGTAAGGA AGAAGGAGAA GGAACCCCCT CCAAAAAAAA CCCATATAAA AAATAATAAA   
  
  
+ GAAAAAACAG GAGTGAGTTA GAGGGTACCC AAATCTCCAA TCTAAAACTA GAATCTAGGG TCTCTTTTCA   
  
  
+ TTTGCTTGAA AAATTTCTGT TCATGGGTTT TGTGTTTTTA GGGGGATTTG TTTGATTTTT GTAGTCTTTG   
  
  
+ TGGATTTCCT GTGTTATAGA TGCAAGCTAT GCTCTTCAAT TTGCAAGGAA CAGCATCTGG GGGGAGAGAA   
  
  
+ TTTGAGGAAT TTCAAGTACC CATTTCAATT TCAACAAGTA AAATCATCAT TGAAGGATAC TCAAACCCAA   
  
  
+ GCCCAGTTGA TAGTGAACCA ACTTCTACTC TGGATTCTAC TCCAAGTCCC AGCCCTCCCA CCCTCTCCTC   
  
  
+ CTCCTCCAAG AACTCCTACC AAAACGCTGC CGTTTTGGCT CACCCTCCTC AGCTTCACCA TGGATCTGAT   
  
  
+ AATGTGTAAG ACCCCAACCC TTTTGCTTTT ATTTTCCTCA TGCTCCATTC TCATGATTTG TACTTCTGTT   
  
  
+ ATGTAATTGG AGATGTGTCT AAATTTGGAA AAGTTCGTGG GTTTCGTGTT TGAGAATTTA GAATGACATT   
  
  
+ CTTTAATTAG TAATTTGAAG TTTGCATGCT TAATTTAGCT AATCACTGAT TAATTAAAAT AAATGAAGTG   
  
  
+ ATGTAGGTAT GGGCTGTTAC ACAATGTCAC CGGTGGTGGT GACAGCGGCG GGGCGGCGGC GGTTGGATTG   
  
  
+ GAAGAATGGG ACAGCGTGTT TCCAAATGGG GATGGAGCTT TACTCCCTTG GATCATGGGG GAAGGTGATG   
  
  
+ ATTTGGGTCT GAATTTGAAG CATCTCTTAC AATCTGGTTA CCCTGTTGAG TATGAAGGCA ATGCAGGACT   
  
  
+ TGGTGTTGTT GATCAGACAT CTCTTCTTGG AGGTGGTGAT TTGGGGTTTT CTGGTTCTGG GTCTGTGAAT   
  
  
+ AATGGCAAAA TTGGTTCCAT TTTGGGTAAT TGTTCATCTG GGGATTTGGA TTCTAAGGTT TCTAGTGACG   
  
  
+ GGTTGAATTC CAATTGTAGT TCACTGGGGA GTATGAGTAT CCTTGGTTCA ATCCATGGAC CTTTGCCTAA   
  
  
+ TACTGGTGGG TTTGTGTTCC CACAAACACA ACTATTTGAT CTTGGTGATG AGAAGCCTCA GATTTTGAAC   
  
  
+ CCACAATTGA TGACGATGAA TCCAAATCAG GCTCAGAGCA TGGGAAACCC TAGCTTTTTT GTGCCCTCCT   
  
  
+ TGGGTTATTG TCAGTTAGAG CAACATATGG TTCAACCACA GGCGAAACGC CATAGCCCTG GTGTAGTTTT   
  
  
+ GAACTCTGAT GTTATTGCCA AAACCCCGTT TCTCGATCAG GGTCATGAGT TTTTGTTGAG GAAACAACAC   
  
  
+ CATCAACAGA TTTTGCAGCA ACTTCCTATG GGTTTGGCTC ATCAGTCGGT CCCTCAGCAC CTCCAGCAAA   
  
  
+ AGGCGATGAA AGACCAGGTT GTTAAGGACC AGCTTTTGAA GGCAGCTGAC CTGATTCAAA CTGGGAATTT   
  
  
+ CTCACTTGCG CAAGAGATAT TGGCGCGGCT CAATCACCAG CTCTCCCTTC CTGCGAAGCC CCTCATTAGG   
  
  
+ GCAGCTTTGT ATGTCATGGA GGCCTTGCAA ATGCTCATCT TAATGAGCAA TCCTGTAGCA CCTCCACCGA   
  
  
+ TGAAGACGCT TTCCCCTATA GATGTTGTTC ATAAGATGAA TGCCTACAAG GCCTTCTCTG AGGTCTCTCC   
  
  
+ TATTACTCAG TTTACGAATT TCACTTGTGC CCAAGCCATT CTTGAAGCTC TCGATGATGC GGATTGCGTT   
  
  
+ CATGTCATTG ACTTTGATAT TGGTTGTGGG GCTCAATGGG CATCACTGAT TCAGGAGCTT CCATTGAGGA   
  
  
+ AAAAGGGAGC TCCATCCCTG AAAATTACAG CCATAGCTCC CCTGTCAATA AGCCACACTT TTGAACTCAA   
  
  
+ CCTAGCACGG GAAAACCTTG TGCAATTCGC CAATGATGTT GGTGTTGCTT TTGAGCTGCA AGTTGTGAAC   
  
  
+ TTGGATTTAT TTGATCCATC TTCATCTTCA ATGCCAAATG TTGGAACCTC TGGGGATGAG TTGATCGCTG   
  
  
+ TTAGTATACC CATTTGGGCA TGTTCATATC GGCCGTCTAT TCTCCCATCC ATCCTCCGGT TCATTAAGCA   
  
  
+ ACTAGCCCCC AAGATTGTCG TCTCGTTAGA TAGAGGGTCT GATCGTTGCG ACACTCTGTT CCCACAACAT   
  
  
+ CTTATCCATA TCTTAGAGTC CTGCACAAAT TTCTTGGAAT CGCTCGACGG TCTCAATGTA GCATCAGATA   
  
  
+ TTGTAAACAA GGTTGAGAAG TACTTCCTCC AGCCTAAGAT CGAAAACACC GTGTTGGGCC GCGTTCATGC   
  
  
+ CCCCAATAAG ATGCCACATT GGAAGACCCT CTTTGCTTCA GCCGGGTTAT TGCCCTTTCA GTTCAGTAAC   
  
  
+ TTCACAGAAA CACAAGCTGA TTATGTGGTG AAGCGAACCC CAGGGAGGGG ATTTCACATA GAAAAGCGCC   
  
  
+ ACGCATCTTT AGTGCTAAGT TGGCAAAGGC GGGAGCTTGT GACAGCCTCG GCATGGAGGT TTGAGAAGCT   
  
  
+ GTA  

- +Up\_Stream \_Len000TGTAAC CCACCCTAAA TTTAGGAGAT AAAAAAGAAT CCCCTTTTTA TGAACCCTTT   
  
  
- AAAGAGTTAT TGACTTTTAG ATTTATTAAG CACTGAAACA ATCATCCAAA TTACACTAAA TGATGGCACT   
  
  
- CACAGTGAAA ATCTACCATG AAATTCTTTC GTAATATTCA TTTTGTTATA TATAGCATCG TAGTATGATG   
  
  
- AACACATTAT TTAATTGTCG TTGGAGTTAA CATGAATAAA CTAAAGCGTA ACATTAGTTA AAAGGGTTTA   
  
  
- CTAAATGATA GAACCAGAAT GAGTCAACAT GAAGTGTGAG TTATCAAGTT AATAAAAAGT AATCTGATTA   
  
  
- CTCGCTCAGG GAAGATGTTC TCGGATGATG ATGAGATTAT TACATTCCTG ATAACCGATT ACCCCTTGAT   
  
  
- TCGGATGCTG AACAATCCGT ATCCACTTAA AAGGAATGTT CTCTCAAATT AAATGTGAAC TATAATTGAG   
  
  
- CTCTTGCTCT TCGACAGAAT TATCTATGGG TGTATCTTTT ACTTCGTTTA CGGGAACTAA TAACCTCCCA   
  
  
- ACCCGTTTAT ATAAGTCATT GTTACAACAA ACCGTTTATA GCAGACTTTT AACAAAAACT GTTTATAACA   
  
  
- AACCTTCTTA AAAAGCTCTA ATTTATAATT TTTACAATTT TAAAATTAGA TTTATAGTTT GTTTATAACT   
  
  
- TTTTACGAAA TATAATAGAT TTAGAGATTA ATTTTAAACA CATAACAGGT GATCTTTTTA CATATCACTA   
  
  
- TAATGGCTGT ATATACACTA GAGACCACAT TATCGGATGG GATTACAGAT CCTTTTATAA ATTTTAGGGG   
  
  
- TCACCAGTTT TCCTTCAATG CGTGTGATCC CGTTCTGTGA AGTAATCCGA TCAACCACTT TCGATTCAGT   
  
  
- ACTTTAAATC TGGTACCGGT ACAGATGAAC ATCTAGGTTG ATGGTTGTTA GGTAAAAATA AAACCCCTTT   
  
  
- CGAACTTCCC ATTAAACCAT TTACATGTGC CCTTGGAAAA CGATATTTTC TTCACGAAGA ACTTTTTTGT   
  
  
- GGGACATCAT TTTGTCCCGT TTTTTCGGTT GTGAAGACAA AGACCGTCTC TTTGTCATCA CATGGTGAAC   
  
  
- CTCAACCTCA TCTGTGCTAT GTGTTTGGGT CATCTCTCTC TTTGTCCCCT CTCTTTCTCA TGTGTTTAAA   
  
  
- CACGTCCTCT TCCCATTCCT TCTTCCTCTT CCTTGGGGGA GGTTTTTTTT GGGTATATTT TTTATTATTT   
  
  
- CTTTTTTGTC CTCACTCAAT CTCCCATGGG TTTAGAGGTT AGATTTTGAT CTTAGATCCC AGAGAAAAGT   
  
  
- AAACGAACTT TTTAAAGACA AGTACCCAAA ACACAAAAAT CCCCCTAAAC AAACTAAAAA CATCAGAAAC   
  
  
- ACCTAAAGGA CACAATATCT ACGTTCGATA CGAGAAGTTA AACGTTCCTT GTCGTAGACC CCCCTCTCTT   
  
  
- AAACTCCTTA AAGTTCATGG GTAAAGTTAA AGTTGTTCAT TTTAGTAGTA ACTTCCTATG AGTTTGGGTT   
  
  
- CGGGTCAACT ATCACTTGGT TGAAGATGAG ACCTAAGATG AGGTTCAGGG TCGGGAGGGT GGGAGAGGAG   
  
  
- GAGGAGGTTC TTGAGGATGG TTTTGCGACG GCAAAACCGA GTGGGAGGAG TCGAAGTGGT ACCTAGACTA   
  
  
- TTACACATTC TGGGGTTGGG AAAACGAAAA TAAAAGGAGT ACGAGGTAAG AGTACTAAAC ATGAAGACAA   
  
  
- TACATTAACC TCTACACAGA TTTAAACCTT TTCAAGCACC CAAAGCACAA ACTCTTAAAT CTTACTGTAA   
  
  
- GAAATTAATC ATTAAACTTC AAACGTACGA ATTAAATCGA TTAGTGACTA ATTAATTTTA TTTACTTCAC   
  
  
- TACATCCATA CCCGACAATG TGTTACAGTG GCCACCACCA CTGTCGCCGC CCCGCCGCCG CCAACCTAAC   
  
  
- CTTCTTACCC TGTCGCACAA AGGTTTACCC CTACCTCGAA ATGAGGGAAC CTAGTACCCC CTTCCACTAC   
  
  
- TAAACCCAGA CTTAAACTTC GTAGAGAATG TTAGACCAAT GGGACAACTC ATACTTCCGT TACGTCCTGA   
  
  
- ACCACAACAA CTAGTCTGTA GAGAAGAACC TCCACCACTA AACCCCAAAA GACCAAGACC CAGACACTTA   
  
  
- TTACCGTTTT AACCAAGGTA AAACCCATTA ACAAGTAGAC CCCTAAACCT AAGATTCCAA AGATCACTGC   
  
  
- CCAACTTAAG GTTAACATCA AGTGACCCCT CATACTCATA GGAACCAAGT TAGGTACCTG GAAACGGATT   
  
  
- ATGACCACCC AAACACAAGG GTGTTTGTGT TGATAAACTA GAACCACTAC TCTTCGGAGT CTAAAACTTG   
  
  
- GGTGTTAACT ACTGCTACTT AGGTTTAGTC CGAGTCTCGT ACCCTTTGGG ATCGAAAAAA CACGGGAGGA   
  
  
- ACCCAATAAC AGTCAATCTC GTTGTATACC AAGTTGGTGT CCGCTTTGCG GTATCGGGAC CACATCAAAA   
  
  
- CTTGAGACTA CAATAACGGT TTTGGGGCAA AGAGCTAGTC CCAGTACTCA AAAACAACTC CTTTGTTGTG   
  
  
- GTAGTTGTCT AAAACGTCGT TGAAGGATAC CCAAACCGAG TAGTCAGCCA GGGAGTCGTG GAGGTCGTTT   
  
  
- TCCGCTACTT TCTGGTCCAA CAATTCCTGG TCGAAAACTT CCGTCGACTG GACTAAGTTT GACCCTTAAA   
  
  
- GAGTGAACGC GTTCTCTATA ACCGCGCCGA GTTAGTGGTC GAGAGGGAAG GACGCTTCGG GGAGTAATCC   
  
  
- CGTCGAAACA TACAGTACCT CCGGAACGTT TACGAGTAGA ATTACTCGTT AGGACATCGT GGAGGTGGCT   
  
  
- ACTTCTGCGA AAGGGGATAT CTACAACAAG TATTCTACTT ACGGATGTTC CGGAAGAGAC TCCAGAGAGG   
  
  
- ATAATGAGTC AAATGCTTAA AGTGAACACG GGTTCGGTAA GAACTTCGAG AGCTACTACG CCTAACGCAA   
  
  
- GTACAGTAAC TGAAACTATA ACCAACACCC CGAGTTACCC GTAGTGACTA AGTCCTCGAA GGTAACTCCT   
  
  
- TTTTCCCTCG AGGTAGGGAC TTTTAATGTC GGTATCGAGG GGACAGTTAT TCGGTGTGAA AACTTGAGTT   
  
  
- GGATCGTGCC CTTTTGGAAC ACGTTAAGCG GTTACTACAA CCACAACGAA AACTCGACGT TCAACACTTG   
  
  
- AACCTAAATA AACTAGGTAG AAGTAGAAGT TACGGTTTAC AACCTTGGAG ACCCCTACTC AACTAGCGAC   
  
  
- AATCATATGG GTAAACCCGT ACAAGTATAG CCGGCAGATA AGAGGGTAGG TAGGAGGCCA AGTAATTCGT   
  
  
- TGATCGGGGG TTCTAACAGC AGAGCAATCT ATCTCCCAGA CTAGCAACGC TGTGAGACAA GGGTGTTGTA   
  
  
- GAATAGGTAT AGAATCTCAG GACGTGTTTA AAGAACCTTA GCGAGCTGCC AGAGTTACAT CGTAGTCTAT   
  
  
- AACATTTGTT CCAACTCTTC ATGAAGGAGG TCGGATTCTA GCTTTTGTGG CACAACCCGG CGCAAGTACG   
  
  
- GGGGTTATTC TACGGTGTAA CCTTCTGGGA GAAACGAAGT CGGCCCAATA ACGGGAAAGT CAAGTCATTG   
  
  
- AAGTGTCTTT GTGTTCGACT AATACACCAC TTCGCTTGGG GTCCCTCCCC TAAAGTGTAT CTTTTCGCGG   
  
  
- TGCGTAGAAA TCACGATTCA ACCGTTTCCG CCCTCGAACA CTGTCGGAGC CGTACCTCCA AACTCTTCGA   
  
  
- CAT

+     Gap-box

| Site Name | Organism | Position | Strand | Matrix score. | sequence | function |
| --- | --- | --- | --- | --- | --- | --- |
| Gap-box | Arabidopsis thaliana | 1329 | - | 9.5 | CAAATGAA(A/G)A | part of a light responsive element |

>HU05G00466.1   
+ +Up\_Stream \_Len000ACATTG GGTGGGATTT AAATCCTCTA TTTTTTCTTA GGGGAAAAAT ACTTGGGAAA   
  
  
+ TTTCTCAATA ACTGAAAATC TAAATAATTC GTGACTTTGT TAGTAGGTTT AATGTGATTT ACTACCGTGA   
  
  
+ GTGTCACTTT TAGATGGTAC TTTAAGAAAG CATTATAAGT AAAACAATAT ATATCGTAGC ATCATACTAC   
  
  
+ TTGTGTAATA AATTAACAGC AACCTCAATT GTACTTATTT GATTTCGCAT TGTAATCAAT TTTCCCAAAT   
  
  
+ GATTTACTAT CTTGGTCTTA CTCAGTTGTA CTTCACACTC AATAGTTCAA TTATTTTTCA TTAGACTAAT   
  
  
+ GAGCGAGTCC CTTCTACAAG AGCCTACTAC TACTCTAATA ATGTAAGGAC TATTGGCTAA TGGGGAACTA   
  
  
+ AGCCTACGAC TTGTTAGGCA TAGGTGAATT TTCCTTACAA GAGAGTTTAA TTTACACTTG ATATTAACTC   
  
  
+ GAGAACGAGA AGCTGTCTTA ATAGATACCC ACATAGAAAA TGAAGCAAAT GCCCTTGATT ATTGGAGGGT   
  
  
+ TGGGCAAATA TATTCAGTAA CAATGTTGTT TGGCAAATAT CGTCTGAAAA TTGTTTTTGA CAAATATTGT   
  
  
+ TTGGAAGAAT TTTTCGAGAT TAAATATTAA AAATGTTAAA ATTTTAATCT AAATATCAAA CAAATATTGA   
  
  
+ AAAATGCTTT ATATTATCTA AATCTCTAAT TAAAATTTGT GTATTGTCCA CTAGAAAAAT GTATAGTGAT   
  
  
+ ATTACCGACA TATATGTGAT CTCTGGTGTA ATAGCCTACC CTAATGTCTA GGAAAATATT TAAAATCCCC   
  
  
+ AGTGGTCAAA AGGAAGTTAC GCACACTAGG GCAAGACACT TCATTAGGCT AGTTGGTGAA AGCTAAGTCA   
  
  
+ TGAAATTTAG ACCATGGCCA TGTCTACTTG TAGATCCAAC TACCAACAAT CCATTTTTAT TTTGGGGAAA   
  
  
+ GCTTGAAGGG TAATTTGGTA AATGTACACG GGAACCTTTT GCTATAAAAG AAGTGCTTCT TGAAAAAACA   
  
  
+ CCCTGTAGTA AAACAGGGCA AAAAAGCCAA CACTTCTGTT TCTGGCAGAG AAACAGTAGT GTACCACTTG   
  
  
+ GAGTTGGAGT AGACACGATA CACAAACCCA GTAGAGAGAG AAACAGGGGA GAGAAAGAGT ACACAAATTT   
  
  
+ GTGCAGGAGA AGGGTAAGGA AGAAGGAGAA GGAACCCCCT CCAAAAAAAA CCCATATAAA AAATAATAAA   
  
  
+ GAAAAAACAG GAGTGAGTTA GAGGGTACCC AAATCTCCAA TCTAAAACTA GAATCTAGGG TCTCTTTTCA   
  
  
+ TTTGCTTGAA AAATTTCTGT TCATGGGTTT TGTGTTTTTA GGGGGATTTG TTTGATTTTT GTAGTCTTTG   
  
  
+ TGGATTTCCT GTGTTATAGA TGCAAGCTAT GCTCTTCAAT TTGCAAGGAA CAGCATCTGG GGGGAGAGAA   
  
  
+ TTTGAGGAAT TTCAAGTACC CATTTCAATT TCAACAAGTA AAATCATCAT TGAAGGATAC TCAAACCCAA   
  
  
+ GCCCAGTTGA TAGTGAACCA ACTTCTACTC TGGATTCTAC TCCAAGTCCC AGCCCTCCCA CCCTCTCCTC   
  
  
+ CTCCTCCAAG AACTCCTACC AAAACGCTGC CGTTTTGGCT CACCCTCCTC AGCTTCACCA TGGATCTGAT   
  
  
+ AATGTGTAAG ACCCCAACCC TTTTGCTTTT ATTTTCCTCA TGCTCCATTC TCATGATTTG TACTTCTGTT   
  
  
+ ATGTAATTGG AGATGTGTCT AAATTTGGAA AAGTTCGTGG GTTTCGTGTT TGAGAATTTA GAATGACATT   
  
  
+ CTTTAATTAG TAATTTGAAG TTTGCATGCT TAATTTAGCT AATCACTGAT TAATTAAAAT AAATGAAGTG   
  
  
+ ATGTAGGTAT GGGCTGTTAC ACAATGTCAC CGGTGGTGGT GACAGCGGCG GGGCGGCGGC GGTTGGATTG   
  
  
+ GAAGAATGGG ACAGCGTGTT TCCAAATGGG GATGGAGCTT TACTCCCTTG GATCATGGGG GAAGGTGATG   
  
  
+ ATTTGGGTCT GAATTTGAAG CATCTCTTAC AATCTGGTTA CCCTGTTGAG TATGAAGGCA ATGCAGGACT   
  
  
+ TGGTGTTGTT GATCAGACAT CTCTTCTTGG AGGTGGTGAT TTGGGGTTTT CTGGTTCTGG GTCTGTGAAT   
  
  
+ AATGGCAAAA TTGGTTCCAT TTTGGGTAAT TGTTCATCTG GGGATTTGGA TTCTAAGGTT TCTAGTGACG   
  
  
+ GGTTGAATTC CAATTGTAGT TCACTGGGGA GTATGAGTAT CCTTGGTTCA ATCCATGGAC CTTTGCCTAA   
  
  
+ TACTGGTGGG TTTGTGTTCC CACAAACACA ACTATTTGAT CTTGGTGATG AGAAGCCTCA GATTTTGAAC   
  
  
+ CCACAATTGA TGACGATGAA TCCAAATCAG GCTCAGAGCA TGGGAAACCC TAGCTTTTTT GTGCCCTCCT   
  
  
+ TGGGTTATTG TCAGTTAGAG CAACATATGG TTCAACCACA GGCGAAACGC CATAGCCCTG GTGTAGTTTT   
  
  
+ GAACTCTGAT GTTATTGCCA AAACCCCGTT TCTCGATCAG GGTCATGAGT TTTTGTTGAG GAAACAACAC   
  
  
+ CATCAACAGA TTTTGCAGCA ACTTCCTATG GGTTTGGCTC ATCAGTCGGT CCCTCAGCAC CTCCAGCAAA   
  
  
+ AGGCGATGAA AGACCAGGTT GTTAAGGACC AGCTTTTGAA GGCAGCTGAC CTGATTCAAA CTGGGAATTT   
  
  
+ CTCACTTGCG CAAGAGATAT TGGCGCGGCT CAATCACCAG CTCTCCCTTC CTGCGAAGCC CCTCATTAGG   
  
  
+ GCAGCTTTGT ATGTCATGGA GGCCTTGCAA ATGCTCATCT TAATGAGCAA TCCTGTAGCA CCTCCACCGA   
  
  
+ TGAAGACGCT TTCCCCTATA GATGTTGTTC ATAAGATGAA TGCCTACAAG GCCTTCTCTG AGGTCTCTCC   
  
  
+ TATTACTCAG TTTACGAATT TCACTTGTGC CCAAGCCATT CTTGAAGCTC TCGATGATGC GGATTGCGTT   
  
  
+ CATGTCATTG ACTTTGATAT TGGTTGTGGG GCTCAATGGG CATCACTGAT TCAGGAGCTT CCATTGAGGA   
  
  
+ AAAAGGGAGC TCCATCCCTG AAAATTACAG CCATAGCTCC CCTGTCAATA AGCCACACTT TTGAACTCAA   
  
  
+ CCTAGCACGG GAAAACCTTG TGCAATTCGC CAATGATGTT GGTGTTGCTT TTGAGCTGCA AGTTGTGAAC   
  
  
+ TTGGATTTAT TTGATCCATC TTCATCTTCA ATGCCAAATG TTGGAACCTC TGGGGATGAG TTGATCGCTG   
  
  
+ TTAGTATACC CATTTGGGCA TGTTCATATC GGCCGTCTAT TCTCCCATCC ATCCTCCGGT TCATTAAGCA   
  
  
+ ACTAGCCCCC AAGATTGTCG TCTCGTTAGA TAGAGGGTCT GATCGTTGCG ACACTCTGTT CCCACAACAT   
  
  
+ CTTATCCATA TCTTAGAGTC CTGCACAAAT TTCTTGGAAT CGCTCGACGG TCTCAATGTA GCATCAGATA   
  
  
+ TTGTAAACAA GGTTGAGAAG TACTTCCTCC AGCCTAAGAT CGAAAACACC GTGTTGGGCC GCGTTCATGC   
  
  
+ CCCCAATAAG ATGCCACATT GGAAGACCCT CTTTGCTTCA GCCGGGTTAT TGCCCTTTCA GTTCAGTAAC   
  
  
+ TTCACAGAAA CACAAGCTGA TTATGTGGTG AAGCGAACCC CAGGGAGGGG ATTTCACATA GAAAAGCGCC   
  
  
+ ACGCATCTTT AGTGCTAAGT TGGCAAAGGC GGGAGCTTGT GACAGCCTCG GCATGGAGGT TTGAGAAGCT   
  
  
+ GTA  

- +Up\_Stream \_Len000TGTAAC CCACCCTAAA TTTAGGAGAT AAAAAAGAAT CCCCTTTTTA TGAACCCTTT   
  
  
- AAAGAGTTAT TGACTTTTAG ATTTATTAAG CACTGAAACA ATCATCCAAA TTACACTAAA TGATGGCACT   
  
  
- CACAGTGAAA ATCTACCATG AAATTCTTTC GTAATATTCA TTTTGTTATA TATAGCATCG TAGTATGATG   
  
  
- AACACATTAT TTAATTGTCG TTGGAGTTAA CATGAATAAA CTAAAGCGTA ACATTAGTTA AAAGGGTTTA   
  
  
- CTAAATGATA GAACCAGAAT GAGTCAACAT GAAGTGTGAG TTATCAAGTT AATAAAAAGT AATCTGATTA   
  
  
- CTCGCTCAGG GAAGATGTTC TCGGATGATG ATGAGATTAT TACATTCCTG ATAACCGATT ACCCCTTGAT   
  
  
- TCGGATGCTG AACAATCCGT ATCCACTTAA AAGGAATGTT CTCTCAAATT AAATGTGAAC TATAATTGAG   
  
  
- CTCTTGCTCT TCGACAGAAT TATCTATGGG TGTATCTTTT ACTTCGTTTA CGGGAACTAA TAACCTCCCA   
  
  
- ACCCGTTTAT ATAAGTCATT GTTACAACAA ACCGTTTATA GCAGACTTTT AACAAAAACT GTTTATAACA   
  
  
- AACCTTCTTA AAAAGCTCTA ATTTATAATT TTTACAATTT TAAAATTAGA TTTATAGTTT GTTTATAACT   
  
  
- TTTTACGAAA TATAATAGAT TTAGAGATTA ATTTTAAACA CATAACAGGT GATCTTTTTA CATATCACTA   
  
  
- TAATGGCTGT ATATACACTA GAGACCACAT TATCGGATGG GATTACAGAT CCTTTTATAA ATTTTAGGGG   
  
  
- TCACCAGTTT TCCTTCAATG CGTGTGATCC CGTTCTGTGA AGTAATCCGA TCAACCACTT TCGATTCAGT   
  
  
- ACTTTAAATC TGGTACCGGT ACAGATGAAC ATCTAGGTTG ATGGTTGTTA GGTAAAAATA AAACCCCTTT   
  
  
- CGAACTTCCC ATTAAACCAT TTACATGTGC CCTTGGAAAA CGATATTTTC TTCACGAAGA ACTTTTTTGT   
  
  
- GGGACATCAT TTTGTCCCGT TTTTTCGGTT GTGAAGACAA AGACCGTCTC TTTGTCATCA CATGGTGAAC   
  
  
- CTCAACCTCA TCTGTGCTAT GTGTTTGGGT CATCTCTCTC TTTGTCCCCT CTCTTTCTCA TGTGTTTAAA   
  
  
- CACGTCCTCT TCCCATTCCT TCTTCCTCTT CCTTGGGGGA GGTTTTTTTT GGGTATATTT TTTATTATTT   
  
  
- CTTTTTTGTC CTCACTCAAT CTCCCATGGG TTTAGAGGTT AGATTTTGAT CTTAGATCCC AGAGAAAAGT   
  
  
- AAACGAACTT TTTAAAGACA AGTACCCAAA ACACAAAAAT CCCCCTAAAC AAACTAAAAA CATCAGAAAC   
  
  
- ACCTAAAGGA CACAATATCT ACGTTCGATA CGAGAAGTTA AACGTTCCTT GTCGTAGACC CCCCTCTCTT   
  
  
- AAACTCCTTA AAGTTCATGG GTAAAGTTAA AGTTGTTCAT TTTAGTAGTA ACTTCCTATG AGTTTGGGTT   
  
  
- CGGGTCAACT ATCACTTGGT TGAAGATGAG ACCTAAGATG AGGTTCAGGG TCGGGAGGGT GGGAGAGGAG   
  
  
- GAGGAGGTTC TTGAGGATGG TTTTGCGACG GCAAAACCGA GTGGGAGGAG TCGAAGTGGT ACCTAGACTA   
  
  
- TTACACATTC TGGGGTTGGG AAAACGAAAA TAAAAGGAGT ACGAGGTAAG AGTACTAAAC ATGAAGACAA   
  
  
- TACATTAACC TCTACACAGA TTTAAACCTT TTCAAGCACC CAAAGCACAA ACTCTTAAAT CTTACTGTAA   
  
  
- GAAATTAATC ATTAAACTTC AAACGTACGA ATTAAATCGA TTAGTGACTA ATTAATTTTA TTTACTTCAC   
  
  
- TACATCCATA CCCGACAATG TGTTACAGTG GCCACCACCA CTGTCGCCGC CCCGCCGCCG CCAACCTAAC   
  
  
- CTTCTTACCC TGTCGCACAA AGGTTTACCC CTACCTCGAA ATGAGGGAAC CTAGTACCCC CTTCCACTAC   
  
  
- TAAACCCAGA CTTAAACTTC GTAGAGAATG TTAGACCAAT GGGACAACTC ATACTTCCGT TACGTCCTGA   
  
  
- ACCACAACAA CTAGTCTGTA GAGAAGAACC TCCACCACTA AACCCCAAAA GACCAAGACC CAGACACTTA   
  
  
- TTACCGTTTT AACCAAGGTA AAACCCATTA ACAAGTAGAC CCCTAAACCT AAGATTCCAA AGATCACTGC   
  
  
- CCAACTTAAG GTTAACATCA AGTGACCCCT CATACTCATA GGAACCAAGT TAGGTACCTG GAAACGGATT   
  
  
- ATGACCACCC AAACACAAGG GTGTTTGTGT TGATAAACTA GAACCACTAC TCTTCGGAGT CTAAAACTTG   
  
  
- GGTGTTAACT ACTGCTACTT AGGTTTAGTC CGAGTCTCGT ACCCTTTGGG ATCGAAAAAA CACGGGAGGA   
  
  
- ACCCAATAAC AGTCAATCTC GTTGTATACC AAGTTGGTGT CCGCTTTGCG GTATCGGGAC CACATCAAAA   
  
  
- CTTGAGACTA CAATAACGGT TTTGGGGCAA AGAGCTAGTC CCAGTACTCA AAAACAACTC CTTTGTTGTG   
  
  
- GTAGTTGTCT AAAACGTCGT TGAAGGATAC CCAAACCGAG TAGTCAGCCA GGGAGTCGTG GAGGTCGTTT   
  
  
- TCCGCTACTT TCTGGTCCAA CAATTCCTGG TCGAAAACTT CCGTCGACTG GACTAAGTTT GACCCTTAAA   
  
  
- GAGTGAACGC GTTCTCTATA ACCGCGCCGA GTTAGTGGTC GAGAGGGAAG GACGCTTCGG GGAGTAATCC   
  
  
- CGTCGAAACA TACAGTACCT CCGGAACGTT TACGAGTAGA ATTACTCGTT AGGACATCGT GGAGGTGGCT   
  
  
- ACTTCTGCGA AAGGGGATAT CTACAACAAG TATTCTACTT ACGGATGTTC CGGAAGAGAC TCCAGAGAGG   
  
  
- ATAATGAGTC AAATGCTTAA AGTGAACACG GGTTCGGTAA GAACTTCGAG AGCTACTACG CCTAACGCAA   
  
  
- GTACAGTAAC TGAAACTATA ACCAACACCC CGAGTTACCC GTAGTGACTA AGTCCTCGAA GGTAACTCCT   
  
  
- TTTTCCCTCG AGGTAGGGAC TTTTAATGTC GGTATCGAGG GGACAGTTAT TCGGTGTGAA AACTTGAGTT   
  
  
- GGATCGTGCC CTTTTGGAAC ACGTTAAGCG GTTACTACAA CCACAACGAA AACTCGACGT TCAACACTTG   
  
  
- AACCTAAATA AACTAGGTAG AAGTAGAAGT TACGGTTTAC AACCTTGGAG ACCCCTACTC AACTAGCGAC   
  
  
- AATCATATGG GTAAACCCGT ACAAGTATAG CCGGCAGATA AGAGGGTAGG TAGGAGGCCA AGTAATTCGT   
  
  
- TGATCGGGGG TTCTAACAGC AGAGCAATCT ATCTCCCAGA CTAGCAACGC TGTGAGACAA GGGTGTTGTA   
  
  
- GAATAGGTAT AGAATCTCAG GACGTGTTTA AAGAACCTTA GCGAGCTGCC AGAGTTACAT CGTAGTCTAT   
  
  
- AACATTTGTT CCAACTCTTC ATGAAGGAGG TCGGATTCTA GCTTTTGTGG CACAACCCGG CGCAAGTACG   
  
  
- GGGGTTATTC TACGGTGTAA CCTTCTGGGA GAAACGAAGT CGGCCCAATA ACGGGAAAGT CAAGTCATTG   
  
  
- AAGTGTCTTT GTGTTCGACT AATACACCAC TTCGCTTGGG GTCCCTCCCC TAAAGTGTAT CTTTTCGCGG   
  
  
- TGCGTAGAAA TCACGATTCA ACCGTTTCCG CCCTCGAACA CTGTCGGAGC CGTACCTCCA AACTCTTCGA   
  
  
- CAT

+     H-box

| Site Name | Organism | Position | Strand | Matrix score. | sequence | function |
| --- | --- | --- | --- | --- | --- | --- |
| H-box | Phaseolus vulgaris | 809 | + | 9 | CCTACCNNNNNNNCTNNNNA |  |

>HU05G00466.1   
+ +Up\_Stream \_Len000ACATTG GGTGGGATTT AAATCCTCTA TTTTTTCTTA GGGGAAAAAT ACTTGGGAAA   
  
  
+ TTTCTCAATA ACTGAAAATC TAAATAATTC GTGACTTTGT TAGTAGGTTT AATGTGATTT ACTACCGTGA   
  
  
+ GTGTCACTTT TAGATGGTAC TTTAAGAAAG CATTATAAGT AAAACAATAT ATATCGTAGC ATCATACTAC   
  
  
+ TTGTGTAATA AATTAACAGC AACCTCAATT GTACTTATTT GATTTCGCAT TGTAATCAAT TTTCCCAAAT   
  
  
+ GATTTACTAT CTTGGTCTTA CTCAGTTGTA CTTCACACTC AATAGTTCAA TTATTTTTCA TTAGACTAAT   
  
  
+ GAGCGAGTCC CTTCTACAAG AGCCTACTAC TACTCTAATA ATGTAAGGAC TATTGGCTAA TGGGGAACTA   
  
  
+ AGCCTACGAC TTGTTAGGCA TAGGTGAATT TTCCTTACAA GAGAGTTTAA TTTACACTTG ATATTAACTC   
  
  
+ GAGAACGAGA AGCTGTCTTA ATAGATACCC ACATAGAAAA TGAAGCAAAT GCCCTTGATT ATTGGAGGGT   
  
  
+ TGGGCAAATA TATTCAGTAA CAATGTTGTT TGGCAAATAT CGTCTGAAAA TTGTTTTTGA CAAATATTGT   
  
  
+ TTGGAAGAAT TTTTCGAGAT TAAATATTAA AAATGTTAAA ATTTTAATCT AAATATCAAA CAAATATTGA   
  
  
+ AAAATGCTTT ATATTATCTA AATCTCTAAT TAAAATTTGT GTATTGTCCA CTAGAAAAAT GTATAGTGAT   
  
  
+ ATTACCGACA TATATGTGAT CTCTGGTGTA ATAGCCTACC CTAATGTCTA GGAAAATATT TAAAATCCCC   
  
  
+ AGTGGTCAAA AGGAAGTTAC GCACACTAGG GCAAGACACT TCATTAGGCT AGTTGGTGAA AGCTAAGTCA   
  
  
+ TGAAATTTAG ACCATGGCCA TGTCTACTTG TAGATCCAAC TACCAACAAT CCATTTTTAT TTTGGGGAAA   
  
  
+ GCTTGAAGGG TAATTTGGTA AATGTACACG GGAACCTTTT GCTATAAAAG AAGTGCTTCT TGAAAAAACA   
  
  
+ CCCTGTAGTA AAACAGGGCA AAAAAGCCAA CACTTCTGTT TCTGGCAGAG AAACAGTAGT GTACCACTTG   
  
  
+ GAGTTGGAGT AGACACGATA CACAAACCCA GTAGAGAGAG AAACAGGGGA GAGAAAGAGT ACACAAATTT   
  
  
+ GTGCAGGAGA AGGGTAAGGA AGAAGGAGAA GGAACCCCCT CCAAAAAAAA CCCATATAAA AAATAATAAA   
  
  
+ GAAAAAACAG GAGTGAGTTA GAGGGTACCC AAATCTCCAA TCTAAAACTA GAATCTAGGG TCTCTTTTCA   
  
  
+ TTTGCTTGAA AAATTTCTGT TCATGGGTTT TGTGTTTTTA GGGGGATTTG TTTGATTTTT GTAGTCTTTG   
  
  
+ TGGATTTCCT GTGTTATAGA TGCAAGCTAT GCTCTTCAAT TTGCAAGGAA CAGCATCTGG GGGGAGAGAA   
  
  
+ TTTGAGGAAT TTCAAGTACC CATTTCAATT TCAACAAGTA AAATCATCAT TGAAGGATAC TCAAACCCAA   
  
  
+ GCCCAGTTGA TAGTGAACCA ACTTCTACTC TGGATTCTAC TCCAAGTCCC AGCCCTCCCA CCCTCTCCTC   
  
  
+ CTCCTCCAAG AACTCCTACC AAAACGCTGC CGTTTTGGCT CACCCTCCTC AGCTTCACCA TGGATCTGAT   
  
  
+ AATGTGTAAG ACCCCAACCC TTTTGCTTTT ATTTTCCTCA TGCTCCATTC TCATGATTTG TACTTCTGTT   
  
  
+ ATGTAATTGG AGATGTGTCT AAATTTGGAA AAGTTCGTGG GTTTCGTGTT TGAGAATTTA GAATGACATT   
  
  
+ CTTTAATTAG TAATTTGAAG TTTGCATGCT TAATTTAGCT AATCACTGAT TAATTAAAAT AAATGAAGTG   
  
  
+ ATGTAGGTAT GGGCTGTTAC ACAATGTCAC CGGTGGTGGT GACAGCGGCG GGGCGGCGGC GGTTGGATTG   
  
  
+ GAAGAATGGG ACAGCGTGTT TCCAAATGGG GATGGAGCTT TACTCCCTTG GATCATGGGG GAAGGTGATG   
  
  
+ ATTTGGGTCT GAATTTGAAG CATCTCTTAC AATCTGGTTA CCCTGTTGAG TATGAAGGCA ATGCAGGACT   
  
  
+ TGGTGTTGTT GATCAGACAT CTCTTCTTGG AGGTGGTGAT TTGGGGTTTT CTGGTTCTGG GTCTGTGAAT   
  
  
+ AATGGCAAAA TTGGTTCCAT TTTGGGTAAT TGTTCATCTG GGGATTTGGA TTCTAAGGTT TCTAGTGACG   
  
  
+ GGTTGAATTC CAATTGTAGT TCACTGGGGA GTATGAGTAT CCTTGGTTCA ATCCATGGAC CTTTGCCTAA   
  
  
+ TACTGGTGGG TTTGTGTTCC CACAAACACA ACTATTTGAT CTTGGTGATG AGAAGCCTCA GATTTTGAAC   
  
  
+ CCACAATTGA TGACGATGAA TCCAAATCAG GCTCAGAGCA TGGGAAACCC TAGCTTTTTT GTGCCCTCCT   
  
  
+ TGGGTTATTG TCAGTTAGAG CAACATATGG TTCAACCACA GGCGAAACGC CATAGCCCTG GTGTAGTTTT   
  
  
+ GAACTCTGAT GTTATTGCCA AAACCCCGTT TCTCGATCAG GGTCATGAGT TTTTGTTGAG GAAACAACAC   
  
  
+ CATCAACAGA TTTTGCAGCA ACTTCCTATG GGTTTGGCTC ATCAGTCGGT CCCTCAGCAC CTCCAGCAAA   
  
  
+ AGGCGATGAA AGACCAGGTT GTTAAGGACC AGCTTTTGAA GGCAGCTGAC CTGATTCAAA CTGGGAATTT   
  
  
+ CTCACTTGCG CAAGAGATAT TGGCGCGGCT CAATCACCAG CTCTCCCTTC CTGCGAAGCC CCTCATTAGG   
  
  
+ GCAGCTTTGT ATGTCATGGA GGCCTTGCAA ATGCTCATCT TAATGAGCAA TCCTGTAGCA CCTCCACCGA   
  
  
+ TGAAGACGCT TTCCCCTATA GATGTTGTTC ATAAGATGAA TGCCTACAAG GCCTTCTCTG AGGTCTCTCC   
  
  
+ TATTACTCAG TTTACGAATT TCACTTGTGC CCAAGCCATT CTTGAAGCTC TCGATGATGC GGATTGCGTT   
  
  
+ CATGTCATTG ACTTTGATAT TGGTTGTGGG GCTCAATGGG CATCACTGAT TCAGGAGCTT CCATTGAGGA   
  
  
+ AAAAGGGAGC TCCATCCCTG AAAATTACAG CCATAGCTCC CCTGTCAATA AGCCACACTT TTGAACTCAA   
  
  
+ CCTAGCACGG GAAAACCTTG TGCAATTCGC CAATGATGTT GGTGTTGCTT TTGAGCTGCA AGTTGTGAAC   
  
  
+ TTGGATTTAT TTGATCCATC TTCATCTTCA ATGCCAAATG TTGGAACCTC TGGGGATGAG TTGATCGCTG   
  
  
+ TTAGTATACC CATTTGGGCA TGTTCATATC GGCCGTCTAT TCTCCCATCC ATCCTCCGGT TCATTAAGCA   
  
  
+ ACTAGCCCCC AAGATTGTCG TCTCGTTAGA TAGAGGGTCT GATCGTTGCG ACACTCTGTT CCCACAACAT   
  
  
+ CTTATCCATA TCTTAGAGTC CTGCACAAAT TTCTTGGAAT CGCTCGACGG TCTCAATGTA GCATCAGATA   
  
  
+ TTGTAAACAA GGTTGAGAAG TACTTCCTCC AGCCTAAGAT CGAAAACACC GTGTTGGGCC GCGTTCATGC   
  
  
+ CCCCAATAAG ATGCCACATT GGAAGACCCT CTTTGCTTCA GCCGGGTTAT TGCCCTTTCA GTTCAGTAAC   
  
  
+ TTCACAGAAA CACAAGCTGA TTATGTGGTG AAGCGAACCC CAGGGAGGGG ATTTCACATA GAAAAGCGCC   
  
  
+ ACGCATCTTT AGTGCTAAGT TGGCAAAGGC GGGAGCTTGT GACAGCCTCG GCATGGAGGT TTGAGAAGCT   
  
  
+ GTA  

- +Up\_Stream \_Len000TGTAAC CCACCCTAAA TTTAGGAGAT AAAAAAGAAT CCCCTTTTTA TGAACCCTTT   
  
  
- AAAGAGTTAT TGACTTTTAG ATTTATTAAG CACTGAAACA ATCATCCAAA TTACACTAAA TGATGGCACT   
  
  
- CACAGTGAAA ATCTACCATG AAATTCTTTC GTAATATTCA TTTTGTTATA TATAGCATCG TAGTATGATG   
  
  
- AACACATTAT TTAATTGTCG TTGGAGTTAA CATGAATAAA CTAAAGCGTA ACATTAGTTA AAAGGGTTTA   
  
  
- CTAAATGATA GAACCAGAAT GAGTCAACAT GAAGTGTGAG TTATCAAGTT AATAAAAAGT AATCTGATTA   
  
  
- CTCGCTCAGG GAAGATGTTC TCGGATGATG ATGAGATTAT TACATTCCTG ATAACCGATT ACCCCTTGAT   
  
  
- TCGGATGCTG AACAATCCGT ATCCACTTAA AAGGAATGTT CTCTCAAATT AAATGTGAAC TATAATTGAG   
  
  
- CTCTTGCTCT TCGACAGAAT TATCTATGGG TGTATCTTTT ACTTCGTTTA CGGGAACTAA TAACCTCCCA   
  
  
- ACCCGTTTAT ATAAGTCATT GTTACAACAA ACCGTTTATA GCAGACTTTT AACAAAAACT GTTTATAACA   
  
  
- AACCTTCTTA AAAAGCTCTA ATTTATAATT TTTACAATTT TAAAATTAGA TTTATAGTTT GTTTATAACT   
  
  
- TTTTACGAAA TATAATAGAT TTAGAGATTA ATTTTAAACA CATAACAGGT GATCTTTTTA CATATCACTA   
  
  
- TAATGGCTGT ATATACACTA GAGACCACAT TATCGGATGG GATTACAGAT CCTTTTATAA ATTTTAGGGG   
  
  
- TCACCAGTTT TCCTTCAATG CGTGTGATCC CGTTCTGTGA AGTAATCCGA TCAACCACTT TCGATTCAGT   
  
  
- ACTTTAAATC TGGTACCGGT ACAGATGAAC ATCTAGGTTG ATGGTTGTTA GGTAAAAATA AAACCCCTTT   
  
  
- CGAACTTCCC ATTAAACCAT TTACATGTGC CCTTGGAAAA CGATATTTTC TTCACGAAGA ACTTTTTTGT   
  
  
- GGGACATCAT TTTGTCCCGT TTTTTCGGTT GTGAAGACAA AGACCGTCTC TTTGTCATCA CATGGTGAAC   
  
  
- CTCAACCTCA TCTGTGCTAT GTGTTTGGGT CATCTCTCTC TTTGTCCCCT CTCTTTCTCA TGTGTTTAAA   
  
  
- CACGTCCTCT TCCCATTCCT TCTTCCTCTT CCTTGGGGGA GGTTTTTTTT GGGTATATTT TTTATTATTT   
  
  
- CTTTTTTGTC CTCACTCAAT CTCCCATGGG TTTAGAGGTT AGATTTTGAT CTTAGATCCC AGAGAAAAGT   
  
  
- AAACGAACTT TTTAAAGACA AGTACCCAAA ACACAAAAAT CCCCCTAAAC AAACTAAAAA CATCAGAAAC   
  
  
- ACCTAAAGGA CACAATATCT ACGTTCGATA CGAGAAGTTA AACGTTCCTT GTCGTAGACC CCCCTCTCTT   
  
  
- AAACTCCTTA AAGTTCATGG GTAAAGTTAA AGTTGTTCAT TTTAGTAGTA ACTTCCTATG AGTTTGGGTT   
  
  
- CGGGTCAACT ATCACTTGGT TGAAGATGAG ACCTAAGATG AGGTTCAGGG TCGGGAGGGT GGGAGAGGAG   
  
  
- GAGGAGGTTC TTGAGGATGG TTTTGCGACG GCAAAACCGA GTGGGAGGAG TCGAAGTGGT ACCTAGACTA   
  
  
- TTACACATTC TGGGGTTGGG AAAACGAAAA TAAAAGGAGT ACGAGGTAAG AGTACTAAAC ATGAAGACAA   
  
  
- TACATTAACC TCTACACAGA TTTAAACCTT TTCAAGCACC CAAAGCACAA ACTCTTAAAT CTTACTGTAA   
  
  
- GAAATTAATC ATTAAACTTC AAACGTACGA ATTAAATCGA TTAGTGACTA ATTAATTTTA TTTACTTCAC   
  
  
- TACATCCATA CCCGACAATG TGTTACAGTG GCCACCACCA CTGTCGCCGC CCCGCCGCCG CCAACCTAAC   
  
  
- CTTCTTACCC TGTCGCACAA AGGTTTACCC CTACCTCGAA ATGAGGGAAC CTAGTACCCC CTTCCACTAC   
  
  
- TAAACCCAGA CTTAAACTTC GTAGAGAATG TTAGACCAAT GGGACAACTC ATACTTCCGT TACGTCCTGA   
  
  
- ACCACAACAA CTAGTCTGTA GAGAAGAACC TCCACCACTA AACCCCAAAA GACCAAGACC CAGACACTTA   
  
  
- TTACCGTTTT AACCAAGGTA AAACCCATTA ACAAGTAGAC CCCTAAACCT AAGATTCCAA AGATCACTGC   
  
  
- CCAACTTAAG GTTAACATCA AGTGACCCCT CATACTCATA GGAACCAAGT TAGGTACCTG GAAACGGATT   
  
  
- ATGACCACCC AAACACAAGG GTGTTTGTGT TGATAAACTA GAACCACTAC TCTTCGGAGT CTAAAACTTG   
  
  
- GGTGTTAACT ACTGCTACTT AGGTTTAGTC CGAGTCTCGT ACCCTTTGGG ATCGAAAAAA CACGGGAGGA   
  
  
- ACCCAATAAC AGTCAATCTC GTTGTATACC AAGTTGGTGT CCGCTTTGCG GTATCGGGAC CACATCAAAA   
  
  
- CTTGAGACTA CAATAACGGT TTTGGGGCAA AGAGCTAGTC CCAGTACTCA AAAACAACTC CTTTGTTGTG   
  
  
- GTAGTTGTCT AAAACGTCGT TGAAGGATAC CCAAACCGAG TAGTCAGCCA GGGAGTCGTG GAGGTCGTTT   
  
  
- TCCGCTACTT TCTGGTCCAA CAATTCCTGG TCGAAAACTT CCGTCGACTG GACTAAGTTT GACCCTTAAA   
  
  
- GAGTGAACGC GTTCTCTATA ACCGCGCCGA GTTAGTGGTC GAGAGGGAAG GACGCTTCGG GGAGTAATCC   
  
  
- CGTCGAAACA TACAGTACCT CCGGAACGTT TACGAGTAGA ATTACTCGTT AGGACATCGT GGAGGTGGCT   
  
  
- ACTTCTGCGA AAGGGGATAT CTACAACAAG TATTCTACTT ACGGATGTTC CGGAAGAGAC TCCAGAGAGG   
  
  
- ATAATGAGTC AAATGCTTAA AGTGAACACG GGTTCGGTAA GAACTTCGAG AGCTACTACG CCTAACGCAA   
  
  
- GTACAGTAAC TGAAACTATA ACCAACACCC CGAGTTACCC GTAGTGACTA AGTCCTCGAA GGTAACTCCT   
  
  
- TTTTCCCTCG AGGTAGGGAC TTTTAATGTC GGTATCGAGG GGACAGTTAT TCGGTGTGAA AACTTGAGTT   
  
  
- GGATCGTGCC CTTTTGGAAC ACGTTAAGCG GTTACTACAA CCACAACGAA AACTCGACGT TCAACACTTG   
  
  
- AACCTAAATA AACTAGGTAG AAGTAGAAGT TACGGTTTAC AACCTTGGAG ACCCCTACTC AACTAGCGAC   
  
  
- AATCATATGG GTAAACCCGT ACAAGTATAG CCGGCAGATA AGAGGGTAGG TAGGAGGCCA AGTAATTCGT   
  
  
- TGATCGGGGG TTCTAACAGC AGAGCAATCT ATCTCCCAGA CTAGCAACGC TGTGAGACAA GGGTGTTGTA   
  
  
- GAATAGGTAT AGAATCTCAG GACGTGTTTA AAGAACCTTA GCGAGCTGCC AGAGTTACAT CGTAGTCTAT   
  
  
- AACATTTGTT CCAACTCTTC ATGAAGGAGG TCGGATTCTA GCTTTTGTGG CACAACCCGG CGCAAGTACG   
  
  
- GGGGTTATTC TACGGTGTAA CCTTCTGGGA GAAACGAAGT CGGCCCAATA ACGGGAAAGT CAAGTCATTG   
  
  
- AAGTGTCTTT GTGTTCGACT AATACACCAC TTCGCTTGGG GTCCCTCCCC TAAAGTGTAT CTTTTCGCGG   
  
  
- TGCGTAGAAA TCACGATTCA ACCGTTTCCG CCCTCGAACA CTGTCGGAGC CGTACCTCCA AACTCTTCGA   
  
  
- CAT

+     I-box

| Site Name | Organism | Position | Strand | Matrix score. | sequence | function |
| --- | --- | --- | --- | --- | --- | --- |
| I-box | Solanum tuberosum | 1681 | + | 9 | TGATAATGT | part of a light responsive element |
| I-box | Zea mays | 3432 | - | 9 | gGATAAGGTG | part of a light responsive element |

>HU05G00466.1   
+ +Up\_Stream \_Len000ACATTG GGTGGGATTT AAATCCTCTA TTTTTTCTTA GGGGAAAAAT ACTTGGGAAA   
  
  
+ TTTCTCAATA ACTGAAAATC TAAATAATTC GTGACTTTGT TAGTAGGTTT AATGTGATTT ACTACCGTGA   
  
  
+ GTGTCACTTT TAGATGGTAC TTTAAGAAAG CATTATAAGT AAAACAATAT ATATCGTAGC ATCATACTAC   
  
  
+ TTGTGTAATA AATTAACAGC AACCTCAATT GTACTTATTT GATTTCGCAT TGTAATCAAT TTTCCCAAAT   
  
  
+ GATTTACTAT CTTGGTCTTA CTCAGTTGTA CTTCACACTC AATAGTTCAA TTATTTTTCA TTAGACTAAT   
  
  
+ GAGCGAGTCC CTTCTACAAG AGCCTACTAC TACTCTAATA ATGTAAGGAC TATTGGCTAA TGGGGAACTA   
  
  
+ AGCCTACGAC TTGTTAGGCA TAGGTGAATT TTCCTTACAA GAGAGTTTAA TTTACACTTG ATATTAACTC   
  
  
+ GAGAACGAGA AGCTGTCTTA ATAGATACCC ACATAGAAAA TGAAGCAAAT GCCCTTGATT ATTGGAGGGT   
  
  
+ TGGGCAAATA TATTCAGTAA CAATGTTGTT TGGCAAATAT CGTCTGAAAA TTGTTTTTGA CAAATATTGT   
  
  
+ TTGGAAGAAT TTTTCGAGAT TAAATATTAA AAATGTTAAA ATTTTAATCT AAATATCAAA CAAATATTGA   
  
  
+ AAAATGCTTT ATATTATCTA AATCTCTAAT TAAAATTTGT GTATTGTCCA CTAGAAAAAT GTATAGTGAT   
  
  
+ ATTACCGACA TATATGTGAT CTCTGGTGTA ATAGCCTACC CTAATGTCTA GGAAAATATT TAAAATCCCC   
  
  
+ AGTGGTCAAA AGGAAGTTAC GCACACTAGG GCAAGACACT TCATTAGGCT AGTTGGTGAA AGCTAAGTCA   
  
  
+ TGAAATTTAG ACCATGGCCA TGTCTACTTG TAGATCCAAC TACCAACAAT CCATTTTTAT TTTGGGGAAA   
  
  
+ GCTTGAAGGG TAATTTGGTA AATGTACACG GGAACCTTTT GCTATAAAAG AAGTGCTTCT TGAAAAAACA   
  
  
+ CCCTGTAGTA AAACAGGGCA AAAAAGCCAA CACTTCTGTT TCTGGCAGAG AAACAGTAGT GTACCACTTG   
  
  
+ GAGTTGGAGT AGACACGATA CACAAACCCA GTAGAGAGAG AAACAGGGGA GAGAAAGAGT ACACAAATTT   
  
  
+ GTGCAGGAGA AGGGTAAGGA AGAAGGAGAA GGAACCCCCT CCAAAAAAAA CCCATATAAA AAATAATAAA   
  
  
+ GAAAAAACAG GAGTGAGTTA GAGGGTACCC AAATCTCCAA TCTAAAACTA GAATCTAGGG TCTCTTTTCA   
  
  
+ TTTGCTTGAA AAATTTCTGT TCATGGGTTT TGTGTTTTTA GGGGGATTTG TTTGATTTTT GTAGTCTTTG   
  
  
+ TGGATTTCCT GTGTTATAGA TGCAAGCTAT GCTCTTCAAT TTGCAAGGAA CAGCATCTGG GGGGAGAGAA   
  
  
+ TTTGAGGAAT TTCAAGTACC CATTTCAATT TCAACAAGTA AAATCATCAT TGAAGGATAC TCAAACCCAA   
  
  
+ GCCCAGTTGA TAGTGAACCA ACTTCTACTC TGGATTCTAC TCCAAGTCCC AGCCCTCCCA CCCTCTCCTC   
  
  
+ CTCCTCCAAG AACTCCTACC AAAACGCTGC CGTTTTGGCT CACCCTCCTC AGCTTCACCA TGGATCTGAT   
  
  
+ AATGTGTAAG ACCCCAACCC TTTTGCTTTT ATTTTCCTCA TGCTCCATTC TCATGATTTG TACTTCTGTT   
  
  
+ ATGTAATTGG AGATGTGTCT AAATTTGGAA AAGTTCGTGG GTTTCGTGTT TGAGAATTTA GAATGACATT   
  
  
+ CTTTAATTAG TAATTTGAAG TTTGCATGCT TAATTTAGCT AATCACTGAT TAATTAAAAT AAATGAAGTG   
  
  
+ ATGTAGGTAT GGGCTGTTAC ACAATGTCAC CGGTGGTGGT GACAGCGGCG GGGCGGCGGC GGTTGGATTG   
  
  
+ GAAGAATGGG ACAGCGTGTT TCCAAATGGG GATGGAGCTT TACTCCCTTG GATCATGGGG GAAGGTGATG   
  
  
+ ATTTGGGTCT GAATTTGAAG CATCTCTTAC AATCTGGTTA CCCTGTTGAG TATGAAGGCA ATGCAGGACT   
  
  
+ TGGTGTTGTT GATCAGACAT CTCTTCTTGG AGGTGGTGAT TTGGGGTTTT CTGGTTCTGG GTCTGTGAAT   
  
  
+ AATGGCAAAA TTGGTTCCAT TTTGGGTAAT TGTTCATCTG GGGATTTGGA TTCTAAGGTT TCTAGTGACG   
  
  
+ GGTTGAATTC CAATTGTAGT TCACTGGGGA GTATGAGTAT CCTTGGTTCA ATCCATGGAC CTTTGCCTAA   
  
  
+ TACTGGTGGG TTTGTGTTCC CACAAACACA ACTATTTGAT CTTGGTGATG AGAAGCCTCA GATTTTGAAC   
  
  
+ CCACAATTGA TGACGATGAA TCCAAATCAG GCTCAGAGCA TGGGAAACCC TAGCTTTTTT GTGCCCTCCT   
  
  
+ TGGGTTATTG TCAGTTAGAG CAACATATGG TTCAACCACA GGCGAAACGC CATAGCCCTG GTGTAGTTTT   
  
  
+ GAACTCTGAT GTTATTGCCA AAACCCCGTT TCTCGATCAG GGTCATGAGT TTTTGTTGAG GAAACAACAC   
  
  
+ CATCAACAGA TTTTGCAGCA ACTTCCTATG GGTTTGGCTC ATCAGTCGGT CCCTCAGCAC CTCCAGCAAA   
  
  
+ AGGCGATGAA AGACCAGGTT GTTAAGGACC AGCTTTTGAA GGCAGCTGAC CTGATTCAAA CTGGGAATTT   
  
  
+ CTCACTTGCG CAAGAGATAT TGGCGCGGCT CAATCACCAG CTCTCCCTTC CTGCGAAGCC CCTCATTAGG   
  
  
+ GCAGCTTTGT ATGTCATGGA GGCCTTGCAA ATGCTCATCT TAATGAGCAA TCCTGTAGCA CCTCCACCGA   
  
  
+ TGAAGACGCT TTCCCCTATA GATGTTGTTC ATAAGATGAA TGCCTACAAG GCCTTCTCTG AGGTCTCTCC   
  
  
+ TATTACTCAG TTTACGAATT TCACTTGTGC CCAAGCCATT CTTGAAGCTC TCGATGATGC GGATTGCGTT   
  
  
+ CATGTCATTG ACTTTGATAT TGGTTGTGGG GCTCAATGGG CATCACTGAT TCAGGAGCTT CCATTGAGGA   
  
  
+ AAAAGGGAGC TCCATCCCTG AAAATTACAG CCATAGCTCC CCTGTCAATA AGCCACACTT TTGAACTCAA   
  
  
+ CCTAGCACGG GAAAACCTTG TGCAATTCGC CAATGATGTT GGTGTTGCTT TTGAGCTGCA AGTTGTGAAC   
  
  
+ TTGGATTTAT TTGATCCATC TTCATCTTCA ATGCCAAATG TTGGAACCTC TGGGGATGAG TTGATCGCTG   
  
  
+ TTAGTATACC CATTTGGGCA TGTTCATATC GGCCGTCTAT TCTCCCATCC ATCCTCCGGT TCATTAAGCA   
  
  
+ ACTAGCCCCC AAGATTGTCG TCTCGTTAGA TAGAGGGTCT GATCGTTGCG ACACTCTGTT CCCACAACAT   
  
  
+ CTTATCCATA TCTTAGAGTC CTGCACAAAT TTCTTGGAAT CGCTCGACGG TCTCAATGTA GCATCAGATA   
  
  
+ TTGTAAACAA GGTTGAGAAG TACTTCCTCC AGCCTAAGAT CGAAAACACC GTGTTGGGCC GCGTTCATGC   
  
  
+ CCCCAATAAG ATGCCACATT GGAAGACCCT CTTTGCTTCA GCCGGGTTAT TGCCCTTTCA GTTCAGTAAC   
  
  
+ TTCACAGAAA CACAAGCTGA TTATGTGGTG AAGCGAACCC CAGGGAGGGG ATTTCACATA GAAAAGCGCC   
  
  
+ ACGCATCTTT AGTGCTAAGT TGGCAAAGGC GGGAGCTTGT GACAGCCTCG GCATGGAGGT TTGAGAAGCT   
  
  
+ GTA  

- +Up\_Stream \_Len000TGTAAC CCACCCTAAA TTTAGGAGAT AAAAAAGAAT CCCCTTTTTA TGAACCCTTT   
  
  
- AAAGAGTTAT TGACTTTTAG ATTTATTAAG CACTGAAACA ATCATCCAAA TTACACTAAA TGATGGCACT   
  
  
- CACAGTGAAA ATCTACCATG AAATTCTTTC GTAATATTCA TTTTGTTATA TATAGCATCG TAGTATGATG   
  
  
- AACACATTAT TTAATTGTCG TTGGAGTTAA CATGAATAAA CTAAAGCGTA ACATTAGTTA AAAGGGTTTA   
  
  
- CTAAATGATA GAACCAGAAT GAGTCAACAT GAAGTGTGAG TTATCAAGTT AATAAAAAGT AATCTGATTA   
  
  
- CTCGCTCAGG GAAGATGTTC TCGGATGATG ATGAGATTAT TACATTCCTG ATAACCGATT ACCCCTTGAT   
  
  
- TCGGATGCTG AACAATCCGT ATCCACTTAA AAGGAATGTT CTCTCAAATT AAATGTGAAC TATAATTGAG   
  
  
- CTCTTGCTCT TCGACAGAAT TATCTATGGG TGTATCTTTT ACTTCGTTTA CGGGAACTAA TAACCTCCCA   
  
  
- ACCCGTTTAT ATAAGTCATT GTTACAACAA ACCGTTTATA GCAGACTTTT AACAAAAACT GTTTATAACA   
  
  
- AACCTTCTTA AAAAGCTCTA ATTTATAATT TTTACAATTT TAAAATTAGA TTTATAGTTT GTTTATAACT   
  
  
- TTTTACGAAA TATAATAGAT TTAGAGATTA ATTTTAAACA CATAACAGGT GATCTTTTTA CATATCACTA   
  
  
- TAATGGCTGT ATATACACTA GAGACCACAT TATCGGATGG GATTACAGAT CCTTTTATAA ATTTTAGGGG   
  
  
- TCACCAGTTT TCCTTCAATG CGTGTGATCC CGTTCTGTGA AGTAATCCGA TCAACCACTT TCGATTCAGT   
  
  
- ACTTTAAATC TGGTACCGGT ACAGATGAAC ATCTAGGTTG ATGGTTGTTA GGTAAAAATA AAACCCCTTT   
  
  
- CGAACTTCCC ATTAAACCAT TTACATGTGC CCTTGGAAAA CGATATTTTC TTCACGAAGA ACTTTTTTGT   
  
  
- GGGACATCAT TTTGTCCCGT TTTTTCGGTT GTGAAGACAA AGACCGTCTC TTTGTCATCA CATGGTGAAC   
  
  
- CTCAACCTCA TCTGTGCTAT GTGTTTGGGT CATCTCTCTC TTTGTCCCCT CTCTTTCTCA TGTGTTTAAA   
  
  
- CACGTCCTCT TCCCATTCCT TCTTCCTCTT CCTTGGGGGA GGTTTTTTTT GGGTATATTT TTTATTATTT   
  
  
- CTTTTTTGTC CTCACTCAAT CTCCCATGGG TTTAGAGGTT AGATTTTGAT CTTAGATCCC AGAGAAAAGT   
  
  
- AAACGAACTT TTTAAAGACA AGTACCCAAA ACACAAAAAT CCCCCTAAAC AAACTAAAAA CATCAGAAAC   
  
  
- ACCTAAAGGA CACAATATCT ACGTTCGATA CGAGAAGTTA AACGTTCCTT GTCGTAGACC CCCCTCTCTT   
  
  
- AAACTCCTTA AAGTTCATGG GTAAAGTTAA AGTTGTTCAT TTTAGTAGTA ACTTCCTATG AGTTTGGGTT   
  
  
- CGGGTCAACT ATCACTTGGT TGAAGATGAG ACCTAAGATG AGGTTCAGGG TCGGGAGGGT GGGAGAGGAG   
  
  
- GAGGAGGTTC TTGAGGATGG TTTTGCGACG GCAAAACCGA GTGGGAGGAG TCGAAGTGGT ACCTAGACTA   
  
  
- TTACACATTC TGGGGTTGGG AAAACGAAAA TAAAAGGAGT ACGAGGTAAG AGTACTAAAC ATGAAGACAA   
  
  
- TACATTAACC TCTACACAGA TTTAAACCTT TTCAAGCACC CAAAGCACAA ACTCTTAAAT CTTACTGTAA   
  
  
- GAAATTAATC ATTAAACTTC AAACGTACGA ATTAAATCGA TTAGTGACTA ATTAATTTTA TTTACTTCAC   
  
  
- TACATCCATA CCCGACAATG TGTTACAGTG GCCACCACCA CTGTCGCCGC CCCGCCGCCG CCAACCTAAC   
  
  
- CTTCTTACCC TGTCGCACAA AGGTTTACCC CTACCTCGAA ATGAGGGAAC CTAGTACCCC CTTCCACTAC   
  
  
- TAAACCCAGA CTTAAACTTC GTAGAGAATG TTAGACCAAT GGGACAACTC ATACTTCCGT TACGTCCTGA   
  
  
- ACCACAACAA CTAGTCTGTA GAGAAGAACC TCCACCACTA AACCCCAAAA GACCAAGACC CAGACACTTA   
  
  
- TTACCGTTTT AACCAAGGTA AAACCCATTA ACAAGTAGAC CCCTAAACCT AAGATTCCAA AGATCACTGC   
  
  
- CCAACTTAAG GTTAACATCA AGTGACCCCT CATACTCATA GGAACCAAGT TAGGTACCTG GAAACGGATT   
  
  
- ATGACCACCC AAACACAAGG GTGTTTGTGT TGATAAACTA GAACCACTAC TCTTCGGAGT CTAAAACTTG   
  
  
- GGTGTTAACT ACTGCTACTT AGGTTTAGTC CGAGTCTCGT ACCCTTTGGG ATCGAAAAAA CACGGGAGGA   
  
  
- ACCCAATAAC AGTCAATCTC GTTGTATACC AAGTTGGTGT CCGCTTTGCG GTATCGGGAC CACATCAAAA   
  
  
- CTTGAGACTA CAATAACGGT TTTGGGGCAA AGAGCTAGTC CCAGTACTCA AAAACAACTC CTTTGTTGTG   
  
  
- GTAGTTGTCT AAAACGTCGT TGAAGGATAC CCAAACCGAG TAGTCAGCCA GGGAGTCGTG GAGGTCGTTT   
  
  
- TCCGCTACTT TCTGGTCCAA CAATTCCTGG TCGAAAACTT CCGTCGACTG GACTAAGTTT GACCCTTAAA   
  
  
- GAGTGAACGC GTTCTCTATA ACCGCGCCGA GTTAGTGGTC GAGAGGGAAG GACGCTTCGG GGAGTAATCC   
  
  
- CGTCGAAACA TACAGTACCT CCGGAACGTT TACGAGTAGA ATTACTCGTT AGGACATCGT GGAGGTGGCT   
  
  
- ACTTCTGCGA AAGGGGATAT CTACAACAAG TATTCTACTT ACGGATGTTC CGGAAGAGAC TCCAGAGAGG   
  
  
- ATAATGAGTC AAATGCTTAA AGTGAACACG GGTTCGGTAA GAACTTCGAG AGCTACTACG CCTAACGCAA   
  
  
- GTACAGTAAC TGAAACTATA ACCAACACCC CGAGTTACCC GTAGTGACTA AGTCCTCGAA GGTAACTCCT   
  
  
- TTTTCCCTCG AGGTAGGGAC TTTTAATGTC GGTATCGAGG GGACAGTTAT TCGGTGTGAA AACTTGAGTT   
  
  
- GGATCGTGCC CTTTTGGAAC ACGTTAAGCG GTTACTACAA CCACAACGAA AACTCGACGT TCAACACTTG   
  
  
- AACCTAAATA AACTAGGTAG AAGTAGAAGT TACGGTTTAC AACCTTGGAG ACCCCTACTC AACTAGCGAC   
  
  
- AATCATATGG GTAAACCCGT ACAAGTATAG CCGGCAGATA AGAGGGTAGG TAGGAGGCCA AGTAATTCGT   
  
  
- TGATCGGGGG TTCTAACAGC AGAGCAATCT ATCTCCCAGA CTAGCAACGC TGTGAGACAA GGGTGTTGTA   
  
  
- GAATAGGTAT AGAATCTCAG GACGTGTTTA AAGAACCTTA GCGAGCTGCC AGAGTTACAT CGTAGTCTAT   
  
  
- AACATTTGTT CCAACTCTTC ATGAAGGAGG TCGGATTCTA GCTTTTGTGG CACAACCCGG CGCAAGTACG   
  
  
- GGGGTTATTC TACGGTGTAA CCTTCTGGGA GAAACGAAGT CGGCCCAATA ACGGGAAAGT CAAGTCATTG   
  
  
- AAGTGTCTTT GTGTTCGACT AATACACCAC TTCGCTTGGG GTCCCTCCCC TAAAGTGTAT CTTTTCGCGG   
  
  
- TGCGTAGAAA TCACGATTCA ACCGTTTCCG CCCTCGAACA CTGTCGGAGC CGTACCTCCA AACTCTTCGA   
  
  
- CAT

+     MBS

| Site Name | Organism | Position | Strand | Matrix score. | sequence | function |
| --- | --- | --- | --- | --- | --- | --- |
| MBS | Arabidopsis thaliana | 1548 | - | 6 | CAACTG | MYB binding site involved in drought-inducibility |
| MBS | Arabidopsis thaliana | 307 | - | 6 | CAACTG | MYB binding site involved in drought-inducibility |

>HU05G00466.1   
+ +Up\_Stream \_Len000ACATTG GGTGGGATTT AAATCCTCTA TTTTTTCTTA GGGGAAAAAT ACTTGGGAAA   
  
  
+ TTTCTCAATA ACTGAAAATC TAAATAATTC GTGACTTTGT TAGTAGGTTT AATGTGATTT ACTACCGTGA   
  
  
+ GTGTCACTTT TAGATGGTAC TTTAAGAAAG CATTATAAGT AAAACAATAT ATATCGTAGC ATCATACTAC   
  
  
+ TTGTGTAATA AATTAACAGC AACCTCAATT GTACTTATTT GATTTCGCAT TGTAATCAAT TTTCCCAAAT   
  
  
+ GATTTACTAT CTTGGTCTTA CTCAGTTGTA CTTCACACTC AATAGTTCAA TTATTTTTCA TTAGACTAAT   
  
  
+ GAGCGAGTCC CTTCTACAAG AGCCTACTAC TACTCTAATA ATGTAAGGAC TATTGGCTAA TGGGGAACTA   
  
  
+ AGCCTACGAC TTGTTAGGCA TAGGTGAATT TTCCTTACAA GAGAGTTTAA TTTACACTTG ATATTAACTC   
  
  
+ GAGAACGAGA AGCTGTCTTA ATAGATACCC ACATAGAAAA TGAAGCAAAT GCCCTTGATT ATTGGAGGGT   
  
  
+ TGGGCAAATA TATTCAGTAA CAATGTTGTT TGGCAAATAT CGTCTGAAAA TTGTTTTTGA CAAATATTGT   
  
  
+ TTGGAAGAAT TTTTCGAGAT TAAATATTAA AAATGTTAAA ATTTTAATCT AAATATCAAA CAAATATTGA   
  
  
+ AAAATGCTTT ATATTATCTA AATCTCTAAT TAAAATTTGT GTATTGTCCA CTAGAAAAAT GTATAGTGAT   
  
  
+ ATTACCGACA TATATGTGAT CTCTGGTGTA ATAGCCTACC CTAATGTCTA GGAAAATATT TAAAATCCCC   
  
  
+ AGTGGTCAAA AGGAAGTTAC GCACACTAGG GCAAGACACT TCATTAGGCT AGTTGGTGAA AGCTAAGTCA   
  
  
+ TGAAATTTAG ACCATGGCCA TGTCTACTTG TAGATCCAAC TACCAACAAT CCATTTTTAT TTTGGGGAAA   
  
  
+ GCTTGAAGGG TAATTTGGTA AATGTACACG GGAACCTTTT GCTATAAAAG AAGTGCTTCT TGAAAAAACA   
  
  
+ CCCTGTAGTA AAACAGGGCA AAAAAGCCAA CACTTCTGTT TCTGGCAGAG AAACAGTAGT GTACCACTTG   
  
  
+ GAGTTGGAGT AGACACGATA CACAAACCCA GTAGAGAGAG AAACAGGGGA GAGAAAGAGT ACACAAATTT   
  
  
+ GTGCAGGAGA AGGGTAAGGA AGAAGGAGAA GGAACCCCCT CCAAAAAAAA CCCATATAAA AAATAATAAA   
  
  
+ GAAAAAACAG GAGTGAGTTA GAGGGTACCC AAATCTCCAA TCTAAAACTA GAATCTAGGG TCTCTTTTCA   
  
  
+ TTTGCTTGAA AAATTTCTGT TCATGGGTTT TGTGTTTTTA GGGGGATTTG TTTGATTTTT GTAGTCTTTG   
  
  
+ TGGATTTCCT GTGTTATAGA TGCAAGCTAT GCTCTTCAAT TTGCAAGGAA CAGCATCTGG GGGGAGAGAA   
  
  
+ TTTGAGGAAT TTCAAGTACC CATTTCAATT TCAACAAGTA AAATCATCAT TGAAGGATAC TCAAACCCAA   
  
  
+ GCCCAGTTGA TAGTGAACCA ACTTCTACTC TGGATTCTAC TCCAAGTCCC AGCCCTCCCA CCCTCTCCTC   
  
  
+ CTCCTCCAAG AACTCCTACC AAAACGCTGC CGTTTTGGCT CACCCTCCTC AGCTTCACCA TGGATCTGAT   
  
  
+ AATGTGTAAG ACCCCAACCC TTTTGCTTTT ATTTTCCTCA TGCTCCATTC TCATGATTTG TACTTCTGTT   
  
  
+ ATGTAATTGG AGATGTGTCT AAATTTGGAA AAGTTCGTGG GTTTCGTGTT TGAGAATTTA GAATGACATT   
  
  
+ CTTTAATTAG TAATTTGAAG TTTGCATGCT TAATTTAGCT AATCACTGAT TAATTAAAAT AAATGAAGTG   
  
  
+ ATGTAGGTAT GGGCTGTTAC ACAATGTCAC CGGTGGTGGT GACAGCGGCG GGGCGGCGGC GGTTGGATTG   
  
  
+ GAAGAATGGG ACAGCGTGTT TCCAAATGGG GATGGAGCTT TACTCCCTTG GATCATGGGG GAAGGTGATG   
  
  
+ ATTTGGGTCT GAATTTGAAG CATCTCTTAC AATCTGGTTA CCCTGTTGAG TATGAAGGCA ATGCAGGACT   
  
  
+ TGGTGTTGTT GATCAGACAT CTCTTCTTGG AGGTGGTGAT TTGGGGTTTT CTGGTTCTGG GTCTGTGAAT   
  
  
+ AATGGCAAAA TTGGTTCCAT TTTGGGTAAT TGTTCATCTG GGGATTTGGA TTCTAAGGTT TCTAGTGACG   
  
  
+ GGTTGAATTC CAATTGTAGT TCACTGGGGA GTATGAGTAT CCTTGGTTCA ATCCATGGAC CTTTGCCTAA   
  
  
+ TACTGGTGGG TTTGTGTTCC CACAAACACA ACTATTTGAT CTTGGTGATG AGAAGCCTCA GATTTTGAAC   
  
  
+ CCACAATTGA TGACGATGAA TCCAAATCAG GCTCAGAGCA TGGGAAACCC TAGCTTTTTT GTGCCCTCCT   
  
  
+ TGGGTTATTG TCAGTTAGAG CAACATATGG TTCAACCACA GGCGAAACGC CATAGCCCTG GTGTAGTTTT   
  
  
+ GAACTCTGAT GTTATTGCCA AAACCCCGTT TCTCGATCAG GGTCATGAGT TTTTGTTGAG GAAACAACAC   
  
  
+ CATCAACAGA TTTTGCAGCA ACTTCCTATG GGTTTGGCTC ATCAGTCGGT CCCTCAGCAC CTCCAGCAAA   
  
  
+ AGGCGATGAA AGACCAGGTT GTTAAGGACC AGCTTTTGAA GGCAGCTGAC CTGATTCAAA CTGGGAATTT   
  
  
+ CTCACTTGCG CAAGAGATAT TGGCGCGGCT CAATCACCAG CTCTCCCTTC CTGCGAAGCC CCTCATTAGG   
  
  
+ GCAGCTTTGT ATGTCATGGA GGCCTTGCAA ATGCTCATCT TAATGAGCAA TCCTGTAGCA CCTCCACCGA   
  
  
+ TGAAGACGCT TTCCCCTATA GATGTTGTTC ATAAGATGAA TGCCTACAAG GCCTTCTCTG AGGTCTCTCC   
  
  
+ TATTACTCAG TTTACGAATT TCACTTGTGC CCAAGCCATT CTTGAAGCTC TCGATGATGC GGATTGCGTT   
  
  
+ CATGTCATTG ACTTTGATAT TGGTTGTGGG GCTCAATGGG CATCACTGAT TCAGGAGCTT CCATTGAGGA   
  
  
+ AAAAGGGAGC TCCATCCCTG AAAATTACAG CCATAGCTCC CCTGTCAATA AGCCACACTT TTGAACTCAA   
  
  
+ CCTAGCACGG GAAAACCTTG TGCAATTCGC CAATGATGTT GGTGTTGCTT TTGAGCTGCA AGTTGTGAAC   
  
  
+ TTGGATTTAT TTGATCCATC TTCATCTTCA ATGCCAAATG TTGGAACCTC TGGGGATGAG TTGATCGCTG   
  
  
+ TTAGTATACC CATTTGGGCA TGTTCATATC GGCCGTCTAT TCTCCCATCC ATCCTCCGGT TCATTAAGCA   
  
  
+ ACTAGCCCCC AAGATTGTCG TCTCGTTAGA TAGAGGGTCT GATCGTTGCG ACACTCTGTT CCCACAACAT   
  
  
+ CTTATCCATA TCTTAGAGTC CTGCACAAAT TTCTTGGAAT CGCTCGACGG TCTCAATGTA GCATCAGATA   
  
  
+ TTGTAAACAA GGTTGAGAAG TACTTCCTCC AGCCTAAGAT CGAAAACACC GTGTTGGGCC GCGTTCATGC   
  
  
+ CCCCAATAAG ATGCCACATT GGAAGACCCT CTTTGCTTCA GCCGGGTTAT TGCCCTTTCA GTTCAGTAAC   
  
  
+ TTCACAGAAA CACAAGCTGA TTATGTGGTG AAGCGAACCC CAGGGAGGGG ATTTCACATA GAAAAGCGCC   
  
  
+ ACGCATCTTT AGTGCTAAGT TGGCAAAGGC GGGAGCTTGT GACAGCCTCG GCATGGAGGT TTGAGAAGCT   
  
  
+ GTA  

- +Up\_Stream \_Len000TGTAAC CCACCCTAAA TTTAGGAGAT AAAAAAGAAT CCCCTTTTTA TGAACCCTTT   
  
  
- AAAGAGTTAT TGACTTTTAG ATTTATTAAG CACTGAAACA ATCATCCAAA TTACACTAAA TGATGGCACT   
  
  
- CACAGTGAAA ATCTACCATG AAATTCTTTC GTAATATTCA TTTTGTTATA TATAGCATCG TAGTATGATG   
  
  
- AACACATTAT TTAATTGTCG TTGGAGTTAA CATGAATAAA CTAAAGCGTA ACATTAGTTA AAAGGGTTTA   
  
  
- CTAAATGATA GAACCAGAAT GAGTCAACAT GAAGTGTGAG TTATCAAGTT AATAAAAAGT AATCTGATTA   
  
  
- CTCGCTCAGG GAAGATGTTC TCGGATGATG ATGAGATTAT TACATTCCTG ATAACCGATT ACCCCTTGAT   
  
  
- TCGGATGCTG AACAATCCGT ATCCACTTAA AAGGAATGTT CTCTCAAATT AAATGTGAAC TATAATTGAG   
  
  
- CTCTTGCTCT TCGACAGAAT TATCTATGGG TGTATCTTTT ACTTCGTTTA CGGGAACTAA TAACCTCCCA   
  
  
- ACCCGTTTAT ATAAGTCATT GTTACAACAA ACCGTTTATA GCAGACTTTT AACAAAAACT GTTTATAACA   
  
  
- AACCTTCTTA AAAAGCTCTA ATTTATAATT TTTACAATTT TAAAATTAGA TTTATAGTTT GTTTATAACT   
  
  
- TTTTACGAAA TATAATAGAT TTAGAGATTA ATTTTAAACA CATAACAGGT GATCTTTTTA CATATCACTA   
  
  
- TAATGGCTGT ATATACACTA GAGACCACAT TATCGGATGG GATTACAGAT CCTTTTATAA ATTTTAGGGG   
  
  
- TCACCAGTTT TCCTTCAATG CGTGTGATCC CGTTCTGTGA AGTAATCCGA TCAACCACTT TCGATTCAGT   
  
  
- ACTTTAAATC TGGTACCGGT ACAGATGAAC ATCTAGGTTG ATGGTTGTTA GGTAAAAATA AAACCCCTTT   
  
  
- CGAACTTCCC ATTAAACCAT TTACATGTGC CCTTGGAAAA CGATATTTTC TTCACGAAGA ACTTTTTTGT   
  
  
- GGGACATCAT TTTGTCCCGT TTTTTCGGTT GTGAAGACAA AGACCGTCTC TTTGTCATCA CATGGTGAAC   
  
  
- CTCAACCTCA TCTGTGCTAT GTGTTTGGGT CATCTCTCTC TTTGTCCCCT CTCTTTCTCA TGTGTTTAAA   
  
  
- CACGTCCTCT TCCCATTCCT TCTTCCTCTT CCTTGGGGGA GGTTTTTTTT GGGTATATTT TTTATTATTT   
  
  
- CTTTTTTGTC CTCACTCAAT CTCCCATGGG TTTAGAGGTT AGATTTTGAT CTTAGATCCC AGAGAAAAGT   
  
  
- AAACGAACTT TTTAAAGACA AGTACCCAAA ACACAAAAAT CCCCCTAAAC AAACTAAAAA CATCAGAAAC   
  
  
- ACCTAAAGGA CACAATATCT ACGTTCGATA CGAGAAGTTA AACGTTCCTT GTCGTAGACC CCCCTCTCTT   
  
  
- AAACTCCTTA AAGTTCATGG GTAAAGTTAA AGTTGTTCAT TTTAGTAGTA ACTTCCTATG AGTTTGGGTT   
  
  
- CGGGTCAACT ATCACTTGGT TGAAGATGAG ACCTAAGATG AGGTTCAGGG TCGGGAGGGT GGGAGAGGAG   
  
  
- GAGGAGGTTC TTGAGGATGG TTTTGCGACG GCAAAACCGA GTGGGAGGAG TCGAAGTGGT ACCTAGACTA   
  
  
- TTACACATTC TGGGGTTGGG AAAACGAAAA TAAAAGGAGT ACGAGGTAAG AGTACTAAAC ATGAAGACAA   
  
  
- TACATTAACC TCTACACAGA TTTAAACCTT TTCAAGCACC CAAAGCACAA ACTCTTAAAT CTTACTGTAA   
  
  
- GAAATTAATC ATTAAACTTC AAACGTACGA ATTAAATCGA TTAGTGACTA ATTAATTTTA TTTACTTCAC   
  
  
- TACATCCATA CCCGACAATG TGTTACAGTG GCCACCACCA CTGTCGCCGC CCCGCCGCCG CCAACCTAAC   
  
  
- CTTCTTACCC TGTCGCACAA AGGTTTACCC CTACCTCGAA ATGAGGGAAC CTAGTACCCC CTTCCACTAC   
  
  
- TAAACCCAGA CTTAAACTTC GTAGAGAATG TTAGACCAAT GGGACAACTC ATACTTCCGT TACGTCCTGA   
  
  
- ACCACAACAA CTAGTCTGTA GAGAAGAACC TCCACCACTA AACCCCAAAA GACCAAGACC CAGACACTTA   
  
  
- TTACCGTTTT AACCAAGGTA AAACCCATTA ACAAGTAGAC CCCTAAACCT AAGATTCCAA AGATCACTGC   
  
  
- CCAACTTAAG GTTAACATCA AGTGACCCCT CATACTCATA GGAACCAAGT TAGGTACCTG GAAACGGATT   
  
  
- ATGACCACCC AAACACAAGG GTGTTTGTGT TGATAAACTA GAACCACTAC TCTTCGGAGT CTAAAACTTG   
  
  
- GGTGTTAACT ACTGCTACTT AGGTTTAGTC CGAGTCTCGT ACCCTTTGGG ATCGAAAAAA CACGGGAGGA   
  
  
- ACCCAATAAC AGTCAATCTC GTTGTATACC AAGTTGGTGT CCGCTTTGCG GTATCGGGAC CACATCAAAA   
  
  
- CTTGAGACTA CAATAACGGT TTTGGGGCAA AGAGCTAGTC CCAGTACTCA AAAACAACTC CTTTGTTGTG   
  
  
- GTAGTTGTCT AAAACGTCGT TGAAGGATAC CCAAACCGAG TAGTCAGCCA GGGAGTCGTG GAGGTCGTTT   
  
  
- TCCGCTACTT TCTGGTCCAA CAATTCCTGG TCGAAAACTT CCGTCGACTG GACTAAGTTT GACCCTTAAA   
  
  
- GAGTGAACGC GTTCTCTATA ACCGCGCCGA GTTAGTGGTC GAGAGGGAAG GACGCTTCGG GGAGTAATCC   
  
  
- CGTCGAAACA TACAGTACCT CCGGAACGTT TACGAGTAGA ATTACTCGTT AGGACATCGT GGAGGTGGCT   
  
  
- ACTTCTGCGA AAGGGGATAT CTACAACAAG TATTCTACTT ACGGATGTTC CGGAAGAGAC TCCAGAGAGG   
  
  
- ATAATGAGTC AAATGCTTAA AGTGAACACG GGTTCGGTAA GAACTTCGAG AGCTACTACG CCTAACGCAA   
  
  
- GTACAGTAAC TGAAACTATA ACCAACACCC CGAGTTACCC GTAGTGACTA AGTCCTCGAA GGTAACTCCT   
  
  
- TTTTCCCTCG AGGTAGGGAC TTTTAATGTC GGTATCGAGG GGACAGTTAT TCGGTGTGAA AACTTGAGTT   
  
  
- GGATCGTGCC CTTTTGGAAC ACGTTAAGCG GTTACTACAA CCACAACGAA AACTCGACGT TCAACACTTG   
  
  
- AACCTAAATA AACTAGGTAG AAGTAGAAGT TACGGTTTAC AACCTTGGAG ACCCCTACTC AACTAGCGAC   
  
  
- AATCATATGG GTAAACCCGT ACAAGTATAG CCGGCAGATA AGAGGGTAGG TAGGAGGCCA AGTAATTCGT   
  
  
- TGATCGGGGG TTCTAACAGC AGAGCAATCT ATCTCCCAGA CTAGCAACGC TGTGAGACAA GGGTGTTGTA   
  
  
- GAATAGGTAT AGAATCTCAG GACGTGTTTA AAGAACCTTA GCGAGCTGCC AGAGTTACAT CGTAGTCTAT   
  
  
- AACATTTGTT CCAACTCTTC ATGAAGGAGG TCGGATTCTA GCTTTTGTGG CACAACCCGG CGCAAGTACG   
  
  
- GGGGTTATTC TACGGTGTAA CCTTCTGGGA GAAACGAAGT CGGCCCAATA ACGGGAAAGT CAAGTCATTG   
  
  
- AAGTGTCTTT GTGTTCGACT AATACACCAC TTCGCTTGGG GTCCCTCCCC TAAAGTGTAT CTTTTCGCGG   
  
  
- TGCGTAGAAA TCACGATTCA ACCGTTTCCG CCCTCGAACA CTGTCGGAGC CGTACCTCCA AACTCTTCGA   
  
  
- CAT

+     MYB

| Site Name | Organism | Position | Strand | Matrix score. | sequence | function |
| --- | --- | --- | --- | --- | --- | --- |
| MYB | Arabidopsis thaliana | 2487 | + | 6 | CAACCA |  |
| MYB | Arabidopsis thaliana | 3035 | - | 6 | CAACCA |  |
| MYB | Arabidopsis thaliana | 2077 | - | 6 | CAACAG |  |
| MYB | Arabidopsis thaliana | 2069 | - | 6 | TAACCA |  |
| MYB | Arabidopsis thaliana | 2598 | + | 6 | CAACAG |  |

>HU05G00466.1   
+ +Up\_Stream \_Len000ACATTG GGTGGGATTT AAATCCTCTA TTTTTTCTTA GGGGAAAAAT ACTTGGGAAA   
  
  
+ TTTCTCAATA ACTGAAAATC TAAATAATTC GTGACTTTGT TAGTAGGTTT AATGTGATTT ACTACCGTGA   
  
  
+ GTGTCACTTT TAGATGGTAC TTTAAGAAAG CATTATAAGT AAAACAATAT ATATCGTAGC ATCATACTAC   
  
  
+ TTGTGTAATA AATTAACAGC AACCTCAATT GTACTTATTT GATTTCGCAT TGTAATCAAT TTTCCCAAAT   
  
  
+ GATTTACTAT CTTGGTCTTA CTCAGTTGTA CTTCACACTC AATAGTTCAA TTATTTTTCA TTAGACTAAT   
  
  
+ GAGCGAGTCC CTTCTACAAG AGCCTACTAC TACTCTAATA ATGTAAGGAC TATTGGCTAA TGGGGAACTA   
  
  
+ AGCCTACGAC TTGTTAGGCA TAGGTGAATT TTCCTTACAA GAGAGTTTAA TTTACACTTG ATATTAACTC   
  
  
+ GAGAACGAGA AGCTGTCTTA ATAGATACCC ACATAGAAAA TGAAGCAAAT GCCCTTGATT ATTGGAGGGT   
  
  
+ TGGGCAAATA TATTCAGTAA CAATGTTGTT TGGCAAATAT CGTCTGAAAA TTGTTTTTGA CAAATATTGT   
  
  
+ TTGGAAGAAT TTTTCGAGAT TAAATATTAA AAATGTTAAA ATTTTAATCT AAATATCAAA CAAATATTGA   
  
  
+ AAAATGCTTT ATATTATCTA AATCTCTAAT TAAAATTTGT GTATTGTCCA CTAGAAAAAT GTATAGTGAT   
  
  
+ ATTACCGACA TATATGTGAT CTCTGGTGTA ATAGCCTACC CTAATGTCTA GGAAAATATT TAAAATCCCC   
  
  
+ AGTGGTCAAA AGGAAGTTAC GCACACTAGG GCAAGACACT TCATTAGGCT AGTTGGTGAA AGCTAAGTCA   
  
  
+ TGAAATTTAG ACCATGGCCA TGTCTACTTG TAGATCCAAC TACCAACAAT CCATTTTTAT TTTGGGGAAA   
  
  
+ GCTTGAAGGG TAATTTGGTA AATGTACACG GGAACCTTTT GCTATAAAAG AAGTGCTTCT TGAAAAAACA   
  
  
+ CCCTGTAGTA AAACAGGGCA AAAAAGCCAA CACTTCTGTT TCTGGCAGAG AAACAGTAGT GTACCACTTG   
  
  
+ GAGTTGGAGT AGACACGATA CACAAACCCA GTAGAGAGAG AAACAGGGGA GAGAAAGAGT ACACAAATTT   
  
  
+ GTGCAGGAGA AGGGTAAGGA AGAAGGAGAA GGAACCCCCT CCAAAAAAAA CCCATATAAA AAATAATAAA   
  
  
+ GAAAAAACAG GAGTGAGTTA GAGGGTACCC AAATCTCCAA TCTAAAACTA GAATCTAGGG TCTCTTTTCA   
  
  
+ TTTGCTTGAA AAATTTCTGT TCATGGGTTT TGTGTTTTTA GGGGGATTTG TTTGATTTTT GTAGTCTTTG   
  
  
+ TGGATTTCCT GTGTTATAGA TGCAAGCTAT GCTCTTCAAT TTGCAAGGAA CAGCATCTGG GGGGAGAGAA   
  
  
+ TTTGAGGAAT TTCAAGTACC CATTTCAATT TCAACAAGTA AAATCATCAT TGAAGGATAC TCAAACCCAA   
  
  
+ GCCCAGTTGA TAGTGAACCA ACTTCTACTC TGGATTCTAC TCCAAGTCCC AGCCCTCCCA CCCTCTCCTC   
  
  
+ CTCCTCCAAG AACTCCTACC AAAACGCTGC CGTTTTGGCT CACCCTCCTC AGCTTCACCA TGGATCTGAT   
  
  
+ AATGTGTAAG ACCCCAACCC TTTTGCTTTT ATTTTCCTCA TGCTCCATTC TCATGATTTG TACTTCTGTT   
  
  
+ ATGTAATTGG AGATGTGTCT AAATTTGGAA AAGTTCGTGG GTTTCGTGTT TGAGAATTTA GAATGACATT   
  
  
+ CTTTAATTAG TAATTTGAAG TTTGCATGCT TAATTTAGCT AATCACTGAT TAATTAAAAT AAATGAAGTG   
  
  
+ ATGTAGGTAT GGGCTGTTAC ACAATGTCAC CGGTGGTGGT GACAGCGGCG GGGCGGCGGC GGTTGGATTG   
  
  
+ GAAGAATGGG ACAGCGTGTT TCCAAATGGG GATGGAGCTT TACTCCCTTG GATCATGGGG GAAGGTGATG   
  
  
+ ATTTGGGTCT GAATTTGAAG CATCTCTTAC AATCTGGTTA CCCTGTTGAG TATGAAGGCA ATGCAGGACT   
  
  
+ TGGTGTTGTT GATCAGACAT CTCTTCTTGG AGGTGGTGAT TTGGGGTTTT CTGGTTCTGG GTCTGTGAAT   
  
  
+ AATGGCAAAA TTGGTTCCAT TTTGGGTAAT TGTTCATCTG GGGATTTGGA TTCTAAGGTT TCTAGTGACG   
  
  
+ GGTTGAATTC CAATTGTAGT TCACTGGGGA GTATGAGTAT CCTTGGTTCA ATCCATGGAC CTTTGCCTAA   
  
  
+ TACTGGTGGG TTTGTGTTCC CACAAACACA ACTATTTGAT CTTGGTGATG AGAAGCCTCA GATTTTGAAC   
  
  
+ CCACAATTGA TGACGATGAA TCCAAATCAG GCTCAGAGCA TGGGAAACCC TAGCTTTTTT GTGCCCTCCT   
  
  
+ TGGGTTATTG TCAGTTAGAG CAACATATGG TTCAACCACA GGCGAAACGC CATAGCCCTG GTGTAGTTTT   
  
  
+ GAACTCTGAT GTTATTGCCA AAACCCCGTT TCTCGATCAG GGTCATGAGT TTTTGTTGAG GAAACAACAC   
  
  
+ CATCAACAGA TTTTGCAGCA ACTTCCTATG GGTTTGGCTC ATCAGTCGGT CCCTCAGCAC CTCCAGCAAA   
  
  
+ AGGCGATGAA AGACCAGGTT GTTAAGGACC AGCTTTTGAA GGCAGCTGAC CTGATTCAAA CTGGGAATTT   
  
  
+ CTCACTTGCG CAAGAGATAT TGGCGCGGCT CAATCACCAG CTCTCCCTTC CTGCGAAGCC CCTCATTAGG   
  
  
+ GCAGCTTTGT ATGTCATGGA GGCCTTGCAA ATGCTCATCT TAATGAGCAA TCCTGTAGCA CCTCCACCGA   
  
  
+ TGAAGACGCT TTCCCCTATA GATGTTGTTC ATAAGATGAA TGCCTACAAG GCCTTCTCTG AGGTCTCTCC   
  
  
+ TATTACTCAG TTTACGAATT TCACTTGTGC CCAAGCCATT CTTGAAGCTC TCGATGATGC GGATTGCGTT   
  
  
+ CATGTCATTG ACTTTGATAT TGGTTGTGGG GCTCAATGGG CATCACTGAT TCAGGAGCTT CCATTGAGGA   
  
  
+ AAAAGGGAGC TCCATCCCTG AAAATTACAG CCATAGCTCC CCTGTCAATA AGCCACACTT TTGAACTCAA   
  
  
+ CCTAGCACGG GAAAACCTTG TGCAATTCGC CAATGATGTT GGTGTTGCTT TTGAGCTGCA AGTTGTGAAC   
  
  
+ TTGGATTTAT TTGATCCATC TTCATCTTCA ATGCCAAATG TTGGAACCTC TGGGGATGAG TTGATCGCTG   
  
  
+ TTAGTATACC CATTTGGGCA TGTTCATATC GGCCGTCTAT TCTCCCATCC ATCCTCCGGT TCATTAAGCA   
  
  
+ ACTAGCCCCC AAGATTGTCG TCTCGTTAGA TAGAGGGTCT GATCGTTGCG ACACTCTGTT CCCACAACAT   
  
  
+ CTTATCCATA TCTTAGAGTC CTGCACAAAT TTCTTGGAAT CGCTCGACGG TCTCAATGTA GCATCAGATA   
  
  
+ TTGTAAACAA GGTTGAGAAG TACTTCCTCC AGCCTAAGAT CGAAAACACC GTGTTGGGCC GCGTTCATGC   
  
  
+ CCCCAATAAG ATGCCACATT GGAAGACCCT CTTTGCTTCA GCCGGGTTAT TGCCCTTTCA GTTCAGTAAC   
  
  
+ TTCACAGAAA CACAAGCTGA TTATGTGGTG AAGCGAACCC CAGGGAGGGG ATTTCACATA GAAAAGCGCC   
  
  
+ ACGCATCTTT AGTGCTAAGT TGGCAAAGGC GGGAGCTTGT GACAGCCTCG GCATGGAGGT TTGAGAAGCT   
  
  
+ GTA  

- +Up\_Stream \_Len000TGTAAC CCACCCTAAA TTTAGGAGAT AAAAAAGAAT CCCCTTTTTA TGAACCCTTT   
  
  
- AAAGAGTTAT TGACTTTTAG ATTTATTAAG CACTGAAACA ATCATCCAAA TTACACTAAA TGATGGCACT   
  
  
- CACAGTGAAA ATCTACCATG AAATTCTTTC GTAATATTCA TTTTGTTATA TATAGCATCG TAGTATGATG   
  
  
- AACACATTAT TTAATTGTCG TTGGAGTTAA CATGAATAAA CTAAAGCGTA ACATTAGTTA AAAGGGTTTA   
  
  
- CTAAATGATA GAACCAGAAT GAGTCAACAT GAAGTGTGAG TTATCAAGTT AATAAAAAGT AATCTGATTA   
  
  
- CTCGCTCAGG GAAGATGTTC TCGGATGATG ATGAGATTAT TACATTCCTG ATAACCGATT ACCCCTTGAT   
  
  
- TCGGATGCTG AACAATCCGT ATCCACTTAA AAGGAATGTT CTCTCAAATT AAATGTGAAC TATAATTGAG   
  
  
- CTCTTGCTCT TCGACAGAAT TATCTATGGG TGTATCTTTT ACTTCGTTTA CGGGAACTAA TAACCTCCCA   
  
  
- ACCCGTTTAT ATAAGTCATT GTTACAACAA ACCGTTTATA GCAGACTTTT AACAAAAACT GTTTATAACA   
  
  
- AACCTTCTTA AAAAGCTCTA ATTTATAATT TTTACAATTT TAAAATTAGA TTTATAGTTT GTTTATAACT   
  
  
- TTTTACGAAA TATAATAGAT TTAGAGATTA ATTTTAAACA CATAACAGGT GATCTTTTTA CATATCACTA   
  
  
- TAATGGCTGT ATATACACTA GAGACCACAT TATCGGATGG GATTACAGAT CCTTTTATAA ATTTTAGGGG   
  
  
- TCACCAGTTT TCCTTCAATG CGTGTGATCC CGTTCTGTGA AGTAATCCGA TCAACCACTT TCGATTCAGT   
  
  
- ACTTTAAATC TGGTACCGGT ACAGATGAAC ATCTAGGTTG ATGGTTGTTA GGTAAAAATA AAACCCCTTT   
  
  
- CGAACTTCCC ATTAAACCAT TTACATGTGC CCTTGGAAAA CGATATTTTC TTCACGAAGA ACTTTTTTGT   
  
  
- GGGACATCAT TTTGTCCCGT TTTTTCGGTT GTGAAGACAA AGACCGTCTC TTTGTCATCA CATGGTGAAC   
  
  
- CTCAACCTCA TCTGTGCTAT GTGTTTGGGT CATCTCTCTC TTTGTCCCCT CTCTTTCTCA TGTGTTTAAA   
  
  
- CACGTCCTCT TCCCATTCCT TCTTCCTCTT CCTTGGGGGA GGTTTTTTTT GGGTATATTT TTTATTATTT   
  
  
- CTTTTTTGTC CTCACTCAAT CTCCCATGGG TTTAGAGGTT AGATTTTGAT CTTAGATCCC AGAGAAAAGT   
  
  
- AAACGAACTT TTTAAAGACA AGTACCCAAA ACACAAAAAT CCCCCTAAAC AAACTAAAAA CATCAGAAAC   
  
  
- ACCTAAAGGA CACAATATCT ACGTTCGATA CGAGAAGTTA AACGTTCCTT GTCGTAGACC CCCCTCTCTT   
  
  
- AAACTCCTTA AAGTTCATGG GTAAAGTTAA AGTTGTTCAT TTTAGTAGTA ACTTCCTATG AGTTTGGGTT   
  
  
- CGGGTCAACT ATCACTTGGT TGAAGATGAG ACCTAAGATG AGGTTCAGGG TCGGGAGGGT GGGAGAGGAG   
  
  
- GAGGAGGTTC TTGAGGATGG TTTTGCGACG GCAAAACCGA GTGGGAGGAG TCGAAGTGGT ACCTAGACTA   
  
  
- TTACACATTC TGGGGTTGGG AAAACGAAAA TAAAAGGAGT ACGAGGTAAG AGTACTAAAC ATGAAGACAA   
  
  
- TACATTAACC TCTACACAGA TTTAAACCTT TTCAAGCACC CAAAGCACAA ACTCTTAAAT CTTACTGTAA   
  
  
- GAAATTAATC ATTAAACTTC AAACGTACGA ATTAAATCGA TTAGTGACTA ATTAATTTTA TTTACTTCAC   
  
  
- TACATCCATA CCCGACAATG TGTTACAGTG GCCACCACCA CTGTCGCCGC CCCGCCGCCG CCAACCTAAC   
  
  
- CTTCTTACCC TGTCGCACAA AGGTTTACCC CTACCTCGAA ATGAGGGAAC CTAGTACCCC CTTCCACTAC   
  
  
- TAAACCCAGA CTTAAACTTC GTAGAGAATG TTAGACCAAT GGGACAACTC ATACTTCCGT TACGTCCTGA   
  
  
- ACCACAACAA CTAGTCTGTA GAGAAGAACC TCCACCACTA AACCCCAAAA GACCAAGACC CAGACACTTA   
  
  
- TTACCGTTTT AACCAAGGTA AAACCCATTA ACAAGTAGAC CCCTAAACCT AAGATTCCAA AGATCACTGC   
  
  
- CCAACTTAAG GTTAACATCA AGTGACCCCT CATACTCATA GGAACCAAGT TAGGTACCTG GAAACGGATT   
  
  
- ATGACCACCC AAACACAAGG GTGTTTGTGT TGATAAACTA GAACCACTAC TCTTCGGAGT CTAAAACTTG   
  
  
- GGTGTTAACT ACTGCTACTT AGGTTTAGTC CGAGTCTCGT ACCCTTTGGG ATCGAAAAAA CACGGGAGGA   
  
  
- ACCCAATAAC AGTCAATCTC GTTGTATACC AAGTTGGTGT CCGCTTTGCG GTATCGGGAC CACATCAAAA   
  
  
- CTTGAGACTA CAATAACGGT TTTGGGGCAA AGAGCTAGTC CCAGTACTCA AAAACAACTC CTTTGTTGTG   
  
  
- GTAGTTGTCT AAAACGTCGT TGAAGGATAC CCAAACCGAG TAGTCAGCCA GGGAGTCGTG GAGGTCGTTT   
  
  
- TCCGCTACTT TCTGGTCCAA CAATTCCTGG TCGAAAACTT CCGTCGACTG GACTAAGTTT GACCCTTAAA   
  
  
- GAGTGAACGC GTTCTCTATA ACCGCGCCGA GTTAGTGGTC GAGAGGGAAG GACGCTTCGG GGAGTAATCC   
  
  
- CGTCGAAACA TACAGTACCT CCGGAACGTT TACGAGTAGA ATTACTCGTT AGGACATCGT GGAGGTGGCT   
  
  
- ACTTCTGCGA AAGGGGATAT CTACAACAAG TATTCTACTT ACGGATGTTC CGGAAGAGAC TCCAGAGAGG   
  
  
- ATAATGAGTC AAATGCTTAA AGTGAACACG GGTTCGGTAA GAACTTCGAG AGCTACTACG CCTAACGCAA   
  
  
- GTACAGTAAC TGAAACTATA ACCAACACCC CGAGTTACCC GTAGTGACTA AGTCCTCGAA GGTAACTCCT   
  
  
- TTTTCCCTCG AGGTAGGGAC TTTTAATGTC GGTATCGAGG GGACAGTTAT TCGGTGTGAA AACTTGAGTT   
  
  
- GGATCGTGCC CTTTTGGAAC ACGTTAAGCG GTTACTACAA CCACAACGAA AACTCGACGT TCAACACTTG   
  
  
- AACCTAAATA AACTAGGTAG AAGTAGAAGT TACGGTTTAC AACCTTGGAG ACCCCTACTC AACTAGCGAC   
  
  
- AATCATATGG GTAAACCCGT ACAAGTATAG CCGGCAGATA AGAGGGTAGG TAGGAGGCCA AGTAATTCGT   
  
  
- TGATCGGGGG TTCTAACAGC AGAGCAATCT ATCTCCCAGA CTAGCAACGC TGTGAGACAA GGGTGTTGTA   
  
  
- GAATAGGTAT AGAATCTCAG GACGTGTTTA AAGAACCTTA GCGAGCTGCC AGAGTTACAT CGTAGTCTAT   
  
  
- AACATTTGTT CCAACTCTTC ATGAAGGAGG TCGGATTCTA GCTTTTGTGG CACAACCCGG CGCAAGTACG   
  
  
- GGGGTTATTC TACGGTGTAA CCTTCTGGGA GAAACGAAGT CGGCCCAATA ACGGGAAAGT CAAGTCATTG   
  
  
- AAGTGTCTTT GTGTTCGACT AATACACCAC TTCGCTTGGG GTCCCTCCCC TAAAGTGTAT CTTTTCGCGG   
  
  
- TGCGTAGAAA TCACGATTCA ACCGTTTCCG CCCTCGAACA CTGTCGGAGC CGTACCTCCA AACTCTTCGA   
  
  
- CAT

+     MYB-like sequence

| Site Name | Organism | Position | Strand | Matrix score. | sequence | function |
| --- | --- | --- | --- | --- | --- | --- |
| MYB-like sequence | Arabidopsis thaliana | 2069 | - | 6 | TAACCA |  |

>HU05G00466.1   
+ +Up\_Stream \_Len000ACATTG GGTGGGATTT AAATCCTCTA TTTTTTCTTA GGGGAAAAAT ACTTGGGAAA   
  
  
+ TTTCTCAATA ACTGAAAATC TAAATAATTC GTGACTTTGT TAGTAGGTTT AATGTGATTT ACTACCGTGA   
  
  
+ GTGTCACTTT TAGATGGTAC TTTAAGAAAG CATTATAAGT AAAACAATAT ATATCGTAGC ATCATACTAC   
  
  
+ TTGTGTAATA AATTAACAGC AACCTCAATT GTACTTATTT GATTTCGCAT TGTAATCAAT TTTCCCAAAT   
  
  
+ GATTTACTAT CTTGGTCTTA CTCAGTTGTA CTTCACACTC AATAGTTCAA TTATTTTTCA TTAGACTAAT   
  
  
+ GAGCGAGTCC CTTCTACAAG AGCCTACTAC TACTCTAATA ATGTAAGGAC TATTGGCTAA TGGGGAACTA   
  
  
+ AGCCTACGAC TTGTTAGGCA TAGGTGAATT TTCCTTACAA GAGAGTTTAA TTTACACTTG ATATTAACTC   
  
  
+ GAGAACGAGA AGCTGTCTTA ATAGATACCC ACATAGAAAA TGAAGCAAAT GCCCTTGATT ATTGGAGGGT   
  
  
+ TGGGCAAATA TATTCAGTAA CAATGTTGTT TGGCAAATAT CGTCTGAAAA TTGTTTTTGA CAAATATTGT   
  
  
+ TTGGAAGAAT TTTTCGAGAT TAAATATTAA AAATGTTAAA ATTTTAATCT AAATATCAAA CAAATATTGA   
  
  
+ AAAATGCTTT ATATTATCTA AATCTCTAAT TAAAATTTGT GTATTGTCCA CTAGAAAAAT GTATAGTGAT   
  
  
+ ATTACCGACA TATATGTGAT CTCTGGTGTA ATAGCCTACC CTAATGTCTA GGAAAATATT TAAAATCCCC   
  
  
+ AGTGGTCAAA AGGAAGTTAC GCACACTAGG GCAAGACACT TCATTAGGCT AGTTGGTGAA AGCTAAGTCA   
  
  
+ TGAAATTTAG ACCATGGCCA TGTCTACTTG TAGATCCAAC TACCAACAAT CCATTTTTAT TTTGGGGAAA   
  
  
+ GCTTGAAGGG TAATTTGGTA AATGTACACG GGAACCTTTT GCTATAAAAG AAGTGCTTCT TGAAAAAACA   
  
  
+ CCCTGTAGTA AAACAGGGCA AAAAAGCCAA CACTTCTGTT TCTGGCAGAG AAACAGTAGT GTACCACTTG   
  
  
+ GAGTTGGAGT AGACACGATA CACAAACCCA GTAGAGAGAG AAACAGGGGA GAGAAAGAGT ACACAAATTT   
  
  
+ GTGCAGGAGA AGGGTAAGGA AGAAGGAGAA GGAACCCCCT CCAAAAAAAA CCCATATAAA AAATAATAAA   
  
  
+ GAAAAAACAG GAGTGAGTTA GAGGGTACCC AAATCTCCAA TCTAAAACTA GAATCTAGGG TCTCTTTTCA   
  
  
+ TTTGCTTGAA AAATTTCTGT TCATGGGTTT TGTGTTTTTA GGGGGATTTG TTTGATTTTT GTAGTCTTTG   
  
  
+ TGGATTTCCT GTGTTATAGA TGCAAGCTAT GCTCTTCAAT TTGCAAGGAA CAGCATCTGG GGGGAGAGAA   
  
  
+ TTTGAGGAAT TTCAAGTACC CATTTCAATT TCAACAAGTA AAATCATCAT TGAAGGATAC TCAAACCCAA   
  
  
+ GCCCAGTTGA TAGTGAACCA ACTTCTACTC TGGATTCTAC TCCAAGTCCC AGCCCTCCCA CCCTCTCCTC   
  
  
+ CTCCTCCAAG AACTCCTACC AAAACGCTGC CGTTTTGGCT CACCCTCCTC AGCTTCACCA TGGATCTGAT   
  
  
+ AATGTGTAAG ACCCCAACCC TTTTGCTTTT ATTTTCCTCA TGCTCCATTC TCATGATTTG TACTTCTGTT   
  
  
+ ATGTAATTGG AGATGTGTCT AAATTTGGAA AAGTTCGTGG GTTTCGTGTT TGAGAATTTA GAATGACATT   
  
  
+ CTTTAATTAG TAATTTGAAG TTTGCATGCT TAATTTAGCT AATCACTGAT TAATTAAAAT AAATGAAGTG   
  
  
+ ATGTAGGTAT GGGCTGTTAC ACAATGTCAC CGGTGGTGGT GACAGCGGCG GGGCGGCGGC GGTTGGATTG   
  
  
+ GAAGAATGGG ACAGCGTGTT TCCAAATGGG GATGGAGCTT TACTCCCTTG GATCATGGGG GAAGGTGATG   
  
  
+ ATTTGGGTCT GAATTTGAAG CATCTCTTAC AATCTGGTTA CCCTGTTGAG TATGAAGGCA ATGCAGGACT   
  
  
+ TGGTGTTGTT GATCAGACAT CTCTTCTTGG AGGTGGTGAT TTGGGGTTTT CTGGTTCTGG GTCTGTGAAT   
  
  
+ AATGGCAAAA TTGGTTCCAT TTTGGGTAAT TGTTCATCTG GGGATTTGGA TTCTAAGGTT TCTAGTGACG   
  
  
+ GGTTGAATTC CAATTGTAGT TCACTGGGGA GTATGAGTAT CCTTGGTTCA ATCCATGGAC CTTTGCCTAA   
  
  
+ TACTGGTGGG TTTGTGTTCC CACAAACACA ACTATTTGAT CTTGGTGATG AGAAGCCTCA GATTTTGAAC   
  
  
+ CCACAATTGA TGACGATGAA TCCAAATCAG GCTCAGAGCA TGGGAAACCC TAGCTTTTTT GTGCCCTCCT   
  
  
+ TGGGTTATTG TCAGTTAGAG CAACATATGG TTCAACCACA GGCGAAACGC CATAGCCCTG GTGTAGTTTT   
  
  
+ GAACTCTGAT GTTATTGCCA AAACCCCGTT TCTCGATCAG GGTCATGAGT TTTTGTTGAG GAAACAACAC   
  
  
+ CATCAACAGA TTTTGCAGCA ACTTCCTATG GGTTTGGCTC ATCAGTCGGT CCCTCAGCAC CTCCAGCAAA   
  
  
+ AGGCGATGAA AGACCAGGTT GTTAAGGACC AGCTTTTGAA GGCAGCTGAC CTGATTCAAA CTGGGAATTT   
  
  
+ CTCACTTGCG CAAGAGATAT TGGCGCGGCT CAATCACCAG CTCTCCCTTC CTGCGAAGCC CCTCATTAGG   
  
  
+ GCAGCTTTGT ATGTCATGGA GGCCTTGCAA ATGCTCATCT TAATGAGCAA TCCTGTAGCA CCTCCACCGA   
  
  
+ TGAAGACGCT TTCCCCTATA GATGTTGTTC ATAAGATGAA TGCCTACAAG GCCTTCTCTG AGGTCTCTCC   
  
  
+ TATTACTCAG TTTACGAATT TCACTTGTGC CCAAGCCATT CTTGAAGCTC TCGATGATGC GGATTGCGTT   
  
  
+ CATGTCATTG ACTTTGATAT TGGTTGTGGG GCTCAATGGG CATCACTGAT TCAGGAGCTT CCATTGAGGA   
  
  
+ AAAAGGGAGC TCCATCCCTG AAAATTACAG CCATAGCTCC CCTGTCAATA AGCCACACTT TTGAACTCAA   
  
  
+ CCTAGCACGG GAAAACCTTG TGCAATTCGC CAATGATGTT GGTGTTGCTT TTGAGCTGCA AGTTGTGAAC   
  
  
+ TTGGATTTAT TTGATCCATC TTCATCTTCA ATGCCAAATG TTGGAACCTC TGGGGATGAG TTGATCGCTG   
  
  
+ TTAGTATACC CATTTGGGCA TGTTCATATC GGCCGTCTAT TCTCCCATCC ATCCTCCGGT TCATTAAGCA   
  
  
+ ACTAGCCCCC AAGATTGTCG TCTCGTTAGA TAGAGGGTCT GATCGTTGCG ACACTCTGTT CCCACAACAT   
  
  
+ CTTATCCATA TCTTAGAGTC CTGCACAAAT TTCTTGGAAT CGCTCGACGG TCTCAATGTA GCATCAGATA   
  
  
+ TTGTAAACAA GGTTGAGAAG TACTTCCTCC AGCCTAAGAT CGAAAACACC GTGTTGGGCC GCGTTCATGC   
  
  
+ CCCCAATAAG ATGCCACATT GGAAGACCCT CTTTGCTTCA GCCGGGTTAT TGCCCTTTCA GTTCAGTAAC   
  
  
+ TTCACAGAAA CACAAGCTGA TTATGTGGTG AAGCGAACCC CAGGGAGGGG ATTTCACATA GAAAAGCGCC   
  
  
+ ACGCATCTTT AGTGCTAAGT TGGCAAAGGC GGGAGCTTGT GACAGCCTCG GCATGGAGGT TTGAGAAGCT   
  
  
+ GTA  

- +Up\_Stream \_Len000TGTAAC CCACCCTAAA TTTAGGAGAT AAAAAAGAAT CCCCTTTTTA TGAACCCTTT   
  
  
- AAAGAGTTAT TGACTTTTAG ATTTATTAAG CACTGAAACA ATCATCCAAA TTACACTAAA TGATGGCACT   
  
  
- CACAGTGAAA ATCTACCATG AAATTCTTTC GTAATATTCA TTTTGTTATA TATAGCATCG TAGTATGATG   
  
  
- AACACATTAT TTAATTGTCG TTGGAGTTAA CATGAATAAA CTAAAGCGTA ACATTAGTTA AAAGGGTTTA   
  
  
- CTAAATGATA GAACCAGAAT GAGTCAACAT GAAGTGTGAG TTATCAAGTT AATAAAAAGT AATCTGATTA   
  
  
- CTCGCTCAGG GAAGATGTTC TCGGATGATG ATGAGATTAT TACATTCCTG ATAACCGATT ACCCCTTGAT   
  
  
- TCGGATGCTG AACAATCCGT ATCCACTTAA AAGGAATGTT CTCTCAAATT AAATGTGAAC TATAATTGAG   
  
  
- CTCTTGCTCT TCGACAGAAT TATCTATGGG TGTATCTTTT ACTTCGTTTA CGGGAACTAA TAACCTCCCA   
  
  
- ACCCGTTTAT ATAAGTCATT GTTACAACAA ACCGTTTATA GCAGACTTTT AACAAAAACT GTTTATAACA   
  
  
- AACCTTCTTA AAAAGCTCTA ATTTATAATT TTTACAATTT TAAAATTAGA TTTATAGTTT GTTTATAACT   
  
  
- TTTTACGAAA TATAATAGAT TTAGAGATTA ATTTTAAACA CATAACAGGT GATCTTTTTA CATATCACTA   
  
  
- TAATGGCTGT ATATACACTA GAGACCACAT TATCGGATGG GATTACAGAT CCTTTTATAA ATTTTAGGGG   
  
  
- TCACCAGTTT TCCTTCAATG CGTGTGATCC CGTTCTGTGA AGTAATCCGA TCAACCACTT TCGATTCAGT   
  
  
- ACTTTAAATC TGGTACCGGT ACAGATGAAC ATCTAGGTTG ATGGTTGTTA GGTAAAAATA AAACCCCTTT   
  
  
- CGAACTTCCC ATTAAACCAT TTACATGTGC CCTTGGAAAA CGATATTTTC TTCACGAAGA ACTTTTTTGT   
  
  
- GGGACATCAT TTTGTCCCGT TTTTTCGGTT GTGAAGACAA AGACCGTCTC TTTGTCATCA CATGGTGAAC   
  
  
- CTCAACCTCA TCTGTGCTAT GTGTTTGGGT CATCTCTCTC TTTGTCCCCT CTCTTTCTCA TGTGTTTAAA   
  
  
- CACGTCCTCT TCCCATTCCT TCTTCCTCTT CCTTGGGGGA GGTTTTTTTT GGGTATATTT TTTATTATTT   
  
  
- CTTTTTTGTC CTCACTCAAT CTCCCATGGG TTTAGAGGTT AGATTTTGAT CTTAGATCCC AGAGAAAAGT   
  
  
- AAACGAACTT TTTAAAGACA AGTACCCAAA ACACAAAAAT CCCCCTAAAC AAACTAAAAA CATCAGAAAC   
  
  
- ACCTAAAGGA CACAATATCT ACGTTCGATA CGAGAAGTTA AACGTTCCTT GTCGTAGACC CCCCTCTCTT   
  
  
- AAACTCCTTA AAGTTCATGG GTAAAGTTAA AGTTGTTCAT TTTAGTAGTA ACTTCCTATG AGTTTGGGTT   
  
  
- CGGGTCAACT ATCACTTGGT TGAAGATGAG ACCTAAGATG AGGTTCAGGG TCGGGAGGGT GGGAGAGGAG   
  
  
- GAGGAGGTTC TTGAGGATGG TTTTGCGACG GCAAAACCGA GTGGGAGGAG TCGAAGTGGT ACCTAGACTA   
  
  
- TTACACATTC TGGGGTTGGG AAAACGAAAA TAAAAGGAGT ACGAGGTAAG AGTACTAAAC ATGAAGACAA   
  
  
- TACATTAACC TCTACACAGA TTTAAACCTT TTCAAGCACC CAAAGCACAA ACTCTTAAAT CTTACTGTAA   
  
  
- GAAATTAATC ATTAAACTTC AAACGTACGA ATTAAATCGA TTAGTGACTA ATTAATTTTA TTTACTTCAC   
  
  
- TACATCCATA CCCGACAATG TGTTACAGTG GCCACCACCA CTGTCGCCGC CCCGCCGCCG CCAACCTAAC   
  
  
- CTTCTTACCC TGTCGCACAA AGGTTTACCC CTACCTCGAA ATGAGGGAAC CTAGTACCCC CTTCCACTAC   
  
  
- TAAACCCAGA CTTAAACTTC GTAGAGAATG TTAGACCAAT GGGACAACTC ATACTTCCGT TACGTCCTGA   
  
  
- ACCACAACAA CTAGTCTGTA GAGAAGAACC TCCACCACTA AACCCCAAAA GACCAAGACC CAGACACTTA   
  
  
- TTACCGTTTT AACCAAGGTA AAACCCATTA ACAAGTAGAC CCCTAAACCT AAGATTCCAA AGATCACTGC   
  
  
- CCAACTTAAG GTTAACATCA AGTGACCCCT CATACTCATA GGAACCAAGT TAGGTACCTG GAAACGGATT   
  
  
- ATGACCACCC AAACACAAGG GTGTTTGTGT TGATAAACTA GAACCACTAC TCTTCGGAGT CTAAAACTTG   
  
  
- GGTGTTAACT ACTGCTACTT AGGTTTAGTC CGAGTCTCGT ACCCTTTGGG ATCGAAAAAA CACGGGAGGA   
  
  
- ACCCAATAAC AGTCAATCTC GTTGTATACC AAGTTGGTGT CCGCTTTGCG GTATCGGGAC CACATCAAAA   
  
  
- CTTGAGACTA CAATAACGGT TTTGGGGCAA AGAGCTAGTC CCAGTACTCA AAAACAACTC CTTTGTTGTG   
  
  
- GTAGTTGTCT AAAACGTCGT TGAAGGATAC CCAAACCGAG TAGTCAGCCA GGGAGTCGTG GAGGTCGTTT   
  
  
- TCCGCTACTT TCTGGTCCAA CAATTCCTGG TCGAAAACTT CCGTCGACTG GACTAAGTTT GACCCTTAAA   
  
  
- GAGTGAACGC GTTCTCTATA ACCGCGCCGA GTTAGTGGTC GAGAGGGAAG GACGCTTCGG GGAGTAATCC   
  
  
- CGTCGAAACA TACAGTACCT CCGGAACGTT TACGAGTAGA ATTACTCGTT AGGACATCGT GGAGGTGGCT   
  
  
- ACTTCTGCGA AAGGGGATAT CTACAACAAG TATTCTACTT ACGGATGTTC CGGAAGAGAC TCCAGAGAGG   
  
  
- ATAATGAGTC AAATGCTTAA AGTGAACACG GGTTCGGTAA GAACTTCGAG AGCTACTACG CCTAACGCAA   
  
  
- GTACAGTAAC TGAAACTATA ACCAACACCC CGAGTTACCC GTAGTGACTA AGTCCTCGAA GGTAACTCCT   
  
  
- TTTTCCCTCG AGGTAGGGAC TTTTAATGTC GGTATCGAGG GGACAGTTAT TCGGTGTGAA AACTTGAGTT   
  
  
- GGATCGTGCC CTTTTGGAAC ACGTTAAGCG GTTACTACAA CCACAACGAA AACTCGACGT TCAACACTTG   
  
  
- AACCTAAATA AACTAGGTAG AAGTAGAAGT TACGGTTTAC AACCTTGGAG ACCCCTACTC AACTAGCGAC   
  
  
- AATCATATGG GTAAACCCGT ACAAGTATAG CCGGCAGATA AGAGGGTAGG TAGGAGGCCA AGTAATTCGT   
  
  
- TGATCGGGGG TTCTAACAGC AGAGCAATCT ATCTCCCAGA CTAGCAACGC TGTGAGACAA GGGTGTTGTA   
  
  
- GAATAGGTAT AGAATCTCAG GACGTGTTTA AAGAACCTTA GCGAGCTGCC AGAGTTACAT CGTAGTCTAT   
  
  
- AACATTTGTT CCAACTCTTC ATGAAGGAGG TCGGATTCTA GCTTTTGTGG CACAACCCGG CGCAAGTACG   
  
  
- GGGGTTATTC TACGGTGTAA CCTTCTGGGA GAAACGAAGT CGGCCCAATA ACGGGAAAGT CAAGTCATTG   
  
  
- AAGTGTCTTT GTGTTCGACT AATACACCAC TTCGCTTGGG GTCCCTCCCC TAAAGTGTAT CTTTTCGCGG   
  
  
- TGCGTAGAAA TCACGATTCA ACCGTTTCCG CCCTCGAACA CTGTCGGAGC CGTACCTCCA AACTCTTCGA   
  
  
- CAT

+     MYC

| Site Name | Organism | Position | Strand | Matrix score. | sequence | function |
| --- | --- | --- | --- | --- | --- | --- |
| MYC | Arabidopsis thaliana | 2388 | - | 6 | CAATTG |  |
| MYC | Arabidopsis thaliana | 1987 | - | 6 | CATTTG |  |
| MYC | Arabidopsis thaliana | 540 | - | 6 | CATTTG |  |
| MYC | Arabidopsis thaliana | 3259 | - | 6 | CATTTG |  |
| MYC | Arabidopsis thaliana | 3305 | + | 6 | CATTTG |  |
| MYC | Arabidopsis thaliana | 2255 | - | 6 | CAATTG |  |
| MYC | Arabidopsis thaliana | 1333 | + | 6 | CATTTG |  |
| MYC | Arabidopsis thaliana | 2832 | - | 6 | CATTTG |  |
| MYC | Arabidopsis thaliana | 280 | - | 6 | CATTTG |  |
| MYC | Arabidopsis thaliana | 240 | + | 6 | CAATTG |  |

>HU05G00466.1   
+ +Up\_Stream \_Len000ACATTG GGTGGGATTT AAATCCTCTA TTTTTTCTTA GGGGAAAAAT ACTTGGGAAA   
  
  
+ TTTCTCAATA ACTGAAAATC TAAATAATTC GTGACTTTGT TAGTAGGTTT AATGTGATTT ACTACCGTGA   
  
  
+ GTGTCACTTT TAGATGGTAC TTTAAGAAAG CATTATAAGT AAAACAATAT ATATCGTAGC ATCATACTAC   
  
  
+ TTGTGTAATA AATTAACAGC AACCTCAATT GTACTTATTT GATTTCGCAT TGTAATCAAT TTTCCCAAAT   
  
  
+ GATTTACTAT CTTGGTCTTA CTCAGTTGTA CTTCACACTC AATAGTTCAA TTATTTTTCA TTAGACTAAT   
  
  
+ GAGCGAGTCC CTTCTACAAG AGCCTACTAC TACTCTAATA ATGTAAGGAC TATTGGCTAA TGGGGAACTA   
  
  
+ AGCCTACGAC TTGTTAGGCA TAGGTGAATT TTCCTTACAA GAGAGTTTAA TTTACACTTG ATATTAACTC   
  
  
+ GAGAACGAGA AGCTGTCTTA ATAGATACCC ACATAGAAAA TGAAGCAAAT GCCCTTGATT ATTGGAGGGT   
  
  
+ TGGGCAAATA TATTCAGTAA CAATGTTGTT TGGCAAATAT CGTCTGAAAA TTGTTTTTGA CAAATATTGT   
  
  
+ TTGGAAGAAT TTTTCGAGAT TAAATATTAA AAATGTTAAA ATTTTAATCT AAATATCAAA CAAATATTGA   
  
  
+ AAAATGCTTT ATATTATCTA AATCTCTAAT TAAAATTTGT GTATTGTCCA CTAGAAAAAT GTATAGTGAT   
  
  
+ ATTACCGACA TATATGTGAT CTCTGGTGTA ATAGCCTACC CTAATGTCTA GGAAAATATT TAAAATCCCC   
  
  
+ AGTGGTCAAA AGGAAGTTAC GCACACTAGG GCAAGACACT TCATTAGGCT AGTTGGTGAA AGCTAAGTCA   
  
  
+ TGAAATTTAG ACCATGGCCA TGTCTACTTG TAGATCCAAC TACCAACAAT CCATTTTTAT TTTGGGGAAA   
  
  
+ GCTTGAAGGG TAATTTGGTA AATGTACACG GGAACCTTTT GCTATAAAAG AAGTGCTTCT TGAAAAAACA   
  
  
+ CCCTGTAGTA AAACAGGGCA AAAAAGCCAA CACTTCTGTT TCTGGCAGAG AAACAGTAGT GTACCACTTG   
  
  
+ GAGTTGGAGT AGACACGATA CACAAACCCA GTAGAGAGAG AAACAGGGGA GAGAAAGAGT ACACAAATTT   
  
  
+ GTGCAGGAGA AGGGTAAGGA AGAAGGAGAA GGAACCCCCT CCAAAAAAAA CCCATATAAA AAATAATAAA   
  
  
+ GAAAAAACAG GAGTGAGTTA GAGGGTACCC AAATCTCCAA TCTAAAACTA GAATCTAGGG TCTCTTTTCA   
  
  
+ TTTGCTTGAA AAATTTCTGT TCATGGGTTT TGTGTTTTTA GGGGGATTTG TTTGATTTTT GTAGTCTTTG   
  
  
+ TGGATTTCCT GTGTTATAGA TGCAAGCTAT GCTCTTCAAT TTGCAAGGAA CAGCATCTGG GGGGAGAGAA   
  
  
+ TTTGAGGAAT TTCAAGTACC CATTTCAATT TCAACAAGTA AAATCATCAT TGAAGGATAC TCAAACCCAA   
  
  
+ GCCCAGTTGA TAGTGAACCA ACTTCTACTC TGGATTCTAC TCCAAGTCCC AGCCCTCCCA CCCTCTCCTC   
  
  
+ CTCCTCCAAG AACTCCTACC AAAACGCTGC CGTTTTGGCT CACCCTCCTC AGCTTCACCA TGGATCTGAT   
  
  
+ AATGTGTAAG ACCCCAACCC TTTTGCTTTT ATTTTCCTCA TGCTCCATTC TCATGATTTG TACTTCTGTT   
  
  
+ ATGTAATTGG AGATGTGTCT AAATTTGGAA AAGTTCGTGG GTTTCGTGTT TGAGAATTTA GAATGACATT   
  
  
+ CTTTAATTAG TAATTTGAAG TTTGCATGCT TAATTTAGCT AATCACTGAT TAATTAAAAT AAATGAAGTG   
  
  
+ ATGTAGGTAT GGGCTGTTAC ACAATGTCAC CGGTGGTGGT GACAGCGGCG GGGCGGCGGC GGTTGGATTG   
  
  
+ GAAGAATGGG ACAGCGTGTT TCCAAATGGG GATGGAGCTT TACTCCCTTG GATCATGGGG GAAGGTGATG   
  
  
+ ATTTGGGTCT GAATTTGAAG CATCTCTTAC AATCTGGTTA CCCTGTTGAG TATGAAGGCA ATGCAGGACT   
  
  
+ TGGTGTTGTT GATCAGACAT CTCTTCTTGG AGGTGGTGAT TTGGGGTTTT CTGGTTCTGG GTCTGTGAAT   
  
  
+ AATGGCAAAA TTGGTTCCAT TTTGGGTAAT TGTTCATCTG GGGATTTGGA TTCTAAGGTT TCTAGTGACG   
  
  
+ GGTTGAATTC CAATTGTAGT TCACTGGGGA GTATGAGTAT CCTTGGTTCA ATCCATGGAC CTTTGCCTAA   
  
  
+ TACTGGTGGG TTTGTGTTCC CACAAACACA ACTATTTGAT CTTGGTGATG AGAAGCCTCA GATTTTGAAC   
  
  
+ CCACAATTGA TGACGATGAA TCCAAATCAG GCTCAGAGCA TGGGAAACCC TAGCTTTTTT GTGCCCTCCT   
  
  
+ TGGGTTATTG TCAGTTAGAG CAACATATGG TTCAACCACA GGCGAAACGC CATAGCCCTG GTGTAGTTTT   
  
  
+ GAACTCTGAT GTTATTGCCA AAACCCCGTT TCTCGATCAG GGTCATGAGT TTTTGTTGAG GAAACAACAC   
  
  
+ CATCAACAGA TTTTGCAGCA ACTTCCTATG GGTTTGGCTC ATCAGTCGGT CCCTCAGCAC CTCCAGCAAA   
  
  
+ AGGCGATGAA AGACCAGGTT GTTAAGGACC AGCTTTTGAA GGCAGCTGAC CTGATTCAAA CTGGGAATTT   
  
  
+ CTCACTTGCG CAAGAGATAT TGGCGCGGCT CAATCACCAG CTCTCCCTTC CTGCGAAGCC CCTCATTAGG   
  
  
+ GCAGCTTTGT ATGTCATGGA GGCCTTGCAA ATGCTCATCT TAATGAGCAA TCCTGTAGCA CCTCCACCGA   
  
  
+ TGAAGACGCT TTCCCCTATA GATGTTGTTC ATAAGATGAA TGCCTACAAG GCCTTCTCTG AGGTCTCTCC   
  
  
+ TATTACTCAG TTTACGAATT TCACTTGTGC CCAAGCCATT CTTGAAGCTC TCGATGATGC GGATTGCGTT   
  
  
+ CATGTCATTG ACTTTGATAT TGGTTGTGGG GCTCAATGGG CATCACTGAT TCAGGAGCTT CCATTGAGGA   
  
  
+ AAAAGGGAGC TCCATCCCTG AAAATTACAG CCATAGCTCC CCTGTCAATA AGCCACACTT TTGAACTCAA   
  
  
+ CCTAGCACGG GAAAACCTTG TGCAATTCGC CAATGATGTT GGTGTTGCTT TTGAGCTGCA AGTTGTGAAC   
  
  
+ TTGGATTTAT TTGATCCATC TTCATCTTCA ATGCCAAATG TTGGAACCTC TGGGGATGAG TTGATCGCTG   
  
  
+ TTAGTATACC CATTTGGGCA TGTTCATATC GGCCGTCTAT TCTCCCATCC ATCCTCCGGT TCATTAAGCA   
  
  
+ ACTAGCCCCC AAGATTGTCG TCTCGTTAGA TAGAGGGTCT GATCGTTGCG ACACTCTGTT CCCACAACAT   
  
  
+ CTTATCCATA TCTTAGAGTC CTGCACAAAT TTCTTGGAAT CGCTCGACGG TCTCAATGTA GCATCAGATA   
  
  
+ TTGTAAACAA GGTTGAGAAG TACTTCCTCC AGCCTAAGAT CGAAAACACC GTGTTGGGCC GCGTTCATGC   
  
  
+ CCCCAATAAG ATGCCACATT GGAAGACCCT CTTTGCTTCA GCCGGGTTAT TGCCCTTTCA GTTCAGTAAC   
  
  
+ TTCACAGAAA CACAAGCTGA TTATGTGGTG AAGCGAACCC CAGGGAGGGG ATTTCACATA GAAAAGCGCC   
  
  
+ ACGCATCTTT AGTGCTAAGT TGGCAAAGGC GGGAGCTTGT GACAGCCTCG GCATGGAGGT TTGAGAAGCT   
  
  
+ GTA  

- +Up\_Stream \_Len000TGTAAC CCACCCTAAA TTTAGGAGAT AAAAAAGAAT CCCCTTTTTA TGAACCCTTT   
  
  
- AAAGAGTTAT TGACTTTTAG ATTTATTAAG CACTGAAACA ATCATCCAAA TTACACTAAA TGATGGCACT   
  
  
- CACAGTGAAA ATCTACCATG AAATTCTTTC GTAATATTCA TTTTGTTATA TATAGCATCG TAGTATGATG   
  
  
- AACACATTAT TTAATTGTCG TTGGAGTTAA CATGAATAAA CTAAAGCGTA ACATTAGTTA AAAGGGTTTA   
  
  
- CTAAATGATA GAACCAGAAT GAGTCAACAT GAAGTGTGAG TTATCAAGTT AATAAAAAGT AATCTGATTA   
  
  
- CTCGCTCAGG GAAGATGTTC TCGGATGATG ATGAGATTAT TACATTCCTG ATAACCGATT ACCCCTTGAT   
  
  
- TCGGATGCTG AACAATCCGT ATCCACTTAA AAGGAATGTT CTCTCAAATT AAATGTGAAC TATAATTGAG   
  
  
- CTCTTGCTCT TCGACAGAAT TATCTATGGG TGTATCTTTT ACTTCGTTTA CGGGAACTAA TAACCTCCCA   
  
  
- ACCCGTTTAT ATAAGTCATT GTTACAACAA ACCGTTTATA GCAGACTTTT AACAAAAACT GTTTATAACA   
  
  
- AACCTTCTTA AAAAGCTCTA ATTTATAATT TTTACAATTT TAAAATTAGA TTTATAGTTT GTTTATAACT   
  
  
- TTTTACGAAA TATAATAGAT TTAGAGATTA ATTTTAAACA CATAACAGGT GATCTTTTTA CATATCACTA   
  
  
- TAATGGCTGT ATATACACTA GAGACCACAT TATCGGATGG GATTACAGAT CCTTTTATAA ATTTTAGGGG   
  
  
- TCACCAGTTT TCCTTCAATG CGTGTGATCC CGTTCTGTGA AGTAATCCGA TCAACCACTT TCGATTCAGT   
  
  
- ACTTTAAATC TGGTACCGGT ACAGATGAAC ATCTAGGTTG ATGGTTGTTA GGTAAAAATA AAACCCCTTT   
  
  
- CGAACTTCCC ATTAAACCAT TTACATGTGC CCTTGGAAAA CGATATTTTC TTCACGAAGA ACTTTTTTGT   
  
  
- GGGACATCAT TTTGTCCCGT TTTTTCGGTT GTGAAGACAA AGACCGTCTC TTTGTCATCA CATGGTGAAC   
  
  
- CTCAACCTCA TCTGTGCTAT GTGTTTGGGT CATCTCTCTC TTTGTCCCCT CTCTTTCTCA TGTGTTTAAA   
  
  
- CACGTCCTCT TCCCATTCCT TCTTCCTCTT CCTTGGGGGA GGTTTTTTTT GGGTATATTT TTTATTATTT   
  
  
- CTTTTTTGTC CTCACTCAAT CTCCCATGGG TTTAGAGGTT AGATTTTGAT CTTAGATCCC AGAGAAAAGT   
  
  
- AAACGAACTT TTTAAAGACA AGTACCCAAA ACACAAAAAT CCCCCTAAAC AAACTAAAAA CATCAGAAAC   
  
  
- ACCTAAAGGA CACAATATCT ACGTTCGATA CGAGAAGTTA AACGTTCCTT GTCGTAGACC CCCCTCTCTT   
  
  
- AAACTCCTTA AAGTTCATGG GTAAAGTTAA AGTTGTTCAT TTTAGTAGTA ACTTCCTATG AGTTTGGGTT   
  
  
- CGGGTCAACT ATCACTTGGT TGAAGATGAG ACCTAAGATG AGGTTCAGGG TCGGGAGGGT GGGAGAGGAG   
  
  
- GAGGAGGTTC TTGAGGATGG TTTTGCGACG GCAAAACCGA GTGGGAGGAG TCGAAGTGGT ACCTAGACTA   
  
  
- TTACACATTC TGGGGTTGGG AAAACGAAAA TAAAAGGAGT ACGAGGTAAG AGTACTAAAC ATGAAGACAA   
  
  
- TACATTAACC TCTACACAGA TTTAAACCTT TTCAAGCACC CAAAGCACAA ACTCTTAAAT CTTACTGTAA   
  
  
- GAAATTAATC ATTAAACTTC AAACGTACGA ATTAAATCGA TTAGTGACTA ATTAATTTTA TTTACTTCAC   
  
  
- TACATCCATA CCCGACAATG TGTTACAGTG GCCACCACCA CTGTCGCCGC CCCGCCGCCG CCAACCTAAC   
  
  
- CTTCTTACCC TGTCGCACAA AGGTTTACCC CTACCTCGAA ATGAGGGAAC CTAGTACCCC CTTCCACTAC   
  
  
- TAAACCCAGA CTTAAACTTC GTAGAGAATG TTAGACCAAT GGGACAACTC ATACTTCCGT TACGTCCTGA   
  
  
- ACCACAACAA CTAGTCTGTA GAGAAGAACC TCCACCACTA AACCCCAAAA GACCAAGACC CAGACACTTA   
  
  
- TTACCGTTTT AACCAAGGTA AAACCCATTA ACAAGTAGAC CCCTAAACCT AAGATTCCAA AGATCACTGC   
  
  
- CCAACTTAAG GTTAACATCA AGTGACCCCT CATACTCATA GGAACCAAGT TAGGTACCTG GAAACGGATT   
  
  
- ATGACCACCC AAACACAAGG GTGTTTGTGT TGATAAACTA GAACCACTAC TCTTCGGAGT CTAAAACTTG   
  
  
- GGTGTTAACT ACTGCTACTT AGGTTTAGTC CGAGTCTCGT ACCCTTTGGG ATCGAAAAAA CACGGGAGGA   
  
  
- ACCCAATAAC AGTCAATCTC GTTGTATACC AAGTTGGTGT CCGCTTTGCG GTATCGGGAC CACATCAAAA   
  
  
- CTTGAGACTA CAATAACGGT TTTGGGGCAA AGAGCTAGTC CCAGTACTCA AAAACAACTC CTTTGTTGTG   
  
  
- GTAGTTGTCT AAAACGTCGT TGAAGGATAC CCAAACCGAG TAGTCAGCCA GGGAGTCGTG GAGGTCGTTT   
  
  
- TCCGCTACTT TCTGGTCCAA CAATTCCTGG TCGAAAACTT CCGTCGACTG GACTAAGTTT GACCCTTAAA   
  
  
- GAGTGAACGC GTTCTCTATA ACCGCGCCGA GTTAGTGGTC GAGAGGGAAG GACGCTTCGG GGAGTAATCC   
  
  
- CGTCGAAACA TACAGTACCT CCGGAACGTT TACGAGTAGA ATTACTCGTT AGGACATCGT GGAGGTGGCT   
  
  
- ACTTCTGCGA AAGGGGATAT CTACAACAAG TATTCTACTT ACGGATGTTC CGGAAGAGAC TCCAGAGAGG   
  
  
- ATAATGAGTC AAATGCTTAA AGTGAACACG GGTTCGGTAA GAACTTCGAG AGCTACTACG CCTAACGCAA   
  
  
- GTACAGTAAC TGAAACTATA ACCAACACCC CGAGTTACCC GTAGTGACTA AGTCCTCGAA GGTAACTCCT   
  
  
- TTTTCCCTCG AGGTAGGGAC TTTTAATGTC GGTATCGAGG GGACAGTTAT TCGGTGTGAA AACTTGAGTT   
  
  
- GGATCGTGCC CTTTTGGAAC ACGTTAAGCG GTTACTACAA CCACAACGAA AACTCGACGT TCAACACTTG   
  
  
- AACCTAAATA AACTAGGTAG AAGTAGAAGT TACGGTTTAC AACCTTGGAG ACCCCTACTC AACTAGCGAC   
  
  
- AATCATATGG GTAAACCCGT ACAAGTATAG CCGGCAGATA AGAGGGTAGG TAGGAGGCCA AGTAATTCGT   
  
  
- TGATCGGGGG TTCTAACAGC AGAGCAATCT ATCTCCCAGA CTAGCAACGC TGTGAGACAA GGGTGTTGTA   
  
  
- GAATAGGTAT AGAATCTCAG GACGTGTTTA AAGAACCTTA GCGAGCTGCC AGAGTTACAT CGTAGTCTAT   
  
  
- AACATTTGTT CCAACTCTTC ATGAAGGAGG TCGGATTCTA GCTTTTGTGG CACAACCCGG CGCAAGTACG   
  
  
- GGGGTTATTC TACGGTGTAA CCTTCTGGGA GAAACGAAGT CGGCCCAATA ACGGGAAAGT CAAGTCATTG   
  
  
- AAGTGTCTTT GTGTTCGACT AATACACCAC TTCGCTTGGG GTCCCTCCCC TAAAGTGTAT CTTTTCGCGG   
  
  
- TGCGTAGAAA TCACGATTCA ACCGTTTCCG CCCTCGAACA CTGTCGGAGC CGTACCTCCA AACTCTTCGA   
  
  
- CAT

+     Myb

| Site Name | Organism | Position | Strand | Matrix score. | sequence | function |
| --- | --- | --- | --- | --- | --- | --- |
| Myb | Arabidopsis thaliana | 1548 | - | 6 | CAACTG |  |
| Myb | Arabidopsis thaliana | 2466 | - | 6 | TAACTG |  |
| Myb | Arabidopsis thaliana | 307 | - | 6 | CAACTG |  |
| Myb | Arabidopsis thaliana | 83 | + | 6 | TAACTG |  |

>HU05G00466.1   
+ +Up\_Stream \_Len000ACATTG GGTGGGATTT AAATCCTCTA TTTTTTCTTA GGGGAAAAAT ACTTGGGAAA   
  
  
+ TTTCTCAATA ACTGAAAATC TAAATAATTC GTGACTTTGT TAGTAGGTTT AATGTGATTT ACTACCGTGA   
  
  
+ GTGTCACTTT TAGATGGTAC TTTAAGAAAG CATTATAAGT AAAACAATAT ATATCGTAGC ATCATACTAC   
  
  
+ TTGTGTAATA AATTAACAGC AACCTCAATT GTACTTATTT GATTTCGCAT TGTAATCAAT TTTCCCAAAT   
  
  
+ GATTTACTAT CTTGGTCTTA CTCAGTTGTA CTTCACACTC AATAGTTCAA TTATTTTTCA TTAGACTAAT   
  
  
+ GAGCGAGTCC CTTCTACAAG AGCCTACTAC TACTCTAATA ATGTAAGGAC TATTGGCTAA TGGGGAACTA   
  
  
+ AGCCTACGAC TTGTTAGGCA TAGGTGAATT TTCCTTACAA GAGAGTTTAA TTTACACTTG ATATTAACTC   
  
  
+ GAGAACGAGA AGCTGTCTTA ATAGATACCC ACATAGAAAA TGAAGCAAAT GCCCTTGATT ATTGGAGGGT   
  
  
+ TGGGCAAATA TATTCAGTAA CAATGTTGTT TGGCAAATAT CGTCTGAAAA TTGTTTTTGA CAAATATTGT   
  
  
+ TTGGAAGAAT TTTTCGAGAT TAAATATTAA AAATGTTAAA ATTTTAATCT AAATATCAAA CAAATATTGA   
  
  
+ AAAATGCTTT ATATTATCTA AATCTCTAAT TAAAATTTGT GTATTGTCCA CTAGAAAAAT GTATAGTGAT   
  
  
+ ATTACCGACA TATATGTGAT CTCTGGTGTA ATAGCCTACC CTAATGTCTA GGAAAATATT TAAAATCCCC   
  
  
+ AGTGGTCAAA AGGAAGTTAC GCACACTAGG GCAAGACACT TCATTAGGCT AGTTGGTGAA AGCTAAGTCA   
  
  
+ TGAAATTTAG ACCATGGCCA TGTCTACTTG TAGATCCAAC TACCAACAAT CCATTTTTAT TTTGGGGAAA   
  
  
+ GCTTGAAGGG TAATTTGGTA AATGTACACG GGAACCTTTT GCTATAAAAG AAGTGCTTCT TGAAAAAACA   
  
  
+ CCCTGTAGTA AAACAGGGCA AAAAAGCCAA CACTTCTGTT TCTGGCAGAG AAACAGTAGT GTACCACTTG   
  
  
+ GAGTTGGAGT AGACACGATA CACAAACCCA GTAGAGAGAG AAACAGGGGA GAGAAAGAGT ACACAAATTT   
  
  
+ GTGCAGGAGA AGGGTAAGGA AGAAGGAGAA GGAACCCCCT CCAAAAAAAA CCCATATAAA AAATAATAAA   
  
  
+ GAAAAAACAG GAGTGAGTTA GAGGGTACCC AAATCTCCAA TCTAAAACTA GAATCTAGGG TCTCTTTTCA   
  
  
+ TTTGCTTGAA AAATTTCTGT TCATGGGTTT TGTGTTTTTA GGGGGATTTG TTTGATTTTT GTAGTCTTTG   
  
  
+ TGGATTTCCT GTGTTATAGA TGCAAGCTAT GCTCTTCAAT TTGCAAGGAA CAGCATCTGG GGGGAGAGAA   
  
  
+ TTTGAGGAAT TTCAAGTACC CATTTCAATT TCAACAAGTA AAATCATCAT TGAAGGATAC TCAAACCCAA   
  
  
+ GCCCAGTTGA TAGTGAACCA ACTTCTACTC TGGATTCTAC TCCAAGTCCC AGCCCTCCCA CCCTCTCCTC   
  
  
+ CTCCTCCAAG AACTCCTACC AAAACGCTGC CGTTTTGGCT CACCCTCCTC AGCTTCACCA TGGATCTGAT   
  
  
+ AATGTGTAAG ACCCCAACCC TTTTGCTTTT ATTTTCCTCA TGCTCCATTC TCATGATTTG TACTTCTGTT   
  
  
+ ATGTAATTGG AGATGTGTCT AAATTTGGAA AAGTTCGTGG GTTTCGTGTT TGAGAATTTA GAATGACATT   
  
  
+ CTTTAATTAG TAATTTGAAG TTTGCATGCT TAATTTAGCT AATCACTGAT TAATTAAAAT AAATGAAGTG   
  
  
+ ATGTAGGTAT GGGCTGTTAC ACAATGTCAC CGGTGGTGGT GACAGCGGCG GGGCGGCGGC GGTTGGATTG   
  
  
+ GAAGAATGGG ACAGCGTGTT TCCAAATGGG GATGGAGCTT TACTCCCTTG GATCATGGGG GAAGGTGATG   
  
  
+ ATTTGGGTCT GAATTTGAAG CATCTCTTAC AATCTGGTTA CCCTGTTGAG TATGAAGGCA ATGCAGGACT   
  
  
+ TGGTGTTGTT GATCAGACAT CTCTTCTTGG AGGTGGTGAT TTGGGGTTTT CTGGTTCTGG GTCTGTGAAT   
  
  
+ AATGGCAAAA TTGGTTCCAT TTTGGGTAAT TGTTCATCTG GGGATTTGGA TTCTAAGGTT TCTAGTGACG   
  
  
+ GGTTGAATTC CAATTGTAGT TCACTGGGGA GTATGAGTAT CCTTGGTTCA ATCCATGGAC CTTTGCCTAA   
  
  
+ TACTGGTGGG TTTGTGTTCC CACAAACACA ACTATTTGAT CTTGGTGATG AGAAGCCTCA GATTTTGAAC   
  
  
+ CCACAATTGA TGACGATGAA TCCAAATCAG GCTCAGAGCA TGGGAAACCC TAGCTTTTTT GTGCCCTCCT   
  
  
+ TGGGTTATTG TCAGTTAGAG CAACATATGG TTCAACCACA GGCGAAACGC CATAGCCCTG GTGTAGTTTT   
  
  
+ GAACTCTGAT GTTATTGCCA AAACCCCGTT TCTCGATCAG GGTCATGAGT TTTTGTTGAG GAAACAACAC   
  
  
+ CATCAACAGA TTTTGCAGCA ACTTCCTATG GGTTTGGCTC ATCAGTCGGT CCCTCAGCAC CTCCAGCAAA   
  
  
+ AGGCGATGAA AGACCAGGTT GTTAAGGACC AGCTTTTGAA GGCAGCTGAC CTGATTCAAA CTGGGAATTT   
  
  
+ CTCACTTGCG CAAGAGATAT TGGCGCGGCT CAATCACCAG CTCTCCCTTC CTGCGAAGCC CCTCATTAGG   
  
  
+ GCAGCTTTGT ATGTCATGGA GGCCTTGCAA ATGCTCATCT TAATGAGCAA TCCTGTAGCA CCTCCACCGA   
  
  
+ TGAAGACGCT TTCCCCTATA GATGTTGTTC ATAAGATGAA TGCCTACAAG GCCTTCTCTG AGGTCTCTCC   
  
  
+ TATTACTCAG TTTACGAATT TCACTTGTGC CCAAGCCATT CTTGAAGCTC TCGATGATGC GGATTGCGTT   
  
  
+ CATGTCATTG ACTTTGATAT TGGTTGTGGG GCTCAATGGG CATCACTGAT TCAGGAGCTT CCATTGAGGA   
  
  
+ AAAAGGGAGC TCCATCCCTG AAAATTACAG CCATAGCTCC CCTGTCAATA AGCCACACTT TTGAACTCAA   
  
  
+ CCTAGCACGG GAAAACCTTG TGCAATTCGC CAATGATGTT GGTGTTGCTT TTGAGCTGCA AGTTGTGAAC   
  
  
+ TTGGATTTAT TTGATCCATC TTCATCTTCA ATGCCAAATG TTGGAACCTC TGGGGATGAG TTGATCGCTG   
  
  
+ TTAGTATACC CATTTGGGCA TGTTCATATC GGCCGTCTAT TCTCCCATCC ATCCTCCGGT TCATTAAGCA   
  
  
+ ACTAGCCCCC AAGATTGTCG TCTCGTTAGA TAGAGGGTCT GATCGTTGCG ACACTCTGTT CCCACAACAT   
  
  
+ CTTATCCATA TCTTAGAGTC CTGCACAAAT TTCTTGGAAT CGCTCGACGG TCTCAATGTA GCATCAGATA   
  
  
+ TTGTAAACAA GGTTGAGAAG TACTTCCTCC AGCCTAAGAT CGAAAACACC GTGTTGGGCC GCGTTCATGC   
  
  
+ CCCCAATAAG ATGCCACATT GGAAGACCCT CTTTGCTTCA GCCGGGTTAT TGCCCTTTCA GTTCAGTAAC   
  
  
+ TTCACAGAAA CACAAGCTGA TTATGTGGTG AAGCGAACCC CAGGGAGGGG ATTTCACATA GAAAAGCGCC   
  
  
+ ACGCATCTTT AGTGCTAAGT TGGCAAAGGC GGGAGCTTGT GACAGCCTCG GCATGGAGGT TTGAGAAGCT   
  
  
+ GTA  

- +Up\_Stream \_Len000TGTAAC CCACCCTAAA TTTAGGAGAT AAAAAAGAAT CCCCTTTTTA TGAACCCTTT   
  
  
- AAAGAGTTAT TGACTTTTAG ATTTATTAAG CACTGAAACA ATCATCCAAA TTACACTAAA TGATGGCACT   
  
  
- CACAGTGAAA ATCTACCATG AAATTCTTTC GTAATATTCA TTTTGTTATA TATAGCATCG TAGTATGATG   
  
  
- AACACATTAT TTAATTGTCG TTGGAGTTAA CATGAATAAA CTAAAGCGTA ACATTAGTTA AAAGGGTTTA   
  
  
- CTAAATGATA GAACCAGAAT GAGTCAACAT GAAGTGTGAG TTATCAAGTT AATAAAAAGT AATCTGATTA   
  
  
- CTCGCTCAGG GAAGATGTTC TCGGATGATG ATGAGATTAT TACATTCCTG ATAACCGATT ACCCCTTGAT   
  
  
- TCGGATGCTG AACAATCCGT ATCCACTTAA AAGGAATGTT CTCTCAAATT AAATGTGAAC TATAATTGAG   
  
  
- CTCTTGCTCT TCGACAGAAT TATCTATGGG TGTATCTTTT ACTTCGTTTA CGGGAACTAA TAACCTCCCA   
  
  
- ACCCGTTTAT ATAAGTCATT GTTACAACAA ACCGTTTATA GCAGACTTTT AACAAAAACT GTTTATAACA   
  
  
- AACCTTCTTA AAAAGCTCTA ATTTATAATT TTTACAATTT TAAAATTAGA TTTATAGTTT GTTTATAACT   
  
  
- TTTTACGAAA TATAATAGAT TTAGAGATTA ATTTTAAACA CATAACAGGT GATCTTTTTA CATATCACTA   
  
  
- TAATGGCTGT ATATACACTA GAGACCACAT TATCGGATGG GATTACAGAT CCTTTTATAA ATTTTAGGGG   
  
  
- TCACCAGTTT TCCTTCAATG CGTGTGATCC CGTTCTGTGA AGTAATCCGA TCAACCACTT TCGATTCAGT   
  
  
- ACTTTAAATC TGGTACCGGT ACAGATGAAC ATCTAGGTTG ATGGTTGTTA GGTAAAAATA AAACCCCTTT   
  
  
- CGAACTTCCC ATTAAACCAT TTACATGTGC CCTTGGAAAA CGATATTTTC TTCACGAAGA ACTTTTTTGT   
  
  
- GGGACATCAT TTTGTCCCGT TTTTTCGGTT GTGAAGACAA AGACCGTCTC TTTGTCATCA CATGGTGAAC   
  
  
- CTCAACCTCA TCTGTGCTAT GTGTTTGGGT CATCTCTCTC TTTGTCCCCT CTCTTTCTCA TGTGTTTAAA   
  
  
- CACGTCCTCT TCCCATTCCT TCTTCCTCTT CCTTGGGGGA GGTTTTTTTT GGGTATATTT TTTATTATTT   
  
  
- CTTTTTTGTC CTCACTCAAT CTCCCATGGG TTTAGAGGTT AGATTTTGAT CTTAGATCCC AGAGAAAAGT   
  
  
- AAACGAACTT TTTAAAGACA AGTACCCAAA ACACAAAAAT CCCCCTAAAC AAACTAAAAA CATCAGAAAC   
  
  
- ACCTAAAGGA CACAATATCT ACGTTCGATA CGAGAAGTTA AACGTTCCTT GTCGTAGACC CCCCTCTCTT   
  
  
- AAACTCCTTA AAGTTCATGG GTAAAGTTAA AGTTGTTCAT TTTAGTAGTA ACTTCCTATG AGTTTGGGTT   
  
  
- CGGGTCAACT ATCACTTGGT TGAAGATGAG ACCTAAGATG AGGTTCAGGG TCGGGAGGGT GGGAGAGGAG   
  
  
- GAGGAGGTTC TTGAGGATGG TTTTGCGACG GCAAAACCGA GTGGGAGGAG TCGAAGTGGT ACCTAGACTA   
  
  
- TTACACATTC TGGGGTTGGG AAAACGAAAA TAAAAGGAGT ACGAGGTAAG AGTACTAAAC ATGAAGACAA   
  
  
- TACATTAACC TCTACACAGA TTTAAACCTT TTCAAGCACC CAAAGCACAA ACTCTTAAAT CTTACTGTAA   
  
  
- GAAATTAATC ATTAAACTTC AAACGTACGA ATTAAATCGA TTAGTGACTA ATTAATTTTA TTTACTTCAC   
  
  
- TACATCCATA CCCGACAATG TGTTACAGTG GCCACCACCA CTGTCGCCGC CCCGCCGCCG CCAACCTAAC   
  
  
- CTTCTTACCC TGTCGCACAA AGGTTTACCC CTACCTCGAA ATGAGGGAAC CTAGTACCCC CTTCCACTAC   
  
  
- TAAACCCAGA CTTAAACTTC GTAGAGAATG TTAGACCAAT GGGACAACTC ATACTTCCGT TACGTCCTGA   
  
  
- ACCACAACAA CTAGTCTGTA GAGAAGAACC TCCACCACTA AACCCCAAAA GACCAAGACC CAGACACTTA   
  
  
- TTACCGTTTT AACCAAGGTA AAACCCATTA ACAAGTAGAC CCCTAAACCT AAGATTCCAA AGATCACTGC   
  
  
- CCAACTTAAG GTTAACATCA AGTGACCCCT CATACTCATA GGAACCAAGT TAGGTACCTG GAAACGGATT   
  
  
- ATGACCACCC AAACACAAGG GTGTTTGTGT TGATAAACTA GAACCACTAC TCTTCGGAGT CTAAAACTTG   
  
  
- GGTGTTAACT ACTGCTACTT AGGTTTAGTC CGAGTCTCGT ACCCTTTGGG ATCGAAAAAA CACGGGAGGA   
  
  
- ACCCAATAAC AGTCAATCTC GTTGTATACC AAGTTGGTGT CCGCTTTGCG GTATCGGGAC CACATCAAAA   
  
  
- CTTGAGACTA CAATAACGGT TTTGGGGCAA AGAGCTAGTC CCAGTACTCA AAAACAACTC CTTTGTTGTG   
  
  
- GTAGTTGTCT AAAACGTCGT TGAAGGATAC CCAAACCGAG TAGTCAGCCA GGGAGTCGTG GAGGTCGTTT   
  
  
- TCCGCTACTT TCTGGTCCAA CAATTCCTGG TCGAAAACTT CCGTCGACTG GACTAAGTTT GACCCTTAAA   
  
  
- GAGTGAACGC GTTCTCTATA ACCGCGCCGA GTTAGTGGTC GAGAGGGAAG GACGCTTCGG GGAGTAATCC   
  
  
- CGTCGAAACA TACAGTACCT CCGGAACGTT TACGAGTAGA ATTACTCGTT AGGACATCGT GGAGGTGGCT   
  
  
- ACTTCTGCGA AAGGGGATAT CTACAACAAG TATTCTACTT ACGGATGTTC CGGAAGAGAC TCCAGAGAGG   
  
  
- ATAATGAGTC AAATGCTTAA AGTGAACACG GGTTCGGTAA GAACTTCGAG AGCTACTACG CCTAACGCAA   
  
  
- GTACAGTAAC TGAAACTATA ACCAACACCC CGAGTTACCC GTAGTGACTA AGTCCTCGAA GGTAACTCCT   
  
  
- TTTTCCCTCG AGGTAGGGAC TTTTAATGTC GGTATCGAGG GGACAGTTAT TCGGTGTGAA AACTTGAGTT   
  
  
- GGATCGTGCC CTTTTGGAAC ACGTTAAGCG GTTACTACAA CCACAACGAA AACTCGACGT TCAACACTTG   
  
  
- AACCTAAATA AACTAGGTAG AAGTAGAAGT TACGGTTTAC AACCTTGGAG ACCCCTACTC AACTAGCGAC   
  
  
- AATCATATGG GTAAACCCGT ACAAGTATAG CCGGCAGATA AGAGGGTAGG TAGGAGGCCA AGTAATTCGT   
  
  
- TGATCGGGGG TTCTAACAGC AGAGCAATCT ATCTCCCAGA CTAGCAACGC TGTGAGACAA GGGTGTTGTA   
  
  
- GAATAGGTAT AGAATCTCAG GACGTGTTTA AAGAACCTTA GCGAGCTGCC AGAGTTACAT CGTAGTCTAT   
  
  
- AACATTTGTT CCAACTCTTC ATGAAGGAGG TCGGATTCTA GCTTTTGTGG CACAACCCGG CGCAAGTACG   
  
  
- GGGGTTATTC TACGGTGTAA CCTTCTGGGA GAAACGAAGT CGGCCCAATA ACGGGAAAGT CAAGTCATTG   
  
  
- AAGTGTCTTT GTGTTCGACT AATACACCAC TTCGCTTGGG GTCCCTCCCC TAAAGTGTAT CTTTTCGCGG   
  
  
- TGCGTAGAAA TCACGATTCA ACCGTTTCCG CCCTCGAACA CTGTCGGAGC CGTACCTCCA AACTCTTCGA   
  
  
- CAT

+     Myb-binding site

| Site Name | Organism | Position | Strand | Matrix score. | sequence | function |
| --- | --- | --- | --- | --- | --- | --- |
| Myb-binding site | Nicotiana tabacum | 2598 | + | 6 | CAACAG |  |
| Myb-binding site | Nicotiana tabacum | 2077 | - | 6 | CAACAG |  |

>HU05G00466.1   
+ +Up\_Stream \_Len000ACATTG GGTGGGATTT AAATCCTCTA TTTTTTCTTA GGGGAAAAAT ACTTGGGAAA   
  
  
+ TTTCTCAATA ACTGAAAATC TAAATAATTC GTGACTTTGT TAGTAGGTTT AATGTGATTT ACTACCGTGA   
  
  
+ GTGTCACTTT TAGATGGTAC TTTAAGAAAG CATTATAAGT AAAACAATAT ATATCGTAGC ATCATACTAC   
  
  
+ TTGTGTAATA AATTAACAGC AACCTCAATT GTACTTATTT GATTTCGCAT TGTAATCAAT TTTCCCAAAT   
  
  
+ GATTTACTAT CTTGGTCTTA CTCAGTTGTA CTTCACACTC AATAGTTCAA TTATTTTTCA TTAGACTAAT   
  
  
+ GAGCGAGTCC CTTCTACAAG AGCCTACTAC TACTCTAATA ATGTAAGGAC TATTGGCTAA TGGGGAACTA   
  
  
+ AGCCTACGAC TTGTTAGGCA TAGGTGAATT TTCCTTACAA GAGAGTTTAA TTTACACTTG ATATTAACTC   
  
  
+ GAGAACGAGA AGCTGTCTTA ATAGATACCC ACATAGAAAA TGAAGCAAAT GCCCTTGATT ATTGGAGGGT   
  
  
+ TGGGCAAATA TATTCAGTAA CAATGTTGTT TGGCAAATAT CGTCTGAAAA TTGTTTTTGA CAAATATTGT   
  
  
+ TTGGAAGAAT TTTTCGAGAT TAAATATTAA AAATGTTAAA ATTTTAATCT AAATATCAAA CAAATATTGA   
  
  
+ AAAATGCTTT ATATTATCTA AATCTCTAAT TAAAATTTGT GTATTGTCCA CTAGAAAAAT GTATAGTGAT   
  
  
+ ATTACCGACA TATATGTGAT CTCTGGTGTA ATAGCCTACC CTAATGTCTA GGAAAATATT TAAAATCCCC   
  
  
+ AGTGGTCAAA AGGAAGTTAC GCACACTAGG GCAAGACACT TCATTAGGCT AGTTGGTGAA AGCTAAGTCA   
  
  
+ TGAAATTTAG ACCATGGCCA TGTCTACTTG TAGATCCAAC TACCAACAAT CCATTTTTAT TTTGGGGAAA   
  
  
+ GCTTGAAGGG TAATTTGGTA AATGTACACG GGAACCTTTT GCTATAAAAG AAGTGCTTCT TGAAAAAACA   
  
  
+ CCCTGTAGTA AAACAGGGCA AAAAAGCCAA CACTTCTGTT TCTGGCAGAG AAACAGTAGT GTACCACTTG   
  
  
+ GAGTTGGAGT AGACACGATA CACAAACCCA GTAGAGAGAG AAACAGGGGA GAGAAAGAGT ACACAAATTT   
  
  
+ GTGCAGGAGA AGGGTAAGGA AGAAGGAGAA GGAACCCCCT CCAAAAAAAA CCCATATAAA AAATAATAAA   
  
  
+ GAAAAAACAG GAGTGAGTTA GAGGGTACCC AAATCTCCAA TCTAAAACTA GAATCTAGGG TCTCTTTTCA   
  
  
+ TTTGCTTGAA AAATTTCTGT TCATGGGTTT TGTGTTTTTA GGGGGATTTG TTTGATTTTT GTAGTCTTTG   
  
  
+ TGGATTTCCT GTGTTATAGA TGCAAGCTAT GCTCTTCAAT TTGCAAGGAA CAGCATCTGG GGGGAGAGAA   
  
  
+ TTTGAGGAAT TTCAAGTACC CATTTCAATT TCAACAAGTA AAATCATCAT TGAAGGATAC TCAAACCCAA   
  
  
+ GCCCAGTTGA TAGTGAACCA ACTTCTACTC TGGATTCTAC TCCAAGTCCC AGCCCTCCCA CCCTCTCCTC   
  
  
+ CTCCTCCAAG AACTCCTACC AAAACGCTGC CGTTTTGGCT CACCCTCCTC AGCTTCACCA TGGATCTGAT   
  
  
+ AATGTGTAAG ACCCCAACCC TTTTGCTTTT ATTTTCCTCA TGCTCCATTC TCATGATTTG TACTTCTGTT   
  
  
+ ATGTAATTGG AGATGTGTCT AAATTTGGAA AAGTTCGTGG GTTTCGTGTT TGAGAATTTA GAATGACATT   
  
  
+ CTTTAATTAG TAATTTGAAG TTTGCATGCT TAATTTAGCT AATCACTGAT TAATTAAAAT AAATGAAGTG   
  
  
+ ATGTAGGTAT GGGCTGTTAC ACAATGTCAC CGGTGGTGGT GACAGCGGCG GGGCGGCGGC GGTTGGATTG   
  
  
+ GAAGAATGGG ACAGCGTGTT TCCAAATGGG GATGGAGCTT TACTCCCTTG GATCATGGGG GAAGGTGATG   
  
  
+ ATTTGGGTCT GAATTTGAAG CATCTCTTAC AATCTGGTTA CCCTGTTGAG TATGAAGGCA ATGCAGGACT   
  
  
+ TGGTGTTGTT GATCAGACAT CTCTTCTTGG AGGTGGTGAT TTGGGGTTTT CTGGTTCTGG GTCTGTGAAT   
  
  
+ AATGGCAAAA TTGGTTCCAT TTTGGGTAAT TGTTCATCTG GGGATTTGGA TTCTAAGGTT TCTAGTGACG   
  
  
+ GGTTGAATTC CAATTGTAGT TCACTGGGGA GTATGAGTAT CCTTGGTTCA ATCCATGGAC CTTTGCCTAA   
  
  
+ TACTGGTGGG TTTGTGTTCC CACAAACACA ACTATTTGAT CTTGGTGATG AGAAGCCTCA GATTTTGAAC   
  
  
+ CCACAATTGA TGACGATGAA TCCAAATCAG GCTCAGAGCA TGGGAAACCC TAGCTTTTTT GTGCCCTCCT   
  
  
+ TGGGTTATTG TCAGTTAGAG CAACATATGG TTCAACCACA GGCGAAACGC CATAGCCCTG GTGTAGTTTT   
  
  
+ GAACTCTGAT GTTATTGCCA AAACCCCGTT TCTCGATCAG GGTCATGAGT TTTTGTTGAG GAAACAACAC   
  
  
+ CATCAACAGA TTTTGCAGCA ACTTCCTATG GGTTTGGCTC ATCAGTCGGT CCCTCAGCAC CTCCAGCAAA   
  
  
+ AGGCGATGAA AGACCAGGTT GTTAAGGACC AGCTTTTGAA GGCAGCTGAC CTGATTCAAA CTGGGAATTT   
  
  
+ CTCACTTGCG CAAGAGATAT TGGCGCGGCT CAATCACCAG CTCTCCCTTC CTGCGAAGCC CCTCATTAGG   
  
  
+ GCAGCTTTGT ATGTCATGGA GGCCTTGCAA ATGCTCATCT TAATGAGCAA TCCTGTAGCA CCTCCACCGA   
  
  
+ TGAAGACGCT TTCCCCTATA GATGTTGTTC ATAAGATGAA TGCCTACAAG GCCTTCTCTG AGGTCTCTCC   
  
  
+ TATTACTCAG TTTACGAATT TCACTTGTGC CCAAGCCATT CTTGAAGCTC TCGATGATGC GGATTGCGTT   
  
  
+ CATGTCATTG ACTTTGATAT TGGTTGTGGG GCTCAATGGG CATCACTGAT TCAGGAGCTT CCATTGAGGA   
  
  
+ AAAAGGGAGC TCCATCCCTG AAAATTACAG CCATAGCTCC CCTGTCAATA AGCCACACTT TTGAACTCAA   
  
  
+ CCTAGCACGG GAAAACCTTG TGCAATTCGC CAATGATGTT GGTGTTGCTT TTGAGCTGCA AGTTGTGAAC   
  
  
+ TTGGATTTAT TTGATCCATC TTCATCTTCA ATGCCAAATG TTGGAACCTC TGGGGATGAG TTGATCGCTG   
  
  
+ TTAGTATACC CATTTGGGCA TGTTCATATC GGCCGTCTAT TCTCCCATCC ATCCTCCGGT TCATTAAGCA   
  
  
+ ACTAGCCCCC AAGATTGTCG TCTCGTTAGA TAGAGGGTCT GATCGTTGCG ACACTCTGTT CCCACAACAT   
  
  
+ CTTATCCATA TCTTAGAGTC CTGCACAAAT TTCTTGGAAT CGCTCGACGG TCTCAATGTA GCATCAGATA   
  
  
+ TTGTAAACAA GGTTGAGAAG TACTTCCTCC AGCCTAAGAT CGAAAACACC GTGTTGGGCC GCGTTCATGC   
  
  
+ CCCCAATAAG ATGCCACATT GGAAGACCCT CTTTGCTTCA GCCGGGTTAT TGCCCTTTCA GTTCAGTAAC   
  
  
+ TTCACAGAAA CACAAGCTGA TTATGTGGTG AAGCGAACCC CAGGGAGGGG ATTTCACATA GAAAAGCGCC   
  
  
+ ACGCATCTTT AGTGCTAAGT TGGCAAAGGC GGGAGCTTGT GACAGCCTCG GCATGGAGGT TTGAGAAGCT   
  
  
+ GTA  

- +Up\_Stream \_Len000TGTAAC CCACCCTAAA TTTAGGAGAT AAAAAAGAAT CCCCTTTTTA TGAACCCTTT   
  
  
- AAAGAGTTAT TGACTTTTAG ATTTATTAAG CACTGAAACA ATCATCCAAA TTACACTAAA TGATGGCACT   
  
  
- CACAGTGAAA ATCTACCATG AAATTCTTTC GTAATATTCA TTTTGTTATA TATAGCATCG TAGTATGATG   
  
  
- AACACATTAT TTAATTGTCG TTGGAGTTAA CATGAATAAA CTAAAGCGTA ACATTAGTTA AAAGGGTTTA   
  
  
- CTAAATGATA GAACCAGAAT GAGTCAACAT GAAGTGTGAG TTATCAAGTT AATAAAAAGT AATCTGATTA   
  
  
- CTCGCTCAGG GAAGATGTTC TCGGATGATG ATGAGATTAT TACATTCCTG ATAACCGATT ACCCCTTGAT   
  
  
- TCGGATGCTG AACAATCCGT ATCCACTTAA AAGGAATGTT CTCTCAAATT AAATGTGAAC TATAATTGAG   
  
  
- CTCTTGCTCT TCGACAGAAT TATCTATGGG TGTATCTTTT ACTTCGTTTA CGGGAACTAA TAACCTCCCA   
  
  
- ACCCGTTTAT ATAAGTCATT GTTACAACAA ACCGTTTATA GCAGACTTTT AACAAAAACT GTTTATAACA   
  
  
- AACCTTCTTA AAAAGCTCTA ATTTATAATT TTTACAATTT TAAAATTAGA TTTATAGTTT GTTTATAACT   
  
  
- TTTTACGAAA TATAATAGAT TTAGAGATTA ATTTTAAACA CATAACAGGT GATCTTTTTA CATATCACTA   
  
  
- TAATGGCTGT ATATACACTA GAGACCACAT TATCGGATGG GATTACAGAT CCTTTTATAA ATTTTAGGGG   
  
  
- TCACCAGTTT TCCTTCAATG CGTGTGATCC CGTTCTGTGA AGTAATCCGA TCAACCACTT TCGATTCAGT   
  
  
- ACTTTAAATC TGGTACCGGT ACAGATGAAC ATCTAGGTTG ATGGTTGTTA GGTAAAAATA AAACCCCTTT   
  
  
- CGAACTTCCC ATTAAACCAT TTACATGTGC CCTTGGAAAA CGATATTTTC TTCACGAAGA ACTTTTTTGT   
  
  
- GGGACATCAT TTTGTCCCGT TTTTTCGGTT GTGAAGACAA AGACCGTCTC TTTGTCATCA CATGGTGAAC   
  
  
- CTCAACCTCA TCTGTGCTAT GTGTTTGGGT CATCTCTCTC TTTGTCCCCT CTCTTTCTCA TGTGTTTAAA   
  
  
- CACGTCCTCT TCCCATTCCT TCTTCCTCTT CCTTGGGGGA GGTTTTTTTT GGGTATATTT TTTATTATTT   
  
  
- CTTTTTTGTC CTCACTCAAT CTCCCATGGG TTTAGAGGTT AGATTTTGAT CTTAGATCCC AGAGAAAAGT   
  
  
- AAACGAACTT TTTAAAGACA AGTACCCAAA ACACAAAAAT CCCCCTAAAC AAACTAAAAA CATCAGAAAC   
  
  
- ACCTAAAGGA CACAATATCT ACGTTCGATA CGAGAAGTTA AACGTTCCTT GTCGTAGACC CCCCTCTCTT   
  
  
- AAACTCCTTA AAGTTCATGG GTAAAGTTAA AGTTGTTCAT TTTAGTAGTA ACTTCCTATG AGTTTGGGTT   
  
  
- CGGGTCAACT ATCACTTGGT TGAAGATGAG ACCTAAGATG AGGTTCAGGG TCGGGAGGGT GGGAGAGGAG   
  
  
- GAGGAGGTTC TTGAGGATGG TTTTGCGACG GCAAAACCGA GTGGGAGGAG TCGAAGTGGT ACCTAGACTA   
  
  
- TTACACATTC TGGGGTTGGG AAAACGAAAA TAAAAGGAGT ACGAGGTAAG AGTACTAAAC ATGAAGACAA   
  
  
- TACATTAACC TCTACACAGA TTTAAACCTT TTCAAGCACC CAAAGCACAA ACTCTTAAAT CTTACTGTAA   
  
  
- GAAATTAATC ATTAAACTTC AAACGTACGA ATTAAATCGA TTAGTGACTA ATTAATTTTA TTTACTTCAC   
  
  
- TACATCCATA CCCGACAATG TGTTACAGTG GCCACCACCA CTGTCGCCGC CCCGCCGCCG CCAACCTAAC   
  
  
- CTTCTTACCC TGTCGCACAA AGGTTTACCC CTACCTCGAA ATGAGGGAAC CTAGTACCCC CTTCCACTAC   
  
  
- TAAACCCAGA CTTAAACTTC GTAGAGAATG TTAGACCAAT GGGACAACTC ATACTTCCGT TACGTCCTGA   
  
  
- ACCACAACAA CTAGTCTGTA GAGAAGAACC TCCACCACTA AACCCCAAAA GACCAAGACC CAGACACTTA   
  
  
- TTACCGTTTT AACCAAGGTA AAACCCATTA ACAAGTAGAC CCCTAAACCT AAGATTCCAA AGATCACTGC   
  
  
- CCAACTTAAG GTTAACATCA AGTGACCCCT CATACTCATA GGAACCAAGT TAGGTACCTG GAAACGGATT   
  
  
- ATGACCACCC AAACACAAGG GTGTTTGTGT TGATAAACTA GAACCACTAC TCTTCGGAGT CTAAAACTTG   
  
  
- GGTGTTAACT ACTGCTACTT AGGTTTAGTC CGAGTCTCGT ACCCTTTGGG ATCGAAAAAA CACGGGAGGA   
  
  
- ACCCAATAAC AGTCAATCTC GTTGTATACC AAGTTGGTGT CCGCTTTGCG GTATCGGGAC CACATCAAAA   
  
  
- CTTGAGACTA CAATAACGGT TTTGGGGCAA AGAGCTAGTC CCAGTACTCA AAAACAACTC CTTTGTTGTG   
  
  
- GTAGTTGTCT AAAACGTCGT TGAAGGATAC CCAAACCGAG TAGTCAGCCA GGGAGTCGTG GAGGTCGTTT   
  
  
- TCCGCTACTT TCTGGTCCAA CAATTCCTGG TCGAAAACTT CCGTCGACTG GACTAAGTTT GACCCTTAAA   
  
  
- GAGTGAACGC GTTCTCTATA ACCGCGCCGA GTTAGTGGTC GAGAGGGAAG GACGCTTCGG GGAGTAATCC   
  
  
- CGTCGAAACA TACAGTACCT CCGGAACGTT TACGAGTAGA ATTACTCGTT AGGACATCGT GGAGGTGGCT   
  
  
- ACTTCTGCGA AAGGGGATAT CTACAACAAG TATTCTACTT ACGGATGTTC CGGAAGAGAC TCCAGAGAGG   
  
  
- ATAATGAGTC AAATGCTTAA AGTGAACACG GGTTCGGTAA GAACTTCGAG AGCTACTACG CCTAACGCAA   
  
  
- GTACAGTAAC TGAAACTATA ACCAACACCC CGAGTTACCC GTAGTGACTA AGTCCTCGAA GGTAACTCCT   
  
  
- TTTTCCCTCG AGGTAGGGAC TTTTAATGTC GGTATCGAGG GGACAGTTAT TCGGTGTGAA AACTTGAGTT   
  
  
- GGATCGTGCC CTTTTGGAAC ACGTTAAGCG GTTACTACAA CCACAACGAA AACTCGACGT TCAACACTTG   
  
  
- AACCTAAATA AACTAGGTAG AAGTAGAAGT TACGGTTTAC AACCTTGGAG ACCCCTACTC AACTAGCGAC   
  
  
- AATCATATGG GTAAACCCGT ACAAGTATAG CCGGCAGATA AGAGGGTAGG TAGGAGGCCA AGTAATTCGT   
  
  
- TGATCGGGGG TTCTAACAGC AGAGCAATCT ATCTCCCAGA CTAGCAACGC TGTGAGACAA GGGTGTTGTA   
  
  
- GAATAGGTAT AGAATCTCAG GACGTGTTTA AAGAACCTTA GCGAGCTGCC AGAGTTACAT CGTAGTCTAT   
  
  
- AACATTTGTT CCAACTCTTC ATGAAGGAGG TCGGATTCTA GCTTTTGTGG CACAACCCGG CGCAAGTACG   
  
  
- GGGGTTATTC TACGGTGTAA CCTTCTGGGA GAAACGAAGT CGGCCCAATA ACGGGAAAGT CAAGTCATTG   
  
  
- AAGTGTCTTT GTGTTCGACT AATACACCAC TTCGCTTGGG GTCCCTCCCC TAAAGTGTAT CTTTTCGCGG   
  
  
- TGCGTAGAAA TCACGATTCA ACCGTTTCCG CCCTCGAACA CTGTCGGAGC CGTACCTCCA AACTCTTCGA   
  
  
- CAT

+     Myc

| Site Name | Organism | Position | Strand | Matrix score. | sequence | function |
| --- | --- | --- | --- | --- | --- | --- |
| Myc | Arabidopsis thaliana | 2057 | + | 7 | TCTCTTA |  |

>HU05G00466.1   
+ +Up\_Stream \_Len000ACATTG GGTGGGATTT AAATCCTCTA TTTTTTCTTA GGGGAAAAAT ACTTGGGAAA   
  
  
+ TTTCTCAATA ACTGAAAATC TAAATAATTC GTGACTTTGT TAGTAGGTTT AATGTGATTT ACTACCGTGA   
  
  
+ GTGTCACTTT TAGATGGTAC TTTAAGAAAG CATTATAAGT AAAACAATAT ATATCGTAGC ATCATACTAC   
  
  
+ TTGTGTAATA AATTAACAGC AACCTCAATT GTACTTATTT GATTTCGCAT TGTAATCAAT TTTCCCAAAT   
  
  
+ GATTTACTAT CTTGGTCTTA CTCAGTTGTA CTTCACACTC AATAGTTCAA TTATTTTTCA TTAGACTAAT   
  
  
+ GAGCGAGTCC CTTCTACAAG AGCCTACTAC TACTCTAATA ATGTAAGGAC TATTGGCTAA TGGGGAACTA   
  
  
+ AGCCTACGAC TTGTTAGGCA TAGGTGAATT TTCCTTACAA GAGAGTTTAA TTTACACTTG ATATTAACTC   
  
  
+ GAGAACGAGA AGCTGTCTTA ATAGATACCC ACATAGAAAA TGAAGCAAAT GCCCTTGATT ATTGGAGGGT   
  
  
+ TGGGCAAATA TATTCAGTAA CAATGTTGTT TGGCAAATAT CGTCTGAAAA TTGTTTTTGA CAAATATTGT   
  
  
+ TTGGAAGAAT TTTTCGAGAT TAAATATTAA AAATGTTAAA ATTTTAATCT AAATATCAAA CAAATATTGA   
  
  
+ AAAATGCTTT ATATTATCTA AATCTCTAAT TAAAATTTGT GTATTGTCCA CTAGAAAAAT GTATAGTGAT   
  
  
+ ATTACCGACA TATATGTGAT CTCTGGTGTA ATAGCCTACC CTAATGTCTA GGAAAATATT TAAAATCCCC   
  
  
+ AGTGGTCAAA AGGAAGTTAC GCACACTAGG GCAAGACACT TCATTAGGCT AGTTGGTGAA AGCTAAGTCA   
  
  
+ TGAAATTTAG ACCATGGCCA TGTCTACTTG TAGATCCAAC TACCAACAAT CCATTTTTAT TTTGGGGAAA   
  
  
+ GCTTGAAGGG TAATTTGGTA AATGTACACG GGAACCTTTT GCTATAAAAG AAGTGCTTCT TGAAAAAACA   
  
  
+ CCCTGTAGTA AAACAGGGCA AAAAAGCCAA CACTTCTGTT TCTGGCAGAG AAACAGTAGT GTACCACTTG   
  
  
+ GAGTTGGAGT AGACACGATA CACAAACCCA GTAGAGAGAG AAACAGGGGA GAGAAAGAGT ACACAAATTT   
  
  
+ GTGCAGGAGA AGGGTAAGGA AGAAGGAGAA GGAACCCCCT CCAAAAAAAA CCCATATAAA AAATAATAAA   
  
  
+ GAAAAAACAG GAGTGAGTTA GAGGGTACCC AAATCTCCAA TCTAAAACTA GAATCTAGGG TCTCTTTTCA   
  
  
+ TTTGCTTGAA AAATTTCTGT TCATGGGTTT TGTGTTTTTA GGGGGATTTG TTTGATTTTT GTAGTCTTTG   
  
  
+ TGGATTTCCT GTGTTATAGA TGCAAGCTAT GCTCTTCAAT TTGCAAGGAA CAGCATCTGG GGGGAGAGAA   
  
  
+ TTTGAGGAAT TTCAAGTACC CATTTCAATT TCAACAAGTA AAATCATCAT TGAAGGATAC TCAAACCCAA   
  
  
+ GCCCAGTTGA TAGTGAACCA ACTTCTACTC TGGATTCTAC TCCAAGTCCC AGCCCTCCCA CCCTCTCCTC   
  
  
+ CTCCTCCAAG AACTCCTACC AAAACGCTGC CGTTTTGGCT CACCCTCCTC AGCTTCACCA TGGATCTGAT   
  
  
+ AATGTGTAAG ACCCCAACCC TTTTGCTTTT ATTTTCCTCA TGCTCCATTC TCATGATTTG TACTTCTGTT   
  
  
+ ATGTAATTGG AGATGTGTCT AAATTTGGAA AAGTTCGTGG GTTTCGTGTT TGAGAATTTA GAATGACATT   
  
  
+ CTTTAATTAG TAATTTGAAG TTTGCATGCT TAATTTAGCT AATCACTGAT TAATTAAAAT AAATGAAGTG   
  
  
+ ATGTAGGTAT GGGCTGTTAC ACAATGTCAC CGGTGGTGGT GACAGCGGCG GGGCGGCGGC GGTTGGATTG   
  
  
+ GAAGAATGGG ACAGCGTGTT TCCAAATGGG GATGGAGCTT TACTCCCTTG GATCATGGGG GAAGGTGATG   
  
  
+ ATTTGGGTCT GAATTTGAAG CATCTCTTAC AATCTGGTTA CCCTGTTGAG TATGAAGGCA ATGCAGGACT   
  
  
+ TGGTGTTGTT GATCAGACAT CTCTTCTTGG AGGTGGTGAT TTGGGGTTTT CTGGTTCTGG GTCTGTGAAT   
  
  
+ AATGGCAAAA TTGGTTCCAT TTTGGGTAAT TGTTCATCTG GGGATTTGGA TTCTAAGGTT TCTAGTGACG   
  
  
+ GGTTGAATTC CAATTGTAGT TCACTGGGGA GTATGAGTAT CCTTGGTTCA ATCCATGGAC CTTTGCCTAA   
  
  
+ TACTGGTGGG TTTGTGTTCC CACAAACACA ACTATTTGAT CTTGGTGATG AGAAGCCTCA GATTTTGAAC   
  
  
+ CCACAATTGA TGACGATGAA TCCAAATCAG GCTCAGAGCA TGGGAAACCC TAGCTTTTTT GTGCCCTCCT   
  
  
+ TGGGTTATTG TCAGTTAGAG CAACATATGG TTCAACCACA GGCGAAACGC CATAGCCCTG GTGTAGTTTT   
  
  
+ GAACTCTGAT GTTATTGCCA AAACCCCGTT TCTCGATCAG GGTCATGAGT TTTTGTTGAG GAAACAACAC   
  
  
+ CATCAACAGA TTTTGCAGCA ACTTCCTATG GGTTTGGCTC ATCAGTCGGT CCCTCAGCAC CTCCAGCAAA   
  
  
+ AGGCGATGAA AGACCAGGTT GTTAAGGACC AGCTTTTGAA GGCAGCTGAC CTGATTCAAA CTGGGAATTT   
  
  
+ CTCACTTGCG CAAGAGATAT TGGCGCGGCT CAATCACCAG CTCTCCCTTC CTGCGAAGCC CCTCATTAGG   
  
  
+ GCAGCTTTGT ATGTCATGGA GGCCTTGCAA ATGCTCATCT TAATGAGCAA TCCTGTAGCA CCTCCACCGA   
  
  
+ TGAAGACGCT TTCCCCTATA GATGTTGTTC ATAAGATGAA TGCCTACAAG GCCTTCTCTG AGGTCTCTCC   
  
  
+ TATTACTCAG TTTACGAATT TCACTTGTGC CCAAGCCATT CTTGAAGCTC TCGATGATGC GGATTGCGTT   
  
  
+ CATGTCATTG ACTTTGATAT TGGTTGTGGG GCTCAATGGG CATCACTGAT TCAGGAGCTT CCATTGAGGA   
  
  
+ AAAAGGGAGC TCCATCCCTG AAAATTACAG CCATAGCTCC CCTGTCAATA AGCCACACTT TTGAACTCAA   
  
  
+ CCTAGCACGG GAAAACCTTG TGCAATTCGC CAATGATGTT GGTGTTGCTT TTGAGCTGCA AGTTGTGAAC   
  
  
+ TTGGATTTAT TTGATCCATC TTCATCTTCA ATGCCAAATG TTGGAACCTC TGGGGATGAG TTGATCGCTG   
  
  
+ TTAGTATACC CATTTGGGCA TGTTCATATC GGCCGTCTAT TCTCCCATCC ATCCTCCGGT TCATTAAGCA   
  
  
+ ACTAGCCCCC AAGATTGTCG TCTCGTTAGA TAGAGGGTCT GATCGTTGCG ACACTCTGTT CCCACAACAT   
  
  
+ CTTATCCATA TCTTAGAGTC CTGCACAAAT TTCTTGGAAT CGCTCGACGG TCTCAATGTA GCATCAGATA   
  
  
+ TTGTAAACAA GGTTGAGAAG TACTTCCTCC AGCCTAAGAT CGAAAACACC GTGTTGGGCC GCGTTCATGC   
  
  
+ CCCCAATAAG ATGCCACATT GGAAGACCCT CTTTGCTTCA GCCGGGTTAT TGCCCTTTCA GTTCAGTAAC   
  
  
+ TTCACAGAAA CACAAGCTGA TTATGTGGTG AAGCGAACCC CAGGGAGGGG ATTTCACATA GAAAAGCGCC   
  
  
+ ACGCATCTTT AGTGCTAAGT TGGCAAAGGC GGGAGCTTGT GACAGCCTCG GCATGGAGGT TTGAGAAGCT   
  
  
+ GTA  

- +Up\_Stream \_Len000TGTAAC CCACCCTAAA TTTAGGAGAT AAAAAAGAAT CCCCTTTTTA TGAACCCTTT   
  
  
- AAAGAGTTAT TGACTTTTAG ATTTATTAAG CACTGAAACA ATCATCCAAA TTACACTAAA TGATGGCACT   
  
  
- CACAGTGAAA ATCTACCATG AAATTCTTTC GTAATATTCA TTTTGTTATA TATAGCATCG TAGTATGATG   
  
  
- AACACATTAT TTAATTGTCG TTGGAGTTAA CATGAATAAA CTAAAGCGTA ACATTAGTTA AAAGGGTTTA   
  
  
- CTAAATGATA GAACCAGAAT GAGTCAACAT GAAGTGTGAG TTATCAAGTT AATAAAAAGT AATCTGATTA   
  
  
- CTCGCTCAGG GAAGATGTTC TCGGATGATG ATGAGATTAT TACATTCCTG ATAACCGATT ACCCCTTGAT   
  
  
- TCGGATGCTG AACAATCCGT ATCCACTTAA AAGGAATGTT CTCTCAAATT AAATGTGAAC TATAATTGAG   
  
  
- CTCTTGCTCT TCGACAGAAT TATCTATGGG TGTATCTTTT ACTTCGTTTA CGGGAACTAA TAACCTCCCA   
  
  
- ACCCGTTTAT ATAAGTCATT GTTACAACAA ACCGTTTATA GCAGACTTTT AACAAAAACT GTTTATAACA   
  
  
- AACCTTCTTA AAAAGCTCTA ATTTATAATT TTTACAATTT TAAAATTAGA TTTATAGTTT GTTTATAACT   
  
  
- TTTTACGAAA TATAATAGAT TTAGAGATTA ATTTTAAACA CATAACAGGT GATCTTTTTA CATATCACTA   
  
  
- TAATGGCTGT ATATACACTA GAGACCACAT TATCGGATGG GATTACAGAT CCTTTTATAA ATTTTAGGGG   
  
  
- TCACCAGTTT TCCTTCAATG CGTGTGATCC CGTTCTGTGA AGTAATCCGA TCAACCACTT TCGATTCAGT   
  
  
- ACTTTAAATC TGGTACCGGT ACAGATGAAC ATCTAGGTTG ATGGTTGTTA GGTAAAAATA AAACCCCTTT   
  
  
- CGAACTTCCC ATTAAACCAT TTACATGTGC CCTTGGAAAA CGATATTTTC TTCACGAAGA ACTTTTTTGT   
  
  
- GGGACATCAT TTTGTCCCGT TTTTTCGGTT GTGAAGACAA AGACCGTCTC TTTGTCATCA CATGGTGAAC   
  
  
- CTCAACCTCA TCTGTGCTAT GTGTTTGGGT CATCTCTCTC TTTGTCCCCT CTCTTTCTCA TGTGTTTAAA   
  
  
- CACGTCCTCT TCCCATTCCT TCTTCCTCTT CCTTGGGGGA GGTTTTTTTT GGGTATATTT TTTATTATTT   
  
  
- CTTTTTTGTC CTCACTCAAT CTCCCATGGG TTTAGAGGTT AGATTTTGAT CTTAGATCCC AGAGAAAAGT   
  
  
- AAACGAACTT TTTAAAGACA AGTACCCAAA ACACAAAAAT CCCCCTAAAC AAACTAAAAA CATCAGAAAC   
  
  
- ACCTAAAGGA CACAATATCT ACGTTCGATA CGAGAAGTTA AACGTTCCTT GTCGTAGACC CCCCTCTCTT   
  
  
- AAACTCCTTA AAGTTCATGG GTAAAGTTAA AGTTGTTCAT TTTAGTAGTA ACTTCCTATG AGTTTGGGTT   
  
  
- CGGGTCAACT ATCACTTGGT TGAAGATGAG ACCTAAGATG AGGTTCAGGG TCGGGAGGGT GGGAGAGGAG   
  
  
- GAGGAGGTTC TTGAGGATGG TTTTGCGACG GCAAAACCGA GTGGGAGGAG TCGAAGTGGT ACCTAGACTA   
  
  
- TTACACATTC TGGGGTTGGG AAAACGAAAA TAAAAGGAGT ACGAGGTAAG AGTACTAAAC ATGAAGACAA   
  
  
- TACATTAACC TCTACACAGA TTTAAACCTT TTCAAGCACC CAAAGCACAA ACTCTTAAAT CTTACTGTAA   
  
  
- GAAATTAATC ATTAAACTTC AAACGTACGA ATTAAATCGA TTAGTGACTA ATTAATTTTA TTTACTTCAC   
  
  
- TACATCCATA CCCGACAATG TGTTACAGTG GCCACCACCA CTGTCGCCGC CCCGCCGCCG CCAACCTAAC   
  
  
- CTTCTTACCC TGTCGCACAA AGGTTTACCC CTACCTCGAA ATGAGGGAAC CTAGTACCCC CTTCCACTAC   
  
  
- TAAACCCAGA CTTAAACTTC GTAGAGAATG TTAGACCAAT GGGACAACTC ATACTTCCGT TACGTCCTGA   
  
  
- ACCACAACAA CTAGTCTGTA GAGAAGAACC TCCACCACTA AACCCCAAAA GACCAAGACC CAGACACTTA   
  
  
- TTACCGTTTT AACCAAGGTA AAACCCATTA ACAAGTAGAC CCCTAAACCT AAGATTCCAA AGATCACTGC   
  
  
- CCAACTTAAG GTTAACATCA AGTGACCCCT CATACTCATA GGAACCAAGT TAGGTACCTG GAAACGGATT   
  
  
- ATGACCACCC AAACACAAGG GTGTTTGTGT TGATAAACTA GAACCACTAC TCTTCGGAGT CTAAAACTTG   
  
  
- GGTGTTAACT ACTGCTACTT AGGTTTAGTC CGAGTCTCGT ACCCTTTGGG ATCGAAAAAA CACGGGAGGA   
  
  
- ACCCAATAAC AGTCAATCTC GTTGTATACC AAGTTGGTGT CCGCTTTGCG GTATCGGGAC CACATCAAAA   
  
  
- CTTGAGACTA CAATAACGGT TTTGGGGCAA AGAGCTAGTC CCAGTACTCA AAAACAACTC CTTTGTTGTG   
  
  
- GTAGTTGTCT AAAACGTCGT TGAAGGATAC CCAAACCGAG TAGTCAGCCA GGGAGTCGTG GAGGTCGTTT   
  
  
- TCCGCTACTT TCTGGTCCAA CAATTCCTGG TCGAAAACTT CCGTCGACTG GACTAAGTTT GACCCTTAAA   
  
  
- GAGTGAACGC GTTCTCTATA ACCGCGCCGA GTTAGTGGTC GAGAGGGAAG GACGCTTCGG GGAGTAATCC   
  
  
- CGTCGAAACA TACAGTACCT CCGGAACGTT TACGAGTAGA ATTACTCGTT AGGACATCGT GGAGGTGGCT   
  
  
- ACTTCTGCGA AAGGGGATAT CTACAACAAG TATTCTACTT ACGGATGTTC CGGAAGAGAC TCCAGAGAGG   
  
  
- ATAATGAGTC AAATGCTTAA AGTGAACACG GGTTCGGTAA GAACTTCGAG AGCTACTACG CCTAACGCAA   
  
  
- GTACAGTAAC TGAAACTATA ACCAACACCC CGAGTTACCC GTAGTGACTA AGTCCTCGAA GGTAACTCCT   
  
  
- TTTTCCCTCG AGGTAGGGAC TTTTAATGTC GGTATCGAGG GGACAGTTAT TCGGTGTGAA AACTTGAGTT   
  
  
- GGATCGTGCC CTTTTGGAAC ACGTTAAGCG GTTACTACAA CCACAACGAA AACTCGACGT TCAACACTTG   
  
  
- AACCTAAATA AACTAGGTAG AAGTAGAAGT TACGGTTTAC AACCTTGGAG ACCCCTACTC AACTAGCGAC   
  
  
- AATCATATGG GTAAACCCGT ACAAGTATAG CCGGCAGATA AGAGGGTAGG TAGGAGGCCA AGTAATTCGT   
  
  
- TGATCGGGGG TTCTAACAGC AGAGCAATCT ATCTCCCAGA CTAGCAACGC TGTGAGACAA GGGTGTTGTA   
  
  
- GAATAGGTAT AGAATCTCAG GACGTGTTTA AAGAACCTTA GCGAGCTGCC AGAGTTACAT CGTAGTCTAT   
  
  
- AACATTTGTT CCAACTCTTC ATGAAGGAGG TCGGATTCTA GCTTTTGTGG CACAACCCGG CGCAAGTACG   
  
  
- GGGGTTATTC TACGGTGTAA CCTTCTGGGA GAAACGAAGT CGGCCCAATA ACGGGAAAGT CAAGTCATTG   
  
  
- AAGTGTCTTT GTGTTCGACT AATACACCAC TTCGCTTGGG GTCCCTCCCC TAAAGTGTAT CTTTTCGCGG   
  
  
- TGCGTAGAAA TCACGATTCA ACCGTTTCCG CCCTCGAACA CTGTCGGAGC CGTACCTCCA AACTCTTCGA   
  
  
- CAT

+     O2-site

| Site Name | Organism | Position | Strand | Matrix score. | sequence | function |
| --- | --- | --- | --- | --- | --- | --- |
| O2-site | Zea mays | 2997 | + | 9 | GATGATGTGG | cis-acting regulatory element involved in zein metabolism regulation |
| O2-site | Zea mays | 3426 | - | 9 | GATGATGTGG | cis-acting regulatory element involved in zein metabolism regulation |
| O2-site | Zea mays | 3663 | + | 9 | GATGATGTGG | cis-acting regulatory element involved in zein metabolism regulation |
| O2-site | Zea mays | 2031 | + | 9 | GATGATGTGG | cis-acting regulatory element involved in zein metabolism regulation |

>HU05G00466.1   
+ +Up\_Stream \_Len000ACATTG GGTGGGATTT AAATCCTCTA TTTTTTCTTA GGGGAAAAAT ACTTGGGAAA   
  
  
+ TTTCTCAATA ACTGAAAATC TAAATAATTC GTGACTTTGT TAGTAGGTTT AATGTGATTT ACTACCGTGA   
  
  
+ GTGTCACTTT TAGATGGTAC TTTAAGAAAG CATTATAAGT AAAACAATAT ATATCGTAGC ATCATACTAC   
  
  
+ TTGTGTAATA AATTAACAGC AACCTCAATT GTACTTATTT GATTTCGCAT TGTAATCAAT TTTCCCAAAT   
  
  
+ GATTTACTAT CTTGGTCTTA CTCAGTTGTA CTTCACACTC AATAGTTCAA TTATTTTTCA TTAGACTAAT   
  
  
+ GAGCGAGTCC CTTCTACAAG AGCCTACTAC TACTCTAATA ATGTAAGGAC TATTGGCTAA TGGGGAACTA   
  
  
+ AGCCTACGAC TTGTTAGGCA TAGGTGAATT TTCCTTACAA GAGAGTTTAA TTTACACTTG ATATTAACTC   
  
  
+ GAGAACGAGA AGCTGTCTTA ATAGATACCC ACATAGAAAA TGAAGCAAAT GCCCTTGATT ATTGGAGGGT   
  
  
+ TGGGCAAATA TATTCAGTAA CAATGTTGTT TGGCAAATAT CGTCTGAAAA TTGTTTTTGA CAAATATTGT   
  
  
+ TTGGAAGAAT TTTTCGAGAT TAAATATTAA AAATGTTAAA ATTTTAATCT AAATATCAAA CAAATATTGA   
  
  
+ AAAATGCTTT ATATTATCTA AATCTCTAAT TAAAATTTGT GTATTGTCCA CTAGAAAAAT GTATAGTGAT   
  
  
+ ATTACCGACA TATATGTGAT CTCTGGTGTA ATAGCCTACC CTAATGTCTA GGAAAATATT TAAAATCCCC   
  
  
+ AGTGGTCAAA AGGAAGTTAC GCACACTAGG GCAAGACACT TCATTAGGCT AGTTGGTGAA AGCTAAGTCA   
  
  
+ TGAAATTTAG ACCATGGCCA TGTCTACTTG TAGATCCAAC TACCAACAAT CCATTTTTAT TTTGGGGAAA   
  
  
+ GCTTGAAGGG TAATTTGGTA AATGTACACG GGAACCTTTT GCTATAAAAG AAGTGCTTCT TGAAAAAACA   
  
  
+ CCCTGTAGTA AAACAGGGCA AAAAAGCCAA CACTTCTGTT TCTGGCAGAG AAACAGTAGT GTACCACTTG   
  
  
+ GAGTTGGAGT AGACACGATA CACAAACCCA GTAGAGAGAG AAACAGGGGA GAGAAAGAGT ACACAAATTT   
  
  
+ GTGCAGGAGA AGGGTAAGGA AGAAGGAGAA GGAACCCCCT CCAAAAAAAA CCCATATAAA AAATAATAAA   
  
  
+ GAAAAAACAG GAGTGAGTTA GAGGGTACCC AAATCTCCAA TCTAAAACTA GAATCTAGGG TCTCTTTTCA   
  
  
+ TTTGCTTGAA AAATTTCTGT TCATGGGTTT TGTGTTTTTA GGGGGATTTG TTTGATTTTT GTAGTCTTTG   
  
  
+ TGGATTTCCT GTGTTATAGA TGCAAGCTAT GCTCTTCAAT TTGCAAGGAA CAGCATCTGG GGGGAGAGAA   
  
  
+ TTTGAGGAAT TTCAAGTACC CATTTCAATT TCAACAAGTA AAATCATCAT TGAAGGATAC TCAAACCCAA   
  
  
+ GCCCAGTTGA TAGTGAACCA ACTTCTACTC TGGATTCTAC TCCAAGTCCC AGCCCTCCCA CCCTCTCCTC   
  
  
+ CTCCTCCAAG AACTCCTACC AAAACGCTGC CGTTTTGGCT CACCCTCCTC AGCTTCACCA TGGATCTGAT   
  
  
+ AATGTGTAAG ACCCCAACCC TTTTGCTTTT ATTTTCCTCA TGCTCCATTC TCATGATTTG TACTTCTGTT   
  
  
+ ATGTAATTGG AGATGTGTCT AAATTTGGAA AAGTTCGTGG GTTTCGTGTT TGAGAATTTA GAATGACATT   
  
  
+ CTTTAATTAG TAATTTGAAG TTTGCATGCT TAATTTAGCT AATCACTGAT TAATTAAAAT AAATGAAGTG   
  
  
+ ATGTAGGTAT GGGCTGTTAC ACAATGTCAC CGGTGGTGGT GACAGCGGCG GGGCGGCGGC GGTTGGATTG   
  
  
+ GAAGAATGGG ACAGCGTGTT TCCAAATGGG GATGGAGCTT TACTCCCTTG GATCATGGGG GAAGGTGATG   
  
  
+ ATTTGGGTCT GAATTTGAAG CATCTCTTAC AATCTGGTTA CCCTGTTGAG TATGAAGGCA ATGCAGGACT   
  
  
+ TGGTGTTGTT GATCAGACAT CTCTTCTTGG AGGTGGTGAT TTGGGGTTTT CTGGTTCTGG GTCTGTGAAT   
  
  
+ AATGGCAAAA TTGGTTCCAT TTTGGGTAAT TGTTCATCTG GGGATTTGGA TTCTAAGGTT TCTAGTGACG   
  
  
+ GGTTGAATTC CAATTGTAGT TCACTGGGGA GTATGAGTAT CCTTGGTTCA ATCCATGGAC CTTTGCCTAA   
  
  
+ TACTGGTGGG TTTGTGTTCC CACAAACACA ACTATTTGAT CTTGGTGATG AGAAGCCTCA GATTTTGAAC   
  
  
+ CCACAATTGA TGACGATGAA TCCAAATCAG GCTCAGAGCA TGGGAAACCC TAGCTTTTTT GTGCCCTCCT   
  
  
+ TGGGTTATTG TCAGTTAGAG CAACATATGG TTCAACCACA GGCGAAACGC CATAGCCCTG GTGTAGTTTT   
  
  
+ GAACTCTGAT GTTATTGCCA AAACCCCGTT TCTCGATCAG GGTCATGAGT TTTTGTTGAG GAAACAACAC   
  
  
+ CATCAACAGA TTTTGCAGCA ACTTCCTATG GGTTTGGCTC ATCAGTCGGT CCCTCAGCAC CTCCAGCAAA   
  
  
+ AGGCGATGAA AGACCAGGTT GTTAAGGACC AGCTTTTGAA GGCAGCTGAC CTGATTCAAA CTGGGAATTT   
  
  
+ CTCACTTGCG CAAGAGATAT TGGCGCGGCT CAATCACCAG CTCTCCCTTC CTGCGAAGCC CCTCATTAGG   
  
  
+ GCAGCTTTGT ATGTCATGGA GGCCTTGCAA ATGCTCATCT TAATGAGCAA TCCTGTAGCA CCTCCACCGA   
  
  
+ TGAAGACGCT TTCCCCTATA GATGTTGTTC ATAAGATGAA TGCCTACAAG GCCTTCTCTG AGGTCTCTCC   
  
  
+ TATTACTCAG TTTACGAATT TCACTTGTGC CCAAGCCATT CTTGAAGCTC TCGATGATGC GGATTGCGTT   
  
  
+ CATGTCATTG ACTTTGATAT TGGTTGTGGG GCTCAATGGG CATCACTGAT TCAGGAGCTT CCATTGAGGA   
  
  
+ AAAAGGGAGC TCCATCCCTG AAAATTACAG CCATAGCTCC CCTGTCAATA AGCCACACTT TTGAACTCAA   
  
  
+ CCTAGCACGG GAAAACCTTG TGCAATTCGC CAATGATGTT GGTGTTGCTT TTGAGCTGCA AGTTGTGAAC   
  
  
+ TTGGATTTAT TTGATCCATC TTCATCTTCA ATGCCAAATG TTGGAACCTC TGGGGATGAG TTGATCGCTG   
  
  
+ TTAGTATACC CATTTGGGCA TGTTCATATC GGCCGTCTAT TCTCCCATCC ATCCTCCGGT TCATTAAGCA   
  
  
+ ACTAGCCCCC AAGATTGTCG TCTCGTTAGA TAGAGGGTCT GATCGTTGCG ACACTCTGTT CCCACAACAT   
  
  
+ CTTATCCATA TCTTAGAGTC CTGCACAAAT TTCTTGGAAT CGCTCGACGG TCTCAATGTA GCATCAGATA   
  
  
+ TTGTAAACAA GGTTGAGAAG TACTTCCTCC AGCCTAAGAT CGAAAACACC GTGTTGGGCC GCGTTCATGC   
  
  
+ CCCCAATAAG ATGCCACATT GGAAGACCCT CTTTGCTTCA GCCGGGTTAT TGCCCTTTCA GTTCAGTAAC   
  
  
+ TTCACAGAAA CACAAGCTGA TTATGTGGTG AAGCGAACCC CAGGGAGGGG ATTTCACATA GAAAAGCGCC   
  
  
+ ACGCATCTTT AGTGCTAAGT TGGCAAAGGC GGGAGCTTGT GACAGCCTCG GCATGGAGGT TTGAGAAGCT   
  
  
+ GTA  

- +Up\_Stream \_Len000TGTAAC CCACCCTAAA TTTAGGAGAT AAAAAAGAAT CCCCTTTTTA TGAACCCTTT   
  
  
- AAAGAGTTAT TGACTTTTAG ATTTATTAAG CACTGAAACA ATCATCCAAA TTACACTAAA TGATGGCACT   
  
  
- CACAGTGAAA ATCTACCATG AAATTCTTTC GTAATATTCA TTTTGTTATA TATAGCATCG TAGTATGATG   
  
  
- AACACATTAT TTAATTGTCG TTGGAGTTAA CATGAATAAA CTAAAGCGTA ACATTAGTTA AAAGGGTTTA   
  
  
- CTAAATGATA GAACCAGAAT GAGTCAACAT GAAGTGTGAG TTATCAAGTT AATAAAAAGT AATCTGATTA   
  
  
- CTCGCTCAGG GAAGATGTTC TCGGATGATG ATGAGATTAT TACATTCCTG ATAACCGATT ACCCCTTGAT   
  
  
- TCGGATGCTG AACAATCCGT ATCCACTTAA AAGGAATGTT CTCTCAAATT AAATGTGAAC TATAATTGAG   
  
  
- CTCTTGCTCT TCGACAGAAT TATCTATGGG TGTATCTTTT ACTTCGTTTA CGGGAACTAA TAACCTCCCA   
  
  
- ACCCGTTTAT ATAAGTCATT GTTACAACAA ACCGTTTATA GCAGACTTTT AACAAAAACT GTTTATAACA   
  
  
- AACCTTCTTA AAAAGCTCTA ATTTATAATT TTTACAATTT TAAAATTAGA TTTATAGTTT GTTTATAACT   
  
  
- TTTTACGAAA TATAATAGAT TTAGAGATTA ATTTTAAACA CATAACAGGT GATCTTTTTA CATATCACTA   
  
  
- TAATGGCTGT ATATACACTA GAGACCACAT TATCGGATGG GATTACAGAT CCTTTTATAA ATTTTAGGGG   
  
  
- TCACCAGTTT TCCTTCAATG CGTGTGATCC CGTTCTGTGA AGTAATCCGA TCAACCACTT TCGATTCAGT   
  
  
- ACTTTAAATC TGGTACCGGT ACAGATGAAC ATCTAGGTTG ATGGTTGTTA GGTAAAAATA AAACCCCTTT   
  
  
- CGAACTTCCC ATTAAACCAT TTACATGTGC CCTTGGAAAA CGATATTTTC TTCACGAAGA ACTTTTTTGT   
  
  
- GGGACATCAT TTTGTCCCGT TTTTTCGGTT GTGAAGACAA AGACCGTCTC TTTGTCATCA CATGGTGAAC   
  
  
- CTCAACCTCA TCTGTGCTAT GTGTTTGGGT CATCTCTCTC TTTGTCCCCT CTCTTTCTCA TGTGTTTAAA   
  
  
- CACGTCCTCT TCCCATTCCT TCTTCCTCTT CCTTGGGGGA GGTTTTTTTT GGGTATATTT TTTATTATTT   
  
  
- CTTTTTTGTC CTCACTCAAT CTCCCATGGG TTTAGAGGTT AGATTTTGAT CTTAGATCCC AGAGAAAAGT   
  
  
- AAACGAACTT TTTAAAGACA AGTACCCAAA ACACAAAAAT CCCCCTAAAC AAACTAAAAA CATCAGAAAC   
  
  
- ACCTAAAGGA CACAATATCT ACGTTCGATA CGAGAAGTTA AACGTTCCTT GTCGTAGACC CCCCTCTCTT   
  
  
- AAACTCCTTA AAGTTCATGG GTAAAGTTAA AGTTGTTCAT TTTAGTAGTA ACTTCCTATG AGTTTGGGTT   
  
  
- CGGGTCAACT ATCACTTGGT TGAAGATGAG ACCTAAGATG AGGTTCAGGG TCGGGAGGGT GGGAGAGGAG   
  
  
- GAGGAGGTTC TTGAGGATGG TTTTGCGACG GCAAAACCGA GTGGGAGGAG TCGAAGTGGT ACCTAGACTA   
  
  
- TTACACATTC TGGGGTTGGG AAAACGAAAA TAAAAGGAGT ACGAGGTAAG AGTACTAAAC ATGAAGACAA   
  
  
- TACATTAACC TCTACACAGA TTTAAACCTT TTCAAGCACC CAAAGCACAA ACTCTTAAAT CTTACTGTAA   
  
  
- GAAATTAATC ATTAAACTTC AAACGTACGA ATTAAATCGA TTAGTGACTA ATTAATTTTA TTTACTTCAC   
  
  
- TACATCCATA CCCGACAATG TGTTACAGTG GCCACCACCA CTGTCGCCGC CCCGCCGCCG CCAACCTAAC   
  
  
- CTTCTTACCC TGTCGCACAA AGGTTTACCC CTACCTCGAA ATGAGGGAAC CTAGTACCCC CTTCCACTAC   
  
  
- TAAACCCAGA CTTAAACTTC GTAGAGAATG TTAGACCAAT GGGACAACTC ATACTTCCGT TACGTCCTGA   
  
  
- ACCACAACAA CTAGTCTGTA GAGAAGAACC TCCACCACTA AACCCCAAAA GACCAAGACC CAGACACTTA   
  
  
- TTACCGTTTT AACCAAGGTA AAACCCATTA ACAAGTAGAC CCCTAAACCT AAGATTCCAA AGATCACTGC   
  
  
- CCAACTTAAG GTTAACATCA AGTGACCCCT CATACTCATA GGAACCAAGT TAGGTACCTG GAAACGGATT   
  
  
- ATGACCACCC AAACACAAGG GTGTTTGTGT TGATAAACTA GAACCACTAC TCTTCGGAGT CTAAAACTTG   
  
  
- GGTGTTAACT ACTGCTACTT AGGTTTAGTC CGAGTCTCGT ACCCTTTGGG ATCGAAAAAA CACGGGAGGA   
  
  
- ACCCAATAAC AGTCAATCTC GTTGTATACC AAGTTGGTGT CCGCTTTGCG GTATCGGGAC CACATCAAAA   
  
  
- CTTGAGACTA CAATAACGGT TTTGGGGCAA AGAGCTAGTC CCAGTACTCA AAAACAACTC CTTTGTTGTG   
  
  
- GTAGTTGTCT AAAACGTCGT TGAAGGATAC CCAAACCGAG TAGTCAGCCA GGGAGTCGTG GAGGTCGTTT   
  
  
- TCCGCTACTT TCTGGTCCAA CAATTCCTGG TCGAAAACTT CCGTCGACTG GACTAAGTTT GACCCTTAAA   
  
  
- GAGTGAACGC GTTCTCTATA ACCGCGCCGA GTTAGTGGTC GAGAGGGAAG GACGCTTCGG GGAGTAATCC   
  
  
- CGTCGAAACA TACAGTACCT CCGGAACGTT TACGAGTAGA ATTACTCGTT AGGACATCGT GGAGGTGGCT   
  
  
- ACTTCTGCGA AAGGGGATAT CTACAACAAG TATTCTACTT ACGGATGTTC CGGAAGAGAC TCCAGAGAGG   
  
  
- ATAATGAGTC AAATGCTTAA AGTGAACACG GGTTCGGTAA GAACTTCGAG AGCTACTACG CCTAACGCAA   
  
  
- GTACAGTAAC TGAAACTATA ACCAACACCC CGAGTTACCC GTAGTGACTA AGTCCTCGAA GGTAACTCCT   
  
  
- TTTTCCCTCG AGGTAGGGAC TTTTAATGTC GGTATCGAGG GGACAGTTAT TCGGTGTGAA AACTTGAGTT   
  
  
- GGATCGTGCC CTTTTGGAAC ACGTTAAGCG GTTACTACAA CCACAACGAA AACTCGACGT TCAACACTTG   
  
  
- AACCTAAATA AACTAGGTAG AAGTAGAAGT TACGGTTTAC AACCTTGGAG ACCCCTACTC AACTAGCGAC   
  
  
- AATCATATGG GTAAACCCGT ACAAGTATAG CCGGCAGATA AGAGGGTAGG TAGGAGGCCA AGTAATTCGT   
  
  
- TGATCGGGGG TTCTAACAGC AGAGCAATCT ATCTCCCAGA CTAGCAACGC TGTGAGACAA GGGTGTTGTA   
  
  
- GAATAGGTAT AGAATCTCAG GACGTGTTTA AAGAACCTTA GCGAGCTGCC AGAGTTACAT CGTAGTCTAT   
  
  
- AACATTTGTT CCAACTCTTC ATGAAGGAGG TCGGATTCTA GCTTTTGTGG CACAACCCGG CGCAAGTACG   
  
  
- GGGGTTATTC TACGGTGTAA CCTTCTGGGA GAAACGAAGT CGGCCCAATA ACGGGAAAGT CAAGTCATTG   
  
  
- AAGTGTCTTT GTGTTCGACT AATACACCAC TTCGCTTGGG GTCCCTCCCC TAAAGTGTAT CTTTTCGCGG   
  
  
- TGCGTAGAAA TCACGATTCA ACCGTTTCCG CCCTCGAACA CTGTCGGAGC CGTACCTCCA AACTCTTCGA   
  
  
- CAT

+     P-box

| Site Name | Organism | Position | Strand | Matrix score. | sequence | function |
| --- | --- | --- | --- | --- | --- | --- |
| P-box | Oryza sativa | 1019 | + | 7 | CCTTTTG | gibberellin-responsive element |
| P-box | Oryza sativa | 2661 | - | 7 | CCTTTTG | gibberellin-responsive element |
| P-box | Oryza sativa | 851 | - | 7 | CCTTTTG | gibberellin-responsive element |
| P-box | Oryza sativa | 1703 | + | 7 | CCTTTTG | gibberellin-responsive element |

>HU05G00466.1   
+ +Up\_Stream \_Len000ACATTG GGTGGGATTT AAATCCTCTA TTTTTTCTTA GGGGAAAAAT ACTTGGGAAA   
  
  
+ TTTCTCAATA ACTGAAAATC TAAATAATTC GTGACTTTGT TAGTAGGTTT AATGTGATTT ACTACCGTGA   
  
  
+ GTGTCACTTT TAGATGGTAC TTTAAGAAAG CATTATAAGT AAAACAATAT ATATCGTAGC ATCATACTAC   
  
  
+ TTGTGTAATA AATTAACAGC AACCTCAATT GTACTTATTT GATTTCGCAT TGTAATCAAT TTTCCCAAAT   
  
  
+ GATTTACTAT CTTGGTCTTA CTCAGTTGTA CTTCACACTC AATAGTTCAA TTATTTTTCA TTAGACTAAT   
  
  
+ GAGCGAGTCC CTTCTACAAG AGCCTACTAC TACTCTAATA ATGTAAGGAC TATTGGCTAA TGGGGAACTA   
  
  
+ AGCCTACGAC TTGTTAGGCA TAGGTGAATT TTCCTTACAA GAGAGTTTAA TTTACACTTG ATATTAACTC   
  
  
+ GAGAACGAGA AGCTGTCTTA ATAGATACCC ACATAGAAAA TGAAGCAAAT GCCCTTGATT ATTGGAGGGT   
  
  
+ TGGGCAAATA TATTCAGTAA CAATGTTGTT TGGCAAATAT CGTCTGAAAA TTGTTTTTGA CAAATATTGT   
  
  
+ TTGGAAGAAT TTTTCGAGAT TAAATATTAA AAATGTTAAA ATTTTAATCT AAATATCAAA CAAATATTGA   
  
  
+ AAAATGCTTT ATATTATCTA AATCTCTAAT TAAAATTTGT GTATTGTCCA CTAGAAAAAT GTATAGTGAT   
  
  
+ ATTACCGACA TATATGTGAT CTCTGGTGTA ATAGCCTACC CTAATGTCTA GGAAAATATT TAAAATCCCC   
  
  
+ AGTGGTCAAA AGGAAGTTAC GCACACTAGG GCAAGACACT TCATTAGGCT AGTTGGTGAA AGCTAAGTCA   
  
  
+ TGAAATTTAG ACCATGGCCA TGTCTACTTG TAGATCCAAC TACCAACAAT CCATTTTTAT TTTGGGGAAA   
  
  
+ GCTTGAAGGG TAATTTGGTA AATGTACACG GGAACCTTTT GCTATAAAAG AAGTGCTTCT TGAAAAAACA   
  
  
+ CCCTGTAGTA AAACAGGGCA AAAAAGCCAA CACTTCTGTT TCTGGCAGAG AAACAGTAGT GTACCACTTG   
  
  
+ GAGTTGGAGT AGACACGATA CACAAACCCA GTAGAGAGAG AAACAGGGGA GAGAAAGAGT ACACAAATTT   
  
  
+ GTGCAGGAGA AGGGTAAGGA AGAAGGAGAA GGAACCCCCT CCAAAAAAAA CCCATATAAA AAATAATAAA   
  
  
+ GAAAAAACAG GAGTGAGTTA GAGGGTACCC AAATCTCCAA TCTAAAACTA GAATCTAGGG TCTCTTTTCA   
  
  
+ TTTGCTTGAA AAATTTCTGT TCATGGGTTT TGTGTTTTTA GGGGGATTTG TTTGATTTTT GTAGTCTTTG   
  
  
+ TGGATTTCCT GTGTTATAGA TGCAAGCTAT GCTCTTCAAT TTGCAAGGAA CAGCATCTGG GGGGAGAGAA   
  
  
+ TTTGAGGAAT TTCAAGTACC CATTTCAATT TCAACAAGTA AAATCATCAT TGAAGGATAC TCAAACCCAA   
  
  
+ GCCCAGTTGA TAGTGAACCA ACTTCTACTC TGGATTCTAC TCCAAGTCCC AGCCCTCCCA CCCTCTCCTC   
  
  
+ CTCCTCCAAG AACTCCTACC AAAACGCTGC CGTTTTGGCT CACCCTCCTC AGCTTCACCA TGGATCTGAT   
  
  
+ AATGTGTAAG ACCCCAACCC TTTTGCTTTT ATTTTCCTCA TGCTCCATTC TCATGATTTG TACTTCTGTT   
  
  
+ ATGTAATTGG AGATGTGTCT AAATTTGGAA AAGTTCGTGG GTTTCGTGTT TGAGAATTTA GAATGACATT   
  
  
+ CTTTAATTAG TAATTTGAAG TTTGCATGCT TAATTTAGCT AATCACTGAT TAATTAAAAT AAATGAAGTG   
  
  
+ ATGTAGGTAT GGGCTGTTAC ACAATGTCAC CGGTGGTGGT GACAGCGGCG GGGCGGCGGC GGTTGGATTG   
  
  
+ GAAGAATGGG ACAGCGTGTT TCCAAATGGG GATGGAGCTT TACTCCCTTG GATCATGGGG GAAGGTGATG   
  
  
+ ATTTGGGTCT GAATTTGAAG CATCTCTTAC AATCTGGTTA CCCTGTTGAG TATGAAGGCA ATGCAGGACT   
  
  
+ TGGTGTTGTT GATCAGACAT CTCTTCTTGG AGGTGGTGAT TTGGGGTTTT CTGGTTCTGG GTCTGTGAAT   
  
  
+ AATGGCAAAA TTGGTTCCAT TTTGGGTAAT TGTTCATCTG GGGATTTGGA TTCTAAGGTT TCTAGTGACG   
  
  
+ GGTTGAATTC CAATTGTAGT TCACTGGGGA GTATGAGTAT CCTTGGTTCA ATCCATGGAC CTTTGCCTAA   
  
  
+ TACTGGTGGG TTTGTGTTCC CACAAACACA ACTATTTGAT CTTGGTGATG AGAAGCCTCA GATTTTGAAC   
  
  
+ CCACAATTGA TGACGATGAA TCCAAATCAG GCTCAGAGCA TGGGAAACCC TAGCTTTTTT GTGCCCTCCT   
  
  
+ TGGGTTATTG TCAGTTAGAG CAACATATGG TTCAACCACA GGCGAAACGC CATAGCCCTG GTGTAGTTTT   
  
  
+ GAACTCTGAT GTTATTGCCA AAACCCCGTT TCTCGATCAG GGTCATGAGT TTTTGTTGAG GAAACAACAC   
  
  
+ CATCAACAGA TTTTGCAGCA ACTTCCTATG GGTTTGGCTC ATCAGTCGGT CCCTCAGCAC CTCCAGCAAA   
  
  
+ AGGCGATGAA AGACCAGGTT GTTAAGGACC AGCTTTTGAA GGCAGCTGAC CTGATTCAAA CTGGGAATTT   
  
  
+ CTCACTTGCG CAAGAGATAT TGGCGCGGCT CAATCACCAG CTCTCCCTTC CTGCGAAGCC CCTCATTAGG   
  
  
+ GCAGCTTTGT ATGTCATGGA GGCCTTGCAA ATGCTCATCT TAATGAGCAA TCCTGTAGCA CCTCCACCGA   
  
  
+ TGAAGACGCT TTCCCCTATA GATGTTGTTC ATAAGATGAA TGCCTACAAG GCCTTCTCTG AGGTCTCTCC   
  
  
+ TATTACTCAG TTTACGAATT TCACTTGTGC CCAAGCCATT CTTGAAGCTC TCGATGATGC GGATTGCGTT   
  
  
+ CATGTCATTG ACTTTGATAT TGGTTGTGGG GCTCAATGGG CATCACTGAT TCAGGAGCTT CCATTGAGGA   
  
  
+ AAAAGGGAGC TCCATCCCTG AAAATTACAG CCATAGCTCC CCTGTCAATA AGCCACACTT TTGAACTCAA   
  
  
+ CCTAGCACGG GAAAACCTTG TGCAATTCGC CAATGATGTT GGTGTTGCTT TTGAGCTGCA AGTTGTGAAC   
  
  
+ TTGGATTTAT TTGATCCATC TTCATCTTCA ATGCCAAATG TTGGAACCTC TGGGGATGAG TTGATCGCTG   
  
  
+ TTAGTATACC CATTTGGGCA TGTTCATATC GGCCGTCTAT TCTCCCATCC ATCCTCCGGT TCATTAAGCA   
  
  
+ ACTAGCCCCC AAGATTGTCG TCTCGTTAGA TAGAGGGTCT GATCGTTGCG ACACTCTGTT CCCACAACAT   
  
  
+ CTTATCCATA TCTTAGAGTC CTGCACAAAT TTCTTGGAAT CGCTCGACGG TCTCAATGTA GCATCAGATA   
  
  
+ TTGTAAACAA GGTTGAGAAG TACTTCCTCC AGCCTAAGAT CGAAAACACC GTGTTGGGCC GCGTTCATGC   
  
  
+ CCCCAATAAG ATGCCACATT GGAAGACCCT CTTTGCTTCA GCCGGGTTAT TGCCCTTTCA GTTCAGTAAC   
  
  
+ TTCACAGAAA CACAAGCTGA TTATGTGGTG AAGCGAACCC CAGGGAGGGG ATTTCACATA GAAAAGCGCC   
  
  
+ ACGCATCTTT AGTGCTAAGT TGGCAAAGGC GGGAGCTTGT GACAGCCTCG GCATGGAGGT TTGAGAAGCT   
  
  
+ GTA  

- +Up\_Stream \_Len000TGTAAC CCACCCTAAA TTTAGGAGAT AAAAAAGAAT CCCCTTTTTA TGAACCCTTT   
  
  
- AAAGAGTTAT TGACTTTTAG ATTTATTAAG CACTGAAACA ATCATCCAAA TTACACTAAA TGATGGCACT   
  
  
- CACAGTGAAA ATCTACCATG AAATTCTTTC GTAATATTCA TTTTGTTATA TATAGCATCG TAGTATGATG   
  
  
- AACACATTAT TTAATTGTCG TTGGAGTTAA CATGAATAAA CTAAAGCGTA ACATTAGTTA AAAGGGTTTA   
  
  
- CTAAATGATA GAACCAGAAT GAGTCAACAT GAAGTGTGAG TTATCAAGTT AATAAAAAGT AATCTGATTA   
  
  
- CTCGCTCAGG GAAGATGTTC TCGGATGATG ATGAGATTAT TACATTCCTG ATAACCGATT ACCCCTTGAT   
  
  
- TCGGATGCTG AACAATCCGT ATCCACTTAA AAGGAATGTT CTCTCAAATT AAATGTGAAC TATAATTGAG   
  
  
- CTCTTGCTCT TCGACAGAAT TATCTATGGG TGTATCTTTT ACTTCGTTTA CGGGAACTAA TAACCTCCCA   
  
  
- ACCCGTTTAT ATAAGTCATT GTTACAACAA ACCGTTTATA GCAGACTTTT AACAAAAACT GTTTATAACA   
  
  
- AACCTTCTTA AAAAGCTCTA ATTTATAATT TTTACAATTT TAAAATTAGA TTTATAGTTT GTTTATAACT   
  
  
- TTTTACGAAA TATAATAGAT TTAGAGATTA ATTTTAAACA CATAACAGGT GATCTTTTTA CATATCACTA   
  
  
- TAATGGCTGT ATATACACTA GAGACCACAT TATCGGATGG GATTACAGAT CCTTTTATAA ATTTTAGGGG   
  
  
- TCACCAGTTT TCCTTCAATG CGTGTGATCC CGTTCTGTGA AGTAATCCGA TCAACCACTT TCGATTCAGT   
  
  
- ACTTTAAATC TGGTACCGGT ACAGATGAAC ATCTAGGTTG ATGGTTGTTA GGTAAAAATA AAACCCCTTT   
  
  
- CGAACTTCCC ATTAAACCAT TTACATGTGC CCTTGGAAAA CGATATTTTC TTCACGAAGA ACTTTTTTGT   
  
  
- GGGACATCAT TTTGTCCCGT TTTTTCGGTT GTGAAGACAA AGACCGTCTC TTTGTCATCA CATGGTGAAC   
  
  
- CTCAACCTCA TCTGTGCTAT GTGTTTGGGT CATCTCTCTC TTTGTCCCCT CTCTTTCTCA TGTGTTTAAA   
  
  
- CACGTCCTCT TCCCATTCCT TCTTCCTCTT CCTTGGGGGA GGTTTTTTTT GGGTATATTT TTTATTATTT   
  
  
- CTTTTTTGTC CTCACTCAAT CTCCCATGGG TTTAGAGGTT AGATTTTGAT CTTAGATCCC AGAGAAAAGT   
  
  
- AAACGAACTT TTTAAAGACA AGTACCCAAA ACACAAAAAT CCCCCTAAAC AAACTAAAAA CATCAGAAAC   
  
  
- ACCTAAAGGA CACAATATCT ACGTTCGATA CGAGAAGTTA AACGTTCCTT GTCGTAGACC CCCCTCTCTT   
  
  
- AAACTCCTTA AAGTTCATGG GTAAAGTTAA AGTTGTTCAT TTTAGTAGTA ACTTCCTATG AGTTTGGGTT   
  
  
- CGGGTCAACT ATCACTTGGT TGAAGATGAG ACCTAAGATG AGGTTCAGGG TCGGGAGGGT GGGAGAGGAG   
  
  
- GAGGAGGTTC TTGAGGATGG TTTTGCGACG GCAAAACCGA GTGGGAGGAG TCGAAGTGGT ACCTAGACTA   
  
  
- TTACACATTC TGGGGTTGGG AAAACGAAAA TAAAAGGAGT ACGAGGTAAG AGTACTAAAC ATGAAGACAA   
  
  
- TACATTAACC TCTACACAGA TTTAAACCTT TTCAAGCACC CAAAGCACAA ACTCTTAAAT CTTACTGTAA   
  
  
- GAAATTAATC ATTAAACTTC AAACGTACGA ATTAAATCGA TTAGTGACTA ATTAATTTTA TTTACTTCAC   
  
  
- TACATCCATA CCCGACAATG TGTTACAGTG GCCACCACCA CTGTCGCCGC CCCGCCGCCG CCAACCTAAC   
  
  
- CTTCTTACCC TGTCGCACAA AGGTTTACCC CTACCTCGAA ATGAGGGAAC CTAGTACCCC CTTCCACTAC   
  
  
- TAAACCCAGA CTTAAACTTC GTAGAGAATG TTAGACCAAT GGGACAACTC ATACTTCCGT TACGTCCTGA   
  
  
- ACCACAACAA CTAGTCTGTA GAGAAGAACC TCCACCACTA AACCCCAAAA GACCAAGACC CAGACACTTA   
  
  
- TTACCGTTTT AACCAAGGTA AAACCCATTA ACAAGTAGAC CCCTAAACCT AAGATTCCAA AGATCACTGC   
  
  
- CCAACTTAAG GTTAACATCA AGTGACCCCT CATACTCATA GGAACCAAGT TAGGTACCTG GAAACGGATT   
  
  
- ATGACCACCC AAACACAAGG GTGTTTGTGT TGATAAACTA GAACCACTAC TCTTCGGAGT CTAAAACTTG   
  
  
- GGTGTTAACT ACTGCTACTT AGGTTTAGTC CGAGTCTCGT ACCCTTTGGG ATCGAAAAAA CACGGGAGGA   
  
  
- ACCCAATAAC AGTCAATCTC GTTGTATACC AAGTTGGTGT CCGCTTTGCG GTATCGGGAC CACATCAAAA   
  
  
- CTTGAGACTA CAATAACGGT TTTGGGGCAA AGAGCTAGTC CCAGTACTCA AAAACAACTC CTTTGTTGTG   
  
  
- GTAGTTGTCT AAAACGTCGT TGAAGGATAC CCAAACCGAG TAGTCAGCCA GGGAGTCGTG GAGGTCGTTT   
  
  
- TCCGCTACTT TCTGGTCCAA CAATTCCTGG TCGAAAACTT CCGTCGACTG GACTAAGTTT GACCCTTAAA   
  
  
- GAGTGAACGC GTTCTCTATA ACCGCGCCGA GTTAGTGGTC GAGAGGGAAG GACGCTTCGG GGAGTAATCC   
  
  
- CGTCGAAACA TACAGTACCT CCGGAACGTT TACGAGTAGA ATTACTCGTT AGGACATCGT GGAGGTGGCT   
  
  
- ACTTCTGCGA AAGGGGATAT CTACAACAAG TATTCTACTT ACGGATGTTC CGGAAGAGAC TCCAGAGAGG   
  
  
- ATAATGAGTC AAATGCTTAA AGTGAACACG GGTTCGGTAA GAACTTCGAG AGCTACTACG CCTAACGCAA   
  
  
- GTACAGTAAC TGAAACTATA ACCAACACCC CGAGTTACCC GTAGTGACTA AGTCCTCGAA GGTAACTCCT   
  
  
- TTTTCCCTCG AGGTAGGGAC TTTTAATGTC GGTATCGAGG GGACAGTTAT TCGGTGTGAA AACTTGAGTT   
  
  
- GGATCGTGCC CTTTTGGAAC ACGTTAAGCG GTTACTACAA CCACAACGAA AACTCGACGT TCAACACTTG   
  
  
- AACCTAAATA AACTAGGTAG AAGTAGAAGT TACGGTTTAC AACCTTGGAG ACCCCTACTC AACTAGCGAC   
  
  
- AATCATATGG GTAAACCCGT ACAAGTATAG CCGGCAGATA AGAGGGTAGG TAGGAGGCCA AGTAATTCGT   
  
  
- TGATCGGGGG TTCTAACAGC AGAGCAATCT ATCTCCCAGA CTAGCAACGC TGTGAGACAA GGGTGTTGTA   
  
  
- GAATAGGTAT AGAATCTCAG GACGTGTTTA AAGAACCTTA GCGAGCTGCC AGAGTTACAT CGTAGTCTAT   
  
  
- AACATTTGTT CCAACTCTTC ATGAAGGAGG TCGGATTCTA GCTTTTGTGG CACAACCCGG CGCAAGTACG   
  
  
- GGGGTTATTC TACGGTGTAA CCTTCTGGGA GAAACGAAGT CGGCCCAATA ACGGGAAAGT CAAGTCATTG   
  
  
- AAGTGTCTTT GTGTTCGACT AATACACCAC TTCGCTTGGG GTCCCTCCCC TAAAGTGTAT CTTTTCGCGG   
  
  
- TGCGTAGAAA TCACGATTCA ACCGTTTCCG CCCTCGAACA CTGTCGGAGC CGTACCTCCA AACTCTTCGA   
  
  
- CAT

+     STRE

| Site Name | Organism | Position | Strand | Matrix score. | sequence | function |
| --- | --- | --- | --- | --- | --- | --- |
| STRE | Arabidopsis thaliana | 1169 | + | 5 | AGGGG |  |
| STRE | Arabidopsis thaliana | 3123 | - | 5 | AGGGG |  |
| STRE | Arabidopsis thaliana | 54 | + | 5 | AGGGG |  |
| STRE | Arabidopsis thaliana | 2793 | - | 5 | AGGGG |  |
| STRE | Arabidopsis thaliana | 1230 | - | 5 | AGGGG |  |
| STRE | Arabidopsis thaliana | 2887 | - | 5 | AGGGG |  |
| STRE | Arabidopsis thaliana | 3690 | + | 5 | AGGGG |  |
| STRE | Arabidopsis thaliana | 1374 | + | 5 | AGGGG |  |

>HU05G00466.1   
+ +Up\_Stream \_Len000ACATTG GGTGGGATTT AAATCCTCTA TTTTTTCTTA GGGGAAAAAT ACTTGGGAAA   
  
  
+ TTTCTCAATA ACTGAAAATC TAAATAATTC GTGACTTTGT TAGTAGGTTT AATGTGATTT ACTACCGTGA   
  
  
+ GTGTCACTTT TAGATGGTAC TTTAAGAAAG CATTATAAGT AAAACAATAT ATATCGTAGC ATCATACTAC   
  
  
+ TTGTGTAATA AATTAACAGC AACCTCAATT GTACTTATTT GATTTCGCAT TGTAATCAAT TTTCCCAAAT   
  
  
+ GATTTACTAT CTTGGTCTTA CTCAGTTGTA CTTCACACTC AATAGTTCAA TTATTTTTCA TTAGACTAAT   
  
  
+ GAGCGAGTCC CTTCTACAAG AGCCTACTAC TACTCTAATA ATGTAAGGAC TATTGGCTAA TGGGGAACTA   
  
  
+ AGCCTACGAC TTGTTAGGCA TAGGTGAATT TTCCTTACAA GAGAGTTTAA TTTACACTTG ATATTAACTC   
  
  
+ GAGAACGAGA AGCTGTCTTA ATAGATACCC ACATAGAAAA TGAAGCAAAT GCCCTTGATT ATTGGAGGGT   
  
  
+ TGGGCAAATA TATTCAGTAA CAATGTTGTT TGGCAAATAT CGTCTGAAAA TTGTTTTTGA CAAATATTGT   
  
  
+ TTGGAAGAAT TTTTCGAGAT TAAATATTAA AAATGTTAAA ATTTTAATCT AAATATCAAA CAAATATTGA   
  
  
+ AAAATGCTTT ATATTATCTA AATCTCTAAT TAAAATTTGT GTATTGTCCA CTAGAAAAAT GTATAGTGAT   
  
  
+ ATTACCGACA TATATGTGAT CTCTGGTGTA ATAGCCTACC CTAATGTCTA GGAAAATATT TAAAATCCCC   
  
  
+ AGTGGTCAAA AGGAAGTTAC GCACACTAGG GCAAGACACT TCATTAGGCT AGTTGGTGAA AGCTAAGTCA   
  
  
+ TGAAATTTAG ACCATGGCCA TGTCTACTTG TAGATCCAAC TACCAACAAT CCATTTTTAT TTTGGGGAAA   
  
  
+ GCTTGAAGGG TAATTTGGTA AATGTACACG GGAACCTTTT GCTATAAAAG AAGTGCTTCT TGAAAAAACA   
  
  
+ CCCTGTAGTA AAACAGGGCA AAAAAGCCAA CACTTCTGTT TCTGGCAGAG AAACAGTAGT GTACCACTTG   
  
  
+ GAGTTGGAGT AGACACGATA CACAAACCCA GTAGAGAGAG AAACAGGGGA GAGAAAGAGT ACACAAATTT   
  
  
+ GTGCAGGAGA AGGGTAAGGA AGAAGGAGAA GGAACCCCCT CCAAAAAAAA CCCATATAAA AAATAATAAA   
  
  
+ GAAAAAACAG GAGTGAGTTA GAGGGTACCC AAATCTCCAA TCTAAAACTA GAATCTAGGG TCTCTTTTCA   
  
  
+ TTTGCTTGAA AAATTTCTGT TCATGGGTTT TGTGTTTTTA GGGGGATTTG TTTGATTTTT GTAGTCTTTG   
  
  
+ TGGATTTCCT GTGTTATAGA TGCAAGCTAT GCTCTTCAAT TTGCAAGGAA CAGCATCTGG GGGGAGAGAA   
  
  
+ TTTGAGGAAT TTCAAGTACC CATTTCAATT TCAACAAGTA AAATCATCAT TGAAGGATAC TCAAACCCAA   
  
  
+ GCCCAGTTGA TAGTGAACCA ACTTCTACTC TGGATTCTAC TCCAAGTCCC AGCCCTCCCA CCCTCTCCTC   
  
  
+ CTCCTCCAAG AACTCCTACC AAAACGCTGC CGTTTTGGCT CACCCTCCTC AGCTTCACCA TGGATCTGAT   
  
  
+ AATGTGTAAG ACCCCAACCC TTTTGCTTTT ATTTTCCTCA TGCTCCATTC TCATGATTTG TACTTCTGTT   
  
  
+ ATGTAATTGG AGATGTGTCT AAATTTGGAA AAGTTCGTGG GTTTCGTGTT TGAGAATTTA GAATGACATT   
  
  
+ CTTTAATTAG TAATTTGAAG TTTGCATGCT TAATTTAGCT AATCACTGAT TAATTAAAAT AAATGAAGTG   
  
  
+ ATGTAGGTAT GGGCTGTTAC ACAATGTCAC CGGTGGTGGT GACAGCGGCG GGGCGGCGGC GGTTGGATTG   
  
  
+ GAAGAATGGG ACAGCGTGTT TCCAAATGGG GATGGAGCTT TACTCCCTTG GATCATGGGG GAAGGTGATG   
  
  
+ ATTTGGGTCT GAATTTGAAG CATCTCTTAC AATCTGGTTA CCCTGTTGAG TATGAAGGCA ATGCAGGACT   
  
  
+ TGGTGTTGTT GATCAGACAT CTCTTCTTGG AGGTGGTGAT TTGGGGTTTT CTGGTTCTGG GTCTGTGAAT   
  
  
+ AATGGCAAAA TTGGTTCCAT TTTGGGTAAT TGTTCATCTG GGGATTTGGA TTCTAAGGTT TCTAGTGACG   
  
  
+ GGTTGAATTC CAATTGTAGT TCACTGGGGA GTATGAGTAT CCTTGGTTCA ATCCATGGAC CTTTGCCTAA   
  
  
+ TACTGGTGGG TTTGTGTTCC CACAAACACA ACTATTTGAT CTTGGTGATG AGAAGCCTCA GATTTTGAAC   
  
  
+ CCACAATTGA TGACGATGAA TCCAAATCAG GCTCAGAGCA TGGGAAACCC TAGCTTTTTT GTGCCCTCCT   
  
  
+ TGGGTTATTG TCAGTTAGAG CAACATATGG TTCAACCACA GGCGAAACGC CATAGCCCTG GTGTAGTTTT   
  
  
+ GAACTCTGAT GTTATTGCCA AAACCCCGTT TCTCGATCAG GGTCATGAGT TTTTGTTGAG GAAACAACAC   
  
  
+ CATCAACAGA TTTTGCAGCA ACTTCCTATG GGTTTGGCTC ATCAGTCGGT CCCTCAGCAC CTCCAGCAAA   
  
  
+ AGGCGATGAA AGACCAGGTT GTTAAGGACC AGCTTTTGAA GGCAGCTGAC CTGATTCAAA CTGGGAATTT   
  
  
+ CTCACTTGCG CAAGAGATAT TGGCGCGGCT CAATCACCAG CTCTCCCTTC CTGCGAAGCC CCTCATTAGG   
  
  
+ GCAGCTTTGT ATGTCATGGA GGCCTTGCAA ATGCTCATCT TAATGAGCAA TCCTGTAGCA CCTCCACCGA   
  
  
+ TGAAGACGCT TTCCCCTATA GATGTTGTTC ATAAGATGAA TGCCTACAAG GCCTTCTCTG AGGTCTCTCC   
  
  
+ TATTACTCAG TTTACGAATT TCACTTGTGC CCAAGCCATT CTTGAAGCTC TCGATGATGC GGATTGCGTT   
  
  
+ CATGTCATTG ACTTTGATAT TGGTTGTGGG GCTCAATGGG CATCACTGAT TCAGGAGCTT CCATTGAGGA   
  
  
+ AAAAGGGAGC TCCATCCCTG AAAATTACAG CCATAGCTCC CCTGTCAATA AGCCACACTT TTGAACTCAA   
  
  
+ CCTAGCACGG GAAAACCTTG TGCAATTCGC CAATGATGTT GGTGTTGCTT TTGAGCTGCA AGTTGTGAAC   
  
  
+ TTGGATTTAT TTGATCCATC TTCATCTTCA ATGCCAAATG TTGGAACCTC TGGGGATGAG TTGATCGCTG   
  
  
+ TTAGTATACC CATTTGGGCA TGTTCATATC GGCCGTCTAT TCTCCCATCC ATCCTCCGGT TCATTAAGCA   
  
  
+ ACTAGCCCCC AAGATTGTCG TCTCGTTAGA TAGAGGGTCT GATCGTTGCG ACACTCTGTT CCCACAACAT   
  
  
+ CTTATCCATA TCTTAGAGTC CTGCACAAAT TTCTTGGAAT CGCTCGACGG TCTCAATGTA GCATCAGATA   
  
  
+ TTGTAAACAA GGTTGAGAAG TACTTCCTCC AGCCTAAGAT CGAAAACACC GTGTTGGGCC GCGTTCATGC   
  
  
+ CCCCAATAAG ATGCCACATT GGAAGACCCT CTTTGCTTCA GCCGGGTTAT TGCCCTTTCA GTTCAGTAAC   
  
  
+ TTCACAGAAA CACAAGCTGA TTATGTGGTG AAGCGAACCC CAGGGAGGGG ATTTCACATA GAAAAGCGCC   
  
  
+ ACGCATCTTT AGTGCTAAGT TGGCAAAGGC GGGAGCTTGT GACAGCCTCG GCATGGAGGT TTGAGAAGCT   
  
  
+ GTA  

- +Up\_Stream \_Len000TGTAAC CCACCCTAAA TTTAGGAGAT AAAAAAGAAT CCCCTTTTTA TGAACCCTTT   
  
  
- AAAGAGTTAT TGACTTTTAG ATTTATTAAG CACTGAAACA ATCATCCAAA TTACACTAAA TGATGGCACT   
  
  
- CACAGTGAAA ATCTACCATG AAATTCTTTC GTAATATTCA TTTTGTTATA TATAGCATCG TAGTATGATG   
  
  
- AACACATTAT TTAATTGTCG TTGGAGTTAA CATGAATAAA CTAAAGCGTA ACATTAGTTA AAAGGGTTTA   
  
  
- CTAAATGATA GAACCAGAAT GAGTCAACAT GAAGTGTGAG TTATCAAGTT AATAAAAAGT AATCTGATTA   
  
  
- CTCGCTCAGG GAAGATGTTC TCGGATGATG ATGAGATTAT TACATTCCTG ATAACCGATT ACCCCTTGAT   
  
  
- TCGGATGCTG AACAATCCGT ATCCACTTAA AAGGAATGTT CTCTCAAATT AAATGTGAAC TATAATTGAG   
  
  
- CTCTTGCTCT TCGACAGAAT TATCTATGGG TGTATCTTTT ACTTCGTTTA CGGGAACTAA TAACCTCCCA   
  
  
- ACCCGTTTAT ATAAGTCATT GTTACAACAA ACCGTTTATA GCAGACTTTT AACAAAAACT GTTTATAACA   
  
  
- AACCTTCTTA AAAAGCTCTA ATTTATAATT TTTACAATTT TAAAATTAGA TTTATAGTTT GTTTATAACT   
  
  
- TTTTACGAAA TATAATAGAT TTAGAGATTA ATTTTAAACA CATAACAGGT GATCTTTTTA CATATCACTA   
  
  
- TAATGGCTGT ATATACACTA GAGACCACAT TATCGGATGG GATTACAGAT CCTTTTATAA ATTTTAGGGG   
  
  
- TCACCAGTTT TCCTTCAATG CGTGTGATCC CGTTCTGTGA AGTAATCCGA TCAACCACTT TCGATTCAGT   
  
  
- ACTTTAAATC TGGTACCGGT ACAGATGAAC ATCTAGGTTG ATGGTTGTTA GGTAAAAATA AAACCCCTTT   
  
  
- CGAACTTCCC ATTAAACCAT TTACATGTGC CCTTGGAAAA CGATATTTTC TTCACGAAGA ACTTTTTTGT   
  
  
- GGGACATCAT TTTGTCCCGT TTTTTCGGTT GTGAAGACAA AGACCGTCTC TTTGTCATCA CATGGTGAAC   
  
  
- CTCAACCTCA TCTGTGCTAT GTGTTTGGGT CATCTCTCTC TTTGTCCCCT CTCTTTCTCA TGTGTTTAAA   
  
  
- CACGTCCTCT TCCCATTCCT TCTTCCTCTT CCTTGGGGGA GGTTTTTTTT GGGTATATTT TTTATTATTT   
  
  
- CTTTTTTGTC CTCACTCAAT CTCCCATGGG TTTAGAGGTT AGATTTTGAT CTTAGATCCC AGAGAAAAGT   
  
  
- AAACGAACTT TTTAAAGACA AGTACCCAAA ACACAAAAAT CCCCCTAAAC AAACTAAAAA CATCAGAAAC   
  
  
- ACCTAAAGGA CACAATATCT ACGTTCGATA CGAGAAGTTA AACGTTCCTT GTCGTAGACC CCCCTCTCTT   
  
  
- AAACTCCTTA AAGTTCATGG GTAAAGTTAA AGTTGTTCAT TTTAGTAGTA ACTTCCTATG AGTTTGGGTT   
  
  
- CGGGTCAACT ATCACTTGGT TGAAGATGAG ACCTAAGATG AGGTTCAGGG TCGGGAGGGT GGGAGAGGAG   
  
  
- GAGGAGGTTC TTGAGGATGG TTTTGCGACG GCAAAACCGA GTGGGAGGAG TCGAAGTGGT ACCTAGACTA   
  
  
- TTACACATTC TGGGGTTGGG AAAACGAAAA TAAAAGGAGT ACGAGGTAAG AGTACTAAAC ATGAAGACAA   
  
  
- TACATTAACC TCTACACAGA TTTAAACCTT TTCAAGCACC CAAAGCACAA ACTCTTAAAT CTTACTGTAA   
  
  
- GAAATTAATC ATTAAACTTC AAACGTACGA ATTAAATCGA TTAGTGACTA ATTAATTTTA TTTACTTCAC   
  
  
- TACATCCATA CCCGACAATG TGTTACAGTG GCCACCACCA CTGTCGCCGC CCCGCCGCCG CCAACCTAAC   
  
  
- CTTCTTACCC TGTCGCACAA AGGTTTACCC CTACCTCGAA ATGAGGGAAC CTAGTACCCC CTTCCACTAC   
  
  
- TAAACCCAGA CTTAAACTTC GTAGAGAATG TTAGACCAAT GGGACAACTC ATACTTCCGT TACGTCCTGA   
  
  
- ACCACAACAA CTAGTCTGTA GAGAAGAACC TCCACCACTA AACCCCAAAA GACCAAGACC CAGACACTTA   
  
  
- TTACCGTTTT AACCAAGGTA AAACCCATTA ACAAGTAGAC CCCTAAACCT AAGATTCCAA AGATCACTGC   
  
  
- CCAACTTAAG GTTAACATCA AGTGACCCCT CATACTCATA GGAACCAAGT TAGGTACCTG GAAACGGATT   
  
  
- ATGACCACCC AAACACAAGG GTGTTTGTGT TGATAAACTA GAACCACTAC TCTTCGGAGT CTAAAACTTG   
  
  
- GGTGTTAACT ACTGCTACTT AGGTTTAGTC CGAGTCTCGT ACCCTTTGGG ATCGAAAAAA CACGGGAGGA   
  
  
- ACCCAATAAC AGTCAATCTC GTTGTATACC AAGTTGGTGT CCGCTTTGCG GTATCGGGAC CACATCAAAA   
  
  
- CTTGAGACTA CAATAACGGT TTTGGGGCAA AGAGCTAGTC CCAGTACTCA AAAACAACTC CTTTGTTGTG   
  
  
- GTAGTTGTCT AAAACGTCGT TGAAGGATAC CCAAACCGAG TAGTCAGCCA GGGAGTCGTG GAGGTCGTTT   
  
  
- TCCGCTACTT TCTGGTCCAA CAATTCCTGG TCGAAAACTT CCGTCGACTG GACTAAGTTT GACCCTTAAA   
  
  
- GAGTGAACGC GTTCTCTATA ACCGCGCCGA GTTAGTGGTC GAGAGGGAAG GACGCTTCGG GGAGTAATCC   
  
  
- CGTCGAAACA TACAGTACCT CCGGAACGTT TACGAGTAGA ATTACTCGTT AGGACATCGT GGAGGTGGCT   
  
  
- ACTTCTGCGA AAGGGGATAT CTACAACAAG TATTCTACTT ACGGATGTTC CGGAAGAGAC TCCAGAGAGG   
  
  
- ATAATGAGTC AAATGCTTAA AGTGAACACG GGTTCGGTAA GAACTTCGAG AGCTACTACG CCTAACGCAA   
  
  
- GTACAGTAAC TGAAACTATA ACCAACACCC CGAGTTACCC GTAGTGACTA AGTCCTCGAA GGTAACTCCT   
  
  
- TTTTCCCTCG AGGTAGGGAC TTTTAATGTC GGTATCGAGG GGACAGTTAT TCGGTGTGAA AACTTGAGTT   
  
  
- GGATCGTGCC CTTTTGGAAC ACGTTAAGCG GTTACTACAA CCACAACGAA AACTCGACGT TCAACACTTG   
  
  
- AACCTAAATA AACTAGGTAG AAGTAGAAGT TACGGTTTAC AACCTTGGAG ACCCCTACTC AACTAGCGAC   
  
  
- AATCATATGG GTAAACCCGT ACAAGTATAG CCGGCAGATA AGAGGGTAGG TAGGAGGCCA AGTAATTCGT   
  
  
- TGATCGGGGG TTCTAACAGC AGAGCAATCT ATCTCCCAGA CTAGCAACGC TGTGAGACAA GGGTGTTGTA   
  
  
- GAATAGGTAT AGAATCTCAG GACGTGTTTA AAGAACCTTA GCGAGCTGCC AGAGTTACAT CGTAGTCTAT   
  
  
- AACATTTGTT CCAACTCTTC ATGAAGGAGG TCGGATTCTA GCTTTTGTGG CACAACCCGG CGCAAGTACG   
  
  
- GGGGTTATTC TACGGTGTAA CCTTCTGGGA GAAACGAAGT CGGCCCAATA ACGGGAAAGT CAAGTCATTG   
  
  
- AAGTGTCTTT GTGTTCGACT AATACACCAC TTCGCTTGGG GTCCCTCCCC TAAAGTGTAT CTTTTCGCGG   
  
  
- TGCGTAGAAA TCACGATTCA ACCGTTTCCG CCCTCGAACA CTGTCGGAGC CGTACCTCCA AACTCTTCGA   
  
  
- CAT

+     Sp1

| Site Name | Organism | Position | Strand | Matrix score. | sequence | function |
| --- | --- | --- | --- | --- | --- | --- |
| Sp1 | Oryza sativa | 1945 | + | 6 | GGGCGG | light responsive element |

>HU05G00466.1   
+ +Up\_Stream \_Len000ACATTG GGTGGGATTT AAATCCTCTA TTTTTTCTTA GGGGAAAAAT ACTTGGGAAA   
  
  
+ TTTCTCAATA ACTGAAAATC TAAATAATTC GTGACTTTGT TAGTAGGTTT AATGTGATTT ACTACCGTGA   
  
  
+ GTGTCACTTT TAGATGGTAC TTTAAGAAAG CATTATAAGT AAAACAATAT ATATCGTAGC ATCATACTAC   
  
  
+ TTGTGTAATA AATTAACAGC AACCTCAATT GTACTTATTT GATTTCGCAT TGTAATCAAT TTTCCCAAAT   
  
  
+ GATTTACTAT CTTGGTCTTA CTCAGTTGTA CTTCACACTC AATAGTTCAA TTATTTTTCA TTAGACTAAT   
  
  
+ GAGCGAGTCC CTTCTACAAG AGCCTACTAC TACTCTAATA ATGTAAGGAC TATTGGCTAA TGGGGAACTA   
  
  
+ AGCCTACGAC TTGTTAGGCA TAGGTGAATT TTCCTTACAA GAGAGTTTAA TTTACACTTG ATATTAACTC   
  
  
+ GAGAACGAGA AGCTGTCTTA ATAGATACCC ACATAGAAAA TGAAGCAAAT GCCCTTGATT ATTGGAGGGT   
  
  
+ TGGGCAAATA TATTCAGTAA CAATGTTGTT TGGCAAATAT CGTCTGAAAA TTGTTTTTGA CAAATATTGT   
  
  
+ TTGGAAGAAT TTTTCGAGAT TAAATATTAA AAATGTTAAA ATTTTAATCT AAATATCAAA CAAATATTGA   
  
  
+ AAAATGCTTT ATATTATCTA AATCTCTAAT TAAAATTTGT GTATTGTCCA CTAGAAAAAT GTATAGTGAT   
  
  
+ ATTACCGACA TATATGTGAT CTCTGGTGTA ATAGCCTACC CTAATGTCTA GGAAAATATT TAAAATCCCC   
  
  
+ AGTGGTCAAA AGGAAGTTAC GCACACTAGG GCAAGACACT TCATTAGGCT AGTTGGTGAA AGCTAAGTCA   
  
  
+ TGAAATTTAG ACCATGGCCA TGTCTACTTG TAGATCCAAC TACCAACAAT CCATTTTTAT TTTGGGGAAA   
  
  
+ GCTTGAAGGG TAATTTGGTA AATGTACACG GGAACCTTTT GCTATAAAAG AAGTGCTTCT TGAAAAAACA   
  
  
+ CCCTGTAGTA AAACAGGGCA AAAAAGCCAA CACTTCTGTT TCTGGCAGAG AAACAGTAGT GTACCACTTG   
  
  
+ GAGTTGGAGT AGACACGATA CACAAACCCA GTAGAGAGAG AAACAGGGGA GAGAAAGAGT ACACAAATTT   
  
  
+ GTGCAGGAGA AGGGTAAGGA AGAAGGAGAA GGAACCCCCT CCAAAAAAAA CCCATATAAA AAATAATAAA   
  
  
+ GAAAAAACAG GAGTGAGTTA GAGGGTACCC AAATCTCCAA TCTAAAACTA GAATCTAGGG TCTCTTTTCA   
  
  
+ TTTGCTTGAA AAATTTCTGT TCATGGGTTT TGTGTTTTTA GGGGGATTTG TTTGATTTTT GTAGTCTTTG   
  
  
+ TGGATTTCCT GTGTTATAGA TGCAAGCTAT GCTCTTCAAT TTGCAAGGAA CAGCATCTGG GGGGAGAGAA   
  
  
+ TTTGAGGAAT TTCAAGTACC CATTTCAATT TCAACAAGTA AAATCATCAT TGAAGGATAC TCAAACCCAA   
  
  
+ GCCCAGTTGA TAGTGAACCA ACTTCTACTC TGGATTCTAC TCCAAGTCCC AGCCCTCCCA CCCTCTCCTC   
  
  
+ CTCCTCCAAG AACTCCTACC AAAACGCTGC CGTTTTGGCT CACCCTCCTC AGCTTCACCA TGGATCTGAT   
  
  
+ AATGTGTAAG ACCCCAACCC TTTTGCTTTT ATTTTCCTCA TGCTCCATTC TCATGATTTG TACTTCTGTT   
  
  
+ ATGTAATTGG AGATGTGTCT AAATTTGGAA AAGTTCGTGG GTTTCGTGTT TGAGAATTTA GAATGACATT   
  
  
+ CTTTAATTAG TAATTTGAAG TTTGCATGCT TAATTTAGCT AATCACTGAT TAATTAAAAT AAATGAAGTG   
  
  
+ ATGTAGGTAT GGGCTGTTAC ACAATGTCAC CGGTGGTGGT GACAGCGGCG GGGCGGCGGC GGTTGGATTG   
  
  
+ GAAGAATGGG ACAGCGTGTT TCCAAATGGG GATGGAGCTT TACTCCCTTG GATCATGGGG GAAGGTGATG   
  
  
+ ATTTGGGTCT GAATTTGAAG CATCTCTTAC AATCTGGTTA CCCTGTTGAG TATGAAGGCA ATGCAGGACT   
  
  
+ TGGTGTTGTT GATCAGACAT CTCTTCTTGG AGGTGGTGAT TTGGGGTTTT CTGGTTCTGG GTCTGTGAAT   
  
  
+ AATGGCAAAA TTGGTTCCAT TTTGGGTAAT TGTTCATCTG GGGATTTGGA TTCTAAGGTT TCTAGTGACG   
  
  
+ GGTTGAATTC CAATTGTAGT TCACTGGGGA GTATGAGTAT CCTTGGTTCA ATCCATGGAC CTTTGCCTAA   
  
  
+ TACTGGTGGG TTTGTGTTCC CACAAACACA ACTATTTGAT CTTGGTGATG AGAAGCCTCA GATTTTGAAC   
  
  
+ CCACAATTGA TGACGATGAA TCCAAATCAG GCTCAGAGCA TGGGAAACCC TAGCTTTTTT GTGCCCTCCT   
  
  
+ TGGGTTATTG TCAGTTAGAG CAACATATGG TTCAACCACA GGCGAAACGC CATAGCCCTG GTGTAGTTTT   
  
  
+ GAACTCTGAT GTTATTGCCA AAACCCCGTT TCTCGATCAG GGTCATGAGT TTTTGTTGAG GAAACAACAC   
  
  
+ CATCAACAGA TTTTGCAGCA ACTTCCTATG GGTTTGGCTC ATCAGTCGGT CCCTCAGCAC CTCCAGCAAA   
  
  
+ AGGCGATGAA AGACCAGGTT GTTAAGGACC AGCTTTTGAA GGCAGCTGAC CTGATTCAAA CTGGGAATTT   
  
  
+ CTCACTTGCG CAAGAGATAT TGGCGCGGCT CAATCACCAG CTCTCCCTTC CTGCGAAGCC CCTCATTAGG   
  
  
+ GCAGCTTTGT ATGTCATGGA GGCCTTGCAA ATGCTCATCT TAATGAGCAA TCCTGTAGCA CCTCCACCGA   
  
  
+ TGAAGACGCT TTCCCCTATA GATGTTGTTC ATAAGATGAA TGCCTACAAG GCCTTCTCTG AGGTCTCTCC   
  
  
+ TATTACTCAG TTTACGAATT TCACTTGTGC CCAAGCCATT CTTGAAGCTC TCGATGATGC GGATTGCGTT   
  
  
+ CATGTCATTG ACTTTGATAT TGGTTGTGGG GCTCAATGGG CATCACTGAT TCAGGAGCTT CCATTGAGGA   
  
  
+ AAAAGGGAGC TCCATCCCTG AAAATTACAG CCATAGCTCC CCTGTCAATA AGCCACACTT TTGAACTCAA   
  
  
+ CCTAGCACGG GAAAACCTTG TGCAATTCGC CAATGATGTT GGTGTTGCTT TTGAGCTGCA AGTTGTGAAC   
  
  
+ TTGGATTTAT TTGATCCATC TTCATCTTCA ATGCCAAATG TTGGAACCTC TGGGGATGAG TTGATCGCTG   
  
  
+ TTAGTATACC CATTTGGGCA TGTTCATATC GGCCGTCTAT TCTCCCATCC ATCCTCCGGT TCATTAAGCA   
  
  
+ ACTAGCCCCC AAGATTGTCG TCTCGTTAGA TAGAGGGTCT GATCGTTGCG ACACTCTGTT CCCACAACAT   
  
  
+ CTTATCCATA TCTTAGAGTC CTGCACAAAT TTCTTGGAAT CGCTCGACGG TCTCAATGTA GCATCAGATA   
  
  
+ TTGTAAACAA GGTTGAGAAG TACTTCCTCC AGCCTAAGAT CGAAAACACC GTGTTGGGCC GCGTTCATGC   
  
  
+ CCCCAATAAG ATGCCACATT GGAAGACCCT CTTTGCTTCA GCCGGGTTAT TGCCCTTTCA GTTCAGTAAC   
  
  
+ TTCACAGAAA CACAAGCTGA TTATGTGGTG AAGCGAACCC CAGGGAGGGG ATTTCACATA GAAAAGCGCC   
  
  
+ ACGCATCTTT AGTGCTAAGT TGGCAAAGGC GGGAGCTTGT GACAGCCTCG GCATGGAGGT TTGAGAAGCT   
  
  
+ GTA  

- +Up\_Stream \_Len000TGTAAC CCACCCTAAA TTTAGGAGAT AAAAAAGAAT CCCCTTTTTA TGAACCCTTT   
  
  
- AAAGAGTTAT TGACTTTTAG ATTTATTAAG CACTGAAACA ATCATCCAAA TTACACTAAA TGATGGCACT   
  
  
- CACAGTGAAA ATCTACCATG AAATTCTTTC GTAATATTCA TTTTGTTATA TATAGCATCG TAGTATGATG   
  
  
- AACACATTAT TTAATTGTCG TTGGAGTTAA CATGAATAAA CTAAAGCGTA ACATTAGTTA AAAGGGTTTA   
  
  
- CTAAATGATA GAACCAGAAT GAGTCAACAT GAAGTGTGAG TTATCAAGTT AATAAAAAGT AATCTGATTA   
  
  
- CTCGCTCAGG GAAGATGTTC TCGGATGATG ATGAGATTAT TACATTCCTG ATAACCGATT ACCCCTTGAT   
  
  
- TCGGATGCTG AACAATCCGT ATCCACTTAA AAGGAATGTT CTCTCAAATT AAATGTGAAC TATAATTGAG   
  
  
- CTCTTGCTCT TCGACAGAAT TATCTATGGG TGTATCTTTT ACTTCGTTTA CGGGAACTAA TAACCTCCCA   
  
  
- ACCCGTTTAT ATAAGTCATT GTTACAACAA ACCGTTTATA GCAGACTTTT AACAAAAACT GTTTATAACA   
  
  
- AACCTTCTTA AAAAGCTCTA ATTTATAATT TTTACAATTT TAAAATTAGA TTTATAGTTT GTTTATAACT   
  
  
- TTTTACGAAA TATAATAGAT TTAGAGATTA ATTTTAAACA CATAACAGGT GATCTTTTTA CATATCACTA   
  
  
- TAATGGCTGT ATATACACTA GAGACCACAT TATCGGATGG GATTACAGAT CCTTTTATAA ATTTTAGGGG   
  
  
- TCACCAGTTT TCCTTCAATG CGTGTGATCC CGTTCTGTGA AGTAATCCGA TCAACCACTT TCGATTCAGT   
  
  
- ACTTTAAATC TGGTACCGGT ACAGATGAAC ATCTAGGTTG ATGGTTGTTA GGTAAAAATA AAACCCCTTT   
  
  
- CGAACTTCCC ATTAAACCAT TTACATGTGC CCTTGGAAAA CGATATTTTC TTCACGAAGA ACTTTTTTGT   
  
  
- GGGACATCAT TTTGTCCCGT TTTTTCGGTT GTGAAGACAA AGACCGTCTC TTTGTCATCA CATGGTGAAC   
  
  
- CTCAACCTCA TCTGTGCTAT GTGTTTGGGT CATCTCTCTC TTTGTCCCCT CTCTTTCTCA TGTGTTTAAA   
  
  
- CACGTCCTCT TCCCATTCCT TCTTCCTCTT CCTTGGGGGA GGTTTTTTTT GGGTATATTT TTTATTATTT   
  
  
- CTTTTTTGTC CTCACTCAAT CTCCCATGGG TTTAGAGGTT AGATTTTGAT CTTAGATCCC AGAGAAAAGT   
  
  
- AAACGAACTT TTTAAAGACA AGTACCCAAA ACACAAAAAT CCCCCTAAAC AAACTAAAAA CATCAGAAAC   
  
  
- ACCTAAAGGA CACAATATCT ACGTTCGATA CGAGAAGTTA AACGTTCCTT GTCGTAGACC CCCCTCTCTT   
  
  
- AAACTCCTTA AAGTTCATGG GTAAAGTTAA AGTTGTTCAT TTTAGTAGTA ACTTCCTATG AGTTTGGGTT   
  
  
- CGGGTCAACT ATCACTTGGT TGAAGATGAG ACCTAAGATG AGGTTCAGGG TCGGGAGGGT GGGAGAGGAG   
  
  
- GAGGAGGTTC TTGAGGATGG TTTTGCGACG GCAAAACCGA GTGGGAGGAG TCGAAGTGGT ACCTAGACTA   
  
  
- TTACACATTC TGGGGTTGGG AAAACGAAAA TAAAAGGAGT ACGAGGTAAG AGTACTAAAC ATGAAGACAA   
  
  
- TACATTAACC TCTACACAGA TTTAAACCTT TTCAAGCACC CAAAGCACAA ACTCTTAAAT CTTACTGTAA   
  
  
- GAAATTAATC ATTAAACTTC AAACGTACGA ATTAAATCGA TTAGTGACTA ATTAATTTTA TTTACTTCAC   
  
  
- TACATCCATA CCCGACAATG TGTTACAGTG GCCACCACCA CTGTCGCCGC CCCGCCGCCG CCAACCTAAC   
  
  
- CTTCTTACCC TGTCGCACAA AGGTTTACCC CTACCTCGAA ATGAGGGAAC CTAGTACCCC CTTCCACTAC   
  
  
- TAAACCCAGA CTTAAACTTC GTAGAGAATG TTAGACCAAT GGGACAACTC ATACTTCCGT TACGTCCTGA   
  
  
- ACCACAACAA CTAGTCTGTA GAGAAGAACC TCCACCACTA AACCCCAAAA GACCAAGACC CAGACACTTA   
  
  
- TTACCGTTTT AACCAAGGTA AAACCCATTA ACAAGTAGAC CCCTAAACCT AAGATTCCAA AGATCACTGC   
  
  
- CCAACTTAAG GTTAACATCA AGTGACCCCT CATACTCATA GGAACCAAGT TAGGTACCTG GAAACGGATT   
  
  
- ATGACCACCC AAACACAAGG GTGTTTGTGT TGATAAACTA GAACCACTAC TCTTCGGAGT CTAAAACTTG   
  
  
- GGTGTTAACT ACTGCTACTT AGGTTTAGTC CGAGTCTCGT ACCCTTTGGG ATCGAAAAAA CACGGGAGGA   
  
  
- ACCCAATAAC AGTCAATCTC GTTGTATACC AAGTTGGTGT CCGCTTTGCG GTATCGGGAC CACATCAAAA   
  
  
- CTTGAGACTA CAATAACGGT TTTGGGGCAA AGAGCTAGTC CCAGTACTCA AAAACAACTC CTTTGTTGTG   
  
  
- GTAGTTGTCT AAAACGTCGT TGAAGGATAC CCAAACCGAG TAGTCAGCCA GGGAGTCGTG GAGGTCGTTT   
  
  
- TCCGCTACTT TCTGGTCCAA CAATTCCTGG TCGAAAACTT CCGTCGACTG GACTAAGTTT GACCCTTAAA   
  
  
- GAGTGAACGC GTTCTCTATA ACCGCGCCGA GTTAGTGGTC GAGAGGGAAG GACGCTTCGG GGAGTAATCC   
  
  
- CGTCGAAACA TACAGTACCT CCGGAACGTT TACGAGTAGA ATTACTCGTT AGGACATCGT GGAGGTGGCT   
  
  
- ACTTCTGCGA AAGGGGATAT CTACAACAAG TATTCTACTT ACGGATGTTC CGGAAGAGAC TCCAGAGAGG   
  
  
- ATAATGAGTC AAATGCTTAA AGTGAACACG GGTTCGGTAA GAACTTCGAG AGCTACTACG CCTAACGCAA   
  
  
- GTACAGTAAC TGAAACTATA ACCAACACCC CGAGTTACCC GTAGTGACTA AGTCCTCGAA GGTAACTCCT   
  
  
- TTTTCCCTCG AGGTAGGGAC TTTTAATGTC GGTATCGAGG GGACAGTTAT TCGGTGTGAA AACTTGAGTT   
  
  
- GGATCGTGCC CTTTTGGAAC ACGTTAAGCG GTTACTACAA CCACAACGAA AACTCGACGT TCAACACTTG   
  
  
- AACCTAAATA AACTAGGTAG AAGTAGAAGT TACGGTTTAC AACCTTGGAG ACCCCTACTC AACTAGCGAC   
  
  
- AATCATATGG GTAAACCCGT ACAAGTATAG CCGGCAGATA AGAGGGTAGG TAGGAGGCCA AGTAATTCGT   
  
  
- TGATCGGGGG TTCTAACAGC AGAGCAATCT ATCTCCCAGA CTAGCAACGC TGTGAGACAA GGGTGTTGTA   
  
  
- GAATAGGTAT AGAATCTCAG GACGTGTTTA AAGAACCTTA GCGAGCTGCC AGAGTTACAT CGTAGTCTAT   
  
  
- AACATTTGTT CCAACTCTTC ATGAAGGAGG TCGGATTCTA GCTTTTGTGG CACAACCCGG CGCAAGTACG   
  
  
- GGGGTTATTC TACGGTGTAA CCTTCTGGGA GAAACGAAGT CGGCCCAATA ACGGGAAAGT CAAGTCATTG   
  
  
- AAGTGTCTTT GTGTTCGACT AATACACCAC TTCGCTTGGG GTCCCTCCCC TAAAGTGTAT CTTTTCGCGG   
  
  
- TGCGTAGAAA TCACGATTCA ACCGTTTCCG CCCTCGAACA CTGTCGGAGC CGTACCTCCA AACTCTTCGA   
  
  
- CAT

+     TATA-box

| Site Name | Organism | Position | Strand | Matrix score. | sequence | function |
| --- | --- | --- | --- | --- | --- | --- |
| TATA-box | Arabidopsis thaliana | 178 | + | 4 | TATA | core promoter element around -30 of transcription start |
| TATA-box | Arabidopsis thaliana | 3299 | - | 4 | TATA | core promoter element around -30 of transcription start |
| TATA-box | Arabidopsis thaliana | 1418 | - | 5 | TATAA | core promoter element around -30 of transcription start |
| TATA-box | Arabidopsis thaliana | 1027 | + | 4 | TATA | core promoter element around -30 of transcription start |
| TATA-box | Arabidopsis thaliana | 785 | + | 4 | TATA | core promoter element around -30 of transcription start |
| TATA-box | Arabidopsis thaliana | 831 | + | 8 | TATTTAAA | core promoter element around -30 of transcription start |
| TATA-box | Arabidopsis thaliana | 2891 | - | 4 | TATA | core promoter element around -30 of transcription start |
| TATA-box | Helianthus annuus | 764 | - | 6 | TATACA | core promoter element around -30 of transcription start |
| TATA-box | Arabidopsis thaliana | 573 | + | 4 | TATA | core promoter element around -30 of transcription start |
| TATA-box | Oryza sativa | 1391 | - | 7 | TACAAAA | core promoter element around -30 of transcription start |
| TATA-box | Arabidopsis thaliana | 719 | + | 9 | taTATAAAtc | core promoter element around -30 of transcription start |
| TATA-box | Arabidopsis thaliana | 1419 | + | 4 | TATA | core promoter element around -30 of transcription start |
| TATA-box | Arabidopsis thaliana | 177 | - | 5 | TATAA | core promoter element around -30 of transcription start |
| TATA-box | Brassica oleracea | 1248 | + | 6 | ATATAA | core promoter element around -30 of transcription start |
| TATA-box | Arabidopsis thaliana | 766 | + | 4 | TATA | core promoter element around -30 of transcription start |
| TATA-box | Helianthus annuus | 712 | - | 6 | TATAAA | core promoter element around -30 of transcription start |
| TATA-box | Brassica napus | 572 | + | 6 | ATATAT | core promoter element around -30 of transcription start |
| TATA-box | Arabidopsis thaliana | 1247 | + | 9 | ccTATAAAaa | core promoter element around -30 of transcription start |
| TATA-box | Brassica napus | 176 | + | 6 | ATTATA | core promoter element around -30 of transcription start |
| TATA-box | Arabidopsis thaliana | 714 | + | 4 | TATA | core promoter element around -30 of transcription start |
| TATA-box | Brassica napus | 784 | + | 6 | ATATAT | core promoter element around -30 of transcription start |
| TATA-box | Arabidopsis thaliana | 713 | - | 5 | TATAA | core promoter element around -30 of transcription start |
| TATA-box | Arabidopsis thaliana | 1249 | + | 4 | TATA | core promoter element around -30 of transcription start |
| TATA-box | Arabidopsis thaliana | 194 | + | 4 | TATA | core promoter element around -30 of transcription start |
| TATA-box | Brassica napus | 193 | + | 6 | ATATAT | core promoter element around -30 of transcription start |
| TATA-box | Brassica napus | 191 | + | 6 | ATATAT | core promoter element around -30 of transcription start |
| TATA-box | Arabidopsis thaliana | 192 | + | 6 | TATATA | core promoter element around -30 of transcription start |

>HU05G00466.1   
+ +Up\_Stream \_Len000ACATTG GGTGGGATTT AAATCCTCTA TTTTTTCTTA GGGGAAAAAT ACTTGGGAAA   
  
  
+ TTTCTCAATA ACTGAAAATC TAAATAATTC GTGACTTTGT TAGTAGGTTT AATGTGATTT ACTACCGTGA   
  
  
+ GTGTCACTTT TAGATGGTAC TTTAAGAAAG CATTATAAGT AAAACAATAT ATATCGTAGC ATCATACTAC   
  
  
+ TTGTGTAATA AATTAACAGC AACCTCAATT GTACTTATTT GATTTCGCAT TGTAATCAAT TTTCCCAAAT   
  
  
+ GATTTACTAT CTTGGTCTTA CTCAGTTGTA CTTCACACTC AATAGTTCAA TTATTTTTCA TTAGACTAAT   
  
  
+ GAGCGAGTCC CTTCTACAAG AGCCTACTAC TACTCTAATA ATGTAAGGAC TATTGGCTAA TGGGGAACTA   
  
  
+ AGCCTACGAC TTGTTAGGCA TAGGTGAATT TTCCTTACAA GAGAGTTTAA TTTACACTTG ATATTAACTC   
  
  
+ GAGAACGAGA AGCTGTCTTA ATAGATACCC ACATAGAAAA TGAAGCAAAT GCCCTTGATT ATTGGAGGGT   
  
  
+ TGGGCAAATA TATTCAGTAA CAATGTTGTT TGGCAAATAT CGTCTGAAAA TTGTTTTTGA CAAATATTGT   
  
  
+ TTGGAAGAAT TTTTCGAGAT TAAATATTAA AAATGTTAAA ATTTTAATCT AAATATCAAA CAAATATTGA   
  
  
+ AAAATGCTTT ATATTATCTA AATCTCTAAT TAAAATTTGT GTATTGTCCA CTAGAAAAAT GTATAGTGAT   
  
  
+ ATTACCGACA TATATGTGAT CTCTGGTGTA ATAGCCTACC CTAATGTCTA GGAAAATATT TAAAATCCCC   
  
  
+ AGTGGTCAAA AGGAAGTTAC GCACACTAGG GCAAGACACT TCATTAGGCT AGTTGGTGAA AGCTAAGTCA   
  
  
+ TGAAATTTAG ACCATGGCCA TGTCTACTTG TAGATCCAAC TACCAACAAT CCATTTTTAT TTTGGGGAAA   
  
  
+ GCTTGAAGGG TAATTTGGTA AATGTACACG GGAACCTTTT GCTATAAAAG AAGTGCTTCT TGAAAAAACA   
  
  
+ CCCTGTAGTA AAACAGGGCA AAAAAGCCAA CACTTCTGTT TCTGGCAGAG AAACAGTAGT GTACCACTTG   
  
  
+ GAGTTGGAGT AGACACGATA CACAAACCCA GTAGAGAGAG AAACAGGGGA GAGAAAGAGT ACACAAATTT   
  
  
+ GTGCAGGAGA AGGGTAAGGA AGAAGGAGAA GGAACCCCCT CCAAAAAAAA CCCATATAAA AAATAATAAA   
  
  
+ GAAAAAACAG GAGTGAGTTA GAGGGTACCC AAATCTCCAA TCTAAAACTA GAATCTAGGG TCTCTTTTCA   
  
  
+ TTTGCTTGAA AAATTTCTGT TCATGGGTTT TGTGTTTTTA GGGGGATTTG TTTGATTTTT GTAGTCTTTG   
  
  
+ TGGATTTCCT GTGTTATAGA TGCAAGCTAT GCTCTTCAAT TTGCAAGGAA CAGCATCTGG GGGGAGAGAA   
  
  
+ TTTGAGGAAT TTCAAGTACC CATTTCAATT TCAACAAGTA AAATCATCAT TGAAGGATAC TCAAACCCAA   
  
  
+ GCCCAGTTGA TAGTGAACCA ACTTCTACTC TGGATTCTAC TCCAAGTCCC AGCCCTCCCA CCCTCTCCTC   
  
  
+ CTCCTCCAAG AACTCCTACC AAAACGCTGC CGTTTTGGCT CACCCTCCTC AGCTTCACCA TGGATCTGAT   
  
  
+ AATGTGTAAG ACCCCAACCC TTTTGCTTTT ATTTTCCTCA TGCTCCATTC TCATGATTTG TACTTCTGTT   
  
  
+ ATGTAATTGG AGATGTGTCT AAATTTGGAA AAGTTCGTGG GTTTCGTGTT TGAGAATTTA GAATGACATT   
  
  
+ CTTTAATTAG TAATTTGAAG TTTGCATGCT TAATTTAGCT AATCACTGAT TAATTAAAAT AAATGAAGTG   
  
  
+ ATGTAGGTAT GGGCTGTTAC ACAATGTCAC CGGTGGTGGT GACAGCGGCG GGGCGGCGGC GGTTGGATTG   
  
  
+ GAAGAATGGG ACAGCGTGTT TCCAAATGGG GATGGAGCTT TACTCCCTTG GATCATGGGG GAAGGTGATG   
  
  
+ ATTTGGGTCT GAATTTGAAG CATCTCTTAC AATCTGGTTA CCCTGTTGAG TATGAAGGCA ATGCAGGACT   
  
  
+ TGGTGTTGTT GATCAGACAT CTCTTCTTGG AGGTGGTGAT TTGGGGTTTT CTGGTTCTGG GTCTGTGAAT   
  
  
+ AATGGCAAAA TTGGTTCCAT TTTGGGTAAT TGTTCATCTG GGGATTTGGA TTCTAAGGTT TCTAGTGACG   
  
  
+ GGTTGAATTC CAATTGTAGT TCACTGGGGA GTATGAGTAT CCTTGGTTCA ATCCATGGAC CTTTGCCTAA   
  
  
+ TACTGGTGGG TTTGTGTTCC CACAAACACA ACTATTTGAT CTTGGTGATG AGAAGCCTCA GATTTTGAAC   
  
  
+ CCACAATTGA TGACGATGAA TCCAAATCAG GCTCAGAGCA TGGGAAACCC TAGCTTTTTT GTGCCCTCCT   
  
  
+ TGGGTTATTG TCAGTTAGAG CAACATATGG TTCAACCACA GGCGAAACGC CATAGCCCTG GTGTAGTTTT   
  
  
+ GAACTCTGAT GTTATTGCCA AAACCCCGTT TCTCGATCAG GGTCATGAGT TTTTGTTGAG GAAACAACAC   
  
  
+ CATCAACAGA TTTTGCAGCA ACTTCCTATG GGTTTGGCTC ATCAGTCGGT CCCTCAGCAC CTCCAGCAAA   
  
  
+ AGGCGATGAA AGACCAGGTT GTTAAGGACC AGCTTTTGAA GGCAGCTGAC CTGATTCAAA CTGGGAATTT   
  
  
+ CTCACTTGCG CAAGAGATAT TGGCGCGGCT CAATCACCAG CTCTCCCTTC CTGCGAAGCC CCTCATTAGG   
  
  
+ GCAGCTTTGT ATGTCATGGA GGCCTTGCAA ATGCTCATCT TAATGAGCAA TCCTGTAGCA CCTCCACCGA   
  
  
+ TGAAGACGCT TTCCCCTATA GATGTTGTTC ATAAGATGAA TGCCTACAAG GCCTTCTCTG AGGTCTCTCC   
  
  
+ TATTACTCAG TTTACGAATT TCACTTGTGC CCAAGCCATT CTTGAAGCTC TCGATGATGC GGATTGCGTT   
  
  
+ CATGTCATTG ACTTTGATAT TGGTTGTGGG GCTCAATGGG CATCACTGAT TCAGGAGCTT CCATTGAGGA   
  
  
+ AAAAGGGAGC TCCATCCCTG AAAATTACAG CCATAGCTCC CCTGTCAATA AGCCACACTT TTGAACTCAA   
  
  
+ CCTAGCACGG GAAAACCTTG TGCAATTCGC CAATGATGTT GGTGTTGCTT TTGAGCTGCA AGTTGTGAAC   
  
  
+ TTGGATTTAT TTGATCCATC TTCATCTTCA ATGCCAAATG TTGGAACCTC TGGGGATGAG TTGATCGCTG   
  
  
+ TTAGTATACC CATTTGGGCA TGTTCATATC GGCCGTCTAT TCTCCCATCC ATCCTCCGGT TCATTAAGCA   
  
  
+ ACTAGCCCCC AAGATTGTCG TCTCGTTAGA TAGAGGGTCT GATCGTTGCG ACACTCTGTT CCCACAACAT   
  
  
+ CTTATCCATA TCTTAGAGTC CTGCACAAAT TTCTTGGAAT CGCTCGACGG TCTCAATGTA GCATCAGATA   
  
  
+ TTGTAAACAA GGTTGAGAAG TACTTCCTCC AGCCTAAGAT CGAAAACACC GTGTTGGGCC GCGTTCATGC   
  
  
+ CCCCAATAAG ATGCCACATT GGAAGACCCT CTTTGCTTCA GCCGGGTTAT TGCCCTTTCA GTTCAGTAAC   
  
  
+ TTCACAGAAA CACAAGCTGA TTATGTGGTG AAGCGAACCC CAGGGAGGGG ATTTCACATA GAAAAGCGCC   
  
  
+ ACGCATCTTT AGTGCTAAGT TGGCAAAGGC GGGAGCTTGT GACAGCCTCG GCATGGAGGT TTGAGAAGCT   
  
  
+ GTA  

- +Up\_Stream \_Len000TGTAAC CCACCCTAAA TTTAGGAGAT AAAAAAGAAT CCCCTTTTTA TGAACCCTTT   
  
  
- AAAGAGTTAT TGACTTTTAG ATTTATTAAG CACTGAAACA ATCATCCAAA TTACACTAAA TGATGGCACT   
  
  
- CACAGTGAAA ATCTACCATG AAATTCTTTC GTAATATTCA TTTTGTTATA TATAGCATCG TAGTATGATG   
  
  
- AACACATTAT TTAATTGTCG TTGGAGTTAA CATGAATAAA CTAAAGCGTA ACATTAGTTA AAAGGGTTTA   
  
  
- CTAAATGATA GAACCAGAAT GAGTCAACAT GAAGTGTGAG TTATCAAGTT AATAAAAAGT AATCTGATTA   
  
  
- CTCGCTCAGG GAAGATGTTC TCGGATGATG ATGAGATTAT TACATTCCTG ATAACCGATT ACCCCTTGAT   
  
  
- TCGGATGCTG AACAATCCGT ATCCACTTAA AAGGAATGTT CTCTCAAATT AAATGTGAAC TATAATTGAG   
  
  
- CTCTTGCTCT TCGACAGAAT TATCTATGGG TGTATCTTTT ACTTCGTTTA CGGGAACTAA TAACCTCCCA   
  
  
- ACCCGTTTAT ATAAGTCATT GTTACAACAA ACCGTTTATA GCAGACTTTT AACAAAAACT GTTTATAACA   
  
  
- AACCTTCTTA AAAAGCTCTA ATTTATAATT TTTACAATTT TAAAATTAGA TTTATAGTTT GTTTATAACT   
  
  
- TTTTACGAAA TATAATAGAT TTAGAGATTA ATTTTAAACA CATAACAGGT GATCTTTTTA CATATCACTA   
  
  
- TAATGGCTGT ATATACACTA GAGACCACAT TATCGGATGG GATTACAGAT CCTTTTATAA ATTTTAGGGG   
  
  
- TCACCAGTTT TCCTTCAATG CGTGTGATCC CGTTCTGTGA AGTAATCCGA TCAACCACTT TCGATTCAGT   
  
  
- ACTTTAAATC TGGTACCGGT ACAGATGAAC ATCTAGGTTG ATGGTTGTTA GGTAAAAATA AAACCCCTTT   
  
  
- CGAACTTCCC ATTAAACCAT TTACATGTGC CCTTGGAAAA CGATATTTTC TTCACGAAGA ACTTTTTTGT   
  
  
- GGGACATCAT TTTGTCCCGT TTTTTCGGTT GTGAAGACAA AGACCGTCTC TTTGTCATCA CATGGTGAAC   
  
  
- CTCAACCTCA TCTGTGCTAT GTGTTTGGGT CATCTCTCTC TTTGTCCCCT CTCTTTCTCA TGTGTTTAAA   
  
  
- CACGTCCTCT TCCCATTCCT TCTTCCTCTT CCTTGGGGGA GGTTTTTTTT GGGTATATTT TTTATTATTT   
  
  
- CTTTTTTGTC CTCACTCAAT CTCCCATGGG TTTAGAGGTT AGATTTTGAT CTTAGATCCC AGAGAAAAGT   
  
  
- AAACGAACTT TTTAAAGACA AGTACCCAAA ACACAAAAAT CCCCCTAAAC AAACTAAAAA CATCAGAAAC   
  
  
- ACCTAAAGGA CACAATATCT ACGTTCGATA CGAGAAGTTA AACGTTCCTT GTCGTAGACC CCCCTCTCTT   
  
  
- AAACTCCTTA AAGTTCATGG GTAAAGTTAA AGTTGTTCAT TTTAGTAGTA ACTTCCTATG AGTTTGGGTT   
  
  
- CGGGTCAACT ATCACTTGGT TGAAGATGAG ACCTAAGATG AGGTTCAGGG TCGGGAGGGT GGGAGAGGAG   
  
  
- GAGGAGGTTC TTGAGGATGG TTTTGCGACG GCAAAACCGA GTGGGAGGAG TCGAAGTGGT ACCTAGACTA   
  
  
- TTACACATTC TGGGGTTGGG AAAACGAAAA TAAAAGGAGT ACGAGGTAAG AGTACTAAAC ATGAAGACAA   
  
  
- TACATTAACC TCTACACAGA TTTAAACCTT TTCAAGCACC CAAAGCACAA ACTCTTAAAT CTTACTGTAA   
  
  
- GAAATTAATC ATTAAACTTC AAACGTACGA ATTAAATCGA TTAGTGACTA ATTAATTTTA TTTACTTCAC   
  
  
- TACATCCATA CCCGACAATG TGTTACAGTG GCCACCACCA CTGTCGCCGC CCCGCCGCCG CCAACCTAAC   
  
  
- CTTCTTACCC TGTCGCACAA AGGTTTACCC CTACCTCGAA ATGAGGGAAC CTAGTACCCC CTTCCACTAC   
  
  
- TAAACCCAGA CTTAAACTTC GTAGAGAATG TTAGACCAAT GGGACAACTC ATACTTCCGT TACGTCCTGA   
  
  
- ACCACAACAA CTAGTCTGTA GAGAAGAACC TCCACCACTA AACCCCAAAA GACCAAGACC CAGACACTTA   
  
  
- TTACCGTTTT AACCAAGGTA AAACCCATTA ACAAGTAGAC CCCTAAACCT AAGATTCCAA AGATCACTGC   
  
  
- CCAACTTAAG GTTAACATCA AGTGACCCCT CATACTCATA GGAACCAAGT TAGGTACCTG GAAACGGATT   
  
  
- ATGACCACCC AAACACAAGG GTGTTTGTGT TGATAAACTA GAACCACTAC TCTTCGGAGT CTAAAACTTG   
  
  
- GGTGTTAACT ACTGCTACTT AGGTTTAGTC CGAGTCTCGT ACCCTTTGGG ATCGAAAAAA CACGGGAGGA   
  
  
- ACCCAATAAC AGTCAATCTC GTTGTATACC AAGTTGGTGT CCGCTTTGCG GTATCGGGAC CACATCAAAA   
  
  
- CTTGAGACTA CAATAACGGT TTTGGGGCAA AGAGCTAGTC CCAGTACTCA AAAACAACTC CTTTGTTGTG   
  
  
- GTAGTTGTCT AAAACGTCGT TGAAGGATAC CCAAACCGAG TAGTCAGCCA GGGAGTCGTG GAGGTCGTTT   
  
  
- TCCGCTACTT TCTGGTCCAA CAATTCCTGG TCGAAAACTT CCGTCGACTG GACTAAGTTT GACCCTTAAA   
  
  
- GAGTGAACGC GTTCTCTATA ACCGCGCCGA GTTAGTGGTC GAGAGGGAAG GACGCTTCGG GGAGTAATCC   
  
  
- CGTCGAAACA TACAGTACCT CCGGAACGTT TACGAGTAGA ATTACTCGTT AGGACATCGT GGAGGTGGCT   
  
  
- ACTTCTGCGA AAGGGGATAT CTACAACAAG TATTCTACTT ACGGATGTTC CGGAAGAGAC TCCAGAGAGG   
  
  
- ATAATGAGTC AAATGCTTAA AGTGAACACG GGTTCGGTAA GAACTTCGAG AGCTACTACG CCTAACGCAA   
  
  
- GTACAGTAAC TGAAACTATA ACCAACACCC CGAGTTACCC GTAGTGACTA AGTCCTCGAA GGTAACTCCT   
  
  
- TTTTCCCTCG AGGTAGGGAC TTTTAATGTC GGTATCGAGG GGACAGTTAT TCGGTGTGAA AACTTGAGTT   
  
  
- GGATCGTGCC CTTTTGGAAC ACGTTAAGCG GTTACTACAA CCACAACGAA AACTCGACGT TCAACACTTG   
  
  
- AACCTAAATA AACTAGGTAG AAGTAGAAGT TACGGTTTAC AACCTTGGAG ACCCCTACTC AACTAGCGAC   
  
  
- AATCATATGG GTAAACCCGT ACAAGTATAG CCGGCAGATA AGAGGGTAGG TAGGAGGCCA AGTAATTCGT   
  
  
- TGATCGGGGG TTCTAACAGC AGAGCAATCT ATCTCCCAGA CTAGCAACGC TGTGAGACAA GGGTGTTGTA   
  
  
- GAATAGGTAT AGAATCTCAG GACGTGTTTA AAGAACCTTA GCGAGCTGCC AGAGTTACAT CGTAGTCTAT   
  
  
- AACATTTGTT CCAACTCTTC ATGAAGGAGG TCGGATTCTA GCTTTTGTGG CACAACCCGG CGCAAGTACG   
  
  
- GGGGTTATTC TACGGTGTAA CCTTCTGGGA GAAACGAAGT CGGCCCAATA ACGGGAAAGT CAAGTCATTG   
  
  
- AAGTGTCTTT GTGTTCGACT AATACACCAC TTCGCTTGGG GTCCCTCCCC TAAAGTGTAT CTTTTCGCGG   
  
  
- TGCGTAGAAA TCACGATTCA ACCGTTTCCG CCCTCGAACA CTGTCGGAGC CGTACCTCCA AACTCTTCGA   
  
  
- CAT

+     TC-rich repeats

| Site Name | Organism | Position | Strand | Matrix score. | sequence | function |
| --- | --- | --- | --- | --- | --- | --- |
| TC-rich repeats | Nicotiana tabacum | 1802 | - | 9 | ATTCTCTAAC | cis-acting element involved in defense and stress responsiveness |

>HU05G00466.1   
+ +Up\_Stream \_Len000ACATTG GGTGGGATTT AAATCCTCTA TTTTTTCTTA GGGGAAAAAT ACTTGGGAAA   
  
  
+ TTTCTCAATA ACTGAAAATC TAAATAATTC GTGACTTTGT TAGTAGGTTT AATGTGATTT ACTACCGTGA   
  
  
+ GTGTCACTTT TAGATGGTAC TTTAAGAAAG CATTATAAGT AAAACAATAT ATATCGTAGC ATCATACTAC   
  
  
+ TTGTGTAATA AATTAACAGC AACCTCAATT GTACTTATTT GATTTCGCAT TGTAATCAAT TTTCCCAAAT   
  
  
+ GATTTACTAT CTTGGTCTTA CTCAGTTGTA CTTCACACTC AATAGTTCAA TTATTTTTCA TTAGACTAAT   
  
  
+ GAGCGAGTCC CTTCTACAAG AGCCTACTAC TACTCTAATA ATGTAAGGAC TATTGGCTAA TGGGGAACTA   
  
  
+ AGCCTACGAC TTGTTAGGCA TAGGTGAATT TTCCTTACAA GAGAGTTTAA TTTACACTTG ATATTAACTC   
  
  
+ GAGAACGAGA AGCTGTCTTA ATAGATACCC ACATAGAAAA TGAAGCAAAT GCCCTTGATT ATTGGAGGGT   
  
  
+ TGGGCAAATA TATTCAGTAA CAATGTTGTT TGGCAAATAT CGTCTGAAAA TTGTTTTTGA CAAATATTGT   
  
  
+ TTGGAAGAAT TTTTCGAGAT TAAATATTAA AAATGTTAAA ATTTTAATCT AAATATCAAA CAAATATTGA   
  
  
+ AAAATGCTTT ATATTATCTA AATCTCTAAT TAAAATTTGT GTATTGTCCA CTAGAAAAAT GTATAGTGAT   
  
  
+ ATTACCGACA TATATGTGAT CTCTGGTGTA ATAGCCTACC CTAATGTCTA GGAAAATATT TAAAATCCCC   
  
  
+ AGTGGTCAAA AGGAAGTTAC GCACACTAGG GCAAGACACT TCATTAGGCT AGTTGGTGAA AGCTAAGTCA   
  
  
+ TGAAATTTAG ACCATGGCCA TGTCTACTTG TAGATCCAAC TACCAACAAT CCATTTTTAT TTTGGGGAAA   
  
  
+ GCTTGAAGGG TAATTTGGTA AATGTACACG GGAACCTTTT GCTATAAAAG AAGTGCTTCT TGAAAAAACA   
  
  
+ CCCTGTAGTA AAACAGGGCA AAAAAGCCAA CACTTCTGTT TCTGGCAGAG AAACAGTAGT GTACCACTTG   
  
  
+ GAGTTGGAGT AGACACGATA CACAAACCCA GTAGAGAGAG AAACAGGGGA GAGAAAGAGT ACACAAATTT   
  
  
+ GTGCAGGAGA AGGGTAAGGA AGAAGGAGAA GGAACCCCCT CCAAAAAAAA CCCATATAAA AAATAATAAA   
  
  
+ GAAAAAACAG GAGTGAGTTA GAGGGTACCC AAATCTCCAA TCTAAAACTA GAATCTAGGG TCTCTTTTCA   
  
  
+ TTTGCTTGAA AAATTTCTGT TCATGGGTTT TGTGTTTTTA GGGGGATTTG TTTGATTTTT GTAGTCTTTG   
  
  
+ TGGATTTCCT GTGTTATAGA TGCAAGCTAT GCTCTTCAAT TTGCAAGGAA CAGCATCTGG GGGGAGAGAA   
  
  
+ TTTGAGGAAT TTCAAGTACC CATTTCAATT TCAACAAGTA AAATCATCAT TGAAGGATAC TCAAACCCAA   
  
  
+ GCCCAGTTGA TAGTGAACCA ACTTCTACTC TGGATTCTAC TCCAAGTCCC AGCCCTCCCA CCCTCTCCTC   
  
  
+ CTCCTCCAAG AACTCCTACC AAAACGCTGC CGTTTTGGCT CACCCTCCTC AGCTTCACCA TGGATCTGAT   
  
  
+ AATGTGTAAG ACCCCAACCC TTTTGCTTTT ATTTTCCTCA TGCTCCATTC TCATGATTTG TACTTCTGTT   
  
  
+ ATGTAATTGG AGATGTGTCT AAATTTGGAA AAGTTCGTGG GTTTCGTGTT TGAGAATTTA GAATGACATT   
  
  
+ CTTTAATTAG TAATTTGAAG TTTGCATGCT TAATTTAGCT AATCACTGAT TAATTAAAAT AAATGAAGTG   
  
  
+ ATGTAGGTAT GGGCTGTTAC ACAATGTCAC CGGTGGTGGT GACAGCGGCG GGGCGGCGGC GGTTGGATTG   
  
  
+ GAAGAATGGG ACAGCGTGTT TCCAAATGGG GATGGAGCTT TACTCCCTTG GATCATGGGG GAAGGTGATG   
  
  
+ ATTTGGGTCT GAATTTGAAG CATCTCTTAC AATCTGGTTA CCCTGTTGAG TATGAAGGCA ATGCAGGACT   
  
  
+ TGGTGTTGTT GATCAGACAT CTCTTCTTGG AGGTGGTGAT TTGGGGTTTT CTGGTTCTGG GTCTGTGAAT   
  
  
+ AATGGCAAAA TTGGTTCCAT TTTGGGTAAT TGTTCATCTG GGGATTTGGA TTCTAAGGTT TCTAGTGACG   
  
  
+ GGTTGAATTC CAATTGTAGT TCACTGGGGA GTATGAGTAT CCTTGGTTCA ATCCATGGAC CTTTGCCTAA   
  
  
+ TACTGGTGGG TTTGTGTTCC CACAAACACA ACTATTTGAT CTTGGTGATG AGAAGCCTCA GATTTTGAAC   
  
  
+ CCACAATTGA TGACGATGAA TCCAAATCAG GCTCAGAGCA TGGGAAACCC TAGCTTTTTT GTGCCCTCCT   
  
  
+ TGGGTTATTG TCAGTTAGAG CAACATATGG TTCAACCACA GGCGAAACGC CATAGCCCTG GTGTAGTTTT   
  
  
+ GAACTCTGAT GTTATTGCCA AAACCCCGTT TCTCGATCAG GGTCATGAGT TTTTGTTGAG GAAACAACAC   
  
  
+ CATCAACAGA TTTTGCAGCA ACTTCCTATG GGTTTGGCTC ATCAGTCGGT CCCTCAGCAC CTCCAGCAAA   
  
  
+ AGGCGATGAA AGACCAGGTT GTTAAGGACC AGCTTTTGAA GGCAGCTGAC CTGATTCAAA CTGGGAATTT   
  
  
+ CTCACTTGCG CAAGAGATAT TGGCGCGGCT CAATCACCAG CTCTCCCTTC CTGCGAAGCC CCTCATTAGG   
  
  
+ GCAGCTTTGT ATGTCATGGA GGCCTTGCAA ATGCTCATCT TAATGAGCAA TCCTGTAGCA CCTCCACCGA   
  
  
+ TGAAGACGCT TTCCCCTATA GATGTTGTTC ATAAGATGAA TGCCTACAAG GCCTTCTCTG AGGTCTCTCC   
  
  
+ TATTACTCAG TTTACGAATT TCACTTGTGC CCAAGCCATT CTTGAAGCTC TCGATGATGC GGATTGCGTT   
  
  
+ CATGTCATTG ACTTTGATAT TGGTTGTGGG GCTCAATGGG CATCACTGAT TCAGGAGCTT CCATTGAGGA   
  
  
+ AAAAGGGAGC TCCATCCCTG AAAATTACAG CCATAGCTCC CCTGTCAATA AGCCACACTT TTGAACTCAA   
  
  
+ CCTAGCACGG GAAAACCTTG TGCAATTCGC CAATGATGTT GGTGTTGCTT TTGAGCTGCA AGTTGTGAAC   
  
  
+ TTGGATTTAT TTGATCCATC TTCATCTTCA ATGCCAAATG TTGGAACCTC TGGGGATGAG TTGATCGCTG   
  
  
+ TTAGTATACC CATTTGGGCA TGTTCATATC GGCCGTCTAT TCTCCCATCC ATCCTCCGGT TCATTAAGCA   
  
  
+ ACTAGCCCCC AAGATTGTCG TCTCGTTAGA TAGAGGGTCT GATCGTTGCG ACACTCTGTT CCCACAACAT   
  
  
+ CTTATCCATA TCTTAGAGTC CTGCACAAAT TTCTTGGAAT CGCTCGACGG TCTCAATGTA GCATCAGATA   
  
  
+ TTGTAAACAA GGTTGAGAAG TACTTCCTCC AGCCTAAGAT CGAAAACACC GTGTTGGGCC GCGTTCATGC   
  
  
+ CCCCAATAAG ATGCCACATT GGAAGACCCT CTTTGCTTCA GCCGGGTTAT TGCCCTTTCA GTTCAGTAAC   
  
  
+ TTCACAGAAA CACAAGCTGA TTATGTGGTG AAGCGAACCC CAGGGAGGGG ATTTCACATA GAAAAGCGCC   
  
  
+ ACGCATCTTT AGTGCTAAGT TGGCAAAGGC GGGAGCTTGT GACAGCCTCG GCATGGAGGT TTGAGAAGCT   
  
  
+ GTA  

- +Up\_Stream \_Len000TGTAAC CCACCCTAAA TTTAGGAGAT AAAAAAGAAT CCCCTTTTTA TGAACCCTTT   
  
  
- AAAGAGTTAT TGACTTTTAG ATTTATTAAG CACTGAAACA ATCATCCAAA TTACACTAAA TGATGGCACT   
  
  
- CACAGTGAAA ATCTACCATG AAATTCTTTC GTAATATTCA TTTTGTTATA TATAGCATCG TAGTATGATG   
  
  
- AACACATTAT TTAATTGTCG TTGGAGTTAA CATGAATAAA CTAAAGCGTA ACATTAGTTA AAAGGGTTTA   
  
  
- CTAAATGATA GAACCAGAAT GAGTCAACAT GAAGTGTGAG TTATCAAGTT AATAAAAAGT AATCTGATTA   
  
  
- CTCGCTCAGG GAAGATGTTC TCGGATGATG ATGAGATTAT TACATTCCTG ATAACCGATT ACCCCTTGAT   
  
  
- TCGGATGCTG AACAATCCGT ATCCACTTAA AAGGAATGTT CTCTCAAATT AAATGTGAAC TATAATTGAG   
  
  
- CTCTTGCTCT TCGACAGAAT TATCTATGGG TGTATCTTTT ACTTCGTTTA CGGGAACTAA TAACCTCCCA   
  
  
- ACCCGTTTAT ATAAGTCATT GTTACAACAA ACCGTTTATA GCAGACTTTT AACAAAAACT GTTTATAACA   
  
  
- AACCTTCTTA AAAAGCTCTA ATTTATAATT TTTACAATTT TAAAATTAGA TTTATAGTTT GTTTATAACT   
  
  
- TTTTACGAAA TATAATAGAT TTAGAGATTA ATTTTAAACA CATAACAGGT GATCTTTTTA CATATCACTA   
  
  
- TAATGGCTGT ATATACACTA GAGACCACAT TATCGGATGG GATTACAGAT CCTTTTATAA ATTTTAGGGG   
  
  
- TCACCAGTTT TCCTTCAATG CGTGTGATCC CGTTCTGTGA AGTAATCCGA TCAACCACTT TCGATTCAGT   
  
  
- ACTTTAAATC TGGTACCGGT ACAGATGAAC ATCTAGGTTG ATGGTTGTTA GGTAAAAATA AAACCCCTTT   
  
  
- CGAACTTCCC ATTAAACCAT TTACATGTGC CCTTGGAAAA CGATATTTTC TTCACGAAGA ACTTTTTTGT   
  
  
- GGGACATCAT TTTGTCCCGT TTTTTCGGTT GTGAAGACAA AGACCGTCTC TTTGTCATCA CATGGTGAAC   
  
  
- CTCAACCTCA TCTGTGCTAT GTGTTTGGGT CATCTCTCTC TTTGTCCCCT CTCTTTCTCA TGTGTTTAAA   
  
  
- CACGTCCTCT TCCCATTCCT TCTTCCTCTT CCTTGGGGGA GGTTTTTTTT GGGTATATTT TTTATTATTT   
  
  
- CTTTTTTGTC CTCACTCAAT CTCCCATGGG TTTAGAGGTT AGATTTTGAT CTTAGATCCC AGAGAAAAGT   
  
  
- AAACGAACTT TTTAAAGACA AGTACCCAAA ACACAAAAAT CCCCCTAAAC AAACTAAAAA CATCAGAAAC   
  
  
- ACCTAAAGGA CACAATATCT ACGTTCGATA CGAGAAGTTA AACGTTCCTT GTCGTAGACC CCCCTCTCTT   
  
  
- AAACTCCTTA AAGTTCATGG GTAAAGTTAA AGTTGTTCAT TTTAGTAGTA ACTTCCTATG AGTTTGGGTT   
  
  
- CGGGTCAACT ATCACTTGGT TGAAGATGAG ACCTAAGATG AGGTTCAGGG TCGGGAGGGT GGGAGAGGAG   
  
  
- GAGGAGGTTC TTGAGGATGG TTTTGCGACG GCAAAACCGA GTGGGAGGAG TCGAAGTGGT ACCTAGACTA   
  
  
- TTACACATTC TGGGGTTGGG AAAACGAAAA TAAAAGGAGT ACGAGGTAAG AGTACTAAAC ATGAAGACAA   
  
  
- TACATTAACC TCTACACAGA TTTAAACCTT TTCAAGCACC CAAAGCACAA ACTCTTAAAT CTTACTGTAA   
  
  
- GAAATTAATC ATTAAACTTC AAACGTACGA ATTAAATCGA TTAGTGACTA ATTAATTTTA TTTACTTCAC   
  
  
- TACATCCATA CCCGACAATG TGTTACAGTG GCCACCACCA CTGTCGCCGC CCCGCCGCCG CCAACCTAAC   
  
  
- CTTCTTACCC TGTCGCACAA AGGTTTACCC CTACCTCGAA ATGAGGGAAC CTAGTACCCC CTTCCACTAC   
  
  
- TAAACCCAGA CTTAAACTTC GTAGAGAATG TTAGACCAAT GGGACAACTC ATACTTCCGT TACGTCCTGA   
  
  
- ACCACAACAA CTAGTCTGTA GAGAAGAACC TCCACCACTA AACCCCAAAA GACCAAGACC CAGACACTTA   
  
  
- TTACCGTTTT AACCAAGGTA AAACCCATTA ACAAGTAGAC CCCTAAACCT AAGATTCCAA AGATCACTGC   
  
  
- CCAACTTAAG GTTAACATCA AGTGACCCCT CATACTCATA GGAACCAAGT TAGGTACCTG GAAACGGATT   
  
  
- ATGACCACCC AAACACAAGG GTGTTTGTGT TGATAAACTA GAACCACTAC TCTTCGGAGT CTAAAACTTG   
  
  
- GGTGTTAACT ACTGCTACTT AGGTTTAGTC CGAGTCTCGT ACCCTTTGGG ATCGAAAAAA CACGGGAGGA   
  
  
- ACCCAATAAC AGTCAATCTC GTTGTATACC AAGTTGGTGT CCGCTTTGCG GTATCGGGAC CACATCAAAA   
  
  
- CTTGAGACTA CAATAACGGT TTTGGGGCAA AGAGCTAGTC CCAGTACTCA AAAACAACTC CTTTGTTGTG   
  
  
- GTAGTTGTCT AAAACGTCGT TGAAGGATAC CCAAACCGAG TAGTCAGCCA GGGAGTCGTG GAGGTCGTTT   
  
  
- TCCGCTACTT TCTGGTCCAA CAATTCCTGG TCGAAAACTT CCGTCGACTG GACTAAGTTT GACCCTTAAA   
  
  
- GAGTGAACGC GTTCTCTATA ACCGCGCCGA GTTAGTGGTC GAGAGGGAAG GACGCTTCGG GGAGTAATCC   
  
  
- CGTCGAAACA TACAGTACCT CCGGAACGTT TACGAGTAGA ATTACTCGTT AGGACATCGT GGAGGTGGCT   
  
  
- ACTTCTGCGA AAGGGGATAT CTACAACAAG TATTCTACTT ACGGATGTTC CGGAAGAGAC TCCAGAGAGG   
  
  
- ATAATGAGTC AAATGCTTAA AGTGAACACG GGTTCGGTAA GAACTTCGAG AGCTACTACG CCTAACGCAA   
  
  
- GTACAGTAAC TGAAACTATA ACCAACACCC CGAGTTACCC GTAGTGACTA AGTCCTCGAA GGTAACTCCT   
  
  
- TTTTCCCTCG AGGTAGGGAC TTTTAATGTC GGTATCGAGG GGACAGTTAT TCGGTGTGAA AACTTGAGTT   
  
  
- GGATCGTGCC CTTTTGGAAC ACGTTAAGCG GTTACTACAA CCACAACGAA AACTCGACGT TCAACACTTG   
  
  
- AACCTAAATA AACTAGGTAG AAGTAGAAGT TACGGTTTAC AACCTTGGAG ACCCCTACTC AACTAGCGAC   
  
  
- AATCATATGG GTAAACCCGT ACAAGTATAG CCGGCAGATA AGAGGGTAGG TAGGAGGCCA AGTAATTCGT   
  
  
- TGATCGGGGG TTCTAACAGC AGAGCAATCT ATCTCCCAGA CTAGCAACGC TGTGAGACAA GGGTGTTGTA   
  
  
- GAATAGGTAT AGAATCTCAG GACGTGTTTA AAGAACCTTA GCGAGCTGCC AGAGTTACAT CGTAGTCTAT   
  
  
- AACATTTGTT CCAACTCTTC ATGAAGGAGG TCGGATTCTA GCTTTTGTGG CACAACCCGG CGCAAGTACG   
  
  
- GGGGTTATTC TACGGTGTAA CCTTCTGGGA GAAACGAAGT CGGCCCAATA ACGGGAAAGT CAAGTCATTG   
  
  
- AAGTGTCTTT GTGTTCGACT AATACACCAC TTCGCTTGGG GTCCCTCCCC TAAAGTGTAT CTTTTCGCGG   
  
  
- TGCGTAGAAA TCACGATTCA ACCGTTTCCG CCCTCGAACA CTGTCGGAGC CGTACCTCCA AACTCTTCGA   
  
  
- CAT

+     TCA

| Site Name | Organism | Position | Strand | Matrix score. | sequence | function |
| --- | --- | --- | --- | --- | --- | --- |
| TCA | Pisum sativum | 3246 | + | 9 | TCATCTTCAT |  |
| TCA | Pisum sativum | 2839 | + | 9 | TCATCTTCAT |  |
| TCA | Pisum sativum | 2394 | - | 9 | TCATCTTCAT |  |
| TCA | Pisum sativum | 3240 | + | 9 | TCATCTTCAT |  |

>HU05G00466.1   
+ +Up\_Stream \_Len000ACATTG GGTGGGATTT AAATCCTCTA TTTTTTCTTA GGGGAAAAAT ACTTGGGAAA   
  
  
+ TTTCTCAATA ACTGAAAATC TAAATAATTC GTGACTTTGT TAGTAGGTTT AATGTGATTT ACTACCGTGA   
  
  
+ GTGTCACTTT TAGATGGTAC TTTAAGAAAG CATTATAAGT AAAACAATAT ATATCGTAGC ATCATACTAC   
  
  
+ TTGTGTAATA AATTAACAGC AACCTCAATT GTACTTATTT GATTTCGCAT TGTAATCAAT TTTCCCAAAT   
  
  
+ GATTTACTAT CTTGGTCTTA CTCAGTTGTA CTTCACACTC AATAGTTCAA TTATTTTTCA TTAGACTAAT   
  
  
+ GAGCGAGTCC CTTCTACAAG AGCCTACTAC TACTCTAATA ATGTAAGGAC TATTGGCTAA TGGGGAACTA   
  
  
+ AGCCTACGAC TTGTTAGGCA TAGGTGAATT TTCCTTACAA GAGAGTTTAA TTTACACTTG ATATTAACTC   
  
  
+ GAGAACGAGA AGCTGTCTTA ATAGATACCC ACATAGAAAA TGAAGCAAAT GCCCTTGATT ATTGGAGGGT   
  
  
+ TGGGCAAATA TATTCAGTAA CAATGTTGTT TGGCAAATAT CGTCTGAAAA TTGTTTTTGA CAAATATTGT   
  
  
+ TTGGAAGAAT TTTTCGAGAT TAAATATTAA AAATGTTAAA ATTTTAATCT AAATATCAAA CAAATATTGA   
  
  
+ AAAATGCTTT ATATTATCTA AATCTCTAAT TAAAATTTGT GTATTGTCCA CTAGAAAAAT GTATAGTGAT   
  
  
+ ATTACCGACA TATATGTGAT CTCTGGTGTA ATAGCCTACC CTAATGTCTA GGAAAATATT TAAAATCCCC   
  
  
+ AGTGGTCAAA AGGAAGTTAC GCACACTAGG GCAAGACACT TCATTAGGCT AGTTGGTGAA AGCTAAGTCA   
  
  
+ TGAAATTTAG ACCATGGCCA TGTCTACTTG TAGATCCAAC TACCAACAAT CCATTTTTAT TTTGGGGAAA   
  
  
+ GCTTGAAGGG TAATTTGGTA AATGTACACG GGAACCTTTT GCTATAAAAG AAGTGCTTCT TGAAAAAACA   
  
  
+ CCCTGTAGTA AAACAGGGCA AAAAAGCCAA CACTTCTGTT TCTGGCAGAG AAACAGTAGT GTACCACTTG   
  
  
+ GAGTTGGAGT AGACACGATA CACAAACCCA GTAGAGAGAG AAACAGGGGA GAGAAAGAGT ACACAAATTT   
  
  
+ GTGCAGGAGA AGGGTAAGGA AGAAGGAGAA GGAACCCCCT CCAAAAAAAA CCCATATAAA AAATAATAAA   
  
  
+ GAAAAAACAG GAGTGAGTTA GAGGGTACCC AAATCTCCAA TCTAAAACTA GAATCTAGGG TCTCTTTTCA   
  
  
+ TTTGCTTGAA AAATTTCTGT TCATGGGTTT TGTGTTTTTA GGGGGATTTG TTTGATTTTT GTAGTCTTTG   
  
  
+ TGGATTTCCT GTGTTATAGA TGCAAGCTAT GCTCTTCAAT TTGCAAGGAA CAGCATCTGG GGGGAGAGAA   
  
  
+ TTTGAGGAAT TTCAAGTACC CATTTCAATT TCAACAAGTA AAATCATCAT TGAAGGATAC TCAAACCCAA   
  
  
+ GCCCAGTTGA TAGTGAACCA ACTTCTACTC TGGATTCTAC TCCAAGTCCC AGCCCTCCCA CCCTCTCCTC   
  
  
+ CTCCTCCAAG AACTCCTACC AAAACGCTGC CGTTTTGGCT CACCCTCCTC AGCTTCACCA TGGATCTGAT   
  
  
+ AATGTGTAAG ACCCCAACCC TTTTGCTTTT ATTTTCCTCA TGCTCCATTC TCATGATTTG TACTTCTGTT   
  
  
+ ATGTAATTGG AGATGTGTCT AAATTTGGAA AAGTTCGTGG GTTTCGTGTT TGAGAATTTA GAATGACATT   
  
  
+ CTTTAATTAG TAATTTGAAG TTTGCATGCT TAATTTAGCT AATCACTGAT TAATTAAAAT AAATGAAGTG   
  
  
+ ATGTAGGTAT GGGCTGTTAC ACAATGTCAC CGGTGGTGGT GACAGCGGCG GGGCGGCGGC GGTTGGATTG   
  
  
+ GAAGAATGGG ACAGCGTGTT TCCAAATGGG GATGGAGCTT TACTCCCTTG GATCATGGGG GAAGGTGATG   
  
  
+ ATTTGGGTCT GAATTTGAAG CATCTCTTAC AATCTGGTTA CCCTGTTGAG TATGAAGGCA ATGCAGGACT   
  
  
+ TGGTGTTGTT GATCAGACAT CTCTTCTTGG AGGTGGTGAT TTGGGGTTTT CTGGTTCTGG GTCTGTGAAT   
  
  
+ AATGGCAAAA TTGGTTCCAT TTTGGGTAAT TGTTCATCTG GGGATTTGGA TTCTAAGGTT TCTAGTGACG   
  
  
+ GGTTGAATTC CAATTGTAGT TCACTGGGGA GTATGAGTAT CCTTGGTTCA ATCCATGGAC CTTTGCCTAA   
  
  
+ TACTGGTGGG TTTGTGTTCC CACAAACACA ACTATTTGAT CTTGGTGATG AGAAGCCTCA GATTTTGAAC   
  
  
+ CCACAATTGA TGACGATGAA TCCAAATCAG GCTCAGAGCA TGGGAAACCC TAGCTTTTTT GTGCCCTCCT   
  
  
+ TGGGTTATTG TCAGTTAGAG CAACATATGG TTCAACCACA GGCGAAACGC CATAGCCCTG GTGTAGTTTT   
  
  
+ GAACTCTGAT GTTATTGCCA AAACCCCGTT TCTCGATCAG GGTCATGAGT TTTTGTTGAG GAAACAACAC   
  
  
+ CATCAACAGA TTTTGCAGCA ACTTCCTATG GGTTTGGCTC ATCAGTCGGT CCCTCAGCAC CTCCAGCAAA   
  
  
+ AGGCGATGAA AGACCAGGTT GTTAAGGACC AGCTTTTGAA GGCAGCTGAC CTGATTCAAA CTGGGAATTT   
  
  
+ CTCACTTGCG CAAGAGATAT TGGCGCGGCT CAATCACCAG CTCTCCCTTC CTGCGAAGCC CCTCATTAGG   
  
  
+ GCAGCTTTGT ATGTCATGGA GGCCTTGCAA ATGCTCATCT TAATGAGCAA TCCTGTAGCA CCTCCACCGA   
  
  
+ TGAAGACGCT TTCCCCTATA GATGTTGTTC ATAAGATGAA TGCCTACAAG GCCTTCTCTG AGGTCTCTCC   
  
  
+ TATTACTCAG TTTACGAATT TCACTTGTGC CCAAGCCATT CTTGAAGCTC TCGATGATGC GGATTGCGTT   
  
  
+ CATGTCATTG ACTTTGATAT TGGTTGTGGG GCTCAATGGG CATCACTGAT TCAGGAGCTT CCATTGAGGA   
  
  
+ AAAAGGGAGC TCCATCCCTG AAAATTACAG CCATAGCTCC CCTGTCAATA AGCCACACTT TTGAACTCAA   
  
  
+ CCTAGCACGG GAAAACCTTG TGCAATTCGC CAATGATGTT GGTGTTGCTT TTGAGCTGCA AGTTGTGAAC   
  
  
+ TTGGATTTAT TTGATCCATC TTCATCTTCA ATGCCAAATG TTGGAACCTC TGGGGATGAG TTGATCGCTG   
  
  
+ TTAGTATACC CATTTGGGCA TGTTCATATC GGCCGTCTAT TCTCCCATCC ATCCTCCGGT TCATTAAGCA   
  
  
+ ACTAGCCCCC AAGATTGTCG TCTCGTTAGA TAGAGGGTCT GATCGTTGCG ACACTCTGTT CCCACAACAT   
  
  
+ CTTATCCATA TCTTAGAGTC CTGCACAAAT TTCTTGGAAT CGCTCGACGG TCTCAATGTA GCATCAGATA   
  
  
+ TTGTAAACAA GGTTGAGAAG TACTTCCTCC AGCCTAAGAT CGAAAACACC GTGTTGGGCC GCGTTCATGC   
  
  
+ CCCCAATAAG ATGCCACATT GGAAGACCCT CTTTGCTTCA GCCGGGTTAT TGCCCTTTCA GTTCAGTAAC   
  
  
+ TTCACAGAAA CACAAGCTGA TTATGTGGTG AAGCGAACCC CAGGGAGGGG ATTTCACATA GAAAAGCGCC   
  
  
+ ACGCATCTTT AGTGCTAAGT TGGCAAAGGC GGGAGCTTGT GACAGCCTCG GCATGGAGGT TTGAGAAGCT   
  
  
+ GTA  

- +Up\_Stream \_Len000TGTAAC CCACCCTAAA TTTAGGAGAT AAAAAAGAAT CCCCTTTTTA TGAACCCTTT   
  
  
- AAAGAGTTAT TGACTTTTAG ATTTATTAAG CACTGAAACA ATCATCCAAA TTACACTAAA TGATGGCACT   
  
  
- CACAGTGAAA ATCTACCATG AAATTCTTTC GTAATATTCA TTTTGTTATA TATAGCATCG TAGTATGATG   
  
  
- AACACATTAT TTAATTGTCG TTGGAGTTAA CATGAATAAA CTAAAGCGTA ACATTAGTTA AAAGGGTTTA   
  
  
- CTAAATGATA GAACCAGAAT GAGTCAACAT GAAGTGTGAG TTATCAAGTT AATAAAAAGT AATCTGATTA   
  
  
- CTCGCTCAGG GAAGATGTTC TCGGATGATG ATGAGATTAT TACATTCCTG ATAACCGATT ACCCCTTGAT   
  
  
- TCGGATGCTG AACAATCCGT ATCCACTTAA AAGGAATGTT CTCTCAAATT AAATGTGAAC TATAATTGAG   
  
  
- CTCTTGCTCT TCGACAGAAT TATCTATGGG TGTATCTTTT ACTTCGTTTA CGGGAACTAA TAACCTCCCA   
  
  
- ACCCGTTTAT ATAAGTCATT GTTACAACAA ACCGTTTATA GCAGACTTTT AACAAAAACT GTTTATAACA   
  
  
- AACCTTCTTA AAAAGCTCTA ATTTATAATT TTTACAATTT TAAAATTAGA TTTATAGTTT GTTTATAACT   
  
  
- TTTTACGAAA TATAATAGAT TTAGAGATTA ATTTTAAACA CATAACAGGT GATCTTTTTA CATATCACTA   
  
  
- TAATGGCTGT ATATACACTA GAGACCACAT TATCGGATGG GATTACAGAT CCTTTTATAA ATTTTAGGGG   
  
  
- TCACCAGTTT TCCTTCAATG CGTGTGATCC CGTTCTGTGA AGTAATCCGA TCAACCACTT TCGATTCAGT   
  
  
- ACTTTAAATC TGGTACCGGT ACAGATGAAC ATCTAGGTTG ATGGTTGTTA GGTAAAAATA AAACCCCTTT   
  
  
- CGAACTTCCC ATTAAACCAT TTACATGTGC CCTTGGAAAA CGATATTTTC TTCACGAAGA ACTTTTTTGT   
  
  
- GGGACATCAT TTTGTCCCGT TTTTTCGGTT GTGAAGACAA AGACCGTCTC TTTGTCATCA CATGGTGAAC   
  
  
- CTCAACCTCA TCTGTGCTAT GTGTTTGGGT CATCTCTCTC TTTGTCCCCT CTCTTTCTCA TGTGTTTAAA   
  
  
- CACGTCCTCT TCCCATTCCT TCTTCCTCTT CCTTGGGGGA GGTTTTTTTT GGGTATATTT TTTATTATTT   
  
  
- CTTTTTTGTC CTCACTCAAT CTCCCATGGG TTTAGAGGTT AGATTTTGAT CTTAGATCCC AGAGAAAAGT   
  
  
- AAACGAACTT TTTAAAGACA AGTACCCAAA ACACAAAAAT CCCCCTAAAC AAACTAAAAA CATCAGAAAC   
  
  
- ACCTAAAGGA CACAATATCT ACGTTCGATA CGAGAAGTTA AACGTTCCTT GTCGTAGACC CCCCTCTCTT   
  
  
- AAACTCCTTA AAGTTCATGG GTAAAGTTAA AGTTGTTCAT TTTAGTAGTA ACTTCCTATG AGTTTGGGTT   
  
  
- CGGGTCAACT ATCACTTGGT TGAAGATGAG ACCTAAGATG AGGTTCAGGG TCGGGAGGGT GGGAGAGGAG   
  
  
- GAGGAGGTTC TTGAGGATGG TTTTGCGACG GCAAAACCGA GTGGGAGGAG TCGAAGTGGT ACCTAGACTA   
  
  
- TTACACATTC TGGGGTTGGG AAAACGAAAA TAAAAGGAGT ACGAGGTAAG AGTACTAAAC ATGAAGACAA   
  
  
- TACATTAACC TCTACACAGA TTTAAACCTT TTCAAGCACC CAAAGCACAA ACTCTTAAAT CTTACTGTAA   
  
  
- GAAATTAATC ATTAAACTTC AAACGTACGA ATTAAATCGA TTAGTGACTA ATTAATTTTA TTTACTTCAC   
  
  
- TACATCCATA CCCGACAATG TGTTACAGTG GCCACCACCA CTGTCGCCGC CCCGCCGCCG CCAACCTAAC   
  
  
- CTTCTTACCC TGTCGCACAA AGGTTTACCC CTACCTCGAA ATGAGGGAAC CTAGTACCCC CTTCCACTAC   
  
  
- TAAACCCAGA CTTAAACTTC GTAGAGAATG TTAGACCAAT GGGACAACTC ATACTTCCGT TACGTCCTGA   
  
  
- ACCACAACAA CTAGTCTGTA GAGAAGAACC TCCACCACTA AACCCCAAAA GACCAAGACC CAGACACTTA   
  
  
- TTACCGTTTT AACCAAGGTA AAACCCATTA ACAAGTAGAC CCCTAAACCT AAGATTCCAA AGATCACTGC   
  
  
- CCAACTTAAG GTTAACATCA AGTGACCCCT CATACTCATA GGAACCAAGT TAGGTACCTG GAAACGGATT   
  
  
- ATGACCACCC AAACACAAGG GTGTTTGTGT TGATAAACTA GAACCACTAC TCTTCGGAGT CTAAAACTTG   
  
  
- GGTGTTAACT ACTGCTACTT AGGTTTAGTC CGAGTCTCGT ACCCTTTGGG ATCGAAAAAA CACGGGAGGA   
  
  
- ACCCAATAAC AGTCAATCTC GTTGTATACC AAGTTGGTGT CCGCTTTGCG GTATCGGGAC CACATCAAAA   
  
  
- CTTGAGACTA CAATAACGGT TTTGGGGCAA AGAGCTAGTC CCAGTACTCA AAAACAACTC CTTTGTTGTG   
  
  
- GTAGTTGTCT AAAACGTCGT TGAAGGATAC CCAAACCGAG TAGTCAGCCA GGGAGTCGTG GAGGTCGTTT   
  
  
- TCCGCTACTT TCTGGTCCAA CAATTCCTGG TCGAAAACTT CCGTCGACTG GACTAAGTTT GACCCTTAAA   
  
  
- GAGTGAACGC GTTCTCTATA ACCGCGCCGA GTTAGTGGTC GAGAGGGAAG GACGCTTCGG GGAGTAATCC   
  
  
- CGTCGAAACA TACAGTACCT CCGGAACGTT TACGAGTAGA ATTACTCGTT AGGACATCGT GGAGGTGGCT   
  
  
- ACTTCTGCGA AAGGGGATAT CTACAACAAG TATTCTACTT ACGGATGTTC CGGAAGAGAC TCCAGAGAGG   
  
  
- ATAATGAGTC AAATGCTTAA AGTGAACACG GGTTCGGTAA GAACTTCGAG AGCTACTACG CCTAACGCAA   
  
  
- GTACAGTAAC TGAAACTATA ACCAACACCC CGAGTTACCC GTAGTGACTA AGTCCTCGAA GGTAACTCCT   
  
  
- TTTTCCCTCG AGGTAGGGAC TTTTAATGTC GGTATCGAGG GGACAGTTAT TCGGTGTGAA AACTTGAGTT   
  
  
- GGATCGTGCC CTTTTGGAAC ACGTTAAGCG GTTACTACAA CCACAACGAA AACTCGACGT TCAACACTTG   
  
  
- AACCTAAATA AACTAGGTAG AAGTAGAAGT TACGGTTTAC AACCTTGGAG ACCCCTACTC AACTAGCGAC   
  
  
- AATCATATGG GTAAACCCGT ACAAGTATAG CCGGCAGATA AGAGGGTAGG TAGGAGGCCA AGTAATTCGT   
  
  
- TGATCGGGGG TTCTAACAGC AGAGCAATCT ATCTCCCAGA CTAGCAACGC TGTGAGACAA GGGTGTTGTA   
  
  
- GAATAGGTAT AGAATCTCAG GACGTGTTTA AAGAACCTTA GCGAGCTGCC AGAGTTACAT CGTAGTCTAT   
  
  
- AACATTTGTT CCAACTCTTC ATGAAGGAGG TCGGATTCTA GCTTTTGTGG CACAACCCGG CGCAAGTACG   
  
  
- GGGGTTATTC TACGGTGTAA CCTTCTGGGA GAAACGAAGT CGGCCCAATA ACGGGAAAGT CAAGTCATTG   
  
  
- AAGTGTCTTT GTGTTCGACT AATACACCAC TTCGCTTGGG GTCCCTCCCC TAAAGTGTAT CTTTTCGCGG   
  
  
- TGCGTAGAAA TCACGATTCA ACCGTTTCCG CCCTCGAACA CTGTCGGAGC CGTACCTCCA AACTCTTCGA   
  
  
- CAT

+     TCCC-motif

| Site Name | Organism | Position | Strand | Matrix score. | sequence | function |
| --- | --- | --- | --- | --- | --- | --- |
| TCCC-motif | Spinacia oleracea | 2776 | + | 7 | TCTCCCT | part of a light responsive element |

>HU05G00466.1   
+ +Up\_Stream \_Len000ACATTG GGTGGGATTT AAATCCTCTA TTTTTTCTTA GGGGAAAAAT ACTTGGGAAA   
  
  
+ TTTCTCAATA ACTGAAAATC TAAATAATTC GTGACTTTGT TAGTAGGTTT AATGTGATTT ACTACCGTGA   
  
  
+ GTGTCACTTT TAGATGGTAC TTTAAGAAAG CATTATAAGT AAAACAATAT ATATCGTAGC ATCATACTAC   
  
  
+ TTGTGTAATA AATTAACAGC AACCTCAATT GTACTTATTT GATTTCGCAT TGTAATCAAT TTTCCCAAAT   
  
  
+ GATTTACTAT CTTGGTCTTA CTCAGTTGTA CTTCACACTC AATAGTTCAA TTATTTTTCA TTAGACTAAT   
  
  
+ GAGCGAGTCC CTTCTACAAG AGCCTACTAC TACTCTAATA ATGTAAGGAC TATTGGCTAA TGGGGAACTA   
  
  
+ AGCCTACGAC TTGTTAGGCA TAGGTGAATT TTCCTTACAA GAGAGTTTAA TTTACACTTG ATATTAACTC   
  
  
+ GAGAACGAGA AGCTGTCTTA ATAGATACCC ACATAGAAAA TGAAGCAAAT GCCCTTGATT ATTGGAGGGT   
  
  
+ TGGGCAAATA TATTCAGTAA CAATGTTGTT TGGCAAATAT CGTCTGAAAA TTGTTTTTGA CAAATATTGT   
  
  
+ TTGGAAGAAT TTTTCGAGAT TAAATATTAA AAATGTTAAA ATTTTAATCT AAATATCAAA CAAATATTGA   
  
  
+ AAAATGCTTT ATATTATCTA AATCTCTAAT TAAAATTTGT GTATTGTCCA CTAGAAAAAT GTATAGTGAT   
  
  
+ ATTACCGACA TATATGTGAT CTCTGGTGTA ATAGCCTACC CTAATGTCTA GGAAAATATT TAAAATCCCC   
  
  
+ AGTGGTCAAA AGGAAGTTAC GCACACTAGG GCAAGACACT TCATTAGGCT AGTTGGTGAA AGCTAAGTCA   
  
  
+ TGAAATTTAG ACCATGGCCA TGTCTACTTG TAGATCCAAC TACCAACAAT CCATTTTTAT TTTGGGGAAA   
  
  
+ GCTTGAAGGG TAATTTGGTA AATGTACACG GGAACCTTTT GCTATAAAAG AAGTGCTTCT TGAAAAAACA   
  
  
+ CCCTGTAGTA AAACAGGGCA AAAAAGCCAA CACTTCTGTT TCTGGCAGAG AAACAGTAGT GTACCACTTG   
  
  
+ GAGTTGGAGT AGACACGATA CACAAACCCA GTAGAGAGAG AAACAGGGGA GAGAAAGAGT ACACAAATTT   
  
  
+ GTGCAGGAGA AGGGTAAGGA AGAAGGAGAA GGAACCCCCT CCAAAAAAAA CCCATATAAA AAATAATAAA   
  
  
+ GAAAAAACAG GAGTGAGTTA GAGGGTACCC AAATCTCCAA TCTAAAACTA GAATCTAGGG TCTCTTTTCA   
  
  
+ TTTGCTTGAA AAATTTCTGT TCATGGGTTT TGTGTTTTTA GGGGGATTTG TTTGATTTTT GTAGTCTTTG   
  
  
+ TGGATTTCCT GTGTTATAGA TGCAAGCTAT GCTCTTCAAT TTGCAAGGAA CAGCATCTGG GGGGAGAGAA   
  
  
+ TTTGAGGAAT TTCAAGTACC CATTTCAATT TCAACAAGTA AAATCATCAT TGAAGGATAC TCAAACCCAA   
  
  
+ GCCCAGTTGA TAGTGAACCA ACTTCTACTC TGGATTCTAC TCCAAGTCCC AGCCCTCCCA CCCTCTCCTC   
  
  
+ CTCCTCCAAG AACTCCTACC AAAACGCTGC CGTTTTGGCT CACCCTCCTC AGCTTCACCA TGGATCTGAT   
  
  
+ AATGTGTAAG ACCCCAACCC TTTTGCTTTT ATTTTCCTCA TGCTCCATTC TCATGATTTG TACTTCTGTT   
  
  
+ ATGTAATTGG AGATGTGTCT AAATTTGGAA AAGTTCGTGG GTTTCGTGTT TGAGAATTTA GAATGACATT   
  
  
+ CTTTAATTAG TAATTTGAAG TTTGCATGCT TAATTTAGCT AATCACTGAT TAATTAAAAT AAATGAAGTG   
  
  
+ ATGTAGGTAT GGGCTGTTAC ACAATGTCAC CGGTGGTGGT GACAGCGGCG GGGCGGCGGC GGTTGGATTG   
  
  
+ GAAGAATGGG ACAGCGTGTT TCCAAATGGG GATGGAGCTT TACTCCCTTG GATCATGGGG GAAGGTGATG   
  
  
+ ATTTGGGTCT GAATTTGAAG CATCTCTTAC AATCTGGTTA CCCTGTTGAG TATGAAGGCA ATGCAGGACT   
  
  
+ TGGTGTTGTT GATCAGACAT CTCTTCTTGG AGGTGGTGAT TTGGGGTTTT CTGGTTCTGG GTCTGTGAAT   
  
  
+ AATGGCAAAA TTGGTTCCAT TTTGGGTAAT TGTTCATCTG GGGATTTGGA TTCTAAGGTT TCTAGTGACG   
  
  
+ GGTTGAATTC CAATTGTAGT TCACTGGGGA GTATGAGTAT CCTTGGTTCA ATCCATGGAC CTTTGCCTAA   
  
  
+ TACTGGTGGG TTTGTGTTCC CACAAACACA ACTATTTGAT CTTGGTGATG AGAAGCCTCA GATTTTGAAC   
  
  
+ CCACAATTGA TGACGATGAA TCCAAATCAG GCTCAGAGCA TGGGAAACCC TAGCTTTTTT GTGCCCTCCT   
  
  
+ TGGGTTATTG TCAGTTAGAG CAACATATGG TTCAACCACA GGCGAAACGC CATAGCCCTG GTGTAGTTTT   
  
  
+ GAACTCTGAT GTTATTGCCA AAACCCCGTT TCTCGATCAG GGTCATGAGT TTTTGTTGAG GAAACAACAC   
  
  
+ CATCAACAGA TTTTGCAGCA ACTTCCTATG GGTTTGGCTC ATCAGTCGGT CCCTCAGCAC CTCCAGCAAA   
  
  
+ AGGCGATGAA AGACCAGGTT GTTAAGGACC AGCTTTTGAA GGCAGCTGAC CTGATTCAAA CTGGGAATTT   
  
  
+ CTCACTTGCG CAAGAGATAT TGGCGCGGCT CAATCACCAG CTCTCCCTTC CTGCGAAGCC CCTCATTAGG   
  
  
+ GCAGCTTTGT ATGTCATGGA GGCCTTGCAA ATGCTCATCT TAATGAGCAA TCCTGTAGCA CCTCCACCGA   
  
  
+ TGAAGACGCT TTCCCCTATA GATGTTGTTC ATAAGATGAA TGCCTACAAG GCCTTCTCTG AGGTCTCTCC   
  
  
+ TATTACTCAG TTTACGAATT TCACTTGTGC CCAAGCCATT CTTGAAGCTC TCGATGATGC GGATTGCGTT   
  
  
+ CATGTCATTG ACTTTGATAT TGGTTGTGGG GCTCAATGGG CATCACTGAT TCAGGAGCTT CCATTGAGGA   
  
  
+ AAAAGGGAGC TCCATCCCTG AAAATTACAG CCATAGCTCC CCTGTCAATA AGCCACACTT TTGAACTCAA   
  
  
+ CCTAGCACGG GAAAACCTTG TGCAATTCGC CAATGATGTT GGTGTTGCTT TTGAGCTGCA AGTTGTGAAC   
  
  
+ TTGGATTTAT TTGATCCATC TTCATCTTCA ATGCCAAATG TTGGAACCTC TGGGGATGAG TTGATCGCTG   
  
  
+ TTAGTATACC CATTTGGGCA TGTTCATATC GGCCGTCTAT TCTCCCATCC ATCCTCCGGT TCATTAAGCA   
  
  
+ ACTAGCCCCC AAGATTGTCG TCTCGTTAGA TAGAGGGTCT GATCGTTGCG ACACTCTGTT CCCACAACAT   
  
  
+ CTTATCCATA TCTTAGAGTC CTGCACAAAT TTCTTGGAAT CGCTCGACGG TCTCAATGTA GCATCAGATA   
  
  
+ TTGTAAACAA GGTTGAGAAG TACTTCCTCC AGCCTAAGAT CGAAAACACC GTGTTGGGCC GCGTTCATGC   
  
  
+ CCCCAATAAG ATGCCACATT GGAAGACCCT CTTTGCTTCA GCCGGGTTAT TGCCCTTTCA GTTCAGTAAC   
  
  
+ TTCACAGAAA CACAAGCTGA TTATGTGGTG AAGCGAACCC CAGGGAGGGG ATTTCACATA GAAAAGCGCC   
  
  
+ ACGCATCTTT AGTGCTAAGT TGGCAAAGGC GGGAGCTTGT GACAGCCTCG GCATGGAGGT TTGAGAAGCT   
  
  
+ GTA  

- +Up\_Stream \_Len000TGTAAC CCACCCTAAA TTTAGGAGAT AAAAAAGAAT CCCCTTTTTA TGAACCCTTT   
  
  
- AAAGAGTTAT TGACTTTTAG ATTTATTAAG CACTGAAACA ATCATCCAAA TTACACTAAA TGATGGCACT   
  
  
- CACAGTGAAA ATCTACCATG AAATTCTTTC GTAATATTCA TTTTGTTATA TATAGCATCG TAGTATGATG   
  
  
- AACACATTAT TTAATTGTCG TTGGAGTTAA CATGAATAAA CTAAAGCGTA ACATTAGTTA AAAGGGTTTA   
  
  
- CTAAATGATA GAACCAGAAT GAGTCAACAT GAAGTGTGAG TTATCAAGTT AATAAAAAGT AATCTGATTA   
  
  
- CTCGCTCAGG GAAGATGTTC TCGGATGATG ATGAGATTAT TACATTCCTG ATAACCGATT ACCCCTTGAT   
  
  
- TCGGATGCTG AACAATCCGT ATCCACTTAA AAGGAATGTT CTCTCAAATT AAATGTGAAC TATAATTGAG   
  
  
- CTCTTGCTCT TCGACAGAAT TATCTATGGG TGTATCTTTT ACTTCGTTTA CGGGAACTAA TAACCTCCCA   
  
  
- ACCCGTTTAT ATAAGTCATT GTTACAACAA ACCGTTTATA GCAGACTTTT AACAAAAACT GTTTATAACA   
  
  
- AACCTTCTTA AAAAGCTCTA ATTTATAATT TTTACAATTT TAAAATTAGA TTTATAGTTT GTTTATAACT   
  
  
- TTTTACGAAA TATAATAGAT TTAGAGATTA ATTTTAAACA CATAACAGGT GATCTTTTTA CATATCACTA   
  
  
- TAATGGCTGT ATATACACTA GAGACCACAT TATCGGATGG GATTACAGAT CCTTTTATAA ATTTTAGGGG   
  
  
- TCACCAGTTT TCCTTCAATG CGTGTGATCC CGTTCTGTGA AGTAATCCGA TCAACCACTT TCGATTCAGT   
  
  
- ACTTTAAATC TGGTACCGGT ACAGATGAAC ATCTAGGTTG ATGGTTGTTA GGTAAAAATA AAACCCCTTT   
  
  
- CGAACTTCCC ATTAAACCAT TTACATGTGC CCTTGGAAAA CGATATTTTC TTCACGAAGA ACTTTTTTGT   
  
  
- GGGACATCAT TTTGTCCCGT TTTTTCGGTT GTGAAGACAA AGACCGTCTC TTTGTCATCA CATGGTGAAC   
  
  
- CTCAACCTCA TCTGTGCTAT GTGTTTGGGT CATCTCTCTC TTTGTCCCCT CTCTTTCTCA TGTGTTTAAA   
  
  
- CACGTCCTCT TCCCATTCCT TCTTCCTCTT CCTTGGGGGA GGTTTTTTTT GGGTATATTT TTTATTATTT   
  
  
- CTTTTTTGTC CTCACTCAAT CTCCCATGGG TTTAGAGGTT AGATTTTGAT CTTAGATCCC AGAGAAAAGT   
  
  
- AAACGAACTT TTTAAAGACA AGTACCCAAA ACACAAAAAT CCCCCTAAAC AAACTAAAAA CATCAGAAAC   
  
  
- ACCTAAAGGA CACAATATCT ACGTTCGATA CGAGAAGTTA AACGTTCCTT GTCGTAGACC CCCCTCTCTT   
  
  
- AAACTCCTTA AAGTTCATGG GTAAAGTTAA AGTTGTTCAT TTTAGTAGTA ACTTCCTATG AGTTTGGGTT   
  
  
- CGGGTCAACT ATCACTTGGT TGAAGATGAG ACCTAAGATG AGGTTCAGGG TCGGGAGGGT GGGAGAGGAG   
  
  
- GAGGAGGTTC TTGAGGATGG TTTTGCGACG GCAAAACCGA GTGGGAGGAG TCGAAGTGGT ACCTAGACTA   
  
  
- TTACACATTC TGGGGTTGGG AAAACGAAAA TAAAAGGAGT ACGAGGTAAG AGTACTAAAC ATGAAGACAA   
  
  
- TACATTAACC TCTACACAGA TTTAAACCTT TTCAAGCACC CAAAGCACAA ACTCTTAAAT CTTACTGTAA   
  
  
- GAAATTAATC ATTAAACTTC AAACGTACGA ATTAAATCGA TTAGTGACTA ATTAATTTTA TTTACTTCAC   
  
  
- TACATCCATA CCCGACAATG TGTTACAGTG GCCACCACCA CTGTCGCCGC CCCGCCGCCG CCAACCTAAC   
  
  
- CTTCTTACCC TGTCGCACAA AGGTTTACCC CTACCTCGAA ATGAGGGAAC CTAGTACCCC CTTCCACTAC   
  
  
- TAAACCCAGA CTTAAACTTC GTAGAGAATG TTAGACCAAT GGGACAACTC ATACTTCCGT TACGTCCTGA   
  
  
- ACCACAACAA CTAGTCTGTA GAGAAGAACC TCCACCACTA AACCCCAAAA GACCAAGACC CAGACACTTA   
  
  
- TTACCGTTTT AACCAAGGTA AAACCCATTA ACAAGTAGAC CCCTAAACCT AAGATTCCAA AGATCACTGC   
  
  
- CCAACTTAAG GTTAACATCA AGTGACCCCT CATACTCATA GGAACCAAGT TAGGTACCTG GAAACGGATT   
  
  
- ATGACCACCC AAACACAAGG GTGTTTGTGT TGATAAACTA GAACCACTAC TCTTCGGAGT CTAAAACTTG   
  
  
- GGTGTTAACT ACTGCTACTT AGGTTTAGTC CGAGTCTCGT ACCCTTTGGG ATCGAAAAAA CACGGGAGGA   
  
  
- ACCCAATAAC AGTCAATCTC GTTGTATACC AAGTTGGTGT CCGCTTTGCG GTATCGGGAC CACATCAAAA   
  
  
- CTTGAGACTA CAATAACGGT TTTGGGGCAA AGAGCTAGTC CCAGTACTCA AAAACAACTC CTTTGTTGTG   
  
  
- GTAGTTGTCT AAAACGTCGT TGAAGGATAC CCAAACCGAG TAGTCAGCCA GGGAGTCGTG GAGGTCGTTT   
  
  
- TCCGCTACTT TCTGGTCCAA CAATTCCTGG TCGAAAACTT CCGTCGACTG GACTAAGTTT GACCCTTAAA   
  
  
- GAGTGAACGC GTTCTCTATA ACCGCGCCGA GTTAGTGGTC GAGAGGGAAG GACGCTTCGG GGAGTAATCC   
  
  
- CGTCGAAACA TACAGTACCT CCGGAACGTT TACGAGTAGA ATTACTCGTT AGGACATCGT GGAGGTGGCT   
  
  
- ACTTCTGCGA AAGGGGATAT CTACAACAAG TATTCTACTT ACGGATGTTC CGGAAGAGAC TCCAGAGAGG   
  
  
- ATAATGAGTC AAATGCTTAA AGTGAACACG GGTTCGGTAA GAACTTCGAG AGCTACTACG CCTAACGCAA   
  
  
- GTACAGTAAC TGAAACTATA ACCAACACCC CGAGTTACCC GTAGTGACTA AGTCCTCGAA GGTAACTCCT   
  
  
- TTTTCCCTCG AGGTAGGGAC TTTTAATGTC GGTATCGAGG GGACAGTTAT TCGGTGTGAA AACTTGAGTT   
  
  
- GGATCGTGCC CTTTTGGAAC ACGTTAAGCG GTTACTACAA CCACAACGAA AACTCGACGT TCAACACTTG   
  
  
- AACCTAAATA AACTAGGTAG AAGTAGAAGT TACGGTTTAC AACCTTGGAG ACCCCTACTC AACTAGCGAC   
  
  
- AATCATATGG GTAAACCCGT ACAAGTATAG CCGGCAGATA AGAGGGTAGG TAGGAGGCCA AGTAATTCGT   
  
  
- TGATCGGGGG TTCTAACAGC AGAGCAATCT ATCTCCCAGA CTAGCAACGC TGTGAGACAA GGGTGTTGTA   
  
  
- GAATAGGTAT AGAATCTCAG GACGTGTTTA AAGAACCTTA GCGAGCTGCC AGAGTTACAT CGTAGTCTAT   
  
  
- AACATTTGTT CCAACTCTTC ATGAAGGAGG TCGGATTCTA GCTTTTGTGG CACAACCCGG CGCAAGTACG   
  
  
- GGGGTTATTC TACGGTGTAA CCTTCTGGGA GAAACGAAGT CGGCCCAATA ACGGGAAAGT CAAGTCATTG   
  
  
- AAGTGTCTTT GTGTTCGACT AATACACCAC TTCGCTTGGG GTCCCTCCCC TAAAGTGTAT CTTTTCGCGG   
  
  
- TGCGTAGAAA TCACGATTCA ACCGTTTCCG CCCTCGAACA CTGTCGGAGC CGTACCTCCA AACTCTTCGA   
  
  
- CAT

+     TCT-motif

| Site Name | Organism | Position | Strand | Matrix score. | sequence | function |
| --- | --- | --- | --- | --- | --- | --- |
| TCT-motif | Arabidopsis thaliana | 1690 | - | 6 | TCTTAC | part of a light responsive element |
| TCT-motif | Arabidopsis thaliana | 300 | + | 6 | TCTTAC | part of a light responsive element |
| TCT-motif | Arabidopsis thaliana | 2059 | + | 6 | TCTTAC | part of a light responsive element |

>HU05G00466.1   
+ +Up\_Stream \_Len000ACATTG GGTGGGATTT AAATCCTCTA TTTTTTCTTA GGGGAAAAAT ACTTGGGAAA   
  
  
+ TTTCTCAATA ACTGAAAATC TAAATAATTC GTGACTTTGT TAGTAGGTTT AATGTGATTT ACTACCGTGA   
  
  
+ GTGTCACTTT TAGATGGTAC TTTAAGAAAG CATTATAAGT AAAACAATAT ATATCGTAGC ATCATACTAC   
  
  
+ TTGTGTAATA AATTAACAGC AACCTCAATT GTACTTATTT GATTTCGCAT TGTAATCAAT TTTCCCAAAT   
  
  
+ GATTTACTAT CTTGGTCTTA CTCAGTTGTA CTTCACACTC AATAGTTCAA TTATTTTTCA TTAGACTAAT   
  
  
+ GAGCGAGTCC CTTCTACAAG AGCCTACTAC TACTCTAATA ATGTAAGGAC TATTGGCTAA TGGGGAACTA   
  
  
+ AGCCTACGAC TTGTTAGGCA TAGGTGAATT TTCCTTACAA GAGAGTTTAA TTTACACTTG ATATTAACTC   
  
  
+ GAGAACGAGA AGCTGTCTTA ATAGATACCC ACATAGAAAA TGAAGCAAAT GCCCTTGATT ATTGGAGGGT   
  
  
+ TGGGCAAATA TATTCAGTAA CAATGTTGTT TGGCAAATAT CGTCTGAAAA TTGTTTTTGA CAAATATTGT   
  
  
+ TTGGAAGAAT TTTTCGAGAT TAAATATTAA AAATGTTAAA ATTTTAATCT AAATATCAAA CAAATATTGA   
  
  
+ AAAATGCTTT ATATTATCTA AATCTCTAAT TAAAATTTGT GTATTGTCCA CTAGAAAAAT GTATAGTGAT   
  
  
+ ATTACCGACA TATATGTGAT CTCTGGTGTA ATAGCCTACC CTAATGTCTA GGAAAATATT TAAAATCCCC   
  
  
+ AGTGGTCAAA AGGAAGTTAC GCACACTAGG GCAAGACACT TCATTAGGCT AGTTGGTGAA AGCTAAGTCA   
  
  
+ TGAAATTTAG ACCATGGCCA TGTCTACTTG TAGATCCAAC TACCAACAAT CCATTTTTAT TTTGGGGAAA   
  
  
+ GCTTGAAGGG TAATTTGGTA AATGTACACG GGAACCTTTT GCTATAAAAG AAGTGCTTCT TGAAAAAACA   
  
  
+ CCCTGTAGTA AAACAGGGCA AAAAAGCCAA CACTTCTGTT TCTGGCAGAG AAACAGTAGT GTACCACTTG   
  
  
+ GAGTTGGAGT AGACACGATA CACAAACCCA GTAGAGAGAG AAACAGGGGA GAGAAAGAGT ACACAAATTT   
  
  
+ GTGCAGGAGA AGGGTAAGGA AGAAGGAGAA GGAACCCCCT CCAAAAAAAA CCCATATAAA AAATAATAAA   
  
  
+ GAAAAAACAG GAGTGAGTTA GAGGGTACCC AAATCTCCAA TCTAAAACTA GAATCTAGGG TCTCTTTTCA   
  
  
+ TTTGCTTGAA AAATTTCTGT TCATGGGTTT TGTGTTTTTA GGGGGATTTG TTTGATTTTT GTAGTCTTTG   
  
  
+ TGGATTTCCT GTGTTATAGA TGCAAGCTAT GCTCTTCAAT TTGCAAGGAA CAGCATCTGG GGGGAGAGAA   
  
  
+ TTTGAGGAAT TTCAAGTACC CATTTCAATT TCAACAAGTA AAATCATCAT TGAAGGATAC TCAAACCCAA   
  
  
+ GCCCAGTTGA TAGTGAACCA ACTTCTACTC TGGATTCTAC TCCAAGTCCC AGCCCTCCCA CCCTCTCCTC   
  
  
+ CTCCTCCAAG AACTCCTACC AAAACGCTGC CGTTTTGGCT CACCCTCCTC AGCTTCACCA TGGATCTGAT   
  
  
+ AATGTGTAAG ACCCCAACCC TTTTGCTTTT ATTTTCCTCA TGCTCCATTC TCATGATTTG TACTTCTGTT   
  
  
+ ATGTAATTGG AGATGTGTCT AAATTTGGAA AAGTTCGTGG GTTTCGTGTT TGAGAATTTA GAATGACATT   
  
  
+ CTTTAATTAG TAATTTGAAG TTTGCATGCT TAATTTAGCT AATCACTGAT TAATTAAAAT AAATGAAGTG   
  
  
+ ATGTAGGTAT GGGCTGTTAC ACAATGTCAC CGGTGGTGGT GACAGCGGCG GGGCGGCGGC GGTTGGATTG   
  
  
+ GAAGAATGGG ACAGCGTGTT TCCAAATGGG GATGGAGCTT TACTCCCTTG GATCATGGGG GAAGGTGATG   
  
  
+ ATTTGGGTCT GAATTTGAAG CATCTCTTAC AATCTGGTTA CCCTGTTGAG TATGAAGGCA ATGCAGGACT   
  
  
+ TGGTGTTGTT GATCAGACAT CTCTTCTTGG AGGTGGTGAT TTGGGGTTTT CTGGTTCTGG GTCTGTGAAT   
  
  
+ AATGGCAAAA TTGGTTCCAT TTTGGGTAAT TGTTCATCTG GGGATTTGGA TTCTAAGGTT TCTAGTGACG   
  
  
+ GGTTGAATTC CAATTGTAGT TCACTGGGGA GTATGAGTAT CCTTGGTTCA ATCCATGGAC CTTTGCCTAA   
  
  
+ TACTGGTGGG TTTGTGTTCC CACAAACACA ACTATTTGAT CTTGGTGATG AGAAGCCTCA GATTTTGAAC   
  
  
+ CCACAATTGA TGACGATGAA TCCAAATCAG GCTCAGAGCA TGGGAAACCC TAGCTTTTTT GTGCCCTCCT   
  
  
+ TGGGTTATTG TCAGTTAGAG CAACATATGG TTCAACCACA GGCGAAACGC CATAGCCCTG GTGTAGTTTT   
  
  
+ GAACTCTGAT GTTATTGCCA AAACCCCGTT TCTCGATCAG GGTCATGAGT TTTTGTTGAG GAAACAACAC   
  
  
+ CATCAACAGA TTTTGCAGCA ACTTCCTATG GGTTTGGCTC ATCAGTCGGT CCCTCAGCAC CTCCAGCAAA   
  
  
+ AGGCGATGAA AGACCAGGTT GTTAAGGACC AGCTTTTGAA GGCAGCTGAC CTGATTCAAA CTGGGAATTT   
  
  
+ CTCACTTGCG CAAGAGATAT TGGCGCGGCT CAATCACCAG CTCTCCCTTC CTGCGAAGCC CCTCATTAGG   
  
  
+ GCAGCTTTGT ATGTCATGGA GGCCTTGCAA ATGCTCATCT TAATGAGCAA TCCTGTAGCA CCTCCACCGA   
  
  
+ TGAAGACGCT TTCCCCTATA GATGTTGTTC ATAAGATGAA TGCCTACAAG GCCTTCTCTG AGGTCTCTCC   
  
  
+ TATTACTCAG TTTACGAATT TCACTTGTGC CCAAGCCATT CTTGAAGCTC TCGATGATGC GGATTGCGTT   
  
  
+ CATGTCATTG ACTTTGATAT TGGTTGTGGG GCTCAATGGG CATCACTGAT TCAGGAGCTT CCATTGAGGA   
  
  
+ AAAAGGGAGC TCCATCCCTG AAAATTACAG CCATAGCTCC CCTGTCAATA AGCCACACTT TTGAACTCAA   
  
  
+ CCTAGCACGG GAAAACCTTG TGCAATTCGC CAATGATGTT GGTGTTGCTT TTGAGCTGCA AGTTGTGAAC   
  
  
+ TTGGATTTAT TTGATCCATC TTCATCTTCA ATGCCAAATG TTGGAACCTC TGGGGATGAG TTGATCGCTG   
  
  
+ TTAGTATACC CATTTGGGCA TGTTCATATC GGCCGTCTAT TCTCCCATCC ATCCTCCGGT TCATTAAGCA   
  
  
+ ACTAGCCCCC AAGATTGTCG TCTCGTTAGA TAGAGGGTCT GATCGTTGCG ACACTCTGTT CCCACAACAT   
  
  
+ CTTATCCATA TCTTAGAGTC CTGCACAAAT TTCTTGGAAT CGCTCGACGG TCTCAATGTA GCATCAGATA   
  
  
+ TTGTAAACAA GGTTGAGAAG TACTTCCTCC AGCCTAAGAT CGAAAACACC GTGTTGGGCC GCGTTCATGC   
  
  
+ CCCCAATAAG ATGCCACATT GGAAGACCCT CTTTGCTTCA GCCGGGTTAT TGCCCTTTCA GTTCAGTAAC   
  
  
+ TTCACAGAAA CACAAGCTGA TTATGTGGTG AAGCGAACCC CAGGGAGGGG ATTTCACATA GAAAAGCGCC   
  
  
+ ACGCATCTTT AGTGCTAAGT TGGCAAAGGC GGGAGCTTGT GACAGCCTCG GCATGGAGGT TTGAGAAGCT   
  
  
+ GTA  

- +Up\_Stream \_Len000TGTAAC CCACCCTAAA TTTAGGAGAT AAAAAAGAAT CCCCTTTTTA TGAACCCTTT   
  
  
- AAAGAGTTAT TGACTTTTAG ATTTATTAAG CACTGAAACA ATCATCCAAA TTACACTAAA TGATGGCACT   
  
  
- CACAGTGAAA ATCTACCATG AAATTCTTTC GTAATATTCA TTTTGTTATA TATAGCATCG TAGTATGATG   
  
  
- AACACATTAT TTAATTGTCG TTGGAGTTAA CATGAATAAA CTAAAGCGTA ACATTAGTTA AAAGGGTTTA   
  
  
- CTAAATGATA GAACCAGAAT GAGTCAACAT GAAGTGTGAG TTATCAAGTT AATAAAAAGT AATCTGATTA   
  
  
- CTCGCTCAGG GAAGATGTTC TCGGATGATG ATGAGATTAT TACATTCCTG ATAACCGATT ACCCCTTGAT   
  
  
- TCGGATGCTG AACAATCCGT ATCCACTTAA AAGGAATGTT CTCTCAAATT AAATGTGAAC TATAATTGAG   
  
  
- CTCTTGCTCT TCGACAGAAT TATCTATGGG TGTATCTTTT ACTTCGTTTA CGGGAACTAA TAACCTCCCA   
  
  
- ACCCGTTTAT ATAAGTCATT GTTACAACAA ACCGTTTATA GCAGACTTTT AACAAAAACT GTTTATAACA   
  
  
- AACCTTCTTA AAAAGCTCTA ATTTATAATT TTTACAATTT TAAAATTAGA TTTATAGTTT GTTTATAACT   
  
  
- TTTTACGAAA TATAATAGAT TTAGAGATTA ATTTTAAACA CATAACAGGT GATCTTTTTA CATATCACTA   
  
  
- TAATGGCTGT ATATACACTA GAGACCACAT TATCGGATGG GATTACAGAT CCTTTTATAA ATTTTAGGGG   
  
  
- TCACCAGTTT TCCTTCAATG CGTGTGATCC CGTTCTGTGA AGTAATCCGA TCAACCACTT TCGATTCAGT   
  
  
- ACTTTAAATC TGGTACCGGT ACAGATGAAC ATCTAGGTTG ATGGTTGTTA GGTAAAAATA AAACCCCTTT   
  
  
- CGAACTTCCC ATTAAACCAT TTACATGTGC CCTTGGAAAA CGATATTTTC TTCACGAAGA ACTTTTTTGT   
  
  
- GGGACATCAT TTTGTCCCGT TTTTTCGGTT GTGAAGACAA AGACCGTCTC TTTGTCATCA CATGGTGAAC   
  
  
- CTCAACCTCA TCTGTGCTAT GTGTTTGGGT CATCTCTCTC TTTGTCCCCT CTCTTTCTCA TGTGTTTAAA   
  
  
- CACGTCCTCT TCCCATTCCT TCTTCCTCTT CCTTGGGGGA GGTTTTTTTT GGGTATATTT TTTATTATTT   
  
  
- CTTTTTTGTC CTCACTCAAT CTCCCATGGG TTTAGAGGTT AGATTTTGAT CTTAGATCCC AGAGAAAAGT   
  
  
- AAACGAACTT TTTAAAGACA AGTACCCAAA ACACAAAAAT CCCCCTAAAC AAACTAAAAA CATCAGAAAC   
  
  
- ACCTAAAGGA CACAATATCT ACGTTCGATA CGAGAAGTTA AACGTTCCTT GTCGTAGACC CCCCTCTCTT   
  
  
- AAACTCCTTA AAGTTCATGG GTAAAGTTAA AGTTGTTCAT TTTAGTAGTA ACTTCCTATG AGTTTGGGTT   
  
  
- CGGGTCAACT ATCACTTGGT TGAAGATGAG ACCTAAGATG AGGTTCAGGG TCGGGAGGGT GGGAGAGGAG   
  
  
- GAGGAGGTTC TTGAGGATGG TTTTGCGACG GCAAAACCGA GTGGGAGGAG TCGAAGTGGT ACCTAGACTA   
  
  
- TTACACATTC TGGGGTTGGG AAAACGAAAA TAAAAGGAGT ACGAGGTAAG AGTACTAAAC ATGAAGACAA   
  
  
- TACATTAACC TCTACACAGA TTTAAACCTT TTCAAGCACC CAAAGCACAA ACTCTTAAAT CTTACTGTAA   
  
  
- GAAATTAATC ATTAAACTTC AAACGTACGA ATTAAATCGA TTAGTGACTA ATTAATTTTA TTTACTTCAC   
  
  
- TACATCCATA CCCGACAATG TGTTACAGTG GCCACCACCA CTGTCGCCGC CCCGCCGCCG CCAACCTAAC   
  
  
- CTTCTTACCC TGTCGCACAA AGGTTTACCC CTACCTCGAA ATGAGGGAAC CTAGTACCCC CTTCCACTAC   
  
  
- TAAACCCAGA CTTAAACTTC GTAGAGAATG TTAGACCAAT GGGACAACTC ATACTTCCGT TACGTCCTGA   
  
  
- ACCACAACAA CTAGTCTGTA GAGAAGAACC TCCACCACTA AACCCCAAAA GACCAAGACC CAGACACTTA   
  
  
- TTACCGTTTT AACCAAGGTA AAACCCATTA ACAAGTAGAC CCCTAAACCT AAGATTCCAA AGATCACTGC   
  
  
- CCAACTTAAG GTTAACATCA AGTGACCCCT CATACTCATA GGAACCAAGT TAGGTACCTG GAAACGGATT   
  
  
- ATGACCACCC AAACACAAGG GTGTTTGTGT TGATAAACTA GAACCACTAC TCTTCGGAGT CTAAAACTTG   
  
  
- GGTGTTAACT ACTGCTACTT AGGTTTAGTC CGAGTCTCGT ACCCTTTGGG ATCGAAAAAA CACGGGAGGA   
  
  
- ACCCAATAAC AGTCAATCTC GTTGTATACC AAGTTGGTGT CCGCTTTGCG GTATCGGGAC CACATCAAAA   
  
  
- CTTGAGACTA CAATAACGGT TTTGGGGCAA AGAGCTAGTC CCAGTACTCA AAAACAACTC CTTTGTTGTG   
  
  
- GTAGTTGTCT AAAACGTCGT TGAAGGATAC CCAAACCGAG TAGTCAGCCA GGGAGTCGTG GAGGTCGTTT   
  
  
- TCCGCTACTT TCTGGTCCAA CAATTCCTGG TCGAAAACTT CCGTCGACTG GACTAAGTTT GACCCTTAAA   
  
  
- GAGTGAACGC GTTCTCTATA ACCGCGCCGA GTTAGTGGTC GAGAGGGAAG GACGCTTCGG GGAGTAATCC   
  
  
- CGTCGAAACA TACAGTACCT CCGGAACGTT TACGAGTAGA ATTACTCGTT AGGACATCGT GGAGGTGGCT   
  
  
- ACTTCTGCGA AAGGGGATAT CTACAACAAG TATTCTACTT ACGGATGTTC CGGAAGAGAC TCCAGAGAGG   
  
  
- ATAATGAGTC AAATGCTTAA AGTGAACACG GGTTCGGTAA GAACTTCGAG AGCTACTACG CCTAACGCAA   
  
  
- GTACAGTAAC TGAAACTATA ACCAACACCC CGAGTTACCC GTAGTGACTA AGTCCTCGAA GGTAACTCCT   
  
  
- TTTTCCCTCG AGGTAGGGAC TTTTAATGTC GGTATCGAGG GGACAGTTAT TCGGTGTGAA AACTTGAGTT   
  
  
- GGATCGTGCC CTTTTGGAAC ACGTTAAGCG GTTACTACAA CCACAACGAA AACTCGACGT TCAACACTTG   
  
  
- AACCTAAATA AACTAGGTAG AAGTAGAAGT TACGGTTTAC AACCTTGGAG ACCCCTACTC AACTAGCGAC   
  
  
- AATCATATGG GTAAACCCGT ACAAGTATAG CCGGCAGATA AGAGGGTAGG TAGGAGGCCA AGTAATTCGT   
  
  
- TGATCGGGGG TTCTAACAGC AGAGCAATCT ATCTCCCAGA CTAGCAACGC TGTGAGACAA GGGTGTTGTA   
  
  
- GAATAGGTAT AGAATCTCAG GACGTGTTTA AAGAACCTTA GCGAGCTGCC AGAGTTACAT CGTAGTCTAT   
  
  
- AACATTTGTT CCAACTCTTC ATGAAGGAGG TCGGATTCTA GCTTTTGTGG CACAACCCGG CGCAAGTACG   
  
  
- GGGGTTATTC TACGGTGTAA CCTTCTGGGA GAAACGAAGT CGGCCCAATA ACGGGAAAGT CAAGTCATTG   
  
  
- AAGTGTCTTT GTGTTCGACT AATACACCAC TTCGCTTGGG GTCCCTCCCC TAAAGTGTAT CTTTTCGCGG   
  
  
- TGCGTAGAAA TCACGATTCA ACCGTTTCCG CCCTCGAACA CTGTCGGAGC CGTACCTCCA AACTCTTCGA   
  
  
- CAT

+     TGACG-motif

| Site Name | Organism | Position | Strand | Matrix score. | sequence | function |
| --- | --- | --- | --- | --- | --- | --- |
| TGACG-motif | Hordeum vulgare | 2395 | + | 5 | TGACG | cis-acting regulatory element involved in the MeJA-responsiveness |
| TGACG-motif | Hordeum vulgare | 2240 | + | 5 | TGACG | cis-acting regulatory element involved in the MeJA-responsiveness |

>HU05G00466.1   
+ +Up\_Stream \_Len000ACATTG GGTGGGATTT AAATCCTCTA TTTTTTCTTA GGGGAAAAAT ACTTGGGAAA   
  
  
+ TTTCTCAATA ACTGAAAATC TAAATAATTC GTGACTTTGT TAGTAGGTTT AATGTGATTT ACTACCGTGA   
  
  
+ GTGTCACTTT TAGATGGTAC TTTAAGAAAG CATTATAAGT AAAACAATAT ATATCGTAGC ATCATACTAC   
  
  
+ TTGTGTAATA AATTAACAGC AACCTCAATT GTACTTATTT GATTTCGCAT TGTAATCAAT TTTCCCAAAT   
  
  
+ GATTTACTAT CTTGGTCTTA CTCAGTTGTA CTTCACACTC AATAGTTCAA TTATTTTTCA TTAGACTAAT   
  
  
+ GAGCGAGTCC CTTCTACAAG AGCCTACTAC TACTCTAATA ATGTAAGGAC TATTGGCTAA TGGGGAACTA   
  
  
+ AGCCTACGAC TTGTTAGGCA TAGGTGAATT TTCCTTACAA GAGAGTTTAA TTTACACTTG ATATTAACTC   
  
  
+ GAGAACGAGA AGCTGTCTTA ATAGATACCC ACATAGAAAA TGAAGCAAAT GCCCTTGATT ATTGGAGGGT   
  
  
+ TGGGCAAATA TATTCAGTAA CAATGTTGTT TGGCAAATAT CGTCTGAAAA TTGTTTTTGA CAAATATTGT   
  
  
+ TTGGAAGAAT TTTTCGAGAT TAAATATTAA AAATGTTAAA ATTTTAATCT AAATATCAAA CAAATATTGA   
  
  
+ AAAATGCTTT ATATTATCTA AATCTCTAAT TAAAATTTGT GTATTGTCCA CTAGAAAAAT GTATAGTGAT   
  
  
+ ATTACCGACA TATATGTGAT CTCTGGTGTA ATAGCCTACC CTAATGTCTA GGAAAATATT TAAAATCCCC   
  
  
+ AGTGGTCAAA AGGAAGTTAC GCACACTAGG GCAAGACACT TCATTAGGCT AGTTGGTGAA AGCTAAGTCA   
  
  
+ TGAAATTTAG ACCATGGCCA TGTCTACTTG TAGATCCAAC TACCAACAAT CCATTTTTAT TTTGGGGAAA   
  
  
+ GCTTGAAGGG TAATTTGGTA AATGTACACG GGAACCTTTT GCTATAAAAG AAGTGCTTCT TGAAAAAACA   
  
  
+ CCCTGTAGTA AAACAGGGCA AAAAAGCCAA CACTTCTGTT TCTGGCAGAG AAACAGTAGT GTACCACTTG   
  
  
+ GAGTTGGAGT AGACACGATA CACAAACCCA GTAGAGAGAG AAACAGGGGA GAGAAAGAGT ACACAAATTT   
  
  
+ GTGCAGGAGA AGGGTAAGGA AGAAGGAGAA GGAACCCCCT CCAAAAAAAA CCCATATAAA AAATAATAAA   
  
  
+ GAAAAAACAG GAGTGAGTTA GAGGGTACCC AAATCTCCAA TCTAAAACTA GAATCTAGGG TCTCTTTTCA   
  
  
+ TTTGCTTGAA AAATTTCTGT TCATGGGTTT TGTGTTTTTA GGGGGATTTG TTTGATTTTT GTAGTCTTTG   
  
  
+ TGGATTTCCT GTGTTATAGA TGCAAGCTAT GCTCTTCAAT TTGCAAGGAA CAGCATCTGG GGGGAGAGAA   
  
  
+ TTTGAGGAAT TTCAAGTACC CATTTCAATT TCAACAAGTA AAATCATCAT TGAAGGATAC TCAAACCCAA   
  
  
+ GCCCAGTTGA TAGTGAACCA ACTTCTACTC TGGATTCTAC TCCAAGTCCC AGCCCTCCCA CCCTCTCCTC   
  
  
+ CTCCTCCAAG AACTCCTACC AAAACGCTGC CGTTTTGGCT CACCCTCCTC AGCTTCACCA TGGATCTGAT   
  
  
+ AATGTGTAAG ACCCCAACCC TTTTGCTTTT ATTTTCCTCA TGCTCCATTC TCATGATTTG TACTTCTGTT   
  
  
+ ATGTAATTGG AGATGTGTCT AAATTTGGAA AAGTTCGTGG GTTTCGTGTT TGAGAATTTA GAATGACATT   
  
  
+ CTTTAATTAG TAATTTGAAG TTTGCATGCT TAATTTAGCT AATCACTGAT TAATTAAAAT AAATGAAGTG   
  
  
+ ATGTAGGTAT GGGCTGTTAC ACAATGTCAC CGGTGGTGGT GACAGCGGCG GGGCGGCGGC GGTTGGATTG   
  
  
+ GAAGAATGGG ACAGCGTGTT TCCAAATGGG GATGGAGCTT TACTCCCTTG GATCATGGGG GAAGGTGATG   
  
  
+ ATTTGGGTCT GAATTTGAAG CATCTCTTAC AATCTGGTTA CCCTGTTGAG TATGAAGGCA ATGCAGGACT   
  
  
+ TGGTGTTGTT GATCAGACAT CTCTTCTTGG AGGTGGTGAT TTGGGGTTTT CTGGTTCTGG GTCTGTGAAT   
  
  
+ AATGGCAAAA TTGGTTCCAT TTTGGGTAAT TGTTCATCTG GGGATTTGGA TTCTAAGGTT TCTAGTGACG   
  
  
+ GGTTGAATTC CAATTGTAGT TCACTGGGGA GTATGAGTAT CCTTGGTTCA ATCCATGGAC CTTTGCCTAA   
  
  
+ TACTGGTGGG TTTGTGTTCC CACAAACACA ACTATTTGAT CTTGGTGATG AGAAGCCTCA GATTTTGAAC   
  
  
+ CCACAATTGA TGACGATGAA TCCAAATCAG GCTCAGAGCA TGGGAAACCC TAGCTTTTTT GTGCCCTCCT   
  
  
+ TGGGTTATTG TCAGTTAGAG CAACATATGG TTCAACCACA GGCGAAACGC CATAGCCCTG GTGTAGTTTT   
  
  
+ GAACTCTGAT GTTATTGCCA AAACCCCGTT TCTCGATCAG GGTCATGAGT TTTTGTTGAG GAAACAACAC   
  
  
+ CATCAACAGA TTTTGCAGCA ACTTCCTATG GGTTTGGCTC ATCAGTCGGT CCCTCAGCAC CTCCAGCAAA   
  
  
+ AGGCGATGAA AGACCAGGTT GTTAAGGACC AGCTTTTGAA GGCAGCTGAC CTGATTCAAA CTGGGAATTT   
  
  
+ CTCACTTGCG CAAGAGATAT TGGCGCGGCT CAATCACCAG CTCTCCCTTC CTGCGAAGCC CCTCATTAGG   
  
  
+ GCAGCTTTGT ATGTCATGGA GGCCTTGCAA ATGCTCATCT TAATGAGCAA TCCTGTAGCA CCTCCACCGA   
  
  
+ TGAAGACGCT TTCCCCTATA GATGTTGTTC ATAAGATGAA TGCCTACAAG GCCTTCTCTG AGGTCTCTCC   
  
  
+ TATTACTCAG TTTACGAATT TCACTTGTGC CCAAGCCATT CTTGAAGCTC TCGATGATGC GGATTGCGTT   
  
  
+ CATGTCATTG ACTTTGATAT TGGTTGTGGG GCTCAATGGG CATCACTGAT TCAGGAGCTT CCATTGAGGA   
  
  
+ AAAAGGGAGC TCCATCCCTG AAAATTACAG CCATAGCTCC CCTGTCAATA AGCCACACTT TTGAACTCAA   
  
  
+ CCTAGCACGG GAAAACCTTG TGCAATTCGC CAATGATGTT GGTGTTGCTT TTGAGCTGCA AGTTGTGAAC   
  
  
+ TTGGATTTAT TTGATCCATC TTCATCTTCA ATGCCAAATG TTGGAACCTC TGGGGATGAG TTGATCGCTG   
  
  
+ TTAGTATACC CATTTGGGCA TGTTCATATC GGCCGTCTAT TCTCCCATCC ATCCTCCGGT TCATTAAGCA   
  
  
+ ACTAGCCCCC AAGATTGTCG TCTCGTTAGA TAGAGGGTCT GATCGTTGCG ACACTCTGTT CCCACAACAT   
  
  
+ CTTATCCATA TCTTAGAGTC CTGCACAAAT TTCTTGGAAT CGCTCGACGG TCTCAATGTA GCATCAGATA   
  
  
+ TTGTAAACAA GGTTGAGAAG TACTTCCTCC AGCCTAAGAT CGAAAACACC GTGTTGGGCC GCGTTCATGC   
  
  
+ CCCCAATAAG ATGCCACATT GGAAGACCCT CTTTGCTTCA GCCGGGTTAT TGCCCTTTCA GTTCAGTAAC   
  
  
+ TTCACAGAAA CACAAGCTGA TTATGTGGTG AAGCGAACCC CAGGGAGGGG ATTTCACATA GAAAAGCGCC   
  
  
+ ACGCATCTTT AGTGCTAAGT TGGCAAAGGC GGGAGCTTGT GACAGCCTCG GCATGGAGGT TTGAGAAGCT   
  
  
+ GTA  

- +Up\_Stream \_Len000TGTAAC CCACCCTAAA TTTAGGAGAT AAAAAAGAAT CCCCTTTTTA TGAACCCTTT   
  
  
- AAAGAGTTAT TGACTTTTAG ATTTATTAAG CACTGAAACA ATCATCCAAA TTACACTAAA TGATGGCACT   
  
  
- CACAGTGAAA ATCTACCATG AAATTCTTTC GTAATATTCA TTTTGTTATA TATAGCATCG TAGTATGATG   
  
  
- AACACATTAT TTAATTGTCG TTGGAGTTAA CATGAATAAA CTAAAGCGTA ACATTAGTTA AAAGGGTTTA   
  
  
- CTAAATGATA GAACCAGAAT GAGTCAACAT GAAGTGTGAG TTATCAAGTT AATAAAAAGT AATCTGATTA   
  
  
- CTCGCTCAGG GAAGATGTTC TCGGATGATG ATGAGATTAT TACATTCCTG ATAACCGATT ACCCCTTGAT   
  
  
- TCGGATGCTG AACAATCCGT ATCCACTTAA AAGGAATGTT CTCTCAAATT AAATGTGAAC TATAATTGAG   
  
  
- CTCTTGCTCT TCGACAGAAT TATCTATGGG TGTATCTTTT ACTTCGTTTA CGGGAACTAA TAACCTCCCA   
  
  
- ACCCGTTTAT ATAAGTCATT GTTACAACAA ACCGTTTATA GCAGACTTTT AACAAAAACT GTTTATAACA   
  
  
- AACCTTCTTA AAAAGCTCTA ATTTATAATT TTTACAATTT TAAAATTAGA TTTATAGTTT GTTTATAACT   
  
  
- TTTTACGAAA TATAATAGAT TTAGAGATTA ATTTTAAACA CATAACAGGT GATCTTTTTA CATATCACTA   
  
  
- TAATGGCTGT ATATACACTA GAGACCACAT TATCGGATGG GATTACAGAT CCTTTTATAA ATTTTAGGGG   
  
  
- TCACCAGTTT TCCTTCAATG CGTGTGATCC CGTTCTGTGA AGTAATCCGA TCAACCACTT TCGATTCAGT   
  
  
- ACTTTAAATC TGGTACCGGT ACAGATGAAC ATCTAGGTTG ATGGTTGTTA GGTAAAAATA AAACCCCTTT   
  
  
- CGAACTTCCC ATTAAACCAT TTACATGTGC CCTTGGAAAA CGATATTTTC TTCACGAAGA ACTTTTTTGT   
  
  
- GGGACATCAT TTTGTCCCGT TTTTTCGGTT GTGAAGACAA AGACCGTCTC TTTGTCATCA CATGGTGAAC   
  
  
- CTCAACCTCA TCTGTGCTAT GTGTTTGGGT CATCTCTCTC TTTGTCCCCT CTCTTTCTCA TGTGTTTAAA   
  
  
- CACGTCCTCT TCCCATTCCT TCTTCCTCTT CCTTGGGGGA GGTTTTTTTT GGGTATATTT TTTATTATTT   
  
  
- CTTTTTTGTC CTCACTCAAT CTCCCATGGG TTTAGAGGTT AGATTTTGAT CTTAGATCCC AGAGAAAAGT   
  
  
- AAACGAACTT TTTAAAGACA AGTACCCAAA ACACAAAAAT CCCCCTAAAC AAACTAAAAA CATCAGAAAC   
  
  
- ACCTAAAGGA CACAATATCT ACGTTCGATA CGAGAAGTTA AACGTTCCTT GTCGTAGACC CCCCTCTCTT   
  
  
- AAACTCCTTA AAGTTCATGG GTAAAGTTAA AGTTGTTCAT TTTAGTAGTA ACTTCCTATG AGTTTGGGTT   
  
  
- CGGGTCAACT ATCACTTGGT TGAAGATGAG ACCTAAGATG AGGTTCAGGG TCGGGAGGGT GGGAGAGGAG   
  
  
- GAGGAGGTTC TTGAGGATGG TTTTGCGACG GCAAAACCGA GTGGGAGGAG TCGAAGTGGT ACCTAGACTA   
  
  
- TTACACATTC TGGGGTTGGG AAAACGAAAA TAAAAGGAGT ACGAGGTAAG AGTACTAAAC ATGAAGACAA   
  
  
- TACATTAACC TCTACACAGA TTTAAACCTT TTCAAGCACC CAAAGCACAA ACTCTTAAAT CTTACTGTAA   
  
  
- GAAATTAATC ATTAAACTTC AAACGTACGA ATTAAATCGA TTAGTGACTA ATTAATTTTA TTTACTTCAC   
  
  
- TACATCCATA CCCGACAATG TGTTACAGTG GCCACCACCA CTGTCGCCGC CCCGCCGCCG CCAACCTAAC   
  
  
- CTTCTTACCC TGTCGCACAA AGGTTTACCC CTACCTCGAA ATGAGGGAAC CTAGTACCCC CTTCCACTAC   
  
  
- TAAACCCAGA CTTAAACTTC GTAGAGAATG TTAGACCAAT GGGACAACTC ATACTTCCGT TACGTCCTGA   
  
  
- ACCACAACAA CTAGTCTGTA GAGAAGAACC TCCACCACTA AACCCCAAAA GACCAAGACC CAGACACTTA   
  
  
- TTACCGTTTT AACCAAGGTA AAACCCATTA ACAAGTAGAC CCCTAAACCT AAGATTCCAA AGATCACTGC   
  
  
- CCAACTTAAG GTTAACATCA AGTGACCCCT CATACTCATA GGAACCAAGT TAGGTACCTG GAAACGGATT   
  
  
- ATGACCACCC AAACACAAGG GTGTTTGTGT TGATAAACTA GAACCACTAC TCTTCGGAGT CTAAAACTTG   
  
  
- GGTGTTAACT ACTGCTACTT AGGTTTAGTC CGAGTCTCGT ACCCTTTGGG ATCGAAAAAA CACGGGAGGA   
  
  
- ACCCAATAAC AGTCAATCTC GTTGTATACC AAGTTGGTGT CCGCTTTGCG GTATCGGGAC CACATCAAAA   
  
  
- CTTGAGACTA CAATAACGGT TTTGGGGCAA AGAGCTAGTC CCAGTACTCA AAAACAACTC CTTTGTTGTG   
  
  
- GTAGTTGTCT AAAACGTCGT TGAAGGATAC CCAAACCGAG TAGTCAGCCA GGGAGTCGTG GAGGTCGTTT   
  
  
- TCCGCTACTT TCTGGTCCAA CAATTCCTGG TCGAAAACTT CCGTCGACTG GACTAAGTTT GACCCTTAAA   
  
  
- GAGTGAACGC GTTCTCTATA ACCGCGCCGA GTTAGTGGTC GAGAGGGAAG GACGCTTCGG GGAGTAATCC   
  
  
- CGTCGAAACA TACAGTACCT CCGGAACGTT TACGAGTAGA ATTACTCGTT AGGACATCGT GGAGGTGGCT   
  
  
- ACTTCTGCGA AAGGGGATAT CTACAACAAG TATTCTACTT ACGGATGTTC CGGAAGAGAC TCCAGAGAGG   
  
  
- ATAATGAGTC AAATGCTTAA AGTGAACACG GGTTCGGTAA GAACTTCGAG AGCTACTACG CCTAACGCAA   
  
  
- GTACAGTAAC TGAAACTATA ACCAACACCC CGAGTTACCC GTAGTGACTA AGTCCTCGAA GGTAACTCCT   
  
  
- TTTTCCCTCG AGGTAGGGAC TTTTAATGTC GGTATCGAGG GGACAGTTAT TCGGTGTGAA AACTTGAGTT   
  
  
- GGATCGTGCC CTTTTGGAAC ACGTTAAGCG GTTACTACAA CCACAACGAA AACTCGACGT TCAACACTTG   
  
  
- AACCTAAATA AACTAGGTAG AAGTAGAAGT TACGGTTTAC AACCTTGGAG ACCCCTACTC AACTAGCGAC   
  
  
- AATCATATGG GTAAACCCGT ACAAGTATAG CCGGCAGATA AGAGGGTAGG TAGGAGGCCA AGTAATTCGT   
  
  
- TGATCGGGGG TTCTAACAGC AGAGCAATCT ATCTCCCAGA CTAGCAACGC TGTGAGACAA GGGTGTTGTA   
  
  
- GAATAGGTAT AGAATCTCAG GACGTGTTTA AAGAACCTTA GCGAGCTGCC AGAGTTACAT CGTAGTCTAT   
  
  
- AACATTTGTT CCAACTCTTC ATGAAGGAGG TCGGATTCTA GCTTTTGTGG CACAACCCGG CGCAAGTACG   
  
  
- GGGGTTATTC TACGGTGTAA CCTTCTGGGA GAAACGAAGT CGGCCCAATA ACGGGAAAGT CAAGTCATTG   
  
  
- AAGTGTCTTT GTGTTCGACT AATACACCAC TTCGCTTGGG GTCCCTCCCC TAAAGTGTAT CTTTTCGCGG   
  
  
- TGCGTAGAAA TCACGATTCA ACCGTTTCCG CCCTCGAACA CTGTCGGAGC CGTACCTCCA AACTCTTCGA   
  
  
- CAT

+     Unnamed\_\_1

| Site Name | Organism | Position | Strand | Matrix score. | sequence | function |
| --- | --- | --- | --- | --- | --- | --- |
| Unnamed\_\_1 | Zea mays | 3713 | - | 5 | CGTGG |  |
| Unnamed\_\_1 | Zea mays | 1790 | + | 5 | CGTGG |  |

>HU05G00466.1   
+ +Up\_Stream \_Len000ACATTG GGTGGGATTT AAATCCTCTA TTTTTTCTTA GGGGAAAAAT ACTTGGGAAA   
  
  
+ TTTCTCAATA ACTGAAAATC TAAATAATTC GTGACTTTGT TAGTAGGTTT AATGTGATTT ACTACCGTGA   
  
  
+ GTGTCACTTT TAGATGGTAC TTTAAGAAAG CATTATAAGT AAAACAATAT ATATCGTAGC ATCATACTAC   
  
  
+ TTGTGTAATA AATTAACAGC AACCTCAATT GTACTTATTT GATTTCGCAT TGTAATCAAT TTTCCCAAAT   
  
  
+ GATTTACTAT CTTGGTCTTA CTCAGTTGTA CTTCACACTC AATAGTTCAA TTATTTTTCA TTAGACTAAT   
  
  
+ GAGCGAGTCC CTTCTACAAG AGCCTACTAC TACTCTAATA ATGTAAGGAC TATTGGCTAA TGGGGAACTA   
  
  
+ AGCCTACGAC TTGTTAGGCA TAGGTGAATT TTCCTTACAA GAGAGTTTAA TTTACACTTG ATATTAACTC   
  
  
+ GAGAACGAGA AGCTGTCTTA ATAGATACCC ACATAGAAAA TGAAGCAAAT GCCCTTGATT ATTGGAGGGT   
  
  
+ TGGGCAAATA TATTCAGTAA CAATGTTGTT TGGCAAATAT CGTCTGAAAA TTGTTTTTGA CAAATATTGT   
  
  
+ TTGGAAGAAT TTTTCGAGAT TAAATATTAA AAATGTTAAA ATTTTAATCT AAATATCAAA CAAATATTGA   
  
  
+ AAAATGCTTT ATATTATCTA AATCTCTAAT TAAAATTTGT GTATTGTCCA CTAGAAAAAT GTATAGTGAT   
  
  
+ ATTACCGACA TATATGTGAT CTCTGGTGTA ATAGCCTACC CTAATGTCTA GGAAAATATT TAAAATCCCC   
  
  
+ AGTGGTCAAA AGGAAGTTAC GCACACTAGG GCAAGACACT TCATTAGGCT AGTTGGTGAA AGCTAAGTCA   
  
  
+ TGAAATTTAG ACCATGGCCA TGTCTACTTG TAGATCCAAC TACCAACAAT CCATTTTTAT TTTGGGGAAA   
  
  
+ GCTTGAAGGG TAATTTGGTA AATGTACACG GGAACCTTTT GCTATAAAAG AAGTGCTTCT TGAAAAAACA   
  
  
+ CCCTGTAGTA AAACAGGGCA AAAAAGCCAA CACTTCTGTT TCTGGCAGAG AAACAGTAGT GTACCACTTG   
  
  
+ GAGTTGGAGT AGACACGATA CACAAACCCA GTAGAGAGAG AAACAGGGGA GAGAAAGAGT ACACAAATTT   
  
  
+ GTGCAGGAGA AGGGTAAGGA AGAAGGAGAA GGAACCCCCT CCAAAAAAAA CCCATATAAA AAATAATAAA   
  
  
+ GAAAAAACAG GAGTGAGTTA GAGGGTACCC AAATCTCCAA TCTAAAACTA GAATCTAGGG TCTCTTTTCA   
  
  
+ TTTGCTTGAA AAATTTCTGT TCATGGGTTT TGTGTTTTTA GGGGGATTTG TTTGATTTTT GTAGTCTTTG   
  
  
+ TGGATTTCCT GTGTTATAGA TGCAAGCTAT GCTCTTCAAT TTGCAAGGAA CAGCATCTGG GGGGAGAGAA   
  
  
+ TTTGAGGAAT TTCAAGTACC CATTTCAATT TCAACAAGTA AAATCATCAT TGAAGGATAC TCAAACCCAA   
  
  
+ GCCCAGTTGA TAGTGAACCA ACTTCTACTC TGGATTCTAC TCCAAGTCCC AGCCCTCCCA CCCTCTCCTC   
  
  
+ CTCCTCCAAG AACTCCTACC AAAACGCTGC CGTTTTGGCT CACCCTCCTC AGCTTCACCA TGGATCTGAT   
  
  
+ AATGTGTAAG ACCCCAACCC TTTTGCTTTT ATTTTCCTCA TGCTCCATTC TCATGATTTG TACTTCTGTT   
  
  
+ ATGTAATTGG AGATGTGTCT AAATTTGGAA AAGTTCGTGG GTTTCGTGTT TGAGAATTTA GAATGACATT   
  
  
+ CTTTAATTAG TAATTTGAAG TTTGCATGCT TAATTTAGCT AATCACTGAT TAATTAAAAT AAATGAAGTG   
  
  
+ ATGTAGGTAT GGGCTGTTAC ACAATGTCAC CGGTGGTGGT GACAGCGGCG GGGCGGCGGC GGTTGGATTG   
  
  
+ GAAGAATGGG ACAGCGTGTT TCCAAATGGG GATGGAGCTT TACTCCCTTG GATCATGGGG GAAGGTGATG   
  
  
+ ATTTGGGTCT GAATTTGAAG CATCTCTTAC AATCTGGTTA CCCTGTTGAG TATGAAGGCA ATGCAGGACT   
  
  
+ TGGTGTTGTT GATCAGACAT CTCTTCTTGG AGGTGGTGAT TTGGGGTTTT CTGGTTCTGG GTCTGTGAAT   
  
  
+ AATGGCAAAA TTGGTTCCAT TTTGGGTAAT TGTTCATCTG GGGATTTGGA TTCTAAGGTT TCTAGTGACG   
  
  
+ GGTTGAATTC CAATTGTAGT TCACTGGGGA GTATGAGTAT CCTTGGTTCA ATCCATGGAC CTTTGCCTAA   
  
  
+ TACTGGTGGG TTTGTGTTCC CACAAACACA ACTATTTGAT CTTGGTGATG AGAAGCCTCA GATTTTGAAC   
  
  
+ CCACAATTGA TGACGATGAA TCCAAATCAG GCTCAGAGCA TGGGAAACCC TAGCTTTTTT GTGCCCTCCT   
  
  
+ TGGGTTATTG TCAGTTAGAG CAACATATGG TTCAACCACA GGCGAAACGC CATAGCCCTG GTGTAGTTTT   
  
  
+ GAACTCTGAT GTTATTGCCA AAACCCCGTT TCTCGATCAG GGTCATGAGT TTTTGTTGAG GAAACAACAC   
  
  
+ CATCAACAGA TTTTGCAGCA ACTTCCTATG GGTTTGGCTC ATCAGTCGGT CCCTCAGCAC CTCCAGCAAA   
  
  
+ AGGCGATGAA AGACCAGGTT GTTAAGGACC AGCTTTTGAA GGCAGCTGAC CTGATTCAAA CTGGGAATTT   
  
  
+ CTCACTTGCG CAAGAGATAT TGGCGCGGCT CAATCACCAG CTCTCCCTTC CTGCGAAGCC CCTCATTAGG   
  
  
+ GCAGCTTTGT ATGTCATGGA GGCCTTGCAA ATGCTCATCT TAATGAGCAA TCCTGTAGCA CCTCCACCGA   
  
  
+ TGAAGACGCT TTCCCCTATA GATGTTGTTC ATAAGATGAA TGCCTACAAG GCCTTCTCTG AGGTCTCTCC   
  
  
+ TATTACTCAG TTTACGAATT TCACTTGTGC CCAAGCCATT CTTGAAGCTC TCGATGATGC GGATTGCGTT   
  
  
+ CATGTCATTG ACTTTGATAT TGGTTGTGGG GCTCAATGGG CATCACTGAT TCAGGAGCTT CCATTGAGGA   
  
  
+ AAAAGGGAGC TCCATCCCTG AAAATTACAG CCATAGCTCC CCTGTCAATA AGCCACACTT TTGAACTCAA   
  
  
+ CCTAGCACGG GAAAACCTTG TGCAATTCGC CAATGATGTT GGTGTTGCTT TTGAGCTGCA AGTTGTGAAC   
  
  
+ TTGGATTTAT TTGATCCATC TTCATCTTCA ATGCCAAATG TTGGAACCTC TGGGGATGAG TTGATCGCTG   
  
  
+ TTAGTATACC CATTTGGGCA TGTTCATATC GGCCGTCTAT TCTCCCATCC ATCCTCCGGT TCATTAAGCA   
  
  
+ ACTAGCCCCC AAGATTGTCG TCTCGTTAGA TAGAGGGTCT GATCGTTGCG ACACTCTGTT CCCACAACAT   
  
  
+ CTTATCCATA TCTTAGAGTC CTGCACAAAT TTCTTGGAAT CGCTCGACGG TCTCAATGTA GCATCAGATA   
  
  
+ TTGTAAACAA GGTTGAGAAG TACTTCCTCC AGCCTAAGAT CGAAAACACC GTGTTGGGCC GCGTTCATGC   
  
  
+ CCCCAATAAG ATGCCACATT GGAAGACCCT CTTTGCTTCA GCCGGGTTAT TGCCCTTTCA GTTCAGTAAC   
  
  
+ TTCACAGAAA CACAAGCTGA TTATGTGGTG AAGCGAACCC CAGGGAGGGG ATTTCACATA GAAAAGCGCC   
  
  
+ ACGCATCTTT AGTGCTAAGT TGGCAAAGGC GGGAGCTTGT GACAGCCTCG GCATGGAGGT TTGAGAAGCT   
  
  
+ GTA  

- +Up\_Stream \_Len000TGTAAC CCACCCTAAA TTTAGGAGAT AAAAAAGAAT CCCCTTTTTA TGAACCCTTT   
  
  
- AAAGAGTTAT TGACTTTTAG ATTTATTAAG CACTGAAACA ATCATCCAAA TTACACTAAA TGATGGCACT   
  
  
- CACAGTGAAA ATCTACCATG AAATTCTTTC GTAATATTCA TTTTGTTATA TATAGCATCG TAGTATGATG   
  
  
- AACACATTAT TTAATTGTCG TTGGAGTTAA CATGAATAAA CTAAAGCGTA ACATTAGTTA AAAGGGTTTA   
  
  
- CTAAATGATA GAACCAGAAT GAGTCAACAT GAAGTGTGAG TTATCAAGTT AATAAAAAGT AATCTGATTA   
  
  
- CTCGCTCAGG GAAGATGTTC TCGGATGATG ATGAGATTAT TACATTCCTG ATAACCGATT ACCCCTTGAT   
  
  
- TCGGATGCTG AACAATCCGT ATCCACTTAA AAGGAATGTT CTCTCAAATT AAATGTGAAC TATAATTGAG   
  
  
- CTCTTGCTCT TCGACAGAAT TATCTATGGG TGTATCTTTT ACTTCGTTTA CGGGAACTAA TAACCTCCCA   
  
  
- ACCCGTTTAT ATAAGTCATT GTTACAACAA ACCGTTTATA GCAGACTTTT AACAAAAACT GTTTATAACA   
  
  
- AACCTTCTTA AAAAGCTCTA ATTTATAATT TTTACAATTT TAAAATTAGA TTTATAGTTT GTTTATAACT   
  
  
- TTTTACGAAA TATAATAGAT TTAGAGATTA ATTTTAAACA CATAACAGGT GATCTTTTTA CATATCACTA   
  
  
- TAATGGCTGT ATATACACTA GAGACCACAT TATCGGATGG GATTACAGAT CCTTTTATAA ATTTTAGGGG   
  
  
- TCACCAGTTT TCCTTCAATG CGTGTGATCC CGTTCTGTGA AGTAATCCGA TCAACCACTT TCGATTCAGT   
  
  
- ACTTTAAATC TGGTACCGGT ACAGATGAAC ATCTAGGTTG ATGGTTGTTA GGTAAAAATA AAACCCCTTT   
  
  
- CGAACTTCCC ATTAAACCAT TTACATGTGC CCTTGGAAAA CGATATTTTC TTCACGAAGA ACTTTTTTGT   
  
  
- GGGACATCAT TTTGTCCCGT TTTTTCGGTT GTGAAGACAA AGACCGTCTC TTTGTCATCA CATGGTGAAC   
  
  
- CTCAACCTCA TCTGTGCTAT GTGTTTGGGT CATCTCTCTC TTTGTCCCCT CTCTTTCTCA TGTGTTTAAA   
  
  
- CACGTCCTCT TCCCATTCCT TCTTCCTCTT CCTTGGGGGA GGTTTTTTTT GGGTATATTT TTTATTATTT   
  
  
- CTTTTTTGTC CTCACTCAAT CTCCCATGGG TTTAGAGGTT AGATTTTGAT CTTAGATCCC AGAGAAAAGT   
  
  
- AAACGAACTT TTTAAAGACA AGTACCCAAA ACACAAAAAT CCCCCTAAAC AAACTAAAAA CATCAGAAAC   
  
  
- ACCTAAAGGA CACAATATCT ACGTTCGATA CGAGAAGTTA AACGTTCCTT GTCGTAGACC CCCCTCTCTT   
  
  
- AAACTCCTTA AAGTTCATGG GTAAAGTTAA AGTTGTTCAT TTTAGTAGTA ACTTCCTATG AGTTTGGGTT   
  
  
- CGGGTCAACT ATCACTTGGT TGAAGATGAG ACCTAAGATG AGGTTCAGGG TCGGGAGGGT GGGAGAGGAG   
  
  
- GAGGAGGTTC TTGAGGATGG TTTTGCGACG GCAAAACCGA GTGGGAGGAG TCGAAGTGGT ACCTAGACTA   
  
  
- TTACACATTC TGGGGTTGGG AAAACGAAAA TAAAAGGAGT ACGAGGTAAG AGTACTAAAC ATGAAGACAA   
  
  
- TACATTAACC TCTACACAGA TTTAAACCTT TTCAAGCACC CAAAGCACAA ACTCTTAAAT CTTACTGTAA   
  
  
- GAAATTAATC ATTAAACTTC AAACGTACGA ATTAAATCGA TTAGTGACTA ATTAATTTTA TTTACTTCAC   
  
  
- TACATCCATA CCCGACAATG TGTTACAGTG GCCACCACCA CTGTCGCCGC CCCGCCGCCG CCAACCTAAC   
  
  
- CTTCTTACCC TGTCGCACAA AGGTTTACCC CTACCTCGAA ATGAGGGAAC CTAGTACCCC CTTCCACTAC   
  
  
- TAAACCCAGA CTTAAACTTC GTAGAGAATG TTAGACCAAT GGGACAACTC ATACTTCCGT TACGTCCTGA   
  
  
- ACCACAACAA CTAGTCTGTA GAGAAGAACC TCCACCACTA AACCCCAAAA GACCAAGACC CAGACACTTA   
  
  
- TTACCGTTTT AACCAAGGTA AAACCCATTA ACAAGTAGAC CCCTAAACCT AAGATTCCAA AGATCACTGC   
  
  
- CCAACTTAAG GTTAACATCA AGTGACCCCT CATACTCATA GGAACCAAGT TAGGTACCTG GAAACGGATT   
  
  
- ATGACCACCC AAACACAAGG GTGTTTGTGT TGATAAACTA GAACCACTAC TCTTCGGAGT CTAAAACTTG   
  
  
- GGTGTTAACT ACTGCTACTT AGGTTTAGTC CGAGTCTCGT ACCCTTTGGG ATCGAAAAAA CACGGGAGGA   
  
  
- ACCCAATAAC AGTCAATCTC GTTGTATACC AAGTTGGTGT CCGCTTTGCG GTATCGGGAC CACATCAAAA   
  
  
- CTTGAGACTA CAATAACGGT TTTGGGGCAA AGAGCTAGTC CCAGTACTCA AAAACAACTC CTTTGTTGTG   
  
  
- GTAGTTGTCT AAAACGTCGT TGAAGGATAC CCAAACCGAG TAGTCAGCCA GGGAGTCGTG GAGGTCGTTT   
  
  
- TCCGCTACTT TCTGGTCCAA CAATTCCTGG TCGAAAACTT CCGTCGACTG GACTAAGTTT GACCCTTAAA   
  
  
- GAGTGAACGC GTTCTCTATA ACCGCGCCGA GTTAGTGGTC GAGAGGGAAG GACGCTTCGG GGAGTAATCC   
  
  
- CGTCGAAACA TACAGTACCT CCGGAACGTT TACGAGTAGA ATTACTCGTT AGGACATCGT GGAGGTGGCT   
  
  
- ACTTCTGCGA AAGGGGATAT CTACAACAAG TATTCTACTT ACGGATGTTC CGGAAGAGAC TCCAGAGAGG   
  
  
- ATAATGAGTC AAATGCTTAA AGTGAACACG GGTTCGGTAA GAACTTCGAG AGCTACTACG CCTAACGCAA   
  
  
- GTACAGTAAC TGAAACTATA ACCAACACCC CGAGTTACCC GTAGTGACTA AGTCCTCGAA GGTAACTCCT   
  
  
- TTTTCCCTCG AGGTAGGGAC TTTTAATGTC GGTATCGAGG GGACAGTTAT TCGGTGTGAA AACTTGAGTT   
  
  
- GGATCGTGCC CTTTTGGAAC ACGTTAAGCG GTTACTACAA CCACAACGAA AACTCGACGT TCAACACTTG   
  
  
- AACCTAAATA AACTAGGTAG AAGTAGAAGT TACGGTTTAC AACCTTGGAG ACCCCTACTC AACTAGCGAC   
  
  
- AATCATATGG GTAAACCCGT ACAAGTATAG CCGGCAGATA AGAGGGTAGG TAGGAGGCCA AGTAATTCGT   
  
  
- TGATCGGGGG TTCTAACAGC AGAGCAATCT ATCTCCCAGA CTAGCAACGC TGTGAGACAA GGGTGTTGTA   
  
  
- GAATAGGTAT AGAATCTCAG GACGTGTTTA AAGAACCTTA GCGAGCTGCC AGAGTTACAT CGTAGTCTAT   
  
  
- AACATTTGTT CCAACTCTTC ATGAAGGAGG TCGGATTCTA GCTTTTGTGG CACAACCCGG CGCAAGTACG   
  
  
- GGGGTTATTC TACGGTGTAA CCTTCTGGGA GAAACGAAGT CGGCCCAATA ACGGGAAAGT CAAGTCATTG   
  
  
- AAGTGTCTTT GTGTTCGACT AATACACCAC TTCGCTTGGG GTCCCTCCCC TAAAGTGTAT CTTTTCGCGG   
  
  
- TGCGTAGAAA TCACGATTCA ACCGTTTCCG CCCTCGAACA CTGTCGGAGC CGTACCTCCA AACTCTTCGA   
  
  
- CAT

+     Unnamed\_\_4

| Site Name | Organism | Position | Strand | Matrix score. | sequence | function |
| --- | --- | --- | --- | --- | --- | --- |
| Unnamed\_\_4 | Petroselinum hortense | 3336 | + | 4 | CTCC |  |
| Unnamed\_\_4 | Petroselinum hortense | 3348 | + | 4 | CTCC |  |
| Unnamed\_\_4 | Petroselinum hortense | 3121 | + | 4 | CTCC |  |
| Unnamed\_\_4 | Petroselinum hortense | 3090 | - | 4 | CTCC |  |
| Unnamed\_\_4 | Petroselinum hortense | 3094 | + | 4 | CTCC |  |
| Unnamed\_\_4 | Petroselinum hortense | 2866 | + | 4 | CTCC |  |
| Unnamed\_\_4 | Petroselinum hortense | 3769 | - | 4 | CTCC |  |
| Unnamed\_\_4 | Petroselinum hortense | 3688 | - | 4 | CTCC |  |
| Unnamed\_\_4 | Petroselinum hortense | 2272 | - | 4 | CTCC |  |
| Unnamed\_\_4 | Petroselinum hortense | 3531 | + | 4 | CTCC |  |
| Unnamed\_\_4 | Petroselinum hortense | 2655 | + | 4 | CTCC |  |
| Unnamed\_\_4 | Petroselinum hortense | 1627 | + | 4 | CTCC |  |
| Unnamed\_\_4 | Petroselinum hortense | 1615 | + | 4 | CTCC |  |
| Unnamed\_\_4 | Petroselinum hortense | 1618 | + | 4 | CTCC |  |
| Unnamed\_\_4 | Petroselinum hortense | 3068 | - | 4 | CTCC |  |
| Unnamed\_\_4 | Petroselinum hortense | 1727 | + | 4 | CTCC |  |
| Unnamed\_\_4 | Petroselinum hortense | 1763 | - | 4 | CTCC |  |
| Unnamed\_\_4 | Petroselinum hortense | 1599 | + | 4 | CTCC |  |
| Unnamed\_\_4 | Petroselinum hortense | 2777 | + | 4 | CTCC |  |
| Unnamed\_\_4 | Petroselinum hortense | 3746 | - | 4 | CTCC |  |
| Unnamed\_\_4 | Petroselinum hortense | 1274 | - | 4 | CTCC |  |
| Unnamed\_\_4 | Petroselinum hortense | 1233 | + | 4 | CTCC |  |
| Unnamed\_\_4 | Petroselinum hortense | 1200 | - | 4 | CTCC |  |
| Unnamed\_\_4 | Petroselinum hortense | 1130 | - | 4 | CTCC |  |
| Unnamed\_\_4 | Petroselinum hortense | 1659 | + | 4 | CTCC |  |
| Unnamed\_\_4 | Petroselinum hortense | 1584 | + | 4 | CTCC |  |
| Unnamed\_\_4 | Petroselinum hortense | 1998 | - | 4 | CTCC |  |
| Unnamed\_\_4 | Petroselinum hortense | 2133 | - | 4 | CTCC |  |
| Unnamed\_\_4 | Petroselinum hortense | 2822 | - | 4 | CTCC |  |
| Unnamed\_\_4 | Petroselinum hortense | 1612 | + | 4 | CTCC |  |
| Unnamed\_\_4 | Petroselinum hortense | 1609 | + | 4 | CTCC |  |
| Unnamed\_\_4 | Petroselinum hortense | 2450 | + | 4 | CTCC |  |
| Unnamed\_\_4 | Petroselinum hortense | 1299 | + | 4 | CTCC |  |
| Unnamed\_\_4 | Petroselinum hortense | 1467 | - | 4 | CTCC |  |
| Unnamed\_\_4 | Petroselinum hortense | 1172 | - | 4 | CTCC |  |
| Unnamed\_\_4 | Petroselinum hortense | 1124 | - | 4 | CTCC |  |
| Unnamed\_\_4 | Petroselinum hortense | 558 | - | 4 | CTCC |  |
| Unnamed\_\_4 | Petroselinum hortense | 2941 | + | 4 | CTCC |  |
| Unnamed\_\_4 | Petroselinum hortense | 1219 | - | 4 | CTCC |  |
| Unnamed\_\_4 | Petroselinum hortense | 2007 | + | 4 | CTCC |  |

>HU05G00466.1   
+ +Up\_Stream \_Len000ACATTG GGTGGGATTT AAATCCTCTA TTTTTTCTTA GGGGAAAAAT ACTTGGGAAA   
  
  
+ TTTCTCAATA ACTGAAAATC TAAATAATTC GTGACTTTGT TAGTAGGTTT AATGTGATTT ACTACCGTGA   
  
  
+ GTGTCACTTT TAGATGGTAC TTTAAGAAAG CATTATAAGT AAAACAATAT ATATCGTAGC ATCATACTAC   
  
  
+ TTGTGTAATA AATTAACAGC AACCTCAATT GTACTTATTT GATTTCGCAT TGTAATCAAT TTTCCCAAAT   
  
  
+ GATTTACTAT CTTGGTCTTA CTCAGTTGTA CTTCACACTC AATAGTTCAA TTATTTTTCA TTAGACTAAT   
  
  
+ GAGCGAGTCC CTTCTACAAG AGCCTACTAC TACTCTAATA ATGTAAGGAC TATTGGCTAA TGGGGAACTA   
  
  
+ AGCCTACGAC TTGTTAGGCA TAGGTGAATT TTCCTTACAA GAGAGTTTAA TTTACACTTG ATATTAACTC   
  
  
+ GAGAACGAGA AGCTGTCTTA ATAGATACCC ACATAGAAAA TGAAGCAAAT GCCCTTGATT ATTGGAGGGT   
  
  
+ TGGGCAAATA TATTCAGTAA CAATGTTGTT TGGCAAATAT CGTCTGAAAA TTGTTTTTGA CAAATATTGT   
  
  
+ TTGGAAGAAT TTTTCGAGAT TAAATATTAA AAATGTTAAA ATTTTAATCT AAATATCAAA CAAATATTGA   
  
  
+ AAAATGCTTT ATATTATCTA AATCTCTAAT TAAAATTTGT GTATTGTCCA CTAGAAAAAT GTATAGTGAT   
  
  
+ ATTACCGACA TATATGTGAT CTCTGGTGTA ATAGCCTACC CTAATGTCTA GGAAAATATT TAAAATCCCC   
  
  
+ AGTGGTCAAA AGGAAGTTAC GCACACTAGG GCAAGACACT TCATTAGGCT AGTTGGTGAA AGCTAAGTCA   
  
  
+ TGAAATTTAG ACCATGGCCA TGTCTACTTG TAGATCCAAC TACCAACAAT CCATTTTTAT TTTGGGGAAA   
  
  
+ GCTTGAAGGG TAATTTGGTA AATGTACACG GGAACCTTTT GCTATAAAAG AAGTGCTTCT TGAAAAAACA   
  
  
+ CCCTGTAGTA AAACAGGGCA AAAAAGCCAA CACTTCTGTT TCTGGCAGAG AAACAGTAGT GTACCACTTG   
  
  
+ GAGTTGGAGT AGACACGATA CACAAACCCA GTAGAGAGAG AAACAGGGGA GAGAAAGAGT ACACAAATTT   
  
  
+ GTGCAGGAGA AGGGTAAGGA AGAAGGAGAA GGAACCCCCT CCAAAAAAAA CCCATATAAA AAATAATAAA   
  
  
+ GAAAAAACAG GAGTGAGTTA GAGGGTACCC AAATCTCCAA TCTAAAACTA GAATCTAGGG TCTCTTTTCA   
  
  
+ TTTGCTTGAA AAATTTCTGT TCATGGGTTT TGTGTTTTTA GGGGGATTTG TTTGATTTTT GTAGTCTTTG   
  
  
+ TGGATTTCCT GTGTTATAGA TGCAAGCTAT GCTCTTCAAT TTGCAAGGAA CAGCATCTGG GGGGAGAGAA   
  
  
+ TTTGAGGAAT TTCAAGTACC CATTTCAATT TCAACAAGTA AAATCATCAT TGAAGGATAC TCAAACCCAA   
  
  
+ GCCCAGTTGA TAGTGAACCA ACTTCTACTC TGGATTCTAC TCCAAGTCCC AGCCCTCCCA CCCTCTCCTC   
  
  
+ CTCCTCCAAG AACTCCTACC AAAACGCTGC CGTTTTGGCT CACCCTCCTC AGCTTCACCA TGGATCTGAT   
  
  
+ AATGTGTAAG ACCCCAACCC TTTTGCTTTT ATTTTCCTCA TGCTCCATTC TCATGATTTG TACTTCTGTT   
  
  
+ ATGTAATTGG AGATGTGTCT AAATTTGGAA AAGTTCGTGG GTTTCGTGTT TGAGAATTTA GAATGACATT   
  
  
+ CTTTAATTAG TAATTTGAAG TTTGCATGCT TAATTTAGCT AATCACTGAT TAATTAAAAT AAATGAAGTG   
  
  
+ ATGTAGGTAT GGGCTGTTAC ACAATGTCAC CGGTGGTGGT GACAGCGGCG GGGCGGCGGC GGTTGGATTG   
  
  
+ GAAGAATGGG ACAGCGTGTT TCCAAATGGG GATGGAGCTT TACTCCCTTG GATCATGGGG GAAGGTGATG   
  
  
+ ATTTGGGTCT GAATTTGAAG CATCTCTTAC AATCTGGTTA CCCTGTTGAG TATGAAGGCA ATGCAGGACT   
  
  
+ TGGTGTTGTT GATCAGACAT CTCTTCTTGG AGGTGGTGAT TTGGGGTTTT CTGGTTCTGG GTCTGTGAAT   
  
  
+ AATGGCAAAA TTGGTTCCAT TTTGGGTAAT TGTTCATCTG GGGATTTGGA TTCTAAGGTT TCTAGTGACG   
  
  
+ GGTTGAATTC CAATTGTAGT TCACTGGGGA GTATGAGTAT CCTTGGTTCA ATCCATGGAC CTTTGCCTAA   
  
  
+ TACTGGTGGG TTTGTGTTCC CACAAACACA ACTATTTGAT CTTGGTGATG AGAAGCCTCA GATTTTGAAC   
  
  
+ CCACAATTGA TGACGATGAA TCCAAATCAG GCTCAGAGCA TGGGAAACCC TAGCTTTTTT GTGCCCTCCT   
  
  
+ TGGGTTATTG TCAGTTAGAG CAACATATGG TTCAACCACA GGCGAAACGC CATAGCCCTG GTGTAGTTTT   
  
  
+ GAACTCTGAT GTTATTGCCA AAACCCCGTT TCTCGATCAG GGTCATGAGT TTTTGTTGAG GAAACAACAC   
  
  
+ CATCAACAGA TTTTGCAGCA ACTTCCTATG GGTTTGGCTC ATCAGTCGGT CCCTCAGCAC CTCCAGCAAA   
  
  
+ AGGCGATGAA AGACCAGGTT GTTAAGGACC AGCTTTTGAA GGCAGCTGAC CTGATTCAAA CTGGGAATTT   
  
  
+ CTCACTTGCG CAAGAGATAT TGGCGCGGCT CAATCACCAG CTCTCCCTTC CTGCGAAGCC CCTCATTAGG   
  
  
+ GCAGCTTTGT ATGTCATGGA GGCCTTGCAA ATGCTCATCT TAATGAGCAA TCCTGTAGCA CCTCCACCGA   
  
  
+ TGAAGACGCT TTCCCCTATA GATGTTGTTC ATAAGATGAA TGCCTACAAG GCCTTCTCTG AGGTCTCTCC   
  
  
+ TATTACTCAG TTTACGAATT TCACTTGTGC CCAAGCCATT CTTGAAGCTC TCGATGATGC GGATTGCGTT   
  
  
+ CATGTCATTG ACTTTGATAT TGGTTGTGGG GCTCAATGGG CATCACTGAT TCAGGAGCTT CCATTGAGGA   
  
  
+ AAAAGGGAGC TCCATCCCTG AAAATTACAG CCATAGCTCC CCTGTCAATA AGCCACACTT TTGAACTCAA   
  
  
+ CCTAGCACGG GAAAACCTTG TGCAATTCGC CAATGATGTT GGTGTTGCTT TTGAGCTGCA AGTTGTGAAC   
  
  
+ TTGGATTTAT TTGATCCATC TTCATCTTCA ATGCCAAATG TTGGAACCTC TGGGGATGAG TTGATCGCTG   
  
  
+ TTAGTATACC CATTTGGGCA TGTTCATATC GGCCGTCTAT TCTCCCATCC ATCCTCCGGT TCATTAAGCA   
  
  
+ ACTAGCCCCC AAGATTGTCG TCTCGTTAGA TAGAGGGTCT GATCGTTGCG ACACTCTGTT CCCACAACAT   
  
  
+ CTTATCCATA TCTTAGAGTC CTGCACAAAT TTCTTGGAAT CGCTCGACGG TCTCAATGTA GCATCAGATA   
  
  
+ TTGTAAACAA GGTTGAGAAG TACTTCCTCC AGCCTAAGAT CGAAAACACC GTGTTGGGCC GCGTTCATGC   
  
  
+ CCCCAATAAG ATGCCACATT GGAAGACCCT CTTTGCTTCA GCCGGGTTAT TGCCCTTTCA GTTCAGTAAC   
  
  
+ TTCACAGAAA CACAAGCTGA TTATGTGGTG AAGCGAACCC CAGGGAGGGG ATTTCACATA GAAAAGCGCC   
  
  
+ ACGCATCTTT AGTGCTAAGT TGGCAAAGGC GGGAGCTTGT GACAGCCTCG GCATGGAGGT TTGAGAAGCT   
  
  
+ GTA  

- +Up\_Stream \_Len000TGTAAC CCACCCTAAA TTTAGGAGAT AAAAAAGAAT CCCCTTTTTA TGAACCCTTT   
  
  
- AAAGAGTTAT TGACTTTTAG ATTTATTAAG CACTGAAACA ATCATCCAAA TTACACTAAA TGATGGCACT   
  
  
- CACAGTGAAA ATCTACCATG AAATTCTTTC GTAATATTCA TTTTGTTATA TATAGCATCG TAGTATGATG   
  
  
- AACACATTAT TTAATTGTCG TTGGAGTTAA CATGAATAAA CTAAAGCGTA ACATTAGTTA AAAGGGTTTA   
  
  
- CTAAATGATA GAACCAGAAT GAGTCAACAT GAAGTGTGAG TTATCAAGTT AATAAAAAGT AATCTGATTA   
  
  
- CTCGCTCAGG GAAGATGTTC TCGGATGATG ATGAGATTAT TACATTCCTG ATAACCGATT ACCCCTTGAT   
  
  
- TCGGATGCTG AACAATCCGT ATCCACTTAA AAGGAATGTT CTCTCAAATT AAATGTGAAC TATAATTGAG   
  
  
- CTCTTGCTCT TCGACAGAAT TATCTATGGG TGTATCTTTT ACTTCGTTTA CGGGAACTAA TAACCTCCCA   
  
  
- ACCCGTTTAT ATAAGTCATT GTTACAACAA ACCGTTTATA GCAGACTTTT AACAAAAACT GTTTATAACA   
  
  
- AACCTTCTTA AAAAGCTCTA ATTTATAATT TTTACAATTT TAAAATTAGA TTTATAGTTT GTTTATAACT   
  
  
- TTTTACGAAA TATAATAGAT TTAGAGATTA ATTTTAAACA CATAACAGGT GATCTTTTTA CATATCACTA   
  
  
- TAATGGCTGT ATATACACTA GAGACCACAT TATCGGATGG GATTACAGAT CCTTTTATAA ATTTTAGGGG   
  
  
- TCACCAGTTT TCCTTCAATG CGTGTGATCC CGTTCTGTGA AGTAATCCGA TCAACCACTT TCGATTCAGT   
  
  
- ACTTTAAATC TGGTACCGGT ACAGATGAAC ATCTAGGTTG ATGGTTGTTA GGTAAAAATA AAACCCCTTT   
  
  
- CGAACTTCCC ATTAAACCAT TTACATGTGC CCTTGGAAAA CGATATTTTC TTCACGAAGA ACTTTTTTGT   
  
  
- GGGACATCAT TTTGTCCCGT TTTTTCGGTT GTGAAGACAA AGACCGTCTC TTTGTCATCA CATGGTGAAC   
  
  
- CTCAACCTCA TCTGTGCTAT GTGTTTGGGT CATCTCTCTC TTTGTCCCCT CTCTTTCTCA TGTGTTTAAA   
  
  
- CACGTCCTCT TCCCATTCCT TCTTCCTCTT CCTTGGGGGA GGTTTTTTTT GGGTATATTT TTTATTATTT   
  
  
- CTTTTTTGTC CTCACTCAAT CTCCCATGGG TTTAGAGGTT AGATTTTGAT CTTAGATCCC AGAGAAAAGT   
  
  
- AAACGAACTT TTTAAAGACA AGTACCCAAA ACACAAAAAT CCCCCTAAAC AAACTAAAAA CATCAGAAAC   
  
  
- ACCTAAAGGA CACAATATCT ACGTTCGATA CGAGAAGTTA AACGTTCCTT GTCGTAGACC CCCCTCTCTT   
  
  
- AAACTCCTTA AAGTTCATGG GTAAAGTTAA AGTTGTTCAT TTTAGTAGTA ACTTCCTATG AGTTTGGGTT   
  
  
- CGGGTCAACT ATCACTTGGT TGAAGATGAG ACCTAAGATG AGGTTCAGGG TCGGGAGGGT GGGAGAGGAG   
  
  
- GAGGAGGTTC TTGAGGATGG TTTTGCGACG GCAAAACCGA GTGGGAGGAG TCGAAGTGGT ACCTAGACTA   
  
  
- TTACACATTC TGGGGTTGGG AAAACGAAAA TAAAAGGAGT ACGAGGTAAG AGTACTAAAC ATGAAGACAA   
  
  
- TACATTAACC TCTACACAGA TTTAAACCTT TTCAAGCACC CAAAGCACAA ACTCTTAAAT CTTACTGTAA   
  
  
- GAAATTAATC ATTAAACTTC AAACGTACGA ATTAAATCGA TTAGTGACTA ATTAATTTTA TTTACTTCAC   
  
  
- TACATCCATA CCCGACAATG TGTTACAGTG GCCACCACCA CTGTCGCCGC CCCGCCGCCG CCAACCTAAC   
  
  
- CTTCTTACCC TGTCGCACAA AGGTTTACCC CTACCTCGAA ATGAGGGAAC CTAGTACCCC CTTCCACTAC   
  
  
- TAAACCCAGA CTTAAACTTC GTAGAGAATG TTAGACCAAT GGGACAACTC ATACTTCCGT TACGTCCTGA   
  
  
- ACCACAACAA CTAGTCTGTA GAGAAGAACC TCCACCACTA AACCCCAAAA GACCAAGACC CAGACACTTA   
  
  
- TTACCGTTTT AACCAAGGTA AAACCCATTA ACAAGTAGAC CCCTAAACCT AAGATTCCAA AGATCACTGC   
  
  
- CCAACTTAAG GTTAACATCA AGTGACCCCT CATACTCATA GGAACCAAGT TAGGTACCTG GAAACGGATT   
  
  
- ATGACCACCC AAACACAAGG GTGTTTGTGT TGATAAACTA GAACCACTAC TCTTCGGAGT CTAAAACTTG   
  
  
- GGTGTTAACT ACTGCTACTT AGGTTTAGTC CGAGTCTCGT ACCCTTTGGG ATCGAAAAAA CACGGGAGGA   
  
  
- ACCCAATAAC AGTCAATCTC GTTGTATACC AAGTTGGTGT CCGCTTTGCG GTATCGGGAC CACATCAAAA   
  
  
- CTTGAGACTA CAATAACGGT TTTGGGGCAA AGAGCTAGTC CCAGTACTCA AAAACAACTC CTTTGTTGTG   
  
  
- GTAGTTGTCT AAAACGTCGT TGAAGGATAC CCAAACCGAG TAGTCAGCCA GGGAGTCGTG GAGGTCGTTT   
  
  
- TCCGCTACTT TCTGGTCCAA CAATTCCTGG TCGAAAACTT CCGTCGACTG GACTAAGTTT GACCCTTAAA   
  
  
- GAGTGAACGC GTTCTCTATA ACCGCGCCGA GTTAGTGGTC GAGAGGGAAG GACGCTTCGG GGAGTAATCC   
  
  
- CGTCGAAACA TACAGTACCT CCGGAACGTT TACGAGTAGA ATTACTCGTT AGGACATCGT GGAGGTGGCT   
  
  
- ACTTCTGCGA AAGGGGATAT CTACAACAAG TATTCTACTT ACGGATGTTC CGGAAGAGAC TCCAGAGAGG   
  
  
- ATAATGAGTC AAATGCTTAA AGTGAACACG GGTTCGGTAA GAACTTCGAG AGCTACTACG CCTAACGCAA   
  
  
- GTACAGTAAC TGAAACTATA ACCAACACCC CGAGTTACCC GTAGTGACTA AGTCCTCGAA GGTAACTCCT   
  
  
- TTTTCCCTCG AGGTAGGGAC TTTTAATGTC GGTATCGAGG GGACAGTTAT TCGGTGTGAA AACTTGAGTT   
  
  
- GGATCGTGCC CTTTTGGAAC ACGTTAAGCG GTTACTACAA CCACAACGAA AACTCGACGT TCAACACTTG   
  
  
- AACCTAAATA AACTAGGTAG AAGTAGAAGT TACGGTTTAC AACCTTGGAG ACCCCTACTC AACTAGCGAC   
  
  
- AATCATATGG GTAAACCCGT ACAAGTATAG CCGGCAGATA AGAGGGTAGG TAGGAGGCCA AGTAATTCGT   
  
  
- TGATCGGGGG TTCTAACAGC AGAGCAATCT ATCTCCCAGA CTAGCAACGC TGTGAGACAA GGGTGTTGTA   
  
  
- GAATAGGTAT AGAATCTCAG GACGTGTTTA AAGAACCTTA GCGAGCTGCC AGAGTTACAT CGTAGTCTAT   
  
  
- AACATTTGTT CCAACTCTTC ATGAAGGAGG TCGGATTCTA GCTTTTGTGG CACAACCCGG CGCAAGTACG   
  
  
- GGGGTTATTC TACGGTGTAA CCTTCTGGGA GAAACGAAGT CGGCCCAATA ACGGGAAAGT CAAGTCATTG   
  
  
- AAGTGTCTTT GTGTTCGACT AATACACCAC TTCGCTTGGG GTCCCTCCCC TAAAGTGTAT CTTTTCGCGG   
  
  
- TGCGTAGAAA TCACGATTCA ACCGTTTCCG CCCTCGAACA CTGTCGGAGC CGTACCTCCA AACTCTTCGA   
  
  
- CAT

+     W box

| Site Name | Organism | Position | Strand | Matrix score. | sequence | function |
| --- | --- | --- | --- | --- | --- | --- |
| W box | Arabidopsis thaliana | 848 | - | 6 | TTGACC |  |

>HU05G00466.1   
+ +Up\_Stream \_Len000ACATTG GGTGGGATTT AAATCCTCTA TTTTTTCTTA GGGGAAAAAT ACTTGGGAAA   
  
  
+ TTTCTCAATA ACTGAAAATC TAAATAATTC GTGACTTTGT TAGTAGGTTT AATGTGATTT ACTACCGTGA   
  
  
+ GTGTCACTTT TAGATGGTAC TTTAAGAAAG CATTATAAGT AAAACAATAT ATATCGTAGC ATCATACTAC   
  
  
+ TTGTGTAATA AATTAACAGC AACCTCAATT GTACTTATTT GATTTCGCAT TGTAATCAAT TTTCCCAAAT   
  
  
+ GATTTACTAT CTTGGTCTTA CTCAGTTGTA CTTCACACTC AATAGTTCAA TTATTTTTCA TTAGACTAAT   
  
  
+ GAGCGAGTCC CTTCTACAAG AGCCTACTAC TACTCTAATA ATGTAAGGAC TATTGGCTAA TGGGGAACTA   
  
  
+ AGCCTACGAC TTGTTAGGCA TAGGTGAATT TTCCTTACAA GAGAGTTTAA TTTACACTTG ATATTAACTC   
  
  
+ GAGAACGAGA AGCTGTCTTA ATAGATACCC ACATAGAAAA TGAAGCAAAT GCCCTTGATT ATTGGAGGGT   
  
  
+ TGGGCAAATA TATTCAGTAA CAATGTTGTT TGGCAAATAT CGTCTGAAAA TTGTTTTTGA CAAATATTGT   
  
  
+ TTGGAAGAAT TTTTCGAGAT TAAATATTAA AAATGTTAAA ATTTTAATCT AAATATCAAA CAAATATTGA   
  
  
+ AAAATGCTTT ATATTATCTA AATCTCTAAT TAAAATTTGT GTATTGTCCA CTAGAAAAAT GTATAGTGAT   
  
  
+ ATTACCGACA TATATGTGAT CTCTGGTGTA ATAGCCTACC CTAATGTCTA GGAAAATATT TAAAATCCCC   
  
  
+ AGTGGTCAAA AGGAAGTTAC GCACACTAGG GCAAGACACT TCATTAGGCT AGTTGGTGAA AGCTAAGTCA   
  
  
+ TGAAATTTAG ACCATGGCCA TGTCTACTTG TAGATCCAAC TACCAACAAT CCATTTTTAT TTTGGGGAAA   
  
  
+ GCTTGAAGGG TAATTTGGTA AATGTACACG GGAACCTTTT GCTATAAAAG AAGTGCTTCT TGAAAAAACA   
  
  
+ CCCTGTAGTA AAACAGGGCA AAAAAGCCAA CACTTCTGTT TCTGGCAGAG AAACAGTAGT GTACCACTTG   
  
  
+ GAGTTGGAGT AGACACGATA CACAAACCCA GTAGAGAGAG AAACAGGGGA GAGAAAGAGT ACACAAATTT   
  
  
+ GTGCAGGAGA AGGGTAAGGA AGAAGGAGAA GGAACCCCCT CCAAAAAAAA CCCATATAAA AAATAATAAA   
  
  
+ GAAAAAACAG GAGTGAGTTA GAGGGTACCC AAATCTCCAA TCTAAAACTA GAATCTAGGG TCTCTTTTCA   
  
  
+ TTTGCTTGAA AAATTTCTGT TCATGGGTTT TGTGTTTTTA GGGGGATTTG TTTGATTTTT GTAGTCTTTG   
  
  
+ TGGATTTCCT GTGTTATAGA TGCAAGCTAT GCTCTTCAAT TTGCAAGGAA CAGCATCTGG GGGGAGAGAA   
  
  
+ TTTGAGGAAT TTCAAGTACC CATTTCAATT TCAACAAGTA AAATCATCAT TGAAGGATAC TCAAACCCAA   
  
  
+ GCCCAGTTGA TAGTGAACCA ACTTCTACTC TGGATTCTAC TCCAAGTCCC AGCCCTCCCA CCCTCTCCTC   
  
  
+ CTCCTCCAAG AACTCCTACC AAAACGCTGC CGTTTTGGCT CACCCTCCTC AGCTTCACCA TGGATCTGAT   
  
  
+ AATGTGTAAG ACCCCAACCC TTTTGCTTTT ATTTTCCTCA TGCTCCATTC TCATGATTTG TACTTCTGTT   
  
  
+ ATGTAATTGG AGATGTGTCT AAATTTGGAA AAGTTCGTGG GTTTCGTGTT TGAGAATTTA GAATGACATT   
  
  
+ CTTTAATTAG TAATTTGAAG TTTGCATGCT TAATTTAGCT AATCACTGAT TAATTAAAAT AAATGAAGTG   
  
  
+ ATGTAGGTAT GGGCTGTTAC ACAATGTCAC CGGTGGTGGT GACAGCGGCG GGGCGGCGGC GGTTGGATTG   
  
  
+ GAAGAATGGG ACAGCGTGTT TCCAAATGGG GATGGAGCTT TACTCCCTTG GATCATGGGG GAAGGTGATG   
  
  
+ ATTTGGGTCT GAATTTGAAG CATCTCTTAC AATCTGGTTA CCCTGTTGAG TATGAAGGCA ATGCAGGACT   
  
  
+ TGGTGTTGTT GATCAGACAT CTCTTCTTGG AGGTGGTGAT TTGGGGTTTT CTGGTTCTGG GTCTGTGAAT   
  
  
+ AATGGCAAAA TTGGTTCCAT TTTGGGTAAT TGTTCATCTG GGGATTTGGA TTCTAAGGTT TCTAGTGACG   
  
  
+ GGTTGAATTC CAATTGTAGT TCACTGGGGA GTATGAGTAT CCTTGGTTCA ATCCATGGAC CTTTGCCTAA   
  
  
+ TACTGGTGGG TTTGTGTTCC CACAAACACA ACTATTTGAT CTTGGTGATG AGAAGCCTCA GATTTTGAAC   
  
  
+ CCACAATTGA TGACGATGAA TCCAAATCAG GCTCAGAGCA TGGGAAACCC TAGCTTTTTT GTGCCCTCCT   
  
  
+ TGGGTTATTG TCAGTTAGAG CAACATATGG TTCAACCACA GGCGAAACGC CATAGCCCTG GTGTAGTTTT   
  
  
+ GAACTCTGAT GTTATTGCCA AAACCCCGTT TCTCGATCAG GGTCATGAGT TTTTGTTGAG GAAACAACAC   
  
  
+ CATCAACAGA TTTTGCAGCA ACTTCCTATG GGTTTGGCTC ATCAGTCGGT CCCTCAGCAC CTCCAGCAAA   
  
  
+ AGGCGATGAA AGACCAGGTT GTTAAGGACC AGCTTTTGAA GGCAGCTGAC CTGATTCAAA CTGGGAATTT   
  
  
+ CTCACTTGCG CAAGAGATAT TGGCGCGGCT CAATCACCAG CTCTCCCTTC CTGCGAAGCC CCTCATTAGG   
  
  
+ GCAGCTTTGT ATGTCATGGA GGCCTTGCAA ATGCTCATCT TAATGAGCAA TCCTGTAGCA CCTCCACCGA   
  
  
+ TGAAGACGCT TTCCCCTATA GATGTTGTTC ATAAGATGAA TGCCTACAAG GCCTTCTCTG AGGTCTCTCC   
  
  
+ TATTACTCAG TTTACGAATT TCACTTGTGC CCAAGCCATT CTTGAAGCTC TCGATGATGC GGATTGCGTT   
  
  
+ CATGTCATTG ACTTTGATAT TGGTTGTGGG GCTCAATGGG CATCACTGAT TCAGGAGCTT CCATTGAGGA   
  
  
+ AAAAGGGAGC TCCATCCCTG AAAATTACAG CCATAGCTCC CCTGTCAATA AGCCACACTT TTGAACTCAA   
  
  
+ CCTAGCACGG GAAAACCTTG TGCAATTCGC CAATGATGTT GGTGTTGCTT TTGAGCTGCA AGTTGTGAAC   
  
  
+ TTGGATTTAT TTGATCCATC TTCATCTTCA ATGCCAAATG TTGGAACCTC TGGGGATGAG TTGATCGCTG   
  
  
+ TTAGTATACC CATTTGGGCA TGTTCATATC GGCCGTCTAT TCTCCCATCC ATCCTCCGGT TCATTAAGCA   
  
  
+ ACTAGCCCCC AAGATTGTCG TCTCGTTAGA TAGAGGGTCT GATCGTTGCG ACACTCTGTT CCCACAACAT   
  
  
+ CTTATCCATA TCTTAGAGTC CTGCACAAAT TTCTTGGAAT CGCTCGACGG TCTCAATGTA GCATCAGATA   
  
  
+ TTGTAAACAA GGTTGAGAAG TACTTCCTCC AGCCTAAGAT CGAAAACACC GTGTTGGGCC GCGTTCATGC   
  
  
+ CCCCAATAAG ATGCCACATT GGAAGACCCT CTTTGCTTCA GCCGGGTTAT TGCCCTTTCA GTTCAGTAAC   
  
  
+ TTCACAGAAA CACAAGCTGA TTATGTGGTG AAGCGAACCC CAGGGAGGGG ATTTCACATA GAAAAGCGCC   
  
  
+ ACGCATCTTT AGTGCTAAGT TGGCAAAGGC GGGAGCTTGT GACAGCCTCG GCATGGAGGT TTGAGAAGCT   
  
  
+ GTA  

- +Up\_Stream \_Len000TGTAAC CCACCCTAAA TTTAGGAGAT AAAAAAGAAT CCCCTTTTTA TGAACCCTTT   
  
  
- AAAGAGTTAT TGACTTTTAG ATTTATTAAG CACTGAAACA ATCATCCAAA TTACACTAAA TGATGGCACT   
  
  
- CACAGTGAAA ATCTACCATG AAATTCTTTC GTAATATTCA TTTTGTTATA TATAGCATCG TAGTATGATG   
  
  
- AACACATTAT TTAATTGTCG TTGGAGTTAA CATGAATAAA CTAAAGCGTA ACATTAGTTA AAAGGGTTTA   
  
  
- CTAAATGATA GAACCAGAAT GAGTCAACAT GAAGTGTGAG TTATCAAGTT AATAAAAAGT AATCTGATTA   
  
  
- CTCGCTCAGG GAAGATGTTC TCGGATGATG ATGAGATTAT TACATTCCTG ATAACCGATT ACCCCTTGAT   
  
  
- TCGGATGCTG AACAATCCGT ATCCACTTAA AAGGAATGTT CTCTCAAATT AAATGTGAAC TATAATTGAG   
  
  
- CTCTTGCTCT TCGACAGAAT TATCTATGGG TGTATCTTTT ACTTCGTTTA CGGGAACTAA TAACCTCCCA   
  
  
- ACCCGTTTAT ATAAGTCATT GTTACAACAA ACCGTTTATA GCAGACTTTT AACAAAAACT GTTTATAACA   
  
  
- AACCTTCTTA AAAAGCTCTA ATTTATAATT TTTACAATTT TAAAATTAGA TTTATAGTTT GTTTATAACT   
  
  
- TTTTACGAAA TATAATAGAT TTAGAGATTA ATTTTAAACA CATAACAGGT GATCTTTTTA CATATCACTA   
  
  
- TAATGGCTGT ATATACACTA GAGACCACAT TATCGGATGG GATTACAGAT CCTTTTATAA ATTTTAGGGG   
  
  
- TCACCAGTTT TCCTTCAATG CGTGTGATCC CGTTCTGTGA AGTAATCCGA TCAACCACTT TCGATTCAGT   
  
  
- ACTTTAAATC TGGTACCGGT ACAGATGAAC ATCTAGGTTG ATGGTTGTTA GGTAAAAATA AAACCCCTTT   
  
  
- CGAACTTCCC ATTAAACCAT TTACATGTGC CCTTGGAAAA CGATATTTTC TTCACGAAGA ACTTTTTTGT   
  
  
- GGGACATCAT TTTGTCCCGT TTTTTCGGTT GTGAAGACAA AGACCGTCTC TTTGTCATCA CATGGTGAAC   
  
  
- CTCAACCTCA TCTGTGCTAT GTGTTTGGGT CATCTCTCTC TTTGTCCCCT CTCTTTCTCA TGTGTTTAAA   
  
  
- CACGTCCTCT TCCCATTCCT TCTTCCTCTT CCTTGGGGGA GGTTTTTTTT GGGTATATTT TTTATTATTT   
  
  
- CTTTTTTGTC CTCACTCAAT CTCCCATGGG TTTAGAGGTT AGATTTTGAT CTTAGATCCC AGAGAAAAGT   
  
  
- AAACGAACTT TTTAAAGACA AGTACCCAAA ACACAAAAAT CCCCCTAAAC AAACTAAAAA CATCAGAAAC   
  
  
- ACCTAAAGGA CACAATATCT ACGTTCGATA CGAGAAGTTA AACGTTCCTT GTCGTAGACC CCCCTCTCTT   
  
  
- AAACTCCTTA AAGTTCATGG GTAAAGTTAA AGTTGTTCAT TTTAGTAGTA ACTTCCTATG AGTTTGGGTT   
  
  
- CGGGTCAACT ATCACTTGGT TGAAGATGAG ACCTAAGATG AGGTTCAGGG TCGGGAGGGT GGGAGAGGAG   
  
  
- GAGGAGGTTC TTGAGGATGG TTTTGCGACG GCAAAACCGA GTGGGAGGAG TCGAAGTGGT ACCTAGACTA   
  
  
- TTACACATTC TGGGGTTGGG AAAACGAAAA TAAAAGGAGT ACGAGGTAAG AGTACTAAAC ATGAAGACAA   
  
  
- TACATTAACC TCTACACAGA TTTAAACCTT TTCAAGCACC CAAAGCACAA ACTCTTAAAT CTTACTGTAA   
  
  
- GAAATTAATC ATTAAACTTC AAACGTACGA ATTAAATCGA TTAGTGACTA ATTAATTTTA TTTACTTCAC   
  
  
- TACATCCATA CCCGACAATG TGTTACAGTG GCCACCACCA CTGTCGCCGC CCCGCCGCCG CCAACCTAAC   
  
  
- CTTCTTACCC TGTCGCACAA AGGTTTACCC CTACCTCGAA ATGAGGGAAC CTAGTACCCC CTTCCACTAC   
  
  
- TAAACCCAGA CTTAAACTTC GTAGAGAATG TTAGACCAAT GGGACAACTC ATACTTCCGT TACGTCCTGA   
  
  
- ACCACAACAA CTAGTCTGTA GAGAAGAACC TCCACCACTA AACCCCAAAA GACCAAGACC CAGACACTTA   
  
  
- TTACCGTTTT AACCAAGGTA AAACCCATTA ACAAGTAGAC CCCTAAACCT AAGATTCCAA AGATCACTGC   
  
  
- CCAACTTAAG GTTAACATCA AGTGACCCCT CATACTCATA GGAACCAAGT TAGGTACCTG GAAACGGATT   
  
  
- ATGACCACCC AAACACAAGG GTGTTTGTGT TGATAAACTA GAACCACTAC TCTTCGGAGT CTAAAACTTG   
  
  
- GGTGTTAACT ACTGCTACTT AGGTTTAGTC CGAGTCTCGT ACCCTTTGGG ATCGAAAAAA CACGGGAGGA   
  
  
- ACCCAATAAC AGTCAATCTC GTTGTATACC AAGTTGGTGT CCGCTTTGCG GTATCGGGAC CACATCAAAA   
  
  
- CTTGAGACTA CAATAACGGT TTTGGGGCAA AGAGCTAGTC CCAGTACTCA AAAACAACTC CTTTGTTGTG   
  
  
- GTAGTTGTCT AAAACGTCGT TGAAGGATAC CCAAACCGAG TAGTCAGCCA GGGAGTCGTG GAGGTCGTTT   
  
  
- TCCGCTACTT TCTGGTCCAA CAATTCCTGG TCGAAAACTT CCGTCGACTG GACTAAGTTT GACCCTTAAA   
  
  
- GAGTGAACGC GTTCTCTATA ACCGCGCCGA GTTAGTGGTC GAGAGGGAAG GACGCTTCGG GGAGTAATCC   
  
  
- CGTCGAAACA TACAGTACCT CCGGAACGTT TACGAGTAGA ATTACTCGTT AGGACATCGT GGAGGTGGCT   
  
  
- ACTTCTGCGA AAGGGGATAT CTACAACAAG TATTCTACTT ACGGATGTTC CGGAAGAGAC TCCAGAGAGG   
  
  
- ATAATGAGTC AAATGCTTAA AGTGAACACG GGTTCGGTAA GAACTTCGAG AGCTACTACG CCTAACGCAA   
  
  
- GTACAGTAAC TGAAACTATA ACCAACACCC CGAGTTACCC GTAGTGACTA AGTCCTCGAA GGTAACTCCT   
  
  
- TTTTCCCTCG AGGTAGGGAC TTTTAATGTC GGTATCGAGG GGACAGTTAT TCGGTGTGAA AACTTGAGTT   
  
  
- GGATCGTGCC CTTTTGGAAC ACGTTAAGCG GTTACTACAA CCACAACGAA AACTCGACGT TCAACACTTG   
  
  
- AACCTAAATA AACTAGGTAG AAGTAGAAGT TACGGTTTAC AACCTTGGAG ACCCCTACTC AACTAGCGAC   
  
  
- AATCATATGG GTAAACCCGT ACAAGTATAG CCGGCAGATA AGAGGGTAGG TAGGAGGCCA AGTAATTCGT   
  
  
- TGATCGGGGG TTCTAACAGC AGAGCAATCT ATCTCCCAGA CTAGCAACGC TGTGAGACAA GGGTGTTGTA   
  
  
- GAATAGGTAT AGAATCTCAG GACGTGTTTA AAGAACCTTA GCGAGCTGCC AGAGTTACAT CGTAGTCTAT   
  
  
- AACATTTGTT CCAACTCTTC ATGAAGGAGG TCGGATTCTA GCTTTTGTGG CACAACCCGG CGCAAGTACG   
  
  
- GGGGTTATTC TACGGTGTAA CCTTCTGGGA GAAACGAAGT CGGCCCAATA ACGGGAAAGT CAAGTCATTG   
  
  
- AAGTGTCTTT GTGTTCGACT AATACACCAC TTCGCTTGGG GTCCCTCCCC TAAAGTGTAT CTTTTCGCGG   
  
  
- TGCGTAGAAA TCACGATTCA ACCGTTTCCG CCCTCGAACA CTGTCGGAGC CGTACCTCCA AACTCTTCGA   
  
  
- CAT

+     WRE3

| Site Name | Organism | Position | Strand | Matrix score. | sequence | function |
| --- | --- | --- | --- | --- | --- | --- |
| WRE3 | Pisum sativum | 2135 | - | 6 | CCACCT |  |

>HU05G00466.1   
+ +Up\_Stream \_Len000ACATTG GGTGGGATTT AAATCCTCTA TTTTTTCTTA GGGGAAAAAT ACTTGGGAAA   
  
  
+ TTTCTCAATA ACTGAAAATC TAAATAATTC GTGACTTTGT TAGTAGGTTT AATGTGATTT ACTACCGTGA   
  
  
+ GTGTCACTTT TAGATGGTAC TTTAAGAAAG CATTATAAGT AAAACAATAT ATATCGTAGC ATCATACTAC   
  
  
+ TTGTGTAATA AATTAACAGC AACCTCAATT GTACTTATTT GATTTCGCAT TGTAATCAAT TTTCCCAAAT   
  
  
+ GATTTACTAT CTTGGTCTTA CTCAGTTGTA CTTCACACTC AATAGTTCAA TTATTTTTCA TTAGACTAAT   
  
  
+ GAGCGAGTCC CTTCTACAAG AGCCTACTAC TACTCTAATA ATGTAAGGAC TATTGGCTAA TGGGGAACTA
[truncated: 28,820 more chars]
