# Supplementary material for: Genome-Wide Identification and Expression Pattern of the GRAS Gene Family in Pitaya (Selenicereus undatus L.)
Source: Biology (Basel). 2022 Dec 21;12(1):11. doi: 10.3390/biology12010011 (PMC9854919; doi:10.3390/biology12010011)
Supplement: Supplementary file 1 [file biology-12-00011-s001.zip › Supplementary file S5/HU05G01267.1_plantcare.html]

Content-Type: text/html; charset=ISO-8859-1


PlantCARE


Webmaster Firefox specific output  
To save the result:
click on the frame with the right mouse button and save the source code as a text file with extension .html  
REFERENCE:PlantCARE: a database of plant cis-acting regulatory elements and a portal to tools for in silico analysis of promoter sequences.  
Lescot, M., Déhais, P., Moreau, Y., De Moor, B., Rouzé ,P.,and Rombauts, S.  
Nucleic Acids Res., Database issue(2002), 30(1):325-327.   


---

>HU05G01267.1   
+ +Up\_Stream \_Len000TTACAA AAACGATGCT CTAAACCTAT TAGGTCGTTC CGAAACCCGC AAAATTGAAA   
  
  
+ TATGATCGCC CGTATTTTAC CCAAACCTGA AATACACTTA ATATGTATTT TACCTGAACC CAAATACACC   
  
  
+ GGACCTGTAT TTAACACGGG CACCCGAAAT GATAGGTCTA ACTCTCACCA CCACCGCATT TGGCACCCGA   
  
  
+ ACGGTAAAAA ATGTTCATCT ATTATGCACT TTGGCTTCAT CCATAACCCT ATGATCTATC TGTGCCAATA   
  
  
+ TTTGGAGCAC CCAGCGAAGT AGCGCATGCT CTAAACTCTT GCCTCAAAAC AAAACAAATA GTGGGTCATA   
  
  
+ TTCCCTTCAA AGCACTAACA AACACACACT CTCTCTCTCT CAGTAGCCAA CAACTTAAGG CGGTGTTTGC   
  
  
+ TTGGTTTTGG AGAGGGCGTG TTTGGATGAG GTGCAAAACA CATAGATGAA ATCATAAACA ACTTGATTCC   
  
  
+ CAGCCACCCA TGACCTACTC AGATAACAAG GGACAAAAAC AAAGAGGTTT TCTGAAACAG AGAAAACAGG   
  
  
+ GGCGTTGTGT TGCGCTGTTG CTCACAAAAT TCCAATATTC CTCTAAAAGC TCCCAAACGG AGACAAATCA   
  
  
+ AAGAGGACAA AGACATGTGG GATTTAATAC AAAATTTGAC TAACTCTGCA ACGGAAAACA ACCCCTAGGG   
  
  
+ TGTTTTTCTG CGTGTTGTAC ACACTTCACT TTTAACCTGC TCTGCCTCTG CCTTTGCCAC GGCTTTTAAT   
  
  
+ GGATAAGACC AATTAAGAAA CCCAAATATC TCGTAAATTT GTTGAGATTC CTTTCTGCGT TCTCAAGTGT   
  
  
+ TTCAAAAACC CAGTTTGGAG GTTGCACTTA TTCATGCTTT TCTTCAAGTC AGTGAGCTTA GTGTCTGGAA   
  
  
+ CTGAAGCTCC TCCTGGTAAT GCTCTGTTTT TCATTTCTTG TTGTGCGTTT ATAAAAATTC GGCCTATTGA   
  
  
+ TGGAAGTTTG TTTGATGTTT TTGGGTAAGT TTTTGATTAA TAGAGATGAA TTAGTAAAGT AAAATTTGAG   
  
  
+ TGGTATTTAA TGATCCTTAA GCAGTCTATG CTTTGTTGTC ACATGCGTTT CTTGGCTTTT GACTGAAACA   
  
  
+ TTTCTGTGTT AGATGAAAAA CTCATTTTGA TGCATGAGAT GATAAAATCG CTTATTTTTG CCTTTATTTG   
  
  
+ GTGTGTGGGG GGGGGGGGGG GGGGGCGCAG GTCTGAGGTA AAAGATATGT GAATTCATTT GGAGTCTCGA   
  
  
+ TAATTCGTTG GCTATGTTGA CAATCACGAG TGGTCGTTAT CGCTCATCTC ACAGGTTGAT AATTCACTCA   
  
  
+ AACTTCAAGC GGCCTGAATC ATTCAGTCCG GGTTGTTAAA ATCTCAACAG TCATTTCACA CGAGTGGAGG   
  
  
+ GCCCCAAAAG ACACCATTCT TTCGAACTTT TTTTTTTTGA AGGTCATTTT TTCAAGAACA AAACTAATGT   
  
  
+ GAACATGCGA CTTCTATGAG TAAAGTGTAA AGATGCATAA AGTCTTTTTT TCTTTTATAT AAAAAGTACT   
  
  
+ CAAAAAAAAA AGAGTTGGAG AGCTGATTCC CCATGCATCC TCTAAGCAAA GGTGGTGGAC GGCAGAAAAT   
  
  
+ TAGTAGAGTT ATTGTGAGAT TAAAGTTTGC GCACCTGAAC GGCTGACCTC TAGTTTGCTA TCAACGTTAC   
  
  
+ ATTTATGTGT CATAATTTTT GCATGAAACA ATTTCACGCA ATGTTTTCTG ATGGCAGTTA TGTTTAGAAA   
  
  
+ TAAGTTTAAT CGGACCTTAT TCCACTGATT TATATTTGAT TCTTCCCTCT TTACTACGGC TGTCCATTCA   
  
  
+ CAAGCCAAAG AATTCTCCCC CCAAAAAAAA GAAAAAACAG TATAGAACCA TGTTTATTAT CTCATTTTTT   
  
  
+ TCTCTCCCAT TTGATCTGAA ACACAAACTT GCAGGGGTGC AAGTTGGACA CTTAGTAGCT GCAGGAATGA   
  
  
+ TTCAATAGGA ATCCTCATCT TTGACAACAT CACCACTTGA ATACATATCA CTTGATGGCA CTCTCCCCTG   
  
  
+ GTTTAGGCTT CCCATATCCG TGGCTTAGGG AGCTAAAACC TGAGCAAAGA GGACTCTGTC TAATCCATCT   
  
  
+ CCTCCTTTCA TGTGCTAATC AAGTTGCTAC TGGGAGCATC GACAATGCTA ATGTCAGCCT TGAGCATATT   
  
  
+ TCCCACCTTG CCTCTCCCAC CGGAGATACA ATGCAGCGAA TTGCTGCTTA CTTTGCTGAA GCCTTTGCTG   
  
  
+ ACCGTTTACT AAGGGCTTGG CAGCCCGGTC TTCTCAAAGC CTTGAATTGT ACCAAGATGT CATCTGTTTC   
  
  
+ CGAACAAATT CTTGTTCAAA AGTTGTTCTT TGATCTTCTT CCGTTCTTGA AGCTTTCATA TCTTGTGACG   
  
  
+ AACCAGGCAA TCACGGAGGC CATAGAAGGA GAAAAGATGG TTCATATAAT TGATCTCCAT TCTTGTGAAT   
  
  
+ CGGCTCTGTG GATTAGTCTC CTCCAGGCAT TGAGTGTTCG ACCTGAAGGC CCACCCCATT TGAGGATAAC   
  
  
+ CGGTATACAT GAGAAGAAAG AAGTGTTGGA TCAAATGGCT ATGCAACTAA ACAAAGAGGC TGAAAAATTG   
  
  
+ GACATCCCAT TTCAATTCAA TCCTATCGCA AGCAAACTAG ACGACCTTGA TGTCGAAAGC TTGAGTGTCA   
  
  
+ AGACCGGAGA AGCACTTGTT ATTAGTTCTG TGCTCCAACT ACATTCTCTT TTGGCATTGG ATGAGGGATC   
  
  
+ AATGCCTAAG AACGCAGGCA TGGCTTACTT GCAAAGGGTG TTTTATATGA AACCACGAAA ATTGGAGGAC   
  
  
+ TTGCCCAACA AGGATTTGAT GAAAATGTTG AACTCAAATG AAGATTCTAC ATCATCATCA TCTTCATCTC   
  
  
+ CTATCCCTTC ATCAAAACTT GATGCCTTTT TAAAAGCCCT CCTTGGGCTT TCGCCAAAAC TCATGGTTGT   
  
  
+ AACCGAGCAA GAATCAAACC ACAATGGAAG TGCCCTAATA GAAAGAGTGA TGGAGTCATT GAACTTCTAT   
  
  
+ GCAGCATTGT TTGATTGCTT GGAATCCACT ATATCGAGGA CATCGATAGA GAGACAGAAG CTCGAGAAGT   
  
  
+ TGATGTTTGG AGAGGAGATC AAGAACCTCA CAGCTTGTGA AGGGGCAGAG AGAAAGGCAA GGCACGAGAA   
  
  
+ GCTCAGTGAA TGGGTTCAAA GATTTGAGTC AGTAGGATTT AAAAGGGAGC CATTGAGCTA CCATGGTTTC   
  
  
+ TTGCTTGCTA GGAGGTTTTT ACATACCAAT AATTATGAGG GGTATAACAT CAAGGAAGTC AATGGTTTTC   
  
  
+ TTGTTATCTG TTGGCAAGAT AGACCCCTGT ATTCTGTTTC AGCTTGGAGA TTTTA  

- +Up\_Stream \_Len000AATGTT TTTGCTACGA GATTTGGATA ATCCAGCAAG GCTTTGGGCG TTTTAACTTT   
  
  
- ATACTAGCGG GCATAAAATG GGTTTGGACT TTATGTGAAT TATACATAAA ATGGACTTGG GTTTATGTGG   
  
  
- CCTGGACATA AATTGTGCCC GTGGGCTTTA CTATCCAGAT TGAGAGTGGT GGTGGCGTAA ACCGTGGGCT   
  
  
- TGCCATTTTT TACAAGTAGA TAATACGTGA AACCGAAGTA GGTATTGGGA TACTAGATAG ACACGGTTAT   
  
  
- AAACCTCGTG GGTCGCTTCA TCGCGTACGA GATTTGAGAA CGGAGTTTTG TTTTGTTTAT CACCCAGTAT   
  
  
- AAGGGAAGTT TCGTGATTGT TTGTGTGTGA GAGAGAGAGA GTCATCGGTT GTTGAATTCC GCCACAAACG   
  
  
- AACCAAAACC TCTCCCGCAC AAACCTACTC CACGTTTTGT GTATCTACTT TAGTATTTGT TGAACTAAGG   
  
  
- GTCGGTGGGT ACTGGATGAG TCTATTGTTC CCTGTTTTTG TTTCTCCAAA AGACTTTGTC TCTTTTGTCC   
  
  
- CCGCAACACA ACGCGACAAC GAGTGTTTTA AGGTTATAAG GAGATTTTCG AGGGTTTGCC TCTGTTTAGT   
  
  
- TTCTCCTGTT TCTGTACACC CTAAATTATG TTTTAAACTG ATTGAGACGT TGCCTTTTGT TGGGGATCCC   
  
  
- ACAAAAAGAC GCACAACATG TGTGAAGTGA AAATTGGACG AGACGGAGAC GGAAACGGTG CCGAAAATTA   
  
  
- CCTATTCTGG TTAATTCTTT GGGTTTATAG AGCATTTAAA CAACTCTAAG GAAAGACGCA AGAGTTCACA   
  
  
- AAGTTTTTGG GTCAAACCTC CAACGTGAAT AAGTACGAAA AGAAGTTCAG TCACTCGAAT CACAGACCTT   
  
  
- GACTTCGAGG AGGACCATTA CGAGACAAAA AGTAAAGAAC AACACGCAAA TATTTTTAAG CCGGATAACT   
  
  
- ACCTTCAAAC AAACTACAAA AACCCATTCA AAAACTAATT ATCTCTACTT AATCATTTCA TTTTAAACTC   
  
  
- ACCATAAATT ACTAGGAATT CGTCAGATAC GAAACAACAG TGTACGCAAA GAACCGAAAA CTGACTTTGT   
  
  
- AAAGACACAA TCTACTTTTT GAGTAAAACT ACGTACTCTA CTATTTTAGC GAATAAAAAC GGAAATAAAC   
  
  
- CACACACCCC CCCCCCCCCC CCCCCGCGTC CAGACTCCAT TTTCTATACA CTTAAGTAAA CCTCAGAGCT   
  
  
- ATTAAGCAAC CGATACAACT GTTAGTGCTC ACCAGCAATA GCGAGTAGAG TGTCCAACTA TTAAGTGAGT   
  
  
- TTGAAGTTCG CCGGACTTAG TAAGTCAGGC CCAACAATTT TAGAGTTGTC AGTAAAGTGT GCTCACCTCC   
  
  
- CGGGGTTTTC TGTGGTAAGA AAGCTTGAAA AAAAAAAACT TCCAGTAAAA AAGTTCTTGT TTTGATTACA   
  
  
- CTTGTACGCT GAAGATACTC ATTTCACATT TCTACGTATT TCAGAAAAAA AGAAAATATA TTTTTCATGA   
  
  
- GTTTTTTTTT TCTCAACCTC TCGACTAAGG GGTACGTAGG AGATTCGTTT CCACCACCTG CCGTCTTTTA   
  
  
- ATCATCTCAA TAACACTCTA ATTTCAAACG CGTGGACTTG CCGACTGGAG ATCAAACGAT AGTTGCAATG   
  
  
- TAAATACACA GTATTAAAAA CGTACTTTGT TAAAGTGCGT TACAAAAGAC TACCGTCAAT ACAAATCTTT   
  
  
- ATTCAAATTA GCCTGGAATA AGGTGACTAA ATATAAACTA AGAAGGGAGA AATGATGCCG ACAGGTAAGT   
  
  
- GTTCGGTTTC TTAAGAGGGG GGTTTTTTTT CTTTTTTGTC ATATCTTGGT ACAAATAATA GAGTAAAAAA   
  
  
- AGAGAGGGTA AACTAGACTT TGTGTTTGAA CGTCCCCACG TTCAACCTGT GAATCATCGA CGTCCTTACT   
  
  
- AAGTTATCCT TAGGAGTAGA AACTGTTGTA GTGGTGAACT TATGTATAGT GAACTACCGT GAGAGGGGAC   
  
  
- CAAATCCGAA GGGTATAGGC ACCGAATCCC TCGATTTTGG ACTCGTTTCT CCTGAGACAG ATTAGGTAGA   
  
  
- GGAGGAAAGT ACACGATTAG TTCAACGATG ACCCTCGTAG CTGTTACGAT TACAGTCGGA ACTCGTATAA   
  
  
- AGGGTGGAAC GGAGAGGGTG GCCTCTATGT TACGTCGCTT AACGACGAAT GAAACGACTT CGGAAACGAC   
  
  
- TGGCAAATGA TTCCCGAACC GTCGGGCCAG AAGAGTTTCG GAACTTAACA TGGTTCTACA GTAGACAAAG   
  
  
- GCTTGTTTAA GAACAAGTTT TCAACAAGAA ACTAGAAGAA GGCAAGAACT TCGAAAGTAT AGAACACTGC   
  
  
- TTGGTCCGTT AGTGCCTCCG GTATCTTCCT CTTTTCTACC AAGTATATTA ACTAGAGGTA AGAACACTTA   
  
  
- GCCGAGACAC CTAATCAGAG GAGGTCCGTA ACTCACAAGC TGGACTTCCG GGTGGGGTAA ACTCCTATTG   
  
  
- GCCATATGTA CTCTTCTTTC TTCACAACCT AGTTTACCGA TACGTTGATT TGTTTCTCCG ACTTTTTAAC   
  
  
- CTGTAGGGTA AAGTTAAGTT AGGATAGCGT TCGTTTGATC TGCTGGAACT ACAGCTTTCG AACTCACAGT   
  
  
- TCTGGCCTCT TCGTGAACAA TAATCAAGAC ACGAGGTTGA TGTAAGAGAA AACCGTAACC TACTCCCTAG   
  
  
- TTACGGATTC TTGCGTCCGT ACCGAATGAA CGTTTCCCAC AAAATATACT TTGGTGCTTT TAACCTCCTG   
  
  
- AACGGGTTGT TCCTAAACTA CTTTTACAAC TTGAGTTTAC TTCTAAGATG TAGTAGTAGT AGAAGTAGAG   
  
  
- GATAGGGAAG TAGTTTTGAA CTACGGAAAA ATTTTCGGGA GGAACCCGAA AGCGGTTTTG AGTACCAACA   
  
  
- TTGGCTCGTT CTTAGTTTGG TGTTACCTTC ACGGGATTAT CTTTCTCACT ACCTCAGTAA CTTGAAGATA   
  
  
- CGTCGTAACA AACTAACGAA CCTTAGGTGA TATAGCTCCT GTAGCTATCT CTCTGTCTTC GAGCTCTTCA   
  
  
- ACTACAAACC TCTCCTCTAG TTCTTGGAGT GTCGAACACT TCCCCGTCTC TCTTTCCGTT CCGTGCTCTT   
  
  
- CGAGTCACTT ACCCAAGTTT CTAAACTCAG TCATCCTAAA TTTTCCCTCG GTAACTCGAT GGTACCAAAG   
  
  
- AACGAACGAT CCTCCAAAAA TGTATGGTTA TTAATACTCC CCATATTGTA GTTCCTTCAG TTACCAAAAG   
  
  
- AACAATAGAC AACCGTTCTA TCTGGGGACA TAAGACAAAG TCGAACCTCT AAAAT

  
  
Motifs Found  

+   

| Site Name | Organism | Position | Strand | Matrix score. | sequence | function |
| --- | --- | --- | --- | --- | --- | --- |
|  | organism | 3093 | - | 4 | motif\_sequence | short\_function |
|  | organism | 1475 | - | 4 | motif\_sequence | short\_function |
|  | organism | 2114 | + | 4 | motif\_sequence | short\_function |
|  | organism | 2025 | + | 4 | motif\_sequence | short\_function |
|  | organism | 1617 | - | 4 | motif\_sequence | short\_function |
|  | organism | 1896 | + | 4 | motif\_sequence | short\_function |
|  | organism | 744 | + | 4 | motif\_sequence | short\_function |
|  | organism | 1814 | + | 4 | motif\_sequence | short\_function |
|  | organism | 433 | - | 4 | motif\_sequence | short\_function |
|  | organism | 3340 | - | 4 | motif\_sequence | short\_function |
|  | organism | 2101 | + | 4 | motif\_sequence | short\_function |
|  | organism | 1561 | - | 4 | motif\_sequence | short\_function |
|  | organism | 678 | + | 4 | motif\_sequence | short\_function |
|  | organism | 3129 | - | 4 | motif\_sequence | short\_function |
|  | organism | 2594 | - | 4 | motif\_sequence | short\_function |
|  | organism | 225 | + | 4 | motif\_sequence | short\_function |
|  | organism | 274 | + | 4 | motif\_sequence | short\_function |
|  | organism | 2692 | + | 4 | motif\_sequence | short\_function |
|  | organism | 750 | + | 4 | motif\_sequence | short\_function |
|  | organism | 2196 | - | 4 | motif\_sequence | short\_function |
|  | organism | 2870 | + | 4 | motif\_sequence | short\_function |
|  | organism | 3052 | - | 4 | motif\_sequence | short\_function |
|  | organism | 2186 | + | 4 | motif\_sequence | short\_function |
|  | organism | 2844 | - | 4 | motif\_sequence | short\_function |
|  | organism | 2437 | + | 4 | motif\_sequence | short\_function |
|  | organism | 3098 | - | 4 | motif\_sequence | short\_function |
|  | organism | 2864 | + | 4 | motif\_sequence | short\_function |
|  | organism | 2347 | + | 4 | motif\_sequence | short\_function |

>HU05G01267.1   
+ +Up\_Stream \_Len000TTACAA AAACGATGCT CTAAACCTAT TAGGTCGTTC CGAAACCCGC AAAATTGAAA   
  
  
+ TATGATCGCC CGTATTTTAC CCAAACCTGA AATACACTTA ATATGTATTT TACCTGAACC CAAATACACC   
  
  
+ GGACCTGTAT TTAACACGGG CACCCGAAAT GATAGGTCTA ACTCTCACCA CCACCGCATT TGGCACCCGA   
  
  
+ ACGGTAAAAA ATGTTCATCT ATTATGCACT TTGGCTTCAT CCATAACCCT ATGATCTATC TGTGCCAATA   
  
  
+ TTTGGAGCAC CCAGCGAAGT AGCGCATGCT CTAAACTCTT GCCTCAAAAC AAAACAAATA GTGGGTCATA   
  
  
+ TTCCCTTCAA AGCACTAACA AACACACACT CTCTCTCTCT CAGTAGCCAA CAACTTAAGG CGGTGTTTGC   
  
  
+ TTGGTTTTGG AGAGGGCGTG TTTGGATGAG GTGCAAAACA CATAGATGAA ATCATAAACA ACTTGATTCC   
  
  
+ CAGCCACCCA TGACCTACTC AGATAACAAG GGACAAAAAC AAAGAGGTTT TCTGAAACAG AGAAAACAGG   
  
  
+ GGCGTTGTGT TGCGCTGTTG CTCACAAAAT TCCAATATTC CTCTAAAAGC TCCCAAACGG AGACAAATCA   
  
  
+ AAGAGGACAA AGACATGTGG GATTTAATAC AAAATTTGAC TAACTCTGCA ACGGAAAACA ACCCCTAGGG   
  
  
+ TGTTTTTCTG CGTGTTGTAC ACACTTCACT TTTAACCTGC TCTGCCTCTG CCTTTGCCAC GGCTTTTAAT   
  
  
+ GGATAAGACC AATTAAGAAA CCCAAATATC TCGTAAATTT GTTGAGATTC CTTTCTGCGT TCTCAAGTGT   
  
  
+ TTCAAAAACC CAGTTTGGAG GTTGCACTTA TTCATGCTTT TCTTCAAGTC AGTGAGCTTA GTGTCTGGAA   
  
  
+ CTGAAGCTCC TCCTGGTAAT GCTCTGTTTT TCATTTCTTG TTGTGCGTTT ATAAAAATTC GGCCTATTGA   
  
  
+ TGGAAGTTTG TTTGATGTTT TTGGGTAAGT TTTTGATTAA TAGAGATGAA TTAGTAAAGT AAAATTTGAG   
  
  
+ TGGTATTTAA TGATCCTTAA GCAGTCTATG CTTTGTTGTC ACATGCGTTT CTTGGCTTTT GACTGAAACA   
  
  
+ TTTCTGTGTT AGATGAAAAA CTCATTTTGA TGCATGAGAT GATAAAATCG CTTATTTTTG CCTTTATTTG   
  
  
+ GTGTGTGGGG GGGGGGGGGG GGGGGCGCAG GTCTGAGGTA AAAGATATGT GAATTCATTT GGAGTCTCGA   
  
  
+ TAATTCGTTG GCTATGTTGA CAATCACGAG TGGTCGTTAT CGCTCATCTC ACAGGTTGAT AATTCACTCA   
  
  
+ AACTTCAAGC GGCCTGAATC ATTCAGTCCG GGTTGTTAAA ATCTCAACAG TCATTTCACA CGAGTGGAGG   
  
  
+ GCCCCAAAAG ACACCATTCT TTCGAACTTT TTTTTTTTGA AGGTCATTTT TTCAAGAACA AAACTAATGT   
  
  
+ GAACATGCGA CTTCTATGAG TAAAGTGTAA AGATGCATAA AGTCTTTTTT TCTTTTATAT AAAAAGTACT   
  
  
+ CAAAAAAAAA AGAGTTGGAG AGCTGATTCC CCATGCATCC TCTAAGCAAA GGTGGTGGAC GGCAGAAAAT   
  
  
+ TAGTAGAGTT ATTGTGAGAT TAAAGTTTGC GCACCTGAAC GGCTGACCTC TAGTTTGCTA TCAACGTTAC   
  
  
+ ATTTATGTGT CATAATTTTT GCATGAAACA ATTTCACGCA ATGTTTTCTG ATGGCAGTTA TGTTTAGAAA   
  
  
+ TAAGTTTAAT CGGACCTTAT TCCACTGATT TATATTTGAT TCTTCCCTCT TTACTACGGC TGTCCATTCA   
  
  
+ CAAGCCAAAG AATTCTCCCC CCAAAAAAAA GAAAAAACAG TATAGAACCA TGTTTATTAT CTCATTTTTT   
  
  
+ TCTCTCCCAT TTGATCTGAA ACACAAACTT GCAGGGGTGC AAGTTGGACA CTTAGTAGCT GCAGGAATGA   
  
  
+ TTCAATAGGA ATCCTCATCT TTGACAACAT CACCACTTGA ATACATATCA CTTGATGGCA CTCTCCCCTG   
  
  
+ GTTTAGGCTT CCCATATCCG TGGCTTAGGG AGCTAAAACC TGAGCAAAGA GGACTCTGTC TAATCCATCT   
  
  
+ CCTCCTTTCA TGTGCTAATC AAGTTGCTAC TGGGAGCATC GACAATGCTA ATGTCAGCCT TGAGCATATT   
  
  
+ TCCCACCTTG CCTCTCCCAC CGGAGATACA ATGCAGCGAA TTGCTGCTTA CTTTGCTGAA GCCTTTGCTG   
  
  
+ ACCGTTTACT AAGGGCTTGG CAGCCCGGTC TTCTCAAAGC CTTGAATTGT ACCAAGATGT CATCTGTTTC   
  
  
+ CGAACAAATT CTTGTTCAAA AGTTGTTCTT TGATCTTCTT CCGTTCTTGA AGCTTTCATA TCTTGTGACG   
  
  
+ AACCAGGCAA TCACGGAGGC CATAGAAGGA GAAAAGATGG TTCATATAAT TGATCTCCAT TCTTGTGAAT   
  
  
+ CGGCTCTGTG GATTAGTCTC CTCCAGGCAT TGAGTGTTCG ACCTGAAGGC CCACCCCATT TGAGGATAAC   
  
  
+ CGGTATACAT GAGAAGAAAG AAGTGTTGGA TCAAATGGCT ATGCAACTAA ACAAAGAGGC TGAAAAATTG   
  
  
+ GACATCCCAT TTCAATTCAA TCCTATCGCA AGCAAACTAG ACGACCTTGA TGTCGAAAGC TTGAGTGTCA   
  
  
+ AGACCGGAGA AGCACTTGTT ATTAGTTCTG TGCTCCAACT ACATTCTCTT TTGGCATTGG ATGAGGGATC   
  
  
+ AATGCCTAAG AACGCAGGCA TGGCTTACTT GCAAAGGGTG TTTTATATGA AACCACGAAA ATTGGAGGAC   
  
  
+ TTGCCCAACA AGGATTTGAT GAAAATGTTG AACTCAAATG AAGATTCTAC ATCATCATCA TCTTCATCTC   
  
  
+ CTATCCCTTC ATCAAAACTT GATGCCTTTT TAAAAGCCCT CCTTGGGCTT TCGCCAAAAC TCATGGTTGT   
  
  
+ AACCGAGCAA GAATCAAACC ACAATGGAAG TGCCCTAATA GAAAGAGTGA TGGAGTCATT GAACTTCTAT   
  
  
+ GCAGCATTGT TTGATTGCTT GGAATCCACT ATATCGAGGA CATCGATAGA GAGACAGAAG CTCGAGAAGT   
  
  
+ TGATGTTTGG AGAGGAGATC AAGAACCTCA CAGCTTGTGA AGGGGCAGAG AGAAAGGCAA GGCACGAGAA   
  
  
+ GCTCAGTGAA TGGGTTCAAA GATTTGAGTC AGTAGGATTT AAAAGGGAGC CATTGAGCTA CCATGGTTTC   
  
  
+ TTGCTTGCTA GGAGGTTTTT ACATACCAAT AATTATGAGG GGTATAACAT CAAGGAAGTC AATGGTTTTC   
  
  
+ TTGTTATCTG TTGGCAAGAT AGACCCCTGT ATTCTGTTTC AGCTTGGAGA TTTTA  

- +Up\_Stream \_Len000AATGTT TTTGCTACGA GATTTGGATA ATCCAGCAAG GCTTTGGGCG TTTTAACTTT   
  
  
- ATACTAGCGG GCATAAAATG GGTTTGGACT TTATGTGAAT TATACATAAA ATGGACTTGG GTTTATGTGG   
  
  
- CCTGGACATA AATTGTGCCC GTGGGCTTTA CTATCCAGAT TGAGAGTGGT GGTGGCGTAA ACCGTGGGCT   
  
  
- TGCCATTTTT TACAAGTAGA TAATACGTGA AACCGAAGTA GGTATTGGGA TACTAGATAG ACACGGTTAT   
  
  
- AAACCTCGTG GGTCGCTTCA TCGCGTACGA GATTTGAGAA CGGAGTTTTG TTTTGTTTAT CACCCAGTAT   
  
  
- AAGGGAAGTT TCGTGATTGT TTGTGTGTGA GAGAGAGAGA GTCATCGGTT GTTGAATTCC GCCACAAACG   
  
  
- AACCAAAACC TCTCCCGCAC AAACCTACTC CACGTTTTGT GTATCTACTT TAGTATTTGT TGAACTAAGG   
  
  
- GTCGGTGGGT ACTGGATGAG TCTATTGTTC CCTGTTTTTG TTTCTCCAAA AGACTTTGTC TCTTTTGTCC   
  
  
- CCGCAACACA ACGCGACAAC GAGTGTTTTA AGGTTATAAG GAGATTTTCG AGGGTTTGCC TCTGTTTAGT   
  
  
- TTCTCCTGTT TCTGTACACC CTAAATTATG TTTTAAACTG ATTGAGACGT TGCCTTTTGT TGGGGATCCC   
  
  
- ACAAAAAGAC GCACAACATG TGTGAAGTGA AAATTGGACG AGACGGAGAC GGAAACGGTG CCGAAAATTA   
  
  
- CCTATTCTGG TTAATTCTTT GGGTTTATAG AGCATTTAAA CAACTCTAAG GAAAGACGCA AGAGTTCACA   
  
  
- AAGTTTTTGG GTCAAACCTC CAACGTGAAT AAGTACGAAA AGAAGTTCAG TCACTCGAAT CACAGACCTT   
  
  
- GACTTCGAGG AGGACCATTA CGAGACAAAA AGTAAAGAAC AACACGCAAA TATTTTTAAG CCGGATAACT   
  
  
- ACCTTCAAAC AAACTACAAA AACCCATTCA AAAACTAATT ATCTCTACTT AATCATTTCA TTTTAAACTC   
  
  
- ACCATAAATT ACTAGGAATT CGTCAGATAC GAAACAACAG TGTACGCAAA GAACCGAAAA CTGACTTTGT   
  
  
- AAAGACACAA TCTACTTTTT GAGTAAAACT ACGTACTCTA CTATTTTAGC GAATAAAAAC GGAAATAAAC   
  
  
- CACACACCCC CCCCCCCCCC CCCCCGCGTC CAGACTCCAT TTTCTATACA CTTAAGTAAA CCTCAGAGCT   
  
  
- ATTAAGCAAC CGATACAACT GTTAGTGCTC ACCAGCAATA GCGAGTAGAG TGTCCAACTA TTAAGTGAGT   
  
  
- TTGAAGTTCG CCGGACTTAG TAAGTCAGGC CCAACAATTT TAGAGTTGTC AGTAAAGTGT GCTCACCTCC   
  
  
- CGGGGTTTTC TGTGGTAAGA AAGCTTGAAA AAAAAAAACT TCCAGTAAAA AAGTTCTTGT TTTGATTACA   
  
  
- CTTGTACGCT GAAGATACTC ATTTCACATT TCTACGTATT TCAGAAAAAA AGAAAATATA TTTTTCATGA   
  
  
- GTTTTTTTTT TCTCAACCTC TCGACTAAGG GGTACGTAGG AGATTCGTTT CCACCACCTG CCGTCTTTTA   
  
  
- ATCATCTCAA TAACACTCTA ATTTCAAACG CGTGGACTTG CCGACTGGAG ATCAAACGAT AGTTGCAATG   
  
  
- TAAATACACA GTATTAAAAA CGTACTTTGT TAAAGTGCGT TACAAAAGAC TACCGTCAAT ACAAATCTTT   
  
  
- ATTCAAATTA GCCTGGAATA AGGTGACTAA ATATAAACTA AGAAGGGAGA AATGATGCCG ACAGGTAAGT   
  
  
- GTTCGGTTTC TTAAGAGGGG GGTTTTTTTT CTTTTTTGTC ATATCTTGGT ACAAATAATA GAGTAAAAAA   
  
  
- AGAGAGGGTA AACTAGACTT TGTGTTTGAA CGTCCCCACG TTCAACCTGT GAATCATCGA CGTCCTTACT   
  
  
- AAGTTATCCT TAGGAGTAGA AACTGTTGTA GTGGTGAACT TATGTATAGT GAACTACCGT GAGAGGGGAC   
  
  
- CAAATCCGAA GGGTATAGGC ACCGAATCCC TCGATTTTGG ACTCGTTTCT CCTGAGACAG ATTAGGTAGA   
  
  
- GGAGGAAAGT ACACGATTAG TTCAACGATG ACCCTCGTAG CTGTTACGAT TACAGTCGGA ACTCGTATAA   
  
  
- AGGGTGGAAC GGAGAGGGTG GCCTCTATGT TACGTCGCTT AACGACGAAT GAAACGACTT CGGAAACGAC   
  
  
- TGGCAAATGA TTCCCGAACC GTCGGGCCAG AAGAGTTTCG GAACTTAACA TGGTTCTACA GTAGACAAAG   
  
  
- GCTTGTTTAA GAACAAGTTT TCAACAAGAA ACTAGAAGAA GGCAAGAACT TCGAAAGTAT AGAACACTGC   
  
  
- TTGGTCCGTT AGTGCCTCCG GTATCTTCCT CTTTTCTACC AAGTATATTA ACTAGAGGTA AGAACACTTA   
  
  
- GCCGAGACAC CTAATCAGAG GAGGTCCGTA ACTCACAAGC TGGACTTCCG GGTGGGGTAA ACTCCTATTG   
  
  
- GCCATATGTA CTCTTCTTTC TTCACAACCT AGTTTACCGA TACGTTGATT TGTTTCTCCG ACTTTTTAAC   
  
  
- CTGTAGGGTA AAGTTAAGTT AGGATAGCGT TCGTTTGATC TGCTGGAACT ACAGCTTTCG AACTCACAGT   
  
  
- TCTGGCCTCT TCGTGAACAA TAATCAAGAC ACGAGGTTGA TGTAAGAGAA AACCGTAACC TACTCCCTAG   
  
  
- TTACGGATTC TTGCGTCCGT ACCGAATGAA CGTTTCCCAC AAAATATACT TTGGTGCTTT TAACCTCCTG   
  
  
- AACGGGTTGT TCCTAAACTA CTTTTACAAC TTGAGTTTAC TTCTAAGATG TAGTAGTAGT AGAAGTAGAG   
  
  
- GATAGGGAAG TAGTTTTGAA CTACGGAAAA ATTTTCGGGA GGAACCCGAA AGCGGTTTTG AGTACCAACA   
  
  
- TTGGCTCGTT CTTAGTTTGG TGTTACCTTC ACGGGATTAT CTTTCTCACT ACCTCAGTAA CTTGAAGATA   
  
  
- CGTCGTAACA AACTAACGAA CCTTAGGTGA TATAGCTCCT GTAGCTATCT CTCTGTCTTC GAGCTCTTCA   
  
  
- ACTACAAACC TCTCCTCTAG TTCTTGGAGT GTCGAACACT TCCCCGTCTC TCTTTCCGTT CCGTGCTCTT   
  
  
- CGAGTCACTT ACCCAAGTTT CTAAACTCAG TCATCCTAAA TTTTCCCTCG GTAACTCGAT GGTACCAAAG   
  
  
- AACGAACGAT CCTCCAAAAA TGTATGGTTA TTAATACTCC CCATATTGTA GTTCCTTCAG TTACCAAAAG   
  
  
- AACAATAGAC AACCGTTCTA TCTGGGGACA TAAGACAAAG TCGAACCTCT AAAATC

+     A-box

| Site Name | Organism | Position | Strand | Matrix score. | sequence | function |
| --- | --- | --- | --- | --- | --- | --- |
| A-box | Petroselinum crispum | 1601 | - | 6 | CCGTCC | cis-acting regulatory element |

>HU05G01267.1   
+ +Up\_Stream \_Len000TTACAA AAACGATGCT CTAAACCTAT TAGGTCGTTC CGAAACCCGC AAAATTGAAA   
  
  
+ TATGATCGCC CGTATTTTAC CCAAACCTGA AATACACTTA ATATGTATTT TACCTGAACC CAAATACACC   
  
  
+ GGACCTGTAT TTAACACGGG CACCCGAAAT GATAGGTCTA ACTCTCACCA CCACCGCATT TGGCACCCGA   
  
  
+ ACGGTAAAAA ATGTTCATCT ATTATGCACT TTGGCTTCAT CCATAACCCT ATGATCTATC TGTGCCAATA   
  
  
+ TTTGGAGCAC CCAGCGAAGT AGCGCATGCT CTAAACTCTT GCCTCAAAAC AAAACAAATA GTGGGTCATA   
  
  
+ TTCCCTTCAA AGCACTAACA AACACACACT CTCTCTCTCT CAGTAGCCAA CAACTTAAGG CGGTGTTTGC   
  
  
+ TTGGTTTTGG AGAGGGCGTG TTTGGATGAG GTGCAAAACA CATAGATGAA ATCATAAACA ACTTGATTCC   
  
  
+ CAGCCACCCA TGACCTACTC AGATAACAAG GGACAAAAAC AAAGAGGTTT TCTGAAACAG AGAAAACAGG   
  
  
+ GGCGTTGTGT TGCGCTGTTG CTCACAAAAT TCCAATATTC CTCTAAAAGC TCCCAAACGG AGACAAATCA   
  
  
+ AAGAGGACAA AGACATGTGG GATTTAATAC AAAATTTGAC TAACTCTGCA ACGGAAAACA ACCCCTAGGG   
  
  
+ TGTTTTTCTG CGTGTTGTAC ACACTTCACT TTTAACCTGC TCTGCCTCTG CCTTTGCCAC GGCTTTTAAT   
  
  
+ GGATAAGACC AATTAAGAAA CCCAAATATC TCGTAAATTT GTTGAGATTC CTTTCTGCGT TCTCAAGTGT   
  
  
+ TTCAAAAACC CAGTTTGGAG GTTGCACTTA TTCATGCTTT TCTTCAAGTC AGTGAGCTTA GTGTCTGGAA   
  
  
+ CTGAAGCTCC TCCTGGTAAT GCTCTGTTTT TCATTTCTTG TTGTGCGTTT ATAAAAATTC GGCCTATTGA   
  
  
+ TGGAAGTTTG TTTGATGTTT TTGGGTAAGT TTTTGATTAA TAGAGATGAA TTAGTAAAGT AAAATTTGAG   
  
  
+ TGGTATTTAA TGATCCTTAA GCAGTCTATG CTTTGTTGTC ACATGCGTTT CTTGGCTTTT GACTGAAACA   
  
  
+ TTTCTGTGTT AGATGAAAAA CTCATTTTGA TGCATGAGAT GATAAAATCG CTTATTTTTG CCTTTATTTG   
  
  
+ GTGTGTGGGG GGGGGGGGGG GGGGGCGCAG GTCTGAGGTA AAAGATATGT GAATTCATTT GGAGTCTCGA   
  
  
+ TAATTCGTTG GCTATGTTGA CAATCACGAG TGGTCGTTAT CGCTCATCTC ACAGGTTGAT AATTCACTCA   
  
  
+ AACTTCAAGC GGCCTGAATC ATTCAGTCCG GGTTGTTAAA ATCTCAACAG TCATTTCACA CGAGTGGAGG   
  
  
+ GCCCCAAAAG ACACCATTCT TTCGAACTTT TTTTTTTTGA AGGTCATTTT TTCAAGAACA AAACTAATGT   
  
  
+ GAACATGCGA CTTCTATGAG TAAAGTGTAA AGATGCATAA AGTCTTTTTT TCTTTTATAT AAAAAGTACT   
  
  
+ CAAAAAAAAA AGAGTTGGAG AGCTGATTCC CCATGCATCC TCTAAGCAAA GGTGGTGGAC GGCAGAAAAT   
  
  
+ TAGTAGAGTT ATTGTGAGAT TAAAGTTTGC GCACCTGAAC GGCTGACCTC TAGTTTGCTA TCAACGTTAC   
  
  
+ ATTTATGTGT CATAATTTTT GCATGAAACA ATTTCACGCA ATGTTTTCTG ATGGCAGTTA TGTTTAGAAA   
  
  
+ TAAGTTTAAT CGGACCTTAT TCCACTGATT TATATTTGAT TCTTCCCTCT TTACTACGGC TGTCCATTCA   
  
  
+ CAAGCCAAAG AATTCTCCCC CCAAAAAAAA GAAAAAACAG TATAGAACCA TGTTTATTAT CTCATTTTTT   
  
  
+ TCTCTCCCAT TTGATCTGAA ACACAAACTT GCAGGGGTGC AAGTTGGACA CTTAGTAGCT GCAGGAATGA   
  
  
+ TTCAATAGGA ATCCTCATCT TTGACAACAT CACCACTTGA ATACATATCA CTTGATGGCA CTCTCCCCTG   
  
  
+ GTTTAGGCTT CCCATATCCG TGGCTTAGGG AGCTAAAACC TGAGCAAAGA GGACTCTGTC TAATCCATCT   
  
  
+ CCTCCTTTCA TGTGCTAATC AAGTTGCTAC TGGGAGCATC GACAATGCTA ATGTCAGCCT TGAGCATATT   
  
  
+ TCCCACCTTG CCTCTCCCAC CGGAGATACA ATGCAGCGAA TTGCTGCTTA CTTTGCTGAA GCCTTTGCTG   
  
  
+ ACCGTTTACT AAGGGCTTGG CAGCCCGGTC TTCTCAAAGC CTTGAATTGT ACCAAGATGT CATCTGTTTC   
  
  
+ CGAACAAATT CTTGTTCAAA AGTTGTTCTT TGATCTTCTT CCGTTCTTGA AGCTTTCATA TCTTGTGACG   
  
  
+ AACCAGGCAA TCACGGAGGC CATAGAAGGA GAAAAGATGG TTCATATAAT TGATCTCCAT TCTTGTGAAT   
  
  
+ CGGCTCTGTG GATTAGTCTC CTCCAGGCAT TGAGTGTTCG ACCTGAAGGC CCACCCCATT TGAGGATAAC   
  
  
+ CGGTATACAT GAGAAGAAAG AAGTGTTGGA TCAAATGGCT ATGCAACTAA ACAAAGAGGC TGAAAAATTG   
  
  
+ GACATCCCAT TTCAATTCAA TCCTATCGCA AGCAAACTAG ACGACCTTGA TGTCGAAAGC TTGAGTGTCA   
  
  
+ AGACCGGAGA AGCACTTGTT ATTAGTTCTG TGCTCCAACT ACATTCTCTT TTGGCATTGG ATGAGGGATC   
  
  
+ AATGCCTAAG AACGCAGGCA TGGCTTACTT GCAAAGGGTG TTTTATATGA AACCACGAAA ATTGGAGGAC   
  
  
+ TTGCCCAACA AGGATTTGAT GAAAATGTTG AACTCAAATG AAGATTCTAC ATCATCATCA TCTTCATCTC   
  
  
+ CTATCCCTTC ATCAAAACTT GATGCCTTTT TAAAAGCCCT CCTTGGGCTT TCGCCAAAAC TCATGGTTGT   
  
  
+ AACCGAGCAA GAATCAAACC ACAATGGAAG TGCCCTAATA GAAAGAGTGA TGGAGTCATT GAACTTCTAT   
  
  
+ GCAGCATTGT TTGATTGCTT GGAATCCACT ATATCGAGGA CATCGATAGA GAGACAGAAG CTCGAGAAGT   
  
  
+ TGATGTTTGG AGAGGAGATC AAGAACCTCA CAGCTTGTGA AGGGGCAGAG AGAAAGGCAA GGCACGAGAA   
  
  
+ GCTCAGTGAA TGGGTTCAAA GATTTGAGTC AGTAGGATTT AAAAGGGAGC CATTGAGCTA CCATGGTTTC   
  
  
+ TTGCTTGCTA GGAGGTTTTT ACATACCAAT AATTATGAGG GGTATAACAT CAAGGAAGTC AATGGTTTTC   
  
  
+ TTGTTATCTG TTGGCAAGAT AGACCCCTGT ATTCTGTTTC AGCTTGGAGA TTTTA  

- +Up\_Stream \_Len000AATGTT TTTGCTACGA GATTTGGATA ATCCAGCAAG GCTTTGGGCG TTTTAACTTT   
  
  
- ATACTAGCGG GCATAAAATG GGTTTGGACT TTATGTGAAT TATACATAAA ATGGACTTGG GTTTATGTGG   
  
  
- CCTGGACATA AATTGTGCCC GTGGGCTTTA CTATCCAGAT TGAGAGTGGT GGTGGCGTAA ACCGTGGGCT   
  
  
- TGCCATTTTT TACAAGTAGA TAATACGTGA AACCGAAGTA GGTATTGGGA TACTAGATAG ACACGGTTAT   
  
  
- AAACCTCGTG GGTCGCTTCA TCGCGTACGA GATTTGAGAA CGGAGTTTTG TTTTGTTTAT CACCCAGTAT   
  
  
- AAGGGAAGTT TCGTGATTGT TTGTGTGTGA GAGAGAGAGA GTCATCGGTT GTTGAATTCC GCCACAAACG   
  
  
- AACCAAAACC TCTCCCGCAC AAACCTACTC CACGTTTTGT GTATCTACTT TAGTATTTGT TGAACTAAGG   
  
  
- GTCGGTGGGT ACTGGATGAG TCTATTGTTC CCTGTTTTTG TTTCTCCAAA AGACTTTGTC TCTTTTGTCC   
  
  
- CCGCAACACA ACGCGACAAC GAGTGTTTTA AGGTTATAAG GAGATTTTCG AGGGTTTGCC TCTGTTTAGT   
  
  
- TTCTCCTGTT TCTGTACACC CTAAATTATG TTTTAAACTG ATTGAGACGT TGCCTTTTGT TGGGGATCCC   
  
  
- ACAAAAAGAC GCACAACATG TGTGAAGTGA AAATTGGACG AGACGGAGAC GGAAACGGTG CCGAAAATTA   
  
  
- CCTATTCTGG TTAATTCTTT GGGTTTATAG AGCATTTAAA CAACTCTAAG GAAAGACGCA AGAGTTCACA   
  
  
- AAGTTTTTGG GTCAAACCTC CAACGTGAAT AAGTACGAAA AGAAGTTCAG TCACTCGAAT CACAGACCTT   
  
  
- GACTTCGAGG AGGACCATTA CGAGACAAAA AGTAAAGAAC AACACGCAAA TATTTTTAAG CCGGATAACT   
  
  
- ACCTTCAAAC AAACTACAAA AACCCATTCA AAAACTAATT ATCTCTACTT AATCATTTCA TTTTAAACTC   
  
  
- ACCATAAATT ACTAGGAATT CGTCAGATAC GAAACAACAG TGTACGCAAA GAACCGAAAA CTGACTTTGT   
  
  
- AAAGACACAA TCTACTTTTT GAGTAAAACT ACGTACTCTA CTATTTTAGC GAATAAAAAC GGAAATAAAC   
  
  
- CACACACCCC CCCCCCCCCC CCCCCGCGTC CAGACTCCAT TTTCTATACA CTTAAGTAAA CCTCAGAGCT   
  
  
- ATTAAGCAAC CGATACAACT GTTAGTGCTC ACCAGCAATA GCGAGTAGAG TGTCCAACTA TTAAGTGAGT   
  
  
- TTGAAGTTCG CCGGACTTAG TAAGTCAGGC CCAACAATTT TAGAGTTGTC AGTAAAGTGT GCTCACCTCC   
  
  
- CGGGGTTTTC TGTGGTAAGA AAGCTTGAAA AAAAAAAACT TCCAGTAAAA AAGTTCTTGT TTTGATTACA   
  
  
- CTTGTACGCT GAAGATACTC ATTTCACATT TCTACGTATT TCAGAAAAAA AGAAAATATA TTTTTCATGA   
  
  
- GTTTTTTTTT TCTCAACCTC TCGACTAAGG GGTACGTAGG AGATTCGTTT CCACCACCTG CCGTCTTTTA   
  
  
- ATCATCTCAA TAACACTCTA ATTTCAAACG CGTGGACTTG CCGACTGGAG ATCAAACGAT AGTTGCAATG   
  
  
- TAAATACACA GTATTAAAAA CGTACTTTGT TAAAGTGCGT TACAAAAGAC TACCGTCAAT ACAAATCTTT   
  
  
- ATTCAAATTA GCCTGGAATA AGGTGACTAA ATATAAACTA AGAAGGGAGA AATGATGCCG ACAGGTAAGT   
  
  
- GTTCGGTTTC TTAAGAGGGG GGTTTTTTTT CTTTTTTGTC ATATCTTGGT ACAAATAATA GAGTAAAAAA   
  
  
- AGAGAGGGTA AACTAGACTT TGTGTTTGAA CGTCCCCACG TTCAACCTGT GAATCATCGA CGTCCTTACT   
  
  
- AAGTTATCCT TAGGAGTAGA AACTGTTGTA GTGGTGAACT TATGTATAGT GAACTACCGT GAGAGGGGAC   
  
  
- CAAATCCGAA GGGTATAGGC ACCGAATCCC TCGATTTTGG ACTCGTTTCT CCTGAGACAG ATTAGGTAGA   
  
  
- GGAGGAAAGT ACACGATTAG TTCAACGATG ACCCTCGTAG CTGTTACGAT TACAGTCGGA ACTCGTATAA   
  
  
- AGGGTGGAAC GGAGAGGGTG GCCTCTATGT TACGTCGCTT AACGACGAAT GAAACGACTT CGGAAACGAC   
  
  
- TGGCAAATGA TTCCCGAACC GTCGGGCCAG AAGAGTTTCG GAACTTAACA TGGTTCTACA GTAGACAAAG   
  
  
- GCTTGTTTAA GAACAAGTTT TCAACAAGAA ACTAGAAGAA GGCAAGAACT TCGAAAGTAT AGAACACTGC   
  
  
- TTGGTCCGTT AGTGCCTCCG GTATCTTCCT CTTTTCTACC AAGTATATTA ACTAGAGGTA AGAACACTTA   
  
  
- GCCGAGACAC CTAATCAGAG GAGGTCCGTA ACTCACAAGC TGGACTTCCG GGTGGGGTAA ACTCCTATTG   
  
  
- GCCATATGTA CTCTTCTTTC TTCACAACCT AGTTTACCGA TACGTTGATT TGTTTCTCCG ACTTTTTAAC   
  
  
- CTGTAGGGTA AAGTTAAGTT AGGATAGCGT TCGTTTGATC TGCTGGAACT ACAGCTTTCG AACTCACAGT   
  
  
- TCTGGCCTCT TCGTGAACAA TAATCAAGAC ACGAGGTTGA TGTAAGAGAA AACCGTAACC TACTCCCTAG   
  
  
- TTACGGATTC TTGCGTCCGT ACCGAATGAA CGTTTCCCAC AAAATATACT TTGGTGCTTT TAACCTCCTG   
  
  
- AACGGGTTGT TCCTAAACTA CTTTTACAAC TTGAGTTTAC TTCTAAGATG TAGTAGTAGT AGAAGTAGAG   
  
  
- GATAGGGAAG TAGTTTTGAA CTACGGAAAA ATTTTCGGGA GGAACCCGAA AGCGGTTTTG AGTACCAACA   
  
  
- TTGGCTCGTT CTTAGTTTGG TGTTACCTTC ACGGGATTAT CTTTCTCACT ACCTCAGTAA CTTGAAGATA   
  
  
- CGTCGTAACA AACTAACGAA CCTTAGGTGA TATAGCTCCT GTAGCTATCT CTCTGTCTTC GAGCTCTTCA   
  
  
- ACTACAAACC TCTCCTCTAG TTCTTGGAGT GTCGAACACT TCCCCGTCTC TCTTTCCGTT CCGTGCTCTT   
  
  
- CGAGTCACTT ACCCAAGTTT CTAAACTCAG TCATCCTAAA TTTTCCCTCG GTAACTCGAT GGTACCAAAG   
  
  
- AACGAACGAT CCTCCAAAAA TGTATGGTTA TTAATACTCC CCATATTGTA GTTCCTTCAG TTACCAAAAG   
  
  
- AACAATAGAC AACCGTTCTA TCTGGGGACA TAAGACAAAG TCGAACCTCT AAAAT

+     AAGAA-motif

| Site Name | Organism | Position | Strand | Matrix score. | sequence | function |
| --- | --- | --- | --- | --- | --- | --- |
| AAGAA-motif | Avena sativa | 2540 | + | 7 | GAAAGAA |  |
| AAGAA-motif | Avena sativa | 1421 | - | 7 | GAAAGAA |  |

>HU05G01267.1   
+ +Up\_Stream \_Len000TTACAA AAACGATGCT CTAAACCTAT TAGGTCGTTC CGAAACCCGC AAAATTGAAA   
  
  
+ TATGATCGCC CGTATTTTAC CCAAACCTGA AATACACTTA ATATGTATTT TACCTGAACC CAAATACACC   
  
  
+ GGACCTGTAT TTAACACGGG CACCCGAAAT GATAGGTCTA ACTCTCACCA CCACCGCATT TGGCACCCGA   
  
  
+ ACGGTAAAAA ATGTTCATCT ATTATGCACT TTGGCTTCAT CCATAACCCT ATGATCTATC TGTGCCAATA   
  
  
+ TTTGGAGCAC CCAGCGAAGT AGCGCATGCT CTAAACTCTT GCCTCAAAAC AAAACAAATA GTGGGTCATA   
  
  
+ TTCCCTTCAA AGCACTAACA AACACACACT CTCTCTCTCT CAGTAGCCAA CAACTTAAGG CGGTGTTTGC   
  
  
+ TTGGTTTTGG AGAGGGCGTG TTTGGATGAG GTGCAAAACA CATAGATGAA ATCATAAACA ACTTGATTCC   
  
  
+ CAGCCACCCA TGACCTACTC AGATAACAAG GGACAAAAAC AAAGAGGTTT TCTGAAACAG AGAAAACAGG   
  
  
+ GGCGTTGTGT TGCGCTGTTG CTCACAAAAT TCCAATATTC CTCTAAAAGC TCCCAAACGG AGACAAATCA   
  
  
+ AAGAGGACAA AGACATGTGG GATTTAATAC AAAATTTGAC TAACTCTGCA ACGGAAAACA ACCCCTAGGG   
  
  
+ TGTTTTTCTG CGTGTTGTAC ACACTTCACT TTTAACCTGC TCTGCCTCTG CCTTTGCCAC GGCTTTTAAT   
  
  
+ GGATAAGACC AATTAAGAAA CCCAAATATC TCGTAAATTT GTTGAGATTC CTTTCTGCGT TCTCAAGTGT   
  
  
+ TTCAAAAACC CAGTTTGGAG GTTGCACTTA TTCATGCTTT TCTTCAAGTC AGTGAGCTTA GTGTCTGGAA   
  
  
+ CTGAAGCTCC TCCTGGTAAT GCTCTGTTTT TCATTTCTTG TTGTGCGTTT ATAAAAATTC GGCCTATTGA   
  
  
+ TGGAAGTTTG TTTGATGTTT TTGGGTAAGT TTTTGATTAA TAGAGATGAA TTAGTAAAGT AAAATTTGAG   
  
  
+ TGGTATTTAA TGATCCTTAA GCAGTCTATG CTTTGTTGTC ACATGCGTTT CTTGGCTTTT GACTGAAACA   
  
  
+ TTTCTGTGTT AGATGAAAAA CTCATTTTGA TGCATGAGAT GATAAAATCG CTTATTTTTG CCTTTATTTG   
  
  
+ GTGTGTGGGG GGGGGGGGGG GGGGGCGCAG GTCTGAGGTA AAAGATATGT GAATTCATTT GGAGTCTCGA   
  
  
+ TAATTCGTTG GCTATGTTGA CAATCACGAG TGGTCGTTAT CGCTCATCTC ACAGGTTGAT AATTCACTCA   
  
  
+ AACTTCAAGC GGCCTGAATC ATTCAGTCCG GGTTGTTAAA ATCTCAACAG TCATTTCACA CGAGTGGAGG   
  
  
+ GCCCCAAAAG ACACCATTCT TTCGAACTTT TTTTTTTTGA AGGTCATTTT TTCAAGAACA AAACTAATGT   
  
  
+ GAACATGCGA CTTCTATGAG TAAAGTGTAA AGATGCATAA AGTCTTTTTT TCTTTTATAT AAAAAGTACT   
  
  
+ CAAAAAAAAA AGAGTTGGAG AGCTGATTCC CCATGCATCC TCTAAGCAAA GGTGGTGGAC GGCAGAAAAT   
  
  
+ TAGTAGAGTT ATTGTGAGAT TAAAGTTTGC GCACCTGAAC GGCTGACCTC TAGTTTGCTA TCAACGTTAC   
  
  
+ ATTTATGTGT CATAATTTTT GCATGAAACA ATTTCACGCA ATGTTTTCTG ATGGCAGTTA TGTTTAGAAA   
  
  
+ TAAGTTTAAT CGGACCTTAT TCCACTGATT TATATTTGAT TCTTCCCTCT TTACTACGGC TGTCCATTCA   
  
  
+ CAAGCCAAAG AATTCTCCCC CCAAAAAAAA GAAAAAACAG TATAGAACCA TGTTTATTAT CTCATTTTTT   
  
  
+ TCTCTCCCAT TTGATCTGAA ACACAAACTT GCAGGGGTGC AAGTTGGACA CTTAGTAGCT GCAGGAATGA   
  
  
+ TTCAATAGGA ATCCTCATCT TTGACAACAT CACCACTTGA ATACATATCA CTTGATGGCA CTCTCCCCTG   
  
  
+ GTTTAGGCTT CCCATATCCG TGGCTTAGGG AGCTAAAACC TGAGCAAAGA GGACTCTGTC TAATCCATCT   
  
  
+ CCTCCTTTCA TGTGCTAATC AAGTTGCTAC TGGGAGCATC GACAATGCTA ATGTCAGCCT TGAGCATATT   
  
  
+ TCCCACCTTG CCTCTCCCAC CGGAGATACA ATGCAGCGAA TTGCTGCTTA CTTTGCTGAA GCCTTTGCTG   
  
  
+ ACCGTTTACT AAGGGCTTGG CAGCCCGGTC TTCTCAAAGC CTTGAATTGT ACCAAGATGT CATCTGTTTC   
  
  
+ CGAACAAATT CTTGTTCAAA AGTTGTTCTT TGATCTTCTT CCGTTCTTGA AGCTTTCATA TCTTGTGACG   
  
  
+ AACCAGGCAA TCACGGAGGC CATAGAAGGA GAAAAGATGG TTCATATAAT TGATCTCCAT TCTTGTGAAT   
  
  
+ CGGCTCTGTG GATTAGTCTC CTCCAGGCAT TGAGTGTTCG ACCTGAAGGC CCACCCCATT TGAGGATAAC   
  
  
+ CGGTATACAT GAGAAGAAAG AAGTGTTGGA TCAAATGGCT ATGCAACTAA ACAAAGAGGC TGAAAAATTG   
  
  
+ GACATCCCAT TTCAATTCAA TCCTATCGCA AGCAAACTAG ACGACCTTGA TGTCGAAAGC TTGAGTGTCA   
  
  
+ AGACCGGAGA AGCACTTGTT ATTAGTTCTG TGCTCCAACT ACATTCTCTT TTGGCATTGG ATGAGGGATC   
  
  
+ AATGCCTAAG AACGCAGGCA TGGCTTACTT GCAAAGGGTG TTTTATATGA AACCACGAAA ATTGGAGGAC   
  
  
+ TTGCCCAACA AGGATTTGAT GAAAATGTTG AACTCAAATG AAGATTCTAC ATCATCATCA TCTTCATCTC   
  
  
+ CTATCCCTTC ATCAAAACTT GATGCCTTTT TAAAAGCCCT CCTTGGGCTT TCGCCAAAAC TCATGGTTGT   
  
  
+ AACCGAGCAA GAATCAAACC ACAATGGAAG TGCCCTAATA GAAAGAGTGA TGGAGTCATT GAACTTCTAT   
  
  
+ GCAGCATTGT TTGATTGCTT GGAATCCACT ATATCGAGGA CATCGATAGA GAGACAGAAG CTCGAGAAGT   
  
  
+ TGATGTTTGG AGAGGAGATC AAGAACCTCA CAGCTTGTGA AGGGGCAGAG AGAAAGGCAA GGCACGAGAA   
  
  
+ GCTCAGTGAA TGGGTTCAAA GATTTGAGTC AGTAGGATTT AAAAGGGAGC CATTGAGCTA CCATGGTTTC   
  
  
+ TTGCTTGCTA GGAGGTTTTT ACATACCAAT AATTATGAGG GGTATAACAT CAAGGAAGTC AATGGTTTTC   
  
  
+ TTGTTATCTG TTGGCAAGAT AGACCCCTGT ATTCTGTTTC AGCTTGGAGA TTTTA  

- +Up\_Stream \_Len000AATGTT TTTGCTACGA GATTTGGATA ATCCAGCAAG GCTTTGGGCG TTTTAACTTT   
  
  
- ATACTAGCGG GCATAAAATG GGTTTGGACT TTATGTGAAT TATACATAAA ATGGACTTGG GTTTATGTGG   
  
  
- CCTGGACATA AATTGTGCCC GTGGGCTTTA CTATCCAGAT TGAGAGTGGT GGTGGCGTAA ACCGTGGGCT   
  
  
- TGCCATTTTT TACAAGTAGA TAATACGTGA AACCGAAGTA GGTATTGGGA TACTAGATAG ACACGGTTAT   
  
  
- AAACCTCGTG GGTCGCTTCA TCGCGTACGA GATTTGAGAA CGGAGTTTTG TTTTGTTTAT CACCCAGTAT   
  
  
- AAGGGAAGTT TCGTGATTGT TTGTGTGTGA GAGAGAGAGA GTCATCGGTT GTTGAATTCC GCCACAAACG   
  
  
- AACCAAAACC TCTCCCGCAC AAACCTACTC CACGTTTTGT GTATCTACTT TAGTATTTGT TGAACTAAGG   
  
  
- GTCGGTGGGT ACTGGATGAG TCTATTGTTC CCTGTTTTTG TTTCTCCAAA AGACTTTGTC TCTTTTGTCC   
  
  
- CCGCAACACA ACGCGACAAC GAGTGTTTTA AGGTTATAAG GAGATTTTCG AGGGTTTGCC TCTGTTTAGT   
  
  
- TTCTCCTGTT TCTGTACACC CTAAATTATG TTTTAAACTG ATTGAGACGT TGCCTTTTGT TGGGGATCCC   
  
  
- ACAAAAAGAC GCACAACATG TGTGAAGTGA AAATTGGACG AGACGGAGAC GGAAACGGTG CCGAAAATTA   
  
  
- CCTATTCTGG TTAATTCTTT GGGTTTATAG AGCATTTAAA CAACTCTAAG GAAAGACGCA AGAGTTCACA   
  
  
- AAGTTTTTGG GTCAAACCTC CAACGTGAAT AAGTACGAAA AGAAGTTCAG TCACTCGAAT CACAGACCTT   
  
  
- GACTTCGAGG AGGACCATTA CGAGACAAAA AGTAAAGAAC AACACGCAAA TATTTTTAAG CCGGATAACT   
  
  
- ACCTTCAAAC AAACTACAAA AACCCATTCA AAAACTAATT ATCTCTACTT AATCATTTCA TTTTAAACTC   
  
  
- ACCATAAATT ACTAGGAATT CGTCAGATAC GAAACAACAG TGTACGCAAA GAACCGAAAA CTGACTTTGT   
  
  
- AAAGACACAA TCTACTTTTT GAGTAAAACT ACGTACTCTA CTATTTTAGC GAATAAAAAC GGAAATAAAC   
  
  
- CACACACCCC CCCCCCCCCC CCCCCGCGTC CAGACTCCAT TTTCTATACA CTTAAGTAAA CCTCAGAGCT   
  
  
- ATTAAGCAAC CGATACAACT GTTAGTGCTC ACCAGCAATA GCGAGTAGAG TGTCCAACTA TTAAGTGAGT   
  
  
- TTGAAGTTCG CCGGACTTAG TAAGTCAGGC CCAACAATTT TAGAGTTGTC AGTAAAGTGT GCTCACCTCC   
  
  
- CGGGGTTTTC TGTGGTAAGA AAGCTTGAAA AAAAAAAACT TCCAGTAAAA AAGTTCTTGT TTTGATTACA   
  
  
- CTTGTACGCT GAAGATACTC ATTTCACATT TCTACGTATT TCAGAAAAAA AGAAAATATA TTTTTCATGA   
  
  
- GTTTTTTTTT TCTCAACCTC TCGACTAAGG GGTACGTAGG AGATTCGTTT CCACCACCTG CCGTCTTTTA   
  
  
- ATCATCTCAA TAACACTCTA ATTTCAAACG CGTGGACTTG CCGACTGGAG ATCAAACGAT AGTTGCAATG   
  
  
- TAAATACACA GTATTAAAAA CGTACTTTGT TAAAGTGCGT TACAAAAGAC TACCGTCAAT ACAAATCTTT   
  
  
- ATTCAAATTA GCCTGGAATA AGGTGACTAA ATATAAACTA AGAAGGGAGA AATGATGCCG ACAGGTAAGT   
  
  
- GTTCGGTTTC TTAAGAGGGG GGTTTTTTTT CTTTTTTGTC ATATCTTGGT ACAAATAATA GAGTAAAAAA   
  
  
- AGAGAGGGTA AACTAGACTT TGTGTTTGAA CGTCCCCACG TTCAACCTGT GAATCATCGA CGTCCTTACT   
  
  
- AAGTTATCCT TAGGAGTAGA AACTGTTGTA GTGGTGAACT TATGTATAGT GAACTACCGT GAGAGGGGAC   
  
  
- CAAATCCGAA GGGTATAGGC ACCGAATCCC TCGATTTTGG ACTCGTTTCT CCTGAGACAG ATTAGGTAGA   
  
  
- GGAGGAAAGT ACACGATTAG TTCAACGATG ACCCTCGTAG CTGTTACGAT TACAGTCGGA ACTCGTATAA   
  
  
- AGGGTGGAAC GGAGAGGGTG GCCTCTATGT TACGTCGCTT AACGACGAAT GAAACGACTT CGGAAACGAC   
  
  
- TGGCAAATGA TTCCCGAACC GTCGGGCCAG AAGAGTTTCG GAACTTAACA TGGTTCTACA GTAGACAAAG   
  
  
- GCTTGTTTAA GAACAAGTTT TCAACAAGAA ACTAGAAGAA GGCAAGAACT TCGAAAGTAT AGAACACTGC   
  
  
- TTGGTCCGTT AGTGCCTCCG GTATCTTCCT CTTTTCTACC AAGTATATTA ACTAGAGGTA AGAACACTTA   
  
  
- GCCGAGACAC CTAATCAGAG GAGGTCCGTA ACTCACAAGC TGGACTTCCG GGTGGGGTAA ACTCCTATTG   
  
  
- GCCATATGTA CTCTTCTTTC TTCACAACCT AGTTTACCGA TACGTTGATT TGTTTCTCCG ACTTTTTAAC   
  
  
- CTGTAGGGTA AAGTTAAGTT AGGATAGCGT TCGTTTGATC TGCTGGAACT ACAGCTTTCG AACTCACAGT   
  
  
- TCTGGCCTCT TCGTGAACAA TAATCAAGAC ACGAGGTTGA TGTAAGAGAA AACCGTAACC TACTCCCTAG   
  
  
- TTACGGATTC TTGCGTCCGT ACCGAATGAA CGTTTCCCAC AAAATATACT TTGGTGCTTT TAACCTCCTG   
  
  
- AACGGGTTGT TCCTAAACTA CTTTTACAAC TTGAGTTTAC TTCTAAGATG TAGTAGTAGT AGAAGTAGAG   
  
  
- GATAGGGAAG TAGTTTTGAA CTACGGAAAA ATTTTCGGGA GGAACCCGAA AGCGGTTTTG AGTACCAACA   
  
  
- TTGGCTCGTT CTTAGTTTGG TGTTACCTTC ACGGGATTAT CTTTCTCACT ACCTCAGTAA CTTGAAGATA   
  
  
- CGTCGTAACA AACTAACGAA CCTTAGGTGA TATAGCTCCT GTAGCTATCT CTCTGTCTTC GAGCTCTTCA   
  
  
- ACTACAAACC TCTCCTCTAG TTCTTGGAGT GTCGAACACT TCCCCGTCTC TCTTTCCGTT CCGTGCTCTT   
  
  
- CGAGTCACTT ACCCAAGTTT CTAAACTCAG TCATCCTAAA TTTTCCCTCG GTAACTCGAT GGTACCAAAG   
  
  
- AACGAACGAT CCTCCAAAAA TGTATGGTTA TTAATACTCC CCATATTGTA GTTCCTTCAG TTACCAAAAG   
  
  
- AACAATAGAC AACCGTTCTA TCTGGGGACA TAAGACAAAG TCGAACCTCT AAAAT

+     ABRE

| Site Name | Organism | Position | Strand | Matrix score. | sequence | function |
| --- | --- | --- | --- | --- | --- | --- |
| ABRE | Arabidopsis thaliana | 1362 | - | 7 | AACCCGG | cis-acting element involved in the abscisic acid responsiveness |

>HU05G01267.1   
+ +Up\_Stream \_Len000TTACAA AAACGATGCT CTAAACCTAT TAGGTCGTTC CGAAACCCGC AAAATTGAAA   
  
  
+ TATGATCGCC CGTATTTTAC CCAAACCTGA AATACACTTA ATATGTATTT TACCTGAACC CAAATACACC   
  
  
+ GGACCTGTAT TTAACACGGG CACCCGAAAT GATAGGTCTA ACTCTCACCA CCACCGCATT TGGCACCCGA   
  
  
+ ACGGTAAAAA ATGTTCATCT ATTATGCACT TTGGCTTCAT CCATAACCCT ATGATCTATC TGTGCCAATA   
  
  
+ TTTGGAGCAC CCAGCGAAGT AGCGCATGCT CTAAACTCTT GCCTCAAAAC AAAACAAATA GTGGGTCATA   
  
  
+ TTCCCTTCAA AGCACTAACA AACACACACT CTCTCTCTCT CAGTAGCCAA CAACTTAAGG CGGTGTTTGC   
  
  
+ TTGGTTTTGG AGAGGGCGTG TTTGGATGAG GTGCAAAACA CATAGATGAA ATCATAAACA ACTTGATTCC   
  
  
+ CAGCCACCCA TGACCTACTC AGATAACAAG GGACAAAAAC AAAGAGGTTT TCTGAAACAG AGAAAACAGG   
  
  
+ GGCGTTGTGT TGCGCTGTTG CTCACAAAAT TCCAATATTC CTCTAAAAGC TCCCAAACGG AGACAAATCA   
  
  
+ AAGAGGACAA AGACATGTGG GATTTAATAC AAAATTTGAC TAACTCTGCA ACGGAAAACA ACCCCTAGGG   
  
  
+ TGTTTTTCTG CGTGTTGTAC ACACTTCACT TTTAACCTGC TCTGCCTCTG CCTTTGCCAC GGCTTTTAAT   
  
  
+ GGATAAGACC AATTAAGAAA CCCAAATATC TCGTAAATTT GTTGAGATTC CTTTCTGCGT TCTCAAGTGT   
  
  
+ TTCAAAAACC CAGTTTGGAG GTTGCACTTA TTCATGCTTT TCTTCAAGTC AGTGAGCTTA GTGTCTGGAA   
  
  
+ CTGAAGCTCC TCCTGGTAAT GCTCTGTTTT TCATTTCTTG TTGTGCGTTT ATAAAAATTC GGCCTATTGA   
  
  
+ TGGAAGTTTG TTTGATGTTT TTGGGTAAGT TTTTGATTAA TAGAGATGAA TTAGTAAAGT AAAATTTGAG   
  
  
+ TGGTATTTAA TGATCCTTAA GCAGTCTATG CTTTGTTGTC ACATGCGTTT CTTGGCTTTT GACTGAAACA   
  
  
+ TTTCTGTGTT AGATGAAAAA CTCATTTTGA TGCATGAGAT GATAAAATCG CTTATTTTTG CCTTTATTTG   
  
  
+ GTGTGTGGGG GGGGGGGGGG GGGGGCGCAG GTCTGAGGTA AAAGATATGT GAATTCATTT GGAGTCTCGA   
  
  
+ TAATTCGTTG GCTATGTTGA CAATCACGAG TGGTCGTTAT CGCTCATCTC ACAGGTTGAT AATTCACTCA   
  
  
+ AACTTCAAGC GGCCTGAATC ATTCAGTCCG GGTTGTTAAA ATCTCAACAG TCATTTCACA CGAGTGGAGG   
  
  
+ GCCCCAAAAG ACACCATTCT TTCGAACTTT TTTTTTTTGA AGGTCATTTT TTCAAGAACA AAACTAATGT   
  
  
+ GAACATGCGA CTTCTATGAG TAAAGTGTAA AGATGCATAA AGTCTTTTTT TCTTTTATAT AAAAAGTACT   
  
  
+ CAAAAAAAAA AGAGTTGGAG AGCTGATTCC CCATGCATCC TCTAAGCAAA GGTGGTGGAC GGCAGAAAAT   
  
  
+ TAGTAGAGTT ATTGTGAGAT TAAAGTTTGC GCACCTGAAC GGCTGACCTC TAGTTTGCTA TCAACGTTAC   
  
  
+ ATTTATGTGT CATAATTTTT GCATGAAACA ATTTCACGCA ATGTTTTCTG ATGGCAGTTA TGTTTAGAAA   
  
  
+ TAAGTTTAAT CGGACCTTAT TCCACTGATT TATATTTGAT TCTTCCCTCT TTACTACGGC TGTCCATTCA   
  
  
+ CAAGCCAAAG AATTCTCCCC CCAAAAAAAA GAAAAAACAG TATAGAACCA TGTTTATTAT CTCATTTTTT   
  
  
+ TCTCTCCCAT TTGATCTGAA ACACAAACTT GCAGGGGTGC AAGTTGGACA CTTAGTAGCT GCAGGAATGA   
  
  
+ TTCAATAGGA ATCCTCATCT TTGACAACAT CACCACTTGA ATACATATCA CTTGATGGCA CTCTCCCCTG   
  
  
+ GTTTAGGCTT CCCATATCCG TGGCTTAGGG AGCTAAAACC TGAGCAAAGA GGACTCTGTC TAATCCATCT   
  
  
+ CCTCCTTTCA TGTGCTAATC AAGTTGCTAC TGGGAGCATC GACAATGCTA ATGTCAGCCT TGAGCATATT   
  
  
+ TCCCACCTTG CCTCTCCCAC CGGAGATACA ATGCAGCGAA TTGCTGCTTA CTTTGCTGAA GCCTTTGCTG   
  
  
+ ACCGTTTACT AAGGGCTTGG CAGCCCGGTC TTCTCAAAGC CTTGAATTGT ACCAAGATGT CATCTGTTTC   
  
  
+ CGAACAAATT CTTGTTCAAA AGTTGTTCTT TGATCTTCTT CCGTTCTTGA AGCTTTCATA TCTTGTGACG   
  
  
+ AACCAGGCAA TCACGGAGGC CATAGAAGGA GAAAAGATGG TTCATATAAT TGATCTCCAT TCTTGTGAAT   
  
  
+ CGGCTCTGTG GATTAGTCTC CTCCAGGCAT TGAGTGTTCG ACCTGAAGGC CCACCCCATT TGAGGATAAC   
  
  
+ CGGTATACAT GAGAAGAAAG AAGTGTTGGA TCAAATGGCT ATGCAACTAA ACAAAGAGGC TGAAAAATTG   
  
  
+ GACATCCCAT TTCAATTCAA TCCTATCGCA AGCAAACTAG ACGACCTTGA TGTCGAAAGC TTGAGTGTCA   
  
  
+ AGACCGGAGA AGCACTTGTT ATTAGTTCTG TGCTCCAACT ACATTCTCTT TTGGCATTGG ATGAGGGATC   
  
  
+ AATGCCTAAG AACGCAGGCA TGGCTTACTT GCAAAGGGTG TTTTATATGA AACCACGAAA ATTGGAGGAC   
  
  
+ TTGCCCAACA AGGATTTGAT GAAAATGTTG AACTCAAATG AAGATTCTAC ATCATCATCA TCTTCATCTC   
  
  
+ CTATCCCTTC ATCAAAACTT GATGCCTTTT TAAAAGCCCT CCTTGGGCTT TCGCCAAAAC TCATGGTTGT   
  
  
+ AACCGAGCAA GAATCAAACC ACAATGGAAG TGCCCTAATA GAAAGAGTGA TGGAGTCATT GAACTTCTAT   
  
  
+ GCAGCATTGT TTGATTGCTT GGAATCCACT ATATCGAGGA CATCGATAGA GAGACAGAAG CTCGAGAAGT   
  
  
+ TGATGTTTGG AGAGGAGATC AAGAACCTCA CAGCTTGTGA AGGGGCAGAG AGAAAGGCAA GGCACGAGAA   
  
  
+ GCTCAGTGAA TGGGTTCAAA GATTTGAGTC AGTAGGATTT AAAAGGGAGC CATTGAGCTA CCATGGTTTC   
  
  
+ TTGCTTGCTA GGAGGTTTTT ACATACCAAT AATTATGAGG GGTATAACAT CAAGGAAGTC AATGGTTTTC   
  
  
+ TTGTTATCTG TTGGCAAGAT AGACCCCTGT ATTCTGTTTC AGCTTGGAGA TTTTA  

- +Up\_Stream \_Len000AATGTT TTTGCTACGA GATTTGGATA ATCCAGCAAG GCTTTGGGCG TTTTAACTTT   
  
  
- ATACTAGCGG GCATAAAATG GGTTTGGACT TTATGTGAAT TATACATAAA ATGGACTTGG GTTTATGTGG   
  
  
- CCTGGACATA AATTGTGCCC GTGGGCTTTA CTATCCAGAT TGAGAGTGGT GGTGGCGTAA ACCGTGGGCT   
  
  
- TGCCATTTTT TACAAGTAGA TAATACGTGA AACCGAAGTA GGTATTGGGA TACTAGATAG ACACGGTTAT   
  
  
- AAACCTCGTG GGTCGCTTCA TCGCGTACGA GATTTGAGAA CGGAGTTTTG TTTTGTTTAT CACCCAGTAT   
  
  
- AAGGGAAGTT TCGTGATTGT TTGTGTGTGA GAGAGAGAGA GTCATCGGTT GTTGAATTCC GCCACAAACG   
  
  
- AACCAAAACC TCTCCCGCAC AAACCTACTC CACGTTTTGT GTATCTACTT TAGTATTTGT TGAACTAAGG   
  
  
- GTCGGTGGGT ACTGGATGAG TCTATTGTTC CCTGTTTTTG TTTCTCCAAA AGACTTTGTC TCTTTTGTCC   
  
  
- CCGCAACACA ACGCGACAAC GAGTGTTTTA AGGTTATAAG GAGATTTTCG AGGGTTTGCC TCTGTTTAGT   
  
  
- TTCTCCTGTT TCTGTACACC CTAAATTATG TTTTAAACTG ATTGAGACGT TGCCTTTTGT TGGGGATCCC   
  
  
- ACAAAAAGAC GCACAACATG TGTGAAGTGA AAATTGGACG AGACGGAGAC GGAAACGGTG CCGAAAATTA   
  
  
- CCTATTCTGG TTAATTCTTT GGGTTTATAG AGCATTTAAA CAACTCTAAG GAAAGACGCA AGAGTTCACA   
  
  
- AAGTTTTTGG GTCAAACCTC CAACGTGAAT AAGTACGAAA AGAAGTTCAG TCACTCGAAT CACAGACCTT   
  
  
- GACTTCGAGG AGGACCATTA CGAGACAAAA AGTAAAGAAC AACACGCAAA TATTTTTAAG CCGGATAACT   
  
  
- ACCTTCAAAC AAACTACAAA AACCCATTCA AAAACTAATT ATCTCTACTT AATCATTTCA TTTTAAACTC   
  
  
- ACCATAAATT ACTAGGAATT CGTCAGATAC GAAACAACAG TGTACGCAAA GAACCGAAAA CTGACTTTGT   
  
  
- AAAGACACAA TCTACTTTTT GAGTAAAACT ACGTACTCTA CTATTTTAGC GAATAAAAAC GGAAATAAAC   
  
  
- CACACACCCC CCCCCCCCCC CCCCCGCGTC CAGACTCCAT TTTCTATACA CTTAAGTAAA CCTCAGAGCT   
  
  
- ATTAAGCAAC CGATACAACT GTTAGTGCTC ACCAGCAATA GCGAGTAGAG TGTCCAACTA TTAAGTGAGT   
  
  
- TTGAAGTTCG CCGGACTTAG TAAGTCAGGC CCAACAATTT TAGAGTTGTC AGTAAAGTGT GCTCACCTCC   
  
  
- CGGGGTTTTC TGTGGTAAGA AAGCTTGAAA AAAAAAAACT TCCAGTAAAA AAGTTCTTGT TTTGATTACA   
  
  
- CTTGTACGCT GAAGATACTC ATTTCACATT TCTACGTATT TCAGAAAAAA AGAAAATATA TTTTTCATGA   
  
  
- GTTTTTTTTT TCTCAACCTC TCGACTAAGG GGTACGTAGG AGATTCGTTT CCACCACCTG CCGTCTTTTA   
  
  
- ATCATCTCAA TAACACTCTA ATTTCAAACG CGTGGACTTG CCGACTGGAG ATCAAACGAT AGTTGCAATG   
  
  
- TAAATACACA GTATTAAAAA CGTACTTTGT TAAAGTGCGT TACAAAAGAC TACCGTCAAT ACAAATCTTT   
  
  
- ATTCAAATTA GCCTGGAATA AGGTGACTAA ATATAAACTA AGAAGGGAGA AATGATGCCG ACAGGTAAGT   
  
  
- GTTCGGTTTC TTAAGAGGGG GGTTTTTTTT CTTTTTTGTC ATATCTTGGT ACAAATAATA GAGTAAAAAA   
  
  
- AGAGAGGGTA AACTAGACTT TGTGTTTGAA CGTCCCCACG TTCAACCTGT GAATCATCGA CGTCCTTACT   
  
  
- AAGTTATCCT TAGGAGTAGA AACTGTTGTA GTGGTGAACT TATGTATAGT GAACTACCGT GAGAGGGGAC   
  
  
- CAAATCCGAA GGGTATAGGC ACCGAATCCC TCGATTTTGG ACTCGTTTCT CCTGAGACAG ATTAGGTAGA   
  
  
- GGAGGAAAGT ACACGATTAG TTCAACGATG ACCCTCGTAG CTGTTACGAT TACAGTCGGA ACTCGTATAA   
  
  
- AGGGTGGAAC GGAGAGGGTG GCCTCTATGT TACGTCGCTT AACGACGAAT GAAACGACTT CGGAAACGAC   
  
  
- TGGCAAATGA TTCCCGAACC GTCGGGCCAG AAGAGTTTCG GAACTTAACA TGGTTCTACA GTAGACAAAG   
  
  
- GCTTGTTTAA GAACAAGTTT TCAACAAGAA ACTAGAAGAA GGCAAGAACT TCGAAAGTAT AGAACACTGC   
  
  
- TTGGTCCGTT AGTGCCTCCG GTATCTTCCT CTTTTCTACC AAGTATATTA ACTAGAGGTA AGAACACTTA   
  
  
- GCCGAGACAC CTAATCAGAG GAGGTCCGTA ACTCACAAGC TGGACTTCCG GGTGGGGTAA ACTCCTATTG   
  
  
- GCCATATGTA CTCTTCTTTC TTCACAACCT AGTTTACCGA TACGTTGATT TGTTTCTCCG ACTTTTTAAC   
  
  
- CTGTAGGGTA AAGTTAAGTT AGGATAGCGT TCGTTTGATC TGCTGGAACT ACAGCTTTCG AACTCACAGT   
  
  
- TCTGGCCTCT TCGTGAACAA TAATCAAGAC ACGAGGTTGA TGTAAGAGAA AACCGTAACC TACTCCCTAG   
  
  
- TTACGGATTC TTGCGTCCGT ACCGAATGAA CGTTTCCCAC AAAATATACT TTGGTGCTTT TAACCTCCTG   
  
  
- AACGGGTTGT TCCTAAACTA CTTTTACAAC TTGAGTTTAC TTCTAAGATG TAGTAGTAGT AGAAGTAGAG   
  
  
- GATAGGGAAG TAGTTTTGAA CTACGGAAAA ATTTTCGGGA GGAACCCGAA AGCGGTTTTG AGTACCAACA   
  
  
- TTGGCTCGTT CTTAGTTTGG TGTTACCTTC ACGGGATTAT CTTTCTCACT ACCTCAGTAA CTTGAAGATA   
  
  
- CGTCGTAACA AACTAACGAA CCTTAGGTGA TATAGCTCCT GTAGCTATCT CTCTGTCTTC GAGCTCTTCA   
  
  
- ACTACAAACC TCTCCTCTAG TTCTTGGAGT GTCGAACACT TCCCCGTCTC TCTTTCCGTT CCGTGCTCTT   
  
  
- CGAGTCACTT ACCCAAGTTT CTAAACTCAG TCATCCTAAA TTTTCCCTCG GTAACTCGAT GGTACCAAAG   
  
  
- AACGAACGAT CCTCCAAAAA TGTATGGTTA TTAATACTCC CCATATTGTA GTTCCTTCAG TTACCAAAAG   
  
  
- AACAATAGAC AACCGTTCTA TCTGGGGACA TAAGACAAAG TCGAACCTCT AAAAT

+     ARE

| Site Name | Organism | Position | Strand | Matrix score. | sequence | function |
| --- | --- | --- | --- | --- | --- | --- |
| ARE | Zea mays | 3287 | - | 6 | AAACCA | cis-acting regulatory element essential for the anaerobic induction |
| ARE | Zea mays | 2960 | + | 6 | AAACCA | cis-acting regulatory element essential for the anaerobic induction |
| ARE | Zea mays | 3218 | - | 6 | AAACCA | cis-acting regulatory element essential for the anaerobic induction |
| ARE | Zea mays | 2784 | + | 6 | AAACCA | cis-acting regulatory element essential for the anaerobic induction |
| ARE | Zea mays | 426 | - | 6 | AAACCA | cis-acting regulatory element essential for the anaerobic induction |
| ARE | Zea mays | 2033 | - | 6 | AAACCA | cis-acting regulatory element essential for the anaerobic induction |

>HU05G01267.1   
+ +Up\_Stream \_Len000TTACAA AAACGATGCT CTAAACCTAT TAGGTCGTTC CGAAACCCGC AAAATTGAAA   
  
  
+ TATGATCGCC CGTATTTTAC CCAAACCTGA AATACACTTA ATATGTATTT TACCTGAACC CAAATACACC   
  
  
+ GGACCTGTAT TTAACACGGG CACCCGAAAT GATAGGTCTA ACTCTCACCA CCACCGCATT TGGCACCCGA   
  
  
+ ACGGTAAAAA ATGTTCATCT ATTATGCACT TTGGCTTCAT CCATAACCCT ATGATCTATC TGTGCCAATA   
  
  
+ TTTGGAGCAC CCAGCGAAGT AGCGCATGCT CTAAACTCTT GCCTCAAAAC AAAACAAATA GTGGGTCATA   
  
  
+ TTCCCTTCAA AGCACTAACA AACACACACT CTCTCTCTCT CAGTAGCCAA CAACTTAAGG CGGTGTTTGC   
  
  
+ TTGGTTTTGG AGAGGGCGTG TTTGGATGAG GTGCAAAACA CATAGATGAA ATCATAAACA ACTTGATTCC   
  
  
+ CAGCCACCCA TGACCTACTC AGATAACAAG GGACAAAAAC AAAGAGGTTT TCTGAAACAG AGAAAACAGG   
  
  
+ GGCGTTGTGT TGCGCTGTTG CTCACAAAAT TCCAATATTC CTCTAAAAGC TCCCAAACGG AGACAAATCA   
  
  
+ AAGAGGACAA AGACATGTGG GATTTAATAC AAAATTTGAC TAACTCTGCA ACGGAAAACA ACCCCTAGGG   
  
  
+ TGTTTTTCTG CGTGTTGTAC ACACTTCACT TTTAACCTGC TCTGCCTCTG CCTTTGCCAC GGCTTTTAAT   
  
  
+ GGATAAGACC AATTAAGAAA CCCAAATATC TCGTAAATTT GTTGAGATTC CTTTCTGCGT TCTCAAGTGT   
  
  
+ TTCAAAAACC CAGTTTGGAG GTTGCACTTA TTCATGCTTT TCTTCAAGTC AGTGAGCTTA GTGTCTGGAA   
  
  
+ CTGAAGCTCC TCCTGGTAAT GCTCTGTTTT TCATTTCTTG TTGTGCGTTT ATAAAAATTC GGCCTATTGA   
  
  
+ TGGAAGTTTG TTTGATGTTT TTGGGTAAGT TTTTGATTAA TAGAGATGAA TTAGTAAAGT AAAATTTGAG   
  
  
+ TGGTATTTAA TGATCCTTAA GCAGTCTATG CTTTGTTGTC ACATGCGTTT CTTGGCTTTT GACTGAAACA   
  
  
+ TTTCTGTGTT AGATGAAAAA CTCATTTTGA TGCATGAGAT GATAAAATCG CTTATTTTTG CCTTTATTTG   
  
  
+ GTGTGTGGGG GGGGGGGGGG GGGGGCGCAG GTCTGAGGTA AAAGATATGT GAATTCATTT GGAGTCTCGA   
  
  
+ TAATTCGTTG GCTATGTTGA CAATCACGAG TGGTCGTTAT CGCTCATCTC ACAGGTTGAT AATTCACTCA   
  
  
+ AACTTCAAGC GGCCTGAATC ATTCAGTCCG GGTTGTTAAA ATCTCAACAG TCATTTCACA CGAGTGGAGG   
  
  
+ GCCCCAAAAG ACACCATTCT TTCGAACTTT TTTTTTTTGA AGGTCATTTT TTCAAGAACA AAACTAATGT   
  
  
+ GAACATGCGA CTTCTATGAG TAAAGTGTAA AGATGCATAA AGTCTTTTTT TCTTTTATAT AAAAAGTACT   
  
  
+ CAAAAAAAAA AGAGTTGGAG AGCTGATTCC CCATGCATCC TCTAAGCAAA GGTGGTGGAC GGCAGAAAAT   
  
  
+ TAGTAGAGTT ATTGTGAGAT TAAAGTTTGC GCACCTGAAC GGCTGACCTC TAGTTTGCTA TCAACGTTAC   
  
  
+ ATTTATGTGT CATAATTTTT GCATGAAACA ATTTCACGCA ATGTTTTCTG ATGGCAGTTA TGTTTAGAAA   
  
  
+ TAAGTTTAAT CGGACCTTAT TCCACTGATT TATATTTGAT TCTTCCCTCT TTACTACGGC TGTCCATTCA   
  
  
+ CAAGCCAAAG AATTCTCCCC CCAAAAAAAA GAAAAAACAG TATAGAACCA TGTTTATTAT CTCATTTTTT   
  
  
+ TCTCTCCCAT TTGATCTGAA ACACAAACTT GCAGGGGTGC AAGTTGGACA CTTAGTAGCT GCAGGAATGA   
  
  
+ TTCAATAGGA ATCCTCATCT TTGACAACAT CACCACTTGA ATACATATCA CTTGATGGCA CTCTCCCCTG   
  
  
+ GTTTAGGCTT CCCATATCCG TGGCTTAGGG AGCTAAAACC TGAGCAAAGA GGACTCTGTC TAATCCATCT   
  
  
+ CCTCCTTTCA TGTGCTAATC AAGTTGCTAC TGGGAGCATC GACAATGCTA ATGTCAGCCT TGAGCATATT   
  
  
+ TCCCACCTTG CCTCTCCCAC CGGAGATACA ATGCAGCGAA TTGCTGCTTA CTTTGCTGAA GCCTTTGCTG   
  
  
+ ACCGTTTACT AAGGGCTTGG CAGCCCGGTC TTCTCAAAGC CTTGAATTGT ACCAAGATGT CATCTGTTTC   
  
  
+ CGAACAAATT CTTGTTCAAA AGTTGTTCTT TGATCTTCTT CCGTTCTTGA AGCTTTCATA TCTTGTGACG   
  
  
+ AACCAGGCAA TCACGGAGGC CATAGAAGGA GAAAAGATGG TTCATATAAT TGATCTCCAT TCTTGTGAAT   
  
  
+ CGGCTCTGTG GATTAGTCTC CTCCAGGCAT TGAGTGTTCG ACCTGAAGGC CCACCCCATT TGAGGATAAC   
  
  
+ CGGTATACAT GAGAAGAAAG AAGTGTTGGA TCAAATGGCT ATGCAACTAA ACAAAGAGGC TGAAAAATTG   
  
  
+ GACATCCCAT TTCAATTCAA TCCTATCGCA AGCAAACTAG ACGACCTTGA TGTCGAAAGC TTGAGTGTCA   
  
  
+ AGACCGGAGA AGCACTTGTT ATTAGTTCTG TGCTCCAACT ACATTCTCTT TTGGCATTGG ATGAGGGATC   
  
  
+ AATGCCTAAG AACGCAGGCA TGGCTTACTT GCAAAGGGTG TTTTATATGA AACCACGAAA ATTGGAGGAC   
  
  
+ TTGCCCAACA AGGATTTGAT GAAAATGTTG AACTCAAATG AAGATTCTAC ATCATCATCA TCTTCATCTC   
  
  
+ CTATCCCTTC ATCAAAACTT GATGCCTTTT TAAAAGCCCT CCTTGGGCTT TCGCCAAAAC TCATGGTTGT   
  
  
+ AACCGAGCAA GAATCAAACC ACAATGGAAG TGCCCTAATA GAAAGAGTGA TGGAGTCATT GAACTTCTAT   
  
  
+ GCAGCATTGT TTGATTGCTT GGAATCCACT ATATCGAGGA CATCGATAGA GAGACAGAAG CTCGAGAAGT   
  
  
+ TGATGTTTGG AGAGGAGATC AAGAACCTCA CAGCTTGTGA AGGGGCAGAG AGAAAGGCAA GGCACGAGAA   
  
  
+ GCTCAGTGAA TGGGTTCAAA GATTTGAGTC AGTAGGATTT AAAAGGGAGC CATTGAGCTA CCATGGTTTC   
  
  
+ TTGCTTGCTA GGAGGTTTTT ACATACCAAT AATTATGAGG GGTATAACAT CAAGGAAGTC AATGGTTTTC   
  
  
+ TTGTTATCTG TTGGCAAGAT AGACCCCTGT ATTCTGTTTC AGCTTGGAGA TTTTA  

- +Up\_Stream \_Len000AATGTT TTTGCTACGA GATTTGGATA ATCCAGCAAG GCTTTGGGCG TTTTAACTTT   
  
  
- ATACTAGCGG GCATAAAATG GGTTTGGACT TTATGTGAAT TATACATAAA ATGGACTTGG GTTTATGTGG   
  
  
- CCTGGACATA AATTGTGCCC GTGGGCTTTA CTATCCAGAT TGAGAGTGGT GGTGGCGTAA ACCGTGGGCT   
  
  
- TGCCATTTTT TACAAGTAGA TAATACGTGA AACCGAAGTA GGTATTGGGA TACTAGATAG ACACGGTTAT   
  
  
- AAACCTCGTG GGTCGCTTCA TCGCGTACGA GATTTGAGAA CGGAGTTTTG TTTTGTTTAT CACCCAGTAT   
  
  
- AAGGGAAGTT TCGTGATTGT TTGTGTGTGA GAGAGAGAGA GTCATCGGTT GTTGAATTCC GCCACAAACG   
  
  
- AACCAAAACC TCTCCCGCAC AAACCTACTC CACGTTTTGT GTATCTACTT TAGTATTTGT TGAACTAAGG   
  
  
- GTCGGTGGGT ACTGGATGAG TCTATTGTTC CCTGTTTTTG TTTCTCCAAA AGACTTTGTC TCTTTTGTCC   
  
  
- CCGCAACACA ACGCGACAAC GAGTGTTTTA AGGTTATAAG GAGATTTTCG AGGGTTTGCC TCTGTTTAGT   
  
  
- TTCTCCTGTT TCTGTACACC CTAAATTATG TTTTAAACTG ATTGAGACGT TGCCTTTTGT TGGGGATCCC   
  
  
- ACAAAAAGAC GCACAACATG TGTGAAGTGA AAATTGGACG AGACGGAGAC GGAAACGGTG CCGAAAATTA   
  
  
- CCTATTCTGG TTAATTCTTT GGGTTTATAG AGCATTTAAA CAACTCTAAG GAAAGACGCA AGAGTTCACA   
  
  
- AAGTTTTTGG GTCAAACCTC CAACGTGAAT AAGTACGAAA AGAAGTTCAG TCACTCGAAT CACAGACCTT   
  
  
- GACTTCGAGG AGGACCATTA CGAGACAAAA AGTAAAGAAC AACACGCAAA TATTTTTAAG CCGGATAACT   
  
  
- ACCTTCAAAC AAACTACAAA AACCCATTCA AAAACTAATT ATCTCTACTT AATCATTTCA TTTTAAACTC   
  
  
- ACCATAAATT ACTAGGAATT CGTCAGATAC GAAACAACAG TGTACGCAAA GAACCGAAAA CTGACTTTGT   
  
  
- AAAGACACAA TCTACTTTTT GAGTAAAACT ACGTACTCTA CTATTTTAGC GAATAAAAAC GGAAATAAAC   
  
  
- CACACACCCC CCCCCCCCCC CCCCCGCGTC CAGACTCCAT TTTCTATACA CTTAAGTAAA CCTCAGAGCT   
  
  
- ATTAAGCAAC CGATACAACT GTTAGTGCTC ACCAGCAATA GCGAGTAGAG TGTCCAACTA TTAAGTGAGT   
  
  
- TTGAAGTTCG CCGGACTTAG TAAGTCAGGC CCAACAATTT TAGAGTTGTC AGTAAAGTGT GCTCACCTCC   
  
  
- CGGGGTTTTC TGTGGTAAGA AAGCTTGAAA AAAAAAAACT TCCAGTAAAA AAGTTCTTGT TTTGATTACA   
  
  
- CTTGTACGCT GAAGATACTC ATTTCACATT TCTACGTATT TCAGAAAAAA AGAAAATATA TTTTTCATGA   
  
  
- GTTTTTTTTT TCTCAACCTC TCGACTAAGG GGTACGTAGG AGATTCGTTT CCACCACCTG CCGTCTTTTA   
  
  
- ATCATCTCAA TAACACTCTA ATTTCAAACG CGTGGACTTG CCGACTGGAG ATCAAACGAT AGTTGCAATG   
  
  
- TAAATACACA GTATTAAAAA CGTACTTTGT TAAAGTGCGT TACAAAAGAC TACCGTCAAT ACAAATCTTT   
  
  
- ATTCAAATTA GCCTGGAATA AGGTGACTAA ATATAAACTA AGAAGGGAGA AATGATGCCG ACAGGTAAGT   
  
  
- GTTCGGTTTC TTAAGAGGGG GGTTTTTTTT CTTTTTTGTC ATATCTTGGT ACAAATAATA GAGTAAAAAA   
  
  
- AGAGAGGGTA AACTAGACTT TGTGTTTGAA CGTCCCCACG TTCAACCTGT GAATCATCGA CGTCCTTACT   
  
  
- AAGTTATCCT TAGGAGTAGA AACTGTTGTA GTGGTGAACT TATGTATAGT GAACTACCGT GAGAGGGGAC   
  
  
- CAAATCCGAA GGGTATAGGC ACCGAATCCC TCGATTTTGG ACTCGTTTCT CCTGAGACAG ATTAGGTAGA   
  
  
- GGAGGAAAGT ACACGATTAG TTCAACGATG ACCCTCGTAG CTGTTACGAT TACAGTCGGA ACTCGTATAA   
  
  
- AGGGTGGAAC GGAGAGGGTG GCCTCTATGT TACGTCGCTT AACGACGAAT GAAACGACTT CGGAAACGAC   
  
  
- TGGCAAATGA TTCCCGAACC GTCGGGCCAG AAGAGTTTCG GAACTTAACA TGGTTCTACA GTAGACAAAG   
  
  
- GCTTGTTTAA GAACAAGTTT TCAACAAGAA ACTAGAAGAA GGCAAGAACT TCGAAAGTAT AGAACACTGC   
  
  
- TTGGTCCGTT AGTGCCTCCG GTATCTTCCT CTTTTCTACC AAGTATATTA ACTAGAGGTA AGAACACTTA   
  
  
- GCCGAGACAC CTAATCAGAG GAGGTCCGTA ACTCACAAGC TGGACTTCCG GGTGGGGTAA ACTCCTATTG   
  
  
- GCCATATGTA CTCTTCTTTC TTCACAACCT AGTTTACCGA TACGTTGATT TGTTTCTCCG ACTTTTTAAC   
  
  
- CTGTAGGGTA AAGTTAAGTT AGGATAGCGT TCGTTTGATC TGCTGGAACT ACAGCTTTCG AACTCACAGT   
  
  
- TCTGGCCTCT TCGTGAACAA TAATCAAGAC ACGAGGTTGA TGTAAGAGAA AACCGTAACC TACTCCCTAG   
  
  
- TTACGGATTC TTGCGTCCGT ACCGAATGAA CGTTTCCCAC AAAATATACT TTGGTGCTTT TAACCTCCTG   
  
  
- AACGGGTTGT TCCTAAACTA CTTTTACAAC TTGAGTTTAC TTCTAAGATG TAGTAGTAGT AGAAGTAGAG   
  
  
- GATAGGGAAG TAGTTTTGAA CTACGGAAAA ATTTTCGGGA GGAACCCGAA AGCGGTTTTG AGTACCAACA   
  
  
- TTGGCTCGTT CTTAGTTTGG TGTTACCTTC ACGGGATTAT CTTTCTCACT ACCTCAGTAA CTTGAAGATA   
  
  
- CGTCGTAACA AACTAACGAA CCTTAGGTGA TATAGCTCCT GTAGCTATCT CTCTGTCTTC GAGCTCTTCA   
  
  
- ACTACAAACC TCTCCTCTAG TTCTTGGAGT GTCGAACACT TCCCCGTCTC TCTTTCCGTT CCGTGCTCTT   
  
  
- CGAGTCACTT ACCCAAGTTT CTAAACTCAG TCATCCTAAA TTTTCCCTCG GTAACTCGAT GGTACCAAAG   
  
  
- AACGAACGAT CCTCCAAAAA TGTATGGTTA TTAATACTCC CCATATTGTA GTTCCTTCAG TTACCAAAAG   
  
  
- AACAATAGAC AACCGTTCTA TCTGGGGACA TAAGACAAAG TCGAACCTCT AAAAT

+     AT~TATA-box

| Site Name | Organism | Position | Strand | Matrix score. | sequence | function |
| --- | --- | --- | --- | --- | --- | --- |
| AT~TATA-box | Arabidopsis thaliana | 1530 | + | 6 | TATATA |  |
| AT~TATA-box | Arabidopsis thaliana | 1528 | - | 8 | TATATAAA |  |

>HU05G01267.1   
+ +Up\_Stream \_Len000TTACAA AAACGATGCT CTAAACCTAT TAGGTCGTTC CGAAACCCGC AAAATTGAAA   
  
  
+ TATGATCGCC CGTATTTTAC CCAAACCTGA AATACACTTA ATATGTATTT TACCTGAACC CAAATACACC   
  
  
+ GGACCTGTAT TTAACACGGG CACCCGAAAT GATAGGTCTA ACTCTCACCA CCACCGCATT TGGCACCCGA   
  
  
+ ACGGTAAAAA ATGTTCATCT ATTATGCACT TTGGCTTCAT CCATAACCCT ATGATCTATC TGTGCCAATA   
  
  
+ TTTGGAGCAC CCAGCGAAGT AGCGCATGCT CTAAACTCTT GCCTCAAAAC AAAACAAATA GTGGGTCATA   
  
  
+ TTCCCTTCAA AGCACTAACA AACACACACT CTCTCTCTCT CAGTAGCCAA CAACTTAAGG CGGTGTTTGC   
  
  
+ TTGGTTTTGG AGAGGGCGTG TTTGGATGAG GTGCAAAACA CATAGATGAA ATCATAAACA ACTTGATTCC   
  
  
+ CAGCCACCCA TGACCTACTC AGATAACAAG GGACAAAAAC AAAGAGGTTT TCTGAAACAG AGAAAACAGG   
  
  
+ GGCGTTGTGT TGCGCTGTTG CTCACAAAAT TCCAATATTC CTCTAAAAGC TCCCAAACGG AGACAAATCA   
  
  
+ AAGAGGACAA AGACATGTGG GATTTAATAC AAAATTTGAC TAACTCTGCA ACGGAAAACA ACCCCTAGGG   
  
  
+ TGTTTTTCTG CGTGTTGTAC ACACTTCACT TTTAACCTGC TCTGCCTCTG CCTTTGCCAC GGCTTTTAAT   
  
  
+ GGATAAGACC AATTAAGAAA CCCAAATATC TCGTAAATTT GTTGAGATTC CTTTCTGCGT TCTCAAGTGT   
  
  
+ TTCAAAAACC CAGTTTGGAG GTTGCACTTA TTCATGCTTT TCTTCAAGTC AGTGAGCTTA GTGTCTGGAA   
  
  
+ CTGAAGCTCC TCCTGGTAAT GCTCTGTTTT TCATTTCTTG TTGTGCGTTT ATAAAAATTC GGCCTATTGA   
  
  
+ TGGAAGTTTG TTTGATGTTT TTGGGTAAGT TTTTGATTAA TAGAGATGAA TTAGTAAAGT AAAATTTGAG   
  
  
+ TGGTATTTAA TGATCCTTAA GCAGTCTATG CTTTGTTGTC ACATGCGTTT CTTGGCTTTT GACTGAAACA   
  
  
+ TTTCTGTGTT AGATGAAAAA CTCATTTTGA TGCATGAGAT GATAAAATCG CTTATTTTTG CCTTTATTTG   
  
  
+ GTGTGTGGGG GGGGGGGGGG GGGGGCGCAG GTCTGAGGTA AAAGATATGT GAATTCATTT GGAGTCTCGA   
  
  
+ TAATTCGTTG GCTATGTTGA CAATCACGAG TGGTCGTTAT CGCTCATCTC ACAGGTTGAT AATTCACTCA   
  
  
+ AACTTCAAGC GGCCTGAATC ATTCAGTCCG GGTTGTTAAA ATCTCAACAG TCATTTCACA CGAGTGGAGG   
  
  
+ GCCCCAAAAG ACACCATTCT TTCGAACTTT TTTTTTTTGA AGGTCATTTT TTCAAGAACA AAACTAATGT   
  
  
+ GAACATGCGA CTTCTATGAG TAAAGTGTAA AGATGCATAA AGTCTTTTTT TCTTTTATAT AAAAAGTACT   
  
  
+ CAAAAAAAAA AGAGTTGGAG AGCTGATTCC CCATGCATCC TCTAAGCAAA GGTGGTGGAC GGCAGAAAAT   
  
  
+ TAGTAGAGTT ATTGTGAGAT TAAAGTTTGC GCACCTGAAC GGCTGACCTC TAGTTTGCTA TCAACGTTAC   
  
  
+ ATTTATGTGT CATAATTTTT GCATGAAACA ATTTCACGCA ATGTTTTCTG ATGGCAGTTA TGTTTAGAAA   
  
  
+ TAAGTTTAAT CGGACCTTAT TCCACTGATT TATATTTGAT TCTTCCCTCT TTACTACGGC TGTCCATTCA   
  
  
+ CAAGCCAAAG AATTCTCCCC CCAAAAAAAA GAAAAAACAG TATAGAACCA TGTTTATTAT CTCATTTTTT   
  
  
+ TCTCTCCCAT TTGATCTGAA ACACAAACTT GCAGGGGTGC AAGTTGGACA CTTAGTAGCT GCAGGAATGA   
  
  
+ TTCAATAGGA ATCCTCATCT TTGACAACAT CACCACTTGA ATACATATCA CTTGATGGCA CTCTCCCCTG   
  
  
+ GTTTAGGCTT CCCATATCCG TGGCTTAGGG AGCTAAAACC TGAGCAAAGA GGACTCTGTC TAATCCATCT   
  
  
+ CCTCCTTTCA TGTGCTAATC AAGTTGCTAC TGGGAGCATC GACAATGCTA ATGTCAGCCT TGAGCATATT   
  
  
+ TCCCACCTTG CCTCTCCCAC CGGAGATACA ATGCAGCGAA TTGCTGCTTA CTTTGCTGAA GCCTTTGCTG   
  
  
+ ACCGTTTACT AAGGGCTTGG CAGCCCGGTC TTCTCAAAGC CTTGAATTGT ACCAAGATGT CATCTGTTTC   
  
  
+ CGAACAAATT CTTGTTCAAA AGTTGTTCTT TGATCTTCTT CCGTTCTTGA AGCTTTCATA TCTTGTGACG   
  
  
+ AACCAGGCAA TCACGGAGGC CATAGAAGGA GAAAAGATGG TTCATATAAT TGATCTCCAT TCTTGTGAAT   
  
  
+ CGGCTCTGTG GATTAGTCTC CTCCAGGCAT TGAGTGTTCG ACCTGAAGGC CCACCCCATT TGAGGATAAC   
  
  
+ CGGTATACAT GAGAAGAAAG AAGTGTTGGA TCAAATGGCT ATGCAACTAA ACAAAGAGGC TGAAAAATTG   
  
  
+ GACATCCCAT TTCAATTCAA TCCTATCGCA AGCAAACTAG ACGACCTTGA TGTCGAAAGC TTGAGTGTCA   
  
  
+ AGACCGGAGA AGCACTTGTT ATTAGTTCTG TGCTCCAACT ACATTCTCTT TTGGCATTGG ATGAGGGATC   
  
  
+ AATGCCTAAG AACGCAGGCA TGGCTTACTT GCAAAGGGTG TTTTATATGA AACCACGAAA ATTGGAGGAC   
  
  
+ TTGCCCAACA AGGATTTGAT GAAAATGTTG AACTCAAATG AAGATTCTAC ATCATCATCA TCTTCATCTC   
  
  
+ CTATCCCTTC ATCAAAACTT GATGCCTTTT TAAAAGCCCT CCTTGGGCTT TCGCCAAAAC TCATGGTTGT   
  
  
+ AACCGAGCAA GAATCAAACC ACAATGGAAG TGCCCTAATA GAAAGAGTGA TGGAGTCATT GAACTTCTAT   
  
  
+ GCAGCATTGT TTGATTGCTT GGAATCCACT ATATCGAGGA CATCGATAGA GAGACAGAAG CTCGAGAAGT   
  
  
+ TGATGTTTGG AGAGGAGATC AAGAACCTCA CAGCTTGTGA AGGGGCAGAG AGAAAGGCAA GGCACGAGAA   
  
  
+ GCTCAGTGAA TGGGTTCAAA GATTTGAGTC AGTAGGATTT AAAAGGGAGC CATTGAGCTA CCATGGTTTC   
  
  
+ TTGCTTGCTA GGAGGTTTTT ACATACCAAT AATTATGAGG GGTATAACAT CAAGGAAGTC AATGGTTTTC   
  
  
+ TTGTTATCTG TTGGCAAGAT AGACCCCTGT ATTCTGTTTC AGCTTGGAGA TTTTA  

- +Up\_Stream \_Len000AATGTT TTTGCTACGA GATTTGGATA ATCCAGCAAG GCTTTGGGCG TTTTAACTTT   
  
  
- ATACTAGCGG GCATAAAATG GGTTTGGACT TTATGTGAAT TATACATAAA ATGGACTTGG GTTTATGTGG   
  
  
- CCTGGACATA AATTGTGCCC GTGGGCTTTA CTATCCAGAT TGAGAGTGGT GGTGGCGTAA ACCGTGGGCT   
  
  
- TGCCATTTTT TACAAGTAGA TAATACGTGA AACCGAAGTA GGTATTGGGA TACTAGATAG ACACGGTTAT   
  
  
- AAACCTCGTG GGTCGCTTCA TCGCGTACGA GATTTGAGAA CGGAGTTTTG TTTTGTTTAT CACCCAGTAT   
  
  
- AAGGGAAGTT TCGTGATTGT TTGTGTGTGA GAGAGAGAGA GTCATCGGTT GTTGAATTCC GCCACAAACG   
  
  
- AACCAAAACC TCTCCCGCAC AAACCTACTC CACGTTTTGT GTATCTACTT TAGTATTTGT TGAACTAAGG   
  
  
- GTCGGTGGGT ACTGGATGAG TCTATTGTTC CCTGTTTTTG TTTCTCCAAA AGACTTTGTC TCTTTTGTCC   
  
  
- CCGCAACACA ACGCGACAAC GAGTGTTTTA AGGTTATAAG GAGATTTTCG AGGGTTTGCC TCTGTTTAGT   
  
  
- TTCTCCTGTT TCTGTACACC CTAAATTATG TTTTAAACTG ATTGAGACGT TGCCTTTTGT TGGGGATCCC   
  
  
- ACAAAAAGAC GCACAACATG TGTGAAGTGA AAATTGGACG AGACGGAGAC GGAAACGGTG CCGAAAATTA   
  
  
- CCTATTCTGG TTAATTCTTT GGGTTTATAG AGCATTTAAA CAACTCTAAG GAAAGACGCA AGAGTTCACA   
  
  
- AAGTTTTTGG GTCAAACCTC CAACGTGAAT AAGTACGAAA AGAAGTTCAG TCACTCGAAT CACAGACCTT   
  
  
- GACTTCGAGG AGGACCATTA CGAGACAAAA AGTAAAGAAC AACACGCAAA TATTTTTAAG CCGGATAACT   
  
  
- ACCTTCAAAC AAACTACAAA AACCCATTCA AAAACTAATT ATCTCTACTT AATCATTTCA TTTTAAACTC   
  
  
- ACCATAAATT ACTAGGAATT CGTCAGATAC GAAACAACAG TGTACGCAAA GAACCGAAAA CTGACTTTGT   
  
  
- AAAGACACAA TCTACTTTTT GAGTAAAACT ACGTACTCTA CTATTTTAGC GAATAAAAAC GGAAATAAAC   
  
  
- CACACACCCC CCCCCCCCCC CCCCCGCGTC CAGACTCCAT TTTCTATACA CTTAAGTAAA CCTCAGAGCT   
  
  
- ATTAAGCAAC CGATACAACT GTTAGTGCTC ACCAGCAATA GCGAGTAGAG TGTCCAACTA TTAAGTGAGT   
  
  
- TTGAAGTTCG CCGGACTTAG TAAGTCAGGC CCAACAATTT TAGAGTTGTC AGTAAAGTGT GCTCACCTCC   
  
  
- CGGGGTTTTC TGTGGTAAGA AAGCTTGAAA AAAAAAAACT TCCAGTAAAA AAGTTCTTGT TTTGATTACA   
  
  
- CTTGTACGCT GAAGATACTC ATTTCACATT TCTACGTATT TCAGAAAAAA AGAAAATATA TTTTTCATGA   
  
  
- GTTTTTTTTT TCTCAACCTC TCGACTAAGG GGTACGTAGG AGATTCGTTT CCACCACCTG CCGTCTTTTA   
  
  
- ATCATCTCAA TAACACTCTA ATTTCAAACG CGTGGACTTG CCGACTGGAG ATCAAACGAT AGTTGCAATG   
  
  
- TAAATACACA GTATTAAAAA CGTACTTTGT TAAAGTGCGT TACAAAAGAC TACCGTCAAT ACAAATCTTT   
  
  
- ATTCAAATTA GCCTGGAATA AGGTGACTAA ATATAAACTA AGAAGGGAGA AATGATGCCG ACAGGTAAGT   
  
  
- GTTCGGTTTC TTAAGAGGGG GGTTTTTTTT CTTTTTTGTC ATATCTTGGT ACAAATAATA GAGTAAAAAA   
  
  
- AGAGAGGGTA AACTAGACTT TGTGTTTGAA CGTCCCCACG TTCAACCTGT GAATCATCGA CGTCCTTACT   
  
  
- AAGTTATCCT TAGGAGTAGA AACTGTTGTA GTGGTGAACT TATGTATAGT GAACTACCGT GAGAGGGGAC   
  
  
- CAAATCCGAA GGGTATAGGC ACCGAATCCC TCGATTTTGG ACTCGTTTCT CCTGAGACAG ATTAGGTAGA   
  
  
- GGAGGAAAGT ACACGATTAG TTCAACGATG ACCCTCGTAG CTGTTACGAT TACAGTCGGA ACTCGTATAA   
  
  
- AGGGTGGAAC GGAGAGGGTG GCCTCTATGT TACGTCGCTT AACGACGAAT GAAACGACTT CGGAAACGAC   
  
  
- TGGCAAATGA TTCCCGAACC GTCGGGCCAG AAGAGTTTCG GAACTTAACA TGGTTCTACA GTAGACAAAG   
  
  
- GCTTGTTTAA GAACAAGTTT TCAACAAGAA ACTAGAAGAA GGCAAGAACT TCGAAAGTAT AGAACACTGC   
  
  
- TTGGTCCGTT AGTGCCTCCG GTATCTTCCT CTTTTCTACC AAGTATATTA ACTAGAGGTA AGAACACTTA   
  
  
- GCCGAGACAC CTAATCAGAG GAGGTCCGTA ACTCACAAGC TGGACTTCCG GGTGGGGTAA ACTCCTATTG   
  
  
- GCCATATGTA CTCTTCTTTC TTCACAACCT AGTTTACCGA TACGTTGATT TGTTTCTCCG ACTTTTTAAC   
  
  
- CTGTAGGGTA AAGTTAAGTT AGGATAGCGT TCGTTTGATC TGCTGGAACT ACAGCTTTCG AACTCACAGT   
  
  
- TCTGGCCTCT TCGTGAACAA TAATCAAGAC ACGAGGTTGA TGTAAGAGAA AACCGTAACC TACTCCCTAG   
  
  
- TTACGGATTC TTGCGTCCGT ACCGAATGAA CGTTTCCCAC AAAATATACT TTGGTGCTTT TAACCTCCTG   
  
  
- AACGGGTTGT TCCTAAACTA CTTTTACAAC TTGAGTTTAC TTCTAAGATG TAGTAGTAGT AGAAGTAGAG   
  
  
- GATAGGGAAG TAGTTTTGAA CTACGGAAAA ATTTTCGGGA GGAACCCGAA AGCGGTTTTG AGTACCAACA   
  
  
- TTGGCTCGTT CTTAGTTTGG TGTTACCTTC ACGGGATTAT CTTTCTCACT ACCTCAGTAA CTTGAAGATA   
  
  
- CGTCGTAACA AACTAACGAA CCTTAGGTGA TATAGCTCCT GTAGCTATCT CTCTGTCTTC GAGCTCTTCA   
  
  
- ACTACAAACC TCTCCTCTAG TTCTTGGAGT GTCGAACACT TCCCCGTCTC TCTTTCCGTT CCGTGCTCTT   
  
  
- CGAGTCACTT ACCCAAGTTT CTAAACTCAG TCATCCTAAA TTTTCCCTCG GTAACTCGAT GGTACCAAAG   
  
  
- AACGAACGAT CCTCCAAAAA TGTATGGTTA TTAATACTCC CCATATTGTA GTTCCTTCAG TTACCAAAAG   
  
  
- AACAATAGAC AACCGTTCTA TCTGGGGACA TAAGACAAAG TCGAACCTCT AAAAT

+     Box 4

| Site Name | Organism | Position | Strand | Matrix score. | sequence | function |
| --- | --- | --- | --- | --- | --- | --- |
| Box 4 | Petroselinum crispum | 1020 | + | 6 | ATTAAT | part of a conserved DNA module involved in light responsiveness |

>HU05G01267.1   
+ +Up\_Stream \_Len000TTACAA AAACGATGCT CTAAACCTAT TAGGTCGTTC CGAAACCCGC AAAATTGAAA   
  
  
+ TATGATCGCC CGTATTTTAC CCAAACCTGA AATACACTTA ATATGTATTT TACCTGAACC CAAATACACC   
  
  
+ GGACCTGTAT TTAACACGGG CACCCGAAAT GATAGGTCTA ACTCTCACCA CCACCGCATT TGGCACCCGA   
  
  
+ ACGGTAAAAA ATGTTCATCT ATTATGCACT TTGGCTTCAT CCATAACCCT ATGATCTATC TGTGCCAATA   
  
  
+ TTTGGAGCAC CCAGCGAAGT AGCGCATGCT CTAAACTCTT GCCTCAAAAC AAAACAAATA GTGGGTCATA   
  
  
+ TTCCCTTCAA AGCACTAACA AACACACACT CTCTCTCTCT CAGTAGCCAA CAACTTAAGG CGGTGTTTGC   
  
  
+ TTGGTTTTGG AGAGGGCGTG TTTGGATGAG GTGCAAAACA CATAGATGAA ATCATAAACA ACTTGATTCC   
  
  
+ CAGCCACCCA TGACCTACTC AGATAACAAG GGACAAAAAC AAAGAGGTTT TCTGAAACAG AGAAAACAGG   
  
  
+ GGCGTTGTGT TGCGCTGTTG CTCACAAAAT TCCAATATTC CTCTAAAAGC TCCCAAACGG AGACAAATCA   
  
  
+ AAGAGGACAA AGACATGTGG GATTTAATAC AAAATTTGAC TAACTCTGCA ACGGAAAACA ACCCCTAGGG   
  
  
+ TGTTTTTCTG CGTGTTGTAC ACACTTCACT TTTAACCTGC TCTGCCTCTG CCTTTGCCAC GGCTTTTAAT   
  
  
+ GGATAAGACC AATTAAGAAA CCCAAATATC TCGTAAATTT GTTGAGATTC CTTTCTGCGT TCTCAAGTGT   
  
  
+ TTCAAAAACC CAGTTTGGAG GTTGCACTTA TTCATGCTTT TCTTCAAGTC AGTGAGCTTA GTGTCTGGAA   
  
  
+ CTGAAGCTCC TCCTGGTAAT GCTCTGTTTT TCATTTCTTG TTGTGCGTTT ATAAAAATTC GGCCTATTGA   
  
  
+ TGGAAGTTTG TTTGATGTTT TTGGGTAAGT TTTTGATTAA TAGAGATGAA TTAGTAAAGT AAAATTTGAG   
  
  
+ TGGTATTTAA TGATCCTTAA GCAGTCTATG CTTTGTTGTC ACATGCGTTT CTTGGCTTTT GACTGAAACA   
  
  
+ TTTCTGTGTT AGATGAAAAA CTCATTTTGA TGCATGAGAT GATAAAATCG CTTATTTTTG CCTTTATTTG   
  
  
+ GTGTGTGGGG GGGGGGGGGG GGGGGCGCAG GTCTGAGGTA AAAGATATGT GAATTCATTT GGAGTCTCGA   
  
  
+ TAATTCGTTG GCTATGTTGA CAATCACGAG TGGTCGTTAT CGCTCATCTC ACAGGTTGAT AATTCACTCA   
  
  
+ AACTTCAAGC GGCCTGAATC ATTCAGTCCG GGTTGTTAAA ATCTCAACAG TCATTTCACA CGAGTGGAGG   
  
  
+ GCCCCAAAAG ACACCATTCT TTCGAACTTT TTTTTTTTGA AGGTCATTTT TTCAAGAACA AAACTAATGT   
  
  
+ GAACATGCGA CTTCTATGAG TAAAGTGTAA AGATGCATAA AGTCTTTTTT TCTTTTATAT AAAAAGTACT   
  
  
+ CAAAAAAAAA AGAGTTGGAG AGCTGATTCC CCATGCATCC TCTAAGCAAA GGTGGTGGAC GGCAGAAAAT   
  
  
+ TAGTAGAGTT ATTGTGAGAT TAAAGTTTGC GCACCTGAAC GGCTGACCTC TAGTTTGCTA TCAACGTTAC   
  
  
+ ATTTATGTGT CATAATTTTT GCATGAAACA ATTTCACGCA ATGTTTTCTG ATGGCAGTTA TGTTTAGAAA   
  
  
+ TAAGTTTAAT CGGACCTTAT TCCACTGATT TATATTTGAT TCTTCCCTCT TTACTACGGC TGTCCATTCA   
  
  
+ CAAGCCAAAG AATTCTCCCC CCAAAAAAAA GAAAAAACAG TATAGAACCA TGTTTATTAT CTCATTTTTT   
  
  
+ TCTCTCCCAT TTGATCTGAA ACACAAACTT GCAGGGGTGC AAGTTGGACA CTTAGTAGCT GCAGGAATGA   
  
  
+ TTCAATAGGA ATCCTCATCT TTGACAACAT CACCACTTGA ATACATATCA CTTGATGGCA CTCTCCCCTG   
  
  
+ GTTTAGGCTT CCCATATCCG TGGCTTAGGG AGCTAAAACC TGAGCAAAGA GGACTCTGTC TAATCCATCT   
  
  
+ CCTCCTTTCA TGTGCTAATC AAGTTGCTAC TGGGAGCATC GACAATGCTA ATGTCAGCCT TGAGCATATT   
  
  
+ TCCCACCTTG CCTCTCCCAC CGGAGATACA ATGCAGCGAA TTGCTGCTTA CTTTGCTGAA GCCTTTGCTG   
  
  
+ ACCGTTTACT AAGGGCTTGG CAGCCCGGTC TTCTCAAAGC CTTGAATTGT ACCAAGATGT CATCTGTTTC   
  
  
+ CGAACAAATT CTTGTTCAAA AGTTGTTCTT TGATCTTCTT CCGTTCTTGA AGCTTTCATA TCTTGTGACG   
  
  
+ AACCAGGCAA TCACGGAGGC CATAGAAGGA GAAAAGATGG TTCATATAAT TGATCTCCAT TCTTGTGAAT   
  
  
+ CGGCTCTGTG GATTAGTCTC CTCCAGGCAT TGAGTGTTCG ACCTGAAGGC CCACCCCATT TGAGGATAAC   
  
  
+ CGGTATACAT GAGAAGAAAG AAGTGTTGGA TCAAATGGCT ATGCAACTAA ACAAAGAGGC TGAAAAATTG   
  
  
+ GACATCCCAT TTCAATTCAA TCCTATCGCA AGCAAACTAG ACGACCTTGA TGTCGAAAGC TTGAGTGTCA   
  
  
+ AGACCGGAGA AGCACTTGTT ATTAGTTCTG TGCTCCAACT ACATTCTCTT TTGGCATTGG ATGAGGGATC   
  
  
+ AATGCCTAAG AACGCAGGCA TGGCTTACTT GCAAAGGGTG TTTTATATGA AACCACGAAA ATTGGAGGAC   
  
  
+ TTGCCCAACA AGGATTTGAT GAAAATGTTG AACTCAAATG AAGATTCTAC ATCATCATCA TCTTCATCTC   
  
  
+ CTATCCCTTC ATCAAAACTT GATGCCTTTT TAAAAGCCCT CCTTGGGCTT TCGCCAAAAC TCATGGTTGT   
  
  
+ AACCGAGCAA GAATCAAACC ACAATGGAAG TGCCCTAATA GAAAGAGTGA TGGAGTCATT GAACTTCTAT   
  
  
+ GCAGCATTGT TTGATTGCTT GGAATCCACT ATATCGAGGA CATCGATAGA GAGACAGAAG CTCGAGAAGT   
  
  
+ TGATGTTTGG AGAGGAGATC AAGAACCTCA CAGCTTGTGA AGGGGCAGAG AGAAAGGCAA GGCACGAGAA   
  
  
+ GCTCAGTGAA TGGGTTCAAA GATTTGAGTC AGTAGGATTT AAAAGGGAGC CATTGAGCTA CCATGGTTTC   
  
  
+ TTGCTTGCTA GGAGGTTTTT ACATACCAAT AATTATGAGG GGTATAACAT CAAGGAAGTC AATGGTTTTC   
  
  
+ TTGTTATCTG TTGGCAAGAT AGACCCCTGT ATTCTGTTTC AGCTTGGAGA TTTTA  

- +Up\_Stream \_Len000AATGTT TTTGCTACGA GATTTGGATA ATCCAGCAAG GCTTTGGGCG TTTTAACTTT   
  
  
- ATACTAGCGG GCATAAAATG GGTTTGGACT TTATGTGAAT TATACATAAA ATGGACTTGG GTTTATGTGG   
  
  
- CCTGGACATA AATTGTGCCC GTGGGCTTTA CTATCCAGAT TGAGAGTGGT GGTGGCGTAA ACCGTGGGCT   
  
  
- TGCCATTTTT TACAAGTAGA TAATACGTGA AACCGAAGTA GGTATTGGGA TACTAGATAG ACACGGTTAT   
  
  
- AAACCTCGTG GGTCGCTTCA TCGCGTACGA GATTTGAGAA CGGAGTTTTG TTTTGTTTAT CACCCAGTAT   
  
  
- AAGGGAAGTT TCGTGATTGT TTGTGTGTGA GAGAGAGAGA GTCATCGGTT GTTGAATTCC GCCACAAACG   
  
  
- AACCAAAACC TCTCCCGCAC AAACCTACTC CACGTTTTGT GTATCTACTT TAGTATTTGT TGAACTAAGG   
  
  
- GTCGGTGGGT ACTGGATGAG TCTATTGTTC CCTGTTTTTG TTTCTCCAAA AGACTTTGTC TCTTTTGTCC   
  
  
- CCGCAACACA ACGCGACAAC GAGTGTTTTA AGGTTATAAG GAGATTTTCG AGGGTTTGCC TCTGTTTAGT   
  
  
- TTCTCCTGTT TCTGTACACC CTAAATTATG TTTTAAACTG ATTGAGACGT TGCCTTTTGT TGGGGATCCC   
  
  
- ACAAAAAGAC GCACAACATG TGTGAAGTGA AAATTGGACG AGACGGAGAC GGAAACGGTG CCGAAAATTA   
  
  
- CCTATTCTGG TTAATTCTTT GGGTTTATAG AGCATTTAAA CAACTCTAAG GAAAGACGCA AGAGTTCACA   
  
  
- AAGTTTTTGG GTCAAACCTC CAACGTGAAT AAGTACGAAA AGAAGTTCAG TCACTCGAAT CACAGACCTT   
  
  
- GACTTCGAGG AGGACCATTA CGAGACAAAA AGTAAAGAAC AACACGCAAA TATTTTTAAG CCGGATAACT   
  
  
- ACCTTCAAAC AAACTACAAA AACCCATTCA AAAACTAATT ATCTCTACTT AATCATTTCA TTTTAAACTC   
  
  
- ACCATAAATT ACTAGGAATT CGTCAGATAC GAAACAACAG TGTACGCAAA GAACCGAAAA CTGACTTTGT   
  
  
- AAAGACACAA TCTACTTTTT GAGTAAAACT ACGTACTCTA CTATTTTAGC GAATAAAAAC GGAAATAAAC   
  
  
- CACACACCCC CCCCCCCCCC CCCCCGCGTC CAGACTCCAT TTTCTATACA CTTAAGTAAA CCTCAGAGCT   
  
  
- ATTAAGCAAC CGATACAACT GTTAGTGCTC ACCAGCAATA GCGAGTAGAG TGTCCAACTA TTAAGTGAGT   
  
  
- TTGAAGTTCG CCGGACTTAG TAAGTCAGGC CCAACAATTT TAGAGTTGTC AGTAAAGTGT GCTCACCTCC   
  
  
- CGGGGTTTTC TGTGGTAAGA AAGCTTGAAA AAAAAAAACT TCCAGTAAAA AAGTTCTTGT TTTGATTACA   
  
  
- CTTGTACGCT GAAGATACTC ATTTCACATT TCTACGTATT TCAGAAAAAA AGAAAATATA TTTTTCATGA   
  
  
- GTTTTTTTTT TCTCAACCTC TCGACTAAGG GGTACGTAGG AGATTCGTTT CCACCACCTG CCGTCTTTTA   
  
  
- ATCATCTCAA TAACACTCTA ATTTCAAACG CGTGGACTTG CCGACTGGAG ATCAAACGAT AGTTGCAATG   
  
  
- TAAATACACA GTATTAAAAA CGTACTTTGT TAAAGTGCGT TACAAAAGAC TACCGTCAAT ACAAATCTTT   
  
  
- ATTCAAATTA GCCTGGAATA AGGTGACTAA ATATAAACTA AGAAGGGAGA AATGATGCCG ACAGGTAAGT   
  
  
- GTTCGGTTTC TTAAGAGGGG GGTTTTTTTT CTTTTTTGTC ATATCTTGGT ACAAATAATA GAGTAAAAAA   
  
  
- AGAGAGGGTA AACTAGACTT TGTGTTTGAA CGTCCCCACG TTCAACCTGT GAATCATCGA CGTCCTTACT   
  
  
- AAGTTATCCT TAGGAGTAGA AACTGTTGTA GTGGTGAACT TATGTATAGT GAACTACCGT GAGAGGGGAC   
  
  
- CAAATCCGAA GGGTATAGGC ACCGAATCCC TCGATTTTGG ACTCGTTTCT CCTGAGACAG ATTAGGTAGA   
  
  
- GGAGGAAAGT ACACGATTAG TTCAACGATG ACCCTCGTAG CTGTTACGAT TACAGTCGGA ACTCGTATAA   
  
  
- AGGGTGGAAC GGAGAGGGTG GCCTCTATGT TACGTCGCTT AACGACGAAT GAAACGACTT CGGAAACGAC   
  
  
- TGGCAAATGA TTCCCGAACC GTCGGGCCAG AAGAGTTTCG GAACTTAACA TGGTTCTACA GTAGACAAAG   
  
  
- GCTTGTTTAA GAACAAGTTT TCAACAAGAA ACTAGAAGAA GGCAAGAACT TCGAAAGTAT AGAACACTGC   
  
  
- TTGGTCCGTT AGTGCCTCCG GTATCTTCCT CTTTTCTACC AAGTATATTA ACTAGAGGTA AGAACACTTA   
  
  
- GCCGAGACAC CTAATCAGAG GAGGTCCGTA ACTCACAAGC TGGACTTCCG GGTGGGGTAA ACTCCTATTG   
  
  
- GCCATATGTA CTCTTCTTTC TTCACAACCT AGTTTACCGA TACGTTGATT TGTTTCTCCG ACTTTTTAAC   
  
  
- CTGTAGGGTA AAGTTAAGTT AGGATAGCGT TCGTTTGATC TGCTGGAACT ACAGCTTTCG AACTCACAGT   
  
  
- TCTGGCCTCT TCGTGAACAA TAATCAAGAC ACGAGGTTGA TGTAAGAGAA AACCGTAACC TACTCCCTAG   
  
  
- TTACGGATTC TTGCGTCCGT ACCGAATGAA CGTTTCCCAC AAAATATACT TTGGTGCTTT TAACCTCCTG   
  
  
- AACGGGTTGT TCCTAAACTA CTTTTACAAC TTGAGTTTAC TTCTAAGATG TAGTAGTAGT AGAAGTAGAG   
  
  
- GATAGGGAAG TAGTTTTGAA CTACGGAAAA ATTTTCGGGA GGAACCCGAA AGCGGTTTTG AGTACCAACA   
  
  
- TTGGCTCGTT CTTAGTTTGG TGTTACCTTC ACGGGATTAT CTTTCTCACT ACCTCAGTAA CTTGAAGATA   
  
  
- CGTCGTAACA AACTAACGAA CCTTAGGTGA TATAGCTCCT GTAGCTATCT CTCTGTCTTC GAGCTCTTCA   
  
  
- ACTACAAACC TCTCCTCTAG TTCTTGGAGT GTCGAACACT TCCCCGTCTC TCTTTCCGTT CCGTGCTCTT   
  
  
- CGAGTCACTT ACCCAAGTTT CTAAACTCAG TCATCCTAAA TTTTCCCTCG GTAACTCGAT GGTACCAAAG   
  
  
- AACGAACGAT CCTCCAAAAA TGTATGGTTA TTAATACTCC CCATATTGTA GTTCCTTCAG TTACCAAAAG   
  
  
- AACAATAGAC AACCGTTCTA TCTGGGGACA TAAGACAAAG TCGAACCTCT AAAAT

+     CAAT-box

| Site Name | Organism | Position | Strand | Matrix score. | sequence | function |
| --- | --- | --- | --- | --- | --- | --- |
| CAAT-box | Nicotiana glutinosa | 2966 | + | 4 | CAAT |  |
| CAAT-box | Pisum sativum | 2839 | + | 5 | CAAAT | common cis-acting element in promoter and enhancer regions |
| CAAT-box | Petunia hybrida | 3304 | - | 7 | TGCCAAC | common cis-acting element in promoter and enhancer regions |
| CAAT-box | Nicotiana glutinosa | 3284 | + | 4 | CAAT |  |
| CAAT-box | Pisum sativum | 2556 | + | 5 | CAAAT | common cis-acting element in promoter and enhancer regions |
| CAAT-box | Pisum sativum | 2512 | - | 5 | CAAAT | common cis-acting element in promoter and enhancer regions |
| CAAT-box | Nicotiana glutinosa | 2147 | + | 4 | CAAT |  |
| CAAT-box | Pisum sativum | 135 | + | 5 | CAAAT | common cis-acting element in promoter and enhancer regions |
| CAAT-box | Nicotiana glutinosa | 3206 | - | 4 | CAAT |  |
| CAAT-box | Pisum sativum | 628 | + | 5 | CAAAT | common cis-acting element in promoter and enhancer regions |
| CAAT-box | Arabidopsis thaliana | 2795 | - | 5 | CCAAT | common cis-acting element in promoter and enhancer regions |
| CAAT-box | Nicotiana glutinosa | 2734 | + | 4 | CAAT |  |
| CAAT-box | Nicotiana glutinosa | 2612 | + | 4 | CAAT |  |
| CAAT-box | Nicotiana glutinosa | 2607 | + | 4 | CAAT |  |
| CAAT-box | Pisum sativum | 1048 | - | 5 | CAAAT | common cis-acting element in promoter and enhancer regions |
| CAAT-box | Pisum sativum | 284 | - | 5 | CAAAT | common cis-acting element in promoter and enhancer regions |
| CAAT-box | Arabidopsis thaliana | 3250 | + | 5 | CCAAT | common cis-acting element in promoter and enhancer regions |
| CAAT-box | Nicotiana glutinosa | 1723 | + | 4 | CAAT |  |
| CAAT-box | Pisum sativum | 1903 | - | 5 | CAAAT | common cis-acting element in promoter and enhancer regions |
| CAAT-box | Arabidopsis thaliana | 2591 | - | 5 | CCAAT | common cis-acting element in promoter and enhancer regions |
| CAAT-box | Nicotiana glutinosa | 784 | + | 4 | CAAT |  |
| CAAT-box | Nicotiana glutinosa | 3020 | - | 4 | CAAT |  |
| CAAT-box | Arabidopsis thaliana | 2720 | - | 5 | CCAAT | common cis-acting element in promoter and enhancer regions |
| CAAT-box | Pisum sativum | 1190 | - | 5 | CAAAT | common cis-acting element in promoter and enhancer regions |
| CAAT-box | Nicotiana glutinosa | 1285 | + | 4 | CAAT |  |
| CAAT-box | Pisum sativum | 2818 | - | 5 | CAAAT | common cis-acting element in promoter and enhancer regions |
| CAAT-box | Nicotiana glutinosa | 980 | - | 4 | CAAT |  |
| CAAT-box | Pisum sativum | 668 | - | 5 | CAAAT | common cis-acting element in promoter and enhancer regions |
| CAAT-box | Nicotiana glutinosa | 68 | - | 4 | CAAT |  |
| CAAT-box | Nicotiana glutinosa | 2433 | - | 4 | CAAT |  |
| CAAT-box | Nicotiana glutinosa | 2203 | + | 4 | CAAT |  |
| CAAT-box | Pisum sativum | 3176 | - | 5 | CAAAT | common cis-acting element in promoter and enhancer regions |
| CAAT-box | Nicotiana glutinosa | 2214 | - | 4 | CAAT |  |
| CAAT-box | Pisum sativum | 202 | - | 5 | CAAAT | common cis-acting element in promoter and enhancer regions |
| CAAT-box | Nicotiana glutinosa | 3028 | - | 4 | CAAT |  |
| CAAT-box | Pisum sativum | 811 | - | 5 | CAAAT | common cis-acting element in promoter and enhancer regions |
| CAAT-box | Pisum sativum | 797 | + | 5 | CAAAT | common cis-acting element in promoter and enhancer regions |
| CAAT-box | Nicotiana glutinosa | 2483 | - | 4 | CAAT |  |
| CAAT-box | Nicotiana glutinosa | 2392 | + | 4 | CAAT |  |
| CAAT-box | Nicotiana glutinosa | 2290 | - | 4 | CAAT |  |
| CAAT-box | Nicotiana glutinosa | 1625 | - | 4 | CAAT |  |
| CAAT-box | Pisum sativum | 339 | + | 5 | CAAAT | common cis-acting element in promoter and enhancer regions |
| CAAT-box | Pisum sativum | 2319 | + | 5 | CAAAT | common cis-acting element in promoter and enhancer regions |
| CAAT-box | Pisum sativum | 1788 | - | 5 | CAAAT | common cis-acting element in promoter and enhancer regions |
| CAAT-box | Nicotiana glutinosa | 3251 | + | 4 | CAAT |  |
| CAAT-box | Nicotiana glutinosa | 3002 | - | 4 | CAAT |  |
| CAAT-box | Nicotiana glutinosa | 597 | + | 4 | CAAT |  |
| CAAT-box | Pisum sativum | 1251 | - | 5 | CAAAT | common cis-acting element in promoter and enhancer regions |
| CAAT-box | Arabidopsis thaliana | 279 | + | 5 | CCAAT | common cis-acting element in promoter and enhancer regions |
| CAAT-box | Nicotiana glutinosa | 1967 | + | 4 | CAAT |  |
| CAAT-box | Arabidopsis thaliana | 783 | + | 5 | CCAAT | common cis-acting element in promoter and enhancer regions |
| CAAT-box | Nicotiana glutinosa | 1713 | + | 4 | CAAT |  |
| CAAT-box | Arabidopsis thaliana | 596 | + | 5 | CCAAT | common cis-acting element in promoter and enhancer regions |
| CAAT-box | Nicotiana glutinosa | 280 | + | 4 | CAAT |  |

>HU05G01267.1   
+ +Up\_Stream \_Len000TTACAA AAACGATGCT CTAAACCTAT TAGGTCGTTC CGAAACCCGC AAAATTGAAA   
  
  
+ TATGATCGCC CGTATTTTAC CCAAACCTGA AATACACTTA ATATGTATTT TACCTGAACC CAAATACACC   
  
  
+ GGACCTGTAT TTAACACGGG CACCCGAAAT GATAGGTCTA ACTCTCACCA CCACCGCATT TGGCACCCGA   
  
  
+ ACGGTAAAAA ATGTTCATCT ATTATGCACT TTGGCTTCAT CCATAACCCT ATGATCTATC TGTGCCAATA   
  
  
+ TTTGGAGCAC CCAGCGAAGT AGCGCATGCT CTAAACTCTT GCCTCAAAAC AAAACAAATA GTGGGTCATA   
  
  
+ TTCCCTTCAA AGCACTAACA AACACACACT CTCTCTCTCT CAGTAGCCAA CAACTTAAGG CGGTGTTTGC   
  
  
+ TTGGTTTTGG AGAGGGCGTG TTTGGATGAG GTGCAAAACA CATAGATGAA ATCATAAACA ACTTGATTCC   
  
  
+ CAGCCACCCA TGACCTACTC AGATAACAAG GGACAAAAAC AAAGAGGTTT TCTGAAACAG AGAAAACAGG   
  
  
+ GGCGTTGTGT TGCGCTGTTG CTCACAAAAT TCCAATATTC CTCTAAAAGC TCCCAAACGG AGACAAATCA   
  
  
+ AAGAGGACAA AGACATGTGG GATTTAATAC AAAATTTGAC TAACTCTGCA ACGGAAAACA ACCCCTAGGG   
  
  
+ TGTTTTTCTG CGTGTTGTAC ACACTTCACT TTTAACCTGC TCTGCCTCTG CCTTTGCCAC GGCTTTTAAT   
  
  
+ GGATAAGACC AATTAAGAAA CCCAAATATC TCGTAAATTT GTTGAGATTC CTTTCTGCGT TCTCAAGTGT   
  
  
+ TTCAAAAACC CAGTTTGGAG GTTGCACTTA TTCATGCTTT TCTTCAAGTC AGTGAGCTTA GTGTCTGGAA   
  
  
+ CTGAAGCTCC TCCTGGTAAT GCTCTGTTTT TCATTTCTTG TTGTGCGTTT ATAAAAATTC GGCCTATTGA   
  
  
+ TGGAAGTTTG TTTGATGTTT TTGGGTAAGT TTTTGATTAA TAGAGATGAA TTAGTAAAGT AAAATTTGAG   
  
  
+ TGGTATTTAA TGATCCTTAA GCAGTCTATG CTTTGTTGTC ACATGCGTTT CTTGGCTTTT GACTGAAACA   
  
  
+ TTTCTGTGTT AGATGAAAAA CTCATTTTGA TGCATGAGAT GATAAAATCG CTTATTTTTG CCTTTATTTG   
  
  
+ GTGTGTGGGG GGGGGGGGGG GGGGGCGCAG GTCTGAGGTA AAAGATATGT GAATTCATTT GGAGTCTCGA   
  
  
+ TAATTCGTTG GCTATGTTGA CAATCACGAG TGGTCGTTAT CGCTCATCTC ACAGGTTGAT AATTCACTCA   
  
  
+ AACTTCAAGC GGCCTGAATC ATTCAGTCCG GGTTGTTAAA ATCTCAACAG TCATTTCACA CGAGTGGAGG   
  
  
+ GCCCCAAAAG ACACCATTCT TTCGAACTTT TTTTTTTTGA AGGTCATTTT TTCAAGAACA AAACTAATGT   
  
  
+ GAACATGCGA CTTCTATGAG TAAAGTGTAA AGATGCATAA AGTCTTTTTT TCTTTTATAT AAAAAGTACT   
  
  
+ CAAAAAAAAA AGAGTTGGAG AGCTGATTCC CCATGCATCC TCTAAGCAAA GGTGGTGGAC GGCAGAAAAT   
  
  
+ TAGTAGAGTT ATTGTGAGAT TAAAGTTTGC GCACCTGAAC GGCTGACCTC TAGTTTGCTA TCAACGTTAC   
  
  
+ ATTTATGTGT CATAATTTTT GCATGAAACA ATTTCACGCA ATGTTTTCTG ATGGCAGTTA TGTTTAGAAA   
  
  
+ TAAGTTTAAT CGGACCTTAT TCCACTGATT TATATTTGAT TCTTCCCTCT TTACTACGGC TGTCCATTCA   
  
  
+ CAAGCCAAAG AATTCTCCCC CCAAAAAAAA GAAAAAACAG TATAGAACCA TGTTTATTAT CTCATTTTTT   
  
  
+ TCTCTCCCAT TTGATCTGAA ACACAAACTT GCAGGGGTGC AAGTTGGACA CTTAGTAGCT GCAGGAATGA   
  
  
+ TTCAATAGGA ATCCTCATCT TTGACAACAT CACCACTTGA ATACATATCA CTTGATGGCA CTCTCCCCTG   
  
  
+ GTTTAGGCTT CCCATATCCG TGGCTTAGGG AGCTAAAACC TGAGCAAAGA GGACTCTGTC TAATCCATCT   
  
  
+ CCTCCTTTCA TGTGCTAATC AAGTTGCTAC TGGGAGCATC GACAATGCTA ATGTCAGCCT TGAGCATATT   
  
  
+ TCCCACCTTG CCTCTCCCAC CGGAGATACA ATGCAGCGAA TTGCTGCTTA CTTTGCTGAA GCCTTTGCTG   
  
  
+ ACCGTTTACT AAGGGCTTGG CAGCCCGGTC TTCTCAAAGC CTTGAATTGT ACCAAGATGT CATCTGTTTC   
  
  
+ CGAACAAATT CTTGTTCAAA AGTTGTTCTT TGATCTTCTT CCGTTCTTGA AGCTTTCATA TCTTGTGACG   
  
  
+ AACCAGGCAA TCACGGAGGC CATAGAAGGA GAAAAGATGG TTCATATAAT TGATCTCCAT TCTTGTGAAT   
  
  
+ CGGCTCTGTG GATTAGTCTC CTCCAGGCAT TGAGTGTTCG ACCTGAAGGC CCACCCCATT TGAGGATAAC   
  
  
+ CGGTATACAT GAGAAGAAAG AAGTGTTGGA TCAAATGGCT ATGCAACTAA ACAAAGAGGC TGAAAAATTG   
  
  
+ GACATCCCAT TTCAATTCAA TCCTATCGCA AGCAAACTAG ACGACCTTGA TGTCGAAAGC TTGAGTGTCA   
  
  
+ AGACCGGAGA AGCACTTGTT ATTAGTTCTG TGCTCCAACT ACATTCTCTT TTGGCATTGG ATGAGGGATC   
  
  
+ AATGCCTAAG AACGCAGGCA TGGCTTACTT GCAAAGGGTG TTTTATATGA AACCACGAAA ATTGGAGGAC   
  
  
+ TTGCCCAACA AGGATTTGAT GAAAATGTTG AACTCAAATG AAGATTCTAC ATCATCATCA TCTTCATCTC   
  
  
+ CTATCCCTTC ATCAAAACTT GATGCCTTTT TAAAAGCCCT CCTTGGGCTT TCGCCAAAAC TCATGGTTGT   
  
  
+ AACCGAGCAA GAATCAAACC ACAATGGAAG TGCCCTAATA GAAAGAGTGA TGGAGTCATT GAACTTCTAT   
  
  
+ GCAGCATTGT TTGATTGCTT GGAATCCACT ATATCGAGGA CATCGATAGA GAGACAGAAG CTCGAGAAGT   
  
  
+ TGATGTTTGG AGAGGAGATC AAGAACCTCA CAGCTTGTGA AGGGGCAGAG AGAAAGGCAA GGCACGAGAA   
  
  
+ GCTCAGTGAA TGGGTTCAAA GATTTGAGTC AGTAGGATTT AAAAGGGAGC CATTGAGCTA CCATGGTTTC   
  
  
+ TTGCTTGCTA GGAGGTTTTT ACATACCAAT AATTATGAGG GGTATAACAT CAAGGAAGTC AATGGTTTTC   
  
  
+ TTGTTATCTG TTGGCAAGAT AGACCCCTGT ATTCTGTTTC AGCTTGGAGA TTTTA  

- +Up\_Stream \_Len000AATGTT TTTGCTACGA GATTTGGATA ATCCAGCAAG GCTTTGGGCG TTTTAACTTT   
  
  
- ATACTAGCGG GCATAAAATG GGTTTGGACT TTATGTGAAT TATACATAAA ATGGACTTGG GTTTATGTGG   
  
  
- CCTGGACATA AATTGTGCCC GTGGGCTTTA CTATCCAGAT TGAGAGTGGT GGTGGCGTAA ACCGTGGGCT   
  
  
- TGCCATTTTT TACAAGTAGA TAATACGTGA AACCGAAGTA GGTATTGGGA TACTAGATAG ACACGGTTAT   
  
  
- AAACCTCGTG GGTCGCTTCA TCGCGTACGA GATTTGAGAA CGGAGTTTTG TTTTGTTTAT CACCCAGTAT   
  
  
- AAGGGAAGTT TCGTGATTGT TTGTGTGTGA GAGAGAGAGA GTCATCGGTT GTTGAATTCC GCCACAAACG   
  
  
- AACCAAAACC TCTCCCGCAC AAACCTACTC CACGTTTTGT GTATCTACTT TAGTATTTGT TGAACTAAGG   
  
  
- GTCGGTGGGT ACTGGATGAG TCTATTGTTC CCTGTTTTTG TTTCTCCAAA AGACTTTGTC TCTTTTGTCC   
  
  
- CCGCAACACA ACGCGACAAC GAGTGTTTTA AGGTTATAAG GAGATTTTCG AGGGTTTGCC TCTGTTTAGT   
  
  
- TTCTCCTGTT TCTGTACACC CTAAATTATG TTTTAAACTG ATTGAGACGT TGCCTTTTGT TGGGGATCCC   
  
  
- ACAAAAAGAC GCACAACATG TGTGAAGTGA AAATTGGACG AGACGGAGAC GGAAACGGTG CCGAAAATTA   
  
  
- CCTATTCTGG TTAATTCTTT GGGTTTATAG AGCATTTAAA CAACTCTAAG GAAAGACGCA AGAGTTCACA   
  
  
- AAGTTTTTGG GTCAAACCTC CAACGTGAAT AAGTACGAAA AGAAGTTCAG TCACTCGAAT CACAGACCTT   
  
  
- GACTTCGAGG AGGACCATTA CGAGACAAAA AGTAAAGAAC AACACGCAAA TATTTTTAAG CCGGATAACT   
  
  
- ACCTTCAAAC AAACTACAAA AACCCATTCA AAAACTAATT ATCTCTACTT AATCATTTCA TTTTAAACTC   
  
  
- ACCATAAATT ACTAGGAATT CGTCAGATAC GAAACAACAG TGTACGCAAA GAACCGAAAA CTGACTTTGT   
  
  
- AAAGACACAA TCTACTTTTT GAGTAAAACT ACGTACTCTA CTATTTTAGC GAATAAAAAC GGAAATAAAC   
  
  
- CACACACCCC CCCCCCCCCC CCCCCGCGTC CAGACTCCAT TTTCTATACA CTTAAGTAAA CCTCAGAGCT   
  
  
- ATTAAGCAAC CGATACAACT GTTAGTGCTC ACCAGCAATA GCGAGTAGAG TGTCCAACTA TTAAGTGAGT   
  
  
- TTGAAGTTCG CCGGACTTAG TAAGTCAGGC CCAACAATTT TAGAGTTGTC AGTAAAGTGT GCTCACCTCC   
  
  
- CGGGGTTTTC TGTGGTAAGA AAGCTTGAAA AAAAAAAACT TCCAGTAAAA AAGTTCTTGT TTTGATTACA   
  
  
- CTTGTACGCT GAAGATACTC ATTTCACATT TCTACGTATT TCAGAAAAAA AGAAAATATA TTTTTCATGA   
  
  
- GTTTTTTTTT TCTCAACCTC TCGACTAAGG GGTACGTAGG AGATTCGTTT CCACCACCTG CCGTCTTTTA   
  
  
- ATCATCTCAA TAACACTCTA ATTTCAAACG CGTGGACTTG CCGACTGGAG ATCAAACGAT AGTTGCAATG   
  
  
- TAAATACACA GTATTAAAAA CGTACTTTGT TAAAGTGCGT TACAAAAGAC TACCGTCAAT ACAAATCTTT   
  
  
- ATTCAAATTA GCCTGGAATA AGGTGACTAA ATATAAACTA AGAAGGGAGA AATGATGCCG ACAGGTAAGT   
  
  
- GTTCGGTTTC TTAAGAGGGG GGTTTTTTTT CTTTTTTGTC ATATCTTGGT ACAAATAATA GAGTAAAAAA   
  
  
- AGAGAGGGTA AACTAGACTT TGTGTTTGAA CGTCCCCACG TTCAACCTGT GAATCATCGA CGTCCTTACT   
  
  
- AAGTTATCCT TAGGAGTAGA AACTGTTGTA GTGGTGAACT TATGTATAGT GAACTACCGT GAGAGGGGAC   
  
  
- CAAATCCGAA GGGTATAGGC ACCGAATCCC TCGATTTTGG ACTCGTTTCT CCTGAGACAG ATTAGGTAGA   
  
  
- GGAGGAAAGT ACACGATTAG TTCAACGATG ACCCTCGTAG CTGTTACGAT TACAGTCGGA ACTCGTATAA   
  
  
- AGGGTGGAAC GGAGAGGGTG GCCTCTATGT TACGTCGCTT AACGACGAAT GAAACGACTT CGGAAACGAC   
  
  
- TGGCAAATGA TTCCCGAACC GTCGGGCCAG AAGAGTTTCG GAACTTAACA TGGTTCTACA GTAGACAAAG   
  
  
- GCTTGTTTAA GAACAAGTTT TCAACAAGAA ACTAGAAGAA GGCAAGAACT TCGAAAGTAT AGAACACTGC   
  
  
- TTGGTCCGTT AGTGCCTCCG GTATCTTCCT CTTTTCTACC AAGTATATTA ACTAGAGGTA AGAACACTTA   
  
  
- GCCGAGACAC CTAATCAGAG GAGGTCCGTA ACTCACAAGC TGGACTTCCG GGTGGGGTAA ACTCCTATTG   
  
  
- GCCATATGTA CTCTTCTTTC TTCACAACCT AGTTTACCGA TACGTTGATT TGTTTCTCCG ACTTTTTAAC   
  
  
- CTGTAGGGTA AAGTTAAGTT AGGATAGCGT TCGTTTGATC TGCTGGAACT ACAGCTTTCG AACTCACAGT   
  
  
- TCTGGCCTCT TCGTGAACAA TAATCAAGAC ACGAGGTTGA TGTAAGAGAA AACCGTAACC TACTCCCTAG   
  
  
- TTACGGATTC TTGCGTCCGT ACCGAATGAA CGTTTCCCAC AAAATATACT TTGGTGCTTT TAACCTCCTG   
  
  
- AACGGGTTGT TCCTAAACTA CTTTTACAAC TTGAGTTTAC TTCTAAGATG TAGTAGTAGT AGAAGTAGAG   
  
  
- GATAGGGAAG TAGTTTTGAA CTACGGAAAA ATTTTCGGGA GGAACCCGAA AGCGGTTTTG AGTACCAACA   
  
  
- TTGGCTCGTT CTTAGTTTGG TGTTACCTTC ACGGGATTAT CTTTCTCACT ACCTCAGTAA CTTGAAGATA   
  
  
- CGTCGTAACA AACTAACGAA CCTTAGGTGA TATAGCTCCT GTAGCTATCT CTCTGTCTTC GAGCTCTTCA   
  
  
- ACTACAAACC TCTCCTCTAG TTCTTGGAGT GTCGAACACT TCCCCGTCTC TCTTTCCGTT CCGTGCTCTT   
  
  
- CGAGTCACTT ACCCAAGTTT CTAAACTCAG TCATCCTAAA TTTTCCCTCG GTAACTCGAT GGTACCAAAG   
  
  
- AACGAACGAT CCTCCAAAAA TGTATGGTTA TTAATACTCC CCATATTGTA GTTCCTTCAG TTACCAAAAG   
  
  
- AACAATAGAC AACCGTTCTA TCTGGGGACA TAAGACAAAG TCGAACCTCT AAAAT

+     CCAAT-box

| Site Name | Organism | Position | Strand | Matrix score. | sequence | function |
| --- | --- | --- | --- | --- | --- | --- |
| CCAAT-box | Hordeum vulgare | 683 | + | 6 | CAACGG | MYBHv1 binding site |

>HU05G01267.1   
+ +Up\_Stream \_Len000TTACAA AAACGATGCT CTAAACCTAT TAGGTCGTTC CGAAACCCGC AAAATTGAAA   
  
  
+ TATGATCGCC CGTATTTTAC CCAAACCTGA AATACACTTA ATATGTATTT TACCTGAACC CAAATACACC   
  
  
+ GGACCTGTAT TTAACACGGG CACCCGAAAT GATAGGTCTA ACTCTCACCA CCACCGCATT TGGCACCCGA   
  
  
+ ACGGTAAAAA ATGTTCATCT ATTATGCACT TTGGCTTCAT CCATAACCCT ATGATCTATC TGTGCCAATA   
  
  
+ TTTGGAGCAC CCAGCGAAGT AGCGCATGCT CTAAACTCTT GCCTCAAAAC AAAACAAATA GTGGGTCATA   
  
  
+ TTCCCTTCAA AGCACTAACA AACACACACT CTCTCTCTCT CAGTAGCCAA CAACTTAAGG CGGTGTTTGC   
  
  
+ TTGGTTTTGG AGAGGGCGTG TTTGGATGAG GTGCAAAACA CATAGATGAA ATCATAAACA ACTTGATTCC   
  
  
+ CAGCCACCCA TGACCTACTC AGATAACAAG GGACAAAAAC AAAGAGGTTT TCTGAAACAG AGAAAACAGG   
  
  
+ GGCGTTGTGT TGCGCTGTTG CTCACAAAAT TCCAATATTC CTCTAAAAGC TCCCAAACGG AGACAAATCA   
  
  
+ AAGAGGACAA AGACATGTGG GATTTAATAC AAAATTTGAC TAACTCTGCA ACGGAAAACA ACCCCTAGGG   
  
  
+ TGTTTTTCTG CGTGTTGTAC ACACTTCACT TTTAACCTGC TCTGCCTCTG CCTTTGCCAC GGCTTTTAAT   
  
  
+ GGATAAGACC AATTAAGAAA CCCAAATATC TCGTAAATTT GTTGAGATTC CTTTCTGCGT TCTCAAGTGT   
  
  
+ TTCAAAAACC CAGTTTGGAG GTTGCACTTA TTCATGCTTT TCTTCAAGTC AGTGAGCTTA GTGTCTGGAA   
  
  
+ CTGAAGCTCC TCCTGGTAAT GCTCTGTTTT TCATTTCTTG TTGTGCGTTT ATAAAAATTC GGCCTATTGA   
  
  
+ TGGAAGTTTG TTTGATGTTT TTGGGTAAGT TTTTGATTAA TAGAGATGAA TTAGTAAAGT AAAATTTGAG   
  
  
+ TGGTATTTAA TGATCCTTAA GCAGTCTATG CTTTGTTGTC ACATGCGTTT CTTGGCTTTT GACTGAAACA   
  
  
+ TTTCTGTGTT AGATGAAAAA CTCATTTTGA TGCATGAGAT GATAAAATCG CTTATTTTTG CCTTTATTTG   
  
  
+ GTGTGTGGGG GGGGGGGGGG GGGGGCGCAG GTCTGAGGTA AAAGATATGT GAATTCATTT GGAGTCTCGA   
  
  
+ TAATTCGTTG GCTATGTTGA CAATCACGAG TGGTCGTTAT CGCTCATCTC ACAGGTTGAT AATTCACTCA   
  
  
+ AACTTCAAGC GGCCTGAATC ATTCAGTCCG GGTTGTTAAA ATCTCAACAG TCATTTCACA CGAGTGGAGG   
  
  
+ GCCCCAAAAG ACACCATTCT TTCGAACTTT TTTTTTTTGA AGGTCATTTT TTCAAGAACA AAACTAATGT   
  
  
+ GAACATGCGA CTTCTATGAG TAAAGTGTAA AGATGCATAA AGTCTTTTTT TCTTTTATAT AAAAAGTACT   
  
  
+ CAAAAAAAAA AGAGTTGGAG AGCTGATTCC CCATGCATCC TCTAAGCAAA GGTGGTGGAC GGCAGAAAAT   
  
  
+ TAGTAGAGTT ATTGTGAGAT TAAAGTTTGC GCACCTGAAC GGCTGACCTC TAGTTTGCTA TCAACGTTAC   
  
  
+ ATTTATGTGT CATAATTTTT GCATGAAACA ATTTCACGCA ATGTTTTCTG ATGGCAGTTA TGTTTAGAAA   
  
  
+ TAAGTTTAAT CGGACCTTAT TCCACTGATT TATATTTGAT TCTTCCCTCT TTACTACGGC TGTCCATTCA   
  
  
+ CAAGCCAAAG AATTCTCCCC CCAAAAAAAA GAAAAAACAG TATAGAACCA TGTTTATTAT CTCATTTTTT   
  
  
+ TCTCTCCCAT TTGATCTGAA ACACAAACTT GCAGGGGTGC AAGTTGGACA CTTAGTAGCT GCAGGAATGA   
  
  
+ TTCAATAGGA ATCCTCATCT TTGACAACAT CACCACTTGA ATACATATCA CTTGATGGCA CTCTCCCCTG   
  
  
+ GTTTAGGCTT CCCATATCCG TGGCTTAGGG AGCTAAAACC TGAGCAAAGA GGACTCTGTC TAATCCATCT   
  
  
+ CCTCCTTTCA TGTGCTAATC AAGTTGCTAC TGGGAGCATC GACAATGCTA ATGTCAGCCT TGAGCATATT   
  
  
+ TCCCACCTTG CCTCTCCCAC CGGAGATACA ATGCAGCGAA TTGCTGCTTA CTTTGCTGAA GCCTTTGCTG   
  
  
+ ACCGTTTACT AAGGGCTTGG CAGCCCGGTC TTCTCAAAGC CTTGAATTGT ACCAAGATGT CATCTGTTTC   
  
  
+ CGAACAAATT CTTGTTCAAA AGTTGTTCTT TGATCTTCTT CCGTTCTTGA AGCTTTCATA TCTTGTGACG   
  
  
+ AACCAGGCAA TCACGGAGGC CATAGAAGGA GAAAAGATGG TTCATATAAT TGATCTCCAT TCTTGTGAAT   
  
  
+ CGGCTCTGTG GATTAGTCTC CTCCAGGCAT TGAGTGTTCG ACCTGAAGGC CCACCCCATT TGAGGATAAC   
  
  
+ CGGTATACAT GAGAAGAAAG AAGTGTTGGA TCAAATGGCT ATGCAACTAA ACAAAGAGGC TGAAAAATTG   
  
  
+ GACATCCCAT TTCAATTCAA TCCTATCGCA AGCAAACTAG ACGACCTTGA TGTCGAAAGC TTGAGTGTCA   
  
  
+ AGACCGGAGA AGCACTTGTT ATTAGTTCTG TGCTCCAACT ACATTCTCTT TTGGCATTGG ATGAGGGATC   
  
  
+ AATGCCTAAG AACGCAGGCA TGGCTTACTT GCAAAGGGTG TTTTATATGA AACCACGAAA ATTGGAGGAC   
  
  
+ TTGCCCAACA AGGATTTGAT GAAAATGTTG AACTCAAATG AAGATTCTAC ATCATCATCA TCTTCATCTC   
  
  
+ CTATCCCTTC ATCAAAACTT GATGCCTTTT TAAAAGCCCT CCTTGGGCTT TCGCCAAAAC TCATGGTTGT   
  
  
+ AACCGAGCAA GAATCAAACC ACAATGGAAG TGCCCTAATA GAAAGAGTGA TGGAGTCATT GAACTTCTAT   
  
  
+ GCAGCATTGT TTGATTGCTT GGAATCCACT ATATCGAGGA CATCGATAGA GAGACAGAAG CTCGAGAAGT   
  
  
+ TGATGTTTGG AGAGGAGATC AAGAACCTCA CAGCTTGTGA AGGGGCAGAG AGAAAGGCAA GGCACGAGAA   
  
  
+ GCTCAGTGAA TGGGTTCAAA GATTTGAGTC AGTAGGATTT AAAAGGGAGC CATTGAGCTA CCATGGTTTC   
  
  
+ TTGCTTGCTA GGAGGTTTTT ACATACCAAT AATTATGAGG GGTATAACAT CAAGGAAGTC AATGGTTTTC   
  
  
+ TTGTTATCTG TTGGCAAGAT AGACCCCTGT ATTCTGTTTC AGCTTGGAGA TTTTA  

- +Up\_Stream \_Len000AATGTT TTTGCTACGA GATTTGGATA ATCCAGCAAG GCTTTGGGCG TTTTAACTTT   
  
  
- ATACTAGCGG GCATAAAATG GGTTTGGACT TTATGTGAAT TATACATAAA ATGGACTTGG GTTTATGTGG   
  
  
- CCTGGACATA AATTGTGCCC GTGGGCTTTA CTATCCAGAT TGAGAGTGGT GGTGGCGTAA ACCGTGGGCT   
  
  
- TGCCATTTTT TACAAGTAGA TAATACGTGA AACCGAAGTA GGTATTGGGA TACTAGATAG ACACGGTTAT   
  
  
- AAACCTCGTG GGTCGCTTCA TCGCGTACGA GATTTGAGAA CGGAGTTTTG TTTTGTTTAT CACCCAGTAT   
  
  
- AAGGGAAGTT TCGTGATTGT TTGTGTGTGA GAGAGAGAGA GTCATCGGTT GTTGAATTCC GCCACAAACG   
  
  
- AACCAAAACC TCTCCCGCAC AAACCTACTC CACGTTTTGT GTATCTACTT TAGTATTTGT TGAACTAAGG   
  
  
- GTCGGTGGGT ACTGGATGAG TCTATTGTTC CCTGTTTTTG TTTCTCCAAA AGACTTTGTC TCTTTTGTCC   
  
  
- CCGCAACACA ACGCGACAAC GAGTGTTTTA AGGTTATAAG GAGATTTTCG AGGGTTTGCC TCTGTTTAGT   
  
  
- TTCTCCTGTT TCTGTACACC CTAAATTATG TTTTAAACTG ATTGAGACGT TGCCTTTTGT TGGGGATCCC   
  
  
- ACAAAAAGAC GCACAACATG TGTGAAGTGA AAATTGGACG AGACGGAGAC GGAAACGGTG CCGAAAATTA   
  
  
- CCTATTCTGG TTAATTCTTT GGGTTTATAG AGCATTTAAA CAACTCTAAG GAAAGACGCA AGAGTTCACA   
  
  
- AAGTTTTTGG GTCAAACCTC CAACGTGAAT AAGTACGAAA AGAAGTTCAG TCACTCGAAT CACAGACCTT   
  
  
- GACTTCGAGG AGGACCATTA CGAGACAAAA AGTAAAGAAC AACACGCAAA TATTTTTAAG CCGGATAACT   
  
  
- ACCTTCAAAC AAACTACAAA AACCCATTCA AAAACTAATT ATCTCTACTT AATCATTTCA TTTTAAACTC   
  
  
- ACCATAAATT ACTAGGAATT CGTCAGATAC GAAACAACAG TGTACGCAAA GAACCGAAAA CTGACTTTGT   
  
  
- AAAGACACAA TCTACTTTTT GAGTAAAACT ACGTACTCTA CTATTTTAGC GAATAAAAAC GGAAATAAAC   
  
  
- CACACACCCC CCCCCCCCCC CCCCCGCGTC CAGACTCCAT TTTCTATACA CTTAAGTAAA CCTCAGAGCT   
  
  
- ATTAAGCAAC CGATACAACT GTTAGTGCTC ACCAGCAATA GCGAGTAGAG TGTCCAACTA TTAAGTGAGT   
  
  
- TTGAAGTTCG CCGGACTTAG TAAGTCAGGC CCAACAATTT TAGAGTTGTC AGTAAAGTGT GCTCACCTCC   
  
  
- CGGGGTTTTC TGTGGTAAGA AAGCTTGAAA AAAAAAAACT TCCAGTAAAA AAGTTCTTGT TTTGATTACA   
  
  
- CTTGTACGCT GAAGATACTC ATTTCACATT TCTACGTATT TCAGAAAAAA AGAAAATATA TTTTTCATGA   
  
  
- GTTTTTTTTT TCTCAACCTC TCGACTAAGG GGTACGTAGG AGATTCGTTT CCACCACCTG CCGTCTTTTA   
  
  
- ATCATCTCAA TAACACTCTA ATTTCAAACG CGTGGACTTG CCGACTGGAG ATCAAACGAT AGTTGCAATG   
  
  
- TAAATACACA GTATTAAAAA CGTACTTTGT TAAAGTGCGT TACAAAAGAC TACCGTCAAT ACAAATCTTT   
  
  
- ATTCAAATTA GCCTGGAATA AGGTGACTAA ATATAAACTA AGAAGGGAGA AATGATGCCG ACAGGTAAGT   
  
  
- GTTCGGTTTC TTAAGAGGGG GGTTTTTTTT CTTTTTTGTC ATATCTTGGT ACAAATAATA GAGTAAAAAA   
  
  
- AGAGAGGGTA AACTAGACTT TGTGTTTGAA CGTCCCCACG TTCAACCTGT GAATCATCGA CGTCCTTACT   
  
  
- AAGTTATCCT TAGGAGTAGA AACTGTTGTA GTGGTGAACT TATGTATAGT GAACTACCGT GAGAGGGGAC   
  
  
- CAAATCCGAA GGGTATAGGC ACCGAATCCC TCGATTTTGG ACTCGTTTCT CCTGAGACAG ATTAGGTAGA   
  
  
- GGAGGAAAGT ACACGATTAG TTCAACGATG ACCCTCGTAG CTGTTACGAT TACAGTCGGA ACTCGTATAA   
  
  
- AGGGTGGAAC GGAGAGGGTG GCCTCTATGT TACGTCGCTT AACGACGAAT GAAACGACTT CGGAAACGAC   
  
  
- TGGCAAATGA TTCCCGAACC GTCGGGCCAG AAGAGTTTCG GAACTTAACA TGGTTCTACA GTAGACAAAG   
  
  
- GCTTGTTTAA GAACAAGTTT TCAACAAGAA ACTAGAAGAA GGCAAGAACT TCGAAAGTAT AGAACACTGC   
  
  
- TTGGTCCGTT AGTGCCTCCG GTATCTTCCT CTTTTCTACC AAGTATATTA ACTAGAGGTA AGAACACTTA   
  
  
- GCCGAGACAC CTAATCAGAG GAGGTCCGTA ACTCACAAGC TGGACTTCCG GGTGGGGTAA ACTCCTATTG   
  
  
- GCCATATGTA CTCTTCTTTC TTCACAACCT AGTTTACCGA TACGTTGATT TGTTTCTCCG ACTTTTTAAC   
  
  
- CTGTAGGGTA AAGTTAAGTT AGGATAGCGT TCGTTTGATC TGCTGGAACT ACAGCTTTCG AACTCACAGT   
  
  
- TCTGGCCTCT TCGTGAACAA TAATCAAGAC ACGAGGTTGA TGTAAGAGAA AACCGTAACC TACTCCCTAG   
  
  
- TTACGGATTC TTGCGTCCGT ACCGAATGAA CGTTTCCCAC AAAATATACT TTGGTGCTTT TAACCTCCTG   
  
  
- AACGGGTTGT TCCTAAACTA CTTTTACAAC TTGAGTTTAC TTCTAAGATG TAGTAGTAGT AGAAGTAGAG   
  
  
- GATAGGGAAG TAGTTTTGAA CTACGGAAAA ATTTTCGGGA GGAACCCGAA AGCGGTTTTG AGTACCAACA   
  
  
- TTGGCTCGTT CTTAGTTTGG TGTTACCTTC ACGGGATTAT CTTTCTCACT ACCTCAGTAA CTTGAAGATA   
  
  
- CGTCGTAACA AACTAACGAA CCTTAGGTGA TATAGCTCCT GTAGCTATCT CTCTGTCTTC GAGCTCTTCA   
  
  
- ACTACAAACC TCTCCTCTAG TTCTTGGAGT GTCGAACACT TCCCCGTCTC TCTTTCCGTT CCGTGCTCTT   
  
  
- CGAGTCACTT ACCCAAGTTT CTAAACTCAG TCATCCTAAA TTTTCCCTCG GTAACTCGAT GGTACCAAAG   
  
  
- AACGAACGAT CCTCCAAAAA TGTATGGTTA TTAATACTCC CCATATTGTA GTTCCTTCAG TTACCAAAAG   
  
  
- AACAATAGAC AACCGTTCTA TCTGGGGACA TAAGACAAAG TCGAACCTCT AAAAT

+     CCGTCC motif

| Site Name | Organism | Position | Strand | Matrix score. | sequence | function |
| --- | --- | --- | --- | --- | --- | --- |
| CCGTCC motif | Nicotiana tabacum | 1601 | - | 6 | CCGTCC |  |

>HU05G01267.1   
+ +Up\_Stream \_Len000TTACAA AAACGATGCT CTAAACCTAT TAGGTCGTTC CGAAACCCGC AAAATTGAAA   
  
  
+ TATGATCGCC CGTATTTTAC CCAAACCTGA AATACACTTA ATATGTATTT TACCTGAACC CAAATACACC   
  
  
+ GGACCTGTAT TTAACACGGG CACCCGAAAT GATAGGTCTA ACTCTCACCA CCACCGCATT TGGCACCCGA   
  
  
+ ACGGTAAAAA ATGTTCATCT ATTATGCACT TTGGCTTCAT CCATAACCCT ATGATCTATC TGTGCCAATA   
  
  
+ TTTGGAGCAC CCAGCGAAGT AGCGCATGCT CTAAACTCTT GCCTCAAAAC AAAACAAATA GTGGGTCATA   
  
  
+ TTCCCTTCAA AGCACTAACA AACACACACT CTCTCTCTCT CAGTAGCCAA CAACTTAAGG CGGTGTTTGC   
  
  
+ TTGGTTTTGG AGAGGGCGTG TTTGGATGAG GTGCAAAACA CATAGATGAA ATCATAAACA ACTTGATTCC   
  
  
+ CAGCCACCCA TGACCTACTC AGATAACAAG GGACAAAAAC AAAGAGGTTT TCTGAAACAG AGAAAACAGG   
  
  
+ GGCGTTGTGT TGCGCTGTTG CTCACAAAAT TCCAATATTC CTCTAAAAGC TCCCAAACGG AGACAAATCA   
  
  
+ AAGAGGACAA AGACATGTGG GATTTAATAC AAAATTTGAC TAACTCTGCA ACGGAAAACA ACCCCTAGGG   
  
  
+ TGTTTTTCTG CGTGTTGTAC ACACTTCACT TTTAACCTGC TCTGCCTCTG CCTTTGCCAC GGCTTTTAAT   
  
  
+ GGATAAGACC AATTAAGAAA CCCAAATATC TCGTAAATTT GTTGAGATTC CTTTCTGCGT TCTCAAGTGT   
  
  
+ TTCAAAAACC CAGTTTGGAG GTTGCACTTA TTCATGCTTT TCTTCAAGTC AGTGAGCTTA GTGTCTGGAA   
  
  
+ CTGAAGCTCC TCCTGGTAAT GCTCTGTTTT TCATTTCTTG TTGTGCGTTT ATAAAAATTC GGCCTATTGA   
  
  
+ TGGAAGTTTG TTTGATGTTT TTGGGTAAGT TTTTGATTAA TAGAGATGAA TTAGTAAAGT AAAATTTGAG   
  
  
+ TGGTATTTAA TGATCCTTAA GCAGTCTATG CTTTGTTGTC ACATGCGTTT CTTGGCTTTT GACTGAAACA   
  
  
+ TTTCTGTGTT AGATGAAAAA CTCATTTTGA TGCATGAGAT GATAAAATCG CTTATTTTTG CCTTTATTTG   
  
  
+ GTGTGTGGGG GGGGGGGGGG GGGGGCGCAG GTCTGAGGTA AAAGATATGT GAATTCATTT GGAGTCTCGA   
  
  
+ TAATTCGTTG GCTATGTTGA CAATCACGAG TGGTCGTTAT CGCTCATCTC ACAGGTTGAT AATTCACTCA   
  
  
+ AACTTCAAGC GGCCTGAATC ATTCAGTCCG GGTTGTTAAA ATCTCAACAG TCATTTCACA CGAGTGGAGG   
  
  
+ GCCCCAAAAG ACACCATTCT TTCGAACTTT TTTTTTTTGA AGGTCATTTT TTCAAGAACA AAACTAATGT   
  
  
+ GAACATGCGA CTTCTATGAG TAAAGTGTAA AGATGCATAA AGTCTTTTTT TCTTTTATAT AAAAAGTACT   
  
  
+ CAAAAAAAAA AGAGTTGGAG AGCTGATTCC CCATGCATCC TCTAAGCAAA GGTGGTGGAC GGCAGAAAAT   
  
  
+ TAGTAGAGTT ATTGTGAGAT TAAAGTTTGC GCACCTGAAC GGCTGACCTC TAGTTTGCTA TCAACGTTAC   
  
  
+ ATTTATGTGT CATAATTTTT GCATGAAACA ATTTCACGCA ATGTTTTCTG ATGGCAGTTA TGTTTAGAAA   
  
  
+ TAAGTTTAAT CGGACCTTAT TCCACTGATT TATATTTGAT TCTTCCCTCT TTACTACGGC TGTCCATTCA   
  
  
+ CAAGCCAAAG AATTCTCCCC CCAAAAAAAA GAAAAAACAG TATAGAACCA TGTTTATTAT CTCATTTTTT   
  
  
+ TCTCTCCCAT TTGATCTGAA ACACAAACTT GCAGGGGTGC AAGTTGGACA CTTAGTAGCT GCAGGAATGA   
  
  
+ TTCAATAGGA ATCCTCATCT TTGACAACAT CACCACTTGA ATACATATCA CTTGATGGCA CTCTCCCCTG   
  
  
+ GTTTAGGCTT CCCATATCCG TGGCTTAGGG AGCTAAAACC TGAGCAAAGA GGACTCTGTC TAATCCATCT   
  
  
+ CCTCCTTTCA TGTGCTAATC AAGTTGCTAC TGGGAGCATC GACAATGCTA ATGTCAGCCT TGAGCATATT   
  
  
+ TCCCACCTTG CCTCTCCCAC CGGAGATACA ATGCAGCGAA TTGCTGCTTA CTTTGCTGAA GCCTTTGCTG   
  
  
+ ACCGTTTACT AAGGGCTTGG CAGCCCGGTC TTCTCAAAGC CTTGAATTGT ACCAAGATGT CATCTGTTTC   
  
  
+ CGAACAAATT CTTGTTCAAA AGTTGTTCTT TGATCTTCTT CCGTTCTTGA AGCTTTCATA TCTTGTGACG   
  
  
+ AACCAGGCAA TCACGGAGGC CATAGAAGGA GAAAAGATGG TTCATATAAT TGATCTCCAT TCTTGTGAAT   
  
  
+ CGGCTCTGTG GATTAGTCTC CTCCAGGCAT TGAGTGTTCG ACCTGAAGGC CCACCCCATT TGAGGATAAC   
  
  
+ CGGTATACAT GAGAAGAAAG AAGTGTTGGA TCAAATGGCT ATGCAACTAA ACAAAGAGGC TGAAAAATTG   
  
  
+ GACATCCCAT TTCAATTCAA TCCTATCGCA AGCAAACTAG ACGACCTTGA TGTCGAAAGC TTGAGTGTCA   
  
  
+ AGACCGGAGA AGCACTTGTT ATTAGTTCTG TGCTCCAACT ACATTCTCTT TTGGCATTGG ATGAGGGATC   
  
  
+ AATGCCTAAG AACGCAGGCA TGGCTTACTT GCAAAGGGTG TTTTATATGA AACCACGAAA ATTGGAGGAC   
  
  
+ TTGCCCAACA AGGATTTGAT GAAAATGTTG AACTCAAATG AAGATTCTAC ATCATCATCA TCTTCATCTC   
  
  
+ CTATCCCTTC ATCAAAACTT GATGCCTTTT TAAAAGCCCT CCTTGGGCTT TCGCCAAAAC TCATGGTTGT   
  
  
+ AACCGAGCAA GAATCAAACC ACAATGGAAG TGCCCTAATA GAAAGAGTGA TGGAGTCATT GAACTTCTAT   
  
  
+ GCAGCATTGT TTGATTGCTT GGAATCCACT ATATCGAGGA CATCGATAGA GAGACAGAAG CTCGAGAAGT   
  
  
+ TGATGTTTGG AGAGGAGATC AAGAACCTCA CAGCTTGTGA AGGGGCAGAG AGAAAGGCAA GGCACGAGAA   
  
  
+ GCTCAGTGAA TGGGTTCAAA GATTTGAGTC AGTAGGATTT AAAAGGGAGC CATTGAGCTA CCATGGTTTC   
  
  
+ TTGCTTGCTA GGAGGTTTTT ACATACCAAT AATTATGAGG GGTATAACAT CAAGGAAGTC AATGGTTTTC   
  
  
+ TTGTTATCTG TTGGCAAGAT AGACCCCTGT ATTCTGTTTC AGCTTGGAGA TTTTA  

- +Up\_Stream \_Len000AATGTT TTTGCTACGA GATTTGGATA ATCCAGCAAG GCTTTGGGCG TTTTAACTTT   
  
  
- ATACTAGCGG GCATAAAATG GGTTTGGACT TTATGTGAAT TATACATAAA ATGGACTTGG GTTTATGTGG   
  
  
- CCTGGACATA AATTGTGCCC GTGGGCTTTA CTATCCAGAT TGAGAGTGGT GGTGGCGTAA ACCGTGGGCT   
  
  
- TGCCATTTTT TACAAGTAGA TAATACGTGA AACCGAAGTA GGTATTGGGA TACTAGATAG ACACGGTTAT   
  
  
- AAACCTCGTG GGTCGCTTCA TCGCGTACGA GATTTGAGAA CGGAGTTTTG TTTTGTTTAT CACCCAGTAT   
  
  
- AAGGGAAGTT TCGTGATTGT TTGTGTGTGA GAGAGAGAGA GTCATCGGTT GTTGAATTCC GCCACAAACG   
  
  
- AACCAAAACC TCTCCCGCAC AAACCTACTC CACGTTTTGT GTATCTACTT TAGTATTTGT TGAACTAAGG   
  
  
- GTCGGTGGGT ACTGGATGAG TCTATTGTTC CCTGTTTTTG TTTCTCCAAA AGACTTTGTC TCTTTTGTCC   
  
  
- CCGCAACACA ACGCGACAAC GAGTGTTTTA AGGTTATAAG GAGATTTTCG AGGGTTTGCC TCTGTTTAGT   
  
  
- TTCTCCTGTT TCTGTACACC CTAAATTATG TTTTAAACTG ATTGAGACGT TGCCTTTTGT TGGGGATCCC   
  
  
- ACAAAAAGAC GCACAACATG TGTGAAGTGA AAATTGGACG AGACGGAGAC GGAAACGGTG CCGAAAATTA   
  
  
- CCTATTCTGG TTAATTCTTT GGGTTTATAG AGCATTTAAA CAACTCTAAG GAAAGACGCA AGAGTTCACA   
  
  
- AAGTTTTTGG GTCAAACCTC CAACGTGAAT AAGTACGAAA AGAAGTTCAG TCACTCGAAT CACAGACCTT   
  
  
- GACTTCGAGG AGGACCATTA CGAGACAAAA AGTAAAGAAC AACACGCAAA TATTTTTAAG CCGGATAACT   
  
  
- ACCTTCAAAC AAACTACAAA AACCCATTCA AAAACTAATT ATCTCTACTT AATCATTTCA TTTTAAACTC   
  
  
- ACCATAAATT ACTAGGAATT CGTCAGATAC GAAACAACAG TGTACGCAAA GAACCGAAAA CTGACTTTGT   
  
  
- AAAGACACAA TCTACTTTTT GAGTAAAACT ACGTACTCTA CTATTTTAGC GAATAAAAAC GGAAATAAAC   
  
  
- CACACACCCC CCCCCCCCCC CCCCCGCGTC CAGACTCCAT TTTCTATACA CTTAAGTAAA CCTCAGAGCT   
  
  
- ATTAAGCAAC CGATACAACT GTTAGTGCTC ACCAGCAATA GCGAGTAGAG TGTCCAACTA TTAAGTGAGT   
  
  
- TTGAAGTTCG CCGGACTTAG TAAGTCAGGC CCAACAATTT TAGAGTTGTC AGTAAAGTGT GCTCACCTCC   
  
  
- CGGGGTTTTC TGTGGTAAGA AAGCTTGAAA AAAAAAAACT TCCAGTAAAA AAGTTCTTGT TTTGATTACA   
  
  
- CTTGTACGCT GAAGATACTC ATTTCACATT TCTACGTATT TCAGAAAAAA AGAAAATATA TTTTTCATGA   
  
  
- GTTTTTTTTT TCTCAACCTC TCGACTAAGG GGTACGTAGG AGATTCGTTT CCACCACCTG CCGTCTTTTA   
  
  
- ATCATCTCAA TAACACTCTA ATTTCAAACG CGTGGACTTG CCGACTGGAG ATCAAACGAT AGTTGCAATG   
  
  
- TAAATACACA GTATTAAAAA CGTACTTTGT TAAAGTGCGT TACAAAAGAC TACCGTCAAT ACAAATCTTT   
  
  
- ATTCAAATTA GCCTGGAATA AGGTGACTAA ATATAAACTA AGAAGGGAGA AATGATGCCG ACAGGTAAGT   
  
  
- GTTCGGTTTC TTAAGAGGGG GGTTTTTTTT CTTTTTTGTC ATATCTTGGT ACAAATAATA GAGTAAAAAA   
  
  
- AGAGAGGGTA AACTAGACTT TGTGTTTGAA CGTCCCCACG TTCAACCTGT GAATCATCGA CGTCCTTACT   
  
  
- AAGTTATCCT TAGGAGTAGA AACTGTTGTA GTGGTGAACT TATGTATAGT GAACTACCGT GAGAGGGGAC   
  
  
- CAAATCCGAA GGGTATAGGC ACCGAATCCC TCGATTTTGG ACTCGTTTCT CCTGAGACAG ATTAGGTAGA   
  
  
- GGAGGAAAGT ACACGATTAG TTCAACGATG ACCCTCGTAG CTGTTACGAT TACAGTCGGA ACTCGTATAA   
  
  
- AGGGTGGAAC GGAGAGGGTG GCCTCTATGT TACGTCGCTT AACGACGAAT GAAACGACTT CGGAAACGAC   
  
  
- TGGCAAATGA TTCCCGAACC GTCGGGCCAG AAGAGTTTCG GAACTTAACA TGGTTCTACA GTAGACAAAG   
  
  
- GCTTGTTTAA GAACAAGTTT TCAACAAGAA ACTAGAAGAA GGCAAGAACT TCGAAAGTAT AGAACACTGC   
  
  
- TTGGTCCGTT AGTGCCTCCG GTATCTTCCT CTTTTCTACC AAGTATATTA ACTAGAGGTA AGAACACTTA   
  
  
- GCCGAGACAC CTAATCAGAG GAGGTCCGTA ACTCACAAGC TGGACTTCCG GGTGGGGTAA ACTCCTATTG   
  
  
- GCCATATGTA CTCTTCTTTC TTCACAACCT AGTTTACCGA TACGTTGATT TGTTTCTCCG ACTTTTTAAC   
  
  
- CTGTAGGGTA AAGTTAAGTT AGGATAGCGT TCGTTTGATC TGCTGGAACT ACAGCTTTCG AACTCACAGT   
  
  
- TCTGGCCTCT TCGTGAACAA TAATCAAGAC ACGAGGTTGA TGTAAGAGAA AACCGTAACC TACTCCCTAG   
  
  
- TTACGGATTC TTGCGTCCGT ACCGAATGAA CGTTTCCCAC AAAATATACT TTGGTGCTTT TAACCTCCTG   
  
  
- AACGGGTTGT TCCTAAACTA CTTTTACAAC TTGAGTTTAC TTCTAAGATG TAGTAGTAGT AGAAGTAGAG   
  
  
- GATAGGGAAG TAGTTTTGAA CTACGGAAAA ATTTTCGGGA GGAACCCGAA AGCGGTTTTG AGTACCAACA   
  
  
- TTGGCTCGTT CTTAGTTTGG TGTTACCTTC ACGGGATTAT CTTTCTCACT ACCTCAGTAA CTTGAAGATA   
  
  
- CGTCGTAACA AACTAACGAA CCTTAGGTGA TATAGCTCCT GTAGCTATCT CTCTGTCTTC GAGCTCTTCA   
  
  
- ACTACAAACC TCTCCTCTAG TTCTTGGAGT GTCGAACACT TCCCCGTCTC TCTTTCCGTT CCGTGCTCTT   
  
  
- CGAGTCACTT ACCCAAGTTT CTAAACTCAG TCATCCTAAA TTTTCCCTCG GTAACTCGAT GGTACCAAAG   
  
  
- AACGAACGAT CCTCCAAAAA TGTATGGTTA TTAATACTCC CCATATTGTA GTTCCTTCAG TTACCAAAAG   
  
  
- AACAATAGAC AACCGTTCTA TCTGGGGACA TAAGACAAAG TCGAACCTCT AAAAT

+     CCGTCC-box

| Site Name | Organism | Position | Strand | Matrix score. | sequence | function |
| --- | --- | --- | --- | --- | --- | --- |
| CCGTCC-box | Petroselinum hortense | 1601 | - | 6 | CCGTCC |  |

>HU05G01267.1   
+ +Up\_Stream \_Len000TTACAA AAACGATGCT CTAAACCTAT TAGGTCGTTC CGAAACCCGC AAAATTGAAA   
  
  
+ TATGATCGCC CGTATTTTAC CCAAACCTGA AATACACTTA ATATGTATTT TACCTGAACC CAAATACACC   
  
  
+ GGACCTGTAT TTAACACGGG CACCCGAAAT GATAGGTCTA ACTCTCACCA CCACCGCATT TGGCACCCGA   
  
  
+ ACGGTAAAAA ATGTTCATCT ATTATGCACT TTGGCTTCAT CCATAACCCT ATGATCTATC TGTGCCAATA   
  
  
+ TTTGGAGCAC CCAGCGAAGT AGCGCATGCT CTAAACTCTT GCCTCAAAAC AAAACAAATA GTGGGTCATA   
  
  
+ TTCCCTTCAA AGCACTAACA AACACACACT CTCTCTCTCT CAGTAGCCAA CAACTTAAGG CGGTGTTTGC   
  
  
+ TTGGTTTTGG AGAGGGCGTG TTTGGATGAG GTGCAAAACA CATAGATGAA ATCATAAACA ACTTGATTCC   
  
  
+ CAGCCACCCA TGACCTACTC AGATAACAAG GGACAAAAAC AAAGAGGTTT TCTGAAACAG AGAAAACAGG   
  
  
+ GGCGTTGTGT TGCGCTGTTG CTCACAAAAT TCCAATATTC CTCTAAAAGC TCCCAAACGG AGACAAATCA   
  
  
+ AAGAGGACAA AGACATGTGG GATTTAATAC AAAATTTGAC TAACTCTGCA ACGGAAAACA ACCCCTAGGG   
  
  
+ TGTTTTTCTG CGTGTTGTAC ACACTTCACT TTTAACCTGC TCTGCCTCTG CCTTTGCCAC GGCTTTTAAT   
  
  
+ GGATAAGACC AATTAAGAAA CCCAAATATC TCGTAAATTT GTTGAGATTC CTTTCTGCGT TCTCAAGTGT   
  
  
+ TTCAAAAACC CAGTTTGGAG GTTGCACTTA TTCATGCTTT TCTTCAAGTC AGTGAGCTTA GTGTCTGGAA   
  
  
+ CTGAAGCTCC TCCTGGTAAT GCTCTGTTTT TCATTTCTTG TTGTGCGTTT ATAAAAATTC GGCCTATTGA   
  
  
+ TGGAAGTTTG TTTGATGTTT TTGGGTAAGT TTTTGATTAA TAGAGATGAA TTAGTAAAGT AAAATTTGAG   
  
  
+ TGGTATTTAA TGATCCTTAA GCAGTCTATG CTTTGTTGTC ACATGCGTTT CTTGGCTTTT GACTGAAACA   
  
  
+ TTTCTGTGTT AGATGAAAAA CTCATTTTGA TGCATGAGAT GATAAAATCG CTTATTTTTG CCTTTATTTG   
  
  
+ GTGTGTGGGG GGGGGGGGGG GGGGGCGCAG GTCTGAGGTA AAAGATATGT GAATTCATTT GGAGTCTCGA   
  
  
+ TAATTCGTTG GCTATGTTGA CAATCACGAG TGGTCGTTAT CGCTCATCTC ACAGGTTGAT AATTCACTCA   
  
  
+ AACTTCAAGC GGCCTGAATC ATTCAGTCCG GGTTGTTAAA ATCTCAACAG TCATTTCACA CGAGTGGAGG   
  
  
+ GCCCCAAAAG ACACCATTCT TTCGAACTTT TTTTTTTTGA AGGTCATTTT TTCAAGAACA AAACTAATGT   
  
  
+ GAACATGCGA CTTCTATGAG TAAAGTGTAA AGATGCATAA AGTCTTTTTT TCTTTTATAT AAAAAGTACT   
  
  
+ CAAAAAAAAA AGAGTTGGAG AGCTGATTCC CCATGCATCC TCTAAGCAAA GGTGGTGGAC GGCAGAAAAT   
  
  
+ TAGTAGAGTT ATTGTGAGAT TAAAGTTTGC GCACCTGAAC GGCTGACCTC TAGTTTGCTA TCAACGTTAC   
  
  
+ ATTTATGTGT CATAATTTTT GCATGAAACA ATTTCACGCA ATGTTTTCTG ATGGCAGTTA TGTTTAGAAA   
  
  
+ TAAGTTTAAT CGGACCTTAT TCCACTGATT TATATTTGAT TCTTCCCTCT TTACTACGGC TGTCCATTCA   
  
  
+ CAAGCCAAAG AATTCTCCCC CCAAAAAAAA GAAAAAACAG TATAGAACCA TGTTTATTAT CTCATTTTTT   
  
  
+ TCTCTCCCAT TTGATCTGAA ACACAAACTT GCAGGGGTGC AAGTTGGACA CTTAGTAGCT GCAGGAATGA   
  
  
+ TTCAATAGGA ATCCTCATCT TTGACAACAT CACCACTTGA ATACATATCA CTTGATGGCA CTCTCCCCTG   
  
  
+ GTTTAGGCTT CCCATATCCG TGGCTTAGGG AGCTAAAACC TGAGCAAAGA GGACTCTGTC TAATCCATCT   
  
  
+ CCTCCTTTCA TGTGCTAATC AAGTTGCTAC TGGGAGCATC GACAATGCTA ATGTCAGCCT TGAGCATATT   
  
  
+ TCCCACCTTG CCTCTCCCAC CGGAGATACA ATGCAGCGAA TTGCTGCTTA CTTTGCTGAA GCCTTTGCTG   
  
  
+ ACCGTTTACT AAGGGCTTGG CAGCCCGGTC TTCTCAAAGC CTTGAATTGT ACCAAGATGT CATCTGTTTC   
  
  
+ CGAACAAATT CTTGTTCAAA AGTTGTTCTT TGATCTTCTT CCGTTCTTGA AGCTTTCATA TCTTGTGACG   
  
  
+ AACCAGGCAA TCACGGAGGC CATAGAAGGA GAAAAGATGG TTCATATAAT TGATCTCCAT TCTTGTGAAT   
  
  
+ CGGCTCTGTG GATTAGTCTC CTCCAGGCAT TGAGTGTTCG ACCTGAAGGC CCACCCCATT TGAGGATAAC   
  
  
+ CGGTATACAT GAGAAGAAAG AAGTGTTGGA TCAAATGGCT ATGCAACTAA ACAAAGAGGC TGAAAAATTG   
  
  
+ GACATCCCAT TTCAATTCAA TCCTATCGCA AGCAAACTAG ACGACCTTGA TGTCGAAAGC TTGAGTGTCA   
  
  
+ AGACCGGAGA AGCACTTGTT ATTAGTTCTG TGCTCCAACT ACATTCTCTT TTGGCATTGG ATGAGGGATC   
  
  
+ AATGCCTAAG AACGCAGGCA TGGCTTACTT GCAAAGGGTG TTTTATATGA AACCACGAAA ATTGGAGGAC   
  
  
+ TTGCCCAACA AGGATTTGAT GAAAATGTTG AACTCAAATG AAGATTCTAC ATCATCATCA TCTTCATCTC   
  
  
+ CTATCCCTTC ATCAAAACTT GATGCCTTTT TAAAAGCCCT CCTTGGGCTT TCGCCAAAAC TCATGGTTGT   
  
  
+ AACCGAGCAA GAATCAAACC ACAATGGAAG TGCCCTAATA GAAAGAGTGA TGGAGTCATT GAACTTCTAT   
  
  
+ GCAGCATTGT TTGATTGCTT GGAATCCACT ATATCGAGGA CATCGATAGA GAGACAGAAG CTCGAGAAGT   
  
  
+ TGATGTTTGG AGAGGAGATC AAGAACCTCA CAGCTTGTGA AGGGGCAGAG AGAAAGGCAA GGCACGAGAA   
  
  
+ GCTCAGTGAA TGGGTTCAAA GATTTGAGTC AGTAGGATTT AAAAGGGAGC CATTGAGCTA CCATGGTTTC   
  
  
+ TTGCTTGCTA GGAGGTTTTT ACATACCAAT AATTATGAGG GGTATAACAT CAAGGAAGTC AATGGTTTTC   
  
  
+ TTGTTATCTG TTGGCAAGAT AGACCCCTGT ATTCTGTTTC AGCTTGGAGA TTTTA  

- +Up\_Stream \_Len000AATGTT TTTGCTACGA GATTTGGATA ATCCAGCAAG GCTTTGGGCG TTTTAACTTT   
  
  
- ATACTAGCGG GCATAAAATG GGTTTGGACT TTATGTGAAT TATACATAAA ATGGACTTGG GTTTATGTGG   
  
  
- CCTGGACATA AATTGTGCCC GTGGGCTTTA CTATCCAGAT TGAGAGTGGT GGTGGCGTAA ACCGTGGGCT   
  
  
- TGCCATTTTT TACAAGTAGA TAATACGTGA AACCGAAGTA GGTATTGGGA TACTAGATAG ACACGGTTAT   
  
  
- AAACCTCGTG GGTCGCTTCA TCGCGTACGA GATTTGAGAA CGGAGTTTTG TTTTGTTTAT CACCCAGTAT   
  
  
- AAGGGAAGTT TCGTGATTGT TTGTGTGTGA GAGAGAGAGA GTCATCGGTT GTTGAATTCC GCCACAAACG   
  
  
- AACCAAAACC TCTCCCGCAC AAACCTACTC CACGTTTTGT GTATCTACTT TAGTATTTGT TGAACTAAGG   
  
  
- GTCGGTGGGT ACTGGATGAG TCTATTGTTC CCTGTTTTTG TTTCTCCAAA AGACTTTGTC TCTTTTGTCC   
  
  
- CCGCAACACA ACGCGACAAC GAGTGTTTTA AGGTTATAAG GAGATTTTCG AGGGTTTGCC TCTGTTTAGT   
  
  
- TTCTCCTGTT TCTGTACACC CTAAATTATG TTTTAAACTG ATTGAGACGT TGCCTTTTGT TGGGGATCCC   
  
  
- ACAAAAAGAC GCACAACATG TGTGAAGTGA AAATTGGACG AGACGGAGAC GGAAACGGTG CCGAAAATTA   
  
  
- CCTATTCTGG TTAATTCTTT GGGTTTATAG AGCATTTAAA CAACTCTAAG GAAAGACGCA AGAGTTCACA   
  
  
- AAGTTTTTGG GTCAAACCTC CAACGTGAAT AAGTACGAAA AGAAGTTCAG TCACTCGAAT CACAGACCTT   
  
  
- GACTTCGAGG AGGACCATTA CGAGACAAAA AGTAAAGAAC AACACGCAAA TATTTTTAAG CCGGATAACT   
  
  
- ACCTTCAAAC AAACTACAAA AACCCATTCA AAAACTAATT ATCTCTACTT AATCATTTCA TTTTAAACTC   
  
  
- ACCATAAATT ACTAGGAATT CGTCAGATAC GAAACAACAG TGTACGCAAA GAACCGAAAA CTGACTTTGT   
  
  
- AAAGACACAA TCTACTTTTT GAGTAAAACT ACGTACTCTA CTATTTTAGC GAATAAAAAC GGAAATAAAC   
  
  
- CACACACCCC CCCCCCCCCC CCCCCGCGTC CAGACTCCAT TTTCTATACA CTTAAGTAAA CCTCAGAGCT   
  
  
- ATTAAGCAAC CGATACAACT GTTAGTGCTC ACCAGCAATA GCGAGTAGAG TGTCCAACTA TTAAGTGAGT   
  
  
- TTGAAGTTCG CCGGACTTAG TAAGTCAGGC CCAACAATTT TAGAGTTGTC AGTAAAGTGT GCTCACCTCC   
  
  
- CGGGGTTTTC TGTGGTAAGA AAGCTTGAAA AAAAAAAACT TCCAGTAAAA AAGTTCTTGT TTTGATTACA   
  
  
- CTTGTACGCT GAAGATACTC ATTTCACATT TCTACGTATT TCAGAAAAAA AGAAAATATA TTTTTCATGA   
  
  
- GTTTTTTTTT TCTCAACCTC TCGACTAAGG GGTACGTAGG AGATTCGTTT CCACCACCTG CCGTCTTTTA   
  
  
- ATCATCTCAA TAACACTCTA ATTTCAAACG CGTGGACTTG CCGACTGGAG ATCAAACGAT AGTTGCAATG   
  
  
- TAAATACACA GTATTAAAAA CGTACTTTGT TAAAGTGCGT TACAAAAGAC TACCGTCAAT ACAAATCTTT   
  
  
- ATTCAAATTA GCCTGGAATA AGGTGACTAA ATATAAACTA AGAAGGGAGA AATGATGCCG ACAGGTAAGT   
  
  
- GTTCGGTTTC TTAAGAGGGG GGTTTTTTTT CTTTTTTGTC ATATCTTGGT ACAAATAATA GAGTAAAAAA   
  
  
- AGAGAGGGTA AACTAGACTT TGTGTTTGAA CGTCCCCACG TTCAACCTGT GAATCATCGA CGTCCTTACT   
  
  
- AAGTTATCCT TAGGAGTAGA AACTGTTGTA GTGGTGAACT TATGTATAGT GAACTACCGT GAGAGGGGAC   
  
  
- CAAATCCGAA GGGTATAGGC ACCGAATCCC TCGATTTTGG ACTCGTTTCT CCTGAGACAG ATTAGGTAGA   
  
  
- GGAGGAAAGT ACACGATTAG TTCAACGATG ACCCTCGTAG CTGTTACGAT TACAGTCGGA ACTCGTATAA   
  
  
- AGGGTGGAAC GGAGAGGGTG GCCTCTATGT TACGTCGCTT AACGACGAAT GAAACGACTT CGGAAACGAC   
  
  
- TGGCAAATGA TTCCCGAACC GTCGGGCCAG AAGAGTTTCG GAACTTAACA TGGTTCTACA GTAGACAAAG   
  
  
- GCTTGTTTAA GAACAAGTTT TCAACAAGAA ACTAGAAGAA GGCAAGAACT TCGAAAGTAT AGAACACTGC   
  
  
- TTGGTCCGTT AGTGCCTCCG GTATCTTCCT CTTTTCTACC AAGTATATTA ACTAGAGGTA AGAACACTTA   
  
  
- GCCGAGACAC CTAATCAGAG GAGGTCCGTA ACTCACAAGC TGGACTTCCG GGTGGGGTAA ACTCCTATTG   
  
  
- GCCATATGTA CTCTTCTTTC TTCACAACCT AGTTTACCGA TACGTTGATT TGTTTCTCCG ACTTTTTAAC   
  
  
- CTGTAGGGTA AAGTTAAGTT AGGATAGCGT TCGTTTGATC TGCTGGAACT ACAGCTTTCG AACTCACAGT   
  
  
- TCTGGCCTCT TCGTGAACAA TAATCAAGAC ACGAGGTTGA TGTAAGAGAA AACCGTAACC TACTCCCTAG   
  
  
- TTACGGATTC TTGCGTCCGT ACCGAATGAA CGTTTCCCAC AAAATATACT TTGGTGCTTT TAACCTCCTG   
  
  
- AACGGGTTGT TCCTAAACTA CTTTTACAAC TTGAGTTTAC TTCTAAGATG TAGTAGTAGT AGAAGTAGAG   
  
  
- GATAGGGAAG TAGTTTTGAA CTACGGAAAA ATTTTCGGGA GGAACCCGAA AGCGGTTTTG AGTACCAACA   
  
  
- TTGGCTCGTT CTTAGTTTGG TGTTACCTTC ACGGGATTAT CTTTCTCACT ACCTCAGTAA CTTGAAGATA   
  
  
- CGTCGTAACA AACTAACGAA CCTTAGGTGA TATAGCTCCT GTAGCTATCT CTCTGTCTTC GAGCTCTTCA   
  
  
- ACTACAAACC TCTCCTCTAG TTCTTGGAGT GTCGAACACT TCCCCGTCTC TCTTTCCGTT CCGTGCTCTT   
  
  
- CGAGTCACTT ACCCAAGTTT CTAAACTCAG TCATCCTAAA TTTTCCCTCG GTAACTCGAT GGTACCAAAG   
  
  
- AACGAACGAT CCTCCAAAAA TGTATGGTTA TTAATACTCC CCATATTGTA GTTCCTTCAG TTACCAAAAG   
  
  
- AACAATAGAC AACCGTTCTA TCTGGGGACA TAAGACAAAG TCGAACCTCT AAAAT

+     CGTCA-motif

| Site Name | Organism | Position | Strand | Matrix score. | sequence | function |
| --- | --- | --- | --- | --- | --- | --- |
| CGTCA-motif | Hordeum vulgare | 2380 | - | 5 | CGTCA | cis-acting regulatory element involved in the MeJA-responsiveness |

>HU05G01267.1   
+ +Up\_Stream \_Len000TTACAA AAACGATGCT CTAAACCTAT TAGGTCGTTC CGAAACCCGC AAAATTGAAA   
  
  
+ TATGATCGCC CGTATTTTAC CCAAACCTGA AATACACTTA ATATGTATTT TACCTGAACC CAAATACACC   
  
  
+ GGACCTGTAT TTAACACGGG CACCCGAAAT GATAGGTCTA ACTCTCACCA CCACCGCATT TGGCACCCGA   
  
  
+ ACGGTAAAAA ATGTTCATCT ATTATGCACT TTGGCTTCAT CCATAACCCT ATGATCTATC TGTGCCAATA   
  
  
+ TTTGGAGCAC CCAGCGAAGT AGCGCATGCT CTAAACTCTT GCCTCAAAAC AAAACAAATA GTGGGTCATA   
  
  
+ TTCCCTTCAA AGCACTAACA AACACACACT CTCTCTCTCT CAGTAGCCAA CAACTTAAGG CGGTGTTTGC   
  
  
+ TTGGTTTTGG AGAGGGCGTG TTTGGATGAG GTGCAAAACA CATAGATGAA ATCATAAACA ACTTGATTCC   
  
  
+ CAGCCACCCA TGACCTACTC AGATAACAAG GGACAAAAAC AAAGAGGTTT TCTGAAACAG AGAAAACAGG   
  
  
+ GGCGTTGTGT TGCGCTGTTG CTCACAAAAT TCCAATATTC CTCTAAAAGC TCCCAAACGG AGACAAATCA   
  
  
+ AAGAGGACAA AGACATGTGG GATTTAATAC AAAATTTGAC TAACTCTGCA ACGGAAAACA ACCCCTAGGG   
  
  
+ TGTTTTTCTG CGTGTTGTAC ACACTTCACT TTTAACCTGC TCTGCCTCTG CCTTTGCCAC GGCTTTTAAT   
  
  
+ GGATAAGACC AATTAAGAAA CCCAAATATC TCGTAAATTT GTTGAGATTC CTTTCTGCGT TCTCAAGTGT   
  
  
+ TTCAAAAACC CAGTTTGGAG GTTGCACTTA TTCATGCTTT TCTTCAAGTC AGTGAGCTTA GTGTCTGGAA   
  
  
+ CTGAAGCTCC TCCTGGTAAT GCTCTGTTTT TCATTTCTTG TTGTGCGTTT ATAAAAATTC GGCCTATTGA   
  
  
+ TGGAAGTTTG TTTGATGTTT TTGGGTAAGT TTTTGATTAA TAGAGATGAA TTAGTAAAGT AAAATTTGAG   
  
  
+ TGGTATTTAA TGATCCTTAA GCAGTCTATG CTTTGTTGTC ACATGCGTTT CTTGGCTTTT GACTGAAACA   
  
  
+ TTTCTGTGTT AGATGAAAAA CTCATTTTGA TGCATGAGAT GATAAAATCG CTTATTTTTG CCTTTATTTG   
  
  
+ GTGTGTGGGG GGGGGGGGGG GGGGGCGCAG GTCTGAGGTA AAAGATATGT GAATTCATTT GGAGTCTCGA   
  
  
+ TAATTCGTTG GCTATGTTGA CAATCACGAG TGGTCGTTAT CGCTCATCTC ACAGGTTGAT AATTCACTCA   
  
  
+ AACTTCAAGC GGCCTGAATC ATTCAGTCCG GGTTGTTAAA ATCTCAACAG TCATTTCACA CGAGTGGAGG   
  
  
+ GCCCCAAAAG ACACCATTCT TTCGAACTTT TTTTTTTTGA AGGTCATTTT TTCAAGAACA AAACTAATGT   
  
  
+ GAACATGCGA CTTCTATGAG TAAAGTGTAA AGATGCATAA AGTCTTTTTT TCTTTTATAT AAAAAGTACT   
  
  
+ CAAAAAAAAA AGAGTTGGAG AGCTGATTCC CCATGCATCC TCTAAGCAAA GGTGGTGGAC GGCAGAAAAT   
  
  
+ TAGTAGAGTT ATTGTGAGAT TAAAGTTTGC GCACCTGAAC GGCTGACCTC TAGTTTGCTA TCAACGTTAC   
  
  
+ ATTTATGTGT CATAATTTTT GCATGAAACA ATTTCACGCA ATGTTTTCTG ATGGCAGTTA TGTTTAGAAA   
  
  
+ TAAGTTTAAT CGGACCTTAT TCCACTGATT TATATTTGAT TCTTCCCTCT TTACTACGGC TGTCCATTCA   
  
  
+ CAAGCCAAAG AATTCTCCCC CCAAAAAAAA GAAAAAACAG TATAGAACCA TGTTTATTAT CTCATTTTTT   
  
  
+ TCTCTCCCAT TTGATCTGAA ACACAAACTT GCAGGGGTGC AAGTTGGACA CTTAGTAGCT GCAGGAATGA   
  
  
+ TTCAATAGGA ATCCTCATCT TTGACAACAT CACCACTTGA ATACATATCA CTTGATGGCA CTCTCCCCTG   
  
  
+ GTTTAGGCTT CCCATATCCG TGGCTTAGGG AGCTAAAACC TGAGCAAAGA GGACTCTGTC TAATCCATCT   
  
  
+ CCTCCTTTCA TGTGCTAATC AAGTTGCTAC TGGGAGCATC GACAATGCTA ATGTCAGCCT TGAGCATATT   
  
  
+ TCCCACCTTG CCTCTCCCAC CGGAGATACA ATGCAGCGAA TTGCTGCTTA CTTTGCTGAA GCCTTTGCTG   
  
  
+ ACCGTTTACT AAGGGCTTGG CAGCCCGGTC TTCTCAAAGC CTTGAATTGT ACCAAGATGT CATCTGTTTC   
  
  
+ CGAACAAATT CTTGTTCAAA AGTTGTTCTT TGATCTTCTT CCGTTCTTGA AGCTTTCATA TCTTGTGACG   
  
  
+ AACCAGGCAA TCACGGAGGC CATAGAAGGA GAAAAGATGG TTCATATAAT TGATCTCCAT TCTTGTGAAT   
  
  
+ CGGCTCTGTG GATTAGTCTC CTCCAGGCAT TGAGTGTTCG ACCTGAAGGC CCACCCCATT TGAGGATAAC   
  
  
+ CGGTATACAT GAGAAGAAAG AAGTGTTGGA TCAAATGGCT ATGCAACTAA ACAAAGAGGC TGAAAAATTG   
  
  
+ GACATCCCAT TTCAATTCAA TCCTATCGCA AGCAAACTAG ACGACCTTGA TGTCGAAAGC TTGAGTGTCA   
  
  
+ AGACCGGAGA AGCACTTGTT ATTAGTTCTG TGCTCCAACT ACATTCTCTT TTGGCATTGG ATGAGGGATC   
  
  
+ AATGCCTAAG AACGCAGGCA TGGCTTACTT GCAAAGGGTG TTTTATATGA AACCACGAAA ATTGGAGGAC   
  
  
+ TTGCCCAACA AGGATTTGAT GAAAATGTTG AACTCAAATG AAGATTCTAC ATCATCATCA TCTTCATCTC   
  
  
+ CTATCCCTTC ATCAAAACTT GATGCCTTTT TAAAAGCCCT CCTTGGGCTT TCGCCAAAAC TCATGGTTGT   
  
  
+ AACCGAGCAA GAATCAAACC ACAATGGAAG TGCCCTAATA GAAAGAGTGA TGGAGTCATT GAACTTCTAT   
  
  
+ GCAGCATTGT TTGATTGCTT GGAATCCACT ATATCGAGGA CATCGATAGA GAGACAGAAG CTCGAGAAGT   
  
  
+ TGATGTTTGG AGAGGAGATC AAGAACCTCA CAGCTTGTGA AGGGGCAGAG AGAAAGGCAA GGCACGAGAA   
  
  
+ GCTCAGTGAA TGGGTTCAAA GATTTGAGTC AGTAGGATTT AAAAGGGAGC CATTGAGCTA CCATGGTTTC   
  
  
+ TTGCTTGCTA GGAGGTTTTT ACATACCAAT AATTATGAGG GGTATAACAT CAAGGAAGTC AATGGTTTTC   
  
  
+ TTGTTATCTG TTGGCAAGAT AGACCCCTGT ATTCTGTTTC AGCTTGGAGA TTTTA  

- +Up\_Stream \_Len000AATGTT TTTGCTACGA GATTTGGATA ATCCAGCAAG GCTTTGGGCG TTTTAACTTT   
  
  
- ATACTAGCGG GCATAAAATG GGTTTGGACT TTATGTGAAT TATACATAAA ATGGACTTGG GTTTATGTGG   
  
  
- CCTGGACATA AATTGTGCCC GTGGGCTTTA CTATCCAGAT TGAGAGTGGT GGTGGCGTAA ACCGTGGGCT   
  
  
- TGCCATTTTT TACAAGTAGA TAATACGTGA AACCGAAGTA GGTATTGGGA TACTAGATAG ACACGGTTAT   
  
  
- AAACCTCGTG GGTCGCTTCA TCGCGTACGA GATTTGAGAA CGGAGTTTTG TTTTGTTTAT CACCCAGTAT   
  
  
- AAGGGAAGTT TCGTGATTGT TTGTGTGTGA GAGAGAGAGA GTCATCGGTT GTTGAATTCC GCCACAAACG   
  
  
- AACCAAAACC TCTCCCGCAC AAACCTACTC CACGTTTTGT GTATCTACTT TAGTATTTGT TGAACTAAGG   
  
  
- GTCGGTGGGT ACTGGATGAG TCTATTGTTC CCTGTTTTTG TTTCTCCAAA AGACTTTGTC TCTTTTGTCC   
  
  
- CCGCAACACA ACGCGACAAC GAGTGTTTTA AGGTTATAAG GAGATTTTCG AGGGTTTGCC TCTGTTTAGT   
  
  
- TTCTCCTGTT TCTGTACACC CTAAATTATG TTTTAAACTG ATTGAGACGT TGCCTTTTGT TGGGGATCCC   
  
  
- ACAAAAAGAC GCACAACATG TGTGAAGTGA AAATTGGACG AGACGGAGAC GGAAACGGTG CCGAAAATTA   
  
  
- CCTATTCTGG TTAATTCTTT GGGTTTATAG AGCATTTAAA CAACTCTAAG GAAAGACGCA AGAGTTCACA   
  
  
- AAGTTTTTGG GTCAAACCTC CAACGTGAAT AAGTACGAAA AGAAGTTCAG TCACTCGAAT CACAGACCTT   
  
  
- GACTTCGAGG AGGACCATTA CGAGACAAAA AGTAAAGAAC AACACGCAAA TATTTTTAAG CCGGATAACT   
  
  
- ACCTTCAAAC AAACTACAAA AACCCATTCA AAAACTAATT ATCTCTACTT AATCATTTCA TTTTAAACTC   
  
  
- ACCATAAATT ACTAGGAATT CGTCAGATAC GAAACAACAG TGTACGCAAA GAACCGAAAA CTGACTTTGT   
  
  
- AAAGACACAA TCTACTTTTT GAGTAAAACT ACGTACTCTA CTATTTTAGC GAATAAAAAC GGAAATAAAC   
  
  
- CACACACCCC CCCCCCCCCC CCCCCGCGTC CAGACTCCAT TTTCTATACA CTTAAGTAAA CCTCAGAGCT   
  
  
- ATTAAGCAAC CGATACAACT GTTAGTGCTC ACCAGCAATA GCGAGTAGAG TGTCCAACTA TTAAGTGAGT   
  
  
- TTGAAGTTCG CCGGACTTAG TAAGTCAGGC CCAACAATTT TAGAGTTGTC AGTAAAGTGT GCTCACCTCC   
  
  
- CGGGGTTTTC TGTGGTAAGA AAGCTTGAAA AAAAAAAACT TCCAGTAAAA AAGTTCTTGT TTTGATTACA   
  
  
- CTTGTACGCT GAAGATACTC ATTTCACATT TCTACGTATT TCAGAAAAAA AGAAAATATA TTTTTCATGA   
  
  
- GTTTTTTTTT TCTCAACCTC TCGACTAAGG GGTACGTAGG AGATTCGTTT CCACCACCTG CCGTCTTTTA   
  
  
- ATCATCTCAA TAACACTCTA ATTTCAAACG CGTGGACTTG CCGACTGGAG ATCAAACGAT AGTTGCAATG   
  
  
- TAAATACACA GTATTAAAAA CGTACTTTGT TAAAGTGCGT TACAAAAGAC TACCGTCAAT ACAAATCTTT   
  
  
- ATTCAAATTA GCCTGGAATA AGGTGACTAA ATATAAACTA AGAAGGGAGA AATGATGCCG ACAGGTAAGT   
  
  
- GTTCGGTTTC TTAAGAGGGG GGTTTTTTTT CTTTTTTGTC ATATCTTGGT ACAAATAATA GAGTAAAAAA   
  
  
- AGAGAGGGTA AACTAGACTT TGTGTTTGAA CGTCCCCACG TTCAACCTGT GAATCATCGA CGTCCTTACT   
  
  
- AAGTTATCCT TAGGAGTAGA AACTGTTGTA GTGGTGAACT TATGTATAGT GAACTACCGT GAGAGGGGAC   
  
  
- CAAATCCGAA GGGTATAGGC ACCGAATCCC TCGATTTTGG ACTCGTTTCT CCTGAGACAG ATTAGGTAGA   
  
  
- GGAGGAAAGT ACACGATTAG TTCAACGATG ACCCTCGTAG CTGTTACGAT TACAGTCGGA ACTCGTATAA   
  
  
- AGGGTGGAAC GGAGAGGGTG GCCTCTATGT TACGTCGCTT AACGACGAAT GAAACGACTT CGGAAACGAC   
  
  
- TGGCAAATGA TTCCCGAACC GTCGGGCCAG AAGAGTTTCG GAACTTAACA TGGTTCTACA GTAGACAAAG   
  
  
- GCTTGTTTAA GAACAAGTTT TCAACAAGAA ACTAGAAGAA GGCAAGAACT TCGAAAGTAT AGAACACTGC   
  
  
- TTGGTCCGTT AGTGCCTCCG GTATCTTCCT CTTTTCTACC AAGTATATTA ACTAGAGGTA AGAACACTTA   
  
  
- GCCGAGACAC CTAATCAGAG GAGGTCCGTA ACTCACAAGC TGGACTTCCG GGTGGGGTAA ACTCCTATTG   
  
  
- GCCATATGTA CTCTTCTTTC TTCACAACCT AGTTTACCGA TACGTTGATT TGTTTCTCCG ACTTTTTAAC   
  
  
- CTGTAGGGTA AAGTTAAGTT AGGATAGCGT TCGTTTGATC TGCTGGAACT ACAGCTTTCG AACTCACAGT   
  
  
- TCTGGCCTCT TCGTGAACAA TAATCAAGAC ACGAGGTTGA TGTAAGAGAA AACCGTAACC TACTCCCTAG   
  
  
- TTACGGATTC TTGCGTCCGT ACCGAATGAA CGTTTCCCAC AAAATATACT TTGGTGCTTT TAACCTCCTG   
  
  
- AACGGGTTGT TCCTAAACTA CTTTTACAAC TTGAGTTTAC TTCTAAGATG TAGTAGTAGT AGAAGTAGAG   
  
  
- GATAGGGAAG TAGTTTTGAA CTACGGAAAA ATTTTCGGGA GGAACCCGAA AGCGGTTTTG AGTACCAACA   
  
  
- TTGGCTCGTT CTTAGTTTGG TGTTACCTTC ACGGGATTAT CTTTCTCACT ACCTCAGTAA CTTGAAGATA   
  
  
- CGTCGTAACA AACTAACGAA CCTTAGGTGA TATAGCTCCT GTAGCTATCT CTCTGTCTTC GAGCTCTTCA   
  
  
- ACTACAAACC TCTCCTCTAG TTCTTGGAGT GTCGAACACT TCCCCGTCTC TCTTTCCGTT CCGTGCTCTT   
  
  
- CGAGTCACTT ACCCAAGTTT CTAAACTCAG TCATCCTAAA TTTTCCCTCG GTAACTCGAT GGTACCAAAG   
  
  
- AACGAACGAT CCTCCAAAAA TGTATGGTTA TTAATACTCC CCATATTGTA GTTCCTTCAG TTACCAAAAG   
  
  
- AACAATAGAC AACCGTTCTA TCTGGGGACA TAAGACAAAG TCGAACCTCT AAAAT

+     GARE-motif

| Site Name | Organism | Position | Strand | Matrix score. | sequence | function |
| --- | --- | --- | --- | --- | --- | --- |
| GARE-motif | Brassica oleracea | 3301 | + | 7 | TCTGTTG | gibberellin-responsive element |

>HU05G01267.1   
+ +Up\_Stream \_Len000TTACAA AAACGATGCT CTAAACCTAT TAGGTCGTTC CGAAACCCGC AAAATTGAAA   
  
  
+ TATGATCGCC CGTATTTTAC CCAAACCTGA AATACACTTA ATATGTATTT TACCTGAACC CAAATACACC   
  
  
+ GGACCTGTAT TTAACACGGG CACCCGAAAT GATAGGTCTA ACTCTCACCA CCACCGCATT TGGCACCCGA   
  
  
+ ACGGTAAAAA ATGTTCATCT ATTATGCACT TTGGCTTCAT CCATAACCCT ATGATCTATC TGTGCCAATA   
  
  
+ TTTGGAGCAC CCAGCGAAGT AGCGCATGCT CTAAACTCTT GCCTCAAAAC AAAACAAATA GTGGGTCATA   
  
  
+ TTCCCTTCAA AGCACTAACA AACACACACT CTCTCTCTCT CAGTAGCCAA CAACTTAAGG CGGTGTTTGC   
  
  
+ TTGGTTTTGG AGAGGGCGTG TTTGGATGAG GTGCAAAACA CATAGATGAA ATCATAAACA ACTTGATTCC   
  
  
+ CAGCCACCCA TGACCTACTC AGATAACAAG GGACAAAAAC AAAGAGGTTT TCTGAAACAG AGAAAACAGG   
  
  
+ GGCGTTGTGT TGCGCTGTTG CTCACAAAAT TCCAATATTC CTCTAAAAGC TCCCAAACGG AGACAAATCA   
  
  
+ AAGAGGACAA AGACATGTGG GATTTAATAC AAAATTTGAC TAACTCTGCA ACGGAAAACA ACCCCTAGGG   
  
  
+ TGTTTTTCTG CGTGTTGTAC ACACTTCACT TTTAACCTGC TCTGCCTCTG CCTTTGCCAC GGCTTTTAAT   
  
  
+ GGATAAGACC AATTAAGAAA CCCAAATATC TCGTAAATTT GTTGAGATTC CTTTCTGCGT TCTCAAGTGT   
  
  
+ TTCAAAAACC CAGTTTGGAG GTTGCACTTA TTCATGCTTT TCTTCAAGTC AGTGAGCTTA GTGTCTGGAA   
  
  
+ CTGAAGCTCC TCCTGGTAAT GCTCTGTTTT TCATTTCTTG TTGTGCGTTT ATAAAAATTC GGCCTATTGA   
  
  
+ TGGAAGTTTG TTTGATGTTT TTGGGTAAGT TTTTGATTAA TAGAGATGAA TTAGTAAAGT AAAATTTGAG   
  
  
+ TGGTATTTAA TGATCCTTAA GCAGTCTATG CTTTGTTGTC ACATGCGTTT CTTGGCTTTT GACTGAAACA   
  
  
+ TTTCTGTGTT AGATGAAAAA CTCATTTTGA TGCATGAGAT GATAAAATCG CTTATTTTTG CCTTTATTTG   
  
  
+ GTGTGTGGGG GGGGGGGGGG GGGGGCGCAG GTCTGAGGTA AAAGATATGT GAATTCATTT GGAGTCTCGA   
  
  
+ TAATTCGTTG GCTATGTTGA CAATCACGAG TGGTCGTTAT CGCTCATCTC ACAGGTTGAT AATTCACTCA   
  
  
+ AACTTCAAGC GGCCTGAATC ATTCAGTCCG GGTTGTTAAA ATCTCAACAG TCATTTCACA CGAGTGGAGG   
  
  
+ GCCCCAAAAG ACACCATTCT TTCGAACTTT TTTTTTTTGA AGGTCATTTT TTCAAGAACA AAACTAATGT   
  
  
+ GAACATGCGA CTTCTATGAG TAAAGTGTAA AGATGCATAA AGTCTTTTTT TCTTTTATAT AAAAAGTACT   
  
  
+ CAAAAAAAAA AGAGTTGGAG AGCTGATTCC CCATGCATCC TCTAAGCAAA GGTGGTGGAC GGCAGAAAAT   
  
  
+ TAGTAGAGTT ATTGTGAGAT TAAAGTTTGC GCACCTGAAC GGCTGACCTC TAGTTTGCTA TCAACGTTAC   
  
  
+ ATTTATGTGT CATAATTTTT GCATGAAACA ATTTCACGCA ATGTTTTCTG ATGGCAGTTA TGTTTAGAAA   
  
  
+ TAAGTTTAAT CGGACCTTAT TCCACTGATT TATATTTGAT TCTTCCCTCT TTACTACGGC TGTCCATTCA   
  
  
+ CAAGCCAAAG AATTCTCCCC CCAAAAAAAA GAAAAAACAG TATAGAACCA TGTTTATTAT CTCATTTTTT   
  
  
+ TCTCTCCCAT TTGATCTGAA ACACAAACTT GCAGGGGTGC AAGTTGGACA CTTAGTAGCT GCAGGAATGA   
  
  
+ TTCAATAGGA ATCCTCATCT TTGACAACAT CACCACTTGA ATACATATCA CTTGATGGCA CTCTCCCCTG   
  
  
+ GTTTAGGCTT CCCATATCCG TGGCTTAGGG AGCTAAAACC TGAGCAAAGA GGACTCTGTC TAATCCATCT   
  
  
+ CCTCCTTTCA TGTGCTAATC AAGTTGCTAC TGGGAGCATC GACAATGCTA ATGTCAGCCT TGAGCATATT   
  
  
+ TCCCACCTTG CCTCTCCCAC CGGAGATACA ATGCAGCGAA TTGCTGCTTA CTTTGCTGAA GCCTTTGCTG   
  
  
+ ACCGTTTACT AAGGGCTTGG CAGCCCGGTC TTCTCAAAGC CTTGAATTGT ACCAAGATGT CATCTGTTTC   
  
  
+ CGAACAAATT CTTGTTCAAA AGTTGTTCTT TGATCTTCTT CCGTTCTTGA AGCTTTCATA TCTTGTGACG   
  
  
+ AACCAGGCAA TCACGGAGGC CATAGAAGGA GAAAAGATGG TTCATATAAT TGATCTCCAT TCTTGTGAAT   
  
  
+ CGGCTCTGTG GATTAGTCTC CTCCAGGCAT TGAGTGTTCG ACCTGAAGGC CCACCCCATT TGAGGATAAC   
  
  
+ CGGTATACAT GAGAAGAAAG AAGTGTTGGA TCAAATGGCT ATGCAACTAA ACAAAGAGGC TGAAAAATTG   
  
  
+ GACATCCCAT TTCAATTCAA TCCTATCGCA AGCAAACTAG ACGACCTTGA TGTCGAAAGC TTGAGTGTCA   
  
  
+ AGACCGGAGA AGCACTTGTT ATTAGTTCTG TGCTCCAACT ACATTCTCTT TTGGCATTGG ATGAGGGATC   
  
  
+ AATGCCTAAG AACGCAGGCA TGGCTTACTT GCAAAGGGTG TTTTATATGA AACCACGAAA ATTGGAGGAC   
  
  
+ TTGCCCAACA AGGATTTGAT GAAAATGTTG AACTCAAATG AAGATTCTAC ATCATCATCA TCTTCATCTC   
  
  
+ CTATCCCTTC ATCAAAACTT GATGCCTTTT TAAAAGCCCT CCTTGGGCTT TCGCCAAAAC TCATGGTTGT   
  
  
+ AACCGAGCAA GAATCAAACC ACAATGGAAG TGCCCTAATA GAAAGAGTGA TGGAGTCATT GAACTTCTAT   
  
  
+ GCAGCATTGT TTGATTGCTT GGAATCCACT ATATCGAGGA CATCGATAGA GAGACAGAAG CTCGAGAAGT   
  
  
+ TGATGTTTGG AGAGGAGATC AAGAACCTCA CAGCTTGTGA AGGGGCAGAG AGAAAGGCAA GGCACGAGAA   
  
  
+ GCTCAGTGAA TGGGTTCAAA GATTTGAGTC AGTAGGATTT AAAAGGGAGC CATTGAGCTA CCATGGTTTC   
  
  
+ TTGCTTGCTA GGAGGTTTTT ACATACCAAT AATTATGAGG GGTATAACAT CAAGGAAGTC AATGGTTTTC   
  
  
+ TTGTTATCTG TTGGCAAGAT AGACCCCTGT ATTCTGTTTC AGCTTGGAGA TTTTA  

- +Up\_Stream \_Len000AATGTT TTTGCTACGA GATTTGGATA ATCCAGCAAG GCTTTGGGCG TTTTAACTTT   
  
  
- ATACTAGCGG GCATAAAATG GGTTTGGACT TTATGTGAAT TATACATAAA ATGGACTTGG GTTTATGTGG   
  
  
- CCTGGACATA AATTGTGCCC GTGGGCTTTA CTATCCAGAT TGAGAGTGGT GGTGGCGTAA ACCGTGGGCT   
  
  
- TGCCATTTTT TACAAGTAGA TAATACGTGA AACCGAAGTA GGTATTGGGA TACTAGATAG ACACGGTTAT   
  
  
- AAACCTCGTG GGTCGCTTCA TCGCGTACGA GATTTGAGAA CGGAGTTTTG TTTTGTTTAT CACCCAGTAT   
  
  
- AAGGGAAGTT TCGTGATTGT TTGTGTGTGA GAGAGAGAGA GTCATCGGTT GTTGAATTCC GCCACAAACG   
  
  
- AACCAAAACC TCTCCCGCAC AAACCTACTC CACGTTTTGT GTATCTACTT TAGTATTTGT TGAACTAAGG   
  
  
- GTCGGTGGGT ACTGGATGAG TCTATTGTTC CCTGTTTTTG TTTCTCCAAA AGACTTTGTC TCTTTTGTCC   
  
  
- CCGCAACACA ACGCGACAAC GAGTGTTTTA AGGTTATAAG GAGATTTTCG AGGGTTTGCC TCTGTTTAGT   
  
  
- TTCTCCTGTT TCTGTACACC CTAAATTATG TTTTAAACTG ATTGAGACGT TGCCTTTTGT TGGGGATCCC   
  
  
- ACAAAAAGAC GCACAACATG TGTGAAGTGA AAATTGGACG AGACGGAGAC GGAAACGGTG CCGAAAATTA   
  
  
- CCTATTCTGG TTAATTCTTT GGGTTTATAG AGCATTTAAA CAACTCTAAG GAAAGACGCA AGAGTTCACA   
  
  
- AAGTTTTTGG GTCAAACCTC CAACGTGAAT AAGTACGAAA AGAAGTTCAG TCACTCGAAT CACAGACCTT   
  
  
- GACTTCGAGG AGGACCATTA CGAGACAAAA AGTAAAGAAC AACACGCAAA TATTTTTAAG CCGGATAACT   
  
  
- ACCTTCAAAC AAACTACAAA AACCCATTCA AAAACTAATT ATCTCTACTT AATCATTTCA TTTTAAACTC   
  
  
- ACCATAAATT ACTAGGAATT CGTCAGATAC GAAACAACAG TGTACGCAAA GAACCGAAAA CTGACTTTGT   
  
  
- AAAGACACAA TCTACTTTTT GAGTAAAACT ACGTACTCTA CTATTTTAGC GAATAAAAAC GGAAATAAAC   
  
  
- CACACACCCC CCCCCCCCCC CCCCCGCGTC CAGACTCCAT TTTCTATACA CTTAAGTAAA CCTCAGAGCT   
  
  
- ATTAAGCAAC CGATACAACT GTTAGTGCTC ACCAGCAATA GCGAGTAGAG TGTCCAACTA TTAAGTGAGT   
  
  
- TTGAAGTTCG CCGGACTTAG TAAGTCAGGC CCAACAATTT TAGAGTTGTC AGTAAAGTGT GCTCACCTCC   
  
  
- CGGGGTTTTC TGTGGTAAGA AAGCTTGAAA AAAAAAAACT TCCAGTAAAA AAGTTCTTGT TTTGATTACA   
  
  
- CTTGTACGCT GAAGATACTC ATTTCACATT TCTACGTATT TCAGAAAAAA AGAAAATATA TTTTTCATGA   
  
  
- GTTTTTTTTT TCTCAACCTC TCGACTAAGG GGTACGTAGG AGATTCGTTT CCACCACCTG CCGTCTTTTA   
  
  
- ATCATCTCAA TAACACTCTA ATTTCAAACG CGTGGACTTG CCGACTGGAG ATCAAACGAT AGTTGCAATG   
  
  
- TAAATACACA GTATTAAAAA CGTACTTTGT TAAAGTGCGT TACAAAAGAC TACCGTCAAT ACAAATCTTT   
  
  
- ATTCAAATTA GCCTGGAATA AGGTGACTAA ATATAAACTA AGAAGGGAGA AATGATGCCG ACAGGTAAGT   
  
  
- GTTCGGTTTC TTAAGAGGGG GGTTTTTTTT CTTTTTTGTC ATATCTTGGT ACAAATAATA GAGTAAAAAA   
  
  
- AGAGAGGGTA AACTAGACTT TGTGTTTGAA CGTCCCCACG TTCAACCTGT GAATCATCGA CGTCCTTACT   
  
  
- AAGTTATCCT TAGGAGTAGA AACTGTTGTA GTGGTGAACT TATGTATAGT GAACTACCGT GAGAGGGGAC   
  
  
- CAAATCCGAA GGGTATAGGC ACCGAATCCC TCGATTTTGG ACTCGTTTCT CCTGAGACAG ATTAGGTAGA   
  
  
- GGAGGAAAGT ACACGATTAG TTCAACGATG ACCCTCGTAG CTGTTACGAT TACAGTCGGA ACTCGTATAA   
  
  
- AGGGTGGAAC GGAGAGGGTG GCCTCTATGT TACGTCGCTT AACGACGAAT GAAACGACTT CGGAAACGAC   
  
  
- TGGCAAATGA TTCCCGAACC GTCGGGCCAG AAGAGTTTCG GAACTTAACA TGGTTCTACA GTAGACAAAG   
  
  
- GCTTGTTTAA GAACAAGTTT TCAACAAGAA ACTAGAAGAA GGCAAGAACT TCGAAAGTAT AGAACACTGC   
  
  
- TTGGTCCGTT AGTGCCTCCG GTATCTTCCT CTTTTCTACC AAGTATATTA ACTAGAGGTA AGAACACTTA   
  
  
- GCCGAGACAC CTAATCAGAG GAGGTCCGTA ACTCACAAGC TGGACTTCCG GGTGGGGTAA ACTCCTATTG   
  
  
- GCCATATGTA CTCTTCTTTC TTCACAACCT AGTTTACCGA TACGTTGATT TGTTTCTCCG ACTTTTTAAC   
  
  
- CTGTAGGGTA AAGTTAAGTT AGGATAGCGT TCGTTTGATC TGCTGGAACT ACAGCTTTCG AACTCACAGT   
  
  
- TCTGGCCTCT TCGTGAACAA TAATCAAGAC ACGAGGTTGA TGTAAGAGAA AACCGTAACC TACTCCCTAG   
  
  
- TTACGGATTC TTGCGTCCGT ACCGAATGAA CGTTTCCCAC AAAATATACT TTGGTGCTTT TAACCTCCTG   
  
  
- AACGGGTTGT TCCTAAACTA CTTTTACAAC TTGAGTTTAC TTCTAAGATG TAGTAGTAGT AGAAGTAGAG   
  
  
- GATAGGGAAG TAGTTTTGAA CTACGGAAAA ATTTTCGGGA GGAACCCGAA AGCGGTTTTG AGTACCAACA   
  
  
- TTGGCTCGTT CTTAGTTTGG TGTTACCTTC ACGGGATTAT CTTTCTCACT ACCTCAGTAA CTTGAAGATA   
  
  
- CGTCGTAACA AACTAACGAA CCTTAGGTGA TATAGCTCCT GTAGCTATCT CTCTGTCTTC GAGCTCTTCA   
  
  
- ACTACAAACC TCTCCTCTAG TTCTTGGAGT GTCGAACACT TCCCCGTCTC TCTTTCCGTT CCGTGCTCTT   
  
  
- CGAGTCACTT ACCCAAGTTT CTAAACTCAG TCATCCTAAA TTTTCCCTCG GTAACTCGAT GGTACCAAAG   
  
  
- AACGAACGAT CCTCCAAAAA TGTATGGTTA TTAATACTCC CCATATTGTA GTTCCTTCAG TTACCAAAAG   
  
  
- AACAATAGAC AACCGTTCTA TCTGGGGACA TAAGACAAAG TCGAACCTCT AAAAT

+     GATA-motif

| Site Name | Organism | Position | Strand | Matrix score. | sequence | function |
| --- | --- | --- | --- | --- | --- | --- |
| GATA-motif | Arabidopsis thaliana | 2873 | - | 7 | GATAGGA | part of a light responsive element |
| GATA-motif | Arabidopsis thaliana | 2615 | - | 7 | GATAGGA | part of a light responsive element |

>HU05G01267.1   
+ +Up\_Stream \_Len000TTACAA AAACGATGCT CTAAACCTAT TAGGTCGTTC CGAAACCCGC AAAATTGAAA   
  
  
+ TATGATCGCC CGTATTTTAC CCAAACCTGA AATACACTTA ATATGTATTT TACCTGAACC CAAATACACC   
  
  
+ GGACCTGTAT TTAACACGGG CACCCGAAAT GATAGGTCTA ACTCTCACCA CCACCGCATT TGGCACCCGA   
  
  
+ ACGGTAAAAA ATGTTCATCT ATTATGCACT TTGGCTTCAT CCATAACCCT ATGATCTATC TGTGCCAATA   
  
  
+ TTTGGAGCAC CCAGCGAAGT AGCGCATGCT CTAAACTCTT GCCTCAAAAC AAAACAAATA GTGGGTCATA   
  
  
+ TTCCCTTCAA AGCACTAACA AACACACACT CTCTCTCTCT CAGTAGCCAA CAACTTAAGG CGGTGTTTGC   
  
  
+ TTGGTTTTGG AGAGGGCGTG TTTGGATGAG GTGCAAAACA CATAGATGAA ATCATAAACA ACTTGATTCC   
  
  
+ CAGCCACCCA TGACCTACTC AGATAACAAG GGACAAAAAC AAAGAGGTTT TCTGAAACAG AGAAAACAGG   
  
  
+ GGCGTTGTGT TGCGCTGTTG CTCACAAAAT TCCAATATTC CTCTAAAAGC TCCCAAACGG AGACAAATCA   
  
  
+ AAGAGGACAA AGACATGTGG GATTTAATAC AAAATTTGAC TAACTCTGCA ACGGAAAACA ACCCCTAGGG   
  
  
+ TGTTTTTCTG CGTGTTGTAC ACACTTCACT TTTAACCTGC TCTGCCTCTG CCTTTGCCAC GGCTTTTAAT   
  
  
+ GGATAAGACC AATTAAGAAA CCCAAATATC TCGTAAATTT GTTGAGATTC CTTTCTGCGT TCTCAAGTGT   
  
  
+ TTCAAAAACC CAGTTTGGAG GTTGCACTTA TTCATGCTTT TCTTCAAGTC AGTGAGCTTA GTGTCTGGAA   
  
  
+ CTGAAGCTCC TCCTGGTAAT GCTCTGTTTT TCATTTCTTG TTGTGCGTTT ATAAAAATTC GGCCTATTGA   
  
  
+ TGGAAGTTTG TTTGATGTTT TTGGGTAAGT TTTTGATTAA TAGAGATGAA TTAGTAAAGT AAAATTTGAG   
  
  
+ TGGTATTTAA TGATCCTTAA GCAGTCTATG CTTTGTTGTC ACATGCGTTT CTTGGCTTTT GACTGAAACA   
  
  
+ TTTCTGTGTT AGATGAAAAA CTCATTTTGA TGCATGAGAT GATAAAATCG CTTATTTTTG CCTTTATTTG   
  
  
+ GTGTGTGGGG GGGGGGGGGG GGGGGCGCAG GTCTGAGGTA AAAGATATGT GAATTCATTT GGAGTCTCGA   
  
  
+ TAATTCGTTG GCTATGTTGA CAATCACGAG TGGTCGTTAT CGCTCATCTC ACAGGTTGAT AATTCACTCA   
  
  
+ AACTTCAAGC GGCCTGAATC ATTCAGTCCG GGTTGTTAAA ATCTCAACAG TCATTTCACA CGAGTGGAGG   
  
  
+ GCCCCAAAAG ACACCATTCT TTCGAACTTT TTTTTTTTGA AGGTCATTTT TTCAAGAACA AAACTAATGT   
  
  
+ GAACATGCGA CTTCTATGAG TAAAGTGTAA AGATGCATAA AGTCTTTTTT TCTTTTATAT AAAAAGTACT   
  
  
+ CAAAAAAAAA AGAGTTGGAG AGCTGATTCC CCATGCATCC TCTAAGCAAA GGTGGTGGAC GGCAGAAAAT   
  
  
+ TAGTAGAGTT ATTGTGAGAT TAAAGTTTGC GCACCTGAAC GGCTGACCTC TAGTTTGCTA TCAACGTTAC   
  
  
+ ATTTATGTGT CATAATTTTT GCATGAAACA ATTTCACGCA ATGTTTTCTG ATGGCAGTTA TGTTTAGAAA   
  
  
+ TAAGTTTAAT CGGACCTTAT TCCACTGATT TATATTTGAT TCTTCCCTCT TTACTACGGC TGTCCATTCA   
  
  
+ CAAGCCAAAG AATTCTCCCC CCAAAAAAAA GAAAAAACAG TATAGAACCA TGTTTATTAT CTCATTTTTT   
  
  
+ TCTCTCCCAT TTGATCTGAA ACACAAACTT GCAGGGGTGC AAGTTGGACA CTTAGTAGCT GCAGGAATGA   
  
  
+ TTCAATAGGA ATCCTCATCT TTGACAACAT CACCACTTGA ATACATATCA CTTGATGGCA CTCTCCCCTG   
  
  
+ GTTTAGGCTT CCCATATCCG TGGCTTAGGG AGCTAAAACC TGAGCAAAGA GGACTCTGTC TAATCCATCT   
  
  
+ CCTCCTTTCA TGTGCTAATC AAGTTGCTAC TGGGAGCATC GACAATGCTA ATGTCAGCCT TGAGCATATT   
  
  
+ TCCCACCTTG CCTCTCCCAC CGGAGATACA ATGCAGCGAA TTGCTGCTTA CTTTGCTGAA GCCTTTGCTG   
  
  
+ ACCGTTTACT AAGGGCTTGG CAGCCCGGTC TTCTCAAAGC CTTGAATTGT ACCAAGATGT CATCTGTTTC   
  
  
+ CGAACAAATT CTTGTTCAAA AGTTGTTCTT TGATCTTCTT CCGTTCTTGA AGCTTTCATA TCTTGTGACG   
  
  
+ AACCAGGCAA TCACGGAGGC CATAGAAGGA GAAAAGATGG TTCATATAAT TGATCTCCAT TCTTGTGAAT   
  
  
+ CGGCTCTGTG GATTAGTCTC CTCCAGGCAT TGAGTGTTCG ACCTGAAGGC CCACCCCATT TGAGGATAAC   
  
  
+ CGGTATACAT GAGAAGAAAG AAGTGTTGGA TCAAATGGCT ATGCAACTAA ACAAAGAGGC TGAAAAATTG   
  
  
+ GACATCCCAT TTCAATTCAA TCCTATCGCA AGCAAACTAG ACGACCTTGA TGTCGAAAGC TTGAGTGTCA   
  
  
+ AGACCGGAGA AGCACTTGTT ATTAGTTCTG TGCTCCAACT ACATTCTCTT TTGGCATTGG ATGAGGGATC   
  
  
+ AATGCCTAAG AACGCAGGCA TGGCTTACTT GCAAAGGGTG TTTTATATGA AACCACGAAA ATTGGAGGAC   
  
  
+ TTGCCCAACA AGGATTTGAT GAAAATGTTG AACTCAAATG AAGATTCTAC ATCATCATCA TCTTCATCTC   
  
  
+ CTATCCCTTC ATCAAAACTT GATGCCTTTT TAAAAGCCCT CCTTGGGCTT TCGCCAAAAC TCATGGTTGT   
  
  
+ AACCGAGCAA GAATCAAACC ACAATGGAAG TGCCCTAATA GAAAGAGTGA TGGAGTCATT GAACTTCTAT   
  
  
+ GCAGCATTGT TTGATTGCTT GGAATCCACT ATATCGAGGA CATCGATAGA GAGACAGAAG CTCGAGAAGT   
  
  
+ TGATGTTTGG AGAGGAGATC AAGAACCTCA CAGCTTGTGA AGGGGCAGAG AGAAAGGCAA GGCACGAGAA   
  
  
+ GCTCAGTGAA TGGGTTCAAA GATTTGAGTC AGTAGGATTT AAAAGGGAGC CATTGAGCTA CCATGGTTTC   
  
  
+ TTGCTTGCTA GGAGGTTTTT ACATACCAAT AATTATGAGG GGTATAACAT CAAGGAAGTC AATGGTTTTC   
  
  
+ TTGTTATCTG TTGGCAAGAT AGACCCCTGT ATTCTGTTTC AGCTTGGAGA TTTTA  

- +Up\_Stream \_Len000AATGTT TTTGCTACGA GATTTGGATA ATCCAGCAAG GCTTTGGGCG TTTTAACTTT   
  
  
- ATACTAGCGG GCATAAAATG GGTTTGGACT TTATGTGAAT TATACATAAA ATGGACTTGG GTTTATGTGG   
  
  
- CCTGGACATA AATTGTGCCC GTGGGCTTTA CTATCCAGAT TGAGAGTGGT GGTGGCGTAA ACCGTGGGCT   
  
  
- TGCCATTTTT TACAAGTAGA TAATACGTGA AACCGAAGTA GGTATTGGGA TACTAGATAG ACACGGTTAT   
  
  
- AAACCTCGTG GGTCGCTTCA TCGCGTACGA GATTTGAGAA CGGAGTTTTG TTTTGTTTAT CACCCAGTAT   
  
  
- AAGGGAAGTT TCGTGATTGT TTGTGTGTGA GAGAGAGAGA GTCATCGGTT GTTGAATTCC GCCACAAACG   
  
  
- AACCAAAACC TCTCCCGCAC AAACCTACTC CACGTTTTGT GTATCTACTT TAGTATTTGT TGAACTAAGG   
  
  
- GTCGGTGGGT ACTGGATGAG TCTATTGTTC CCTGTTTTTG TTTCTCCAAA AGACTTTGTC TCTTTTGTCC   
  
  
- CCGCAACACA ACGCGACAAC GAGTGTTTTA AGGTTATAAG GAGATTTTCG AGGGTTTGCC TCTGTTTAGT   
  
  
- TTCTCCTGTT TCTGTACACC CTAAATTATG TTTTAAACTG ATTGAGACGT TGCCTTTTGT TGGGGATCCC   
  
  
- ACAAAAAGAC GCACAACATG TGTGAAGTGA AAATTGGACG AGACGGAGAC GGAAACGGTG CCGAAAATTA   
  
  
- CCTATTCTGG TTAATTCTTT GGGTTTATAG AGCATTTAAA CAACTCTAAG GAAAGACGCA AGAGTTCACA   
  
  
- AAGTTTTTGG GTCAAACCTC CAACGTGAAT AAGTACGAAA AGAAGTTCAG TCACTCGAAT CACAGACCTT   
  
  
- GACTTCGAGG AGGACCATTA CGAGACAAAA AGTAAAGAAC AACACGCAAA TATTTTTAAG CCGGATAACT   
  
  
- ACCTTCAAAC AAACTACAAA AACCCATTCA AAAACTAATT ATCTCTACTT AATCATTTCA TTTTAAACTC   
  
  
- ACCATAAATT ACTAGGAATT CGTCAGATAC GAAACAACAG TGTACGCAAA GAACCGAAAA CTGACTTTGT   
  
  
- AAAGACACAA TCTACTTTTT GAGTAAAACT ACGTACTCTA CTATTTTAGC GAATAAAAAC GGAAATAAAC   
  
  
- CACACACCCC CCCCCCCCCC CCCCCGCGTC CAGACTCCAT TTTCTATACA CTTAAGTAAA CCTCAGAGCT   
  
  
- ATTAAGCAAC CGATACAACT GTTAGTGCTC ACCAGCAATA GCGAGTAGAG TGTCCAACTA TTAAGTGAGT   
  
  
- TTGAAGTTCG CCGGACTTAG TAAGTCAGGC CCAACAATTT TAGAGTTGTC AGTAAAGTGT GCTCACCTCC   
  
  
- CGGGGTTTTC TGTGGTAAGA AAGCTTGAAA AAAAAAAACT TCCAGTAAAA AAGTTCTTGT TTTGATTACA   
  
  
- CTTGTACGCT GAAGATACTC ATTTCACATT TCTACGTATT TCAGAAAAAA AGAAAATATA TTTTTCATGA   
  
  
- GTTTTTTTTT TCTCAACCTC TCGACTAAGG GGTACGTAGG AGATTCGTTT CCACCACCTG CCGTCTTTTA   
  
  
- ATCATCTCAA TAACACTCTA ATTTCAAACG CGTGGACTTG CCGACTGGAG ATCAAACGAT AGTTGCAATG   
  
  
- TAAATACACA GTATTAAAAA CGTACTTTGT TAAAGTGCGT TACAAAAGAC TACCGTCAAT ACAAATCTTT   
  
  
- ATTCAAATTA GCCTGGAATA AGGTGACTAA ATATAAACTA AGAAGGGAGA AATGATGCCG ACAGGTAAGT   
  
  
- GTTCGGTTTC TTAAGAGGGG GGTTTTTTTT CTTTTTTGTC ATATCTTGGT ACAAATAATA GAGTAAAAAA   
  
  
- AGAGAGGGTA AACTAGACTT TGTGTTTGAA CGTCCCCACG TTCAACCTGT GAATCATCGA CGTCCTTACT   
  
  
- AAGTTATCCT TAGGAGTAGA AACTGTTGTA GTGGTGAACT TATGTATAGT GAACTACCGT GAGAGGGGAC   
  
  
- CAAATCCGAA GGGTATAGGC ACCGAATCCC TCGATTTTGG ACTCGTTTCT CCTGAGACAG ATTAGGTAGA   
  
  
- GGAGGAAAGT ACACGATTAG TTCAACGATG ACCCTCGTAG CTGTTACGAT TACAGTCGGA ACTCGTATAA   
  
  
- AGGGTGGAAC GGAGAGGGTG GCCTCTATGT TACGTCGCTT AACGACGAAT GAAACGACTT CGGAAACGAC   
  
  
- TGGCAAATGA TTCCCGAACC GTCGGGCCAG AAGAGTTTCG GAACTTAACA TGGTTCTACA GTAGACAAAG   
  
  
- GCTTGTTTAA GAACAAGTTT TCAACAAGAA ACTAGAAGAA GGCAAGAACT TCGAAAGTAT AGAACACTGC   
  
  
- TTGGTCCGTT AGTGCCTCCG GTATCTTCCT CTTTTCTACC AAGTATATTA ACTAGAGGTA AGAACACTTA   
  
  
- GCCGAGACAC CTAATCAGAG GAGGTCCGTA ACTCACAAGC TGGACTTCCG GGTGGGGTAA ACTCCTATTG   
  
  
- GCCATATGTA CTCTTCTTTC TTCACAACCT AGTTTACCGA TACGTTGATT TGTTTCTCCG ACTTTTTAAC   
  
  
- CTGTAGGGTA AAGTTAAGTT AGGATAGCGT TCGTTTGATC TGCTGGAACT ACAGCTTTCG AACTCACAGT   
  
  
- TCTGGCCTCT TCGTGAACAA TAATCAAGAC ACGAGGTTGA TGTAAGAGAA AACCGTAACC TACTCCCTAG   
  
  
- TTACGGATTC TTGCGTCCGT ACCGAATGAA CGTTTCCCAC AAAATATACT TTGGTGCTTT TAACCTCCTG   
  
  
- AACGGGTTGT TCCTAAACTA CTTTTACAAC TTGAGTTTAC TTCTAAGATG TAGTAGTAGT AGAAGTAGAG   
  
  
- GATAGGGAAG TAGTTTTGAA CTACGGAAAA ATTTTCGGGA GGAACCCGAA AGCGGTTTTG AGTACCAACA   
  
  
- TTGGCTCGTT CTTAGTTTGG TGTTACCTTC ACGGGATTAT CTTTCTCACT ACCTCAGTAA CTTGAAGATA   
  
  
- CGTCGTAACA AACTAACGAA CCTTAGGTGA TATAGCTCCT GTAGCTATCT CTCTGTCTTC GAGCTCTTCA   
  
  
- ACTACAAACC TCTCCTCTAG TTCTTGGAGT GTCGAACACT TCCCCGTCTC TCTTTCCGTT CCGTGCTCTT   
  
  
- CGAGTCACTT ACCCAAGTTT CTAAACTCAG TCATCCTAAA TTTTCCCTCG GTAACTCGAT GGTACCAAAG   
  
  
- AACGAACGAT CCTCCAAAAA TGTATGGTTA TTAATACTCC CCATATTGTA GTTCCTTCAG TTACCAAAAG   
  
  
- AACAATAGAC AACCGTTCTA TCTGGGGACA TAAGACAAAG TCGAACCTCT AAAAT

+     GCN4\_motif

| Site Name | Organism | Position | Strand | Matrix score. | sequence | function |
| --- | --- | --- | --- | --- | --- | --- |
| GCN4\_motif | Oryza sativa | 3179 | + | 7 | TGAGTCA | cis-regulatory element involved in endosperm expression |

>HU05G01267.1   
+ +Up\_Stream \_Len000TTACAA AAACGATGCT CTAAACCTAT TAGGTCGTTC CGAAACCCGC AAAATTGAAA   
  
  
+ TATGATCGCC CGTATTTTAC CCAAACCTGA AATACACTTA ATATGTATTT TACCTGAACC CAAATACACC   
  
  
+ GGACCTGTAT TTAACACGGG CACCCGAAAT GATAGGTCTA ACTCTCACCA CCACCGCATT TGGCACCCGA   
  
  
+ ACGGTAAAAA ATGTTCATCT ATTATGCACT TTGGCTTCAT CCATAACCCT ATGATCTATC TGTGCCAATA   
  
  
+ TTTGGAGCAC CCAGCGAAGT AGCGCATGCT CTAAACTCTT GCCTCAAAAC AAAACAAATA GTGGGTCATA   
  
  
+ TTCCCTTCAA AGCACTAACA AACACACACT CTCTCTCTCT CAGTAGCCAA CAACTTAAGG CGGTGTTTGC   
  
  
+ TTGGTTTTGG AGAGGGCGTG TTTGGATGAG GTGCAAAACA CATAGATGAA ATCATAAACA ACTTGATTCC   
  
  
+ CAGCCACCCA TGACCTACTC AGATAACAAG GGACAAAAAC AAAGAGGTTT TCTGAAACAG AGAAAACAGG   
  
  
+ GGCGTTGTGT TGCGCTGTTG CTCACAAAAT TCCAATATTC CTCTAAAAGC TCCCAAACGG AGACAAATCA   
  
  
+ AAGAGGACAA AGACATGTGG GATTTAATAC AAAATTTGAC TAACTCTGCA ACGGAAAACA ACCCCTAGGG   
  
  
+ TGTTTTTCTG CGTGTTGTAC ACACTTCACT TTTAACCTGC TCTGCCTCTG CCTTTGCCAC GGCTTTTAAT   
  
  
+ GGATAAGACC AATTAAGAAA CCCAAATATC TCGTAAATTT GTTGAGATTC CTTTCTGCGT TCTCAAGTGT   
  
  
+ TTCAAAAACC CAGTTTGGAG GTTGCACTTA TTCATGCTTT TCTTCAAGTC AGTGAGCTTA GTGTCTGGAA   
  
  
+ CTGAAGCTCC TCCTGGTAAT GCTCTGTTTT TCATTTCTTG TTGTGCGTTT ATAAAAATTC GGCCTATTGA   
  
  
+ TGGAAGTTTG TTTGATGTTT TTGGGTAAGT TTTTGATTAA TAGAGATGAA TTAGTAAAGT AAAATTTGAG   
  
  
+ TGGTATTTAA TGATCCTTAA GCAGTCTATG CTTTGTTGTC ACATGCGTTT CTTGGCTTTT GACTGAAACA   
  
  
+ TTTCTGTGTT AGATGAAAAA CTCATTTTGA TGCATGAGAT GATAAAATCG CTTATTTTTG CCTTTATTTG   
  
  
+ GTGTGTGGGG GGGGGGGGGG GGGGGCGCAG GTCTGAGGTA AAAGATATGT GAATTCATTT GGAGTCTCGA   
  
  
+ TAATTCGTTG GCTATGTTGA CAATCACGAG TGGTCGTTAT CGCTCATCTC ACAGGTTGAT AATTCACTCA   
  
  
+ AACTTCAAGC GGCCTGAATC ATTCAGTCCG GGTTGTTAAA ATCTCAACAG TCATTTCACA CGAGTGGAGG   
  
  
+ GCCCCAAAAG ACACCATTCT TTCGAACTTT TTTTTTTTGA AGGTCATTTT TTCAAGAACA AAACTAATGT   
  
  
+ GAACATGCGA CTTCTATGAG TAAAGTGTAA AGATGCATAA AGTCTTTTTT TCTTTTATAT AAAAAGTACT   
  
  
+ CAAAAAAAAA AGAGTTGGAG AGCTGATTCC CCATGCATCC TCTAAGCAAA GGTGGTGGAC GGCAGAAAAT   
  
  
+ TAGTAGAGTT ATTGTGAGAT TAAAGTTTGC GCACCTGAAC GGCTGACCTC TAGTTTGCTA TCAACGTTAC   
  
  
+ ATTTATGTGT CATAATTTTT GCATGAAACA ATTTCACGCA ATGTTTTCTG ATGGCAGTTA TGTTTAGAAA   
  
  
+ TAAGTTTAAT CGGACCTTAT TCCACTGATT TATATTTGAT TCTTCCCTCT TTACTACGGC TGTCCATTCA   
  
  
+ CAAGCCAAAG AATTCTCCCC CCAAAAAAAA GAAAAAACAG TATAGAACCA TGTTTATTAT CTCATTTTTT   
  
  
+ TCTCTCCCAT TTGATCTGAA ACACAAACTT GCAGGGGTGC AAGTTGGACA CTTAGTAGCT GCAGGAATGA   
  
  
+ TTCAATAGGA ATCCTCATCT TTGACAACAT CACCACTTGA ATACATATCA CTTGATGGCA CTCTCCCCTG   
  
  
+ GTTTAGGCTT CCCATATCCG TGGCTTAGGG AGCTAAAACC TGAGCAAAGA GGACTCTGTC TAATCCATCT   
  
  
+ CCTCCTTTCA TGTGCTAATC AAGTTGCTAC TGGGAGCATC GACAATGCTA ATGTCAGCCT TGAGCATATT   
  
  
+ TCCCACCTTG CCTCTCCCAC CGGAGATACA ATGCAGCGAA TTGCTGCTTA CTTTGCTGAA GCCTTTGCTG   
  
  
+ ACCGTTTACT AAGGGCTTGG CAGCCCGGTC TTCTCAAAGC CTTGAATTGT ACCAAGATGT CATCTGTTTC   
  
  
+ CGAACAAATT CTTGTTCAAA AGTTGTTCTT TGATCTTCTT CCGTTCTTGA AGCTTTCATA TCTTGTGACG   
  
  
+ AACCAGGCAA TCACGGAGGC CATAGAAGGA GAAAAGATGG TTCATATAAT TGATCTCCAT TCTTGTGAAT   
  
  
+ CGGCTCTGTG GATTAGTCTC CTCCAGGCAT TGAGTGTTCG ACCTGAAGGC CCACCCCATT TGAGGATAAC   
  
  
+ CGGTATACAT GAGAAGAAAG AAGTGTTGGA TCAAATGGCT ATGCAACTAA ACAAAGAGGC TGAAAAATTG   
  
  
+ GACATCCCAT TTCAATTCAA TCCTATCGCA AGCAAACTAG ACGACCTTGA TGTCGAAAGC TTGAGTGTCA   
  
  
+ AGACCGGAGA AGCACTTGTT ATTAGTTCTG TGCTCCAACT ACATTCTCTT TTGGCATTGG ATGAGGGATC   
  
  
+ AATGCCTAAG AACGCAGGCA TGGCTTACTT GCAAAGGGTG TTTTATATGA AACCACGAAA ATTGGAGGAC   
  
  
+ TTGCCCAACA AGGATTTGAT GAAAATGTTG AACTCAAATG AAGATTCTAC ATCATCATCA TCTTCATCTC   
  
  
+ CTATCCCTTC ATCAAAACTT GATGCCTTTT TAAAAGCCCT CCTTGGGCTT TCGCCAAAAC TCATGGTTGT   
  
  
+ AACCGAGCAA GAATCAAACC ACAATGGAAG TGCCCTAATA GAAAGAGTGA TGGAGTCATT GAACTTCTAT   
  
  
+ GCAGCATTGT TTGATTGCTT GGAATCCACT ATATCGAGGA CATCGATAGA GAGACAGAAG CTCGAGAAGT   
  
  
+ TGATGTTTGG AGAGGAGATC AAGAACCTCA CAGCTTGTGA AGGGGCAGAG AGAAAGGCAA GGCACGAGAA   
  
  
+ GCTCAGTGAA TGGGTTCAAA GATTTGAGTC AGTAGGATTT AAAAGGGAGC CATTGAGCTA CCATGGTTTC   
  
  
+ TTGCTTGCTA GGAGGTTTTT ACATACCAAT AATTATGAGG GGTATAACAT CAAGGAAGTC AATGGTTTTC   
  
  
+ TTGTTATCTG TTGGCAAGAT AGACCCCTGT ATTCTGTTTC AGCTTGGAGA TTTTA  

- +Up\_Stream \_Len000AATGTT TTTGCTACGA GATTTGGATA ATCCAGCAAG GCTTTGGGCG TTTTAACTTT   
  
  
- ATACTAGCGG GCATAAAATG GGTTTGGACT TTATGTGAAT TATACATAAA ATGGACTTGG GTTTATGTGG   
  
  
- CCTGGACATA AATTGTGCCC GTGGGCTTTA CTATCCAGAT TGAGAGTGGT GGTGGCGTAA ACCGTGGGCT   
  
  
- TGCCATTTTT TACAAGTAGA TAATACGTGA AACCGAAGTA GGTATTGGGA TACTAGATAG ACACGGTTAT   
  
  
- AAACCTCGTG GGTCGCTTCA TCGCGTACGA GATTTGAGAA CGGAGTTTTG TTTTGTTTAT CACCCAGTAT   
  
  
- AAGGGAAGTT TCGTGATTGT TTGTGTGTGA GAGAGAGAGA GTCATCGGTT GTTGAATTCC GCCACAAACG   
  
  
- AACCAAAACC TCTCCCGCAC AAACCTACTC CACGTTTTGT GTATCTACTT TAGTATTTGT TGAACTAAGG   
  
  
- GTCGGTGGGT ACTGGATGAG TCTATTGTTC CCTGTTTTTG TTTCTCCAAA AGACTTTGTC TCTTTTGTCC   
  
  
- CCGCAACACA ACGCGACAAC GAGTGTTTTA AGGTTATAAG GAGATTTTCG AGGGTTTGCC TCTGTTTAGT   
  
  
- TTCTCCTGTT TCTGTACACC CTAAATTATG TTTTAAACTG ATTGAGACGT TGCCTTTTGT TGGGGATCCC   
  
  
- ACAAAAAGAC GCACAACATG TGTGAAGTGA AAATTGGACG AGACGGAGAC GGAAACGGTG CCGAAAATTA   
  
  
- CCTATTCTGG TTAATTCTTT GGGTTTATAG AGCATTTAAA CAACTCTAAG GAAAGACGCA AGAGTTCACA   
  
  
- AAGTTTTTGG GTCAAACCTC CAACGTGAAT AAGTACGAAA AGAAGTTCAG TCACTCGAAT CACAGACCTT   
  
  
- GACTTCGAGG AGGACCATTA CGAGACAAAA AGTAAAGAAC AACACGCAAA TATTTTTAAG CCGGATAACT   
  
  
- ACCTTCAAAC AAACTACAAA AACCCATTCA AAAACTAATT ATCTCTACTT AATCATTTCA TTTTAAACTC   
  
  
- ACCATAAATT ACTAGGAATT CGTCAGATAC GAAACAACAG TGTACGCAAA GAACCGAAAA CTGACTTTGT   
  
  
- AAAGACACAA TCTACTTTTT GAGTAAAACT ACGTACTCTA CTATTTTAGC GAATAAAAAC GGAAATAAAC   
  
  
- CACACACCCC CCCCCCCCCC CCCCCGCGTC CAGACTCCAT TTTCTATACA CTTAAGTAAA CCTCAGAGCT   
  
  
- ATTAAGCAAC CGATACAACT GTTAGTGCTC ACCAGCAATA GCGAGTAGAG TGTCCAACTA TTAAGTGAGT   
  
  
- TTGAAGTTCG CCGGACTTAG TAAGTCAGGC CCAACAATTT TAGAGTTGTC AGTAAAGTGT GCTCACCTCC   
  
  
- CGGGGTTTTC TGTGGTAAGA AAGCTTGAAA AAAAAAAACT TCCAGTAAAA AAGTTCTTGT TTTGATTACA   
  
  
- CTTGTACGCT GAAGATACTC ATTTCACATT TCTACGTATT TCAGAAAAAA AGAAAATATA TTTTTCATGA   
  
  
- GTTTTTTTTT TCTCAACCTC TCGACTAAGG GGTACGTAGG AGATTCGTTT CCACCACCTG CCGTCTTTTA   
  
  
- ATCATCTCAA TAACACTCTA ATTTCAAACG CGTGGACTTG CCGACTGGAG ATCAAACGAT AGTTGCAATG   
  
  
- TAAATACACA GTATTAAAAA CGTACTTTGT TAAAGTGCGT TACAAAAGAC TACCGTCAAT ACAAATCTTT   
  
  
- ATTCAAATTA GCCTGGAATA AGGTGACTAA ATATAAACTA AGAAGGGAGA AATGATGCCG ACAGGTAAGT   
  
  
- GTTCGGTTTC TTAAGAGGGG GGTTTTTTTT CTTTTTTGTC ATATCTTGGT ACAAATAATA GAGTAAAAAA   
  
  
- AGAGAGGGTA AACTAGACTT TGTGTTTGAA CGTCCCCACG TTCAACCTGT GAATCATCGA CGTCCTTACT   
  
  
- AAGTTATCCT TAGGAGTAGA AACTGTTGTA GTGGTGAACT TATGTATAGT GAACTACCGT GAGAGGGGAC   
  
  
- CAAATCCGAA GGGTATAGGC ACCGAATCCC TCGATTTTGG ACTCGTTTCT CCTGAGACAG ATTAGGTAGA   
  
  
- GGAGGAAAGT ACACGATTAG TTCAACGATG ACCCTCGTAG CTGTTACGAT TACAGTCGGA ACTCGTATAA   
  
  
- AGGGTGGAAC GGAGAGGGTG GCCTCTATGT TACGTCGCTT AACGACGAAT GAAACGACTT CGGAAACGAC   
  
  
- TGGCAAATGA TTCCCGAACC GTCGGGCCAG AAGAGTTTCG GAACTTAACA TGGTTCTACA GTAGACAAAG   
  
  
- GCTTGTTTAA GAACAAGTTT TCAACAAGAA ACTAGAAGAA GGCAAGAACT TCGAAAGTAT AGAACACTGC   
  
  
- TTGGTCCGTT AGTGCCTCCG GTATCTTCCT CTTTTCTACC AAGTATATTA ACTAGAGGTA AGAACACTTA   
  
  
- GCCGAGACAC CTAATCAGAG GAGGTCCGTA ACTCACAAGC TGGACTTCCG GGTGGGGTAA ACTCCTATTG   
  
  
- GCCATATGTA CTCTTCTTTC TTCACAACCT AGTTTACCGA TACGTTGATT TGTTTCTCCG ACTTTTTAAC   
  
  
- CTGTAGGGTA AAGTTAAGTT AGGATAGCGT TCGTTTGATC TGCTGGAACT ACAGCTTTCG AACTCACAGT   
  
  
- TCTGGCCTCT TCGTGAACAA TAATCAAGAC ACGAGGTTGA TGTAAGAGAA AACCGTAACC TACTCCCTAG   
  
  
- TTACGGATTC TTGCGTCCGT ACCGAATGAA CGTTTCCCAC AAAATATACT TTGGTGCTTT TAACCTCCTG   
  
  
- AACGGGTTGT TCCTAAACTA CTTTTACAAC TTGAGTTTAC TTCTAAGATG TAGTAGTAGT AGAAGTAGAG   
  
  
- GATAGGGAAG TAGTTTTGAA CTACGGAAAA ATTTTCGGGA GGAACCCGAA AGCGGTTTTG AGTACCAACA   
  
  
- TTGGCTCGTT CTTAGTTTGG TGTTACCTTC ACGGGATTAT CTTTCTCACT ACCTCAGTAA CTTGAAGATA   
  
  
- CGTCGTAACA AACTAACGAA CCTTAGGTGA TATAGCTCCT GTAGCTATCT CTCTGTCTTC GAGCTCTTCA   
  
  
- ACTACAAACC TCTCCTCTAG TTCTTGGAGT GTCGAACACT TCCCCGTCTC TCTTTCCGTT CCGTGCTCTT   
  
  
- CGAGTCACTT ACCCAAGTTT CTAAACTCAG TCATCCTAAA TTTTCCCTCG GTAACTCGAT GGTACCAAAG   
  
  
- AACGAACGAT CCTCCAAAAA TGTATGGTTA TTAATACTCC CCATATTGTA GTTCCTTCAG TTACCAAAAG   
  
  
- AACAATAGAC AACCGTTCTA TCTGGGGACA TAAGACAAAG TCGAACCTCT AAAAT

+     GT1-motif

| Site Name | Organism | Position | Strand | Matrix score. | sequence | function |
| --- | --- | --- | --- | --- | --- | --- |
| GT1-motif | Arabidopsis thaliana | 736 | - | 6 | GGTTAA | light responsive element |

>HU05G01267.1   
+ +Up\_Stream \_Len000TTACAA AAACGATGCT CTAAACCTAT TAGGTCGTTC CGAAACCCGC AAAATTGAAA   
  
  
+ TATGATCGCC CGTATTTTAC CCAAACCTGA AATACACTTA ATATGTATTT TACCTGAACC CAAATACACC   
  
  
+ GGACCTGTAT TTAACACGGG CACCCGAAAT GATAGGTCTA ACTCTCACCA CCACCGCATT TGGCACCCGA   
  
  
+ ACGGTAAAAA ATGTTCATCT ATTATGCACT TTGGCTTCAT CCATAACCCT ATGATCTATC TGTGCCAATA   
  
  
+ TTTGGAGCAC CCAGCGAAGT AGCGCATGCT CTAAACTCTT GCCTCAAAAC AAAACAAATA GTGGGTCATA   
  
  
+ TTCCCTTCAA AGCACTAACA AACACACACT CTCTCTCTCT CAGTAGCCAA CAACTTAAGG CGGTGTTTGC   
  
  
+ TTGGTTTTGG AGAGGGCGTG TTTGGATGAG GTGCAAAACA CATAGATGAA ATCATAAACA ACTTGATTCC   
  
  
+ CAGCCACCCA TGACCTACTC AGATAACAAG GGACAAAAAC AAAGAGGTTT TCTGAAACAG AGAAAACAGG   
  
  
+ GGCGTTGTGT TGCGCTGTTG CTCACAAAAT TCCAATATTC CTCTAAAAGC TCCCAAACGG AGACAAATCA   
  
  
+ AAGAGGACAA AGACATGTGG GATTTAATAC AAAATTTGAC TAACTCTGCA ACGGAAAACA ACCCCTAGGG   
  
  
+ TGTTTTTCTG CGTGTTGTAC ACACTTCACT TTTAACCTGC TCTGCCTCTG CCTTTGCCAC GGCTTTTAAT   
  
  
+ GGATAAGACC AATTAAGAAA CCCAAATATC TCGTAAATTT GTTGAGATTC CTTTCTGCGT TCTCAAGTGT   
  
  
+ TTCAAAAACC CAGTTTGGAG GTTGCACTTA TTCATGCTTT TCTTCAAGTC AGTGAGCTTA GTGTCTGGAA   
  
  
+ CTGAAGCTCC TCCTGGTAAT GCTCTGTTTT TCATTTCTTG TTGTGCGTTT ATAAAAATTC GGCCTATTGA   
  
  
+ TGGAAGTTTG TTTGATGTTT TTGGGTAAGT TTTTGATTAA TAGAGATGAA TTAGTAAAGT AAAATTTGAG   
  
  
+ TGGTATTTAA TGATCCTTAA GCAGTCTATG CTTTGTTGTC ACATGCGTTT CTTGGCTTTT GACTGAAACA   
  
  
+ TTTCTGTGTT AGATGAAAAA CTCATTTTGA TGCATGAGAT GATAAAATCG CTTATTTTTG CCTTTATTTG   
  
  
+ GTGTGTGGGG GGGGGGGGGG GGGGGCGCAG GTCTGAGGTA AAAGATATGT GAATTCATTT GGAGTCTCGA   
  
  
+ TAATTCGTTG GCTATGTTGA CAATCACGAG TGGTCGTTAT CGCTCATCTC ACAGGTTGAT AATTCACTCA   
  
  
+ AACTTCAAGC GGCCTGAATC ATTCAGTCCG GGTTGTTAAA ATCTCAACAG TCATTTCACA CGAGTGGAGG   
  
  
+ GCCCCAAAAG ACACCATTCT TTCGAACTTT TTTTTTTTGA AGGTCATTTT TTCAAGAACA AAACTAATGT   
  
  
+ GAACATGCGA CTTCTATGAG TAAAGTGTAA AGATGCATAA AGTCTTTTTT TCTTTTATAT AAAAAGTACT   
  
  
+ CAAAAAAAAA AGAGTTGGAG AGCTGATTCC CCATGCATCC TCTAAGCAAA GGTGGTGGAC GGCAGAAAAT   
  
  
+ TAGTAGAGTT ATTGTGAGAT TAAAGTTTGC GCACCTGAAC GGCTGACCTC TAGTTTGCTA TCAACGTTAC   
  
  
+ ATTTATGTGT CATAATTTTT GCATGAAACA ATTTCACGCA ATGTTTTCTG ATGGCAGTTA TGTTTAGAAA   
  
  
+ TAAGTTTAAT CGGACCTTAT TCCACTGATT TATATTTGAT TCTTCCCTCT TTACTACGGC TGTCCATTCA   
  
  
+ CAAGCCAAAG AATTCTCCCC CCAAAAAAAA GAAAAAACAG TATAGAACCA TGTTTATTAT CTCATTTTTT   
  
  
+ TCTCTCCCAT TTGATCTGAA ACACAAACTT GCAGGGGTGC AAGTTGGACA CTTAGTAGCT GCAGGAATGA   
  
  
+ TTCAATAGGA ATCCTCATCT TTGACAACAT CACCACTTGA ATACATATCA CTTGATGGCA CTCTCCCCTG   
  
  
+ GTTTAGGCTT CCCATATCCG TGGCTTAGGG AGCTAAAACC TGAGCAAAGA GGACTCTGTC TAATCCATCT   
  
  
+ CCTCCTTTCA TGTGCTAATC AAGTTGCTAC TGGGAGCATC GACAATGCTA ATGTCAGCCT TGAGCATATT   
  
  
+ TCCCACCTTG CCTCTCCCAC CGGAGATACA ATGCAGCGAA TTGCTGCTTA CTTTGCTGAA GCCTTTGCTG   
  
  
+ ACCGTTTACT AAGGGCTTGG CAGCCCGGTC TTCTCAAAGC CTTGAATTGT ACCAAGATGT CATCTGTTTC   
  
  
+ CGAACAAATT CTTGTTCAAA AGTTGTTCTT TGATCTTCTT CCGTTCTTGA AGCTTTCATA TCTTGTGACG   
  
  
+ AACCAGGCAA TCACGGAGGC CATAGAAGGA GAAAAGATGG TTCATATAAT TGATCTCCAT TCTTGTGAAT   
  
  
+ CGGCTCTGTG GATTAGTCTC CTCCAGGCAT TGAGTGTTCG ACCTGAAGGC CCACCCCATT TGAGGATAAC   
  
  
+ CGGTATACAT GAGAAGAAAG AAGTGTTGGA TCAAATGGCT ATGCAACTAA ACAAAGAGGC TGAAAAATTG   
  
  
+ GACATCCCAT TTCAATTCAA TCCTATCGCA AGCAAACTAG ACGACCTTGA TGTCGAAAGC TTGAGTGTCA   
  
  
+ AGACCGGAGA AGCACTTGTT ATTAGTTCTG TGCTCCAACT ACATTCTCTT TTGGCATTGG ATGAGGGATC   
  
  
+ AATGCCTAAG AACGCAGGCA TGGCTTACTT GCAAAGGGTG TTTTATATGA AACCACGAAA ATTGGAGGAC   
  
  
+ TTGCCCAACA AGGATTTGAT GAAAATGTTG AACTCAAATG AAGATTCTAC ATCATCATCA TCTTCATCTC   
  
  
+ CTATCCCTTC ATCAAAACTT GATGCCTTTT TAAAAGCCCT CCTTGGGCTT TCGCCAAAAC TCATGGTTGT   
  
  
+ AACCGAGCAA GAATCAAACC ACAATGGAAG TGCCCTAATA GAAAGAGTGA TGGAGTCATT GAACTTCTAT   
  
  
+ GCAGCATTGT TTGATTGCTT GGAATCCACT ATATCGAGGA CATCGATAGA GAGACAGAAG CTCGAGAAGT   
  
  
+ TGATGTTTGG AGAGGAGATC AAGAACCTCA CAGCTTGTGA AGGGGCAGAG AGAAAGGCAA GGCACGAGAA   
  
  
+ GCTCAGTGAA TGGGTTCAAA GATTTGAGTC AGTAGGATTT AAAAGGGAGC CATTGAGCTA CCATGGTTTC   
  
  
+ TTGCTTGCTA GGAGGTTTTT ACATACCAAT AATTATGAGG GGTATAACAT CAAGGAAGTC AATGGTTTTC   
  
  
+ TTGTTATCTG TTGGCAAGAT AGACCCCTGT ATTCTGTTTC AGCTTGGAGA TTTTA  

- +Up\_Stream \_Len000AATGTT TTTGCTACGA GATTTGGATA ATCCAGCAAG GCTTTGGGCG TTTTAACTTT   
  
  
- ATACTAGCGG GCATAAAATG GGTTTGGACT TTATGTGAAT TATACATAAA ATGGACTTGG GTTTATGTGG   
  
  
- CCTGGACATA AATTGTGCCC GTGGGCTTTA CTATCCAGAT TGAGAGTGGT GGTGGCGTAA ACCGTGGGCT   
  
  
- TGCCATTTTT TACAAGTAGA TAATACGTGA AACCGAAGTA GGTATTGGGA TACTAGATAG ACACGGTTAT   
  
  
- AAACCTCGTG GGTCGCTTCA TCGCGTACGA GATTTGAGAA CGGAGTTTTG TTTTGTTTAT CACCCAGTAT   
  
  
- AAGGGAAGTT TCGTGATTGT TTGTGTGTGA GAGAGAGAGA GTCATCGGTT GTTGAATTCC GCCACAAACG   
  
  
- AACCAAAACC TCTCCCGCAC AAACCTACTC CACGTTTTGT GTATCTACTT TAGTATTTGT TGAACTAAGG   
  
  
- GTCGGTGGGT ACTGGATGAG TCTATTGTTC CCTGTTTTTG TTTCTCCAAA AGACTTTGTC TCTTTTGTCC   
  
  
- CCGCAACACA ACGCGACAAC GAGTGTTTTA AGGTTATAAG GAGATTTTCG AGGGTTTGCC TCTGTTTAGT   
  
  
- TTCTCCTGTT TCTGTACACC CTAAATTATG TTTTAAACTG ATTGAGACGT TGCCTTTTGT TGGGGATCCC   
  
  
- ACAAAAAGAC GCACAACATG TGTGAAGTGA AAATTGGACG AGACGGAGAC GGAAACGGTG CCGAAAATTA   
  
  
- CCTATTCTGG TTAATTCTTT GGGTTTATAG AGCATTTAAA CAACTCTAAG GAAAGACGCA AGAGTTCACA   
  
  
- AAGTTTTTGG GTCAAACCTC CAACGTGAAT AAGTACGAAA AGAAGTTCAG TCACTCGAAT CACAGACCTT   
  
  
- GACTTCGAGG AGGACCATTA CGAGACAAAA AGTAAAGAAC AACACGCAAA TATTTTTAAG CCGGATAACT   
  
  
- ACCTTCAAAC AAACTACAAA AACCCATTCA AAAACTAATT ATCTCTACTT AATCATTTCA TTTTAAACTC   
  
  
- ACCATAAATT ACTAGGAATT CGTCAGATAC GAAACAACAG TGTACGCAAA GAACCGAAAA CTGACTTTGT   
  
  
- AAAGACACAA TCTACTTTTT GAGTAAAACT ACGTACTCTA CTATTTTAGC GAATAAAAAC GGAAATAAAC   
  
  
- CACACACCCC CCCCCCCCCC CCCCCGCGTC CAGACTCCAT TTTCTATACA CTTAAGTAAA CCTCAGAGCT   
  
  
- ATTAAGCAAC CGATACAACT GTTAGTGCTC ACCAGCAATA GCGAGTAGAG TGTCCAACTA TTAAGTGAGT   
  
  
- TTGAAGTTCG CCGGACTTAG TAAGTCAGGC CCAACAATTT TAGAGTTGTC AGTAAAGTGT GCTCACCTCC   
  
  
- CGGGGTTTTC TGTGGTAAGA AAGCTTGAAA AAAAAAAACT TCCAGTAAAA AAGTTCTTGT TTTGATTACA   
  
  
- CTTGTACGCT GAAGATACTC ATTTCACATT TCTACGTATT TCAGAAAAAA AGAAAATATA TTTTTCATGA   
  
  
- GTTTTTTTTT TCTCAACCTC TCGACTAAGG GGTACGTAGG AGATTCGTTT CCACCACCTG CCGTCTTTTA   
  
  
- ATCATCTCAA TAACACTCTA ATTTCAAACG CGTGGACTTG CCGACTGGAG ATCAAACGAT AGTTGCAATG   
  
  
- TAAATACACA GTATTAAAAA CGTACTTTGT TAAAGTGCGT TACAAAAGAC TACCGTCAAT ACAAATCTTT   
  
  
- ATTCAAATTA GCCTGGAATA AGGTGACTAA ATATAAACTA AGAAGGGAGA AATGATGCCG ACAGGTAAGT   
  
  
- GTTCGGTTTC TTAAGAGGGG GGTTTTTTTT CTTTTTTGTC ATATCTTGGT ACAAATAATA GAGTAAAAAA   
  
  
- AGAGAGGGTA AACTAGACTT TGTGTTTGAA CGTCCCCACG TTCAACCTGT GAATCATCGA CGTCCTTACT   
  
  
- AAGTTATCCT TAGGAGTAGA AACTGTTGTA GTGGTGAACT TATGTATAGT GAACTACCGT GAGAGGGGAC   
  
  
- CAAATCCGAA GGGTATAGGC ACCGAATCCC TCGATTTTGG ACTCGTTTCT CCTGAGACAG ATTAGGTAGA   
  
  
- GGAGGAAAGT ACACGATTAG TTCAACGATG ACCCTCGTAG CTGTTACGAT TACAGTCGGA ACTCGTATAA   
  
  
- AGGGTGGAAC GGAGAGGGTG GCCTCTATGT TACGTCGCTT AACGACGAAT GAAACGACTT CGGAAACGAC   
  
  
- TGGCAAATGA TTCCCGAACC GTCGGGCCAG AAGAGTTTCG GAACTTAACA TGGTTCTACA GTAGACAAAG   
  
  
- GCTTGTTTAA GAACAAGTTT TCAACAAGAA ACTAGAAGAA GGCAAGAACT TCGAAAGTAT AGAACACTGC   
  
  
- TTGGTCCGTT AGTGCCTCCG GTATCTTCCT CTTTTCTACC AAGTATATTA ACTAGAGGTA AGAACACTTA   
  
  
- GCCGAGACAC CTAATCAGAG GAGGTCCGTA ACTCACAAGC TGGACTTCCG GGTGGGGTAA ACTCCTATTG   
  
  
- GCCATATGTA CTCTTCTTTC TTCACAACCT AGTTTACCGA TACGTTGATT TGTTTCTCCG ACTTTTTAAC   
  
  
- CTGTAGGGTA AAGTTAAGTT AGGATAGCGT TCGTTTGATC TGCTGGAACT ACAGCTTTCG AACTCACAGT   
  
  
- TCTGGCCTCT TCGTGAACAA TAATCAAGAC ACGAGGTTGA TGTAAGAGAA AACCGTAACC TACTCCCTAG   
  
  
- TTACGGATTC TTGCGTCCGT ACCGAATGAA CGTTTCCCAC AAAATATACT TTGGTGCTTT TAACCTCCTG   
  
  
- AACGGGTTGT TCCTAAACTA CTTTTACAAC TTGAGTTTAC TTCTAAGATG TAGTAGTAGT AGAAGTAGAG   
  
  
- GATAGGGAAG TAGTTTTGAA CTACGGAAAA ATTTTCGGGA GGAACCCGAA AGCGGTTTTG AGTACCAACA   
  
  
- TTGGCTCGTT CTTAGTTTGG TGTTACCTTC ACGGGATTAT CTTTCTCACT ACCTCAGTAA CTTGAAGATA   
  
  
- CGTCGTAACA AACTAACGAA CCTTAGGTGA TATAGCTCCT GTAGCTATCT CTCTGTCTTC GAGCTCTTCA   
  
  
- ACTACAAACC TCTCCTCTAG TTCTTGGAGT GTCGAACACT TCCCCGTCTC TCTTTCCGTT CCGTGCTCTT   
  
  
- CGAGTCACTT ACCCAAGTTT CTAAACTCAG TCATCCTAAA TTTTCCCTCG GTAACTCGAT GGTACCAAAG   
  
  
- AACGAACGAT CCTCCAAAAA TGTATGGTTA TTAATACTCC CCATATTGTA GTTCCTTCAG TTACCAAAAG   
  
  
- AACAATAGAC AACCGTTCTA TCTGGGGACA TAAGACAAAG TCGAACCTCT AAAAT

+     Gap-box

| Site Name | Organism | Position | Strand | Matrix score. | sequence | function |
| --- | --- | --- | --- | --- | --- | --- |
| Gap-box | Arabidopsis thaliana | 2839 | + | 9.5 | CAAATGAA(A/G)A | part of a light responsive element |

>HU05G01267.1   
+ +Up\_Stream \_Len000TTACAA AAACGATGCT CTAAACCTAT TAGGTCGTTC CGAAACCCGC AAAATTGAAA   
  
  
+ TATGATCGCC CGTATTTTAC CCAAACCTGA AATACACTTA ATATGTATTT TACCTGAACC CAAATACACC   
  
  
+ GGACCTGTAT TTAACACGGG CACCCGAAAT GATAGGTCTA ACTCTCACCA CCACCGCATT TGGCACCCGA   
  
  
+ ACGGTAAAAA ATGTTCATCT ATTATGCACT TTGGCTTCAT CCATAACCCT ATGATCTATC TGTGCCAATA   
  
  
+ TTTGGAGCAC CCAGCGAAGT AGCGCATGCT CTAAACTCTT GCCTCAAAAC AAAACAAATA GTGGGTCATA   
  
  
+ TTCCCTTCAA AGCACTAACA AACACACACT CTCTCTCTCT CAGTAGCCAA CAACTTAAGG CGGTGTTTGC   
  
  
+ TTGGTTTTGG AGAGGGCGTG TTTGGATGAG GTGCAAAACA CATAGATGAA ATCATAAACA ACTTGATTCC   
  
  
+ CAGCCACCCA TGACCTACTC AGATAACAAG GGACAAAAAC AAAGAGGTTT TCTGAAACAG AGAAAACAGG   
  
  
+ GGCGTTGTGT TGCGCTGTTG CTCACAAAAT TCCAATATTC CTCTAAAAGC TCCCAAACGG AGACAAATCA   
  
  
+ AAGAGGACAA AGACATGTGG GATTTAATAC AAAATTTGAC TAACTCTGCA ACGGAAAACA ACCCCTAGGG   
  
  
+ TGTTTTTCTG CGTGTTGTAC ACACTTCACT TTTAACCTGC TCTGCCTCTG CCTTTGCCAC GGCTTTTAAT   
  
  
+ GGATAAGACC AATTAAGAAA CCCAAATATC TCGTAAATTT GTTGAGATTC CTTTCTGCGT TCTCAAGTGT   
  
  
+ TTCAAAAACC CAGTTTGGAG GTTGCACTTA TTCATGCTTT TCTTCAAGTC AGTGAGCTTA GTGTCTGGAA   
  
  
+ CTGAAGCTCC TCCTGGTAAT GCTCTGTTTT TCATTTCTTG TTGTGCGTTT ATAAAAATTC GGCCTATTGA   
  
  
+ TGGAAGTTTG TTTGATGTTT TTGGGTAAGT TTTTGATTAA TAGAGATGAA TTAGTAAAGT AAAATTTGAG   
  
  
+ TGGTATTTAA TGATCCTTAA GCAGTCTATG CTTTGTTGTC ACATGCGTTT CTTGGCTTTT GACTGAAACA   
  
  
+ TTTCTGTGTT AGATGAAAAA CTCATTTTGA TGCATGAGAT GATAAAATCG CTTATTTTTG CCTTTATTTG   
  
  
+ GTGTGTGGGG GGGGGGGGGG GGGGGCGCAG GTCTGAGGTA AAAGATATGT GAATTCATTT GGAGTCTCGA   
  
  
+ TAATTCGTTG GCTATGTTGA CAATCACGAG TGGTCGTTAT CGCTCATCTC ACAGGTTGAT AATTCACTCA   
  
  
+ AACTTCAAGC GGCCTGAATC ATTCAGTCCG GGTTGTTAAA ATCTCAACAG TCATTTCACA CGAGTGGAGG   
  
  
+ GCCCCAAAAG ACACCATTCT TTCGAACTTT TTTTTTTTGA AGGTCATTTT TTCAAGAACA AAACTAATGT   
  
  
+ GAACATGCGA CTTCTATGAG TAAAGTGTAA AGATGCATAA AGTCTTTTTT TCTTTTATAT AAAAAGTACT   
  
  
+ CAAAAAAAAA AGAGTTGGAG AGCTGATTCC CCATGCATCC TCTAAGCAAA GGTGGTGGAC GGCAGAAAAT   
  
  
+ TAGTAGAGTT ATTGTGAGAT TAAAGTTTGC GCACCTGAAC GGCTGACCTC TAGTTTGCTA TCAACGTTAC   
  
  
+ ATTTATGTGT CATAATTTTT GCATGAAACA ATTTCACGCA ATGTTTTCTG ATGGCAGTTA TGTTTAGAAA   
  
  
+ TAAGTTTAAT CGGACCTTAT TCCACTGATT TATATTTGAT TCTTCCCTCT TTACTACGGC TGTCCATTCA   
  
  
+ CAAGCCAAAG AATTCTCCCC CCAAAAAAAA GAAAAAACAG TATAGAACCA TGTTTATTAT CTCATTTTTT   
  
  
+ TCTCTCCCAT TTGATCTGAA ACACAAACTT GCAGGGGTGC AAGTTGGACA CTTAGTAGCT GCAGGAATGA   
  
  
+ TTCAATAGGA ATCCTCATCT TTGACAACAT CACCACTTGA ATACATATCA CTTGATGGCA CTCTCCCCTG   
  
  
+ GTTTAGGCTT CCCATATCCG TGGCTTAGGG AGCTAAAACC TGAGCAAAGA GGACTCTGTC TAATCCATCT   
  
  
+ CCTCCTTTCA TGTGCTAATC AAGTTGCTAC TGGGAGCATC GACAATGCTA ATGTCAGCCT TGAGCATATT   
  
  
+ TCCCACCTTG CCTCTCCCAC CGGAGATACA ATGCAGCGAA TTGCTGCTTA CTTTGCTGAA GCCTTTGCTG   
  
  
+ ACCGTTTACT AAGGGCTTGG CAGCCCGGTC TTCTCAAAGC CTTGAATTGT ACCAAGATGT CATCTGTTTC   
  
  
+ CGAACAAATT CTTGTTCAAA AGTTGTTCTT TGATCTTCTT CCGTTCTTGA AGCTTTCATA TCTTGTGACG   
  
  
+ AACCAGGCAA TCACGGAGGC CATAGAAGGA GAAAAGATGG TTCATATAAT TGATCTCCAT TCTTGTGAAT   
  
  
+ CGGCTCTGTG GATTAGTCTC CTCCAGGCAT TGAGTGTTCG ACCTGAAGGC CCACCCCATT TGAGGATAAC   
  
  
+ CGGTATACAT GAGAAGAAAG AAGTGTTGGA TCAAATGGCT ATGCAACTAA ACAAAGAGGC TGAAAAATTG   
  
  
+ GACATCCCAT TTCAATTCAA TCCTATCGCA AGCAAACTAG ACGACCTTGA TGTCGAAAGC TTGAGTGTCA   
  
  
+ AGACCGGAGA AGCACTTGTT ATTAGTTCTG TGCTCCAACT ACATTCTCTT TTGGCATTGG ATGAGGGATC   
  
  
+ AATGCCTAAG AACGCAGGCA TGGCTTACTT GCAAAGGGTG TTTTATATGA AACCACGAAA ATTGGAGGAC   
  
  
+ TTGCCCAACA AGGATTTGAT GAAAATGTTG AACTCAAATG AAGATTCTAC ATCATCATCA TCTTCATCTC   
  
  
+ CTATCCCTTC ATCAAAACTT GATGCCTTTT TAAAAGCCCT CCTTGGGCTT TCGCCAAAAC TCATGGTTGT   
  
  
+ AACCGAGCAA GAATCAAACC ACAATGGAAG TGCCCTAATA GAAAGAGTGA TGGAGTCATT GAACTTCTAT   
  
  
+ GCAGCATTGT TTGATTGCTT GGAATCCACT ATATCGAGGA CATCGATAGA GAGACAGAAG CTCGAGAAGT   
  
  
+ TGATGTTTGG AGAGGAGATC AAGAACCTCA CAGCTTGTGA AGGGGCAGAG AGAAAGGCAA GGCACGAGAA   
  
  
+ GCTCAGTGAA TGGGTTCAAA GATTTGAGTC AGTAGGATTT AAAAGGGAGC CATTGAGCTA CCATGGTTTC   
  
  
+ TTGCTTGCTA GGAGGTTTTT ACATACCAAT AATTATGAGG GGTATAACAT CAAGGAAGTC AATGGTTTTC   
  
  
+ TTGTTATCTG TTGGCAAGAT AGACCCCTGT ATTCTGTTTC AGCTTGGAGA TTTTA  

- +Up\_Stream \_Len000AATGTT TTTGCTACGA GATTTGGATA ATCCAGCAAG GCTTTGGGCG TTTTAACTTT   
  
  
- ATACTAGCGG GCATAAAATG GGTTTGGACT TTATGTGAAT TATACATAAA ATGGACTTGG GTTTATGTGG   
  
  
- CCTGGACATA AATTGTGCCC GTGGGCTTTA CTATCCAGAT TGAGAGTGGT GGTGGCGTAA ACCGTGGGCT   
  
  
- TGCCATTTTT TACAAGTAGA TAATACGTGA AACCGAAGTA GGTATTGGGA TACTAGATAG ACACGGTTAT   
  
  
- AAACCTCGTG GGTCGCTTCA TCGCGTACGA GATTTGAGAA CGGAGTTTTG TTTTGTTTAT CACCCAGTAT   
  
  
- AAGGGAAGTT TCGTGATTGT TTGTGTGTGA GAGAGAGAGA GTCATCGGTT GTTGAATTCC GCCACAAACG   
  
  
- AACCAAAACC TCTCCCGCAC AAACCTACTC CACGTTTTGT GTATCTACTT TAGTATTTGT TGAACTAAGG   
  
  
- GTCGGTGGGT ACTGGATGAG TCTATTGTTC CCTGTTTTTG TTTCTCCAAA AGACTTTGTC TCTTTTGTCC   
  
  
- CCGCAACACA ACGCGACAAC GAGTGTTTTA AGGTTATAAG GAGATTTTCG AGGGTTTGCC TCTGTTTAGT   
  
  
- TTCTCCTGTT TCTGTACACC CTAAATTATG TTTTAAACTG ATTGAGACGT TGCCTTTTGT TGGGGATCCC   
  
  
- ACAAAAAGAC GCACAACATG TGTGAAGTGA AAATTGGACG AGACGGAGAC GGAAACGGTG CCGAAAATTA   
  
  
- CCTATTCTGG TTAATTCTTT GGGTTTATAG AGCATTTAAA CAACTCTAAG GAAAGACGCA AGAGTTCACA   
  
  
- AAGTTTTTGG GTCAAACCTC CAACGTGAAT AAGTACGAAA AGAAGTTCAG TCACTCGAAT CACAGACCTT   
  
  
- GACTTCGAGG AGGACCATTA CGAGACAAAA AGTAAAGAAC AACACGCAAA TATTTTTAAG CCGGATAACT   
  
  
- ACCTTCAAAC AAACTACAAA AACCCATTCA AAAACTAATT ATCTCTACTT AATCATTTCA TTTTAAACTC   
  
  
- ACCATAAATT ACTAGGAATT CGTCAGATAC GAAACAACAG TGTACGCAAA GAACCGAAAA CTGACTTTGT   
  
  
- AAAGACACAA TCTACTTTTT GAGTAAAACT ACGTACTCTA CTATTTTAGC GAATAAAAAC GGAAATAAAC   
  
  
- CACACACCCC CCCCCCCCCC CCCCCGCGTC CAGACTCCAT TTTCTATACA CTTAAGTAAA CCTCAGAGCT   
  
  
- ATTAAGCAAC CGATACAACT GTTAGTGCTC ACCAGCAATA GCGAGTAGAG TGTCCAACTA TTAAGTGAGT   
  
  
- TTGAAGTTCG CCGGACTTAG TAAGTCAGGC CCAACAATTT TAGAGTTGTC AGTAAAGTGT GCTCACCTCC   
  
  
- CGGGGTTTTC TGTGGTAAGA AAGCTTGAAA AAAAAAAACT TCCAGTAAAA AAGTTCTTGT TTTGATTACA   
  
  
- CTTGTACGCT GAAGATACTC ATTTCACATT TCTACGTATT TCAGAAAAAA AGAAAATATA TTTTTCATGA   
  
  
- GTTTTTTTTT TCTCAACCTC TCGACTAAGG GGTACGTAGG AGATTCGTTT CCACCACCTG CCGTCTTTTA   
  
  
- ATCATCTCAA TAACACTCTA ATTTCAAACG CGTGGACTTG CCGACTGGAG ATCAAACGAT AGTTGCAATG   
  
  
- TAAATACACA GTATTAAAAA CGTACTTTGT TAAAGTGCGT TACAAAAGAC TACCGTCAAT ACAAATCTTT   
  
  
- ATTCAAATTA GCCTGGAATA AGGTGACTAA ATATAAACTA AGAAGGGAGA AATGATGCCG ACAGGTAAGT   
  
  
- GTTCGGTTTC TTAAGAGGGG GGTTTTTTTT CTTTTTTGTC ATATCTTGGT ACAAATAATA GAGTAAAAAA   
  
  
- AGAGAGGGTA AACTAGACTT TGTGTTTGAA CGTCCCCACG TTCAACCTGT GAATCATCGA CGTCCTTACT   
  
  
- AAGTTATCCT TAGGAGTAGA AACTGTTGTA GTGGTGAACT TATGTATAGT GAACTACCGT GAGAGGGGAC   
  
  
- CAAATCCGAA GGGTATAGGC ACCGAATCCC TCGATTTTGG ACTCGTTTCT CCTGAGACAG ATTAGGTAGA   
  
  
- GGAGGAAAGT ACACGATTAG TTCAACGATG ACCCTCGTAG CTGTTACGAT TACAGTCGGA ACTCGTATAA   
  
  
- AGGGTGGAAC GGAGAGGGTG GCCTCTATGT TACGTCGCTT AACGACGAAT GAAACGACTT CGGAAACGAC   
  
  
- TGGCAAATGA TTCCCGAACC GTCGGGCCAG AAGAGTTTCG GAACTTAACA TGGTTCTACA GTAGACAAAG   
  
  
- GCTTGTTTAA GAACAAGTTT TCAACAAGAA ACTAGAAGAA GGCAAGAACT TCGAAAGTAT AGAACACTGC   
  
  
- TTGGTCCGTT AGTGCCTCCG GTATCTTCCT CTTTTCTACC AAGTATATTA ACTAGAGGTA AGAACACTTA   
  
  
- GCCGAGACAC CTAATCAGAG GAGGTCCGTA ACTCACAAGC TGGACTTCCG GGTGGGGTAA ACTCCTATTG   
  
  
- GCCATATGTA CTCTTCTTTC TTCACAACCT AGTTTACCGA TACGTTGATT TGTTTCTCCG ACTTTTTAAC   
  
  
- CTGTAGGGTA AAGTTAAGTT AGGATAGCGT TCGTTTGATC TGCTGGAACT ACAGCTTTCG AACTCACAGT   
  
  
- TCTGGCCTCT TCGTGAACAA TAATCAAGAC ACGAGGTTGA TGTAAGAGAA AACCGTAACC TACTCCCTAG   
  
  
- TTACGGATTC TTGCGTCCGT ACCGAATGAA CGTTTCCCAC AAAATATACT TTGGTGCTTT TAACCTCCTG   
  
  
- AACGGGTTGT TCCTAAACTA CTTTTACAAC TTGAGTTTAC TTCTAAGATG TAGTAGTAGT AGAAGTAGAG   
  
  
- GATAGGGAAG TAGTTTTGAA CTACGGAAAA ATTTTCGGGA GGAACCCGAA AGCGGTTTTG AGTACCAACA   
  
  
- TTGGCTCGTT CTTAGTTTGG TGTTACCTTC ACGGGATTAT CTTTCTCACT ACCTCAGTAA CTTGAAGATA   
  
  
- CGTCGTAACA AACTAACGAA CCTTAGGTGA TATAGCTCCT GTAGCTATCT CTCTGTCTTC GAGCTCTTCA   
  
  
- ACTACAAACC TCTCCTCTAG TTCTTGGAGT GTCGAACACT TCCCCGTCTC TCTTTCCGTT CCGTGCTCTT   
  
  
- CGAGTCACTT ACCCAAGTTT CTAAACTCAG TCATCCTAAA TTTTCCCTCG GTAACTCGAT GGTACCAAAG   
  
  
- AACGAACGAT CCTCCAAAAA TGTATGGTTA TTAATACTCC CCATATTGTA GTTCCTTCAG TTACCAAAAG   
  
  
- AACAATAGAC AACCGTTCTA TCTGGGGACA TAAGACAAAG TCGAACCTCT AAAAT

+     I-box

| Site Name | Organism | Position | Strand | Matrix score. | sequence | function |
| --- | --- | --- | --- | --- | --- | --- |
| I-box | Zea mays | 448 | + | 9 | gGATAAGGTG | part of a light responsive element |

>HU05G01267.1   
+ +Up\_Stream \_Len000TTACAA AAACGATGCT CTAAACCTAT TAGGTCGTTC CGAAACCCGC AAAATTGAAA   
  
  
+ TATGATCGCC CGTATTTTAC CCAAACCTGA AATACACTTA ATATGTATTT TACCTGAACC CAAATACACC   
  
  
+ GGACCTGTAT TTAACACGGG CACCCGAAAT GATAGGTCTA ACTCTCACCA CCACCGCATT TGGCACCCGA   
  
  
+ ACGGTAAAAA ATGTTCATCT ATTATGCACT TTGGCTTCAT CCATAACCCT ATGATCTATC TGTGCCAATA   
  
  
+ TTTGGAGCAC CCAGCGAAGT AGCGCATGCT CTAAACTCTT GCCTCAAAAC AAAACAAATA GTGGGTCATA   
  
  
+ TTCCCTTCAA AGCACTAACA AACACACACT CTCTCTCTCT CAGTAGCCAA CAACTTAAGG CGGTGTTTGC   
  
  
+ TTGGTTTTGG AGAGGGCGTG TTTGGATGAG GTGCAAAACA CATAGATGAA ATCATAAACA ACTTGATTCC   
  
  
+ CAGCCACCCA TGACCTACTC AGATAACAAG GGACAAAAAC AAAGAGGTTT TCTGAAACAG AGAAAACAGG   
  
  
+ GGCGTTGTGT TGCGCTGTTG CTCACAAAAT TCCAATATTC CTCTAAAAGC TCCCAAACGG AGACAAATCA   
  
  
+ AAGAGGACAA AGACATGTGG GATTTAATAC AAAATTTGAC TAACTCTGCA ACGGAAAACA ACCCCTAGGG   
  
  
+ TGTTTTTCTG CGTGTTGTAC ACACTTCACT TTTAACCTGC TCTGCCTCTG CCTTTGCCAC GGCTTTTAAT   
  
  
+ GGATAAGACC AATTAAGAAA CCCAAATATC TCGTAAATTT GTTGAGATTC CTTTCTGCGT TCTCAAGTGT   
  
  
+ TTCAAAAACC CAGTTTGGAG GTTGCACTTA TTCATGCTTT TCTTCAAGTC AGTGAGCTTA GTGTCTGGAA   
  
  
+ CTGAAGCTCC TCCTGGTAAT GCTCTGTTTT TCATTTCTTG TTGTGCGTTT ATAAAAATTC GGCCTATTGA   
  
  
+ TGGAAGTTTG TTTGATGTTT TTGGGTAAGT TTTTGATTAA TAGAGATGAA TTAGTAAAGT AAAATTTGAG   
  
  
+ TGGTATTTAA TGATCCTTAA GCAGTCTATG CTTTGTTGTC ACATGCGTTT CTTGGCTTTT GACTGAAACA   
  
  
+ TTTCTGTGTT AGATGAAAAA CTCATTTTGA TGCATGAGAT GATAAAATCG CTTATTTTTG CCTTTATTTG   
  
  
+ GTGTGTGGGG GGGGGGGGGG GGGGGCGCAG GTCTGAGGTA AAAGATATGT GAATTCATTT GGAGTCTCGA   
  
  
+ TAATTCGTTG GCTATGTTGA CAATCACGAG TGGTCGTTAT CGCTCATCTC ACAGGTTGAT AATTCACTCA   
  
  
+ AACTTCAAGC GGCCTGAATC ATTCAGTCCG GGTTGTTAAA ATCTCAACAG TCATTTCACA CGAGTGGAGG   
  
  
+ GCCCCAAAAG ACACCATTCT TTCGAACTTT TTTTTTTTGA AGGTCATTTT TTCAAGAACA AAACTAATGT   
  
  
+ GAACATGCGA CTTCTATGAG TAAAGTGTAA AGATGCATAA AGTCTTTTTT TCTTTTATAT AAAAAGTACT   
  
  
+ CAAAAAAAAA AGAGTTGGAG AGCTGATTCC CCATGCATCC TCTAAGCAAA GGTGGTGGAC GGCAGAAAAT   
  
  
+ TAGTAGAGTT ATTGTGAGAT TAAAGTTTGC GCACCTGAAC GGCTGACCTC TAGTTTGCTA TCAACGTTAC   
  
  
+ ATTTATGTGT CATAATTTTT GCATGAAACA ATTTCACGCA ATGTTTTCTG ATGGCAGTTA TGTTTAGAAA   
  
  
+ TAAGTTTAAT CGGACCTTAT TCCACTGATT TATATTTGAT TCTTCCCTCT TTACTACGGC TGTCCATTCA   
  
  
+ CAAGCCAAAG AATTCTCCCC CCAAAAAAAA GAAAAAACAG TATAGAACCA TGTTTATTAT CTCATTTTTT   
  
  
+ TCTCTCCCAT TTGATCTGAA ACACAAACTT GCAGGGGTGC AAGTTGGACA CTTAGTAGCT GCAGGAATGA   
  
  
+ TTCAATAGGA ATCCTCATCT TTGACAACAT CACCACTTGA ATACATATCA CTTGATGGCA CTCTCCCCTG   
  
  
+ GTTTAGGCTT CCCATATCCG TGGCTTAGGG AGCTAAAACC TGAGCAAAGA GGACTCTGTC TAATCCATCT   
  
  
+ CCTCCTTTCA TGTGCTAATC AAGTTGCTAC TGGGAGCATC GACAATGCTA ATGTCAGCCT TGAGCATATT   
  
  
+ TCCCACCTTG CCTCTCCCAC CGGAGATACA ATGCAGCGAA TTGCTGCTTA CTTTGCTGAA GCCTTTGCTG   
  
  
+ ACCGTTTACT AAGGGCTTGG CAGCCCGGTC TTCTCAAAGC CTTGAATTGT ACCAAGATGT CATCTGTTTC   
  
  
+ CGAACAAATT CTTGTTCAAA AGTTGTTCTT TGATCTTCTT CCGTTCTTGA AGCTTTCATA TCTTGTGACG   
  
  
+ AACCAGGCAA TCACGGAGGC CATAGAAGGA GAAAAGATGG TTCATATAAT TGATCTCCAT TCTTGTGAAT   
  
  
+ CGGCTCTGTG GATTAGTCTC CTCCAGGCAT TGAGTGTTCG ACCTGAAGGC CCACCCCATT TGAGGATAAC   
  
  
+ CGGTATACAT GAGAAGAAAG AAGTGTTGGA TCAAATGGCT ATGCAACTAA ACAAAGAGGC TGAAAAATTG   
  
  
+ GACATCCCAT TTCAATTCAA TCCTATCGCA AGCAAACTAG ACGACCTTGA TGTCGAAAGC TTGAGTGTCA   
  
  
+ AGACCGGAGA AGCACTTGTT ATTAGTTCTG TGCTCCAACT ACATTCTCTT TTGGCATTGG ATGAGGGATC   
  
  
+ AATGCCTAAG AACGCAGGCA TGGCTTACTT GCAAAGGGTG TTTTATATGA AACCACGAAA ATTGGAGGAC   
  
  
+ TTGCCCAACA AGGATTTGAT GAAAATGTTG AACTCAAATG AAGATTCTAC ATCATCATCA TCTTCATCTC   
  
  
+ CTATCCCTTC ATCAAAACTT GATGCCTTTT TAAAAGCCCT CCTTGGGCTT TCGCCAAAAC TCATGGTTGT   
  
  
+ AACCGAGCAA GAATCAAACC ACAATGGAAG TGCCCTAATA GAAAGAGTGA TGGAGTCATT GAACTTCTAT   
  
  
+ GCAGCATTGT TTGATTGCTT GGAATCCACT ATATCGAGGA CATCGATAGA GAGACAGAAG CTCGAGAAGT   
  
  
+ TGATGTTTGG AGAGGAGATC AAGAACCTCA CAGCTTGTGA AGGGGCAGAG AGAAAGGCAA GGCACGAGAA   
  
  
+ GCTCAGTGAA TGGGTTCAAA GATTTGAGTC AGTAGGATTT AAAAGGGAGC CATTGAGCTA CCATGGTTTC   
  
  
+ TTGCTTGCTA GGAGGTTTTT ACATACCAAT AATTATGAGG GGTATAACAT CAAGGAAGTC AATGGTTTTC   
  
  
+ TTGTTATCTG TTGGCAAGAT AGACCCCTGT ATTCTGTTTC AGCTTGGAGA TTTTA  

- +Up\_Stream \_Len000AATGTT TTTGCTACGA GATTTGGATA ATCCAGCAAG GCTTTGGGCG TTTTAACTTT   
  
  
- ATACTAGCGG GCATAAAATG GGTTTGGACT TTATGTGAAT TATACATAAA ATGGACTTGG GTTTATGTGG   
  
  
- CCTGGACATA AATTGTGCCC GTGGGCTTTA CTATCCAGAT TGAGAGTGGT GGTGGCGTAA ACCGTGGGCT   
  
  
- TGCCATTTTT TACAAGTAGA TAATACGTGA AACCGAAGTA GGTATTGGGA TACTAGATAG ACACGGTTAT   
  
  
- AAACCTCGTG GGTCGCTTCA TCGCGTACGA GATTTGAGAA CGGAGTTTTG TTTTGTTTAT CACCCAGTAT   
  
  
- AAGGGAAGTT TCGTGATTGT TTGTGTGTGA GAGAGAGAGA GTCATCGGTT GTTGAATTCC GCCACAAACG   
  
  
- AACCAAAACC TCTCCCGCAC AAACCTACTC CACGTTTTGT GTATCTACTT TAGTATTTGT TGAACTAAGG   
  
  
- GTCGGTGGGT ACTGGATGAG TCTATTGTTC CCTGTTTTTG TTTCTCCAAA AGACTTTGTC TCTTTTGTCC   
  
  
- CCGCAACACA ACGCGACAAC GAGTGTTTTA AGGTTATAAG GAGATTTTCG AGGGTTTGCC TCTGTTTAGT   
  
  
- TTCTCCTGTT TCTGTACACC CTAAATTATG TTTTAAACTG ATTGAGACGT TGCCTTTTGT TGGGGATCCC   
  
  
- ACAAAAAGAC GCACAACATG TGTGAAGTGA AAATTGGACG AGACGGAGAC GGAAACGGTG CCGAAAATTA   
  
  
- CCTATTCTGG TTAATTCTTT GGGTTTATAG AGCATTTAAA CAACTCTAAG GAAAGACGCA AGAGTTCACA   
  
  
- AAGTTTTTGG GTCAAACCTC CAACGTGAAT AAGTACGAAA AGAAGTTCAG TCACTCGAAT CACAGACCTT   
  
  
- GACTTCGAGG AGGACCATTA CGAGACAAAA AGTAAAGAAC AACACGCAAA TATTTTTAAG CCGGATAACT   
  
  
- ACCTTCAAAC AAACTACAAA AACCCATTCA AAAACTAATT ATCTCTACTT AATCATTTCA TTTTAAACTC   
  
  
- ACCATAAATT ACTAGGAATT CGTCAGATAC GAAACAACAG TGTACGCAAA GAACCGAAAA CTGACTTTGT   
  
  
- AAAGACACAA TCTACTTTTT GAGTAAAACT ACGTACTCTA CTATTTTAGC GAATAAAAAC GGAAATAAAC   
  
  
- CACACACCCC CCCCCCCCCC CCCCCGCGTC CAGACTCCAT TTTCTATACA CTTAAGTAAA CCTCAGAGCT   
  
  
- ATTAAGCAAC CGATACAACT GTTAGTGCTC ACCAGCAATA GCGAGTAGAG TGTCCAACTA TTAAGTGAGT   
  
  
- TTGAAGTTCG CCGGACTTAG TAAGTCAGGC CCAACAATTT TAGAGTTGTC AGTAAAGTGT GCTCACCTCC   
  
  
- CGGGGTTTTC TGTGGTAAGA AAGCTTGAAA AAAAAAAACT TCCAGTAAAA AAGTTCTTGT TTTGATTACA   
  
  
- CTTGTACGCT GAAGATACTC ATTTCACATT TCTACGTATT TCAGAAAAAA AGAAAATATA TTTTTCATGA   
  
  
- GTTTTTTTTT TCTCAACCTC TCGACTAAGG GGTACGTAGG AGATTCGTTT CCACCACCTG CCGTCTTTTA   
  
  
- ATCATCTCAA TAACACTCTA ATTTCAAACG CGTGGACTTG CCGACTGGAG ATCAAACGAT AGTTGCAATG   
  
  
- TAAATACACA GTATTAAAAA CGTACTTTGT TAAAGTGCGT TACAAAAGAC TACCGTCAAT ACAAATCTTT   
  
  
- ATTCAAATTA GCCTGGAATA AGGTGACTAA ATATAAACTA AGAAGGGAGA AATGATGCCG ACAGGTAAGT   
  
  
- GTTCGGTTTC TTAAGAGGGG GGTTTTTTTT CTTTTTTGTC ATATCTTGGT ACAAATAATA GAGTAAAAAA   
  
  
- AGAGAGGGTA AACTAGACTT TGTGTTTGAA CGTCCCCACG TTCAACCTGT GAATCATCGA CGTCCTTACT   
  
  
- AAGTTATCCT TAGGAGTAGA AACTGTTGTA GTGGTGAACT TATGTATAGT GAACTACCGT GAGAGGGGAC   
  
  
- CAAATCCGAA GGGTATAGGC ACCGAATCCC TCGATTTTGG ACTCGTTTCT CCTGAGACAG ATTAGGTAGA   
  
  
- GGAGGAAAGT ACACGATTAG TTCAACGATG ACCCTCGTAG CTGTTACGAT TACAGTCGGA ACTCGTATAA   
  
  
- AGGGTGGAAC GGAGAGGGTG GCCTCTATGT TACGTCGCTT AACGACGAAT GAAACGACTT CGGAAACGAC   
  
  
- TGGCAAATGA TTCCCGAACC GTCGGGCCAG AAGAGTTTCG GAACTTAACA TGGTTCTACA GTAGACAAAG   
  
  
- GCTTGTTTAA GAACAAGTTT TCAACAAGAA ACTAGAAGAA GGCAAGAACT TCGAAAGTAT AGAACACTGC   
  
  
- TTGGTCCGTT AGTGCCTCCG GTATCTTCCT CTTTTCTACC AAGTATATTA ACTAGAGGTA AGAACACTTA   
  
  
- GCCGAGACAC CTAATCAGAG GAGGTCCGTA ACTCACAAGC TGGACTTCCG GGTGGGGTAA ACTCCTATTG   
  
  
- GCCATATGTA CTCTTCTTTC TTCACAACCT AGTTTACCGA TACGTTGATT TGTTTCTCCG ACTTTTTAAC   
  
  
- CTGTAGGGTA AAGTTAAGTT AGGATAGCGT TCGTTTGATC TGCTGGAACT ACAGCTTTCG AACTCACAGT   
  
  
- TCTGGCCTCT TCGTGAACAA TAATCAAGAC ACGAGGTTGA TGTAAGAGAA AACCGTAACC TACTCCCTAG   
  
  
- TTACGGATTC TTGCGTCCGT ACCGAATGAA CGTTTCCCAC AAAATATACT TTGGTGCTTT TAACCTCCTG   
  
  
- AACGGGTTGT TCCTAAACTA CTTTTACAAC TTGAGTTTAC TTCTAAGATG TAGTAGTAGT AGAAGTAGAG   
  
  
- GATAGGGAAG TAGTTTTGAA CTACGGAAAA ATTTTCGGGA GGAACCCGAA AGCGGTTTTG AGTACCAACA   
  
  
- TTGGCTCGTT CTTAGTTTGG TGTTACCTTC ACGGGATTAT CTTTCTCACT ACCTCAGTAA CTTGAAGATA   
  
  
- CGTCGTAACA AACTAACGAA CCTTAGGTGA TATAGCTCCT GTAGCTATCT CTCTGTCTTC GAGCTCTTCA   
  
  
- ACTACAAACC TCTCCTCTAG TTCTTGGAGT GTCGAACACT TCCCCGTCTC TCTTTCCGTT CCGTGCTCTT   
  
  
- CGAGTCACTT ACCCAAGTTT CTAAACTCAG TCATCCTAAA TTTTCCCTCG GTAACTCGAT GGTACCAAAG   
  
  
- AACGAACGAT CCTCCAAAAA TGTATGGTTA TTAATACTCC CCATATTGTA GTTCCTTCAG TTACCAAAAG   
  
  
- AACAATAGAC AACCGTTCTA TCTGGGGACA TAAGACAAAG TCGAACCTCT AAAAT

+     LTR

| Site Name | Organism | Position | Strand | Matrix score. | sequence | function |
| --- | --- | --- | --- | --- | --- | --- |
| LTR | Hordeum vulgare | 54 | + | 6 | CCGAAA | cis-acting element involved in low-temperature responsiveness |
| LTR | Hordeum vulgare | 168 | + | 6 | CCGAAA | cis-acting element involved in low-temperature responsiveness |

>HU05G01267.1   
+ +Up\_Stream \_Len000TTACAA AAACGATGCT CTAAACCTAT TAGGTCGTTC CGAAACCCGC AAAATTGAAA   
  
  
+ TATGATCGCC CGTATTTTAC CCAAACCTGA AATACACTTA ATATGTATTT TACCTGAACC CAAATACACC   
  
  
+ GGACCTGTAT TTAACACGGG CACCCGAAAT GATAGGTCTA ACTCTCACCA CCACCGCATT TGGCACCCGA   
  
  
+ ACGGTAAAAA ATGTTCATCT ATTATGCACT TTGGCTTCAT CCATAACCCT ATGATCTATC TGTGCCAATA   
  
  
+ TTTGGAGCAC CCAGCGAAGT AGCGCATGCT CTAAACTCTT GCCTCAAAAC AAAACAAATA GTGGGTCATA   
  
  
+ TTCCCTTCAA AGCACTAACA AACACACACT CTCTCTCTCT CAGTAGCCAA CAACTTAAGG CGGTGTTTGC   
  
  
+ TTGGTTTTGG AGAGGGCGTG TTTGGATGAG GTGCAAAACA CATAGATGAA ATCATAAACA ACTTGATTCC   
  
  
+ CAGCCACCCA TGACCTACTC AGATAACAAG GGACAAAAAC AAAGAGGTTT TCTGAAACAG AGAAAACAGG   
  
  
+ GGCGTTGTGT TGCGCTGTTG CTCACAAAAT TCCAATATTC CTCTAAAAGC TCCCAAACGG AGACAAATCA   
  
  
+ AAGAGGACAA AGACATGTGG GATTTAATAC AAAATTTGAC TAACTCTGCA ACGGAAAACA ACCCCTAGGG   
  
  
+ TGTTTTTCTG CGTGTTGTAC ACACTTCACT TTTAACCTGC TCTGCCTCTG CCTTTGCCAC GGCTTTTAAT   
  
  
+ GGATAAGACC AATTAAGAAA CCCAAATATC TCGTAAATTT GTTGAGATTC CTTTCTGCGT TCTCAAGTGT   
  
  
+ TTCAAAAACC CAGTTTGGAG GTTGCACTTA TTCATGCTTT TCTTCAAGTC AGTGAGCTTA GTGTCTGGAA   
  
  
+ CTGAAGCTCC TCCTGGTAAT GCTCTGTTTT TCATTTCTTG TTGTGCGTTT ATAAAAATTC GGCCTATTGA   
  
  
+ TGGAAGTTTG TTTGATGTTT TTGGGTAAGT TTTTGATTAA TAGAGATGAA TTAGTAAAGT AAAATTTGAG   
  
  
+ TGGTATTTAA TGATCCTTAA GCAGTCTATG CTTTGTTGTC ACATGCGTTT CTTGGCTTTT GACTGAAACA   
  
  
+ TTTCTGTGTT AGATGAAAAA CTCATTTTGA TGCATGAGAT GATAAAATCG CTTATTTTTG CCTTTATTTG   
  
  
+ GTGTGTGGGG GGGGGGGGGG GGGGGCGCAG GTCTGAGGTA AAAGATATGT GAATTCATTT GGAGTCTCGA   
  
  
+ TAATTCGTTG GCTATGTTGA CAATCACGAG TGGTCGTTAT CGCTCATCTC ACAGGTTGAT AATTCACTCA   
  
  
+ AACTTCAAGC GGCCTGAATC ATTCAGTCCG GGTTGTTAAA ATCTCAACAG TCATTTCACA CGAGTGGAGG   
  
  
+ GCCCCAAAAG ACACCATTCT TTCGAACTTT TTTTTTTTGA AGGTCATTTT TTCAAGAACA AAACTAATGT   
  
  
+ GAACATGCGA CTTCTATGAG TAAAGTGTAA AGATGCATAA AGTCTTTTTT TCTTTTATAT AAAAAGTACT   
  
  
+ CAAAAAAAAA AGAGTTGGAG AGCTGATTCC CCATGCATCC TCTAAGCAAA GGTGGTGGAC GGCAGAAAAT   
  
  
+ TAGTAGAGTT ATTGTGAGAT TAAAGTTTGC GCACCTGAAC GGCTGACCTC TAGTTTGCTA TCAACGTTAC   
  
  
+ ATTTATGTGT CATAATTTTT GCATGAAACA ATTTCACGCA ATGTTTTCTG ATGGCAGTTA TGTTTAGAAA   
  
  
+ TAAGTTTAAT CGGACCTTAT TCCACTGATT TATATTTGAT TCTTCCCTCT TTACTACGGC TGTCCATTCA   
  
  
+ CAAGCCAAAG AATTCTCCCC CCAAAAAAAA GAAAAAACAG TATAGAACCA TGTTTATTAT CTCATTTTTT   
  
  
+ TCTCTCCCAT TTGATCTGAA ACACAAACTT GCAGGGGTGC AAGTTGGACA CTTAGTAGCT GCAGGAATGA   
  
  
+ TTCAATAGGA ATCCTCATCT TTGACAACAT CACCACTTGA ATACATATCA CTTGATGGCA CTCTCCCCTG   
  
  
+ GTTTAGGCTT CCCATATCCG TGGCTTAGGG AGCTAAAACC TGAGCAAAGA GGACTCTGTC TAATCCATCT   
  
  
+ CCTCCTTTCA TGTGCTAATC AAGTTGCTAC TGGGAGCATC GACAATGCTA ATGTCAGCCT TGAGCATATT   
  
  
+ TCCCACCTTG CCTCTCCCAC CGGAGATACA ATGCAGCGAA TTGCTGCTTA CTTTGCTGAA GCCTTTGCTG   
  
  
+ ACCGTTTACT AAGGGCTTGG CAGCCCGGTC TTCTCAAAGC CTTGAATTGT ACCAAGATGT CATCTGTTTC   
  
  
+ CGAACAAATT CTTGTTCAAA AGTTGTTCTT TGATCTTCTT CCGTTCTTGA AGCTTTCATA TCTTGTGACG   
  
  
+ AACCAGGCAA TCACGGAGGC CATAGAAGGA GAAAAGATGG TTCATATAAT TGATCTCCAT TCTTGTGAAT   
  
  
+ CGGCTCTGTG GATTAGTCTC CTCCAGGCAT TGAGTGTTCG ACCTGAAGGC CCACCCCATT TGAGGATAAC   
  
  
+ CGGTATACAT GAGAAGAAAG AAGTGTTGGA TCAAATGGCT ATGCAACTAA ACAAAGAGGC TGAAAAATTG   
  
  
+ GACATCCCAT TTCAATTCAA TCCTATCGCA AGCAAACTAG ACGACCTTGA TGTCGAAAGC TTGAGTGTCA   
  
  
+ AGACCGGAGA AGCACTTGTT ATTAGTTCTG TGCTCCAACT ACATTCTCTT TTGGCATTGG ATGAGGGATC   
  
  
+ AATGCCTAAG AACGCAGGCA TGGCTTACTT GCAAAGGGTG TTTTATATGA AACCACGAAA ATTGGAGGAC   
  
  
+ TTGCCCAACA AGGATTTGAT GAAAATGTTG AACTCAAATG AAGATTCTAC ATCATCATCA TCTTCATCTC   
  
  
+ CTATCCCTTC ATCAAAACTT GATGCCTTTT TAAAAGCCCT CCTTGGGCTT TCGCCAAAAC TCATGGTTGT   
  
  
+ AACCGAGCAA GAATCAAACC ACAATGGAAG TGCCCTAATA GAAAGAGTGA TGGAGTCATT GAACTTCTAT   
  
  
+ GCAGCATTGT TTGATTGCTT GGAATCCACT ATATCGAGGA CATCGATAGA GAGACAGAAG CTCGAGAAGT   
  
  
+ TGATGTTTGG AGAGGAGATC AAGAACCTCA CAGCTTGTGA AGGGGCAGAG AGAAAGGCAA GGCACGAGAA   
  
  
+ GCTCAGTGAA TGGGTTCAAA GATTTGAGTC AGTAGGATTT AAAAGGGAGC CATTGAGCTA CCATGGTTTC   
  
  
+ TTGCTTGCTA GGAGGTTTTT ACATACCAAT AATTATGAGG GGTATAACAT CAAGGAAGTC AATGGTTTTC   
  
  
+ TTGTTATCTG TTGGCAAGAT AGACCCCTGT ATTCTGTTTC AGCTTGGAGA TTTTA  

- +Up\_Stream \_Len000AATGTT TTTGCTACGA GATTTGGATA ATCCAGCAAG GCTTTGGGCG TTTTAACTTT   
  
  
- ATACTAGCGG GCATAAAATG GGTTTGGACT TTATGTGAAT TATACATAAA ATGGACTTGG GTTTATGTGG   
  
  
- CCTGGACATA AATTGTGCCC GTGGGCTTTA CTATCCAGAT TGAGAGTGGT GGTGGCGTAA ACCGTGGGCT   
  
  
- TGCCATTTTT TACAAGTAGA TAATACGTGA AACCGAAGTA GGTATTGGGA TACTAGATAG ACACGGTTAT   
  
  
- AAACCTCGTG GGTCGCTTCA TCGCGTACGA GATTTGAGAA CGGAGTTTTG TTTTGTTTAT CACCCAGTAT   
  
  
- AAGGGAAGTT TCGTGATTGT TTGTGTGTGA GAGAGAGAGA GTCATCGGTT GTTGAATTCC GCCACAAACG   
  
  
- AACCAAAACC TCTCCCGCAC AAACCTACTC CACGTTTTGT GTATCTACTT TAGTATTTGT TGAACTAAGG   
  
  
- GTCGGTGGGT ACTGGATGAG TCTATTGTTC CCTGTTTTTG TTTCTCCAAA AGACTTTGTC TCTTTTGTCC   
  
  
- CCGCAACACA ACGCGACAAC GAGTGTTTTA AGGTTATAAG GAGATTTTCG AGGGTTTGCC TCTGTTTAGT   
  
  
- TTCTCCTGTT TCTGTACACC CTAAATTATG TTTTAAACTG ATTGAGACGT TGCCTTTTGT TGGGGATCCC   
  
  
- ACAAAAAGAC GCACAACATG TGTGAAGTGA AAATTGGACG AGACGGAGAC GGAAACGGTG CCGAAAATTA   
  
  
- CCTATTCTGG TTAATTCTTT GGGTTTATAG AGCATTTAAA CAACTCTAAG GAAAGACGCA AGAGTTCACA   
  
  
- AAGTTTTTGG GTCAAACCTC CAACGTGAAT AAGTACGAAA AGAAGTTCAG TCACTCGAAT CACAGACCTT   
  
  
- GACTTCGAGG AGGACCATTA CGAGACAAAA AGTAAAGAAC AACACGCAAA TATTTTTAAG CCGGATAACT   
  
  
- ACCTTCAAAC AAACTACAAA AACCCATTCA AAAACTAATT ATCTCTACTT AATCATTTCA TTTTAAACTC   
  
  
- ACCATAAATT ACTAGGAATT CGTCAGATAC GAAACAACAG TGTACGCAAA GAACCGAAAA CTGACTTTGT   
  
  
- AAAGACACAA TCTACTTTTT GAGTAAAACT ACGTACTCTA CTATTTTAGC GAATAAAAAC GGAAATAAAC   
  
  
- CACACACCCC CCCCCCCCCC CCCCCGCGTC CAGACTCCAT TTTCTATACA CTTAAGTAAA CCTCAGAGCT   
  
  
- ATTAAGCAAC CGATACAACT GTTAGTGCTC ACCAGCAATA GCGAGTAGAG TGTCCAACTA TTAAGTGAGT   
  
  
- TTGAAGTTCG CCGGACTTAG TAAGTCAGGC CCAACAATTT TAGAGTTGTC AGTAAAGTGT GCTCACCTCC   
  
  
- CGGGGTTTTC TGTGGTAAGA AAGCTTGAAA AAAAAAAACT TCCAGTAAAA AAGTTCTTGT TTTGATTACA   
  
  
- CTTGTACGCT GAAGATACTC ATTTCACATT TCTACGTATT TCAGAAAAAA AGAAAATATA TTTTTCATGA   
  
  
- GTTTTTTTTT TCTCAACCTC TCGACTAAGG GGTACGTAGG AGATTCGTTT CCACCACCTG CCGTCTTTTA   
  
  
- ATCATCTCAA TAACACTCTA ATTTCAAACG CGTGGACTTG CCGACTGGAG ATCAAACGAT AGTTGCAATG   
  
  
- TAAATACACA GTATTAAAAA CGTACTTTGT TAAAGTGCGT TACAAAAGAC TACCGTCAAT ACAAATCTTT   
  
  
- ATTCAAATTA GCCTGGAATA AGGTGACTAA ATATAAACTA AGAAGGGAGA AATGATGCCG ACAGGTAAGT   
  
  
- GTTCGGTTTC TTAAGAGGGG GGTTTTTTTT CTTTTTTGTC ATATCTTGGT ACAAATAATA GAGTAAAAAA   
  
  
- AGAGAGGGTA AACTAGACTT TGTGTTTGAA CGTCCCCACG TTCAACCTGT GAATCATCGA CGTCCTTACT   
  
  
- AAGTTATCCT TAGGAGTAGA AACTGTTGTA GTGGTGAACT TATGTATAGT GAACTACCGT GAGAGGGGAC   
  
  
- CAAATCCGAA GGGTATAGGC ACCGAATCCC TCGATTTTGG ACTCGTTTCT CCTGAGACAG ATTAGGTAGA   
  
  
- GGAGGAAAGT ACACGATTAG TTCAACGATG ACCCTCGTAG CTGTTACGAT TACAGTCGGA ACTCGTATAA   
  
  
- AGGGTGGAAC GGAGAGGGTG GCCTCTATGT TACGTCGCTT AACGACGAAT GAAACGACTT CGGAAACGAC   
  
  
- TGGCAAATGA TTCCCGAACC GTCGGGCCAG AAGAGTTTCG GAACTTAACA TGGTTCTACA GTAGACAAAG   
  
  
- GCTTGTTTAA GAACAAGTTT TCAACAAGAA ACTAGAAGAA GGCAAGAACT TCGAAAGTAT AGAACACTGC   
  
  
- TTGGTCCGTT AGTGCCTCCG GTATCTTCCT CTTTTCTACC AAGTATATTA ACTAGAGGTA AGAACACTTA   
  
  
- GCCGAGACAC CTAATCAGAG GAGGTCCGTA ACTCACAAGC TGGACTTCCG GGTGGGGTAA ACTCCTATTG   
  
  
- GCCATATGTA CTCTTCTTTC TTCACAACCT AGTTTACCGA TACGTTGATT TGTTTCTCCG ACTTTTTAAC   
  
  
- CTGTAGGGTA AAGTTAAGTT AGGATAGCGT TCGTTTGATC TGCTGGAACT ACAGCTTTCG AACTCACAGT   
  
  
- TCTGGCCTCT TCGTGAACAA TAATCAAGAC ACGAGGTTGA TGTAAGAGAA AACCGTAACC TACTCCCTAG   
  
  
- TTACGGATTC TTGCGTCCGT ACCGAATGAA CGTTTCCCAC AAAATATACT TTGGTGCTTT TAACCTCCTG   
  
  
- AACGGGTTGT TCCTAAACTA CTTTTACAAC TTGAGTTTAC TTCTAAGATG TAGTAGTAGT AGAAGTAGAG   
  
  
- GATAGGGAAG TAGTTTTGAA CTACGGAAAA ATTTTCGGGA GGAACCCGAA AGCGGTTTTG AGTACCAACA   
  
  
- TTGGCTCGTT CTTAGTTTGG TGTTACCTTC ACGGGATTAT CTTTCTCACT ACCTCAGTAA CTTGAAGATA   
  
  
- CGTCGTAACA AACTAACGAA CCTTAGGTGA TATAGCTCCT GTAGCTATCT CTCTGTCTTC GAGCTCTTCA   
  
  
- ACTACAAACC TCTCCTCTAG TTCTTGGAGT GTCGAACACT TCCCCGTCTC TCTTTCCGTT CCGTGCTCTT   
  
  
- CGAGTCACTT ACCCAAGTTT CTAAACTCAG TCATCCTAAA TTTTCCCTCG GTAACTCGAT GGTACCAAAG   
  
  
- AACGAACGAT CCTCCAAAAA TGTATGGTTA TTAATACTCC CCATATTGTA GTTCCTTCAG TTACCAAAAG   
  
  
- AACAATAGAC AACCGTTCTA TCTGGGGACA TAAGACAAAG TCGAACCTCT AAAAT

+     MYB

| Site Name | Organism | Position | Strand | Matrix score. | sequence | function |
| --- | --- | --- | --- | --- | --- | --- |
| MYB | Arabidopsis thaliana | 579 | - | 6 | CAACAG |  |
| MYB | Arabidopsis thaliana | 3302 | - | 6 | CAACAG |  |
| MYB | Arabidopsis thaliana | 2938 | - | 6 | CAACCA |  |
| MYB | Arabidopsis thaliana | 1379 | + | 6 | CAACAG |  |

>HU05G01267.1   
+ +Up\_Stream \_Len000TTACAA AAACGATGCT CTAAACCTAT TAGGTCGTTC CGAAACCCGC AAAATTGAAA   
  
  
+ TATGATCGCC CGTATTTTAC CCAAACCTGA AATACACTTA ATATGTATTT TACCTGAACC CAAATACACC   
  
  
+ GGACCTGTAT TTAACACGGG CACCCGAAAT GATAGGTCTA ACTCTCACCA CCACCGCATT TGGCACCCGA   
  
  
+ ACGGTAAAAA ATGTTCATCT ATTATGCACT TTGGCTTCAT CCATAACCCT ATGATCTATC TGTGCCAATA   
  
  
+ TTTGGAGCAC CCAGCGAAGT AGCGCATGCT CTAAACTCTT GCCTCAAAAC AAAACAAATA GTGGGTCATA   
  
  
+ TTCCCTTCAA AGCACTAACA AACACACACT CTCTCTCTCT CAGTAGCCAA CAACTTAAGG CGGTGTTTGC   
  
  
+ TTGGTTTTGG AGAGGGCGTG TTTGGATGAG GTGCAAAACA CATAGATGAA ATCATAAACA ACTTGATTCC   
  
  
+ CAGCCACCCA TGACCTACTC AGATAACAAG GGACAAAAAC AAAGAGGTTT TCTGAAACAG AGAAAACAGG   
  
  
+ GGCGTTGTGT TGCGCTGTTG CTCACAAAAT TCCAATATTC CTCTAAAAGC TCCCAAACGG AGACAAATCA   
  
  
+ AAGAGGACAA AGACATGTGG GATTTAATAC AAAATTTGAC TAACTCTGCA ACGGAAAACA ACCCCTAGGG   
  
  
+ TGTTTTTCTG CGTGTTGTAC ACACTTCACT TTTAACCTGC TCTGCCTCTG CCTTTGCCAC GGCTTTTAAT   
  
  
+ GGATAAGACC AATTAAGAAA CCCAAATATC TCGTAAATTT GTTGAGATTC CTTTCTGCGT TCTCAAGTGT   
  
  
+ TTCAAAAACC CAGTTTGGAG GTTGCACTTA TTCATGCTTT TCTTCAAGTC AGTGAGCTTA GTGTCTGGAA   
  
  
+ CTGAAGCTCC TCCTGGTAAT GCTCTGTTTT TCATTTCTTG TTGTGCGTTT ATAAAAATTC GGCCTATTGA   
  
  
+ TGGAAGTTTG TTTGATGTTT TTGGGTAAGT TTTTGATTAA TAGAGATGAA TTAGTAAAGT AAAATTTGAG   
  
  
+ TGGTATTTAA TGATCCTTAA GCAGTCTATG CTTTGTTGTC ACATGCGTTT CTTGGCTTTT GACTGAAACA   
  
  
+ TTTCTGTGTT AGATGAAAAA CTCATTTTGA TGCATGAGAT GATAAAATCG CTTATTTTTG CCTTTATTTG   
  
  
+ GTGTGTGGGG GGGGGGGGGG GGGGGCGCAG GTCTGAGGTA AAAGATATGT GAATTCATTT GGAGTCTCGA   
  
  
+ TAATTCGTTG GCTATGTTGA CAATCACGAG TGGTCGTTAT CGCTCATCTC ACAGGTTGAT AATTCACTCA   
  
  
+ AACTTCAAGC GGCCTGAATC ATTCAGTCCG GGTTGTTAAA ATCTCAACAG TCATTTCACA CGAGTGGAGG   
  
  
+ GCCCCAAAAG ACACCATTCT TTCGAACTTT TTTTTTTTGA AGGTCATTTT TTCAAGAACA AAACTAATGT   
  
  
+ GAACATGCGA CTTCTATGAG TAAAGTGTAA AGATGCATAA AGTCTTTTTT TCTTTTATAT AAAAAGTACT   
  
  
+ CAAAAAAAAA AGAGTTGGAG AGCTGATTCC CCATGCATCC TCTAAGCAAA GGTGGTGGAC GGCAGAAAAT   
  
  
+ TAGTAGAGTT ATTGTGAGAT TAAAGTTTGC GCACCTGAAC GGCTGACCTC TAGTTTGCTA TCAACGTTAC   
  
  
+ ATTTATGTGT CATAATTTTT GCATGAAACA ATTTCACGCA ATGTTTTCTG ATGGCAGTTA TGTTTAGAAA   
  
  
+ TAAGTTTAAT CGGACCTTAT TCCACTGATT TATATTTGAT TCTTCCCTCT TTACTACGGC TGTCCATTCA   
  
  
+ CAAGCCAAAG AATTCTCCCC CCAAAAAAAA GAAAAAACAG TATAGAACCA TGTTTATTAT CTCATTTTTT   
  
  
+ TCTCTCCCAT TTGATCTGAA ACACAAACTT GCAGGGGTGC AAGTTGGACA CTTAGTAGCT GCAGGAATGA   
  
  
+ TTCAATAGGA ATCCTCATCT TTGACAACAT CACCACTTGA ATACATATCA CTTGATGGCA CTCTCCCCTG   
  
  
+ GTTTAGGCTT CCCATATCCG TGGCTTAGGG AGCTAAAACC TGAGCAAAGA GGACTCTGTC TAATCCATCT   
  
  
+ CCTCCTTTCA TGTGCTAATC AAGTTGCTAC TGGGAGCATC GACAATGCTA ATGTCAGCCT TGAGCATATT   
  
  
+ TCCCACCTTG CCTCTCCCAC CGGAGATACA ATGCAGCGAA TTGCTGCTTA CTTTGCTGAA GCCTTTGCTG   
  
  
+ ACCGTTTACT AAGGGCTTGG CAGCCCGGTC TTCTCAAAGC CTTGAATTGT ACCAAGATGT CATCTGTTTC   
  
  
+ CGAACAAATT CTTGTTCAAA AGTTGTTCTT TGATCTTCTT CCGTTCTTGA AGCTTTCATA TCTTGTGACG   
  
  
+ AACCAGGCAA TCACGGAGGC CATAGAAGGA GAAAAGATGG TTCATATAAT TGATCTCCAT TCTTGTGAAT   
  
  
+ CGGCTCTGTG GATTAGTCTC CTCCAGGCAT TGAGTGTTCG ACCTGAAGGC CCACCCCATT TGAGGATAAC   
  
  
+ CGGTATACAT GAGAAGAAAG AAGTGTTGGA TCAAATGGCT ATGCAACTAA ACAAAGAGGC TGAAAAATTG   
  
  
+ GACATCCCAT TTCAATTCAA TCCTATCGCA AGCAAACTAG ACGACCTTGA TGTCGAAAGC TTGAGTGTCA   
  
  
+ AGACCGGAGA AGCACTTGTT ATTAGTTCTG TGCTCCAACT ACATTCTCTT TTGGCATTGG ATGAGGGATC   
  
  
+ AATGCCTAAG AACGCAGGCA TGGCTTACTT GCAAAGGGTG TTTTATATGA AACCACGAAA ATTGGAGGAC   
  
  
+ TTGCCCAACA AGGATTTGAT GAAAATGTTG AACTCAAATG AAGATTCTAC ATCATCATCA TCTTCATCTC   
  
  
+ CTATCCCTTC ATCAAAACTT GATGCCTTTT TAAAAGCCCT CCTTGGGCTT TCGCCAAAAC TCATGGTTGT   
  
  
+ AACCGAGCAA GAATCAAACC ACAATGGAAG TGCCCTAATA GAAAGAGTGA TGGAGTCATT GAACTTCTAT   
  
  
+ GCAGCATTGT TTGATTGCTT GGAATCCACT ATATCGAGGA CATCGATAGA GAGACAGAAG CTCGAGAAGT   
  
  
+ TGATGTTTGG AGAGGAGATC AAGAACCTCA CAGCTTGTGA AGGGGCAGAG AGAAAGGCAA GGCACGAGAA   
  
  
+ GCTCAGTGAA TGGGTTCAAA GATTTGAGTC AGTAGGATTT AAAAGGGAGC CATTGAGCTA CCATGGTTTC   
  
  
+ TTGCTTGCTA GGAGGTTTTT ACATACCAAT AATTATGAGG GGTATAACAT CAAGGAAGTC AATGGTTTTC   
  
  
+ TTGTTATCTG TTGGCAAGAT AGACCCCTGT ATTCTGTTTC AGCTTGGAGA TTTTA  

- +Up\_Stream \_Len000AATGTT TTTGCTACGA GATTTGGATA ATCCAGCAAG GCTTTGGGCG TTTTAACTTT   
  
  
- ATACTAGCGG GCATAAAATG GGTTTGGACT TTATGTGAAT TATACATAAA ATGGACTTGG GTTTATGTGG   
  
  
- CCTGGACATA AATTGTGCCC GTGGGCTTTA CTATCCAGAT TGAGAGTGGT GGTGGCGTAA ACCGTGGGCT   
  
  
- TGCCATTTTT TACAAGTAGA TAATACGTGA AACCGAAGTA GGTATTGGGA TACTAGATAG ACACGGTTAT   
  
  
- AAACCTCGTG GGTCGCTTCA TCGCGTACGA GATTTGAGAA CGGAGTTTTG TTTTGTTTAT CACCCAGTAT   
  
  
- AAGGGAAGTT TCGTGATTGT TTGTGTGTGA GAGAGAGAGA GTCATCGGTT GTTGAATTCC GCCACAAACG   
  
  
- AACCAAAACC TCTCCCGCAC AAACCTACTC CACGTTTTGT GTATCTACTT TAGTATTTGT TGAACTAAGG   
  
  
- GTCGGTGGGT ACTGGATGAG TCTATTGTTC CCTGTTTTTG TTTCTCCAAA AGACTTTGTC TCTTTTGTCC   
  
  
- CCGCAACACA ACGCGACAAC GAGTGTTTTA AGGTTATAAG GAGATTTTCG AGGGTTTGCC TCTGTTTAGT   
  
  
- TTCTCCTGTT TCTGTACACC CTAAATTATG TTTTAAACTG ATTGAGACGT TGCCTTTTGT TGGGGATCCC   
  
  
- ACAAAAAGAC GCACAACATG TGTGAAGTGA AAATTGGACG AGACGGAGAC GGAAACGGTG CCGAAAATTA   
  
  
- CCTATTCTGG TTAATTCTTT GGGTTTATAG AGCATTTAAA CAACTCTAAG GAAAGACGCA AGAGTTCACA   
  
  
- AAGTTTTTGG GTCAAACCTC CAACGTGAAT AAGTACGAAA AGAAGTTCAG TCACTCGAAT CACAGACCTT   
  
  
- GACTTCGAGG AGGACCATTA CGAGACAAAA AGTAAAGAAC AACACGCAAA TATTTTTAAG CCGGATAACT   
  
  
- ACCTTCAAAC AAACTACAAA AACCCATTCA AAAACTAATT ATCTCTACTT AATCATTTCA TTTTAAACTC   
  
  
- ACCATAAATT ACTAGGAATT CGTCAGATAC GAAACAACAG TGTACGCAAA GAACCGAAAA CTGACTTTGT   
  
  
- AAAGACACAA TCTACTTTTT GAGTAAAACT ACGTACTCTA CTATTTTAGC GAATAAAAAC GGAAATAAAC   
  
  
- CACACACCCC CCCCCCCCCC CCCCCGCGTC CAGACTCCAT TTTCTATACA CTTAAGTAAA CCTCAGAGCT   
  
  
- ATTAAGCAAC CGATACAACT GTTAGTGCTC ACCAGCAATA GCGAGTAGAG TGTCCAACTA TTAAGTGAGT   
  
  
- TTGAAGTTCG CCGGACTTAG TAAGTCAGGC CCAACAATTT TAGAGTTGTC AGTAAAGTGT GCTCACCTCC   
  
  
- CGGGGTTTTC TGTGGTAAGA AAGCTTGAAA AAAAAAAACT TCCAGTAAAA AAGTTCTTGT TTTGATTACA   
  
  
- CTTGTACGCT GAAGATACTC ATTTCACATT TCTACGTATT TCAGAAAAAA AGAAAATATA TTTTTCATGA   
  
  
- GTTTTTTTTT TCTCAACCTC TCGACTAAGG GGTACGTAGG AGATTCGTTT CCACCACCTG CCGTCTTTTA   
  
  
- ATCATCTCAA TAACACTCTA ATTTCAAACG CGTGGACTTG CCGACTGGAG ATCAAACGAT AGTTGCAATG   
  
  
- TAAATACACA GTATTAAAAA CGTACTTTGT TAAAGTGCGT TACAAAAGAC TACCGTCAAT ACAAATCTTT   
  
  
- ATTCAAATTA GCCTGGAATA AGGTGACTAA ATATAAACTA AGAAGGGAGA AATGATGCCG ACAGGTAAGT   
  
  
- GTTCGGTTTC TTAAGAGGGG GGTTTTTTTT CTTTTTTGTC ATATCTTGGT ACAAATAATA GAGTAAAAAA   
  
  
- AGAGAGGGTA AACTAGACTT TGTGTTTGAA CGTCCCCACG TTCAACCTGT GAATCATCGA CGTCCTTACT   
  
  
- AAGTTATCCT TAGGAGTAGA AACTGTTGTA GTGGTGAACT TATGTATAGT GAACTACCGT GAGAGGGGAC   
  
  
- CAAATCCGAA GGGTATAGGC ACCGAATCCC TCGATTTTGG ACTCGTTTCT CCTGAGACAG ATTAGGTAGA   
  
  
- GGAGGAAAGT ACACGATTAG TTCAACGATG ACCCTCGTAG CTGTTACGAT TACAGTCGGA ACTCGTATAA   
  
  
- AGGGTGGAAC GGAGAGGGTG GCCTCTATGT TACGTCGCTT AACGACGAAT GAAACGACTT CGGAAACGAC   
  
  
- TGGCAAATGA TTCCCGAACC GTCGGGCCAG AAGAGTTTCG GAACTTAACA TGGTTCTACA GTAGACAAAG   
  
  
- GCTTGTTTAA GAACAAGTTT TCAACAAGAA ACTAGAAGAA GGCAAGAACT TCGAAAGTAT AGAACACTGC   
  
  
- TTGGTCCGTT AGTGCCTCCG GTATCTTCCT CTTTTCTACC AAGTATATTA ACTAGAGGTA AGAACACTTA   
  
  
- GCCGAGACAC CTAATCAGAG GAGGTCCGTA ACTCACAAGC TGGACTTCCG GGTGGGGTAA ACTCCTATTG   
  
  
- GCCATATGTA CTCTTCTTTC TTCACAACCT AGTTTACCGA TACGTTGATT TGTTTCTCCG ACTTTTTAAC   
  
  
- CTGTAGGGTA AAGTTAAGTT AGGATAGCGT TCGTTTGATC TGCTGGAACT ACAGCTTTCG AACTCACAGT   
  
  
- TCTGGCCTCT TCGTGAACAA TAATCAAGAC ACGAGGTTGA TGTAAGAGAA AACCGTAACC TACTCCCTAG   
  
  
- TTACGGATTC TTGCGTCCGT ACCGAATGAA CGTTTCCCAC AAAATATACT TTGGTGCTTT TAACCTCCTG   
  
  
- AACGGGTTGT TCCTAAACTA CTTTTACAAC TTGAGTTTAC TTCTAAGATG TAGTAGTAGT AGAAGTAGAG   
  
  
- GATAGGGAAG TAGTTTTGAA CTACGGAAAA ATTTTCGGGA GGAACCCGAA AGCGGTTTTG AGTACCAACA   
  
  
- TTGGCTCGTT CTTAGTTTGG TGTTACCTTC ACGGGATTAT CTTTCTCACT ACCTCAGTAA CTTGAAGATA   
  
  
- CGTCGTAACA AACTAACGAA CCTTAGGTGA TATAGCTCCT GTAGCTATCT CTCTGTCTTC GAGCTCTTCA   
  
  
- ACTACAAACC TCTCCTCTAG TTCTTGGAGT GTCGAACACT TCCCCGTCTC TCTTTCCGTT CCGTGCTCTT   
  
  
- CGAGTCACTT ACCCAAGTTT CTAAACTCAG TCATCCTAAA TTTTCCCTCG GTAACTCGAT GGTACCAAAG   
  
  
- AACGAACGAT CCTCCAAAAA TGTATGGTTA TTAATACTCC CCATATTGTA GTTCCTTCAG TTACCAAAAG   
  
  
- AACAATAGAC AACCGTTCTA TCTGGGGACA TAAGACAAAG TCGAACCTCT AAAAT

+     MYB recognition site

| Site Name | Organism | Position | Strand | Matrix score. | sequence | function |
| --- | --- | --- | --- | --- | --- | --- |
| MYB recognition site | Arabidopsis thaliana | 683 | - | 6 | CCGTTG |  |

>HU05G01267.1   
+ +Up\_Stream \_Len000TTACAA AAACGATGCT CTAAACCTAT TAGGTCGTTC CGAAACCCGC AAAATTGAAA   
  
  
+ TATGATCGCC CGTATTTTAC CCAAACCTGA AATACACTTA ATATGTATTT TACCTGAACC CAAATACACC   
  
  
+ GGACCTGTAT TTAACACGGG CACCCGAAAT GATAGGTCTA ACTCTCACCA CCACCGCATT TGGCACCCGA   
  
  
+ ACGGTAAAAA ATGTTCATCT ATTATGCACT TTGGCTTCAT CCATAACCCT ATGATCTATC TGTGCCAATA   
  
  
+ TTTGGAGCAC CCAGCGAAGT AGCGCATGCT CTAAACTCTT GCCTCAAAAC AAAACAAATA GTGGGTCATA   
  
  
+ TTCCCTTCAA AGCACTAACA AACACACACT CTCTCTCTCT CAGTAGCCAA CAACTTAAGG CGGTGTTTGC   
  
  
+ TTGGTTTTGG AGAGGGCGTG TTTGGATGAG GTGCAAAACA CATAGATGAA ATCATAAACA ACTTGATTCC   
  
  
+ CAGCCACCCA TGACCTACTC AGATAACAAG GGACAAAAAC AAAGAGGTTT TCTGAAACAG AGAAAACAGG   
  
  
+ GGCGTTGTGT TGCGCTGTTG CTCACAAAAT TCCAATATTC CTCTAAAAGC TCCCAAACGG AGACAAATCA   
  
  
+ AAGAGGACAA AGACATGTGG GATTTAATAC AAAATTTGAC TAACTCTGCA ACGGAAAACA ACCCCTAGGG   
  
  
+ TGTTTTTCTG CGTGTTGTAC ACACTTCACT TTTAACCTGC TCTGCCTCTG CCTTTGCCAC GGCTTTTAAT   
  
  
+ GGATAAGACC AATTAAGAAA CCCAAATATC TCGTAAATTT GTTGAGATTC CTTTCTGCGT TCTCAAGTGT   
  
  
+ TTCAAAAACC CAGTTTGGAG GTTGCACTTA TTCATGCTTT TCTTCAAGTC AGTGAGCTTA GTGTCTGGAA   
  
  
+ CTGAAGCTCC TCCTGGTAAT GCTCTGTTTT TCATTTCTTG TTGTGCGTTT ATAAAAATTC GGCCTATTGA   
  
  
+ TGGAAGTTTG TTTGATGTTT TTGGGTAAGT TTTTGATTAA TAGAGATGAA TTAGTAAAGT AAAATTTGAG   
  
  
+ TGGTATTTAA TGATCCTTAA GCAGTCTATG CTTTGTTGTC ACATGCGTTT CTTGGCTTTT GACTGAAACA   
  
  
+ TTTCTGTGTT AGATGAAAAA CTCATTTTGA TGCATGAGAT GATAAAATCG CTTATTTTTG CCTTTATTTG   
  
  
+ GTGTGTGGGG GGGGGGGGGG GGGGGCGCAG GTCTGAGGTA AAAGATATGT GAATTCATTT GGAGTCTCGA   
  
  
+ TAATTCGTTG GCTATGTTGA CAATCACGAG TGGTCGTTAT CGCTCATCTC ACAGGTTGAT AATTCACTCA   
  
  
+ AACTTCAAGC GGCCTGAATC ATTCAGTCCG GGTTGTTAAA ATCTCAACAG TCATTTCACA CGAGTGGAGG   
  
  
+ GCCCCAAAAG ACACCATTCT TTCGAACTTT TTTTTTTTGA AGGTCATTTT TTCAAGAACA AAACTAATGT   
  
  
+ GAACATGCGA CTTCTATGAG TAAAGTGTAA AGATGCATAA AGTCTTTTTT TCTTTTATAT AAAAAGTACT   
  
  
+ CAAAAAAAAA AGAGTTGGAG AGCTGATTCC CCATGCATCC TCTAAGCAAA GGTGGTGGAC GGCAGAAAAT   
  
  
+ TAGTAGAGTT ATTGTGAGAT TAAAGTTTGC GCACCTGAAC GGCTGACCTC TAGTTTGCTA TCAACGTTAC   
  
  
+ ATTTATGTGT CATAATTTTT GCATGAAACA ATTTCACGCA ATGTTTTCTG ATGGCAGTTA TGTTTAGAAA   
  
  
+ TAAGTTTAAT CGGACCTTAT TCCACTGATT TATATTTGAT TCTTCCCTCT TTACTACGGC TGTCCATTCA   
  
  
+ CAAGCCAAAG AATTCTCCCC CCAAAAAAAA GAAAAAACAG TATAGAACCA TGTTTATTAT CTCATTTTTT   
  
  
+ TCTCTCCCAT TTGATCTGAA ACACAAACTT GCAGGGGTGC AAGTTGGACA CTTAGTAGCT GCAGGAATGA   
  
  
+ TTCAATAGGA ATCCTCATCT TTGACAACAT CACCACTTGA ATACATATCA CTTGATGGCA CTCTCCCCTG   
  
  
+ GTTTAGGCTT CCCATATCCG TGGCTTAGGG AGCTAAAACC TGAGCAAAGA GGACTCTGTC TAATCCATCT   
  
  
+ CCTCCTTTCA TGTGCTAATC AAGTTGCTAC TGGGAGCATC GACAATGCTA ATGTCAGCCT TGAGCATATT   
  
  
+ TCCCACCTTG CCTCTCCCAC CGGAGATACA ATGCAGCGAA TTGCTGCTTA CTTTGCTGAA GCCTTTGCTG   
  
  
+ ACCGTTTACT AAGGGCTTGG CAGCCCGGTC TTCTCAAAGC CTTGAATTGT ACCAAGATGT CATCTGTTTC   
  
  
+ CGAACAAATT CTTGTTCAAA AGTTGTTCTT TGATCTTCTT CCGTTCTTGA AGCTTTCATA TCTTGTGACG   
  
  
+ AACCAGGCAA TCACGGAGGC CATAGAAGGA GAAAAGATGG TTCATATAAT TGATCTCCAT TCTTGTGAAT   
  
  
+ CGGCTCTGTG GATTAGTCTC CTCCAGGCAT TGAGTGTTCG ACCTGAAGGC CCACCCCATT TGAGGATAAC   
  
  
+ CGGTATACAT GAGAAGAAAG AAGTGTTGGA TCAAATGGCT ATGCAACTAA ACAAAGAGGC TGAAAAATTG   
  
  
+ GACATCCCAT TTCAATTCAA TCCTATCGCA AGCAAACTAG ACGACCTTGA TGTCGAAAGC TTGAGTGTCA   
  
  
+ AGACCGGAGA AGCACTTGTT ATTAGTTCTG TGCTCCAACT ACATTCTCTT TTGGCATTGG ATGAGGGATC   
  
  
+ AATGCCTAAG AACGCAGGCA TGGCTTACTT GCAAAGGGTG TTTTATATGA AACCACGAAA ATTGGAGGAC   
  
  
+ TTGCCCAACA AGGATTTGAT GAAAATGTTG AACTCAAATG AAGATTCTAC ATCATCATCA TCTTCATCTC   
  
  
+ CTATCCCTTC ATCAAAACTT GATGCCTTTT TAAAAGCCCT CCTTGGGCTT TCGCCAAAAC TCATGGTTGT   
  
  
+ AACCGAGCAA GAATCAAACC ACAATGGAAG TGCCCTAATA GAAAGAGTGA TGGAGTCATT GAACTTCTAT   
  
  
+ GCAGCATTGT TTGATTGCTT GGAATCCACT ATATCGAGGA CATCGATAGA GAGACAGAAG CTCGAGAAGT   
  
  
+ TGATGTTTGG AGAGGAGATC AAGAACCTCA CAGCTTGTGA AGGGGCAGAG AGAAAGGCAA GGCACGAGAA   
  
  
+ GCTCAGTGAA TGGGTTCAAA GATTTGAGTC AGTAGGATTT AAAAGGGAGC CATTGAGCTA CCATGGTTTC   
  
  
+ TTGCTTGCTA GGAGGTTTTT ACATACCAAT AATTATGAGG GGTATAACAT CAAGGAAGTC AATGGTTTTC   
  
  
+ TTGTTATCTG TTGGCAAGAT AGACCCCTGT ATTCTGTTTC AGCTTGGAGA TTTTA  

- +Up\_Stream \_Len000AATGTT TTTGCTACGA GATTTGGATA ATCCAGCAAG GCTTTGGGCG TTTTAACTTT   
  
  
- ATACTAGCGG GCATAAAATG GGTTTGGACT TTATGTGAAT TATACATAAA ATGGACTTGG GTTTATGTGG   
  
  
- CCTGGACATA AATTGTGCCC GTGGGCTTTA CTATCCAGAT TGAGAGTGGT GGTGGCGTAA ACCGTGGGCT   
  
  
- TGCCATTTTT TACAAGTAGA TAATACGTGA AACCGAAGTA GGTATTGGGA TACTAGATAG ACACGGTTAT   
  
  
- AAACCTCGTG GGTCGCTTCA TCGCGTACGA GATTTGAGAA CGGAGTTTTG TTTTGTTTAT CACCCAGTAT   
  
  
- AAGGGAAGTT TCGTGATTGT TTGTGTGTGA GAGAGAGAGA GTCATCGGTT GTTGAATTCC GCCACAAACG   
  
  
- AACCAAAACC TCTCCCGCAC AAACCTACTC CACGTTTTGT GTATCTACTT TAGTATTTGT TGAACTAAGG   
  
  
- GTCGGTGGGT ACTGGATGAG TCTATTGTTC CCTGTTTTTG TTTCTCCAAA AGACTTTGTC TCTTTTGTCC   
  
  
- CCGCAACACA ACGCGACAAC GAGTGTTTTA AGGTTATAAG GAGATTTTCG AGGGTTTGCC TCTGTTTAGT   
  
  
- TTCTCCTGTT TCTGTACACC CTAAATTATG TTTTAAACTG ATTGAGACGT TGCCTTTTGT TGGGGATCCC   
  
  
- ACAAAAAGAC GCACAACATG TGTGAAGTGA AAATTGGACG AGACGGAGAC GGAAACGGTG CCGAAAATTA   
  
  
- CCTATTCTGG TTAATTCTTT GGGTTTATAG AGCATTTAAA CAACTCTAAG GAAAGACGCA AGAGTTCACA   
  
  
- AAGTTTTTGG GTCAAACCTC CAACGTGAAT AAGTACGAAA AGAAGTTCAG TCACTCGAAT CACAGACCTT   
  
  
- GACTTCGAGG AGGACCATTA CGAGACAAAA AGTAAAGAAC AACACGCAAA TATTTTTAAG CCGGATAACT   
  
  
- ACCTTCAAAC AAACTACAAA AACCCATTCA AAAACTAATT ATCTCTACTT AATCATTTCA TTTTAAACTC   
  
  
- ACCATAAATT ACTAGGAATT CGTCAGATAC GAAACAACAG TGTACGCAAA GAACCGAAAA CTGACTTTGT   
  
  
- AAAGACACAA TCTACTTTTT GAGTAAAACT ACGTACTCTA CTATTTTAGC GAATAAAAAC GGAAATAAAC   
  
  
- CACACACCCC CCCCCCCCCC CCCCCGCGTC CAGACTCCAT TTTCTATACA CTTAAGTAAA CCTCAGAGCT   
  
  
- ATTAAGCAAC CGATACAACT GTTAGTGCTC ACCAGCAATA GCGAGTAGAG TGTCCAACTA TTAAGTGAGT   
  
  
- TTGAAGTTCG CCGGACTTAG TAAGTCAGGC CCAACAATTT TAGAGTTGTC AGTAAAGTGT GCTCACCTCC   
  
  
- CGGGGTTTTC TGTGGTAAGA AAGCTTGAAA AAAAAAAACT TCCAGTAAAA AAGTTCTTGT TTTGATTACA   
  
  
- CTTGTACGCT GAAGATACTC ATTTCACATT TCTACGTATT TCAGAAAAAA AGAAAATATA TTTTTCATGA   
  
  
- GTTTTTTTTT TCTCAACCTC TCGACTAAGG GGTACGTAGG AGATTCGTTT CCACCACCTG CCGTCTTTTA   
  
  
- ATCATCTCAA TAACACTCTA ATTTCAAACG CGTGGACTTG CCGACTGGAG ATCAAACGAT AGTTGCAATG   
  
  
- TAAATACACA GTATTAAAAA CGTACTTTGT TAAAGTGCGT TACAAAAGAC TACCGTCAAT ACAAATCTTT   
  
  
- ATTCAAATTA GCCTGGAATA AGGTGACTAA ATATAAACTA AGAAGGGAGA AATGATGCCG ACAGGTAAGT   
  
  
- GTTCGGTTTC TTAAGAGGGG GGTTTTTTTT CTTTTTTGTC ATATCTTGGT ACAAATAATA GAGTAAAAAA   
  
  
- AGAGAGGGTA AACTAGACTT TGTGTTTGAA CGTCCCCACG TTCAACCTGT GAATCATCGA CGTCCTTACT   
  
  
- AAGTTATCCT TAGGAGTAGA AACTGTTGTA GTGGTGAACT TATGTATAGT GAACTACCGT GAGAGGGGAC   
  
  
- CAAATCCGAA GGGTATAGGC ACCGAATCCC TCGATTTTGG ACTCGTTTCT CCTGAGACAG ATTAGGTAGA   
  
  
- GGAGGAAAGT ACACGATTAG TTCAACGATG ACCCTCGTAG CTGTTACGAT TACAGTCGGA ACTCGTATAA   
  
  
- AGGGTGGAAC GGAGAGGGTG GCCTCTATGT TACGTCGCTT AACGACGAAT GAAACGACTT CGGAAACGAC   
  
  
- TGGCAAATGA TTCCCGAACC GTCGGGCCAG AAGAGTTTCG GAACTTAACA TGGTTCTACA GTAGACAAAG   
  
  
- GCTTGTTTAA GAACAAGTTT TCAACAAGAA ACTAGAAGAA GGCAAGAACT TCGAAAGTAT AGAACACTGC   
  
  
- TTGGTCCGTT AGTGCCTCCG GTATCTTCCT CTTTTCTACC AAGTATATTA ACTAGAGGTA AGAACACTTA   
  
  
- GCCGAGACAC CTAATCAGAG GAGGTCCGTA ACTCACAAGC TGGACTTCCG GGTGGGGTAA ACTCCTATTG   
  
  
- GCCATATGTA CTCTTCTTTC TTCACAACCT AGTTTACCGA TACGTTGATT TGTTTCTCCG ACTTTTTAAC   
  
  
- CTGTAGGGTA AAGTTAAGTT AGGATAGCGT TCGTTTGATC TGCTGGAACT ACAGCTTTCG AACTCACAGT   
  
  
- TCTGGCCTCT TCGTGAACAA TAATCAAGAC ACGAGGTTGA TGTAAGAGAA AACCGTAACC TACTCCCTAG   
  
  
- TTACGGATTC TTGCGTCCGT ACCGAATGAA CGTTTCCCAC AAAATATACT TTGGTGCTTT TAACCTCCTG   
  
  
- AACGGGTTGT TCCTAAACTA CTTTTACAAC TTGAGTTTAC TTCTAAGATG TAGTAGTAGT AGAAGTAGAG   
  
  
- GATAGGGAAG TAGTTTTGAA CTACGGAAAA ATTTTCGGGA GGAACCCGAA AGCGGTTTTG AGTACCAACA   
  
  
- TTGGCTCGTT CTTAGTTTGG TGTTACCTTC ACGGGATTAT CTTTCTCACT ACCTCAGTAA CTTGAAGATA   
  
  
- CGTCGTAACA AACTAACGAA CCTTAGGTGA TATAGCTCCT GTAGCTATCT CTCTGTCTTC GAGCTCTTCA   
  
  
- ACTACAAACC TCTCCTCTAG TTCTTGGAGT GTCGAACACT TCCCCGTCTC TCTTTCCGTT CCGTGCTCTT   
  
  
- CGAGTCACTT ACCCAAGTTT CTAAACTCAG TCATCCTAAA TTTTCCCTCG GTAACTCGAT GGTACCAAAG   
  
  
- AACGAACGAT CCTCCAAAAA TGTATGGTTA TTAATACTCC CCATATTGTA GTTCCTTCAG TTACCAAAAG   
  
  
- AACAATAGAC AACCGTTCTA TCTGGGGACA TAAGACAAAG TCGAACCTCT AAAAT

+     MYC

| Site Name | Organism | Position | Strand | Matrix score. | sequence | function |
| --- | --- | --- | --- | --- | --- | --- |
| MYC | Arabidopsis thaliana | 2839 | - | 6 | CATTTG |  |
| MYC | Arabidopsis thaliana | 648 | + | 6 | CATGTG |  |
| MYC | Arabidopsis thaliana | 2511 | + | 6 | CATTTG |  |
| MYC | Arabidopsis thaliana | 1902 | + | 6 | CATTTG |  |
| MYC | Arabidopsis thaliana | 2556 | - | 6 | CATTTG |  |
| MYC | Arabidopsis thaliana | 1094 | - | 6 | CATGTG |  |
| MYC | Arabidopsis thaliana | 1250 | + | 6 | CATTTG |  |
| MYC | Arabidopsis thaliana | 2113 | + | 6 | CATGTG |  |
| MYC | Arabidopsis thaliana | 201 | + | 6 | CATTTG |  |

>HU05G01267.1   
+ +Up\_Stream \_Len000TTACAA AAACGATGCT CTAAACCTAT TAGGTCGTTC CGAAACCCGC AAAATTGAAA   
  
  
+ TATGATCGCC CGTATTTTAC CCAAACCTGA AATACACTTA ATATGTATTT TACCTGAACC CAAATACACC   
  
  
+ GGACCTGTAT TTAACACGGG CACCCGAAAT GATAGGTCTA ACTCTCACCA CCACCGCATT TGGCACCCGA   
  
  
+ ACGGTAAAAA ATGTTCATCT ATTATGCACT TTGGCTTCAT CCATAACCCT ATGATCTATC TGTGCCAATA   
  
  
+ TTTGGAGCAC CCAGCGAAGT AGCGCATGCT CTAAACTCTT GCCTCAAAAC AAAACAAATA GTGGGTCATA   
  
  
+ TTCCCTTCAA AGCACTAACA AACACACACT CTCTCTCTCT CAGTAGCCAA CAACTTAAGG CGGTGTTTGC   
  
  
+ TTGGTTTTGG AGAGGGCGTG TTTGGATGAG GTGCAAAACA CATAGATGAA ATCATAAACA ACTTGATTCC   
  
  
+ CAGCCACCCA TGACCTACTC AGATAACAAG GGACAAAAAC AAAGAGGTTT TCTGAAACAG AGAAAACAGG   
  
  
+ GGCGTTGTGT TGCGCTGTTG CTCACAAAAT TCCAATATTC CTCTAAAAGC TCCCAAACGG AGACAAATCA   
  
  
+ AAGAGGACAA AGACATGTGG GATTTAATAC AAAATTTGAC TAACTCTGCA ACGGAAAACA ACCCCTAGGG   
  
  
+ TGTTTTTCTG CGTGTTGTAC ACACTTCACT TTTAACCTGC TCTGCCTCTG CCTTTGCCAC GGCTTTTAAT   
  
  
+ GGATAAGACC AATTAAGAAA CCCAAATATC TCGTAAATTT GTTGAGATTC CTTTCTGCGT TCTCAAGTGT   
  
  
+ TTCAAAAACC CAGTTTGGAG GTTGCACTTA TTCATGCTTT TCTTCAAGTC AGTGAGCTTA GTGTCTGGAA   
  
  
+ CTGAAGCTCC TCCTGGTAAT GCTCTGTTTT TCATTTCTTG TTGTGCGTTT ATAAAAATTC GGCCTATTGA   
  
  
+ TGGAAGTTTG TTTGATGTTT TTGGGTAAGT TTTTGATTAA TAGAGATGAA TTAGTAAAGT AAAATTTGAG   
  
  
+ TGGTATTTAA TGATCCTTAA GCAGTCTATG CTTTGTTGTC ACATGCGTTT CTTGGCTTTT GACTGAAACA   
  
  
+ TTTCTGTGTT AGATGAAAAA CTCATTTTGA TGCATGAGAT GATAAAATCG CTTATTTTTG CCTTTATTTG   
  
  
+ GTGTGTGGGG GGGGGGGGGG GGGGGCGCAG GTCTGAGGTA AAAGATATGT GAATTCATTT GGAGTCTCGA   
  
  
+ TAATTCGTTG GCTATGTTGA CAATCACGAG TGGTCGTTAT CGCTCATCTC ACAGGTTGAT AATTCACTCA   
  
  
+ AACTTCAAGC GGCCTGAATC ATTCAGTCCG GGTTGTTAAA ATCTCAACAG TCATTTCACA CGAGTGGAGG   
  
  
+ GCCCCAAAAG ACACCATTCT TTCGAACTTT TTTTTTTTGA AGGTCATTTT TTCAAGAACA AAACTAATGT   
  
  
+ GAACATGCGA CTTCTATGAG TAAAGTGTAA AGATGCATAA AGTCTTTTTT TCTTTTATAT AAAAAGTACT   
  
  
+ CAAAAAAAAA AGAGTTGGAG AGCTGATTCC CCATGCATCC TCTAAGCAAA GGTGGTGGAC GGCAGAAAAT   
  
  
+ TAGTAGAGTT ATTGTGAGAT TAAAGTTTGC GCACCTGAAC GGCTGACCTC TAGTTTGCTA TCAACGTTAC   
  
  
+ ATTTATGTGT CATAATTTTT GCATGAAACA ATTTCACGCA ATGTTTTCTG ATGGCAGTTA TGTTTAGAAA   
  
  
+ TAAGTTTAAT CGGACCTTAT TCCACTGATT TATATTTGAT TCTTCCCTCT TTACTACGGC TGTCCATTCA   
  
  
+ CAAGCCAAAG AATTCTCCCC CCAAAAAAAA GAAAAAACAG TATAGAACCA TGTTTATTAT CTCATTTTTT   
  
  
+ TCTCTCCCAT TTGATCTGAA ACACAAACTT GCAGGGGTGC AAGTTGGACA CTTAGTAGCT GCAGGAATGA   
  
  
+ TTCAATAGGA ATCCTCATCT TTGACAACAT CACCACTTGA ATACATATCA CTTGATGGCA CTCTCCCCTG   
  
  
+ GTTTAGGCTT CCCATATCCG TGGCTTAGGG AGCTAAAACC TGAGCAAAGA GGACTCTGTC TAATCCATCT   
  
  
+ CCTCCTTTCA TGTGCTAATC AAGTTGCTAC TGGGAGCATC GACAATGCTA ATGTCAGCCT TGAGCATATT   
  
  
+ TCCCACCTTG CCTCTCCCAC CGGAGATACA ATGCAGCGAA TTGCTGCTTA CTTTGCTGAA GCCTTTGCTG   
  
  
+ ACCGTTTACT AAGGGCTTGG CAGCCCGGTC TTCTCAAAGC CTTGAATTGT ACCAAGATGT CATCTGTTTC   
  
  
+ CGAACAAATT CTTGTTCAAA AGTTGTTCTT TGATCTTCTT CCGTTCTTGA AGCTTTCATA TCTTGTGACG   
  
  
+ AACCAGGCAA TCACGGAGGC CATAGAAGGA GAAAAGATGG TTCATATAAT TGATCTCCAT TCTTGTGAAT   
  
  
+ CGGCTCTGTG GATTAGTCTC CTCCAGGCAT TGAGTGTTCG ACCTGAAGGC CCACCCCATT TGAGGATAAC   
  
  
+ CGGTATACAT GAGAAGAAAG AAGTGTTGGA TCAAATGGCT ATGCAACTAA ACAAAGAGGC TGAAAAATTG   
  
  
+ GACATCCCAT TTCAATTCAA TCCTATCGCA AGCAAACTAG ACGACCTTGA TGTCGAAAGC TTGAGTGTCA   
  
  
+ AGACCGGAGA AGCACTTGTT ATTAGTTCTG TGCTCCAACT ACATTCTCTT TTGGCATTGG ATGAGGGATC   
  
  
+ AATGCCTAAG AACGCAGGCA TGGCTTACTT GCAAAGGGTG TTTTATATGA AACCACGAAA ATTGGAGGAC   
  
  
+ TTGCCCAACA AGGATTTGAT GAAAATGTTG AACTCAAATG AAGATTCTAC ATCATCATCA TCTTCATCTC   
  
  
+ CTATCCCTTC ATCAAAACTT GATGCCTTTT TAAAAGCCCT CCTTGGGCTT TCGCCAAAAC TCATGGTTGT   
  
  
+ AACCGAGCAA GAATCAAACC ACAATGGAAG TGCCCTAATA GAAAGAGTGA TGGAGTCATT GAACTTCTAT   
  
  
+ GCAGCATTGT TTGATTGCTT GGAATCCACT ATATCGAGGA CATCGATAGA GAGACAGAAG CTCGAGAAGT   
  
  
+ TGATGTTTGG AGAGGAGATC AAGAACCTCA CAGCTTGTGA AGGGGCAGAG AGAAAGGCAA GGCACGAGAA   
  
  
+ GCTCAGTGAA TGGGTTCAAA GATTTGAGTC AGTAGGATTT AAAAGGGAGC CATTGAGCTA CCATGGTTTC   
  
  
+ TTGCTTGCTA GGAGGTTTTT ACATACCAAT AATTATGAGG GGTATAACAT CAAGGAAGTC AATGGTTTTC   
  
  
+ TTGTTATCTG TTGGCAAGAT AGACCCCTGT ATTCTGTTTC AGCTTGGAGA TTTTA  

- +Up\_Stream \_Len000AATGTT TTTGCTACGA GATTTGGATA ATCCAGCAAG GCTTTGGGCG TTTTAACTTT   
  
  
- ATACTAGCGG GCATAAAATG GGTTTGGACT TTATGTGAAT TATACATAAA ATGGACTTGG GTTTATGTGG   
  
  
- CCTGGACATA AATTGTGCCC GTGGGCTTTA CTATCCAGAT TGAGAGTGGT GGTGGCGTAA ACCGTGGGCT   
  
  
- TGCCATTTTT TACAAGTAGA TAATACGTGA AACCGAAGTA GGTATTGGGA TACTAGATAG ACACGGTTAT   
  
  
- AAACCTCGTG GGTCGCTTCA TCGCGTACGA GATTTGAGAA CGGAGTTTTG TTTTGTTTAT CACCCAGTAT   
  
  
- AAGGGAAGTT TCGTGATTGT TTGTGTGTGA GAGAGAGAGA GTCATCGGTT GTTGAATTCC GCCACAAACG   
  
  
- AACCAAAACC TCTCCCGCAC AAACCTACTC CACGTTTTGT GTATCTACTT TAGTATTTGT TGAACTAAGG   
  
  
- GTCGGTGGGT ACTGGATGAG TCTATTGTTC CCTGTTTTTG TTTCTCCAAA AGACTTTGTC TCTTTTGTCC   
  
  
- CCGCAACACA ACGCGACAAC GAGTGTTTTA AGGTTATAAG GAGATTTTCG AGGGTTTGCC TCTGTTTAGT   
  
  
- TTCTCCTGTT TCTGTACACC CTAAATTATG TTTTAAACTG ATTGAGACGT TGCCTTTTGT TGGGGATCCC   
  
  
- ACAAAAAGAC GCACAACATG TGTGAAGTGA AAATTGGACG AGACGGAGAC GGAAACGGTG CCGAAAATTA   
  
  
- CCTATTCTGG TTAATTCTTT GGGTTTATAG AGCATTTAAA CAACTCTAAG GAAAGACGCA AGAGTTCACA   
  
  
- AAGTTTTTGG GTCAAACCTC CAACGTGAAT AAGTACGAAA AGAAGTTCAG TCACTCGAAT CACAGACCTT   
  
  
- GACTTCGAGG AGGACCATTA CGAGACAAAA AGTAAAGAAC AACACGCAAA TATTTTTAAG CCGGATAACT   
  
  
- ACCTTCAAAC AAACTACAAA AACCCATTCA AAAACTAATT ATCTCTACTT AATCATTTCA TTTTAAACTC   
  
  
- ACCATAAATT ACTAGGAATT CGTCAGATAC GAAACAACAG TGTACGCAAA GAACCGAAAA CTGACTTTGT   
  
  
- AAAGACACAA TCTACTTTTT GAGTAAAACT ACGTACTCTA CTATTTTAGC GAATAAAAAC GGAAATAAAC   
  
  
- CACACACCCC CCCCCCCCCC CCCCCGCGTC CAGACTCCAT TTTCTATACA CTTAAGTAAA CCTCAGAGCT   
  
  
- ATTAAGCAAC CGATACAACT GTTAGTGCTC ACCAGCAATA GCGAGTAGAG TGTCCAACTA TTAAGTGAGT   
  
  
- TTGAAGTTCG CCGGACTTAG TAAGTCAGGC CCAACAATTT TAGAGTTGTC AGTAAAGTGT GCTCACCTCC   
  
  
- CGGGGTTTTC TGTGGTAAGA AAGCTTGAAA AAAAAAAACT TCCAGTAAAA AAGTTCTTGT TTTGATTACA   
  
  
- CTTGTACGCT GAAGATACTC ATTTCACATT TCTACGTATT TCAGAAAAAA AGAAAATATA TTTTTCATGA   
  
  
- GTTTTTTTTT TCTCAACCTC TCGACTAAGG GGTACGTAGG AGATTCGTTT CCACCACCTG CCGTCTTTTA   
  
  
- ATCATCTCAA TAACACTCTA ATTTCAAACG CGTGGACTTG CCGACTGGAG ATCAAACGAT AGTTGCAATG   
  
  
- TAAATACACA GTATTAAAAA CGTACTTTGT TAAAGTGCGT TACAAAAGAC TACCGTCAAT ACAAATCTTT   
  
  
- ATTCAAATTA GCCTGGAATA AGGTGACTAA ATATAAACTA AGAAGGGAGA AATGATGCCG ACAGGTAAGT   
  
  
- GTTCGGTTTC TTAAGAGGGG GGTTTTTTTT CTTTTTTGTC ATATCTTGGT ACAAATAATA GAGTAAAAAA   
  
  
- AGAGAGGGTA AACTAGACTT TGTGTTTGAA CGTCCCCACG TTCAACCTGT GAATCATCGA CGTCCTTACT   
  
  
- AAGTTATCCT TAGGAGTAGA AACTGTTGTA GTGGTGAACT TATGTATAGT GAACTACCGT GAGAGGGGAC   
  
  
- CAAATCCGAA GGGTATAGGC ACCGAATCCC TCGATTTTGG ACTCGTTTCT CCTGAGACAG ATTAGGTAGA   
  
  
- GGAGGAAAGT ACACGATTAG TTCAACGATG ACCCTCGTAG CTGTTACGAT TACAGTCGGA ACTCGTATAA   
  
  
- AGGGTGGAAC GGAGAGGGTG GCCTCTATGT TACGTCGCTT AACGACGAAT GAAACGACTT CGGAAACGAC   
  
  
- TGGCAAATGA TTCCCGAACC GTCGGGCCAG AAGAGTTTCG GAACTTAACA TGGTTCTACA GTAGACAAAG   
  
  
- GCTTGTTTAA GAACAAGTTT TCAACAAGAA ACTAGAAGAA GGCAAGAACT TCGAAAGTAT AGAACACTGC   
  
  
- TTGGTCCGTT AGTGCCTCCG GTATCTTCCT CTTTTCTACC AAGTATATTA ACTAGAGGTA AGAACACTTA   
  
  
- GCCGAGACAC CTAATCAGAG GAGGTCCGTA ACTCACAAGC TGGACTTCCG GGTGGGGTAA ACTCCTATTG   
  
  
- GCCATATGTA CTCTTCTTTC TTCACAACCT AGTTTACCGA TACGTTGATT TGTTTCTCCG ACTTTTTAAC   
  
  
- CTGTAGGGTA AAGTTAAGTT AGGATAGCGT TCGTTTGATC TGCTGGAACT ACAGCTTTCG AACTCACAGT   
  
  
- TCTGGCCTCT TCGTGAACAA TAATCAAGAC ACGAGGTTGA TGTAAGAGAA AACCGTAACC TACTCCCTAG   
  
  
- TTACGGATTC TTGCGTCCGT ACCGAATGAA CGTTTCCCAC AAAATATACT TTGGTGCTTT TAACCTCCTG   
  
  
- AACGGGTTGT TCCTAAACTA CTTTTACAAC TTGAGTTTAC TTCTAAGATG TAGTAGTAGT AGAAGTAGAG   
  
  
- GATAGGGAAG TAGTTTTGAA CTACGGAAAA ATTTTCGGGA GGAACCCGAA AGCGGTTTTG AGTACCAACA   
  
  
- TTGGCTCGTT CTTAGTTTGG TGTTACCTTC ACGGGATTAT CTTTCTCACT ACCTCAGTAA CTTGAAGATA   
  
  
- CGTCGTAACA AACTAACGAA CCTTAGGTGA TATAGCTCCT GTAGCTATCT CTCTGTCTTC GAGCTCTTCA   
  
  
- ACTACAAACC TCTCCTCTAG TTCTTGGAGT GTCGAACACT TCCCCGTCTC TCTTTCCGTT CCGTGCTCTT   
  
  
- CGAGTCACTT ACCCAAGTTT CTAAACTCAG TCATCCTAAA TTTTCCCTCG GTAACTCGAT GGTACCAAAG   
  
  
- AACGAACGAT CCTCCAAAAA TGTATGGTTA TTAATACTCC CCATATTGTA GTTCCTTCAG TTACCAAAAG   
  
  
- AACAATAGAC AACCGTTCTA TCTGGGGACA TAAGACAAAG TCGAACCTCT AAAAT

+     Myb

| Site Name | Organism | Position | Strand | Matrix score. | sequence | function |
| --- | --- | --- | --- | --- | --- | --- |
| Myb | Arabidopsis thaliana | 1739 | - | 6 | TAACTG |  |

>HU05G01267.1   
+ +Up\_Stream \_Len000TTACAA AAACGATGCT CTAAACCTAT TAGGTCGTTC CGAAACCCGC AAAATTGAAA   
  
  
+ TATGATCGCC CGTATTTTAC CCAAACCTGA AATACACTTA ATATGTATTT TACCTGAACC CAAATACACC   
  
  
+ GGACCTGTAT TTAACACGGG CACCCGAAAT GATAGGTCTA ACTCTCACCA CCACCGCATT TGGCACCCGA   
  
  
+ ACGGTAAAAA ATGTTCATCT ATTATGCACT TTGGCTTCAT CCATAACCCT ATGATCTATC TGTGCCAATA   
  
  
+ TTTGGAGCAC CCAGCGAAGT AGCGCATGCT CTAAACTCTT GCCTCAAAAC AAAACAAATA GTGGGTCATA   
  
  
+ TTCCCTTCAA AGCACTAACA AACACACACT CTCTCTCTCT CAGTAGCCAA CAACTTAAGG CGGTGTTTGC   
  
  
+ TTGGTTTTGG AGAGGGCGTG TTTGGATGAG GTGCAAAACA CATAGATGAA ATCATAAACA ACTTGATTCC   
  
  
+ CAGCCACCCA TGACCTACTC AGATAACAAG GGACAAAAAC AAAGAGGTTT TCTGAAACAG AGAAAACAGG   
  
  
+ GGCGTTGTGT TGCGCTGTTG CTCACAAAAT TCCAATATTC CTCTAAAAGC TCCCAAACGG AGACAAATCA   
  
  
+ AAGAGGACAA AGACATGTGG GATTTAATAC AAAATTTGAC TAACTCTGCA ACGGAAAACA ACCCCTAGGG   
  
  
+ TGTTTTTCTG CGTGTTGTAC ACACTTCACT TTTAACCTGC TCTGCCTCTG CCTTTGCCAC GGCTTTTAAT   
  
  
+ GGATAAGACC AATTAAGAAA CCCAAATATC TCGTAAATTT GTTGAGATTC CTTTCTGCGT TCTCAAGTGT   
  
  
+ TTCAAAAACC CAGTTTGGAG GTTGCACTTA TTCATGCTTT TCTTCAAGTC AGTGAGCTTA GTGTCTGGAA   
  
  
+ CTGAAGCTCC TCCTGGTAAT GCTCTGTTTT TCATTTCTTG TTGTGCGTTT ATAAAAATTC GGCCTATTGA   
  
  
+ TGGAAGTTTG TTTGATGTTT TTGGGTAAGT TTTTGATTAA TAGAGATGAA TTAGTAAAGT AAAATTTGAG   
  
  
+ TGGTATTTAA TGATCCTTAA GCAGTCTATG CTTTGTTGTC ACATGCGTTT CTTGGCTTTT GACTGAAACA   
  
  
+ TTTCTGTGTT AGATGAAAAA CTCATTTTGA TGCATGAGAT GATAAAATCG CTTATTTTTG CCTTTATTTG   
  
  
+ GTGTGTGGGG GGGGGGGGGG GGGGGCGCAG GTCTGAGGTA AAAGATATGT GAATTCATTT GGAGTCTCGA   
  
  
+ TAATTCGTTG GCTATGTTGA CAATCACGAG TGGTCGTTAT CGCTCATCTC ACAGGTTGAT AATTCACTCA   
  
  
+ AACTTCAAGC GGCCTGAATC ATTCAGTCCG GGTTGTTAAA ATCTCAACAG TCATTTCACA CGAGTGGAGG   
  
  
+ GCCCCAAAAG ACACCATTCT TTCGAACTTT TTTTTTTTGA AGGTCATTTT TTCAAGAACA AAACTAATGT   
  
  
+ GAACATGCGA CTTCTATGAG TAAAGTGTAA AGATGCATAA AGTCTTTTTT TCTTTTATAT AAAAAGTACT   
  
  
+ CAAAAAAAAA AGAGTTGGAG AGCTGATTCC CCATGCATCC TCTAAGCAAA GGTGGTGGAC GGCAGAAAAT   
  
  
+ TAGTAGAGTT ATTGTGAGAT TAAAGTTTGC GCACCTGAAC GGCTGACCTC TAGTTTGCTA TCAACGTTAC   
  
  
+ ATTTATGTGT CATAATTTTT GCATGAAACA ATTTCACGCA ATGTTTTCTG ATGGCAGTTA TGTTTAGAAA   
  
  
+ TAAGTTTAAT CGGACCTTAT TCCACTGATT TATATTTGAT TCTTCCCTCT TTACTACGGC TGTCCATTCA   
  
  
+ CAAGCCAAAG AATTCTCCCC CCAAAAAAAA GAAAAAACAG TATAGAACCA TGTTTATTAT CTCATTTTTT   
  
  
+ TCTCTCCCAT TTGATCTGAA ACACAAACTT GCAGGGGTGC AAGTTGGACA CTTAGTAGCT GCAGGAATGA   
  
  
+ TTCAATAGGA ATCCTCATCT TTGACAACAT CACCACTTGA ATACATATCA CTTGATGGCA CTCTCCCCTG   
  
  
+ GTTTAGGCTT CCCATATCCG TGGCTTAGGG AGCTAAAACC TGAGCAAAGA GGACTCTGTC TAATCCATCT   
  
  
+ CCTCCTTTCA TGTGCTAATC AAGTTGCTAC TGGGAGCATC GACAATGCTA ATGTCAGCCT TGAGCATATT   
  
  
+ TCCCACCTTG CCTCTCCCAC CGGAGATACA ATGCAGCGAA TTGCTGCTTA CTTTGCTGAA GCCTTTGCTG   
  
  
+ ACCGTTTACT AAGGGCTTGG CAGCCCGGTC TTCTCAAAGC CTTGAATTGT ACCAAGATGT CATCTGTTTC   
  
  
+ CGAACAAATT CTTGTTCAAA AGTTGTTCTT TGATCTTCTT CCGTTCTTGA AGCTTTCATA TCTTGTGACG   
  
  
+ AACCAGGCAA TCACGGAGGC CATAGAAGGA GAAAAGATGG TTCATATAAT TGATCTCCAT TCTTGTGAAT   
  
  
+ CGGCTCTGTG GATTAGTCTC CTCCAGGCAT TGAGTGTTCG ACCTGAAGGC CCACCCCATT TGAGGATAAC   
  
  
+ CGGTATACAT GAGAAGAAAG AAGTGTTGGA TCAAATGGCT ATGCAACTAA ACAAAGAGGC TGAAAAATTG   
  
  
+ GACATCCCAT TTCAATTCAA TCCTATCGCA AGCAAACTAG ACGACCTTGA TGTCGAAAGC TTGAGTGTCA   
  
  
+ AGACCGGAGA AGCACTTGTT ATTAGTTCTG TGCTCCAACT ACATTCTCTT TTGGCATTGG ATGAGGGATC   
  
  
+ AATGCCTAAG AACGCAGGCA TGGCTTACTT GCAAAGGGTG TTTTATATGA AACCACGAAA ATTGGAGGAC   
  
  
+ TTGCCCAACA AGGATTTGAT GAAAATGTTG AACTCAAATG AAGATTCTAC ATCATCATCA TCTTCATCTC   
  
  
+ CTATCCCTTC ATCAAAACTT GATGCCTTTT TAAAAGCCCT CCTTGGGCTT TCGCCAAAAC TCATGGTTGT   
  
  
+ AACCGAGCAA GAATCAAACC ACAATGGAAG TGCCCTAATA GAAAGAGTGA TGGAGTCATT GAACTTCTAT   
  
  
+ GCAGCATTGT TTGATTGCTT GGAATCCACT ATATCGAGGA CATCGATAGA GAGACAGAAG CTCGAGAAGT   
  
  
+ TGATGTTTGG AGAGGAGATC AAGAACCTCA CAGCTTGTGA AGGGGCAGAG AGAAAGGCAA GGCACGAGAA   
  
  
+ GCTCAGTGAA TGGGTTCAAA GATTTGAGTC AGTAGGATTT AAAAGGGAGC CATTGAGCTA CCATGGTTTC   
  
  
+ TTGCTTGCTA GGAGGTTTTT ACATACCAAT AATTATGAGG GGTATAACAT CAAGGAAGTC AATGGTTTTC   
  
  
+ TTGTTATCTG TTGGCAAGAT AGACCCCTGT ATTCTGTTTC AGCTTGGAGA TTTTA  

- +Up\_Stream \_Len000AATGTT TTTGCTACGA GATTTGGATA ATCCAGCAAG GCTTTGGGCG TTTTAACTTT   
  
  
- ATACTAGCGG GCATAAAATG GGTTTGGACT TTATGTGAAT TATACATAAA ATGGACTTGG GTTTATGTGG   
  
  
- CCTGGACATA AATTGTGCCC GTGGGCTTTA CTATCCAGAT TGAGAGTGGT GGTGGCGTAA ACCGTGGGCT   
  
  
- TGCCATTTTT TACAAGTAGA TAATACGTGA AACCGAAGTA GGTATTGGGA TACTAGATAG ACACGGTTAT   
  
  
- AAACCTCGTG GGTCGCTTCA TCGCGTACGA GATTTGAGAA CGGAGTTTTG TTTTGTTTAT CACCCAGTAT   
  
  
- AAGGGAAGTT TCGTGATTGT TTGTGTGTGA GAGAGAGAGA GTCATCGGTT GTTGAATTCC GCCACAAACG   
  
  
- AACCAAAACC TCTCCCGCAC AAACCTACTC CACGTTTTGT GTATCTACTT TAGTATTTGT TGAACTAAGG   
  
  
- GTCGGTGGGT ACTGGATGAG TCTATTGTTC CCTGTTTTTG TTTCTCCAAA AGACTTTGTC TCTTTTGTCC   
  
  
- CCGCAACACA ACGCGACAAC GAGTGTTTTA AGGTTATAAG GAGATTTTCG AGGGTTTGCC TCTGTTTAGT   
  
  
- TTCTCCTGTT TCTGTACACC CTAAATTATG TTTTAAACTG ATTGAGACGT TGCCTTTTGT TGGGGATCCC   
  
  
- ACAAAAAGAC GCACAACATG TGTGAAGTGA AAATTGGACG AGACGGAGAC GGAAACGGTG CCGAAAATTA   
  
  
- CCTATTCTGG TTAATTCTTT GGGTTTATAG AGCATTTAAA CAACTCTAAG GAAAGACGCA AGAGTTCACA   
  
  
- AAGTTTTTGG GTCAAACCTC CAACGTGAAT AAGTACGAAA AGAAGTTCAG TCACTCGAAT CACAGACCTT   
  
  
- GACTTCGAGG AGGACCATTA CGAGACAAAA AGTAAAGAAC AACACGCAAA TATTTTTAAG CCGGATAACT   
  
  
- ACCTTCAAAC AAACTACAAA AACCCATTCA AAAACTAATT ATCTCTACTT AATCATTTCA TTTTAAACTC   
  
  
- ACCATAAATT ACTAGGAATT CGTCAGATAC GAAACAACAG TGTACGCAAA GAACCGAAAA CTGACTTTGT   
  
  
- AAAGACACAA TCTACTTTTT GAGTAAAACT ACGTACTCTA CTATTTTAGC GAATAAAAAC GGAAATAAAC   
  
  
- CACACACCCC CCCCCCCCCC CCCCCGCGTC CAGACTCCAT TTTCTATACA CTTAAGTAAA CCTCAGAGCT   
  
  
- ATTAAGCAAC CGATACAACT GTTAGTGCTC ACCAGCAATA GCGAGTAGAG TGTCCAACTA TTAAGTGAGT   
  
  
- TTGAAGTTCG CCGGACTTAG TAAGTCAGGC CCAACAATTT TAGAGTTGTC AGTAAAGTGT GCTCACCTCC   
  
  
- CGGGGTTTTC TGTGGTAAGA AAGCTTGAAA AAAAAAAACT TCCAGTAAAA AAGTTCTTGT TTTGATTACA   
  
  
- CTTGTACGCT GAAGATACTC ATTTCACATT TCTACGTATT TCAGAAAAAA AGAAAATATA TTTTTCATGA   
  
  
- GTTTTTTTTT TCTCAACCTC TCGACTAAGG GGTACGTAGG AGATTCGTTT CCACCACCTG CCGTCTTTTA   
  
  
- ATCATCTCAA TAACACTCTA ATTTCAAACG CGTGGACTTG CCGACTGGAG ATCAAACGAT AGTTGCAATG   
  
  
- TAAATACACA GTATTAAAAA CGTACTTTGT TAAAGTGCGT TACAAAAGAC TACCGTCAAT ACAAATCTTT   
  
  
- ATTCAAATTA GCCTGGAATA AGGTGACTAA ATATAAACTA AGAAGGGAGA AATGATGCCG ACAGGTAAGT   
  
  
- GTTCGGTTTC TTAAGAGGGG GGTTTTTTTT CTTTTTTGTC ATATCTTGGT ACAAATAATA GAGTAAAAAA   
  
  
- AGAGAGGGTA AACTAGACTT TGTGTTTGAA CGTCCCCACG TTCAACCTGT GAATCATCGA CGTCCTTACT   
  
  
- AAGTTATCCT TAGGAGTAGA AACTGTTGTA GTGGTGAACT TATGTATAGT GAACTACCGT GAGAGGGGAC   
  
  
- CAAATCCGAA GGGTATAGGC ACCGAATCCC TCGATTTTGG ACTCGTTTCT CCTGAGACAG ATTAGGTAGA   
  
  
- GGAGGAAAGT ACACGATTAG TTCAACGATG ACCCTCGTAG CTGTTACGAT TACAGTCGGA ACTCGTATAA   
  
  
- AGGGTGGAAC GGAGAGGGTG GCCTCTATGT TACGTCGCTT AACGACGAAT GAAACGACTT CGGAAACGAC   
  
  
- TGGCAAATGA TTCCCGAACC GTCGGGCCAG AAGAGTTTCG GAACTTAACA TGGTTCTACA GTAGACAAAG   
  
  
- GCTTGTTTAA GAACAAGTTT TCAACAAGAA ACTAGAAGAA GGCAAGAACT TCGAAAGTAT AGAACACTGC   
  
  
- TTGGTCCGTT AGTGCCTCCG GTATCTTCCT CTTTTCTACC AAGTATATTA ACTAGAGGTA AGAACACTTA   
  
  
- GCCGAGACAC CTAATCAGAG GAGGTCCGTA ACTCACAAGC TGGACTTCCG GGTGGGGTAA ACTCCTATTG   
  
  
- GCCATATGTA CTCTTCTTTC TTCACAACCT AGTTTACCGA TACGTTGATT TGTTTCTCCG ACTTTTTAAC   
  
  
- CTGTAGGGTA AAGTTAAGTT AGGATAGCGT TCGTTTGATC TGCTGGAACT ACAGCTTTCG AACTCACAGT   
  
  
- TCTGGCCTCT TCGTGAACAA TAATCAAGAC ACGAGGTTGA TGTAAGAGAA AACCGTAACC TACTCCCTAG   
  
  
- TTACGGATTC TTGCGTCCGT ACCGAATGAA CGTTTCCCAC AAAATATACT TTGGTGCTTT TAACCTCCTG   
  
  
- AACGGGTTGT TCCTAAACTA CTTTTACAAC TTGAGTTTAC TTCTAAGATG TAGTAGTAGT AGAAGTAGAG   
  
  
- GATAGGGAAG TAGTTTTGAA CTACGGAAAA ATTTTCGGGA GGAACCCGAA AGCGGTTTTG AGTACCAACA   
  
  
- TTGGCTCGTT CTTAGTTTGG TGTTACCTTC ACGGGATTAT CTTTCTCACT ACCTCAGTAA CTTGAAGATA   
  
  
- CGTCGTAACA AACTAACGAA CCTTAGGTGA TATAGCTCCT GTAGCTATCT CTCTGTCTTC GAGCTCTTCA   
  
  
- ACTACAAACC TCTCCTCTAG TTCTTGGAGT GTCGAACACT TCCCCGTCTC TCTTTCCGTT CCGTGCTCTT   
  
  
- CGAGTCACTT ACCCAAGTTT CTAAACTCAG TCATCCTAAA TTTTCCCTCG GTAACTCGAT GGTACCAAAG   
  
  
- AACGAACGAT CCTCCAAAAA TGTATGGTTA TTAATACTCC CCATATTGTA GTTCCTTCAG TTACCAAAAG   
  
  
- AACAATAGAC AACCGTTCTA TCTGGGGACA TAAGACAAAG TCGAACCTCT AAAAT

+     Myb-binding site

| Site Name | Organism | Position | Strand | Matrix score. | sequence | function |
| --- | --- | --- | --- | --- | --- | --- |
| Myb-binding site | Nicotiana tabacum | 3302 | - | 6 | CAACAG |  |
| Myb-binding site | Nicotiana tabacum | 579 | - | 6 | CAACAG |  |
| Myb-binding site | Nicotiana tabacum | 1379 | + | 6 | CAACAG |  |

>HU05G01267.1   
+ +Up\_Stream \_Len000TTACAA AAACGATGCT CTAAACCTAT TAGGTCGTTC CGAAACCCGC AAAATTGAAA   
  
  
+ TATGATCGCC CGTATTTTAC CCAAACCTGA AATACACTTA ATATGTATTT TACCTGAACC CAAATACACC   
  
  
+ GGACCTGTAT TTAACACGGG CACCCGAAAT GATAGGTCTA ACTCTCACCA CCACCGCATT TGGCACCCGA   
  
  
+ ACGGTAAAAA ATGTTCATCT ATTATGCACT TTGGCTTCAT CCATAACCCT ATGATCTATC TGTGCCAATA   
  
  
+ TTTGGAGCAC CCAGCGAAGT AGCGCATGCT CTAAACTCTT GCCTCAAAAC AAAACAAATA GTGGGTCATA   
  
  
+ TTCCCTTCAA AGCACTAACA AACACACACT CTCTCTCTCT CAGTAGCCAA CAACTTAAGG CGGTGTTTGC   
  
  
+ TTGGTTTTGG AGAGGGCGTG TTTGGATGAG GTGCAAAACA CATAGATGAA ATCATAAACA ACTTGATTCC   
  
  
+ CAGCCACCCA TGACCTACTC AGATAACAAG GGACAAAAAC AAAGAGGTTT TCTGAAACAG AGAAAACAGG   
  
  
+ GGCGTTGTGT TGCGCTGTTG CTCACAAAAT TCCAATATTC CTCTAAAAGC TCCCAAACGG AGACAAATCA   
  
  
+ AAGAGGACAA AGACATGTGG GATTTAATAC AAAATTTGAC TAACTCTGCA ACGGAAAACA ACCCCTAGGG   
  
  
+ TGTTTTTCTG CGTGTTGTAC ACACTTCACT TTTAACCTGC TCTGCCTCTG CCTTTGCCAC GGCTTTTAAT   
  
  
+ GGATAAGACC AATTAAGAAA CCCAAATATC TCGTAAATTT GTTGAGATTC CTTTCTGCGT TCTCAAGTGT   
  
  
+ TTCAAAAACC CAGTTTGGAG GTTGCACTTA TTCATGCTTT TCTTCAAGTC AGTGAGCTTA GTGTCTGGAA   
  
  
+ CTGAAGCTCC TCCTGGTAAT GCTCTGTTTT TCATTTCTTG TTGTGCGTTT ATAAAAATTC GGCCTATTGA   
  
  
+ TGGAAGTTTG TTTGATGTTT TTGGGTAAGT TTTTGATTAA TAGAGATGAA TTAGTAAAGT AAAATTTGAG   
  
  
+ TGGTATTTAA TGATCCTTAA GCAGTCTATG CTTTGTTGTC ACATGCGTTT CTTGGCTTTT GACTGAAACA   
  
  
+ TTTCTGTGTT AGATGAAAAA CTCATTTTGA TGCATGAGAT GATAAAATCG CTTATTTTTG CCTTTATTTG   
  
  
+ GTGTGTGGGG GGGGGGGGGG GGGGGCGCAG GTCTGAGGTA AAAGATATGT GAATTCATTT GGAGTCTCGA   
  
  
+ TAATTCGTTG GCTATGTTGA CAATCACGAG TGGTCGTTAT CGCTCATCTC ACAGGTTGAT AATTCACTCA   
  
  
+ AACTTCAAGC GGCCTGAATC ATTCAGTCCG GGTTGTTAAA ATCTCAACAG TCATTTCACA CGAGTGGAGG   
  
  
+ GCCCCAAAAG ACACCATTCT TTCGAACTTT TTTTTTTTGA AGGTCATTTT TTCAAGAACA AAACTAATGT   
  
  
+ GAACATGCGA CTTCTATGAG TAAAGTGTAA AGATGCATAA AGTCTTTTTT TCTTTTATAT AAAAAGTACT   
  
  
+ CAAAAAAAAA AGAGTTGGAG AGCTGATTCC CCATGCATCC TCTAAGCAAA GGTGGTGGAC GGCAGAAAAT   
  
  
+ TAGTAGAGTT ATTGTGAGAT TAAAGTTTGC GCACCTGAAC GGCTGACCTC TAGTTTGCTA TCAACGTTAC   
  
  
+ ATTTATGTGT CATAATTTTT GCATGAAACA ATTTCACGCA ATGTTTTCTG ATGGCAGTTA TGTTTAGAAA   
  
  
+ TAAGTTTAAT CGGACCTTAT TCCACTGATT TATATTTGAT TCTTCCCTCT TTACTACGGC TGTCCATTCA   
  
  
+ CAAGCCAAAG AATTCTCCCC CCAAAAAAAA GAAAAAACAG TATAGAACCA TGTTTATTAT CTCATTTTTT   
  
  
+ TCTCTCCCAT TTGATCTGAA ACACAAACTT GCAGGGGTGC AAGTTGGACA CTTAGTAGCT GCAGGAATGA   
  
  
+ TTCAATAGGA ATCCTCATCT TTGACAACAT CACCACTTGA ATACATATCA CTTGATGGCA CTCTCCCCTG   
  
  
+ GTTTAGGCTT CCCATATCCG TGGCTTAGGG AGCTAAAACC TGAGCAAAGA GGACTCTGTC TAATCCATCT   
  
  
+ CCTCCTTTCA TGTGCTAATC AAGTTGCTAC TGGGAGCATC GACAATGCTA ATGTCAGCCT TGAGCATATT   
  
  
+ TCCCACCTTG CCTCTCCCAC CGGAGATACA ATGCAGCGAA TTGCTGCTTA CTTTGCTGAA GCCTTTGCTG   
  
  
+ ACCGTTTACT AAGGGCTTGG CAGCCCGGTC TTCTCAAAGC CTTGAATTGT ACCAAGATGT CATCTGTTTC   
  
  
+ CGAACAAATT CTTGTTCAAA AGTTGTTCTT TGATCTTCTT CCGTTCTTGA AGCTTTCATA TCTTGTGACG   
  
  
+ AACCAGGCAA TCACGGAGGC CATAGAAGGA GAAAAGATGG TTCATATAAT TGATCTCCAT TCTTGTGAAT   
  
  
+ CGGCTCTGTG GATTAGTCTC CTCCAGGCAT TGAGTGTTCG ACCTGAAGGC CCACCCCATT TGAGGATAAC   
  
  
+ CGGTATACAT GAGAAGAAAG AAGTGTTGGA TCAAATGGCT ATGCAACTAA ACAAAGAGGC TGAAAAATTG   
  
  
+ GACATCCCAT TTCAATTCAA TCCTATCGCA AGCAAACTAG ACGACCTTGA TGTCGAAAGC TTGAGTGTCA   
  
  
+ AGACCGGAGA AGCACTTGTT ATTAGTTCTG TGCTCCAACT ACATTCTCTT TTGGCATTGG ATGAGGGATC   
  
  
+ AATGCCTAAG AACGCAGGCA TGGCTTACTT GCAAAGGGTG TTTTATATGA AACCACGAAA ATTGGAGGAC   
  
  
+ TTGCCCAACA AGGATTTGAT GAAAATGTTG AACTCAAATG AAGATTCTAC ATCATCATCA TCTTCATCTC   
  
  
+ CTATCCCTTC ATCAAAACTT GATGCCTTTT TAAAAGCCCT CCTTGGGCTT TCGCCAAAAC TCATGGTTGT   
  
  
+ AACCGAGCAA GAATCAAACC ACAATGGAAG TGCCCTAATA GAAAGAGTGA TGGAGTCATT GAACTTCTAT   
  
  
+ GCAGCATTGT TTGATTGCTT GGAATCCACT ATATCGAGGA CATCGATAGA GAGACAGAAG CTCGAGAAGT   
  
  
+ TGATGTTTGG AGAGGAGATC AAGAACCTCA CAGCTTGTGA AGGGGCAGAG AGAAAGGCAA GGCACGAGAA   
  
  
+ GCTCAGTGAA TGGGTTCAAA GATTTGAGTC AGTAGGATTT AAAAGGGAGC CATTGAGCTA CCATGGTTTC   
  
  
+ TTGCTTGCTA GGAGGTTTTT ACATACCAAT AATTATGAGG GGTATAACAT CAAGGAAGTC AATGGTTTTC   
  
  
+ TTGTTATCTG TTGGCAAGAT AGACCCCTGT ATTCTGTTTC AGCTTGGAGA TTTTA  

- +Up\_Stream \_Len000AATGTT TTTGCTACGA GATTTGGATA ATCCAGCAAG GCTTTGGGCG TTTTAACTTT   
  
  
- ATACTAGCGG GCATAAAATG GGTTTGGACT TTATGTGAAT TATACATAAA ATGGACTTGG GTTTATGTGG   
  
  
- CCTGGACATA AATTGTGCCC GTGGGCTTTA CTATCCAGAT TGAGAGTGGT GGTGGCGTAA ACCGTGGGCT   
  
  
- TGCCATTTTT TACAAGTAGA TAATACGTGA AACCGAAGTA GGTATTGGGA TACTAGATAG ACACGGTTAT   
  
  
- AAACCTCGTG GGTCGCTTCA TCGCGTACGA GATTTGAGAA CGGAGTTTTG TTTTGTTTAT CACCCAGTAT   
  
  
- AAGGGAAGTT TCGTGATTGT TTGTGTGTGA GAGAGAGAGA GTCATCGGTT GTTGAATTCC GCCACAAACG   
  
  
- AACCAAAACC TCTCCCGCAC AAACCTACTC CACGTTTTGT GTATCTACTT TAGTATTTGT TGAACTAAGG   
  
  
- GTCGGTGGGT ACTGGATGAG TCTATTGTTC CCTGTTTTTG TTTCTCCAAA AGACTTTGTC TCTTTTGTCC   
  
  
- CCGCAACACA ACGCGACAAC GAGTGTTTTA AGGTTATAAG GAGATTTTCG AGGGTTTGCC TCTGTTTAGT   
  
  
- TTCTCCTGTT TCTGTACACC CTAAATTATG TTTTAAACTG ATTGAGACGT TGCCTTTTGT TGGGGATCCC   
  
  
- ACAAAAAGAC GCACAACATG TGTGAAGTGA AAATTGGACG AGACGGAGAC GGAAACGGTG CCGAAAATTA   
  
  
- CCTATTCTGG TTAATTCTTT GGGTTTATAG AGCATTTAAA CAACTCTAAG GAAAGACGCA AGAGTTCACA   
  
  
- AAGTTTTTGG GTCAAACCTC CAACGTGAAT AAGTACGAAA AGAAGTTCAG TCACTCGAAT CACAGACCTT   
  
  
- GACTTCGAGG AGGACCATTA CGAGACAAAA AGTAAAGAAC AACACGCAAA TATTTTTAAG CCGGATAACT   
  
  
- ACCTTCAAAC AAACTACAAA AACCCATTCA AAAACTAATT ATCTCTACTT AATCATTTCA TTTTAAACTC   
  
  
- ACCATAAATT ACTAGGAATT CGTCAGATAC GAAACAACAG TGTACGCAAA GAACCGAAAA CTGACTTTGT   
  
  
- AAAGACACAA TCTACTTTTT GAGTAAAACT ACGTACTCTA CTATTTTAGC GAATAAAAAC GGAAATAAAC   
  
  
- CACACACCCC CCCCCCCCCC CCCCCGCGTC CAGACTCCAT TTTCTATACA CTTAAGTAAA CCTCAGAGCT   
  
  
- ATTAAGCAAC CGATACAACT GTTAGTGCTC ACCAGCAATA GCGAGTAGAG TGTCCAACTA TTAAGTGAGT   
  
  
- TTGAAGTTCG CCGGACTTAG TAAGTCAGGC CCAACAATTT TAGAGTTGTC AGTAAAGTGT GCTCACCTCC   
  
  
- CGGGGTTTTC TGTGGTAAGA AAGCTTGAAA AAAAAAAACT TCCAGTAAAA AAGTTCTTGT TTTGATTACA   
  
  
- CTTGTACGCT GAAGATACTC ATTTCACATT TCTACGTATT TCAGAAAAAA AGAAAATATA TTTTTCATGA   
  
  
- GTTTTTTTTT TCTCAACCTC TCGACTAAGG GGTACGTAGG AGATTCGTTT CCACCACCTG CCGTCTTTTA   
  
  
- ATCATCTCAA TAACACTCTA ATTTCAAACG CGTGGACTTG CCGACTGGAG ATCAAACGAT AGTTGCAATG   
  
  
- TAAATACACA GTATTAAAAA CGTACTTTGT TAAAGTGCGT TACAAAAGAC TACCGTCAAT ACAAATCTTT   
  
  
- ATTCAAATTA GCCTGGAATA AGGTGACTAA ATATAAACTA AGAAGGGAGA AATGATGCCG ACAGGTAAGT   
  
  
- GTTCGGTTTC TTAAGAGGGG GGTTTTTTTT CTTTTTTGTC ATATCTTGGT ACAAATAATA GAGTAAAAAA   
  
  
- AGAGAGGGTA AACTAGACTT TGTGTTTGAA CGTCCCCACG TTCAACCTGT GAATCATCGA CGTCCTTACT   
  
  
- AAGTTATCCT TAGGAGTAGA AACTGTTGTA GTGGTGAACT TATGTATAGT GAACTACCGT GAGAGGGGAC   
  
  
- CAAATCCGAA GGGTATAGGC ACCGAATCCC TCGATTTTGG ACTCGTTTCT CCTGAGACAG ATTAGGTAGA   
  
  
- GGAGGAAAGT ACACGATTAG TTCAACGATG ACCCTCGTAG CTGTTACGAT TACAGTCGGA ACTCGTATAA   
  
  
- AGGGTGGAAC GGAGAGGGTG GCCTCTATGT TACGTCGCTT AACGACGAAT GAAACGACTT CGGAAACGAC   
  
  
- TGGCAAATGA TTCCCGAACC GTCGGGCCAG AAGAGTTTCG GAACTTAACA TGGTTCTACA GTAGACAAAG   
  
  
- GCTTGTTTAA GAACAAGTTT TCAACAAGAA ACTAGAAGAA GGCAAGAACT TCGAAAGTAT AGAACACTGC   
  
  
- TTGGTCCGTT AGTGCCTCCG GTATCTTCCT CTTTTCTACC AAGTATATTA ACTAGAGGTA AGAACACTTA   
  
  
- GCCGAGACAC CTAATCAGAG GAGGTCCGTA ACTCACAAGC TGGACTTCCG GGTGGGGTAA ACTCCTATTG   
  
  
- GCCATATGTA CTCTTCTTTC TTCACAACCT AGTTTACCGA TACGTTGATT TGTTTCTCCG ACTTTTTAAC   
  
  
- CTGTAGGGTA AAGTTAAGTT AGGATAGCGT TCGTTTGATC TGCTGGAACT ACAGCTTTCG AACTCACAGT   
  
  
- TCTGGCCTCT TCGTGAACAA TAATCAAGAC ACGAGGTTGA TGTAAGAGAA AACCGTAACC TACTCCCTAG   
  
  
- TTACGGATTC TTGCGTCCGT ACCGAATGAA CGTTTCCCAC AAAATATACT TTGGTGCTTT TAACCTCCTG   
  
  
- AACGGGTTGT TCCTAAACTA CTTTTACAAC TTGAGTTTAC TTCTAAGATG TAGTAGTAGT AGAAGTAGAG   
  
  
- GATAGGGAAG TAGTTTTGAA CTACGGAAAA ATTTTCGGGA GGAACCCGAA AGCGGTTTTG AGTACCAACA   
  
  
- TTGGCTCGTT CTTAGTTTGG TGTTACCTTC ACGGGATTAT CTTTCTCACT ACCTCAGTAA CTTGAAGATA   
  
  
- CGTCGTAACA AACTAACGAA CCTTAGGTGA TATAGCTCCT GTAGCTATCT CTCTGTCTTC GAGCTCTTCA   
  
  
- ACTACAAACC TCTCCTCTAG TTCTTGGAGT GTCGAACACT TCCCCGTCTC TCTTTCCGTT CCGTGCTCTT   
  
  
- CGAGTCACTT ACCCAAGTTT CTAAACTCAG TCATCCTAAA TTTTCCCTCG GTAACTCGAT GGTACCAAAG   
  
  
- AACGAACGAT CCTCCAAAAA TGTATGGTTA TTAATACTCC CCATATTGTA GTTCCTTCAG TTACCAAAAG   
  
  
- AACAATAGAC AACCGTTCTA TCTGGGGACA TAAGACAAAG TCGAACCTCT AAAAT

+     O2-site

| Site Name | Organism | Position | Strand | Matrix score. | sequence | function |
| --- | --- | --- | --- | --- | --- | --- |
| O2-site | Zea mays | 2851 | - | 9 | GATGATGTGG | cis-acting regulatory element involved in zein metabolism regulation |

>HU05G01267.1   
+ +Up\_Stream \_Len000TTACAA AAACGATGCT CTAAACCTAT TAGGTCGTTC CGAAACCCGC AAAATTGAAA   
  
  
+ TATGATCGCC CGTATTTTAC CCAAACCTGA AATACACTTA ATATGTATTT TACCTGAACC CAAATACACC   
  
  
+ GGACCTGTAT TTAACACGGG CACCCGAAAT GATAGGTCTA ACTCTCACCA CCACCGCATT TGGCACCCGA   
  
  
+ ACGGTAAAAA ATGTTCATCT ATTATGCACT TTGGCTTCAT CCATAACCCT ATGATCTATC TGTGCCAATA   
  
  
+ TTTGGAGCAC CCAGCGAAGT AGCGCATGCT CTAAACTCTT GCCTCAAAAC AAAACAAATA GTGGGTCATA   
  
  
+ TTCCCTTCAA AGCACTAACA AACACACACT CTCTCTCTCT CAGTAGCCAA CAACTTAAGG CGGTGTTTGC   
  
  
+ TTGGTTTTGG AGAGGGCGTG TTTGGATGAG GTGCAAAACA CATAGATGAA ATCATAAACA ACTTGATTCC   
  
  
+ CAGCCACCCA TGACCTACTC AGATAACAAG GGACAAAAAC AAAGAGGTTT TCTGAAACAG AGAAAACAGG   
  
  
+ GGCGTTGTGT TGCGCTGTTG CTCACAAAAT TCCAATATTC CTCTAAAAGC TCCCAAACGG AGACAAATCA   
  
  
+ AAGAGGACAA AGACATGTGG GATTTAATAC AAAATTTGAC TAACTCTGCA ACGGAAAACA ACCCCTAGGG   
  
  
+ TGTTTTTCTG CGTGTTGTAC ACACTTCACT TTTAACCTGC TCTGCCTCTG CCTTTGCCAC GGCTTTTAAT   
  
  
+ GGATAAGACC AATTAAGAAA CCCAAATATC TCGTAAATTT GTTGAGATTC CTTTCTGCGT TCTCAAGTGT   
  
  
+ TTCAAAAACC CAGTTTGGAG GTTGCACTTA TTCATGCTTT TCTTCAAGTC AGTGAGCTTA GTGTCTGGAA   
  
  
+ CTGAAGCTCC TCCTGGTAAT GCTCTGTTTT TCATTTCTTG TTGTGCGTTT ATAAAAATTC GGCCTATTGA   
  
  
+ TGGAAGTTTG TTTGATGTTT TTGGGTAAGT TTTTGATTAA TAGAGATGAA TTAGTAAAGT AAAATTTGAG   
  
  
+ TGGTATTTAA TGATCCTTAA GCAGTCTATG CTTTGTTGTC ACATGCGTTT CTTGGCTTTT GACTGAAACA   
  
  
+ TTTCTGTGTT AGATGAAAAA CTCATTTTGA TGCATGAGAT GATAAAATCG CTTATTTTTG CCTTTATTTG   
  
  
+ GTGTGTGGGG GGGGGGGGGG GGGGGCGCAG GTCTGAGGTA AAAGATATGT GAATTCATTT GGAGTCTCGA   
  
  
+ TAATTCGTTG GCTATGTTGA CAATCACGAG TGGTCGTTAT CGCTCATCTC ACAGGTTGAT AATTCACTCA   
  
  
+ AACTTCAAGC GGCCTGAATC ATTCAGTCCG GGTTGTTAAA ATCTCAACAG TCATTTCACA CGAGTGGAGG   
  
  
+ GCCCCAAAAG ACACCATTCT TTCGAACTTT TTTTTTTTGA AGGTCATTTT TTCAAGAACA AAACTAATGT   
  
  
+ GAACATGCGA CTTCTATGAG TAAAGTGTAA AGATGCATAA AGTCTTTTTT TCTTTTATAT AAAAAGTACT   
  
  
+ CAAAAAAAAA AGAGTTGGAG AGCTGATTCC CCATGCATCC TCTAAGCAAA GGTGGTGGAC GGCAGAAAAT   
  
  
+ TAGTAGAGTT ATTGTGAGAT TAAAGTTTGC GCACCTGAAC GGCTGACCTC TAGTTTGCTA TCAACGTTAC   
  
  
+ ATTTATGTGT CATAATTTTT GCATGAAACA ATTTCACGCA ATGTTTTCTG ATGGCAGTTA TGTTTAGAAA   
  
  
+ TAAGTTTAAT CGGACCTTAT TCCACTGATT TATATTTGAT TCTTCCCTCT TTACTACGGC TGTCCATTCA   
  
  
+ CAAGCCAAAG AATTCTCCCC CCAAAAAAAA GAAAAAACAG TATAGAACCA TGTTTATTAT CTCATTTTTT   
  
  
+ TCTCTCCCAT TTGATCTGAA ACACAAACTT GCAGGGGTGC AAGTTGGACA CTTAGTAGCT GCAGGAATGA   
  
  
+ TTCAATAGGA ATCCTCATCT TTGACAACAT CACCACTTGA ATACATATCA CTTGATGGCA CTCTCCCCTG   
  
  
+ GTTTAGGCTT CCCATATCCG TGGCTTAGGG AGCTAAAACC TGAGCAAAGA GGACTCTGTC TAATCCATCT   
  
  
+ CCTCCTTTCA TGTGCTAATC AAGTTGCTAC TGGGAGCATC GACAATGCTA ATGTCAGCCT TGAGCATATT   
  
  
+ TCCCACCTTG CCTCTCCCAC CGGAGATACA ATGCAGCGAA TTGCTGCTTA CTTTGCTGAA GCCTTTGCTG   
  
  
+ ACCGTTTACT AAGGGCTTGG CAGCCCGGTC TTCTCAAAGC CTTGAATTGT ACCAAGATGT CATCTGTTTC   
  
  
+ CGAACAAATT CTTGTTCAAA AGTTGTTCTT TGATCTTCTT CCGTTCTTGA AGCTTTCATA TCTTGTGACG   
  
  
+ AACCAGGCAA TCACGGAGGC CATAGAAGGA GAAAAGATGG TTCATATAAT TGATCTCCAT TCTTGTGAAT   
  
  
+ CGGCTCTGTG GATTAGTCTC CTCCAGGCAT TGAGTGTTCG ACCTGAAGGC CCACCCCATT TGAGGATAAC   
  
  
+ CGGTATACAT GAGAAGAAAG AAGTGTTGGA TCAAATGGCT ATGCAACTAA ACAAAGAGGC TGAAAAATTG   
  
  
+ GACATCCCAT TTCAATTCAA TCCTATCGCA AGCAAACTAG ACGACCTTGA TGTCGAAAGC TTGAGTGTCA   
  
  
+ AGACCGGAGA AGCACTTGTT ATTAGTTCTG TGCTCCAACT ACATTCTCTT TTGGCATTGG ATGAGGGATC   
  
  
+ AATGCCTAAG AACGCAGGCA TGGCTTACTT GCAAAGGGTG TTTTATATGA AACCACGAAA ATTGGAGGAC   
  
  
+ TTGCCCAACA AGGATTTGAT GAAAATGTTG AACTCAAATG AAGATTCTAC ATCATCATCA TCTTCATCTC   
  
  
+ CTATCCCTTC ATCAAAACTT GATGCCTTTT TAAAAGCCCT CCTTGGGCTT TCGCCAAAAC TCATGGTTGT   
  
  
+ AACCGAGCAA GAATCAAACC ACAATGGAAG TGCCCTAATA GAAAGAGTGA TGGAGTCATT GAACTTCTAT   
  
  
+ GCAGCATTGT TTGATTGCTT GGAATCCACT ATATCGAGGA CATCGATAGA GAGACAGAAG CTCGAGAAGT   
  
  
+ TGATGTTTGG AGAGGAGATC AAGAACCTCA CAGCTTGTGA AGGGGCAGAG AGAAAGGCAA GGCACGAGAA   
  
  
+ GCTCAGTGAA TGGGTTCAAA GATTTGAGTC AGTAGGATTT AAAAGGGAGC CATTGAGCTA CCATGGTTTC   
  
  
+ TTGCTTGCTA GGAGGTTTTT ACATACCAAT AATTATGAGG GGTATAACAT CAAGGAAGTC AATGGTTTTC   
  
  
+ TTGTTATCTG TTGGCAAGAT AGACCCCTGT ATTCTGTTTC AGCTTGGAGA TTTTA  

- +Up\_Stream \_Len000AATGTT TTTGCTACGA GATTTGGATA ATCCAGCAAG GCTTTGGGCG TTTTAACTTT   
  
  
- ATACTAGCGG GCATAAAATG GGTTTGGACT TTATGTGAAT TATACATAAA ATGGACTTGG GTTTATGTGG   
  
  
- CCTGGACATA AATTGTGCCC GTGGGCTTTA CTATCCAGAT TGAGAGTGGT GGTGGCGTAA ACCGTGGGCT   
  
  
- TGCCATTTTT TACAAGTAGA TAATACGTGA AACCGAAGTA GGTATTGGGA TACTAGATAG ACACGGTTAT   
  
  
- AAACCTCGTG GGTCGCTTCA TCGCGTACGA GATTTGAGAA CGGAGTTTTG TTTTGTTTAT CACCCAGTAT   
  
  
- AAGGGAAGTT TCGTGATTGT TTGTGTGTGA GAGAGAGAGA GTCATCGGTT GTTGAATTCC GCCACAAACG   
  
  
- AACCAAAACC TCTCCCGCAC AAACCTACTC CACGTTTTGT GTATCTACTT TAGTATTTGT TGAACTAAGG   
  
  
- GTCGGTGGGT ACTGGATGAG TCTATTGTTC CCTGTTTTTG TTTCTCCAAA AGACTTTGTC TCTTTTGTCC   
  
  
- CCGCAACACA ACGCGACAAC GAGTGTTTTA AGGTTATAAG GAGATTTTCG AGGGTTTGCC TCTGTTTAGT   
  
  
- TTCTCCTGTT TCTGTACACC CTAAATTATG TTTTAAACTG ATTGAGACGT TGCCTTTTGT TGGGGATCCC   
  
  
- ACAAAAAGAC GCACAACATG TGTGAAGTGA AAATTGGACG AGACGGAGAC GGAAACGGTG CCGAAAATTA   
  
  
- CCTATTCTGG TTAATTCTTT GGGTTTATAG AGCATTTAAA CAACTCTAAG GAAAGACGCA AGAGTTCACA   
  
  
- AAGTTTTTGG GTCAAACCTC CAACGTGAAT AAGTACGAAA AGAAGTTCAG TCACTCGAAT CACAGACCTT   
  
  
- GACTTCGAGG AGGACCATTA CGAGACAAAA AGTAAAGAAC AACACGCAAA TATTTTTAAG CCGGATAACT   
  
  
- ACCTTCAAAC AAACTACAAA AACCCATTCA AAAACTAATT ATCTCTACTT AATCATTTCA TTTTAAACTC   
  
  
- ACCATAAATT ACTAGGAATT CGTCAGATAC GAAACAACAG TGTACGCAAA GAACCGAAAA CTGACTTTGT   
  
  
- AAAGACACAA TCTACTTTTT GAGTAAAACT ACGTACTCTA CTATTTTAGC GAATAAAAAC GGAAATAAAC   
  
  
- CACACACCCC CCCCCCCCCC CCCCCGCGTC CAGACTCCAT TTTCTATACA CTTAAGTAAA CCTCAGAGCT   
  
  
- ATTAAGCAAC CGATACAACT GTTAGTGCTC ACCAGCAATA GCGAGTAGAG TGTCCAACTA TTAAGTGAGT   
  
  
- TTGAAGTTCG CCGGACTTAG TAAGTCAGGC CCAACAATTT TAGAGTTGTC AGTAAAGTGT GCTCACCTCC   
  
  
- CGGGGTTTTC TGTGGTAAGA AAGCTTGAAA AAAAAAAACT TCCAGTAAAA AAGTTCTTGT TTTGATTACA   
  
  
- CTTGTACGCT GAAGATACTC ATTTCACATT TCTACGTATT TCAGAAAAAA AGAAAATATA TTTTTCATGA   
  
  
- GTTTTTTTTT TCTCAACCTC TCGACTAAGG GGTACGTAGG AGATTCGTTT CCACCACCTG CCGTCTTTTA   
  
  
- ATCATCTCAA TAACACTCTA ATTTCAAACG CGTGGACTTG CCGACTGGAG ATCAAACGAT AGTTGCAATG   
  
  
- TAAATACACA GTATTAAAAA CGTACTTTGT TAAAGTGCGT TACAAAAGAC TACCGTCAAT ACAAATCTTT   
  
  
- ATTCAAATTA GCCTGGAATA AGGTGACTAA ATATAAACTA AGAAGGGAGA AATGATGCCG ACAGGTAAGT   
  
  
- GTTCGGTTTC TTAAGAGGGG GGTTTTTTTT CTTTTTTGTC ATATCTTGGT ACAAATAATA GAGTAAAAAA   
  
  
- AGAGAGGGTA AACTAGACTT TGTGTTTGAA CGTCCCCACG TTCAACCTGT GAATCATCGA CGTCCTTACT   
  
  
- AAGTTATCCT TAGGAGTAGA AACTGTTGTA GTGGTGAACT TATGTATAGT GAACTACCGT GAGAGGGGAC   
  
  
- CAAATCCGAA GGGTATAGGC ACCGAATCCC TCGATTTTGG ACTCGTTTCT CCTGAGACAG ATTAGGTAGA   
  
  
- GGAGGAAAGT ACACGATTAG TTCAACGATG ACCCTCGTAG CTGTTACGAT TACAGTCGGA ACTCGTATAA   
  
  
- AGGGTGGAAC GGAGAGGGTG GCCTCTATGT TACGTCGCTT AACGACGAAT GAAACGACTT CGGAAACGAC   
  
  
- TGGCAAATGA TTCCCGAACC GTCGGGCCAG AAGAGTTTCG GAACTTAACA TGGTTCTACA GTAGACAAAG   
  
  
- GCTTGTTTAA GAACAAGTTT TCAACAAGAA ACTAGAAGAA GGCAAGAACT TCGAAAGTAT AGAACACTGC   
  
  
- TTGGTCCGTT AGTGCCTCCG GTATCTTCCT CTTTTCTACC AAGTATATTA ACTAGAGGTA AGAACACTTA   
  
  
- GCCGAGACAC CTAATCAGAG GAGGTCCGTA ACTCACAAGC TGGACTTCCG GGTGGGGTAA ACTCCTATTG   
  
  
- GCCATATGTA CTCTTCTTTC TTCACAACCT AGTTTACCGA TACGTTGATT TGTTTCTCCG ACTTTTTAAC   
  
  
- CTGTAGGGTA AAGTTAAGTT AGGATAGCGT TCGTTTGATC TGCTGGAACT ACAGCTTTCG AACTCACAGT   
  
  
- TCTGGCCTCT TCGTGAACAA TAATCAAGAC ACGAGGTTGA TGTAAGAGAA AACCGTAACC TACTCCCTAG   
  
  
- TTACGGATTC TTGCGTCCGT ACCGAATGAA CGTTTCCCAC AAAATATACT TTGGTGCTTT TAACCTCCTG   
  
  
- AACGGGTTGT TCCTAAACTA CTTTTACAAC TTGAGTTTAC TTCTAAGATG TAGTAGTAGT AGAAGTAGAG   
  
  
- GATAGGGAAG TAGTTTTGAA CTACGGAAAA ATTTTCGGGA GGAACCCGAA AGCGGTTTTG AGTACCAACA   
  
  
- TTGGCTCGTT CTTAGTTTGG TGTTACCTTC ACGGGATTAT CTTTCTCACT ACCTCAGTAA CTTGAAGATA   
  
  
- CGTCGTAACA AACTAACGAA CCTTAGGTGA TATAGCTCCT GTAGCTATCT CTCTGTCTTC GAGCTCTTCA   
  
  
- ACTACAAACC TCTCCTCTAG TTCTTGGAGT GTCGAACACT TCCCCGTCTC TCTTTCCGTT CCGTGCTCTT   
  
  
- CGAGTCACTT ACCCAAGTTT CTAAACTCAG TCATCCTAAA TTTTCCCTCG GTAACTCGAT GGTACCAAAG   
  
  
- AACGAACGAT CCTCCAAAAA TGTATGGTTA TTAATACTCC CCATATTGTA GTTCCTTCAG TTACCAAAAG   
  
  
- AACAATAGAC AACCGTTCTA TCTGGGGACA TAAGACAAAG TCGAACCTCT AAAAT

+     STRE

| Site Name | Organism | Position | Strand | Matrix score. | sequence | function |
| --- | --- | --- | --- | --- | --- | --- |
| STRE | Arabidopsis thaliana | 3125 | + | 5 | AGGGG |  |
| STRE | Arabidopsis thaliana | 562 | + | 5 | AGGGG |  |
| STRE | Arabidopsis thaliana | 696 | - | 5 | AGGGG |  |
| STRE | Arabidopsis thaliana | 3318 | - | 5 | AGGGG |  |
| STRE | Arabidopsis thaliana | 2029 | - | 5 | AGGGG |  |
| STRE | Arabidopsis thaliana | 1927 | + | 5 | AGGGG |  |
| STRE | Arabidopsis thaliana | 3262 | + | 5 | AGGGG |  |

>HU05G01267.1   
+ +Up\_Stream \_Len000TTACAA AAACGATGCT CTAAACCTAT TAGGTCGTTC CGAAACCCGC AAAATTGAAA   
  
  
+ TATGATCGCC CGTATTTTAC CCAAACCTGA AATACACTTA ATATGTATTT TACCTGAACC CAAATACACC   
  
  
+ GGACCTGTAT TTAACACGGG CACCCGAAAT GATAGGTCTA ACTCTCACCA CCACCGCATT TGGCACCCGA   
  
  
+ ACGGTAAAAA ATGTTCATCT ATTATGCACT TTGGCTTCAT CCATAACCCT ATGATCTATC TGTGCCAATA   
  
  
+ TTTGGAGCAC CCAGCGAAGT AGCGCATGCT CTAAACTCTT GCCTCAAAAC AAAACAAATA GTGGGTCATA   
  
  
+ TTCCCTTCAA AGCACTAACA AACACACACT CTCTCTCTCT CAGTAGCCAA CAACTTAAGG CGGTGTTTGC   
  
  
+ TTGGTTTTGG AGAGGGCGTG TTTGGATGAG GTGCAAAACA CATAGATGAA ATCATAAACA ACTTGATTCC   
  
  
+ CAGCCACCCA TGACCTACTC AGATAACAAG GGACAAAAAC AAAGAGGTTT TCTGAAACAG AGAAAACAGG   
  
  
+ GGCGTTGTGT TGCGCTGTTG CTCACAAAAT TCCAATATTC CTCTAAAAGC TCCCAAACGG AGACAAATCA   
  
  
+ AAGAGGACAA AGACATGTGG GATTTAATAC AAAATTTGAC TAACTCTGCA ACGGAAAACA ACCCCTAGGG   
  
  
+ TGTTTTTCTG CGTGTTGTAC ACACTTCACT TTTAACCTGC TCTGCCTCTG CCTTTGCCAC GGCTTTTAAT   
  
  
+ GGATAAGACC AATTAAGAAA CCCAAATATC TCGTAAATTT GTTGAGATTC CTTTCTGCGT TCTCAAGTGT   
  
  
+ TTCAAAAACC CAGTTTGGAG GTTGCACTTA TTCATGCTTT TCTTCAAGTC AGTGAGCTTA GTGTCTGGAA   
  
  
+ CTGAAGCTCC TCCTGGTAAT GCTCTGTTTT TCATTTCTTG TTGTGCGTTT ATAAAAATTC GGCCTATTGA   
  
  
+ TGGAAGTTTG TTTGATGTTT TTGGGTAAGT TTTTGATTAA TAGAGATGAA TTAGTAAAGT AAAATTTGAG   
  
  
+ TGGTATTTAA TGATCCTTAA GCAGTCTATG CTTTGTTGTC ACATGCGTTT CTTGGCTTTT GACTGAAACA   
  
  
+ TTTCTGTGTT AGATGAAAAA CTCATTTTGA TGCATGAGAT GATAAAATCG CTTATTTTTG CCTTTATTTG   
  
  
+ GTGTGTGGGG GGGGGGGGGG GGGGGCGCAG GTCTGAGGTA AAAGATATGT GAATTCATTT GGAGTCTCGA   
  
  
+ TAATTCGTTG GCTATGTTGA CAATCACGAG TGGTCGTTAT CGCTCATCTC ACAGGTTGAT AATTCACTCA   
  
  
+ AACTTCAAGC GGCCTGAATC ATTCAGTCCG GGTTGTTAAA ATCTCAACAG TCATTTCACA CGAGTGGAGG   
  
  
+ GCCCCAAAAG ACACCATTCT TTCGAACTTT TTTTTTTTGA AGGTCATTTT TTCAAGAACA AAACTAATGT   
  
  
+ GAACATGCGA CTTCTATGAG TAAAGTGTAA AGATGCATAA AGTCTTTTTT TCTTTTATAT AAAAAGTACT   
  
  
+ CAAAAAAAAA AGAGTTGGAG AGCTGATTCC CCATGCATCC TCTAAGCAAA GGTGGTGGAC GGCAGAAAAT   
  
  
+ TAGTAGAGTT ATTGTGAGAT TAAAGTTTGC GCACCTGAAC GGCTGACCTC TAGTTTGCTA TCAACGTTAC   
  
  
+ ATTTATGTGT CATAATTTTT GCATGAAACA ATTTCACGCA ATGTTTTCTG ATGGCAGTTA TGTTTAGAAA   
  
  
+ TAAGTTTAAT CGGACCTTAT TCCACTGATT TATATTTGAT TCTTCCCTCT TTACTACGGC TGTCCATTCA   
  
  
+ CAAGCCAAAG AATTCTCCCC CCAAAAAAAA GAAAAAACAG TATAGAACCA TGTTTATTAT CTCATTTTTT   
  
  
+ TCTCTCCCAT TTGATCTGAA ACACAAACTT GCAGGGGTGC AAGTTGGACA CTTAGTAGCT GCAGGAATGA   
  
  
+ TTCAATAGGA ATCCTCATCT TTGACAACAT CACCACTTGA ATACATATCA CTTGATGGCA CTCTCCCCTG   
  
  
+ GTTTAGGCTT CCCATATCCG TGGCTTAGGG AGCTAAAACC TGAGCAAAGA GGACTCTGTC TAATCCATCT   
  
  
+ CCTCCTTTCA TGTGCTAATC AAGTTGCTAC TGGGAGCATC GACAATGCTA ATGTCAGCCT TGAGCATATT   
  
  
+ TCCCACCTTG CCTCTCCCAC CGGAGATACA ATGCAGCGAA TTGCTGCTTA CTTTGCTGAA GCCTTTGCTG   
  
  
+ ACCGTTTACT AAGGGCTTGG CAGCCCGGTC TTCTCAAAGC CTTGAATTGT ACCAAGATGT CATCTGTTTC   
  
  
+ CGAACAAATT CTTGTTCAAA AGTTGTTCTT TGATCTTCTT CCGTTCTTGA AGCTTTCATA TCTTGTGACG   
  
  
+ AACCAGGCAA TCACGGAGGC CATAGAAGGA GAAAAGATGG TTCATATAAT TGATCTCCAT TCTTGTGAAT   
  
  
+ CGGCTCTGTG GATTAGTCTC CTCCAGGCAT TGAGTGTTCG ACCTGAAGGC CCACCCCATT TGAGGATAAC   
  
  
+ CGGTATACAT GAGAAGAAAG AAGTGTTGGA TCAAATGGCT ATGCAACTAA ACAAAGAGGC TGAAAAATTG   
  
  
+ GACATCCCAT TTCAATTCAA TCCTATCGCA AGCAAACTAG ACGACCTTGA TGTCGAAAGC TTGAGTGTCA   
  
  
+ AGACCGGAGA AGCACTTGTT ATTAGTTCTG TGCTCCAACT ACATTCTCTT TTGGCATTGG ATGAGGGATC   
  
  
+ AATGCCTAAG AACGCAGGCA TGGCTTACTT GCAAAGGGTG TTTTATATGA AACCACGAAA ATTGGAGGAC   
  
  
+ TTGCCCAACA AGGATTTGAT GAAAATGTTG AACTCAAATG AAGATTCTAC ATCATCATCA TCTTCATCTC   
  
  
+ CTATCCCTTC ATCAAAACTT GATGCCTTTT TAAAAGCCCT CCTTGGGCTT TCGCCAAAAC TCATGGTTGT   
  
  
+ AACCGAGCAA GAATCAAACC ACAATGGAAG TGCCCTAATA GAAAGAGTGA TGGAGTCATT GAACTTCTAT   
  
  
+ GCAGCATTGT TTGATTGCTT GGAATCCACT ATATCGAGGA CATCGATAGA GAGACAGAAG CTCGAGAAGT   
  
  
+ TGATGTTTGG AGAGGAGATC AAGAACCTCA CAGCTTGTGA AGGGGCAGAG AGAAAGGCAA GGCACGAGAA   
  
  
+ GCTCAGTGAA TGGGTTCAAA GATTTGAGTC AGTAGGATTT AAAAGGGAGC CATTGAGCTA CCATGGTTTC   
  
  
+ TTGCTTGCTA GGAGGTTTTT ACATACCAAT AATTATGAGG GGTATAACAT CAAGGAAGTC AATGGTTTTC   
  
  
+ TTGTTATCTG TTGGCAAGAT AGACCCCTGT ATTCTGTTTC AGCTTGGAGA TTTTA  

- +Up\_Stream \_Len000AATGTT TTTGCTACGA GATTTGGATA ATCCAGCAAG GCTTTGGGCG TTTTAACTTT   
  
  
- ATACTAGCGG GCATAAAATG GGTTTGGACT TTATGTGAAT TATACATAAA ATGGACTTGG GTTTATGTGG   
  
  
- CCTGGACATA AATTGTGCCC GTGGGCTTTA CTATCCAGAT TGAGAGTGGT GGTGGCGTAA ACCGTGGGCT   
  
  
- TGCCATTTTT TACAAGTAGA TAATACGTGA AACCGAAGTA GGTATTGGGA TACTAGATAG ACACGGTTAT   
  
  
- AAACCTCGTG GGTCGCTTCA TCGCGTACGA GATTTGAGAA CGGAGTTTTG TTTTGTTTAT CACCCAGTAT   
  
  
- AAGGGAAGTT TCGTGATTGT TTGTGTGTGA GAGAGAGAGA GTCATCGGTT GTTGAATTCC GCCACAAACG   
  
  
- AACCAAAACC TCTCCCGCAC AAACCTACTC CACGTTTTGT GTATCTACTT TAGTATTTGT TGAACTAAGG   
  
  
- GTCGGTGGGT ACTGGATGAG TCTATTGTTC CCTGTTTTTG TTTCTCCAAA AGACTTTGTC TCTTTTGTCC   
  
  
- CCGCAACACA ACGCGACAAC GAGTGTTTTA AGGTTATAAG GAGATTTTCG AGGGTTTGCC TCTGTTTAGT   
  
  
- TTCTCCTGTT TCTGTACACC CTAAATTATG TTTTAAACTG ATTGAGACGT TGCCTTTTGT TGGGGATCCC   
  
  
- ACAAAAAGAC GCACAACATG TGTGAAGTGA AAATTGGACG AGACGGAGAC GGAAACGGTG CCGAAAATTA   
  
  
- CCTATTCTGG TTAATTCTTT GGGTTTATAG AGCATTTAAA CAACTCTAAG GAAAGACGCA AGAGTTCACA   
  
  
- AAGTTTTTGG GTCAAACCTC CAACGTGAAT AAGTACGAAA AGAAGTTCAG TCACTCGAAT CACAGACCTT   
  
  
- GACTTCGAGG AGGACCATTA CGAGACAAAA AGTAAAGAAC AACACGCAAA TATTTTTAAG CCGGATAACT   
  
  
- ACCTTCAAAC AAACTACAAA AACCCATTCA AAAACTAATT ATCTCTACTT AATCATTTCA TTTTAAACTC   
  
  
- ACCATAAATT ACTAGGAATT CGTCAGATAC GAAACAACAG TGTACGCAAA GAACCGAAAA CTGACTTTGT   
  
  
- AAAGACACAA TCTACTTTTT GAGTAAAACT ACGTACTCTA CTATTTTAGC GAATAAAAAC GGAAATAAAC   
  
  
- CACACACCCC CCCCCCCCCC CCCCCGCGTC CAGACTCCAT TTTCTATACA CTTAAGTAAA CCTCAGAGCT   
  
  
- ATTAAGCAAC CGATACAACT GTTAGTGCTC ACCAGCAATA GCGAGTAGAG TGTCCAACTA TTAAGTGAGT   
  
  
- TTGAAGTTCG CCGGACTTAG TAAGTCAGGC CCAACAATTT TAGAGTTGTC AGTAAAGTGT GCTCACCTCC   
  
  
- CGGGGTTTTC TGTGGTAAGA AAGCTTGAAA AAAAAAAACT TCCAGTAAAA AAGTTCTTGT TTTGATTACA   
  
  
- CTTGTACGCT GAAGATACTC ATTTCACATT TCTACGTATT TCAGAAAAAA AGAAAATATA TTTTTCATGA   
  
  
- GTTTTTTTTT TCTCAACCTC TCGACTAAGG GGTACGTAGG AGATTCGTTT CCACCACCTG CCGTCTTTTA   
  
  
- ATCATCTCAA TAACACTCTA ATTTCAAACG CGTGGACTTG CCGACTGGAG ATCAAACGAT AGTTGCAATG   
  
  
- TAAATACACA GTATTAAAAA CGTACTTTGT TAAAGTGCGT TACAAAAGAC TACCGTCAAT ACAAATCTTT   
  
  
- ATTCAAATTA GCCTGGAATA AGGTGACTAA ATATAAACTA AGAAGGGAGA AATGATGCCG ACAGGTAAGT   
  
  
- GTTCGGTTTC TTAAGAGGGG GGTTTTTTTT CTTTTTTGTC ATATCTTGGT ACAAATAATA GAGTAAAAAA   
  
  
- AGAGAGGGTA AACTAGACTT TGTGTTTGAA CGTCCCCACG TTCAACCTGT GAATCATCGA CGTCCTTACT   
  
  
- AAGTTATCCT TAGGAGTAGA AACTGTTGTA GTGGTGAACT TATGTATAGT GAACTACCGT GAGAGGGGAC   
  
  
- CAAATCCGAA GGGTATAGGC ACCGAATCCC TCGATTTTGG ACTCGTTTCT CCTGAGACAG ATTAGGTAGA   
  
  
- GGAGGAAAGT ACACGATTAG TTCAACGATG ACCCTCGTAG CTGTTACGAT TACAGTCGGA ACTCGTATAA   
  
  
- AGGGTGGAAC GGAGAGGGTG GCCTCTATGT TACGTCGCTT AACGACGAAT GAAACGACTT CGGAAACGAC   
  
  
- TGGCAAATGA TTCCCGAACC GTCGGGCCAG AAGAGTTTCG GAACTTAACA TGGTTCTACA GTAGACAAAG   
  
  
- GCTTGTTTAA GAACAAGTTT TCAACAAGAA ACTAGAAGAA GGCAAGAACT TCGAAAGTAT AGAACACTGC   
  
  
- TTGGTCCGTT AGTGCCTCCG GTATCTTCCT CTTTTCTACC AAGTATATTA ACTAGAGGTA AGAACACTTA   
  
  
- GCCGAGACAC CTAATCAGAG GAGGTCCGTA ACTCACAAGC TGGACTTCCG GGTGGGGTAA ACTCCTATTG   
  
  
- GCCATATGTA CTCTTCTTTC TTCACAACCT AGTTTACCGA TACGTTGATT TGTTTCTCCG ACTTTTTAAC   
  
  
- CTGTAGGGTA AAGTTAAGTT AGGATAGCGT TCGTTTGATC TGCTGGAACT ACAGCTTTCG AACTCACAGT   
  
  
- TCTGGCCTCT TCGTGAACAA TAATCAAGAC ACGAGGTTGA TGTAAGAGAA AACCGTAACC TACTCCCTAG   
  
  
- TTACGGATTC TTGCGTCCGT ACCGAATGAA CGTTTCCCAC AAAATATACT TTGGTGCTTT TAACCTCCTG   
  
  
- AACGGGTTGT TCCTAAACTA CTTTTACAAC TTGAGTTTAC TTCTAAGATG TAGTAGTAGT AGAAGTAGAG   
  
  
- GATAGGGAAG TAGTTTTGAA CTACGGAAAA ATTTTCGGGA GGAACCCGAA AGCGGTTTTG AGTACCAACA   
  
  
- TTGGCTCGTT CTTAGTTTGG TGTTACCTTC ACGGGATTAT CTTTCTCACT ACCTCAGTAA CTTGAAGATA   
  
  
- CGTCGTAACA AACTAACGAA CCTTAGGTGA TATAGCTCCT GTAGCTATCT CTCTGTCTTC GAGCTCTTCA   
  
  
- ACTACAAACC TCTCCTCTAG TTCTTGGAGT GTCGAACACT TCCCCGTCTC TCTTTCCGTT CCGTGCTCTT   
  
  
- CGAGTCACTT ACCCAAGTTT CTAAACTCAG TCATCCTAAA TTTTCCCTCG GTAACTCGAT GGTACCAAAG   
  
  
- AACGAACGAT CCTCCAAAAA TGTATGGTTA TTAATACTCC CCATATTGTA GTTCCTTCAG TTACCAAAAG   
  
  
- AACAATAGAC AACCGTTCTA TCTGGGGACA TAAGACAAAG TCGAACCTCT AAAAT

+     TATA-box

| Site Name | Organism | Position | Strand | Matrix score. | sequence | function |
| --- | --- | --- | --- | --- | --- | --- |
| TATA-box | Helianthus annuus | 1783 | - | 6 | TATAAA | core promoter element around -30 of transcription start |
| TATA-box | Arabidopsis thaliana | 1532 | + | 4 | TATA | core promoter element around -30 of transcription start |
| TATA-box | Arabidopsis thaliana | 1530 | + | 6 | TATATA | core promoter element around -30 of transcription start |
| TATA-box | Brassica oleracea | 1531 | + | 6 | ATATAA | core promoter element around -30 of transcription start |
| TATA-box | Arabidopsis thaliana | 963 | - | 5 | TATAA | core promoter element around -30 of transcription start |
| TATA-box | Helianthus annuus | 962 | - | 6 | TATAAA | core promoter element around -30 of transcription start |
| TATA-box | Arabidopsis thaliana | 1865 | - | 4 | TATA | core promoter element around -30 of transcription start |
| TATA-box | Brassica juncea | 1782 | - | 7 | TATAAAT | core promoter element around -30 of transcription start |
| TATA-box | Arabidopsis thaliana | 3267 | - | 4 | TATA | core promoter element around -30 of transcription start |
| TATA-box | Helianthus annuus | 1528 | - | 6 | TATAAA | core promoter element around -30 of transcription start |
| TATA-box | Arabidopsis thaliana | 3044 | - | 4 | TATA | core promoter element around -30 of transcription start |
| TATA-box | Arabidopsis thaliana | 1781 | - | 9 | taTATAAAtc | core promoter element around -30 of transcription start |
| TATA-box | Arabidopsis thaliana | 2429 | - | 4 | TATA | core promoter element around -30 of transcription start |
| TATA-box | Arabidopsis thaliana | 1784 | - | 5 | TATAA | core promoter element around -30 of transcription start |
| TATA-box | Oryza sativa | 20 | + | 7 | TACAAAA | core promoter element around -30 of transcription start |
| TATA-box | Oryza sativa | 662 | + | 7 | TACAAAA | core promoter element around -30 of transcription start |
| TATA-box | Pisum sativum | 2775 | - | 7 | TATAAAA | core promoter element around -30 of transcription start |
| TATA-box | Pisum sativum | 1527 | - | 7 | TATAAAA | core promoter element around -30 of transcription start |
| TATA-box | Brassica oleracea | 2428 | + | 6 | ATATAA | core promoter element around -30 of transcription start |
| TATA-box | Arabidopsis thaliana | 1529 | - | 7 | TATATAA | core promoter element around -30 of transcription start |
| TATA-box | Arabidopsis thaliana | 964 | + | 4 | TATA | core promoter element around -30 of transcription start |
| TATA-box | Arabidopsis thaliana | 2777 | - | 5 | TATAA | core promoter element around -30 of transcription start |
| TATA-box | Arabidopsis thaliana | 2778 | - | 4 | TATA | core promoter element around -30 of transcription start |
| TATA-box | Arabidopsis thaliana | 2528 | - | 4 | TATA | core promoter element around -30 of transcription start |
| TATA-box | Arabidopsis thaliana | 1785 | - | 4 | TATA | core promoter element around -30 of transcription start |
| TATA-box | Helianthus annuus | 2776 | - | 6 | TATAAA | core promoter element around -30 of transcription start |

>HU05G01267.1   
+ +Up\_Stream \_Len000TTACAA AAACGATGCT CTAAACCTAT TAGGTCGTTC CGAAACCCGC AAAATTGAAA   
  
  
+ TATGATCGCC CGTATTTTAC CCAAACCTGA AATACACTTA ATATGTATTT TACCTGAACC CAAATACACC   
  
  
+ GGACCTGTAT TTAACACGGG CACCCGAAAT GATAGGTCTA ACTCTCACCA CCACCGCATT TGGCACCCGA   
  
  
+ ACGGTAAAAA ATGTTCATCT ATTATGCACT TTGGCTTCAT CCATAACCCT ATGATCTATC TGTGCCAATA   
  
  
+ TTTGGAGCAC CCAGCGAAGT AGCGCATGCT CTAAACTCTT GCCTCAAAAC AAAACAAATA GTGGGTCATA   
  
  
+ TTCCCTTCAA AGCACTAACA AACACACACT CTCTCTCTCT CAGTAGCCAA CAACTTAAGG CGGTGTTTGC   
  
  
+ TTGGTTTTGG AGAGGGCGTG TTTGGATGAG GTGCAAAACA CATAGATGAA ATCATAAACA ACTTGATTCC   
  
  
+ CAGCCACCCA TGACCTACTC AGATAACAAG GGACAAAAAC AAAGAGGTTT TCTGAAACAG AGAAAACAGG   
  
  
+ GGCGTTGTGT TGCGCTGTTG CTCACAAAAT TCCAATATTC CTCTAAAAGC TCCCAAACGG AGACAAATCA   
  
  
+ AAGAGGACAA AGACATGTGG GATTTAATAC AAAATTTGAC TAACTCTGCA ACGGAAAACA ACCCCTAGGG   
  
  
+ TGTTTTTCTG CGTGTTGTAC ACACTTCACT TTTAACCTGC TCTGCCTCTG CCTTTGCCAC GGCTTTTAAT   
  
  
+ GGATAAGACC AATTAAGAAA CCCAAATATC TCGTAAATTT GTTGAGATTC CTTTCTGCGT TCTCAAGTGT   
  
  
+ TTCAAAAACC CAGTTTGGAG GTTGCACTTA TTCATGCTTT TCTTCAAGTC AGTGAGCTTA GTGTCTGGAA   
  
  
+ CTGAAGCTCC TCCTGGTAAT GCTCTGTTTT TCATTTCTTG TTGTGCGTTT ATAAAAATTC GGCCTATTGA   
  
  
+ TGGAAGTTTG TTTGATGTTT TTGGGTAAGT TTTTGATTAA TAGAGATGAA TTAGTAAAGT AAAATTTGAG   
  
  
+ TGGTATTTAA TGATCCTTAA GCAGTCTATG CTTTGTTGTC ACATGCGTTT CTTGGCTTTT GACTGAAACA   
  
  
+ TTTCTGTGTT AGATGAAAAA CTCATTTTGA TGCATGAGAT GATAAAATCG CTTATTTTTG CCTTTATTTG   
  
  
+ GTGTGTGGGG GGGGGGGGGG GGGGGCGCAG GTCTGAGGTA AAAGATATGT GAATTCATTT GGAGTCTCGA   
  
  
+ TAATTCGTTG GCTATGTTGA CAATCACGAG TGGTCGTTAT CGCTCATCTC ACAGGTTGAT AATTCACTCA   
  
  
+ AACTTCAAGC GGCCTGAATC ATTCAGTCCG GGTTGTTAAA ATCTCAACAG TCATTTCACA CGAGTGGAGG   
  
  
+ GCCCCAAAAG ACACCATTCT TTCGAACTTT TTTTTTTTGA AGGTCATTTT TTCAAGAACA AAACTAATGT   
  
  
+ GAACATGCGA CTTCTATGAG TAAAGTGTAA AGATGCATAA AGTCTTTTTT TCTTTTATAT AAAAAGTACT   
  
  
+ CAAAAAAAAA AGAGTTGGAG AGCTGATTCC CCATGCATCC TCTAAGCAAA GGTGGTGGAC GGCAGAAAAT   
  
  
+ TAGTAGAGTT ATTGTGAGAT TAAAGTTTGC GCACCTGAAC GGCTGACCTC TAGTTTGCTA TCAACGTTAC   
  
  
+ ATTTATGTGT CATAATTTTT GCATGAAACA ATTTCACGCA ATGTTTTCTG ATGGCAGTTA TGTTTAGAAA   
  
  
+ TAAGTTTAAT CGGACCTTAT TCCACTGATT TATATTTGAT TCTTCCCTCT TTACTACGGC TGTCCATTCA   
  
  
+ CAAGCCAAAG AATTCTCCCC CCAAAAAAAA GAAAAAACAG TATAGAACCA TGTTTATTAT CTCATTTTTT   
  
  
+ TCTCTCCCAT TTGATCTGAA ACACAAACTT GCAGGGGTGC AAGTTGGACA CTTAGTAGCT GCAGGAATGA   
  
  
+ TTCAATAGGA ATCCTCATCT TTGACAACAT CACCACTTGA ATACATATCA CTTGATGGCA CTCTCCCCTG   
  
  
+ GTTTAGGCTT CCCATATCCG TGGCTTAGGG AGCTAAAACC TGAGCAAAGA GGACTCTGTC TAATCCATCT   
  
  
+ CCTCCTTTCA TGTGCTAATC AAGTTGCTAC TGGGAGCATC GACAATGCTA ATGTCAGCCT TGAGCATATT   
  
  
+ TCCCACCTTG CCTCTCCCAC CGGAGATACA ATGCAGCGAA TTGCTGCTTA CTTTGCTGAA GCCTTTGCTG   
  
  
+ ACCGTTTACT AAGGGCTTGG CAGCCCGGTC TTCTCAAAGC CTTGAATTGT ACCAAGATGT CATCTGTTTC   
  
  
+ CGAACAAATT CTTGTTCAAA AGTTGTTCTT TGATCTTCTT CCGTTCTTGA AGCTTTCATA TCTTGTGACG   
  
  
+ AACCAGGCAA TCACGGAGGC CATAGAAGGA GAAAAGATGG TTCATATAAT TGATCTCCAT TCTTGTGAAT   
  
  
+ CGGCTCTGTG GATTAGTCTC CTCCAGGCAT TGAGTGTTCG ACCTGAAGGC CCACCCCATT TGAGGATAAC   
  
  
+ CGGTATACAT GAGAAGAAAG AAGTGTTGGA TCAAATGGCT ATGCAACTAA ACAAAGAGGC TGAAAAATTG   
  
  
+ GACATCCCAT TTCAATTCAA TCCTATCGCA AGCAAACTAG ACGACCTTGA TGTCGAAAGC TTGAGTGTCA   
  
  
+ AGACCGGAGA AGCACTTGTT ATTAGTTCTG TGCTCCAACT ACATTCTCTT TTGGCATTGG ATGAGGGATC   
  
  
+ AATGCCTAAG AACGCAGGCA TGGCTTACTT GCAAAGGGTG TTTTATATGA AACCACGAAA ATTGGAGGAC   
  
  
+ TTGCCCAACA AGGATTTGAT GAAAATGTTG AACTCAAATG AAGATTCTAC ATCATCATCA TCTTCATCTC   
  
  
+ CTATCCCTTC ATCAAAACTT GATGCCTTTT TAAAAGCCCT CCTTGGGCTT TCGCCAAAAC TCATGGTTGT   
  
  
+ AACCGAGCAA GAATCAAACC ACAATGGAAG TGCCCTAATA GAAAGAGTGA TGGAGTCATT GAACTTCTAT   
  
  
+ GCAGCATTGT TTGATTGCTT GGAATCCACT ATATCGAGGA CATCGATAGA GAGACAGAAG CTCGAGAAGT   
  
  
+ TGATGTTTGG AGAGGAGATC AAGAACCTCA CAGCTTGTGA AGGGGCAGAG AGAAAGGCAA GGCACGAGAA   
  
  
+ GCTCAGTGAA TGGGTTCAAA GATTTGAGTC AGTAGGATTT AAAAGGGAGC CATTGAGCTA CCATGGTTTC   
  
  
+ TTGCTTGCTA GGAGGTTTTT ACATACCAAT AATTATGAGG GGTATAACAT CAAGGAAGTC AATGGTTTTC   
  
  
+ TTGTTATCTG TTGGCAAGAT AGACCCCTGT ATTCTGTTTC AGCTTGGAGA TTTTA  

- +Up\_Stream \_Len000AATGTT TTTGCTACGA GATTTGGATA ATCCAGCAAG GCTTTGGGCG TTTTAACTTT   
  
  
- ATACTAGCGG GCATAAAATG GGTTTGGACT TTATGTGAAT TATACATAAA ATGGACTTGG GTTTATGTGG   
  
  
- CCTGGACATA AATTGTGCCC GTGGGCTTTA CTATCCAGAT TGAGAGTGGT GGTGGCGTAA ACCGTGGGCT   
  
  
- TGCCATTTTT TACAAGTAGA TAATACGTGA AACCGAAGTA GGTATTGGGA TACTAGATAG ACACGGTTAT   
  
  
- AAACCTCGTG GGTCGCTTCA TCGCGTACGA GATTTGAGAA CGGAGTTTTG TTTTGTTTAT CACCCAGTAT   
  
  
- AAGGGAAGTT TCGTGATTGT TTGTGTGTGA GAGAGAGAGA GTCATCGGTT GTTGAATTCC GCCACAAACG   
  
  
- AACCAAAACC TCTCCCGCAC AAACCTACTC CACGTTTTGT GTATCTACTT TAGTATTTGT TGAACTAAGG   
  
  
- GTCGGTGGGT ACTGGATGAG TCTATTGTTC CCTGTTTTTG TTTCTCCAAA AGACTTTGTC TCTTTTGTCC   
  
  
- CCGCAACACA ACGCGACAAC GAGTGTTTTA AGGTTATAAG GAGATTTTCG AGGGTTTGCC TCTGTTTAGT   
  
  
- TTCTCCTGTT TCTGTACACC CTAAATTATG TTTTAAACTG ATTGAGACGT TGCCTTTTGT TGGGGATCCC   
  
  
- ACAAAAAGAC GCACAACATG TGTGAAGTGA AAATTGGACG AGACGGAGAC GGAAACGGTG CCGAAAATTA   
  
  
- CCTATTCTGG TTAATTCTTT GGGTTTATAG AGCATTTAAA CAACTCTAAG GAAAGACGCA AGAGTTCACA   
  
  
- AAGTTTTTGG GTCAAACCTC CAACGTGAAT AAGTACGAAA AGAAGTTCAG TCACTCGAAT CACAGACCTT   
  
  
- GACTTCGAGG AGGACCATTA CGAGACAAAA AGTAAAGAAC AACACGCAAA TATTTTTAAG CCGGATAACT   
  
  
- ACCTTCAAAC AAACTACAAA AACCCATTCA AAAACTAATT ATCTCTACTT AATCATTTCA TTTTAAACTC   
  
  
- ACCATAAATT ACTAGGAATT CGTCAGATAC GAAACAACAG TGTACGCAAA GAACCGAAAA CTGACTTTGT   
  
  
- AAAGACACAA TCTACTTTTT GAGTAAAACT ACGTACTCTA CTATTTTAGC GAATAAAAAC GGAAATAAAC   
  
  
- CACACACCCC CCCCCCCCCC CCCCCGCGTC CAGACTCCAT TTTCTATACA CTTAAGTAAA CCTCAGAGCT   
  
  
- ATTAAGCAAC CGATACAACT GTTAGTGCTC ACCAGCAATA GCGAGTAGAG TGTCCAACTA TTAAGTGAGT   
  
  
- TTGAAGTTCG CCGGACTTAG TAAGTCAGGC CCAACAATTT TAGAGTTGTC AGTAAAGTGT GCTCACCTCC   
  
  
- CGGGGTTTTC TGTGGTAAGA AAGCTTGAAA AAAAAAAACT TCCAGTAAAA AAGTTCTTGT TTTGATTACA   
  
  
- CTTGTACGCT GAAGATACTC ATTTCACATT TCTACGTATT TCAGAAAAAA AGAAAATATA TTTTTCATGA   
  
  
- GTTTTTTTTT TCTCAACCTC TCGACTAAGG GGTACGTAGG AGATTCGTTT CCACCACCTG CCGTCTTTTA   
  
  
- ATCATCTCAA TAACACTCTA ATTTCAAACG CGTGGACTTG CCGACTGGAG ATCAAACGAT AGTTGCAATG   
  
  
- TAAATACACA GTATTAAAAA CGTACTTTGT TAAAGTGCGT TACAAAAGAC TACCGTCAAT ACAAATCTTT   
  
  
- ATTCAAATTA GCCTGGAATA AGGTGACTAA ATATAAACTA AGAAGGGAGA AATGATGCCG ACAGGTAAGT   
  
  
- GTTCGGTTTC TTAAGAGGGG GGTTTTTTTT CTTTTTTGTC ATATCTTGGT ACAAATAATA GAGTAAAAAA   
  
  
- AGAGAGGGTA AACTAGACTT TGTGTTTGAA CGTCCCCACG TTCAACCTGT GAATCATCGA CGTCCTTACT   
  
  
- AAGTTATCCT TAGGAGTAGA AACTGTTGTA GTGGTGAACT TATGTATAGT GAACTACCGT GAGAGGGGAC   
  
  
- CAAATCCGAA GGGTATAGGC ACCGAATCCC TCGATTTTGG ACTCGTTTCT CCTGAGACAG ATTAGGTAGA   
  
  
- GGAGGAAAGT ACACGATTAG TTCAACGATG ACCCTCGTAG CTGTTACGAT TACAGTCGGA ACTCGTATAA   
  
  
- AGGGTGGAAC GGAGAGGGTG GCCTCTATGT TACGTCGCTT AACGACGAAT GAAACGACTT CGGAAACGAC   
  
  
- TGGCAAATGA TTCCCGAACC GTCGGGCCAG AAGAGTTTCG GAACTTAACA TGGTTCTACA GTAGACAAAG   
  
  
- GCTTGTTTAA GAACAAGTTT TCAACAAGAA ACTAGAAGAA GGCAAGAACT TCGAAAGTAT AGAACACTGC   
  
  
- TTGGTCCGTT AGTGCCTCCG GTATCTTCCT CTTTTCTACC AAGTATATTA ACTAGAGGTA AGAACACTTA   
  
  
- GCCGAGACAC CTAATCAGAG GAGGTCCGTA ACTCACAAGC TGGACTTCCG GGTGGGGTAA ACTCCTATTG   
  
  
- GCCATATGTA CTCTTCTTTC TTCACAACCT AGTTTACCGA TACGTTGATT TGTTTCTCCG ACTTTTTAAC   
  
  
- CTGTAGGGTA AAGTTAAGTT AGGATAGCGT TCGTTTGATC TGCTGGAACT ACAGCTTTCG AACTCACAGT   
  
  
- TCTGGCCTCT TCGTGAACAA TAATCAAGAC ACGAGGTTGA TGTAAGAGAA AACCGTAACC TACTCCCTAG   
  
  
- TTACGGATTC TTGCGTCCGT ACCGAATGAA CGTTTCCCAC AAAATATACT TTGGTGCTTT TAACCTCCTG   
  
  
- AACGGGTTGT TCCTAAACTA CTTTTACAAC TTGAGTTTAC TTCTAAGATG TAGTAGTAGT AGAAGTAGAG   
  
  
- GATAGGGAAG TAGTTTTGAA CTACGGAAAA ATTTTCGGGA GGAACCCGAA AGCGGTTTTG AGTACCAACA   
  
  
- TTGGCTCGTT CTTAGTTTGG TGTTACCTTC ACGGGATTAT CTTTCTCACT ACCTCAGTAA CTTGAAGATA   
  
  
- CGTCGTAACA AACTAACGAA CCTTAGGTGA TATAGCTCCT GTAGCTATCT CTCTGTCTTC GAGCTCTTCA   
  
  
- ACTACAAACC TCTCCTCTAG TTCTTGGAGT GTCGAACACT TCCCCGTCTC TCTTTCCGTT CCGTGCTCTT   
  
  
- CGAGTCACTT ACCCAAGTTT CTAAACTCAG TCATCCTAAA TTTTCCCTCG GTAACTCGAT GGTACCAAAG   
  
  
- AACGAACGAT CCTCCAAAAA TGTATGGTTA TTAATACTCC CCATATTGTA GTTCCTTCAG TTACCAAAAG   
  
  
- AACAATAGAC AACCGTTCTA TCTGGGGACA TAAGACAAAG TCGAACCTCT AAAAT

+     TCA

| Site Name | Organism | Position | Strand | Matrix score. | sequence | function |
| --- | --- | --- | --- | --- | --- | --- |
| TCA | Pisum sativum | 2856 | + | 9 | TCATCTTCAT |  |
| TCA | Pisum sativum | 2862 | + | 10 | TCATCTTCAT |  |

>HU05G01267.1   
+ +Up\_Stream \_Len000TTACAA AAACGATGCT CTAAACCTAT TAGGTCGTTC CGAAACCCGC AAAATTGAAA   
  
  
+ TATGATCGCC CGTATTTTAC CCAAACCTGA AATACACTTA ATATGTATTT TACCTGAACC CAAATACACC   
  
  
+ GGACCTGTAT TTAACACGGG CACCCGAAAT GATAGGTCTA ACTCTCACCA CCACCGCATT TGGCACCCGA   
  
  
+ ACGGTAAAAA ATGTTCATCT ATTATGCACT TTGGCTTCAT CCATAACCCT ATGATCTATC TGTGCCAATA   
  
  
+ TTTGGAGCAC CCAGCGAAGT AGCGCATGCT CTAAACTCTT GCCTCAAAAC AAAACAAATA GTGGGTCATA   
  
  
+ TTCCCTTCAA AGCACTAACA AACACACACT CTCTCTCTCT CAGTAGCCAA CAACTTAAGG CGGTGTTTGC   
  
  
+ TTGGTTTTGG AGAGGGCGTG TTTGGATGAG GTGCAAAACA CATAGATGAA ATCATAAACA ACTTGATTCC   
  
  
+ CAGCCACCCA TGACCTACTC AGATAACAAG GGACAAAAAC AAAGAGGTTT TCTGAAACAG AGAAAACAGG   
  
  
+ GGCGTTGTGT TGCGCTGTTG CTCACAAAAT TCCAATATTC CTCTAAAAGC TCCCAAACGG AGACAAATCA   
  
  
+ AAGAGGACAA AGACATGTGG GATTTAATAC AAAATTTGAC TAACTCTGCA ACGGAAAACA ACCCCTAGGG   
  
  
+ TGTTTTTCTG CGTGTTGTAC ACACTTCACT TTTAACCTGC TCTGCCTCTG CCTTTGCCAC GGCTTTTAAT   
  
  
+ GGATAAGACC AATTAAGAAA CCCAAATATC TCGTAAATTT GTTGAGATTC CTTTCTGCGT TCTCAAGTGT   
  
  
+ TTCAAAAACC CAGTTTGGAG GTTGCACTTA TTCATGCTTT TCTTCAAGTC AGTGAGCTTA GTGTCTGGAA   
  
  
+ CTGAAGCTCC TCCTGGTAAT GCTCTGTTTT TCATTTCTTG TTGTGCGTTT ATAAAAATTC GGCCTATTGA   
  
  
+ TGGAAGTTTG TTTGATGTTT TTGGGTAAGT TTTTGATTAA TAGAGATGAA TTAGTAAAGT AAAATTTGAG   
  
  
+ TGGTATTTAA TGATCCTTAA GCAGTCTATG CTTTGTTGTC ACATGCGTTT CTTGGCTTTT GACTGAAACA   
  
  
+ TTTCTGTGTT AGATGAAAAA CTCATTTTGA TGCATGAGAT GATAAAATCG CTTATTTTTG CCTTTATTTG   
  
  
+ GTGTGTGGGG GGGGGGGGGG GGGGGCGCAG GTCTGAGGTA AAAGATATGT GAATTCATTT GGAGTCTCGA   
  
  
+ TAATTCGTTG GCTATGTTGA CAATCACGAG TGGTCGTTAT CGCTCATCTC ACAGGTTGAT AATTCACTCA   
  
  
+ AACTTCAAGC GGCCTGAATC ATTCAGTCCG GGTTGTTAAA ATCTCAACAG TCATTTCACA CGAGTGGAGG   
  
  
+ GCCCCAAAAG ACACCATTCT TTCGAACTTT TTTTTTTTGA AGGTCATTTT TTCAAGAACA AAACTAATGT   
  
  
+ GAACATGCGA CTTCTATGAG TAAAGTGTAA AGATGCATAA AGTCTTTTTT TCTTTTATAT AAAAAGTACT   
  
  
+ CAAAAAAAAA AGAGTTGGAG AGCTGATTCC CCATGCATCC TCTAAGCAAA GGTGGTGGAC GGCAGAAAAT   
  
  
+ TAGTAGAGTT ATTGTGAGAT TAAAGTTTGC GCACCTGAAC GGCTGACCTC TAGTTTGCTA TCAACGTTAC   
  
  
+ ATTTATGTGT CATAATTTTT GCATGAAACA ATTTCACGCA ATGTTTTCTG ATGGCAGTTA TGTTTAGAAA   
  
  
+ TAAGTTTAAT CGGACCTTAT TCCACTGATT TATATTTGAT TCTTCCCTCT TTACTACGGC TGTCCATTCA   
  
  
+ CAAGCCAAAG AATTCTCCCC CCAAAAAAAA GAAAAAACAG TATAGAACCA TGTTTATTAT CTCATTTTTT   
  
  
+ TCTCTCCCAT TTGATCTGAA ACACAAACTT GCAGGGGTGC AAGTTGGACA CTTAGTAGCT GCAGGAATGA   
  
  
+ TTCAATAGGA ATCCTCATCT TTGACAACAT CACCACTTGA ATACATATCA CTTGATGGCA CTCTCCCCTG   
  
  
+ GTTTAGGCTT CCCATATCCG TGGCTTAGGG AGCTAAAACC TGAGCAAAGA GGACTCTGTC TAATCCATCT   
  
  
+ CCTCCTTTCA TGTGCTAATC AAGTTGCTAC TGGGAGCATC GACAATGCTA ATGTCAGCCT TGAGCATATT   
  
  
+ TCCCACCTTG CCTCTCCCAC CGGAGATACA ATGCAGCGAA TTGCTGCTTA CTTTGCTGAA GCCTTTGCTG   
  
  
+ ACCGTTTACT AAGGGCTTGG CAGCCCGGTC TTCTCAAAGC CTTGAATTGT ACCAAGATGT CATCTGTTTC   
  
  
+ CGAACAAATT CTTGTTCAAA AGTTGTTCTT TGATCTTCTT CCGTTCTTGA AGCTTTCATA TCTTGTGACG   
  
  
+ AACCAGGCAA TCACGGAGGC CATAGAAGGA GAAAAGATGG TTCATATAAT TGATCTCCAT TCTTGTGAAT   
  
  
+ CGGCTCTGTG GATTAGTCTC CTCCAGGCAT TGAGTGTTCG ACCTGAAGGC CCACCCCATT TGAGGATAAC   
  
  
+ CGGTATACAT GAGAAGAAAG AAGTGTTGGA TCAAATGGCT ATGCAACTAA ACAAAGAGGC TGAAAAATTG   
  
  
+ GACATCCCAT TTCAATTCAA TCCTATCGCA AGCAAACTAG ACGACCTTGA TGTCGAAAGC TTGAGTGTCA   
  
  
+ AGACCGGAGA AGCACTTGTT ATTAGTTCTG TGCTCCAACT ACATTCTCTT TTGGCATTGG ATGAGGGATC   
  
  
+ AATGCCTAAG AACGCAGGCA TGGCTTACTT GCAAAGGGTG TTTTATATGA AACCACGAAA ATTGGAGGAC   
  
  
+ TTGCCCAACA AGGATTTGAT GAAAATGTTG AACTCAAATG AAGATTCTAC ATCATCATCA TCTTCATCTC   
  
  
+ CTATCCCTTC ATCAAAACTT GATGCCTTTT TAAAAGCCCT CCTTGGGCTT TCGCCAAAAC TCATGGTTGT   
  
  
+ AACCGAGCAA GAATCAAACC ACAATGGAAG TGCCCTAATA GAAAGAGTGA TGGAGTCATT GAACTTCTAT   
  
  
+ GCAGCATTGT TTGATTGCTT GGAATCCACT ATATCGAGGA CATCGATAGA GAGACAGAAG CTCGAGAAGT   
  
  
+ TGATGTTTGG AGAGGAGATC AAGAACCTCA CAGCTTGTGA AGGGGCAGAG AGAAAGGCAA GGCACGAGAA   
  
  
+ GCTCAGTGAA TGGGTTCAAA GATTTGAGTC AGTAGGATTT AAAAGGGAGC CATTGAGCTA CCATGGTTTC   
  
  
+ TTGCTTGCTA GGAGGTTTTT ACATACCAAT AATTATGAGG GGTATAACAT CAAGGAAGTC AATGGTTTTC   
  
  
+ TTGTTATCTG TTGGCAAGAT AGACCCCTGT ATTCTGTTTC AGCTTGGAGA TTTTA  

- +Up\_Stream \_Len000AATGTT TTTGCTACGA GATTTGGATA ATCCAGCAAG GCTTTGGGCG TTTTAACTTT   
  
  
- ATACTAGCGG GCATAAAATG GGTTTGGACT TTATGTGAAT TATACATAAA ATGGACTTGG GTTTATGTGG   
  
  
- CCTGGACATA AATTGTGCCC GTGGGCTTTA CTATCCAGAT TGAGAGTGGT GGTGGCGTAA ACCGTGGGCT   
  
  
- TGCCATTTTT TACAAGTAGA TAATACGTGA AACCGAAGTA GGTATTGGGA TACTAGATAG ACACGGTTAT   
  
  
- AAACCTCGTG GGTCGCTTCA TCGCGTACGA GATTTGAGAA CGGAGTTTTG TTTTGTTTAT CACCCAGTAT   
  
  
- AAGGGAAGTT TCGTGATTGT TTGTGTGTGA GAGAGAGAGA GTCATCGGTT GTTGAATTCC GCCACAAACG   
  
  
- AACCAAAACC TCTCCCGCAC AAACCTACTC CACGTTTTGT GTATCTACTT TAGTATTTGT TGAACTAAGG   
  
  
- GTCGGTGGGT ACTGGATGAG TCTATTGTTC CCTGTTTTTG TTTCTCCAAA AGACTTTGTC TCTTTTGTCC   
  
  
- CCGCAACACA ACGCGACAAC GAGTGTTTTA AGGTTATAAG GAGATTTTCG AGGGTTTGCC TCTGTTTAGT   
  
  
- TTCTCCTGTT TCTGTACACC CTAAATTATG TTTTAAACTG ATTGAGACGT TGCCTTTTGT TGGGGATCCC   
  
  
- ACAAAAAGAC GCACAACATG TGTGAAGTGA AAATTGGACG AGACGGAGAC GGAAACGGTG CCGAAAATTA   
  
  
- CCTATTCTGG TTAATTCTTT GGGTTTATAG AGCATTTAAA CAACTCTAAG GAAAGACGCA AGAGTTCACA   
  
  
- AAGTTTTTGG GTCAAACCTC CAACGTGAAT AAGTACGAAA AGAAGTTCAG TCACTCGAAT CACAGACCTT   
  
  
- GACTTCGAGG AGGACCATTA CGAGACAAAA AGTAAAGAAC AACACGCAAA TATTTTTAAG CCGGATAACT   
  
  
- ACCTTCAAAC AAACTACAAA AACCCATTCA AAAACTAATT ATCTCTACTT AATCATTTCA TTTTAAACTC   
  
  
- ACCATAAATT ACTAGGAATT CGTCAGATAC GAAACAACAG TGTACGCAAA GAACCGAAAA CTGACTTTGT   
  
  
- AAAGACACAA TCTACTTTTT GAGTAAAACT ACGTACTCTA CTATTTTAGC GAATAAAAAC GGAAATAAAC   
  
  
- CACACACCCC CCCCCCCCCC CCCCCGCGTC CAGACTCCAT TTTCTATACA CTTAAGTAAA CCTCAGAGCT   
  
  
- ATTAAGCAAC CGATACAACT GTTAGTGCTC ACCAGCAATA GCGAGTAGAG TGTCCAACTA TTAAGTGAGT   
  
  
- TTGAAGTTCG CCGGACTTAG TAAGTCAGGC CCAACAATTT TAGAGTTGTC AGTAAAGTGT GCTCACCTCC   
  
  
- CGGGGTTTTC TGTGGTAAGA AAGCTTGAAA AAAAAAAACT TCCAGTAAAA AAGTTCTTGT TTTGATTACA   
  
  
- CTTGTACGCT GAAGATACTC ATTTCACATT TCTACGTATT TCAGAAAAAA AGAAAATATA TTTTTCATGA   
  
  
- GTTTTTTTTT TCTCAACCTC TCGACTAAGG GGTACGTAGG AGATTCGTTT CCACCACCTG CCGTCTTTTA   
  
  
- ATCATCTCAA TAACACTCTA ATTTCAAACG CGTGGACTTG CCGACTGGAG ATCAAACGAT AGTTGCAATG   
  
  
- TAAATACACA GTATTAAAAA CGTACTTTGT TAAAGTGCGT TACAAAAGAC TACCGTCAAT ACAAATCTTT   
  
  
- ATTCAAATTA GCCTGGAATA AGGTGACTAA ATATAAACTA AGAAGGGAGA AATGATGCCG ACAGGTAAGT   
  
  
- GTTCGGTTTC TTAAGAGGGG GGTTTTTTTT CTTTTTTGTC ATATCTTGGT ACAAATAATA GAGTAAAAAA   
  
  
- AGAGAGGGTA AACTAGACTT TGTGTTTGAA CGTCCCCACG TTCAACCTGT GAATCATCGA CGTCCTTACT   
  
  
- AAGTTATCCT TAGGAGTAGA AACTGTTGTA GTGGTGAACT TATGTATAGT GAACTACCGT GAGAGGGGAC   
  
  
- CAAATCCGAA GGGTATAGGC ACCGAATCCC TCGATTTTGG ACTCGTTTCT CCTGAGACAG ATTAGGTAGA   
  
  
- GGAGGAAAGT ACACGATTAG TTCAACGATG ACCCTCGTAG CTGTTACGAT TACAGTCGGA ACTCGTATAA   
  
  
- AGGGTGGAAC GGAGAGGGTG GCCTCTATGT TACGTCGCTT AACGACGAAT GAAACGACTT CGGAAACGAC   
  
  
- TGGCAAATGA TTCCCGAACC GTCGGGCCAG AAGAGTTTCG GAACTTAACA TGGTTCTACA GTAGACAAAG   
  
  
- GCTTGTTTAA GAACAAGTTT TCAACAAGAA ACTAGAAGAA GGCAAGAACT TCGAAAGTAT AGAACACTGC   
  
  
- TTGGTCCGTT AGTGCCTCCG GTATCTTCCT CTTTTCTACC AAGTATATTA ACTAGAGGTA AGAACACTTA   
  
  
- GCCGAGACAC CTAATCAGAG GAGGTCCGTA ACTCACAAGC TGGACTTCCG GGTGGGGTAA ACTCCTATTG   
  
  
- GCCATATGTA CTCTTCTTTC TTCACAACCT AGTTTACCGA TACGTTGATT TGTTTCTCCG ACTTTTTAAC   
  
  
- CTGTAGGGTA AAGTTAAGTT AGGATAGCGT TCGTTTGATC TGCTGGAACT ACAGCTTTCG AACTCACAGT   
  
  
- TCTGGCCTCT TCGTGAACAA TAATCAAGAC ACGAGGTTGA TGTAAGAGAA AACCGTAACC TACTCCCTAG   
  
  
- TTACGGATTC TTGCGTCCGT ACCGAATGAA CGTTTCCCAC AAAATATACT TTGGTGCTTT TAACCTCCTG   
  
  
- AACGGGTTGT TCCTAAACTA CTTTTACAAC TTGAGTTTAC TTCTAAGATG TAGTAGTAGT AGAAGTAGAG   
  
  
- GATAGGGAAG TAGTTTTGAA CTACGGAAAA ATTTTCGGGA GGAACCCGAA AGCGGTTTTG AGTACCAACA   
  
  
- TTGGCTCGTT CTTAGTTTGG TGTTACCTTC ACGGGATTAT CTTTCTCACT ACCTCAGTAA CTTGAAGATA   
  
  
- CGTCGTAACA AACTAACGAA CCTTAGGTGA TATAGCTCCT GTAGCTATCT CTCTGTCTTC GAGCTCTTCA   
  
  
- ACTACAAACC TCTCCTCTAG TTCTTGGAGT GTCGAACACT TCCCCGTCTC TCTTTCCGTT CCGTGCTCTT   
  
  
- CGAGTCACTT ACCCAAGTTT CTAAACTCAG TCATCCTAAA TTTTCCCTCG GTAACTCGAT GGTACCAAAG   
  
  
- AACGAACGAT CCTCCAAAAA TGTATGGTTA TTAATACTCC CCATATTGTA GTTCCTTCAG TTACCAAAAG   
  
  
- AACAATAGAC AACCGTTCTA TCTGGGGACA TAAGACAAAG TCGAACCTCT AAAAT

+     TCA-element

| Site Name | Organism | Position | Strand | Matrix score. | sequence | function |
| --- | --- | --- | --- | --- | --- | --- |
| TCA-element | Nicotiana tabacum | 2415 | - | 9 | CCATCTTTTT | cis-acting element involved in salicylic acid responsiveness |

>HU05G01267.1   
+ +Up\_Stream \_Len000TTACAA AAACGATGCT CTAAACCTAT TAGGTCGTTC CGAAACCCGC AAAATTGAAA   
  
  
+ TATGATCGCC CGTATTTTAC CCAAACCTGA AATACACTTA ATATGTATTT TACCTGAACC CAAATACACC   
  
  
+ GGACCTGTAT TTAACACGGG CACCCGAAAT GATAGGTCTA ACTCTCACCA CCACCGCATT TGGCACCCGA   
  
  
+ ACGGTAAAAA ATGTTCATCT ATTATGCACT TTGGCTTCAT CCATAACCCT ATGATCTATC TGTGCCAATA   
  
  
+ TTTGGAGCAC CCAGCGAAGT AGCGCATGCT CTAAACTCTT GCCTCAAAAC AAAACAAATA GTGGGTCATA   
  
  
+ TTCCCTTCAA AGCACTAACA AACACACACT CTCTCTCTCT CAGTAGCCAA CAACTTAAGG CGGTGTTTGC   
  
  
+ TTGGTTTTGG AGAGGGCGTG TTTGGATGAG GTGCAAAACA CATAGATGAA ATCATAAACA ACTTGATTCC   
  
  
+ CAGCCACCCA TGACCTACTC AGATAACAAG GGACAAAAAC AAAGAGGTTT TCTGAAACAG AGAAAACAGG   
  
  
+ GGCGTTGTGT TGCGCTGTTG CTCACAAAAT TCCAATATTC CTCTAAAAGC TCCCAAACGG AGACAAATCA   
  
  
+ AAGAGGACAA AGACATGTGG GATTTAATAC AAAATTTGAC TAACTCTGCA ACGGAAAACA ACCCCTAGGG   
  
  
+ TGTTTTTCTG CGTGTTGTAC ACACTTCACT TTTAACCTGC TCTGCCTCTG CCTTTGCCAC GGCTTTTAAT   
  
  
+ GGATAAGACC AATTAAGAAA CCCAAATATC TCGTAAATTT GTTGAGATTC CTTTCTGCGT TCTCAAGTGT   
  
  
+ TTCAAAAACC CAGTTTGGAG GTTGCACTTA TTCATGCTTT TCTTCAAGTC AGTGAGCTTA GTGTCTGGAA   
  
  
+ CTGAAGCTCC TCCTGGTAAT GCTCTGTTTT TCATTTCTTG TTGTGCGTTT ATAAAAATTC GGCCTATTGA   
  
  
+ TGGAAGTTTG TTTGATGTTT TTGGGTAAGT TTTTGATTAA TAGAGATGAA TTAGTAAAGT AAAATTTGAG   
  
  
+ TGGTATTTAA TGATCCTTAA GCAGTCTATG CTTTGTTGTC ACATGCGTTT CTTGGCTTTT GACTGAAACA   
  
  
+ TTTCTGTGTT AGATGAAAAA CTCATTTTGA TGCATGAGAT GATAAAATCG CTTATTTTTG CCTTTATTTG   
  
  
+ GTGTGTGGGG GGGGGGGGGG GGGGGCGCAG GTCTGAGGTA AAAGATATGT GAATTCATTT GGAGTCTCGA   
  
  
+ TAATTCGTTG GCTATGTTGA CAATCACGAG TGGTCGTTAT CGCTCATCTC ACAGGTTGAT AATTCACTCA   
  
  
+ AACTTCAAGC GGCCTGAATC ATTCAGTCCG GGTTGTTAAA ATCTCAACAG TCATTTCACA CGAGTGGAGG   
  
  
+ GCCCCAAAAG ACACCATTCT TTCGAACTTT TTTTTTTTGA AGGTCATTTT TTCAAGAACA AAACTAATGT   
  
  
+ GAACATGCGA CTTCTATGAG TAAAGTGTAA AGATGCATAA AGTCTTTTTT TCTTTTATAT AAAAAGTACT   
  
  
+ CAAAAAAAAA AGAGTTGGAG AGCTGATTCC CCATGCATCC TCTAAGCAAA GGTGGTGGAC GGCAGAAAAT   
  
  
+ TAGTAGAGTT ATTGTGAGAT TAAAGTTTGC GCACCTGAAC GGCTGACCTC TAGTTTGCTA TCAACGTTAC   
  
  
+ ATTTATGTGT CATAATTTTT GCATGAAACA ATTTCACGCA ATGTTTTCTG ATGGCAGTTA TGTTTAGAAA   
  
  
+ TAAGTTTAAT CGGACCTTAT TCCACTGATT TATATTTGAT TCTTCCCTCT TTACTACGGC TGTCCATTCA   
  
  
+ CAAGCCAAAG AATTCTCCCC CCAAAAAAAA GAAAAAACAG TATAGAACCA TGTTTATTAT CTCATTTTTT   
  
  
+ TCTCTCCCAT TTGATCTGAA ACACAAACTT GCAGGGGTGC AAGTTGGACA CTTAGTAGCT GCAGGAATGA   
  
  
+ TTCAATAGGA ATCCTCATCT TTGACAACAT CACCACTTGA ATACATATCA CTTGATGGCA CTCTCCCCTG   
  
  
+ GTTTAGGCTT CCCATATCCG TGGCTTAGGG AGCTAAAACC TGAGCAAAGA GGACTCTGTC TAATCCATCT   
  
  
+ CCTCCTTTCA TGTGCTAATC AAGTTGCTAC TGGGAGCATC GACAATGCTA ATGTCAGCCT TGAGCATATT   
  
  
+ TCCCACCTTG CCTCTCCCAC CGGAGATACA ATGCAGCGAA TTGCTGCTTA CTTTGCTGAA GCCTTTGCTG   
  
  
+ ACCGTTTACT AAGGGCTTGG CAGCCCGGTC TTCTCAAAGC CTTGAATTGT ACCAAGATGT CATCTGTTTC   
  
  
+ CGAACAAATT CTTGTTCAAA AGTTGTTCTT TGATCTTCTT CCGTTCTTGA AGCTTTCATA TCTTGTGACG   
  
  
+ AACCAGGCAA TCACGGAGGC CATAGAAGGA GAAAAGATGG TTCATATAAT TGATCTCCAT TCTTGTGAAT   
  
  
+ CGGCTCTGTG GATTAGTCTC CTCCAGGCAT TGAGTGTTCG ACCTGAAGGC CCACCCCATT TGAGGATAAC   
  
  
+ CGGTATACAT GAGAAGAAAG AAGTGTTGGA TCAAATGGCT ATGCAACTAA ACAAAGAGGC TGAAAAATTG   
  
  
+ GACATCCCAT TTCAATTCAA TCCTATCGCA AGCAAACTAG ACGACCTTGA TGTCGAAAGC TTGAGTGTCA   
  
  
+ AGACCGGAGA AGCACTTGTT ATTAGTTCTG TGCTCCAACT ACATTCTCTT TTGGCATTGG ATGAGGGATC   
  
  
+ AATGCCTAAG AACGCAGGCA TGGCTTACTT GCAAAGGGTG TTTTATATGA AACCACGAAA ATTGGAGGAC   
  
  
+ TTGCCCAACA AGGATTTGAT GAAAATGTTG AACTCAAATG AAGATTCTAC ATCATCATCA TCTTCATCTC   
  
  
+ CTATCCCTTC ATCAAAACTT GATGCCTTTT TAAAAGCCCT CCTTGGGCTT TCGCCAAAAC TCATGGTTGT   
  
  
+ AACCGAGCAA GAATCAAACC ACAATGGAAG TGCCCTAATA GAAAGAGTGA TGGAGTCATT GAACTTCTAT   
  
  
+ GCAGCATTGT TTGATTGCTT GGAATCCACT ATATCGAGGA CATCGATAGA GAGACAGAAG CTCGAGAAGT   
  
  
+ TGATGTTTGG AGAGGAGATC AAGAACCTCA CAGCTTGTGA AGGGGCAGAG AGAAAGGCAA GGCACGAGAA   
  
  
+ GCTCAGTGAA TGGGTTCAAA GATTTGAGTC AGTAGGATTT AAAAGGGAGC CATTGAGCTA CCATGGTTTC   
  
  
+ TTGCTTGCTA GGAGGTTTTT ACATACCAAT AATTATGAGG GGTATAACAT CAAGGAAGTC AATGGTTTTC   
  
  
+ TTGTTATCTG TTGGCAAGAT AGACCCCTGT ATTCTGTTTC AGCTTGGAGA TTTTA  

- +Up\_Stream \_Len000AATGTT TTTGCTACGA GATTTGGATA ATCCAGCAAG GCTTTGGGCG TTTTAACTTT   
  
  
- ATACTAGCGG GCATAAAATG GGTTTGGACT TTATGTGAAT TATACATAAA ATGGACTTGG GTTTATGTGG   
  
  
- CCTGGACATA AATTGTGCCC GTGGGCTTTA CTATCCAGAT TGAGAGTGGT GGTGGCGTAA ACCGTGGGCT   
  
  
- TGCCATTTTT TACAAGTAGA TAATACGTGA AACCGAAGTA GGTATTGGGA TACTAGATAG ACACGGTTAT   
  
  
- AAACCTCGTG GGTCGCTTCA TCGCGTACGA GATTTGAGAA CGGAGTTTTG TTTTGTTTAT CACCCAGTAT   
  
  
- AAGGGAAGTT TCGTGATTGT TTGTGTGTGA GAGAGAGAGA GTCATCGGTT GTTGAATTCC GCCACAAACG   
  
  
- AACCAAAACC TCTCCCGCAC AAACCTACTC CACGTTTTGT GTATCTACTT TAGTATTTGT TGAACTAAGG   
  
  
- GTCGGTGGGT ACTGGATGAG TCTATTGTTC CCTGTTTTTG TTTCTCCAAA AGACTTTGTC TCTTTTGTCC   
  
  
- CCGCAACACA ACGCGACAAC GAGTGTTTTA AGGTTATAAG GAGATTTTCG AGGGTTTGCC TCTGTTTAGT   
  
  
- TTCTCCTGTT TCTGTACACC CTAAATTATG TTTTAAACTG ATTGAGACGT TGCCTTTTGT TGGGGATCCC   
  
  
- ACAAAAAGAC GCACAACATG TGTGAAGTGA AAATTGGACG AGACGGAGAC GGAAACGGTG CCGAAAATTA   
  
  
- CCTATTCTGG TTAATTCTTT GGGTTTATAG AGCATTTAAA CAACTCTAAG GAAAGACGCA AGAGTTCACA   
  
  
- AAGTTTTTGG GTCAAACCTC CAACGTGAAT AAGTACGAAA AGAAGTTCAG TCACTCGAAT CACAGACCTT   
  
  
- GACTTCGAGG AGGACCATTA CGAGACAAAA AGTAAAGAAC AACACGCAAA TATTTTTAAG CCGGATAACT   
  
  
- ACCTTCAAAC AAACTACAAA AACCCATTCA AAAACTAATT ATCTCTACTT AATCATTTCA TTTTAAACTC   
  
  
- ACCATAAATT ACTAGGAATT CGTCAGATAC GAAACAACAG TGTACGCAAA GAACCGAAAA CTGACTTTGT   
  
  
- AAAGACACAA TCTACTTTTT GAGTAAAACT ACGTACTCTA CTATTTTAGC GAATAAAAAC GGAAATAAAC   
  
  
- CACACACCCC CCCCCCCCCC CCCCCGCGTC CAGACTCCAT TTTCTATACA CTTAAGTAAA CCTCAGAGCT   
  
  
- ATTAAGCAAC CGATACAACT GTTAGTGCTC ACCAGCAATA GCGAGTAGAG TGTCCAACTA TTAAGTGAGT   
  
  
- TTGAAGTTCG CCGGACTTAG TAAGTCAGGC CCAACAATTT TAGAGTTGTC AGTAAAGTGT GCTCACCTCC   
  
  
- CGGGGTTTTC TGTGGTAAGA AAGCTTGAAA AAAAAAAACT TCCAGTAAAA AAGTTCTTGT TTTGATTACA   
  
  
- CTTGTACGCT GAAGATACTC ATTTCACATT TCTACGTATT TCAGAAAAAA AGAAAATATA TTTTTCATGA   
  
  
- GTTTTTTTTT TCTCAACCTC TCGACTAAGG GGTACGTAGG AGATTCGTTT CCACCACCTG CCGTCTTTTA   
  
  
- ATCATCTCAA TAACACTCTA ATTTCAAACG CGTGGACTTG CCGACTGGAG ATCAAACGAT AGTTGCAATG   
  
  
- TAAATACACA GTATTAAAAA CGTACTTTGT TAAAGTGCGT TACAAAAGAC TACCGTCAAT ACAAATCTTT   
  
  
- ATTCAAATTA GCCTGGAATA AGGTGACTAA ATATAAACTA AGAAGGGAGA AATGATGCCG ACAGGTAAGT   
  
  
- GTTCGGTTTC TTAAGAGGGG GGTTTTTTTT CTTTTTTGTC ATATCTTGGT ACAAATAATA GAGTAAAAAA   
  
  
- AGAGAGGGTA AACTAGACTT TGTGTTTGAA CGTCCCCACG TTCAACCTGT GAATCATCGA CGTCCTTACT   
  
  
- AAGTTATCCT TAGGAGTAGA AACTGTTGTA GTGGTGAACT TATGTATAGT GAACTACCGT GAGAGGGGAC   
  
  
- CAAATCCGAA GGGTATAGGC ACCGAATCCC TCGATTTTGG ACTCGTTTCT CCTGAGACAG ATTAGGTAGA   
  
  
- GGAGGAAAGT ACACGATTAG TTCAACGATG ACCCTCGTAG CTGTTACGAT TACAGTCGGA ACTCGTATAA   
  
  
- AGGGTGGAAC GGAGAGGGTG GCCTCTATGT TACGTCGCTT AACGACGAAT GAAACGACTT CGGAAACGAC   
  
  
- TGGCAAATGA TTCCCGAACC GTCGGGCCAG AAGAGTTTCG GAACTTAACA TGGTTCTACA GTAGACAAAG   
  
  
- GCTTGTTTAA GAACAAGTTT TCAACAAGAA ACTAGAAGAA GGCAAGAACT TCGAAAGTAT AGAACACTGC   
  
  
- TTGGTCCGTT AGTGCCTCCG GTATCTTCCT CTTTTCTACC AAGTATATTA ACTAGAGGTA AGAACACTTA   
  
  
- GCCGAGACAC CTAATCAGAG GAGGTCCGTA ACTCACAAGC TGGACTTCCG GGTGGGGTAA ACTCCTATTG   
  
  
- GCCATATGTA CTCTTCTTTC TTCACAACCT AGTTTACCGA TACGTTGATT TGTTTCTCCG ACTTTTTAAC   
  
  
- CTGTAGGGTA AAGTTAAGTT AGGATAGCGT TCGTTTGATC TGCTGGAACT ACAGCTTTCG AACTCACAGT   
  
  
- TCTGGCCTCT TCGTGAACAA TAATCAAGAC ACGAGGTTGA TGTAAGAGAA AACCGTAACC TACTCCCTAG   
  
  
- TTACGGATTC TTGCGTCCGT ACCGAATGAA CGTTTCCCAC AAAATATACT TTGGTGCTTT TAACCTCCTG   
  
  
- AACGGGTTGT TCCTAAACTA CTTTTACAAC TTGAGTTTAC TTCTAAGATG TAGTAGTAGT AGAAGTAGAG   
  
  
- GATAGGGAAG TAGTTTTGAA CTACGGAAAA ATTTTCGGGA GGAACCCGAA AGCGGTTTTG AGTACCAACA   
  
  
- TTGGCTCGTT CTTAGTTTGG TGTTACCTTC ACGGGATTAT CTTTCTCACT ACCTCAGTAA CTTGAAGATA   
  
  
- CGTCGTAACA AACTAACGAA CCTTAGGTGA TATAGCTCCT GTAGCTATCT CTCTGTCTTC GAGCTCTTCA   
  
  
- ACTACAAACC TCTCCTCTAG TTCTTGGAGT GTCGAACACT TCCCCGTCTC TCTTTCCGTT CCGTGCTCTT   
  
  
- CGAGTCACTT ACCCAAGTTT CTAAACTCAG TCATCCTAAA TTTTCCCTCG GTAACTCGAT GGTACCAAAG   
  
  
- AACGAACGAT CCTCCAAAAA TGTATGGTTA TTAATACTCC CCATATTGTA GTTCCTTCAG TTACCAAAAG   
  
  
- AACAATAGAC AACCGTTCTA TCTGGGGACA TAAGACAAAG TCGAACCTCT AAAAT

+     TGA-element

| Site Name | Organism | Position | Strand | Matrix score. | sequence | function |
| --- | --- | --- | --- | --- | --- | --- |
| TGA-element | Brassica oleracea | 48 | - | 6 | AACGAC | auxin-responsive element |
| TGA-element | Brassica oleracea | 1297 | - | 6 | AACGAC | auxin-responsive element |

>HU05G01267.1   
+ +Up\_Stream \_Len000TTACAA AAACGATGCT CTAAACCTAT TAGGTCGTTC CGAAACCCGC AAAATTGAAA   
  
  
+ TATGATCGCC CGTATTTTAC CCAAACCTGA AATACACTTA ATATGTATTT TACCTGAACC CAAATACACC   
  
  
+ GGACCTGTAT TTAACACGGG CACCCGAAAT GATAGGTCTA ACTCTCACCA CCACCGCATT TGGCACCCGA   
  
  
+ ACGGTAAAAA ATGTTCATCT ATTATGCACT TTGGCTTCAT CCATAACCCT ATGATCTATC TGTGCCAATA   
  
  
+ TTTGGAGCAC CCAGCGAAGT AGCGCATGCT CTAAACTCTT GCCTCAAAAC AAAACAAATA GTGGGTCATA   
  
  
+ TTCCCTTCAA AGCACTAACA AACACACACT CTCTCTCTCT CAGTAGCCAA CAACTTAAGG CGGTGTTTGC   
  
  
+ TTGGTTTTGG AGAGGGCGTG TTTGGATGAG GTGCAAAACA CATAGATGAA ATCATAAACA ACTTGATTCC   
  
  
+ CAGCCACCCA TGACCTACTC AGATAACAAG GGACAAAAAC AAAGAGGTTT TCTGAAACAG AGAAAACAGG   
  
  
+ GGCGTTGTGT TGCGCTGTTG CTCACAAAAT TCCAATATTC CTCTAAAAGC TCCCAAACGG AGACAAATCA   
  
  
+ AAGAGGACAA AGACATGTGG GATTTAATAC AAAATTTGAC TAACTCTGCA ACGGAAAACA ACCCCTAGGG   
  
  
+ TGTTTTTCTG CGTGTTGTAC ACACTTCACT TTTAACCTGC TCTGCCTCTG CCTTTGCCAC GGCTTTTAAT   
  
  
+ GGATAAGACC AATTAAGAAA CCCAAATATC TCGTAAATTT GTTGAGATTC CTTTCTGCGT TCTCAAGTGT   
  
  
+ TTCAAAAACC CAGTTTGGAG GTTGCACTTA TTCATGCTTT TCTTCAAGTC AGTGAGCTTA GTGTCTGGAA   
  
  
+ CTGAAGCTCC TCCTGGTAAT GCTCTGTTTT TCATTTCTTG TTGTGCGTTT ATAAAAATTC GGCCTATTGA   
  
  
+ TGGAAGTTTG TTTGATGTTT TTGGGTAAGT TTTTGATTAA TAGAGATGAA TTAGTAAAGT AAAATTTGAG   
  
  
+ TGGTATTTAA TGATCCTTAA GCAGTCTATG CTTTGTTGTC ACATGCGTTT CTTGGCTTTT GACTGAAACA   
  
  
+ TTTCTGTGTT AGATGAAAAA CTCATTTTGA TGCATGAGAT GATAAAATCG CTTATTTTTG CCTTTATTTG   
  
  
+ GTGTGTGGGG GGGGGGGGGG GGGGGCGCAG GTCTGAGGTA AAAGATATGT GAATTCATTT GGAGTCTCGA   
  
  
+ TAATTCGTTG GCTATGTTGA CAATCACGAG TGGTCGTTAT CGCTCATCTC ACAGGTTGAT AATTCACTCA   
  
  
+ AACTTCAAGC GGCCTGAATC ATTCAGTCCG GGTTGTTAAA ATCTCAACAG TCATTTCACA CGAGTGGAGG   
  
  
+ GCCCCAAAAG ACACCATTCT TTCGAACTTT TTTTTTTTGA AGGTCATTTT TTCAAGAACA AAACTAATGT   
  
  
+ GAACATGCGA CTTCTATGAG TAAAGTGTAA AGATGCATAA AGTCTTTTTT TCTTTTATAT AAAAAGTACT   
  
  
+ CAAAAAAAAA AGAGTTGGAG AGCTGATTCC CCATGCATCC TCTAAGCAAA GGTGGTGGAC GGCAGAAAAT   
  
  
+ TAGTAGAGTT ATTGTGAGAT TAAAGTTTGC GCACCTGAAC GGCTGACCTC TAGTTTGCTA TCAACGTTAC   
  
  
+ ATTTATGTGT CATAATTTTT GCATGAAACA ATTTCACGCA ATGTTTTCTG ATGGCAGTTA TGTTTAGAAA   
  
  
+ TAAGTTTAAT CGGACCTTAT TCCACTGATT TATATTTGAT TCTTCCCTCT TTACTACGGC TGTCCATTCA   
  
  
+ CAAGCCAAAG AATTCTCCCC CCAAAAAAAA GAAAAAACAG TATAGAACCA TGTTTATTAT CTCATTTTTT   
  
  
+ TCTCTCCCAT TTGATCTGAA ACACAAACTT GCAGGGGTGC AAGTTGGACA CTTAGTAGCT GCAGGAATGA   
  
  
+ TTCAATAGGA ATCCTCATCT TTGACAACAT CACCACTTGA ATACATATCA CTTGATGGCA CTCTCCCCTG   
  
  
+ GTTTAGGCTT CCCATATCCG TGGCTTAGGG AGCTAAAACC TGAGCAAAGA GGACTCTGTC TAATCCATCT   
  
  
+ CCTCCTTTCA TGTGCTAATC AAGTTGCTAC TGGGAGCATC GACAATGCTA ATGTCAGCCT TGAGCATATT   
  
  
+ TCCCACCTTG CCTCTCCCAC CGGAGATACA ATGCAGCGAA TTGCTGCTTA CTTTGCTGAA GCCTTTGCTG   
  
  
+ ACCGTTTACT AAGGGCTTGG CAGCCCGGTC TTCTCAAAGC CTTGAATTGT ACCAAGATGT CATCTGTTTC   
  
  
+ CGAACAAATT CTTGTTCAAA AGTTGTTCTT TGATCTTCTT CCGTTCTTGA AGCTTTCATA TCTTGTGACG   
  
  
+ AACCAGGCAA TCACGGAGGC CATAGAAGGA GAAAAGATGG TTCATATAAT TGATCTCCAT TCTTGTGAAT   
  
  
+ CGGCTCTGTG GATTAGTCTC CTCCAGGCAT TGAGTGTTCG ACCTGAAGGC CCACCCCATT TGAGGATAAC   
  
  
+ CGGTATACAT GAGAAGAAAG AAGTGTTGGA TCAAATGGCT ATGCAACTAA ACAAAGAGGC TGAAAAATTG   
  
  
+ GACATCCCAT TTCAATTCAA TCCTATCGCA AGCAAACTAG ACGACCTTGA TGTCGAAAGC TTGAGTGTCA   
  
  
+ AGACCGGAGA AGCACTTGTT ATTAGTTCTG TGCTCCAACT ACATTCTCTT TTGGCATTGG ATGAGGGATC   
  
  
+ AATGCCTAAG AACGCAGGCA TGGCTTACTT GCAAAGGGTG TTTTATATGA AACCACGAAA ATTGGAGGAC   
  
  
+ TTGCCCAACA AGGATTTGAT GAAAATGTTG AACTCAAATG AAGATTCTAC ATCATCATCA TCTTCATCTC   
  
  
+ CTATCCCTTC ATCAAAACTT GATGCCTTTT TAAAAGCCCT CCTTGGGCTT TCGCCAAAAC TCATGGTTGT   
  
  
+ AACCGAGCAA GAATCAAACC ACAATGGAAG TGCCCTAATA GAAAGAGTGA TGGAGTCATT GAACTTCTAT   
  
  
+ GCAGCATTGT TTGATTGCTT GGAATCCACT ATATCGAGGA CATCGATAGA GAGACAGAAG CTCGAGAAGT   
  
  
+ TGATGTTTGG AGAGGAGATC AAGAACCTCA CAGCTTGTGA AGGGGCAGAG AGAAAGGCAA GGCACGAGAA   
  
  
+ GCTCAGTGAA TGGGTTCAAA GATTTGAGTC AGTAGGATTT AAAAGGGAGC CATTGAGCTA CCATGGTTTC   
  
  
+ TTGCTTGCTA GGAGGTTTTT ACATACCAAT AATTATGAGG GGTATAACAT CAAGGAAGTC AATGGTTTTC   
  
  
+ TTGTTATCTG TTGGCAAGAT AGACCCCTGT ATTCTGTTTC AGCTTGGAGA TTTTA  

- +Up\_Stream \_Len000AATGTT TTTGCTACGA GATTTGGATA ATCCAGCAAG GCTTTGGGCG TTTTAACTTT   
  
  
- ATACTAGCGG GCATAAAATG GGTTTGGACT TTATGTGAAT TATACATAAA ATGGACTTGG GTTTATGTGG   
  
  
- CCTGGACATA AATTGTGCCC GTGGGCTTTA CTATCCAGAT TGAGAGTGGT GGTGGCGTAA ACCGTGGGCT   
  
  
- TGCCATTTTT TACAAGTAGA TAATACGTGA AACCGAAGTA GGTATTGGGA TACTAGATAG ACACGGTTAT   
  
  
- AAACCTCGTG GGTCGCTTCA TCGCGTACGA GATTTGAGAA CGGAGTTTTG TTTTGTTTAT CACCCAGTAT   
  
  
- AAGGGAAGTT TCGTGATTGT TTGTGTGTGA GAGAGAGAGA GTCATCGGTT GTTGAATTCC GCCACAAACG   
  
  
- AACCAAAACC TCTCCCGCAC AAACCTACTC CACGTTTTGT GTATCTACTT TAGTATTTGT TGAACTAAGG   
  
  
- GTCGGTGGGT ACTGGATGAG TCTATTGTTC CCTGTTTTTG TTTCTCCAAA AGACTTTGTC TCTTTTGTCC   
  
  
- CCGCAACACA ACGCGACAAC GAGTGTTTTA AGGTTATAAG GAGATTTTCG AGGGTTTGCC TCTGTTTAGT   
  
  
- TTCTCCTGTT TCTGTACACC CTAAATTATG TTTTAAACTG ATTGAGACGT TGCCTTTTGT TGGGGATCCC   
  
  
- ACAAAAAGAC GCACAACATG TGTGAAGTGA AAATTGGACG AGACGGAGAC GGAAACGGTG CCGAAAATTA   
  
  
- CCTATTCTGG TTAATTCTTT GGGTTTATAG AGCATTTAAA CAACTCTAAG GAAAGACGCA AGAGTTCACA   
  
  
- AAGTTTTTGG GTCAAACCTC CAACGTGAAT AAGTACGAAA AGAAGTTCAG TCACTCGAAT CACAGACCTT   
  
  
- GACTTCGAGG AGGACCATTA CGAGACAAAA AGTAAAGAAC AACACGCAAA TATTTTTAAG CCGGATAACT   
  
  
- ACCTTCAAAC AAACTACAAA AACCCATTCA AAAACTAATT ATCTCTACTT AATCATTTCA TTTTAAACTC   
  
  
- ACCATAAATT ACTAGGAATT CGTCAGATAC GAAACAACAG TGTACGCAAA GAACCGAAAA CTGACTTTGT   
  
  
- AAAGACACAA TCTACTTTTT GAGTAAAACT ACGTACTCTA CTATTTTAGC GAATAAAAAC GGAAATAAAC   
  
  
- CACACACCCC CCCCCCCCCC CCCCCGCGTC CAGACTCCAT TTTCTATACA CTTAAGTAAA CCTCAGAGCT   
  
  
- ATTAAGCAAC CGATACAACT GTTAGTGCTC ACCAGCAATA GCGAGTAGAG TGTCCAACTA TTAAGTGAGT   
  
  
- TTGAAGTTCG CCGGACTTAG TAAGTCAGGC CCAACAATTT TAGAGTTGTC AGTAAAGTGT GCTCACCTCC   
  
  
- CGGGGTTTTC TGTGGTAAGA AAGCTTGAAA AAAAAAAACT TCCAGTAAAA AAGTTCTTGT TTTGATTACA   
  
  
- CTTGTACGCT GAAGATACTC ATTTCACATT TCTACGTATT TCAGAAAAAA AGAAAATATA TTTTTCATGA   
  
  
- GTTTTTTTTT TCTCAACCTC TCGACTAAGG GGTACGTAGG AGATTCGTTT CCACCACCTG CCGTCTTTTA   
  
  
- ATCATCTCAA TAACACTCTA ATTTCAAACG CGTGGACTTG CCGACTGGAG ATCAAACGAT AGTTGCAATG   
  
  
- TAAATACACA GTATTAAAAA CGTACTTTGT TAAAGTGCGT TACAAAAGAC TACCGTCAAT ACAAATCTTT   
  
  
- ATTCAAATTA GCCTGGAATA AGGTGACTAA ATATAAACTA AGAAGGGAGA AATGATGCCG ACAGGTAAGT   
  
  
- GTTCGGTTTC TTAAGAGGGG GGTTTTTTTT CTTTTTTGTC ATATCTTGGT ACAAATAATA GAGTAAAAAA   
  
  
- AGAGAGGGTA AACTAGACTT TGTGTTTGAA CGTCCCCACG TTCAACCTGT GAATCATCGA CGTCCTTACT   
  
  
- AAGTTATCCT TAGGAGTAGA AACTGTTGTA GTGGTGAACT TATGTATAGT GAACTACCGT GAGAGGGGAC   
  
  
- CAAATCCGAA GGGTATAGGC ACCGAATCCC TCGATTTTGG ACTCGTTTCT CCTGAGACAG ATTAGGTAGA   
  
  
- GGAGGAAAGT ACACGATTAG TTCAACGATG ACCCTCGTAG CTGTTACGAT TACAGTCGGA ACTCGTATAA   
  
  
- AGGGTGGAAC GGAGAGGGTG GCCTCTATGT TACGTCGCTT AACGACGAAT GAAACGACTT CGGAAACGAC   
  
  
- TGGCAAATGA TTCCCGAACC GTCGGGCCAG AAGAGTTTCG GAACTTAACA TGGTTCTACA GTAGACAAAG   
  
  
- GCTTGTTTAA GAACAAGTTT TCAACAAGAA ACTAGAAGAA GGCAAGAACT TCGAAAGTAT AGAACACTGC   
  
  
- TTGGTCCGTT AGTGCCTCCG GTATCTTCCT CTTTTCTACC AAGTATATTA ACTAGAGGTA AGAACACTTA   
  
  
- GCCGAGACAC CTAATCAGAG GAGGTCCGTA ACTCACAAGC TGGACTTCCG GGTGGGGTAA ACTCCTATTG   
  
  
- GCCATATGTA CTCTTCTTTC TTCACAACCT AGTTTACCGA TACGTTGATT TGTTTCTCCG ACTTTTTAAC   
  
  
- CTGTAGGGTA AAGTTAAGTT AGGATAGCGT TCGTTTGATC TGCTGGAACT ACAGCTTTCG AACTCACAGT   
  
  
- TCTGGCCTCT TCGTGAACAA TAATCAAGAC ACGAGGTTGA TGTAAGAGAA AACCGTAACC TACTCCCTAG   
  
  
- TTACGGATTC TTGCGTCCGT ACCGAATGAA CGTTTCCCAC AAAATATACT TTGGTGCTTT TAACCTCCTG   
  
  
- AACGGGTTGT TCCTAAACTA CTTTTACAAC TTGAGTTTAC TTCTAAGATG TAGTAGTAGT AGAAGTAGAG   
  
  
- GATAGGGAAG TAGTTTTGAA CTACGGAAAA ATTTTCGGGA GGAACCCGAA AGCGGTTTTG AGTACCAACA   
  
  
- TTGGCTCGTT CTTAGTTTGG TGTTACCTTC ACGGGATTAT CTTTCTCACT ACCTCAGTAA CTTGAAGATA   
  
  
- CGTCGTAACA AACTAACGAA CCTTAGGTGA TATAGCTCCT GTAGCTATCT CTCTGTCTTC GAGCTCTTCA   
  
  
- ACTACAAACC TCTCCTCTAG TTCTTGGAGT GTCGAACACT TCCCCGTCTC TCTTTCCGTT CCGTGCTCTT   
  
  
- CGAGTCACTT ACCCAAGTTT CTAAACTCAG TCATCCTAAA TTTTCCCTCG GTAACTCGAT GGTACCAAAG   
  
  
- AACGAACGAT CCTCCAAAAA TGTATGGTTA TTAATACTCC CCATATTGTA GTTCCTTCAG TTACCAAAAG   
  
  
- AACAATAGAC AACCGTTCTA TCTGGGGACA TAAGACAAAG TCGAACCTCT AAAAT

+     TGACG-motif

| Site Name | Organism | Position | Strand | Matrix score. | sequence | function |
| --- | --- | --- | --- | --- | --- | --- |
| TGACG-motif | Hordeum vulgare | 2380 | + | 5 | TGACG | cis-acting regulatory element involved in the MeJA-responsiveness |

>HU05G01267.1   
+ +Up\_Stream \_Len000TTACAA AAACGATGCT CTAAACCTAT TAGGTCGTTC CGAAACCCGC AAAATTGAAA   
  
  
+ TATGATCGCC CGTATTTTAC CCAAACCTGA AATACACTTA ATATGTATTT TACCTGAACC CAAATACACC   
  
  
+ GGACCTGTAT TTAACACGGG CACCCGAAAT GATAGGTCTA ACTCTCACCA CCACCGCATT TGGCACCCGA   
  
  
+ ACGGTAAAAA ATGTTCATCT ATTATGCACT TTGGCTTCAT CCATAACCCT ATGATCTATC TGTGCCAATA   
  
  
+ TTTGGAGCAC CCAGCGAAGT AGCGCATGCT CTAAACTCTT GCCTCAAAAC AAAACAAATA GTGGGTCATA   
  
  
+ TTCCCTTCAA AGCACTAACA AACACACACT CTCTCTCTCT CAGTAGCCAA CAACTTAAGG CGGTGTTTGC   
  
  
+ TTGGTTTTGG AGAGGGCGTG TTTGGATGAG GTGCAAAACA CATAGATGAA ATCATAAACA ACTTGATTCC   
  
  
+ CAGCCACCCA TGACCTACTC AGATAACAAG GGACAAAAAC AAAGAGGTTT TCTGAAACAG AGAAAACAGG   
  
  
+ GGCGTTGTGT TGCGCTGTTG CTCACAAAAT TCCAATATTC CTCTAAAAGC TCCCAAACGG AGACAAATCA   
  
  
+ AAGAGGACAA AGACATGTGG GATTTAATAC AAAATTTGAC TAACTCTGCA ACGGAAAACA ACCCCTAGGG   
  
  
+ TGTTTTTCTG CGTGTTGTAC ACACTTCACT TTTAACCTGC TCTGCCTCTG CCTTTGCCAC GGCTTTTAAT   
  
  
+ GGATAAGACC AATTAAGAAA CCCAAATATC TCGTAAATTT GTTGAGATTC CTTTCTGCGT TCTCAAGTGT   
  
  
+ TTCAAAAACC CAGTTTGGAG GTTGCACTTA TTCATGCTTT TCTTCAAGTC AGTGAGCTTA GTGTCTGGAA   
  
  
+ CTGAAGCTCC TCCTGGTAAT GCTCTGTTTT TCATTTCTTG TTGTGCGTTT ATAAAAATTC GGCCTATTGA   
  
  
+ TGGAAGTTTG TTTGATGTTT TTGGGTAAGT TTTTGATTAA TAGAGATGAA TTAGTAAAGT AAAATTTGAG   
  
  
+ TGGTATTTAA TGATCCTTAA GCAGTCTATG CTTTGTTGTC ACATGCGTTT CTTGGCTTTT GACTGAAACA   
  
  
+ TTTCTGTGTT AGATGAAAAA CTCATTTTGA TGCATGAGAT GATAAAATCG CTTATTTTTG CCTTTATTTG   
  
  
+ GTGTGTGGGG GGGGGGGGGG GGGGGCGCAG GTCTGAGGTA AAAGATATGT GAATTCATTT GGAGTCTCGA   
  
  
+ TAATTCGTTG GCTATGTTGA CAATCACGAG TGGTCGTTAT CGCTCATCTC ACAGGTTGAT AATTCACTCA   
  
  
+ AACTTCAAGC GGCCTGAATC ATTCAGTCCG GGTTGTTAAA ATCTCAACAG TCATTTCACA CGAGTGGAGG   
  
  
+ GCCCCAAAAG ACACCATTCT TTCGAACTTT TTTTTTTTGA AGGTCATTTT TTCAAGAACA AAACTAATGT   
  
  
+ GAACATGCGA CTTCTATGAG TAAAGTGTAA AGATGCATAA AGTCTTTTTT TCTTTTATAT AAAAAGTACT   
  
  
+ CAAAAAAAAA AGAGTTGGAG AGCTGATTCC CCATGCATCC TCTAAGCAAA GGTGGTGGAC GGCAGAAAAT   
  
  
+ TAGTAGAGTT ATTGTGAGAT TAAAGTTTGC GCACCTGAAC GGCTGACCTC TAGTTTGCTA TCAACGTTAC   
  
  
+ ATTTATGTGT CATAATTTTT GCATGAAACA ATTTCACGCA ATGTTTTCTG ATGGCAGTTA TGTTTAGAAA   
  
  
+ TAAGTTTAAT CGGACCTTAT TCCACTGATT TATATTTGAT TCTTCCCTCT TTACTACGGC TGTCCATTCA   
  
  
+ CAAGCCAAAG AATTCTCCCC CCAAAAAAAA GAAAAAACAG TATAGAACCA TGTTTATTAT CTCATTTTTT   
  
  
+ TCTCTCCCAT TTGATCTGAA ACACAAACTT GCAGGGGTGC AAGTTGGACA CTTAGTAGCT GCAGGAATGA   
  
  
+ TTCAATAGGA ATCCTCATCT TTGACAACAT CACCACTTGA ATACATATCA CTTGATGGCA CTCTCCCCTG   
  
  
+ GTTTAGGCTT CCCATATCCG TGGCTTAGGG AGCTAAAACC TGAGCAAAGA GGACTCTGTC TAATCCATCT   
  
  
+ CCTCCTTTCA TGTGCTAATC AAGTTGCTAC TGGGAGCATC GACAATGCTA ATGTCAGCCT TGAGCATATT   
  
  
+ TCCCACCTTG CCTCTCCCAC CGGAGATACA ATGCAGCGAA TTGCTGCTTA CTTTGCTGAA GCCTTTGCTG   
  
  
+ ACCGTTTACT AAGGGCTTGG CAGCCCGGTC TTCTCAAAGC CTTGAATTGT ACCAAGATGT CATCTGTTTC   
  
  
+ CGAACAAATT CTTGTTCAAA AGTTGTTCTT TGATCTTCTT CCGTTCTTGA AGCTTTCATA TCTTGTGACG   
  
  
+ AACCAGGCAA TCACGGAGGC CATAGAAGGA GAAAAGATGG TTCATATAAT TGATCTCCAT TCTTGTGAAT   
  
  
+ CGGCTCTGTG GATTAGTCTC CTCCAGGCAT TGAGTGTTCG ACCTGAAGGC CCACCCCATT TGAGGATAAC   
  
  
+ CGGTATACAT GAGAAGAAAG AAGTGTTGGA TCAAATGGCT ATGCAACTAA ACAAAGAGGC TGAAAAATTG   
  
  
+ GACATCCCAT TTCAATTCAA TCCTATCGCA AGCAAACTAG ACGACCTTGA TGTCGAAAGC TTGAGTGTCA   
  
  
+ AGACCGGAGA AGCACTTGTT ATTAGTTCTG TGCTCCAACT ACATTCTCTT TTGGCATTGG ATGAGGGATC   
  
  
+ AATGCCTAAG AACGCAGGCA TGGCTTACTT GCAAAGGGTG TTTTATATGA AACCACGAAA ATTGGAGGAC   
  
  
+ TTGCCCAACA AGGATTTGAT GAAAATGTTG AACTCAAATG AAGATTCTAC ATCATCATCA TCTTCATCTC   
  
  
+ CTATCCCTTC ATCAAAACTT GATGCCTTTT TAAAAGCCCT CCTTGGGCTT TCGCCAAAAC TCATGGTTGT   
  
  
+ AACCGAGCAA GAATCAAACC ACAATGGAAG TGCCCTAATA GAAAGAGTGA TGGAGTCATT GAACTTCTAT   
  
  
+ GCAGCATTGT TTGATTGCTT GGAATCCACT ATATCGAGGA CATCGATAGA GAGACAGAAG CTCGAGAAGT   
  
  
+ TGATGTTTGG AGAGGAGATC AAGAACCTCA CAGCTTGTGA AGGGGCAGAG AGAAAGGCAA GGCACGAGAA   
  
  
+ GCTCAGTGAA TGGGTTCAAA GATTTGAGTC AGTAGGATTT AAAAGGGAGC CATTGAGCTA CCATGGTTTC   
  
  
+ TTGCTTGCTA GGAGGTTTTT ACATACCAAT AATTATGAGG GGTATAACAT CAAGGAAGTC AATGGTTTTC   
  
  
+ TTGTTATCTG TTGGCAAGAT AGACCCCTGT ATTCTGTTTC AGCTTGGAGA TTTTA  

- +Up\_Stream \_Len000AATGTT TTTGCTACGA GATTTGGATA ATCCAGCAAG GCTTTGGGCG TTTTAACTTT   
  
  
- ATACTAGCGG GCATAAAATG GGTTTGGACT TTATGTGAAT TATACATAAA ATGGACTTGG GTTTATGTGG   
  
  
- CCTGGACATA AATTGTGCCC GTGGGCTTTA CTATCCAGAT TGAGAGTGGT GGTGGCGTAA ACCGTGGGCT   
  
  
- TGCCATTTTT TACAAGTAGA TAATACGTGA AACCGAAGTA GGTATTGGGA TACTAGATAG ACACGGTTAT   
  
  
- AAACCTCGTG GGTCGCTTCA TCGCGTACGA GATTTGAGAA CGGAGTTTTG TTTTGTTTAT CACCCAGTAT   
  
  
- AAGGGAAGTT TCGTGATTGT TTGTGTGTGA GAGAGAGAGA GTCATCGGTT GTTGAATTCC GCCACAAACG   
  
  
- AACCAAAACC TCTCCCGCAC AAACCTACTC CACGTTTTGT GTATCTACTT TAGTATTTGT TGAACTAAGG   
  
  
- GTCGGTGGGT ACTGGATGAG TCTATTGTTC CCTGTTTTTG TTTCTCCAAA AGACTTTGTC TCTTTTGTCC   
  
  
- CCGCAACACA ACGCGACAAC GAGTGTTTTA AGGTTATAAG GAGATTTTCG AGGGTTTGCC TCTGTTTAGT   
  
  
- TTCTCCTGTT TCTGTACACC CTAAATTATG TTTTAAACTG ATTGAGACGT TGCCTTTTGT TGGGGATCCC   
  
  
- ACAAAAAGAC GCACAACATG TGTGAAGTGA AAATTGGACG AGACGGAGAC GGAAACGGTG CCGAAAATTA   
  
  
- CCTATTCTGG TTAATTCTTT GGGTTTATAG AGCATTTAAA CAACTCTAAG GAAAGACGCA AGAGTTCACA   
  
  
- AAGTTTTTGG GTCAAACCTC CAACGTGAAT AAGTACGAAA AGAAGTTCAG TCACTCGAAT CACAGACCTT   
  
  
- GACTTCGAGG AGGACCATTA CGAGACAAAA AGTAAAGAAC AACACGCAAA TATTTTTAAG CCGGATAACT   
  
  
- ACCTTCAAAC AAACTACAAA AACCCATTCA AAAACTAATT ATCTCTACTT AATCATTTCA TTTTAAACTC   
  
  
- ACCATAAATT ACTAGGAATT CGTCAGATAC GAAACAACAG TGTACGCAAA GAACCGAAAA CTGACTTTGT   
  
  
- AAAGACACAA TCTACTTTTT GAGTAAAACT ACGTACTCTA CTATTTTAGC GAATAAAAAC GGAAATAAAC   
  
  
- CACACACCCC CCCCCCCCCC CCCCCGCGTC CAGACTCCAT TTTCTATACA CTTAAGTAAA CCTCAGAGCT   
  
  
- ATTAAGCAAC CGATACAACT GTTAGTGCTC ACCAGCAATA GCGAGTAGAG TGTCCAACTA TTAAGTGAGT   
  
  
- TTGAAGTTCG CCGGACTTAG TAAGTCAGGC CCAACAATTT TAGAGTTGTC AGTAAAGTGT GCTCACCTCC   
  
  
- CGGGGTTTTC TGTGGTAAGA AAGCTTGAAA AAAAAAAACT TCCAGTAAAA AAGTTCTTGT TTTGATTACA   
  
  
- CTTGTACGCT GAAGATACTC ATTTCACATT TCTACGTATT TCAGAAAAAA AGAAAATATA TTTTTCATGA   
  
  
- GTTTTTTTTT TCTCAACCTC TCGACTAAGG GGTACGTAGG AGATTCGTTT CCACCACCTG CCGTCTTTTA   
  
  
- ATCATCTCAA TAACACTCTA ATTTCAAACG CGTGGACTTG CCGACTGGAG ATCAAACGAT AGTTGCAATG   
  
  
- TAAATACACA GTATTAAAAA CGTACTTTGT TAAAGTGCGT TACAAAAGAC TACCGTCAAT ACAAATCTTT   
  
  
- ATTCAAATTA GCCTGGAATA AGGTGACTAA ATATAAACTA AGAAGGGAGA AATGATGCCG ACAGGTAAGT   
  
  
- GTTCGGTTTC TTAAGAGGGG GGTTTTTTTT CTTTTTTGTC ATATCTTGGT ACAAATAATA GAGTAAAAAA   
  
  
- AGAGAGGGTA AACTAGACTT TGTGTTTGAA CGTCCCCACG TTCAACCTGT GAATCATCGA CGTCCTTACT   
  
  
- AAGTTATCCT TAGGAGTAGA AACTGTTGTA GTGGTGAACT TATGTATAGT GAACTACCGT GAGAGGGGAC   
  
  
- CAAATCCGAA GGGTATAGGC ACCGAATCCC TCGATTTTGG ACTCGTTTCT CCTGAGACAG ATTAGGTAGA   
  
  
- GGAGGAAAGT ACACGATTAG TTCAACGATG ACCCTCGTAG CTGTTACGAT TACAGTCGGA ACTCGTATAA   
  
  
- AGGGTGGAAC GGAGAGGGTG GCCTCTATGT TACGTCGCTT AACGACGAAT GAAACGACTT CGGAAACGAC   
  
  
- TGGCAAATGA TTCCCGAACC GTCGGGCCAG AAGAGTTTCG GAACTTAACA TGGTTCTACA GTAGACAAAG   
  
  
- GCTTGTTTAA GAACAAGTTT TCAACAAGAA ACTAGAAGAA GGCAAGAACT TCGAAAGTAT AGAACACTGC   
  
  
- TTGGTCCGTT AGTGCCTCCG GTATCTTCCT CTTTTCTACC AAGTATATTA ACTAGAGGTA AGAACACTTA   
  
  
- GCCGAGACAC CTAATCAGAG GAGGTCCGTA ACTCACAAGC TGGACTTCCG GGTGGGGTAA ACTCCTATTG   
  
  
- GCCATATGTA CTCTTCTTTC TTCACAACCT AGTTTACCGA TACGTTGATT TGTTTCTCCG ACTTTTTAAC   
  
  
- CTGTAGGGTA AAGTTAAGTT AGGATAGCGT TCGTTTGATC TGCTGGAACT ACAGCTTTCG AACTCACAGT   
  
  
- TCTGGCCTCT TCGTGAACAA TAATCAAGAC ACGAGGTTGA TGTAAGAGAA AACCGTAACC TACTCCCTAG   
  
  
- TTACGGATTC TTGCGTCCGT ACCGAATGAA CGTTTCCCAC AAAATATACT TTGGTGCTTT TAACCTCCTG   
  
  
- AACGGGTTGT TCCTAAACTA CTTTTACAAC TTGAGTTTAC TTCTAAGATG TAGTAGTAGT AGAAGTAGAG   
  
  
- GATAGGGAAG TAGTTTTGAA CTACGGAAAA ATTTTCGGGA GGAACCCGAA AGCGGTTTTG AGTACCAACA   
  
  
- TTGGCTCGTT CTTAGTTTGG TGTTACCTTC ACGGGATTAT CTTTCTCACT ACCTCAGTAA CTTGAAGATA   
  
  
- CGTCGTAACA AACTAACGAA CCTTAGGTGA TATAGCTCCT GTAGCTATCT CTCTGTCTTC GAGCTCTTCA   
  
  
- ACTACAAACC TCTCCTCTAG TTCTTGGAGT GTCGAACACT TCCCCGTCTC TCTTTCCGTT CCGTGCTCTT   
  
  
- CGAGTCACTT ACCCAAGTTT CTAAACTCAG TCATCCTAAA TTTTCCCTCG GTAACTCGAT GGTACCAAAG   
  
  
- AACGAACGAT CCTCCAAAAA TGTATGGTTA TTAATACTCC CCATATTGTA GTTCCTTCAG TTACCAAAAG   
  
  
- AACAATAGAC AACCGTTCTA TCTGGGGACA TAAGACAAAG TCGAACCTCT AAAAT

+     Unnamed\_\_1

| Site Name | Organism | Position | Strand | Matrix score. | sequence | function |
| --- | --- | --- | --- | --- | --- | --- |
| Unnamed\_\_1 | Zea mays | 761 | - | 5 | CGTGG |  |
| Unnamed\_\_1 | Zea mays | 2053 | + | 5 | CGTGG |  |
| Unnamed\_\_1 | Zea mays | 2787 | - | 5 | CGTGG |  |

>HU05G01267.1   
+ +Up\_Stream \_Len000TTACAA AAACGATGCT CTAAACCTAT TAGGTCGTTC CGAAACCCGC AAAATTGAAA   
  
  
+ TATGATCGCC CGTATTTTAC CCAAACCTGA AATACACTTA ATATGTATTT TACCTGAACC CAAATACACC   
  
  
+ GGACCTGTAT TTAACACGGG CACCCGAAAT GATAGGTCTA ACTCTCACCA CCACCGCATT TGGCACCCGA   
  
  
+ ACGGTAAAAA ATGTTCATCT ATTATGCACT TTGGCTTCAT CCATAACCCT ATGATCTATC TGTGCCAATA   
  
  
+ TTTGGAGCAC CCAGCGAAGT AGCGCATGCT CTAAACTCTT GCCTCAAAAC AAAACAAATA GTGGGTCATA   
  
  
+ TTCCCTTCAA AGCACTAACA AACACACACT CTCTCTCTCT CAGTAGCCAA CAACTTAAGG CGGTGTTTGC   
  
  
+ TTGGTTTTGG AGAGGGCGTG TTTGGATGAG GTGCAAAACA CATAGATGAA ATCATAAACA ACTTGATTCC   
  
  
+ CAGCCACCCA TGACCTACTC AGATAACAAG GGACAAAAAC AAAGAGGTTT TCTGAAACAG AGAAAACAGG   
  
  
+ GGCGTTGTGT TGCGCTGTTG CTCACAAAAT TCCAATATTC CTCTAAAAGC TCCCAAACGG AGACAAATCA   
  
  
+ AAGAGGACAA AGACATGTGG GATTTAATAC AAAATTTGAC TAACTCTGCA ACGGAAAACA ACCCCTAGGG   
  
  
+ TGTTTTTCTG CGTGTTGTAC ACACTTCACT TTTAACCTGC TCTGCCTCTG CCTTTGCCAC GGCTTTTAAT   
  
  
+ GGATAAGACC AATTAAGAAA CCCAAATATC TCGTAAATTT GTTGAGATTC CTTTCTGCGT TCTCAAGTGT   
  
  
+ TTCAAAAACC CAGTTTGGAG GTTGCACTTA TTCATGCTTT TCTTCAAGTC AGTGAGCTTA GTGTCTGGAA   
  
  
+ CTGAAGCTCC TCCTGGTAAT GCTCTGTTTT TCATTTCTTG TTGTGCGTTT ATAAAAATTC GGCCTATTGA   
  
  
+ TGGAAGTTTG TTTGATGTTT TTGGGTAAGT TTTTGATTAA TAGAGATGAA TTAGTAAAGT AAAATTTGAG   
  
  
+ TGGTATTTAA TGATCCTTAA GCAGTCTATG CTTTGTTGTC ACATGCGTTT CTTGGCTTTT GACTGAAACA   
  
  
+ TTTCTGTGTT AGATGAAAAA CTCATTTTGA TGCATGAGAT GATAAAATCG CTTATTTTTG CCTTTATTTG   
  
  
+ GTGTGTGGGG GGGGGGGGGG GGGGGCGCAG GTCTGAGGTA AAAGATATGT GAATTCATTT GGAGTCTCGA   
  
  
+ TAATTCGTTG GCTATGTTGA CAATCACGAG TGGTCGTTAT CGCTCATCTC ACAGGTTGAT AATTCACTCA   
  
  
+ AACTTCAAGC GGCCTGAATC ATTCAGTCCG GGTTGTTAAA ATCTCAACAG TCATTTCACA CGAGTGGAGG   
  
  
+ GCCCCAAAAG ACACCATTCT TTCGAACTTT TTTTTTTTGA AGGTCATTTT TTCAAGAACA AAACTAATGT   
  
  
+ GAACATGCGA CTTCTATGAG TAAAGTGTAA AGATGCATAA AGTCTTTTTT TCTTTTATAT AAAAAGTACT   
  
  
+ CAAAAAAAAA AGAGTTGGAG AGCTGATTCC CCATGCATCC TCTAAGCAAA GGTGGTGGAC GGCAGAAAAT   
  
  
+ TAGTAGAGTT ATTGTGAGAT TAAAGTTTGC GCACCTGAAC GGCTGACCTC TAGTTTGCTA TCAACGTTAC   
  
  
+ ATTTATGTGT CATAATTTTT GCATGAAACA ATTTCACGCA ATGTTTTCTG ATGGCAGTTA TGTTTAGAAA   
  
  
+ TAAGTTTAAT CGGACCTTAT TCCACTGATT TATATTTGAT TCTTCCCTCT TTACTACGGC TGTCCATTCA   
  
  
+ CAAGCCAAAG AATTCTCCCC CCAAAAAAAA GAAAAAACAG TATAGAACCA TGTTTATTAT CTCATTTTTT   
  
  
+ TCTCTCCCAT TTGATCTGAA ACACAAACTT GCAGGGGTGC AAGTTGGACA CTTAGTAGCT GCAGGAATGA   
  
  
+ TTCAATAGGA ATCCTCATCT TTGACAACAT CACCACTTGA ATACATATCA CTTGATGGCA CTCTCCCCTG   
  
  
+ GTTTAGGCTT CCCATATCCG TGGCTTAGGG AGCTAAAACC TGAGCAAAGA GGACTCTGTC TAATCCATCT   
  
  
+ CCTCCTTTCA TGTGCTAATC AAGTTGCTAC TGGGAGCATC GACAATGCTA ATGTCAGCCT TGAGCATATT   
  
  
+ TCCCACCTTG CCTCTCCCAC CGGAGATACA ATGCAGCGAA TTGCTGCTTA CTTTGCTGAA GCCTTTGCTG   
  
  
+ ACCGTTTACT AAGGGCTTGG CAGCCCGGTC TTCTCAAAGC CTTGAATTGT ACCAAGATGT CATCTGTTTC   
  
  
+ CGAACAAATT CTTGTTCAAA AGTTGTTCTT TGATCTTCTT CCGTTCTTGA AGCTTTCATA TCTTGTGACG   
  
  
+ AACCAGGCAA TCACGGAGGC CATAGAAGGA GAAAAGATGG TTCATATAAT TGATCTCCAT TCTTGTGAAT   
  
  
+ CGGCTCTGTG GATTAGTCTC CTCCAGGCAT TGAGTGTTCG ACCTGAAGGC CCACCCCATT TGAGGATAAC   
  
  
+ CGGTATACAT GAGAAGAAAG AAGTGTTGGA TCAAATGGCT ATGCAACTAA ACAAAGAGGC TGAAAAATTG   
  
  
+ GACATCCCAT TTCAATTCAA TCCTATCGCA AGCAAACTAG ACGACCTTGA TGTCGAAAGC TTGAGTGTCA   
  
  
+ AGACCGGAGA AGCACTTGTT ATTAGTTCTG TGCTCCAACT ACATTCTCTT TTGGCATTGG ATGAGGGATC   
  
  
+ AATGCCTAAG AACGCAGGCA TGGCTTACTT GCAAAGGGTG TTTTATATGA AACCACGAAA ATTGGAGGAC   
  
  
+ TTGCCCAACA AGGATTTGAT GAAAATGTTG AACTCAAATG AAGATTCTAC ATCATCATCA TCTTCATCTC   
  
  
+ CTATCCCTTC ATCAAAACTT GATGCCTTTT TAAAAGCCCT CCTTGGGCTT TCGCCAAAAC TCATGGTTGT   
  
  
+ AACCGAGCAA GAATCAAACC ACAATGGAAG TGCCCTAATA GAAAGAGTGA TGGAGTCATT GAACTTCTAT   
  
  
+ GCAGCATTGT TTGATTGCTT GGAATCCACT ATATCGAGGA CATCGATAGA GAGACAGAAG CTCGAGAAGT   
  
  
+ TGATGTTTGG AGAGGAGATC AAGAACCTCA CAGCTTGTGA AGGGGCAGAG AGAAAGGCAA GGCACGAGAA   
  
  
+ GCTCAGTGAA TGGGTTCAAA GATTTGAGTC AGTAGGATTT AAAAGGGAGC CATTGAGCTA CCATGGTTTC   
  
  
+ TTGCTTGCTA GGAGGTTTTT ACATACCAAT AATTATGAGG GGTATAACAT CAAGGAAGTC AATGGTTTTC   
  
  
+ TTGTTATCTG TTGGCAAGAT AGACCCCTGT ATTCTGTTTC AGCTTGGAGA TTTTA  

- +Up\_Stream \_Len000AATGTT TTTGCTACGA GATTTGGATA ATCCAGCAAG GCTTTGGGCG TTTTAACTTT   
  
  
- ATACTAGCGG GCATAAAATG GGTTTGGACT TTATGTGAAT TATACATAAA ATGGACTTGG GTTTATGTGG   
  
  
- CCTGGACATA AATTGTGCCC GTGGGCTTTA CTATCCAGAT TGAGAGTGGT GGTGGCGTAA ACCGTGGGCT   
  
  
- TGCCATTTTT TACAAGTAGA TAATACGTGA AACCGAAGTA GGTATTGGGA TACTAGATAG ACACGGTTAT   
  
  
- AAACCTCGTG GGTCGCTTCA TCGCGTACGA GATTTGAGAA CGGAGTTTTG TTTTGTTTAT CACCCAGTAT   
  
  
- AAGGGAAGTT TCGTGATTGT TTGTGTGTGA GAGAGAGAGA GTCATCGGTT GTTGAATTCC GCCACAAACG   
  
  
- AACCAAAACC TCTCCCGCAC AAACCTACTC CACGTTTTGT GTATCTACTT TAGTATTTGT TGAACTAAGG   
  
  
- GTCGGTGGGT ACTGGATGAG TCTATTGTTC CCTGTTTTTG TTTCTCCAAA AGACTTTGTC TCTTTTGTCC   
  
  
- CCGCAACACA ACGCGACAAC GAGTGTTTTA AGGTTATAAG GAGATTTTCG AGGGTTTGCC TCTGTTTAGT   
  
  
- TTCTCCTGTT TCTGTACACC CTAAATTATG TTTTAAACTG ATTGAGACGT TGCCTTTTGT TGGGGATCCC   
  
  
- ACAAAAAGAC GCACAACATG TGTGAAGTGA AAATTGGACG AGACGGAGAC GGAAACGGTG CCGAAAATTA   
  
  
- CCTATTCTGG TTAATTCTTT GGGTTTATAG AGCATTTAAA CAACTCTAAG GAAAGACGCA AGAGTTCACA   
  
  
- AAGTTTTTGG GTCAAACCTC CAACGTGAAT AAGTACGAAA AGAAGTTCAG TCACTCGAAT CACAGACCTT   
  
  
- GACTTCGAGG AGGACCATTA CGAGACAAAA AGTAAAGAAC AACACGCAAA TATTTTTAAG CCGGATAACT   
  
  
- ACCTTCAAAC AAACTACAAA AACCCATTCA AAAACTAATT ATCTCTACTT AATCATTTCA TTTTAAACTC   
  
  
- ACCATAAATT ACTAGGAATT CGTCAGATAC GAAACAACAG TGTACGCAAA GAACCGAAAA CTGACTTTGT   
  
  
- AAAGACACAA TCTACTTTTT GAGTAAAACT ACGTACTCTA CTATTTTAGC GAATAAAAAC GGAAATAAAC   
  
  
- CACACACCCC CCCCCCCCCC CCCCCGCGTC CAGACTCCAT TTTCTATACA CTTAAGTAAA CCTCAGAGCT   
  
  
- ATTAAGCAAC CGATACAACT GTTAGTGCTC ACCAGCAATA GCGAGTAGAG TGTCCAACTA TTAAGTGAGT   
  
  
- TTGAAGTTCG CCGGACTTAG TAAGTCAGGC CCAACAATTT TAGAGTTGTC AGTAAAGTGT GCTCACCTCC   
  
  
- CGGGGTTTTC TGTGGTAAGA AAGCTTGAAA AAAAAAAACT TCCAGTAAAA AAGTTCTTGT TTTGATTACA   
  
  
- CTTGTACGCT GAAGATACTC ATTTCACATT TCTACGTATT TCAGAAAAAA AGAAAATATA TTTTTCATGA   
  
  
- GTTTTTTTTT TCTCAACCTC TCGACTAAGG GGTACGTAGG AGATTCGTTT CCACCACCTG CCGTCTTTTA   
  
  
- ATCATCTCAA TAACACTCTA ATTTCAAACG CGTGGACTTG CCGACTGGAG ATCAAACGAT AGTTGCAATG   
  
  
- TAAATACACA GTATTAAAAA CGTACTTTGT TAAAGTGCGT TACAAAAGAC TACCGTCAAT ACAAATCTTT   
  
  
- ATTCAAATTA GCCTGGAATA AGGTGACTAA ATATAAACTA AGAAGGGAGA AATGATGCCG ACAGGTAAGT   
  
  
- GTTCGGTTTC TTAAGAGGGG GGTTTTTTTT CTTTTTTGTC ATATCTTGGT ACAAATAATA GAGTAAAAAA   
  
  
- AGAGAGGGTA AACTAGACTT TGTGTTTGAA CGTCCCCACG TTCAACCTGT GAATCATCGA CGTCCTTACT   
  
  
- AAGTTATCCT TAGGAGTAGA AACTGTTGTA GTGGTGAACT TATGTATAGT GAACTACCGT GAGAGGGGAC   
  
  
- CAAATCCGAA GGGTATAGGC ACCGAATCCC TCGATTTTGG ACTCGTTTCT CCTGAGACAG ATTAGGTAGA   
  
  
- GGAGGAAAGT ACACGATTAG TTCAACGATG ACCCTCGTAG CTGTTACGAT TACAGTCGGA ACTCGTATAA   
  
  
- AGGGTGGAAC GGAGAGGGTG GCCTCTATGT TACGTCGCTT AACGACGAAT GAAACGACTT CGGAAACGAC   
  
  
- TGGCAAATGA TTCCCGAACC GTCGGGCCAG AAGAGTTTCG GAACTTAACA TGGTTCTACA GTAGACAAAG   
  
  
- GCTTGTTTAA GAACAAGTTT TCAACAAGAA ACTAGAAGAA GGCAAGAACT TCGAAAGTAT AGAACACTGC   
  
  
- TTGGTCCGTT AGTGCCTCCG GTATCTTCCT CTTTTCTACC AAGTATATTA ACTAGAGGTA AGAACACTTA   
  
  
- GCCGAGACAC CTAATCAGAG GAGGTCCGTA ACTCACAAGC TGGACTTCCG GGTGGGGTAA ACTCCTATTG   
  
  
- GCCATATGTA CTCTTCTTTC TTCACAACCT AGTTTACCGA TACGTTGATT TGTTTCTCCG ACTTTTTAAC   
  
  
- CTGTAGGGTA AAGTTAAGTT AGGATAGCGT TCGTTTGATC TGCTGGAACT ACAGCTTTCG AACTCACAGT   
  
  
- TCTGGCCTCT TCGTGAACAA TAATCAAGAC ACGAGGTTGA TGTAAGAGAA AACCGTAACC TACTCCCTAG   
  
  
- TTACGGATTC TTGCGTCCGT ACCGAATGAA CGTTTCCCAC AAAATATACT TTGGTGCTTT TAACCTCCTG   
  
  
- AACGGGTTGT TCCTAAACTA CTTTTACAAC TTGAGTTTAC TTCTAAGATG TAGTAGTAGT AGAAGTAGAG   
  
  
- GATAGGGAAG TAGTTTTGAA CTACGGAAAA ATTTTCGGGA GGAACCCGAA AGCGGTTTTG AGTACCAACA   
  
  
- TTGGCTCGTT CTTAGTTTGG TGTTACCTTC ACGGGATTAT CTTTCTCACT ACCTCAGTAA CTTGAAGATA   
  
  
- CGTCGTAACA AACTAACGAA CCTTAGGTGA TATAGCTCCT GTAGCTATCT CTCTGTCTTC GAGCTCTTCA   
  
  
- ACTACAAACC TCTCCTCTAG TTCTTGGAGT GTCGAACACT TCCCCGTCTC TCTTTCCGTT CCGTGCTCTT   
  
  
- CGAGTCACTT ACCCAAGTTT CTAAACTCAG TCATCCTAAA TTTTCCCTCG GTAACTCGAT GGTACCAAAG   
  
  
- AACGAACGAT CCTCCAAAAA TGTATGGTTA TTAATACTCC CCATATTGTA GTTCCTTCAG TTACCAAAAG   
  
  
- AACAATAGAC AACCGTTCTA TCTGGGGACA TAAGACAAAG TCGAACCTCT AAAAT

+     Unnamed\_\_4

| Site Name | Organism | Position | Strand | Matrix score. | sequence | function |
| --- | --- | --- | --- | --- | --- | --- |
| Unnamed\_\_4 | Petroselinum hortense | 2063 | - | 4 | CTCC |  |
| Unnamed\_\_4 | Petroselinum hortense | 2913 | + | 4 | CTCC |  |
| Unnamed\_\_4 | Petroselinum hortense | 2670 | - | 4 | CTCC |  |
| Unnamed\_\_4 | Petroselinum hortense | 3340 | - | 4 | CTCC |  |
| Unnamed\_\_4 | Petroselinum hortense | 1898 | + | 4 | CTCC |  |
| Unnamed\_\_4 | Petroselinum hortense | 2475 | + | 4 | CTCC |  |
| Unnamed\_\_4 | Petroselinum hortense | 2472 | + | 4 | CTCC |  |
| Unnamed\_\_4 | Petroselinum hortense | 2439 | + | 4 | CTCC |  |
| Unnamed\_\_4 | Petroselinum hortense | 2412 | - | 4 | CTCC |  |
| Unnamed\_\_4 | Petroselinum hortense | 924 | + | 4 | CTCC |  |
| Unnamed\_\_4 | Petroselinum hortense | 2027 | + | 4 | CTCC |  |
| Unnamed\_\_4 | Petroselinum hortense | 1561 | - | 4 | CTCC |  |
| Unnamed\_\_4 | Petroselinum hortense | 1400 | - | 4 | CTCC |  |
| Unnamed\_\_4 | Petroselinum hortense | 2103 | + | 4 | CTCC |  |
| Unnamed\_\_4 | Petroselinum hortense | 861 | - | 4 | CTCC |  |
| Unnamed\_\_4 | Petroselinum hortense | 3093 | - | 4 | CTCC |  |
| Unnamed\_\_4 | Petroselinum hortense | 2137 | - | 4 | CTCC |  |
| Unnamed\_\_4 | Petroselinum hortense | 2872 | + | 4 | CTCC |  |
| Unnamed\_\_4 | Petroselinum hortense | 1255 | - | 4 | CTCC |  |
| Unnamed\_\_4 | Petroselinum hortense | 1839 | + | 4 | CTCC |  |
| Unnamed\_\_4 | Petroselinum hortense | 433 | - | 4 | CTCC |  |
| Unnamed\_\_4 | Petroselinum hortense | 921 | + | 4 | CTCC |  |
| Unnamed\_\_4 | Petroselinum hortense | 2196 | - | 4 | CTCC |  |
| Unnamed\_\_4 | Petroselinum hortense | 614 | + | 4 | CTCC |  |
| Unnamed\_\_4 | Petroselinum hortense | 2996 | - | 4 | CTCC |  |
| Unnamed\_\_4 | Petroselinum hortense | 2188 | + | 4 | CTCC |  |
| Unnamed\_\_4 | Petroselinum hortense | 3200 | - | 4 | CTCC |  |
| Unnamed\_\_4 | Petroselinum hortense | 3235 | - | 4 | CTCC |  |
| Unnamed\_\_4 | Petroselinum hortense | 2106 | + | 4 | CTCC |  |
| Unnamed\_\_4 | Petroselinum hortense | 288 | - | 4 | CTCC |  |
| Unnamed\_\_4 | Petroselinum hortense | 623 | - | 4 | CTCC |  |
| Unnamed\_\_4 | Petroselinum hortense | 2697 | + | 4 | CTCC |  |
| Unnamed\_\_4 | Petroselinum hortense | 2399 | - | 4 | CTCC |  |
| Unnamed\_\_4 | Petroselinum hortense | 3098 | - | 4 | CTCC |  |
| Unnamed\_\_4 | Petroselinum hortense | 2798 | - | 4 | CTCC |  |

>HU05G01267.1   
+ +Up\_Stream \_Len000TTACAA AAACGATGCT CTAAACCTAT TAGGTCGTTC CGAAACCCGC AAAATTGAAA   
  
  
+ TATGATCGCC CGTATTTTAC CCAAACCTGA AATACACTTA ATATGTATTT TACCTGAACC CAAATACACC   
  
  
+ GGACCTGTAT TTAACACGGG CACCCGAAAT GATAGGTCTA ACTCTCACCA CCACCGCATT TGGCACCCGA   
  
  
+ ACGGTAAAAA ATGTTCATCT ATTATGCACT TTGGCTTCAT CCATAACCCT ATGATCTATC TGTGCCAATA   
  
  
+ TTTGGAGCAC CCAGCGAAGT AGCGCATGCT CTAAACTCTT GCCTCAAAAC AAAACAAATA GTGGGTCATA   
  
  
+ TTCCCTTCAA AGCACTAACA AACACACACT CTCTCTCTCT CAGTAGCCAA CAACTTAAGG CGGTGTTTGC   
  
  
+ TTGGTTTTGG AGAGGGCGTG TTTGGATGAG GTGCAAAACA CATAGATGAA ATCATAAACA ACTTGATTCC   
  
  
+ CAGCCACCCA TGACCTACTC AGATAACAAG GGACAAAAAC AAAGAGGTTT TCTGAAACAG AGAAAACAGG   
  
  
+ GGCGTTGTGT TGCGCTGTTG CTCACAAAAT TCCAATATTC CTCTAAAAGC TCCCAAACGG AGACAAATCA   
  
  
+ AAGAGGACAA AGACATGTGG GATTTAATAC AAAATTTGAC TAACTCTGCA ACGGAAAACA ACCCCTAGGG   
  
  
+ TGTTTTTCTG CGTGTTGTAC ACACTTCACT TTTAACCTGC TCTGCCTCTG CCTTTGCCAC GGCTTTTAAT   
  
  
+ GGATAAGACC AATTAAGAAA CCCAAATATC TCGTAAATTT GTTGAGATTC CTTTCTGCGT TCTCAAGTGT   
  
  
+ TTCAAAAACC CAGTTTGGAG GTTGCACTTA TTCATGCTTT TCTTCAAGTC AGTGAGCTTA GTGTCTGGAA   
  
  
+ CTGAAGCTCC TCCTGGTAAT GCTCTGTTTT TCATTTCTTG TTGTGCGTTT ATAAAAATTC GGCCTATTGA   
  
  
+ TGGAAGTTTG TTTGATGTTT TTGGGTAAGT TTTTGATTAA TAGAGATGAA TTAGTAAAGT AAAATTTGAG   
  
  
+ TGGTATTTAA TGATCCTTAA GCAGTCTATG CTTTGTTGTC ACATGCGTTT CTTGGCTTTT GACTGAAACA   
  
  
+ TTTCTGTGTT AGATGAAAAA CTCATTTTGA TGCATGAGAT GATAAAATCG CTTATTTTTG CCTTTATTTG   
  
  
+ GTGTGTGGGG GGGGGGGGGG GGGGGCGCAG GTCTGAGGTA AAAGATATGT GAATTCATTT GGAGTCTCGA   
  
  
+ TAATTCGTTG GCTATGTTGA CAATCACGAG TGGTCGTTAT CGCTCATCTC ACAGGTTGAT AATTCACTCA   
  
  
+ AACTTCAAGC GGCCTGAATC ATTCAGTCCG GGTTGTTAAA ATCTCAACAG TCATTTCACA CGAGTGGAGG   
  
  
+ GCCCCAAAAG ACACCATTCT TTCGAACTTT TTTTTTTTGA AGGTCATTTT TTCAAGAACA AAACTAATGT   
  
  
+ GAACATGCGA CTTCTATGAG TAAAGTGTAA AGATGCATAA AGTCTTTTTT TCTTTTATAT AAAAAGTACT   
  
  
+ CAAAAAAAAA AGAGTTGGAG AGCTGATTCC CCATGCATCC TCTAAGCAAA GGTGGTGGAC GGCAGAAAAT   
  
  
+ TAGTAGAGTT ATTGTGAGAT TAAAGTTTGC GCACCTGAAC GGCTGACCTC TAGTTTGCTA TCAACGTTAC   
  
  
+ ATTTATGTGT CATAATTTTT GCATGAAACA ATTTCACGCA ATGTTTTCTG ATGGCAGTTA TGTTTAGAAA   
  
  
+ TAAGTTTAAT CGGACCTTAT TCCACTGATT TATATTTGAT TCTTCCCTCT TTACTACGGC TGTCCATTCA   
  
  
+ CAAGCCAAAG AATTCTCCCC CCAAAAAAAA GAAAAAACAG TATAGAACCA TGTTTATTAT CTCATTTTTT   
  
  
+ TCTCTCCCAT TTGATCTGAA ACACAAACTT GCAGGGGTGC AAGTTGGACA CTTAGTAGCT GCAGGAATGA   
  
  
+ TTCAATAGGA ATCCTCATCT TTGACAACAT CACCACTTGA ATACATATCA CTTGATGGCA CTCTCCCCTG   
  
  
+ GTTTAGGCTT CCCATATCCG TGGCTTAGGG AGCTAAAACC TGAGCAAAGA GGACTCTGTC TAATCCATCT   
  
  
+ CCTCCTTTCA TGTGCTAATC AAGTTGCTAC TGGGAGCATC GACAATGCTA ATGTCAGCCT TGAGCATATT   
  
  
+ TCCCACCTTG CCTCTCCCAC CGGAGATACA ATGCAGCGAA TTGCTGCTTA CTTTGCTGAA GCCTTTGCTG   
  
  
+ ACCGTTTACT AAGGGCTTGG CAGCCCGGTC TTCTCAAAGC CTTGAATTGT ACCAAGATGT CATCTGTTTC   
  
  
+ CGAACAAATT CTTGTTCAAA AGTTGTTCTT TGATCTTCTT CCGTTCTTGA AGCTTTCATA TCTTGTGACG   
  
  
+ AACCAGGCAA TCACGGAGGC CATAGAAGGA GAAAAGATGG TTCATATAAT TGATCTCCAT TCTTGTGAAT   
  
  
+ CGGCTCTGTG GATTAGTCTC CTCCAGGCAT TGAGTGTTCG ACCTGAAGGC CCACCCCATT TGAGGATAAC   
  
  
+ CGGTATACAT GAGAAGAAAG AAGTGTTGGA TCAAATGGCT ATGCAACTAA ACAAAGAGGC TGAAAAATTG   
  
  
+ GACATCCCAT TTCAATTCAA TCCTATCGCA AGCAAACTAG ACGACCTTGA TGTCGAAAGC TTGAGTGTCA   
  
  
+ AGACCGGAGA AGCACTTGTT ATTAGTTCTG TGCTCCAACT ACATTCTCTT TTGGCATTGG ATGAGGGATC   
  
  
+ AATGCCTAAG AACGCAGGCA TGGCTTACTT GCAAAGGGTG TTTTATATGA AACCACGAAA ATTGGAGGAC   
  
  
+ TTGCCCAACA AGGATTTGAT GAAAATGTTG AACTCAAATG AAGATTCTAC ATCATCATCA TCTTCATCTC   
  
  
+ CTATCCCTTC ATCAAAACTT GATGCCTTTT TAAAAGCCCT CCTTGGGCTT TCGCCAAAAC TCATGGTTGT   
  
  
+ AACCGAGCAA GAATCAAACC ACAATGGAAG TGCCCTAATA GAAAGAGTGA TGGAGTCATT GAACTTCTAT   
  
  
+ GCAGCATTGT TTGATTGCTT GGAATCCACT ATATCGAGGA CATCGATAGA GAGACAGAAG CTCGAGAAGT   
  
  
+ TGATGTTTGG AGAGGAGATC AAGAACCTCA CAGCTTGTGA AGGGGCAGAG AGAAAGGCAA GGCACGAGAA   
  
  
+ GCTCAGTGAA TGGGTTCAAA GATTTGAGTC AGTAGGATTT AAAAGGGAGC CATTGAGCTA CCATGGTTTC   
  
  
+ TTGCTTGCTA GGAGGTTTTT ACATACCAAT AATTATGAGG GGTATAACAT CAAGGAAGTC AATGGTTTTC   
  
  
+ TTGTTATCTG TTGGCAAGAT AGACCCCTGT ATTCTGTTTC AGCTTGGAGA TTTTA  

- +Up\_Stream \_Len000AATGTT TTTGCTACGA GATTTGGATA ATCCAGCAAG GCTTTGGGCG TTTTAACTTT   
  
  
- ATACTAGCGG GCATAAAATG GGTTTGGACT TTATGTGAAT TATACATAAA ATGGACTTGG GTTTATGTGG   
  
  
- CCTGGACATA AATTGTGCCC GTGGGCTTTA CTATCCAGAT TGAGAGTGGT GGTGGCGTAA ACCGTGGGCT   
  
  
- TGCCATTTTT TACAAGTAGA TAATACGTGA AACCGAAGTA GGTATTGGGA TACTAGATAG ACACGGTTAT   
  
  
- AAACCTCGTG GGTCGCTTCA TCGCGTACGA GATTTGAGAA CGGAGTTTTG TTTTGTTTAT CACCCAGTAT   
  
  
- AAGGGAAGTT TCGTGATTGT TTGTGTGTGA GAGAGAGAGA GTCATCGGTT GTTGAATTCC GCCACAAACG   
  
  
- AACCAAAACC TCTCCCGCAC AAACCTACTC CACGTTTTGT GTATCTACTT TAGTATTTGT TGAACTAAGG   
  
  
- GTCGGTGGGT ACTGGATGAG TCTATTGTTC CCTGTTTTTG TTTCTCCAAA AGACTTTGTC TCTTTTGTCC   
  
  
- CCGCAACACA ACGCGACAAC GAGTGTTTTA AGGTTATAAG GAGATTTTCG AGGGTTTGCC TCTGTTTAGT   
  
  
- TTCTCCTGTT TCTGTACACC CTAAATTATG TTTTAAACTG ATTGAGACGT TGCCTTTTGT TGGGGATCCC   
  
  
- ACAAAAAGAC GCACAACATG TGTGAAGTGA AAATTGGACG AGACGGAGAC GGAAACGGTG CCGAAAATTA   
  
  
- CCTATTCTGG TTAATTCTTT GGGTTTATAG AGCATTTAAA CAACTCTAAG GAAAGACGCA AGAGTTCACA   
  
  
- AAGTTTTTGG GTCAAACCTC CAACGTGAAT AAGTACGAAA AGAAGTTCAG TCACTCGAAT CACAGACCTT   
  
  
- GACTTCGAGG AGGACCATTA CGAGACAAAA AGTAAAGAAC AACACGCAAA TATTTTTAAG CCGGATAACT   
  
  
- ACCTTCAAAC AAACTACAAA AACCCATTCA AAAACTAATT ATCTCTACTT AATCATTTCA TTTTAAACTC   
  
  
- ACCATAAATT ACTAGGAATT CGTCAGATAC GAAACAACAG TGTACGCAAA GAACCGAAAA CTGACTTTGT   
  
  
- AAAGACACAA TCTACTTTTT GAGTAAAACT ACGTACTCTA CTATTTTAGC GAATAAAAAC GGAAATAAAC   
  
  
- CACACACCCC CCCCCCCCCC CCCCCGCGTC CAGACTCCAT TTTCTATACA CTTAAGTAAA CCTCAGAGCT   
  
  
- ATTAAGCAAC CGATACAACT GTTAGTGCTC ACCAGCAATA GCGAGTAGAG TGTCCAACTA TTAAGTGAGT   
  
  
- TTGAAGTTCG CCGGACTTAG TAAGTCAGGC CCAACAATTT TAGAGTTGTC AGTAAAGTGT GCTCACCTCC   
  
  
- CGGGGTTTTC TGTGGTAAGA AAGCTTGAAA AAAAAAAACT TCCAGTAAAA AAGTTCTTGT TTTGATTACA   
  
  
- CTTGTACGCT GAAGATACTC ATTTCACATT TCTACGTATT TCAGAAAAAA AGAAAATATA TTTTTCATGA   
  
  
- GTTTTTTTTT TCTCAACCTC TCGACTAAGG GGTACGTAGG AGATTCGTTT CCACCACCTG CCGTCTTTTA   
  
  
- ATCATCTCAA TAACACTCTA ATTTCAAACG CGTGGACTTG CCGACTGGAG ATCAAACGAT AGTTGCAATG   
  
  
- TAAATACACA GTATTAAAAA CGTACTTTGT TAAAGTGCGT TACAAAAGAC TACCGTCAAT ACAAATCTTT   
  
  
- ATTCAAATTA GCCTGGAATA AGGTGACTAA ATATAAACTA AGAAGGGAGA AATGATGCCG ACAGGTAAGT   
  
  
- GTTCGGTTTC TTAAGAGGGG GGTTTTTTTT CTTTTTTGTC ATATCTTGGT ACAAATAATA GAGTAAAAAA   
  
  
- AGAGAGGGTA AACTAGACTT TGTGTTTGAA CGTCCCCACG TTCAACCTGT GAATCATCGA CGTCCTTACT   
  
  
- AAGTTATCCT TAGGAGTAGA AACTGTTGTA GTGGTGAACT TATGTATAGT GAACTACCGT GAGAGGGGAC   
  
  
- CAAATCCGAA GGGTATAGGC ACCGAATCCC TCGATTTTGG ACTCGTTTCT CCTGAGACAG ATTAGGTAGA   
  
  
- GGAGGAAAGT ACACGATTAG TTCAACGATG ACCCTCGTAG CTGTTACGAT TACAGTCGGA ACTCGTATAA   
  
  
- AGGGTGGAAC GGAGAGGGTG GCCTCTATGT TACGTCGCTT AACGACGAAT GAAACGACTT CGGAAACGAC   
  
  
- TGGCAAATGA TTCCCGAACC GTCGGGCCAG AAGAGTTTCG GAACTTAACA TGGTTCTACA GTAGACAAAG   
  
  
- GCTTGTTTAA GAACAAGTTT TCAACAAGAA ACTAGAAGAA GGCAAGAACT TCGAAAGTAT AGAACACTGC   
  
  
- TTGGTCCGTT AGTGCCTCCG GTATCTTCCT CTTTTCTACC AAGTATATTA ACTAGAGGTA AGAACACTTA   
  
  
- GCCGAGACAC CTAATCAGAG GAGGTCCGTA ACTCACAAGC TGGACTTCCG GGTGGGGTAA ACTCCTATTG   
  
  
- GCCATATGTA CTCTTCTTTC TTCACAACCT AGTTTACCGA TACGTTGATT TGTTTCTCCG ACTTTTTAAC   
  
  
- CTGTAGGGTA AAGTTAAGTT AGGATAGCGT TCGTTTGATC TGCTGGAACT ACAGCTTTCG AACTCACAGT   
  
  
- TCTGGCCTCT TCGTGAACAA TAATCAAGAC ACGAGGTTGA TGTAAGAGAA AACCGTAACC TACTCCCTAG   
  
  
- TTACGGATTC TTGCGTCCGT ACCGAATGAA CGTTTCCCAC AAAATATACT TTGGTGCTTT TAACCTCCTG   
  
  
- AACGGGTTGT TCCTAAACTA CTTTTACAAC TTGAGTTTAC TTCTAAGATG TAGTAGTAGT AGAAGTAGAG   
  
  
- GATAGGGAAG TAGTTTTGAA CTACGGAAAA ATTTTCGGGA GGAACCCGAA AGCGGTTTTG AGTACCAACA   
  
  
- TTGGCTCGTT CTTAGTTTGG TGTTACCTTC ACGGGATTAT CTTTCTCACT ACCTCAGTAA CTTGAAGATA   
  
  
- CGTCGTAACA AACTAACGAA CCTTAGGTGA TATAGCTCCT GTAGCTATCT CTCTGTCTTC GAGCTCTTCA   
  
  
- ACTACAAACC TCTCCTCTAG TTCTTGGAGT GTCGAACACT TCCCCGTCTC TCTTTCCGTT CCGTGCTCTT   
  
  
- CGAGTCACTT ACCCAAGTTT CTAAACTCAG TCATCCTAAA TTTTCCCTCG GTAACTCGAT GGTACCAAAG   
  
  
- AACGAACGAT CCTCCAAAAA TGTATGGTTA TTAATACTCC CCATATTGTA GTTCCTTCAG TTACCAAAAG   
  
  
- AACAATAGAC AACCGTTCTA TCTGGGGACA TAAGACAAAG TCGAACCTCT AAAAT

+     WRE3

| Site Name | Organism | Position | Strand | Matrix score. | sequence | function |
| --- | --- | --- | --- | --- | --- | --- |
| WRE3 | Pisum sativum | 1594 | - | 6 | CCACCT |  |
| WRE3 | Pisum sativum | 2177 | + | 6 | CCACCT |  |

>HU05G01267.1   
+ +Up\_Stream \_Len000TTACAA AAACGATGCT CTAAACCTAT TAGGTCGTTC CGAAACCCGC AAAATTGAAA   
  
  
+ TATGATCGCC CGTATTTTAC CCAAACCTGA AATACACTTA ATATGTATTT TACCTGAACC CAAATACACC   
  
  
+ GGACCTGTAT TTAACACGGG CACCCGAAAT GATAGGTCTA ACTCTCACCA CCACCGCATT TGGCACCCGA   
  
  
+ ACGGTAAAAA ATGTTCATCT ATTATGCACT TTGGCTTCAT CCATAACCCT ATGATCTATC TGTGCCAATA   
  
  
+ TTTGGAGCAC CCAGCGAAGT AGCGCATGCT CTAAACTCTT GCCTCAAAAC AAAACAAATA GTGGGTCATA   
  
  
+ TTCCCTTCAA AGCACTAACA AACACACACT CTCTCTCTCT CAGTAGCCAA CAACTTAAGG CGGTGTTTGC   
  
  
+ TTGGTTTTGG AGAGGGCGTG TTTGGATGAG GTGCAAAACA CATAGATGAA ATCATAAACA ACTTGATTCC   
  
  
+ CAGCCACCCA TGACCTACTC AGATAACAAG GGACAAAAAC AAAGAGGTTT TCTGAAACAG AGAAAACAGG   
  
  
+ GGCGTTGTGT TGCGCTGTTG CTCACAAAAT TCCAATATTC CTCTAAAAGC TCCCAAACGG AGACAAATCA   
  
  
+ AAGAGGACAA AGACATGTGG GATTTAATAC AAAATTTGAC TAACTCTGCA ACGGAAAACA ACCCCTAGGG   
  
  
+ TGTTTTTCTG CGTGTTGTAC ACACTTCACT TTTAACCTGC TCTGCCTCTG CCTTTGCCAC GGCTTTTAAT   
  
  
+ GGATAAGACC AATTAAGAAA CCCAAATATC TCGTAAATTT GTTGAGATTC CTTTCTGCGT TCTCAAGTGT   
  
  
+ TTCAAAAACC CAGTTTGGAG GTTGCACTTA TTCATGCTTT TCTTCAAGTC AGTGAGCTTA GTGTCTGGAA   
  
  
+ CTGAAGCTCC TCCTGGTAAT GCTCTGTTTT TCATTTCTTG TTGTGCGTTT ATAAAAATTC GGCCTATTGA   
  
  
+ TGGAAGTTTG TTTGATGTTT TTGGGTAAGT TTTTGATTAA TAGAGATGAA TTAGTAAAGT AAAATTTGAG   
  
  
+ TGGTATTTAA TGATCCTTAA GCAGTCTATG CTTTGTTGTC ACATGCGTTT CTTGGCTTTT GACTGAAACA   
  
  
+ TTTCTGTGTT AGATGAAAAA CTCATTTTGA TGCATGAGAT GATAAAATCG CTTATTTTTG CCTTTATTTG   
  
  
+ GTGTGTGGGG GGGGGGGGGG GGGGGCGCAG GTCTGAGGTA AAAGATATGT GAATTCATTT GGAGTCTCGA   
  
  
+ TAATTCGTTG GCTATGTTGA CAATCACGAG TGGTCGTTAT CGCTCATCTC ACAGGTTGAT AATTCACTCA   
  
  
+ AACTTCAAGC GGCCTGAATC ATTCAGTCCG GGTTGTTAAA ATCTCAACAG TCATTTCACA CGAGTGGAGG   
  
  
+ GCCCCAAAAG ACACCATTCT TTCGAACTTT TTTTTTTTGA AGGTCATTTT TTCAAGAACA AAACTAATGT   
  
  
+ GAACATGCGA CTTCTATGAG TAAAGTGTAA AGATGCATAA AGTCTTTTTT TCTTTTATAT AAAAAGTACT   
  
  
+ CAAAAAAAAA AGAGTTGGAG AGCTGATTCC CCATGCATCC TCTAAGCAAA GGTGGTGGAC GGCAGAAAAT   
  
  
+ TAGTAGAGTT ATTGTGAGAT TAAAGTTTGC GCACCTGAAC GGCTGACCTC TAGTTTGCTA TCAACGTTAC   
  
  
+ ATTTATGTGT CATAATTTTT GCATGAAACA ATTTCACGCA ATGTTTTCTG ATGGCAGTTA TGTTTAGAAA   
  
  
+ TAAGTTTAAT CGGACCTTAT TCCACTGATT TATATTTGAT TCTTCCCTCT TTACTACGGC TGTCCATTCA   
  
  
+ CAAGCCAAAG AATTCTCCCC CCAAAAAAAA GAAAAAACAG TATAGAACCA TGTTTATTAT CTCATTTTTT   
  
  
+ TCTCTCCCAT TTGATCTGAA ACACAAACTT GCAGGGGTGC AAGTTGGACA CTTAGTAGCT GCAGGAATGA   
  
  
+ TTCAATAGGA ATCCTCATCT TTGACAACAT CACCACTTGA ATACATATCA CTTGATGGCA CTCTCCCCTG   
  
  
+ GTTTAGGCTT CCCATATCCG TGGCTTAGGG AGCTAAAACC TGAGCAAAGA GGACTCTGTC TAATCCATCT   
  
  
+ CCTCCTTTCA TGTGCTAATC AAGTTGCTAC TGGGAGCATC GACAATGCTA ATGTCAGCCT TGAGCATATT   
  
  
+ TCCCACCTTG CCTCTCCCAC CGGAGATACA ATGCAGCGAA TTGCTGCTTA CTTTGCTGAA GCCTTTGCTG   
  
  
+ ACCGTTTACT AAGGGCTTGG CAGCCCGGTC TTCTCAAAGC CTTGAATTGT ACCAAGATGT CATCTGTTTC   
  
  
+ CGAACAAATT CTTGTTCAAA AGTTGTTCTT TGATCTTCTT CCGTTCTTGA AGCTTTCATA TCTTGTGACG   
  
  
+ AACCAGGCAA TCACGGAGGC CATAGAAGGA GAAAAGATGG TTCATATAAT TGATCTCCAT TCTTGTGAAT   
  
  
+ CGGCTCTGTG GATTAGTCTC CTCCAGGCAT TGAGTGTTCG ACCTGAAGGC CCACCCCATT TGAGGATAAC   
  
  
+ CGGTATACAT GAGAAGAAAG AAGTGTTGGA TCAAATGGCT ATGCAACTAA ACAAAGAGGC TGAAAAATTG   
  
  
+ GACATCCCAT TTCAATTCAA TCCTATCGCA AGCAAACTAG ACGACCTTGA TGTCGAAAGC TTGAGTGTCA   
  
  
+ AGACCGGAGA AGCACTTGTT ATTAGTTCTG TGCTCCAACT ACATTCTCTT TTGGCATTGG ATGAGGGATC   
  
  
+ AATGCCTAAG AACGCAGGCA TGGCTTACTT GCAAAGGGTG TTTTATATGA AACCACGAAA ATTGGAGGAC   
  
  
+ TTGCCCAACA AGGATTTGAT GAAAATGTTG AACTCAAATG AAGATTCTAC ATCATCATCA TCTTCATCTC   
  
  
+ CTATCCCTTC ATCAAAACTT GATGCCTTTT TAAAAGCCCT CCTTGGGCTT TCGCCAAAAC TCATGGTTGT   
  
  
+ AACCGAGCAA GAATCAAACC ACAATGGAAG TGCCCTAATA GAAAGAGTGA TGGAGTCATT GAACTTCTAT   
  
  
+ GCAGCATTGT TTGATTGCTT GGAATCCACT ATATCGAGGA CATCGATAGA GAGACAGAAG CTCGAGAAGT   
  
  
+ TGATGTTTGG AGAGGAGATC AAGAACCTCA CAGCTTGTGA AGGGGCAGAG AGAAAGGCAA GGCACGAGAA   
  
  
+ GCTCAGTGAA TGGGTTCAAA GATTTGAGTC AGTAGGATTT AAAAGGGAGC CATTGAGCTA CCATGGTTTC   
  
  
+ TTGCTTGCTA GGAGGTTTTT ACATACCAAT AATTATGAGG GGTATAACAT CAAGGAAGTC AATGGTTTTC   
  
  
+ TTGTTATCTG TTGGCAAGAT AGACCCCTGT ATTCTGTTTC AGCTTGGAGA TTTTA  

- +Up\_Stream \_Len000AATGTT TTTGCTACGA GATTTGGATA ATCCAGCAAG GCTTTGGGCG TTTTAACTTT   
  
  
- ATACTAGCGG GCATAAAATG GGTTTGGACT TTATGTGAAT TATACATAAA ATGGACTTGG GTTTATGTGG   
  
  
- CCTGGACATA AATTGTGCCC GTGGGCTTTA CTATCCAGAT TGAGAGTGGT GGTGGCGTAA ACCGTGGGCT   
  
  
- TGCCATTTTT TACAAGTAGA TAATACGTGA AACCGAAGTA GGTATTGGGA TACTAGATAG ACACGGTTAT   
  
  
- AAACCTCGTG GGTCGCTTCA TCGCGTACGA GATTTGAGAA CGGAGTTTTG TTTTGTTTAT CACCCAGTAT   
  
  
- AAGGGAAGTT TCGTGATTGT TTGTGTGTGA GAGAGAGAGA GTCATCGGTT GTTGAATTCC GCCACAAACG   
  
  
- AACCAAAACC TCTCCCGCAC AAACCTACTC CACGTTTTGT GTATCTACTT TAGTATTTGT TGAACTAAGG   
  
  
- GTCGGTGGGT ACTGGATGAG TCTATTGTTC CCTGTTTTTG TTTCTCCAAA AGACTTTGTC TCTTTTGTCC   
  
  
- CCGCAACACA ACGCGACAAC GAGTGTTTTA AGGTTATAAG GAGATTTTCG AGGGTTTGCC TCTGTTTAGT   
  
  
- TTCTCCTGTT TCTGTACACC CTAAATTATG TTTTAAACTG ATTGAGACGT TGCCTTTTGT TGGGGATCCC   
  
  
- ACAAAAAGAC GCACAACATG TGTGAAGTGA AAATTGGACG AGACGGAGAC GGAAACGGTG CCGAAAATTA   
  
  
- CCTATTCTGG TTAATTCTTT GGGTTTATAG AGCATTTAAA CAACTCTAAG GAAAGACGCA AGAGTTCACA   
  
  
- AAGTTTTTGG GTCAAACCTC CAACGTGAAT AAGTACGAAA AGAAGTTCAG TCACTCGAAT CACAGACCTT   
  
  
- GACTTCGAGG AGGACCATTA CGAGACAAAA AGTAAAGAAC AACACGCAAA TATTTTTAAG CCGGATAACT   
  
  
- ACCTTCAAAC AAACTACAAA AACCCATTCA AAAACTAATT ATCTCTACTT AATCATTTCA TTTTAAACTC   
  
  
- ACCATAAATT ACTAGGAATT CGTCAGATAC GAAACAACAG TGTACGCAAA GAACCGAAAA CTGACTTTGT   
  
  
- AAAGACACAA TCTACTTTTT GAGTAAAACT ACGTACTCTA CTATTTTAGC GAATAAAAAC GGAAATAAAC   
  
  
- CACACACCCC CCCCCCCCCC CCCCCGCGTC CAGACTCCAT TTTCTATACA CTTAAGTAAA CCTCAGAGCT   
  
  
- ATTAAGCAAC CGATACAACT GTTAGTGCTC ACCAGCAATA GCGAGTAGAG TGTCCAACTA TTAAGTGAGT   
  
  
- TTGAAGTTCG CCGGACTTAG TAAGTCAGGC CCAACAATTT TAGAGTTGTC AGTAAAGTGT GCTCACCTCC   
  
  
- CGGGGTTTTC TGTGGTAAGA AAGCTTGAAA AAAAAAAACT TCCAGTAAAA AAGTTCTTGT TTTGATTACA   
  
  
- CTTGTACGCT GAAGATACTC ATTTCACATT TCTACGTATT TCAGAAAAAA AGAAAATATA TTTTTCATGA   
  
  
- GTTTTTTTTT TCTCAACCTC TCGACTAAGG GGTACGTAGG AGATTCGTTT CCACCACCTG CCGTCTTTTA   
  
  
- ATCATCTCAA TAACACTCTA ATTTCAAACG CGTGGACTTG CCGACTGGAG ATCAAACGAT AGTTGCAATG   
  
  
- TAAATACACA GTATTAAAAA CGTACTTTGT TAAAGTGCGT TACAAAAGAC TACCGTCAAT ACAAATCTTT   
  
  
- ATTCAAATTA GCCTGGAATA AGGTGACTAA ATATAAACTA AGAAGGGAGA AATGATGCCG ACAGGTAAGT   
  
  
- GTTCGGTTTC TTAAGAGGGG GGTTTTTTTT CTTTTTTGTC ATATCTTGGT ACAAATAATA GAGTAAAAAA   
  
  
- AGAGAGGGTA AACTAGACTT TGTGTTTGAA CGTCCCCACG TTCAACCTGT GAATCATCGA CGTCCTTACT   
  
  
- AAGTTATCCT TAGGAGTAGA AACTGTTGTA GTGGTGAACT TATGTATAGT GAACTACCGT GAGAGGGGAC   
  
  
- CAAATCCGAA GGGTATAGGC ACCGAATCCC TCGATTTTGG ACTCGTTTCT CCTGAGACAG ATTAGGTAGA   
  
  
- GGAGGAAAGT ACACGATTAG TTCAACGATG ACCCTCGTAG CTGTTACGAT TACAGTCGGA ACTCGTATAA   
  
  
- AGGGTGGAAC GGAGAGGGTG GCCTCTATGT TACGTCGCTT AACGACGAAT GAAACGACTT CGGAAACGAC   
  
  
- TGGCAAATGA TTCCCGAACC GTCGGGCCAG AAGAGTTTCG GAACTTAACA TGGTTCTACA GTAGACAAAG   
  
  
- GCTTGTTTAA GAACAAGTTT TCAACAAGAA ACTAGAAGAA GGCAAGAACT TCGAAAGTAT AGAACACTGC   
  
  
- TTGGTCCGTT AGTGCCTCCG GTATCTTCCT CTTTTCTACC AAGTATATTA ACTAGAGGTA AGAACACTTA   
  
  
- GCCGAGACAC CTAATCAGAG GAGGTCCGTA ACTCACAAGC TGGACTTCCG GGTGGGGTAA ACTCCTATTG   
  
  
- GCCATATGTA CTCTTCTTTC TTCACAACCT AGTTTACCGA TACGTTGATT TGTTTCTCCG ACTTTTTAAC   
  
  
- CTGTAGGGTA AAGTTAAGTT AGGATAGCGT TCGTTTGATC TGCTGGAACT ACAGCTTTCG AACTCACAGT   
  
  
- TCTGGCCTCT TCGTGAACAA TAATCAAGAC ACGAGGTTGA TGTAAGAGAA AACCGTAACC TACTCCCTAG   
  
  
- TTACGGATTC TTGCGTCCGT ACCGAATGAA CGTTTCCCAC AAAATATACT TTGGTGCTTT TAACCTCCTG   
  
  
- AACGGGTTGT TCCTAAACTA CTTTTACAAC TTGAGTTTAC TTCTAAGATG TAGTAGTAGT AGAAGTAGAG   
  
  
- GATAGGGAAG TAGTTTTGAA CTACGGAAAA ATTTTCGGGA GGAACCCGAA AGCGGTTTTG AGTACCAACA   
  
  
- TTGGCTCGTT CTTAGTTTGG TGTTACCTTC ACGGGATTAT CTTTCTCACT ACCTCAGTAA CTTGAAGATA   
  
  
- CGTCGTAACA AACTAACGAA CCTTAGGTGA TATAGCTCCT GTAGCTATCT CTCTGTCTTC GAGCTCTTCA   
  
  
- ACTACAAACC TCTCCTCTAG TTCTTGGAGT GTCGAACACT TCCCCGTCTC TCTTTCCGTT CCGTGCTCTT   
  
  
- CGAGTCACTT ACCCAAGTTT CTAAACTCAG TCATCCTAAA TTTTCCCTCG GTAACTCGAT GGTACCAAAG   
  
  
- AACGAACGAT CCTCCAAAAA TGTATGGTTA TTAATACTCC CCATATTGTA GTTCCTTCAG TTACCAAAAG   
  
  
- AACAATAGAC AACCGTTCTA TCTGGGGACA TAAGACAAAG TCGAACCTCT AAAAT

+     WUN-motif

| Site Name | Organism | Position | Strand | Matrix score. | sequence | function |
| --- | --- | --- | --- | --- | --- | --- |
| WUN-motif | Nicotiana glutinosa | 2601 | + | 9 | CCATTTCAA |  |

>HU05G01267.1   
+ +Up\_Stream \_Len000TTACAA AAACGATGCT CTAAACCTAT TAGGTCGTTC CGAAACCCGC AAAATTGAAA   
  
  
+ TATGATCGCC CGTATTTTAC CCAAACCTGA AATACACTTA ATATGTATTT TACCTGAACC CAAATACACC   
  
  
+ GGACCTGTAT TTAACACGGG CACCCGAAAT GATAGGTCTA ACTCTCACCA CCACCGCATT TGGCACCCGA   
  
  
+ ACGGTAAAAA ATGTTCATCT ATTATGCACT TTGGCTTCAT CCATAACCCT ATGATCTATC TGTGCCAATA   
  
  
+ TTTGGAGCAC CCAGCGAAGT AGCGCATGCT CTAAACTCTT GCCTCAAAAC AAAACAAATA GTGGGTCATA   
  
  
+ TTCCCTTCAA AGCACTAACA AACACACACT CTCTCTCTCT CAGTAGCCAA CAACTTAAGG CGGTGTTTGC   
  
  
+ TTGGTTTTGG AGAGGGCGTG TTTGGATGAG GTGCAAAACA CATAGATGAA ATCATAAACA ACTTGATTCC   
  
  
+ CAGCCACCCA TGACCTACTC AGATAACAAG GGACAAAAAC AAAGAGGTTT TCTGAAACAG AGAAAACAGG   
  
  
+ GGCGTTGTGT TGCGCTGTTG CTCACAAAAT TCCAATATTC CTCTAAAAGC TCCCAAACGG AGACAAATCA   
  
  
+ AAGAGGACAA AGACATGTGG GATTTAATAC AAAATTTGAC TAACTCTGCA ACGGAAAACA ACCCCTAGGG   
  
  
+ TGTTTTTCTG CGTGTTGTAC ACACTTCACT TTTAACCTGC TCTGCCTCTG CCTTTGCCAC GGCTTTTAAT   
  
  
+ GGATAAGACC AATTAAGAAA CCCAAATATC TCGTAAATTT GTTGAGATTC CTTTCTGCGT TCTCAAGTGT   
  
  
+ TTCAAAAACC CAGTTTGGAG GTTGCACTTA TTCATGCTTT TCTTCAAGTC AGTGAGCTTA GTGTCTGGAA   
  
  
+ CTGAAGCTCC TCCTGGTAAT GCTCTGTTTT TCATTTCTTG TTGTGCGTTT ATAAAAATTC GGCCTATTGA   
  
  
+ TGGAAGTTTG TTTGATGTTT TTGGGTAAGT TTTTGATTAA TAGAGATGAA TTAGTAAAGT AAAATTTGAG   
  
  
+ TGGTATTTAA TGATCCTTAA GCAGTCTATG CTTTGTTGTC ACATGCGTTT CTTGGCTTTT GACTGAAACA   
  
  
+ TTTCTGTGTT AGATGAAAAA CTCATTTTGA TGCATGAGAT GATAAAATCG CTTATTTTTG CCTTTATTTG   
  
  
+ GTGTGTGGGG GGGGGGGGGG GGGGGCGCAG GTCTGAGGTA AAAGATATGT GAATTCATTT GGAGTCTCGA   
  
  
+ TAATTCGTTG GCTATGTTGA CAATCACGAG TGGTCGTTAT CGCTCATCTC ACAGGTTGAT AATTCACTCA   
  
  
+ AACTTCAAGC GGCCTGAATC ATTCAGTCCG GGTTGTTAAA ATCTCAACAG TCATTTCACA CGAGTGGAGG   
  
  
+ GCCCCAAAAG ACACCATTCT TTCGAACTTT TTTTTTTTGA AGGTCATTTT TTCAAGAACA AAACTAATGT   
  
  
+ GAACATGCGA CTTCTATGAG TAAAGTGTAA AGATGCATAA AGTCTTTTTT TCTTTTATAT AAAAAGTACT   
  
  
+ CAAAAAAAAA AGAGTTGGAG AGCTGATTCC CCATGCATCC TCTAAGCAAA GGTGGTGGAC GGCAGAAAAT   
  
  
+ TAGTAGAGTT ATTGTGAGAT TAAAGTTTGC GCACCTGAAC GGCTGACCTC TAGTTTGCTA TCAACGTTAC   
  
  
+ ATTTATGTGT CATAATTTTT GCATGAAACA ATTTCACGCA ATGTTTTCTG ATGGCAGTTA TGTTTAGAAA   
  
  
+ TAAGTTTAAT CGGACCTTAT TCCACTGATT TATATTTGAT TCTTCCCTCT TTACTACGGC TGTCCATTCA   
  
  
+ CAAGCCAAAG AATTCTCCCC CCAAAAAAAA GAAAAAACAG TATAGAACCA TGTTTATTAT CTCATTTTTT   
  
  
+ TCTCTCCCAT TTGATCTGAA ACACAAACTT GCAGGGGTGC AAGTTGGACA CTTAGTAGCT GCAGGAATGA   
  
  
+ TTCAATAGGA ATCCTCATCT TTGACAACAT CACCACTTGA ATACATATCA CTTGATGGCA CTCTCCCCTG   
  
  
+ GTTTAGGCTT CCCATATCCG TGGCTTAGGG AGCTAAAACC TGAGCAAAGA GGACTCTGTC TAATCCATCT   
  
  
+ CCTCCTTTCA TGTGCTAATC AAGTTGCTAC TGGGAGCATC GACAATGCTA ATGTCAGCCT TGAGCATATT   
  
  
+ TCCCACCTTG CCTCTCCCAC CGGAGATACA ATGCAGCGAA TTGCTGCTTA CTTTGCTGAA GCCTTTGCTG   
  
  
+ ACCGTTTACT AAGGGCTTGG CAGCCCGGTC TTCTCAAAGC CTTGAATTGT ACCAAGATGT CATCTGTTTC   
  
  
+ CGAACAAATT CTTGTTCAAA AGTTGTTCTT TGATCTTCTT CCGTTCTTGA AGCTTTCATA TCTTGTGACG   
  
  
+ AACCAGGCAA TCACGGAGGC CATAGAAGGA GAAAAGATGG TTCATATAAT TGATCTCCAT TCTTGTGAAT   
  
  
+ CGGCTCTGTG GATTAGTCTC CTCCAGGCAT TGAGTGTTCG ACCTGAAGGC CCACCCCATT TGAGGATAAC   
  
  
+ CGGTATACAT GAGAAGAAAG AAGTGTTGGA TCAAATGGCT ATGCAACTAA ACAAAGAGGC TGAAAAATTG   
  
  
+ GACATCCCAT TTCAATTCAA TCCTATCGCA AGCAAACTAG ACGACCTTGA TGTCGAAAGC TTGAGTGTCA   
  
  
+ AGACCGGAGA AGCACTTGTT ATTAGTTCTG TGCTCCAACT ACATTCTCTT TTGGCATTGG ATGAGGGATC   
  
  
+ AATGCCTAAG AACGCAGGCA TGGCTTACTT GCAAAGGGTG TTTTATATGA AACCACGAAA ATTGGAGGAC   
  
  
+ TTGCCCAACA AGGATTTGAT GAAAATGTTG AACTCAAATG AAGATTCTAC ATCATCATCA TCTTCATCTC   
  
  
+ CTATCCCTTC ATCAAAACTT GATGCCTTTT TAAAAGCCCT CCTTGGGCTT TCGCCAAAAC TCATGGTTGT   
  
  
+ AACCGAGCAA GAATCAAACC ACAATGGAAG TGCCCTAATA GAAAGAGTGA TGGAGTCATT GAACTTCTAT   
  
  
+ GCAGCATTGT TTGATTGCTT GGAATCCACT ATATCGAGGA CATCGATAGA GAGACAGAAG CTCGAGAAGT   
  
  
+ TGATGTTTGG AGAGGAGATC AAGAACCTCA CAGCTTGTGA AGGGGCAGAG AGAAAGGCAA GGCACGAGAA   
  
  
+ GCTCAGTGAA TGGGTTCAAA GATTTGAGTC AGTAGGATTT AAAAGGGAGC CATTGAGCTA CCATGGTTTC   
  
  
+ TTGCTTGCTA GGAGGTTTTT ACATACCAAT AATTATGAGG GGTATAACAT CAAGGAAGTC AATGGTTTTC   
  
  
+ TTGTTATCTG TTGGCAAGAT AGACCCCTGT ATTCTGTTTC AGCTTGGAGA TTTTA  

- +Up\_Stream \_Len000AATGTT TTTGCTACGA GATTTGGATA ATCCAGCAAG GCTTTGGGCG TTTTAACTTT   
  
  
- ATACTAGCGG GCATAAAATG GGTTTGGACT TTATGTGAAT TATACATAAA ATGGACTTGG GTTTATGTGG   
  
  
- CCTGGACATA AATTGTGCCC GTGGGCTTTA CTATCCAGAT TGAGAGTGGT GGTGGCGTAA ACCGTGGGCT   
  
  
- TGCCATTTTT TACAAGTAGA TAATACGTGA AACCGAAGTA GGTATTGGGA TACTAGATAG ACACGGTTAT   
  
  
- AAACCTCGTG GGTCGCTTCA TCGCGTACGA GATTTGAGAA CGGAGTTTTG TTTTGTTTAT CACCCAGTAT   
  
  
- AAGGGAAGTT TCGTGATTGT TTGTGTGTGA GAGAGAGAGA GTCATCGGTT GTTGAATTCC GCCACAAACG   
  
  
- AACCAAAACC TCTCCCGCAC AAACCTACTC CACGTTTTGT GTATCTACTT TAGTATTTGT TGAACTAAGG   
  
  
- GTCGGTGGGT ACTGGATGAG TCTATTGTTC CCTGTTTTTG TTTCTCCAAA AGACTTTGTC TCTTTTGTCC   
  
  
- CCGCAACACA ACGCGACAAC GAGTGTTTTA AGGTTATAAG GAGATTTTCG AGGGTTTGCC TCTGTTTAGT   
  
  
- TTCTCCTGTT TCTGTACACC CTAAATTATG TTTTAAACTG ATTGAGACGT TGCCTTTTGT TGGGGATCCC   
  
  
- ACAAAAAGAC GCACAACATG TGTGAAGTGA AAATTGGACG AGACGGAGAC GGAAACGGTG CCGAAAATTA   
  
  
- CCTATTCTGG TTAATTCTTT GGGTTTATAG AGCATTTAAA CAACTCTAAG GAAAGACGCA AGAGTTCACA   
  
  
- AAGTTTTTGG GTCAAACCTC CAACGTGAAT AAGTACGAAA AGAAGTTCAG TCACTCGAAT CACAGACCTT   
  
  
- GACTTCGAGG AGGACCATTA CGAGACAAAA AGTAAAGAAC AACACGCAAA TATTTTTAAG CCGGATAACT   
  
  
- ACCTTCAAAC AAACTACAAA AACCCATTCA AAAACTAATT ATCTCTACTT AATCATTTCA TTTTAAACTC   
  
  
- ACCATAAATT ACTAGGAATT CGTCAGATAC GAAACAACAG TGTACGCAAA GAACCGAAAA CTGACTTTGT   
  
  
- AAAGACACAA TCTACTTTTT GAGTAAAACT ACGTACTCTA CTATTTTAGC GAATAAAAAC GGAAATAAAC   
  
  
- CACACACCCC CCCCCCCCCC CCCCCGCGTC CAGACTCCAT TTTCTATACA CTTAAGTAAA CCTCAGAGCT   
  
  
- ATTAAGCAAC CGATACAACT GTTAGTGCTC ACCAGCAATA GCGAGTAGAG TGTCCAACTA TTAAGTGAGT   
  
  
- TTGAAGTTCG CCGGACTTAG TAAGTCAGGC CCAACAATTT TAGAGTTGTC AGTAAAGTGT GCTCACCTCC   
  
  
- CGGGGTTTTC TGTGGTAAGA AAGCTTGAAA AAAAAAAACT TCCAGTAAAA AAGTTCTTGT TTTGATTACA   
  
  
- CTTGTACGCT GAAGATACTC ATTTCACATT TCTACGTATT TCAGAAAAAA AGAAAATATA TTTTTCATGA   
  
  
- GTTTTTTTTT TCTCAACCTC TCGACTAAGG GGTACGTAGG AGATTCGTTT CCACCACCTG CCGTCTTTTA   
  
  
- ATCATCTCAA TAACACTCTA ATTTCAAACG CGTGGACTTG CCGACTGGAG ATCAAACGAT AGTTGCAATG   
  
  
- TAAATACACA GTATTAAAAA CGTACTTTGT TAAAGTGCGT TACAAAAGAC TACCGTCAAT ACAAATCTTT   
  
  
- ATTCAAATTA GCCTGGAATA AGGTGACTAA ATATAAACTA AGAAGGGAGA AATGATGCCG ACAGGTAAGT   
  
  
- GTTCGGTTTC TTAAGAGGGG GGTTTTTTTT CTTTTTTGTC ATATCTTGGT ACAAATAATA GAGTAAAAAA   
  
  
- AGAGAGGGTA AACTAGACTT TGTGTTTGAA CGTCCCCACG TTCAACCTGT GAATCATCGA CGTCCTTACT   
  
  
- AAGTTATCCT TAGGAGTAGA AACTGTTGTA GTGGTGAACT TATGTATAGT GAACTACCGT GAGAGGGGAC   
  
  
- CAAATCCGAA GGGTATAGGC ACCGAATCCC TCGATTTTGG ACTCGTTTCT CCTGAGACAG ATTAGGTAGA   
  
  
- GGAGGAAAGT ACACGATTAG TTCAACGATG ACCCTCGTAG CTGTTACGAT TACAGTCGGA ACTCGTATAA   
  
  
- AGGGTGGAAC GGAGAGGGTG GCCTCTATGT TACGTCGCTT AACGACGAAT GAAACGACTT CGGAAACGAC   
  
  
- TGGCAAATGA TTCCCGAACC GTCGGGCCAG AAGAGTTTCG GAACTTAACA TGGTTCTACA GTAGACAAAG   
  
  
- GCTTGTTTAA GAACAAGTTT TCAACAAGAA ACTAGAAGAA GGCAAGAACT TCGAAAGTAT AGAACACTGC   
  
  
- TTGGTCCGTT AGTGCCTCCG GTATCTTCCT CTTTTCTACC AAGTATATTA ACTAGAGGTA AGAACACTTA   
  
  
- GCCGAGACAC CTAATCAGAG GAGGTCCGTA ACTCACAAGC TGGACTTCCG GGTGGGGTAA ACTCCTATTG   
  
  
- GCCATATGTA CTCTTCTTTC TTCACAACCT AGTTTACCGA TACGTTGATT TGTTTCTCCG ACTTTTTAAC   
  
  
- CTGTAGGGTA AAGTTAAGTT AGGATAGCGT TCGTTTGATC TGCTGGAACT ACAGCTTTCG AACTCACAGT   
  
  
- TCTGGCCTCT TCGTGAACAA TAATCAAGAC ACGAGGTTGA TGTAAGAGAA AACCGTAACC TACTCCCTAG   
  
  
- TTACGGATTC TTGCGTCCGT ACCGAATGAA CGTTTCCCAC AAAATATACT TTGGTGCTTT TAACCTCCTG   
  
  
- AACGGGTTGT TCCTAAACTA CTTTTACAAC TTGAGTTTAC TTCTAAGATG TAGTAGTAGT AGAAGTAGAG   
  
  
- GATAGGGAAG TAGTTTTGAA CTACGGAAAA ATTTTCGGGA GGAACCCGAA AGCGGTTTTG AGTACCAACA   
  
  
- TTGGCTCGTT CTTAGTTTGG TGTTACCTTC ACGGGATTAT CTTTCTCACT ACCTCAGTAA CTTGAAGATA   
  
  
- CGTCGTAACA AACTAACGAA CCTTAGGTGA TATAGCTCCT GTAGCTATCT CTCTGTCTTC GAGCTCTTCA   
  
  
- ACTACAAACC TCTCCTCTAG TTCTTGGAGT GTCGAACACT TCCCCGTCTC TCTTTCCGTT CCGTGCTCTT   
  
  
- CGAGTCACTT ACCCAAGTTT CTAAACTCAG TCATCCTAAA TTTTCCCTCG GTAACTCGAT GGTACCAAAG   
  
  
- AACGAACGAT CCTCCAAAAA TGTATGGTTA TTAATACTCC CCATATTGTA GTTCCTTCAG TTACCAAAAG   
  
  
- AACAATAGAC AACCGTTCTA TCTGGGGACA TAAGACAAAG TCGAACCTCT AAAAT

+     as-1

| Site Name | Organism | Position | Strand | Matrix score. | sequence | function |
| --- | --- | --- | --- | --- | --- | --- |
| as-1 | Arabidopsis thaliana | 2380 | + | 5 | TGACG |  |

>HU05G01267.1   
+ +Up\_Stream \_Len000TTACAA AAACGATGCT CTAAACCTAT TAGGTCGTTC CGAAACCCGC AAAATTGAAA   
  
  
+ TATGATCGCC CGTATTTTAC CCAAACCTGA AATACACTTA ATATGTATTT TACCTGAACC CAAATACACC   
  
  
+ GGACCTGTAT TTAACACGGG CACCCGAAAT GATAGGTCTA ACTCTCACCA CCACCGCATT TGGCACCCGA   
  
  
+ ACGGTAAAAA ATGTTCATCT ATTATGCACT TTGGCTTCAT CCATAACCCT ATGATCTATC TGTGCCAATA   
  
  
+ TTTGGAGCAC CCAGCGAAGT AGCGCATGCT CTAAACTCTT GCCTCAAAAC AAAACAAATA GTGGGTCATA   
  
  
+ TTCCCTTCAA AGCACTAACA AACACACACT CTCTCTCTCT CAGTAGCCAA CAACTTAAGG CGGTGTTTGC   
  
  
+ TTGGTTTTGG AGAGGGCGTG TTTGGATGAG GTGCAAAACA CATAGATGAA ATCATAAACA ACTTGATTCC   
  
  
+ CAGCCACCCA TGACCTACTC AGATAACAAG GGACAAAAAC AAAGAGGTTT TCTGAAACAG AGAAAACAGG   
  
  
+ GGCGTTGTGT TGCGCTGTTG CTCACAAAAT TCCAATATTC CTCTAAAAGC TCCCAAACGG AGACAAATCA   
  
  
+ AAGAGGACAA AGACATGTGG GATTTAATAC AAAATTTGAC TAACTCTGCA ACGGAAAACA ACCCCTAGGG   
  
  
+ TGTTTTTCTG CGTGTTGTAC ACACTTCACT TTTAACCTGC TCTGCCTCTG CCTTTGCCAC GGCTTTTAAT   
  
  
+ GGATAAGACC AATTAAGAAA CCCAAATATC TCGTAAATTT GTTGAGATTC CTTTCTGCGT TCTCAAGTGT   
  
  
+ TTCAAAAACC CAGTTTGGAG GTTGCACTTA TTCATGCTTT TCTTCAAGTC AGTGAGCTTA GTGTCTGGAA   
  
  
+ CTGAAGCTCC TCCTGGTAAT GCTCTGTTTT TCATTTCTTG TTGTGCGTTT ATAAAAATTC GGCCTATTGA   
  
  
+ TGGAAGTTTG TTTGATGTTT TTGGGTAAGT TTTTGATTAA TAGAGATGAA TTAGTAAAGT AAAATTTGAG   
  
  
+ TGGTATTTAA TGATCCTTAA GCAGTCTATG CTTTGTTGTC ACATGCGTTT CTTGGCTTTT GACTGAAACA   
  
  
+ TTTCTGTGTT AGATGAAAAA CTCATTTTGA TGCATGAGAT GATAAAATCG CTTATTTTTG CCTTTATTTG   
  
  
+ GTGTGTGGGG GGGGGGGGGG GGGGGCGCAG GTCTGAGGTA AAAGATATGT GAATTCATTT GGAGTCTCGA   
  
  
+ TAATTCGTTG GCTATGTTGA CAATCACGAG TGGTCGTTAT CGCTCATCTC ACAGGTTGAT AATTCACTCA   
  
  
+ AACTTCAAGC GGCCTGAATC ATTCAGTCCG GGTTGTTAAA ATCTCAACAG TCATTTCACA CGAGTGGAGG   
  
  
+ GCCCCAAAAG ACACCATTCT TTCGAACTTT TTTTTTTTGA AGGTCATTTT TTCAAGAACA AAACTAATGT   
  
  
+ GAACATGCGA CTTCTATGAG TAAAGTGTAA AGATGCATAA AGTCTTTTTT TCTTTTATAT AAAAAGTACT   
  
  
+ CAAAAAAAAA AGAGTTGGAG AGCTGATTCC CCATGCATCC TCTAAGCAAA GGTGGTGGAC GGCAGAAAAT   
  
  
+ TAGTAGAGTT ATTGTGAGAT TAAAGTTTGC GCACCTGAAC GGCTGACCTC TAGTTTGCTA TCAACGTTAC   
  
  
+ ATTTATGTGT CATAATTTTT GCATGAAACA ATTTCACGCA ATGTTTTCTG ATGGCAGTTA TGTTTAGAAA   
  
  
+ TAAGTTTAAT CGGACCTTAT TCCACTGATT TATATTTGAT TCTTCCCTCT TTACTACGGC TGTCCATTCA   
  
  
+ CAAGCCAAAG AATTCTCCCC CCAAAAAAAA GAAAAAACAG TATAGAACCA TGTTTATTAT CTCATTTTTT   
  
  
+ TCTCTCCCAT TTGATCTGAA ACACAAACTT GCAGGGGTGC AAGTTGGACA CTTAGTAGCT GCAGGAATGA   
  
  
+ TTCAATAGGA ATCCTCATCT TTGACAACAT CACCACTTGA ATACATATCA CTTGATGGCA CTCTCCCCTG   
  
  
+ GTTTAGGCTT CCCATATCCG TGGCTTAGGG AGCTAAAACC TGAGCAAAGA GGACTCTGTC TAATCCATCT   
  
  
+ CCTCCTTTCA TGTGCTAATC AAGTTGCTAC TGGGAGCATC GACAATGCTA ATGTCAGCCT TGAGCATATT   
  
  
+ TCCCACCTTG CCTCTCCCAC CGGAGATACA ATGCAGCGAA TTGCTGCTTA CTTTGCTGAA GCCTTTGCTG   
  
  
+ ACCGTTTACT AAGGGCTTGG CAGCCCGGTC TTCTCAAAGC CTTGAATTGT ACCAAGATGT CATCTGTTTC   
  
  
+ CGAACAAATT CTTGTTCAAA AGTTGTTCTT TGATCTTCTT CCGTTCTTGA AGCTTTCATA TCTTGTGACG   
  
  
+ AACCAGGCAA TCACGGAGGC CATAGAAGGA GAAAAGATGG TTCATATAAT TGATCTCCAT TCTTGTGAAT   
  
  
+ CGGCTCTGTG GATTAGTCTC CTCCAGGCAT TGAGTGTTCG ACCTGAAGGC CCACCCCATT TGAGGATAAC   
  
  
+ CGGTATACAT GAGAAGAAAG AAGTGTTGGA TCAAATGGCT ATGCAACTAA ACAAAGAGGC TGAAAAATTG   
  
  
+ GACATCCCAT TTCAATTCAA TCCTATCGCA AGCAAACTAG ACGACCTTGA TGTCGAAAGC TTGAGTGTCA   
  
  
+ AGACCGGAGA AGCACTTGTT ATTAGTTCTG TGCTCCAACT ACATTCTCTT TTGGCATTGG ATGAGGGATC   
  
  
+ AATGCCTAAG AACGCAGGCA TGGCTTACTT GCAAAGGGTG TTTTATATGA AACCACGAAA ATTGGAGGAC   
  
  
+ TTGCCCAACA AGGATTTGAT GAAAATGTTG AACTCAAATG AAGATTCTAC ATCATCATCA TCTTCATCTC   
  
  
+ CTATCCCTTC ATCAAAACTT GATGCCTTTT TAAAAGCCCT CCTTGGGCTT TCGCCAAAAC TCATGGTTGT   
  
  
+ AACCGAGCAA GAATCAAACC ACAATGGAAG TGCCCTAATA GAAAGAGTGA TGGAGTCATT GAACTTCTAT   
  
  
+ GCAGCATTGT TTGATTGCTT GGAATCCACT ATATCGAGGA CATCGATAGA GAGACAGAAG CTCGAGAAGT   
  
  
+ TGATGTTTGG AGAGGAGATC AAGAACCTCA CAGCTTGTGA AGGGGCAGAG AGAAAGGCAA GGCACGAGAA   
  
  
+ GCTCAGTGAA TGGGTTCAAA GATTTGAGTC AGTAGGATTT AAAAGGGAGC CATTGAGCTA CCATGGTTTC   
  
  
+ TTGCTTGCTA GGAGGTTTTT ACATACCAAT AATTATGAGG GGTATAACAT CAAGGAAGTC AATGGTTTTC   
  
  
+ TTGTTATCTG TTGGCAAGAT AGACCCCTGT ATTCTGTTTC AGCTTGGAGA TTTTA  

- +Up\_Stream \_Len000AATGTT TTTGCTACGA GATTTGGATA ATCCAGCAAG GCTTTGGGCG TTTTAACTTT   
  
  
- ATACTAGCGG GCATAAAATG GGTTTGGACT TTATGTGAAT TATACATAAA ATGGACTTGG GTTTATGTGG   
  
  
- CCTGGACATA AATTGTGCCC GTGGGCTTTA CTATCCAGAT TGAGAGTGGT GGTGGCGTAA ACCGTGGGCT   
  
  
- TGCCATTTTT TACAAGTAGA TAATACGTGA AACCGAAGTA GGTATTGGGA TACTAGATAG ACACGGTTAT   
  
  
- AAACCTCGTG GGTCGCTTCA TCGCGTACGA GATTTGAGAA CGGAGTTTTG TTTTGTTTAT CACCCAGTAT   
  
  
- AAGGGAAGTT TCGTGATTGT TTGTGTGTGA GAGAGAGAGA GTCATCGGTT GTTGAATTCC GCCACAAACG   
  
  
- AACCAAAACC TCTCCCGCAC AAACCTACTC CACGTTTTGT GTATCTACTT TAGTATTTGT TGAACTAAGG   
  
  
- GTCGGTGGGT ACTGGATGAG TCTATTGTTC CCTGTTTTTG TTTCTCCAAA AGACTTTGTC TCTTTTGTCC   
  
  
- CCGCAACACA ACGCGACAAC GAGTGTTTTA AGGTTATAAG GAGATTTTCG AGGGTTTGCC TCTGTTTAGT   
  
  
- TTCTCCTGTT TCTGTACACC CTAAATTATG TTTTAAACTG ATTGAGACGT TGCCTTTTGT TGGGGATCCC   
  
  
- ACAAAAAGAC GCACAACATG TGTGAAGTGA AAATTGGACG AGACGGAGAC GGAAACGGTG CCGAAAATTA   
  
  
- CCTATTCTGG TTAATTCTTT GGGTTTATAG AGCATTTAAA CAACTCTAAG GAAAGACGCA AGAGTTCACA   
  
  
- AAGTTTTTGG GTCAAACCTC CAACGTGAAT AAGTACGAAA AGAAGTTCAG TCACTCGAAT CACAGACCTT   
  
  
- GACTTCGAGG AGGACCATTA CGAGACAAAA AGTAAAGAAC AACACGCAAA TATTTTTAAG CCGGATAACT   
  
  
- ACCTTCAAAC AAACTACAAA AACCCATTCA AAAACTAATT ATCTCTACTT AATCATTTCA TTTTAAACTC   
  
  
- ACCATAAATT ACTAGGAATT CGTCAGATAC GAAACAACAG TGTACGCAAA GAACCGAAAA CTGACTTTGT   
  
  
- AAAGACACAA TCTACTTTTT GAGTAAAACT ACGTACTCTA CTATTTTAGC GAATAAAAAC GGAAATAAAC   
  
  
- CACACACCCC CCCCCCCCCC CCCCCGCGTC CAGACTCCAT TTTCTATACA CTTAAGTAAA CCTCAGAGCT   
  
  
- ATTAAGCAAC CGATACAACT GTTAGTGCTC ACCAGCAATA GCGAGTAGAG TGTCCAACTA TTAAGTGAGT   
  
  
- TTGAAGTTCG CCGGACTTAG TAAGTCAGGC CCAACAATTT TAGAGTTGTC AGTAAAGTGT GCTCACCTCC   
  
  
- CGGGGTTTTC TGTGGTAAGA AAGCTTGAAA AAAAAAAACT TCCAGTAAAA AAGTTCTTGT TTTGATTACA   
  
  
- CTTGTACGCT GAAGATACTC ATTTCACATT TCTACGTATT TCAGAAAAAA AGAAAATATA TTTTTCATGA   
  
  
- GTTTTTTTTT TCTCAACCTC TCGACTAAGG GGTACGTAGG AGATTCGTTT CCACCACCTG CCGTCTTTTA   
  
  
- ATCATCTCAA TAACACTCTA ATTTCAAACG CGTGGACTTG CCGACTGGAG ATCAAACGAT AGTTGCAATG   
  
  
- TAAATACACA GTATTAAAAA CGTACTTTGT TAAAGTGCGT TACAAAAGAC TACCGTCAAT ACAAATCTTT   
  
  
- ATTCAAATTA GCCTGGAATA AGGTGACTAA ATATAAACTA AGAAGGGAGA AATGATGCCG ACAGGTAAGT   
  
  
- GTTCGGTTTC TTAAGAGGGG GGTTTTTTTT CTTTTTTGTC ATATCTTGGT ACAAATAATA GAGTAAAAAA   
  
  
- AGAGAGGGTA AACTAGACTT TGTGTTTGAA CGTCCCCACG TTCAACCTGT GAATCATCGA CGTCCTTACT   
  
  
- AAGTTATCCT TAGGAGTAGA AACTGTTGTA GTGGTGAACT TATGTATAGT GAACTACCGT GAGAGGGGAC   
  
  
- CAAATCCGAA GGGTATAGGC ACCGAATCCC TCGATTTTGG ACTCGTTTCT CCTGAGACAG ATTAGGTAGA   
  
  
- GGAGGAAAGT ACACGATTAG TTCAACGATG ACCCTCGTAG CTGTTACGAT TACAGTCGGA ACTCGTATAA   
  
  
- AGGGTGGAAC GGAGAGGGTG GCCTCTATGT TACGTCGCTT AACGACGAAT GAAACGACTT CGGAAACGAC   
  
  
- TGGCAAATGA TTCCCGAACC GTCGGGCCAG AAGAGTTTCG GAACTTAACA TGGTTCTACA GTAGACAAAG   
  
  
- GCTTGTTTAA GAACAAGTTT TCAACAAGAA ACTAGAAGAA GGCAAGAACT TCGAAAGTAT AGAACACTGC   
  
  
- TTGGTCCGTT AGTGCCTCCG GTATCTTCCT CTTTTCTACC AAGTATATTA ACTAGAGGTA AGAACACTTA   
  
  
- GCCGAGACAC CTAATCAGAG GAGGTCCGTA ACTCACAAGC TGGACTTCCG GGTGGGGTAA ACTCCTATTG   
  
  
- GCCATATGTA CTCTTCTTTC TTCACAACCT AGTTTACCGA TACGTTGATT TGTTTCTCCG ACTTTTTAAC   
  
  
- CTGTAGGGTA AAGTTAAGTT AGGATAGCGT TCGTTTGATC TGCTGGAACT ACAGCTTTCG AACTCACAGT   
  
  
- TCTGGCCTCT TCGTGAACAA TAATCAAGAC ACGAGGTTGA TGTAAGAGAA AACCGTAACC TACTCCCTAG   
  
  
- TTACGGATTC TTGCGTCCGT ACCGAATGAA CGTTTCCCAC AAAATATACT TTGGTGCTTT TAACCTCCTG   
  
  
- AACGGGTTGT TCCTAAACTA CTTTTACAAC TTGAGTTTAC TTCTAAGATG TAGTAGTAGT AGAAGTAGAG   
  
  
- GATAGGGAAG TAGTTTTGAA CTACGGAAAA ATTTTCGGGA GGAACCCGAA AGCGGTTTTG AGTACCAACA   
  
  
- TTGGCTCGTT CTTAGTTTGG TGTTACCTTC ACGGGATTAT CTTTCTCACT ACCTCAGTAA CTTGAAGATA   
  
  
- CGTCGTAACA AACTAACGAA CCTTAGGTGA TATAGCTCCT GTAGCTATCT CTCTGTCTTC GAGCTCTTCA   
  
  
- ACTACAAACC TCTCCTCTAG TTCTTGGAGT GTCGAACACT TCCCCGTCTC TCTTTCCGTT CCGTGCTCTT   
  
  
- CGAGTCACTT ACCCAAGTTT CTAAACTCAG TCATCCTAAA TTTTCCCTCG GTAACTCGAT GGTACCAAAG   
  
  
- AACGAACGAT CCTCCAAAAA TGTATGGTTA TTAATACTCC CCATATTGTA GTTCCTTCAG TTACCAAAAG   
  
  
- AACAATAGAC AACCGTTCTA TCTGGGGACA TAAGACAAAG TCGAACCTCT AAAAT

+     box S

| Site Name | Organism | Position | Strand | Matrix score. | sequence | function |
| --- | --- | --- | --- | --- | --- | --- |
| box S | Arabidopsis thaliana | 496 | + | 7 | AGCCACC |  |

>HU05G01267.1   
+ +Up\_Stream \_Len000TTACAA AAACGATGCT CTAAACCTAT TAGGTCGTTC CGAAACCCGC AAAATTGAAA   
  
  
+ TATGATCGCC CGTATTTTAC CCAAACCTGA AATACACTTA ATATGTATTT TACCTGAACC CAAATACACC   
  
  
+ GGACCTGTAT TTAACACGGG CACCCGAAAT GATAGGTCTA ACTCTCACCA CCACCGCATT TGGCACCCGA   
  
  
+ ACGGTAAAAA ATGTTCATCT ATTATGCACT TTGGCTTCAT CCATAACCCT ATGATCTATC TGTGCCAATA   
  
  
+ TTTGGAGCAC CCAGCGAAGT AGCGCATGCT CTAAACTCTT GCCTCAAAAC AAAACAAATA GTGGGTCATA   
  
  
+ TTCCCTTCAA AGCACTAACA AACACACACT CTCTCTCTCT CAGTAGCCAA CAACTTAAGG CGGTGTTTGC   
  
  
+ TTGGTTTTGG AGAGGGCGTG TTTGGATGAG GTGCAAAACA CATAGATGAA ATCATAAACA ACTTGATTCC   
  
  
+ CAGCCACCCA TGACCTACTC AGATAACAAG GGACAAAAAC AAAGAGGTTT TCTGAAACAG AGAAAACAGG   
  
  
+ GGCGTTGTGT TGCGCTGTTG CTCACAAAAT TCCAATATTC CTCTAAAAGC TCCCAAACGG AGACAAATCA   
  
  
+ AAGAGGACAA AGACATGTGG GATTTAATAC AAAATTTGAC TAACTCTGCA ACGGAAAACA ACCCCTAGGG   
  
  
+ TGTTTTTCTG CGTGTTGTAC ACACTTCACT TTTAACCTGC TCTGCCTCTG CCTTTGCCAC GGCTTTTAAT   
  
  
+ GGATAAGACC AATTAAGAAA CCCAAATATC TCGTAAATTT GTTGAGATTC CTTTCTGCGT TCTCAAGTGT   
  
  
+ TTCAAAAACC CAGTTTGGAG GTTGCACTTA TTCATGCTTT TCTTCAAGTC AGTGAGCTTA GTGTCTGGAA   
  
  
+ CTGAAGCTCC TCCTGGTAAT GCTCTGTTTT TCATTTCTTG TTGTGCGTTT ATAAAAATTC GGCCTATTGA   
  
  
+ TGGAAGTTTG TTTGATGTTT TTGGGTAAGT TTTTGATTAA TAGAGATGAA TTAGTAAAGT AAAATTTGAG   
  
  
+ TGGTATTTAA TGATCCTTAA GCAGTCTATG CTTTGTTGTC ACATGCGTTT CTTGGCTTTT GACTGAAACA   
  
  
+ TTTCTGTGTT AGATGAAAAA CTCATTTTGA TGCATGAGAT GATAAAATCG CTTATTTTTG CCTTTATTTG   
  
  
+ GTGTGTGGGG GGGGGGGGGG GGGGGCGCAG GTCTGAGGTA AAAGATATGT GAATTCATTT GGAGTCTCGA   
  
  
+ TAATTCGTTG GCTATGTTGA CAATCACGAG TGGTCGTTAT CGCTCATCTC ACAGGTTGAT AATTCACTCA   
  
  
+ AACTTCAAGC GGCCTGAATC ATTCAGTCCG GGTTGTTAAA ATCTCAACAG TCATTTCACA CGAGTGGAGG   
  
  
+ GCCCCAAAAG ACACCATTCT TTCGAACTTT TTTTTTTTGA AGGTCATTTT TTCAAGAACA AAACTAATGT   
  
  
+ GAACATGCGA CTTCTATGAG TAAAGTGTAA AGATGCATAA AGTCTTTTTT TCTTTTATAT AAAAAGTACT   
  
  
+ CAAAAAAAAA AGAGTTGGAG AGCTGATTCC CCATGCATCC TCTAAGCAAA GGTGGTGGAC GGCAGAAAAT   
  
  
+ TAGTAGAGTT ATTGTGAGAT TAAAGTTTGC GCACCTGAAC GGCTGACCTC TAGTTTGCTA TCAACGTTAC   
  
  
+ ATTTATGTGT CATAATTTTT GCATGAAACA ATTTCACGCA ATGTTTTCTG ATGGCAGTTA TGTTTAGAAA   
  
  
+ TAAGTTTAAT CGGACCTTAT TCCACTGATT TATATTTGAT TCTTCCCTCT TTACTACGGC TGTCCATTCA   
  
  
+ CAAGCCAAAG AATTCTCCCC CCAAAAAAAA GAAAAAACAG TATAGAACCA TGTTTATTAT CTCATTTTTT   
  
  
+ TCTCTCCCAT TTGATCTGAA ACACAAACTT GCAGGGGTGC AAGTTGGACA CTTAGTAGCT GCAGGAATGA   
  
  
+ TTCAATAGGA ATCCTCATCT TTGACAACAT CACCACTTGA ATACATATCA CTTGATGGCA CTCTCCCCTG   
  
  
+ GTTTAGGCTT CCCATATCCG TGGCTTAGGG AGCTAAAACC TGAGCAAAGA GGACTCTGTC TAATCCATCT   
  
  
+ CCTCCTTTCA TGTGCTAATC AAGTTGCTAC TGGGAGCATC GACAATGCTA ATGTCAGCCT TGAGCATATT   
  
  
+ TCCCACCTTG CCTCTCCCAC CGGAGATACA ATGCAGCGAA TTGCTGCTTA CTTTGCTGAA GCCTTTGCTG   
  
  
+ ACCGTTTACT AAGGGCTTGG CAGCCCGGTC TTCTCAAAGC CTTGAATTGT ACCAAGATGT CATCTGTTTC   
  
  
+ CGAACAAATT CTTGTTCAAA AGTTGTTCTT TGATCTTCTT CCGTTCTTGA AGCTTTCATA TCTTGTGACG   
  
  
+ AACCAGGCAA TCACGGAGGC CATAGAAGGA GAAAAGATGG TTCATATAAT TGATCTCCAT TCTTGTGAAT   
  
  
+ CGGCTCTGTG GATTAGTCTC CTCCAGGCAT TGAGTGTTCG ACCTGAAGGC CCACCCCATT TGAGGATAAC   
  
  
+ CGGTATACAT GAGAAGAAAG AAGTGTTGGA TCAAATGGCT ATGCAACTAA ACAAAGAGGC TGAAAAATTG   
  
  
+ GACATCCCAT TTCAATTCAA TCCTATCGCA AGCAAACTAG ACGACCTTGA TGTCGAAAGC TTGAGTGTCA   
  
  
+ AGACCGGAGA AGCACTTGTT ATTAGTTCTG TGCTCCAACT ACATTCTCTT TTGGCATTGG ATGAGGGATC   
  
  
+ AATGCCTAAG AACGCAGGCA TGGCTTACTT GCAAAGGGTG TTTTATATGA AACCACGAAA ATTGGAGGAC   
  
  
+ TTGCCCAACA AGGATTTGAT GAAAATGTTG AACTCAAATG AAGATTCTAC ATCATCATCA TCTTCATCTC   
  
  
+ CTATCCCTTC ATCAAAACTT GATGCCTTTT TAAAAGCCCT CCTTGGGCTT TCGCCAAAAC TCATGGTTGT   
  
  
+ AACCGAGCAA GAATCAAACC ACAATGGAAG TGCCCTAATA GAAAGAGTGA TGGAGTCATT GAACTTCTAT   
  
  
+ GCAGCATTGT TTGATTGCTT GGAATCCACT ATATCGAGGA CATCGATAGA GAGACAGAAG CTCGAGAAGT   
  
  
+ TGATGTTTGG AGAGGAGATC AAGAACCTCA CAGCTTGTGA AGGGGCAGAG AGAAAGGCAA GGCACGAGAA   
  
  
+ GCTCAGTGAA TGGGTTCAAA GATTTGAGTC AGTAGGATTT AAAAGGGAGC CATTGAGCTA CCATGGTTTC   
  
  
+ TTGCTTGCTA GGAGGTTTTT ACATACCAAT AATTATGAGG GGTATAACAT CAAGGAAGTC AATGGTTTTC   
  
  
+ TTGTTATCTG TTGGCAAGAT AGACCCCTGT ATTCTGTTTC AGCTTGGAGA TTTTA  

- +Up\_Stream \_Len000AATGTT TTTGCTACGA GATTTGGATA ATCCAGCAAG GCTTTGGGCG TTTTAACTTT   
  
  
- ATACTAGCGG GCATAAAATG GGTTTGGACT TTATGTGAAT TATACATAAA ATGGACTTGG GTTTATGTGG   
  
  
- CCTGGACATA AATTGTGCCC GTGGGCTTTA CTATCCAGAT TGAGAGTGGT GGTGGCGTAA ACCGTGGGCT   
  
  
- TGCCATTTTT TACAAGTAGA TAATACGTGA AACCGAAGTA GGTATTGGGA TACTAGATAG ACACGGTTAT   
  
  
- AAACCTCGTG GGTCGCTTCA TCGCGTACGA GATTTGAGAA CGGAGTTTTG TTTTGTTTAT CACCCAGTAT   
  
  
- AAGGGAAGTT TCGTGATTGT TTGTGTGTGA GAGAGAGAGA GTCATCGGTT GTTGAATTCC GCCACAAACG   
  
  
- AACCAAAACC TCTCCCGCAC AAACCTACTC CACGTTTTGT GTATCTACTT TAGTATTTGT TGAACTAAGG   
  
  
- GTCGGTGGGT ACTGGATGAG TCTATTGTTC CCTGTTTTTG TTTCTCCAAA AGACTTTGTC TCTTTTGTCC   
  
  
- CCGCAACACA ACGCGACAAC GAGTGTTTTA AGGTTATAAG GAGATTTTCG AGGGTTTGCC TCTGTTTAGT   
  
  
- TTCTCCTGTT TCTGTACACC CTAAATTATG TTTTAAACTG ATTGAGACGT TGCCTTTTGT TGGGGATCCC   
  
  
- ACAAAAAGAC GCACAACATG TGTGAAGTGA AAATTGGACG AGACGGAGAC GGAAACGGTG CCGAAAATTA   
  
  
- CCTATTCTGG TTAATTCTTT GGGTTTATAG AGCATTTAAA CAACTCTAAG GAAAGACGCA AGAGTTCACA   
  
  
- AAGTTTTTGG GTCAAACCTC CAACGTGAAT AAGTACGAAA AGAAGTTCAG TCACTCGAAT CACAGACCTT   
  
  
- GACTTCGAGG AGGACCATTA CGAGACAAAA AGTAAAGAAC AACACGCAAA TATTTTTAAG CCGGATAACT   
  
  
- ACCTTCAAAC AAACTACAAA AACCCATTCA AAAACTAATT ATCTCTACTT AATCATTTCA TTTTAAACTC   
  
  
- ACCATAAATT ACTAGGAATT CGTCAGATAC GAAACAACAG TGTACGCAAA GAACCGAAAA CTGACTTTGT   
  
  
- AAAGACACAA TCTACTTTTT GAGTAAAACT ACGTACTCTA CTATTTTAGC GAATAAAAAC GGAAATAAAC   
  
  
- CACACACCCC CCCCCCCCCC CCCCCGCGTC CAGACTCCAT TTTCTATACA CTTAAGTAAA CCTCAGAGCT   
  
  
- ATTAAGCAAC CGATACAACT GTTAGTGCTC ACCAGCAATA GCGAGTAGAG TGTCCAACTA TTAAGTGAGT   
  
  
- TTGAAGTTCG CCGGACTTAG TAAGTCAGGC CCAACAATTT TAGAGTTGTC AGTAAAGTGT GCTCACCTCC   
  
  
- CGGGGTTTTC TGTGGTAAGA AAGCTTGAAA AAAAAAAACT TCCAGTAAAA AAGTTCTTGT TTTGATTACA   
  
  
- CTTGTACGCT GAAGATACTC ATTTCACATT TCTACGTATT TCAGAAAAAA AGAAAATATA TTTTTCATGA   
  
  
- GTTTTTTTTT TCTCAACCTC TCGACTAAGG GGTACGTAGG AGATTCGTTT CCACCACCTG CCGTCTTTTA   
  
  
- ATCATCTCAA TAACACTCTA ATTTCAAACG CGTGGACTTG CCGACTGGAG ATCAAACGAT AGTTGCAATG   
  
  
- TAAATACACA GTATTAAAAA CGTACTTTGT TAAAGTGCGT TACAAAAGAC TACCGTCAAT ACAAATCTTT   
  
  
- ATTCAAATTA GCCTGGAATA AGGTGACTAA ATATAAACTA AGAAGGGAGA AATGATGCCG ACAGGTAAGT   
  
  
- GTTCGGTTTC TTAAGAGGGG GGTTTTTTTT CTTTTTTGTC ATATCTTGGT ACAAATAATA GAGTAAAAAA   
  
  
- AGAGAGGGTA AACTAGACTT TGTGTTTGAA CGTCCCCACG TTCAACCTGT GAATCATCGA CGTCCTTACT   
  
  
- AAGTTATCCT TAGGAGTAGA AACTGTTGTA GTGGTGAACT TATGTATAGT GAACTACCGT GAGAGGGGAC   
  
  
- CAAATCCGAA GGGTATAGGC ACCGAATCCC TCGATTTTGG ACTCGTTTCT CCTGAGACAG ATTAGGTAGA   
  
  
- GGAGGAAAGT ACACGATTAG TTCAACGATG ACCCTCGTAG CTGTTACGAT TACAGTCGGA ACTCGTATAA   
  
  
- AGGGTGGAAC GGAGAGGGTG GCCTCTATGT TACGTCGCTT AACGACGAAT GAAACGACTT CGGAAACGAC   
  
  
- TGGCAAATGA TTCCCGAACC GTCGGGCCAG AAGAGTTTCG GAACTTAACA TGGTTCTACA GTAGACAAAG   
  
  
- GCTTGTTTAA GAACAAGTTT TCAACAAGAA ACTAGAAGAA GGCAAGAACT TCGAAAGTAT AGAACACTGC   
  
  
- TTGGTCCGTT AGTGCCTCCG GTATCTTCCT CTTTTCTACC AAGTATATTA ACTAGAGGTA AGAACACTTA   
  
  
- GCCGAGACAC CTAATCAGAG GAGGTCCGTA ACTCACAAGC TGGACTTCCG GGTGGGGTAA ACTCCTATTG   
  
  
- GCCATATGTA CTCTTCTTTC TTCACAACCT AGTTTACCGA TACGTTGATT TGTTTCTCCG ACTTTTTAAC   
  
  
- CTGTAGGGTA AAGTTAAGTT AGGATAGCGT TCGTTTGATC TGCTGGAACT ACAGCTTTCG AACTCACAGT   
  
  
- TCTGGCCTCT TCGTGAACAA TAATCAAGAC ACGAGGTTGA TGTAAGAGAA AACCGTAACC TACTCCCTAG   
  
  
- TTACGGATTC TTGCGTCCGT ACCGAATGAA CGTTTCCCAC AAAATATACT TTGGTGCTTT TAACCTCCTG   
  
  
- AACGGGTTGT TCCTAAACTA CTTTTACAAC TTGAGTTTAC TTCTAAGATG TAGTAGTAGT AGAAGTAGAG   
  
  
- GATAGGGAAG TAGTTTTGAA CTACGGAAAA ATTTTCGGGA GGAACCCGAA AGCGGTTTTG AGTACCAACA   
  
  
- TTGGCTCGTT CTTAGTTTGG TGTTACCTTC ACGGGATTAT CTTTCTCACT ACCTCAGTAA CTTGAAGATA   
  
  
- CGTCGTAACA AACTAACGAA CCTTAGGTGA TATAGCTCCT GTAGCTATCT CTCTGTCTTC GAGCTCTTCA   
  
  
- ACTACAAACC TCTCCTCTAG TTCTTGGAGT GTCGAACACT TCCCCGTCTC TCTTTCCGTT CCGTGCTCTT   
  
  
- CGAGTCACTT ACCCAAGTTT CTAAACTCAG TCATCCTAAA TTTTCCCTCG GTAACTCGAT GGTACCAAAG   
  
  
- AACGAACGAT CCTCCAAAAA TGTATGGTTA TTAATACTCC CCATATTGTA GTTCCTTCAG TTACCAAAAG   
  
  
- AACAATAGAC AACCGTTCTA TCTGGGGACA TAAGACAAAG TCGAACCTCT AAAAT

+     chs-CMA2a

| Site Name | Organism | Position | Strand | Matrix score. | sequence | function |
| --- | --- | --- | --- | --- | --- | --- |
| chs-CMA2a | Petroselinum crispum | 2012 | + | 8 | TCACTTGA | part of a light responsive element |

>HU05G01267.1   
+ +Up\_Stream \_Len000TTACAA AAACGATGCT CTAAACCTAT TAGGTCGTTC CGAAACCCGC AAAATTGAAA   
  
  
+ TATGATCGCC CGTATTTTAC CCAAACCTGA AATACACTTA ATATGTATTT TACCTGAACC CAAATACACC   
  
  
+ GGACCTGTAT TTAACACGGG CACCCGAAAT GATAGGTCTA ACTCTCACCA CCACCGCATT TGGCACCCGA   
  
  
+ ACGGTAAAAA ATGTTCATCT ATTATGCACT TTGGCTTCAT CCATAACCCT ATGATCTATC TGTGCCAATA   
  
  
+ TTTGGAGCAC CCAGCGAAGT AGCGCATGCT CTAAACTCTT GCCTCAAAAC AAAACAAATA GTGGGTCATA   
  
  
+ TTCCCTTCAA AGCACTAACA AACACACACT CTCTCTCTCT CAGTAGCCAA CAACTTAAGG CGGTGTTTGC   
  
  
+ TTGGTTTTGG AGAGGGCGTG TTTGGATGAG GTGCAAAACA CATAGATGAA ATCATAAACA ACTTGATTCC   
  
  
+ CAGCCACCCA TGACCTACTC AGATAACAAG GGACAAAAAC AAAGAGGTTT TCTGAAACAG AGAAAACAGG   
  
  
+ GGCGTTGTGT TGCGCTGTTG CTCACAAAAT TCCAATATTC CTCTAAAAGC TCCCAAACGG AGACAAATCA   
  
  
+ AAGAGGACAA AGACATGTGG GATTTAATAC AAAATTTGAC TAACTCTGCA ACGGAAAACA ACCCCTAGGG   
  
  
+ TGTTTTTCTG CGTGTTGTAC ACACTTCACT TTTAACCTGC TCTGCCTCTG CCTTTGCCAC GGCTTTTAAT   
  
  
+ GGATAAGACC AATTAAGAAA CCCAAATATC TCGTAAATTT GTTGAGATTC CTTTCTGCGT TCTCAAGTGT   
  
  
+ TTCAAAAACC CAGTTTGGAG GTTGCACTTA TTCATGCTTT TCTTCAAGTC AGTGAGCTTA GTGTCTGGAA   
  
  
+ CTGAAGCTCC TCCTGGTAAT GCTCTGTTTT TCATTTCTTG TTGTGCGTTT ATAAAAATTC GGCCTATTGA   
  
  
+ TGGAAGTTTG TTTGATGTTT TTGGGTAAGT TTTTGATTAA TAGAGATGAA TTAGTAAAGT AAAATTTGAG   
  
  
+ TGGTATTTAA TGATCCTTAA GCAGTCTATG CTTTGTTGTC ACATGCGTTT CTTGGCTTTT GACTGAAACA   
  
  
+ TTTCTGTGTT AGATGAAAAA CTCATTTTGA TGCATGAGAT GATAAAATCG CTTATTTTTG CCTTTATTTG   
  
  
+ GTGTGTGGGG GGGGGGGGGG GGGGGCGCAG GTCTGAGGTA AAAGATATGT GAATTCATTT GGAGTCTCGA   
  
  
+ TAATTCGTTG GCTATGTTGA CAATCACGAG TGGTCGTTAT CGCTCATCTC ACAGGTTGAT AATTCACTCA   
  
  
+ AACTTCAAGC GGCCTGAATC ATTCAGTCCG GGTTGTTAAA ATCTCAACAG TCATTTCACA CGAGTGGAGG   
  
  
+ GCCCCAAAAG ACACCATTCT TTCGAACTTT TTTTTTTTGA AGGTCATTTT TTCAAGAACA AAACTAATGT   
  
  
+ GAACATGCGA CTTCTATGAG TAAAGTGTAA AGATGCATAA AGTCTTTTTT TCTTTTATAT AAAAAGTACT   
  
  
+ CAAAAAAAAA AGAGTTGGAG AGCTGATTCC CCATGCATCC TCTAAGCAAA GGTGGTGGAC GGCAGAAAAT   
  
  
+ TAGTAGAGTT ATTGTGAGAT TAAAGTTTGC GCACCTGAAC GGCTGACCTC TAGTTTGCTA TCAACGTTAC   
  
  
+ ATTTATGTGT CATAATTTTT GCATGAAACA ATTTCACGCA ATGTTTTCTG ATGGCAGTTA TGTTTAGAAA   
  
  
+ TAAGTTTAAT CGGACCTTAT TCCACTGATT TATATTTGAT TCTTCCCTCT TTACTACGGC TGTCCATTCA   
  
  
+ CAAGCCAAAG AATTCTCCCC CCAAAAAAAA GAAAAAACAG TATAGAACCA TGTTTATTAT CTCATTTTTT   
  
  
+ TCTCTCCCAT TTGATCTGAA ACACAAACTT GCAGGGGTGC AAGTTGGACA CTTAGTAGCT GCAGGAATGA   
  
  
+ TTCAATAGGA ATCCTCATCT TTGACAACAT CACCACTTGA ATACATATCA CTTGATGGCA CTCTCCCCTG   
  
  
+ GTTTAGGCTT CCCATATCCG TGGCTTAGGG AGCTAAAACC TGAGCAAAGA GGACTCTGTC TAATCCATCT   
  
  
+ CCTCCTTTCA TGTGCTAATC AAGTTGCTAC TGGGAGCATC GACAATGCTA ATGTCAGCCT TGAGCATATT   
  
  
+ TCCCACCTTG CCTCTCCCAC CGGAGATACA ATGCAGCGAA TTGCTGCTTA CTTTGCTGAA GCCTTTGCTG   
  
  
+ ACCGTTTACT AAGGGCTTGG CAGCCCGGTC TTCTCAAAGC CTTGAATTGT ACCAAGATGT CATCTGTTTC   
  
  
+ CGAACAAATT CTTGTTCAAA AGTTGTTCTT TGATCTTCTT CCGTTCTTGA AGCTTTCATA TCTTGTGACG   
  
  
+ AACCAGGCAA TCACGGAGGC CATAGAAGGA GAAAAGATGG TTCATATAAT TGATCTCCAT TCTTGTGAAT   
  
  
+ CGGCTCTGTG GATTAGTCTC CTCCAGGCAT TGAGTGTTCG ACCTGAAGGC CCACCCCATT TGAGGATAAC   
  
  
+ CGGTATACAT GAGAAGAAAG AAGTGTTGGA TCAAATGGCT ATGCAACTAA ACAAAGAGGC TGAAAAATTG   
  
  
+ GACATCCCAT TTCAATTCAA TCCTATCGCA AGCAAACTAG ACGACCTTGA TGTCGAAAGC TTGAGTGTCA   
  
  
+ AGACCGGAGA AGCACTTGTT ATTAGTTCTG TGCTCCAACT ACATTCTCTT TTGGCATTGG ATGAGGGATC   
  
  
+ AATGCCTAAG AACGCAGGCA TGGCTTACTT GCAAAGGGTG TTTTATATGA AACCACGAAA ATTGGAGGAC   
  
  
+ TTGCCCAACA AGGATTTGAT GAAAATGTTG AACTCAAATG AAGATTCTAC ATCATCATCA TCTTCATCTC   
  
  
+ CTATCCCTTC ATCAAAACTT GATGCCTTTT TAAAAGCCCT CCTTGGGCTT TCGCCAAAAC TCATGGTTGT   
  
  
+ AACCGAGCAA GAATCAAACC ACAATGGAAG TGCCCTAATA GAAAGAGTGA TGGAGTCATT GAACTTCTAT   
  
  
+ GCAGCATTGT TTGATTGCTT GGAATCCACT ATATCGAGGA CATCGATAGA GAGACAGAAG CTCGAGAAGT   
  
  
+ TGATGTTTGG AGAGGAGATC AAGAACCTCA CAGCTTGTGA AGGGGCAGAG AGAAAGGCAA GGCACGAGAA   
  
  
+ GCTCAGTGAA TGGGTTCAAA GATTTGAGTC AGTAGGATTT AAAAGGGAGC CATTGAGCTA CCATGGTTTC   
  
  
+ TTGCTTGCTA GGAGGTTTTT ACATACCAAT AATTATGAGG GGTATAACAT CAAGGAAGTC AATGGTTTTC   
  
  
+ TTGTTATCTG TTGGCAAGAT AGACCCCTGT ATTCTGTTTC AGCTTGGAGA TTTTA  

- +Up\_Stream \_Len000AATGTT TTTGCTACGA GATTTGGATA ATCCAGCAAG GCTTTGGGCG TTTTAACTTT   
  
  
- ATACTAGCGG GCATAAAATG GGTTTGGACT TTATGTGAAT TATACATAAA ATGGACTTGG GTTTATGTGG   
  
  
- CCTGGACATA AATTGTGCCC GTGGGCTTTA CTATCCAGAT TGAGAGTGGT GGTGGCGTAA ACCGTGGGCT   
  
  
- TGCCATTTTT TACAAGTAGA TAATACGTGA AACCGAAGTA GGTATTGGGA TACTAGATAG ACACGGTTAT   
  
  
- AAACCTCGTG GGTCGCTTCA TCGCGTACGA GATTTGAGAA CGGAGTTTTG TTTTGTTTAT CACCCAGTAT   
  
  
- AAGGGAAGTT TCGTGATTGT TTGTGTGTGA GAGAGAGAGA GTCATCGGTT GTTGAATTCC GCCACAAACG   
  
  
- AACCAAAACC TCTCCCGCAC AAACCTACTC CACGTTTTGT GTATCTACTT TAGTATTTGT TGAACTAAGG   
  
  
- GTCGGTGGGT ACTGGATGAG TCTATTGTTC CCTGTTTTTG TTTCTCCAAA AGACTTTGTC TCTTTTGTCC   
  
  
- CCGCAACACA ACGCGACAAC GAGTGTTTTA AGGTTATAAG GAGATTTTCG AGGGTTTGCC TCTGTTTAGT   
  
  
- TTCTCCTGTT TCTGTACACC CTAAATTATG TTTTAAACTG ATTGAGACGT TGCCTTTTGT TGGGGATCCC   
  
  
- ACAAAAAGAC GCACAACATG TGTGAAGTGA AAATTGGACG AGACGGAGAC GGAAACGGTG CCGAAAATTA   
  
  
- CCTATTCTGG TTAATTCTTT GGGTTTATAG AGCATTTAAA CAACTCTAAG GAAAGACGCA AGAGTTCACA   
  
  
- AAGTTTTTGG GTCAAACCTC CAACGTGAAT AAGTACGAAA AGAAGTTCAG TCACTCGAAT CACAGACCTT   
  
  
- GACTTCGAGG AGGACCATTA CGAGACAAAA AGTAAAGAAC AACACGCAAA TATTTTTAAG CCGGATAACT   
  
  
- ACCTTCAAAC AAACTACAAA AACCCATTCA AAAACTAATT ATCTCTACTT AATCATTTCA TTTTAAACTC   
  
  
- ACCATAAATT ACTAGGAATT CGTCAGATAC GAAACAACAG TGTACGCAAA GAACCGAAAA CTGACTTTGT   
  
  
- AAAGACACAA TCTACTTTTT GAGTAAAACT ACGTACTCTA CTATTTTAGC GAATAAAAAC GGAAATAAAC   
  
  
- CACACACCCC CCCCCCCCCC CCCCCGCGTC CAGACTCCAT TTTCTATACA CTTAAGTAAA CCTCAGAGCT   
  
  
- ATTAAGCAAC CGATACAACT GTTAGTGCTC ACCAGCAATA GCGAGTAGAG TGTCCAACTA TTAAGTGAGT   
  
  
- TTGAAGTTCG CCGGACTTAG TAAGTCAGGC CCAACAATTT TAGAGTTGTC AGTAAAGTGT GCTCACCTCC   
  
  
- CGGGGTTTTC TGTGGTAAGA AAGCTTGAAA AAAAAAAACT TCCAGTAAAA AAGTTCTTGT TTTGATTACA   
  
  
- CTTGTACGCT GAAGATACTC ATTTCACATT TCTACGTATT TCAGAAAAAA AGAAAATATA TTTTTCATGA   
  
  
- GTTTTTTTTT TCTCAACCTC TCGACTAAGG GGTACGTAGG AGATTCGTTT CCACCACCTG CCGTCTTTTA   
  
  
- ATCATCTCAA TAACACTCTA ATTTCAAACG CGTGGACTTG CCGACTGGAG ATCAAACGAT AGTTGCAATG   
  
  
- TAAATACACA GTATTAAAAA CGTACTTTGT TAAAGTGCGT TACAAAAGAC TACCGTCAAT ACAAATCTTT   
  
  
- ATTCAAATTA GCCTGGAATA AGGTGACTAA ATATAAACTA AGAAGGGAGA AATGATGCCG ACAGGTAAGT   
  
  
- GTTCGGTTTC TTAAGAGGGG GGTTTTTTTT CTTTTTTGTC ATATCTTGGT ACAAATAATA GAGTAAAAAA   
  
  
- AGAGAGGGTA AACTAGACTT TGTGTTTGAA CGTCCCCACG TTCAACCTGT GAATCATCGA CGTCCTTACT   
  
  
- AAGTTATCCT TAGGAGTAGA AACTGTTGTA GTGGTGAACT TATGTATAGT GAACTACCGT GAGAGGGGAC   
  
  
- CAAATCCGAA GGGTATAGGC ACCGAATCCC TCGATTTTGG ACTCGTTTCT CCTGAGACAG ATTAGGTAGA   
  
  
- GGAGGAAAGT ACACGATTAG TTCAACGATG ACCCTCGTAG CTGTTACGAT TACAGTCGGA ACTCGTATAA   
  
  
- AGGGTGGAAC GGAGAGGGTG GCCTCTATGT TACGTCGCTT AACGACGAAT GAAACGACTT CGGAAACGAC   
  
  
- TGGCAAATGA TTCCCGAACC GTCGGGCCAG AAGAGTTTCG GAACTTAACA TGGTTCTACA GTAGACAAAG   
  
  
- GCTTGTTTAA GAACAAGTTT TCAACAAGAA ACTAGAAGAA GGCAAGAACT TCGAAAGTAT AGAACACTGC   
  
  
- TTGGTCCGTT AGTGCCTCCG GTATCTTCCT CTTTTCTACC AAGTATATTA ACTAGAGGTA AGAACACTTA   
  
  
- GCCGAGACAC CTAATCAGAG GAGGTCCGTA ACTCACAAGC TGGACTTCCG GGTGGGGTAA ACTCCTATTG   
  
  
- GCCATATGTA CTCTTCTTTC TTCACAACCT AGTTTACCGA TACGTTGATT TGTTTCTCCG ACTTTTTAAC   
  
  
- CTGTAGGGTA AAGTTAAGTT AGGATAGCGT TCGTTTGATC TGCTGGAACT ACAGCTTTCG AACTCACAGT   
  
  
- TCTGGCCTCT TCGTGAACAA TAATCAAGAC ACGAGGTTGA TGTAAGAGAA AACCGTAACC TACTCCCTAG   
  
  
- TTACGGATTC TTGCGTCCGT ACCGAATGAA CGTTTCCCAC AAAATATACT TTGGTGCTTT TAACCTCCTG   
  
  
- AACGGGTTGT TCCTAAACTA CTTTTACAAC TTGAGTTTAC TTCTAAGATG TAGTAGTAGT AGAAGTAGAG   
  
  
- GATAGGGAAG TAGTTTTGAA CTACGGAAAA ATTTTCGGGA GGAACCCGAA AGCGGTTTTG AGTACCAACA   
  
  
- TTGGCTCGTT CTTAGTTTGG TGTTACCTTC ACGGGATTAT CTTTCTCACT ACCTCAGTAA CTTGAAGATA   
  
  
- CGTCGTAACA AACTAACGAA CCTTAGGTGA TATAGCTCCT GTAGCTATCT CTCTGTCTTC GAGCTCTTCA   
  
  
- ACTACAAACC TCTCCTCTAG TTCTTGGAGT GTCGAACACT TCCCCGTCTC TCTTTCCGTT CCGTGCTCTT   
  
  
- CGAGTCACTT ACCCAAGTTT CTAAACTCAG TCATCCTAAA TTTTCCCTCG GTAACTCGAT GGTACCAAAG   
  
  
- AACGAACGAT CCTCCAAAAA TGTATGGTTA TTAATACTCC CCATATTGTA GTTCCTTCAG TTACCAAAAG   
  
  
- AACAATAGAC AACCGTTCTA TCTGGGGACA TAAGACAAAG TCGAACCTCT AAAAT
