# Supplementary material for: Genome-Wide Identification and Expression Pattern of the GRAS Gene Family in Pitaya (Selenicereus undatus L.)
Source: Biology (Basel). 2022 Dec 21;12(1):11. doi: 10.3390/biology12010011 (PMC9854919; doi:10.3390/biology12010011)
Supplement: Supplementary file 1 [file biology-12-00011-s001.zip › Supplementary file S5/HU05G01597.1_plantcare.html]

Content-Type: text/html; charset=ISO-8859-1


PlantCARE


Webmaster Firefox specific output  
To save the result:
click on the frame with the right mouse button and save the source code as a text file with extension .html  
REFERENCE:PlantCARE: a database of plant cis-acting regulatory elements and a portal to tools for in silico analysis of promoter sequences.  
Lescot, M., Déhais, P., Moreau, Y., De Moor, B., Rouzé ,P.,and Rombauts, S.  
Nucleic Acids Res., Database issue(2002), 30(1):325-327.   


---

>HU05G01597.1   
+ -Up\_Stream \_Len000TTTGTA TTTCAATATT GTAATTAGGT GGGATATAGA GTTGAAGGGC AAACTCTACC   
  
  
+ AACGACTTGT TGAAGGATCA ACTTGTAGCT TGGAACTTAA TGTATGTGTA CTATAGAGTT GTGTAATCTC   
  
  
+ TCCTAATAAA ATTGGGTTAA TCCTAGAATT AGAGAACTTT AAGCGGGGAC TAAGCTTGGT TGAGGCTAAC   
  
  
+ TTCGTTAAAA AGTTCTTGTG TCATGTCCAC GTTTATCTTT TCACTATTTA TTTCAATTAA TTTGCGATAT   
  
  
+ TTATCATCAC CCTATTCACC GTCTTTTCGG ATGATTTAAC TAGTCAAGCC GGACCAACAT TGCTTTTTAT   
  
  
+ AGTGTATTTG CAACTCATGT TGATTATTAG ATGTATCAAC AAAAAATGTT TCTCAAAATA AATAGTAGCT   
  
  
+ CTTTAAATTT TATTCCACAT AAATTCAAAA TCAATATCGA TATCGTTCCA TAATATAGCA GCATTATGAA   
  
  
+ AACAATATTT GCTAATGTTT ACATTGTGCT CTTTTCACTC AAAAATTGTA AGGCTTCAAA TACTATTAAA   
  
  
+ GAAACCATTA TTTCACTCGA CTATTTAGCT TGCATATATA GACAATCTTG CAAATAGTAA CATGTCTTCA   
  
  
+ TTCGACTATT TGCTTCCAAA TGAATAATCT CGTAAACATA TATACATACA TACATATATA TATATATATA   
  
  
+ TATATATATA TATATATATA GTAATATGTA TTTAACTTCA CATATACATG AATATAAAAA TATAAGAGGG   
  
  
+ AAAAAAGACG CTAGGTAGAT GGACTACAAA AGCCATTCAT GTATTGAGAC GGCTCATAAA ATCATACGGG   
  
  
+ GAAAGGAAAA AAAAAGATCT CTACATAATG TTCATAGATT TATAGAAAGC ATATGATGGA TTACCGCATA   
  
  
+ AGGTGTTATA TATGGGAGTG TTTGTAGAAG AAAGATGTAT ATCCGAACTA TCAAAGATAC ATATGAGGGA   
  
  
+ GTAAAAACAA GTGTAAGAAC CTCAGGAGGA GACATAAAAG ATTTTTCTAT TGGCATTGGA CACCGTCAAG   
  
  
+ TATCCGCTTT AAACCTGTTC TTTTTCACTA TTATCATGGA TGTATTCACA AAAGGAATTC AATACGAAGT   
  
  
+ ACCATGATGT ATGCTTTTTA CTGATAGTAT TGGTCTCATC GATGAGACTA AGGATGGTCT TAATAGCAAA   
  
  
+ TTAGAGATTT CATTAGATAA AATCTACTGT ACATGACAAA ACAATGATAT ATAGCATCCT ATTTAAAAAG   
  
  
+ CTACGTGTCA GTCATATATG AATTGCATCT AATTTTTAAT AGATATATAA TATGATATGA TATAATAGGA   
  
  
+ TATGATGTAG ATTGAATTCA AACTCATAAT TCATTAAGCA TGGTGCAATG TTCATTTTCT TGATTTTAAA   
  
  
+ CCGACTATTA CTTCAACTGT ATATAATTCG AATTGCTTTG GTATTTTAAA ATTTAAACTA GTAATTACTG   
  
  
+ CCTCTATTAC TGATGTTTAT ATTAAGTGGT GCTTAACTGC TTATTGATGA AACCATTTTT ATTTTCGTGT   
  
  
+ AAAAGAGGGC ATAATCCATC CTCGTGTATT GGCGGGGCTC GGCGCCCTAT TCAGCTATTC TCTGTATGTC   
  
  
+ AAACAAAACC GCTTCTATTA CCCATTGGTC TCTAAACACG CAGGGGTTTT AGCCCGGAGA GTTAATTCTT   
  
  
+ TATTGAGGTC CCCTGTTCTC CATTGCAGGA TTTTTGCTCA GGTACTTCTC TGTTTCTTCT CTTTAATCTT   
  
  
+ CTGTATCTTT CTGCATCAAG TTTAATGCTT TCTGGGATTT TCAGCTACGA TTTATGTGAA ATTGTAAGAT   
  
  
+ TTCTGAACTG GGCATTCAGG GCTTGGTTCA TATGTATTCT TTTCTGCTTG AAATGTTTTA ATTTCATAGT   
  
  
+ TCCAATGGGG TTATTCAAAG ATAGAATTAA GCTATAATCA CCGAGAAGAC TTGCTGACCT TTGTTTCTTT   
  
  
+ TTTGTTTCTT TTTTTTAATT AAATCTGGCT ATTACTTGGC TATAGTTTTT GCTAATGGAT CCACAAATAG   
  
  
+ ACGAATTATA TGGGCCTTTG CACCAAATGA AGTTCAATGA TCAGAGGAGA CCCATTTTAT CAAATGAAAA   
  
  
+ TTTTGTTAAT GTGTTGAAAC CGCCAAATTC CAATTGGAAT TCACCTTTTG GGAACCCTAC TGTGATTACC   
  
  
+ ACAAACCCAA ATTTTGATTT GGTTGTGCCA TCACTAGGTC CCAATGTTGA GGGAGCTCTC CTTGAAGATT   
  
  
+ ATGATTTTAG TGATGTTGTT CTTAAGTACA TCAATCAAGT TCTTATGGAC GAGGATGAGG GAGAGAAAAT   
  
  
+ CCATTTGGGC CACGGGCCTC TGGCTCTTGA AGCGGCCGAG AAGTCGTTAT ATGAGGTGCT TGGGCAGAGC   
  
  
+ CATTCTCCAC AAAATCAATA CTCTCCAGAG GACAAGCGTG ATGACCTGAG CAAGAGTAGT GTTAGAAGCT   
  
  
+ GTGTCAGTTA TAGCTGTGAC GGTGCAAGTA GTGGCAGTGG CATCGTGGAG CAAAGTTGGT CGAGTTATCC   
  
  
+ TCCGGAGTAC AATCCCATGC GGATGGCAGA TTTTCCTGCC TTGCCCTCAA AACCCTTGTT GAGAACATCT   
  
  
+ TATAGCTTAT CAAATAGCTT GACAGGTGAG GGTAATGGTT TGGTGGATAG ACCGGCAGAC TCACCTGTAA   
  
  
+ GCGCTCTTAG TGTGTCTGAT ATATTTAATG ATAGTCAGTC AGTGATACAA TTTCAGAAAG GGATGGAGGA   
  
  
+ AGCGAGCAAA TTTCTTCCCA AGGCTTCTTT GTGTTTGGAT TTGAACACTG GACCCTGCCT TCGGGAGTCG   
  
  
+ GTCAATAAGG CACGAGGTGG GCTGATTTTG GTGGAGACTA GCCATGAAAA TGAGGTTGCA GTGCTAGCTA   
  
  
+ GTAGGGGAAA GAAGCATCGC CATCCTGAGG AATTACGTTC AGAAGTTGGG AGGGGCAGTA AGCAGCCAGT   
  
  
+ TGTTTTCCAG TTTTCAGACG AGGCAGTTGT GAGATCAGAA ATGTTTGATA GGGTATTGCT GTGCAGTGGT   
  
  
+ GGCAAAAATG ATGCTGACCT ACGCCAAACT TTACAGAGTG AACTAAATAA AACTATGCAA AATGCTCAAA   
  
  
+ TGAAAGATTC TAATGGTGGA AAAGGCCGTG GTAGGAAGCA AGTTGGCAGG AGGGATGTGG TGGATCTAAG   
  
  
+ ATCTCTTTTA CGCCTTTGTG CACAAGCAGT TGGATCCAAT GACCAAAGAA GCGCAAATGA CTTAATTAGG   
  
  
+ CAAATTAGGA AGCATTCTTC TCCAACCGGG GATGGGAATC AAAGAATGGC TCATTATTTT GCTGATGGTC   
  
  
+ TTGAGGCTCG GCTTGCTGGT GTTGGGACCC CAATATACAA GTCTCTCGTG ACTGGCCCAG CTTCTGCTGT   
  
  
+ TGATATCTTG AGAGCTTACC ATATGTTTCT TGCCACATGC CCATTCAAGA AAATAGGGAA TTTCTTCTCT   
  
  
+ AATAGAACAA TTATGGATGT AGCTCAGAAA GCAAATCGAC TTCATATTAT TGATCTTGGT ATTGTCTATG   
  
  
+ GTTTCCAATG GCCTTGTCTA ATTCAGCGCC TTTCATCTAG GCCTGGCGGG CCTCCCAAAC TTCGGATTAC   
  
  
+ AGGCGTTGAT CTCCCCCAAC CCGGTTTCCG ACCAGCCAAA AGAGTCGAGG AGACTGGGCG ACGCTTGAGG   
  
  
+ AACTATGCTG AGTCATTCAA TGTGCCATTT GAGTTCAATG CCATAGCAAA GAGCTGGGAA ACACTTACTG   
  
  
+ TTAAAGATCT CAAGATTGAT CCCGATGAGG TGCTTGTTGT GAACTGTCTG TTCAGGTTTA AATACATTCC   
  
  
+ TGAGGAAACA GTAATTGCAG AATGCCCAAG AGATATTGTT CTTAATCTGA TCAGACAGAT AAAACCAGCT   
  
  
+ GTTTTCATAC ATGGTGTAGT CAATGGAGCC TTCAATTCTC CTTTTTTCAT TATTCGGTTC CGTGAGGCTC   
  
  
+ TCTTCCACTT CTCCACTCTA TTTGATGTGT TGGAGGCAAA TCTTCCCCGG GACATCGAGG AGAGGATACT   
  
  
+ GATAGAGCGA GACATCTTTG GGCGACAGGC AATGAATGTG ATCGCTTGTG AGGGTTTGGA GAGGATAGAA   
  
  
+ AGGCCAGAGA AATACAAACA GTGGCGGGTC CGAAATGAGA GGGCTGGGTT GAAGCAGCTG CCTTTGAATC   
  
  
+ AGGAGACTGT GGAAATGGCC AAAAAACGGG TGACAGCTGT CTATCACAAA GATTTCTCCA TTGATGACGA   
  
  
+ TGGGCACTGG TTGTTACAGG GATGGAAAGG GAGAATTGTC TATGCACTCA CTACTTGGAA GCCTGCTGAC   
  
  
+ TA  

- -Up\_Stream \_Len000AAACAT AAAGTTATAA CATTAATCCA CCCTATATCT CAACTTCCCG TTTGAGATGG   
  
  
- TTGCTGAACA ACTTCCTAGT TGAACATCGA ACCTTGAATT ACATACACAT GATATCTCAA CACATTAGAG   
  
  
- AGGATTATTT TAACCCAATT AGGATCTTAA TCTCTTGAAA TTCGCCCCTG ATTCGAACCA ACTCCGATTG   
  
  
- AAGCAATTTT TCAAGAACAC AGTACAGGTG CAAATAGAAA AGTGATAAAT AAAGTTAATT AAACGCTATA   
  
  
- AATAGTAGTG GGATAAGTGG CAGAAAAGCC TACTAAATTG ATCAGTTCGG CCTGGTTGTA ACGAAAAATA   
  
  
- TCACATAAAC GTTGAGTACA ACTAATAATC TACATAGTTG TTTTTTACAA AGAGTTTTAT TTATCATCGA   
  
  
- GAAATTTAAA ATAAGGTGTA TTTAAGTTTT AGTTATAGCT ATAGCAAGGT ATTATATCGT CGTAATACTT   
  
  
- TTGTTATAAA CGATTACAAA TGTAACACGA GAAAAGTGAG TTTTTAACAT TCCGAAGTTT ATGATAATTT   
  
  
- CTTTGGTAAT AAAGTGAGCT GATAAATCGA ACGTATATAT CTGTTAGAAC GTTTATCATT GTACAGAAGT   
  
  
- AAGCTGATAA ACGAAGGTTT ACTTATTAGA GCATTTGTAT ATATGTATGT ATGTATATAT ATATATATAT   
  
  
- ATATATATAT ATATATATAT CATTATACAT AAATTGAAGT GTATATGTAC TTATATTTTT ATATTCTCCC   
  
  
- TTTTTTCTGC GATCCATCTA CCTGATGTTT TCGGTAAGTA CATAACTCTG CCGAGTATTT TAGTATGCCC   
  
  
- CTTTCCTTTT TTTTTCTAGA GATGTATTAC AAGTATCTAA ATATCTTTCG TATACTACCT AATGGCGTAT   
  
  
- TCCACAATAT ATACCCTCAC AAACATCTTC TTTCTACATA TAGGCTTGAT AGTTTCTATG TATACTCCCT   
  
  
- CATTTTTGTT CACATTCTTG GAGTCCTCCT CTGTATTTTC TAAAAAGATA ACCGTAACCT GTGGCAGTTC   
  
  
- ATAGGCGAAA TTTGGACAAG AAAAAGTGAT AATAGTACCT ACATAAGTGT TTTCCTTAAG TTATGCTTCA   
  
  
- TGGTACTACA TACGAAAAAT GACTATCATA ACCAGAGTAG CTACTCTGAT TCCTACCAGA ATTATCGTTT   
  
  
- AATCTCTAAA GTAATCTATT TTAGATGACA TGTACTGTTT TGTTACTATA TATCGTAGGA TAAATTTTTC   
  
  
- GATGCACAGT CAGTATATAC TTAACGTAGA TTAAAAATTA TCTATATATT ATACTATACT ATATTATCCT   
  
  
- ATACTACATC TAACTTAAGT TTGAGTATTA AGTAATTCGT ACCACGTTAC AAGTAAAAGA ACTAAAATTT   
  
  
- GGCTGATAAT GAAGTTGACA TATATTAAGC TTAACGAAAC CATAAAATTT TAAATTTGAT CATTAATGAC   
  
  
- GGAGATAATG ACTACAAATA TAATTCACCA CGAATTGACG AATAACTACT TTGGTAAAAA TAAAAGCACA   
  
  
- TTTTCTCCCG TATTAGGTAG GAGCACATAA CCGCCCCGAG CCGCGGGATA AGTCGATAAG AGACATACAG   
  
  
- TTTGTTTTGG CGAAGATAAT GGGTAACCAG AGATTTGTGC GTCCCCAAAA TCGGGCCTCT CAATTAAGAA   
  
  
- ATAACTCCAG GGGACAAGAG GTAACGTCCT AAAAACGAGT CCATGAAGAG ACAAAGAAGA GAAATTAGAA   
  
  
- GACATAGAAA GACGTAGTTC AAATTACGAA AGACCCTAAA AGTCGATGCT AAATACACTT TAACATTCTA   
  
  
- AAGACTTGAC CCGTAAGTCC CGAACCAAGT ATACATAAGA AAAGACGAAC TTTACAAAAT TAAAGTATCA   
  
  
- AGGTTACCCC AATAAGTTTC TATCTTAATT CGATATTAGT GGCTCTTCTG AACGACTGGA AACAAAGAAA   
  
  
- AAACAAAGAA AAAAAATTAA TTTAGACCGA TAATGAACCG ATATCAAAAA CGATTACCTA GGTGTTTATC   
  
  
- TGCTTAATAT ACCCGGAAAC GTGGTTTACT TCAAGTTACT AGTCTCCTCT GGGTAAAATA GTTTACTTTT   
  
  
- AAAACAATTA CACAACTTTG GCGGTTTAAG GTTAACCTTA AGTGGAAAAC CCTTGGGATG ACACTAATGG   
  
  
- TGTTTGGGTT TAAAACTAAA CCAACACGGT AGTGATCCAG GGTTACAACT CCCTCGAGAG GAACTTCTAA   
  
  
- TACTAAAATC ACTACAACAA GAATTCATGT AGTTAGTTCA AGAATACCTG CTCCTACTCC CTCTCTTTTA   
  
  
- GGTAAACCCG GTGCCCGGAG ACCGAGAACT TCGCCGGCTC TTCAGCAATA TACTCCACGA ACCCGTCTCG   
  
  
- GTAAGAGGTG TTTTAGTTAT GAGAGGTCTC CTGTTCGCAC TACTGGACTC GTTCTCATCA CAATCTTCGA   
  
  
- CACAGTCAAT ATCGACACTG CCACGTTCAT CACCGTCACC GTAGCACCTC GTTTCAACCA GCTCAATAGG   
  
  
- AGGCCTCATG TTAGGGTACG CCTACCGTCT AAAAGGACGG AACGGGAGTT TTGGGAACAA CTCTTGTAGA   
  
  
- ATATCGAATA GTTTATCGAA CTGTCCACTC CCATTACCAA ACCACCTATC TGGCCGTCTG AGTGGACATT   
  
  
- CGCGAGAATC ACACAGACTA TATAAATTAC TATCAGTCAG TCACTATGTT AAAGTCTTTC CCTACCTCCT   
  
  
- TCGCTCGTTT AAAGAAGGGT TCCGAAGAAA CACAAACCTA AACTTGTGAC CTGGGACGGA AGCCCTCAGC   
  
  
- CAGTTATTCC GTGCTCCACC CGACTAAAAC CACCTCTGAT CGGTACTTTT ACTCCAACGT CACGATCGAT   
  
  
- CATCCCCTTT CTTCGTAGCG GTAGGACTCC TTAATGCAAG TCTTCAACCC TCCCCGTCAT TCGTCGGTCA   
  
  
- ACAAAAGGTC AAAAGTCTGC TCCGTCAACA CTCTAGTCTT TACAAACTAT CCCATAACGA CACGTCACCA   
  
  
- CCGTTTTTAC TACGACTGGA TGCGGTTTGA AATGTCTCAC TTGATTTATT TTGATACGTT TTACGAGTTT   
  
  
- ACTTTCTAAG ATTACCACCT TTTCCGGCAC CATCCTTCGT TCAACCGTCC TCCCTACACC ACCTAGATTC   
  
  
- TAGAGAAAAT GCGGAAACAC GTGTTCGTCA ACCTAGGTTA CTGGTTTCTT CGCGTTTACT GAATTAATCC   
  
  
- GTTTAATCCT TCGTAAGAAG AGGTTGGCCC CTACCCTTAG TTTCTTACCG AGTAATAAAA CGACTACCAG   
  
  
- AACTCCGAGC CGAACGACCA CAACCCTGGG GTTATATGTT CAGAGAGCAC TGACCGGGTC GAAGACGACA   
  
  
- ACTATAGAAC TCTCGAATGG TATACAAAGA ACGGTGTACG GGTAAGTTCT TTTATCCCTT AAAGAAGAGA   
  
  
- TTATCTTGTT AATACCTACA TCGAGTCTTT CGTTTAGCTG AAGTATAATA ACTAGAACCA TAACAGATAC   
  
  
- CAAAGGTTAC CGGAACAGAT TAAGTCGCGG AAAGTAGATC CGGACCGCCC GGAGGGTTTG AAGCCTAATG   
  
  
- TCCGCAACTA GAGGGGGTTG GGCCAAAGGC TGGTCGGTTT TCTCAGCTCC TCTGACCCGC TGCGAACTCC   
  
  
- TTGATACGAC TCAGTAAGTT ACACGGTAAA CTCAAGTTAC GGTATCGTTT CTCGACCCTT TGTGAATGAC   
  
  
- AATTTCTAGA GTTCTAACTA GGGCTACTCC ACGAACAACA CTTGACAGAC AAGTCCAAAT TTATGTAAGG   
  
  
- ACTCCTTTGT CATTAACGTC TTACGGGTTC TCTATAACAA GAATTAGACT AGTCTGTCTA TTTTGGTCGA   
  
  
- CAAAAGTATG TACCACATCA GTTACCTCGG AAGTTAAGAG GAAAAAAGTA ATAAGCCAAG GCACTCCGAG   
  
  
- AGAAGGTGAA GAGGTGAGAT AAACTACACA ACCTCCGTTT AGAAGGGGCC CTGTAGCTCC TCTCCTATGA   
  
  
- CTATCTCGCT CTGTAGAAAC CCGCTGTCCG TTACTTACAC TAGCGAACAC TCCCAAACCT CTCCTATCTT   
  
  
- TCCGGTCTCT TTATGTTTGT CACCGCCCAG GCTTTACTCT CCCGACCCAA CTTCGTCGAC GGAAACTTAG   
  
  
- TCCTCTGACA CCTTTACCGG TTTTTTGCCC ACTGTCGACA GATAGTGTTT CTAAAGAGGT AACTACTGCT   
  
  
- ACCCGTGACC AACAATGTCC CTACCTTTCC CTCTTAACAG ATACGTGAGT GATGAACCTT CGGACGACTG   
  
  
- AT

  
  
Motifs Found  

+   

| Site Name | Organism | Position | Strand | Matrix score. | sequence | function |
| --- | --- | --- | --- | --- | --- | --- |
|  | organism | 3924 | + | 4 | motif\_sequence | short\_function |
|  | organism | 3583 | + | 4 | motif\_sequence | short\_function |
|  | organism | 1670 | - | 4 | motif\_sequence | short\_function |
|  | organism | 1697 | + | 4 | motif\_sequence | short\_function |
|  | organism | 872 | + | 4 | motif\_sequence | short\_function |
|  | organism | 1341 | - | 4 | motif\_sequence | short\_function |
|  | organism | 142 | + | 4 | motif\_sequence | short\_function |
|  | organism | 2304 | - | 4 | motif\_sequence | short\_function |
|  | organism | 863 | + | 4 | motif\_sequence | short\_function |
|  | organism | 1221 | + | 4 | motif\_sequence | short\_function |
|  | organism | 237 | + | 4 | motif\_sequence | short\_function |
|  | organism | 3664 | + | 4 | motif\_sequence | short\_function |
|  | organism | 1750 | + | 4 | motif\_sequence | short\_function |
|  | organism | 2230 | + | 4 | motif\_sequence | short\_function |
|  | organism | 3964 | + | 4 | motif\_sequence | short\_function |
|  | organism | 2587 | - | 4 | motif\_sequence | short\_function |
|  | organism | 68 | + | 4 | motif\_sequence | short\_function |
|  | organism | 789 | - | 4 | motif\_sequence | short\_function |
|  | organism | 2378 | - | 4 | motif\_sequence | short\_function |
|  | organism | 1223 | - | 4 | motif\_sequence | short\_function |
|  | organism | 2270 | - | 4 | motif\_sequence | short\_function |
|  | organism | 1382 | + | 4 | motif\_sequence | short\_function |
|  | organism | 3762 | + | 4 | motif\_sequence | short\_function |
|  | organism | 1069 | + | 4 | motif\_sequence | short\_function |
|  | organism | 3983 | - | 4 | motif\_sequence | short\_function |
|  | organism | 1216 | + | 4 | motif\_sequence | short\_function |
|  | organism | 3974 | - | 4 | motif\_sequence | short\_function |
|  | organism | 2405 | + | 4 | motif\_sequence | short\_function |
|  | organism | 2238 | - | 4 | motif\_sequence | short\_function |
|  | organism | 4052 | - | 4 | motif\_sequence | short\_function |
|  | organism | 2550 | - | 4 | motif\_sequence | short\_function |
|  | organism | 3003 | + | 4 | motif\_sequence | short\_function |

>HU05G01597.1   
+ -Up\_Stream \_Len000TTTGTA TTTCAATATT GTAATTAGGT GGGATATAGA GTTGAAGGGC AAACTCTACC   
  
  
+ AACGACTTGT TGAAGGATCA ACTTGTAGCT TGGAACTTAA TGTATGTGTA CTATAGAGTT GTGTAATCTC   
  
  
+ TCCTAATAAA ATTGGGTTAA TCCTAGAATT AGAGAACTTT AAGCGGGGAC TAAGCTTGGT TGAGGCTAAC   
  
  
+ TTCGTTAAAA AGTTCTTGTG TCATGTCCAC GTTTATCTTT TCACTATTTA TTTCAATTAA TTTGCGATAT   
  
  
+ TTATCATCAC CCTATTCACC GTCTTTTCGG ATGATTTAAC TAGTCAAGCC GGACCAACAT TGCTTTTTAT   
  
  
+ AGTGTATTTG CAACTCATGT TGATTATTAG ATGTATCAAC AAAAAATGTT TCTCAAAATA AATAGTAGCT   
  
  
+ CTTTAAATTT TATTCCACAT AAATTCAAAA TCAATATCGA TATCGTTCCA TAATATAGCA GCATTATGAA   
  
  
+ AACAATATTT GCTAATGTTT ACATTGTGCT CTTTTCACTC AAAAATTGTA AGGCTTCAAA TACTATTAAA   
  
  
+ GAAACCATTA TTTCACTCGA CTATTTAGCT TGCATATATA GACAATCTTG CAAATAGTAA CATGTCTTCA   
  
  
+ TTCGACTATT TGCTTCCAAA TGAATAATCT CGTAAACATA TATACATACA TACATATATA TATATATATA   
  
  
+ TATATATATA TATATATATA GTAATATGTA TTTAACTTCA CATATACATG AATATAAAAA TATAAGAGGG   
  
  
+ AAAAAAGACG CTAGGTAGAT GGACTACAAA AGCCATTCAT GTATTGAGAC GGCTCATAAA ATCATACGGG   
  
  
+ GAAAGGAAAA AAAAAGATCT CTACATAATG TTCATAGATT TATAGAAAGC ATATGATGGA TTACCGCATA   
  
  
+ AGGTGTTATA TATGGGAGTG TTTGTAGAAG AAAGATGTAT ATCCGAACTA TCAAAGATAC ATATGAGGGA   
  
  
+ GTAAAAACAA GTGTAAGAAC CTCAGGAGGA GACATAAAAG ATTTTTCTAT TGGCATTGGA CACCGTCAAG   
  
  
+ TATCCGCTTT AAACCTGTTC TTTTTCACTA TTATCATGGA TGTATTCACA AAAGGAATTC AATACGAAGT   
  
  
+ ACCATGATGT ATGCTTTTTA CTGATAGTAT TGGTCTCATC GATGAGACTA AGGATGGTCT TAATAGCAAA   
  
  
+ TTAGAGATTT CATTAGATAA AATCTACTGT ACATGACAAA ACAATGATAT ATAGCATCCT ATTTAAAAAG   
  
  
+ CTACGTGTCA GTCATATATG AATTGCATCT AATTTTTAAT AGATATATAA TATGATATGA TATAATAGGA   
  
  
+ TATGATGTAG ATTGAATTCA AACTCATAAT TCATTAAGCA TGGTGCAATG TTCATTTTCT TGATTTTAAA   
  
  
+ CCGACTATTA CTTCAACTGT ATATAATTCG AATTGCTTTG GTATTTTAAA ATTTAAACTA GTAATTACTG   
  
  
+ CCTCTATTAC TGATGTTTAT ATTAAGTGGT GCTTAACTGC TTATTGATGA AACCATTTTT ATTTTCGTGT   
  
  
+ AAAAGAGGGC ATAATCCATC CTCGTGTATT GGCGGGGCTC GGCGCCCTAT TCAGCTATTC TCTGTATGTC   
  
  
+ AAACAAAACC GCTTCTATTA CCCATTGGTC TCTAAACACG CAGGGGTTTT AGCCCGGAGA GTTAATTCTT   
  
  
+ TATTGAGGTC CCCTGTTCTC CATTGCAGGA TTTTTGCTCA GGTACTTCTC TGTTTCTTCT CTTTAATCTT   
  
  
+ CTGTATCTTT CTGCATCAAG TTTAATGCTT TCTGGGATTT TCAGCTACGA TTTATGTGAA ATTGTAAGAT   
  
  
+ TTCTGAACTG GGCATTCAGG GCTTGGTTCA TATGTATTCT TTTCTGCTTG AAATGTTTTA ATTTCATAGT   
  
  
+ TCCAATGGGG TTATTCAAAG ATAGAATTAA GCTATAATCA CCGAGAAGAC TTGCTGACCT TTGTTTCTTT   
  
  
+ TTTGTTTCTT TTTTTTAATT AAATCTGGCT ATTACTTGGC TATAGTTTTT GCTAATGGAT CCACAAATAG   
  
  
+ ACGAATTATA TGGGCCTTTG CACCAAATGA AGTTCAATGA TCAGAGGAGA CCCATTTTAT CAAATGAAAA   
  
  
+ TTTTGTTAAT GTGTTGAAAC CGCCAAATTC CAATTGGAAT TCACCTTTTG GGAACCCTAC TGTGATTACC   
  
  
+ ACAAACCCAA ATTTTGATTT GGTTGTGCCA TCACTAGGTC CCAATGTTGA GGGAGCTCTC CTTGAAGATT   
  
  
+ ATGATTTTAG TGATGTTGTT CTTAAGTACA TCAATCAAGT TCTTATGGAC GAGGATGAGG GAGAGAAAAT   
  
  
+ CCATTTGGGC CACGGGCCTC TGGCTCTTGA AGCGGCCGAG AAGTCGTTAT ATGAGGTGCT TGGGCAGAGC   
  
  
+ CATTCTCCAC AAAATCAATA CTCTCCAGAG GACAAGCGTG ATGACCTGAG CAAGAGTAGT GTTAGAAGCT   
  
  
+ GTGTCAGTTA TAGCTGTGAC GGTGCAAGTA GTGGCAGTGG CATCGTGGAG CAAAGTTGGT CGAGTTATCC   
  
  
+ TCCGGAGTAC AATCCCATGC GGATGGCAGA TTTTCCTGCC TTGCCCTCAA AACCCTTGTT GAGAACATCT   
  
  
+ TATAGCTTAT CAAATAGCTT GACAGGTGAG GGTAATGGTT TGGTGGATAG ACCGGCAGAC TCACCTGTAA   
  
  
+ GCGCTCTTAG TGTGTCTGAT ATATTTAATG ATAGTCAGTC AGTGATACAA TTTCAGAAAG GGATGGAGGA   
  
  
+ AGCGAGCAAA TTTCTTCCCA AGGCTTCTTT GTGTTTGGAT TTGAACACTG GACCCTGCCT TCGGGAGTCG   
  
  
+ GTCAATAAGG CACGAGGTGG GCTGATTTTG GTGGAGACTA GCCATGAAAA TGAGGTTGCA GTGCTAGCTA   
  
  
+ GTAGGGGAAA GAAGCATCGC CATCCTGAGG AATTACGTTC AGAAGTTGGG AGGGGCAGTA AGCAGCCAGT   
  
  
+ TGTTTTCCAG TTTTCAGACG AGGCAGTTGT GAGATCAGAA ATGTTTGATA GGGTATTGCT GTGCAGTGGT   
  
  
+ GGCAAAAATG ATGCTGACCT ACGCCAAACT TTACAGAGTG AACTAAATAA AACTATGCAA AATGCTCAAA   
  
  
+ TGAAAGATTC TAATGGTGGA AAAGGCCGTG GTAGGAAGCA AGTTGGCAGG AGGGATGTGG TGGATCTAAG   
  
  
+ ATCTCTTTTA CGCCTTTGTG CACAAGCAGT TGGATCCAAT GACCAAAGAA GCGCAAATGA CTTAATTAGG   
  
  
+ CAAATTAGGA AGCATTCTTC TCCAACCGGG GATGGGAATC AAAGAATGGC TCATTATTTT GCTGATGGTC   
  
  
+ TTGAGGCTCG GCTTGCTGGT GTTGGGACCC CAATATACAA GTCTCTCGTG ACTGGCCCAG CTTCTGCTGT   
  
  
+ TGATATCTTG AGAGCTTACC ATATGTTTCT TGCCACATGC CCATTCAAGA AAATAGGGAA TTTCTTCTCT   
  
  
+ AATAGAACAA TTATGGATGT AGCTCAGAAA GCAAATCGAC TTCATATTAT TGATCTTGGT ATTGTCTATG   
  
  
+ GTTTCCAATG GCCTTGTCTA ATTCAGCGCC TTTCATCTAG GCCTGGCGGG CCTCCCAAAC TTCGGATTAC   
  
  
+ AGGCGTTGAT CTCCCCCAAC CCGGTTTCCG ACCAGCCAAA AGAGTCGAGG AGACTGGGCG ACGCTTGAGG   
  
  
+ AACTATGCTG AGTCATTCAA TGTGCCATTT GAGTTCAATG CCATAGCAAA GAGCTGGGAA ACACTTACTG   
  
  
+ TTAAAGATCT CAAGATTGAT CCCGATGAGG TGCTTGTTGT GAACTGTCTG TTCAGGTTTA AATACATTCC   
  
  
+ TGAGGAAACA GTAATTGCAG AATGCCCAAG AGATATTGTT CTTAATCTGA TCAGACAGAT AAAACCAGCT   
  
  
+ GTTTTCATAC ATGGTGTAGT CAATGGAGCC TTCAATTCTC CTTTTTTCAT TATTCGGTTC CGTGAGGCTC   
  
  
+ TCTTCCACTT CTCCACTCTA TTTGATGTGT TGGAGGCAAA TCTTCCCCGG GACATCGAGG AGAGGATACT   
  
  
+ GATAGAGCGA GACATCTTTG GGCGACAGGC AATGAATGTG ATCGCTTGTG AGGGTTTGGA GAGGATAGAA   
  
  
+ AGGCCAGAGA AATACAAACA GTGGCGGGTC CGAAATGAGA GGGCTGGGTT GAAGCAGCTG CCTTTGAATC   
  
  
+ AGGAGACTGT GGAAATGGCC AAAAAACGGG TGACAGCTGT CTATCACAAA GATTTCTCCA TTGATGACGA   
  
  
+ TGGGCACTGG TTGTTACAGG GATGGAAAGG GAGAATTGTC TATGCACTCA CTACTTGGAA GCCTGCTGAC   
  
  
+ TA  

- -Up\_Stream \_Len000AAACAT AAAGTTATAA CATTAATCCA CCCTATATCT CAACTTCCCG TTTGAGATGG   
  
  
- TTGCTGAACA ACTTCCTAGT TGAACATCGA ACCTTGAATT ACATACACAT GATATCTCAA CACATTAGAG   
  
  
- AGGATTATTT TAACCCAATT AGGATCTTAA TCTCTTGAAA TTCGCCCCTG ATTCGAACCA ACTCCGATTG   
  
  
- AAGCAATTTT TCAAGAACAC AGTACAGGTG CAAATAGAAA AGTGATAAAT AAAGTTAATT AAACGCTATA   
  
  
- AATAGTAGTG GGATAAGTGG CAGAAAAGCC TACTAAATTG ATCAGTTCGG CCTGGTTGTA ACGAAAAATA   
  
  
- TCACATAAAC GTTGAGTACA ACTAATAATC TACATAGTTG TTTTTTACAA AGAGTTTTAT TTATCATCGA   
  
  
- GAAATTTAAA ATAAGGTGTA TTTAAGTTTT AGTTATAGCT ATAGCAAGGT ATTATATCGT CGTAATACTT   
  
  
- TTGTTATAAA CGATTACAAA TGTAACACGA GAAAAGTGAG TTTTTAACAT TCCGAAGTTT ATGATAATTT   
  
  
- CTTTGGTAAT AAAGTGAGCT GATAAATCGA ACGTATATAT CTGTTAGAAC GTTTATCATT GTACAGAAGT   
  
  
- AAGCTGATAA ACGAAGGTTT ACTTATTAGA GCATTTGTAT ATATGTATGT ATGTATATAT ATATATATAT   
  
  
- ATATATATAT ATATATATAT CATTATACAT AAATTGAAGT GTATATGTAC TTATATTTTT ATATTCTCCC   
  
  
- TTTTTTCTGC GATCCATCTA CCTGATGTTT TCGGTAAGTA CATAACTCTG CCGAGTATTT TAGTATGCCC   
  
  
- CTTTCCTTTT TTTTTCTAGA GATGTATTAC AAGTATCTAA ATATCTTTCG TATACTACCT AATGGCGTAT   
  
  
- TCCACAATAT ATACCCTCAC AAACATCTTC TTTCTACATA TAGGCTTGAT AGTTTCTATG TATACTCCCT   
  
  
- CATTTTTGTT CACATTCTTG GAGTCCTCCT CTGTATTTTC TAAAAAGATA ACCGTAACCT GTGGCAGTTC   
  
  
- ATAGGCGAAA TTTGGACAAG AAAAAGTGAT AATAGTACCT ACATAAGTGT TTTCCTTAAG TTATGCTTCA   
  
  
- TGGTACTACA TACGAAAAAT GACTATCATA ACCAGAGTAG CTACTCTGAT TCCTACCAGA ATTATCGTTT   
  
  
- AATCTCTAAA GTAATCTATT TTAGATGACA TGTACTGTTT TGTTACTATA TATCGTAGGA TAAATTTTTC   
  
  
- GATGCACAGT CAGTATATAC TTAACGTAGA TTAAAAATTA TCTATATATT ATACTATACT ATATTATCCT   
  
  
- ATACTACATC TAACTTAAGT TTGAGTATTA AGTAATTCGT ACCACGTTAC AAGTAAAAGA ACTAAAATTT   
  
  
- GGCTGATAAT GAAGTTGACA TATATTAAGC TTAACGAAAC CATAAAATTT TAAATTTGAT CATTAATGAC   
  
  
- GGAGATAATG ACTACAAATA TAATTCACCA CGAATTGACG AATAACTACT TTGGTAAAAA TAAAAGCACA   
  
  
- TTTTCTCCCG TATTAGGTAG GAGCACATAA CCGCCCCGAG CCGCGGGATA AGTCGATAAG AGACATACAG   
  
  
- TTTGTTTTGG CGAAGATAAT GGGTAACCAG AGATTTGTGC GTCCCCAAAA TCGGGCCTCT CAATTAAGAA   
  
  
- ATAACTCCAG GGGACAAGAG GTAACGTCCT AAAAACGAGT CCATGAAGAG ACAAAGAAGA GAAATTAGAA   
  
  
- GACATAGAAA GACGTAGTTC AAATTACGAA AGACCCTAAA AGTCGATGCT AAATACACTT TAACATTCTA   
  
  
- AAGACTTGAC CCGTAAGTCC CGAACCAAGT ATACATAAGA AAAGACGAAC TTTACAAAAT TAAAGTATCA   
  
  
- AGGTTACCCC AATAAGTTTC TATCTTAATT CGATATTAGT GGCTCTTCTG AACGACTGGA AACAAAGAAA   
  
  
- AAACAAAGAA AAAAAATTAA TTTAGACCGA TAATGAACCG ATATCAAAAA CGATTACCTA GGTGTTTATC   
  
  
- TGCTTAATAT ACCCGGAAAC GTGGTTTACT TCAAGTTACT AGTCTCCTCT GGGTAAAATA GTTTACTTTT   
  
  
- AAAACAATTA CACAACTTTG GCGGTTTAAG GTTAACCTTA AGTGGAAAAC CCTTGGGATG ACACTAATGG   
  
  
- TGTTTGGGTT TAAAACTAAA CCAACACGGT AGTGATCCAG GGTTACAACT CCCTCGAGAG GAACTTCTAA   
  
  
- TACTAAAATC ACTACAACAA GAATTCATGT AGTTAGTTCA AGAATACCTG CTCCTACTCC CTCTCTTTTA   
  
  
- GGTAAACCCG GTGCCCGGAG ACCGAGAACT TCGCCGGCTC TTCAGCAATA TACTCCACGA ACCCGTCTCG   
  
  
- GTAAGAGGTG TTTTAGTTAT GAGAGGTCTC CTGTTCGCAC TACTGGACTC GTTCTCATCA CAATCTTCGA   
  
  
- CACAGTCAAT ATCGACACTG CCACGTTCAT CACCGTCACC GTAGCACCTC GTTTCAACCA GCTCAATAGG   
  
  
- AGGCCTCATG TTAGGGTACG CCTACCGTCT AAAAGGACGG AACGGGAGTT TTGGGAACAA CTCTTGTAGA   
  
  
- ATATCGAATA GTTTATCGAA CTGTCCACTC CCATTACCAA ACCACCTATC TGGCCGTCTG AGTGGACATT   
  
  
- CGCGAGAATC ACACAGACTA TATAAATTAC TATCAGTCAG TCACTATGTT AAAGTCTTTC CCTACCTCCT   
  
  
- TCGCTCGTTT AAAGAAGGGT TCCGAAGAAA CACAAACCTA AACTTGTGAC CTGGGACGGA AGCCCTCAGC   
  
  
- CAGTTATTCC GTGCTCCACC CGACTAAAAC CACCTCTGAT CGGTACTTTT ACTCCAACGT CACGATCGAT   
  
  
- CATCCCCTTT CTTCGTAGCG GTAGGACTCC TTAATGCAAG TCTTCAACCC TCCCCGTCAT TCGTCGGTCA   
  
  
- ACAAAAGGTC AAAAGTCTGC TCCGTCAACA CTCTAGTCTT TACAAACTAT CCCATAACGA CACGTCACCA   
  
  
- CCGTTTTTAC TACGACTGGA TGCGGTTTGA AATGTCTCAC TTGATTTATT TTGATACGTT TTACGAGTTT   
  
  
- ACTTTCTAAG ATTACCACCT TTTCCGGCAC CATCCTTCGT TCAACCGTCC TCCCTACACC ACCTAGATTC   
  
  
- TAGAGAAAAT GCGGAAACAC GTGTTCGTCA ACCTAGGTTA CTGGTTTCTT CGCGTTTACT GAATTAATCC   
  
  
- GTTTAATCCT TCGTAAGAAG AGGTTGGCCC CTACCCTTAG TTTCTTACCG AGTAATAAAA CGACTACCAG   
  
  
- AACTCCGAGC CGAACGACCA CAACCCTGGG GTTATATGTT CAGAGAGCAC TGACCGGGTC GAAGACGACA   
  
  
- ACTATAGAAC TCTCGAATGG TATACAAAGA ACGGTGTACG GGTAAGTTCT TTTATCCCTT AAAGAAGAGA   
  
  
- TTATCTTGTT AATACCTACA TCGAGTCTTT CGTTTAGCTG AAGTATAATA ACTAGAACCA TAACAGATAC   
  
  
- CAAAGGTTAC CGGAACAGAT TAAGTCGCGG AAAGTAGATC CGGACCGCCC GGAGGGTTTG AAGCCTAATG   
  
  
- TCCGCAACTA GAGGGGGTTG GGCCAAAGGC TGGTCGGTTT TCTCAGCTCC TCTGACCCGC TGCGAACTCC   
  
  
- TTGATACGAC TCAGTAAGTT ACACGGTAAA CTCAAGTTAC GGTATCGTTT CTCGACCCTT TGTGAATGAC   
  
  
- AATTTCTAGA GTTCTAACTA GGGCTACTCC ACGAACAACA CTTGACAGAC AAGTCCAAAT TTATGTAAGG   
  
  
- ACTCCTTTGT CATTAACGTC TTACGGGTTC TCTATAACAA GAATTAGACT AGTCTGTCTA TTTTGGTCGA   
  
  
- CAAAAGTATG TACCACATCA GTTACCTCGG AAGTTAAGAG GAAAAAAGTA ATAAGCCAAG GCACTCCGAG   
  
  
- AGAAGGTGAA GAGGTGAGAT AAACTACACA ACCTCCGTTT AGAAGGGGCC CTGTAGCTCC TCTCCTATGA   
  
  
- CTATCTCGCT CTGTAGAAAC CCGCTGTCCG TTACTTACAC TAGCGAACAC TCCCAAACCT CTCCTATCTT   
  
  
- TCCGGTCTCT TTATGTTTGT CACCGCCCAG GCTTTACTCT CCCGACCCAA CTTCGTCGAC GGAAACTTAG   
  
  
- TCCTCTGACA CCTTTACCGG TTTTTTGCCC ACTGTCGACA GATAGTGTTT CTAAAGAGGT AACTACTGCT   
  
  
- ACCCGTGACC AACAATGTCC CTACCTTTCC CTCTTAACAG ATACGTGAGT GATGAACCTT CGGACGACTG   
  
  
- AT

+     AAGAA-motif

| Site Name | Organism | Position | Strand | Matrix score. | sequence | function |
| --- | --- | --- | --- | --- | --- | --- |
| AAGAA-motif | Avena sativa | 2881 | + | 7 | GAAAGAA |  |

>HU05G01597.1   
+ -Up\_Stream \_Len000TTTGTA TTTCAATATT GTAATTAGGT GGGATATAGA GTTGAAGGGC AAACTCTACC   
  
  
+ AACGACTTGT TGAAGGATCA ACTTGTAGCT TGGAACTTAA TGTATGTGTA CTATAGAGTT GTGTAATCTC   
  
  
+ TCCTAATAAA ATTGGGTTAA TCCTAGAATT AGAGAACTTT AAGCGGGGAC TAAGCTTGGT TGAGGCTAAC   
  
  
+ TTCGTTAAAA AGTTCTTGTG TCATGTCCAC GTTTATCTTT TCACTATTTA TTTCAATTAA TTTGCGATAT   
  
  
+ TTATCATCAC CCTATTCACC GTCTTTTCGG ATGATTTAAC TAGTCAAGCC GGACCAACAT TGCTTTTTAT   
  
  
+ AGTGTATTTG CAACTCATGT TGATTATTAG ATGTATCAAC AAAAAATGTT TCTCAAAATA AATAGTAGCT   
  
  
+ CTTTAAATTT TATTCCACAT AAATTCAAAA TCAATATCGA TATCGTTCCA TAATATAGCA GCATTATGAA   
  
  
+ AACAATATTT GCTAATGTTT ACATTGTGCT CTTTTCACTC AAAAATTGTA AGGCTTCAAA TACTATTAAA   
  
  
+ GAAACCATTA TTTCACTCGA CTATTTAGCT TGCATATATA GACAATCTTG CAAATAGTAA CATGTCTTCA   
  
  
+ TTCGACTATT TGCTTCCAAA TGAATAATCT CGTAAACATA TATACATACA TACATATATA TATATATATA   
  
  
+ TATATATATA TATATATATA GTAATATGTA TTTAACTTCA CATATACATG AATATAAAAA TATAAGAGGG   
  
  
+ AAAAAAGACG CTAGGTAGAT GGACTACAAA AGCCATTCAT GTATTGAGAC GGCTCATAAA ATCATACGGG   
  
  
+ GAAAGGAAAA AAAAAGATCT CTACATAATG TTCATAGATT TATAGAAAGC ATATGATGGA TTACCGCATA   
  
  
+ AGGTGTTATA TATGGGAGTG TTTGTAGAAG AAAGATGTAT ATCCGAACTA TCAAAGATAC ATATGAGGGA   
  
  
+ GTAAAAACAA GTGTAAGAAC CTCAGGAGGA GACATAAAAG ATTTTTCTAT TGGCATTGGA CACCGTCAAG   
  
  
+ TATCCGCTTT AAACCTGTTC TTTTTCACTA TTATCATGGA TGTATTCACA AAAGGAATTC AATACGAAGT   
  
  
+ ACCATGATGT ATGCTTTTTA CTGATAGTAT TGGTCTCATC GATGAGACTA AGGATGGTCT TAATAGCAAA   
  
  
+ TTAGAGATTT CATTAGATAA AATCTACTGT ACATGACAAA ACAATGATAT ATAGCATCCT ATTTAAAAAG   
  
  
+ CTACGTGTCA GTCATATATG AATTGCATCT AATTTTTAAT AGATATATAA TATGATATGA TATAATAGGA   
  
  
+ TATGATGTAG ATTGAATTCA AACTCATAAT TCATTAAGCA TGGTGCAATG TTCATTTTCT TGATTTTAAA   
  
  
+ CCGACTATTA CTTCAACTGT ATATAATTCG AATTGCTTTG GTATTTTAAA ATTTAAACTA GTAATTACTG   
  
  
+ CCTCTATTAC TGATGTTTAT ATTAAGTGGT GCTTAACTGC TTATTGATGA AACCATTTTT ATTTTCGTGT   
  
  
+ AAAAGAGGGC ATAATCCATC CTCGTGTATT GGCGGGGCTC GGCGCCCTAT TCAGCTATTC TCTGTATGTC   
  
  
+ AAACAAAACC GCTTCTATTA CCCATTGGTC TCTAAACACG CAGGGGTTTT AGCCCGGAGA GTTAATTCTT   
  
  
+ TATTGAGGTC CCCTGTTCTC CATTGCAGGA TTTTTGCTCA GGTACTTCTC TGTTTCTTCT CTTTAATCTT   
  
  
+ CTGTATCTTT CTGCATCAAG TTTAATGCTT TCTGGGATTT TCAGCTACGA TTTATGTGAA ATTGTAAGAT   
  
  
+ TTCTGAACTG GGCATTCAGG GCTTGGTTCA TATGTATTCT TTTCTGCTTG AAATGTTTTA ATTTCATAGT   
  
  
+ TCCAATGGGG TTATTCAAAG ATAGAATTAA GCTATAATCA CCGAGAAGAC TTGCTGACCT TTGTTTCTTT   
  
  
+ TTTGTTTCTT TTTTTTAATT AAATCTGGCT ATTACTTGGC TATAGTTTTT GCTAATGGAT CCACAAATAG   
  
  
+ ACGAATTATA TGGGCCTTTG CACCAAATGA AGTTCAATGA TCAGAGGAGA CCCATTTTAT CAAATGAAAA   
  
  
+ TTTTGTTAAT GTGTTGAAAC CGCCAAATTC CAATTGGAAT TCACCTTTTG GGAACCCTAC TGTGATTACC   
  
  
+ ACAAACCCAA ATTTTGATTT GGTTGTGCCA TCACTAGGTC CCAATGTTGA GGGAGCTCTC CTTGAAGATT   
  
  
+ ATGATTTTAG TGATGTTGTT CTTAAGTACA TCAATCAAGT TCTTATGGAC GAGGATGAGG GAGAGAAAAT   
  
  
+ CCATTTGGGC CACGGGCCTC TGGCTCTTGA AGCGGCCGAG AAGTCGTTAT ATGAGGTGCT TGGGCAGAGC   
  
  
+ CATTCTCCAC AAAATCAATA CTCTCCAGAG GACAAGCGTG ATGACCTGAG CAAGAGTAGT GTTAGAAGCT   
  
  
+ GTGTCAGTTA TAGCTGTGAC GGTGCAAGTA GTGGCAGTGG CATCGTGGAG CAAAGTTGGT CGAGTTATCC   
  
  
+ TCCGGAGTAC AATCCCATGC GGATGGCAGA TTTTCCTGCC TTGCCCTCAA AACCCTTGTT GAGAACATCT   
  
  
+ TATAGCTTAT CAAATAGCTT GACAGGTGAG GGTAATGGTT TGGTGGATAG ACCGGCAGAC TCACCTGTAA   
  
  
+ GCGCTCTTAG TGTGTCTGAT ATATTTAATG ATAGTCAGTC AGTGATACAA TTTCAGAAAG GGATGGAGGA   
  
  
+ AGCGAGCAAA TTTCTTCCCA AGGCTTCTTT GTGTTTGGAT TTGAACACTG GACCCTGCCT TCGGGAGTCG   
  
  
+ GTCAATAAGG CACGAGGTGG GCTGATTTTG GTGGAGACTA GCCATGAAAA TGAGGTTGCA GTGCTAGCTA   
  
  
+ GTAGGGGAAA GAAGCATCGC CATCCTGAGG AATTACGTTC AGAAGTTGGG AGGGGCAGTA AGCAGCCAGT   
  
  
+ TGTTTTCCAG TTTTCAGACG AGGCAGTTGT GAGATCAGAA ATGTTTGATA GGGTATTGCT GTGCAGTGGT   
  
  
+ GGCAAAAATG ATGCTGACCT ACGCCAAACT TTACAGAGTG AACTAAATAA AACTATGCAA AATGCTCAAA   
  
  
+ TGAAAGATTC TAATGGTGGA AAAGGCCGTG GTAGGAAGCA AGTTGGCAGG AGGGATGTGG TGGATCTAAG   
  
  
+ ATCTCTTTTA CGCCTTTGTG CACAAGCAGT TGGATCCAAT GACCAAAGAA GCGCAAATGA CTTAATTAGG   
  
  
+ CAAATTAGGA AGCATTCTTC TCCAACCGGG GATGGGAATC AAAGAATGGC TCATTATTTT GCTGATGGTC   
  
  
+ TTGAGGCTCG GCTTGCTGGT GTTGGGACCC CAATATACAA GTCTCTCGTG ACTGGCCCAG CTTCTGCTGT   
  
  
+ TGATATCTTG AGAGCTTACC ATATGTTTCT TGCCACATGC CCATTCAAGA AAATAGGGAA TTTCTTCTCT   
  
  
+ AATAGAACAA TTATGGATGT AGCTCAGAAA GCAAATCGAC TTCATATTAT TGATCTTGGT ATTGTCTATG   
  
  
+ GTTTCCAATG GCCTTGTCTA ATTCAGCGCC TTTCATCTAG GCCTGGCGGG CCTCCCAAAC TTCGGATTAC   
  
  
+ AGGCGTTGAT CTCCCCCAAC CCGGTTTCCG ACCAGCCAAA AGAGTCGAGG AGACTGGGCG ACGCTTGAGG   
  
  
+ AACTATGCTG AGTCATTCAA TGTGCCATTT GAGTTCAATG CCATAGCAAA GAGCTGGGAA ACACTTACTG   
  
  
+ TTAAAGATCT CAAGATTGAT CCCGATGAGG TGCTTGTTGT GAACTGTCTG TTCAGGTTTA AATACATTCC   
  
  
+ TGAGGAAACA GTAATTGCAG AATGCCCAAG AGATATTGTT CTTAATCTGA TCAGACAGAT AAAACCAGCT   
  
  
+ GTTTTCATAC ATGGTGTAGT CAATGGAGCC TTCAATTCTC CTTTTTTCAT TATTCGGTTC CGTGAGGCTC   
  
  
+ TCTTCCACTT CTCCACTCTA TTTGATGTGT TGGAGGCAAA TCTTCCCCGG GACATCGAGG AGAGGATACT   
  
  
+ GATAGAGCGA GACATCTTTG GGCGACAGGC AATGAATGTG ATCGCTTGTG AGGGTTTGGA GAGGATAGAA   
  
  
+ AGGCCAGAGA AATACAAACA GTGGCGGGTC CGAAATGAGA GGGCTGGGTT GAAGCAGCTG CCTTTGAATC   
  
  
+ AGGAGACTGT GGAAATGGCC AAAAAACGGG TGACAGCTGT CTATCACAAA GATTTCTCCA TTGATGACGA   
  
  
+ TGGGCACTGG TTGTTACAGG GATGGAAAGG GAGAATTGTC TATGCACTCA CTACTTGGAA GCCTGCTGAC   
  
  
+ TA  

- -Up\_Stream \_Len000AAACAT AAAGTTATAA CATTAATCCA CCCTATATCT CAACTTCCCG TTTGAGATGG   
  
  
- TTGCTGAACA ACTTCCTAGT TGAACATCGA ACCTTGAATT ACATACACAT GATATCTCAA CACATTAGAG   
  
  
- AGGATTATTT TAACCCAATT AGGATCTTAA TCTCTTGAAA TTCGCCCCTG ATTCGAACCA ACTCCGATTG   
  
  
- AAGCAATTTT TCAAGAACAC AGTACAGGTG CAAATAGAAA AGTGATAAAT AAAGTTAATT AAACGCTATA   
  
  
- AATAGTAGTG GGATAAGTGG CAGAAAAGCC TACTAAATTG ATCAGTTCGG CCTGGTTGTA ACGAAAAATA   
  
  
- TCACATAAAC GTTGAGTACA ACTAATAATC TACATAGTTG TTTTTTACAA AGAGTTTTAT TTATCATCGA   
  
  
- GAAATTTAAA ATAAGGTGTA TTTAAGTTTT AGTTATAGCT ATAGCAAGGT ATTATATCGT CGTAATACTT   
  
  
- TTGTTATAAA CGATTACAAA TGTAACACGA GAAAAGTGAG TTTTTAACAT TCCGAAGTTT ATGATAATTT   
  
  
- CTTTGGTAAT AAAGTGAGCT GATAAATCGA ACGTATATAT CTGTTAGAAC GTTTATCATT GTACAGAAGT   
  
  
- AAGCTGATAA ACGAAGGTTT ACTTATTAGA GCATTTGTAT ATATGTATGT ATGTATATAT ATATATATAT   
  
  
- ATATATATAT ATATATATAT CATTATACAT AAATTGAAGT GTATATGTAC TTATATTTTT ATATTCTCCC   
  
  
- TTTTTTCTGC GATCCATCTA CCTGATGTTT TCGGTAAGTA CATAACTCTG CCGAGTATTT TAGTATGCCC   
  
  
- CTTTCCTTTT TTTTTCTAGA GATGTATTAC AAGTATCTAA ATATCTTTCG TATACTACCT AATGGCGTAT   
  
  
- TCCACAATAT ATACCCTCAC AAACATCTTC TTTCTACATA TAGGCTTGAT AGTTTCTATG TATACTCCCT   
  
  
- CATTTTTGTT CACATTCTTG GAGTCCTCCT CTGTATTTTC TAAAAAGATA ACCGTAACCT GTGGCAGTTC   
  
  
- ATAGGCGAAA TTTGGACAAG AAAAAGTGAT AATAGTACCT ACATAAGTGT TTTCCTTAAG TTATGCTTCA   
  
  
- TGGTACTACA TACGAAAAAT GACTATCATA ACCAGAGTAG CTACTCTGAT TCCTACCAGA ATTATCGTTT   
  
  
- AATCTCTAAA GTAATCTATT TTAGATGACA TGTACTGTTT TGTTACTATA TATCGTAGGA TAAATTTTTC   
  
  
- GATGCACAGT CAGTATATAC TTAACGTAGA TTAAAAATTA TCTATATATT ATACTATACT ATATTATCCT   
  
  
- ATACTACATC TAACTTAAGT TTGAGTATTA AGTAATTCGT ACCACGTTAC AAGTAAAAGA ACTAAAATTT   
  
  
- GGCTGATAAT GAAGTTGACA TATATTAAGC TTAACGAAAC CATAAAATTT TAAATTTGAT CATTAATGAC   
  
  
- GGAGATAATG ACTACAAATA TAATTCACCA CGAATTGACG AATAACTACT TTGGTAAAAA TAAAAGCACA   
  
  
- TTTTCTCCCG TATTAGGTAG GAGCACATAA CCGCCCCGAG CCGCGGGATA AGTCGATAAG AGACATACAG   
  
  
- TTTGTTTTGG CGAAGATAAT GGGTAACCAG AGATTTGTGC GTCCCCAAAA TCGGGCCTCT CAATTAAGAA   
  
  
- ATAACTCCAG GGGACAAGAG GTAACGTCCT AAAAACGAGT CCATGAAGAG ACAAAGAAGA GAAATTAGAA   
  
  
- GACATAGAAA GACGTAGTTC AAATTACGAA AGACCCTAAA AGTCGATGCT AAATACACTT TAACATTCTA   
  
  
- AAGACTTGAC CCGTAAGTCC CGAACCAAGT ATACATAAGA AAAGACGAAC TTTACAAAAT TAAAGTATCA   
  
  
- AGGTTACCCC AATAAGTTTC TATCTTAATT CGATATTAGT GGCTCTTCTG AACGACTGGA AACAAAGAAA   
  
  
- AAACAAAGAA AAAAAATTAA TTTAGACCGA TAATGAACCG ATATCAAAAA CGATTACCTA GGTGTTTATC   
  
  
- TGCTTAATAT ACCCGGAAAC GTGGTTTACT TCAAGTTACT AGTCTCCTCT GGGTAAAATA GTTTACTTTT   
  
  
- AAAACAATTA CACAACTTTG GCGGTTTAAG GTTAACCTTA AGTGGAAAAC CCTTGGGATG ACACTAATGG   
  
  
- TGTTTGGGTT TAAAACTAAA CCAACACGGT AGTGATCCAG GGTTACAACT CCCTCGAGAG GAACTTCTAA   
  
  
- TACTAAAATC ACTACAACAA GAATTCATGT AGTTAGTTCA AGAATACCTG CTCCTACTCC CTCTCTTTTA   
  
  
- GGTAAACCCG GTGCCCGGAG ACCGAGAACT TCGCCGGCTC TTCAGCAATA TACTCCACGA ACCCGTCTCG   
  
  
- GTAAGAGGTG TTTTAGTTAT GAGAGGTCTC CTGTTCGCAC TACTGGACTC GTTCTCATCA CAATCTTCGA   
  
  
- CACAGTCAAT ATCGACACTG CCACGTTCAT CACCGTCACC GTAGCACCTC GTTTCAACCA GCTCAATAGG   
  
  
- AGGCCTCATG TTAGGGTACG CCTACCGTCT AAAAGGACGG AACGGGAGTT TTGGGAACAA CTCTTGTAGA   
  
  
- ATATCGAATA GTTTATCGAA CTGTCCACTC CCATTACCAA ACCACCTATC TGGCCGTCTG AGTGGACATT   
  
  
- CGCGAGAATC ACACAGACTA TATAAATTAC TATCAGTCAG TCACTATGTT AAAGTCTTTC CCTACCTCCT   
  
  
- TCGCTCGTTT AAAGAAGGGT TCCGAAGAAA CACAAACCTA AACTTGTGAC CTGGGACGGA AGCCCTCAGC   
  
  
- CAGTTATTCC GTGCTCCACC CGACTAAAAC CACCTCTGAT CGGTACTTTT ACTCCAACGT CACGATCGAT   
  
  
- CATCCCCTTT CTTCGTAGCG GTAGGACTCC TTAATGCAAG TCTTCAACCC TCCCCGTCAT TCGTCGGTCA   
  
  
- ACAAAAGGTC AAAAGTCTGC TCCGTCAACA CTCTAGTCTT TACAAACTAT CCCATAACGA CACGTCACCA   
  
  
- CCGTTTTTAC TACGACTGGA TGCGGTTTGA AATGTCTCAC TTGATTTATT TTGATACGTT TTACGAGTTT   
  
  
- ACTTTCTAAG ATTACCACCT TTTCCGGCAC CATCCTTCGT TCAACCGTCC TCCCTACACC ACCTAGATTC   
  
  
- TAGAGAAAAT GCGGAAACAC GTGTTCGTCA ACCTAGGTTA CTGGTTTCTT CGCGTTTACT GAATTAATCC   
  
  
- GTTTAATCCT TCGTAAGAAG AGGTTGGCCC CTACCCTTAG TTTCTTACCG AGTAATAAAA CGACTACCAG   
  
  
- AACTCCGAGC CGAACGACCA CAACCCTGGG GTTATATGTT CAGAGAGCAC TGACCGGGTC GAAGACGACA   
  
  
- ACTATAGAAC TCTCGAATGG TATACAAAGA ACGGTGTACG GGTAAGTTCT TTTATCCCTT AAAGAAGAGA   
  
  
- TTATCTTGTT AATACCTACA TCGAGTCTTT CGTTTAGCTG AAGTATAATA ACTAGAACCA TAACAGATAC   
  
  
- CAAAGGTTAC CGGAACAGAT TAAGTCGCGG AAAGTAGATC CGGACCGCCC GGAGGGTTTG AAGCCTAATG   
  
  
- TCCGCAACTA GAGGGGGTTG GGCCAAAGGC TGGTCGGTTT TCTCAGCTCC TCTGACCCGC TGCGAACTCC   
  
  
- TTGATACGAC TCAGTAAGTT ACACGGTAAA CTCAAGTTAC GGTATCGTTT CTCGACCCTT TGTGAATGAC   
  
  
- AATTTCTAGA GTTCTAACTA GGGCTACTCC ACGAACAACA CTTGACAGAC AAGTCCAAAT TTATGTAAGG   
  
  
- ACTCCTTTGT CATTAACGTC TTACGGGTTC TCTATAACAA GAATTAGACT AGTCTGTCTA TTTTGGTCGA   
  
  
- CAAAAGTATG TACCACATCA GTTACCTCGG AAGTTAAGAG GAAAAAAGTA ATAAGCCAAG GCACTCCGAG   
  
  
- AGAAGGTGAA GAGGTGAGAT AAACTACACA ACCTCCGTTT AGAAGGGGCC CTGTAGCTCC TCTCCTATGA   
  
  
- CTATCTCGCT CTGTAGAAAC CCGCTGTCCG TTACTTACAC TAGCGAACAC TCCCAAACCT CTCCTATCTT   
  
  
- TCCGGTCTCT TTATGTTTGT CACCGCCCAG GCTTTACTCT CCCGACCCAA CTTCGTCGAC GGAAACTTAG   
  
  
- TCCTCTGACA CCTTTACCGG TTTTTTGCCC ACTGTCGACA GATAGTGTTT CTAAAGAGGT AACTACTGCT   
  
  
- ACCCGTGACC AACAATGTCC CTACCTTTCC CTCTTAACAG ATACGTGAGT GATGAACCTT CGGACGACTG   
  
  
- AT

+     ABRE

| Site Name | Organism | Position | Strand | Matrix score. | sequence | function |
| --- | --- | --- | --- | --- | --- | --- |
| ABRE | Arabidopsis thaliana | 1267 | + | 5 | ACGTG | cis-acting element involved in the abscisic acid responsiveness |
| ABRE | Oryza sativa | 1266 | + | 8 | TACGTGTC | cis-acting element involved in the abscisic acid responsiveness |
| ABRE | Arabidopsis thaliana | 242 | - | 5 | ACGTG | cis-acting element involved in the abscisic acid responsiveness |
| ABRE | Hordeum vulgare | 1264 | + | 9 | GCAACGTGTC | cis-acting element involved in the abscisic acid responsiveness |
| ABRE | Arabidopsis thaliana | 3592 | + | 7 | AACCCGG | cis-acting element involved in the abscisic acid responsiveness |

>HU05G01597.1   
+ -Up\_Stream \_Len000TTTGTA TTTCAATATT GTAATTAGGT GGGATATAGA GTTGAAGGGC AAACTCTACC   
  
  
+ AACGACTTGT TGAAGGATCA ACTTGTAGCT TGGAACTTAA TGTATGTGTA CTATAGAGTT GTGTAATCTC   
  
  
+ TCCTAATAAA ATTGGGTTAA TCCTAGAATT AGAGAACTTT AAGCGGGGAC TAAGCTTGGT TGAGGCTAAC   
  
  
+ TTCGTTAAAA AGTTCTTGTG TCATGTCCAC GTTTATCTTT TCACTATTTA TTTCAATTAA TTTGCGATAT   
  
  
+ TTATCATCAC CCTATTCACC GTCTTTTCGG ATGATTTAAC TAGTCAAGCC GGACCAACAT TGCTTTTTAT   
  
  
+ AGTGTATTTG CAACTCATGT TGATTATTAG ATGTATCAAC AAAAAATGTT TCTCAAAATA AATAGTAGCT   
  
  
+ CTTTAAATTT TATTCCACAT AAATTCAAAA TCAATATCGA TATCGTTCCA TAATATAGCA GCATTATGAA   
  
  
+ AACAATATTT GCTAATGTTT ACATTGTGCT CTTTTCACTC AAAAATTGTA AGGCTTCAAA TACTATTAAA   
  
  
+ GAAACCATTA TTTCACTCGA CTATTTAGCT TGCATATATA GACAATCTTG CAAATAGTAA CATGTCTTCA   
  
  
+ TTCGACTATT TGCTTCCAAA TGAATAATCT CGTAAACATA TATACATACA TACATATATA TATATATATA   
  
  
+ TATATATATA TATATATATA GTAATATGTA TTTAACTTCA CATATACATG AATATAAAAA TATAAGAGGG   
  
  
+ AAAAAAGACG CTAGGTAGAT GGACTACAAA AGCCATTCAT GTATTGAGAC GGCTCATAAA ATCATACGGG   
  
  
+ GAAAGGAAAA AAAAAGATCT CTACATAATG TTCATAGATT TATAGAAAGC ATATGATGGA TTACCGCATA   
  
  
+ AGGTGTTATA TATGGGAGTG TTTGTAGAAG AAAGATGTAT ATCCGAACTA TCAAAGATAC ATATGAGGGA   
  
  
+ GTAAAAACAA GTGTAAGAAC CTCAGGAGGA GACATAAAAG ATTTTTCTAT TGGCATTGGA CACCGTCAAG   
  
  
+ TATCCGCTTT AAACCTGTTC TTTTTCACTA TTATCATGGA TGTATTCACA AAAGGAATTC AATACGAAGT   
  
  
+ ACCATGATGT ATGCTTTTTA CTGATAGTAT TGGTCTCATC GATGAGACTA AGGATGGTCT TAATAGCAAA   
  
  
+ TTAGAGATTT CATTAGATAA AATCTACTGT ACATGACAAA ACAATGATAT ATAGCATCCT ATTTAAAAAG   
  
  
+ CTACGTGTCA GTCATATATG AATTGCATCT AATTTTTAAT AGATATATAA TATGATATGA TATAATAGGA   
  
  
+ TATGATGTAG ATTGAATTCA AACTCATAAT TCATTAAGCA TGGTGCAATG TTCATTTTCT TGATTTTAAA   
  
  
+ CCGACTATTA CTTCAACTGT ATATAATTCG AATTGCTTTG GTATTTTAAA ATTTAAACTA GTAATTACTG   
  
  
+ CCTCTATTAC TGATGTTTAT ATTAAGTGGT GCTTAACTGC TTATTGATGA AACCATTTTT ATTTTCGTGT   
  
  
+ AAAAGAGGGC ATAATCCATC CTCGTGTATT GGCGGGGCTC GGCGCCCTAT TCAGCTATTC TCTGTATGTC   
  
  
+ AAACAAAACC GCTTCTATTA CCCATTGGTC TCTAAACACG CAGGGGTTTT AGCCCGGAGA GTTAATTCTT   
  
  
+ TATTGAGGTC CCCTGTTCTC CATTGCAGGA TTTTTGCTCA GGTACTTCTC TGTTTCTTCT CTTTAATCTT   
  
  
+ CTGTATCTTT CTGCATCAAG TTTAATGCTT TCTGGGATTT TCAGCTACGA TTTATGTGAA ATTGTAAGAT   
  
  
+ TTCTGAACTG GGCATTCAGG GCTTGGTTCA TATGTATTCT TTTCTGCTTG AAATGTTTTA ATTTCATAGT   
  
  
+ TCCAATGGGG TTATTCAAAG ATAGAATTAA GCTATAATCA CCGAGAAGAC TTGCTGACCT TTGTTTCTTT   
  
  
+ TTTGTTTCTT TTTTTTAATT AAATCTGGCT ATTACTTGGC TATAGTTTTT GCTAATGGAT CCACAAATAG   
  
  
+ ACGAATTATA TGGGCCTTTG CACCAAATGA AGTTCAATGA TCAGAGGAGA CCCATTTTAT CAAATGAAAA   
  
  
+ TTTTGTTAAT GTGTTGAAAC CGCCAAATTC CAATTGGAAT TCACCTTTTG GGAACCCTAC TGTGATTACC   
  
  
+ ACAAACCCAA ATTTTGATTT GGTTGTGCCA TCACTAGGTC CCAATGTTGA GGGAGCTCTC CTTGAAGATT   
  
  
+ ATGATTTTAG TGATGTTGTT CTTAAGTACA TCAATCAAGT TCTTATGGAC GAGGATGAGG GAGAGAAAAT   
  
  
+ CCATTTGGGC CACGGGCCTC TGGCTCTTGA AGCGGCCGAG AAGTCGTTAT ATGAGGTGCT TGGGCAGAGC   
  
  
+ CATTCTCCAC AAAATCAATA CTCTCCAGAG GACAAGCGTG ATGACCTGAG CAAGAGTAGT GTTAGAAGCT   
  
  
+ GTGTCAGTTA TAGCTGTGAC GGTGCAAGTA GTGGCAGTGG CATCGTGGAG CAAAGTTGGT CGAGTTATCC   
  
  
+ TCCGGAGTAC AATCCCATGC GGATGGCAGA TTTTCCTGCC TTGCCCTCAA AACCCTTGTT GAGAACATCT   
  
  
+ TATAGCTTAT CAAATAGCTT GACAGGTGAG GGTAATGGTT TGGTGGATAG ACCGGCAGAC TCACCTGTAA   
  
  
+ GCGCTCTTAG TGTGTCTGAT ATATTTAATG ATAGTCAGTC AGTGATACAA TTTCAGAAAG GGATGGAGGA   
  
  
+ AGCGAGCAAA TTTCTTCCCA AGGCTTCTTT GTGTTTGGAT TTGAACACTG GACCCTGCCT TCGGGAGTCG   
  
  
+ GTCAATAAGG CACGAGGTGG GCTGATTTTG GTGGAGACTA GCCATGAAAA TGAGGTTGCA GTGCTAGCTA   
  
  
+ GTAGGGGAAA GAAGCATCGC CATCCTGAGG AATTACGTTC AGAAGTTGGG AGGGGCAGTA AGCAGCCAGT   
  
  
+ TGTTTTCCAG TTTTCAGACG AGGCAGTTGT GAGATCAGAA ATGTTTGATA GGGTATTGCT GTGCAGTGGT   
  
  
+ GGCAAAAATG ATGCTGACCT ACGCCAAACT TTACAGAGTG AACTAAATAA AACTATGCAA AATGCTCAAA   
  
  
+ TGAAAGATTC TAATGGTGGA AAAGGCCGTG GTAGGAAGCA AGTTGGCAGG AGGGATGTGG TGGATCTAAG   
  
  
+ ATCTCTTTTA CGCCTTTGTG CACAAGCAGT TGGATCCAAT GACCAAAGAA GCGCAAATGA CTTAATTAGG   
  
  
+ CAAATTAGGA AGCATTCTTC TCCAACCGGG GATGGGAATC AAAGAATGGC TCATTATTTT GCTGATGGTC   
  
  
+ TTGAGGCTCG GCTTGCTGGT GTTGGGACCC CAATATACAA GTCTCTCGTG ACTGGCCCAG CTTCTGCTGT   
  
  
+ TGATATCTTG AGAGCTTACC ATATGTTTCT TGCCACATGC CCATTCAAGA AAATAGGGAA TTTCTTCTCT   
  
  
+ AATAGAACAA TTATGGATGT AGCTCAGAAA GCAAATCGAC TTCATATTAT TGATCTTGGT ATTGTCTATG   
  
  
+ GTTTCCAATG GCCTTGTCTA ATTCAGCGCC TTTCATCTAG GCCTGGCGGG CCTCCCAAAC TTCGGATTAC   
  
  
+ AGGCGTTGAT CTCCCCCAAC CCGGTTTCCG ACCAGCCAAA AGAGTCGAGG AGACTGGGCG ACGCTTGAGG   
  
  
+ AACTATGCTG AGTCATTCAA TGTGCCATTT GAGTTCAATG CCATAGCAAA GAGCTGGGAA ACACTTACTG   
  
  
+ TTAAAGATCT CAAGATTGAT CCCGATGAGG TGCTTGTTGT GAACTGTCTG TTCAGGTTTA AATACATTCC   
  
  
+ TGAGGAAACA GTAATTGCAG AATGCCCAAG AGATATTGTT CTTAATCTGA TCAGACAGAT AAAACCAGCT   
  
  
+ GTTTTCATAC ATGGTGTAGT CAATGGAGCC TTCAATTCTC CTTTTTTCAT TATTCGGTTC CGTGAGGCTC   
  
  
+ TCTTCCACTT CTCCACTCTA TTTGATGTGT TGGAGGCAAA TCTTCCCCGG GACATCGAGG AGAGGATACT   
  
  
+ GATAGAGCGA GACATCTTTG GGCGACAGGC AATGAATGTG ATCGCTTGTG AGGGTTTGGA GAGGATAGAA   
  
  
+ AGGCCAGAGA AATACAAACA GTGGCGGGTC CGAAATGAGA GGGCTGGGTT GAAGCAGCTG CCTTTGAATC   
  
  
+ AGGAGACTGT GGAAATGGCC AAAAAACGGG TGACAGCTGT CTATCACAAA GATTTCTCCA TTGATGACGA   
  
  
+ TGGGCACTGG TTGTTACAGG GATGGAAAGG GAGAATTGTC TATGCACTCA CTACTTGGAA GCCTGCTGAC   
  
  
+ TA  

- -Up\_Stream \_Len000AAACAT AAAGTTATAA CATTAATCCA CCCTATATCT CAACTTCCCG TTTGAGATGG   
  
  
- TTGCTGAACA ACTTCCTAGT TGAACATCGA ACCTTGAATT ACATACACAT GATATCTCAA CACATTAGAG   
  
  
- AGGATTATTT TAACCCAATT AGGATCTTAA TCTCTTGAAA TTCGCCCCTG ATTCGAACCA ACTCCGATTG   
  
  
- AAGCAATTTT TCAAGAACAC AGTACAGGTG CAAATAGAAA AGTGATAAAT AAAGTTAATT AAACGCTATA   
  
  
- AATAGTAGTG GGATAAGTGG CAGAAAAGCC TACTAAATTG ATCAGTTCGG CCTGGTTGTA ACGAAAAATA   
  
  
- TCACATAAAC GTTGAGTACA ACTAATAATC TACATAGTTG TTTTTTACAA AGAGTTTTAT TTATCATCGA   
  
  
- GAAATTTAAA ATAAGGTGTA TTTAAGTTTT AGTTATAGCT ATAGCAAGGT ATTATATCGT CGTAATACTT   
  
  
- TTGTTATAAA CGATTACAAA TGTAACACGA GAAAAGTGAG TTTTTAACAT TCCGAAGTTT ATGATAATTT   
  
  
- CTTTGGTAAT AAAGTGAGCT GATAAATCGA ACGTATATAT CTGTTAGAAC GTTTATCATT GTACAGAAGT   
  
  
- AAGCTGATAA ACGAAGGTTT ACTTATTAGA GCATTTGTAT ATATGTATGT ATGTATATAT ATATATATAT   
  
  
- ATATATATAT ATATATATAT CATTATACAT AAATTGAAGT GTATATGTAC TTATATTTTT ATATTCTCCC   
  
  
- TTTTTTCTGC GATCCATCTA CCTGATGTTT TCGGTAAGTA CATAACTCTG CCGAGTATTT TAGTATGCCC   
  
  
- CTTTCCTTTT TTTTTCTAGA GATGTATTAC AAGTATCTAA ATATCTTTCG TATACTACCT AATGGCGTAT   
  
  
- TCCACAATAT ATACCCTCAC AAACATCTTC TTTCTACATA TAGGCTTGAT AGTTTCTATG TATACTCCCT   
  
  
- CATTTTTGTT CACATTCTTG GAGTCCTCCT CTGTATTTTC TAAAAAGATA ACCGTAACCT GTGGCAGTTC   
  
  
- ATAGGCGAAA TTTGGACAAG AAAAAGTGAT AATAGTACCT ACATAAGTGT TTTCCTTAAG TTATGCTTCA   
  
  
- TGGTACTACA TACGAAAAAT GACTATCATA ACCAGAGTAG CTACTCTGAT TCCTACCAGA ATTATCGTTT   
  
  
- AATCTCTAAA GTAATCTATT TTAGATGACA TGTACTGTTT TGTTACTATA TATCGTAGGA TAAATTTTTC   
  
  
- GATGCACAGT CAGTATATAC TTAACGTAGA TTAAAAATTA TCTATATATT ATACTATACT ATATTATCCT   
  
  
- ATACTACATC TAACTTAAGT TTGAGTATTA AGTAATTCGT ACCACGTTAC AAGTAAAAGA ACTAAAATTT   
  
  
- GGCTGATAAT GAAGTTGACA TATATTAAGC TTAACGAAAC CATAAAATTT TAAATTTGAT CATTAATGAC   
  
  
- GGAGATAATG ACTACAAATA TAATTCACCA CGAATTGACG AATAACTACT TTGGTAAAAA TAAAAGCACA   
  
  
- TTTTCTCCCG TATTAGGTAG GAGCACATAA CCGCCCCGAG CCGCGGGATA AGTCGATAAG AGACATACAG   
  
  
- TTTGTTTTGG CGAAGATAAT GGGTAACCAG AGATTTGTGC GTCCCCAAAA TCGGGCCTCT CAATTAAGAA   
  
  
- ATAACTCCAG GGGACAAGAG GTAACGTCCT AAAAACGAGT CCATGAAGAG ACAAAGAAGA GAAATTAGAA   
  
  
- GACATAGAAA GACGTAGTTC AAATTACGAA AGACCCTAAA AGTCGATGCT AAATACACTT TAACATTCTA   
  
  
- AAGACTTGAC CCGTAAGTCC CGAACCAAGT ATACATAAGA AAAGACGAAC TTTACAAAAT TAAAGTATCA   
  
  
- AGGTTACCCC AATAAGTTTC TATCTTAATT CGATATTAGT GGCTCTTCTG AACGACTGGA AACAAAGAAA   
  
  
- AAACAAAGAA AAAAAATTAA TTTAGACCGA TAATGAACCG ATATCAAAAA CGATTACCTA GGTGTTTATC   
  
  
- TGCTTAATAT ACCCGGAAAC GTGGTTTACT TCAAGTTACT AGTCTCCTCT GGGTAAAATA GTTTACTTTT   
  
  
- AAAACAATTA CACAACTTTG GCGGTTTAAG GTTAACCTTA AGTGGAAAAC CCTTGGGATG ACACTAATGG   
  
  
- TGTTTGGGTT TAAAACTAAA CCAACACGGT AGTGATCCAG GGTTACAACT CCCTCGAGAG GAACTTCTAA   
  
  
- TACTAAAATC ACTACAACAA GAATTCATGT AGTTAGTTCA AGAATACCTG CTCCTACTCC CTCTCTTTTA   
  
  
- GGTAAACCCG GTGCCCGGAG ACCGAGAACT TCGCCGGCTC TTCAGCAATA TACTCCACGA ACCCGTCTCG   
  
  
- GTAAGAGGTG TTTTAGTTAT GAGAGGTCTC CTGTTCGCAC TACTGGACTC GTTCTCATCA CAATCTTCGA   
  
  
- CACAGTCAAT ATCGACACTG CCACGTTCAT CACCGTCACC GTAGCACCTC GTTTCAACCA GCTCAATAGG   
  
  
- AGGCCTCATG TTAGGGTACG CCTACCGTCT AAAAGGACGG AACGGGAGTT TTGGGAACAA CTCTTGTAGA   
  
  
- ATATCGAATA GTTTATCGAA CTGTCCACTC CCATTACCAA ACCACCTATC TGGCCGTCTG AGTGGACATT   
  
  
- CGCGAGAATC ACACAGACTA TATAAATTAC TATCAGTCAG TCACTATGTT AAAGTCTTTC CCTACCTCCT   
  
  
- TCGCTCGTTT AAAGAAGGGT TCCGAAGAAA CACAAACCTA AACTTGTGAC CTGGGACGGA AGCCCTCAGC   
  
  
- CAGTTATTCC GTGCTCCACC CGACTAAAAC CACCTCTGAT CGGTACTTTT ACTCCAACGT CACGATCGAT   
  
  
- CATCCCCTTT CTTCGTAGCG GTAGGACTCC TTAATGCAAG TCTTCAACCC TCCCCGTCAT TCGTCGGTCA   
  
  
- ACAAAAGGTC AAAAGTCTGC TCCGTCAACA CTCTAGTCTT TACAAACTAT CCCATAACGA CACGTCACCA   
  
  
- CCGTTTTTAC TACGACTGGA TGCGGTTTGA AATGTCTCAC TTGATTTATT TTGATACGTT TTACGAGTTT   
  
  
- ACTTTCTAAG ATTACCACCT TTTCCGGCAC CATCCTTCGT TCAACCGTCC TCCCTACACC ACCTAGATTC   
  
  
- TAGAGAAAAT GCGGAAACAC GTGTTCGTCA ACCTAGGTTA CTGGTTTCTT CGCGTTTACT GAATTAATCC   
  
  
- GTTTAATCCT TCGTAAGAAG AGGTTGGCCC CTACCCTTAG TTTCTTACCG AGTAATAAAA CGACTACCAG   
  
  
- AACTCCGAGC CGAACGACCA CAACCCTGGG GTTATATGTT CAGAGAGCAC TGACCGGGTC GAAGACGACA   
  
  
- ACTATAGAAC TCTCGAATGG TATACAAAGA ACGGTGTACG GGTAAGTTCT TTTATCCCTT AAAGAAGAGA   
  
  
- TTATCTTGTT AATACCTACA TCGAGTCTTT CGTTTAGCTG AAGTATAATA ACTAGAACCA TAACAGATAC   
  
  
- CAAAGGTTAC CGGAACAGAT TAAGTCGCGG AAAGTAGATC CGGACCGCCC GGAGGGTTTG AAGCCTAATG   
  
  
- TCCGCAACTA GAGGGGGTTG GGCCAAAGGC TGGTCGGTTT TCTCAGCTCC TCTGACCCGC TGCGAACTCC   
  
  
- TTGATACGAC TCAGTAAGTT ACACGGTAAA CTCAAGTTAC GGTATCGTTT CTCGACCCTT TGTGAATGAC   
  
  
- AATTTCTAGA GTTCTAACTA GGGCTACTCC ACGAACAACA CTTGACAGAC AAGTCCAAAT TTATGTAAGG   
  
  
- ACTCCTTTGT CATTAACGTC TTACGGGTTC TCTATAACAA GAATTAGACT AGTCTGTCTA TTTTGGTCGA   
  
  
- CAAAAGTATG TACCACATCA GTTACCTCGG AAGTTAAGAG GAAAAAAGTA ATAAGCCAAG GCACTCCGAG   
  
  
- AGAAGGTGAA GAGGTGAGAT AAACTACACA ACCTCCGTTT AGAAGGGGCC CTGTAGCTCC TCTCCTATGA   
  
  
- CTATCTCGCT CTGTAGAAAC CCGCTGTCCG TTACTTACAC TAGCGAACAC TCCCAAACCT CTCCTATCTT   
  
  
- TCCGGTCTCT TTATGTTTGT CACCGCCCAG GCTTTACTCT CCCGACCCAA CTTCGTCGAC GGAAACTTAG   
  
  
- TCCTCTGACA CCTTTACCGG TTTTTTGCCC ACTGTCGACA GATAGTGTTT CTAAAGAGGT AACTACTGCT   
  
  
- ACCCGTGACC AACAATGTCC CTACCTTTCC CTCTTAACAG ATACGTGAGT GATGAACCTT CGGACGACTG   
  
  
- AT

+     ABRE3a

| Site Name | Organism | Position | Strand | Matrix score. | sequence | function |
| --- | --- | --- | --- | --- | --- | --- |
| ABRE3a | Zea mays | 1266 | + | 6 | TACGTG |  |

>HU05G01597.1   
+ -Up\_Stream \_Len000TTTGTA TTTCAATATT GTAATTAGGT GGGATATAGA GTTGAAGGGC AAACTCTACC   
  
  
+ AACGACTTGT TGAAGGATCA ACTTGTAGCT TGGAACTTAA TGTATGTGTA CTATAGAGTT GTGTAATCTC   
  
  
+ TCCTAATAAA ATTGGGTTAA TCCTAGAATT AGAGAACTTT AAGCGGGGAC TAAGCTTGGT TGAGGCTAAC   
  
  
+ TTCGTTAAAA AGTTCTTGTG TCATGTCCAC GTTTATCTTT TCACTATTTA TTTCAATTAA TTTGCGATAT   
  
  
+ TTATCATCAC CCTATTCACC GTCTTTTCGG ATGATTTAAC TAGTCAAGCC GGACCAACAT TGCTTTTTAT   
  
  
+ AGTGTATTTG CAACTCATGT TGATTATTAG ATGTATCAAC AAAAAATGTT TCTCAAAATA AATAGTAGCT   
  
  
+ CTTTAAATTT TATTCCACAT AAATTCAAAA TCAATATCGA TATCGTTCCA TAATATAGCA GCATTATGAA   
  
  
+ AACAATATTT GCTAATGTTT ACATTGTGCT CTTTTCACTC AAAAATTGTA AGGCTTCAAA TACTATTAAA   
  
  
+ GAAACCATTA TTTCACTCGA CTATTTAGCT TGCATATATA GACAATCTTG CAAATAGTAA CATGTCTTCA   
  
  
+ TTCGACTATT TGCTTCCAAA TGAATAATCT CGTAAACATA TATACATACA TACATATATA TATATATATA   
  
  
+ TATATATATA TATATATATA GTAATATGTA TTTAACTTCA CATATACATG AATATAAAAA TATAAGAGGG   
  
  
+ AAAAAAGACG CTAGGTAGAT GGACTACAAA AGCCATTCAT GTATTGAGAC GGCTCATAAA ATCATACGGG   
  
  
+ GAAAGGAAAA AAAAAGATCT CTACATAATG TTCATAGATT TATAGAAAGC ATATGATGGA TTACCGCATA   
  
  
+ AGGTGTTATA TATGGGAGTG TTTGTAGAAG AAAGATGTAT ATCCGAACTA TCAAAGATAC ATATGAGGGA   
  
  
+ GTAAAAACAA GTGTAAGAAC CTCAGGAGGA GACATAAAAG ATTTTTCTAT TGGCATTGGA CACCGTCAAG   
  
  
+ TATCCGCTTT AAACCTGTTC TTTTTCACTA TTATCATGGA TGTATTCACA AAAGGAATTC AATACGAAGT   
  
  
+ ACCATGATGT ATGCTTTTTA CTGATAGTAT TGGTCTCATC GATGAGACTA AGGATGGTCT TAATAGCAAA   
  
  
+ TTAGAGATTT CATTAGATAA AATCTACTGT ACATGACAAA ACAATGATAT ATAGCATCCT ATTTAAAAAG   
  
  
+ CTACGTGTCA GTCATATATG AATTGCATCT AATTTTTAAT AGATATATAA TATGATATGA TATAATAGGA   
  
  
+ TATGATGTAG ATTGAATTCA AACTCATAAT TCATTAAGCA TGGTGCAATG TTCATTTTCT TGATTTTAAA   
  
  
+ CCGACTATTA CTTCAACTGT ATATAATTCG AATTGCTTTG GTATTTTAAA ATTTAAACTA GTAATTACTG   
  
  
+ CCTCTATTAC TGATGTTTAT ATTAAGTGGT GCTTAACTGC TTATTGATGA AACCATTTTT ATTTTCGTGT   
  
  
+ AAAAGAGGGC ATAATCCATC CTCGTGTATT GGCGGGGCTC GGCGCCCTAT TCAGCTATTC TCTGTATGTC   
  
  
+ AAACAAAACC GCTTCTATTA CCCATTGGTC TCTAAACACG CAGGGGTTTT AGCCCGGAGA GTTAATTCTT   
  
  
+ TATTGAGGTC CCCTGTTCTC CATTGCAGGA TTTTTGCTCA GGTACTTCTC TGTTTCTTCT CTTTAATCTT   
  
  
+ CTGTATCTTT CTGCATCAAG TTTAATGCTT TCTGGGATTT TCAGCTACGA TTTATGTGAA ATTGTAAGAT   
  
  
+ TTCTGAACTG GGCATTCAGG GCTTGGTTCA TATGTATTCT TTTCTGCTTG AAATGTTTTA ATTTCATAGT   
  
  
+ TCCAATGGGG TTATTCAAAG ATAGAATTAA GCTATAATCA CCGAGAAGAC TTGCTGACCT TTGTTTCTTT   
  
  
+ TTTGTTTCTT TTTTTTAATT AAATCTGGCT ATTACTTGGC TATAGTTTTT GCTAATGGAT CCACAAATAG   
  
  
+ ACGAATTATA TGGGCCTTTG CACCAAATGA AGTTCAATGA TCAGAGGAGA CCCATTTTAT CAAATGAAAA   
  
  
+ TTTTGTTAAT GTGTTGAAAC CGCCAAATTC CAATTGGAAT TCACCTTTTG GGAACCCTAC TGTGATTACC   
  
  
+ ACAAACCCAA ATTTTGATTT GGTTGTGCCA TCACTAGGTC CCAATGTTGA GGGAGCTCTC CTTGAAGATT   
  
  
+ ATGATTTTAG TGATGTTGTT CTTAAGTACA TCAATCAAGT TCTTATGGAC GAGGATGAGG GAGAGAAAAT   
  
  
+ CCATTTGGGC CACGGGCCTC TGGCTCTTGA AGCGGCCGAG AAGTCGTTAT ATGAGGTGCT TGGGCAGAGC   
  
  
+ CATTCTCCAC AAAATCAATA CTCTCCAGAG GACAAGCGTG ATGACCTGAG CAAGAGTAGT GTTAGAAGCT   
  
  
+ GTGTCAGTTA TAGCTGTGAC GGTGCAAGTA GTGGCAGTGG CATCGTGGAG CAAAGTTGGT CGAGTTATCC   
  
  
+ TCCGGAGTAC AATCCCATGC GGATGGCAGA TTTTCCTGCC TTGCCCTCAA AACCCTTGTT GAGAACATCT   
  
  
+ TATAGCTTAT CAAATAGCTT GACAGGTGAG GGTAATGGTT TGGTGGATAG ACCGGCAGAC TCACCTGTAA   
  
  
+ GCGCTCTTAG TGTGTCTGAT ATATTTAATG ATAGTCAGTC AGTGATACAA TTTCAGAAAG GGATGGAGGA   
  
  
+ AGCGAGCAAA TTTCTTCCCA AGGCTTCTTT GTGTTTGGAT TTGAACACTG GACCCTGCCT TCGGGAGTCG   
  
  
+ GTCAATAAGG CACGAGGTGG GCTGATTTTG GTGGAGACTA GCCATGAAAA TGAGGTTGCA GTGCTAGCTA   
  
  
+ GTAGGGGAAA GAAGCATCGC CATCCTGAGG AATTACGTTC AGAAGTTGGG AGGGGCAGTA AGCAGCCAGT   
  
  
+ TGTTTTCCAG TTTTCAGACG AGGCAGTTGT GAGATCAGAA ATGTTTGATA GGGTATTGCT GTGCAGTGGT   
  
  
+ GGCAAAAATG ATGCTGACCT ACGCCAAACT TTACAGAGTG AACTAAATAA AACTATGCAA AATGCTCAAA   
  
  
+ TGAAAGATTC TAATGGTGGA AAAGGCCGTG GTAGGAAGCA AGTTGGCAGG AGGGATGTGG TGGATCTAAG   
  
  
+ ATCTCTTTTA CGCCTTTGTG CACAAGCAGT TGGATCCAAT GACCAAAGAA GCGCAAATGA CTTAATTAGG   
  
  
+ CAAATTAGGA AGCATTCTTC TCCAACCGGG GATGGGAATC AAAGAATGGC TCATTATTTT GCTGATGGTC   
  
  
+ TTGAGGCTCG GCTTGCTGGT GTTGGGACCC CAATATACAA GTCTCTCGTG ACTGGCCCAG CTTCTGCTGT   
  
  
+ TGATATCTTG AGAGCTTACC ATATGTTTCT TGCCACATGC CCATTCAAGA AAATAGGGAA TTTCTTCTCT   
  
  
+ AATAGAACAA TTATGGATGT AGCTCAGAAA GCAAATCGAC TTCATATTAT TGATCTTGGT ATTGTCTATG   
  
  
+ GTTTCCAATG GCCTTGTCTA ATTCAGCGCC TTTCATCTAG GCCTGGCGGG CCTCCCAAAC TTCGGATTAC   
  
  
+ AGGCGTTGAT CTCCCCCAAC CCGGTTTCCG ACCAGCCAAA AGAGTCGAGG AGACTGGGCG ACGCTTGAGG   
  
  
+ AACTATGCTG AGTCATTCAA TGTGCCATTT GAGTTCAATG CCATAGCAAA GAGCTGGGAA ACACTTACTG   
  
  
+ TTAAAGATCT CAAGATTGAT CCCGATGAGG TGCTTGTTGT GAACTGTCTG TTCAGGTTTA AATACATTCC   
  
  
+ TGAGGAAACA GTAATTGCAG AATGCCCAAG AGATATTGTT CTTAATCTGA TCAGACAGAT AAAACCAGCT   
  
  
+ GTTTTCATAC ATGGTGTAGT CAATGGAGCC TTCAATTCTC CTTTTTTCAT TATTCGGTTC CGTGAGGCTC   
  
  
+ TCTTCCACTT CTCCACTCTA TTTGATGTGT TGGAGGCAAA TCTTCCCCGG GACATCGAGG AGAGGATACT   
  
  
+ GATAGAGCGA GACATCTTTG GGCGACAGGC AATGAATGTG ATCGCTTGTG AGGGTTTGGA GAGGATAGAA   
  
  
+ AGGCCAGAGA AATACAAACA GTGGCGGGTC CGAAATGAGA GGGCTGGGTT GAAGCAGCTG CCTTTGAATC   
  
  
+ AGGAGACTGT GGAAATGGCC AAAAAACGGG TGACAGCTGT CTATCACAAA GATTTCTCCA TTGATGACGA   
  
  
+ TGGGCACTGG TTGTTACAGG GATGGAAAGG GAGAATTGTC TATGCACTCA CTACTTGGAA GCCTGCTGAC   
  
  
+ TA  

- -Up\_Stream \_Len000AAACAT AAAGTTATAA CATTAATCCA CCCTATATCT CAACTTCCCG TTTGAGATGG   
  
  
- TTGCTGAACA ACTTCCTAGT TGAACATCGA ACCTTGAATT ACATACACAT GATATCTCAA CACATTAGAG   
  
  
- AGGATTATTT TAACCCAATT AGGATCTTAA TCTCTTGAAA TTCGCCCCTG ATTCGAACCA ACTCCGATTG   
  
  
- AAGCAATTTT TCAAGAACAC AGTACAGGTG CAAATAGAAA AGTGATAAAT AAAGTTAATT AAACGCTATA   
  
  
- AATAGTAGTG GGATAAGTGG CAGAAAAGCC TACTAAATTG ATCAGTTCGG CCTGGTTGTA ACGAAAAATA   
  
  
- TCACATAAAC GTTGAGTACA ACTAATAATC TACATAGTTG TTTTTTACAA AGAGTTTTAT TTATCATCGA   
  
  
- GAAATTTAAA ATAAGGTGTA TTTAAGTTTT AGTTATAGCT ATAGCAAGGT ATTATATCGT CGTAATACTT   
  
  
- TTGTTATAAA CGATTACAAA TGTAACACGA GAAAAGTGAG TTTTTAACAT TCCGAAGTTT ATGATAATTT   
  
  
- CTTTGGTAAT AAAGTGAGCT GATAAATCGA ACGTATATAT CTGTTAGAAC GTTTATCATT GTACAGAAGT   
  
  
- AAGCTGATAA ACGAAGGTTT ACTTATTAGA GCATTTGTAT ATATGTATGT ATGTATATAT ATATATATAT   
  
  
- ATATATATAT ATATATATAT CATTATACAT AAATTGAAGT GTATATGTAC TTATATTTTT ATATTCTCCC   
  
  
- TTTTTTCTGC GATCCATCTA CCTGATGTTT TCGGTAAGTA CATAACTCTG CCGAGTATTT TAGTATGCCC   
  
  
- CTTTCCTTTT TTTTTCTAGA GATGTATTAC AAGTATCTAA ATATCTTTCG TATACTACCT AATGGCGTAT   
  
  
- TCCACAATAT ATACCCTCAC AAACATCTTC TTTCTACATA TAGGCTTGAT AGTTTCTATG TATACTCCCT   
  
  
- CATTTTTGTT CACATTCTTG GAGTCCTCCT CTGTATTTTC TAAAAAGATA ACCGTAACCT GTGGCAGTTC   
  
  
- ATAGGCGAAA TTTGGACAAG AAAAAGTGAT AATAGTACCT ACATAAGTGT TTTCCTTAAG TTATGCTTCA   
  
  
- TGGTACTACA TACGAAAAAT GACTATCATA ACCAGAGTAG CTACTCTGAT TCCTACCAGA ATTATCGTTT   
  
  
- AATCTCTAAA GTAATCTATT TTAGATGACA TGTACTGTTT TGTTACTATA TATCGTAGGA TAAATTTTTC   
  
  
- GATGCACAGT CAGTATATAC TTAACGTAGA TTAAAAATTA TCTATATATT ATACTATACT ATATTATCCT   
  
  
- ATACTACATC TAACTTAAGT TTGAGTATTA AGTAATTCGT ACCACGTTAC AAGTAAAAGA ACTAAAATTT   
  
  
- GGCTGATAAT GAAGTTGACA TATATTAAGC TTAACGAAAC CATAAAATTT TAAATTTGAT CATTAATGAC   
  
  
- GGAGATAATG ACTACAAATA TAATTCACCA CGAATTGACG AATAACTACT TTGGTAAAAA TAAAAGCACA   
  
  
- TTTTCTCCCG TATTAGGTAG GAGCACATAA CCGCCCCGAG CCGCGGGATA AGTCGATAAG AGACATACAG   
  
  
- TTTGTTTTGG CGAAGATAAT GGGTAACCAG AGATTTGTGC GTCCCCAAAA TCGGGCCTCT CAATTAAGAA   
  
  
- ATAACTCCAG GGGACAAGAG GTAACGTCCT AAAAACGAGT CCATGAAGAG ACAAAGAAGA GAAATTAGAA   
  
  
- GACATAGAAA GACGTAGTTC AAATTACGAA AGACCCTAAA AGTCGATGCT AAATACACTT TAACATTCTA   
  
  
- AAGACTTGAC CCGTAAGTCC CGAACCAAGT ATACATAAGA AAAGACGAAC TTTACAAAAT TAAAGTATCA   
  
  
- AGGTTACCCC AATAAGTTTC TATCTTAATT CGATATTAGT GGCTCTTCTG AACGACTGGA AACAAAGAAA   
  
  
- AAACAAAGAA AAAAAATTAA TTTAGACCGA TAATGAACCG ATATCAAAAA CGATTACCTA GGTGTTTATC   
  
  
- TGCTTAATAT ACCCGGAAAC GTGGTTTACT TCAAGTTACT AGTCTCCTCT GGGTAAAATA GTTTACTTTT   
  
  
- AAAACAATTA CACAACTTTG GCGGTTTAAG GTTAACCTTA AGTGGAAAAC CCTTGGGATG ACACTAATGG   
  
  
- TGTTTGGGTT TAAAACTAAA CCAACACGGT AGTGATCCAG GGTTACAACT CCCTCGAGAG GAACTTCTAA   
  
  
- TACTAAAATC ACTACAACAA GAATTCATGT AGTTAGTTCA AGAATACCTG CTCCTACTCC CTCTCTTTTA   
  
  
- GGTAAACCCG GTGCCCGGAG ACCGAGAACT TCGCCGGCTC TTCAGCAATA TACTCCACGA ACCCGTCTCG   
  
  
- GTAAGAGGTG TTTTAGTTAT GAGAGGTCTC CTGTTCGCAC TACTGGACTC GTTCTCATCA CAATCTTCGA   
  
  
- CACAGTCAAT ATCGACACTG CCACGTTCAT CACCGTCACC GTAGCACCTC GTTTCAACCA GCTCAATAGG   
  
  
- AGGCCTCATG TTAGGGTACG CCTACCGTCT AAAAGGACGG AACGGGAGTT TTGGGAACAA CTCTTGTAGA   
  
  
- ATATCGAATA GTTTATCGAA CTGTCCACTC CCATTACCAA ACCACCTATC TGGCCGTCTG AGTGGACATT   
  
  
- CGCGAGAATC ACACAGACTA TATAAATTAC TATCAGTCAG TCACTATGTT AAAGTCTTTC CCTACCTCCT   
  
  
- TCGCTCGTTT AAAGAAGGGT TCCGAAGAAA CACAAACCTA AACTTGTGAC CTGGGACGGA AGCCCTCAGC   
  
  
- CAGTTATTCC GTGCTCCACC CGACTAAAAC CACCTCTGAT CGGTACTTTT ACTCCAACGT CACGATCGAT   
  
  
- CATCCCCTTT CTTCGTAGCG GTAGGACTCC TTAATGCAAG TCTTCAACCC TCCCCGTCAT TCGTCGGTCA   
  
  
- ACAAAAGGTC AAAAGTCTGC TCCGTCAACA CTCTAGTCTT TACAAACTAT CCCATAACGA CACGTCACCA   
  
  
- CCGTTTTTAC TACGACTGGA TGCGGTTTGA AATGTCTCAC TTGATTTATT TTGATACGTT TTACGAGTTT   
  
  
- ACTTTCTAAG ATTACCACCT TTTCCGGCAC CATCCTTCGT TCAACCGTCC TCCCTACACC ACCTAGATTC   
  
  
- TAGAGAAAAT GCGGAAACAC GTGTTCGTCA ACCTAGGTTA CTGGTTTCTT CGCGTTTACT GAATTAATCC   
  
  
- GTTTAATCCT TCGTAAGAAG AGGTTGGCCC CTACCCTTAG TTTCTTACCG AGTAATAAAA CGACTACCAG   
  
  
- AACTCCGAGC CGAACGACCA CAACCCTGGG GTTATATGTT CAGAGAGCAC TGACCGGGTC GAAGACGACA   
  
  
- ACTATAGAAC TCTCGAATGG TATACAAAGA ACGGTGTACG GGTAAGTTCT TTTATCCCTT AAAGAAGAGA   
  
  
- TTATCTTGTT AATACCTACA TCGAGTCTTT CGTTTAGCTG AAGTATAATA ACTAGAACCA TAACAGATAC   
  
  
- CAAAGGTTAC CGGAACAGAT TAAGTCGCGG AAAGTAGATC CGGACCGCCC GGAGGGTTTG AAGCCTAATG   
  
  
- TCCGCAACTA GAGGGGGTTG GGCCAAAGGC TGGTCGGTTT TCTCAGCTCC TCTGACCCGC TGCGAACTCC   
  
  
- TTGATACGAC TCAGTAAGTT ACACGGTAAA CTCAAGTTAC GGTATCGTTT CTCGACCCTT TGTGAATGAC   
  
  
- AATTTCTAGA GTTCTAACTA GGGCTACTCC ACGAACAACA CTTGACAGAC AAGTCCAAAT TTATGTAAGG   
  
  
- ACTCCTTTGT CATTAACGTC TTACGGGTTC TCTATAACAA GAATTAGACT AGTCTGTCTA TTTTGGTCGA   
  
  
- CAAAAGTATG TACCACATCA GTTACCTCGG AAGTTAAGAG GAAAAAAGTA ATAAGCCAAG GCACTCCGAG   
  
  
- AGAAGGTGAA GAGGTGAGAT AAACTACACA ACCTCCGTTT AGAAGGGGCC CTGTAGCTCC TCTCCTATGA   
  
  
- CTATCTCGCT CTGTAGAAAC CCGCTGTCCG TTACTTACAC TAGCGAACAC TCCCAAACCT CTCCTATCTT   
  
  
- TCCGGTCTCT TTATGTTTGT CACCGCCCAG GCTTTACTCT CCCGACCCAA CTTCGTCGAC GGAAACTTAG   
  
  
- TCCTCTGACA CCTTTACCGG TTTTTTGCCC ACTGTCGACA GATAGTGTTT CTAAAGAGGT AACTACTGCT   
  
  
- ACCCGTGACC AACAATGTCC CTACCTTTCC CTCTTAACAG ATACGTGAGT GATGAACCTT CGGACGACTG   
  
  
- AT

+     ABRE4

| Site Name | Organism | Position | Strand | Matrix score. | sequence | function |
| --- | --- | --- | --- | --- | --- | --- |
| ABRE4 | Zea mays | 1266 | - | 6 | CACGTA |  |

>HU05G01597.1   
+ -Up\_Stream \_Len000TTTGTA TTTCAATATT GTAATTAGGT GGGATATAGA GTTGAAGGGC AAACTCTACC   
  
  
+ AACGACTTGT TGAAGGATCA ACTTGTAGCT TGGAACTTAA TGTATGTGTA CTATAGAGTT GTGTAATCTC   
  
  
+ TCCTAATAAA ATTGGGTTAA TCCTAGAATT AGAGAACTTT AAGCGGGGAC TAAGCTTGGT TGAGGCTAAC   
  
  
+ TTCGTTAAAA AGTTCTTGTG TCATGTCCAC GTTTATCTTT TCACTATTTA TTTCAATTAA TTTGCGATAT   
  
  
+ TTATCATCAC CCTATTCACC GTCTTTTCGG ATGATTTAAC TAGTCAAGCC GGACCAACAT TGCTTTTTAT   
  
  
+ AGTGTATTTG CAACTCATGT TGATTATTAG ATGTATCAAC AAAAAATGTT TCTCAAAATA AATAGTAGCT   
  
  
+ CTTTAAATTT TATTCCACAT AAATTCAAAA TCAATATCGA TATCGTTCCA TAATATAGCA GCATTATGAA   
  
  
+ AACAATATTT GCTAATGTTT ACATTGTGCT CTTTTCACTC AAAAATTGTA AGGCTTCAAA TACTATTAAA   
  
  
+ GAAACCATTA TTTCACTCGA CTATTTAGCT TGCATATATA GACAATCTTG CAAATAGTAA CATGTCTTCA   
  
  
+ TTCGACTATT TGCTTCCAAA TGAATAATCT CGTAAACATA TATACATACA TACATATATA TATATATATA   
  
  
+ TATATATATA TATATATATA GTAATATGTA TTTAACTTCA CATATACATG AATATAAAAA TATAAGAGGG   
  
  
+ AAAAAAGACG CTAGGTAGAT GGACTACAAA AGCCATTCAT GTATTGAGAC GGCTCATAAA ATCATACGGG   
  
  
+ GAAAGGAAAA AAAAAGATCT CTACATAATG TTCATAGATT TATAGAAAGC ATATGATGGA TTACCGCATA   
  
  
+ AGGTGTTATA TATGGGAGTG TTTGTAGAAG AAAGATGTAT ATCCGAACTA TCAAAGATAC ATATGAGGGA   
  
  
+ GTAAAAACAA GTGTAAGAAC CTCAGGAGGA GACATAAAAG ATTTTTCTAT TGGCATTGGA CACCGTCAAG   
  
  
+ TATCCGCTTT AAACCTGTTC TTTTTCACTA TTATCATGGA TGTATTCACA AAAGGAATTC AATACGAAGT   
  
  
+ ACCATGATGT ATGCTTTTTA CTGATAGTAT TGGTCTCATC GATGAGACTA AGGATGGTCT TAATAGCAAA   
  
  
+ TTAGAGATTT CATTAGATAA AATCTACTGT ACATGACAAA ACAATGATAT ATAGCATCCT ATTTAAAAAG   
  
  
+ CTACGTGTCA GTCATATATG AATTGCATCT AATTTTTAAT AGATATATAA TATGATATGA TATAATAGGA   
  
  
+ TATGATGTAG ATTGAATTCA AACTCATAAT TCATTAAGCA TGGTGCAATG TTCATTTTCT TGATTTTAAA   
  
  
+ CCGACTATTA CTTCAACTGT ATATAATTCG AATTGCTTTG GTATTTTAAA ATTTAAACTA GTAATTACTG   
  
  
+ CCTCTATTAC TGATGTTTAT ATTAAGTGGT GCTTAACTGC TTATTGATGA AACCATTTTT ATTTTCGTGT   
  
  
+ AAAAGAGGGC ATAATCCATC CTCGTGTATT GGCGGGGCTC GGCGCCCTAT TCAGCTATTC TCTGTATGTC   
  
  
+ AAACAAAACC GCTTCTATTA CCCATTGGTC TCTAAACACG CAGGGGTTTT AGCCCGGAGA GTTAATTCTT   
  
  
+ TATTGAGGTC CCCTGTTCTC CATTGCAGGA TTTTTGCTCA GGTACTTCTC TGTTTCTTCT CTTTAATCTT   
  
  
+ CTGTATCTTT CTGCATCAAG TTTAATGCTT TCTGGGATTT TCAGCTACGA TTTATGTGAA ATTGTAAGAT   
  
  
+ TTCTGAACTG GGCATTCAGG GCTTGGTTCA TATGTATTCT TTTCTGCTTG AAATGTTTTA ATTTCATAGT   
  
  
+ TCCAATGGGG TTATTCAAAG ATAGAATTAA GCTATAATCA CCGAGAAGAC TTGCTGACCT TTGTTTCTTT   
  
  
+ TTTGTTTCTT TTTTTTAATT AAATCTGGCT ATTACTTGGC TATAGTTTTT GCTAATGGAT CCACAAATAG   
  
  
+ ACGAATTATA TGGGCCTTTG CACCAAATGA AGTTCAATGA TCAGAGGAGA CCCATTTTAT CAAATGAAAA   
  
  
+ TTTTGTTAAT GTGTTGAAAC CGCCAAATTC CAATTGGAAT TCACCTTTTG GGAACCCTAC TGTGATTACC   
  
  
+ ACAAACCCAA ATTTTGATTT GGTTGTGCCA TCACTAGGTC CCAATGTTGA GGGAGCTCTC CTTGAAGATT   
  
  
+ ATGATTTTAG TGATGTTGTT CTTAAGTACA TCAATCAAGT TCTTATGGAC GAGGATGAGG GAGAGAAAAT   
  
  
+ CCATTTGGGC CACGGGCCTC TGGCTCTTGA AGCGGCCGAG AAGTCGTTAT ATGAGGTGCT TGGGCAGAGC   
  
  
+ CATTCTCCAC AAAATCAATA CTCTCCAGAG GACAAGCGTG ATGACCTGAG CAAGAGTAGT GTTAGAAGCT   
  
  
+ GTGTCAGTTA TAGCTGTGAC GGTGCAAGTA GTGGCAGTGG CATCGTGGAG CAAAGTTGGT CGAGTTATCC   
  
  
+ TCCGGAGTAC AATCCCATGC GGATGGCAGA TTTTCCTGCC TTGCCCTCAA AACCCTTGTT GAGAACATCT   
  
  
+ TATAGCTTAT CAAATAGCTT GACAGGTGAG GGTAATGGTT TGGTGGATAG ACCGGCAGAC TCACCTGTAA   
  
  
+ GCGCTCTTAG TGTGTCTGAT ATATTTAATG ATAGTCAGTC AGTGATACAA TTTCAGAAAG GGATGGAGGA   
  
  
+ AGCGAGCAAA TTTCTTCCCA AGGCTTCTTT GTGTTTGGAT TTGAACACTG GACCCTGCCT TCGGGAGTCG   
  
  
+ GTCAATAAGG CACGAGGTGG GCTGATTTTG GTGGAGACTA GCCATGAAAA TGAGGTTGCA GTGCTAGCTA   
  
  
+ GTAGGGGAAA GAAGCATCGC CATCCTGAGG AATTACGTTC AGAAGTTGGG AGGGGCAGTA AGCAGCCAGT   
  
  
+ TGTTTTCCAG TTTTCAGACG AGGCAGTTGT GAGATCAGAA ATGTTTGATA GGGTATTGCT GTGCAGTGGT   
  
  
+ GGCAAAAATG ATGCTGACCT ACGCCAAACT TTACAGAGTG AACTAAATAA AACTATGCAA AATGCTCAAA   
  
  
+ TGAAAGATTC TAATGGTGGA AAAGGCCGTG GTAGGAAGCA AGTTGGCAGG AGGGATGTGG TGGATCTAAG   
  
  
+ ATCTCTTTTA CGCCTTTGTG CACAAGCAGT TGGATCCAAT GACCAAAGAA GCGCAAATGA CTTAATTAGG   
  
  
+ CAAATTAGGA AGCATTCTTC TCCAACCGGG GATGGGAATC AAAGAATGGC TCATTATTTT GCTGATGGTC   
  
  
+ TTGAGGCTCG GCTTGCTGGT GTTGGGACCC CAATATACAA GTCTCTCGTG ACTGGCCCAG CTTCTGCTGT   
  
  
+ TGATATCTTG AGAGCTTACC ATATGTTTCT TGCCACATGC CCATTCAAGA AAATAGGGAA TTTCTTCTCT   
  
  
+ AATAGAACAA TTATGGATGT AGCTCAGAAA GCAAATCGAC TTCATATTAT TGATCTTGGT ATTGTCTATG   
  
  
+ GTTTCCAATG GCCTTGTCTA ATTCAGCGCC TTTCATCTAG GCCTGGCGGG CCTCCCAAAC TTCGGATTAC   
  
  
+ AGGCGTTGAT CTCCCCCAAC CCGGTTTCCG ACCAGCCAAA AGAGTCGAGG AGACTGGGCG ACGCTTGAGG   
  
  
+ AACTATGCTG AGTCATTCAA TGTGCCATTT GAGTTCAATG CCATAGCAAA GAGCTGGGAA ACACTTACTG   
  
  
+ TTAAAGATCT CAAGATTGAT CCCGATGAGG TGCTTGTTGT GAACTGTCTG TTCAGGTTTA AATACATTCC   
  
  
+ TGAGGAAACA GTAATTGCAG AATGCCCAAG AGATATTGTT CTTAATCTGA TCAGACAGAT AAAACCAGCT   
  
  
+ GTTTTCATAC ATGGTGTAGT CAATGGAGCC TTCAATTCTC CTTTTTTCAT TATTCGGTTC CGTGAGGCTC   
  
  
+ TCTTCCACTT CTCCACTCTA TTTGATGTGT TGGAGGCAAA TCTTCCCCGG GACATCGAGG AGAGGATACT   
  
  
+ GATAGAGCGA GACATCTTTG GGCGACAGGC AATGAATGTG ATCGCTTGTG AGGGTTTGGA GAGGATAGAA   
  
  
+ AGGCCAGAGA AATACAAACA GTGGCGGGTC CGAAATGAGA GGGCTGGGTT GAAGCAGCTG CCTTTGAATC   
  
  
+ AGGAGACTGT GGAAATGGCC AAAAAACGGG TGACAGCTGT CTATCACAAA GATTTCTCCA TTGATGACGA   
  
  
+ TGGGCACTGG TTGTTACAGG GATGGAAAGG GAGAATTGTC TATGCACTCA CTACTTGGAA GCCTGCTGAC   
  
  
+ TA  

- -Up\_Stream \_Len000AAACAT AAAGTTATAA CATTAATCCA CCCTATATCT CAACTTCCCG TTTGAGATGG   
  
  
- TTGCTGAACA ACTTCCTAGT TGAACATCGA ACCTTGAATT ACATACACAT GATATCTCAA CACATTAGAG   
  
  
- AGGATTATTT TAACCCAATT AGGATCTTAA TCTCTTGAAA TTCGCCCCTG ATTCGAACCA ACTCCGATTG   
  
  
- AAGCAATTTT TCAAGAACAC AGTACAGGTG CAAATAGAAA AGTGATAAAT AAAGTTAATT AAACGCTATA   
  
  
- AATAGTAGTG GGATAAGTGG CAGAAAAGCC TACTAAATTG ATCAGTTCGG CCTGGTTGTA ACGAAAAATA   
  
  
- TCACATAAAC GTTGAGTACA ACTAATAATC TACATAGTTG TTTTTTACAA AGAGTTTTAT TTATCATCGA   
  
  
- GAAATTTAAA ATAAGGTGTA TTTAAGTTTT AGTTATAGCT ATAGCAAGGT ATTATATCGT CGTAATACTT   
  
  
- TTGTTATAAA CGATTACAAA TGTAACACGA GAAAAGTGAG TTTTTAACAT TCCGAAGTTT ATGATAATTT   
  
  
- CTTTGGTAAT AAAGTGAGCT GATAAATCGA ACGTATATAT CTGTTAGAAC GTTTATCATT GTACAGAAGT   
  
  
- AAGCTGATAA ACGAAGGTTT ACTTATTAGA GCATTTGTAT ATATGTATGT ATGTATATAT ATATATATAT   
  
  
- ATATATATAT ATATATATAT CATTATACAT AAATTGAAGT GTATATGTAC TTATATTTTT ATATTCTCCC   
  
  
- TTTTTTCTGC GATCCATCTA CCTGATGTTT TCGGTAAGTA CATAACTCTG CCGAGTATTT TAGTATGCCC   
  
  
- CTTTCCTTTT TTTTTCTAGA GATGTATTAC AAGTATCTAA ATATCTTTCG TATACTACCT AATGGCGTAT   
  
  
- TCCACAATAT ATACCCTCAC AAACATCTTC TTTCTACATA TAGGCTTGAT AGTTTCTATG TATACTCCCT   
  
  
- CATTTTTGTT CACATTCTTG GAGTCCTCCT CTGTATTTTC TAAAAAGATA ACCGTAACCT GTGGCAGTTC   
  
  
- ATAGGCGAAA TTTGGACAAG AAAAAGTGAT AATAGTACCT ACATAAGTGT TTTCCTTAAG TTATGCTTCA   
  
  
- TGGTACTACA TACGAAAAAT GACTATCATA ACCAGAGTAG CTACTCTGAT TCCTACCAGA ATTATCGTTT   
  
  
- AATCTCTAAA GTAATCTATT TTAGATGACA TGTACTGTTT TGTTACTATA TATCGTAGGA TAAATTTTTC   
  
  
- GATGCACAGT CAGTATATAC TTAACGTAGA TTAAAAATTA TCTATATATT ATACTATACT ATATTATCCT   
  
  
- ATACTACATC TAACTTAAGT TTGAGTATTA AGTAATTCGT ACCACGTTAC AAGTAAAAGA ACTAAAATTT   
  
  
- GGCTGATAAT GAAGTTGACA TATATTAAGC TTAACGAAAC CATAAAATTT TAAATTTGAT CATTAATGAC   
  
  
- GGAGATAATG ACTACAAATA TAATTCACCA CGAATTGACG AATAACTACT TTGGTAAAAA TAAAAGCACA   
  
  
- TTTTCTCCCG TATTAGGTAG GAGCACATAA CCGCCCCGAG CCGCGGGATA AGTCGATAAG AGACATACAG   
  
  
- TTTGTTTTGG CGAAGATAAT GGGTAACCAG AGATTTGTGC GTCCCCAAAA TCGGGCCTCT CAATTAAGAA   
  
  
- ATAACTCCAG GGGACAAGAG GTAACGTCCT AAAAACGAGT CCATGAAGAG ACAAAGAAGA GAAATTAGAA   
  
  
- GACATAGAAA GACGTAGTTC AAATTACGAA AGACCCTAAA AGTCGATGCT AAATACACTT TAACATTCTA   
  
  
- AAGACTTGAC CCGTAAGTCC CGAACCAAGT ATACATAAGA AAAGACGAAC TTTACAAAAT TAAAGTATCA   
  
  
- AGGTTACCCC AATAAGTTTC TATCTTAATT CGATATTAGT GGCTCTTCTG AACGACTGGA AACAAAGAAA   
  
  
- AAACAAAGAA AAAAAATTAA TTTAGACCGA TAATGAACCG ATATCAAAAA CGATTACCTA GGTGTTTATC   
  
  
- TGCTTAATAT ACCCGGAAAC GTGGTTTACT TCAAGTTACT AGTCTCCTCT GGGTAAAATA GTTTACTTTT   
  
  
- AAAACAATTA CACAACTTTG GCGGTTTAAG GTTAACCTTA AGTGGAAAAC CCTTGGGATG ACACTAATGG   
  
  
- TGTTTGGGTT TAAAACTAAA CCAACACGGT AGTGATCCAG GGTTACAACT CCCTCGAGAG GAACTTCTAA   
  
  
- TACTAAAATC ACTACAACAA GAATTCATGT AGTTAGTTCA AGAATACCTG CTCCTACTCC CTCTCTTTTA   
  
  
- GGTAAACCCG GTGCCCGGAG ACCGAGAACT TCGCCGGCTC TTCAGCAATA TACTCCACGA ACCCGTCTCG   
  
  
- GTAAGAGGTG TTTTAGTTAT GAGAGGTCTC CTGTTCGCAC TACTGGACTC GTTCTCATCA CAATCTTCGA   
  
  
- CACAGTCAAT ATCGACACTG CCACGTTCAT CACCGTCACC GTAGCACCTC GTTTCAACCA GCTCAATAGG   
  
  
- AGGCCTCATG TTAGGGTACG CCTACCGTCT AAAAGGACGG AACGGGAGTT TTGGGAACAA CTCTTGTAGA   
  
  
- ATATCGAATA GTTTATCGAA CTGTCCACTC CCATTACCAA ACCACCTATC TGGCCGTCTG AGTGGACATT   
  
  
- CGCGAGAATC ACACAGACTA TATAAATTAC TATCAGTCAG TCACTATGTT AAAGTCTTTC CCTACCTCCT   
  
  
- TCGCTCGTTT AAAGAAGGGT TCCGAAGAAA CACAAACCTA AACTTGTGAC CTGGGACGGA AGCCCTCAGC   
  
  
- CAGTTATTCC GTGCTCCACC CGACTAAAAC CACCTCTGAT CGGTACTTTT ACTCCAACGT CACGATCGAT   
  
  
- CATCCCCTTT CTTCGTAGCG GTAGGACTCC TTAATGCAAG TCTTCAACCC TCCCCGTCAT TCGTCGGTCA   
  
  
- ACAAAAGGTC AAAAGTCTGC TCCGTCAACA CTCTAGTCTT TACAAACTAT CCCATAACGA CACGTCACCA   
  
  
- CCGTTTTTAC TACGACTGGA TGCGGTTTGA AATGTCTCAC TTGATTTATT TTGATACGTT TTACGAGTTT   
  
  
- ACTTTCTAAG ATTACCACCT TTTCCGGCAC CATCCTTCGT TCAACCGTCC TCCCTACACC ACCTAGATTC   
  
  
- TAGAGAAAAT GCGGAAACAC GTGTTCGTCA ACCTAGGTTA CTGGTTTCTT CGCGTTTACT GAATTAATCC   
  
  
- GTTTAATCCT TCGTAAGAAG AGGTTGGCCC CTACCCTTAG TTTCTTACCG AGTAATAAAA CGACTACCAG   
  
  
- AACTCCGAGC CGAACGACCA CAACCCTGGG GTTATATGTT CAGAGAGCAC TGACCGGGTC GAAGACGACA   
  
  
- ACTATAGAAC TCTCGAATGG TATACAAAGA ACGGTGTACG GGTAAGTTCT TTTATCCCTT AAAGAAGAGA   
  
  
- TTATCTTGTT AATACCTACA TCGAGTCTTT CGTTTAGCTG AAGTATAATA ACTAGAACCA TAACAGATAC   
  
  
- CAAAGGTTAC CGGAACAGAT TAAGTCGCGG AAAGTAGATC CGGACCGCCC GGAGGGTTTG AAGCCTAATG   
  
  
- TCCGCAACTA GAGGGGGTTG GGCCAAAGGC TGGTCGGTTT TCTCAGCTCC TCTGACCCGC TGCGAACTCC   
  
  
- TTGATACGAC TCAGTAAGTT ACACGGTAAA CTCAAGTTAC GGTATCGTTT CTCGACCCTT TGTGAATGAC   
  
  
- AATTTCTAGA GTTCTAACTA GGGCTACTCC ACGAACAACA CTTGACAGAC AAGTCCAAAT TTATGTAAGG   
  
  
- ACTCCTTTGT CATTAACGTC TTACGGGTTC TCTATAACAA GAATTAGACT AGTCTGTCTA TTTTGGTCGA   
  
  
- CAAAAGTATG TACCACATCA GTTACCTCGG AAGTTAAGAG GAAAAAAGTA ATAAGCCAAG GCACTCCGAG   
  
  
- AGAAGGTGAA GAGGTGAGAT AAACTACACA ACCTCCGTTT AGAAGGGGCC CTGTAGCTCC TCTCCTATGA   
  
  
- CTATCTCGCT CTGTAGAAAC CCGCTGTCCG TTACTTACAC TAGCGAACAC TCCCAAACCT CTCCTATCTT   
  
  
- TCCGGTCTCT TTATGTTTGT CACCGCCCAG GCTTTACTCT CCCGACCCAA CTTCGTCGAC GGAAACTTAG   
  
  
- TCCTCTGACA CCTTTACCGG TTTTTTGCCC ACTGTCGACA GATAGTGTTT CTAAAGAGGT AACTACTGCT   
  
  
- ACCCGTGACC AACAATGTCC CTACCTTTCC CTCTTAACAG ATACGTGAGT GATGAACCTT CGGACGACTG   
  
  
- AT

+     AE-box

| Site Name | Organism | Position | Strand | Matrix score. | sequence | function |
| --- | --- | --- | --- | --- | --- | --- |
| AE-box | Arabidopsis thaliana | 1966 | - | 8 | AGAAACAA | part of a module for light response |
| AE-box | Arabidopsis thaliana | 1955 | - | 8 | AGAAACAA | part of a module for light response |

>HU05G01597.1   
+ -Up\_Stream \_Len000TTTGTA TTTCAATATT GTAATTAGGT GGGATATAGA GTTGAAGGGC AAACTCTACC   
  
  
+ AACGACTTGT TGAAGGATCA ACTTGTAGCT TGGAACTTAA TGTATGTGTA CTATAGAGTT GTGTAATCTC   
  
  
+ TCCTAATAAA ATTGGGTTAA TCCTAGAATT AGAGAACTTT AAGCGGGGAC TAAGCTTGGT TGAGGCTAAC   
  
  
+ TTCGTTAAAA AGTTCTTGTG TCATGTCCAC GTTTATCTTT TCACTATTTA TTTCAATTAA TTTGCGATAT   
  
  
+ TTATCATCAC CCTATTCACC GTCTTTTCGG ATGATTTAAC TAGTCAAGCC GGACCAACAT TGCTTTTTAT   
  
  
+ AGTGTATTTG CAACTCATGT TGATTATTAG ATGTATCAAC AAAAAATGTT TCTCAAAATA AATAGTAGCT   
  
  
+ CTTTAAATTT TATTCCACAT AAATTCAAAA TCAATATCGA TATCGTTCCA TAATATAGCA GCATTATGAA   
  
  
+ AACAATATTT GCTAATGTTT ACATTGTGCT CTTTTCACTC AAAAATTGTA AGGCTTCAAA TACTATTAAA   
  
  
+ GAAACCATTA TTTCACTCGA CTATTTAGCT TGCATATATA GACAATCTTG CAAATAGTAA CATGTCTTCA   
  
  
+ TTCGACTATT TGCTTCCAAA TGAATAATCT CGTAAACATA TATACATACA TACATATATA TATATATATA   
  
  
+ TATATATATA TATATATATA GTAATATGTA TTTAACTTCA CATATACATG AATATAAAAA TATAAGAGGG   
  
  
+ AAAAAAGACG CTAGGTAGAT GGACTACAAA AGCCATTCAT GTATTGAGAC GGCTCATAAA ATCATACGGG   
  
  
+ GAAAGGAAAA AAAAAGATCT CTACATAATG TTCATAGATT TATAGAAAGC ATATGATGGA TTACCGCATA   
  
  
+ AGGTGTTATA TATGGGAGTG TTTGTAGAAG AAAGATGTAT ATCCGAACTA TCAAAGATAC ATATGAGGGA   
  
  
+ GTAAAAACAA GTGTAAGAAC CTCAGGAGGA GACATAAAAG ATTTTTCTAT TGGCATTGGA CACCGTCAAG   
  
  
+ TATCCGCTTT AAACCTGTTC TTTTTCACTA TTATCATGGA TGTATTCACA AAAGGAATTC AATACGAAGT   
  
  
+ ACCATGATGT ATGCTTTTTA CTGATAGTAT TGGTCTCATC GATGAGACTA AGGATGGTCT TAATAGCAAA   
  
  
+ TTAGAGATTT CATTAGATAA AATCTACTGT ACATGACAAA ACAATGATAT ATAGCATCCT ATTTAAAAAG   
  
  
+ CTACGTGTCA GTCATATATG AATTGCATCT AATTTTTAAT AGATATATAA TATGATATGA TATAATAGGA   
  
  
+ TATGATGTAG ATTGAATTCA AACTCATAAT TCATTAAGCA TGGTGCAATG TTCATTTTCT TGATTTTAAA   
  
  
+ CCGACTATTA CTTCAACTGT ATATAATTCG AATTGCTTTG GTATTTTAAA ATTTAAACTA GTAATTACTG   
  
  
+ CCTCTATTAC TGATGTTTAT ATTAAGTGGT GCTTAACTGC TTATTGATGA AACCATTTTT ATTTTCGTGT   
  
  
+ AAAAGAGGGC ATAATCCATC CTCGTGTATT GGCGGGGCTC GGCGCCCTAT TCAGCTATTC TCTGTATGTC   
  
  
+ AAACAAAACC GCTTCTATTA CCCATTGGTC TCTAAACACG CAGGGGTTTT AGCCCGGAGA GTTAATTCTT   
  
  
+ TATTGAGGTC CCCTGTTCTC CATTGCAGGA TTTTTGCTCA GGTACTTCTC TGTTTCTTCT CTTTAATCTT   
  
  
+ CTGTATCTTT CTGCATCAAG TTTAATGCTT TCTGGGATTT TCAGCTACGA TTTATGTGAA ATTGTAAGAT   
  
  
+ TTCTGAACTG GGCATTCAGG GCTTGGTTCA TATGTATTCT TTTCTGCTTG AAATGTTTTA ATTTCATAGT   
  
  
+ TCCAATGGGG TTATTCAAAG ATAGAATTAA GCTATAATCA CCGAGAAGAC TTGCTGACCT TTGTTTCTTT   
  
  
+ TTTGTTTCTT TTTTTTAATT AAATCTGGCT ATTACTTGGC TATAGTTTTT GCTAATGGAT CCACAAATAG   
  
  
+ ACGAATTATA TGGGCCTTTG CACCAAATGA AGTTCAATGA TCAGAGGAGA CCCATTTTAT CAAATGAAAA   
  
  
+ TTTTGTTAAT GTGTTGAAAC CGCCAAATTC CAATTGGAAT TCACCTTTTG GGAACCCTAC TGTGATTACC   
  
  
+ ACAAACCCAA ATTTTGATTT GGTTGTGCCA TCACTAGGTC CCAATGTTGA GGGAGCTCTC CTTGAAGATT   
  
  
+ ATGATTTTAG TGATGTTGTT CTTAAGTACA TCAATCAAGT TCTTATGGAC GAGGATGAGG GAGAGAAAAT   
  
  
+ CCATTTGGGC CACGGGCCTC TGGCTCTTGA AGCGGCCGAG AAGTCGTTAT ATGAGGTGCT TGGGCAGAGC   
  
  
+ CATTCTCCAC AAAATCAATA CTCTCCAGAG GACAAGCGTG ATGACCTGAG CAAGAGTAGT GTTAGAAGCT   
  
  
+ GTGTCAGTTA TAGCTGTGAC GGTGCAAGTA GTGGCAGTGG CATCGTGGAG CAAAGTTGGT CGAGTTATCC   
  
  
+ TCCGGAGTAC AATCCCATGC GGATGGCAGA TTTTCCTGCC TTGCCCTCAA AACCCTTGTT GAGAACATCT   
  
  
+ TATAGCTTAT CAAATAGCTT GACAGGTGAG GGTAATGGTT TGGTGGATAG ACCGGCAGAC TCACCTGTAA   
  
  
+ GCGCTCTTAG TGTGTCTGAT ATATTTAATG ATAGTCAGTC AGTGATACAA TTTCAGAAAG GGATGGAGGA   
  
  
+ AGCGAGCAAA TTTCTTCCCA AGGCTTCTTT GTGTTTGGAT TTGAACACTG GACCCTGCCT TCGGGAGTCG   
  
  
+ GTCAATAAGG CACGAGGTGG GCTGATTTTG GTGGAGACTA GCCATGAAAA TGAGGTTGCA GTGCTAGCTA   
  
  
+ GTAGGGGAAA GAAGCATCGC CATCCTGAGG AATTACGTTC AGAAGTTGGG AGGGGCAGTA AGCAGCCAGT   
  
  
+ TGTTTTCCAG TTTTCAGACG AGGCAGTTGT GAGATCAGAA ATGTTTGATA GGGTATTGCT GTGCAGTGGT   
  
  
+ GGCAAAAATG ATGCTGACCT ACGCCAAACT TTACAGAGTG AACTAAATAA AACTATGCAA AATGCTCAAA   
  
  
+ TGAAAGATTC TAATGGTGGA AAAGGCCGTG GTAGGAAGCA AGTTGGCAGG AGGGATGTGG TGGATCTAAG   
  
  
+ ATCTCTTTTA CGCCTTTGTG CACAAGCAGT TGGATCCAAT GACCAAAGAA GCGCAAATGA CTTAATTAGG   
  
  
+ CAAATTAGGA AGCATTCTTC TCCAACCGGG GATGGGAATC AAAGAATGGC TCATTATTTT GCTGATGGTC   
  
  
+ TTGAGGCTCG GCTTGCTGGT GTTGGGACCC CAATATACAA GTCTCTCGTG ACTGGCCCAG CTTCTGCTGT   
  
  
+ TGATATCTTG AGAGCTTACC ATATGTTTCT TGCCACATGC CCATTCAAGA AAATAGGGAA TTTCTTCTCT   
  
  
+ AATAGAACAA TTATGGATGT AGCTCAGAAA GCAAATCGAC TTCATATTAT TGATCTTGGT ATTGTCTATG   
  
  
+ GTTTCCAATG GCCTTGTCTA ATTCAGCGCC TTTCATCTAG GCCTGGCGGG CCTCCCAAAC TTCGGATTAC   
  
  
+ AGGCGTTGAT CTCCCCCAAC CCGGTTTCCG ACCAGCCAAA AGAGTCGAGG AGACTGGGCG ACGCTTGAGG   
  
  
+ AACTATGCTG AGTCATTCAA TGTGCCATTT GAGTTCAATG CCATAGCAAA GAGCTGGGAA ACACTTACTG   
  
  
+ TTAAAGATCT CAAGATTGAT CCCGATGAGG TGCTTGTTGT GAACTGTCTG TTCAGGTTTA AATACATTCC   
  
  
+ TGAGGAAACA GTAATTGCAG AATGCCCAAG AGATATTGTT CTTAATCTGA TCAGACAGAT AAAACCAGCT   
  
  
+ GTTTTCATAC ATGGTGTAGT CAATGGAGCC TTCAATTCTC CTTTTTTCAT TATTCGGTTC CGTGAGGCTC   
  
  
+ TCTTCCACTT CTCCACTCTA TTTGATGTGT TGGAGGCAAA TCTTCCCCGG GACATCGAGG AGAGGATACT   
  
  
+ GATAGAGCGA GACATCTTTG GGCGACAGGC AATGAATGTG ATCGCTTGTG AGGGTTTGGA GAGGATAGAA   
  
  
+ AGGCCAGAGA AATACAAACA GTGGCGGGTC CGAAATGAGA GGGCTGGGTT GAAGCAGCTG CCTTTGAATC   
  
  
+ AGGAGACTGT GGAAATGGCC AAAAAACGGG TGACAGCTGT CTATCACAAA GATTTCTCCA TTGATGACGA   
  
  
+ TGGGCACTGG TTGTTACAGG GATGGAAAGG GAGAATTGTC TATGCACTCA CTACTTGGAA GCCTGCTGAC   
  
  
+ TA  

- -Up\_Stream \_Len000AAACAT AAAGTTATAA CATTAATCCA CCCTATATCT CAACTTCCCG TTTGAGATGG   
  
  
- TTGCTGAACA ACTTCCTAGT TGAACATCGA ACCTTGAATT ACATACACAT GATATCTCAA CACATTAGAG   
  
  
- AGGATTATTT TAACCCAATT AGGATCTTAA TCTCTTGAAA TTCGCCCCTG ATTCGAACCA ACTCCGATTG   
  
  
- AAGCAATTTT TCAAGAACAC AGTACAGGTG CAAATAGAAA AGTGATAAAT AAAGTTAATT AAACGCTATA   
  
  
- AATAGTAGTG GGATAAGTGG CAGAAAAGCC TACTAAATTG ATCAGTTCGG CCTGGTTGTA ACGAAAAATA   
  
  
- TCACATAAAC GTTGAGTACA ACTAATAATC TACATAGTTG TTTTTTACAA AGAGTTTTAT TTATCATCGA   
  
  
- GAAATTTAAA ATAAGGTGTA TTTAAGTTTT AGTTATAGCT ATAGCAAGGT ATTATATCGT CGTAATACTT   
  
  
- TTGTTATAAA CGATTACAAA TGTAACACGA GAAAAGTGAG TTTTTAACAT TCCGAAGTTT ATGATAATTT   
  
  
- CTTTGGTAAT AAAGTGAGCT GATAAATCGA ACGTATATAT CTGTTAGAAC GTTTATCATT GTACAGAAGT   
  
  
- AAGCTGATAA ACGAAGGTTT ACTTATTAGA GCATTTGTAT ATATGTATGT ATGTATATAT ATATATATAT   
  
  
- ATATATATAT ATATATATAT CATTATACAT AAATTGAAGT GTATATGTAC TTATATTTTT ATATTCTCCC   
  
  
- TTTTTTCTGC GATCCATCTA CCTGATGTTT TCGGTAAGTA CATAACTCTG CCGAGTATTT TAGTATGCCC   
  
  
- CTTTCCTTTT TTTTTCTAGA GATGTATTAC AAGTATCTAA ATATCTTTCG TATACTACCT AATGGCGTAT   
  
  
- TCCACAATAT ATACCCTCAC AAACATCTTC TTTCTACATA TAGGCTTGAT AGTTTCTATG TATACTCCCT   
  
  
- CATTTTTGTT CACATTCTTG GAGTCCTCCT CTGTATTTTC TAAAAAGATA ACCGTAACCT GTGGCAGTTC   
  
  
- ATAGGCGAAA TTTGGACAAG AAAAAGTGAT AATAGTACCT ACATAAGTGT TTTCCTTAAG TTATGCTTCA   
  
  
- TGGTACTACA TACGAAAAAT GACTATCATA ACCAGAGTAG CTACTCTGAT TCCTACCAGA ATTATCGTTT   
  
  
- AATCTCTAAA GTAATCTATT TTAGATGACA TGTACTGTTT TGTTACTATA TATCGTAGGA TAAATTTTTC   
  
  
- GATGCACAGT CAGTATATAC TTAACGTAGA TTAAAAATTA TCTATATATT ATACTATACT ATATTATCCT   
  
  
- ATACTACATC TAACTTAAGT TTGAGTATTA AGTAATTCGT ACCACGTTAC AAGTAAAAGA ACTAAAATTT   
  
  
- GGCTGATAAT GAAGTTGACA TATATTAAGC TTAACGAAAC CATAAAATTT TAAATTTGAT CATTAATGAC   
  
  
- GGAGATAATG ACTACAAATA TAATTCACCA CGAATTGACG AATAACTACT TTGGTAAAAA TAAAAGCACA   
  
  
- TTTTCTCCCG TATTAGGTAG GAGCACATAA CCGCCCCGAG CCGCGGGATA AGTCGATAAG AGACATACAG   
  
  
- TTTGTTTTGG CGAAGATAAT GGGTAACCAG AGATTTGTGC GTCCCCAAAA TCGGGCCTCT CAATTAAGAA   
  
  
- ATAACTCCAG GGGACAAGAG GTAACGTCCT AAAAACGAGT CCATGAAGAG ACAAAGAAGA GAAATTAGAA   
  
  
- GACATAGAAA GACGTAGTTC AAATTACGAA AGACCCTAAA AGTCGATGCT AAATACACTT TAACATTCTA   
  
  
- AAGACTTGAC CCGTAAGTCC CGAACCAAGT ATACATAAGA AAAGACGAAC TTTACAAAAT TAAAGTATCA   
  
  
- AGGTTACCCC AATAAGTTTC TATCTTAATT CGATATTAGT GGCTCTTCTG AACGACTGGA AACAAAGAAA   
  
  
- AAACAAAGAA AAAAAATTAA TTTAGACCGA TAATGAACCG ATATCAAAAA CGATTACCTA GGTGTTTATC   
  
  
- TGCTTAATAT ACCCGGAAAC GTGGTTTACT TCAAGTTACT AGTCTCCTCT GGGTAAAATA GTTTACTTTT   
  
  
- AAAACAATTA CACAACTTTG GCGGTTTAAG GTTAACCTTA AGTGGAAAAC CCTTGGGATG ACACTAATGG   
  
  
- TGTTTGGGTT TAAAACTAAA CCAACACGGT AGTGATCCAG GGTTACAACT CCCTCGAGAG GAACTTCTAA   
  
  
- TACTAAAATC ACTACAACAA GAATTCATGT AGTTAGTTCA AGAATACCTG CTCCTACTCC CTCTCTTTTA   
  
  
- GGTAAACCCG GTGCCCGGAG ACCGAGAACT TCGCCGGCTC TTCAGCAATA TACTCCACGA ACCCGTCTCG   
  
  
- GTAAGAGGTG TTTTAGTTAT GAGAGGTCTC CTGTTCGCAC TACTGGACTC GTTCTCATCA CAATCTTCGA   
  
  
- CACAGTCAAT ATCGACACTG CCACGTTCAT CACCGTCACC GTAGCACCTC GTTTCAACCA GCTCAATAGG   
  
  
- AGGCCTCATG TTAGGGTACG CCTACCGTCT AAAAGGACGG AACGGGAGTT TTGGGAACAA CTCTTGTAGA   
  
  
- ATATCGAATA GTTTATCGAA CTGTCCACTC CCATTACCAA ACCACCTATC TGGCCGTCTG AGTGGACATT   
  
  
- CGCGAGAATC ACACAGACTA TATAAATTAC TATCAGTCAG TCACTATGTT AAAGTCTTTC CCTACCTCCT   
  
  
- TCGCTCGTTT AAAGAAGGGT TCCGAAGAAA CACAAACCTA AACTTGTGAC CTGGGACGGA AGCCCTCAGC   
  
  
- CAGTTATTCC GTGCTCCACC CGACTAAAAC CACCTCTGAT CGGTACTTTT ACTCCAACGT CACGATCGAT   
  
  
- CATCCCCTTT CTTCGTAGCG GTAGGACTCC TTAATGCAAG TCTTCAACCC TCCCCGTCAT TCGTCGGTCA   
  
  
- ACAAAAGGTC AAAAGTCTGC TCCGTCAACA CTCTAGTCTT TACAAACTAT CCCATAACGA CACGTCACCA   
  
  
- CCGTTTTTAC TACGACTGGA TGCGGTTTGA AATGTCTCAC TTGATTTATT TTGATACGTT TTACGAGTTT   
  
  
- ACTTTCTAAG ATTACCACCT TTTCCGGCAC CATCCTTCGT TCAACCGTCC TCCCTACACC ACCTAGATTC   
  
  
- TAGAGAAAAT GCGGAAACAC GTGTTCGTCA ACCTAGGTTA CTGGTTTCTT CGCGTTTACT GAATTAATCC   
  
  
- GTTTAATCCT TCGTAAGAAG AGGTTGGCCC CTACCCTTAG TTTCTTACCG AGTAATAAAA CGACTACCAG   
  
  
- AACTCCGAGC CGAACGACCA CAACCCTGGG GTTATATGTT CAGAGAGCAC TGACCGGGTC GAAGACGACA   
  
  
- ACTATAGAAC TCTCGAATGG TATACAAAGA ACGGTGTACG GGTAAGTTCT TTTATCCCTT AAAGAAGAGA   
  
  
- TTATCTTGTT AATACCTACA TCGAGTCTTT CGTTTAGCTG AAGTATAATA ACTAGAACCA TAACAGATAC   
  
  
- CAAAGGTTAC CGGAACAGAT TAAGTCGCGG AAAGTAGATC CGGACCGCCC GGAGGGTTTG AAGCCTAATG   
  
  
- TCCGCAACTA GAGGGGGTTG GGCCAAAGGC TGGTCGGTTT TCTCAGCTCC TCTGACCCGC TGCGAACTCC   
  
  
- TTGATACGAC TCAGTAAGTT ACACGGTAAA CTCAAGTTAC GGTATCGTTT CTCGACCCTT TGTGAATGAC   
  
  
- AATTTCTAGA GTTCTAACTA GGGCTACTCC ACGAACAACA CTTGACAGAC AAGTCCAAAT TTATGTAAGG   
  
  
- ACTCCTTTGT CATTAACGTC TTACGGGTTC TCTATAACAA GAATTAGACT AGTCTGTCTA TTTTGGTCGA   
  
  
- CAAAAGTATG TACCACATCA GTTACCTCGG AAGTTAAGAG GAAAAAAGTA ATAAGCCAAG GCACTCCGAG   
  
  
- AGAAGGTGAA GAGGTGAGAT AAACTACACA ACCTCCGTTT AGAAGGGGCC CTGTAGCTCC TCTCCTATGA   
  
  
- CTATCTCGCT CTGTAGAAAC CCGCTGTCCG TTACTTACAC TAGCGAACAC TCCCAAACCT CTCCTATCTT   
  
  
- TCCGGTCTCT TTATGTTTGT CACCGCCCAG GCTTTACTCT CCCGACCCAA CTTCGTCGAC GGAAACTTAG   
  
  
- TCCTCTGACA CCTTTACCGG TTTTTTGCCC ACTGTCGACA GATAGTGTTT CTAAAGAGGT AACTACTGCT   
  
  
- ACCCGTGACC AACAATGTCC CTACCTTTCC CTCTTAACAG ATACGTGAGT GATGAACCTT CGGACGACTG   
  
  
- AT

+     ARE

| Site Name | Organism | Position | Strand | Matrix score. | sequence | function |
| --- | --- | --- | --- | --- | --- | --- |
| ARE | Zea mays | 3846 | + | 6 | AAACCA | cis-acting regulatory element essential for the anaerobic induction |
| ARE | Zea mays | 2630 | - | 6 | AAACCA | cis-acting regulatory element essential for the anaerobic induction |
| ARE | Zea mays | 566 | + | 6 | AAACCA | cis-acting regulatory element essential for the anaerobic induction |
| ARE | Zea mays | 1524 | + | 6 | AAACCA | cis-acting regulatory element essential for the anaerobic induction |
| ARE | Zea mays | 3503 | - | 6 | AAACCA | cis-acting regulatory element essential for the anaerobic induction |

>HU05G01597.1   
+ -Up\_Stream \_Len000TTTGTA TTTCAATATT GTAATTAGGT GGGATATAGA GTTGAAGGGC AAACTCTACC   
  
  
+ AACGACTTGT TGAAGGATCA ACTTGTAGCT TGGAACTTAA TGTATGTGTA CTATAGAGTT GTGTAATCTC   
  
  
+ TCCTAATAAA ATTGGGTTAA TCCTAGAATT AGAGAACTTT AAGCGGGGAC TAAGCTTGGT TGAGGCTAAC   
  
  
+ TTCGTTAAAA AGTTCTTGTG TCATGTCCAC GTTTATCTTT TCACTATTTA TTTCAATTAA TTTGCGATAT   
  
  
+ TTATCATCAC CCTATTCACC GTCTTTTCGG ATGATTTAAC TAGTCAAGCC GGACCAACAT TGCTTTTTAT   
  
  
+ AGTGTATTTG CAACTCATGT TGATTATTAG ATGTATCAAC AAAAAATGTT TCTCAAAATA AATAGTAGCT   
  
  
+ CTTTAAATTT TATTCCACAT AAATTCAAAA TCAATATCGA TATCGTTCCA TAATATAGCA GCATTATGAA   
  
  
+ AACAATATTT GCTAATGTTT ACATTGTGCT CTTTTCACTC AAAAATTGTA AGGCTTCAAA TACTATTAAA   
  
  
+ GAAACCATTA TTTCACTCGA CTATTTAGCT TGCATATATA GACAATCTTG CAAATAGTAA CATGTCTTCA   
  
  
+ TTCGACTATT TGCTTCCAAA TGAATAATCT CGTAAACATA TATACATACA TACATATATA TATATATATA   
  
  
+ TATATATATA TATATATATA GTAATATGTA TTTAACTTCA CATATACATG AATATAAAAA TATAAGAGGG   
  
  
+ AAAAAAGACG CTAGGTAGAT GGACTACAAA AGCCATTCAT GTATTGAGAC GGCTCATAAA ATCATACGGG   
  
  
+ GAAAGGAAAA AAAAAGATCT CTACATAATG TTCATAGATT TATAGAAAGC ATATGATGGA TTACCGCATA   
  
  
+ AGGTGTTATA TATGGGAGTG TTTGTAGAAG AAAGATGTAT ATCCGAACTA TCAAAGATAC ATATGAGGGA   
  
  
+ GTAAAAACAA GTGTAAGAAC CTCAGGAGGA GACATAAAAG ATTTTTCTAT TGGCATTGGA CACCGTCAAG   
  
  
+ TATCCGCTTT AAACCTGTTC TTTTTCACTA TTATCATGGA TGTATTCACA AAAGGAATTC AATACGAAGT   
  
  
+ ACCATGATGT ATGCTTTTTA CTGATAGTAT TGGTCTCATC GATGAGACTA AGGATGGTCT TAATAGCAAA   
  
  
+ TTAGAGATTT CATTAGATAA AATCTACTGT ACATGACAAA ACAATGATAT ATAGCATCCT ATTTAAAAAG   
  
  
+ CTACGTGTCA GTCATATATG AATTGCATCT AATTTTTAAT AGATATATAA TATGATATGA TATAATAGGA   
  
  
+ TATGATGTAG ATTGAATTCA AACTCATAAT TCATTAAGCA TGGTGCAATG TTCATTTTCT TGATTTTAAA   
  
  
+ CCGACTATTA CTTCAACTGT ATATAATTCG AATTGCTTTG GTATTTTAAA ATTTAAACTA GTAATTACTG   
  
  
+ CCTCTATTAC TGATGTTTAT ATTAAGTGGT GCTTAACTGC TTATTGATGA AACCATTTTT ATTTTCGTGT   
  
  
+ AAAAGAGGGC ATAATCCATC CTCGTGTATT GGCGGGGCTC GGCGCCCTAT TCAGCTATTC TCTGTATGTC   
  
  
+ AAACAAAACC GCTTCTATTA CCCATTGGTC TCTAAACACG CAGGGGTTTT AGCCCGGAGA GTTAATTCTT   
  
  
+ TATTGAGGTC CCCTGTTCTC CATTGCAGGA TTTTTGCTCA GGTACTTCTC TGTTTCTTCT CTTTAATCTT   
  
  
+ CTGTATCTTT CTGCATCAAG TTTAATGCTT TCTGGGATTT TCAGCTACGA TTTATGTGAA ATTGTAAGAT   
  
  
+ TTCTGAACTG GGCATTCAGG GCTTGGTTCA TATGTATTCT TTTCTGCTTG AAATGTTTTA ATTTCATAGT   
  
  
+ TCCAATGGGG TTATTCAAAG ATAGAATTAA GCTATAATCA CCGAGAAGAC TTGCTGACCT TTGTTTCTTT   
  
  
+ TTTGTTTCTT TTTTTTAATT AAATCTGGCT ATTACTTGGC TATAGTTTTT GCTAATGGAT CCACAAATAG   
  
  
+ ACGAATTATA TGGGCCTTTG CACCAAATGA AGTTCAATGA TCAGAGGAGA CCCATTTTAT CAAATGAAAA   
  
  
+ TTTTGTTAAT GTGTTGAAAC CGCCAAATTC CAATTGGAAT TCACCTTTTG GGAACCCTAC TGTGATTACC   
  
  
+ ACAAACCCAA ATTTTGATTT GGTTGTGCCA TCACTAGGTC CCAATGTTGA GGGAGCTCTC CTTGAAGATT   
  
  
+ ATGATTTTAG TGATGTTGTT CTTAAGTACA TCAATCAAGT TCTTATGGAC GAGGATGAGG GAGAGAAAAT   
  
  
+ CCATTTGGGC CACGGGCCTC TGGCTCTTGA AGCGGCCGAG AAGTCGTTAT ATGAGGTGCT TGGGCAGAGC   
  
  
+ CATTCTCCAC AAAATCAATA CTCTCCAGAG GACAAGCGTG ATGACCTGAG CAAGAGTAGT GTTAGAAGCT   
  
  
+ GTGTCAGTTA TAGCTGTGAC GGTGCAAGTA GTGGCAGTGG CATCGTGGAG CAAAGTTGGT CGAGTTATCC   
  
  
+ TCCGGAGTAC AATCCCATGC GGATGGCAGA TTTTCCTGCC TTGCCCTCAA AACCCTTGTT GAGAACATCT   
  
  
+ TATAGCTTAT CAAATAGCTT GACAGGTGAG GGTAATGGTT TGGTGGATAG ACCGGCAGAC TCACCTGTAA   
  
  
+ GCGCTCTTAG TGTGTCTGAT ATATTTAATG ATAGTCAGTC AGTGATACAA TTTCAGAAAG GGATGGAGGA   
  
  
+ AGCGAGCAAA TTTCTTCCCA AGGCTTCTTT GTGTTTGGAT TTGAACACTG GACCCTGCCT TCGGGAGTCG   
  
  
+ GTCAATAAGG CACGAGGTGG GCTGATTTTG GTGGAGACTA GCCATGAAAA TGAGGTTGCA GTGCTAGCTA   
  
  
+ GTAGGGGAAA GAAGCATCGC CATCCTGAGG AATTACGTTC AGAAGTTGGG AGGGGCAGTA AGCAGCCAGT   
  
  
+ TGTTTTCCAG TTTTCAGACG AGGCAGTTGT GAGATCAGAA ATGTTTGATA GGGTATTGCT GTGCAGTGGT   
  
  
+ GGCAAAAATG ATGCTGACCT ACGCCAAACT TTACAGAGTG AACTAAATAA AACTATGCAA AATGCTCAAA   
  
  
+ TGAAAGATTC TAATGGTGGA AAAGGCCGTG GTAGGAAGCA AGTTGGCAGG AGGGATGTGG TGGATCTAAG   
  
  
+ ATCTCTTTTA CGCCTTTGTG CACAAGCAGT TGGATCCAAT GACCAAAGAA GCGCAAATGA CTTAATTAGG   
  
  
+ CAAATTAGGA AGCATTCTTC TCCAACCGGG GATGGGAATC AAAGAATGGC TCATTATTTT GCTGATGGTC   
  
  
+ TTGAGGCTCG GCTTGCTGGT GTTGGGACCC CAATATACAA GTCTCTCGTG ACTGGCCCAG CTTCTGCTGT   
  
  
+ TGATATCTTG AGAGCTTACC ATATGTTTCT TGCCACATGC CCATTCAAGA AAATAGGGAA TTTCTTCTCT   
  
  
+ AATAGAACAA TTATGGATGT AGCTCAGAAA GCAAATCGAC TTCATATTAT TGATCTTGGT ATTGTCTATG   
  
  
+ GTTTCCAATG GCCTTGTCTA ATTCAGCGCC TTTCATCTAG GCCTGGCGGG CCTCCCAAAC TTCGGATTAC   
  
  
+ AGGCGTTGAT CTCCCCCAAC CCGGTTTCCG ACCAGCCAAA AGAGTCGAGG AGACTGGGCG ACGCTTGAGG   
  
  
+ AACTATGCTG AGTCATTCAA TGTGCCATTT GAGTTCAATG CCATAGCAAA GAGCTGGGAA ACACTTACTG   
  
  
+ TTAAAGATCT CAAGATTGAT CCCGATGAGG TGCTTGTTGT GAACTGTCTG TTCAGGTTTA AATACATTCC   
  
  
+ TGAGGAAACA GTAATTGCAG AATGCCCAAG AGATATTGTT CTTAATCTGA TCAGACAGAT AAAACCAGCT   
  
  
+ GTTTTCATAC ATGGTGTAGT CAATGGAGCC TTCAATTCTC CTTTTTTCAT TATTCGGTTC CGTGAGGCTC   
  
  
+ TCTTCCACTT CTCCACTCTA TTTGATGTGT TGGAGGCAAA TCTTCCCCGG GACATCGAGG AGAGGATACT   
  
  
+ GATAGAGCGA GACATCTTTG GGCGACAGGC AATGAATGTG ATCGCTTGTG AGGGTTTGGA GAGGATAGAA   
  
  
+ AGGCCAGAGA AATACAAACA GTGGCGGGTC CGAAATGAGA GGGCTGGGTT GAAGCAGCTG CCTTTGAATC   
  
  
+ AGGAGACTGT GGAAATGGCC AAAAAACGGG TGACAGCTGT CTATCACAAA GATTTCTCCA TTGATGACGA   
  
  
+ TGGGCACTGG TTGTTACAGG GATGGAAAGG GAGAATTGTC TATGCACTCA CTACTTGGAA GCCTGCTGAC   
  
  
+ TA  

- -Up\_Stream \_Len000AAACAT AAAGTTATAA CATTAATCCA CCCTATATCT CAACTTCCCG TTTGAGATGG   
  
  
- TTGCTGAACA ACTTCCTAGT TGAACATCGA ACCTTGAATT ACATACACAT GATATCTCAA CACATTAGAG   
  
  
- AGGATTATTT TAACCCAATT AGGATCTTAA TCTCTTGAAA TTCGCCCCTG ATTCGAACCA ACTCCGATTG   
  
  
- AAGCAATTTT TCAAGAACAC AGTACAGGTG CAAATAGAAA AGTGATAAAT AAAGTTAATT AAACGCTATA   
  
  
- AATAGTAGTG GGATAAGTGG CAGAAAAGCC TACTAAATTG ATCAGTTCGG CCTGGTTGTA ACGAAAAATA   
  
  
- TCACATAAAC GTTGAGTACA ACTAATAATC TACATAGTTG TTTTTTACAA AGAGTTTTAT TTATCATCGA   
  
  
- GAAATTTAAA ATAAGGTGTA TTTAAGTTTT AGTTATAGCT ATAGCAAGGT ATTATATCGT CGTAATACTT   
  
  
- TTGTTATAAA CGATTACAAA TGTAACACGA GAAAAGTGAG TTTTTAACAT TCCGAAGTTT ATGATAATTT   
  
  
- CTTTGGTAAT AAAGTGAGCT GATAAATCGA ACGTATATAT CTGTTAGAAC GTTTATCATT GTACAGAAGT   
  
  
- AAGCTGATAA ACGAAGGTTT ACTTATTAGA GCATTTGTAT ATATGTATGT ATGTATATAT ATATATATAT   
  
  
- ATATATATAT ATATATATAT CATTATACAT AAATTGAAGT GTATATGTAC TTATATTTTT ATATTCTCCC   
  
  
- TTTTTTCTGC GATCCATCTA CCTGATGTTT TCGGTAAGTA CATAACTCTG CCGAGTATTT TAGTATGCCC   
  
  
- CTTTCCTTTT TTTTTCTAGA GATGTATTAC AAGTATCTAA ATATCTTTCG TATACTACCT AATGGCGTAT   
  
  
- TCCACAATAT ATACCCTCAC AAACATCTTC TTTCTACATA TAGGCTTGAT AGTTTCTATG TATACTCCCT   
  
  
- CATTTTTGTT CACATTCTTG GAGTCCTCCT CTGTATTTTC TAAAAAGATA ACCGTAACCT GTGGCAGTTC   
  
  
- ATAGGCGAAA TTTGGACAAG AAAAAGTGAT AATAGTACCT ACATAAGTGT TTTCCTTAAG TTATGCTTCA   
  
  
- TGGTACTACA TACGAAAAAT GACTATCATA ACCAGAGTAG CTACTCTGAT TCCTACCAGA ATTATCGTTT   
  
  
- AATCTCTAAA GTAATCTATT TTAGATGACA TGTACTGTTT TGTTACTATA TATCGTAGGA TAAATTTTTC   
  
  
- GATGCACAGT CAGTATATAC TTAACGTAGA TTAAAAATTA TCTATATATT ATACTATACT ATATTATCCT   
  
  
- ATACTACATC TAACTTAAGT TTGAGTATTA AGTAATTCGT ACCACGTTAC AAGTAAAAGA ACTAAAATTT   
  
  
- GGCTGATAAT GAAGTTGACA TATATTAAGC TTAACGAAAC CATAAAATTT TAAATTTGAT CATTAATGAC   
  
  
- GGAGATAATG ACTACAAATA TAATTCACCA CGAATTGACG AATAACTACT TTGGTAAAAA TAAAAGCACA   
  
  
- TTTTCTCCCG TATTAGGTAG GAGCACATAA CCGCCCCGAG CCGCGGGATA AGTCGATAAG AGACATACAG   
  
  
- TTTGTTTTGG CGAAGATAAT GGGTAACCAG AGATTTGTGC GTCCCCAAAA TCGGGCCTCT CAATTAAGAA   
  
  
- ATAACTCCAG GGGACAAGAG GTAACGTCCT AAAAACGAGT CCATGAAGAG ACAAAGAAGA GAAATTAGAA   
  
  
- GACATAGAAA GACGTAGTTC AAATTACGAA AGACCCTAAA AGTCGATGCT AAATACACTT TAACATTCTA   
  
  
- AAGACTTGAC CCGTAAGTCC CGAACCAAGT ATACATAAGA AAAGACGAAC TTTACAAAAT TAAAGTATCA   
  
  
- AGGTTACCCC AATAAGTTTC TATCTTAATT CGATATTAGT GGCTCTTCTG AACGACTGGA AACAAAGAAA   
  
  
- AAACAAAGAA AAAAAATTAA TTTAGACCGA TAATGAACCG ATATCAAAAA CGATTACCTA GGTGTTTATC   
  
  
- TGCTTAATAT ACCCGGAAAC GTGGTTTACT TCAAGTTACT AGTCTCCTCT GGGTAAAATA GTTTACTTTT   
  
  
- AAAACAATTA CACAACTTTG GCGGTTTAAG GTTAACCTTA AGTGGAAAAC CCTTGGGATG ACACTAATGG   
  
  
- TGTTTGGGTT TAAAACTAAA CCAACACGGT AGTGATCCAG GGTTACAACT CCCTCGAGAG GAACTTCTAA   
  
  
- TACTAAAATC ACTACAACAA GAATTCATGT AGTTAGTTCA AGAATACCTG CTCCTACTCC CTCTCTTTTA   
  
  
- GGTAAACCCG GTGCCCGGAG ACCGAGAACT TCGCCGGCTC TTCAGCAATA TACTCCACGA ACCCGTCTCG   
  
  
- GTAAGAGGTG TTTTAGTTAT GAGAGGTCTC CTGTTCGCAC TACTGGACTC GTTCTCATCA CAATCTTCGA   
  
  
- CACAGTCAAT ATCGACACTG CCACGTTCAT CACCGTCACC GTAGCACCTC GTTTCAACCA GCTCAATAGG   
  
  
- AGGCCTCATG TTAGGGTACG CCTACCGTCT AAAAGGACGG AACGGGAGTT TTGGGAACAA CTCTTGTAGA   
  
  
- ATATCGAATA GTTTATCGAA CTGTCCACTC CCATTACCAA ACCACCTATC TGGCCGTCTG AGTGGACATT   
  
  
- CGCGAGAATC ACACAGACTA TATAAATTAC TATCAGTCAG TCACTATGTT AAAGTCTTTC CCTACCTCCT   
  
  
- TCGCTCGTTT AAAGAAGGGT TCCGAAGAAA CACAAACCTA AACTTGTGAC CTGGGACGGA AGCCCTCAGC   
  
  
- CAGTTATTCC GTGCTCCACC CGACTAAAAC CACCTCTGAT CGGTACTTTT ACTCCAACGT CACGATCGAT   
  
  
- CATCCCCTTT CTTCGTAGCG GTAGGACTCC TTAATGCAAG TCTTCAACCC TCCCCGTCAT TCGTCGGTCA   
  
  
- ACAAAAGGTC AAAAGTCTGC TCCGTCAACA CTCTAGTCTT TACAAACTAT CCCATAACGA CACGTCACCA   
  
  
- CCGTTTTTAC TACGACTGGA TGCGGTTTGA AATGTCTCAC TTGATTTATT TTGATACGTT TTACGAGTTT   
  
  
- ACTTTCTAAG ATTACCACCT TTTCCGGCAC CATCCTTCGT TCAACCGTCC TCCCTACACC ACCTAGATTC   
  
  
- TAGAGAAAAT GCGGAAACAC GTGTTCGTCA ACCTAGGTTA CTGGTTTCTT CGCGTTTACT GAATTAATCC   
  
  
- GTTTAATCCT TCGTAAGAAG AGGTTGGCCC CTACCCTTAG TTTCTTACCG AGTAATAAAA CGACTACCAG   
  
  
- AACTCCGAGC CGAACGACCA CAACCCTGGG GTTATATGTT CAGAGAGCAC TGACCGGGTC GAAGACGACA   
  
  
- ACTATAGAAC TCTCGAATGG TATACAAAGA ACGGTGTACG GGTAAGTTCT TTTATCCCTT AAAGAAGAGA   
  
  
- TTATCTTGTT AATACCTACA TCGAGTCTTT CGTTTAGCTG AAGTATAATA ACTAGAACCA TAACAGATAC   
  
  
- CAAAGGTTAC CGGAACAGAT TAAGTCGCGG AAAGTAGATC CGGACCGCCC GGAGGGTTTG AAGCCTAATG   
  
  
- TCCGCAACTA GAGGGGGTTG GGCCAAAGGC TGGTCGGTTT TCTCAGCTCC TCTGACCCGC TGCGAACTCC   
  
  
- TTGATACGAC TCAGTAAGTT ACACGGTAAA CTCAAGTTAC GGTATCGTTT CTCGACCCTT TGTGAATGAC   
  
  
- AATTTCTAGA GTTCTAACTA GGGCTACTCC ACGAACAACA CTTGACAGAC AAGTCCAAAT TTATGTAAGG   
  
  
- ACTCCTTTGT CATTAACGTC TTACGGGTTC TCTATAACAA GAATTAGACT AGTCTGTCTA TTTTGGTCGA   
  
  
- CAAAAGTATG TACCACATCA GTTACCTCGG AAGTTAAGAG GAAAAAAGTA ATAAGCCAAG GCACTCCGAG   
  
  
- AGAAGGTGAA GAGGTGAGAT AAACTACACA ACCTCCGTTT AGAAGGGGCC CTGTAGCTCC TCTCCTATGA   
  
  
- CTATCTCGCT CTGTAGAAAC CCGCTGTCCG TTACTTACAC TAGCGAACAC TCCCAAACCT CTCCTATCTT   
  
  
- TCCGGTCTCT TTATGTTTGT CACCGCCCAG GCTTTACTCT CCCGACCCAA CTTCGTCGAC GGAAACTTAG   
  
  
- TCCTCTGACA CCTTTACCGG TTTTTTGCCC ACTGTCGACA GATAGTGTTT CTAAAGAGGT AACTACTGCT   
  
  
- ACCCGTGACC AACAATGTCC CTACCTTTCC CTCTTAACAG ATACGTGAGT GATGAACCTT CGGACGACTG   
  
  
- AT

+     AT~ABRE

| Site Name | Organism | Position | Strand | Matrix score. | sequence | function |
| --- | --- | --- | --- | --- | --- | --- |
| AT~ABRE | Arabidopsis thaliana | 1266 | + | 8 | TACGTGTC |  |

>HU05G01597.1   
+ -Up\_Stream \_Len000TTTGTA TTTCAATATT GTAATTAGGT GGGATATAGA GTTGAAGGGC AAACTCTACC   
  
  
+ AACGACTTGT TGAAGGATCA ACTTGTAGCT TGGAACTTAA TGTATGTGTA CTATAGAGTT GTGTAATCTC   
  
  
+ TCCTAATAAA ATTGGGTTAA TCCTAGAATT AGAGAACTTT AAGCGGGGAC TAAGCTTGGT TGAGGCTAAC   
  
  
+ TTCGTTAAAA AGTTCTTGTG TCATGTCCAC GTTTATCTTT TCACTATTTA TTTCAATTAA TTTGCGATAT   
  
  
+ TTATCATCAC CCTATTCACC GTCTTTTCGG ATGATTTAAC TAGTCAAGCC GGACCAACAT TGCTTTTTAT   
  
  
+ AGTGTATTTG CAACTCATGT TGATTATTAG ATGTATCAAC AAAAAATGTT TCTCAAAATA AATAGTAGCT   
  
  
+ CTTTAAATTT TATTCCACAT AAATTCAAAA TCAATATCGA TATCGTTCCA TAATATAGCA GCATTATGAA   
  
  
+ AACAATATTT GCTAATGTTT ACATTGTGCT CTTTTCACTC AAAAATTGTA AGGCTTCAAA TACTATTAAA   
  
  
+ GAAACCATTA TTTCACTCGA CTATTTAGCT TGCATATATA GACAATCTTG CAAATAGTAA CATGTCTTCA   
  
  
+ TTCGACTATT TGCTTCCAAA TGAATAATCT CGTAAACATA TATACATACA TACATATATA TATATATATA   
  
  
+ TATATATATA TATATATATA GTAATATGTA TTTAACTTCA CATATACATG AATATAAAAA TATAAGAGGG   
  
  
+ AAAAAAGACG CTAGGTAGAT GGACTACAAA AGCCATTCAT GTATTGAGAC GGCTCATAAA ATCATACGGG   
  
  
+ GAAAGGAAAA AAAAAGATCT CTACATAATG TTCATAGATT TATAGAAAGC ATATGATGGA TTACCGCATA   
  
  
+ AGGTGTTATA TATGGGAGTG TTTGTAGAAG AAAGATGTAT ATCCGAACTA TCAAAGATAC ATATGAGGGA   
  
  
+ GTAAAAACAA GTGTAAGAAC CTCAGGAGGA GACATAAAAG ATTTTTCTAT TGGCATTGGA CACCGTCAAG   
  
  
+ TATCCGCTTT AAACCTGTTC TTTTTCACTA TTATCATGGA TGTATTCACA AAAGGAATTC AATACGAAGT   
  
  
+ ACCATGATGT ATGCTTTTTA CTGATAGTAT TGGTCTCATC GATGAGACTA AGGATGGTCT TAATAGCAAA   
  
  
+ TTAGAGATTT CATTAGATAA AATCTACTGT ACATGACAAA ACAATGATAT ATAGCATCCT ATTTAAAAAG   
  
  
+ CTACGTGTCA GTCATATATG AATTGCATCT AATTTTTAAT AGATATATAA TATGATATGA TATAATAGGA   
  
  
+ TATGATGTAG ATTGAATTCA AACTCATAAT TCATTAAGCA TGGTGCAATG TTCATTTTCT TGATTTTAAA   
  
  
+ CCGACTATTA CTTCAACTGT ATATAATTCG AATTGCTTTG GTATTTTAAA ATTTAAACTA GTAATTACTG   
  
  
+ CCTCTATTAC TGATGTTTAT ATTAAGTGGT GCTTAACTGC TTATTGATGA AACCATTTTT ATTTTCGTGT   
  
  
+ AAAAGAGGGC ATAATCCATC CTCGTGTATT GGCGGGGCTC GGCGCCCTAT TCAGCTATTC TCTGTATGTC   
  
  
+ AAACAAAACC GCTTCTATTA CCCATTGGTC TCTAAACACG CAGGGGTTTT AGCCCGGAGA GTTAATTCTT   
  
  
+ TATTGAGGTC CCCTGTTCTC CATTGCAGGA TTTTTGCTCA GGTACTTCTC TGTTTCTTCT CTTTAATCTT   
  
  
+ CTGTATCTTT CTGCATCAAG TTTAATGCTT TCTGGGATTT TCAGCTACGA TTTATGTGAA ATTGTAAGAT   
  
  
+ TTCTGAACTG GGCATTCAGG GCTTGGTTCA TATGTATTCT TTTCTGCTTG AAATGTTTTA ATTTCATAGT   
  
  
+ TCCAATGGGG TTATTCAAAG ATAGAATTAA GCTATAATCA CCGAGAAGAC TTGCTGACCT TTGTTTCTTT   
  
  
+ TTTGTTTCTT TTTTTTAATT AAATCTGGCT ATTACTTGGC TATAGTTTTT GCTAATGGAT CCACAAATAG   
  
  
+ ACGAATTATA TGGGCCTTTG CACCAAATGA AGTTCAATGA TCAGAGGAGA CCCATTTTAT CAAATGAAAA   
  
  
+ TTTTGTTAAT GTGTTGAAAC CGCCAAATTC CAATTGGAAT TCACCTTTTG GGAACCCTAC TGTGATTACC   
  
  
+ ACAAACCCAA ATTTTGATTT GGTTGTGCCA TCACTAGGTC CCAATGTTGA GGGAGCTCTC CTTGAAGATT   
  
  
+ ATGATTTTAG TGATGTTGTT CTTAAGTACA TCAATCAAGT TCTTATGGAC GAGGATGAGG GAGAGAAAAT   
  
  
+ CCATTTGGGC CACGGGCCTC TGGCTCTTGA AGCGGCCGAG AAGTCGTTAT ATGAGGTGCT TGGGCAGAGC   
  
  
+ CATTCTCCAC AAAATCAATA CTCTCCAGAG GACAAGCGTG ATGACCTGAG CAAGAGTAGT GTTAGAAGCT   
  
  
+ GTGTCAGTTA TAGCTGTGAC GGTGCAAGTA GTGGCAGTGG CATCGTGGAG CAAAGTTGGT CGAGTTATCC   
  
  
+ TCCGGAGTAC AATCCCATGC GGATGGCAGA TTTTCCTGCC TTGCCCTCAA AACCCTTGTT GAGAACATCT   
  
  
+ TATAGCTTAT CAAATAGCTT GACAGGTGAG GGTAATGGTT TGGTGGATAG ACCGGCAGAC TCACCTGTAA   
  
  
+ GCGCTCTTAG TGTGTCTGAT ATATTTAATG ATAGTCAGTC AGTGATACAA TTTCAGAAAG GGATGGAGGA   
  
  
+ AGCGAGCAAA TTTCTTCCCA AGGCTTCTTT GTGTTTGGAT TTGAACACTG GACCCTGCCT TCGGGAGTCG   
  
  
+ GTCAATAAGG CACGAGGTGG GCTGATTTTG GTGGAGACTA GCCATGAAAA TGAGGTTGCA GTGCTAGCTA   
  
  
+ GTAGGGGAAA GAAGCATCGC CATCCTGAGG AATTACGTTC AGAAGTTGGG AGGGGCAGTA AGCAGCCAGT   
  
  
+ TGTTTTCCAG TTTTCAGACG AGGCAGTTGT GAGATCAGAA ATGTTTGATA GGGTATTGCT GTGCAGTGGT   
  
  
+ GGCAAAAATG ATGCTGACCT ACGCCAAACT TTACAGAGTG AACTAAATAA AACTATGCAA AATGCTCAAA   
  
  
+ TGAAAGATTC TAATGGTGGA AAAGGCCGTG GTAGGAAGCA AGTTGGCAGG AGGGATGTGG TGGATCTAAG   
  
  
+ ATCTCTTTTA CGCCTTTGTG CACAAGCAGT TGGATCCAAT GACCAAAGAA GCGCAAATGA CTTAATTAGG   
  
  
+ CAAATTAGGA AGCATTCTTC TCCAACCGGG GATGGGAATC AAAGAATGGC TCATTATTTT GCTGATGGTC   
  
  
+ TTGAGGCTCG GCTTGCTGGT GTTGGGACCC CAATATACAA GTCTCTCGTG ACTGGCCCAG CTTCTGCTGT   
  
  
+ TGATATCTTG AGAGCTTACC ATATGTTTCT TGCCACATGC CCATTCAAGA AAATAGGGAA TTTCTTCTCT   
  
  
+ AATAGAACAA TTATGGATGT AGCTCAGAAA GCAAATCGAC TTCATATTAT TGATCTTGGT ATTGTCTATG   
  
  
+ GTTTCCAATG GCCTTGTCTA ATTCAGCGCC TTTCATCTAG GCCTGGCGGG CCTCCCAAAC TTCGGATTAC   
  
  
+ AGGCGTTGAT CTCCCCCAAC CCGGTTTCCG ACCAGCCAAA AGAGTCGAGG AGACTGGGCG ACGCTTGAGG   
  
  
+ AACTATGCTG AGTCATTCAA TGTGCCATTT GAGTTCAATG CCATAGCAAA GAGCTGGGAA ACACTTACTG   
  
  
+ TTAAAGATCT CAAGATTGAT CCCGATGAGG TGCTTGTTGT GAACTGTCTG TTCAGGTTTA AATACATTCC   
  
  
+ TGAGGAAACA GTAATTGCAG AATGCCCAAG AGATATTGTT CTTAATCTGA TCAGACAGAT AAAACCAGCT   
  
  
+ GTTTTCATAC ATGGTGTAGT CAATGGAGCC TTCAATTCTC CTTTTTTCAT TATTCGGTTC CGTGAGGCTC   
  
  
+ TCTTCCACTT CTCCACTCTA TTTGATGTGT TGGAGGCAAA TCTTCCCCGG GACATCGAGG AGAGGATACT   
  
  
+ GATAGAGCGA GACATCTTTG GGCGACAGGC AATGAATGTG ATCGCTTGTG AGGGTTTGGA GAGGATAGAA   
  
  
+ AGGCCAGAGA AATACAAACA GTGGCGGGTC CGAAATGAGA GGGCTGGGTT GAAGCAGCTG CCTTTGAATC   
  
  
+ AGGAGACTGT GGAAATGGCC AAAAAACGGG TGACAGCTGT CTATCACAAA GATTTCTCCA TTGATGACGA   
  
  
+ TGGGCACTGG TTGTTACAGG GATGGAAAGG GAGAATTGTC TATGCACTCA CTACTTGGAA GCCTGCTGAC   
  
  
+ TA  

- -Up\_Stream \_Len000AAACAT AAAGTTATAA CATTAATCCA CCCTATATCT CAACTTCCCG TTTGAGATGG   
  
  
- TTGCTGAACA ACTTCCTAGT TGAACATCGA ACCTTGAATT ACATACACAT GATATCTCAA CACATTAGAG   
  
  
- AGGATTATTT TAACCCAATT AGGATCTTAA TCTCTTGAAA TTCGCCCCTG ATTCGAACCA ACTCCGATTG   
  
  
- AAGCAATTTT TCAAGAACAC AGTACAGGTG CAAATAGAAA AGTGATAAAT AAAGTTAATT AAACGCTATA   
  
  
- AATAGTAGTG GGATAAGTGG CAGAAAAGCC TACTAAATTG ATCAGTTCGG CCTGGTTGTA ACGAAAAATA   
  
  
- TCACATAAAC GTTGAGTACA ACTAATAATC TACATAGTTG TTTTTTACAA AGAGTTTTAT TTATCATCGA   
  
  
- GAAATTTAAA ATAAGGTGTA TTTAAGTTTT AGTTATAGCT ATAGCAAGGT ATTATATCGT CGTAATACTT   
  
  
- TTGTTATAAA CGATTACAAA TGTAACACGA GAAAAGTGAG TTTTTAACAT TCCGAAGTTT ATGATAATTT   
  
  
- CTTTGGTAAT AAAGTGAGCT GATAAATCGA ACGTATATAT CTGTTAGAAC GTTTATCATT GTACAGAAGT   
  
  
- AAGCTGATAA ACGAAGGTTT ACTTATTAGA GCATTTGTAT ATATGTATGT ATGTATATAT ATATATATAT   
  
  
- ATATATATAT ATATATATAT CATTATACAT AAATTGAAGT GTATATGTAC TTATATTTTT ATATTCTCCC   
  
  
- TTTTTTCTGC GATCCATCTA CCTGATGTTT TCGGTAAGTA CATAACTCTG CCGAGTATTT TAGTATGCCC   
  
  
- CTTTCCTTTT TTTTTCTAGA GATGTATTAC AAGTATCTAA ATATCTTTCG TATACTACCT AATGGCGTAT   
  
  
- TCCACAATAT ATACCCTCAC AAACATCTTC TTTCTACATA TAGGCTTGAT AGTTTCTATG TATACTCCCT   
  
  
- CATTTTTGTT CACATTCTTG GAGTCCTCCT CTGTATTTTC TAAAAAGATA ACCGTAACCT GTGGCAGTTC   
  
  
- ATAGGCGAAA TTTGGACAAG AAAAAGTGAT AATAGTACCT ACATAAGTGT TTTCCTTAAG TTATGCTTCA   
  
  
- TGGTACTACA TACGAAAAAT GACTATCATA ACCAGAGTAG CTACTCTGAT TCCTACCAGA ATTATCGTTT   
  
  
- AATCTCTAAA GTAATCTATT TTAGATGACA TGTACTGTTT TGTTACTATA TATCGTAGGA TAAATTTTTC   
  
  
- GATGCACAGT CAGTATATAC TTAACGTAGA TTAAAAATTA TCTATATATT ATACTATACT ATATTATCCT   
  
  
- ATACTACATC TAACTTAAGT TTGAGTATTA AGTAATTCGT ACCACGTTAC AAGTAAAAGA ACTAAAATTT   
  
  
- GGCTGATAAT GAAGTTGACA TATATTAAGC TTAACGAAAC CATAAAATTT TAAATTTGAT CATTAATGAC   
  
  
- GGAGATAATG ACTACAAATA TAATTCACCA CGAATTGACG AATAACTACT TTGGTAAAAA TAAAAGCACA   
  
  
- TTTTCTCCCG TATTAGGTAG GAGCACATAA CCGCCCCGAG CCGCGGGATA AGTCGATAAG AGACATACAG   
  
  
- TTTGTTTTGG CGAAGATAAT GGGTAACCAG AGATTTGTGC GTCCCCAAAA TCGGGCCTCT CAATTAAGAA   
  
  
- ATAACTCCAG GGGACAAGAG GTAACGTCCT AAAAACGAGT CCATGAAGAG ACAAAGAAGA GAAATTAGAA   
  
  
- GACATAGAAA GACGTAGTTC AAATTACGAA AGACCCTAAA AGTCGATGCT AAATACACTT TAACATTCTA   
  
  
- AAGACTTGAC CCGTAAGTCC CGAACCAAGT ATACATAAGA AAAGACGAAC TTTACAAAAT TAAAGTATCA   
  
  
- AGGTTACCCC AATAAGTTTC TATCTTAATT CGATATTAGT GGCTCTTCTG AACGACTGGA AACAAAGAAA   
  
  
- AAACAAAGAA AAAAAATTAA TTTAGACCGA TAATGAACCG ATATCAAAAA CGATTACCTA GGTGTTTATC   
  
  
- TGCTTAATAT ACCCGGAAAC GTGGTTTACT TCAAGTTACT AGTCTCCTCT GGGTAAAATA GTTTACTTTT   
  
  
- AAAACAATTA CACAACTTTG GCGGTTTAAG GTTAACCTTA AGTGGAAAAC CCTTGGGATG ACACTAATGG   
  
  
- TGTTTGGGTT TAAAACTAAA CCAACACGGT AGTGATCCAG GGTTACAACT CCCTCGAGAG GAACTTCTAA   
  
  
- TACTAAAATC ACTACAACAA GAATTCATGT AGTTAGTTCA AGAATACCTG CTCCTACTCC CTCTCTTTTA   
  
  
- GGTAAACCCG GTGCCCGGAG ACCGAGAACT TCGCCGGCTC TTCAGCAATA TACTCCACGA ACCCGTCTCG   
  
  
- GTAAGAGGTG TTTTAGTTAT GAGAGGTCTC CTGTTCGCAC TACTGGACTC GTTCTCATCA CAATCTTCGA   
  
  
- CACAGTCAAT ATCGACACTG CCACGTTCAT CACCGTCACC GTAGCACCTC GTTTCAACCA GCTCAATAGG   
  
  
- AGGCCTCATG TTAGGGTACG CCTACCGTCT AAAAGGACGG AACGGGAGTT TTGGGAACAA CTCTTGTAGA   
  
  
- ATATCGAATA GTTTATCGAA CTGTCCACTC CCATTACCAA ACCACCTATC TGGCCGTCTG AGTGGACATT   
  
  
- CGCGAGAATC ACACAGACTA TATAAATTAC TATCAGTCAG TCACTATGTT AAAGTCTTTC CCTACCTCCT   
  
  
- TCGCTCGTTT AAAGAAGGGT TCCGAAGAAA CACAAACCTA AACTTGTGAC CTGGGACGGA AGCCCTCAGC   
  
  
- CAGTTATTCC GTGCTCCACC CGACTAAAAC CACCTCTGAT CGGTACTTTT ACTCCAACGT CACGATCGAT   
  
  
- CATCCCCTTT CTTCGTAGCG GTAGGACTCC TTAATGCAAG TCTTCAACCC TCCCCGTCAT TCGTCGGTCA   
  
  
- ACAAAAGGTC AAAAGTCTGC TCCGTCAACA CTCTAGTCTT TACAAACTAT CCCATAACGA CACGTCACCA   
  
  
- CCGTTTTTAC TACGACTGGA TGCGGTTTGA AATGTCTCAC TTGATTTATT TTGATACGTT TTACGAGTTT   
  
  
- ACTTTCTAAG ATTACCACCT TTTCCGGCAC CATCCTTCGT TCAACCGTCC TCCCTACACC ACCTAGATTC   
  
  
- TAGAGAAAAT GCGGAAACAC GTGTTCGTCA ACCTAGGTTA CTGGTTTCTT CGCGTTTACT GAATTAATCC   
  
  
- GTTTAATCCT TCGTAAGAAG AGGTTGGCCC CTACCCTTAG TTTCTTACCG AGTAATAAAA CGACTACCAG   
  
  
- AACTCCGAGC CGAACGACCA CAACCCTGGG GTTATATGTT CAGAGAGCAC TGACCGGGTC GAAGACGACA   
  
  
- ACTATAGAAC TCTCGAATGG TATACAAAGA ACGGTGTACG GGTAAGTTCT TTTATCCCTT AAAGAAGAGA   
  
  
- TTATCTTGTT AATACCTACA TCGAGTCTTT CGTTTAGCTG AAGTATAATA ACTAGAACCA TAACAGATAC   
  
  
- CAAAGGTTAC CGGAACAGAT TAAGTCGCGG AAAGTAGATC CGGACCGCCC GGAGGGTTTG AAGCCTAATG   
  
  
- TCCGCAACTA GAGGGGGTTG GGCCAAAGGC TGGTCGGTTT TCTCAGCTCC TCTGACCCGC TGCGAACTCC   
  
  
- TTGATACGAC TCAGTAAGTT ACACGGTAAA CTCAAGTTAC GGTATCGTTT CTCGACCCTT TGTGAATGAC   
  
  
- AATTTCTAGA GTTCTAACTA GGGCTACTCC ACGAACAACA CTTGACAGAC AAGTCCAAAT TTATGTAAGG   
  
  
- ACTCCTTTGT CATTAACGTC TTACGGGTTC TCTATAACAA GAATTAGACT AGTCTGTCTA TTTTGGTCGA   
  
  
- CAAAAGTATG TACCACATCA GTTACCTCGG AAGTTAAGAG GAAAAAAGTA ATAAGCCAAG GCACTCCGAG   
  
  
- AGAAGGTGAA GAGGTGAGAT AAACTACACA ACCTCCGTTT AGAAGGGGCC CTGTAGCTCC TCTCCTATGA   
  
  
- CTATCTCGCT CTGTAGAAAC CCGCTGTCCG TTACTTACAC TAGCGAACAC TCCCAAACCT CTCCTATCTT   
  
  
- TCCGGTCTCT TTATGTTTGT CACCGCCCAG GCTTTACTCT CCCGACCCAA CTTCGTCGAC GGAAACTTAG   
  
  
- TCCTCTGACA CCTTTACCGG TTTTTTGCCC ACTGTCGACA GATAGTGTTT CTAAAGAGGT AACTACTGCT   
  
  
- ACCCGTGACC AACAATGTCC CTACCTTTCC CTCTTAACAG ATACGTGAGT GATGAACCTT CGGACGACTG   
  
  
- AT

+     AT~TATA-box

| Site Name | Organism | Position | Strand | Matrix score. | sequence | function |
| --- | --- | --- | --- | --- | --- | --- |
| AT~TATA-box | Arabidopsis thaliana | 1424 | + | 6 | TATATA |  |
| AT~TATA-box | Arabidopsis thaliana | 1308 | + | 6 | TATATA |  |
| AT~TATA-box | Arabidopsis thaliana | 1242 | + | 6 | TATATA |  |
| AT~TATA-box | Arabidopsis thaliana | 707 | + | 6 | TATATA |  |
| AT~TATA-box | Arabidopsis thaliana | 705 | + | 6 | TATATA |  |
| AT~TATA-box | Arabidopsis thaliana | 703 | + | 6 | TATATA |  |
| AT~TATA-box | Arabidopsis thaliana | 701 | + | 6 | TATATA |  |
| AT~TATA-box | Arabidopsis thaliana | 921 | + | 6 | TATATA |  |
| AT~TATA-box | Arabidopsis thaliana | 717 | + | 6 | TATATA |  |
| AT~TATA-box | Arabidopsis thaliana | 719 | + | 6 | TATATA |  |
| AT~TATA-box | Arabidopsis thaliana | 715 | + | 6 | TATATA |  |
| AT~TATA-box | Arabidopsis thaliana | 711 | + | 6 | TATATA |  |
| AT~TATA-box | Arabidopsis thaliana | 689 | + | 6 | TATATA |  |
| AT~TATA-box | Arabidopsis thaliana | 699 | + | 6 | TATATA |  |
| AT~TATA-box | Arabidopsis thaliana | 709 | + | 6 | TATATA |  |
| AT~TATA-box | Arabidopsis thaliana | 713 | + | 6 | TATATA |  |
| AT~TATA-box | Arabidopsis thaliana | 693 | + | 6 | TATATA |  |
| AT~TATA-box | Arabidopsis thaliana | 695 | + | 6 | TATATA |  |
| AT~TATA-box | Arabidopsis thaliana | 697 | + | 6 | TATATA |  |
| AT~TATA-box | Arabidopsis thaliana | 691 | + | 6 | TATATA |  |
| AT~TATA-box | Arabidopsis thaliana | 673 | + | 6 | TATATA |  |
| AT~TATA-box | Arabidopsis thaliana | 599 | + | 6 | TATATA |  |

>HU05G01597.1   
+ -Up\_Stream \_Len000TTTGTA TTTCAATATT GTAATTAGGT GGGATATAGA GTTGAAGGGC AAACTCTACC   
  
  
+ AACGACTTGT TGAAGGATCA ACTTGTAGCT TGGAACTTAA TGTATGTGTA CTATAGAGTT GTGTAATCTC   
  
  
+ TCCTAATAAA ATTGGGTTAA TCCTAGAATT AGAGAACTTT AAGCGGGGAC TAAGCTTGGT TGAGGCTAAC   
  
  
+ TTCGTTAAAA AGTTCTTGTG TCATGTCCAC GTTTATCTTT TCACTATTTA TTTCAATTAA TTTGCGATAT   
  
  
+ TTATCATCAC CCTATTCACC GTCTTTTCGG ATGATTTAAC TAGTCAAGCC GGACCAACAT TGCTTTTTAT   
  
  
+ AGTGTATTTG CAACTCATGT TGATTATTAG ATGTATCAAC AAAAAATGTT TCTCAAAATA AATAGTAGCT   
  
  
+ CTTTAAATTT TATTCCACAT AAATTCAAAA TCAATATCGA TATCGTTCCA TAATATAGCA GCATTATGAA   
  
  
+ AACAATATTT GCTAATGTTT ACATTGTGCT CTTTTCACTC AAAAATTGTA AGGCTTCAAA TACTATTAAA   
  
  
+ GAAACCATTA TTTCACTCGA CTATTTAGCT TGCATATATA GACAATCTTG CAAATAGTAA CATGTCTTCA   
  
  
+ TTCGACTATT TGCTTCCAAA TGAATAATCT CGTAAACATA TATACATACA TACATATATA TATATATATA   
  
  
+ TATATATATA TATATATATA GTAATATGTA TTTAACTTCA CATATACATG AATATAAAAA TATAAGAGGG   
  
  
+ AAAAAAGACG CTAGGTAGAT GGACTACAAA AGCCATTCAT GTATTGAGAC GGCTCATAAA ATCATACGGG   
  
  
+ GAAAGGAAAA AAAAAGATCT CTACATAATG TTCATAGATT TATAGAAAGC ATATGATGGA TTACCGCATA   
  
  
+ AGGTGTTATA TATGGGAGTG TTTGTAGAAG AAAGATGTAT ATCCGAACTA TCAAAGATAC ATATGAGGGA   
  
  
+ GTAAAAACAA GTGTAAGAAC CTCAGGAGGA GACATAAAAG ATTTTTCTAT TGGCATTGGA CACCGTCAAG   
  
  
+ TATCCGCTTT AAACCTGTTC TTTTTCACTA TTATCATGGA TGTATTCACA AAAGGAATTC AATACGAAGT   
  
  
+ ACCATGATGT ATGCTTTTTA CTGATAGTAT TGGTCTCATC GATGAGACTA AGGATGGTCT TAATAGCAAA   
  
  
+ TTAGAGATTT CATTAGATAA AATCTACTGT ACATGACAAA ACAATGATAT ATAGCATCCT ATTTAAAAAG   
  
  
+ CTACGTGTCA GTCATATATG AATTGCATCT AATTTTTAAT AGATATATAA TATGATATGA TATAATAGGA   
  
  
+ TATGATGTAG ATTGAATTCA AACTCATAAT TCATTAAGCA TGGTGCAATG TTCATTTTCT TGATTTTAAA   
  
  
+ CCGACTATTA CTTCAACTGT ATATAATTCG AATTGCTTTG GTATTTTAAA ATTTAAACTA GTAATTACTG   
  
  
+ CCTCTATTAC TGATGTTTAT ATTAAGTGGT GCTTAACTGC TTATTGATGA AACCATTTTT ATTTTCGTGT   
  
  
+ AAAAGAGGGC ATAATCCATC CTCGTGTATT GGCGGGGCTC GGCGCCCTAT TCAGCTATTC TCTGTATGTC   
  
  
+ AAACAAAACC GCTTCTATTA CCCATTGGTC TCTAAACACG CAGGGGTTTT AGCCCGGAGA GTTAATTCTT   
  
  
+ TATTGAGGTC CCCTGTTCTC CATTGCAGGA TTTTTGCTCA GGTACTTCTC TGTTTCTTCT CTTTAATCTT   
  
  
+ CTGTATCTTT CTGCATCAAG TTTAATGCTT TCTGGGATTT TCAGCTACGA TTTATGTGAA ATTGTAAGAT   
  
  
+ TTCTGAACTG GGCATTCAGG GCTTGGTTCA TATGTATTCT TTTCTGCTTG AAATGTTTTA ATTTCATAGT   
  
  
+ TCCAATGGGG TTATTCAAAG ATAGAATTAA GCTATAATCA CCGAGAAGAC TTGCTGACCT TTGTTTCTTT   
  
  
+ TTTGTTTCTT TTTTTTAATT AAATCTGGCT ATTACTTGGC TATAGTTTTT GCTAATGGAT CCACAAATAG   
  
  
+ ACGAATTATA TGGGCCTTTG CACCAAATGA AGTTCAATGA TCAGAGGAGA CCCATTTTAT CAAATGAAAA   
  
  
+ TTTTGTTAAT GTGTTGAAAC CGCCAAATTC CAATTGGAAT TCACCTTTTG GGAACCCTAC TGTGATTACC   
  
  
+ ACAAACCCAA ATTTTGATTT GGTTGTGCCA TCACTAGGTC CCAATGTTGA GGGAGCTCTC CTTGAAGATT   
  
  
+ ATGATTTTAG TGATGTTGTT CTTAAGTACA TCAATCAAGT TCTTATGGAC GAGGATGAGG GAGAGAAAAT   
  
  
+ CCATTTGGGC CACGGGCCTC TGGCTCTTGA AGCGGCCGAG AAGTCGTTAT ATGAGGTGCT TGGGCAGAGC   
  
  
+ CATTCTCCAC AAAATCAATA CTCTCCAGAG GACAAGCGTG ATGACCTGAG CAAGAGTAGT GTTAGAAGCT   
  
  
+ GTGTCAGTTA TAGCTGTGAC GGTGCAAGTA GTGGCAGTGG CATCGTGGAG CAAAGTTGGT CGAGTTATCC   
  
  
+ TCCGGAGTAC AATCCCATGC GGATGGCAGA TTTTCCTGCC TTGCCCTCAA AACCCTTGTT GAGAACATCT   
  
  
+ TATAGCTTAT CAAATAGCTT GACAGGTGAG GGTAATGGTT TGGTGGATAG ACCGGCAGAC TCACCTGTAA   
  
  
+ GCGCTCTTAG TGTGTCTGAT ATATTTAATG ATAGTCAGTC AGTGATACAA TTTCAGAAAG GGATGGAGGA   
  
  
+ AGCGAGCAAA TTTCTTCCCA AGGCTTCTTT GTGTTTGGAT TTGAACACTG GACCCTGCCT TCGGGAGTCG   
  
  
+ GTCAATAAGG CACGAGGTGG GCTGATTTTG GTGGAGACTA GCCATGAAAA TGAGGTTGCA GTGCTAGCTA   
  
  
+ GTAGGGGAAA GAAGCATCGC CATCCTGAGG AATTACGTTC AGAAGTTGGG AGGGGCAGTA AGCAGCCAGT   
  
  
+ TGTTTTCCAG TTTTCAGACG AGGCAGTTGT GAGATCAGAA ATGTTTGATA GGGTATTGCT GTGCAGTGGT   
  
  
+ GGCAAAAATG ATGCTGACCT ACGCCAAACT TTACAGAGTG AACTAAATAA AACTATGCAA AATGCTCAAA   
  
  
+ TGAAAGATTC TAATGGTGGA AAAGGCCGTG GTAGGAAGCA AGTTGGCAGG AGGGATGTGG TGGATCTAAG   
  
  
+ ATCTCTTTTA CGCCTTTGTG CACAAGCAGT TGGATCCAAT GACCAAAGAA GCGCAAATGA CTTAATTAGG   
  
  
+ CAAATTAGGA AGCATTCTTC TCCAACCGGG GATGGGAATC AAAGAATGGC TCATTATTTT GCTGATGGTC   
  
  
+ TTGAGGCTCG GCTTGCTGGT GTTGGGACCC CAATATACAA GTCTCTCGTG ACTGGCCCAG CTTCTGCTGT   
  
  
+ TGATATCTTG AGAGCTTACC ATATGTTTCT TGCCACATGC CCATTCAAGA AAATAGGGAA TTTCTTCTCT   
  
  
+ AATAGAACAA TTATGGATGT AGCTCAGAAA GCAAATCGAC TTCATATTAT TGATCTTGGT ATTGTCTATG   
  
  
+ GTTTCCAATG GCCTTGTCTA ATTCAGCGCC TTTCATCTAG GCCTGGCGGG CCTCCCAAAC TTCGGATTAC   
  
  
+ AGGCGTTGAT CTCCCCCAAC CCGGTTTCCG ACCAGCCAAA AGAGTCGAGG AGACTGGGCG ACGCTTGAGG   
  
  
+ AACTATGCTG AGTCATTCAA TGTGCCATTT GAGTTCAATG CCATAGCAAA GAGCTGGGAA ACACTTACTG   
  
  
+ TTAAAGATCT CAAGATTGAT CCCGATGAGG TGCTTGTTGT GAACTGTCTG TTCAGGTTTA AATACATTCC   
  
  
+ TGAGGAAACA GTAATTGCAG AATGCCCAAG AGATATTGTT CTTAATCTGA TCAGACAGAT AAAACCAGCT   
  
  
+ GTTTTCATAC ATGGTGTAGT CAATGGAGCC TTCAATTCTC CTTTTTTCAT TATTCGGTTC CGTGAGGCTC   
  
  
+ TCTTCCACTT CTCCACTCTA TTTGATGTGT TGGAGGCAAA TCTTCCCCGG GACATCGAGG AGAGGATACT   
  
  
+ GATAGAGCGA GACATCTTTG GGCGACAGGC AATGAATGTG ATCGCTTGTG AGGGTTTGGA GAGGATAGAA   
  
  
+ AGGCCAGAGA AATACAAACA GTGGCGGGTC CGAAATGAGA GGGCTGGGTT GAAGCAGCTG CCTTTGAATC   
  
  
+ AGGAGACTGT GGAAATGGCC AAAAAACGGG TGACAGCTGT CTATCACAAA GATTTCTCCA TTGATGACGA   
  
  
+ TGGGCACTGG TTGTTACAGG GATGGAAAGG GAGAATTGTC TATGCACTCA CTACTTGGAA GCCTGCTGAC   
  
  
+ TA  

- -Up\_Stream \_Len000AAACAT AAAGTTATAA CATTAATCCA CCCTATATCT CAACTTCCCG TTTGAGATGG   
  
  
- TTGCTGAACA ACTTCCTAGT TGAACATCGA ACCTTGAATT ACATACACAT GATATCTCAA CACATTAGAG   
  
  
- AGGATTATTT TAACCCAATT AGGATCTTAA TCTCTTGAAA TTCGCCCCTG ATTCGAACCA ACTCCGATTG   
  
  
- AAGCAATTTT TCAAGAACAC AGTACAGGTG CAAATAGAAA AGTGATAAAT AAAGTTAATT AAACGCTATA   
  
  
- AATAGTAGTG GGATAAGTGG CAGAAAAGCC TACTAAATTG ATCAGTTCGG CCTGGTTGTA ACGAAAAATA   
  
  
- TCACATAAAC GTTGAGTACA ACTAATAATC TACATAGTTG TTTTTTACAA AGAGTTTTAT TTATCATCGA   
  
  
- GAAATTTAAA ATAAGGTGTA TTTAAGTTTT AGTTATAGCT ATAGCAAGGT ATTATATCGT CGTAATACTT   
  
  
- TTGTTATAAA CGATTACAAA TGTAACACGA GAAAAGTGAG TTTTTAACAT TCCGAAGTTT ATGATAATTT   
  
  
- CTTTGGTAAT AAAGTGAGCT GATAAATCGA ACGTATATAT CTGTTAGAAC GTTTATCATT GTACAGAAGT   
  
  
- AAGCTGATAA ACGAAGGTTT ACTTATTAGA GCATTTGTAT ATATGTATGT ATGTATATAT ATATATATAT   
  
  
- ATATATATAT ATATATATAT CATTATACAT AAATTGAAGT GTATATGTAC TTATATTTTT ATATTCTCCC   
  
  
- TTTTTTCTGC GATCCATCTA CCTGATGTTT TCGGTAAGTA CATAACTCTG CCGAGTATTT TAGTATGCCC   
  
  
- CTTTCCTTTT TTTTTCTAGA GATGTATTAC AAGTATCTAA ATATCTTTCG TATACTACCT AATGGCGTAT   
  
  
- TCCACAATAT ATACCCTCAC AAACATCTTC TTTCTACATA TAGGCTTGAT AGTTTCTATG TATACTCCCT   
  
  
- CATTTTTGTT CACATTCTTG GAGTCCTCCT CTGTATTTTC TAAAAAGATA ACCGTAACCT GTGGCAGTTC   
  
  
- ATAGGCGAAA TTTGGACAAG AAAAAGTGAT AATAGTACCT ACATAAGTGT TTTCCTTAAG TTATGCTTCA   
  
  
- TGGTACTACA TACGAAAAAT GACTATCATA ACCAGAGTAG CTACTCTGAT TCCTACCAGA ATTATCGTTT   
  
  
- AATCTCTAAA GTAATCTATT TTAGATGACA TGTACTGTTT TGTTACTATA TATCGTAGGA TAAATTTTTC   
  
  
- GATGCACAGT CAGTATATAC TTAACGTAGA TTAAAAATTA TCTATATATT ATACTATACT ATATTATCCT   
  
  
- ATACTACATC TAACTTAAGT TTGAGTATTA AGTAATTCGT ACCACGTTAC AAGTAAAAGA ACTAAAATTT   
  
  
- GGCTGATAAT GAAGTTGACA TATATTAAGC TTAACGAAAC CATAAAATTT TAAATTTGAT CATTAATGAC   
  
  
- GGAGATAATG ACTACAAATA TAATTCACCA CGAATTGACG AATAACTACT TTGGTAAAAA TAAAAGCACA   
  
  
- TTTTCTCCCG TATTAGGTAG GAGCACATAA CCGCCCCGAG CCGCGGGATA AGTCGATAAG AGACATACAG   
  
  
- TTTGTTTTGG CGAAGATAAT GGGTAACCAG AGATTTGTGC GTCCCCAAAA TCGGGCCTCT CAATTAAGAA   
  
  
- ATAACTCCAG GGGACAAGAG GTAACGTCCT AAAAACGAGT CCATGAAGAG ACAAAGAAGA GAAATTAGAA   
  
  
- GACATAGAAA GACGTAGTTC AAATTACGAA AGACCCTAAA AGTCGATGCT AAATACACTT TAACATTCTA   
  
  
- AAGACTTGAC CCGTAAGTCC CGAACCAAGT ATACATAAGA AAAGACGAAC TTTACAAAAT TAAAGTATCA   
  
  
- AGGTTACCCC AATAAGTTTC TATCTTAATT CGATATTAGT GGCTCTTCTG AACGACTGGA AACAAAGAAA   
  
  
- AAACAAAGAA AAAAAATTAA TTTAGACCGA TAATGAACCG ATATCAAAAA CGATTACCTA GGTGTTTATC   
  
  
- TGCTTAATAT ACCCGGAAAC GTGGTTTACT TCAAGTTACT AGTCTCCTCT GGGTAAAATA GTTTACTTTT   
  
  
- AAAACAATTA CACAACTTTG GCGGTTTAAG GTTAACCTTA AGTGGAAAAC CCTTGGGATG ACACTAATGG   
  
  
- TGTTTGGGTT TAAAACTAAA CCAACACGGT AGTGATCCAG GGTTACAACT CCCTCGAGAG GAACTTCTAA   
  
  
- TACTAAAATC ACTACAACAA GAATTCATGT AGTTAGTTCA AGAATACCTG CTCCTACTCC CTCTCTTTTA   
  
  
- GGTAAACCCG GTGCCCGGAG ACCGAGAACT TCGCCGGCTC TTCAGCAATA TACTCCACGA ACCCGTCTCG   
  
  
- GTAAGAGGTG TTTTAGTTAT GAGAGGTCTC CTGTTCGCAC TACTGGACTC GTTCTCATCA CAATCTTCGA   
  
  
- CACAGTCAAT ATCGACACTG CCACGTTCAT CACCGTCACC GTAGCACCTC GTTTCAACCA GCTCAATAGG   
  
  
- AGGCCTCATG TTAGGGTACG CCTACCGTCT AAAAGGACGG AACGGGAGTT TTGGGAACAA CTCTTGTAGA   
  
  
- ATATCGAATA GTTTATCGAA CTGTCCACTC CCATTACCAA ACCACCTATC TGGCCGTCTG AGTGGACATT   
  
  
- CGCGAGAATC ACACAGACTA TATAAATTAC TATCAGTCAG TCACTATGTT AAAGTCTTTC CCTACCTCCT   
  
  
- TCGCTCGTTT AAAGAAGGGT TCCGAAGAAA CACAAACCTA AACTTGTGAC CTGGGACGGA AGCCCTCAGC   
  
  
- CAGTTATTCC GTGCTCCACC CGACTAAAAC CACCTCTGAT CGGTACTTTT ACTCCAACGT CACGATCGAT   
  
  
- CATCCCCTTT CTTCGTAGCG GTAGGACTCC TTAATGCAAG TCTTCAACCC TCCCCGTCAT TCGTCGGTCA   
  
  
- ACAAAAGGTC AAAAGTCTGC TCCGTCAACA CTCTAGTCTT TACAAACTAT CCCATAACGA CACGTCACCA   
  
  
- CCGTTTTTAC TACGACTGGA TGCGGTTTGA AATGTCTCAC TTGATTTATT TTGATACGTT TTACGAGTTT   
  
  
- ACTTTCTAAG ATTACCACCT TTTCCGGCAC CATCCTTCGT TCAACCGTCC TCCCTACACC ACCTAGATTC   
  
  
- TAGAGAAAAT GCGGAAACAC GTGTTCGTCA ACCTAGGTTA CTGGTTTCTT CGCGTTTACT GAATTAATCC   
  
  
- GTTTAATCCT TCGTAAGAAG AGGTTGGCCC CTACCCTTAG TTTCTTACCG AGTAATAAAA CGACTACCAG   
  
  
- AACTCCGAGC CGAACGACCA CAACCCTGGG GTTATATGTT CAGAGAGCAC TGACCGGGTC GAAGACGACA   
  
  
- ACTATAGAAC TCTCGAATGG TATACAAAGA ACGGTGTACG GGTAAGTTCT TTTATCCCTT AAAGAAGAGA   
  
  
- TTATCTTGTT AATACCTACA TCGAGTCTTT CGTTTAGCTG AAGTATAATA ACTAGAACCA TAACAGATAC   
  
  
- CAAAGGTTAC CGGAACAGAT TAAGTCGCGG AAAGTAGATC CGGACCGCCC GGAGGGTTTG AAGCCTAATG   
  
  
- TCCGCAACTA GAGGGGGTTG GGCCAAAGGC TGGTCGGTTT TCTCAGCTCC TCTGACCCGC TGCGAACTCC   
  
  
- TTGATACGAC TCAGTAAGTT ACACGGTAAA CTCAAGTTAC GGTATCGTTT CTCGACCCTT TGTGAATGAC   
  
  
- AATTTCTAGA GTTCTAACTA GGGCTACTCC ACGAACAACA CTTGACAGAC AAGTCCAAAT TTATGTAAGG   
  
  
- ACTCCTTTGT CATTAACGTC TTACGGGTTC TCTATAACAA GAATTAGACT AGTCTGTCTA TTTTGGTCGA   
  
  
- CAAAAGTATG TACCACATCA GTTACCTCGG AAGTTAAGAG GAAAAAAGTA ATAAGCCAAG GCACTCCGAG   
  
  
- AGAAGGTGAA GAGGTGAGAT AAACTACACA ACCTCCGTTT AGAAGGGGCC CTGTAGCTCC TCTCCTATGA   
  
  
- CTATCTCGCT CTGTAGAAAC CCGCTGTCCG TTACTTACAC TAGCGAACAC TCCCAAACCT CTCCTATCTT   
  
  
- TCCGGTCTCT TTATGTTTGT CACCGCCCAG GCTTTACTCT CCCGACCCAA CTTCGTCGAC GGAAACTTAG   
  
  
- TCCTCTGACA CCTTTACCGG TTTTTTGCCC ACTGTCGACA GATAGTGTTT CTAAAGAGGT AACTACTGCT   
  
  
- ACCCGTGACC AACAATGTCC CTACCTTTCC CTCTTAACAG ATACGTGAGT GATGAACCTT CGGACGACTG   
  
  
- AT

+     Box 4

| Site Name | Organism | Position | Strand | Matrix score. | sequence | function |
| --- | --- | --- | --- | --- | --- | --- |
| Box 4 | Petroselinum crispum | 270 | + | 6 | ATTAAT | part of a conserved DNA module involved in light responsiveness |

>HU05G01597.1   
+ -Up\_Stream \_Len000TTTGTA TTTCAATATT GTAATTAGGT GGGATATAGA GTTGAAGGGC AAACTCTACC   
  
  
+ AACGACTTGT TGAAGGATCA ACTTGTAGCT TGGAACTTAA TGTATGTGTA CTATAGAGTT GTGTAATCTC   
  
  
+ TCCTAATAAA ATTGGGTTAA TCCTAGAATT AGAGAACTTT AAGCGGGGAC TAAGCTTGGT TGAGGCTAAC   
  
  
+ TTCGTTAAAA AGTTCTTGTG TCATGTCCAC GTTTATCTTT TCACTATTTA TTTCAATTAA TTTGCGATAT   
  
  
+ TTATCATCAC CCTATTCACC GTCTTTTCGG ATGATTTAAC TAGTCAAGCC GGACCAACAT TGCTTTTTAT   
  
  
+ AGTGTATTTG CAACTCATGT TGATTATTAG ATGTATCAAC AAAAAATGTT TCTCAAAATA AATAGTAGCT   
  
  
+ CTTTAAATTT TATTCCACAT AAATTCAAAA TCAATATCGA TATCGTTCCA TAATATAGCA GCATTATGAA   
  
  
+ AACAATATTT GCTAATGTTT ACATTGTGCT CTTTTCACTC AAAAATTGTA AGGCTTCAAA TACTATTAAA   
  
  
+ GAAACCATTA TTTCACTCGA CTATTTAGCT TGCATATATA GACAATCTTG CAAATAGTAA CATGTCTTCA   
  
  
+ TTCGACTATT TGCTTCCAAA TGAATAATCT CGTAAACATA TATACATACA TACATATATA TATATATATA   
  
  
+ TATATATATA TATATATATA GTAATATGTA TTTAACTTCA CATATACATG AATATAAAAA TATAAGAGGG   
  
  
+ AAAAAAGACG CTAGGTAGAT GGACTACAAA AGCCATTCAT GTATTGAGAC GGCTCATAAA ATCATACGGG   
  
  
+ GAAAGGAAAA AAAAAGATCT CTACATAATG TTCATAGATT TATAGAAAGC ATATGATGGA TTACCGCATA   
  
  
+ AGGTGTTATA TATGGGAGTG TTTGTAGAAG AAAGATGTAT ATCCGAACTA TCAAAGATAC ATATGAGGGA   
  
  
+ GTAAAAACAA GTGTAAGAAC CTCAGGAGGA GACATAAAAG ATTTTTCTAT TGGCATTGGA CACCGTCAAG   
  
  
+ TATCCGCTTT AAACCTGTTC TTTTTCACTA TTATCATGGA TGTATTCACA AAAGGAATTC AATACGAAGT   
  
  
+ ACCATGATGT ATGCTTTTTA CTGATAGTAT TGGTCTCATC GATGAGACTA AGGATGGTCT TAATAGCAAA   
  
  
+ TTAGAGATTT CATTAGATAA AATCTACTGT ACATGACAAA ACAATGATAT ATAGCATCCT ATTTAAAAAG   
  
  
+ CTACGTGTCA GTCATATATG AATTGCATCT AATTTTTAAT AGATATATAA TATGATATGA TATAATAGGA   
  
  
+ TATGATGTAG ATTGAATTCA AACTCATAAT TCATTAAGCA TGGTGCAATG TTCATTTTCT TGATTTTAAA   
  
  
+ CCGACTATTA CTTCAACTGT ATATAATTCG AATTGCTTTG GTATTTTAAA ATTTAAACTA GTAATTACTG   
  
  
+ CCTCTATTAC TGATGTTTAT ATTAAGTGGT GCTTAACTGC TTATTGATGA AACCATTTTT ATTTTCGTGT   
  
  
+ AAAAGAGGGC ATAATCCATC CTCGTGTATT GGCGGGGCTC GGCGCCCTAT TCAGCTATTC TCTGTATGTC   
  
  
+ AAACAAAACC GCTTCTATTA CCCATTGGTC TCTAAACACG CAGGGGTTTT AGCCCGGAGA GTTAATTCTT   
  
  
+ TATTGAGGTC CCCTGTTCTC CATTGCAGGA TTTTTGCTCA GGTACTTCTC TGTTTCTTCT CTTTAATCTT   
  
  
+ CTGTATCTTT CTGCATCAAG TTTAATGCTT TCTGGGATTT TCAGCTACGA TTTATGTGAA ATTGTAAGAT   
  
  
+ TTCTGAACTG GGCATTCAGG GCTTGGTTCA TATGTATTCT TTTCTGCTTG AAATGTTTTA ATTTCATAGT   
  
  
+ TCCAATGGGG TTATTCAAAG ATAGAATTAA GCTATAATCA CCGAGAAGAC TTGCTGACCT TTGTTTCTTT   
  
  
+ TTTGTTTCTT TTTTTTAATT AAATCTGGCT ATTACTTGGC TATAGTTTTT GCTAATGGAT CCACAAATAG   
  
  
+ ACGAATTATA TGGGCCTTTG CACCAAATGA AGTTCAATGA TCAGAGGAGA CCCATTTTAT CAAATGAAAA   
  
  
+ TTTTGTTAAT GTGTTGAAAC CGCCAAATTC CAATTGGAAT TCACCTTTTG GGAACCCTAC TGTGATTACC   
  
  
+ ACAAACCCAA ATTTTGATTT GGTTGTGCCA TCACTAGGTC CCAATGTTGA GGGAGCTCTC CTTGAAGATT   
  
  
+ ATGATTTTAG TGATGTTGTT CTTAAGTACA TCAATCAAGT TCTTATGGAC GAGGATGAGG GAGAGAAAAT   
  
  
+ CCATTTGGGC CACGGGCCTC TGGCTCTTGA AGCGGCCGAG AAGTCGTTAT ATGAGGTGCT TGGGCAGAGC   
  
  
+ CATTCTCCAC AAAATCAATA CTCTCCAGAG GACAAGCGTG ATGACCTGAG CAAGAGTAGT GTTAGAAGCT   
  
  
+ GTGTCAGTTA TAGCTGTGAC GGTGCAAGTA GTGGCAGTGG CATCGTGGAG CAAAGTTGGT CGAGTTATCC   
  
  
+ TCCGGAGTAC AATCCCATGC GGATGGCAGA TTTTCCTGCC TTGCCCTCAA AACCCTTGTT GAGAACATCT   
  
  
+ TATAGCTTAT CAAATAGCTT GACAGGTGAG GGTAATGGTT TGGTGGATAG ACCGGCAGAC TCACCTGTAA   
  
  
+ GCGCTCTTAG TGTGTCTGAT ATATTTAATG ATAGTCAGTC AGTGATACAA TTTCAGAAAG GGATGGAGGA   
  
  
+ AGCGAGCAAA TTTCTTCCCA AGGCTTCTTT GTGTTTGGAT TTGAACACTG GACCCTGCCT TCGGGAGTCG   
  
  
+ GTCAATAAGG CACGAGGTGG GCTGATTTTG GTGGAGACTA GCCATGAAAA TGAGGTTGCA GTGCTAGCTA   
  
  
+ GTAGGGGAAA GAAGCATCGC CATCCTGAGG AATTACGTTC AGAAGTTGGG AGGGGCAGTA AGCAGCCAGT   
  
  
+ TGTTTTCCAG TTTTCAGACG AGGCAGTTGT GAGATCAGAA ATGTTTGATA GGGTATTGCT GTGCAGTGGT   
  
  
+ GGCAAAAATG ATGCTGACCT ACGCCAAACT TTACAGAGTG AACTAAATAA AACTATGCAA AATGCTCAAA   
  
  
+ TGAAAGATTC TAATGGTGGA AAAGGCCGTG GTAGGAAGCA AGTTGGCAGG AGGGATGTGG TGGATCTAAG   
  
  
+ ATCTCTTTTA CGCCTTTGTG CACAAGCAGT TGGATCCAAT GACCAAAGAA GCGCAAATGA CTTAATTAGG   
  
  
+ CAAATTAGGA AGCATTCTTC TCCAACCGGG GATGGGAATC AAAGAATGGC TCATTATTTT GCTGATGGTC   
  
  
+ TTGAGGCTCG GCTTGCTGGT GTTGGGACCC CAATATACAA GTCTCTCGTG ACTGGCCCAG CTTCTGCTGT   
  
  
+ TGATATCTTG AGAGCTTACC ATATGTTTCT TGCCACATGC CCATTCAAGA AAATAGGGAA TTTCTTCTCT   
  
  
+ AATAGAACAA TTATGGATGT AGCTCAGAAA GCAAATCGAC TTCATATTAT TGATCTTGGT ATTGTCTATG   
  
  
+ GTTTCCAATG GCCTTGTCTA ATTCAGCGCC TTTCATCTAG GCCTGGCGGG CCTCCCAAAC TTCGGATTAC   
  
  
+ AGGCGTTGAT CTCCCCCAAC CCGGTTTCCG ACCAGCCAAA AGAGTCGAGG AGACTGGGCG ACGCTTGAGG   
  
  
+ AACTATGCTG AGTCATTCAA TGTGCCATTT GAGTTCAATG CCATAGCAAA GAGCTGGGAA ACACTTACTG   
  
  
+ TTAAAGATCT CAAGATTGAT CCCGATGAGG TGCTTGTTGT GAACTGTCTG TTCAGGTTTA AATACATTCC   
  
  
+ TGAGGAAACA GTAATTGCAG AATGCCCAAG AGATATTGTT CTTAATCTGA TCAGACAGAT AAAACCAGCT   
  
  
+ GTTTTCATAC ATGGTGTAGT CAATGGAGCC TTCAATTCTC CTTTTTTCAT TATTCGGTTC CGTGAGGCTC   
  
  
+ TCTTCCACTT CTCCACTCTA TTTGATGTGT TGGAGGCAAA TCTTCCCCGG GACATCGAGG AGAGGATACT   
  
  
+ GATAGAGCGA GACATCTTTG GGCGACAGGC AATGAATGTG ATCGCTTGTG AGGGTTTGGA GAGGATAGAA   
  
  
+ AGGCCAGAGA AATACAAACA GTGGCGGGTC CGAAATGAGA GGGCTGGGTT GAAGCAGCTG CCTTTGAATC   
  
  
+ AGGAGACTGT GGAAATGGCC AAAAAACGGG TGACAGCTGT CTATCACAAA GATTTCTCCA TTGATGACGA   
  
  
+ TGGGCACTGG TTGTTACAGG GATGGAAAGG GAGAATTGTC TATGCACTCA CTACTTGGAA GCCTGCTGAC   
  
  
+ TA  

- -Up\_Stream \_Len000AAACAT AAAGTTATAA CATTAATCCA CCCTATATCT CAACTTCCCG TTTGAGATGG   
  
  
- TTGCTGAACA ACTTCCTAGT TGAACATCGA ACCTTGAATT ACATACACAT GATATCTCAA CACATTAGAG   
  
  
- AGGATTATTT TAACCCAATT AGGATCTTAA TCTCTTGAAA TTCGCCCCTG ATTCGAACCA ACTCCGATTG   
  
  
- AAGCAATTTT TCAAGAACAC AGTACAGGTG CAAATAGAAA AGTGATAAAT AAAGTTAATT AAACGCTATA   
  
  
- AATAGTAGTG GGATAAGTGG CAGAAAAGCC TACTAAATTG ATCAGTTCGG CCTGGTTGTA ACGAAAAATA   
  
  
- TCACATAAAC GTTGAGTACA ACTAATAATC TACATAGTTG TTTTTTACAA AGAGTTTTAT TTATCATCGA   
  
  
- GAAATTTAAA ATAAGGTGTA TTTAAGTTTT AGTTATAGCT ATAGCAAGGT ATTATATCGT CGTAATACTT   
  
  
- TTGTTATAAA CGATTACAAA TGTAACACGA GAAAAGTGAG TTTTTAACAT TCCGAAGTTT ATGATAATTT   
  
  
- CTTTGGTAAT AAAGTGAGCT GATAAATCGA ACGTATATAT CTGTTAGAAC GTTTATCATT GTACAGAAGT   
  
  
- AAGCTGATAA ACGAAGGTTT ACTTATTAGA GCATTTGTAT ATATGTATGT ATGTATATAT ATATATATAT   
  
  
- ATATATATAT ATATATATAT CATTATACAT AAATTGAAGT GTATATGTAC TTATATTTTT ATATTCTCCC   
  
  
- TTTTTTCTGC GATCCATCTA CCTGATGTTT TCGGTAAGTA CATAACTCTG CCGAGTATTT TAGTATGCCC   
  
  
- CTTTCCTTTT TTTTTCTAGA GATGTATTAC AAGTATCTAA ATATCTTTCG TATACTACCT AATGGCGTAT   
  
  
- TCCACAATAT ATACCCTCAC AAACATCTTC TTTCTACATA TAGGCTTGAT AGTTTCTATG TATACTCCCT   
  
  
- CATTTTTGTT CACATTCTTG GAGTCCTCCT CTGTATTTTC TAAAAAGATA ACCGTAACCT GTGGCAGTTC   
  
  
- ATAGGCGAAA TTTGGACAAG AAAAAGTGAT AATAGTACCT ACATAAGTGT TTTCCTTAAG TTATGCTTCA   
  
  
- TGGTACTACA TACGAAAAAT GACTATCATA ACCAGAGTAG CTACTCTGAT TCCTACCAGA ATTATCGTTT   
  
  
- AATCTCTAAA GTAATCTATT TTAGATGACA TGTACTGTTT TGTTACTATA TATCGTAGGA TAAATTTTTC   
  
  
- GATGCACAGT CAGTATATAC TTAACGTAGA TTAAAAATTA TCTATATATT ATACTATACT ATATTATCCT   
  
  
- ATACTACATC TAACTTAAGT TTGAGTATTA AGTAATTCGT ACCACGTTAC AAGTAAAAGA ACTAAAATTT   
  
  
- GGCTGATAAT GAAGTTGACA TATATTAAGC TTAACGAAAC CATAAAATTT TAAATTTGAT CATTAATGAC   
  
  
- GGAGATAATG ACTACAAATA TAATTCACCA CGAATTGACG AATAACTACT TTGGTAAAAA TAAAAGCACA   
  
  
- TTTTCTCCCG TATTAGGTAG GAGCACATAA CCGCCCCGAG CCGCGGGATA AGTCGATAAG AGACATACAG   
  
  
- TTTGTTTTGG CGAAGATAAT GGGTAACCAG AGATTTGTGC GTCCCCAAAA TCGGGCCTCT CAATTAAGAA   
  
  
- ATAACTCCAG GGGACAAGAG GTAACGTCCT AAAAACGAGT CCATGAAGAG ACAAAGAAGA GAAATTAGAA   
  
  
- GACATAGAAA GACGTAGTTC AAATTACGAA AGACCCTAAA AGTCGATGCT AAATACACTT TAACATTCTA   
  
  
- AAGACTTGAC CCGTAAGTCC CGAACCAAGT ATACATAAGA AAAGACGAAC TTTACAAAAT TAAAGTATCA   
  
  
- AGGTTACCCC AATAAGTTTC TATCTTAATT CGATATTAGT GGCTCTTCTG AACGACTGGA AACAAAGAAA   
  
  
- AAACAAAGAA AAAAAATTAA TTTAGACCGA TAATGAACCG ATATCAAAAA CGATTACCTA GGTGTTTATC   
  
  
- TGCTTAATAT ACCCGGAAAC GTGGTTTACT TCAAGTTACT AGTCTCCTCT GGGTAAAATA GTTTACTTTT   
  
  
- AAAACAATTA CACAACTTTG GCGGTTTAAG GTTAACCTTA AGTGGAAAAC CCTTGGGATG ACACTAATGG   
  
  
- TGTTTGGGTT TAAAACTAAA CCAACACGGT AGTGATCCAG GGTTACAACT CCCTCGAGAG GAACTTCTAA   
  
  
- TACTAAAATC ACTACAACAA GAATTCATGT AGTTAGTTCA AGAATACCTG CTCCTACTCC CTCTCTTTTA   
  
  
- GGTAAACCCG GTGCCCGGAG ACCGAGAACT TCGCCGGCTC TTCAGCAATA TACTCCACGA ACCCGTCTCG   
  
  
- GTAAGAGGTG TTTTAGTTAT GAGAGGTCTC CTGTTCGCAC TACTGGACTC GTTCTCATCA CAATCTTCGA   
  
  
- CACAGTCAAT ATCGACACTG CCACGTTCAT CACCGTCACC GTAGCACCTC GTTTCAACCA GCTCAATAGG   
  
  
- AGGCCTCATG TTAGGGTACG CCTACCGTCT AAAAGGACGG AACGGGAGTT TTGGGAACAA CTCTTGTAGA   
  
  
- ATATCGAATA GTTTATCGAA CTGTCCACTC CCATTACCAA ACCACCTATC TGGCCGTCTG AGTGGACATT   
  
  
- CGCGAGAATC ACACAGACTA TATAAATTAC TATCAGTCAG TCACTATGTT AAAGTCTTTC CCTACCTCCT   
  
  
- TCGCTCGTTT AAAGAAGGGT TCCGAAGAAA CACAAACCTA AACTTGTGAC CTGGGACGGA AGCCCTCAGC   
  
  
- CAGTTATTCC GTGCTCCACC CGACTAAAAC CACCTCTGAT CGGTACTTTT ACTCCAACGT CACGATCGAT   
  
  
- CATCCCCTTT CTTCGTAGCG GTAGGACTCC TTAATGCAAG TCTTCAACCC TCCCCGTCAT TCGTCGGTCA   
  
  
- ACAAAAGGTC AAAAGTCTGC TCCGTCAACA CTCTAGTCTT TACAAACTAT CCCATAACGA CACGTCACCA   
  
  
- CCGTTTTTAC TACGACTGGA TGCGGTTTGA AATGTCTCAC TTGATTTATT TTGATACGTT TTACGAGTTT   
  
  
- ACTTTCTAAG ATTACCACCT TTTCCGGCAC CATCCTTCGT TCAACCGTCC TCCCTACACC ACCTAGATTC   
  
  
- TAGAGAAAAT GCGGAAACAC GTGTTCGTCA ACCTAGGTTA CTGGTTTCTT CGCGTTTACT GAATTAATCC   
  
  
- GTTTAATCCT TCGTAAGAAG AGGTTGGCCC CTACCCTTAG TTTCTTACCG AGTAATAAAA CGACTACCAG   
  
  
- AACTCCGAGC CGAACGACCA CAACCCTGGG GTTATATGTT CAGAGAGCAC TGACCGGGTC GAAGACGACA   
  
  
- ACTATAGAAC TCTCGAATGG TATACAAAGA ACGGTGTACG GGTAAGTTCT TTTATCCCTT AAAGAAGAGA   
  
  
- TTATCTTGTT AATACCTACA TCGAGTCTTT CGTTTAGCTG AAGTATAATA ACTAGAACCA TAACAGATAC   
  
  
- CAAAGGTTAC CGGAACAGAT TAAGTCGCGG AAAGTAGATC CGGACCGCCC GGAGGGTTTG AAGCCTAATG   
  
  
- TCCGCAACTA GAGGGGGTTG GGCCAAAGGC TGGTCGGTTT TCTCAGCTCC TCTGACCCGC TGCGAACTCC   
  
  
- TTGATACGAC TCAGTAAGTT ACACGGTAAA CTCAAGTTAC GGTATCGTTT CTCGACCCTT TGTGAATGAC   
  
  
- AATTTCTAGA GTTCTAACTA GGGCTACTCC ACGAACAACA CTTGACAGAC AAGTCCAAAT TTATGTAAGG   
  
  
- ACTCCTTTGT CATTAACGTC TTACGGGTTC TCTATAACAA GAATTAGACT AGTCTGTCTA TTTTGGTCGA   
  
  
- CAAAAGTATG TACCACATCA GTTACCTCGG AAGTTAAGAG GAAAAAAGTA ATAAGCCAAG GCACTCCGAG   
  
  
- AGAAGGTGAA GAGGTGAGAT AAACTACACA ACCTCCGTTT AGAAGGGGCC CTGTAGCTCC TCTCCTATGA   
  
  
- CTATCTCGCT CTGTAGAAAC CCGCTGTCCG TTACTTACAC TAGCGAACAC TCCCAAACCT CTCCTATCTT   
  
  
- TCCGGTCTCT TTATGTTTGT CACCGCCCAG GCTTTACTCT CCCGACCCAA CTTCGTCGAC GGAAACTTAG   
  
  
- TCCTCTGACA CCTTTACCGG TTTTTTGCCC ACTGTCGACA GATAGTGTTT CTAAAGAGGT AACTACTGCT   
  
  
- ACCCGTGACC AACAATGTCC CTACCTTTCC CTCTTAACAG ATACGTGAGT GATGAACCTT CGGACGACTG   
  
  
- AT

+     CAAT-box

| Site Name | Organism | Position | Strand | Matrix score. | sequence | function |
| --- | --- | --- | --- | --- | --- | --- |
| CAAT-box | Nicotiana glutinosa | 4194 | - | 4 | CAAT |  |
| CAAT-box | Nicotiana glutinosa | 3680 | + | 4 | CAAT |  |
| CAAT-box | Nicotiana glutinosa | 3819 | - | 4 | CAAT |  |
| CAAT-box | Nicotiana glutinosa | 3495 | - | 4 | CAAT |  |
| CAAT-box | Nicotiana glutinosa | 3325 | + | 4 | CAAT |  |
| CAAT-box | Nicotiana glutinosa | 3662 | + | 4 | CAAT |  |
| CAAT-box | Nicotiana glutinosa | 1436 | - | 4 | CAAT |  |
| CAAT-box | Nicotiana glutinosa | 817 | - | 4 | CAAT |  |
| CAAT-box | Nicotiana glutinosa | 1517 | - | 4 | CAAT |  |
| CAAT-box | Nicotiana glutinosa | 2216 | + | 4 | CAAT |  |
| CAAT-box | Pisum sativum | 651 | + | 5 | CAAAT | common cis-acting element in promoter and enhancer regions |
| CAAT-box | Pisum sativum | 642 | - | 5 | CAAAT | common cis-acting element in promoter and enhancer regions |
| CAAT-box | Nicotiana glutinosa | 607 | + | 4 | CAAT |  |
| CAAT-box | Nicotiana glutinosa | 28 | + | 4 | CAAT |  |
| CAAT-box | Pisum sativum | 3225 | + | 5 | CAAAT | common cis-acting element in promoter and enhancer regions |
| CAAT-box | Pisum sativum | 3208 | + | 5 | CAAAT | common cis-acting element in promoter and enhancer regions |
| CAAT-box | Arabidopsis thaliana | 3190 | + | 5 | CCAAT | common cis-acting element in promoter and enhancer regions |
| CAAT-box | Pisum sativum | 2095 | + | 5 | CAAAT | common cis-acting element in promoter and enhancer regions |
| CAAT-box | Nicotiana glutinosa | 4239 | - | 4 | CAAT |  |
| CAAT-box | Pisum sativum | 274 | - | 5 | CAAAT | common cis-acting element in promoter and enhancer regions |
| CAAT-box | Nicotiana glutinosa | 268 | + | 4 | CAAT |  |
| CAAT-box | Nicotiana glutinosa | 2276 | + | 4 | CAAT |  |
| CAAT-box | Nicotiana glutinosa | 2400 | + | 4 | CAAT |  |
| CAAT-box | Nicotiana glutinosa | 1236 | + | 4 | CAAT |  |
| CAAT-box | Nicotiana glutinosa | 1686 | - | 4 | CAAT |  |
| CAAT-box | Nicotiana glutinosa | 1286 | - | 4 | CAAT |  |
| CAAT-box | Nicotiana glutinosa | 3442 | + | 4 | CAAT |  |
| CAAT-box | Nicotiana glutinosa | 3875 | + | 4 | CAAT |  |
| CAAT-box | Pisum sativum | 3961 | + | 5 | CAAAT | common cis-acting element in promoter and enhancer regions |
| CAAT-box | Arabidopsis thaliana | 1039 | - | 5 | CCAAT | common cis-acting element in promoter and enhancer regions |
| CAAT-box | Nicotiana glutinosa | 1114 | + | 4 | CAAT |  |
| CAAT-box | Pisum sativum | 1191 | + | 5 | CAAAT | common cis-acting element in promoter and enhancer regions |
| CAAT-box | Nicotiana glutinosa | 1380 | + | 4 | CAAT |  |
| CAAT-box | Nicotiana glutinosa | 1345 | - | 4 | CAAT |  |
| CAAT-box | Pisum sativum | 551 | + | 5 | CAAAT | common cis-acting element in promoter and enhancer regions |
| CAAT-box | Pisum sativum | 501 | - | 5 | CAAAT | common cis-acting element in promoter and enhancer regions |
| CAAT-box | Pisum sativum | 360 | - | 5 | CAAAT | common cis-acting element in promoter and enhancer regions |
| CAAT-box | Arabidopsis thaliana | 2134 | + | 5 | CCAAT | common cis-acting element in promoter and enhancer regions |
| CAAT-box | Nicotiana glutinosa | 2135 | + | 4 | CAAT |  |
| CAAT-box | Nicotiana glutinosa | 2069 | + | 4 | CAAT |  |
| CAAT-box | Nicotiana glutinosa | 456 | + | 4 | CAAT |  |
| CAAT-box | Pisum sativum | 2128 | + | 5 | CAAAT | common cis-acting element in promoter and enhancer regions |
| CAAT-box | Nicotiana glutinosa | 1897 | + | 4 | CAAT |  |
| CAAT-box | Nicotiana glutinosa | 539 | - | 4 | CAAT |  |
| CAAT-box | Arabidopsis thaliana | 1153 | - | 5 | CCAAT | common cis-acting element in promoter and enhancer regions |
| CAAT-box | Arabidopsis thaliana | 153 | - | 8 | CCCAATTT | common cis-acting element in promoter and enhancer regions |
| CAAT-box | Pisum sativum | 2058 | + | 5 | CAAAT | common cis-acting element in promoter and enhancer regions |
| CAAT-box | Pisum sativum | 2028 | + | 5 | CAAAT | common cis-acting element in promoter and enhancer regions |
| CAAT-box | Arabidopsis thaliana | 1896 | + | 5 | CCAAT | common cis-acting element in promoter and enhancer regions |
| CAAT-box | Arabidopsis thaliana | 1572 | - | 5 | CCAAT | common cis-acting element in promoter and enhancer regions |
| CAAT-box | Nicotiana glutinosa | 1706 | - | 4 | CAAT |  |
| CAAT-box | Nicotiana glutinosa | 497 | + | 4 | CAAT |  |
| CAAT-box | Nicotiana glutinosa | 517 | - | 4 | CAAT |  |
| CAAT-box | Nicotiana glutinosa | 1815 | - | 4 | CAAT |  |
| CAAT-box | Pisum sativum | 615 | + | 5 | CAAAT | common cis-acting element in promoter and enhancer regions |
| CAAT-box | Arabidopsis thaliana | 1638 | - | 5 | CCAAT | common cis-acting element in promoter and enhancer regions |
| CAAT-box | Nicotiana glutinosa | 3191 | + | 4 | CAAT |  |
| CAAT-box | Nicotiana glutinosa | 4024 | + | 4 | CAAT |  |
| CAAT-box | Pisum sativum | 3944 | - | 5 | CAAAT | common cis-acting element in promoter and enhancer regions |
| CAAT-box | Nicotiana glutinosa | 32 | - | 4 | CAAT |  |
| CAAT-box | Arabidopsis thaliana | 1033 | - | 5 | CCAAT | common cis-acting element in promoter and enhancer regions |
| CAAT-box | Arabidopsis thaliana | 2137 | - | 5 | CCAAT | common cis-acting element in promoter and enhancer regions |
| CAAT-box | Nicotiana glutinosa | 3887 | + | 4 | CAAT |  |
| CAAT-box | Pisum sativum | 2182 | + | 5 | CAAAT | common cis-acting element in promoter and enhancer regions |
| CAAT-box | Arabidopsis thaliana | 155 | - | 5 | CCAAT | common cis-acting element in promoter and enhancer regions |
| CAAT-box | Nicotiana glutinosa | 343 | - | 4 | CAAT |  |
| CAAT-box | Nicotiana glutinosa | 2712 | + | 4 | CAAT |  |
| CAAT-box | Nicotiana glutinosa | 3483 | - | 4 | CAAT |  |
| CAAT-box | Nicotiana glutinosa | 2999 | - | 4 | CAAT |  |
| CAAT-box | Pisum sativum | 3466 | + | 5 | CAAAT | common cis-acting element in promoter and enhancer regions |
| CAAT-box | Pisum sativum | 3671 | - | 5 | CAAAT | common cis-acting element in promoter and enhancer regions |
| CAAT-box | Nicotiana glutinosa | 3510 | + | 4 | CAAT |  |
| CAAT-box | Nicotiana glutinosa | 3798 | - | 4 | CAAT |  |
| CAAT-box | Nicotiana glutinosa | 3729 | - | 4 | CAAT |  |
| CAAT-box | Pisum sativum | 2191 | - | 5 | CAAAT | common cis-acting element in promoter and enhancer regions |
| CAAT-box | Petunia hybrida | 3126 | - | 7 | TGCCAAC | common cis-acting element in promoter and enhancer regions |
| CAAT-box | Arabidopsis thaliana | 3509 | + | 5 | CCAAT | common cis-acting element in promoter and enhancer regions |
| CAAT-box | Arabidopsis thaliana | 2215 | + | 5 | CCAAT | common cis-acting element in promoter and enhancer regions |
| CAAT-box | Arabidopsis thaliana | 3324 | + | 5 | CCAAT | common cis-acting element in promoter and enhancer regions |
| CAAT-box | Pisum sativum | 3081 | + | 5 | CAAAT | common cis-acting element in promoter and enhancer regions |
| CAAT-box | Nicotiana glutinosa | 2534 | + | 4 | CAAT |  |
| CAAT-box | Pisum sativum | 2741 | + | 5 | CAAAT | common cis-acting element in promoter and enhancer regions |
| CAAT-box | Pisum sativum | 2317 | - | 5 | CAAAT | common cis-acting element in promoter and enhancer regions |
| CAAT-box | Pisum sativum | 2605 | + | 5 | CAAAT | common cis-acting element in promoter and enhancer regions |
| CAAT-box | Pisum sativum | 2773 | - | 5 | CAAAT | common cis-acting element in promoter and enhancer regions |
| CAAT-box | Nicotiana glutinosa | 2807 | + | 4 | CAAT |  |

>HU05G01597.1   
+ -Up\_Stream \_Len000TTTGTA TTTCAATATT GTAATTAGGT GGGATATAGA GTTGAAGGGC AAACTCTACC   
  
  
+ AACGACTTGT TGAAGGATCA ACTTGTAGCT TGGAACTTAA TGTATGTGTA CTATAGAGTT GTGTAATCTC   
  
  
+ TCCTAATAAA ATTGGGTTAA TCCTAGAATT AGAGAACTTT AAGCGGGGAC TAAGCTTGGT TGAGGCTAAC   
  
  
+ TTCGTTAAAA AGTTCTTGTG TCATGTCCAC GTTTATCTTT TCACTATTTA TTTCAATTAA TTTGCGATAT   
  
  
+ TTATCATCAC CCTATTCACC GTCTTTTCGG ATGATTTAAC TAGTCAAGCC GGACCAACAT TGCTTTTTAT   
  
  
+ AGTGTATTTG CAACTCATGT TGATTATTAG ATGTATCAAC AAAAAATGTT TCTCAAAATA AATAGTAGCT   
  
  
+ CTTTAAATTT TATTCCACAT AAATTCAAAA TCAATATCGA TATCGTTCCA TAATATAGCA GCATTATGAA   
  
  
+ AACAATATTT GCTAATGTTT ACATTGTGCT CTTTTCACTC AAAAATTGTA AGGCTTCAAA TACTATTAAA   
  
  
+ GAAACCATTA TTTCACTCGA CTATTTAGCT TGCATATATA GACAATCTTG CAAATAGTAA CATGTCTTCA   
  
  
+ TTCGACTATT TGCTTCCAAA TGAATAATCT CGTAAACATA TATACATACA TACATATATA TATATATATA   
  
  
+ TATATATATA TATATATATA GTAATATGTA TTTAACTTCA CATATACATG AATATAAAAA TATAAGAGGG   
  
  
+ AAAAAAGACG CTAGGTAGAT GGACTACAAA AGCCATTCAT GTATTGAGAC GGCTCATAAA ATCATACGGG   
  
  
+ GAAAGGAAAA AAAAAGATCT CTACATAATG TTCATAGATT TATAGAAAGC ATATGATGGA TTACCGCATA   
  
  
+ AGGTGTTATA TATGGGAGTG TTTGTAGAAG AAAGATGTAT ATCCGAACTA TCAAAGATAC ATATGAGGGA   
  
  
+ GTAAAAACAA GTGTAAGAAC CTCAGGAGGA GACATAAAAG ATTTTTCTAT TGGCATTGGA CACCGTCAAG   
  
  
+ TATCCGCTTT AAACCTGTTC TTTTTCACTA TTATCATGGA TGTATTCACA AAAGGAATTC AATACGAAGT   
  
  
+ ACCATGATGT ATGCTTTTTA CTGATAGTAT TGGTCTCATC GATGAGACTA AGGATGGTCT TAATAGCAAA   
  
  
+ TTAGAGATTT CATTAGATAA AATCTACTGT ACATGACAAA ACAATGATAT ATAGCATCCT ATTTAAAAAG   
  
  
+ CTACGTGTCA GTCATATATG AATTGCATCT AATTTTTAAT AGATATATAA TATGATATGA TATAATAGGA   
  
  
+ TATGATGTAG ATTGAATTCA AACTCATAAT TCATTAAGCA TGGTGCAATG TTCATTTTCT TGATTTTAAA   
  
  
+ CCGACTATTA CTTCAACTGT ATATAATTCG AATTGCTTTG GTATTTTAAA ATTTAAACTA GTAATTACTG   
  
  
+ CCTCTATTAC TGATGTTTAT ATTAAGTGGT GCTTAACTGC TTATTGATGA AACCATTTTT ATTTTCGTGT   
  
  
+ AAAAGAGGGC ATAATCCATC CTCGTGTATT GGCGGGGCTC GGCGCCCTAT TCAGCTATTC TCTGTATGTC   
  
  
+ AAACAAAACC GCTTCTATTA CCCATTGGTC TCTAAACACG CAGGGGTTTT AGCCCGGAGA GTTAATTCTT   
  
  
+ TATTGAGGTC CCCTGTTCTC CATTGCAGGA TTTTTGCTCA GGTACTTCTC TGTTTCTTCT CTTTAATCTT   
  
  
+ CTGTATCTTT CTGCATCAAG TTTAATGCTT TCTGGGATTT TCAGCTACGA TTTATGTGAA ATTGTAAGAT   
  
  
+ TTCTGAACTG GGCATTCAGG GCTTGGTTCA TATGTATTCT TTTCTGCTTG AAATGTTTTA ATTTCATAGT   
  
  
+ TCCAATGGGG TTATTCAAAG ATAGAATTAA GCTATAATCA CCGAGAAGAC TTGCTGACCT TTGTTTCTTT   
  
  
+ TTTGTTTCTT TTTTTTAATT AAATCTGGCT ATTACTTGGC TATAGTTTTT GCTAATGGAT CCACAAATAG   
  
  
+ ACGAATTATA TGGGCCTTTG CACCAAATGA AGTTCAATGA TCAGAGGAGA CCCATTTTAT CAAATGAAAA   
  
  
+ TTTTGTTAAT GTGTTGAAAC CGCCAAATTC CAATTGGAAT TCACCTTTTG GGAACCCTAC TGTGATTACC   
  
  
+ ACAAACCCAA ATTTTGATTT GGTTGTGCCA TCACTAGGTC CCAATGTTGA GGGAGCTCTC CTTGAAGATT   
  
  
+ ATGATTTTAG TGATGTTGTT CTTAAGTACA TCAATCAAGT TCTTATGGAC GAGGATGAGG GAGAGAAAAT   
  
  
+ CCATTTGGGC CACGGGCCTC TGGCTCTTGA AGCGGCCGAG AAGTCGTTAT ATGAGGTGCT TGGGCAGAGC   
  
  
+ CATTCTCCAC AAAATCAATA CTCTCCAGAG GACAAGCGTG ATGACCTGAG CAAGAGTAGT GTTAGAAGCT   
  
  
+ GTGTCAGTTA TAGCTGTGAC GGTGCAAGTA GTGGCAGTGG CATCGTGGAG CAAAGTTGGT CGAGTTATCC   
  
  
+ TCCGGAGTAC AATCCCATGC GGATGGCAGA TTTTCCTGCC TTGCCCTCAA AACCCTTGTT GAGAACATCT   
  
  
+ TATAGCTTAT CAAATAGCTT GACAGGTGAG GGTAATGGTT TGGTGGATAG ACCGGCAGAC TCACCTGTAA   
  
  
+ GCGCTCTTAG TGTGTCTGAT ATATTTAATG ATAGTCAGTC AGTGATACAA TTTCAGAAAG GGATGGAGGA   
  
  
+ AGCGAGCAAA TTTCTTCCCA AGGCTTCTTT GTGTTTGGAT TTGAACACTG GACCCTGCCT TCGGGAGTCG   
  
  
+ GTCAATAAGG CACGAGGTGG GCTGATTTTG GTGGAGACTA GCCATGAAAA TGAGGTTGCA GTGCTAGCTA   
  
  
+ GTAGGGGAAA GAAGCATCGC CATCCTGAGG AATTACGTTC AGAAGTTGGG AGGGGCAGTA AGCAGCCAGT   
  
  
+ TGTTTTCCAG TTTTCAGACG AGGCAGTTGT GAGATCAGAA ATGTTTGATA GGGTATTGCT GTGCAGTGGT   
  
  
+ GGCAAAAATG ATGCTGACCT ACGCCAAACT TTACAGAGTG AACTAAATAA AACTATGCAA AATGCTCAAA   
  
  
+ TGAAAGATTC TAATGGTGGA AAAGGCCGTG GTAGGAAGCA AGTTGGCAGG AGGGATGTGG TGGATCTAAG   
  
  
+ ATCTCTTTTA CGCCTTTGTG CACAAGCAGT TGGATCCAAT GACCAAAGAA GCGCAAATGA CTTAATTAGG   
  
  
+ CAAATTAGGA AGCATTCTTC TCCAACCGGG GATGGGAATC AAAGAATGGC TCATTATTTT GCTGATGGTC   
  
  
+ TTGAGGCTCG GCTTGCTGGT GTTGGGACCC CAATATACAA GTCTCTCGTG ACTGGCCCAG CTTCTGCTGT   
  
  
+ TGATATCTTG AGAGCTTACC ATATGTTTCT TGCCACATGC CCATTCAAGA AAATAGGGAA TTTCTTCTCT   
  
  
+ AATAGAACAA TTATGGATGT AGCTCAGAAA GCAAATCGAC TTCATATTAT TGATCTTGGT ATTGTCTATG   
  
  
+ GTTTCCAATG GCCTTGTCTA ATTCAGCGCC TTTCATCTAG GCCTGGCGGG CCTCCCAAAC TTCGGATTAC   
  
  
+ AGGCGTTGAT CTCCCCCAAC CCGGTTTCCG ACCAGCCAAA AGAGTCGAGG AGACTGGGCG ACGCTTGAGG   
  
  
+ AACTATGCTG AGTCATTCAA TGTGCCATTT GAGTTCAATG CCATAGCAAA GAGCTGGGAA ACACTTACTG   
  
  
+ TTAAAGATCT CAAGATTGAT CCCGATGAGG TGCTTGTTGT GAACTGTCTG TTCAGGTTTA AATACATTCC   
  
  
+ TGAGGAAACA GTAATTGCAG AATGCCCAAG AGATATTGTT CTTAATCTGA TCAGACAGAT AAAACCAGCT   
  
  
+ GTTTTCATAC ATGGTGTAGT CAATGGAGCC TTCAATTCTC CTTTTTTCAT TATTCGGTTC CGTGAGGCTC   
  
  
+ TCTTCCACTT CTCCACTCTA TTTGATGTGT TGGAGGCAAA TCTTCCCCGG GACATCGAGG AGAGGATACT   
  
  
+ GATAGAGCGA GACATCTTTG GGCGACAGGC AATGAATGTG ATCGCTTGTG AGGGTTTGGA GAGGATAGAA   
  
  
+ AGGCCAGAGA AATACAAACA GTGGCGGGTC CGAAATGAGA GGGCTGGGTT GAAGCAGCTG CCTTTGAATC   
  
  
+ AGGAGACTGT GGAAATGGCC AAAAAACGGG TGACAGCTGT CTATCACAAA GATTTCTCCA TTGATGACGA   
  
  
+ TGGGCACTGG TTGTTACAGG GATGGAAAGG GAGAATTGTC TATGCACTCA CTACTTGGAA GCCTGCTGAC   
  
  
+ TA  

- -Up\_Stream \_Len000AAACAT AAAGTTATAA CATTAATCCA CCCTATATCT CAACTTCCCG TTTGAGATGG   
  
  
- TTGCTGAACA ACTTCCTAGT TGAACATCGA ACCTTGAATT ACATACACAT GATATCTCAA CACATTAGAG   
  
  
- AGGATTATTT TAACCCAATT AGGATCTTAA TCTCTTGAAA TTCGCCCCTG ATTCGAACCA ACTCCGATTG   
  
  
- AAGCAATTTT TCAAGAACAC AGTACAGGTG CAAATAGAAA AGTGATAAAT AAAGTTAATT AAACGCTATA   
  
  
- AATAGTAGTG GGATAAGTGG CAGAAAAGCC TACTAAATTG ATCAGTTCGG CCTGGTTGTA ACGAAAAATA   
  
  
- TCACATAAAC GTTGAGTACA ACTAATAATC TACATAGTTG TTTTTTACAA AGAGTTTTAT TTATCATCGA   
  
  
- GAAATTTAAA ATAAGGTGTA TTTAAGTTTT AGTTATAGCT ATAGCAAGGT ATTATATCGT CGTAATACTT   
  
  
- TTGTTATAAA CGATTACAAA TGTAACACGA GAAAAGTGAG TTTTTAACAT TCCGAAGTTT ATGATAATTT   
  
  
- CTTTGGTAAT AAAGTGAGCT GATAAATCGA ACGTATATAT CTGTTAGAAC GTTTATCATT GTACAGAAGT   
  
  
- AAGCTGATAA ACGAAGGTTT ACTTATTAGA GCATTTGTAT ATATGTATGT ATGTATATAT ATATATATAT   
  
  
- ATATATATAT ATATATATAT CATTATACAT AAATTGAAGT GTATATGTAC TTATATTTTT ATATTCTCCC   
  
  
- TTTTTTCTGC GATCCATCTA CCTGATGTTT TCGGTAAGTA CATAACTCTG CCGAGTATTT TAGTATGCCC   
  
  
- CTTTCCTTTT TTTTTCTAGA GATGTATTAC AAGTATCTAA ATATCTTTCG TATACTACCT AATGGCGTAT   
  
  
- TCCACAATAT ATACCCTCAC AAACATCTTC TTTCTACATA TAGGCTTGAT AGTTTCTATG TATACTCCCT   
  
  
- CATTTTTGTT CACATTCTTG GAGTCCTCCT CTGTATTTTC TAAAAAGATA ACCGTAACCT GTGGCAGTTC   
  
  
- ATAGGCGAAA TTTGGACAAG AAAAAGTGAT AATAGTACCT ACATAAGTGT TTTCCTTAAG TTATGCTTCA   
  
  
- TGGTACTACA TACGAAAAAT GACTATCATA ACCAGAGTAG CTACTCTGAT TCCTACCAGA ATTATCGTTT   
  
  
- AATCTCTAAA GTAATCTATT TTAGATGACA TGTACTGTTT TGTTACTATA TATCGTAGGA TAAATTTTTC   
  
  
- GATGCACAGT CAGTATATAC TTAACGTAGA TTAAAAATTA TCTATATATT ATACTATACT ATATTATCCT   
  
  
- ATACTACATC TAACTTAAGT TTGAGTATTA AGTAATTCGT ACCACGTTAC AAGTAAAAGA ACTAAAATTT   
  
  
- GGCTGATAAT GAAGTTGACA TATATTAAGC TTAACGAAAC CATAAAATTT TAAATTTGAT CATTAATGAC   
  
  
- GGAGATAATG ACTACAAATA TAATTCACCA CGAATTGACG AATAACTACT TTGGTAAAAA TAAAAGCACA   
  
  
- TTTTCTCCCG TATTAGGTAG GAGCACATAA CCGCCCCGAG CCGCGGGATA AGTCGATAAG AGACATACAG   
  
  
- TTTGTTTTGG CGAAGATAAT GGGTAACCAG AGATTTGTGC GTCCCCAAAA TCGGGCCTCT CAATTAAGAA   
  
  
- ATAACTCCAG GGGACAAGAG GTAACGTCCT AAAAACGAGT CCATGAAGAG ACAAAGAAGA GAAATTAGAA   
  
  
- GACATAGAAA GACGTAGTTC AAATTACGAA AGACCCTAAA AGTCGATGCT AAATACACTT TAACATTCTA   
  
  
- AAGACTTGAC CCGTAAGTCC CGAACCAAGT ATACATAAGA AAAGACGAAC TTTACAAAAT TAAAGTATCA   
  
  
- AGGTTACCCC AATAAGTTTC TATCTTAATT CGATATTAGT GGCTCTTCTG AACGACTGGA AACAAAGAAA   
  
  
- AAACAAAGAA AAAAAATTAA TTTAGACCGA TAATGAACCG ATATCAAAAA CGATTACCTA GGTGTTTATC   
  
  
- TGCTTAATAT ACCCGGAAAC GTGGTTTACT TCAAGTTACT AGTCTCCTCT GGGTAAAATA GTTTACTTTT   
  
  
- AAAACAATTA CACAACTTTG GCGGTTTAAG GTTAACCTTA AGTGGAAAAC CCTTGGGATG ACACTAATGG   
  
  
- TGTTTGGGTT TAAAACTAAA CCAACACGGT AGTGATCCAG GGTTACAACT CCCTCGAGAG GAACTTCTAA   
  
  
- TACTAAAATC ACTACAACAA GAATTCATGT AGTTAGTTCA AGAATACCTG CTCCTACTCC CTCTCTTTTA   
  
  
- GGTAAACCCG GTGCCCGGAG ACCGAGAACT TCGCCGGCTC TTCAGCAATA TACTCCACGA ACCCGTCTCG   
  
  
- GTAAGAGGTG TTTTAGTTAT GAGAGGTCTC CTGTTCGCAC TACTGGACTC GTTCTCATCA CAATCTTCGA   
  
  
- CACAGTCAAT ATCGACACTG CCACGTTCAT CACCGTCACC GTAGCACCTC GTTTCAACCA GCTCAATAGG   
  
  
- AGGCCTCATG TTAGGGTACG CCTACCGTCT AAAAGGACGG AACGGGAGTT TTGGGAACAA CTCTTGTAGA   
  
  
- ATATCGAATA GTTTATCGAA CTGTCCACTC CCATTACCAA ACCACCTATC TGGCCGTCTG AGTGGACATT   
  
  
- CGCGAGAATC ACACAGACTA TATAAATTAC TATCAGTCAG TCACTATGTT AAAGTCTTTC CCTACCTCCT   
  
  
- TCGCTCGTTT AAAGAAGGGT TCCGAAGAAA CACAAACCTA AACTTGTGAC CTGGGACGGA AGCCCTCAGC   
  
  
- CAGTTATTCC GTGCTCCACC CGACTAAAAC CACCTCTGAT CGGTACTTTT ACTCCAACGT CACGATCGAT   
  
  
- CATCCCCTTT CTTCGTAGCG GTAGGACTCC TTAATGCAAG TCTTCAACCC TCCCCGTCAT TCGTCGGTCA   
  
  
- ACAAAAGGTC AAAAGTCTGC TCCGTCAACA CTCTAGTCTT TACAAACTAT CCCATAACGA CACGTCACCA   
  
  
- CCGTTTTTAC TACGACTGGA TGCGGTTTGA AATGTCTCAC TTGATTTATT TTGATACGTT TTACGAGTTT   
  
  
- ACTTTCTAAG ATTACCACCT TTTCCGGCAC CATCCTTCGT TCAACCGTCC TCCCTACACC ACCTAGATTC   
  
  
- TAGAGAAAAT GCGGAAACAC GTGTTCGTCA ACCTAGGTTA CTGGTTTCTT CGCGTTTACT GAATTAATCC   
  
  
- GTTTAATCCT TCGTAAGAAG AGGTTGGCCC CTACCCTTAG TTTCTTACCG AGTAATAAAA CGACTACCAG   
  
  
- AACTCCGAGC CGAACGACCA CAACCCTGGG GTTATATGTT CAGAGAGCAC TGACCGGGTC GAAGACGACA   
  
  
- ACTATAGAAC TCTCGAATGG TATACAAAGA ACGGTGTACG GGTAAGTTCT TTTATCCCTT AAAGAAGAGA   
  
  
- TTATCTTGTT AATACCTACA TCGAGTCTTT CGTTTAGCTG AAGTATAATA ACTAGAACCA TAACAGATAC   
  
  
- CAAAGGTTAC CGGAACAGAT TAAGTCGCGG AAAGTAGATC CGGACCGCCC GGAGGGTTTG AAGCCTAATG   
  
  
- TCCGCAACTA GAGGGGGTTG GGCCAAAGGC TGGTCGGTTT TCTCAGCTCC TCTGACCCGC TGCGAACTCC   
  
  
- TTGATACGAC TCAGTAAGTT ACACGGTAAA CTCAAGTTAC GGTATCGTTT CTCGACCCTT TGTGAATGAC   
  
  
- AATTTCTAGA GTTCTAACTA GGGCTACTCC ACGAACAACA CTTGACAGAC AAGTCCAAAT TTATGTAAGG   
  
  
- ACTCCTTTGT CATTAACGTC TTACGGGTTC TCTATAACAA GAATTAGACT AGTCTGTCTA TTTTGGTCGA   
  
  
- CAAAAGTATG TACCACATCA GTTACCTCGG AAGTTAAGAG GAAAAAAGTA ATAAGCCAAG GCACTCCGAG   
  
  
- AGAAGGTGAA GAGGTGAGAT AAACTACACA ACCTCCGTTT AGAAGGGGCC CTGTAGCTCC TCTCCTATGA   
  
  
- CTATCTCGCT CTGTAGAAAC CCGCTGTCCG TTACTTACAC TAGCGAACAC TCCCAAACCT CTCCTATCTT   
  
  
- TCCGGTCTCT TTATGTTTGT CACCGCCCAG GCTTTACTCT CCCGACCCAA CTTCGTCGAC GGAAACTTAG   
  
  
- TCCTCTGACA CCTTTACCGG TTTTTTGCCC ACTGTCGACA GATAGTGTTT CTAAAGAGGT AACTACTGCT   
  
  
- ACCCGTGACC AACAATGTCC CTACCTTTCC CTCTTAACAG ATACGTGAGT GATGAACCTT CGGACGACTG   
  
  
- AT

+     CAT-box

| Site Name | Organism | Position | Strand | Matrix score. | sequence | function |
| --- | --- | --- | --- | --- | --- | --- |
| CAT-box | Arabidopsis thaliana | 2490 | - | 6 | GCCACT | cis-acting regulatory element related to meristem expression |
| CAT-box | Arabidopsis thaliana | 4084 | - | 6 | GCCACT | cis-acting regulatory element related to meristem expression |
| CAT-box | Arabidopsis thaliana | 2484 | - | 6 | GCCACT | cis-acting regulatory element related to meristem expression |

>HU05G01597.1   
+ -Up\_Stream \_Len000TTTGTA TTTCAATATT GTAATTAGGT GGGATATAGA GTTGAAGGGC AAACTCTACC   
  
  
+ AACGACTTGT TGAAGGATCA ACTTGTAGCT TGGAACTTAA TGTATGTGTA CTATAGAGTT GTGTAATCTC   
  
  
+ TCCTAATAAA ATTGGGTTAA TCCTAGAATT AGAGAACTTT AAGCGGGGAC TAAGCTTGGT TGAGGCTAAC   
  
  
+ TTCGTTAAAA AGTTCTTGTG TCATGTCCAC GTTTATCTTT TCACTATTTA TTTCAATTAA TTTGCGATAT   
  
  
+ TTATCATCAC CCTATTCACC GTCTTTTCGG ATGATTTAAC TAGTCAAGCC GGACCAACAT TGCTTTTTAT   
  
  
+ AGTGTATTTG CAACTCATGT TGATTATTAG ATGTATCAAC AAAAAATGTT TCTCAAAATA AATAGTAGCT   
  
  
+ CTTTAAATTT TATTCCACAT AAATTCAAAA TCAATATCGA TATCGTTCCA TAATATAGCA GCATTATGAA   
  
  
+ AACAATATTT GCTAATGTTT ACATTGTGCT CTTTTCACTC AAAAATTGTA AGGCTTCAAA TACTATTAAA   
  
  
+ GAAACCATTA TTTCACTCGA CTATTTAGCT TGCATATATA GACAATCTTG CAAATAGTAA CATGTCTTCA   
  
  
+ TTCGACTATT TGCTTCCAAA TGAATAATCT CGTAAACATA TATACATACA TACATATATA TATATATATA   
  
  
+ TATATATATA TATATATATA GTAATATGTA TTTAACTTCA CATATACATG AATATAAAAA TATAAGAGGG   
  
  
+ AAAAAAGACG CTAGGTAGAT GGACTACAAA AGCCATTCAT GTATTGAGAC GGCTCATAAA ATCATACGGG   
  
  
+ GAAAGGAAAA AAAAAGATCT CTACATAATG TTCATAGATT TATAGAAAGC ATATGATGGA TTACCGCATA   
  
  
+ AGGTGTTATA TATGGGAGTG TTTGTAGAAG AAAGATGTAT ATCCGAACTA TCAAAGATAC ATATGAGGGA   
  
  
+ GTAAAAACAA GTGTAAGAAC CTCAGGAGGA GACATAAAAG ATTTTTCTAT TGGCATTGGA CACCGTCAAG   
  
  
+ TATCCGCTTT AAACCTGTTC TTTTTCACTA TTATCATGGA TGTATTCACA AAAGGAATTC AATACGAAGT   
  
  
+ ACCATGATGT ATGCTTTTTA CTGATAGTAT TGGTCTCATC GATGAGACTA AGGATGGTCT TAATAGCAAA   
  
  
+ TTAGAGATTT CATTAGATAA AATCTACTGT ACATGACAAA ACAATGATAT ATAGCATCCT ATTTAAAAAG   
  
  
+ CTACGTGTCA GTCATATATG AATTGCATCT AATTTTTAAT AGATATATAA TATGATATGA TATAATAGGA   
  
  
+ TATGATGTAG ATTGAATTCA AACTCATAAT TCATTAAGCA TGGTGCAATG TTCATTTTCT TGATTTTAAA   
  
  
+ CCGACTATTA CTTCAACTGT ATATAATTCG AATTGCTTTG GTATTTTAAA ATTTAAACTA GTAATTACTG   
  
  
+ CCTCTATTAC TGATGTTTAT ATTAAGTGGT GCTTAACTGC TTATTGATGA AACCATTTTT ATTTTCGTGT   
  
  
+ AAAAGAGGGC ATAATCCATC CTCGTGTATT GGCGGGGCTC GGCGCCCTAT TCAGCTATTC TCTGTATGTC   
  
  
+ AAACAAAACC GCTTCTATTA CCCATTGGTC TCTAAACACG CAGGGGTTTT AGCCCGGAGA GTTAATTCTT   
  
  
+ TATTGAGGTC CCCTGTTCTC CATTGCAGGA TTTTTGCTCA GGTACTTCTC TGTTTCTTCT CTTTAATCTT   
  
  
+ CTGTATCTTT CTGCATCAAG TTTAATGCTT TCTGGGATTT TCAGCTACGA TTTATGTGAA ATTGTAAGAT   
  
  
+ TTCTGAACTG GGCATTCAGG GCTTGGTTCA TATGTATTCT TTTCTGCTTG AAATGTTTTA ATTTCATAGT   
  
  
+ TCCAATGGGG TTATTCAAAG ATAGAATTAA GCTATAATCA CCGAGAAGAC TTGCTGACCT TTGTTTCTTT   
  
  
+ TTTGTTTCTT TTTTTTAATT AAATCTGGCT ATTACTTGGC TATAGTTTTT GCTAATGGAT CCACAAATAG   
  
  
+ ACGAATTATA TGGGCCTTTG CACCAAATGA AGTTCAATGA TCAGAGGAGA CCCATTTTAT CAAATGAAAA   
  
  
+ TTTTGTTAAT GTGTTGAAAC CGCCAAATTC CAATTGGAAT TCACCTTTTG GGAACCCTAC TGTGATTACC   
  
  
+ ACAAACCCAA ATTTTGATTT GGTTGTGCCA TCACTAGGTC CCAATGTTGA GGGAGCTCTC CTTGAAGATT   
  
  
+ ATGATTTTAG TGATGTTGTT CTTAAGTACA TCAATCAAGT TCTTATGGAC GAGGATGAGG GAGAGAAAAT   
  
  
+ CCATTTGGGC CACGGGCCTC TGGCTCTTGA AGCGGCCGAG AAGTCGTTAT ATGAGGTGCT TGGGCAGAGC   
  
  
+ CATTCTCCAC AAAATCAATA CTCTCCAGAG GACAAGCGTG ATGACCTGAG CAAGAGTAGT GTTAGAAGCT   
  
  
+ GTGTCAGTTA TAGCTGTGAC GGTGCAAGTA GTGGCAGTGG CATCGTGGAG CAAAGTTGGT CGAGTTATCC   
  
  
+ TCCGGAGTAC AATCCCATGC GGATGGCAGA TTTTCCTGCC TTGCCCTCAA AACCCTTGTT GAGAACATCT   
  
  
+ TATAGCTTAT CAAATAGCTT GACAGGTGAG GGTAATGGTT TGGTGGATAG ACCGGCAGAC TCACCTGTAA   
  
  
+ GCGCTCTTAG TGTGTCTGAT ATATTTAATG ATAGTCAGTC AGTGATACAA TTTCAGAAAG GGATGGAGGA   
  
  
+ AGCGAGCAAA TTTCTTCCCA AGGCTTCTTT GTGTTTGGAT TTGAACACTG GACCCTGCCT TCGGGAGTCG   
  
  
+ GTCAATAAGG CACGAGGTGG GCTGATTTTG GTGGAGACTA GCCATGAAAA TGAGGTTGCA GTGCTAGCTA   
  
  
+ GTAGGGGAAA GAAGCATCGC CATCCTGAGG AATTACGTTC AGAAGTTGGG AGGGGCAGTA AGCAGCCAGT   
  
  
+ TGTTTTCCAG TTTTCAGACG AGGCAGTTGT GAGATCAGAA ATGTTTGATA GGGTATTGCT GTGCAGTGGT   
  
  
+ GGCAAAAATG ATGCTGACCT ACGCCAAACT TTACAGAGTG AACTAAATAA AACTATGCAA AATGCTCAAA   
  
  
+ TGAAAGATTC TAATGGTGGA AAAGGCCGTG GTAGGAAGCA AGTTGGCAGG AGGGATGTGG TGGATCTAAG   
  
  
+ ATCTCTTTTA CGCCTTTGTG CACAAGCAGT TGGATCCAAT GACCAAAGAA GCGCAAATGA CTTAATTAGG   
  
  
+ CAAATTAGGA AGCATTCTTC TCCAACCGGG GATGGGAATC AAAGAATGGC TCATTATTTT GCTGATGGTC   
  
  
+ TTGAGGCTCG GCTTGCTGGT GTTGGGACCC CAATATACAA GTCTCTCGTG ACTGGCCCAG CTTCTGCTGT   
  
  
+ TGATATCTTG AGAGCTTACC ATATGTTTCT TGCCACATGC CCATTCAAGA AAATAGGGAA TTTCTTCTCT   
  
  
+ AATAGAACAA TTATGGATGT AGCTCAGAAA GCAAATCGAC TTCATATTAT TGATCTTGGT ATTGTCTATG   
  
  
+ GTTTCCAATG GCCTTGTCTA ATTCAGCGCC TTTCATCTAG GCCTGGCGGG CCTCCCAAAC TTCGGATTAC   
  
  
+ AGGCGTTGAT CTCCCCCAAC CCGGTTTCCG ACCAGCCAAA AGAGTCGAGG AGACTGGGCG ACGCTTGAGG   
  
  
+ AACTATGCTG AGTCATTCAA TGTGCCATTT GAGTTCAATG CCATAGCAAA GAGCTGGGAA ACACTTACTG   
  
  
+ TTAAAGATCT CAAGATTGAT CCCGATGAGG TGCTTGTTGT GAACTGTCTG TTCAGGTTTA AATACATTCC   
  
  
+ TGAGGAAACA GTAATTGCAG AATGCCCAAG AGATATTGTT CTTAATCTGA TCAGACAGAT AAAACCAGCT   
  
  
+ GTTTTCATAC ATGGTGTAGT CAATGGAGCC TTCAATTCTC CTTTTTTCAT TATTCGGTTC CGTGAGGCTC   
  
  
+ TCTTCCACTT CTCCACTCTA TTTGATGTGT TGGAGGCAAA TCTTCCCCGG GACATCGAGG AGAGGATACT   
  
  
+ GATAGAGCGA GACATCTTTG GGCGACAGGC AATGAATGTG ATCGCTTGTG AGGGTTTGGA GAGGATAGAA   
  
  
+ AGGCCAGAGA AATACAAACA GTGGCGGGTC CGAAATGAGA GGGCTGGGTT GAAGCAGCTG CCTTTGAATC   
  
  
+ AGGAGACTGT GGAAATGGCC AAAAAACGGG TGACAGCTGT CTATCACAAA GATTTCTCCA TTGATGACGA   
  
  
+ TGGGCACTGG TTGTTACAGG GATGGAAAGG GAGAATTGTC TATGCACTCA CTACTTGGAA GCCTGCTGAC   
  
  
+ TA  

- -Up\_Stream \_Len000AAACAT AAAGTTATAA CATTAATCCA CCCTATATCT CAACTTCCCG TTTGAGATGG   
  
  
- TTGCTGAACA ACTTCCTAGT TGAACATCGA ACCTTGAATT ACATACACAT GATATCTCAA CACATTAGAG   
  
  
- AGGATTATTT TAACCCAATT AGGATCTTAA TCTCTTGAAA TTCGCCCCTG ATTCGAACCA ACTCCGATTG   
  
  
- AAGCAATTTT TCAAGAACAC AGTACAGGTG CAAATAGAAA AGTGATAAAT AAAGTTAATT AAACGCTATA   
  
  
- AATAGTAGTG GGATAAGTGG CAGAAAAGCC TACTAAATTG ATCAGTTCGG CCTGGTTGTA ACGAAAAATA   
  
  
- TCACATAAAC GTTGAGTACA ACTAATAATC TACATAGTTG TTTTTTACAA AGAGTTTTAT TTATCATCGA   
  
  
- GAAATTTAAA ATAAGGTGTA TTTAAGTTTT AGTTATAGCT ATAGCAAGGT ATTATATCGT CGTAATACTT   
  
  
- TTGTTATAAA CGATTACAAA TGTAACACGA GAAAAGTGAG TTTTTAACAT TCCGAAGTTT ATGATAATTT   
  
  
- CTTTGGTAAT AAAGTGAGCT GATAAATCGA ACGTATATAT CTGTTAGAAC GTTTATCATT GTACAGAAGT   
  
  
- AAGCTGATAA ACGAAGGTTT ACTTATTAGA GCATTTGTAT ATATGTATGT ATGTATATAT ATATATATAT   
  
  
- ATATATATAT ATATATATAT CATTATACAT AAATTGAAGT GTATATGTAC TTATATTTTT ATATTCTCCC   
  
  
- TTTTTTCTGC GATCCATCTA CCTGATGTTT TCGGTAAGTA CATAACTCTG CCGAGTATTT TAGTATGCCC   
  
  
- CTTTCCTTTT TTTTTCTAGA GATGTATTAC AAGTATCTAA ATATCTTTCG TATACTACCT AATGGCGTAT   
  
  
- TCCACAATAT ATACCCTCAC AAACATCTTC TTTCTACATA TAGGCTTGAT AGTTTCTATG TATACTCCCT   
  
  
- CATTTTTGTT CACATTCTTG GAGTCCTCCT CTGTATTTTC TAAAAAGATA ACCGTAACCT GTGGCAGTTC   
  
  
- ATAGGCGAAA TTTGGACAAG AAAAAGTGAT AATAGTACCT ACATAAGTGT TTTCCTTAAG TTATGCTTCA   
  
  
- TGGTACTACA TACGAAAAAT GACTATCATA ACCAGAGTAG CTACTCTGAT TCCTACCAGA ATTATCGTTT   
  
  
- AATCTCTAAA GTAATCTATT TTAGATGACA TGTACTGTTT TGTTACTATA TATCGTAGGA TAAATTTTTC   
  
  
- GATGCACAGT CAGTATATAC TTAACGTAGA TTAAAAATTA TCTATATATT ATACTATACT ATATTATCCT   
  
  
- ATACTACATC TAACTTAAGT TTGAGTATTA AGTAATTCGT ACCACGTTAC AAGTAAAAGA ACTAAAATTT   
  
  
- GGCTGATAAT GAAGTTGACA TATATTAAGC TTAACGAAAC CATAAAATTT TAAATTTGAT CATTAATGAC   
  
  
- GGAGATAATG ACTACAAATA TAATTCACCA CGAATTGACG AATAACTACT TTGGTAAAAA TAAAAGCACA   
  
  
- TTTTCTCCCG TATTAGGTAG GAGCACATAA CCGCCCCGAG CCGCGGGATA AGTCGATAAG AGACATACAG   
  
  
- TTTGTTTTGG CGAAGATAAT GGGTAACCAG AGATTTGTGC GTCCCCAAAA TCGGGCCTCT CAATTAAGAA   
  
  
- ATAACTCCAG GGGACAAGAG GTAACGTCCT AAAAACGAGT CCATGAAGAG ACAAAGAAGA GAAATTAGAA   
  
  
- GACATAGAAA GACGTAGTTC AAATTACGAA AGACCCTAAA AGTCGATGCT AAATACACTT TAACATTCTA   
  
  
- AAGACTTGAC CCGTAAGTCC CGAACCAAGT ATACATAAGA AAAGACGAAC TTTACAAAAT TAAAGTATCA   
  
  
- AGGTTACCCC AATAAGTTTC TATCTTAATT CGATATTAGT GGCTCTTCTG AACGACTGGA AACAAAGAAA   
  
  
- AAACAAAGAA AAAAAATTAA TTTAGACCGA TAATGAACCG ATATCAAAAA CGATTACCTA GGTGTTTATC   
  
  
- TGCTTAATAT ACCCGGAAAC GTGGTTTACT TCAAGTTACT AGTCTCCTCT GGGTAAAATA GTTTACTTTT   
  
  
- AAAACAATTA CACAACTTTG GCGGTTTAAG GTTAACCTTA AGTGGAAAAC CCTTGGGATG ACACTAATGG   
  
  
- TGTTTGGGTT TAAAACTAAA CCAACACGGT AGTGATCCAG GGTTACAACT CCCTCGAGAG GAACTTCTAA   
  
  
- TACTAAAATC ACTACAACAA GAATTCATGT AGTTAGTTCA AGAATACCTG CTCCTACTCC CTCTCTTTTA   
  
  
- GGTAAACCCG GTGCCCGGAG ACCGAGAACT TCGCCGGCTC TTCAGCAATA TACTCCACGA ACCCGTCTCG   
  
  
- GTAAGAGGTG TTTTAGTTAT GAGAGGTCTC CTGTTCGCAC TACTGGACTC GTTCTCATCA CAATCTTCGA   
  
  
- CACAGTCAAT ATCGACACTG CCACGTTCAT CACCGTCACC GTAGCACCTC GTTTCAACCA GCTCAATAGG   
  
  
- AGGCCTCATG TTAGGGTACG CCTACCGTCT AAAAGGACGG AACGGGAGTT TTGGGAACAA CTCTTGTAGA   
  
  
- ATATCGAATA GTTTATCGAA CTGTCCACTC CCATTACCAA ACCACCTATC TGGCCGTCTG AGTGGACATT   
  
  
- CGCGAGAATC ACACAGACTA TATAAATTAC TATCAGTCAG TCACTATGTT AAAGTCTTTC CCTACCTCCT   
  
  
- TCGCTCGTTT AAAGAAGGGT TCCGAAGAAA CACAAACCTA AACTTGTGAC CTGGGACGGA AGCCCTCAGC   
  
  
- CAGTTATTCC GTGCTCCACC CGACTAAAAC CACCTCTGAT CGGTACTTTT ACTCCAACGT CACGATCGAT   
  
  
- CATCCCCTTT CTTCGTAGCG GTAGGACTCC TTAATGCAAG TCTTCAACCC TCCCCGTCAT TCGTCGGTCA   
  
  
- ACAAAAGGTC AAAAGTCTGC TCCGTCAACA CTCTAGTCTT TACAAACTAT CCCATAACGA CACGTCACCA   
  
  
- CCGTTTTTAC TACGACTGGA TGCGGTTTGA AATGTCTCAC TTGATTTATT TTGATACGTT TTACGAGTTT   
  
  
- ACTTTCTAAG ATTACCACCT TTTCCGGCAC CATCCTTCGT TCAACCGTCC TCCCTACACC ACCTAGATTC   
  
  
- TAGAGAAAAT GCGGAAACAC GTGTTCGTCA ACCTAGGTTA CTGGTTTCTT CGCGTTTACT GAATTAATCC   
  
  
- GTTTAATCCT TCGTAAGAAG AGGTTGGCCC CTACCCTTAG TTTCTTACCG AGTAATAAAA CGACTACCAG   
  
  
- AACTCCGAGC CGAACGACCA CAACCCTGGG GTTATATGTT CAGAGAGCAC TGACCGGGTC GAAGACGACA   
  
  
- ACTATAGAAC TCTCGAATGG TATACAAAGA ACGGTGTACG GGTAAGTTCT TTTATCCCTT AAAGAAGAGA   
  
  
- TTATCTTGTT AATACCTACA TCGAGTCTTT CGTTTAGCTG AAGTATAATA ACTAGAACCA TAACAGATAC   
  
  
- CAAAGGTTAC CGGAACAGAT TAAGTCGCGG AAAGTAGATC CGGACCGCCC GGAGGGTTTG AAGCCTAATG   
  
  
- TCCGCAACTA GAGGGGGTTG GGCCAAAGGC TGGTCGGTTT TCTCAGCTCC TCTGACCCGC TGCGAACTCC   
  
  
- TTGATACGAC TCAGTAAGTT ACACGGTAAA CTCAAGTTAC GGTATCGTTT CTCGACCCTT TGTGAATGAC   
  
  
- AATTTCTAGA GTTCTAACTA GGGCTACTCC ACGAACAACA CTTGACAGAC AAGTCCAAAT TTATGTAAGG   
  
  
- ACTCCTTTGT CATTAACGTC TTACGGGTTC TCTATAACAA GAATTAGACT AGTCTGTCTA TTTTGGTCGA   
  
  
- CAAAAGTATG TACCACATCA GTTACCTCGG AAGTTAAGAG GAAAAAAGTA ATAAGCCAAG GCACTCCGAG   
  
  
- AGAAGGTGAA GAGGTGAGAT AAACTACACA ACCTCCGTTT AGAAGGGGCC CTGTAGCTCC TCTCCTATGA   
  
  
- CTATCTCGCT CTGTAGAAAC CCGCTGTCCG TTACTTACAC TAGCGAACAC TCCCAAACCT CTCCTATCTT   
  
  
- TCCGGTCTCT TTATGTTTGT CACCGCCCAG GCTTTACTCT CCCGACCCAA CTTCGTCGAC GGAAACTTAG   
  
  
- TCCTCTGACA CCTTTACCGG TTTTTTGCCC ACTGTCGACA GATAGTGTTT CTAAAGAGGT AACTACTGCT   
  
  
- ACCCGTGACC AACAATGTCC CTACCTTTCC CTCTTAACAG ATACGTGAGT GATGAACCTT CGGACGACTG   
  
  
- AT

+     CGTCA-motif

| Site Name | Organism | Position | Strand | Matrix score. | sequence | function |
| --- | --- | --- | --- | --- | --- | --- |
| CGTCA-motif | Hordeum vulgare | 2471 | - | 5 | CGTCA | cis-acting regulatory element involved in the MeJA-responsiveness |
| CGTCA-motif | Hordeum vulgare | 1048 | + | 5 | CGTCA | cis-acting regulatory element involved in the MeJA-responsiveness |
| CGTCA-motif | Hordeum vulgare | 4199 | - | 5 | CGTCA | cis-acting regulatory element involved in the MeJA-responsiveness |

>HU05G01597.1   
+ -Up\_Stream \_Len000TTTGTA TTTCAATATT GTAATTAGGT GGGATATAGA GTTGAAGGGC AAACTCTACC   
  
  
+ AACGACTTGT TGAAGGATCA ACTTGTAGCT TGGAACTTAA TGTATGTGTA CTATAGAGTT GTGTAATCTC   
  
  
+ TCCTAATAAA ATTGGGTTAA TCCTAGAATT AGAGAACTTT AAGCGGGGAC TAAGCTTGGT TGAGGCTAAC   
  
  
+ TTCGTTAAAA AGTTCTTGTG TCATGTCCAC GTTTATCTTT TCACTATTTA TTTCAATTAA TTTGCGATAT   
  
  
+ TTATCATCAC CCTATTCACC GTCTTTTCGG ATGATTTAAC TAGTCAAGCC GGACCAACAT TGCTTTTTAT   
  
  
+ AGTGTATTTG CAACTCATGT TGATTATTAG ATGTATCAAC AAAAAATGTT TCTCAAAATA AATAGTAGCT   
  
  
+ CTTTAAATTT TATTCCACAT AAATTCAAAA TCAATATCGA TATCGTTCCA TAATATAGCA GCATTATGAA   
  
  
+ AACAATATTT GCTAATGTTT ACATTGTGCT CTTTTCACTC AAAAATTGTA AGGCTTCAAA TACTATTAAA   
  
  
+ GAAACCATTA TTTCACTCGA CTATTTAGCT TGCATATATA GACAATCTTG CAAATAGTAA CATGTCTTCA   
  
  
+ TTCGACTATT TGCTTCCAAA TGAATAATCT CGTAAACATA TATACATACA TACATATATA TATATATATA   
  
  
+ TATATATATA TATATATATA GTAATATGTA TTTAACTTCA CATATACATG AATATAAAAA TATAAGAGGG   
  
  
+ AAAAAAGACG CTAGGTAGAT GGACTACAAA AGCCATTCAT GTATTGAGAC GGCTCATAAA ATCATACGGG   
  
  
+ GAAAGGAAAA AAAAAGATCT CTACATAATG TTCATAGATT TATAGAAAGC ATATGATGGA TTACCGCATA   
  
  
+ AGGTGTTATA TATGGGAGTG TTTGTAGAAG AAAGATGTAT ATCCGAACTA TCAAAGATAC ATATGAGGGA   
  
  
+ GTAAAAACAA GTGTAAGAAC CTCAGGAGGA GACATAAAAG ATTTTTCTAT TGGCATTGGA CACCGTCAAG   
  
  
+ TATCCGCTTT AAACCTGTTC TTTTTCACTA TTATCATGGA TGTATTCACA AAAGGAATTC AATACGAAGT   
  
  
+ ACCATGATGT ATGCTTTTTA CTGATAGTAT TGGTCTCATC GATGAGACTA AGGATGGTCT TAATAGCAAA   
  
  
+ TTAGAGATTT CATTAGATAA AATCTACTGT ACATGACAAA ACAATGATAT ATAGCATCCT ATTTAAAAAG   
  
  
+ CTACGTGTCA GTCATATATG AATTGCATCT AATTTTTAAT AGATATATAA TATGATATGA TATAATAGGA   
  
  
+ TATGATGTAG ATTGAATTCA AACTCATAAT TCATTAAGCA TGGTGCAATG TTCATTTTCT TGATTTTAAA   
  
  
+ CCGACTATTA CTTCAACTGT ATATAATTCG AATTGCTTTG GTATTTTAAA ATTTAAACTA GTAATTACTG   
  
  
+ CCTCTATTAC TGATGTTTAT ATTAAGTGGT GCTTAACTGC TTATTGATGA AACCATTTTT ATTTTCGTGT   
  
  
+ AAAAGAGGGC ATAATCCATC CTCGTGTATT GGCGGGGCTC GGCGCCCTAT TCAGCTATTC TCTGTATGTC   
  
  
+ AAACAAAACC GCTTCTATTA CCCATTGGTC TCTAAACACG CAGGGGTTTT AGCCCGGAGA GTTAATTCTT   
  
  
+ TATTGAGGTC CCCTGTTCTC CATTGCAGGA TTTTTGCTCA GGTACTTCTC TGTTTCTTCT CTTTAATCTT   
  
  
+ CTGTATCTTT CTGCATCAAG TTTAATGCTT TCTGGGATTT TCAGCTACGA TTTATGTGAA ATTGTAAGAT   
  
  
+ TTCTGAACTG GGCATTCAGG GCTTGGTTCA TATGTATTCT TTTCTGCTTG AAATGTTTTA ATTTCATAGT   
  
  
+ TCCAATGGGG TTATTCAAAG ATAGAATTAA GCTATAATCA CCGAGAAGAC TTGCTGACCT TTGTTTCTTT   
  
  
+ TTTGTTTCTT TTTTTTAATT AAATCTGGCT ATTACTTGGC TATAGTTTTT GCTAATGGAT CCACAAATAG   
  
  
+ ACGAATTATA TGGGCCTTTG CACCAAATGA AGTTCAATGA TCAGAGGAGA CCCATTTTAT CAAATGAAAA   
  
  
+ TTTTGTTAAT GTGTTGAAAC CGCCAAATTC CAATTGGAAT TCACCTTTTG GGAACCCTAC TGTGATTACC   
  
  
+ ACAAACCCAA ATTTTGATTT GGTTGTGCCA TCACTAGGTC CCAATGTTGA GGGAGCTCTC CTTGAAGATT   
  
  
+ ATGATTTTAG TGATGTTGTT CTTAAGTACA TCAATCAAGT TCTTATGGAC GAGGATGAGG GAGAGAAAAT   
  
  
+ CCATTTGGGC CACGGGCCTC TGGCTCTTGA AGCGGCCGAG AAGTCGTTAT ATGAGGTGCT TGGGCAGAGC   
  
  
+ CATTCTCCAC AAAATCAATA CTCTCCAGAG GACAAGCGTG ATGACCTGAG CAAGAGTAGT GTTAGAAGCT   
  
  
+ GTGTCAGTTA TAGCTGTGAC GGTGCAAGTA GTGGCAGTGG CATCGTGGAG CAAAGTTGGT CGAGTTATCC   
  
  
+ TCCGGAGTAC AATCCCATGC GGATGGCAGA TTTTCCTGCC TTGCCCTCAA AACCCTTGTT GAGAACATCT   
  
  
+ TATAGCTTAT CAAATAGCTT GACAGGTGAG GGTAATGGTT TGGTGGATAG ACCGGCAGAC TCACCTGTAA   
  
  
+ GCGCTCTTAG TGTGTCTGAT ATATTTAATG ATAGTCAGTC AGTGATACAA TTTCAGAAAG GGATGGAGGA   
  
  
+ AGCGAGCAAA TTTCTTCCCA AGGCTTCTTT GTGTTTGGAT TTGAACACTG GACCCTGCCT TCGGGAGTCG   
  
  
+ GTCAATAAGG CACGAGGTGG GCTGATTTTG GTGGAGACTA GCCATGAAAA TGAGGTTGCA GTGCTAGCTA   
  
  
+ GTAGGGGAAA GAAGCATCGC CATCCTGAGG AATTACGTTC AGAAGTTGGG AGGGGCAGTA AGCAGCCAGT   
  
  
+ TGTTTTCCAG TTTTCAGACG AGGCAGTTGT GAGATCAGAA ATGTTTGATA GGGTATTGCT GTGCAGTGGT   
  
  
+ GGCAAAAATG ATGCTGACCT ACGCCAAACT TTACAGAGTG AACTAAATAA AACTATGCAA AATGCTCAAA   
  
  
+ TGAAAGATTC TAATGGTGGA AAAGGCCGTG GTAGGAAGCA AGTTGGCAGG AGGGATGTGG TGGATCTAAG   
  
  
+ ATCTCTTTTA CGCCTTTGTG CACAAGCAGT TGGATCCAAT GACCAAAGAA GCGCAAATGA CTTAATTAGG   
  
  
+ CAAATTAGGA AGCATTCTTC TCCAACCGGG GATGGGAATC AAAGAATGGC TCATTATTTT GCTGATGGTC   
  
  
+ TTGAGGCTCG GCTTGCTGGT GTTGGGACCC CAATATACAA GTCTCTCGTG ACTGGCCCAG CTTCTGCTGT   
  
  
+ TGATATCTTG AGAGCTTACC ATATGTTTCT TGCCACATGC CCATTCAAGA AAATAGGGAA TTTCTTCTCT   
  
  
+ AATAGAACAA TTATGGATGT AGCTCAGAAA GCAAATCGAC TTCATATTAT TGATCTTGGT ATTGTCTATG   
  
  
+ GTTTCCAATG GCCTTGTCTA ATTCAGCGCC TTTCATCTAG GCCTGGCGGG CCTCCCAAAC TTCGGATTAC   
  
  
+ AGGCGTTGAT CTCCCCCAAC CCGGTTTCCG ACCAGCCAAA AGAGTCGAGG AGACTGGGCG ACGCTTGAGG   
  
  
+ AACTATGCTG AGTCATTCAA TGTGCCATTT GAGTTCAATG CCATAGCAAA GAGCTGGGAA ACACTTACTG   
  
  
+ TTAAAGATCT CAAGATTGAT CCCGATGAGG TGCTTGTTGT GAACTGTCTG TTCAGGTTTA AATACATTCC   
  
  
+ TGAGGAAACA GTAATTGCAG AATGCCCAAG AGATATTGTT CTTAATCTGA TCAGACAGAT AAAACCAGCT   
  
  
+ GTTTTCATAC ATGGTGTAGT CAATGGAGCC TTCAATTCTC CTTTTTTCAT TATTCGGTTC CGTGAGGCTC   
  
  
+ TCTTCCACTT CTCCACTCTA TTTGATGTGT TGGAGGCAAA TCTTCCCCGG GACATCGAGG AGAGGATACT   
  
  
+ GATAGAGCGA GACATCTTTG GGCGACAGGC AATGAATGTG ATCGCTTGTG AGGGTTTGGA GAGGATAGAA   
  
  
+ AGGCCAGAGA AATACAAACA GTGGCGGGTC CGAAATGAGA GGGCTGGGTT GAAGCAGCTG CCTTTGAATC   
  
  
+ AGGAGACTGT GGAAATGGCC AAAAAACGGG TGACAGCTGT CTATCACAAA GATTTCTCCA TTGATGACGA   
  
  
+ TGGGCACTGG TTGTTACAGG GATGGAAAGG GAGAATTGTC TATGCACTCA CTACTTGGAA GCCTGCTGAC   
  
  
+ TA  

- -Up\_Stream \_Len000AAACAT AAAGTTATAA CATTAATCCA CCCTATATCT CAACTTCCCG TTTGAGATGG   
  
  
- TTGCTGAACA ACTTCCTAGT TGAACATCGA ACCTTGAATT ACATACACAT GATATCTCAA CACATTAGAG   
  
  
- AGGATTATTT TAACCCAATT AGGATCTTAA TCTCTTGAAA TTCGCCCCTG ATTCGAACCA ACTCCGATTG   
  
  
- AAGCAATTTT TCAAGAACAC AGTACAGGTG CAAATAGAAA AGTGATAAAT AAAGTTAATT AAACGCTATA   
  
  
- AATAGTAGTG GGATAAGTGG CAGAAAAGCC TACTAAATTG ATCAGTTCGG CCTGGTTGTA ACGAAAAATA   
  
  
- TCACATAAAC GTTGAGTACA ACTAATAATC TACATAGTTG TTTTTTACAA AGAGTTTTAT TTATCATCGA   
  
  
- GAAATTTAAA ATAAGGTGTA TTTAAGTTTT AGTTATAGCT ATAGCAAGGT ATTATATCGT CGTAATACTT   
  
  
- TTGTTATAAA CGATTACAAA TGTAACACGA GAAAAGTGAG TTTTTAACAT TCCGAAGTTT ATGATAATTT   
  
  
- CTTTGGTAAT AAAGTGAGCT GATAAATCGA ACGTATATAT CTGTTAGAAC GTTTATCATT GTACAGAAGT   
  
  
- AAGCTGATAA ACGAAGGTTT ACTTATTAGA GCATTTGTAT ATATGTATGT ATGTATATAT ATATATATAT   
  
  
- ATATATATAT ATATATATAT CATTATACAT AAATTGAAGT GTATATGTAC TTATATTTTT ATATTCTCCC   
  
  
- TTTTTTCTGC GATCCATCTA CCTGATGTTT TCGGTAAGTA CATAACTCTG CCGAGTATTT TAGTATGCCC   
  
  
- CTTTCCTTTT TTTTTCTAGA GATGTATTAC AAGTATCTAA ATATCTTTCG TATACTACCT AATGGCGTAT   
  
  
- TCCACAATAT ATACCCTCAC AAACATCTTC TTTCTACATA TAGGCTTGAT AGTTTCTATG TATACTCCCT   
  
  
- CATTTTTGTT CACATTCTTG GAGTCCTCCT CTGTATTTTC TAAAAAGATA ACCGTAACCT GTGGCAGTTC   
  
  
- ATAGGCGAAA TTTGGACAAG AAAAAGTGAT AATAGTACCT ACATAAGTGT TTTCCTTAAG TTATGCTTCA   
  
  
- TGGTACTACA TACGAAAAAT GACTATCATA ACCAGAGTAG CTACTCTGAT TCCTACCAGA ATTATCGTTT   
  
  
- AATCTCTAAA GTAATCTATT TTAGATGACA TGTACTGTTT TGTTACTATA TATCGTAGGA TAAATTTTTC   
  
  
- GATGCACAGT CAGTATATAC TTAACGTAGA TTAAAAATTA TCTATATATT ATACTATACT ATATTATCCT   
  
  
- ATACTACATC TAACTTAAGT TTGAGTATTA AGTAATTCGT ACCACGTTAC AAGTAAAAGA ACTAAAATTT   
  
  
- GGCTGATAAT GAAGTTGACA TATATTAAGC TTAACGAAAC CATAAAATTT TAAATTTGAT CATTAATGAC   
  
  
- GGAGATAATG ACTACAAATA TAATTCACCA CGAATTGACG AATAACTACT TTGGTAAAAA TAAAAGCACA   
  
  
- TTTTCTCCCG TATTAGGTAG GAGCACATAA CCGCCCCGAG CCGCGGGATA AGTCGATAAG AGACATACAG   
  
  
- TTTGTTTTGG CGAAGATAAT GGGTAACCAG AGATTTGTGC GTCCCCAAAA TCGGGCCTCT CAATTAAGAA   
  
  
- ATAACTCCAG GGGACAAGAG GTAACGTCCT AAAAACGAGT CCATGAAGAG ACAAAGAAGA GAAATTAGAA   
  
  
- GACATAGAAA GACGTAGTTC AAATTACGAA AGACCCTAAA AGTCGATGCT AAATACACTT TAACATTCTA   
  
  
- AAGACTTGAC CCGTAAGTCC CGAACCAAGT ATACATAAGA AAAGACGAAC TTTACAAAAT TAAAGTATCA   
  
  
- AGGTTACCCC AATAAGTTTC TATCTTAATT CGATATTAGT GGCTCTTCTG AACGACTGGA AACAAAGAAA   
  
  
- AAACAAAGAA AAAAAATTAA TTTAGACCGA TAATGAACCG ATATCAAAAA CGATTACCTA GGTGTTTATC   
  
  
- TGCTTAATAT ACCCGGAAAC GTGGTTTACT TCAAGTTACT AGTCTCCTCT GGGTAAAATA GTTTACTTTT   
  
  
- AAAACAATTA CACAACTTTG GCGGTTTAAG GTTAACCTTA AGTGGAAAAC CCTTGGGATG ACACTAATGG   
  
  
- TGTTTGGGTT TAAAACTAAA CCAACACGGT AGTGATCCAG GGTTACAACT CCCTCGAGAG GAACTTCTAA   
  
  
- TACTAAAATC ACTACAACAA GAATTCATGT AGTTAGTTCA AGAATACCTG CTCCTACTCC CTCTCTTTTA   
  
  
- GGTAAACCCG GTGCCCGGAG ACCGAGAACT TCGCCGGCTC TTCAGCAATA TACTCCACGA ACCCGTCTCG   
  
  
- GTAAGAGGTG TTTTAGTTAT GAGAGGTCTC CTGTTCGCAC TACTGGACTC GTTCTCATCA CAATCTTCGA   
  
  
- CACAGTCAAT ATCGACACTG CCACGTTCAT CACCGTCACC GTAGCACCTC GTTTCAACCA GCTCAATAGG   
  
  
- AGGCCTCATG TTAGGGTACG CCTACCGTCT AAAAGGACGG AACGGGAGTT TTGGGAACAA CTCTTGTAGA   
  
  
- ATATCGAATA GTTTATCGAA CTGTCCACTC CCATTACCAA ACCACCTATC TGGCCGTCTG AGTGGACATT   
  
  
- CGCGAGAATC ACACAGACTA TATAAATTAC TATCAGTCAG TCACTATGTT AAAGTCTTTC CCTACCTCCT   
  
  
- TCGCTCGTTT AAAGAAGGGT TCCGAAGAAA CACAAACCTA AACTTGTGAC CTGGGACGGA AGCCCTCAGC   
  
  
- CAGTTATTCC GTGCTCCACC CGACTAAAAC CACCTCTGAT CGGTACTTTT ACTCCAACGT CACGATCGAT   
  
  
- CATCCCCTTT CTTCGTAGCG GTAGGACTCC TTAATGCAAG TCTTCAACCC TCCCCGTCAT TCGTCGGTCA   
  
  
- ACAAAAGGTC AAAAGTCTGC TCCGTCAACA CTCTAGTCTT TACAAACTAT CCCATAACGA CACGTCACCA   
  
  
- CCGTTTTTAC TACGACTGGA TGCGGTTTGA AATGTCTCAC TTGATTTATT TTGATACGTT TTACGAGTTT   
  
  
- ACTTTCTAAG ATTACCACCT TTTCCGGCAC CATCCTTCGT TCAACCGTCC TCCCTACACC ACCTAGATTC   
  
  
- TAGAGAAAAT GCGGAAACAC GTGTTCGTCA ACCTAGGTTA CTGGTTTCTT CGCGTTTACT GAATTAATCC   
  
  
- GTTTAATCCT TCGTAAGAAG AGGTTGGCCC CTACCCTTAG TTTCTTACCG AGTAATAAAA CGACTACCAG   
  
  
- AACTCCGAGC CGAACGACCA CAACCCTGGG GTTATATGTT CAGAGAGCAC TGACCGGGTC GAAGACGACA   
  
  
- ACTATAGAAC TCTCGAATGG TATACAAAGA ACGGTGTACG GGTAAGTTCT TTTATCCCTT AAAGAAGAGA   
  
  
- TTATCTTGTT AATACCTACA TCGAGTCTTT CGTTTAGCTG AAGTATAATA ACTAGAACCA TAACAGATAC   
  
  
- CAAAGGTTAC CGGAACAGAT TAAGTCGCGG AAAGTAGATC CGGACCGCCC GGAGGGTTTG AAGCCTAATG   
  
  
- TCCGCAACTA GAGGGGGTTG GGCCAAAGGC TGGTCGGTTT TCTCAGCTCC TCTGACCCGC TGCGAACTCC   
  
  
- TTGATACGAC TCAGTAAGTT ACACGGTAAA CTCAAGTTAC GGTATCGTTT CTCGACCCTT TGTGAATGAC   
  
  
- AATTTCTAGA GTTCTAACTA GGGCTACTCC ACGAACAACA CTTGACAGAC AAGTCCAAAT TTATGTAAGG   
  
  
- ACTCCTTTGT CATTAACGTC TTACGGGTTC TCTATAACAA GAATTAGACT AGTCTGTCTA TTTTGGTCGA   
  
  
- CAAAAGTATG TACCACATCA GTTACCTCGG AAGTTAAGAG GAAAAAAGTA ATAAGCCAAG GCACTCCGAG   
  
  
- AGAAGGTGAA GAGGTGAGAT AAACTACACA ACCTCCGTTT AGAAGGGGCC CTGTAGCTCC TCTCCTATGA   
  
  
- CTATCTCGCT CTGTAGAAAC CCGCTGTCCG TTACTTACAC TAGCGAACAC TCCCAAACCT CTCCTATCTT   
  
  
- TCCGGTCTCT TTATGTTTGT CACCGCCCAG GCTTTACTCT CCCGACCCAA CTTCGTCGAC GGAAACTTAG   
  
  
- TCCTCTGACA CCTTTACCGG TTTTTTGCCC ACTGTCGACA GATAGTGTTT CTAAAGAGGT AACTACTGCT   
  
  
- ACCCGTGACC AACAATGTCC CTACCTTTCC CTCTTAACAG ATACGTGAGT GATGAACCTT CGGACGACTG   
  
  
- AT

+     DRE1

| Site Name | Organism | Position | Strand | Matrix score. | sequence | function |
| --- | --- | --- | --- | --- | --- | --- |
| DRE1 | Zea mays | 1934 | + | 7 | ACCGAGA |  |

>HU05G01597.1   
+ -Up\_Stream \_Len000TTTGTA TTTCAATATT GTAATTAGGT GGGATATAGA GTTGAAGGGC AAACTCTACC   
  
  
+ AACGACTTGT TGAAGGATCA ACTTGTAGCT TGGAACTTAA TGTATGTGTA CTATAGAGTT GTGTAATCTC   
  
  
+ TCCTAATAAA ATTGGGTTAA TCCTAGAATT AGAGAACTTT AAGCGGGGAC TAAGCTTGGT TGAGGCTAAC   
  
  
+ TTCGTTAAAA AGTTCTTGTG TCATGTCCAC GTTTATCTTT TCACTATTTA TTTCAATTAA TTTGCGATAT   
  
  
+ TTATCATCAC CCTATTCACC GTCTTTTCGG ATGATTTAAC TAGTCAAGCC GGACCAACAT TGCTTTTTAT   
  
  
+ AGTGTATTTG CAACTCATGT TGATTATTAG ATGTATCAAC AAAAAATGTT TCTCAAAATA AATAGTAGCT   
  
  
+ CTTTAAATTT TATTCCACAT AAATTCAAAA TCAATATCGA TATCGTTCCA TAATATAGCA GCATTATGAA   
  
  
+ AACAATATTT GCTAATGTTT ACATTGTGCT CTTTTCACTC AAAAATTGTA AGGCTTCAAA TACTATTAAA   
  
  
+ GAAACCATTA TTTCACTCGA CTATTTAGCT TGCATATATA GACAATCTTG CAAATAGTAA CATGTCTTCA   
  
  
+ TTCGACTATT TGCTTCCAAA TGAATAATCT CGTAAACATA TATACATACA TACATATATA TATATATATA   
  
  
+ TATATATATA TATATATATA GTAATATGTA TTTAACTTCA CATATACATG AATATAAAAA TATAAGAGGG   
  
  
+ AAAAAAGACG CTAGGTAGAT GGACTACAAA AGCCATTCAT GTATTGAGAC GGCTCATAAA ATCATACGGG   
  
  
+ GAAAGGAAAA AAAAAGATCT CTACATAATG TTCATAGATT TATAGAAAGC ATATGATGGA TTACCGCATA   
  
  
+ AGGTGTTATA TATGGGAGTG TTTGTAGAAG AAAGATGTAT ATCCGAACTA TCAAAGATAC ATATGAGGGA   
  
  
+ GTAAAAACAA GTGTAAGAAC CTCAGGAGGA GACATAAAAG ATTTTTCTAT TGGCATTGGA CACCGTCAAG   
  
  
+ TATCCGCTTT AAACCTGTTC TTTTTCACTA TTATCATGGA TGTATTCACA AAAGGAATTC AATACGAAGT   
  
  
+ ACCATGATGT ATGCTTTTTA CTGATAGTAT TGGTCTCATC GATGAGACTA AGGATGGTCT TAATAGCAAA   
  
  
+ TTAGAGATTT CATTAGATAA AATCTACTGT ACATGACAAA ACAATGATAT ATAGCATCCT ATTTAAAAAG   
  
  
+ CTACGTGTCA GTCATATATG AATTGCATCT AATTTTTAAT AGATATATAA TATGATATGA TATAATAGGA   
  
  
+ TATGATGTAG ATTGAATTCA AACTCATAAT TCATTAAGCA TGGTGCAATG TTCATTTTCT TGATTTTAAA   
  
  
+ CCGACTATTA CTTCAACTGT ATATAATTCG AATTGCTTTG GTATTTTAAA ATTTAAACTA GTAATTACTG   
  
  
+ CCTCTATTAC TGATGTTTAT ATTAAGTGGT GCTTAACTGC TTATTGATGA AACCATTTTT ATTTTCGTGT   
  
  
+ AAAAGAGGGC ATAATCCATC CTCGTGTATT GGCGGGGCTC GGCGCCCTAT TCAGCTATTC TCTGTATGTC   
  
  
+ AAACAAAACC GCTTCTATTA CCCATTGGTC TCTAAACACG CAGGGGTTTT AGCCCGGAGA GTTAATTCTT   
  
  
+ TATTGAGGTC CCCTGTTCTC CATTGCAGGA TTTTTGCTCA GGTACTTCTC TGTTTCTTCT CTTTAATCTT   
  
  
+ CTGTATCTTT CTGCATCAAG TTTAATGCTT TCTGGGATTT TCAGCTACGA TTTATGTGAA ATTGTAAGAT   
  
  
+ TTCTGAACTG GGCATTCAGG GCTTGGTTCA TATGTATTCT TTTCTGCTTG AAATGTTTTA ATTTCATAGT   
  
  
+ TCCAATGGGG TTATTCAAAG ATAGAATTAA GCTATAATCA CCGAGAAGAC TTGCTGACCT TTGTTTCTTT   
  
  
+ TTTGTTTCTT TTTTTTAATT AAATCTGGCT ATTACTTGGC TATAGTTTTT GCTAATGGAT CCACAAATAG   
  
  
+ ACGAATTATA TGGGCCTTTG CACCAAATGA AGTTCAATGA TCAGAGGAGA CCCATTTTAT CAAATGAAAA   
  
  
+ TTTTGTTAAT GTGTTGAAAC CGCCAAATTC CAATTGGAAT TCACCTTTTG GGAACCCTAC TGTGATTACC   
  
  
+ ACAAACCCAA ATTTTGATTT GGTTGTGCCA TCACTAGGTC CCAATGTTGA GGGAGCTCTC CTTGAAGATT   
  
  
+ ATGATTTTAG TGATGTTGTT CTTAAGTACA TCAATCAAGT TCTTATGGAC GAGGATGAGG GAGAGAAAAT   
  
  
+ CCATTTGGGC CACGGGCCTC TGGCTCTTGA AGCGGCCGAG AAGTCGTTAT ATGAGGTGCT TGGGCAGAGC   
  
  
+ CATTCTCCAC AAAATCAATA CTCTCCAGAG GACAAGCGTG ATGACCTGAG CAAGAGTAGT GTTAGAAGCT   
  
  
+ GTGTCAGTTA TAGCTGTGAC GGTGCAAGTA GTGGCAGTGG CATCGTGGAG CAAAGTTGGT CGAGTTATCC   
  
  
+ TCCGGAGTAC AATCCCATGC GGATGGCAGA TTTTCCTGCC TTGCCCTCAA AACCCTTGTT GAGAACATCT   
  
  
+ TATAGCTTAT CAAATAGCTT GACAGGTGAG GGTAATGGTT TGGTGGATAG ACCGGCAGAC TCACCTGTAA   
  
  
+ GCGCTCTTAG TGTGTCTGAT ATATTTAATG ATAGTCAGTC AGTGATACAA TTTCAGAAAG GGATGGAGGA   
  
  
+ AGCGAGCAAA TTTCTTCCCA AGGCTTCTTT GTGTTTGGAT TTGAACACTG GACCCTGCCT TCGGGAGTCG   
  
  
+ GTCAATAAGG CACGAGGTGG GCTGATTTTG GTGGAGACTA GCCATGAAAA TGAGGTTGCA GTGCTAGCTA   
  
  
+ GTAGGGGAAA GAAGCATCGC CATCCTGAGG AATTACGTTC AGAAGTTGGG AGGGGCAGTA AGCAGCCAGT   
  
  
+ TGTTTTCCAG TTTTCAGACG AGGCAGTTGT GAGATCAGAA ATGTTTGATA GGGTATTGCT GTGCAGTGGT   
  
  
+ GGCAAAAATG ATGCTGACCT ACGCCAAACT TTACAGAGTG AACTAAATAA AACTATGCAA AATGCTCAAA   
  
  
+ TGAAAGATTC TAATGGTGGA AAAGGCCGTG GTAGGAAGCA AGTTGGCAGG AGGGATGTGG TGGATCTAAG   
  
  
+ ATCTCTTTTA CGCCTTTGTG CACAAGCAGT TGGATCCAAT GACCAAAGAA GCGCAAATGA CTTAATTAGG   
  
  
+ CAAATTAGGA AGCATTCTTC TCCAACCGGG GATGGGAATC AAAGAATGGC TCATTATTTT GCTGATGGTC   
  
  
+ TTGAGGCTCG GCTTGCTGGT GTTGGGACCC CAATATACAA GTCTCTCGTG ACTGGCCCAG CTTCTGCTGT   
  
  
+ TGATATCTTG AGAGCTTACC ATATGTTTCT TGCCACATGC CCATTCAAGA AAATAGGGAA TTTCTTCTCT   
  
  
+ AATAGAACAA TTATGGATGT AGCTCAGAAA GCAAATCGAC TTCATATTAT TGATCTTGGT ATTGTCTATG   
  
  
+ GTTTCCAATG GCCTTGTCTA ATTCAGCGCC TTTCATCTAG GCCTGGCGGG CCTCCCAAAC TTCGGATTAC   
  
  
+ AGGCGTTGAT CTCCCCCAAC CCGGTTTCCG ACCAGCCAAA AGAGTCGAGG AGACTGGGCG ACGCTTGAGG   
  
  
+ AACTATGCTG AGTCATTCAA TGTGCCATTT GAGTTCAATG CCATAGCAAA GAGCTGGGAA ACACTTACTG   
  
  
+ TTAAAGATCT CAAGATTGAT CCCGATGAGG TGCTTGTTGT GAACTGTCTG TTCAGGTTTA AATACATTCC   
  
  
+ TGAGGAAACA GTAATTGCAG AATGCCCAAG AGATATTGTT CTTAATCTGA TCAGACAGAT AAAACCAGCT   
  
  
+ GTTTTCATAC ATGGTGTAGT CAATGGAGCC TTCAATTCTC CTTTTTTCAT TATTCGGTTC CGTGAGGCTC   
  
  
+ TCTTCCACTT CTCCACTCTA TTTGATGTGT TGGAGGCAAA TCTTCCCCGG GACATCGAGG AGAGGATACT   
  
  
+ GATAGAGCGA GACATCTTTG GGCGACAGGC AATGAATGTG ATCGCTTGTG AGGGTTTGGA GAGGATAGAA   
  
  
+ AGGCCAGAGA AATACAAACA GTGGCGGGTC CGAAATGAGA GGGCTGGGTT GAAGCAGCTG CCTTTGAATC   
  
  
+ AGGAGACTGT GGAAATGGCC AAAAAACGGG TGACAGCTGT CTATCACAAA GATTTCTCCA TTGATGACGA   
  
  
+ TGGGCACTGG TTGTTACAGG GATGGAAAGG GAGAATTGTC TATGCACTCA CTACTTGGAA GCCTGCTGAC   
  
  
+ TA  

- -Up\_Stream \_Len000AAACAT AAAGTTATAA CATTAATCCA CCCTATATCT CAACTTCCCG TTTGAGATGG   
  
  
- TTGCTGAACA ACTTCCTAGT TGAACATCGA ACCTTGAATT ACATACACAT GATATCTCAA CACATTAGAG   
  
  
- AGGATTATTT TAACCCAATT AGGATCTTAA TCTCTTGAAA TTCGCCCCTG ATTCGAACCA ACTCCGATTG   
  
  
- AAGCAATTTT TCAAGAACAC AGTACAGGTG CAAATAGAAA AGTGATAAAT AAAGTTAATT AAACGCTATA   
  
  
- AATAGTAGTG GGATAAGTGG CAGAAAAGCC TACTAAATTG ATCAGTTCGG CCTGGTTGTA ACGAAAAATA   
  
  
- TCACATAAAC GTTGAGTACA ACTAATAATC TACATAGTTG TTTTTTACAA AGAGTTTTAT TTATCATCGA   
  
  
- GAAATTTAAA ATAAGGTGTA TTTAAGTTTT AGTTATAGCT ATAGCAAGGT ATTATATCGT CGTAATACTT   
  
  
- TTGTTATAAA CGATTACAAA TGTAACACGA GAAAAGTGAG TTTTTAACAT TCCGAAGTTT ATGATAATTT   
  
  
- CTTTGGTAAT AAAGTGAGCT GATAAATCGA ACGTATATAT CTGTTAGAAC GTTTATCATT GTACAGAAGT   
  
  
- AAGCTGATAA ACGAAGGTTT ACTTATTAGA GCATTTGTAT ATATGTATGT ATGTATATAT ATATATATAT   
  
  
- ATATATATAT ATATATATAT CATTATACAT AAATTGAAGT GTATATGTAC TTATATTTTT ATATTCTCCC   
  
  
- TTTTTTCTGC GATCCATCTA CCTGATGTTT TCGGTAAGTA CATAACTCTG CCGAGTATTT TAGTATGCCC   
  
  
- CTTTCCTTTT TTTTTCTAGA GATGTATTAC AAGTATCTAA ATATCTTTCG TATACTACCT AATGGCGTAT   
  
  
- TCCACAATAT ATACCCTCAC AAACATCTTC TTTCTACATA TAGGCTTGAT AGTTTCTATG TATACTCCCT   
  
  
- CATTTTTGTT CACATTCTTG GAGTCCTCCT CTGTATTTTC TAAAAAGATA ACCGTAACCT GTGGCAGTTC   
  
  
- ATAGGCGAAA TTTGGACAAG AAAAAGTGAT AATAGTACCT ACATAAGTGT TTTCCTTAAG TTATGCTTCA   
  
  
- TGGTACTACA TACGAAAAAT GACTATCATA ACCAGAGTAG CTACTCTGAT TCCTACCAGA ATTATCGTTT   
  
  
- AATCTCTAAA GTAATCTATT TTAGATGACA TGTACTGTTT TGTTACTATA TATCGTAGGA TAAATTTTTC   
  
  
- GATGCACAGT CAGTATATAC TTAACGTAGA TTAAAAATTA TCTATATATT ATACTATACT ATATTATCCT   
  
  
- ATACTACATC TAACTTAAGT TTGAGTATTA AGTAATTCGT ACCACGTTAC AAGTAAAAGA ACTAAAATTT   
  
  
- GGCTGATAAT GAAGTTGACA TATATTAAGC TTAACGAAAC CATAAAATTT TAAATTTGAT CATTAATGAC   
  
  
- GGAGATAATG ACTACAAATA TAATTCACCA CGAATTGACG AATAACTACT TTGGTAAAAA TAAAAGCACA   
  
  
- TTTTCTCCCG TATTAGGTAG GAGCACATAA CCGCCCCGAG CCGCGGGATA AGTCGATAAG AGACATACAG   
  
  
- TTTGTTTTGG CGAAGATAAT GGGTAACCAG AGATTTGTGC GTCCCCAAAA TCGGGCCTCT CAATTAAGAA   
  
  
- ATAACTCCAG GGGACAAGAG GTAACGTCCT AAAAACGAGT CCATGAAGAG ACAAAGAAGA GAAATTAGAA   
  
  
- GACATAGAAA GACGTAGTTC AAATTACGAA AGACCCTAAA AGTCGATGCT AAATACACTT TAACATTCTA   
  
  
- AAGACTTGAC CCGTAAGTCC CGAACCAAGT ATACATAAGA AAAGACGAAC TTTACAAAAT TAAAGTATCA   
  
  
- AGGTTACCCC AATAAGTTTC TATCTTAATT CGATATTAGT GGCTCTTCTG AACGACTGGA AACAAAGAAA   
  
  
- AAACAAAGAA AAAAAATTAA TTTAGACCGA TAATGAACCG ATATCAAAAA CGATTACCTA GGTGTTTATC   
  
  
- TGCTTAATAT ACCCGGAAAC GTGGTTTACT TCAAGTTACT AGTCTCCTCT GGGTAAAATA GTTTACTTTT   
  
  
- AAAACAATTA CACAACTTTG GCGGTTTAAG GTTAACCTTA AGTGGAAAAC CCTTGGGATG ACACTAATGG   
  
  
- TGTTTGGGTT TAAAACTAAA CCAACACGGT AGTGATCCAG GGTTACAACT CCCTCGAGAG GAACTTCTAA   
  
  
- TACTAAAATC ACTACAACAA GAATTCATGT AGTTAGTTCA AGAATACCTG CTCCTACTCC CTCTCTTTTA   
  
  
- GGTAAACCCG GTGCCCGGAG ACCGAGAACT TCGCCGGCTC TTCAGCAATA TACTCCACGA ACCCGTCTCG   
  
  
- GTAAGAGGTG TTTTAGTTAT GAGAGGTCTC CTGTTCGCAC TACTGGACTC GTTCTCATCA CAATCTTCGA   
  
  
- CACAGTCAAT ATCGACACTG CCACGTTCAT CACCGTCACC GTAGCACCTC GTTTCAACCA GCTCAATAGG   
  
  
- AGGCCTCATG TTAGGGTACG CCTACCGTCT AAAAGGACGG AACGGGAGTT TTGGGAACAA CTCTTGTAGA   
  
  
- ATATCGAATA GTTTATCGAA CTGTCCACTC CCATTACCAA ACCACCTATC TGGCCGTCTG AGTGGACATT   
  
  
- CGCGAGAATC ACACAGACTA TATAAATTAC TATCAGTCAG TCACTATGTT AAAGTCTTTC CCTACCTCCT   
  
  
- TCGCTCGTTT AAAGAAGGGT TCCGAAGAAA CACAAACCTA AACTTGTGAC CTGGGACGGA AGCCCTCAGC   
  
  
- CAGTTATTCC GTGCTCCACC CGACTAAAAC CACCTCTGAT CGGTACTTTT ACTCCAACGT CACGATCGAT   
  
  
- CATCCCCTTT CTTCGTAGCG GTAGGACTCC TTAATGCAAG TCTTCAACCC TCCCCGTCAT TCGTCGGTCA   
  
  
- ACAAAAGGTC AAAAGTCTGC TCCGTCAACA CTCTAGTCTT TACAAACTAT CCCATAACGA CACGTCACCA   
  
  
- CCGTTTTTAC TACGACTGGA TGCGGTTTGA AATGTCTCAC TTGATTTATT TTGATACGTT TTACGAGTTT   
  
  
- ACTTTCTAAG ATTACCACCT TTTCCGGCAC CATCCTTCGT TCAACCGTCC TCCCTACACC ACCTAGATTC   
  
  
- TAGAGAAAAT GCGGAAACAC GTGTTCGTCA ACCTAGGTTA CTGGTTTCTT CGCGTTTACT GAATTAATCC   
  
  
- GTTTAATCCT TCGTAAGAAG AGGTTGGCCC CTACCCTTAG TTTCTTACCG AGTAATAAAA CGACTACCAG   
  
  
- AACTCCGAGC CGAACGACCA CAACCCTGGG GTTATATGTT CAGAGAGCAC TGACCGGGTC GAAGACGACA   
  
  
- ACTATAGAAC TCTCGAATGG TATACAAAGA ACGGTGTACG GGTAAGTTCT TTTATCCCTT AAAGAAGAGA   
  
  
- TTATCTTGTT AATACCTACA TCGAGTCTTT CGTTTAGCTG AAGTATAATA ACTAGAACCA TAACAGATAC   
  
  
- CAAAGGTTAC CGGAACAGAT TAAGTCGCGG AAAGTAGATC CGGACCGCCC GGAGGGTTTG AAGCCTAATG   
  
  
- TCCGCAACTA GAGGGGGTTG GGCCAAAGGC TGGTCGGTTT TCTCAGCTCC TCTGACCCGC TGCGAACTCC   
  
  
- TTGATACGAC TCAGTAAGTT ACACGGTAAA CTCAAGTTAC GGTATCGTTT CTCGACCCTT TGTGAATGAC   
  
  
- AATTTCTAGA GTTCTAACTA GGGCTACTCC ACGAACAACA CTTGACAGAC AAGTCCAAAT TTATGTAAGG   
  
  
- ACTCCTTTGT CATTAACGTC TTACGGGTTC TCTATAACAA GAATTAGACT AGTCTGTCTA TTTTGGTCGA   
  
  
- CAAAAGTATG TACCACATCA GTTACCTCGG AAGTTAAGAG GAAAAAAGTA ATAAGCCAAG GCACTCCGAG   
  
  
- AGAAGGTGAA GAGGTGAGAT AAACTACACA ACCTCCGTTT AGAAGGGGCC CTGTAGCTCC TCTCCTATGA   
  
  
- CTATCTCGCT CTGTAGAAAC CCGCTGTCCG TTACTTACAC TAGCGAACAC TCCCAAACCT CTCCTATCTT   
  
  
- TCCGGTCTCT TTATGTTTGT CACCGCCCAG GCTTTACTCT CCCGACCCAA CTTCGTCGAC GGAAACTTAG   
  
  
- TCCTCTGACA CCTTTACCGG TTTTTTGCCC ACTGTCGACA GATAGTGTTT CTAAAGAGGT AACTACTGCT   
  
  
- ACCCGTGACC AACAATGTCC CTACCTTTCC CTCTTAACAG ATACGTGAGT GATGAACCTT CGGACGACTG   
  
  
- AT

+     ERE

| Site Name | Organism | Position | Strand | Matrix score. | sequence | function |
| --- | --- | --- | --- | --- | --- | --- |
| ERE | Nicotiana glutinos | 1397 | + | 8 | ATTTTAAA |  |
| ERE | Nicotiana glutinos | 1885 | + | 8 | ATTTCATA |  |
| ERE | Nicotiana glutinos | 1447 | + | 8 | ATTTTAAA |  |
| ERE | Nicotiana glutinos | 1449 | - | 8 | ATTTTAAA |  |

>HU05G01597.1   
+ -Up\_Stream \_Len000TTTGTA TTTCAATATT GTAATTAGGT GGGATATAGA GTTGAAGGGC AAACTCTACC   
  
  
+ AACGACTTGT TGAAGGATCA ACTTGTAGCT TGGAACTTAA TGTATGTGTA CTATAGAGTT GTGTAATCTC   
  
  
+ TCCTAATAAA ATTGGGTTAA TCCTAGAATT AGAGAACTTT AAGCGGGGAC TAAGCTTGGT TGAGGCTAAC   
  
  
+ TTCGTTAAAA AGTTCTTGTG TCATGTCCAC GTTTATCTTT TCACTATTTA TTTCAATTAA TTTGCGATAT   
  
  
+ TTATCATCAC CCTATTCACC GTCTTTTCGG ATGATTTAAC TAGTCAAGCC GGACCAACAT TGCTTTTTAT   
  
  
+ AGTGTATTTG CAACTCATGT TGATTATTAG ATGTATCAAC AAAAAATGTT TCTCAAAATA AATAGTAGCT   
  
  
+ CTTTAAATTT TATTCCACAT AAATTCAAAA TCAATATCGA TATCGTTCCA TAATATAGCA GCATTATGAA   
  
  
+ AACAATATTT GCTAATGTTT ACATTGTGCT CTTTTCACTC AAAAATTGTA AGGCTTCAAA TACTATTAAA   
  
  
+ GAAACCATTA TTTCACTCGA CTATTTAGCT TGCATATATA GACAATCTTG CAAATAGTAA CATGTCTTCA   
  
  
+ TTCGACTATT TGCTTCCAAA TGAATAATCT CGTAAACATA TATACATACA TACATATATA TATATATATA   
  
  
+ TATATATATA TATATATATA GTAATATGTA TTTAACTTCA CATATACATG AATATAAAAA TATAAGAGGG   
  
  
+ AAAAAAGACG CTAGGTAGAT GGACTACAAA AGCCATTCAT GTATTGAGAC GGCTCATAAA ATCATACGGG   
  
  
+ GAAAGGAAAA AAAAAGATCT CTACATAATG TTCATAGATT TATAGAAAGC ATATGATGGA TTACCGCATA   
  
  
+ AGGTGTTATA TATGGGAGTG TTTGTAGAAG AAAGATGTAT ATCCGAACTA TCAAAGATAC ATATGAGGGA   
  
  
+ GTAAAAACAA GTGTAAGAAC CTCAGGAGGA GACATAAAAG ATTTTTCTAT TGGCATTGGA CACCGTCAAG   
  
  
+ TATCCGCTTT AAACCTGTTC TTTTTCACTA TTATCATGGA TGTATTCACA AAAGGAATTC AATACGAAGT   
  
  
+ ACCATGATGT ATGCTTTTTA CTGATAGTAT TGGTCTCATC GATGAGACTA AGGATGGTCT TAATAGCAAA   
  
  
+ TTAGAGATTT CATTAGATAA AATCTACTGT ACATGACAAA ACAATGATAT ATAGCATCCT ATTTAAAAAG   
  
  
+ CTACGTGTCA GTCATATATG AATTGCATCT AATTTTTAAT AGATATATAA TATGATATGA TATAATAGGA   
  
  
+ TATGATGTAG ATTGAATTCA AACTCATAAT TCATTAAGCA TGGTGCAATG TTCATTTTCT TGATTTTAAA   
  
  
+ CCGACTATTA CTTCAACTGT ATATAATTCG AATTGCTTTG GTATTTTAAA ATTTAAACTA GTAATTACTG   
  
  
+ CCTCTATTAC TGATGTTTAT ATTAAGTGGT GCTTAACTGC TTATTGATGA AACCATTTTT ATTTTCGTGT   
  
  
+ AAAAGAGGGC ATAATCCATC CTCGTGTATT GGCGGGGCTC GGCGCCCTAT TCAGCTATTC TCTGTATGTC   
  
  
+ AAACAAAACC GCTTCTATTA CCCATTGGTC TCTAAACACG CAGGGGTTTT AGCCCGGAGA GTTAATTCTT   
  
  
+ TATTGAGGTC CCCTGTTCTC CATTGCAGGA TTTTTGCTCA GGTACTTCTC TGTTTCTTCT CTTTAATCTT   
  
  
+ CTGTATCTTT CTGCATCAAG TTTAATGCTT TCTGGGATTT TCAGCTACGA TTTATGTGAA ATTGTAAGAT   
  
  
+ TTCTGAACTG GGCATTCAGG GCTTGGTTCA TATGTATTCT TTTCTGCTTG AAATGTTTTA ATTTCATAGT   
  
  
+ TCCAATGGGG TTATTCAAAG ATAGAATTAA GCTATAATCA CCGAGAAGAC TTGCTGACCT TTGTTTCTTT   
  
  
+ TTTGTTTCTT TTTTTTAATT AAATCTGGCT ATTACTTGGC TATAGTTTTT GCTAATGGAT CCACAAATAG   
  
  
+ ACGAATTATA TGGGCCTTTG CACCAAATGA AGTTCAATGA TCAGAGGAGA CCCATTTTAT CAAATGAAAA   
  
  
+ TTTTGTTAAT GTGTTGAAAC CGCCAAATTC CAATTGGAAT TCACCTTTTG GGAACCCTAC TGTGATTACC   
  
  
+ ACAAACCCAA ATTTTGATTT GGTTGTGCCA TCACTAGGTC CCAATGTTGA GGGAGCTCTC CTTGAAGATT   
  
  
+ ATGATTTTAG TGATGTTGTT CTTAAGTACA TCAATCAAGT TCTTATGGAC GAGGATGAGG GAGAGAAAAT   
  
  
+ CCATTTGGGC CACGGGCCTC TGGCTCTTGA AGCGGCCGAG AAGTCGTTAT ATGAGGTGCT TGGGCAGAGC   
  
  
+ CATTCTCCAC AAAATCAATA CTCTCCAGAG GACAAGCGTG ATGACCTGAG CAAGAGTAGT GTTAGAAGCT   
  
  
+ GTGTCAGTTA TAGCTGTGAC GGTGCAAGTA GTGGCAGTGG CATCGTGGAG CAAAGTTGGT CGAGTTATCC   
  
  
+ TCCGGAGTAC AATCCCATGC GGATGGCAGA TTTTCCTGCC TTGCCCTCAA AACCCTTGTT GAGAACATCT   
  
  
+ TATAGCTTAT CAAATAGCTT GACAGGTGAG GGTAATGGTT TGGTGGATAG ACCGGCAGAC TCACCTGTAA   
  
  
+ GCGCTCTTAG TGTGTCTGAT ATATTTAATG ATAGTCAGTC AGTGATACAA TTTCAGAAAG GGATGGAGGA   
  
  
+ AGCGAGCAAA TTTCTTCCCA AGGCTTCTTT GTGTTTGGAT TTGAACACTG GACCCTGCCT TCGGGAGTCG   
  
  
+ GTCAATAAGG CACGAGGTGG GCTGATTTTG GTGGAGACTA GCCATGAAAA TGAGGTTGCA GTGCTAGCTA   
  
  
+ GTAGGGGAAA GAAGCATCGC CATCCTGAGG AATTACGTTC AGAAGTTGGG AGGGGCAGTA AGCAGCCAGT   
  
  
+ TGTTTTCCAG TTTTCAGACG AGGCAGTTGT GAGATCAGAA ATGTTTGATA GGGTATTGCT GTGCAGTGGT   
  
  
+ GGCAAAAATG ATGCTGACCT ACGCCAAACT TTACAGAGTG AACTAAATAA AACTATGCAA AATGCTCAAA   
  
  
+ TGAAAGATTC TAATGGTGGA AAAGGCCGTG GTAGGAAGCA AGTTGGCAGG AGGGATGTGG TGGATCTAAG   
  
  
+ ATCTCTTTTA CGCCTTTGTG CACAAGCAGT TGGATCCAAT GACCAAAGAA GCGCAAATGA CTTAATTAGG   
  
  
+ CAAATTAGGA AGCATTCTTC TCCAACCGGG GATGGGAATC AAAGAATGGC TCATTATTTT GCTGATGGTC   
  
  
+ TTGAGGCTCG GCTTGCTGGT GTTGGGACCC CAATATACAA GTCTCTCGTG ACTGGCCCAG CTTCTGCTGT   
  
  
+ TGATATCTTG AGAGCTTACC ATATGTTTCT TGCCACATGC CCATTCAAGA AAATAGGGAA TTTCTTCTCT   
  
  
+ AATAGAACAA TTATGGATGT AGCTCAGAAA GCAAATCGAC TTCATATTAT TGATCTTGGT ATTGTCTATG   
  
  
+ GTTTCCAATG GCCTTGTCTA ATTCAGCGCC TTTCATCTAG GCCTGGCGGG CCTCCCAAAC TTCGGATTAC   
  
  
+ AGGCGTTGAT CTCCCCCAAC CCGGTTTCCG ACCAGCCAAA AGAGTCGAGG AGACTGGGCG ACGCTTGAGG   
  
  
+ AACTATGCTG AGTCATTCAA TGTGCCATTT GAGTTCAATG CCATAGCAAA GAGCTGGGAA ACACTTACTG   
  
  
+ TTAAAGATCT CAAGATTGAT CCCGATGAGG TGCTTGTTGT GAACTGTCTG TTCAGGTTTA AATACATTCC   
  
  
+ TGAGGAAACA GTAATTGCAG AATGCCCAAG AGATATTGTT CTTAATCTGA TCAGACAGAT AAAACCAGCT   
  
  
+ GTTTTCATAC ATGGTGTAGT CAATGGAGCC TTCAATTCTC CTTTTTTCAT TATTCGGTTC CGTGAGGCTC   
  
  
+ TCTTCCACTT CTCCACTCTA TTTGATGTGT TGGAGGCAAA TCTTCCCCGG GACATCGAGG AGAGGATACT   
  
  
+ GATAGAGCGA GACATCTTTG GGCGACAGGC AATGAATGTG ATCGCTTGTG AGGGTTTGGA GAGGATAGAA   
  
  
+ AGGCCAGAGA AATACAAACA GTGGCGGGTC CGAAATGAGA GGGCTGGGTT GAAGCAGCTG CCTTTGAATC   
  
  
+ AGGAGACTGT GGAAATGGCC AAAAAACGGG TGACAGCTGT CTATCACAAA GATTTCTCCA TTGATGACGA   
  
  
+ TGGGCACTGG TTGTTACAGG GATGGAAAGG GAGAATTGTC TATGCACTCA CTACTTGGAA GCCTGCTGAC   
  
  
+ TA  

- -Up\_Stream \_Len000AAACAT AAAGTTATAA CATTAATCCA CCCTATATCT CAACTTCCCG TTTGAGATGG   
  
  
- TTGCTGAACA ACTTCCTAGT TGAACATCGA ACCTTGAATT ACATACACAT GATATCTCAA CACATTAGAG   
  
  
- AGGATTATTT TAACCCAATT AGGATCTTAA TCTCTTGAAA TTCGCCCCTG ATTCGAACCA ACTCCGATTG   
  
  
- AAGCAATTTT TCAAGAACAC AGTACAGGTG CAAATAGAAA AGTGATAAAT AAAGTTAATT AAACGCTATA   
  
  
- AATAGTAGTG GGATAAGTGG CAGAAAAGCC TACTAAATTG ATCAGTTCGG CCTGGTTGTA ACGAAAAATA   
  
  
- TCACATAAAC GTTGAGTACA ACTAATAATC TACATAGTTG TTTTTTACAA AGAGTTTTAT TTATCATCGA   
  
  
- GAAATTTAAA ATAAGGTGTA TTTAAGTTTT AGTTATAGCT ATAGCAAGGT ATTATATCGT CGTAATACTT   
  
  
- TTGTTATAAA CGATTACAAA TGTAACACGA GAAAAGTGAG TTTTTAACAT TCCGAAGTTT ATGATAATTT   
  
  
- CTTTGGTAAT AAAGTGAGCT GATAAATCGA ACGTATATAT CTGTTAGAAC GTTTATCATT GTACAGAAGT   
  
  
- AAGCTGATAA ACGAAGGTTT ACTTATTAGA GCATTTGTAT ATATGTATGT ATGTATATAT ATATATATAT   
  
  
- ATATATATAT ATATATATAT CATTATACAT AAATTGAAGT GTATATGTAC TTATATTTTT ATATTCTCCC   
  
  
- TTTTTTCTGC GATCCATCTA CCTGATGTTT TCGGTAAGTA CATAACTCTG CCGAGTATTT TAGTATGCCC   
  
  
- CTTTCCTTTT TTTTTCTAGA GATGTATTAC AAGTATCTAA ATATCTTTCG TATACTACCT AATGGCGTAT   
  
  
- TCCACAATAT ATACCCTCAC AAACATCTTC TTTCTACATA TAGGCTTGAT AGTTTCTATG TATACTCCCT   
  
  
- CATTTTTGTT CACATTCTTG GAGTCCTCCT CTGTATTTTC TAAAAAGATA ACCGTAACCT GTGGCAGTTC   
  
  
- ATAGGCGAAA TTTGGACAAG AAAAAGTGAT AATAGTACCT ACATAAGTGT TTTCCTTAAG TTATGCTTCA   
  
  
- TGGTACTACA TACGAAAAAT GACTATCATA ACCAGAGTAG CTACTCTGAT TCCTACCAGA ATTATCGTTT   
  
  
- AATCTCTAAA GTAATCTATT TTAGATGACA TGTACTGTTT TGTTACTATA TATCGTAGGA TAAATTTTTC   
  
  
- GATGCACAGT CAGTATATAC TTAACGTAGA TTAAAAATTA TCTATATATT ATACTATACT ATATTATCCT   
  
  
- ATACTACATC TAACTTAAGT TTGAGTATTA AGTAATTCGT ACCACGTTAC AAGTAAAAGA ACTAAAATTT   
  
  
- GGCTGATAAT GAAGTTGACA TATATTAAGC TTAACGAAAC CATAAAATTT TAAATTTGAT CATTAATGAC   
  
  
- GGAGATAATG ACTACAAATA TAATTCACCA CGAATTGACG AATAACTACT TTGGTAAAAA TAAAAGCACA   
  
  
- TTTTCTCCCG TATTAGGTAG GAGCACATAA CCGCCCCGAG CCGCGGGATA AGTCGATAAG AGACATACAG   
  
  
- TTTGTTTTGG CGAAGATAAT GGGTAACCAG AGATTTGTGC GTCCCCAAAA TCGGGCCTCT CAATTAAGAA   
  
  
- ATAACTCCAG GGGACAAGAG GTAACGTCCT AAAAACGAGT CCATGAAGAG ACAAAGAAGA GAAATTAGAA   
  
  
- GACATAGAAA GACGTAGTTC AAATTACGAA AGACCCTAAA AGTCGATGCT AAATACACTT TAACATTCTA   
  
  
- AAGACTTGAC CCGTAAGTCC CGAACCAAGT ATACATAAGA AAAGACGAAC TTTACAAAAT TAAAGTATCA   
  
  
- AGGTTACCCC AATAAGTTTC TATCTTAATT CGATATTAGT GGCTCTTCTG AACGACTGGA AACAAAGAAA   
  
  
- AAACAAAGAA AAAAAATTAA TTTAGACCGA TAATGAACCG ATATCAAAAA CGATTACCTA GGTGTTTATC   
  
  
- TGCTTAATAT ACCCGGAAAC GTGGTTTACT TCAAGTTACT AGTCTCCTCT GGGTAAAATA GTTTACTTTT   
  
  
- AAAACAATTA CACAACTTTG GCGGTTTAAG GTTAACCTTA AGTGGAAAAC CCTTGGGATG ACACTAATGG   
  
  
- TGTTTGGGTT TAAAACTAAA CCAACACGGT AGTGATCCAG GGTTACAACT CCCTCGAGAG GAACTTCTAA   
  
  
- TACTAAAATC ACTACAACAA GAATTCATGT AGTTAGTTCA AGAATACCTG CTCCTACTCC CTCTCTTTTA   
  
  
- GGTAAACCCG GTGCCCGGAG ACCGAGAACT TCGCCGGCTC TTCAGCAATA TACTCCACGA ACCCGTCTCG   
  
  
- GTAAGAGGTG TTTTAGTTAT GAGAGGTCTC CTGTTCGCAC TACTGGACTC GTTCTCATCA CAATCTTCGA   
  
  
- CACAGTCAAT ATCGACACTG CCACGTTCAT CACCGTCACC GTAGCACCTC GTTTCAACCA GCTCAATAGG   
  
  
- AGGCCTCATG TTAGGGTACG CCTACCGTCT AAAAGGACGG AACGGGAGTT TTGGGAACAA CTCTTGTAGA   
  
  
- ATATCGAATA GTTTATCGAA CTGTCCACTC CCATTACCAA ACCACCTATC TGGCCGTCTG AGTGGACATT   
  
  
- CGCGAGAATC ACACAGACTA TATAAATTAC TATCAGTCAG TCACTATGTT AAAGTCTTTC CCTACCTCCT   
  
  
- TCGCTCGTTT AAAGAAGGGT TCCGAAGAAA CACAAACCTA AACTTGTGAC CTGGGACGGA AGCCCTCAGC   
  
  
- CAGTTATTCC GTGCTCCACC CGACTAAAAC CACCTCTGAT CGGTACTTTT ACTCCAACGT CACGATCGAT   
  
  
- CATCCCCTTT CTTCGTAGCG GTAGGACTCC TTAATGCAAG TCTTCAACCC TCCCCGTCAT TCGTCGGTCA   
  
  
- ACAAAAGGTC AAAAGTCTGC TCCGTCAACA CTCTAGTCTT TACAAACTAT CCCATAACGA CACGTCACCA   
  
  
- CCGTTTTTAC TACGACTGGA TGCGGTTTGA AATGTCTCAC TTGATTTATT TTGATACGTT TTACGAGTTT   
  
  
- ACTTTCTAAG ATTACCACCT TTTCCGGCAC CATCCTTCGT TCAACCGTCC TCCCTACACC ACCTAGATTC   
  
  
- TAGAGAAAAT GCGGAAACAC GTGTTCGTCA ACCTAGGTTA CTGGTTTCTT CGCGTTTACT GAATTAATCC   
  
  
- GTTTAATCCT TCGTAAGAAG AGGTTGGCCC CTACCCTTAG TTTCTTACCG AGTAATAAAA CGACTACCAG   
  
  
- AACTCCGAGC CGAACGACCA CAACCCTGGG GTTATATGTT CAGAGAGCAC TGACCGGGTC GAAGACGACA   
  
  
- ACTATAGAAC TCTCGAATGG TATACAAAGA ACGGTGTACG GGTAAGTTCT TTTATCCCTT AAAGAAGAGA   
  
  
- TTATCTTGTT AATACCTACA TCGAGTCTTT CGTTTAGCTG AAGTATAATA ACTAGAACCA TAACAGATAC   
  
  
- CAAAGGTTAC CGGAACAGAT TAAGTCGCGG AAAGTAGATC CGGACCGCCC GGAGGGTTTG AAGCCTAATG   
  
  
- TCCGCAACTA GAGGGGGTTG GGCCAAAGGC TGGTCGGTTT TCTCAGCTCC TCTGACCCGC TGCGAACTCC   
  
  
- TTGATACGAC TCAGTAAGTT ACACGGTAAA CTCAAGTTAC GGTATCGTTT CTCGACCCTT TGTGAATGAC   
  
  
- AATTTCTAGA GTTCTAACTA GGGCTACTCC ACGAACAACA CTTGACAGAC AAGTCCAAAT TTATGTAAGG   
  
  
- ACTCCTTTGT CATTAACGTC TTACGGGTTC TCTATAACAA GAATTAGACT AGTCTGTCTA TTTTGGTCGA   
  
  
- CAAAAGTATG TACCACATCA GTTACCTCGG AAGTTAAGAG GAAAAAAGTA ATAAGCCAAG GCACTCCGAG   
  
  
- AGAAGGTGAA GAGGTGAGAT AAACTACACA ACCTCCGTTT AGAAGGGGCC CTGTAGCTCC TCTCCTATGA   
  
  
- CTATCTCGCT CTGTAGAAAC CCGCTGTCCG TTACTTACAC TAGCGAACAC TCCCAAACCT CTCCTATCTT   
  
  
- TCCGGTCTCT TTATGTTTGT CACCGCCCAG GCTTTACTCT CCCGACCCAA CTTCGTCGAC GGAAACTTAG   
  
  
- TCCTCTGACA CCTTTACCGG TTTTTTGCCC ACTGTCGACA GATAGTGTTT CTAAAGAGGT AACTACTGCT   
  
  
- ACCCGTGACC AACAATGTCC CTACCTTTCC CTCTTAACAG ATACGTGAGT GATGAACCTT CGGACGACTG   
  
  
- AT

+     G-Box

| Site Name | Organism | Position | Strand | Matrix score. | sequence | function |
| --- | --- | --- | --- | --- | --- | --- |
| G-Box | Pisum sativum | 242 | + | 6 | CACGTT | cis-acting regulatory element involved in light responsiveness |

>HU05G01597.1   
+ -Up\_Stream \_Len000TTTGTA TTTCAATATT GTAATTAGGT GGGATATAGA GTTGAAGGGC AAACTCTACC   
  
  
+ AACGACTTGT TGAAGGATCA ACTTGTAGCT TGGAACTTAA TGTATGTGTA CTATAGAGTT GTGTAATCTC   
  
  
+ TCCTAATAAA ATTGGGTTAA TCCTAGAATT AGAGAACTTT AAGCGGGGAC TAAGCTTGGT TGAGGCTAAC   
  
  
+ TTCGTTAAAA AGTTCTTGTG TCATGTCCAC GTTTATCTTT TCACTATTTA TTTCAATTAA TTTGCGATAT   
  
  
+ TTATCATCAC CCTATTCACC GTCTTTTCGG ATGATTTAAC TAGTCAAGCC GGACCAACAT TGCTTTTTAT   
  
  
+ AGTGTATTTG CAACTCATGT TGATTATTAG ATGTATCAAC AAAAAATGTT TCTCAAAATA AATAGTAGCT   
  
  
+ CTTTAAATTT TATTCCACAT AAATTCAAAA TCAATATCGA TATCGTTCCA TAATATAGCA GCATTATGAA   
  
  
+ AACAATATTT GCTAATGTTT ACATTGTGCT CTTTTCACTC AAAAATTGTA AGGCTTCAAA TACTATTAAA   
  
  
+ GAAACCATTA TTTCACTCGA CTATTTAGCT TGCATATATA GACAATCTTG CAAATAGTAA CATGTCTTCA   
  
  
+ TTCGACTATT TGCTTCCAAA TGAATAATCT CGTAAACATA TATACATACA TACATATATA TATATATATA   
  
  
+ TATATATATA TATATATATA GTAATATGTA TTTAACTTCA CATATACATG AATATAAAAA TATAAGAGGG   
  
  
+ AAAAAAGACG CTAGGTAGAT GGACTACAAA AGCCATTCAT GTATTGAGAC GGCTCATAAA ATCATACGGG   
  
  
+ GAAAGGAAAA AAAAAGATCT CTACATAATG TTCATAGATT TATAGAAAGC ATATGATGGA TTACCGCATA   
  
  
+ AGGTGTTATA TATGGGAGTG TTTGTAGAAG AAAGATGTAT ATCCGAACTA TCAAAGATAC ATATGAGGGA   
  
  
+ GTAAAAACAA GTGTAAGAAC CTCAGGAGGA GACATAAAAG ATTTTTCTAT TGGCATTGGA CACCGTCAAG   
  
  
+ TATCCGCTTT AAACCTGTTC TTTTTCACTA TTATCATGGA TGTATTCACA AAAGGAATTC AATACGAAGT   
  
  
+ ACCATGATGT ATGCTTTTTA CTGATAGTAT TGGTCTCATC GATGAGACTA AGGATGGTCT TAATAGCAAA   
  
  
+ TTAGAGATTT CATTAGATAA AATCTACTGT ACATGACAAA ACAATGATAT ATAGCATCCT ATTTAAAAAG   
  
  
+ CTACGTGTCA GTCATATATG AATTGCATCT AATTTTTAAT AGATATATAA TATGATATGA TATAATAGGA   
  
  
+ TATGATGTAG ATTGAATTCA AACTCATAAT TCATTAAGCA TGGTGCAATG TTCATTTTCT TGATTTTAAA   
  
  
+ CCGACTATTA CTTCAACTGT ATATAATTCG AATTGCTTTG GTATTTTAAA ATTTAAACTA GTAATTACTG   
  
  
+ CCTCTATTAC TGATGTTTAT ATTAAGTGGT GCTTAACTGC TTATTGATGA AACCATTTTT ATTTTCGTGT   
  
  
+ AAAAGAGGGC ATAATCCATC CTCGTGTATT GGCGGGGCTC GGCGCCCTAT TCAGCTATTC TCTGTATGTC   
  
  
+ AAACAAAACC GCTTCTATTA CCCATTGGTC TCTAAACACG CAGGGGTTTT AGCCCGGAGA GTTAATTCTT   
  
  
+ TATTGAGGTC CCCTGTTCTC CATTGCAGGA TTTTTGCTCA GGTACTTCTC TGTTTCTTCT CTTTAATCTT   
  
  
+ CTGTATCTTT CTGCATCAAG TTTAATGCTT TCTGGGATTT TCAGCTACGA TTTATGTGAA ATTGTAAGAT   
  
  
+ TTCTGAACTG GGCATTCAGG GCTTGGTTCA TATGTATTCT TTTCTGCTTG AAATGTTTTA ATTTCATAGT   
  
  
+ TCCAATGGGG TTATTCAAAG ATAGAATTAA GCTATAATCA CCGAGAAGAC TTGCTGACCT TTGTTTCTTT   
  
  
+ TTTGTTTCTT TTTTTTAATT AAATCTGGCT ATTACTTGGC TATAGTTTTT GCTAATGGAT CCACAAATAG   
  
  
+ ACGAATTATA TGGGCCTTTG CACCAAATGA AGTTCAATGA TCAGAGGAGA CCCATTTTAT CAAATGAAAA   
  
  
+ TTTTGTTAAT GTGTTGAAAC CGCCAAATTC CAATTGGAAT TCACCTTTTG GGAACCCTAC TGTGATTACC   
  
  
+ ACAAACCCAA ATTTTGATTT GGTTGTGCCA TCACTAGGTC CCAATGTTGA GGGAGCTCTC CTTGAAGATT   
  
  
+ ATGATTTTAG TGATGTTGTT CTTAAGTACA TCAATCAAGT TCTTATGGAC GAGGATGAGG GAGAGAAAAT   
  
  
+ CCATTTGGGC CACGGGCCTC TGGCTCTTGA AGCGGCCGAG AAGTCGTTAT ATGAGGTGCT TGGGCAGAGC   
  
  
+ CATTCTCCAC AAAATCAATA CTCTCCAGAG GACAAGCGTG ATGACCTGAG CAAGAGTAGT GTTAGAAGCT   
  
  
+ GTGTCAGTTA TAGCTGTGAC GGTGCAAGTA GTGGCAGTGG CATCGTGGAG CAAAGTTGGT CGAGTTATCC   
  
  
+ TCCGGAGTAC AATCCCATGC GGATGGCAGA TTTTCCTGCC TTGCCCTCAA AACCCTTGTT GAGAACATCT   
  
  
+ TATAGCTTAT CAAATAGCTT GACAGGTGAG GGTAATGGTT TGGTGGATAG ACCGGCAGAC TCACCTGTAA   
  
  
+ GCGCTCTTAG TGTGTCTGAT ATATTTAATG ATAGTCAGTC AGTGATACAA TTTCAGAAAG GGATGGAGGA   
  
  
+ AGCGAGCAAA TTTCTTCCCA AGGCTTCTTT GTGTTTGGAT TTGAACACTG GACCCTGCCT TCGGGAGTCG   
  
  
+ GTCAATAAGG CACGAGGTGG GCTGATTTTG GTGGAGACTA GCCATGAAAA TGAGGTTGCA GTGCTAGCTA   
  
  
+ GTAGGGGAAA GAAGCATCGC CATCCTGAGG AATTACGTTC AGAAGTTGGG AGGGGCAGTA AGCAGCCAGT   
  
  
+ TGTTTTCCAG TTTTCAGACG AGGCAGTTGT GAGATCAGAA ATGTTTGATA GGGTATTGCT GTGCAGTGGT   
  
  
+ GGCAAAAATG ATGCTGACCT ACGCCAAACT TTACAGAGTG AACTAAATAA AACTATGCAA AATGCTCAAA   
  
  
+ TGAAAGATTC TAATGGTGGA AAAGGCCGTG GTAGGAAGCA AGTTGGCAGG AGGGATGTGG TGGATCTAAG   
  
  
+ ATCTCTTTTA CGCCTTTGTG CACAAGCAGT TGGATCCAAT GACCAAAGAA GCGCAAATGA CTTAATTAGG   
  
  
+ CAAATTAGGA AGCATTCTTC TCCAACCGGG GATGGGAATC AAAGAATGGC TCATTATTTT GCTGATGGTC   
  
  
+ TTGAGGCTCG GCTTGCTGGT GTTGGGACCC CAATATACAA GTCTCTCGTG ACTGGCCCAG CTTCTGCTGT   
  
  
+ TGATATCTTG AGAGCTTACC ATATGTTTCT TGCCACATGC CCATTCAAGA AAATAGGGAA TTTCTTCTCT   
  
  
+ AATAGAACAA TTATGGATGT AGCTCAGAAA GCAAATCGAC TTCATATTAT TGATCTTGGT ATTGTCTATG   
  
  
+ GTTTCCAATG GCCTTGTCTA ATTCAGCGCC TTTCATCTAG GCCTGGCGGG CCTCCCAAAC TTCGGATTAC   
  
  
+ AGGCGTTGAT CTCCCCCAAC CCGGTTTCCG ACCAGCCAAA AGAGTCGAGG AGACTGGGCG ACGCTTGAGG   
  
  
+ AACTATGCTG AGTCATTCAA TGTGCCATTT GAGTTCAATG CCATAGCAAA GAGCTGGGAA ACACTTACTG   
  
  
+ TTAAAGATCT CAAGATTGAT CCCGATGAGG TGCTTGTTGT GAACTGTCTG TTCAGGTTTA AATACATTCC   
  
  
+ TGAGGAAACA GTAATTGCAG AATGCCCAAG AGATATTGTT CTTAATCTGA TCAGACAGAT AAAACCAGCT   
  
  
+ GTTTTCATAC ATGGTGTAGT CAATGGAGCC TTCAATTCTC CTTTTTTCAT TATTCGGTTC CGTGAGGCTC   
  
  
+ TCTTCCACTT CTCCACTCTA TTTGATGTGT TGGAGGCAAA TCTTCCCCGG GACATCGAGG AGAGGATACT   
  
  
+ GATAGAGCGA GACATCTTTG GGCGACAGGC AATGAATGTG ATCGCTTGTG AGGGTTTGGA GAGGATAGAA   
  
  
+ AGGCCAGAGA AATACAAACA GTGGCGGGTC CGAAATGAGA GGGCTGGGTT GAAGCAGCTG CCTTTGAATC   
  
  
+ AGGAGACTGT GGAAATGGCC AAAAAACGGG TGACAGCTGT CTATCACAAA GATTTCTCCA TTGATGACGA   
  
  
+ TGGGCACTGG TTGTTACAGG GATGGAAAGG GAGAATTGTC TATGCACTCA CTACTTGGAA GCCTGCTGAC   
  
  
+ TA  

- -Up\_Stream \_Len000AAACAT AAAGTTATAA CATTAATCCA CCCTATATCT CAACTTCCCG TTTGAGATGG   
  
  
- TTGCTGAACA ACTTCCTAGT TGAACATCGA ACCTTGAATT ACATACACAT GATATCTCAA CACATTAGAG   
  
  
- AGGATTATTT TAACCCAATT AGGATCTTAA TCTCTTGAAA TTCGCCCCTG ATTCGAACCA ACTCCGATTG   
  
  
- AAGCAATTTT TCAAGAACAC AGTACAGGTG CAAATAGAAA AGTGATAAAT AAAGTTAATT AAACGCTATA   
  
  
- AATAGTAGTG GGATAAGTGG CAGAAAAGCC TACTAAATTG ATCAGTTCGG CCTGGTTGTA ACGAAAAATA   
  
  
- TCACATAAAC GTTGAGTACA ACTAATAATC TACATAGTTG TTTTTTACAA AGAGTTTTAT TTATCATCGA   
  
  
- GAAATTTAAA ATAAGGTGTA TTTAAGTTTT AGTTATAGCT ATAGCAAGGT ATTATATCGT CGTAATACTT   
  
  
- TTGTTATAAA CGATTACAAA TGTAACACGA GAAAAGTGAG TTTTTAACAT TCCGAAGTTT ATGATAATTT   
  
  
- CTTTGGTAAT AAAGTGAGCT GATAAATCGA ACGTATATAT CTGTTAGAAC GTTTATCATT GTACAGAAGT   
  
  
- AAGCTGATAA ACGAAGGTTT ACTTATTAGA GCATTTGTAT ATATGTATGT ATGTATATAT ATATATATAT   
  
  
- ATATATATAT ATATATATAT CATTATACAT AAATTGAAGT GTATATGTAC TTATATTTTT ATATTCTCCC   
  
  
- TTTTTTCTGC GATCCATCTA CCTGATGTTT TCGGTAAGTA CATAACTCTG CCGAGTATTT TAGTATGCCC   
  
  
- CTTTCCTTTT TTTTTCTAGA GATGTATTAC AAGTATCTAA ATATCTTTCG TATACTACCT AATGGCGTAT   
  
  
- TCCACAATAT ATACCCTCAC AAACATCTTC TTTCTACATA TAGGCTTGAT AGTTTCTATG TATACTCCCT   
  
  
- CATTTTTGTT CACATTCTTG GAGTCCTCCT CTGTATTTTC TAAAAAGATA ACCGTAACCT GTGGCAGTTC   
  
  
- ATAGGCGAAA TTTGGACAAG AAAAAGTGAT AATAGTACCT ACATAAGTGT TTTCCTTAAG TTATGCTTCA   
  
  
- TGGTACTACA TACGAAAAAT GACTATCATA ACCAGAGTAG CTACTCTGAT TCCTACCAGA ATTATCGTTT   
  
  
- AATCTCTAAA GTAATCTATT TTAGATGACA TGTACTGTTT TGTTACTATA TATCGTAGGA TAAATTTTTC   
  
  
- GATGCACAGT CAGTATATAC TTAACGTAGA TTAAAAATTA TCTATATATT ATACTATACT ATATTATCCT   
  
  
- ATACTACATC TAACTTAAGT TTGAGTATTA AGTAATTCGT ACCACGTTAC AAGTAAAAGA ACTAAAATTT   
  
  
- GGCTGATAAT GAAGTTGACA TATATTAAGC TTAACGAAAC CATAAAATTT TAAATTTGAT CATTAATGAC   
  
  
- GGAGATAATG ACTACAAATA TAATTCACCA CGAATTGACG AATAACTACT TTGGTAAAAA TAAAAGCACA   
  
  
- TTTTCTCCCG TATTAGGTAG GAGCACATAA CCGCCCCGAG CCGCGGGATA AGTCGATAAG AGACATACAG   
  
  
- TTTGTTTTGG CGAAGATAAT GGGTAACCAG AGATTTGTGC GTCCCCAAAA TCGGGCCTCT CAATTAAGAA   
  
  
- ATAACTCCAG GGGACAAGAG GTAACGTCCT AAAAACGAGT CCATGAAGAG ACAAAGAAGA GAAATTAGAA   
  
  
- GACATAGAAA GACGTAGTTC AAATTACGAA AGACCCTAAA AGTCGATGCT AAATACACTT TAACATTCTA   
  
  
- AAGACTTGAC CCGTAAGTCC CGAACCAAGT ATACATAAGA AAAGACGAAC TTTACAAAAT TAAAGTATCA   
  
  
- AGGTTACCCC AATAAGTTTC TATCTTAATT CGATATTAGT GGCTCTTCTG AACGACTGGA AACAAAGAAA   
  
  
- AAACAAAGAA AAAAAATTAA TTTAGACCGA TAATGAACCG ATATCAAAAA CGATTACCTA GGTGTTTATC   
  
  
- TGCTTAATAT ACCCGGAAAC GTGGTTTACT TCAAGTTACT AGTCTCCTCT GGGTAAAATA GTTTACTTTT   
  
  
- AAAACAATTA CACAACTTTG GCGGTTTAAG GTTAACCTTA AGTGGAAAAC CCTTGGGATG ACACTAATGG   
  
  
- TGTTTGGGTT TAAAACTAAA CCAACACGGT AGTGATCCAG GGTTACAACT CCCTCGAGAG GAACTTCTAA   
  
  
- TACTAAAATC ACTACAACAA GAATTCATGT AGTTAGTTCA AGAATACCTG CTCCTACTCC CTCTCTTTTA   
  
  
- GGTAAACCCG GTGCCCGGAG ACCGAGAACT TCGCCGGCTC TTCAGCAATA TACTCCACGA ACCCGTCTCG   
  
  
- GTAAGAGGTG TTTTAGTTAT GAGAGGTCTC CTGTTCGCAC TACTGGACTC GTTCTCATCA CAATCTTCGA   
  
  
- CACAGTCAAT ATCGACACTG CCACGTTCAT CACCGTCACC GTAGCACCTC GTTTCAACCA GCTCAATAGG   
  
  
- AGGCCTCATG TTAGGGTACG CCTACCGTCT AAAAGGACGG AACGGGAGTT TTGGGAACAA CTCTTGTAGA   
  
  
- ATATCGAATA GTTTATCGAA CTGTCCACTC CCATTACCAA ACCACCTATC TGGCCGTCTG AGTGGACATT   
  
  
- CGCGAGAATC ACACAGACTA TATAAATTAC TATCAGTCAG TCACTATGTT AAAGTCTTTC CCTACCTCCT   
  
  
- TCGCTCGTTT AAAGAAGGGT TCCGAAGAAA CACAAACCTA AACTTGTGAC CTGGGACGGA AGCCCTCAGC   
  
  
- CAGTTATTCC GTGCTCCACC CGACTAAAAC CACCTCTGAT CGGTACTTTT ACTCCAACGT CACGATCGAT   
  
  
- CATCCCCTTT CTTCGTAGCG GTAGGACTCC TTAATGCAAG TCTTCAACCC TCCCCGTCAT TCGTCGGTCA   
  
  
- ACAAAAGGTC AAAAGTCTGC TCCGTCAACA CTCTAGTCTT TACAAACTAT CCCATAACGA CACGTCACCA   
  
  
- CCGTTTTTAC TACGACTGGA TGCGGTTTGA AATGTCTCAC TTGATTTATT TTGATACGTT TTACGAGTTT   
  
  
- ACTTTCTAAG ATTACCACCT TTTCCGGCAC CATCCTTCGT TCAACCGTCC TCCCTACACC ACCTAGATTC   
  
  
- TAGAGAAAAT GCGGAAACAC GTGTTCGTCA ACCTAGGTTA CTGGTTTCTT CGCGTTTACT GAATTAATCC   
  
  
- GTTTAATCCT TCGTAAGAAG AGGTTGGCCC CTACCCTTAG TTTCTTACCG AGTAATAAAA CGACTACCAG   
  
  
- AACTCCGAGC CGAACGACCA CAACCCTGGG GTTATATGTT CAGAGAGCAC TGACCGGGTC GAAGACGACA   
  
  
- ACTATAGAAC TCTCGAATGG TATACAAAGA ACGGTGTACG GGTAAGTTCT TTTATCCCTT AAAGAAGAGA   
  
  
- TTATCTTGTT AATACCTACA TCGAGTCTTT CGTTTAGCTG AAGTATAATA ACTAGAACCA TAACAGATAC   
  
  
- CAAAGGTTAC CGGAACAGAT TAAGTCGCGG AAAGTAGATC CGGACCGCCC GGAGGGTTTG AAGCCTAATG   
  
  
- TCCGCAACTA GAGGGGGTTG GGCCAAAGGC TGGTCGGTTT TCTCAGCTCC TCTGACCCGC TGCGAACTCC   
  
  
- TTGATACGAC TCAGTAAGTT ACACGGTAAA CTCAAGTTAC GGTATCGTTT CTCGACCCTT TGTGAATGAC   
  
  
- AATTTCTAGA GTTCTAACTA GGGCTACTCC ACGAACAACA CTTGACAGAC AAGTCCAAAT TTATGTAAGG   
  
  
- ACTCCTTTGT CATTAACGTC TTACGGGTTC TCTATAACAA GAATTAGACT AGTCTGTCTA TTTTGGTCGA   
  
  
- CAAAAGTATG TACCACATCA GTTACCTCGG AAGTTAAGAG GAAAAAAGTA ATAAGCCAAG GCACTCCGAG   
  
  
- AGAAGGTGAA GAGGTGAGAT AAACTACACA ACCTCCGTTT AGAAGGGGCC CTGTAGCTCC TCTCCTATGA   
  
  
- CTATCTCGCT CTGTAGAAAC CCGCTGTCCG TTACTTACAC TAGCGAACAC TCCCAAACCT CTCCTATCTT   
  
  
- TCCGGTCTCT TTATGTTTGT CACCGCCCAG GCTTTACTCT CCCGACCCAA CTTCGTCGAC GGAAACTTAG   
  
  
- TCCTCTGACA CCTTTACCGG TTTTTTGCCC ACTGTCGACA GATAGTGTTT CTAAAGAGGT AACTACTGCT   
  
  
- ACCCGTGACC AACAATGTCC CTACCTTTCC CTCTTAACAG ATACGTGAGT GATGAACCTT CGGACGACTG   
  
  
- AT

+     G-box

| Site Name | Organism | Position | Strand | Matrix score. | sequence | function |
| --- | --- | --- | --- | --- | --- | --- |
| G-box | Arabidopsis thaliana | 1266 | + | 6 | TACGTG | cis-acting regulatory element involved in light responsiveness |
| G-box | Brassica oleracea | 1265 | - | 9 | TAACACGTAG | cis-acting regulatory element involved in light responsiveness |
| G-box | Nicotiana plumbaginifolia | 2959 | + | 10 | CAGACGTGGCA | cis-acting regulatory element involved in light responsiveness |
| G-box | Brassica oleracea | 242 | - | 8 | TAAACGTG | cis-acting regulatory element involved in light responsiveness |

>HU05G01597.1   
+ -Up\_Stream \_Len000TTTGTA TTTCAATATT GTAATTAGGT GGGATATAGA GTTGAAGGGC AAACTCTACC   
  
  
+ AACGACTTGT TGAAGGATCA ACTTGTAGCT TGGAACTTAA TGTATGTGTA CTATAGAGTT GTGTAATCTC   
  
  
+ TCCTAATAAA ATTGGGTTAA TCCTAGAATT AGAGAACTTT AAGCGGGGAC TAAGCTTGGT TGAGGCTAAC   
  
  
+ TTCGTTAAAA AGTTCTTGTG TCATGTCCAC GTTTATCTTT TCACTATTTA TTTCAATTAA TTTGCGATAT   
  
  
+ TTATCATCAC CCTATTCACC GTCTTTTCGG ATGATTTAAC TAGTCAAGCC GGACCAACAT TGCTTTTTAT   
  
  
+ AGTGTATTTG CAACTCATGT TGATTATTAG ATGTATCAAC AAAAAATGTT TCTCAAAATA AATAGTAGCT   
  
  
+ CTTTAAATTT TATTCCACAT AAATTCAAAA TCAATATCGA TATCGTTCCA TAATATAGCA GCATTATGAA   
  
  
+ AACAATATTT GCTAATGTTT ACATTGTGCT CTTTTCACTC AAAAATTGTA AGGCTTCAAA TACTATTAAA   
  
  
+ GAAACCATTA TTTCACTCGA CTATTTAGCT TGCATATATA GACAATCTTG CAAATAGTAA CATGTCTTCA   
  
  
+ TTCGACTATT TGCTTCCAAA TGAATAATCT CGTAAACATA TATACATACA TACATATATA TATATATATA   
  
  
+ TATATATATA TATATATATA GTAATATGTA TTTAACTTCA CATATACATG AATATAAAAA TATAAGAGGG   
  
  
+ AAAAAAGACG CTAGGTAGAT GGACTACAAA AGCCATTCAT GTATTGAGAC GGCTCATAAA ATCATACGGG   
  
  
+ GAAAGGAAAA AAAAAGATCT CTACATAATG TTCATAGATT TATAGAAAGC ATATGATGGA TTACCGCATA   
  
  
+ AGGTGTTATA TATGGGAGTG TTTGTAGAAG AAAGATGTAT ATCCGAACTA TCAAAGATAC ATATGAGGGA   
  
  
+ GTAAAAACAA GTGTAAGAAC CTCAGGAGGA GACATAAAAG ATTTTTCTAT TGGCATTGGA CACCGTCAAG   
  
  
+ TATCCGCTTT AAACCTGTTC TTTTTCACTA TTATCATGGA TGTATTCACA AAAGGAATTC AATACGAAGT   
  
  
+ ACCATGATGT ATGCTTTTTA CTGATAGTAT TGGTCTCATC GATGAGACTA AGGATGGTCT TAATAGCAAA   
  
  
+ TTAGAGATTT CATTAGATAA AATCTACTGT ACATGACAAA ACAATGATAT ATAGCATCCT ATTTAAAAAG   
  
  
+ CTACGTGTCA GTCATATATG AATTGCATCT AATTTTTAAT AGATATATAA TATGATATGA TATAATAGGA   
  
  
+ TATGATGTAG ATTGAATTCA AACTCATAAT TCATTAAGCA TGGTGCAATG TTCATTTTCT TGATTTTAAA   
  
  
+ CCGACTATTA CTTCAACTGT ATATAATTCG AATTGCTTTG GTATTTTAAA ATTTAAACTA GTAATTACTG   
  
  
+ CCTCTATTAC TGATGTTTAT ATTAAGTGGT GCTTAACTGC TTATTGATGA AACCATTTTT ATTTTCGTGT   
  
  
+ AAAAGAGGGC ATAATCCATC CTCGTGTATT GGCGGGGCTC GGCGCCCTAT TCAGCTATTC TCTGTATGTC   
  
  
+ AAACAAAACC GCTTCTATTA CCCATTGGTC TCTAAACACG CAGGGGTTTT AGCCCGGAGA GTTAATTCTT   
  
  
+ TATTGAGGTC CCCTGTTCTC CATTGCAGGA TTTTTGCTCA GGTACTTCTC TGTTTCTTCT CTTTAATCTT   
  
  
+ CTGTATCTTT CTGCATCAAG TTTAATGCTT TCTGGGATTT TCAGCTACGA TTTATGTGAA ATTGTAAGAT   
  
  
+ TTCTGAACTG GGCATTCAGG GCTTGGTTCA TATGTATTCT TTTCTGCTTG AAATGTTTTA ATTTCATAGT   
  
  
+ TCCAATGGGG TTATTCAAAG ATAGAATTAA GCTATAATCA CCGAGAAGAC TTGCTGACCT TTGTTTCTTT   
  
  
+ TTTGTTTCTT TTTTTTAATT AAATCTGGCT ATTACTTGGC TATAGTTTTT GCTAATGGAT CCACAAATAG   
  
  
+ ACGAATTATA TGGGCCTTTG CACCAAATGA AGTTCAATGA TCAGAGGAGA CCCATTTTAT CAAATGAAAA   
  
  
+ TTTTGTTAAT GTGTTGAAAC CGCCAAATTC CAATTGGAAT TCACCTTTTG GGAACCCTAC TGTGATTACC   
  
  
+ ACAAACCCAA ATTTTGATTT GGTTGTGCCA TCACTAGGTC CCAATGTTGA GGGAGCTCTC CTTGAAGATT   
  
  
+ ATGATTTTAG TGATGTTGTT CTTAAGTACA TCAATCAAGT TCTTATGGAC GAGGATGAGG GAGAGAAAAT   
  
  
+ CCATTTGGGC CACGGGCCTC TGGCTCTTGA AGCGGCCGAG AAGTCGTTAT ATGAGGTGCT TGGGCAGAGC   
  
  
+ CATTCTCCAC AAAATCAATA CTCTCCAGAG GACAAGCGTG ATGACCTGAG CAAGAGTAGT GTTAGAAGCT   
  
  
+ GTGTCAGTTA TAGCTGTGAC GGTGCAAGTA GTGGCAGTGG CATCGTGGAG CAAAGTTGGT CGAGTTATCC   
  
  
+ TCCGGAGTAC AATCCCATGC GGATGGCAGA TTTTCCTGCC TTGCCCTCAA AACCCTTGTT GAGAACATCT   
  
  
+ TATAGCTTAT CAAATAGCTT GACAGGTGAG GGTAATGGTT TGGTGGATAG ACCGGCAGAC TCACCTGTAA   
  
  
+ GCGCTCTTAG TGTGTCTGAT ATATTTAATG ATAGTCAGTC AGTGATACAA TTTCAGAAAG GGATGGAGGA   
  
  
+ AGCGAGCAAA TTTCTTCCCA AGGCTTCTTT GTGTTTGGAT TTGAACACTG GACCCTGCCT TCGGGAGTCG   
  
  
+ GTCAATAAGG CACGAGGTGG GCTGATTTTG GTGGAGACTA GCCATGAAAA TGAGGTTGCA GTGCTAGCTA   
  
  
+ GTAGGGGAAA GAAGCATCGC CATCCTGAGG AATTACGTTC AGAAGTTGGG AGGGGCAGTA AGCAGCCAGT   
  
  
+ TGTTTTCCAG TTTTCAGACG AGGCAGTTGT GAGATCAGAA ATGTTTGATA GGGTATTGCT GTGCAGTGGT   
  
  
+ GGCAAAAATG ATGCTGACCT ACGCCAAACT TTACAGAGTG AACTAAATAA AACTATGCAA AATGCTCAAA   
  
  
+ TGAAAGATTC TAATGGTGGA AAAGGCCGTG GTAGGAAGCA AGTTGGCAGG AGGGATGTGG TGGATCTAAG   
  
  
+ ATCTCTTTTA CGCCTTTGTG CACAAGCAGT TGGATCCAAT GACCAAAGAA GCGCAAATGA CTTAATTAGG   
  
  
+ CAAATTAGGA AGCATTCTTC TCCAACCGGG GATGGGAATC AAAGAATGGC TCATTATTTT GCTGATGGTC   
  
  
+ TTGAGGCTCG GCTTGCTGGT GTTGGGACCC CAATATACAA GTCTCTCGTG ACTGGCCCAG CTTCTGCTGT   
  
  
+ TGATATCTTG AGAGCTTACC ATATGTTTCT TGCCACATGC CCATTCAAGA AAATAGGGAA TTTCTTCTCT   
  
  
+ AATAGAACAA TTATGGATGT AGCTCAGAAA GCAAATCGAC TTCATATTAT TGATCTTGGT ATTGTCTATG   
  
  
+ GTTTCCAATG GCCTTGTCTA ATTCAGCGCC TTTCATCTAG GCCTGGCGGG CCTCCCAAAC TTCGGATTAC   
  
  
+ AGGCGTTGAT CTCCCCCAAC CCGGTTTCCG ACCAGCCAAA AGAGTCGAGG AGACTGGGCG ACGCTTGAGG   
  
  
+ AACTATGCTG AGTCATTCAA TGTGCCATTT GAGTTCAATG CCATAGCAAA GAGCTGGGAA ACACTTACTG   
  
  
+ TTAAAGATCT CAAGATTGAT CCCGATGAGG TGCTTGTTGT GAACTGTCTG TTCAGGTTTA AATACATTCC   
  
  
+ TGAGGAAACA GTAATTGCAG AATGCCCAAG AGATATTGTT CTTAATCTGA TCAGACAGAT AAAACCAGCT   
  
  
+ GTTTTCATAC ATGGTGTAGT CAATGGAGCC TTCAATTCTC CTTTTTTCAT TATTCGGTTC CGTGAGGCTC   
  
  
+ TCTTCCACTT CTCCACTCTA TTTGATGTGT TGGAGGCAAA TCTTCCCCGG GACATCGAGG AGAGGATACT   
  
  
+ GATAGAGCGA GACATCTTTG GGCGACAGGC AATGAATGTG ATCGCTTGTG AGGGTTTGGA GAGGATAGAA   
  
  
+ AGGCCAGAGA AATACAAACA GTGGCGGGTC CGAAATGAGA GGGCTGGGTT GAAGCAGCTG CCTTTGAATC   
  
  
+ AGGAGACTGT GGAAATGGCC AAAAAACGGG TGACAGCTGT CTATCACAAA GATTTCTCCA TTGATGACGA   
  
  
+ TGGGCACTGG TTGTTACAGG GATGGAAAGG GAGAATTGTC TATGCACTCA CTACTTGGAA GCCTGCTGAC   
  
  
+ TA  

- -Up\_Stream \_Len000AAACAT AAAGTTATAA CATTAATCCA CCCTATATCT CAACTTCCCG TTTGAGATGG   
  
  
- TTGCTGAACA ACTTCCTAGT TGAACATCGA ACCTTGAATT ACATACACAT GATATCTCAA CACATTAGAG   
  
  
- AGGATTATTT TAACCCAATT AGGATCTTAA TCTCTTGAAA TTCGCCCCTG ATTCGAACCA ACTCCGATTG   
  
  
- AAGCAATTTT TCAAGAACAC AGTACAGGTG CAAATAGAAA AGTGATAAAT AAAGTTAATT AAACGCTATA   
  
  
- AATAGTAGTG GGATAAGTGG CAGAAAAGCC TACTAAATTG ATCAGTTCGG CCTGGTTGTA ACGAAAAATA   
  
  
- TCACATAAAC GTTGAGTACA ACTAATAATC TACATAGTTG TTTTTTACAA AGAGTTTTAT TTATCATCGA   
  
  
- GAAATTTAAA ATAAGGTGTA TTTAAGTTTT AGTTATAGCT ATAGCAAGGT ATTATATCGT CGTAATACTT   
  
  
- TTGTTATAAA CGATTACAAA TGTAACACGA GAAAAGTGAG TTTTTAACAT TCCGAAGTTT ATGATAATTT   
  
  
- CTTTGGTAAT AAAGTGAGCT GATAAATCGA ACGTATATAT CTGTTAGAAC GTTTATCATT GTACAGAAGT   
  
  
- AAGCTGATAA ACGAAGGTTT ACTTATTAGA GCATTTGTAT ATATGTATGT ATGTATATAT ATATATATAT   
  
  
- ATATATATAT ATATATATAT CATTATACAT AAATTGAAGT GTATATGTAC TTATATTTTT ATATTCTCCC   
  
  
- TTTTTTCTGC GATCCATCTA CCTGATGTTT TCGGTAAGTA CATAACTCTG CCGAGTATTT TAGTATGCCC   
  
  
- CTTTCCTTTT TTTTTCTAGA GATGTATTAC AAGTATCTAA ATATCTTTCG TATACTACCT AATGGCGTAT   
  
  
- TCCACAATAT ATACCCTCAC AAACATCTTC TTTCTACATA TAGGCTTGAT AGTTTCTATG TATACTCCCT   
  
  
- CATTTTTGTT CACATTCTTG GAGTCCTCCT CTGTATTTTC TAAAAAGATA ACCGTAACCT GTGGCAGTTC   
  
  
- ATAGGCGAAA TTTGGACAAG AAAAAGTGAT AATAGTACCT ACATAAGTGT TTTCCTTAAG TTATGCTTCA   
  
  
- TGGTACTACA TACGAAAAAT GACTATCATA ACCAGAGTAG CTACTCTGAT TCCTACCAGA ATTATCGTTT   
  
  
- AATCTCTAAA GTAATCTATT TTAGATGACA TGTACTGTTT TGTTACTATA TATCGTAGGA TAAATTTTTC   
  
  
- GATGCACAGT CAGTATATAC TTAACGTAGA TTAAAAATTA TCTATATATT ATACTATACT ATATTATCCT   
  
  
- ATACTACATC TAACTTAAGT TTGAGTATTA AGTAATTCGT ACCACGTTAC AAGTAAAAGA ACTAAAATTT   
  
  
- GGCTGATAAT GAAGTTGACA TATATTAAGC TTAACGAAAC CATAAAATTT TAAATTTGAT CATTAATGAC   
  
  
- GGAGATAATG ACTACAAATA TAATTCACCA CGAATTGACG AATAACTACT TTGGTAAAAA TAAAAGCACA   
  
  
- TTTTCTCCCG TATTAGGTAG GAGCACATAA CCGCCCCGAG CCGCGGGATA AGTCGATAAG AGACATACAG   
  
  
- TTTGTTTTGG CGAAGATAAT GGGTAACCAG AGATTTGTGC GTCCCCAAAA TCGGGCCTCT CAATTAAGAA   
  
  
- ATAACTCCAG GGGACAAGAG GTAACGTCCT AAAAACGAGT CCATGAAGAG ACAAAGAAGA GAAATTAGAA   
  
  
- GACATAGAAA GACGTAGTTC AAATTACGAA AGACCCTAAA AGTCGATGCT AAATACACTT TAACATTCTA   
  
  
- AAGACTTGAC CCGTAAGTCC CGAACCAAGT ATACATAAGA AAAGACGAAC TTTACAAAAT TAAAGTATCA   
  
  
- AGGTTACCCC AATAAGTTTC TATCTTAATT CGATATTAGT GGCTCTTCTG AACGACTGGA AACAAAGAAA   
  
  
- AAACAAAGAA AAAAAATTAA TTTAGACCGA TAATGAACCG ATATCAAAAA CGATTACCTA GGTGTTTATC   
  
  
- TGCTTAATAT ACCCGGAAAC GTGGTTTACT TCAAGTTACT AGTCTCCTCT GGGTAAAATA GTTTACTTTT   
  
  
- AAAACAATTA CACAACTTTG GCGGTTTAAG GTTAACCTTA AGTGGAAAAC CCTTGGGATG ACACTAATGG   
  
  
- TGTTTGGGTT TAAAACTAAA CCAACACGGT AGTGATCCAG GGTTACAACT CCCTCGAGAG GAACTTCTAA   
  
  
- TACTAAAATC ACTACAACAA GAATTCATGT AGTTAGTTCA AGAATACCTG CTCCTACTCC CTCTCTTTTA   
  
  
- GGTAAACCCG GTGCCCGGAG ACCGAGAACT TCGCCGGCTC TTCAGCAATA TACTCCACGA ACCCGTCTCG   
  
  
- GTAAGAGGTG TTTTAGTTAT GAGAGGTCTC CTGTTCGCAC TACTGGACTC GTTCTCATCA CAATCTTCGA   
  
  
- CACAGTCAAT ATCGACACTG CCACGTTCAT CACCGTCACC GTAGCACCTC GTTTCAACCA GCTCAATAGG   
  
  
- AGGCCTCATG TTAGGGTACG CCTACCGTCT AAAAGGACGG AACGGGAGTT TTGGGAACAA CTCTTGTAGA   
  
  
- ATATCGAATA GTTTATCGAA CTGTCCACTC CCATTACCAA ACCACCTATC TGGCCGTCTG AGTGGACATT   
  
  
- CGCGAGAATC ACACAGACTA TATAAATTAC TATCAGTCAG TCACTATGTT AAAGTCTTTC CCTACCTCCT   
  
  
- TCGCTCGTTT AAAGAAGGGT TCCGAAGAAA CACAAACCTA AACTTGTGAC CTGGGACGGA AGCCCTCAGC   
  
  
- CAGTTATTCC GTGCTCCACC CGACTAAAAC CACCTCTGAT CGGTACTTTT ACTCCAACGT CACGATCGAT   
  
  
- CATCCCCTTT CTTCGTAGCG GTAGGACTCC TTAATGCAAG TCTTCAACCC TCCCCGTCAT TCGTCGGTCA   
  
  
- ACAAAAGGTC AAAAGTCTGC TCCGTCAACA CTCTAGTCTT TACAAACTAT CCCATAACGA CACGTCACCA   
  
  
- CCGTTTTTAC TACGACTGGA TGCGGTTTGA AATGTCTCAC TTGATTTATT TTGATACGTT TTACGAGTTT   
  
  
- ACTTTCTAAG ATTACCACCT TTTCCGGCAC CATCCTTCGT TCAACCGTCC TCCCTACACC ACCTAGATTC   
  
  
- TAGAGAAAAT GCGGAAACAC GTGTTCGTCA ACCTAGGTTA CTGGTTTCTT CGCGTTTACT GAATTAATCC   
  
  
- GTTTAATCCT TCGTAAGAAG AGGTTGGCCC CTACCCTTAG TTTCTTACCG AGTAATAAAA CGACTACCAG   
  
  
- AACTCCGAGC CGAACGACCA CAACCCTGGG GTTATATGTT CAGAGAGCAC TGACCGGGTC GAAGACGACA   
  
  
- ACTATAGAAC TCTCGAATGG TATACAAAGA ACGGTGTACG GGTAAGTTCT TTTATCCCTT AAAGAAGAGA   
  
  
- TTATCTTGTT AATACCTACA TCGAGTCTTT CGTTTAGCTG AAGTATAATA ACTAGAACCA TAACAGATAC   
  
  
- CAAAGGTTAC CGGAACAGAT TAAGTCGCGG AAAGTAGATC CGGACCGCCC GGAGGGTTTG AAGCCTAATG   
  
  
- TCCGCAACTA GAGGGGGTTG GGCCAAAGGC TGGTCGGTTT TCTCAGCTCC TCTGACCCGC TGCGAACTCC   
  
  
- TTGATACGAC TCAGTAAGTT ACACGGTAAA CTCAAGTTAC GGTATCGTTT CTCGACCCTT TGTGAATGAC   
  
  
- AATTTCTAGA GTTCTAACTA GGGCTACTCC ACGAACAACA CTTGACAGAC AAGTCCAAAT TTATGTAAGG   
  
  
- ACTCCTTTGT CATTAACGTC TTACGGGTTC TCTATAACAA GAATTAGACT AGTCTGTCTA TTTTGGTCGA   
  
  
- CAAAAGTATG TACCACATCA GTTACCTCGG AAGTTAAGAG GAAAAAAGTA ATAAGCCAAG GCACTCCGAG   
  
  
- AGAAGGTGAA GAGGTGAGAT AAACTACACA ACCTCCGTTT AGAAGGGGCC CTGTAGCTCC TCTCCTATGA   
  
  
- CTATCTCGCT CTGTAGAAAC CCGCTGTCCG TTACTTACAC TAGCGAACAC TCCCAAACCT CTCCTATCTT   
  
  
- TCCGGTCTCT TTATGTTTGT CACCGCCCAG GCTTTACTCT CCCGACCCAA CTTCGTCGAC GGAAACTTAG   
  
  
- TCCTCTGACA CCTTTACCGG TTTTTTGCCC ACTGTCGACA GATAGTGTTT CTAAAGAGGT AACTACTGCT   
  
  
- ACCCGTGACC AACAATGTCC CTACCTTTCC CTCTTAACAG ATACGTGAGT GATGAACCTT CGGACGACTG   
  
  
- AT

+     GATA-motif

| Site Name | Organism | Position | Strand | Matrix score. | sequence | function |
| --- | --- | --- | --- | --- | --- | --- |
| GATA-motif | Pisum sativum | 2991 | + | 7 | GATAGGG | part of a light responsive element |

>HU05G01597.1   
+ -Up\_Stream \_Len000TTTGTA TTTCAATATT GTAATTAGGT GGGATATAGA GTTGAAGGGC AAACTCTACC   
  
  
+ AACGACTTGT TGAAGGATCA ACTTGTAGCT TGGAACTTAA TGTATGTGTA CTATAGAGTT GTGTAATCTC   
  
  
+ TCCTAATAAA ATTGGGTTAA TCCTAGAATT AGAGAACTTT AAGCGGGGAC TAAGCTTGGT TGAGGCTAAC   
  
  
+ TTCGTTAAAA AGTTCTTGTG TCATGTCCAC GTTTATCTTT TCACTATTTA TTTCAATTAA TTTGCGATAT   
  
  
+ TTATCATCAC CCTATTCACC GTCTTTTCGG ATGATTTAAC TAGTCAAGCC GGACCAACAT TGCTTTTTAT   
  
  
+ AGTGTATTTG CAACTCATGT TGATTATTAG ATGTATCAAC AAAAAATGTT TCTCAAAATA AATAGTAGCT   
  
  
+ CTTTAAATTT TATTCCACAT AAATTCAAAA TCAATATCGA TATCGTTCCA TAATATAGCA GCATTATGAA   
  
  
+ AACAATATTT GCTAATGTTT ACATTGTGCT CTTTTCACTC AAAAATTGTA AGGCTTCAAA TACTATTAAA   
  
  
+ GAAACCATTA TTTCACTCGA CTATTTAGCT TGCATATATA GACAATCTTG CAAATAGTAA CATGTCTTCA   
  
  
+ TTCGACTATT TGCTTCCAAA TGAATAATCT CGTAAACATA TATACATACA TACATATATA TATATATATA   
  
  
+ TATATATATA TATATATATA GTAATATGTA TTTAACTTCA CATATACATG AATATAAAAA TATAAGAGGG   
  
  
+ AAAAAAGACG CTAGGTAGAT GGACTACAAA AGCCATTCAT GTATTGAGAC GGCTCATAAA ATCATACGGG   
  
  
+ GAAAGGAAAA AAAAAGATCT CTACATAATG TTCATAGATT TATAGAAAGC ATATGATGGA TTACCGCATA   
  
  
+ AGGTGTTATA TATGGGAGTG TTTGTAGAAG AAAGATGTAT ATCCGAACTA TCAAAGATAC ATATGAGGGA   
  
  
+ GTAAAAACAA GTGTAAGAAC CTCAGGAGGA GACATAAAAG ATTTTTCTAT TGGCATTGGA CACCGTCAAG   
  
  
+ TATCCGCTTT AAACCTGTTC TTTTTCACTA TTATCATGGA TGTATTCACA AAAGGAATTC AATACGAAGT   
  
  
+ ACCATGATGT ATGCTTTTTA CTGATAGTAT TGGTCTCATC GATGAGACTA AGGATGGTCT TAATAGCAAA   
  
  
+ TTAGAGATTT CATTAGATAA AATCTACTGT ACATGACAAA ACAATGATAT ATAGCATCCT ATTTAAAAAG   
  
  
+ CTACGTGTCA GTCATATATG AATTGCATCT AATTTTTAAT AGATATATAA TATGATATGA TATAATAGGA   
  
  
+ TATGATGTAG ATTGAATTCA AACTCATAAT TCATTAAGCA TGGTGCAATG TTCATTTTCT TGATTTTAAA   
  
  
+ CCGACTATTA CTTCAACTGT ATATAATTCG AATTGCTTTG GTATTTTAAA ATTTAAACTA GTAATTACTG   
  
  
+ CCTCTATTAC TGATGTTTAT ATTAAGTGGT GCTTAACTGC TTATTGATGA AACCATTTTT ATTTTCGTGT   
  
  
+ AAAAGAGGGC ATAATCCATC CTCGTGTATT GGCGGGGCTC GGCGCCCTAT TCAGCTATTC TCTGTATGTC   
  
  
+ AAACAAAACC GCTTCTATTA CCCATTGGTC TCTAAACACG CAGGGGTTTT AGCCCGGAGA GTTAATTCTT   
  
  
+ TATTGAGGTC CCCTGTTCTC CATTGCAGGA TTTTTGCTCA GGTACTTCTC TGTTTCTTCT CTTTAATCTT   
  
  
+ CTGTATCTTT CTGCATCAAG TTTAATGCTT TCTGGGATTT TCAGCTACGA TTTATGTGAA ATTGTAAGAT   
  
  
+ TTCTGAACTG GGCATTCAGG GCTTGGTTCA TATGTATTCT TTTCTGCTTG AAATGTTTTA ATTTCATAGT   
  
  
+ TCCAATGGGG TTATTCAAAG ATAGAATTAA GCTATAATCA CCGAGAAGAC TTGCTGACCT TTGTTTCTTT   
  
  
+ TTTGTTTCTT TTTTTTAATT AAATCTGGCT ATTACTTGGC TATAGTTTTT GCTAATGGAT CCACAAATAG   
  
  
+ ACGAATTATA TGGGCCTTTG CACCAAATGA AGTTCAATGA TCAGAGGAGA CCCATTTTAT CAAATGAAAA   
  
  
+ TTTTGTTAAT GTGTTGAAAC CGCCAAATTC CAATTGGAAT TCACCTTTTG GGAACCCTAC TGTGATTACC   
  
  
+ ACAAACCCAA ATTTTGATTT GGTTGTGCCA TCACTAGGTC CCAATGTTGA GGGAGCTCTC CTTGAAGATT   
  
  
+ ATGATTTTAG TGATGTTGTT CTTAAGTACA TCAATCAAGT TCTTATGGAC GAGGATGAGG GAGAGAAAAT   
  
  
+ CCATTTGGGC CACGGGCCTC TGGCTCTTGA AGCGGCCGAG AAGTCGTTAT ATGAGGTGCT TGGGCAGAGC   
  
  
+ CATTCTCCAC AAAATCAATA CTCTCCAGAG GACAAGCGTG ATGACCTGAG CAAGAGTAGT GTTAGAAGCT   
  
  
+ GTGTCAGTTA TAGCTGTGAC GGTGCAAGTA GTGGCAGTGG CATCGTGGAG CAAAGTTGGT CGAGTTATCC   
  
  
+ TCCGGAGTAC AATCCCATGC GGATGGCAGA TTTTCCTGCC TTGCCCTCAA AACCCTTGTT GAGAACATCT   
  
  
+ TATAGCTTAT CAAATAGCTT GACAGGTGAG GGTAATGGTT TGGTGGATAG ACCGGCAGAC TCACCTGTAA   
  
  
+ GCGCTCTTAG TGTGTCTGAT ATATTTAATG ATAGTCAGTC AGTGATACAA TTTCAGAAAG GGATGGAGGA   
  
  
+ AGCGAGCAAA TTTCTTCCCA AGGCTTCTTT GTGTTTGGAT TTGAACACTG GACCCTGCCT TCGGGAGTCG   
  
  
+ GTCAATAAGG CACGAGGTGG GCTGATTTTG GTGGAGACTA GCCATGAAAA TGAGGTTGCA GTGCTAGCTA   
  
  
+ GTAGGGGAAA GAAGCATCGC CATCCTGAGG AATTACGTTC AGAAGTTGGG AGGGGCAGTA AGCAGCCAGT   
  
  
+ TGTTTTCCAG TTTTCAGACG AGGCAGTTGT GAGATCAGAA ATGTTTGATA GGGTATTGCT GTGCAGTGGT   
  
  
+ GGCAAAAATG ATGCTGACCT ACGCCAAACT TTACAGAGTG AACTAAATAA AACTATGCAA AATGCTCAAA   
  
  
+ TGAAAGATTC TAATGGTGGA AAAGGCCGTG GTAGGAAGCA AGTTGGCAGG AGGGATGTGG TGGATCTAAG   
  
  
+ ATCTCTTTTA CGCCTTTGTG CACAAGCAGT TGGATCCAAT GACCAAAGAA GCGCAAATGA CTTAATTAGG   
  
  
+ CAAATTAGGA AGCATTCTTC TCCAACCGGG GATGGGAATC AAAGAATGGC TCATTATTTT GCTGATGGTC   
  
  
+ TTGAGGCTCG GCTTGCTGGT GTTGGGACCC CAATATACAA GTCTCTCGTG ACTGGCCCAG CTTCTGCTGT   
  
  
+ TGATATCTTG AGAGCTTACC ATATGTTTCT TGCCACATGC CCATTCAAGA AAATAGGGAA TTTCTTCTCT   
  
  
+ AATAGAACAA TTATGGATGT AGCTCAGAAA GCAAATCGAC TTCATATTAT TGATCTTGGT ATTGTCTATG   
  
  
+ GTTTCCAATG GCCTTGTCTA ATTCAGCGCC TTTCATCTAG GCCTGGCGGG CCTCCCAAAC TTCGGATTAC   
  
  
+ AGGCGTTGAT CTCCCCCAAC CCGGTTTCCG ACCAGCCAAA AGAGTCGAGG AGACTGGGCG ACGCTTGAGG   
  
  
+ AACTATGCTG AGTCATTCAA TGTGCCATTT GAGTTCAATG CCATAGCAAA GAGCTGGGAA ACACTTACTG   
  
  
+ TTAAAGATCT CAAGATTGAT CCCGATGAGG TGCTTGTTGT GAACTGTCTG TTCAGGTTTA AATACATTCC   
  
  
+ TGAGGAAACA GTAATTGCAG AATGCCCAAG AGATATTGTT CTTAATCTGA TCAGACAGAT AAAACCAGCT   
  
  
+ GTTTTCATAC ATGGTGTAGT CAATGGAGCC TTCAATTCTC CTTTTTTCAT TATTCGGTTC CGTGAGGCTC   
  
  
+ TCTTCCACTT CTCCACTCTA TTTGATGTGT TGGAGGCAAA TCTTCCCCGG GACATCGAGG AGAGGATACT   
  
  
+ GATAGAGCGA GACATCTTTG GGCGACAGGC AATGAATGTG ATCGCTTGTG AGGGTTTGGA GAGGATAGAA   
  
  
+ AGGCCAGAGA AATACAAACA GTGGCGGGTC CGAAATGAGA GGGCTGGGTT GAAGCAGCTG CCTTTGAATC   
  
  
+ AGGAGACTGT GGAAATGGCC AAAAAACGGG TGACAGCTGT CTATCACAAA GATTTCTCCA TTGATGACGA   
  
  
+ TGGGCACTGG TTGTTACAGG GATGGAAAGG GAGAATTGTC TATGCACTCA CTACTTGGAA GCCTGCTGAC   
  
  
+ TA  

- -Up\_Stream \_Len000AAACAT AAAGTTATAA CATTAATCCA CCCTATATCT CAACTTCCCG TTTGAGATGG   
  
  
- TTGCTGAACA ACTTCCTAGT TGAACATCGA ACCTTGAATT ACATACACAT GATATCTCAA CACATTAGAG   
  
  
- AGGATTATTT TAACCCAATT AGGATCTTAA TCTCTTGAAA TTCGCCCCTG ATTCGAACCA ACTCCGATTG   
  
  
- AAGCAATTTT TCAAGAACAC AGTACAGGTG CAAATAGAAA AGTGATAAAT AAAGTTAATT AAACGCTATA   
  
  
- AATAGTAGTG GGATAAGTGG CAGAAAAGCC TACTAAATTG ATCAGTTCGG CCTGGTTGTA ACGAAAAATA   
  
  
- TCACATAAAC GTTGAGTACA ACTAATAATC TACATAGTTG TTTTTTACAA AGAGTTTTAT TTATCATCGA   
  
  
- GAAATTTAAA ATAAGGTGTA TTTAAGTTTT AGTTATAGCT ATAGCAAGGT ATTATATCGT CGTAATACTT   
  
  
- TTGTTATAAA CGATTACAAA TGTAACACGA GAAAAGTGAG TTTTTAACAT TCCGAAGTTT ATGATAATTT   
  
  
- CTTTGGTAAT AAAGTGAGCT GATAAATCGA ACGTATATAT CTGTTAGAAC GTTTATCATT GTACAGAAGT   
  
  
- AAGCTGATAA ACGAAGGTTT ACTTATTAGA GCATTTGTAT ATATGTATGT ATGTATATAT ATATATATAT   
  
  
- ATATATATAT ATATATATAT CATTATACAT AAATTGAAGT GTATATGTAC TTATATTTTT ATATTCTCCC   
  
  
- TTTTTTCTGC GATCCATCTA CCTGATGTTT TCGGTAAGTA CATAACTCTG CCGAGTATTT TAGTATGCCC   
  
  
- CTTTCCTTTT TTTTTCTAGA GATGTATTAC AAGTATCTAA ATATCTTTCG TATACTACCT AATGGCGTAT   
  
  
- TCCACAATAT ATACCCTCAC AAACATCTTC TTTCTACATA TAGGCTTGAT AGTTTCTATG TATACTCCCT   
  
  
- CATTTTTGTT CACATTCTTG GAGTCCTCCT CTGTATTTTC TAAAAAGATA ACCGTAACCT GTGGCAGTTC   
  
  
- ATAGGCGAAA TTTGGACAAG AAAAAGTGAT AATAGTACCT ACATAAGTGT TTTCCTTAAG TTATGCTTCA   
  
  
- TGGTACTACA TACGAAAAAT GACTATCATA ACCAGAGTAG CTACTCTGAT TCCTACCAGA ATTATCGTTT   
  
  
- AATCTCTAAA GTAATCTATT TTAGATGACA TGTACTGTTT TGTTACTATA TATCGTAGGA TAAATTTTTC   
  
  
- GATGCACAGT CAGTATATAC TTAACGTAGA TTAAAAATTA TCTATATATT ATACTATACT ATATTATCCT   
  
  
- ATACTACATC TAACTTAAGT TTGAGTATTA AGTAATTCGT ACCACGTTAC AAGTAAAAGA ACTAAAATTT   
  
  
- GGCTGATAAT GAAGTTGACA TATATTAAGC TTAACGAAAC CATAAAATTT TAAATTTGAT CATTAATGAC   
  
  
- GGAGATAATG ACTACAAATA TAATTCACCA CGAATTGACG AATAACTACT TTGGTAAAAA TAAAAGCACA   
  
  
- TTTTCTCCCG TATTAGGTAG GAGCACATAA CCGCCCCGAG CCGCGGGATA AGTCGATAAG AGACATACAG   
  
  
- TTTGTTTTGG CGAAGATAAT GGGTAACCAG AGATTTGTGC GTCCCCAAAA TCGGGCCTCT CAATTAAGAA   
  
  
- ATAACTCCAG GGGACAAGAG GTAACGTCCT AAAAACGAGT CCATGAAGAG ACAAAGAAGA GAAATTAGAA   
  
  
- GACATAGAAA GACGTAGTTC AAATTACGAA AGACCCTAAA AGTCGATGCT AAATACACTT TAACATTCTA   
  
  
- AAGACTTGAC CCGTAAGTCC CGAACCAAGT ATACATAAGA AAAGACGAAC TTTACAAAAT TAAAGTATCA   
  
  
- AGGTTACCCC AATAAGTTTC TATCTTAATT CGATATTAGT GGCTCTTCTG AACGACTGGA AACAAAGAAA   
  
  
- AAACAAAGAA AAAAAATTAA TTTAGACCGA TAATGAACCG ATATCAAAAA CGATTACCTA GGTGTTTATC   
  
  
- TGCTTAATAT ACCCGGAAAC GTGGTTTACT TCAAGTTACT AGTCTCCTCT GGGTAAAATA GTTTACTTTT   
  
  
- AAAACAATTA CACAACTTTG GCGGTTTAAG GTTAACCTTA AGTGGAAAAC CCTTGGGATG ACACTAATGG   
  
  
- TGTTTGGGTT TAAAACTAAA CCAACACGGT AGTGATCCAG GGTTACAACT CCCTCGAGAG GAACTTCTAA   
  
  
- TACTAAAATC ACTACAACAA GAATTCATGT AGTTAGTTCA AGAATACCTG CTCCTACTCC CTCTCTTTTA   
  
  
- GGTAAACCCG GTGCCCGGAG ACCGAGAACT TCGCCGGCTC TTCAGCAATA TACTCCACGA ACCCGTCTCG   
  
  
- GTAAGAGGTG TTTTAGTTAT GAGAGGTCTC CTGTTCGCAC TACTGGACTC GTTCTCATCA CAATCTTCGA   
  
  
- CACAGTCAAT ATCGACACTG CCACGTTCAT CACCGTCACC GTAGCACCTC GTTTCAACCA GCTCAATAGG   
  
  
- AGGCCTCATG TTAGGGTACG CCTACCGTCT AAAAGGACGG AACGGGAGTT TTGGGAACAA CTCTTGTAGA   
  
  
- ATATCGAATA GTTTATCGAA CTGTCCACTC CCATTACCAA ACCACCTATC TGGCCGTCTG AGTGGACATT   
  
  
- CGCGAGAATC ACACAGACTA TATAAATTAC TATCAGTCAG TCACTATGTT AAAGTCTTTC CCTACCTCCT   
  
  
- TCGCTCGTTT AAAGAAGGGT TCCGAAGAAA CACAAACCTA AACTTGTGAC CTGGGACGGA AGCCCTCAGC   
  
  
- CAGTTATTCC GTGCTCCACC CGACTAAAAC CACCTCTGAT CGGTACTTTT ACTCCAACGT CACGATCGAT   
  
  
- CATCCCCTTT CTTCGTAGCG GTAGGACTCC TTAATGCAAG TCTTCAACCC TCCCCGTCAT TCGTCGGTCA   
  
  
- ACAAAAGGTC AAAAGTCTGC TCCGTCAACA CTCTAGTCTT TACAAACTAT CCCATAACGA CACGTCACCA   
  
  
- CCGTTTTTAC TACGACTGGA TGCGGTTTGA AATGTCTCAC TTGATTTATT TTGATACGTT TTACGAGTTT   
  
  
- ACTTTCTAAG ATTACCACCT TTTCCGGCAC CATCCTTCGT TCAACCGTCC TCCCTACACC ACCTAGATTC   
  
  
- TAGAGAAAAT GCGGAAACAC GTGTTCGTCA ACCTAGGTTA CTGGTTTCTT CGCGTTTACT GAATTAATCC   
  
  
- GTTTAATCCT TCGTAAGAAG AGGTTGGCCC CTACCCTTAG TTTCTTACCG AGTAATAAAA CGACTACCAG   
  
  
- AACTCCGAGC CGAACGACCA CAACCCTGGG GTTATATGTT CAGAGAGCAC TGACCGGGTC GAAGACGACA   
  
  
- ACTATAGAAC TCTCGAATGG TATACAAAGA ACGGTGTACG GGTAAGTTCT TTTATCCCTT AAAGAAGAGA   
  
  
- TTATCTTGTT AATACCTACA TCGAGTCTTT CGTTTAGCTG AAGTATAATA ACTAGAACCA TAACAGATAC   
  
  
- CAAAGGTTAC CGGAACAGAT TAAGTCGCGG AAAGTAGATC CGGACCGCCC GGAGGGTTTG AAGCCTAATG   
  
  
- TCCGCAACTA GAGGGGGTTG GGCCAAAGGC TGGTCGGTTT TCTCAGCTCC TCTGACCCGC TGCGAACTCC   
  
  
- TTGATACGAC TCAGTAAGTT ACACGGTAAA CTCAAGTTAC GGTATCGTTT CTCGACCCTT TGTGAATGAC   
  
  
- AATTTCTAGA GTTCTAACTA GGGCTACTCC ACGAACAACA CTTGACAGAC AAGTCCAAAT TTATGTAAGG   
  
  
- ACTCCTTTGT CATTAACGTC TTACGGGTTC TCTATAACAA GAATTAGACT AGTCTGTCTA TTTTGGTCGA   
  
  
- CAAAAGTATG TACCACATCA GTTACCTCGG AAGTTAAGAG GAAAAAAGTA ATAAGCCAAG GCACTCCGAG   
  
  
- AGAAGGTGAA GAGGTGAGAT AAACTACACA ACCTCCGTTT AGAAGGGGCC CTGTAGCTCC TCTCCTATGA   
  
  
- CTATCTCGCT CTGTAGAAAC CCGCTGTCCG TTACTTACAC TAGCGAACAC TCCCAAACCT CTCCTATCTT   
  
  
- TCCGGTCTCT TTATGTTTGT CACCGCCCAG GCTTTACTCT CCCGACCCAA CTTCGTCGAC GGAAACTTAG   
  
  
- TCCTCTGACA CCTTTACCGG TTTTTTGCCC ACTGTCGACA GATAGTGTTT CTAAAGAGGT AACTACTGCT   
  
  
- ACCCGTGACC AACAATGTCC CTACCTTTCC CTCTTAACAG ATACGTGAGT GATGAACCTT CGGACGACTG   
  
  
- AT

+     GC-motif

| Site Name | Organism | Position | Strand | Matrix score. | sequence | function |
| --- | --- | --- | --- | --- | --- | --- |
| GC-motif | Oryza sativa | 1584 | + | 9 | CGGCGCCCT | ? |

>HU05G01597.1   
+ -Up\_Stream \_Len000TTTGTA TTTCAATATT GTAATTAGGT GGGATATAGA GTTGAAGGGC AAACTCTACC   
  
  
+ AACGACTTGT TGAAGGATCA ACTTGTAGCT TGGAACTTAA TGTATGTGTA CTATAGAGTT GTGTAATCTC   
  
  
+ TCCTAATAAA ATTGGGTTAA TCCTAGAATT AGAGAACTTT AAGCGGGGAC TAAGCTTGGT TGAGGCTAAC   
  
  
+ TTCGTTAAAA AGTTCTTGTG TCATGTCCAC GTTTATCTTT TCACTATTTA TTTCAATTAA TTTGCGATAT   
  
  
+ TTATCATCAC CCTATTCACC GTCTTTTCGG ATGATTTAAC TAGTCAAGCC GGACCAACAT TGCTTTTTAT   
  
  
+ AGTGTATTTG CAACTCATGT TGATTATTAG ATGTATCAAC AAAAAATGTT TCTCAAAATA AATAGTAGCT   
  
  
+ CTTTAAATTT TATTCCACAT AAATTCAAAA TCAATATCGA TATCGTTCCA TAATATAGCA GCATTATGAA   
  
  
+ AACAATATTT GCTAATGTTT ACATTGTGCT CTTTTCACTC AAAAATTGTA AGGCTTCAAA TACTATTAAA   
  
  
+ GAAACCATTA TTTCACTCGA CTATTTAGCT TGCATATATA GACAATCTTG CAAATAGTAA CATGTCTTCA   
  
  
+ TTCGACTATT TGCTTCCAAA TGAATAATCT CGTAAACATA TATACATACA TACATATATA TATATATATA   
  
  
+ TATATATATA TATATATATA GTAATATGTA TTTAACTTCA CATATACATG AATATAAAAA TATAAGAGGG   
  
  
+ AAAAAAGACG CTAGGTAGAT GGACTACAAA AGCCATTCAT GTATTGAGAC GGCTCATAAA ATCATACGGG   
  
  
+ GAAAGGAAAA AAAAAGATCT CTACATAATG TTCATAGATT TATAGAAAGC ATATGATGGA TTACCGCATA   
  
  
+ AGGTGTTATA TATGGGAGTG TTTGTAGAAG AAAGATGTAT ATCCGAACTA TCAAAGATAC ATATGAGGGA   
  
  
+ GTAAAAACAA GTGTAAGAAC CTCAGGAGGA GACATAAAAG ATTTTTCTAT TGGCATTGGA CACCGTCAAG   
  
  
+ TATCCGCTTT AAACCTGTTC TTTTTCACTA TTATCATGGA TGTATTCACA AAAGGAATTC AATACGAAGT   
  
  
+ ACCATGATGT ATGCTTTTTA CTGATAGTAT TGGTCTCATC GATGAGACTA AGGATGGTCT TAATAGCAAA   
  
  
+ TTAGAGATTT CATTAGATAA AATCTACTGT ACATGACAAA ACAATGATAT ATAGCATCCT ATTTAAAAAG   
  
  
+ CTACGTGTCA GTCATATATG AATTGCATCT AATTTTTAAT AGATATATAA TATGATATGA TATAATAGGA   
  
  
+ TATGATGTAG ATTGAATTCA AACTCATAAT TCATTAAGCA TGGTGCAATG TTCATTTTCT TGATTTTAAA   
  
  
+ CCGACTATTA CTTCAACTGT ATATAATTCG AATTGCTTTG GTATTTTAAA ATTTAAACTA GTAATTACTG   
  
  
+ CCTCTATTAC TGATGTTTAT ATTAAGTGGT GCTTAACTGC TTATTGATGA AACCATTTTT ATTTTCGTGT   
  
  
+ AAAAGAGGGC ATAATCCATC CTCGTGTATT GGCGGGGCTC GGCGCCCTAT TCAGCTATTC TCTGTATGTC   
  
  
+ AAACAAAACC GCTTCTATTA CCCATTGGTC TCTAAACACG CAGGGGTTTT AGCCCGGAGA GTTAATTCTT   
  
  
+ TATTGAGGTC CCCTGTTCTC CATTGCAGGA TTTTTGCTCA GGTACTTCTC TGTTTCTTCT CTTTAATCTT   
  
  
+ CTGTATCTTT CTGCATCAAG TTTAATGCTT TCTGGGATTT TCAGCTACGA TTTATGTGAA ATTGTAAGAT   
  
  
+ TTCTGAACTG GGCATTCAGG GCTTGGTTCA TATGTATTCT TTTCTGCTTG AAATGTTTTA ATTTCATAGT   
  
  
+ TCCAATGGGG TTATTCAAAG ATAGAATTAA GCTATAATCA CCGAGAAGAC TTGCTGACCT TTGTTTCTTT   
  
  
+ TTTGTTTCTT TTTTTTAATT AAATCTGGCT ATTACTTGGC TATAGTTTTT GCTAATGGAT CCACAAATAG   
  
  
+ ACGAATTATA TGGGCCTTTG CACCAAATGA AGTTCAATGA TCAGAGGAGA CCCATTTTAT CAAATGAAAA   
  
  
+ TTTTGTTAAT GTGTTGAAAC CGCCAAATTC CAATTGGAAT TCACCTTTTG GGAACCCTAC TGTGATTACC   
  
  
+ ACAAACCCAA ATTTTGATTT GGTTGTGCCA TCACTAGGTC CCAATGTTGA GGGAGCTCTC CTTGAAGATT   
  
  
+ ATGATTTTAG TGATGTTGTT CTTAAGTACA TCAATCAAGT TCTTATGGAC GAGGATGAGG GAGAGAAAAT   
  
  
+ CCATTTGGGC CACGGGCCTC TGGCTCTTGA AGCGGCCGAG AAGTCGTTAT ATGAGGTGCT TGGGCAGAGC   
  
  
+ CATTCTCCAC AAAATCAATA CTCTCCAGAG GACAAGCGTG ATGACCTGAG CAAGAGTAGT GTTAGAAGCT   
  
  
+ GTGTCAGTTA TAGCTGTGAC GGTGCAAGTA GTGGCAGTGG CATCGTGGAG CAAAGTTGGT CGAGTTATCC   
  
  
+ TCCGGAGTAC AATCCCATGC GGATGGCAGA TTTTCCTGCC TTGCCCTCAA AACCCTTGTT GAGAACATCT   
  
  
+ TATAGCTTAT CAAATAGCTT GACAGGTGAG GGTAATGGTT TGGTGGATAG ACCGGCAGAC TCACCTGTAA   
  
  
+ GCGCTCTTAG TGTGTCTGAT ATATTTAATG ATAGTCAGTC AGTGATACAA TTTCAGAAAG GGATGGAGGA   
  
  
+ AGCGAGCAAA TTTCTTCCCA AGGCTTCTTT GTGTTTGGAT TTGAACACTG GACCCTGCCT TCGGGAGTCG   
  
  
+ GTCAATAAGG CACGAGGTGG GCTGATTTTG GTGGAGACTA GCCATGAAAA TGAGGTTGCA GTGCTAGCTA   
  
  
+ GTAGGGGAAA GAAGCATCGC CATCCTGAGG AATTACGTTC AGAAGTTGGG AGGGGCAGTA AGCAGCCAGT   
  
  
+ TGTTTTCCAG TTTTCAGACG AGGCAGTTGT GAGATCAGAA ATGTTTGATA GGGTATTGCT GTGCAGTGGT   
  
  
+ GGCAAAAATG ATGCTGACCT ACGCCAAACT TTACAGAGTG AACTAAATAA AACTATGCAA AATGCTCAAA   
  
  
+ TGAAAGATTC TAATGGTGGA AAAGGCCGTG GTAGGAAGCA AGTTGGCAGG AGGGATGTGG TGGATCTAAG   
  
  
+ ATCTCTTTTA CGCCTTTGTG CACAAGCAGT TGGATCCAAT GACCAAAGAA GCGCAAATGA CTTAATTAGG   
  
  
+ CAAATTAGGA AGCATTCTTC TCCAACCGGG GATGGGAATC AAAGAATGGC TCATTATTTT GCTGATGGTC   
  
  
+ TTGAGGCTCG GCTTGCTGGT GTTGGGACCC CAATATACAA GTCTCTCGTG ACTGGCCCAG CTTCTGCTGT   
  
  
+ TGATATCTTG AGAGCTTACC ATATGTTTCT TGCCACATGC CCATTCAAGA AAATAGGGAA TTTCTTCTCT   
  
  
+ AATAGAACAA TTATGGATGT AGCTCAGAAA GCAAATCGAC TTCATATTAT TGATCTTGGT ATTGTCTATG   
  
  
+ GTTTCCAATG GCCTTGTCTA ATTCAGCGCC TTTCATCTAG GCCTGGCGGG CCTCCCAAAC TTCGGATTAC   
  
  
+ AGGCGTTGAT CTCCCCCAAC CCGGTTTCCG ACCAGCCAAA AGAGTCGAGG AGACTGGGCG ACGCTTGAGG   
  
  
+ AACTATGCTG AGTCATTCAA TGTGCCATTT GAGTTCAATG CCATAGCAAA GAGCTGGGAA ACACTTACTG   
  
  
+ TTAAAGATCT CAAGATTGAT CCCGATGAGG TGCTTGTTGT GAACTGTCTG TTCAGGTTTA AATACATTCC   
  
  
+ TGAGGAAACA GTAATTGCAG AATGCCCAAG AGATATTGTT CTTAATCTGA TCAGACAGAT AAAACCAGCT   
  
  
+ GTTTTCATAC ATGGTGTAGT CAATGGAGCC TTCAATTCTC CTTTTTTCAT TATTCGGTTC CGTGAGGCTC   
  
  
+ TCTTCCACTT CTCCACTCTA TTTGATGTGT TGGAGGCAAA TCTTCCCCGG GACATCGAGG AGAGGATACT   
  
  
+ GATAGAGCGA GACATCTTTG GGCGACAGGC AATGAATGTG ATCGCTTGTG AGGGTTTGGA GAGGATAGAA   
  
  
+ AGGCCAGAGA AATACAAACA GTGGCGGGTC CGAAATGAGA GGGCTGGGTT GAAGCAGCTG CCTTTGAATC   
  
  
+ AGGAGACTGT GGAAATGGCC AAAAAACGGG TGACAGCTGT CTATCACAAA GATTTCTCCA TTGATGACGA   
  
  
+ TGGGCACTGG TTGTTACAGG GATGGAAAGG GAGAATTGTC TATGCACTCA CTACTTGGAA GCCTGCTGAC   
  
  
+ TA  

- -Up\_Stream \_Len000AAACAT AAAGTTATAA CATTAATCCA CCCTATATCT CAACTTCCCG TTTGAGATGG   
  
  
- TTGCTGAACA ACTTCCTAGT TGAACATCGA ACCTTGAATT ACATACACAT GATATCTCAA CACATTAGAG   
  
  
- AGGATTATTT TAACCCAATT AGGATCTTAA TCTCTTGAAA TTCGCCCCTG ATTCGAACCA ACTCCGATTG   
  
  
- AAGCAATTTT TCAAGAACAC AGTACAGGTG CAAATAGAAA AGTGATAAAT AAAGTTAATT AAACGCTATA   
  
  
- AATAGTAGTG GGATAAGTGG CAGAAAAGCC TACTAAATTG ATCAGTTCGG CCTGGTTGTA ACGAAAAATA   
  
  
- TCACATAAAC GTTGAGTACA ACTAATAATC TACATAGTTG TTTTTTACAA AGAGTTTTAT TTATCATCGA   
  
  
- GAAATTTAAA ATAAGGTGTA TTTAAGTTTT AGTTATAGCT ATAGCAAGGT ATTATATCGT CGTAATACTT   
  
  
- TTGTTATAAA CGATTACAAA TGTAACACGA GAAAAGTGAG TTTTTAACAT TCCGAAGTTT ATGATAATTT   
  
  
- CTTTGGTAAT AAAGTGAGCT GATAAATCGA ACGTATATAT CTGTTAGAAC GTTTATCATT GTACAGAAGT   
  
  
- AAGCTGATAA ACGAAGGTTT ACTTATTAGA GCATTTGTAT ATATGTATGT ATGTATATAT ATATATATAT   
  
  
- ATATATATAT ATATATATAT CATTATACAT AAATTGAAGT GTATATGTAC TTATATTTTT ATATTCTCCC   
  
  
- TTTTTTCTGC GATCCATCTA CCTGATGTTT TCGGTAAGTA CATAACTCTG CCGAGTATTT TAGTATGCCC   
  
  
- CTTTCCTTTT TTTTTCTAGA GATGTATTAC AAGTATCTAA ATATCTTTCG TATACTACCT AATGGCGTAT   
  
  
- TCCACAATAT ATACCCTCAC AAACATCTTC TTTCTACATA TAGGCTTGAT AGTTTCTATG TATACTCCCT   
  
  
- CATTTTTGTT CACATTCTTG GAGTCCTCCT CTGTATTTTC TAAAAAGATA ACCGTAACCT GTGGCAGTTC   
  
  
- ATAGGCGAAA TTTGGACAAG AAAAAGTGAT AATAGTACCT ACATAAGTGT TTTCCTTAAG TTATGCTTCA   
  
  
- TGGTACTACA TACGAAAAAT GACTATCATA ACCAGAGTAG CTACTCTGAT TCCTACCAGA ATTATCGTTT   
  
  
- AATCTCTAAA GTAATCTATT TTAGATGACA TGTACTGTTT TGTTACTATA TATCGTAGGA TAAATTTTTC   
  
  
- GATGCACAGT CAGTATATAC TTAACGTAGA TTAAAAATTA TCTATATATT ATACTATACT ATATTATCCT   
  
  
- ATACTACATC TAACTTAAGT TTGAGTATTA AGTAATTCGT ACCACGTTAC AAGTAAAAGA ACTAAAATTT   
  
  
- GGCTGATAAT GAAGTTGACA TATATTAAGC TTAACGAAAC CATAAAATTT TAAATTTGAT CATTAATGAC   
  
  
- GGAGATAATG ACTACAAATA TAATTCACCA CGAATTGACG AATAACTACT TTGGTAAAAA TAAAAGCACA   
  
  
- TTTTCTCCCG TATTAGGTAG GAGCACATAA CCGCCCCGAG CCGCGGGATA AGTCGATAAG AGACATACAG   
  
  
- TTTGTTTTGG CGAAGATAAT GGGTAACCAG AGATTTGTGC GTCCCCAAAA TCGGGCCTCT CAATTAAGAA   
  
  
- ATAACTCCAG GGGACAAGAG GTAACGTCCT AAAAACGAGT CCATGAAGAG ACAAAGAAGA GAAATTAGAA   
  
  
- GACATAGAAA GACGTAGTTC AAATTACGAA AGACCCTAAA AGTCGATGCT AAATACACTT TAACATTCTA   
  
  
- AAGACTTGAC CCGTAAGTCC CGAACCAAGT ATACATAAGA AAAGACGAAC TTTACAAAAT TAAAGTATCA   
  
  
- AGGTTACCCC AATAAGTTTC TATCTTAATT CGATATTAGT GGCTCTTCTG AACGACTGGA AACAAAGAAA   
  
  
- AAACAAAGAA AAAAAATTAA TTTAGACCGA TAATGAACCG ATATCAAAAA CGATTACCTA GGTGTTTATC   
  
  
- TGCTTAATAT ACCCGGAAAC GTGGTTTACT TCAAGTTACT AGTCTCCTCT GGGTAAAATA GTTTACTTTT   
  
  
- AAAACAATTA CACAACTTTG GCGGTTTAAG GTTAACCTTA AGTGGAAAAC CCTTGGGATG ACACTAATGG   
  
  
- TGTTTGGGTT TAAAACTAAA CCAACACGGT AGTGATCCAG GGTTACAACT CCCTCGAGAG GAACTTCTAA   
  
  
- TACTAAAATC ACTACAACAA GAATTCATGT AGTTAGTTCA AGAATACCTG CTCCTACTCC CTCTCTTTTA   
  
  
- GGTAAACCCG GTGCCCGGAG ACCGAGAACT TCGCCGGCTC TTCAGCAATA TACTCCACGA ACCCGTCTCG   
  
  
- GTAAGAGGTG TTTTAGTTAT GAGAGGTCTC CTGTTCGCAC TACTGGACTC GTTCTCATCA CAATCTTCGA   
  
  
- CACAGTCAAT ATCGACACTG CCACGTTCAT CACCGTCACC GTAGCACCTC GTTTCAACCA GCTCAATAGG   
  
  
- AGGCCTCATG TTAGGGTACG CCTACCGTCT AAAAGGACGG AACGGGAGTT TTGGGAACAA CTCTTGTAGA   
  
  
- ATATCGAATA GTTTATCGAA CTGTCCACTC CCATTACCAA ACCACCTATC TGGCCGTCTG AGTGGACATT   
  
  
- CGCGAGAATC ACACAGACTA TATAAATTAC TATCAGTCAG TCACTATGTT AAAGTCTTTC CCTACCTCCT   
  
  
- TCGCTCGTTT AAAGAAGGGT TCCGAAGAAA CACAAACCTA AACTTGTGAC CTGGGACGGA AGCCCTCAGC   
  
  
- CAGTTATTCC GTGCTCCACC CGACTAAAAC CACCTCTGAT CGGTACTTTT ACTCCAACGT CACGATCGAT   
  
  
- CATCCCCTTT CTTCGTAGCG GTAGGACTCC TTAATGCAAG TCTTCAACCC TCCCCGTCAT TCGTCGGTCA   
  
  
- ACAAAAGGTC AAAAGTCTGC TCCGTCAACA CTCTAGTCTT TACAAACTAT CCCATAACGA CACGTCACCA   
  
  
- CCGTTTTTAC TACGACTGGA TGCGGTTTGA AATGTCTCAC TTGATTTATT TTGATACGTT TTACGAGTTT   
  
  
- ACTTTCTAAG ATTACCACCT TTTCCGGCAC CATCCTTCGT TCAACCGTCC TCCCTACACC ACCTAGATTC   
  
  
- TAGAGAAAAT GCGGAAACAC GTGTTCGTCA ACCTAGGTTA CTGGTTTCTT CGCGTTTACT GAATTAATCC   
  
  
- GTTTAATCCT TCGTAAGAAG AGGTTGGCCC CTACCCTTAG TTTCTTACCG AGTAATAAAA CGACTACCAG   
  
  
- AACTCCGAGC CGAACGACCA CAACCCTGGG GTTATATGTT CAGAGAGCAC TGACCGGGTC GAAGACGACA   
  
  
- ACTATAGAAC TCTCGAATGG TATACAAAGA ACGGTGTACG GGTAAGTTCT TTTATCCCTT AAAGAAGAGA   
  
  
- TTATCTTGTT AATACCTACA TCGAGTCTTT CGTTTAGCTG AAGTATAATA ACTAGAACCA TAACAGATAC   
  
  
- CAAAGGTTAC CGGAACAGAT TAAGTCGCGG AAAGTAGATC CGGACCGCCC GGAGGGTTTG AAGCCTAATG   
  
  
- TCCGCAACTA GAGGGGGTTG GGCCAAAGGC TGGTCGGTTT TCTCAGCTCC TCTGACCCGC TGCGAACTCC   
  
  
- TTGATACGAC TCAGTAAGTT ACACGGTAAA CTCAAGTTAC GGTATCGTTT CTCGACCCTT TGTGAATGAC   
  
  
- AATTTCTAGA GTTCTAACTA GGGCTACTCC ACGAACAACA CTTGACAGAC AAGTCCAAAT TTATGTAAGG   
  
  
- ACTCCTTTGT CATTAACGTC TTACGGGTTC TCTATAACAA GAATTAGACT AGTCTGTCTA TTTTGGTCGA   
  
  
- CAAAAGTATG TACCACATCA GTTACCTCGG AAGTTAAGAG GAAAAAAGTA ATAAGCCAAG GCACTCCGAG   
  
  
- AGAAGGTGAA GAGGTGAGAT AAACTACACA ACCTCCGTTT AGAAGGGGCC CTGTAGCTCC TCTCCTATGA   
  
  
- CTATCTCGCT CTGTAGAAAC CCGCTGTCCG TTACTTACAC TAGCGAACAC TCCCAAACCT CTCCTATCTT   
  
  
- TCCGGTCTCT TTATGTTTGT CACCGCCCAG GCTTTACTCT CCCGACCCAA CTTCGTCGAC GGAAACTTAG   
  
  
- TCCTCTGACA CCTTTACCGG TTTTTTGCCC ACTGTCGACA GATAGTGTTT CTAAAGAGGT AACTACTGCT   
  
  
- ACCCGTGACC AACAATGTCC CTACCTTTCC CTCTTAACAG ATACGTGAGT GATGAACCTT CGGACGACTG   
  
  
- AT

+     GCN4\_motif

| Site Name | Organism | Position | Strand | Matrix score. | sequence | function |
| --- | --- | --- | --- | --- | --- | --- |
| GCN4\_motif | Oryza sativa | 3653 | + | 7 | TGAGTCA | cis-regulatory element involved in endosperm expression |

>HU05G01597.1   
+ -Up\_Stream \_Len000TTTGTA TTTCAATATT GTAATTAGGT GGGATATAGA GTTGAAGGGC AAACTCTACC   
  
  
+ AACGACTTGT TGAAGGATCA ACTTGTAGCT TGGAACTTAA TGTATGTGTA CTATAGAGTT GTGTAATCTC   
  
  
+ TCCTAATAAA ATTGGGTTAA TCCTAGAATT AGAGAACTTT AAGCGGGGAC TAAGCTTGGT TGAGGCTAAC   
  
  
+ TTCGTTAAAA AGTTCTTGTG TCATGTCCAC GTTTATCTTT TCACTATTTA TTTCAATTAA TTTGCGATAT   
  
  
+ TTATCATCAC CCTATTCACC GTCTTTTCGG ATGATTTAAC TAGTCAAGCC GGACCAACAT TGCTTTTTAT   
  
  
+ AGTGTATTTG CAACTCATGT TGATTATTAG ATGTATCAAC AAAAAATGTT TCTCAAAATA AATAGTAGCT   
  
  
+ CTTTAAATTT TATTCCACAT AAATTCAAAA TCAATATCGA TATCGTTCCA TAATATAGCA GCATTATGAA   
  
  
+ AACAATATTT GCTAATGTTT ACATTGTGCT CTTTTCACTC AAAAATTGTA AGGCTTCAAA TACTATTAAA   
  
  
+ GAAACCATTA TTTCACTCGA CTATTTAGCT TGCATATATA GACAATCTTG CAAATAGTAA CATGTCTTCA   
  
  
+ TTCGACTATT TGCTTCCAAA TGAATAATCT CGTAAACATA TATACATACA TACATATATA TATATATATA   
  
  
+ TATATATATA TATATATATA GTAATATGTA TTTAACTTCA CATATACATG AATATAAAAA TATAAGAGGG   
  
  
+ AAAAAAGACG CTAGGTAGAT GGACTACAAA AGCCATTCAT GTATTGAGAC GGCTCATAAA ATCATACGGG   
  
  
+ GAAAGGAAAA AAAAAGATCT CTACATAATG TTCATAGATT TATAGAAAGC ATATGATGGA TTACCGCATA   
  
  
+ AGGTGTTATA TATGGGAGTG TTTGTAGAAG AAAGATGTAT ATCCGAACTA TCAAAGATAC ATATGAGGGA   
  
  
+ GTAAAAACAA GTGTAAGAAC CTCAGGAGGA GACATAAAAG ATTTTTCTAT TGGCATTGGA CACCGTCAAG   
  
  
+ TATCCGCTTT AAACCTGTTC TTTTTCACTA TTATCATGGA TGTATTCACA AAAGGAATTC AATACGAAGT   
  
  
+ ACCATGATGT ATGCTTTTTA CTGATAGTAT TGGTCTCATC GATGAGACTA AGGATGGTCT TAATAGCAAA   
  
  
+ TTAGAGATTT CATTAGATAA AATCTACTGT ACATGACAAA ACAATGATAT ATAGCATCCT ATTTAAAAAG   
  
  
+ CTACGTGTCA GTCATATATG AATTGCATCT AATTTTTAAT AGATATATAA TATGATATGA TATAATAGGA   
  
  
+ TATGATGTAG ATTGAATTCA AACTCATAAT TCATTAAGCA TGGTGCAATG TTCATTTTCT TGATTTTAAA   
  
  
+ CCGACTATTA CTTCAACTGT ATATAATTCG AATTGCTTTG GTATTTTAAA ATTTAAACTA GTAATTACTG   
  
  
+ CCTCTATTAC TGATGTTTAT ATTAAGTGGT GCTTAACTGC TTATTGATGA AACCATTTTT ATTTTCGTGT   
  
  
+ AAAAGAGGGC ATAATCCATC CTCGTGTATT GGCGGGGCTC GGCGCCCTAT TCAGCTATTC TCTGTATGTC   
  
  
+ AAACAAAACC GCTTCTATTA CCCATTGGTC TCTAAACACG CAGGGGTTTT AGCCCGGAGA GTTAATTCTT   
  
  
+ TATTGAGGTC CCCTGTTCTC CATTGCAGGA TTTTTGCTCA GGTACTTCTC TGTTTCTTCT CTTTAATCTT   
  
  
+ CTGTATCTTT CTGCATCAAG TTTAATGCTT TCTGGGATTT TCAGCTACGA TTTATGTGAA ATTGTAAGAT   
  
  
+ TTCTGAACTG GGCATTCAGG GCTTGGTTCA TATGTATTCT TTTCTGCTTG AAATGTTTTA ATTTCATAGT   
  
  
+ TCCAATGGGG TTATTCAAAG ATAGAATTAA GCTATAATCA CCGAGAAGAC TTGCTGACCT TTGTTTCTTT   
  
  
+ TTTGTTTCTT TTTTTTAATT AAATCTGGCT ATTACTTGGC TATAGTTTTT GCTAATGGAT CCACAAATAG   
  
  
+ ACGAATTATA TGGGCCTTTG CACCAAATGA AGTTCAATGA TCAGAGGAGA CCCATTTTAT CAAATGAAAA   
  
  
+ TTTTGTTAAT GTGTTGAAAC CGCCAAATTC CAATTGGAAT TCACCTTTTG GGAACCCTAC TGTGATTACC   
  
  
+ ACAAACCCAA ATTTTGATTT GGTTGTGCCA TCACTAGGTC CCAATGTTGA GGGAGCTCTC CTTGAAGATT   
  
  
+ ATGATTTTAG TGATGTTGTT CTTAAGTACA TCAATCAAGT TCTTATGGAC GAGGATGAGG GAGAGAAAAT   
  
  
+ CCATTTGGGC CACGGGCCTC TGGCTCTTGA AGCGGCCGAG AAGTCGTTAT ATGAGGTGCT TGGGCAGAGC   
  
  
+ CATTCTCCAC AAAATCAATA CTCTCCAGAG GACAAGCGTG ATGACCTGAG CAAGAGTAGT GTTAGAAGCT   
  
  
+ GTGTCAGTTA TAGCTGTGAC GGTGCAAGTA GTGGCAGTGG CATCGTGGAG CAAAGTTGGT CGAGTTATCC   
  
  
+ TCCGGAGTAC AATCCCATGC GGATGGCAGA TTTTCCTGCC TTGCCCTCAA AACCCTTGTT GAGAACATCT   
  
  
+ TATAGCTTAT CAAATAGCTT GACAGGTGAG GGTAATGGTT TGGTGGATAG ACCGGCAGAC TCACCTGTAA   
  
  
+ GCGCTCTTAG TGTGTCTGAT ATATTTAATG ATAGTCAGTC AGTGATACAA TTTCAGAAAG GGATGGAGGA   
  
  
+ AGCGAGCAAA TTTCTTCCCA AGGCTTCTTT GTGTTTGGAT TTGAACACTG GACCCTGCCT TCGGGAGTCG   
  
  
+ GTCAATAAGG CACGAGGTGG GCTGATTTTG GTGGAGACTA GCCATGAAAA TGAGGTTGCA GTGCTAGCTA   
  
  
+ GTAGGGGAAA GAAGCATCGC CATCCTGAGG AATTACGTTC AGAAGTTGGG AGGGGCAGTA AGCAGCCAGT   
  
  
+ TGTTTTCCAG TTTTCAGACG AGGCAGTTGT GAGATCAGAA ATGTTTGATA GGGTATTGCT GTGCAGTGGT   
  
  
+ GGCAAAAATG ATGCTGACCT ACGCCAAACT TTACAGAGTG AACTAAATAA AACTATGCAA AATGCTCAAA   
  
  
+ TGAAAGATTC TAATGGTGGA AAAGGCCGTG GTAGGAAGCA AGTTGGCAGG AGGGATGTGG TGGATCTAAG   
  
  
+ ATCTCTTTTA CGCCTTTGTG CACAAGCAGT TGGATCCAAT GACCAAAGAA GCGCAAATGA CTTAATTAGG   
  
  
+ CAAATTAGGA AGCATTCTTC TCCAACCGGG GATGGGAATC AAAGAATGGC TCATTATTTT GCTGATGGTC   
  
  
+ TTGAGGCTCG GCTTGCTGGT GTTGGGACCC CAATATACAA GTCTCTCGTG ACTGGCCCAG CTTCTGCTGT   
  
  
+ TGATATCTTG AGAGCTTACC ATATGTTTCT TGCCACATGC CCATTCAAGA AAATAGGGAA TTTCTTCTCT   
  
  
+ AATAGAACAA TTATGGATGT AGCTCAGAAA GCAAATCGAC TTCATATTAT TGATCTTGGT ATTGTCTATG   
  
  
+ GTTTCCAATG GCCTTGTCTA ATTCAGCGCC TTTCATCTAG GCCTGGCGGG CCTCCCAAAC TTCGGATTAC   
  
  
+ AGGCGTTGAT CTCCCCCAAC CCGGTTTCCG ACCAGCCAAA AGAGTCGAGG AGACTGGGCG ACGCTTGAGG   
  
  
+ AACTATGCTG AGTCATTCAA TGTGCCATTT GAGTTCAATG CCATAGCAAA GAGCTGGGAA ACACTTACTG   
  
  
+ TTAAAGATCT CAAGATTGAT CCCGATGAGG TGCTTGTTGT GAACTGTCTG TTCAGGTTTA AATACATTCC   
  
  
+ TGAGGAAACA GTAATTGCAG AATGCCCAAG AGATATTGTT CTTAATCTGA TCAGACAGAT AAAACCAGCT   
  
  
+ GTTTTCATAC ATGGTGTAGT CAATGGAGCC TTCAATTCTC CTTTTTTCAT TATTCGGTTC CGTGAGGCTC   
  
  
+ TCTTCCACTT CTCCACTCTA TTTGATGTGT TGGAGGCAAA TCTTCCCCGG GACATCGAGG AGAGGATACT   
  
  
+ GATAGAGCGA GACATCTTTG GGCGACAGGC AATGAATGTG ATCGCTTGTG AGGGTTTGGA GAGGATAGAA   
  
  
+ AGGCCAGAGA AATACAAACA GTGGCGGGTC CGAAATGAGA GGGCTGGGTT GAAGCAGCTG CCTTTGAATC   
  
  
+ AGGAGACTGT GGAAATGGCC AAAAAACGGG TGACAGCTGT CTATCACAAA GATTTCTCCA TTGATGACGA   
  
  
+ TGGGCACTGG TTGTTACAGG GATGGAAAGG GAGAATTGTC TATGCACTCA CTACTTGGAA GCCTGCTGAC   
  
  
+ TA  

- -Up\_Stream \_Len000AAACAT AAAGTTATAA CATTAATCCA CCCTATATCT CAACTTCCCG TTTGAGATGG   
  
  
- TTGCTGAACA ACTTCCTAGT TGAACATCGA ACCTTGAATT ACATACACAT GATATCTCAA CACATTAGAG   
  
  
- AGGATTATTT TAACCCAATT AGGATCTTAA TCTCTTGAAA TTCGCCCCTG ATTCGAACCA ACTCCGATTG   
  
  
- AAGCAATTTT TCAAGAACAC AGTACAGGTG CAAATAGAAA AGTGATAAAT AAAGTTAATT AAACGCTATA   
  
  
- AATAGTAGTG GGATAAGTGG CAGAAAAGCC TACTAAATTG ATCAGTTCGG CCTGGTTGTA ACGAAAAATA   
  
  
- TCACATAAAC GTTGAGTACA ACTAATAATC TACATAGTTG TTTTTTACAA AGAGTTTTAT TTATCATCGA   
  
  
- GAAATTTAAA ATAAGGTGTA TTTAAGTTTT AGTTATAGCT ATAGCAAGGT ATTATATCGT CGTAATACTT   
  
  
- TTGTTATAAA CGATTACAAA TGTAACACGA GAAAAGTGAG TTTTTAACAT TCCGAAGTTT ATGATAATTT   
  
  
- CTTTGGTAAT AAAGTGAGCT GATAAATCGA ACGTATATAT CTGTTAGAAC GTTTATCATT GTACAGAAGT   
  
  
- AAGCTGATAA ACGAAGGTTT ACTTATTAGA GCATTTGTAT ATATGTATGT ATGTATATAT ATATATATAT   
  
  
- ATATATATAT ATATATATAT CATTATACAT AAATTGAAGT GTATATGTAC TTATATTTTT ATATTCTCCC   
  
  
- TTTTTTCTGC GATCCATCTA CCTGATGTTT TCGGTAAGTA CATAACTCTG CCGAGTATTT TAGTATGCCC   
  
  
- CTTTCCTTTT TTTTTCTAGA GATGTATTAC AAGTATCTAA ATATCTTTCG TATACTACCT AATGGCGTAT   
  
  
- TCCACAATAT ATACCCTCAC AAACATCTTC TTTCTACATA TAGGCTTGAT AGTTTCTATG TATACTCCCT   
  
  
- CATTTTTGTT CACATTCTTG GAGTCCTCCT CTGTATTTTC TAAAAAGATA ACCGTAACCT GTGGCAGTTC   
  
  
- ATAGGCGAAA TTTGGACAAG AAAAAGTGAT AATAGTACCT ACATAAGTGT TTTCCTTAAG TTATGCTTCA   
  
  
- TGGTACTACA TACGAAAAAT GACTATCATA ACCAGAGTAG CTACTCTGAT TCCTACCAGA ATTATCGTTT   
  
  
- AATCTCTAAA GTAATCTATT TTAGATGACA TGTACTGTTT TGTTACTATA TATCGTAGGA TAAATTTTTC   
  
  
- GATGCACAGT CAGTATATAC TTAACGTAGA TTAAAAATTA TCTATATATT ATACTATACT ATATTATCCT   
  
  
- ATACTACATC TAACTTAAGT TTGAGTATTA AGTAATTCGT ACCACGTTAC AAGTAAAAGA ACTAAAATTT   
  
  
- GGCTGATAAT GAAGTTGACA TATATTAAGC TTAACGAAAC CATAAAATTT TAAATTTGAT CATTAATGAC   
  
  
- GGAGATAATG ACTACAAATA TAATTCACCA CGAATTGACG AATAACTACT TTGGTAAAAA TAAAAGCACA   
  
  
- TTTTCTCCCG TATTAGGTAG GAGCACATAA CCGCCCCGAG CCGCGGGATA AGTCGATAAG AGACATACAG   
  
  
- TTTGTTTTGG CGAAGATAAT GGGTAACCAG AGATTTGTGC GTCCCCAAAA TCGGGCCTCT CAATTAAGAA   
  
  
- ATAACTCCAG GGGACAAGAG GTAACGTCCT AAAAACGAGT CCATGAAGAG ACAAAGAAGA GAAATTAGAA   
  
  
- GACATAGAAA GACGTAGTTC AAATTACGAA AGACCCTAAA AGTCGATGCT AAATACACTT TAACATTCTA   
  
  
- AAGACTTGAC CCGTAAGTCC CGAACCAAGT ATACATAAGA AAAGACGAAC TTTACAAAAT TAAAGTATCA   
  
  
- AGGTTACCCC AATAAGTTTC TATCTTAATT CGATATTAGT GGCTCTTCTG AACGACTGGA AACAAAGAAA   
  
  
- AAACAAAGAA AAAAAATTAA TTTAGACCGA TAATGAACCG ATATCAAAAA CGATTACCTA GGTGTTTATC   
  
  
- TGCTTAATAT ACCCGGAAAC GTGGTTTACT TCAAGTTACT AGTCTCCTCT GGGTAAAATA GTTTACTTTT   
  
  
- AAAACAATTA CACAACTTTG GCGGTTTAAG GTTAACCTTA AGTGGAAAAC CCTTGGGATG ACACTAATGG   
  
  
- TGTTTGGGTT TAAAACTAAA CCAACACGGT AGTGATCCAG GGTTACAACT CCCTCGAGAG GAACTTCTAA   
  
  
- TACTAAAATC ACTACAACAA GAATTCATGT AGTTAGTTCA AGAATACCTG CTCCTACTCC CTCTCTTTTA   
  
  
- GGTAAACCCG GTGCCCGGAG ACCGAGAACT TCGCCGGCTC TTCAGCAATA TACTCCACGA ACCCGTCTCG   
  
  
- GTAAGAGGTG TTTTAGTTAT GAGAGGTCTC CTGTTCGCAC TACTGGACTC GTTCTCATCA CAATCTTCGA   
  
  
- CACAGTCAAT ATCGACACTG CCACGTTCAT CACCGTCACC GTAGCACCTC GTTTCAACCA GCTCAATAGG   
  
  
- AGGCCTCATG TTAGGGTACG CCTACCGTCT AAAAGGACGG AACGGGAGTT TTGGGAACAA CTCTTGTAGA   
  
  
- ATATCGAATA GTTTATCGAA CTGTCCACTC CCATTACCAA ACCACCTATC TGGCCGTCTG AGTGGACATT   
  
  
- CGCGAGAATC ACACAGACTA TATAAATTAC TATCAGTCAG TCACTATGTT AAAGTCTTTC CCTACCTCCT   
  
  
- TCGCTCGTTT AAAGAAGGGT TCCGAAGAAA CACAAACCTA AACTTGTGAC CTGGGACGGA AGCCCTCAGC   
  
  
- CAGTTATTCC GTGCTCCACC CGACTAAAAC CACCTCTGAT CGGTACTTTT ACTCCAACGT CACGATCGAT   
  
  
- CATCCCCTTT CTTCGTAGCG GTAGGACTCC TTAATGCAAG TCTTCAACCC TCCCCGTCAT TCGTCGGTCA   
  
  
- ACAAAAGGTC AAAAGTCTGC TCCGTCAACA CTCTAGTCTT TACAAACTAT CCCATAACGA CACGTCACCA   
  
  
- CCGTTTTTAC TACGACTGGA TGCGGTTTGA AATGTCTCAC TTGATTTATT TTGATACGTT TTACGAGTTT   
  
  
- ACTTTCTAAG ATTACCACCT TTTCCGGCAC CATCCTTCGT TCAACCGTCC TCCCTACACC ACCTAGATTC   
  
  
- TAGAGAAAAT GCGGAAACAC GTGTTCGTCA ACCTAGGTTA CTGGTTTCTT CGCGTTTACT GAATTAATCC   
  
  
- GTTTAATCCT TCGTAAGAAG AGGTTGGCCC CTACCCTTAG TTTCTTACCG AGTAATAAAA CGACTACCAG   
  
  
- AACTCCGAGC CGAACGACCA CAACCCTGGG GTTATATGTT CAGAGAGCAC TGACCGGGTC GAAGACGACA   
  
  
- ACTATAGAAC TCTCGAATGG TATACAAAGA ACGGTGTACG GGTAAGTTCT TTTATCCCTT AAAGAAGAGA   
  
  
- TTATCTTGTT AATACCTACA TCGAGTCTTT CGTTTAGCTG AAGTATAATA ACTAGAACCA TAACAGATAC   
  
  
- CAAAGGTTAC CGGAACAGAT TAAGTCGCGG AAAGTAGATC CGGACCGCCC GGAGGGTTTG AAGCCTAATG   
  
  
- TCCGCAACTA GAGGGGGTTG GGCCAAAGGC TGGTCGGTTT TCTCAGCTCC TCTGACCCGC TGCGAACTCC   
  
  
- TTGATACGAC TCAGTAAGTT ACACGGTAAA CTCAAGTTAC GGTATCGTTT CTCGACCCTT TGTGAATGAC   
  
  
- AATTTCTAGA GTTCTAACTA GGGCTACTCC ACGAACAACA CTTGACAGAC AAGTCCAAAT TTATGTAAGG   
  
  
- ACTCCTTTGT CATTAACGTC TTACGGGTTC TCTATAACAA GAATTAGACT AGTCTGTCTA TTTTGGTCGA   
  
  
- CAAAAGTATG TACCACATCA GTTACCTCGG AAGTTAAGAG GAAAAAAGTA ATAAGCCAAG GCACTCCGAG   
  
  
- AGAAGGTGAA GAGGTGAGAT AAACTACACA ACCTCCGTTT AGAAGGGGCC CTGTAGCTCC TCTCCTATGA   
  
  
- CTATCTCGCT CTGTAGAAAC CCGCTGTCCG TTACTTACAC TAGCGAACAC TCCCAAACCT CTCCTATCTT   
  
  
- TCCGGTCTCT TTATGTTTGT CACCGCCCAG GCTTTACTCT CCCGACCCAA CTTCGTCGAC GGAAACTTAG   
  
  
- TCCTCTGACA CCTTTACCGG TTTTTTGCCC ACTGTCGACA GATAGTGTTT CTAAAGAGGT AACTACTGCT   
  
  
- ACCCGTGACC AACAATGTCC CTACCTTTCC CTCTTAACAG ATACGTGAGT GATGAACCTT CGGACGACTG   
  
  
- AT

+     GT1-motif

| Site Name | Organism | Position | Strand | Matrix score. | sequence | function |
| --- | --- | --- | --- | --- | --- | --- |
| GT1-motif | Avena sativa | 159 | + | 7 | GGTTAAT | light responsive element |

>HU05G01597.1   
+ -Up\_Stream \_Len000TTTGTA TTTCAATATT GTAATTAGGT GGGATATAGA GTTGAAGGGC AAACTCTACC   
  
  
+ AACGACTTGT TGAAGGATCA ACTTGTAGCT TGGAACTTAA TGTATGTGTA CTATAGAGTT GTGTAATCTC   
  
  
+ TCCTAATAAA ATTGGGTTAA TCCTAGAATT AGAGAACTTT AAGCGGGGAC TAAGCTTGGT TGAGGCTAAC   
  
  
+ TTCGTTAAAA AGTTCTTGTG TCATGTCCAC GTTTATCTTT TCACTATTTA TTTCAATTAA TTTGCGATAT   
  
  
+ TTATCATCAC CCTATTCACC GTCTTTTCGG ATGATTTAAC TAGTCAAGCC GGACCAACAT TGCTTTTTAT   
  
  
+ AGTGTATTTG CAACTCATGT TGATTATTAG ATGTATCAAC AAAAAATGTT TCTCAAAATA AATAGTAGCT   
  
  
+ CTTTAAATTT TATTCCACAT AAATTCAAAA TCAATATCGA TATCGTTCCA TAATATAGCA GCATTATGAA   
  
  
+ AACAATATTT GCTAATGTTT ACATTGTGCT CTTTTCACTC AAAAATTGTA AGGCTTCAAA TACTATTAAA   
  
  
+ GAAACCATTA TTTCACTCGA CTATTTAGCT TGCATATATA GACAATCTTG CAAATAGTAA CATGTCTTCA   
  
  
+ TTCGACTATT TGCTTCCAAA TGAATAATCT CGTAAACATA TATACATACA TACATATATA TATATATATA   
  
  
+ TATATATATA TATATATATA GTAATATGTA TTTAACTTCA CATATACATG AATATAAAAA TATAAGAGGG   
  
  
+ AAAAAAGACG CTAGGTAGAT GGACTACAAA AGCCATTCAT GTATTGAGAC GGCTCATAAA ATCATACGGG   
  
  
+ GAAAGGAAAA AAAAAGATCT CTACATAATG TTCATAGATT TATAGAAAGC ATATGATGGA TTACCGCATA   
  
  
+ AGGTGTTATA TATGGGAGTG TTTGTAGAAG AAAGATGTAT ATCCGAACTA TCAAAGATAC ATATGAGGGA   
  
  
+ GTAAAAACAA GTGTAAGAAC CTCAGGAGGA GACATAAAAG ATTTTTCTAT TGGCATTGGA CACCGTCAAG   
  
  
+ TATCCGCTTT AAACCTGTTC TTTTTCACTA TTATCATGGA TGTATTCACA AAAGGAATTC AATACGAAGT   
  
  
+ ACCATGATGT ATGCTTTTTA CTGATAGTAT TGGTCTCATC GATGAGACTA AGGATGGTCT TAATAGCAAA   
  
  
+ TTAGAGATTT CATTAGATAA AATCTACTGT ACATGACAAA ACAATGATAT ATAGCATCCT ATTTAAAAAG   
  
  
+ CTACGTGTCA GTCATATATG AATTGCATCT AATTTTTAAT AGATATATAA TATGATATGA TATAATAGGA   
  
  
+ TATGATGTAG ATTGAATTCA AACTCATAAT TCATTAAGCA TGGTGCAATG TTCATTTTCT TGATTTTAAA   
  
  
+ CCGACTATTA CTTCAACTGT ATATAATTCG AATTGCTTTG GTATTTTAAA ATTTAAACTA GTAATTACTG   
  
  
+ CCTCTATTAC TGATGTTTAT ATTAAGTGGT GCTTAACTGC TTATTGATGA AACCATTTTT ATTTTCGTGT   
  
  
+ AAAAGAGGGC ATAATCCATC CTCGTGTATT GGCGGGGCTC GGCGCCCTAT TCAGCTATTC TCTGTATGTC   
  
  
+ AAACAAAACC GCTTCTATTA CCCATTGGTC TCTAAACACG CAGGGGTTTT AGCCCGGAGA GTTAATTCTT   
  
  
+ TATTGAGGTC CCCTGTTCTC CATTGCAGGA TTTTTGCTCA GGTACTTCTC TGTTTCTTCT CTTTAATCTT   
  
  
+ CTGTATCTTT CTGCATCAAG TTTAATGCTT TCTGGGATTT TCAGCTACGA TTTATGTGAA ATTGTAAGAT   
  
  
+ TTCTGAACTG GGCATTCAGG GCTTGGTTCA TATGTATTCT TTTCTGCTTG AAATGTTTTA ATTTCATAGT   
  
  
+ TCCAATGGGG TTATTCAAAG ATAGAATTAA GCTATAATCA CCGAGAAGAC TTGCTGACCT TTGTTTCTTT   
  
  
+ TTTGTTTCTT TTTTTTAATT AAATCTGGCT ATTACTTGGC TATAGTTTTT GCTAATGGAT CCACAAATAG   
  
  
+ ACGAATTATA TGGGCCTTTG CACCAAATGA AGTTCAATGA TCAGAGGAGA CCCATTTTAT CAAATGAAAA   
  
  
+ TTTTGTTAAT GTGTTGAAAC CGCCAAATTC CAATTGGAAT TCACCTTTTG GGAACCCTAC TGTGATTACC   
  
  
+ ACAAACCCAA ATTTTGATTT GGTTGTGCCA TCACTAGGTC CCAATGTTGA GGGAGCTCTC CTTGAAGATT   
  
  
+ ATGATTTTAG TGATGTTGTT CTTAAGTACA TCAATCAAGT TCTTATGGAC GAGGATGAGG GAGAGAAAAT   
  
  
+ CCATTTGGGC CACGGGCCTC TGGCTCTTGA AGCGGCCGAG AAGTCGTTAT ATGAGGTGCT TGGGCAGAGC   
  
  
+ CATTCTCCAC AAAATCAATA CTCTCCAGAG GACAAGCGTG ATGACCTGAG CAAGAGTAGT GTTAGAAGCT   
  
  
+ GTGTCAGTTA TAGCTGTGAC GGTGCAAGTA GTGGCAGTGG CATCGTGGAG CAAAGTTGGT CGAGTTATCC   
  
  
+ TCCGGAGTAC AATCCCATGC GGATGGCAGA TTTTCCTGCC TTGCCCTCAA AACCCTTGTT GAGAACATCT   
  
  
+ TATAGCTTAT CAAATAGCTT GACAGGTGAG GGTAATGGTT TGGTGGATAG ACCGGCAGAC TCACCTGTAA   
  
  
+ GCGCTCTTAG TGTGTCTGAT ATATTTAATG ATAGTCAGTC AGTGATACAA TTTCAGAAAG GGATGGAGGA   
  
  
+ AGCGAGCAAA TTTCTTCCCA AGGCTTCTTT GTGTTTGGAT TTGAACACTG GACCCTGCCT TCGGGAGTCG   
  
  
+ GTCAATAAGG CACGAGGTGG GCTGATTTTG GTGGAGACTA GCCATGAAAA TGAGGTTGCA GTGCTAGCTA   
  
  
+ GTAGGGGAAA GAAGCATCGC CATCCTGAGG AATTACGTTC AGAAGTTGGG AGGGGCAGTA AGCAGCCAGT   
  
  
+ TGTTTTCCAG TTTTCAGACG AGGCAGTTGT GAGATCAGAA ATGTTTGATA GGGTATTGCT GTGCAGTGGT   
  
  
+ GGCAAAAATG ATGCTGACCT ACGCCAAACT TTACAGAGTG AACTAAATAA AACTATGCAA AATGCTCAAA   
  
  
+ TGAAAGATTC TAATGGTGGA AAAGGCCGTG GTAGGAAGCA AGTTGGCAGG AGGGATGTGG TGGATCTAAG   
  
  
+ ATCTCTTTTA CGCCTTTGTG CACAAGCAGT TGGATCCAAT GACCAAAGAA GCGCAAATGA CTTAATTAGG   
  
  
+ CAAATTAGGA AGCATTCTTC TCCAACCGGG GATGGGAATC AAAGAATGGC TCATTATTTT GCTGATGGTC   
  
  
+ TTGAGGCTCG GCTTGCTGGT GTTGGGACCC CAATATACAA GTCTCTCGTG ACTGGCCCAG CTTCTGCTGT   
  
  
+ TGATATCTTG AGAGCTTACC ATATGTTTCT TGCCACATGC CCATTCAAGA AAATAGGGAA TTTCTTCTCT   
  
  
+ AATAGAACAA TTATGGATGT AGCTCAGAAA GCAAATCGAC TTCATATTAT TGATCTTGGT ATTGTCTATG   
  
  
+ GTTTCCAATG GCCTTGTCTA ATTCAGCGCC TTTCATCTAG GCCTGGCGGG CCTCCCAAAC TTCGGATTAC   
  
  
+ AGGCGTTGAT CTCCCCCAAC CCGGTTTCCG ACCAGCCAAA AGAGTCGAGG AGACTGGGCG ACGCTTGAGG   
  
  
+ AACTATGCTG AGTCATTCAA TGTGCCATTT GAGTTCAATG CCATAGCAAA GAGCTGGGAA ACACTTACTG   
  
  
+ TTAAAGATCT CAAGATTGAT CCCGATGAGG TGCTTGTTGT GAACTGTCTG TTCAGGTTTA AATACATTCC   
  
  
+ TGAGGAAACA GTAATTGCAG AATGCCCAAG AGATATTGTT CTTAATCTGA TCAGACAGAT AAAACCAGCT   
  
  
+ GTTTTCATAC ATGGTGTAGT CAATGGAGCC TTCAATTCTC CTTTTTTCAT TATTCGGTTC CGTGAGGCTC   
  
  
+ TCTTCCACTT CTCCACTCTA TTTGATGTGT TGGAGGCAAA TCTTCCCCGG GACATCGAGG AGAGGATACT   
  
  
+ GATAGAGCGA GACATCTTTG GGCGACAGGC AATGAATGTG ATCGCTTGTG AGGGTTTGGA GAGGATAGAA   
  
  
+ AGGCCAGAGA AATACAAACA GTGGCGGGTC CGAAATGAGA GGGCTGGGTT GAAGCAGCTG CCTTTGAATC   
  
  
+ AGGAGACTGT GGAAATGGCC AAAAAACGGG TGACAGCTGT CTATCACAAA GATTTCTCCA TTGATGACGA   
  
  
+ TGGGCACTGG TTGTTACAGG GATGGAAAGG GAGAATTGTC TATGCACTCA CTACTTGGAA GCCTGCTGAC   
  
  
+ TA  

- -Up\_Stream \_Len000AAACAT AAAGTTATAA CATTAATCCA CCCTATATCT CAACTTCCCG TTTGAGATGG   
  
  
- TTGCTGAACA ACTTCCTAGT TGAACATCGA ACCTTGAATT ACATACACAT GATATCTCAA CACATTAGAG   
  
  
- AGGATTATTT TAACCCAATT AGGATCTTAA TCTCTTGAAA TTCGCCCCTG ATTCGAACCA ACTCCGATTG   
  
  
- AAGCAATTTT TCAAGAACAC AGTACAGGTG CAAATAGAAA AGTGATAAAT AAAGTTAATT AAACGCTATA   
  
  
- AATAGTAGTG GGATAAGTGG CAGAAAAGCC TACTAAATTG ATCAGTTCGG CCTGGTTGTA ACGAAAAATA   
  
  
- TCACATAAAC GTTGAGTACA ACTAATAATC TACATAGTTG TTTTTTACAA AGAGTTTTAT TTATCATCGA   
  
  
- GAAATTTAAA ATAAGGTGTA TTTAAGTTTT AGTTATAGCT ATAGCAAGGT ATTATATCGT CGTAATACTT   
  
  
- TTGTTATAAA CGATTACAAA TGTAACACGA GAAAAGTGAG TTTTTAACAT TCCGAAGTTT ATGATAATTT   
  
  
- CTTTGGTAAT AAAGTGAGCT GATAAATCGA ACGTATATAT CTGTTAGAAC GTTTATCATT GTACAGAAGT   
  
  
- AAGCTGATAA ACGAAGGTTT ACTTATTAGA GCATTTGTAT ATATGTATGT ATGTATATAT ATATATATAT   
  
  
- ATATATATAT ATATATATAT CATTATACAT AAATTGAAGT GTATATGTAC TTATATTTTT ATATTCTCCC   
  
  
- TTTTTTCTGC GATCCATCTA CCTGATGTTT TCGGTAAGTA CATAACTCTG CCGAGTATTT TAGTATGCCC   
  
  
- CTTTCCTTTT TTTTTCTAGA GATGTATTAC AAGTATCTAA ATATCTTTCG TATACTACCT AATGGCGTAT   
  
  
- TCCACAATAT ATACCCTCAC AAACATCTTC TTTCTACATA TAGGCTTGAT AGTTTCTATG TATACTCCCT   
  
  
- CATTTTTGTT CACATTCTTG GAGTCCTCCT CTGTATTTTC TAAAAAGATA ACCGTAACCT GTGGCAGTTC   
  
  
- ATAGGCGAAA TTTGGACAAG AAAAAGTGAT AATAGTACCT ACATAAGTGT TTTCCTTAAG TTATGCTTCA   
  
  
- TGGTACTACA TACGAAAAAT GACTATCATA ACCAGAGTAG CTACTCTGAT TCCTACCAGA ATTATCGTTT   
  
  
- AATCTCTAAA GTAATCTATT TTAGATGACA TGTACTGTTT TGTTACTATA TATCGTAGGA TAAATTTTTC   
  
  
- GATGCACAGT CAGTATATAC TTAACGTAGA TTAAAAATTA TCTATATATT ATACTATACT ATATTATCCT   
  
  
- ATACTACATC TAACTTAAGT TTGAGTATTA AGTAATTCGT ACCACGTTAC AAGTAAAAGA ACTAAAATTT   
  
  
- GGCTGATAAT GAAGTTGACA TATATTAAGC TTAACGAAAC CATAAAATTT TAAATTTGAT CATTAATGAC   
  
  
- GGAGATAATG ACTACAAATA TAATTCACCA CGAATTGACG AATAACTACT TTGGTAAAAA TAAAAGCACA   
  
  
- TTTTCTCCCG TATTAGGTAG GAGCACATAA CCGCCCCGAG CCGCGGGATA AGTCGATAAG AGACATACAG   
  
  
- TTTGTTTTGG CGAAGATAAT GGGTAACCAG AGATTTGTGC GTCCCCAAAA TCGGGCCTCT CAATTAAGAA   
  
  
- ATAACTCCAG GGGACAAGAG GTAACGTCCT AAAAACGAGT CCATGAAGAG ACAAAGAAGA GAAATTAGAA   
  
  
- GACATAGAAA GACGTAGTTC AAATTACGAA AGACCCTAAA AGTCGATGCT AAATACACTT TAACATTCTA   
  
  
- AAGACTTGAC CCGTAAGTCC CGAACCAAGT ATACATAAGA AAAGACGAAC TTTACAAAAT TAAAGTATCA   
  
  
- AGGTTACCCC AATAAGTTTC TATCTTAATT CGATATTAGT GGCTCTTCTG AACGACTGGA AACAAAGAAA   
  
  
- AAACAAAGAA AAAAAATTAA TTTAGACCGA TAATGAACCG ATATCAAAAA CGATTACCTA GGTGTTTATC   
  
  
- TGCTTAATAT ACCCGGAAAC GTGGTTTACT TCAAGTTACT AGTCTCCTCT GGGTAAAATA GTTTACTTTT   
  
  
- AAAACAATTA CACAACTTTG GCGGTTTAAG GTTAACCTTA AGTGGAAAAC CCTTGGGATG ACACTAATGG   
  
  
- TGTTTGGGTT TAAAACTAAA CCAACACGGT AGTGATCCAG GGTTACAACT CCCTCGAGAG GAACTTCTAA   
  
  
- TACTAAAATC ACTACAACAA GAATTCATGT AGTTAGTTCA AGAATACCTG CTCCTACTCC CTCTCTTTTA   
  
  
- GGTAAACCCG GTGCCCGGAG ACCGAGAACT TCGCCGGCTC TTCAGCAATA TACTCCACGA ACCCGTCTCG   
  
  
- GTAAGAGGTG TTTTAGTTAT GAGAGGTCTC CTGTTCGCAC TACTGGACTC GTTCTCATCA CAATCTTCGA   
  
  
- CACAGTCAAT ATCGACACTG CCACGTTCAT CACCGTCACC GTAGCACCTC GTTTCAACCA GCTCAATAGG   
  
  
- AGGCCTCATG TTAGGGTACG CCTACCGTCT AAAAGGACGG AACGGGAGTT TTGGGAACAA CTCTTGTAGA   
  
  
- ATATCGAATA GTTTATCGAA CTGTCCACTC CCATTACCAA ACCACCTATC TGGCCGTCTG AGTGGACATT   
  
  
- CGCGAGAATC ACACAGACTA TATAAATTAC TATCAGTCAG TCACTATGTT AAAGTCTTTC CCTACCTCCT   
  
  
- TCGCTCGTTT AAAGAAGGGT TCCGAAGAAA CACAAACCTA AACTTGTGAC CTGGGACGGA AGCCCTCAGC   
  
  
- CAGTTATTCC GTGCTCCACC CGACTAAAAC CACCTCTGAT CGGTACTTTT ACTCCAACGT CACGATCGAT   
  
  
- CATCCCCTTT CTTCGTAGCG GTAGGACTCC TTAATGCAAG TCTTCAACCC TCCCCGTCAT TCGTCGGTCA   
  
  
- ACAAAAGGTC AAAAGTCTGC TCCGTCAACA CTCTAGTCTT TACAAACTAT CCCATAACGA CACGTCACCA   
  
  
- CCGTTTTTAC TACGACTGGA TGCGGTTTGA AATGTCTCAC TTGATTTATT TTGATACGTT TTACGAGTTT   
  
  
- ACTTTCTAAG ATTACCACCT TTTCCGGCAC CATCCTTCGT TCAACCGTCC TCCCTACACC ACCTAGATTC   
  
  
- TAGAGAAAAT GCGGAAACAC GTGTTCGTCA ACCTAGGTTA CTGGTTTCTT CGCGTTTACT GAATTAATCC   
  
  
- GTTTAATCCT TCGTAAGAAG AGGTTGGCCC CTACCCTTAG TTTCTTACCG AGTAATAAAA CGACTACCAG   
  
  
- AACTCCGAGC CGAACGACCA CAACCCTGGG GTTATATGTT CAGAGAGCAC TGACCGGGTC GAAGACGACA   
  
  
- ACTATAGAAC TCTCGAATGG TATACAAAGA ACGGTGTACG GGTAAGTTCT TTTATCCCTT AAAGAAGAGA   
  
  
- TTATCTTGTT AATACCTACA TCGAGTCTTT CGTTTAGCTG AAGTATAATA ACTAGAACCA TAACAGATAC   
  
  
- CAAAGGTTAC CGGAACAGAT TAAGTCGCGG AAAGTAGATC CGGACCGCCC GGAGGGTTTG AAGCCTAATG   
  
  
- TCCGCAACTA GAGGGGGTTG GGCCAAAGGC TGGTCGGTTT TCTCAGCTCC TCTGACCCGC TGCGAACTCC   
  
  
- TTGATACGAC TCAGTAAGTT ACACGGTAAA CTCAAGTTAC GGTATCGTTT CTCGACCCTT TGTGAATGAC   
  
  
- AATTTCTAGA GTTCTAACTA GGGCTACTCC ACGAACAACA CTTGACAGAC AAGTCCAAAT TTATGTAAGG   
  
  
- ACTCCTTTGT CATTAACGTC TTACGGGTTC TCTATAACAA GAATTAGACT AGTCTGTCTA TTTTGGTCGA   
  
  
- CAAAAGTATG TACCACATCA GTTACCTCGG AAGTTAAGAG GAAAAAAGTA ATAAGCCAAG GCACTCCGAG   
  
  
- AGAAGGTGAA GAGGTGAGAT AAACTACACA ACCTCCGTTT AGAAGGGGCC CTGTAGCTCC TCTCCTATGA   
  
  
- CTATCTCGCT CTGTAGAAAC CCGCTGTCCG TTACTTACAC TAGCGAACAC TCCCAAACCT CTCCTATCTT   
  
  
- TCCGGTCTCT TTATGTTTGT CACCGCCCAG GCTTTACTCT CCCGACCCAA CTTCGTCGAC GGAAACTTAG   
  
  
- TCCTCTGACA CCTTTACCGG TTTTTTGCCC ACTGTCGACA GATAGTGTTT CTAAAGAGGT AACTACTGCT   
  
  
- ACCCGTGACC AACAATGTCC CTACCTTTCC CTCTTAACAG ATACGTGAGT GATGAACCTT CGGACGACTG   
  
  
- AT

+     Gap-box

| Site Name | Organism | Position | Strand | Matrix score. | sequence | function |
| --- | --- | --- | --- | --- | --- | --- |
| Gap-box | Arabidopsis thaliana | 2095 | + | 9.5 | CAAATGAA(A/G)A | part of a light responsive element |
| Gap-box | Arabidopsis thaliana | 651 | + | 9 | CAAATGAA(A/G)A | part of a light responsive element |

>HU05G01597.1   
+ -Up\_Stream \_Len000TTTGTA TTTCAATATT GTAATTAGGT GGGATATAGA GTTGAAGGGC AAACTCTACC   
  
  
+ AACGACTTGT TGAAGGATCA ACTTGTAGCT TGGAACTTAA TGTATGTGTA CTATAGAGTT GTGTAATCTC   
  
  
+ TCCTAATAAA ATTGGGTTAA TCCTAGAATT AGAGAACTTT AAGCGGGGAC TAAGCTTGGT TGAGGCTAAC   
  
  
+ TTCGTTAAAA AGTTCTTGTG TCATGTCCAC GTTTATCTTT TCACTATTTA TTTCAATTAA TTTGCGATAT   
  
  
+ TTATCATCAC CCTATTCACC GTCTTTTCGG ATGATTTAAC TAGTCAAGCC GGACCAACAT TGCTTTTTAT   
  
  
+ AGTGTATTTG CAACTCATGT TGATTATTAG ATGTATCAAC AAAAAATGTT TCTCAAAATA AATAGTAGCT   
  
  
+ CTTTAAATTT TATTCCACAT AAATTCAAAA TCAATATCGA TATCGTTCCA TAATATAGCA GCATTATGAA   
  
  
+ AACAATATTT GCTAATGTTT ACATTGTGCT CTTTTCACTC AAAAATTGTA AGGCTTCAAA TACTATTAAA   
  
  
+ GAAACCATTA TTTCACTCGA CTATTTAGCT TGCATATATA GACAATCTTG CAAATAGTAA CATGTCTTCA   
  
  
+ TTCGACTATT TGCTTCCAAA TGAATAATCT CGTAAACATA TATACATACA TACATATATA TATATATATA   
  
  
+ TATATATATA TATATATATA GTAATATGTA TTTAACTTCA CATATACATG AATATAAAAA TATAAGAGGG   
  
  
+ AAAAAAGACG CTAGGTAGAT GGACTACAAA AGCCATTCAT GTATTGAGAC GGCTCATAAA ATCATACGGG   
  
  
+ GAAAGGAAAA AAAAAGATCT CTACATAATG TTCATAGATT TATAGAAAGC ATATGATGGA TTACCGCATA   
  
  
+ AGGTGTTATA TATGGGAGTG TTTGTAGAAG AAAGATGTAT ATCCGAACTA TCAAAGATAC ATATGAGGGA   
  
  
+ GTAAAAACAA GTGTAAGAAC CTCAGGAGGA GACATAAAAG ATTTTTCTAT TGGCATTGGA CACCGTCAAG   
  
  
+ TATCCGCTTT AAACCTGTTC TTTTTCACTA TTATCATGGA TGTATTCACA AAAGGAATTC AATACGAAGT   
  
  
+ ACCATGATGT ATGCTTTTTA CTGATAGTAT TGGTCTCATC GATGAGACTA AGGATGGTCT TAATAGCAAA   
  
  
+ TTAGAGATTT CATTAGATAA AATCTACTGT ACATGACAAA ACAATGATAT ATAGCATCCT ATTTAAAAAG   
  
  
+ CTACGTGTCA GTCATATATG AATTGCATCT AATTTTTAAT AGATATATAA TATGATATGA TATAATAGGA   
  
  
+ TATGATGTAG ATTGAATTCA AACTCATAAT TCATTAAGCA TGGTGCAATG TTCATTTTCT TGATTTTAAA   
  
  
+ CCGACTATTA CTTCAACTGT ATATAATTCG AATTGCTTTG GTATTTTAAA ATTTAAACTA GTAATTACTG   
  
  
+ CCTCTATTAC TGATGTTTAT ATTAAGTGGT GCTTAACTGC TTATTGATGA AACCATTTTT ATTTTCGTGT   
  
  
+ AAAAGAGGGC ATAATCCATC CTCGTGTATT GGCGGGGCTC GGCGCCCTAT TCAGCTATTC TCTGTATGTC   
  
  
+ AAACAAAACC GCTTCTATTA CCCATTGGTC TCTAAACACG CAGGGGTTTT AGCCCGGAGA GTTAATTCTT   
  
  
+ TATTGAGGTC CCCTGTTCTC CATTGCAGGA TTTTTGCTCA GGTACTTCTC TGTTTCTTCT CTTTAATCTT   
  
  
+ CTGTATCTTT CTGCATCAAG TTTAATGCTT TCTGGGATTT TCAGCTACGA TTTATGTGAA ATTGTAAGAT   
  
  
+ TTCTGAACTG GGCATTCAGG GCTTGGTTCA TATGTATTCT TTTCTGCTTG AAATGTTTTA ATTTCATAGT   
  
  
+ TCCAATGGGG TTATTCAAAG ATAGAATTAA GCTATAATCA CCGAGAAGAC TTGCTGACCT TTGTTTCTTT   
  
  
+ TTTGTTTCTT TTTTTTAATT AAATCTGGCT ATTACTTGGC TATAGTTTTT GCTAATGGAT CCACAAATAG   
  
  
+ ACGAATTATA TGGGCCTTTG CACCAAATGA AGTTCAATGA TCAGAGGAGA CCCATTTTAT CAAATGAAAA   
  
  
+ TTTTGTTAAT GTGTTGAAAC CGCCAAATTC CAATTGGAAT TCACCTTTTG GGAACCCTAC TGTGATTACC   
  
  
+ ACAAACCCAA ATTTTGATTT GGTTGTGCCA TCACTAGGTC CCAATGTTGA GGGAGCTCTC CTTGAAGATT   
  
  
+ ATGATTTTAG TGATGTTGTT CTTAAGTACA TCAATCAAGT TCTTATGGAC GAGGATGAGG GAGAGAAAAT   
  
  
+ CCATTTGGGC CACGGGCCTC TGGCTCTTGA AGCGGCCGAG AAGTCGTTAT ATGAGGTGCT TGGGCAGAGC   
  
  
+ CATTCTCCAC AAAATCAATA CTCTCCAGAG GACAAGCGTG ATGACCTGAG CAAGAGTAGT GTTAGAAGCT   
  
  
+ GTGTCAGTTA TAGCTGTGAC GGTGCAAGTA GTGGCAGTGG CATCGTGGAG CAAAGTTGGT CGAGTTATCC   
  
  
+ TCCGGAGTAC AATCCCATGC GGATGGCAGA TTTTCCTGCC TTGCCCTCAA AACCCTTGTT GAGAACATCT   
  
  
+ TATAGCTTAT CAAATAGCTT GACAGGTGAG GGTAATGGTT TGGTGGATAG ACCGGCAGAC TCACCTGTAA   
  
  
+ GCGCTCTTAG TGTGTCTGAT ATATTTAATG ATAGTCAGTC AGTGATACAA TTTCAGAAAG GGATGGAGGA   
  
  
+ AGCGAGCAAA TTTCTTCCCA AGGCTTCTTT GTGTTTGGAT TTGAACACTG GACCCTGCCT TCGGGAGTCG   
  
  
+ GTCAATAAGG CACGAGGTGG GCTGATTTTG GTGGAGACTA GCCATGAAAA TGAGGTTGCA GTGCTAGCTA   
  
  
+ GTAGGGGAAA GAAGCATCGC CATCCTGAGG AATTACGTTC AGAAGTTGGG AGGGGCAGTA AGCAGCCAGT   
  
  
+ TGTTTTCCAG TTTTCAGACG AGGCAGTTGT GAGATCAGAA ATGTTTGATA GGGTATTGCT GTGCAGTGGT   
  
  
+ GGCAAAAATG ATGCTGACCT ACGCCAAACT TTACAGAGTG AACTAAATAA AACTATGCAA AATGCTCAAA   
  
  
+ TGAAAGATTC TAATGGTGGA AAAGGCCGTG GTAGGAAGCA AGTTGGCAGG AGGGATGTGG TGGATCTAAG   
  
  
+ ATCTCTTTTA CGCCTTTGTG CACAAGCAGT TGGATCCAAT GACCAAAGAA GCGCAAATGA CTTAATTAGG   
  
  
+ CAAATTAGGA AGCATTCTTC TCCAACCGGG GATGGGAATC AAAGAATGGC TCATTATTTT GCTGATGGTC   
  
  
+ TTGAGGCTCG GCTTGCTGGT GTTGGGACCC CAATATACAA GTCTCTCGTG ACTGGCCCAG CTTCTGCTGT   
  
  
+ TGATATCTTG AGAGCTTACC ATATGTTTCT TGCCACATGC CCATTCAAGA AAATAGGGAA TTTCTTCTCT   
  
  
+ AATAGAACAA TTATGGATGT AGCTCAGAAA GCAAATCGAC TTCATATTAT TGATCTTGGT ATTGTCTATG   
  
  
+ GTTTCCAATG GCCTTGTCTA ATTCAGCGCC TTTCATCTAG GCCTGGCGGG CCTCCCAAAC TTCGGATTAC   
  
  
+ AGGCGTTGAT CTCCCCCAAC CCGGTTTCCG ACCAGCCAAA AGAGTCGAGG AGACTGGGCG ACGCTTGAGG   
  
  
+ AACTATGCTG AGTCATTCAA TGTGCCATTT GAGTTCAATG CCATAGCAAA GAGCTGGGAA ACACTTACTG   
  
  
+ TTAAAGATCT CAAGATTGAT CCCGATGAGG TGCTTGTTGT GAACTGTCTG TTCAGGTTTA AATACATTCC   
  
  
+ TGAGGAAACA GTAATTGCAG AATGCCCAAG AGATATTGTT CTTAATCTGA TCAGACAGAT AAAACCAGCT   
  
  
+ GTTTTCATAC ATGGTGTAGT CAATGGAGCC TTCAATTCTC CTTTTTTCAT TATTCGGTTC CGTGAGGCTC   
  
  
+ TCTTCCACTT CTCCACTCTA TTTGATGTGT TGGAGGCAAA TCTTCCCCGG GACATCGAGG AGAGGATACT   
  
  
+ GATAGAGCGA GACATCTTTG GGCGACAGGC AATGAATGTG ATCGCTTGTG AGGGTTTGGA GAGGATAGAA   
  
  
+ AGGCCAGAGA AATACAAACA GTGGCGGGTC CGAAATGAGA GGGCTGGGTT GAAGCAGCTG CCTTTGAATC   
  
  
+ AGGAGACTGT GGAAATGGCC AAAAAACGGG TGACAGCTGT CTATCACAAA GATTTCTCCA TTGATGACGA   
  
  
+ TGGGCACTGG TTGTTACAGG GATGGAAAGG GAGAATTGTC TATGCACTCA CTACTTGGAA GCCTGCTGAC   
  
  
+ TA  

- -Up\_Stream \_Len000AAACAT AAAGTTATAA CATTAATCCA CCCTATATCT CAACTTCCCG TTTGAGATGG   
  
  
- TTGCTGAACA ACTTCCTAGT TGAACATCGA ACCTTGAATT ACATACACAT GATATCTCAA CACATTAGAG   
  
  
- AGGATTATTT TAACCCAATT AGGATCTTAA TCTCTTGAAA TTCGCCCCTG ATTCGAACCA ACTCCGATTG   
  
  
- AAGCAATTTT TCAAGAACAC AGTACAGGTG CAAATAGAAA AGTGATAAAT AAAGTTAATT AAACGCTATA   
  
  
- AATAGTAGTG GGATAAGTGG CAGAAAAGCC TACTAAATTG ATCAGTTCGG CCTGGTTGTA ACGAAAAATA   
  
  
- TCACATAAAC GTTGAGTACA ACTAATAATC TACATAGTTG TTTTTTACAA AGAGTTTTAT TTATCATCGA   
  
  
- GAAATTTAAA ATAAGGTGTA TTTAAGTTTT AGTTATAGCT ATAGCAAGGT ATTATATCGT CGTAATACTT   
  
  
- TTGTTATAAA CGATTACAAA TGTAACACGA GAAAAGTGAG TTTTTAACAT TCCGAAGTTT ATGATAATTT   
  
  
- CTTTGGTAAT AAAGTGAGCT GATAAATCGA ACGTATATAT CTGTTAGAAC GTTTATCATT GTACAGAAGT   
  
  
- AAGCTGATAA ACGAAGGTTT ACTTATTAGA GCATTTGTAT ATATGTATGT ATGTATATAT ATATATATAT   
  
  
- ATATATATAT ATATATATAT CATTATACAT AAATTGAAGT GTATATGTAC TTATATTTTT ATATTCTCCC   
  
  
- TTTTTTCTGC GATCCATCTA CCTGATGTTT TCGGTAAGTA CATAACTCTG CCGAGTATTT TAGTATGCCC   
  
  
- CTTTCCTTTT TTTTTCTAGA GATGTATTAC AAGTATCTAA ATATCTTTCG TATACTACCT AATGGCGTAT   
  
  
- TCCACAATAT ATACCCTCAC AAACATCTTC TTTCTACATA TAGGCTTGAT AGTTTCTATG TATACTCCCT   
  
  
- CATTTTTGTT CACATTCTTG GAGTCCTCCT CTGTATTTTC TAAAAAGATA ACCGTAACCT GTGGCAGTTC   
  
  
- ATAGGCGAAA TTTGGACAAG AAAAAGTGAT AATAGTACCT ACATAAGTGT TTTCCTTAAG TTATGCTTCA   
  
  
- TGGTACTACA TACGAAAAAT GACTATCATA ACCAGAGTAG CTACTCTGAT TCCTACCAGA ATTATCGTTT   
  
  
- AATCTCTAAA GTAATCTATT TTAGATGACA TGTACTGTTT TGTTACTATA TATCGTAGGA TAAATTTTTC   
  
  
- GATGCACAGT CAGTATATAC TTAACGTAGA TTAAAAATTA TCTATATATT ATACTATACT ATATTATCCT   
  
  
- ATACTACATC TAACTTAAGT TTGAGTATTA AGTAATTCGT ACCACGTTAC AAGTAAAAGA ACTAAAATTT   
  
  
- GGCTGATAAT GAAGTTGACA TATATTAAGC TTAACGAAAC CATAAAATTT TAAATTTGAT CATTAATGAC   
  
  
- GGAGATAATG ACTACAAATA TAATTCACCA CGAATTGACG AATAACTACT TTGGTAAAAA TAAAAGCACA   
  
  
- TTTTCTCCCG TATTAGGTAG GAGCACATAA CCGCCCCGAG CCGCGGGATA AGTCGATAAG AGACATACAG   
  
  
- TTTGTTTTGG CGAAGATAAT GGGTAACCAG AGATTTGTGC GTCCCCAAAA TCGGGCCTCT CAATTAAGAA   
  
  
- ATAACTCCAG GGGACAAGAG GTAACGTCCT AAAAACGAGT CCATGAAGAG ACAAAGAAGA GAAATTAGAA   
  
  
- GACATAGAAA GACGTAGTTC AAATTACGAA AGACCCTAAA AGTCGATGCT AAATACACTT TAACATTCTA   
  
  
- AAGACTTGAC CCGTAAGTCC CGAACCAAGT ATACATAAGA AAAGACGAAC TTTACAAAAT TAAAGTATCA   
  
  
- AGGTTACCCC AATAAGTTTC TATCTTAATT CGATATTAGT GGCTCTTCTG AACGACTGGA AACAAAGAAA   
  
  
- AAACAAAGAA AAAAAATTAA TTTAGACCGA TAATGAACCG ATATCAAAAA CGATTACCTA GGTGTTTATC   
  
  
- TGCTTAATAT ACCCGGAAAC GTGGTTTACT TCAAGTTACT AGTCTCCTCT GGGTAAAATA GTTTACTTTT   
  
  
- AAAACAATTA CACAACTTTG GCGGTTTAAG GTTAACCTTA AGTGGAAAAC CCTTGGGATG ACACTAATGG   
  
  
- TGTTTGGGTT TAAAACTAAA CCAACACGGT AGTGATCCAG GGTTACAACT CCCTCGAGAG GAACTTCTAA   
  
  
- TACTAAAATC ACTACAACAA GAATTCATGT AGTTAGTTCA AGAATACCTG CTCCTACTCC CTCTCTTTTA   
  
  
- GGTAAACCCG GTGCCCGGAG ACCGAGAACT TCGCCGGCTC TTCAGCAATA TACTCCACGA ACCCGTCTCG   
  
  
- GTAAGAGGTG TTTTAGTTAT GAGAGGTCTC CTGTTCGCAC TACTGGACTC GTTCTCATCA CAATCTTCGA   
  
  
- CACAGTCAAT ATCGACACTG CCACGTTCAT CACCGTCACC GTAGCACCTC GTTTCAACCA GCTCAATAGG   
  
  
- AGGCCTCATG TTAGGGTACG CCTACCGTCT AAAAGGACGG AACGGGAGTT TTGGGAACAA CTCTTGTAGA   
  
  
- ATATCGAATA GTTTATCGAA CTGTCCACTC CCATTACCAA ACCACCTATC TGGCCGTCTG AGTGGACATT   
  
  
- CGCGAGAATC ACACAGACTA TATAAATTAC TATCAGTCAG TCACTATGTT AAAGTCTTTC CCTACCTCCT   
  
  
- TCGCTCGTTT AAAGAAGGGT TCCGAAGAAA CACAAACCTA AACTTGTGAC CTGGGACGGA AGCCCTCAGC   
  
  
- CAGTTATTCC GTGCTCCACC CGACTAAAAC CACCTCTGAT CGGTACTTTT ACTCCAACGT CACGATCGAT   
  
  
- CATCCCCTTT CTTCGTAGCG GTAGGACTCC TTAATGCAAG TCTTCAACCC TCCCCGTCAT TCGTCGGTCA   
  
  
- ACAAAAGGTC AAAAGTCTGC TCCGTCAACA CTCTAGTCTT TACAAACTAT CCCATAACGA CACGTCACCA   
  
  
- CCGTTTTTAC TACGACTGGA TGCGGTTTGA AATGTCTCAC TTGATTTATT TTGATACGTT TTACGAGTTT   
  
  
- ACTTTCTAAG ATTACCACCT TTTCCGGCAC CATCCTTCGT TCAACCGTCC TCCCTACACC ACCTAGATTC   
  
  
- TAGAGAAAAT GCGGAAACAC GTGTTCGTCA ACCTAGGTTA CTGGTTTCTT CGCGTTTACT GAATTAATCC   
  
  
- GTTTAATCCT TCGTAAGAAG AGGTTGGCCC CTACCCTTAG TTTCTTACCG AGTAATAAAA CGACTACCAG   
  
  
- AACTCCGAGC CGAACGACCA CAACCCTGGG GTTATATGTT CAGAGAGCAC TGACCGGGTC GAAGACGACA   
  
  
- ACTATAGAAC TCTCGAATGG TATACAAAGA ACGGTGTACG GGTAAGTTCT TTTATCCCTT AAAGAAGAGA   
  
  
- TTATCTTGTT AATACCTACA TCGAGTCTTT CGTTTAGCTG AAGTATAATA ACTAGAACCA TAACAGATAC   
  
  
- CAAAGGTTAC CGGAACAGAT TAAGTCGCGG AAAGTAGATC CGGACCGCCC GGAGGGTTTG AAGCCTAATG   
  
  
- TCCGCAACTA GAGGGGGTTG GGCCAAAGGC TGGTCGGTTT TCTCAGCTCC TCTGACCCGC TGCGAACTCC   
  
  
- TTGATACGAC TCAGTAAGTT ACACGGTAAA CTCAAGTTAC GGTATCGTTT CTCGACCCTT TGTGAATGAC   
  
  
- AATTTCTAGA GTTCTAACTA GGGCTACTCC ACGAACAACA CTTGACAGAC AAGTCCAAAT TTATGTAAGG   
  
  
- ACTCCTTTGT CATTAACGTC TTACGGGTTC TCTATAACAA GAATTAGACT AGTCTGTCTA TTTTGGTCGA   
  
  
- CAAAAGTATG TACCACATCA GTTACCTCGG AAGTTAAGAG GAAAAAAGTA ATAAGCCAAG GCACTCCGAG   
  
  
- AGAAGGTGAA GAGGTGAGAT AAACTACACA ACCTCCGTTT AGAAGGGGCC CTGTAGCTCC TCTCCTATGA   
  
  
- CTATCTCGCT CTGTAGAAAC CCGCTGTCCG TTACTTACAC TAGCGAACAC TCCCAAACCT CTCCTATCTT   
  
  
- TCCGGTCTCT TTATGTTTGT CACCGCCCAG GCTTTACTCT CCCGACCCAA CTTCGTCGAC GGAAACTTAG   
  
  
- TCCTCTGACA CCTTTACCGG TTTTTTGCCC ACTGTCGACA GATAGTGTTT CTAAAGAGGT AACTACTGCT   
  
  
- ACCCGTGACC AACAATGTCC CTACCTTTCC CTCTTAACAG ATACGTGAGT GATGAACCTT CGGACGACTG   
  
  
- AT

+     I-box

| Site Name | Organism | Position | Strand | Matrix score. | sequence | function |
| --- | --- | --- | --- | --- | --- | --- |
| I-box | Zea mays | 910 | + | 9 | gGATAAGGTG | part of a light responsive element |

>HU05G01597.1   
+ -Up\_Stream \_Len000TTTGTA TTTCAATATT GTAATTAGGT GGGATATAGA GTTGAAGGGC AAACTCTACC   
  
  
+ AACGACTTGT TGAAGGATCA ACTTGTAGCT TGGAACTTAA TGTATGTGTA CTATAGAGTT GTGTAATCTC   
  
  
+ TCCTAATAAA ATTGGGTTAA TCCTAGAATT AGAGAACTTT AAGCGGGGAC TAAGCTTGGT TGAGGCTAAC   
  
  
+ TTCGTTAAAA AGTTCTTGTG TCATGTCCAC GTTTATCTTT TCACTATTTA TTTCAATTAA TTTGCGATAT   
  
  
+ TTATCATCAC CCTATTCACC GTCTTTTCGG ATGATTTAAC TAGTCAAGCC GGACCAACAT TGCTTTTTAT   
  
  
+ AGTGTATTTG CAACTCATGT TGATTATTAG ATGTATCAAC AAAAAATGTT TCTCAAAATA AATAGTAGCT   
  
  
+ CTTTAAATTT TATTCCACAT AAATTCAAAA TCAATATCGA TATCGTTCCA TAATATAGCA GCATTATGAA   
  
  
+ AACAATATTT GCTAATGTTT ACATTGTGCT CTTTTCACTC AAAAATTGTA AGGCTTCAAA TACTATTAAA   
  
  
+ GAAACCATTA TTTCACTCGA CTATTTAGCT TGCATATATA GACAATCTTG CAAATAGTAA CATGTCTTCA   
  
  
+ TTCGACTATT TGCTTCCAAA TGAATAATCT CGTAAACATA TATACATACA TACATATATA TATATATATA   
  
  
+ TATATATATA TATATATATA GTAATATGTA TTTAACTTCA CATATACATG AATATAAAAA TATAAGAGGG   
  
  
+ AAAAAAGACG CTAGGTAGAT GGACTACAAA AGCCATTCAT GTATTGAGAC GGCTCATAAA ATCATACGGG   
  
  
+ GAAAGGAAAA AAAAAGATCT CTACATAATG TTCATAGATT TATAGAAAGC ATATGATGGA TTACCGCATA   
  
  
+ AGGTGTTATA TATGGGAGTG TTTGTAGAAG AAAGATGTAT ATCCGAACTA TCAAAGATAC ATATGAGGGA   
  
  
+ GTAAAAACAA GTGTAAGAAC CTCAGGAGGA GACATAAAAG ATTTTTCTAT TGGCATTGGA CACCGTCAAG   
  
  
+ TATCCGCTTT AAACCTGTTC TTTTTCACTA TTATCATGGA TGTATTCACA AAAGGAATTC AATACGAAGT   
  
  
+ ACCATGATGT ATGCTTTTTA CTGATAGTAT TGGTCTCATC GATGAGACTA AGGATGGTCT TAATAGCAAA   
  
  
+ TTAGAGATTT CATTAGATAA AATCTACTGT ACATGACAAA ACAATGATAT ATAGCATCCT ATTTAAAAAG   
  
  
+ CTACGTGTCA GTCATATATG AATTGCATCT AATTTTTAAT AGATATATAA TATGATATGA TATAATAGGA   
  
  
+ TATGATGTAG ATTGAATTCA AACTCATAAT TCATTAAGCA TGGTGCAATG TTCATTTTCT TGATTTTAAA   
  
  
+ CCGACTATTA CTTCAACTGT ATATAATTCG AATTGCTTTG GTATTTTAAA ATTTAAACTA GTAATTACTG   
  
  
+ CCTCTATTAC TGATGTTTAT ATTAAGTGGT GCTTAACTGC TTATTGATGA AACCATTTTT ATTTTCGTGT   
  
  
+ AAAAGAGGGC ATAATCCATC CTCGTGTATT GGCGGGGCTC GGCGCCCTAT TCAGCTATTC TCTGTATGTC   
  
  
+ AAACAAAACC GCTTCTATTA CCCATTGGTC TCTAAACACG CAGGGGTTTT AGCCCGGAGA GTTAATTCTT   
  
  
+ TATTGAGGTC CCCTGTTCTC CATTGCAGGA TTTTTGCTCA GGTACTTCTC TGTTTCTTCT CTTTAATCTT   
  
  
+ CTGTATCTTT CTGCATCAAG TTTAATGCTT TCTGGGATTT TCAGCTACGA TTTATGTGAA ATTGTAAGAT   
  
  
+ TTCTGAACTG GGCATTCAGG GCTTGGTTCA TATGTATTCT TTTCTGCTTG AAATGTTTTA ATTTCATAGT   
  
  
+ TCCAATGGGG TTATTCAAAG ATAGAATTAA GCTATAATCA CCGAGAAGAC TTGCTGACCT TTGTTTCTTT   
  
  
+ TTTGTTTCTT TTTTTTAATT AAATCTGGCT ATTACTTGGC TATAGTTTTT GCTAATGGAT CCACAAATAG   
  
  
+ ACGAATTATA TGGGCCTTTG CACCAAATGA AGTTCAATGA TCAGAGGAGA CCCATTTTAT CAAATGAAAA   
  
  
+ TTTTGTTAAT GTGTTGAAAC CGCCAAATTC CAATTGGAAT TCACCTTTTG GGAACCCTAC TGTGATTACC   
  
  
+ ACAAACCCAA ATTTTGATTT GGTTGTGCCA TCACTAGGTC CCAATGTTGA GGGAGCTCTC CTTGAAGATT   
  
  
+ ATGATTTTAG TGATGTTGTT CTTAAGTACA TCAATCAAGT TCTTATGGAC GAGGATGAGG GAGAGAAAAT   
  
  
+ CCATTTGGGC CACGGGCCTC TGGCTCTTGA AGCGGCCGAG AAGTCGTTAT ATGAGGTGCT TGGGCAGAGC   
  
  
+ CATTCTCCAC AAAATCAATA CTCTCCAGAG GACAAGCGTG ATGACCTGAG CAAGAGTAGT GTTAGAAGCT   
  
  
+ GTGTCAGTTA TAGCTGTGAC GGTGCAAGTA GTGGCAGTGG CATCGTGGAG CAAAGTTGGT CGAGTTATCC   
  
  
+ TCCGGAGTAC AATCCCATGC GGATGGCAGA TTTTCCTGCC TTGCCCTCAA AACCCTTGTT GAGAACATCT   
  
  
+ TATAGCTTAT CAAATAGCTT GACAGGTGAG GGTAATGGTT TGGTGGATAG ACCGGCAGAC TCACCTGTAA   
  
  
+ GCGCTCTTAG TGTGTCTGAT ATATTTAATG ATAGTCAGTC AGTGATACAA TTTCAGAAAG GGATGGAGGA   
  
  
+ AGCGAGCAAA TTTCTTCCCA AGGCTTCTTT GTGTTTGGAT TTGAACACTG GACCCTGCCT TCGGGAGTCG   
  
  
+ GTCAATAAGG CACGAGGTGG GCTGATTTTG GTGGAGACTA GCCATGAAAA TGAGGTTGCA GTGCTAGCTA   
  
  
+ GTAGGGGAAA GAAGCATCGC CATCCTGAGG AATTACGTTC AGAAGTTGGG AGGGGCAGTA AGCAGCCAGT   
  
  
+ TGTTTTCCAG TTTTCAGACG AGGCAGTTGT GAGATCAGAA ATGTTTGATA GGGTATTGCT GTGCAGTGGT   
  
  
+ GGCAAAAATG ATGCTGACCT ACGCCAAACT TTACAGAGTG AACTAAATAA AACTATGCAA AATGCTCAAA   
  
  
+ TGAAAGATTC TAATGGTGGA AAAGGCCGTG GTAGGAAGCA AGTTGGCAGG AGGGATGTGG TGGATCTAAG   
  
  
+ ATCTCTTTTA CGCCTTTGTG CACAAGCAGT TGGATCCAAT GACCAAAGAA GCGCAAATGA CTTAATTAGG   
  
  
+ CAAATTAGGA AGCATTCTTC TCCAACCGGG GATGGGAATC AAAGAATGGC TCATTATTTT GCTGATGGTC   
  
  
+ TTGAGGCTCG GCTTGCTGGT GTTGGGACCC CAATATACAA GTCTCTCGTG ACTGGCCCAG CTTCTGCTGT   
  
  
+ TGATATCTTG AGAGCTTACC ATATGTTTCT TGCCACATGC CCATTCAAGA AAATAGGGAA TTTCTTCTCT   
  
  
+ AATAGAACAA TTATGGATGT AGCTCAGAAA GCAAATCGAC TTCATATTAT TGATCTTGGT ATTGTCTATG   
  
  
+ GTTTCCAATG GCCTTGTCTA ATTCAGCGCC TTTCATCTAG GCCTGGCGGG CCTCCCAAAC TTCGGATTAC   
  
  
+ AGGCGTTGAT CTCCCCCAAC CCGGTTTCCG ACCAGCCAAA AGAGTCGAGG AGACTGGGCG ACGCTTGAGG   
  
  
+ AACTATGCTG AGTCATTCAA TGTGCCATTT GAGTTCAATG CCATAGCAAA GAGCTGGGAA ACACTTACTG   
  
  
+ TTAAAGATCT CAAGATTGAT CCCGATGAGG TGCTTGTTGT GAACTGTCTG TTCAGGTTTA AATACATTCC   
  
  
+ TGAGGAAACA GTAATTGCAG AATGCCCAAG AGATATTGTT CTTAATCTGA TCAGACAGAT AAAACCAGCT   
  
  
+ GTTTTCATAC ATGGTGTAGT CAATGGAGCC TTCAATTCTC CTTTTTTCAT TATTCGGTTC CGTGAGGCTC   
  
  
+ TCTTCCACTT CTCCACTCTA TTTGATGTGT TGGAGGCAAA TCTTCCCCGG GACATCGAGG AGAGGATACT   
  
  
+ GATAGAGCGA GACATCTTTG GGCGACAGGC AATGAATGTG ATCGCTTGTG AGGGTTTGGA GAGGATAGAA   
  
  
+ AGGCCAGAGA AATACAAACA GTGGCGGGTC CGAAATGAGA GGGCTGGGTT GAAGCAGCTG CCTTTGAATC   
  
  
+ AGGAGACTGT GGAAATGGCC AAAAAACGGG TGACAGCTGT CTATCACAAA GATTTCTCCA TTGATGACGA   
  
  
+ TGGGCACTGG TTGTTACAGG GATGGAAAGG GAGAATTGTC TATGCACTCA CTACTTGGAA GCCTGCTGAC   
  
  
+ TA  

- -Up\_Stream \_Len000AAACAT AAAGTTATAA CATTAATCCA CCCTATATCT CAACTTCCCG TTTGAGATGG   
  
  
- TTGCTGAACA ACTTCCTAGT TGAACATCGA ACCTTGAATT ACATACACAT GATATCTCAA CACATTAGAG   
  
  
- AGGATTATTT TAACCCAATT AGGATCTTAA TCTCTTGAAA TTCGCCCCTG ATTCGAACCA ACTCCGATTG   
  
  
- AAGCAATTTT TCAAGAACAC AGTACAGGTG CAAATAGAAA AGTGATAAAT AAAGTTAATT AAACGCTATA   
  
  
- AATAGTAGTG GGATAAGTGG CAGAAAAGCC TACTAAATTG ATCAGTTCGG CCTGGTTGTA ACGAAAAATA   
  
  
- TCACATAAAC GTTGAGTACA ACTAATAATC TACATAGTTG TTTTTTACAA AGAGTTTTAT TTATCATCGA   
  
  
- GAAATTTAAA ATAAGGTGTA TTTAAGTTTT AGTTATAGCT ATAGCAAGGT ATTATATCGT CGTAATACTT   
  
  
- TTGTTATAAA CGATTACAAA TGTAACACGA GAAAAGTGAG TTTTTAACAT TCCGAAGTTT ATGATAATTT   
  
  
- CTTTGGTAAT AAAGTGAGCT GATAAATCGA ACGTATATAT CTGTTAGAAC GTTTATCATT GTACAGAAGT   
  
  
- AAGCTGATAA ACGAAGGTTT ACTTATTAGA GCATTTGTAT ATATGTATGT ATGTATATAT ATATATATAT   
  
  
- ATATATATAT ATATATATAT CATTATACAT AAATTGAAGT GTATATGTAC TTATATTTTT ATATTCTCCC   
  
  
- TTTTTTCTGC GATCCATCTA CCTGATGTTT TCGGTAAGTA CATAACTCTG CCGAGTATTT TAGTATGCCC   
  
  
- CTTTCCTTTT TTTTTCTAGA GATGTATTAC AAGTATCTAA ATATCTTTCG TATACTACCT AATGGCGTAT   
  
  
- TCCACAATAT ATACCCTCAC AAACATCTTC TTTCTACATA TAGGCTTGAT AGTTTCTATG TATACTCCCT   
  
  
- CATTTTTGTT CACATTCTTG GAGTCCTCCT CTGTATTTTC TAAAAAGATA ACCGTAACCT GTGGCAGTTC   
  
  
- ATAGGCGAAA TTTGGACAAG AAAAAGTGAT AATAGTACCT ACATAAGTGT TTTCCTTAAG TTATGCTTCA   
  
  
- TGGTACTACA TACGAAAAAT GACTATCATA ACCAGAGTAG CTACTCTGAT TCCTACCAGA ATTATCGTTT   
  
  
- AATCTCTAAA GTAATCTATT TTAGATGACA TGTACTGTTT TGTTACTATA TATCGTAGGA TAAATTTTTC   
  
  
- GATGCACAGT CAGTATATAC TTAACGTAGA TTAAAAATTA TCTATATATT ATACTATACT ATATTATCCT   
  
  
- ATACTACATC TAACTTAAGT TTGAGTATTA AGTAATTCGT ACCACGTTAC AAGTAAAAGA ACTAAAATTT   
  
  
- GGCTGATAAT GAAGTTGACA TATATTAAGC TTAACGAAAC CATAAAATTT TAAATTTGAT CATTAATGAC   
  
  
- GGAGATAATG ACTACAAATA TAATTCACCA CGAATTGACG AATAACTACT TTGGTAAAAA TAAAAGCACA   
  
  
- TTTTCTCCCG TATTAGGTAG GAGCACATAA CCGCCCCGAG CCGCGGGATA AGTCGATAAG AGACATACAG   
  
  
- TTTGTTTTGG CGAAGATAAT GGGTAACCAG AGATTTGTGC GTCCCCAAAA TCGGGCCTCT CAATTAAGAA   
  
  
- ATAACTCCAG GGGACAAGAG GTAACGTCCT AAAAACGAGT CCATGAAGAG ACAAAGAAGA GAAATTAGAA   
  
  
- GACATAGAAA GACGTAGTTC AAATTACGAA AGACCCTAAA AGTCGATGCT AAATACACTT TAACATTCTA   
  
  
- AAGACTTGAC CCGTAAGTCC CGAACCAAGT ATACATAAGA AAAGACGAAC TTTACAAAAT TAAAGTATCA   
  
  
- AGGTTACCCC AATAAGTTTC TATCTTAATT CGATATTAGT GGCTCTTCTG AACGACTGGA AACAAAGAAA   
  
  
- AAACAAAGAA AAAAAATTAA TTTAGACCGA TAATGAACCG ATATCAAAAA CGATTACCTA GGTGTTTATC   
  
  
- TGCTTAATAT ACCCGGAAAC GTGGTTTACT TCAAGTTACT AGTCTCCTCT GGGTAAAATA GTTTACTTTT   
  
  
- AAAACAATTA CACAACTTTG GCGGTTTAAG GTTAACCTTA AGTGGAAAAC CCTTGGGATG ACACTAATGG   
  
  
- TGTTTGGGTT TAAAACTAAA CCAACACGGT AGTGATCCAG GGTTACAACT CCCTCGAGAG GAACTTCTAA   
  
  
- TACTAAAATC ACTACAACAA GAATTCATGT AGTTAGTTCA AGAATACCTG CTCCTACTCC CTCTCTTTTA   
  
  
- GGTAAACCCG GTGCCCGGAG ACCGAGAACT TCGCCGGCTC TTCAGCAATA TACTCCACGA ACCCGTCTCG   
  
  
- GTAAGAGGTG TTTTAGTTAT GAGAGGTCTC CTGTTCGCAC TACTGGACTC GTTCTCATCA CAATCTTCGA   
  
  
- CACAGTCAAT ATCGACACTG CCACGTTCAT CACCGTCACC GTAGCACCTC GTTTCAACCA GCTCAATAGG   
  
  
- AGGCCTCATG TTAGGGTACG CCTACCGTCT AAAAGGACGG AACGGGAGTT TTGGGAACAA CTCTTGTAGA   
  
  
- ATATCGAATA GTTTATCGAA CTGTCCACTC CCATTACCAA ACCACCTATC TGGCCGTCTG AGTGGACATT   
  
  
- CGCGAGAATC ACACAGACTA TATAAATTAC TATCAGTCAG TCACTATGTT AAAGTCTTTC CCTACCTCCT   
  
  
- TCGCTCGTTT AAAGAAGGGT TCCGAAGAAA CACAAACCTA AACTTGTGAC CTGGGACGGA AGCCCTCAGC   
  
  
- CAGTTATTCC GTGCTCCACC CGACTAAAAC CACCTCTGAT CGGTACTTTT ACTCCAACGT CACGATCGAT   
  
  
- CATCCCCTTT CTTCGTAGCG GTAGGACTCC TTAATGCAAG TCTTCAACCC TCCCCGTCAT TCGTCGGTCA   
  
  
- ACAAAAGGTC AAAAGTCTGC TCCGTCAACA CTCTAGTCTT TACAAACTAT CCCATAACGA CACGTCACCA   
  
  
- CCGTTTTTAC TACGACTGGA TGCGGTTTGA AATGTCTCAC TTGATTTATT TTGATACGTT TTACGAGTTT   
  
  
- ACTTTCTAAG ATTACCACCT TTTCCGGCAC CATCCTTCGT TCAACCGTCC TCCCTACACC ACCTAGATTC   
  
  
- TAGAGAAAAT GCGGAAACAC GTGTTCGTCA ACCTAGGTTA CTGGTTTCTT CGCGTTTACT GAATTAATCC   
  
  
- GTTTAATCCT TCGTAAGAAG AGGTTGGCCC CTACCCTTAG TTTCTTACCG AGTAATAAAA CGACTACCAG   
  
  
- AACTCCGAGC CGAACGACCA CAACCCTGGG GTTATATGTT CAGAGAGCAC TGACCGGGTC GAAGACGACA   
  
  
- ACTATAGAAC TCTCGAATGG TATACAAAGA ACGGTGTACG GGTAAGTTCT TTTATCCCTT AAAGAAGAGA   
  
  
- TTATCTTGTT AATACCTACA TCGAGTCTTT CGTTTAGCTG AAGTATAATA ACTAGAACCA TAACAGATAC   
  
  
- CAAAGGTTAC CGGAACAGAT TAAGTCGCGG AAAGTAGATC CGGACCGCCC GGAGGGTTTG AAGCCTAATG   
  
  
- TCCGCAACTA GAGGGGGTTG GGCCAAAGGC TGGTCGGTTT TCTCAGCTCC TCTGACCCGC TGCGAACTCC   
  
  
- TTGATACGAC TCAGTAAGTT ACACGGTAAA CTCAAGTTAC GGTATCGTTT CTCGACCCTT TGTGAATGAC   
  
  
- AATTTCTAGA GTTCTAACTA GGGCTACTCC ACGAACAACA CTTGACAGAC AAGTCCAAAT TTATGTAAGG   
  
  
- ACTCCTTTGT CATTAACGTC TTACGGGTTC TCTATAACAA GAATTAGACT AGTCTGTCTA TTTTGGTCGA   
  
  
- CAAAAGTATG TACCACATCA GTTACCTCGG AAGTTAAGAG GAAAAAAGTA ATAAGCCAAG GCACTCCGAG   
  
  
- AGAAGGTGAA GAGGTGAGAT AAACTACACA ACCTCCGTTT AGAAGGGGCC CTGTAGCTCC TCTCCTATGA   
  
  
- CTATCTCGCT CTGTAGAAAC CCGCTGTCCG TTACTTACAC TAGCGAACAC TCCCAAACCT CTCCTATCTT   
  
  
- TCCGGTCTCT TTATGTTTGT CACCGCCCAG GCTTTACTCT CCCGACCCAA CTTCGTCGAC GGAAACTTAG   
  
  
- TCCTCTGACA CCTTTACCGG TTTTTTGCCC ACTGTCGACA GATAGTGTTT CTAAAGAGGT AACTACTGCT   
  
  
- ACCCGTGACC AACAATGTCC CTACCTTTCC CTCTTAACAG ATACGTGAGT GATGAACCTT CGGACGACTG   
  
  
- AT

+     L-box

| Site Name | Organism | Position | Strand | Matrix score. | sequence | function |
| --- | --- | --- | --- | --- | --- | --- |
| L-box | Petroselinum crispum | 39 | - | 10 | ATCCCACCTAC | part of a light responsive element |

>HU05G01597.1   
+ -Up\_Stream \_Len000TTTGTA TTTCAATATT GTAATTAGGT GGGATATAGA GTTGAAGGGC AAACTCTACC   
  
  
+ AACGACTTGT TGAAGGATCA ACTTGTAGCT TGGAACTTAA TGTATGTGTA CTATAGAGTT GTGTAATCTC   
  
  
+ TCCTAATAAA ATTGGGTTAA TCCTAGAATT AGAGAACTTT AAGCGGGGAC TAAGCTTGGT TGAGGCTAAC   
  
  
+ TTCGTTAAAA AGTTCTTGTG TCATGTCCAC GTTTATCTTT TCACTATTTA TTTCAATTAA TTTGCGATAT   
  
  
+ TTATCATCAC CCTATTCACC GTCTTTTCGG ATGATTTAAC TAGTCAAGCC GGACCAACAT TGCTTTTTAT   
  
  
+ AGTGTATTTG CAACTCATGT TGATTATTAG ATGTATCAAC AAAAAATGTT TCTCAAAATA AATAGTAGCT   
  
  
+ CTTTAAATTT TATTCCACAT AAATTCAAAA TCAATATCGA TATCGTTCCA TAATATAGCA GCATTATGAA   
  
  
+ AACAATATTT GCTAATGTTT ACATTGTGCT CTTTTCACTC AAAAATTGTA AGGCTTCAAA TACTATTAAA   
  
  
+ GAAACCATTA TTTCACTCGA CTATTTAGCT TGCATATATA GACAATCTTG CAAATAGTAA CATGTCTTCA   
  
  
+ TTCGACTATT TGCTTCCAAA TGAATAATCT CGTAAACATA TATACATACA TACATATATA TATATATATA   
  
  
+ TATATATATA TATATATATA GTAATATGTA TTTAACTTCA CATATACATG AATATAAAAA TATAAGAGGG   
  
  
+ AAAAAAGACG CTAGGTAGAT GGACTACAAA AGCCATTCAT GTATTGAGAC GGCTCATAAA ATCATACGGG   
  
  
+ GAAAGGAAAA AAAAAGATCT CTACATAATG TTCATAGATT TATAGAAAGC ATATGATGGA TTACCGCATA   
  
  
+ AGGTGTTATA TATGGGAGTG TTTGTAGAAG AAAGATGTAT ATCCGAACTA TCAAAGATAC ATATGAGGGA   
  
  
+ GTAAAAACAA GTGTAAGAAC CTCAGGAGGA GACATAAAAG ATTTTTCTAT TGGCATTGGA CACCGTCAAG   
  
  
+ TATCCGCTTT AAACCTGTTC TTTTTCACTA TTATCATGGA TGTATTCACA AAAGGAATTC AATACGAAGT   
  
  
+ ACCATGATGT ATGCTTTTTA CTGATAGTAT TGGTCTCATC GATGAGACTA AGGATGGTCT TAATAGCAAA   
  
  
+ TTAGAGATTT CATTAGATAA AATCTACTGT ACATGACAAA ACAATGATAT ATAGCATCCT ATTTAAAAAG   
  
  
+ CTACGTGTCA GTCATATATG AATTGCATCT AATTTTTAAT AGATATATAA TATGATATGA TATAATAGGA   
  
  
+ TATGATGTAG ATTGAATTCA AACTCATAAT TCATTAAGCA TGGTGCAATG TTCATTTTCT TGATTTTAAA   
  
  
+ CCGACTATTA CTTCAACTGT ATATAATTCG AATTGCTTTG GTATTTTAAA ATTTAAACTA GTAATTACTG   
  
  
+ CCTCTATTAC TGATGTTTAT ATTAAGTGGT GCTTAACTGC TTATTGATGA AACCATTTTT ATTTTCGTGT   
  
  
+ AAAAGAGGGC ATAATCCATC CTCGTGTATT GGCGGGGCTC GGCGCCCTAT TCAGCTATTC TCTGTATGTC   
  
  
+ AAACAAAACC GCTTCTATTA CCCATTGGTC TCTAAACACG CAGGGGTTTT AGCCCGGAGA GTTAATTCTT   
  
  
+ TATTGAGGTC CCCTGTTCTC CATTGCAGGA TTTTTGCTCA GGTACTTCTC TGTTTCTTCT CTTTAATCTT   
  
  
+ CTGTATCTTT CTGCATCAAG TTTAATGCTT TCTGGGATTT TCAGCTACGA TTTATGTGAA ATTGTAAGAT   
  
  
+ TTCTGAACTG GGCATTCAGG GCTTGGTTCA TATGTATTCT TTTCTGCTTG AAATGTTTTA ATTTCATAGT   
  
  
+ TCCAATGGGG TTATTCAAAG ATAGAATTAA GCTATAATCA CCGAGAAGAC TTGCTGACCT TTGTTTCTTT   
  
  
+ TTTGTTTCTT TTTTTTAATT AAATCTGGCT ATTACTTGGC TATAGTTTTT GCTAATGGAT CCACAAATAG   
  
  
+ ACGAATTATA TGGGCCTTTG CACCAAATGA AGTTCAATGA TCAGAGGAGA CCCATTTTAT CAAATGAAAA   
  
  
+ TTTTGTTAAT GTGTTGAAAC CGCCAAATTC CAATTGGAAT TCACCTTTTG GGAACCCTAC TGTGATTACC   
  
  
+ ACAAACCCAA ATTTTGATTT GGTTGTGCCA TCACTAGGTC CCAATGTTGA GGGAGCTCTC CTTGAAGATT   
  
  
+ ATGATTTTAG TGATGTTGTT CTTAAGTACA TCAATCAAGT TCTTATGGAC GAGGATGAGG GAGAGAAAAT   
  
  
+ CCATTTGGGC CACGGGCCTC TGGCTCTTGA AGCGGCCGAG AAGTCGTTAT ATGAGGTGCT TGGGCAGAGC   
  
  
+ CATTCTCCAC AAAATCAATA CTCTCCAGAG GACAAGCGTG ATGACCTGAG CAAGAGTAGT GTTAGAAGCT   
  
  
+ GTGTCAGTTA TAGCTGTGAC GGTGCAAGTA GTGGCAGTGG CATCGTGGAG CAAAGTTGGT CGAGTTATCC   
  
  
+ TCCGGAGTAC AATCCCATGC GGATGGCAGA TTTTCCTGCC TTGCCCTCAA AACCCTTGTT GAGAACATCT   
  
  
+ TATAGCTTAT CAAATAGCTT GACAGGTGAG GGTAATGGTT TGGTGGATAG ACCGGCAGAC TCACCTGTAA   
  
  
+ GCGCTCTTAG TGTGTCTGAT ATATTTAATG ATAGTCAGTC AGTGATACAA TTTCAGAAAG GGATGGAGGA   
  
  
+ AGCGAGCAAA TTTCTTCCCA AGGCTTCTTT GTGTTTGGAT TTGAACACTG GACCCTGCCT TCGGGAGTCG   
  
  
+ GTCAATAAGG CACGAGGTGG GCTGATTTTG GTGGAGACTA GCCATGAAAA TGAGGTTGCA GTGCTAGCTA   
  
  
+ GTAGGGGAAA GAAGCATCGC CATCCTGAGG AATTACGTTC AGAAGTTGGG AGGGGCAGTA AGCAGCCAGT   
  
  
+ TGTTTTCCAG TTTTCAGACG AGGCAGTTGT GAGATCAGAA ATGTTTGATA GGGTATTGCT GTGCAGTGGT   
  
  
+ GGCAAAAATG ATGCTGACCT ACGCCAAACT TTACAGAGTG AACTAAATAA AACTATGCAA AATGCTCAAA   
  
  
+ TGAAAGATTC TAATGGTGGA AAAGGCCGTG GTAGGAAGCA AGTTGGCAGG AGGGATGTGG TGGATCTAAG   
  
  
+ ATCTCTTTTA CGCCTTTGTG CACAAGCAGT TGGATCCAAT GACCAAAGAA GCGCAAATGA CTTAATTAGG   
  
  
+ CAAATTAGGA AGCATTCTTC TCCAACCGGG GATGGGAATC AAAGAATGGC TCATTATTTT GCTGATGGTC   
  
  
+ TTGAGGCTCG GCTTGCTGGT GTTGGGACCC CAATATACAA GTCTCTCGTG ACTGGCCCAG CTTCTGCTGT   
  
  
+ TGATATCTTG AGAGCTTACC ATATGTTTCT TGCCACATGC CCATTCAAGA AAATAGGGAA TTTCTTCTCT   
  
  
+ AATAGAACAA TTATGGATGT AGCTCAGAAA GCAAATCGAC TTCATATTAT TGATCTTGGT ATTGTCTATG   
  
  
+ GTTTCCAATG GCCTTGTCTA ATTCAGCGCC TTTCATCTAG GCCTGGCGGG CCTCCCAAAC TTCGGATTAC   
  
  
+ AGGCGTTGAT CTCCCCCAAC CCGGTTTCCG ACCAGCCAAA AGAGTCGAGG AGACTGGGCG ACGCTTGAGG   
  
  
+ AACTATGCTG AGTCATTCAA TGTGCCATTT GAGTTCAATG CCATAGCAAA GAGCTGGGAA ACACTTACTG   
  
  
+ TTAAAGATCT CAAGATTGAT CCCGATGAGG TGCTTGTTGT GAACTGTCTG TTCAGGTTTA AATACATTCC   
  
  
+ TGAGGAAACA GTAATTGCAG AATGCCCAAG AGATATTGTT CTTAATCTGA TCAGACAGAT AAAACCAGCT   
  
  
+ GTTTTCATAC ATGGTGTAGT CAATGGAGCC TTCAATTCTC CTTTTTTCAT TATTCGGTTC CGTGAGGCTC   
  
  
+ TCTTCCACTT CTCCACTCTA TTTGATGTGT TGGAGGCAAA TCTTCCCCGG GACATCGAGG AGAGGATACT   
  
  
+ GATAGAGCGA GACATCTTTG GGCGACAGGC AATGAATGTG ATCGCTTGTG AGGGTTTGGA GAGGATAGAA   
  
  
+ AGGCCAGAGA AATACAAACA GTGGCGGGTC CGAAATGAGA GGGCTGGGTT GAAGCAGCTG CCTTTGAATC   
  
  
+ AGGAGACTGT GGAAATGGCC AAAAAACGGG TGACAGCTGT CTATCACAAA GATTTCTCCA TTGATGACGA   
  
  
+ TGGGCACTGG TTGTTACAGG GATGGAAAGG GAGAATTGTC TATGCACTCA CTACTTGGAA GCCTGCTGAC   
  
  
+ TA  

- -Up\_Stream \_Len000AAACAT AAAGTTATAA CATTAATCCA CCCTATATCT CAACTTCCCG TTTGAGATGG   
  
  
- TTGCTGAACA ACTTCCTAGT TGAACATCGA ACCTTGAATT ACATACACAT GATATCTCAA CACATTAGAG   
  
  
- AGGATTATTT TAACCCAATT AGGATCTTAA TCTCTTGAAA TTCGCCCCTG ATTCGAACCA ACTCCGATTG   
  
  
- AAGCAATTTT TCAAGAACAC AGTACAGGTG CAAATAGAAA AGTGATAAAT AAAGTTAATT AAACGCTATA   
  
  
- AATAGTAGTG GGATAAGTGG CAGAAAAGCC TACTAAATTG ATCAGTTCGG CCTGGTTGTA ACGAAAAATA   
  
  
- TCACATAAAC GTTGAGTACA ACTAATAATC TACATAGTTG TTTTTTACAA AGAGTTTTAT TTATCATCGA   
  
  
- GAAATTTAAA ATAAGGTGTA TTTAAGTTTT AGTTATAGCT ATAGCAAGGT ATTATATCGT CGTAATACTT   
  
  
- TTGTTATAAA CGATTACAAA TGTAACACGA GAAAAGTGAG TTTTTAACAT TCCGAAGTTT ATGATAATTT   
  
  
- CTTTGGTAAT AAAGTGAGCT GATAAATCGA ACGTATATAT CTGTTAGAAC GTTTATCATT GTACAGAAGT   
  
  
- AAGCTGATAA ACGAAGGTTT ACTTATTAGA GCATTTGTAT ATATGTATGT ATGTATATAT ATATATATAT   
  
  
- ATATATATAT ATATATATAT CATTATACAT AAATTGAAGT GTATATGTAC TTATATTTTT ATATTCTCCC   
  
  
- TTTTTTCTGC GATCCATCTA CCTGATGTTT TCGGTAAGTA CATAACTCTG CCGAGTATTT TAGTATGCCC   
  
  
- CTTTCCTTTT TTTTTCTAGA GATGTATTAC AAGTATCTAA ATATCTTTCG TATACTACCT AATGGCGTAT   
  
  
- TCCACAATAT ATACCCTCAC AAACATCTTC TTTCTACATA TAGGCTTGAT AGTTTCTATG TATACTCCCT   
  
  
- CATTTTTGTT CACATTCTTG GAGTCCTCCT CTGTATTTTC TAAAAAGATA ACCGTAACCT GTGGCAGTTC   
  
  
- ATAGGCGAAA TTTGGACAAG AAAAAGTGAT AATAGTACCT ACATAAGTGT TTTCCTTAAG TTATGCTTCA   
  
  
- TGGTACTACA TACGAAAAAT GACTATCATA ACCAGAGTAG CTACTCTGAT TCCTACCAGA ATTATCGTTT   
  
  
- AATCTCTAAA GTAATCTATT TTAGATGACA TGTACTGTTT TGTTACTATA TATCGTAGGA TAAATTTTTC   
  
  
- GATGCACAGT CAGTATATAC TTAACGTAGA TTAAAAATTA TCTATATATT ATACTATACT ATATTATCCT   
  
  
- ATACTACATC TAACTTAAGT TTGAGTATTA AGTAATTCGT ACCACGTTAC AAGTAAAAGA ACTAAAATTT   
  
  
- GGCTGATAAT GAAGTTGACA TATATTAAGC TTAACGAAAC CATAAAATTT TAAATTTGAT CATTAATGAC   
  
  
- GGAGATAATG ACTACAAATA TAATTCACCA CGAATTGACG AATAACTACT TTGGTAAAAA TAAAAGCACA   
  
  
- TTTTCTCCCG TATTAGGTAG GAGCACATAA CCGCCCCGAG CCGCGGGATA AGTCGATAAG AGACATACAG   
  
  
- TTTGTTTTGG CGAAGATAAT GGGTAACCAG AGATTTGTGC GTCCCCAAAA TCGGGCCTCT CAATTAAGAA   
  
  
- ATAACTCCAG GGGACAAGAG GTAACGTCCT AAAAACGAGT CCATGAAGAG ACAAAGAAGA GAAATTAGAA   
  
  
- GACATAGAAA GACGTAGTTC AAATTACGAA AGACCCTAAA AGTCGATGCT AAATACACTT TAACATTCTA   
  
  
- AAGACTTGAC CCGTAAGTCC CGAACCAAGT ATACATAAGA AAAGACGAAC TTTACAAAAT TAAAGTATCA   
  
  
- AGGTTACCCC AATAAGTTTC TATCTTAATT CGATATTAGT GGCTCTTCTG AACGACTGGA AACAAAGAAA   
  
  
- AAACAAAGAA AAAAAATTAA TTTAGACCGA TAATGAACCG ATATCAAAAA CGATTACCTA GGTGTTTATC   
  
  
- TGCTTAATAT ACCCGGAAAC GTGGTTTACT TCAAGTTACT AGTCTCCTCT GGGTAAAATA GTTTACTTTT   
  
  
- AAAACAATTA CACAACTTTG GCGGTTTAAG GTTAACCTTA AGTGGAAAAC CCTTGGGATG ACACTAATGG   
  
  
- TGTTTGGGTT TAAAACTAAA CCAACACGGT AGTGATCCAG GGTTACAACT CCCTCGAGAG GAACTTCTAA   
  
  
- TACTAAAATC ACTACAACAA GAATTCATGT AGTTAGTTCA AGAATACCTG CTCCTACTCC CTCTCTTTTA   
  
  
- GGTAAACCCG GTGCCCGGAG ACCGAGAACT TCGCCGGCTC TTCAGCAATA TACTCCACGA ACCCGTCTCG   
  
  
- GTAAGAGGTG TTTTAGTTAT GAGAGGTCTC CTGTTCGCAC TACTGGACTC GTTCTCATCA CAATCTTCGA   
  
  
- CACAGTCAAT ATCGACACTG CCACGTTCAT CACCGTCACC GTAGCACCTC GTTTCAACCA GCTCAATAGG   
  
  
- AGGCCTCATG TTAGGGTACG CCTACCGTCT AAAAGGACGG AACGGGAGTT TTGGGAACAA CTCTTGTAGA   
  
  
- ATATCGAATA GTTTATCGAA CTGTCCACTC CCATTACCAA ACCACCTATC TGGCCGTCTG AGTGGACATT   
  
  
- CGCGAGAATC ACACAGACTA TATAAATTAC TATCAGTCAG TCACTATGTT AAAGTCTTTC CCTACCTCCT   
  
  
- TCGCTCGTTT AAAGAAGGGT TCCGAAGAAA CACAAACCTA AACTTGTGAC CTGGGACGGA AGCCCTCAGC   
  
  
- CAGTTATTCC GTGCTCCACC CGACTAAAAC CACCTCTGAT CGGTACTTTT ACTCCAACGT CACGATCGAT   
  
  
- CATCCCCTTT CTTCGTAGCG GTAGGACTCC TTAATGCAAG TCTTCAACCC TCCCCGTCAT TCGTCGGTCA   
  
  
- ACAAAAGGTC AAAAGTCTGC TCCGTCAACA CTCTAGTCTT TACAAACTAT CCCATAACGA CACGTCACCA   
  
  
- CCGTTTTTAC TACGACTGGA TGCGGTTTGA AATGTCTCAC TTGATTTATT TTGATACGTT TTACGAGTTT   
  
  
- ACTTTCTAAG ATTACCACCT TTTCCGGCAC CATCCTTCGT TCAACCGTCC TCCCTACACC ACCTAGATTC   
  
  
- TAGAGAAAAT GCGGAAACAC GTGTTCGTCA ACCTAGGTTA CTGGTTTCTT CGCGTTTACT GAATTAATCC   
  
  
- GTTTAATCCT TCGTAAGAAG AGGTTGGCCC CTACCCTTAG TTTCTTACCG AGTAATAAAA CGACTACCAG   
  
  
- AACTCCGAGC CGAACGACCA CAACCCTGGG GTTATATGTT CAGAGAGCAC TGACCGGGTC GAAGACGACA   
  
  
- ACTATAGAAC TCTCGAATGG TATACAAAGA ACGGTGTACG GGTAAGTTCT TTTATCCCTT AAAGAAGAGA   
  
  
- TTATCTTGTT AATACCTACA TCGAGTCTTT CGTTTAGCTG AAGTATAATA ACTAGAACCA TAACAGATAC   
  
  
- CAAAGGTTAC CGGAACAGAT TAAGTCGCGG AAAGTAGATC CGGACCGCCC GGAGGGTTTG AAGCCTAATG   
  
  
- TCCGCAACTA GAGGGGGTTG GGCCAAAGGC TGGTCGGTTT TCTCAGCTCC TCTGACCCGC TGCGAACTCC   
  
  
- TTGATACGAC TCAGTAAGTT ACACGGTAAA CTCAAGTTAC GGTATCGTTT CTCGACCCTT TGTGAATGAC   
  
  
- AATTTCTAGA GTTCTAACTA GGGCTACTCC ACGAACAACA CTTGACAGAC AAGTCCAAAT TTATGTAAGG   
  
  
- ACTCCTTTGT CATTAACGTC TTACGGGTTC TCTATAACAA GAATTAGACT AGTCTGTCTA TTTTGGTCGA   
  
  
- CAAAAGTATG TACCACATCA GTTACCTCGG AAGTTAAGAG GAAAAAAGTA ATAAGCCAAG GCACTCCGAG   
  
  
- AGAAGGTGAA GAGGTGAGAT AAACTACACA ACCTCCGTTT AGAAGGGGCC CTGTAGCTCC TCTCCTATGA   
  
  
- CTATCTCGCT CTGTAGAAAC CCGCTGTCCG TTACTTACAC TAGCGAACAC TCCCAAACCT CTCCTATCTT   
  
  
- TCCGGTCTCT TTATGTTTGT CACCGCCCAG GCTTTACTCT CCCGACCCAA CTTCGTCGAC GGAAACTTAG   
  
  
- TCCTCTGACA CCTTTACCGG TTTTTTGCCC ACTGTCGACA GATAGTGTTT CTAAAGAGGT AACTACTGCT   
  
  
- ACCCGTGACC AACAATGTCC CTACCTTTCC CTCTTAACAG ATACGTGAGT GATGAACCTT CGGACGACTG   
  
  
- AT

+     LTR

| Site Name | Organism | Position | Strand | Matrix score. | sequence | function |
| --- | --- | --- | --- | --- | --- | --- |
| LTR | Hordeum vulgare | 4094 | + | 6 | CCGAAA | cis-acting element involved in low-temperature responsiveness |
| LTR | Hordeum vulgare | 309 | - | 6 | CCGAAA | cis-acting element involved in low-temperature responsiveness |

>HU05G01597.1   
+ -Up\_Stream \_Len000TTTGTA TTTCAATATT GTAATTAGGT GGGATATAGA GTTGAAGGGC AAACTCTACC   
  
  
+ AACGACTTGT TGAAGGATCA ACTTGTAGCT TGGAACTTAA TGTATGTGTA CTATAGAGTT GTGTAATCTC   
  
  
+ TCCTAATAAA ATTGGGTTAA TCCTAGAATT AGAGAACTTT AAGCGGGGAC TAAGCTTGGT TGAGGCTAAC   
  
  
+ TTCGTTAAAA AGTTCTTGTG TCATGTCCAC GTTTATCTTT TCACTATTTA TTTCAATTAA TTTGCGATAT   
  
  
+ TTATCATCAC CCTATTCACC GTCTTTTCGG ATGATTTAAC TAGTCAAGCC GGACCAACAT TGCTTTTTAT   
  
  
+ AGTGTATTTG CAACTCATGT TGATTATTAG ATGTATCAAC AAAAAATGTT TCTCAAAATA AATAGTAGCT   
  
  
+ CTTTAAATTT TATTCCACAT AAATTCAAAA TCAATATCGA TATCGTTCCA TAATATAGCA GCATTATGAA   
  
  
+ AACAATATTT GCTAATGTTT ACATTGTGCT CTTTTCACTC AAAAATTGTA AGGCTTCAAA TACTATTAAA   
  
  
+ GAAACCATTA TTTCACTCGA CTATTTAGCT TGCATATATA GACAATCTTG CAAATAGTAA CATGTCTTCA   
  
  
+ TTCGACTATT TGCTTCCAAA TGAATAATCT CGTAAACATA TATACATACA TACATATATA TATATATATA   
  
  
+ TATATATATA TATATATATA GTAATATGTA TTTAACTTCA CATATACATG AATATAAAAA TATAAGAGGG   
  
  
+ AAAAAAGACG CTAGGTAGAT GGACTACAAA AGCCATTCAT GTATTGAGAC GGCTCATAAA ATCATACGGG   
  
  
+ GAAAGGAAAA AAAAAGATCT CTACATAATG TTCATAGATT TATAGAAAGC ATATGATGGA TTACCGCATA   
  
  
+ AGGTGTTATA TATGGGAGTG TTTGTAGAAG AAAGATGTAT ATCCGAACTA TCAAAGATAC ATATGAGGGA   
  
  
+ GTAAAAACAA GTGTAAGAAC CTCAGGAGGA GACATAAAAG ATTTTTCTAT TGGCATTGGA CACCGTCAAG   
  
  
+ TATCCGCTTT AAACCTGTTC TTTTTCACTA TTATCATGGA TGTATTCACA AAAGGAATTC AATACGAAGT   
  
  
+ ACCATGATGT ATGCTTTTTA CTGATAGTAT TGGTCTCATC GATGAGACTA AGGATGGTCT TAATAGCAAA   
  
  
+ TTAGAGATTT CATTAGATAA AATCTACTGT ACATGACAAA ACAATGATAT ATAGCATCCT ATTTAAAAAG   
  
  
+ CTACGTGTCA GTCATATATG AATTGCATCT AATTTTTAAT AGATATATAA TATGATATGA TATAATAGGA   
  
  
+ TATGATGTAG ATTGAATTCA AACTCATAAT TCATTAAGCA TGGTGCAATG TTCATTTTCT TGATTTTAAA   
  
  
+ CCGACTATTA CTTCAACTGT ATATAATTCG AATTGCTTTG GTATTTTAAA ATTTAAACTA GTAATTACTG   
  
  
+ CCTCTATTAC TGATGTTTAT ATTAAGTGGT GCTTAACTGC TTATTGATGA AACCATTTTT ATTTTCGTGT   
  
  
+ AAAAGAGGGC ATAATCCATC CTCGTGTATT GGCGGGGCTC GGCGCCCTAT TCAGCTATTC TCTGTATGTC   
  
  
+ AAACAAAACC GCTTCTATTA CCCATTGGTC TCTAAACACG CAGGGGTTTT AGCCCGGAGA GTTAATTCTT   
  
  
+ TATTGAGGTC CCCTGTTCTC CATTGCAGGA TTTTTGCTCA GGTACTTCTC TGTTTCTTCT CTTTAATCTT   
  
  
+ CTGTATCTTT CTGCATCAAG TTTAATGCTT TCTGGGATTT TCAGCTACGA TTTATGTGAA ATTGTAAGAT   
  
  
+ TTCTGAACTG GGCATTCAGG GCTTGGTTCA TATGTATTCT TTTCTGCTTG AAATGTTTTA ATTTCATAGT   
  
  
+ TCCAATGGGG TTATTCAAAG ATAGAATTAA GCTATAATCA CCGAGAAGAC TTGCTGACCT TTGTTTCTTT   
  
  
+ TTTGTTTCTT TTTTTTAATT AAATCTGGCT ATTACTTGGC TATAGTTTTT GCTAATGGAT CCACAAATAG   
  
  
+ ACGAATTATA TGGGCCTTTG CACCAAATGA AGTTCAATGA TCAGAGGAGA CCCATTTTAT CAAATGAAAA   
  
  
+ TTTTGTTAAT GTGTTGAAAC CGCCAAATTC CAATTGGAAT TCACCTTTTG GGAACCCTAC TGTGATTACC   
  
  
+ ACAAACCCAA ATTTTGATTT GGTTGTGCCA TCACTAGGTC CCAATGTTGA GGGAGCTCTC CTTGAAGATT   
  
  
+ ATGATTTTAG TGATGTTGTT CTTAAGTACA TCAATCAAGT TCTTATGGAC GAGGATGAGG GAGAGAAAAT   
  
  
+ CCATTTGGGC CACGGGCCTC TGGCTCTTGA AGCGGCCGAG AAGTCGTTAT ATGAGGTGCT TGGGCAGAGC   
  
  
+ CATTCTCCAC AAAATCAATA CTCTCCAGAG GACAAGCGTG ATGACCTGAG CAAGAGTAGT GTTAGAAGCT   
  
  
+ GTGTCAGTTA TAGCTGTGAC GGTGCAAGTA GTGGCAGTGG CATCGTGGAG CAAAGTTGGT CGAGTTATCC   
  
  
+ TCCGGAGTAC AATCCCATGC GGATGGCAGA TTTTCCTGCC TTGCCCTCAA AACCCTTGTT GAGAACATCT   
  
  
+ TATAGCTTAT CAAATAGCTT GACAGGTGAG GGTAATGGTT TGGTGGATAG ACCGGCAGAC TCACCTGTAA   
  
  
+ GCGCTCTTAG TGTGTCTGAT ATATTTAATG ATAGTCAGTC AGTGATACAA TTTCAGAAAG GGATGGAGGA   
  
  
+ AGCGAGCAAA TTTCTTCCCA AGGCTTCTTT GTGTTTGGAT TTGAACACTG GACCCTGCCT TCGGGAGTCG   
  
  
+ GTCAATAAGG CACGAGGTGG GCTGATTTTG GTGGAGACTA GCCATGAAAA TGAGGTTGCA GTGCTAGCTA   
  
  
+ GTAGGGGAAA GAAGCATCGC CATCCTGAGG AATTACGTTC AGAAGTTGGG AGGGGCAGTA AGCAGCCAGT   
  
  
+ TGTTTTCCAG TTTTCAGACG AGGCAGTTGT GAGATCAGAA ATGTTTGATA GGGTATTGCT GTGCAGTGGT   
  
  
+ GGCAAAAATG ATGCTGACCT ACGCCAAACT TTACAGAGTG AACTAAATAA AACTATGCAA AATGCTCAAA   
  
  
+ TGAAAGATTC TAATGGTGGA AAAGGCCGTG GTAGGAAGCA AGTTGGCAGG AGGGATGTGG TGGATCTAAG   
  
  
+ ATCTCTTTTA CGCCTTTGTG CACAAGCAGT TGGATCCAAT GACCAAAGAA GCGCAAATGA CTTAATTAGG   
  
  
+ CAAATTAGGA AGCATTCTTC TCCAACCGGG GATGGGAATC AAAGAATGGC TCATTATTTT GCTGATGGTC   
  
  
+ TTGAGGCTCG GCTTGCTGGT GTTGGGACCC CAATATACAA GTCTCTCGTG ACTGGCCCAG CTTCTGCTGT   
  
  
+ TGATATCTTG AGAGCTTACC ATATGTTTCT TGCCACATGC CCATTCAAGA AAATAGGGAA TTTCTTCTCT   
  
  
+ AATAGAACAA TTATGGATGT AGCTCAGAAA GCAAATCGAC TTCATATTAT TGATCTTGGT ATTGTCTATG   
  
  
+ GTTTCCAATG GCCTTGTCTA ATTCAGCGCC TTTCATCTAG GCCTGGCGGG CCTCCCAAAC TTCGGATTAC   
  
  
+ AGGCGTTGAT CTCCCCCAAC CCGGTTTCCG ACCAGCCAAA AGAGTCGAGG AGACTGGGCG ACGCTTGAGG   
  
  
+ AACTATGCTG AGTCATTCAA TGTGCCATTT GAGTTCAATG CCATAGCAAA GAGCTGGGAA ACACTTACTG   
  
  
+ TTAAAGATCT CAAGATTGAT CCCGATGAGG TGCTTGTTGT GAACTGTCTG TTCAGGTTTA AATACATTCC   
  
  
+ TGAGGAAACA GTAATTGCAG AATGCCCAAG AGATATTGTT CTTAATCTGA TCAGACAGAT AAAACCAGCT   
  
  
+ GTTTTCATAC ATGGTGTAGT CAATGGAGCC TTCAATTCTC CTTTTTTCAT TATTCGGTTC CGTGAGGCTC   
  
  
+ TCTTCCACTT CTCCACTCTA TTTGATGTGT TGGAGGCAAA TCTTCCCCGG GACATCGAGG AGAGGATACT   
  
  
+ GATAGAGCGA GACATCTTTG GGCGACAGGC AATGAATGTG ATCGCTTGTG AGGGTTTGGA GAGGATAGAA   
  
  
+ AGGCCAGAGA AATACAAACA GTGGCGGGTC CGAAATGAGA GGGCTGGGTT GAAGCAGCTG CCTTTGAATC   
  
  
+ AGGAGACTGT GGAAATGGCC AAAAAACGGG TGACAGCTGT CTATCACAAA GATTTCTCCA TTGATGACGA   
  
  
+ TGGGCACTGG TTGTTACAGG GATGGAAAGG GAGAATTGTC TATGCACTCA CTACTTGGAA GCCTGCTGAC   
  
  
+ TA  

- -Up\_Stream \_Len000AAACAT AAAGTTATAA CATTAATCCA CCCTATATCT CAACTTCCCG TTTGAGATGG   
  
  
- TTGCTGAACA ACTTCCTAGT TGAACATCGA ACCTTGAATT ACATACACAT GATATCTCAA CACATTAGAG   
  
  
- AGGATTATTT TAACCCAATT AGGATCTTAA TCTCTTGAAA TTCGCCCCTG ATTCGAACCA ACTCCGATTG   
  
  
- AAGCAATTTT TCAAGAACAC AGTACAGGTG CAAATAGAAA AGTGATAAAT AAAGTTAATT AAACGCTATA   
  
  
- AATAGTAGTG GGATAAGTGG CAGAAAAGCC TACTAAATTG ATCAGTTCGG CCTGGTTGTA ACGAAAAATA   
  
  
- TCACATAAAC GTTGAGTACA ACTAATAATC TACATAGTTG TTTTTTACAA AGAGTTTTAT TTATCATCGA   
  
  
- GAAATTTAAA ATAAGGTGTA TTTAAGTTTT AGTTATAGCT ATAGCAAGGT ATTATATCGT CGTAATACTT   
  
  
- TTGTTATAAA CGATTACAAA TGTAACACGA GAAAAGTGAG TTTTTAACAT TCCGAAGTTT ATGATAATTT   
  
  
- CTTTGGTAAT AAAGTGAGCT GATAAATCGA ACGTATATAT CTGTTAGAAC GTTTATCATT GTACAGAAGT   
  
  
- AAGCTGATAA ACGAAGGTTT ACTTATTAGA GCATTTGTAT ATATGTATGT ATGTATATAT ATATATATAT   
  
  
- ATATATATAT ATATATATAT CATTATACAT AAATTGAAGT GTATATGTAC TTATATTTTT ATATTCTCCC   
  
  
- TTTTTTCTGC GATCCATCTA CCTGATGTTT TCGGTAAGTA CATAACTCTG CCGAGTATTT TAGTATGCCC   
  
  
- CTTTCCTTTT TTTTTCTAGA GATGTATTAC AAGTATCTAA ATATCTTTCG TATACTACCT AATGGCGTAT   
  
  
- TCCACAATAT ATACCCTCAC AAACATCTTC TTTCTACATA TAGGCTTGAT AGTTTCTATG TATACTCCCT   
  
  
- CATTTTTGTT CACATTCTTG GAGTCCTCCT CTGTATTTTC TAAAAAGATA ACCGTAACCT GTGGCAGTTC   
  
  
- ATAGGCGAAA TTTGGACAAG AAAAAGTGAT AATAGTACCT ACATAAGTGT TTTCCTTAAG TTATGCTTCA   
  
  
- TGGTACTACA TACGAAAAAT GACTATCATA ACCAGAGTAG CTACTCTGAT TCCTACCAGA ATTATCGTTT   
  
  
- AATCTCTAAA GTAATCTATT TTAGATGACA TGTACTGTTT TGTTACTATA TATCGTAGGA TAAATTTTTC   
  
  
- GATGCACAGT CAGTATATAC TTAACGTAGA TTAAAAATTA TCTATATATT ATACTATACT ATATTATCCT   
  
  
- ATACTACATC TAACTTAAGT TTGAGTATTA AGTAATTCGT ACCACGTTAC AAGTAAAAGA ACTAAAATTT   
  
  
- GGCTGATAAT GAAGTTGACA TATATTAAGC TTAACGAAAC CATAAAATTT TAAATTTGAT CATTAATGAC   
  
  
- GGAGATAATG ACTACAAATA TAATTCACCA CGAATTGACG AATAACTACT TTGGTAAAAA TAAAAGCACA   
  
  
- TTTTCTCCCG TATTAGGTAG GAGCACATAA CCGCCCCGAG CCGCGGGATA AGTCGATAAG AGACATACAG   
  
  
- TTTGTTTTGG CGAAGATAAT GGGTAACCAG AGATTTGTGC GTCCCCAAAA TCGGGCCTCT CAATTAAGAA   
  
  
- ATAACTCCAG GGGACAAGAG GTAACGTCCT AAAAACGAGT CCATGAAGAG ACAAAGAAGA GAAATTAGAA   
  
  
- GACATAGAAA GACGTAGTTC AAATTACGAA AGACCCTAAA AGTCGATGCT AAATACACTT TAACATTCTA   
  
  
- AAGACTTGAC CCGTAAGTCC CGAACCAAGT ATACATAAGA AAAGACGAAC TTTACAAAAT TAAAGTATCA   
  
  
- AGGTTACCCC AATAAGTTTC TATCTTAATT CGATATTAGT GGCTCTTCTG AACGACTGGA AACAAAGAAA   
  
  
- AAACAAAGAA AAAAAATTAA TTTAGACCGA TAATGAACCG ATATCAAAAA CGATTACCTA GGTGTTTATC   
  
  
- TGCTTAATAT ACCCGGAAAC GTGGTTTACT TCAAGTTACT AGTCTCCTCT GGGTAAAATA GTTTACTTTT   
  
  
- AAAACAATTA CACAACTTTG GCGGTTTAAG GTTAACCTTA AGTGGAAAAC CCTTGGGATG ACACTAATGG   
  
  
- TGTTTGGGTT TAAAACTAAA CCAACACGGT AGTGATCCAG GGTTACAACT CCCTCGAGAG GAACTTCTAA   
  
  
- TACTAAAATC ACTACAACAA GAATTCATGT AGTTAGTTCA AGAATACCTG CTCCTACTCC CTCTCTTTTA   
  
  
- GGTAAACCCG GTGCCCGGAG ACCGAGAACT TCGCCGGCTC TTCAGCAATA TACTCCACGA ACCCGTCTCG   
  
  
- GTAAGAGGTG TTTTAGTTAT GAGAGGTCTC CTGTTCGCAC TACTGGACTC GTTCTCATCA CAATCTTCGA   
  
  
- CACAGTCAAT ATCGACACTG CCACGTTCAT CACCGTCACC GTAGCACCTC GTTTCAACCA GCTCAATAGG   
  
  
- AGGCCTCATG TTAGGGTACG CCTACCGTCT AAAAGGACGG AACGGGAGTT TTGGGAACAA CTCTTGTAGA   
  
  
- ATATCGAATA GTTTATCGAA CTGTCCACTC CCATTACCAA ACCACCTATC TGGCCGTCTG AGTGGACATT   
  
  
- CGCGAGAATC ACACAGACTA TATAAATTAC TATCAGTCAG TCACTATGTT AAAGTCTTTC CCTACCTCCT   
  
  
- TCGCTCGTTT AAAGAAGGGT TCCGAAGAAA CACAAACCTA AACTTGTGAC CTGGGACGGA AGCCCTCAGC   
  
  
- CAGTTATTCC GTGCTCCACC CGACTAAAAC CACCTCTGAT CGGTACTTTT ACTCCAACGT CACGATCGAT   
  
  
- CATCCCCTTT CTTCGTAGCG GTAGGACTCC TTAATGCAAG TCTTCAACCC TCCCCGTCAT TCGTCGGTCA   
  
  
- ACAAAAGGTC AAAAGTCTGC TCCGTCAACA CTCTAGTCTT TACAAACTAT CCCATAACGA CACGTCACCA   
  
  
- CCGTTTTTAC TACGACTGGA TGCGGTTTGA AATGTCTCAC TTGATTTATT TTGATACGTT TTACGAGTTT   
  
  
- ACTTTCTAAG ATTACCACCT TTTCCGGCAC CATCCTTCGT TCAACCGTCC TCCCTACACC ACCTAGATTC   
  
  
- TAGAGAAAAT GCGGAAACAC GTGTTCGTCA ACCTAGGTTA CTGGTTTCTT CGCGTTTACT GAATTAATCC   
  
  
- GTTTAATCCT TCGTAAGAAG AGGTTGGCCC CTACCCTTAG TTTCTTACCG AGTAATAAAA CGACTACCAG   
  
  
- AACTCCGAGC CGAACGACCA CAACCCTGGG GTTATATGTT CAGAGAGCAC TGACCGGGTC GAAGACGACA   
  
  
- ACTATAGAAC TCTCGAATGG TATACAAAGA ACGGTGTACG GGTAAGTTCT TTTATCCCTT AAAGAAGAGA   
  
  
- TTATCTTGTT AATACCTACA TCGAGTCTTT CGTTTAGCTG AAGTATAATA ACTAGAACCA TAACAGATAC   
  
  
- CAAAGGTTAC CGGAACAGAT TAAGTCGCGG AAAGTAGATC CGGACCGCCC GGAGGGTTTG AAGCCTAATG   
  
  
- TCCGCAACTA GAGGGGGTTG GGCCAAAGGC TGGTCGGTTT TCTCAGCTCC TCTGACCCGC TGCGAACTCC   
  
  
- TTGATACGAC TCAGTAAGTT ACACGGTAAA CTCAAGTTAC GGTATCGTTT CTCGACCCTT TGTGAATGAC   
  
  
- AATTTCTAGA GTTCTAACTA GGGCTACTCC ACGAACAACA CTTGACAGAC AAGTCCAAAT TTATGTAAGG   
  
  
- ACTCCTTTGT CATTAACGTC TTACGGGTTC TCTATAACAA GAATTAGACT AGTCTGTCTA TTTTGGTCGA   
  
  
- CAAAAGTATG TACCACATCA GTTACCTCGG AAGTTAAGAG GAAAAAAGTA ATAAGCCAAG GCACTCCGAG   
  
  
- AGAAGGTGAA GAGGTGAGAT AAACTACACA ACCTCCGTTT AGAAGGGGCC CTGTAGCTCC TCTCCTATGA   
  
  
- CTATCTCGCT CTGTAGAAAC CCGCTGTCCG TTACTTACAC TAGCGAACAC TCCCAAACCT CTCCTATCTT   
  
  
- TCCGGTCTCT TTATGTTTGT CACCGCCCAG GCTTTACTCT CCCGACCCAA CTTCGTCGAC GGAAACTTAG   
  
  
- TCCTCTGACA CCTTTACCGG TTTTTTGCCC ACTGTCGACA GATAGTGTTT CTAAAGAGGT AACTACTGCT   
  
  
- ACCCGTGACC AACAATGTCC CTACCTTTCC CTCTTAACAG ATACGTGAGT GATGAACCTT CGGACGACTG   
  
  
- AT

+     MBS

| Site Name | Organism | Position | Strand | Matrix score. | sequence | function |
| --- | --- | --- | --- | --- | --- | --- |
| MBS | Arabidopsis thaliana | 1418 | + | 6 | CAACTG | MYB binding site involved in drought-inducibility |
| MBS | Arabidopsis thaliana | 2941 | - | 6 | CAACTG | MYB binding site involved in drought-inducibility |
| MBS | Arabidopsis thaliana | 2968 | - | 6 | CAACTG | MYB binding site involved in drought-inducibility |
| MBS | Arabidopsis thaliana | 3181 | - | 6 | CAACTG | MYB binding site involved in drought-inducibility |

>HU05G01597.1   
+ -Up\_Stream \_Len000TTTGTA TTTCAATATT GTAATTAGGT GGGATATAGA GTTGAAGGGC AAACTCTACC   
  
  
+ AACGACTTGT TGAAGGATCA ACTTGTAGCT TGGAACTTAA TGTATGTGTA CTATAGAGTT GTGTAATCTC   
  
  
+ TCCTAATAAA ATTGGGTTAA TCCTAGAATT AGAGAACTTT AAGCGGGGAC TAAGCTTGGT TGAGGCTAAC   
  
  
+ TTCGTTAAAA AGTTCTTGTG TCATGTCCAC GTTTATCTTT TCACTATTTA TTTCAATTAA TTTGCGATAT   
  
  
+ TTATCATCAC CCTATTCACC GTCTTTTCGG ATGATTTAAC TAGTCAAGCC GGACCAACAT TGCTTTTTAT   
  
  
+ AGTGTATTTG CAACTCATGT TGATTATTAG ATGTATCAAC AAAAAATGTT TCTCAAAATA AATAGTAGCT   
  
  
+ CTTTAAATTT TATTCCACAT AAATTCAAAA TCAATATCGA TATCGTTCCA TAATATAGCA GCATTATGAA   
  
  
+ AACAATATTT GCTAATGTTT ACATTGTGCT CTTTTCACTC AAAAATTGTA AGGCTTCAAA TACTATTAAA   
  
  
+ GAAACCATTA TTTCACTCGA CTATTTAGCT TGCATATATA GACAATCTTG CAAATAGTAA CATGTCTTCA   
  
  
+ TTCGACTATT TGCTTCCAAA TGAATAATCT CGTAAACATA TATACATACA TACATATATA TATATATATA   
  
  
+ TATATATATA TATATATATA GTAATATGTA TTTAACTTCA CATATACATG AATATAAAAA TATAAGAGGG   
  
  
+ AAAAAAGACG CTAGGTAGAT GGACTACAAA AGCCATTCAT GTATTGAGAC GGCTCATAAA ATCATACGGG   
  
  
+ GAAAGGAAAA AAAAAGATCT CTACATAATG TTCATAGATT TATAGAAAGC ATATGATGGA TTACCGCATA   
  
  
+ AGGTGTTATA TATGGGAGTG TTTGTAGAAG AAAGATGTAT ATCCGAACTA TCAAAGATAC ATATGAGGGA   
  
  
+ GTAAAAACAA GTGTAAGAAC CTCAGGAGGA GACATAAAAG ATTTTTCTAT TGGCATTGGA CACCGTCAAG   
  
  
+ TATCCGCTTT AAACCTGTTC TTTTTCACTA TTATCATGGA TGTATTCACA AAAGGAATTC AATACGAAGT   
  
  
+ ACCATGATGT ATGCTTTTTA CTGATAGTAT TGGTCTCATC GATGAGACTA AGGATGGTCT TAATAGCAAA   
  
  
+ TTAGAGATTT CATTAGATAA AATCTACTGT ACATGACAAA ACAATGATAT ATAGCATCCT ATTTAAAAAG   
  
  
+ CTACGTGTCA GTCATATATG AATTGCATCT AATTTTTAAT AGATATATAA TATGATATGA TATAATAGGA   
  
  
+ TATGATGTAG ATTGAATTCA AACTCATAAT TCATTAAGCA TGGTGCAATG TTCATTTTCT TGATTTTAAA   
  
  
+ CCGACTATTA CTTCAACTGT ATATAATTCG AATTGCTTTG GTATTTTAAA ATTTAAACTA GTAATTACTG   
  
  
+ CCTCTATTAC TGATGTTTAT ATTAAGTGGT GCTTAACTGC TTATTGATGA AACCATTTTT ATTTTCGTGT   
  
  
+ AAAAGAGGGC ATAATCCATC CTCGTGTATT GGCGGGGCTC GGCGCCCTAT TCAGCTATTC TCTGTATGTC   
  
  
+ AAACAAAACC GCTTCTATTA CCCATTGGTC TCTAAACACG CAGGGGTTTT AGCCCGGAGA GTTAATTCTT   
  
  
+ TATTGAGGTC CCCTGTTCTC CATTGCAGGA TTTTTGCTCA GGTACTTCTC TGTTTCTTCT CTTTAATCTT   
  
  
+ CTGTATCTTT CTGCATCAAG TTTAATGCTT TCTGGGATTT TCAGCTACGA TTTATGTGAA ATTGTAAGAT   
  
  
+ TTCTGAACTG GGCATTCAGG GCTTGGTTCA TATGTATTCT TTTCTGCTTG AAATGTTTTA ATTTCATAGT   
  
  
+ TCCAATGGGG TTATTCAAAG ATAGAATTAA GCTATAATCA CCGAGAAGAC TTGCTGACCT TTGTTTCTTT   
  
  
+ TTTGTTTCTT TTTTTTAATT AAATCTGGCT ATTACTTGGC TATAGTTTTT GCTAATGGAT CCACAAATAG   
  
  
+ ACGAATTATA TGGGCCTTTG CACCAAATGA AGTTCAATGA TCAGAGGAGA CCCATTTTAT CAAATGAAAA   
  
  
+ TTTTGTTAAT GTGTTGAAAC CGCCAAATTC CAATTGGAAT TCACCTTTTG GGAACCCTAC TGTGATTACC   
  
  
+ ACAAACCCAA ATTTTGATTT GGTTGTGCCA TCACTAGGTC CCAATGTTGA GGGAGCTCTC CTTGAAGATT   
  
  
+ ATGATTTTAG TGATGTTGTT CTTAAGTACA TCAATCAAGT TCTTATGGAC GAGGATGAGG GAGAGAAAAT   
  
  
+ CCATTTGGGC CACGGGCCTC TGGCTCTTGA AGCGGCCGAG AAGTCGTTAT ATGAGGTGCT TGGGCAGAGC   
  
  
+ CATTCTCCAC AAAATCAATA CTCTCCAGAG GACAAGCGTG ATGACCTGAG CAAGAGTAGT GTTAGAAGCT   
  
  
+ GTGTCAGTTA TAGCTGTGAC GGTGCAAGTA GTGGCAGTGG CATCGTGGAG CAAAGTTGGT CGAGTTATCC   
  
  
+ TCCGGAGTAC AATCCCATGC GGATGGCAGA TTTTCCTGCC TTGCCCTCAA AACCCTTGTT GAGAACATCT   
  
  
+ TATAGCTTAT CAAATAGCTT GACAGGTGAG GGTAATGGTT TGGTGGATAG ACCGGCAGAC TCACCTGTAA   
  
  
+ GCGCTCTTAG TGTGTCTGAT ATATTTAATG ATAGTCAGTC AGTGATACAA TTTCAGAAAG GGATGGAGGA   
  
  
+ AGCGAGCAAA TTTCTTCCCA AGGCTTCTTT GTGTTTGGAT TTGAACACTG GACCCTGCCT TCGGGAGTCG   
  
  
+ GTCAATAAGG CACGAGGTGG GCTGATTTTG GTGGAGACTA GCCATGAAAA TGAGGTTGCA GTGCTAGCTA   
  
  
+ GTAGGGGAAA GAAGCATCGC CATCCTGAGG AATTACGTTC AGAAGTTGGG AGGGGCAGTA AGCAGCCAGT   
  
  
+ TGTTTTCCAG TTTTCAGACG AGGCAGTTGT GAGATCAGAA ATGTTTGATA GGGTATTGCT GTGCAGTGGT   
  
  
+ GGCAAAAATG ATGCTGACCT ACGCCAAACT TTACAGAGTG AACTAAATAA AACTATGCAA AATGCTCAAA   
  
  
+ TGAAAGATTC TAATGGTGGA AAAGGCCGTG GTAGGAAGCA AGTTGGCAGG AGGGATGTGG TGGATCTAAG   
  
  
+ ATCTCTTTTA CGCCTTTGTG CACAAGCAGT TGGATCCAAT GACCAAAGAA GCGCAAATGA CTTAATTAGG   
  
  
+ CAAATTAGGA AGCATTCTTC TCCAACCGGG GATGGGAATC AAAGAATGGC TCATTATTTT GCTGATGGTC   
  
  
+ TTGAGGCTCG GCTTGCTGGT GTTGGGACCC CAATATACAA GTCTCTCGTG ACTGGCCCAG CTTCTGCTGT   
  
  
+ TGATATCTTG AGAGCTTACC ATATGTTTCT TGCCACATGC CCATTCAAGA AAATAGGGAA TTTCTTCTCT   
  
  
+ AATAGAACAA TTATGGATGT AGCTCAGAAA GCAAATCGAC TTCATATTAT TGATCTTGGT ATTGTCTATG   
  
  
+ GTTTCCAATG GCCTTGTCTA ATTCAGCGCC TTTCATCTAG GCCTGGCGGG CCTCCCAAAC TTCGGATTAC   
  
  
+ AGGCGTTGAT CTCCCCCAAC CCGGTTTCCG ACCAGCCAAA AGAGTCGAGG AGACTGGGCG ACGCTTGAGG   
  
  
+ AACTATGCTG AGTCATTCAA TGTGCCATTT GAGTTCAATG CCATAGCAAA GAGCTGGGAA ACACTTACTG   
  
  
+ TTAAAGATCT CAAGATTGAT CCCGATGAGG TGCTTGTTGT GAACTGTCTG TTCAGGTTTA AATACATTCC   
  
  
+ TGAGGAAACA GTAATTGCAG AATGCCCAAG AGATATTGTT CTTAATCTGA TCAGACAGAT AAAACCAGCT   
  
  
+ GTTTTCATAC ATGGTGTAGT CAATGGAGCC TTCAATTCTC CTTTTTTCAT TATTCGGTTC CGTGAGGCTC   
  
  
+ TCTTCCACTT CTCCACTCTA TTTGATGTGT TGGAGGCAAA TCTTCCCCGG GACATCGAGG AGAGGATACT   
  
  
+ GATAGAGCGA GACATCTTTG GGCGACAGGC AATGAATGTG ATCGCTTGTG AGGGTTTGGA GAGGATAGAA   
  
  
+ AGGCCAGAGA AATACAAACA GTGGCGGGTC CGAAATGAGA GGGCTGGGTT GAAGCAGCTG CCTTTGAATC   
  
  
+ AGGAGACTGT GGAAATGGCC AAAAAACGGG TGACAGCTGT CTATCACAAA GATTTCTCCA TTGATGACGA   
  
  
+ TGGGCACTGG TTGTTACAGG GATGGAAAGG GAGAATTGTC TATGCACTCA CTACTTGGAA GCCTGCTGAC   
  
  
+ TA  

- -Up\_Stream \_Len000AAACAT AAAGTTATAA CATTAATCCA CCCTATATCT CAACTTCCCG TTTGAGATGG   
  
  
- TTGCTGAACA ACTTCCTAGT TGAACATCGA ACCTTGAATT ACATACACAT GATATCTCAA CACATTAGAG   
  
  
- AGGATTATTT TAACCCAATT AGGATCTTAA TCTCTTGAAA TTCGCCCCTG ATTCGAACCA ACTCCGATTG   
  
  
- AAGCAATTTT TCAAGAACAC AGTACAGGTG CAAATAGAAA AGTGATAAAT AAAGTTAATT AAACGCTATA   
  
  
- AATAGTAGTG GGATAAGTGG CAGAAAAGCC TACTAAATTG ATCAGTTCGG CCTGGTTGTA ACGAAAAATA   
  
  
- TCACATAAAC GTTGAGTACA ACTAATAATC TACATAGTTG TTTTTTACAA AGAGTTTTAT TTATCATCGA   
  
  
- GAAATTTAAA ATAAGGTGTA TTTAAGTTTT AGTTATAGCT ATAGCAAGGT ATTATATCGT CGTAATACTT   
  
  
- TTGTTATAAA CGATTACAAA TGTAACACGA GAAAAGTGAG TTTTTAACAT TCCGAAGTTT ATGATAATTT   
  
  
- CTTTGGTAAT AAAGTGAGCT GATAAATCGA ACGTATATAT CTGTTAGAAC GTTTATCATT GTACAGAAGT   
  
  
- AAGCTGATAA ACGAAGGTTT ACTTATTAGA GCATTTGTAT ATATGTATGT ATGTATATAT ATATATATAT   
  
  
- ATATATATAT ATATATATAT CATTATACAT AAATTGAAGT GTATATGTAC TTATATTTTT ATATTCTCCC   
  
  
- TTTTTTCTGC GATCCATCTA CCTGATGTTT TCGGTAAGTA CATAACTCTG CCGAGTATTT TAGTATGCCC   
  
  
- CTTTCCTTTT TTTTTCTAGA GATGTATTAC AAGTATCTAA ATATCTTTCG TATACTACCT AATGGCGTAT   
  
  
- TCCACAATAT ATACCCTCAC AAACATCTTC TTTCTACATA TAGGCTTGAT AGTTTCTATG TATACTCCCT   
  
  
- CATTTTTGTT CACATTCTTG GAGTCCTCCT CTGTATTTTC TAAAAAGATA ACCGTAACCT GTGGCAGTTC   
  
  
- ATAGGCGAAA TTTGGACAAG AAAAAGTGAT AATAGTACCT ACATAAGTGT TTTCCTTAAG TTATGCTTCA   
  
  
- TGGTACTACA TACGAAAAAT GACTATCATA ACCAGAGTAG CTACTCTGAT TCCTACCAGA ATTATCGTTT   
  
  
- AATCTCTAAA GTAATCTATT TTAGATGACA TGTACTGTTT TGTTACTATA TATCGTAGGA TAAATTTTTC   
  
  
- GATGCACAGT CAGTATATAC TTAACGTAGA TTAAAAATTA TCTATATATT ATACTATACT ATATTATCCT   
  
  
- ATACTACATC TAACTTAAGT TTGAGTATTA AGTAATTCGT ACCACGTTAC AAGTAAAAGA ACTAAAATTT   
  
  
- GGCTGATAAT GAAGTTGACA TATATTAAGC TTAACGAAAC CATAAAATTT TAAATTTGAT CATTAATGAC   
  
  
- GGAGATAATG ACTACAAATA TAATTCACCA CGAATTGACG AATAACTACT TTGGTAAAAA TAAAAGCACA   
  
  
- TTTTCTCCCG TATTAGGTAG GAGCACATAA CCGCCCCGAG CCGCGGGATA AGTCGATAAG AGACATACAG   
  
  
- TTTGTTTTGG CGAAGATAAT GGGTAACCAG AGATTTGTGC GTCCCCAAAA TCGGGCCTCT CAATTAAGAA   
  
  
- ATAACTCCAG GGGACAAGAG GTAACGTCCT AAAAACGAGT CCATGAAGAG ACAAAGAAGA GAAATTAGAA   
  
  
- GACATAGAAA GACGTAGTTC AAATTACGAA AGACCCTAAA AGTCGATGCT AAATACACTT TAACATTCTA   
  
  
- AAGACTTGAC CCGTAAGTCC CGAACCAAGT ATACATAAGA AAAGACGAAC TTTACAAAAT TAAAGTATCA   
  
  
- AGGTTACCCC AATAAGTTTC TATCTTAATT CGATATTAGT GGCTCTTCTG AACGACTGGA AACAAAGAAA   
  
  
- AAACAAAGAA AAAAAATTAA TTTAGACCGA TAATGAACCG ATATCAAAAA CGATTACCTA GGTGTTTATC   
  
  
- TGCTTAATAT ACCCGGAAAC GTGGTTTACT TCAAGTTACT AGTCTCCTCT GGGTAAAATA GTTTACTTTT   
  
  
- AAAACAATTA CACAACTTTG GCGGTTTAAG GTTAACCTTA AGTGGAAAAC CCTTGGGATG ACACTAATGG   
  
  
- TGTTTGGGTT TAAAACTAAA CCAACACGGT AGTGATCCAG GGTTACAACT CCCTCGAGAG GAACTTCTAA   
  
  
- TACTAAAATC ACTACAACAA GAATTCATGT AGTTAGTTCA AGAATACCTG CTCCTACTCC CTCTCTTTTA   
  
  
- GGTAAACCCG GTGCCCGGAG ACCGAGAACT TCGCCGGCTC TTCAGCAATA TACTCCACGA ACCCGTCTCG   
  
  
- GTAAGAGGTG TTTTAGTTAT GAGAGGTCTC CTGTTCGCAC TACTGGACTC GTTCTCATCA CAATCTTCGA   
  
  
- CACAGTCAAT ATCGACACTG CCACGTTCAT CACCGTCACC GTAGCACCTC GTTTCAACCA GCTCAATAGG   
  
  
- AGGCCTCATG TTAGGGTACG CCTACCGTCT AAAAGGACGG AACGGGAGTT TTGGGAACAA CTCTTGTAGA   
  
  
- ATATCGAATA GTTTATCGAA CTGTCCACTC CCATTACCAA ACCACCTATC TGGCCGTCTG AGTGGACATT   
  
  
- CGCGAGAATC ACACAGACTA TATAAATTAC TATCAGTCAG TCACTATGTT AAAGTCTTTC CCTACCTCCT   
  
  
- TCGCTCGTTT AAAGAAGGGT TCCGAAGAAA CACAAACCTA AACTTGTGAC CTGGGACGGA AGCCCTCAGC   
  
  
- CAGTTATTCC GTGCTCCACC CGACTAAAAC CACCTCTGAT CGGTACTTTT ACTCCAACGT CACGATCGAT   
  
  
- CATCCCCTTT CTTCGTAGCG GTAGGACTCC TTAATGCAAG TCTTCAACCC TCCCCGTCAT TCGTCGGTCA   
  
  
- ACAAAAGGTC AAAAGTCTGC TCCGTCAACA CTCTAGTCTT TACAAACTAT CCCATAACGA CACGTCACCA   
  
  
- CCGTTTTTAC TACGACTGGA TGCGGTTTGA AATGTCTCAC TTGATTTATT TTGATACGTT TTACGAGTTT   
  
  
- ACTTTCTAAG ATTACCACCT TTTCCGGCAC CATCCTTCGT TCAACCGTCC TCCCTACACC ACCTAGATTC   
  
  
- TAGAGAAAAT GCGGAAACAC GTGTTCGTCA ACCTAGGTTA CTGGTTTCTT CGCGTTTACT GAATTAATCC   
  
  
- GTTTAATCCT TCGTAAGAAG AGGTTGGCCC CTACCCTTAG TTTCTTACCG AGTAATAAAA CGACTACCAG   
  
  
- AACTCCGAGC CGAACGACCA CAACCCTGGG GTTATATGTT CAGAGAGCAC TGACCGGGTC GAAGACGACA   
  
  
- ACTATAGAAC TCTCGAATGG TATACAAAGA ACGGTGTACG GGTAAGTTCT TTTATCCCTT AAAGAAGAGA   
  
  
- TTATCTTGTT AATACCTACA TCGAGTCTTT CGTTTAGCTG AAGTATAATA ACTAGAACCA TAACAGATAC   
  
  
- CAAAGGTTAC CGGAACAGAT TAAGTCGCGG AAAGTAGATC CGGACCGCCC GGAGGGTTTG AAGCCTAATG   
  
  
- TCCGCAACTA GAGGGGGTTG GGCCAAAGGC TGGTCGGTTT TCTCAGCTCC TCTGACCCGC TGCGAACTCC   
  
  
- TTGATACGAC TCAGTAAGTT ACACGGTAAA CTCAAGTTAC GGTATCGTTT CTCGACCCTT TGTGAATGAC   
  
  
- AATTTCTAGA GTTCTAACTA GGGCTACTCC ACGAACAACA CTTGACAGAC AAGTCCAAAT TTATGTAAGG   
  
  
- ACTCCTTTGT CATTAACGTC TTACGGGTTC TCTATAACAA GAATTAGACT AGTCTGTCTA TTTTGGTCGA   
  
  
- CAAAAGTATG TACCACATCA GTTACCTCGG AAGTTAAGAG GAAAAAAGTA ATAAGCCAAG GCACTCCGAG   
  
  
- AGAAGGTGAA GAGGTGAGAT AAACTACACA ACCTCCGTTT AGAAGGGGCC CTGTAGCTCC TCTCCTATGA   
  
  
- CTATCTCGCT CTGTAGAAAC CCGCTGTCCG TTACTTACAC TAGCGAACAC TCCCAAACCT CTCCTATCTT   
  
  
- TCCGGTCTCT TTATGTTTGT CACCGCCCAG GCTTTACTCT CCCGACCCAA CTTCGTCGAC GGAAACTTAG   
  
  
- TCCTCTGACA CCTTTACCGG TTTTTTGCCC ACTGTCGACA GATAGTGTTT CTAAAGAGGT AACTACTGCT   
  
  
- ACCCGTGACC AACAATGTCC CTACCTTTCC CTCTTAACAG ATACGTGAGT GATGAACCTT CGGACGACTG   
  
  
- AT

+     MYB

| Site Name | Organism | Position | Strand | Matrix score. | sequence | function |
| --- | --- | --- | --- | --- | --- | --- |
| MYB | Arabidopsis thaliana | 201 | - | 6 | CAACCA |  |
| MYB | Arabidopsis thaliana | 3361 | - | 6 | CAACAG |  |
| MYB | Arabidopsis thaliana | 2194 | - | 6 | CAACCA |  |
| MYB | Arabidopsis thaliana | 4212 | - | 6 | CAACCA |  |

>HU05G01597.1   
+ -Up\_Stream \_Len000TTTGTA TTTCAATATT GTAATTAGGT GGGATATAGA GTTGAAGGGC AAACTCTACC   
  
  
+ AACGACTTGT TGAAGGATCA ACTTGTAGCT TGGAACTTAA TGTATGTGTA CTATAGAGTT GTGTAATCTC   
  
  
+ TCCTAATAAA ATTGGGTTAA TCCTAGAATT AGAGAACTTT AAGCGGGGAC TAAGCTTGGT TGAGGCTAAC   
  
  
+ TTCGTTAAAA AGTTCTTGTG TCATGTCCAC GTTTATCTTT TCACTATTTA TTTCAATTAA TTTGCGATAT   
  
  
+ TTATCATCAC CCTATTCACC GTCTTTTCGG ATGATTTAAC TAGTCAAGCC GGACCAACAT TGCTTTTTAT   
  
  
+ AGTGTATTTG CAACTCATGT TGATTATTAG ATGTATCAAC AAAAAATGTT TCTCAAAATA AATAGTAGCT   
  
  
+ CTTTAAATTT TATTCCACAT AAATTCAAAA TCAATATCGA TATCGTTCCA TAATATAGCA GCATTATGAA   
  
  
+ AACAATATTT GCTAATGTTT ACATTGTGCT CTTTTCACTC AAAAATTGTA AGGCTTCAAA TACTATTAAA   
  
  
+ GAAACCATTA TTTCACTCGA CTATTTAGCT TGCATATATA GACAATCTTG CAAATAGTAA CATGTCTTCA   
  
  
+ TTCGACTATT TGCTTCCAAA TGAATAATCT CGTAAACATA TATACATACA TACATATATA TATATATATA   
  
  
+ TATATATATA TATATATATA GTAATATGTA TTTAACTTCA CATATACATG AATATAAAAA TATAAGAGGG   
  
  
+ AAAAAAGACG CTAGGTAGAT GGACTACAAA AGCCATTCAT GTATTGAGAC GGCTCATAAA ATCATACGGG   
  
  
+ GAAAGGAAAA AAAAAGATCT CTACATAATG TTCATAGATT TATAGAAAGC ATATGATGGA TTACCGCATA   
  
  
+ AGGTGTTATA TATGGGAGTG TTTGTAGAAG AAAGATGTAT ATCCGAACTA TCAAAGATAC ATATGAGGGA   
  
  
+ GTAAAAACAA GTGTAAGAAC CTCAGGAGGA GACATAAAAG ATTTTTCTAT TGGCATTGGA CACCGTCAAG   
  
  
+ TATCCGCTTT AAACCTGTTC TTTTTCACTA TTATCATGGA TGTATTCACA AAAGGAATTC AATACGAAGT   
  
  
+ ACCATGATGT ATGCTTTTTA CTGATAGTAT TGGTCTCATC GATGAGACTA AGGATGGTCT TAATAGCAAA   
  
  
+ TTAGAGATTT CATTAGATAA AATCTACTGT ACATGACAAA ACAATGATAT ATAGCATCCT ATTTAAAAAG   
  
  
+ CTACGTGTCA GTCATATATG AATTGCATCT AATTTTTAAT AGATATATAA TATGATATGA TATAATAGGA   
  
  
+ TATGATGTAG ATTGAATTCA AACTCATAAT TCATTAAGCA TGGTGCAATG TTCATTTTCT TGATTTTAAA   
  
  
+ CCGACTATTA CTTCAACTGT ATATAATTCG AATTGCTTTG GTATTTTAAA ATTTAAACTA GTAATTACTG   
  
  
+ CCTCTATTAC TGATGTTTAT ATTAAGTGGT GCTTAACTGC TTATTGATGA AACCATTTTT ATTTTCGTGT   
  
  
+ AAAAGAGGGC ATAATCCATC CTCGTGTATT GGCGGGGCTC GGCGCCCTAT TCAGCTATTC TCTGTATGTC   
  
  
+ AAACAAAACC GCTTCTATTA CCCATTGGTC TCTAAACACG CAGGGGTTTT AGCCCGGAGA GTTAATTCTT   
  
  
+ TATTGAGGTC CCCTGTTCTC CATTGCAGGA TTTTTGCTCA GGTACTTCTC TGTTTCTTCT CTTTAATCTT   
  
  
+ CTGTATCTTT CTGCATCAAG TTTAATGCTT TCTGGGATTT TCAGCTACGA TTTATGTGAA ATTGTAAGAT   
  
  
+ TTCTGAACTG GGCATTCAGG GCTTGGTTCA TATGTATTCT TTTCTGCTTG AAATGTTTTA ATTTCATAGT   
  
  
+ TCCAATGGGG TTATTCAAAG ATAGAATTAA GCTATAATCA CCGAGAAGAC TTGCTGACCT TTGTTTCTTT   
  
  
+ TTTGTTTCTT TTTTTTAATT AAATCTGGCT ATTACTTGGC TATAGTTTTT GCTAATGGAT CCACAAATAG   
  
  
+ ACGAATTATA TGGGCCTTTG CACCAAATGA AGTTCAATGA TCAGAGGAGA CCCATTTTAT CAAATGAAAA   
  
  
+ TTTTGTTAAT GTGTTGAAAC CGCCAAATTC CAATTGGAAT TCACCTTTTG GGAACCCTAC TGTGATTACC   
  
  
+ ACAAACCCAA ATTTTGATTT GGTTGTGCCA TCACTAGGTC CCAATGTTGA GGGAGCTCTC CTTGAAGATT   
  
  
+ ATGATTTTAG TGATGTTGTT CTTAAGTACA TCAATCAAGT TCTTATGGAC GAGGATGAGG GAGAGAAAAT   
  
  
+ CCATTTGGGC CACGGGCCTC TGGCTCTTGA AGCGGCCGAG AAGTCGTTAT ATGAGGTGCT TGGGCAGAGC   
  
  
+ CATTCTCCAC AAAATCAATA CTCTCCAGAG GACAAGCGTG ATGACCTGAG CAAGAGTAGT GTTAGAAGCT   
  
  
+ GTGTCAGTTA TAGCTGTGAC GGTGCAAGTA GTGGCAGTGG CATCGTGGAG CAAAGTTGGT CGAGTTATCC   
  
  
+ TCCGGAGTAC AATCCCATGC GGATGGCAGA TTTTCCTGCC TTGCCCTCAA AACCCTTGTT GAGAACATCT   
  
  
+ TATAGCTTAT CAAATAGCTT GACAGGTGAG GGTAATGGTT TGGTGGATAG ACCGGCAGAC TCACCTGTAA   
  
  
+ GCGCTCTTAG TGTGTCTGAT ATATTTAATG ATAGTCAGTC AGTGATACAA TTTCAGAAAG GGATGGAGGA   
  
  
+ AGCGAGCAAA TTTCTTCCCA AGGCTTCTTT GTGTTTGGAT TTGAACACTG GACCCTGCCT TCGGGAGTCG   
  
  
+ GTCAATAAGG CACGAGGTGG GCTGATTTTG GTGGAGACTA GCCATGAAAA TGAGGTTGCA GTGCTAGCTA   
  
  
+ GTAGGGGAAA GAAGCATCGC CATCCTGAGG AATTACGTTC AGAAGTTGGG AGGGGCAGTA AGCAGCCAGT   
  
  
+ TGTTTTCCAG TTTTCAGACG AGGCAGTTGT GAGATCAGAA ATGTTTGATA GGGTATTGCT GTGCAGTGGT   
  
  
+ GGCAAAAATG ATGCTGACCT ACGCCAAACT TTACAGAGTG AACTAAATAA AACTATGCAA AATGCTCAAA   
  
  
+ TGAAAGATTC TAATGGTGGA AAAGGCCGTG GTAGGAAGCA AGTTGGCAGG AGGGATGTGG TGGATCTAAG   
  
  
+ ATCTCTTTTA CGCCTTTGTG CACAAGCAGT TGGATCCAAT GACCAAAGAA GCGCAAATGA CTTAATTAGG   
  
  
+ CAAATTAGGA AGCATTCTTC TCCAACCGGG GATGGGAATC AAAGAATGGC TCATTATTTT GCTGATGGTC   
  
  
+ TTGAGGCTCG GCTTGCTGGT GTTGGGACCC CAATATACAA GTCTCTCGTG ACTGGCCCAG CTTCTGCTGT   
  
  
+ TGATATCTTG AGAGCTTACC ATATGTTTCT TGCCACATGC CCATTCAAGA AAATAGGGAA TTTCTTCTCT   
  
  
+ AATAGAACAA TTATGGATGT AGCTCAGAAA GCAAATCGAC TTCATATTAT TGATCTTGGT ATTGTCTATG   
  
  
+ GTTTCCAATG GCCTTGTCTA ATTCAGCGCC TTTCATCTAG GCCTGGCGGG CCTCCCAAAC TTCGGATTAC   
  
  
+ AGGCGTTGAT CTCCCCCAAC CCGGTTTCCG ACCAGCCAAA AGAGTCGAGG AGACTGGGCG ACGCTTGAGG   
  
  
+ AACTATGCTG AGTCATTCAA TGTGCCATTT GAGTTCAATG CCATAGCAAA GAGCTGGGAA ACACTTACTG   
  
  
+ TTAAAGATCT CAAGATTGAT CCCGATGAGG TGCTTGTTGT GAACTGTCTG TTCAGGTTTA AATACATTCC   
  
  
+ TGAGGAAACA GTAATTGCAG AATGCCCAAG AGATATTGTT CTTAATCTGA TCAGACAGAT AAAACCAGCT   
  
  
+ GTTTTCATAC ATGGTGTAGT CAATGGAGCC TTCAATTCTC CTTTTTTCAT TATTCGGTTC CGTGAGGCTC   
  
  
+ TCTTCCACTT CTCCACTCTA TTTGATGTGT TGGAGGCAAA TCTTCCCCGG GACATCGAGG AGAGGATACT   
  
  
+ GATAGAGCGA GACATCTTTG GGCGACAGGC AATGAATGTG ATCGCTTGTG AGGGTTTGGA GAGGATAGAA   
  
  
+ AGGCCAGAGA AATACAAACA GTGGCGGGTC CGAAATGAGA GGGCTGGGTT GAAGCAGCTG CCTTTGAATC   
  
  
+ AGGAGACTGT GGAAATGGCC AAAAAACGGG TGACAGCTGT CTATCACAAA GATTTCTCCA TTGATGACGA   
  
  
+ TGGGCACTGG TTGTTACAGG GATGGAAAGG GAGAATTGTC TATGCACTCA CTACTTGGAA GCCTGCTGAC   
  
  
+ TA  

- -Up\_Stream \_Len000AAACAT AAAGTTATAA CATTAATCCA CCCTATATCT CAACTTCCCG TTTGAGATGG   
  
  
- TTGCTGAACA ACTTCCTAGT TGAACATCGA ACCTTGAATT ACATACACAT GATATCTCAA CACATTAGAG   
  
  
- AGGATTATTT TAACCCAATT AGGATCTTAA TCTCTTGAAA TTCGCCCCTG ATTCGAACCA ACTCCGATTG   
  
  
- AAGCAATTTT TCAAGAACAC AGTACAGGTG CAAATAGAAA AGTGATAAAT AAAGTTAATT AAACGCTATA   
  
  
- AATAGTAGTG GGATAAGTGG CAGAAAAGCC TACTAAATTG ATCAGTTCGG CCTGGTTGTA ACGAAAAATA   
  
  
- TCACATAAAC GTTGAGTACA ACTAATAATC TACATAGTTG TTTTTTACAA AGAGTTTTAT TTATCATCGA   
  
  
- GAAATTTAAA ATAAGGTGTA TTTAAGTTTT AGTTATAGCT ATAGCAAGGT ATTATATCGT CGTAATACTT   
  
  
- TTGTTATAAA CGATTACAAA TGTAACACGA GAAAAGTGAG TTTTTAACAT TCCGAAGTTT ATGATAATTT   
  
  
- CTTTGGTAAT AAAGTGAGCT GATAAATCGA ACGTATATAT CTGTTAGAAC GTTTATCATT GTACAGAAGT   
  
  
- AAGCTGATAA ACGAAGGTTT ACTTATTAGA GCATTTGTAT ATATGTATGT ATGTATATAT ATATATATAT   
  
  
- ATATATATAT ATATATATAT CATTATACAT AAATTGAAGT GTATATGTAC TTATATTTTT ATATTCTCCC   
  
  
- TTTTTTCTGC GATCCATCTA CCTGATGTTT TCGGTAAGTA CATAACTCTG CCGAGTATTT TAGTATGCCC   
  
  
- CTTTCCTTTT TTTTTCTAGA GATGTATTAC AAGTATCTAA ATATCTTTCG TATACTACCT AATGGCGTAT   
  
  
- TCCACAATAT ATACCCTCAC AAACATCTTC TTTCTACATA TAGGCTTGAT AGTTTCTATG TATACTCCCT   
  
  
- CATTTTTGTT CACATTCTTG GAGTCCTCCT CTGTATTTTC TAAAAAGATA ACCGTAACCT GTGGCAGTTC   
  
  
- ATAGGCGAAA TTTGGACAAG AAAAAGTGAT AATAGTACCT ACATAAGTGT TTTCCTTAAG TTATGCTTCA   
  
  
- TGGTACTACA TACGAAAAAT GACTATCATA ACCAGAGTAG CTACTCTGAT TCCTACCAGA ATTATCGTTT   
  
  
- AATCTCTAAA GTAATCTATT TTAGATGACA TGTACTGTTT TGTTACTATA TATCGTAGGA TAAATTTTTC   
  
  
- GATGCACAGT CAGTATATAC TTAACGTAGA TTAAAAATTA TCTATATATT ATACTATACT ATATTATCCT   
  
  
- ATACTACATC TAACTTAAGT TTGAGTATTA AGTAATTCGT ACCACGTTAC AAGTAAAAGA ACTAAAATTT   
  
  
- GGCTGATAAT GAAGTTGACA TATATTAAGC TTAACGAAAC CATAAAATTT TAAATTTGAT CATTAATGAC   
  
  
- GGAGATAATG ACTACAAATA TAATTCACCA CGAATTGACG AATAACTACT TTGGTAAAAA TAAAAGCACA   
  
  
- TTTTCTCCCG TATTAGGTAG GAGCACATAA CCGCCCCGAG CCGCGGGATA AGTCGATAAG AGACATACAG   
  
  
- TTTGTTTTGG CGAAGATAAT GGGTAACCAG AGATTTGTGC GTCCCCAAAA TCGGGCCTCT CAATTAAGAA   
  
  
- ATAACTCCAG GGGACAAGAG GTAACGTCCT AAAAACGAGT CCATGAAGAG ACAAAGAAGA GAAATTAGAA   
  
  
- GACATAGAAA GACGTAGTTC AAATTACGAA AGACCCTAAA AGTCGATGCT AAATACACTT TAACATTCTA   
  
  
- AAGACTTGAC CCGTAAGTCC CGAACCAAGT ATACATAAGA AAAGACGAAC TTTACAAAAT TAAAGTATCA   
  
  
- AGGTTACCCC AATAAGTTTC TATCTTAATT CGATATTAGT GGCTCTTCTG AACGACTGGA AACAAAGAAA   
  
  
- AAACAAAGAA AAAAAATTAA TTTAGACCGA TAATGAACCG ATATCAAAAA CGATTACCTA GGTGTTTATC   
  
  
- TGCTTAATAT ACCCGGAAAC GTGGTTTACT TCAAGTTACT AGTCTCCTCT GGGTAAAATA GTTTACTTTT   
  
  
- AAAACAATTA CACAACTTTG GCGGTTTAAG GTTAACCTTA AGTGGAAAAC CCTTGGGATG ACACTAATGG   
  
  
- TGTTTGGGTT TAAAACTAAA CCAACACGGT AGTGATCCAG GGTTACAACT CCCTCGAGAG GAACTTCTAA   
  
  
- TACTAAAATC ACTACAACAA GAATTCATGT AGTTAGTTCA AGAATACCTG CTCCTACTCC CTCTCTTTTA   
  
  
- GGTAAACCCG GTGCCCGGAG ACCGAGAACT TCGCCGGCTC TTCAGCAATA TACTCCACGA ACCCGTCTCG   
  
  
- GTAAGAGGTG TTTTAGTTAT GAGAGGTCTC CTGTTCGCAC TACTGGACTC GTTCTCATCA CAATCTTCGA   
  
  
- CACAGTCAAT ATCGACACTG CCACGTTCAT CACCGTCACC GTAGCACCTC GTTTCAACCA GCTCAATAGG   
  
  
- AGGCCTCATG TTAGGGTACG CCTACCGTCT AAAAGGACGG AACGGGAGTT TTGGGAACAA CTCTTGTAGA   
  
  
- ATATCGAATA GTTTATCGAA CTGTCCACTC CCATTACCAA ACCACCTATC TGGCCGTCTG AGTGGACATT   
  
  
- CGCGAGAATC ACACAGACTA TATAAATTAC TATCAGTCAG TCACTATGTT AAAGTCTTTC CCTACCTCCT   
  
  
- TCGCTCGTTT AAAGAAGGGT TCCGAAGAAA CACAAACCTA AACTTGTGAC CTGGGACGGA AGCCCTCAGC   
  
  
- CAGTTATTCC GTGCTCCACC CGACTAAAAC CACCTCTGAT CGGTACTTTT ACTCCAACGT CACGATCGAT   
  
  
- CATCCCCTTT CTTCGTAGCG GTAGGACTCC TTAATGCAAG TCTTCAACCC TCCCCGTCAT TCGTCGGTCA   
  
  
- ACAAAAGGTC AAAAGTCTGC TCCGTCAACA CTCTAGTCTT TACAAACTAT CCCATAACGA CACGTCACCA   
  
  
- CCGTTTTTAC TACGACTGGA TGCGGTTTGA AATGTCTCAC TTGATTTATT TTGATACGTT TTACGAGTTT   
  
  
- ACTTTCTAAG ATTACCACCT TTTCCGGCAC CATCCTTCGT TCAACCGTCC TCCCTACACC ACCTAGATTC   
  
  
- TAGAGAAAAT GCGGAAACAC GTGTTCGTCA ACCTAGGTTA CTGGTTTCTT CGCGTTTACT GAATTAATCC   
  
  
- GTTTAATCCT TCGTAAGAAG AGGTTGGCCC CTACCCTTAG TTTCTTACCG AGTAATAAAA CGACTACCAG   
  
  
- AACTCCGAGC CGAACGACCA CAACCCTGGG GTTATATGTT CAGAGAGCAC TGACCGGGTC GAAGACGACA   
  
  
- ACTATAGAAC TCTCGAATGG TATACAAAGA ACGGTGTACG GGTAAGTTCT TTTATCCCTT AAAGAAGAGA   
  
  
- TTATCTTGTT AATACCTACA TCGAGTCTTT CGTTTAGCTG AAGTATAATA ACTAGAACCA TAACAGATAC   
  
  
- CAAAGGTTAC CGGAACAGAT TAAGTCGCGG AAAGTAGATC CGGACCGCCC GGAGGGTTTG AAGCCTAATG   
  
  
- TCCGCAACTA GAGGGGGTTG GGCCAAAGGC TGGTCGGTTT TCTCAGCTCC TCTGACCCGC TGCGAACTCC   
  
  
- TTGATACGAC TCAGTAAGTT ACACGGTAAA CTCAAGTTAC GGTATCGTTT CTCGACCCTT TGTGAATGAC   
  
  
- AATTTCTAGA GTTCTAACTA GGGCTACTCC ACGAACAACA CTTGACAGAC AAGTCCAAAT TTATGTAAGG   
  
  
- ACTCCTTTGT CATTAACGTC TTACGGGTTC TCTATAACAA GAATTAGACT AGTCTGTCTA TTTTGGTCGA   
  
  
- CAAAAGTATG TACCACATCA GTTACCTCGG AAGTTAAGAG GAAAAAAGTA ATAAGCCAAG GCACTCCGAG   
  
  
- AGAAGGTGAA GAGGTGAGAT AAACTACACA ACCTCCGTTT AGAAGGGGCC CTGTAGCTCC TCTCCTATGA   
  
  
- CTATCTCGCT CTGTAGAAAC CCGCTGTCCG TTACTTACAC TAGCGAACAC TCCCAAACCT CTCCTATCTT   
  
  
- TCCGGTCTCT TTATGTTTGT CACCGCCCAG GCTTTACTCT CCCGACCCAA CTTCGTCGAC GGAAACTTAG   
  
  
- TCCTCTGACA CCTTTACCGG TTTTTTGCCC ACTGTCGACA GATAGTGTTT CTAAAGAGGT AACTACTGCT   
  
  
- ACCCGTGACC AACAATGTCC CTACCTTTCC CTCTTAACAG ATACGTGAGT GATGAACCTT CGGACGACTG   
  
  
- AT

+     MYC

| Site Name | Organism | Position | Strand | Matrix score. | sequence | function |
| --- | --- | --- | --- | --- | --- | --- |
| MYC | Arabidopsis thaliana | 2316 | + | 6 | CATTTG |  |
| MYC | Arabidopsis thaliana | 3081 | - | 6 | CATTTG |  |
| MYC | Arabidopsis thaliana | 3208 | - | 6 | CATTTG |  |
| MYC | Arabidopsis thaliana | 2058 | - | 6 | CATTTG |  |
| MYC | Arabidopsis thaliana | 651 | - | 6 | CATTTG |  |
| MYC | Arabidopsis thaliana | 3670 | + | 6 | CATTTG |  |
| MYC | Arabidopsis thaliana | 3398 | - | 6 | CATGTG |  |
| MYC | Arabidopsis thaliana | 2135 | + | 6 | CAATTG |  |
| MYC | Arabidopsis thaliana | 2095 | - | 6 | CATTTG |  |

>HU05G01597.1   
+ -Up\_Stream \_Len000TTTGTA TTTCAATATT GTAATTAGGT GGGATATAGA GTTGAAGGGC AAACTCTACC   
  
  
+ AACGACTTGT TGAAGGATCA ACTTGTAGCT TGGAACTTAA TGTATGTGTA CTATAGAGTT GTGTAATCTC   
  
  
+ TCCTAATAAA ATTGGGTTAA TCCTAGAATT AGAGAACTTT AAGCGGGGAC TAAGCTTGGT TGAGGCTAAC   
  
  
+ TTCGTTAAAA AGTTCTTGTG TCATGTCCAC GTTTATCTTT TCACTATTTA TTTCAATTAA TTTGCGATAT   
  
  
+ TTATCATCAC CCTATTCACC GTCTTTTCGG ATGATTTAAC TAGTCAAGCC GGACCAACAT TGCTTTTTAT   
  
  
+ AGTGTATTTG CAACTCATGT TGATTATTAG ATGTATCAAC AAAAAATGTT TCTCAAAATA AATAGTAGCT   
  
  
+ CTTTAAATTT TATTCCACAT AAATTCAAAA TCAATATCGA TATCGTTCCA TAATATAGCA GCATTATGAA   
  
  
+ AACAATATTT GCTAATGTTT ACATTGTGCT CTTTTCACTC AAAAATTGTA AGGCTTCAAA TACTATTAAA   
  
  
+ GAAACCATTA TTTCACTCGA CTATTTAGCT TGCATATATA GACAATCTTG CAAATAGTAA CATGTCTTCA   
  
  
+ TTCGACTATT TGCTTCCAAA TGAATAATCT CGTAAACATA TATACATACA TACATATATA TATATATATA   
  
  
+ TATATATATA TATATATATA GTAATATGTA TTTAACTTCA CATATACATG AATATAAAAA TATAAGAGGG   
  
  
+ AAAAAAGACG CTAGGTAGAT GGACTACAAA AGCCATTCAT GTATTGAGAC GGCTCATAAA ATCATACGGG   
  
  
+ GAAAGGAAAA AAAAAGATCT CTACATAATG TTCATAGATT TATAGAAAGC ATATGATGGA TTACCGCATA   
  
  
+ AGGTGTTATA TATGGGAGTG TTTGTAGAAG AAAGATGTAT ATCCGAACTA TCAAAGATAC ATATGAGGGA   
  
  
+ GTAAAAACAA GTGTAAGAAC CTCAGGAGGA GACATAAAAG ATTTTTCTAT TGGCATTGGA CACCGTCAAG   
  
  
+ TATCCGCTTT AAACCTGTTC TTTTTCACTA TTATCATGGA TGTATTCACA AAAGGAATTC AATACGAAGT   
  
  
+ ACCATGATGT ATGCTTTTTA CTGATAGTAT TGGTCTCATC GATGAGACTA AGGATGGTCT TAATAGCAAA   
  
  
+ TTAGAGATTT CATTAGATAA AATCTACTGT ACATGACAAA ACAATGATAT ATAGCATCCT ATTTAAAAAG   
  
  
+ CTACGTGTCA GTCATATATG AATTGCATCT AATTTTTAAT AGATATATAA TATGATATGA TATAATAGGA   
  
  
+ TATGATGTAG ATTGAATTCA AACTCATAAT TCATTAAGCA TGGTGCAATG TTCATTTTCT TGATTTTAAA   
  
  
+ CCGACTATTA CTTCAACTGT ATATAATTCG AATTGCTTTG GTATTTTAAA ATTTAAACTA GTAATTACTG   
  
  
+ CCTCTATTAC TGATGTTTAT ATTAAGTGGT GCTTAACTGC TTATTGATGA AACCATTTTT ATTTTCGTGT   
  
  
+ AAAAGAGGGC ATAATCCATC CTCGTGTATT GGCGGGGCTC GGCGCCCTAT TCAGCTATTC TCTGTATGTC   
  
  
+ AAACAAAACC GCTTCTATTA CCCATTGGTC TCTAAACACG CAGGGGTTTT AGCCCGGAGA GTTAATTCTT   
  
  
+ TATTGAGGTC CCCTGTTCTC CATTGCAGGA TTTTTGCTCA GGTACTTCTC TGTTTCTTCT CTTTAATCTT   
  
  
+ CTGTATCTTT CTGCATCAAG TTTAATGCTT TCTGGGATTT TCAGCTACGA TTTATGTGAA ATTGTAAGAT   
  
  
+ TTCTGAACTG GGCATTCAGG GCTTGGTTCA TATGTATTCT TTTCTGCTTG AAATGTTTTA ATTTCATAGT   
  
  
+ TCCAATGGGG TTATTCAAAG ATAGAATTAA GCTATAATCA CCGAGAAGAC TTGCTGACCT TTGTTTCTTT   
  
  
+ TTTGTTTCTT TTTTTTAATT AAATCTGGCT ATTACTTGGC TATAGTTTTT GCTAATGGAT CCACAAATAG   
  
  
+ ACGAATTATA TGGGCCTTTG CACCAAATGA AGTTCAATGA TCAGAGGAGA CCCATTTTAT CAAATGAAAA   
  
  
+ TTTTGTTAAT GTGTTGAAAC CGCCAAATTC CAATTGGAAT TCACCTTTTG GGAACCCTAC TGTGATTACC   
  
  
+ ACAAACCCAA ATTTTGATTT GGTTGTGCCA TCACTAGGTC CCAATGTTGA GGGAGCTCTC CTTGAAGATT   
  
  
+ ATGATTTTAG TGATGTTGTT CTTAAGTACA TCAATCAAGT TCTTATGGAC GAGGATGAGG GAGAGAAAAT   
  
  
+ CCATTTGGGC CACGGGCCTC TGGCTCTTGA AGCGGCCGAG AAGTCGTTAT ATGAGGTGCT TGGGCAGAGC   
  
  
+ CATTCTCCAC AAAATCAATA CTCTCCAGAG GACAAGCGTG ATGACCTGAG CAAGAGTAGT GTTAGAAGCT   
  
  
+ GTGTCAGTTA TAGCTGTGAC GGTGCAAGTA GTGGCAGTGG CATCGTGGAG CAAAGTTGGT CGAGTTATCC   
  
  
+ TCCGGAGTAC AATCCCATGC GGATGGCAGA TTTTCCTGCC TTGCCCTCAA AACCCTTGTT GAGAACATCT   
  
  
+ TATAGCTTAT CAAATAGCTT GACAGGTGAG GGTAATGGTT TGGTGGATAG ACCGGCAGAC TCACCTGTAA   
  
  
+ GCGCTCTTAG TGTGTCTGAT ATATTTAATG ATAGTCAGTC AGTGATACAA TTTCAGAAAG GGATGGAGGA   
  
  
+ AGCGAGCAAA TTTCTTCCCA AGGCTTCTTT GTGTTTGGAT TTGAACACTG GACCCTGCCT TCGGGAGTCG   
  
  
+ GTCAATAAGG CACGAGGTGG GCTGATTTTG GTGGAGACTA GCCATGAAAA TGAGGTTGCA GTGCTAGCTA   
  
  
+ GTAGGGGAAA GAAGCATCGC CATCCTGAGG AATTACGTTC AGAAGTTGGG AGGGGCAGTA AGCAGCCAGT   
  
  
+ TGTTTTCCAG TTTTCAGACG AGGCAGTTGT GAGATCAGAA ATGTTTGATA GGGTATTGCT GTGCAGTGGT   
  
  
+ GGCAAAAATG ATGCTGACCT ACGCCAAACT TTACAGAGTG AACTAAATAA AACTATGCAA AATGCTCAAA   
  
  
+ TGAAAGATTC TAATGGTGGA AAAGGCCGTG GTAGGAAGCA AGTTGGCAGG AGGGATGTGG TGGATCTAAG   
  
  
+ ATCTCTTTTA CGCCTTTGTG CACAAGCAGT TGGATCCAAT GACCAAAGAA GCGCAAATGA CTTAATTAGG   
  
  
+ CAAATTAGGA AGCATTCTTC TCCAACCGGG GATGGGAATC AAAGAATGGC TCATTATTTT GCTGATGGTC   
  
  
+ TTGAGGCTCG GCTTGCTGGT GTTGGGACCC CAATATACAA GTCTCTCGTG ACTGGCCCAG CTTCTGCTGT   
  
  
+ TGATATCTTG AGAGCTTACC ATATGTTTCT TGCCACATGC CCATTCAAGA AAATAGGGAA TTTCTTCTCT   
  
  
+ AATAGAACAA TTATGGATGT AGCTCAGAAA GCAAATCGAC TTCATATTAT TGATCTTGGT ATTGTCTATG   
  
  
+ GTTTCCAATG GCCTTGTCTA ATTCAGCGCC TTTCATCTAG GCCTGGCGGG CCTCCCAAAC TTCGGATTAC   
  
  
+ AGGCGTTGAT CTCCCCCAAC CCGGTTTCCG ACCAGCCAAA AGAGTCGAGG AGACTGGGCG ACGCTTGAGG   
  
  
+ AACTATGCTG AGTCATTCAA TGTGCCATTT GAGTTCAATG CCATAGCAAA GAGCTGGGAA ACACTTACTG   
  
  
+ TTAAAGATCT CAAGATTGAT CCCGATGAGG TGCTTGTTGT GAACTGTCTG TTCAGGTTTA AATACATTCC   
  
  
+ TGAGGAAACA GTAATTGCAG AATGCCCAAG AGATATTGTT CTTAATCTGA TCAGACAGAT AAAACCAGCT   
  
  
+ GTTTTCATAC ATGGTGTAGT CAATGGAGCC TTCAATTCTC CTTTTTTCAT TATTCGGTTC CGTGAGGCTC   
  
  
+ TCTTCCACTT CTCCACTCTA TTTGATGTGT TGGAGGCAAA TCTTCCCCGG GACATCGAGG AGAGGATACT   
  
  
+ GATAGAGCGA GACATCTTTG GGCGACAGGC AATGAATGTG ATCGCTTGTG AGGGTTTGGA GAGGATAGAA   
  
  
+ AGGCCAGAGA AATACAAACA GTGGCGGGTC CGAAATGAGA GGGCTGGGTT GAAGCAGCTG CCTTTGAATC   
  
  
+ AGGAGACTGT GGAAATGGCC AAAAAACGGG TGACAGCTGT CTATCACAAA GATTTCTCCA TTGATGACGA   
  
  
+ TGGGCACTGG TTGTTACAGG GATGGAAAGG GAGAATTGTC TATGCACTCA CTACTTGGAA GCCTGCTGAC   
  
  
+ TA  

- -Up\_Stream \_Len000AAACAT AAAGTTATAA CATTAATCCA CCCTATATCT CAACTTCCCG TTTGAGATGG   
  
  
- TTGCTGAACA ACTTCCTAGT TGAACATCGA ACCTTGAATT ACATACACAT GATATCTCAA CACATTAGAG   
  
  
- AGGATTATTT TAACCCAATT AGGATCTTAA TCTCTTGAAA TTCGCCCCTG ATTCGAACCA ACTCCGATTG   
  
  
- AAGCAATTTT TCAAGAACAC AGTACAGGTG CAAATAGAAA AGTGATAAAT AAAGTTAATT AAACGCTATA   
  
  
- AATAGTAGTG GGATAAGTGG CAGAAAAGCC TACTAAATTG ATCAGTTCGG CCTGGTTGTA ACGAAAAATA   
  
  
- TCACATAAAC GTTGAGTACA ACTAATAATC TACATAGTTG TTTTTTACAA AGAGTTTTAT TTATCATCGA   
  
  
- GAAATTTAAA ATAAGGTGTA TTTAAGTTTT AGTTATAGCT ATAGCAAGGT ATTATATCGT CGTAATACTT   
  
  
- TTGTTATAAA CGATTACAAA TGTAACACGA GAAAAGTGAG TTTTTAACAT TCCGAAGTTT ATGATAATTT   
  
  
- CTTTGGTAAT AAAGTGAGCT GATAAATCGA ACGTATATAT CTGTTAGAAC GTTTATCATT GTACAGAAGT   
  
  
- AAGCTGATAA ACGAAGGTTT ACTTATTAGA GCATTTGTAT ATATGTATGT ATGTATATAT ATATATATAT   
  
  
- ATATATATAT ATATATATAT CATTATACAT AAATTGAAGT GTATATGTAC TTATATTTTT ATATTCTCCC   
  
  
- TTTTTTCTGC GATCCATCTA CCTGATGTTT TCGGTAAGTA CATAACTCTG CCGAGTATTT TAGTATGCCC   
  
  
- CTTTCCTTTT TTTTTCTAGA GATGTATTAC AAGTATCTAA ATATCTTTCG TATACTACCT AATGGCGTAT   
  
  
- TCCACAATAT ATACCCTCAC AAACATCTTC TTTCTACATA TAGGCTTGAT AGTTTCTATG TATACTCCCT   
  
  
- CATTTTTGTT CACATTCTTG GAGTCCTCCT CTGTATTTTC TAAAAAGATA ACCGTAACCT GTGGCAGTTC   
  
  
- ATAGGCGAAA TTTGGACAAG AAAAAGTGAT AATAGTACCT ACATAAGTGT TTTCCTTAAG TTATGCTTCA   
  
  
- TGGTACTACA TACGAAAAAT GACTATCATA ACCAGAGTAG CTACTCTGAT TCCTACCAGA ATTATCGTTT   
  
  
- AATCTCTAAA GTAATCTATT TTAGATGACA TGTACTGTTT TGTTACTATA TATCGTAGGA TAAATTTTTC   
  
  
- GATGCACAGT CAGTATATAC TTAACGTAGA TTAAAAATTA TCTATATATT ATACTATACT ATATTATCCT   
  
  
- ATACTACATC TAACTTAAGT TTGAGTATTA AGTAATTCGT ACCACGTTAC AAGTAAAAGA ACTAAAATTT   
  
  
- GGCTGATAAT GAAGTTGACA TATATTAAGC TTAACGAAAC CATAAAATTT TAAATTTGAT CATTAATGAC   
  
  
- GGAGATAATG ACTACAAATA TAATTCACCA CGAATTGACG AATAACTACT TTGGTAAAAA TAAAAGCACA   
  
  
- TTTTCTCCCG TATTAGGTAG GAGCACATAA CCGCCCCGAG CCGCGGGATA AGTCGATAAG AGACATACAG   
  
  
- TTTGTTTTGG CGAAGATAAT GGGTAACCAG AGATTTGTGC GTCCCCAAAA TCGGGCCTCT CAATTAAGAA   
  
  
- ATAACTCCAG GGGACAAGAG GTAACGTCCT AAAAACGAGT CCATGAAGAG ACAAAGAAGA GAAATTAGAA   
  
  
- GACATAGAAA GACGTAGTTC AAATTACGAA AGACCCTAAA AGTCGATGCT AAATACACTT TAACATTCTA   
  
  
- AAGACTTGAC CCGTAAGTCC CGAACCAAGT ATACATAAGA AAAGACGAAC TTTACAAAAT TAAAGTATCA   
  
  
- AGGTTACCCC AATAAGTTTC TATCTTAATT CGATATTAGT GGCTCTTCTG AACGACTGGA AACAAAGAAA   
  
  
- AAACAAAGAA AAAAAATTAA TTTAGACCGA TAATGAACCG ATATCAAAAA CGATTACCTA GGTGTTTATC   
  
  
- TGCTTAATAT ACCCGGAAAC GTGGTTTACT TCAAGTTACT AGTCTCCTCT GGGTAAAATA GTTTACTTTT   
  
  
- AAAACAATTA CACAACTTTG GCGGTTTAAG GTTAACCTTA AGTGGAAAAC CCTTGGGATG ACACTAATGG   
  
  
- TGTTTGGGTT TAAAACTAAA CCAACACGGT AGTGATCCAG GGTTACAACT CCCTCGAGAG GAACTTCTAA   
  
  
- TACTAAAATC ACTACAACAA GAATTCATGT AGTTAGTTCA AGAATACCTG CTCCTACTCC CTCTCTTTTA   
  
  
- GGTAAACCCG GTGCCCGGAG ACCGAGAACT TCGCCGGCTC TTCAGCAATA TACTCCACGA ACCCGTCTCG   
  
  
- GTAAGAGGTG TTTTAGTTAT GAGAGGTCTC CTGTTCGCAC TACTGGACTC GTTCTCATCA CAATCTTCGA   
  
  
- CACAGTCAAT ATCGACACTG CCACGTTCAT CACCGTCACC GTAGCACCTC GTTTCAACCA GCTCAATAGG   
  
  
- AGGCCTCATG TTAGGGTACG CCTACCGTCT AAAAGGACGG AACGGGAGTT TTGGGAACAA CTCTTGTAGA   
  
  
- ATATCGAATA GTTTATCGAA CTGTCCACTC CCATTACCAA ACCACCTATC TGGCCGTCTG AGTGGACATT   
  
  
- CGCGAGAATC ACACAGACTA TATAAATTAC TATCAGTCAG TCACTATGTT AAAGTCTTTC CCTACCTCCT   
  
  
- TCGCTCGTTT AAAGAAGGGT TCCGAAGAAA CACAAACCTA AACTTGTGAC CTGGGACGGA AGCCCTCAGC   
  
  
- CAGTTATTCC GTGCTCCACC CGACTAAAAC CACCTCTGAT CGGTACTTTT ACTCCAACGT CACGATCGAT   
  
  
- CATCCCCTTT CTTCGTAGCG GTAGGACTCC TTAATGCAAG TCTTCAACCC TCCCCGTCAT TCGTCGGTCA   
  
  
- ACAAAAGGTC AAAAGTCTGC TCCGTCAACA CTCTAGTCTT TACAAACTAT CCCATAACGA CACGTCACCA   
  
  
- CCGTTTTTAC TACGACTGGA TGCGGTTTGA AATGTCTCAC TTGATTTATT TTGATACGTT TTACGAGTTT   
  
  
- ACTTTCTAAG ATTACCACCT TTTCCGGCAC CATCCTTCGT TCAACCGTCC TCCCTACACC ACCTAGATTC   
  
  
- TAGAGAAAAT GCGGAAACAC GTGTTCGTCA ACCTAGGTTA CTGGTTTCTT CGCGTTTACT GAATTAATCC   
  
  
- GTTTAATCCT TCGTAAGAAG AGGTTGGCCC CTACCCTTAG TTTCTTACCG AGTAATAAAA CGACTACCAG   
  
  
- AACTCCGAGC CGAACGACCA CAACCCTGGG GTTATATGTT CAGAGAGCAC TGACCGGGTC GAAGACGACA   
  
  
- ACTATAGAAC TCTCGAATGG TATACAAAGA ACGGTGTACG GGTAAGTTCT TTTATCCCTT AAAGAAGAGA   
  
  
- TTATCTTGTT AATACCTACA TCGAGTCTTT CGTTTAGCTG AAGTATAATA ACTAGAACCA TAACAGATAC   
  
  
- CAAAGGTTAC CGGAACAGAT TAAGTCGCGG AAAGTAGATC CGGACCGCCC GGAGGGTTTG AAGCCTAATG   
  
  
- TCCGCAACTA GAGGGGGTTG GGCCAAAGGC TGGTCGGTTT TCTCAGCTCC TCTGACCCGC TGCGAACTCC   
  
  
- TTGATACGAC TCAGTAAGTT ACACGGTAAA CTCAAGTTAC GGTATCGTTT CTCGACCCTT TGTGAATGAC   
  
  
- AATTTCTAGA GTTCTAACTA GGGCTACTCC ACGAACAACA CTTGACAGAC AAGTCCAAAT TTATGTAAGG   
  
  
- ACTCCTTTGT CATTAACGTC TTACGGGTTC TCTATAACAA GAATTAGACT AGTCTGTCTA TTTTGGTCGA   
  
  
- CAAAAGTATG TACCACATCA GTTACCTCGG AAGTTAAGAG GAAAAAAGTA ATAAGCCAAG GCACTCCGAG   
  
  
- AGAAGGTGAA GAGGTGAGAT AAACTACACA ACCTCCGTTT AGAAGGGGCC CTGTAGCTCC TCTCCTATGA   
  
  
- CTATCTCGCT CTGTAGAAAC CCGCTGTCCG TTACTTACAC TAGCGAACAC TCCCAAACCT CTCCTATCTT   
  
  
- TCCGGTCTCT TTATGTTTGT CACCGCCCAG GCTTTACTCT CCCGACCCAA CTTCGTCGAC GGAAACTTAG   
  
  
- TCCTCTGACA CCTTTACCGG TTTTTTGCCC ACTGTCGACA GATAGTGTTT CTAAAGAGGT AACTACTGCT   
  
  
- ACCCGTGACC AACAATGTCC CTACCTTTCC CTCTTAACAG ATACGTGAGT GATGAACCTT CGGACGACTG   
  
  
- AT

+     Myb

| Site Name | Organism | Position | Strand | Matrix score. | sequence | function |
| --- | --- | --- | --- | --- | --- | --- |
| Myb | Arabidopsis thaliana | 3181 | - | 6 | CAACTG |  |
| Myb | Arabidopsis thaliana | 2968 | - | 6 | CAACTG |  |
| Myb | Arabidopsis thaliana | 2459 | - | 6 | TAACTG |  |
| Myb | Arabidopsis thaliana | 1418 | + | 6 | CAACTG |  |
| Myb | Arabidopsis thaliana | 1508 | + | 6 | TAACTG |  |
| Myb | Arabidopsis thaliana | 2941 | - | 6 | CAACTG |  |

>HU05G01597.1   
+ -Up\_Stream \_Len000TTTGTA TTTCAATATT GTAATTAGGT GGGATATAGA GTTGAAGGGC AAACTCTACC   
  
  
+ AACGACTTGT TGAAGGATCA ACTTGTAGCT TGGAACTTAA TGTATGTGTA CTATAGAGTT GTGTAATCTC   
  
  
+ TCCTAATAAA ATTGGGTTAA TCCTAGAATT AGAGAACTTT AAGCGGGGAC TAAGCTTGGT TGAGGCTAAC   
  
  
+ TTCGTTAAAA AGTTCTTGTG TCATGTCCAC GTTTATCTTT TCACTATTTA TTTCAATTAA TTTGCGATAT   
  
  
+ TTATCATCAC CCTATTCACC GTCTTTTCGG ATGATTTAAC TAGTCAAGCC GGACCAACAT TGCTTTTTAT   
  
  
+ AGTGTATTTG CAACTCATGT TGATTATTAG ATGTATCAAC AAAAAATGTT TCTCAAAATA AATAGTAGCT   
  
  
+ CTTTAAATTT TATTCCACAT AAATTCAAAA TCAATATCGA TATCGTTCCA TAATATAGCA GCATTATGAA   
  
  
+ AACAATATTT GCTAATGTTT ACATTGTGCT CTTTTCACTC AAAAATTGTA AGGCTTCAAA TACTATTAAA   
  
  
+ GAAACCATTA TTTCACTCGA CTATTTAGCT TGCATATATA GACAATCTTG CAAATAGTAA CATGTCTTCA   
  
  
+ TTCGACTATT TGCTTCCAAA TGAATAATCT CGTAAACATA TATACATACA TACATATATA TATATATATA   
  
  
+ TATATATATA TATATATATA GTAATATGTA TTTAACTTCA CATATACATG AATATAAAAA TATAAGAGGG   
  
  
+ AAAAAAGACG CTAGGTAGAT GGACTACAAA AGCCATTCAT GTATTGAGAC GGCTCATAAA ATCATACGGG   
  
  
+ GAAAGGAAAA AAAAAGATCT CTACATAATG TTCATAGATT TATAGAAAGC ATATGATGGA TTACCGCATA   
  
  
+ AGGTGTTATA TATGGGAGTG TTTGTAGAAG AAAGATGTAT ATCCGAACTA TCAAAGATAC ATATGAGGGA   
  
  
+ GTAAAAACAA GTGTAAGAAC CTCAGGAGGA GACATAAAAG ATTTTTCTAT TGGCATTGGA CACCGTCAAG   
  
  
+ TATCCGCTTT AAACCTGTTC TTTTTCACTA TTATCATGGA TGTATTCACA AAAGGAATTC AATACGAAGT   
  
  
+ ACCATGATGT ATGCTTTTTA CTGATAGTAT TGGTCTCATC GATGAGACTA AGGATGGTCT TAATAGCAAA   
  
  
+ TTAGAGATTT CATTAGATAA AATCTACTGT ACATGACAAA ACAATGATAT ATAGCATCCT ATTTAAAAAG   
  
  
+ CTACGTGTCA GTCATATATG AATTGCATCT AATTTTTAAT AGATATATAA TATGATATGA TATAATAGGA   
  
  
+ TATGATGTAG ATTGAATTCA AACTCATAAT TCATTAAGCA TGGTGCAATG TTCATTTTCT TGATTTTAAA   
  
  
+ CCGACTATTA CTTCAACTGT ATATAATTCG AATTGCTTTG GTATTTTAAA ATTTAAACTA GTAATTACTG   
  
  
+ CCTCTATTAC TGATGTTTAT ATTAAGTGGT GCTTAACTGC TTATTGATGA AACCATTTTT ATTTTCGTGT   
  
  
+ AAAAGAGGGC ATAATCCATC CTCGTGTATT GGCGGGGCTC GGCGCCCTAT TCAGCTATTC TCTGTATGTC   
  
  
+ AAACAAAACC GCTTCTATTA CCCATTGGTC TCTAAACACG CAGGGGTTTT AGCCCGGAGA GTTAATTCTT   
  
  
+ TATTGAGGTC CCCTGTTCTC CATTGCAGGA TTTTTGCTCA GGTACTTCTC TGTTTCTTCT CTTTAATCTT   
  
  
+ CTGTATCTTT CTGCATCAAG TTTAATGCTT TCTGGGATTT TCAGCTACGA TTTATGTGAA ATTGTAAGAT   
  
  
+ TTCTGAACTG GGCATTCAGG GCTTGGTTCA TATGTATTCT TTTCTGCTTG AAATGTTTTA ATTTCATAGT   
  
  
+ TCCAATGGGG TTATTCAAAG ATAGAATTAA GCTATAATCA CCGAGAAGAC TTGCTGACCT TTGTTTCTTT   
  
  
+ TTTGTTTCTT TTTTTTAATT AAATCTGGCT ATTACTTGGC TATAGTTTTT GCTAATGGAT CCACAAATAG   
  
  
+ ACGAATTATA TGGGCCTTTG CACCAAATGA AGTTCAATGA TCAGAGGAGA CCCATTTTAT CAAATGAAAA   
  
  
+ TTTTGTTAAT GTGTTGAAAC CGCCAAATTC CAATTGGAAT TCACCTTTTG GGAACCCTAC TGTGATTACC   
  
  
+ ACAAACCCAA ATTTTGATTT GGTTGTGCCA TCACTAGGTC CCAATGTTGA GGGAGCTCTC CTTGAAGATT   
  
  
+ ATGATTTTAG TGATGTTGTT CTTAAGTACA TCAATCAAGT TCTTATGGAC GAGGATGAGG GAGAGAAAAT   
  
  
+ CCATTTGGGC CACGGGCCTC TGGCTCTTGA AGCGGCCGAG AAGTCGTTAT ATGAGGTGCT TGGGCAGAGC   
  
  
+ CATTCTCCAC AAAATCAATA CTCTCCAGAG GACAAGCGTG ATGACCTGAG CAAGAGTAGT GTTAGAAGCT   
  
  
+ GTGTCAGTTA TAGCTGTGAC GGTGCAAGTA GTGGCAGTGG CATCGTGGAG CAAAGTTGGT CGAGTTATCC   
  
  
+ TCCGGAGTAC AATCCCATGC GGATGGCAGA TTTTCCTGCC TTGCCCTCAA AACCCTTGTT GAGAACATCT   
  
  
+ TATAGCTTAT CAAATAGCTT GACAGGTGAG GGTAATGGTT TGGTGGATAG ACCGGCAGAC TCACCTGTAA   
  
  
+ GCGCTCTTAG TGTGTCTGAT ATATTTAATG ATAGTCAGTC AGTGATACAA TTTCAGAAAG GGATGGAGGA   
  
  
+ AGCGAGCAAA TTTCTTCCCA AGGCTTCTTT GTGTTTGGAT TTGAACACTG GACCCTGCCT TCGGGAGTCG   
  
  
+ GTCAATAAGG CACGAGGTGG GCTGATTTTG GTGGAGACTA GCCATGAAAA TGAGGTTGCA GTGCTAGCTA   
  
  
+ GTAGGGGAAA GAAGCATCGC CATCCTGAGG AATTACGTTC AGAAGTTGGG AGGGGCAGTA AGCAGCCAGT   
  
  
+ TGTTTTCCAG TTTTCAGACG AGGCAGTTGT GAGATCAGAA ATGTTTGATA GGGTATTGCT GTGCAGTGGT   
  
  
+ GGCAAAAATG ATGCTGACCT ACGCCAAACT TTACAGAGTG AACTAAATAA AACTATGCAA AATGCTCAAA   
  
  
+ TGAAAGATTC TAATGGTGGA AAAGGCCGTG GTAGGAAGCA AGTTGGCAGG AGGGATGTGG TGGATCTAAG   
  
  
+ ATCTCTTTTA CGCCTTTGTG CACAAGCAGT TGGATCCAAT GACCAAAGAA GCGCAAATGA CTTAATTAGG   
  
  
+ CAAATTAGGA AGCATTCTTC TCCAACCGGG GATGGGAATC AAAGAATGGC TCATTATTTT GCTGATGGTC   
  
  
+ TTGAGGCTCG GCTTGCTGGT GTTGGGACCC CAATATACAA GTCTCTCGTG ACTGGCCCAG CTTCTGCTGT   
  
  
+ TGATATCTTG AGAGCTTACC ATATGTTTCT TGCCACATGC CCATTCAAGA AAATAGGGAA TTTCTTCTCT   
  
  
+ AATAGAACAA TTATGGATGT AGCTCAGAAA GCAAATCGAC TTCATATTAT TGATCTTGGT ATTGTCTATG   
  
  
+ GTTTCCAATG GCCTTGTCTA ATTCAGCGCC TTTCATCTAG GCCTGGCGGG CCTCCCAAAC TTCGGATTAC   
  
  
+ AGGCGTTGAT CTCCCCCAAC CCGGTTTCCG ACCAGCCAAA AGAGTCGAGG AGACTGGGCG ACGCTTGAGG   
  
  
+ AACTATGCTG AGTCATTCAA TGTGCCATTT GAGTTCAATG CCATAGCAAA GAGCTGGGAA ACACTTACTG   
  
  
+ TTAAAGATCT CAAGATTGAT CCCGATGAGG TGCTTGTTGT GAACTGTCTG TTCAGGTTTA AATACATTCC   
  
  
+ TGAGGAAACA GTAATTGCAG AATGCCCAAG AGATATTGTT CTTAATCTGA TCAGACAGAT AAAACCAGCT   
  
  
+ GTTTTCATAC ATGGTGTAGT CAATGGAGCC TTCAATTCTC CTTTTTTCAT TATTCGGTTC CGTGAGGCTC   
  
  
+ TCTTCCACTT CTCCACTCTA TTTGATGTGT TGGAGGCAAA TCTTCCCCGG GACATCGAGG AGAGGATACT   
  
  
+ GATAGAGCGA GACATCTTTG GGCGACAGGC AATGAATGTG ATCGCTTGTG AGGGTTTGGA GAGGATAGAA   
  
  
+ AGGCCAGAGA AATACAAACA GTGGCGGGTC CGAAATGAGA GGGCTGGGTT GAAGCAGCTG CCTTTGAATC   
  
  
+ AGGAGACTGT GGAAATGGCC AAAAAACGGG TGACAGCTGT CTATCACAAA GATTTCTCCA TTGATGACGA   
  
  
+ TGGGCACTGG TTGTTACAGG GATGGAAAGG GAGAATTGTC TATGCACTCA CTACTTGGAA GCCTGCTGAC   
  
  
+ TA  

- -Up\_Stream \_Len000AAACAT AAAGTTATAA CATTAATCCA CCCTATATCT CAACTTCCCG TTTGAGATGG   
  
  
- TTGCTGAACA ACTTCCTAGT TGAACATCGA ACCTTGAATT ACATACACAT GATATCTCAA CACATTAGAG   
  
  
- AGGATTATTT TAACCCAATT AGGATCTTAA TCTCTTGAAA TTCGCCCCTG ATTCGAACCA ACTCCGATTG   
  
  
- AAGCAATTTT TCAAGAACAC AGTACAGGTG CAAATAGAAA AGTGATAAAT AAAGTTAATT AAACGCTATA   
  
  
- AATAGTAGTG GGATAAGTGG CAGAAAAGCC TACTAAATTG ATCAGTTCGG CCTGGTTGTA ACGAAAAATA   
  
  
- TCACATAAAC GTTGAGTACA ACTAATAATC TACATAGTTG TTTTTTACAA AGAGTTTTAT TTATCATCGA   
  
  
- GAAATTTAAA ATAAGGTGTA TTTAAGTTTT AGTTATAGCT ATAGCAAGGT ATTATATCGT CGTAATACTT   
  
  
- TTGTTATAAA CGATTACAAA TGTAACACGA GAAAAGTGAG TTTTTAACAT TCCGAAGTTT ATGATAATTT   
  
  
- CTTTGGTAAT AAAGTGAGCT GATAAATCGA ACGTATATAT CTGTTAGAAC GTTTATCATT GTACAGAAGT   
  
  
- AAGCTGATAA ACGAAGGTTT ACTTATTAGA GCATTTGTAT ATATGTATGT ATGTATATAT ATATATATAT   
  
  
- ATATATATAT ATATATATAT CATTATACAT AAATTGAAGT GTATATGTAC TTATATTTTT ATATTCTCCC   
  
  
- TTTTTTCTGC GATCCATCTA CCTGATGTTT TCGGTAAGTA CATAACTCTG CCGAGTATTT TAGTATGCCC   
  
  
- CTTTCCTTTT TTTTTCTAGA GATGTATTAC AAGTATCTAA ATATCTTTCG TATACTACCT AATGGCGTAT   
  
  
- TCCACAATAT ATACCCTCAC AAACATCTTC TTTCTACATA TAGGCTTGAT AGTTTCTATG TATACTCCCT   
  
  
- CATTTTTGTT CACATTCTTG GAGTCCTCCT CTGTATTTTC TAAAAAGATA ACCGTAACCT GTGGCAGTTC   
  
  
- ATAGGCGAAA TTTGGACAAG AAAAAGTGAT AATAGTACCT ACATAAGTGT TTTCCTTAAG TTATGCTTCA   
  
  
- TGGTACTACA TACGAAAAAT GACTATCATA ACCAGAGTAG CTACTCTGAT TCCTACCAGA ATTATCGTTT   
  
  
- AATCTCTAAA GTAATCTATT TTAGATGACA TGTACTGTTT TGTTACTATA TATCGTAGGA TAAATTTTTC   
  
  
- GATGCACAGT CAGTATATAC TTAACGTAGA TTAAAAATTA TCTATATATT ATACTATACT ATATTATCCT   
  
  
- ATACTACATC TAACTTAAGT TTGAGTATTA AGTAATTCGT ACCACGTTAC AAGTAAAAGA ACTAAAATTT   
  
  
- GGCTGATAAT GAAGTTGACA TATATTAAGC TTAACGAAAC CATAAAATTT TAAATTTGAT CATTAATGAC   
  
  
- GGAGATAATG ACTACAAATA TAATTCACCA CGAATTGACG AATAACTACT TTGGTAAAAA TAAAAGCACA   
  
  
- TTTTCTCCCG TATTAGGTAG GAGCACATAA CCGCCCCGAG CCGCGGGATA AGTCGATAAG AGACATACAG   
  
  
- TTTGTTTTGG CGAAGATAAT GGGTAACCAG AGATTTGTGC GTCCCCAAAA TCGGGCCTCT CAATTAAGAA   
  
  
- ATAACTCCAG GGGACAAGAG GTAACGTCCT AAAAACGAGT CCATGAAGAG ACAAAGAAGA GAAATTAGAA   
  
  
- GACATAGAAA GACGTAGTTC AAATTACGAA AGACCCTAAA AGTCGATGCT AAATACACTT TAACATTCTA   
  
  
- AAGACTTGAC CCGTAAGTCC CGAACCAAGT ATACATAAGA AAAGACGAAC TTTACAAAAT TAAAGTATCA   
  
  
- AGGTTACCCC AATAAGTTTC TATCTTAATT CGATATTAGT GGCTCTTCTG AACGACTGGA AACAAAGAAA   
  
  
- AAACAAAGAA AAAAAATTAA TTTAGACCGA TAATGAACCG ATATCAAAAA CGATTACCTA GGTGTTTATC   
  
  
- TGCTTAATAT ACCCGGAAAC GTGGTTTACT TCAAGTTACT AGTCTCCTCT GGGTAAAATA GTTTACTTTT   
  
  
- AAAACAATTA CACAACTTTG GCGGTTTAAG GTTAACCTTA AGTGGAAAAC CCTTGGGATG ACACTAATGG   
  
  
- TGTTTGGGTT TAAAACTAAA CCAACACGGT AGTGATCCAG GGTTACAACT CCCTCGAGAG GAACTTCTAA   
  
  
- TACTAAAATC ACTACAACAA GAATTCATGT AGTTAGTTCA AGAATACCTG CTCCTACTCC CTCTCTTTTA   
  
  
- GGTAAACCCG GTGCCCGGAG ACCGAGAACT TCGCCGGCTC TTCAGCAATA TACTCCACGA ACCCGTCTCG   
  
  
- GTAAGAGGTG TTTTAGTTAT GAGAGGTCTC CTGTTCGCAC TACTGGACTC GTTCTCATCA CAATCTTCGA   
  
  
- CACAGTCAAT ATCGACACTG CCACGTTCAT CACCGTCACC GTAGCACCTC GTTTCAACCA GCTCAATAGG   
  
  
- AGGCCTCATG TTAGGGTACG CCTACCGTCT AAAAGGACGG AACGGGAGTT TTGGGAACAA CTCTTGTAGA   
  
  
- ATATCGAATA GTTTATCGAA CTGTCCACTC CCATTACCAA ACCACCTATC TGGCCGTCTG AGTGGACATT   
  
  
- CGCGAGAATC ACACAGACTA TATAAATTAC TATCAGTCAG TCACTATGTT AAAGTCTTTC CCTACCTCCT   
  
  
- TCGCTCGTTT AAAGAAGGGT TCCGAAGAAA CACAAACCTA AACTTGTGAC CTGGGACGGA AGCCCTCAGC   
  
  
- CAGTTATTCC GTGCTCCACC CGACTAAAAC CACCTCTGAT CGGTACTTTT ACTCCAACGT CACGATCGAT   
  
  
- CATCCCCTTT CTTCGTAGCG GTAGGACTCC TTAATGCAAG TCTTCAACCC TCCCCGTCAT TCGTCGGTCA   
  
  
- ACAAAAGGTC AAAAGTCTGC TCCGTCAACA CTCTAGTCTT TACAAACTAT CCCATAACGA CACGTCACCA   
  
  
- CCGTTTTTAC TACGACTGGA TGCGGTTTGA AATGTCTCAC TTGATTTATT TTGATACGTT TTACGAGTTT   
  
  
- ACTTTCTAAG ATTACCACCT TTTCCGGCAC CATCCTTCGT TCAACCGTCC TCCCTACACC ACCTAGATTC   
  
  
- TAGAGAAAAT GCGGAAACAC GTGTTCGTCA ACCTAGGTTA CTGGTTTCTT CGCGTTTACT GAATTAATCC   
  
  
- GTTTAATCCT TCGTAAGAAG AGGTTGGCCC CTACCCTTAG TTTCTTACCG AGTAATAAAA CGACTACCAG   
  
  
- AACTCCGAGC CGAACGACCA CAACCCTGGG GTTATATGTT CAGAGAGCAC TGACCGGGTC GAAGACGACA   
  
  
- ACTATAGAAC TCTCGAATGG TATACAAAGA ACGGTGTACG GGTAAGTTCT TTTATCCCTT AAAGAAGAGA   
  
  
- TTATCTTGTT AATACCTACA TCGAGTCTTT CGTTTAGCTG AAGTATAATA ACTAGAACCA TAACAGATAC   
  
  
- CAAAGGTTAC CGGAACAGAT TAAGTCGCGG AAAGTAGATC CGGACCGCCC GGAGGGTTTG AAGCCTAATG   
  
  
- TCCGCAACTA GAGGGGGTTG GGCCAAAGGC TGGTCGGTTT TCTCAGCTCC TCTGACCCGC TGCGAACTCC   
  
  
- TTGATACGAC TCAGTAAGTT ACACGGTAAA CTCAAGTTAC GGTATCGTTT CTCGACCCTT TGTGAATGAC   
  
  
- AATTTCTAGA GTTCTAACTA GGGCTACTCC ACGAACAACA CTTGACAGAC AAGTCCAAAT TTATGTAAGG   
  
  
- ACTCCTTTGT CATTAACGTC TTACGGGTTC TCTATAACAA GAATTAGACT AGTCTGTCTA TTTTGGTCGA   
  
  
- CAAAAGTATG TACCACATCA GTTACCTCGG AAGTTAAGAG GAAAAAAGTA ATAAGCCAAG GCACTCCGAG   
  
  
- AGAAGGTGAA GAGGTGAGAT AAACTACACA ACCTCCGTTT AGAAGGGGCC CTGTAGCTCC TCTCCTATGA   
  
  
- CTATCTCGCT CTGTAGAAAC CCGCTGTCCG TTACTTACAC TAGCGAACAC TCCCAAACCT CTCCTATCTT   
  
  
- TCCGGTCTCT TTATGTTTGT CACCGCCCAG GCTTTACTCT CCCGACCCAA CTTCGTCGAC GGAAACTTAG   
  
  
- TCCTCTGACA CCTTTACCGG TTTTTTGCCC ACTGTCGACA GATAGTGTTT CTAAAGAGGT AACTACTGCT   
  
  
- ACCCGTGACC AACAATGTCC CTACCTTTCC CTCTTAACAG ATACGTGAGT GATGAACCTT CGGACGACTG   
  
  
- AT

+     Myb-binding site

| Site Name | Organism | Position | Strand | Matrix score. | sequence | function |
| --- | --- | --- | --- | --- | --- | --- |
| Myb-binding site | Nicotiana tabacum | 3361 | - | 6 | CAACAG |  |

>HU05G01597.1   
+ -Up\_Stream \_Len000TTTGTA TTTCAATATT GTAATTAGGT GGGATATAGA GTTGAAGGGC AAACTCTACC   
  
  
+ AACGACTTGT TGAAGGATCA ACTTGTAGCT TGGAACTTAA TGTATGTGTA CTATAGAGTT GTGTAATCTC   
  
  
+ TCCTAATAAA ATTGGGTTAA TCCTAGAATT AGAGAACTTT AAGCGGGGAC TAAGCTTGGT TGAGGCTAAC   
  
  
+ TTCGTTAAAA AGTTCTTGTG TCATGTCCAC GTTTATCTTT TCACTATTTA TTTCAATTAA TTTGCGATAT   
  
  
+ TTATCATCAC CCTATTCACC GTCTTTTCGG ATGATTTAAC TAGTCAAGCC GGACCAACAT TGCTTTTTAT   
  
  
+ AGTGTATTTG CAACTCATGT TGATTATTAG ATGTATCAAC AAAAAATGTT TCTCAAAATA AATAGTAGCT   
  
  
+ CTTTAAATTT TATTCCACAT AAATTCAAAA TCAATATCGA TATCGTTCCA TAATATAGCA GCATTATGAA   
  
  
+ AACAATATTT GCTAATGTTT ACATTGTGCT CTTTTCACTC AAAAATTGTA AGGCTTCAAA TACTATTAAA   
  
  
+ GAAACCATTA TTTCACTCGA CTATTTAGCT TGCATATATA GACAATCTTG CAAATAGTAA CATGTCTTCA   
  
  
+ TTCGACTATT TGCTTCCAAA TGAATAATCT CGTAAACATA TATACATACA TACATATATA TATATATATA   
  
  
+ TATATATATA TATATATATA GTAATATGTA TTTAACTTCA CATATACATG AATATAAAAA TATAAGAGGG   
  
  
+ AAAAAAGACG CTAGGTAGAT GGACTACAAA AGCCATTCAT GTATTGAGAC GGCTCATAAA ATCATACGGG   
  
  
+ GAAAGGAAAA AAAAAGATCT CTACATAATG TTCATAGATT TATAGAAAGC ATATGATGGA TTACCGCATA   
  
  
+ AGGTGTTATA TATGGGAGTG TTTGTAGAAG AAAGATGTAT ATCCGAACTA TCAAAGATAC ATATGAGGGA   
  
  
+ GTAAAAACAA GTGTAAGAAC CTCAGGAGGA GACATAAAAG ATTTTTCTAT TGGCATTGGA CACCGTCAAG   
  
  
+ TATCCGCTTT AAACCTGTTC TTTTTCACTA TTATCATGGA TGTATTCACA AAAGGAATTC AATACGAAGT   
  
  
+ ACCATGATGT ATGCTTTTTA CTGATAGTAT TGGTCTCATC GATGAGACTA AGGATGGTCT TAATAGCAAA   
  
  
+ TTAGAGATTT CATTAGATAA AATCTACTGT ACATGACAAA ACAATGATAT ATAGCATCCT ATTTAAAAAG   
  
  
+ CTACGTGTCA GTCATATATG AATTGCATCT AATTTTTAAT AGATATATAA TATGATATGA TATAATAGGA   
  
  
+ TATGATGTAG ATTGAATTCA AACTCATAAT TCATTAAGCA TGGTGCAATG TTCATTTTCT TGATTTTAAA   
  
  
+ CCGACTATTA CTTCAACTGT ATATAATTCG AATTGCTTTG GTATTTTAAA ATTTAAACTA GTAATTACTG   
  
  
+ CCTCTATTAC TGATGTTTAT ATTAAGTGGT GCTTAACTGC TTATTGATGA AACCATTTTT ATTTTCGTGT   
  
  
+ AAAAGAGGGC ATAATCCATC CTCGTGTATT GGCGGGGCTC GGCGCCCTAT TCAGCTATTC TCTGTATGTC   
  
  
+ AAACAAAACC GCTTCTATTA CCCATTGGTC TCTAAACACG CAGGGGTTTT AGCCCGGAGA GTTAATTCTT   
  
  
+ TATTGAGGTC CCCTGTTCTC CATTGCAGGA TTTTTGCTCA GGTACTTCTC TGTTTCTTCT CTTTAATCTT   
  
  
+ CTGTATCTTT CTGCATCAAG TTTAATGCTT TCTGGGATTT TCAGCTACGA TTTATGTGAA ATTGTAAGAT   
  
  
+ TTCTGAACTG GGCATTCAGG GCTTGGTTCA TATGTATTCT TTTCTGCTTG AAATGTTTTA ATTTCATAGT   
  
  
+ TCCAATGGGG TTATTCAAAG ATAGAATTAA GCTATAATCA CCGAGAAGAC TTGCTGACCT TTGTTTCTTT   
  
  
+ TTTGTTTCTT TTTTTTAATT AAATCTGGCT ATTACTTGGC TATAGTTTTT GCTAATGGAT CCACAAATAG   
  
  
+ ACGAATTATA TGGGCCTTTG CACCAAATGA AGTTCAATGA TCAGAGGAGA CCCATTTTAT CAAATGAAAA   
  
  
+ TTTTGTTAAT GTGTTGAAAC CGCCAAATTC CAATTGGAAT TCACCTTTTG GGAACCCTAC TGTGATTACC   
  
  
+ ACAAACCCAA ATTTTGATTT GGTTGTGCCA TCACTAGGTC CCAATGTTGA GGGAGCTCTC CTTGAAGATT   
  
  
+ ATGATTTTAG TGATGTTGTT CTTAAGTACA TCAATCAAGT TCTTATGGAC GAGGATGAGG GAGAGAAAAT   
  
  
+ CCATTTGGGC CACGGGCCTC TGGCTCTTGA AGCGGCCGAG AAGTCGTTAT ATGAGGTGCT TGGGCAGAGC   
  
  
+ CATTCTCCAC AAAATCAATA CTCTCCAGAG GACAAGCGTG ATGACCTGAG CAAGAGTAGT GTTAGAAGCT   
  
  
+ GTGTCAGTTA TAGCTGTGAC GGTGCAAGTA GTGGCAGTGG CATCGTGGAG CAAAGTTGGT CGAGTTATCC   
  
  
+ TCCGGAGTAC AATCCCATGC GGATGGCAGA TTTTCCTGCC TTGCCCTCAA AACCCTTGTT GAGAACATCT   
  
  
+ TATAGCTTAT CAAATAGCTT GACAGGTGAG GGTAATGGTT TGGTGGATAG ACCGGCAGAC TCACCTGTAA   
  
  
+ GCGCTCTTAG TGTGTCTGAT ATATTTAATG ATAGTCAGTC AGTGATACAA TTTCAGAAAG GGATGGAGGA   
  
  
+ AGCGAGCAAA TTTCTTCCCA AGGCTTCTTT GTGTTTGGAT TTGAACACTG GACCCTGCCT TCGGGAGTCG   
  
  
+ GTCAATAAGG CACGAGGTGG GCTGATTTTG GTGGAGACTA GCCATGAAAA TGAGGTTGCA GTGCTAGCTA   
  
  
+ GTAGGGGAAA GAAGCATCGC CATCCTGAGG AATTACGTTC AGAAGTTGGG AGGGGCAGTA AGCAGCCAGT   
  
  
+ TGTTTTCCAG TTTTCAGACG AGGCAGTTGT GAGATCAGAA ATGTTTGATA GGGTATTGCT GTGCAGTGGT   
  
  
+ GGCAAAAATG ATGCTGACCT ACGCCAAACT TTACAGAGTG AACTAAATAA AACTATGCAA AATGCTCAAA   
  
  
+ TGAAAGATTC TAATGGTGGA AAAGGCCGTG GTAGGAAGCA AGTTGGCAGG AGGGATGTGG TGGATCTAAG   
  
  
+ ATCTCTTTTA CGCCTTTGTG CACAAGCAGT TGGATCCAAT GACCAAAGAA GCGCAAATGA CTTAATTAGG   
  
  
+ CAAATTAGGA AGCATTCTTC TCCAACCGGG GATGGGAATC AAAGAATGGC TCATTATTTT GCTGATGGTC   
  
  
+ TTGAGGCTCG GCTTGCTGGT GTTGGGACCC CAATATACAA GTCTCTCGTG ACTGGCCCAG CTTCTGCTGT   
  
  
+ TGATATCTTG AGAGCTTACC ATATGTTTCT TGCCACATGC CCATTCAAGA AAATAGGGAA TTTCTTCTCT   
  
  
+ AATAGAACAA TTATGGATGT AGCTCAGAAA GCAAATCGAC TTCATATTAT TGATCTTGGT ATTGTCTATG   
  
  
+ GTTTCCAATG GCCTTGTCTA ATTCAGCGCC TTTCATCTAG GCCTGGCGGG CCTCCCAAAC TTCGGATTAC   
  
  
+ AGGCGTTGAT CTCCCCCAAC CCGGTTTCCG ACCAGCCAAA AGAGTCGAGG AGACTGGGCG ACGCTTGAGG   
  
  
+ AACTATGCTG AGTCATTCAA TGTGCCATTT GAGTTCAATG CCATAGCAAA GAGCTGGGAA ACACTTACTG   
  
  
+ TTAAAGATCT CAAGATTGAT CCCGATGAGG TGCTTGTTGT GAACTGTCTG TTCAGGTTTA AATACATTCC   
  
  
+ TGAGGAAACA GTAATTGCAG AATGCCCAAG AGATATTGTT CTTAATCTGA TCAGACAGAT AAAACCAGCT   
  
  
+ GTTTTCATAC ATGGTGTAGT CAATGGAGCC TTCAATTCTC CTTTTTTCAT TATTCGGTTC CGTGAGGCTC   
  
  
+ TCTTCCACTT CTCCACTCTA TTTGATGTGT TGGAGGCAAA TCTTCCCCGG GACATCGAGG AGAGGATACT   
  
  
+ GATAGAGCGA GACATCTTTG GGCGACAGGC AATGAATGTG ATCGCTTGTG AGGGTTTGGA GAGGATAGAA   
  
  
+ AGGCCAGAGA AATACAAACA GTGGCGGGTC CGAAATGAGA GGGCTGGGTT GAAGCAGCTG CCTTTGAATC   
  
  
+ AGGAGACTGT GGAAATGGCC AAAAAACGGG TGACAGCTGT CTATCACAAA GATTTCTCCA TTGATGACGA   
  
  
+ TGGGCACTGG TTGTTACAGG GATGGAAAGG GAGAATTGTC TATGCACTCA CTACTTGGAA GCCTGCTGAC   
  
  
+ TA  

- -Up\_Stream \_Len000AAACAT AAAGTTATAA CATTAATCCA CCCTATATCT CAACTTCCCG TTTGAGATGG   
  
  
- TTGCTGAACA ACTTCCTAGT TGAACATCGA ACCTTGAATT ACATACACAT GATATCTCAA CACATTAGAG   
  
  
- AGGATTATTT TAACCCAATT AGGATCTTAA TCTCTTGAAA TTCGCCCCTG ATTCGAACCA ACTCCGATTG   
  
  
- AAGCAATTTT TCAAGAACAC AGTACAGGTG CAAATAGAAA AGTGATAAAT AAAGTTAATT AAACGCTATA   
  
  
- AATAGTAGTG GGATAAGTGG CAGAAAAGCC TACTAAATTG ATCAGTTCGG CCTGGTTGTA ACGAAAAATA   
  
  
- TCACATAAAC GTTGAGTACA ACTAATAATC TACATAGTTG TTTTTTACAA AGAGTTTTAT TTATCATCGA   
  
  
- GAAATTTAAA ATAAGGTGTA TTTAAGTTTT AGTTATAGCT ATAGCAAGGT ATTATATCGT CGTAATACTT   
  
  
- TTGTTATAAA CGATTACAAA TGTAACACGA GAAAAGTGAG TTTTTAACAT TCCGAAGTTT ATGATAATTT   
  
  
- CTTTGGTAAT AAAGTGAGCT GATAAATCGA ACGTATATAT CTGTTAGAAC GTTTATCATT GTACAGAAGT   
  
  
- AAGCTGATAA ACGAAGGTTT ACTTATTAGA GCATTTGTAT ATATGTATGT ATGTATATAT ATATATATAT   
  
  
- ATATATATAT ATATATATAT CATTATACAT AAATTGAAGT GTATATGTAC TTATATTTTT ATATTCTCCC   
  
  
- TTTTTTCTGC GATCCATCTA CCTGATGTTT TCGGTAAGTA CATAACTCTG CCGAGTATTT TAGTATGCCC   
  
  
- CTTTCCTTTT TTTTTCTAGA GATGTATTAC AAGTATCTAA ATATCTTTCG TATACTACCT AATGGCGTAT   
  
  
- TCCACAATAT ATACCCTCAC AAACATCTTC TTTCTACATA TAGGCTTGAT AGTTTCTATG TATACTCCCT   
  
  
- CATTTTTGTT CACATTCTTG GAGTCCTCCT CTGTATTTTC TAAAAAGATA ACCGTAACCT GTGGCAGTTC   
  
  
- ATAGGCGAAA TTTGGACAAG AAAAAGTGAT AATAGTACCT ACATAAGTGT TTTCCTTAAG TTATGCTTCA   
  
  
- TGGTACTACA TACGAAAAAT GACTATCATA ACCAGAGTAG CTACTCTGAT TCCTACCAGA ATTATCGTTT   
  
  
- AATCTCTAAA GTAATCTATT TTAGATGACA TGTACTGTTT TGTTACTATA TATCGTAGGA TAAATTTTTC   
  
  
- GATGCACAGT CAGTATATAC TTAACGTAGA TTAAAAATTA TCTATATATT ATACTATACT ATATTATCCT   
  
  
- ATACTACATC TAACTTAAGT TTGAGTATTA AGTAATTCGT ACCACGTTAC AAGTAAAAGA ACTAAAATTT   
  
  
- GGCTGATAAT GAAGTTGACA TATATTAAGC TTAACGAAAC CATAAAATTT TAAATTTGAT CATTAATGAC   
  
  
- GGAGATAATG ACTACAAATA TAATTCACCA CGAATTGACG AATAACTACT TTGGTAAAAA TAAAAGCACA   
  
  
- TTTTCTCCCG TATTAGGTAG GAGCACATAA CCGCCCCGAG CCGCGGGATA AGTCGATAAG AGACATACAG   
  
  
- TTTGTTTTGG CGAAGATAAT GGGTAACCAG AGATTTGTGC GTCCCCAAAA TCGGGCCTCT CAATTAAGAA   
  
  
- ATAACTCCAG GGGACAAGAG GTAACGTCCT AAAAACGAGT CCATGAAGAG ACAAAGAAGA GAAATTAGAA   
  
  
- GACATAGAAA GACGTAGTTC AAATTACGAA AGACCCTAAA AGTCGATGCT AAATACACTT TAACATTCTA   
  
  
- AAGACTTGAC CCGTAAGTCC CGAACCAAGT ATACATAAGA AAAGACGAAC TTTACAAAAT TAAAGTATCA   
  
  
- AGGTTACCCC AATAAGTTTC TATCTTAATT CGATATTAGT GGCTCTTCTG AACGACTGGA AACAAAGAAA   
  
  
- AAACAAAGAA AAAAAATTAA TTTAGACCGA TAATGAACCG ATATCAAAAA CGATTACCTA GGTGTTTATC   
  
  
- TGCTTAATAT ACCCGGAAAC GTGGTTTACT TCAAGTTACT AGTCTCCTCT GGGTAAAATA GTTTACTTTT   
  
  
- AAAACAATTA CACAACTTTG GCGGTTTAAG GTTAACCTTA AGTGGAAAAC CCTTGGGATG ACACTAATGG   
  
  
- TGTTTGGGTT TAAAACTAAA CCAACACGGT AGTGATCCAG GGTTACAACT CCCTCGAGAG GAACTTCTAA   
  
  
- TACTAAAATC ACTACAACAA GAATTCATGT AGTTAGTTCA AGAATACCTG CTCCTACTCC CTCTCTTTTA   
  
  
- GGTAAACCCG GTGCCCGGAG ACCGAGAACT TCGCCGGCTC TTCAGCAATA TACTCCACGA ACCCGTCTCG   
  
  
- GTAAGAGGTG TTTTAGTTAT GAGAGGTCTC CTGTTCGCAC TACTGGACTC GTTCTCATCA CAATCTTCGA   
  
  
- CACAGTCAAT ATCGACACTG CCACGTTCAT CACCGTCACC GTAGCACCTC GTTTCAACCA GCTCAATAGG   
  
  
- AGGCCTCATG TTAGGGTACG CCTACCGTCT AAAAGGACGG AACGGGAGTT TTGGGAACAA CTCTTGTAGA   
  
  
- ATATCGAATA GTTTATCGAA CTGTCCACTC CCATTACCAA ACCACCTATC TGGCCGTCTG AGTGGACATT   
  
  
- CGCGAGAATC ACACAGACTA TATAAATTAC TATCAGTCAG TCACTATGTT AAAGTCTTTC CCTACCTCCT   
  
  
- TCGCTCGTTT AAAGAAGGGT TCCGAAGAAA CACAAACCTA AACTTGTGAC CTGGGACGGA AGCCCTCAGC   
  
  
- CAGTTATTCC GTGCTCCACC CGACTAAAAC CACCTCTGAT CGGTACTTTT ACTCCAACGT CACGATCGAT   
  
  
- CATCCCCTTT CTTCGTAGCG GTAGGACTCC TTAATGCAAG TCTTCAACCC TCCCCGTCAT TCGTCGGTCA   
  
  
- ACAAAAGGTC AAAAGTCTGC TCCGTCAACA CTCTAGTCTT TACAAACTAT CCCATAACGA CACGTCACCA   
  
  
- CCGTTTTTAC TACGACTGGA TGCGGTTTGA AATGTCTCAC TTGATTTATT TTGATACGTT TTACGAGTTT   
  
  
- ACTTTCTAAG ATTACCACCT TTTCCGGCAC CATCCTTCGT TCAACCGTCC TCCCTACACC ACCTAGATTC   
  
  
- TAGAGAAAAT GCGGAAACAC GTGTTCGTCA ACCTAGGTTA CTGGTTTCTT CGCGTTTACT GAATTAATCC   
  
  
- GTTTAATCCT TCGTAAGAAG AGGTTGGCCC CTACCCTTAG TTTCTTACCG AGTAATAAAA CGACTACCAG   
  
  
- AACTCCGAGC CGAACGACCA CAACCCTGGG GTTATATGTT CAGAGAGCAC TGACCGGGTC GAAGACGACA   
  
  
- ACTATAGAAC TCTCGAATGG TATACAAAGA ACGGTGTACG GGTAAGTTCT TTTATCCCTT AAAGAAGAGA   
  
  
- TTATCTTGTT AATACCTACA TCGAGTCTTT CGTTTAGCTG AAGTATAATA ACTAGAACCA TAACAGATAC   
  
  
- CAAAGGTTAC CGGAACAGAT TAAGTCGCGG AAAGTAGATC CGGACCGCCC GGAGGGTTTG AAGCCTAATG   
  
  
- TCCGCAACTA GAGGGGGTTG GGCCAAAGGC TGGTCGGTTT TCTCAGCTCC TCTGACCCGC TGCGAACTCC   
  
  
- TTGATACGAC TCAGTAAGTT ACACGGTAAA CTCAAGTTAC GGTATCGTTT CTCGACCCTT TGTGAATGAC   
  
  
- AATTTCTAGA GTTCTAACTA GGGCTACTCC ACGAACAACA CTTGACAGAC AAGTCCAAAT TTATGTAAGG   
  
  
- ACTCCTTTGT CATTAACGTC TTACGGGTTC TCTATAACAA GAATTAGACT AGTCTGTCTA TTTTGGTCGA   
  
  
- CAAAAGTATG TACCACATCA GTTACCTCGG AAGTTAAGAG GAAAAAAGTA ATAAGCCAAG GCACTCCGAG   
  
  
- AGAAGGTGAA GAGGTGAGAT AAACTACACA ACCTCCGTTT AGAAGGGGCC CTGTAGCTCC TCTCCTATGA   
  
  
- CTATCTCGCT CTGTAGAAAC CCGCTGTCCG TTACTTACAC TAGCGAACAC TCCCAAACCT CTCCTATCTT   
  
  
- TCCGGTCTCT TTATGTTTGT CACCGCCCAG GCTTTACTCT CCCGACCCAA CTTCGTCGAC GGAAACTTAG   
  
  
- TCCTCTGACA CCTTTACCGG TTTTTTGCCC ACTGTCGACA GATAGTGTTT CTAAAGAGGT AACTACTGCT   
  
  
- ACCCGTGACC AACAATGTCC CTACCTTTCC CTCTTAACAG ATACGTGAGT GATGAACCTT CGGACGACTG   
  
  
- AT

+     O2-site

| Site Name | Organism | Position | Strand | Matrix score. | sequence | function |
| --- | --- | --- | --- | --- | --- | --- |
| O2-site | Zea mays | 2424 | + | 8 | GATGA(C/T)(A/G)TG(A/G) | cis-acting regulatory element involved in zein metabolism regulation |

>HU05G01597.1   
+ -Up\_Stream \_Len000TTTGTA TTTCAATATT GTAATTAGGT GGGATATAGA GTTGAAGGGC AAACTCTACC   
  
  
+ AACGACTTGT TGAAGGATCA ACTTGTAGCT TGGAACTTAA TGTATGTGTA CTATAGAGTT GTGTAATCTC   
  
  
+ TCCTAATAAA ATTGGGTTAA TCCTAGAATT AGAGAACTTT AAGCGGGGAC TAAGCTTGGT TGAGGCTAAC   
  
  
+ TTCGTTAAAA AGTTCTTGTG TCATGTCCAC GTTTATCTTT TCACTATTTA TTTCAATTAA TTTGCGATAT   
  
  
+ TTATCATCAC CCTATTCACC GTCTTTTCGG ATGATTTAAC TAGTCAAGCC GGACCAACAT TGCTTTTTAT   
  
  
+ AGTGTATTTG CAACTCATGT TGATTATTAG ATGTATCAAC AAAAAATGTT TCTCAAAATA AATAGTAGCT   
  
  
+ CTTTAAATTT TATTCCACAT AAATTCAAAA TCAATATCGA TATCGTTCCA TAATATAGCA GCATTATGAA   
  
  
+ AACAATATTT GCTAATGTTT ACATTGTGCT CTTTTCACTC AAAAATTGTA AGGCTTCAAA TACTATTAAA   
  
  
+ GAAACCATTA TTTCACTCGA CTATTTAGCT TGCATATATA GACAATCTTG CAAATAGTAA CATGTCTTCA   
  
  
+ TTCGACTATT TGCTTCCAAA TGAATAATCT CGTAAACATA TATACATACA TACATATATA TATATATATA   
  
  
+ TATATATATA TATATATATA GTAATATGTA TTTAACTTCA CATATACATG AATATAAAAA TATAAGAGGG   
  
  
+ AAAAAAGACG CTAGGTAGAT GGACTACAAA AGCCATTCAT GTATTGAGAC GGCTCATAAA ATCATACGGG   
  
  
+ GAAAGGAAAA AAAAAGATCT CTACATAATG TTCATAGATT TATAGAAAGC ATATGATGGA TTACCGCATA   
  
  
+ AGGTGTTATA TATGGGAGTG TTTGTAGAAG AAAGATGTAT ATCCGAACTA TCAAAGATAC ATATGAGGGA   
  
  
+ GTAAAAACAA GTGTAAGAAC CTCAGGAGGA GACATAAAAG ATTTTTCTAT TGGCATTGGA CACCGTCAAG   
  
  
+ TATCCGCTTT AAACCTGTTC TTTTTCACTA TTATCATGGA TGTATTCACA AAAGGAATTC AATACGAAGT   
  
  
+ ACCATGATGT ATGCTTTTTA CTGATAGTAT TGGTCTCATC GATGAGACTA AGGATGGTCT TAATAGCAAA   
  
  
+ TTAGAGATTT CATTAGATAA AATCTACTGT ACATGACAAA ACAATGATAT ATAGCATCCT ATTTAAAAAG   
  
  
+ CTACGTGTCA GTCATATATG AATTGCATCT AATTTTTAAT AGATATATAA TATGATATGA TATAATAGGA   
  
  
+ TATGATGTAG ATTGAATTCA AACTCATAAT TCATTAAGCA TGGTGCAATG TTCATTTTCT TGATTTTAAA   
  
  
+ CCGACTATTA CTTCAACTGT ATATAATTCG AATTGCTTTG GTATTTTAAA ATTTAAACTA GTAATTACTG   
  
  
+ CCTCTATTAC TGATGTTTAT ATTAAGTGGT GCTTAACTGC TTATTGATGA AACCATTTTT ATTTTCGTGT   
  
  
+ AAAAGAGGGC ATAATCCATC CTCGTGTATT GGCGGGGCTC GGCGCCCTAT TCAGCTATTC TCTGTATGTC   
  
  
+ AAACAAAACC GCTTCTATTA CCCATTGGTC TCTAAACACG CAGGGGTTTT AGCCCGGAGA GTTAATTCTT   
  
  
+ TATTGAGGTC CCCTGTTCTC CATTGCAGGA TTTTTGCTCA GGTACTTCTC TGTTTCTTCT CTTTAATCTT   
  
  
+ CTGTATCTTT CTGCATCAAG TTTAATGCTT TCTGGGATTT TCAGCTACGA TTTATGTGAA ATTGTAAGAT   
  
  
+ TTCTGAACTG GGCATTCAGG GCTTGGTTCA TATGTATTCT TTTCTGCTTG AAATGTTTTA ATTTCATAGT   
  
  
+ TCCAATGGGG TTATTCAAAG ATAGAATTAA GCTATAATCA CCGAGAAGAC TTGCTGACCT TTGTTTCTTT   
  
  
+ TTTGTTTCTT TTTTTTAATT AAATCTGGCT ATTACTTGGC TATAGTTTTT GCTAATGGAT CCACAAATAG   
  
  
+ ACGAATTATA TGGGCCTTTG CACCAAATGA AGTTCAATGA TCAGAGGAGA CCCATTTTAT CAAATGAAAA   
  
  
+ TTTTGTTAAT GTGTTGAAAC CGCCAAATTC CAATTGGAAT TCACCTTTTG GGAACCCTAC TGTGATTACC   
  
  
+ ACAAACCCAA ATTTTGATTT GGTTGTGCCA TCACTAGGTC CCAATGTTGA GGGAGCTCTC CTTGAAGATT   
  
  
+ ATGATTTTAG TGATGTTGTT CTTAAGTACA TCAATCAAGT TCTTATGGAC GAGGATGAGG GAGAGAAAAT   
  
  
+ CCATTTGGGC CACGGGCCTC TGGCTCTTGA AGCGGCCGAG AAGTCGTTAT ATGAGGTGCT TGGGCAGAGC   
  
  
+ CATTCTCCAC AAAATCAATA CTCTCCAGAG GACAAGCGTG ATGACCTGAG CAAGAGTAGT GTTAGAAGCT   
  
  
+ GTGTCAGTTA TAGCTGTGAC GGTGCAAGTA GTGGCAGTGG CATCGTGGAG CAAAGTTGGT CGAGTTATCC   
  
  
+ TCCGGAGTAC AATCCCATGC GGATGGCAGA TTTTCCTGCC TTGCCCTCAA AACCCTTGTT GAGAACATCT   
  
  
+ TATAGCTTAT CAAATAGCTT GACAGGTGAG GGTAATGGTT TGGTGGATAG ACCGGCAGAC TCACCTGTAA   
  
  
+ GCGCTCTTAG TGTGTCTGAT ATATTTAATG ATAGTCAGTC AGTGATACAA TTTCAGAAAG GGATGGAGGA   
  
  
+ AGCGAGCAAA TTTCTTCCCA AGGCTTCTTT GTGTTTGGAT TTGAACACTG GACCCTGCCT TCGGGAGTCG   
  
  
+ GTCAATAAGG CACGAGGTGG GCTGATTTTG GTGGAGACTA GCCATGAAAA TGAGGTTGCA GTGCTAGCTA   
  
  
+ GTAGGGGAAA GAAGCATCGC CATCCTGAGG AATTACGTTC AGAAGTTGGG AGGGGCAGTA AGCAGCCAGT   
  
  
+ TGTTTTCCAG TTTTCAGACG AGGCAGTTGT GAGATCAGAA ATGTTTGATA GGGTATTGCT GTGCAGTGGT   
  
  
+ GGCAAAAATG ATGCTGACCT ACGCCAAACT TTACAGAGTG AACTAAATAA AACTATGCAA AATGCTCAAA   
  
  
+ TGAAAGATTC TAATGGTGGA AAAGGCCGTG GTAGGAAGCA AGTTGGCAGG AGGGATGTGG TGGATCTAAG   
  
  
+ ATCTCTTTTA CGCCTTTGTG CACAAGCAGT TGGATCCAAT GACCAAAGAA GCGCAAATGA CTTAATTAGG   
  
  
+ CAAATTAGGA AGCATTCTTC TCCAACCGGG GATGGGAATC AAAGAATGGC TCATTATTTT GCTGATGGTC   
  
  
+ TTGAGGCTCG GCTTGCTGGT GTTGGGACCC CAATATACAA GTCTCTCGTG ACTGGCCCAG CTTCTGCTGT   
  
  
+ TGATATCTTG AGAGCTTACC ATATGTTTCT TGCCACATGC CCATTCAAGA AAATAGGGAA TTTCTTCTCT   
  
  
+ AATAGAACAA TTATGGATGT AGCTCAGAAA GCAAATCGAC TTCATATTAT TGATCTTGGT ATTGTCTATG   
  
  
+ GTTTCCAATG GCCTTGTCTA ATTCAGCGCC TTTCATCTAG GCCTGGCGGG CCTCCCAAAC TTCGGATTAC   
  
  
+ AGGCGTTGAT CTCCCCCAAC CCGGTTTCCG ACCAGCCAAA AGAGTCGAGG AGACTGGGCG ACGCTTGAGG   
  
  
+ AACTATGCTG AGTCATTCAA TGTGCCATTT GAGTTCAATG CCATAGCAAA GAGCTGGGAA ACACTTACTG   
  
  
+ TTAAAGATCT CAAGATTGAT CCCGATGAGG TGCTTGTTGT GAACTGTCTG TTCAGGTTTA AATACATTCC   
  
  
+ TGAGGAAACA GTAATTGCAG AATGCCCAAG AGATATTGTT CTTAATCTGA TCAGACAGAT AAAACCAGCT   
  
  
+ GTTTTCATAC ATGGTGTAGT CAATGGAGCC TTCAATTCTC CTTTTTTCAT TATTCGGTTC CGTGAGGCTC   
  
  
+ TCTTCCACTT CTCCACTCTA TTTGATGTGT TGGAGGCAAA TCTTCCCCGG GACATCGAGG AGAGGATACT   
  
  
+ GATAGAGCGA GACATCTTTG GGCGACAGGC AATGAATGTG ATCGCTTGTG AGGGTTTGGA GAGGATAGAA   
  
  
+ AGGCCAGAGA AATACAAACA GTGGCGGGTC CGAAATGAGA GGGCTGGGTT GAAGCAGCTG CCTTTGAATC   
  
  
+ AGGAGACTGT GGAAATGGCC AAAAAACGGG TGACAGCTGT CTATCACAAA GATTTCTCCA TTGATGACGA   
  
  
+ TGGGCACTGG TTGTTACAGG GATGGAAAGG GAGAATTGTC TATGCACTCA CTACTTGGAA GCCTGCTGAC   
  
  
+ TA  

- -Up\_Stream \_Len000AAACAT AAAGTTATAA CATTAATCCA CCCTATATCT CAACTTCCCG TTTGAGATGG   
  
  
- TTGCTGAACA ACTTCCTAGT TGAACATCGA ACCTTGAATT ACATACACAT GATATCTCAA CACATTAGAG   
  
  
- AGGATTATTT TAACCCAATT AGGATCTTAA TCTCTTGAAA TTCGCCCCTG ATTCGAACCA ACTCCGATTG   
  
  
- AAGCAATTTT TCAAGAACAC AGTACAGGTG CAAATAGAAA AGTGATAAAT AAAGTTAATT AAACGCTATA   
  
  
- AATAGTAGTG GGATAAGTGG CAGAAAAGCC TACTAAATTG ATCAGTTCGG CCTGGTTGTA ACGAAAAATA   
  
  
- TCACATAAAC GTTGAGTACA ACTAATAATC TACATAGTTG TTTTTTACAA AGAGTTTTAT TTATCATCGA   
  
  
- GAAATTTAAA ATAAGGTGTA TTTAAGTTTT AGTTATAGCT ATAGCAAGGT ATTATATCGT CGTAATACTT   
  
  
- TTGTTATAAA CGATTACAAA TGTAACACGA GAAAAGTGAG TTTTTAACAT TCCGAAGTTT ATGATAATTT   
  
  
- CTTTGGTAAT AAAGTGAGCT GATAAATCGA ACGTATATAT CTGTTAGAAC GTTTATCATT GTACAGAAGT   
  
  
- AAGCTGATAA ACGAAGGTTT ACTTATTAGA GCATTTGTAT ATATGTATGT ATGTATATAT ATATATATAT   
  
  
- ATATATATAT ATATATATAT CATTATACAT AAATTGAAGT GTATATGTAC TTATATTTTT ATATTCTCCC   
  
  
- TTTTTTCTGC GATCCATCTA CCTGATGTTT TCGGTAAGTA CATAACTCTG CCGAGTATTT TAGTATGCCC   
  
  
- CTTTCCTTTT TTTTTCTAGA GATGTATTAC AAGTATCTAA ATATCTTTCG TATACTACCT AATGGCGTAT   
  
  
- TCCACAATAT ATACCCTCAC AAACATCTTC TTTCTACATA TAGGCTTGAT AGTTTCTATG TATACTCCCT   
  
  
- CATTTTTGTT CACATTCTTG GAGTCCTCCT CTGTATTTTC TAAAAAGATA ACCGTAACCT GTGGCAGTTC   
  
  
- ATAGGCGAAA TTTGGACAAG AAAAAGTGAT AATAGTACCT ACATAAGTGT TTTCCTTAAG TTATGCTTCA   
  
  
- TGGTACTACA TACGAAAAAT GACTATCATA ACCAGAGTAG CTACTCTGAT TCCTACCAGA ATTATCGTTT   
  
  
- AATCTCTAAA GTAATCTATT TTAGATGACA TGTACTGTTT TGTTACTATA TATCGTAGGA TAAATTTTTC   
  
  
- GATGCACAGT CAGTATATAC TTAACGTAGA TTAAAAATTA TCTATATATT ATACTATACT ATATTATCCT   
  
  
- ATACTACATC TAACTTAAGT TTGAGTATTA AGTAATTCGT ACCACGTTAC AAGTAAAAGA ACTAAAATTT   
  
  
- GGCTGATAAT GAAGTTGACA TATATTAAGC TTAACGAAAC CATAAAATTT TAAATTTGAT CATTAATGAC   
  
  
- GGAGATAATG ACTACAAATA TAATTCACCA CGAATTGACG AATAACTACT TTGGTAAAAA TAAAAGCACA   
  
  
- TTTTCTCCCG TATTAGGTAG GAGCACATAA CCGCCCCGAG CCGCGGGATA AGTCGATAAG AGACATACAG   
  
  
- TTTGTTTTGG CGAAGATAAT GGGTAACCAG AGATTTGTGC GTCCCCAAAA TCGGGCCTCT CAATTAAGAA   
  
  
- ATAACTCCAG GGGACAAGAG GTAACGTCCT AAAAACGAGT CCATGAAGAG ACAAAGAAGA GAAATTAGAA   
  
  
- GACATAGAAA GACGTAGTTC AAATTACGAA AGACCCTAAA AGTCGATGCT AAATACACTT TAACATTCTA   
  
  
- AAGACTTGAC CCGTAAGTCC CGAACCAAGT ATACATAAGA AAAGACGAAC TTTACAAAAT TAAAGTATCA   
  
  
- AGGTTACCCC AATAAGTTTC TATCTTAATT CGATATTAGT GGCTCTTCTG AACGACTGGA AACAAAGAAA   
  
  
- AAACAAAGAA AAAAAATTAA TTTAGACCGA TAATGAACCG ATATCAAAAA CGATTACCTA GGTGTTTATC   
  
  
- TGCTTAATAT ACCCGGAAAC GTGGTTTACT TCAAGTTACT AGTCTCCTCT GGGTAAAATA GTTTACTTTT   
  
  
- AAAACAATTA CACAACTTTG GCGGTTTAAG GTTAACCTTA AGTGGAAAAC CCTTGGGATG ACACTAATGG   
  
  
- TGTTTGGGTT TAAAACTAAA CCAACACGGT AGTGATCCAG GGTTACAACT CCCTCGAGAG GAACTTCTAA   
  
  
- TACTAAAATC ACTACAACAA GAATTCATGT AGTTAGTTCA AGAATACCTG CTCCTACTCC CTCTCTTTTA   
  
  
- GGTAAACCCG GTGCCCGGAG ACCGAGAACT TCGCCGGCTC TTCAGCAATA TACTCCACGA ACCCGTCTCG   
  
  
- GTAAGAGGTG TTTTAGTTAT GAGAGGTCTC CTGTTCGCAC TACTGGACTC GTTCTCATCA CAATCTTCGA   
  
  
- CACAGTCAAT ATCGACACTG CCACGTTCAT CACCGTCACC GTAGCACCTC GTTTCAACCA GCTCAATAGG   
  
  
- AGGCCTCATG TTAGGGTACG CCTACCGTCT AAAAGGACGG AACGGGAGTT TTGGGAACAA CTCTTGTAGA   
  
  
- ATATCGAATA GTTTATCGAA CTGTCCACTC CCATTACCAA ACCACCTATC TGGCCGTCTG AGTGGACATT   
  
  
- CGCGAGAATC ACACAGACTA TATAAATTAC TATCAGTCAG TCACTATGTT AAAGTCTTTC CCTACCTCCT   
  
  
- TCGCTCGTTT AAAGAAGGGT TCCGAAGAAA CACAAACCTA AACTTGTGAC CTGGGACGGA AGCCCTCAGC   
  
  
- CAGTTATTCC GTGCTCCACC CGACTAAAAC CACCTCTGAT CGGTACTTTT ACTCCAACGT CACGATCGAT   
  
  
- CATCCCCTTT CTTCGTAGCG GTAGGACTCC TTAATGCAAG TCTTCAACCC TCCCCGTCAT TCGTCGGTCA   
  
  
- ACAAAAGGTC AAAAGTCTGC TCCGTCAACA CTCTAGTCTT TACAAACTAT CCCATAACGA CACGTCACCA   
  
  
- CCGTTTTTAC TACGACTGGA TGCGGTTTGA AATGTCTCAC TTGATTTATT TTGATACGTT TTACGAGTTT   
  
  
- ACTTTCTAAG ATTACCACCT TTTCCGGCAC CATCCTTCGT TCAACCGTCC TCCCTACACC ACCTAGATTC   
  
  
- TAGAGAAAAT GCGGAAACAC GTGTTCGTCA ACCTAGGTTA CTGGTTTCTT CGCGTTTACT GAATTAATCC   
  
  
- GTTTAATCCT TCGTAAGAAG AGGTTGGCCC CTACCCTTAG TTTCTTACCG AGTAATAAAA CGACTACCAG   
  
  
- AACTCCGAGC CGAACGACCA CAACCCTGGG GTTATATGTT CAGAGAGCAC TGACCGGGTC GAAGACGACA   
  
  
- ACTATAGAAC TCTCGAATGG TATACAAAGA ACGGTGTACG GGTAAGTTCT TTTATCCCTT AAAGAAGAGA   
  
  
- TTATCTTGTT AATACCTACA TCGAGTCTTT CGTTTAGCTG AAGTATAATA ACTAGAACCA TAACAGATAC   
  
  
- CAAAGGTTAC CGGAACAGAT TAAGTCGCGG AAAGTAGATC CGGACCGCCC GGAGGGTTTG AAGCCTAATG   
  
  
- TCCGCAACTA GAGGGGGTTG GGCCAAAGGC TGGTCGGTTT TCTCAGCTCC TCTGACCCGC TGCGAACTCC   
  
  
- TTGATACGAC TCAGTAAGTT ACACGGTAAA CTCAAGTTAC GGTATCGTTT CTCGACCCTT TGTGAATGAC   
  
  
- AATTTCTAGA GTTCTAACTA GGGCTACTCC ACGAACAACA CTTGACAGAC AAGTCCAAAT TTATGTAAGG   
  
  
- ACTCCTTTGT CATTAACGTC TTACGGGTTC TCTATAACAA GAATTAGACT AGTCTGTCTA TTTTGGTCGA   
  
  
- CAAAAGTATG TACCACATCA GTTACCTCGG AAGTTAAGAG GAAAAAAGTA ATAAGCCAAG GCACTCCGAG   
  
  
- AGAAGGTGAA GAGGTGAGAT AAACTACACA ACCTCCGTTT AGAAGGGGCC CTGTAGCTCC TCTCCTATGA   
  
  
- CTATCTCGCT CTGTAGAAAC CCGCTGTCCG TTACTTACAC TAGCGAACAC TCCCAAACCT CTCCTATCTT   
  
  
- TCCGGTCTCT TTATGTTTGT CACCGCCCAG GCTTTACTCT CCCGACCCAA CTTCGTCGAC GGAAACTTAG   
  
  
- TCCTCTGACA CCTTTACCGG TTTTTTGCCC ACTGTCGACA GATAGTGTTT CTAAAGAGGT AACTACTGCT   
  
  
- ACCCGTGACC AACAATGTCC CTACCTTTCC CTCTTAACAG ATACGTGAGT GATGAACCTT CGGACGACTG   
  
  
- AT

+     P-box

| Site Name | Organism | Position | Strand | Matrix score. | sequence | function |
| --- | --- | --- | --- | --- | --- | --- |
| P-box | Oryza sativa | 1103 | - | 7 | CCTTTTG | gibberellin-responsive element |
| P-box | Oryza sativa | 2148 | + | 7 | CCTTTTG | gibberellin-responsive element |

>HU05G01597.1   
+ -Up\_Stream \_Len000TTTGTA TTTCAATATT GTAATTAGGT GGGATATAGA GTTGAAGGGC AAACTCTACC   
  
  
+ AACGACTTGT TGAAGGATCA ACTTGTAGCT TGGAACTTAA TGTATGTGTA CTATAGAGTT GTGTAATCTC   
  
  
+ TCCTAATAAA ATTGGGTTAA TCCTAGAATT AGAGAACTTT AAGCGGGGAC TAAGCTTGGT TGAGGCTAAC   
  
  
+ TTCGTTAAAA AGTTCTTGTG TCATGTCCAC GTTTATCTTT TCACTATTTA TTTCAATTAA TTTGCGATAT   
  
  
+ TTATCATCAC CCTATTCACC GTCTTTTCGG ATGATTTAAC TAGTCAAGCC GGACCAACAT TGCTTTTTAT   
  
  
+ AGTGTATTTG CAACTCATGT TGATTATTAG ATGTATCAAC AAAAAATGTT TCTCAAAATA AATAGTAGCT   
  
  
+ CTTTAAATTT TATTCCACAT AAATTCAAAA TCAATATCGA TATCGTTCCA TAATATAGCA GCATTATGAA   
  
  
+ AACAATATTT GCTAATGTTT ACATTGTGCT CTTTTCACTC AAAAATTGTA AGGCTTCAAA TACTATTAAA   
  
  
+ GAAACCATTA TTTCACTCGA CTATTTAGCT TGCATATATA GACAATCTTG CAAATAGTAA CATGTCTTCA   
  
  
+ TTCGACTATT TGCTTCCAAA TGAATAATCT CGTAAACATA TATACATACA TACATATATA TATATATATA   
  
  
+ TATATATATA TATATATATA GTAATATGTA TTTAACTTCA CATATACATG AATATAAAAA TATAAGAGGG   
  
  
+ AAAAAAGACG CTAGGTAGAT GGACTACAAA AGCCATTCAT GTATTGAGAC GGCTCATAAA ATCATACGGG   
  
  
+ GAAAGGAAAA AAAAAGATCT CTACATAATG TTCATAGATT TATAGAAAGC ATATGATGGA TTACCGCATA   
  
  
+ AGGTGTTATA TATGGGAGTG TTTGTAGAAG AAAGATGTAT ATCCGAACTA TCAAAGATAC ATATGAGGGA   
  
  
+ GTAAAAACAA GTGTAAGAAC CTCAGGAGGA GACATAAAAG ATTTTTCTAT TGGCATTGGA CACCGTCAAG   
  
  
+ TATCCGCTTT AAACCTGTTC TTTTTCACTA TTATCATGGA TGTATTCACA AAAGGAATTC AATACGAAGT   
  
  
+ ACCATGATGT ATGCTTTTTA CTGATAGTAT TGGTCTCATC GATGAGACTA AGGATGGTCT TAATAGCAAA   
  
  
+ TTAGAGATTT CATTAGATAA AATCTACTGT ACATGACAAA ACAATGATAT ATAGCATCCT ATTTAAAAAG   
  
  
+ CTACGTGTCA GTCATATATG AATTGCATCT AATTTTTAAT AGATATATAA TATGATATGA TATAATAGGA   
  
  
+ TATGATGTAG ATTGAATTCA AACTCATAAT TCATTAAGCA TGGTGCAATG TTCATTTTCT TGATTTTAAA   
  
  
+ CCGACTATTA CTTCAACTGT ATATAATTCG AATTGCTTTG GTATTTTAAA ATTTAAACTA GTAATTACTG   
  
  
+ CCTCTATTAC TGATGTTTAT ATTAAGTGGT GCTTAACTGC TTATTGATGA AACCATTTTT ATTTTCGTGT   
  
  
+ AAAAGAGGGC ATAATCCATC CTCGTGTATT GGCGGGGCTC GGCGCCCTAT TCAGCTATTC TCTGTATGTC   
  
  
+ AAACAAAACC GCTTCTATTA CCCATTGGTC TCTAAACACG CAGGGGTTTT AGCCCGGAGA GTTAATTCTT   
  
  
+ TATTGAGGTC CCCTGTTCTC CATTGCAGGA TTTTTGCTCA GGTACTTCTC TGTTTCTTCT CTTTAATCTT   
  
  
+ CTGTATCTTT CTGCATCAAG TTTAATGCTT TCTGGGATTT TCAGCTACGA TTTATGTGAA ATTGTAAGAT   
  
  
+ TTCTGAACTG GGCATTCAGG GCTTGGTTCA TATGTATTCT TTTCTGCTTG AAATGTTTTA ATTTCATAGT   
  
  
+ TCCAATGGGG TTATTCAAAG ATAGAATTAA GCTATAATCA CCGAGAAGAC TTGCTGACCT TTGTTTCTTT   
  
  
+ TTTGTTTCTT TTTTTTAATT AAATCTGGCT ATTACTTGGC TATAGTTTTT GCTAATGGAT CCACAAATAG   
  
  
+ ACGAATTATA TGGGCCTTTG CACCAAATGA AGTTCAATGA TCAGAGGAGA CCCATTTTAT CAAATGAAAA   
  
  
+ TTTTGTTAAT GTGTTGAAAC CGCCAAATTC CAATTGGAAT TCACCTTTTG GGAACCCTAC TGTGATTACC   
  
  
+ ACAAACCCAA ATTTTGATTT GGTTGTGCCA TCACTAGGTC CCAATGTTGA GGGAGCTCTC CTTGAAGATT   
  
  
+ ATGATTTTAG TGATGTTGTT CTTAAGTACA TCAATCAAGT TCTTATGGAC GAGGATGAGG GAGAGAAAAT   
  
  
+ CCATTTGGGC CACGGGCCTC TGGCTCTTGA AGCGGCCGAG AAGTCGTTAT ATGAGGTGCT TGGGCAGAGC   
  
  
+ CATTCTCCAC AAAATCAATA CTCTCCAGAG GACAAGCGTG ATGACCTGAG CAAGAGTAGT GTTAGAAGCT   
  
  
+ GTGTCAGTTA TAGCTGTGAC GGTGCAAGTA GTGGCAGTGG CATCGTGGAG CAAAGTTGGT CGAGTTATCC   
  
  
+ TCCGGAGTAC AATCCCATGC GGATGGCAGA TTTTCCTGCC TTGCCCTCAA AACCCTTGTT GAGAACATCT   
  
  
+ TATAGCTTAT CAAATAGCTT GACAGGTGAG GGTAATGGTT TGGTGGATAG ACCGGCAGAC TCACCTGTAA   
  
  
+ GCGCTCTTAG TGTGTCTGAT ATATTTAATG ATAGTCAGTC AGTGATACAA TTTCAGAAAG GGATGGAGGA   
  
  
+ AGCGAGCAAA TTTCTTCCCA AGGCTTCTTT GTGTTTGGAT TTGAACACTG GACCCTGCCT TCGGGAGTCG   
  
  
+ GTCAATAAGG CACGAGGTGG GCTGATTTTG GTGGAGACTA GCCATGAAAA TGAGGTTGCA GTGCTAGCTA   
  
  
+ GTAGGGGAAA GAAGCATCGC CATCCTGAGG AATTACGTTC AGAAGTTGGG AGGGGCAGTA AGCAGCCAGT   
  
  
+ TGTTTTCCAG TTTTCAGACG AGGCAGTTGT GAGATCAGAA ATGTTTGATA GGGTATTGCT GTGCAGTGGT   
  
  
+ GGCAAAAATG ATGCTGACCT ACGCCAAACT TTACAGAGTG AACTAAATAA AACTATGCAA AATGCTCAAA   
  
  
+ TGAAAGATTC TAATGGTGGA AAAGGCCGTG GTAGGAAGCA AGTTGGCAGG AGGGATGTGG TGGATCTAAG   
  
  
+ ATCTCTTTTA CGCCTTTGTG CACAAGCAGT TGGATCCAAT GACCAAAGAA GCGCAAATGA CTTAATTAGG   
  
  
+ CAAATTAGGA AGCATTCTTC TCCAACCGGG GATGGGAATC AAAGAATGGC TCATTATTTT GCTGATGGTC   
  
  
+ TTGAGGCTCG GCTTGCTGGT GTTGGGACCC CAATATACAA GTCTCTCGTG ACTGGCCCAG CTTCTGCTGT   
  
  
+ TGATATCTTG AGAGCTTACC ATATGTTTCT TGCCACATGC CCATTCAAGA AAATAGGGAA TTTCTTCTCT   
  
  
+ AATAGAACAA TTATGGATGT AGCTCAGAAA GCAAATCGAC TTCATATTAT TGATCTTGGT ATTGTCTATG   
  
  
+ GTTTCCAATG GCCTTGTCTA ATTCAGCGCC TTTCATCTAG GCCTGGCGGG CCTCCCAAAC TTCGGATTAC   
  
  
+ AGGCGTTGAT CTCCCCCAAC CCGGTTTCCG ACCAGCCAAA AGAGTCGAGG AGACTGGGCG ACGCTTGAGG   
  
  
+ AACTATGCTG AGTCATTCAA TGTGCCATTT GAGTTCAATG CCATAGCAAA GAGCTGGGAA ACACTTACTG   
  
  
+ TTAAAGATCT CAAGATTGAT CCCGATGAGG TGCTTGTTGT GAACTGTCTG TTCAGGTTTA AATACATTCC   
  
  
+ TGAGGAAACA GTAATTGCAG AATGCCCAAG AGATATTGTT CTTAATCTGA TCAGACAGAT AAAACCAGCT   
  
  
+ GTTTTCATAC ATGGTGTAGT CAATGGAGCC TTCAATTCTC CTTTTTTCAT TATTCGGTTC CGTGAGGCTC   
  
  
+ TCTTCCACTT CTCCACTCTA TTTGATGTGT TGGAGGCAAA TCTTCCCCGG GACATCGAGG AGAGGATACT   
  
  
+ GATAGAGCGA GACATCTTTG GGCGACAGGC AATGAATGTG ATCGCTTGTG AGGGTTTGGA GAGGATAGAA   
  
  
+ AGGCCAGAGA AATACAAACA GTGGCGGGTC CGAAATGAGA GGGCTGGGTT GAAGCAGCTG CCTTTGAATC   
  
  
+ AGGAGACTGT GGAAATGGCC AAAAAACGGG TGACAGCTGT CTATCACAAA GATTTCTCCA TTGATGACGA   
  
  
+ TGGGCACTGG TTGTTACAGG GATGGAAAGG GAGAATTGTC TATGCACTCA CTACTTGGAA GCCTGCTGAC   
  
  
+ TA  

- -Up\_Stream \_Len000AAACAT AAAGTTATAA CATTAATCCA CCCTATATCT CAACTTCCCG TTTGAGATGG   
  
  
- TTGCTGAACA ACTTCCTAGT TGAACATCGA ACCTTGAATT ACATACACAT GATATCTCAA CACATTAGAG   
  
  
- AGGATTATTT TAACCCAATT AGGATCTTAA TCTCTTGAAA TTCGCCCCTG ATTCGAACCA ACTCCGATTG   
  
  
- AAGCAATTTT TCAAGAACAC AGTACAGGTG CAAATAGAAA AGTGATAAAT AAAGTTAATT AAACGCTATA   
  
  
- AATAGTAGTG GGATAAGTGG CAGAAAAGCC TACTAAATTG ATCAGTTCGG CCTGGTTGTA ACGAAAAATA   
  
  
- TCACATAAAC GTTGAGTACA ACTAATAATC TACATAGTTG TTTTTTACAA AGAGTTTTAT TTATCATCGA   
  
  
- GAAATTTAAA ATAAGGTGTA TTTAAGTTTT AGTTATAGCT ATAGCAAGGT ATTATATCGT CGTAATACTT   
  
  
- TTGTTATAAA CGATTACAAA TGTAACACGA GAAAAGTGAG TTTTTAACAT TCCGAAGTTT ATGATAATTT   
  
  
- CTTTGGTAAT AAAGTGAGCT GATAAATCGA ACGTATATAT CTGTTAGAAC GTTTATCATT GTACAGAAGT   
  
  
- AAGCTGATAA ACGAAGGTTT ACTTATTAGA GCATTTGTAT ATATGTATGT ATGTATATAT ATATATATAT   
  
  
- ATATATATAT ATATATATAT CATTATACAT AAATTGAAGT GTATATGTAC TTATATTTTT ATATTCTCCC   
  
  
- TTTTTTCTGC GATCCATCTA CCTGATGTTT TCGGTAAGTA CATAACTCTG CCGAGTATTT TAGTATGCCC   
  
  
- CTTTCCTTTT TTTTTCTAGA GATGTATTAC AAGTATCTAA ATATCTTTCG TATACTACCT AATGGCGTAT   
  
  
- TCCACAATAT ATACCCTCAC AAACATCTTC TTTCTACATA TAGGCTTGAT AGTTTCTATG TATACTCCCT   
  
  
- CATTTTTGTT CACATTCTTG GAGTCCTCCT CTGTATTTTC TAAAAAGATA ACCGTAACCT GTGGCAGTTC   
  
  
- ATAGGCGAAA TTTGGACAAG AAAAAGTGAT AATAGTACCT ACATAAGTGT TTTCCTTAAG TTATGCTTCA   
  
  
- TGGTACTACA TACGAAAAAT GACTATCATA ACCAGAGTAG CTACTCTGAT TCCTACCAGA ATTATCGTTT   
  
  
- AATCTCTAAA GTAATCTATT TTAGATGACA TGTACTGTTT TGTTACTATA TATCGTAGGA TAAATTTTTC   
  
  
- GATGCACAGT CAGTATATAC TTAACGTAGA TTAAAAATTA TCTATATATT ATACTATACT ATATTATCCT   
  
  
- ATACTACATC TAACTTAAGT TTGAGTATTA AGTAATTCGT ACCACGTTAC AAGTAAAAGA ACTAAAATTT   
  
  
- GGCTGATAAT GAAGTTGACA TATATTAAGC TTAACGAAAC CATAAAATTT TAAATTTGAT CATTAATGAC   
  
  
- GGAGATAATG ACTACAAATA TAATTCACCA CGAATTGACG AATAACTACT TTGGTAAAAA TAAAAGCACA   
  
  
- TTTTCTCCCG TATTAGGTAG GAGCACATAA CCGCCCCGAG CCGCGGGATA AGTCGATAAG AGACATACAG   
  
  
- TTTGTTTTGG CGAAGATAAT GGGTAACCAG AGATTTGTGC GTCCCCAAAA TCGGGCCTCT CAATTAAGAA   
  
  
- ATAACTCCAG GGGACAAGAG GTAACGTCCT AAAAACGAGT CCATGAAGAG ACAAAGAAGA GAAATTAGAA   
  
  
- GACATAGAAA GACGTAGTTC AAATTACGAA AGACCCTAAA AGTCGATGCT AAATACACTT TAACATTCTA   
  
  
- AAGACTTGAC CCGTAAGTCC CGAACCAAGT ATACATAAGA AAAGACGAAC TTTACAAAAT TAAAGTATCA   
  
  
- AGGTTACCCC AATAAGTTTC TATCTTAATT CGATATTAGT GGCTCTTCTG AACGACTGGA AACAAAGAAA   
  
  
- AAACAAAGAA AAAAAATTAA TTTAGACCGA TAATGAACCG ATATCAAAAA CGATTACCTA GGTGTTTATC   
  
  
- TGCTTAATAT ACCCGGAAAC GTGGTTTACT TCAAGTTACT AGTCTCCTCT GGGTAAAATA GTTTACTTTT   
  
  
- AAAACAATTA CACAACTTTG GCGGTTTAAG GTTAACCTTA AGTGGAAAAC CCTTGGGATG ACACTAATGG   
  
  
- TGTTTGGGTT TAAAACTAAA CCAACACGGT AGTGATCCAG GGTTACAACT CCCTCGAGAG GAACTTCTAA   
  
  
- TACTAAAATC ACTACAACAA GAATTCATGT AGTTAGTTCA AGAATACCTG CTCCTACTCC CTCTCTTTTA   
  
  
- GGTAAACCCG GTGCCCGGAG ACCGAGAACT TCGCCGGCTC TTCAGCAATA TACTCCACGA ACCCGTCTCG   
  
  
- GTAAGAGGTG TTTTAGTTAT GAGAGGTCTC CTGTTCGCAC TACTGGACTC GTTCTCATCA CAATCTTCGA   
  
  
- CACAGTCAAT ATCGACACTG CCACGTTCAT CACCGTCACC GTAGCACCTC GTTTCAACCA GCTCAATAGG   
  
  
- AGGCCTCATG TTAGGGTACG CCTACCGTCT AAAAGGACGG AACGGGAGTT TTGGGAACAA CTCTTGTAGA   
  
  
- ATATCGAATA GTTTATCGAA CTGTCCACTC CCATTACCAA ACCACCTATC TGGCCGTCTG AGTGGACATT   
  
  
- CGCGAGAATC ACACAGACTA TATAAATTAC TATCAGTCAG TCACTATGTT AAAGTCTTTC CCTACCTCCT   
  
  
- TCGCTCGTTT AAAGAAGGGT TCCGAAGAAA CACAAACCTA AACTTGTGAC CTGGGACGGA AGCCCTCAGC   
  
  
- CAGTTATTCC GTGCTCCACC CGACTAAAAC CACCTCTGAT CGGTACTTTT ACTCCAACGT CACGATCGAT   
  
  
- CATCCCCTTT CTTCGTAGCG GTAGGACTCC TTAATGCAAG TCTTCAACCC TCCCCGTCAT TCGTCGGTCA   
  
  
- ACAAAAGGTC AAAAGTCTGC TCCGTCAACA CTCTAGTCTT TACAAACTAT CCCATAACGA CACGTCACCA   
  
  
- CCGTTTTTAC TACGACTGGA TGCGGTTTGA AATGTCTCAC TTGATTTATT TTGATACGTT TTACGAGTTT   
  
  
- ACTTTCTAAG ATTACCACCT TTTCCGGCAC CATCCTTCGT TCAACCGTCC TCCCTACACC ACCTAGATTC   
  
  
- TAGAGAAAAT GCGGAAACAC GTGTTCGTCA ACCTAGGTTA CTGGTTTCTT CGCGTTTACT GAATTAATCC   
  
  
- GTTTAATCCT TCGTAAGAAG AGGTTGGCCC CTACCCTTAG TTTCTTACCG AGTAATAAAA CGACTACCAG   
  
  
- AACTCCGAGC CGAACGACCA CAACCCTGGG GTTATATGTT CAGAGAGCAC TGACCGGGTC GAAGACGACA   
  
  
- ACTATAGAAC TCTCGAATGG TATACAAAGA ACGGTGTACG GGTAAGTTCT TTTATCCCTT AAAGAAGAGA   
  
  
- TTATCTTGTT AATACCTACA TCGAGTCTTT CGTTTAGCTG AAGTATAATA ACTAGAACCA TAACAGATAC   
  
  
- CAAAGGTTAC CGGAACAGAT TAAGTCGCGG AAAGTAGATC CGGACCGCCC GGAGGGTTTG AAGCCTAATG   
  
  
- TCCGCAACTA GAGGGGGTTG GGCCAAAGGC TGGTCGGTTT TCTCAGCTCC TCTGACCCGC TGCGAACTCC   
  
  
- TTGATACGAC TCAGTAAGTT ACACGGTAAA CTCAAGTTAC GGTATCGTTT CTCGACCCTT TGTGAATGAC   
  
  
- AATTTCTAGA GTTCTAACTA GGGCTACTCC ACGAACAACA CTTGACAGAC AAGTCCAAAT TTATGTAAGG   
  
  
- ACTCCTTTGT CATTAACGTC TTACGGGTTC TCTATAACAA GAATTAGACT AGTCTGTCTA TTTTGGTCGA   
  
  
- CAAAAGTATG TACCACATCA GTTACCTCGG AAGTTAAGAG GAAAAAAGTA ATAAGCCAAG GCACTCCGAG   
  
  
- AGAAGGTGAA GAGGTGAGAT AAACTACACA ACCTCCGTTT AGAAGGGGCC CTGTAGCTCC TCTCCTATGA   
  
  
- CTATCTCGCT CTGTAGAAAC CCGCTGTCCG TTACTTACAC TAGCGAACAC TCCCAAACCT CTCCTATCTT   
  
  
- TCCGGTCTCT TTATGTTTGT CACCGCCCAG GCTTTACTCT CCCGACCCAA CTTCGTCGAC GGAAACTTAG   
  
  
- TCCTCTGACA CCTTTACCGG TTTTTTGCCC ACTGTCGACA GATAGTGTTT CTAAAGAGGT AACTACTGCT   
  
  
- ACCCGTGACC AACAATGTCC CTACCTTTCC CTCTTAACAG ATACGTGAGT GATGAACCTT CGGACGACTG   
  
  
- AT

+     STRE

| Site Name | Organism | Position | Strand | Matrix score. | sequence | function |
| --- | --- | --- | --- | --- | --- | --- |
| STRE | Arabidopsis thaliana | 1656 | + | 5 | AGGGG |  |
| STRE | Arabidopsis thaliana | 2877 | + | 5 | AGGGG |  |
| STRE | Arabidopsis thaliana | 1694 | - | 5 | AGGGG |  |
| STRE | Arabidopsis thaliana | 2925 | + | 5 | AGGGG |  |

>HU05G01597.1   
+ -Up\_Stream \_Len000TTTGTA TTTCAATATT GTAATTAGGT GGGATATAGA GTTGAAGGGC AAACTCTACC   
  
  
+ AACGACTTGT TGAAGGATCA ACTTGTAGCT TGGAACTTAA TGTATGTGTA CTATAGAGTT GTGTAATCTC   
  
  
+ TCCTAATAAA ATTGGGTTAA TCCTAGAATT AGAGAACTTT AAGCGGGGAC TAAGCTTGGT TGAGGCTAAC   
  
  
+ TTCGTTAAAA AGTTCTTGTG TCATGTCCAC GTTTATCTTT TCACTATTTA TTTCAATTAA TTTGCGATAT   
  
  
+ TTATCATCAC CCTATTCACC GTCTTTTCGG ATGATTTAAC TAGTCAAGCC GGACCAACAT TGCTTTTTAT   
  
  
+ AGTGTATTTG CAACTCATGT TGATTATTAG ATGTATCAAC AAAAAATGTT TCTCAAAATA AATAGTAGCT   
  
  
+ CTTTAAATTT TATTCCACAT AAATTCAAAA TCAATATCGA TATCGTTCCA TAATATAGCA GCATTATGAA   
  
  
+ AACAATATTT GCTAATGTTT ACATTGTGCT CTTTTCACTC AAAAATTGTA AGGCTTCAAA TACTATTAAA   
  
  
+ GAAACCATTA TTTCACTCGA CTATTTAGCT TGCATATATA GACAATCTTG CAAATAGTAA CATGTCTTCA   
  
  
+ TTCGACTATT TGCTTCCAAA TGAATAATCT CGTAAACATA TATACATACA TACATATATA TATATATATA   
  
  
+ TATATATATA TATATATATA GTAATATGTA TTTAACTTCA CATATACATG AATATAAAAA TATAAGAGGG   
  
  
+ AAAAAAGACG CTAGGTAGAT GGACTACAAA AGCCATTCAT GTATTGAGAC GGCTCATAAA ATCATACGGG   
  
  
+ GAAAGGAAAA AAAAAGATCT CTACATAATG TTCATAGATT TATAGAAAGC ATATGATGGA TTACCGCATA   
  
  
+ AGGTGTTATA TATGGGAGTG TTTGTAGAAG AAAGATGTAT ATCCGAACTA TCAAAGATAC ATATGAGGGA   
  
  
+ GTAAAAACAA GTGTAAGAAC CTCAGGAGGA GACATAAAAG ATTTTTCTAT TGGCATTGGA CACCGTCAAG   
  
  
+ TATCCGCTTT AAACCTGTTC TTTTTCACTA TTATCATGGA TGTATTCACA AAAGGAATTC AATACGAAGT   
  
  
+ ACCATGATGT ATGCTTTTTA CTGATAGTAT TGGTCTCATC GATGAGACTA AGGATGGTCT TAATAGCAAA   
  
  
+ TTAGAGATTT CATTAGATAA AATCTACTGT ACATGACAAA ACAATGATAT ATAGCATCCT ATTTAAAAAG   
  
  
+ CTACGTGTCA GTCATATATG AATTGCATCT AATTTTTAAT AGATATATAA TATGATATGA TATAATAGGA   
  
  
+ TATGATGTAG ATTGAATTCA AACTCATAAT TCATTAAGCA TGGTGCAATG TTCATTTTCT TGATTTTAAA   
  
  
+ CCGACTATTA CTTCAACTGT ATATAATTCG AATTGCTTTG GTATTTTAAA ATTTAAACTA GTAATTACTG   
  
  
+ CCTCTATTAC TGATGTTTAT ATTAAGTGGT GCTTAACTGC TTATTGATGA AACCATTTTT ATTTTCGTGT   
  
  
+ AAAAGAGGGC ATAATCCATC CTCGTGTATT GGCGGGGCTC GGCGCCCTAT TCAGCTATTC TCTGTATGTC   
  
  
+ AAACAAAACC GCTTCTATTA CCCATTGGTC TCTAAACACG CAGGGGTTTT AGCCCGGAGA GTTAATTCTT   
  
  
+ TATTGAGGTC CCCTGTTCTC CATTGCAGGA TTTTTGCTCA GGTACTTCTC TGTTTCTTCT CTTTAATCTT   
  
  
+ CTGTATCTTT CTGCATCAAG TTTAATGCTT TCTGGGATTT TCAGCTACGA TTTATGTGAA ATTGTAAGAT   
  
  
+ TTCTGAACTG GGCATTCAGG GCTTGGTTCA TATGTATTCT TTTCTGCTTG AAATGTTTTA ATTTCATAGT   
  
  
+ TCCAATGGGG TTATTCAAAG ATAGAATTAA GCTATAATCA CCGAGAAGAC TTGCTGACCT TTGTTTCTTT   
  
  
+ TTTGTTTCTT TTTTTTAATT AAATCTGGCT ATTACTTGGC TATAGTTTTT GCTAATGGAT CCACAAATAG   
  
  
+ ACGAATTATA TGGGCCTTTG CACCAAATGA AGTTCAATGA TCAGAGGAGA CCCATTTTAT CAAATGAAAA   
  
  
+ TTTTGTTAAT GTGTTGAAAC CGCCAAATTC CAATTGGAAT TCACCTTTTG GGAACCCTAC TGTGATTACC   
  
  
+ ACAAACCCAA ATTTTGATTT GGTTGTGCCA TCACTAGGTC CCAATGTTGA GGGAGCTCTC CTTGAAGATT   
  
  
+ ATGATTTTAG TGATGTTGTT CTTAAGTACA TCAATCAAGT TCTTATGGAC GAGGATGAGG GAGAGAAAAT   
  
  
+ CCATTTGGGC CACGGGCCTC TGGCTCTTGA AGCGGCCGAG AAGTCGTTAT ATGAGGTGCT TGGGCAGAGC   
  
  
+ CATTCTCCAC AAAATCAATA CTCTCCAGAG GACAAGCGTG ATGACCTGAG CAAGAGTAGT GTTAGAAGCT   
  
  
+ GTGTCAGTTA TAGCTGTGAC GGTGCAAGTA GTGGCAGTGG CATCGTGGAG CAAAGTTGGT CGAGTTATCC   
  
  
+ TCCGGAGTAC AATCCCATGC GGATGGCAGA TTTTCCTGCC TTGCCCTCAA AACCCTTGTT GAGAACATCT   
  
  
+ TATAGCTTAT CAAATAGCTT GACAGGTGAG GGTAATGGTT TGGTGGATAG ACCGGCAGAC TCACCTGTAA   
  
  
+ GCGCTCTTAG TGTGTCTGAT ATATTTAATG ATAGTCAGTC AGTGATACAA TTTCAGAAAG GGATGGAGGA   
  
  
+ AGCGAGCAAA TTTCTTCCCA AGGCTTCTTT GTGTTTGGAT TTGAACACTG GACCCTGCCT TCGGGAGTCG   
  
  
+ GTCAATAAGG CACGAGGTGG GCTGATTTTG GTGGAGACTA GCCATGAAAA TGAGGTTGCA GTGCTAGCTA   
  
  
+ GTAGGGGAAA GAAGCATCGC CATCCTGAGG AATTACGTTC AGAAGTTGGG AGGGGCAGTA AGCAGCCAGT   
  
  
+ TGTTTTCCAG TTTTCAGACG AGGCAGTTGT GAGATCAGAA ATGTTTGATA GGGTATTGCT GTGCAGTGGT   
  
  
+ GGCAAAAATG ATGCTGACCT ACGCCAAACT TTACAGAGTG AACTAAATAA AACTATGCAA AATGCTCAAA   
  
  
+ TGAAAGATTC TAATGGTGGA AAAGGCCGTG GTAGGAAGCA AGTTGGCAGG AGGGATGTGG TGGATCTAAG   
  
  
+ ATCTCTTTTA CGCCTTTGTG CACAAGCAGT TGGATCCAAT GACCAAAGAA GCGCAAATGA CTTAATTAGG   
  
  
+ CAAATTAGGA AGCATTCTTC TCCAACCGGG GATGGGAATC AAAGAATGGC TCATTATTTT GCTGATGGTC   
  
  
+ TTGAGGCTCG GCTTGCTGGT GTTGGGACCC CAATATACAA GTCTCTCGTG ACTGGCCCAG CTTCTGCTGT   
  
  
+ TGATATCTTG AGAGCTTACC ATATGTTTCT TGCCACATGC CCATTCAAGA AAATAGGGAA TTTCTTCTCT   
  
  
+ AATAGAACAA TTATGGATGT AGCTCAGAAA GCAAATCGAC TTCATATTAT TGATCTTGGT ATTGTCTATG   
  
  
+ GTTTCCAATG GCCTTGTCTA ATTCAGCGCC TTTCATCTAG GCCTGGCGGG CCTCCCAAAC TTCGGATTAC   
  
  
+ AGGCGTTGAT CTCCCCCAAC CCGGTTTCCG ACCAGCCAAA AGAGTCGAGG AGACTGGGCG ACGCTTGAGG   
  
  
+ AACTATGCTG AGTCATTCAA TGTGCCATTT GAGTTCAATG CCATAGCAAA GAGCTGGGAA ACACTTACTG   
  
  
+ TTAAAGATCT CAAGATTGAT CCCGATGAGG TGCTTGTTGT GAACTGTCTG TTCAGGTTTA AATACATTCC   
  
  
+ TGAGGAAACA GTAATTGCAG AATGCCCAAG AGATATTGTT CTTAATCTGA TCAGACAGAT AAAACCAGCT   
  
  
+ GTTTTCATAC ATGGTGTAGT CAATGGAGCC TTCAATTCTC CTTTTTTCAT TATTCGGTTC CGTGAGGCTC   
  
  
+ TCTTCCACTT CTCCACTCTA TTTGATGTGT TGGAGGCAAA TCTTCCCCGG GACATCGAGG AGAGGATACT   
  
  
+ GATAGAGCGA GACATCTTTG GGCGACAGGC AATGAATGTG ATCGCTTGTG AGGGTTTGGA GAGGATAGAA   
  
  
+ AGGCCAGAGA AATACAAACA GTGGCGGGTC CGAAATGAGA GGGCTGGGTT GAAGCAGCTG CCTTTGAATC   
  
  
+ AGGAGACTGT GGAAATGGCC AAAAAACGGG TGACAGCTGT CTATCACAAA GATTTCTCCA TTGATGACGA   
  
  
+ TGGGCACTGG TTGTTACAGG GATGGAAAGG GAGAATTGTC TATGCACTCA CTACTTGGAA GCCTGCTGAC   
  
  
+ TA  

- -Up\_Stream \_Len000AAACAT AAAGTTATAA CATTAATCCA CCCTATATCT CAACTTCCCG TTTGAGATGG   
  
  
- TTGCTGAACA ACTTCCTAGT TGAACATCGA ACCTTGAATT ACATACACAT GATATCTCAA CACATTAGAG   
  
  
- AGGATTATTT TAACCCAATT AGGATCTTAA TCTCTTGAAA TTCGCCCCTG ATTCGAACCA ACTCCGATTG   
  
  
- AAGCAATTTT TCAAGAACAC AGTACAGGTG CAAATAGAAA AGTGATAAAT AAAGTTAATT AAACGCTATA   
  
  
- AATAGTAGTG GGATAAGTGG CAGAAAAGCC TACTAAATTG ATCAGTTCGG CCTGGTTGTA ACGAAAAATA   
  
  
- TCACATAAAC GTTGAGTACA ACTAATAATC TACATAGTTG TTTTTTACAA AGAGTTTTAT TTATCATCGA   
  
  
- GAAATTTAAA ATAAGGTGTA TTTAAGTTTT AGTTATAGCT ATAGCAAGGT ATTATATCGT CGTAATACTT   
  
  
- TTGTTATAAA CGATTACAAA TGTAACACGA GAAAAGTGAG TTTTTAACAT TCCGAAGTTT ATGATAATTT   
  
  
- CTTTGGTAAT AAAGTGAGCT GATAAATCGA ACGTATATAT CTGTTAGAAC GTTTATCATT GTACAGAAGT   
  
  
- AAGCTGATAA ACGAAGGTTT ACTTATTAGA GCATTTGTAT ATATGTATGT ATGTATATAT ATATATATAT   
  
  
- ATATATATAT ATATATATAT CATTATACAT AAATTGAAGT GTATATGTAC TTATATTTTT ATATTCTCCC   
  
  
- TTTTTTCTGC GATCCATCTA CCTGATGTTT TCGGTAAGTA CATAACTCTG CCGAGTATTT TAGTATGCCC   
  
  
- CTTTCCTTTT TTTTTCTAGA GATGTATTAC AAGTATCTAA ATATCTTTCG TATACTACCT AATGGCGTAT   
  
  
- TCCACAATAT ATACCCTCAC AAACATCTTC TTTCTACATA TAGGCTTGAT AGTTTCTATG TATACTCCCT   
  
  
- CATTTTTGTT CACATTCTTG GAGTCCTCCT CTGTATTTTC TAAAAAGATA ACCGTAACCT GTGGCAGTTC   
  
  
- ATAGGCGAAA TTTGGACAAG AAAAAGTGAT AATAGTACCT ACATAAGTGT TTTCCTTAAG TTATGCTTCA   
  
  
- TGGTACTACA TACGAAAAAT GACTATCATA ACCAGAGTAG CTACTCTGAT TCCTACCAGA ATTATCGTTT   
  
  
- AATCTCTAAA GTAATCTATT TTAGATGACA TGTACTGTTT TGTTACTATA TATCGTAGGA TAAATTTTTC   
  
  
- GATGCACAGT CAGTATATAC TTAACGTAGA TTAAAAATTA TCTATATATT ATACTATACT ATATTATCCT   
  
  
- ATACTACATC TAACTTAAGT TTGAGTATTA AGTAATTCGT ACCACGTTAC AAGTAAAAGA ACTAAAATTT   
  
  
- GGCTGATAAT GAAGTTGACA TATATTAAGC TTAACGAAAC CATAAAATTT TAAATTTGAT CATTAATGAC   
  
  
- GGAGATAATG ACTACAAATA TAATTCACCA CGAATTGACG AATAACTACT TTGGTAAAAA TAAAAGCACA   
  
  
- TTTTCTCCCG TATTAGGTAG GAGCACATAA CCGCCCCGAG CCGCGGGATA AGTCGATAAG AGACATACAG   
  
  
- TTTGTTTTGG CGAAGATAAT GGGTAACCAG AGATTTGTGC GTCCCCAAAA TCGGGCCTCT CAATTAAGAA   
  
  
- ATAACTCCAG GGGACAAGAG GTAACGTCCT AAAAACGAGT CCATGAAGAG ACAAAGAAGA GAAATTAGAA   
  
  
- GACATAGAAA GACGTAGTTC AAATTACGAA AGACCCTAAA AGTCGATGCT AAATACACTT TAACATTCTA   
  
  
- AAGACTTGAC CCGTAAGTCC CGAACCAAGT ATACATAAGA AAAGACGAAC TTTACAAAAT TAAAGTATCA   
  
  
- AGGTTACCCC AATAAGTTTC TATCTTAATT CGATATTAGT GGCTCTTCTG AACGACTGGA AACAAAGAAA   
  
  
- AAACAAAGAA AAAAAATTAA TTTAGACCGA TAATGAACCG ATATCAAAAA CGATTACCTA GGTGTTTATC   
  
  
- TGCTTAATAT ACCCGGAAAC GTGGTTTACT TCAAGTTACT AGTCTCCTCT GGGTAAAATA GTTTACTTTT   
  
  
- AAAACAATTA CACAACTTTG GCGGTTTAAG GTTAACCTTA AGTGGAAAAC CCTTGGGATG ACACTAATGG   
  
  
- TGTTTGGGTT TAAAACTAAA CCAACACGGT AGTGATCCAG GGTTACAACT CCCTCGAGAG GAACTTCTAA   
  
  
- TACTAAAATC ACTACAACAA GAATTCATGT AGTTAGTTCA AGAATACCTG CTCCTACTCC CTCTCTTTTA   
  
  
- GGTAAACCCG GTGCCCGGAG ACCGAGAACT TCGCCGGCTC TTCAGCAATA TACTCCACGA ACCCGTCTCG   
  
  
- GTAAGAGGTG TTTTAGTTAT GAGAGGTCTC CTGTTCGCAC TACTGGACTC GTTCTCATCA CAATCTTCGA   
  
  
- CACAGTCAAT ATCGACACTG CCACGTTCAT CACCGTCACC GTAGCACCTC GTTTCAACCA GCTCAATAGG   
  
  
- AGGCCTCATG TTAGGGTACG CCTACCGTCT AAAAGGACGG AACGGGAGTT TTGGGAACAA CTCTTGTAGA   
  
  
- ATATCGAATA GTTTATCGAA CTGTCCACTC CCATTACCAA ACCACCTATC TGGCCGTCTG AGTGGACATT   
  
  
- CGCGAGAATC ACACAGACTA TATAAATTAC TATCAGTCAG TCACTATGTT AAAGTCTTTC CCTACCTCCT   
  
  
- TCGCTCGTTT AAAGAAGGGT TCCGAAGAAA CACAAACCTA AACTTGTGAC CTGGGACGGA AGCCCTCAGC   
  
  
- CAGTTATTCC GTGCTCCACC CGACTAAAAC CACCTCTGAT CGGTACTTTT ACTCCAACGT CACGATCGAT   
  
  
- CATCCCCTTT CTTCGTAGCG GTAGGACTCC TTAATGCAAG TCTTCAACCC TCCCCGTCAT TCGTCGGTCA   
  
  
- ACAAAAGGTC AAAAGTCTGC TCCGTCAACA CTCTAGTCTT TACAAACTAT CCCATAACGA CACGTCACCA   
  
  
- CCGTTTTTAC TACGACTGGA TGCGGTTTGA AATGTCTCAC TTGATTTATT TTGATACGTT TTACGAGTTT   
  
  
- ACTTTCTAAG ATTACCACCT TTTCCGGCAC CATCCTTCGT TCAACCGTCC TCCCTACACC ACCTAGATTC   
  
  
- TAGAGAAAAT GCGGAAACAC GTGTTCGTCA ACCTAGGTTA CTGGTTTCTT CGCGTTTACT GAATTAATCC   
  
  
- GTTTAATCCT TCGTAAGAAG AGGTTGGCCC CTACCCTTAG TTTCTTACCG AGTAATAAAA CGACTACCAG   
  
  
- AACTCCGAGC CGAACGACCA CAACCCTGGG GTTATATGTT CAGAGAGCAC TGACCGGGTC GAAGACGACA   
  
  
- ACTATAGAAC TCTCGAATGG TATACAAAGA ACGGTGTACG GGTAAGTTCT TTTATCCCTT AAAGAAGAGA   
  
  
- TTATCTTGTT AATACCTACA TCGAGTCTTT CGTTTAGCTG AAGTATAATA ACTAGAACCA TAACAGATAC   
  
  
- CAAAGGTTAC CGGAACAGAT TAAGTCGCGG AAAGTAGATC CGGACCGCCC GGAGGGTTTG AAGCCTAATG   
  
  
- TCCGCAACTA GAGGGGGTTG GGCCAAAGGC TGGTCGGTTT TCTCAGCTCC TCTGACCCGC TGCGAACTCC   
  
  
- TTGATACGAC TCAGTAAGTT ACACGGTAAA CTCAAGTTAC GGTATCGTTT CTCGACCCTT TGTGAATGAC   
  
  
- AATTTCTAGA GTTCTAACTA GGGCTACTCC ACGAACAACA CTTGACAGAC AAGTCCAAAT TTATGTAAGG   
  
  
- ACTCCTTTGT CATTAACGTC TTACGGGTTC TCTATAACAA GAATTAGACT AGTCTGTCTA TTTTGGTCGA   
  
  
- CAAAAGTATG TACCACATCA GTTACCTCGG AAGTTAAGAG GAAAAAAGTA ATAAGCCAAG GCACTCCGAG   
  
  
- AGAAGGTGAA GAGGTGAGAT AAACTACACA ACCTCCGTTT AGAAGGGGCC CTGTAGCTCC TCTCCTATGA   
  
  
- CTATCTCGCT CTGTAGAAAC CCGCTGTCCG TTACTTACAC TAGCGAACAC TCCCAAACCT CTCCTATCTT   
  
  
- TCCGGTCTCT TTATGTTTGT CACCGCCCAG GCTTTACTCT CCCGACCCAA CTTCGTCGAC GGAAACTTAG   
  
  
- TCCTCTGACA CCTTTACCGG TTTTTTGCCC ACTGTCGACA GATAGTGTTT CTAAAGAGGT AACTACTGCT   
  
  
- ACCCGTGACC AACAATGTCC CTACCTTTCC CTCTTAACAG ATACGTGAGT GATGAACCTT CGGACGACTG   
  
  
- AT

+     TATA-box

| Site Name | Organism | Position | Strand | Matrix score. | sequence | function |
| --- | --- | --- | --- | --- | --- | --- |
| TATA-box | Brassica napus | 2683 | - | 6 | ATATAT | core promoter element around -30 of transcription start |
| TATA-box | Arabidopsis thaliana | 2595 | - | 4 | TATA | core promoter element around -30 of transcription start |
| TATA-box | Arabidopsis thaliana | 2362 | - | 4 | TATA | core promoter element around -30 of transcription start |
| TATA-box | Arabidopsis thaliana | 1491 | - | 5 | TATAA | core promoter element around -30 of transcription start |
| TATA-box | Arabidopsis thaliana | 1426 | + | 4 | TATA | core promoter element around -30 of transcription start |
| TATA-box | Brassica napus | 1307 | + | 6 | ATATAT | core promoter element around -30 of transcription start |
| TATA-box | Brassica oleracea | 1425 | + | 6 | ATATAA | core promoter element around -30 of transcription start |
| TATA-box | Brassica napus | 698 | + | 6 | ATATAT | core promoter element around -30 of transcription start |
| TATA-box | Arabidopsis thaliana | 1325 | + | 4 | TATA | core promoter element around -30 of transcription start |
| TATA-box | Arabidopsis thaliana | 1492 | + | 4 | TATA | core promoter element around -30 of transcription start |
| TATA-box | Brassica napus | 2039 | + | 6 | ATTATA | core promoter element around -30 of transcription start |
| TATA-box | Brassica juncea | 882 | - | 7 | TATAAAT | core promoter element around -30 of transcription start |
| TATA-box | Brassica napus | 704 | + | 6 | ATATAT | core promoter element around -30 of transcription start |
| TATA-box | Arabidopsis thaliana | 923 | + | 4 | TATA | core promoter element around -30 of transcription start |
| TATA-box | Arabidopsis thaliana | 2005 | + | 4 | TATA | core promoter element around -30 of transcription start |
| TATA-box | Helianthus annuus | 1422 | - | 6 | TATACA | core promoter element around -30 of transcription start |
| TATA-box | Brassica oleracea | 1324 | + | 6 | ATATAA | core promoter element around -30 of transcription start |
| TATA-box | Brassica napus | 714 | + | 6 | ATATAT | core promoter element around -30 of transcription start |
| TATA-box | Arabidopsis thaliana | 1279 | + | 4 | TATA | core promoter element around -30 of transcription start |
| TATA-box | Arabidopsis thaliana | 881 | - | 9 | taTATAAAtc | core promoter element around -30 of transcription start |
| TATA-box | Brassica napus | 690 | + | 6 | ATATAT | core promoter element around -30 of transcription start |
| TATA-box | Arabidopsis thaliana | 691 | + | 6 | TATATA | core promoter element around -30 of transcription start |
| TATA-box | Arabidopsis thaliana | 1927 | + | 4 | TATA | core promoter element around -30 of transcription start |
| TATA-box | Arabidopsis thaliana | 711 | + | 6 | TATATA | core promoter element around -30 of transcription start |
| TATA-box | Arabidopsis thaliana | 1244 | + | 4 | TATA | core promoter element around -30 of transcription start |
| TATA-box | Brassica napus | 1241 | + | 6 | ATATAT | core promoter element around -30 of transcription start |
| TATA-box | Arabidopsis thaliana | 1308 | + | 6 | TATATA | core promoter element around -30 of transcription start |
| TATA-box | Brassica napus | 710 | + | 6 | ATATAT | core promoter element around -30 of transcription start |
| TATA-box | Arabidopsis thaliana | 1424 | + | 6 | TATATA | core promoter element around -30 of transcription start |
| TATA-box | Arabidopsis thaliana | 1310 | + | 4 | TATA | core promoter element around -30 of transcription start |
| TATA-box | Oryza sativa | 799 | + | 7 | TACAAAA | core promoter element around -30 of transcription start |
| TATA-box | Brassica oleracea | 1309 | + | 6 | ATATAA | core promoter element around -30 of transcription start |
| TATA-box | Arabidopsis thaliana | 1242 | + | 6 | TATATA | core promoter element around -30 of transcription start |
| TATA-box | Arabidopsis thaliana | 952 | + | 4 | TATA | core promoter element around -30 of transcription start |
| TATA-box | Arabidopsis thaliana | 765 | + | 4 | TATA | core promoter element around -30 of transcription start |
| TATA-box | Helianthus annuus | 950 | - | 6 | TATACA | core promoter element around -30 of transcription start |
| TATA-box | Brassica oleracea | 764 | + | 6 | ATATAA | core promoter element around -30 of transcription start |
| TATA-box | Arabidopsis thaliana | 2462 | - | 5 | TATAA | core promoter element around -30 of transcription start |
| TATA-box | Arabidopsis thaliana | 921 | + | 6 | TATATA | core promoter element around -30 of transcription start |
| TATA-box | Arabidopsis thaliana | 885 | + | 4 | TATA | core promoter element around -30 of transcription start |
| TATA-box | Brassica napus | 708 | + | 6 | ATATAT | core promoter element around -30 of transcription start |
| TATA-box | Arabidopsis thaliana | 719 | + | 6 | TATATA | core promoter element around -30 of transcription start |
| TATA-box | Brassica napus | 712 | + | 6 | ATATAT | core promoter element around -30 of transcription start |
| TATA-box | Arabidopsis thaliana | 920 | - | 7 | TATATAA | core promoter element around -30 of transcription start |
| TATA-box | Helianthus annuus | 1490 | - | 6 | TATAAA | core promoter element around -30 of transcription start |
| TATA-box | Arabidopsis thaliana | 2040 | - | 5 | TATAA | core promoter element around -30 of transcription start |
| TATA-box | Brassica napus | 688 | + | 6 | ATATAT | core promoter element around -30 of transcription start |
| TATA-box | Arabidopsis thaliana | 2463 | - | 4 | TATA | core promoter element around -30 of transcription start |
| TATA-box | Arabidopsis thaliana | 689 | + | 6 | TATATA | core promoter element around -30 of transcription start |
| TATA-box | Arabidopsis thaliana | 699 | + | 6 | TATATA | core promoter element around -30 of transcription start |
| TATA-box | Brassica napus | 696 | + | 6 | ATATAT | core promoter element around -30 of transcription start |
| TATA-box | Brassica napus | 598 | + | 6 | ATATAT | core promoter element around -30 of transcription start |
| TATA-box | Brassica oleracea | 756 | + | 6 | ATATAA | core promoter element around -30 of transcription start |
| TATA-box | Brassica napus | 1278 | + | 6 | ATATAT | core promoter element around -30 of transcription start |
| TATA-box | Arabidopsis thaliana | 352 | + | 4 | TATA | core promoter element around -30 of transcription start |
| TATA-box | Arabidopsis thaliana | 884 | - | 5 | TATAA | core promoter element around -30 of transcription start |
| TATA-box | Arabidopsis thaliana | 715 | + | 6 | TATATA | core promoter element around -30 of transcription start |
| TATA-box | Arabidopsis thaliana | 3328 | - | 4 | TATA | core promoter element around -30 of transcription start |
| TATA-box | Arabidopsis thaliana | 701 | + | 6 | TATATA | core promoter element around -30 of transcription start |
| TATA-box | Brassica napus | 692 | + | 6 | ATATAT | core promoter element around -30 of transcription start |
| TATA-box | Arabidopsis thaliana | 1254 | + | 8 | TATTTAAA | core promoter element around -30 of transcription start |
| TATA-box | Arabidopsis thaliana | 757 | + | 4 | TATA | core promoter element around -30 of transcription start |
| TATA-box | Brassica napus | 718 | + | 6 | ATATAT | core promoter element around -30 of transcription start |
| TATA-box | Arabidopsis thaliana | 705 | + | 6 | TATATA | core promoter element around -30 of transcription start |
| TATA-box | Brassica napus | 700 | + | 6 | ATATAT | core promoter element around -30 of transcription start |
| TATA-box | Arabidopsis thaliana | 2041 | + | 4 | TATA | core promoter element around -30 of transcription start |
| TATA-box | Arabidopsis thaliana | 707 | + | 6 | TATATA | core promoter element around -30 of transcription start |
| TATA-box | Arabidopsis thaliana | 747 | + | 4 | TATA | core promoter element around -30 of transcription start |
| TATA-box | Helianthus annuus | 883 | - | 6 | TATAAA | core promoter element around -30 of transcription start |
| TATA-box | Arabidopsis thaliana | 693 | + | 6 | TATATA | core promoter element around -30 of transcription start |
| TATA-box | Arabidopsis thaliana | 351 | - | 5 | TATAA | core promoter element around -30 of transcription start |
| TATA-box | Arabidopsis thaliana | 2594 | - | 5 | TATAA | core promoter element around -30 of transcription start |
| TATA-box | Arabidopsis thaliana | 3771 | - | 8 | TATTTAAA | core promoter element around -30 of transcription start |
| TATA-box | Arabidopsis thaliana | 697 | + | 6 | TATATA | core promoter element around -30 of transcription start |
| TATA-box | Arabidopsis thaliana | 709 | + | 6 | TATATA | core promoter element around -30 of transcription start |
| TATA-box | Brassica napus | 706 | + | 6 | ATATAT | core promoter element around -30 of transcription start |
| TATA-box | Brassica napus | 922 | + | 6 | ATATAT | core promoter element around -30 of transcription start |
| TATA-box | Arabidopsis thaliana | 713 | + | 6 | TATATA | core promoter element around -30 of transcription start |
| TATA-box | Brassica napus | 694 | + | 6 | ATATAT | core promoter element around -30 of transcription start |
| TATA-box | Arabidopsis thaliana | 721 | + | 4 | TATA | core promoter element around -30 of transcription start |
| TATA-box | Arabidopsis thaliana | 695 | + | 6 | TATATA | core promoter element around -30 of transcription start |
| TATA-box | Arabidopsis thaliana | 2361 | - | 5 | TATAA | core promoter element around -30 of transcription start |
| TATA-box | Arabidopsis thaliana | 675 | + | 4 | TATA | core promoter element around -30 of transcription start |
| TATA-box | Arabidopsis thaliana | 478 | + | 4 | TATA | core promoter element around -30 of transcription start |
| TATA-box | Pisum sativum | 349 | - | 7 | TATAAAA | core promoter element around -30 of transcription start |
| TATA-box | Arabidopsis thaliana | 2684 | - | 4 | TATA | core promoter element around -30 of transcription start |
| TATA-box | Helianthus annuus | 350 | - | 6 | TATAAA | core promoter element around -30 of transcription start |
| TATA-box | Brassica napus | 702 | + | 6 | ATATAT | core promoter element around -30 of transcription start |
| TATA-box | Brassica napus | 716 | + | 6 | ATATAT | core promoter element around -30 of transcription start |
| TATA-box | Arabidopsis thaliana | 703 | + | 6 | TATATA | core promoter element around -30 of transcription start |
| TATA-box | Arabidopsis thaliana | 717 | + | 6 | TATATA | core promoter element around -30 of transcription start |
| TATA-box | Arabidopsis thaliana | 601 | + | 4 | TATA | core promoter element around -30 of transcription start |
| TATA-box | Brassica napus | 672 | + | 6 | ATATAT | core promoter element around -30 of transcription start |
| TATA-box | Arabidopsis thaliana | 673 | + | 6 | TATATA | core promoter element around -30 of transcription start |
[truncated: 190,589 more chars]
