# Supplementary material for: Genome-Wide Identification and Expression Pattern of the GRAS Gene Family in Pitaya (Selenicereus undatus L.)
Source: Biology (Basel). 2022 Dec 21;12(1):11. doi: 10.3390/biology12010011 (PMC9854919; doi:10.3390/biology12010011)
Supplement: Supplementary file 1 [file biology-12-00011-s001.zip › Supplementary file S5/HU05G01983.1_plantcare.html]

Content-Type: text/html; charset=ISO-8859-1


PlantCARE


Webmaster Firefox specific output  
To save the result:
click on the frame with the right mouse button and save the source code as a text file with extension .html  
REFERENCE:PlantCARE: a database of plant cis-acting regulatory elements and a portal to tools for in silico analysis of promoter sequences.  
Lescot, M., Déhais, P., Moreau, Y., De Moor, B., Rouzé ,P.,and Rombauts, S.  
Nucleic Acids Res., Database issue(2002), 30(1):325-327.   


---

>HU05G01983.1   
+ +Up\_Stream \_Len000AAATCA AACTCATAAA CAATAATTTT TTAAAACTTA AATGGTAATT ATTAGATTAA   
  
  
+ TAAAAATCCT AATGGTTGTT TAATGTTACA TCAAGACATG CTTTTTGGTG TATATATAAT GGCAGCATGT   
  
  
+ GGGTTAAGCG GCAACAAATT ATTCTCAAAT GCATGCAGTA ATTAATTTAA TTAATTAATC ACCTGCATAA   
  
  
+ TCTCAATACT AATCCTTTCA TTCTGTGCCC CACCCAACCC CGAAAATAGT AATCTTTCAA ACGTTGGAAG   
  
  
+ TGGGATTAGG TATAGAGTAG TCCTACAGAT GATGATCTAC CTACATATAA CATAATCTGA CTTTTCTTTT   
  
  
+ TTAAATAACT TGTATTGATG GCACAGAATT TGACATGTTG GCATATGGTA CTAAGAAATT AATTAACGTT   
  
  
+ GTGCTAAGAT ATAGGTCATT CTTTTTGGAG ACATAAGGCC CGCGTTCACG CAGGGTTCGA GAAGGGCCAC   
  
  
+ ATTCAATGGA TGAATTGTAG GTAGTTTAAT CTGACTTTGT CAGTGACTGA TTCCACGGCT TGAACCCGCG   
  
  
+ ACATTAATAT AGGTTGACTA AAGTCGTTTG ATACTTATTT TTCTTAAGTA AAGTCTTGAA TTCAAATCTT   
  
  
+ ATAAATAAAT AAAAAAATCT ATAATAAAAG AATTCTACCA TTTATTAAGT CAACAACTCA ATTCGAATGA   
  
  
+ AATAAAAAAT CAAGATCACC AAAAAAAATA AATAAAAGGA AAGGTAGATT GCATTTGGAT TTTGAGTGGC   
  
  
+ GTGGTGGTGG GTTTGATGCT GGTGCTGCAT ACACCGCAGC TTTTCTCTGT CCTTTTACCT CTCTTTGTCT   
  
  
+ GCAAGAACCC CATTTGGCAC GCCTGCTCTC TTTTCTATGT TTTCTTTTCT ATCTTTTAAG AAACAATGGA   
  
  
+ AAGCTAACAC ACTAACACTA ACCCATTGGT CCTTTTTTCT TATCTGCAAA ATTAAAGTAA CAATATTTTT   
  
  
+ CTTATCCCCT TTTTCGTTCC ACCCATTTCT TTATCCTTCT GTTTGAACCT AATCTGTATA TATGCATGCA   
  
  
+ TGTGTATTTT ACTTAGAATC TGTTTTGATT CTTTGATGTA ATTAATTAGT AGTCTTTTTA ATTGAATTTT   
  
  
+ TAACAGTAGT TTGAATTAGA CATTTATCGA TATTATATTT TTTATATATA ATTAAAAAAT ACAAAATGAT   
  
  
+ AAGTGTCTTA CTACAATACA CTTCATAAGT GTCGTGTTTT AACTTAAAAA TCATACTATT ATGTTTCTAA   
  
  
+ ATTGAAAAAA AAGTGAGATT AAGTAGCAGA AGCATATTAC AACTTATAAA TTATATAAAA TCATCTTTTT   
  
  
+ ATCCATAATT TATCACATTT CTAAAATACG CATATCATAT CTACTTATGG ATAGGGTTAA TAAGATTAGG   
  
  
+ GCATCATTTA AGATTTTATC AGCGTAACCA ACGAATGTTT AAGGTTAGCA GTTCATAATT AAGTGTAATC   
  
  
+ ATTAAAAAAT TTATGATTTT AAATCATAAA ATATGTTAAT CTAGGATGAG GCAAGCGGCA AGACATATAT   
  
  
+ CACTATCCTT AAAAAATATA ATATATTGAT TTCAAAGAAT TATGAAGTAT ATTAATTTCA TATATAATGA   
  
  
+ CGATTATGTA TTTTTTTACT AAATTACTTG TACCCATAAT AAATAGAATC GATGCATCAT TCATGGCCTT   
  
  
+ TCATTCTGTA ATATGGTTGA CTCCAAAAAT TCTCAAAGTA TTTTTATATT TTGGGAGGGG AGATTTTAGT   
  
  
+ TTGATACTTT TGTTGAACTA GTGAACAATT TCCAACAACC TCCACCACCC CCAAACATCA ACAATCTGAT   
  
  
+ TGATGTATGC ATCCCAAGCT TGAATTGAAT AATAATTTCA ATAACAACAA TAATCGTTTT ACCTTGACAA   
  
  
+ GCTCCACAGC AACTCAACGT CTTCCAAACC AACCCATCAC TCATCATCAT CATCCCCACT ATCCCATTCA   
  
  
+ CCCCCACCAA AAGGAGCCTA AAGACAGTAA CAAGAAGAAT GCTTGCCTTT TTTCATGGAT GAAGAAGACT   
  
  
+ TCTCTTCTTC CTCTACTTCT TCCCACCACT ATGATACACA CCACCTCCAA CATCATTACC CCACCAATTA   
  
  
+ TAATTCCCGT TATACCCTGG ACCCTCCCTC CACCACCACC ACAGCCACAA CCACCACCTC ATCCTCCACT   
  
  
+ CCCACCCACC ATGGCTTCGG TGGTGGGCTT GACTCCTCCT CCCCGTCTGC CGCTGTGGAA TTATCTTTCT   
  
  
+ CCCCGGACAT CCTCCTCTCC TCCCCCACCG GAAAGTGGGC GTCTGACATT CTTATGGAGG CGGCTAGGGC   
  
  
+ TTTTTCAGAC ACGAACACCA CCAAGCTCCA CCAGCTCCTC TGGATGCTGA ACGAGCTCGG CTCCCCCTAC   
  
  
+ GGTGATGTCG AGCAGAAGCT GGTGGCGTAC TTTAACCAAG CCCTTTTTGC CCGCCTCACT GCCACCGGAC   
  
  
+ CCCGCCACCG AGCCGCTATG CTCGCCGCTG CGGACAAGAC CTCCTCCTTC GACTCCACGC TCAGGTTGCT   
  
  
+ CCTCAAGTTC CAGGAAGTCA GCCCGTGGAC CACCTTCGGC CACGTGGCTT GCAACGGTGC AATCATCGAG   
  
  
+ GCCTTAGAGA GGGACCCACA TGAAAAGAGT AAGCTCCACG TCATCGATAT CAGCACCACC TTCTGCACCC   
  
  
+ AGTGGCCCAC CTTGCTTGAG GCTTTAGCCA CCCGCATGGA CGACACTCCC CACCTCAGCC TCACTGCCGT   
  
  
+ CGTCGTCAAC AAGTACGCCG CCGTGAGCGG TGGCGAGGAC GACGGGGGAG AAGGATCAAA GAGGGTGATG   
  
  
+ AGAGAGATTG GTCTTCGTCT TGAGAAGTTC GCTAGGTTAA TGGGGGTTCC CTTCAAGTTC AACGTGGTTT   
  
  
+ ACCACGTAGG TGATCTATCC CAGTTGGATT TTTCTCGTTT GGATATTAAA GATGATGAAG CGGTGGCGAT   
  
  
+ TAACTGTGTC AACTCGCTCC ACTCGGTGGA CCTCCGATAT CGGGAGGTAG TGCTCGCGGC GTTCAGGCGG   
  
  
+ CTGCGGCCGA GGGTGGTGAC GGTGGTGGAG GAAGAGGCGG AGCTGACTGA GGTGGGTGAG GGTCAGTATG   
  
  
+ AATTTTTTAG GCGGTTTGAG GAGTGTCTTA GGTGGTTTAG GGTTTACTTT GAGGCCGTAG GAGATTGTTT   
  
  
+ TCCTAGGACT AGCAATGAGA AATTGATGTT AGAGCGGGCC GCGGGCCGGG CCATGGTGGA TCTCCTTGCA   
  
  
+ TGTCCCGAGC CTGGTGGGTC GGCTGAGGGG AGGGAGACGG CGAGACGGTG GTCGGGTCGG ATGCATGGGG   
  
  
+ CGGGGTTCGA GCACGTGGGG TTCAGTGACG AAGTGTGTGA TGATGTGAGG GCCTTGTTGA GGAGGTACAA   
  
  
+ GGAAGGATGG TCAATGACAC AGTGCACCAT GGATGGCGGT AATCACCCTG GAATACTGTT GTGGTGGCGG   
  
  
+ GATCAACCGG TGGTTTGGGC CAGTGCATGG CGGCCTTG  

- +Up\_Stream \_Len000TTTAGT TTGAGTATTT GTTATTAAAA AATTTTGAAT TTACCATTAA TAATCTAATT   
  
  
- ATTTTTAGGA TTACCAACAA ATTACAATGT AGTTCTGTAC GAAAAACCAC ATATATATTA CCGTCGTACA   
  
  
- CCCAATTCGC CGTTGTTTAA TAAGAGTTTA CGTACGTCAT TAATTAAATT AATTAATTAG TGGACGTATT   
  
  
- AGAGTTATGA TTAGGAAAGT AAGACACGGG GTGGGTTGGG GCTTTTATCA TTAGAAAGTT TGCAACCTTC   
  
  
- ACCCTAATCC ATATCTCATC AGGATGTCTA CTACTAGATG GATGTATATT GTATTAGACT GAAAAGAAAA   
  
  
- AATTTATTGA ACATAACTAC CGTGTCTTAA ACTGTACAAC CGTATACCAT GATTCTTTAA TTAATTGCAA   
  
  
- CACGATTCTA TATCCAGTAA GAAAAACCTC TGTATTCCGG GCGCAAGTGC GTCCCAAGCT CTTCCCGGTG   
  
  
- TAAGTTACCT ACTTAACATC CATCAAATTA GACTGAAACA GTCACTGACT AAGGTGCCGA ACTTGGGCGC   
  
  
- TGTAATTATA TCCAACTGAT TTCAGCAAAC TATGAATAAA AAGAATTCAT TTCAGAACTT AAGTTTAGAA   
  
  
- TATTTATTTA TTTTTTTAGA TATTATTTTC TTAAGATGGT AAATAATTCA GTTGTTGAGT TAAGCTTACT   
  
  
- TTATTTTTTA GTTCTAGTGG TTTTTTTTAT TTATTTTCCT TTCCATCTAA CGTAAACCTA AAACTCACCG   
  
  
- CACCACCACC CAAACTACGA CCACGACGTA TGTGGCGTCG AAAAGAGACA GGAAAATGGA GAGAAACAGA   
  
  
- CGTTCTTGGG GTAAACCGTG CGGACGAGAG AAAAGATACA AAAGAAAAGA TAGAAAATTC TTTGTTACCT   
  
  
- TTCGATTGTG TGATTGTGAT TGGGTAACCA GGAAAAAAGA ATAGACGTTT TAATTTCATT GTTATAAAAA   
  
  
- GAATAGGGGA AAAAGCAAGG TGGGTAAAGA AATAGGAAGA CAAACTTGGA TTAGACATAT ATACGTACGT   
  
  
- ACACATAAAA TGAATCTTAG ACAAAACTAA GAAACTACAT TAATTAATCA TCAGAAAAAT TAACTTAAAA   
  
  
- ATTGTCATCA AACTTAATCT GTAAATAGCT ATAATATAAA AAATATATAT TAATTTTTTA TGTTTTACTA   
  
  
- TTCACAGAAT GATGTTATGT GAAGTATTCA CAGCACAAAA TTGAATTTTT AGTATGATAA TACAAAGATT   
  
  
- TAACTTTTTT TTCACTCTAA TTCATCGTCT TCGTATAATG TTGAATATTT AATATATTTT AGTAGAAAAA   
  
  
- TAGGTATTAA ATAGTGTAAA GATTTTATGC GTATAGTATA GATGAATACC TATCCCAATT ATTCTAATCC   
  
  
- CGTAGTAAAT TCTAAAATAG TCGCATTGGT TGCTTACAAA TTCCAATCGT CAAGTATTAA TTCACATTAG   
  
  
- TAATTTTTTA AATACTAAAA TTTAGTATTT TATACAATTA GATCCTACTC CGTTCGCCGT TCTGTATATA   
  
  
- GTGATAGGAA TTTTTTATAT TATATAACTA AAGTTTCTTA ATACTTCATA TAATTAAAGT ATATATTACT   
  
  
- GCTAATACAT AAAAAAATGA TTTAATGAAC ATGGGTATTA TTTATCTTAG CTACGTAGTA AGTACCGGAA   
  
  
- AGTAAGACAT TATACCAACT GAGGTTTTTA AGAGTTTCAT AAAAATATAA AACCCTCCCC TCTAAAATCA   
  
  
- AACTATGAAA ACAACTTGAT CACTTGTTAA AGGTTGTTGG AGGTGGTGGG GGTTTGTAGT TGTTAGACTA   
  
  
- ACTACATACG TAGGGTTCGA ACTTAACTTA TTATTAAAGT TATTGTTGTT ATTAGCAAAA TGGAACTGTT   
  
  
- CGAGGTGTCG TTGAGTTGCA GAAGGTTTGG TTGGGTAGTG AGTAGTAGTA GTAGGGGTGA TAGGGTAAGT   
  
  
- GGGGGTGGTT TTCCTCGGAT TTCTGTCATT GTTCTTCTTA CGAACGGAAA AAAGTACCTA CTTCTTCTGA   
  
  
- AGAGAAGAAG GAGATGAAGA AGGGTGGTGA TACTATGTGT GGTGGAGGTT GTAGTAATGG GGTGGTTAAT   
  
  
- ATTAAGGGCA ATATGGGACC TGGGAGGGAG GTGGTGGTGG TGTCGGTGTT GGTGGTGGAG TAGGAGGTGA   
  
  
- GGGTGGGTGG TACCGAAGCC ACCACCCGAA CTGAGGAGGA GGGGCAGACG GCGACACCTT AATAGAAAGA   
  
  
- GGGGCCTGTA GGAGGAGAGG AGGGGGTGGC CTTTCACCCG CAGACTGTAA GAATACCTCC GCCGATCCCG   
  
  
- AAAAAGTCTG TGCTTGTGGT GGTTCGAGGT GGTCGAGGAG ACCTACGACT TGCTCGAGCC GAGGGGGATG   
  
  
- CCACTACAGC TCGTCTTCGA CCACCGCATG AAATTGGTTC GGGAAAAACG GGCGGAGTGA CGGTGGCCTG   
  
  
- GGGCGGTGGC TCGGCGATAC GAGCGGCGAC GCCTGTTCTG GAGGAGGAAG CTGAGGTGCG AGTCCAACGA   
  
  
- GGAGTTCAAG GTCCTTCAGT CGGGCACCTG GTGGAAGCCG GTGCACCGAA CGTTGCCACG TTAGTAGCTC   
  
  
- CGGAATCTCT CCCTGGGTGT ACTTTTCTCA TTCGAGGTGC AGTAGCTATA GTCGTGGTGG AAGACGTGGG   
  
  
- TCACCGGGTG GAACGAACTC CGAAATCGGT GGGCGTACCT GCTGTGAGGG GTGGAGTCGG AGTGACGGCA   
  
  
- GCAGCAGTTG TTCATGCGGC GGCACTCGCC ACCGCTCCTG CTGCCCCCTC TTCCTAGTTT CTCCCACTAC   
  
  
- TCTCTCTAAC CAGAAGCAGA ACTCTTCAAG CGATCCAATT ACCCCCAAGG GAAGTTCAAG TTGCACCAAA   
  
  
- TGGTGCATCC ACTAGATAGG GTCAACCTAA AAAGAGCAAA CCTATAATTT CTACTACTTC GCCACCGCTA   
  
  
- ATTGACACAG TTGAGCGAGG TGAGCCACCT GGAGGCTATA GCCCTCCATC ACGAGCGCCG CAAGTCCGCC   
  
  
- GACGCCGGCT CCCACCACTG CCACCACCTC CTTCTCCGCC TCGACTGACT CCACCCACTC CCAGTCATAC   
  
  
- TTAAAAAATC CGCCAAACTC CTCACAGAAT CCACCAAATC CCAAATGAAA CTCCGGCATC CTCTAACAAA   
  
  
- AGGATCCTGA TCGTTACTCT TTAACTACAA TCTCGCCCGG CGCCCGGCCC GGTACCACCT AGAGGAACGT   
  
  
- ACAGGGCTCG GACCACCCAG CCGACTCCCC TCCCTCTGCC GCTCTGCCAC CAGCCCAGCC TACGTACCCC   
  
  
- GCCCCAAGCT CGTGCACCCC AAGTCACTGC TTCACACACT ACTACACTCC CGGAACAACT CCTCCATGTT   
  
  
- CCTTCCTACC AGTTACTGTG TCACGTGGTA CCTACCGCCA TTAGTGGGAC CTTATGACAA CACCACCGCC   
  
  
- CTAGTTGGCC ACCAAACCCG GTCACGTACC GCCGGAAC

  
  
Motifs Found  

+   

| Site Name | Organism | Position | Strand | Matrix score. | sequence | function |
| --- | --- | --- | --- | --- | --- | --- |
|  | organism | 3214 | + | 4 | motif\_sequence | short\_function |
|  | organism | 3144 | - | 4 | motif\_sequence | short\_function |
|  | organism | 375 | - | 4 | motif\_sequence | short\_function |
|  | organism | 319 | + | 4 | motif\_sequence | short\_function |
|  | organism | 3045 | - | 4 | motif\_sequence | short\_function |
|  | organism | 2249 | - | 4 | motif\_sequence | short\_function |
|  | organism | 237 | + | 4 | motif\_sequence | short\_function |
|  | organism | 1373 | + | 4 | motif\_sequence | short\_function |
|  | organism | 3224 | + | 4 | motif\_sequence | short\_function |
|  | organism | 2259 | + | 4 | motif\_sequence | short\_function |
|  | organism | 2036 | + | 4 | motif\_sequence | short\_function |
|  | organism | 748 | - | 4 | motif\_sequence | short\_function |
|  | organism | 2045 | + | 4 | motif\_sequence | short\_function |
|  | organism | 956 | + | 4 | motif\_sequence | short\_function |
|  | organism | 821 | + | 4 | motif\_sequence | short\_function |
|  | organism | 1743 | - | 4 | motif\_sequence | short\_function |

>HU05G01983.1   
+ +Up\_Stream \_Len000AAATCA AACTCATAAA CAATAATTTT TTAAAACTTA AATGGTAATT ATTAGATTAA   
  
  
+ TAAAAATCCT AATGGTTGTT TAATGTTACA TCAAGACATG CTTTTTGGTG TATATATAAT GGCAGCATGT   
  
  
+ GGGTTAAGCG GCAACAAATT ATTCTCAAAT GCATGCAGTA ATTAATTTAA TTAATTAATC ACCTGCATAA   
  
  
+ TCTCAATACT AATCCTTTCA TTCTGTGCCC CACCCAACCC CGAAAATAGT AATCTTTCAA ACGTTGGAAG   
  
  
+ TGGGATTAGG TATAGAGTAG TCCTACAGAT GATGATCTAC CTACATATAA CATAATCTGA CTTTTCTTTT   
  
  
+ TTAAATAACT TGTATTGATG GCACAGAATT TGACATGTTG GCATATGGTA CTAAGAAATT AATTAACGTT   
  
  
+ GTGCTAAGAT ATAGGTCATT CTTTTTGGAG ACATAAGGCC CGCGTTCACG CAGGGTTCGA GAAGGGCCAC   
  
  
+ ATTCAATGGA TGAATTGTAG GTAGTTTAAT CTGACTTTGT CAGTGACTGA TTCCACGGCT TGAACCCGCG   
  
  
+ ACATTAATAT AGGTTGACTA AAGTCGTTTG ATACTTATTT TTCTTAAGTA AAGTCTTGAA TTCAAATCTT   
  
  
+ ATAAATAAAT AAAAAAATCT ATAATAAAAG AATTCTACCA TTTATTAAGT CAACAACTCA ATTCGAATGA   
  
  
+ AATAAAAAAT CAAGATCACC AAAAAAAATA AATAAAAGGA AAGGTAGATT GCATTTGGAT TTTGAGTGGC   
  
  
+ GTGGTGGTGG GTTTGATGCT GGTGCTGCAT ACACCGCAGC TTTTCTCTGT CCTTTTACCT CTCTTTGTCT   
  
  
+ GCAAGAACCC CATTTGGCAC GCCTGCTCTC TTTTCTATGT TTTCTTTTCT ATCTTTTAAG AAACAATGGA   
  
  
+ AAGCTAACAC ACTAACACTA ACCCATTGGT CCTTTTTTCT TATCTGCAAA ATTAAAGTAA CAATATTTTT   
  
  
+ CTTATCCCCT TTTTCGTTCC ACCCATTTCT TTATCCTTCT GTTTGAACCT AATCTGTATA TATGCATGCA   
  
  
+ TGTGTATTTT ACTTAGAATC TGTTTTGATT CTTTGATGTA ATTAATTAGT AGTCTTTTTA ATTGAATTTT   
  
  
+ TAACAGTAGT TTGAATTAGA CATTTATCGA TATTATATTT TTTATATATA ATTAAAAAAT ACAAAATGAT   
  
  
+ AAGTGTCTTA CTACAATACA CTTCATAAGT GTCGTGTTTT AACTTAAAAA TCATACTATT ATGTTTCTAA   
  
  
+ ATTGAAAAAA AAGTGAGATT AAGTAGCAGA AGCATATTAC AACTTATAAA TTATATAAAA TCATCTTTTT   
  
  
+ ATCCATAATT TATCACATTT CTAAAATACG CATATCATAT CTACTTATGG ATAGGGTTAA TAAGATTAGG   
  
  
+ GCATCATTTA AGATTTTATC AGCGTAACCA ACGAATGTTT AAGGTTAGCA GTTCATAATT AAGTGTAATC   
  
  
+ ATTAAAAAAT TTATGATTTT AAATCATAAA ATATGTTAAT CTAGGATGAG GCAAGCGGCA AGACATATAT   
  
  
+ CACTATCCTT AAAAAATATA ATATATTGAT TTCAAAGAAT TATGAAGTAT ATTAATTTCA TATATAATGA   
  
  
+ CGATTATGTA TTTTTTTACT AAATTACTTG TACCCATAAT AAATAGAATC GATGCATCAT TCATGGCCTT   
  
  
+ TCATTCTGTA ATATGGTTGA CTCCAAAAAT TCTCAAAGTA TTTTTATATT TTGGGAGGGG AGATTTTAGT   
  
  
+ TTGATACTTT TGTTGAACTA GTGAACAATT TCCAACAACC TCCACCACCC CCAAACATCA ACAATCTGAT   
  
  
+ TGATGTATGC ATCCCAAGCT TGAATTGAAT AATAATTTCA ATAACAACAA TAATCGTTTT ACCTTGACAA   
  
  
+ GCTCCACAGC AACTCAACGT CTTCCAAACC AACCCATCAC TCATCATCAT CATCCCCACT ATCCCATTCA   
  
  
+ CCCCCACCAA AAGGAGCCTA AAGACAGTAA CAAGAAGAAT GCTTGCCTTT TTTCATGGAT GAAGAAGACT   
  
  
+ TCTCTTCTTC CTCTACTTCT TCCCACCACT ATGATACACA CCACCTCCAA CATCATTACC CCACCAATTA   
  
  
+ TAATTCCCGT TATACCCTGG ACCCTCCCTC CACCACCACC ACAGCCACAA CCACCACCTC ATCCTCCACT   
  
  
+ CCCACCCACC ATGGCTTCGG TGGTGGGCTT GACTCCTCCT CCCCGTCTGC CGCTGTGGAA TTATCTTTCT   
  
  
+ CCCCGGACAT CCTCCTCTCC TCCCCCACCG GAAAGTGGGC GTCTGACATT CTTATGGAGG CGGCTAGGGC   
  
  
+ TTTTTCAGAC ACGAACACCA CCAAGCTCCA CCAGCTCCTC TGGATGCTGA ACGAGCTCGG CTCCCCCTAC   
  
  
+ GGTGATGTCG AGCAGAAGCT GGTGGCGTAC TTTAACCAAG CCCTTTTTGC CCGCCTCACT GCCACCGGAC   
  
  
+ CCCGCCACCG AGCCGCTATG CTCGCCGCTG CGGACAAGAC CTCCTCCTTC GACTCCACGC TCAGGTTGCT   
  
  
+ CCTCAAGTTC CAGGAAGTCA GCCCGTGGAC CACCTTCGGC CACGTGGCTT GCAACGGTGC AATCATCGAG   
  
  
+ GCCTTAGAGA GGGACCCACA TGAAAAGAGT AAGCTCCACG TCATCGATAT CAGCACCACC TTCTGCACCC   
  
  
+ AGTGGCCCAC CTTGCTTGAG GCTTTAGCCA CCCGCATGGA CGACACTCCC CACCTCAGCC TCACTGCCGT   
  
  
+ CGTCGTCAAC AAGTACGCCG CCGTGAGCGG TGGCGAGGAC GACGGGGGAG AAGGATCAAA GAGGGTGATG   
  
  
+ AGAGAGATTG GTCTTCGTCT TGAGAAGTTC GCTAGGTTAA TGGGGGTTCC CTTCAAGTTC AACGTGGTTT   
  
  
+ ACCACGTAGG TGATCTATCC CAGTTGGATT TTTCTCGTTT GGATATTAAA GATGATGAAG CGGTGGCGAT   
  
  
+ TAACTGTGTC AACTCGCTCC ACTCGGTGGA CCTCCGATAT CGGGAGGTAG TGCTCGCGGC GTTCAGGCGG   
  
  
+ CTGCGGCCGA GGGTGGTGAC GGTGGTGGAG GAAGAGGCGG AGCTGACTGA GGTGGGTGAG GGTCAGTATG   
  
  
+ AATTTTTTAG GCGGTTTGAG GAGTGTCTTA GGTGGTTTAG GGTTTACTTT GAGGCCGTAG GAGATTGTTT   
  
  
+ TCCTAGGACT AGCAATGAGA AATTGATGTT AGAGCGGGCC GCGGGCCGGG CCATGGTGGA TCTCCTTGCA   
  
  
+ TGTCCCGAGC CTGGTGGGTC GGCTGAGGGG AGGGAGACGG CGAGACGGTG GTCGGGTCGG ATGCATGGGG   
  
  
+ CGGGGTTCGA GCACGTGGGG TTCAGTGACG AAGTGTGTGA TGATGTGAGG GCCTTGTTGA GGAGGTACAA   
  
  
+ GGAAGGATGG TCAATGACAC AGTGCACCAT GGATGGCGGT AATCACCCTG GAATACTGTT GTGGTGGCGG   
  
  
+ GATCAACCGG TGGTTTGGGC CAGTGCATGG CGGCCTTG  

- +Up\_Stream \_Len000TTTAGT TTGAGTATTT GTTATTAAAA AATTTTGAAT TTACCATTAA TAATCTAATT   
  
  
- ATTTTTAGGA TTACCAACAA ATTACAATGT AGTTCTGTAC GAAAAACCAC ATATATATTA CCGTCGTACA   
  
  
- CCCAATTCGC CGTTGTTTAA TAAGAGTTTA CGTACGTCAT TAATTAAATT AATTAATTAG TGGACGTATT   
  
  
- AGAGTTATGA TTAGGAAAGT AAGACACGGG GTGGGTTGGG GCTTTTATCA TTAGAAAGTT TGCAACCTTC   
  
  
- ACCCTAATCC ATATCTCATC AGGATGTCTA CTACTAGATG GATGTATATT GTATTAGACT GAAAAGAAAA   
  
  
- AATTTATTGA ACATAACTAC CGTGTCTTAA ACTGTACAAC CGTATACCAT GATTCTTTAA TTAATTGCAA   
  
  
- CACGATTCTA TATCCAGTAA GAAAAACCTC TGTATTCCGG GCGCAAGTGC GTCCCAAGCT CTTCCCGGTG   
  
  
- TAAGTTACCT ACTTAACATC CATCAAATTA GACTGAAACA GTCACTGACT AAGGTGCCGA ACTTGGGCGC   
  
  
- TGTAATTATA TCCAACTGAT TTCAGCAAAC TATGAATAAA AAGAATTCAT TTCAGAACTT AAGTTTAGAA   
  
  
- TATTTATTTA TTTTTTTAGA TATTATTTTC TTAAGATGGT AAATAATTCA GTTGTTGAGT TAAGCTTACT   
  
  
- TTATTTTTTA GTTCTAGTGG TTTTTTTTAT TTATTTTCCT TTCCATCTAA CGTAAACCTA AAACTCACCG   
  
  
- CACCACCACC CAAACTACGA CCACGACGTA TGTGGCGTCG AAAAGAGACA GGAAAATGGA GAGAAACAGA   
  
  
- CGTTCTTGGG GTAAACCGTG CGGACGAGAG AAAAGATACA AAAGAAAAGA TAGAAAATTC TTTGTTACCT   
  
  
- TTCGATTGTG TGATTGTGAT TGGGTAACCA GGAAAAAAGA ATAGACGTTT TAATTTCATT GTTATAAAAA   
  
  
- GAATAGGGGA AAAAGCAAGG TGGGTAAAGA AATAGGAAGA CAAACTTGGA TTAGACATAT ATACGTACGT   
  
  
- ACACATAAAA TGAATCTTAG ACAAAACTAA GAAACTACAT TAATTAATCA TCAGAAAAAT TAACTTAAAA   
  
  
- ATTGTCATCA AACTTAATCT GTAAATAGCT ATAATATAAA AAATATATAT TAATTTTTTA TGTTTTACTA   
  
  
- TTCACAGAAT GATGTTATGT GAAGTATTCA CAGCACAAAA TTGAATTTTT AGTATGATAA TACAAAGATT   
  
  
- TAACTTTTTT TTCACTCTAA TTCATCGTCT TCGTATAATG TTGAATATTT AATATATTTT AGTAGAAAAA   
  
  
- TAGGTATTAA ATAGTGTAAA GATTTTATGC GTATAGTATA GATGAATACC TATCCCAATT ATTCTAATCC   
  
  
- CGTAGTAAAT TCTAAAATAG TCGCATTGGT TGCTTACAAA TTCCAATCGT CAAGTATTAA TTCACATTAG   
  
  
- TAATTTTTTA AATACTAAAA TTTAGTATTT TATACAATTA GATCCTACTC CGTTCGCCGT TCTGTATATA   
  
  
- GTGATAGGAA TTTTTTATAT TATATAACTA AAGTTTCTTA ATACTTCATA TAATTAAAGT ATATATTACT   
  
  
- GCTAATACAT AAAAAAATGA TTTAATGAAC ATGGGTATTA TTTATCTTAG CTACGTAGTA AGTACCGGAA   
  
  
- AGTAAGACAT TATACCAACT GAGGTTTTTA AGAGTTTCAT AAAAATATAA AACCCTCCCC TCTAAAATCA   
  
  
- AACTATGAAA ACAACTTGAT CACTTGTTAA AGGTTGTTGG AGGTGGTGGG GGTTTGTAGT TGTTAGACTA   
  
  
- ACTACATACG TAGGGTTCGA ACTTAACTTA TTATTAAAGT TATTGTTGTT ATTAGCAAAA TGGAACTGTT   
  
  
- CGAGGTGTCG TTGAGTTGCA GAAGGTTTGG TTGGGTAGTG AGTAGTAGTA GTAGGGGTGA TAGGGTAAGT   
  
  
- GGGGGTGGTT TTCCTCGGAT TTCTGTCATT GTTCTTCTTA CGAACGGAAA AAAGTACCTA CTTCTTCTGA   
  
  
- AGAGAAGAAG GAGATGAAGA AGGGTGGTGA TACTATGTGT GGTGGAGGTT GTAGTAATGG GGTGGTTAAT   
  
  
- ATTAAGGGCA ATATGGGACC TGGGAGGGAG GTGGTGGTGG TGTCGGTGTT GGTGGTGGAG TAGGAGGTGA   
  
  
- GGGTGGGTGG TACCGAAGCC ACCACCCGAA CTGAGGAGGA GGGGCAGACG GCGACACCTT AATAGAAAGA   
  
  
- GGGGCCTGTA GGAGGAGAGG AGGGGGTGGC CTTTCACCCG CAGACTGTAA GAATACCTCC GCCGATCCCG   
  
  
- AAAAAGTCTG TGCTTGTGGT GGTTCGAGGT GGTCGAGGAG ACCTACGACT TGCTCGAGCC GAGGGGGATG   
  
  
- CCACTACAGC TCGTCTTCGA CCACCGCATG AAATTGGTTC GGGAAAAACG GGCGGAGTGA CGGTGGCCTG   
  
  
- GGGCGGTGGC TCGGCGATAC GAGCGGCGAC GCCTGTTCTG GAGGAGGAAG CTGAGGTGCG AGTCCAACGA   
  
  
- GGAGTTCAAG GTCCTTCAGT CGGGCACCTG GTGGAAGCCG GTGCACCGAA CGTTGCCACG TTAGTAGCTC   
  
  
- CGGAATCTCT CCCTGGGTGT ACTTTTCTCA TTCGAGGTGC AGTAGCTATA GTCGTGGTGG AAGACGTGGG   
  
  
- TCACCGGGTG GAACGAACTC CGAAATCGGT GGGCGTACCT GCTGTGAGGG GTGGAGTCGG AGTGACGGCA   
  
  
- GCAGCAGTTG TTCATGCGGC GGCACTCGCC ACCGCTCCTG CTGCCCCCTC TTCCTAGTTT CTCCCACTAC   
  
  
- TCTCTCTAAC CAGAAGCAGA ACTCTTCAAG CGATCCAATT ACCCCCAAGG GAAGTTCAAG TTGCACCAAA   
  
  
- TGGTGCATCC ACTAGATAGG GTCAACCTAA AAAGAGCAAA CCTATAATTT CTACTACTTC GCCACCGCTA   
  
  
- ATTGACACAG TTGAGCGAGG TGAGCCACCT GGAGGCTATA GCCCTCCATC ACGAGCGCCG CAAGTCCGCC   
  
  
- GACGCCGGCT CCCACCACTG CCACCACCTC CTTCTCCGCC TCGACTGACT CCACCCACTC CCAGTCATAC   
  
  
- TTAAAAAATC CGCCAAACTC CTCACAGAAT CCACCAAATC CCAAATGAAA CTCCGGCATC CTCTAACAAA   
  
  
- AGGATCCTGA TCGTTACTCT TTAACTACAA TCTCGCCCGG CGCCCGGCCC GGTACCACCT AGAGGAACGT   
  
  
- ACAGGGCTCG GACCACCCAG CCGACTCCCC TCCCTCTGCC GCTCTGCCAC CAGCCCAGCC TACGTACCCC   
  
  
- GCCCCAAGCT CGTGCACCCC AAGTCACTGC TTCACACACT ACTACACTCC CGGAACAACT CCTCCATGTT   
  
  
- CCTTCCTACC AGTTACTGTG TCACGTGGTA CCTACCGCCA TTAGTGGGAC CTTATGACAA CACCACCGCC   
  
  
- CTAGTTGGCC ACCAAACCCG GTCACGTACC GCCGGAAC

+     AAGAA-motif

| Site Name | Organism | Position | Strand | Matrix score. | sequence | function |
| --- | --- | --- | --- | --- | --- | --- |
| AAGAA-motif | Avena sativa | 1010 | - | 9 | gGTAAAGAAA |  |

>HU05G01983.1   
+ +Up\_Stream \_Len000AAATCA AACTCATAAA CAATAATTTT TTAAAACTTA AATGGTAATT ATTAGATTAA   
  
  
+ TAAAAATCCT AATGGTTGTT TAATGTTACA TCAAGACATG CTTTTTGGTG TATATATAAT GGCAGCATGT   
  
  
+ GGGTTAAGCG GCAACAAATT ATTCTCAAAT GCATGCAGTA ATTAATTTAA TTAATTAATC ACCTGCATAA   
  
  
+ TCTCAATACT AATCCTTTCA TTCTGTGCCC CACCCAACCC CGAAAATAGT AATCTTTCAA ACGTTGGAAG   
  
  
+ TGGGATTAGG TATAGAGTAG TCCTACAGAT GATGATCTAC CTACATATAA CATAATCTGA CTTTTCTTTT   
  
  
+ TTAAATAACT TGTATTGATG GCACAGAATT TGACATGTTG GCATATGGTA CTAAGAAATT AATTAACGTT   
  
  
+ GTGCTAAGAT ATAGGTCATT CTTTTTGGAG ACATAAGGCC CGCGTTCACG CAGGGTTCGA GAAGGGCCAC   
  
  
+ ATTCAATGGA TGAATTGTAG GTAGTTTAAT CTGACTTTGT CAGTGACTGA TTCCACGGCT TGAACCCGCG   
  
  
+ ACATTAATAT AGGTTGACTA AAGTCGTTTG ATACTTATTT TTCTTAAGTA AAGTCTTGAA TTCAAATCTT   
  
  
+ ATAAATAAAT AAAAAAATCT ATAATAAAAG AATTCTACCA TTTATTAAGT CAACAACTCA ATTCGAATGA   
  
  
+ AATAAAAAAT CAAGATCACC AAAAAAAATA AATAAAAGGA AAGGTAGATT GCATTTGGAT TTTGAGTGGC   
  
  
+ GTGGTGGTGG GTTTGATGCT GGTGCTGCAT ACACCGCAGC TTTTCTCTGT CCTTTTACCT CTCTTTGTCT   
  
  
+ GCAAGAACCC CATTTGGCAC GCCTGCTCTC TTTTCTATGT TTTCTTTTCT ATCTTTTAAG AAACAATGGA   
  
  
+ AAGCTAACAC ACTAACACTA ACCCATTGGT CCTTTTTTCT TATCTGCAAA ATTAAAGTAA CAATATTTTT   
  
  
+ CTTATCCCCT TTTTCGTTCC ACCCATTTCT TTATCCTTCT GTTTGAACCT AATCTGTATA TATGCATGCA   
  
  
+ TGTGTATTTT ACTTAGAATC TGTTTTGATT CTTTGATGTA ATTAATTAGT AGTCTTTTTA ATTGAATTTT   
  
  
+ TAACAGTAGT TTGAATTAGA CATTTATCGA TATTATATTT TTTATATATA ATTAAAAAAT ACAAAATGAT   
  
  
+ AAGTGTCTTA CTACAATACA CTTCATAAGT GTCGTGTTTT AACTTAAAAA TCATACTATT ATGTTTCTAA   
  
  
+ ATTGAAAAAA AAGTGAGATT AAGTAGCAGA AGCATATTAC AACTTATAAA TTATATAAAA TCATCTTTTT   
  
  
+ ATCCATAATT TATCACATTT CTAAAATACG CATATCATAT CTACTTATGG ATAGGGTTAA TAAGATTAGG   
  
  
+ GCATCATTTA AGATTTTATC AGCGTAACCA ACGAATGTTT AAGGTTAGCA GTTCATAATT AAGTGTAATC   
  
  
+ ATTAAAAAAT TTATGATTTT AAATCATAAA ATATGTTAAT CTAGGATGAG GCAAGCGGCA AGACATATAT   
  
  
+ CACTATCCTT AAAAAATATA ATATATTGAT TTCAAAGAAT TATGAAGTAT ATTAATTTCA TATATAATGA   
  
  
+ CGATTATGTA TTTTTTTACT AAATTACTTG TACCCATAAT AAATAGAATC GATGCATCAT TCATGGCCTT   
  
  
+ TCATTCTGTA ATATGGTTGA CTCCAAAAAT TCTCAAAGTA TTTTTATATT TTGGGAGGGG AGATTTTAGT   
  
  
+ TTGATACTTT TGTTGAACTA GTGAACAATT TCCAACAACC TCCACCACCC CCAAACATCA ACAATCTGAT   
  
  
+ TGATGTATGC ATCCCAAGCT TGAATTGAAT AATAATTTCA ATAACAACAA TAATCGTTTT ACCTTGACAA   
  
  
+ GCTCCACAGC AACTCAACGT CTTCCAAACC AACCCATCAC TCATCATCAT CATCCCCACT ATCCCATTCA   
  
  
+ CCCCCACCAA AAGGAGCCTA AAGACAGTAA CAAGAAGAAT GCTTGCCTTT TTTCATGGAT GAAGAAGACT   
  
  
+ TCTCTTCTTC CTCTACTTCT TCCCACCACT ATGATACACA CCACCTCCAA CATCATTACC CCACCAATTA   
  
  
+ TAATTCCCGT TATACCCTGG ACCCTCCCTC CACCACCACC ACAGCCACAA CCACCACCTC ATCCTCCACT   
  
  
+ CCCACCCACC ATGGCTTCGG TGGTGGGCTT GACTCCTCCT CCCCGTCTGC CGCTGTGGAA TTATCTTTCT   
  
  
+ CCCCGGACAT CCTCCTCTCC TCCCCCACCG GAAAGTGGGC GTCTGACATT CTTATGGAGG CGGCTAGGGC   
  
  
+ TTTTTCAGAC ACGAACACCA CCAAGCTCCA CCAGCTCCTC TGGATGCTGA ACGAGCTCGG CTCCCCCTAC   
  
  
+ GGTGATGTCG AGCAGAAGCT GGTGGCGTAC TTTAACCAAG CCCTTTTTGC CCGCCTCACT GCCACCGGAC   
  
  
+ CCCGCCACCG AGCCGCTATG CTCGCCGCTG CGGACAAGAC CTCCTCCTTC GACTCCACGC TCAGGTTGCT   
  
  
+ CCTCAAGTTC CAGGAAGTCA GCCCGTGGAC CACCTTCGGC CACGTGGCTT GCAACGGTGC AATCATCGAG   
  
  
+ GCCTTAGAGA GGGACCCACA TGAAAAGAGT AAGCTCCACG TCATCGATAT CAGCACCACC TTCTGCACCC   
  
  
+ AGTGGCCCAC CTTGCTTGAG GCTTTAGCCA CCCGCATGGA CGACACTCCC CACCTCAGCC TCACTGCCGT   
  
  
+ CGTCGTCAAC AAGTACGCCG CCGTGAGCGG TGGCGAGGAC GACGGGGGAG AAGGATCAAA GAGGGTGATG   
  
  
+ AGAGAGATTG GTCTTCGTCT TGAGAAGTTC GCTAGGTTAA TGGGGGTTCC CTTCAAGTTC AACGTGGTTT   
  
  
+ ACCACGTAGG TGATCTATCC CAGTTGGATT TTTCTCGTTT GGATATTAAA GATGATGAAG CGGTGGCGAT   
  
  
+ TAACTGTGTC AACTCGCTCC ACTCGGTGGA CCTCCGATAT CGGGAGGTAG TGCTCGCGGC GTTCAGGCGG   
  
  
+ CTGCGGCCGA GGGTGGTGAC GGTGGTGGAG GAAGAGGCGG AGCTGACTGA GGTGGGTGAG GGTCAGTATG   
  
  
+ AATTTTTTAG GCGGTTTGAG GAGTGTCTTA GGTGGTTTAG GGTTTACTTT GAGGCCGTAG GAGATTGTTT   
  
  
+ TCCTAGGACT AGCAATGAGA AATTGATGTT AGAGCGGGCC GCGGGCCGGG CCATGGTGGA TCTCCTTGCA   
  
  
+ TGTCCCGAGC CTGGTGGGTC GGCTGAGGGG AGGGAGACGG CGAGACGGTG GTCGGGTCGG ATGCATGGGG   
  
  
+ CGGGGTTCGA GCACGTGGGG TTCAGTGACG AAGTGTGTGA TGATGTGAGG GCCTTGTTGA GGAGGTACAA   
  
  
+ GGAAGGATGG TCAATGACAC AGTGCACCAT GGATGGCGGT AATCACCCTG GAATACTGTT GTGGTGGCGG   
  
  
+ GATCAACCGG TGGTTTGGGC CAGTGCATGG CGGCCTTG  

- +Up\_Stream \_Len000TTTAGT TTGAGTATTT GTTATTAAAA AATTTTGAAT TTACCATTAA TAATCTAATT   
  
  
- ATTTTTAGGA TTACCAACAA ATTACAATGT AGTTCTGTAC GAAAAACCAC ATATATATTA CCGTCGTACA   
  
  
- CCCAATTCGC CGTTGTTTAA TAAGAGTTTA CGTACGTCAT TAATTAAATT AATTAATTAG TGGACGTATT   
  
  
- AGAGTTATGA TTAGGAAAGT AAGACACGGG GTGGGTTGGG GCTTTTATCA TTAGAAAGTT TGCAACCTTC   
  
  
- ACCCTAATCC ATATCTCATC AGGATGTCTA CTACTAGATG GATGTATATT GTATTAGACT GAAAAGAAAA   
  
  
- AATTTATTGA ACATAACTAC CGTGTCTTAA ACTGTACAAC CGTATACCAT GATTCTTTAA TTAATTGCAA   
  
  
- CACGATTCTA TATCCAGTAA GAAAAACCTC TGTATTCCGG GCGCAAGTGC GTCCCAAGCT CTTCCCGGTG   
  
  
- TAAGTTACCT ACTTAACATC CATCAAATTA GACTGAAACA GTCACTGACT AAGGTGCCGA ACTTGGGCGC   
  
  
- TGTAATTATA TCCAACTGAT TTCAGCAAAC TATGAATAAA AAGAATTCAT TTCAGAACTT AAGTTTAGAA   
  
  
- TATTTATTTA TTTTTTTAGA TATTATTTTC TTAAGATGGT AAATAATTCA GTTGTTGAGT TAAGCTTACT   
  
  
- TTATTTTTTA GTTCTAGTGG TTTTTTTTAT TTATTTTCCT TTCCATCTAA CGTAAACCTA AAACTCACCG   
  
  
- CACCACCACC CAAACTACGA CCACGACGTA TGTGGCGTCG AAAAGAGACA GGAAAATGGA GAGAAACAGA   
  
  
- CGTTCTTGGG GTAAACCGTG CGGACGAGAG AAAAGATACA AAAGAAAAGA TAGAAAATTC TTTGTTACCT   
  
  
- TTCGATTGTG TGATTGTGAT TGGGTAACCA GGAAAAAAGA ATAGACGTTT TAATTTCATT GTTATAAAAA   
  
  
- GAATAGGGGA AAAAGCAAGG TGGGTAAAGA AATAGGAAGA CAAACTTGGA TTAGACATAT ATACGTACGT   
  
  
- ACACATAAAA TGAATCTTAG ACAAAACTAA GAAACTACAT TAATTAATCA TCAGAAAAAT TAACTTAAAA   
  
  
- ATTGTCATCA AACTTAATCT GTAAATAGCT ATAATATAAA AAATATATAT TAATTTTTTA TGTTTTACTA   
  
  
- TTCACAGAAT GATGTTATGT GAAGTATTCA CAGCACAAAA TTGAATTTTT AGTATGATAA TACAAAGATT   
  
  
- TAACTTTTTT TTCACTCTAA TTCATCGTCT TCGTATAATG TTGAATATTT AATATATTTT AGTAGAAAAA   
  
  
- TAGGTATTAA ATAGTGTAAA GATTTTATGC GTATAGTATA GATGAATACC TATCCCAATT ATTCTAATCC   
  
  
- CGTAGTAAAT TCTAAAATAG TCGCATTGGT TGCTTACAAA TTCCAATCGT CAAGTATTAA TTCACATTAG   
  
  
- TAATTTTTTA AATACTAAAA TTTAGTATTT TATACAATTA GATCCTACTC CGTTCGCCGT TCTGTATATA   
  
  
- GTGATAGGAA TTTTTTATAT TATATAACTA AAGTTTCTTA ATACTTCATA TAATTAAAGT ATATATTACT   
  
  
- GCTAATACAT AAAAAAATGA TTTAATGAAC ATGGGTATTA TTTATCTTAG CTACGTAGTA AGTACCGGAA   
  
  
- AGTAAGACAT TATACCAACT GAGGTTTTTA AGAGTTTCAT AAAAATATAA AACCCTCCCC TCTAAAATCA   
  
  
- AACTATGAAA ACAACTTGAT CACTTGTTAA AGGTTGTTGG AGGTGGTGGG GGTTTGTAGT TGTTAGACTA   
  
  
- ACTACATACG TAGGGTTCGA ACTTAACTTA TTATTAAAGT TATTGTTGTT ATTAGCAAAA TGGAACTGTT   
  
  
- CGAGGTGTCG TTGAGTTGCA GAAGGTTTGG TTGGGTAGTG AGTAGTAGTA GTAGGGGTGA TAGGGTAAGT   
  
  
- GGGGGTGGTT TTCCTCGGAT TTCTGTCATT GTTCTTCTTA CGAACGGAAA AAAGTACCTA CTTCTTCTGA   
  
  
- AGAGAAGAAG GAGATGAAGA AGGGTGGTGA TACTATGTGT GGTGGAGGTT GTAGTAATGG GGTGGTTAAT   
  
  
- ATTAAGGGCA ATATGGGACC TGGGAGGGAG GTGGTGGTGG TGTCGGTGTT GGTGGTGGAG TAGGAGGTGA   
  
  
- GGGTGGGTGG TACCGAAGCC ACCACCCGAA CTGAGGAGGA GGGGCAGACG GCGACACCTT AATAGAAAGA   
  
  
- GGGGCCTGTA GGAGGAGAGG AGGGGGTGGC CTTTCACCCG CAGACTGTAA GAATACCTCC GCCGATCCCG   
  
  
- AAAAAGTCTG TGCTTGTGGT GGTTCGAGGT GGTCGAGGAG ACCTACGACT TGCTCGAGCC GAGGGGGATG   
  
  
- CCACTACAGC TCGTCTTCGA CCACCGCATG AAATTGGTTC GGGAAAAACG GGCGGAGTGA CGGTGGCCTG   
  
  
- GGGCGGTGGC TCGGCGATAC GAGCGGCGAC GCCTGTTCTG GAGGAGGAAG CTGAGGTGCG AGTCCAACGA   
  
  
- GGAGTTCAAG GTCCTTCAGT CGGGCACCTG GTGGAAGCCG GTGCACCGAA CGTTGCCACG TTAGTAGCTC   
  
  
- CGGAATCTCT CCCTGGGTGT ACTTTTCTCA TTCGAGGTGC AGTAGCTATA GTCGTGGTGG AAGACGTGGG   
  
  
- TCACCGGGTG GAACGAACTC CGAAATCGGT GGGCGTACCT GCTGTGAGGG GTGGAGTCGG AGTGACGGCA   
  
  
- GCAGCAGTTG TTCATGCGGC GGCACTCGCC ACCGCTCCTG CTGCCCCCTC TTCCTAGTTT CTCCCACTAC   
  
  
- TCTCTCTAAC CAGAAGCAGA ACTCTTCAAG CGATCCAATT ACCCCCAAGG GAAGTTCAAG TTGCACCAAA   
  
  
- TGGTGCATCC ACTAGATAGG GTCAACCTAA AAAGAGCAAA CCTATAATTT CTACTACTTC GCCACCGCTA   
  
  
- ATTGACACAG TTGAGCGAGG TGAGCCACCT GGAGGCTATA GCCCTCCATC ACGAGCGCCG CAAGTCCGCC   
  
  
- GACGCCGGCT CCCACCACTG CCACCACCTC CTTCTCCGCC TCGACTGACT CCACCCACTC CCAGTCATAC   
  
  
- TTAAAAAATC CGCCAAACTC CTCACAGAAT CCACCAAATC CCAAATGAAA CTCCGGCATC CTCTAACAAA   
  
  
- AGGATCCTGA TCGTTACTCT TTAACTACAA TCTCGCCCGG CGCCCGGCCC GGTACCACCT AGAGGAACGT   
  
  
- ACAGGGCTCG GACCACCCAG CCGACTCCCC TCCCTCTGCC GCTCTGCCAC CAGCCCAGCC TACGTACCCC   
  
  
- GCCCCAAGCT CGTGCACCCC AAGTCACTGC TTCACACACT ACTACACTCC CGGAACAACT CCTCCATGTT   
  
  
- CCTTCCTACC AGTTACTGTG TCACGTGGTA CCTACCGCCA TTAGTGGGAC CTTATGACAA CACCACCGCC   
  
  
- CTAGTTGGCC ACCAAACCCG GTCACGTACC GCCGGAAC

+     ABRE

| Site Name | Organism | Position | Strand | Matrix score. | sequence | function |
| --- | --- | --- | --- | --- | --- | --- |
| ABRE | Arabidopsis thaliana | 3306 | - | 6 | CACGTG | cis-acting element involved in the abscisic acid responsiveness |
| ABRE | Arabidopsis thaliana | 3307 | + | 5 | ACGTG | cis-acting element involved in the abscisic acid responsiveness |
| ABRE | Arabidopsis thaliana | 2566 | + | 5 | ACGTG | cis-acting element involved in the abscisic acid responsiveness |
| ABRE | Arabidopsis thaliana | 2565 | - | 6 | CACGTG | cis-acting element involved in the abscisic acid responsiveness |
| ABRE | Arabidopsis thaliana | 2877 | - | 5 | ACGTG | cis-acting element involved in the abscisic acid responsiveness |
| ABRE | Oryza sativa | 2563 | - | 9 | GCCGCGTGGC | cis-acting element involved in the abscisic acid responsiveness |
| ABRE | Arabidopsis thaliana | 2866 | + | 5 | ACGTG | cis-acting element involved in the abscisic acid responsiveness |
| ABRE | Arabidopsis thaliana | 2631 | - | 5 | ACGTG | cis-acting element involved in the abscisic acid responsiveness |

>HU05G01983.1   
+ +Up\_Stream \_Len000AAATCA AACTCATAAA CAATAATTTT TTAAAACTTA AATGGTAATT ATTAGATTAA   
  
  
+ TAAAAATCCT AATGGTTGTT TAATGTTACA TCAAGACATG CTTTTTGGTG TATATATAAT GGCAGCATGT   
  
  
+ GGGTTAAGCG GCAACAAATT ATTCTCAAAT GCATGCAGTA ATTAATTTAA TTAATTAATC ACCTGCATAA   
  
  
+ TCTCAATACT AATCCTTTCA TTCTGTGCCC CACCCAACCC CGAAAATAGT AATCTTTCAA ACGTTGGAAG   
  
  
+ TGGGATTAGG TATAGAGTAG TCCTACAGAT GATGATCTAC CTACATATAA CATAATCTGA CTTTTCTTTT   
  
  
+ TTAAATAACT TGTATTGATG GCACAGAATT TGACATGTTG GCATATGGTA CTAAGAAATT AATTAACGTT   
  
  
+ GTGCTAAGAT ATAGGTCATT CTTTTTGGAG ACATAAGGCC CGCGTTCACG CAGGGTTCGA GAAGGGCCAC   
  
  
+ ATTCAATGGA TGAATTGTAG GTAGTTTAAT CTGACTTTGT CAGTGACTGA TTCCACGGCT TGAACCCGCG   
  
  
+ ACATTAATAT AGGTTGACTA AAGTCGTTTG ATACTTATTT TTCTTAAGTA AAGTCTTGAA TTCAAATCTT   
  
  
+ ATAAATAAAT AAAAAAATCT ATAATAAAAG AATTCTACCA TTTATTAAGT CAACAACTCA ATTCGAATGA   
  
  
+ AATAAAAAAT CAAGATCACC AAAAAAAATA AATAAAAGGA AAGGTAGATT GCATTTGGAT TTTGAGTGGC   
  
  
+ GTGGTGGTGG GTTTGATGCT GGTGCTGCAT ACACCGCAGC TTTTCTCTGT CCTTTTACCT CTCTTTGTCT   
  
  
+ GCAAGAACCC CATTTGGCAC GCCTGCTCTC TTTTCTATGT TTTCTTTTCT ATCTTTTAAG AAACAATGGA   
  
  
+ AAGCTAACAC ACTAACACTA ACCCATTGGT CCTTTTTTCT TATCTGCAAA ATTAAAGTAA CAATATTTTT   
  
  
+ CTTATCCCCT TTTTCGTTCC ACCCATTTCT TTATCCTTCT GTTTGAACCT AATCTGTATA TATGCATGCA   
  
  
+ TGTGTATTTT ACTTAGAATC TGTTTTGATT CTTTGATGTA ATTAATTAGT AGTCTTTTTA ATTGAATTTT   
  
  
+ TAACAGTAGT TTGAATTAGA CATTTATCGA TATTATATTT TTTATATATA ATTAAAAAAT ACAAAATGAT   
  
  
+ AAGTGTCTTA CTACAATACA CTTCATAAGT GTCGTGTTTT AACTTAAAAA TCATACTATT ATGTTTCTAA   
  
  
+ ATTGAAAAAA AAGTGAGATT AAGTAGCAGA AGCATATTAC AACTTATAAA TTATATAAAA TCATCTTTTT   
  
  
+ ATCCATAATT TATCACATTT CTAAAATACG CATATCATAT CTACTTATGG ATAGGGTTAA TAAGATTAGG   
  
  
+ GCATCATTTA AGATTTTATC AGCGTAACCA ACGAATGTTT AAGGTTAGCA GTTCATAATT AAGTGTAATC   
  
  
+ ATTAAAAAAT TTATGATTTT AAATCATAAA ATATGTTAAT CTAGGATGAG GCAAGCGGCA AGACATATAT   
  
  
+ CACTATCCTT AAAAAATATA ATATATTGAT TTCAAAGAAT TATGAAGTAT ATTAATTTCA TATATAATGA   
  
  
+ CGATTATGTA TTTTTTTACT AAATTACTTG TACCCATAAT AAATAGAATC GATGCATCAT TCATGGCCTT   
  
  
+ TCATTCTGTA ATATGGTTGA CTCCAAAAAT TCTCAAAGTA TTTTTATATT TTGGGAGGGG AGATTTTAGT   
  
  
+ TTGATACTTT TGTTGAACTA GTGAACAATT TCCAACAACC TCCACCACCC CCAAACATCA ACAATCTGAT   
  
  
+ TGATGTATGC ATCCCAAGCT TGAATTGAAT AATAATTTCA ATAACAACAA TAATCGTTTT ACCTTGACAA   
  
  
+ GCTCCACAGC AACTCAACGT CTTCCAAACC AACCCATCAC TCATCATCAT CATCCCCACT ATCCCATTCA   
  
  
+ CCCCCACCAA AAGGAGCCTA AAGACAGTAA CAAGAAGAAT GCTTGCCTTT TTTCATGGAT GAAGAAGACT   
  
  
+ TCTCTTCTTC CTCTACTTCT TCCCACCACT ATGATACACA CCACCTCCAA CATCATTACC CCACCAATTA   
  
  
+ TAATTCCCGT TATACCCTGG ACCCTCCCTC CACCACCACC ACAGCCACAA CCACCACCTC ATCCTCCACT   
  
  
+ CCCACCCACC ATGGCTTCGG TGGTGGGCTT GACTCCTCCT CCCCGTCTGC CGCTGTGGAA TTATCTTTCT   
  
  
+ CCCCGGACAT CCTCCTCTCC TCCCCCACCG GAAAGTGGGC GTCTGACATT CTTATGGAGG CGGCTAGGGC   
  
  
+ TTTTTCAGAC ACGAACACCA CCAAGCTCCA CCAGCTCCTC TGGATGCTGA ACGAGCTCGG CTCCCCCTAC   
  
  
+ GGTGATGTCG AGCAGAAGCT GGTGGCGTAC TTTAACCAAG CCCTTTTTGC CCGCCTCACT GCCACCGGAC   
  
  
+ CCCGCCACCG AGCCGCTATG CTCGCCGCTG CGGACAAGAC CTCCTCCTTC GACTCCACGC TCAGGTTGCT   
  
  
+ CCTCAAGTTC CAGGAAGTCA GCCCGTGGAC CACCTTCGGC CACGTGGCTT GCAACGGTGC AATCATCGAG   
  
  
+ GCCTTAGAGA GGGACCCACA TGAAAAGAGT AAGCTCCACG TCATCGATAT CAGCACCACC TTCTGCACCC   
  
  
+ AGTGGCCCAC CTTGCTTGAG GCTTTAGCCA CCCGCATGGA CGACACTCCC CACCTCAGCC TCACTGCCGT   
  
  
+ CGTCGTCAAC AAGTACGCCG CCGTGAGCGG TGGCGAGGAC GACGGGGGAG AAGGATCAAA GAGGGTGATG   
  
  
+ AGAGAGATTG GTCTTCGTCT TGAGAAGTTC GCTAGGTTAA TGGGGGTTCC CTTCAAGTTC AACGTGGTTT   
  
  
+ ACCACGTAGG TGATCTATCC CAGTTGGATT TTTCTCGTTT GGATATTAAA GATGATGAAG CGGTGGCGAT   
  
  
+ TAACTGTGTC AACTCGCTCC ACTCGGTGGA CCTCCGATAT CGGGAGGTAG TGCTCGCGGC GTTCAGGCGG   
  
  
+ CTGCGGCCGA GGGTGGTGAC GGTGGTGGAG GAAGAGGCGG AGCTGACTGA GGTGGGTGAG GGTCAGTATG   
  
  
+ AATTTTTTAG GCGGTTTGAG GAGTGTCTTA GGTGGTTTAG GGTTTACTTT GAGGCCGTAG GAGATTGTTT   
  
  
+ TCCTAGGACT AGCAATGAGA AATTGATGTT AGAGCGGGCC GCGGGCCGGG CCATGGTGGA TCTCCTTGCA   
  
  
+ TGTCCCGAGC CTGGTGGGTC GGCTGAGGGG AGGGAGACGG CGAGACGGTG GTCGGGTCGG ATGCATGGGG   
  
  
+ CGGGGTTCGA GCACGTGGGG TTCAGTGACG AAGTGTGTGA TGATGTGAGG GCCTTGTTGA GGAGGTACAA   
  
  
+ GGAAGGATGG TCAATGACAC AGTGCACCAT GGATGGCGGT AATCACCCTG GAATACTGTT GTGGTGGCGG   
  
  
+ GATCAACCGG TGGTTTGGGC CAGTGCATGG CGGCCTTG  

- +Up\_Stream \_Len000TTTAGT TTGAGTATTT GTTATTAAAA AATTTTGAAT TTACCATTAA TAATCTAATT   
  
  
- ATTTTTAGGA TTACCAACAA ATTACAATGT AGTTCTGTAC GAAAAACCAC ATATATATTA CCGTCGTACA   
  
  
- CCCAATTCGC CGTTGTTTAA TAAGAGTTTA CGTACGTCAT TAATTAAATT AATTAATTAG TGGACGTATT   
  
  
- AGAGTTATGA TTAGGAAAGT AAGACACGGG GTGGGTTGGG GCTTTTATCA TTAGAAAGTT TGCAACCTTC   
  
  
- ACCCTAATCC ATATCTCATC AGGATGTCTA CTACTAGATG GATGTATATT GTATTAGACT GAAAAGAAAA   
  
  
- AATTTATTGA ACATAACTAC CGTGTCTTAA ACTGTACAAC CGTATACCAT GATTCTTTAA TTAATTGCAA   
  
  
- CACGATTCTA TATCCAGTAA GAAAAACCTC TGTATTCCGG GCGCAAGTGC GTCCCAAGCT CTTCCCGGTG   
  
  
- TAAGTTACCT ACTTAACATC CATCAAATTA GACTGAAACA GTCACTGACT AAGGTGCCGA ACTTGGGCGC   
  
  
- TGTAATTATA TCCAACTGAT TTCAGCAAAC TATGAATAAA AAGAATTCAT TTCAGAACTT AAGTTTAGAA   
  
  
- TATTTATTTA TTTTTTTAGA TATTATTTTC TTAAGATGGT AAATAATTCA GTTGTTGAGT TAAGCTTACT   
  
  
- TTATTTTTTA GTTCTAGTGG TTTTTTTTAT TTATTTTCCT TTCCATCTAA CGTAAACCTA AAACTCACCG   
  
  
- CACCACCACC CAAACTACGA CCACGACGTA TGTGGCGTCG AAAAGAGACA GGAAAATGGA GAGAAACAGA   
  
  
- CGTTCTTGGG GTAAACCGTG CGGACGAGAG AAAAGATACA AAAGAAAAGA TAGAAAATTC TTTGTTACCT   
  
  
- TTCGATTGTG TGATTGTGAT TGGGTAACCA GGAAAAAAGA ATAGACGTTT TAATTTCATT GTTATAAAAA   
  
  
- GAATAGGGGA AAAAGCAAGG TGGGTAAAGA AATAGGAAGA CAAACTTGGA TTAGACATAT ATACGTACGT   
  
  
- ACACATAAAA TGAATCTTAG ACAAAACTAA GAAACTACAT TAATTAATCA TCAGAAAAAT TAACTTAAAA   
  
  
- ATTGTCATCA AACTTAATCT GTAAATAGCT ATAATATAAA AAATATATAT TAATTTTTTA TGTTTTACTA   
  
  
- TTCACAGAAT GATGTTATGT GAAGTATTCA CAGCACAAAA TTGAATTTTT AGTATGATAA TACAAAGATT   
  
  
- TAACTTTTTT TTCACTCTAA TTCATCGTCT TCGTATAATG TTGAATATTT AATATATTTT AGTAGAAAAA   
  
  
- TAGGTATTAA ATAGTGTAAA GATTTTATGC GTATAGTATA GATGAATACC TATCCCAATT ATTCTAATCC   
  
  
- CGTAGTAAAT TCTAAAATAG TCGCATTGGT TGCTTACAAA TTCCAATCGT CAAGTATTAA TTCACATTAG   
  
  
- TAATTTTTTA AATACTAAAA TTTAGTATTT TATACAATTA GATCCTACTC CGTTCGCCGT TCTGTATATA   
  
  
- GTGATAGGAA TTTTTTATAT TATATAACTA AAGTTTCTTA ATACTTCATA TAATTAAAGT ATATATTACT   
  
  
- GCTAATACAT AAAAAAATGA TTTAATGAAC ATGGGTATTA TTTATCTTAG CTACGTAGTA AGTACCGGAA   
  
  
- AGTAAGACAT TATACCAACT GAGGTTTTTA AGAGTTTCAT AAAAATATAA AACCCTCCCC TCTAAAATCA   
  
  
- AACTATGAAA ACAACTTGAT CACTTGTTAA AGGTTGTTGG AGGTGGTGGG GGTTTGTAGT TGTTAGACTA   
  
  
- ACTACATACG TAGGGTTCGA ACTTAACTTA TTATTAAAGT TATTGTTGTT ATTAGCAAAA TGGAACTGTT   
  
  
- CGAGGTGTCG TTGAGTTGCA GAAGGTTTGG TTGGGTAGTG AGTAGTAGTA GTAGGGGTGA TAGGGTAAGT   
  
  
- GGGGGTGGTT TTCCTCGGAT TTCTGTCATT GTTCTTCTTA CGAACGGAAA AAAGTACCTA CTTCTTCTGA   
  
  
- AGAGAAGAAG GAGATGAAGA AGGGTGGTGA TACTATGTGT GGTGGAGGTT GTAGTAATGG GGTGGTTAAT   
  
  
- ATTAAGGGCA ATATGGGACC TGGGAGGGAG GTGGTGGTGG TGTCGGTGTT GGTGGTGGAG TAGGAGGTGA   
  
  
- GGGTGGGTGG TACCGAAGCC ACCACCCGAA CTGAGGAGGA GGGGCAGACG GCGACACCTT AATAGAAAGA   
  
  
- GGGGCCTGTA GGAGGAGAGG AGGGGGTGGC CTTTCACCCG CAGACTGTAA GAATACCTCC GCCGATCCCG   
  
  
- AAAAAGTCTG TGCTTGTGGT GGTTCGAGGT GGTCGAGGAG ACCTACGACT TGCTCGAGCC GAGGGGGATG   
  
  
- CCACTACAGC TCGTCTTCGA CCACCGCATG AAATTGGTTC GGGAAAAACG GGCGGAGTGA CGGTGGCCTG   
  
  
- GGGCGGTGGC TCGGCGATAC GAGCGGCGAC GCCTGTTCTG GAGGAGGAAG CTGAGGTGCG AGTCCAACGA   
  
  
- GGAGTTCAAG GTCCTTCAGT CGGGCACCTG GTGGAAGCCG GTGCACCGAA CGTTGCCACG TTAGTAGCTC   
  
  
- CGGAATCTCT CCCTGGGTGT ACTTTTCTCA TTCGAGGTGC AGTAGCTATA GTCGTGGTGG AAGACGTGGG   
  
  
- TCACCGGGTG GAACGAACTC CGAAATCGGT GGGCGTACCT GCTGTGAGGG GTGGAGTCGG AGTGACGGCA   
  
  
- GCAGCAGTTG TTCATGCGGC GGCACTCGCC ACCGCTCCTG CTGCCCCCTC TTCCTAGTTT CTCCCACTAC   
  
  
- TCTCTCTAAC CAGAAGCAGA ACTCTTCAAG CGATCCAATT ACCCCCAAGG GAAGTTCAAG TTGCACCAAA   
  
  
- TGGTGCATCC ACTAGATAGG GTCAACCTAA AAAGAGCAAA CCTATAATTT CTACTACTTC GCCACCGCTA   
  
  
- ATTGACACAG TTGAGCGAGG TGAGCCACCT GGAGGCTATA GCCCTCCATC ACGAGCGCCG CAAGTCCGCC   
  
  
- GACGCCGGCT CCCACCACTG CCACCACCTC CTTCTCCGCC TCGACTGACT CCACCCACTC CCAGTCATAC   
  
  
- TTAAAAAATC CGCCAAACTC CTCACAGAAT CCACCAAATC CCAAATGAAA CTCCGGCATC CTCTAACAAA   
  
  
- AGGATCCTGA TCGTTACTCT TTAACTACAA TCTCGCCCGG CGCCCGGCCC GGTACCACCT AGAGGAACGT   
  
  
- ACAGGGCTCG GACCACCCAG CCGACTCCCC TCCCTCTGCC GCTCTGCCAC CAGCCCAGCC TACGTACCCC   
  
  
- GCCCCAAGCT CGTGCACCCC AAGTCACTGC TTCACACACT ACTACACTCC CGGAACAACT CCTCCATGTT   
  
  
- CCTTCCTACC AGTTACTGTG TCACGTGGTA CCTACCGCCA TTAGTGGGAC CTTATGACAA CACCACCGCC   
  
  
- CTAGTTGGCC ACCAAACCCG GTCACGTACC GCCGGAAC

+     ABRE2

| Site Name | Organism | Position | Strand | Matrix score. | sequence | function |
| --- | --- | --- | --- | --- | --- | --- |
| ABRE2 | Zea mays | 2564 | - | 8 | CCACGTGG |  |

>HU05G01983.1   
+ +Up\_Stream \_Len000AAATCA AACTCATAAA CAATAATTTT TTAAAACTTA AATGGTAATT ATTAGATTAA   
  
  
+ TAAAAATCCT AATGGTTGTT TAATGTTACA TCAAGACATG CTTTTTGGTG TATATATAAT GGCAGCATGT   
  
  
+ GGGTTAAGCG GCAACAAATT ATTCTCAAAT GCATGCAGTA ATTAATTTAA TTAATTAATC ACCTGCATAA   
  
  
+ TCTCAATACT AATCCTTTCA TTCTGTGCCC CACCCAACCC CGAAAATAGT AATCTTTCAA ACGTTGGAAG   
  
  
+ TGGGATTAGG TATAGAGTAG TCCTACAGAT GATGATCTAC CTACATATAA CATAATCTGA CTTTTCTTTT   
  
  
+ TTAAATAACT TGTATTGATG GCACAGAATT TGACATGTTG GCATATGGTA CTAAGAAATT AATTAACGTT   
  
  
+ GTGCTAAGAT ATAGGTCATT CTTTTTGGAG ACATAAGGCC CGCGTTCACG CAGGGTTCGA GAAGGGCCAC   
  
  
+ ATTCAATGGA TGAATTGTAG GTAGTTTAAT CTGACTTTGT CAGTGACTGA TTCCACGGCT TGAACCCGCG   
  
  
+ ACATTAATAT AGGTTGACTA AAGTCGTTTG ATACTTATTT TTCTTAAGTA AAGTCTTGAA TTCAAATCTT   
  
  
+ ATAAATAAAT AAAAAAATCT ATAATAAAAG AATTCTACCA TTTATTAAGT CAACAACTCA ATTCGAATGA   
  
  
+ AATAAAAAAT CAAGATCACC AAAAAAAATA AATAAAAGGA AAGGTAGATT GCATTTGGAT TTTGAGTGGC   
  
  
+ GTGGTGGTGG GTTTGATGCT GGTGCTGCAT ACACCGCAGC TTTTCTCTGT CCTTTTACCT CTCTTTGTCT   
  
  
+ GCAAGAACCC CATTTGGCAC GCCTGCTCTC TTTTCTATGT TTTCTTTTCT ATCTTTTAAG AAACAATGGA   
  
  
+ AAGCTAACAC ACTAACACTA ACCCATTGGT CCTTTTTTCT TATCTGCAAA ATTAAAGTAA CAATATTTTT   
  
  
+ CTTATCCCCT TTTTCGTTCC ACCCATTTCT TTATCCTTCT GTTTGAACCT AATCTGTATA TATGCATGCA   
  
  
+ TGTGTATTTT ACTTAGAATC TGTTTTGATT CTTTGATGTA ATTAATTAGT AGTCTTTTTA ATTGAATTTT   
  
  
+ TAACAGTAGT TTGAATTAGA CATTTATCGA TATTATATTT TTTATATATA ATTAAAAAAT ACAAAATGAT   
  
  
+ AAGTGTCTTA CTACAATACA CTTCATAAGT GTCGTGTTTT AACTTAAAAA TCATACTATT ATGTTTCTAA   
  
  
+ ATTGAAAAAA AAGTGAGATT AAGTAGCAGA AGCATATTAC AACTTATAAA TTATATAAAA TCATCTTTTT   
  
  
+ ATCCATAATT TATCACATTT CTAAAATACG CATATCATAT CTACTTATGG ATAGGGTTAA TAAGATTAGG   
  
  
+ GCATCATTTA AGATTTTATC AGCGTAACCA ACGAATGTTT AAGGTTAGCA GTTCATAATT AAGTGTAATC   
  
  
+ ATTAAAAAAT TTATGATTTT AAATCATAAA ATATGTTAAT CTAGGATGAG GCAAGCGGCA AGACATATAT   
  
  
+ CACTATCCTT AAAAAATATA ATATATTGAT TTCAAAGAAT TATGAAGTAT ATTAATTTCA TATATAATGA   
  
  
+ CGATTATGTA TTTTTTTACT AAATTACTTG TACCCATAAT AAATAGAATC GATGCATCAT TCATGGCCTT   
  
  
+ TCATTCTGTA ATATGGTTGA CTCCAAAAAT TCTCAAAGTA TTTTTATATT TTGGGAGGGG AGATTTTAGT   
  
  
+ TTGATACTTT TGTTGAACTA GTGAACAATT TCCAACAACC TCCACCACCC CCAAACATCA ACAATCTGAT   
  
  
+ TGATGTATGC ATCCCAAGCT TGAATTGAAT AATAATTTCA ATAACAACAA TAATCGTTTT ACCTTGACAA   
  
  
+ GCTCCACAGC AACTCAACGT CTTCCAAACC AACCCATCAC TCATCATCAT CATCCCCACT ATCCCATTCA   
  
  
+ CCCCCACCAA AAGGAGCCTA AAGACAGTAA CAAGAAGAAT GCTTGCCTTT TTTCATGGAT GAAGAAGACT   
  
  
+ TCTCTTCTTC CTCTACTTCT TCCCACCACT ATGATACACA CCACCTCCAA CATCATTACC CCACCAATTA   
  
  
+ TAATTCCCGT TATACCCTGG ACCCTCCCTC CACCACCACC ACAGCCACAA CCACCACCTC ATCCTCCACT   
  
  
+ CCCACCCACC ATGGCTTCGG TGGTGGGCTT GACTCCTCCT CCCCGTCTGC CGCTGTGGAA TTATCTTTCT   
  
  
+ CCCCGGACAT CCTCCTCTCC TCCCCCACCG GAAAGTGGGC GTCTGACATT CTTATGGAGG CGGCTAGGGC   
  
  
+ TTTTTCAGAC ACGAACACCA CCAAGCTCCA CCAGCTCCTC TGGATGCTGA ACGAGCTCGG CTCCCCCTAC   
  
  
+ GGTGATGTCG AGCAGAAGCT GGTGGCGTAC TTTAACCAAG CCCTTTTTGC CCGCCTCACT GCCACCGGAC   
  
  
+ CCCGCCACCG AGCCGCTATG CTCGCCGCTG CGGACAAGAC CTCCTCCTTC GACTCCACGC TCAGGTTGCT   
  
  
+ CCTCAAGTTC CAGGAAGTCA GCCCGTGGAC CACCTTCGGC CACGTGGCTT GCAACGGTGC AATCATCGAG   
  
  
+ GCCTTAGAGA GGGACCCACA TGAAAAGAGT AAGCTCCACG TCATCGATAT CAGCACCACC TTCTGCACCC   
  
  
+ AGTGGCCCAC CTTGCTTGAG GCTTTAGCCA CCCGCATGGA CGACACTCCC CACCTCAGCC TCACTGCCGT   
  
  
+ CGTCGTCAAC AAGTACGCCG CCGTGAGCGG TGGCGAGGAC GACGGGGGAG AAGGATCAAA GAGGGTGATG   
  
  
+ AGAGAGATTG GTCTTCGTCT TGAGAAGTTC GCTAGGTTAA TGGGGGTTCC CTTCAAGTTC AACGTGGTTT   
  
  
+ ACCACGTAGG TGATCTATCC CAGTTGGATT TTTCTCGTTT GGATATTAAA GATGATGAAG CGGTGGCGAT   
  
  
+ TAACTGTGTC AACTCGCTCC ACTCGGTGGA CCTCCGATAT CGGGAGGTAG TGCTCGCGGC GTTCAGGCGG   
  
  
+ CTGCGGCCGA GGGTGGTGAC GGTGGTGGAG GAAGAGGCGG AGCTGACTGA GGTGGGTGAG GGTCAGTATG   
  
  
+ AATTTTTTAG GCGGTTTGAG GAGTGTCTTA GGTGGTTTAG GGTTTACTTT GAGGCCGTAG GAGATTGTTT   
  
  
+ TCCTAGGACT AGCAATGAGA AATTGATGTT AGAGCGGGCC GCGGGCCGGG CCATGGTGGA TCTCCTTGCA   
  
  
+ TGTCCCGAGC CTGGTGGGTC GGCTGAGGGG AGGGAGACGG CGAGACGGTG GTCGGGTCGG ATGCATGGGG   
  
  
+ CGGGGTTCGA GCACGTGGGG TTCAGTGACG AAGTGTGTGA TGATGTGAGG GCCTTGTTGA GGAGGTACAA   
  
  
+ GGAAGGATGG TCAATGACAC AGTGCACCAT GGATGGCGGT AATCACCCTG GAATACTGTT GTGGTGGCGG   
  
  
+ GATCAACCGG TGGTTTGGGC CAGTGCATGG CGGCCTTG  

- +Up\_Stream \_Len000TTTAGT TTGAGTATTT GTTATTAAAA AATTTTGAAT TTACCATTAA TAATCTAATT   
  
  
- ATTTTTAGGA TTACCAACAA ATTACAATGT AGTTCTGTAC GAAAAACCAC ATATATATTA CCGTCGTACA   
  
  
- CCCAATTCGC CGTTGTTTAA TAAGAGTTTA CGTACGTCAT TAATTAAATT AATTAATTAG TGGACGTATT   
  
  
- AGAGTTATGA TTAGGAAAGT AAGACACGGG GTGGGTTGGG GCTTTTATCA TTAGAAAGTT TGCAACCTTC   
  
  
- ACCCTAATCC ATATCTCATC AGGATGTCTA CTACTAGATG GATGTATATT GTATTAGACT GAAAAGAAAA   
  
  
- AATTTATTGA ACATAACTAC CGTGTCTTAA ACTGTACAAC CGTATACCAT GATTCTTTAA TTAATTGCAA   
  
  
- CACGATTCTA TATCCAGTAA GAAAAACCTC TGTATTCCGG GCGCAAGTGC GTCCCAAGCT CTTCCCGGTG   
  
  
- TAAGTTACCT ACTTAACATC CATCAAATTA GACTGAAACA GTCACTGACT AAGGTGCCGA ACTTGGGCGC   
  
  
- TGTAATTATA TCCAACTGAT TTCAGCAAAC TATGAATAAA AAGAATTCAT TTCAGAACTT AAGTTTAGAA   
  
  
- TATTTATTTA TTTTTTTAGA TATTATTTTC TTAAGATGGT AAATAATTCA GTTGTTGAGT TAAGCTTACT   
  
  
- TTATTTTTTA GTTCTAGTGG TTTTTTTTAT TTATTTTCCT TTCCATCTAA CGTAAACCTA AAACTCACCG   
  
  
- CACCACCACC CAAACTACGA CCACGACGTA TGTGGCGTCG AAAAGAGACA GGAAAATGGA GAGAAACAGA   
  
  
- CGTTCTTGGG GTAAACCGTG CGGACGAGAG AAAAGATACA AAAGAAAAGA TAGAAAATTC TTTGTTACCT   
  
  
- TTCGATTGTG TGATTGTGAT TGGGTAACCA GGAAAAAAGA ATAGACGTTT TAATTTCATT GTTATAAAAA   
  
  
- GAATAGGGGA AAAAGCAAGG TGGGTAAAGA AATAGGAAGA CAAACTTGGA TTAGACATAT ATACGTACGT   
  
  
- ACACATAAAA TGAATCTTAG ACAAAACTAA GAAACTACAT TAATTAATCA TCAGAAAAAT TAACTTAAAA   
  
  
- ATTGTCATCA AACTTAATCT GTAAATAGCT ATAATATAAA AAATATATAT TAATTTTTTA TGTTTTACTA   
  
  
- TTCACAGAAT GATGTTATGT GAAGTATTCA CAGCACAAAA TTGAATTTTT AGTATGATAA TACAAAGATT   
  
  
- TAACTTTTTT TTCACTCTAA TTCATCGTCT TCGTATAATG TTGAATATTT AATATATTTT AGTAGAAAAA   
  
  
- TAGGTATTAA ATAGTGTAAA GATTTTATGC GTATAGTATA GATGAATACC TATCCCAATT ATTCTAATCC   
  
  
- CGTAGTAAAT TCTAAAATAG TCGCATTGGT TGCTTACAAA TTCCAATCGT CAAGTATTAA TTCACATTAG   
  
  
- TAATTTTTTA AATACTAAAA TTTAGTATTT TATACAATTA GATCCTACTC CGTTCGCCGT TCTGTATATA   
  
  
- GTGATAGGAA TTTTTTATAT TATATAACTA AAGTTTCTTA ATACTTCATA TAATTAAAGT ATATATTACT   
  
  
- GCTAATACAT AAAAAAATGA TTTAATGAAC ATGGGTATTA TTTATCTTAG CTACGTAGTA AGTACCGGAA   
  
  
- AGTAAGACAT TATACCAACT GAGGTTTTTA AGAGTTTCAT AAAAATATAA AACCCTCCCC TCTAAAATCA   
  
  
- AACTATGAAA ACAACTTGAT CACTTGTTAA AGGTTGTTGG AGGTGGTGGG GGTTTGTAGT TGTTAGACTA   
  
  
- ACTACATACG TAGGGTTCGA ACTTAACTTA TTATTAAAGT TATTGTTGTT ATTAGCAAAA TGGAACTGTT   
  
  
- CGAGGTGTCG TTGAGTTGCA GAAGGTTTGG TTGGGTAGTG AGTAGTAGTA GTAGGGGTGA TAGGGTAAGT   
  
  
- GGGGGTGGTT TTCCTCGGAT TTCTGTCATT GTTCTTCTTA CGAACGGAAA AAAGTACCTA CTTCTTCTGA   
  
  
- AGAGAAGAAG GAGATGAAGA AGGGTGGTGA TACTATGTGT GGTGGAGGTT GTAGTAATGG GGTGGTTAAT   
  
  
- ATTAAGGGCA ATATGGGACC TGGGAGGGAG GTGGTGGTGG TGTCGGTGTT GGTGGTGGAG TAGGAGGTGA   
  
  
- GGGTGGGTGG TACCGAAGCC ACCACCCGAA CTGAGGAGGA GGGGCAGACG GCGACACCTT AATAGAAAGA   
  
  
- GGGGCCTGTA GGAGGAGAGG AGGGGGTGGC CTTTCACCCG CAGACTGTAA GAATACCTCC GCCGATCCCG   
  
  
- AAAAAGTCTG TGCTTGTGGT GGTTCGAGGT GGTCGAGGAG ACCTACGACT TGCTCGAGCC GAGGGGGATG   
  
  
- CCACTACAGC TCGTCTTCGA CCACCGCATG AAATTGGTTC GGGAAAAACG GGCGGAGTGA CGGTGGCCTG   
  
  
- GGGCGGTGGC TCGGCGATAC GAGCGGCGAC GCCTGTTCTG GAGGAGGAAG CTGAGGTGCG AGTCCAACGA   
  
  
- GGAGTTCAAG GTCCTTCAGT CGGGCACCTG GTGGAAGCCG GTGCACCGAA CGTTGCCACG TTAGTAGCTC   
  
  
- CGGAATCTCT CCCTGGGTGT ACTTTTCTCA TTCGAGGTGC AGTAGCTATA GTCGTGGTGG AAGACGTGGG   
  
  
- TCACCGGGTG GAACGAACTC CGAAATCGGT GGGCGTACCT GCTGTGAGGG GTGGAGTCGG AGTGACGGCA   
  
  
- GCAGCAGTTG TTCATGCGGC GGCACTCGCC ACCGCTCCTG CTGCCCCCTC TTCCTAGTTT CTCCCACTAC   
  
  
- TCTCTCTAAC CAGAAGCAGA ACTCTTCAAG CGATCCAATT ACCCCCAAGG GAAGTTCAAG TTGCACCAAA   
  
  
- TGGTGCATCC ACTAGATAGG GTCAACCTAA AAAGAGCAAA CCTATAATTT CTACTACTTC GCCACCGCTA   
  
  
- ATTGACACAG TTGAGCGAGG TGAGCCACCT GGAGGCTATA GCCCTCCATC ACGAGCGCCG CAAGTCCGCC   
  
  
- GACGCCGGCT CCCACCACTG CCACCACCTC CTTCTCCGCC TCGACTGACT CCACCCACTC CCAGTCATAC   
  
  
- TTAAAAAATC CGCCAAACTC CTCACAGAAT CCACCAAATC CCAAATGAAA CTCCGGCATC CTCTAACAAA   
  
  
- AGGATCCTGA TCGTTACTCT TTAACTACAA TCTCGCCCGG CGCCCGGCCC GGTACCACCT AGAGGAACGT   
  
  
- ACAGGGCTCG GACCACCCAG CCGACTCCCC TCCCTCTGCC GCTCTGCCAC CAGCCCAGCC TACGTACCCC   
  
  
- GCCCCAAGCT CGTGCACCCC AAGTCACTGC TTCACACACT ACTACACTCC CGGAACAACT CCTCCATGTT   
  
  
- CCTTCCTACC AGTTACTGTG TCACGTGGTA CCTACCGCCA TTAGTGGGAC CTTATGACAA CACCACCGCC   
  
  
- CTAGTTGGCC ACCAAACCCG GTCACGTACC GCCGGAAC

+     ABRE3a

| Site Name | Organism | Position | Strand | Matrix score. | sequence | function |
| --- | --- | --- | --- | --- | --- | --- |
| ABRE3a | Zea mays | 2877 | - | 6 | TACGTG |  |

>HU05G01983.1   
+ +Up\_Stream \_Len000AAATCA AACTCATAAA CAATAATTTT TTAAAACTTA AATGGTAATT ATTAGATTAA   
  
  
+ TAAAAATCCT AATGGTTGTT TAATGTTACA TCAAGACATG CTTTTTGGTG TATATATAAT GGCAGCATGT   
  
  
+ GGGTTAAGCG GCAACAAATT ATTCTCAAAT GCATGCAGTA ATTAATTTAA TTAATTAATC ACCTGCATAA   
  
  
+ TCTCAATACT AATCCTTTCA TTCTGTGCCC CACCCAACCC CGAAAATAGT AATCTTTCAA ACGTTGGAAG   
  
  
+ TGGGATTAGG TATAGAGTAG TCCTACAGAT GATGATCTAC CTACATATAA CATAATCTGA CTTTTCTTTT   
  
  
+ TTAAATAACT TGTATTGATG GCACAGAATT TGACATGTTG GCATATGGTA CTAAGAAATT AATTAACGTT   
  
  
+ GTGCTAAGAT ATAGGTCATT CTTTTTGGAG ACATAAGGCC CGCGTTCACG CAGGGTTCGA GAAGGGCCAC   
  
  
+ ATTCAATGGA TGAATTGTAG GTAGTTTAAT CTGACTTTGT CAGTGACTGA TTCCACGGCT TGAACCCGCG   
  
  
+ ACATTAATAT AGGTTGACTA AAGTCGTTTG ATACTTATTT TTCTTAAGTA AAGTCTTGAA TTCAAATCTT   
  
  
+ ATAAATAAAT AAAAAAATCT ATAATAAAAG AATTCTACCA TTTATTAAGT CAACAACTCA ATTCGAATGA   
  
  
+ AATAAAAAAT CAAGATCACC AAAAAAAATA AATAAAAGGA AAGGTAGATT GCATTTGGAT TTTGAGTGGC   
  
  
+ GTGGTGGTGG GTTTGATGCT GGTGCTGCAT ACACCGCAGC TTTTCTCTGT CCTTTTACCT CTCTTTGTCT   
  
  
+ GCAAGAACCC CATTTGGCAC GCCTGCTCTC TTTTCTATGT TTTCTTTTCT ATCTTTTAAG AAACAATGGA   
  
  
+ AAGCTAACAC ACTAACACTA ACCCATTGGT CCTTTTTTCT TATCTGCAAA ATTAAAGTAA CAATATTTTT   
  
  
+ CTTATCCCCT TTTTCGTTCC ACCCATTTCT TTATCCTTCT GTTTGAACCT AATCTGTATA TATGCATGCA   
  
  
+ TGTGTATTTT ACTTAGAATC TGTTTTGATT CTTTGATGTA ATTAATTAGT AGTCTTTTTA ATTGAATTTT   
  
  
+ TAACAGTAGT TTGAATTAGA CATTTATCGA TATTATATTT TTTATATATA ATTAAAAAAT ACAAAATGAT   
  
  
+ AAGTGTCTTA CTACAATACA CTTCATAAGT GTCGTGTTTT AACTTAAAAA TCATACTATT ATGTTTCTAA   
  
  
+ ATTGAAAAAA AAGTGAGATT AAGTAGCAGA AGCATATTAC AACTTATAAA TTATATAAAA TCATCTTTTT   
  
  
+ ATCCATAATT TATCACATTT CTAAAATACG CATATCATAT CTACTTATGG ATAGGGTTAA TAAGATTAGG   
  
  
+ GCATCATTTA AGATTTTATC AGCGTAACCA ACGAATGTTT AAGGTTAGCA GTTCATAATT AAGTGTAATC   
  
  
+ ATTAAAAAAT TTATGATTTT AAATCATAAA ATATGTTAAT CTAGGATGAG GCAAGCGGCA AGACATATAT   
  
  
+ CACTATCCTT AAAAAATATA ATATATTGAT TTCAAAGAAT TATGAAGTAT ATTAATTTCA TATATAATGA   
  
  
+ CGATTATGTA TTTTTTTACT AAATTACTTG TACCCATAAT AAATAGAATC GATGCATCAT TCATGGCCTT   
  
  
+ TCATTCTGTA ATATGGTTGA CTCCAAAAAT TCTCAAAGTA TTTTTATATT TTGGGAGGGG AGATTTTAGT   
  
  
+ TTGATACTTT TGTTGAACTA GTGAACAATT TCCAACAACC TCCACCACCC CCAAACATCA ACAATCTGAT   
  
  
+ TGATGTATGC ATCCCAAGCT TGAATTGAAT AATAATTTCA ATAACAACAA TAATCGTTTT ACCTTGACAA   
  
  
+ GCTCCACAGC AACTCAACGT CTTCCAAACC AACCCATCAC TCATCATCAT CATCCCCACT ATCCCATTCA   
  
  
+ CCCCCACCAA AAGGAGCCTA AAGACAGTAA CAAGAAGAAT GCTTGCCTTT TTTCATGGAT GAAGAAGACT   
  
  
+ TCTCTTCTTC CTCTACTTCT TCCCACCACT ATGATACACA CCACCTCCAA CATCATTACC CCACCAATTA   
  
  
+ TAATTCCCGT TATACCCTGG ACCCTCCCTC CACCACCACC ACAGCCACAA CCACCACCTC ATCCTCCACT   
  
  
+ CCCACCCACC ATGGCTTCGG TGGTGGGCTT GACTCCTCCT CCCCGTCTGC CGCTGTGGAA TTATCTTTCT   
  
  
+ CCCCGGACAT CCTCCTCTCC TCCCCCACCG GAAAGTGGGC GTCTGACATT CTTATGGAGG CGGCTAGGGC   
  
  
+ TTTTTCAGAC ACGAACACCA CCAAGCTCCA CCAGCTCCTC TGGATGCTGA ACGAGCTCGG CTCCCCCTAC   
  
  
+ GGTGATGTCG AGCAGAAGCT GGTGGCGTAC TTTAACCAAG CCCTTTTTGC CCGCCTCACT GCCACCGGAC   
  
  
+ CCCGCCACCG AGCCGCTATG CTCGCCGCTG CGGACAAGAC CTCCTCCTTC GACTCCACGC TCAGGTTGCT   
  
  
+ CCTCAAGTTC CAGGAAGTCA GCCCGTGGAC CACCTTCGGC CACGTGGCTT GCAACGGTGC AATCATCGAG   
  
  
+ GCCTTAGAGA GGGACCCACA TGAAAAGAGT AAGCTCCACG TCATCGATAT CAGCACCACC TTCTGCACCC   
  
  
+ AGTGGCCCAC CTTGCTTGAG GCTTTAGCCA CCCGCATGGA CGACACTCCC CACCTCAGCC TCACTGCCGT   
  
  
+ CGTCGTCAAC AAGTACGCCG CCGTGAGCGG TGGCGAGGAC GACGGGGGAG AAGGATCAAA GAGGGTGATG   
  
  
+ AGAGAGATTG GTCTTCGTCT TGAGAAGTTC GCTAGGTTAA TGGGGGTTCC CTTCAAGTTC AACGTGGTTT   
  
  
+ ACCACGTAGG TGATCTATCC CAGTTGGATT TTTCTCGTTT GGATATTAAA GATGATGAAG CGGTGGCGAT   
  
  
+ TAACTGTGTC AACTCGCTCC ACTCGGTGGA CCTCCGATAT CGGGAGGTAG TGCTCGCGGC GTTCAGGCGG   
  
  
+ CTGCGGCCGA GGGTGGTGAC GGTGGTGGAG GAAGAGGCGG AGCTGACTGA GGTGGGTGAG GGTCAGTATG   
  
  
+ AATTTTTTAG GCGGTTTGAG GAGTGTCTTA GGTGGTTTAG GGTTTACTTT GAGGCCGTAG GAGATTGTTT   
  
  
+ TCCTAGGACT AGCAATGAGA AATTGATGTT AGAGCGGGCC GCGGGCCGGG CCATGGTGGA TCTCCTTGCA   
  
  
+ TGTCCCGAGC CTGGTGGGTC GGCTGAGGGG AGGGAGACGG CGAGACGGTG GTCGGGTCGG ATGCATGGGG   
  
  
+ CGGGGTTCGA GCACGTGGGG TTCAGTGACG AAGTGTGTGA TGATGTGAGG GCCTTGTTGA GGAGGTACAA   
  
  
+ GGAAGGATGG TCAATGACAC AGTGCACCAT GGATGGCGGT AATCACCCTG GAATACTGTT GTGGTGGCGG   
  
  
+ GATCAACCGG TGGTTTGGGC CAGTGCATGG CGGCCTTG  

- +Up\_Stream \_Len000TTTAGT TTGAGTATTT GTTATTAAAA AATTTTGAAT TTACCATTAA TAATCTAATT   
  
  
- ATTTTTAGGA TTACCAACAA ATTACAATGT AGTTCTGTAC GAAAAACCAC ATATATATTA CCGTCGTACA   
  
  
- CCCAATTCGC CGTTGTTTAA TAAGAGTTTA CGTACGTCAT TAATTAAATT AATTAATTAG TGGACGTATT   
  
  
- AGAGTTATGA TTAGGAAAGT AAGACACGGG GTGGGTTGGG GCTTTTATCA TTAGAAAGTT TGCAACCTTC   
  
  
- ACCCTAATCC ATATCTCATC AGGATGTCTA CTACTAGATG GATGTATATT GTATTAGACT GAAAAGAAAA   
  
  
- AATTTATTGA ACATAACTAC CGTGTCTTAA ACTGTACAAC CGTATACCAT GATTCTTTAA TTAATTGCAA   
  
  
- CACGATTCTA TATCCAGTAA GAAAAACCTC TGTATTCCGG GCGCAAGTGC GTCCCAAGCT CTTCCCGGTG   
  
  
- TAAGTTACCT ACTTAACATC CATCAAATTA GACTGAAACA GTCACTGACT AAGGTGCCGA ACTTGGGCGC   
  
  
- TGTAATTATA TCCAACTGAT TTCAGCAAAC TATGAATAAA AAGAATTCAT TTCAGAACTT AAGTTTAGAA   
  
  
- TATTTATTTA TTTTTTTAGA TATTATTTTC TTAAGATGGT AAATAATTCA GTTGTTGAGT TAAGCTTACT   
  
  
- TTATTTTTTA GTTCTAGTGG TTTTTTTTAT TTATTTTCCT TTCCATCTAA CGTAAACCTA AAACTCACCG   
  
  
- CACCACCACC CAAACTACGA CCACGACGTA TGTGGCGTCG AAAAGAGACA GGAAAATGGA GAGAAACAGA   
  
  
- CGTTCTTGGG GTAAACCGTG CGGACGAGAG AAAAGATACA AAAGAAAAGA TAGAAAATTC TTTGTTACCT   
  
  
- TTCGATTGTG TGATTGTGAT TGGGTAACCA GGAAAAAAGA ATAGACGTTT TAATTTCATT GTTATAAAAA   
  
  
- GAATAGGGGA AAAAGCAAGG TGGGTAAAGA AATAGGAAGA CAAACTTGGA TTAGACATAT ATACGTACGT   
  
  
- ACACATAAAA TGAATCTTAG ACAAAACTAA GAAACTACAT TAATTAATCA TCAGAAAAAT TAACTTAAAA   
  
  
- ATTGTCATCA AACTTAATCT GTAAATAGCT ATAATATAAA AAATATATAT TAATTTTTTA TGTTTTACTA   
  
  
- TTCACAGAAT GATGTTATGT GAAGTATTCA CAGCACAAAA TTGAATTTTT AGTATGATAA TACAAAGATT   
  
  
- TAACTTTTTT TTCACTCTAA TTCATCGTCT TCGTATAATG TTGAATATTT AATATATTTT AGTAGAAAAA   
  
  
- TAGGTATTAA ATAGTGTAAA GATTTTATGC GTATAGTATA GATGAATACC TATCCCAATT ATTCTAATCC   
  
  
- CGTAGTAAAT TCTAAAATAG TCGCATTGGT TGCTTACAAA TTCCAATCGT CAAGTATTAA TTCACATTAG   
  
  
- TAATTTTTTA AATACTAAAA TTTAGTATTT TATACAATTA GATCCTACTC CGTTCGCCGT TCTGTATATA   
  
  
- GTGATAGGAA TTTTTTATAT TATATAACTA AAGTTTCTTA ATACTTCATA TAATTAAAGT ATATATTACT   
  
  
- GCTAATACAT AAAAAAATGA TTTAATGAAC ATGGGTATTA TTTATCTTAG CTACGTAGTA AGTACCGGAA   
  
  
- AGTAAGACAT TATACCAACT GAGGTTTTTA AGAGTTTCAT AAAAATATAA AACCCTCCCC TCTAAAATCA   
  
  
- AACTATGAAA ACAACTTGAT CACTTGTTAA AGGTTGTTGG AGGTGGTGGG GGTTTGTAGT TGTTAGACTA   
  
  
- ACTACATACG TAGGGTTCGA ACTTAACTTA TTATTAAAGT TATTGTTGTT ATTAGCAAAA TGGAACTGTT   
  
  
- CGAGGTGTCG TTGAGTTGCA GAAGGTTTGG TTGGGTAGTG AGTAGTAGTA GTAGGGGTGA TAGGGTAAGT   
  
  
- GGGGGTGGTT TTCCTCGGAT TTCTGTCATT GTTCTTCTTA CGAACGGAAA AAAGTACCTA CTTCTTCTGA   
  
  
- AGAGAAGAAG GAGATGAAGA AGGGTGGTGA TACTATGTGT GGTGGAGGTT GTAGTAATGG GGTGGTTAAT   
  
  
- ATTAAGGGCA ATATGGGACC TGGGAGGGAG GTGGTGGTGG TGTCGGTGTT GGTGGTGGAG TAGGAGGTGA   
  
  
- GGGTGGGTGG TACCGAAGCC ACCACCCGAA CTGAGGAGGA GGGGCAGACG GCGACACCTT AATAGAAAGA   
  
  
- GGGGCCTGTA GGAGGAGAGG AGGGGGTGGC CTTTCACCCG CAGACTGTAA GAATACCTCC GCCGATCCCG   
  
  
- AAAAAGTCTG TGCTTGTGGT GGTTCGAGGT GGTCGAGGAG ACCTACGACT TGCTCGAGCC GAGGGGGATG   
  
  
- CCACTACAGC TCGTCTTCGA CCACCGCATG AAATTGGTTC GGGAAAAACG GGCGGAGTGA CGGTGGCCTG   
  
  
- GGGCGGTGGC TCGGCGATAC GAGCGGCGAC GCCTGTTCTG GAGGAGGAAG CTGAGGTGCG AGTCCAACGA   
  
  
- GGAGTTCAAG GTCCTTCAGT CGGGCACCTG GTGGAAGCCG GTGCACCGAA CGTTGCCACG TTAGTAGCTC   
  
  
- CGGAATCTCT CCCTGGGTGT ACTTTTCTCA TTCGAGGTGC AGTAGCTATA GTCGTGGTGG AAGACGTGGG   
  
  
- TCACCGGGTG GAACGAACTC CGAAATCGGT GGGCGTACCT GCTGTGAGGG GTGGAGTCGG AGTGACGGCA   
  
  
- GCAGCAGTTG TTCATGCGGC GGCACTCGCC ACCGCTCCTG CTGCCCCCTC TTCCTAGTTT CTCCCACTAC   
  
  
- TCTCTCTAAC CAGAAGCAGA ACTCTTCAAG CGATCCAATT ACCCCCAAGG GAAGTTCAAG TTGCACCAAA   
  
  
- TGGTGCATCC ACTAGATAGG GTCAACCTAA AAAGAGCAAA CCTATAATTT CTACTACTTC GCCACCGCTA   
  
  
- ATTGACACAG TTGAGCGAGG TGAGCCACCT GGAGGCTATA GCCCTCCATC ACGAGCGCCG CAAGTCCGCC   
  
  
- GACGCCGGCT CCCACCACTG CCACCACCTC CTTCTCCGCC TCGACTGACT CCACCCACTC CCAGTCATAC   
  
  
- TTAAAAAATC CGCCAAACTC CTCACAGAAT CCACCAAATC CCAAATGAAA CTCCGGCATC CTCTAACAAA   
  
  
- AGGATCCTGA TCGTTACTCT TTAACTACAA TCTCGCCCGG CGCCCGGCCC GGTACCACCT AGAGGAACGT   
  
  
- ACAGGGCTCG GACCACCCAG CCGACTCCCC TCCCTCTGCC GCTCTGCCAC CAGCCCAGCC TACGTACCCC   
  
  
- GCCCCAAGCT CGTGCACCCC AAGTCACTGC TTCACACACT ACTACACTCC CGGAACAACT CCTCCATGTT   
  
  
- CCTTCCTACC AGTTACTGTG TCACGTGGTA CCTACCGCCA TTAGTGGGAC CTTATGACAA CACCACCGCC   
  
  
- CTAGTTGGCC ACCAAACCCG GTCACGTACC GCCGGAAC

+     ABRE4

| Site Name | Organism | Position | Strand | Matrix score. | sequence | function |
| --- | --- | --- | --- | --- | --- | --- |
| ABRE4 | Zea mays | 2877 | + | 6 | CACGTA |  |

>HU05G01983.1   
+ +Up\_Stream \_Len000AAATCA AACTCATAAA CAATAATTTT TTAAAACTTA AATGGTAATT ATTAGATTAA   
  
  
+ TAAAAATCCT AATGGTTGTT TAATGTTACA TCAAGACATG CTTTTTGGTG TATATATAAT GGCAGCATGT   
  
  
+ GGGTTAAGCG GCAACAAATT ATTCTCAAAT GCATGCAGTA ATTAATTTAA TTAATTAATC ACCTGCATAA   
  
  
+ TCTCAATACT AATCCTTTCA TTCTGTGCCC CACCCAACCC CGAAAATAGT AATCTTTCAA ACGTTGGAAG   
  
  
+ TGGGATTAGG TATAGAGTAG TCCTACAGAT GATGATCTAC CTACATATAA CATAATCTGA CTTTTCTTTT   
  
  
+ TTAAATAACT TGTATTGATG GCACAGAATT TGACATGTTG GCATATGGTA CTAAGAAATT AATTAACGTT   
  
  
+ GTGCTAAGAT ATAGGTCATT CTTTTTGGAG ACATAAGGCC CGCGTTCACG CAGGGTTCGA GAAGGGCCAC   
  
  
+ ATTCAATGGA TGAATTGTAG GTAGTTTAAT CTGACTTTGT CAGTGACTGA TTCCACGGCT TGAACCCGCG   
  
  
+ ACATTAATAT AGGTTGACTA AAGTCGTTTG ATACTTATTT TTCTTAAGTA AAGTCTTGAA TTCAAATCTT   
  
  
+ ATAAATAAAT AAAAAAATCT ATAATAAAAG AATTCTACCA TTTATTAAGT CAACAACTCA ATTCGAATGA   
  
  
+ AATAAAAAAT CAAGATCACC AAAAAAAATA AATAAAAGGA AAGGTAGATT GCATTTGGAT TTTGAGTGGC   
  
  
+ GTGGTGGTGG GTTTGATGCT GGTGCTGCAT ACACCGCAGC TTTTCTCTGT CCTTTTACCT CTCTTTGTCT   
  
  
+ GCAAGAACCC CATTTGGCAC GCCTGCTCTC TTTTCTATGT TTTCTTTTCT ATCTTTTAAG AAACAATGGA   
  
  
+ AAGCTAACAC ACTAACACTA ACCCATTGGT CCTTTTTTCT TATCTGCAAA ATTAAAGTAA CAATATTTTT   
  
  
+ CTTATCCCCT TTTTCGTTCC ACCCATTTCT TTATCCTTCT GTTTGAACCT AATCTGTATA TATGCATGCA   
  
  
+ TGTGTATTTT ACTTAGAATC TGTTTTGATT CTTTGATGTA ATTAATTAGT AGTCTTTTTA ATTGAATTTT   
  
  
+ TAACAGTAGT TTGAATTAGA CATTTATCGA TATTATATTT TTTATATATA ATTAAAAAAT ACAAAATGAT   
  
  
+ AAGTGTCTTA CTACAATACA CTTCATAAGT GTCGTGTTTT AACTTAAAAA TCATACTATT ATGTTTCTAA   
  
  
+ ATTGAAAAAA AAGTGAGATT AAGTAGCAGA AGCATATTAC AACTTATAAA TTATATAAAA TCATCTTTTT   
  
  
+ ATCCATAATT TATCACATTT CTAAAATACG CATATCATAT CTACTTATGG ATAGGGTTAA TAAGATTAGG   
  
  
+ GCATCATTTA AGATTTTATC AGCGTAACCA ACGAATGTTT AAGGTTAGCA GTTCATAATT AAGTGTAATC   
  
  
+ ATTAAAAAAT TTATGATTTT AAATCATAAA ATATGTTAAT CTAGGATGAG GCAAGCGGCA AGACATATAT   
  
  
+ CACTATCCTT AAAAAATATA ATATATTGAT TTCAAAGAAT TATGAAGTAT ATTAATTTCA TATATAATGA   
  
  
+ CGATTATGTA TTTTTTTACT AAATTACTTG TACCCATAAT AAATAGAATC GATGCATCAT TCATGGCCTT   
  
  
+ TCATTCTGTA ATATGGTTGA CTCCAAAAAT TCTCAAAGTA TTTTTATATT TTGGGAGGGG AGATTTTAGT   
  
  
+ TTGATACTTT TGTTGAACTA GTGAACAATT TCCAACAACC TCCACCACCC CCAAACATCA ACAATCTGAT   
  
  
+ TGATGTATGC ATCCCAAGCT TGAATTGAAT AATAATTTCA ATAACAACAA TAATCGTTTT ACCTTGACAA   
  
  
+ GCTCCACAGC AACTCAACGT CTTCCAAACC AACCCATCAC TCATCATCAT CATCCCCACT ATCCCATTCA   
  
  
+ CCCCCACCAA AAGGAGCCTA AAGACAGTAA CAAGAAGAAT GCTTGCCTTT TTTCATGGAT GAAGAAGACT   
  
  
+ TCTCTTCTTC CTCTACTTCT TCCCACCACT ATGATACACA CCACCTCCAA CATCATTACC CCACCAATTA   
  
  
+ TAATTCCCGT TATACCCTGG ACCCTCCCTC CACCACCACC ACAGCCACAA CCACCACCTC ATCCTCCACT   
  
  
+ CCCACCCACC ATGGCTTCGG TGGTGGGCTT GACTCCTCCT CCCCGTCTGC CGCTGTGGAA TTATCTTTCT   
  
  
+ CCCCGGACAT CCTCCTCTCC TCCCCCACCG GAAAGTGGGC GTCTGACATT CTTATGGAGG CGGCTAGGGC   
  
  
+ TTTTTCAGAC ACGAACACCA CCAAGCTCCA CCAGCTCCTC TGGATGCTGA ACGAGCTCGG CTCCCCCTAC   
  
  
+ GGTGATGTCG AGCAGAAGCT GGTGGCGTAC TTTAACCAAG CCCTTTTTGC CCGCCTCACT GCCACCGGAC   
  
  
+ CCCGCCACCG AGCCGCTATG CTCGCCGCTG CGGACAAGAC CTCCTCCTTC GACTCCACGC TCAGGTTGCT   
  
  
+ CCTCAAGTTC CAGGAAGTCA GCCCGTGGAC CACCTTCGGC CACGTGGCTT GCAACGGTGC AATCATCGAG   
  
  
+ GCCTTAGAGA GGGACCCACA TGAAAAGAGT AAGCTCCACG TCATCGATAT CAGCACCACC TTCTGCACCC   
  
  
+ AGTGGCCCAC CTTGCTTGAG GCTTTAGCCA CCCGCATGGA CGACACTCCC CACCTCAGCC TCACTGCCGT   
  
  
+ CGTCGTCAAC AAGTACGCCG CCGTGAGCGG TGGCGAGGAC GACGGGGGAG AAGGATCAAA GAGGGTGATG   
  
  
+ AGAGAGATTG GTCTTCGTCT TGAGAAGTTC GCTAGGTTAA TGGGGGTTCC CTTCAAGTTC AACGTGGTTT   
  
  
+ ACCACGTAGG TGATCTATCC CAGTTGGATT TTTCTCGTTT GGATATTAAA GATGATGAAG CGGTGGCGAT   
  
  
+ TAACTGTGTC AACTCGCTCC ACTCGGTGGA CCTCCGATAT CGGGAGGTAG TGCTCGCGGC GTTCAGGCGG   
  
  
+ CTGCGGCCGA GGGTGGTGAC GGTGGTGGAG GAAGAGGCGG AGCTGACTGA GGTGGGTGAG GGTCAGTATG   
  
  
+ AATTTTTTAG GCGGTTTGAG GAGTGTCTTA GGTGGTTTAG GGTTTACTTT GAGGCCGTAG GAGATTGTTT   
  
  
+ TCCTAGGACT AGCAATGAGA AATTGATGTT AGAGCGGGCC GCGGGCCGGG CCATGGTGGA TCTCCTTGCA   
  
  
+ TGTCCCGAGC CTGGTGGGTC GGCTGAGGGG AGGGAGACGG CGAGACGGTG GTCGGGTCGG ATGCATGGGG   
  
  
+ CGGGGTTCGA GCACGTGGGG TTCAGTGACG AAGTGTGTGA TGATGTGAGG GCCTTGTTGA GGAGGTACAA   
  
  
+ GGAAGGATGG TCAATGACAC AGTGCACCAT GGATGGCGGT AATCACCCTG GAATACTGTT GTGGTGGCGG   
  
  
+ GATCAACCGG TGGTTTGGGC CAGTGCATGG CGGCCTTG  

- +Up\_Stream \_Len000TTTAGT TTGAGTATTT GTTATTAAAA AATTTTGAAT TTACCATTAA TAATCTAATT   
  
  
- ATTTTTAGGA TTACCAACAA ATTACAATGT AGTTCTGTAC GAAAAACCAC ATATATATTA CCGTCGTACA   
  
  
- CCCAATTCGC CGTTGTTTAA TAAGAGTTTA CGTACGTCAT TAATTAAATT AATTAATTAG TGGACGTATT   
  
  
- AGAGTTATGA TTAGGAAAGT AAGACACGGG GTGGGTTGGG GCTTTTATCA TTAGAAAGTT TGCAACCTTC   
  
  
- ACCCTAATCC ATATCTCATC AGGATGTCTA CTACTAGATG GATGTATATT GTATTAGACT GAAAAGAAAA   
  
  
- AATTTATTGA ACATAACTAC CGTGTCTTAA ACTGTACAAC CGTATACCAT GATTCTTTAA TTAATTGCAA   
  
  
- CACGATTCTA TATCCAGTAA GAAAAACCTC TGTATTCCGG GCGCAAGTGC GTCCCAAGCT CTTCCCGGTG   
  
  
- TAAGTTACCT ACTTAACATC CATCAAATTA GACTGAAACA GTCACTGACT AAGGTGCCGA ACTTGGGCGC   
  
  
- TGTAATTATA TCCAACTGAT TTCAGCAAAC TATGAATAAA AAGAATTCAT TTCAGAACTT AAGTTTAGAA   
  
  
- TATTTATTTA TTTTTTTAGA TATTATTTTC TTAAGATGGT AAATAATTCA GTTGTTGAGT TAAGCTTACT   
  
  
- TTATTTTTTA GTTCTAGTGG TTTTTTTTAT TTATTTTCCT TTCCATCTAA CGTAAACCTA AAACTCACCG   
  
  
- CACCACCACC CAAACTACGA CCACGACGTA TGTGGCGTCG AAAAGAGACA GGAAAATGGA GAGAAACAGA   
  
  
- CGTTCTTGGG GTAAACCGTG CGGACGAGAG AAAAGATACA AAAGAAAAGA TAGAAAATTC TTTGTTACCT   
  
  
- TTCGATTGTG TGATTGTGAT TGGGTAACCA GGAAAAAAGA ATAGACGTTT TAATTTCATT GTTATAAAAA   
  
  
- GAATAGGGGA AAAAGCAAGG TGGGTAAAGA AATAGGAAGA CAAACTTGGA TTAGACATAT ATACGTACGT   
  
  
- ACACATAAAA TGAATCTTAG ACAAAACTAA GAAACTACAT TAATTAATCA TCAGAAAAAT TAACTTAAAA   
  
  
- ATTGTCATCA AACTTAATCT GTAAATAGCT ATAATATAAA AAATATATAT TAATTTTTTA TGTTTTACTA   
  
  
- TTCACAGAAT GATGTTATGT GAAGTATTCA CAGCACAAAA TTGAATTTTT AGTATGATAA TACAAAGATT   
  
  
- TAACTTTTTT TTCACTCTAA TTCATCGTCT TCGTATAATG TTGAATATTT AATATATTTT AGTAGAAAAA   
  
  
- TAGGTATTAA ATAGTGTAAA GATTTTATGC GTATAGTATA GATGAATACC TATCCCAATT ATTCTAATCC   
  
  
- CGTAGTAAAT TCTAAAATAG TCGCATTGGT TGCTTACAAA TTCCAATCGT CAAGTATTAA TTCACATTAG   
  
  
- TAATTTTTTA AATACTAAAA TTTAGTATTT TATACAATTA GATCCTACTC CGTTCGCCGT TCTGTATATA   
  
  
- GTGATAGGAA TTTTTTATAT TATATAACTA AAGTTTCTTA ATACTTCATA TAATTAAAGT ATATATTACT   
  
  
- GCTAATACAT AAAAAAATGA TTTAATGAAC ATGGGTATTA TTTATCTTAG CTACGTAGTA AGTACCGGAA   
  
  
- AGTAAGACAT TATACCAACT GAGGTTTTTA AGAGTTTCAT AAAAATATAA AACCCTCCCC TCTAAAATCA   
  
  
- AACTATGAAA ACAACTTGAT CACTTGTTAA AGGTTGTTGG AGGTGGTGGG GGTTTGTAGT TGTTAGACTA   
  
  
- ACTACATACG TAGGGTTCGA ACTTAACTTA TTATTAAAGT TATTGTTGTT ATTAGCAAAA TGGAACTGTT   
  
  
- CGAGGTGTCG TTGAGTTGCA GAAGGTTTGG TTGGGTAGTG AGTAGTAGTA GTAGGGGTGA TAGGGTAAGT   
  
  
- GGGGGTGGTT TTCCTCGGAT TTCTGTCATT GTTCTTCTTA CGAACGGAAA AAAGTACCTA CTTCTTCTGA   
  
  
- AGAGAAGAAG GAGATGAAGA AGGGTGGTGA TACTATGTGT GGTGGAGGTT GTAGTAATGG GGTGGTTAAT   
  
  
- ATTAAGGGCA ATATGGGACC TGGGAGGGAG GTGGTGGTGG TGTCGGTGTT GGTGGTGGAG TAGGAGGTGA   
  
  
- GGGTGGGTGG TACCGAAGCC ACCACCCGAA CTGAGGAGGA GGGGCAGACG GCGACACCTT AATAGAAAGA   
  
  
- GGGGCCTGTA GGAGGAGAGG AGGGGGTGGC CTTTCACCCG CAGACTGTAA GAATACCTCC GCCGATCCCG   
  
  
- AAAAAGTCTG TGCTTGTGGT GGTTCGAGGT GGTCGAGGAG ACCTACGACT TGCTCGAGCC GAGGGGGATG   
  
  
- CCACTACAGC TCGTCTTCGA CCACCGCATG AAATTGGTTC GGGAAAAACG GGCGGAGTGA CGGTGGCCTG   
  
  
- GGGCGGTGGC TCGGCGATAC GAGCGGCGAC GCCTGTTCTG GAGGAGGAAG CTGAGGTGCG AGTCCAACGA   
  
  
- GGAGTTCAAG GTCCTTCAGT CGGGCACCTG GTGGAAGCCG GTGCACCGAA CGTTGCCACG TTAGTAGCTC   
  
  
- CGGAATCTCT CCCTGGGTGT ACTTTTCTCA TTCGAGGTGC AGTAGCTATA GTCGTGGTGG AAGACGTGGG   
  
  
- TCACCGGGTG GAACGAACTC CGAAATCGGT GGGCGTACCT GCTGTGAGGG GTGGAGTCGG AGTGACGGCA   
  
  
- GCAGCAGTTG TTCATGCGGC GGCACTCGCC ACCGCTCCTG CTGCCCCCTC TTCCTAGTTT CTCCCACTAC   
  
  
- TCTCTCTAAC CAGAAGCAGA ACTCTTCAAG CGATCCAATT ACCCCCAAGG GAAGTTCAAG TTGCACCAAA   
  
  
- TGGTGCATCC ACTAGATAGG GTCAACCTAA AAAGAGCAAA CCTATAATTT CTACTACTTC GCCACCGCTA   
  
  
- ATTGACACAG TTGAGCGAGG TGAGCCACCT GGAGGCTATA GCCCTCCATC ACGAGCGCCG CAAGTCCGCC   
  
  
- GACGCCGGCT CCCACCACTG CCACCACCTC CTTCTCCGCC TCGACTGACT CCACCCACTC CCAGTCATAC   
  
  
- TTAAAAAATC CGCCAAACTC CTCACAGAAT CCACCAAATC CCAAATGAAA CTCCGGCATC CTCTAACAAA   
  
  
- AGGATCCTGA TCGTTACTCT TTAACTACAA TCTCGCCCGG CGCCCGGCCC GGTACCACCT AGAGGAACGT   
  
  
- ACAGGGCTCG GACCACCCAG CCGACTCCCC TCCCTCTGCC GCTCTGCCAC CAGCCCAGCC TACGTACCCC   
  
  
- GCCCCAAGCT CGTGCACCCC AAGTCACTGC TTCACACACT ACTACACTCC CGGAACAACT CCTCCATGTT   
  
  
- CCTTCCTACC AGTTACTGTG TCACGTGGTA CCTACCGCCA TTAGTGGGAC CTTATGACAA CACCACCGCC   
  
  
- CTAGTTGGCC ACCAAACCCG GTCACGTACC GCCGGAAC

+     AC-I

| Site Name | Organism | Position | Strand | Matrix score. | sequence | function |
| --- | --- | --- | --- | --- | --- | --- |
| AC-I | Phaseolus vulgaris | 3065 | - | 9 | (T/C)C(T/C)(C/T)ACC(T/C)ACC |  |
| AC-I | Phaseolus vulgaris | 2174 | + | 9 | (T/C)C(T/C)(C/T)ACC(T/C)ACC |  |

>HU05G01983.1   
+ +Up\_Stream \_Len000AAATCA AACTCATAAA CAATAATTTT TTAAAACTTA AATGGTAATT ATTAGATTAA   
  
  
+ TAAAAATCCT AATGGTTGTT TAATGTTACA TCAAGACATG CTTTTTGGTG TATATATAAT GGCAGCATGT   
  
  
+ GGGTTAAGCG GCAACAAATT ATTCTCAAAT GCATGCAGTA ATTAATTTAA TTAATTAATC ACCTGCATAA   
  
  
+ TCTCAATACT AATCCTTTCA TTCTGTGCCC CACCCAACCC CGAAAATAGT AATCTTTCAA ACGTTGGAAG   
  
  
+ TGGGATTAGG TATAGAGTAG TCCTACAGAT GATGATCTAC CTACATATAA CATAATCTGA CTTTTCTTTT   
  
  
+ TTAAATAACT TGTATTGATG GCACAGAATT TGACATGTTG GCATATGGTA CTAAGAAATT AATTAACGTT   
  
  
+ GTGCTAAGAT ATAGGTCATT CTTTTTGGAG ACATAAGGCC CGCGTTCACG CAGGGTTCGA GAAGGGCCAC   
  
  
+ ATTCAATGGA TGAATTGTAG GTAGTTTAAT CTGACTTTGT CAGTGACTGA TTCCACGGCT TGAACCCGCG   
  
  
+ ACATTAATAT AGGTTGACTA AAGTCGTTTG ATACTTATTT TTCTTAAGTA AAGTCTTGAA TTCAAATCTT   
  
  
+ ATAAATAAAT AAAAAAATCT ATAATAAAAG AATTCTACCA TTTATTAAGT CAACAACTCA ATTCGAATGA   
  
  
+ AATAAAAAAT CAAGATCACC AAAAAAAATA AATAAAAGGA AAGGTAGATT GCATTTGGAT TTTGAGTGGC   
  
  
+ GTGGTGGTGG GTTTGATGCT GGTGCTGCAT ACACCGCAGC TTTTCTCTGT CCTTTTACCT CTCTTTGTCT   
  
  
+ GCAAGAACCC CATTTGGCAC GCCTGCTCTC TTTTCTATGT TTTCTTTTCT ATCTTTTAAG AAACAATGGA   
  
  
+ AAGCTAACAC ACTAACACTA ACCCATTGGT CCTTTTTTCT TATCTGCAAA ATTAAAGTAA CAATATTTTT   
  
  
+ CTTATCCCCT TTTTCGTTCC ACCCATTTCT TTATCCTTCT GTTTGAACCT AATCTGTATA TATGCATGCA   
  
  
+ TGTGTATTTT ACTTAGAATC TGTTTTGATT CTTTGATGTA ATTAATTAGT AGTCTTTTTA ATTGAATTTT   
  
  
+ TAACAGTAGT TTGAATTAGA CATTTATCGA TATTATATTT TTTATATATA ATTAAAAAAT ACAAAATGAT   
  
  
+ AAGTGTCTTA CTACAATACA CTTCATAAGT GTCGTGTTTT AACTTAAAAA TCATACTATT ATGTTTCTAA   
  
  
+ ATTGAAAAAA AAGTGAGATT AAGTAGCAGA AGCATATTAC AACTTATAAA TTATATAAAA TCATCTTTTT   
  
  
+ ATCCATAATT TATCACATTT CTAAAATACG CATATCATAT CTACTTATGG ATAGGGTTAA TAAGATTAGG   
  
  
+ GCATCATTTA AGATTTTATC AGCGTAACCA ACGAATGTTT AAGGTTAGCA GTTCATAATT AAGTGTAATC   
  
  
+ ATTAAAAAAT TTATGATTTT AAATCATAAA ATATGTTAAT CTAGGATGAG GCAAGCGGCA AGACATATAT   
  
  
+ CACTATCCTT AAAAAATATA ATATATTGAT TTCAAAGAAT TATGAAGTAT ATTAATTTCA TATATAATGA   
  
  
+ CGATTATGTA TTTTTTTACT AAATTACTTG TACCCATAAT AAATAGAATC GATGCATCAT TCATGGCCTT   
  
  
+ TCATTCTGTA ATATGGTTGA CTCCAAAAAT TCTCAAAGTA TTTTTATATT TTGGGAGGGG AGATTTTAGT   
  
  
+ TTGATACTTT TGTTGAACTA GTGAACAATT TCCAACAACC TCCACCACCC CCAAACATCA ACAATCTGAT   
  
  
+ TGATGTATGC ATCCCAAGCT TGAATTGAAT AATAATTTCA ATAACAACAA TAATCGTTTT ACCTTGACAA   
  
  
+ GCTCCACAGC AACTCAACGT CTTCCAAACC AACCCATCAC TCATCATCAT CATCCCCACT ATCCCATTCA   
  
  
+ CCCCCACCAA AAGGAGCCTA AAGACAGTAA CAAGAAGAAT GCTTGCCTTT TTTCATGGAT GAAGAAGACT   
  
  
+ TCTCTTCTTC CTCTACTTCT TCCCACCACT ATGATACACA CCACCTCCAA CATCATTACC CCACCAATTA   
  
  
+ TAATTCCCGT TATACCCTGG ACCCTCCCTC CACCACCACC ACAGCCACAA CCACCACCTC ATCCTCCACT   
  
  
+ CCCACCCACC ATGGCTTCGG TGGTGGGCTT GACTCCTCCT CCCCGTCTGC CGCTGTGGAA TTATCTTTCT   
  
  
+ CCCCGGACAT CCTCCTCTCC TCCCCCACCG GAAAGTGGGC GTCTGACATT CTTATGGAGG CGGCTAGGGC   
  
  
+ TTTTTCAGAC ACGAACACCA CCAAGCTCCA CCAGCTCCTC TGGATGCTGA ACGAGCTCGG CTCCCCCTAC   
  
  
+ GGTGATGTCG AGCAGAAGCT GGTGGCGTAC TTTAACCAAG CCCTTTTTGC CCGCCTCACT GCCACCGGAC   
  
  
+ CCCGCCACCG AGCCGCTATG CTCGCCGCTG CGGACAAGAC CTCCTCCTTC GACTCCACGC TCAGGTTGCT   
  
  
+ CCTCAAGTTC CAGGAAGTCA GCCCGTGGAC CACCTTCGGC CACGTGGCTT GCAACGGTGC AATCATCGAG   
  
  
+ GCCTTAGAGA GGGACCCACA TGAAAAGAGT AAGCTCCACG TCATCGATAT CAGCACCACC TTCTGCACCC   
  
  
+ AGTGGCCCAC CTTGCTTGAG GCTTTAGCCA CCCGCATGGA CGACACTCCC CACCTCAGCC TCACTGCCGT   
  
  
+ CGTCGTCAAC AAGTACGCCG CCGTGAGCGG TGGCGAGGAC GACGGGGGAG AAGGATCAAA GAGGGTGATG   
  
  
+ AGAGAGATTG GTCTTCGTCT TGAGAAGTTC GCTAGGTTAA TGGGGGTTCC CTTCAAGTTC AACGTGGTTT   
  
  
+ ACCACGTAGG TGATCTATCC CAGTTGGATT TTTCTCGTTT GGATATTAAA GATGATGAAG CGGTGGCGAT   
  
  
+ TAACTGTGTC AACTCGCTCC ACTCGGTGGA CCTCCGATAT CGGGAGGTAG TGCTCGCGGC GTTCAGGCGG   
  
  
+ CTGCGGCCGA GGGTGGTGAC GGTGGTGGAG GAAGAGGCGG AGCTGACTGA GGTGGGTGAG GGTCAGTATG   
  
  
+ AATTTTTTAG GCGGTTTGAG GAGTGTCTTA GGTGGTTTAG GGTTTACTTT GAGGCCGTAG GAGATTGTTT   
  
  
+ TCCTAGGACT AGCAATGAGA AATTGATGTT AGAGCGGGCC GCGGGCCGGG CCATGGTGGA TCTCCTTGCA   
  
  
+ TGTCCCGAGC CTGGTGGGTC GGCTGAGGGG AGGGAGACGG CGAGACGGTG GTCGGGTCGG ATGCATGGGG   
  
  
+ CGGGGTTCGA GCACGTGGGG TTCAGTGACG AAGTGTGTGA TGATGTGAGG GCCTTGTTGA GGAGGTACAA   
  
  
+ GGAAGGATGG TCAATGACAC AGTGCACCAT GGATGGCGGT AATCACCCTG GAATACTGTT GTGGTGGCGG   
  
  
+ GATCAACCGG TGGTTTGGGC CAGTGCATGG CGGCCTTG  

- +Up\_Stream \_Len000TTTAGT TTGAGTATTT GTTATTAAAA AATTTTGAAT TTACCATTAA TAATCTAATT   
  
  
- ATTTTTAGGA TTACCAACAA ATTACAATGT AGTTCTGTAC GAAAAACCAC ATATATATTA CCGTCGTACA   
  
  
- CCCAATTCGC CGTTGTTTAA TAAGAGTTTA CGTACGTCAT TAATTAAATT AATTAATTAG TGGACGTATT   
  
  
- AGAGTTATGA TTAGGAAAGT AAGACACGGG GTGGGTTGGG GCTTTTATCA TTAGAAAGTT TGCAACCTTC   
  
  
- ACCCTAATCC ATATCTCATC AGGATGTCTA CTACTAGATG GATGTATATT GTATTAGACT GAAAAGAAAA   
  
  
- AATTTATTGA ACATAACTAC CGTGTCTTAA ACTGTACAAC CGTATACCAT GATTCTTTAA TTAATTGCAA   
  
  
- CACGATTCTA TATCCAGTAA GAAAAACCTC TGTATTCCGG GCGCAAGTGC GTCCCAAGCT CTTCCCGGTG   
  
  
- TAAGTTACCT ACTTAACATC CATCAAATTA GACTGAAACA GTCACTGACT AAGGTGCCGA ACTTGGGCGC   
  
  
- TGTAATTATA TCCAACTGAT TTCAGCAAAC TATGAATAAA AAGAATTCAT TTCAGAACTT AAGTTTAGAA   
  
  
- TATTTATTTA TTTTTTTAGA TATTATTTTC TTAAGATGGT AAATAATTCA GTTGTTGAGT TAAGCTTACT   
  
  
- TTATTTTTTA GTTCTAGTGG TTTTTTTTAT TTATTTTCCT TTCCATCTAA CGTAAACCTA AAACTCACCG   
  
  
- CACCACCACC CAAACTACGA CCACGACGTA TGTGGCGTCG AAAAGAGACA GGAAAATGGA GAGAAACAGA   
  
  
- CGTTCTTGGG GTAAACCGTG CGGACGAGAG AAAAGATACA AAAGAAAAGA TAGAAAATTC TTTGTTACCT   
  
  
- TTCGATTGTG TGATTGTGAT TGGGTAACCA GGAAAAAAGA ATAGACGTTT TAATTTCATT GTTATAAAAA   
  
  
- GAATAGGGGA AAAAGCAAGG TGGGTAAAGA AATAGGAAGA CAAACTTGGA TTAGACATAT ATACGTACGT   
  
  
- ACACATAAAA TGAATCTTAG ACAAAACTAA GAAACTACAT TAATTAATCA TCAGAAAAAT TAACTTAAAA   
  
  
- ATTGTCATCA AACTTAATCT GTAAATAGCT ATAATATAAA AAATATATAT TAATTTTTTA TGTTTTACTA   
  
  
- TTCACAGAAT GATGTTATGT GAAGTATTCA CAGCACAAAA TTGAATTTTT AGTATGATAA TACAAAGATT   
  
  
- TAACTTTTTT TTCACTCTAA TTCATCGTCT TCGTATAATG TTGAATATTT AATATATTTT AGTAGAAAAA   
  
  
- TAGGTATTAA ATAGTGTAAA GATTTTATGC GTATAGTATA GATGAATACC TATCCCAATT ATTCTAATCC   
  
  
- CGTAGTAAAT TCTAAAATAG TCGCATTGGT TGCTTACAAA TTCCAATCGT CAAGTATTAA TTCACATTAG   
  
  
- TAATTTTTTA AATACTAAAA TTTAGTATTT TATACAATTA GATCCTACTC CGTTCGCCGT TCTGTATATA   
  
  
- GTGATAGGAA TTTTTTATAT TATATAACTA AAGTTTCTTA ATACTTCATA TAATTAAAGT ATATATTACT   
  
  
- GCTAATACAT AAAAAAATGA TTTAATGAAC ATGGGTATTA TTTATCTTAG CTACGTAGTA AGTACCGGAA   
  
  
- AGTAAGACAT TATACCAACT GAGGTTTTTA AGAGTTTCAT AAAAATATAA AACCCTCCCC TCTAAAATCA   
  
  
- AACTATGAAA ACAACTTGAT CACTTGTTAA AGGTTGTTGG AGGTGGTGGG GGTTTGTAGT TGTTAGACTA   
  
  
- ACTACATACG TAGGGTTCGA ACTTAACTTA TTATTAAAGT TATTGTTGTT ATTAGCAAAA TGGAACTGTT   
  
  
- CGAGGTGTCG TTGAGTTGCA GAAGGTTTGG TTGGGTAGTG AGTAGTAGTA GTAGGGGTGA TAGGGTAAGT   
  
  
- GGGGGTGGTT TTCCTCGGAT TTCTGTCATT GTTCTTCTTA CGAACGGAAA AAAGTACCTA CTTCTTCTGA   
  
  
- AGAGAAGAAG GAGATGAAGA AGGGTGGTGA TACTATGTGT GGTGGAGGTT GTAGTAATGG GGTGGTTAAT   
  
  
- ATTAAGGGCA ATATGGGACC TGGGAGGGAG GTGGTGGTGG TGTCGGTGTT GGTGGTGGAG TAGGAGGTGA   
  
  
- GGGTGGGTGG TACCGAAGCC ACCACCCGAA CTGAGGAGGA GGGGCAGACG GCGACACCTT AATAGAAAGA   
  
  
- GGGGCCTGTA GGAGGAGAGG AGGGGGTGGC CTTTCACCCG CAGACTGTAA GAATACCTCC GCCGATCCCG   
  
  
- AAAAAGTCTG TGCTTGTGGT GGTTCGAGGT GGTCGAGGAG ACCTACGACT TGCTCGAGCC GAGGGGGATG   
  
  
- CCACTACAGC TCGTCTTCGA CCACCGCATG AAATTGGTTC GGGAAAAACG GGCGGAGTGA CGGTGGCCTG   
  
  
- GGGCGGTGGC TCGGCGATAC GAGCGGCGAC GCCTGTTCTG GAGGAGGAAG CTGAGGTGCG AGTCCAACGA   
  
  
- GGAGTTCAAG GTCCTTCAGT CGGGCACCTG GTGGAAGCCG GTGCACCGAA CGTTGCCACG TTAGTAGCTC   
  
  
- CGGAATCTCT CCCTGGGTGT ACTTTTCTCA TTCGAGGTGC AGTAGCTATA GTCGTGGTGG AAGACGTGGG   
  
  
- TCACCGGGTG GAACGAACTC CGAAATCGGT GGGCGTACCT GCTGTGAGGG GTGGAGTCGG AGTGACGGCA   
  
  
- GCAGCAGTTG TTCATGCGGC GGCACTCGCC ACCGCTCCTG CTGCCCCCTC TTCCTAGTTT CTCCCACTAC   
  
  
- TCTCTCTAAC CAGAAGCAGA ACTCTTCAAG CGATCCAATT ACCCCCAAGG GAAGTTCAAG TTGCACCAAA   
  
  
- TGGTGCATCC ACTAGATAGG GTCAACCTAA AAAGAGCAAA CCTATAATTT CTACTACTTC GCCACCGCTA   
  
  
- ATTGACACAG TTGAGCGAGG TGAGCCACCT GGAGGCTATA GCCCTCCATC ACGAGCGCCG CAAGTCCGCC   
  
  
- GACGCCGGCT CCCACCACTG CCACCACCTC CTTCTCCGCC TCGACTGACT CCACCCACTC CCAGTCATAC   
  
  
- TTAAAAAATC CGCCAAACTC CTCACAGAAT CCACCAAATC CCAAATGAAA CTCCGGCATC CTCTAACAAA   
  
  
- AGGATCCTGA TCGTTACTCT TTAACTACAA TCTCGCCCGG CGCCCGGCCC GGTACCACCT AGAGGAACGT   
  
  
- ACAGGGCTCG GACCACCCAG CCGACTCCCC TCCCTCTGCC GCTCTGCCAC CAGCCCAGCC TACGTACCCC   
  
  
- GCCCCAAGCT CGTGCACCCC AAGTCACTGC TTCACACACT ACTACACTCC CGGAACAACT CCTCCATGTT   
  
  
- CCTTCCTACC AGTTACTGTG TCACGTGGTA CCTACCGCCA TTAGTGGGAC CTTATGACAA CACCACCGCC   
  
  
- CTAGTTGGCC ACCAAACCCG GTCACGTACC GCCGGAAC

+     ACE

| Site Name | Organism | Position | Strand | Matrix score. | sequence | function |
| --- | --- | --- | --- | --- | --- | --- |
| ACE | Petroselinum crispum | 1218 | - | 9 | GACACGTATG | cis-acting element involved in light responsiveness |

>HU05G01983.1   
+ +Up\_Stream \_Len000AAATCA AACTCATAAA CAATAATTTT TTAAAACTTA AATGGTAATT ATTAGATTAA   
  
  
+ TAAAAATCCT AATGGTTGTT TAATGTTACA TCAAGACATG CTTTTTGGTG TATATATAAT GGCAGCATGT   
  
  
+ GGGTTAAGCG GCAACAAATT ATTCTCAAAT GCATGCAGTA ATTAATTTAA TTAATTAATC ACCTGCATAA   
  
  
+ TCTCAATACT AATCCTTTCA TTCTGTGCCC CACCCAACCC CGAAAATAGT AATCTTTCAA ACGTTGGAAG   
  
  
+ TGGGATTAGG TATAGAGTAG TCCTACAGAT GATGATCTAC CTACATATAA CATAATCTGA CTTTTCTTTT   
  
  
+ TTAAATAACT TGTATTGATG GCACAGAATT TGACATGTTG GCATATGGTA CTAAGAAATT AATTAACGTT   
  
  
+ GTGCTAAGAT ATAGGTCATT CTTTTTGGAG ACATAAGGCC CGCGTTCACG CAGGGTTCGA GAAGGGCCAC   
  
  
+ ATTCAATGGA TGAATTGTAG GTAGTTTAAT CTGACTTTGT CAGTGACTGA TTCCACGGCT TGAACCCGCG   
  
  
+ ACATTAATAT AGGTTGACTA AAGTCGTTTG ATACTTATTT TTCTTAAGTA AAGTCTTGAA TTCAAATCTT   
  
  
+ ATAAATAAAT AAAAAAATCT ATAATAAAAG AATTCTACCA TTTATTAAGT CAACAACTCA ATTCGAATGA   
  
  
+ AATAAAAAAT CAAGATCACC AAAAAAAATA AATAAAAGGA AAGGTAGATT GCATTTGGAT TTTGAGTGGC   
  
  
+ GTGGTGGTGG GTTTGATGCT GGTGCTGCAT ACACCGCAGC TTTTCTCTGT CCTTTTACCT CTCTTTGTCT   
  
  
+ GCAAGAACCC CATTTGGCAC GCCTGCTCTC TTTTCTATGT TTTCTTTTCT ATCTTTTAAG AAACAATGGA   
  
  
+ AAGCTAACAC ACTAACACTA ACCCATTGGT CCTTTTTTCT TATCTGCAAA ATTAAAGTAA CAATATTTTT   
  
  
+ CTTATCCCCT TTTTCGTTCC ACCCATTTCT TTATCCTTCT GTTTGAACCT AATCTGTATA TATGCATGCA   
  
  
+ TGTGTATTTT ACTTAGAATC TGTTTTGATT CTTTGATGTA ATTAATTAGT AGTCTTTTTA ATTGAATTTT   
  
  
+ TAACAGTAGT TTGAATTAGA CATTTATCGA TATTATATTT TTTATATATA ATTAAAAAAT ACAAAATGAT   
  
  
+ AAGTGTCTTA CTACAATACA CTTCATAAGT GTCGTGTTTT AACTTAAAAA TCATACTATT ATGTTTCTAA   
  
  
+ ATTGAAAAAA AAGTGAGATT AAGTAGCAGA AGCATATTAC AACTTATAAA TTATATAAAA TCATCTTTTT   
  
  
+ ATCCATAATT TATCACATTT CTAAAATACG CATATCATAT CTACTTATGG ATAGGGTTAA TAAGATTAGG   
  
  
+ GCATCATTTA AGATTTTATC AGCGTAACCA ACGAATGTTT AAGGTTAGCA GTTCATAATT AAGTGTAATC   
  
  
+ ATTAAAAAAT TTATGATTTT AAATCATAAA ATATGTTAAT CTAGGATGAG GCAAGCGGCA AGACATATAT   
  
  
+ CACTATCCTT AAAAAATATA ATATATTGAT TTCAAAGAAT TATGAAGTAT ATTAATTTCA TATATAATGA   
  
  
+ CGATTATGTA TTTTTTTACT AAATTACTTG TACCCATAAT AAATAGAATC GATGCATCAT TCATGGCCTT   
  
  
+ TCATTCTGTA ATATGGTTGA CTCCAAAAAT TCTCAAAGTA TTTTTATATT TTGGGAGGGG AGATTTTAGT   
  
  
+ TTGATACTTT TGTTGAACTA GTGAACAATT TCCAACAACC TCCACCACCC CCAAACATCA ACAATCTGAT   
  
  
+ TGATGTATGC ATCCCAAGCT TGAATTGAAT AATAATTTCA ATAACAACAA TAATCGTTTT ACCTTGACAA   
  
  
+ GCTCCACAGC AACTCAACGT CTTCCAAACC AACCCATCAC TCATCATCAT CATCCCCACT ATCCCATTCA   
  
  
+ CCCCCACCAA AAGGAGCCTA AAGACAGTAA CAAGAAGAAT GCTTGCCTTT TTTCATGGAT GAAGAAGACT   
  
  
+ TCTCTTCTTC CTCTACTTCT TCCCACCACT ATGATACACA CCACCTCCAA CATCATTACC CCACCAATTA   
  
  
+ TAATTCCCGT TATACCCTGG ACCCTCCCTC CACCACCACC ACAGCCACAA CCACCACCTC ATCCTCCACT   
  
  
+ CCCACCCACC ATGGCTTCGG TGGTGGGCTT GACTCCTCCT CCCCGTCTGC CGCTGTGGAA TTATCTTTCT   
  
  
+ CCCCGGACAT CCTCCTCTCC TCCCCCACCG GAAAGTGGGC GTCTGACATT CTTATGGAGG CGGCTAGGGC   
  
  
+ TTTTTCAGAC ACGAACACCA CCAAGCTCCA CCAGCTCCTC TGGATGCTGA ACGAGCTCGG CTCCCCCTAC   
  
  
+ GGTGATGTCG AGCAGAAGCT GGTGGCGTAC TTTAACCAAG CCCTTTTTGC CCGCCTCACT GCCACCGGAC   
  
  
+ CCCGCCACCG AGCCGCTATG CTCGCCGCTG CGGACAAGAC CTCCTCCTTC GACTCCACGC TCAGGTTGCT   
  
  
+ CCTCAAGTTC CAGGAAGTCA GCCCGTGGAC CACCTTCGGC CACGTGGCTT GCAACGGTGC AATCATCGAG   
  
  
+ GCCTTAGAGA GGGACCCACA TGAAAAGAGT AAGCTCCACG TCATCGATAT CAGCACCACC TTCTGCACCC   
  
  
+ AGTGGCCCAC CTTGCTTGAG GCTTTAGCCA CCCGCATGGA CGACACTCCC CACCTCAGCC TCACTGCCGT   
  
  
+ CGTCGTCAAC AAGTACGCCG CCGTGAGCGG TGGCGAGGAC GACGGGGGAG AAGGATCAAA GAGGGTGATG   
  
  
+ AGAGAGATTG GTCTTCGTCT TGAGAAGTTC GCTAGGTTAA TGGGGGTTCC CTTCAAGTTC AACGTGGTTT   
  
  
+ ACCACGTAGG TGATCTATCC CAGTTGGATT TTTCTCGTTT GGATATTAAA GATGATGAAG CGGTGGCGAT   
  
  
+ TAACTGTGTC AACTCGCTCC ACTCGGTGGA CCTCCGATAT CGGGAGGTAG TGCTCGCGGC GTTCAGGCGG   
  
  
+ CTGCGGCCGA GGGTGGTGAC GGTGGTGGAG GAAGAGGCGG AGCTGACTGA GGTGGGTGAG GGTCAGTATG   
  
  
+ AATTTTTTAG GCGGTTTGAG GAGTGTCTTA GGTGGTTTAG GGTTTACTTT GAGGCCGTAG GAGATTGTTT   
  
  
+ TCCTAGGACT AGCAATGAGA AATTGATGTT AGAGCGGGCC GCGGGCCGGG CCATGGTGGA TCTCCTTGCA   
  
  
+ TGTCCCGAGC CTGGTGGGTC GGCTGAGGGG AGGGAGACGG CGAGACGGTG GTCGGGTCGG ATGCATGGGG   
  
  
+ CGGGGTTCGA GCACGTGGGG TTCAGTGACG AAGTGTGTGA TGATGTGAGG GCCTTGTTGA GGAGGTACAA   
  
  
+ GGAAGGATGG TCAATGACAC AGTGCACCAT GGATGGCGGT AATCACCCTG GAATACTGTT GTGGTGGCGG   
  
  
+ GATCAACCGG TGGTTTGGGC CAGTGCATGG CGGCCTTG  

- +Up\_Stream \_Len000TTTAGT TTGAGTATTT GTTATTAAAA AATTTTGAAT TTACCATTAA TAATCTAATT   
  
  
- ATTTTTAGGA TTACCAACAA ATTACAATGT AGTTCTGTAC GAAAAACCAC ATATATATTA CCGTCGTACA   
  
  
- CCCAATTCGC CGTTGTTTAA TAAGAGTTTA CGTACGTCAT TAATTAAATT AATTAATTAG TGGACGTATT   
  
  
- AGAGTTATGA TTAGGAAAGT AAGACACGGG GTGGGTTGGG GCTTTTATCA TTAGAAAGTT TGCAACCTTC   
  
  
- ACCCTAATCC ATATCTCATC AGGATGTCTA CTACTAGATG GATGTATATT GTATTAGACT GAAAAGAAAA   
  
  
- AATTTATTGA ACATAACTAC CGTGTCTTAA ACTGTACAAC CGTATACCAT GATTCTTTAA TTAATTGCAA   
  
  
- CACGATTCTA TATCCAGTAA GAAAAACCTC TGTATTCCGG GCGCAAGTGC GTCCCAAGCT CTTCCCGGTG   
  
  
- TAAGTTACCT ACTTAACATC CATCAAATTA GACTGAAACA GTCACTGACT AAGGTGCCGA ACTTGGGCGC   
  
  
- TGTAATTATA TCCAACTGAT TTCAGCAAAC TATGAATAAA AAGAATTCAT TTCAGAACTT AAGTTTAGAA   
  
  
- TATTTATTTA TTTTTTTAGA TATTATTTTC TTAAGATGGT AAATAATTCA GTTGTTGAGT TAAGCTTACT   
  
  
- TTATTTTTTA GTTCTAGTGG TTTTTTTTAT TTATTTTCCT TTCCATCTAA CGTAAACCTA AAACTCACCG   
  
  
- CACCACCACC CAAACTACGA CCACGACGTA TGTGGCGTCG AAAAGAGACA GGAAAATGGA GAGAAACAGA   
  
  
- CGTTCTTGGG GTAAACCGTG CGGACGAGAG AAAAGATACA AAAGAAAAGA TAGAAAATTC TTTGTTACCT   
  
  
- TTCGATTGTG TGATTGTGAT TGGGTAACCA GGAAAAAAGA ATAGACGTTT TAATTTCATT GTTATAAAAA   
  
  
- GAATAGGGGA AAAAGCAAGG TGGGTAAAGA AATAGGAAGA CAAACTTGGA TTAGACATAT ATACGTACGT   
  
  
- ACACATAAAA TGAATCTTAG ACAAAACTAA GAAACTACAT TAATTAATCA TCAGAAAAAT TAACTTAAAA   
  
  
- ATTGTCATCA AACTTAATCT GTAAATAGCT ATAATATAAA AAATATATAT TAATTTTTTA TGTTTTACTA   
  
  
- TTCACAGAAT GATGTTATGT GAAGTATTCA CAGCACAAAA TTGAATTTTT AGTATGATAA TACAAAGATT   
  
  
- TAACTTTTTT TTCACTCTAA TTCATCGTCT TCGTATAATG TTGAATATTT AATATATTTT AGTAGAAAAA   
  
  
- TAGGTATTAA ATAGTGTAAA GATTTTATGC GTATAGTATA GATGAATACC TATCCCAATT ATTCTAATCC   
  
  
- CGTAGTAAAT TCTAAAATAG TCGCATTGGT TGCTTACAAA TTCCAATCGT CAAGTATTAA TTCACATTAG   
  
  
- TAATTTTTTA AATACTAAAA TTTAGTATTT TATACAATTA GATCCTACTC CGTTCGCCGT TCTGTATATA   
  
  
- GTGATAGGAA TTTTTTATAT TATATAACTA AAGTTTCTTA ATACTTCATA TAATTAAAGT ATATATTACT   
  
  
- GCTAATACAT AAAAAAATGA TTTAATGAAC ATGGGTATTA TTTATCTTAG CTACGTAGTA AGTACCGGAA   
  
  
- AGTAAGACAT TATACCAACT GAGGTTTTTA AGAGTTTCAT AAAAATATAA AACCCTCCCC TCTAAAATCA   
  
  
- AACTATGAAA ACAACTTGAT CACTTGTTAA AGGTTGTTGG AGGTGGTGGG GGTTTGTAGT TGTTAGACTA   
  
  
- ACTACATACG TAGGGTTCGA ACTTAACTTA TTATTAAAGT TATTGTTGTT ATTAGCAAAA TGGAACTGTT   
  
  
- CGAGGTGTCG TTGAGTTGCA GAAGGTTTGG TTGGGTAGTG AGTAGTAGTA GTAGGGGTGA TAGGGTAAGT   
  
  
- GGGGGTGGTT TTCCTCGGAT TTCTGTCATT GTTCTTCTTA CGAACGGAAA AAAGTACCTA CTTCTTCTGA   
  
  
- AGAGAAGAAG GAGATGAAGA AGGGTGGTGA TACTATGTGT GGTGGAGGTT GTAGTAATGG GGTGGTTAAT   
  
  
- ATTAAGGGCA ATATGGGACC TGGGAGGGAG GTGGTGGTGG TGTCGGTGTT GGTGGTGGAG TAGGAGGTGA   
  
  
- GGGTGGGTGG TACCGAAGCC ACCACCCGAA CTGAGGAGGA GGGGCAGACG GCGACACCTT AATAGAAAGA   
  
  
- GGGGCCTGTA GGAGGAGAGG AGGGGGTGGC CTTTCACCCG CAGACTGTAA GAATACCTCC GCCGATCCCG   
  
  
- AAAAAGTCTG TGCTTGTGGT GGTTCGAGGT GGTCGAGGAG ACCTACGACT TGCTCGAGCC GAGGGGGATG   
  
  
- CCACTACAGC TCGTCTTCGA CCACCGCATG AAATTGGTTC GGGAAAAACG GGCGGAGTGA CGGTGGCCTG   
  
  
- GGGCGGTGGC TCGGCGATAC GAGCGGCGAC GCCTGTTCTG GAGGAGGAAG CTGAGGTGCG AGTCCAACGA   
  
  
- GGAGTTCAAG GTCCTTCAGT CGGGCACCTG GTGGAAGCCG GTGCACCGAA CGTTGCCACG TTAGTAGCTC   
  
  
- CGGAATCTCT CCCTGGGTGT ACTTTTCTCA TTCGAGGTGC AGTAGCTATA GTCGTGGTGG AAGACGTGGG   
  
  
- TCACCGGGTG GAACGAACTC CGAAATCGGT GGGCGTACCT GCTGTGAGGG GTGGAGTCGG AGTGACGGCA   
  
  
- GCAGCAGTTG TTCATGCGGC GGCACTCGCC ACCGCTCCTG CTGCCCCCTC TTCCTAGTTT CTCCCACTAC   
  
  
- TCTCTCTAAC CAGAAGCAGA ACTCTTCAAG CGATCCAATT ACCCCCAAGG GAAGTTCAAG TTGCACCAAA   
  
  
- TGGTGCATCC ACTAGATAGG GTCAACCTAA AAAGAGCAAA CCTATAATTT CTACTACTTC GCCACCGCTA   
  
  
- ATTGACACAG TTGAGCGAGG TGAGCCACCT GGAGGCTATA GCCCTCCATC ACGAGCGCCG CAAGTCCGCC   
  
  
- GACGCCGGCT CCCACCACTG CCACCACCTC CTTCTCCGCC TCGACTGACT CCACCCACTC CCAGTCATAC   
  
  
- TTAAAAAATC CGCCAAACTC CTCACAGAAT CCACCAAATC CCAAATGAAA CTCCGGCATC CTCTAACAAA   
  
  
- AGGATCCTGA TCGTTACTCT TTAACTACAA TCTCGCCCGG CGCCCGGCCC GGTACCACCT AGAGGAACGT   
  
  
- ACAGGGCTCG GACCACCCAG CCGACTCCCC TCCCTCTGCC GCTCTGCCAC CAGCCCAGCC TACGTACCCC   
  
  
- GCCCCAAGCT CGTGCACCCC AAGTCACTGC TTCACACACT ACTACACTCC CGGAACAACT CCTCCATGTT   
  
  
- CCTTCCTACC AGTTACTGTG TCACGTGGTA CCTACCGCCA TTAGTGGGAC CTTATGACAA CACCACCGCC   
  
  
- CTAGTTGGCC ACCAAACCCG GTCACGTACC GCCGGAAC

+     AE-box

| Site Name | Organism | Position | Strand | Matrix score. | sequence | function |
| --- | --- | --- | --- | --- | --- | --- |
| AE-box | Arabidopsis thaliana | 903 | + | 8 | AGAAACAA | part of a module for light response |

>HU05G01983.1   
+ +Up\_Stream \_Len000AAATCA AACTCATAAA CAATAATTTT TTAAAACTTA AATGGTAATT ATTAGATTAA   
  
  
+ TAAAAATCCT AATGGTTGTT TAATGTTACA TCAAGACATG CTTTTTGGTG TATATATAAT GGCAGCATGT   
  
  
+ GGGTTAAGCG GCAACAAATT ATTCTCAAAT GCATGCAGTA ATTAATTTAA TTAATTAATC ACCTGCATAA   
  
  
+ TCTCAATACT AATCCTTTCA TTCTGTGCCC CACCCAACCC CGAAAATAGT AATCTTTCAA ACGTTGGAAG   
  
  
+ TGGGATTAGG TATAGAGTAG TCCTACAGAT GATGATCTAC CTACATATAA CATAATCTGA CTTTTCTTTT   
  
  
+ TTAAATAACT TGTATTGATG GCACAGAATT TGACATGTTG GCATATGGTA CTAAGAAATT AATTAACGTT   
  
  
+ GTGCTAAGAT ATAGGTCATT CTTTTTGGAG ACATAAGGCC CGCGTTCACG CAGGGTTCGA GAAGGGCCAC   
  
  
+ ATTCAATGGA TGAATTGTAG GTAGTTTAAT CTGACTTTGT CAGTGACTGA TTCCACGGCT TGAACCCGCG   
  
  
+ ACATTAATAT AGGTTGACTA AAGTCGTTTG ATACTTATTT TTCTTAAGTA AAGTCTTGAA TTCAAATCTT   
  
  
+ ATAAATAAAT AAAAAAATCT ATAATAAAAG AATTCTACCA TTTATTAAGT CAACAACTCA ATTCGAATGA   
  
  
+ AATAAAAAAT CAAGATCACC AAAAAAAATA AATAAAAGGA AAGGTAGATT GCATTTGGAT TTTGAGTGGC   
  
  
+ GTGGTGGTGG GTTTGATGCT GGTGCTGCAT ACACCGCAGC TTTTCTCTGT CCTTTTACCT CTCTTTGTCT   
  
  
+ GCAAGAACCC CATTTGGCAC GCCTGCTCTC TTTTCTATGT TTTCTTTTCT ATCTTTTAAG AAACAATGGA   
  
  
+ AAGCTAACAC ACTAACACTA ACCCATTGGT CCTTTTTTCT TATCTGCAAA ATTAAAGTAA CAATATTTTT   
  
  
+ CTTATCCCCT TTTTCGTTCC ACCCATTTCT TTATCCTTCT GTTTGAACCT AATCTGTATA TATGCATGCA   
  
  
+ TGTGTATTTT ACTTAGAATC TGTTTTGATT CTTTGATGTA ATTAATTAGT AGTCTTTTTA ATTGAATTTT   
  
  
+ TAACAGTAGT TTGAATTAGA CATTTATCGA TATTATATTT TTTATATATA ATTAAAAAAT ACAAAATGAT   
  
  
+ AAGTGTCTTA CTACAATACA CTTCATAAGT GTCGTGTTTT AACTTAAAAA TCATACTATT ATGTTTCTAA   
  
  
+ ATTGAAAAAA AAGTGAGATT AAGTAGCAGA AGCATATTAC AACTTATAAA TTATATAAAA TCATCTTTTT   
  
  
+ ATCCATAATT TATCACATTT CTAAAATACG CATATCATAT CTACTTATGG ATAGGGTTAA TAAGATTAGG   
  
  
+ GCATCATTTA AGATTTTATC AGCGTAACCA ACGAATGTTT AAGGTTAGCA GTTCATAATT AAGTGTAATC   
  
  
+ ATTAAAAAAT TTATGATTTT AAATCATAAA ATATGTTAAT CTAGGATGAG GCAAGCGGCA AGACATATAT   
  
  
+ CACTATCCTT AAAAAATATA ATATATTGAT TTCAAAGAAT TATGAAGTAT ATTAATTTCA TATATAATGA   
  
  
+ CGATTATGTA TTTTTTTACT AAATTACTTG TACCCATAAT AAATAGAATC GATGCATCAT TCATGGCCTT   
  
  
+ TCATTCTGTA ATATGGTTGA CTCCAAAAAT TCTCAAAGTA TTTTTATATT TTGGGAGGGG AGATTTTAGT   
  
  
+ TTGATACTTT TGTTGAACTA GTGAACAATT TCCAACAACC TCCACCACCC CCAAACATCA ACAATCTGAT   
  
  
+ TGATGTATGC ATCCCAAGCT TGAATTGAAT AATAATTTCA ATAACAACAA TAATCGTTTT ACCTTGACAA   
  
  
+ GCTCCACAGC AACTCAACGT CTTCCAAACC AACCCATCAC TCATCATCAT CATCCCCACT ATCCCATTCA   
  
  
+ CCCCCACCAA AAGGAGCCTA AAGACAGTAA CAAGAAGAAT GCTTGCCTTT TTTCATGGAT GAAGAAGACT   
  
  
+ TCTCTTCTTC CTCTACTTCT TCCCACCACT ATGATACACA CCACCTCCAA CATCATTACC CCACCAATTA   
  
  
+ TAATTCCCGT TATACCCTGG ACCCTCCCTC CACCACCACC ACAGCCACAA CCACCACCTC ATCCTCCACT   
  
  
+ CCCACCCACC ATGGCTTCGG TGGTGGGCTT GACTCCTCCT CCCCGTCTGC CGCTGTGGAA TTATCTTTCT   
  
  
+ CCCCGGACAT CCTCCTCTCC TCCCCCACCG GAAAGTGGGC GTCTGACATT CTTATGGAGG CGGCTAGGGC   
  
  
+ TTTTTCAGAC ACGAACACCA CCAAGCTCCA CCAGCTCCTC TGGATGCTGA ACGAGCTCGG CTCCCCCTAC   
  
  
+ GGTGATGTCG AGCAGAAGCT GGTGGCGTAC TTTAACCAAG CCCTTTTTGC CCGCCTCACT GCCACCGGAC   
  
  
+ CCCGCCACCG AGCCGCTATG CTCGCCGCTG CGGACAAGAC CTCCTCCTTC GACTCCACGC TCAGGTTGCT   
  
  
+ CCTCAAGTTC CAGGAAGTCA GCCCGTGGAC CACCTTCGGC CACGTGGCTT GCAACGGTGC AATCATCGAG   
  
  
+ GCCTTAGAGA GGGACCCACA TGAAAAGAGT AAGCTCCACG TCATCGATAT CAGCACCACC TTCTGCACCC   
  
  
+ AGTGGCCCAC CTTGCTTGAG GCTTTAGCCA CCCGCATGGA CGACACTCCC CACCTCAGCC TCACTGCCGT   
  
  
+ CGTCGTCAAC AAGTACGCCG CCGTGAGCGG TGGCGAGGAC GACGGGGGAG AAGGATCAAA GAGGGTGATG   
  
  
+ AGAGAGATTG GTCTTCGTCT TGAGAAGTTC GCTAGGTTAA TGGGGGTTCC CTTCAAGTTC AACGTGGTTT   
  
  
+ ACCACGTAGG TGATCTATCC CAGTTGGATT TTTCTCGTTT GGATATTAAA GATGATGAAG CGGTGGCGAT   
  
  
+ TAACTGTGTC AACTCGCTCC ACTCGGTGGA CCTCCGATAT CGGGAGGTAG TGCTCGCGGC GTTCAGGCGG   
  
  
+ CTGCGGCCGA GGGTGGTGAC GGTGGTGGAG GAAGAGGCGG AGCTGACTGA GGTGGGTGAG GGTCAGTATG   
  
  
+ AATTTTTTAG GCGGTTTGAG GAGTGTCTTA GGTGGTTTAG GGTTTACTTT GAGGCCGTAG GAGATTGTTT   
  
  
+ TCCTAGGACT AGCAATGAGA AATTGATGTT AGAGCGGGCC GCGGGCCGGG CCATGGTGGA TCTCCTTGCA   
  
  
+ TGTCCCGAGC CTGGTGGGTC GGCTGAGGGG AGGGAGACGG CGAGACGGTG GTCGGGTCGG ATGCATGGGG   
  
  
+ CGGGGTTCGA GCACGTGGGG TTCAGTGACG AAGTGTGTGA TGATGTGAGG GCCTTGTTGA GGAGGTACAA   
  
  
+ GGAAGGATGG TCAATGACAC AGTGCACCAT GGATGGCGGT AATCACCCTG GAATACTGTT GTGGTGGCGG   
  
  
+ GATCAACCGG TGGTTTGGGC CAGTGCATGG CGGCCTTG  

- +Up\_Stream \_Len000TTTAGT TTGAGTATTT GTTATTAAAA AATTTTGAAT TTACCATTAA TAATCTAATT   
  
  
- ATTTTTAGGA TTACCAACAA ATTACAATGT AGTTCTGTAC GAAAAACCAC ATATATATTA CCGTCGTACA   
  
  
- CCCAATTCGC CGTTGTTTAA TAAGAGTTTA CGTACGTCAT TAATTAAATT AATTAATTAG TGGACGTATT   
  
  
- AGAGTTATGA TTAGGAAAGT AAGACACGGG GTGGGTTGGG GCTTTTATCA TTAGAAAGTT TGCAACCTTC   
  
  
- ACCCTAATCC ATATCTCATC AGGATGTCTA CTACTAGATG GATGTATATT GTATTAGACT GAAAAGAAAA   
  
  
- AATTTATTGA ACATAACTAC CGTGTCTTAA ACTGTACAAC CGTATACCAT GATTCTTTAA TTAATTGCAA   
  
  
- CACGATTCTA TATCCAGTAA GAAAAACCTC TGTATTCCGG GCGCAAGTGC GTCCCAAGCT CTTCCCGGTG   
  
  
- TAAGTTACCT ACTTAACATC CATCAAATTA GACTGAAACA GTCACTGACT AAGGTGCCGA ACTTGGGCGC   
  
  
- TGTAATTATA TCCAACTGAT TTCAGCAAAC TATGAATAAA AAGAATTCAT TTCAGAACTT AAGTTTAGAA   
  
  
- TATTTATTTA TTTTTTTAGA TATTATTTTC TTAAGATGGT AAATAATTCA GTTGTTGAGT TAAGCTTACT   
  
  
- TTATTTTTTA GTTCTAGTGG TTTTTTTTAT TTATTTTCCT TTCCATCTAA CGTAAACCTA AAACTCACCG   
  
  
- CACCACCACC CAAACTACGA CCACGACGTA TGTGGCGTCG AAAAGAGACA GGAAAATGGA GAGAAACAGA   
  
  
- CGTTCTTGGG GTAAACCGTG CGGACGAGAG AAAAGATACA AAAGAAAAGA TAGAAAATTC TTTGTTACCT   
  
  
- TTCGATTGTG TGATTGTGAT TGGGTAACCA GGAAAAAAGA ATAGACGTTT TAATTTCATT GTTATAAAAA   
  
  
- GAATAGGGGA AAAAGCAAGG TGGGTAAAGA AATAGGAAGA CAAACTTGGA TTAGACATAT ATACGTACGT   
  
  
- ACACATAAAA TGAATCTTAG ACAAAACTAA GAAACTACAT TAATTAATCA TCAGAAAAAT TAACTTAAAA   
  
  
- ATTGTCATCA AACTTAATCT GTAAATAGCT ATAATATAAA AAATATATAT TAATTTTTTA TGTTTTACTA   
  
  
- TTCACAGAAT GATGTTATGT GAAGTATTCA CAGCACAAAA TTGAATTTTT AGTATGATAA TACAAAGATT   
  
  
- TAACTTTTTT TTCACTCTAA TTCATCGTCT TCGTATAATG TTGAATATTT AATATATTTT AGTAGAAAAA   
  
  
- TAGGTATTAA ATAGTGTAAA GATTTTATGC GTATAGTATA GATGAATACC TATCCCAATT ATTCTAATCC   
  
  
- CGTAGTAAAT TCTAAAATAG TCGCATTGGT TGCTTACAAA TTCCAATCGT CAAGTATTAA TTCACATTAG   
  
  
- TAATTTTTTA AATACTAAAA TTTAGTATTT TATACAATTA GATCCTACTC CGTTCGCCGT TCTGTATATA   
  
  
- GTGATAGGAA TTTTTTATAT TATATAACTA AAGTTTCTTA ATACTTCATA TAATTAAAGT ATATATTACT   
  
  
- GCTAATACAT AAAAAAATGA TTTAATGAAC ATGGGTATTA TTTATCTTAG CTACGTAGTA AGTACCGGAA   
  
  
- AGTAAGACAT TATACCAACT GAGGTTTTTA AGAGTTTCAT AAAAATATAA AACCCTCCCC TCTAAAATCA   
  
  
- AACTATGAAA ACAACTTGAT CACTTGTTAA AGGTTGTTGG AGGTGGTGGG GGTTTGTAGT TGTTAGACTA   
  
  
- ACTACATACG TAGGGTTCGA ACTTAACTTA TTATTAAAGT TATTGTTGTT ATTAGCAAAA TGGAACTGTT   
  
  
- CGAGGTGTCG TTGAGTTGCA GAAGGTTTGG TTGGGTAGTG AGTAGTAGTA GTAGGGGTGA TAGGGTAAGT   
  
  
- GGGGGTGGTT TTCCTCGGAT TTCTGTCATT GTTCTTCTTA CGAACGGAAA AAAGTACCTA CTTCTTCTGA   
  
  
- AGAGAAGAAG GAGATGAAGA AGGGTGGTGA TACTATGTGT GGTGGAGGTT GTAGTAATGG GGTGGTTAAT   
  
  
- ATTAAGGGCA ATATGGGACC TGGGAGGGAG GTGGTGGTGG TGTCGGTGTT GGTGGTGGAG TAGGAGGTGA   
  
  
- GGGTGGGTGG TACCGAAGCC ACCACCCGAA CTGAGGAGGA GGGGCAGACG GCGACACCTT AATAGAAAGA   
  
  
- GGGGCCTGTA GGAGGAGAGG AGGGGGTGGC CTTTCACCCG CAGACTGTAA GAATACCTCC GCCGATCCCG   
  
  
- AAAAAGTCTG TGCTTGTGGT GGTTCGAGGT GGTCGAGGAG ACCTACGACT TGCTCGAGCC GAGGGGGATG   
  
  
- CCACTACAGC TCGTCTTCGA CCACCGCATG AAATTGGTTC GGGAAAAACG GGCGGAGTGA CGGTGGCCTG   
  
  
- GGGCGGTGGC TCGGCGATAC GAGCGGCGAC GCCTGTTCTG GAGGAGGAAG CTGAGGTGCG AGTCCAACGA   
  
  
- GGAGTTCAAG GTCCTTCAGT CGGGCACCTG GTGGAAGCCG GTGCACCGAA CGTTGCCACG TTAGTAGCTC   
  
  
- CGGAATCTCT CCCTGGGTGT ACTTTTCTCA TTCGAGGTGC AGTAGCTATA GTCGTGGTGG AAGACGTGGG   
  
  
- TCACCGGGTG GAACGAACTC CGAAATCGGT GGGCGTACCT GCTGTGAGGG GTGGAGTCGG AGTGACGGCA   
  
  
- GCAGCAGTTG TTCATGCGGC GGCACTCGCC ACCGCTCCTG CTGCCCCCTC TTCCTAGTTT CTCCCACTAC   
  
  
- TCTCTCTAAC CAGAAGCAGA ACTCTTCAAG CGATCCAATT ACCCCCAAGG GAAGTTCAAG TTGCACCAAA   
  
  
- TGGTGCATCC ACTAGATAGG GTCAACCTAA AAAGAGCAAA CCTATAATTT CTACTACTTC GCCACCGCTA   
  
  
- ATTGACACAG TTGAGCGAGG TGAGCCACCT GGAGGCTATA GCCCTCCATC ACGAGCGCCG CAAGTCCGCC   
  
  
- GACGCCGGCT CCCACCACTG CCACCACCTC CTTCTCCGCC TCGACTGACT CCACCCACTC CCAGTCATAC   
  
  
- TTAAAAAATC CGCCAAACTC CTCACAGAAT CCACCAAATC CCAAATGAAA CTCCGGCATC CTCTAACAAA   
  
  
- AGGATCCTGA TCGTTACTCT TTAACTACAA TCTCGCCCGG CGCCCGGCCC GGTACCACCT AGAGGAACGT   
  
  
- ACAGGGCTCG GACCACCCAG CCGACTCCCC TCCCTCTGCC GCTCTGCCAC CAGCCCAGCC TACGTACCCC   
  
  
- GCCCCAAGCT CGTGCACCCC AAGTCACTGC TTCACACACT ACTACACTCC CGGAACAACT CCTCCATGTT   
  
  
- CCTTCCTACC AGTTACTGTG TCACGTGGTA CCTACCGCCA TTAGTGGGAC CTTATGACAA CACCACCGCC   
  
  
- CTAGTTGGCC ACCAAACCCG GTCACGTACC GCCGGAAC

+     ARE

| Site Name | Organism | Position | Strand | Matrix score. | sequence | function |
| --- | --- | --- | --- | --- | --- | --- |
| ARE | Zea mays | 2869 | - | 6 | AAACCA | cis-acting regulatory element essential for the anaerobic induction |
| ARE | Zea mays | 3117 | - | 6 | AAACCA | cis-acting regulatory element essential for the anaerobic induction |
| ARE | Zea mays | 3445 | - | 6 | AAACCA | cis-acting regulatory element essential for the anaerobic induction |
| ARE | Zea mays | 1920 | + | 6 | AAACCA | cis-acting regulatory element essential for the anaerobic induction |

>HU05G01983.1   
+ +Up\_Stream \_Len000AAATCA AACTCATAAA CAATAATTTT TTAAAACTTA AATGGTAATT ATTAGATTAA   
  
  
+ TAAAAATCCT AATGGTTGTT TAATGTTACA TCAAGACATG CTTTTTGGTG TATATATAAT GGCAGCATGT   
  
  
+ GGGTTAAGCG GCAACAAATT ATTCTCAAAT GCATGCAGTA ATTAATTTAA TTAATTAATC ACCTGCATAA   
  
  
+ TCTCAATACT AATCCTTTCA TTCTGTGCCC CACCCAACCC CGAAAATAGT AATCTTTCAA ACGTTGGAAG   
  
  
+ TGGGATTAGG TATAGAGTAG TCCTACAGAT GATGATCTAC CTACATATAA CATAATCTGA CTTTTCTTTT   
  
  
+ TTAAATAACT TGTATTGATG GCACAGAATT TGACATGTTG GCATATGGTA CTAAGAAATT AATTAACGTT   
  
  
+ GTGCTAAGAT ATAGGTCATT CTTTTTGGAG ACATAAGGCC CGCGTTCACG CAGGGTTCGA GAAGGGCCAC   
  
  
+ ATTCAATGGA TGAATTGTAG GTAGTTTAAT CTGACTTTGT CAGTGACTGA TTCCACGGCT TGAACCCGCG   
  
  
+ ACATTAATAT AGGTTGACTA AAGTCGTTTG ATACTTATTT TTCTTAAGTA AAGTCTTGAA TTCAAATCTT   
  
  
+ ATAAATAAAT AAAAAAATCT ATAATAAAAG AATTCTACCA TTTATTAAGT CAACAACTCA ATTCGAATGA   
  
  
+ AATAAAAAAT CAAGATCACC AAAAAAAATA AATAAAAGGA AAGGTAGATT GCATTTGGAT TTTGAGTGGC   
  
  
+ GTGGTGGTGG GTTTGATGCT GGTGCTGCAT ACACCGCAGC TTTTCTCTGT CCTTTTACCT CTCTTTGTCT   
  
  
+ GCAAGAACCC CATTTGGCAC GCCTGCTCTC TTTTCTATGT TTTCTTTTCT ATCTTTTAAG AAACAATGGA   
  
  
+ AAGCTAACAC ACTAACACTA ACCCATTGGT CCTTTTTTCT TATCTGCAAA ATTAAAGTAA CAATATTTTT   
  
  
+ CTTATCCCCT TTTTCGTTCC ACCCATTTCT TTATCCTTCT GTTTGAACCT AATCTGTATA TATGCATGCA   
  
  
+ TGTGTATTTT ACTTAGAATC TGTTTTGATT CTTTGATGTA ATTAATTAGT AGTCTTTTTA ATTGAATTTT   
  
  
+ TAACAGTAGT TTGAATTAGA CATTTATCGA TATTATATTT TTTATATATA ATTAAAAAAT ACAAAATGAT   
  
  
+ AAGTGTCTTA CTACAATACA CTTCATAAGT GTCGTGTTTT AACTTAAAAA TCATACTATT ATGTTTCTAA   
  
  
+ ATTGAAAAAA AAGTGAGATT AAGTAGCAGA AGCATATTAC AACTTATAAA TTATATAAAA TCATCTTTTT   
  
  
+ ATCCATAATT TATCACATTT CTAAAATACG CATATCATAT CTACTTATGG ATAGGGTTAA TAAGATTAGG   
  
  
+ GCATCATTTA AGATTTTATC AGCGTAACCA ACGAATGTTT AAGGTTAGCA GTTCATAATT AAGTGTAATC   
  
  
+ ATTAAAAAAT TTATGATTTT AAATCATAAA ATATGTTAAT CTAGGATGAG GCAAGCGGCA AGACATATAT   
  
  
+ CACTATCCTT AAAAAATATA ATATATTGAT TTCAAAGAAT TATGAAGTAT ATTAATTTCA TATATAATGA   
  
  
+ CGATTATGTA TTTTTTTACT AAATTACTTG TACCCATAAT AAATAGAATC GATGCATCAT TCATGGCCTT   
  
  
+ TCATTCTGTA ATATGGTTGA CTCCAAAAAT TCTCAAAGTA TTTTTATATT TTGGGAGGGG AGATTTTAGT   
  
  
+ TTGATACTTT TGTTGAACTA GTGAACAATT TCCAACAACC TCCACCACCC CCAAACATCA ACAATCTGAT   
  
  
+ TGATGTATGC ATCCCAAGCT TGAATTGAAT AATAATTTCA ATAACAACAA TAATCGTTTT ACCTTGACAA   
  
  
+ GCTCCACAGC AACTCAACGT CTTCCAAACC AACCCATCAC TCATCATCAT CATCCCCACT ATCCCATTCA   
  
  
+ CCCCCACCAA AAGGAGCCTA AAGACAGTAA CAAGAAGAAT GCTTGCCTTT TTTCATGGAT GAAGAAGACT   
  
  
+ TCTCTTCTTC CTCTACTTCT TCCCACCACT ATGATACACA CCACCTCCAA CATCATTACC CCACCAATTA   
  
  
+ TAATTCCCGT TATACCCTGG ACCCTCCCTC CACCACCACC ACAGCCACAA CCACCACCTC ATCCTCCACT   
  
  
+ CCCACCCACC ATGGCTTCGG TGGTGGGCTT GACTCCTCCT CCCCGTCTGC CGCTGTGGAA TTATCTTTCT   
  
  
+ CCCCGGACAT CCTCCTCTCC TCCCCCACCG GAAAGTGGGC GTCTGACATT CTTATGGAGG CGGCTAGGGC   
  
  
+ TTTTTCAGAC ACGAACACCA CCAAGCTCCA CCAGCTCCTC TGGATGCTGA ACGAGCTCGG CTCCCCCTAC   
  
  
+ GGTGATGTCG AGCAGAAGCT GGTGGCGTAC TTTAACCAAG CCCTTTTTGC CCGCCTCACT GCCACCGGAC   
  
  
+ CCCGCCACCG AGCCGCTATG CTCGCCGCTG CGGACAAGAC CTCCTCCTTC GACTCCACGC TCAGGTTGCT   
  
  
+ CCTCAAGTTC CAGGAAGTCA GCCCGTGGAC CACCTTCGGC CACGTGGCTT GCAACGGTGC AATCATCGAG   
  
  
+ GCCTTAGAGA GGGACCCACA TGAAAAGAGT AAGCTCCACG TCATCGATAT CAGCACCACC TTCTGCACCC   
  
  
+ AGTGGCCCAC CTTGCTTGAG GCTTTAGCCA CCCGCATGGA CGACACTCCC CACCTCAGCC TCACTGCCGT   
  
  
+ CGTCGTCAAC AAGTACGCCG CCGTGAGCGG TGGCGAGGAC GACGGGGGAG AAGGATCAAA GAGGGTGATG   
  
  
+ AGAGAGATTG GTCTTCGTCT TGAGAAGTTC GCTAGGTTAA TGGGGGTTCC CTTCAAGTTC AACGTGGTTT   
  
  
+ ACCACGTAGG TGATCTATCC CAGTTGGATT TTTCTCGTTT GGATATTAAA GATGATGAAG CGGTGGCGAT   
  
  
+ TAACTGTGTC AACTCGCTCC ACTCGGTGGA CCTCCGATAT CGGGAGGTAG TGCTCGCGGC GTTCAGGCGG   
  
  
+ CTGCGGCCGA GGGTGGTGAC GGTGGTGGAG GAAGAGGCGG AGCTGACTGA GGTGGGTGAG GGTCAGTATG   
  
  
+ AATTTTTTAG GCGGTTTGAG GAGTGTCTTA GGTGGTTTAG GGTTTACTTT GAGGCCGTAG GAGATTGTTT   
  
  
+ TCCTAGGACT AGCAATGAGA AATTGATGTT AGAGCGGGCC GCGGGCCGGG CCATGGTGGA TCTCCTTGCA   
  
  
+ TGTCCCGAGC CTGGTGGGTC GGCTGAGGGG AGGGAGACGG CGAGACGGTG GTCGGGTCGG ATGCATGGGG   
  
  
+ CGGGGTTCGA GCACGTGGGG TTCAGTGACG AAGTGTGTGA TGATGTGAGG GCCTTGTTGA GGAGGTACAA   
  
  
+ GGAAGGATGG TCAATGACAC AGTGCACCAT GGATGGCGGT AATCACCCTG GAATACTGTT GTGGTGGCGG   
  
  
+ GATCAACCGG TGGTTTGGGC CAGTGCATGG CGGCCTTG  

- +Up\_Stream \_Len000TTTAGT TTGAGTATTT GTTATTAAAA AATTTTGAAT TTACCATTAA TAATCTAATT   
  
  
- ATTTTTAGGA TTACCAACAA ATTACAATGT AGTTCTGTAC GAAAAACCAC ATATATATTA CCGTCGTACA   
  
  
- CCCAATTCGC CGTTGTTTAA TAAGAGTTTA CGTACGTCAT TAATTAAATT AATTAATTAG TGGACGTATT   
  
  
- AGAGTTATGA TTAGGAAAGT AAGACACGGG GTGGGTTGGG GCTTTTATCA TTAGAAAGTT TGCAACCTTC   
  
  
- ACCCTAATCC ATATCTCATC AGGATGTCTA CTACTAGATG GATGTATATT GTATTAGACT GAAAAGAAAA   
  
  
- AATTTATTGA ACATAACTAC CGTGTCTTAA ACTGTACAAC CGTATACCAT GATTCTTTAA TTAATTGCAA   
  
  
- CACGATTCTA TATCCAGTAA GAAAAACCTC TGTATTCCGG GCGCAAGTGC GTCCCAAGCT CTTCCCGGTG   
  
  
- TAAGTTACCT ACTTAACATC CATCAAATTA GACTGAAACA GTCACTGACT AAGGTGCCGA ACTTGGGCGC   
  
  
- TGTAATTATA TCCAACTGAT TTCAGCAAAC TATGAATAAA AAGAATTCAT TTCAGAACTT AAGTTTAGAA   
  
  
- TATTTATTTA TTTTTTTAGA TATTATTTTC TTAAGATGGT AAATAATTCA GTTGTTGAGT TAAGCTTACT   
  
  
- TTATTTTTTA GTTCTAGTGG TTTTTTTTAT TTATTTTCCT TTCCATCTAA CGTAAACCTA AAACTCACCG   
  
  
- CACCACCACC CAAACTACGA CCACGACGTA TGTGGCGTCG AAAAGAGACA GGAAAATGGA GAGAAACAGA   
  
  
- CGTTCTTGGG GTAAACCGTG CGGACGAGAG AAAAGATACA AAAGAAAAGA TAGAAAATTC TTTGTTACCT   
  
  
- TTCGATTGTG TGATTGTGAT TGGGTAACCA GGAAAAAAGA ATAGACGTTT TAATTTCATT GTTATAAAAA   
  
  
- GAATAGGGGA AAAAGCAAGG TGGGTAAAGA AATAGGAAGA CAAACTTGGA TTAGACATAT ATACGTACGT   
  
  
- ACACATAAAA TGAATCTTAG ACAAAACTAA GAAACTACAT TAATTAATCA TCAGAAAAAT TAACTTAAAA   
  
  
- ATTGTCATCA AACTTAATCT GTAAATAGCT ATAATATAAA AAATATATAT TAATTTTTTA TGTTTTACTA   
  
  
- TTCACAGAAT GATGTTATGT GAAGTATTCA CAGCACAAAA TTGAATTTTT AGTATGATAA TACAAAGATT   
  
  
- TAACTTTTTT TTCACTCTAA TTCATCGTCT TCGTATAATG TTGAATATTT AATATATTTT AGTAGAAAAA   
  
  
- TAGGTATTAA ATAGTGTAAA GATTTTATGC GTATAGTATA GATGAATACC TATCCCAATT ATTCTAATCC   
  
  
- CGTAGTAAAT TCTAAAATAG TCGCATTGGT TGCTTACAAA TTCCAATCGT CAAGTATTAA TTCACATTAG   
  
  
- TAATTTTTTA AATACTAAAA TTTAGTATTT TATACAATTA GATCCTACTC CGTTCGCCGT TCTGTATATA   
  
  
- GTGATAGGAA TTTTTTATAT TATATAACTA AAGTTTCTTA ATACTTCATA TAATTAAAGT ATATATTACT   
  
  
- GCTAATACAT AAAAAAATGA TTTAATGAAC ATGGGTATTA TTTATCTTAG CTACGTAGTA AGTACCGGAA   
  
  
- AGTAAGACAT TATACCAACT GAGGTTTTTA AGAGTTTCAT AAAAATATAA AACCCTCCCC TCTAAAATCA   
  
  
- AACTATGAAA ACAACTTGAT CACTTGTTAA AGGTTGTTGG AGGTGGTGGG GGTTTGTAGT TGTTAGACTA   
  
  
- ACTACATACG TAGGGTTCGA ACTTAACTTA TTATTAAAGT TATTGTTGTT ATTAGCAAAA TGGAACTGTT   
  
  
- CGAGGTGTCG TTGAGTTGCA GAAGGTTTGG TTGGGTAGTG AGTAGTAGTA GTAGGGGTGA TAGGGTAAGT   
  
  
- GGGGGTGGTT TTCCTCGGAT TTCTGTCATT GTTCTTCTTA CGAACGGAAA AAAGTACCTA CTTCTTCTGA   
  
  
- AGAGAAGAAG GAGATGAAGA AGGGTGGTGA TACTATGTGT GGTGGAGGTT GTAGTAATGG GGTGGTTAAT   
  
  
- ATTAAGGGCA ATATGGGACC TGGGAGGGAG GTGGTGGTGG TGTCGGTGTT GGTGGTGGAG TAGGAGGTGA   
  
  
- GGGTGGGTGG TACCGAAGCC ACCACCCGAA CTGAGGAGGA GGGGCAGACG GCGACACCTT AATAGAAAGA   
  
  
- GGGGCCTGTA GGAGGAGAGG AGGGGGTGGC CTTTCACCCG CAGACTGTAA GAATACCTCC GCCGATCCCG   
  
  
- AAAAAGTCTG TGCTTGTGGT GGTTCGAGGT GGTCGAGGAG ACCTACGACT TGCTCGAGCC GAGGGGGATG   
  
  
- CCACTACAGC TCGTCTTCGA CCACCGCATG AAATTGGTTC GGGAAAAACG GGCGGAGTGA CGGTGGCCTG   
  
  
- GGGCGGTGGC TCGGCGATAC GAGCGGCGAC GCCTGTTCTG GAGGAGGAAG CTGAGGTGCG AGTCCAACGA   
  
  
- GGAGTTCAAG GTCCTTCAGT CGGGCACCTG GTGGAAGCCG GTGCACCGAA CGTTGCCACG TTAGTAGCTC   
  
  
- CGGAATCTCT CCCTGGGTGT ACTTTTCTCA TTCGAGGTGC AGTAGCTATA GTCGTGGTGG AAGACGTGGG   
  
  
- TCACCGGGTG GAACGAACTC CGAAATCGGT GGGCGTACCT GCTGTGAGGG GTGGAGTCGG AGTGACGGCA   
  
  
- GCAGCAGTTG TTCATGCGGC GGCACTCGCC ACCGCTCCTG CTGCCCCCTC TTCCTAGTTT CTCCCACTAC   
  
  
- TCTCTCTAAC CAGAAGCAGA ACTCTTCAAG CGATCCAATT ACCCCCAAGG GAAGTTCAAG TTGCACCAAA   
  
  
- TGGTGCATCC ACTAGATAGG GTCAACCTAA AAAGAGCAAA CCTATAATTT CTACTACTTC GCCACCGCTA   
  
  
- ATTGACACAG TTGAGCGAGG TGAGCCACCT GGAGGCTATA GCCCTCCATC ACGAGCGCCG CAAGTCCGCC   
  
  
- GACGCCGGCT CCCACCACTG CCACCACCTC CTTCTCCGCC TCGACTGACT CCACCCACTC CCAGTCATAC   
  
  
- TTAAAAAATC CGCCAAACTC CTCACAGAAT CCACCAAATC CCAAATGAAA CTCCGGCATC CTCTAACAAA   
  
  
- AGGATCCTGA TCGTTACTCT TTAACTACAA TCTCGCCCGG CGCCCGGCCC GGTACCACCT AGAGGAACGT   
  
  
- ACAGGGCTCG GACCACCCAG CCGACTCCCC TCCCTCTGCC GCTCTGCCAC CAGCCCAGCC TACGTACCCC   
  
  
- GCCCCAAGCT CGTGCACCCC AAGTCACTGC TTCACACACT ACTACACTCC CGGAACAACT CCTCCATGTT   
  
  
- CCTTCCTACC AGTTACTGTG TCACGTGGTA CCTACCGCCA TTAGTGGGAC CTTATGACAA CACCACCGCC   
  
  
- CTAGTTGGCC ACCAAACCCG GTCACGTACC GCCGGAAC

+     ATC-motif

| Site Name | Organism | Position | Strand | Matrix score. | sequence | function |
| --- | --- | --- | --- | --- | --- | --- |
| ATC-motif | Spinacia oleracea | 262 | + | 8 | AGTAATCT | part of a conserved DNA module involved in light responsiveness |

>HU05G01983.1   
+ +Up\_Stream \_Len000AAATCA AACTCATAAA CAATAATTTT TTAAAACTTA AATGGTAATT ATTAGATTAA   
  
  
+ TAAAAATCCT AATGGTTGTT TAATGTTACA TCAAGACATG CTTTTTGGTG TATATATAAT GGCAGCATGT   
  
  
+ GGGTTAAGCG GCAACAAATT ATTCTCAAAT GCATGCAGTA ATTAATTTAA TTAATTAATC ACCTGCATAA   
  
  
+ TCTCAATACT AATCCTTTCA TTCTGTGCCC CACCCAACCC CGAAAATAGT AATCTTTCAA ACGTTGGAAG   
  
  
+ TGGGATTAGG TATAGAGTAG TCCTACAGAT GATGATCTAC CTACATATAA CATAATCTGA CTTTTCTTTT   
  
  
+ TTAAATAACT TGTATTGATG GCACAGAATT TGACATGTTG GCATATGGTA CTAAGAAATT AATTAACGTT   
  
  
+ GTGCTAAGAT ATAGGTCATT CTTTTTGGAG ACATAAGGCC CGCGTTCACG CAGGGTTCGA GAAGGGCCAC   
  
  
+ ATTCAATGGA TGAATTGTAG GTAGTTTAAT CTGACTTTGT CAGTGACTGA TTCCACGGCT TGAACCCGCG   
  
  
+ ACATTAATAT AGGTTGACTA AAGTCGTTTG ATACTTATTT TTCTTAAGTA AAGTCTTGAA TTCAAATCTT   
  
  
+ ATAAATAAAT AAAAAAATCT ATAATAAAAG AATTCTACCA TTTATTAAGT CAACAACTCA ATTCGAATGA   
  
  
+ AATAAAAAAT CAAGATCACC AAAAAAAATA AATAAAAGGA AAGGTAGATT GCATTTGGAT TTTGAGTGGC   
  
  
+ GTGGTGGTGG GTTTGATGCT GGTGCTGCAT ACACCGCAGC TTTTCTCTGT CCTTTTACCT CTCTTTGTCT   
  
  
+ GCAAGAACCC CATTTGGCAC GCCTGCTCTC TTTTCTATGT TTTCTTTTCT ATCTTTTAAG AAACAATGGA   
  
  
+ AAGCTAACAC ACTAACACTA ACCCATTGGT CCTTTTTTCT TATCTGCAAA ATTAAAGTAA CAATATTTTT   
  
  
+ CTTATCCCCT TTTTCGTTCC ACCCATTTCT TTATCCTTCT GTTTGAACCT AATCTGTATA TATGCATGCA   
  
  
+ TGTGTATTTT ACTTAGAATC TGTTTTGATT CTTTGATGTA ATTAATTAGT AGTCTTTTTA ATTGAATTTT   
  
  
+ TAACAGTAGT TTGAATTAGA CATTTATCGA TATTATATTT TTTATATATA ATTAAAAAAT ACAAAATGAT   
  
  
+ AAGTGTCTTA CTACAATACA CTTCATAAGT GTCGTGTTTT AACTTAAAAA TCATACTATT ATGTTTCTAA   
  
  
+ ATTGAAAAAA AAGTGAGATT AAGTAGCAGA AGCATATTAC AACTTATAAA TTATATAAAA TCATCTTTTT   
  
  
+ ATCCATAATT TATCACATTT CTAAAATACG CATATCATAT CTACTTATGG ATAGGGTTAA TAAGATTAGG   
  
  
+ GCATCATTTA AGATTTTATC AGCGTAACCA ACGAATGTTT AAGGTTAGCA GTTCATAATT AAGTGTAATC   
  
  
+ ATTAAAAAAT TTATGATTTT AAATCATAAA ATATGTTAAT CTAGGATGAG GCAAGCGGCA AGACATATAT   
  
  
+ CACTATCCTT AAAAAATATA ATATATTGAT TTCAAAGAAT TATGAAGTAT ATTAATTTCA TATATAATGA   
  
  
+ CGATTATGTA TTTTTTTACT AAATTACTTG TACCCATAAT AAATAGAATC GATGCATCAT TCATGGCCTT   
  
  
+ TCATTCTGTA ATATGGTTGA CTCCAAAAAT TCTCAAAGTA TTTTTATATT TTGGGAGGGG AGATTTTAGT   
  
  
+ TTGATACTTT TGTTGAACTA GTGAACAATT TCCAACAACC TCCACCACCC CCAAACATCA ACAATCTGAT   
  
  
+ TGATGTATGC ATCCCAAGCT TGAATTGAAT AATAATTTCA ATAACAACAA TAATCGTTTT ACCTTGACAA   
  
  
+ GCTCCACAGC AACTCAACGT CTTCCAAACC AACCCATCAC TCATCATCAT CATCCCCACT ATCCCATTCA   
  
  
+ CCCCCACCAA AAGGAGCCTA AAGACAGTAA CAAGAAGAAT GCTTGCCTTT TTTCATGGAT GAAGAAGACT   
  
  
+ TCTCTTCTTC CTCTACTTCT TCCCACCACT ATGATACACA CCACCTCCAA CATCATTACC CCACCAATTA   
  
  
+ TAATTCCCGT TATACCCTGG ACCCTCCCTC CACCACCACC ACAGCCACAA CCACCACCTC ATCCTCCACT   
  
  
+ CCCACCCACC ATGGCTTCGG TGGTGGGCTT GACTCCTCCT CCCCGTCTGC CGCTGTGGAA TTATCTTTCT   
  
  
+ CCCCGGACAT CCTCCTCTCC TCCCCCACCG GAAAGTGGGC GTCTGACATT CTTATGGAGG CGGCTAGGGC   
  
  
+ TTTTTCAGAC ACGAACACCA CCAAGCTCCA CCAGCTCCTC TGGATGCTGA ACGAGCTCGG CTCCCCCTAC   
  
  
+ GGTGATGTCG AGCAGAAGCT GGTGGCGTAC TTTAACCAAG CCCTTTTTGC CCGCCTCACT GCCACCGGAC   
  
  
+ CCCGCCACCG AGCCGCTATG CTCGCCGCTG CGGACAAGAC CTCCTCCTTC GACTCCACGC TCAGGTTGCT   
  
  
+ CCTCAAGTTC CAGGAAGTCA GCCCGTGGAC CACCTTCGGC CACGTGGCTT GCAACGGTGC AATCATCGAG   
  
  
+ GCCTTAGAGA GGGACCCACA TGAAAAGAGT AAGCTCCACG TCATCGATAT CAGCACCACC TTCTGCACCC   
  
  
+ AGTGGCCCAC CTTGCTTGAG GCTTTAGCCA CCCGCATGGA CGACACTCCC CACCTCAGCC TCACTGCCGT   
  
  
+ CGTCGTCAAC AAGTACGCCG CCGTGAGCGG TGGCGAGGAC GACGGGGGAG AAGGATCAAA GAGGGTGATG   
  
  
+ AGAGAGATTG GTCTTCGTCT TGAGAAGTTC GCTAGGTTAA TGGGGGTTCC CTTCAAGTTC AACGTGGTTT   
  
  
+ ACCACGTAGG TGATCTATCC CAGTTGGATT TTTCTCGTTT GGATATTAAA GATGATGAAG CGGTGGCGAT   
  
  
+ TAACTGTGTC AACTCGCTCC ACTCGGTGGA CCTCCGATAT CGGGAGGTAG TGCTCGCGGC GTTCAGGCGG   
  
  
+ CTGCGGCCGA GGGTGGTGAC GGTGGTGGAG GAAGAGGCGG AGCTGACTGA GGTGGGTGAG GGTCAGTATG   
  
  
+ AATTTTTTAG GCGGTTTGAG GAGTGTCTTA GGTGGTTTAG GGTTTACTTT GAGGCCGTAG GAGATTGTTT   
  
  
+ TCCTAGGACT AGCAATGAGA AATTGATGTT AGAGCGGGCC GCGGGCCGGG CCATGGTGGA TCTCCTTGCA   
  
  
+ TGTCCCGAGC CTGGTGGGTC GGCTGAGGGG AGGGAGACGG CGAGACGGTG GTCGGGTCGG ATGCATGGGG   
  
  
+ CGGGGTTCGA GCACGTGGGG TTCAGTGACG AAGTGTGTGA TGATGTGAGG GCCTTGTTGA GGAGGTACAA   
  
  
+ GGAAGGATGG TCAATGACAC AGTGCACCAT GGATGGCGGT AATCACCCTG GAATACTGTT GTGGTGGCGG   
  
  
+ GATCAACCGG TGGTTTGGGC CAGTGCATGG CGGCCTTG  

- +Up\_Stream \_Len000TTTAGT TTGAGTATTT GTTATTAAAA AATTTTGAAT TTACCATTAA TAATCTAATT   
  
  
- ATTTTTAGGA TTACCAACAA ATTACAATGT AGTTCTGTAC GAAAAACCAC ATATATATTA CCGTCGTACA   
  
  
- CCCAATTCGC CGTTGTTTAA TAAGAGTTTA CGTACGTCAT TAATTAAATT AATTAATTAG TGGACGTATT   
  
  
- AGAGTTATGA TTAGGAAAGT AAGACACGGG GTGGGTTGGG GCTTTTATCA TTAGAAAGTT TGCAACCTTC   
  
  
- ACCCTAATCC ATATCTCATC AGGATGTCTA CTACTAGATG GATGTATATT GTATTAGACT GAAAAGAAAA   
  
  
- AATTTATTGA ACATAACTAC CGTGTCTTAA ACTGTACAAC CGTATACCAT GATTCTTTAA TTAATTGCAA   
  
  
- CACGATTCTA TATCCAGTAA GAAAAACCTC TGTATTCCGG GCGCAAGTGC GTCCCAAGCT CTTCCCGGTG   
  
  
- TAAGTTACCT ACTTAACATC CATCAAATTA GACTGAAACA GTCACTGACT AAGGTGCCGA ACTTGGGCGC   
  
  
- TGTAATTATA TCCAACTGAT TTCAGCAAAC TATGAATAAA AAGAATTCAT TTCAGAACTT AAGTTTAGAA   
  
  
- TATTTATTTA TTTTTTTAGA TATTATTTTC TTAAGATGGT AAATAATTCA GTTGTTGAGT TAAGCTTACT   
  
  
- TTATTTTTTA GTTCTAGTGG TTTTTTTTAT TTATTTTCCT TTCCATCTAA CGTAAACCTA AAACTCACCG   
  
  
- CACCACCACC CAAACTACGA CCACGACGTA TGTGGCGTCG AAAAGAGACA GGAAAATGGA GAGAAACAGA   
  
  
- CGTTCTTGGG GTAAACCGTG CGGACGAGAG AAAAGATACA AAAGAAAAGA TAGAAAATTC TTTGTTACCT   
  
  
- TTCGATTGTG TGATTGTGAT TGGGTAACCA GGAAAAAAGA ATAGACGTTT TAATTTCATT GTTATAAAAA   
  
  
- GAATAGGGGA AAAAGCAAGG TGGGTAAAGA AATAGGAAGA CAAACTTGGA TTAGACATAT ATACGTACGT   
  
  
- ACACATAAAA TGAATCTTAG ACAAAACTAA GAAACTACAT TAATTAATCA TCAGAAAAAT TAACTTAAAA   
  
  
- ATTGTCATCA AACTTAATCT GTAAATAGCT ATAATATAAA AAATATATAT TAATTTTTTA TGTTTTACTA   
  
  
- TTCACAGAAT GATGTTATGT GAAGTATTCA CAGCACAAAA TTGAATTTTT AGTATGATAA TACAAAGATT   
  
  
- TAACTTTTTT TTCACTCTAA TTCATCGTCT TCGTATAATG TTGAATATTT AATATATTTT AGTAGAAAAA   
  
  
- TAGGTATTAA ATAGTGTAAA GATTTTATGC GTATAGTATA GATGAATACC TATCCCAATT ATTCTAATCC   
  
  
- CGTAGTAAAT TCTAAAATAG TCGCATTGGT TGCTTACAAA TTCCAATCGT CAAGTATTAA TTCACATTAG   
  
  
- TAATTTTTTA AATACTAAAA TTTAGTATTT TATACAATTA GATCCTACTC CGTTCGCCGT TCTGTATATA   
  
  
- GTGATAGGAA TTTTTTATAT TATATAACTA AAGTTTCTTA ATACTTCATA TAATTAAAGT ATATATTACT   
  
  
- GCTAATACAT AAAAAAATGA TTTAATGAAC ATGGGTATTA TTTATCTTAG CTACGTAGTA AGTACCGGAA   
  
  
- AGTAAGACAT TATACCAACT GAGGTTTTTA AGAGTTTCAT AAAAATATAA AACCCTCCCC TCTAAAATCA   
  
  
- AACTATGAAA ACAACTTGAT CACTTGTTAA AGGTTGTTGG AGGTGGTGGG GGTTTGTAGT TGTTAGACTA   
  
  
- ACTACATACG TAGGGTTCGA ACTTAACTTA TTATTAAAGT TATTGTTGTT ATTAGCAAAA TGGAACTGTT   
  
  
- CGAGGTGTCG TTGAGTTGCA GAAGGTTTGG TTGGGTAGTG AGTAGTAGTA GTAGGGGTGA TAGGGTAAGT   
  
  
- GGGGGTGGTT TTCCTCGGAT TTCTGTCATT GTTCTTCTTA CGAACGGAAA AAAGTACCTA CTTCTTCTGA   
  
  
- AGAGAAGAAG GAGATGAAGA AGGGTGGTGA TACTATGTGT GGTGGAGGTT GTAGTAATGG GGTGGTTAAT   
  
  
- ATTAAGGGCA ATATGGGACC TGGGAGGGAG GTGGTGGTGG TGTCGGTGTT GGTGGTGGAG TAGGAGGTGA   
  
  
- GGGTGGGTGG TACCGAAGCC ACCACCCGAA CTGAGGAGGA GGGGCAGACG GCGACACCTT AATAGAAAGA   
  
  
- GGGGCCTGTA GGAGGAGAGG AGGGGGTGGC CTTTCACCCG CAGACTGTAA GAATACCTCC GCCGATCCCG   
  
  
- AAAAAGTCTG TGCTTGTGGT GGTTCGAGGT GGTCGAGGAG ACCTACGACT TGCTCGAGCC GAGGGGGATG   
  
  
- CCACTACAGC TCGTCTTCGA CCACCGCATG AAATTGGTTC GGGAAAAACG GGCGGAGTGA CGGTGGCCTG   
  
  
- GGGCGGTGGC TCGGCGATAC GAGCGGCGAC GCCTGTTCTG GAGGAGGAAG CTGAGGTGCG AGTCCAACGA   
  
  
- GGAGTTCAAG GTCCTTCAGT CGGGCACCTG GTGGAAGCCG GTGCACCGAA CGTTGCCACG TTAGTAGCTC   
  
  
- CGGAATCTCT CCCTGGGTGT ACTTTTCTCA TTCGAGGTGC AGTAGCTATA GTCGTGGTGG AAGACGTGGG   
  
  
- TCACCGGGTG GAACGAACTC CGAAATCGGT GGGCGTACCT GCTGTGAGGG GTGGAGTCGG AGTGACGGCA   
  
  
- GCAGCAGTTG TTCATGCGGC GGCACTCGCC ACCGCTCCTG CTGCCCCCTC TTCCTAGTTT CTCCCACTAC   
  
  
- TCTCTCTAAC CAGAAGCAGA ACTCTTCAAG CGATCCAATT ACCCCCAAGG GAAGTTCAAG TTGCACCAAA   
  
  
- TGGTGCATCC ACTAGATAGG GTCAACCTAA AAAGAGCAAA CCTATAATTT CTACTACTTC GCCACCGCTA   
  
  
- ATTGACACAG TTGAGCGAGG TGAGCCACCT GGAGGCTATA GCCCTCCATC ACGAGCGCCG CAAGTCCGCC   
  
  
- GACGCCGGCT CCCACCACTG CCACCACCTC CTTCTCCGCC TCGACTGACT CCACCCACTC CCAGTCATAC   
  
  
- TTAAAAAATC CGCCAAACTC CTCACAGAAT CCACCAAATC CCAAATGAAA CTCCGGCATC CTCTAACAAA   
  
  
- AGGATCCTGA TCGTTACTCT TTAACTACAA TCTCGCCCGG CGCCCGGCCC GGTACCACCT AGAGGAACGT   
  
  
- ACAGGGCTCG GACCACCCAG CCGACTCCCC TCCCTCTGCC GCTCTGCCAC CAGCCCAGCC TACGTACCCC   
  
  
- GCCCCAAGCT CGTGCACCCC AAGTCACTGC TTCACACACT ACTACACTCC CGGAACAACT CCTCCATGTT   
  
  
- CCTTCCTACC AGTTACTGTG TCACGTGGTA CCTACCGCCA TTAGTGGGAC CTTATGACAA CACCACCGCC   
  
  
- CTAGTTGGCC ACCAAACCCG GTCACGTACC GCCGGAAC

+     AT~TATA-box

| Site Name | Organism | Position | Strand | Matrix score. | sequence | function |
| --- | --- | --- | --- | --- | --- | --- |
| AT~TATA-box | Arabidopsis thaliana | 1316 | + | 6 | TATATA |  |
| AT~TATA-box | Arabidopsis thaliana | 127 | + | 6 | TATATA |  |
| AT~TATA-box | Arabidopsis thaliana | 1167 | + | 6 | TATATA |  |
| AT~TATA-box | Arabidopsis thaliana | 125 | + | 6 | TATATA |  |
| AT~TATA-box | Arabidopsis thaliana | 1041 | + | 6 | TATATA |  |
| AT~TATA-box | Arabidopsis thaliana | 1169 | + | 6 | TATATA |  |
| AT~TATA-box | Arabidopsis thaliana | 1165 | - | 8 | TATATAAA |  |
| AT~TATA-box | Arabidopsis thaliana | 1605 | + | 6 | TATATA |  |

>HU05G01983.1   
+ +Up\_Stream \_Len000AAATCA AACTCATAAA CAATAATTTT TTAAAACTTA AATGGTAATT ATTAGATTAA   
  
  
+ TAAAAATCCT AATGGTTGTT TAATGTTACA TCAAGACATG CTTTTTGGTG TATATATAAT GGCAGCATGT   
  
  
+ GGGTTAAGCG GCAACAAATT ATTCTCAAAT GCATGCAGTA ATTAATTTAA TTAATTAATC ACCTGCATAA   
  
  
+ TCTCAATACT AATCCTTTCA TTCTGTGCCC CACCCAACCC CGAAAATAGT AATCTTTCAA ACGTTGGAAG   
  
  
+ TGGGATTAGG TATAGAGTAG TCCTACAGAT GATGATCTAC CTACATATAA CATAATCTGA CTTTTCTTTT   
  
  
+ TTAAATAACT TGTATTGATG GCACAGAATT TGACATGTTG GCATATGGTA CTAAGAAATT AATTAACGTT   
  
  
+ GTGCTAAGAT ATAGGTCATT CTTTTTGGAG ACATAAGGCC CGCGTTCACG CAGGGTTCGA GAAGGGCCAC   
  
  
+ ATTCAATGGA TGAATTGTAG GTAGTTTAAT CTGACTTTGT CAGTGACTGA TTCCACGGCT TGAACCCGCG   
  
  
+ ACATTAATAT AGGTTGACTA AAGTCGTTTG ATACTTATTT TTCTTAAGTA AAGTCTTGAA TTCAAATCTT   
  
  
+ ATAAATAAAT AAAAAAATCT ATAATAAAAG AATTCTACCA TTTATTAAGT CAACAACTCA ATTCGAATGA   
  
  
+ AATAAAAAAT CAAGATCACC AAAAAAAATA AATAAAAGGA AAGGTAGATT GCATTTGGAT TTTGAGTGGC   
  
  
+ GTGGTGGTGG GTTTGATGCT GGTGCTGCAT ACACCGCAGC TTTTCTCTGT CCTTTTACCT CTCTTTGTCT   
  
  
+ GCAAGAACCC CATTTGGCAC GCCTGCTCTC TTTTCTATGT TTTCTTTTCT ATCTTTTAAG AAACAATGGA   
  
  
+ AAGCTAACAC ACTAACACTA ACCCATTGGT CCTTTTTTCT TATCTGCAAA ATTAAAGTAA CAATATTTTT   
  
  
+ CTTATCCCCT TTTTCGTTCC ACCCATTTCT TTATCCTTCT GTTTGAACCT AATCTGTATA TATGCATGCA   
  
  
+ TGTGTATTTT ACTTAGAATC TGTTTTGATT CTTTGATGTA ATTAATTAGT AGTCTTTTTA ATTGAATTTT   
  
  
+ TAACAGTAGT TTGAATTAGA CATTTATCGA TATTATATTT TTTATATATA ATTAAAAAAT ACAAAATGAT   
  
  
+ AAGTGTCTTA CTACAATACA CTTCATAAGT GTCGTGTTTT AACTTAAAAA TCATACTATT ATGTTTCTAA   
  
  
+ ATTGAAAAAA AAGTGAGATT AAGTAGCAGA AGCATATTAC AACTTATAAA TTATATAAAA TCATCTTTTT   
  
  
+ ATCCATAATT TATCACATTT CTAAAATACG CATATCATAT CTACTTATGG ATAGGGTTAA TAAGATTAGG   
  
  
+ GCATCATTTA AGATTTTATC AGCGTAACCA ACGAATGTTT AAGGTTAGCA GTTCATAATT AAGTGTAATC   
  
  
+ ATTAAAAAAT TTATGATTTT AAATCATAAA ATATGTTAAT CTAGGATGAG GCAAGCGGCA AGACATATAT   
  
  
+ CACTATCCTT AAAAAATATA ATATATTGAT TTCAAAGAAT TATGAAGTAT ATTAATTTCA TATATAATGA   
  
  
+ CGATTATGTA TTTTTTTACT AAATTACTTG TACCCATAAT AAATAGAATC GATGCATCAT TCATGGCCTT   
  
  
+ TCATTCTGTA ATATGGTTGA CTCCAAAAAT TCTCAAAGTA TTTTTATATT TTGGGAGGGG AGATTTTAGT   
  
  
+ TTGATACTTT TGTTGAACTA GTGAACAATT TCCAACAACC TCCACCACCC CCAAACATCA ACAATCTGAT   
  
  
+ TGATGTATGC ATCCCAAGCT TGAATTGAAT AATAATTTCA ATAACAACAA TAATCGTTTT ACCTTGACAA   
  
  
+ GCTCCACAGC AACTCAACGT CTTCCAAACC AACCCATCAC TCATCATCAT CATCCCCACT ATCCCATTCA   
  
  
+ CCCCCACCAA AAGGAGCCTA AAGACAGTAA CAAGAAGAAT GCTTGCCTTT TTTCATGGAT GAAGAAGACT   
  
  
+ TCTCTTCTTC CTCTACTTCT TCCCACCACT ATGATACACA CCACCTCCAA CATCATTACC CCACCAATTA   
  
  
+ TAATTCCCGT TATACCCTGG ACCCTCCCTC CACCACCACC ACAGCCACAA CCACCACCTC ATCCTCCACT   
  
  
+ CCCACCCACC ATGGCTTCGG TGGTGGGCTT GACTCCTCCT CCCCGTCTGC CGCTGTGGAA TTATCTTTCT   
  
  
+ CCCCGGACAT CCTCCTCTCC TCCCCCACCG GAAAGTGGGC GTCTGACATT CTTATGGAGG CGGCTAGGGC   
  
  
+ TTTTTCAGAC ACGAACACCA CCAAGCTCCA CCAGCTCCTC TGGATGCTGA ACGAGCTCGG CTCCCCCTAC   
  
  
+ GGTGATGTCG AGCAGAAGCT GGTGGCGTAC TTTAACCAAG CCCTTTTTGC CCGCCTCACT GCCACCGGAC   
  
  
+ CCCGCCACCG AGCCGCTATG CTCGCCGCTG CGGACAAGAC CTCCTCCTTC GACTCCACGC TCAGGTTGCT   
  
  
+ CCTCAAGTTC CAGGAAGTCA GCCCGTGGAC CACCTTCGGC CACGTGGCTT GCAACGGTGC AATCATCGAG   
  
  
+ GCCTTAGAGA GGGACCCACA TGAAAAGAGT AAGCTCCACG TCATCGATAT CAGCACCACC TTCTGCACCC   
  
  
+ AGTGGCCCAC CTTGCTTGAG GCTTTAGCCA CCCGCATGGA CGACACTCCC CACCTCAGCC TCACTGCCGT   
  
  
+ CGTCGTCAAC AAGTACGCCG CCGTGAGCGG TGGCGAGGAC GACGGGGGAG AAGGATCAAA GAGGGTGATG   
  
  
+ AGAGAGATTG GTCTTCGTCT TGAGAAGTTC GCTAGGTTAA TGGGGGTTCC CTTCAAGTTC AACGTGGTTT   
  
  
+ ACCACGTAGG TGATCTATCC CAGTTGGATT TTTCTCGTTT GGATATTAAA GATGATGAAG CGGTGGCGAT   
  
  
+ TAACTGTGTC AACTCGCTCC ACTCGGTGGA CCTCCGATAT CGGGAGGTAG TGCTCGCGGC GTTCAGGCGG   
  
  
+ CTGCGGCCGA GGGTGGTGAC GGTGGTGGAG GAAGAGGCGG AGCTGACTGA GGTGGGTGAG GGTCAGTATG   
  
  
+ AATTTTTTAG GCGGTTTGAG GAGTGTCTTA GGTGGTTTAG GGTTTACTTT GAGGCCGTAG GAGATTGTTT   
  
  
+ TCCTAGGACT AGCAATGAGA AATTGATGTT AGAGCGGGCC GCGGGCCGGG CCATGGTGGA TCTCCTTGCA   
  
  
+ TGTCCCGAGC CTGGTGGGTC GGCTGAGGGG AGGGAGACGG CGAGACGGTG GTCGGGTCGG ATGCATGGGG   
  
  
+ CGGGGTTCGA GCACGTGGGG TTCAGTGACG AAGTGTGTGA TGATGTGAGG GCCTTGTTGA GGAGGTACAA   
  
  
+ GGAAGGATGG TCAATGACAC AGTGCACCAT GGATGGCGGT AATCACCCTG GAATACTGTT GTGGTGGCGG   
  
  
+ GATCAACCGG TGGTTTGGGC CAGTGCATGG CGGCCTTG  

- +Up\_Stream \_Len000TTTAGT TTGAGTATTT GTTATTAAAA AATTTTGAAT TTACCATTAA TAATCTAATT   
  
  
- ATTTTTAGGA TTACCAACAA ATTACAATGT AGTTCTGTAC GAAAAACCAC ATATATATTA CCGTCGTACA   
  
  
- CCCAATTCGC CGTTGTTTAA TAAGAGTTTA CGTACGTCAT TAATTAAATT AATTAATTAG TGGACGTATT   
  
  
- AGAGTTATGA TTAGGAAAGT AAGACACGGG GTGGGTTGGG GCTTTTATCA TTAGAAAGTT TGCAACCTTC   
  
  
- ACCCTAATCC ATATCTCATC AGGATGTCTA CTACTAGATG GATGTATATT GTATTAGACT GAAAAGAAAA   
  
  
- AATTTATTGA ACATAACTAC CGTGTCTTAA ACTGTACAAC CGTATACCAT GATTCTTTAA TTAATTGCAA   
  
  
- CACGATTCTA TATCCAGTAA GAAAAACCTC TGTATTCCGG GCGCAAGTGC GTCCCAAGCT CTTCCCGGTG   
  
  
- TAAGTTACCT ACTTAACATC CATCAAATTA GACTGAAACA GTCACTGACT AAGGTGCCGA ACTTGGGCGC   
  
  
- TGTAATTATA TCCAACTGAT TTCAGCAAAC TATGAATAAA AAGAATTCAT TTCAGAACTT AAGTTTAGAA   
  
  
- TATTTATTTA TTTTTTTAGA TATTATTTTC TTAAGATGGT AAATAATTCA GTTGTTGAGT TAAGCTTACT   
  
  
- TTATTTTTTA GTTCTAGTGG TTTTTTTTAT TTATTTTCCT TTCCATCTAA CGTAAACCTA AAACTCACCG   
  
  
- CACCACCACC CAAACTACGA CCACGACGTA TGTGGCGTCG AAAAGAGACA GGAAAATGGA GAGAAACAGA   
  
  
- CGTTCTTGGG GTAAACCGTG CGGACGAGAG AAAAGATACA AAAGAAAAGA TAGAAAATTC TTTGTTACCT   
  
  
- TTCGATTGTG TGATTGTGAT TGGGTAACCA GGAAAAAAGA ATAGACGTTT TAATTTCATT GTTATAAAAA   
  
  
- GAATAGGGGA AAAAGCAAGG TGGGTAAAGA AATAGGAAGA CAAACTTGGA TTAGACATAT ATACGTACGT   
  
  
- ACACATAAAA TGAATCTTAG ACAAAACTAA GAAACTACAT TAATTAATCA TCAGAAAAAT TAACTTAAAA   
  
  
- ATTGTCATCA AACTTAATCT GTAAATAGCT ATAATATAAA AAATATATAT TAATTTTTTA TGTTTTACTA   
  
  
- TTCACAGAAT GATGTTATGT GAAGTATTCA CAGCACAAAA TTGAATTTTT AGTATGATAA TACAAAGATT   
  
  
- TAACTTTTTT TTCACTCTAA TTCATCGTCT TCGTATAATG TTGAATATTT AATATATTTT AGTAGAAAAA   
  
  
- TAGGTATTAA ATAGTGTAAA GATTTTATGC GTATAGTATA GATGAATACC TATCCCAATT ATTCTAATCC   
  
  
- CGTAGTAAAT TCTAAAATAG TCGCATTGGT TGCTTACAAA TTCCAATCGT CAAGTATTAA TTCACATTAG   
  
  
- TAATTTTTTA AATACTAAAA TTTAGTATTT TATACAATTA GATCCTACTC CGTTCGCCGT TCTGTATATA   
  
  
- GTGATAGGAA TTTTTTATAT TATATAACTA AAGTTTCTTA ATACTTCATA TAATTAAAGT ATATATTACT   
  
  
- GCTAATACAT AAAAAAATGA TTTAATGAAC ATGGGTATTA TTTATCTTAG CTACGTAGTA AGTACCGGAA   
  
  
- AGTAAGACAT TATACCAACT GAGGTTTTTA AGAGTTTCAT AAAAATATAA AACCCTCCCC TCTAAAATCA   
  
  
- AACTATGAAA ACAACTTGAT CACTTGTTAA AGGTTGTTGG AGGTGGTGGG GGTTTGTAGT TGTTAGACTA   
  
  
- ACTACATACG TAGGGTTCGA ACTTAACTTA TTATTAAAGT TATTGTTGTT ATTAGCAAAA TGGAACTGTT   
  
  
- CGAGGTGTCG TTGAGTTGCA GAAGGTTTGG TTGGGTAGTG AGTAGTAGTA GTAGGGGTGA TAGGGTAAGT   
  
  
- GGGGGTGGTT TTCCTCGGAT TTCTGTCATT GTTCTTCTTA CGAACGGAAA AAAGTACCTA CTTCTTCTGA   
  
  
- AGAGAAGAAG GAGATGAAGA AGGGTGGTGA TACTATGTGT GGTGGAGGTT GTAGTAATGG GGTGGTTAAT   
  
  
- ATTAAGGGCA ATATGGGACC TGGGAGGGAG GTGGTGGTGG TGTCGGTGTT GGTGGTGGAG TAGGAGGTGA   
  
  
- GGGTGGGTGG TACCGAAGCC ACCACCCGAA CTGAGGAGGA GGGGCAGACG GCGACACCTT AATAGAAAGA   
  
  
- GGGGCCTGTA GGAGGAGAGG AGGGGGTGGC CTTTCACCCG CAGACTGTAA GAATACCTCC GCCGATCCCG   
  
  
- AAAAAGTCTG TGCTTGTGGT GGTTCGAGGT GGTCGAGGAG ACCTACGACT TGCTCGAGCC GAGGGGGATG   
  
  
- CCACTACAGC TCGTCTTCGA CCACCGCATG AAATTGGTTC GGGAAAAACG GGCGGAGTGA CGGTGGCCTG   
  
  
- GGGCGGTGGC TCGGCGATAC GAGCGGCGAC GCCTGTTCTG GAGGAGGAAG CTGAGGTGCG AGTCCAACGA   
  
  
- GGAGTTCAAG GTCCTTCAGT CGGGCACCTG GTGGAAGCCG GTGCACCGAA CGTTGCCACG TTAGTAGCTC   
  
  
- CGGAATCTCT CCCTGGGTGT ACTTTTCTCA TTCGAGGTGC AGTAGCTATA GTCGTGGTGG AAGACGTGGG   
  
  
- TCACCGGGTG GAACGAACTC CGAAATCGGT GGGCGTACCT GCTGTGAGGG GTGGAGTCGG AGTGACGGCA   
  
  
- GCAGCAGTTG TTCATGCGGC GGCACTCGCC ACCGCTCCTG CTGCCCCCTC TTCCTAGTTT CTCCCACTAC   
  
  
- TCTCTCTAAC CAGAAGCAGA ACTCTTCAAG CGATCCAATT ACCCCCAAGG GAAGTTCAAG TTGCACCAAA   
  
  
- TGGTGCATCC ACTAGATAGG GTCAACCTAA AAAGAGCAAA CCTATAATTT CTACTACTTC GCCACCGCTA   
  
  
- ATTGACACAG TTGAGCGAGG TGAGCCACCT GGAGGCTATA GCCCTCCATC ACGAGCGCCG CAAGTCCGCC   
  
  
- GACGCCGGCT CCCACCACTG CCACCACCTC CTTCTCCGCC TCGACTGACT CCACCCACTC CCAGTCATAC   
  
  
- TTAAAAAATC CGCCAAACTC CTCACAGAAT CCACCAAATC CCAAATGAAA CTCCGGCATC CTCTAACAAA   
  
  
- AGGATCCTGA TCGTTACTCT TTAACTACAA TCTCGCCCGG CGCCCGGCCC GGTACCACCT AGAGGAACGT   
  
  
- ACAGGGCTCG GACCACCCAG CCGACTCCCC TCCCTCTGCC GCTCTGCCAC CAGCCCAGCC TACGTACCCC   
  
  
- GCCCCAAGCT CGTGCACCCC AAGTCACTGC TTCACACACT ACTACACTCC CGGAACAACT CCTCCATGTT   
  
  
- CCTTCCTACC AGTTACTGTG TCACGTGGTA CCTACCGCCA TTAGTGGGAC CTTATGACAA CACCACCGCC   
  
  
- CTAGTTGGCC ACCAAACCCG GTCACGTACC GCCGGAAC

+     Box 4

| Site Name | Organism | Position | Strand | Matrix score. | sequence | function |
| --- | --- | --- | --- | --- | --- | --- |
| Box 4 | Petroselinum crispum | 194 | + | 6 | ATTAAT | part of a conserved DNA module involved in light responsiveness |
| Box 4 | Petroselinum crispum | 1095 | + | 6 | ATTAAT | part of a conserved DNA module involved in light responsiveness |
| Box 4 | Petroselinum crispum | 412 | + | 6 | ATTAAT | part of a conserved DNA module involved in light responsiveness |
| Box 4 | Petroselinum crispum | 567 | + | 6 | ATTAAT | part of a conserved DNA module involved in light responsiveness |
| Box 4 | Petroselinum crispum | 185 | + | 6 | ATTAAT | part of a conserved DNA module involved in light responsiveness |
| Box 4 | Petroselinum crispum | 70 | + | 6 | ATTAAT | part of a conserved DNA module involved in light responsiveness |
| Box 4 | Petroselinum crispum | 1595 | + | 6 | ATTAAT | part of a conserved DNA module involved in light responsiveness |
| Box 4 | Petroselinum crispum | 198 | + | 6 | ATTAAT | part of a conserved DNA module involved in light responsiveness |

>HU05G01983.1   
+ +Up\_Stream \_Len000AAATCA AACTCATAAA CAATAATTTT TTAAAACTTA AATGGTAATT ATTAGATTAA   
  
  
+ TAAAAATCCT AATGGTTGTT TAATGTTACA TCAAGACATG CTTTTTGGTG TATATATAAT GGCAGCATGT   
  
  
+ GGGTTAAGCG GCAACAAATT ATTCTCAAAT GCATGCAGTA ATTAATTTAA TTAATTAATC ACCTGCATAA   
  
  
+ TCTCAATACT AATCCTTTCA TTCTGTGCCC CACCCAACCC CGAAAATAGT AATCTTTCAA ACGTTGGAAG   
  
  
+ TGGGATTAGG TATAGAGTAG TCCTACAGAT GATGATCTAC CTACATATAA CATAATCTGA CTTTTCTTTT   
  
  
+ TTAAATAACT TGTATTGATG GCACAGAATT TGACATGTTG GCATATGGTA CTAAGAAATT AATTAACGTT   
  
  
+ GTGCTAAGAT ATAGGTCATT CTTTTTGGAG ACATAAGGCC CGCGTTCACG CAGGGTTCGA GAAGGGCCAC   
  
  
+ ATTCAATGGA TGAATTGTAG GTAGTTTAAT CTGACTTTGT CAGTGACTGA TTCCACGGCT TGAACCCGCG   
  
  
+ ACATTAATAT AGGTTGACTA AAGTCGTTTG ATACTTATTT TTCTTAAGTA AAGTCTTGAA TTCAAATCTT   
  
  
+ ATAAATAAAT AAAAAAATCT ATAATAAAAG AATTCTACCA TTTATTAAGT CAACAACTCA ATTCGAATGA   
  
  
+ AATAAAAAAT CAAGATCACC AAAAAAAATA AATAAAAGGA AAGGTAGATT GCATTTGGAT TTTGAGTGGC   
  
  
+ GTGGTGGTGG GTTTGATGCT GGTGCTGCAT ACACCGCAGC TTTTCTCTGT CCTTTTACCT CTCTTTGTCT   
  
  
+ GCAAGAACCC CATTTGGCAC GCCTGCTCTC TTTTCTATGT TTTCTTTTCT ATCTTTTAAG AAACAATGGA   
  
  
+ AAGCTAACAC ACTAACACTA ACCCATTGGT CCTTTTTTCT TATCTGCAAA ATTAAAGTAA CAATATTTTT   
  
  
+ CTTATCCCCT TTTTCGTTCC ACCCATTTCT TTATCCTTCT GTTTGAACCT AATCTGTATA TATGCATGCA   
  
  
+ TGTGTATTTT ACTTAGAATC TGTTTTGATT CTTTGATGTA ATTAATTAGT AGTCTTTTTA ATTGAATTTT   
  
  
+ TAACAGTAGT TTGAATTAGA CATTTATCGA TATTATATTT TTTATATATA ATTAAAAAAT ACAAAATGAT   
  
  
+ AAGTGTCTTA CTACAATACA CTTCATAAGT GTCGTGTTTT AACTTAAAAA TCATACTATT ATGTTTCTAA   
  
  
+ ATTGAAAAAA AAGTGAGATT AAGTAGCAGA AGCATATTAC AACTTATAAA TTATATAAAA TCATCTTTTT   
  
  
+ ATCCATAATT TATCACATTT CTAAAATACG CATATCATAT CTACTTATGG ATAGGGTTAA TAAGATTAGG   
  
  
+ GCATCATTTA AGATTTTATC AGCGTAACCA ACGAATGTTT AAGGTTAGCA GTTCATAATT AAGTGTAATC   
  
  
+ ATTAAAAAAT TTATGATTTT AAATCATAAA ATATGTTAAT CTAGGATGAG GCAAGCGGCA AGACATATAT   
  
  
+ CACTATCCTT AAAAAATATA ATATATTGAT TTCAAAGAAT TATGAAGTAT ATTAATTTCA TATATAATGA   
  
  
+ CGATTATGTA TTTTTTTACT AAATTACTTG TACCCATAAT AAATAGAATC GATGCATCAT TCATGGCCTT   
  
  
+ TCATTCTGTA ATATGGTTGA CTCCAAAAAT TCTCAAAGTA TTTTTATATT TTGGGAGGGG AGATTTTAGT   
  
  
+ TTGATACTTT TGTTGAACTA GTGAACAATT TCCAACAACC TCCACCACCC CCAAACATCA ACAATCTGAT   
  
  
+ TGATGTATGC ATCCCAAGCT TGAATTGAAT AATAATTTCA ATAACAACAA TAATCGTTTT ACCTTGACAA   
  
  
+ GCTCCACAGC AACTCAACGT CTTCCAAACC AACCCATCAC TCATCATCAT CATCCCCACT ATCCCATTCA   
  
  
+ CCCCCACCAA AAGGAGCCTA AAGACAGTAA CAAGAAGAAT GCTTGCCTTT TTTCATGGAT GAAGAAGACT   
  
  
+ TCTCTTCTTC CTCTACTTCT TCCCACCACT ATGATACACA CCACCTCCAA CATCATTACC CCACCAATTA   
  
  
+ TAATTCCCGT TATACCCTGG ACCCTCCCTC CACCACCACC ACAGCCACAA CCACCACCTC ATCCTCCACT   
  
  
+ CCCACCCACC ATGGCTTCGG TGGTGGGCTT GACTCCTCCT CCCCGTCTGC CGCTGTGGAA TTATCTTTCT   
  
  
+ CCCCGGACAT CCTCCTCTCC TCCCCCACCG GAAAGTGGGC GTCTGACATT CTTATGGAGG CGGCTAGGGC   
  
  
+ TTTTTCAGAC ACGAACACCA CCAAGCTCCA CCAGCTCCTC TGGATGCTGA ACGAGCTCGG CTCCCCCTAC   
  
  
+ GGTGATGTCG AGCAGAAGCT GGTGGCGTAC TTTAACCAAG CCCTTTTTGC CCGCCTCACT GCCACCGGAC   
  
  
+ CCCGCCACCG AGCCGCTATG CTCGCCGCTG CGGACAAGAC CTCCTCCTTC GACTCCACGC TCAGGTTGCT   
  
  
+ CCTCAAGTTC CAGGAAGTCA GCCCGTGGAC CACCTTCGGC CACGTGGCTT GCAACGGTGC AATCATCGAG   
  
  
+ GCCTTAGAGA GGGACCCACA TGAAAAGAGT AAGCTCCACG TCATCGATAT CAGCACCACC TTCTGCACCC   
  
  
+ AGTGGCCCAC CTTGCTTGAG GCTTTAGCCA CCCGCATGGA CGACACTCCC CACCTCAGCC TCACTGCCGT   
  
  
+ CGTCGTCAAC AAGTACGCCG CCGTGAGCGG TGGCGAGGAC GACGGGGGAG AAGGATCAAA GAGGGTGATG   
  
  
+ AGAGAGATTG GTCTTCGTCT TGAGAAGTTC GCTAGGTTAA TGGGGGTTCC CTTCAAGTTC AACGTGGTTT   
  
  
+ ACCACGTAGG TGATCTATCC CAGTTGGATT TTTCTCGTTT GGATATTAAA GATGATGAAG CGGTGGCGAT   
  
  
+ TAACTGTGTC AACTCGCTCC ACTCGGTGGA CCTCCGATAT CGGGAGGTAG TGCTCGCGGC GTTCAGGCGG   
  
  
+ CTGCGGCCGA GGGTGGTGAC GGTGGTGGAG GAAGAGGCGG AGCTGACTGA GGTGGGTGAG GGTCAGTATG   
  
  
+ AATTTTTTAG GCGGTTTGAG GAGTGTCTTA GGTGGTTTAG GGTTTACTTT GAGGCCGTAG GAGATTGTTT   
  
  
+ TCCTAGGACT AGCAATGAGA AATTGATGTT AGAGCGGGCC GCGGGCCGGG CCATGGTGGA TCTCCTTGCA   
  
  
+ TGTCCCGAGC CTGGTGGGTC GGCTGAGGGG AGGGAGACGG CGAGACGGTG GTCGGGTCGG ATGCATGGGG   
  
  
+ CGGGGTTCGA GCACGTGGGG TTCAGTGACG AAGTGTGTGA TGATGTGAGG GCCTTGTTGA GGAGGTACAA   
  
  
+ GGAAGGATGG TCAATGACAC AGTGCACCAT GGATGGCGGT AATCACCCTG GAATACTGTT GTGGTGGCGG   
  
  
+ GATCAACCGG TGGTTTGGGC CAGTGCATGG CGGCCTTG  

- +Up\_Stream \_Len000TTTAGT TTGAGTATTT GTTATTAAAA AATTTTGAAT TTACCATTAA TAATCTAATT   
  
  
- ATTTTTAGGA TTACCAACAA ATTACAATGT AGTTCTGTAC GAAAAACCAC ATATATATTA CCGTCGTACA   
  
  
- CCCAATTCGC CGTTGTTTAA TAAGAGTTTA CGTACGTCAT TAATTAAATT AATTAATTAG TGGACGTATT   
  
  
- AGAGTTATGA TTAGGAAAGT AAGACACGGG GTGGGTTGGG GCTTTTATCA TTAGAAAGTT TGCAACCTTC   
  
  
- ACCCTAATCC ATATCTCATC AGGATGTCTA CTACTAGATG GATGTATATT GTATTAGACT GAAAAGAAAA   
  
  
- AATTTATTGA ACATAACTAC CGTGTCTTAA ACTGTACAAC CGTATACCAT GATTCTTTAA TTAATTGCAA   
  
  
- CACGATTCTA TATCCAGTAA GAAAAACCTC TGTATTCCGG GCGCAAGTGC GTCCCAAGCT CTTCCCGGTG   
  
  
- TAAGTTACCT ACTTAACATC CATCAAATTA GACTGAAACA GTCACTGACT AAGGTGCCGA ACTTGGGCGC   
  
  
- TGTAATTATA TCCAACTGAT TTCAGCAAAC TATGAATAAA AAGAATTCAT TTCAGAACTT AAGTTTAGAA   
  
  
- TATTTATTTA TTTTTTTAGA TATTATTTTC TTAAGATGGT AAATAATTCA GTTGTTGAGT TAAGCTTACT   
  
  
- TTATTTTTTA GTTCTAGTGG TTTTTTTTAT TTATTTTCCT TTCCATCTAA CGTAAACCTA AAACTCACCG   
  
  
- CACCACCACC CAAACTACGA CCACGACGTA TGTGGCGTCG AAAAGAGACA GGAAAATGGA GAGAAACAGA   
  
  
- CGTTCTTGGG GTAAACCGTG CGGACGAGAG AAAAGATACA AAAGAAAAGA TAGAAAATTC TTTGTTACCT   
  
  
- TTCGATTGTG TGATTGTGAT TGGGTAACCA GGAAAAAAGA ATAGACGTTT TAATTTCATT GTTATAAAAA   
  
  
- GAATAGGGGA AAAAGCAAGG TGGGTAAAGA AATAGGAAGA CAAACTTGGA TTAGACATAT ATACGTACGT   
  
  
- ACACATAAAA TGAATCTTAG ACAAAACTAA GAAACTACAT TAATTAATCA TCAGAAAAAT TAACTTAAAA   
  
  
- ATTGTCATCA AACTTAATCT GTAAATAGCT ATAATATAAA AAATATATAT TAATTTTTTA TGTTTTACTA   
  
  
- TTCACAGAAT GATGTTATGT GAAGTATTCA CAGCACAAAA TTGAATTTTT AGTATGATAA TACAAAGATT   
  
  
- TAACTTTTTT TTCACTCTAA TTCATCGTCT TCGTATAATG TTGAATATTT AATATATTTT AGTAGAAAAA   
  
  
- TAGGTATTAA ATAGTGTAAA GATTTTATGC GTATAGTATA GATGAATACC TATCCCAATT ATTCTAATCC   
  
  
- CGTAGTAAAT TCTAAAATAG TCGCATTGGT TGCTTACAAA TTCCAATCGT CAAGTATTAA TTCACATTAG   
  
  
- TAATTTTTTA AATACTAAAA TTTAGTATTT TATACAATTA GATCCTACTC CGTTCGCCGT TCTGTATATA   
  
  
- GTGATAGGAA TTTTTTATAT TATATAACTA AAGTTTCTTA ATACTTCATA TAATTAAAGT ATATATTACT   
  
  
- GCTAATACAT AAAAAAATGA TTTAATGAAC ATGGGTATTA TTTATCTTAG CTACGTAGTA AGTACCGGAA   
  
  
- AGTAAGACAT TATACCAACT GAGGTTTTTA AGAGTTTCAT AAAAATATAA AACCCTCCCC TCTAAAATCA   
  
  
- AACTATGAAA ACAACTTGAT CACTTGTTAA AGGTTGTTGG AGGTGGTGGG GGTTTGTAGT TGTTAGACTA   
  
  
- ACTACATACG TAGGGTTCGA ACTTAACTTA TTATTAAAGT TATTGTTGTT ATTAGCAAAA TGGAACTGTT   
  
  
- CGAGGTGTCG TTGAGTTGCA GAAGGTTTGG TTGGGTAGTG AGTAGTAGTA GTAGGGGTGA TAGGGTAAGT   
  
  
- GGGGGTGGTT TTCCTCGGAT TTCTGTCATT GTTCTTCTTA CGAACGGAAA AAAGTACCTA CTTCTTCTGA   
  
  
- AGAGAAGAAG GAGATGAAGA AGGGTGGTGA TACTATGTGT GGTGGAGGTT GTAGTAATGG GGTGGTTAAT   
  
  
- ATTAAGGGCA ATATGGGACC TGGGAGGGAG GTGGTGGTGG TGTCGGTGTT GGTGGTGGAG TAGGAGGTGA   
  
  
- GGGTGGGTGG TACCGAAGCC ACCACCCGAA CTGAGGAGGA GGGGCAGACG GCGACACCTT AATAGAAAGA   
  
  
- GGGGCCTGTA GGAGGAGAGG AGGGGGTGGC CTTTCACCCG CAGACTGTAA GAATACCTCC GCCGATCCCG   
  
  
- AAAAAGTCTG TGCTTGTGGT GGTTCGAGGT GGTCGAGGAG ACCTACGACT TGCTCGAGCC GAGGGGGATG   
  
  
- CCACTACAGC TCGTCTTCGA CCACCGCATG AAATTGGTTC GGGAAAAACG GGCGGAGTGA CGGTGGCCTG   
  
  
- GGGCGGTGGC TCGGCGATAC GAGCGGCGAC GCCTGTTCTG GAGGAGGAAG CTGAGGTGCG AGTCCAACGA   
  
  
- GGAGTTCAAG GTCCTTCAGT CGGGCACCTG GTGGAAGCCG GTGCACCGAA CGTTGCCACG TTAGTAGCTC   
  
  
- CGGAATCTCT CCCTGGGTGT ACTTTTCTCA TTCGAGGTGC AGTAGCTATA GTCGTGGTGG AAGACGTGGG   
  
  
- TCACCGGGTG GAACGAACTC CGAAATCGGT GGGCGTACCT GCTGTGAGGG GTGGAGTCGG AGTGACGGCA   
  
  
- GCAGCAGTTG TTCATGCGGC GGCACTCGCC ACCGCTCCTG CTGCCCCCTC TTCCTAGTTT CTCCCACTAC   
  
  
- TCTCTCTAAC CAGAAGCAGA ACTCTTCAAG CGATCCAATT ACCCCCAAGG GAAGTTCAAG TTGCACCAAA   
  
  
- TGGTGCATCC ACTAGATAGG GTCAACCTAA AAAGAGCAAA CCTATAATTT CTACTACTTC GCCACCGCTA   
  
  
- ATTGACACAG TTGAGCGAGG TGAGCCACCT GGAGGCTATA GCCCTCCATC ACGAGCGCCG CAAGTCCGCC   
  
  
- GACGCCGGCT CCCACCACTG CCACCACCTC CTTCTCCGCC TCGACTGACT CCACCCACTC CCAGTCATAC   
  
  
- TTAAAAAATC CGCCAAACTC CTCACAGAAT CCACCAAATC CCAAATGAAA CTCCGGCATC CTCTAACAAA   
  
  
- AGGATCCTGA TCGTTACTCT TTAACTACAA TCTCGCCCGG CGCCCGGCCC GGTACCACCT AGAGGAACGT   
  
  
- ACAGGGCTCG GACCACCCAG CCGACTCCCC TCCCTCTGCC GCTCTGCCAC CAGCCCAGCC TACGTACCCC   
  
  
- GCCCCAAGCT CGTGCACCCC AAGTCACTGC TTCACACACT ACTACACTCC CGGAACAACT CCTCCATGTT   
  
  
- CCTTCCTACC AGTTACTGTG TCACGTGGTA CCTACCGCCA TTAGTGGGAC CTTATGACAA CACCACCGCC   
  
  
- CTAGTTGGCC ACCAAACCCG GTCACGTACC GCCGGAAC

+     Box II

| Site Name | Organism | Position | Strand | Matrix score. | sequence | function |
| --- | --- | --- | --- | --- | --- | --- |
| Box II | Petroselinum crispum | 2563 | - | 9 | CCACGTGGC | part of a light responsive element |
| Box II | Petroselinum crispum | 2564 | + | 9 | CCACGTGGC | part of a light responsive element |

>HU05G01983.1   
+ +Up\_Stream \_Len000AAATCA AACTCATAAA CAATAATTTT TTAAAACTTA AATGGTAATT ATTAGATTAA   
  
  
+ TAAAAATCCT AATGGTTGTT TAATGTTACA TCAAGACATG CTTTTTGGTG TATATATAAT GGCAGCATGT   
  
  
+ GGGTTAAGCG GCAACAAATT ATTCTCAAAT GCATGCAGTA ATTAATTTAA TTAATTAATC ACCTGCATAA   
  
  
+ TCTCAATACT AATCCTTTCA TTCTGTGCCC CACCCAACCC CGAAAATAGT AATCTTTCAA ACGTTGGAAG   
  
  
+ TGGGATTAGG TATAGAGTAG TCCTACAGAT GATGATCTAC CTACATATAA CATAATCTGA CTTTTCTTTT   
  
  
+ TTAAATAACT TGTATTGATG GCACAGAATT TGACATGTTG GCATATGGTA CTAAGAAATT AATTAACGTT   
  
  
+ GTGCTAAGAT ATAGGTCATT CTTTTTGGAG ACATAAGGCC CGCGTTCACG CAGGGTTCGA GAAGGGCCAC   
  
  
+ ATTCAATGGA TGAATTGTAG GTAGTTTAAT CTGACTTTGT CAGTGACTGA TTCCACGGCT TGAACCCGCG   
  
  
+ ACATTAATAT AGGTTGACTA AAGTCGTTTG ATACTTATTT TTCTTAAGTA AAGTCTTGAA TTCAAATCTT   
  
  
+ ATAAATAAAT AAAAAAATCT ATAATAAAAG AATTCTACCA TTTATTAAGT CAACAACTCA ATTCGAATGA   
  
  
+ AATAAAAAAT CAAGATCACC AAAAAAAATA AATAAAAGGA AAGGTAGATT GCATTTGGAT TTTGAGTGGC   
  
  
+ GTGGTGGTGG GTTTGATGCT GGTGCTGCAT ACACCGCAGC TTTTCTCTGT CCTTTTACCT CTCTTTGTCT   
  
  
+ GCAAGAACCC CATTTGGCAC GCCTGCTCTC TTTTCTATGT TTTCTTTTCT ATCTTTTAAG AAACAATGGA   
  
  
+ AAGCTAACAC ACTAACACTA ACCCATTGGT CCTTTTTTCT TATCTGCAAA ATTAAAGTAA CAATATTTTT   
  
  
+ CTTATCCCCT TTTTCGTTCC ACCCATTTCT TTATCCTTCT GTTTGAACCT AATCTGTATA TATGCATGCA   
  
  
+ TGTGTATTTT ACTTAGAATC TGTTTTGATT CTTTGATGTA ATTAATTAGT AGTCTTTTTA ATTGAATTTT   
  
  
+ TAACAGTAGT TTGAATTAGA CATTTATCGA TATTATATTT TTTATATATA ATTAAAAAAT ACAAAATGAT   
  
  
+ AAGTGTCTTA CTACAATACA CTTCATAAGT GTCGTGTTTT AACTTAAAAA TCATACTATT ATGTTTCTAA   
  
  
+ ATTGAAAAAA AAGTGAGATT AAGTAGCAGA AGCATATTAC AACTTATAAA TTATATAAAA TCATCTTTTT   
  
  
+ ATCCATAATT TATCACATTT CTAAAATACG CATATCATAT CTACTTATGG ATAGGGTTAA TAAGATTAGG   
  
  
+ GCATCATTTA AGATTTTATC AGCGTAACCA ACGAATGTTT AAGGTTAGCA GTTCATAATT AAGTGTAATC   
  
  
+ ATTAAAAAAT TTATGATTTT AAATCATAAA ATATGTTAAT CTAGGATGAG GCAAGCGGCA AGACATATAT   
  
  
+ CACTATCCTT AAAAAATATA ATATATTGAT TTCAAAGAAT TATGAAGTAT ATTAATTTCA TATATAATGA   
  
  
+ CGATTATGTA TTTTTTTACT AAATTACTTG TACCCATAAT AAATAGAATC GATGCATCAT TCATGGCCTT   
  
  
+ TCATTCTGTA ATATGGTTGA CTCCAAAAAT TCTCAAAGTA TTTTTATATT TTGGGAGGGG AGATTTTAGT   
  
  
+ TTGATACTTT TGTTGAACTA GTGAACAATT TCCAACAACC TCCACCACCC CCAAACATCA ACAATCTGAT   
  
  
+ TGATGTATGC ATCCCAAGCT TGAATTGAAT AATAATTTCA ATAACAACAA TAATCGTTTT ACCTTGACAA   
  
  
+ GCTCCACAGC AACTCAACGT CTTCCAAACC AACCCATCAC TCATCATCAT CATCCCCACT ATCCCATTCA   
  
  
+ CCCCCACCAA AAGGAGCCTA AAGACAGTAA CAAGAAGAAT GCTTGCCTTT TTTCATGGAT GAAGAAGACT   
  
  
+ TCTCTTCTTC CTCTACTTCT TCCCACCACT ATGATACACA CCACCTCCAA CATCATTACC CCACCAATTA   
  
  
+ TAATTCCCGT TATACCCTGG ACCCTCCCTC CACCACCACC ACAGCCACAA CCACCACCTC ATCCTCCACT   
  
  
+ CCCACCCACC ATGGCTTCGG TGGTGGGCTT GACTCCTCCT CCCCGTCTGC CGCTGTGGAA TTATCTTTCT   
  
  
+ CCCCGGACAT CCTCCTCTCC TCCCCCACCG GAAAGTGGGC GTCTGACATT CTTATGGAGG CGGCTAGGGC   
  
  
+ TTTTTCAGAC ACGAACACCA CCAAGCTCCA CCAGCTCCTC TGGATGCTGA ACGAGCTCGG CTCCCCCTAC   
  
  
+ GGTGATGTCG AGCAGAAGCT GGTGGCGTAC TTTAACCAAG CCCTTTTTGC CCGCCTCACT GCCACCGGAC   
  
  
+ CCCGCCACCG AGCCGCTATG CTCGCCGCTG CGGACAAGAC CTCCTCCTTC GACTCCACGC TCAGGTTGCT   
  
  
+ CCTCAAGTTC CAGGAAGTCA GCCCGTGGAC CACCTTCGGC CACGTGGCTT GCAACGGTGC AATCATCGAG   
  
  
+ GCCTTAGAGA GGGACCCACA TGAAAAGAGT AAGCTCCACG TCATCGATAT CAGCACCACC TTCTGCACCC   
  
  
+ AGTGGCCCAC CTTGCTTGAG GCTTTAGCCA CCCGCATGGA CGACACTCCC CACCTCAGCC TCACTGCCGT   
  
  
+ CGTCGTCAAC AAGTACGCCG CCGTGAGCGG TGGCGAGGAC GACGGGGGAG AAGGATCAAA GAGGGTGATG   
  
  
+ AGAGAGATTG GTCTTCGTCT TGAGAAGTTC GCTAGGTTAA TGGGGGTTCC CTTCAAGTTC AACGTGGTTT   
  
  
+ ACCACGTAGG TGATCTATCC CAGTTGGATT TTTCTCGTTT GGATATTAAA GATGATGAAG CGGTGGCGAT   
  
  
+ TAACTGTGTC AACTCGCTCC ACTCGGTGGA CCTCCGATAT CGGGAGGTAG TGCTCGCGGC GTTCAGGCGG   
  
  
+ CTGCGGCCGA GGGTGGTGAC GGTGGTGGAG GAAGAGGCGG AGCTGACTGA GGTGGGTGAG GGTCAGTATG   
  
  
+ AATTTTTTAG GCGGTTTGAG GAGTGTCTTA GGTGGTTTAG GGTTTACTTT GAGGCCGTAG GAGATTGTTT   
  
  
+ TCCTAGGACT AGCAATGAGA AATTGATGTT AGAGCGGGCC GCGGGCCGGG CCATGGTGGA TCTCCTTGCA   
  
  
+ TGTCCCGAGC CTGGTGGGTC GGCTGAGGGG AGGGAGACGG CGAGACGGTG GTCGGGTCGG ATGCATGGGG   
  
  
+ CGGGGTTCGA GCACGTGGGG TTCAGTGACG AAGTGTGTGA TGATGTGAGG GCCTTGTTGA GGAGGTACAA   
  
  
+ GGAAGGATGG TCAATGACAC AGTGCACCAT GGATGGCGGT AATCACCCTG GAATACTGTT GTGGTGGCGG   
  
  
+ GATCAACCGG TGGTTTGGGC CAGTGCATGG CGGCCTTG  

- +Up\_Stream \_Len000TTTAGT TTGAGTATTT GTTATTAAAA AATTTTGAAT TTACCATTAA TAATCTAATT   
  
  
- ATTTTTAGGA TTACCAACAA ATTACAATGT AGTTCTGTAC GAAAAACCAC ATATATATTA CCGTCGTACA   
  
  
- CCCAATTCGC CGTTGTTTAA TAAGAGTTTA CGTACGTCAT TAATTAAATT AATTAATTAG TGGACGTATT   
  
  
- AGAGTTATGA TTAGGAAAGT AAGACACGGG GTGGGTTGGG GCTTTTATCA TTAGAAAGTT TGCAACCTTC   
  
  
- ACCCTAATCC ATATCTCATC AGGATGTCTA CTACTAGATG GATGTATATT GTATTAGACT GAAAAGAAAA   
  
  
- AATTTATTGA ACATAACTAC CGTGTCTTAA ACTGTACAAC CGTATACCAT GATTCTTTAA TTAATTGCAA   
  
  
- CACGATTCTA TATCCAGTAA GAAAAACCTC TGTATTCCGG GCGCAAGTGC GTCCCAAGCT CTTCCCGGTG   
  
  
- TAAGTTACCT ACTTAACATC CATCAAATTA GACTGAAACA GTCACTGACT AAGGTGCCGA ACTTGGGCGC   
  
  
- TGTAATTATA TCCAACTGAT TTCAGCAAAC TATGAATAAA AAGAATTCAT TTCAGAACTT AAGTTTAGAA   
  
  
- TATTTATTTA TTTTTTTAGA TATTATTTTC TTAAGATGGT AAATAATTCA GTTGTTGAGT TAAGCTTACT   
  
  
- TTATTTTTTA GTTCTAGTGG TTTTTTTTAT TTATTTTCCT TTCCATCTAA CGTAAACCTA AAACTCACCG   
  
  
- CACCACCACC CAAACTACGA CCACGACGTA TGTGGCGTCG AAAAGAGACA GGAAAATGGA GAGAAACAGA   
  
  
- CGTTCTTGGG GTAAACCGTG CGGACGAGAG AAAAGATACA AAAGAAAAGA TAGAAAATTC TTTGTTACCT   
  
  
- TTCGATTGTG TGATTGTGAT TGGGTAACCA GGAAAAAAGA ATAGACGTTT TAATTTCATT GTTATAAAAA   
  
  
- GAATAGGGGA AAAAGCAAGG TGGGTAAAGA AATAGGAAGA CAAACTTGGA TTAGACATAT ATACGTACGT   
  
  
- ACACATAAAA TGAATCTTAG ACAAAACTAA GAAACTACAT TAATTAATCA TCAGAAAAAT TAACTTAAAA   
  
  
- ATTGTCATCA AACTTAATCT GTAAATAGCT ATAATATAAA AAATATATAT TAATTTTTTA TGTTTTACTA   
  
  
- TTCACAGAAT GATGTTATGT GAAGTATTCA CAGCACAAAA TTGAATTTTT AGTATGATAA TACAAAGATT   
  
  
- TAACTTTTTT TTCACTCTAA TTCATCGTCT TCGTATAATG TTGAATATTT AATATATTTT AGTAGAAAAA   
  
  
- TAGGTATTAA ATAGTGTAAA GATTTTATGC GTATAGTATA GATGAATACC TATCCCAATT ATTCTAATCC   
  
  
- CGTAGTAAAT TCTAAAATAG TCGCATTGGT TGCTTACAAA TTCCAATCGT CAAGTATTAA TTCACATTAG   
  
  
- TAATTTTTTA AATACTAAAA TTTAGTATTT TATACAATTA GATCCTACTC CGTTCGCCGT TCTGTATATA   
  
  
- GTGATAGGAA TTTTTTATAT TATATAACTA AAGTTTCTTA ATACTTCATA TAATTAAAGT ATATATTACT   
  
  
- GCTAATACAT AAAAAAATGA TTTAATGAAC ATGGGTATTA TTTATCTTAG CTACGTAGTA AGTACCGGAA   
  
  
- AGTAAGACAT TATACCAACT GAGGTTTTTA AGAGTTTCAT AAAAATATAA AACCCTCCCC TCTAAAATCA   
  
  
- AACTATGAAA ACAACTTGAT CACTTGTTAA AGGTTGTTGG AGGTGGTGGG GGTTTGTAGT TGTTAGACTA   
  
  
- ACTACATACG TAGGGTTCGA ACTTAACTTA TTATTAAAGT TATTGTTGTT ATTAGCAAAA TGGAACTGTT   
  
  
- CGAGGTGTCG TTGAGTTGCA GAAGGTTTGG TTGGGTAGTG AGTAGTAGTA GTAGGGGTGA TAGGGTAAGT   
  
  
- GGGGGTGGTT TTCCTCGGAT TTCTGTCATT GTTCTTCTTA CGAACGGAAA AAAGTACCTA CTTCTTCTGA   
  
  
- AGAGAAGAAG GAGATGAAGA AGGGTGGTGA TACTATGTGT GGTGGAGGTT GTAGTAATGG GGTGGTTAAT   
  
  
- ATTAAGGGCA ATATGGGACC TGGGAGGGAG GTGGTGGTGG TGTCGGTGTT GGTGGTGGAG TAGGAGGTGA   
  
  
- GGGTGGGTGG TACCGAAGCC ACCACCCGAA CTGAGGAGGA GGGGCAGACG GCGACACCTT AATAGAAAGA   
  
  
- GGGGCCTGTA GGAGGAGAGG AGGGGGTGGC CTTTCACCCG CAGACTGTAA GAATACCTCC GCCGATCCCG   
  
  
- AAAAAGTCTG TGCTTGTGGT GGTTCGAGGT GGTCGAGGAG ACCTACGACT TGCTCGAGCC GAGGGGGATG   
  
  
- CCACTACAGC TCGTCTTCGA CCACCGCATG AAATTGGTTC GGGAAAAACG GGCGGAGTGA CGGTGGCCTG   
  
  
- GGGCGGTGGC TCGGCGATAC GAGCGGCGAC GCCTGTTCTG GAGGAGGAAG CTGAGGTGCG AGTCCAACGA   
  
  
- GGAGTTCAAG GTCCTTCAGT CGGGCACCTG GTGGAAGCCG GTGCACCGAA CGTTGCCACG TTAGTAGCTC   
  
  
- CGGAATCTCT CCCTGGGTGT ACTTTTCTCA TTCGAGGTGC AGTAGCTATA GTCGTGGTGG AAGACGTGGG   
  
  
- TCACCGGGTG GAACGAACTC CGAAATCGGT GGGCGTACCT GCTGTGAGGG GTGGAGTCGG AGTGACGGCA   
  
  
- GCAGCAGTTG TTCATGCGGC GGCACTCGCC ACCGCTCCTG CTGCCCCCTC TTCCTAGTTT CTCCCACTAC   
  
  
- TCTCTCTAAC CAGAAGCAGA ACTCTTCAAG CGATCCAATT ACCCCCAAGG GAAGTTCAAG TTGCACCAAA   
  
  
- TGGTGCATCC ACTAGATAGG GTCAACCTAA AAAGAGCAAA CCTATAATTT CTACTACTTC GCCACCGCTA   
  
  
- ATTGACACAG TTGAGCGAGG TGAGCCACCT GGAGGCTATA GCCCTCCATC ACGAGCGCCG CAAGTCCGCC   
  
  
- GACGCCGGCT CCCACCACTG CCACCACCTC CTTCTCCGCC TCGACTGACT CCACCCACTC CCAGTCATAC   
  
  
- TTAAAAAATC CGCCAAACTC CTCACAGAAT CCACCAAATC CCAAATGAAA CTCCGGCATC CTCTAACAAA   
  
  
- AGGATCCTGA TCGTTACTCT TTAACTACAA TCTCGCCCGG CGCCCGGCCC GGTACCACCT AGAGGAACGT   
  
  
- ACAGGGCTCG GACCACCCAG CCGACTCCCC TCCCTCTGCC GCTCTGCCAC CAGCCCAGCC TACGTACCCC   
  
  
- GCCCCAAGCT CGTGCACCCC AAGTCACTGC TTCACACACT ACTACACTCC CGGAACAACT CCTCCATGTT   
  
  
- CCTTCCTACC AGTTACTGTG TCACGTGGTA CCTACCGCCA TTAGTGGGAC CTTATGACAA CACCACCGCC   
  
  
- CTAGTTGGCC ACCAAACCCG GTCACGTACC GCCGGAAC

+     CAAT-box

| Site Name | Organism | Position | Strand | Matrix score. | sequence | function |
| --- | --- | --- | --- | --- | --- | --- |
| CAAT-box | Nicotiana glutinosa | 3167 | + | 4 | CAAT |  |
| CAAT-box | Arabidopsis thaliana | 2811 | - | 5 | CCAAT | common cis-acting element in promoter and enhancer regions |
| CAAT-box | Nicotiana glutinosa | 1872 | + | 4 | CAAT |  |
| CAAT-box | Nicotiana glutinosa | 1816 | + | 4 | CAAT |  |
| CAAT-box | Nicotiana glutinosa | 1848 | - | 4 | CAAT |  |
| CAAT-box | Nicotiana glutinosa | 1823 | - | 4 | CAAT |  |
| CAAT-box | Nicotiana glutinosa | 1780 | + | 4 | CAAT |  |
| CAAT-box | Nicotiana glutinosa | 1115 | - | 4 | CAAT |  |
| CAAT-box | Nicotiana glutinosa | 908 | + | 4 | CAAT |  |
| CAAT-box | Nicotiana glutinosa | 2584 | + | 4 | CAAT |  |
| CAAT-box | Pisum sativum | 382 | - | 5 | CAAAT | common cis-acting element in promoter and enhancer regions |
| CAAT-box | Nicotiana glutinosa | 1863 | + | 4 | CAAT |  |
| CAAT-box | Nicotiana glutinosa | 368 | - | 4 | CAAT |  |
| CAAT-box | Nicotiana glutinosa | 975 | + | 4 | CAAT |  |
| CAAT-box | Arabidopsis thaliana | 939 | - | 5 | CCAAT | common cis-acting element in promoter and enhancer regions |
| CAAT-box | Nicotiana glutinosa | 3376 | + | 4 | CAAT |  |
| CAAT-box | Nicotiana glutinosa | 1208 | + | 4 | CAAT |  |
| CAAT-box | Petunia hybrida | 391 | - | 7 | TGCCAAC | common cis-acting element in promoter and enhancer regions |
| CAAT-box | Pisum sativum | 757 | - | 5 | CAAAT | common cis-acting element in promoter and enhancer regions |
| CAAT-box | Nicotiana glutinosa | 498 | + | 4 | CAAT |  |
| CAAT-box | Pisum sativum | 856 | - | 5 | CAAAT | common cis-acting element in promoter and enhancer regions |
| CAAT-box | Nicotiana glutinosa | 2099 | + | 4 | CAAT |  |
| CAAT-box | Pisum sativum | 627 | + | 5 | CAAAT | common cis-acting element in promoter and enhancer regions |
| CAAT-box | Nicotiana glutinosa | 1265 | - | 4 | CAAT |  |
| CAAT-box | Nicotiana glutinosa | 1569 | - | 4 | CAAT |  |
| CAAT-box | Nicotiana glutinosa | 693 | + | 4 | CAAT |  |
| CAAT-box | Nicotiana glutinosa | 752 | - | 4 | CAAT |  |
| CAAT-box | Nicotiana glutinosa | 508 | - | 4 | CAAT |  |
| CAAT-box | Nicotiana glutinosa | 3148 | - | 4 | CAAT |  |
| CAAT-box | Arabidopsis thaliana | 2098 | + | 5 | CCAAT | common cis-acting element in promoter and enhancer regions |
| CAAT-box | Nicotiana glutinosa | 3176 | - | 4 | CAAT |  |
| CAAT-box | Pisum sativum | 159 | + | 5 | CAAAT | common cis-acting element in promoter and enhancer regions |
| CAAT-box | Nicotiana glutinosa | 35 | + | 4 | CAAT |  |
| CAAT-box | Pisum sativum | 170 | + | 5 | CAAAT | common cis-acting element in promoter and enhancer regions |
| CAAT-box | Nicotiana glutinosa | 218 | + | 4 | CAAT |  |

>HU05G01983.1   
+ +Up\_Stream \_Len000AAATCA AACTCATAAA CAATAATTTT TTAAAACTTA AATGGTAATT ATTAGATTAA   
  
  
+ TAAAAATCCT AATGGTTGTT TAATGTTACA TCAAGACATG CTTTTTGGTG TATATATAAT GGCAGCATGT   
  
  
+ GGGTTAAGCG GCAACAAATT ATTCTCAAAT GCATGCAGTA ATTAATTTAA TTAATTAATC ACCTGCATAA   
  
  
+ TCTCAATACT AATCCTTTCA TTCTGTGCCC CACCCAACCC CGAAAATAGT AATCTTTCAA ACGTTGGAAG   
  
  
+ TGGGATTAGG TATAGAGTAG TCCTACAGAT GATGATCTAC CTACATATAA CATAATCTGA CTTTTCTTTT   
  
  
+ TTAAATAACT TGTATTGATG GCACAGAATT TGACATGTTG GCATATGGTA CTAAGAAATT AATTAACGTT   
  
  
+ GTGCTAAGAT ATAGGTCATT CTTTTTGGAG ACATAAGGCC CGCGTTCACG CAGGGTTCGA GAAGGGCCAC   
  
  
+ ATTCAATGGA TGAATTGTAG GTAGTTTAAT CTGACTTTGT CAGTGACTGA TTCCACGGCT TGAACCCGCG   
  
  
+ ACATTAATAT AGGTTGACTA AAGTCGTTTG ATACTTATTT TTCTTAAGTA AAGTCTTGAA TTCAAATCTT   
  
  
+ ATAAATAAAT AAAAAAATCT ATAATAAAAG AATTCTACCA TTTATTAAGT CAACAACTCA ATTCGAATGA   
  
  
+ AATAAAAAAT CAAGATCACC AAAAAAAATA AATAAAAGGA AAGGTAGATT GCATTTGGAT TTTGAGTGGC   
  
  
+ GTGGTGGTGG GTTTGATGCT GGTGCTGCAT ACACCGCAGC TTTTCTCTGT CCTTTTACCT CTCTTTGTCT   
  
  
+ GCAAGAACCC CATTTGGCAC GCCTGCTCTC TTTTCTATGT TTTCTTTTCT ATCTTTTAAG AAACAATGGA   
  
  
+ AAGCTAACAC ACTAACACTA ACCCATTGGT CCTTTTTTCT TATCTGCAAA ATTAAAGTAA CAATATTTTT   
  
  
+ CTTATCCCCT TTTTCGTTCC ACCCATTTCT TTATCCTTCT GTTTGAACCT AATCTGTATA TATGCATGCA   
  
  
+ TGTGTATTTT ACTTAGAATC TGTTTTGATT CTTTGATGTA ATTAATTAGT AGTCTTTTTA ATTGAATTTT   
  
  
+ TAACAGTAGT TTGAATTAGA CATTTATCGA TATTATATTT TTTATATATA ATTAAAAAAT ACAAAATGAT   
  
  
+ AAGTGTCTTA CTACAATACA CTTCATAAGT GTCGTGTTTT AACTTAAAAA TCATACTATT ATGTTTCTAA   
  
  
+ ATTGAAAAAA AAGTGAGATT AAGTAGCAGA AGCATATTAC AACTTATAAA TTATATAAAA TCATCTTTTT   
  
  
+ ATCCATAATT TATCACATTT CTAAAATACG CATATCATAT CTACTTATGG ATAGGGTTAA TAAGATTAGG   
  
  
+ GCATCATTTA AGATTTTATC AGCGTAACCA ACGAATGTTT AAGGTTAGCA GTTCATAATT AAGTGTAATC   
  
  
+ ATTAAAAAAT TTATGATTTT AAATCATAAA ATATGTTAAT CTAGGATGAG GCAAGCGGCA AGACATATAT   
  
  
+ CACTATCCTT AAAAAATATA ATATATTGAT TTCAAAGAAT TATGAAGTAT ATTAATTTCA TATATAATGA   
  
  
+ CGATTATGTA TTTTTTTACT AAATTACTTG TACCCATAAT AAATAGAATC GATGCATCAT TCATGGCCTT   
  
  
+ TCATTCTGTA ATATGGTTGA CTCCAAAAAT TCTCAAAGTA TTTTTATATT TTGGGAGGGG AGATTTTAGT   
  
  
+ TTGATACTTT TGTTGAACTA GTGAACAATT TCCAACAACC TCCACCACCC CCAAACATCA ACAATCTGAT   
  
  
+ TGATGTATGC ATCCCAAGCT TGAATTGAAT AATAATTTCA ATAACAACAA TAATCGTTTT ACCTTGACAA   
  
  
+ GCTCCACAGC AACTCAACGT CTTCCAAACC AACCCATCAC TCATCATCAT CATCCCCACT ATCCCATTCA   
  
  
+ CCCCCACCAA AAGGAGCCTA AAGACAGTAA CAAGAAGAAT GCTTGCCTTT TTTCATGGAT GAAGAAGACT   
  
  
+ TCTCTTCTTC CTCTACTTCT TCCCACCACT ATGATACACA CCACCTCCAA CATCATTACC CCACCAATTA   
  
  
+ TAATTCCCGT TATACCCTGG ACCCTCCCTC CACCACCACC ACAGCCACAA CCACCACCTC ATCCTCCACT   
  
  
+ CCCACCCACC ATGGCTTCGG TGGTGGGCTT GACTCCTCCT CCCCGTCTGC CGCTGTGGAA TTATCTTTCT   
  
  
+ CCCCGGACAT CCTCCTCTCC TCCCCCACCG GAAAGTGGGC GTCTGACATT CTTATGGAGG CGGCTAGGGC   
  
  
+ TTTTTCAGAC ACGAACACCA CCAAGCTCCA CCAGCTCCTC TGGATGCTGA ACGAGCTCGG CTCCCCCTAC   
  
  
+ GGTGATGTCG AGCAGAAGCT GGTGGCGTAC TTTAACCAAG CCCTTTTTGC CCGCCTCACT GCCACCGGAC   
  
  
+ CCCGCCACCG AGCCGCTATG CTCGCCGCTG CGGACAAGAC CTCCTCCTTC GACTCCACGC TCAGGTTGCT   
  
  
+ CCTCAAGTTC CAGGAAGTCA GCCCGTGGAC CACCTTCGGC CACGTGGCTT GCAACGGTGC AATCATCGAG   
  
  
+ GCCTTAGAGA GGGACCCACA TGAAAAGAGT AAGCTCCACG TCATCGATAT CAGCACCACC TTCTGCACCC   
  
  
+ AGTGGCCCAC CTTGCTTGAG GCTTTAGCCA CCCGCATGGA CGACACTCCC CACCTCAGCC TCACTGCCGT   
  
  
+ CGTCGTCAAC AAGTACGCCG CCGTGAGCGG TGGCGAGGAC GACGGGGGAG AAGGATCAAA GAGGGTGATG   
  
  
+ AGAGAGATTG GTCTTCGTCT TGAGAAGTTC GCTAGGTTAA TGGGGGTTCC CTTCAAGTTC AACGTGGTTT   
  
  
+ ACCACGTAGG TGATCTATCC CAGTTGGATT TTTCTCGTTT GGATATTAAA GATGATGAAG CGGTGGCGAT   
  
  
+ TAACTGTGTC AACTCGCTCC ACTCGGTGGA CCTCCGATAT CGGGAGGTAG TGCTCGCGGC GTTCAGGCGG   
  
  
+ CTGCGGCCGA GGGTGGTGAC GGTGGTGGAG GAAGAGGCGG AGCTGACTGA GGTGGGTGAG GGTCAGTATG   
  
  
+ AATTTTTTAG GCGGTTTGAG GAGTGTCTTA GGTGGTTTAG GGTTTACTTT GAGGCCGTAG GAGATTGTTT   
  
  
+ TCCTAGGACT AGCAATGAGA AATTGATGTT AGAGCGGGCC GCGGGCCGGG CCATGGTGGA TCTCCTTGCA   
  
  
+ TGTCCCGAGC CTGGTGGGTC GGCTGAGGGG AGGGAGACGG CGAGACGGTG GTCGGGTCGG ATGCATGGGG   
  
  
+ CGGGGTTCGA GCACGTGGGG TTCAGTGACG AAGTGTGTGA TGATGTGAGG GCCTTGTTGA GGAGGTACAA   
  
  
+ GGAAGGATGG TCAATGACAC AGTGCACCAT GGATGGCGGT AATCACCCTG GAATACTGTT GTGGTGGCGG   
  
  
+ GATCAACCGG TGGTTTGGGC CAGTGCATGG CGGCCTTG  

- +Up\_Stream \_Len000TTTAGT TTGAGTATTT GTTATTAAAA AATTTTGAAT TTACCATTAA TAATCTAATT   
  
  
- ATTTTTAGGA TTACCAACAA ATTACAATGT AGTTCTGTAC GAAAAACCAC ATATATATTA CCGTCGTACA   
  
  
- CCCAATTCGC CGTTGTTTAA TAAGAGTTTA CGTACGTCAT TAATTAAATT AATTAATTAG TGGACGTATT   
  
  
- AGAGTTATGA TTAGGAAAGT AAGACACGGG GTGGGTTGGG GCTTTTATCA TTAGAAAGTT TGCAACCTTC   
  
  
- ACCCTAATCC ATATCTCATC AGGATGTCTA CTACTAGATG GATGTATATT GTATTAGACT GAAAAGAAAA   
  
  
- AATTTATTGA ACATAACTAC CGTGTCTTAA ACTGTACAAC CGTATACCAT GATTCTTTAA TTAATTGCAA   
  
  
- CACGATTCTA TATCCAGTAA GAAAAACCTC TGTATTCCGG GCGCAAGTGC GTCCCAAGCT CTTCCCGGTG   
  
  
- TAAGTTACCT ACTTAACATC CATCAAATTA GACTGAAACA GTCACTGACT AAGGTGCCGA ACTTGGGCGC   
  
  
- TGTAATTATA TCCAACTGAT TTCAGCAAAC TATGAATAAA AAGAATTCAT TTCAGAACTT AAGTTTAGAA   
  
  
- TATTTATTTA TTTTTTTAGA TATTATTTTC TTAAGATGGT AAATAATTCA GTTGTTGAGT TAAGCTTACT   
  
  
- TTATTTTTTA GTTCTAGTGG TTTTTTTTAT TTATTTTCCT TTCCATCTAA CGTAAACCTA AAACTCACCG   
  
  
- CACCACCACC CAAACTACGA CCACGACGTA TGTGGCGTCG AAAAGAGACA GGAAAATGGA GAGAAACAGA   
  
  
- CGTTCTTGGG GTAAACCGTG CGGACGAGAG AAAAGATACA AAAGAAAAGA TAGAAAATTC TTTGTTACCT   
  
  
- TTCGATTGTG TGATTGTGAT TGGGTAACCA GGAAAAAAGA ATAGACGTTT TAATTTCATT GTTATAAAAA   
  
  
- GAATAGGGGA AAAAGCAAGG TGGGTAAAGA AATAGGAAGA CAAACTTGGA TTAGACATAT ATACGTACGT   
  
  
- ACACATAAAA TGAATCTTAG ACAAAACTAA GAAACTACAT TAATTAATCA TCAGAAAAAT TAACTTAAAA   
  
  
- ATTGTCATCA AACTTAATCT GTAAATAGCT ATAATATAAA AAATATATAT TAATTTTTTA TGTTTTACTA   
  
  
- TTCACAGAAT GATGTTATGT GAAGTATTCA CAGCACAAAA TTGAATTTTT AGTATGATAA TACAAAGATT   
  
  
- TAACTTTTTT TTCACTCTAA TTCATCGTCT TCGTATAATG TTGAATATTT AATATATTTT AGTAGAAAAA   
  
  
- TAGGTATTAA ATAGTGTAAA GATTTTATGC GTATAGTATA GATGAATACC TATCCCAATT ATTCTAATCC   
  
  
- CGTAGTAAAT TCTAAAATAG TCGCATTGGT TGCTTACAAA TTCCAATCGT CAAGTATTAA TTCACATTAG   
  
  
- TAATTTTTTA AATACTAAAA TTTAGTATTT TATACAATTA GATCCTACTC CGTTCGCCGT TCTGTATATA   
  
  
- GTGATAGGAA TTTTTTATAT TATATAACTA AAGTTTCTTA ATACTTCATA TAATTAAAGT ATATATTACT   
  
  
- GCTAATACAT AAAAAAATGA TTTAATGAAC ATGGGTATTA TTTATCTTAG CTACGTAGTA AGTACCGGAA   
  
  
- AGTAAGACAT TATACCAACT GAGGTTTTTA AGAGTTTCAT AAAAATATAA AACCCTCCCC TCTAAAATCA   
  
  
- AACTATGAAA ACAACTTGAT CACTTGTTAA AGGTTGTTGG AGGTGGTGGG GGTTTGTAGT TGTTAGACTA   
  
  
- ACTACATACG TAGGGTTCGA ACTTAACTTA TTATTAAAGT TATTGTTGTT ATTAGCAAAA TGGAACTGTT   
  
  
- CGAGGTGTCG TTGAGTTGCA GAAGGTTTGG TTGGGTAGTG AGTAGTAGTA GTAGGGGTGA TAGGGTAAGT   
  
  
- GGGGGTGGTT TTCCTCGGAT TTCTGTCATT GTTCTTCTTA CGAACGGAAA AAAGTACCTA CTTCTTCTGA   
  
  
- AGAGAAGAAG GAGATGAAGA AGGGTGGTGA TACTATGTGT GGTGGAGGTT GTAGTAATGG GGTGGTTAAT   
  
  
- ATTAAGGGCA ATATGGGACC TGGGAGGGAG GTGGTGGTGG TGTCGGTGTT GGTGGTGGAG TAGGAGGTGA   
  
  
- GGGTGGGTGG TACCGAAGCC ACCACCCGAA CTGAGGAGGA GGGGCAGACG GCGACACCTT AATAGAAAGA   
  
  
- GGGGCCTGTA GGAGGAGAGG AGGGGGTGGC CTTTCACCCG CAGACTGTAA GAATACCTCC GCCGATCCCG   
  
  
- AAAAAGTCTG TGCTTGTGGT GGTTCGAGGT GGTCGAGGAG ACCTACGACT TGCTCGAGCC GAGGGGGATG   
  
  
- CCACTACAGC TCGTCTTCGA CCACCGCATG AAATTGGTTC GGGAAAAACG GGCGGAGTGA CGGTGGCCTG   
  
  
- GGGCGGTGGC TCGGCGATAC GAGCGGCGAC GCCTGTTCTG GAGGAGGAAG CTGAGGTGCG AGTCCAACGA   
  
  
- GGAGTTCAAG GTCCTTCAGT CGGGCACCTG GTGGAAGCCG GTGCACCGAA CGTTGCCACG TTAGTAGCTC   
  
  
- CGGAATCTCT CCCTGGGTGT ACTTTTCTCA TTCGAGGTGC AGTAGCTATA GTCGTGGTGG AAGACGTGGG   
  
  
- TCACCGGGTG GAACGAACTC CGAAATCGGT GGGCGTACCT GCTGTGAGGG GTGGAGTCGG AGTGACGGCA   
  
  
- GCAGCAGTTG TTCATGCGGC GGCACTCGCC ACCGCTCCTG CTGCCCCCTC TTCCTAGTTT CTCCCACTAC   
  
  
- TCTCTCTAAC CAGAAGCAGA ACTCTTCAAG CGATCCAATT ACCCCCAAGG GAAGTTCAAG TTGCACCAAA   
  
  
- TGGTGCATCC ACTAGATAGG GTCAACCTAA AAAGAGCAAA CCTATAATTT CTACTACTTC GCCACCGCTA   
  
  
- ATTGACACAG TTGAGCGAGG TGAGCCACCT GGAGGCTATA GCCCTCCATC ACGAGCGCCG CAAGTCCGCC   
  
  
- GACGCCGGCT CCCACCACTG CCACCACCTC CTTCTCCGCC TCGACTGACT CCACCCACTC CCAGTCATAC   
  
  
- TTAAAAAATC CGCCAAACTC CTCACAGAAT CCACCAAATC CCAAATGAAA CTCCGGCATC CTCTAACAAA   
  
  
- AGGATCCTGA TCGTTACTCT TTAACTACAA TCTCGCCCGG CGCCCGGCCC GGTACCACCT AGAGGAACGT   
  
  
- ACAGGGCTCG GACCACCCAG CCGACTCCCC TCCCTCTGCC GCTCTGCCAC CAGCCCAGCC TACGTACCCC   
  
  
- GCCCCAAGCT CGTGCACCCC AAGTCACTGC TTCACACACT ACTACACTCC CGGAACAACT CCTCCATGTT   
  
  
- CCTTCCTACC AGTTACTGTG TCACGTGGTA CCTACCGCCA TTAGTGGGAC CTTATGACAA CACCACCGCC   
  
  
- CTAGTTGGCC ACCAAACCCG GTCACGTACC GCCGGAAC

+     CAT-box

| Site Name | Organism | Position | Strand | Matrix score. | sequence | function |
| --- | --- | --- | --- | --- | --- | --- |
| CAT-box | Arabidopsis thaliana | 769 | - | 6 | GCCACT | cis-acting regulatory element related to meristem expression |
| CAT-box | Arabidopsis thaliana | 2665 | - | 6 | GCCACT | cis-acting regulatory element related to meristem expression |

>HU05G01983.1   
+ +Up\_Stream \_Len000AAATCA AACTCATAAA CAATAATTTT TTAAAACTTA AATGGTAATT ATTAGATTAA   
  
  
+ TAAAAATCCT AATGGTTGTT TAATGTTACA TCAAGACATG CTTTTTGGTG TATATATAAT GGCAGCATGT   
  
  
+ GGGTTAAGCG GCAACAAATT ATTCTCAAAT GCATGCAGTA ATTAATTTAA TTAATTAATC ACCTGCATAA   
  
  
+ TCTCAATACT AATCCTTTCA TTCTGTGCCC CACCCAACCC CGAAAATAGT AATCTTTCAA ACGTTGGAAG   
  
  
+ TGGGATTAGG TATAGAGTAG TCCTACAGAT GATGATCTAC CTACATATAA CATAATCTGA CTTTTCTTTT   
  
  
+ TTAAATAACT TGTATTGATG GCACAGAATT TGACATGTTG GCATATGGTA CTAAGAAATT AATTAACGTT   
  
  
+ GTGCTAAGAT ATAGGTCATT CTTTTTGGAG ACATAAGGCC CGCGTTCACG CAGGGTTCGA GAAGGGCCAC   
  
  
+ ATTCAATGGA TGAATTGTAG GTAGTTTAAT CTGACTTTGT CAGTGACTGA TTCCACGGCT TGAACCCGCG   
  
  
+ ACATTAATAT AGGTTGACTA AAGTCGTTTG ATACTTATTT TTCTTAAGTA AAGTCTTGAA TTCAAATCTT   
  
  
+ ATAAATAAAT AAAAAAATCT ATAATAAAAG AATTCTACCA TTTATTAAGT CAACAACTCA ATTCGAATGA   
  
  
+ AATAAAAAAT CAAGATCACC AAAAAAAATA AATAAAAGGA AAGGTAGATT GCATTTGGAT TTTGAGTGGC   
  
  
+ GTGGTGGTGG GTTTGATGCT GGTGCTGCAT ACACCGCAGC TTTTCTCTGT CCTTTTACCT CTCTTTGTCT   
  
  
+ GCAAGAACCC CATTTGGCAC GCCTGCTCTC TTTTCTATGT TTTCTTTTCT ATCTTTTAAG AAACAATGGA   
  
  
+ AAGCTAACAC ACTAACACTA ACCCATTGGT CCTTTTTTCT TATCTGCAAA ATTAAAGTAA CAATATTTTT   
  
  
+ CTTATCCCCT TTTTCGTTCC ACCCATTTCT TTATCCTTCT GTTTGAACCT AATCTGTATA TATGCATGCA   
  
  
+ TGTGTATTTT ACTTAGAATC TGTTTTGATT CTTTGATGTA ATTAATTAGT AGTCTTTTTA ATTGAATTTT   
  
  
+ TAACAGTAGT TTGAATTAGA CATTTATCGA TATTATATTT TTTATATATA ATTAAAAAAT ACAAAATGAT   
  
  
+ AAGTGTCTTA CTACAATACA CTTCATAAGT GTCGTGTTTT AACTTAAAAA TCATACTATT ATGTTTCTAA   
  
  
+ ATTGAAAAAA AAGTGAGATT AAGTAGCAGA AGCATATTAC AACTTATAAA TTATATAAAA TCATCTTTTT   
  
  
+ ATCCATAATT TATCACATTT CTAAAATACG CATATCATAT CTACTTATGG ATAGGGTTAA TAAGATTAGG   
  
  
+ GCATCATTTA AGATTTTATC AGCGTAACCA ACGAATGTTT AAGGTTAGCA GTTCATAATT AAGTGTAATC   
  
  
+ ATTAAAAAAT TTATGATTTT AAATCATAAA ATATGTTAAT CTAGGATGAG GCAAGCGGCA AGACATATAT   
  
  
+ CACTATCCTT AAAAAATATA ATATATTGAT TTCAAAGAAT TATGAAGTAT ATTAATTTCA TATATAATGA   
  
  
+ CGATTATGTA TTTTTTTACT AAATTACTTG TACCCATAAT AAATAGAATC GATGCATCAT TCATGGCCTT   
  
  
+ TCATTCTGTA ATATGGTTGA CTCCAAAAAT TCTCAAAGTA TTTTTATATT TTGGGAGGGG AGATTTTAGT   
  
  
+ TTGATACTTT TGTTGAACTA GTGAACAATT TCCAACAACC TCCACCACCC CCAAACATCA ACAATCTGAT   
  
  
+ TGATGTATGC ATCCCAAGCT TGAATTGAAT AATAATTTCA ATAACAACAA TAATCGTTTT ACCTTGACAA   
  
  
+ GCTCCACAGC AACTCAACGT CTTCCAAACC AACCCATCAC TCATCATCAT CATCCCCACT ATCCCATTCA   
  
  
+ CCCCCACCAA AAGGAGCCTA AAGACAGTAA CAAGAAGAAT GCTTGCCTTT TTTCATGGAT GAAGAAGACT   
  
  
+ TCTCTTCTTC CTCTACTTCT TCCCACCACT ATGATACACA CCACCTCCAA CATCATTACC CCACCAATTA   
  
  
+ TAATTCCCGT TATACCCTGG ACCCTCCCTC CACCACCACC ACAGCCACAA CCACCACCTC ATCCTCCACT   
  
  
+ CCCACCCACC ATGGCTTCGG TGGTGGGCTT GACTCCTCCT CCCCGTCTGC CGCTGTGGAA TTATCTTTCT   
  
  
+ CCCCGGACAT CCTCCTCTCC TCCCCCACCG GAAAGTGGGC GTCTGACATT CTTATGGAGG CGGCTAGGGC   
  
  
+ TTTTTCAGAC ACGAACACCA CCAAGCTCCA CCAGCTCCTC TGGATGCTGA ACGAGCTCGG CTCCCCCTAC   
  
  
+ GGTGATGTCG AGCAGAAGCT GGTGGCGTAC TTTAACCAAG CCCTTTTTGC CCGCCTCACT GCCACCGGAC   
  
  
+ CCCGCCACCG AGCCGCTATG CTCGCCGCTG CGGACAAGAC CTCCTCCTTC GACTCCACGC TCAGGTTGCT   
  
  
+ CCTCAAGTTC CAGGAAGTCA GCCCGTGGAC CACCTTCGGC CACGTGGCTT GCAACGGTGC AATCATCGAG   
  
  
+ GCCTTAGAGA GGGACCCACA TGAAAAGAGT AAGCTCCACG TCATCGATAT CAGCACCACC TTCTGCACCC   
  
  
+ AGTGGCCCAC CTTGCTTGAG GCTTTAGCCA CCCGCATGGA CGACACTCCC CACCTCAGCC TCACTGCCGT   
  
  
+ CGTCGTCAAC AAGTACGCCG CCGTGAGCGG TGGCGAGGAC GACGGGGGAG AAGGATCAAA GAGGGTGATG   
  
  
+ AGAGAGATTG GTCTTCGTCT TGAGAAGTTC GCTAGGTTAA TGGGGGTTCC CTTCAAGTTC AACGTGGTTT   
  
  
+ ACCACGTAGG TGATCTATCC CAGTTGGATT TTTCTCGTTT GGATATTAAA GATGATGAAG CGGTGGCGAT   
  
  
+ TAACTGTGTC AACTCGCTCC ACTCGGTGGA CCTCCGATAT CGGGAGGTAG TGCTCGCGGC GTTCAGGCGG   
  
  
+ CTGCGGCCGA GGGTGGTGAC GGTGGTGGAG GAAGAGGCGG AGCTGACTGA GGTGGGTGAG GGTCAGTATG   
  
  
+ AATTTTTTAG GCGGTTTGAG GAGTGTCTTA GGTGGTTTAG GGTTTACTTT GAGGCCGTAG GAGATTGTTT   
  
  
+ TCCTAGGACT AGCAATGAGA AATTGATGTT AGAGCGGGCC GCGGGCCGGG CCATGGTGGA TCTCCTTGCA   
  
  
+ TGTCCCGAGC CTGGTGGGTC GGCTGAGGGG AGGGAGACGG CGAGACGGTG GTCGGGTCGG ATGCATGGGG   
  
  
+ CGGGGTTCGA GCACGTGGGG TTCAGTGACG AAGTGTGTGA TGATGTGAGG GCCTTGTTGA GGAGGTACAA   
  
  
+ GGAAGGATGG TCAATGACAC AGTGCACCAT GGATGGCGGT AATCACCCTG GAATACTGTT GTGGTGGCGG   
  
  
+ GATCAACCGG TGGTTTGGGC CAGTGCATGG CGGCCTTG  

- +Up\_Stream \_Len000TTTAGT TTGAGTATTT GTTATTAAAA AATTTTGAAT TTACCATTAA TAATCTAATT   
  
  
- ATTTTTAGGA TTACCAACAA ATTACAATGT AGTTCTGTAC GAAAAACCAC ATATATATTA CCGTCGTACA   
  
  
- CCCAATTCGC CGTTGTTTAA TAAGAGTTTA CGTACGTCAT TAATTAAATT AATTAATTAG TGGACGTATT   
  
  
- AGAGTTATGA TTAGGAAAGT AAGACACGGG GTGGGTTGGG GCTTTTATCA TTAGAAAGTT TGCAACCTTC   
  
  
- ACCCTAATCC ATATCTCATC AGGATGTCTA CTACTAGATG GATGTATATT GTATTAGACT GAAAAGAAAA   
  
  
- AATTTATTGA ACATAACTAC CGTGTCTTAA ACTGTACAAC CGTATACCAT GATTCTTTAA TTAATTGCAA   
  
  
- CACGATTCTA TATCCAGTAA GAAAAACCTC TGTATTCCGG GCGCAAGTGC GTCCCAAGCT CTTCCCGGTG   
  
  
- TAAGTTACCT ACTTAACATC CATCAAATTA GACTGAAACA GTCACTGACT AAGGTGCCGA ACTTGGGCGC   
  
  
- TGTAATTATA TCCAACTGAT TTCAGCAAAC TATGAATAAA AAGAATTCAT TTCAGAACTT AAGTTTAGAA   
  
  
- TATTTATTTA TTTTTTTAGA TATTATTTTC TTAAGATGGT AAATAATTCA GTTGTTGAGT TAAGCTTACT   
  
  
- TTATTTTTTA GTTCTAGTGG TTTTTTTTAT TTATTTTCCT TTCCATCTAA CGTAAACCTA AAACTCACCG   
  
  
- CACCACCACC CAAACTACGA CCACGACGTA TGTGGCGTCG AAAAGAGACA GGAAAATGGA GAGAAACAGA   
  
  
- CGTTCTTGGG GTAAACCGTG CGGACGAGAG AAAAGATACA AAAGAAAAGA TAGAAAATTC TTTGTTACCT   
  
  
- TTCGATTGTG TGATTGTGAT TGGGTAACCA GGAAAAAAGA ATAGACGTTT TAATTTCATT GTTATAAAAA   
  
  
- GAATAGGGGA AAAAGCAAGG TGGGTAAAGA AATAGGAAGA CAAACTTGGA TTAGACATAT ATACGTACGT   
  
  
- ACACATAAAA TGAATCTTAG ACAAAACTAA GAAACTACAT TAATTAATCA TCAGAAAAAT TAACTTAAAA   
  
  
- ATTGTCATCA AACTTAATCT GTAAATAGCT ATAATATAAA AAATATATAT TAATTTTTTA TGTTTTACTA   
  
  
- TTCACAGAAT GATGTTATGT GAAGTATTCA CAGCACAAAA TTGAATTTTT AGTATGATAA TACAAAGATT   
  
  
- TAACTTTTTT TTCACTCTAA TTCATCGTCT TCGTATAATG TTGAATATTT AATATATTTT AGTAGAAAAA   
  
  
- TAGGTATTAA ATAGTGTAAA GATTTTATGC GTATAGTATA GATGAATACC TATCCCAATT ATTCTAATCC   
  
  
- CGTAGTAAAT TCTAAAATAG TCGCATTGGT TGCTTACAAA TTCCAATCGT CAAGTATTAA TTCACATTAG   
  
  
- TAATTTTTTA AATACTAAAA TTTAGTATTT TATACAATTA GATCCTACTC CGTTCGCCGT TCTGTATATA   
  
  
- GTGATAGGAA TTTTTTATAT TATATAACTA AAGTTTCTTA ATACTTCATA TAATTAAAGT ATATATTACT   
  
  
- GCTAATACAT AAAAAAATGA TTTAATGAAC ATGGGTATTA TTTATCTTAG CTACGTAGTA AGTACCGGAA   
  
  
- AGTAAGACAT TATACCAACT GAGGTTTTTA AGAGTTTCAT AAAAATATAA AACCCTCCCC TCTAAAATCA   
  
  
- AACTATGAAA ACAACTTGAT CACTTGTTAA AGGTTGTTGG AGGTGGTGGG GGTTTGTAGT TGTTAGACTA   
  
  
- ACTACATACG TAGGGTTCGA ACTTAACTTA TTATTAAAGT TATTGTTGTT ATTAGCAAAA TGGAACTGTT   
  
  
- CGAGGTGTCG TTGAGTTGCA GAAGGTTTGG TTGGGTAGTG AGTAGTAGTA GTAGGGGTGA TAGGGTAAGT   
  
  
- GGGGGTGGTT TTCCTCGGAT TTCTGTCATT GTTCTTCTTA CGAACGGAAA AAAGTACCTA CTTCTTCTGA   
  
  
- AGAGAAGAAG GAGATGAAGA AGGGTGGTGA TACTATGTGT GGTGGAGGTT GTAGTAATGG GGTGGTTAAT   
  
  
- ATTAAGGGCA ATATGGGACC TGGGAGGGAG GTGGTGGTGG TGTCGGTGTT GGTGGTGGAG TAGGAGGTGA   
  
  
- GGGTGGGTGG TACCGAAGCC ACCACCCGAA CTGAGGAGGA GGGGCAGACG GCGACACCTT AATAGAAAGA   
  
  
- GGGGCCTGTA GGAGGAGAGG AGGGGGTGGC CTTTCACCCG CAGACTGTAA GAATACCTCC GCCGATCCCG   
  
  
- AAAAAGTCTG TGCTTGTGGT GGTTCGAGGT GGTCGAGGAG ACCTACGACT TGCTCGAGCC GAGGGGGATG   
  
  
- CCACTACAGC TCGTCTTCGA CCACCGCATG AAATTGGTTC GGGAAAAACG GGCGGAGTGA CGGTGGCCTG   
  
  
- GGGCGGTGGC TCGGCGATAC GAGCGGCGAC GCCTGTTCTG GAGGAGGAAG CTGAGGTGCG AGTCCAACGA   
  
  
- GGAGTTCAAG GTCCTTCAGT CGGGCACCTG GTGGAAGCCG GTGCACCGAA CGTTGCCACG TTAGTAGCTC   
  
  
- CGGAATCTCT CCCTGGGTGT ACTTTTCTCA TTCGAGGTGC AGTAGCTATA GTCGTGGTGG AAGACGTGGG   
  
  
- TCACCGGGTG GAACGAACTC CGAAATCGGT GGGCGTACCT GCTGTGAGGG GTGGAGTCGG AGTGACGGCA   
  
  
- GCAGCAGTTG TTCATGCGGC GGCACTCGCC ACCGCTCCTG CTGCCCCCTC TTCCTAGTTT CTCCCACTAC   
  
  
- TCTCTCTAAC CAGAAGCAGA ACTCTTCAAG CGATCCAATT ACCCCCAAGG GAAGTTCAAG TTGCACCAAA   
  
  
- TGGTGCATCC ACTAGATAGG GTCAACCTAA AAAGAGCAAA CCTATAATTT CTACTACTTC GCCACCGCTA   
  
  
- ATTGACACAG TTGAGCGAGG TGAGCCACCT GGAGGCTATA GCCCTCCATC ACGAGCGCCG CAAGTCCGCC   
  
  
- GACGCCGGCT CCCACCACTG CCACCACCTC CTTCTCCGCC TCGACTGACT CCACCCACTC CCAGTCATAC   
  
  
- TTAAAAAATC CGCCAAACTC CTCACAGAAT CCACCAAATC CCAAATGAAA CTCCGGCATC CTCTAACAAA   
  
  
- AGGATCCTGA TCGTTACTCT TTAACTACAA TCTCGCCCGG CGCCCGGCCC GGTACCACCT AGAGGAACGT   
  
  
- ACAGGGCTCG GACCACCCAG CCGACTCCCC TCCCTCTGCC GCTCTGCCAC CAGCCCAGCC TACGTACCCC   
  
  
- GCCCCAAGCT CGTGCACCCC AAGTCACTGC TTCACACACT ACTACACTCC CGGAACAACT CCTCCATGTT   
  
  
- CCTTCCTACC AGTTACTGTG TCACGTGGTA CCTACCGCCA TTAGTGGGAC CTTATGACAA CACCACCGCC   
  
  
- CTAGTTGGCC ACCAAACCCG GTCACGTACC GCCGGAAC

+     CCAAT-box

| Site Name | Organism | Position | Strand | Matrix score. | sequence | function |
| --- | --- | --- | --- | --- | --- | --- |
| CCAAT-box | Hordeum vulgare | 2576 | + | 6 | CAACGG | MYBHv1 binding site |

>HU05G01983.1   
+ +Up\_Stream \_Len000AAATCA AACTCATAAA CAATAATTTT TTAAAACTTA AATGGTAATT ATTAGATTAA   
  
  
+ TAAAAATCCT AATGGTTGTT TAATGTTACA TCAAGACATG CTTTTTGGTG TATATATAAT GGCAGCATGT   
  
  
+ GGGTTAAGCG GCAACAAATT ATTCTCAAAT GCATGCAGTA ATTAATTTAA TTAATTAATC ACCTGCATAA   
  
  
+ TCTCAATACT AATCCTTTCA TTCTGTGCCC CACCCAACCC CGAAAATAGT AATCTTTCAA ACGTTGGAAG   
  
  
+ TGGGATTAGG TATAGAGTAG TCCTACAGAT GATGATCTAC CTACATATAA CATAATCTGA CTTTTCTTTT   
  
  
+ TTAAATAACT TGTATTGATG GCACAGAATT TGACATGTTG GCATATGGTA CTAAGAAATT AATTAACGTT   
  
  
+ GTGCTAAGAT ATAGGTCATT CTTTTTGGAG ACATAAGGCC CGCGTTCACG CAGGGTTCGA GAAGGGCCAC   
  
  
+ ATTCAATGGA TGAATTGTAG GTAGTTTAAT CTGACTTTGT CAGTGACTGA TTCCACGGCT TGAACCCGCG   
  
  
+ ACATTAATAT AGGTTGACTA AAGTCGTTTG ATACTTATTT TTCTTAAGTA AAGTCTTGAA TTCAAATCTT   
  
  
+ ATAAATAAAT AAAAAAATCT ATAATAAAAG AATTCTACCA TTTATTAAGT CAACAACTCA ATTCGAATGA   
  
  
+ AATAAAAAAT CAAGATCACC AAAAAAAATA AATAAAAGGA AAGGTAGATT GCATTTGGAT TTTGAGTGGC   
  
  
+ GTGGTGGTGG GTTTGATGCT GGTGCTGCAT ACACCGCAGC TTTTCTCTGT CCTTTTACCT CTCTTTGTCT   
  
  
+ GCAAGAACCC CATTTGGCAC GCCTGCTCTC TTTTCTATGT TTTCTTTTCT ATCTTTTAAG AAACAATGGA   
  
  
+ AAGCTAACAC ACTAACACTA ACCCATTGGT CCTTTTTTCT TATCTGCAAA ATTAAAGTAA CAATATTTTT   
  
  
+ CTTATCCCCT TTTTCGTTCC ACCCATTTCT TTATCCTTCT GTTTGAACCT AATCTGTATA TATGCATGCA   
  
  
+ TGTGTATTTT ACTTAGAATC TGTTTTGATT CTTTGATGTA ATTAATTAGT AGTCTTTTTA ATTGAATTTT   
  
  
+ TAACAGTAGT TTGAATTAGA CATTTATCGA TATTATATTT TTTATATATA ATTAAAAAAT ACAAAATGAT   
  
  
+ AAGTGTCTTA CTACAATACA CTTCATAAGT GTCGTGTTTT AACTTAAAAA TCATACTATT ATGTTTCTAA   
  
  
+ ATTGAAAAAA AAGTGAGATT AAGTAGCAGA AGCATATTAC AACTTATAAA TTATATAAAA TCATCTTTTT   
  
  
+ ATCCATAATT TATCACATTT CTAAAATACG CATATCATAT CTACTTATGG ATAGGGTTAA TAAGATTAGG   
  
  
+ GCATCATTTA AGATTTTATC AGCGTAACCA ACGAATGTTT AAGGTTAGCA GTTCATAATT AAGTGTAATC   
  
  
+ ATTAAAAAAT TTATGATTTT AAATCATAAA ATATGTTAAT CTAGGATGAG GCAAGCGGCA AGACATATAT   
  
  
+ CACTATCCTT AAAAAATATA ATATATTGAT TTCAAAGAAT TATGAAGTAT ATTAATTTCA TATATAATGA   
  
  
+ CGATTATGTA TTTTTTTACT AAATTACTTG TACCCATAAT AAATAGAATC GATGCATCAT TCATGGCCTT   
  
  
+ TCATTCTGTA ATATGGTTGA CTCCAAAAAT TCTCAAAGTA TTTTTATATT TTGGGAGGGG AGATTTTAGT   
  
  
+ TTGATACTTT TGTTGAACTA GTGAACAATT TCCAACAACC TCCACCACCC CCAAACATCA ACAATCTGAT   
  
  
+ TGATGTATGC ATCCCAAGCT TGAATTGAAT AATAATTTCA ATAACAACAA TAATCGTTTT ACCTTGACAA   
  
  
+ GCTCCACAGC AACTCAACGT CTTCCAAACC AACCCATCAC TCATCATCAT CATCCCCACT ATCCCATTCA   
  
  
+ CCCCCACCAA AAGGAGCCTA AAGACAGTAA CAAGAAGAAT GCTTGCCTTT TTTCATGGAT GAAGAAGACT   
  
  
+ TCTCTTCTTC CTCTACTTCT TCCCACCACT ATGATACACA CCACCTCCAA CATCATTACC CCACCAATTA   
  
  
+ TAATTCCCGT TATACCCTGG ACCCTCCCTC CACCACCACC ACAGCCACAA CCACCACCTC ATCCTCCACT   
  
  
+ CCCACCCACC ATGGCTTCGG TGGTGGGCTT GACTCCTCCT CCCCGTCTGC CGCTGTGGAA TTATCTTTCT   
  
  
+ CCCCGGACAT CCTCCTCTCC TCCCCCACCG GAAAGTGGGC GTCTGACATT CTTATGGAGG CGGCTAGGGC   
  
  
+ TTTTTCAGAC ACGAACACCA CCAAGCTCCA CCAGCTCCTC TGGATGCTGA ACGAGCTCGG CTCCCCCTAC   
  
  
+ GGTGATGTCG AGCAGAAGCT GGTGGCGTAC TTTAACCAAG CCCTTTTTGC CCGCCTCACT GCCACCGGAC   
  
  
+ CCCGCCACCG AGCCGCTATG CTCGCCGCTG CGGACAAGAC CTCCTCCTTC GACTCCACGC TCAGGTTGCT   
  
  
+ CCTCAAGTTC CAGGAAGTCA GCCCGTGGAC CACCTTCGGC CACGTGGCTT GCAACGGTGC AATCATCGAG   
  
  
+ GCCTTAGAGA GGGACCCACA TGAAAAGAGT AAGCTCCACG TCATCGATAT CAGCACCACC TTCTGCACCC   
  
  
+ AGTGGCCCAC CTTGCTTGAG GCTTTAGCCA CCCGCATGGA CGACACTCCC CACCTCAGCC TCACTGCCGT   
  
  
+ CGTCGTCAAC AAGTACGCCG CCGTGAGCGG TGGCGAGGAC GACGGGGGAG AAGGATCAAA GAGGGTGATG   
  
  
+ AGAGAGATTG GTCTTCGTCT TGAGAAGTTC GCTAGGTTAA TGGGGGTTCC CTTCAAGTTC AACGTGGTTT   
  
  
+ ACCACGTAGG TGATCTATCC CAGTTGGATT TTTCTCGTTT GGATATTAAA GATGATGAAG CGGTGGCGAT   
  
  
+ TAACTGTGTC AACTCGCTCC ACTCGGTGGA CCTCCGATAT CGGGAGGTAG TGCTCGCGGC GTTCAGGCGG   
  
  
+ CTGCGGCCGA GGGTGGTGAC GGTGGTGGAG GAAGAGGCGG AGCTGACTGA GGTGGGTGAG GGTCAGTATG   
  
  
+ AATTTTTTAG GCGGTTTGAG GAGTGTCTTA GGTGGTTTAG GGTTTACTTT GAGGCCGTAG GAGATTGTTT   
  
  
+ TCCTAGGACT AGCAATGAGA AATTGATGTT AGAGCGGGCC GCGGGCCGGG CCATGGTGGA TCTCCTTGCA   
  
  
+ TGTCCCGAGC CTGGTGGGTC GGCTGAGGGG AGGGAGACGG CGAGACGGTG GTCGGGTCGG ATGCATGGGG   
  
  
+ CGGGGTTCGA GCACGTGGGG TTCAGTGACG AAGTGTGTGA TGATGTGAGG GCCTTGTTGA GGAGGTACAA   
  
  
+ GGAAGGATGG TCAATGACAC AGTGCACCAT GGATGGCGGT AATCACCCTG GAATACTGTT GTGGTGGCGG   
  
  
+ GATCAACCGG TGGTTTGGGC CAGTGCATGG CGGCCTTG  

- +Up\_Stream \_Len000TTTAGT TTGAGTATTT GTTATTAAAA AATTTTGAAT TTACCATTAA TAATCTAATT   
  
  
- ATTTTTAGGA TTACCAACAA ATTACAATGT AGTTCTGTAC GAAAAACCAC ATATATATTA CCGTCGTACA   
  
  
- CCCAATTCGC CGTTGTTTAA TAAGAGTTTA CGTACGTCAT TAATTAAATT AATTAATTAG TGGACGTATT   
  
  
- AGAGTTATGA TTAGGAAAGT AAGACACGGG GTGGGTTGGG GCTTTTATCA TTAGAAAGTT TGCAACCTTC   
  
  
- ACCCTAATCC ATATCTCATC AGGATGTCTA CTACTAGATG GATGTATATT GTATTAGACT GAAAAGAAAA   
  
  
- AATTTATTGA ACATAACTAC CGTGTCTTAA ACTGTACAAC CGTATACCAT GATTCTTTAA TTAATTGCAA   
  
  
- CACGATTCTA TATCCAGTAA GAAAAACCTC TGTATTCCGG GCGCAAGTGC GTCCCAAGCT CTTCCCGGTG   
  
  
- TAAGTTACCT ACTTAACATC CATCAAATTA GACTGAAACA GTCACTGACT AAGGTGCCGA ACTTGGGCGC   
  
  
- TGTAATTATA TCCAACTGAT TTCAGCAAAC TATGAATAAA AAGAATTCAT TTCAGAACTT AAGTTTAGAA   
  
  
- TATTTATTTA TTTTTTTAGA TATTATTTTC TTAAGATGGT AAATAATTCA GTTGTTGAGT TAAGCTTACT   
  
  
- TTATTTTTTA GTTCTAGTGG TTTTTTTTAT TTATTTTCCT TTCCATCTAA CGTAAACCTA AAACTCACCG   
  
  
- CACCACCACC CAAACTACGA CCACGACGTA TGTGGCGTCG AAAAGAGACA GGAAAATGGA GAGAAACAGA   
  
  
- CGTTCTTGGG GTAAACCGTG CGGACGAGAG AAAAGATACA AAAGAAAAGA TAGAAAATTC TTTGTTACCT   
  
  
- TTCGATTGTG TGATTGTGAT TGGGTAACCA GGAAAAAAGA ATAGACGTTT TAATTTCATT GTTATAAAAA   
  
  
- GAATAGGGGA AAAAGCAAGG TGGGTAAAGA AATAGGAAGA CAAACTTGGA TTAGACATAT ATACGTACGT   
  
  
- ACACATAAAA TGAATCTTAG ACAAAACTAA GAAACTACAT TAATTAATCA TCAGAAAAAT TAACTTAAAA   
  
  
- ATTGTCATCA AACTTAATCT GTAAATAGCT ATAATATAAA AAATATATAT TAATTTTTTA TGTTTTACTA   
  
  
- TTCACAGAAT GATGTTATGT GAAGTATTCA CAGCACAAAA TTGAATTTTT AGTATGATAA TACAAAGATT   
  
  
- TAACTTTTTT TTCACTCTAA TTCATCGTCT TCGTATAATG TTGAATATTT AATATATTTT AGTAGAAAAA   
  
  
- TAGGTATTAA ATAGTGTAAA GATTTTATGC GTATAGTATA GATGAATACC TATCCCAATT ATTCTAATCC   
  
  
- CGTAGTAAAT TCTAAAATAG TCGCATTGGT TGCTTACAAA TTCCAATCGT CAAGTATTAA TTCACATTAG   
  
  
- TAATTTTTTA AATACTAAAA TTTAGTATTT TATACAATTA GATCCTACTC CGTTCGCCGT TCTGTATATA   
  
  
- GTGATAGGAA TTTTTTATAT TATATAACTA AAGTTTCTTA ATACTTCATA TAATTAAAGT ATATATTACT   
  
  
- GCTAATACAT AAAAAAATGA TTTAATGAAC ATGGGTATTA TTTATCTTAG CTACGTAGTA AGTACCGGAA   
  
  
- AGTAAGACAT TATACCAACT GAGGTTTTTA AGAGTTTCAT AAAAATATAA AACCCTCCCC TCTAAAATCA   
  
  
- AACTATGAAA ACAACTTGAT CACTTGTTAA AGGTTGTTGG AGGTGGTGGG GGTTTGTAGT TGTTAGACTA   
  
  
- ACTACATACG TAGGGTTCGA ACTTAACTTA TTATTAAAGT TATTGTTGTT ATTAGCAAAA TGGAACTGTT   
  
  
- CGAGGTGTCG TTGAGTTGCA GAAGGTTTGG TTGGGTAGTG AGTAGTAGTA GTAGGGGTGA TAGGGTAAGT   
  
  
- GGGGGTGGTT TTCCTCGGAT TTCTGTCATT GTTCTTCTTA CGAACGGAAA AAAGTACCTA CTTCTTCTGA   
  
  
- AGAGAAGAAG GAGATGAAGA AGGGTGGTGA TACTATGTGT GGTGGAGGTT GTAGTAATGG GGTGGTTAAT   
  
  
- ATTAAGGGCA ATATGGGACC TGGGAGGGAG GTGGTGGTGG TGTCGGTGTT GGTGGTGGAG TAGGAGGTGA   
  
  
- GGGTGGGTGG TACCGAAGCC ACCACCCGAA CTGAGGAGGA GGGGCAGACG GCGACACCTT AATAGAAAGA   
  
  
- GGGGCCTGTA GGAGGAGAGG AGGGGGTGGC CTTTCACCCG CAGACTGTAA GAATACCTCC GCCGATCCCG   
  
  
- AAAAAGTCTG TGCTTGTGGT GGTTCGAGGT GGTCGAGGAG ACCTACGACT TGCTCGAGCC GAGGGGGATG   
  
  
- CCACTACAGC TCGTCTTCGA CCACCGCATG AAATTGGTTC GGGAAAAACG GGCGGAGTGA CGGTGGCCTG   
  
  
- GGGCGGTGGC TCGGCGATAC GAGCGGCGAC GCCTGTTCTG GAGGAGGAAG CTGAGGTGCG AGTCCAACGA   
  
  
- GGAGTTCAAG GTCCTTCAGT CGGGCACCTG GTGGAAGCCG GTGCACCGAA CGTTGCCACG TTAGTAGCTC   
  
  
- CGGAATCTCT CCCTGGGTGT ACTTTTCTCA TTCGAGGTGC AGTAGCTATA GTCGTGGTGG AAGACGTGGG   
  
  
- TCACCGGGTG GAACGAACTC CGAAATCGGT GGGCGTACCT GCTGTGAGGG GTGGAGTCGG AGTGACGGCA   
  
  
- GCAGCAGTTG TTCATGCGGC GGCACTCGCC ACCGCTCCTG CTGCCCCCTC TTCCTAGTTT CTCCCACTAC   
  
  
- TCTCTCTAAC CAGAAGCAGA ACTCTTCAAG CGATCCAATT ACCCCCAAGG GAAGTTCAAG TTGCACCAAA   
  
  
- TGGTGCATCC ACTAGATAGG GTCAACCTAA AAAGAGCAAA CCTATAATTT CTACTACTTC GCCACCGCTA   
  
  
- ATTGACACAG TTGAGCGAGG TGAGCCACCT GGAGGCTATA GCCCTCCATC ACGAGCGCCG CAAGTCCGCC   
  
  
- GACGCCGGCT CCCACCACTG CCACCACCTC CTTCTCCGCC TCGACTGACT CCACCCACTC CCAGTCATAC   
  
  
- TTAAAAAATC CGCCAAACTC CTCACAGAAT CCACCAAATC CCAAATGAAA CTCCGGCATC CTCTAACAAA   
  
  
- AGGATCCTGA TCGTTACTCT TTAACTACAA TCTCGCCCGG CGCCCGGCCC GGTACCACCT AGAGGAACGT   
  
  
- ACAGGGCTCG GACCACCCAG CCGACTCCCC TCCCTCTGCC GCTCTGCCAC CAGCCCAGCC TACGTACCCC   
  
  
- GCCCCAAGCT CGTGCACCCC AAGTCACTGC TTCACACACT ACTACACTCC CGGAACAACT CCTCCATGTT   
  
  
- CCTTCCTACC AGTTACTGTG TCACGTGGTA CCTACCGCCA TTAGTGGGAC CTTATGACAA CACCACCGCC   
  
  
- CTAGTTGGCC ACCAAACCCG GTCACGTACC GCCGGAAC

+     CGTCA-motif

| Site Name | Organism | Position | Strand | Matrix score. | sequence | function |
| --- | --- | --- | --- | --- | --- | --- |
| CGTCA-motif | Hordeum vulgare | 3031 | - | 5 | CGTCA | cis-acting regulatory element involved in the MeJA-responsiveness |
| CGTCA-motif | Hordeum vulgare | 2633 | + | 5 | CGTCA | cis-acting regulatory element involved in the MeJA-responsiveness |
| CGTCA-motif | Hordeum vulgare | 3320 | - | 5 | CGTCA | cis-acting regulatory element involved in the MeJA-responsiveness |
| CGTCA-motif | Hordeum vulgare | 1612 | - | 5 | CGTCA | cis-acting regulatory element involved in the MeJA-responsiveness |
| CGTCA-motif | Hordeum vulgare | 2738 | + | 5 | CGTCA | cis-acting regulatory element involved in the MeJA-responsiveness |

>HU05G01983.1   
+ +Up\_Stream \_Len000AAATCA AACTCATAAA CAATAATTTT TTAAAACTTA AATGGTAATT ATTAGATTAA   
  
  
+ TAAAAATCCT AATGGTTGTT TAATGTTACA TCAAGACATG CTTTTTGGTG TATATATAAT GGCAGCATGT   
  
  
+ GGGTTAAGCG GCAACAAATT ATTCTCAAAT GCATGCAGTA ATTAATTTAA TTAATTAATC ACCTGCATAA   
  
  
+ TCTCAATACT AATCCTTTCA TTCTGTGCCC CACCCAACCC CGAAAATAGT AATCTTTCAA ACGTTGGAAG   
  
  
+ TGGGATTAGG TATAGAGTAG TCCTACAGAT GATGATCTAC CTACATATAA CATAATCTGA CTTTTCTTTT   
  
  
+ TTAAATAACT TGTATTGATG GCACAGAATT TGACATGTTG GCATATGGTA CTAAGAAATT AATTAACGTT   
  
  
+ GTGCTAAGAT ATAGGTCATT CTTTTTGGAG ACATAAGGCC CGCGTTCACG CAGGGTTCGA GAAGGGCCAC   
  
  
+ ATTCAATGGA TGAATTGTAG GTAGTTTAAT CTGACTTTGT CAGTGACTGA TTCCACGGCT TGAACCCGCG   
  
  
+ ACATTAATAT AGGTTGACTA AAGTCGTTTG ATACTTATTT TTCTTAAGTA AAGTCTTGAA TTCAAATCTT   
  
  
+ ATAAATAAAT AAAAAAATCT ATAATAAAAG AATTCTACCA TTTATTAAGT CAACAACTCA ATTCGAATGA   
  
  
+ AATAAAAAAT CAAGATCACC AAAAAAAATA AATAAAAGGA AAGGTAGATT GCATTTGGAT TTTGAGTGGC   
  
  
+ GTGGTGGTGG GTTTGATGCT GGTGCTGCAT ACACCGCAGC TTTTCTCTGT CCTTTTACCT CTCTTTGTCT   
  
  
+ GCAAGAACCC CATTTGGCAC GCCTGCTCTC TTTTCTATGT TTTCTTTTCT ATCTTTTAAG AAACAATGGA   
  
  
+ AAGCTAACAC ACTAACACTA ACCCATTGGT CCTTTTTTCT TATCTGCAAA ATTAAAGTAA CAATATTTTT   
  
  
+ CTTATCCCCT TTTTCGTTCC ACCCATTTCT TTATCCTTCT GTTTGAACCT AATCTGTATA TATGCATGCA   
  
  
+ TGTGTATTTT ACTTAGAATC TGTTTTGATT CTTTGATGTA ATTAATTAGT AGTCTTTTTA ATTGAATTTT   
  
  
+ TAACAGTAGT TTGAATTAGA CATTTATCGA TATTATATTT TTTATATATA ATTAAAAAAT ACAAAATGAT   
  
  
+ AAGTGTCTTA CTACAATACA CTTCATAAGT GTCGTGTTTT AACTTAAAAA TCATACTATT ATGTTTCTAA   
  
  
+ ATTGAAAAAA AAGTGAGATT AAGTAGCAGA AGCATATTAC AACTTATAAA TTATATAAAA TCATCTTTTT   
  
  
+ ATCCATAATT TATCACATTT CTAAAATACG CATATCATAT CTACTTATGG ATAGGGTTAA TAAGATTAGG   
  
  
+ GCATCATTTA AGATTTTATC AGCGTAACCA ACGAATGTTT AAGGTTAGCA GTTCATAATT AAGTGTAATC   
  
  
+ ATTAAAAAAT TTATGATTTT AAATCATAAA ATATGTTAAT CTAGGATGAG GCAAGCGGCA AGACATATAT   
  
  
+ CACTATCCTT AAAAAATATA ATATATTGAT TTCAAAGAAT TATGAAGTAT ATTAATTTCA TATATAATGA   
  
  
+ CGATTATGTA TTTTTTTACT AAATTACTTG TACCCATAAT AAATAGAATC GATGCATCAT TCATGGCCTT   
  
  
+ TCATTCTGTA ATATGGTTGA CTCCAAAAAT TCTCAAAGTA TTTTTATATT TTGGGAGGGG AGATTTTAGT   
  
  
+ TTGATACTTT TGTTGAACTA GTGAACAATT TCCAACAACC TCCACCACCC CCAAACATCA ACAATCTGAT   
  
  
+ TGATGTATGC ATCCCAAGCT TGAATTGAAT AATAATTTCA ATAACAACAA TAATCGTTTT ACCTTGACAA   
  
  
+ GCTCCACAGC AACTCAACGT CTTCCAAACC AACCCATCAC TCATCATCAT CATCCCCACT ATCCCATTCA   
  
  
+ CCCCCACCAA AAGGAGCCTA AAGACAGTAA CAAGAAGAAT GCTTGCCTTT TTTCATGGAT GAAGAAGACT   
  
  
+ TCTCTTCTTC CTCTACTTCT TCCCACCACT ATGATACACA CCACCTCCAA CATCATTACC CCACCAATTA   
  
  
+ TAATTCCCGT TATACCCTGG ACCCTCCCTC CACCACCACC ACAGCCACAA CCACCACCTC ATCCTCCACT   
  
  
+ CCCACCCACC ATGGCTTCGG TGGTGGGCTT GACTCCTCCT CCCCGTCTGC CGCTGTGGAA TTATCTTTCT   
  
  
+ CCCCGGACAT CCTCCTCTCC TCCCCCACCG GAAAGTGGGC GTCTGACATT CTTATGGAGG CGGCTAGGGC   
  
  
+ TTTTTCAGAC ACGAACACCA CCAAGCTCCA CCAGCTCCTC TGGATGCTGA ACGAGCTCGG CTCCCCCTAC   
  
  
+ GGTGATGTCG AGCAGAAGCT GGTGGCGTAC TTTAACCAAG CCCTTTTTGC CCGCCTCACT GCCACCGGAC   
  
  
+ CCCGCCACCG AGCCGCTATG CTCGCCGCTG CGGACAAGAC CTCCTCCTTC GACTCCACGC TCAGGTTGCT   
  
  
+ CCTCAAGTTC CAGGAAGTCA GCCCGTGGAC CACCTTCGGC CACGTGGCTT GCAACGGTGC AATCATCGAG   
  
  
+ GCCTTAGAGA GGGACCCACA TGAAAAGAGT AAGCTCCACG TCATCGATAT CAGCACCACC TTCTGCACCC   
  
  
+ AGTGGCCCAC CTTGCTTGAG GCTTTAGCCA CCCGCATGGA CGACACTCCC CACCTCAGCC TCACTGCCGT   
  
  
+ CGTCGTCAAC AAGTACGCCG CCGTGAGCGG TGGCGAGGAC GACGGGGGAG AAGGATCAAA GAGGGTGATG   
  
  
+ AGAGAGATTG GTCTTCGTCT TGAGAAGTTC GCTAGGTTAA TGGGGGTTCC CTTCAAGTTC AACGTGGTTT   
  
  
+ ACCACGTAGG TGATCTATCC CAGTTGGATT TTTCTCGTTT GGATATTAAA GATGATGAAG CGGTGGCGAT   
  
  
+ TAACTGTGTC AACTCGCTCC ACTCGGTGGA CCTCCGATAT CGGGAGGTAG TGCTCGCGGC GTTCAGGCGG   
  
  
+ CTGCGGCCGA GGGTGGTGAC GGTGGTGGAG GAAGAGGCGG AGCTGACTGA GGTGGGTGAG GGTCAGTATG   
  
  
+ AATTTTTTAG GCGGTTTGAG GAGTGTCTTA GGTGGTTTAG GGTTTACTTT GAGGCCGTAG GAGATTGTTT   
  
  
+ TCCTAGGACT AGCAATGAGA AATTGATGTT AGAGCGGGCC GCGGGCCGGG CCATGGTGGA TCTCCTTGCA   
  
  
+ TGTCCCGAGC CTGGTGGGTC GGCTGAGGGG AGGGAGACGG CGAGACGGTG GTCGGGTCGG ATGCATGGGG   
  
  
+ CGGGGTTCGA GCACGTGGGG TTCAGTGACG AAGTGTGTGA TGATGTGAGG GCCTTGTTGA GGAGGTACAA   
  
  
+ GGAAGGATGG TCAATGACAC AGTGCACCAT GGATGGCGGT AATCACCCTG GAATACTGTT GTGGTGGCGG   
  
  
+ GATCAACCGG TGGTTTGGGC CAGTGCATGG CGGCCTTG  

- +Up\_Stream \_Len000TTTAGT TTGAGTATTT GTTATTAAAA AATTTTGAAT TTACCATTAA TAATCTAATT   
  
  
- ATTTTTAGGA TTACCAACAA ATTACAATGT AGTTCTGTAC GAAAAACCAC ATATATATTA CCGTCGTACA   
  
  
- CCCAATTCGC CGTTGTTTAA TAAGAGTTTA CGTACGTCAT TAATTAAATT AATTAATTAG TGGACGTATT   
  
  
- AGAGTTATGA TTAGGAAAGT AAGACACGGG GTGGGTTGGG GCTTTTATCA TTAGAAAGTT TGCAACCTTC   
  
  
- ACCCTAATCC ATATCTCATC AGGATGTCTA CTACTAGATG GATGTATATT GTATTAGACT GAAAAGAAAA   
  
  
- AATTTATTGA ACATAACTAC CGTGTCTTAA ACTGTACAAC CGTATACCAT GATTCTTTAA TTAATTGCAA   
  
  
- CACGATTCTA TATCCAGTAA GAAAAACCTC TGTATTCCGG GCGCAAGTGC GTCCCAAGCT CTTCCCGGTG   
  
  
- TAAGTTACCT ACTTAACATC CATCAAATTA GACTGAAACA GTCACTGACT AAGGTGCCGA ACTTGGGCGC   
  
  
- TGTAATTATA TCCAACTGAT TTCAGCAAAC TATGAATAAA AAGAATTCAT TTCAGAACTT AAGTTTAGAA   
  
  
- TATTTATTTA TTTTTTTAGA TATTATTTTC TTAAGATGGT AAATAATTCA GTTGTTGAGT TAAGCTTACT   
  
  
- TTATTTTTTA GTTCTAGTGG TTTTTTTTAT TTATTTTCCT TTCCATCTAA CGTAAACCTA AAACTCACCG   
  
  
- CACCACCACC CAAACTACGA CCACGACGTA TGTGGCGTCG AAAAGAGACA GGAAAATGGA GAGAAACAGA   
  
  
- CGTTCTTGGG GTAAACCGTG CGGACGAGAG AAAAGATACA AAAGAAAAGA TAGAAAATTC TTTGTTACCT   
  
  
- TTCGATTGTG TGATTGTGAT TGGGTAACCA GGAAAAAAGA ATAGACGTTT TAATTTCATT GTTATAAAAA   
  
  
- GAATAGGGGA AAAAGCAAGG TGGGTAAAGA AATAGGAAGA CAAACTTGGA TTAGACATAT ATACGTACGT   
  
  
- ACACATAAAA TGAATCTTAG ACAAAACTAA GAAACTACAT TAATTAATCA TCAGAAAAAT TAACTTAAAA   
  
  
- ATTGTCATCA AACTTAATCT GTAAATAGCT ATAATATAAA AAATATATAT TAATTTTTTA TGTTTTACTA   
  
  
- TTCACAGAAT GATGTTATGT GAAGTATTCA CAGCACAAAA TTGAATTTTT AGTATGATAA TACAAAGATT   
  
  
- TAACTTTTTT TTCACTCTAA TTCATCGTCT TCGTATAATG TTGAATATTT AATATATTTT AGTAGAAAAA   
  
  
- TAGGTATTAA ATAGTGTAAA GATTTTATGC GTATAGTATA GATGAATACC TATCCCAATT ATTCTAATCC   
  
  
- CGTAGTAAAT TCTAAAATAG TCGCATTGGT TGCTTACAAA TTCCAATCGT CAAGTATTAA TTCACATTAG   
  
  
- TAATTTTTTA AATACTAAAA TTTAGTATTT TATACAATTA GATCCTACTC CGTTCGCCGT TCTGTATATA   
  
  
- GTGATAGGAA TTTTTTATAT TATATAACTA AAGTTTCTTA ATACTTCATA TAATTAAAGT ATATATTACT   
  
  
- GCTAATACAT AAAAAAATGA TTTAATGAAC ATGGGTATTA TTTATCTTAG CTACGTAGTA AGTACCGGAA   
  
  
- AGTAAGACAT TATACCAACT GAGGTTTTTA AGAGTTTCAT AAAAATATAA AACCCTCCCC TCTAAAATCA   
  
  
- AACTATGAAA ACAACTTGAT CACTTGTTAA AGGTTGTTGG AGGTGGTGGG GGTTTGTAGT TGTTAGACTA   
  
  
- ACTACATACG TAGGGTTCGA ACTTAACTTA TTATTAAAGT TATTGTTGTT ATTAGCAAAA TGGAACTGTT   
  
  
- CGAGGTGTCG TTGAGTTGCA GAAGGTTTGG TTGGGTAGTG AGTAGTAGTA GTAGGGGTGA TAGGGTAAGT   
  
  
- GGGGGTGGTT TTCCTCGGAT TTCTGTCATT GTTCTTCTTA CGAACGGAAA AAAGTACCTA CTTCTTCTGA   
  
  
- AGAGAAGAAG GAGATGAAGA AGGGTGGTGA TACTATGTGT GGTGGAGGTT GTAGTAATGG GGTGGTTAAT   
  
  
- ATTAAGGGCA ATATGGGACC TGGGAGGGAG GTGGTGGTGG TGTCGGTGTT GGTGGTGGAG TAGGAGGTGA   
  
  
- GGGTGGGTGG TACCGAAGCC ACCACCCGAA CTGAGGAGGA GGGGCAGACG GCGACACCTT AATAGAAAGA   
  
  
- GGGGCCTGTA GGAGGAGAGG AGGGGGTGGC CTTTCACCCG CAGACTGTAA GAATACCTCC GCCGATCCCG   
  
  
- AAAAAGTCTG TGCTTGTGGT GGTTCGAGGT GGTCGAGGAG ACCTACGACT TGCTCGAGCC GAGGGGGATG   
  
  
- CCACTACAGC TCGTCTTCGA CCACCGCATG AAATTGGTTC GGGAAAAACG GGCGGAGTGA CGGTGGCCTG   
  
  
- GGGCGGTGGC TCGGCGATAC GAGCGGCGAC GCCTGTTCTG GAGGAGGAAG CTGAGGTGCG AGTCCAACGA   
  
  
- GGAGTTCAAG GTCCTTCAGT CGGGCACCTG GTGGAAGCCG GTGCACCGAA CGTTGCCACG TTAGTAGCTC   
  
  
- CGGAATCTCT CCCTGGGTGT ACTTTTCTCA TTCGAGGTGC AGTAGCTATA GTCGTGGTGG AAGACGTGGG   
  
  
- TCACCGGGTG GAACGAACTC CGAAATCGGT GGGCGTACCT GCTGTGAGGG GTGGAGTCGG AGTGACGGCA   
  
  
- GCAGCAGTTG TTCATGCGGC GGCACTCGCC ACCGCTCCTG CTGCCCCCTC TTCCTAGTTT CTCCCACTAC   
  
  
- TCTCTCTAAC CAGAAGCAGA ACTCTTCAAG CGATCCAATT ACCCCCAAGG GAAGTTCAAG TTGCACCAAA   
  
  
- TGGTGCATCC ACTAGATAGG GTCAACCTAA AAAGAGCAAA CCTATAATTT CTACTACTTC GCCACCGCTA   
  
  
- ATTGACACAG TTGAGCGAGG TGAGCCACCT GGAGGCTATA GCCCTCCATC ACGAGCGCCG CAAGTCCGCC   
  
  
- GACGCCGGCT CCCACCACTG CCACCACCTC CTTCTCCGCC TCGACTGACT CCACCCACTC CCAGTCATAC   
  
  
- TTAAAAAATC CGCCAAACTC CTCACAGAAT CCACCAAATC CCAAATGAAA CTCCGGCATC CTCTAACAAA   
  
  
- AGGATCCTGA TCGTTACTCT TTAACTACAA TCTCGCCCGG CGCCCGGCCC GGTACCACCT AGAGGAACGT   
  
  
- ACAGGGCTCG GACCACCCAG CCGACTCCCC TCCCTCTGCC GCTCTGCCAC CAGCCCAGCC TACGTACCCC   
  
  
- GCCCCAAGCT CGTGCACCCC AAGTCACTGC TTCACACACT ACTACACTCC CGGAACAACT CCTCCATGTT   
  
  
- CCTTCCTACC AGTTACTGTG TCACGTGGTA CCTACCGCCA TTAGTGGGAC CTTATGACAA CACCACCGCC   
  
  
- CTAGTTGGCC ACCAAACCCG GTCACGTACC GCCGGAAC

+     CTAG-motif

| Site Name | Organism | Position | Strand | Matrix score. | sequence | function |
| --- | --- | --- | --- | --- | --- | --- |
| CTAG-motif | Avena sativa | 1286 | + | 9 | ACTAGCAGAA |  |

>HU05G01983.1   
+ +Up\_Stream \_Len000AAATCA AACTCATAAA CAATAATTTT TTAAAACTTA AATGGTAATT ATTAGATTAA   
  
  
+ TAAAAATCCT AATGGTTGTT TAATGTTACA TCAAGACATG CTTTTTGGTG TATATATAAT GGCAGCATGT   
  
  
+ GGGTTAAGCG GCAACAAATT ATTCTCAAAT GCATGCAGTA ATTAATTTAA TTAATTAATC ACCTGCATAA   
  
  
+ TCTCAATACT AATCCTTTCA TTCTGTGCCC CACCCAACCC CGAAAATAGT AATCTTTCAA ACGTTGGAAG   
  
  
+ TGGGATTAGG TATAGAGTAG TCCTACAGAT GATGATCTAC CTACATATAA CATAATCTGA CTTTTCTTTT   
  
  
+ TTAAATAACT TGTATTGATG GCACAGAATT TGACATGTTG GCATATGGTA CTAAGAAATT AATTAACGTT   
  
  
+ GTGCTAAGAT ATAGGTCATT CTTTTTGGAG ACATAAGGCC CGCGTTCACG CAGGGTTCGA GAAGGGCCAC   
  
  
+ ATTCAATGGA TGAATTGTAG GTAGTTTAAT CTGACTTTGT CAGTGACTGA TTCCACGGCT TGAACCCGCG   
  
  
+ ACATTAATAT AGGTTGACTA AAGTCGTTTG ATACTTATTT TTCTTAAGTA AAGTCTTGAA TTCAAATCTT   
  
  
+ ATAAATAAAT AAAAAAATCT ATAATAAAAG AATTCTACCA TTTATTAAGT CAACAACTCA ATTCGAATGA   
  
  
+ AATAAAAAAT CAAGATCACC AAAAAAAATA AATAAAAGGA AAGGTAGATT GCATTTGGAT TTTGAGTGGC   
  
  
+ GTGGTGGTGG GTTTGATGCT GGTGCTGCAT ACACCGCAGC TTTTCTCTGT CCTTTTACCT CTCTTTGTCT   
  
  
+ GCAAGAACCC CATTTGGCAC GCCTGCTCTC TTTTCTATGT TTTCTTTTCT ATCTTTTAAG AAACAATGGA   
  
  
+ AAGCTAACAC ACTAACACTA ACCCATTGGT CCTTTTTTCT TATCTGCAAA ATTAAAGTAA CAATATTTTT   
  
  
+ CTTATCCCCT TTTTCGTTCC ACCCATTTCT TTATCCTTCT GTTTGAACCT AATCTGTATA TATGCATGCA   
  
  
+ TGTGTATTTT ACTTAGAATC TGTTTTGATT CTTTGATGTA ATTAATTAGT AGTCTTTTTA ATTGAATTTT   
  
  
+ TAACAGTAGT TTGAATTAGA CATTTATCGA TATTATATTT TTTATATATA ATTAAAAAAT ACAAAATGAT   
  
  
+ AAGTGTCTTA CTACAATACA CTTCATAAGT GTCGTGTTTT AACTTAAAAA TCATACTATT ATGTTTCTAA   
  
  
+ ATTGAAAAAA AAGTGAGATT AAGTAGCAGA AGCATATTAC AACTTATAAA TTATATAAAA TCATCTTTTT   
  
  
+ ATCCATAATT TATCACATTT CTAAAATACG CATATCATAT CTACTTATGG ATAGGGTTAA TAAGATTAGG   
  
  
+ GCATCATTTA AGATTTTATC AGCGTAACCA ACGAATGTTT AAGGTTAGCA GTTCATAATT AAGTGTAATC   
  
  
+ ATTAAAAAAT TTATGATTTT AAATCATAAA ATATGTTAAT CTAGGATGAG GCAAGCGGCA AGACATATAT   
  
  
+ CACTATCCTT AAAAAATATA ATATATTGAT TTCAAAGAAT TATGAAGTAT ATTAATTTCA TATATAATGA   
  
  
+ CGATTATGTA TTTTTTTACT AAATTACTTG TACCCATAAT AAATAGAATC GATGCATCAT TCATGGCCTT   
  
  
+ TCATTCTGTA ATATGGTTGA CTCCAAAAAT TCTCAAAGTA TTTTTATATT TTGGGAGGGG AGATTTTAGT   
  
  
+ TTGATACTTT TGTTGAACTA GTGAACAATT TCCAACAACC TCCACCACCC CCAAACATCA ACAATCTGAT   
  
  
+ TGATGTATGC ATCCCAAGCT TGAATTGAAT AATAATTTCA ATAACAACAA TAATCGTTTT ACCTTGACAA   
  
  
+ GCTCCACAGC AACTCAACGT CTTCCAAACC AACCCATCAC TCATCATCAT CATCCCCACT ATCCCATTCA   
  
  
+ CCCCCACCAA AAGGAGCCTA AAGACAGTAA CAAGAAGAAT GCTTGCCTTT TTTCATGGAT GAAGAAGACT   
  
  
+ TCTCTTCTTC CTCTACTTCT TCCCACCACT ATGATACACA CCACCTCCAA CATCATTACC CCACCAATTA   
  
  
+ TAATTCCCGT TATACCCTGG ACCCTCCCTC CACCACCACC ACAGCCACAA CCACCACCTC ATCCTCCACT   
  
  
+ CCCACCCACC ATGGCTTCGG TGGTGGGCTT GACTCCTCCT CCCCGTCTGC CGCTGTGGAA TTATCTTTCT   
  
  
+ CCCCGGACAT CCTCCTCTCC TCCCCCACCG GAAAGTGGGC GTCTGACATT CTTATGGAGG CGGCTAGGGC   
  
  
+ TTTTTCAGAC ACGAACACCA CCAAGCTCCA CCAGCTCCTC TGGATGCTGA ACGAGCTCGG CTCCCCCTAC   
  
  
+ GGTGATGTCG AGCAGAAGCT GGTGGCGTAC TTTAACCAAG CCCTTTTTGC CCGCCTCACT GCCACCGGAC   
  
  
+ CCCGCCACCG AGCCGCTATG CTCGCCGCTG CGGACAAGAC CTCCTCCTTC GACTCCACGC TCAGGTTGCT   
  
  
+ CCTCAAGTTC CAGGAAGTCA GCCCGTGGAC CACCTTCGGC CACGTGGCTT GCAACGGTGC AATCATCGAG   
  
  
+ GCCTTAGAGA GGGACCCACA TGAAAAGAGT AAGCTCCACG TCATCGATAT CAGCACCACC TTCTGCACCC   
  
  
+ AGTGGCCCAC CTTGCTTGAG GCTTTAGCCA CCCGCATGGA CGACACTCCC CACCTCAGCC TCACTGCCGT   
  
  
+ CGTCGTCAAC AAGTACGCCG CCGTGAGCGG TGGCGAGGAC GACGGGGGAG AAGGATCAAA GAGGGTGATG   
  
  
+ AGAGAGATTG GTCTTCGTCT TGAGAAGTTC GCTAGGTTAA TGGGGGTTCC CTTCAAGTTC AACGTGGTTT   
  
  
+ ACCACGTAGG TGATCTATCC CAGTTGGATT TTTCTCGTTT GGATATTAAA GATGATGAAG CGGTGGCGAT   
  
  
+ TAACTGTGTC AACTCGCTCC ACTCGGTGGA CCTCCGATAT CGGGAGGTAG TGCTCGCGGC GTTCAGGCGG   
  
  
+ CTGCGGCCGA GGGTGGTGAC GGTGGTGGAG GAAGAGGCGG AGCTGACTGA GGTGGGTGAG GGTCAGTATG   
  
  
+ AATTTTTTAG GCGGTTTGAG GAGTGTCTTA GGTGGTTTAG GGTTTACTTT GAGGCCGTAG GAGATTGTTT   
  
  
+ TCCTAGGACT AGCAATGAGA AATTGATGTT AGAGCGGGCC GCGGGCCGGG CCATGGTGGA TCTCCTTGCA   
  
  
+ TGTCCCGAGC CTGGTGGGTC GGCTGAGGGG AGGGAGACGG CGAGACGGTG GTCGGGTCGG ATGCATGGGG   
  
  
+ CGGGGTTCGA GCACGTGGGG TTCAGTGACG AAGTGTGTGA TGATGTGAGG GCCTTGTTGA GGAGGTACAA   
  
  
+ GGAAGGATGG TCAATGACAC AGTGCACCAT GGATGGCGGT AATCACCCTG GAATACTGTT GTGGTGGCGG   
  
  
+ GATCAACCGG TGGTTTGGGC CAGTGCATGG CGGCCTTG  

- +Up\_Stream \_Len000TTTAGT TTGAGTATTT GTTATTAAAA AATTTTGAAT TTACCATTAA TAATCTAATT   
  
  
- ATTTTTAGGA TTACCAACAA ATTACAATGT AGTTCTGTAC GAAAAACCAC ATATATATTA CCGTCGTACA   
  
  
- CCCAATTCGC CGTTGTTTAA TAAGAGTTTA CGTACGTCAT TAATTAAATT AATTAATTAG TGGACGTATT   
  
  
- AGAGTTATGA TTAGGAAAGT AAGACACGGG GTGGGTTGGG GCTTTTATCA TTAGAAAGTT TGCAACCTTC   
  
  
- ACCCTAATCC ATATCTCATC AGGATGTCTA CTACTAGATG GATGTATATT GTATTAGACT GAAAAGAAAA   
  
  
- AATTTATTGA ACATAACTAC CGTGTCTTAA ACTGTACAAC CGTATACCAT GATTCTTTAA TTAATTGCAA   
  
  
- CACGATTCTA TATCCAGTAA GAAAAACCTC TGTATTCCGG GCGCAAGTGC GTCCCAAGCT CTTCCCGGTG   
  
  
- TAAGTTACCT ACTTAACATC CATCAAATTA GACTGAAACA GTCACTGACT AAGGTGCCGA ACTTGGGCGC   
  
  
- TGTAATTATA TCCAACTGAT TTCAGCAAAC TATGAATAAA AAGAATTCAT TTCAGAACTT AAGTTTAGAA   
  
  
- TATTTATTTA TTTTTTTAGA TATTATTTTC TTAAGATGGT AAATAATTCA GTTGTTGAGT TAAGCTTACT   
  
  
- TTATTTTTTA GTTCTAGTGG TTTTTTTTAT TTATTTTCCT TTCCATCTAA CGTAAACCTA AAACTCACCG   
  
  
- CACCACCACC CAAACTACGA CCACGACGTA TGTGGCGTCG AAAAGAGACA GGAAAATGGA GAGAAACAGA   
  
  
- CGTTCTTGGG GTAAACCGTG CGGACGAGAG AAAAGATACA AAAGAAAAGA TAGAAAATTC TTTGTTACCT   
  
  
- TTCGATTGTG TGATTGTGAT TGGGTAACCA GGAAAAAAGA ATAGACGTTT TAATTTCATT GTTATAAAAA   
  
  
- GAATAGGGGA AAAAGCAAGG TGGGTAAAGA AATAGGAAGA CAAACTTGGA TTAGACATAT ATACGTACGT   
  
  
- ACACATAAAA TGAATCTTAG ACAAAACTAA GAAACTACAT TAATTAATCA TCAGAAAAAT TAACTTAAAA   
  
  
- ATTGTCATCA AACTTAATCT GTAAATAGCT ATAATATAAA AAATATATAT TAATTTTTTA TGTTTTACTA   
  
  
- TTCACAGAAT GATGTTATGT GAAGTATTCA CAGCACAAAA TTGAATTTTT AGTATGATAA TACAAAGATT   
  
  
- TAACTTTTTT TTCACTCTAA TTCATCGTCT TCGTATAATG TTGAATATTT AATATATTTT AGTAGAAAAA   
  
  
- TAGGTATTAA ATAGTGTAAA GATTTTATGC GTATAGTATA GATGAATACC TATCCCAATT ATTCTAATCC   
  
  
- CGTAGTAAAT TCTAAAATAG TCGCATTGGT TGCTTACAAA TTCCAATCGT CAAGTATTAA TTCACATTAG   
  
  
- TAATTTTTTA AATACTAAAA TTTAGTATTT TATACAATTA GATCCTACTC CGTTCGCCGT TCTGTATATA   
  
  
- GTGATAGGAA TTTTTTATAT TATATAACTA AAGTTTCTTA ATACTTCATA TAATTAAAGT ATATATTACT   
  
  
- GCTAATACAT AAAAAAATGA TTTAATGAAC ATGGGTATTA TTTATCTTAG CTACGTAGTA AGTACCGGAA   
  
  
- AGTAAGACAT TATACCAACT GAGGTTTTTA AGAGTTTCAT AAAAATATAA AACCCTCCCC TCTAAAATCA   
  
  
- AACTATGAAA ACAACTTGAT CACTTGTTAA AGGTTGTTGG AGGTGGTGGG GGTTTGTAGT TGTTAGACTA   
  
  
- ACTACATACG TAGGGTTCGA ACTTAACTTA TTATTAAAGT TATTGTTGTT ATTAGCAAAA TGGAACTGTT   
  
  
- CGAGGTGTCG TTGAGTTGCA GAAGGTTTGG TTGGGTAGTG AGTAGTAGTA GTAGGGGTGA TAGGGTAAGT   
  
  
- GGGGGTGGTT TTCCTCGGAT TTCTGTCATT GTTCTTCTTA CGAACGGAAA AAAGTACCTA CTTCTTCTGA   
  
  
- AGAGAAGAAG GAGATGAAGA AGGGTGGTGA TACTATGTGT GGTGGAGGTT GTAGTAATGG GGTGGTTAAT   
  
  
- ATTAAGGGCA ATATGGGACC TGGGAGGGAG GTGGTGGTGG TGTCGGTGTT GGTGGTGGAG TAGGAGGTGA   
  
  
- GGGTGGGTGG TACCGAAGCC ACCACCCGAA CTGAGGAGGA GGGGCAGACG GCGACACCTT AATAGAAAGA   
  
  
- GGGGCCTGTA GGAGGAGAGG AGGGGGTGGC CTTTCACCCG CAGACTGTAA GAATACCTCC GCCGATCCCG   
  
  
- AAAAAGTCTG TGCTTGTGGT GGTTCGAGGT GGTCGAGGAG ACCTACGACT TGCTCGAGCC GAGGGGGATG   
  
  
- CCACTACAGC TCGTCTTCGA CCACCGCATG AAATTGGTTC GGGAAAAACG GGCGGAGTGA CGGTGGCCTG   
  
  
- GGGCGGTGGC TCGGCGATAC GAGCGGCGAC GCCTGTTCTG GAGGAGGAAG CTGAGGTGCG AGTCCAACGA   
  
  
- GGAGTTCAAG GTCCTTCAGT CGGGCACCTG GTGGAAGCCG GTGCACCGAA CGTTGCCACG TTAGTAGCTC   
  
  
- CGGAATCTCT CCCTGGGTGT ACTTTTCTCA TTCGAGGTGC AGTAGCTATA GTCGTGGTGG AAGACGTGGG   
  
  
- TCACCGGGTG GAACGAACTC CGAAATCGGT GGGCGTACCT GCTGTGAGGG GTGGAGTCGG AGTGACGGCA   
  
  
- GCAGCAGTTG TTCATGCGGC GGCACTCGCC ACCGCTCCTG CTGCCCCCTC TTCCTAGTTT CTCCCACTAC   
  
  
- TCTCTCTAAC CAGAAGCAGA ACTCTTCAAG CGATCCAATT ACCCCCAAGG GAAGTTCAAG TTGCACCAAA   
  
  
- TGGTGCATCC ACTAGATAGG GTCAACCTAA AAAGAGCAAA CCTATAATTT CTACTACTTC GCCACCGCTA   
  
  
- ATTGACACAG TTGAGCGAGG TGAGCCACCT GGAGGCTATA GCCCTCCATC ACGAGCGCCG CAAGTCCGCC   
  
  
- GACGCCGGCT CCCACCACTG CCACCACCTC CTTCTCCGCC TCGACTGACT CCACCCACTC CCAGTCATAC   
  
  
- TTAAAAAATC CGCCAAACTC CTCACAGAAT CCACCAAATC CCAAATGAAA CTCCGGCATC CTCTAACAAA   
  
  
- AGGATCCTGA TCGTTACTCT TTAACTACAA TCTCGCCCGG CGCCCGGCCC GGTACCACCT AGAGGAACGT   
  
  
- ACAGGGCTCG GACCACCCAG CCGACTCCCC TCCCTCTGCC GCTCTGCCAC CAGCCCAGCC TACGTACCCC   
  
  
- GCCCCAAGCT CGTGCACCCC AAGTCACTGC TTCACACACT ACTACACTCC CGGAACAACT CCTCCATGTT   
  
  
- CCTTCCTACC AGTTACTGTG TCACGTGGTA CCTACCGCCA TTAGTGGGAC CTTATGACAA CACCACCGCC   
  
  
- CTAGTTGGCC ACCAAACCCG GTCACGTACC GCCGGAAC

+     DRE core

| Site Name | Organism | Position | Strand | Matrix score. | sequence | function |
| --- | --- | --- | --- | --- | --- | --- |
| DRE core | Arabidopsis thaliana | 3242 | - | 6 | GCCGAC |  |

>HU05G01983.1   
+ +Up\_Stream \_Len000AAATCA AACTCATAAA CAATAATTTT TTAAAACTTA AATGGTAATT ATTAGATTAA   
  
  
+ TAAAAATCCT AATGGTTGTT TAATGTTACA TCAAGACATG CTTTTTGGTG TATATATAAT GGCAGCATGT   
  
  
+ GGGTTAAGCG GCAACAAATT ATTCTCAAAT GCATGCAGTA ATTAATTTAA TTAATTAATC ACCTGCATAA   
  
  
+ TCTCAATACT AATCCTTTCA TTCTGTGCCC CACCCAACCC CGAAAATAGT AATCTTTCAA ACGTTGGAAG   
  
  
+ TGGGATTAGG TATAGAGTAG TCCTACAGAT GATGATCTAC CTACATATAA CATAATCTGA CTTTTCTTTT   
  
  
+ TTAAATAACT TGTATTGATG GCACAGAATT TGACATGTTG GCATATGGTA CTAAGAAATT AATTAACGTT   
  
  
+ GTGCTAAGAT ATAGGTCATT CTTTTTGGAG ACATAAGGCC CGCGTTCACG CAGGGTTCGA GAAGGGCCAC   
  
  
+ ATTCAATGGA TGAATTGTAG GTAGTTTAAT CTGACTTTGT CAGTGACTGA TTCCACGGCT TGAACCCGCG   
  
  
+ ACATTAATAT AGGTTGACTA AAGTCGTTTG ATACTTATTT TTCTTAAGTA AAGTCTTGAA TTCAAATCTT   
  
  
+ ATAAATAAAT AAAAAAATCT ATAATAAAAG AATTCTACCA TTTATTAAGT CAACAACTCA ATTCGAATGA   
  
  
+ AATAAAAAAT CAAGATCACC AAAAAAAATA AATAAAAGGA AAGGTAGATT GCATTTGGAT TTTGAGTGGC   
  
  
+ GTGGTGGTGG GTTTGATGCT GGTGCTGCAT ACACCGCAGC TTTTCTCTGT CCTTTTACCT CTCTTTGTCT   
  
  
+ GCAAGAACCC CATTTGGCAC GCCTGCTCTC TTTTCTATGT TTTCTTTTCT ATCTTTTAAG AAACAATGGA   
  
  
+ AAGCTAACAC ACTAACACTA ACCCATTGGT CCTTTTTTCT TATCTGCAAA ATTAAAGTAA CAATATTTTT   
  
  
+ CTTATCCCCT TTTTCGTTCC ACCCATTTCT TTATCCTTCT GTTTGAACCT AATCTGTATA TATGCATGCA   
  
  
+ TGTGTATTTT ACTTAGAATC TGTTTTGATT CTTTGATGTA ATTAATTAGT AGTCTTTTTA ATTGAATTTT   
  
  
+ TAACAGTAGT TTGAATTAGA CATTTATCGA TATTATATTT TTTATATATA ATTAAAAAAT ACAAAATGAT   
  
  
+ AAGTGTCTTA CTACAATACA CTTCATAAGT GTCGTGTTTT AACTTAAAAA TCATACTATT ATGTTTCTAA   
  
  
+ ATTGAAAAAA AAGTGAGATT AAGTAGCAGA AGCATATTAC AACTTATAAA TTATATAAAA TCATCTTTTT   
  
  
+ ATCCATAATT TATCACATTT CTAAAATACG CATATCATAT CTACTTATGG ATAGGGTTAA TAAGATTAGG   
  
  
+ GCATCATTTA AGATTTTATC AGCGTAACCA ACGAATGTTT AAGGTTAGCA GTTCATAATT AAGTGTAATC   
  
  
+ ATTAAAAAAT TTATGATTTT AAATCATAAA ATATGTTAAT CTAGGATGAG GCAAGCGGCA AGACATATAT   
  
  
+ CACTATCCTT AAAAAATATA ATATATTGAT TTCAAAGAAT TATGAAGTAT ATTAATTTCA TATATAATGA   
  
  
+ CGATTATGTA TTTTTTTACT AAATTACTTG TACCCATAAT AAATAGAATC GATGCATCAT TCATGGCCTT   
  
  
+ TCATTCTGTA ATATGGTTGA CTCCAAAAAT TCTCAAAGTA TTTTTATATT TTGGGAGGGG AGATTTTAGT   
  
  
+ TTGATACTTT TGTTGAACTA GTGAACAATT TCCAACAACC TCCACCACCC CCAAACATCA ACAATCTGAT   
  
  
+ TGATGTATGC ATCCCAAGCT TGAATTGAAT AATAATTTCA ATAACAACAA TAATCGTTTT ACCTTGACAA   
  
  
+ GCTCCACAGC AACTCAACGT CTTCCAAACC AACCCATCAC TCATCATCAT CATCCCCACT ATCCCATTCA   
  
  
+ CCCCCACCAA AAGGAGCCTA AAGACAGTAA CAAGAAGAAT GCTTGCCTTT TTTCATGGAT GAAGAAGACT   
  
  
+ TCTCTTCTTC CTCTACTTCT TCCCACCACT ATGATACACA CCACCTCCAA CATCATTACC CCACCAATTA   
  
  
+ TAATTCCCGT TATACCCTGG ACCCTCCCTC CACCACCACC ACAGCCACAA CCACCACCTC ATCCTCCACT   
  
  
+ CCCACCCACC ATGGCTTCGG TGGTGGGCTT GACTCCTCCT CCCCGTCTGC CGCTGTGGAA TTATCTTTCT   
  
  
+ CCCCGGACAT CCTCCTCTCC TCCCCCACCG GAAAGTGGGC GTCTGACATT CTTATGGAGG CGGCTAGGGC   
  
  
+ TTTTTCAGAC ACGAACACCA CCAAGCTCCA CCAGCTCCTC TGGATGCTGA ACGAGCTCGG CTCCCCCTAC   
  
  
+ GGTGATGTCG AGCAGAAGCT GGTGGCGTAC TTTAACCAAG CCCTTTTTGC CCGCCTCACT GCCACCGGAC   
  
  
+ CCCGCCACCG AGCCGCTATG CTCGCCGCTG CGGACAAGAC CTCCTCCTTC GACTCCACGC TCAGGTTGCT   
  
  
+ CCTCAAGTTC CAGGAAGTCA GCCCGTGGAC CACCTTCGGC CACGTGGCTT GCAACGGTGC AATCATCGAG   
  
  
+ GCCTTAGAGA GGGACCCACA TGAAAAGAGT AAGCTCCACG TCATCGATAT CAGCACCACC TTCTGCACCC   
  
  
+ AGTGGCCCAC CTTGCTTGAG GCTTTAGCCA CCCGCATGGA CGACACTCCC CACCTCAGCC TCACTGCCGT   
  
  
+ CGTCGTCAAC AAGTACGCCG CCGTGAGCGG TGGCGAGGAC GACGGGGGAG AAGGATCAAA GAGGGTGATG   
  
  
+ AGAGAGATTG GTCTTCGTCT TGAGAAGTTC GCTAGGTTAA TGGGGGTTCC CTTCAAGTTC AACGTGGTTT   
  
  
+ ACCACGTAGG TGATCTATCC CAGTTGGATT TTTCTCGTTT GGATATTAAA GATGATGAAG CGGTGGCGAT   
  
  
+ TAACTGTGTC AACTCGCTCC ACTCGGTGGA CCTCCGATAT CGGGAGGTAG TGCTCGCGGC GTTCAGGCGG   
  
  
+ CTGCGGCCGA GGGTGGTGAC GGTGGTGGAG GAAGAGGCGG AGCTGACTGA GGTGGGTGAG GGTCAGTATG   
  
  
+ AATTTTTTAG GCGGTTTGAG GAGTGTCTTA GGTGGTTTAG GGTTTACTTT GAGGCCGTAG GAGATTGTTT   
  
  
+ TCCTAGGACT AGCAATGAGA AATTGATGTT AGAGCGGGCC GCGGGCCGGG CCATGGTGGA TCTCCTTGCA   
  
  
+ TGTCCCGAGC CTGGTGGGTC GGCTGAGGGG AGGGAGACGG CGAGACGGTG GTCGGGTCGG ATGCATGGGG   
  
  
+ CGGGGTTCGA GCACGTGGGG TTCAGTGACG AAGTGTGTGA TGATGTGAGG GCCTTGTTGA GGAGGTACAA   
  
  
+ GGAAGGATGG TCAATGACAC AGTGCACCAT GGATGGCGGT AATCACCCTG GAATACTGTT GTGGTGGCGG   
  
  
+ GATCAACCGG TGGTTTGGGC CAGTGCATGG CGGCCTTG  

- +Up\_Stream \_Len000TTTAGT TTGAGTATTT GTTATTAAAA AATTTTGAAT TTACCATTAA TAATCTAATT   
  
  
- ATTTTTAGGA TTACCAACAA ATTACAATGT AGTTCTGTAC GAAAAACCAC ATATATATTA CCGTCGTACA   
  
  
- CCCAATTCGC CGTTGTTTAA TAAGAGTTTA CGTACGTCAT TAATTAAATT AATTAATTAG TGGACGTATT   
  
  
- AGAGTTATGA TTAGGAAAGT AAGACACGGG GTGGGTTGGG GCTTTTATCA TTAGAAAGTT TGCAACCTTC   
  
  
- ACCCTAATCC ATATCTCATC AGGATGTCTA CTACTAGATG GATGTATATT GTATTAGACT GAAAAGAAAA   
  
  
- AATTTATTGA ACATAACTAC CGTGTCTTAA ACTGTACAAC CGTATACCAT GATTCTTTAA TTAATTGCAA   
  
  
- CACGATTCTA TATCCAGTAA GAAAAACCTC TGTATTCCGG GCGCAAGTGC GTCCCAAGCT CTTCCCGGTG   
  
  
- TAAGTTACCT ACTTAACATC CATCAAATTA GACTGAAACA GTCACTGACT AAGGTGCCGA ACTTGGGCGC   
  
  
- TGTAATTATA TCCAACTGAT TTCAGCAAAC TATGAATAAA AAGAATTCAT TTCAGAACTT AAGTTTAGAA   
  
  
- TATTTATTTA TTTTTTTAGA TATTATTTTC TTAAGATGGT AAATAATTCA GTTGTTGAGT TAAGCTTACT   
  
  
- TTATTTTTTA GTTCTAGTGG TTTTTTTTAT TTATTTTCCT TTCCATCTAA CGTAAACCTA AAACTCACCG   
  
  
- CACCACCACC CAAACTACGA CCACGACGTA TGTGGCGTCG AAAAGAGACA GGAAAATGGA GAGAAACAGA   
  
  
- CGTTCTTGGG GTAAACCGTG CGGACGAGAG AAAAGATACA AAAGAAAAGA TAGAAAATTC TTTGTTACCT   
  
  
- TTCGATTGTG TGATTGTGAT TGGGTAACCA GGAAAAAAGA ATAGACGTTT TAATTTCATT GTTATAAAAA   
  
  
- GAATAGGGGA AAAAGCAAGG TGGGTAAAGA AATAGGAAGA CAAACTTGGA TTAGACATAT ATACGTACGT   
  
  
- ACACATAAAA TGAATCTTAG ACAAAACTAA GAAACTACAT TAATTAATCA TCAGAAAAAT TAACTTAAAA   
  
  
- ATTGTCATCA AACTTAATCT GTAAATAGCT ATAATATAAA AAATATATAT TAATTTTTTA TGTTTTACTA   
  
  
- TTCACAGAAT GATGTTATGT GAAGTATTCA CAGCACAAAA TTGAATTTTT AGTATGATAA TACAAAGATT   
  
  
- TAACTTTTTT TTCACTCTAA TTCATCGTCT TCGTATAATG TTGAATATTT AATATATTTT AGTAGAAAAA   
  
  
- TAGGTATTAA ATAGTGTAAA GATTTTATGC GTATAGTATA GATGAATACC TATCCCAATT ATTCTAATCC   
  
  
- CGTAGTAAAT TCTAAAATAG TCGCATTGGT TGCTTACAAA TTCCAATCGT CAAGTATTAA TTCACATTAG   
  
  
- TAATTTTTTA AATACTAAAA TTTAGTATTT TATACAATTA GATCCTACTC CGTTCGCCGT TCTGTATATA   
  
  
- GTGATAGGAA TTTTTTATAT TATATAACTA AAGTTTCTTA ATACTTCATA TAATTAAAGT ATATATTACT   
  
  
- GCTAATACAT AAAAAAATGA TTTAATGAAC ATGGGTATTA TTTATCTTAG CTACGTAGTA AGTACCGGAA   
  
  
- AGTAAGACAT TATACCAACT GAGGTTTTTA AGAGTTTCAT AAAAATATAA AACCCTCCCC TCTAAAATCA   
  
  
- AACTATGAAA ACAACTTGAT CACTTGTTAA AGGTTGTTGG AGGTGGTGGG GGTTTGTAGT TGTTAGACTA   
  
  
- ACTACATACG TAGGGTTCGA ACTTAACTTA TTATTAAAGT TATTGTTGTT ATTAGCAAAA TGGAACTGTT   
  
  
- CGAGGTGTCG TTGAGTTGCA GAAGGTTTGG TTGGGTAGTG AGTAGTAGTA GTAGGGGTGA TAGGGTAAGT   
  
  
- GGGGGTGGTT TTCCTCGGAT TTCTGTCATT GTTCTTCTTA CGAACGGAAA AAAGTACCTA CTTCTTCTGA   
  
  
- AGAGAAGAAG GAGATGAAGA AGGGTGGTGA TACTATGTGT GGTGGAGGTT GTAGTAATGG GGTGGTTAAT   
  
  
- ATTAAGGGCA ATATGGGACC TGGGAGGGAG GTGGTGGTGG TGTCGGTGTT GGTGGTGGAG TAGGAGGTGA   
  
  
- GGGTGGGTGG TACCGAAGCC ACCACCCGAA CTGAGGAGGA GGGGCAGACG GCGACACCTT AATAGAAAGA   
  
  
- GGGGCCTGTA GGAGGAGAGG AGGGGGTGGC CTTTCACCCG CAGACTGTAA GAATACCTCC GCCGATCCCG   
  
  
- AAAAAGTCTG TGCTTGTGGT GGTTCGAGGT GGTCGAGGAG ACCTACGACT TGCTCGAGCC GAGGGGGATG   
  
  
- CCACTACAGC TCGTCTTCGA CCACCGCATG AAATTGGTTC GGGAAAAACG GGCGGAGTGA CGGTGGCCTG   
  
  
- GGGCGGTGGC TCGGCGATAC GAGCGGCGAC GCCTGTTCTG GAGGAGGAAG CTGAGGTGCG AGTCCAACGA   
  
  
- GGAGTTCAAG GTCCTTCAGT CGGGCACCTG GTGGAAGCCG GTGCACCGAA CGTTGCCACG TTAGTAGCTC   
  
  
- CGGAATCTCT CCCTGGGTGT ACTTTTCTCA TTCGAGGTGC AGTAGCTATA GTCGTGGTGG AAGACGTGGG   
  
  
- TCACCGGGTG GAACGAACTC CGAAATCGGT GGGCGTACCT GCTGTGAGGG GTGGAGTCGG AGTGACGGCA   
  
  
- GCAGCAGTTG TTCATGCGGC GGCACTCGCC ACCGCTCCTG CTGCCCCCTC TTCCTAGTTT CTCCCACTAC   
  
  
- TCTCTCTAAC CAGAAGCAGA ACTCTTCAAG CGATCCAATT ACCCCCAAGG GAAGTTCAAG TTGCACCAAA   
  
  
- TGGTGCATCC ACTAGATAGG GTCAACCTAA AAAGAGCAAA CCTATAATTT CTACTACTTC GCCACCGCTA   
  
  
- ATTGACACAG TTGAGCGAGG TGAGCCACCT GGAGGCTATA GCCCTCCATC ACGAGCGCCG CAAGTCCGCC   
  
  
- GACGCCGGCT CCCACCACTG CCACCACCTC CTTCTCCGCC TCGACTGACT CCACCCACTC CCAGTCATAC   
  
  
- TTAAAAAATC CGCCAAACTC CTCACAGAAT CCACCAAATC CCAAATGAAA CTCCGGCATC CTCTAACAAA   
  
  
- AGGATCCTGA TCGTTACTCT TTAACTACAA TCTCGCCCGG CGCCCGGCCC GGTACCACCT AGAGGAACGT   
  
  
- ACAGGGCTCG GACCACCCAG CCGACTCCCC TCCCTCTGCC GCTCTGCCAC CAGCCCAGCC TACGTACCCC   
  
  
- GCCCCAAGCT CGTGCACCCC AAGTCACTGC TTCACACACT ACTACACTCC CGGAACAACT CCTCCATGTT   
  
  
- CCTTCCTACC AGTTACTGTG TCACGTGGTA CCTACCGCCA TTAGTGGGAC CTTATGACAA CACCACCGCC   
  
  
- CTAGTTGGCC ACCAAACCCG GTCACGTACC GCCGGAAC

+     ERE

| Site Name | Organism | Position | Strand | Matrix score. | sequence | function |
| --- | --- | --- | --- | --- | --- | --- |
| ERE | Nicotiana glutinos | 1599 | + | 8 | ATTTCATA |  |
| ERE | Nicotiana glutinos | 1490 | + | 8 | ATTTTAAA |  |

>HU05G01983.1   
+ +Up\_Stream \_Len000AAATCA AACTCATAAA CAATAATTTT TTAAAACTTA AATGGTAATT ATTAGATTAA   
  
  
+ TAAAAATCCT AATGGTTGTT TAATGTTACA TCAAGACATG CTTTTTGGTG TATATATAAT GGCAGCATGT   
  
  
+ GGGTTAAGCG GCAACAAATT ATTCTCAAAT GCATGCAGTA ATTAATTTAA TTAATTAATC ACCTGCATAA   
  
  
+ TCTCAATACT AATCCTTTCA TTCTGTGCCC CACCCAACCC CGAAAATAGT AATCTTTCAA ACGTTGGAAG   
  
  
+ TGGGATTAGG TATAGAGTAG TCCTACAGAT GATGATCTAC CTACATATAA CATAATCTGA CTTTTCTTTT   
  
  
+ TTAAATAACT TGTATTGATG GCACAGAATT TGACATGTTG GCATATGGTA CTAAGAAATT AATTAACGTT   
  
  
+ GTGCTAAGAT ATAGGTCATT CTTTTTGGAG ACATAAGGCC CGCGTTCACG CAGGGTTCGA GAAGGGCCAC   
  
  
+ ATTCAATGGA TGAATTGTAG GTAGTTTAAT CTGACTTTGT CAGTGACTGA TTCCACGGCT TGAACCCGCG   
  
  
+ ACATTAATAT AGGTTGACTA AAGTCGTTTG ATACTTATTT TTCTTAAGTA AAGTCTTGAA TTCAAATCTT   
  
  
+ ATAAATAAAT AAAAAAATCT ATAATAAAAG AATTCTACCA TTTATTAAGT CAACAACTCA ATTCGAATGA   
  
  
+ AATAAAAAAT CAAGATCACC AAAAAAAATA AATAAAAGGA AAGGTAGATT GCATTTGGAT TTTGAGTGGC   
  
  
+ GTGGTGGTGG GTTTGATGCT GGTGCTGCAT ACACCGCAGC TTTTCTCTGT CCTTTTACCT CTCTTTGTCT   
  
  
+ GCAAGAACCC CATTTGGCAC GCCTGCTCTC TTTTCTATGT TTTCTTTTCT ATCTTTTAAG AAACAATGGA   
  
  
+ AAGCTAACAC ACTAACACTA ACCCATTGGT CCTTTTTTCT TATCTGCAAA ATTAAAGTAA CAATATTTTT   
  
  
+ CTTATCCCCT TTTTCGTTCC ACCCATTTCT TTATCCTTCT GTTTGAACCT AATCTGTATA TATGCATGCA   
  
  
+ TGTGTATTTT ACTTAGAATC TGTTTTGATT CTTTGATGTA ATTAATTAGT AGTCTTTTTA ATTGAATTTT   
  
  
+ TAACAGTAGT TTGAATTAGA CATTTATCGA TATTATATTT TTTATATATA ATTAAAAAAT ACAAAATGAT   
  
  
+ AAGTGTCTTA CTACAATACA CTTCATAAGT GTCGTGTTTT AACTTAAAAA TCATACTATT ATGTTTCTAA   
  
  
+ ATTGAAAAAA AAGTGAGATT AAGTAGCAGA AGCATATTAC AACTTATAAA TTATATAAAA TCATCTTTTT   
  
  
+ ATCCATAATT TATCACATTT CTAAAATACG CATATCATAT CTACTTATGG ATAGGGTTAA TAAGATTAGG   
  
  
+ GCATCATTTA AGATTTTATC AGCGTAACCA ACGAATGTTT AAGGTTAGCA GTTCATAATT AAGTGTAATC   
  
  
+ ATTAAAAAAT TTATGATTTT AAATCATAAA ATATGTTAAT CTAGGATGAG GCAAGCGGCA AGACATATAT   
  
  
+ CACTATCCTT AAAAAATATA ATATATTGAT TTCAAAGAAT TATGAAGTAT ATTAATTTCA TATATAATGA   
  
  
+ CGATTATGTA TTTTTTTACT AAATTACTTG TACCCATAAT AAATAGAATC GATGCATCAT TCATGGCCTT   
  
  
+ TCATTCTGTA ATATGGTTGA CTCCAAAAAT TCTCAAAGTA TTTTTATATT TTGGGAGGGG AGATTTTAGT   
  
  
+ TTGATACTTT TGTTGAACTA GTGAACAATT TCCAACAACC TCCACCACCC CCAAACATCA ACAATCTGAT   
  
  
+ TGATGTATGC ATCCCAAGCT TGAATTGAAT AATAATTTCA ATAACAACAA TAATCGTTTT ACCTTGACAA   
  
  
+ GCTCCACAGC AACTCAACGT CTTCCAAACC AACCCATCAC TCATCATCAT CATCCCCACT ATCCCATTCA   
  
  
+ CCCCCACCAA AAGGAGCCTA AAGACAGTAA CAAGAAGAAT GCTTGCCTTT TTTCATGGAT GAAGAAGACT   
  
  
+ TCTCTTCTTC CTCTACTTCT TCCCACCACT ATGATACACA CCACCTCCAA CATCATTACC CCACCAATTA   
  
  
+ TAATTCCCGT TATACCCTGG ACCCTCCCTC CACCACCACC ACAGCCACAA CCACCACCTC ATCCTCCACT   
  
  
+ CCCACCCACC ATGGCTTCGG TGGTGGGCTT GACTCCTCCT CCCCGTCTGC CGCTGTGGAA TTATCTTTCT   
  
  
+ CCCCGGACAT CCTCCTCTCC TCCCCCACCG GAAAGTGGGC GTCTGACATT CTTATGGAGG CGGCTAGGGC   
  
  
+ TTTTTCAGAC ACGAACACCA CCAAGCTCCA CCAGCTCCTC TGGATGCTGA ACGAGCTCGG CTCCCCCTAC   
  
  
+ GGTGATGTCG AGCAGAAGCT GGTGGCGTAC TTTAACCAAG CCCTTTTTGC CCGCCTCACT GCCACCGGAC   
  
  
+ CCCGCCACCG AGCCGCTATG CTCGCCGCTG CGGACAAGAC CTCCTCCTTC GACTCCACGC TCAGGTTGCT   
  
  
+ CCTCAAGTTC CAGGAAGTCA GCCCGTGGAC CACCTTCGGC CACGTGGCTT GCAACGGTGC AATCATCGAG   
  
  
+ GCCTTAGAGA GGGACCCACA TGAAAAGAGT AAGCTCCACG TCATCGATAT CAGCACCACC TTCTGCACCC   
  
  
+ AGTGGCCCAC CTTGCTTGAG GCTTTAGCCA CCCGCATGGA CGACACTCCC CACCTCAGCC TCACTGCCGT   
  
  
+ CGTCGTCAAC AAGTACGCCG CCGTGAGCGG TGGCGAGGAC GACGGGGGAG AAGGATCAAA GAGGGTGATG   
  
  
+ AGAGAGATTG GTCTTCGTCT TGAGAAGTTC GCTAGGTTAA TGGGGGTTCC CTTCAAGTTC AACGTGGTTT   
  
  
+ ACCACGTAGG TGATCTATCC CAGTTGGATT TTTCTCGTTT GGATATTAAA GATGATGAAG CGGTGGCGAT   
  
  
+ TAACTGTGTC AACTCGCTCC ACTCGGTGGA CCTCCGATAT CGGGAGGTAG TGCTCGCGGC GTTCAGGCGG   
  
  
+ CTGCGGCCGA GGGTGGTGAC GGTGGTGGAG GAAGAGGCGG AGCTGACTGA GGTGGGTGAG GGTCAGTATG   
  
  
+ AATTTTTTAG GCGGTTTGAG GAGTGTCTTA GGTGGTTTAG GGTTTACTTT GAGGCCGTAG GAGATTGTTT   
  
  
+ TCCTAGGACT AGCAATGAGA AATTGATGTT AGAGCGGGCC GCGGGCCGGG CCATGGTGGA TCTCCTTGCA   
  
  
+ TGTCCCGAGC CTGGTGGGTC GGCTGAGGGG AGGGAGACGG CGAGACGGTG GTCGGGTCGG ATGCATGGGG   
  
  
+ CGGGGTTCGA GCACGTGGGG TTCAGTGACG AAGTGTGTGA TGATGTGAGG GCCTTGTTGA GGAGGTACAA   
  
  
+ GGAAGGATGG TCAATGACAC AGTGCACCAT GGATGGCGGT AATCACCCTG GAATACTGTT GTGGTGGCGG   
  
  
+ GATCAACCGG TGGTTTGGGC CAGTGCATGG CGGCCTTG  

- +Up\_Stream \_Len000TTTAGT TTGAGTATTT GTTATTAAAA AATTTTGAAT TTACCATTAA TAATCTAATT   
  
  
- ATTTTTAGGA TTACCAACAA ATTACAATGT AGTTCTGTAC GAAAAACCAC ATATATATTA CCGTCGTACA   
  
  
- CCCAATTCGC CGTTGTTTAA TAAGAGTTTA CGTACGTCAT TAATTAAATT AATTAATTAG TGGACGTATT   
  
  
- AGAGTTATGA TTAGGAAAGT AAGACACGGG GTGGGTTGGG GCTTTTATCA TTAGAAAGTT TGCAACCTTC   
  
  
- ACCCTAATCC ATATCTCATC AGGATGTCTA CTACTAGATG GATGTATATT GTATTAGACT GAAAAGAAAA   
  
  
- AATTTATTGA ACATAACTAC CGTGTCTTAA ACTGTACAAC CGTATACCAT GATTCTTTAA TTAATTGCAA   
  
  
- CACGATTCTA TATCCAGTAA GAAAAACCTC TGTATTCCGG GCGCAAGTGC GTCCCAAGCT CTTCCCGGTG   
  
  
- TAAGTTACCT ACTTAACATC CATCAAATTA GACTGAAACA GTCACTGACT AAGGTGCCGA ACTTGGGCGC   
  
  
- TGTAATTATA TCCAACTGAT TTCAGCAAAC TATGAATAAA AAGAATTCAT TTCAGAACTT AAGTTTAGAA   
  
  
- TATTTATTTA TTTTTTTAGA TATTATTTTC TTAAGATGGT AAATAATTCA GTTGTTGAGT TAAGCTTACT   
  
  
- TTATTTTTTA GTTCTAGTGG TTTTTTTTAT TTATTTTCCT TTCCATCTAA CGTAAACCTA AAACTCACCG   
  
  
- CACCACCACC CAAACTACGA CCACGACGTA TGTGGCGTCG AAAAGAGACA GGAAAATGGA GAGAAACAGA   
  
  
- CGTTCTTGGG GTAAACCGTG CGGACGAGAG AAAAGATACA AAAGAAAAGA TAGAAAATTC TTTGTTACCT   
  
  
- TTCGATTGTG TGATTGTGAT TGGGTAACCA GGAAAAAAGA ATAGACGTTT TAATTTCATT GTTATAAAAA   
  
  
- GAATAGGGGA AAAAGCAAGG TGGGTAAAGA AATAGGAAGA CAAACTTGGA TTAGACATAT ATACGTACGT   
  
  
- ACACATAAAA TGAATCTTAG ACAAAACTAA GAAACTACAT TAATTAATCA TCAGAAAAAT TAACTTAAAA   
  
  
- ATTGTCATCA AACTTAATCT GTAAATAGCT ATAATATAAA AAATATATAT TAATTTTTTA TGTTTTACTA   
  
  
- TTCACAGAAT GATGTTATGT GAAGTATTCA CAGCACAAAA TTGAATTTTT AGTATGATAA TACAAAGATT   
  
  
- TAACTTTTTT TTCACTCTAA TTCATCGTCT TCGTATAATG TTGAATATTT AATATATTTT AGTAGAAAAA   
  
  
- TAGGTATTAA ATAGTGTAAA GATTTTATGC GTATAGTATA GATGAATACC TATCCCAATT ATTCTAATCC   
  
  
- CGTAGTAAAT TCTAAAATAG TCGCATTGGT TGCTTACAAA TTCCAATCGT CAAGTATTAA TTCACATTAG   
  
  
- TAATTTTTTA AATACTAAAA TTTAGTATTT TATACAATTA GATCCTACTC CGTTCGCCGT TCTGTATATA   
  
  
- GTGATAGGAA TTTTTTATAT TATATAACTA AAGTTTCTTA ATACTTCATA TAATTAAAGT ATATATTACT   
  
  
- GCTAATACAT AAAAAAATGA TTTAATGAAC ATGGGTATTA TTTATCTTAG CTACGTAGTA AGTACCGGAA   
  
  
- AGTAAGACAT TATACCAACT GAGGTTTTTA AGAGTTTCAT AAAAATATAA AACCCTCCCC TCTAAAATCA   
  
  
- AACTATGAAA ACAACTTGAT CACTTGTTAA AGGTTGTTGG AGGTGGTGGG GGTTTGTAGT TGTTAGACTA   
  
  
- ACTACATACG TAGGGTTCGA ACTTAACTTA TTATTAAAGT TATTGTTGTT ATTAGCAAAA TGGAACTGTT   
  
  
- CGAGGTGTCG TTGAGTTGCA GAAGGTTTGG TTGGGTAGTG AGTAGTAGTA GTAGGGGTGA TAGGGTAAGT   
  
  
- GGGGGTGGTT TTCCTCGGAT TTCTGTCATT GTTCTTCTTA CGAACGGAAA AAAGTACCTA CTTCTTCTGA   
  
  
- AGAGAAGAAG GAGATGAAGA AGGGTGGTGA TACTATGTGT GGTGGAGGTT GTAGTAATGG GGTGGTTAAT   
  
  
- ATTAAGGGCA ATATGGGACC TGGGAGGGAG GTGGTGGTGG TGTCGGTGTT GGTGGTGGAG TAGGAGGTGA   
  
  
- GGGTGGGTGG TACCGAAGCC ACCACCCGAA CTGAGGAGGA GGGGCAGACG GCGACACCTT AATAGAAAGA   
  
  
- GGGGCCTGTA GGAGGAGAGG AGGGGGTGGC CTTTCACCCG CAGACTGTAA GAATACCTCC GCCGATCCCG   
  
  
- AAAAAGTCTG TGCTTGTGGT GGTTCGAGGT GGTCGAGGAG ACCTACGACT TGCTCGAGCC GAGGGGGATG   
  
  
- CCACTACAGC TCGTCTTCGA CCACCGCATG AAATTGGTTC GGGAAAAACG GGCGGAGTGA CGGTGGCCTG   
  
  
- GGGCGGTGGC TCGGCGATAC GAGCGGCGAC GCCTGTTCTG GAGGAGGAAG CTGAGGTGCG AGTCCAACGA   
  
  
- GGAGTTCAAG GTCCTTCAGT CGGGCACCTG GTGGAAGCCG GTGCACCGAA CGTTGCCACG TTAGTAGCTC   
  
  
- CGGAATCTCT CCCTGGGTGT ACTTTTCTCA TTCGAGGTGC AGTAGCTATA GTCGTGGTGG AAGACGTGGG   
  
  
- TCACCGGGTG GAACGAACTC CGAAATCGGT GGGCGTACCT GCTGTGAGGG GTGGAGTCGG AGTGACGGCA   
  
  
- GCAGCAGTTG TTCATGCGGC GGCACTCGCC ACCGCTCCTG CTGCCCCCTC TTCCTAGTTT CTCCCACTAC   
  
  
- TCTCTCTAAC CAGAAGCAGA ACTCTTCAAG CGATCCAATT ACCCCCAAGG GAAGTTCAAG TTGCACCAAA   
  
  
- TGGTGCATCC ACTAGATAGG GTCAACCTAA AAAGAGCAAA CCTATAATTT CTACTACTTC GCCACCGCTA   
  
  
- ATTGACACAG TTGAGCGAGG TGAGCCACCT GGAGGCTATA GCCCTCCATC ACGAGCGCCG CAAGTCCGCC   
  
  
- GACGCCGGCT CCCACCACTG CCACCACCTC CTTCTCCGCC TCGACTGACT CCACCCACTC CCAGTCATAC   
  
  
- TTAAAAAATC CGCCAAACTC CTCACAGAAT CCACCAAATC CCAAATGAAA CTCCGGCATC CTCTAACAAA   
  
  
- AGGATCCTGA TCGTTACTCT TTAACTACAA TCTCGCCCGG CGCCCGGCCC GGTACCACCT AGAGGAACGT   
  
  
- ACAGGGCTCG GACCACCCAG CCGACTCCCC TCCCTCTGCC GCTCTGCCAC CAGCCCAGCC TACGTACCCC   
  
  
- GCCCCAAGCT CGTGCACCCC AAGTCACTGC TTCACACACT ACTACACTCC CGGAACAACT CCTCCATGTT   
  
  
- CCTTCCTACC AGTTACTGTG TCACGTGGTA CCTACCGCCA TTAGTGGGAC CTTATGACAA CACCACCGCC   
  
  
- CTAGTTGGCC ACCAAACCCG GTCACGTACC GCCGGAAC

+     G-Box

| Site Name | Organism | Position | Strand | Matrix score. | sequence | function |
| --- | --- | --- | --- | --- | --- | --- |
| G-Box | Pisum sativum | 2565 | - | 6 | CACGTG | cis-acting regulatory element involved in light responsiveness |
| G-Box | Pisum sativum | 3306 | - | 6 | CACGTG | cis-acting regulatory element involved in light responsiveness |
| G-Box | Pisum sativum | 2865 | - | 6 | CACGTT | cis-acting regulatory element involved in light responsiveness |

>HU05G01983.1   
+ +Up\_Stream \_Len000AAATCA AACTCATAAA CAATAATTTT TTAAAACTTA AATGGTAATT ATTAGATTAA   
  
  
+ TAAAAATCCT AATGGTTGTT TAATGTTACA TCAAGACATG CTTTTTGGTG TATATATAAT GGCAGCATGT   
  
  
+ GGGTTAAGCG GCAACAAATT ATTCTCAAAT GCATGCAGTA ATTAATTTAA TTAATTAATC ACCTGCATAA   
  
  
+ TCTCAATACT AATCCTTTCA TTCTGTGCCC CACCCAACCC CGAAAATAGT AATCTTTCAA ACGTTGGAAG   
  
  
+ TGGGATTAGG TATAGAGTAG TCCTACAGAT GATGATCTAC CTACATATAA CATAATCTGA CTTTTCTTTT   
  
  
+ TTAAATAACT TGTATTGATG GCACAGAATT TGACATGTTG GCATATGGTA CTAAGAAATT AATTAACGTT   
  
  
+ GTGCTAAGAT ATAGGTCATT CTTTTTGGAG ACATAAGGCC CGCGTTCACG CAGGGTTCGA GAAGGGCCAC   
  
  
+ ATTCAATGGA TGAATTGTAG GTAGTTTAAT CTGACTTTGT CAGTGACTGA TTCCACGGCT TGAACCCGCG   
  
  
+ ACATTAATAT AGGTTGACTA AAGTCGTTTG ATACTTATTT TTCTTAAGTA AAGTCTTGAA TTCAAATCTT   
  
  
+ ATAAATAAAT AAAAAAATCT ATAATAAAAG AATTCTACCA TTTATTAAGT CAACAACTCA ATTCGAATGA   
  
  
+ AATAAAAAAT CAAGATCACC AAAAAAAATA AATAAAAGGA AAGGTAGATT GCATTTGGAT TTTGAGTGGC   
  
  
+ GTGGTGGTGG GTTTGATGCT GGTGCTGCAT ACACCGCAGC TTTTCTCTGT CCTTTTACCT CTCTTTGTCT   
  
  
+ GCAAGAACCC CATTTGGCAC GCCTGCTCTC TTTTCTATGT TTTCTTTTCT ATCTTTTAAG AAACAATGGA   
  
  
+ AAGCTAACAC ACTAACACTA ACCCATTGGT CCTTTTTTCT TATCTGCAAA ATTAAAGTAA CAATATTTTT   
  
  
+ CTTATCCCCT TTTTCGTTCC ACCCATTTCT TTATCCTTCT GTTTGAACCT AATCTGTATA TATGCATGCA   
  
  
+ TGTGTATTTT ACTTAGAATC TGTTTTGATT CTTTGATGTA ATTAATTAGT AGTCTTTTTA ATTGAATTTT   
  
  
+ TAACAGTAGT TTGAATTAGA CATTTATCGA TATTATATTT TTTATATATA ATTAAAAAAT ACAAAATGAT   
  
  
+ AAGTGTCTTA CTACAATACA CTTCATAAGT GTCGTGTTTT AACTTAAAAA TCATACTATT ATGTTTCTAA   
  
  
+ ATTGAAAAAA AAGTGAGATT AAGTAGCAGA AGCATATTAC AACTTATAAA TTATATAAAA TCATCTTTTT   
  
  
+ ATCCATAATT TATCACATTT CTAAAATACG CATATCATAT CTACTTATGG ATAGGGTTAA TAAGATTAGG   
  
  
+ GCATCATTTA AGATTTTATC AGCGTAACCA ACGAATGTTT AAGGTTAGCA GTTCATAATT AAGTGTAATC   
  
  
+ ATTAAAAAAT TTATGATTTT AAATCATAAA ATATGTTAAT CTAGGATGAG GCAAGCGGCA AGACATATAT   
  
  
+ CACTATCCTT AAAAAATATA ATATATTGAT TTCAAAGAAT TATGAAGTAT ATTAATTTCA TATATAATGA   
  
  
+ CGATTATGTA TTTTTTTACT AAATTACTTG TACCCATAAT AAATAGAATC GATGCATCAT TCATGGCCTT   
  
  
+ TCATTCTGTA ATATGGTTGA CTCCAAAAAT TCTCAAAGTA TTTTTATATT TTGGGAGGGG AGATTTTAGT   
  
  
+ TTGATACTTT TGTTGAACTA GTGAACAATT TCCAACAACC TCCACCACCC CCAAACATCA ACAATCTGAT   
  
  
+ TGATGTATGC ATCCCAAGCT TGAATTGAAT AATAATTTCA ATAACAACAA TAATCGTTTT ACCTTGACAA   
  
  
+ GCTCCACAGC AACTCAACGT CTTCCAAACC AACCCATCAC TCATCATCAT CATCCCCACT ATCCCATTCA   
  
  
+ CCCCCACCAA AAGGAGCCTA AAGACAGTAA CAAGAAGAAT GCTTGCCTTT TTTCATGGAT GAAGAAGACT   
  
  
+ TCTCTTCTTC CTCTACTTCT TCCCACCACT ATGATACACA CCACCTCCAA CATCATTACC CCACCAATTA   
  
  
+ TAATTCCCGT TATACCCTGG ACCCTCCCTC CACCACCACC ACAGCCACAA CCACCACCTC ATCCTCCACT   
  
  
+ CCCACCCACC ATGGCTTCGG TGGTGGGCTT GACTCCTCCT CCCCGTCTGC CGCTGTGGAA TTATCTTTCT   
  
  
+ CCCCGGACAT CCTCCTCTCC TCCCCCACCG GAAAGTGGGC GTCTGACATT CTTATGGAGG CGGCTAGGGC   
  
  
+ TTTTTCAGAC ACGAACACCA CCAAGCTCCA CCAGCTCCTC TGGATGCTGA ACGAGCTCGG CTCCCCCTAC   
  
  
+ GGTGATGTCG AGCAGAAGCT GGTGGCGTAC TTTAACCAAG CCCTTTTTGC CCGCCTCACT GCCACCGGAC   
  
  
+ CCCGCCACCG AGCCGCTATG CTCGCCGCTG CGGACAAGAC CTCCTCCTTC GACTCCACGC TCAGGTTGCT   
  
  
+ CCTCAAGTTC CAGGAAGTCA GCCCGTGGAC CACCTTCGGC CACGTGGCTT GCAACGGTGC AATCATCGAG   
  
  
+ GCCTTAGAGA GGGACCCACA TGAAAAGAGT AAGCTCCACG TCATCGATAT CAGCACCACC TTCTGCACCC   
  
  
+ AGTGGCCCAC CTTGCTTGAG GCTTTAGCCA CCCGCATGGA CGACACTCCC CACCTCAGCC TCACTGCCGT   
  
  
+ CGTCGTCAAC AAGTACGCCG CCGTGAGCGG TGGCGAGGAC GACGGGGGAG AAGGATCAAA GAGGGTGATG   
  
  
+ AGAGAGATTG GTCTTCGTCT TGAGAAGTTC GCTAGGTTAA TGGGGGTTCC CTTCAAGTTC AACGTGGTTT   
  
  
+ ACCACGTAGG TGATCTATCC CAGTTGGATT TTTCTCGTTT GGATATTAAA GATGATGAAG CGGTGGCGAT   
  
  
+ TAACTGTGTC AACTCGCTCC ACTCGGTGGA CCTCCGATAT CGGGAGGTAG TGCTCGCGGC GTTCAGGCGG   
  
  
+ CTGCGGCCGA GGGTGGTGAC GGTGGTGGAG GAAGAGGCGG AGCTGACTGA GGTGGGTGAG GGTCAGTATG   
  
  
+ AATTTTTTAG GCGGTTTGAG GAGTGTCTTA GGTGGTTTAG GGTTTACTTT GAGGCCGTAG GAGATTGTTT   
  
  
+ TCCTAGGACT AGCAATGAGA AATTGATGTT AGAGCGGGCC GCGGGCCGGG CCATGGTGGA TCTCCTTGCA   
  
  
+ TGTCCCGAGC CTGGTGGGTC GGCTGAGGGG AGGGAGACGG CGAGACGGTG GTCGGGTCGG ATGCATGGGG   
  
  
+ CGGGGTTCGA GCACGTGGGG TTCAGTGACG AAGTGTGTGA TGATGTGAGG GCCTTGTTGA GGAGGTACAA   
  
  
+ GGAAGGATGG TCAATGACAC AGTGCACCAT GGATGGCGGT AATCACCCTG GAATACTGTT GTGGTGGCGG   
  
  
+ GATCAACCGG TGGTTTGGGC CAGTGCATGG CGGCCTTG  

- +Up\_Stream \_Len000TTTAGT TTGAGTATTT GTTATTAAAA AATTTTGAAT TTACCATTAA TAATCTAATT   
  
  
- ATTTTTAGGA TTACCAACAA ATTACAATGT AGTTCTGTAC GAAAAACCAC ATATATATTA CCGTCGTACA   
  
  
- CCCAATTCGC CGTTGTTTAA TAAGAGTTTA CGTACGTCAT TAATTAAATT AATTAATTAG TGGACGTATT   
  
  
- AGAGTTATGA TTAGGAAAGT AAGACACGGG GTGGGTTGGG GCTTTTATCA TTAGAAAGTT TGCAACCTTC   
  
  
- ACCCTAATCC ATATCTCATC AGGATGTCTA CTACTAGATG GATGTATATT GTATTAGACT GAAAAGAAAA   
  
  
- AATTTATTGA ACATAACTAC CGTGTCTTAA ACTGTACAAC CGTATACCAT GATTCTTTAA TTAATTGCAA   
  
  
- CACGATTCTA TATCCAGTAA GAAAAACCTC TGTATTCCGG GCGCAAGTGC GTCCCAAGCT CTTCCCGGTG   
  
  
- TAAGTTACCT ACTTAACATC CATCAAATTA GACTGAAACA GTCACTGACT AAGGTGCCGA ACTTGGGCGC   
  
  
- TGTAATTATA TCCAACTGAT TTCAGCAAAC TATGAATAAA AAGAATTCAT TTCAGAACTT AAGTTTAGAA   
  
  
- TATTTATTTA TTTTTTTAGA TATTATTTTC TTAAGATGGT AAATAATTCA GTTGTTGAGT TAAGCTTACT   
  
  
- TTATTTTTTA GTTCTAGTGG TTTTTTTTAT TTATTTTCCT TTCCATCTAA CGTAAACCTA AAACTCACCG   
  
  
- CACCACCACC CAAACTACGA CCACGACGTA TGTGGCGTCG AAAAGAGACA GGAAAATGGA GAGAAACAGA   
  
  
- CGTTCTTGGG GTAAACCGTG CGGACGAGAG AAAAGATACA AAAGAAAAGA TAGAAAATTC TTTGTTACCT   
  
  
- TTCGATTGTG TGATTGTGAT TGGGTAACCA GGAAAAAAGA ATAGACGTTT TAATTTCATT GTTATAAAAA   
  
  
- GAATAGGGGA AAAAGCAAGG TGGGTAAAGA AATAGGAAGA CAAACTTGGA TTAGACATAT ATACGTACGT   
  
  
- ACACATAAAA TGAATCTTAG ACAAAACTAA GAAACTACAT TAATTAATCA TCAGAAAAAT TAACTTAAAA   
  
  
- ATTGTCATCA AACTTAATCT GTAAATAGCT ATAATATAAA AAATATATAT TAATTTTTTA TGTTTTACTA   
  
  
- TTCACAGAAT GATGTTATGT GAAGTATTCA CAGCACAAAA TTGAATTTTT AGTATGATAA TACAAAGATT   
  
  
- TAACTTTTTT TTCACTCTAA TTCATCGTCT TCGTATAATG TTGAATATTT AATATATTTT AGTAGAAAAA   
  
  
- TAGGTATTAA ATAGTGTAAA GATTTTATGC GTATAGTATA GATGAATACC TATCCCAATT ATTCTAATCC   
  
  
- CGTAGTAAAT TCTAAAATAG TCGCATTGGT TGCTTACAAA TTCCAATCGT CAAGTATTAA TTCACATTAG   
  
  
- TAATTTTTTA AATACTAAAA TTTAGTATTT TATACAATTA GATCCTACTC CGTTCGCCGT TCTGTATATA   
  
  
- GTGATAGGAA TTTTTTATAT TATATAACTA AAGTTTCTTA ATACTTCATA TAATTAAAGT ATATATTACT   
  
  
- GCTAATACAT AAAAAAATGA TTTAATGAAC ATGGGTATTA TTTATCTTAG CTACGTAGTA AGTACCGGAA   
  
  
- AGTAAGACAT TATACCAACT GAGGTTTTTA AGAGTTTCAT AAAAATATAA AACCCTCCCC TCTAAAATCA   
  
  
- AACTATGAAA ACAACTTGAT CACTTGTTAA AGGTTGTTGG AGGTGGTGGG GGTTTGTAGT TGTTAGACTA   
  
  
- ACTACATACG TAGGGTTCGA ACTTAACTTA TTATTAAAGT TATTGTTGTT ATTAGCAAAA TGGAACTGTT   
  
  
- CGAGGTGTCG TTGAGTTGCA GAAGGTTTGG TTGGGTAGTG AGTAGTAGTA GTAGGGGTGA TAGGGTAAGT   
  
  
- GGGGGTGGTT TTCCTCGGAT TTCTGTCATT GTTCTTCTTA CGAACGGAAA AAAGTACCTA CTTCTTCTGA   
  
  
- AGAGAAGAAG GAGATGAAGA AGGGTGGTGA TACTATGTGT GGTGGAGGTT GTAGTAATGG GGTGGTTAAT   
  
  
- ATTAAGGGCA ATATGGGACC TGGGAGGGAG GTGGTGGTGG TGTCGGTGTT GGTGGTGGAG TAGGAGGTGA   
  
  
- GGGTGGGTGG TACCGAAGCC ACCACCCGAA CTGAGGAGGA GGGGCAGACG GCGACACCTT AATAGAAAGA   
  
  
- GGGGCCTGTA GGAGGAGAGG AGGGGGTGGC CTTTCACCCG CAGACTGTAA GAATACCTCC GCCGATCCCG   
  
  
- AAAAAGTCTG TGCTTGTGGT GGTTCGAGGT GGTCGAGGAG ACCTACGACT TGCTCGAGCC GAGGGGGATG   
  
  
- CCACTACAGC TCGTCTTCGA CCACCGCATG AAATTGGTTC GGGAAAAACG GGCGGAGTGA CGGTGGCCTG   
  
  
- GGGCGGTGGC TCGGCGATAC GAGCGGCGAC GCCTGTTCTG GAGGAGGAAG CTGAGGTGCG AGTCCAACGA   
  
  
- GGAGTTCAAG GTCCTTCAGT CGGGCACCTG GTGGAAGCCG GTGCACCGAA CGTTGCCACG TTAGTAGCTC   
  
  
- CGGAATCTCT CCCTGGGTGT ACTTTTCTCA TTCGAGGTGC AGTAGCTATA GTCGTGGTGG AAGACGTGGG   
  
  
- TCACCGGGTG GAACGAACTC CGAAATCGGT GGGCGTACCT GCTGTGAGGG GTGGAGTCGG AGTGACGGCA   
  
  
- GCAGCAGTTG TTCATGCGGC GGCACTCGCC ACCGCTCCTG CTGCCCCCTC TTCCTAGTTT CTCCCACTAC   
  
  
- TCTCTCTAAC CAGAAGCAGA ACTCTTCAAG CGATCCAATT ACCCCCAAGG GAAGTTCAAG TTGCACCAAA   
  
  
- TGGTGCATCC ACTAGATAGG GTCAACCTAA AAAGAGCAAA CCTATAATTT CTACTACTTC GCCACCGCTA   
  
  
- ATTGACACAG TTGAGCGAGG TGAGCCACCT GGAGGCTATA GCCCTCCATC ACGAGCGCCG CAAGTCCGCC   
  
  
- GACGCCGGCT CCCACCACTG CCACCACCTC CTTCTCCGCC TCGACTGACT CCACCCACTC CCAGTCATAC   
  
  
- TTAAAAAATC CGCCAAACTC CTCACAGAAT CCACCAAATC CCAAATGAAA CTCCGGCATC CTCTAACAAA   
  
  
- AGGATCCTGA TCGTTACTCT TTAACTACAA TCTCGCCCGG CGCCCGGCCC GGTACCACCT AGAGGAACGT   
  
  
- ACAGGGCTCG GACCACCCAG CCGACTCCCC TCCCTCTGCC GCTCTGCCAC CAGCCCAGCC TACGTACCCC   
  
  
- GCCCCAAGCT CGTGCACCCC AAGTCACTGC TTCACACACT ACTACACTCC CGGAACAACT CCTCCATGTT   
  
  
- CCTTCCTACC AGTTACTGTG TCACGTGGTA CCTACCGCCA TTAGTGGGAC CTTATGACAA CACCACCGCC   
  
  
- CTAGTTGGCC ACCAAACCCG GTCACGTACC GCCGGAAC

+     G-box

| Site Name | Organism | Position | Strand | Matrix score. | sequence | function |
| --- | --- | --- | --- | --- | --- | --- |
| G-box | Brassica oleracea | 2874 | + | 9 | TAACACGTAG | cis-acting regulatory element involved in light responsiveness |
| G-box | Arabidopsis thaliana | 2565 | - | 6 | CACGTG | cis-acting regulatory element involved in light responsiveness |
| G-box | Zea mays | 2631 | + | 6 | CACGTC | cis-acting regulatory element involved in light responsiveness |
| G-box | Arabidopsis thaliana | 2563 | - | 9 | GCCACGTGGA | cis-acting regulatory element involved in light responsiveness |
| G-box | Zea mays | 1225 | - | 6 | CACGAC | cis-acting regulatory element involved in light responsiveness |
| G-box | Arabidopsis thaliana | 3306 | - | 6 | CACGTG | cis-acting regulatory element involved in light responsiveness |
| G-box | Arabidopsis thaliana | 2877 | - | 6 | TACGTG | cis-acting regulatory element involved in light responsiveness |

>HU05G01983.1   
+ +Up\_Stream \_Len000AAATCA AACTCATAAA CAATAATTTT TTAAAACTTA AATGGTAATT ATTAGATTAA   
  
  
+ TAAAAATCCT AATGGTTGTT TAATGTTACA TCAAGACATG CTTTTTGGTG TATATATAAT GGCAGCATGT   
  
  
+ GGGTTAAGCG GCAACAAATT ATTCTCAAAT GCATGCAGTA ATTAATTTAA TTAATTAATC ACCTGCATAA   
  
  
+ TCTCAATACT AATCCTTTCA TTCTGTGCCC CACCCAACCC CGAAAATAGT AATCTTTCAA ACGTTGGAAG   
  
  
+ TGGGATTAGG TATAGAGTAG TCCTACAGAT GATGATCTAC CTACATATAA CATAATCTGA CTTTTCTTTT   
  
  
+ TTAAATAACT TGTATTGATG GCACAGAATT TGACATGTTG GCATATGGTA CTAAGAAATT AATTAACGTT   
  
  
+ GTGCTAAGAT ATAGGTCATT CTTTTTGGAG ACATAAGGCC CGCGTTCACG CAGGGTTCGA GAAGGGCCAC   
  
  
+ ATTCAATGGA TGAATTGTAG GTAGTTTAAT CTGACTTTGT CAGTGACTGA TTCCACGGCT TGAACCCGCG   
  
  
+ ACATTAATAT AGGTTGACTA AAGTCGTTTG ATACTTATTT TTCTTAAGTA AAGTCTTGAA TTCAAATCTT   
  
  
+ ATAAATAAAT AAAAAAATCT ATAATAAAAG AATTCTACCA TTTATTAAGT CAACAACTCA ATTCGAATGA   
  
  
+ AATAAAAAAT CAAGATCACC AAAAAAAATA AATAAAAGGA AAGGTAGATT GCATTTGGAT TTTGAGTGGC   
  
  
+ GTGGTGGTGG GTTTGATGCT GGTGCTGCAT ACACCGCAGC TTTTCTCTGT CCTTTTACCT CTCTTTGTCT   
  
  
+ GCAAGAACCC CATTTGGCAC GCCTGCTCTC TTTTCTATGT TTTCTTTTCT ATCTTTTAAG AAACAATGGA   
  
  
+ AAGCTAACAC ACTAACACTA ACCCATTGGT CCTTTTTTCT TATCTGCAAA ATTAAAGTAA CAATATTTTT   
  
  
+ CTTATCCCCT TTTTCGTTCC ACCCATTTCT TTATCCTTCT GTTTGAACCT AATCTGTATA TATGCATGCA   
  
  
+ TGTGTATTTT ACTTAGAATC TGTTTTGATT CTTTGATGTA ATTAATTAGT AGTCTTTTTA ATTGAATTTT   
  
  
+ TAACAGTAGT TTGAATTAGA CATTTATCGA TATTATATTT TTTATATATA ATTAAAAAAT ACAAAATGAT   
  
  
+ AAGTGTCTTA CTACAATACA CTTCATAAGT GTCGTGTTTT AACTTAAAAA TCATACTATT ATGTTTCTAA   
  
  
+ ATTGAAAAAA AAGTGAGATT AAGTAGCAGA AGCATATTAC AACTTATAAA TTATATAAAA TCATCTTTTT   
  
  
+ ATCCATAATT TATCACATTT CTAAAATACG CATATCATAT CTACTTATGG ATAGGGTTAA TAAGATTAGG   
  
  
+ GCATCATTTA AGATTTTATC AGCGTAACCA ACGAATGTTT AAGGTTAGCA GTTCATAATT AAGTGTAATC   
  
  
+ ATTAAAAAAT TTATGATTTT AAATCATAAA ATATGTTAAT CTAGGATGAG GCAAGCGGCA AGACATATAT   
  
  
+ CACTATCCTT AAAAAATATA ATATATTGAT TTCAAAGAAT TATGAAGTAT ATTAATTTCA TATATAATGA   
  
  
+ CGATTATGTA TTTTTTTACT AAATTACTTG TACCCATAAT AAATAGAATC GATGCATCAT TCATGGCCTT   
  
  
+ TCATTCTGTA ATATGGTTGA CTCCAAAAAT TCTCAAAGTA TTTTTATATT TTGGGAGGGG AGATTTTAGT   
  
  
+ TTGATACTTT TGTTGAACTA GTGAACAATT TCCAACAACC TCCACCACCC CCAAACATCA ACAATCTGAT   
  
  
+ TGATGTATGC ATCCCAAGCT TGAATTGAAT AATAATTTCA ATAACAACAA TAATCGTTTT ACCTTGACAA   
  
  
+ GCTCCACAGC AACTCAACGT CTTCCAAACC AACCCATCAC TCATCATCAT CATCCCCACT ATCCCATTCA   
  
  
+ CCCCCACCAA AAGGAGCCTA AAGACAGTAA CAAGAAGAAT GCTTGCCTTT TTTCATGGAT GAAGAAGACT   
  
  
+ TCTCTTCTTC CTCTACTTCT TCCCACCACT ATGATACACA CCACCTCCAA CATCATTACC CCACCAATTA   
  
  
+ TAATTCCCGT TATACCCTGG ACCCTCCCTC CACCACCACC ACAGCCACAA CCACCACCTC ATCCTCCACT   
  
  
+ CCCACCCACC ATGGCTTCGG TGGTGGGCTT GACTCCTCCT CCCCGTCTGC CGCTGTGGAA TTATCTTTCT   
  
  
+ CCCCGGACAT CCTCCTCTCC TCCCCCACCG GAAAGTGGGC GTCTGACATT CTTATGGAGG CGGCTAGGGC   
  
  
+ TTTTTCAGAC ACGAACACCA CCAAGCTCCA CCAGCTCCTC TGGATGCTGA ACGAGCTCGG CTCCCCCTAC   
  
  
+ GGTGATGTCG AGCAGAAGCT GGTGGCGTAC TTTAACCAAG CCCTTTTTGC CCGCCTCACT GCCACCGGAC   
  
  
+ CCCGCCACCG AGCCGCTATG CTCGCCGCTG CGGACAAGAC CTCCTCCTTC GACTCCACGC TCAGGTTGCT   
  
  
+ CCTCAAGTTC CAGGAAGTCA GCCCGTGGAC CACCTTCGGC CACGTGGCTT GCAACGGTGC AATCATCGAG   
  
  
+ GCCTTAGAGA GGGACCCACA TGAAAAGAGT AAGCTCCACG TCATCGATAT CAGCACCACC TTCTGCACCC   
  
  
+ AGTGGCCCAC CTTGCTTGAG GCTTTAGCCA CCCGCATGGA CGACACTCCC CACCTCAGCC TCACTGCCGT   
  
  
+ CGTCGTCAAC AAGTACGCCG CCGTGAGCGG TGGCGAGGAC GACGGGGGAG AAGGATCAAA GAGGGTGATG   
  
  
+ AGAGAGATTG GTCTTCGTCT TGAGAAGTTC GCTAGGTTAA TGGGGGTTCC CTTCAAGTTC AACGTGGTTT   
  
  
+ ACCACGTAGG TGATCTATCC CAGTTGGATT TTTCTCGTTT GGATATTAAA GATGATGAAG CGGTGGCGAT   
  
  
+ TAACTGTGTC AACTCGCTCC ACTCGGTGGA CCTCCGATAT CGGGAGGTAG TGCTCGCGGC GTTCAGGCGG   
  
  
+ CTGCGGCCGA GGGTGGTGAC GGTGGTGGAG GAAGAGGCGG AGCTGACTGA GGTGGGTGAG GGTCAGTATG   
  
  
+ AATTTTTTAG GCGGTTTGAG GAGTGTCTTA GGTGGTTTAG GGTTTACTTT GAGGCCGTAG GAGATTGTTT   
  
  
+ TCCTAGGACT AGCAATGAGA AATTGATGTT AGAGCGGGCC GCGGGCCGGG CCATGGTGGA TCTCCTTGCA   
  
  
+ TGTCCCGAGC CTGGTGGGTC GGCTGAGGGG AGGGAGACGG CGAGACGGTG GTCGGGTCGG ATGCATGGGG   
  
  
+ CGGGGTTCGA GCACGTGGGG TTCAGTGACG AAGTGTGTGA TGATGTGAGG GCCTTGTTGA GGAGGTACAA   
  
  
+ GGAAGGATGG TCAATGACAC AGTGCACCAT GGATGGCGGT AATCACCCTG GAATACTGTT GTGGTGGCGG   
  
  
+ GATCAACCGG TGGTTTGGGC CAGTGCATGG CGGCCTTG  

- +Up\_Stream \_Len000TTTAGT TTGAGTATTT GTTATTAAAA AATTTTGAAT TTACCATTAA TAATCTAATT   
  
  
- ATTTTTAGGA TTACCAACAA ATTACAATGT AGTTCTGTAC GAAAAACCAC ATATATATTA CCGTCGTACA   
  
  
- CCCAATTCGC CGTTGTTTAA TAAGAGTTTA CGTACGTCAT TAATTAAATT AATTAATTAG TGGACGTATT   
  
  
- AGAGTTATGA TTAGGAAAGT AAGACACGGG GTGGGTTGGG GCTTTTATCA TTAGAAAGTT TGCAACCTTC   
  
  
- ACCCTAATCC ATATCTCATC AGGATGTCTA CTACTAGATG GATGTATATT GTATTAGACT GAAAAGAAAA   
  
  
- AATTTATTGA ACATAACTAC CGTGTCTTAA ACTGTACAAC CGTATACCAT GATTCTTTAA TTAATTGCAA   
  
  
- CACGATTCTA TATCCAGTAA GAAAAACCTC TGTATTCCGG GCGCAAGTGC GTCCCAAGCT CTTCCCGGTG   
  
  
- TAAGTTACCT ACTTAACATC CATCAAATTA GACTGAAACA GTCACTGACT AAGGTGCCGA ACTTGGGCGC   
  
  
- TGTAATTATA TCCAACTGAT TTCAGCAAAC TATGAATAAA AAGAATTCAT TTCAGAACTT AAGTTTAGAA   
  
  
- TATTTATTTA TTTTTTTAGA TATTATTTTC TTAAGATGGT AAATAATTCA GTTGTTGAGT TAAGCTTACT   
  
  
- TTATTTTTTA GTTCTAGTGG TTTTTTTTAT TTATTTTCCT TTCCATCTAA CGTAAACCTA AAACTCACCG   
  
  
- CACCACCACC CAAACTACGA CCACGACGTA TGTGGCGTCG AAAAGAGACA GGAAAATGGA GAGAAACAGA   
  
  
- CGTTCTTGGG GTAAACCGTG CGGACGAGAG AAAAGATACA AAAGAAAAGA TAGAAAATTC TTTGTTACCT   
  
  
- TTCGATTGTG TGATTGTGAT TGGGTAACCA GGAAAAAAGA ATAGACGTTT TAATTTCATT GTTATAAAAA   
  
  
- GAATAGGGGA AAAAGCAAGG TGGGTAAAGA AATAGGAAGA CAAACTTGGA TTAGACATAT ATACGTACGT   
  
  
- ACACATAAAA TGAATCTTAG ACAAAACTAA GAAACTACAT TAATTAATCA TCAGAAAAAT TAACTTAAAA   
  
  
- ATTGTCATCA AACTTAATCT GTAAATAGCT ATAATATAAA AAATATATAT TAATTTTTTA TGTTTTACTA   
  
  
- TTCACAGAAT GATGTTATGT GAAGTATTCA CAGCACAAAA TTGAATTTTT AGTATGATAA TACAAAGATT   
  
  
- TAACTTTTTT TTCACTCTAA TTCATCGTCT TCGTATAATG TTGAATATTT AATATATTTT AGTAGAAAAA   
  
  
- TAGGTATTAA ATAGTGTAAA GATTTTATGC GTATAGTATA GATGAATACC TATCCCAATT ATTCTAATCC   
  
  
- CGTAGTAAAT TCTAAAATAG TCGCATTGGT TGCTTACAAA TTCCAATCGT CAAGTATTAA TTCACATTAG   
  
  
- TAATTTTTTA AATACTAAAA TTTAGTATTT TATACAATTA GATCCTACTC CGTTCGCCGT TCTGTATATA   
  
  
- GTGATAGGAA TTTTTTATAT TATATAACTA AAGTTTCTTA ATACTTCATA TAATTAAAGT ATATATTACT   
  
  
- GCTAATACAT AAAAAAATGA TTTAATGAAC ATGGGTATTA TTTATCTTAG CTACGTAGTA AGTACCGGAA   
  
  
- AGTAAGACAT TATACCAACT GAGGTTTTTA AGAGTTTCAT AAAAATATAA AACCCTCCCC TCTAAAATCA   
  
  
- AACTATGAAA ACAACTTGAT CACTTGTTAA AGGTTGTTGG AGGTGGTGGG GGTTTGTAGT TGTTAGACTA   
  
  
- ACTACATACG TAGGGTTCGA ACTTAACTTA TTATTAAAGT TATTGTTGTT ATTAGCAAAA TGGAACTGTT   
  
  
- CGAGGTGTCG TTGAGTTGCA GAAGGTTTGG TTGGGTAGTG AGTAGTAGTA GTAGGGGTGA TAGGGTAAGT   
  
  
- GGGGGTGGTT TTCCTCGGAT TTCTGTCATT GTTCTTCTTA CGAACGGAAA AAAGTACCTA CTTCTTCTGA   
  
  
- AGAGAAGAAG GAGATGAAGA AGGGTGGTGA TACTATGTGT GGTGGAGGTT GTAGTAATGG GGTGGTTAAT   
  
  
- ATTAAGGGCA ATATGGGACC TGGGAGGGAG GTGGTGGTGG TGTCGGTGTT GGTGGTGGAG TAGGAGGTGA   
  
  
- GGGTGGGTGG TACCGAAGCC ACCACCCGAA CTGAGGAGGA GGGGCAGACG GCGACACCTT AATAGAAAGA   
  
  
- GGGGCCTGTA GGAGGAGAGG AGGGGGTGGC CTTTCACCCG CAGACTGTAA GAATACCTCC GCCGATCCCG   
  
  
- AAAAAGTCTG TGCTTGTGGT GGTTCGAGGT GGTCGAGGAG ACCTACGACT TGCTCGAGCC GAGGGGGATG   
  
  
- CCACTACAGC TCGTCTTCGA CCACCGCATG AAATTGGTTC GGGAAAAACG GGCGGAGTGA CGGTGGCCTG   
  
  
- GGGCGGTGGC TCGGCGATAC GAGCGGCGAC GCCTGTTCTG GAGGAGGAAG CTGAGGTGCG AGTCCAACGA   
  
  
- GGAGTTCAAG GTCCTTCAGT CGGGCACCTG GTGGAAGCCG GTGCACCGAA CGTTGCCACG TTAGTAGCTC   
  
  
- CGGAATCTCT CCCTGGGTGT ACTTTTCTCA TTCGAGGTGC AGTAGCTATA GTCGTGGTGG AAGACGTGGG   
  
  
- TCACCGGGTG GAACGAACTC CGAAATCGGT GGGCGTACCT GCTGTGAGGG GTGGAGTCGG AGTGACGGCA   
  
  
- GCAGCAGTTG TTCATGCGGC GGCACTCGCC ACCGCTCCTG CTGCCCCCTC TTCCTAGTTT CTCCCACTAC   
  
  
- TCTCTCTAAC CAGAAGCAGA ACTCTTCAAG CGATCCAATT ACCCCCAAGG GAAGTTCAAG TTGCACCAAA   
  
  
- TGGTGCATCC ACTAGATAGG GTCAACCTAA AAAGAGCAAA CCTATAATTT CTACTACTTC GCCACCGCTA   
  
  
- ATTGACACAG TTGAGCGAGG TGAGCCACCT GGAGGCTATA GCCCTCCATC ACGAGCGCCG CAAGTCCGCC   
  
  
- GACGCCGGCT CCCACCACTG CCACCACCTC CTTCTCCGCC TCGACTGACT CCACCCACTC CCAGTCATAC   
  
  
- TTAAAAAATC CGCCAAACTC CTCACAGAAT CCACCAAATC CCAAATGAAA CTCCGGCATC CTCTAACAAA   
  
  
- AGGATCCTGA TCGTTACTCT TTAACTACAA TCTCGCCCGG CGCCCGGCCC GGTACCACCT AGAGGAACGT   
  
  
- ACAGGGCTCG GACCACCCAG CCGACTCCCC TCCCTCTGCC GCTCTGCCAC CAGCCCAGCC TACGTACCCC   
  
  
- GCCCCAAGCT CGTGCACCCC AAGTCACTGC TTCACACACT ACTACACTCC CGGAACAACT CCTCCATGTT   
  
  
- CCTTCCTACC AGTTACTGTG TCACGTGGTA CCTACCGCCA TTAGTGGGAC CTTATGACAA CACCACCGCC   
  
  
- CTAGTTGGCC ACCAAACCCG GTCACGTACC GCCGGAAC

+     GATA-motif

| Site Name | Organism | Position | Strand | Matrix score. | sequence | function |
| --- | --- | --- | --- | --- | --- | --- |
| GATA-motif | Solanum tuberosum | 1013 | - | 9 | AAGGATAAGG | part of a light responsive element |
| GATA-motif | Pisum sativum | 1384 | + | 7 | GATAGGG | part of a light responsive element |

>HU05G01983.1   
+ +Up\_Stream \_Len000AAATCA AACTCATAAA CAATAATTTT TTAAAACTTA AATGGTAATT ATTAGATTAA   
  
  
+ TAAAAATCCT AATGGTTGTT TAATGTTACA TCAAGACATG CTTTTTGGTG TATATATAAT GGCAGCATGT   
  
  
+ GGGTTAAGCG GCAACAAATT ATTCTCAAAT GCATGCAGTA ATTAATTTAA TTAATTAATC ACCTGCATAA   
  
  
+ TCTCAATACT AATCCTTTCA TTCTGTGCCC CACCCAACCC CGAAAATAGT AATCTTTCAA ACGTTGGAAG   
  
  
+ TGGGATTAGG TATAGAGTAG TCCTACAGAT GATGATCTAC CTACATATAA CATAATCTGA CTTTTCTTTT   
  
  
+ TTAAATAACT TGTATTGATG GCACAGAATT TGACATGTTG GCATATGGTA CTAAGAAATT AATTAACGTT   
  
  
+ GTGCTAAGAT ATAGGTCATT CTTTTTGGAG ACATAAGGCC CGCGTTCACG CAGGGTTCGA GAAGGGCCAC   
  
  
+ ATTCAATGGA TGAATTGTAG GTAGTTTAAT CTGACTTTGT CAGTGACTGA TTCCACGGCT TGAACCCGCG   
  
  
+ ACATTAATAT AGGTTGACTA AAGTCGTTTG ATACTTATTT TTCTTAAGTA AAGTCTTGAA TTCAAATCTT   
  
  
+ ATAAATAAAT AAAAAAATCT ATAATAAAAG AATTCTACCA TTTATTAAGT CAACAACTCA ATTCGAATGA   
  
  
+ AATAAAAAAT CAAGATCACC AAAAAAAATA AATAAAAGGA AAGGTAGATT GCATTTGGAT TTTGAGTGGC   
  
  
+ GTGGTGGTGG GTTTGATGCT GGTGCTGCAT ACACCGCAGC TTTTCTCTGT CCTTTTACCT CTCTTTGTCT   
  
  
+ GCAAGAACCC CATTTGGCAC GCCTGCTCTC TTTTCTATGT TTTCTTTTCT ATCTTTTAAG AAACAATGGA   
  
  
+ AAGCTAACAC ACTAACACTA ACCCATTGGT CCTTTTTTCT TATCTGCAAA ATTAAAGTAA CAATATTTTT   
  
  
+ CTTATCCCCT TTTTCGTTCC ACCCATTTCT TTATCCTTCT GTTTGAACCT AATCTGTATA TATGCATGCA   
  
  
+ TGTGTATTTT ACTTAGAATC TGTTTTGATT CTTTGATGTA ATTAATTAGT AGTCTTTTTA ATTGAATTTT   
  
  
+ TAACAGTAGT TTGAATTAGA CATTTATCGA TATTATATTT TTTATATATA ATTAAAAAAT ACAAAATGAT   
  
  
+ AAGTGTCTTA CTACAATACA CTTCATAAGT GTCGTGTTTT AACTTAAAAA TCATACTATT ATGTTTCTAA   
  
  
+ ATTGAAAAAA AAGTGAGATT AAGTAGCAGA AGCATATTAC AACTTATAAA TTATATAAAA TCATCTTTTT   
  
  
+ ATCCATAATT TATCACATTT CTAAAATACG CATATCATAT CTACTTATGG ATAGGGTTAA TAAGATTAGG   
  
  
+ GCATCATTTA AGATTTTATC AGCGTAACCA ACGAATGTTT AAGGTTAGCA GTTCATAATT AAGTGTAATC   
  
  
+ ATTAAAAAAT TTATGATTTT AAATCATAAA ATATGTTAAT CTAGGATGAG GCAAGCGGCA AGACATATAT   
  
  
+ CACTATCCTT AAAAAATATA ATATATTGAT TTCAAAGAAT TATGAAGTAT ATTAATTTCA TATATAATGA   
  
  
+ CGATTATGTA TTTTTTTACT AAATTACTTG TACCCATAAT AAATAGAATC GATGCATCAT TCATGGCCTT   
  
  
+ TCATTCTGTA ATATGGTTGA CTCCAAAAAT TCTCAAAGTA TTTTTATATT TTGGGAGGGG AGATTTTAGT   
  
  
+ TTGATACTTT TGTTGAACTA GTGAACAATT TCCAACAACC TCCACCACCC CCAAACATCA ACAATCTGAT   
  
  
+ TGATGTATGC ATCCCAAGCT TGAATTGAAT AATAATTTCA ATAACAACAA TAATCGTTTT ACCTTGACAA   
  
  
+ GCTCCACAGC AACTCAACGT CTTCCAAACC AACCCATCAC TCATCATCAT CATCCCCACT ATCCCATTCA   
  
  
+ CCCCCACCAA AAGGAGCCTA AAGACAGTAA CAAGAAGAAT GCTTGCCTTT TTTCATGGAT GAAGAAGACT   
  
  
+ TCTCTTCTTC CTCTACTTCT TCCCACCACT ATGATACACA CCACCTCCAA CATCATTACC CCACCAATTA   
  
  
+ TAATTCCCGT TATACCCTGG ACCCTCCCTC CACCACCACC ACAGCCACAA CCACCACCTC ATCCTCCACT   
  
  
+ CCCACCCACC ATGGCTTCGG TGGTGGGCTT GACTCCTCCT CCCCGTCTGC CGCTGTGGAA TTATCTTTCT   
  
  
+ CCCCGGACAT CCTCCTCTCC TCCCCCACCG GAAAGTGGGC GTCTGACATT CTTATGGAGG CGGCTAGGGC   
  
  
+ TTTTTCAGAC ACGAACACCA CCAAGCTCCA CCAGCTCCTC TGGATGCTGA ACGAGCTCGG CTCCCCCTAC   
  
  
+ GGTGATGTCG AGCAGAAGCT GGTGGCGTAC TTTAACCAAG CCCTTTTTGC CCGCCTCACT GCCACCGGAC   
  
  
+ CCCGCCACCG AGCCGCTATG CTCGCCGCTG CGGACAAGAC CTCCTCCTTC GACTCCACGC TCAGGTTGCT   
  
  
+ CCTCAAGTTC CAGGAAGTCA GCCCGTGGAC CACCTTCGGC CACGTGGCTT GCAACGGTGC AATCATCGAG   
  
  
+ GCCTTAGAGA GGGACCCACA TGAAAAGAGT AAGCTCCACG TCATCGATAT CAGCACCACC TTCTGCACCC   
  
  
+ AGTGGCCCAC CTTGCTTGAG GCTTTAGCCA CCCGCATGGA CGACACTCCC CACCTCAGCC TCACTGCCGT   
  
  
+ CGTCGTCAAC AAGTACGCCG CCGTGAGCGG TGGCGAGGAC GACGGGGGAG AAGGATCAAA GAGGGTGATG   
  
  
+ AGAGAGATTG GTCTTCGTCT TGAGAAGTTC GCTAGGTTAA TGGGGGTTCC CTTCAAGTTC AACGTGGTTT   
  
  
+ ACCACGTAGG TGATCTATCC CAGTTGGATT TTTCTCGTTT GGATATTAAA GATGATGAAG CGGTGGCGAT   
  
  
+ TAACTGTGTC AACTCGCTCC ACTCGGTGGA CCTCCGATAT CGGGAGGTAG TGCTCGCGGC GTTCAGGCGG   
  
  
+ CTGCGGCCGA GGGTGGTGAC GGTGGTGGAG GAAGAGGCGG AGCTGACTGA GGTGGGTGAG GGTCAGTATG   
  
  
+ AATTTTTTAG GCGGTTTGAG GAGTGTCTTA GGTGGTTTAG GGTTTACTTT GAGGCCGTAG GAGATTGTTT   
  
  
+ TCCTAGGACT AGCAATGAGA AATTGATGTT AGAGCGGGCC GCGGGCCGGG CCATGGTGGA TCTCCTTGCA   
  
  
+ TGTCCCGAGC CTGGTGGGTC GGCTGAGGGG AGGGAGACGG CGAGACGGTG GTCGGGTCGG ATGCATGGGG   
  
  
+ CGGGGTTCGA GCACGTGGGG TTCAGTGACG AAGTGTGTGA TGATGTGAGG GCCTTGTTGA GGAGGTACAA   
  
  
+ GGAAGGATGG TCAATGACAC AGTGCACCAT GGATGGCGGT AATCACCCTG GAATACTGTT GTGGTGGCGG   
  
  
+ GATCAACCGG TGGTTTGGGC CAGTGCATGG CGGCCTTG  

- +Up\_Stream \_Len000TTTAGT TTGAGTATTT GTTATTAAAA AATTTTGAAT TTACCATTAA TAATCTAATT   
  
  
- ATTTTTAGGA TTACCAACAA ATTACAATGT AGTTCTGTAC GAAAAACCAC ATATATATTA CCGTCGTACA   
  
  
- CCCAATTCGC CGTTGTTTAA TAAGAGTTTA CGTACGTCAT TAATTAAATT AATTAATTAG TGGACGTATT   
  
  
- AGAGTTATGA TTAGGAAAGT AAGACACGGG GTGGGTTGGG GCTTTTATCA TTAGAAAGTT TGCAACCTTC   
  
  
- ACCCTAATCC ATATCTCATC AGGATGTCTA CTACTAGATG GATGTATATT GTATTAGACT GAAAAGAAAA   
  
  
- AATTTATTGA ACATAACTAC CGTGTCTTAA ACTGTACAAC CGTATACCAT GATTCTTTAA TTAATTGCAA   
  
  
- CACGATTCTA TATCCAGTAA GAAAAACCTC TGTATTCCGG GCGCAAGTGC GTCCCAAGCT CTTCCCGGTG   
  
  
- TAAGTTACCT ACTTAACATC CATCAAATTA GACTGAAACA GTCACTGACT AAGGTGCCGA ACTTGGGCGC   
  
  
- TGTAATTATA TCCAACTGAT TTCAGCAAAC TATGAATAAA AAGAATTCAT TTCAGAACTT AAGTTTAGAA   
  
  
- TATTTATTTA TTTTTTTAGA TATTATTTTC TTAAGATGGT AAATAATTCA GTTGTTGAGT TAAGCTTACT   
  
  
- TTATTTTTTA GTTCTAGTGG TTTTTTTTAT TTATTTTCCT TTCCATCTAA CGTAAACCTA AAACTCACCG   
  
  
- CACCACCACC CAAACTACGA CCACGACGTA TGTGGCGTCG AAAAGAGACA GGAAAATGGA GAGAAACAGA   
  
  
- CGTTCTTGGG GTAAACCGTG CGGACGAGAG AAAAGATACA AAAGAAAAGA TAGAAAATTC TTTGTTACCT   
  
  
- TTCGATTGTG TGATTGTGAT TGGGTAACCA GGAAAAAAGA ATAGACGTTT TAATTTCATT GTTATAAAAA   
  
  
- GAATAGGGGA AAAAGCAAGG TGGGTAAAGA AATAGGAAGA CAAACTTGGA TTAGACATAT ATACGTACGT   
  
  
- ACACATAAAA TGAATCTTAG ACAAAACTAA GAAACTACAT TAATTAATCA TCAGAAAAAT TAACTTAAAA   
  
  
- ATTGTCATCA AACTTAATCT GTAAATAGCT ATAATATAAA AAATATATAT TAATTTTTTA TGTTTTACTA   
  
  
- TTCACAGAAT GATGTTATGT GAAGTATTCA CAGCACAAAA TTGAATTTTT AGTATGATAA TACAAAGATT   
  
  
- TAACTTTTTT TTCACTCTAA TTCATCGTCT TCGTATAATG TTGAATATTT AATATATTTT AGTAGAAAAA   
  
  
- TAGGTATTAA ATAGTGTAAA GATTTTATGC GTATAGTATA GATGAATACC TATCCCAATT ATTCTAATCC   
  
  
- CGTAGTAAAT TCTAAAATAG TCGCATTGGT TGCTTACAAA TTCCAATCGT CAAGTATTAA TTCACATTAG   
  
  
- TAATTTTTTA AATACTAAAA TTTAGTATTT TATACAATTA GATCCTACTC CGTTCGCCGT TCTGTATATA   
  
  
- GTGATAGGAA TTTTTTATAT TATATAACTA AAGTTTCTTA ATACTTCATA TAATTAAAGT ATATATTACT   
  
  
- GCTAATACAT AAAAAAATGA TTTAATGAAC ATGGGTATTA TTTATCTTAG CTACGTAGTA AGTACCGGAA   
  
  
- AGTAAGACAT TATACCAACT GAGGTTTTTA AGAGTTTCAT AAAAATATAA AACCCTCCCC TCTAAAATCA   
  
  
- AACTATGAAA ACAACTTGAT CACTTGTTAA AGGTTGTTGG AGGTGGTGGG GGTTTGTAGT TGTTAGACTA   
  
  
- ACTACATACG TAGGGTTCGA ACTTAACTTA TTATTAAAGT TATTGTTGTT ATTAGCAAAA TGGAACTGTT   
  
  
- CGAGGTGTCG TTGAGTTGCA GAAGGTTTGG TTGGGTAGTG AGTAGTAGTA GTAGGGGTGA TAGGGTAAGT   
  
  
- GGGGGTGGTT TTCCTCGGAT TTCTGTCATT GTTCTTCTTA CGAACGGAAA AAAGTACCTA CTTCTTCTGA   
  
  
- AGAGAAGAAG GAGATGAAGA AGGGTGGTGA TACTATGTGT GGTGGAGGTT GTAGTAATGG GGTGGTTAAT   
  
  
- ATTAAGGGCA ATATGGGACC TGGGAGGGAG GTGGTGGTGG TGTCGGTGTT GGTGGTGGAG TAGGAGGTGA   
  
  
- GGGTGGGTGG TACCGAAGCC ACCACCCGAA CTGAGGAGGA GGGGCAGACG GCGACACCTT AATAGAAAGA   
  
  
- GGGGCCTGTA GGAGGAGAGG AGGGGGTGGC CTTTCACCCG CAGACTGTAA GAATACCTCC GCCGATCCCG   
  
  
- AAAAAGTCTG TGCTTGTGGT GGTTCGAGGT GGTCGAGGAG ACCTACGACT TGCTCGAGCC GAGGGGGATG   
  
  
- CCACTACAGC TCGTCTTCGA CCACCGCATG AAATTGGTTC GGGAAAAACG GGCGGAGTGA CGGTGGCCTG   
  
  
- GGGCGGTGGC TCGGCGATAC GAGCGGCGAC GCCTGTTCTG GAGGAGGAAG CTGAGGTGCG AGTCCAACGA   
  
  
- GGAGTTCAAG GTCCTTCAGT CGGGCACCTG GTGGAAGCCG GTGCACCGAA CGTTGCCACG TTAGTAGCTC   
  
  
- CGGAATCTCT CCCTGGGTGT ACTTTTCTCA TTCGAGGTGC AGTAGCTATA GTCGTGGTGG AAGACGTGGG   
  
  
- TCACCGGGTG GAACGAACTC CGAAATCGGT GGGCGTACCT GCTGTGAGGG GTGGAGTCGG AGTGACGGCA   
  
  
- GCAGCAGTTG TTCATGCGGC GGCACTCGCC ACCGCTCCTG CTGCCCCCTC TTCCTAGTTT CTCCCACTAC   
  
  
- TCTCTCTAAC CAGAAGCAGA ACTCTTCAAG CGATCCAATT ACCCCCAAGG GAAGTTCAAG TTGCACCAAA   
  
  
- TGGTGCATCC ACTAGATAGG GTCAACCTAA AAAGAGCAAA CCTATAATTT CTACTACTTC GCCACCGCTA   
  
  
- ATTGACACAG TTGAGCGAGG TGAGCCACCT GGAGGCTATA GCCCTCCATC ACGAGCGCCG CAAGTCCGCC   
  
  
- GACGCCGGCT CCCACCACTG CCACCACCTC CTTCTCCGCC TCGACTGACT CCACCCACTC CCAGTCATAC   
  
  
- TTAAAAAATC CGCCAAACTC CTCACAGAAT CCACCAAATC CCAAATGAAA CTCCGGCATC CTCTAACAAA   
  
  
- AGGATCCTGA TCGTTACTCT TTAACTACAA TCTCGCCCGG CGCCCGGCCC GGTACCACCT AGAGGAACGT   
  
  
- ACAGGGCTCG GACCACCCAG CCGACTCCCC TCCCTCTGCC GCTCTGCCAC CAGCCCAGCC TACGTACCCC   
  
  
- GCCCCAAGCT CGTGCACCCC AAGTCACTGC TTCACACACT ACTACACTCC CGGAACAACT CCTCCATGTT   
  
  
- CCTTCCTACC AGTTACTGTG TCACGTGGTA CCTACCGCCA TTAGTGGGAC CTTATGACAA CACCACCGCC   
  
  
- CTAGTTGGCC ACCAAACCCG GTCACGTACC GCCGGAAC

+     GC-motif

| Site Name | Organism | Position | Strand | Matrix score. | sequence | function |
| --- | --- | --- | --- | --- | --- | --- |
| GC-motif | Zea mays | 2777 | - | 6 | CCCCCG | enhancer-like element involved in anoxic specific inducibility |

>HU05G01983.1   
+ +Up\_Stream \_Len000AAATCA AACTCATAAA CAATAATTTT TTAAAACTTA AATGGTAATT ATTAGATTAA   
  
  
+ TAAAAATCCT AATGGTTGTT TAATGTTACA TCAAGACATG CTTTTTGGTG TATATATAAT GGCAGCATGT   
  
  
+ GGGTTAAGCG GCAACAAATT ATTCTCAAAT GCATGCAGTA ATTAATTTAA TTAATTAATC ACCTGCATAA   
  
  
+ TCTCAATACT AATCCTTTCA TTCTGTGCCC CACCCAACCC CGAAAATAGT AATCTTTCAA ACGTTGGAAG   
  
  
+ TGGGATTAGG TATAGAGTAG TCCTACAGAT GATGATCTAC CTACATATAA CATAATCTGA CTTTTCTTTT   
  
  
+ TTAAATAACT TGTATTGATG GCACAGAATT TGACATGTTG GCATATGGTA CTAAGAAATT AATTAACGTT   
  
  
+ GTGCTAAGAT ATAGGTCATT CTTTTTGGAG ACATAAGGCC CGCGTTCACG CAGGGTTCGA GAAGGGCCAC   
  
  
+ ATTCAATGGA TGAATTGTAG GTAGTTTAAT CTGACTTTGT CAGTGACTGA TTCCACGGCT TGAACCCGCG   
  
  
+ ACATTAATAT AGGTTGACTA AAGTCGTTTG ATACTTATTT TTCTTAAGTA AAGTCTTGAA TTCAAATCTT   
  
  
+ ATAAATAAAT AAAAAAATCT ATAATAAAAG AATTCTACCA TTTATTAAGT CAACAACTCA ATTCGAATGA   
  
  
+ AATAAAAAAT CAAGATCACC AAAAAAAATA AATAAAAGGA AAGGTAGATT GCATTTGGAT TTTGAGTGGC   
  
  
+ GTGGTGGTGG GTTTGATGCT GGTGCTGCAT ACACCGCAGC TTTTCTCTGT CCTTTTACCT CTCTTTGTCT   
  
  
+ GCAAGAACCC CATTTGGCAC GCCTGCTCTC TTTTCTATGT TTTCTTTTCT ATCTTTTAAG AAACAATGGA   
  
  
+ AAGCTAACAC ACTAACACTA ACCCATTGGT CCTTTTTTCT TATCTGCAAA ATTAAAGTAA CAATATTTTT   
  
  
+ CTTATCCCCT TTTTCGTTCC ACCCATTTCT TTATCCTTCT GTTTGAACCT AATCTGTATA TATGCATGCA   
  
  
+ TGTGTATTTT ACTTAGAATC TGTTTTGATT CTTTGATGTA ATTAATTAGT AGTCTTTTTA ATTGAATTTT   
  
  
+ TAACAGTAGT TTGAATTAGA CATTTATCGA TATTATATTT TTTATATATA ATTAAAAAAT ACAAAATGAT   
  
  
+ AAGTGTCTTA CTACAATACA CTTCATAAGT GTCGTGTTTT AACTTAAAAA TCATACTATT ATGTTTCTAA   
  
  
+ ATTGAAAAAA AAGTGAGATT AAGTAGCAGA AGCATATTAC AACTTATAAA TTATATAAAA TCATCTTTTT   
  
  
+ ATCCATAATT TATCACATTT CTAAAATACG CATATCATAT CTACTTATGG ATAGGGTTAA TAAGATTAGG   
  
  
+ GCATCATTTA AGATTTTATC AGCGTAACCA ACGAATGTTT AAGGTTAGCA GTTCATAATT AAGTGTAATC   
  
  
+ ATTAAAAAAT TTATGATTTT AAATCATAAA ATATGTTAAT CTAGGATGAG GCAAGCGGCA AGACATATAT   
  
  
+ CACTATCCTT AAAAAATATA ATATATTGAT TTCAAAGAAT TATGAAGTAT ATTAATTTCA TATATAATGA   
  
  
+ CGATTATGTA TTTTTTTACT AAATTACTTG TACCCATAAT AAATAGAATC GATGCATCAT TCATGGCCTT   
  
  
+ TCATTCTGTA ATATGGTTGA CTCCAAAAAT TCTCAAAGTA TTTTTATATT TTGGGAGGGG AGATTTTAGT   
  
  
+ TTGATACTTT TGTTGAACTA GTGAACAATT TCCAACAACC TCCACCACCC CCAAACATCA ACAATCTGAT   
  
  
+ TGATGTATGC ATCCCAAGCT TGAATTGAAT AATAATTTCA ATAACAACAA TAATCGTTTT ACCTTGACAA   
  
  
+ GCTCCACAGC AACTCAACGT CTTCCAAACC AACCCATCAC TCATCATCAT CATCCCCACT ATCCCATTCA   
  
  
+ CCCCCACCAA AAGGAGCCTA AAGACAGTAA CAAGAAGAAT GCTTGCCTTT TTTCATGGAT GAAGAAGACT   
  
  
+ TCTCTTCTTC CTCTACTTCT TCCCACCACT ATGATACACA CCACCTCCAA CATCATTACC CCACCAATTA   
  
  
+ TAATTCCCGT TATACCCTGG ACCCTCCCTC CACCACCACC ACAGCCACAA CCACCACCTC ATCCTCCACT   
  
  
+ CCCACCCACC ATGGCTTCGG TGGTGGGCTT GACTCCTCCT CCCCGTCTGC CGCTGTGGAA TTATCTTTCT   
  
  
+ CCCCGGACAT CCTCCTCTCC TCCCCCACCG GAAAGTGGGC GTCTGACATT CTTATGGAGG CGGCTAGGGC   
  
  
+ TTTTTCAGAC ACGAACACCA CCAAGCTCCA CCAGCTCCTC TGGATGCTGA ACGAGCTCGG CTCCCCCTAC   
  
  
+ GGTGATGTCG AGCAGAAGCT GGTGGCGTAC TTTAACCAAG CCCTTTTTGC CCGCCTCACT GCCACCGGAC   
  
  
+ CCCGCCACCG AGCCGCTATG CTCGCCGCTG CGGACAAGAC CTCCTCCTTC GACTCCACGC TCAGGTTGCT   
  
  
+ CCTCAAGTTC CAGGAAGTCA GCCCGTGGAC CACCTTCGGC CACGTGGCTT GCAACGGTGC AATCATCGAG   
  
  
+ GCCTTAGAGA GGGACCCACA TGAAAAGAGT AAGCTCCACG TCATCGATAT CAGCACCACC TTCTGCACCC   
  
  
+ AGTGGCCCAC CTTGCTTGAG GCTTTAGCCA CCCGCATGGA CGACACTCCC CACCTCAGCC TCACTGCCGT   
  
  
+ CGTCGTCAAC AAGTACGCCG CCGTGAGCGG TGGCGAGGAC GACGGGGGAG AAGGATCAAA GAGGGTGATG   
  
  
+ AGAGAGATTG GTCTTCGTCT TGAGAAGTTC GCTAGGTTAA TGGGGGTTCC CTTCAAGTTC AACGTGGTTT   
  
  
+ ACCACGTAGG TGATCTATCC CAGTTGGATT TTTCTCGTTT GGATATTAAA GATGATGAAG CGGTGGCGAT   
  
  
+ TAACTGTGTC AACTCGCTCC ACTCGGTGGA CCTCCGATAT CGGGAGGTAG TGCTCGCGGC GTTCAGGCGG   
  
  
+ CTGCGGCCGA GGGTGGTGAC GGTGGTGGAG GAAGAGGCGG AGCTGACTGA GGTGGGTGAG GGTCAGTATG   
  
  
+ AATTTTTTAG GCGGTTTGAG GAGTGTCTTA GGTGGTTTAG GGTTTACTTT GAGGCCGTAG GAGATTGTTT   
  
  
+ TCCTAGGACT AGCAATGAGA AATTGATGTT AGAGCGGGCC GCGGGCCGGG CCATGGTGGA TCTCCTTGCA   
  
  
+ TGTCCCGAGC CTGGTGGGTC GGCTGAGGGG AGGGAGACGG CGAGACGGTG GTCGGGTCGG ATGCATGGGG   
  
  
+ CGGGGTTCGA GCACGTGGGG TTCAGTGACG AAGTGTGTGA TGATGTGAGG GCCTTGTTGA GGAGGTACAA   
  
  
+ GGAAGGATGG TCAATGACAC AGTGCACCAT GGATGGCGGT AATCACCCTG GAATACTGTT GTGGTGGCGG   
  
  
+ GATCAACCGG TGGTTTGGGC CAGTGCATGG CGGCCTTG  

- +Up\_Stream \_Len000TTTAGT TTGAGTATTT GTTATTAAAA AATTTTGAAT TTACCATTAA TAATCTAATT   
  
  
- ATTTTTAGGA TTACCAACAA ATTACAATGT AGTTCTGTAC GAAAAACCAC ATATATATTA CCGTCGTACA   
  
  
- CCCAATTCGC CGTTGTTTAA TAAGAGTTTA CGTACGTCAT TAATTAAATT AATTAATTAG TGGACGTATT   
  
  
- AGAGTTATGA TTAGGAAAGT AAGACACGGG GTGGGTTGGG GCTTTTATCA TTAGAAAGTT TGCAACCTTC   
  
  
- ACCCTAATCC ATATCTCATC AGGATGTCTA CTACTAGATG GATGTATATT GTATTAGACT GAAAAGAAAA   
  
  
- AATTTATTGA ACATAACTAC CGTGTCTTAA ACTGTACAAC CGTATACCAT GATTCTTTAA TTAATTGCAA   
  
  
- CACGATTCTA TATCCAGTAA GAAAAACCTC TGTATTCCGG GCGCAAGTGC GTCCCAAGCT CTTCCCGGTG   
  
  
- TAAGTTACCT ACTTAACATC CATCAAATTA GACTGAAACA GTCACTGACT AAGGTGCCGA ACTTGGGCGC   
  
  
- TGTAATTATA TCCAACTGAT TTCAGCAAAC TATGAATAAA AAGAATTCAT TTCAGAACTT AAGTTTAGAA   
  
  
- TATTTATTTA TTTTTTTAGA TATTATTTTC TTAAGATGGT AAATAATTCA GTTGTTGAGT TAAGCTTACT   
  
  
- TTATTTTTTA GTTCTAGTGG TTTTTTTTAT TTATTTTCCT TTCCATCTAA CGTAAACCTA AAACTCACCG   
  
  
- CACCACCACC CAAACTACGA CCACGACGTA TGTGGCGTCG AAAAGAGACA GGAAAATGGA GAGAAACAGA   
  
  
- CGTTCTTGGG GTAAACCGTG CGGACGAGAG AAAAGATACA AAAGAAAAGA TAGAAAATTC TTTGTTACCT   
  
  
- TTCGATTGTG TGATTGTGAT TGGGTAACCA GGAAAAAAGA ATAGACGTTT TAATTTCATT GTTATAAAAA   
  
  
- GAATAGGGGA AAAAGCAAGG TGGGTAAAGA AATAGGAAGA CAAACTTGGA TTAGACATAT ATACGTACGT   
  
  
- ACACATAAAA TGAATCTTAG ACAAAACTAA GAAACTACAT TAATTAATCA TCAGAAAAAT TAACTTAAAA   
  
  
- ATTGTCATCA AACTTAATCT GTAAATAGCT ATAATATAAA AAATATATAT TAATTTTTTA TGTTTTACTA   
  
  
- TTCACAGAAT GATGTTATGT GAAGTATTCA CAGCACAAAA TTGAATTTTT AGTATGATAA TACAAAGATT   
  
  
- TAACTTTTTT TTCACTCTAA TTCATCGTCT TCGTATAATG TTGAATATTT AATATATTTT AGTAGAAAAA   
  
  
- TAGGTATTAA ATAGTGTAAA GATTTTATGC GTATAGTATA GATGAATACC TATCCCAATT ATTCTAATCC   
  
  
- CGTAGTAAAT TCTAAAATAG TCGCATTGGT TGCTTACAAA TTCCAATCGT CAAGTATTAA TTCACATTAG   
  
  
- TAATTTTTTA AATACTAAAA TTTAGTATTT TATACAATTA GATCCTACTC CGTTCGCCGT TCTGTATATA   
  
  
- GTGATAGGAA TTTTTTATAT TATATAACTA AAGTTTCTTA ATACTTCATA TAATTAAAGT ATATATTACT   
  
  
- GCTAATACAT AAAAAAATGA TTTAATGAAC ATGGGTATTA TTTATCTTAG CTACGTAGTA AGTACCGGAA   
  
  
- AGTAAGACAT TATACCAACT GAGGTTTTTA AGAGTTTCAT AAAAATATAA AACCCTCCCC TCTAAAATCA   
  
  
- AACTATGAAA ACAACTTGAT CACTTGTTAA AGGTTGTTGG AGGTGGTGGG GGTTTGTAGT TGTTAGACTA   
  
  
- ACTACATACG TAGGGTTCGA ACTTAACTTA TTATTAAAGT TATTGTTGTT ATTAGCAAAA TGGAACTGTT   
  
  
- CGAGGTGTCG TTGAGTTGCA GAAGGTTTGG TTGGGTAGTG AGTAGTAGTA GTAGGGGTGA TAGGGTAAGT   
  
  
- GGGGGTGGTT TTCCTCGGAT TTCTGTCATT GTTCTTCTTA CGAACGGAAA AAAGTACCTA CTTCTTCTGA   
  
  
- AGAGAAGAAG GAGATGAAGA AGGGTGGTGA TACTATGTGT GGTGGAGGTT GTAGTAATGG GGTGGTTAAT   
  
  
- ATTAAGGGCA ATATGGGACC TGGGAGGGAG GTGGTGGTGG TGTCGGTGTT GGTGGTGGAG TAGGAGGTGA   
  
  
- GGGTGGGTGG TACCGAAGCC ACCACCCGAA CTGAGGAGGA GGGGCAGACG GCGACACCTT AATAGAAAGA   
  
  
- GGGGCCTGTA GGAGGAGAGG AGGGGGTGGC CTTTCACCCG CAGACTGTAA GAATACCTCC GCCGATCCCG   
  
  
- AAAAAGTCTG TGCTTGTGGT GGTTCGAGGT GGTCGAGGAG ACCTACGACT TGCTCGAGCC GAGGGGGATG   
  
  
- CCACTACAGC TCGTCTTCGA CCACCGCATG AAATTGGTTC GGGAAAAACG GGCGGAGTGA CGGTGGCCTG   
  
  
- GGGCGGTGGC TCGGCGATAC GAGCGGCGAC GCCTGTTCTG GAGGAGGAAG CTGAGGTGCG AGTCCAACGA   
  
  
- GGAGTTCAAG GTCCTTCAGT CGGGCACCTG GTGGAAGCCG GTGCACCGAA CGTTGCCACG TTAGTAGCTC   
  
  
- CGGAATCTCT CCCTGGGTGT ACTTTTCTCA TTCGAGGTGC AGTAGCTATA GTCGTGGTGG AAGACGTGGG   
  
  
- TCACCGGGTG GAACGAACTC CGAAATCGGT GGGCGTACCT GCTGTGAGGG GTGGAGTCGG AGTGACGGCA   
  
  
- GCAGCAGTTG TTCATGCGGC GGCACTCGCC ACCGCTCCTG CTGCCCCCTC TTCCTAGTTT CTCCCACTAC   
  
  
- TCTCTCTAAC CAGAAGCAGA ACTCTTCAAG CGATCCAATT ACCCCCAAGG GAAGTTCAAG TTGCACCAAA   
  
  
- TGGTGCATCC ACTAGATAGG GTCAACCTAA AAAGAGCAAA CCTATAATTT CTACTACTTC GCCACCGCTA   
  
  
- ATTGACACAG TTGAGCGAGG TGAGCCACCT GGAGGCTATA GCCCTCCATC ACGAGCGCCG CAAGTCCGCC   
  
  
- GACGCCGGCT CCCACCACTG CCACCACCTC CTTCTCCGCC TCGACTGACT CCACCCACTC CCAGTCATAC   
  
  
- TTAAAAAATC CGCCAAACTC CTCACAGAAT CCACCAAATC CCAAATGAAA CTCCGGCATC CTCTAACAAA   
  
  
- AGGATCCTGA TCGTTACTCT TTAACTACAA TCTCGCCCGG CGCCCGGCCC GGTACCACCT AGAGGAACGT   
  
  
- ACAGGGCTCG GACCACCCAG CCGACTCCCC TCCCTCTGCC GCTCTGCCAC CAGCCCAGCC TACGTACCCC   
  
  
- GCCCCAAGCT CGTGCACCCC AAGTCACTGC TTCACACACT ACTACACTCC CGGAACAACT CCTCCATGTT   
  
  
- CCTTCCTACC AGTTACTGTG TCACGTGGTA CCTACCGCCA TTAGTGGGAC CTTATGACAA CACCACCGCC   
  
  
- CTAGTTGGCC ACCAAACCCG GTCACGTACC GCCGGAAC

+     GT1-motif

| Site Name | Organism | Position | Strand | Matrix score. | sequence | function |
| --- | --- | --- | --- | --- | --- | --- |
| GT1-motif | Avena sativa | 1389 | + | 7 | GGTTAAT | light responsive element |
| GT1-motif | Arabidopsis thaliana | 2416 | - | 6 | GGTTAA | light responsive element |
| GT1-motif | Avena sativa | 2839 | + | 7 | GGTTAAT | light responsive element |
| GT1-motif | Arabidopsis thaliana | 146 | + | 6 | GGTTAA | light responsive element |

>HU05G01983.1   
+ +Up\_Stream \_Len000AAATCA AACTCATAAA CAATAATTTT TTAAAACTTA AATGGTAATT ATTAGATTAA   
  
  
+ TAAAAATCCT AATGGTTGTT TAATGTTACA TCAAGACATG CTTTTTGGTG TATATATAAT GGCAGCATGT   
  
  
+ GGGTTAAGCG GCAACAAATT ATTCTCAAAT GCATGCAGTA ATTAATTTAA TTAATTAATC ACCTGCATAA   
  
  
+ TCTCAATACT AATCCTTTCA TTCTGTGCCC CACCCAACCC CGAAAATAGT AATCTTTCAA ACGTTGGAAG   
  
  
+ TGGGATTAGG TATAGAGTAG TCCTACAGAT GATGATCTAC CTACATATAA CATAATCTGA CTTTTCTTTT   
  
  
+ TTAAATAACT TGTATTGATG GCACAGAATT TGACATGTTG GCATATGGTA CTAAGAAATT AATTAACGTT   
  
  
+ GTGCTAAGAT ATAGGTCATT CTTTTTGGAG ACATAAGGCC CGCGTTCACG CAGGGTTCGA GAAGGGCCAC   
  
  
+ ATTCAATGGA TGAATTGTAG GTAGTTTAAT CTGACTTTGT CAGTGACTGA TTCCACGGCT TGAACCCGCG   
  
  
+ ACATTAATAT AGGTTGACTA AAGTCGTTTG ATACTTATTT TTCTTAAGTA AAGTCTTGAA TTCAAATCTT   
  
  
+ ATAAATAAAT AAAAAAATCT ATAATAAAAG AATTCTACCA TTTATTAAGT CAACAACTCA ATTCGAATGA   
  
  
+ AATAAAAAAT CAAGATCACC AAAAAAAATA AATAAAAGGA AAGGTAGATT GCATTTGGAT TTTGAGTGGC   
  
  
+ GTGGTGGTGG GTTTGATGCT GGTGCTGCAT ACACCGCAGC TTTTCTCTGT CCTTTTACCT CTCTTTGTCT   
  
  
+ GCAAGAACCC CATTTGGCAC GCCTGCTCTC TTTTCTATGT TTTCTTTTCT ATCTTTTAAG AAACAATGGA   
  
  
+ AAGCTAACAC ACTAACACTA ACCCATTGGT CCTTTTTTCT TATCTGCAAA ATTAAAGTAA CAATATTTTT   
  
  
+ CTTATCCCCT TTTTCGTTCC ACCCATTTCT TTATCCTTCT GTTTGAACCT AATCTGTATA TATGCATGCA   
  
  
+ TGTGTATTTT ACTTAGAATC TGTTTTGATT CTTTGATGTA ATTAATTAGT AGTCTTTTTA ATTGAATTTT   
  
  
+ TAACAGTAGT TTGAATTAGA CATTTATCGA TATTATATTT TTTATATATA ATTAAAAAAT ACAAAATGAT   
  
  
+ AAGTGTCTTA CTACAATACA CTTCATAAGT GTCGTGTTTT AACTTAAAAA TCATACTATT ATGTTTCTAA   
  
  
+ ATTGAAAAAA AAGTGAGATT AAGTAGCAGA AGCATATTAC AACTTATAAA TTATATAAAA TCATCTTTTT   
  
  
+ ATCCATAATT TATCACATTT CTAAAATACG CATATCATAT CTACTTATGG ATAGGGTTAA TAAGATTAGG   
  
  
+ GCATCATTTA AGATTTTATC AGCGTAACCA ACGAATGTTT AAGGTTAGCA GTTCATAATT AAGTGTAATC   
  
  
+ ATTAAAAAAT TTATGATTTT AAATCATAAA ATATGTTAAT CTAGGATGAG GCAAGCGGCA AGACATATAT   
  
  
+ CACTATCCTT AAAAAATATA ATATATTGAT TTCAAAGAAT TATGAAGTAT ATTAATTTCA TATATAATGA   
  
  
+ CGATTATGTA TTTTTTTACT AAATTACTTG TACCCATAAT AAATAGAATC GATGCATCAT TCATGGCCTT   
  
  
+ TCATTCTGTA ATATGGTTGA CTCCAAAAAT TCTCAAAGTA TTTTTATATT TTGGGAGGGG AGATTTTAGT   
  
  
+ TTGATACTTT TGTTGAACTA GTGAACAATT TCCAACAACC TCCACCACCC CCAAACATCA ACAATCTGAT   
  
  
+ TGATGTATGC ATCCCAAGCT TGAATTGAAT AATAATTTCA ATAACAACAA TAATCGTTTT ACCTTGACAA   
  
  
+ GCTCCACAGC AACTCAACGT CTTCCAAACC AACCCATCAC TCATCATCAT CATCCCCACT ATCCCATTCA   
  
  
+ CCCCCACCAA AAGGAGCCTA AAGACAGTAA CAAGAAGAAT GCTTGCCTTT TTTCATGGAT GAAGAAGACT   
  
  
+ TCTCTTCTTC CTCTACTTCT TCCCACCACT ATGATACACA CCACCTCCAA CATCATTACC CCACCAATTA   
  
  
+ TAATTCCCGT TATACCCTGG ACCCTCCCTC CACCACCACC ACAGCCACAA CCACCACCTC ATCCTCCACT   
  
  
+ CCCACCCACC ATGGCTTCGG TGGTGGGCTT GACTCCTCCT CCCCGTCTGC CGCTGTGGAA TTATCTTTCT   
  
  
+ CCCCGGACAT CCTCCTCTCC TCCCCCACCG GAAAGTGGGC GTCTGACATT CTTATGGAGG CGGCTAGGGC   
  
  
+ TTTTTCAGAC ACGAACACCA CCAAGCTCCA CCAGCTCCTC TGGATGCTGA ACGAGCTCGG CTCCCCCTAC   
  
  
+ GGTGATGTCG AGCAGAAGCT GGTGGCGTAC TTTAACCAAG CCCTTTTTGC CCGCCTCACT GCCACCGGAC   
  
  
+ CCCGCCACCG AGCCGCTATG CTCGCCGCTG CGGACAAGAC CTCCTCCTTC GACTCCACGC TCAGGTTGCT   
  
  
+ CCTCAAGTTC CAGGAAGTCA GCCCGTGGAC CACCTTCGGC CACGTGGCTT GCAACGGTGC AATCATCGAG   
  
  
+ GCCTTAGAGA GGGACCCACA TGAAAAGAGT AAGCTCCACG TCATCGATAT CAGCACCACC TTCTGCACCC   
  
  
+ AGTGGCCCAC CTTGCTTGAG GCTTTAGCCA CCCGCATGGA CGACACTCCC CACCTCAGCC TCACTGCCGT   
  
  
+ CGTCGTCAAC AAGTACGCCG CCGTGAGCGG TGGCGAGGAC GACGGGGGAG AAGGATCAAA GAGGGTGATG   
  
  
+ AGAGAGATTG GTCTTCGTCT TGAGAAGTTC GCTAGGTTAA TGGGGGTTCC CTTCAAGTTC AACGTGGTTT   
  
  
+ ACCACGTAGG TGATCTATCC CAGTTGGATT TTTCTCGTTT GGATATTAAA GATGATGAAG CGGTGGCGAT   
  
  
+ TAACTGTGTC AACTCGCTCC ACTCGGTGGA CCTCCGATAT CGGGAGGTAG TGCTCGCGGC GTTCAGGCGG   
  
  
+ CTGCGGCCGA GGGTGGTGAC GGTGGTGGAG GAAGAGGCGG AGCTGACTGA GGTGGGTGAG GGTCAGTATG   
  
  
+ AATTTTTTAG GCGGTTTGAG GAGTGTCTTA GGTGGTTTAG GGTTTACTTT GAGGCCGTAG GAGATTGTTT   
  
  
+ TCCTAGGACT AGCAATGAGA AATTGATGTT AGAGCGGGCC GCGGGCCGGG CCATGGTGGA TCTCCTTGCA   
  
  
+ TGTCCCGAGC CTGGTGGGTC GGCTGAGGGG AGGGAGACGG CGAGACGGTG GTCGGGTCGG ATGCATGGGG   
  
  
+ CGGGGTTCGA GCACGTGGGG TTCAGTGACG AAGTGTGTGA TGATGTGAGG GCCTTGTTGA GGAGGTACAA   
  
  
+ GGAAGGATGG TCAATGACAC AGTGCACCAT GGATGGCGGT AATCACCCTG GAATACTGTT GTGGTGGCGG   
  
  
+ GATCAACCGG TGGTTTGGGC CAGTGCATGG CGGCCTTG  

- +Up\_Stream \_Len000TTTAGT TTGAGTATTT GTTATTAAAA AATTTTGAAT TTACCATTAA TAATCTAATT   
  
  
- ATTTTTAGGA TTACCAACAA ATTACAATGT AGTTCTGTAC GAAAAACCAC ATATATATTA CCGTCGTACA   
  
  
- CCCAATTCGC CGTTGTTTAA TAAGAGTTTA CGTACGTCAT TAATTAAATT AATTAATTAG TGGACGTATT   
  
  
- AGAGTTATGA TTAGGAAAGT AAGACACGGG GTGGGTTGGG GCTTTTATCA TTAGAAAGTT TGCAACCTTC   
  
  
- ACCCTAATCC ATATCTCATC AGGATGTCTA CTACTAGATG GATGTATATT GTATTAGACT GAAAAGAAAA   
  
  
- AATTTATTGA ACATAACTAC CGTGTCTTAA ACTGTACAAC CGTATACCAT GATTCTTTAA TTAATTGCAA   
  
  
- CACGATTCTA TATCCAGTAA GAAAAACCTC TGTATTCCGG GCGCAAGTGC GTCCCAAGCT CTTCCCGGTG   
  
  
- TAAGTTACCT ACTTAACATC CATCAAATTA GACTGAAACA GTCACTGACT AAGGTGCCGA ACTTGGGCGC   
  
  
- TGTAATTATA TCCAACTGAT TTCAGCAAAC TATGAATAAA AAGAATTCAT TTCAGAACTT AAGTTTAGAA   
  
  
- TATTTATTTA TTTTTTTAGA TATTATTTTC TTAAGATGGT AAATAATTCA GTTGTTGAGT TAAGCTTACT   
  
  
- TTATTTTTTA GTTCTAGTGG TTTTTTTTAT TTATTTTCCT TTCCATCTAA CGTAAACCTA AAACTCACCG   
  
  
- CACCACCACC CAAACTACGA CCACGACGTA TGTGGCGTCG AAAAGAGACA GGAAAATGGA GAGAAACAGA   
  
  
- CGTTCTTGGG GTAAACCGTG CGGACGAGAG AAAAGATACA AAAGAAAAGA TAGAAAATTC TTTGTTACCT   
  
  
- TTCGATTGTG TGATTGTGAT TGGGTAACCA GGAAAAAAGA ATAGACGTTT TAATTTCATT GTTATAAAAA   
  
  
- GAATAGGGGA AAAAGCAAGG TGGGTAAAGA AATAGGAAGA CAAACTTGGA TTAGACATAT ATACGTACGT   
  
  
- ACACATAAAA TGAATCTTAG ACAAAACTAA GAAACTACAT TAATTAATCA TCAGAAAAAT TAACTTAAAA   
  
  
- ATTGTCATCA AACTTAATCT GTAAATAGCT ATAATATAAA AAATATATAT TAATTTTTTA TGTTTTACTA   
  
  
- TTCACAGAAT GATGTTATGT GAAGTATTCA CAGCACAAAA TTGAATTTTT AGTATGATAA TACAAAGATT   
  
  
- TAACTTTTTT TTCACTCTAA TTCATCGTCT TCGTATAATG TTGAATATTT AATATATTTT AGTAGAAAAA   
  
  
- TAGGTATTAA ATAGTGTAAA GATTTTATGC GTATAGTATA GATGAATACC TATCCCAATT ATTCTAATCC   
  
  
- CGTAGTAAAT TCTAAAATAG TCGCATTGGT TGCTTACAAA TTCCAATCGT CAAGTATTAA TTCACATTAG   
  
  
- TAATTTTTTA AATACTAAAA TTTAGTATTT TATACAATTA GATCCTACTC CGTTCGCCGT TCTGTATATA   
  
  
- GTGATAGGAA TTTTTTATAT TATATAACTA AAGTTTCTTA ATACTTCATA TAATTAAAGT ATATATTACT   
  
  
- GCTAATACAT AAAAAAATGA TTTAATGAAC ATGGGTATTA TTTATCTTAG CTACGTAGTA AGTACCGGAA   
  
  
- AGTAAGACAT TATACCAACT GAGGTTTTTA AGAGTTTCAT AAAAATATAA AACCCTCCCC TCTAAAATCA   
  
  
- AACTATGAAA ACAACTTGAT CACTTGTTAA AGGTTGTTGG AGGTGGTGGG GGTTTGTAGT TGTTAGACTA   
  
  
- ACTACATACG TAGGGTTCGA ACTTAACTTA TTATTAAAGT TATTGTTGTT ATTAGCAAAA TGGAACTGTT   
  
  
- CGAGGTGTCG TTGAGTTGCA GAAGGTTTGG TTGGGTAGTG AGTAGTAGTA GTAGGGGTGA TAGGGTAAGT   
  
  
- GGGGGTGGTT TTCCTCGGAT TTCTGTCATT GTTCTTCTTA CGAACGGAAA AAAGTACCTA CTTCTTCTGA   
  
  
- AGAGAAGAAG GAGATGAAGA AGGGTGGTGA TACTATGTGT GGTGGAGGTT GTAGTAATGG GGTGGTTAAT   
  
  
- ATTAAGGGCA ATATGGGACC TGGGAGGGAG GTGGTGGTGG TGTCGGTGTT GGTGGTGGAG TAGGAGGTGA   
  
  
- GGGTGGGTGG TACCGAAGCC ACCACCCGAA CTGAGGAGGA GGGGCAGACG GCGACACCTT AATAGAAAGA   
  
  
- GGGGCCTGTA GGAGGAGAGG AGGGGGTGGC CTTTCACCCG CAGACTGTAA GAATACCTCC GCCGATCCCG   
  
  
- AAAAAGTCTG TGCTTGTGGT GGTTCGAGGT GGTCGAGGAG ACCTACGACT TGCTCGAGCC GAGGGGGATG   
  
  
- CCACTACAGC TCGTCTTCGA CCACCGCATG AAATTGGTTC GGGAAAAACG GGCGGAGTGA CGGTGGCCTG   
  
  
- GGGCGGTGGC TCGGCGATAC GAGCGGCGAC GCCTGTTCTG GAGGAGGAAG CTGAGGTGCG AGTCCAACGA   
  
  
- GGAGTTCAAG GTCCTTCAGT CGGGCACCTG GTGGAAGCCG GTGCACCGAA CGTTGCCACG TTAGTAGCTC   
  
  
- CGGAATCTCT CCCTGGGTGT ACTTTTCTCA TTCGAGGTGC AGTAGCTATA GTCGTGGTGG AAGACGTGGG   
  
  
- TCACCGGGTG GAACGAACTC CGAAATCGGT GGGCGTACCT GCTGTGAGGG GTGGAGTCGG AGTGACGGCA   
  
  
- GCAGCAGTTG TTCATGCGGC GGCACTCGCC ACCGCTCCTG CTGCCCCCTC TTCCTAGTTT CTCCCACTAC   
  
  
- TCTCTCTAAC CAGAAGCAGA ACTCTTCAAG CGATCCAATT ACCCCCAAGG GAAGTTCAAG TTGCACCAAA   
  
  
- TGGTGCATCC ACTAGATAGG GTCAACCTAA AAAGAGCAAA CCTATAATTT CTACTACTTC GCCACCGCTA   
  
  
- ATTGACACAG TTGAGCGAGG TGAGCCACCT GGAGGCTATA GCCCTCCATC ACGAGCGCCG CAAGTCCGCC   
  
  
- GACGCCGGCT CCCACCACTG CCACCACCTC CTTCTCCGCC TCGACTGACT CCACCCACTC CCAGTCATAC   
  
  
- TTAAAAAATC CGCCAAACTC CTCACAGAAT CCACCAAATC CCAAATGAAA CTCCGGCATC CTCTAACAAA   
  
  
- AGGATCCTGA TCGTTACTCT TTAACTACAA TCTCGCCCGG CGCCCGGCCC GGTACCACCT AGAGGAACGT   
  
  
- ACAGGGCTCG GACCACCCAG CCGACTCCCC TCCCTCTGCC GCTCTGCCAC CAGCCCAGCC TACGTACCCC   
  
  
- GCCCCAAGCT CGTGCACCCC AAGTCACTGC TTCACACACT ACTACACTCC CGGAACAACT CCTCCATGTT   
  
  
- CCTTCCTACC AGTTACTGTG TCACGTGGTA CCTACCGCCA TTAGTGGGAC CTTATGACAA CACCACCGCC   
  
  
- CTAGTTGGCC ACCAAACCCG GTCACGTACC GCCGGAAC

+     HD-Zip 3

| Site Name | Organism | Position | Strand | Matrix score. | sequence | function |
| --- | --- | --- | --- | --- | --- | --- |
| HD-Zip 3 | Arabidopsis thaliana | 1469 | + | 9.5 | GTAAT(G/C)ATTAC | protein binding site |

>HU05G01983.1   
+ +Up\_Stream \_Len000AAATCA AACTCATAAA CAATAATTTT TTAAAACTTA AATGGTAATT ATTAGATTAA   
  
  
+ TAAAAATCCT AATGGTTGTT TAATGTTACA TCAAGACATG CTTTTTGGTG TATATATAAT GGCAGCATGT   
  
  
+ GGGTTAAGCG GCAACAAATT ATTCTCAAAT GCATGCAGTA ATTAATTTAA TTAATTAATC ACCTGCATAA   
  
  
+ TCTCAATACT AATCCTTTCA TTCTGTGCCC CACCCAACCC CGAAAATAGT AATCTTTCAA ACGTTGGAAG   
  
  
+ TGGGATTAGG TATAGAGTAG TCCTACAGAT GATGATCTAC CTACATATAA CATAATCTGA CTTTTCTTTT   
  
  
+ TTAAATAACT TGTATTGATG GCACAGAATT TGACATGTTG GCATATGGTA CTAAGAAATT AATTAACGTT   
  
  
+ GTGCTAAGAT ATAGGTCATT CTTTTTGGAG ACATAAGGCC CGCGTTCACG CAGGGTTCGA GAAGGGCCAC   
  
  
+ ATTCAATGGA TGAATTGTAG GTAGTTTAAT CTGACTTTGT CAGTGACTGA TTCCACGGCT TGAACCCGCG   
  
  
+ ACATTAATAT AGGTTGACTA AAGTCGTTTG ATACTTATTT TTCTTAAGTA AAGTCTTGAA TTCAAATCTT   
  
  
+ ATAAATAAAT AAAAAAATCT ATAATAAAAG AATTCTACCA TTTATTAAGT CAACAACTCA ATTCGAATGA   
  
  
+ AATAAAAAAT CAAGATCACC AAAAAAAATA AATAAAAGGA AAGGTAGATT GCATTTGGAT TTTGAGTGGC   
  
  
+ GTGGTGGTGG GTTTGATGCT GGTGCTGCAT ACACCGCAGC TTTTCTCTGT CCTTTTACCT CTCTTTGTCT   
  
  
+ GCAAGAACCC CATTTGGCAC GCCTGCTCTC TTTTCTATGT TTTCTTTTCT ATCTTTTAAG AAACAATGGA   
  
  
+ AAGCTAACAC ACTAACACTA ACCCATTGGT CCTTTTTTCT TATCTGCAAA ATTAAAGTAA CAATATTTTT   
  
  
+ CTTATCCCCT TTTTCGTTCC ACCCATTTCT TTATCCTTCT GTTTGAACCT AATCTGTATA TATGCATGCA   
  
  
+ TGTGTATTTT ACTTAGAATC TGTTTTGATT CTTTGATGTA ATTAATTAGT AGTCTTTTTA ATTGAATTTT   
  
  
+ TAACAGTAGT TTGAATTAGA CATTTATCGA TATTATATTT TTTATATATA ATTAAAAAAT ACAAAATGAT   
  
  
+ AAGTGTCTTA CTACAATACA CTTCATAAGT GTCGTGTTTT AACTTAAAAA TCATACTATT ATGTTTCTAA   
  
  
+ ATTGAAAAAA AAGTGAGATT AAGTAGCAGA AGCATATTAC AACTTATAAA TTATATAAAA TCATCTTTTT   
  
  
+ ATCCATAATT TATCACATTT CTAAAATACG CATATCATAT CTACTTATGG ATAGGGTTAA TAAGATTAGG   
  
  
+ GCATCATTTA AGATTTTATC AGCGTAACCA ACGAATGTTT AAGGTTAGCA GTTCATAATT AAGTGTAATC   
  
  
+ ATTAAAAAAT TTATGATTTT AAATCATAAA ATATGTTAAT CTAGGATGAG GCAAGCGGCA AGACATATAT   
  
  
+ CACTATCCTT AAAAAATATA ATATATTGAT TTCAAAGAAT TATGAAGTAT ATTAATTTCA TATATAATGA   
  
  
+ CGATTATGTA TTTTTTTACT AAATTACTTG TACCCATAAT AAATAGAATC GATGCATCAT TCATGGCCTT   
  
  
+ TCATTCTGTA ATATGGTTGA CTCCAAAAAT TCTCAAAGTA TTTTTATATT TTGGGAGGGG AGATTTTAGT   
  
  
+ TTGATACTTT TGTTGAACTA GTGAACAATT TCCAACAACC TCCACCACCC CCAAACATCA ACAATCTGAT   
  
  
+ TGATGTATGC ATCCCAAGCT TGAATTGAAT AATAATTTCA ATAACAACAA TAATCGTTTT ACCTTGACAA   
  
  
+ GCTCCACAGC AACTCAACGT CTTCCAAACC AACCCATCAC TCATCATCAT CATCCCCACT ATCCCATTCA   
  
  
+ CCCCCACCAA AAGGAGCCTA AAGACAGTAA CAAGAAGAAT GCTTGCCTTT TTTCATGGAT GAAGAAGACT   
  
  
+ TCTCTTCTTC CTCTACTTCT TCCCACCACT ATGATACACA CCACCTCCAA CATCATTACC CCACCAATTA   
  
  
+ TAATTCCCGT TATACCCTGG ACCCTCCCTC CACCACCACC ACAGCCACAA CCACCACCTC ATCCTCCACT   
  
  
+ CCCACCCACC ATGGCTTCGG TGGTGGGCTT GACTCCTCCT CCCCGTCTGC CGCTGTGGAA TTATCTTTCT   
  
  
+ CCCCGGACAT CCTCCTCTCC TCCCCCACCG GAAAGTGGGC GTCTGACATT CTTATGGAGG CGGCTAGGGC   
  
  
+ TTTTTCAGAC ACGAACACCA CCAAGCTCCA CCAGCTCCTC TGGATGCTGA ACGAGCTCGG CTCCCCCTAC   
  
  
+ GGTGATGTCG AGCAGAAGCT GGTGGCGTAC TTTAACCAAG CCCTTTTTGC CCGCCTCACT GCCACCGGAC   
  
  
+ CCCGCCACCG AGCCGCTATG CTCGCCGCTG CGGACAAGAC CTCCTCCTTC GACTCCACGC TCAGGTTGCT   
  
  
+ CCTCAAGTTC CAGGAAGTCA GCCCGTGGAC CACCTTCGGC CACGTGGCTT GCAACGGTGC AATCATCGAG   
  
  
+ GCCTTAGAGA GGGACCCACA TGAAAAGAGT AAGCTCCACG TCATCGATAT CAGCACCACC TTCTGCACCC   
  
  
+ AGTGGCCCAC CTTGCTTGAG GCTTTAGCCA CCCGCATGGA CGACACTCCC CACCTCAGCC TCACTGCCGT   
  
  
+ CGTCGTCAAC AAGTACGCCG CCGTGAGCGG TGGCGAGGAC GACGGGGGAG AAGGATCAAA GAGGGTGATG   
  
  
+ AGAGAGATTG GTCTTCGTCT TGAGAAGTTC GCTAGGTTAA TGGGGGTTCC CTTCAAGTTC AACGTGGTTT   
  
  
+ ACCACGTAGG TGATCTATCC CAGTTGGATT TTTCTCGTTT GGATATTAAA GATGATGAAG CGGTGGCGAT   
  
  
+ TAACTGTGTC AACTCGCTCC ACTCGGTGGA CCTCCGATAT CGGGAGGTAG TGCTCGCGGC GTTCAGGCGG   
  
  
+ CTGCGGCCGA GGGTGGTGAC GGTGGTGGAG GAAGAGGCGG AGCTGACTGA GGTGGGTGAG GGTCAGTATG   
  
  
+ AATTTTTTAG GCGGTTTGAG GAGTGTCTTA GGTGGTTTAG GGTTTACTTT GAGGCCGTAG GAGATTGTTT   
  
  
+ TCCTAGGACT AGCAATGAGA AATTGATGTT AGAGCGGGCC GCGGGCCGGG CCATGGTGGA TCTCCTTGCA   
  
  
+ TGTCCCGAGC CTGGTGGGTC GGCTGAGGGG AGGGAGACGG CGAGACGGTG GTCGGGTCGG ATGCATGGGG   
  
  
+ CGGGGTTCGA GCACGTGGGG TTCAGTGACG AAGTGTGTGA TGATGTGAGG GCCTTGTTGA GGAGGTACAA   
  
  
+ GGAAGGATGG TCAATGACAC AGTGCACCAT GGATGGCGGT AATCACCCTG GAATACTGTT GTGGTGGCGG   
  
  
+ GATCAACCGG TGGTTTGGGC CAGTGCATGG CGGCCTTG  

- +Up\_Stream \_Len000TTTAGT TTGAGTATTT GTTATTAAAA AATTTTGAAT TTACCATTAA TAATCTAATT   
  
  
- ATTTTTAGGA TTACCAACAA ATTACAATGT AGTTCTGTAC GAAAAACCAC ATATATATTA CCGTCGTACA   
  
  
- CCCAATTCGC CGTTGTTTAA TAAGAGTTTA CGTACGTCAT TAATTAAATT AATTAATTAG TGGACGTATT   
  
  
- AGAGTTATGA TTAGGAAAGT AAGACACGGG GTGGGTTGGG GCTTTTATCA TTAGAAAGTT TGCAACCTTC   
  
  
- ACCCTAATCC ATATCTCATC AGGATGTCTA CTACTAGATG GATGTATATT GTATTAGACT GAAAAGAAAA   
  
  
- AATTTATTGA ACATAACTAC CGTGTCTTAA ACTGTACAAC CGTATACCAT GATTCTTTAA TTAATTGCAA   
  
  
- CACGATTCTA TATCCAGTAA GAAAAACCTC TGTATTCCGG GCGCAAGTGC GTCCCAAGCT CTTCCCGGTG   
  
  
- TAAGTTACCT ACTTAACATC CATCAAATTA GACTGAAACA GTCACTGACT AAGGTGCCGA ACTTGGGCGC   
  
  
- TGTAATTATA TCCAACTGAT TTCAGCAAAC TATGAATAAA AAGAATTCAT TTCAGAACTT AAGTTTAGAA   
  
  
- TATTTATTTA TTTTTTTAGA TATTATTTTC TTAAGATGGT AAATAATTCA GTTGTTGAGT TAAGCTTACT   
  
  
- TTATTTTTTA GTTCTAGTGG TTTTTTTTAT TTATTTTCCT TTCCATCTAA CGTAAACCTA AAACTCACCG   
  
  
- CACCACCACC CAAACTACGA CCACGACGTA TGTGGCGTCG AAAAGAGACA GGAAAATGGA GAGAAACAGA   
  
  
- CGTTCTTGGG GTAAACCGTG CGGACGAGAG AAAAGATACA AAAGAAAAGA TAGAAAATTC TTTGTTACCT   
  
  
- TTCGATTGTG TGATTGTGAT TGGGTAACCA GGAAAAAAGA ATAGACGTTT TAATTTCATT GTTATAAAAA   
  
  
- GAATAGGGGA AAAAGCAAGG TGGGTAAAGA AATAGGAAGA CAAACTTGGA TTAGACATAT ATACGTACGT   
  
  
- ACACATAAAA TGAATCTTAG ACAAAACTAA GAAACTACAT TAATTAATCA TCAGAAAAAT TAACTTAAAA   
  
  
- ATTGTCATCA AACTTAATCT GTAAATAGCT ATAATATAAA AAATATATAT TAATTTTTTA TGTTTTACTA   
  
  
- TTCACAGAAT GATGTTATGT GAAGTATTCA CAGCACAAAA TTGAATTTTT AGTATGATAA TACAAAGATT   
  
  
- TAACTTTTTT TTCACTCTAA TTCATCGTCT TCGTATAATG TTGAATATTT AATATATTTT AGTAGAAAAA   
  
  
- TAGGTATTAA ATAGTGTAAA GATTTTATGC GTATAGTATA GATGAATACC TATCCCAATT ATTCTAATCC   
  
  
- CGTAGTAAAT TCTAAAATAG TCGCATTGGT TGCTTACAAA TTCCAATCGT CAAGTATTAA TTCACATTAG   
  
  
- TAATTTTTTA AATACTAAAA TTTAGTATTT TATACAATTA GATCCTACTC CGTTCGCCGT TCTGTATATA   
  
  
- GTGATAGGAA TTTTTTATAT TATATAACTA AAGTTTCTTA ATACTTCATA TAATTAAAGT ATATATTACT   
  
  
- GCTAATACAT AAAAAAATGA TTTAATGAAC ATGGGTATTA TTTATCTTAG CTACGTAGTA AGTACCGGAA   
  
  
- AGTAAGACAT TATACCAACT GAGGTTTTTA AGAGTTTCAT AAAAATATAA AACCCTCCCC TCTAAAATCA   
  
  
- AACTATGAAA ACAACTTGAT CACTTGTTAA AGGTTGTTGG AGGTGGTGGG GGTTTGTAGT TGTTAGACTA   
  
  
- ACTACATACG TAGGGTTCGA ACTTAACTTA TTATTAAAGT TATTGTTGTT ATTAGCAAAA TGGAACTGTT   
  
  
- CGAGGTGTCG TTGAGTTGCA GAAGGTTTGG TTGGGTAGTG AGTAGTAGTA GTAGGGGTGA TAGGGTAAGT   
  
  
- GGGGGTGGTT TTCCTCGGAT TTCTGTCATT GTTCTTCTTA CGAACGGAAA AAAGTACCTA CTTCTTCTGA   
  
  
- AGAGAAGAAG GAGATGAAGA AGGGTGGTGA TACTATGTGT GGTGGAGGTT GTAGTAATGG GGTGGTTAAT   
  
  
- ATTAAGGGCA ATATGGGACC TGGGAGGGAG GTGGTGGTGG TGTCGGTGTT GGTGGTGGAG TAGGAGGTGA   
  
  
- GGGTGGGTGG TACCGAAGCC ACCACCCGAA CTGAGGAGGA GGGGCAGACG GCGACACCTT AATAGAAAGA   
  
  
- GGGGCCTGTA GGAGGAGAGG AGGGGGTGGC CTTTCACCCG CAGACTGTAA GAATACCTCC GCCGATCCCG   
  
  
- AAAAAGTCTG TGCTTGTGGT GGTTCGAGGT GGTCGAGGAG ACCTACGACT TGCTCGAGCC GAGGGGGATG   
  
  
- CCACTACAGC TCGTCTTCGA CCACCGCATG AAATTGGTTC GGGAAAAACG GGCGGAGTGA CGGTGGCCTG   
  
  
- GGGCGGTGGC TCGGCGATAC GAGCGGCGAC GCCTGTTCTG GAGGAGGAAG CTGAGGTGCG AGTCCAACGA   
  
  
- GGAGTTCAAG GTCCTTCAGT CGGGCACCTG GTGGAAGCCG GTGCACCGAA CGTTGCCACG TTAGTAGCTC   
  
  
- CGGAATCTCT CCCTGGGTGT ACTTTTCTCA TTCGAGGTGC AGTAGCTATA GTCGTGGTGG AAGACGTGGG   
  
  
- TCACCGGGTG GAACGAACTC CGAAATCGGT GGGCGTACCT GCTGTGAGGG GTGGAGTCGG AGTGACGGCA   
  
  
- GCAGCAGTTG TTCATGCGGC GGCACTCGCC ACCGCTCCTG CTGCCCCCTC TTCCTAGTTT CTCCCACTAC   
  
  
- TCTCTCTAAC CAGAAGCAGA ACTCTTCAAG CGATCCAATT ACCCCCAAGG GAAGTTCAAG TTGCACCAAA   
  
  
- TGGTGCATCC ACTAGATAGG GTCAACCTAA AAAGAGCAAA CCTATAATTT CTACTACTTC GCCACCGCTA   
  
  
- ATTGACACAG TTGAGCGAGG TGAGCCACCT GGAGGCTATA GCCCTCCATC ACGAGCGCCG CAAGTCCGCC   
  
  
- GACGCCGGCT CCCACCACTG CCACCACCTC CTTCTCCGCC TCGACTGACT CCACCCACTC CCAGTCATAC   
  
  
- TTAAAAAATC CGCCAAACTC CTCACAGAAT CCACCAAATC CCAAATGAAA CTCCGGCATC CTCTAACAAA   
  
  
- AGGATCCTGA TCGTTACTCT TTAACTACAA TCTCGCCCGG CGCCCGGCCC GGTACCACCT AGAGGAACGT   
  
  
- ACAGGGCTCG GACCACCCAG CCGACTCCCC TCCCTCTGCC GCTCTGCCAC CAGCCCAGCC TACGTACCCC   
  
  
- GCCCCAAGCT CGTGCACCCC AAGTCACTGC TTCACACACT ACTACACTCC CGGAACAACT CCTCCATGTT   
  
  
- CCTTCCTACC AGTTACTGTG TCACGTGGTA CCTACCGCCA TTAGTGGGAC CTTATGACAA CACCACCGCC   
  
  
- CTAGTTGGCC ACCAAACCCG GTCACGTACC GCCGGAAC

+     I-box

| Site Name | Organism | Position | Strand | Matrix score. | sequence | function |
| --- | --- | --- | --- | --- | --- | --- |
| I-box | Zea mays | 2159 | - | 9 | gGATAAGGTG | part of a light responsive element |

>HU05G01983.1   
+ +Up\_Stream \_Len000AAATCA AACTCATAAA CAATAATTTT TTAAAACTTA AATGGTAATT ATTAGATTAA   
  
  
+ TAAAAATCCT AATGGTTGTT TAATGTTACA TCAAGACATG CTTTTTGGTG TATATATAAT GGCAGCATGT   
  
  
+ GGGTTAAGCG GCAACAAATT ATTCTCAAAT GCATGCAGTA ATTAATTTAA TTAATTAATC ACCTGCATAA   
  
  
+ TCTCAATACT AATCCTTTCA TTCTGTGCCC CACCCAACCC CGAAAATAGT AATCTTTCAA ACGTTGGAAG   
  
  
+ TGGGATTAGG TATAGAGTAG TCCTACAGAT GATGATCTAC CTACATATAA CATAATCTGA CTTTTCTTTT   
  
  
+ TTAAATAACT TGTATTGATG GCACAGAATT TGACATGTTG GCATATGGTA CTAAGAAATT AATTAACGTT   
  
  
+ GTGCTAAGAT ATAGGTCATT CTTTTTGGAG ACATAAGGCC CGCGTTCACG CAGGGTTCGA GAAGGGCCAC   
  
  
+ ATTCAATGGA TGAATTGTAG GTAGTTTAAT CTGACTTTGT CAGTGACTGA TTCCACGGCT TGAACCCGCG   
  
  
+ ACATTAATAT AGGTTGACTA AAGTCGTTTG ATACTTATTT TTCTTAAGTA AAGTCTTGAA TTCAAATCTT   
  
  
+ ATAAATAAAT AAAAAAATCT ATAATAAAAG AATTCTACCA TTTATTAAGT CAACAACTCA ATTCGAATGA   
  
  
+ AATAAAAAAT CAAGATCACC AAAAAAAATA AATAAAAGGA AAGGTAGATT GCATTTGGAT TTTGAGTGGC   
  
  
+ GTGGTGGTGG GTTTGATGCT GGTGCTGCAT ACACCGCAGC TTTTCTCTGT CCTTTTACCT CTCTTTGTCT   
  
  
+ GCAAGAACCC CATTTGGCAC GCCTGCTCTC TTTTCTATGT TTTCTTTTCT ATCTTTTAAG AAACAATGGA   
  
  
+ AAGCTAACAC ACTAACACTA ACCCATTGGT CCTTTTTTCT TATCTGCAAA ATTAAAGTAA CAATATTTTT   
  
  
+ CTTATCCCCT TTTTCGTTCC ACCCATTTCT TTATCCTTCT GTTTGAACCT AATCTGTATA TATGCATGCA   
  
  
+ TGTGTATTTT ACTTAGAATC TGTTTTGATT CTTTGATGTA ATTAATTAGT AGTCTTTTTA ATTGAATTTT   
  
  
+ TAACAGTAGT TTGAATTAGA CATTTATCGA TATTATATTT TTTATATATA ATTAAAAAAT ACAAAATGAT   
  
  
+ AAGTGTCTTA CTACAATACA CTTCATAAGT GTCGTGTTTT AACTTAAAAA TCATACTATT ATGTTTCTAA   
  
  
+ ATTGAAAAAA AAGTGAGATT AAGTAGCAGA AGCATATTAC AACTTATAAA TTATATAAAA TCATCTTTTT   
  
  
+ ATCCATAATT TATCACATTT CTAAAATACG CATATCATAT CTACTTATGG ATAGGGTTAA TAAGATTAGG   
  
  
+ GCATCATTTA AGATTTTATC AGCGTAACCA ACGAATGTTT AAGGTTAGCA GTTCATAATT AAGTGTAATC   
  
  
+ ATTAAAAAAT TTATGATTTT AAATCATAAA ATATGTTAAT CTAGGATGAG GCAAGCGGCA AGACATATAT   
  
  
+ CACTATCCTT AAAAAATATA ATATATTGAT TTCAAAGAAT TATGAAGTAT ATTAATTTCA TATATAATGA   
  
  
+ CGATTATGTA TTTTTTTACT AAATTACTTG TACCCATAAT AAATAGAATC GATGCATCAT TCATGGCCTT   
  
  
+ TCATTCTGTA ATATGGTTGA CTCCAAAAAT TCTCAAAGTA TTTTTATATT TTGGGAGGGG AGATTTTAGT   
  
  
+ TTGATACTTT TGTTGAACTA GTGAACAATT TCCAACAACC TCCACCACCC CCAAACATCA ACAATCTGAT   
  
  
+ TGATGTATGC ATCCCAAGCT TGAATTGAAT AATAATTTCA ATAACAACAA TAATCGTTTT ACCTTGACAA   
  
  
+ GCTCCACAGC AACTCAACGT CTTCCAAACC AACCCATCAC TCATCATCAT CATCCCCACT ATCCCATTCA   
  
  
+ CCCCCACCAA AAGGAGCCTA AAGACAGTAA CAAGAAGAAT GCTTGCCTTT TTTCATGGAT GAAGAAGACT   
  
  
+ TCTCTTCTTC CTCTACTTCT TCCCACCACT ATGATACACA CCACCTCCAA CATCATTACC CCACCAATTA   
  
  
+ TAATTCCCGT TATACCCTGG ACCCTCCCTC CACCACCACC ACAGCCACAA CCACCACCTC ATCCTCCACT   
  
  
+ CCCACCCACC ATGGCTTCGG TGGTGGGCTT GACTCCTCCT CCCCGTCTGC CGCTGTGGAA TTATCTTTCT   
  
  
+ CCCCGGACAT CCTCCTCTCC TCCCCCACCG GAAAGTGGGC GTCTGACATT CTTATGGAGG CGGCTAGGGC   
  
  
+ TTTTTCAGAC ACGAACACCA CCAAGCTCCA CCAGCTCCTC TGGATGCTGA ACGAGCTCGG CTCCCCCTAC   
  
  
+ GGTGATGTCG AGCAGAAGCT GGTGGCGTAC TTTAACCAAG CCCTTTTTGC CCGCCTCACT GCCACCGGAC   
  
  
+ CCCGCCACCG AGCCGCTATG CTCGCCGCTG CGGACAAGAC CTCCTCCTTC GACTCCACGC TCAGGTTGCT   
  
  
+ CCTCAAGTTC CAGGAAGTCA GCCCGTGGAC CACCTTCGGC CACGTGGCTT GCAACGGTGC AATCATCGAG   
  
  
+ GCCTTAGAGA GGGACCCACA TGAAAAGAGT AAGCTCCACG TCATCGATAT CAGCACCACC TTCTGCACCC   
  
  
+ AGTGGCCCAC CTTGCTTGAG GCTTTAGCCA CCCGCATGGA CGACACTCCC CACCTCAGCC TCACTGCCGT   
  
  
+ CGTCGTCAAC AAGTACGCCG CCGTGAGCGG TGGCGAGGAC GACGGGGGAG AAGGATCAAA GAGGGTGATG   
  
  
+ AGAGAGATTG GTCTTCGTCT TGAGAAGTTC GCTAGGTTAA TGGGGGTTCC CTTCAAGTTC AACGTGGTTT   
  
  
+ ACCACGTAGG TGATCTATCC CAGTTGGATT TTTCTCGTTT GGATATTAAA GATGATGAAG CGGTGGCGAT   
  
  
+ TAACTGTGTC AACTCGCTCC ACTCGGTGGA CCTCCGATAT CGGGAGGTAG TGCTCGCGGC GTTCAGGCGG   
  
  
+ CTGCGGCCGA GGGTGGTGAC GGTGGTGGAG GAAGAGGCGG AGCTGACTGA GGTGGGTGAG GGTCAGTATG   
  
  
+ AATTTTTTAG GCGGTTTGAG GAGTGTCTTA GGTGGTTTAG GGTTTACTTT GAGGCCGTAG GAGATTGTTT   
  
  
+ TCCTAGGACT AGCAATGAGA AATTGATGTT AGAGCGGGCC GCGGGCCGGG CCATGGTGGA TCTCCTTGCA   
  
  
+ TGTCCCGAGC CTGGTGGGTC GGCTGAGGGG AGGGAGACGG CGAGACGGTG GTCGGGTCGG ATGCATGGGG   
  
  
+ CGGGGTTCGA GCACGTGGGG TTCAGTGACG AAGTGTGTGA TGATGTGAGG GCCTTGTTGA GGAGGTACAA   
  
  
+ GGAAGGATGG TCAATGACAC AGTGCACCAT GGATGGCGGT AATCACCCTG GAATACTGTT GTGGTGGCGG   
  
  
+ GATCAACCGG TGGTTTGGGC CAGTGCATGG CGGCCTTG  

- +Up\_Stream \_Len000TTTAGT TTGAGTATTT GTTATTAAAA AATTTTGAAT TTACCATTAA TAATCTAATT   
  
  
- ATTTTTAGGA TTACCAACAA ATTACAATGT AGTTCTGTAC GAAAAACCAC ATATATATTA CCGTCGTACA   
  
  
- CCCAATTCGC CGTTGTTTAA TAAGAGTTTA CGTACGTCAT TAATTAAATT AATTAATTAG TGGACGTATT   
  
  
- AGAGTTATGA TTAGGAAAGT AAGACACGGG GTGGGTTGGG GCTTTTATCA TTAGAAAGTT TGCAACCTTC   
  
  
- ACCCTAATCC ATATCTCATC AGGATGTCTA CTACTAGATG GATGTATATT GTATTAGACT GAAAAGAAAA   
  
  
- AATTTATTGA ACATAACTAC CGTGTCTTAA ACTGTACAAC CGTATACCAT GATTCTTTAA TTAATTGCAA   
  
  
- CACGATTCTA TATCCAGTAA GAAAAACCTC TGTATTCCGG GCGCAAGTGC GTCCCAAGCT CTTCCCGGTG   
  
  
- TAAGTTACCT ACTTAACATC CATCAAATTA GACTGAAACA GTCACTGACT AAGGTGCCGA ACTTGGGCGC   
  
  
- TGTAATTATA TCCAACTGAT TTCAGCAAAC TATGAATAAA AAGAATTCAT TTCAGAACTT AAGTTTAGAA   
  
  
- TATTTATTTA TTTTTTTAGA TATTATTTTC TTAAGATGGT AAATAATTCA GTTGTTGAGT TAAGCTTACT   
  
  
- TTATTTTTTA GTTCTAGTGG TTTTTTTTAT TTATTTTCCT TTCCATCTAA CGTAAACCTA AAACTCACCG   
  
  
- CACCACCACC CAAACTACGA CCACGACGTA TGTGGCGTCG AAAAGAGACA GGAAAATGGA GAGAAACAGA   
  
  
- CGTTCTTGGG GTAAACCGTG CGGACGAGAG AAAAGATACA AAAGAAAAGA TAGAAAATTC TTTGTTACCT   
  
  
- TTCGATTGTG TGATTGTGAT TGGGTAACCA GGAAAAAAGA ATAGACGTTT TAATTTCATT GTTATAAAAA   
  
  
- GAATAGGGGA AAAAGCAAGG TGGGTAAAGA AATAGGAAGA CAAACTTGGA TTAGACATAT ATACGTACGT   
  
  
- ACACATAAAA TGAATCTTAG ACAAAACTAA GAAACTACAT TAATTAATCA TCAGAAAAAT TAACTTAAAA   
  
  
- ATTGTCATCA AACTTAATCT GTAAATAGCT ATAATATAAA AAATATATAT TAATTTTTTA TGTTTTACTA   
  
  
- TTCACAGAAT GATGTTATGT GAAGTATTCA CAGCACAAAA TTGAATTTTT AGTATGATAA TACAAAGATT   
  
  
- TAACTTTTTT TTCACTCTAA TTCATCGTCT TCGTATAATG TTGAATATTT AATATATTTT AGTAGAAAAA   
  
  
- TAGGTATTAA ATAGTGTAAA GATTTTATGC GTATAGTATA GATGAATACC TATCCCAATT ATTCTAATCC   
  
  
- CGTAGTAAAT TCTAAAATAG TCGCATTGGT TGCTTACAAA TTCCAATCGT CAAGTATTAA TTCACATTAG   
  
  
- TAATTTTTTA AATACTAAAA TTTAGTATTT TATACAATTA GATCCTACTC CGTTCGCCGT TCTGTATATA   
  
  
- GTGATAGGAA TTTTTTATAT TATATAACTA AAGTTTCTTA ATACTTCATA TAATTAAAGT ATATATTACT   
  
  
- GCTAATACAT AAAAAAATGA TTTAATGAAC ATGGGTATTA TTTATCTTAG CTACGTAGTA AGTACCGGAA   
  
  
- AGTAAGACAT TATACCAACT GAGGTTTTTA AGAGTTTCAT AAAAATATAA AACCCTCCCC TCTAAAATCA   
  
  
- AACTATGAAA ACAACTTGAT CACTTGTTAA AGGTTGTTGG AGGTGGTGGG GGTTTGTAGT TGTTAGACTA   
  
  
- ACTACATACG TAGGGTTCGA ACTTAACTTA TTATTAAAGT TATTGTTGTT ATTAGCAAAA TGGAACTGTT   
  
  
- CGAGGTGTCG TTGAGTTGCA GAAGGTTTGG TTGGGTAGTG AGTAGTAGTA GTAGGGGTGA TAGGGTAAGT   
  
  
- GGGGGTGGTT TTCCTCGGAT TTCTGTCATT GTTCTTCTTA CGAACGGAAA AAAGTACCTA CTTCTTCTGA   
  
  
- AGAGAAGAAG GAGATGAAGA AGGGTGGTGA TACTATGTGT GGTGGAGGTT GTAGTAATGG GGTGGTTAAT   
  
  
- ATTAAGGGCA ATATGGGACC TGGGAGGGAG GTGGTGGTGG TGTCGGTGTT GGTGGTGGAG TAGGAGGTGA   
  
  
- GGGTGGGTGG TACCGAAGCC ACCACCCGAA CTGAGGAGGA GGGGCAGACG GCGACACCTT AATAGAAAGA   
  
  
- GGGGCCTGTA GGAGGAGAGG AGGGGGTGGC CTTTCACCCG CAGACTGTAA GAATACCTCC GCCGATCCCG   
  
  
- AAAAAGTCTG TGCTTGTGGT GGTTCGAGGT GGTCGAGGAG ACCTACGACT TGCTCGAGCC GAGGGGGATG   
  
  
- CCACTACAGC TCGTCTTCGA CCACCGCATG AAATTGGTTC GGGAAAAACG GGCGGAGTGA CGGTGGCCTG   
  
  
- GGGCGGTGGC TCGGCGATAC GAGCGGCGAC GCCTGTTCTG GAGGAGGAAG CTGAGGTGCG AGTCCAACGA   
  
  
- GGAGTTCAAG GTCCTTCAGT CGGGCACCTG GTGGAAGCCG GTGCACCGAA CGTTGCCACG TTAGTAGCTC   
  
  
- CGGAATCTCT CCCTGGGTGT ACTTTTCTCA TTCGAGGTGC AGTAGCTATA GTCGTGGTGG AAGACGTGGG   
  
  
- TCACCGGGTG GAACGAACTC CGAAATCGGT GGGCGTACCT GCTGTGAGGG GTGGAGTCGG AGTGACGGCA   
  
  
- GCAGCAGTTG TTCATGCGGC GGCACTCGCC ACCGCTCCTG CTGCCCCCTC TTCCTAGTTT CTCCCACTAC   
  
  
- TCTCTCTAAC CAGAAGCAGA ACTCTTCAAG CGATCCAATT ACCCCCAAGG GAAGTTCAAG TTGCACCAAA   
  
  
- TGGTGCATCC ACTAGATAGG GTCAACCTAA AAAGAGCAAA CCTATAATTT CTACTACTTC GCCACCGCTA   
  
  
- ATTGACACAG TTGAGCGAGG TGAGCCACCT GGAGGCTATA GCCCTCCATC ACGAGCGCCG CAAGTCCGCC   
  
  
- GACGCCGGCT CCCACCACTG CCACCACCTC CTTCTCCGCC TCGACTGACT CCACCCACTC CCAGTCATAC   
  
  
- TTAAAAAATC CGCCAAACTC CTCACAGAAT CCACCAAATC CCAAATGAAA CTCCGGCATC CTCTAACAAA   
  
  
- AGGATCCTGA TCGTTACTCT TTAACTACAA TCTCGCCCGG CGCCCGGCCC GGTACCACCT AGAGGAACGT   
  
  
- ACAGGGCTCG GACCACCCAG CCGACTCCCC TCCCTCTGCC GCTCTGCCAC CAGCCCAGCC TACGTACCCC   
  
  
- GCCCCAAGCT CGTGCACCCC AAGTCACTGC TTCACACACT ACTACACTCC CGGAACAACT CCTCCATGTT   
  
  
- CCTTCCTACC AGTTACTGTG TCACGTGGTA CCTACCGCCA TTAGTGGGAC CTTATGACAA CACCACCGCC   
  
  
- CTAGTTGGCC ACCAAACCCG GTCACGTACC GCCGGAAC

+     LTR

| Site Name | Organism | Position | Strand | Matrix score. | sequence | function |
| --- | --- | --- | --- | --- | --- | --- |
| LTR | Hordeum vulgare | 254 | + | 6 | CCGAAA | cis-acting element involved in low-temperature responsiveness |

>HU05G01983.1   
+ +Up\_Stream \_Len000AAATCA AACTCATAAA CAATAATTTT TTAAAACTTA AATGGTAATT ATTAGATTAA   
  
  
+ TAAAAATCCT AATGGTTGTT TAATGTTACA TCAAGACATG CTTTTTGGTG TATATATAAT GGCAGCATGT   
  
  
+ GGGTTAAGCG GCAACAAATT ATTCTCAAAT GCATGCAGTA ATTAATTTAA TTAATTAATC ACCTGCATAA   
  
  
+ TCTCAATACT AATCCTTTCA TTCTGTGCCC CACCCAACCC CGAAAATAGT AATCTTTCAA ACGTTGGAAG   
  
  
+ TGGGATTAGG TATAGAGTAG TCCTACAGAT GATGATCTAC CTACATATAA CATAATCTGA CTTTTCTTTT   
  
  
+ TTAAATAACT TGTATTGATG GCACAGAATT TGACATGTTG GCATATGGTA CTAAGAAATT AATTAACGTT   
  
  
+ GTGCTAAGAT ATAGGTCATT CTTTTTGGAG ACATAAGGCC CGCGTTCACG CAGGGTTCGA GAAGGGCCAC   
  
  
+ ATTCAATGGA TGAATTGTAG GTAGTTTAAT CTGACTTTGT CAGTGACTGA TTCCACGGCT TGAACCCGCG   
  
  
+ ACATTAATAT AGGTTGACTA AAGTCGTTTG ATACTTATTT TTCTTAAGTA AAGTCTTGAA TTCAAATCTT   
  
  
+ ATAAATAAAT AAAAAAATCT ATAATAAAAG AATTCTACCA TTTATTAAGT CAACAACTCA ATTCGAATGA   
  
  
+ AATAAAAAAT CAAGATCACC AAAAAAAATA AATAAAAGGA AAGGTAGATT GCATTTGGAT TTTGAGTGGC   
  
  
+ GTGGTGGTGG GTTTGATGCT GGTGCTGCAT ACACCGCAGC TTTTCTCTGT CCTTTTACCT CTCTTTGTCT   
  
  
+ GCAAGAACCC CATTTGGCAC GCCTGCTCTC TTTTCTATGT TTTCTTTTCT ATCTTTTAAG AAACAATGGA   
  
  
+ AAGCTAACAC ACTAACACTA ACCCATTGGT CCTTTTTTCT TATCTGCAAA ATTAAAGTAA CAATATTTTT   
  
  
+ CTTATCCCCT TTTTCGTTCC ACCCATTTCT TTATCCTTCT GTTTGAACCT AATCTGTATA TATGCATGCA   
  
  
+ TGTGTATTTT ACTTAGAATC TGTTTTGATT CTTTGATGTA ATTAATTAGT AGTCTTTTTA ATTGAATTTT   
  
  
+ TAACAGTAGT TTGAATTAGA CATTTATCGA TATTATATTT TTTATATATA ATTAAAAAAT ACAAAATGAT   
  
  
+ AAGTGTCTTA CTACAATACA CTTCATAAGT GTCGTGTTTT AACTTAAAAA TCATACTATT ATGTTTCTAA   
  
  
+ ATTGAAAAAA AAGTGAGATT AAGTAGCAGA AGCATATTAC AACTTATAAA TTATATAAAA TCATCTTTTT   
  
  
+ ATCCATAATT TATCACATTT CTAAAATACG CATATCATAT CTACTTATGG ATAGGGTTAA TAAGATTAGG   
  
  
+ GCATCATTTA AGATTTTATC AGCGTAACCA ACGAATGTTT AAGGTTAGCA GTTCATAATT AAGTGTAATC   
  
  
+ ATTAAAAAAT TTATGATTTT AAATCATAAA ATATGTTAAT CTAGGATGAG GCAAGCGGCA AGACATATAT   
  
  
+ CACTATCCTT AAAAAATATA ATATATTGAT TTCAAAGAAT TATGAAGTAT ATTAATTTCA TATATAATGA   
  
  
+ CGATTATGTA TTTTTTTACT AAATTACTTG TACCCATAAT AAATAGAATC GATGCATCAT TCATGGCCTT   
  
  
+ TCATTCTGTA ATATGGTTGA CTCCAAAAAT TCTCAAAGTA TTTTTATATT TTGGGAGGGG AGATTTTAGT   
  
  
+ TTGATACTTT TGTTGAACTA GTGAACAATT TCCAACAACC TCCACCACCC CCAAACATCA ACAATCTGAT   
  
  
+ TGATGTATGC ATCCCAAGCT TGAATTGAAT AATAATTTCA ATAACAACAA TAATCGTTTT ACCTTGACAA   
  
  
+ GCTCCACAGC AACTCAACGT CTTCCAAACC AACCCATCAC TCATCATCAT CATCCCCACT ATCCCATTCA   
  
  
+ CCCCCACCAA AAGGAGCCTA AAGACAGTAA CAAGAAGAAT GCTTGCCTTT TTTCATGGAT GAAGAAGACT   
  
  
+ TCTCTTCTTC CTCTACTTCT TCCCACCACT ATGATACACA CCACCTCCAA CATCATTACC CCACCAATTA   
  
  
+ TAATTCCCGT TATACCCTGG ACCCTCCCTC CACCACCACC ACAGCCACAA CCACCACCTC ATCCTCCACT   
  
  
+ CCCACCCACC ATGGCTTCGG TGGTGGGCTT GACTCCTCCT CCCCGTCTGC CGCTGTGGAA TTATCTTTCT   
  
  
+ CCCCGGACAT CCTCCTCTCC TCCCCCACCG GAAAGTGGGC GTCTGACATT CTTATGGAGG CGGCTAGGGC   
  
  
+ TTTTTCAGAC ACGAACACCA CCAAGCTCCA CCAGCTCCTC TGGATGCTGA ACGAGCTCGG CTCCCCCTAC   
  
  
+ GGTGATGTCG AGCAGAAGCT GGTGGCGTAC TTTAACCAAG CCCTTTTTGC CCGCCTCACT GCCACCGGAC   
  
  
+ CCCGCCACCG AGCCGCTATG CTCGCCGCTG CGGACAAGAC CTCCTCCTTC GACTCCACGC TCAGGTTGCT   
  
  
+ CCTCAAGTTC CAGGAAGTCA GCCCGTGGAC CACCTTCGGC CACGTGGCTT GCAACGGTGC AATCATCGAG   
  
  
+ GCCTTAGAGA GGGACCCACA TGAAAAGAGT AAGCTCCACG TCATCGATAT CAGCACCACC TTCTGCACCC   
  
  
+ AGTGGCCCAC CTTGCTTGAG GCTTTAGCCA CCCGCATGGA CGACACTCCC CACCTCAGCC TCACTGCCGT   
  
  
+ CGTCGTCAAC AAGTACGCCG CCGTGAGCGG TGGCGAGGAC GACGGGGGAG AAGGATCAAA GAGGGTGATG   
  
  
+ AGAGAGATTG GTCTTCGTCT TGAGAAGTTC GCTAGGTTAA TGGGGGTTCC CTTCAAGTTC AACGTGGTTT   
  
  
+ ACCACGTAGG TGATCTATCC CAGTTGGATT TTTCTCGTTT GGATATTAAA GATGATGAAG CGGTGGCGAT   
  
  
+ TAACTGTGTC AACTCGCTCC ACTCGGTGGA CCTCCGATAT CGGGAGGTAG TGCTCGCGGC GTTCAGGCGG   
  
  
+ CTGCGGCCGA GGGTGGTGAC GGTGGTGGAG GAAGAGGCGG AGCTGACTGA GGTGGGTGAG GGTCAGTATG   
  
  
+ AATTTTTTAG GCGGTTTGAG GAGTGTCTTA GGTGGTTTAG GGTTTACTTT GAGGCCGTAG GAGATTGTTT   
  
  
+ TCCTAGGACT AGCAATGAGA AATTGATGTT AGAGCGGGCC GCGGGCCGGG CCATGGTGGA TCTCCTTGCA   
  
  
+ TGTCCCGAGC CTGGTGGGTC GGCTGAGGGG AGGGAGACGG CGAGACGGTG GTCGGGTCGG ATGCATGGGG   
  
  
+ CGGGGTTCGA GCACGTGGGG TTCAGTGACG AAGTGTGTGA TGATGTGAGG GCCTTGTTGA GGAGGTACAA   
  
  
+ GGAAGGATGG TCAATGACAC AGTGCACCAT GGATGGCGGT AATCACCCTG GAATACTGTT GTGGTGGCGG   
  
  
+ GATCAACCGG TGGTTTGGGC CAGTGCATGG CGGCCTTG  

- +Up\_Stream \_Len000TTTAGT TTGAGTATTT GTTATTAAAA AATTTTGAAT TTACCATTAA TAATCTAATT   
  
  
- ATTTTTAGGA TTACCAACAA ATTACAATGT AGTTCTGTAC GAAAAACCAC ATATATATTA CCGTCGTACA   
  
  
- CCCAATTCGC CGTTGTTTAA TAAGAGTTTA CGTACGTCAT TAATTAAATT AATTAATTAG TGGACGTATT   
  
  
- AGAGTTATGA TTAGGAAAGT AAGACACGGG GTGGGTTGGG GCTTTTATCA TTAGAAAGTT TGCAACCTTC   
  
  
- ACCCTAATCC ATATCTCATC AGGATGTCTA CTACTAGATG GATGTATATT GTATTAGACT GAAAAGAAAA   
  
  
- AATTTATTGA ACATAACTAC CGTGTCTTAA ACTGTACAAC CGTATACCAT GATTCTTTAA TTAATTGCAA   
  
  
- CACGATTCTA TATCCAGTAA GAAAAACCTC TGTATTCCGG GCGCAAGTGC GTCCCAAGCT CTTCCCGGTG   
  
  
- TAAGTTACCT ACTTAACATC CATCAAATTA GACTGAAACA GTCACTGACT AAGGTGCCGA ACTTGGGCGC   
  
  
- TGTAATTATA TCCAACTGAT TTCAGCAAAC TATGAATAAA AAGAATTCAT TTCAGAACTT AAGTTTAGAA   
  
  
- TATTTATTTA TTTTTTTAGA TATTATTTTC TTAAGATGGT AAATAATTCA GTTGTTGAGT TAAGCTTACT   
  
  
- TTATTTTTTA GTTCTAGTGG TTTTTTTTAT TTATTTTCCT TTCCATCTAA CGTAAACCTA AAACTCACCG   
  
  
- CACCACCACC CAAACTACGA CCACGACGTA TGTGGCGTCG AAAAGAGACA GGAAAATGGA GAGAAACAGA   
  
  
- CGTTCTTGGG GTAAACCGTG CGGACGAGAG AAAAGATACA AAAGAAAAGA TAGAAAATTC TTTGTTACCT   
  
  
- TTCGATTGTG TGATTGTGAT TGGGTAACCA GGAAAAAAGA ATAGACGTTT TAATTTCATT GTTATAAAAA   
  
  
- GAATAGGGGA AAAAGCAAGG TGGGTAAAGA AATAGGAAGA CAAACTTGGA TTAGACATAT ATACGTACGT   
  
  
- ACACATAAAA TGAATCTTAG ACAAAACTAA GAAACTACAT TAATTAATCA TCAGAAAAAT TAACTTAAAA   
  
  
- ATTGTCATCA AACTTAATCT GTAAATAGCT ATAATATAAA AAATATATAT TAATTTTTTA TGTTTTACTA   
  
  
- TTCACAGAAT GATGTTATGT GAAGTATTCA CAGCACAAAA TTGAATTTTT AGTATGATAA TACAAAGATT   
  
  
- TAACTTTTTT TTCACTCTAA TTCATCGTCT TCGTATAATG TTGAATATTT AATATATTTT AGTAGAAAAA   
  
  
- TAGGTATTAA ATAGTGTAAA GATTTTATGC GTATAGTATA GATGAATACC TATCCCAATT ATTCTAATCC   
  
  
- CGTAGTAAAT TCTAAAATAG TCGCATTGGT TGCTTACAAA TTCCAATCGT CAAGTATTAA TTCACATTAG   
  
  
- TAATTTTTTA AATACTAAAA TTTAGTATTT TATACAATTA GATCCTACTC CGTTCGCCGT TCTGTATATA   
  
  
- GTGATAGGAA TTTTTTATAT TATATAACTA AAGTTTCTTA ATACTTCATA TAATTAAAGT ATATATTACT   
  
  
- GCTAATACAT AAAAAAATGA TTTAATGAAC ATGGGTATTA TTTATCTTAG CTACGTAGTA AGTACCGGAA   
  
  
- AGTAAGACAT TATACCAACT GAGGTTTTTA AGAGTTTCAT AAAAATATAA AACCCTCCCC TCTAAAATCA   
  
  
- AACTATGAAA ACAACTTGAT CACTTGTTAA AGGTTGTTGG AGGTGGTGGG GGTTTGTAGT TGTTAGACTA   
  
  
- ACTACATACG TAGGGTTCGA ACTTAACTTA TTATTAAAGT TATTGTTGTT ATTAGCAAAA TGGAACTGTT   
  
  
- CGAGGTGTCG TTGAGTTGCA GAAGGTTTGG TTGGGTAGTG AGTAGTAGTA GTAGGGGTGA TAGGGTAAGT   
  
  
- GGGGGTGGTT TTCCTCGGAT TTCTGTCATT GTTCTTCTTA CGAACGGAAA AAAGTACCTA CTTCTTCTGA   
  
  
- AGAGAAGAAG GAGATGAAGA AGGGTGGTGA TACTATGTGT GGTGGAGGTT GTAGTAATGG GGTGGTTAAT   
  
  
- ATTAAGGGCA ATATGGGACC TGGGAGGGAG GTGGTGGTGG TGTCGGTGTT GGTGGTGGAG TAGGAGGTGA   
  
  
- GGGTGGGTGG TACCGAAGCC ACCACCCGAA CTGAGGAGGA GGGGCAGACG GCGACACCTT AATAGAAAGA   
  
  
- GGGGCCTGTA GGAGGAGAGG AGGGGGTGGC CTTTCACCCG CAGACTGTAA GAATACCTCC GCCGATCCCG   
  
  
- AAAAAGTCTG TGCTTGTGGT GGTTCGAGGT GGTCGAGGAG ACCTACGACT TGCTCGAGCC GAGGGGGATG   
  
  
- CCACTACAGC TCGTCTTCGA CCACCGCATG AAATTGGTTC GGGAAAAACG GGCGGAGTGA CGGTGGCCTG   
  
  
- GGGCGGTGGC TCGGCGATAC GAGCGGCGAC GCCTGTTCTG GAGGAGGAAG CTGAGGTGCG AGTCCAACGA   
  
  
- GGAGTTCAAG GTCCTTCAGT CGGGCACCTG GTGGAAGCCG GTGCACCGAA CGTTGCCACG TTAGTAGCTC   
  
  
- CGGAATCTCT CCCTGGGTGT ACTTTTCTCA TTCGAGGTGC AGTAGCTATA GTCGTGGTGG AAGACGTGGG   
  
  
- TCACCGGGTG GAACGAACTC CGAAATCGGT GGGCGTACCT GCTGTGAGGG GTGGAGTCGG AGTGACGGCA   
  
  
- GCAGCAGTTG TTCATGCGGC GGCACTCGCC ACCGCTCCTG CTGCCCCCTC TTCCTAGTTT CTCCCACTAC   
  
  
- TCTCTCTAAC CAGAAGCAGA ACTCTTCAAG CGATCCAATT ACCCCCAAGG GAAGTTCAAG TTGCACCAAA   
  
  
- TGGTGCATCC ACTAGATAGG GTCAACCTAA AAAGAGCAAA CCTATAATTT CTACTACTTC GCCACCGCTA   
  
  
- ATTGACACAG TTGAGCGAGG TGAGCCACCT GGAGGCTATA GCCCTCCATC ACGAGCGCCG CAAGTCCGCC   
  
  
- GACGCCGGCT CCCACCACTG CCACCACCTC CTTCTCCGCC TCGACTGACT CCACCCACTC CCAGTCATAC   
  
  
- TTAAAAAATC CGCCAAACTC CTCACAGAAT CCACCAAATC CCAAATGAAA CTCCGGCATC CTCTAACAAA   
  
  
- AGGATCCTGA TCGTTACTCT TTAACTACAA TCTCGCCCGG CGCCCGGCCC GGTACCACCT AGAGGAACGT   
  
  
- ACAGGGCTCG GACCACCCAG CCGACTCCCC TCCCTCTGCC GCTCTGCCAC CAGCCCAGCC TACGTACCCC   
  
  
- GCCCCAAGCT CGTGCACCCC AAGTCACTGC TTCACACACT ACTACACTCC CGGAACAACT CCTCCATGTT   
  
  
- CCTTCCTACC AGTTACTGTG TCACGTGGTA CCTACCGCCA TTAGTGGGAC CTTATGACAA CACCACCGCC   
  
  
- CTAGTTGGCC ACCAAACCCG GTCACGTACC GCCGGAAC

+     MBS

| Site Name | Organism | Position | Strand | Matrix score. | sequence | function |
| --- | --- | --- | --- | --- | --- | --- |
| MBS | Arabidopsis thaliana | 2895 | - | 6 | CAACTG | MYB binding site involved in drought-inducibility |

>HU05G01983.1   
+ +Up\_Stream \_Len000AAATCA AACTCATAAA CAATAATTTT TTAAAACTTA AATGGTAATT ATTAGATTAA   
  
  
+ TAAAAATCCT AATGGTTGTT TAATGTTACA TCAAGACATG CTTTTTGGTG TATATATAAT GGCAGCATGT   
  
  
+ GGGTTAAGCG GCAACAAATT ATTCTCAAAT GCATGCAGTA ATTAATTTAA TTAATTAATC ACCTGCATAA   
  
  
+ TCTCAATACT AATCCTTTCA TTCTGTGCCC CACCCAACCC CGAAAATAGT AATCTTTCAA ACGTTGGAAG   
  
  
+ TGGGATTAGG TATAGAGTAG TCCTACAGAT GATGATCTAC CTACATATAA CATAATCTGA CTTTTCTTTT   
  
  
+ TTAAATAACT TGTATTGATG GCACAGAATT TGACATGTTG GCATATGGTA CTAAGAAATT AATTAACGTT   
  
  
+ GTGCTAAGAT ATAGGTCATT CTTTTTGGAG ACATAAGGCC CGCGTTCACG CAGGGTTCGA GAAGGGCCAC   
  
  
+ ATTCAATGGA TGAATTGTAG GTAGTTTAAT CTGACTTTGT CAGTGACTGA TTCCACGGCT TGAACCCGCG   
  
  
+ ACATTAATAT AGGTTGACTA AAGTCGTTTG ATACTTATTT TTCTTAAGTA AAGTCTTGAA TTCAAATCTT   
  
  
+ ATAAATAAAT AAAAAAATCT ATAATAAAAG AATTCTACCA TTTATTAAGT CAACAACTCA ATTCGAATGA   
  
  
+ AATAAAAAAT CAAGATCACC AAAAAAAATA AATAAAAGGA AAGGTAGATT GCATTTGGAT TTTGAGTGGC   
  
  
+ GTGGTGGTGG GTTTGATGCT GGTGCTGCAT ACACCGCAGC TTTTCTCTGT CCTTTTACCT CTCTTTGTCT   
  
  
+ GCAAGAACCC CATTTGGCAC GCCTGCTCTC TTTTCTATGT TTTCTTTTCT ATCTTTTAAG AAACAATGGA   
  
  
+ AAGCTAACAC ACTAACACTA ACCCATTGGT CCTTTTTTCT TATCTGCAAA ATTAAAGTAA CAATATTTTT   
  
  
+ CTTATCCCCT TTTTCGTTCC ACCCATTTCT TTATCCTTCT GTTTGAACCT AATCTGTATA TATGCATGCA   
  
  
+ TGTGTATTTT ACTTAGAATC TGTTTTGATT CTTTGATGTA ATTAATTAGT AGTCTTTTTA ATTGAATTTT   
  
  
+ TAACAGTAGT TTGAATTAGA CATTTATCGA TATTATATTT TTTATATATA ATTAAAAAAT ACAAAATGAT   
  
  
+ AAGTGTCTTA CTACAATACA CTTCATAAGT GTCGTGTTTT AACTTAAAAA TCATACTATT ATGTTTCTAA   
  
  
+ ATTGAAAAAA AAGTGAGATT AAGTAGCAGA AGCATATTAC AACTTATAAA TTATATAAAA TCATCTTTTT   
  
  
+ ATCCATAATT TATCACATTT CTAAAATACG CATATCATAT CTACTTATGG ATAGGGTTAA TAAGATTAGG   
  
  
+ GCATCATTTA AGATTTTATC AGCGTAACCA ACGAATGTTT AAGGTTAGCA GTTCATAATT AAGTGTAATC   
  
  
+ ATTAAAAAAT TTATGATTTT AAATCATAAA ATATGTTAAT CTAGGATGAG GCAAGCGGCA AGACATATAT   
  
  
+ CACTATCCTT AAAAAATATA ATATATTGAT TTCAAAGAAT TATGAAGTAT ATTAATTTCA TATATAATGA   
  
  
+ CGATTATGTA TTTTTTTACT AAATTACTTG TACCCATAAT AAATAGAATC GATGCATCAT TCATGGCCTT   
  
  
+ TCATTCTGTA ATATGGTTGA CTCCAAAAAT TCTCAAAGTA TTTTTATATT TTGGGAGGGG AGATTTTAGT   
  
  
+ TTGATACTTT TGTTGAACTA GTGAACAATT TCCAACAACC TCCACCACCC CCAAACATCA ACAATCTGAT   
  
  
+ TGATGTATGC ATCCCAAGCT TGAATTGAAT AATAATTTCA ATAACAACAA TAATCGTTTT ACCTTGACAA   
  
  
+ GCTCCACAGC AACTCAACGT CTTCCAAACC AACCCATCAC TCATCATCAT CATCCCCACT ATCCCATTCA   
  
  
+ CCCCCACCAA AAGGAGCCTA AAGACAGTAA CAAGAAGAAT GCTTGCCTTT TTTCATGGAT GAAGAAGACT   
  
  
+ TCTCTTCTTC CTCTACTTCT TCCCACCACT ATGATACACA CCACCTCCAA CATCATTACC CCACCAATTA   
  
  
+ TAATTCCCGT TATACCCTGG ACCCTCCCTC CACCACCACC ACAGCCACAA CCACCACCTC ATCCTCCACT   
  
  
+ CCCACCCACC ATGGCTTCGG TGGTGGGCTT GACTCCTCCT CCCCGTCTGC CGCTGTGGAA TTATCTTTCT   
  
  
+ CCCCGGACAT CCTCCTCTCC TCCCCCACCG GAAAGTGGGC GTCTGACATT CTTATGGAGG CGGCTAGGGC   
  
  
+ TTTTTCAGAC ACGAACACCA CCAAGCTCCA CCAGCTCCTC TGGATGCTGA ACGAGCTCGG CTCCCCCTAC   
  
  
+ GGTGATGTCG AGCAGAAGCT GGTGGCGTAC TTTAACCAAG CCCTTTTTGC CCGCCTCACT GCCACCGGAC   
  
  
+ CCCGCCACCG AGCCGCTATG CTCGCCGCTG CGGACAAGAC CTCCTCCTTC GACTCCACGC TCAGGTTGCT   
  
  
+ CCTCAAGTTC CAGGAAGTCA GCCCGTGGAC CACCTTCGGC CACGTGGCTT GCAACGGTGC AATCATCGAG   
  
  
+ GCCTTAGAGA GGGACCCACA TGAAAAGAGT AAGCTCCACG TCATCGATAT CAGCACCACC TTCTGCACCC   
  
  
+ AGTGGCCCAC CTTGCTTGAG GCTTTAGCCA CCCGCATGGA CGACACTCCC CACCTCAGCC TCACTGCCGT   
  
  
+ CGTCGTCAAC AAGTACGCCG CCGTGAGCGG TGGCGAGGAC GACGGGGGAG AAGGATCAAA GAGGGTGATG   
  
  
+ AGAGAGATTG GTCTTCGTCT TGAGAAGTTC GCTAGGTTAA TGGGGGTTCC CTTCAAGTTC AACGTGGTTT   
  
  
+ ACCACGTAGG TGATCTATCC CAGTTGGATT TTTCTCGTTT GGATATTAAA GATGATGAAG CGGTGGCGAT   
  
  
+ TAACTGTGTC AACTCGCTCC ACTCGGTGGA CCTCCGATAT CGGGAGGTAG TGCTCGCGGC GTTCAGGCGG   
  
  
+ CTGCGGCCGA GGGTGGTGAC GGTGGTGGAG GAAGAGGCGG AGCTGACTGA GGTGGGTGAG GGTCAGTATG   
  
  
+ AATTTTTTAG GCGGTTTGAG GAGTGTCTTA GGTGGTTTAG GGTTTACTTT GAGGCCGTAG GAGATTGTTT   
  
  
+ TCCTAGGACT AGCAATGAGA AATTGATGTT AGAGCGGGCC GCGGGCCGGG CCATGGTGGA TCTCCTTGCA   
  
  
+ TGTCCCGAGC CTGGTGGGTC GGCTGAGGGG AGGGAGACGG CGAGACGGTG GTCGGGTCGG ATGCATGGGG   
  
  
+ CGGGGTTCGA GCACGTGGGG TTCAGTGACG AAGTGTGTGA TGATGTGAGG GCCTTGTTGA GGAGGTACAA   
  
  
+ GGAAGGATGG TCAATGACAC AGTGCACCAT GGATGGCGGT AATCACCCTG GAATACTGTT GTGGTGGCGG   
  
  
+ GATCAACCGG TGGTTTGGGC CAGTGCATGG CGGCCTTG  

- +Up\_Stream \_Len000TTTAGT TTGAGTATTT GTTATTAAAA AATTTTGAAT TTACCATTAA TAATCTAATT   
  
  
- ATTTTTAGGA TTACCAACAA ATTACAATGT AGTTCTGTAC GAAAAACCAC ATATATATTA CCGTCGTACA   
  
  
- CCCAATTCGC CGTTGTTTAA TAAGAGTTTA CGTACGTCAT TAATTAAATT AATTAATTAG TGGACGTATT   
  
  
- AGAGTTATGA TTAGGAAAGT AAGACACGGG GTGGGTTGGG GCTTTTATCA TTAGAAAGTT TGCAACCTTC   
  
  
- ACCCTAATCC ATATCTCATC AGGATGTCTA CTACTAGATG GATGTATATT GTATTAGACT GAAAAGAAAA   
  
  
- AATTTATTGA ACATAACTAC CGTGTCTTAA ACTGTACAAC CGTATACCAT GATTCTTTAA TTAATTGCAA   
  
  
- CACGATTCTA TATCCAGTAA GAAAAACCTC TGTATTCCGG GCGCAAGTGC GTCCCAAGCT CTTCCCGGTG   
  
  
- TAAGTTACCT ACTTAACATC CATCAAATTA GACTGAAACA GTCACTGACT AAGGTGCCGA ACTTGGGCGC   
  
  
- TGTAATTATA TCCAACTGAT TTCAGCAAAC TATGAATAAA AAGAATTCAT TTCAGAACTT AAGTTTAGAA   
  
  
- TATTTATTTA TTTTTTTAGA TATTATTTTC TTAAGATGGT AAATAATTCA GTTGTTGAGT TAAGCTTACT   
  
  
- TTATTTTTTA GTTCTAGTGG TTTTTTTTAT TTATTTTCCT TTCCATCTAA CGTAAACCTA AAACTCACCG   
  
  
- CACCACCACC CAAACTACGA CCACGACGTA TGTGGCGTCG AAAAGAGACA GGAAAATGGA GAGAAACAGA   
  
  
- CGTTCTTGGG GTAAACCGTG CGGACGAGAG AAAAGATACA AAAGAAAAGA TAGAAAATTC TTTGTTACCT   
  
  
- TTCGATTGTG TGATTGTGAT TGGGTAACCA GGAAAAAAGA ATAGACGTTT TAATTTCATT GTTATAAAAA   
  
  
- GAATAGGGGA AAAAGCAAGG TGGGTAAAGA AATAGGAAGA CAAACTTGGA TTAGACATAT ATACGTACGT   
  
  
- ACACATAAAA TGAATCTTAG ACAAAACTAA GAAACTACAT TAATTAATCA TCAGAAAAAT TAACTTAAAA   
  
  
- ATTGTCATCA AACTTAATCT GTAAATAGCT ATAATATAAA AAATATATAT TAATTTTTTA TGTTTTACTA   
  
  
- TTCACAGAAT GATGTTATGT GAAGTATTCA CAGCACAAAA TTGAATTTTT AGTATGATAA TACAAAGATT   
  
  
- TAACTTTTTT TTCACTCTAA TTCATCGTCT TCGTATAATG TTGAATATTT AATATATTTT AGTAGAAAAA   
  
  
- TAGGTATTAA ATAGTGTAAA GATTTTATGC GTATAGTATA GATGAATACC TATCCCAATT ATTCTAATCC   
  
  
- CGTAGTAAAT TCTAAAATAG TCGCATTGGT TGCTTACAAA TTCCAATCGT CAAGTATTAA TTCACATTAG   
  
  
- TAATTTTTTA AATACTAAAA TTTAGTATTT TATACAATTA GATCCTACTC CGTTCGCCGT TCTGTATATA   
  
  
- GTGATAGGAA TTTTTTATAT TATATAACTA AAGTTTCTTA ATACTTCATA TAATTAAAGT ATATATTACT   
  
  
- GCTAATACAT AAAAAAATGA TTTAATGAAC ATGGGTATTA TTTATCTTAG CTACGTAGTA AGTACCGGAA   
  
  
- AGTAAGACAT TATACCAACT GAGGTTTTTA AGAGTTTCAT AAAAATATAA AACCCTCCCC TCTAAAATCA   
  
  
- AACTATGAAA ACAACTTGAT CACTTGTTAA AGGTTGTTGG AGGTGGTGGG GGTTTGTAGT TGTTAGACTA   
  
  
- ACTACATACG TAGGGTTCGA ACTTAACTTA TTATTAAAGT TATTGTTGTT ATTAGCAAAA TGGAACTGTT   
  
  
- CGAGGTGTCG TTGAGTTGCA GAAGGTTTGG TTGGGTAGTG AGTAGTAGTA GTAGGGGTGA TAGGGTAAGT   
  
  
- GGGGGTGGTT TTCCTCGGAT TTCTGTCATT GTTCTTCTTA CGAACGGAAA AAAGTACCTA CTTCTTCTGA   
  
  
- AGAGAAGAAG GAGATGAAGA AGGGTGGTGA TACTATGTGT GGTGGAGGTT GTAGTAATGG GGTGGTTAAT   
  
  
- ATTAAGGGCA ATATGGGACC TGGGAGGGAG GTGGTGGTGG TGTCGGTGTT GGTGGTGGAG TAGGAGGTGA   
  
  
- GGGTGGGTGG TACCGAAGCC ACCACCCGAA CTGAGGAGGA GGGGCAGACG GCGACACCTT AATAGAAAGA   
  
  
- GGGGCCTGTA GGAGGAGAGG AGGGGGTGGC CTTTCACCCG CAGACTGTAA GAATACCTCC GCCGATCCCG   
  
  
- AAAAAGTCTG TGCTTGTGGT GGTTCGAGGT GGTCGAGGAG ACCTACGACT TGCTCGAGCC GAGGGGGATG   
  
  
- CCACTACAGC TCGTCTTCGA CCACCGCATG AAATTGGTTC GGGAAAAACG GGCGGAGTGA CGGTGGCCTG   
  
  
- GGGCGGTGGC TCGGCGATAC GAGCGGCGAC GCCTGTTCTG GAGGAGGAAG CTGAGGTGCG AGTCCAACGA   
  
  
- GGAGTTCAAG GTCCTTCAGT CGGGCACCTG GTGGAAGCCG GTGCACCGAA CGTTGCCACG TTAGTAGCTC   
  
  
- CGGAATCTCT CCCTGGGTGT ACTTTTCTCA TTCGAGGTGC AGTAGCTATA GTCGTGGTGG AAGACGTGGG   
  
  
- TCACCGGGTG GAACGAACTC CGAAATCGGT GGGCGTACCT GCTGTGAGGG GTGGAGTCGG AGTGACGGCA   
  
  
- GCAGCAGTTG TTCATGCGGC GGCACTCGCC ACCGCTCCTG CTGCCCCCTC TTCCTAGTTT CTCCCACTAC   
  
  
- TCTCTCTAAC CAGAAGCAGA ACTCTTCAAG CGATCCAATT ACCCCCAAGG GAAGTTCAAG TTGCACCAAA   
  
  
- TGGTGCATCC ACTAGATAGG GTCAACCTAA AAAGAGCAAA CCTATAATTT CTACTACTTC GCCACCGCTA   
  
  
- ATTGACACAG TTGAGCGAGG TGAGCCACCT GGAGGCTATA GCCCTCCATC ACGAGCGCCG CAAGTCCGCC   
  
  
- GACGCCGGCT CCCACCACTG CCACCACCTC CTTCTCCGCC TCGACTGACT CCACCCACTC CCAGTCATAC   
  
  
- TTAAAAAATC CGCCAAACTC CTCACAGAAT CCACCAAATC CCAAATGAAA CTCCGGCATC CTCTAACAAA   
  
  
- AGGATCCTGA TCGTTACTCT TTAACTACAA TCTCGCCCGG CGCCCGGCCC GGTACCACCT AGAGGAACGT   
  
  
- ACAGGGCTCG GACCACCCAG CCGACTCCCC TCCCTCTGCC GCTCTGCCAC CAGCCCAGCC TACGTACCCC   
  
  
- GCCCCAAGCT CGTGCACCCC AAGTCACTGC TTCACACACT ACTACACTCC CGGAACAACT CCTCCATGTT   
  
  
- CCTTCCTACC AGTTACTGTG TCACGTGGTA CCTACCGCCA TTAGTGGGAC CTTATGACAA CACCACCGCC   
  
  
- CTAGTTGGCC ACCAAACCCG GTCACGTACC GCCGGAAC

+     MRE

| Site Name | Organism | Position | Strand | Matrix score. | sequence | function |
| --- | --- | --- | --- | --- | --- | --- |
| MRE | Petroselinum crispum | 1030 | + | 7 | AACCTAA | MYB binding site involved in light responsiveness |

>HU05G01983.1   
+ +Up\_Stream \_Len000AAATCA AACTCATAAA CAATAATTTT TTAAAACTTA AATGGTAATT ATTAGATTAA   
  
  
+ TAAAAATCCT AATGGTTGTT TAATGTTACA TCAAGACATG CTTTTTGGTG TATATATAAT GGCAGCATGT   
  
  
+ GGGTTAAGCG GCAACAAATT ATTCTCAAAT GCATGCAGTA ATTAATTTAA TTAATTAATC ACCTGCATAA   
  
  
+ TCTCAATACT AATCCTTTCA TTCTGTGCCC CACCCAACCC CGAAAATAGT AATCTTTCAA ACGTTGGAAG   
  
  
+ TGGGATTAGG TATAGAGTAG TCCTACAGAT GATGATCTAC CTACATATAA CATAATCTGA CTTTTCTTTT   
  
  
+ TTAAATAACT TGTATTGATG GCACAGAATT TGACATGTTG GCATATGGTA CTAAGAAATT AATTAACGTT   
  
  
+ GTGCTAAGAT ATAGGTCATT CTTTTTGGAG ACATAAGGCC CGCGTTCACG CAGGGTTCGA GAAGGGCCAC   
  
  
+ ATTCAATGGA TGAATTGTAG GTAGTTTAAT CTGACTTTGT CAGTGACTGA TTCCACGGCT TGAACCCGCG   
  
  
+ ACATTAATAT AGGTTGACTA AAGTCGTTTG ATACTTATTT TTCTTAAGTA AAGTCTTGAA TTCAAATCTT   
  
  
+ ATAAATAAAT AAAAAAATCT ATAATAAAAG AATTCTACCA TTTATTAAGT CAACAACTCA ATTCGAATGA   
  
  
+ AATAAAAAAT CAAGATCACC AAAAAAAATA AATAAAAGGA AAGGTAGATT GCATTTGGAT TTTGAGTGGC   
  
  
+ GTGGTGGTGG GTTTGATGCT GGTGCTGCAT ACACCGCAGC TTTTCTCTGT CCTTTTACCT CTCTTTGTCT   
  
  
+ GCAAGAACCC CATTTGGCAC GCCTGCTCTC TTTTCTATGT TTTCTTTTCT ATCTTTTAAG AAACAATGGA   
  
  
+ AAGCTAACAC ACTAACACTA ACCCATTGGT CCTTTTTTCT TATCTGCAAA ATTAAAGTAA CAATATTTTT   
  
  
+ CTTATCCCCT TTTTCGTTCC ACCCATTTCT TTATCCTTCT GTTTGAACCT AATCTGTATA TATGCATGCA   
  
  
+ TGTGTATTTT ACTTAGAATC TGTTTTGATT CTTTGATGTA ATTAATTAGT AGTCTTTTTA ATTGAATTTT   
  
  
+ TAACAGTAGT TTGAATTAGA CATTTATCGA TATTATATTT TTTATATATA ATTAAAAAAT ACAAAATGAT   
  
  
+ AAGTGTCTTA CTACAATACA CTTCATAAGT GTCGTGTTTT AACTTAAAAA TCATACTATT ATGTTTCTAA   
  
  
+ ATTGAAAAAA AAGTGAGATT AAGTAGCAGA AGCATATTAC AACTTATAAA TTATATAAAA TCATCTTTTT   
  
  
+ ATCCATAATT TATCACATTT CTAAAATACG CATATCATAT CTACTTATGG ATAGGGTTAA TAAGATTAGG   
  
  
+ GCATCATTTA AGATTTTATC AGCGTAACCA ACGAATGTTT AAGGTTAGCA GTTCATAATT AAGTGTAATC   
  
  
+ ATTAAAAAAT TTATGATTTT AAATCATAAA ATATGTTAAT CTAGGATGAG GCAAGCGGCA AGACATATAT   
  
  
+ CACTATCCTT AAAAAATATA ATATATTGAT TTCAAAGAAT TATGAAGTAT ATTAATTTCA TATATAATGA   
  
  
+ CGATTATGTA TTTTTTTACT AAATTACTTG TACCCATAAT AAATAGAATC GATGCATCAT TCATGGCCTT   
  
  
+ TCATTCTGTA ATATGGTTGA CTCCAAAAAT TCTCAAAGTA TTTTTATATT TTGGGAGGGG AGATTTTAGT   
  
  
+ TTGATACTTT TGTTGAACTA GTGAACAATT TCCAACAACC TCCACCACCC CCAAACATCA ACAATCTGAT   
  
  
+ TGATGTATGC ATCCCAAGCT TGAATTGAAT AATAATTTCA ATAACAACAA TAATCGTTTT ACCTTGACAA   
  
  
+ GCTCCACAGC AACTCAACGT CTTCCAAACC AACCCATCAC TCATCATCAT CATCCCCACT ATCCCATTCA   
  
  
+ CCCCCACCAA AAGGAGCCTA AAGACAGTAA CAAGAAGAAT GCTTGCCTTT TTTCATGGAT GAAGAAGACT   
  
  
+ TCTCTTCTTC CTCTACTTCT TCCCACCACT ATGATACACA CCACCTCCAA CATCATTACC CCACCAATTA   
  
  
+ TAATTCCCGT TATACCCTGG ACCCTCCCTC CACCACCACC ACAGCCACAA CCACCACCTC ATCCTCCACT   
  
  
+ CCCACCCACC ATGGCTTCGG TGGTGGGCTT GACTCCTCCT CCCCGTCTGC CGCTGTGGAA TTATCTTTCT   
  
  
+ CCCCGGACAT CCTCCTCTCC TCCCCCACCG GAAAGTGGGC GTCTGACATT CTTATGGAGG CGGCTAGGGC   
  
  
+ TTTTTCAGAC ACGAACACCA CCAAGCTCCA CCAGCTCCTC TGGATGCTGA ACGAGCTCGG CTCCCCCTAC   
  
  
+ GGTGATGTCG AGCAGAAGCT GGTGGCGTAC TTTAACCAAG CCCTTTTTGC CCGCCTCACT GCCACCGGAC   
  
  
+ CCCGCCACCG AGCCGCTATG CTCGCCGCTG CGGACAAGAC CTCCTCCTTC GACTCCACGC TCAGGTTGCT   
  
  
+ CCTCAAGTTC CAGGAAGTCA GCCCGTGGAC CACCTTCGGC CACGTGGCTT GCAACGGTGC AATCATCGAG   
  
  
+ GCCTTAGAGA GGGACCCACA TGAAAAGAGT AAGCTCCACG TCATCGATAT CAGCACCACC TTCTGCACCC   
  
  
+ AGTGGCCCAC CTTGCTTGAG GCTTTAGCCA CCCGCATGGA CGACACTCCC CACCTCAGCC TCACTGCCGT   
  
  
+ CGTCGTCAAC AAGTACGCCG CCGTGAGCGG TGGCGAGGAC GACGGGGGAG AAGGATCAAA GAGGGTGATG   
  
  
+ AGAGAGATTG GTCTTCGTCT TGAGAAGTTC GCTAGGTTAA TGGGGGTTCC CTTCAAGTTC AACGTGGTTT   
  
  
+ ACCACGTAGG TGATCTATCC CAGTTGGATT TTTCTCGTTT GGATATTAAA GATGATGAAG CGGTGGCGAT   
  
  
+ TAACTGTGTC AACTCGCTCC ACTCGGTGGA CCTCCGATAT CGGGAGGTAG TGCTCGCGGC GTTCAGGCGG   
  
  
+ CTGCGGCCGA GGGTGGTGAC GGTGGTGGAG GAAGAGGCGG AGCTGACTGA GGTGGGTGAG GGTCAGTATG   
  
  
+ AATTTTTTAG GCGGTTTGAG GAGTGTCTTA GGTGGTTTAG GGTTTACTTT GAGGCCGTAG GAGATTGTTT   
  
  
+ TCCTAGGACT AGCAATGAGA AATTGATGTT AGAGCGGGCC GCGGGCCGGG CCATGGTGGA TCTCCTTGCA   
  
  
+ TGTCCCGAGC CTGGTGGGTC GGCTGAGGGG AGGGAGACGG CGAGACGGTG GTCGGGTCGG ATGCATGGGG   
  
  
+ CGGGGTTCGA GCACGTGGGG TTCAGTGACG AAGTGTGTGA TGATGTGAGG GCCTTGTTGA GGAGGTACAA   
  
  
+ GGAAGGATGG TCAATGACAC AGTGCACCAT GGATGGCGGT AATCACCCTG GAATACTGTT GTGGTGGCGG   
  
  
+ GATCAACCGG TGGTTTGGGC CAGTGCATGG CGGCCTTG  

- +Up\_Stream \_Len000TTTAGT TTGAGTATTT GTTATTAAAA AATTTTGAAT TTACCATTAA TAATCTAATT   
  
  
- ATTTTTAGGA TTACCAACAA ATTACAATGT AGTTCTGTAC GAAAAACCAC ATATATATTA CCGTCGTACA   
  
  
- CCCAATTCGC CGTTGTTTAA TAAGAGTTTA CGTACGTCAT TAATTAAATT AATTAATTAG TGGACGTATT   
  
  
- AGAGTTATGA TTAGGAAAGT AAGACACGGG GTGGGTTGGG GCTTTTATCA TTAGAAAGTT TGCAACCTTC   
  
  
- ACCCTAATCC ATATCTCATC AGGATGTCTA CTACTAGATG GATGTATATT GTATTAGACT GAAAAGAAAA   
  
  
- AATTTATTGA ACATAACTAC CGTGTCTTAA ACTGTACAAC CGTATACCAT GATTCTTTAA TTAATTGCAA   
  
  
- CACGATTCTA TATCCAGTAA GAAAAACCTC TGTATTCCGG GCGCAAGTGC GTCCCAAGCT CTTCCCGGTG   
  
  
- TAAGTTACCT ACTTAACATC CATCAAATTA GACTGAAACA GTCACTGACT AAGGTGCCGA ACTTGGGCGC   
  
  
- TGTAATTATA TCCAACTGAT TTCAGCAAAC TATGAATAAA AAGAATTCAT TTCAGAACTT AAGTTTAGAA   
  
  
- TATTTATTTA TTTTTTTAGA TATTATTTTC TTAAGATGGT AAATAATTCA GTTGTTGAGT TAAGCTTACT   
  
  
- TTATTTTTTA GTTCTAGTGG TTTTTTTTAT TTATTTTCCT TTCCATCTAA CGTAAACCTA AAACTCACCG   
  
  
- CACCACCACC CAAACTACGA CCACGACGTA TGTGGCGTCG AAAAGAGACA GGAAAATGGA GAGAAACAGA   
  
  
- CGTTCTTGGG GTAAACCGTG CGGACGAGAG AAAAGATACA AAAGAAAAGA TAGAAAATTC TTTGTTACCT   
  
  
- TTCGATTGTG TGATTGTGAT TGGGTAACCA GGAAAAAAGA ATAGACGTTT TAATTTCATT GTTATAAAAA   
  
  
- GAATAGGGGA AAAAGCAAGG TGGGTAAAGA AATAGGAAGA CAAACTTGGA TTAGACATAT ATACGTACGT   
  
  
- ACACATAAAA TGAATCTTAG ACAAAACTAA GAAACTACAT TAATTAATCA TCAGAAAAAT TAACTTAAAA   
  
  
- ATTGTCATCA AACTTAATCT GTAAATAGCT ATAATATAAA AAATATATAT TAATTTTTTA TGTTTTACTA   
  
  
- TTCACAGAAT GATGTTATGT GAAGTATTCA CAGCACAAAA TTGAATTTTT AGTATGATAA TACAAAGATT   
  
  
- TAACTTTTTT TTCACTCTAA TTCATCGTCT TCGTATAATG TTGAATATTT AATATATTTT AGTAGAAAAA   
  
  
- TAGGTATTAA ATAGTGTAAA GATTTTATGC GTATAGTATA GATGAATACC TATCCCAATT ATTCTAATCC   
  
  
- CGTAGTAAAT TCTAAAATAG TCGCATTGGT TGCTTACAAA TTCCAATCGT CAAGTATTAA TTCACATTAG   
  
  
- TAATTTTTTA AATACTAAAA TTTAGTATTT TATACAATTA GATCCTACTC CGTTCGCCGT TCTGTATATA   
  
  
- GTGATAGGAA TTTTTTATAT TATATAACTA AAGTTTCTTA ATACTTCATA TAATTAAAGT ATATATTACT   
  
  
- GCTAATACAT AAAAAAATGA TTTAATGAAC ATGGGTATTA TTTATCTTAG CTACGTAGTA AGTACCGGAA   
  
  
- AGTAAGACAT TATACCAACT GAGGTTTTTA AGAGTTTCAT AAAAATATAA AACCCTCCCC TCTAAAATCA   
  
  
- AACTATGAAA ACAACTTGAT CACTTGTTAA AGGTTGTTGG AGGTGGTGGG GGTTTGTAGT TGTTAGACTA   
  
  
- ACTACATACG TAGGGTTCGA ACTTAACTTA TTATTAAAGT TATTGTTGTT ATTAGCAAAA TGGAACTGTT   
  
  
- CGAGGTGTCG TTGAGTTGCA GAAGGTTTGG TTGGGTAGTG AGTAGTAGTA GTAGGGGTGA TAGGGTAAGT   
  
  
- GGGGGTGGTT TTCCTCGGAT TTCTGTCATT GTTCTTCTTA CGAACGGAAA AAAGTACCTA CTTCTTCTGA   
  
  
- AGAGAAGAAG GAGATGAAGA AGGGTGGTGA TACTATGTGT GGTGGAGGTT GTAGTAATGG GGTGGTTAAT   
  
  
- ATTAAGGGCA ATATGGGACC TGGGAGGGAG GTGGTGGTGG TGTCGGTGTT GGTGGTGGAG TAGGAGGTGA   
  
  
- GGGTGGGTGG TACCGAAGCC ACCACCCGAA CTGAGGAGGA GGGGCAGACG GCGACACCTT AATAGAAAGA   
  
  
- GGGGCCTGTA GGAGGAGAGG AGGGGGTGGC CTTTCACCCG CAGACTGTAA GAATACCTCC GCCGATCCCG   
  
  
- AAAAAGTCTG TGCTTGTGGT GGTTCGAGGT GGTCGAGGAG ACCTACGACT TGCTCGAGCC GAGGGGGATG   
  
  
- CCACTACAGC TCGTCTTCGA CCACCGCATG AAATTGGTTC GGGAAAAACG GGCGGAGTGA CGGTGGCCTG   
  
  
- GGGCGGTGGC TCGGCGATAC GAGCGGCGAC GCCTGTTCTG GAGGAGGAAG CTGAGGTGCG AGTCCAACGA   
  
  
- GGAGTTCAAG GTCCTTCAGT CGGGCACCTG GTGGAAGCCG GTGCACCGAA CGTTGCCACG TTAGTAGCTC   
  
  
- CGGAATCTCT CCCTGGGTGT ACTTTTCTCA TTCGAGGTGC AGTAGCTATA GTCGTGGTGG AAGACGTGGG   
  
  
- TCACCGGGTG GAACGAACTC CGAAATCGGT GGGCGTACCT GCTGTGAGGG GTGGAGTCGG AGTGACGGCA   
  
  
- GCAGCAGTTG TTCATGCGGC GGCACTCGCC ACCGCTCCTG CTGCCCCCTC TTCCTAGTTT CTCCCACTAC   
  
  
- TCTCTCTAAC CAGAAGCAGA ACTCTTCAAG CGATCCAATT ACCCCCAAGG GAAGTTCAAG TTGCACCAAA   
  
  
- TGGTGCATCC ACTAGATAGG GTCAACCTAA AAAGAGCAAA CCTATAATTT CTACTACTTC GCCACCGCTA   
  
  
- ATTGACACAG TTGAGCGAGG TGAGCCACCT GGAGGCTATA GCCCTCCATC ACGAGCGCCG CAAGTCCGCC   
  
  
- GACGCCGGCT CCCACCACTG CCACCACCTC CTTCTCCGCC TCGACTGACT CCACCCACTC CCAGTCATAC   
  
  
- TTAAAAAATC CGCCAAACTC CTCACAGAAT CCACCAAATC CCAAATGAAA CTCCGGCATC CTCTAACAAA   
  
  
- AGGATCCTGA TCGTTACTCT TTAACTACAA TCTCGCCCGG CGCCCGGCCC GGTACCACCT AGAGGAACGT   
  
  
- ACAGGGCTCG GACCACCCAG CCGACTCCCC TCCCTCTGCC GCTCTGCCAC CAGCCCAGCC TACGTACCCC   
  
  
- GCCCCAAGCT CGTGCACCCC AAGTCACTGC TTCACACACT ACTACACTCC CGGAACAACT CCTCCATGTT   
  
  
- CCTTCCTACC AGTTACTGTG TCACGTGGTA CCTACCGCCA TTAGTGGGAC CTTATGACAA CACCACCGCC   
  
  
- CTAGTTGGCC ACCAAACCCG GTCACGTACC GCCGGAAC

+     MYB

| Site Name | Organism | Position | Strand | Matrix score. | sequence | function |
| --- | --- | --- | --- | --- | --- | --- |
| MYB | Arabidopsis thaliana | 2417 | + | 6 | TAACCA |  |
| MYB | Arabidopsis thaliana | 1429 | + | 6 | TAACCA |  |
| MYB | Arabidopsis thaliana | 87 | - | 6 | CAACCA |  |
| MYB | Arabidopsis thaliana | 2152 | + | 6 | CAACCA |  |
| MYB | Arabidopsis thaliana | 3420 | - | 6 | CAACAG |  |
| MYB | Arabidopsis thaliana | 1698 | - | 6 | CAACCA |  |

>HU05G01983.1   
+ +Up\_Stream \_Len000AAATCA AACTCATAAA CAATAATTTT TTAAAACTTA AATGGTAATT ATTAGATTAA   
  
  
+ TAAAAATCCT AATGGTTGTT TAATGTTACA TCAAGACATG CTTTTTGGTG TATATATAAT GGCAGCATGT   
  
  
+ GGGTTAAGCG GCAACAAATT ATTCTCAAAT GCATGCAGTA ATTAATTTAA TTAATTAATC ACCTGCATAA   
  
  
+ TCTCAATACT AATCCTTTCA TTCTGTGCCC CACCCAACCC CGAAAATAGT AATCTTTCAA ACGTTGGAAG   
  
  
+ TGGGATTAGG TATAGAGTAG TCCTACAGAT GATGATCTAC CTACATATAA CATAATCTGA CTTTTCTTTT   
  
  
+ TTAAATAACT TGTATTGATG GCACAGAATT TGACATGTTG GCATATGGTA CTAAGAAATT AATTAACGTT   
  
  
+ GTGCTAAGAT ATAGGTCATT CTTTTTGGAG ACATAAGGCC CGCGTTCACG CAGGGTTCGA GAAGGGCCAC   
  
  
+ ATTCAATGGA TGAATTGTAG GTAGTTTAAT CTGACTTTGT CAGTGACTGA TTCCACGGCT TGAACCCGCG   
  
  
+ ACATTAATAT AGGTTGACTA AAGTCGTTTG ATACTTATTT TTCTTAAGTA AAGTCTTGAA TTCAAATCTT   
  
  
+ ATAAATAAAT AAAAAAATCT ATAATAAAAG AATTCTACCA TTTATTAAGT CAACAACTCA ATTCGAATGA   
  
  
+ AATAAAAAAT CAAGATCACC AAAAAAAATA AATAAAAGGA AAGGTAGATT GCATTTGGAT TTTGAGTGGC   
  
  
+ GTGGTGGTGG GTTTGATGCT GGTGCTGCAT ACACCGCAGC TTTTCTCTGT CCTTTTACCT CTCTTTGTCT   
  
  
+ GCAAGAACCC CATTTGGCAC GCCTGCTCTC TTTTCTATGT TTTCTTTTCT ATCTTTTAAG AAACAATGGA   
  
  
+ AAGCTAACAC ACTAACACTA ACCCATTGGT CCTTTTTTCT TATCTGCAAA ATTAAAGTAA CAATATTTTT   
  
  
+ CTTATCCCCT TTTTCGTTCC ACCCATTTCT TTATCCTTCT GTTTGAACCT AATCTGTATA TATGCATGCA   
  
  
+ TGTGTATTTT ACTTAGAATC TGTTTTGATT CTTTGATGTA ATTAATTAGT AGTCTTTTTA ATTGAATTTT   
  
  
+ TAACAGTAGT TTGAATTAGA CATTTATCGA TATTATATTT TTTATATATA ATTAAAAAAT ACAAAATGAT   
  
  
+ AAGTGTCTTA CTACAATACA CTTCATAAGT GTCGTGTTTT AACTTAAAAA TCATACTATT ATGTTTCTAA   
  
  
+ ATTGAAAAAA AAGTGAGATT AAGTAGCAGA AGCATATTAC AACTTATAAA TTATATAAAA TCATCTTTTT   
  
  
+ ATCCATAATT TATCACATTT CTAAAATACG CATATCATAT CTACTTATGG ATAGGGTTAA TAAGATTAGG   
  
  
+ GCATCATTTA AGATTTTATC AGCGTAACCA ACGAATGTTT AAGGTTAGCA GTTCATAATT AAGTGTAATC   
  
  
+ ATTAAAAAAT TTATGATTTT AAATCATAAA ATATGTTAAT CTAGGATGAG GCAAGCGGCA AGACATATAT   
  
  
+ CACTATCCTT AAAAAATATA ATATATTGAT TTCAAAGAAT TATGAAGTAT ATTAATTTCA TATATAATGA   
  
  
+ CGATTATGTA TTTTTTTACT AAATTACTTG TACCCATAAT AAATAGAATC GATGCATCAT TCATGGCCTT   
  
  
+ TCATTCTGTA ATATGGTTGA CTCCAAAAAT TCTCAAAGTA TTTTTATATT TTGGGAGGGG AGATTTTAGT   
  
  
+ TTGATACTTT TGTTGAACTA GTGAACAATT TCCAACAACC TCCACCACCC CCAAACATCA ACAATCTGAT   
  
  
+ TGATGTATGC ATCCCAAGCT TGAATTGAAT AATAATTTCA ATAACAACAA TAATCGTTTT ACCTTGACAA   
  
  
+ GCTCCACAGC AACTCAACGT CTTCCAAACC AACCCATCAC TCATCATCAT CATCCCCACT ATCCCATTCA   
  
  
+ CCCCCACCAA AAGGAGCCTA AAGACAGTAA CAAGAAGAAT GCTTGCCTTT TTTCATGGAT GAAGAAGACT   
  
  
+ TCTCTTCTTC CTCTACTTCT TCCCACCACT ATGATACACA CCACCTCCAA CATCATTACC CCACCAATTA   
  
  
+ TAATTCCCGT TATACCCTGG ACCCTCCCTC CACCACCACC ACAGCCACAA CCACCACCTC ATCCTCCACT   
  
  
+ CCCACCCACC ATGGCTTCGG TGGTGGGCTT GACTCCTCCT CCCCGTCTGC CGCTGTGGAA TTATCTTTCT   
  
  
+ CCCCGGACAT CCTCCTCTCC TCCCCCACCG GAAAGTGGGC GTCTGACATT CTTATGGAGG CGGCTAGGGC   
  
  
+ TTTTTCAGAC ACGAACACCA CCAAGCTCCA CCAGCTCCTC TGGATGCTGA ACGAGCTCGG CTCCCCCTAC   
  
  
+ GGTGATGTCG AGCAGAAGCT GGTGGCGTAC TTTAACCAAG CCCTTTTTGC CCGCCTCACT GCCACCGGAC   
  
  
+ CCCGCCACCG AGCCGCTATG CTCGCCGCTG CGGACAAGAC CTCCTCCTTC GACTCCACGC TCAGGTTGCT   
  
  
+ CCTCAAGTTC CAGGAAGTCA GCCCGTGGAC CACCTTCGGC CACGTGGCTT GCAACGGTGC AATCATCGAG   
  
  
+ GCCTTAGAGA GGGACCCACA TGAAAAGAGT AAGCTCCACG TCATCGATAT CAGCACCACC TTCTGCACCC   
  
  
+ AGTGGCCCAC CTTGCTTGAG GCTTTAGCCA CCCGCATGGA CGACACTCCC CACCTCAGCC TCACTGCCGT   
  
  
+ CGTCGTCAAC AAGTACGCCG CCGTGAGCGG TGGCGAGGAC GACGGGGGAG AAGGATCAAA GAGGGTGATG   
  
  
+ AGAGAGATTG GTCTTCGTCT TGAGAAGTTC GCTAGGTTAA TGGGGGTTCC CTTCAAGTTC AACGTGGTTT   
  
  
+ ACCACGTAGG TGATCTATCC CAGTTGGATT TTTCTCGTTT GGATATTAAA GATGATGAAG CGGTGGCGAT   
  
  
+ TAACTGTGTC AACTCGCTCC ACTCGGTGGA CCTCCGATAT CGGGAGGTAG TGCTCGCGGC GTTCAGGCGG   
  
  
+ CTGCGGCCGA GGGTGGTGAC GGTGGTGGAG GAAGAGGCGG AGCTGACTGA GGTGGGTGAG GGTCAGTATG   
  
  
+ AATTTTTTAG GCGGTTTGAG GAGTGTCTTA GGTGGTTTAG GGTTTACTTT GAGGCCGTAG GAGATTGTTT   
  
  
+ TCCTAGGACT AGCAATGAGA AATTGATGTT AGAGCGGGCC GCGGGCCGGG CCATGGTGGA TCTCCTTGCA   
  
  
+ TGTCCCGAGC CTGGTGGGTC GGCTGAGGGG AGGGAGACGG CGAGACGGTG GTCGGGTCGG ATGCATGGGG   
  
  
+ CGGGGTTCGA GCACGTGGGG TTCAGTGACG AAGTGTGTGA TGATGTGAGG GCCTTGTTGA GGAGGTACAA   
  
  
+ GGAAGGATGG TCAATGACAC AGTGCACCAT GGATGGCGGT AATCACCCTG GAATACTGTT GTGGTGGCGG   
  
  
+ GATCAACCGG TGGTTTGGGC CAGTGCATGG CGGCCTTG  

- +Up\_Stream \_Len000TTTAGT TTGAGTATTT GTTATTAAAA AATTTTGAAT TTACCATTAA TAATCTAATT   
  
  
- ATTTTTAGGA TTACCAACAA ATTACAATGT AGTTCTGTAC GAAAAACCAC ATATATATTA CCGTCGTACA   
  
  
- CCCAATTCGC CGTTGTTTAA TAAGAGTTTA CGTACGTCAT TAATTAAATT AATTAATTAG TGGACGTATT   
  
  
- AGAGTTATGA TTAGGAAAGT AAGACACGGG GTGGGTTGGG GCTTTTATCA TTAGAAAGTT TGCAACCTTC   
  
  
- ACCCTAATCC ATATCTCATC AGGATGTCTA CTACTAGATG GATGTATATT GTATTAGACT GAAAAGAAAA   
  
  
- AATTTATTGA ACATAACTAC CGTGTCTTAA ACTGTACAAC CGTATACCAT GATTCTTTAA TTAATTGCAA   
  
  
- CACGATTCTA TATCCAGTAA GAAAAACCTC TGTATTCCGG GCGCAAGTGC GTCCCAAGCT CTTCCCGGTG   
  
  
- TAAGTTACCT ACTTAACATC CATCAAATTA GACTGAAACA GTCACTGACT AAGGTGCCGA ACTTGGGCGC   
  
  
- TGTAATTATA TCCAACTGAT TTCAGCAAAC TATGAATAAA AAGAATTCAT TTCAGAACTT AAGTTTAGAA   
  
  
- TATTTATTTA TTTTTTTAGA TATTATTTTC TTAAGATGGT AAATAATTCA GTTGTTGAGT TAAGCTTACT   
  
  
- TTATTTTTTA GTTCTAGTGG TTTTTTTTAT TTATTTTCCT TTCCATCTAA CGTAAACCTA AAACTCACCG   
  
  
- CACCACCACC CAAACTACGA CCACGACGTA TGTGGCGTCG AAAAGAGACA GGAAAATGGA GAGAAACAGA   
  
  
- CGTTCTTGGG GTAAACCGTG CGGACGAGAG AAAAGATACA AAAGAAAAGA TAGAAAATTC TTTGTTACCT   
  
  
- TTCGATTGTG TGATTGTGAT TGGGTAACCA GGAAAAAAGA ATAGACGTTT TAATTTCATT GTTATAAAAA   
  
  
- GAATAGGGGA AAAAGCAAGG TGGGTAAAGA AATAGGAAGA CAAACTTGGA TTAGACATAT ATACGTACGT   
  
  
- ACACATAAAA TGAATCTTAG ACAAAACTAA GAAACTACAT TAATTAATCA TCAGAAAAAT TAACTTAAAA   
  
  
- ATTGTCATCA AACTTAATCT GTAAATAGCT ATAATATAAA AAATATATAT TAATTTTTTA TGTTTTACTA   
  
  
- TTCACAGAAT GATGTTATGT GAAGTATTCA CAGCACAAAA TTGAATTTTT AGTATGATAA TACAAAGATT   
  
  
- TAACTTTTTT TTCACTCTAA TTCATCGTCT TCGTATAATG TTGAATATTT AATATATTTT AGTAGAAAAA   
  
  
- TAGGTATTAA ATAGTGTAAA GATTTTATGC GTATAGTATA GATGAATACC TATCCCAATT ATTCTAATCC   
  
  
- CGTAGTAAAT TCTAAAATAG TCGCATTGGT TGCTTACAAA TTCCAATCGT CAAGTATTAA TTCACATTAG   
  
  
- TAATTTTTTA AATACTAAAA TTTAGTATTT TATACAATTA GATCCTACTC CGTTCGCCGT TCTGTATATA   
  
  
- GTGATAGGAA TTTTTTATAT TATATAACTA AAGTTTCTTA ATACTTCATA TAATTAAAGT ATATATTACT   
  
  
- GCTAATACAT AAAAAAATGA TTTAATGAAC ATGGGTATTA TTTATCTTAG CTACGTAGTA AGTACCGGAA   
  
  
- AGTAAGACAT TATACCAACT GAGGTTTTTA AGAGTTTCAT AAAAATATAA AACCCTCCCC TCTAAAATCA   
  
  
- AACTATGAAA ACAACTTGAT CACTTGTTAA AGGTTGTTGG AGGTGGTGGG GGTTTGTAGT TGTTAGACTA   
  
  
- ACTACATACG TAGGGTTCGA ACTTAACTTA TTATTAAAGT TATTGTTGTT ATTAGCAAAA TGGAACTGTT   
  
  
- CGAGGTGTCG TTGAGTTGCA GAAGGTTTGG TTGGGTAGTG AGTAGTAGTA GTAGGGGTGA TAGGGTAAGT   
  
  
- GGGGGTGGTT TTCCTCGGAT TTCTGTCATT GTTCTTCTTA CGAACGGAAA AAAGTACCTA CTTCTTCTGA   
  
  
- AGAGAAGAAG GAGATGAAGA AGGGTGGTGA TACTATGTGT GGTGGAGGTT GTAGTAATGG GGTGGTTAAT   
  
  
- ATTAAGGGCA ATATGGGACC TGGGAGGGAG GTGGTGGTGG TGTCGGTGTT GGTGGTGGAG TAGGAGGTGA   
  
  
- GGGTGGGTGG TACCGAAGCC ACCACCCGAA CTGAGGAGGA GGGGCAGACG GCGACACCTT AATAGAAAGA   
  
  
- GGGGCCTGTA GGAGGAGAGG AGGGGGTGGC CTTTCACCCG CAGACTGTAA GAATACCTCC GCCGATCCCG   
  
  
- AAAAAGTCTG TGCTTGTGGT GGTTCGAGGT GGTCGAGGAG ACCTACGACT TGCTCGAGCC GAGGGGGATG   
  
  
- CCACTACAGC TCGTCTTCGA CCACCGCATG AAATTGGTTC GGGAAAAACG GGCGGAGTGA CGGTGGCCTG   
  
  
- GGGCGGTGGC TCGGCGATAC GAGCGGCGAC GCCTGTTCTG GAGGAGGAAG CTGAGGTGCG AGTCCAACGA   
  
  
- GGAGTTCAAG GTCCTTCAGT CGGGCACCTG GTGGAAGCCG GTGCACCGAA CGTTGCCACG TTAGTAGCTC   
  
  
- CGGAATCTCT CCCTGGGTGT ACTTTTCTCA TTCGAGGTGC AGTAGCTATA GTCGTGGTGG AAGACGTGGG   
  
  
- TCACCGGGTG GAACGAACTC CGAAATCGGT GGGCGTACCT GCTGTGAGGG GTGGAGTCGG AGTGACGGCA   
  
  
- GCAGCAGTTG TTCATGCGGC GGCACTCGCC ACCGCTCCTG CTGCCCCCTC TTCCTAGTTT CTCCCACTAC   
  
  
- TCTCTCTAAC CAGAAGCAGA ACTCTTCAAG CGATCCAATT ACCCCCAAGG GAAGTTCAAG TTGCACCAAA   
  
  
- TGGTGCATCC ACTAGATAGG GTCAACCTAA AAAGAGCAAA CCTATAATTT CTACTACTTC GCCACCGCTA   
  
  
- ATTGACACAG TTGAGCGAGG TGAGCCACCT GGAGGCTATA GCCCTCCATC ACGAGCGCCG CAAGTCCGCC   
  
  
- GACGCCGGCT CCCACCACTG CCACCACCTC CTTCTCCGCC TCGACTGACT CCACCCACTC CCAGTCATAC   
  
  
- TTAAAAAATC CGCCAAACTC CTCACAGAAT CCACCAAATC CCAAATGAAA CTCCGGCATC CTCTAACAAA   
  
  
- AGGATCCTGA TCGTTACTCT TTAACTACAA TCTCGCCCGG CGCCCGGCCC GGTACCACCT AGAGGAACGT   
  
  
- ACAGGGCTCG GACCACCCAG CCGACTCCCC TCCCTCTGCC GCTCTGCCAC CAGCCCAGCC TACGTACCCC   
  
  
- GCCCCAAGCT CGTGCACCCC AAGTCACTGC TTCACACACT ACTACACTCC CGGAACAACT CCTCCATGTT   
  
  
- CCTTCCTACC AGTTACTGTG TCACGTGGTA CCTACCGCCA TTAGTGGGAC CTTATGACAA CACCACCGCC   
  
  
- CTAGTTGGCC ACCAAACCCG GTCACGTACC GCCGGAAC

+     MYB recognition site

| Site Name | Organism | Position | Strand | Matrix score. | sequence | function |
| --- | --- | --- | --- | --- | --- | --- |
| MYB recognition site | Arabidopsis thaliana | 2576 | - | 6 | CCGTTG |  |

>HU05G01983.1   
+ +Up\_Stream \_Len000AAATCA AACTCATAAA CAATAATTTT TTAAAACTTA AATGGTAATT ATTAGATTAA   
  
  
+ TAAAAATCCT AATGGTTGTT TAATGTTACA TCAAGACATG CTTTTTGGTG TATATATAAT GGCAGCATGT   
  
  
+ GGGTTAAGCG GCAACAAATT ATTCTCAAAT GCATGCAGTA ATTAATTTAA TTAATTAATC ACCTGCATAA   
  
  
+ TCTCAATACT AATCCTTTCA TTCTGTGCCC CACCCAACCC CGAAAATAGT AATCTTTCAA ACGTTGGAAG   
  
  
+ TGGGATTAGG TATAGAGTAG TCCTACAGAT GATGATCTAC CTACATATAA CATAATCTGA CTTTTCTTTT   
  
  
+ TTAAATAACT TGTATTGATG GCACAGAATT TGACATGTTG GCATATGGTA CTAAGAAATT AATTAACGTT   
  
  
+ GTGCTAAGAT ATAGGTCATT CTTTTTGGAG ACATAAGGCC CGCGTTCACG CAGGGTTCGA GAAGGGCCAC   
  
  
+ ATTCAATGGA TGAATTGTAG GTAGTTTAAT CTGACTTTGT CAGTGACTGA TTCCACGGCT TGAACCCGCG   
  
  
+ ACATTAATAT AGGTTGACTA AAGTCGTTTG ATACTTATTT TTCTTAAGTA AAGTCTTGAA TTCAAATCTT   
  
  
+ ATAAATAAAT AAAAAAATCT ATAATAAAAG AATTCTACCA TTTATTAAGT CAACAACTCA ATTCGAATGA   
  
  
+ AATAAAAAAT CAAGATCACC AAAAAAAATA AATAAAAGGA AAGGTAGATT GCATTTGGAT TTTGAGTGGC   
  
  
+ GTGGTGGTGG GTTTGATGCT GGTGCTGCAT ACACCGCAGC TTTTCTCTGT CCTTTTACCT CTCTTTGTCT   
  
  
+ GCAAGAACCC CATTTGGCAC GCCTGCTCTC TTTTCTATGT TTTCTTTTCT ATCTTTTAAG AAACAATGGA   
  
  
+ AAGCTAACAC ACTAACACTA ACCCATTGGT CCTTTTTTCT TATCTGCAAA ATTAAAGTAA CAATATTTTT   
  
  
+ CTTATCCCCT TTTTCGTTCC ACCCATTTCT TTATCCTTCT GTTTGAACCT AATCTGTATA TATGCATGCA   
  
  
+ TGTGTATTTT ACTTAGAATC TGTTTTGATT CTTTGATGTA ATTAATTAGT AGTCTTTTTA ATTGAATTTT   
  
  
+ TAACAGTAGT TTGAATTAGA CATTTATCGA TATTATATTT TTTATATATA ATTAAAAAAT ACAAAATGAT   
  
  
+ AAGTGTCTTA CTACAATACA CTTCATAAGT GTCGTGTTTT AACTTAAAAA TCATACTATT ATGTTTCTAA   
  
  
+ ATTGAAAAAA AAGTGAGATT AAGTAGCAGA AGCATATTAC AACTTATAAA TTATATAAAA TCATCTTTTT   
  
  
+ ATCCATAATT TATCACATTT CTAAAATACG CATATCATAT CTACTTATGG ATAGGGTTAA TAAGATTAGG   
  
  
+ GCATCATTTA AGATTTTATC AGCGTAACCA ACGAATGTTT AAGGTTAGCA GTTCATAATT AAGTGTAATC   
  
  
+ ATTAAAAAAT TTATGATTTT AAATCATAAA ATATGTTAAT CTAGGATGAG GCAAGCGGCA AGACATATAT   
  
  
+ CACTATCCTT AAAAAATATA ATATATTGAT TTCAAAGAAT TATGAAGTAT ATTAATTTCA TATATAATGA   
  
  
+ CGATTATGTA TTTTTTTACT AAATTACTTG TACCCATAAT AAATAGAATC GATGCATCAT TCATGGCCTT   
  
  
+ TCATTCTGTA ATATGGTTGA CTCCAAAAAT TCTCAAAGTA TTTTTATATT TTGGGAGGGG AGATTTTAGT   
  
  
+ TTGATACTTT TGTTGAACTA GTGAACAATT TCCAACAACC TCCACCACCC CCAAACATCA ACAATCTGAT   
  
  
+ TGATGTATGC ATCCCAAGCT TGAATTGAAT AATAATTTCA ATAACAACAA TAATCGTTTT ACCTTGACAA   
  
  
+ GCTCCACAGC AACTCAACGT CTTCCAAACC AACCCATCAC TCATCATCAT CATCCCCACT ATCCCATTCA   
  
  
+ CCCCCACCAA AAGGAGCCTA AAGACAGTAA CAAGAAGAAT GCTTGCCTTT TTTCATGGAT GAAGAAGACT   
  
  
+ TCTCTTCTTC CTCTACTTCT TCCCACCACT ATGATACACA CCACCTCCAA CATCATTACC CCACCAATTA   
  
  
+ TAATTCCCGT TATACCCTGG ACCCTCCCTC CACCACCACC ACAGCCACAA CCACCACCTC ATCCTCCACT   
  
  
+ CCCACCCACC ATGGCTTCGG TGGTGGGCTT GACTCCTCCT CCCCGTCTGC CGCTGTGGAA TTATCTTTCT   
  
  
+ CCCCGGACAT CCTCCTCTCC TCCCCCACCG GAAAGTGGGC GTCTGACATT CTTATGGAGG CGGCTAGGGC   
  
  
+ TTTTTCAGAC ACGAACACCA CCAAGCTCCA CCAGCTCCTC TGGATGCTGA ACGAGCTCGG CTCCCCCTAC   
  
  
+ GGTGATGTCG AGCAGAAGCT GGTGGCGTAC TTTAACCAAG CCCTTTTTGC CCGCCTCACT GCCACCGGAC   
  
  
+ CCCGCCACCG AGCCGCTATG CTCGCCGCTG CGGACAAGAC CTCCTCCTTC GACTCCACGC TCAGGTTGCT   
  
  
+ CCTCAAGTTC CAGGAAGTCA GCCCGTGGAC CACCTTCGGC CACGTGGCTT GCAACGGTGC AATCATCGAG   
  
  
+ GCCTTAGAGA GGGACCCACA TGAAAAGAGT AAGCTCCACG TCATCGATAT CAGCACCACC TTCTGCACCC   
  
  
+ AGTGGCCCAC CTTGCTTGAG GCTTTAGCCA CCCGCATGGA CGACACTCCC CACCTCAGCC TCACTGCCGT   
  
  
+ CGTCGTCAAC AAGTACGCCG CCGTGAGCGG TGGCGAGGAC GACGGGGGAG AAGGATCAAA GAGGGTGATG   
  
  
+ AGAGAGATTG GTCTTCGTCT TGAGAAGTTC GCTAGGTTAA TGGGGGTTCC CTTCAAGTTC AACGTGGTTT   
  
  
+ ACCACGTAGG TGATCTATCC CAGTTGGATT TTTCTCGTTT GGATATTAAA GATGATGAAG CGGTGGCGAT   
  
  
+ TAACTGTGTC AACTCGCTCC ACTCGGTGGA CCTCCGATAT CGGGAGGTAG TGCTCGCGGC GTTCAGGCGG   
  
  
+ CTGCGGCCGA GGGTGGTGAC GGTGGTGGAG GAAGAGGCGG AGCTGACTGA GGTGGGTGAG GGTCAGTATG   
  
  
+ AATTTTTTAG GCGGTTTGAG GAGTGTCTTA GGTGGTTTAG GGTTTACTTT GAGGCCGTAG GAGATTGTTT   
  
  
+ TCCTAGGACT AGCAATGAGA AATTGATGTT AGAGCGGGCC GCGGGCCGGG CCATGGTGGA TCTCCTTGCA   
  
  
+ TGTCCCGAGC CTGGTGGGTC GGCTGAGGGG AGGGAGACGG CGAGACGGTG GTCGGGTCGG ATGCATGGGG   
  
  
+ CGGGGTTCGA GCACGTGGGG TTCAGTGACG AAGTGTGTGA TGATGTGAGG GCCTTGTTGA GGAGGTACAA   
  
  
+ GGAAGGATGG TCAATGACAC AGTGCACCAT GGATGGCGGT AATCACCCTG GAATACTGTT GTGGTGGCGG   
  
  
+ GATCAACCGG TGGTTTGGGC CAGTGCATGG CGGCCTTG  

- +Up\_Stream \_Len000TTTAGT TTGAGTATTT GTTATTAAAA AATTTTGAAT TTACCATTAA TAATCTAATT   
  
  
- ATTTTTAGGA TTACCAACAA ATTACAATGT AGTTCTGTAC GAAAAACCAC ATATATATTA CCGTCGTACA   
  
  
- CCCAATTCGC CGTTGTTTAA TAAGAGTTTA CGTACGTCAT TAATTAAATT AATTAATTAG TGGACGTATT   
  
  
- AGAGTTATGA TTAGGAAAGT AAGACACGGG GTGGGTTGGG GCTTTTATCA TTAGAAAGTT TGCAACCTTC   
  
  
- ACCCTAATCC ATATCTCATC AGGATGTCTA CTACTAGATG GATGTATATT GTATTAGACT GAAAAGAAAA   
  
  
- AATTTATTGA ACATAACTAC CGTGTCTTAA ACTGTACAAC CGTATACCAT GATTCTTTAA TTAATTGCAA   
  
  
- CACGATTCTA TATCCAGTAA GAAAAACCTC TGTATTCCGG GCGCAAGTGC GTCCCAAGCT CTTCCCGGTG   
  
  
- TAAGTTACCT ACTTAACATC CATCAAATTA GACTGAAACA GTCACTGACT AAGGTGCCGA ACTTGGGCGC   
  
  
- TGTAATTATA TCCAACTGAT TTCAGCAAAC TATGAATAAA AAGAATTCAT TTCAGAACTT AAGTTTAGAA   
  
  
- TATTTATTTA TTTTTTTAGA TATTATTTTC TTAAGATGGT AAATAATTCA GTTGTTGAGT TAAGCTTACT   
  
  
- TTATTTTTTA GTTCTAGTGG TTTTTTTTAT TTATTTTCCT TTCCATCTAA CGTAAACCTA AAACTCACCG   
  
  
- CACCACCACC CAAACTACGA CCACGACGTA TGTGGCGTCG AAAAGAGACA GGAAAATGGA GAGAAACAGA   
  
  
- CGTTCTTGGG GTAAACCGTG CGGACGAGAG AAAAGATACA AAAGAAAAGA TAGAAAATTC TTTGTTACCT   
  
  
- TTCGATTGTG TGATTGTGAT TGGGTAACCA GGAAAAAAGA ATAGACGTTT TAATTTCATT GTTATAAAAA   
  
  
- GAATAGGGGA AAAAGCAAGG TGGGTAAAGA AATAGGAAGA CAAACTTGGA TTAGACATAT ATACGTACGT   
  
  
- ACACATAAAA TGAATCTTAG ACAAAACTAA GAAACTACAT TAATTAATCA TCAGAAAAAT TAACTTAAAA   
  
  
- ATTGTCATCA AACTTAATCT GTAAATAGCT ATAATATAAA AAATATATAT TAATTTTTTA TGTTTTACTA   
  
  
- TTCACAGAAT GATGTTATGT GAAGTATTCA CAGCACAAAA TTGAATTTTT AGTATGATAA TACAAAGATT   
  
  
- TAACTTTTTT TTCACTCTAA TTCATCGTCT TCGTATAATG TTGAATATTT AATATATTTT AGTAGAAAAA   
  
  
- TAGGTATTAA ATAGTGTAAA GATTTTATGC GTATAGTATA GATGAATACC TATCCCAATT ATTCTAATCC   
  
  
- CGTAGTAAAT TCTAAAATAG TCGCATTGGT TGCTTACAAA TTCCAATCGT CAAGTATTAA TTCACATTAG   
  
  
- TAATTTTTTA AATACTAAAA TTTAGTATTT TATACAATTA GATCCTACTC CGTTCGCCGT TCTGTATATA   
  
  
- GTGATAGGAA TTTTTTATAT TATATAACTA AAGTTTCTTA ATACTTCATA TAATTAAAGT ATATATTACT   
  
  
- GCTAATACAT AAAAAAATGA TTTAATGAAC ATGGGTATTA TTTATCTTAG CTACGTAGTA AGTACCGGAA   
  
  
- AGTAAGACAT TATACCAACT GAGGTTTTTA AGAGTTTCAT AAAAATATAA AACCCTCCCC TCTAAAATCA   
  
  
- AACTATGAAA ACAACTTGAT CACTTGTTAA AGGTTGTTGG AGGTGGTGGG GGTTTGTAGT TGTTAGACTA   
  
  
- ACTACATACG TAGGGTTCGA ACTTAACTTA TTATTAAAGT TATTGTTGTT ATTAGCAAAA TGGAACTGTT   
  
  
- CGAGGTGTCG TTGAGTTGCA GAAGGTTTGG TTGGGTAGTG AGTAGTAGTA GTAGGGGTGA TAGGGTAAGT   
  
  
- GGGGGTGGTT TTCCTCGGAT TTCTGTCATT GTTCTTCTTA CGAACGGAAA AAAGTACCTA CTTCTTCTGA   
  
  
- AGAGAAGAAG GAGATGAAGA AGGGTGGTGA TACTATGTGT GGTGGAGGTT GTAGTAATGG GGTGGTTAAT   
  
  
- ATTAAGGGCA ATATGGGACC TGGGAGGGAG GTGGTGGTGG TGTCGGTGTT GGTGGTGGAG TAGGAGGTGA   
  
  
- GGGTGGGTGG TACCGAAGCC ACCACCCGAA CTGAGGAGGA GGGGCAGACG GCGACACCTT AATAGAAAGA   
  
  
- GGGGCCTGTA GGAGGAGAGG AGGGGGTGGC CTTTCACCCG CAGACTGTAA GAATACCTCC GCCGATCCCG   
  
  
- AAAAAGTCTG TGCTTGTGGT GGTTCGAGGT GGTCGAGGAG ACCTACGACT TGCTCGAGCC GAGGGGGATG   
  
  
- CCACTACAGC TCGTCTTCGA CCACCGCATG AAATTGGTTC GGGAAAAACG GGCGGAGTGA CGGTGGCCTG   
  
  
- GGGCGGTGGC TCGGCGATAC GAGCGGCGAC GCCTGTTCTG GAGGAGGAAG CTGAGGTGCG AGTCCAACGA   
  
  
- GGAGTTCAAG GTCCTTCAGT CGGGCACCTG GTGGAAGCCG GTGCACCGAA CGTTGCCACG TTAGTAGCTC   
  
  
- CGGAATCTCT CCCTGGGTGT ACTTTTCTCA TTCGAGGTGC AGTAGCTATA GTCGTGGTGG AAGACGTGGG   
  
  
- TCACCGGGTG GAACGAACTC CGAAATCGGT GGGCGTACCT GCTGTGAGGG GTGGAGTCGG AGTGACGGCA   
  
  
- GCAGCAGTTG TTCATGCGGC GGCACTCGCC ACCGCTCCTG CTGCCCCCTC TTCCTAGTTT CTCCCACTAC   
  
  
- TCTCTCTAAC CAGAAGCAGA ACTCTTCAAG CGATCCAATT ACCCCCAAGG GAAGTTCAAG TTGCACCAAA   
  
  
- TGGTGCATCC ACTAGATAGG GTCAACCTAA AAAGAGCAAA CCTATAATTT CTACTACTTC GCCACCGCTA   
  
  
- ATTGACACAG TTGAGCGAGG TGAGCCACCT GGAGGCTATA GCCCTCCATC ACGAGCGCCG CAAGTCCGCC   
  
  
- GACGCCGGCT CCCACCACTG CCACCACCTC CTTCTCCGCC TCGACTGACT CCACCCACTC CCAGTCATAC   
  
  
- TTAAAAAATC CGCCAAACTC CTCACAGAAT CCACCAAATC CCAAATGAAA CTCCGGCATC CTCTAACAAA   
  
  
- AGGATCCTGA TCGTTACTCT TTAACTACAA TCTCGCCCGG CGCCCGGCCC GGTACCACCT AGAGGAACGT   
  
  
- ACAGGGCTCG GACCACCCAG CCGACTCCCC TCCCTCTGCC GCTCTGCCAC CAGCCCAGCC TACGTACCCC   
  
  
- GCCCCAAGCT CGTGCACCCC AAGTCACTGC TTCACACACT ACTACACTCC CGGAACAACT CCTCCATGTT   
  
  
- CCTTCCTACC AGTTACTGTG TCACGTGGTA CCTACCGCCA TTAGTGGGAC CTTATGACAA CACCACCGCC   
  
  
- CTAGTTGGCC ACCAAACCCG GTCACGTACC GCCGGAAC

+     MYB-like sequence

| Site Name | Organism | Position | Strand | Matrix score. | sequence | function |
| --- | --- | --- | --- | --- | --- | --- |
| MYB-like sequence | Arabidopsis thaliana | 2417 | + | 6 | TAACCA |  |
| MYB-like sequence | Arabidopsis thaliana | 1429 | + | 6 | TAACCA |  |

>HU05G01983.1   
+ +Up\_Stream \_Len000AAATCA AACTCATAAA CAATAATTTT TTAAAACTTA AATGGTAATT ATTAGATTAA   
  
  
+ TAAAAATCCT AATGGTTGTT TAATGTTACA TCAAGACATG CTTTTTGGTG TATATATAAT GGCAGCATGT   
  
  
+ GGGTTAAGCG GCAACAAATT ATTCTCAAAT GCATGCAGTA ATTAATTTAA TTAATTAATC ACCTGCATAA   
  
  
+ TCTCAATACT AATCCTTTCA TTCTGTGCCC CACCCAACCC CGAAAATAGT AATCTTTCAA ACGTTGGAAG   
  
  
+ TGGGATTAGG TATAGAGTAG TCCTACAGAT GATGATCTAC CTACATATAA CATAATCTGA CTTTTCTTTT   
  
  
+ TTAAATAACT TGTATTGATG GCACAGAATT TGACATGTTG GCATATGGTA CTAAGAAATT AATTAACGTT   
  
  
+ GTGCTAAGAT ATAGGTCATT CTTTTTGGAG ACATAAGGCC CGCGTTCACG CAGGGTTCGA GAAGGGCCAC   
  
  
+ ATTCAATGGA TGAATTGTAG GTAGTTTAAT CTGACTTTGT CAGTGACTGA TTCCACGGCT TGAACCCGCG   
  
  
+ ACATTAATAT AGGTTGACTA AAGTCGTTTG ATACTTATTT TTCTTAAGTA AAGTCTTGAA TTCAAATCTT   
  
  
+ ATAAATAAAT AAAAAAATCT ATAATAAAAG AATTCTACCA TTTATTAAGT CAACAACTCA ATTCGAATGA   
  
  
+ AATAAAAAAT CAAGATCACC AAAAAAAATA AATAAAAGGA AAGGTAGATT GCATTTGGAT TTTGAGTGGC   
  
  
+ GTGGTGGTGG GTTTGATGCT GGTGCTGCAT ACACCGCAGC TTTTCTCTGT CCTTTTACCT CTCTTTGTCT   
  
  
+ GCAAGAACCC CATTTGGCAC GCCTGCTCTC TTTTCTATGT TTTCTTTTCT ATCTTTTAAG AAACAATGGA   
  
  
+ AAGCTAACAC ACTAACACTA ACCCATTGGT CCTTTTTTCT TATCTGCAAA ATTAAAGTAA CAATATTTTT   
  
  
+ CTTATCCCCT TTTTCGTTCC ACCCATTTCT TTATCCTTCT GTTTGAACCT AATCTGTATA TATGCATGCA   
  
  
+ TGTGTATTTT ACTTAGAATC TGTTTTGATT CTTTGATGTA ATTAATTAGT AGTCTTTTTA ATTGAATTTT   
  
  
+ TAACAGTAGT TTGAATTAGA CATTTATCGA TATTATATTT TTTATATATA ATTAAAAAAT ACAAAATGAT   
  
  
+ AAGTGTCTTA CTACAATACA CTTCATAAGT GTCGTGTTTT AACTTAAAAA TCATACTATT ATGTTTCTAA   
  
  
+ ATTGAAAAAA AAGTGAGATT AAGTAGCAGA AGCATATTAC AACTTATAAA TTATATAAAA TCATCTTTTT   
  
  
+ ATCCATAATT TATCACATTT CTAAAATACG CATATCATAT CTACTTATGG ATAGGGTTAA TAAGATTAGG   
  
  
+ GCATCATTTA AGATTTTATC AGCGTAACCA ACGAATGTTT AAGGTTAGCA GTTCATAATT AAGTGTAATC   
  
  
+ ATTAAAAAAT TTATGATTTT AAATCATAAA ATATGTTAAT CTAGGATGAG GCAAGCGGCA AGACATATAT   
  
  
+ CACTATCCTT AAAAAATATA ATATATTGAT TTCAAAGAAT TATGAAGTAT ATTAATTTCA TATATAATGA   
  
  
+ CGATTATGTA TTTTTTTACT AAATTACTTG TACCCATAAT AAATAGAATC GATGCATCAT TCATGGCCTT   
  
  
+ TCATTCTGTA ATATGGTTGA CTCCAAAAAT TCTCAAAGTA TTTTTATATT TTGGGAGGGG AGATTTTAGT   
  
  
+ TTGATACTTT TGTTGAACTA GTGAACAATT TCCAACAACC TCCACCACCC CCAAACATCA ACAATCTGAT   
  
  
+ TGATGTATGC ATCCCAAGCT TGAATTGAAT AATAATTTCA ATAACAACAA TAATCGTTTT ACCTTGACAA   
  
  
+ GCTCCACAGC AACTCAACGT CTTCCAAACC AACCCATCAC TCATCATCAT CATCCCCACT ATCCCATTCA   
  
  
+ CCCCCACCAA AAGGAGCCTA AAGACAGTAA CAAGAAGAAT GCTTGCCTTT TTTCATGGAT GAAGAAGACT   
  
  
+ TCTCTTCTTC CTCTACTTCT TCCCACCACT ATGATACACA CCACCTCCAA CATCATTACC CCACCAATTA   
  
  
+ TAATTCCCGT TATACCCTGG ACCCTCCCTC CACCACCACC ACAGCCACAA CCACCACCTC ATCCTCCACT   
  
  
+ CCCACCCACC ATGGCTTCGG TGGTGGGCTT GACTCCTCCT CCCCGTCTGC CGCTGTGGAA TTATCTTTCT   
  
  
+ CCCCGGACAT CCTCCTCTCC TCCCCCACCG GAAAGTGGGC GTCTGACATT CTTATGGAGG CGGCTAGGGC   
  
  
+ TTTTTCAGAC ACGAACACCA CCAAGCTCCA CCAGCTCCTC TGGATGCTGA ACGAGCTCGG CTCCCCCTAC   
  
  
+ GGTGATGTCG AGCAGAAGCT GGTGGCGTAC TTTAACCAAG CCCTTTTTGC CCGCCTCACT GCCACCGGAC   
  
  
+ CCCGCCACCG AGCCGCTATG CTCGCCGCTG CGGACAAGAC CTCCTCCTTC GACTCCACGC TCAGGTTGCT   
  
  
+ CCTCAAGTTC CAGGAAGTCA GCCCGTGGAC CACCTTCGGC CACGTGGCTT GCAACGGTGC AATCATCGAG   
  
  
+ GCCTTAGAGA GGGACCCACA TGAAAAGAGT AAGCTCCACG TCATCGATAT CAGCACCACC TTCTGCACCC   
  
  
+ AGTGGCCCAC CTTGCTTGAG GCTTTAGCCA CCCGCATGGA CGACACTCCC CACCTCAGCC TCACTGCCGT   
  
  
+ CGTCGTCAAC AAGTACGCCG CCGTGAGCGG TGGCGAGGAC GACGGGGGAG AAGGATCAAA GAGGGTGATG   
  
  
+ AGAGAGATTG GTCTTCGTCT TGAGAAGTTC GCTAGGTTAA TGGGGGTTCC CTTCAAGTTC AACGTGGTTT   
  
  
+ ACCACGTAGG TGATCTATCC CAGTTGGATT TTTCTCGTTT GGATATTAAA GATGATGAAG CGGTGGCGAT   
  
  
+ TAACTGTGTC AACTCGCTCC ACTCGGTGGA CCTCCGATAT CGGGAGGTAG TGCTCGCGGC GTTCAGGCGG   
  
  
+ CTGCGGCCGA GGGTGGTGAC GGTGGTGGAG GAAGAGGCGG AGCTGACTGA GGTGGGTGAG GGTCAGTATG   
  
  
+ AATTTTTTAG GCGGTTTGAG GAGTGTCTTA GGTGGTTTAG GGTTTACTTT GAGGCCGTAG GAGATTGTTT   
  
  
+ TCCTAGGACT AGCAATGAGA AATTGATGTT AGAGCGGGCC GCGGGCCGGG CCATGGTGGA TCTCCTTGCA   
  
  
+ TGTCCCGAGC CTGGTGGGTC GGCTGAGGGG AGGGAGACGG CGAGACGGTG GTCGGGTCGG ATGCATGGGG   
  
  
+ CGGGGTTCGA GCACGTGGGG TTCAGTGACG AAGTGTGTGA TGATGTGAGG GCCTTGTTGA GGAGGTACAA   
  
  
+ GGAAGGATGG TCAATGACAC AGTGCACCAT GGATGGCGGT AATCACCCTG GAATACTGTT GTGGTGGCGG   
  
  
+ GATCAACCGG TGGTTTGGGC CAGTGCATGG CGGCCTTG  

- +Up\_Stream \_Len000TTTAGT TTGAGTATTT GTTATTAAAA AATTTTGAAT TTACCATTAA TAATCTAATT   
  
  
- ATTTTTAGGA TTACCAACAA ATTACAATGT AGTTCTGTAC GAAAAACCAC ATATATATTA CCGTCGTACA   
  
  
- CCCAATTCGC CGTTGTTTAA TAAGAGTTTA CGTACGTCAT TAATTAAATT AATTAATTAG TGGACGTATT   
  
  
- AGAGTTATGA TTAGGAAAGT AAGACACGGG GTGGGTTGGG GCTTTTATCA TTAGAAAGTT TGCAACCTTC   
  
  
- ACCCTAATCC ATATCTCATC AGGATGTCTA CTACTAGATG GATGTATATT GTATTAGACT GAAAAGAAAA   
  
  
- AATTTATTGA ACATAACTAC CGTGTCTTAA ACTGTACAAC CGTATACCAT GATTCTTTAA TTAATTGCAA   
  
  
- CACGATTCTA TATCCAGTAA GAAAAACCTC TGTATTCCGG GCGCAAGTGC GTCCCAAGCT CTTCCCGGTG   
  
  
- TAAGTTACCT ACTTAACATC CATCAAATTA GACTGAAACA GTCACTGACT AAGGTGCCGA ACTTGGGCGC   
  
  
- TGTAATTATA TCCAACTGAT TTCAGCAAAC TATGAATAAA AAGAATTCAT TTCAGAACTT AAGTTTAGAA   
  
  
- TATTTATTTA TTTTTTTAGA TATTATTTTC TTAAGATGGT AAATAATTCA GTTGTTGAGT TAAGCTTACT   
  
  
- TTATTTTTTA GTTCTAGTGG TTTTTTTTAT TTATTTTCCT TTCCATCTAA CGTAAACCTA AAACTCACCG   
  
  
- CACCACCACC CAAACTACGA CCACGACGTA TGTGGCGTCG AAAAGAGACA GGAAAATGGA GAGAAACAGA   
  
  
- CGTTCTTGGG GTAAACCGTG CGGACGAGAG AAAAGATACA AAAGAAAAGA TAGAAAATTC TTTGTTACCT   
  
  
- TTCGATTGTG TGATTGTGAT TGGGTAACCA GGAAAAAAGA ATAGACGTTT TAATTTCATT GTTATAAAAA   
  
  
- GAATAGGGGA AAAAGCAAGG TGGGTAAAGA AATAGGAAGA CAAACTTGGA TTAGACATAT ATACGTACGT   
  
  
- ACACATAAAA TGAATCTTAG ACAAAACTAA GAAACTACAT TAATTAATCA TCAGAAAAAT TAACTTAAAA   
  
  
- ATTGTCATCA AACTTAATCT GTAAATAGCT ATAATATAAA AAATATATAT TAATTTTTTA TGTTTTACTA   
  
  
- TTCACAGAAT GATGTTATGT GAAGTATTCA CAGCACAAAA TTGAATTTTT AGTATGATAA TACAAAGATT   
  
  
- TAACTTTTTT TTCACTCTAA TTCATCGTCT TCGTATAATG TTGAATATTT AATATATTTT AGTAGAAAAA   
  
  
- TAGGTATTAA ATAGTGTAAA GATTTTATGC GTATAGTATA GATGAATACC TATCCCAATT ATTCTAATCC   
  
  
- CGTAGTAAAT TCTAAAATAG TCGCATTGGT TGCTTACAAA TTCCAATCGT CAAGTATTAA TTCACATTAG   
  
  
- TAATTTTTTA AATACTAAAA TTTAGTATTT TATACAATTA GATCCTACTC CGTTCGCCGT TCTGTATATA   
  
  
- GTGATAGGAA TTTTTTATAT TATATAACTA AAGTTTCTTA ATACTTCATA TAATTAAAGT ATATATTACT   
  
  
- GCTAATACAT AAAAAAATGA TTTAATGAAC ATGGGTATTA TTTATCTTAG CTACGTAGTA AGTACCGGAA   
  
  
- AGTAAGACAT TATACCAACT GAGGTTTTTA AGAGTTTCAT AAAAATATAA AACCCTCCCC TCTAAAATCA   
  
  
- AACTATGAAA ACAACTTGAT CACTTGTTAA AGGTTGTTGG AGGTGGTGGG GGTTTGTAGT TGTTAGACTA   
  
  
- ACTACATACG TAGGGTTCGA ACTTAACTTA TTATTAAAGT TATTGTTGTT ATTAGCAAAA TGGAACTGTT   
  
  
- CGAGGTGTCG TTGAGTTGCA GAAGGTTTGG TTGGGTAGTG AGTAGTAGTA GTAGGGGTGA TAGGGTAAGT   
  
  
- GGGGGTGGTT TTCCTCGGAT TTCTGTCATT GTTCTTCTTA CGAACGGAAA AAAGTACCTA CTTCTTCTGA   
  
  
- AGAGAAGAAG GAGATGAAGA AGGGTGGTGA TACTATGTGT GGTGGAGGTT GTAGTAATGG GGTGGTTAAT   
  
  
- ATTAAGGGCA ATATGGGACC TGGGAGGGAG GTGGTGGTGG TGTCGGTGTT GGTGGTGGAG TAGGAGGTGA   
  
  
- GGGTGGGTGG TACCGAAGCC ACCACCCGAA CTGAGGAGGA GGGGCAGACG GCGACACCTT AATAGAAAGA   
  
  
- GGGGCCTGTA GGAGGAGAGG AGGGGGTGGC CTTTCACCCG CAGACTGTAA GAATACCTCC GCCGATCCCG   
  
  
- AAAAAGTCTG TGCTTGTGGT GGTTCGAGGT GGTCGAGGAG ACCTACGACT TGCTCGAGCC GAGGGGGATG   
  
  
- CCACTACAGC TCGTCTTCGA CCACCGCATG AAATTGGTTC GGGAAAAACG GGCGGAGTGA CGGTGGCCTG   
  
  
- GGGCGGTGGC TCGGCGATAC GAGCGGCGAC GCCTGTTCTG GAGGAGGAAG CTGAGGTGCG AGTCCAACGA   
  
  
- GGAGTTCAAG GTCCTTCAGT CGGGCACCTG GTGGAAGCCG GTGCACCGAA CGTTGCCACG TTAGTAGCTC   
  
  
- CGGAATCTCT CCCTGGGTGT ACTTTTCTCA TTCGAGGTGC AGTAGCTATA GTCGTGGTGG AAGACGTGGG   
  
  
- TCACCGGGTG GAACGAACTC CGAAATCGGT GGGCGTACCT GCTGTGAGGG GTGGAGTCGG AGTGACGGCA   
  
  
- GCAGCAGTTG TTCATGCGGC GGCACTCGCC ACCGCTCCTG CTGCCCCCTC TTCCTAGTTT CTCCCACTAC   
  
  
- TCTCTCTAAC CAGAAGCAGA ACTCTTCAAG CGATCCAATT ACCCCCAAGG GAAGTTCAAG TTGCACCAAA   
  
  
- TGGTGCATCC ACTAGATAGG GTCAACCTAA AAAGAGCAAA CCTATAATTT CTACTACTTC GCCACCGCTA   
  
  
- ATTGACACAG TTGAGCGAGG TGAGCCACCT GGAGGCTATA GCCCTCCATC ACGAGCGCCG CAAGTCCGCC   
  
  
- GACGCCGGCT CCCACCACTG CCACCACCTC CTTCTCCGCC TCGACTGACT CCACCCACTC CCAGTCATAC   
  
  
- TTAAAAAATC CGCCAAACTC CTCACAGAAT CCACCAAATC CCAAATGAAA CTCCGGCATC CTCTAACAAA   
  
  
- AGGATCCTGA TCGTTACTCT TTAACTACAA TCTCGCCCGG CGCCCGGCCC GGTACCACCT AGAGGAACGT   
  
  
- ACAGGGCTCG GACCACCCAG CCGACTCCCC TCCCTCTGCC GCTCTGCCAC CAGCCCAGCC TACGTACCCC   
  
  
- GCCCCAAGCT CGTGCACCCC AAGTCACTGC TTCACACACT ACTACACTCC CGGAACAACT CCTCCATGTT   
  
  
- CCTTCCTACC AGTTACTGTG TCACGTGGTA CCTACCGCCA TTAGTGGGAC CTTATGACAA CACCACCGCC   
  
  
- CTAGTTGGCC ACCAAACCCG GTCACGTACC GCCGGAAC

+     MYC

| Site Name | Organism | Position | Strand | Matrix score. | sequence | function |
| --- | --- | --- | --- | --- | --- | --- |
| MYC | Arabidopsis thaliana | 170 | - | 6 | CATTTG |  |
| MYC | Arabidopsis thaliana | 1053 | + | 6 | CATGTG |  |
| MYC | Arabidopsis thaliana | 855 | + | 6 | CATTTG |  |
| MYC | Arabidopsis thaliana | 756 | + | 6 | CATTTG |  |
| MYC | Arabidopsis thaliana | 140 | + | 6 | CATGTG |  |
| MYC | Arabidopsis thaliana | 2611 | - | 6 | CATGTG |  |

>HU05G01983.1   
+ +Up\_Stream \_Len000AAATCA AACTCATAAA CAATAATTTT TTAAAACTTA AATGGTAATT ATTAGATTAA   
  
  
+ TAAAAATCCT AATGGTTGTT TAATGTTACA TCAAGACATG CTTTTTGGTG TATATATAAT GGCAGCATGT   
  
  
+ GGGTTAAGCG GCAACAAATT ATTCTCAAAT GCATGCAGTA ATTAATTTAA TTAATTAATC ACCTGCATAA   
  
  
+ TCTCAATACT AATCCTTTCA TTCTGTGCCC CACCCAACCC CGAAAATAGT AATCTTTCAA ACGTTGGAAG   
  
  
+ TGGGATTAGG TATAGAGTAG TCCTACAGAT GATGATCTAC CTACATATAA CATAATCTGA CTTTTCTTTT   
  
  
+ TTAAATAACT TGTATTGATG GCACAGAATT TGACATGTTG GCATATGGTA CTAAGAAATT AATTAACGTT   
  
  
+ GTGCTAAGAT ATAGGTCATT CTTTTTGGAG ACATAAGGCC CGCGTTCACG CAGGGTTCGA GAAGGGCCAC   
  
  
+ ATTCAATGGA TGAATTGTAG GTAGTTTAAT CTGACTTTGT CAGTGACTGA TTCCACGGCT TGAACCCGCG   
  
  
+ ACATTAATAT AGGTTGACTA AAGTCGTTTG ATACTTATTT TTCTTAAGTA AAGTCTTGAA TTCAAATCTT   
  
  
+ ATAAATAAAT AAAAAAATCT ATAATAAAAG AATTCTACCA TTTATTAAGT CAACAACTCA ATTCGAATGA   
  
  
+ AATAAAAAAT CAAGATCACC AAAAAAAATA AATAAAAGGA AAGGTAGATT GCATTTGGAT TTTGAGTGGC   
  
  
+ GTGGTGGTGG GTTTGATGCT GGTGCTGCAT ACACCGCAGC TTTTCTCTGT CCTTTTACCT CTCTTTGTCT   
  
  
+ GCAAGAACCC CATTTGGCAC GCCTGCTCTC TTTTCTATGT TTTCTTTTCT ATCTTTTAAG AAACAATGGA   
  
  
+ AAGCTAACAC ACTAACACTA ACCCATTGGT CCTTTTTTCT TATCTGCAAA ATTAAAGTAA CAATATTTTT   
  
  
+ CTTATCCCCT TTTTCGTTCC ACCCATTTCT TTATCCTTCT GTTTGAACCT AATCTGTATA TATGCATGCA   
  
  
+ TGTGTATTTT ACTTAGAATC TGTTTTGATT CTTTGATGTA ATTAATTAGT AGTCTTTTTA ATTGAATTTT   
  
  
+ TAACAGTAGT TTGAATTAGA CATTTATCGA TATTATATTT TTTATATATA ATTAAAAAAT ACAAAATGAT   
  
  
+ AAGTGTCTTA CTACAATACA CTTCATAAGT GTCGTGTTTT AACTTAAAAA TCATACTATT ATGTTTCTAA   
  
  
+ ATTGAAAAAA AAGTGAGATT AAGTAGCAGA AGCATATTAC AACTTATAAA TTATATAAAA TCATCTTTTT   
  
  
+ ATCCATAATT TATCACATTT CTAAAATACG CATATCATAT CTACTTATGG ATAGGGTTAA TAAGATTAGG   
  
  
+ GCATCATTTA AGATTTTATC AGCGTAACCA ACGAATGTTT AAGGTTAGCA GTTCATAATT AAGTGTAATC   
  
  
+ ATTAAAAAAT TTATGATTTT AAATCATAAA ATATGTTAAT CTAGGATGAG GCAAGCGGCA AGACATATAT   
  
  
+ CACTATCCTT AAAAAATATA ATATATTGAT TTCAAAGAAT TATGAAGTAT ATTAATTTCA TATATAATGA   
  
  
+ CGATTATGTA TTTTTTTACT AAATTACTTG TACCCATAAT AAATAGAATC GATGCATCAT TCATGGCCTT   
  
  
+ TCATTCTGTA ATATGGTTGA CTCCAAAAAT TCTCAAAGTA TTTTTATATT TTGGGAGGGG AGATTTTAGT   
  
  
+ TTGATACTTT TGTTGAACTA GTGAACAATT TCCAACAACC TCCACCACCC CCAAACATCA ACAATCTGAT   
  
  
+ TGATGTATGC ATCCCAAGCT TGAATTGAAT AATAATTTCA ATAACAACAA TAATCGTTTT ACCTTGACAA   
  
  
+ GCTCCACAGC AACTCAACGT CTTCCAAACC AACCCATCAC TCATCATCAT CATCCCCACT ATCCCATTCA   
  
  
+ CCCCCACCAA AAGGAGCCTA AAGACAGTAA CAAGAAGAAT GCTTGCCTTT TTTCATGGAT GAAGAAGACT   
  
  
+ TCTCTTCTTC CTCTACTTCT TCCCACCACT ATGATACACA CCACCTCCAA CATCATTACC CCACCAATTA   
  
  
+ TAATTCCCGT TATACCCTGG ACCCTCCCTC CACCACCACC ACAGCCACAA CCACCACCTC ATCCTCCACT   
  
  
+ CCCACCCACC ATGGCTTCGG TGGTGGGCTT GACTCCTCCT CCCCGTCTGC CGCTGTGGAA TTATCTTTCT   
  
  
+ CCCCGGACAT CCTCCTCTCC TCCCCCACCG GAAAGTGGGC GTCTGACATT CTTATGGAGG CGGCTAGGGC   
  
  
+ TTTTTCAGAC ACGAACACCA CCAAGCTCCA CCAGCTCCTC TGGATGCTGA ACGAGCTCGG CTCCCCCTAC   
  
  
+ GGTGATGTCG AGCAGAAGCT GGTGGCGTAC TTTAACCAAG CCCTTTTTGC CCGCCTCACT GCCACCGGAC   
  
  
+ CCCGCCACCG AGCCGCTATG CTCGCCGCTG CGGACAAGAC CTCCTCCTTC GACTCCACGC TCAGGTTGCT   
  
  
+ CCTCAAGTTC CAGGAAGTCA GCCCGTGGAC CACCTTCGGC CACGTGGCTT GCAACGGTGC AATCATCGAG   
  
  
+ GCCTTAGAGA GGGACCCACA TGAAAAGAGT AAGCTCCACG TCATCGATAT CAGCACCACC TTCTGCACCC   
  
  
+ AGTGGCCCAC CTTGCTTGAG GCTTTAGCCA CCCGCATGGA CGACACTCCC CACCTCAGCC TCACTGCCGT   
  
  
+ CGTCGTCAAC AAGTACGCCG CCGTGAGCGG TGGCGAGGAC GACGGGGGAG AAGGATCAAA GAGGGTGATG   
  
  
+ AGAGAGATTG GTCTTCGTCT TGAGAAGTTC GCTAGGTTAA TGGGGGTTCC CTTCAAGTTC AACGTGGTTT   
  
  
+ ACCACGTAGG TGATCTATCC CAGTTGGATT TTTCTCGTTT GGATATTAAA GATGATGAAG CGGTGGCGAT   
  
  
+ TAACTGTGTC AACTCGCTCC ACTCGGTGGA CCTCCGATAT CGGGAGGTAG TGCTCGCGGC GTTCAGGCGG   
  
  
+ CTGCGGCCGA GGGTGGTGAC GGTGGTGGAG GAAGAGGCGG AGCTGACTGA GGTGGGTGAG GGTCAGTATG   
  
  
+ AATTTTTTAG GCGGTTTGAG GAGTGTCTTA GGTGGTTTAG GGTTTACTTT GAGGCCGTAG GAGATTGTTT   
  
  
+ TCCTAGGACT AGCAATGAGA AATTGATGTT AGAGCGGGCC GCGGGCCGGG CCATGGTGGA TCTCCTTGCA   
  
  
+ TGTCCCGAGC CTGGTGGGTC GGCTGAGGGG AGGGAGACGG CGAGACGGTG GTCGGGTCGG ATGCATGGGG   
  
  
+ CGGGGTTCGA GCACGTGGGG TTCAGTGACG AAGTGTGTGA TGATGTGAGG GCCTTGTTGA GGAGGTACAA   
  
  
+ GGAAGGATGG TCAATGACAC AGTGCACCAT GGATGGCGGT AATCACCCTG GAATACTGTT GTGGTGGCGG   
  
  
+ GATCAACCGG TGGTTTGGGC CAGTGCATGG CGGCCTTG  

- +Up\_Stream \_Len000TTTAGT TTGAGTATTT GTTATTAAAA AATTTTGAAT TTACCATTAA TAATCTAATT   
  
  
- ATTTTTAGGA TTACCAACAA ATTACAATGT AGTTCTGTAC GAAAAACCAC ATATATATTA CCGTCGTACA   
  
  
- CCCAATTCGC CGTTGTTTAA TAAGAGTTTA CGTACGTCAT TAATTAAATT AATTAATTAG TGGACGTATT   
  
  
- AGAGTTATGA TTAGGAAAGT AAGACACGGG GTGGGTTGGG GCTTTTATCA TTAGAAAGTT TGCAACCTTC   
  
  
- ACCCTAATCC ATATCTCATC AGGATGTCTA CTACTAGATG GATGTATATT GTATTAGACT GAAAAGAAAA   
  
  
- AATTTATTGA ACATAACTAC CGTGTCTTAA ACTGTACAAC CGTATACCAT GATTCTTTAA TTAATTGCAA   
  
  
- CACGATTCTA TATCCAGTAA GAAAAACCTC TGTATTCCGG GCGCAAGTGC GTCCCAAGCT CTTCCCGGTG   
  
  
- TAAGTTACCT ACTTAACATC CATCAAATTA GACTGAAACA GTCACTGACT AAGGTGCCGA ACTTGGGCGC   
  
  
- TGTAATTATA TCCAACTGAT TTCAGCAAAC TATGAATAAA AAGAATTCAT TTCAGAACTT AAGTTTAGAA   
  
  
- TATTTATTTA TTTTTTTAGA TATTATTTTC TTAAGATGGT AAATAATTCA GTTGTTGAGT TAAGCTTACT   
  
  
- TTATTTTTTA GTTCTAGTGG TTTTTTTTAT TTATTTTCCT TTCCATCTAA CGTAAACCTA AAACTCACCG   
  
  
- CACCACCACC CAAACTACGA CCACGACGTA TGTGGCGTCG AAAAGAGACA GGAAAATGGA GAGAAACAGA   
  
  
- CGTTCTTGGG GTAAACCGTG CGGACGAGAG AAAAGATACA AAAGAAAAGA TAGAAAATTC TTTGTTACCT   
  
  
- TTCGATTGTG TGATTGTGAT TGGGTAACCA GGAAAAAAGA ATAGACGTTT TAATTTCATT GTTATAAAAA   
  
  
- GAATAGGGGA AAAAGCAAGG TGGGTAAAGA AATAGGAAGA CAAACTTGGA TTAGACATAT ATACGTACGT   
  
  
- ACACATAAAA TGAATCTTAG ACAAAACTAA GAAACTACAT TAATTAATCA TCAGAAAAAT TAACTTAAAA   
  
  
- ATTGTCATCA AACTTAATCT GTAAATAGCT ATAATATAAA AAATATATAT TAATTTTTTA TGTTTTACTA   
  
  
- TTCACAGAAT GATGTTATGT GAAGTATTCA CAGCACAAAA TTGAATTTTT AGTATGATAA TACAAAGATT   
  
  
- TAACTTTTTT TTCACTCTAA TTCATCGTCT TCGTATAATG TTGAATATTT AATATATTTT AGTAGAAAAA   
  
  
- TAGGTATTAA ATAGTGTAAA GATTTTATGC GTATAGTATA GATGAATACC TATCCCAATT ATTCTAATCC   
  
  
- CGTAGTAAAT TCTAAAATAG TCGCATTGGT TGCTTACAAA TTCCAATCGT CAAGTATTAA TTCACATTAG   
  
  
- TAATTTTTTA AATACTAAAA TTTAGTATTT TATACAATTA GATCCTACTC CGTTCGCCGT TCTGTATATA   
  
  
- GTGATAGGAA TTTTTTATAT TATATAACTA AAGTTTCTTA ATACTTCATA TAATTAAAGT ATATATTACT   
  
  
- GCTAATACAT AAAAAAATGA TTTAATGAAC ATGGGTATTA TTTATCTTAG CTACGTAGTA AGTACCGGAA   
  
  
- AGTAAGACAT TATACCAACT GAGGTTTTTA AGAGTTTCAT AAAAATATAA AACCCTCCCC TCTAAAATCA   
  
  
- AACTATGAAA ACAACTTGAT CACTTGTTAA AGGTTGTTGG AGGTGGTGGG GGTTTGTAGT TGTTAGACTA   
  
  
- ACTACATACG TAGGGTTCGA ACTTAACTTA TTATTAAAGT TATTGTTGTT ATTAGCAAAA TGGAACTGTT   
  
  
- CGAGGTGTCG TTGAGTTGCA GAAGGTTTGG TTGGGTAGTG AGTAGTAGTA GTAGGGGTGA TAGGGTAAGT   
  
  
- GGGGGTGGTT TTCCTCGGAT TTCTGTCATT GTTCTTCTTA CGAACGGAAA AAAGTACCTA CTTCTTCTGA   
  
  
- AGAGAAGAAG GAGATGAAGA AGGGTGGTGA TACTATGTGT GGTGGAGGTT GTAGTAATGG GGTGGTTAAT   
  
  
- ATTAAGGGCA ATATGGGACC TGGGAGGGAG GTGGTGGTGG TGTCGGTGTT GGTGGTGGAG TAGGAGGTGA   
  
  
- GGGTGGGTGG TACCGAAGCC ACCACCCGAA CTGAGGAGGA GGGGCAGACG GCGACACCTT AATAGAAAGA   
  
  
- GGGGCCTGTA GGAGGAGAGG AGGGGGTGGC CTTTCACCCG CAGACTGTAA GAATACCTCC GCCGATCCCG   
  
  
- AAAAAGTCTG TGCTTGTGGT GGTTCGAGGT GGTCGAGGAG ACCTACGACT TGCTCGAGCC GAGGGGGATG   
  
  
- CCACTACAGC TCGTCTTCGA CCACCGCATG AAATTGGTTC GGGAAAAACG GGCGGAGTGA CGGTGGCCTG   
  
  
- GGGCGGTGGC TCGGCGATAC GAGCGGCGAC GCCTGTTCTG GAGGAGGAAG CTGAGGTGCG AGTCCAACGA   
  
  
- GGAGTTCAAG GTCCTTCAGT CGGGCACCTG GTGGAAGCCG GTGCACCGAA CGTTGCCACG TTAGTAGCTC   
  
  
- CGGAATCTCT CCCTGGGTGT ACTTTTCTCA TTCGAGGTGC AGTAGCTATA GTCGTGGTGG AAGACGTGGG   
  
  
- TCACCGGGTG GAACGAACTC CGAAATCGGT GGGCGTACCT GCTGTGAGGG GTGGAGTCGG AGTGACGGCA   
  
  
- GCAGCAGTTG TTCATGCGGC GGCACTCGCC ACCGCTCCTG CTGCCCCCTC TTCCTAGTTT CTCCCACTAC   
  
  
- TCTCTCTAAC CAGAAGCAGA ACTCTTCAAG CGATCCAATT ACCCCCAAGG GAAGTTCAAG TTGCACCAAA   
  
  
- TGGTGCATCC ACTAGATAGG GTCAACCTAA AAAGAGCAAA CCTATAATTT CTACTACTTC GCCACCGCTA   
  
  
- ATTGACACAG TTGAGCGAGG TGAGCCACCT GGAGGCTATA GCCCTCCATC ACGAGCGCCG CAAGTCCGCC   
  
  
- GACGCCGGCT CCCACCACTG CCACCACCTC CTTCTCCGCC TCGACTGACT CCACCCACTC CCAGTCATAC   
  
  
- TTAAAAAATC CGCCAAACTC CTCACAGAAT CCACCAAATC CCAAATGAAA CTCCGGCATC CTCTAACAAA   
  
  
- AGGATCCTGA TCGTTACTCT TTAACTACAA TCTCGCCCGG CGCCCGGCCC GGTACCACCT AGAGGAACGT   
  
  
- ACAGGGCTCG GACCACCCAG CCGACTCCCC TCCCTCTGCC GCTCTGCCAC CAGCCCAGCC TACGTACCCC   
  
  
- GCCCCAAGCT CGTGCACCCC AAGTCACTGC TTCACACACT ACTACACTCC CGGAACAACT CCTCCATGTT   
  
  
- CCTTCCTACC AGTTACTGTG TCACGTGGTA CCTACCGCCA TTAGTGGGAC CTTATGACAA CACCACCGCC   
  
  
- CTAGTTGGCC ACCAAACCCG GTCACGTACC GCCGGAAC

+     Myb

| Site Name | Organism | Position | Strand | Matrix score. | sequence | function |
| --- | --- | --- | --- | --- | --- | --- |
| Myb | Arabidopsis thaliana | 2945 | + | 6 | TAACTG |  |
| Myb | Arabidopsis thaliana | 2895 | - | 6 | CAACTG |  |

>HU05G01983.1   
+ +Up\_Stream \_Len000AAATCA AACTCATAAA CAATAATTTT TTAAAACTTA AATGGTAATT ATTAGATTAA   
  
  
+ TAAAAATCCT AATGGTTGTT TAATGTTACA TCAAGACATG CTTTTTGGTG TATATATAAT GGCAGCATGT   
  
  
+ GGGTTAAGCG GCAACAAATT ATTCTCAAAT GCATGCAGTA ATTAATTTAA TTAATTAATC ACCTGCATAA   
  
  
+ TCTCAATACT AATCCTTTCA TTCTGTGCCC CACCCAACCC CGAAAATAGT AATCTTTCAA ACGTTGGAAG   
  
  
+ TGGGATTAGG TATAGAGTAG TCCTACAGAT GATGATCTAC CTACATATAA CATAATCTGA CTTTTCTTTT   
  
  
+ TTAAATAACT TGTATTGATG GCACAGAATT TGACATGTTG GCATATGGTA CTAAGAAATT AATTAACGTT   
  
  
+ GTGCTAAGAT ATAGGTCATT CTTTTTGGAG ACATAAGGCC CGCGTTCACG CAGGGTTCGA GAAGGGCCAC   
  
  
+ ATTCAATGGA TGAATTGTAG GTAGTTTAAT CTGACTTTGT CAGTGACTGA TTCCACGGCT TGAACCCGCG   
  
  
+ ACATTAATAT AGGTTGACTA AAGTCGTTTG ATACTTATTT TTCTTAAGTA AAGTCTTGAA TTCAAATCTT   
  
  
+ ATAAATAAAT AAAAAAATCT ATAATAAAAG AATTCTACCA TTTATTAAGT CAACAACTCA ATTCGAATGA   
  
  
+ AATAAAAAAT CAAGATCACC AAAAAAAATA AATAAAAGGA AAGGTAGATT GCATTTGGAT TTTGAGTGGC   
  
  
+ GTGGTGGTGG GTTTGATGCT GGTGCTGCAT ACACCGCAGC TTTTCTCTGT CCTTTTACCT CTCTTTGTCT   
  
  
+ GCAAGAACCC CATTTGGCAC GCCTGCTCTC TTTTCTATGT TTTCTTTTCT ATCTTTTAAG AAACAATGGA   
  
  
+ AAGCTAACAC ACTAACACTA ACCCATTGGT CCTTTTTTCT TATCTGCAAA ATTAAAGTAA CAATATTTTT   
  
  
+ CTTATCCCCT TTTTCGTTCC ACCCATTTCT TTATCCTTCT GTTTGAACCT AATCTGTATA TATGCATGCA   
  
  
+ TGTGTATTTT ACTTAGAATC TGTTTTGATT CTTTGATGTA ATTAATTAGT AGTCTTTTTA ATTGAATTTT   
  
  
+ TAACAGTAGT TTGAATTAGA CATTTATCGA TATTATATTT TTTATATATA ATTAAAAAAT ACAAAATGAT   
  
  
+ AAGTGTCTTA CTACAATACA CTTCATAAGT GTCGTGTTTT AACTTAAAAA TCATACTATT ATGTTTCTAA   
  
  
+ ATTGAAAAAA AAGTGAGATT AAGTAGCAGA AGCATATTAC AACTTATAAA TTATATAAAA TCATCTTTTT   
  
  
+ ATCCATAATT TATCACATTT CTAAAATACG CATATCATAT CTACTTATGG ATAGGGTTAA TAAGATTAGG   
  
  
+ GCATCATTTA AGATTTTATC AGCGTAACCA ACGAATGTTT AAGGTTAGCA GTTCATAATT AAGTGTAATC   
  
  
+ ATTAAAAAAT TTATGATTTT AAATCATAAA ATATGTTAAT CTAGGATGAG GCAAGCGGCA AGACATATAT   
  
  
+ CACTATCCTT AAAAAATATA ATATATTGAT TTCAAAGAAT TATGAAGTAT ATTAATTTCA TATATAATGA   
  
  
+ CGATTATGTA TTTTTTTACT AAATTACTTG TACCCATAAT AAATAGAATC GATGCATCAT TCATGGCCTT   
  
  
+ TCATTCTGTA ATATGGTTGA CTCCAAAAAT TCTCAAAGTA TTTTTATATT TTGGGAGGGG AGATTTTAGT   
  
  
+ TTGATACTTT TGTTGAACTA GTGAACAATT TCCAACAACC TCCACCACCC CCAAACATCA ACAATCTGAT   
  
  
+ TGATGTATGC ATCCCAAGCT TGAATTGAAT AATAATTTCA ATAACAACAA TAATCGTTTT ACCTTGACAA   
  
  
+ GCTCCACAGC AACTCAACGT CTTCCAAACC AACCCATCAC TCATCATCAT CATCCCCACT ATCCCATTCA   
  
  
+ CCCCCACCAA AAGGAGCCTA AAGACAGTAA CAAGAAGAAT GCTTGCCTTT TTTCATGGAT GAAGAAGACT   
  
  
+ TCTCTTCTTC CTCTACTTCT TCCCACCACT ATGATACACA CCACCTCCAA CATCATTACC CCACCAATTA   
  
  
+ TAATTCCCGT TATACCCTGG ACCCTCCCTC CACCACCACC ACAGCCACAA CCACCACCTC ATCCTCCACT   
  
  
+ CCCACCCACC ATGGCTTCGG TGGTGGGCTT GACTCCTCCT CCCCGTCTGC CGCTGTGGAA TTATCTTTCT   
  
  
+ CCCCGGACAT CCTCCTCTCC TCCCCCACCG GAAAGTGGGC GTCTGACATT CTTATGGAGG CGGCTAGGGC   
  
  
+ TTTTTCAGAC ACGAACACCA CCAAGCTCCA CCAGCTCCTC TGGATGCTGA ACGAGCTCGG CTCCCCCTAC   
  
  
+ GGTGATGTCG AGCAGAAGCT GGTGGCGTAC TTTAACCAAG CCCTTTTTGC CCGCCTCACT GCCACCGGAC   
  
  
+ CCCGCCACCG AGCCGCTATG CTCGCCGCTG CGGACAAGAC CTCCTCCTTC GACTCCACGC TCAGGTTGCT   
  
  
+ CCTCAAGTTC CAGGAAGTCA GCCCGTGGAC CACCTTCGGC CACGTGGCTT GCAACGGTGC AATCATCGAG   
  
  
+ GCCTTAGAGA GGGACCCACA TGAAAAGAGT AAGCTCCACG TCATCGATAT CAGCACCACC TTCTGCACCC   
  
  
+ AGTGGCCCAC CTTGCTTGAG GCTTTAGCCA CCCGCATGGA CGACACTCCC CACCTCAGCC TCACTGCCGT   
  
  
+ CGTCGTCAAC AAGTACGCCG CCGTGAGCGG TGGCGAGGAC GACGGGGGAG AAGGATCAAA GAGGGTGATG   
  
  
+ AGAGAGATTG GTCTTCGTCT TGAGAAGTTC GCTAGGTTAA TGGGGGTTCC CTTCAAGTTC AACGTGGTTT   
  
  
+ ACCACGTAGG TGATCTATCC CAGTTGGATT TTTCTCGTTT GGATATTAAA GATGATGAAG CGGTGGCGAT   
  
  
+ TAACTGTGTC AACTCGCTCC ACTCGGTGGA CCTCCGATAT CGGGAGGTAG TGCTCGCGGC GTTCAGGCGG   
  
  
+ CTGCGGCCGA GGGTGGTGAC GGTGGTGGAG GAAGAGGCGG AGCTGACTGA GGTGGGTGAG GGTCAGTATG   
  
  
+ AATTTTTTAG GCGGTTTGAG GAGTGTCTTA GGTGGTTTAG GGTTTACTTT GAGGCCGTAG GAGATTGTTT   
  
  
+ TCCTAGGACT AGCAATGAGA AATTGATGTT AGAGCGGGCC GCGGGCCGGG CCATGGTGGA TCTCCTTGCA   
  
  
+ TGTCCCGAGC CTGGTGGGTC GGCTGAGGGG AGGGAGACGG CGAGACGGTG GTCGGGTCGG ATGCATGGGG   
  
  
+ CGGGGTTCGA GCACGTGGGG TTCAGTGACG AAGTGTGTGA TGATGTGAGG GCCTTGTTGA GGAGGTACAA   
  
  
+ GGAAGGATGG TCAATGACAC AGTGCACCAT GGATGGCGGT AATCACCCTG GAATACTGTT GTGGTGGCGG   
  
  
+ GATCAACCGG TGGTTTGGGC CAGTGCATGG CGGCCTTG  

- +Up\_Stream \_Len000TTTAGT TTGAGTATTT GTTATTAAAA AATTTTGAAT TTACCATTAA TAATCTAATT   
  
  
- ATTTTTAGGA TTACCAACAA ATTACAATGT AGTTCTGTAC GAAAAACCAC ATATATATTA CCGTCGTACA   
  
  
- CCCAATTCGC CGTTGTTTAA TAAGAGTTTA CGTACGTCAT TAATTAAATT AATTAATTAG TGGACGTATT   
  
  
- AGAGTTATGA TTAGGAAAGT AAGACACGGG GTGGGTTGGG GCTTTTATCA TTAGAAAGTT TGCAACCTTC   
  
  
- ACCCTAATCC ATATCTCATC AGGATGTCTA CTACTAGATG GATGTATATT GTATTAGACT GAAAAGAAAA   
  
  
- AATTTATTGA ACATAACTAC CGTGTCTTAA ACTGTACAAC CGTATACCAT GATTCTTTAA TTAATTGCAA   
  
  
- CACGATTCTA TATCCAGTAA GAAAAACCTC TGTATTCCGG GCGCAAGTGC GTCCCAAGCT CTTCCCGGTG   
  
  
- TAAGTTACCT ACTTAACATC CATCAAATTA GACTGAAACA GTCACTGACT AAGGTGCCGA ACTTGGGCGC   
  
  
- TGTAATTATA TCCAACTGAT TTCAGCAAAC TATGAATAAA AAGAATTCAT TTCAGAACTT AAGTTTAGAA   
  
  
- TATTTATTTA TTTTTTTAGA TATTATTTTC TTAAGATGGT AAATAATTCA GTTGTTGAGT TAAGCTTACT   
  
  
- TTATTTTTTA GTTCTAGTGG TTTTTTTTAT TTATTTTCCT TTCCATCTAA CGTAAACCTA AAACTCACCG   
  
  
- CACCACCACC CAAACTACGA CCACGACGTA TGTGGCGTCG AAAAGAGACA GGAAAATGGA GAGAAACAGA   
  
  
- CGTTCTTGGG GTAAACCGTG CGGACGAGAG AAAAGATACA AAAGAAAAGA TAGAAAATTC TTTGTTACCT   
  
  
- TTCGATTGTG TGATTGTGAT TGGGTAACCA GGAAAAAAGA ATAGACGTTT TAATTTCATT GTTATAAAAA   
  
  
- GAATAGGGGA AAAAGCAAGG TGGGTAAAGA AATAGGAAGA CAAACTTGGA TTAGACATAT ATACGTACGT   
  
  
- ACACATAAAA TGAATCTTAG ACAAAACTAA GAAACTACAT TAATTAATCA TCAGAAAAAT TAACTTAAAA   
  
  
- ATTGTCATCA AACTTAATCT GTAAATAGCT ATAATATAAA AAATATATAT TAATTTTTTA TGTTTTACTA   
  
  
- TTCACAGAAT GATGTTATGT GAAGTATTCA CAGCACAAAA TTGAATTTTT AGTATGATAA TACAAAGATT   
  
  
- TAACTTTTTT TTCACTCTAA TTCATCGTCT TCGTATAATG TTGAATATTT AATATATTTT AGTAGAAAAA   
  
  
- TAGGTATTAA ATAGTGTAAA GATTTTATGC GTATAGTATA GATGAATACC TATCCCAATT ATTCTAATCC   
  
  
- CGTAGTAAAT TCTAAAATAG TCGCATTGGT TGCTTACAAA TTCCAATCGT CAAGTATTAA TTCACATTAG   
  
  
- TAATTTTTTA AATACTAAAA TTTAGTATTT TATACAATTA GATCCTACTC CGTTCGCCGT TCTGTATATA   
  
  
- GTGATAGGAA TTTTTTATAT TATATAACTA AAGTTTCTTA ATACTTCATA TAATTAAAGT ATATATTACT   
  
  
- GCTAATACAT AAAAAAATGA TTTAATGAAC ATGGGTATTA TTTATCTTAG CTACGTAGTA AGTACCGGAA   
  
  
- AGTAAGACAT TATACCAACT GAGGTTTTTA AGAGTTTCAT AAAAATATAA AACCCTCCCC TCTAAAATCA   
  
  
- AACTATGAAA ACAACTTGAT CACTTGTTAA AGGTTGTTGG AGGTGGTGGG GGTTTGTAGT TGTTAGACTA   
  
  
- ACTACATACG TAGGGTTCGA ACTTAACTTA TTATTAAAGT TATTGTTGTT ATTAGCAAAA TGGAACTGTT   
  
  
- CGAGGTGTCG TTGAGTTGCA GAAGGTTTGG TTGGGTAGTG AGTAGTAGTA GTAGGGGTGA TAGGGTAAGT   
  
  
- GGGGGTGGTT TTCCTCGGAT TTCTGTCATT GTTCTTCTTA CGAACGGAAA AAAGTACCTA CTTCTTCTGA   
  
  
- AGAGAAGAAG GAGATGAAGA AGGGTGGTGA TACTATGTGT GGTGGAGGTT GTAGTAATGG GGTGGTTAAT   
  
  
- ATTAAGGGCA ATATGGGACC TGGGAGGGAG GTGGTGGTGG TGTCGGTGTT GGTGGTGGAG TAGGAGGTGA   
  
  
- GGGTGGGTGG TACCGAAGCC ACCACCCGAA CTGAGGAGGA GGGGCAGACG GCGACACCTT AATAGAAAGA   
  
  
- GGGGCCTGTA GGAGGAGAGG AGGGGGTGGC CTTTCACCCG CAGACTGTAA GAATACCTCC GCCGATCCCG   
  
  
- AAAAAGTCTG TGCTTGTGGT GGTTCGAGGT GGTCGAGGAG ACCTACGACT TGCTCGAGCC GAGGGGGATG   
  
  
- CCACTACAGC TCGTCTTCGA CCACCGCATG AAATTGGTTC GGGAAAAACG GGCGGAGTGA CGGTGGCCTG   
  
  
- GGGCGGTGGC TCGGCGATAC GAGCGGCGAC GCCTGTTCTG GAGGAGGAAG CTGAGGTGCG AGTCCAACGA   
  
  
- GGAGTTCAAG GTCCTTCAGT CGGGCACCTG GTGGAAGCCG GTGCACCGAA CGTTGCCACG TTAGTAGCTC   
  
  
- CGGAATCTCT CCCTGGGTGT ACTTTTCTCA TTCGAGGTGC AGTAGCTATA GTCGTGGTGG AAGACGTGGG   
  
  
- TCACCGGGTG GAACGAACTC CGAAATCGGT GGGCGTACCT GCTGTGAGGG GTGGAGTCGG AGTGACGGCA   
  
  
- GCAGCAGTTG TTCATGCGGC GGCACTCGCC ACCGCTCCTG CTGCCCCCTC TTCCTAGTTT CTCCCACTAC   
  
  
- TCTCTCTAAC CAGAAGCAGA ACTCTTCAAG CGATCCAATT ACCCCCAAGG GAAGTTCAAG TTGCACCAAA   
  
  
- TGGTGCATCC ACTAGATAGG GTCAACCTAA AAAGAGCAAA CCTATAATTT CTACTACTTC GCCACCGCTA   
  
  
- ATTGACACAG TTGAGCGAGG TGAGCCACCT GGAGGCTATA GCCCTCCATC ACGAGCGCCG CAAGTCCGCC   
  
  
- GACGCCGGCT CCCACCACTG CCACCACCTC CTTCTCCGCC TCGACTGACT CCACCCACTC CCAGTCATAC   
  
  
- TTAAAAAATC CGCCAAACTC CTCACAGAAT CCACCAAATC CCAAATGAAA CTCCGGCATC CTCTAACAAA   
  
  
- AGGATCCTGA TCGTTACTCT TTAACTACAA TCTCGCCCGG CGCCCGGCCC GGTACCACCT AGAGGAACGT   
  
  
- ACAGGGCTCG GACCACCCAG CCGACTCCCC TCCCTCTGCC GCTCTGCCAC CAGCCCAGCC TACGTACCCC   
  
  
- GCCCCAAGCT CGTGCACCCC AAGTCACTGC TTCACACACT ACTACACTCC CGGAACAACT CCTCCATGTT   
  
  
- CCTTCCTACC AGTTACTGTG TCACGTGGTA CCTACCGCCA TTAGTGGGAC CTTATGACAA CACCACCGCC   
  
  
- CTAGTTGGCC ACCAAACCCG GTCACGTACC GCCGGAAC

+     Myb-binding site

| Site Name | Organism | Position | Strand | Matrix score. | sequence | function |
| --- | --- | --- | --- | --- | --- | --- |
| Myb-binding site | Nicotiana tabacum | 3420 | - | 6 | CAACAG |  |

>HU05G01983.1   
+ +Up\_Stream \_Len000AAATCA AACTCATAAA CAATAATTTT TTAAAACTTA AATGGTAATT ATTAGATTAA   
  
  
+ TAAAAATCCT AATGGTTGTT TAATGTTACA TCAAGACATG CTTTTTGGTG TATATATAAT GGCAGCATGT   
  
  
+ GGGTTAAGCG GCAACAAATT ATTCTCAAAT GCATGCAGTA ATTAATTTAA TTAATTAATC ACCTGCATAA   
  
  
+ TCTCAATACT AATCCTTTCA TTCTGTGCCC CACCCAACCC CGAAAATAGT AATCTTTCAA ACGTTGGAAG   
  
  
+ TGGGATTAGG TATAGAGTAG TCCTACAGAT GATGATCTAC CTACATATAA CATAATCTGA CTTTTCTTTT   
  
  
+ TTAAATAACT TGTATTGATG GCACAGAATT TGACATGTTG GCATATGGTA CTAAGAAATT AATTAACGTT   
  
  
+ GTGCTAAGAT ATAGGTCATT CTTTTTGGAG ACATAAGGCC CGCGTTCACG CAGGGTTCGA GAAGGGCCAC   
  
  
+ ATTCAATGGA TGAATTGTAG GTAGTTTAAT CTGACTTTGT CAGTGACTGA TTCCACGGCT TGAACCCGCG   
  
  
+ ACATTAATAT AGGTTGACTA AAGTCGTTTG ATACTTATTT TTCTTAAGTA AAGTCTTGAA TTCAAATCTT   
  
  
+ ATAAATAAAT AAAAAAATCT ATAATAAAAG AATTCTACCA TTTATTAAGT CAACAACTCA ATTCGAATGA   
  
  
+ AATAAAAAAT CAAGATCACC AAAAAAAATA AATAAAAGGA AAGGTAGATT GCATTTGGAT TTTGAGTGGC   
  
  
+ GTGGTGGTGG GTTTGATGCT GGTGCTGCAT ACACCGCAGC TTTTCTCTGT CCTTTTACCT CTCTTTGTCT   
  
  
+ GCAAGAACCC CATTTGGCAC GCCTGCTCTC TTTTCTATGT TTTCTTTTCT ATCTTTTAAG AAACAATGGA   
  
  
+ AAGCTAACAC ACTAACACTA ACCCATTGGT CCTTTTTTCT TATCTGCAAA ATTAAAGTAA CAATATTTTT   
  
  
+ CTTATCCCCT TTTTCGTTCC ACCCATTTCT TTATCCTTCT GTTTGAACCT AATCTGTATA TATGCATGCA   
  
  
+ TGTGTATTTT ACTTAGAATC TGTTTTGATT CTTTGATGTA ATTAATTAGT AGTCTTTTTA ATTGAATTTT   
  
  
+ TAACAGTAGT TTGAATTAGA CATTTATCGA TATTATATTT TTTATATATA ATTAAAAAAT ACAAAATGAT   
  
  
+ AAGTGTCTTA CTACAATACA CTTCATAAGT GTCGTGTTTT AACTTAAAAA TCATACTATT ATGTTTCTAA   
  
  
+ ATTGAAAAAA AAGTGAGATT AAGTAGCAGA AGCATATTAC AACTTATAAA TTATATAAAA TCATCTTTTT   
  
  
+ ATCCATAATT TATCACATTT CTAAAATACG CATATCATAT CTACTTATGG ATAGGGTTAA TAAGATTAGG   
  
  
+ GCATCATTTA AGATTTTATC AGCGTAACCA ACGAATGTTT AAGGTTAGCA GTTCATAATT AAGTGTAATC   
  
  
+ ATTAAAAAAT TTATGATTTT AAATCATAAA ATATGTTAAT CTAGGATGAG GCAAGCGGCA AGACATATAT   
  
  
+ CACTATCCTT AAAAAATATA ATATATTGAT TTCAAAGAAT TATGAAGTAT ATTAATTTCA TATATAATGA   
  
  
+ CGATTATGTA TTTTTTTACT AAATTACTTG TACCCATAAT AAATAGAATC GATGCATCAT TCATGGCCTT   
  
  
+ TCATTCTGTA ATATGGTTGA CTCCAAAAAT TCTCAAAGTA TTTTTATATT TTGGGAGGGG AGATTTTAGT   
  
  
+ TTGATACTTT TGTTGAACTA GTGAACAATT TCCAACAACC TCCACCACCC CCAAACATCA ACAATCTGAT   
  
  
+ TGATGTATGC ATCCCAAGCT TGAATTGAAT AATAATTTCA ATAACAACAA TAATCGTTTT ACCTTGACAA   
  
  
+ GCTCCACAGC AACTCAACGT CTTCCAAACC AACCCATCAC TCATCATCAT CATCCCCACT ATCCCATTCA   
  
  
+ CCCCCACCAA AAGGAGCCTA AAGACAGTAA CAAGAAGAAT GCTTGCCTTT TTTCATGGAT GAAGAAGACT   
  
  
+ TCTCTTCTTC CTCTACTTCT TCCCACCACT ATGATACACA CCACCTCCAA CATCATTACC CCACCAATTA   
  
  
+ TAATTCCCGT TATACCCTGG ACCCTCCCTC CACCACCACC ACAGCCACAA CCACCACCTC ATCCTCCACT   
  
  
+ CCCACCCACC ATGGCTTCGG TGGTGGGCTT GACTCCTCCT CCCCGTCTGC CGCTGTGGAA TTATCTTTCT   
  
  
+ CCCCGGACAT CCTCCTCTCC TCCCCCACCG GAAAGTGGGC GTCTGACATT CTTATGGAGG CGGCTAGGGC   
  
  
+ TTTTTCAGAC ACGAACACCA CCAAGCTCCA CCAGCTCCTC TGGATGCTGA ACGAGCTCGG CTCCCCCTAC   
  
  
+ GGTGATGTCG AGCAGAAGCT GGTGGCGTAC TTTAACCAAG CCCTTTTTGC CCGCCTCACT GCCACCGGAC   
  
  
+ CCCGCCACCG AGCCGCTATG CTCGCCGCTG CGGACAAGAC CTCCTCCTTC GACTCCACGC TCAGGTTGCT   
  
  
+ CCTCAAGTTC CAGGAAGTCA GCCCGTGGAC CACCTTCGGC CACGTGGCTT GCAACGGTGC AATCATCGAG   
  
  
+ GCCTTAGAGA GGGACCCACA TGAAAAGAGT AAGCTCCACG TCATCGATAT CAGCACCACC TTCTGCACCC   
  
  
+ AGTGGCCCAC CTTGCTTGAG GCTTTAGCCA CCCGCATGGA CGACACTCCC CACCTCAGCC TCACTGCCGT   
  
  
+ CGTCGTCAAC AAGTACGCCG CCGTGAGCGG TGGCGAGGAC GACGGGGGAG AAGGATCAAA GAGGGTGATG   
  
  
+ AGAGAGATTG GTCTTCGTCT TGAGAAGTTC GCTAGGTTAA TGGGGGTTCC CTTCAAGTTC AACGTGGTTT   
  
  
+ ACCACGTAGG TGATCTATCC CAGTTGGATT TTTCTCGTTT GGATATTAAA GATGATGAAG CGGTGGCGAT   
  
  
+ TAACTGTGTC AACTCGCTCC ACTCGGTGGA CCTCCGATAT CGGGAGGTAG TGCTCGCGGC GTTCAGGCGG   
  
  
+ CTGCGGCCGA GGGTGGTGAC GGTGGTGGAG GAAGAGGCGG AGCTGACTGA GGTGGGTGAG GGTCAGTATG   
  
  
+ AATTTTTTAG GCGGTTTGAG GAGTGTCTTA GGTGGTTTAG GGTTTACTTT GAGGCCGTAG GAGATTGTTT   
  
  
+ TCCTAGGACT AGCAATGAGA AATTGATGTT AGAGCGGGCC GCGGGCCGGG CCATGGTGGA TCTCCTTGCA   
  
  
+ TGTCCCGAGC CTGGTGGGTC GGCTGAGGGG AGGGAGACGG CGAGACGGTG GTCGGGTCGG ATGCATGGGG   
  
  
+ CGGGGTTCGA GCACGTGGGG TTCAGTGACG AAGTGTGTGA TGATGTGAGG GCCTTGTTGA GGAGGTACAA   
  
  
+ GGAAGGATGG TCAATGACAC AGTGCACCAT GGATGGCGGT AATCACCCTG GAATACTGTT GTGGTGGCGG   
  
  
+ GATCAACCGG TGGTTTGGGC CAGTGCATGG CGGCCTTG  

- +Up\_Stream \_Len000TTTAGT TTGAGTATTT GTTATTAAAA AATTTTGAAT TTACCATTAA TAATCTAATT   
  
  
- ATTTTTAGGA TTACCAACAA ATTACAATGT AGTTCTGTAC GAAAAACCAC ATATATATTA CCGTCGTACA   
  
  
- CCCAATTCGC CGTTGTTTAA TAAGAGTTTA CGTACGTCAT TAATTAAATT AATTAATTAG TGGACGTATT   
  
  
- AGAGTTATGA TTAGGAAAGT AAGACACGGG GTGGGTTGGG GCTTTTATCA TTAGAAAGTT TGCAACCTTC   
  
  
- ACCCTAATCC ATATCTCATC AGGATGTCTA CTACTAGATG GATGTATATT GTATTAGACT GAAAAGAAAA   
  
  
- AATTTATTGA ACATAACTAC CGTGTCTTAA ACTGTACAAC CGTATACCAT GATTCTTTAA TTAATTGCAA   
  
  
- CACGATTCTA TATCCAGTAA GAAAAACCTC TGTATTCCGG GCGCAAGTGC GTCCCAAGCT CTTCCCGGTG   
  
  
- TAAGTTACCT ACTTAACATC CATCAAATTA GACTGAAACA GTCACTGACT AAGGTGCCGA ACTTGGGCGC   
  
  
- TGTAATTATA TCCAACTGAT TTCAGCAAAC TATGAATAAA AAGAATTCAT TTCAGAACTT AAGTTTAGAA   
  
  
- TATTTATTTA TTTTTTTAGA TATTATTTTC TTAAGATGGT AAATAATTCA GTTGTTGAGT TAAGCTTACT   
  
  
- TTATTTTTTA GTTCTAGTGG TTTTTTTTAT TTATTTTCCT TTCCATCTAA CGTAAACCTA AAACTCACCG   
  
  
- CACCACCACC CAAACTACGA CCACGACGTA TGTGGCGTCG AAAAGAGACA GGAAAATGGA GAGAAACAGA   
  
  
- CGTTCTTGGG GTAAACCGTG CGGACGAGAG AAAAGATACA AAAGAAAAGA TAGAAAATTC TTTGTTACCT   
  
  
- TTCGATTGTG TGATTGTGAT TGGGTAACCA GGAAAAAAGA ATAGACGTTT TAATTTCATT GTTATAAAAA   
  
  
- GAATAGGGGA AAAAGCAAGG TGGGTAAAGA AATAGGAAGA CAAACTTGGA TTAGACATAT ATACGTACGT   
  
  
- ACACATAAAA TGAATCTTAG ACAAAACTAA GAAACTACAT TAATTAATCA TCAGAAAAAT TAACTTAAAA   
  
  
- ATTGTCATCA AACTTAATCT GTAAATAGCT ATAATATAAA AAATATATAT TAATTTTTTA TGTTTTACTA   
  
  
- TTCACAGAAT GATGTTATGT GAAGTATTCA CAGCACAAAA TTGAATTTTT AGTATGATAA TACAAAGATT   
  
  
- TAACTTTTTT TTCACTCTAA TTCATCGTCT TCGTATAATG TTGAATATTT AATATATTTT AGTAGAAAAA   
  
  
- TAGGTATTAA ATAGTGTAAA GATTTTATGC GTATAGTATA GATGAATACC TATCCCAATT ATTCTAATCC   
  
  
- CGTAGTAAAT TCTAAAATAG TCGCATTGGT TGCTTACAAA TTCCAATCGT CAAGTATTAA TTCACATTAG   
  
  
- TAATTTTTTA AATACTAAAA TTTAGTATTT TATACAATTA GATCCTACTC CGTTCGCCGT TCTGTATATA   
  
  
- GTGATAGGAA TTTTTTATAT TATATAACTA AAGTTTCTTA ATACTTCATA TAATTAAAGT ATATATTACT   
  
  
- GCTAATACAT AAAAAAATGA TTTAATGAAC ATGGGTATTA TTTATCTTAG CTACGTAGTA AGTACCGGAA   
  
  
- AGTAAGACAT TATACCAACT GAGGTTTTTA AGAGTTTCAT AAAAATATAA AACCCTCCCC TCTAAAATCA   
  
  
- AACTATGAAA ACAACTTGAT CACTTGTTAA AGGTTGTTGG AGGTGGTGGG GGTTTGTAGT TGTTAGACTA   
  
  
- ACTACATACG TAGGGTTCGA ACTTAACTTA TTATTAAAGT TATTGTTGTT ATTAGCAAAA TGGAACTGTT   
  
  
- CGAGGTGTCG TTGAGTTGCA GAAGGTTTGG TTGGGTAGTG AGTAGTAGTA GTAGGGGTGA TAGGGTAAGT   
  
  
- GGGGGTGGTT TTCCTCGGAT TTCTGTCATT GTTCTTCTTA CGAACGGAAA AAAGTACCTA CTTCTTCTGA   
  
  
- AGAGAAGAAG GAGATGAAGA AGGGTGGTGA TACTATGTGT GGTGGAGGTT GTAGTAATGG GGTGGTTAAT   
  
  
- ATTAAGGGCA ATATGGGACC TGGGAGGGAG GTGGTGGTGG TGTCGGTGTT GGTGGTGGAG TAGGAGGTGA   
  
  
- GGGTGGGTGG TACCGAAGCC ACCACCCGAA CTGAGGAGGA GGGGCAGACG GCGACACCTT AATAGAAAGA   
  
  
- GGGGCCTGTA GGAGGAGAGG AGGGGGTGGC CTTTCACCCG CAGACTGTAA GAATACCTCC GCCGATCCCG   
  
  
- AAAAAGTCTG TGCTTGTGGT GGTTCGAGGT GGTCGAGGAG ACCTACGACT TGCTCGAGCC GAGGGGGATG   
  
  
- CCACTACAGC TCGTCTTCGA CCACCGCATG AAATTGGTTC GGGAAAAACG GGCGGAGTGA CGGTGGCCTG   
  
  
- GGGCGGTGGC TCGGCGATAC GAGCGGCGAC GCCTGTTCTG GAGGAGGAAG CTGAGGTGCG AGTCCAACGA   
  
  
- GGAGTTCAAG GTCCTTCAGT CGGGCACCTG GTGGAAGCCG GTGCACCGAA CGTTGCCACG TTAGTAGCTC   
  
  
- CGGAATCTCT CCCTGGGTGT ACTTTTCTCA TTCGAGGTGC AGTAGCTATA GTCGTGGTGG AAGACGTGGG   
  
  
- TCACCGGGTG GAACGAACTC CGAAATCGGT GGGCGTACCT GCTGTGAGGG GTGGAGTCGG AGTGACGGCA   
  
  
- GCAGCAGTTG TTCATGCGGC GGCACTCGCC ACCGCTCCTG CTGCCCCCTC TTCCTAGTTT CTCCCACTAC   
  
  
- TCTCTCTAAC CAGAAGCAGA ACTCTTCAAG CGATCCAATT ACCCCCAAGG GAAGTTCAAG TTGCACCAAA   
  
  
- TGGTGCATCC ACTAGATAGG GTCAACCTAA AAAGAGCAAA CCTATAATTT CTACTACTTC GCCACCGCTA   
  
  
- ATTGACACAG TTGAGCGAGG TGAGCCACCT GGAGGCTATA GCCCTCCATC ACGAGCGCCG CAAGTCCGCC   
  
  
- GACGCCGGCT CCCACCACTG CCACCACCTC CTTCTCCGCC TCGACTGACT CCACCCACTC CCAGTCATAC   
  
  
- TTAAAAAATC CGCCAAACTC CTCACAGAAT CCACCAAATC CCAAATGAAA CTCCGGCATC CTCTAACAAA   
  
  
- AGGATCCTGA TCGTTACTCT TTAACTACAA TCTCGCCCGG CGCCCGGCCC GGTACCACCT AGAGGAACGT   
  
  
- ACAGGGCTCG GACCACCCAG CCGACTCCCC TCCCTCTGCC GCTCTGCCAC CAGCCCAGCC TACGTACCCC   
  
  
- GCCCCAAGCT CGTGCACCCC AAGTCACTGC TTCACACACT ACTACACTCC CGGAACAACT CCTCCATGTT   
  
  
- CCTTCCTACC AGTTACTGTG TCACGTGGTA CCTACCGCCA TTAGTGGGAC CTTATGACAA CACCACCGCC   
  
  
- CTAGTTGGCC ACCAAACCCG GTCACGTACC GCCGGAAC

+     O2-site

| Site Name | Organism | Position | Strand | Matrix score. | sequence | function |
| --- | --- | --- | --- | --- | --- | --- |
| O2-site | Zea mays | 2630 | - | 9 | GATGATGTGG | cis-acting regulatory element involved in zein metabolism regulation |
| O2-site | Zea mays | 2158 | - | 9 | GATGATGTGG | cis-acting regulatory element involved in zein metabolism regulation |
| O2-site | Zea mays | 3333 | + | 9 | GATGATGTGG | cis-acting regulatory element involved in zein metabolism regulation |

>HU05G01983.1   
+ +Up\_Stream \_Len000AAATCA AACTCATAAA CAATAATTTT TTAAAACTTA AATGGTAATT ATTAGATTAA   
  
  
+ TAAAAATCCT AATGGTTGTT TAATGTTACA TCAAGACATG CTTTTTGGTG TATATATAAT GGCAGCATGT   
  
  
+ GGGTTAAGCG GCAACAAATT ATTCTCAAAT GCATGCAGTA ATTAATTTAA TTAATTAATC ACCTGCATAA   
  
  
+ TCTCAATACT AATCCTTTCA TTCTGTGCCC CACCCAACCC CGAAAATAGT AATCTTTCAA ACGTTGGAAG   
  
  
+ TGGGATTAGG TATAGAGTAG TCCTACAGAT GATGATCTAC CTACATATAA CATAATCTGA CTTTTCTTTT   
  
  
+ TTAAATAACT TGTATTGATG GCACAGAATT TGACATGTTG GCATATGGTA CTAAGAAATT AATTAACGTT   
  
  
+ GTGCTAAGAT ATAGGTCATT CTTTTTGGAG ACATAAGGCC CGCGTTCACG CAGGGTTCGA GAAGGGCCAC   
  
  
+ ATTCAATGGA TGAATTGTAG GTAGTTTAAT CTGACTTTGT CAGTGACTGA TTCCACGGCT TGAACCCGCG   
  
  
+ ACATTAATAT AGGTTGACTA AAGTCGTTTG ATACTTATTT TTCTTAAGTA AAGTCTTGAA TTCAAATCTT   
  
  
+ ATAAATAAAT AAAAAAATCT ATAATAAAAG AATTCTACCA TTTATTAAGT CAACAACTCA ATTCGAATGA   
  
  
+ AATAAAAAAT CAAGATCACC AAAAAAAATA AATAAAAGGA AAGGTAGATT GCATTTGGAT TTTGAGTGGC   
  
  
+ GTGGTGGTGG GTTTGATGCT GGTGCTGCAT ACACCGCAGC TTTTCTCTGT CCTTTTACCT CTCTTTGTCT   
  
  
+ GCAAGAACCC CATTTGGCAC GCCTGCTCTC TTTTCTATGT TTTCTTTTCT ATCTTTTAAG AAACAATGGA   
  
  
+ AAGCTAACAC ACTAACACTA ACCCATTGGT CCTTTTTTCT TATCTGCAAA ATTAAAGTAA CAATATTTTT   
  
  
+ CTTATCCCCT TTTTCGTTCC ACCCATTTCT TTATCCTTCT GTTTGAACCT AATCTGTATA TATGCATGCA   
  
  
+ TGTGTATTTT ACTTAGAATC TGTTTTGATT CTTTGATGTA ATTAATTAGT AGTCTTTTTA ATTGAATTTT   
  
  
+ TAACAGTAGT TTGAATTAGA CATTTATCGA TATTATATTT TTTATATATA ATTAAAAAAT ACAAAATGAT   
  
  
+ AAGTGTCTTA CTACAATACA CTTCATAAGT GTCGTGTTTT AACTTAAAAA TCATACTATT ATGTTTCTAA   
  
  
+ ATTGAAAAAA AAGTGAGATT AAGTAGCAGA AGCATATTAC AACTTATAAA TTATATAAAA TCATCTTTTT   
  
  
+ ATCCATAATT TATCACATTT CTAAAATACG CATATCATAT CTACTTATGG ATAGGGTTAA TAAGATTAGG   
  
  
+ GCATCATTTA AGATTTTATC AGCGTAACCA ACGAATGTTT AAGGTTAGCA GTTCATAATT AAGTGTAATC   
  
  
+ ATTAAAAAAT TTATGATTTT AAATCATAAA ATATGTTAAT CTAGGATGAG GCAAGCGGCA AGACATATAT   
  
  
+ CACTATCCTT AAAAAATATA ATATATTGAT TTCAAAGAAT TATGAAGTAT ATTAATTTCA TATATAATGA   
  
  
+ CGATTATGTA TTTTTTTACT AAATTACTTG TACCCATAAT AAATAGAATC GATGCATCAT TCATGGCCTT   
  
  
+ TCATTCTGTA ATATGGTTGA CTCCAAAAAT TCTCAAAGTA TTTTTATATT TTGGGAGGGG AGATTTTAGT   
  
  
+ TTGATACTTT TGTTGAACTA GTGAACAATT TCCAACAACC TCCACCACCC CCAAACATCA ACAATCTGAT   
  
  
+ TGATGTATGC ATCCCAAGCT TGAATTGAAT AATAATTTCA ATAACAACAA TAATCGTTTT ACCTTGACAA   
  
  
+ GCTCCACAGC AACTCAACGT CTTCCAAACC AACCCATCAC TCATCATCAT CATCCCCACT ATCCCATTCA   
  
  
+ CCCCCACCAA AAGGAGCCTA AAGACAGTAA CAAGAAGAAT GCTTGCCTTT TTTCATGGAT GAAGAAGACT   
  
  
+ TCTCTTCTTC CTCTACTTCT TCCCACCACT ATGATACACA CCACCTCCAA CATCATTACC CCACCAATTA   
  
  
+ TAATTCCCGT TATACCCTGG ACCCTCCCTC CACCACCACC ACAGCCACAA CCACCACCTC ATCCTCCACT   
  
  
+ CCCACCCACC ATGGCTTCGG TGGTGGGCTT GACTCCTCCT CCCCGTCTGC CGCTGTGGAA TTATCTTTCT   
  
  
+ CCCCGGACAT CCTCCTCTCC TCCCCCACCG GAAAGTGGGC GTCTGACATT CTTATGGAGG CGGCTAGGGC   
  
  
+ TTTTTCAGAC ACGAACACCA CCAAGCTCCA CCAGCTCCTC TGGATGCTGA ACGAGCTCGG CTCCCCCTAC   
  
  
+ GGTGATGTCG AGCAGAAGCT GGTGGCGTAC TTTAACCAAG CCCTTTTTGC CCGCCTCACT GCCACCGGAC   
  
  
+ CCCGCCACCG AGCCGCTATG CTCGCCGCTG CGGACAAGAC CTCCTCCTTC GACTCCACGC TCAGGTTGCT   
  
  
+ CCTCAAGTTC CAGGAAGTCA GCCCGTGGAC CACCTTCGGC CACGTGGCTT GCAACGGTGC AATCATCGAG   
  
  
+ GCCTTAGAGA GGGACCCACA TGAAAAGAGT AAGCTCCACG TCATCGATAT CAGCACCACC TTCTGCACCC   
  
  
+ AGTGGCCCAC CTTGCTTGAG GCTTTAGCCA CCCGCATGGA CGACACTCCC CACCTCAGCC TCACTGCCGT   
  
  
+ CGTCGTCAAC AAGTACGCCG CCGTGAGCGG TGGCGAGGAC GACGGGGGAG AAGGATCAAA GAGGGTGATG   
  
  
+ AGAGAGATTG GTCTTCGTCT TGAGAAGTTC GCTAGGTTAA TGGGGGTTCC CTTCAAGTTC AACGTGGTTT   
  
  
+ ACCACGTAGG TGATCTATCC CAGTTGGATT TTTCTCGTTT GGATATTAAA GATGATGAAG CGGTGGCGAT   
  
  
+ TAACTGTGTC AACTCGCTCC ACTCGGTGGA CCTCCGATAT CGGGAGGTAG TGCTCGCGGC GTTCAGGCGG   
  
  
+ CTGCGGCCGA GGGTGGTGAC GGTGGTGGAG GAAGAGGCGG AGCTGACTGA GGTGGGTGAG GGTCAGTATG   
  
  
+ AATTTTTTAG GCGGTTTGAG GAGTGTCTTA GGTGGTTTAG GGTTTACTTT GAGGCCGTAG GAGATTGTTT   
  
  
+ TCCTAGGACT AGCAATGAGA AATTGATGTT AGAGCGGGCC GCGGGCCGGG CCATGGTGGA TCTCCTTGCA   
  
  
+ TGTCCCGAGC CTGGTGGGTC GGCTGAGGGG AGGGAGACGG CGAGACGGTG GTCGGGTCGG ATGCATGGGG   
  
  
+ CGGGGTTCGA GCACGTGGGG TTCAGTGACG AAGTGTGTGA TGATGTGAGG GCCTTGTTGA GGAGGTACAA   
  
  
+ GGAAGGATGG TCAATGACAC AGTGCACCAT GGATGGCGGT AATCACCCTG GAATACTGTT GTGGTGGCGG   
  
  
+ GATCAACCGG TGGTTTGGGC CAGTGCATGG CGGCCTTG  

- +Up\_Stream \_Len000TTTAGT TTGAGTATTT GTTATTAAAA AATTTTGAAT TTACCATTAA TAATCTAATT   
  
  
- ATTTTTAGGA TTACCAACAA ATTACAATGT AGTTCTGTAC GAAAAACCAC ATATATATTA CCGTCGTACA   
  
  
- CCCAATTCGC CGTTGTTTAA TAAGAGTTTA CGTACGTCAT TAATTAAATT AATTAATTAG TGGACGTATT   
  
  
- AGAGTTATGA TTAGGAAAGT AAGACACGGG GTGGGTTGGG GCTTTTATCA TTAGAAAGTT TGCAACCTTC   
  
  
- ACCCTAATCC ATATCTCATC AGGATGTCTA CTACTAGATG GATGTATATT GTATTAGACT GAAAAGAAAA   
  
  
- AATTTATTGA ACATAACTAC CGTGTCTTAA ACTGTACAAC CGTATACCAT GATTCTTTAA TTAATTGCAA   
  
  
- CACGATTCTA TATCCAGTAA GAAAAACCTC TGTATTCCGG GCGCAAGTGC GTCCCAAGCT CTTCCCGGTG   
  
  
- TAAGTTACCT ACTTAACATC CATCAAATTA GACTGAAACA GTCACTGACT AAGGTGCCGA ACTTGGGCGC   
  
  
- TGTAATTATA TCCAACTGAT TTCAGCAAAC TATGAATAAA AAGAATTCAT TTCAGAACTT AAGTTTAGAA   
  
  
- TATTTATTTA TTTTTTTAGA TATTATTTTC TTAAGATGGT AAATAATTCA GTTGTTGAGT TAAGCTTACT   
  
  
- TTATTTTTTA GTTCTAGTGG TTTTTTTTAT TTATTTTCCT TTCCATCTAA CGTAAACCTA AAACTCACCG   
  
  
- CACCACCACC CAAACTACGA CCACGACGTA TGTGGCGTCG AAAAGAGACA GGAAAATGGA GAGAAACAGA   
  
  
- CGTTCTTGGG GTAAACCGTG CGGACGAGAG AAAAGATACA AAAGAAAAGA TAGAAAATTC TTTGTTACCT   
  
  
- TTCGATTGTG TGATTGTGAT TGGGTAACCA GGAAAAAAGA ATAGACGTTT TAATTTCATT GTTATAAAAA   
  
  
- GAATAGGGGA AAAAGCAAGG TGGGTAAAGA AATAGGAAGA CAAACTTGGA TTAGACATAT ATACGTACGT   
  
  
- ACACATAAAA TGAATCTTAG ACAAAACTAA GAAACTACAT TAATTAATCA TCAGAAAAAT TAACTTAAAA   
  
  
- ATTGTCATCA AACTTAATCT GTAAATAGCT ATAATATAAA AAATATATAT TAATTTTTTA TGTTTTACTA   
  
  
- TTCACAGAAT GATGTTATGT GAAGTATTCA CAGCACAAAA TTGAATTTTT AGTATGATAA TACAAAGATT   
  
  
- TAACTTTTTT TTCACTCTAA TTCATCGTCT TCGTATAATG TTGAATATTT AATATATTTT AGTAGAAAAA   
  
  
- TAGGTATTAA ATAGTGTAAA GATTTTATGC GTATAGTATA GATGAATACC TATCCCAATT ATTCTAATCC   
  
  
- CGTAGTAAAT TCTAAAATAG TCGCATTGGT TGCTTACAAA TTCCAATCGT CAAGTATTAA TTCACATTAG   
  
  
- TAATTTTTTA AATACTAAAA TTTAGTATTT TATACAATTA GATCCTACTC CGTTCGCCGT TCTGTATATA   
  
  
- GTGATAGGAA TTTTTTATAT TATATAACTA AAGTTTCTTA ATACTTCATA TAATTAAAGT ATATATTACT   
  
  
- GCTAATACAT AAAAAAATGA TTTAATGAAC ATGGGTATTA TTTATCTTAG CTACGTAGTA AGTACCGGAA   
  
  
- AGTAAGACAT TATACCAACT GAGGTTTTTA AGAGTTTCAT AAAAATATAA AACCCTCCCC TCTAAAATCA   
  
  
- AACTATGAAA ACAACTTGAT CACTTGTTAA AGGTTGTTGG AGGTGGTGGG GGTTTGTAGT TGTTAGACTA   
  
  
- ACTACATACG TAGGGTTCGA ACTTAACTTA TTATTAAAGT TATTGTTGTT ATTAGCAAAA TGGAACTGTT   
  
  
- CGAGGTGTCG TTGAGTTGCA GAAGGTTTGG TTGGGTAGTG AGTAGTAGTA GTAGGGGTGA TAGGGTAAGT   
  
  
- GGGGGTGGTT TTCCTCGGAT TTCTGTCATT GTTCTTCTTA CGAACGGAAA AAAGTACCTA CTTCTTCTGA   
  
  
- AGAGAAGAAG GAGATGAAGA AGGGTGGTGA TACTATGTGT GGTGGAGGTT GTAGTAATGG GGTGGTTAAT   
  
  
- ATTAAGGGCA ATATGGGACC TGGGAGGGAG GTGGTGGTGG TGTCGGTGTT GGTGGTGGAG TAGGAGGTGA   
  
  
- GGGTGGGTGG TACCGAAGCC ACCACCCGAA CTGAGGAGGA GGGGCAGACG GCGACACCTT AATAGAAAGA   
  
  
- GGGGCCTGTA GGAGGAGAGG AGGGGGTGGC CTTTCACCCG CAGACTGTAA GAATACCTCC GCCGATCCCG   
  
  
- AAAAAGTCTG TGCTTGTGGT GGTTCGAGGT GGTCGAGGAG ACCTACGACT TGCTCGAGCC GAGGGGGATG   
  
  
- CCACTACAGC TCGTCTTCGA CCACCGCATG AAATTGGTTC GGGAAAAACG GGCGGAGTGA CGGTGGCCTG   
  
  
- GGGCGGTGGC TCGGCGATAC GAGCGGCGAC GCCTGTTCTG GAGGAGGAAG CTGAGGTGCG AGTCCAACGA   
  
  
- GGAGTTCAAG GTCCTTCAGT CGGGCACCTG GTGGAAGCCG GTGCACCGAA CGTTGCCACG TTAGTAGCTC   
  
  
- CGGAATCTCT CCCTGGGTGT ACTTTTCTCA TTCGAGGTGC AGTAGCTATA GTCGTGGTGG AAGACGTGGG   
  
  
- TCACCGGGTG GAACGAACTC CGAAATCGGT GGGCGTACCT GCTGTGAGGG GTGGAGTCGG AGTGACGGCA   
  
  
- GCAGCAGTTG TTCATGCGGC GGCACTCGCC ACCGCTCCTG CTGCCCCCTC TTCCTAGTTT CTCCCACTAC   
  
  
- TCTCTCTAAC CAGAAGCAGA ACTCTTCAAG CGATCCAATT ACCCCCAAGG GAAGTTCAAG TTGCACCAAA   
  
  
- TGGTGCATCC ACTAGATAGG GTCAACCTAA AAAGAGCAAA CCTATAATTT CTACTACTTC GCCACCGCTA   
  
  
- ATTGACACAG TTGAGCGAGG TGAGCCACCT GGAGGCTATA GCCCTCCATC ACGAGCGCCG CAAGTCCGCC   
  
  
- GACGCCGGCT CCCACCACTG CCACCACCTC CTTCTCCGCC TCGACTGACT CCACCCACTC CCAGTCATAC   
  
  
- TTAAAAAATC CGCCAAACTC CTCACAGAAT CCACCAAATC CCAAATGAAA CTCCGGCATC CTCTAACAAA   
  
  
- AGGATCCTGA TCGTTACTCT TTAACTACAA TCTCGCCCGG CGCCCGGCCC GGTACCACCT AGAGGAACGT   
  
  
- ACAGGGCTCG GACCACCCAG CCGACTCCCC TCCCTCTGCC GCTCTGCCAC CAGCCCAGCC TACGTACCCC   
  
  
- GCCCCAAGCT CGTGCACCCC AAGTCACTGC TTCACACACT ACTACACTCC CGGAACAACT CCTCCATGTT   
  
  
- CCTTCCTACC AGTTACTGTG TCACGTGGTA CCTACCGCCA TTAGTGGGAC CTTATGACAA CACCACCGCC   
  
  
- CTAGTTGGCC ACCAAACCCG GTCACGTACC GCCGGAAC

+     P-box

| Site Name | Organism | Position | Strand | Matrix score. | sequence | function |
| --- | --- | --- | --- | --- | --- | --- |
| P-box | Oryza sativa | 1972 | - | 7 | CCTTTTG | gibberellin-responsive element |

>HU05G01983.1   
+ +Up\_Stream \_Len000AAATCA AACTCATAAA CAATAATTTT TTAAAACTTA AATGGTAATT ATTAGATTAA   
  
  
+ TAAAAATCCT AATGGTTGTT TAATGTTACA TCAAGACATG CTTTTTGGTG TATATATAAT GGCAGCATGT   
  
  
+ GGGTTAAGCG GCAACAAATT ATTCTCAAAT GCATGCAGTA ATTAATTTAA TTAATTAATC ACCTGCATAA   
  
  
+ TCTCAATACT AATCCTTTCA TTCTGTGCCC CACCCAACCC CGAAAATAGT AATCTTTCAA ACGTTGGAAG   
  
  
+ TGGGATTAGG TATAGAGTAG TCCTACAGAT GATGATCTAC CTACATATAA CATAATCTGA CTTTTCTTTT   
  
  
+ TTAAATAACT TGTATTGATG GCACAGAATT TGACATGTTG GCATATGGTA CTAAGAAATT AATTAACGTT   
  
  
+ GTGCTAAGAT ATAGGTCATT CTTTTTGGAG ACATAAGGCC CGCGTTCACG CAGGGTTCGA GAAGGGCCAC   
  
  
+ ATTCAATGGA TGAATTGTAG GTAGTTTAAT CTGACTTTGT CAGTGACTGA TTCCACGGCT TGAACCCGCG   
  
  
+ ACATTAATAT AGGTTGACTA AAGTCGTTTG ATACTTATTT TTCTTAAGTA AAGTCTTGAA TTCAAATCTT   
  
  
+ ATAAATAAAT AAAAAAATCT ATAATAAAAG AATTCTACCA TTTATTAAGT CAACAACTCA ATTCGAATGA   
  
  
+ AATAAAAAAT CAAGATCACC AAAAAAAATA AATAAAAGGA AAGGTAGATT GCATTTGGAT TTTGAGTGGC   
  
  
+ GTGGTGGTGG GTTTGATGCT GGTGCTGCAT ACACCGCAGC TTTTCTCTGT CCTTTTACCT CTCTTTGTCT   
  
  
+ GCAAGAACCC CATTTGGCAC GCCTGCTCTC TTTTCTATGT TTTCTTTTCT ATCTTTTAAG AAACAATGGA   
  
  
+ AAGCTAACAC ACTAACACTA ACCCATTGGT CCTTTTTTCT TATCTGCAAA ATTAAAGTAA CAATATTTTT   
  
  
+ CTTATCCCCT TTTTCGTTCC ACCCATTTCT TTATCCTTCT GTTTGAACCT AATCTGTATA TATGCATGCA   
  
  
+ TGTGTATTTT ACTTAGAATC TGTTTTGATT CTTTGATGTA ATTAATTAGT AGTCTTTTTA ATTGAATTTT   
  
  
+ TAACAGTAGT TTGAATTAGA CATTTATCGA TATTATATTT TTTATATATA ATTAAAAAAT ACAAAATGAT   
  
  
+ AAGTGTCTTA CTACAATACA CTTCATAAGT GTCGTGTTTT AACTTAAAAA TCATACTATT ATGTTTCTAA   
  
  
+ ATTGAAAAAA AAGTGAGATT AAGTAGCAGA AGCATATTAC AACTTATAAA TTATATAAAA TCATCTTTTT   
  
  
+ ATCCATAATT TATCACATTT CTAAAATACG CATATCATAT CTACTTATGG ATAGGGTTAA TAAGATTAGG   
  
  
+ GCATCATTTA AGATTTTATC AGCGTAACCA ACGAATGTTT AAGGTTAGCA GTTCATAATT AAGTGTAATC   
  
  
+ ATTAAAAAAT TTATGATTTT AAATCATAAA ATATGTTAAT CTAGGATGAG GCAAGCGGCA AGACATATAT   
  
  
+ CACTATCCTT AAAAAATATA ATATATTGAT TTCAAAGAAT TATGAAGTAT ATTAATTTCA TATATAATGA   
  
  
+ CGATTATGTA TTTTTTTACT AAATTACTTG TACCCATAAT AAATAGAATC GATGCATCAT TCATGGCCTT   
  
  
+ TCATTCTGTA ATATGGTTGA CTCCAAAAAT TCTCAAAGTA TTTTTATATT TTGGGAGGGG AGATTTTAGT   
  
  
+ TTGATACTTT TGTTGAACTA GTGAACAATT TCCAACAACC TCCACCACCC CCAAACATCA ACAATCTGAT   
  
  
+ TGATGTATGC ATCCCAAGCT TGAATTGAAT AATAATTTCA ATAACAACAA TAATCGTTTT ACCTTGACAA   
  
  
+ GCTCCACAGC AACTCAACGT CTTCCAAACC AACCCATCAC TCATCATCAT CATCCCCACT ATCCCATTCA   
  
  
+ CCCCCACCAA AAGGAGCCTA AAGACAGTAA CAAGAAGAAT GCTTGCCTTT TTTCATGGAT GAAGAAGACT   
  
  
+ TCTCTTCTTC CTCTACTTCT TCCCACCACT ATGATACACA CCACCTCCAA CATCATTACC CCACCAATTA   
  
  
+ TAATTCCCGT TATACCCTGG ACCCTCCCTC CACCACCACC ACAGCCACAA CCACCACCTC ATCCTCCACT   
  
  
+ CCCACCCACC ATGGCTTCGG TGGTGGGCTT GACTCCTCCT CCCCGTCTGC CGCTGTGGAA TTATCTTTCT   
  
  
+ CCCCGGACAT CCTCCTCTCC TCCCCCACCG GAAAGTGGGC GTCTGACATT CTTATGGAGG CGGCTAGGGC   
  
  
+ TTTTTCAGAC ACGAACACCA CCAAGCTCCA CCAGCTCCTC TGGATGCTGA ACGAGCTCGG CTCCCCCTAC   
  
  
+ GGTGATGTCG AGCAGAAGCT GGTGGCGTAC TTTAACCAAG CCCTTTTTGC CCGCCTCACT GCCACCGGAC   
  
  
+ CCCGCCACCG AGCCGCTATG CTCGCCGCTG CGGACAAGAC CTCCTCCTTC GACTCCACGC TCAGGTTGCT   
  
  
+ CCTCAAGTTC CAGGAAGTCA GCCCGTGGAC CACCTTCGGC CACGTGGCTT GCAACGGTGC AATCATCGAG   
  
  
+ GCCTTAGAGA GGGACCCACA TGAAAAGAGT AAGCTCCACG TCATCGATAT CAGCACCACC TTCTGCACCC   
  
  
+ AGTGGCCCAC CTTGCTTGAG GCTTTAGCCA CCCGCATGGA CGACACTCCC CACCTCAGCC TCACTGCCGT   
  
  
+ CGTCGTCAAC AAGTACGCCG CCGTGAGCGG TGGCGAGGAC GACGGGGGAG AAGGATCAAA GAGGGTGATG   
  
  
+ AGAGAGATTG GTCTTCGTCT TGAGAAGTTC GCTAGGTTAA TGGGGGTTCC CTTCAAGTTC AACGTGGTTT   
  
  
+ ACCACGTAGG TGATCTATCC CAGTTGGATT TTTCTCGTTT GGATATTAAA GATGATGAAG CGGTGGCGAT   
  
  
+ TAACTGTGTC AACTCGCTCC ACTCGGTGGA CCTCCGATAT CGGGAGGTAG TGCTCGCGGC GTTCAGGCGG   
  
  
+ CTGCGGCCGA GGGTGGTGAC GGTGGTGGAG GAAGAGGCGG AGCTGACTGA GGTGGGTGAG GGTCAGTATG   
  
  
+ AATTTTTTAG GCGGTTTGAG GAGTGTCTTA GGTGGTTTAG GGTTTACTTT GAGGCCGTAG GAGATTGTTT   
  
  
+ TCCTAGGACT AGCAATGAGA AATTGATGTT AGAGCGGGCC GCGGGCCGGG CCATGGTGGA TCTCCTTGCA   
  
  
+ TGTCCCGAGC CTGGTGGGTC GGCTGAGGGG AGGGAGACGG CGAGACGGTG GTCGGGTCGG ATGCATGGGG   
  
  
+ CGGGGTTCGA GCACGTGGGG TTCAGTGACG AAGTGTGTGA TGATGTGAGG GCCTTGTTGA GGAGGTACAA   
  
  
+ GGAAGGATGG TCAATGACAC AGTGCACCAT GGATGGCGGT AATCACCCTG GAATACTGTT GTGGTGGCGG   
  
  
+ GATCAACCGG TGGTTTGGGC CAGTGCATGG CGGCCTTG  

- +Up\_Stream \_Len000TTTAGT TTGAGTATTT GTTATTAAAA AATTTTGAAT TTACCATTAA TAATCTAATT   
  
  
- ATTTTTAGGA TTACCAACAA ATTACAATGT AGTTCTGTAC GAAAAACCAC ATATATATTA CCGTCGTACA   
  
  
- CCCAATTCGC CGTTGTTTAA TAAGAGTTTA CGTACGTCAT TAATTAAATT AATTAATTAG TGGACGTATT   
  
  
- AGAGTTATGA TTAGGAAAGT AAGACACGGG GTGGGTTGGG GCTTTTATCA TTAGAAAGTT TGCAACCTTC   
  
  
- ACCCTAATCC ATATCTCATC AGGATGTCTA CTACTAGATG GATGTATATT GTATTAGACT GAAAAGAAAA   
  
  
- AATTTATTGA ACATAACTAC CGTGTCTTAA ACTGTACAAC CGTATACCAT GATTCTTTAA TTAATTGCAA   
  
  
- CACGATTCTA TATCCAGTAA GAAAAACCTC TGTATTCCGG GCGCAAGTGC GTCCCAAGCT CTTCCCGGTG   
  
  
- TAAGTTACCT ACTTAACATC CATCAAATTA GACTGAAACA GTCACTGACT AAGGTGCCGA ACTTGGGCGC   
  
  
- TGTAATTATA TCCAACTGAT TTCAGCAAAC TATGAATAAA AAGAATTCAT TTCAGAACTT AAGTTTAGAA   
  
  
- TATTTATTTA TTTTTTTAGA TATTATTTTC TTAAGATGGT AAATAATTCA GTTGTTGAGT TAAGCTTACT   
  
  
- TTATTTTTTA GTTCTAGTGG TTTTTTTTAT TTATTTTCCT TTCCATCTAA CGTAAACCTA AAACTCACCG   
  
  
- CACCACCACC CAAACTACGA CCACGACGTA TGTGGCGTCG AAAAGAGACA GGAAAATGGA GAGAAACAGA   
  
  
- CGTTCTTGGG GTAAACCGTG CGGACGAGAG AAAAGATACA AAAGAAAAGA TAGAAAATTC TTTGTTACCT   
  
  
- TTCGATTGTG TGATTGTGAT TGGGTAACCA GGAAAAAAGA ATAGACGTTT TAATTTCATT GTTATAAAAA   
  
  
- GAATAGGGGA AAAAGCAAGG TGGGTAAAGA AATAGGAAGA CAAACTTGGA TTAGACATAT ATACGTACGT   
  
  
- ACACATAAAA TGAATCTTAG ACAAAACTAA GAAACTACAT TAATTAATCA TCAGAAAAAT TAACTTAAAA   
  
  
- ATTGTCATCA AACTTAATCT GTAAATAGCT ATAATATAAA AAATATATAT TAATTTTTTA TGTTTTACTA   
  
  
- TTCACAGAAT GATGTTATGT GAAGTATTCA CAGCACAAAA TTGAATTTTT AGTATGATAA TACAAAGATT   
  
  
- TAACTTTTTT TTCACTCTAA TTCATCGTCT TCGTATAATG TTGAATATTT AATATATTTT AGTAGAAAAA   
  
  
- TAGGTATTAA ATAGTGTAAA GATTTTATGC GTATAGTATA GATGAATACC TATCCCAATT ATTCTAATCC   
  
  
- CGTAGTAAAT TCTAAAATAG TCGCATTGGT TGCTTACAAA TTCCAATCGT CAAGTATTAA TTCACATTAG   
  
  
- TAATTTTTTA AATACTAAAA TTTAGTATTT TATACAATTA GATCCTACTC CGTTCGCCGT TCTGTATATA   
  
  
- GTGATAGGAA TTTTTTATAT TATATAACTA AAGTTTCTTA ATACTTCATA TAATTAAAGT ATATATTACT   
  
  
- GCTAATACAT AAAAAAATGA TTTAATGAAC ATGGGTATTA TTTATCTTAG CTACGTAGTA AGTACCGGAA   
  
  
- AGTAAGACAT TATACCAACT GAGGTTTTTA AGAGTTTCAT AAAAATATAA AACCCTCCCC TCTAAAATCA   
  
  
- AACTATGAAA ACAACTTGAT CACTTGTTAA AGGTTGTTGG AGGTGGTGGG GGTTTGTAGT TGTTAGACTA   
  
  
- ACTACATACG TAGGGTTCGA ACTTAACTTA TTATTAAAGT TATTGTTGTT ATTAGCAAAA TGGAACTGTT   
  
  
- CGAGGTGTCG TTGAGTTGCA GAAGGTTTGG TTGGGTAGTG AGTAGTAGTA GTAGGGGTGA TAGGGTAAGT   
  
  
- GGGGGTGGTT TTCCTCGGAT TTCTGTCATT GTTCTTCTTA CGAACGGAAA AAAGTACCTA CTTCTTCTGA   
  
  
- AGAGAAGAAG GAGATGAAGA AGGGTGGTGA TACTATGTGT GGTGGAGGTT GTAGTAATGG GGTGGTTAAT   
  
  
- ATTAAGGGCA ATATGGGACC TGGGAGGGAG GTGGTGGTGG TGTCGGTGTT GGTGGTGGAG TAGGAGGTGA   
  
  
- GGGTGGGTGG TACCGAAGCC ACCACCCGAA CTGAGGAGGA GGGGCAGACG GCGACACCTT AATAGAAAGA   
  
  
- GGGGCCTGTA GGAGGAGAGG AGGGGGTGGC CTTTCACCCG CAGACTGTAA GAATACCTCC GCCGATCCCG   
  
  
- AAAAAGTCTG TGCTTGTGGT GGTTCGAGGT GGTCGAGGAG ACCTACGACT TGCTCGAGCC GAGGGGGATG   
  
  
- CCACTACAGC TCGTCTTCGA CCACCGCATG AAATTGGTTC GGGAAAAACG GGCGGAGTGA CGGTGGCCTG   
  
  
- GGGCGGTGGC TCGGCGATAC GAGCGGCGAC GCCTGTTCTG GAGGAGGAAG CTGAGGTGCG AGTCCAACGA   
  
  
- GGAGTTCAAG GTCCTTCAGT CGGGCACCTG GTGGAAGCCG GTGCACCGAA CGTTGCCACG TTAGTAGCTC   
  
  
- CGGAATCTCT CCCTGGGTGT ACTTTTCTCA TTCGAGGTGC AGTAGCTATA GTCGTGGTGG AAGACGTGGG   
  
  
- TCACCGGGTG GAACGAACTC CGAAATCGGT GGGCGTACCT GCTGTGAGGG GTGGAGTCGG AGTGACGGCA   
  
  
- GCAGCAGTTG TTCATGCGGC GGCACTCGCC ACCGCTCCTG CTGCCCCCTC TTCCTAGTTT CTCCCACTAC   
  
  
- TCTCTCTAAC CAGAAGCAGA ACTCTTCAAG CGATCCAATT ACCCCCAAGG GAAGTTCAAG TTGCACCAAA   
  
  
- TGGTGCATCC ACTAGATAGG GTCAACCTAA AAAGAGCAAA CCTATAATTT CTACTACTTC GCCACCGCTA   
  
  
- ATTGACACAG TTGAGCGAGG TGAGCCACCT GGAGGCTATA GCCCTCCATC ACGAGCGCCG CAAGTCCGCC   
  
  
- GACGCCGGCT CCCACCACTG CCACCACCTC CTTCTCCGCC TCGACTGACT CCACCCACTC CCAGTCATAC   
  
  
- TTAAAAAATC CGCCAAACTC CTCACAGAAT CCACCAAATC CCAAATGAAA CTCCGGCATC CTCTAACAAA   
  
  
- AGGATCCTGA TCGTTACTCT TTAACTACAA TCTCGCCCGG CGCCCGGCCC GGTACCACCT AGAGGAACGT   
  
  
- ACAGGGCTCG GACCACCCAG CCGACTCCCC TCCCTCTGCC GCTCTGCCAC CAGCCCAGCC TACGTACCCC   
  
  
- GCCCCAAGCT CGTGCACCCC AAGTCACTGC TTCACACACT ACTACACTCC CGGAACAACT CCTCCATGTT   
  
  
- CCTTCCTACC AGTTACTGTG TCACGTGGTA CCTACCGCCA TTAGTGGGAC CTTATGACAA CACCACCGCC   
  
  
- CTAGTTGGCC ACCAAACCCG GTCACGTACC GCCGGAAC

+     RY-element

| Site Name | Organism | Position | Strand | Matrix score. | sequence | function |
| --- | --- | --- | --- | --- | --- | --- |
| RY-element | Helianthus annuus | 1049 | + | 8 | CATGCATG | cis-acting regulatory element involved in seed-specific regulation |

>HU05G01983.1   
+ +Up\_Stream \_Len000AAATCA AACTCATAAA CAATAATTTT TTAAAACTTA AATGGTAATT ATTAGATTAA   
  
  
+ TAAAAATCCT AATGGTTGTT TAATGTTACA TCAAGACATG CTTTTTGGTG TATATATAAT GGCAGCATGT   
  
  
+ GGGTTAAGCG GCAACAAATT ATTCTCAAAT GCATGCAGTA ATTAATTTAA TTAATTAATC ACCTGCATAA   
  
  
+ TCTCAATACT AATCCTTTCA TTCTGTGCCC CACCCAACCC CGAAAATAGT AATCTTTCAA ACGTTGGAAG   
  
  
+ TGGGATTAGG TATAGAGTAG TCCTACAGAT GATGATCTAC CTACATATAA CATAATCTGA CTTTTCTTTT   
  
  
+ TTAAATAACT TGTATTGATG GCACAGAATT TGACATGTTG GCATATGGTA CTAAGAAATT AATTAACGTT   
  
  
+ GTGCTAAGAT ATAGGTCATT CTTTTTGGAG ACATAAGGCC CGCGTTCACG CAGGGTTCGA GAAGGGCCAC   
  
  
+ ATTCAATGGA TGAATTGTAG GTAGTTTAAT CTGACTTTGT CAGTGACTGA TTCCACGGCT TGAACCCGCG   
  
  
+ ACATTAATAT AGGTTGACTA AAGTCGTTTG ATACTTATTT TTCTTAAGTA AAGTCTTGAA TTCAAATCTT   
  
  
+ ATAAATAAAT AAAAAAATCT ATAATAAAAG AATTCTACCA TTTATTAAGT CAACAACTCA ATTCGAATGA   
  
  
+ AATAAAAAAT CAAGATCACC AAAAAAAATA AATAAAAGGA AAGGTAGATT GCATTTGGAT TTTGAGTGGC   
  
  
+ GTGGTGGTGG GTTTGATGCT GGTGCTGCAT ACACCGCAGC TTTTCTCTGT CCTTTTACCT CTCTTTGTCT   
  
  
+ GCAAGAACCC CATTTGGCAC GCCTGCTCTC TTTTCTATGT TTTCTTTTCT ATCTTTTAAG AAACAATGGA   
  
  
+ AAGCTAACAC ACTAACACTA ACCCATTGGT CCTTTTTTCT TATCTGCAAA ATTAAAGTAA CAATATTTTT   
  
  
+ CTTATCCCCT TTTTCGTTCC ACCCATTTCT TTATCCTTCT GTTTGAACCT AATCTGTATA TATGCATGCA   
  
  
+ TGTGTATTTT ACTTAGAATC TGTTTTGATT CTTTGATGTA ATTAATTAGT AGTCTTTTTA ATTGAATTTT   
  
  
+ TAACAGTAGT TTGAATTAGA CATTTATCGA TATTATATTT TTTATATATA ATTAAAAAAT ACAAAATGAT   
  
  
+ AAGTGTCTTA CTACAATACA CTTCATAAGT GTCGTGTTTT AACTTAAAAA TCATACTATT ATGTTTCTAA   
  
  
+ ATTGAAAAAA AAGTGAGATT AAGTAGCAGA AGCATATTAC AACTTATAAA TTATATAAAA TCATCTTTTT   
  
  
+ ATCCATAATT TATCACATTT CTAAAATACG CATATCATAT CTACTTATGG ATAGGGTTAA TAAGATTAGG   
  
  
+ GCATCATTTA AGATTTTATC AGCGTAACCA ACGAATGTTT AAGGTTAGCA GTTCATAATT AAGTGTAATC   
  
  
+ ATTAAAAAAT TTATGATTTT AAATCATAAA ATATGTTAAT CTAGGATGAG GCAAGCGGCA AGACATATAT   
  
  
+ CACTATCCTT AAAAAATATA ATATATTGAT TTCAAAGAAT TATGAAGTAT ATTAATTTCA TATATAATGA   
  
  
+ CGATTATGTA TTTTTTTACT AAATTACTTG TACCCATAAT AAATAGAATC GATGCATCAT TCATGGCCTT   
  
  
+ TCATTCTGTA ATATGGTTGA CTCCAAAAAT TCTCAAAGTA TTTTTATATT TTGGGAGGGG AGATTTTAGT   
  
  
+ TTGATACTTT TGTTGAACTA GTGAACAATT TCCAACAACC TCCACCACCC CCAAACATCA ACAATCTGAT   
  
  
+ TGATGTATGC ATCCCAAGCT TGAATTGAAT AATAATTTCA ATAACAACAA TAATCGTTTT ACCTTGACAA   
  
  
+ GCTCCACAGC AACTCAACGT CTTCCAAACC AACCCATCAC TCATCATCAT CATCCCCACT ATCCCATTCA   
  
  
+ CCCCCACCAA AAGGAGCCTA AAGACAGTAA CAAGAAGAAT GCTTGCCTTT TTTCATGGAT GAAGAAGACT   
  
  
+ TCTCTTCTTC CTCTACTTCT TCCCACCACT ATGATACACA CCACCTCCAA CATCATTACC CCACCAATTA   
  
  
+ TAATTCCCGT TATACCCTGG ACCCTCCCTC CACCACCACC ACAGCCACAA CCACCACCTC ATCCTCCACT   
  
  
+ CCCACCCACC ATGGCTTCGG TGGTGGGCTT GACTCCTCCT CCCCGTCTGC CGCTGTGGAA TTATCTTTCT   
  
  
+ CCCCGGACAT CCTCCTCTCC TCCCCCACCG GAAAGTGGGC GTCTGACATT CTTATGGAGG CGGCTAGGGC   
  
  
+ TTTTTCAGAC ACGAACACCA CCAAGCTCCA CCAGCTCCTC TGGATGCTGA ACGAGCTCGG CTCCCCCTAC   
  
  
+ GGTGATGTCG AGCAGAAGCT GGTGGCGTAC TTTAACCAAG CCCTTTTTGC CCGCCTCACT GCCACCGGAC   
  
  
+ CCCGCCACCG AGCCGCTATG CTCGCCGCTG CGGACAAGAC CTCCTCCTTC GACTCCACGC TCAGGTTGCT   
  
  
+ CCTCAAGTTC CAGGAAGTCA GCCCGTGGAC CACCTTCGGC CACGTGGCTT GCAACGGTGC AATCATCGAG   
  
  
+ GCCTTAGAGA GGGACCCACA TGAAAAGAGT AAGCTCCACG TCATCGATAT CAGCACCACC TTCTGCACCC   
  
  
+ AGTGGCCCAC CTTGCTTGAG GCTTTAGCCA CCCGCATGGA CGACACTCCC CACCTCAGCC TCACTGCCGT   
  
  
+ CGTCGTCAAC AAGTACGCCG CCGTGAGCGG TGGCGAGGAC GACGGGGGAG AAGGATCAAA GAGGGTGATG   
  
  
+ AGAGAGATTG GTCTTCGTCT TGAGAAGTTC GCTAGGTTAA TGGGGGTTCC CTTCAAGTTC AACGTGGTTT   
  
  
+ ACCACGTAGG TGATCTATCC CAGTTGGATT TTTCTCGTTT GGATATTAAA GATGATGAAG CGGTGGCGAT   
  
  
+ TAACTGTGTC AACTCGCTCC ACTCGGTGGA CCTCCGATAT CGGGAGGTAG TGCTCGCGGC GTTCAGGCGG   
  
  
+ CTGCGGCCGA GGGTGGTGAC GGTGGTGGAG GAAGAGGCGG AGCTGACTGA GGTGGGTGAG GGTCAGTATG   
  
  
+ AATTTTTTAG GCGGTTTGAG GAGTGTCTTA GGTGGTTTAG GGTTTACTTT GAGGCCGTAG GAGATTGTTT   
  
  
+ TCCTAGGACT AGCAATGAGA AATTGATGTT AGAGCGGGCC GCGGGCCGGG CCATGGTGGA TCTCCTTGCA   
  
  
+ TGTCCCGAGC CTGGTGGGTC GGCTGAGGGG AGGGAGACGG CGAGACGGTG GTCGGGTCGG ATGCATGGGG   
  
  
+ CGGGGTTCGA GCACGTGGGG TTCAGTGACG AAGTGTGTGA TGATGTGAGG GCCTTGTTGA GGAGGTACAA   
  
  
+ GGAAGGATGG TCAATGACAC AGTGCACCAT GGATGGCGGT AATCACCCTG GAATACTGTT GTGGTGGCGG   
  
  
+ GATCAACCGG TGGTTTGGGC CAGTGCATGG CGGCCTTG  

- +Up\_Stream \_Len000TTTAGT TTGAGTATTT GTTATTAAAA AATTTTGAAT TTACCATTAA TAATCTAATT   
  
  
- ATTTTTAGGA TTACCAACAA ATTACAATGT AGTTCTGTAC GAAAAACCAC ATATATATTA CCGTCGTACA   
  
  
- CCCAATTCGC CGTTGTTTAA TAAGAGTTTA CGTACGTCAT TAATTAAATT AATTAATTAG TGGACGTATT   
  
  
- AGAGTTATGA TTAGGAAAGT AAGACACGGG GTGGGTTGGG GCTTTTATCA TTAGAAAGTT TGCAACCTTC   
  
  
- ACCCTAATCC ATATCTCATC AGGATGTCTA CTACTAGATG GATGTATATT GTATTAGACT GAAAAGAAAA   
  
  
- AATTTATTGA ACATAACTAC CGTGTCTTAA ACTGTACAAC CGTATACCAT GATTCTTTAA TTAATTGCAA   
  
  
- CACGATTCTA TATCCAGTAA GAAAAACCTC TGTATTCCGG GCGCAAGTGC GTCCCAAGCT CTTCCCGGTG   
  
  
- TAAGTTACCT ACTTAACATC CATCAAATTA GACTGAAACA GTCACTGACT AAGGTGCCGA ACTTGGGCGC   
  
  
- TGTAATTATA TCCAACTGAT TTCAGCAAAC TATGAATAAA AAGAATTCAT TTCAGAACTT AAGTTTAGAA   
  
  
- TATTTATTTA TTTTTTTAGA TATTATTTTC TTAAGATGGT AAATAATTCA GTTGTTGAGT TAAGCTTACT   
  
  
- TTATTTTTTA GTTCTAGTGG TTTTTTTTAT TTATTTTCCT TTCCATCTAA CGTAAACCTA AAACTCACCG   
  
  
- CACCACCACC CAAACTACGA CCACGACGTA TGTGGCGTCG AAAAGAGACA GGAAAATGGA GAGAAACAGA   
  
  
- CGTTCTTGGG GTAAACCGTG CGGACGAGAG AAAAGATACA AAAGAAAAGA TAGAAAATTC TTTGTTACCT   
  
  
- TTCGATTGTG TGATTGTGAT TGGGTAACCA GGAAAAAAGA ATAGACGTTT TAATTTCATT GTTATAAAAA   
  
  
- GAATAGGGGA AAAAGCAAGG TGGGTAAAGA AATAGGAAGA CAAACTTGGA TTAGACATAT ATACGTACGT   
  
  
- ACACATAAAA TGAATCTTAG ACAAAACTAA GAAACTACAT TAATTAATCA TCAGAAAAAT TAACTTAAAA   
  
  
- ATTGTCATCA AACTTAATCT GTAAATAGCT ATAATATAAA AAATATATAT TAATTTTTTA TGTTTTACTA   
  
  
- TTCACAGAAT GATGTTATGT GAAGTATTCA CAGCACAAAA TTGAATTTTT AGTATGATAA TACAAAGATT   
  
  
- TAACTTTTTT TTCACTCTAA TTCATCGTCT TCGTATAATG TTGAATATTT AATATATTTT AGTAGAAAAA   
  
  
- TAGGTATTAA ATAGTGTAAA GATTTTATGC GTATAGTATA GATGAATACC TATCCCAATT ATTCTAATCC   
  
  
- CGTAGTAAAT TCTAAAATAG TCGCATTGGT TGCTTACAAA TTCCAATCGT CAAGTATTAA TTCACATTAG   
  
  
- TAATTTTTTA AATACTAAAA TTTAGTATTT TATACAATTA GATCCTACTC CGTTCGCCGT TCTGTATATA   
  
  
- GTGATAGGAA TTTTTTATAT TATATAACTA AAGTTTCTTA ATACTTCATA TAATTAAAGT ATATATTACT   
  
  
- GCTAATACAT AAAAAAATGA TTTAATGAAC ATGGGTATTA TTTATCTTAG CTACGTAGTA AGTACCGGAA   
  
  
- AGTAAGACAT TATACCAACT GAGGTTTTTA AGAGTTTCAT AAAAATATAA AACCCTCCCC TCTAAAATCA   
  
  
- AACTATGAAA ACAACTTGAT CACTTGTTAA AGGTTGTTGG AGGTGGTGGG GGTTTGTAGT TGTTAGACTA   
  
  
- ACTACATACG TAGGGTTCGA ACTTAACTTA TTATTAAAGT TATTGTTGTT ATTAGCAAAA TGGAACTGTT   
  
  
- CGAGGTGTCG TTGAGTTGCA GAAGGTTTGG TTGGGTAGTG AGTAGTAGTA GTAGGGGTGA TAGGGTAAGT   
  
  
- GGGGGTGGTT TTCCTCGGAT TTCTGTCATT GTTCTTCTTA CGAACGGAAA AAAGTACCTA CTTCTTCTGA   
  
  
- AGAGAAGAAG GAGATGAAGA AGGGTGGTGA TACTATGTGT GGTGGAGGTT GTAGTAATGG GGTGGTTAAT   
  
  
- ATTAAGGGCA ATATGGGACC TGGGAGGGAG GTGGTGGTGG TGTCGGTGTT GGTGGTGGAG TAGGAGGTGA   
  
  
- GGGTGGGTGG TACCGAAGCC ACCACCCGAA CTGAGGAGGA GGGGCAGACG GCGACACCTT AATAGAAAGA   
  
  
- GGGGCCTGTA GGAGGAGAGG AGGGGGTGGC CTTTCACCCG CAGACTGTAA GAATACCTCC GCCGATCCCG   
  
  
- AAAAAGTCTG TGCTTGTGGT GGTTCGAGGT GGTCGAGGAG ACCTACGACT TGCTCGAGCC GAGGGGGATG   
  
  
- CCACTACAGC TCGTCTTCGA CCACCGCATG AAATTGGTTC GGGAAAAACG GGCGGAGTGA CGGTGGCCTG   
  
  
- GGGCGGTGGC TCGGCGATAC GAGCGGCGAC GCCTGTTCTG GAGGAGGAAG CTGAGGTGCG AGTCCAACGA   
  
  
- GGAGTTCAAG GTCCTTCAGT CGGGCACCTG GTGGAAGCCG GTGCACCGAA CGTTGCCACG TTAGTAGCTC   
  
  
- CGGAATCTCT CCCTGGGTGT ACTTTTCTCA TTCGAGGTGC AGTAGCTATA GTCGTGGTGG AAGACGTGGG   
  
  
- TCACCGGGTG GAACGAACTC CGAAATCGGT GGGCGTACCT GCTGTGAGGG GTGGAGTCGG AGTGACGGCA   
  
  
- GCAGCAGTTG TTCATGCGGC GGCACTCGCC ACCGCTCCTG CTGCCCCCTC TTCCTAGTTT CTCCCACTAC   
  
  
- TCTCTCTAAC CAGAAGCAGA ACTCTTCAAG CGATCCAATT ACCCCCAAGG GAAGTTCAAG TTGCACCAAA   
  
  
- TGGTGCATCC ACTAGATAGG GTCAACCTAA AAAGAGCAAA CCTATAATTT CTACTACTTC GCCACCGCTA   
  
  
- ATTGACACAG TTGAGCGAGG TGAGCCACCT GGAGGCTATA GCCCTCCATC ACGAGCGCCG CAAGTCCGCC   
  
  
- GACGCCGGCT CCCACCACTG CCACCACCTC CTTCTCCGCC TCGACTGACT CCACCCACTC CCAGTCATAC   
  
  
- TTAAAAAATC CGCCAAACTC CTCACAGAAT CCACCAAATC CCAAATGAAA CTCCGGCATC CTCTAACAAA   
  
  
- AGGATCCTGA TCGTTACTCT TTAACTACAA TCTCGCCCGG CGCCCGGCCC GGTACCACCT AGAGGAACGT   
  
  
- ACAGGGCTCG GACCACCCAG CCGACTCCCC TCCCTCTGCC GCTCTGCCAC CAGCCCAGCC TACGTACCCC   
  
  
- GCCCCAAGCT CGTGCACCCC AAGTCACTGC TTCACACACT ACTACACTCC CGGAACAACT CCTCCATGTT   
  
  
- CCTTCCTACC AGTTACTGTG TCACGTGGTA CCTACCGCCA TTAGTGGGAC CTTATGACAA CACCACCGCC   
  
  
- CTAGTTGGCC ACCAAACCCG GTCACGTACC GCCGGAAC

+     STRE

| Site Name | Organism | Position | Strand | Matrix score. | sequence | function |
| --- | --- | --- | --- | --- | --- | --- |
| STRE | Arabidopsis thaliana | 2378 | - | 5 | AGGGG |  |
| STRE | Arabidopsis thaliana | 3250 | + | 5 | AGGGG |  |
| STRE | Arabidopsis thaliana | 990 | - | 5 | AGGGG |  |
| STRE | Arabidopsis thaliana | 1740 | + | 5 | AGGGG |  |

>HU05G01983.1   
+ +Up\_Stream \_Len000AAATCA AACTCATAAA CAATAATTTT TTAAAACTTA AATGGTAATT ATTAGATTAA   
  
  
+ TAAAAATCCT AATGGTTGTT TAATGTTACA TCAAGACATG CTTTTTGGTG TATATATAAT GGCAGCATGT   
  
  
+ GGGTTAAGCG GCAACAAATT ATTCTCAAAT GCATGCAGTA ATTAATTTAA TTAATTAATC ACCTGCATAA   
  
  
+ TCTCAATACT AATCCTTTCA TTCTGTGCCC CACCCAACCC CGAAAATAGT AATCTTTCAA ACGTTGGAAG   
  
  
+ TGGGATTAGG TATAGAGTAG TCCTACAGAT GATGATCTAC CTACATATAA CATAATCTGA CTTTTCTTTT   
  
  
+ TTAAATAACT TGTATTGATG GCACAGAATT TGACATGTTG GCATATGGTA CTAAGAAATT AATTAACGTT   
  
  
+ GTGCTAAGAT ATAGGTCATT CTTTTTGGAG ACATAAGGCC CGCGTTCACG CAGGGTTCGA GAAGGGCCAC   
  
  
+ ATTCAATGGA TGAATTGTAG GTAGTTTAAT CTGACTTTGT CAGTGACTGA TTCCACGGCT TGAACCCGCG   
  
  
+ ACATTAATAT AGGTTGACTA AAGTCGTTTG ATACTTATTT TTCTTAAGTA AAGTCTTGAA TTCAAATCTT   
  
  
+ ATAAATAAAT AAAAAAATCT ATAATAAAAG AATTCTACCA TTTATTAAGT CAACAACTCA ATTCGAATGA   
  
  
+ AATAAAAAAT CAAGATCACC AAAAAAAATA AATAAAAGGA AAGGTAGATT GCATTTGGAT TTTGAGTGGC   
  
  
+ GTGGTGGTGG GTTTGATGCT GGTGCTGCAT ACACCGCAGC TTTTCTCTGT CCTTTTACCT CTCTTTGTCT   
  
  
+ GCAAGAACCC CATTTGGCAC GCCTGCTCTC TTTTCTATGT TTTCTTTTCT ATCTTTTAAG AAACAATGGA   
  
  
+ AAGCTAACAC ACTAACACTA ACCCATTGGT CCTTTTTTCT TATCTGCAAA ATTAAAGTAA CAATATTTTT   
  
  
+ CTTATCCCCT TTTTCGTTCC ACCCATTTCT TTATCCTTCT GTTTGAACCT AATCTGTATA TATGCATGCA   
  
  
+ TGTGTATTTT ACTTAGAATC TGTTTTGATT CTTTGATGTA ATTAATTAGT AGTCTTTTTA ATTGAATTTT   
  
  
+ TAACAGTAGT TTGAATTAGA CATTTATCGA TATTATATTT TTTATATATA ATTAAAAAAT ACAAAATGAT   
  
  
+ AAGTGTCTTA CTACAATACA CTTCATAAGT GTCGTGTTTT AACTTAAAAA TCATACTATT ATGTTTCTAA   
  
  
+ ATTGAAAAAA AAGTGAGATT AAGTAGCAGA AGCATATTAC AACTTATAAA TTATATAAAA TCATCTTTTT   
  
  
+ ATCCATAATT TATCACATTT CTAAAATACG CATATCATAT CTACTTATGG ATAGGGTTAA TAAGATTAGG   
  
  
+ GCATCATTTA AGATTTTATC AGCGTAACCA ACGAATGTTT AAGGTTAGCA GTTCATAATT AAGTGTAATC   
  
  
+ ATTAAAAAAT TTATGATTTT AAATCATAAA ATATGTTAAT CTAGGATGAG GCAAGCGGCA AGACATATAT   
  
  
+ CACTATCCTT AAAAAATATA ATATATTGAT TTCAAAGAAT TATGAAGTAT ATTAATTTCA TATATAATGA   
  
  
+ CGATTATGTA TTTTTTTACT AAATTACTTG TACCCATAAT AAATAGAATC GATGCATCAT TCATGGCCTT   
  
  
+ TCATTCTGTA ATATGGTTGA CTCCAAAAAT TCTCAAAGTA TTTTTATATT TTGGGAGGGG AGATTTTAGT   
  
  
+ TTGATACTTT TGTTGAACTA GTGAACAATT TCCAACAACC TCCACCACCC CCAAACATCA ACAATCTGAT   
  
  
+ TGATGTATGC ATCCCAAGCT TGAATTGAAT AATAATTTCA ATAACAACAA TAATCGTTTT ACCTTGACAA   
  
  
+ GCTCCACAGC AACTCAACGT CTTCCAAACC AACCCATCAC TCATCATCAT CATCCCCACT ATCCCATTCA   
  
  
+ CCCCCACCAA AAGGAGCCTA AAGACAGTAA CAAGAAGAAT GCTTGCCTTT TTTCATGGAT GAAGAAGACT   
  
  
+ TCTCTTCTTC CTCTACTTCT TCCCACCACT ATGATACACA CCACCTCCAA CATCATTACC CCACCAATTA   
  
  
+ TAATTCCCGT TATACCCTGG ACCCTCCCTC CACCACCACC ACAGCCACAA CCACCACCTC ATCCTCCACT   
  
  
+ CCCACCCACC ATGGCTTCGG TGGTGGGCTT GACTCCTCCT CCCCGTCTGC CGCTGTGGAA TTATCTTTCT   
  
  
+ CCCCGGACAT CCTCCTCTCC TCCCCCACCG GAAAGTGGGC GTCTGACATT CTTATGGAGG CGGCTAGGGC   
  
  
+ TTTTTCAGAC ACGAACACCA CCAAGCTCCA CCAGCTCCTC TGGATGCTGA ACGAGCTCGG CTCCCCCTAC   
  
  
+ GGTGATGTCG AGCAGAAGCT GGTGGCGTAC TTTAACCAAG CCCTTTTTGC CCGCCTCACT GCCACCGGAC   
  
  
+ CCCGCCACCG AGCCGCTATG CTCGCCGCTG CGGACAAGAC CTCCTCCTTC GACTCCACGC TCAGGTTGCT   
  
  
+ CCTCAAGTTC CAGGAAGTCA GCCCGTGGAC CACCTTCGGC CACGTGGCTT GCAACGGTGC AATCATCGAG   
  
  
+ GCCTTAGAGA GGGACCCACA TGAAAAGAGT AAGCTCCACG TCATCGATAT CAGCACCACC TTCTGCACCC   
  
  
+ AGTGGCCCAC CTTGCTTGAG GCTTTAGCCA CCCGCATGGA CGACACTCCC CACCTCAGCC TCACTGCCGT   
  
  
+ CGTCGTCAAC AAGTACGCCG CCGTGAGCGG TGGCGAGGAC GACGGGGGAG AAGGATCAAA GAGGGTGATG   
  
  
+ AGAGAGATTG GTCTTCGTCT TGAGAAGTTC GCTAGGTTAA TGGGGGTTCC CTTCAAGTTC AACGTGGTTT   
  
  
+ ACCACGTAGG TGATCTATCC CAGTTGGATT TTTCTCGTTT GGATATTAAA GATGATGAAG CGGTGGCGAT   
  
  
+ TAACTGTGTC AACTCGCTCC ACTCGGTGGA CCTCCGATAT CGGGAGGTAG TGCTCGCGGC GTTCAGGCGG   
  
  
+ CTGCGGCCGA GGGTGGTGAC GGTGGTGGAG GAAGAGGCGG AGCTGACTGA GGTGGGTGAG GGTCAGTATG   
  
  
+ AATTTTTTAG GCGGTTTGAG GAGTGTCTTA GGTGGTTTAG GGTTTACTTT GAGGCCGTAG GAGATTGTTT   
  
  
+ TCCTAGGACT AGCAATGAGA AATTGATGTT AGAGCGGGCC GCGGGCCGGG CCATGGTGGA TCTCCTTGCA   
  
  
+ TGTCCCGAGC CTGGTGGGTC GGCTGAGGGG AGGGAGACGG CGAGACGGTG GTCGGGTCGG ATGCATGGGG   
  
  
+ CGGGGTTCGA GCACGTGGGG TTCAGTGACG AAGTGTGTGA TGATGTGAGG GCCTTGTTGA GGAGGTACAA   
  
  
+ GGAAGGATGG TCAATGACAC AGTGCACCAT GGATGGCGGT AATCACCCTG GAATACTGTT GTGGTGGCGG   
  
  
+ GATCAACCGG TGGTTTGGGC CAGTGCATGG CGGCCTTG  

- +Up\_Stream \_Len000TTTAGT TTGAGTATTT GTTATTAAAA AATTTTGAAT TTACCATTAA TAATCTAATT   
  
  
- ATTTTTAGGA TTACCAACAA ATTACAATGT AGTTCTGTAC GAAAAACCAC ATATATATTA CCGTCGTACA   
  
  
- CCCAATTCGC CGTTGTTTAA TAAGAGTTTA CGTACGTCAT TAATTAAATT AATTAATTAG TGGACGTATT   
  
  
- AGAGTTATGA TTAGGAAAGT AAGACACGGG GTGGGTTGGG GCTTTTATCA TTAGAAAGTT TGCAACCTTC   
  
  
- ACCCTAATCC ATATCTCATC AGGATGTCTA CTACTAGATG GATGTATATT GTATTAGACT GAAAAGAAAA   
  
  
- AATTTATTGA ACATAACTAC CGTGTCTTAA ACTGTACAAC CGTATACCAT GATTCTTTAA TTAATTGCAA   
  
  
- CACGATTCTA TATCCAGTAA GAAAAACCTC TGTATTCCGG GCGCAAGTGC GTCCCAAGCT CTTCCCGGTG   
  
  
- TAAGTTACCT ACTTAACATC CATCAAATTA GACTGAAACA GTCACTGACT AAGGTGCCGA ACTTGGGCGC   
  
  
- TGTAATTATA TCCAACTGAT TTCAGCAAAC TATGAATAAA AAGAATTCAT TTCAGAACTT AAGTTTAGAA   
  
  
- TATTTATTTA TTTTTTTAGA TATTATTTTC TTAAGATGGT AAATAATTCA GTTGTTGAGT TAAGCTTACT   
  
  
- TTATTTTTTA GTTCTAGTGG TTTTTTTTAT TTATTTTCCT TTCCATCTAA CGTAAACCTA AAACTCACCG   
  
  
- CACCACCACC CAAACTACGA CCACGACGTA TGTGGCGTCG AAAAGAGACA GGAAAATGGA GAGAAACAGA   
  
  
- CGTTCTTGGG GTAAACCGTG CGGACGAGAG AAAAGATACA AAAGAAAAGA TAGAAAATTC TTTGTTACCT   
  
  
- TTCGATTGTG TGATTGTGAT TGGGTAACCA GGAAAAAAGA ATAGACGTTT TAATTTCATT GTTATAAAAA   
  
  
- GAATAGGGGA AAAAGCAAGG TGGGTAAAGA AATAGGAAGA CAAACTTGGA TTAGACATAT ATACGTACGT   
  
  
- ACACATAAAA TGAATCTTAG ACAAAACTAA GAAACTACAT TAATTAATCA TCAGAAAAAT TAACTTAAAA   
  
  
- ATTGTCATCA AACTTAATCT GTAAATAGCT ATAATATAAA AAATATATAT TAATTTTTTA TGTTTTACTA   
  
  
- TTCACAGAAT GATGTTATGT GAAGTATTCA CAGCACAAAA TTGAATTTTT AGTATGATAA TACAAAGATT   
  
  
- TAACTTTTTT TTCACTCTAA TTCATCGTCT TCGTATAATG TTGAATATTT AATATATTTT AGTAGAAAAA   
  
  
- TAGGTATTAA ATAGTGTAAA GATTTTATGC GTATAGTATA GATGAATACC TATCCCAATT ATTCTAATCC   
  
  
- CGTAGTAAAT TCTAAAATAG TCGCATTGGT TGCTTACAAA TTCCAATCGT CAAGTATTAA TTCACATTAG   
  
  
- TAATTTTTTA AATACTAAAA TTTAGTATTT TATACAATTA GATCCTACTC CGTTCGCCGT TCTGTATATA   
  
  
- GTGATAGGAA TTTTTTATAT TATATAACTA AAGTTTCTTA ATACTTCATA TAATTAAAGT ATATATTACT   
  
  
- GCTAATACAT AAAAAAATGA TTTAATGAAC ATGGGTATTA TTTATCTTAG CTACGTAGTA AGTACCGGAA   
  
  
- AGTAAGACAT TATACCAACT GAGGTTTTTA AGAGTTTCAT AAAAATATAA AACCCTCCCC TCTAAAATCA   
  
  
- AACTATGAAA ACAACTTGAT CACTTGTTAA AGGTTGTTGG AGGTGGTGGG GGTTTGTAGT TGTTAGACTA   
  
  
- ACTACATACG TAGGGTTCGA ACTTAACTTA TTATTAAAGT TATTGTTGTT ATTAGCAAAA TGGAACTGTT   
  
  
- CGAGGTGTCG TTGAGTTGCA GAAGGTTTGG TTGGGTAGTG AGTAGTAGTA GTAGGGGTGA TAGGGTAAGT   
  
  
- GGGGGTGGTT TTCCTCGGAT TTCTGTCATT GTTCTTCTTA CGAACGGAAA AAAGTACCTA CTTCTTCTGA   
  
  
- AGAGAAGAAG GAGATGAAGA AGGGTGGTGA TACTATGTGT GGTGGAGGTT GTAGTAATGG GGTGGTTAAT   
  
  
- ATTAAGGGCA ATATGGGACC TGGGAGGGAG GTGGTGGTGG TGTCGGTGTT GGTGGTGGAG TAGGAGGTGA   
  
  
- GGGTGGGTGG TACCGAAGCC ACCACCCGAA CTGAGGAGGA GGGGCAGACG GCGACACCTT AATAGAAAGA   
  
  
- GGGGCCTGTA GGAGGAGAGG AGGGGGTGGC CTTTCACCCG CAGACTGTAA GAATACCTCC GCCGATCCCG   
  
  
- AAAAAGTCTG TGCTTGTGGT GGTTCGAGGT GGTCGAGGAG ACCTACGACT TGCTCGAGCC GAGGGGGATG   
  
  
- CCACTACAGC TCGTCTTCGA CCACCGCATG AAATTGGTTC GGGAAAAACG GGCGGAGTGA CGGTGGCCTG   
  
  
- GGGCGGTGGC TCGGCGATAC GAGCGGCGAC GCCTGTTCTG GAGGAGGAAG CTGAGGTGCG AGTCCAACGA   
  
  
- GGAGTTCAAG GTCCTTCAGT CGGGCACCTG GTGGAAGCCG GTGCACCGAA CGTTGCCACG TTAGTAGCTC   
  
  
- CGGAATCTCT CCCTGGGTGT ACTTTTCTCA TTCGAGGTGC AGTAGCTATA GTCGTGGTGG AAGACGTGGG   
  
  
- TCACCGGGTG GAACGAACTC CGAAATCGGT GGGCGTACCT GCTGTGAGGG GTGGAGTCGG AGTGACGGCA   
  
  
- GCAGCAGTTG TTCATGCGGC GGCACTCGCC ACCGCTCCTG CTGCCCCCTC TTCCTAGTTT CTCCCACTAC   
  
  
- TCTCTCTAAC CAGAAGCAGA ACTCTTCAAG CGATCCAATT ACCCCCAAGG GAAGTTCAAG TTGCACCAAA   
  
  
- TGGTGCATCC ACTAGATAGG GTCAACCTAA AAAGAGCAAA CCTATAATTT CTACTACTTC GCCACCGCTA   
  
  
- ATTGACACAG TTGAGCGAGG TGAGCCACCT GGAGGCTATA GCCCTCCATC ACGAGCGCCG CAAGTCCGCC   
  
  
- GACGCCGGCT CCCACCACTG CCACCACCTC CTTCTCCGCC TCGACTGACT CCACCCACTC CCAGTCATAC   
  
  
- TTAAAAAATC CGCCAAACTC CTCACAGAAT CCACCAAATC CCAAATGAAA CTCCGGCATC CTCTAACAAA   
  
  
- AGGATCCTGA TCGTTACTCT TTAACTACAA TCTCGCCCGG CGCCCGGCCC GGTACCACCT AGAGGAACGT   
  
  
- ACAGGGCTCG GACCACCCAG CCGACTCCCC TCCCTCTGCC GCTCTGCCAC CAGCCCAGCC TACGTACCCC   
  
  
- GCCCCAAGCT CGTGCACCCC AAGTCACTGC TTCACACACT ACTACACTCC CGGAACAACT CCTCCATGTT   
  
  
- CCTTCCTACC AGTTACTGTG TCACGTGGTA CCTACCGCCA TTAGTGGGAC CTTATGACAA CACCACCGCC   
  
  
- CTAGTTGGCC ACCAAACCCG GTCACGTACC GCCGGAAC

+     Sp1

| Site Name | Organism | Position | Strand | Matrix score. | sequence | function |
| --- | --- | --- | --- | --- | --- | --- |
| Sp1 | Oryza sativa | 3292 | + | 6 | GGGCGG | light responsive element |

>HU05G01983.1   
+ +Up\_Stream \_Len000AAATCA AACTCATAAA CAATAATTTT TTAAAACTTA AATGGTAATT ATTAGATTAA   
  
  
+ TAAAAATCCT AATGGTTGTT TAATGTTACA TCAAGACATG CTTTTTGGTG TATATATAAT GGCAGCATGT   
  
  
+ GGGTTAAGCG GCAACAAATT ATTCTCAAAT GCATGCAGTA ATTAATTTAA TTAATTAATC ACCTGCATAA   
  
  
+ TCTCAATACT AATCCTTTCA TTCTGTGCCC CACCCAACCC CGAAAATAGT AATCTTTCAA ACGTTGGAAG   
  
  
+ TGGGATTAGG TATAGAGTAG TCCTACAGAT GATGATCTAC CTACATATAA CATAATCTGA CTTTTCTTTT   
  
  
+ TTAAATAACT TGTATTGATG GCACAGAATT TGACATGTTG GCATATGGTA CTAAGAAATT AATTAACGTT   
  
  
+ GTGCTAAGAT ATAGGTCATT CTTTTTGGAG ACATAAGGCC CGCGTTCACG CAGGGTTCGA GAAGGGCCAC   
  
  
+ ATTCAATGGA TGAATTGTAG GTAGTTTAAT CTGACTTTGT CAGTGACTGA TTCCACGGCT TGAACCCGCG   
  
  
+ ACATTAATAT AGGTTGACTA AAGTCGTTTG ATACTTATTT TTCTTAAGTA AAGTCTTGAA TTCAAATCTT   
  
  
+ ATAAATAAAT AAAAAAATCT ATAATAAAAG AATTCTACCA TTTATTAAGT CAACAACTCA ATTCGAATGA   
  
  
+ AATAAAAAAT CAAGATCACC AAAAAAAATA AATAAAAGGA AAGGTAGATT GCATTTGGAT TTTGAGTGGC   
  
  
+ GTGGTGGTGG GTTTGATGCT GGTGCTGCAT ACACCGCAGC TTTTCTCTGT CCTTTTACCT CTCTTTGTCT   
  
  
+ GCAAGAACCC CATTTGGCAC GCCTGCTCTC TTTTCTATGT TTTCTTTTCT ATCTTTTAAG AAACAATGGA   
  
  
+ AAGCTAACAC ACTAACACTA ACCCATTGGT CCTTTTTTCT TATCTGCAAA ATTAAAGTAA CAATATTTTT   
  
  
+ CTTATCCCCT TTTTCGTTCC ACCCATTTCT TTATCCTTCT GTTTGAACCT AATCTGTATA TATGCATGCA   
  
  
+ TGTGTATTTT ACTTAGAATC TGTTTTGATT CTTTGATGTA ATTAATTAGT AGTCTTTTTA ATTGAATTTT   
  
  
+ TAACAGTAGT TTGAATTAGA CATTTATCGA TATTATATTT TTTATATATA ATTAAAAAAT ACAAAATGAT   
  
  
+ AAGTGTCTTA CTACAATACA CTTCATAAGT GTCGTGTTTT AACTTAAAAA TCATACTATT ATGTTTCTAA   
  
  
+ ATTGAAAAAA AAGTGAGATT AAGTAGCAGA AGCATATTAC AACTTATAAA TTATATAAAA TCATCTTTTT   
  
  
+ ATCCATAATT TATCACATTT CTAAAATACG CATATCATAT CTACTTATGG ATAGGGTTAA TAAGATTAGG   
  
  
+ GCATCATTTA AGATTTTATC AGCGTAACCA ACGAATGTTT AAGGTTAGCA GTTCATAATT AAGTGTAATC   
  
  
+ ATTAAAAAAT TTATGATTTT AAATCATAAA ATATGTTAAT CTAGGATGAG GCAAGCGGCA AGACATATAT   
  
  
+ CACTATCCTT AAAAAATATA ATATATTGAT TTCAAAGAAT TATGAAGTAT ATTAATTTCA TATATAATGA   
  
  
+ CGATTATGTA TTTTTTTACT AAATTACTTG TACCCATAAT AAATAGAATC GATGCATCAT TCATGGCCTT   
  
  
+ TCATTCTGTA ATATGGTTGA CTCCAAAAAT TCTCAAAGTA TTTTTATATT TTGGGAGGGG AGATTTTAGT   
  
  
+ TTGATACTTT TGTTGAACTA GTGAACAATT TCCAACAACC TCCACCACCC CCAAACATCA ACAATCTGAT   
  
  
+ TGATGTATGC ATCCCAAGCT TGAATTGAAT AATAATTTCA ATAACAACAA TAATCGTTTT ACCTTGACAA   
  
  
+ GCTCCACAGC AACTCAACGT CTTCCAAACC AACCCATCAC TCATCATCAT CATCCCCACT ATCCCATTCA   
  
  
+ CCCCCACCAA AAGGAGCCTA AAGACAGTAA CAAGAAGAAT GCTTGCCTTT TTTCATGGAT GAAGAAGACT   
  
  
+ TCTCTTCTTC CTCTACTTCT TCCCACCACT ATGATACACA CCACCTCCAA CATCATTACC CCACCAATTA   
  
  
+ TAATTCCCGT TATACCCTGG ACCCTCCCTC CACCACCACC ACAGCCACAA CCACCACCTC ATCCTCCACT   
  
  
+ CCCACCCACC ATGGCTTCGG TGGTGGGCTT GACTCCTCCT CCCCGTCTGC CGCTGTGGAA TTATCTTTCT   
  
  
+ CCCCGGACAT CCTCCTCTCC TCCCCCACCG GAAAGTGGGC GTCTGACATT CTTATGGAGG CGGCTAGGGC   
  
  
+ TTTTTCAGAC ACGAACACCA CCAAGCTCCA CCAGCTCCTC TGGATGCTGA ACGAGCTCGG CTCCCCCTAC   
  
  
+ GGTGATGTCG AGCAGAAGCT GGTGGCGTAC TTTAACCAAG CCCTTTTTGC CCGCCTCACT GCCACCGGAC   
  
  
+ CCCGCCACCG AGCCGCTATG CTCGCCGCTG CGGACAAGAC CTCCTCCTTC GACTCCACGC TCAGGTTGCT   
  
  
+ CCTCAAGTTC CAGGAAGTCA GCCCGTGGAC CACCTTCGGC CACGTGGCTT GCAACGGTGC AATCATCGAG   
  
  
+ GCCTTAGAGA GGGACCCACA TGAAAAGAGT AAGCTCCACG TCATCGATAT CAGCACCACC TTCTGCACCC   
  
  
+ AGTGGCCCAC CTTGCTTGAG GCTTTAGCCA CCCGCATGGA CGACACTCCC CACCTCAGCC TCACTGCCGT   
  
  
+ CGTCGTCAAC AAGTACGCCG CCGTGAGCGG TGGCGAGGAC GACGGGGGAG AAGGATCAAA GAGGGTGATG   
  
  
+ AGAGAGATTG GTCTTCGTCT TGAGAAGTTC GCTAGGTTAA TGGGGGTTCC CTTCAAGTTC AACGTGGTTT   
  
  
+ ACCACGTAGG TGATCTATCC CAGTTGGATT TTTCTCGTTT GGATATTAAA GATGATGAAG CGGTGGCGAT   
  
  
+ TAACTGTGTC AACTCGCTCC ACTCGGTGGA CCTCCGATAT CGGGAGGTAG TGCTCGCGGC GTTCAGGCGG   
  
  
+ CTGCGGCCGA GGGTGGTGAC GGTGGTGGAG GAAGAGGCGG AGCTGACTGA GGTGGGTGAG GGTCAGTATG   
  
  
+ AATTTTTTAG GCGGTTTGAG GAGTGTCTTA GGTGGTTTAG GGTTTACTTT GAGGCCGTAG GAGATTGTTT   
  
  
+ TCCTAGGACT AGCAATGAGA AATTGATGTT AGAGCGGGCC GCGGGCCGGG CCATGGTGGA TCTCCTTGCA   
  
  
+ TGTCCCGAGC CTGGTGGGTC GGCTGAGGGG AGGGAGACGG CGAGACGGTG GTCGGGTCGG ATGCATGGGG   
  
  
+ CGGGGTTCGA GCACGTGGGG TTCAGTGACG AAGTGTGTGA TGATGTGAGG GCCTTGTTGA GGAGGTACAA   
  
  
+ GGAAGGATGG TCAATGACAC AGTGCACCAT GGATGGCGGT AATCACCCTG GAATACTGTT GTGGTGGCGG   
  
  
+ GATCAACCGG TGGTTTGGGC CAGTGCATGG CGGCCTTG  

- +Up\_Stream \_Len000TTTAGT TTGAGTATTT GTTATTAAAA AATTTTGAAT TTACCATTAA TAATCTAATT   
  
  
- ATTTTTAGGA TTACCAACAA ATTACAATGT AGTTCTGTAC GAAAAACCAC ATATATATTA CCGTCGTACA   
  
  
- CCCAATTCGC CGTTGTTTAA TAAGAGTTTA CGTACGTCAT TAATTAAATT AATTAATTAG TGGACGTATT   
  
  
- AGAGTTATGA TTAGGAAAGT AAGACACGGG GTGGGTTGGG GCTTTTATCA TTAGAAAGTT TGCAACCTTC   
  
  
- ACCCTAATCC ATATCTCATC AGGATGTCTA CTACTAGATG GATGTATATT GTATTAGACT GAAAAGAAAA   
  
  
- AATTTATTGA ACATAACTAC CGTGTCTTAA ACTGTACAAC CGTATACCAT GATTCTTTAA TTAATTGCAA   
  
  
- CACGATTCTA TATCCAGTAA GAAAAACCTC TGTATTCCGG GCGCAAGTGC GTCCCAAGCT CTTCCCGGTG   
  
  
- TAAGTTACCT ACTTAACATC CATCAAATTA GACTGAAACA GTCACTGACT AAGGTGCCGA ACTTGGGCGC   
  
  
- TGTAATTATA TCCAACTGAT TTCAGCAAAC TATGAATAAA AAGAATTCAT TTCAGAACTT AAGTTTAGAA   
  
  
- TATTTATTTA TTTTTTTAGA TATTATTTTC TTAAGATGGT AAATAATTCA GTTGTTGAGT TAAGCTTACT   
  
  
- TTATTTTTTA GTTCTAGTGG TTTTTTTTAT TTATTTTCCT TTCCATCTAA CGTAAACCTA AAACTCACCG   
  
  
- CACCACCACC CAAACTACGA CCACGACGTA TGTGGCGTCG AAAAGAGACA GGAAAATGGA GAGAAACAGA   
  
  
- CGTTCTTGGG GTAAACCGTG CGGACGAGAG AAAAGATACA AAAGAAAAGA TAGAAAATTC TTTGTTACCT   
  
  
- TTCGATTGTG TGATTGTGAT TGGGTAACCA GGAAAAAAGA ATAGACGTTT TAATTTCATT GTTATAAAAA   
  
  
- GAATAGGGGA AAAAGCAAGG TGGGTAAAGA AATAGGAAGA CAAACTTGGA TTAGACATAT ATACGTACGT   
  
  
- ACACATAAAA TGAATCTTAG ACAAAACTAA GAAACTACAT TAATTAATCA TCAGAAAAAT TAACTTAAAA   
  
  
- ATTGTCATCA AACTTAATCT GTAAATAGCT ATAATATAAA AAATATATAT TAATTTTTTA TGTTTTACTA   
  
  
- TTCACAGAAT GATGTTATGT GAAGTATTCA CAGCACAAAA TTGAATTTTT AGTATGATAA TACAAAGATT   
  
  
- TAACTTTTTT TTCACTCTAA TTCATCGTCT TCGTATAATG TTGAATATTT AATATATTTT AGTAGAAAAA   
  
  
- TAGGTATTAA ATAGTGTAAA GATTTTATGC GTATAGTATA GATGAATACC TATCCCAATT ATTCTAATCC   
  
  
- CGTAGTAAAT TCTAAAATAG TCGCATTGGT TGCTTACAAA TTCCAATCGT CAAGTATTAA TTCACATTAG   
  
  
- TAATTTTTTA AATACTAAAA TTTAGTATTT TATACAATTA GATCCTACTC CGTTCGCCGT TCTGTATATA   
  
  
- GTGATAGGAA TTTTTTATAT TATATAACTA AAGTTTCTTA ATACTTCATA TAATTAAAGT ATATATTACT   
  
  
- GCTAATACAT AAAAAAATGA TTTAATGAAC ATGGGTATTA TTTATCTTAG CTACGTAGTA AGTACCGGAA   
  
  
- AGTAAGACAT TATACCAACT GAGGTTTTTA AGAGTTTCAT AAAAATATAA AACCCTCCCC TCTAAAATCA   
  
  
- AACTATGAAA ACAACTTGAT CACTTGTTAA AGGTTGTTGG AGGTGGTGGG GGTTTGTAGT TGTTAGACTA   
  
  
- ACTACATACG TAGGGTTCGA ACTTAACTTA TTATTAAAGT TATTGTTGTT ATTAGCAAAA TGGAACTGTT   
  
  
- CGAGGTGTCG TTGAGTTGCA GAAGGTTTGG TTGGGTAGTG AGTAGTAGTA GTAGGGGTGA TAGGGTAAGT   
  
  
- GGGGGTGGTT TTCCTCGGAT TTCTGTCATT GTTCTTCTTA CGAACGGAAA AAAGTACCTA CTTCTTCTGA   
  
  
- AGAGAAGAAG GAGATGAAGA AGGGTGGTGA TACTATGTGT GGTGGAGGTT GTAGTAATGG GGTGGTTAAT   
  
  
- ATTAAGGGCA ATATGGGACC TGGGAGGGAG GTGGTGGTGG TGTCGGTGTT GGTGGTGGAG TAGGAGGTGA   
  
  
- GGGTGGGTGG TACCGAAGCC ACCACCCGAA CTGAGGAGGA GGGGCAGACG GCGACACCTT AATAGAAAGA   
  
  
- GGGGCCTGTA GGAGGAGAGG AGGGGGTGGC CTTTCACCCG CAGACTGTAA GAATACCTCC GCCGATCCCG   
  
  
- AAAAAGTCTG TGCTTGTGGT GGTTCGAGGT GGTCGAGGAG ACCTACGACT TGCTCGAGCC GAGGGGGATG   
  
  
- CCACTACAGC TCGTCTTCGA CCACCGCATG AAATTGGTTC GGGAAAAACG GGCGGAGTGA CGGTGGCCTG   
  
  
- GGGCGGTGGC TCGGCGATAC GAGCGGCGAC GCCTGTTCTG GAGGAGGAAG CTGAGGTGCG AGTCCAACGA   
  
  
- GGAGTTCAAG GTCCTTCAGT CGGGCACCTG GTGGAAGCCG GTGCACCGAA CGTTGCCACG TTAGTAGCTC   
  
  
- CGGAATCTCT CCCTGGGTGT ACTTTTCTCA TTCGAGGTGC AGTAGCTATA GTCGTGGTGG AAGACGTGGG   
  
  
- TCACCGGGTG GAACGAACTC CGAAATCGGT GGGCGTACCT GCTGTGAGGG GTGGAGTCGG AGTGACGGCA   
  
  
- GCAGCAGTTG TTCATGCGGC GGCACTCGCC ACCGCTCCTG CTGCCCCCTC TTCCTAGTTT CTCCCACTAC   
  
  
- TCTCTCTAAC CAGAAGCAGA ACTCTTCAAG CGATCCAATT ACCCCCAAGG GAAGTTCAAG TTGCACCAAA   
  
  
- TGGTGCATCC ACTAGATAGG GTCAACCTAA AAAGAGCAAA CCTATAATTT CTACTACTTC GCCACCGCTA   
  
  
- ATTGACACAG TTGAGCGAGG TGAGCCACCT GGAGGCTATA GCCCTCCATC ACGAGCGCCG CAAGTCCGCC   
  
  
- GACGCCGGCT CCCACCACTG CCACCACCTC CTTCTCCGCC TCGACTGACT CCACCCACTC CCAGTCATAC   
  
  
- TTAAAAAATC CGCCAAACTC CTCACAGAAT CCACCAAATC CCAAATGAAA CTCCGGCATC CTCTAACAAA   
  
  
- AGGATCCTGA TCGTTACTCT TTAACTACAA TCTCGCCCGG CGCCCGGCCC GGTACCACCT AGAGGAACGT   
  
  
- ACAGGGCTCG GACCACCCAG CCGACTCCCC TCCCTCTGCC GCTCTGCCAC CAGCCCAGCC TACGTACCCC   
  
  
- GCCCCAAGCT CGTGCACCCC AAGTCACTGC TTCACACACT ACTACACTCC CGGAACAACT CCTCCATGTT   
  
  
- CCTTCCTACC AGTTACTGTG TCACGTGGTA CCTACCGCCA TTAGTGGGAC CTTATGACAA CACCACCGCC   
  
  
- CTAGTTGGCC ACCAAACCCG GTCACGTACC GCCGGAAC

+     TATA

| Site Name | Organism | Position | Strand | Matrix score. | sequence | function |
| --- | --- | --- | --- | --- | --- | --- |
| TATA | Arabidopsis thaliana | 1318 | + | 8 | TATAAAAT |  |

>HU05G01983.1   
+ +Up\_Stream \_Len000AAATCA AACTCATAAA CAATAATTTT TTAAAACTTA AATGGTAATT ATTAGATTAA   
  
  
+ TAAAAATCCT AATGGTTGTT TAATGTTACA TCAAGACATG CTTTTTGGTG TATATATAAT GGCAGCATGT   
  
  
+ GGGTTAAGCG GCAACAAATT ATTCTCAAAT GCATGCAGTA ATTAATTTAA TTAATTAATC ACCTGCATAA   
  
  
+ TCTCAATACT AATCCTTTCA TTCTGTGCCC CACCCAACCC CGAAAATAGT AATCTTTCAA ACGTTGGAAG   
  
  
+ TGGGATTAGG TATAGAGTAG TCCTACAGAT GATGATCTAC CTACATATAA CATAATCTGA CTTTTCTTTT   
  
  
+ TTAAATAACT TGTATTGATG GCACAGAATT TGACATGTTG GCATATGGTA CTAAGAAATT AATTAACGTT   
  
  
+ GTGCTAAGAT ATAGGTCATT CTTTTTGGAG ACATAAGGCC CGCGTTCACG CAGGGTTCGA GAAGGGCCAC   
  
  
+ ATTCAATGGA TGAATTGTAG GTAGTTTAAT CTGACTTTGT CAGTGACTGA TTCCACGGCT TGAACCCGCG   
  
  
+ ACATTAATAT AGGTTGACTA AAGTCGTTTG ATACTTATTT TTCTTAAGTA AAGTCTTGAA TTCAAATCTT   
  
  
+ ATAAATAAAT AAAAAAATCT ATAATAAAAG AATTCTACCA TTTATTAAGT CAACAACTCA ATTCGAATGA   
  
  
+ AATAAAAAAT CAAGATCACC AAAAAAAATA AATAAAAGGA AAGGTAGATT GCATTTGGAT TTTGAGTGGC   
  
  
+ GTGGTGGTGG GTTTGATGCT GGTGCTGCAT ACACCGCAGC TTTTCTCTGT CCTTTTACCT CTCTTTGTCT   
  
  
+ GCAAGAACCC CATTTGGCAC GCCTGCTCTC TTTTCTATGT TTTCTTTTCT ATCTTTTAAG AAACAATGGA   
  
  
+ AAGCTAACAC ACTAACACTA ACCCATTGGT CCTTTTTTCT TATCTGCAAA ATTAAAGTAA CAATATTTTT   
  
  
+ CTTATCCCCT TTTTCGTTCC ACCCATTTCT TTATCCTTCT GTTTGAACCT AATCTGTATA TATGCATGCA   
  
  
+ TGTGTATTTT ACTTAGAATC TGTTTTGATT CTTTGATGTA ATTAATTAGT AGTCTTTTTA ATTGAATTTT   
  
  
+ TAACAGTAGT TTGAATTAGA CATTTATCGA TATTATATTT TTTATATATA ATTAAAAAAT ACAAAATGAT   
  
  
+ AAGTGTCTTA CTACAATACA CTTCATAAGT GTCGTGTTTT AACTTAAAAA TCATACTATT ATGTTTCTAA   
  
  
+ ATTGAAAAAA AAGTGAGATT AAGTAGCAGA AGCATATTAC AACTTATAAA TTATATAAAA TCATCTTTTT   
  
  
+ ATCCATAATT TATCACATTT CTAAAATACG CATATCATAT CTACTTATGG ATAGGGTTAA TAAGATTAGG   
  
  
+ GCATCATTTA AGATTTTATC AGCGTAACCA ACGAATGTTT AAGGTTAGCA GTTCATAATT AAGTGTAATC   
  
  
+ ATTAAAAAAT TTATGATTTT AAATCATAAA ATATGTTAAT CTAGGATGAG GCAAGCGGCA AGACATATAT   
  
  
+ CACTATCCTT AAAAAATATA ATATATTGAT TTCAAAGAAT TATGAAGTAT ATTAATTTCA TATATAATGA   
  
  
+ CGATTATGTA TTTTTTTACT AAATTACTTG TACCCATAAT AAATAGAATC GATGCATCAT TCATGGCCTT   
  
  
+ TCATTCTGTA ATATGGTTGA CTCCAAAAAT TCTCAAAGTA TTTTTATATT TTGGGAGGGG AGATTTTAGT   
  
  
+ TTGATACTTT TGTTGAACTA GTGAACAATT TCCAACAACC TCCACCACCC CCAAACATCA ACAATCTGAT   
  
  
+ TGATGTATGC ATCCCAAGCT TGAATTGAAT AATAATTTCA ATAACAACAA TAATCGTTTT ACCTTGACAA   
  
  
+ GCTCCACAGC AACTCAACGT CTTCCAAACC AACCCATCAC TCATCATCAT CATCCCCACT ATCCCATTCA   
  
  
+ CCCCCACCAA AAGGAGCCTA AAGACAGTAA CAAGAAGAAT GCTTGCCTTT TTTCATGGAT GAAGAAGACT   
  
  
+ TCTCTTCTTC CTCTACTTCT TCCCACCACT ATGATACACA CCACCTCCAA CATCATTACC CCACCAATTA   
  
  
+ TAATTCCCGT TATACCCTGG ACCCTCCCTC CACCACCACC ACAGCCACAA CCACCACCTC ATCCTCCACT   
  
  
+ CCCACCCACC ATGGCTTCGG TGGTGGGCTT GACTCCTCCT CCCCGTCTGC CGCTGTGGAA TTATCTTTCT   
  
  
+ CCCCGGACAT CCTCCTCTCC TCCCCCACCG GAAAGTGGGC GTCTGACATT CTTATGGAGG CGGCTAGGGC   
  
  
+ TTTTTCAGAC ACGAACACCA CCAAGCTCCA CCAGCTCCTC TGGATGCTGA ACGAGCTCGG CTCCCCCTAC   
  
  
+ GGTGATGTCG AGCAGAAGCT GGTGGCGTAC TTTAACCAAG CCCTTTTTGC CCGCCTCACT GCCACCGGAC   
  
  
+ CCCGCCACCG AGCCGCTATG CTCGCCGCTG CGGACAAGAC CTCCTCCTTC GACTCCACGC TCAGGTTGCT   
  
  
+ CCTCAAGTTC CAGGAAGTCA GCCCGTGGAC CACCTTCGGC CACGTGGCTT GCAACGGTGC AATCATCGAG   
  
  
+ GCCTTAGAGA GGGACCCACA TGAAAAGAGT AAGCTCCACG TCATCGATAT CAGCACCACC TTCTGCACCC   
  
  
+ AGTGGCCCAC CTTGCTTGAG GCTTTAGCCA CCCGCATGGA CGACACTCCC CACCTCAGCC TCACTGCCGT   
  
  
+ CGTCGTCAAC AAGTACGCCG CCGTGAGCGG TGGCGAGGAC GACGGGGGAG AAGGATCAAA GAGGGTGATG   
  
  
+ AGAGAGATTG GTCTTCGTCT TGAGAAGTTC GCTAGGTTAA TGGGGGTTCC CTTCAAGTTC AACGTGGTTT   
  
  
+ ACCACGTAGG TGATCTATCC CAGTTGGATT TTTCTCGTTT GGATATTAAA GATGATGAAG CGGTGGCGAT   
  
  
+ TAACTGTGTC AACTCGCTCC ACTCGGTGGA CCTCCGATAT CGGGAGGTAG TGCTCGCGGC GTTCAGGCGG   
  
  
+ CTGCGGCCGA GGGTGGTGAC GGTGGTGGAG GAAGAGGCGG AGCTGACTGA GGTGGGTGAG GGTCAGTATG   
  
  
+ AATTTTTTAG GCGGTTTGAG GAGTGTCTTA GGTGGTTTAG GGTTTACTTT GAGGCCGTAG GAGATTGTTT   
  
  
+ TCCTAGGACT AGCAATGAGA AATTGATGTT AGAGCGGGCC GCGGGCCGGG CCATGGTGGA TCTCCTTGCA   
  
  
+ TGTCCCGAGC CTGGTGGGTC GGCTGAGGGG AGGGAGACGG CGAGACGGTG GTCGGGTCGG ATGCATGGGG   
  
  
+ CGGGGTTCGA GCACGTGGGG TTCAGTGACG AAGTGTGTGA TGATGTGAGG GCCTTGTTGA GGAGGTACAA
[truncated: 168,401 more chars]
