# Supplementary material for: Genome-Wide Identification and Expression Pattern of the GRAS Gene Family in Pitaya (Selenicereus undatus L.)
Source: Biology (Basel). 2022 Dec 21;12(1):11. doi: 10.3390/biology12010011 (PMC9854919; doi:10.3390/biology12010011)
Supplement: Supplementary file 1 [file biology-12-00011-s001.zip › Supplementary file S5/HU05G02245.1_plantcare.html]

Content-Type: text/html; charset=ISO-8859-1


PlantCARE


Webmaster Firefox specific output  
To save the result:
click on the frame with the right mouse button and save the source code as a text file with extension .html  
REFERENCE:PlantCARE: a database of plant cis-acting regulatory elements and a portal to tools for in silico analysis of promoter sequences.  
Lescot, M., Déhais, P., Moreau, Y., De Moor, B., Rouzé ,P.,and Rombauts, S.  
Nucleic Acids Res., Database issue(2002), 30(1):325-327.   


---

>HU05G02245.1   
+ -Up\_Stream \_Len000ATAATT TATCTTTCTT TAATTTCGTT TCATGTAAAA AATTTCAGTC TCATCTGCGA   
  
  
+ TTGAATCTCT TTTCAATTCG CATCAATAAT TCATCCAATT CAAATGTGCC TTTCTTAATT TCATTACATT   
  
  
+ GGGTTGGTAT AAATGTTAGC ACGATAGATT GGAATAGCAA GGCTCTAATT CAATTATGAC TTGAATGTAT   
  
  
+ GCATGCTTTT ATCATTAGTT AATTAACATT TTCTGTATTG AAATCAGCTC GAAAGAGAAC CATATGTAGT   
  
  
+ TGTACTTGCG ACGATATATA TGTTTAGTTT AGTTAGTTCT TACCTGTTTT GGAATTGTTG GGGAGATCTG   
  
  
+ AGGAAGGAGC CCGCTTCAAT CAAAGTTCAT GGCAATAAGC CAAAGTGAGG TTCCATCCTA AAACCAATTG   
  
  
+ GCAATAGGAA GAGTAGCCCA CTTAACTTGT ATACTATGAC TCTTCTTTTC AAACTCCCAA TGTGGGACTT   
  
  
+ CTTTACTCAT GTGTGATATT CCCAACAGGA ATGATCAGTT CCACATGTTT TCATCTCTTT GGACTCGAAC   
  
  
+ TTCGTGTAAT TGTATATGCC ATGGCACAAA TAGTTTTGAT GGTGCCACCA AGTTGCTCAA TCAATGTAAT   
  
  
+ ACTGCTACTA TCTAACTTGC CTTGGTGGAC ATCAATAAGA TCACGAGATG AGGAGAACTG AATCTTGGCC   
  
  
+ AACCACGTCG TTTGACTTCT CTTACCAATA AAGATTGAAT AGACAGTGTT TCTCTCCCCC TTCCATTCTT   
  
  
+ CTTTTCTAGT GATGTCGAAA AAGACTTTAT ACATGTCCAG CAACGCTAGC CACAAAATAT TCCATTTCTG   
  
  
+ TAATCCACTC ATGTCAATTA AACTTGTATC ATCAAATGGT AGTTTCCGTA CTAATCACTA CATCTAGATA   
  
  
+ TTCCTTGATA AATTTAATTT GACTTTAAAG GCTCGATCTT TTTAAGAAAA GTTTTGTTGC TTACAACCTG   
  
  
+ TAAAGCCTAG AGAACAAAGC CAAAGAAGAA ATTTAAGTTC TATATATAGC ATAATCTCTC AAAAAATATA   
  
  
+ TTTTTTAAAT ATAGTGCGAT ATTGACTGAC CTAATATGGG TTATTACAGG AGATGGGGAG ATATCTACAT   
  
  
+ TAACTACTTG AACAACTTAA ATTTTCATTT TAACTCGGTC ATTAAGAACA CTAAGAACTT TATATATTAG   
  
  
+ TGATATCTCA GGCGTAAAAG AAACTACTTT GAATTAATCC ATGCAATCCT ACTCTTTATT TCCATCCGTT   
  
  
+ AAAATTTTCC TTCAAAACTC CCTTCTGTTC ATTATTAATT CAATATCGTA TTACACTTGT TGGATTATTT   
  
  
+ CTGTGATTTG ATCGTCATTT TCTATATATG AATAACTACA AAAATCATAG TCCGAGTACA TTAATATTAA   
  
  
+ TAAAATAGGA TGAGTGATAA CCTCATATAG TGCGTTTATC CCTACCACTA GGCTGGATTT CTGCATAAAG   
  
  
+ AAGAAATTGA ACATATGGAA AAAGAAATAA AGAGAAGCAA CTTCCTTGAG TGGGAGAAGA ATCGTAGAAA   
  
  
+ GTTAAACTTA TGAAAAAGAG CATTAACAAA AAGGAAATTA TAAGGCTTAC CGTTGAAGCC TCATATAATT   
  
  
+ TTGCAAGAGA CAGACAAAGA AAGTGTCTTT CAAATGTCAT ATTTCTCGAG CATGCATGAA AAATGTACTT   
  
  
+ CGGCTACAAT AGTAATAGTA TATATGTTGG CTTCATACAC TTGACCCCAA TGTATGTCAA CGTGACTAGT   
  
  
+ GACCACACAC ATCGTTGTCC CTGCCCGCCC CACTACCCAA ACTCCCCGCA CGCAAAGATA AAAAGCCCCC   
  
  
+ CAATTTTTAT TTGCCTCCCA AAAAGCCCAC CCGCGCTTTT TTATTTGCCT TCTTTTTTCC TACTTAAACC   
  
  
+ ACCTCCTCAA CGAACTTTGC AACATACATA CTAGTCTATA AGCAATTTAG AGGGAAGGGA AGCAACCTAA   
  
  
+ ACAGAGCGAG ATTGGCGGGG TGTGTTTGAG GGCTTGCGTT TTGGTGCAAT AGGAATGAAA GTGCCCATTT   
  
  
+ CAACCACCAT CCAAACTTGC AACTCCGGCA CCGGCAAACC ACTTTCCTTT GAGAATACTG CCCTACATAC   
  
  
+ CGCCTTTCAA GTTCCCAATA CCACCTACGA ACCCACCTCG GTTCTTGACC TCCAGCGCAG CCCTAGCCCC   
  
  
+ ACCTCCACCC AAAAACCGCC CTTTGCCGCC TCCAACCACC AGCCTGCCTC GGCTGATCTG GACAACTTGG   
  
  
+ ACGGGTGGGA TTCAATCCTA TCAGAGCTGG GGCTTAATGA CGAATTTACC CCTAATTCCA AACTCTGGTC   
  
  
+ CCAAATTAGT CCGTCTGATC CTCACCTCCC CCAACTCCCT GATTTCCCAA CATCTCAGCC GTTTGATCAT   
  
  
+ CACAACCCAC CCCCAATAAA CCTCCCATCA TCAGATTTCA ACCTCTGTGA TTTCTCCTAC AATCACAACC   
  
  
+ CCAATTTTGG GCCGTTCGAT CTTCATCACA ACCCGCATCA GAACAACCCG AACAACAATA ACTACGGCTT   
  
  
+ TGATTTCATA GACGACCTCA TCAAAGCAGC ACAGTCCCTC GAATCCAACG ACTCTCATCA AGTCCACCTG   
  
  
+ ATATTGGCGC GGCTCAATCA ACGGCTCAGA TCACCCACCG GCAAACCGCT CCAGCGGGCC GCCTACTACT   
  
  
+ TCAAGGAAGC CCTCATCGCC GCCGCGGCCG GCCCGCCCCG CCCCGCTCGG CTTTCATCGT ATGAGGTGGT   
  
  
+ GCAGACCATC CGAGCCTACA AGGCGTTTTC AGGAATTTCG CCAGTCTCTC TCTTCTCCAC TTTCGCCGCC   
  
  
+ AACCAGGCGA TTCTTGAGGC GGTGGACACG GCGGCCTTCA TCCACATCAT CGATTTCGAC ATCGGATTCG   
  
  
+ GCGGCCACTG GGCCTCGTTC CTCCGCGAAC TGGTCGACAA AGCTGATCCT GCGAAACTTA GTTCAGTGGT   
  
  
+ GTTACGAATC ACCGCCATCG TTCCCGAAGA ATTCGGGATC GAAAGCAAGC TAGTGAGAGA AAATCTGTCG   
  
  
+ CAATTTGCCC GAGATCTCAA CATCAACTTC CACATCGATT ATGTCTTATT TCAGAGCTTT GAAATCTTAT   
  
  
+ CCTTCAAATC TGTCAAATTC ATCGAAAGGG AGAAATTGGC GCTGCATCTT TCGCCGGCCG TGTTCCACCG   
  
  
+ GCTAGGGAGC GGAATTTCGA AGTTTATCGC TGATCTCCGA TCAATCTCGC CGAGCTCCGT CGTGGTGGTG   
  
  
+ GACAGAGATG TCGGGATTGA CATCGGAACG TCGTCGTTTA GCATGAATTT CGTCGCCGGA ATTGAATTCT   
  
  
+ ACACCGGGAT GCTGGAGTCG CTTGACGCAG CCACCGCCGG GGGGCTCATC GGCGGTGTGG ACTGCGTGAG   
  
  
+ ACGGATTGAG ACGTTTGTTC TCCACCCAAG GATCATGGCG GCGGTAGAGG CGGCGGCGTC GGCGTCGGCC   
  
  
+ GGGCGGAGGA CGGCGTGGAG GGAGGCATTC GCGGCGGCGG GGATTAGGGC GGTGGGGTTT AGCCAGTTTG   
  
  
+ CTGATTTCCA GGCCGAATGT TTGTTGAGGA GAGCTCAGGT TGGTGGCTTC CACGTGGCAA AGCGCCATGG   
  
  
+ AGAGATGATG CTTTACTGGC ATGACCGCCC ACTCGTCGCC ACATCAGCTT GGAGGTGTTA   

- -Up\_Stream \_Len000TATTAA ATAGAAAGAA ATTAAAGCAA AGTACATTTT TTAAAGTCAG AGTAGACGCT   
  
  
- AACTTAGAGA AAAGTTAAGC GTAGTTATTA AGTAGGTTAA GTTTACACGG AAAGAATTAA AGTAATGTAA   
  
  
- CCCAACCATA TTTACAATCG TGCTATCTAA CCTTATCGTT CCGAGATTAA GTTAATACTG AACTTACATA   
  
  
- CGTACGAAAA TAGTAATCAA TTAATTGTAA AAGACATAAC TTTAGTCGAG CTTTCTCTTG GTATACATCA   
  
  
- ACATGAACGC TGCTATATAT ACAAATCAAA TCAATCAAGA ATGGACAAAA CCTTAACAAC CCCTCTAGAC   
  
  
- TCCTTCCTCG GGCGAAGTTA GTTTCAAGTA CCGTTATTCG GTTTCACTCC AAGGTAGGAT TTTGGTTAAC   
  
  
- CGTTATCCTT CTCATCGGGT GAATTGAACA TATGATACTG AGAAGAAAAG TTTGAGGGTT ACACCCTGAA   
  
  
- GAAATGAGTA CACACTATAA GGGTTGTCCT TACTAGTCAA GGTGTACAAA AGTAGAGAAA CCTGAGCTTG   
  
  
- AAGCACATTA ACATATACGG TACCGTGTTT ATCAAAACTA CCACGGTGGT TCAACGAGTT AGTTACATTA   
  
  
- TGACGATGAT AGATTGAACG GAACCACCTG TAGTTATTCT AGTGCTCTAC TCCTCTTGAC TTAGAACCGG   
  
  
- TTGGTGCAGC AAACTGAAGA GAATGGTTAT TTCTAACTTA TCTGTCACAA AGAGAGGGGG AAGGTAAGAA   
  
  
- GAAAAGATCA CTACAGCTTT TTCTGAAATA TGTACAGGTC GTTGCGATCG GTGTTTTATA AGGTAAAGAC   
  
  
- ATTAGGTGAG TACAGTTAAT TTGAACATAG TAGTTTACCA TCAAAGGCAT GATTAGTGAT GTAGATCTAT   
  
  
- AAGGAACTAT TTAAATTAAA CTGAAATTTC CGAGCTAGAA AAATTCTTTT CAAAACAACG AATGTTGGAC   
  
  
- ATTTCGGATC TCTTGTTTCG GTTTCTTCTT TAAATTCAAG ATATATATCG TATTAGAGAG TTTTTTATAT   
  
  
- AAAAAATTTA TATCACGCTA TAACTGACTG GATTATACCC AATAATGTCC TCTACCCCTC TATAGATGTA   
  
  
- ATTGATGAAC TTGTTGAATT TAAAAGTAAA ATTGAGCCAG TAATTCTTGT GATTCTTGAA ATATATAATC   
  
  
- ACTATAGAGT CCGCATTTTC TTTGATGAAA CTTAATTAGG TACGTTAGGA TGAGAAATAA AGGTAGGCAA   
  
  
- TTTTAAAAGG AAGTTTTGAG GGAAGACAAG TAATAATTAA GTTATAGCAT AATGTGAACA ACCTAATAAA   
  
  
- GACACTAAAC TAGCAGTAAA AGATATATAC TTATTGATGT TTTTAGTATC AGGCTCATGT AATTATAATT   
  
  
- ATTTTATCCT ACTCACTATT GGAGTATATC ACGCAAATAG GGATGGTGAT CCGACCTAAA GACGTATTTC   
  
  
- TTCTTTAACT TGTATACCTT TTTCTTTATT TCTCTTCGTT GAAGGAACTC ACCCTCTTCT TAGCATCTTT   
  
  
- CAATTTGAAT ACTTTTTCTC GTAATTGTTT TTCCTTTAAT ATTCCGAATG GCAACTTCGG AGTATATTAA   
  
  
- AACGTTCTCT GTCTGTTTCT TTCACAGAAA GTTTACAGTA TAAAGAGCTC GTACGTACTT TTTACATGAA   
  
  
- GCCGATGTTA TCATTATCAT ATATACAACC GAAGTATGTG AACTGGGGTT ACATACAGTT GCACTGATCA   
  
  
- CTGGTGTGTG TAGCAACAGG GACGGGCGGG GTGATGGGTT TGAGGGGCGT GCGTTTCTAT TTTTCGGGGG   
  
  
- GTTAAAAATA AACGGAGGGT TTTTCGGGTG GGCGCGAAAA AATAAACGGA AGAAAAAAGG ATGAATTTGG   
  
  
- TGGAGGAGTT GCTTGAAACG TTGTATGTAT GATCAGATAT TCGTTAAATC TCCCTTCCCT TCGTTGGATT   
  
  
- TGTCTCGCTC TAACCGCCCC ACACAAACTC CCGAACGCAA AACCACGTTA TCCTTACTTT CACGGGTAAA   
  
  
- GTTGGTGGTA GGTTTGAACG TTGAGGCCGT GGCCGTTTGG TGAAAGGAAA CTCTTATGAC GGGATGTATG   
  
  
- GCGGAAAGTT CAAGGGTTAT GGTGGATGCT TGGGTGGAGC CAAGAACTGG AGGTCGCGTC GGGATCGGGG   
  
  
- TGGAGGTGGG TTTTTGGCGG GAAACGGCGG AGGTTGGTGG TCGGACGGAG CCGACTAGAC CTGTTGAACC   
  
  
- TGCCCACCCT AAGTTAGGAT AGTCTCGACC CCGAATTACT GCTTAAATGG GGATTAAGGT TTGAGACCAG   
  
  
- GGTTTAATCA GGCAGACTAG GAGTGGAGGG GGTTGAGGGA CTAAAGGGTT GTAGAGTCGG CAAACTAGTA   
  
  
- GTGTTGGGTG GGGGTTATTT GGAGGGTAGT AGTCTAAAGT TGGAGACACT AAAGAGGATG TTAGTGTTGG   
  
  
- GGTTAAAACC CGGCAAGCTA GAAGTAGTGT TGGGCGTAGT CTTGTTGGGC TTGTTGTTAT TGATGCCGAA   
  
  
- ACTAAAGTAT CTGCTGGAGT AGTTTCGTCG TGTCAGGGAG CTTAGGTTGC TGAGAGTAGT TCAGGTGGAC   
  
  
- TATAACCGCG CCGAGTTAGT TGCCGAGTCT AGTGGGTGGC CGTTTGGCGA GGTCGCCCGG CGGATGATGA   
  
  
- AGTTCCTTCG GGAGTAGCGG CGGCGCCGGC CGGGCGGGGC GGGGCGAGCC GAAAGTAGCA TACTCCACCA   
  
  
- CGTCTGGTAG GCTCGGATGT TCCGCAAAAG TCCTTAAAGC GGTCAGAGAG AGAAGAGGTG AAAGCGGCGG   
  
  
- TTGGTCCGCT AAGAACTCCG CCACCTGTGC CGCCGGAAGT AGGTGTAGTA GCTAAAGCTG TAGCCTAAGC   
  
  
- CGCCGGTGAC CCGGAGCAAG GAGGCGCTTG ACCAGCTGTT TCGACTAGGA CGCTTTGAAT CAAGTCACCA   
  
  
- CAATGCTTAG TGGCGGTAGC AAGGGCTTCT TAAGCCCTAG CTTTCGTTCG ATCACTCTCT TTTAGACAGC   
  
  
- GTTAAACGGG CTCTAGAGTT GTAGTTGAAG GTGTAGCTAA TACAGAATAA AGTCTCGAAA CTTTAGAATA   
  
  
- GGAAGTTTAG ACAGTTTAAG TAGCTTTCCC TCTTTAACCG CGACGTAGAA AGCGGCCGGC ACAAGGTGGC   
  
  
- CGATCCCTCG CCTTAAAGCT TCAAATAGCG ACTAGAGGCT AGTTAGAGCG GCTCGAGGCA GCACCACCAC   
  
  
- CTGTCTCTAC AGCCCTAACT GTAGCCTTGC AGCAGCAAAT CGTACTTAAA GCAGCGGCCT TAACTTAAGA   
  
  
- TGTGGCCCTA CGACCTCAGC GAACTGCGTC GGTGGCGGCC CCCCGAGTAG CCGCCACACC TGACGCACTC   
  
  
- TGCCTAACTC TGCAAACAAG AGGTGGGTTC CTAGTACCGC CGCCATCTCC GCCGCCGCAG CCGCAGCCGG   
  
  
- CCCGCCTCCT GCCGCACCTC CCTCCGTAAG CGCCGCCGCC CCTAATCCCG CCACCCCAAA TCGGTCAAAC   
  
  
- GACTAAAGGT CCGGCTTACA AACAACTCCT CTCGAGTCCA ACCACCGAAG GTGCACCGTT TCGCGGTACC   
  
  
- TCTCTACTAC GAAATGACCG TACTGGCGGG TGAGCAGCGG TGTAGTCGAA CCTCCACAAT

  
  
Motifs Found  

+   

| Site Name | Organism | Position | Strand | Matrix score. | sequence | function |
| --- | --- | --- | --- | --- | --- | --- |
|  | organism | 2784 | + | 4 | motif\_sequence | short\_function |
|  | organism | 1117 | + | 4 | motif\_sequence | short\_function |
|  | organism | 3573 | - | 4 | motif\_sequence | short\_function |
|  | organism | 2473 | + | 4 | motif\_sequence | short\_function |
|  | organism | 1111 | - | 4 | motif\_sequence | short\_function |
|  | organism | 1103 | - | 4 | motif\_sequence | short\_function |
|  | organism | 464 | + | 4 | motif\_sequence | short\_function |
|  | organism | 67 | + | 4 | motif\_sequence | short\_function |
|  | organism | 1677 | + | 4 | motif\_sequence | short\_function |
|  | organism | 1289 | + | 4 | motif\_sequence | short\_function |
|  | organism | 807 | + | 4 | motif\_sequence | short\_function |
|  | organism | 118 | + | 4 | motif\_sequence | short\_function |
|  | organism | 3532 | - | 4 | motif\_sequence | short\_function |
|  | organism | 2553 | - | 4 | motif\_sequence | short\_function |
|  | organism | 3408 | - | 4 | motif\_sequence | short\_function |
|  | organism | 3224 | - | 4 | motif\_sequence | short\_function |
|  | organism | 661 | - | 4 | motif\_sequence | short\_function |
|  | organism | 1483 | - | 4 | motif\_sequence | short\_function |
|  | organism | 756 | + | 4 | motif\_sequence | short\_function |
|  | organism | 1390 | - | 4 | motif\_sequence | short\_function |
|  | organism | 346 | - | 4 | motif\_sequence | short\_function |
|  | organism | 432 | - | 4 | motif\_sequence | short\_function |
|  | organism | 3187 | + | 4 | motif\_sequence | short\_function |

>HU05G02245.1   
+ -Up\_Stream \_Len000ATAATT TATCTTTCTT TAATTTCGTT TCATGTAAAA AATTTCAGTC TCATCTGCGA   
  
  
+ TTGAATCTCT TTTCAATTCG CATCAATAAT TCATCCAATT CAAATGTGCC TTTCTTAATT TCATTACATT   
  
  
+ GGGTTGGTAT AAATGTTAGC ACGATAGATT GGAATAGCAA GGCTCTAATT CAATTATGAC TTGAATGTAT   
  
  
+ GCATGCTTTT ATCATTAGTT AATTAACATT TTCTGTATTG AAATCAGCTC GAAAGAGAAC CATATGTAGT   
  
  
+ TGTACTTGCG ACGATATATA TGTTTAGTTT AGTTAGTTCT TACCTGTTTT GGAATTGTTG GGGAGATCTG   
  
  
+ AGGAAGGAGC CCGCTTCAAT CAAAGTTCAT GGCAATAAGC CAAAGTGAGG TTCCATCCTA AAACCAATTG   
  
  
+ GCAATAGGAA GAGTAGCCCA CTTAACTTGT ATACTATGAC TCTTCTTTTC AAACTCCCAA TGTGGGACTT   
  
  
+ CTTTACTCAT GTGTGATATT CCCAACAGGA ATGATCAGTT CCACATGTTT TCATCTCTTT GGACTCGAAC   
  
  
+ TTCGTGTAAT TGTATATGCC ATGGCACAAA TAGTTTTGAT GGTGCCACCA AGTTGCTCAA TCAATGTAAT   
  
  
+ ACTGCTACTA TCTAACTTGC CTTGGTGGAC ATCAATAAGA TCACGAGATG AGGAGAACTG AATCTTGGCC   
  
  
+ AACCACGTCG TTTGACTTCT CTTACCAATA AAGATTGAAT AGACAGTGTT TCTCTCCCCC TTCCATTCTT   
  
  
+ CTTTTCTAGT GATGTCGAAA AAGACTTTAT ACATGTCCAG CAACGCTAGC CACAAAATAT TCCATTTCTG   
  
  
+ TAATCCACTC ATGTCAATTA AACTTGTATC ATCAAATGGT AGTTTCCGTA CTAATCACTA CATCTAGATA   
  
  
+ TTCCTTGATA AATTTAATTT GACTTTAAAG GCTCGATCTT TTTAAGAAAA GTTTTGTTGC TTACAACCTG   
  
  
+ TAAAGCCTAG AGAACAAAGC CAAAGAAGAA ATTTAAGTTC TATATATAGC ATAATCTCTC AAAAAATATA   
  
  
+ TTTTTTAAAT ATAGTGCGAT ATTGACTGAC CTAATATGGG TTATTACAGG AGATGGGGAG ATATCTACAT   
  
  
+ TAACTACTTG AACAACTTAA ATTTTCATTT TAACTCGGTC ATTAAGAACA CTAAGAACTT TATATATTAG   
  
  
+ TGATATCTCA GGCGTAAAAG AAACTACTTT GAATTAATCC ATGCAATCCT ACTCTTTATT TCCATCCGTT   
  
  
+ AAAATTTTCC TTCAAAACTC CCTTCTGTTC ATTATTAATT CAATATCGTA TTACACTTGT TGGATTATTT   
  
  
+ CTGTGATTTG ATCGTCATTT TCTATATATG AATAACTACA AAAATCATAG TCCGAGTACA TTAATATTAA   
  
  
+ TAAAATAGGA TGAGTGATAA CCTCATATAG TGCGTTTATC CCTACCACTA GGCTGGATTT CTGCATAAAG   
  
  
+ AAGAAATTGA ACATATGGAA AAAGAAATAA AGAGAAGCAA CTTCCTTGAG TGGGAGAAGA ATCGTAGAAA   
  
  
+ GTTAAACTTA TGAAAAAGAG CATTAACAAA AAGGAAATTA TAAGGCTTAC CGTTGAAGCC TCATATAATT   
  
  
+ TTGCAAGAGA CAGACAAAGA AAGTGTCTTT CAAATGTCAT ATTTCTCGAG CATGCATGAA AAATGTACTT   
  
  
+ CGGCTACAAT AGTAATAGTA TATATGTTGG CTTCATACAC TTGACCCCAA TGTATGTCAA CGTGACTAGT   
  
  
+ GACCACACAC ATCGTTGTCC CTGCCCGCCC CACTACCCAA ACTCCCCGCA CGCAAAGATA AAAAGCCCCC   
  
  
+ CAATTTTTAT TTGCCTCCCA AAAAGCCCAC CCGCGCTTTT TTATTTGCCT TCTTTTTTCC TACTTAAACC   
  
  
+ ACCTCCTCAA CGAACTTTGC AACATACATA CTAGTCTATA AGCAATTTAG AGGGAAGGGA AGCAACCTAA   
  
  
+ ACAGAGCGAG ATTGGCGGGG TGTGTTTGAG GGCTTGCGTT TTGGTGCAAT AGGAATGAAA GTGCCCATTT   
  
  
+ CAACCACCAT CCAAACTTGC AACTCCGGCA CCGGCAAACC ACTTTCCTTT GAGAATACTG CCCTACATAC   
  
  
+ CGCCTTTCAA GTTCCCAATA CCACCTACGA ACCCACCTCG GTTCTTGACC TCCAGCGCAG CCCTAGCCCC   
  
  
+ ACCTCCACCC AAAAACCGCC CTTTGCCGCC TCCAACCACC AGCCTGCCTC GGCTGATCTG GACAACTTGG   
  
  
+ ACGGGTGGGA TTCAATCCTA TCAGAGCTGG GGCTTAATGA CGAATTTACC CCTAATTCCA AACTCTGGTC   
  
  
+ CCAAATTAGT CCGTCTGATC CTCACCTCCC CCAACTCCCT GATTTCCCAA CATCTCAGCC GTTTGATCAT   
  
  
+ CACAACCCAC CCCCAATAAA CCTCCCATCA TCAGATTTCA ACCTCTGTGA TTTCTCCTAC AATCACAACC   
  
  
+ CCAATTTTGG GCCGTTCGAT CTTCATCACA ACCCGCATCA GAACAACCCG AACAACAATA ACTACGGCTT   
  
  
+ TGATTTCATA GACGACCTCA TCAAAGCAGC ACAGTCCCTC GAATCCAACG ACTCTCATCA AGTCCACCTG   
  
  
+ ATATTGGCGC GGCTCAATCA ACGGCTCAGA TCACCCACCG GCAAACCGCT CCAGCGGGCC GCCTACTACT   
  
  
+ TCAAGGAAGC CCTCATCGCC GCCGCGGCCG GCCCGCCCCG CCCCGCTCGG CTTTCATCGT ATGAGGTGGT   
  
  
+ GCAGACCATC CGAGCCTACA AGGCGTTTTC AGGAATTTCG CCAGTCTCTC TCTTCTCCAC TTTCGCCGCC   
  
  
+ AACCAGGCGA TTCTTGAGGC GGTGGACACG GCGGCCTTCA TCCACATCAT CGATTTCGAC ATCGGATTCG   
  
  
+ GCGGCCACTG GGCCTCGTTC CTCCGCGAAC TGGTCGACAA AGCTGATCCT GCGAAACTTA GTTCAGTGGT   
  
  
+ GTTACGAATC ACCGCCATCG TTCCCGAAGA ATTCGGGATC GAAAGCAAGC TAGTGAGAGA AAATCTGTCG   
  
  
+ CAATTTGCCC GAGATCTCAA CATCAACTTC CACATCGATT ATGTCTTATT TCAGAGCTTT GAAATCTTAT   
  
  
+ CCTTCAAATC TGTCAAATTC ATCGAAAGGG AGAAATTGGC GCTGCATCTT TCGCCGGCCG TGTTCCACCG   
  
  
+ GCTAGGGAGC GGAATTTCGA AGTTTATCGC TGATCTCCGA TCAATCTCGC CGAGCTCCGT CGTGGTGGTG   
  
  
+ GACAGAGATG TCGGGATTGA CATCGGAACG TCGTCGTTTA GCATGAATTT CGTCGCCGGA ATTGAATTCT   
  
  
+ ACACCGGGAT GCTGGAGTCG CTTGACGCAG CCACCGCCGG GGGGCTCATC GGCGGTGTGG ACTGCGTGAG   
  
  
+ ACGGATTGAG ACGTTTGTTC TCCACCCAAG GATCATGGCG GCGGTAGAGG CGGCGGCGTC GGCGTCGGCC   
  
  
+ GGGCGGAGGA CGGCGTGGAG GGAGGCATTC GCGGCGGCGG GGATTAGGGC GGTGGGGTTT AGCCAGTTTG   
  
  
+ CTGATTTCCA GGCCGAATGT TTGTTGAGGA GAGCTCAGGT TGGTGGCTTC CACGTGGCAA AGCGCCATGG   
  
  
+ AGAGATGATG CTTTACTGGC ATGACCGCCC ACTCGTCGCC ACATCAGCTT GGAGGTGTTA   

- -Up\_Stream \_Len000TATTAA ATAGAAAGAA ATTAAAGCAA AGTACATTTT TTAAAGTCAG AGTAGACGCT   
  
  
- AACTTAGAGA AAAGTTAAGC GTAGTTATTA AGTAGGTTAA GTTTACACGG AAAGAATTAA AGTAATGTAA   
  
  
- CCCAACCATA TTTACAATCG TGCTATCTAA CCTTATCGTT CCGAGATTAA GTTAATACTG AACTTACATA   
  
  
- CGTACGAAAA TAGTAATCAA TTAATTGTAA AAGACATAAC TTTAGTCGAG CTTTCTCTTG GTATACATCA   
  
  
- ACATGAACGC TGCTATATAT ACAAATCAAA TCAATCAAGA ATGGACAAAA CCTTAACAAC CCCTCTAGAC   
  
  
- TCCTTCCTCG GGCGAAGTTA GTTTCAAGTA CCGTTATTCG GTTTCACTCC AAGGTAGGAT TTTGGTTAAC   
  
  
- CGTTATCCTT CTCATCGGGT GAATTGAACA TATGATACTG AGAAGAAAAG TTTGAGGGTT ACACCCTGAA   
  
  
- GAAATGAGTA CACACTATAA GGGTTGTCCT TACTAGTCAA GGTGTACAAA AGTAGAGAAA CCTGAGCTTG   
  
  
- AAGCACATTA ACATATACGG TACCGTGTTT ATCAAAACTA CCACGGTGGT TCAACGAGTT AGTTACATTA   
  
  
- TGACGATGAT AGATTGAACG GAACCACCTG TAGTTATTCT AGTGCTCTAC TCCTCTTGAC TTAGAACCGG   
  
  
- TTGGTGCAGC AAACTGAAGA GAATGGTTAT TTCTAACTTA TCTGTCACAA AGAGAGGGGG AAGGTAAGAA   
  
  
- GAAAAGATCA CTACAGCTTT TTCTGAAATA TGTACAGGTC GTTGCGATCG GTGTTTTATA AGGTAAAGAC   
  
  
- ATTAGGTGAG TACAGTTAAT TTGAACATAG TAGTTTACCA TCAAAGGCAT GATTAGTGAT GTAGATCTAT   
  
  
- AAGGAACTAT TTAAATTAAA CTGAAATTTC CGAGCTAGAA AAATTCTTTT CAAAACAACG AATGTTGGAC   
  
  
- ATTTCGGATC TCTTGTTTCG GTTTCTTCTT TAAATTCAAG ATATATATCG TATTAGAGAG TTTTTTATAT   
  
  
- AAAAAATTTA TATCACGCTA TAACTGACTG GATTATACCC AATAATGTCC TCTACCCCTC TATAGATGTA   
  
  
- ATTGATGAAC TTGTTGAATT TAAAAGTAAA ATTGAGCCAG TAATTCTTGT GATTCTTGAA ATATATAATC   
  
  
- ACTATAGAGT CCGCATTTTC TTTGATGAAA CTTAATTAGG TACGTTAGGA TGAGAAATAA AGGTAGGCAA   
  
  
- TTTTAAAAGG AAGTTTTGAG GGAAGACAAG TAATAATTAA GTTATAGCAT AATGTGAACA ACCTAATAAA   
  
  
- GACACTAAAC TAGCAGTAAA AGATATATAC TTATTGATGT TTTTAGTATC AGGCTCATGT AATTATAATT   
  
  
- ATTTTATCCT ACTCACTATT GGAGTATATC ACGCAAATAG GGATGGTGAT CCGACCTAAA GACGTATTTC   
  
  
- TTCTTTAACT TGTATACCTT TTTCTTTATT TCTCTTCGTT GAAGGAACTC ACCCTCTTCT TAGCATCTTT   
  
  
- CAATTTGAAT ACTTTTTCTC GTAATTGTTT TTCCTTTAAT ATTCCGAATG GCAACTTCGG AGTATATTAA   
  
  
- AACGTTCTCT GTCTGTTTCT TTCACAGAAA GTTTACAGTA TAAAGAGCTC GTACGTACTT TTTACATGAA   
  
  
- GCCGATGTTA TCATTATCAT ATATACAACC GAAGTATGTG AACTGGGGTT ACATACAGTT GCACTGATCA   
  
  
- CTGGTGTGTG TAGCAACAGG GACGGGCGGG GTGATGGGTT TGAGGGGCGT GCGTTTCTAT TTTTCGGGGG   
  
  
- GTTAAAAATA AACGGAGGGT TTTTCGGGTG GGCGCGAAAA AATAAACGGA AGAAAAAAGG ATGAATTTGG   
  
  
- TGGAGGAGTT GCTTGAAACG TTGTATGTAT GATCAGATAT TCGTTAAATC TCCCTTCCCT TCGTTGGATT   
  
  
- TGTCTCGCTC TAACCGCCCC ACACAAACTC CCGAACGCAA AACCACGTTA TCCTTACTTT CACGGGTAAA   
  
  
- GTTGGTGGTA GGTTTGAACG TTGAGGCCGT GGCCGTTTGG TGAAAGGAAA CTCTTATGAC GGGATGTATG   
  
  
- GCGGAAAGTT CAAGGGTTAT GGTGGATGCT TGGGTGGAGC CAAGAACTGG AGGTCGCGTC GGGATCGGGG   
  
  
- TGGAGGTGGG TTTTTGGCGG GAAACGGCGG AGGTTGGTGG TCGGACGGAG CCGACTAGAC CTGTTGAACC   
  
  
- TGCCCACCCT AAGTTAGGAT AGTCTCGACC CCGAATTACT GCTTAAATGG GGATTAAGGT TTGAGACCAG   
  
  
- GGTTTAATCA GGCAGACTAG GAGTGGAGGG GGTTGAGGGA CTAAAGGGTT GTAGAGTCGG CAAACTAGTA   
  
  
- GTGTTGGGTG GGGGTTATTT GGAGGGTAGT AGTCTAAAGT TGGAGACACT AAAGAGGATG TTAGTGTTGG   
  
  
- GGTTAAAACC CGGCAAGCTA GAAGTAGTGT TGGGCGTAGT CTTGTTGGGC TTGTTGTTAT TGATGCCGAA   
  
  
- ACTAAAGTAT CTGCTGGAGT AGTTTCGTCG TGTCAGGGAG CTTAGGTTGC TGAGAGTAGT TCAGGTGGAC   
  
  
- TATAACCGCG CCGAGTTAGT TGCCGAGTCT AGTGGGTGGC CGTTTGGCGA GGTCGCCCGG CGGATGATGA   
  
  
- AGTTCCTTCG GGAGTAGCGG CGGCGCCGGC CGGGCGGGGC GGGGCGAGCC GAAAGTAGCA TACTCCACCA   
  
  
- CGTCTGGTAG GCTCGGATGT TCCGCAAAAG TCCTTAAAGC GGTCAGAGAG AGAAGAGGTG AAAGCGGCGG   
  
  
- TTGGTCCGCT AAGAACTCCG CCACCTGTGC CGCCGGAAGT AGGTGTAGTA GCTAAAGCTG TAGCCTAAGC   
  
  
- CGCCGGTGAC CCGGAGCAAG GAGGCGCTTG ACCAGCTGTT TCGACTAGGA CGCTTTGAAT CAAGTCACCA   
  
  
- CAATGCTTAG TGGCGGTAGC AAGGGCTTCT TAAGCCCTAG CTTTCGTTCG ATCACTCTCT TTTAGACAGC   
  
  
- GTTAAACGGG CTCTAGAGTT GTAGTTGAAG GTGTAGCTAA TACAGAATAA AGTCTCGAAA CTTTAGAATA   
  
  
- GGAAGTTTAG ACAGTTTAAG TAGCTTTCCC TCTTTAACCG CGACGTAGAA AGCGGCCGGC ACAAGGTGGC   
  
  
- CGATCCCTCG CCTTAAAGCT TCAAATAGCG ACTAGAGGCT AGTTAGAGCG GCTCGAGGCA GCACCACCAC   
  
  
- CTGTCTCTAC AGCCCTAACT GTAGCCTTGC AGCAGCAAAT CGTACTTAAA GCAGCGGCCT TAACTTAAGA   
  
  
- TGTGGCCCTA CGACCTCAGC GAACTGCGTC GGTGGCGGCC CCCCGAGTAG CCGCCACACC TGACGCACTC   
  
  
- TGCCTAACTC TGCAAACAAG AGGTGGGTTC CTAGTACCGC CGCCATCTCC GCCGCCGCAG CCGCAGCCGG   
  
  
- CCCGCCTCCT GCCGCACCTC CCTCCGTAAG CGCCGCCGCC CCTAATCCCG CCACCCCAAA TCGGTCAAAC   
  
  
- GACTAAAGGT CCGGCTTACA AACAACTCCT CTCGAGTCCA ACCACCGAAG GTGCACCGTT TCGCGGTACC   
  
  
- TCTCTACTAC GAAATGACCG TACTGGCGGG TGAGCAGCGG TGTAGTCGAA CCTCCACAAT

+     3-AF3 binding site

| Site Name | Organism | Position | Strand | Matrix score. | sequence | function |
| --- | --- | --- | --- | --- | --- | --- |
| 3-AF3 binding site | Pisum sativum | 640 | + | 10 | CACTATCTAAC | part of a conserved DNA module array (CMA3) |

>HU05G02245.1   
+ -Up\_Stream \_Len000ATAATT TATCTTTCTT TAATTTCGTT TCATGTAAAA AATTTCAGTC TCATCTGCGA   
  
  
+ TTGAATCTCT TTTCAATTCG CATCAATAAT TCATCCAATT CAAATGTGCC TTTCTTAATT TCATTACATT   
  
  
+ GGGTTGGTAT AAATGTTAGC ACGATAGATT GGAATAGCAA GGCTCTAATT CAATTATGAC TTGAATGTAT   
  
  
+ GCATGCTTTT ATCATTAGTT AATTAACATT TTCTGTATTG AAATCAGCTC GAAAGAGAAC CATATGTAGT   
  
  
+ TGTACTTGCG ACGATATATA TGTTTAGTTT AGTTAGTTCT TACCTGTTTT GGAATTGTTG GGGAGATCTG   
  
  
+ AGGAAGGAGC CCGCTTCAAT CAAAGTTCAT GGCAATAAGC CAAAGTGAGG TTCCATCCTA AAACCAATTG   
  
  
+ GCAATAGGAA GAGTAGCCCA CTTAACTTGT ATACTATGAC TCTTCTTTTC AAACTCCCAA TGTGGGACTT   
  
  
+ CTTTACTCAT GTGTGATATT CCCAACAGGA ATGATCAGTT CCACATGTTT TCATCTCTTT GGACTCGAAC   
  
  
+ TTCGTGTAAT TGTATATGCC ATGGCACAAA TAGTTTTGAT GGTGCCACCA AGTTGCTCAA TCAATGTAAT   
  
  
+ ACTGCTACTA TCTAACTTGC CTTGGTGGAC ATCAATAAGA TCACGAGATG AGGAGAACTG AATCTTGGCC   
  
  
+ AACCACGTCG TTTGACTTCT CTTACCAATA AAGATTGAAT AGACAGTGTT TCTCTCCCCC TTCCATTCTT   
  
  
+ CTTTTCTAGT GATGTCGAAA AAGACTTTAT ACATGTCCAG CAACGCTAGC CACAAAATAT TCCATTTCTG   
  
  
+ TAATCCACTC ATGTCAATTA AACTTGTATC ATCAAATGGT AGTTTCCGTA CTAATCACTA CATCTAGATA   
  
  
+ TTCCTTGATA AATTTAATTT GACTTTAAAG GCTCGATCTT TTTAAGAAAA GTTTTGTTGC TTACAACCTG   
  
  
+ TAAAGCCTAG AGAACAAAGC CAAAGAAGAA ATTTAAGTTC TATATATAGC ATAATCTCTC AAAAAATATA   
  
  
+ TTTTTTAAAT ATAGTGCGAT ATTGACTGAC CTAATATGGG TTATTACAGG AGATGGGGAG ATATCTACAT   
  
  
+ TAACTACTTG AACAACTTAA ATTTTCATTT TAACTCGGTC ATTAAGAACA CTAAGAACTT TATATATTAG   
  
  
+ TGATATCTCA GGCGTAAAAG AAACTACTTT GAATTAATCC ATGCAATCCT ACTCTTTATT TCCATCCGTT   
  
  
+ AAAATTTTCC TTCAAAACTC CCTTCTGTTC ATTATTAATT CAATATCGTA TTACACTTGT TGGATTATTT   
  
  
+ CTGTGATTTG ATCGTCATTT TCTATATATG AATAACTACA AAAATCATAG TCCGAGTACA TTAATATTAA   
  
  
+ TAAAATAGGA TGAGTGATAA CCTCATATAG TGCGTTTATC CCTACCACTA GGCTGGATTT CTGCATAAAG   
  
  
+ AAGAAATTGA ACATATGGAA AAAGAAATAA AGAGAAGCAA CTTCCTTGAG TGGGAGAAGA ATCGTAGAAA   
  
  
+ GTTAAACTTA TGAAAAAGAG CATTAACAAA AAGGAAATTA TAAGGCTTAC CGTTGAAGCC TCATATAATT   
  
  
+ TTGCAAGAGA CAGACAAAGA AAGTGTCTTT CAAATGTCAT ATTTCTCGAG CATGCATGAA AAATGTACTT   
  
  
+ CGGCTACAAT AGTAATAGTA TATATGTTGG CTTCATACAC TTGACCCCAA TGTATGTCAA CGTGACTAGT   
  
  
+ GACCACACAC ATCGTTGTCC CTGCCCGCCC CACTACCCAA ACTCCCCGCA CGCAAAGATA AAAAGCCCCC   
  
  
+ CAATTTTTAT TTGCCTCCCA AAAAGCCCAC CCGCGCTTTT TTATTTGCCT TCTTTTTTCC TACTTAAACC   
  
  
+ ACCTCCTCAA CGAACTTTGC AACATACATA CTAGTCTATA AGCAATTTAG AGGGAAGGGA AGCAACCTAA   
  
  
+ ACAGAGCGAG ATTGGCGGGG TGTGTTTGAG GGCTTGCGTT TTGGTGCAAT AGGAATGAAA GTGCCCATTT   
  
  
+ CAACCACCAT CCAAACTTGC AACTCCGGCA CCGGCAAACC ACTTTCCTTT GAGAATACTG CCCTACATAC   
  
  
+ CGCCTTTCAA GTTCCCAATA CCACCTACGA ACCCACCTCG GTTCTTGACC TCCAGCGCAG CCCTAGCCCC   
  
  
+ ACCTCCACCC AAAAACCGCC CTTTGCCGCC TCCAACCACC AGCCTGCCTC GGCTGATCTG GACAACTTGG   
  
  
+ ACGGGTGGGA TTCAATCCTA TCAGAGCTGG GGCTTAATGA CGAATTTACC CCTAATTCCA AACTCTGGTC   
  
  
+ CCAAATTAGT CCGTCTGATC CTCACCTCCC CCAACTCCCT GATTTCCCAA CATCTCAGCC GTTTGATCAT   
  
  
+ CACAACCCAC CCCCAATAAA CCTCCCATCA TCAGATTTCA ACCTCTGTGA TTTCTCCTAC AATCACAACC   
  
  
+ CCAATTTTGG GCCGTTCGAT CTTCATCACA ACCCGCATCA GAACAACCCG AACAACAATA ACTACGGCTT   
  
  
+ TGATTTCATA GACGACCTCA TCAAAGCAGC ACAGTCCCTC GAATCCAACG ACTCTCATCA AGTCCACCTG   
  
  
+ ATATTGGCGC GGCTCAATCA ACGGCTCAGA TCACCCACCG GCAAACCGCT CCAGCGGGCC GCCTACTACT   
  
  
+ TCAAGGAAGC CCTCATCGCC GCCGCGGCCG GCCCGCCCCG CCCCGCTCGG CTTTCATCGT ATGAGGTGGT   
  
  
+ GCAGACCATC CGAGCCTACA AGGCGTTTTC AGGAATTTCG CCAGTCTCTC TCTTCTCCAC TTTCGCCGCC   
  
  
+ AACCAGGCGA TTCTTGAGGC GGTGGACACG GCGGCCTTCA TCCACATCAT CGATTTCGAC ATCGGATTCG   
  
  
+ GCGGCCACTG GGCCTCGTTC CTCCGCGAAC TGGTCGACAA AGCTGATCCT GCGAAACTTA GTTCAGTGGT   
  
  
+ GTTACGAATC ACCGCCATCG TTCCCGAAGA ATTCGGGATC GAAAGCAAGC TAGTGAGAGA AAATCTGTCG   
  
  
+ CAATTTGCCC GAGATCTCAA CATCAACTTC CACATCGATT ATGTCTTATT TCAGAGCTTT GAAATCTTAT   
  
  
+ CCTTCAAATC TGTCAAATTC ATCGAAAGGG AGAAATTGGC GCTGCATCTT TCGCCGGCCG TGTTCCACCG   
  
  
+ GCTAGGGAGC GGAATTTCGA AGTTTATCGC TGATCTCCGA TCAATCTCGC CGAGCTCCGT CGTGGTGGTG   
  
  
+ GACAGAGATG TCGGGATTGA CATCGGAACG TCGTCGTTTA GCATGAATTT CGTCGCCGGA ATTGAATTCT   
  
  
+ ACACCGGGAT GCTGGAGTCG CTTGACGCAG CCACCGCCGG GGGGCTCATC GGCGGTGTGG ACTGCGTGAG   
  
  
+ ACGGATTGAG ACGTTTGTTC TCCACCCAAG GATCATGGCG GCGGTAGAGG CGGCGGCGTC GGCGTCGGCC   
  
  
+ GGGCGGAGGA CGGCGTGGAG GGAGGCATTC GCGGCGGCGG GGATTAGGGC GGTGGGGTTT AGCCAGTTTG   
  
  
+ CTGATTTCCA GGCCGAATGT TTGTTGAGGA GAGCTCAGGT TGGTGGCTTC CACGTGGCAA AGCGCCATGG   
  
  
+ AGAGATGATG CTTTACTGGC ATGACCGCCC ACTCGTCGCC ACATCAGCTT GGAGGTGTTA   

- -Up\_Stream \_Len000TATTAA ATAGAAAGAA ATTAAAGCAA AGTACATTTT TTAAAGTCAG AGTAGACGCT   
  
  
- AACTTAGAGA AAAGTTAAGC GTAGTTATTA AGTAGGTTAA GTTTACACGG AAAGAATTAA AGTAATGTAA   
  
  
- CCCAACCATA TTTACAATCG TGCTATCTAA CCTTATCGTT CCGAGATTAA GTTAATACTG AACTTACATA   
  
  
- CGTACGAAAA TAGTAATCAA TTAATTGTAA AAGACATAAC TTTAGTCGAG CTTTCTCTTG GTATACATCA   
  
  
- ACATGAACGC TGCTATATAT ACAAATCAAA TCAATCAAGA ATGGACAAAA CCTTAACAAC CCCTCTAGAC   
  
  
- TCCTTCCTCG GGCGAAGTTA GTTTCAAGTA CCGTTATTCG GTTTCACTCC AAGGTAGGAT TTTGGTTAAC   
  
  
- CGTTATCCTT CTCATCGGGT GAATTGAACA TATGATACTG AGAAGAAAAG TTTGAGGGTT ACACCCTGAA   
  
  
- GAAATGAGTA CACACTATAA GGGTTGTCCT TACTAGTCAA GGTGTACAAA AGTAGAGAAA CCTGAGCTTG   
  
  
- AAGCACATTA ACATATACGG TACCGTGTTT ATCAAAACTA CCACGGTGGT TCAACGAGTT AGTTACATTA   
  
  
- TGACGATGAT AGATTGAACG GAACCACCTG TAGTTATTCT AGTGCTCTAC TCCTCTTGAC TTAGAACCGG   
  
  
- TTGGTGCAGC AAACTGAAGA GAATGGTTAT TTCTAACTTA TCTGTCACAA AGAGAGGGGG AAGGTAAGAA   
  
  
- GAAAAGATCA CTACAGCTTT TTCTGAAATA TGTACAGGTC GTTGCGATCG GTGTTTTATA AGGTAAAGAC   
  
  
- ATTAGGTGAG TACAGTTAAT TTGAACATAG TAGTTTACCA TCAAAGGCAT GATTAGTGAT GTAGATCTAT   
  
  
- AAGGAACTAT TTAAATTAAA CTGAAATTTC CGAGCTAGAA AAATTCTTTT CAAAACAACG AATGTTGGAC   
  
  
- ATTTCGGATC TCTTGTTTCG GTTTCTTCTT TAAATTCAAG ATATATATCG TATTAGAGAG TTTTTTATAT   
  
  
- AAAAAATTTA TATCACGCTA TAACTGACTG GATTATACCC AATAATGTCC TCTACCCCTC TATAGATGTA   
  
  
- ATTGATGAAC TTGTTGAATT TAAAAGTAAA ATTGAGCCAG TAATTCTTGT GATTCTTGAA ATATATAATC   
  
  
- ACTATAGAGT CCGCATTTTC TTTGATGAAA CTTAATTAGG TACGTTAGGA TGAGAAATAA AGGTAGGCAA   
  
  
- TTTTAAAAGG AAGTTTTGAG GGAAGACAAG TAATAATTAA GTTATAGCAT AATGTGAACA ACCTAATAAA   
  
  
- GACACTAAAC TAGCAGTAAA AGATATATAC TTATTGATGT TTTTAGTATC AGGCTCATGT AATTATAATT   
  
  
- ATTTTATCCT ACTCACTATT GGAGTATATC ACGCAAATAG GGATGGTGAT CCGACCTAAA GACGTATTTC   
  
  
- TTCTTTAACT TGTATACCTT TTTCTTTATT TCTCTTCGTT GAAGGAACTC ACCCTCTTCT TAGCATCTTT   
  
  
- CAATTTGAAT ACTTTTTCTC GTAATTGTTT TTCCTTTAAT ATTCCGAATG GCAACTTCGG AGTATATTAA   
  
  
- AACGTTCTCT GTCTGTTTCT TTCACAGAAA GTTTACAGTA TAAAGAGCTC GTACGTACTT TTTACATGAA   
  
  
- GCCGATGTTA TCATTATCAT ATATACAACC GAAGTATGTG AACTGGGGTT ACATACAGTT GCACTGATCA   
  
  
- CTGGTGTGTG TAGCAACAGG GACGGGCGGG GTGATGGGTT TGAGGGGCGT GCGTTTCTAT TTTTCGGGGG   
  
  
- GTTAAAAATA AACGGAGGGT TTTTCGGGTG GGCGCGAAAA AATAAACGGA AGAAAAAAGG ATGAATTTGG   
  
  
- TGGAGGAGTT GCTTGAAACG TTGTATGTAT GATCAGATAT TCGTTAAATC TCCCTTCCCT TCGTTGGATT   
  
  
- TGTCTCGCTC TAACCGCCCC ACACAAACTC CCGAACGCAA AACCACGTTA TCCTTACTTT CACGGGTAAA   
  
  
- GTTGGTGGTA GGTTTGAACG TTGAGGCCGT GGCCGTTTGG TGAAAGGAAA CTCTTATGAC GGGATGTATG   
  
  
- GCGGAAAGTT CAAGGGTTAT GGTGGATGCT TGGGTGGAGC CAAGAACTGG AGGTCGCGTC GGGATCGGGG   
  
  
- TGGAGGTGGG TTTTTGGCGG GAAACGGCGG AGGTTGGTGG TCGGACGGAG CCGACTAGAC CTGTTGAACC   
  
  
- TGCCCACCCT AAGTTAGGAT AGTCTCGACC CCGAATTACT GCTTAAATGG GGATTAAGGT TTGAGACCAG   
  
  
- GGTTTAATCA GGCAGACTAG GAGTGGAGGG GGTTGAGGGA CTAAAGGGTT GTAGAGTCGG CAAACTAGTA   
  
  
- GTGTTGGGTG GGGGTTATTT GGAGGGTAGT AGTCTAAAGT TGGAGACACT AAAGAGGATG TTAGTGTTGG   
  
  
- GGTTAAAACC CGGCAAGCTA GAAGTAGTGT TGGGCGTAGT CTTGTTGGGC TTGTTGTTAT TGATGCCGAA   
  
  
- ACTAAAGTAT CTGCTGGAGT AGTTTCGTCG TGTCAGGGAG CTTAGGTTGC TGAGAGTAGT TCAGGTGGAC   
  
  
- TATAACCGCG CCGAGTTAGT TGCCGAGTCT AGTGGGTGGC CGTTTGGCGA GGTCGCCCGG CGGATGATGA   
  
  
- AGTTCCTTCG GGAGTAGCGG CGGCGCCGGC CGGGCGGGGC GGGGCGAGCC GAAAGTAGCA TACTCCACCA   
  
  
- CGTCTGGTAG GCTCGGATGT TCCGCAAAAG TCCTTAAAGC GGTCAGAGAG AGAAGAGGTG AAAGCGGCGG   
  
  
- TTGGTCCGCT AAGAACTCCG CCACCTGTGC CGCCGGAAGT AGGTGTAGTA GCTAAAGCTG TAGCCTAAGC   
  
  
- CGCCGGTGAC CCGGAGCAAG GAGGCGCTTG ACCAGCTGTT TCGACTAGGA CGCTTTGAAT CAAGTCACCA   
  
  
- CAATGCTTAG TGGCGGTAGC AAGGGCTTCT TAAGCCCTAG CTTTCGTTCG ATCACTCTCT TTTAGACAGC   
  
  
- GTTAAACGGG CTCTAGAGTT GTAGTTGAAG GTGTAGCTAA TACAGAATAA AGTCTCGAAA CTTTAGAATA   
  
  
- GGAAGTTTAG ACAGTTTAAG TAGCTTTCCC TCTTTAACCG CGACGTAGAA AGCGGCCGGC ACAAGGTGGC   
  
  
- CGATCCCTCG CCTTAAAGCT TCAAATAGCG ACTAGAGGCT AGTTAGAGCG GCTCGAGGCA GCACCACCAC   
  
  
- CTGTCTCTAC AGCCCTAACT GTAGCCTTGC AGCAGCAAAT CGTACTTAAA GCAGCGGCCT TAACTTAAGA   
  
  
- TGTGGCCCTA CGACCTCAGC GAACTGCGTC GGTGGCGGCC CCCCGAGTAG CCGCCACACC TGACGCACTC   
  
  
- TGCCTAACTC TGCAAACAAG AGGTGGGTTC CTAGTACCGC CGCCATCTCC GCCGCCGCAG CCGCAGCCGG   
  
  
- CCCGCCTCCT GCCGCACCTC CCTCCGTAAG CGCCGCCGCC CCTAATCCCG CCACCCCAAA TCGGTCAAAC   
  
  
- GACTAAAGGT CCGGCTTACA AACAACTCCT CTCGAGTCCA ACCACCGAAG GTGCACCGTT TCGCGGTACC   
  
  
- TCTCTACTAC GAAATGACCG TACTGGCGGG TGAGCAGCGG TGTAGTCGAA CCTCCACAAT

+     A-box

| Site Name | Organism | Position | Strand | Matrix score. | sequence | function |
| --- | --- | --- | --- | --- | --- | --- |
| A-box | Petroselinum crispum | 2243 | - | 6 | CCGTCC | cis-acting regulatory element |
| A-box | Petroselinum crispum | 3442 | - | 6 | CCGTCC | cis-acting regulatory element |

>HU05G02245.1   
+ -Up\_Stream \_Len000ATAATT TATCTTTCTT TAATTTCGTT TCATGTAAAA AATTTCAGTC TCATCTGCGA   
  
  
+ TTGAATCTCT TTTCAATTCG CATCAATAAT TCATCCAATT CAAATGTGCC TTTCTTAATT TCATTACATT   
  
  
+ GGGTTGGTAT AAATGTTAGC ACGATAGATT GGAATAGCAA GGCTCTAATT CAATTATGAC TTGAATGTAT   
  
  
+ GCATGCTTTT ATCATTAGTT AATTAACATT TTCTGTATTG AAATCAGCTC GAAAGAGAAC CATATGTAGT   
  
  
+ TGTACTTGCG ACGATATATA TGTTTAGTTT AGTTAGTTCT TACCTGTTTT GGAATTGTTG GGGAGATCTG   
  
  
+ AGGAAGGAGC CCGCTTCAAT CAAAGTTCAT GGCAATAAGC CAAAGTGAGG TTCCATCCTA AAACCAATTG   
  
  
+ GCAATAGGAA GAGTAGCCCA CTTAACTTGT ATACTATGAC TCTTCTTTTC AAACTCCCAA TGTGGGACTT   
  
  
+ CTTTACTCAT GTGTGATATT CCCAACAGGA ATGATCAGTT CCACATGTTT TCATCTCTTT GGACTCGAAC   
  
  
+ TTCGTGTAAT TGTATATGCC ATGGCACAAA TAGTTTTGAT GGTGCCACCA AGTTGCTCAA TCAATGTAAT   
  
  
+ ACTGCTACTA TCTAACTTGC CTTGGTGGAC ATCAATAAGA TCACGAGATG AGGAGAACTG AATCTTGGCC   
  
  
+ AACCACGTCG TTTGACTTCT CTTACCAATA AAGATTGAAT AGACAGTGTT TCTCTCCCCC TTCCATTCTT   
  
  
+ CTTTTCTAGT GATGTCGAAA AAGACTTTAT ACATGTCCAG CAACGCTAGC CACAAAATAT TCCATTTCTG   
  
  
+ TAATCCACTC ATGTCAATTA AACTTGTATC ATCAAATGGT AGTTTCCGTA CTAATCACTA CATCTAGATA   
  
  
+ TTCCTTGATA AATTTAATTT GACTTTAAAG GCTCGATCTT TTTAAGAAAA GTTTTGTTGC TTACAACCTG   
  
  
+ TAAAGCCTAG AGAACAAAGC CAAAGAAGAA ATTTAAGTTC TATATATAGC ATAATCTCTC AAAAAATATA   
  
  
+ TTTTTTAAAT ATAGTGCGAT ATTGACTGAC CTAATATGGG TTATTACAGG AGATGGGGAG ATATCTACAT   
  
  
+ TAACTACTTG AACAACTTAA ATTTTCATTT TAACTCGGTC ATTAAGAACA CTAAGAACTT TATATATTAG   
  
  
+ TGATATCTCA GGCGTAAAAG AAACTACTTT GAATTAATCC ATGCAATCCT ACTCTTTATT TCCATCCGTT   
  
  
+ AAAATTTTCC TTCAAAACTC CCTTCTGTTC ATTATTAATT CAATATCGTA TTACACTTGT TGGATTATTT   
  
  
+ CTGTGATTTG ATCGTCATTT TCTATATATG AATAACTACA AAAATCATAG TCCGAGTACA TTAATATTAA   
  
  
+ TAAAATAGGA TGAGTGATAA CCTCATATAG TGCGTTTATC CCTACCACTA GGCTGGATTT CTGCATAAAG   
  
  
+ AAGAAATTGA ACATATGGAA AAAGAAATAA AGAGAAGCAA CTTCCTTGAG TGGGAGAAGA ATCGTAGAAA   
  
  
+ GTTAAACTTA TGAAAAAGAG CATTAACAAA AAGGAAATTA TAAGGCTTAC CGTTGAAGCC TCATATAATT   
  
  
+ TTGCAAGAGA CAGACAAAGA AAGTGTCTTT CAAATGTCAT ATTTCTCGAG CATGCATGAA AAATGTACTT   
  
  
+ CGGCTACAAT AGTAATAGTA TATATGTTGG CTTCATACAC TTGACCCCAA TGTATGTCAA CGTGACTAGT   
  
  
+ GACCACACAC ATCGTTGTCC CTGCCCGCCC CACTACCCAA ACTCCCCGCA CGCAAAGATA AAAAGCCCCC   
  
  
+ CAATTTTTAT TTGCCTCCCA AAAAGCCCAC CCGCGCTTTT TTATTTGCCT TCTTTTTTCC TACTTAAACC   
  
  
+ ACCTCCTCAA CGAACTTTGC AACATACATA CTAGTCTATA AGCAATTTAG AGGGAAGGGA AGCAACCTAA   
  
  
+ ACAGAGCGAG ATTGGCGGGG TGTGTTTGAG GGCTTGCGTT TTGGTGCAAT AGGAATGAAA GTGCCCATTT   
  
  
+ CAACCACCAT CCAAACTTGC AACTCCGGCA CCGGCAAACC ACTTTCCTTT GAGAATACTG CCCTACATAC   
  
  
+ CGCCTTTCAA GTTCCCAATA CCACCTACGA ACCCACCTCG GTTCTTGACC TCCAGCGCAG CCCTAGCCCC   
  
  
+ ACCTCCACCC AAAAACCGCC CTTTGCCGCC TCCAACCACC AGCCTGCCTC GGCTGATCTG GACAACTTGG   
  
  
+ ACGGGTGGGA TTCAATCCTA TCAGAGCTGG GGCTTAATGA CGAATTTACC CCTAATTCCA AACTCTGGTC   
  
  
+ CCAAATTAGT CCGTCTGATC CTCACCTCCC CCAACTCCCT GATTTCCCAA CATCTCAGCC GTTTGATCAT   
  
  
+ CACAACCCAC CCCCAATAAA CCTCCCATCA TCAGATTTCA ACCTCTGTGA TTTCTCCTAC AATCACAACC   
  
  
+ CCAATTTTGG GCCGTTCGAT CTTCATCACA ACCCGCATCA GAACAACCCG AACAACAATA ACTACGGCTT   
  
  
+ TGATTTCATA GACGACCTCA TCAAAGCAGC ACAGTCCCTC GAATCCAACG ACTCTCATCA AGTCCACCTG   
  
  
+ ATATTGGCGC GGCTCAATCA ACGGCTCAGA TCACCCACCG GCAAACCGCT CCAGCGGGCC GCCTACTACT   
  
  
+ TCAAGGAAGC CCTCATCGCC GCCGCGGCCG GCCCGCCCCG CCCCGCTCGG CTTTCATCGT ATGAGGTGGT   
  
  
+ GCAGACCATC CGAGCCTACA AGGCGTTTTC AGGAATTTCG CCAGTCTCTC TCTTCTCCAC TTTCGCCGCC   
  
  
+ AACCAGGCGA TTCTTGAGGC GGTGGACACG GCGGCCTTCA TCCACATCAT CGATTTCGAC ATCGGATTCG   
  
  
+ GCGGCCACTG GGCCTCGTTC CTCCGCGAAC TGGTCGACAA AGCTGATCCT GCGAAACTTA GTTCAGTGGT   
  
  
+ GTTACGAATC ACCGCCATCG TTCCCGAAGA ATTCGGGATC GAAAGCAAGC TAGTGAGAGA AAATCTGTCG   
  
  
+ CAATTTGCCC GAGATCTCAA CATCAACTTC CACATCGATT ATGTCTTATT TCAGAGCTTT GAAATCTTAT   
  
  
+ CCTTCAAATC TGTCAAATTC ATCGAAAGGG AGAAATTGGC GCTGCATCTT TCGCCGGCCG TGTTCCACCG   
  
  
+ GCTAGGGAGC GGAATTTCGA AGTTTATCGC TGATCTCCGA TCAATCTCGC CGAGCTCCGT CGTGGTGGTG   
  
  
+ GACAGAGATG TCGGGATTGA CATCGGAACG TCGTCGTTTA GCATGAATTT CGTCGCCGGA ATTGAATTCT   
  
  
+ ACACCGGGAT GCTGGAGTCG CTTGACGCAG CCACCGCCGG GGGGCTCATC GGCGGTGTGG ACTGCGTGAG   
  
  
+ ACGGATTGAG ACGTTTGTTC TCCACCCAAG GATCATGGCG GCGGTAGAGG CGGCGGCGTC GGCGTCGGCC   
  
  
+ GGGCGGAGGA CGGCGTGGAG GGAGGCATTC GCGGCGGCGG GGATTAGGGC GGTGGGGTTT AGCCAGTTTG   
  
  
+ CTGATTTCCA GGCCGAATGT TTGTTGAGGA GAGCTCAGGT TGGTGGCTTC CACGTGGCAA AGCGCCATGG   
  
  
+ AGAGATGATG CTTTACTGGC ATGACCGCCC ACTCGTCGCC ACATCAGCTT GGAGGTGTTA   

- -Up\_Stream \_Len000TATTAA ATAGAAAGAA ATTAAAGCAA AGTACATTTT TTAAAGTCAG AGTAGACGCT   
  
  
- AACTTAGAGA AAAGTTAAGC GTAGTTATTA AGTAGGTTAA GTTTACACGG AAAGAATTAA AGTAATGTAA   
  
  
- CCCAACCATA TTTACAATCG TGCTATCTAA CCTTATCGTT CCGAGATTAA GTTAATACTG AACTTACATA   
  
  
- CGTACGAAAA TAGTAATCAA TTAATTGTAA AAGACATAAC TTTAGTCGAG CTTTCTCTTG GTATACATCA   
  
  
- ACATGAACGC TGCTATATAT ACAAATCAAA TCAATCAAGA ATGGACAAAA CCTTAACAAC CCCTCTAGAC   
  
  
- TCCTTCCTCG GGCGAAGTTA GTTTCAAGTA CCGTTATTCG GTTTCACTCC AAGGTAGGAT TTTGGTTAAC   
  
  
- CGTTATCCTT CTCATCGGGT GAATTGAACA TATGATACTG AGAAGAAAAG TTTGAGGGTT ACACCCTGAA   
  
  
- GAAATGAGTA CACACTATAA GGGTTGTCCT TACTAGTCAA GGTGTACAAA AGTAGAGAAA CCTGAGCTTG   
  
  
- AAGCACATTA ACATATACGG TACCGTGTTT ATCAAAACTA CCACGGTGGT TCAACGAGTT AGTTACATTA   
  
  
- TGACGATGAT AGATTGAACG GAACCACCTG TAGTTATTCT AGTGCTCTAC TCCTCTTGAC TTAGAACCGG   
  
  
- TTGGTGCAGC AAACTGAAGA GAATGGTTAT TTCTAACTTA TCTGTCACAA AGAGAGGGGG AAGGTAAGAA   
  
  
- GAAAAGATCA CTACAGCTTT TTCTGAAATA TGTACAGGTC GTTGCGATCG GTGTTTTATA AGGTAAAGAC   
  
  
- ATTAGGTGAG TACAGTTAAT TTGAACATAG TAGTTTACCA TCAAAGGCAT GATTAGTGAT GTAGATCTAT   
  
  
- AAGGAACTAT TTAAATTAAA CTGAAATTTC CGAGCTAGAA AAATTCTTTT CAAAACAACG AATGTTGGAC   
  
  
- ATTTCGGATC TCTTGTTTCG GTTTCTTCTT TAAATTCAAG ATATATATCG TATTAGAGAG TTTTTTATAT   
  
  
- AAAAAATTTA TATCACGCTA TAACTGACTG GATTATACCC AATAATGTCC TCTACCCCTC TATAGATGTA   
  
  
- ATTGATGAAC TTGTTGAATT TAAAAGTAAA ATTGAGCCAG TAATTCTTGT GATTCTTGAA ATATATAATC   
  
  
- ACTATAGAGT CCGCATTTTC TTTGATGAAA CTTAATTAGG TACGTTAGGA TGAGAAATAA AGGTAGGCAA   
  
  
- TTTTAAAAGG AAGTTTTGAG GGAAGACAAG TAATAATTAA GTTATAGCAT AATGTGAACA ACCTAATAAA   
  
  
- GACACTAAAC TAGCAGTAAA AGATATATAC TTATTGATGT TTTTAGTATC AGGCTCATGT AATTATAATT   
  
  
- ATTTTATCCT ACTCACTATT GGAGTATATC ACGCAAATAG GGATGGTGAT CCGACCTAAA GACGTATTTC   
  
  
- TTCTTTAACT TGTATACCTT TTTCTTTATT TCTCTTCGTT GAAGGAACTC ACCCTCTTCT TAGCATCTTT   
  
  
- CAATTTGAAT ACTTTTTCTC GTAATTGTTT TTCCTTTAAT ATTCCGAATG GCAACTTCGG AGTATATTAA   
  
  
- AACGTTCTCT GTCTGTTTCT TTCACAGAAA GTTTACAGTA TAAAGAGCTC GTACGTACTT TTTACATGAA   
  
  
- GCCGATGTTA TCATTATCAT ATATACAACC GAAGTATGTG AACTGGGGTT ACATACAGTT GCACTGATCA   
  
  
- CTGGTGTGTG TAGCAACAGG GACGGGCGGG GTGATGGGTT TGAGGGGCGT GCGTTTCTAT TTTTCGGGGG   
  
  
- GTTAAAAATA AACGGAGGGT TTTTCGGGTG GGCGCGAAAA AATAAACGGA AGAAAAAAGG ATGAATTTGG   
  
  
- TGGAGGAGTT GCTTGAAACG TTGTATGTAT GATCAGATAT TCGTTAAATC TCCCTTCCCT TCGTTGGATT   
  
  
- TGTCTCGCTC TAACCGCCCC ACACAAACTC CCGAACGCAA AACCACGTTA TCCTTACTTT CACGGGTAAA   
  
  
- GTTGGTGGTA GGTTTGAACG TTGAGGCCGT GGCCGTTTGG TGAAAGGAAA CTCTTATGAC GGGATGTATG   
  
  
- GCGGAAAGTT CAAGGGTTAT GGTGGATGCT TGGGTGGAGC CAAGAACTGG AGGTCGCGTC GGGATCGGGG   
  
  
- TGGAGGTGGG TTTTTGGCGG GAAACGGCGG AGGTTGGTGG TCGGACGGAG CCGACTAGAC CTGTTGAACC   
  
  
- TGCCCACCCT AAGTTAGGAT AGTCTCGACC CCGAATTACT GCTTAAATGG GGATTAAGGT TTGAGACCAG   
  
  
- GGTTTAATCA GGCAGACTAG GAGTGGAGGG GGTTGAGGGA CTAAAGGGTT GTAGAGTCGG CAAACTAGTA   
  
  
- GTGTTGGGTG GGGGTTATTT GGAGGGTAGT AGTCTAAAGT TGGAGACACT AAAGAGGATG TTAGTGTTGG   
  
  
- GGTTAAAACC CGGCAAGCTA GAAGTAGTGT TGGGCGTAGT CTTGTTGGGC TTGTTGTTAT TGATGCCGAA   
  
  
- ACTAAAGTAT CTGCTGGAGT AGTTTCGTCG TGTCAGGGAG CTTAGGTTGC TGAGAGTAGT TCAGGTGGAC   
  
  
- TATAACCGCG CCGAGTTAGT TGCCGAGTCT AGTGGGTGGC CGTTTGGCGA GGTCGCCCGG CGGATGATGA   
  
  
- AGTTCCTTCG GGAGTAGCGG CGGCGCCGGC CGGGCGGGGC GGGGCGAGCC GAAAGTAGCA TACTCCACCA   
  
  
- CGTCTGGTAG GCTCGGATGT TCCGCAAAAG TCCTTAAAGC GGTCAGAGAG AGAAGAGGTG AAAGCGGCGG   
  
  
- TTGGTCCGCT AAGAACTCCG CCACCTGTGC CGCCGGAAGT AGGTGTAGTA GCTAAAGCTG TAGCCTAAGC   
  
  
- CGCCGGTGAC CCGGAGCAAG GAGGCGCTTG ACCAGCTGTT TCGACTAGGA CGCTTTGAAT CAAGTCACCA   
  
  
- CAATGCTTAG TGGCGGTAGC AAGGGCTTCT TAAGCCCTAG CTTTCGTTCG ATCACTCTCT TTTAGACAGC   
  
  
- GTTAAACGGG CTCTAGAGTT GTAGTTGAAG GTGTAGCTAA TACAGAATAA AGTCTCGAAA CTTTAGAATA   
  
  
- GGAAGTTTAG ACAGTTTAAG TAGCTTTCCC TCTTTAACCG CGACGTAGAA AGCGGCCGGC ACAAGGTGGC   
  
  
- CGATCCCTCG CCTTAAAGCT TCAAATAGCG ACTAGAGGCT AGTTAGAGCG GCTCGAGGCA GCACCACCAC   
  
  
- CTGTCTCTAC AGCCCTAACT GTAGCCTTGC AGCAGCAAAT CGTACTTAAA GCAGCGGCCT TAACTTAAGA   
  
  
- TGTGGCCCTA CGACCTCAGC GAACTGCGTC GGTGGCGGCC CCCCGAGTAG CCGCCACACC TGACGCACTC   
  
  
- TGCCTAACTC TGCAAACAAG AGGTGGGTTC CTAGTACCGC CGCCATCTCC GCCGCCGCAG CCGCAGCCGG   
  
  
- CCCGCCTCCT GCCGCACCTC CCTCCGTAAG CGCCGCCGCC CCTAATCCCG CCACCCCAAA TCGGTCAAAC   
  
  
- GACTAAAGGT CCGGCTTACA AACAACTCCT CTCGAGTCCA ACCACCGAAG GTGCACCGTT TCGCGGTACC   
  
  
- TCTCTACTAC GAAATGACCG TACTGGCGGG TGAGCAGCGG TGTAGTCGAA CCTCCACAAT

+     ABRE

| Site Name | Organism | Position | Strand | Matrix score. | sequence | function |
| --- | --- | --- | --- | --- | --- | --- |
| ABRE | Arabidopsis thaliana | 3556 | + | 5 | ACGTG | cis-acting element involved in the abscisic acid responsiveness |
| ABRE | Arabidopsis thaliana | 3555 | - | 6 | CACGTG | cis-acting element involved in the abscisic acid responsiveness |
| ABRE | Arabidopsis thaliana | 1744 | + | 5 | ACGTG | cis-acting element involved in the abscisic acid responsiveness |
| ABRE | Arabidopsis thaliana | 708 | - | 5 | ACGTG | cis-acting element involved in the abscisic acid responsiveness |

>HU05G02245.1   
+ -Up\_Stream \_Len000ATAATT TATCTTTCTT TAATTTCGTT TCATGTAAAA AATTTCAGTC TCATCTGCGA   
  
  
+ TTGAATCTCT TTTCAATTCG CATCAATAAT TCATCCAATT CAAATGTGCC TTTCTTAATT TCATTACATT   
  
  
+ GGGTTGGTAT AAATGTTAGC ACGATAGATT GGAATAGCAA GGCTCTAATT CAATTATGAC TTGAATGTAT   
  
  
+ GCATGCTTTT ATCATTAGTT AATTAACATT TTCTGTATTG AAATCAGCTC GAAAGAGAAC CATATGTAGT   
  
  
+ TGTACTTGCG ACGATATATA TGTTTAGTTT AGTTAGTTCT TACCTGTTTT GGAATTGTTG GGGAGATCTG   
  
  
+ AGGAAGGAGC CCGCTTCAAT CAAAGTTCAT GGCAATAAGC CAAAGTGAGG TTCCATCCTA AAACCAATTG   
  
  
+ GCAATAGGAA GAGTAGCCCA CTTAACTTGT ATACTATGAC TCTTCTTTTC AAACTCCCAA TGTGGGACTT   
  
  
+ CTTTACTCAT GTGTGATATT CCCAACAGGA ATGATCAGTT CCACATGTTT TCATCTCTTT GGACTCGAAC   
  
  
+ TTCGTGTAAT TGTATATGCC ATGGCACAAA TAGTTTTGAT GGTGCCACCA AGTTGCTCAA TCAATGTAAT   
  
  
+ ACTGCTACTA TCTAACTTGC CTTGGTGGAC ATCAATAAGA TCACGAGATG AGGAGAACTG AATCTTGGCC   
  
  
+ AACCACGTCG TTTGACTTCT CTTACCAATA AAGATTGAAT AGACAGTGTT TCTCTCCCCC TTCCATTCTT   
  
  
+ CTTTTCTAGT GATGTCGAAA AAGACTTTAT ACATGTCCAG CAACGCTAGC CACAAAATAT TCCATTTCTG   
  
  
+ TAATCCACTC ATGTCAATTA AACTTGTATC ATCAAATGGT AGTTTCCGTA CTAATCACTA CATCTAGATA   
  
  
+ TTCCTTGATA AATTTAATTT GACTTTAAAG GCTCGATCTT TTTAAGAAAA GTTTTGTTGC TTACAACCTG   
  
  
+ TAAAGCCTAG AGAACAAAGC CAAAGAAGAA ATTTAAGTTC TATATATAGC ATAATCTCTC AAAAAATATA   
  
  
+ TTTTTTAAAT ATAGTGCGAT ATTGACTGAC CTAATATGGG TTATTACAGG AGATGGGGAG ATATCTACAT   
  
  
+ TAACTACTTG AACAACTTAA ATTTTCATTT TAACTCGGTC ATTAAGAACA CTAAGAACTT TATATATTAG   
  
  
+ TGATATCTCA GGCGTAAAAG AAACTACTTT GAATTAATCC ATGCAATCCT ACTCTTTATT TCCATCCGTT   
  
  
+ AAAATTTTCC TTCAAAACTC CCTTCTGTTC ATTATTAATT CAATATCGTA TTACACTTGT TGGATTATTT   
  
  
+ CTGTGATTTG ATCGTCATTT TCTATATATG AATAACTACA AAAATCATAG TCCGAGTACA TTAATATTAA   
  
  
+ TAAAATAGGA TGAGTGATAA CCTCATATAG TGCGTTTATC CCTACCACTA GGCTGGATTT CTGCATAAAG   
  
  
+ AAGAAATTGA ACATATGGAA AAAGAAATAA AGAGAAGCAA CTTCCTTGAG TGGGAGAAGA ATCGTAGAAA   
  
  
+ GTTAAACTTA TGAAAAAGAG CATTAACAAA AAGGAAATTA TAAGGCTTAC CGTTGAAGCC TCATATAATT   
  
  
+ TTGCAAGAGA CAGACAAAGA AAGTGTCTTT CAAATGTCAT ATTTCTCGAG CATGCATGAA AAATGTACTT   
  
  
+ CGGCTACAAT AGTAATAGTA TATATGTTGG CTTCATACAC TTGACCCCAA TGTATGTCAA CGTGACTAGT   
  
  
+ GACCACACAC ATCGTTGTCC CTGCCCGCCC CACTACCCAA ACTCCCCGCA CGCAAAGATA AAAAGCCCCC   
  
  
+ CAATTTTTAT TTGCCTCCCA AAAAGCCCAC CCGCGCTTTT TTATTTGCCT TCTTTTTTCC TACTTAAACC   
  
  
+ ACCTCCTCAA CGAACTTTGC AACATACATA CTAGTCTATA AGCAATTTAG AGGGAAGGGA AGCAACCTAA   
  
  
+ ACAGAGCGAG ATTGGCGGGG TGTGTTTGAG GGCTTGCGTT TTGGTGCAAT AGGAATGAAA GTGCCCATTT   
  
  
+ CAACCACCAT CCAAACTTGC AACTCCGGCA CCGGCAAACC ACTTTCCTTT GAGAATACTG CCCTACATAC   
  
  
+ CGCCTTTCAA GTTCCCAATA CCACCTACGA ACCCACCTCG GTTCTTGACC TCCAGCGCAG CCCTAGCCCC   
  
  
+ ACCTCCACCC AAAAACCGCC CTTTGCCGCC TCCAACCACC AGCCTGCCTC GGCTGATCTG GACAACTTGG   
  
  
+ ACGGGTGGGA TTCAATCCTA TCAGAGCTGG GGCTTAATGA CGAATTTACC CCTAATTCCA AACTCTGGTC   
  
  
+ CCAAATTAGT CCGTCTGATC CTCACCTCCC CCAACTCCCT GATTTCCCAA CATCTCAGCC GTTTGATCAT   
  
  
+ CACAACCCAC CCCCAATAAA CCTCCCATCA TCAGATTTCA ACCTCTGTGA TTTCTCCTAC AATCACAACC   
  
  
+ CCAATTTTGG GCCGTTCGAT CTTCATCACA ACCCGCATCA GAACAACCCG AACAACAATA ACTACGGCTT   
  
  
+ TGATTTCATA GACGACCTCA TCAAAGCAGC ACAGTCCCTC GAATCCAACG ACTCTCATCA AGTCCACCTG   
  
  
+ ATATTGGCGC GGCTCAATCA ACGGCTCAGA TCACCCACCG GCAAACCGCT CCAGCGGGCC GCCTACTACT   
  
  
+ TCAAGGAAGC CCTCATCGCC GCCGCGGCCG GCCCGCCCCG CCCCGCTCGG CTTTCATCGT ATGAGGTGGT   
  
  
+ GCAGACCATC CGAGCCTACA AGGCGTTTTC AGGAATTTCG CCAGTCTCTC TCTTCTCCAC TTTCGCCGCC   
  
  
+ AACCAGGCGA TTCTTGAGGC GGTGGACACG GCGGCCTTCA TCCACATCAT CGATTTCGAC ATCGGATTCG   
  
  
+ GCGGCCACTG GGCCTCGTTC CTCCGCGAAC TGGTCGACAA AGCTGATCCT GCGAAACTTA GTTCAGTGGT   
  
  
+ GTTACGAATC ACCGCCATCG TTCCCGAAGA ATTCGGGATC GAAAGCAAGC TAGTGAGAGA AAATCTGTCG   
  
  
+ CAATTTGCCC GAGATCTCAA CATCAACTTC CACATCGATT ATGTCTTATT TCAGAGCTTT GAAATCTTAT   
  
  
+ CCTTCAAATC TGTCAAATTC ATCGAAAGGG AGAAATTGGC GCTGCATCTT TCGCCGGCCG TGTTCCACCG   
  
  
+ GCTAGGGAGC GGAATTTCGA AGTTTATCGC TGATCTCCGA TCAATCTCGC CGAGCTCCGT CGTGGTGGTG   
  
  
+ GACAGAGATG TCGGGATTGA CATCGGAACG TCGTCGTTTA GCATGAATTT CGTCGCCGGA ATTGAATTCT   
  
  
+ ACACCGGGAT GCTGGAGTCG CTTGACGCAG CCACCGCCGG GGGGCTCATC GGCGGTGTGG ACTGCGTGAG   
  
  
+ ACGGATTGAG ACGTTTGTTC TCCACCCAAG GATCATGGCG GCGGTAGAGG CGGCGGCGTC GGCGTCGGCC   
  
  
+ GGGCGGAGGA CGGCGTGGAG GGAGGCATTC GCGGCGGCGG GGATTAGGGC GGTGGGGTTT AGCCAGTTTG   
  
  
+ CTGATTTCCA GGCCGAATGT TTGTTGAGGA GAGCTCAGGT TGGTGGCTTC CACGTGGCAA AGCGCCATGG   
  
  
+ AGAGATGATG CTTTACTGGC ATGACCGCCC ACTCGTCGCC ACATCAGCTT GGAGGTGTTA   

- -Up\_Stream \_Len000TATTAA ATAGAAAGAA ATTAAAGCAA AGTACATTTT TTAAAGTCAG AGTAGACGCT   
  
  
- AACTTAGAGA AAAGTTAAGC GTAGTTATTA AGTAGGTTAA GTTTACACGG AAAGAATTAA AGTAATGTAA   
  
  
- CCCAACCATA TTTACAATCG TGCTATCTAA CCTTATCGTT CCGAGATTAA GTTAATACTG AACTTACATA   
  
  
- CGTACGAAAA TAGTAATCAA TTAATTGTAA AAGACATAAC TTTAGTCGAG CTTTCTCTTG GTATACATCA   
  
  
- ACATGAACGC TGCTATATAT ACAAATCAAA TCAATCAAGA ATGGACAAAA CCTTAACAAC CCCTCTAGAC   
  
  
- TCCTTCCTCG GGCGAAGTTA GTTTCAAGTA CCGTTATTCG GTTTCACTCC AAGGTAGGAT TTTGGTTAAC   
  
  
- CGTTATCCTT CTCATCGGGT GAATTGAACA TATGATACTG AGAAGAAAAG TTTGAGGGTT ACACCCTGAA   
  
  
- GAAATGAGTA CACACTATAA GGGTTGTCCT TACTAGTCAA GGTGTACAAA AGTAGAGAAA CCTGAGCTTG   
  
  
- AAGCACATTA ACATATACGG TACCGTGTTT ATCAAAACTA CCACGGTGGT TCAACGAGTT AGTTACATTA   
  
  
- TGACGATGAT AGATTGAACG GAACCACCTG TAGTTATTCT AGTGCTCTAC TCCTCTTGAC TTAGAACCGG   
  
  
- TTGGTGCAGC AAACTGAAGA GAATGGTTAT TTCTAACTTA TCTGTCACAA AGAGAGGGGG AAGGTAAGAA   
  
  
- GAAAAGATCA CTACAGCTTT TTCTGAAATA TGTACAGGTC GTTGCGATCG GTGTTTTATA AGGTAAAGAC   
  
  
- ATTAGGTGAG TACAGTTAAT TTGAACATAG TAGTTTACCA TCAAAGGCAT GATTAGTGAT GTAGATCTAT   
  
  
- AAGGAACTAT TTAAATTAAA CTGAAATTTC CGAGCTAGAA AAATTCTTTT CAAAACAACG AATGTTGGAC   
  
  
- ATTTCGGATC TCTTGTTTCG GTTTCTTCTT TAAATTCAAG ATATATATCG TATTAGAGAG TTTTTTATAT   
  
  
- AAAAAATTTA TATCACGCTA TAACTGACTG GATTATACCC AATAATGTCC TCTACCCCTC TATAGATGTA   
  
  
- ATTGATGAAC TTGTTGAATT TAAAAGTAAA ATTGAGCCAG TAATTCTTGT GATTCTTGAA ATATATAATC   
  
  
- ACTATAGAGT CCGCATTTTC TTTGATGAAA CTTAATTAGG TACGTTAGGA TGAGAAATAA AGGTAGGCAA   
  
  
- TTTTAAAAGG AAGTTTTGAG GGAAGACAAG TAATAATTAA GTTATAGCAT AATGTGAACA ACCTAATAAA   
  
  
- GACACTAAAC TAGCAGTAAA AGATATATAC TTATTGATGT TTTTAGTATC AGGCTCATGT AATTATAATT   
  
  
- ATTTTATCCT ACTCACTATT GGAGTATATC ACGCAAATAG GGATGGTGAT CCGACCTAAA GACGTATTTC   
  
  
- TTCTTTAACT TGTATACCTT TTTCTTTATT TCTCTTCGTT GAAGGAACTC ACCCTCTTCT TAGCATCTTT   
  
  
- CAATTTGAAT ACTTTTTCTC GTAATTGTTT TTCCTTTAAT ATTCCGAATG GCAACTTCGG AGTATATTAA   
  
  
- AACGTTCTCT GTCTGTTTCT TTCACAGAAA GTTTACAGTA TAAAGAGCTC GTACGTACTT TTTACATGAA   
  
  
- GCCGATGTTA TCATTATCAT ATATACAACC GAAGTATGTG AACTGGGGTT ACATACAGTT GCACTGATCA   
  
  
- CTGGTGTGTG TAGCAACAGG GACGGGCGGG GTGATGGGTT TGAGGGGCGT GCGTTTCTAT TTTTCGGGGG   
  
  
- GTTAAAAATA AACGGAGGGT TTTTCGGGTG GGCGCGAAAA AATAAACGGA AGAAAAAAGG ATGAATTTGG   
  
  
- TGGAGGAGTT GCTTGAAACG TTGTATGTAT GATCAGATAT TCGTTAAATC TCCCTTCCCT TCGTTGGATT   
  
  
- TGTCTCGCTC TAACCGCCCC ACACAAACTC CCGAACGCAA AACCACGTTA TCCTTACTTT CACGGGTAAA   
  
  
- GTTGGTGGTA GGTTTGAACG TTGAGGCCGT GGCCGTTTGG TGAAAGGAAA CTCTTATGAC GGGATGTATG   
  
  
- GCGGAAAGTT CAAGGGTTAT GGTGGATGCT TGGGTGGAGC CAAGAACTGG AGGTCGCGTC GGGATCGGGG   
  
  
- TGGAGGTGGG TTTTTGGCGG GAAACGGCGG AGGTTGGTGG TCGGACGGAG CCGACTAGAC CTGTTGAACC   
  
  
- TGCCCACCCT AAGTTAGGAT AGTCTCGACC CCGAATTACT GCTTAAATGG GGATTAAGGT TTGAGACCAG   
  
  
- GGTTTAATCA GGCAGACTAG GAGTGGAGGG GGTTGAGGGA CTAAAGGGTT GTAGAGTCGG CAAACTAGTA   
  
  
- GTGTTGGGTG GGGGTTATTT GGAGGGTAGT AGTCTAAAGT TGGAGACACT AAAGAGGATG TTAGTGTTGG   
  
  
- GGTTAAAACC CGGCAAGCTA GAAGTAGTGT TGGGCGTAGT CTTGTTGGGC TTGTTGTTAT TGATGCCGAA   
  
  
- ACTAAAGTAT CTGCTGGAGT AGTTTCGTCG TGTCAGGGAG CTTAGGTTGC TGAGAGTAGT TCAGGTGGAC   
  
  
- TATAACCGCG CCGAGTTAGT TGCCGAGTCT AGTGGGTGGC CGTTTGGCGA GGTCGCCCGG CGGATGATGA   
  
  
- AGTTCCTTCG GGAGTAGCGG CGGCGCCGGC CGGGCGGGGC GGGGCGAGCC GAAAGTAGCA TACTCCACCA   
  
  
- CGTCTGGTAG GCTCGGATGT TCCGCAAAAG TCCTTAAAGC GGTCAGAGAG AGAAGAGGTG AAAGCGGCGG   
  
  
- TTGGTCCGCT AAGAACTCCG CCACCTGTGC CGCCGGAAGT AGGTGTAGTA GCTAAAGCTG TAGCCTAAGC   
  
  
- CGCCGGTGAC CCGGAGCAAG GAGGCGCTTG ACCAGCTGTT TCGACTAGGA CGCTTTGAAT CAAGTCACCA   
  
  
- CAATGCTTAG TGGCGGTAGC AAGGGCTTCT TAAGCCCTAG CTTTCGTTCG ATCACTCTCT TTTAGACAGC   
  
  
- GTTAAACGGG CTCTAGAGTT GTAGTTGAAG GTGTAGCTAA TACAGAATAA AGTCTCGAAA CTTTAGAATA   
  
  
- GGAAGTTTAG ACAGTTTAAG TAGCTTTCCC TCTTTAACCG CGACGTAGAA AGCGGCCGGC ACAAGGTGGC   
  
  
- CGATCCCTCG CCTTAAAGCT TCAAATAGCG ACTAGAGGCT AGTTAGAGCG GCTCGAGGCA GCACCACCAC   
  
  
- CTGTCTCTAC AGCCCTAACT GTAGCCTTGC AGCAGCAAAT CGTACTTAAA GCAGCGGCCT TAACTTAAGA   
  
  
- TGTGGCCCTA CGACCTCAGC GAACTGCGTC GGTGGCGGCC CCCCGAGTAG CCGCCACACC TGACGCACTC   
  
  
- TGCCTAACTC TGCAAACAAG AGGTGGGTTC CTAGTACCGC CGCCATCTCC GCCGCCGCAG CCGCAGCCGG   
  
  
- CCCGCCTCCT GCCGCACCTC CCTCCGTAAG CGCCGCCGCC CCTAATCCCG CCACCCCAAA TCGGTCAAAC   
  
  
- GACTAAAGGT CCGGCTTACA AACAACTCCT CTCGAGTCCA ACCACCGAAG GTGCACCGTT TCGCGGTACC   
  
  
- TCTCTACTAC GAAATGACCG TACTGGCGGG TGAGCAGCGG TGTAGTCGAA CCTCCACAAT

+     ABRE2

| Site Name | Organism | Position | Strand | Matrix score. | sequence | function |
| --- | --- | --- | --- | --- | --- | --- |
| ABRE2 | Zea mays | 3554 | - | 8 | CCACGTGG |  |

>HU05G02245.1   
+ -Up\_Stream \_Len000ATAATT TATCTTTCTT TAATTTCGTT TCATGTAAAA AATTTCAGTC TCATCTGCGA   
  
  
+ TTGAATCTCT TTTCAATTCG CATCAATAAT TCATCCAATT CAAATGTGCC TTTCTTAATT TCATTACATT   
  
  
+ GGGTTGGTAT AAATGTTAGC ACGATAGATT GGAATAGCAA GGCTCTAATT CAATTATGAC TTGAATGTAT   
  
  
+ GCATGCTTTT ATCATTAGTT AATTAACATT TTCTGTATTG AAATCAGCTC GAAAGAGAAC CATATGTAGT   
  
  
+ TGTACTTGCG ACGATATATA TGTTTAGTTT AGTTAGTTCT TACCTGTTTT GGAATTGTTG GGGAGATCTG   
  
  
+ AGGAAGGAGC CCGCTTCAAT CAAAGTTCAT GGCAATAAGC CAAAGTGAGG TTCCATCCTA AAACCAATTG   
  
  
+ GCAATAGGAA GAGTAGCCCA CTTAACTTGT ATACTATGAC TCTTCTTTTC AAACTCCCAA TGTGGGACTT   
  
  
+ CTTTACTCAT GTGTGATATT CCCAACAGGA ATGATCAGTT CCACATGTTT TCATCTCTTT GGACTCGAAC   
  
  
+ TTCGTGTAAT TGTATATGCC ATGGCACAAA TAGTTTTGAT GGTGCCACCA AGTTGCTCAA TCAATGTAAT   
  
  
+ ACTGCTACTA TCTAACTTGC CTTGGTGGAC ATCAATAAGA TCACGAGATG AGGAGAACTG AATCTTGGCC   
  
  
+ AACCACGTCG TTTGACTTCT CTTACCAATA AAGATTGAAT AGACAGTGTT TCTCTCCCCC TTCCATTCTT   
  
  
+ CTTTTCTAGT GATGTCGAAA AAGACTTTAT ACATGTCCAG CAACGCTAGC CACAAAATAT TCCATTTCTG   
  
  
+ TAATCCACTC ATGTCAATTA AACTTGTATC ATCAAATGGT AGTTTCCGTA CTAATCACTA CATCTAGATA   
  
  
+ TTCCTTGATA AATTTAATTT GACTTTAAAG GCTCGATCTT TTTAAGAAAA GTTTTGTTGC TTACAACCTG   
  
  
+ TAAAGCCTAG AGAACAAAGC CAAAGAAGAA ATTTAAGTTC TATATATAGC ATAATCTCTC AAAAAATATA   
  
  
+ TTTTTTAAAT ATAGTGCGAT ATTGACTGAC CTAATATGGG TTATTACAGG AGATGGGGAG ATATCTACAT   
  
  
+ TAACTACTTG AACAACTTAA ATTTTCATTT TAACTCGGTC ATTAAGAACA CTAAGAACTT TATATATTAG   
  
  
+ TGATATCTCA GGCGTAAAAG AAACTACTTT GAATTAATCC ATGCAATCCT ACTCTTTATT TCCATCCGTT   
  
  
+ AAAATTTTCC TTCAAAACTC CCTTCTGTTC ATTATTAATT CAATATCGTA TTACACTTGT TGGATTATTT   
  
  
+ CTGTGATTTG ATCGTCATTT TCTATATATG AATAACTACA AAAATCATAG TCCGAGTACA TTAATATTAA   
  
  
+ TAAAATAGGA TGAGTGATAA CCTCATATAG TGCGTTTATC CCTACCACTA GGCTGGATTT CTGCATAAAG   
  
  
+ AAGAAATTGA ACATATGGAA AAAGAAATAA AGAGAAGCAA CTTCCTTGAG TGGGAGAAGA ATCGTAGAAA   
  
  
+ GTTAAACTTA TGAAAAAGAG CATTAACAAA AAGGAAATTA TAAGGCTTAC CGTTGAAGCC TCATATAATT   
  
  
+ TTGCAAGAGA CAGACAAAGA AAGTGTCTTT CAAATGTCAT ATTTCTCGAG CATGCATGAA AAATGTACTT   
  
  
+ CGGCTACAAT AGTAATAGTA TATATGTTGG CTTCATACAC TTGACCCCAA TGTATGTCAA CGTGACTAGT   
  
  
+ GACCACACAC ATCGTTGTCC CTGCCCGCCC CACTACCCAA ACTCCCCGCA CGCAAAGATA AAAAGCCCCC   
  
  
+ CAATTTTTAT TTGCCTCCCA AAAAGCCCAC CCGCGCTTTT TTATTTGCCT TCTTTTTTCC TACTTAAACC   
  
  
+ ACCTCCTCAA CGAACTTTGC AACATACATA CTAGTCTATA AGCAATTTAG AGGGAAGGGA AGCAACCTAA   
  
  
+ ACAGAGCGAG ATTGGCGGGG TGTGTTTGAG GGCTTGCGTT TTGGTGCAAT AGGAATGAAA GTGCCCATTT   
  
  
+ CAACCACCAT CCAAACTTGC AACTCCGGCA CCGGCAAACC ACTTTCCTTT GAGAATACTG CCCTACATAC   
  
  
+ CGCCTTTCAA GTTCCCAATA CCACCTACGA ACCCACCTCG GTTCTTGACC TCCAGCGCAG CCCTAGCCCC   
  
  
+ ACCTCCACCC AAAAACCGCC CTTTGCCGCC TCCAACCACC AGCCTGCCTC GGCTGATCTG GACAACTTGG   
  
  
+ ACGGGTGGGA TTCAATCCTA TCAGAGCTGG GGCTTAATGA CGAATTTACC CCTAATTCCA AACTCTGGTC   
  
  
+ CCAAATTAGT CCGTCTGATC CTCACCTCCC CCAACTCCCT GATTTCCCAA CATCTCAGCC GTTTGATCAT   
  
  
+ CACAACCCAC CCCCAATAAA CCTCCCATCA TCAGATTTCA ACCTCTGTGA TTTCTCCTAC AATCACAACC   
  
  
+ CCAATTTTGG GCCGTTCGAT CTTCATCACA ACCCGCATCA GAACAACCCG AACAACAATA ACTACGGCTT   
  
  
+ TGATTTCATA GACGACCTCA TCAAAGCAGC ACAGTCCCTC GAATCCAACG ACTCTCATCA AGTCCACCTG   
  
  
+ ATATTGGCGC GGCTCAATCA ACGGCTCAGA TCACCCACCG GCAAACCGCT CCAGCGGGCC GCCTACTACT   
  
  
+ TCAAGGAAGC CCTCATCGCC GCCGCGGCCG GCCCGCCCCG CCCCGCTCGG CTTTCATCGT ATGAGGTGGT   
  
  
+ GCAGACCATC CGAGCCTACA AGGCGTTTTC AGGAATTTCG CCAGTCTCTC TCTTCTCCAC TTTCGCCGCC   
  
  
+ AACCAGGCGA TTCTTGAGGC GGTGGACACG GCGGCCTTCA TCCACATCAT CGATTTCGAC ATCGGATTCG   
  
  
+ GCGGCCACTG GGCCTCGTTC CTCCGCGAAC TGGTCGACAA AGCTGATCCT GCGAAACTTA GTTCAGTGGT   
  
  
+ GTTACGAATC ACCGCCATCG TTCCCGAAGA ATTCGGGATC GAAAGCAAGC TAGTGAGAGA AAATCTGTCG   
  
  
+ CAATTTGCCC GAGATCTCAA CATCAACTTC CACATCGATT ATGTCTTATT TCAGAGCTTT GAAATCTTAT   
  
  
+ CCTTCAAATC TGTCAAATTC ATCGAAAGGG AGAAATTGGC GCTGCATCTT TCGCCGGCCG TGTTCCACCG   
  
  
+ GCTAGGGAGC GGAATTTCGA AGTTTATCGC TGATCTCCGA TCAATCTCGC CGAGCTCCGT CGTGGTGGTG   
  
  
+ GACAGAGATG TCGGGATTGA CATCGGAACG TCGTCGTTTA GCATGAATTT CGTCGCCGGA ATTGAATTCT   
  
  
+ ACACCGGGAT GCTGGAGTCG CTTGACGCAG CCACCGCCGG GGGGCTCATC GGCGGTGTGG ACTGCGTGAG   
  
  
+ ACGGATTGAG ACGTTTGTTC TCCACCCAAG GATCATGGCG GCGGTAGAGG CGGCGGCGTC GGCGTCGGCC   
  
  
+ GGGCGGAGGA CGGCGTGGAG GGAGGCATTC GCGGCGGCGG GGATTAGGGC GGTGGGGTTT AGCCAGTTTG   
  
  
+ CTGATTTCCA GGCCGAATGT TTGTTGAGGA GAGCTCAGGT TGGTGGCTTC CACGTGGCAA AGCGCCATGG   
  
  
+ AGAGATGATG CTTTACTGGC ATGACCGCCC ACTCGTCGCC ACATCAGCTT GGAGGTGTTA   

- -Up\_Stream \_Len000TATTAA ATAGAAAGAA ATTAAAGCAA AGTACATTTT TTAAAGTCAG AGTAGACGCT   
  
  
- AACTTAGAGA AAAGTTAAGC GTAGTTATTA AGTAGGTTAA GTTTACACGG AAAGAATTAA AGTAATGTAA   
  
  
- CCCAACCATA TTTACAATCG TGCTATCTAA CCTTATCGTT CCGAGATTAA GTTAATACTG AACTTACATA   
  
  
- CGTACGAAAA TAGTAATCAA TTAATTGTAA AAGACATAAC TTTAGTCGAG CTTTCTCTTG GTATACATCA   
  
  
- ACATGAACGC TGCTATATAT ACAAATCAAA TCAATCAAGA ATGGACAAAA CCTTAACAAC CCCTCTAGAC   
  
  
- TCCTTCCTCG GGCGAAGTTA GTTTCAAGTA CCGTTATTCG GTTTCACTCC AAGGTAGGAT TTTGGTTAAC   
  
  
- CGTTATCCTT CTCATCGGGT GAATTGAACA TATGATACTG AGAAGAAAAG TTTGAGGGTT ACACCCTGAA   
  
  
- GAAATGAGTA CACACTATAA GGGTTGTCCT TACTAGTCAA GGTGTACAAA AGTAGAGAAA CCTGAGCTTG   
  
  
- AAGCACATTA ACATATACGG TACCGTGTTT ATCAAAACTA CCACGGTGGT TCAACGAGTT AGTTACATTA   
  
  
- TGACGATGAT AGATTGAACG GAACCACCTG TAGTTATTCT AGTGCTCTAC TCCTCTTGAC TTAGAACCGG   
  
  
- TTGGTGCAGC AAACTGAAGA GAATGGTTAT TTCTAACTTA TCTGTCACAA AGAGAGGGGG AAGGTAAGAA   
  
  
- GAAAAGATCA CTACAGCTTT TTCTGAAATA TGTACAGGTC GTTGCGATCG GTGTTTTATA AGGTAAAGAC   
  
  
- ATTAGGTGAG TACAGTTAAT TTGAACATAG TAGTTTACCA TCAAAGGCAT GATTAGTGAT GTAGATCTAT   
  
  
- AAGGAACTAT TTAAATTAAA CTGAAATTTC CGAGCTAGAA AAATTCTTTT CAAAACAACG AATGTTGGAC   
  
  
- ATTTCGGATC TCTTGTTTCG GTTTCTTCTT TAAATTCAAG ATATATATCG TATTAGAGAG TTTTTTATAT   
  
  
- AAAAAATTTA TATCACGCTA TAACTGACTG GATTATACCC AATAATGTCC TCTACCCCTC TATAGATGTA   
  
  
- ATTGATGAAC TTGTTGAATT TAAAAGTAAA ATTGAGCCAG TAATTCTTGT GATTCTTGAA ATATATAATC   
  
  
- ACTATAGAGT CCGCATTTTC TTTGATGAAA CTTAATTAGG TACGTTAGGA TGAGAAATAA AGGTAGGCAA   
  
  
- TTTTAAAAGG AAGTTTTGAG GGAAGACAAG TAATAATTAA GTTATAGCAT AATGTGAACA ACCTAATAAA   
  
  
- GACACTAAAC TAGCAGTAAA AGATATATAC TTATTGATGT TTTTAGTATC AGGCTCATGT AATTATAATT   
  
  
- ATTTTATCCT ACTCACTATT GGAGTATATC ACGCAAATAG GGATGGTGAT CCGACCTAAA GACGTATTTC   
  
  
- TTCTTTAACT TGTATACCTT TTTCTTTATT TCTCTTCGTT GAAGGAACTC ACCCTCTTCT TAGCATCTTT   
  
  
- CAATTTGAAT ACTTTTTCTC GTAATTGTTT TTCCTTTAAT ATTCCGAATG GCAACTTCGG AGTATATTAA   
  
  
- AACGTTCTCT GTCTGTTTCT TTCACAGAAA GTTTACAGTA TAAAGAGCTC GTACGTACTT TTTACATGAA   
  
  
- GCCGATGTTA TCATTATCAT ATATACAACC GAAGTATGTG AACTGGGGTT ACATACAGTT GCACTGATCA   
  
  
- CTGGTGTGTG TAGCAACAGG GACGGGCGGG GTGATGGGTT TGAGGGGCGT GCGTTTCTAT TTTTCGGGGG   
  
  
- GTTAAAAATA AACGGAGGGT TTTTCGGGTG GGCGCGAAAA AATAAACGGA AGAAAAAAGG ATGAATTTGG   
  
  
- TGGAGGAGTT GCTTGAAACG TTGTATGTAT GATCAGATAT TCGTTAAATC TCCCTTCCCT TCGTTGGATT   
  
  
- TGTCTCGCTC TAACCGCCCC ACACAAACTC CCGAACGCAA AACCACGTTA TCCTTACTTT CACGGGTAAA   
  
  
- GTTGGTGGTA GGTTTGAACG TTGAGGCCGT GGCCGTTTGG TGAAAGGAAA CTCTTATGAC GGGATGTATG   
  
  
- GCGGAAAGTT CAAGGGTTAT GGTGGATGCT TGGGTGGAGC CAAGAACTGG AGGTCGCGTC GGGATCGGGG   
  
  
- TGGAGGTGGG TTTTTGGCGG GAAACGGCGG AGGTTGGTGG TCGGACGGAG CCGACTAGAC CTGTTGAACC   
  
  
- TGCCCACCCT AAGTTAGGAT AGTCTCGACC CCGAATTACT GCTTAAATGG GGATTAAGGT TTGAGACCAG   
  
  
- GGTTTAATCA GGCAGACTAG GAGTGGAGGG GGTTGAGGGA CTAAAGGGTT GTAGAGTCGG CAAACTAGTA   
  
  
- GTGTTGGGTG GGGGTTATTT GGAGGGTAGT AGTCTAAAGT TGGAGACACT AAAGAGGATG TTAGTGTTGG   
  
  
- GGTTAAAACC CGGCAAGCTA GAAGTAGTGT TGGGCGTAGT CTTGTTGGGC TTGTTGTTAT TGATGCCGAA   
  
  
- ACTAAAGTAT CTGCTGGAGT AGTTTCGTCG TGTCAGGGAG CTTAGGTTGC TGAGAGTAGT TCAGGTGGAC   
  
  
- TATAACCGCG CCGAGTTAGT TGCCGAGTCT AGTGGGTGGC CGTTTGGCGA GGTCGCCCGG CGGATGATGA   
  
  
- AGTTCCTTCG GGAGTAGCGG CGGCGCCGGC CGGGCGGGGC GGGGCGAGCC GAAAGTAGCA TACTCCACCA   
  
  
- CGTCTGGTAG GCTCGGATGT TCCGCAAAAG TCCTTAAAGC GGTCAGAGAG AGAAGAGGTG AAAGCGGCGG   
  
  
- TTGGTCCGCT AAGAACTCCG CCACCTGTGC CGCCGGAAGT AGGTGTAGTA GCTAAAGCTG TAGCCTAAGC   
  
  
- CGCCGGTGAC CCGGAGCAAG GAGGCGCTTG ACCAGCTGTT TCGACTAGGA CGCTTTGAAT CAAGTCACCA   
  
  
- CAATGCTTAG TGGCGGTAGC AAGGGCTTCT TAAGCCCTAG CTTTCGTTCG ATCACTCTCT TTTAGACAGC   
  
  
- GTTAAACGGG CTCTAGAGTT GTAGTTGAAG GTGTAGCTAA TACAGAATAA AGTCTCGAAA CTTTAGAATA   
  
  
- GGAAGTTTAG ACAGTTTAAG TAGCTTTCCC TCTTTAACCG CGACGTAGAA AGCGGCCGGC ACAAGGTGGC   
  
  
- CGATCCCTCG CCTTAAAGCT TCAAATAGCG ACTAGAGGCT AGTTAGAGCG GCTCGAGGCA GCACCACCAC   
  
  
- CTGTCTCTAC AGCCCTAACT GTAGCCTTGC AGCAGCAAAT CGTACTTAAA GCAGCGGCCT TAACTTAAGA   
  
  
- TGTGGCCCTA CGACCTCAGC GAACTGCGTC GGTGGCGGCC CCCCGAGTAG CCGCCACACC TGACGCACTC   
  
  
- TGCCTAACTC TGCAAACAAG AGGTGGGTTC CTAGTACCGC CGCCATCTCC GCCGCCGCAG CCGCAGCCGG   
  
  
- CCCGCCTCCT GCCGCACCTC CCTCCGTAAG CGCCGCCGCC CCTAATCCCG CCACCCCAAA TCGGTCAAAC   
  
  
- GACTAAAGGT CCGGCTTACA AACAACTCCT CTCGAGTCCA ACCACCGAAG GTGCACCGTT TCGCGGTACC   
  
  
- TCTCTACTAC GAAATGACCG TACTGGCGGG TGAGCAGCGG TGTAGTCGAA CCTCCACAAT

+     ACTCATCCT sequence

| Site Name | Organism | Position | Strand | Matrix score. | sequence | function |
| --- | --- | --- | --- | --- | --- | --- |
| ACTCATCCT sequence | Arabidopsis thaliana | 1411 | - | 9 | ACTCATCCT |  |

>HU05G02245.1   
+ -Up\_Stream \_Len000ATAATT TATCTTTCTT TAATTTCGTT TCATGTAAAA AATTTCAGTC TCATCTGCGA   
  
  
+ TTGAATCTCT TTTCAATTCG CATCAATAAT TCATCCAATT CAAATGTGCC TTTCTTAATT TCATTACATT   
  
  
+ GGGTTGGTAT AAATGTTAGC ACGATAGATT GGAATAGCAA GGCTCTAATT CAATTATGAC TTGAATGTAT   
  
  
+ GCATGCTTTT ATCATTAGTT AATTAACATT TTCTGTATTG AAATCAGCTC GAAAGAGAAC CATATGTAGT   
  
  
+ TGTACTTGCG ACGATATATA TGTTTAGTTT AGTTAGTTCT TACCTGTTTT GGAATTGTTG GGGAGATCTG   
  
  
+ AGGAAGGAGC CCGCTTCAAT CAAAGTTCAT GGCAATAAGC CAAAGTGAGG TTCCATCCTA AAACCAATTG   
  
  
+ GCAATAGGAA GAGTAGCCCA CTTAACTTGT ATACTATGAC TCTTCTTTTC AAACTCCCAA TGTGGGACTT   
  
  
+ CTTTACTCAT GTGTGATATT CCCAACAGGA ATGATCAGTT CCACATGTTT TCATCTCTTT GGACTCGAAC   
  
  
+ TTCGTGTAAT TGTATATGCC ATGGCACAAA TAGTTTTGAT GGTGCCACCA AGTTGCTCAA TCAATGTAAT   
  
  
+ ACTGCTACTA TCTAACTTGC CTTGGTGGAC ATCAATAAGA TCACGAGATG AGGAGAACTG AATCTTGGCC   
  
  
+ AACCACGTCG TTTGACTTCT CTTACCAATA AAGATTGAAT AGACAGTGTT TCTCTCCCCC TTCCATTCTT   
  
  
+ CTTTTCTAGT GATGTCGAAA AAGACTTTAT ACATGTCCAG CAACGCTAGC CACAAAATAT TCCATTTCTG   
  
  
+ TAATCCACTC ATGTCAATTA AACTTGTATC ATCAAATGGT AGTTTCCGTA CTAATCACTA CATCTAGATA   
  
  
+ TTCCTTGATA AATTTAATTT GACTTTAAAG GCTCGATCTT TTTAAGAAAA GTTTTGTTGC TTACAACCTG   
  
  
+ TAAAGCCTAG AGAACAAAGC CAAAGAAGAA ATTTAAGTTC TATATATAGC ATAATCTCTC AAAAAATATA   
  
  
+ TTTTTTAAAT ATAGTGCGAT ATTGACTGAC CTAATATGGG TTATTACAGG AGATGGGGAG ATATCTACAT   
  
  
+ TAACTACTTG AACAACTTAA ATTTTCATTT TAACTCGGTC ATTAAGAACA CTAAGAACTT TATATATTAG   
  
  
+ TGATATCTCA GGCGTAAAAG AAACTACTTT GAATTAATCC ATGCAATCCT ACTCTTTATT TCCATCCGTT   
  
  
+ AAAATTTTCC TTCAAAACTC CCTTCTGTTC ATTATTAATT CAATATCGTA TTACACTTGT TGGATTATTT   
  
  
+ CTGTGATTTG ATCGTCATTT TCTATATATG AATAACTACA AAAATCATAG TCCGAGTACA TTAATATTAA   
  
  
+ TAAAATAGGA TGAGTGATAA CCTCATATAG TGCGTTTATC CCTACCACTA GGCTGGATTT CTGCATAAAG   
  
  
+ AAGAAATTGA ACATATGGAA AAAGAAATAA AGAGAAGCAA CTTCCTTGAG TGGGAGAAGA ATCGTAGAAA   
  
  
+ GTTAAACTTA TGAAAAAGAG CATTAACAAA AAGGAAATTA TAAGGCTTAC CGTTGAAGCC TCATATAATT   
  
  
+ TTGCAAGAGA CAGACAAAGA AAGTGTCTTT CAAATGTCAT ATTTCTCGAG CATGCATGAA AAATGTACTT   
  
  
+ CGGCTACAAT AGTAATAGTA TATATGTTGG CTTCATACAC TTGACCCCAA TGTATGTCAA CGTGACTAGT   
  
  
+ GACCACACAC ATCGTTGTCC CTGCCCGCCC CACTACCCAA ACTCCCCGCA CGCAAAGATA AAAAGCCCCC   
  
  
+ CAATTTTTAT TTGCCTCCCA AAAAGCCCAC CCGCGCTTTT TTATTTGCCT TCTTTTTTCC TACTTAAACC   
  
  
+ ACCTCCTCAA CGAACTTTGC AACATACATA CTAGTCTATA AGCAATTTAG AGGGAAGGGA AGCAACCTAA   
  
  
+ ACAGAGCGAG ATTGGCGGGG TGTGTTTGAG GGCTTGCGTT TTGGTGCAAT AGGAATGAAA GTGCCCATTT   
  
  
+ CAACCACCAT CCAAACTTGC AACTCCGGCA CCGGCAAACC ACTTTCCTTT GAGAATACTG CCCTACATAC   
  
  
+ CGCCTTTCAA GTTCCCAATA CCACCTACGA ACCCACCTCG GTTCTTGACC TCCAGCGCAG CCCTAGCCCC   
  
  
+ ACCTCCACCC AAAAACCGCC CTTTGCCGCC TCCAACCACC AGCCTGCCTC GGCTGATCTG GACAACTTGG   
  
  
+ ACGGGTGGGA TTCAATCCTA TCAGAGCTGG GGCTTAATGA CGAATTTACC CCTAATTCCA AACTCTGGTC   
  
  
+ CCAAATTAGT CCGTCTGATC CTCACCTCCC CCAACTCCCT GATTTCCCAA CATCTCAGCC GTTTGATCAT   
  
  
+ CACAACCCAC CCCCAATAAA CCTCCCATCA TCAGATTTCA ACCTCTGTGA TTTCTCCTAC AATCACAACC   
  
  
+ CCAATTTTGG GCCGTTCGAT CTTCATCACA ACCCGCATCA GAACAACCCG AACAACAATA ACTACGGCTT   
  
  
+ TGATTTCATA GACGACCTCA TCAAAGCAGC ACAGTCCCTC GAATCCAACG ACTCTCATCA AGTCCACCTG   
  
  
+ ATATTGGCGC GGCTCAATCA ACGGCTCAGA TCACCCACCG GCAAACCGCT CCAGCGGGCC GCCTACTACT   
  
  
+ TCAAGGAAGC CCTCATCGCC GCCGCGGCCG GCCCGCCCCG CCCCGCTCGG CTTTCATCGT ATGAGGTGGT   
  
  
+ GCAGACCATC CGAGCCTACA AGGCGTTTTC AGGAATTTCG CCAGTCTCTC TCTTCTCCAC TTTCGCCGCC   
  
  
+ AACCAGGCGA TTCTTGAGGC GGTGGACACG GCGGCCTTCA TCCACATCAT CGATTTCGAC ATCGGATTCG   
  
  
+ GCGGCCACTG GGCCTCGTTC CTCCGCGAAC TGGTCGACAA AGCTGATCCT GCGAAACTTA GTTCAGTGGT   
  
  
+ GTTACGAATC ACCGCCATCG TTCCCGAAGA ATTCGGGATC GAAAGCAAGC TAGTGAGAGA AAATCTGTCG   
  
  
+ CAATTTGCCC GAGATCTCAA CATCAACTTC CACATCGATT ATGTCTTATT TCAGAGCTTT GAAATCTTAT   
  
  
+ CCTTCAAATC TGTCAAATTC ATCGAAAGGG AGAAATTGGC GCTGCATCTT TCGCCGGCCG TGTTCCACCG   
  
  
+ GCTAGGGAGC GGAATTTCGA AGTTTATCGC TGATCTCCGA TCAATCTCGC CGAGCTCCGT CGTGGTGGTG   
  
  
+ GACAGAGATG TCGGGATTGA CATCGGAACG TCGTCGTTTA GCATGAATTT CGTCGCCGGA ATTGAATTCT   
  
  
+ ACACCGGGAT GCTGGAGTCG CTTGACGCAG CCACCGCCGG GGGGCTCATC GGCGGTGTGG ACTGCGTGAG   
  
  
+ ACGGATTGAG ACGTTTGTTC TCCACCCAAG GATCATGGCG GCGGTAGAGG CGGCGGCGTC GGCGTCGGCC   
  
  
+ GGGCGGAGGA CGGCGTGGAG GGAGGCATTC GCGGCGGCGG GGATTAGGGC GGTGGGGTTT AGCCAGTTTG   
  
  
+ CTGATTTCCA GGCCGAATGT TTGTTGAGGA GAGCTCAGGT TGGTGGCTTC CACGTGGCAA AGCGCCATGG   
  
  
+ AGAGATGATG CTTTACTGGC ATGACCGCCC ACTCGTCGCC ACATCAGCTT GGAGGTGTTA   

- -Up\_Stream \_Len000TATTAA ATAGAAAGAA ATTAAAGCAA AGTACATTTT TTAAAGTCAG AGTAGACGCT   
  
  
- AACTTAGAGA AAAGTTAAGC GTAGTTATTA AGTAGGTTAA GTTTACACGG AAAGAATTAA AGTAATGTAA   
  
  
- CCCAACCATA TTTACAATCG TGCTATCTAA CCTTATCGTT CCGAGATTAA GTTAATACTG AACTTACATA   
  
  
- CGTACGAAAA TAGTAATCAA TTAATTGTAA AAGACATAAC TTTAGTCGAG CTTTCTCTTG GTATACATCA   
  
  
- ACATGAACGC TGCTATATAT ACAAATCAAA TCAATCAAGA ATGGACAAAA CCTTAACAAC CCCTCTAGAC   
  
  
- TCCTTCCTCG GGCGAAGTTA GTTTCAAGTA CCGTTATTCG GTTTCACTCC AAGGTAGGAT TTTGGTTAAC   
  
  
- CGTTATCCTT CTCATCGGGT GAATTGAACA TATGATACTG AGAAGAAAAG TTTGAGGGTT ACACCCTGAA   
  
  
- GAAATGAGTA CACACTATAA GGGTTGTCCT TACTAGTCAA GGTGTACAAA AGTAGAGAAA CCTGAGCTTG   
  
  
- AAGCACATTA ACATATACGG TACCGTGTTT ATCAAAACTA CCACGGTGGT TCAACGAGTT AGTTACATTA   
  
  
- TGACGATGAT AGATTGAACG GAACCACCTG TAGTTATTCT AGTGCTCTAC TCCTCTTGAC TTAGAACCGG   
  
  
- TTGGTGCAGC AAACTGAAGA GAATGGTTAT TTCTAACTTA TCTGTCACAA AGAGAGGGGG AAGGTAAGAA   
  
  
- GAAAAGATCA CTACAGCTTT TTCTGAAATA TGTACAGGTC GTTGCGATCG GTGTTTTATA AGGTAAAGAC   
  
  
- ATTAGGTGAG TACAGTTAAT TTGAACATAG TAGTTTACCA TCAAAGGCAT GATTAGTGAT GTAGATCTAT   
  
  
- AAGGAACTAT TTAAATTAAA CTGAAATTTC CGAGCTAGAA AAATTCTTTT CAAAACAACG AATGTTGGAC   
  
  
- ATTTCGGATC TCTTGTTTCG GTTTCTTCTT TAAATTCAAG ATATATATCG TATTAGAGAG TTTTTTATAT   
  
  
- AAAAAATTTA TATCACGCTA TAACTGACTG GATTATACCC AATAATGTCC TCTACCCCTC TATAGATGTA   
  
  
- ATTGATGAAC TTGTTGAATT TAAAAGTAAA ATTGAGCCAG TAATTCTTGT GATTCTTGAA ATATATAATC   
  
  
- ACTATAGAGT CCGCATTTTC TTTGATGAAA CTTAATTAGG TACGTTAGGA TGAGAAATAA AGGTAGGCAA   
  
  
- TTTTAAAAGG AAGTTTTGAG GGAAGACAAG TAATAATTAA GTTATAGCAT AATGTGAACA ACCTAATAAA   
  
  
- GACACTAAAC TAGCAGTAAA AGATATATAC TTATTGATGT TTTTAGTATC AGGCTCATGT AATTATAATT   
  
  
- ATTTTATCCT ACTCACTATT GGAGTATATC ACGCAAATAG GGATGGTGAT CCGACCTAAA GACGTATTTC   
  
  
- TTCTTTAACT TGTATACCTT TTTCTTTATT TCTCTTCGTT GAAGGAACTC ACCCTCTTCT TAGCATCTTT   
  
  
- CAATTTGAAT ACTTTTTCTC GTAATTGTTT TTCCTTTAAT ATTCCGAATG GCAACTTCGG AGTATATTAA   
  
  
- AACGTTCTCT GTCTGTTTCT TTCACAGAAA GTTTACAGTA TAAAGAGCTC GTACGTACTT TTTACATGAA   
  
  
- GCCGATGTTA TCATTATCAT ATATACAACC GAAGTATGTG AACTGGGGTT ACATACAGTT GCACTGATCA   
  
  
- CTGGTGTGTG TAGCAACAGG GACGGGCGGG GTGATGGGTT TGAGGGGCGT GCGTTTCTAT TTTTCGGGGG   
  
  
- GTTAAAAATA AACGGAGGGT TTTTCGGGTG GGCGCGAAAA AATAAACGGA AGAAAAAAGG ATGAATTTGG   
  
  
- TGGAGGAGTT GCTTGAAACG TTGTATGTAT GATCAGATAT TCGTTAAATC TCCCTTCCCT TCGTTGGATT   
  
  
- TGTCTCGCTC TAACCGCCCC ACACAAACTC CCGAACGCAA AACCACGTTA TCCTTACTTT CACGGGTAAA   
  
  
- GTTGGTGGTA GGTTTGAACG TTGAGGCCGT GGCCGTTTGG TGAAAGGAAA CTCTTATGAC GGGATGTATG   
  
  
- GCGGAAAGTT CAAGGGTTAT GGTGGATGCT TGGGTGGAGC CAAGAACTGG AGGTCGCGTC GGGATCGGGG   
  
  
- TGGAGGTGGG TTTTTGGCGG GAAACGGCGG AGGTTGGTGG TCGGACGGAG CCGACTAGAC CTGTTGAACC   
  
  
- TGCCCACCCT AAGTTAGGAT AGTCTCGACC CCGAATTACT GCTTAAATGG GGATTAAGGT TTGAGACCAG   
  
  
- GGTTTAATCA GGCAGACTAG GAGTGGAGGG GGTTGAGGGA CTAAAGGGTT GTAGAGTCGG CAAACTAGTA   
  
  
- GTGTTGGGTG GGGGTTATTT GGAGGGTAGT AGTCTAAAGT TGGAGACACT AAAGAGGATG TTAGTGTTGG   
  
  
- GGTTAAAACC CGGCAAGCTA GAAGTAGTGT TGGGCGTAGT CTTGTTGGGC TTGTTGTTAT TGATGCCGAA   
  
  
- ACTAAAGTAT CTGCTGGAGT AGTTTCGTCG TGTCAGGGAG CTTAGGTTGC TGAGAGTAGT TCAGGTGGAC   
  
  
- TATAACCGCG CCGAGTTAGT TGCCGAGTCT AGTGGGTGGC CGTTTGGCGA GGTCGCCCGG CGGATGATGA   
  
  
- AGTTCCTTCG GGAGTAGCGG CGGCGCCGGC CGGGCGGGGC GGGGCGAGCC GAAAGTAGCA TACTCCACCA   
  
  
- CGTCTGGTAG GCTCGGATGT TCCGCAAAAG TCCTTAAAGC GGTCAGAGAG AGAAGAGGTG AAAGCGGCGG   
  
  
- TTGGTCCGCT AAGAACTCCG CCACCTGTGC CGCCGGAAGT AGGTGTAGTA GCTAAAGCTG TAGCCTAAGC   
  
  
- CGCCGGTGAC CCGGAGCAAG GAGGCGCTTG ACCAGCTGTT TCGACTAGGA CGCTTTGAAT CAAGTCACCA   
  
  
- CAATGCTTAG TGGCGGTAGC AAGGGCTTCT TAAGCCCTAG CTTTCGTTCG ATCACTCTCT TTTAGACAGC   
  
  
- GTTAAACGGG CTCTAGAGTT GTAGTTGAAG GTGTAGCTAA TACAGAATAA AGTCTCGAAA CTTTAGAATA   
  
  
- GGAAGTTTAG ACAGTTTAAG TAGCTTTCCC TCTTTAACCG CGACGTAGAA AGCGGCCGGC ACAAGGTGGC   
  
  
- CGATCCCTCG CCTTAAAGCT TCAAATAGCG ACTAGAGGCT AGTTAGAGCG GCTCGAGGCA GCACCACCAC   
  
  
- CTGTCTCTAC AGCCCTAACT GTAGCCTTGC AGCAGCAAAT CGTACTTAAA GCAGCGGCCT TAACTTAAGA   
  
  
- TGTGGCCCTA CGACCTCAGC GAACTGCGTC GGTGGCGGCC CCCCGAGTAG CCGCCACACC TGACGCACTC   
  
  
- TGCCTAACTC TGCAAACAAG AGGTGGGTTC CTAGTACCGC CGCCATCTCC GCCGCCGCAG CCGCAGCCGG   
  
  
- CCCGCCTCCT GCCGCACCTC CCTCCGTAAG CGCCGCCGCC CCTAATCCCG CCACCCCAAA TCGGTCAAAC   
  
  
- GACTAAAGGT CCGGCTTACA AACAACTCCT CTCGAGTCCA ACCACCGAAG GTGCACCGTT TCGCGGTACC   
  
  
- TCTCTACTAC GAAATGACCG TACTGGCGGG TGAGCAGCGG TGTAGTCGAA CCTCCACAAT

+     ARE

| Site Name | Organism | Position | Strand | Matrix score. | sequence | function |
| --- | --- | --- | --- | --- | --- | --- |
| ARE | Zea mays | 2070 | + | 6 | AAACCA | cis-acting regulatory element essential for the anaerobic induction |
| ARE | Zea mays | 1890 | + | 6 | AAACCA | cis-acting regulatory element essential for the anaerobic induction |
| ARE | Zea mays | 415 | + | 6 | AAACCA | cis-acting regulatory element essential for the anaerobic induction |

>HU05G02245.1   
+ -Up\_Stream \_Len000ATAATT TATCTTTCTT TAATTTCGTT TCATGTAAAA AATTTCAGTC TCATCTGCGA   
  
  
+ TTGAATCTCT TTTCAATTCG CATCAATAAT TCATCCAATT CAAATGTGCC TTTCTTAATT TCATTACATT   
  
  
+ GGGTTGGTAT AAATGTTAGC ACGATAGATT GGAATAGCAA GGCTCTAATT CAATTATGAC TTGAATGTAT   
  
  
+ GCATGCTTTT ATCATTAGTT AATTAACATT TTCTGTATTG AAATCAGCTC GAAAGAGAAC CATATGTAGT   
  
  
+ TGTACTTGCG ACGATATATA TGTTTAGTTT AGTTAGTTCT TACCTGTTTT GGAATTGTTG GGGAGATCTG   
  
  
+ AGGAAGGAGC CCGCTTCAAT CAAAGTTCAT GGCAATAAGC CAAAGTGAGG TTCCATCCTA AAACCAATTG   
  
  
+ GCAATAGGAA GAGTAGCCCA CTTAACTTGT ATACTATGAC TCTTCTTTTC AAACTCCCAA TGTGGGACTT   
  
  
+ CTTTACTCAT GTGTGATATT CCCAACAGGA ATGATCAGTT CCACATGTTT TCATCTCTTT GGACTCGAAC   
  
  
+ TTCGTGTAAT TGTATATGCC ATGGCACAAA TAGTTTTGAT GGTGCCACCA AGTTGCTCAA TCAATGTAAT   
  
  
+ ACTGCTACTA TCTAACTTGC CTTGGTGGAC ATCAATAAGA TCACGAGATG AGGAGAACTG AATCTTGGCC   
  
  
+ AACCACGTCG TTTGACTTCT CTTACCAATA AAGATTGAAT AGACAGTGTT TCTCTCCCCC TTCCATTCTT   
  
  
+ CTTTTCTAGT GATGTCGAAA AAGACTTTAT ACATGTCCAG CAACGCTAGC CACAAAATAT TCCATTTCTG   
  
  
+ TAATCCACTC ATGTCAATTA AACTTGTATC ATCAAATGGT AGTTTCCGTA CTAATCACTA CATCTAGATA   
  
  
+ TTCCTTGATA AATTTAATTT GACTTTAAAG GCTCGATCTT TTTAAGAAAA GTTTTGTTGC TTACAACCTG   
  
  
+ TAAAGCCTAG AGAACAAAGC CAAAGAAGAA ATTTAAGTTC TATATATAGC ATAATCTCTC AAAAAATATA   
  
  
+ TTTTTTAAAT ATAGTGCGAT ATTGACTGAC CTAATATGGG TTATTACAGG AGATGGGGAG ATATCTACAT   
  
  
+ TAACTACTTG AACAACTTAA ATTTTCATTT TAACTCGGTC ATTAAGAACA CTAAGAACTT TATATATTAG   
  
  
+ TGATATCTCA GGCGTAAAAG AAACTACTTT GAATTAATCC ATGCAATCCT ACTCTTTATT TCCATCCGTT   
  
  
+ AAAATTTTCC TTCAAAACTC CCTTCTGTTC ATTATTAATT CAATATCGTA TTACACTTGT TGGATTATTT   
  
  
+ CTGTGATTTG ATCGTCATTT TCTATATATG AATAACTACA AAAATCATAG TCCGAGTACA TTAATATTAA   
  
  
+ TAAAATAGGA TGAGTGATAA CCTCATATAG TGCGTTTATC CCTACCACTA GGCTGGATTT CTGCATAAAG   
  
  
+ AAGAAATTGA ACATATGGAA AAAGAAATAA AGAGAAGCAA CTTCCTTGAG TGGGAGAAGA ATCGTAGAAA   
  
  
+ GTTAAACTTA TGAAAAAGAG CATTAACAAA AAGGAAATTA TAAGGCTTAC CGTTGAAGCC TCATATAATT   
  
  
+ TTGCAAGAGA CAGACAAAGA AAGTGTCTTT CAAATGTCAT ATTTCTCGAG CATGCATGAA AAATGTACTT   
  
  
+ CGGCTACAAT AGTAATAGTA TATATGTTGG CTTCATACAC TTGACCCCAA TGTATGTCAA CGTGACTAGT   
  
  
+ GACCACACAC ATCGTTGTCC CTGCCCGCCC CACTACCCAA ACTCCCCGCA CGCAAAGATA AAAAGCCCCC   
  
  
+ CAATTTTTAT TTGCCTCCCA AAAAGCCCAC CCGCGCTTTT TTATTTGCCT TCTTTTTTCC TACTTAAACC   
  
  
+ ACCTCCTCAA CGAACTTTGC AACATACATA CTAGTCTATA AGCAATTTAG AGGGAAGGGA AGCAACCTAA   
  
  
+ ACAGAGCGAG ATTGGCGGGG TGTGTTTGAG GGCTTGCGTT TTGGTGCAAT AGGAATGAAA GTGCCCATTT   
  
  
+ CAACCACCAT CCAAACTTGC AACTCCGGCA CCGGCAAACC ACTTTCCTTT GAGAATACTG CCCTACATAC   
  
  
+ CGCCTTTCAA GTTCCCAATA CCACCTACGA ACCCACCTCG GTTCTTGACC TCCAGCGCAG CCCTAGCCCC   
  
  
+ ACCTCCACCC AAAAACCGCC CTTTGCCGCC TCCAACCACC AGCCTGCCTC GGCTGATCTG GACAACTTGG   
  
  
+ ACGGGTGGGA TTCAATCCTA TCAGAGCTGG GGCTTAATGA CGAATTTACC CCTAATTCCA AACTCTGGTC   
  
  
+ CCAAATTAGT CCGTCTGATC CTCACCTCCC CCAACTCCCT GATTTCCCAA CATCTCAGCC GTTTGATCAT   
  
  
+ CACAACCCAC CCCCAATAAA CCTCCCATCA TCAGATTTCA ACCTCTGTGA TTTCTCCTAC AATCACAACC   
  
  
+ CCAATTTTGG GCCGTTCGAT CTTCATCACA ACCCGCATCA GAACAACCCG AACAACAATA ACTACGGCTT   
  
  
+ TGATTTCATA GACGACCTCA TCAAAGCAGC ACAGTCCCTC GAATCCAACG ACTCTCATCA AGTCCACCTG   
  
  
+ ATATTGGCGC GGCTCAATCA ACGGCTCAGA TCACCCACCG GCAAACCGCT CCAGCGGGCC GCCTACTACT   
  
  
+ TCAAGGAAGC CCTCATCGCC GCCGCGGCCG GCCCGCCCCG CCCCGCTCGG CTTTCATCGT ATGAGGTGGT   
  
  
+ GCAGACCATC CGAGCCTACA AGGCGTTTTC AGGAATTTCG CCAGTCTCTC TCTTCTCCAC TTTCGCCGCC   
  
  
+ AACCAGGCGA TTCTTGAGGC GGTGGACACG GCGGCCTTCA TCCACATCAT CGATTTCGAC ATCGGATTCG   
  
  
+ GCGGCCACTG GGCCTCGTTC CTCCGCGAAC TGGTCGACAA AGCTGATCCT GCGAAACTTA GTTCAGTGGT   
  
  
+ GTTACGAATC ACCGCCATCG TTCCCGAAGA ATTCGGGATC GAAAGCAAGC TAGTGAGAGA AAATCTGTCG   
  
  
+ CAATTTGCCC GAGATCTCAA CATCAACTTC CACATCGATT ATGTCTTATT TCAGAGCTTT GAAATCTTAT   
  
  
+ CCTTCAAATC TGTCAAATTC ATCGAAAGGG AGAAATTGGC GCTGCATCTT TCGCCGGCCG TGTTCCACCG   
  
  
+ GCTAGGGAGC GGAATTTCGA AGTTTATCGC TGATCTCCGA TCAATCTCGC CGAGCTCCGT CGTGGTGGTG   
  
  
+ GACAGAGATG TCGGGATTGA CATCGGAACG TCGTCGTTTA GCATGAATTT CGTCGCCGGA ATTGAATTCT   
  
  
+ ACACCGGGAT GCTGGAGTCG CTTGACGCAG CCACCGCCGG GGGGCTCATC GGCGGTGTGG ACTGCGTGAG   
  
  
+ ACGGATTGAG ACGTTTGTTC TCCACCCAAG GATCATGGCG GCGGTAGAGG CGGCGGCGTC GGCGTCGGCC   
  
  
+ GGGCGGAGGA CGGCGTGGAG GGAGGCATTC GCGGCGGCGG GGATTAGGGC GGTGGGGTTT AGCCAGTTTG   
  
  
+ CTGATTTCCA GGCCGAATGT TTGTTGAGGA GAGCTCAGGT TGGTGGCTTC CACGTGGCAA AGCGCCATGG   
  
  
+ AGAGATGATG CTTTACTGGC ATGACCGCCC ACTCGTCGCC ACATCAGCTT GGAGGTGTTA   

- -Up\_Stream \_Len000TATTAA ATAGAAAGAA ATTAAAGCAA AGTACATTTT TTAAAGTCAG AGTAGACGCT   
  
  
- AACTTAGAGA AAAGTTAAGC GTAGTTATTA AGTAGGTTAA GTTTACACGG AAAGAATTAA AGTAATGTAA   
  
  
- CCCAACCATA TTTACAATCG TGCTATCTAA CCTTATCGTT CCGAGATTAA GTTAATACTG AACTTACATA   
  
  
- CGTACGAAAA TAGTAATCAA TTAATTGTAA AAGACATAAC TTTAGTCGAG CTTTCTCTTG GTATACATCA   
  
  
- ACATGAACGC TGCTATATAT ACAAATCAAA TCAATCAAGA ATGGACAAAA CCTTAACAAC CCCTCTAGAC   
  
  
- TCCTTCCTCG GGCGAAGTTA GTTTCAAGTA CCGTTATTCG GTTTCACTCC AAGGTAGGAT TTTGGTTAAC   
  
  
- CGTTATCCTT CTCATCGGGT GAATTGAACA TATGATACTG AGAAGAAAAG TTTGAGGGTT ACACCCTGAA   
  
  
- GAAATGAGTA CACACTATAA GGGTTGTCCT TACTAGTCAA GGTGTACAAA AGTAGAGAAA CCTGAGCTTG   
  
  
- AAGCACATTA ACATATACGG TACCGTGTTT ATCAAAACTA CCACGGTGGT TCAACGAGTT AGTTACATTA   
  
  
- TGACGATGAT AGATTGAACG GAACCACCTG TAGTTATTCT AGTGCTCTAC TCCTCTTGAC TTAGAACCGG   
  
  
- TTGGTGCAGC AAACTGAAGA GAATGGTTAT TTCTAACTTA TCTGTCACAA AGAGAGGGGG AAGGTAAGAA   
  
  
- GAAAAGATCA CTACAGCTTT TTCTGAAATA TGTACAGGTC GTTGCGATCG GTGTTTTATA AGGTAAAGAC   
  
  
- ATTAGGTGAG TACAGTTAAT TTGAACATAG TAGTTTACCA TCAAAGGCAT GATTAGTGAT GTAGATCTAT   
  
  
- AAGGAACTAT TTAAATTAAA CTGAAATTTC CGAGCTAGAA AAATTCTTTT CAAAACAACG AATGTTGGAC   
  
  
- ATTTCGGATC TCTTGTTTCG GTTTCTTCTT TAAATTCAAG ATATATATCG TATTAGAGAG TTTTTTATAT   
  
  
- AAAAAATTTA TATCACGCTA TAACTGACTG GATTATACCC AATAATGTCC TCTACCCCTC TATAGATGTA   
  
  
- ATTGATGAAC TTGTTGAATT TAAAAGTAAA ATTGAGCCAG TAATTCTTGT GATTCTTGAA ATATATAATC   
  
  
- ACTATAGAGT CCGCATTTTC TTTGATGAAA CTTAATTAGG TACGTTAGGA TGAGAAATAA AGGTAGGCAA   
  
  
- TTTTAAAAGG AAGTTTTGAG GGAAGACAAG TAATAATTAA GTTATAGCAT AATGTGAACA ACCTAATAAA   
  
  
- GACACTAAAC TAGCAGTAAA AGATATATAC TTATTGATGT TTTTAGTATC AGGCTCATGT AATTATAATT   
  
  
- ATTTTATCCT ACTCACTATT GGAGTATATC ACGCAAATAG GGATGGTGAT CCGACCTAAA GACGTATTTC   
  
  
- TTCTTTAACT TGTATACCTT TTTCTTTATT TCTCTTCGTT GAAGGAACTC ACCCTCTTCT TAGCATCTTT   
  
  
- CAATTTGAAT ACTTTTTCTC GTAATTGTTT TTCCTTTAAT ATTCCGAATG GCAACTTCGG AGTATATTAA   
  
  
- AACGTTCTCT GTCTGTTTCT TTCACAGAAA GTTTACAGTA TAAAGAGCTC GTACGTACTT TTTACATGAA   
  
  
- GCCGATGTTA TCATTATCAT ATATACAACC GAAGTATGTG AACTGGGGTT ACATACAGTT GCACTGATCA   
  
  
- CTGGTGTGTG TAGCAACAGG GACGGGCGGG GTGATGGGTT TGAGGGGCGT GCGTTTCTAT TTTTCGGGGG   
  
  
- GTTAAAAATA AACGGAGGGT TTTTCGGGTG GGCGCGAAAA AATAAACGGA AGAAAAAAGG ATGAATTTGG   
  
  
- TGGAGGAGTT GCTTGAAACG TTGTATGTAT GATCAGATAT TCGTTAAATC TCCCTTCCCT TCGTTGGATT   
  
  
- TGTCTCGCTC TAACCGCCCC ACACAAACTC CCGAACGCAA AACCACGTTA TCCTTACTTT CACGGGTAAA   
  
  
- GTTGGTGGTA GGTTTGAACG TTGAGGCCGT GGCCGTTTGG TGAAAGGAAA CTCTTATGAC GGGATGTATG   
  
  
- GCGGAAAGTT CAAGGGTTAT GGTGGATGCT TGGGTGGAGC CAAGAACTGG AGGTCGCGTC GGGATCGGGG   
  
  
- TGGAGGTGGG TTTTTGGCGG GAAACGGCGG AGGTTGGTGG TCGGACGGAG CCGACTAGAC CTGTTGAACC   
  
  
- TGCCCACCCT AAGTTAGGAT AGTCTCGACC CCGAATTACT GCTTAAATGG GGATTAAGGT TTGAGACCAG   
  
  
- GGTTTAATCA GGCAGACTAG GAGTGGAGGG GGTTGAGGGA CTAAAGGGTT GTAGAGTCGG CAAACTAGTA   
  
  
- GTGTTGGGTG GGGGTTATTT GGAGGGTAGT AGTCTAAAGT TGGAGACACT AAAGAGGATG TTAGTGTTGG   
  
  
- GGTTAAAACC CGGCAAGCTA GAAGTAGTGT TGGGCGTAGT CTTGTTGGGC TTGTTGTTAT TGATGCCGAA   
  
  
- ACTAAAGTAT CTGCTGGAGT AGTTTCGTCG TGTCAGGGAG CTTAGGTTGC TGAGAGTAGT TCAGGTGGAC   
  
  
- TATAACCGCG CCGAGTTAGT TGCCGAGTCT AGTGGGTGGC CGTTTGGCGA GGTCGCCCGG CGGATGATGA   
  
  
- AGTTCCTTCG GGAGTAGCGG CGGCGCCGGC CGGGCGGGGC GGGGCGAGCC GAAAGTAGCA TACTCCACCA   
  
  
- CGTCTGGTAG GCTCGGATGT TCCGCAAAAG TCCTTAAAGC GGTCAGAGAG AGAAGAGGTG AAAGCGGCGG   
  
  
- TTGGTCCGCT AAGAACTCCG CCACCTGTGC CGCCGGAAGT AGGTGTAGTA GCTAAAGCTG TAGCCTAAGC   
  
  
- CGCCGGTGAC CCGGAGCAAG GAGGCGCTTG ACCAGCTGTT TCGACTAGGA CGCTTTGAAT CAAGTCACCA   
  
  
- CAATGCTTAG TGGCGGTAGC AAGGGCTTCT TAAGCCCTAG CTTTCGTTCG ATCACTCTCT TTTAGACAGC   
  
  
- GTTAAACGGG CTCTAGAGTT GTAGTTGAAG GTGTAGCTAA TACAGAATAA AGTCTCGAAA CTTTAGAATA   
  
  
- GGAAGTTTAG ACAGTTTAAG TAGCTTTCCC TCTTTAACCG CGACGTAGAA AGCGGCCGGC ACAAGGTGGC   
  
  
- CGATCCCTCG CCTTAAAGCT TCAAATAGCG ACTAGAGGCT AGTTAGAGCG GCTCGAGGCA GCACCACCAC   
  
  
- CTGTCTCTAC AGCCCTAACT GTAGCCTTGC AGCAGCAAAT CGTACTTAAA GCAGCGGCCT TAACTTAAGA   
  
  
- TGTGGCCCTA CGACCTCAGC GAACTGCGTC GGTGGCGGCC CCCCGAGTAG CCGCCACACC TGACGCACTC   
  
  
- TGCCTAACTC TGCAAACAAG AGGTGGGTTC CTAGTACCGC CGCCATCTCC GCCGCCGCAG CCGCAGCCGG   
  
  
- CCCGCCTCCT GCCGCACCTC CCTCCGTAAG CGCCGCCGCC CCTAATCCCG CCACCCCAAA TCGGTCAAAC   
  
  
- GACTAAAGGT CCGGCTTACA AACAACTCCT CTCGAGTCCA ACCACCGAAG GTGCACCGTT TCGCGGTACC   
  
  
- TCTCTACTAC GAAATGACCG TACTGGCGGG TGAGCAGCGG TGTAGTCGAA CCTCCACAAT

+     ATCT-motif

| Site Name | Organism | Position | Strand | Matrix score. | sequence | function |
| --- | --- | --- | --- | --- | --- | --- |
| ATCT-motif | Pisum sativum | 3077 | + | 9 | AATCTAATCC | part of a conserved DNA module involved in light responsiveness |

>HU05G02245.1   
+ -Up\_Stream \_Len000ATAATT TATCTTTCTT TAATTTCGTT TCATGTAAAA AATTTCAGTC TCATCTGCGA   
  
  
+ TTGAATCTCT TTTCAATTCG CATCAATAAT TCATCCAATT CAAATGTGCC TTTCTTAATT TCATTACATT   
  
  
+ GGGTTGGTAT AAATGTTAGC ACGATAGATT GGAATAGCAA GGCTCTAATT CAATTATGAC TTGAATGTAT   
  
  
+ GCATGCTTTT ATCATTAGTT AATTAACATT TTCTGTATTG AAATCAGCTC GAAAGAGAAC CATATGTAGT   
  
  
+ TGTACTTGCG ACGATATATA TGTTTAGTTT AGTTAGTTCT TACCTGTTTT GGAATTGTTG GGGAGATCTG   
  
  
+ AGGAAGGAGC CCGCTTCAAT CAAAGTTCAT GGCAATAAGC CAAAGTGAGG TTCCATCCTA AAACCAATTG   
  
  
+ GCAATAGGAA GAGTAGCCCA CTTAACTTGT ATACTATGAC TCTTCTTTTC AAACTCCCAA TGTGGGACTT   
  
  
+ CTTTACTCAT GTGTGATATT CCCAACAGGA ATGATCAGTT CCACATGTTT TCATCTCTTT GGACTCGAAC   
  
  
+ TTCGTGTAAT TGTATATGCC ATGGCACAAA TAGTTTTGAT GGTGCCACCA AGTTGCTCAA TCAATGTAAT   
  
  
+ ACTGCTACTA TCTAACTTGC CTTGGTGGAC ATCAATAAGA TCACGAGATG AGGAGAACTG AATCTTGGCC   
  
  
+ AACCACGTCG TTTGACTTCT CTTACCAATA AAGATTGAAT AGACAGTGTT TCTCTCCCCC TTCCATTCTT   
  
  
+ CTTTTCTAGT GATGTCGAAA AAGACTTTAT ACATGTCCAG CAACGCTAGC CACAAAATAT TCCATTTCTG   
  
  
+ TAATCCACTC ATGTCAATTA AACTTGTATC ATCAAATGGT AGTTTCCGTA CTAATCACTA CATCTAGATA   
  
  
+ TTCCTTGATA AATTTAATTT GACTTTAAAG GCTCGATCTT TTTAAGAAAA GTTTTGTTGC TTACAACCTG   
  
  
+ TAAAGCCTAG AGAACAAAGC CAAAGAAGAA ATTTAAGTTC TATATATAGC ATAATCTCTC AAAAAATATA   
  
  
+ TTTTTTAAAT ATAGTGCGAT ATTGACTGAC CTAATATGGG TTATTACAGG AGATGGGGAG ATATCTACAT   
  
  
+ TAACTACTTG AACAACTTAA ATTTTCATTT TAACTCGGTC ATTAAGAACA CTAAGAACTT TATATATTAG   
  
  
+ TGATATCTCA GGCGTAAAAG AAACTACTTT GAATTAATCC ATGCAATCCT ACTCTTTATT TCCATCCGTT   
  
  
+ AAAATTTTCC TTCAAAACTC CCTTCTGTTC ATTATTAATT CAATATCGTA TTACACTTGT TGGATTATTT   
  
  
+ CTGTGATTTG ATCGTCATTT TCTATATATG AATAACTACA AAAATCATAG TCCGAGTACA TTAATATTAA   
  
  
+ TAAAATAGGA TGAGTGATAA CCTCATATAG TGCGTTTATC CCTACCACTA GGCTGGATTT CTGCATAAAG   
  
  
+ AAGAAATTGA ACATATGGAA AAAGAAATAA AGAGAAGCAA CTTCCTTGAG TGGGAGAAGA ATCGTAGAAA   
  
  
+ GTTAAACTTA TGAAAAAGAG CATTAACAAA AAGGAAATTA TAAGGCTTAC CGTTGAAGCC TCATATAATT   
  
  
+ TTGCAAGAGA CAGACAAAGA AAGTGTCTTT CAAATGTCAT ATTTCTCGAG CATGCATGAA AAATGTACTT   
  
  
+ CGGCTACAAT AGTAATAGTA TATATGTTGG CTTCATACAC TTGACCCCAA TGTATGTCAA CGTGACTAGT   
  
  
+ GACCACACAC ATCGTTGTCC CTGCCCGCCC CACTACCCAA ACTCCCCGCA CGCAAAGATA AAAAGCCCCC   
  
  
+ CAATTTTTAT TTGCCTCCCA AAAAGCCCAC CCGCGCTTTT TTATTTGCCT TCTTTTTTCC TACTTAAACC   
  
  
+ ACCTCCTCAA CGAACTTTGC AACATACATA CTAGTCTATA AGCAATTTAG AGGGAAGGGA AGCAACCTAA   
  
  
+ ACAGAGCGAG ATTGGCGGGG TGTGTTTGAG GGCTTGCGTT TTGGTGCAAT AGGAATGAAA GTGCCCATTT   
  
  
+ CAACCACCAT CCAAACTTGC AACTCCGGCA CCGGCAAACC ACTTTCCTTT GAGAATACTG CCCTACATAC   
  
  
+ CGCCTTTCAA GTTCCCAATA CCACCTACGA ACCCACCTCG GTTCTTGACC TCCAGCGCAG CCCTAGCCCC   
  
  
+ ACCTCCACCC AAAAACCGCC CTTTGCCGCC TCCAACCACC AGCCTGCCTC GGCTGATCTG GACAACTTGG   
  
  
+ ACGGGTGGGA TTCAATCCTA TCAGAGCTGG GGCTTAATGA CGAATTTACC CCTAATTCCA AACTCTGGTC   
  
  
+ CCAAATTAGT CCGTCTGATC CTCACCTCCC CCAACTCCCT GATTTCCCAA CATCTCAGCC GTTTGATCAT   
  
  
+ CACAACCCAC CCCCAATAAA CCTCCCATCA TCAGATTTCA ACCTCTGTGA TTTCTCCTAC AATCACAACC   
  
  
+ CCAATTTTGG GCCGTTCGAT CTTCATCACA ACCCGCATCA GAACAACCCG AACAACAATA ACTACGGCTT   
  
  
+ TGATTTCATA GACGACCTCA TCAAAGCAGC ACAGTCCCTC GAATCCAACG ACTCTCATCA AGTCCACCTG   
  
  
+ ATATTGGCGC GGCTCAATCA ACGGCTCAGA TCACCCACCG GCAAACCGCT CCAGCGGGCC GCCTACTACT   
  
  
+ TCAAGGAAGC CCTCATCGCC GCCGCGGCCG GCCCGCCCCG CCCCGCTCGG CTTTCATCGT ATGAGGTGGT   
  
  
+ GCAGACCATC CGAGCCTACA AGGCGTTTTC AGGAATTTCG CCAGTCTCTC TCTTCTCCAC TTTCGCCGCC   
  
  
+ AACCAGGCGA TTCTTGAGGC GGTGGACACG GCGGCCTTCA TCCACATCAT CGATTTCGAC ATCGGATTCG   
  
  
+ GCGGCCACTG GGCCTCGTTC CTCCGCGAAC TGGTCGACAA AGCTGATCCT GCGAAACTTA GTTCAGTGGT   
  
  
+ GTTACGAATC ACCGCCATCG TTCCCGAAGA ATTCGGGATC GAAAGCAAGC TAGTGAGAGA AAATCTGTCG   
  
  
+ CAATTTGCCC GAGATCTCAA CATCAACTTC CACATCGATT ATGTCTTATT TCAGAGCTTT GAAATCTTAT   
  
  
+ CCTTCAAATC TGTCAAATTC ATCGAAAGGG AGAAATTGGC GCTGCATCTT TCGCCGGCCG TGTTCCACCG   
  
  
+ GCTAGGGAGC GGAATTTCGA AGTTTATCGC TGATCTCCGA TCAATCTCGC CGAGCTCCGT CGTGGTGGTG   
  
  
+ GACAGAGATG TCGGGATTGA CATCGGAACG TCGTCGTTTA GCATGAATTT CGTCGCCGGA ATTGAATTCT   
  
  
+ ACACCGGGAT GCTGGAGTCG CTTGACGCAG CCACCGCCGG GGGGCTCATC GGCGGTGTGG ACTGCGTGAG   
  
  
+ ACGGATTGAG ACGTTTGTTC TCCACCCAAG GATCATGGCG GCGGTAGAGG CGGCGGCGTC GGCGTCGGCC   
  
  
+ GGGCGGAGGA CGGCGTGGAG GGAGGCATTC GCGGCGGCGG GGATTAGGGC GGTGGGGTTT AGCCAGTTTG   
  
  
+ CTGATTTCCA GGCCGAATGT TTGTTGAGGA GAGCTCAGGT TGGTGGCTTC CACGTGGCAA AGCGCCATGG   
  
  
+ AGAGATGATG CTTTACTGGC ATGACCGCCC ACTCGTCGCC ACATCAGCTT GGAGGTGTTA   

- -Up\_Stream \_Len000TATTAA ATAGAAAGAA ATTAAAGCAA AGTACATTTT TTAAAGTCAG AGTAGACGCT   
  
  
- AACTTAGAGA AAAGTTAAGC GTAGTTATTA AGTAGGTTAA GTTTACACGG AAAGAATTAA AGTAATGTAA   
  
  
- CCCAACCATA TTTACAATCG TGCTATCTAA CCTTATCGTT CCGAGATTAA GTTAATACTG AACTTACATA   
  
  
- CGTACGAAAA TAGTAATCAA TTAATTGTAA AAGACATAAC TTTAGTCGAG CTTTCTCTTG GTATACATCA   
  
  
- ACATGAACGC TGCTATATAT ACAAATCAAA TCAATCAAGA ATGGACAAAA CCTTAACAAC CCCTCTAGAC   
  
  
- TCCTTCCTCG GGCGAAGTTA GTTTCAAGTA CCGTTATTCG GTTTCACTCC AAGGTAGGAT TTTGGTTAAC   
  
  
- CGTTATCCTT CTCATCGGGT GAATTGAACA TATGATACTG AGAAGAAAAG TTTGAGGGTT ACACCCTGAA   
  
  
- GAAATGAGTA CACACTATAA GGGTTGTCCT TACTAGTCAA GGTGTACAAA AGTAGAGAAA CCTGAGCTTG   
  
  
- AAGCACATTA ACATATACGG TACCGTGTTT ATCAAAACTA CCACGGTGGT TCAACGAGTT AGTTACATTA   
  
  
- TGACGATGAT AGATTGAACG GAACCACCTG TAGTTATTCT AGTGCTCTAC TCCTCTTGAC TTAGAACCGG   
  
  
- TTGGTGCAGC AAACTGAAGA GAATGGTTAT TTCTAACTTA TCTGTCACAA AGAGAGGGGG AAGGTAAGAA   
  
  
- GAAAAGATCA CTACAGCTTT TTCTGAAATA TGTACAGGTC GTTGCGATCG GTGTTTTATA AGGTAAAGAC   
  
  
- ATTAGGTGAG TACAGTTAAT TTGAACATAG TAGTTTACCA TCAAAGGCAT GATTAGTGAT GTAGATCTAT   
  
  
- AAGGAACTAT TTAAATTAAA CTGAAATTTC CGAGCTAGAA AAATTCTTTT CAAAACAACG AATGTTGGAC   
  
  
- ATTTCGGATC TCTTGTTTCG GTTTCTTCTT TAAATTCAAG ATATATATCG TATTAGAGAG TTTTTTATAT   
  
  
- AAAAAATTTA TATCACGCTA TAACTGACTG GATTATACCC AATAATGTCC TCTACCCCTC TATAGATGTA   
  
  
- ATTGATGAAC TTGTTGAATT TAAAAGTAAA ATTGAGCCAG TAATTCTTGT GATTCTTGAA ATATATAATC   
  
  
- ACTATAGAGT CCGCATTTTC TTTGATGAAA CTTAATTAGG TACGTTAGGA TGAGAAATAA AGGTAGGCAA   
  
  
- TTTTAAAAGG AAGTTTTGAG GGAAGACAAG TAATAATTAA GTTATAGCAT AATGTGAACA ACCTAATAAA   
  
  
- GACACTAAAC TAGCAGTAAA AGATATATAC TTATTGATGT TTTTAGTATC AGGCTCATGT AATTATAATT   
  
  
- ATTTTATCCT ACTCACTATT GGAGTATATC ACGCAAATAG GGATGGTGAT CCGACCTAAA GACGTATTTC   
  
  
- TTCTTTAACT TGTATACCTT TTTCTTTATT TCTCTTCGTT GAAGGAACTC ACCCTCTTCT TAGCATCTTT   
  
  
- CAATTTGAAT ACTTTTTCTC GTAATTGTTT TTCCTTTAAT ATTCCGAATG GCAACTTCGG AGTATATTAA   
  
  
- AACGTTCTCT GTCTGTTTCT TTCACAGAAA GTTTACAGTA TAAAGAGCTC GTACGTACTT TTTACATGAA   
  
  
- GCCGATGTTA TCATTATCAT ATATACAACC GAAGTATGTG AACTGGGGTT ACATACAGTT GCACTGATCA   
  
  
- CTGGTGTGTG TAGCAACAGG GACGGGCGGG GTGATGGGTT TGAGGGGCGT GCGTTTCTAT TTTTCGGGGG   
  
  
- GTTAAAAATA AACGGAGGGT TTTTCGGGTG GGCGCGAAAA AATAAACGGA AGAAAAAAGG ATGAATTTGG   
  
  
- TGGAGGAGTT GCTTGAAACG TTGTATGTAT GATCAGATAT TCGTTAAATC TCCCTTCCCT TCGTTGGATT   
  
  
- TGTCTCGCTC TAACCGCCCC ACACAAACTC CCGAACGCAA AACCACGTTA TCCTTACTTT CACGGGTAAA   
  
  
- GTTGGTGGTA GGTTTGAACG TTGAGGCCGT GGCCGTTTGG TGAAAGGAAA CTCTTATGAC GGGATGTATG   
  
  
- GCGGAAAGTT CAAGGGTTAT GGTGGATGCT TGGGTGGAGC CAAGAACTGG AGGTCGCGTC GGGATCGGGG   
  
  
- TGGAGGTGGG TTTTTGGCGG GAAACGGCGG AGGTTGGTGG TCGGACGGAG CCGACTAGAC CTGTTGAACC   
  
  
- TGCCCACCCT AAGTTAGGAT AGTCTCGACC CCGAATTACT GCTTAAATGG GGATTAAGGT TTGAGACCAG   
  
  
- GGTTTAATCA GGCAGACTAG GAGTGGAGGG GGTTGAGGGA CTAAAGGGTT GTAGAGTCGG CAAACTAGTA   
  
  
- GTGTTGGGTG GGGGTTATTT GGAGGGTAGT AGTCTAAAGT TGGAGACACT AAAGAGGATG TTAGTGTTGG   
  
  
- GGTTAAAACC CGGCAAGCTA GAAGTAGTGT TGGGCGTAGT CTTGTTGGGC TTGTTGTTAT TGATGCCGAA   
  
  
- ACTAAAGTAT CTGCTGGAGT AGTTTCGTCG TGTCAGGGAG CTTAGGTTGC TGAGAGTAGT TCAGGTGGAC   
  
  
- TATAACCGCG CCGAGTTAGT TGCCGAGTCT AGTGGGTGGC CGTTTGGCGA GGTCGCCCGG CGGATGATGA   
  
  
- AGTTCCTTCG GGAGTAGCGG CGGCGCCGGC CGGGCGGGGC GGGGCGAGCC GAAAGTAGCA TACTCCACCA   
  
  
- CGTCTGGTAG GCTCGGATGT TCCGCAAAAG TCCTTAAAGC GGTCAGAGAG AGAAGAGGTG AAAGCGGCGG   
  
  
- TTGGTCCGCT AAGAACTCCG CCACCTGTGC CGCCGGAAGT AGGTGTAGTA GCTAAAGCTG TAGCCTAAGC   
  
  
- CGCCGGTGAC CCGGAGCAAG GAGGCGCTTG ACCAGCTGTT TCGACTAGGA CGCTTTGAAT CAAGTCACCA   
  
  
- CAATGCTTAG TGGCGGTAGC AAGGGCTTCT TAAGCCCTAG CTTTCGTTCG ATCACTCTCT TTTAGACAGC   
  
  
- GTTAAACGGG CTCTAGAGTT GTAGTTGAAG GTGTAGCTAA TACAGAATAA AGTCTCGAAA CTTTAGAATA   
  
  
- GGAAGTTTAG ACAGTTTAAG TAGCTTTCCC TCTTTAACCG CGACGTAGAA AGCGGCCGGC ACAAGGTGGC   
  
  
- CGATCCCTCG CCTTAAAGCT TCAAATAGCG ACTAGAGGCT AGTTAGAGCG GCTCGAGGCA GCACCACCAC   
  
  
- CTGTCTCTAC AGCCCTAACT GTAGCCTTGC AGCAGCAAAT CGTACTTAAA GCAGCGGCCT TAACTTAAGA   
  
  
- TGTGGCCCTA CGACCTCAGC GAACTGCGTC GGTGGCGGCC CCCCGAGTAG CCGCCACACC TGACGCACTC   
  
  
- TGCCTAACTC TGCAAACAAG AGGTGGGTTC CTAGTACCGC CGCCATCTCC GCCGCCGCAG CCGCAGCCGG   
  
  
- CCCGCCTCCT GCCGCACCTC CCTCCGTAAG CGCCGCCGCC CCTAATCCCG CCACCCCAAA TCGGTCAAAC   
  
  
- GACTAAAGGT CCGGCTTACA AACAACTCCT CTCGAGTCCA ACCACCGAAG GTGCACCGTT TCGCGGTACC   
  
  
- TCTCTACTAC GAAATGACCG TACTGGCGGG TGAGCAGCGG TGTAGTCGAA CCTCCACAAT

+     AT~TATA-box

| Site Name | Organism | Position | Strand | Matrix score. | sequence | function |
| --- | --- | --- | --- | --- | --- | --- |
| AT~TATA-box | Arabidopsis thaliana | 1185 | + | 6 | TATATA |  |
| AT~TATA-box | Arabidopsis thaliana | 1025 | + | 6 | TATATA |  |
| AT~TATA-box | Arabidopsis thaliana | 1027 | + | 6 | TATATA |  |
| AT~TATA-box | Arabidopsis thaliana | 1183 | - | 8 | TATATAAA |  |
| AT~TATA-box | Arabidopsis thaliana | 299 | + | 6 | TATATA |  |
| AT~TATA-box | Arabidopsis thaliana | 1703 | + | 6 | TATATA |  |
| AT~TATA-box | Arabidopsis thaliana | 1357 | + | 6 | TATATA |  |

>HU05G02245.1   
+ -Up\_Stream \_Len000ATAATT TATCTTTCTT TAATTTCGTT TCATGTAAAA AATTTCAGTC TCATCTGCGA   
  
  
+ TTGAATCTCT TTTCAATTCG CATCAATAAT TCATCCAATT CAAATGTGCC TTTCTTAATT TCATTACATT   
  
  
+ GGGTTGGTAT AAATGTTAGC ACGATAGATT GGAATAGCAA GGCTCTAATT CAATTATGAC TTGAATGTAT   
  
  
+ GCATGCTTTT ATCATTAGTT AATTAACATT TTCTGTATTG AAATCAGCTC GAAAGAGAAC CATATGTAGT   
  
  
+ TGTACTTGCG ACGATATATA TGTTTAGTTT AGTTAGTTCT TACCTGTTTT GGAATTGTTG GGGAGATCTG   
  
  
+ AGGAAGGAGC CCGCTTCAAT CAAAGTTCAT GGCAATAAGC CAAAGTGAGG TTCCATCCTA AAACCAATTG   
  
  
+ GCAATAGGAA GAGTAGCCCA CTTAACTTGT ATACTATGAC TCTTCTTTTC AAACTCCCAA TGTGGGACTT   
  
  
+ CTTTACTCAT GTGTGATATT CCCAACAGGA ATGATCAGTT CCACATGTTT TCATCTCTTT GGACTCGAAC   
  
  
+ TTCGTGTAAT TGTATATGCC ATGGCACAAA TAGTTTTGAT GGTGCCACCA AGTTGCTCAA TCAATGTAAT   
  
  
+ ACTGCTACTA TCTAACTTGC CTTGGTGGAC ATCAATAAGA TCACGAGATG AGGAGAACTG AATCTTGGCC   
  
  
+ AACCACGTCG TTTGACTTCT CTTACCAATA AAGATTGAAT AGACAGTGTT TCTCTCCCCC TTCCATTCTT   
  
  
+ CTTTTCTAGT GATGTCGAAA AAGACTTTAT ACATGTCCAG CAACGCTAGC CACAAAATAT TCCATTTCTG   
  
  
+ TAATCCACTC ATGTCAATTA AACTTGTATC ATCAAATGGT AGTTTCCGTA CTAATCACTA CATCTAGATA   
  
  
+ TTCCTTGATA AATTTAATTT GACTTTAAAG GCTCGATCTT TTTAAGAAAA GTTTTGTTGC TTACAACCTG   
  
  
+ TAAAGCCTAG AGAACAAAGC CAAAGAAGAA ATTTAAGTTC TATATATAGC ATAATCTCTC AAAAAATATA   
  
  
+ TTTTTTAAAT ATAGTGCGAT ATTGACTGAC CTAATATGGG TTATTACAGG AGATGGGGAG ATATCTACAT   
  
  
+ TAACTACTTG AACAACTTAA ATTTTCATTT TAACTCGGTC ATTAAGAACA CTAAGAACTT TATATATTAG   
  
  
+ TGATATCTCA GGCGTAAAAG AAACTACTTT GAATTAATCC ATGCAATCCT ACTCTTTATT TCCATCCGTT   
  
  
+ AAAATTTTCC TTCAAAACTC CCTTCTGTTC ATTATTAATT CAATATCGTA TTACACTTGT TGGATTATTT   
  
  
+ CTGTGATTTG ATCGTCATTT TCTATATATG AATAACTACA AAAATCATAG TCCGAGTACA TTAATATTAA   
  
  
+ TAAAATAGGA TGAGTGATAA CCTCATATAG TGCGTTTATC CCTACCACTA GGCTGGATTT CTGCATAAAG   
  
  
+ AAGAAATTGA ACATATGGAA AAAGAAATAA AGAGAAGCAA CTTCCTTGAG TGGGAGAAGA ATCGTAGAAA   
  
  
+ GTTAAACTTA TGAAAAAGAG CATTAACAAA AAGGAAATTA TAAGGCTTAC CGTTGAAGCC TCATATAATT   
  
  
+ TTGCAAGAGA CAGACAAAGA AAGTGTCTTT CAAATGTCAT ATTTCTCGAG CATGCATGAA AAATGTACTT   
  
  
+ CGGCTACAAT AGTAATAGTA TATATGTTGG CTTCATACAC TTGACCCCAA TGTATGTCAA CGTGACTAGT   
  
  
+ GACCACACAC ATCGTTGTCC CTGCCCGCCC CACTACCCAA ACTCCCCGCA CGCAAAGATA AAAAGCCCCC   
  
  
+ CAATTTTTAT TTGCCTCCCA AAAAGCCCAC CCGCGCTTTT TTATTTGCCT TCTTTTTTCC TACTTAAACC   
  
  
+ ACCTCCTCAA CGAACTTTGC AACATACATA CTAGTCTATA AGCAATTTAG AGGGAAGGGA AGCAACCTAA   
  
  
+ ACAGAGCGAG ATTGGCGGGG TGTGTTTGAG GGCTTGCGTT TTGGTGCAAT AGGAATGAAA GTGCCCATTT   
  
  
+ CAACCACCAT CCAAACTTGC AACTCCGGCA CCGGCAAACC ACTTTCCTTT GAGAATACTG CCCTACATAC   
  
  
+ CGCCTTTCAA GTTCCCAATA CCACCTACGA ACCCACCTCG GTTCTTGACC TCCAGCGCAG CCCTAGCCCC   
  
  
+ ACCTCCACCC AAAAACCGCC CTTTGCCGCC TCCAACCACC AGCCTGCCTC GGCTGATCTG GACAACTTGG   
  
  
+ ACGGGTGGGA TTCAATCCTA TCAGAGCTGG GGCTTAATGA CGAATTTACC CCTAATTCCA AACTCTGGTC   
  
  
+ CCAAATTAGT CCGTCTGATC CTCACCTCCC CCAACTCCCT GATTTCCCAA CATCTCAGCC GTTTGATCAT   
  
  
+ CACAACCCAC CCCCAATAAA CCTCCCATCA TCAGATTTCA ACCTCTGTGA TTTCTCCTAC AATCACAACC   
  
  
+ CCAATTTTGG GCCGTTCGAT CTTCATCACA ACCCGCATCA GAACAACCCG AACAACAATA ACTACGGCTT   
  
  
+ TGATTTCATA GACGACCTCA TCAAAGCAGC ACAGTCCCTC GAATCCAACG ACTCTCATCA AGTCCACCTG   
  
  
+ ATATTGGCGC GGCTCAATCA ACGGCTCAGA TCACCCACCG GCAAACCGCT CCAGCGGGCC GCCTACTACT   
  
  
+ TCAAGGAAGC CCTCATCGCC GCCGCGGCCG GCCCGCCCCG CCCCGCTCGG CTTTCATCGT ATGAGGTGGT   
  
  
+ GCAGACCATC CGAGCCTACA AGGCGTTTTC AGGAATTTCG CCAGTCTCTC TCTTCTCCAC TTTCGCCGCC   
  
  
+ AACCAGGCGA TTCTTGAGGC GGTGGACACG GCGGCCTTCA TCCACATCAT CGATTTCGAC ATCGGATTCG   
  
  
+ GCGGCCACTG GGCCTCGTTC CTCCGCGAAC TGGTCGACAA AGCTGATCCT GCGAAACTTA GTTCAGTGGT   
  
  
+ GTTACGAATC ACCGCCATCG TTCCCGAAGA ATTCGGGATC GAAAGCAAGC TAGTGAGAGA AAATCTGTCG   
  
  
+ CAATTTGCCC GAGATCTCAA CATCAACTTC CACATCGATT ATGTCTTATT TCAGAGCTTT GAAATCTTAT   
  
  
+ CCTTCAAATC TGTCAAATTC ATCGAAAGGG AGAAATTGGC GCTGCATCTT TCGCCGGCCG TGTTCCACCG   
  
  
+ GCTAGGGAGC GGAATTTCGA AGTTTATCGC TGATCTCCGA TCAATCTCGC CGAGCTCCGT CGTGGTGGTG   
  
  
+ GACAGAGATG TCGGGATTGA CATCGGAACG TCGTCGTTTA GCATGAATTT CGTCGCCGGA ATTGAATTCT   
  
  
+ ACACCGGGAT GCTGGAGTCG CTTGACGCAG CCACCGCCGG GGGGCTCATC GGCGGTGTGG ACTGCGTGAG   
  
  
+ ACGGATTGAG ACGTTTGTTC TCCACCCAAG GATCATGGCG GCGGTAGAGG CGGCGGCGTC GGCGTCGGCC   
  
  
+ GGGCGGAGGA CGGCGTGGAG GGAGGCATTC GCGGCGGCGG GGATTAGGGC GGTGGGGTTT AGCCAGTTTG   
  
  
+ CTGATTTCCA GGCCGAATGT TTGTTGAGGA GAGCTCAGGT TGGTGGCTTC CACGTGGCAA AGCGCCATGG   
  
  
+ AGAGATGATG CTTTACTGGC ATGACCGCCC ACTCGTCGCC ACATCAGCTT GGAGGTGTTA   

- -Up\_Stream \_Len000TATTAA ATAGAAAGAA ATTAAAGCAA AGTACATTTT TTAAAGTCAG AGTAGACGCT   
  
  
- AACTTAGAGA AAAGTTAAGC GTAGTTATTA AGTAGGTTAA GTTTACACGG AAAGAATTAA AGTAATGTAA   
  
  
- CCCAACCATA TTTACAATCG TGCTATCTAA CCTTATCGTT CCGAGATTAA GTTAATACTG AACTTACATA   
  
  
- CGTACGAAAA TAGTAATCAA TTAATTGTAA AAGACATAAC TTTAGTCGAG CTTTCTCTTG GTATACATCA   
  
  
- ACATGAACGC TGCTATATAT ACAAATCAAA TCAATCAAGA ATGGACAAAA CCTTAACAAC CCCTCTAGAC   
  
  
- TCCTTCCTCG GGCGAAGTTA GTTTCAAGTA CCGTTATTCG GTTTCACTCC AAGGTAGGAT TTTGGTTAAC   
  
  
- CGTTATCCTT CTCATCGGGT GAATTGAACA TATGATACTG AGAAGAAAAG TTTGAGGGTT ACACCCTGAA   
  
  
- GAAATGAGTA CACACTATAA GGGTTGTCCT TACTAGTCAA GGTGTACAAA AGTAGAGAAA CCTGAGCTTG   
  
  
- AAGCACATTA ACATATACGG TACCGTGTTT ATCAAAACTA CCACGGTGGT TCAACGAGTT AGTTACATTA   
  
  
- TGACGATGAT AGATTGAACG GAACCACCTG TAGTTATTCT AGTGCTCTAC TCCTCTTGAC TTAGAACCGG   
  
  
- TTGGTGCAGC AAACTGAAGA GAATGGTTAT TTCTAACTTA TCTGTCACAA AGAGAGGGGG AAGGTAAGAA   
  
  
- GAAAAGATCA CTACAGCTTT TTCTGAAATA TGTACAGGTC GTTGCGATCG GTGTTTTATA AGGTAAAGAC   
  
  
- ATTAGGTGAG TACAGTTAAT TTGAACATAG TAGTTTACCA TCAAAGGCAT GATTAGTGAT GTAGATCTAT   
  
  
- AAGGAACTAT TTAAATTAAA CTGAAATTTC CGAGCTAGAA AAATTCTTTT CAAAACAACG AATGTTGGAC   
  
  
- ATTTCGGATC TCTTGTTTCG GTTTCTTCTT TAAATTCAAG ATATATATCG TATTAGAGAG TTTTTTATAT   
  
  
- AAAAAATTTA TATCACGCTA TAACTGACTG GATTATACCC AATAATGTCC TCTACCCCTC TATAGATGTA   
  
  
- ATTGATGAAC TTGTTGAATT TAAAAGTAAA ATTGAGCCAG TAATTCTTGT GATTCTTGAA ATATATAATC   
  
  
- ACTATAGAGT CCGCATTTTC TTTGATGAAA CTTAATTAGG TACGTTAGGA TGAGAAATAA AGGTAGGCAA   
  
  
- TTTTAAAAGG AAGTTTTGAG GGAAGACAAG TAATAATTAA GTTATAGCAT AATGTGAACA ACCTAATAAA   
  
  
- GACACTAAAC TAGCAGTAAA AGATATATAC TTATTGATGT TTTTAGTATC AGGCTCATGT AATTATAATT   
  
  
- ATTTTATCCT ACTCACTATT GGAGTATATC ACGCAAATAG GGATGGTGAT CCGACCTAAA GACGTATTTC   
  
  
- TTCTTTAACT TGTATACCTT TTTCTTTATT TCTCTTCGTT GAAGGAACTC ACCCTCTTCT TAGCATCTTT   
  
  
- CAATTTGAAT ACTTTTTCTC GTAATTGTTT TTCCTTTAAT ATTCCGAATG GCAACTTCGG AGTATATTAA   
  
  
- AACGTTCTCT GTCTGTTTCT TTCACAGAAA GTTTACAGTA TAAAGAGCTC GTACGTACTT TTTACATGAA   
  
  
- GCCGATGTTA TCATTATCAT ATATACAACC GAAGTATGTG AACTGGGGTT ACATACAGTT GCACTGATCA   
  
  
- CTGGTGTGTG TAGCAACAGG GACGGGCGGG GTGATGGGTT TGAGGGGCGT GCGTTTCTAT TTTTCGGGGG   
  
  
- GTTAAAAATA AACGGAGGGT TTTTCGGGTG GGCGCGAAAA AATAAACGGA AGAAAAAAGG ATGAATTTGG   
  
  
- TGGAGGAGTT GCTTGAAACG TTGTATGTAT GATCAGATAT TCGTTAAATC TCCCTTCCCT TCGTTGGATT   
  
  
- TGTCTCGCTC TAACCGCCCC ACACAAACTC CCGAACGCAA AACCACGTTA TCCTTACTTT CACGGGTAAA   
  
  
- GTTGGTGGTA GGTTTGAACG TTGAGGCCGT GGCCGTTTGG TGAAAGGAAA CTCTTATGAC GGGATGTATG   
  
  
- GCGGAAAGTT CAAGGGTTAT GGTGGATGCT TGGGTGGAGC CAAGAACTGG AGGTCGCGTC GGGATCGGGG   
  
  
- TGGAGGTGGG TTTTTGGCGG GAAACGGCGG AGGTTGGTGG TCGGACGGAG CCGACTAGAC CTGTTGAACC   
  
  
- TGCCCACCCT AAGTTAGGAT AGTCTCGACC CCGAATTACT GCTTAAATGG GGATTAAGGT TTGAGACCAG   
  
  
- GGTTTAATCA GGCAGACTAG GAGTGGAGGG GGTTGAGGGA CTAAAGGGTT GTAGAGTCGG CAAACTAGTA   
  
  
- GTGTTGGGTG GGGGTTATTT GGAGGGTAGT AGTCTAAAGT TGGAGACACT AAAGAGGATG TTAGTGTTGG   
  
  
- GGTTAAAACC CGGCAAGCTA GAAGTAGTGT TGGGCGTAGT CTTGTTGGGC TTGTTGTTAT TGATGCCGAA   
  
  
- ACTAAAGTAT CTGCTGGAGT AGTTTCGTCG TGTCAGGGAG CTTAGGTTGC TGAGAGTAGT TCAGGTGGAC   
  
  
- TATAACCGCG CCGAGTTAGT TGCCGAGTCT AGTGGGTGGC CGTTTGGCGA GGTCGCCCGG CGGATGATGA   
  
  
- AGTTCCTTCG GGAGTAGCGG CGGCGCCGGC CGGGCGGGGC GGGGCGAGCC GAAAGTAGCA TACTCCACCA   
  
  
- CGTCTGGTAG GCTCGGATGT TCCGCAAAAG TCCTTAAAGC GGTCAGAGAG AGAAGAGGTG AAAGCGGCGG   
  
  
- TTGGTCCGCT AAGAACTCCG CCACCTGTGC CGCCGGAAGT AGGTGTAGTA GCTAAAGCTG TAGCCTAAGC   
  
  
- CGCCGGTGAC CCGGAGCAAG GAGGCGCTTG ACCAGCTGTT TCGACTAGGA CGCTTTGAAT CAAGTCACCA   
  
  
- CAATGCTTAG TGGCGGTAGC AAGGGCTTCT TAAGCCCTAG CTTTCGTTCG ATCACTCTCT TTTAGACAGC   
  
  
- GTTAAACGGG CTCTAGAGTT GTAGTTGAAG GTGTAGCTAA TACAGAATAA AGTCTCGAAA CTTTAGAATA   
  
  
- GGAAGTTTAG ACAGTTTAAG TAGCTTTCCC TCTTTAACCG CGACGTAGAA AGCGGCCGGC ACAAGGTGGC   
  
  
- CGATCCCTCG CCTTAAAGCT TCAAATAGCG ACTAGAGGCT AGTTAGAGCG GCTCGAGGCA GCACCACCAC   
  
  
- CTGTCTCTAC AGCCCTAACT GTAGCCTTGC AGCAGCAAAT CGTACTTAAA GCAGCGGCCT TAACTTAAGA   
  
  
- TGTGGCCCTA CGACCTCAGC GAACTGCGTC GGTGGCGGCC CCCCGAGTAG CCGCCACACC TGACGCACTC   
  
  
- TGCCTAACTC TGCAAACAAG AGGTGGGTTC CTAGTACCGC CGCCATCTCC GCCGCCGCAG CCGCAGCCGG   
  
  
- CCCGCCTCCT GCCGCACCTC CCTCCGTAAG CGCCGCCGCC CCTAATCCCG CCACCCCAAA TCGGTCAAAC   
  
  
- GACTAAAGGT CCGGCTTACA AACAACTCCT CTCGAGTCCA ACCACCGAAG GTGCACCGTT TCGCGGTACC   
  
  
- TCTCTACTAC GAAATGACCG TACTGGCGGG TGAGCAGCGG TGTAGTCGAA CCTCCACAAT

+     Box 4

| Site Name | Organism | Position | Strand | Matrix score. | sequence | function |
| --- | --- | --- | --- | --- | --- | --- |
| Box 4 | Petroselinum crispum | 1298 | + | 6 | ATTAAT | part of a conserved DNA module involved in light responsiveness |
| Box 4 | Petroselinum crispum | 1400 | + | 6 | ATTAAT | part of a conserved DNA module involved in light responsiveness |
| Box 4 | Petroselinum crispum | 1394 | + | 6 | ATTAAT | part of a conserved DNA module involved in light responsiveness |
| Box 4 | Petroselinum crispum | 1227 | + | 6 | ATTAAT | part of a conserved DNA module involved in light responsiveness |

>HU05G02245.1   
+ -Up\_Stream \_Len000ATAATT TATCTTTCTT TAATTTCGTT TCATGTAAAA AATTTCAGTC TCATCTGCGA   
  
  
+ TTGAATCTCT TTTCAATTCG CATCAATAAT TCATCCAATT CAAATGTGCC TTTCTTAATT TCATTACATT   
  
  
+ GGGTTGGTAT AAATGTTAGC ACGATAGATT GGAATAGCAA GGCTCTAATT CAATTATGAC TTGAATGTAT   
  
  
+ GCATGCTTTT ATCATTAGTT AATTAACATT TTCTGTATTG AAATCAGCTC GAAAGAGAAC CATATGTAGT   
  
  
+ TGTACTTGCG ACGATATATA TGTTTAGTTT AGTTAGTTCT TACCTGTTTT GGAATTGTTG GGGAGATCTG   
  
  
+ AGGAAGGAGC CCGCTTCAAT CAAAGTTCAT GGCAATAAGC CAAAGTGAGG TTCCATCCTA AAACCAATTG   
  
  
+ GCAATAGGAA GAGTAGCCCA CTTAACTTGT ATACTATGAC TCTTCTTTTC AAACTCCCAA TGTGGGACTT   
  
  
+ CTTTACTCAT GTGTGATATT CCCAACAGGA ATGATCAGTT CCACATGTTT TCATCTCTTT GGACTCGAAC   
  
  
+ TTCGTGTAAT TGTATATGCC ATGGCACAAA TAGTTTTGAT GGTGCCACCA AGTTGCTCAA TCAATGTAAT   
  
  
+ ACTGCTACTA TCTAACTTGC CTTGGTGGAC ATCAATAAGA TCACGAGATG AGGAGAACTG AATCTTGGCC   
  
  
+ AACCACGTCG TTTGACTTCT CTTACCAATA AAGATTGAAT AGACAGTGTT TCTCTCCCCC TTCCATTCTT   
  
  
+ CTTTTCTAGT GATGTCGAAA AAGACTTTAT ACATGTCCAG CAACGCTAGC CACAAAATAT TCCATTTCTG   
  
  
+ TAATCCACTC ATGTCAATTA AACTTGTATC ATCAAATGGT AGTTTCCGTA CTAATCACTA CATCTAGATA   
  
  
+ TTCCTTGATA AATTTAATTT GACTTTAAAG GCTCGATCTT TTTAAGAAAA GTTTTGTTGC TTACAACCTG   
  
  
+ TAAAGCCTAG AGAACAAAGC CAAAGAAGAA ATTTAAGTTC TATATATAGC ATAATCTCTC AAAAAATATA   
  
  
+ TTTTTTAAAT ATAGTGCGAT ATTGACTGAC CTAATATGGG TTATTACAGG AGATGGGGAG ATATCTACAT   
  
  
+ TAACTACTTG AACAACTTAA ATTTTCATTT TAACTCGGTC ATTAAGAACA CTAAGAACTT TATATATTAG   
  
  
+ TGATATCTCA GGCGTAAAAG AAACTACTTT GAATTAATCC ATGCAATCCT ACTCTTTATT TCCATCCGTT   
  
  
+ AAAATTTTCC TTCAAAACTC CCTTCTGTTC ATTATTAATT CAATATCGTA TTACACTTGT TGGATTATTT   
  
  
+ CTGTGATTTG ATCGTCATTT TCTATATATG AATAACTACA AAAATCATAG TCCGAGTACA TTAATATTAA   
  
  
+ TAAAATAGGA TGAGTGATAA CCTCATATAG TGCGTTTATC CCTACCACTA GGCTGGATTT CTGCATAAAG   
  
  
+ AAGAAATTGA ACATATGGAA AAAGAAATAA AGAGAAGCAA CTTCCTTGAG TGGGAGAAGA ATCGTAGAAA   
  
  
+ GTTAAACTTA TGAAAAAGAG CATTAACAAA AAGGAAATTA TAAGGCTTAC CGTTGAAGCC TCATATAATT   
  
  
+ TTGCAAGAGA CAGACAAAGA AAGTGTCTTT CAAATGTCAT ATTTCTCGAG CATGCATGAA AAATGTACTT   
  
  
+ CGGCTACAAT AGTAATAGTA TATATGTTGG CTTCATACAC TTGACCCCAA TGTATGTCAA CGTGACTAGT   
  
  
+ GACCACACAC ATCGTTGTCC CTGCCCGCCC CACTACCCAA ACTCCCCGCA CGCAAAGATA AAAAGCCCCC   
  
  
+ CAATTTTTAT TTGCCTCCCA AAAAGCCCAC CCGCGCTTTT TTATTTGCCT TCTTTTTTCC TACTTAAACC   
  
  
+ ACCTCCTCAA CGAACTTTGC AACATACATA CTAGTCTATA AGCAATTTAG AGGGAAGGGA AGCAACCTAA   
  
  
+ ACAGAGCGAG ATTGGCGGGG TGTGTTTGAG GGCTTGCGTT TTGGTGCAAT AGGAATGAAA GTGCCCATTT   
  
  
+ CAACCACCAT CCAAACTTGC AACTCCGGCA CCGGCAAACC ACTTTCCTTT GAGAATACTG CCCTACATAC   
  
  
+ CGCCTTTCAA GTTCCCAATA CCACCTACGA ACCCACCTCG GTTCTTGACC TCCAGCGCAG CCCTAGCCCC   
  
  
+ ACCTCCACCC AAAAACCGCC CTTTGCCGCC TCCAACCACC AGCCTGCCTC GGCTGATCTG GACAACTTGG   
  
  
+ ACGGGTGGGA TTCAATCCTA TCAGAGCTGG GGCTTAATGA CGAATTTACC CCTAATTCCA AACTCTGGTC   
  
  
+ CCAAATTAGT CCGTCTGATC CTCACCTCCC CCAACTCCCT GATTTCCCAA CATCTCAGCC GTTTGATCAT   
  
  
+ CACAACCCAC CCCCAATAAA CCTCCCATCA TCAGATTTCA ACCTCTGTGA TTTCTCCTAC AATCACAACC   
  
  
+ CCAATTTTGG GCCGTTCGAT CTTCATCACA ACCCGCATCA GAACAACCCG AACAACAATA ACTACGGCTT   
  
  
+ TGATTTCATA GACGACCTCA TCAAAGCAGC ACAGTCCCTC GAATCCAACG ACTCTCATCA AGTCCACCTG   
  
  
+ ATATTGGCGC GGCTCAATCA ACGGCTCAGA TCACCCACCG GCAAACCGCT CCAGCGGGCC GCCTACTACT   
  
  
+ TCAAGGAAGC CCTCATCGCC GCCGCGGCCG GCCCGCCCCG CCCCGCTCGG CTTTCATCGT ATGAGGTGGT   
  
  
+ GCAGACCATC CGAGCCTACA AGGCGTTTTC AGGAATTTCG CCAGTCTCTC TCTTCTCCAC TTTCGCCGCC   
  
  
+ AACCAGGCGA TTCTTGAGGC GGTGGACACG GCGGCCTTCA TCCACATCAT CGATTTCGAC ATCGGATTCG   
  
  
+ GCGGCCACTG GGCCTCGTTC CTCCGCGAAC TGGTCGACAA AGCTGATCCT GCGAAACTTA GTTCAGTGGT   
  
  
+ GTTACGAATC ACCGCCATCG TTCCCGAAGA ATTCGGGATC GAAAGCAAGC TAGTGAGAGA AAATCTGTCG   
  
  
+ CAATTTGCCC GAGATCTCAA CATCAACTTC CACATCGATT ATGTCTTATT TCAGAGCTTT GAAATCTTAT   
  
  
+ CCTTCAAATC TGTCAAATTC ATCGAAAGGG AGAAATTGGC GCTGCATCTT TCGCCGGCCG TGTTCCACCG   
  
  
+ GCTAGGGAGC GGAATTTCGA AGTTTATCGC TGATCTCCGA TCAATCTCGC CGAGCTCCGT CGTGGTGGTG   
  
  
+ GACAGAGATG TCGGGATTGA CATCGGAACG TCGTCGTTTA GCATGAATTT CGTCGCCGGA ATTGAATTCT   
  
  
+ ACACCGGGAT GCTGGAGTCG CTTGACGCAG CCACCGCCGG GGGGCTCATC GGCGGTGTGG ACTGCGTGAG   
  
  
+ ACGGATTGAG ACGTTTGTTC TCCACCCAAG GATCATGGCG GCGGTAGAGG CGGCGGCGTC GGCGTCGGCC   
  
  
+ GGGCGGAGGA CGGCGTGGAG GGAGGCATTC GCGGCGGCGG GGATTAGGGC GGTGGGGTTT AGCCAGTTTG   
  
  
+ CTGATTTCCA GGCCGAATGT TTGTTGAGGA GAGCTCAGGT TGGTGGCTTC CACGTGGCAA AGCGCCATGG   
  
  
+ AGAGATGATG CTTTACTGGC ATGACCGCCC ACTCGTCGCC ACATCAGCTT GGAGGTGTTA   

- -Up\_Stream \_Len000TATTAA ATAGAAAGAA ATTAAAGCAA AGTACATTTT TTAAAGTCAG AGTAGACGCT   
  
  
- AACTTAGAGA AAAGTTAAGC GTAGTTATTA AGTAGGTTAA GTTTACACGG AAAGAATTAA AGTAATGTAA   
  
  
- CCCAACCATA TTTACAATCG TGCTATCTAA CCTTATCGTT CCGAGATTAA GTTAATACTG AACTTACATA   
  
  
- CGTACGAAAA TAGTAATCAA TTAATTGTAA AAGACATAAC TTTAGTCGAG CTTTCTCTTG GTATACATCA   
  
  
- ACATGAACGC TGCTATATAT ACAAATCAAA TCAATCAAGA ATGGACAAAA CCTTAACAAC CCCTCTAGAC   
  
  
- TCCTTCCTCG GGCGAAGTTA GTTTCAAGTA CCGTTATTCG GTTTCACTCC AAGGTAGGAT TTTGGTTAAC   
  
  
- CGTTATCCTT CTCATCGGGT GAATTGAACA TATGATACTG AGAAGAAAAG TTTGAGGGTT ACACCCTGAA   
  
  
- GAAATGAGTA CACACTATAA GGGTTGTCCT TACTAGTCAA GGTGTACAAA AGTAGAGAAA CCTGAGCTTG   
  
  
- AAGCACATTA ACATATACGG TACCGTGTTT ATCAAAACTA CCACGGTGGT TCAACGAGTT AGTTACATTA   
  
  
- TGACGATGAT AGATTGAACG GAACCACCTG TAGTTATTCT AGTGCTCTAC TCCTCTTGAC TTAGAACCGG   
  
  
- TTGGTGCAGC AAACTGAAGA GAATGGTTAT TTCTAACTTA TCTGTCACAA AGAGAGGGGG AAGGTAAGAA   
  
  
- GAAAAGATCA CTACAGCTTT TTCTGAAATA TGTACAGGTC GTTGCGATCG GTGTTTTATA AGGTAAAGAC   
  
  
- ATTAGGTGAG TACAGTTAAT TTGAACATAG TAGTTTACCA TCAAAGGCAT GATTAGTGAT GTAGATCTAT   
  
  
- AAGGAACTAT TTAAATTAAA CTGAAATTTC CGAGCTAGAA AAATTCTTTT CAAAACAACG AATGTTGGAC   
  
  
- ATTTCGGATC TCTTGTTTCG GTTTCTTCTT TAAATTCAAG ATATATATCG TATTAGAGAG TTTTTTATAT   
  
  
- AAAAAATTTA TATCACGCTA TAACTGACTG GATTATACCC AATAATGTCC TCTACCCCTC TATAGATGTA   
  
  
- ATTGATGAAC TTGTTGAATT TAAAAGTAAA ATTGAGCCAG TAATTCTTGT GATTCTTGAA ATATATAATC   
  
  
- ACTATAGAGT CCGCATTTTC TTTGATGAAA CTTAATTAGG TACGTTAGGA TGAGAAATAA AGGTAGGCAA   
  
  
- TTTTAAAAGG AAGTTTTGAG GGAAGACAAG TAATAATTAA GTTATAGCAT AATGTGAACA ACCTAATAAA   
  
  
- GACACTAAAC TAGCAGTAAA AGATATATAC TTATTGATGT TTTTAGTATC AGGCTCATGT AATTATAATT   
  
  
- ATTTTATCCT ACTCACTATT GGAGTATATC ACGCAAATAG GGATGGTGAT CCGACCTAAA GACGTATTTC   
  
  
- TTCTTTAACT TGTATACCTT TTTCTTTATT TCTCTTCGTT GAAGGAACTC ACCCTCTTCT TAGCATCTTT   
  
  
- CAATTTGAAT ACTTTTTCTC GTAATTGTTT TTCCTTTAAT ATTCCGAATG GCAACTTCGG AGTATATTAA   
  
  
- AACGTTCTCT GTCTGTTTCT TTCACAGAAA GTTTACAGTA TAAAGAGCTC GTACGTACTT TTTACATGAA   
  
  
- GCCGATGTTA TCATTATCAT ATATACAACC GAAGTATGTG AACTGGGGTT ACATACAGTT GCACTGATCA   
  
  
- CTGGTGTGTG TAGCAACAGG GACGGGCGGG GTGATGGGTT TGAGGGGCGT GCGTTTCTAT TTTTCGGGGG   
  
  
- GTTAAAAATA AACGGAGGGT TTTTCGGGTG GGCGCGAAAA AATAAACGGA AGAAAAAAGG ATGAATTTGG   
  
  
- TGGAGGAGTT GCTTGAAACG TTGTATGTAT GATCAGATAT TCGTTAAATC TCCCTTCCCT TCGTTGGATT   
  
  
- TGTCTCGCTC TAACCGCCCC ACACAAACTC CCGAACGCAA AACCACGTTA TCCTTACTTT CACGGGTAAA   
  
  
- GTTGGTGGTA GGTTTGAACG TTGAGGCCGT GGCCGTTTGG TGAAAGGAAA CTCTTATGAC GGGATGTATG   
  
  
- GCGGAAAGTT CAAGGGTTAT GGTGGATGCT TGGGTGGAGC CAAGAACTGG AGGTCGCGTC GGGATCGGGG   
  
  
- TGGAGGTGGG TTTTTGGCGG GAAACGGCGG AGGTTGGTGG TCGGACGGAG CCGACTAGAC CTGTTGAACC   
  
  
- TGCCCACCCT AAGTTAGGAT AGTCTCGACC CCGAATTACT GCTTAAATGG GGATTAAGGT TTGAGACCAG   
  
  
- GGTTTAATCA GGCAGACTAG GAGTGGAGGG GGTTGAGGGA CTAAAGGGTT GTAGAGTCGG CAAACTAGTA   
  
  
- GTGTTGGGTG GGGGTTATTT GGAGGGTAGT AGTCTAAAGT TGGAGACACT AAAGAGGATG TTAGTGTTGG   
  
  
- GGTTAAAACC CGGCAAGCTA GAAGTAGTGT TGGGCGTAGT CTTGTTGGGC TTGTTGTTAT TGATGCCGAA   
  
  
- ACTAAAGTAT CTGCTGGAGT AGTTTCGTCG TGTCAGGGAG CTTAGGTTGC TGAGAGTAGT TCAGGTGGAC   
  
  
- TATAACCGCG CCGAGTTAGT TGCCGAGTCT AGTGGGTGGC CGTTTGGCGA GGTCGCCCGG CGGATGATGA   
  
  
- AGTTCCTTCG GGAGTAGCGG CGGCGCCGGC CGGGCGGGGC GGGGCGAGCC GAAAGTAGCA TACTCCACCA   
  
  
- CGTCTGGTAG GCTCGGATGT TCCGCAAAAG TCCTTAAAGC GGTCAGAGAG AGAAGAGGTG AAAGCGGCGG   
  
  
- TTGGTCCGCT AAGAACTCCG CCACCTGTGC CGCCGGAAGT AGGTGTAGTA GCTAAAGCTG TAGCCTAAGC   
  
  
- CGCCGGTGAC CCGGAGCAAG GAGGCGCTTG ACCAGCTGTT TCGACTAGGA CGCTTTGAAT CAAGTCACCA   
  
  
- CAATGCTTAG TGGCGGTAGC AAGGGCTTCT TAAGCCCTAG CTTTCGTTCG ATCACTCTCT TTTAGACAGC   
  
  
- GTTAAACGGG CTCTAGAGTT GTAGTTGAAG GTGTAGCTAA TACAGAATAA AGTCTCGAAA CTTTAGAATA   
  
  
- GGAAGTTTAG ACAGTTTAAG TAGCTTTCCC TCTTTAACCG CGACGTAGAA AGCGGCCGGC ACAAGGTGGC   
  
  
- CGATCCCTCG CCTTAAAGCT TCAAATAGCG ACTAGAGGCT AGTTAGAGCG GCTCGAGGCA GCACCACCAC   
  
  
- CTGTCTCTAC AGCCCTAACT GTAGCCTTGC AGCAGCAAAT CGTACTTAAA GCAGCGGCCT TAACTTAAGA   
  
  
- TGTGGCCCTA CGACCTCAGC GAACTGCGTC GGTGGCGGCC CCCCGAGTAG CCGCCACACC TGACGCACTC   
  
  
- TGCCTAACTC TGCAAACAAG AGGTGGGTTC CTAGTACCGC CGCCATCTCC GCCGCCGCAG CCGCAGCCGG   
  
  
- CCCGCCTCCT GCCGCACCTC CCTCCGTAAG CGCCGCCGCC CCTAATCCCG CCACCCCAAA TCGGTCAAAC   
  
  
- GACTAAAGGT CCGGCTTACA AACAACTCCT CTCGAGTCCA ACCACCGAAG GTGCACCGTT TCGCGGTACC   
  
  
- TCTCTACTAC GAAATGACCG TACTGGCGGG TGAGCAGCGG TGTAGTCGAA CCTCCACAAT

+     Box II

| Site Name | Organism | Position | Strand | Matrix score. | sequence | function |
| --- | --- | --- | --- | --- | --- | --- |
| Box II | Petroselinum crispum | 3554 | + | 9 | CCACGTGGC | part of a light responsive element |

>HU05G02245.1   
+ -Up\_Stream \_Len000ATAATT TATCTTTCTT TAATTTCGTT TCATGTAAAA AATTTCAGTC TCATCTGCGA   
  
  
+ TTGAATCTCT TTTCAATTCG CATCAATAAT TCATCCAATT CAAATGTGCC TTTCTTAATT TCATTACATT   
  
  
+ GGGTTGGTAT AAATGTTAGC ACGATAGATT GGAATAGCAA GGCTCTAATT CAATTATGAC TTGAATGTAT   
  
  
+ GCATGCTTTT ATCATTAGTT AATTAACATT TTCTGTATTG AAATCAGCTC GAAAGAGAAC CATATGTAGT   
  
  
+ TGTACTTGCG ACGATATATA TGTTTAGTTT AGTTAGTTCT TACCTGTTTT GGAATTGTTG GGGAGATCTG   
  
  
+ AGGAAGGAGC CCGCTTCAAT CAAAGTTCAT GGCAATAAGC CAAAGTGAGG TTCCATCCTA AAACCAATTG   
  
  
+ GCAATAGGAA GAGTAGCCCA CTTAACTTGT ATACTATGAC TCTTCTTTTC AAACTCCCAA TGTGGGACTT   
  
  
+ CTTTACTCAT GTGTGATATT CCCAACAGGA ATGATCAGTT CCACATGTTT TCATCTCTTT GGACTCGAAC   
  
  
+ TTCGTGTAAT TGTATATGCC ATGGCACAAA TAGTTTTGAT GGTGCCACCA AGTTGCTCAA TCAATGTAAT   
  
  
+ ACTGCTACTA TCTAACTTGC CTTGGTGGAC ATCAATAAGA TCACGAGATG AGGAGAACTG AATCTTGGCC   
  
  
+ AACCACGTCG TTTGACTTCT CTTACCAATA AAGATTGAAT AGACAGTGTT TCTCTCCCCC TTCCATTCTT   
  
  
+ CTTTTCTAGT GATGTCGAAA AAGACTTTAT ACATGTCCAG CAACGCTAGC CACAAAATAT TCCATTTCTG   
  
  
+ TAATCCACTC ATGTCAATTA AACTTGTATC ATCAAATGGT AGTTTCCGTA CTAATCACTA CATCTAGATA   
  
  
+ TTCCTTGATA AATTTAATTT GACTTTAAAG GCTCGATCTT TTTAAGAAAA GTTTTGTTGC TTACAACCTG   
  
  
+ TAAAGCCTAG AGAACAAAGC CAAAGAAGAA ATTTAAGTTC TATATATAGC ATAATCTCTC AAAAAATATA   
  
  
+ TTTTTTAAAT ATAGTGCGAT ATTGACTGAC CTAATATGGG TTATTACAGG AGATGGGGAG ATATCTACAT   
  
  
+ TAACTACTTG AACAACTTAA ATTTTCATTT TAACTCGGTC ATTAAGAACA CTAAGAACTT TATATATTAG   
  
  
+ TGATATCTCA GGCGTAAAAG AAACTACTTT GAATTAATCC ATGCAATCCT ACTCTTTATT TCCATCCGTT   
  
  
+ AAAATTTTCC TTCAAAACTC CCTTCTGTTC ATTATTAATT CAATATCGTA TTACACTTGT TGGATTATTT   
  
  
+ CTGTGATTTG ATCGTCATTT TCTATATATG AATAACTACA AAAATCATAG TCCGAGTACA TTAATATTAA   
  
  
+ TAAAATAGGA TGAGTGATAA CCTCATATAG TGCGTTTATC CCTACCACTA GGCTGGATTT CTGCATAAAG   
  
  
+ AAGAAATTGA ACATATGGAA AAAGAAATAA AGAGAAGCAA CTTCCTTGAG TGGGAGAAGA ATCGTAGAAA   
  
  
+ GTTAAACTTA TGAAAAAGAG CATTAACAAA AAGGAAATTA TAAGGCTTAC CGTTGAAGCC TCATATAATT   
  
  
+ TTGCAAGAGA CAGACAAAGA AAGTGTCTTT CAAATGTCAT ATTTCTCGAG CATGCATGAA AAATGTACTT   
  
  
+ CGGCTACAAT AGTAATAGTA TATATGTTGG CTTCATACAC TTGACCCCAA TGTATGTCAA CGTGACTAGT   
  
  
+ GACCACACAC ATCGTTGTCC CTGCCCGCCC CACTACCCAA ACTCCCCGCA CGCAAAGATA AAAAGCCCCC   
  
  
+ CAATTTTTAT TTGCCTCCCA AAAAGCCCAC CCGCGCTTTT TTATTTGCCT TCTTTTTTCC TACTTAAACC   
  
  
+ ACCTCCTCAA CGAACTTTGC AACATACATA CTAGTCTATA AGCAATTTAG AGGGAAGGGA AGCAACCTAA   
  
  
+ ACAGAGCGAG ATTGGCGGGG TGTGTTTGAG GGCTTGCGTT TTGGTGCAAT AGGAATGAAA GTGCCCATTT   
  
  
+ CAACCACCAT CCAAACTTGC AACTCCGGCA CCGGCAAACC ACTTTCCTTT GAGAATACTG CCCTACATAC   
  
  
+ CGCCTTTCAA GTTCCCAATA CCACCTACGA ACCCACCTCG GTTCTTGACC TCCAGCGCAG CCCTAGCCCC   
  
  
+ ACCTCCACCC AAAAACCGCC CTTTGCCGCC TCCAACCACC AGCCTGCCTC GGCTGATCTG GACAACTTGG   
  
  
+ ACGGGTGGGA TTCAATCCTA TCAGAGCTGG GGCTTAATGA CGAATTTACC CCTAATTCCA AACTCTGGTC   
  
  
+ CCAAATTAGT CCGTCTGATC CTCACCTCCC CCAACTCCCT GATTTCCCAA CATCTCAGCC GTTTGATCAT   
  
  
+ CACAACCCAC CCCCAATAAA CCTCCCATCA TCAGATTTCA ACCTCTGTGA TTTCTCCTAC AATCACAACC   
  
  
+ CCAATTTTGG GCCGTTCGAT CTTCATCACA ACCCGCATCA GAACAACCCG AACAACAATA ACTACGGCTT   
  
  
+ TGATTTCATA GACGACCTCA TCAAAGCAGC ACAGTCCCTC GAATCCAACG ACTCTCATCA AGTCCACCTG   
  
  
+ ATATTGGCGC GGCTCAATCA ACGGCTCAGA TCACCCACCG GCAAACCGCT CCAGCGGGCC GCCTACTACT   
  
  
+ TCAAGGAAGC CCTCATCGCC GCCGCGGCCG GCCCGCCCCG CCCCGCTCGG CTTTCATCGT ATGAGGTGGT   
  
  
+ GCAGACCATC CGAGCCTACA AGGCGTTTTC AGGAATTTCG CCAGTCTCTC TCTTCTCCAC TTTCGCCGCC   
  
  
+ AACCAGGCGA TTCTTGAGGC GGTGGACACG GCGGCCTTCA TCCACATCAT CGATTTCGAC ATCGGATTCG   
  
  
+ GCGGCCACTG GGCCTCGTTC CTCCGCGAAC TGGTCGACAA AGCTGATCCT GCGAAACTTA GTTCAGTGGT   
  
  
+ GTTACGAATC ACCGCCATCG TTCCCGAAGA ATTCGGGATC GAAAGCAAGC TAGTGAGAGA AAATCTGTCG   
  
  
+ CAATTTGCCC GAGATCTCAA CATCAACTTC CACATCGATT ATGTCTTATT TCAGAGCTTT GAAATCTTAT   
  
  
+ CCTTCAAATC TGTCAAATTC ATCGAAAGGG AGAAATTGGC GCTGCATCTT TCGCCGGCCG TGTTCCACCG   
  
  
+ GCTAGGGAGC GGAATTTCGA AGTTTATCGC TGATCTCCGA TCAATCTCGC CGAGCTCCGT CGTGGTGGTG   
  
  
+ GACAGAGATG TCGGGATTGA CATCGGAACG TCGTCGTTTA GCATGAATTT CGTCGCCGGA ATTGAATTCT   
  
  
+ ACACCGGGAT GCTGGAGTCG CTTGACGCAG CCACCGCCGG GGGGCTCATC GGCGGTGTGG ACTGCGTGAG   
  
  
+ ACGGATTGAG ACGTTTGTTC TCCACCCAAG GATCATGGCG GCGGTAGAGG CGGCGGCGTC GGCGTCGGCC   
  
  
+ GGGCGGAGGA CGGCGTGGAG GGAGGCATTC GCGGCGGCGG GGATTAGGGC GGTGGGGTTT AGCCAGTTTG   
  
  
+ CTGATTTCCA GGCCGAATGT TTGTTGAGGA GAGCTCAGGT TGGTGGCTTC CACGTGGCAA AGCGCCATGG   
  
  
+ AGAGATGATG CTTTACTGGC ATGACCGCCC ACTCGTCGCC ACATCAGCTT GGAGGTGTTA   

- -Up\_Stream \_Len000TATTAA ATAGAAAGAA ATTAAAGCAA AGTACATTTT TTAAAGTCAG AGTAGACGCT   
  
  
- AACTTAGAGA AAAGTTAAGC GTAGTTATTA AGTAGGTTAA GTTTACACGG AAAGAATTAA AGTAATGTAA   
  
  
- CCCAACCATA TTTACAATCG TGCTATCTAA CCTTATCGTT CCGAGATTAA GTTAATACTG AACTTACATA   
  
  
- CGTACGAAAA TAGTAATCAA TTAATTGTAA AAGACATAAC TTTAGTCGAG CTTTCTCTTG GTATACATCA   
  
  
- ACATGAACGC TGCTATATAT ACAAATCAAA TCAATCAAGA ATGGACAAAA CCTTAACAAC CCCTCTAGAC   
  
  
- TCCTTCCTCG GGCGAAGTTA GTTTCAAGTA CCGTTATTCG GTTTCACTCC AAGGTAGGAT TTTGGTTAAC   
  
  
- CGTTATCCTT CTCATCGGGT GAATTGAACA TATGATACTG AGAAGAAAAG TTTGAGGGTT ACACCCTGAA   
  
  
- GAAATGAGTA CACACTATAA GGGTTGTCCT TACTAGTCAA GGTGTACAAA AGTAGAGAAA CCTGAGCTTG   
  
  
- AAGCACATTA ACATATACGG TACCGTGTTT ATCAAAACTA CCACGGTGGT TCAACGAGTT AGTTACATTA   
  
  
- TGACGATGAT AGATTGAACG GAACCACCTG TAGTTATTCT AGTGCTCTAC TCCTCTTGAC TTAGAACCGG   
  
  
- TTGGTGCAGC AAACTGAAGA GAATGGTTAT TTCTAACTTA TCTGTCACAA AGAGAGGGGG AAGGTAAGAA   
  
  
- GAAAAGATCA CTACAGCTTT TTCTGAAATA TGTACAGGTC GTTGCGATCG GTGTTTTATA AGGTAAAGAC   
  
  
- ATTAGGTGAG TACAGTTAAT TTGAACATAG TAGTTTACCA TCAAAGGCAT GATTAGTGAT GTAGATCTAT   
  
  
- AAGGAACTAT TTAAATTAAA CTGAAATTTC CGAGCTAGAA AAATTCTTTT CAAAACAACG AATGTTGGAC   
  
  
- ATTTCGGATC TCTTGTTTCG GTTTCTTCTT TAAATTCAAG ATATATATCG TATTAGAGAG TTTTTTATAT   
  
  
- AAAAAATTTA TATCACGCTA TAACTGACTG GATTATACCC AATAATGTCC TCTACCCCTC TATAGATGTA   
  
  
- ATTGATGAAC TTGTTGAATT TAAAAGTAAA ATTGAGCCAG TAATTCTTGT GATTCTTGAA ATATATAATC   
  
  
- ACTATAGAGT CCGCATTTTC TTTGATGAAA CTTAATTAGG TACGTTAGGA TGAGAAATAA AGGTAGGCAA   
  
  
- TTTTAAAAGG AAGTTTTGAG GGAAGACAAG TAATAATTAA GTTATAGCAT AATGTGAACA ACCTAATAAA   
  
  
- GACACTAAAC TAGCAGTAAA AGATATATAC TTATTGATGT TTTTAGTATC AGGCTCATGT AATTATAATT   
  
  
- ATTTTATCCT ACTCACTATT GGAGTATATC ACGCAAATAG GGATGGTGAT CCGACCTAAA GACGTATTTC   
  
  
- TTCTTTAACT TGTATACCTT TTTCTTTATT TCTCTTCGTT GAAGGAACTC ACCCTCTTCT TAGCATCTTT   
  
  
- CAATTTGAAT ACTTTTTCTC GTAATTGTTT TTCCTTTAAT ATTCCGAATG GCAACTTCGG AGTATATTAA   
  
  
- AACGTTCTCT GTCTGTTTCT TTCACAGAAA GTTTACAGTA TAAAGAGCTC GTACGTACTT TTTACATGAA   
  
  
- GCCGATGTTA TCATTATCAT ATATACAACC GAAGTATGTG AACTGGGGTT ACATACAGTT GCACTGATCA   
  
  
- CTGGTGTGTG TAGCAACAGG GACGGGCGGG GTGATGGGTT TGAGGGGCGT GCGTTTCTAT TTTTCGGGGG   
  
  
- GTTAAAAATA AACGGAGGGT TTTTCGGGTG GGCGCGAAAA AATAAACGGA AGAAAAAAGG ATGAATTTGG   
  
  
- TGGAGGAGTT GCTTGAAACG TTGTATGTAT GATCAGATAT TCGTTAAATC TCCCTTCCCT TCGTTGGATT   
  
  
- TGTCTCGCTC TAACCGCCCC ACACAAACTC CCGAACGCAA AACCACGTTA TCCTTACTTT CACGGGTAAA   
  
  
- GTTGGTGGTA GGTTTGAACG TTGAGGCCGT GGCCGTTTGG TGAAAGGAAA CTCTTATGAC GGGATGTATG   
  
  
- GCGGAAAGTT CAAGGGTTAT GGTGGATGCT TGGGTGGAGC CAAGAACTGG AGGTCGCGTC GGGATCGGGG   
  
  
- TGGAGGTGGG TTTTTGGCGG GAAACGGCGG AGGTTGGTGG TCGGACGGAG CCGACTAGAC CTGTTGAACC   
  
  
- TGCCCACCCT AAGTTAGGAT AGTCTCGACC CCGAATTACT GCTTAAATGG GGATTAAGGT TTGAGACCAG   
  
  
- GGTTTAATCA GGCAGACTAG GAGTGGAGGG GGTTGAGGGA CTAAAGGGTT GTAGAGTCGG CAAACTAGTA   
  
  
- GTGTTGGGTG GGGGTTATTT GGAGGGTAGT AGTCTAAAGT TGGAGACACT AAAGAGGATG TTAGTGTTGG   
  
  
- GGTTAAAACC CGGCAAGCTA GAAGTAGTGT TGGGCGTAGT CTTGTTGGGC TTGTTGTTAT TGATGCCGAA   
  
  
- ACTAAAGTAT CTGCTGGAGT AGTTTCGTCG TGTCAGGGAG CTTAGGTTGC TGAGAGTAGT TCAGGTGGAC   
  
  
- TATAACCGCG CCGAGTTAGT TGCCGAGTCT AGTGGGTGGC CGTTTGGCGA GGTCGCCCGG CGGATGATGA   
  
  
- AGTTCCTTCG GGAGTAGCGG CGGCGCCGGC CGGGCGGGGC GGGGCGAGCC GAAAGTAGCA TACTCCACCA   
  
  
- CGTCTGGTAG GCTCGGATGT TCCGCAAAAG TCCTTAAAGC GGTCAGAGAG AGAAGAGGTG AAAGCGGCGG   
  
  
- TTGGTCCGCT AAGAACTCCG CCACCTGTGC CGCCGGAAGT AGGTGTAGTA GCTAAAGCTG TAGCCTAAGC   
  
  
- CGCCGGTGAC CCGGAGCAAG GAGGCGCTTG ACCAGCTGTT TCGACTAGGA CGCTTTGAAT CAAGTCACCA   
  
  
- CAATGCTTAG TGGCGGTAGC AAGGGCTTCT TAAGCCCTAG CTTTCGTTCG ATCACTCTCT TTTAGACAGC   
  
  
- GTTAAACGGG CTCTAGAGTT GTAGTTGAAG GTGTAGCTAA TACAGAATAA AGTCTCGAAA CTTTAGAATA   
  
  
- GGAAGTTTAG ACAGTTTAAG TAGCTTTCCC TCTTTAACCG CGACGTAGAA AGCGGCCGGC ACAAGGTGGC   
  
  
- CGATCCCTCG CCTTAAAGCT TCAAATAGCG ACTAGAGGCT AGTTAGAGCG GCTCGAGGCA GCACCACCAC   
  
  
- CTGTCTCTAC AGCCCTAACT GTAGCCTTGC AGCAGCAAAT CGTACTTAAA GCAGCGGCCT TAACTTAAGA   
  
  
- TGTGGCCCTA CGACCTCAGC GAACTGCGTC GGTGGCGGCC CCCCGAGTAG CCGCCACACC TGACGCACTC   
  
  
- TGCCTAACTC TGCAAACAAG AGGTGGGTTC CTAGTACCGC CGCCATCTCC GCCGCCGCAG CCGCAGCCGG   
  
  
- CCCGCCTCCT GCCGCACCTC CCTCCGTAAG CGCCGCCGCC CCTAATCCCG CCACCCCAAA TCGGTCAAAC   
  
  
- GACTAAAGGT CCGGCTTACA AACAACTCCT CTCGAGTCCA ACCACCGAAG GTGCACCGTT TCGCGGTACC   
  
  
- TCTCTACTAC GAAATGACCG TACTGGCGGG TGAGCAGCGG TGTAGTCGAA CCTCCACAAT

+     CAAT-box

| Site Name | Organism | Position | Strand | Matrix score. | sequence | function |
| --- | --- | --- | --- | --- | --- | --- |
| CAAT-box | Arabidopsis thaliana | 3119 | - | 5 | CCAAT | common cis-acting element in promoter and enhancer regions |
| CAAT-box | Pisum sativum | 3089 | + | 5 | CAAAT | common cis-acting element in promoter and enhancer regions |
| CAAT-box | Arabidopsis thaliana | 2455 | + | 5 | CCAAT | common cis-acting element in promoter and enhancer regions |
| CAAT-box | Nicotiana glutinosa | 3015 | + | 4 | CAAT |  |
| CAAT-box | Pisum sativum | 591 | + | 5 | CAAAT | common cis-acting element in promoter and enhancer regions |
| CAAT-box | Arabidopsis thaliana | 729 | + | 5 | CCAAT | common cis-acting element in promoter and enhancer regions |
| CAAT-box | Nicotiana glutinosa | 88 | + | 4 | CAAT |  |
| CAAT-box | Nicotiana glutinosa | 3369 | - | 4 | CAAT |  |
| CAAT-box | Nicotiana glutinosa | 2609 | + | 4 | CAAT |  |
| CAAT-box | Pisum sativum | 3098 | + | 5 | CAAAT | common cis-acting element in promoter and enhancer regions |
| CAAT-box | Nicotiana glutinosa | 2510 | + | 4 | CAAT |  |
| CAAT-box | Nicotiana glutinosa | 251 | - | 4 | CAAT |  |
| CAAT-box | Nicotiana glutinosa | 2456 | + | 4 | CAAT |  |
| CAAT-box | Arabidopsis thaliana | 418 | + | 5 | CCAAT | common cis-acting element in promoter and enhancer regions |
| CAAT-box | Pisum sativum | 877 | + | 5 | CAAAT | common cis-acting element in promoter and enhancer regions |
| CAAT-box | Nicotiana glutinosa | 1075 | - | 4 | CAAT |  |
| CAAT-box | Nicotiana glutinosa | 387 | + | 4 | CAAT |  |
| CAAT-box | Nicotiana glutinosa | 738 | - | 4 | CAAT |  |
| CAAT-box | Nicotiana glutinosa | 3285 | - | 4 | CAAT |  |
| CAAT-box | Arabidopsis thaliana | 2454 | + | 8 | CCCAATTT | common cis-acting element in promoter and enhancer regions |
| CAAT-box | Pisum sativum | 2316 | + | 5 | CAAAT | common cis-acting element in promoter and enhancer regions |
| CAAT-box | Arabidopsis thaliana | 1975 | - | 5 | CCAAT | common cis-acting element in promoter and enhancer regions |
| CAAT-box | Nicotiana glutinosa | 2120 | + | 4 | CAAT |  |
| CAAT-box | Nicotiana glutinosa | 2257 | + | 4 | CAAT |  |
| CAAT-box | Arabidopsis thaliana | 1824 | + | 5 | CCAAT | common cis-acting element in promoter and enhancer regions |
| CAAT-box | Nicotiana glutinosa | 3196 | + | 4 | CAAT |  |
| CAAT-box | Nicotiana glutinosa | 1480 | - | 4 | CAAT |  |
| CAAT-box | Nicotiana glutinosa | 1691 | + | 4 | CAAT |  |
| CAAT-box | Arabidopsis thaliana | 1731 | + | 5 | CCAAT | common cis-acting element in promoter and enhancer regions |
| CAAT-box | Nicotiana glutinosa | 1305 | + | 4 | CAAT |  |
| CAAT-box | Nicotiana glutinosa | 371 | + | 4 | CAAT |  |
| CAAT-box | Pisum sativum | 1867 | - | 5 | CAAAT | common cis-acting element in promoter and enhancer regions |
| CAAT-box | Nicotiana glutinosa | 110 | + | 4 | CAAT |  |
| CAAT-box | Nicotiana glutinosa | 74 | - | 4 | CAAT |  |
| CAAT-box | Nicotiana glutinosa | 482 | + | 4 | CAAT |  |
| CAAT-box | Nicotiana glutinosa | 2011 | + | 4 | CAAT |  |
| CAAT-box | Nicotiana glutinosa | 667 | + | 4 | CAAT |  |
| CAAT-box | Arabidopsis thaliana | 421 | - | 5 | CCAAT | common cis-acting element in promoter and enhancer regions |
| CAAT-box | Pisum sativum | 3017 | - | 5 | CAAAT | common cis-acting element in promoter and enhancer regions |
| CAAT-box | Nicotiana glutinosa | 419 | + | 4 | CAAT |  |
| CAAT-box | Nicotiana glutinosa | 1825 | + | 4 | CAAT |  |
| CAAT-box | Pisum sativum | 931 | - | 5 | CAAAT | common cis-acting element in promoter and enhancer regions |
| CAAT-box | Nicotiana glutinosa | 3240 | - | 4 | CAAT |  |
| CAAT-box | Arabidopsis thaliana | 2597 | - | 5 | CCAAT | common cis-acting element in promoter and enhancer regions |
| CAAT-box | Nicotiana glutinosa | 98 | + | 4 | CAAT |  |
| CAAT-box | Arabidopsis thaliana | 109 | + | 5 | CCAAT | common cis-acting element in promoter and enhancer regions |
| CAAT-box | Nicotiana glutinosa | 338 | - | 4 | CAAT |  |
| CAAT-box | Arabidopsis thaliana | 172 | - | 5 | CCAAT | common cis-acting element in promoter and enhancer regions |
| CAAT-box | Arabidopsis thaliana | 142 | - | 5 | CCAAT | common cis-acting element in promoter and enhancer regions |
| CAAT-box | Nicotiana glutinosa | 2398 | + | 4 | CAAT |  |
| CAAT-box | Nicotiana glutinosa | 730 | + | 4 | CAAT |  |
| CAAT-box | Pisum sativum | 1833 | - | 5 | CAAAT | common cis-acting element in promoter and enhancer regions |
| CAAT-box | Arabidopsis thaliana | 481 | + | 5 | CCAAT | common cis-acting element in promoter and enhancer regions |
| CAAT-box | Nicotiana glutinosa | 1238 | + | 4 | CAAT |  |
| CAAT-box | Nicotiana glutinosa | 573 | - | 4 | CAAT |  |
| CAAT-box | Arabidopsis thaliana | 2397 | + | 5 | CCAAT | common cis-acting element in promoter and enhancer regions |
| CAAT-box | Arabidopsis thaliana | 2119 | + | 5 | CCAAT | common cis-acting element in promoter and enhancer regions |
| CAAT-box | Pisum sativum | 115 | + | 5 | CAAAT | common cis-acting element in promoter and enhancer regions |
| CAAT-box | Nicotiana glutinosa | 426 | + | 4 | CAAT |  |
| CAAT-box | Nicotiana glutinosa | 2444 | + | 4 | CAAT |  |
| CAAT-box | Nicotiana glutinosa | 195 | + | 4 | CAAT |  |
| CAAT-box | Nicotiana glutinosa | 1732 | + | 4 | CAAT |  |
| CAAT-box | Pisum sativum | 1340 | - | 5 | CAAAT | common cis-acting element in promoter and enhancer regions |
| CAAT-box | Nicotiana glutinosa | 1937 | + | 4 | CAAT |  |
| CAAT-box | Nicotiana glutinosa | 626 | + | 4 | CAAT |  |
| CAAT-box | Arabidopsis thaliana | 1823 | + | 8 | CCCAATTT | common cis-acting element in promoter and enhancer regions |
| CAAT-box | Pisum sativum | 1645 | + | 5 | CAAAT | common cis-acting element in promoter and enhancer regions |
| CAAT-box | Nicotiana glutinosa | 859 | + | 4 | CAAT |  |
| CAAT-box | Nicotiana glutinosa | 622 | + | 4 | CAAT |  |

>HU05G02245.1   
+ -Up\_Stream \_Len000ATAATT TATCTTTCTT TAATTTCGTT TCATGTAAAA AATTTCAGTC TCATCTGCGA   
  
  
+ TTGAATCTCT TTTCAATTCG CATCAATAAT TCATCCAATT CAAATGTGCC TTTCTTAATT TCATTACATT   
  
  
+ GGGTTGGTAT AAATGTTAGC ACGATAGATT GGAATAGCAA GGCTCTAATT CAATTATGAC TTGAATGTAT   
  
  
+ GCATGCTTTT ATCATTAGTT AATTAACATT TTCTGTATTG AAATCAGCTC GAAAGAGAAC CATATGTAGT   
  
  
+ TGTACTTGCG ACGATATATA TGTTTAGTTT AGTTAGTTCT TACCTGTTTT GGAATTGTTG GGGAGATCTG   
  
  
+ AGGAAGGAGC CCGCTTCAAT CAAAGTTCAT GGCAATAAGC CAAAGTGAGG TTCCATCCTA AAACCAATTG   
  
  
+ GCAATAGGAA GAGTAGCCCA CTTAACTTGT ATACTATGAC TCTTCTTTTC AAACTCCCAA TGTGGGACTT   
  
  
+ CTTTACTCAT GTGTGATATT CCCAACAGGA ATGATCAGTT CCACATGTTT TCATCTCTTT GGACTCGAAC   
  
  
+ TTCGTGTAAT TGTATATGCC ATGGCACAAA TAGTTTTGAT GGTGCCACCA AGTTGCTCAA TCAATGTAAT   
  
  
+ ACTGCTACTA TCTAACTTGC CTTGGTGGAC ATCAATAAGA TCACGAGATG AGGAGAACTG AATCTTGGCC   
  
  
+ AACCACGTCG TTTGACTTCT CTTACCAATA AAGATTGAAT AGACAGTGTT TCTCTCCCCC TTCCATTCTT   
  
  
+ CTTTTCTAGT GATGTCGAAA AAGACTTTAT ACATGTCCAG CAACGCTAGC CACAAAATAT TCCATTTCTG   
  
  
+ TAATCCACTC ATGTCAATTA AACTTGTATC ATCAAATGGT AGTTTCCGTA CTAATCACTA CATCTAGATA   
  
  
+ TTCCTTGATA AATTTAATTT GACTTTAAAG GCTCGATCTT TTTAAGAAAA GTTTTGTTGC TTACAACCTG   
  
  
+ TAAAGCCTAG AGAACAAAGC CAAAGAAGAA ATTTAAGTTC TATATATAGC ATAATCTCTC AAAAAATATA   
  
  
+ TTTTTTAAAT ATAGTGCGAT ATTGACTGAC CTAATATGGG TTATTACAGG AGATGGGGAG ATATCTACAT   
  
  
+ TAACTACTTG AACAACTTAA ATTTTCATTT TAACTCGGTC ATTAAGAACA CTAAGAACTT TATATATTAG   
  
  
+ TGATATCTCA GGCGTAAAAG AAACTACTTT GAATTAATCC ATGCAATCCT ACTCTTTATT TCCATCCGTT   
  
  
+ AAAATTTTCC TTCAAAACTC CCTTCTGTTC ATTATTAATT CAATATCGTA TTACACTTGT TGGATTATTT   
  
  
+ CTGTGATTTG ATCGTCATTT TCTATATATG AATAACTACA AAAATCATAG TCCGAGTACA TTAATATTAA   
  
  
+ TAAAATAGGA TGAGTGATAA CCTCATATAG TGCGTTTATC CCTACCACTA GGCTGGATTT CTGCATAAAG   
  
  
+ AAGAAATTGA ACATATGGAA AAAGAAATAA AGAGAAGCAA CTTCCTTGAG TGGGAGAAGA ATCGTAGAAA   
  
  
+ GTTAAACTTA TGAAAAAGAG CATTAACAAA AAGGAAATTA TAAGGCTTAC CGTTGAAGCC TCATATAATT   
  
  
+ TTGCAAGAGA CAGACAAAGA AAGTGTCTTT CAAATGTCAT ATTTCTCGAG CATGCATGAA AAATGTACTT   
  
  
+ CGGCTACAAT AGTAATAGTA TATATGTTGG CTTCATACAC TTGACCCCAA TGTATGTCAA CGTGACTAGT   
  
  
+ GACCACACAC ATCGTTGTCC CTGCCCGCCC CACTACCCAA ACTCCCCGCA CGCAAAGATA AAAAGCCCCC   
  
  
+ CAATTTTTAT TTGCCTCCCA AAAAGCCCAC CCGCGCTTTT TTATTTGCCT TCTTTTTTCC TACTTAAACC   
  
  
+ ACCTCCTCAA CGAACTTTGC AACATACATA CTAGTCTATA AGCAATTTAG AGGGAAGGGA AGCAACCTAA   
  
  
+ ACAGAGCGAG ATTGGCGGGG TGTGTTTGAG GGCTTGCGTT TTGGTGCAAT AGGAATGAAA GTGCCCATTT   
  
  
+ CAACCACCAT CCAAACTTGC AACTCCGGCA CCGGCAAACC ACTTTCCTTT GAGAATACTG CCCTACATAC   
  
  
+ CGCCTTTCAA GTTCCCAATA CCACCTACGA ACCCACCTCG GTTCTTGACC TCCAGCGCAG CCCTAGCCCC   
  
  
+ ACCTCCACCC AAAAACCGCC CTTTGCCGCC TCCAACCACC AGCCTGCCTC GGCTGATCTG GACAACTTGG   
  
  
+ ACGGGTGGGA TTCAATCCTA TCAGAGCTGG GGCTTAATGA CGAATTTACC CCTAATTCCA AACTCTGGTC   
  
  
+ CCAAATTAGT CCGTCTGATC CTCACCTCCC CCAACTCCCT GATTTCCCAA CATCTCAGCC GTTTGATCAT   
  
  
+ CACAACCCAC CCCCAATAAA CCTCCCATCA TCAGATTTCA ACCTCTGTGA TTTCTCCTAC AATCACAACC   
  
  
+ CCAATTTTGG GCCGTTCGAT CTTCATCACA ACCCGCATCA GAACAACCCG AACAACAATA ACTACGGCTT   
  
  
+ TGATTTCATA GACGACCTCA TCAAAGCAGC ACAGTCCCTC GAATCCAACG ACTCTCATCA AGTCCACCTG   
  
  
+ ATATTGGCGC GGCTCAATCA ACGGCTCAGA TCACCCACCG GCAAACCGCT CCAGCGGGCC GCCTACTACT   
  
  
+ TCAAGGAAGC CCTCATCGCC GCCGCGGCCG GCCCGCCCCG CCCCGCTCGG CTTTCATCGT ATGAGGTGGT   
  
  
+ GCAGACCATC CGAGCCTACA AGGCGTTTTC AGGAATTTCG CCAGTCTCTC TCTTCTCCAC TTTCGCCGCC   
  
  
+ AACCAGGCGA TTCTTGAGGC GGTGGACACG GCGGCCTTCA TCCACATCAT CGATTTCGAC ATCGGATTCG   
  
  
+ GCGGCCACTG GGCCTCGTTC CTCCGCGAAC TGGTCGACAA AGCTGATCCT GCGAAACTTA GTTCAGTGGT   
  
  
+ GTTACGAATC ACCGCCATCG TTCCCGAAGA ATTCGGGATC GAAAGCAAGC TAGTGAGAGA AAATCTGTCG   
  
  
+ CAATTTGCCC GAGATCTCAA CATCAACTTC CACATCGATT ATGTCTTATT TCAGAGCTTT GAAATCTTAT   
  
  
+ CCTTCAAATC TGTCAAATTC ATCGAAAGGG AGAAATTGGC GCTGCATCTT TCGCCGGCCG TGTTCCACCG   
  
  
+ GCTAGGGAGC GGAATTTCGA AGTTTATCGC TGATCTCCGA TCAATCTCGC CGAGCTCCGT CGTGGTGGTG   
  
  
+ GACAGAGATG TCGGGATTGA CATCGGAACG TCGTCGTTTA GCATGAATTT CGTCGCCGGA ATTGAATTCT   
  
  
+ ACACCGGGAT GCTGGAGTCG CTTGACGCAG CCACCGCCGG GGGGCTCATC GGCGGTGTGG ACTGCGTGAG   
  
  
+ ACGGATTGAG ACGTTTGTTC TCCACCCAAG GATCATGGCG GCGGTAGAGG CGGCGGCGTC GGCGTCGGCC   
  
  
+ GGGCGGAGGA CGGCGTGGAG GGAGGCATTC GCGGCGGCGG GGATTAGGGC GGTGGGGTTT AGCCAGTTTG   
  
  
+ CTGATTTCCA GGCCGAATGT TTGTTGAGGA GAGCTCAGGT TGGTGGCTTC CACGTGGCAA AGCGCCATGG   
  
  
+ AGAGATGATG CTTTACTGGC ATGACCGCCC ACTCGTCGCC ACATCAGCTT GGAGGTGTTA   

- -Up\_Stream \_Len000TATTAA ATAGAAAGAA ATTAAAGCAA AGTACATTTT TTAAAGTCAG AGTAGACGCT   
  
  
- AACTTAGAGA AAAGTTAAGC GTAGTTATTA AGTAGGTTAA GTTTACACGG AAAGAATTAA AGTAATGTAA   
  
  
- CCCAACCATA TTTACAATCG TGCTATCTAA CCTTATCGTT CCGAGATTAA GTTAATACTG AACTTACATA   
  
  
- CGTACGAAAA TAGTAATCAA TTAATTGTAA AAGACATAAC TTTAGTCGAG CTTTCTCTTG GTATACATCA   
  
  
- ACATGAACGC TGCTATATAT ACAAATCAAA TCAATCAAGA ATGGACAAAA CCTTAACAAC CCCTCTAGAC   
  
  
- TCCTTCCTCG GGCGAAGTTA GTTTCAAGTA CCGTTATTCG GTTTCACTCC AAGGTAGGAT TTTGGTTAAC   
  
  
- CGTTATCCTT CTCATCGGGT GAATTGAACA TATGATACTG AGAAGAAAAG TTTGAGGGTT ACACCCTGAA   
  
  
- GAAATGAGTA CACACTATAA GGGTTGTCCT TACTAGTCAA GGTGTACAAA AGTAGAGAAA CCTGAGCTTG   
  
  
- AAGCACATTA ACATATACGG TACCGTGTTT ATCAAAACTA CCACGGTGGT TCAACGAGTT AGTTACATTA   
  
  
- TGACGATGAT AGATTGAACG GAACCACCTG TAGTTATTCT AGTGCTCTAC TCCTCTTGAC TTAGAACCGG   
  
  
- TTGGTGCAGC AAACTGAAGA GAATGGTTAT TTCTAACTTA TCTGTCACAA AGAGAGGGGG AAGGTAAGAA   
  
  
- GAAAAGATCA CTACAGCTTT TTCTGAAATA TGTACAGGTC GTTGCGATCG GTGTTTTATA AGGTAAAGAC   
  
  
- ATTAGGTGAG TACAGTTAAT TTGAACATAG TAGTTTACCA TCAAAGGCAT GATTAGTGAT GTAGATCTAT   
  
  
- AAGGAACTAT TTAAATTAAA CTGAAATTTC CGAGCTAGAA AAATTCTTTT CAAAACAACG AATGTTGGAC   
  
  
- ATTTCGGATC TCTTGTTTCG GTTTCTTCTT TAAATTCAAG ATATATATCG TATTAGAGAG TTTTTTATAT   
  
  
- AAAAAATTTA TATCACGCTA TAACTGACTG GATTATACCC AATAATGTCC TCTACCCCTC TATAGATGTA   
  
  
- ATTGATGAAC TTGTTGAATT TAAAAGTAAA ATTGAGCCAG TAATTCTTGT GATTCTTGAA ATATATAATC   
  
  
- ACTATAGAGT CCGCATTTTC TTTGATGAAA CTTAATTAGG TACGTTAGGA TGAGAAATAA AGGTAGGCAA   
  
  
- TTTTAAAAGG AAGTTTTGAG GGAAGACAAG TAATAATTAA GTTATAGCAT AATGTGAACA ACCTAATAAA   
  
  
- GACACTAAAC TAGCAGTAAA AGATATATAC TTATTGATGT TTTTAGTATC AGGCTCATGT AATTATAATT   
  
  
- ATTTTATCCT ACTCACTATT GGAGTATATC ACGCAAATAG GGATGGTGAT CCGACCTAAA GACGTATTTC   
  
  
- TTCTTTAACT TGTATACCTT TTTCTTTATT TCTCTTCGTT GAAGGAACTC ACCCTCTTCT TAGCATCTTT   
  
  
- CAATTTGAAT ACTTTTTCTC GTAATTGTTT TTCCTTTAAT ATTCCGAATG GCAACTTCGG AGTATATTAA   
  
  
- AACGTTCTCT GTCTGTTTCT TTCACAGAAA GTTTACAGTA TAAAGAGCTC GTACGTACTT TTTACATGAA   
  
  
- GCCGATGTTA TCATTATCAT ATATACAACC GAAGTATGTG AACTGGGGTT ACATACAGTT GCACTGATCA   
  
  
- CTGGTGTGTG TAGCAACAGG GACGGGCGGG GTGATGGGTT TGAGGGGCGT GCGTTTCTAT TTTTCGGGGG   
  
  
- GTTAAAAATA AACGGAGGGT TTTTCGGGTG GGCGCGAAAA AATAAACGGA AGAAAAAAGG ATGAATTTGG   
  
  
- TGGAGGAGTT GCTTGAAACG TTGTATGTAT GATCAGATAT TCGTTAAATC TCCCTTCCCT TCGTTGGATT   
  
  
- TGTCTCGCTC TAACCGCCCC ACACAAACTC CCGAACGCAA AACCACGTTA TCCTTACTTT CACGGGTAAA   
  
  
- GTTGGTGGTA GGTTTGAACG TTGAGGCCGT GGCCGTTTGG TGAAAGGAAA CTCTTATGAC GGGATGTATG   
  
  
- GCGGAAAGTT CAAGGGTTAT GGTGGATGCT TGGGTGGAGC CAAGAACTGG AGGTCGCGTC GGGATCGGGG   
  
  
- TGGAGGTGGG TTTTTGGCGG GAAACGGCGG AGGTTGGTGG TCGGACGGAG CCGACTAGAC CTGTTGAACC   
  
  
- TGCCCACCCT AAGTTAGGAT AGTCTCGACC CCGAATTACT GCTTAAATGG GGATTAAGGT TTGAGACCAG   
  
  
- GGTTTAATCA GGCAGACTAG GAGTGGAGGG GGTTGAGGGA CTAAAGGGTT GTAGAGTCGG CAAACTAGTA   
  
  
- GTGTTGGGTG GGGGTTATTT GGAGGGTAGT AGTCTAAAGT TGGAGACACT AAAGAGGATG TTAGTGTTGG   
  
  
- GGTTAAAACC CGGCAAGCTA GAAGTAGTGT TGGGCGTAGT CTTGTTGGGC TTGTTGTTAT TGATGCCGAA   
  
  
- ACTAAAGTAT CTGCTGGAGT AGTTTCGTCG TGTCAGGGAG CTTAGGTTGC TGAGAGTAGT TCAGGTGGAC   
  
  
- TATAACCGCG CCGAGTTAGT TGCCGAGTCT AGTGGGTGGC CGTTTGGCGA GGTCGCCCGG CGGATGATGA   
  
  
- AGTTCCTTCG GGAGTAGCGG CGGCGCCGGC CGGGCGGGGC GGGGCGAGCC GAAAGTAGCA TACTCCACCA   
  
  
- CGTCTGGTAG GCTCGGATGT TCCGCAAAAG TCCTTAAAGC GGTCAGAGAG AGAAGAGGTG AAAGCGGCGG   
  
  
- TTGGTCCGCT AAGAACTCCG CCACCTGTGC CGCCGGAAGT AGGTGTAGTA GCTAAAGCTG TAGCCTAAGC   
  
  
- CGCCGGTGAC CCGGAGCAAG GAGGCGCTTG ACCAGCTGTT TCGACTAGGA CGCTTTGAAT CAAGTCACCA   
  
  
- CAATGCTTAG TGGCGGTAGC AAGGGCTTCT TAAGCCCTAG CTTTCGTTCG ATCACTCTCT TTTAGACAGC   
  
  
- GTTAAACGGG CTCTAGAGTT GTAGTTGAAG GTGTAGCTAA TACAGAATAA AGTCTCGAAA CTTTAGAATA   
  
  
- GGAAGTTTAG ACAGTTTAAG TAGCTTTCCC TCTTTAACCG CGACGTAGAA AGCGGCCGGC ACAAGGTGGC   
  
  
- CGATCCCTCG CCTTAAAGCT TCAAATAGCG ACTAGAGGCT AGTTAGAGCG GCTCGAGGCA GCACCACCAC   
  
  
- CTGTCTCTAC AGCCCTAACT GTAGCCTTGC AGCAGCAAAT CGTACTTAAA GCAGCGGCCT TAACTTAAGA   
  
  
- TGTGGCCCTA CGACCTCAGC GAACTGCGTC GGTGGCGGCC CCCCGAGTAG CCGCCACACC TGACGCACTC   
  
  
- TGCCTAACTC TGCAAACAAG AGGTGGGTTC CTAGTACCGC CGCCATCTCC GCCGCCGCAG CCGCAGCCGG   
  
  
- CCCGCCTCCT GCCGCACCTC CCTCCGTAAG CGCCGCCGCC CCTAATCCCG CCACCCCAAA TCGGTCAAAC   
  
  
- GACTAAAGGT CCGGCTTACA AACAACTCCT CTCGAGTCCA ACCACCGAAG GTGCACCGTT TCGCGGTACC   
  
  
- TCTCTACTAC GAAATGACCG TACTGGCGGG TGAGCAGCGG TGTAGTCGAA CCTCCACAAT

+     CARE

| Site Name | Organism | Position | Strand | Matrix score. | sequence | function |
| --- | --- | --- | --- | --- | --- | --- |
| CARE | Oryza sativa | 2346 | + | 8 | CAACTCCC |  |

>HU05G02245.1   
+ -Up\_Stream \_Len000ATAATT TATCTTTCTT TAATTTCGTT TCATGTAAAA AATTTCAGTC TCATCTGCGA   
  
  
+ TTGAATCTCT TTTCAATTCG CATCAATAAT TCATCCAATT CAAATGTGCC TTTCTTAATT TCATTACATT   
  
  
+ GGGTTGGTAT AAATGTTAGC ACGATAGATT GGAATAGCAA GGCTCTAATT CAATTATGAC TTGAATGTAT   
  
  
+ GCATGCTTTT ATCATTAGTT AATTAACATT TTCTGTATTG AAATCAGCTC GAAAGAGAAC CATATGTAGT   
  
  
+ TGTACTTGCG ACGATATATA TGTTTAGTTT AGTTAGTTCT TACCTGTTTT GGAATTGTTG GGGAGATCTG   
  
  
+ AGGAAGGAGC CCGCTTCAAT CAAAGTTCAT GGCAATAAGC CAAAGTGAGG TTCCATCCTA AAACCAATTG   
  
  
+ GCAATAGGAA GAGTAGCCCA CTTAACTTGT ATACTATGAC TCTTCTTTTC AAACTCCCAA TGTGGGACTT   
  
  
+ CTTTACTCAT GTGTGATATT CCCAACAGGA ATGATCAGTT CCACATGTTT TCATCTCTTT GGACTCGAAC   
  
  
+ TTCGTGTAAT TGTATATGCC ATGGCACAAA TAGTTTTGAT GGTGCCACCA AGTTGCTCAA TCAATGTAAT   
  
  
+ ACTGCTACTA TCTAACTTGC CTTGGTGGAC ATCAATAAGA TCACGAGATG AGGAGAACTG AATCTTGGCC   
  
  
+ AACCACGTCG TTTGACTTCT CTTACCAATA AAGATTGAAT AGACAGTGTT TCTCTCCCCC TTCCATTCTT   
  
  
+ CTTTTCTAGT GATGTCGAAA AAGACTTTAT ACATGTCCAG CAACGCTAGC CACAAAATAT TCCATTTCTG   
  
  
+ TAATCCACTC ATGTCAATTA AACTTGTATC ATCAAATGGT AGTTTCCGTA CTAATCACTA CATCTAGATA   
  
  
+ TTCCTTGATA AATTTAATTT GACTTTAAAG GCTCGATCTT TTTAAGAAAA GTTTTGTTGC TTACAACCTG   
  
  
+ TAAAGCCTAG AGAACAAAGC CAAAGAAGAA ATTTAAGTTC TATATATAGC ATAATCTCTC AAAAAATATA   
  
  
+ TTTTTTAAAT ATAGTGCGAT ATTGACTGAC CTAATATGGG TTATTACAGG AGATGGGGAG ATATCTACAT   
  
  
+ TAACTACTTG AACAACTTAA ATTTTCATTT TAACTCGGTC ATTAAGAACA CTAAGAACTT TATATATTAG   
  
  
+ TGATATCTCA GGCGTAAAAG AAACTACTTT GAATTAATCC ATGCAATCCT ACTCTTTATT TCCATCCGTT   
  
  
+ AAAATTTTCC TTCAAAACTC CCTTCTGTTC ATTATTAATT CAATATCGTA TTACACTTGT TGGATTATTT   
  
  
+ CTGTGATTTG ATCGTCATTT TCTATATATG AATAACTACA AAAATCATAG TCCGAGTACA TTAATATTAA   
  
  
+ TAAAATAGGA TGAGTGATAA CCTCATATAG TGCGTTTATC CCTACCACTA GGCTGGATTT CTGCATAAAG   
  
  
+ AAGAAATTGA ACATATGGAA AAAGAAATAA AGAGAAGCAA CTTCCTTGAG TGGGAGAAGA ATCGTAGAAA   
  
  
+ GTTAAACTTA TGAAAAAGAG CATTAACAAA AAGGAAATTA TAAGGCTTAC CGTTGAAGCC TCATATAATT   
  
  
+ TTGCAAGAGA CAGACAAAGA AAGTGTCTTT CAAATGTCAT ATTTCTCGAG CATGCATGAA AAATGTACTT   
  
  
+ CGGCTACAAT AGTAATAGTA TATATGTTGG CTTCATACAC TTGACCCCAA TGTATGTCAA CGTGACTAGT   
  
  
+ GACCACACAC ATCGTTGTCC CTGCCCGCCC CACTACCCAA ACTCCCCGCA CGCAAAGATA AAAAGCCCCC   
  
  
+ CAATTTTTAT TTGCCTCCCA AAAAGCCCAC CCGCGCTTTT TTATTTGCCT TCTTTTTTCC TACTTAAACC   
  
  
+ ACCTCCTCAA CGAACTTTGC AACATACATA CTAGTCTATA AGCAATTTAG AGGGAAGGGA AGCAACCTAA   
  
  
+ ACAGAGCGAG ATTGGCGGGG TGTGTTTGAG GGCTTGCGTT TTGGTGCAAT AGGAATGAAA GTGCCCATTT   
  
  
+ CAACCACCAT CCAAACTTGC AACTCCGGCA CCGGCAAACC ACTTTCCTTT GAGAATACTG CCCTACATAC   
  
  
+ CGCCTTTCAA GTTCCCAATA CCACCTACGA ACCCACCTCG GTTCTTGACC TCCAGCGCAG CCCTAGCCCC   
  
  
+ ACCTCCACCC AAAAACCGCC CTTTGCCGCC TCCAACCACC AGCCTGCCTC GGCTGATCTG GACAACTTGG   
  
  
+ ACGGGTGGGA TTCAATCCTA TCAGAGCTGG GGCTTAATGA CGAATTTACC CCTAATTCCA AACTCTGGTC   
  
  
+ CCAAATTAGT CCGTCTGATC CTCACCTCCC CCAACTCCCT GATTTCCCAA CATCTCAGCC GTTTGATCAT   
  
  
+ CACAACCCAC CCCCAATAAA CCTCCCATCA TCAGATTTCA ACCTCTGTGA TTTCTCCTAC AATCACAACC   
  
  
+ CCAATTTTGG GCCGTTCGAT CTTCATCACA ACCCGCATCA GAACAACCCG AACAACAATA ACTACGGCTT   
  
  
+ TGATTTCATA GACGACCTCA TCAAAGCAGC ACAGTCCCTC GAATCCAACG ACTCTCATCA AGTCCACCTG   
  
  
+ ATATTGGCGC GGCTCAATCA ACGGCTCAGA TCACCCACCG GCAAACCGCT CCAGCGGGCC GCCTACTACT   
  
  
+ TCAAGGAAGC CCTCATCGCC GCCGCGGCCG GCCCGCCCCG CCCCGCTCGG CTTTCATCGT ATGAGGTGGT   
  
  
+ GCAGACCATC CGAGCCTACA AGGCGTTTTC AGGAATTTCG CCAGTCTCTC TCTTCTCCAC TTTCGCCGCC   
  
  
+ AACCAGGCGA TTCTTGAGGC GGTGGACACG GCGGCCTTCA TCCACATCAT CGATTTCGAC ATCGGATTCG   
  
  
+ GCGGCCACTG GGCCTCGTTC CTCCGCGAAC TGGTCGACAA AGCTGATCCT GCGAAACTTA GTTCAGTGGT   
  
  
+ GTTACGAATC ACCGCCATCG TTCCCGAAGA ATTCGGGATC GAAAGCAAGC TAGTGAGAGA AAATCTGTCG   
  
  
+ CAATTTGCCC GAGATCTCAA CATCAACTTC CACATCGATT ATGTCTTATT TCAGAGCTTT GAAATCTTAT   
  
  
+ CCTTCAAATC TGTCAAATTC ATCGAAAGGG AGAAATTGGC GCTGCATCTT TCGCCGGCCG TGTTCCACCG   
  
  
+ GCTAGGGAGC GGAATTTCGA AGTTTATCGC TGATCTCCGA TCAATCTCGC CGAGCTCCGT CGTGGTGGTG   
  
  
+ GACAGAGATG TCGGGATTGA CATCGGAACG TCGTCGTTTA GCATGAATTT CGTCGCCGGA ATTGAATTCT   
  
  
+ ACACCGGGAT GCTGGAGTCG CTTGACGCAG CCACCGCCGG GGGGCTCATC GGCGGTGTGG ACTGCGTGAG   
  
  
+ ACGGATTGAG ACGTTTGTTC TCCACCCAAG GATCATGGCG GCGGTAGAGG CGGCGGCGTC GGCGTCGGCC   
  
  
+ GGGCGGAGGA CGGCGTGGAG GGAGGCATTC GCGGCGGCGG GGATTAGGGC GGTGGGGTTT AGCCAGTTTG   
  
  
+ CTGATTTCCA GGCCGAATGT TTGTTGAGGA GAGCTCAGGT TGGTGGCTTC CACGTGGCAA AGCGCCATGG   
  
  
+ AGAGATGATG CTTTACTGGC ATGACCGCCC ACTCGTCGCC ACATCAGCTT GGAGGTGTTA   

- -Up\_Stream \_Len000TATTAA ATAGAAAGAA ATTAAAGCAA AGTACATTTT TTAAAGTCAG AGTAGACGCT   
  
  
- AACTTAGAGA AAAGTTAAGC GTAGTTATTA AGTAGGTTAA GTTTACACGG AAAGAATTAA AGTAATGTAA   
  
  
- CCCAACCATA TTTACAATCG TGCTATCTAA CCTTATCGTT CCGAGATTAA GTTAATACTG AACTTACATA   
  
  
- CGTACGAAAA TAGTAATCAA TTAATTGTAA AAGACATAAC TTTAGTCGAG CTTTCTCTTG GTATACATCA   
  
  
- ACATGAACGC TGCTATATAT ACAAATCAAA TCAATCAAGA ATGGACAAAA CCTTAACAAC CCCTCTAGAC   
  
  
- TCCTTCCTCG GGCGAAGTTA GTTTCAAGTA CCGTTATTCG GTTTCACTCC AAGGTAGGAT TTTGGTTAAC   
  
  
- CGTTATCCTT CTCATCGGGT GAATTGAACA TATGATACTG AGAAGAAAAG TTTGAGGGTT ACACCCTGAA   
  
  
- GAAATGAGTA CACACTATAA GGGTTGTCCT TACTAGTCAA GGTGTACAAA AGTAGAGAAA CCTGAGCTTG   
  
  
- AAGCACATTA ACATATACGG TACCGTGTTT ATCAAAACTA CCACGGTGGT TCAACGAGTT AGTTACATTA   
  
  
- TGACGATGAT AGATTGAACG GAACCACCTG TAGTTATTCT AGTGCTCTAC TCCTCTTGAC TTAGAACCGG   
  
  
- TTGGTGCAGC AAACTGAAGA GAATGGTTAT TTCTAACTTA TCTGTCACAA AGAGAGGGGG AAGGTAAGAA   
  
  
- GAAAAGATCA CTACAGCTTT TTCTGAAATA TGTACAGGTC GTTGCGATCG GTGTTTTATA AGGTAAAGAC   
  
  
- ATTAGGTGAG TACAGTTAAT TTGAACATAG TAGTTTACCA TCAAAGGCAT GATTAGTGAT GTAGATCTAT   
  
  
- AAGGAACTAT TTAAATTAAA CTGAAATTTC CGAGCTAGAA AAATTCTTTT CAAAACAACG AATGTTGGAC   
  
  
- ATTTCGGATC TCTTGTTTCG GTTTCTTCTT TAAATTCAAG ATATATATCG TATTAGAGAG TTTTTTATAT   
  
  
- AAAAAATTTA TATCACGCTA TAACTGACTG GATTATACCC AATAATGTCC TCTACCCCTC TATAGATGTA   
  
  
- ATTGATGAAC TTGTTGAATT TAAAAGTAAA ATTGAGCCAG TAATTCTTGT GATTCTTGAA ATATATAATC   
  
  
- ACTATAGAGT CCGCATTTTC TTTGATGAAA CTTAATTAGG TACGTTAGGA TGAGAAATAA AGGTAGGCAA   
  
  
- TTTTAAAAGG AAGTTTTGAG GGAAGACAAG TAATAATTAA GTTATAGCAT AATGTGAACA ACCTAATAAA   
  
  
- GACACTAAAC TAGCAGTAAA AGATATATAC TTATTGATGT TTTTAGTATC AGGCTCATGT AATTATAATT   
  
  
- ATTTTATCCT ACTCACTATT GGAGTATATC ACGCAAATAG GGATGGTGAT CCGACCTAAA GACGTATTTC   
  
  
- TTCTTTAACT TGTATACCTT TTTCTTTATT TCTCTTCGTT GAAGGAACTC ACCCTCTTCT TAGCATCTTT   
  
  
- CAATTTGAAT ACTTTTTCTC GTAATTGTTT TTCCTTTAAT ATTCCGAATG GCAACTTCGG AGTATATTAA   
  
  
- AACGTTCTCT GTCTGTTTCT TTCACAGAAA GTTTACAGTA TAAAGAGCTC GTACGTACTT TTTACATGAA   
  
  
- GCCGATGTTA TCATTATCAT ATATACAACC GAAGTATGTG AACTGGGGTT ACATACAGTT GCACTGATCA   
  
  
- CTGGTGTGTG TAGCAACAGG GACGGGCGGG GTGATGGGTT TGAGGGGCGT GCGTTTCTAT TTTTCGGGGG   
  
  
- GTTAAAAATA AACGGAGGGT TTTTCGGGTG GGCGCGAAAA AATAAACGGA AGAAAAAAGG ATGAATTTGG   
  
  
- TGGAGGAGTT GCTTGAAACG TTGTATGTAT GATCAGATAT TCGTTAAATC TCCCTTCCCT TCGTTGGATT   
  
  
- TGTCTCGCTC TAACCGCCCC ACACAAACTC CCGAACGCAA AACCACGTTA TCCTTACTTT CACGGGTAAA   
  
  
- GTTGGTGGTA GGTTTGAACG TTGAGGCCGT GGCCGTTTGG TGAAAGGAAA CTCTTATGAC GGGATGTATG   
  
  
- GCGGAAAGTT CAAGGGTTAT GGTGGATGCT TGGGTGGAGC CAAGAACTGG AGGTCGCGTC GGGATCGGGG   
  
  
- TGGAGGTGGG TTTTTGGCGG GAAACGGCGG AGGTTGGTGG TCGGACGGAG CCGACTAGAC CTGTTGAACC   
  
  
- TGCCCACCCT AAGTTAGGAT AGTCTCGACC CCGAATTACT GCTTAAATGG GGATTAAGGT TTGAGACCAG   
  
  
- GGTTTAATCA GGCAGACTAG GAGTGGAGGG GGTTGAGGGA CTAAAGGGTT GTAGAGTCGG CAAACTAGTA   
  
  
- GTGTTGGGTG GGGGTTATTT GGAGGGTAGT AGTCTAAAGT TGGAGACACT AAAGAGGATG TTAGTGTTGG   
  
  
- GGTTAAAACC CGGCAAGCTA GAAGTAGTGT TGGGCGTAGT CTTGTTGGGC TTGTTGTTAT TGATGCCGAA   
  
  
- ACTAAAGTAT CTGCTGGAGT AGTTTCGTCG TGTCAGGGAG CTTAGGTTGC TGAGAGTAGT TCAGGTGGAC   
  
  
- TATAACCGCG CCGAGTTAGT TGCCGAGTCT AGTGGGTGGC CGTTTGGCGA GGTCGCCCGG CGGATGATGA   
  
  
- AGTTCCTTCG GGAGTAGCGG CGGCGCCGGC CGGGCGGGGC GGGGCGAGCC GAAAGTAGCA TACTCCACCA   
  
  
- CGTCTGGTAG GCTCGGATGT TCCGCAAAAG TCCTTAAAGC GGTCAGAGAG AGAAGAGGTG AAAGCGGCGG   
  
  
- TTGGTCCGCT AAGAACTCCG CCACCTGTGC CGCCGGAAGT AGGTGTAGTA GCTAAAGCTG TAGCCTAAGC   
  
  
- CGCCGGTGAC CCGGAGCAAG GAGGCGCTTG ACCAGCTGTT TCGACTAGGA CGCTTTGAAT CAAGTCACCA   
  
  
- CAATGCTTAG TGGCGGTAGC AAGGGCTTCT TAAGCCCTAG CTTTCGTTCG ATCACTCTCT TTTAGACAGC   
  
  
- GTTAAACGGG CTCTAGAGTT GTAGTTGAAG GTGTAGCTAA TACAGAATAA AGTCTCGAAA CTTTAGAATA   
  
  
- GGAAGTTTAG ACAGTTTAAG TAGCTTTCCC TCTTTAACCG CGACGTAGAA AGCGGCCGGC ACAAGGTGGC   
  
  
- CGATCCCTCG CCTTAAAGCT TCAAATAGCG ACTAGAGGCT AGTTAGAGCG GCTCGAGGCA GCACCACCAC   
  
  
- CTGTCTCTAC AGCCCTAACT GTAGCCTTGC AGCAGCAAAT CGTACTTAAA GCAGCGGCCT TAACTTAAGA   
  
  
- TGTGGCCCTA CGACCTCAGC GAACTGCGTC GGTGGCGGCC CCCCGAGTAG CCGCCACACC TGACGCACTC   
  
  
- TGCCTAACTC TGCAAACAAG AGGTGGGTTC CTAGTACCGC CGCCATCTCC GCCGCCGCAG CCGCAGCCGG   
  
  
- CCCGCCTCCT GCCGCACCTC CCTCCGTAAG CGCCGCCGCC CCTAATCCCG CCACCCCAAA TCGGTCAAAC   
  
  
- GACTAAAGGT CCGGCTTACA AACAACTCCT CTCGAGTCCA ACCACCGAAG GTGCACCGTT TCGCGGTACC   
  
  
- TCTCTACTAC GAAATGACCG TACTGGCGGG TGAGCAGCGG TGTAGTCGAA CCTCCACAAT

+     CAT-box

| Site Name | Organism | Position | Strand | Matrix score. | sequence | function |
| --- | --- | --- | --- | --- | --- | --- |
| CAT-box | Arabidopsis thaliana | 2878 | + | 6 | GCCACT | cis-acting regulatory element related to meristem expression |

>HU05G02245.1   
+ -Up\_Stream \_Len000ATAATT TATCTTTCTT TAATTTCGTT TCATGTAAAA AATTTCAGTC TCATCTGCGA   
  
  
+ TTGAATCTCT TTTCAATTCG CATCAATAAT TCATCCAATT CAAATGTGCC TTTCTTAATT TCATTACATT   
  
  
+ GGGTTGGTAT AAATGTTAGC ACGATAGATT GGAATAGCAA GGCTCTAATT CAATTATGAC TTGAATGTAT   
  
  
+ GCATGCTTTT ATCATTAGTT AATTAACATT TTCTGTATTG AAATCAGCTC GAAAGAGAAC CATATGTAGT   
  
  
+ TGTACTTGCG ACGATATATA TGTTTAGTTT AGTTAGTTCT TACCTGTTTT GGAATTGTTG GGGAGATCTG   
  
  
+ AGGAAGGAGC CCGCTTCAAT CAAAGTTCAT GGCAATAAGC CAAAGTGAGG TTCCATCCTA AAACCAATTG   
  
  
+ GCAATAGGAA GAGTAGCCCA CTTAACTTGT ATACTATGAC TCTTCTTTTC AAACTCCCAA TGTGGGACTT   
  
  
+ CTTTACTCAT GTGTGATATT CCCAACAGGA ATGATCAGTT CCACATGTTT TCATCTCTTT GGACTCGAAC   
  
  
+ TTCGTGTAAT TGTATATGCC ATGGCACAAA TAGTTTTGAT GGTGCCACCA AGTTGCTCAA TCAATGTAAT   
  
  
+ ACTGCTACTA TCTAACTTGC CTTGGTGGAC ATCAATAAGA TCACGAGATG AGGAGAACTG AATCTTGGCC   
  
  
+ AACCACGTCG TTTGACTTCT CTTACCAATA AAGATTGAAT AGACAGTGTT TCTCTCCCCC TTCCATTCTT   
  
  
+ CTTTTCTAGT GATGTCGAAA AAGACTTTAT ACATGTCCAG CAACGCTAGC CACAAAATAT TCCATTTCTG   
  
  
+ TAATCCACTC ATGTCAATTA AACTTGTATC ATCAAATGGT AGTTTCCGTA CTAATCACTA CATCTAGATA   
  
  
+ TTCCTTGATA AATTTAATTT GACTTTAAAG GCTCGATCTT TTTAAGAAAA GTTTTGTTGC TTACAACCTG   
  
  
+ TAAAGCCTAG AGAACAAAGC CAAAGAAGAA ATTTAAGTTC TATATATAGC ATAATCTCTC AAAAAATATA   
  
  
+ TTTTTTAAAT ATAGTGCGAT ATTGACTGAC CTAATATGGG TTATTACAGG AGATGGGGAG ATATCTACAT   
  
  
+ TAACTACTTG AACAACTTAA ATTTTCATTT TAACTCGGTC ATTAAGAACA CTAAGAACTT TATATATTAG   
  
  
+ TGATATCTCA GGCGTAAAAG AAACTACTTT GAATTAATCC ATGCAATCCT ACTCTTTATT TCCATCCGTT   
  
  
+ AAAATTTTCC TTCAAAACTC CCTTCTGTTC ATTATTAATT CAATATCGTA TTACACTTGT TGGATTATTT   
  
  
+ CTGTGATTTG ATCGTCATTT TCTATATATG AATAACTACA AAAATCATAG TCCGAGTACA TTAATATTAA   
  
  
+ TAAAATAGGA TGAGTGATAA CCTCATATAG TGCGTTTATC CCTACCACTA GGCTGGATTT CTGCATAAAG   
  
  
+ AAGAAATTGA ACATATGGAA AAAGAAATAA AGAGAAGCAA CTTCCTTGAG TGGGAGAAGA ATCGTAGAAA   
  
  
+ GTTAAACTTA TGAAAAAGAG CATTAACAAA AAGGAAATTA TAAGGCTTAC CGTTGAAGCC TCATATAATT   
  
  
+ TTGCAAGAGA CAGACAAAGA AAGTGTCTTT CAAATGTCAT ATTTCTCGAG CATGCATGAA AAATGTACTT   
  
  
+ CGGCTACAAT AGTAATAGTA TATATGTTGG CTTCATACAC TTGACCCCAA TGTATGTCAA CGTGACTAGT   
  
  
+ GACCACACAC ATCGTTGTCC CTGCCCGCCC CACTACCCAA ACTCCCCGCA CGCAAAGATA AAAAGCCCCC   
  
  
+ CAATTTTTAT TTGCCTCCCA AAAAGCCCAC CCGCGCTTTT TTATTTGCCT TCTTTTTTCC TACTTAAACC   
  
  
+ ACCTCCTCAA CGAACTTTGC AACATACATA CTAGTCTATA AGCAATTTAG AGGGAAGGGA AGCAACCTAA   
  
  
+ ACAGAGCGAG ATTGGCGGGG TGTGTTTGAG GGCTTGCGTT TTGGTGCAAT AGGAATGAAA GTGCCCATTT   
  
  
+ CAACCACCAT CCAAACTTGC AACTCCGGCA CCGGCAAACC ACTTTCCTTT GAGAATACTG CCCTACATAC   
  
  
+ CGCCTTTCAA GTTCCCAATA CCACCTACGA ACCCACCTCG GTTCTTGACC TCCAGCGCAG CCCTAGCCCC   
  
  
+ ACCTCCACCC AAAAACCGCC CTTTGCCGCC TCCAACCACC AGCCTGCCTC GGCTGATCTG GACAACTTGG   
  
  
+ ACGGGTGGGA TTCAATCCTA TCAGAGCTGG GGCTTAATGA CGAATTTACC CCTAATTCCA AACTCTGGTC   
  
  
+ CCAAATTAGT CCGTCTGATC CTCACCTCCC CCAACTCCCT GATTTCCCAA CATCTCAGCC GTTTGATCAT   
  
  
+ CACAACCCAC CCCCAATAAA CCTCCCATCA TCAGATTTCA ACCTCTGTGA TTTCTCCTAC AATCACAACC   
  
  
+ CCAATTTTGG GCCGTTCGAT CTTCATCACA ACCCGCATCA GAACAACCCG AACAACAATA ACTACGGCTT   
  
  
+ TGATTTCATA GACGACCTCA TCAAAGCAGC ACAGTCCCTC GAATCCAACG ACTCTCATCA AGTCCACCTG   
  
  
+ ATATTGGCGC GGCTCAATCA ACGGCTCAGA TCACCCACCG GCAAACCGCT CCAGCGGGCC GCCTACTACT   
  
  
+ TCAAGGAAGC CCTCATCGCC GCCGCGGCCG GCCCGCCCCG CCCCGCTCGG CTTTCATCGT ATGAGGTGGT   
  
  
+ GCAGACCATC CGAGCCTACA AGGCGTTTTC AGGAATTTCG CCAGTCTCTC TCTTCTCCAC TTTCGCCGCC   
  
  
+ AACCAGGCGA TTCTTGAGGC GGTGGACACG GCGGCCTTCA TCCACATCAT CGATTTCGAC ATCGGATTCG   
  
  
+ GCGGCCACTG GGCCTCGTTC CTCCGCGAAC TGGTCGACAA AGCTGATCCT GCGAAACTTA GTTCAGTGGT   
  
  
+ GTTACGAATC ACCGCCATCG TTCCCGAAGA ATTCGGGATC GAAAGCAAGC TAGTGAGAGA AAATCTGTCG   
  
  
+ CAATTTGCCC GAGATCTCAA CATCAACTTC CACATCGATT ATGTCTTATT TCAGAGCTTT GAAATCTTAT   
  
  
+ CCTTCAAATC TGTCAAATTC ATCGAAAGGG AGAAATTGGC GCTGCATCTT TCGCCGGCCG TGTTCCACCG   
  
  
+ GCTAGGGAGC GGAATTTCGA AGTTTATCGC TGATCTCCGA TCAATCTCGC CGAGCTCCGT CGTGGTGGTG   
  
  
+ GACAGAGATG TCGGGATTGA CATCGGAACG TCGTCGTTTA GCATGAATTT CGTCGCCGGA ATTGAATTCT   
  
  
+ ACACCGGGAT GCTGGAGTCG CTTGACGCAG CCACCGCCGG GGGGCTCATC GGCGGTGTGG ACTGCGTGAG   
  
  
+ ACGGATTGAG ACGTTTGTTC TCCACCCAAG GATCATGGCG GCGGTAGAGG CGGCGGCGTC GGCGTCGGCC   
  
  
+ GGGCGGAGGA CGGCGTGGAG GGAGGCATTC GCGGCGGCGG GGATTAGGGC GGTGGGGTTT AGCCAGTTTG   
  
  
+ CTGATTTCCA GGCCGAATGT TTGTTGAGGA GAGCTCAGGT TGGTGGCTTC CACGTGGCAA AGCGCCATGG   
  
  
+ AGAGATGATG CTTTACTGGC ATGACCGCCC ACTCGTCGCC ACATCAGCTT GGAGGTGTTA   

- -Up\_Stream \_Len000TATTAA ATAGAAAGAA ATTAAAGCAA AGTACATTTT TTAAAGTCAG AGTAGACGCT   
  
  
- AACTTAGAGA AAAGTTAAGC GTAGTTATTA AGTAGGTTAA GTTTACACGG AAAGAATTAA AGTAATGTAA   
  
  
- CCCAACCATA TTTACAATCG TGCTATCTAA CCTTATCGTT CCGAGATTAA GTTAATACTG AACTTACATA   
  
  
- CGTACGAAAA TAGTAATCAA TTAATTGTAA AAGACATAAC TTTAGTCGAG CTTTCTCTTG GTATACATCA   
  
  
- ACATGAACGC TGCTATATAT ACAAATCAAA TCAATCAAGA ATGGACAAAA CCTTAACAAC CCCTCTAGAC   
  
  
- TCCTTCCTCG GGCGAAGTTA GTTTCAAGTA CCGTTATTCG GTTTCACTCC AAGGTAGGAT TTTGGTTAAC   
  
  
- CGTTATCCTT CTCATCGGGT GAATTGAACA TATGATACTG AGAAGAAAAG TTTGAGGGTT ACACCCTGAA   
  
  
- GAAATGAGTA CACACTATAA GGGTTGTCCT TACTAGTCAA GGTGTACAAA AGTAGAGAAA CCTGAGCTTG   
  
  
- AAGCACATTA ACATATACGG TACCGTGTTT ATCAAAACTA CCACGGTGGT TCAACGAGTT AGTTACATTA   
  
  
- TGACGATGAT AGATTGAACG GAACCACCTG TAGTTATTCT AGTGCTCTAC TCCTCTTGAC TTAGAACCGG   
  
  
- TTGGTGCAGC AAACTGAAGA GAATGGTTAT TTCTAACTTA TCTGTCACAA AGAGAGGGGG AAGGTAAGAA   
  
  
- GAAAAGATCA CTACAGCTTT TTCTGAAATA TGTACAGGTC GTTGCGATCG GTGTTTTATA AGGTAAAGAC   
  
  
- ATTAGGTGAG TACAGTTAAT TTGAACATAG TAGTTTACCA TCAAAGGCAT GATTAGTGAT GTAGATCTAT   
  
  
- AAGGAACTAT TTAAATTAAA CTGAAATTTC CGAGCTAGAA AAATTCTTTT CAAAACAACG AATGTTGGAC   
  
  
- ATTTCGGATC TCTTGTTTCG GTTTCTTCTT TAAATTCAAG ATATATATCG TATTAGAGAG TTTTTTATAT   
  
  
- AAAAAATTTA TATCACGCTA TAACTGACTG GATTATACCC AATAATGTCC TCTACCCCTC TATAGATGTA   
  
  
- ATTGATGAAC TTGTTGAATT TAAAAGTAAA ATTGAGCCAG TAATTCTTGT GATTCTTGAA ATATATAATC   
  
  
- ACTATAGAGT CCGCATTTTC TTTGATGAAA CTTAATTAGG TACGTTAGGA TGAGAAATAA AGGTAGGCAA   
  
  
- TTTTAAAAGG AAGTTTTGAG GGAAGACAAG TAATAATTAA GTTATAGCAT AATGTGAACA ACCTAATAAA   
  
  
- GACACTAAAC TAGCAGTAAA AGATATATAC TTATTGATGT TTTTAGTATC AGGCTCATGT AATTATAATT   
  
  
- ATTTTATCCT ACTCACTATT GGAGTATATC ACGCAAATAG GGATGGTGAT CCGACCTAAA GACGTATTTC   
  
  
- TTCTTTAACT TGTATACCTT TTTCTTTATT TCTCTTCGTT GAAGGAACTC ACCCTCTTCT TAGCATCTTT   
  
  
- CAATTTGAAT ACTTTTTCTC GTAATTGTTT TTCCTTTAAT ATTCCGAATG GCAACTTCGG AGTATATTAA   
  
  
- AACGTTCTCT GTCTGTTTCT TTCACAGAAA GTTTACAGTA TAAAGAGCTC GTACGTACTT TTTACATGAA   
  
  
- GCCGATGTTA TCATTATCAT ATATACAACC GAAGTATGTG AACTGGGGTT ACATACAGTT GCACTGATCA   
  
  
- CTGGTGTGTG TAGCAACAGG GACGGGCGGG GTGATGGGTT TGAGGGGCGT GCGTTTCTAT TTTTCGGGGG   
  
  
- GTTAAAAATA AACGGAGGGT TTTTCGGGTG GGCGCGAAAA AATAAACGGA AGAAAAAAGG ATGAATTTGG   
  
  
- TGGAGGAGTT GCTTGAAACG TTGTATGTAT GATCAGATAT TCGTTAAATC TCCCTTCCCT TCGTTGGATT   
  
  
- TGTCTCGCTC TAACCGCCCC ACACAAACTC CCGAACGCAA AACCACGTTA TCCTTACTTT CACGGGTAAA   
  
  
- GTTGGTGGTA GGTTTGAACG TTGAGGCCGT GGCCGTTTGG TGAAAGGAAA CTCTTATGAC GGGATGTATG   
  
  
- GCGGAAAGTT CAAGGGTTAT GGTGGATGCT TGGGTGGAGC CAAGAACTGG AGGTCGCGTC GGGATCGGGG   
  
  
- TGGAGGTGGG TTTTTGGCGG GAAACGGCGG AGGTTGGTGG TCGGACGGAG CCGACTAGAC CTGTTGAACC   
  
  
- TGCCCACCCT AAGTTAGGAT AGTCTCGACC CCGAATTACT GCTTAAATGG GGATTAAGGT TTGAGACCAG   
  
  
- GGTTTAATCA GGCAGACTAG GAGTGGAGGG GGTTGAGGGA CTAAAGGGTT GTAGAGTCGG CAAACTAGTA   
  
  
- GTGTTGGGTG GGGGTTATTT GGAGGGTAGT AGTCTAAAGT TGGAGACACT AAAGAGGATG TTAGTGTTGG   
  
  
- GGTTAAAACC CGGCAAGCTA GAAGTAGTGT TGGGCGTAGT CTTGTTGGGC TTGTTGTTAT TGATGCCGAA   
  
  
- ACTAAAGTAT CTGCTGGAGT AGTTTCGTCG TGTCAGGGAG CTTAGGTTGC TGAGAGTAGT TCAGGTGGAC   
  
  
- TATAACCGCG CCGAGTTAGT TGCCGAGTCT AGTGGGTGGC CGTTTGGCGA GGTCGCCCGG CGGATGATGA   
  
  
- AGTTCCTTCG GGAGTAGCGG CGGCGCCGGC CGGGCGGGGC GGGGCGAGCC GAAAGTAGCA TACTCCACCA   
  
  
- CGTCTGGTAG GCTCGGATGT TCCGCAAAAG TCCTTAAAGC GGTCAGAGAG AGAAGAGGTG AAAGCGGCGG   
  
  
- TTGGTCCGCT AAGAACTCCG CCACCTGTGC CGCCGGAAGT AGGTGTAGTA GCTAAAGCTG TAGCCTAAGC   
  
  
- CGCCGGTGAC CCGGAGCAAG GAGGCGCTTG ACCAGCTGTT TCGACTAGGA CGCTTTGAAT CAAGTCACCA   
  
  
- CAATGCTTAG TGGCGGTAGC AAGGGCTTCT TAAGCCCTAG CTTTCGTTCG ATCACTCTCT TTTAGACAGC   
  
  
- GTTAAACGGG CTCTAGAGTT GTAGTTGAAG GTGTAGCTAA TACAGAATAA AGTCTCGAAA CTTTAGAATA   
  
  
- GGAAGTTTAG ACAGTTTAAG TAGCTTTCCC TCTTTAACCG CGACGTAGAA AGCGGCCGGC ACAAGGTGGC   
  
  
- CGATCCCTCG CCTTAAAGCT TCAAATAGCG ACTAGAGGCT AGTTAGAGCG GCTCGAGGCA GCACCACCAC   
  
  
- CTGTCTCTAC AGCCCTAACT GTAGCCTTGC AGCAGCAAAT CGTACTTAAA GCAGCGGCCT TAACTTAAGA   
  
  
- TGTGGCCCTA CGACCTCAGC GAACTGCGTC GGTGGCGGCC CCCCGAGTAG CCGCCACACC TGACGCACTC   
  
  
- TGCCTAACTC TGCAAACAAG AGGTGGGTTC CTAGTACCGC CGCCATCTCC GCCGCCGCAG CCGCAGCCGG   
  
  
- CCCGCCTCCT GCCGCACCTC CCTCCGTAAG CGCCGCCGCC CCTAATCCCG CCACCCCAAA TCGGTCAAAC   
  
  
- GACTAAAGGT CCGGCTTACA AACAACTCCT CTCGAGTCCA ACCACCGAAG GTGCACCGTT TCGCGGTACC   
  
  
- TCTCTACTAC GAAATGACCG TACTGGCGGG TGAGCAGCGG TGTAGTCGAA CCTCCACAAT

+     CCAAT-box

| Site Name | Organism | Position | Strand | Matrix score. | sequence | function |
| --- | --- | --- | --- | --- | --- | --- |
| CCAAT-box | Hordeum vulgare | 1594 | - | 6 | CAACGG | MYBHv1 binding site |
| CCAAT-box | Hordeum vulgare | 2613 | + | 6 | CAACGG | MYBHv1 binding site |

>HU05G02245.1   
+ -Up\_Stream \_Len000ATAATT TATCTTTCTT TAATTTCGTT TCATGTAAAA AATTTCAGTC TCATCTGCGA   
  
  
+ TTGAATCTCT TTTCAATTCG CATCAATAAT TCATCCAATT CAAATGTGCC TTTCTTAATT TCATTACATT   
  
  
+ GGGTTGGTAT AAATGTTAGC ACGATAGATT GGAATAGCAA GGCTCTAATT CAATTATGAC TTGAATGTAT   
  
  
+ GCATGCTTTT ATCATTAGTT AATTAACATT TTCTGTATTG AAATCAGCTC GAAAGAGAAC CATATGTAGT   
  
  
+ TGTACTTGCG ACGATATATA TGTTTAGTTT AGTTAGTTCT TACCTGTTTT GGAATTGTTG GGGAGATCTG   
  
  
+ AGGAAGGAGC CCGCTTCAAT CAAAGTTCAT GGCAATAAGC CAAAGTGAGG TTCCATCCTA AAACCAATTG   
  
  
+ GCAATAGGAA GAGTAGCCCA CTTAACTTGT ATACTATGAC TCTTCTTTTC AAACTCCCAA TGTGGGACTT   
  
  
+ CTTTACTCAT GTGTGATATT CCCAACAGGA ATGATCAGTT CCACATGTTT TCATCTCTTT GGACTCGAAC   
  
  
+ TTCGTGTAAT TGTATATGCC ATGGCACAAA TAGTTTTGAT GGTGCCACCA AGTTGCTCAA TCAATGTAAT   
  
  
+ ACTGCTACTA TCTAACTTGC CTTGGTGGAC ATCAATAAGA TCACGAGATG AGGAGAACTG AATCTTGGCC   
  
  
+ AACCACGTCG TTTGACTTCT CTTACCAATA AAGATTGAAT AGACAGTGTT TCTCTCCCCC TTCCATTCTT   
  
  
+ CTTTTCTAGT GATGTCGAAA AAGACTTTAT ACATGTCCAG CAACGCTAGC CACAAAATAT TCCATTTCTG   
  
  
+ TAATCCACTC ATGTCAATTA AACTTGTATC ATCAAATGGT AGTTTCCGTA CTAATCACTA CATCTAGATA   
  
  
+ TTCCTTGATA AATTTAATTT GACTTTAAAG GCTCGATCTT TTTAAGAAAA GTTTTGTTGC TTACAACCTG   
  
  
+ TAAAGCCTAG AGAACAAAGC CAAAGAAGAA ATTTAAGTTC TATATATAGC ATAATCTCTC AAAAAATATA   
  
  
+ TTTTTTAAAT ATAGTGCGAT ATTGACTGAC CTAATATGGG TTATTACAGG AGATGGGGAG ATATCTACAT   
  
  
+ TAACTACTTG AACAACTTAA ATTTTCATTT TAACTCGGTC ATTAAGAACA CTAAGAACTT TATATATTAG   
  
  
+ TGATATCTCA GGCGTAAAAG AAACTACTTT GAATTAATCC ATGCAATCCT ACTCTTTATT TCCATCCGTT   
  
  
+ AAAATTTTCC TTCAAAACTC CCTTCTGTTC ATTATTAATT CAATATCGTA TTACACTTGT TGGATTATTT   
  
  
+ CTGTGATTTG ATCGTCATTT TCTATATATG AATAACTACA AAAATCATAG TCCGAGTACA TTAATATTAA   
  
  
+ TAAAATAGGA TGAGTGATAA CCTCATATAG TGCGTTTATC CCTACCACTA GGCTGGATTT CTGCATAAAG   
  
  
+ AAGAAATTGA ACATATGGAA AAAGAAATAA AGAGAAGCAA CTTCCTTGAG TGGGAGAAGA ATCGTAGAAA   
  
  
+ GTTAAACTTA TGAAAAAGAG CATTAACAAA AAGGAAATTA TAAGGCTTAC CGTTGAAGCC TCATATAATT   
  
  
+ TTGCAAGAGA CAGACAAAGA AAGTGTCTTT CAAATGTCAT ATTTCTCGAG CATGCATGAA AAATGTACTT   
  
  
+ CGGCTACAAT AGTAATAGTA TATATGTTGG CTTCATACAC TTGACCCCAA TGTATGTCAA CGTGACTAGT   
  
  
+ GACCACACAC ATCGTTGTCC CTGCCCGCCC CACTACCCAA ACTCCCCGCA CGCAAAGATA AAAAGCCCCC   
  
  
+ CAATTTTTAT TTGCCTCCCA AAAAGCCCAC CCGCGCTTTT TTATTTGCCT TCTTTTTTCC TACTTAAACC   
  
  
+ ACCTCCTCAA CGAACTTTGC AACATACATA CTAGTCTATA AGCAATTTAG AGGGAAGGGA AGCAACCTAA   
  
  
+ ACAGAGCGAG ATTGGCGGGG TGTGTTTGAG GGCTTGCGTT TTGGTGCAAT AGGAATGAAA GTGCCCATTT   
  
  
+ CAACCACCAT CCAAACTTGC AACTCCGGCA CCGGCAAACC ACTTTCCTTT GAGAATACTG CCCTACATAC   
  
  
+ CGCCTTTCAA GTTCCCAATA CCACCTACGA ACCCACCTCG GTTCTTGACC TCCAGCGCAG CCCTAGCCCC   
  
  
+ ACCTCCACCC AAAAACCGCC CTTTGCCGCC TCCAACCACC AGCCTGCCTC GGCTGATCTG GACAACTTGG   
  
  
+ ACGGGTGGGA TTCAATCCTA TCAGAGCTGG GGCTTAATGA CGAATTTACC CCTAATTCCA AACTCTGGTC   
  
  
+ CCAAATTAGT CCGTCTGATC CTCACCTCCC CCAACTCCCT GATTTCCCAA CATCTCAGCC GTTTGATCAT   
  
  
+ CACAACCCAC CCCCAATAAA CCTCCCATCA TCAGATTTCA ACCTCTGTGA TTTCTCCTAC AATCACAACC   
  
  
+ CCAATTTTGG GCCGTTCGAT CTTCATCACA ACCCGCATCA GAACAACCCG AACAACAATA ACTACGGCTT   
  
  
+ TGATTTCATA GACGACCTCA TCAAAGCAGC ACAGTCCCTC GAATCCAACG ACTCTCATCA AGTCCACCTG   
  
  
+ ATATTGGCGC GGCTCAATCA ACGGCTCAGA TCACCCACCG GCAAACCGCT CCAGCGGGCC GCCTACTACT   
  
  
+ TCAAGGAAGC CCTCATCGCC GCCGCGGCCG GCCCGCCCCG CCCCGCTCGG CTTTCATCGT ATGAGGTGGT   
  
  
+ GCAGACCATC CGAGCCTACA AGGCGTTTTC AGGAATTTCG CCAGTCTCTC TCTTCTCCAC TTTCGCCGCC   
  
  
+ AACCAGGCGA TTCTTGAGGC GGTGGACACG GCGGCCTTCA TCCACATCAT CGATTTCGAC ATCGGATTCG   
  
  
+ GCGGCCACTG GGCCTCGTTC CTCCGCGAAC TGGTCGACAA AGCTGATCCT GCGAAACTTA GTTCAGTGGT   
  
  
+ GTTACGAATC ACCGCCATCG TTCCCGAAGA ATTCGGGATC GAAAGCAAGC TAGTGAGAGA AAATCTGTCG   
  
  
+ CAATTTGCCC GAGATCTCAA CATCAACTTC CACATCGATT ATGTCTTATT TCAGAGCTTT GAAATCTTAT   
  
  
+ CCTTCAAATC TGTCAAATTC ATCGAAAGGG AGAAATTGGC GCTGCATCTT TCGCCGGCCG TGTTCCACCG   
  
  
+ GCTAGGGAGC GGAATTTCGA AGTTTATCGC TGATCTCCGA TCAATCTCGC CGAGCTCCGT CGTGGTGGTG   
  
  
+ GACAGAGATG TCGGGATTGA CATCGGAACG TCGTCGTTTA GCATGAATTT CGTCGCCGGA ATTGAATTCT   
  
  
+ ACACCGGGAT GCTGGAGTCG CTTGACGCAG CCACCGCCGG GGGGCTCATC GGCGGTGTGG ACTGCGTGAG   
  
  
+ ACGGATTGAG ACGTTTGTTC TCCACCCAAG GATCATGGCG GCGGTAGAGG CGGCGGCGTC GGCGTCGGCC   
  
  
+ GGGCGGAGGA CGGCGTGGAG GGAGGCATTC GCGGCGGCGG GGATTAGGGC GGTGGGGTTT AGCCAGTTTG   
  
  
+ CTGATTTCCA GGCCGAATGT TTGTTGAGGA GAGCTCAGGT TGGTGGCTTC CACGTGGCAA AGCGCCATGG   
  
  
+ AGAGATGATG CTTTACTGGC ATGACCGCCC ACTCGTCGCC ACATCAGCTT GGAGGTGTTA   

- -Up\_Stream \_Len000TATTAA ATAGAAAGAA ATTAAAGCAA AGTACATTTT TTAAAGTCAG AGTAGACGCT   
  
  
- AACTTAGAGA AAAGTTAAGC GTAGTTATTA AGTAGGTTAA GTTTACACGG AAAGAATTAA AGTAATGTAA   
  
  
- CCCAACCATA TTTACAATCG TGCTATCTAA CCTTATCGTT CCGAGATTAA GTTAATACTG AACTTACATA   
  
  
- CGTACGAAAA TAGTAATCAA TTAATTGTAA AAGACATAAC TTTAGTCGAG CTTTCTCTTG GTATACATCA   
  
  
- ACATGAACGC TGCTATATAT ACAAATCAAA TCAATCAAGA ATGGACAAAA CCTTAACAAC CCCTCTAGAC   
  
  
- TCCTTCCTCG GGCGAAGTTA GTTTCAAGTA CCGTTATTCG GTTTCACTCC AAGGTAGGAT TTTGGTTAAC   
  
  
- CGTTATCCTT CTCATCGGGT GAATTGAACA TATGATACTG AGAAGAAAAG TTTGAGGGTT ACACCCTGAA   
  
  
- GAAATGAGTA CACACTATAA GGGTTGTCCT TACTAGTCAA GGTGTACAAA AGTAGAGAAA CCTGAGCTTG   
  
  
- AAGCACATTA ACATATACGG TACCGTGTTT ATCAAAACTA CCACGGTGGT TCAACGAGTT AGTTACATTA   
  
  
- TGACGATGAT AGATTGAACG GAACCACCTG TAGTTATTCT AGTGCTCTAC TCCTCTTGAC TTAGAACCGG   
  
  
- TTGGTGCAGC AAACTGAAGA GAATGGTTAT TTCTAACTTA TCTGTCACAA AGAGAGGGGG AAGGTAAGAA   
  
  
- GAAAAGATCA CTACAGCTTT TTCTGAAATA TGTACAGGTC GTTGCGATCG GTGTTTTATA AGGTAAAGAC   
  
  
- ATTAGGTGAG TACAGTTAAT TTGAACATAG TAGTTTACCA TCAAAGGCAT GATTAGTGAT GTAGATCTAT   
  
  
- AAGGAACTAT TTAAATTAAA CTGAAATTTC CGAGCTAGAA AAATTCTTTT CAAAACAACG AATGTTGGAC   
  
  
- ATTTCGGATC TCTTGTTTCG GTTTCTTCTT TAAATTCAAG ATATATATCG TATTAGAGAG TTTTTTATAT   
  
  
- AAAAAATTTA TATCACGCTA TAACTGACTG GATTATACCC AATAATGTCC TCTACCCCTC TATAGATGTA   
  
  
- ATTGATGAAC TTGTTGAATT TAAAAGTAAA ATTGAGCCAG TAATTCTTGT GATTCTTGAA ATATATAATC   
  
  
- ACTATAGAGT CCGCATTTTC TTTGATGAAA CTTAATTAGG TACGTTAGGA TGAGAAATAA AGGTAGGCAA   
  
  
- TTTTAAAAGG AAGTTTTGAG GGAAGACAAG TAATAATTAA GTTATAGCAT AATGTGAACA ACCTAATAAA   
  
  
- GACACTAAAC TAGCAGTAAA AGATATATAC TTATTGATGT TTTTAGTATC AGGCTCATGT AATTATAATT   
  
  
- ATTTTATCCT ACTCACTATT GGAGTATATC ACGCAAATAG GGATGGTGAT CCGACCTAAA GACGTATTTC   
  
  
- TTCTTTAACT TGTATACCTT TTTCTTTATT TCTCTTCGTT GAAGGAACTC ACCCTCTTCT TAGCATCTTT   
  
  
- CAATTTGAAT ACTTTTTCTC GTAATTGTTT TTCCTTTAAT ATTCCGAATG GCAACTTCGG AGTATATTAA   
  
  
- AACGTTCTCT GTCTGTTTCT TTCACAGAAA GTTTACAGTA TAAAGAGCTC GTACGTACTT TTTACATGAA   
  
  
- GCCGATGTTA TCATTATCAT ATATACAACC GAAGTATGTG AACTGGGGTT ACATACAGTT GCACTGATCA   
  
  
- CTGGTGTGTG TAGCAACAGG GACGGGCGGG GTGATGGGTT TGAGGGGCGT GCGTTTCTAT TTTTCGGGGG   
  
  
- GTTAAAAATA AACGGAGGGT TTTTCGGGTG GGCGCGAAAA AATAAACGGA AGAAAAAAGG ATGAATTTGG   
  
  
- TGGAGGAGTT GCTTGAAACG TTGTATGTAT GATCAGATAT TCGTTAAATC TCCCTTCCCT TCGTTGGATT   
  
  
- TGTCTCGCTC TAACCGCCCC ACACAAACTC CCGAACGCAA AACCACGTTA TCCTTACTTT CACGGGTAAA   
  
  
- GTTGGTGGTA GGTTTGAACG TTGAGGCCGT GGCCGTTTGG TGAAAGGAAA CTCTTATGAC GGGATGTATG   
  
  
- GCGGAAAGTT CAAGGGTTAT GGTGGATGCT TGGGTGGAGC CAAGAACTGG AGGTCGCGTC GGGATCGGGG   
  
  
- TGGAGGTGGG TTTTTGGCGG GAAACGGCGG AGGTTGGTGG TCGGACGGAG CCGACTAGAC CTGTTGAACC   
  
  
- TGCCCACCCT AAGTTAGGAT AGTCTCGACC CCGAATTACT GCTTAAATGG GGATTAAGGT TTGAGACCAG   
  
  
- GGTTTAATCA GGCAGACTAG GAGTGGAGGG GGTTGAGGGA CTAAAGGGTT GTAGAGTCGG CAAACTAGTA   
  
  
- GTGTTGGGTG GGGGTTATTT GGAGGGTAGT AGTCTAAAGT TGGAGACACT AAAGAGGATG TTAGTGTTGG   
  
  
- GGTTAAAACC CGGCAAGCTA GAAGTAGTGT TGGGCGTAGT CTTGTTGGGC TTGTTGTTAT TGATGCCGAA   
  
  
- ACTAAAGTAT CTGCTGGAGT AGTTTCGTCG TGTCAGGGAG CTTAGGTTGC TGAGAGTAGT TCAGGTGGAC   
  
  
- TATAACCGCG CCGAGTTAGT TGCCGAGTCT AGTGGGTGGC CGTTTGGCGA GGTCGCCCGG CGGATGATGA   
  
  
- AGTTCCTTCG GGAGTAGCGG CGGCGCCGGC CGGGCGGGGC GGGGCGAGCC GAAAGTAGCA TACTCCACCA   
  
  
- CGTCTGGTAG GCTCGGATGT TCCGCAAAAG TCCTTAAAGC GGTCAGAGAG AGAAGAGGTG AAAGCGGCGG   
  
  
- TTGGTCCGCT AAGAACTCCG CCACCTGTGC CGCCGGAAGT AGGTGTAGTA GCTAAAGCTG TAGCCTAAGC   
  
  
- CGCCGGTGAC CCGGAGCAAG GAGGCGCTTG ACCAGCTGTT TCGACTAGGA CGCTTTGAAT CAAGTCACCA   
  
  
- CAATGCTTAG TGGCGGTAGC AAGGGCTTCT TAAGCCCTAG CTTTCGTTCG ATCACTCTCT TTTAGACAGC   
  
  
- GTTAAACGGG CTCTAGAGTT GTAGTTGAAG GTGTAGCTAA TACAGAATAA AGTCTCGAAA CTTTAGAATA   
  
  
- GGAAGTTTAG ACAGTTTAAG TAGCTTTCCC TCTTTAACCG CGACGTAGAA AGCGGCCGGC ACAAGGTGGC   
  
  
- CGATCCCTCG CCTTAAAGCT TCAAATAGCG ACTAGAGGCT AGTTAGAGCG GCTCGAGGCA GCACCACCAC   
  
  
- CTGTCTCTAC AGCCCTAACT GTAGCCTTGC AGCAGCAAAT CGTACTTAAA GCAGCGGCCT TAACTTAAGA   
  
  
- TGTGGCCCTA CGACCTCAGC GAACTGCGTC GGTGGCGGCC CCCCGAGTAG CCGCCACACC TGACGCACTC   
  
  
- TGCCTAACTC TGCAAACAAG AGGTGGGTTC CTAGTACCGC CGCCATCTCC GCCGCCGCAG CCGCAGCCGG   
  
  
- CCCGCCTCCT GCCGCACCTC CCTCCGTAAG CGCCGCCGCC CCTAATCCCG CCACCCCAAA TCGGTCAAAC   
  
  
- GACTAAAGGT CCGGCTTACA AACAACTCCT CTCGAGTCCA ACCACCGAAG GTGCACCGTT TCGCGGTACC   
  
  
- TCTCTACTAC GAAATGACCG TACTGGCGGG TGAGCAGCGG TGTAGTCGAA CCTCCACAAT

+     CCGTCC motif

| Site Name | Organism | Position | Strand | Matrix score. | sequence | function |
| --- | --- | --- | --- | --- | --- | --- |
| CCGTCC motif | Nicotiana tabacum | 2243 | - | 6 | CCGTCC |  |
| CCGTCC motif | Nicotiana tabacum | 3442 | - | 6 | CCGTCC |  |

>HU05G02245.1   
+ -Up\_Stream \_Len000ATAATT TATCTTTCTT TAATTTCGTT TCATGTAAAA AATTTCAGTC TCATCTGCGA   
  
  
+ TTGAATCTCT TTTCAATTCG CATCAATAAT TCATCCAATT CAAATGTGCC TTTCTTAATT TCATTACATT   
  
  
+ GGGTTGGTAT AAATGTTAGC ACGATAGATT GGAATAGCAA GGCTCTAATT CAATTATGAC TTGAATGTAT   
  
  
+ GCATGCTTTT ATCATTAGTT AATTAACATT TTCTGTATTG AAATCAGCTC GAAAGAGAAC CATATGTAGT   
  
  
+ TGTACTTGCG ACGATATATA TGTTTAGTTT AGTTAGTTCT TACCTGTTTT GGAATTGTTG GGGAGATCTG   
  
  
+ AGGAAGGAGC CCGCTTCAAT CAAAGTTCAT GGCAATAAGC CAAAGTGAGG TTCCATCCTA AAACCAATTG   
  
  
+ GCAATAGGAA GAGTAGCCCA CTTAACTTGT ATACTATGAC TCTTCTTTTC AAACTCCCAA TGTGGGACTT   
  
  
+ CTTTACTCAT GTGTGATATT CCCAACAGGA ATGATCAGTT CCACATGTTT TCATCTCTTT GGACTCGAAC   
  
  
+ TTCGTGTAAT TGTATATGCC ATGGCACAAA TAGTTTTGAT GGTGCCACCA AGTTGCTCAA TCAATGTAAT   
  
  
+ ACTGCTACTA TCTAACTTGC CTTGGTGGAC ATCAATAAGA TCACGAGATG AGGAGAACTG AATCTTGGCC   
  
  
+ AACCACGTCG TTTGACTTCT CTTACCAATA AAGATTGAAT AGACAGTGTT TCTCTCCCCC TTCCATTCTT   
  
  
+ CTTTTCTAGT GATGTCGAAA AAGACTTTAT ACATGTCCAG CAACGCTAGC CACAAAATAT TCCATTTCTG   
  
  
+ TAATCCACTC ATGTCAATTA AACTTGTATC ATCAAATGGT AGTTTCCGTA CTAATCACTA CATCTAGATA   
  
  
+ TTCCTTGATA AATTTAATTT GACTTTAAAG GCTCGATCTT TTTAAGAAAA GTTTTGTTGC TTACAACCTG   
  
  
+ TAAAGCCTAG AGAACAAAGC CAAAGAAGAA ATTTAAGTTC TATATATAGC ATAATCTCTC AAAAAATATA   
  
  
+ TTTTTTAAAT ATAGTGCGAT ATTGACTGAC CTAATATGGG TTATTACAGG AGATGGGGAG ATATCTACAT   
  
  
+ TAACTACTTG AACAACTTAA ATTTTCATTT TAACTCGGTC ATTAAGAACA CTAAGAACTT TATATATTAG   
  
  
+ TGATATCTCA GGCGTAAAAG AAACTACTTT GAATTAATCC ATGCAATCCT ACTCTTTATT TCCATCCGTT   
  
  
+ AAAATTTTCC TTCAAAACTC CCTTCTGTTC ATTATTAATT CAATATCGTA TTACACTTGT TGGATTATTT   
  
  
+ CTGTGATTTG ATCGTCATTT TCTATATATG AATAACTACA AAAATCATAG TCCGAGTACA TTAATATTAA   
  
  
+ TAAAATAGGA TGAGTGATAA CCTCATATAG TGCGTTTATC CCTACCACTA GGCTGGATTT CTGCATAAAG   
  
  
+ AAGAAATTGA ACATATGGAA AAAGAAATAA AGAGAAGCAA CTTCCTTGAG TGGGAGAAGA ATCGTAGAAA   
  
  
+ GTTAAACTTA TGAAAAAGAG CATTAACAAA AAGGAAATTA TAAGGCTTAC CGTTGAAGCC TCATATAATT   
  
  
+ TTGCAAGAGA CAGACAAAGA AAGTGTCTTT CAAATGTCAT ATTTCTCGAG CATGCATGAA AAATGTACTT   
  
  
+ CGGCTACAAT AGTAATAGTA TATATGTTGG CTTCATACAC TTGACCCCAA TGTATGTCAA CGTGACTAGT   
  
  
+ GACCACACAC ATCGTTGTCC CTGCCCGCCC CACTACCCAA ACTCCCCGCA CGCAAAGATA AAAAGCCCCC   
  
  
+ CAATTTTTAT TTGCCTCCCA AAAAGCCCAC CCGCGCTTTT TTATTTGCCT TCTTTTTTCC TACTTAAACC   
  
  
+ ACCTCCTCAA CGAACTTTGC AACATACATA CTAGTCTATA AGCAATTTAG AGGGAAGGGA AGCAACCTAA   
  
  
+ ACAGAGCGAG ATTGGCGGGG TGTGTTTGAG GGCTTGCGTT TTGGTGCAAT AGGAATGAAA GTGCCCATTT   
  
  
+ CAACCACCAT CCAAACTTGC AACTCCGGCA CCGGCAAACC ACTTTCCTTT GAGAATACTG CCCTACATAC   
  
  
+ CGCCTTTCAA GTTCCCAATA CCACCTACGA ACCCACCTCG GTTCTTGACC TCCAGCGCAG CCCTAGCCCC   
  
  
+ ACCTCCACCC AAAAACCGCC CTTTGCCGCC TCCAACCACC AGCCTGCCTC GGCTGATCTG GACAACTTGG   
  
  
+ ACGGGTGGGA TTCAATCCTA TCAGAGCTGG GGCTTAATGA CGAATTTACC CCTAATTCCA AACTCTGGTC   
  
  
+ CCAAATTAGT CCGTCTGATC CTCACCTCCC CCAACTCCCT GATTTCCCAA CATCTCAGCC GTTTGATCAT   
  
  
+ CACAACCCAC CCCCAATAAA CCTCCCATCA TCAGATTTCA ACCTCTGTGA TTTCTCCTAC AATCACAACC   
  
  
+ CCAATTTTGG GCCGTTCGAT CTTCATCACA ACCCGCATCA GAACAACCCG AACAACAATA ACTACGGCTT   
  
  
+ TGATTTCATA GACGACCTCA TCAAAGCAGC ACAGTCCCTC GAATCCAACG ACTCTCATCA AGTCCACCTG   
  
  
+ ATATTGGCGC GGCTCAATCA ACGGCTCAGA TCACCCACCG GCAAACCGCT CCAGCGGGCC GCCTACTACT   
  
  
+ TCAAGGAAGC CCTCATCGCC GCCGCGGCCG GCCCGCCCCG CCCCGCTCGG CTTTCATCGT ATGAGGTGGT   
  
  
+ GCAGACCATC CGAGCCTACA AGGCGTTTTC AGGAATTTCG CCAGTCTCTC TCTTCTCCAC TTTCGCCGCC   
  
  
+ AACCAGGCGA TTCTTGAGGC GGTGGACACG GCGGCCTTCA TCCACATCAT CGATTTCGAC ATCGGATTCG   
  
  
+ GCGGCCACTG GGCCTCGTTC CTCCGCGAAC TGGTCGACAA AGCTGATCCT GCGAAACTTA GTTCAGTGGT   
  
  
+ GTTACGAATC ACCGCCATCG TTCCCGAAGA ATTCGGGATC GAAAGCAAGC TAGTGAGAGA AAATCTGTCG   
  
  
+ CAATTTGCCC GAGATCTCAA CATCAACTTC CACATCGATT ATGTCTTATT TCAGAGCTTT GAAATCTTAT   
  
  
+ CCTTCAAATC TGTCAAATTC ATCGAAAGGG AGAAATTGGC GCTGCATCTT TCGCCGGCCG TGTTCCACCG   
  
  
+ GCTAGGGAGC GGAATTTCGA AGTTTATCGC TGATCTCCGA TCAATCTCGC CGAGCTCCGT CGTGGTGGTG   
  
  
+ GACAGAGATG TCGGGATTGA CATCGGAACG TCGTCGTTTA GCATGAATTT CGTCGCCGGA ATTGAATTCT   
  
  
+ ACACCGGGAT GCTGGAGTCG CTTGACGCAG CCACCGCCGG GGGGCTCATC GGCGGTGTGG ACTGCGTGAG   
  
  
+ ACGGATTGAG ACGTTTGTTC TCCACCCAAG GATCATGGCG GCGGTAGAGG CGGCGGCGTC GGCGTCGGCC   
  
  
+ GGGCGGAGGA CGGCGTGGAG GGAGGCATTC GCGGCGGCGG GGATTAGGGC GGTGGGGTTT AGCCAGTTTG   
  
  
+ CTGATTTCCA GGCCGAATGT TTGTTGAGGA GAGCTCAGGT TGGTGGCTTC CACGTGGCAA AGCGCCATGG   
  
  
+ AGAGATGATG CTTTACTGGC ATGACCGCCC ACTCGTCGCC ACATCAGCTT GGAGGTGTTA   

- -Up\_Stream \_Len000TATTAA ATAGAAAGAA ATTAAAGCAA AGTACATTTT TTAAAGTCAG AGTAGACGCT   
  
  
- AACTTAGAGA AAAGTTAAGC GTAGTTATTA AGTAGGTTAA GTTTACACGG AAAGAATTAA AGTAATGTAA   
  
  
- CCCAACCATA TTTACAATCG TGCTATCTAA CCTTATCGTT CCGAGATTAA GTTAATACTG AACTTACATA   
  
  
- CGTACGAAAA TAGTAATCAA TTAATTGTAA AAGACATAAC TTTAGTCGAG CTTTCTCTTG GTATACATCA   
  
  
- ACATGAACGC TGCTATATAT ACAAATCAAA TCAATCAAGA ATGGACAAAA CCTTAACAAC CCCTCTAGAC   
  
  
- TCCTTCCTCG GGCGAAGTTA GTTTCAAGTA CCGTTATTCG GTTTCACTCC AAGGTAGGAT TTTGGTTAAC   
  
  
- CGTTATCCTT CTCATCGGGT GAATTGAACA TATGATACTG AGAAGAAAAG TTTGAGGGTT ACACCCTGAA   
  
  
- GAAATGAGTA CACACTATAA GGGTTGTCCT TACTAGTCAA GGTGTACAAA AGTAGAGAAA CCTGAGCTTG   
  
  
- AAGCACATTA ACATATACGG TACCGTGTTT ATCAAAACTA CCACGGTGGT TCAACGAGTT AGTTACATTA   
  
  
- TGACGATGAT AGATTGAACG GAACCACCTG TAGTTATTCT AGTGCTCTAC TCCTCTTGAC TTAGAACCGG   
  
  
- TTGGTGCAGC AAACTGAAGA GAATGGTTAT TTCTAACTTA TCTGTCACAA AGAGAGGGGG AAGGTAAGAA   
  
  
- GAAAAGATCA CTACAGCTTT TTCTGAAATA TGTACAGGTC GTTGCGATCG GTGTTTTATA AGGTAAAGAC   
  
  
- ATTAGGTGAG TACAGTTAAT TTGAACATAG TAGTTTACCA TCAAAGGCAT GATTAGTGAT GTAGATCTAT   
  
  
- AAGGAACTAT TTAAATTAAA CTGAAATTTC CGAGCTAGAA AAATTCTTTT CAAAACAACG AATGTTGGAC   
  
  
- ATTTCGGATC TCTTGTTTCG GTTTCTTCTT TAAATTCAAG ATATATATCG TATTAGAGAG TTTTTTATAT   
  
  
- AAAAAATTTA TATCACGCTA TAACTGACTG GATTATACCC AATAATGTCC TCTACCCCTC TATAGATGTA   
  
  
- ATTGATGAAC TTGTTGAATT TAAAAGTAAA ATTGAGCCAG TAATTCTTGT GATTCTTGAA ATATATAATC   
  
  
- ACTATAGAGT CCGCATTTTC TTTGATGAAA CTTAATTAGG TACGTTAGGA TGAGAAATAA AGGTAGGCAA   
  
  
- TTTTAAAAGG AAGTTTTGAG GGAAGACAAG TAATAATTAA GTTATAGCAT AATGTGAACA ACCTAATAAA   
  
  
- GACACTAAAC TAGCAGTAAA AGATATATAC TTATTGATGT TTTTAGTATC AGGCTCATGT AATTATAATT   
  
  
- ATTTTATCCT ACTCACTATT GGAGTATATC ACGCAAATAG GGATGGTGAT CCGACCTAAA GACGTATTTC   
  
  
- TTCTTTAACT TGTATACCTT TTTCTTTATT TCTCTTCGTT GAAGGAACTC ACCCTCTTCT TAGCATCTTT   
  
  
- CAATTTGAAT ACTTTTTCTC GTAATTGTTT TTCCTTTAAT ATTCCGAATG GCAACTTCGG AGTATATTAA   
  
  
- AACGTTCTCT GTCTGTTTCT TTCACAGAAA GTTTACAGTA TAAAGAGCTC GTACGTACTT TTTACATGAA   
  
  
- GCCGATGTTA TCATTATCAT ATATACAACC GAAGTATGTG AACTGGGGTT ACATACAGTT GCACTGATCA   
  
  
- CTGGTGTGTG TAGCAACAGG GACGGGCGGG GTGATGGGTT TGAGGGGCGT GCGTTTCTAT TTTTCGGGGG   
  
  
- GTTAAAAATA AACGGAGGGT TTTTCGGGTG GGCGCGAAAA AATAAACGGA AGAAAAAAGG ATGAATTTGG   
  
  
- TGGAGGAGTT GCTTGAAACG TTGTATGTAT GATCAGATAT TCGTTAAATC TCCCTTCCCT TCGTTGGATT   
  
  
- TGTCTCGCTC TAACCGCCCC ACACAAACTC CCGAACGCAA AACCACGTTA TCCTTACTTT CACGGGTAAA   
  
  
- GTTGGTGGTA GGTTTGAACG TTGAGGCCGT GGCCGTTTGG TGAAAGGAAA CTCTTATGAC GGGATGTATG   
  
  
- GCGGAAAGTT CAAGGGTTAT GGTGGATGCT TGGGTGGAGC CAAGAACTGG AGGTCGCGTC GGGATCGGGG   
  
  
- TGGAGGTGGG TTTTTGGCGG GAAACGGCGG AGGTTGGTGG TCGGACGGAG CCGACTAGAC CTGTTGAACC   
  
  
- TGCCCACCCT AAGTTAGGAT AGTCTCGACC CCGAATTACT GCTTAAATGG GGATTAAGGT TTGAGACCAG   
  
  
- GGTTTAATCA GGCAGACTAG GAGTGGAGGG GGTTGAGGGA CTAAAGGGTT GTAGAGTCGG CAAACTAGTA   
  
  
- GTGTTGGGTG GGGGTTATTT GGAGGGTAGT AGTCTAAAGT TGGAGACACT AAAGAGGATG TTAGTGTTGG   
  
  
- GGTTAAAACC CGGCAAGCTA GAAGTAGTGT TGGGCGTAGT CTTGTTGGGC TTGTTGTTAT TGATGCCGAA   
  
  
- ACTAAAGTAT CTGCTGGAGT AGTTTCGTCG TGTCAGGGAG CTTAGGTTGC TGAGAGTAGT TCAGGTGGAC   
  
  
- TATAACCGCG CCGAGTTAGT TGCCGAGTCT AGTGGGTGGC CGTTTGGCGA GGTCGCCCGG CGGATGATGA   
  
  
- AGTTCCTTCG GGAGTAGCGG CGGCGCCGGC CGGGCGGGGC GGGGCGAGCC GAAAGTAGCA TACTCCACCA   
  
  
- CGTCTGGTAG GCTCGGATGT TCCGCAAAAG TCCTTAAAGC GGTCAGAGAG AGAAGAGGTG AAAGCGGCGG   
  
  
- TTGGTCCGCT AAGAACTCCG CCACCTGTGC CGCCGGAAGT AGGTGTAGTA GCTAAAGCTG TAGCCTAAGC   
  
  
- CGCCGGTGAC CCGGAGCAAG GAGGCGCTTG ACCAGCTGTT TCGACTAGGA CGCTTTGAAT CAAGTCACCA   
  
  
- CAATGCTTAG TGGCGGTAGC AAGGGCTTCT TAAGCCCTAG CTTTCGTTCG ATCACTCTCT TTTAGACAGC   
  
  
- GTTAAACGGG CTCTAGAGTT GTAGTTGAAG GTGTAGCTAA TACAGAATAA AGTCTCGAAA CTTTAGAATA   
  
  
- GGAAGTTTAG ACAGTTTAAG TAGCTTTCCC TCTTTAACCG CGACGTAGAA AGCGGCCGGC ACAAGGTGGC   
  
  
- CGATCCCTCG CCTTAAAGCT TCAAATAGCG ACTAGAGGCT AGTTAGAGCG GCTCGAGGCA GCACCACCAC   
  
  
- CTGTCTCTAC AGCCCTAACT GTAGCCTTGC AGCAGCAAAT CGTACTTAAA GCAGCGGCCT TAACTTAAGA   
  
  
- TGTGGCCCTA CGACCTCAGC GAACTGCGTC GGTGGCGGCC CCCCGAGTAG CCGCCACACC TGACGCACTC   
  
  
- TGCCTAACTC TGCAAACAAG AGGTGGGTTC CTAGTACCGC CGCCATCTCC GCCGCCGCAG CCGCAGCCGG   
  
  
- CCCGCCTCCT GCCGCACCTC CCTCCGTAAG CGCCGCCGCC CCTAATCCCG CCACCCCAAA TCGGTCAAAC   
  
  
- GACTAAAGGT CCGGCTTACA AACAACTCCT CTCGAGTCCA ACCACCGAAG GTGCACCGTT TCGCGGTACC   
  
  
- TCTCTACTAC GAAATGACCG TACTGGCGGG TGAGCAGCGG TGTAGTCGAA CCTCCACAAT

+     CCGTCC-box

| Site Name | Organism | Position | Strand | Matrix score. | sequence | function |
| --- | --- | --- | --- | --- | --- | --- |
| CCGTCC-box | Petroselinum hortense | 2243 | - | 6 | CCGTCC |  |
| CCGTCC-box | Petroselinum hortense | 3442 | - | 6 | CCGTCC |  |

>HU05G02245.1   
+ -Up\_Stream \_Len000ATAATT TATCTTTCTT TAATTTCGTT TCATGTAAAA AATTTCAGTC TCATCTGCGA   
  
  
+ TTGAATCTCT TTTCAATTCG CATCAATAAT TCATCCAATT CAAATGTGCC TTTCTTAATT TCATTACATT   
  
  
+ GGGTTGGTAT AAATGTTAGC ACGATAGATT GGAATAGCAA GGCTCTAATT CAATTATGAC TTGAATGTAT   
  
  
+ GCATGCTTTT ATCATTAGTT AATTAACATT TTCTGTATTG AAATCAGCTC GAAAGAGAAC CATATGTAGT   
  
  
+ TGTACTTGCG ACGATATATA TGTTTAGTTT AGTTAGTTCT TACCTGTTTT GGAATTGTTG GGGAGATCTG   
  
  
+ AGGAAGGAGC CCGCTTCAAT CAAAGTTCAT GGCAATAAGC CAAAGTGAGG TTCCATCCTA AAACCAATTG   
  
  
+ GCAATAGGAA GAGTAGCCCA CTTAACTTGT ATACTATGAC TCTTCTTTTC AAACTCCCAA TGTGGGACTT   
  
  
+ CTTTACTCAT GTGTGATATT CCCAACAGGA ATGATCAGTT CCACATGTTT TCATCTCTTT GGACTCGAAC   
  
  
+ TTCGTGTAAT TGTATATGCC ATGGCACAAA TAGTTTTGAT GGTGCCACCA AGTTGCTCAA TCAATGTAAT   
  
  
+ ACTGCTACTA TCTAACTTGC CTTGGTGGAC ATCAATAAGA TCACGAGATG AGGAGAACTG AATCTTGGCC   
  
  
+ AACCACGTCG TTTGACTTCT CTTACCAATA AAGATTGAAT AGACAGTGTT TCTCTCCCCC TTCCATTCTT   
  
  
+ CTTTTCTAGT GATGTCGAAA AAGACTTTAT ACATGTCCAG CAACGCTAGC CACAAAATAT TCCATTTCTG   
  
  
+ TAATCCACTC ATGTCAATTA AACTTGTATC ATCAAATGGT AGTTTCCGTA CTAATCACTA CATCTAGATA   
  
  
+ TTCCTTGATA AATTTAATTT GACTTTAAAG GCTCGATCTT TTTAAGAAAA GTTTTGTTGC TTACAACCTG   
  
  
+ TAAAGCCTAG AGAACAAAGC CAAAGAAGAA ATTTAAGTTC TATATATAGC ATAATCTCTC AAAAAATATA   
  
  
+ TTTTTTAAAT ATAGTGCGAT ATTGACTGAC CTAATATGGG TTATTACAGG AGATGGGGAG ATATCTACAT   
  
  
+ TAACTACTTG AACAACTTAA ATTTTCATTT TAACTCGGTC ATTAAGAACA CTAAGAACTT TATATATTAG   
  
  
+ TGATATCTCA GGCGTAAAAG AAACTACTTT GAATTAATCC ATGCAATCCT ACTCTTTATT TCCATCCGTT   
  
  
+ AAAATTTTCC TTCAAAACTC CCTTCTGTTC ATTATTAATT CAATATCGTA TTACACTTGT TGGATTATTT   
  
  
+ CTGTGATTTG ATCGTCATTT TCTATATATG AATAACTACA AAAATCATAG TCCGAGTACA TTAATATTAA   
  
  
+ TAAAATAGGA TGAGTGATAA CCTCATATAG TGCGTTTATC CCTACCACTA GGCTGGATTT CTGCATAAAG   
  
  
+ AAGAAATTGA ACATATGGAA AAAGAAATAA AGAGAAGCAA CTTCCTTGAG TGGGAGAAGA ATCGTAGAAA   
  
  
+ GTTAAACTTA TGAAAAAGAG CATTAACAAA AAGGAAATTA TAAGGCTTAC CGTTGAAGCC TCATATAATT   
  
  
+ TTGCAAGAGA CAGACAAAGA AAGTGTCTTT CAAATGTCAT ATTTCTCGAG CATGCATGAA AAATGTACTT   
  
  
+ CGGCTACAAT AGTAATAGTA TATATGTTGG CTTCATACAC TTGACCCCAA TGTATGTCAA CGTGACTAGT   
  
  
+ GACCACACAC ATCGTTGTCC CTGCCCGCCC CACTACCCAA ACTCCCCGCA CGCAAAGATA AAAAGCCCCC   
  
  
+ CAATTTTTAT TTGCCTCCCA AAAAGCCCAC CCGCGCTTTT TTATTTGCCT TCTTTTTTCC TACTTAAACC   
  
  
+ ACCTCCTCAA CGAACTTTGC AACATACATA CTAGTCTATA AGCAATTTAG AGGGAAGGGA AGCAACCTAA   
  
  
+ ACAGAGCGAG ATTGGCGGGG TGTGTTTGAG GGCTTGCGTT TTGGTGCAAT AGGAATGAAA GTGCCCATTT   
  
  
+ CAACCACCAT CCAAACTTGC AACTCCGGCA CCGGCAAACC ACTTTCCTTT GAGAATACTG CCCTACATAC   
  
  
+ CGCCTTTCAA GTTCCCAATA CCACCTACGA ACCCACCTCG GTTCTTGACC TCCAGCGCAG CCCTAGCCCC   
  
  
+ ACCTCCACCC AAAAACCGCC CTTTGCCGCC TCCAACCACC AGCCTGCCTC GGCTGATCTG GACAACTTGG   
  
  
+ ACGGGTGGGA TTCAATCCTA TCAGAGCTGG GGCTTAATGA CGAATTTACC CCTAATTCCA AACTCTGGTC   
  
  
+ CCAAATTAGT CCGTCTGATC CTCACCTCCC CCAACTCCCT GATTTCCCAA CATCTCAGCC GTTTGATCAT   
  
  
+ CACAACCCAC CCCCAATAAA CCTCCCATCA TCAGATTTCA ACCTCTGTGA TTTCTCCTAC AATCACAACC   
  
  
+ CCAATTTTGG GCCGTTCGAT CTTCATCACA ACCCGCATCA GAACAACCCG AACAACAATA ACTACGGCTT   
  
  
+ TGATTTCATA GACGACCTCA TCAAAGCAGC ACAGTCCCTC GAATCCAACG ACTCTCATCA AGTCCACCTG   
  
  
+ ATATTGGCGC GGCTCAATCA ACGGCTCAGA TCACCCACCG GCAAACCGCT CCAGCGGGCC GCCTACTACT   
  
  
+ TCAAGGAAGC CCTCATCGCC GCCGCGGCCG GCCCGCCCCG CCCCGCTCGG CTTTCATCGT ATGAGGTGGT   
  
  
+ GCAGACCATC CGAGCCTACA AGGCGTTTTC AGGAATTTCG CCAGTCTCTC TCTTCTCCAC TTTCGCCGCC   
  
  
+ AACCAGGCGA TTCTTGAGGC GGTGGACACG GCGGCCTTCA TCCACATCAT CGATTTCGAC ATCGGATTCG   
  
  
+ GCGGCCACTG GGCCTCGTTC CTCCGCGAAC TGGTCGACAA AGCTGATCCT GCGAAACTTA GTTCAGTGGT   
  
  
+ GTTACGAATC ACCGCCATCG TTCCCGAAGA ATTCGGGATC GAAAGCAAGC TAGTGAGAGA AAATCTGTCG   
  
  
+ CAATTTGCCC GAGATCTCAA CATCAACTTC CACATCGATT ATGTCTTATT TCAGAGCTTT GAAATCTTAT   
  
  
+ CCTTCAAATC TGTCAAATTC ATCGAAAGGG AGAAATTGGC GCTGCATCTT TCGCCGGCCG TGTTCCACCG   
  
  
+ GCTAGGGAGC GGAATTTCGA AGTTTATCGC TGATCTCCGA TCAATCTCGC CGAGCTCCGT CGTGGTGGTG   
  
  
+ GACAGAGATG TCGGGATTGA CATCGGAACG TCGTCGTTTA GCATGAATTT CGTCGCCGGA ATTGAATTCT   
  
  
+ ACACCGGGAT GCTGGAGTCG CTTGACGCAG CCACCGCCGG GGGGCTCATC GGCGGTGTGG ACTGCGTGAG   
  
  
+ ACGGATTGAG ACGTTTGTTC TCCACCCAAG GATCATGGCG GCGGTAGAGG CGGCGGCGTC GGCGTCGGCC   
  
  
+ GGGCGGAGGA CGGCGTGGAG GGAGGCATTC GCGGCGGCGG GGATTAGGGC GGTGGGGTTT AGCCAGTTTG   
  
  
+ CTGATTTCCA GGCCGAATGT TTGTTGAGGA GAGCTCAGGT TGGTGGCTTC CACGTGGCAA AGCGCCATGG   
  
  
+ AGAGATGATG CTTTACTGGC ATGACCGCCC ACTCGTCGCC ACATCAGCTT GGAGGTGTTA   

- -Up\_Stream \_Len000TATTAA ATAGAAAGAA ATTAAAGCAA AGTACATTTT TTAAAGTCAG AGTAGACGCT   
  
  
- AACTTAGAGA AAAGTTAAGC GTAGTTATTA AGTAGGTTAA GTTTACACGG AAAGAATTAA AGTAATGTAA   
  
  
- CCCAACCATA TTTACAATCG TGCTATCTAA CCTTATCGTT CCGAGATTAA GTTAATACTG AACTTACATA   
  
  
- CGTACGAAAA TAGTAATCAA TTAATTGTAA AAGACATAAC TTTAGTCGAG CTTTCTCTTG GTATACATCA   
  
  
- ACATGAACGC TGCTATATAT ACAAATCAAA TCAATCAAGA ATGGACAAAA CCTTAACAAC CCCTCTAGAC   
  
  
- TCCTTCCTCG GGCGAAGTTA GTTTCAAGTA CCGTTATTCG GTTTCACTCC AAGGTAGGAT TTTGGTTAAC   
  
  
- CGTTATCCTT CTCATCGGGT GAATTGAACA TATGATACTG AGAAGAAAAG TTTGAGGGTT ACACCCTGAA   
  
  
- GAAATGAGTA CACACTATAA GGGTTGTCCT TACTAGTCAA GGTGTACAAA AGTAGAGAAA CCTGAGCTTG   
  
  
- AAGCACATTA ACATATACGG TACCGTGTTT ATCAAAACTA CCACGGTGGT TCAACGAGTT AGTTACATTA   
  
  
- TGACGATGAT AGATTGAACG GAACCACCTG TAGTTATTCT AGTGCTCTAC TCCTCTTGAC TTAGAACCGG   
  
  
- TTGGTGCAGC AAACTGAAGA GAATGGTTAT TTCTAACTTA TCTGTCACAA AGAGAGGGGG AAGGTAAGAA   
  
  
- GAAAAGATCA CTACAGCTTT TTCTGAAATA TGTACAGGTC GTTGCGATCG GTGTTTTATA AGGTAAAGAC   
  
  
- ATTAGGTGAG TACAGTTAAT TTGAACATAG TAGTTTACCA TCAAAGGCAT GATTAGTGAT GTAGATCTAT   
  
  
- AAGGAACTAT TTAAATTAAA CTGAAATTTC CGAGCTAGAA AAATTCTTTT CAAAACAACG AATGTTGGAC   
  
  
- ATTTCGGATC TCTTGTTTCG GTTTCTTCTT TAAATTCAAG ATATATATCG TATTAGAGAG TTTTTTATAT   
  
  
- AAAAAATTTA TATCACGCTA TAACTGACTG GATTATACCC AATAATGTCC TCTACCCCTC TATAGATGTA   
  
  
- ATTGATGAAC TTGTTGAATT TAAAAGTAAA ATTGAGCCAG TAATTCTTGT GATTCTTGAA ATATATAATC   
  
  
- ACTATAGAGT CCGCATTTTC TTTGATGAAA CTTAATTAGG TACGTTAGGA TGAGAAATAA AGGTAGGCAA   
  
  
- TTTTAAAAGG AAGTTTTGAG GGAAGACAAG TAATAATTAA GTTATAGCAT AATGTGAACA ACCTAATAAA   
  
  
- GACACTAAAC TAGCAGTAAA AGATATATAC TTATTGATGT TTTTAGTATC AGGCTCATGT AATTATAATT   
  
  
- ATTTTATCCT ACTCACTATT GGAGTATATC ACGCAAATAG GGATGGTGAT CCGACCTAAA GACGTATTTC   
  
  
- TTCTTTAACT TGTATACCTT TTTCTTTATT TCTCTTCGTT GAAGGAACTC ACCCTCTTCT TAGCATCTTT   
  
  
- CAATTTGAAT ACTTTTTCTC GTAATTGTTT TTCCTTTAAT ATTCCGAATG GCAACTTCGG AGTATATTAA   
  
  
- AACGTTCTCT GTCTGTTTCT TTCACAGAAA GTTTACAGTA TAAAGAGCTC GTACGTACTT TTTACATGAA   
  
  
- GCCGATGTTA TCATTATCAT ATATACAACC GAAGTATGTG AACTGGGGTT ACATACAGTT GCACTGATCA   
  
  
- CTGGTGTGTG TAGCAACAGG GACGGGCGGG GTGATGGGTT TGAGGGGCGT GCGTTTCTAT TTTTCGGGGG   
  
  
- GTTAAAAATA AACGGAGGGT TTTTCGGGTG GGCGCGAAAA AATAAACGGA AGAAAAAAGG ATGAATTTGG   
  
  
- TGGAGGAGTT GCTTGAAACG TTGTATGTAT GATCAGATAT TCGTTAAATC TCCCTTCCCT TCGTTGGATT   
  
  
- TGTCTCGCTC TAACCGCCCC ACACAAACTC CCGAACGCAA AACCACGTTA TCCTTACTTT CACGGGTAAA   
  
  
- GTTGGTGGTA GGTTTGAACG TTGAGGCCGT GGCCGTTTGG TGAAAGGAAA CTCTTATGAC GGGATGTATG   
  
  
- GCGGAAAGTT CAAGGGTTAT GGTGGATGCT TGGGTGGAGC CAAGAACTGG AGGTCGCGTC GGGATCGGGG   
  
  
- TGGAGGTGGG TTTTTGGCGG GAAACGGCGG AGGTTGGTGG TCGGACGGAG CCGACTAGAC CTGTTGAACC   
  
  
- TGCCCACCCT AAGTTAGGAT AGTCTCGACC CCGAATTACT GCTTAAATGG GGATTAAGGT TTGAGACCAG   
  
  
- GGTTTAATCA GGCAGACTAG GAGTGGAGGG GGTTGAGGGA CTAAAGGGTT GTAGAGTCGG CAAACTAGTA   
  
  
- GTGTTGGGTG GGGGTTATTT GGAGGGTAGT AGTCTAAAGT TGGAGACACT AAAGAGGATG TTAGTGTTGG   
  
  
- GGTTAAAACC CGGCAAGCTA GAAGTAGTGT TGGGCGTAGT CTTGTTGGGC TTGTTGTTAT TGATGCCGAA   
  
  
- ACTAAAGTAT CTGCTGGAGT AGTTTCGTCG TGTCAGGGAG CTTAGGTTGC TGAGAGTAGT TCAGGTGGAC   
  
  
- TATAACCGCG CCGAGTTAGT TGCCGAGTCT AGTGGGTGGC CGTTTGGCGA GGTCGCCCGG CGGATGATGA   
  
  
- AGTTCCTTCG GGAGTAGCGG CGGCGCCGGC CGGGCGGGGC GGGGCGAGCC GAAAGTAGCA TACTCCACCA   
  
  
- CGTCTGGTAG GCTCGGATGT TCCGCAAAAG TCCTTAAAGC GGTCAGAGAG AGAAGAGGTG AAAGCGGCGG   
  
  
- TTGGTCCGCT AAGAACTCCG CCACCTGTGC CGCCGGAAGT AGGTGTAGTA GCTAAAGCTG TAGCCTAAGC   
  
  
- CGCCGGTGAC CCGGAGCAAG GAGGCGCTTG ACCAGCTGTT TCGACTAGGA CGCTTTGAAT CAAGTCACCA   
  
  
- CAATGCTTAG TGGCGGTAGC AAGGGCTTCT TAAGCCCTAG CTTTCGTTCG ATCACTCTCT TTTAGACAGC   
  
  
- GTTAAACGGG CTCTAGAGTT GTAGTTGAAG GTGTAGCTAA TACAGAATAA AGTCTCGAAA CTTTAGAATA   
  
  
- GGAAGTTTAG ACAGTTTAAG TAGCTTTCCC TCTTTAACCG CGACGTAGAA AGCGGCCGGC ACAAGGTGGC   
  
  
- CGATCCCTCG CCTTAAAGCT TCAAATAGCG ACTAGAGGCT AGTTAGAGCG GCTCGAGGCA GCACCACCAC   
  
  
- CTGTCTCTAC AGCCCTAACT GTAGCCTTGC AGCAGCAAAT CGTACTTAAA GCAGCGGCCT TAACTTAAGA   
  
  
- TGTGGCCCTA CGACCTCAGC GAACTGCGTC GGTGGCGGCC CCCCGAGTAG CCGCCACACC TGACGCACTC   
  
  
- TGCCTAACTC TGCAAACAAG AGGTGGGTTC CTAGTACCGC CGCCATCTCC GCCGCCGCAG CCGCAGCCGG   
  
  
- CCCGCCTCCT GCCGCACCTC CCTCCGTAAG CGCCGCCGCC CCTAATCCCG CCACCCCAAA TCGGTCAAAC   
  
  
- GACTAAAGGT CCGGCTTACA AACAACTCCT CTCGAGTCCA ACCACCGAAG GTGCACCGTT TCGCGGTACC   
  
  
- TCTCTACTAC GAAATGACCG TACTGGCGGG TGAGCAGCGG TGTAGTCGAA CCTCCACAAT

+     CGTCA-motif

| Site Name | Organism | Position | Strand | Matrix score. | sequence | function |
| --- | --- | --- | --- | --- | --- | --- |
| CGTCA-motif | Hordeum vulgare | 1347 | + | 5 | CGTCA | cis-acting regulatory element involved in the MeJA-responsiveness |
| CGTCA-motif | Hordeum vulgare | 2282 | - | 5 | CGTCA | cis-acting regulatory element involved in the MeJA-responsiveness |
| CGTCA-motif | Hordeum vulgare | 3317 | - | 5 | CGTCA | cis-acting regulatory element involved in the MeJA-responsiveness |

>HU05G02245.1   
+ -Up\_Stream \_Len000ATAATT TATCTTTCTT TAATTTCGTT TCATGTAAAA AATTTCAGTC TCATCTGCGA   
  
  
+ TTGAATCTCT TTTCAATTCG CATCAATAAT TCATCCAATT CAAATGTGCC TTTCTTAATT TCATTACATT   
  
  
+ GGGTTGGTAT AAATGTTAGC ACGATAGATT GGAATAGCAA GGCTCTAATT CAATTATGAC TTGAATGTAT   
  
  
+ GCATGCTTTT ATCATTAGTT AATTAACATT TTCTGTATTG AAATCAGCTC GAAAGAGAAC CATATGTAGT   
  
  
+ TGTACTTGCG ACGATATATA TGTTTAGTTT AGTTAGTTCT TACCTGTTTT GGAATTGTTG GGGAGATCTG   
  
  
+ AGGAAGGAGC CCGCTTCAAT CAAAGTTCAT GGCAATAAGC CAAAGTGAGG TTCCATCCTA AAACCAATTG   
  
  
+ GCAATAGGAA GAGTAGCCCA CTTAACTTGT ATACTATGAC TCTTCTTTTC AAACTCCCAA TGTGGGACTT   
  
  
+ CTTTACTCAT GTGTGATATT CCCAACAGGA ATGATCAGTT CCACATGTTT TCATCTCTTT GGACTCGAAC   
  
  
+ TTCGTGTAAT TGTATATGCC ATGGCACAAA TAGTTTTGAT GGTGCCACCA AGTTGCTCAA TCAATGTAAT   
  
  
+ ACTGCTACTA TCTAACTTGC CTTGGTGGAC ATCAATAAGA TCACGAGATG AGGAGAACTG AATCTTGGCC   
  
  
+ AACCACGTCG TTTGACTTCT CTTACCAATA AAGATTGAAT AGACAGTGTT TCTCTCCCCC TTCCATTCTT   
  
  
+ CTTTTCTAGT GATGTCGAAA AAGACTTTAT ACATGTCCAG CAACGCTAGC CACAAAATAT TCCATTTCTG   
  
  
+ TAATCCACTC ATGTCAATTA AACTTGTATC ATCAAATGGT AGTTTCCGTA CTAATCACTA CATCTAGATA   
  
  
+ TTCCTTGATA AATTTAATTT GACTTTAAAG GCTCGATCTT TTTAAGAAAA GTTTTGTTGC TTACAACCTG   
  
  
+ TAAAGCCTAG AGAACAAAGC CAAAGAAGAA ATTTAAGTTC TATATATAGC ATAATCTCTC AAAAAATATA   
  
  
+ TTTTTTAAAT ATAGTGCGAT ATTGACTGAC CTAATATGGG TTATTACAGG AGATGGGGAG ATATCTACAT   
  
  
+ TAACTACTTG AACAACTTAA ATTTTCATTT TAACTCGGTC ATTAAGAACA CTAAGAACTT TATATATTAG   
  
  
+ TGATATCTCA GGCGTAAAAG AAACTACTTT GAATTAATCC ATGCAATCCT ACTCTTTATT TCCATCCGTT   
  
  
+ AAAATTTTCC TTCAAAACTC CCTTCTGTTC ATTATTAATT CAATATCGTA TTACACTTGT TGGATTATTT   
  
  
+ CTGTGATTTG ATCGTCATTT TCTATATATG AATAACTACA AAAATCATAG TCCGAGTACA TTAATATTAA   
  
  
+ TAAAATAGGA TGAGTGATAA CCTCATATAG TGCGTTTATC CCTACCACTA GGCTGGATTT CTGCATAAAG   
  
  
+ AAGAAATTGA ACATATGGAA AAAGAAATAA AGAGAAGCAA CTTCCTTGAG TGGGAGAAGA ATCGTAGAAA   
  
  
+ GTTAAACTTA TGAAAAAGAG CATTAACAAA AAGGAAATTA TAAGGCTTAC CGTTGAAGCC TCATATAATT   
  
  
+ TTGCAAGAGA CAGACAAAGA AAGTGTCTTT CAAATGTCAT ATTTCTCGAG CATGCATGAA AAATGTACTT   
  
  
+ CGGCTACAAT AGTAATAGTA TATATGTTGG CTTCATACAC TTGACCCCAA TGTATGTCAA CGTGACTAGT   
  
  
+ GACCACACAC ATCGTTGTCC CTGCCCGCCC CACTACCCAA ACTCCCCGCA CGCAAAGATA AAAAGCCCCC   
  
  
+ CAATTTTTAT TTGCCTCCCA AAAAGCCCAC CCGCGCTTTT TTATTTGCCT TCTTTTTTCC TACTTAAACC   
  
  
+ ACCTCCTCAA CGAACTTTGC AACATACATA CTAGTCTATA AGCAATTTAG AGGGAAGGGA AGCAACCTAA   
  
  
+ ACAGAGCGAG ATTGGCGGGG TGTGTTTGAG GGCTTGCGTT TTGGTGCAAT AGGAATGAAA GTGCCCATTT   
  
  
+ CAACCACCAT CCAAACTTGC AACTCCGGCA CCGGCAAACC ACTTTCCTTT GAGAATACTG CCCTACATAC   
  
  
+ CGCCTTTCAA GTTCCCAATA CCACCTACGA ACCCACCTCG GTTCTTGACC TCCAGCGCAG CCCTAGCCCC   
  
  
+ ACCTCCACCC AAAAACCGCC CTTTGCCGCC TCCAACCACC AGCCTGCCTC GGCTGATCTG GACAACTTGG   
  
  
+ ACGGGTGGGA TTCAATCCTA TCAGAGCTGG GGCTTAATGA CGAATTTACC CCTAATTCCA AACTCTGGTC   
  
  
+ CCAAATTAGT CCGTCTGATC CTCACCTCCC CCAACTCCCT GATTTCCCAA CATCTCAGCC GTTTGATCAT   
  
  
+ CACAACCCAC CCCCAATAAA CCTCCCATCA TCAGATTTCA ACCTCTGTGA TTTCTCCTAC AATCACAACC   
  
  
+ CCAATTTTGG GCCGTTCGAT CTTCATCACA ACCCGCATCA GAACAACCCG AACAACAATA ACTACGGCTT   
  
  
+ TGATTTCATA GACGACCTCA TCAAAGCAGC ACAGTCCCTC GAATCCAACG ACTCTCATCA AGTCCACCTG   
  
  
+ ATATTGGCGC GGCTCAATCA ACGGCTCAGA TCACCCACCG GCAAACCGCT CCAGCGGGCC GCCTACTACT   
  
  
+ TCAAGGAAGC CCTCATCGCC GCCGCGGCCG GCCCGCCCCG CCCCGCTCGG CTTTCATCGT ATGAGGTGGT   
  
  
+ GCAGACCATC CGAGCCTACA AGGCGTTTTC AGGAATTTCG CCAGTCTCTC TCTTCTCCAC TTTCGCCGCC   
  
  
+ AACCAGGCGA TTCTTGAGGC GGTGGACACG GCGGCCTTCA TCCACATCAT CGATTTCGAC ATCGGATTCG   
  
  
+ GCGGCCACTG GGCCTCGTTC CTCCGCGAAC TGGTCGACAA AGCTGATCCT GCGAAACTTA GTTCAGTGGT   
  
  
+ GTTACGAATC ACCGCCATCG TTCCCGAAGA ATTCGGGATC GAAAGCAAGC TAGTGAGAGA AAATCTGTCG   
  
  
+ CAATTTGCCC GAGATCTCAA CATCAACTTC CACATCGATT ATGTCTTATT TCAGAGCTTT GAAATCTTAT   
  
  
+ CCTTCAAATC TGTCAAATTC ATCGAAAGGG AGAAATTGGC GCTGCATCTT TCGCCGGCCG TGTTCCACCG   
  
  
+ GCTAGGGAGC GGAATTTCGA AGTTTATCGC TGATCTCCGA TCAATCTCGC CGAGCTCCGT CGTGGTGGTG   
  
  
+ GACAGAGATG TCGGGATTGA CATCGGAACG TCGTCGTTTA GCATGAATTT CGTCGCCGGA ATTGAATTCT   
  
  
+ ACACCGGGAT GCTGGAGTCG CTTGACGCAG CCACCGCCGG GGGGCTCATC GGCGGTGTGG ACTGCGTGAG   
  
  
+ ACGGATTGAG ACGTTTGTTC TCCACCCAAG GATCATGGCG GCGGTAGAGG CGGCGGCGTC GGCGTCGGCC   
  
  
+ GGGCGGAGGA CGGCGTGGAG GGAGGCATTC GCGGCGGCGG GGATTAGGGC GGTGGGGTTT AGCCAGTTTG   
  
  
+ CTGATTTCCA GGCCGAATGT TTGTTGAGGA GAGCTCAGGT TGGTGGCTTC CACGTGGCAA AGCGCCATGG   
  
  
+ AGAGATGATG CTTTACTGGC ATGACCGCCC ACTCGTCGCC ACATCAGCTT GGAGGTGTTA   

- -Up\_Stream \_Len000TATTAA ATAGAAAGAA ATTAAAGCAA AGTACATTTT TTAAAGTCAG AGTAGACGCT   
  
  
- AACTTAGAGA AAAGTTAAGC GTAGTTATTA AGTAGGTTAA GTTTACACGG AAAGAATTAA AGTAATGTAA   
  
  
- CCCAACCATA TTTACAATCG TGCTATCTAA CCTTATCGTT CCGAGATTAA GTTAATACTG AACTTACATA   
  
  
- CGTACGAAAA TAGTAATCAA TTAATTGTAA AAGACATAAC TTTAGTCGAG CTTTCTCTTG GTATACATCA   
  
  
- ACATGAACGC TGCTATATAT ACAAATCAAA TCAATCAAGA ATGGACAAAA CCTTAACAAC CCCTCTAGAC   
  
  
- TCCTTCCTCG GGCGAAGTTA GTTTCAAGTA CCGTTATTCG GTTTCACTCC AAGGTAGGAT TTTGGTTAAC   
  
  
- CGTTATCCTT CTCATCGGGT GAATTGAACA TATGATACTG AGAAGAAAAG TTTGAGGGTT ACACCCTGAA   
  
  
- GAAATGAGTA CACACTATAA GGGTTGTCCT TACTAGTCAA GGTGTACAAA AGTAGAGAAA CCTGAGCTTG   
  
  
- AAGCACATTA ACATATACGG TACCGTGTTT ATCAAAACTA CCACGGTGGT TCAACGAGTT AGTTACATTA   
  
  
- TGACGATGAT AGATTGAACG GAACCACCTG TAGTTATTCT AGTGCTCTAC TCCTCTTGAC TTAGAACCGG   
  
  
- TTGGTGCAGC AAACTGAAGA GAATGGTTAT TTCTAACTTA TCTGTCACAA AGAGAGGGGG AAGGTAAGAA   
  
  
- GAAAAGATCA CTACAGCTTT TTCTGAAATA TGTACAGGTC GTTGCGATCG GTGTTTTATA AGGTAAAGAC   
  
  
- ATTAGGTGAG TACAGTTAAT TTGAACATAG TAGTTTACCA TCAAAGGCAT GATTAGTGAT GTAGATCTAT   
  
  
- AAGGAACTAT TTAAATTAAA CTGAAATTTC CGAGCTAGAA AAATTCTTTT CAAAACAACG AATGTTGGAC   
  
  
- ATTTCGGATC TCTTGTTTCG GTTTCTTCTT TAAATTCAAG ATATATATCG TATTAGAGAG TTTTTTATAT   
  
  
- AAAAAATTTA TATCACGCTA TAACTGACTG GATTATACCC AATAATGTCC TCTACCCCTC TATAGATGTA   
  
  
- ATTGATGAAC TTGTTGAATT TAAAAGTAAA ATTGAGCCAG TAATTCTTGT GATTCTTGAA ATATATAATC   
  
  
- ACTATAGAGT CCGCATTTTC TTTGATGAAA CTTAATTAGG TACGTTAGGA TGAGAAATAA AGGTAGGCAA   
  
  
- TTTTAAAAGG AAGTTTTGAG GGAAGACAAG TAATAATTAA GTTATAGCAT AATGTGAACA ACCTAATAAA   
  
  
- GACACTAAAC TAGCAGTAAA AGATATATAC TTATTGATGT TTTTAGTATC AGGCTCATGT AATTATAATT   
  
  
- ATTTTATCCT ACTCACTATT GGAGTATATC ACGCAAATAG GGATGGTGAT CCGACCTAAA GACGTATTTC   
  
  
- TTCTTTAACT TGTATACCTT TTTCTTTATT TCTCTTCGTT GAAGGAACTC ACCCTCTTCT TAGCATCTTT   
  
  
- CAATTTGAAT ACTTTTTCTC GTAATTGTTT TTCCTTTAAT ATTCCGAATG GCAACTTCGG AGTATATTAA   
  
  
- AACGTTCTCT GTCTGTTTCT TTCACAGAAA GTTTACAGTA TAAAGAGCTC GTACGTACTT TTTACATGAA   
  
  
- GCCGATGTTA TCATTATCAT ATATACAACC GAAGTATGTG AACTGGGGTT ACATACAGTT GCACTGATCA   
  
  
- CTGGTGTGTG TAGCAACAGG GACGGGCGGG GTGATGGGTT TGAGGGGCGT GCGTTTCTAT TTTTCGGGGG   
  
  
- GTTAAAAATA AACGGAGGGT TTTTCGGGTG GGCGCGAAAA AATAAACGGA AGAAAAAAGG ATGAATTTGG   
  
  
- TGGAGGAGTT GCTTGAAACG TTGTATGTAT GATCAGATAT TCGTTAAATC TCCCTTCCCT TCGTTGGATT   
  
  
- TGTCTCGCTC TAACCGCCCC ACACAAACTC CCGAACGCAA AACCACGTTA TCCTTACTTT CACGGGTAAA   
  
  
- GTTGGTGGTA GGTTTGAACG TTGAGGCCGT GGCCGTTTGG TGAAAGGAAA CTCTTATGAC GGGATGTATG   
  
  
- GCGGAAAGTT CAAGGGTTAT GGTGGATGCT TGGGTGGAGC CAAGAACTGG AGGTCGCGTC GGGATCGGGG   
  
  
- TGGAGGTGGG TTTTTGGCGG GAAACGGCGG AGGTTGGTGG TCGGACGGAG CCGACTAGAC CTGTTGAACC   
  
  
- TGCCCACCCT AAGTTAGGAT AGTCTCGACC CCGAATTACT GCTTAAATGG GGATTAAGGT TTGAGACCAG   
  
  
- GGTTTAATCA GGCAGACTAG GAGTGGAGGG GGTTGAGGGA CTAAAGGGTT GTAGAGTCGG CAAACTAGTA   
  
  
- GTGTTGGGTG GGGGTTATTT GGAGGGTAGT AGTCTAAAGT TGGAGACACT AAAGAGGATG TTAGTGTTGG   
  
  
- GGTTAAAACC CGGCAAGCTA GAAGTAGTGT TGGGCGTAGT CTTGTTGGGC TTGTTGTTAT TGATGCCGAA   
  
  
- ACTAAAGTAT CTGCTGGAGT AGTTTCGTCG TGTCAGGGAG CTTAGGTTGC TGAGAGTAGT TCAGGTGGAC   
  
  
- TATAACCGCG CCGAGTTAGT TGCCGAGTCT AGTGGGTGGC CGTTTGGCGA GGTCGCCCGG CGGATGATGA   
  
  
- AGTTCCTTCG GGAGTAGCGG CGGCGCCGGC CGGGCGGGGC GGGGCGAGCC GAAAGTAGCA TACTCCACCA   
  
  
- CGTCTGGTAG GCTCGGATGT TCCGCAAAAG TCCTTAAAGC GGTCAGAGAG AGAAGAGGTG AAAGCGGCGG   
  
  
- TTGGTCCGCT AAGAACTCCG CCACCTGTGC CGCCGGAAGT AGGTGTAGTA GCTAAAGCTG TAGCCTAAGC   
  
  
- CGCCGGTGAC CCGGAGCAAG GAGGCGCTTG ACCAGCTGTT TCGACTAGGA CGCTTTGAAT CAAGTCACCA   
  
  
- CAATGCTTAG TGGCGGTAGC AAGGGCTTCT TAAGCCCTAG CTTTCGTTCG ATCACTCTCT TTTAGACAGC   
  
  
- GTTAAACGGG CTCTAGAGTT GTAGTTGAAG GTGTAGCTAA TACAGAATAA AGTCTCGAAA CTTTAGAATA   
  
  
- GGAAGTTTAG ACAGTTTAAG TAGCTTTCCC TCTTTAACCG CGACGTAGAA AGCGGCCGGC ACAAGGTGGC   
  
  
- CGATCCCTCG CCTTAAAGCT TCAAATAGCG ACTAGAGGCT AGTTAGAGCG GCTCGAGGCA GCACCACCAC   
  
  
- CTGTCTCTAC AGCCCTAACT GTAGCCTTGC AGCAGCAAAT CGTACTTAAA GCAGCGGCCT TAACTTAAGA   
  
  
- TGTGGCCCTA CGACCTCAGC GAACTGCGTC GGTGGCGGCC CCCCGAGTAG CCGCCACACC TGACGCACTC   
  
  
- TGCCTAACTC TGCAAACAAG AGGTGGGTTC CTAGTACCGC CGCCATCTCC GCCGCCGCAG CCGCAGCCGG   
  
  
- CCCGCCTCCT GCCGCACCTC CCTCCGTAAG CGCCGCCGCC CCTAATCCCG CCACCCCAAA TCGGTCAAAC   
  
  
- GACTAAAGGT CCGGCTTACA AACAACTCCT CTCGAGTCCA ACCACCGAAG GTGCACCGTT TCGCGGTACC   
  
  
- TCTCTACTAC GAAATGACCG TACTGGCGGG TGAGCAGCGG TGTAGTCGAA CCTCCACAAT

+     DRE core

| Site Name | Organism | Position | Strand | Matrix score. | sequence | function |
| --- | --- | --- | --- | --- | --- | --- |
| DRE core | Arabidopsis thaliana | 3422 | - | 6 | GCCGAC |  |
| DRE core | Arabidopsis thaliana | 3428 | - | 6 | GCCGAC |  |

>HU05G02245.1   
+ -Up\_Stream \_Len000ATAATT TATCTTTCTT TAATTTCGTT TCATGTAAAA AATTTCAGTC TCATCTGCGA   
  
  
+ TTGAATCTCT TTTCAATTCG CATCAATAAT TCATCCAATT CAAATGTGCC TTTCTTAATT TCATTACATT   
  
  
+ GGGTTGGTAT AAATGTTAGC ACGATAGATT GGAATAGCAA GGCTCTAATT CAATTATGAC TTGAATGTAT   
  
  
+ GCATGCTTTT ATCATTAGTT AATTAACATT TTCTGTATTG AAATCAGCTC GAAAGAGAAC CATATGTAGT   
  
  
+ TGTACTTGCG ACGATATATA TGTTTAGTTT AGTTAGTTCT TACCTGTTTT GGAATTGTTG GGGAGATCTG   
  
  
+ AGGAAGGAGC CCGCTTCAAT CAAAGTTCAT GGCAATAAGC CAAAGTGAGG TTCCATCCTA AAACCAATTG   
  
  
+ GCAATAGGAA GAGTAGCCCA CTTAACTTGT ATACTATGAC TCTTCTTTTC AAACTCCCAA TGTGGGACTT   
  
  
+ CTTTACTCAT GTGTGATATT CCCAACAGGA ATGATCAGTT CCACATGTTT TCATCTCTTT GGACTCGAAC   
  
  
+ TTCGTGTAAT TGTATATGCC ATGGCACAAA TAGTTTTGAT GGTGCCACCA AGTTGCTCAA TCAATGTAAT   
  
  
+ ACTGCTACTA TCTAACTTGC CTTGGTGGAC ATCAATAAGA TCACGAGATG AGGAGAACTG AATCTTGGCC   
  
  
+ AACCACGTCG TTTGACTTCT CTTACCAATA AAGATTGAAT AGACAGTGTT TCTCTCCCCC TTCCATTCTT   
  
  
+ CTTTTCTAGT GATGTCGAAA AAGACTTTAT ACATGTCCAG CAACGCTAGC CACAAAATAT TCCATTTCTG   
  
  
+ TAATCCACTC ATGTCAATTA AACTTGTATC ATCAAATGGT AGTTTCCGTA CTAATCACTA CATCTAGATA   
  
  
+ TTCCTTGATA AATTTAATTT GACTTTAAAG GCTCGATCTT TTTAAGAAAA GTTTTGTTGC TTACAACCTG   
  
  
+ TAAAGCCTAG AGAACAAAGC CAAAGAAGAA ATTTAAGTTC TATATATAGC ATAATCTCTC AAAAAATATA   
  
  
+ TTTTTTAAAT ATAGTGCGAT ATTGACTGAC CTAATATGGG TTATTACAGG AGATGGGGAG ATATCTACAT   
  
  
+ TAACTACTTG AACAACTTAA ATTTTCATTT TAACTCGGTC ATTAAGAACA CTAAGAACTT TATATATTAG   
  
  
+ TGATATCTCA GGCGTAAAAG AAACTACTTT GAATTAATCC ATGCAATCCT ACTCTTTATT TCCATCCGTT   
  
  
+ AAAATTTTCC TTCAAAACTC CCTTCTGTTC ATTATTAATT CAATATCGTA TTACACTTGT TGGATTATTT   
  
  
+ CTGTGATTTG ATCGTCATTT TCTATATATG AATAACTACA AAAATCATAG TCCGAGTACA TTAATATTAA   
  
  
+ TAAAATAGGA TGAGTGATAA CCTCATATAG TGCGTTTATC CCTACCACTA GGCTGGATTT CTGCATAAAG   
  
  
+ AAGAAATTGA ACATATGGAA AAAGAAATAA AGAGAAGCAA CTTCCTTGAG TGGGAGAAGA ATCGTAGAAA   
  
  
+ GTTAAACTTA TGAAAAAGAG CATTAACAAA AAGGAAATTA TAAGGCTTAC CGTTGAAGCC TCATATAATT   
  
  
+ TTGCAAGAGA CAGACAAAGA AAGTGTCTTT CAAATGTCAT ATTTCTCGAG CATGCATGAA AAATGTACTT   
  
  
+ CGGCTACAAT AGTAATAGTA TATATGTTGG CTTCATACAC TTGACCCCAA TGTATGTCAA CGTGACTAGT   
  
  
+ GACCACACAC ATCGTTGTCC CTGCCCGCCC CACTACCCAA ACTCCCCGCA CGCAAAGATA AAAAGCCCCC   
  
  
+ CAATTTTTAT TTGCCTCCCA AAAAGCCCAC CCGCGCTTTT TTATTTGCCT TCTTTTTTCC TACTTAAACC   
  
  
+ ACCTCCTCAA CGAACTTTGC AACATACATA CTAGTCTATA AGCAATTTAG AGGGAAGGGA AGCAACCTAA   
  
  
+ ACAGAGCGAG ATTGGCGGGG TGTGTTTGAG GGCTTGCGTT TTGGTGCAAT AGGAATGAAA GTGCCCATTT   
  
  
+ CAACCACCAT CCAAACTTGC AACTCCGGCA CCGGCAAACC ACTTTCCTTT GAGAATACTG CCCTACATAC   
  
  
+ CGCCTTTCAA GTTCCCAATA CCACCTACGA ACCCACCTCG GTTCTTGACC TCCAGCGCAG CCCTAGCCCC   
  
  
+ ACCTCCACCC AAAAACCGCC CTTTGCCGCC TCCAACCACC AGCCTGCCTC GGCTGATCTG GACAACTTGG   
  
  
+ ACGGGTGGGA TTCAATCCTA TCAGAGCTGG GGCTTAATGA CGAATTTACC CCTAATTCCA AACTCTGGTC   
  
  
+ CCAAATTAGT CCGTCTGATC CTCACCTCCC CCAACTCCCT GATTTCCCAA CATCTCAGCC GTTTGATCAT   
  
  
+ CACAACCCAC CCCCAATAAA CCTCCCATCA TCAGATTTCA ACCTCTGTGA TTTCTCCTAC AATCACAACC   
  
  
+ CCAATTTTGG GCCGTTCGAT CTTCATCACA ACCCGCATCA GAACAACCCG AACAACAATA ACTACGGCTT   
  
  
+ TGATTTCATA GACGACCTCA TCAAAGCAGC ACAGTCCCTC GAATCCAACG ACTCTCATCA AGTCCACCTG   
  
  
+ ATATTGGCGC GGCTCAATCA ACGGCTCAGA TCACCCACCG GCAAACCGCT CCAGCGGGCC GCCTACTACT   
  
  
+ TCAAGGAAGC CCTCATCGCC GCCGCGGCCG GCCCGCCCCG CCCCGCTCGG CTTTCATCGT ATGAGGTGGT   
  
  
+ GCAGACCATC CGAGCCTACA AGGCGTTTTC AGGAATTTCG CCAGTCTCTC TCTTCTCCAC TTTCGCCGCC   
  
  
+ AACCAGGCGA TTCTTGAGGC GGTGGACACG GCGGCCTTCA TCCACATCAT CGATTTCGAC ATCGGATTCG   
  
  
+ GCGGCCACTG GGCCTCGTTC CTCCGCGAAC TGGTCGACAA AGCTGATCCT GCGAAACTTA GTTCAGTGGT   
  
  
+ GTTACGAATC ACCGCCATCG TTCCCGAAGA ATTCGGGATC GAAAGCAAGC TAGTGAGAGA AAATCTGTCG   
  
  
+ CAATTTGCCC GAGATCTCAA CATCAACTTC CACATCGATT ATGTCTTATT TCAGAGCTTT GAAATCTTAT   
  
  
+ CCTTCAAATC TGTCAAATTC ATCGAAAGGG AGAAATTGGC GCTGCATCTT TCGCCGGCCG TGTTCCACCG   
  
  
+ GCTAGGGAGC GGAATTTCGA AGTTTATCGC TGATCTCCGA TCAATCTCGC CGAGCTCCGT CGTGGTGGTG   
  
  
+ GACAGAGATG TCGGGATTGA CATCGGAACG TCGTCGTTTA GCATGAATTT CGTCGCCGGA ATTGAATTCT   
  
  
+ ACACCGGGAT GCTGGAGTCG CTTGACGCAG CCACCGCCGG GGGGCTCATC GGCGGTGTGG ACTGCGTGAG   
  
  
+ ACGGATTGAG ACGTTTGTTC TCCACCCAAG GATCATGGCG GCGGTAGAGG CGGCGGCGTC GGCGTCGGCC   
  
  
+ GGGCGGAGGA CGGCGTGGAG GGAGGCATTC GCGGCGGCGG GGATTAGGGC GGTGGGGTTT AGCCAGTTTG   
  
  
+ CTGATTTCCA GGCCGAATGT TTGTTGAGGA GAGCTCAGGT TGGTGGCTTC CACGTGGCAA AGCGCCATGG   
  
  
+ AGAGATGATG CTTTACTGGC ATGACCGCCC ACTCGTCGCC ACATCAGCTT GGAGGTGTTA   

- -Up\_Stream \_Len000TATTAA ATAGAAAGAA ATTAAAGCAA AGTACATTTT TTAAAGTCAG AGTAGACGCT   
  
  
- AACTTAGAGA AAAGTTAAGC GTAGTTATTA AGTAGGTTAA GTTTACACGG AAAGAATTAA AGTAATGTAA   
  
  
- CCCAACCATA TTTACAATCG TGCTATCTAA CCTTATCGTT CCGAGATTAA GTTAATACTG AACTTACATA   
  
  
- CGTACGAAAA TAGTAATCAA TTAATTGTAA AAGACATAAC TTTAGTCGAG CTTTCTCTTG GTATACATCA   
  
  
- ACATGAACGC TGCTATATAT ACAAATCAAA TCAATCAAGA ATGGACAAAA CCTTAACAAC CCCTCTAGAC   
  
  
- TCCTTCCTCG GGCGAAGTTA GTTTCAAGTA CCGTTATTCG GTTTCACTCC AAGGTAGGAT TTTGGTTAAC   
  
  
- CGTTATCCTT CTCATCGGGT GAATTGAACA TATGATACTG AGAAGAAAAG TTTGAGGGTT ACACCCTGAA   
  
  
- GAAATGAGTA CACACTATAA GGGTTGTCCT TACTAGTCAA GGTGTACAAA AGTAGAGAAA CCTGAGCTTG   
  
  
- AAGCACATTA ACATATACGG TACCGTGTTT ATCAAAACTA CCACGGTGGT TCAACGAGTT AGTTACATTA   
  
  
- TGACGATGAT AGATTGAACG GAACCACCTG TAGTTATTCT AGTGCTCTAC TCCTCTTGAC TTAGAACCGG   
  
  
- TTGGTGCAGC AAACTGAAGA GAATGGTTAT TTCTAACTTA TCTGTCACAA AGAGAGGGGG AAGGTAAGAA   
  
  
- GAAAAGATCA CTACAGCTTT TTCTGAAATA TGTACAGGTC GTTGCGATCG GTGTTTTATA AGGTAAAGAC   
  
  
- ATTAGGTGAG TACAGTTAAT TTGAACATAG TAGTTTACCA TCAAAGGCAT GATTAGTGAT GTAGATCTAT   
  
  
- AAGGAACTAT TTAAATTAAA CTGAAATTTC CGAGCTAGAA AAATTCTTTT CAAAACAACG AATGTTGGAC   
  
  
- ATTTCGGATC TCTTGTTTCG GTTTCTTCTT TAAATTCAAG ATATATATCG TATTAGAGAG TTTTTTATAT   
  
  
- AAAAAATTTA TATCACGCTA TAACTGACTG GATTATACCC AATAATGTCC TCTACCCCTC TATAGATGTA   
  
  
- ATTGATGAAC TTGTTGAATT TAAAAGTAAA ATTGAGCCAG TAATTCTTGT GATTCTTGAA ATATATAATC   
  
  
- ACTATAGAGT CCGCATTTTC TTTGATGAAA CTTAATTAGG TACGTTAGGA TGAGAAATAA AGGTAGGCAA   
  
  
- TTTTAAAAGG AAGTTTTGAG GGAAGACAAG TAATAATTAA GTTATAGCAT AATGTGAACA ACCTAATAAA   
  
  
- GACACTAAAC TAGCAGTAAA AGATATATAC TTATTGATGT TTTTAGTATC AGGCTCATGT AATTATAATT   
  
  
- ATTTTATCCT ACTCACTATT GGAGTATATC ACGCAAATAG GGATGGTGAT CCGACCTAAA GACGTATTTC   
  
  
- TTCTTTAACT TGTATACCTT TTTCTTTATT TCTCTTCGTT GAAGGAACTC ACCCTCTTCT TAGCATCTTT   
  
  
- CAATTTGAAT ACTTTTTCTC GTAATTGTTT TTCCTTTAAT ATTCCGAATG GCAACTTCGG AGTATATTAA   
  
  
- AACGTTCTCT GTCTGTTTCT TTCACAGAAA GTTTACAGTA TAAAGAGCTC GTACGTACTT TTTACATGAA   
  
  
- GCCGATGTTA TCATTATCAT ATATACAACC GAAGTATGTG AACTGGGGTT ACATACAGTT GCACTGATCA   
  
  
- CTGGTGTGTG TAGCAACAGG GACGGGCGGG GTGATGGGTT TGAGGGGCGT GCGTTTCTAT TTTTCGGGGG   
  
  
- GTTAAAAATA AACGGAGGGT TTTTCGGGTG GGCGCGAAAA AATAAACGGA AGAAAAAAGG ATGAATTTGG   
  
  
- TGGAGGAGTT GCTTGAAACG TTGTATGTAT GATCAGATAT TCGTTAAATC TCCCTTCCCT TCGTTGGATT   
  
  
- TGTCTCGCTC TAACCGCCCC ACACAAACTC CCGAACGCAA AACCACGTTA TCCTTACTTT CACGGGTAAA   
  
  
- GTTGGTGGTA GGTTTGAACG TTGAGGCCGT GGCCGTTTGG TGAAAGGAAA CTCTTATGAC GGGATGTATG   
  
  
- GCGGAAAGTT CAAGGGTTAT GGTGGATGCT TGGGTGGAGC CAAGAACTGG AGGTCGCGTC GGGATCGGGG   
  
  
- TGGAGGTGGG TTTTTGGCGG GAAACGGCGG AGGTTGGTGG TCGGACGGAG CCGACTAGAC CTGTTGAACC   
  
  
- TGCCCACCCT AAGTTAGGAT AGTCTCGACC CCGAATTACT GCTTAAATGG GGATTAAGGT TTGAGACCAG   
  
  
- GGTTTAATCA GGCAGACTAG GAGTGGAGGG GGTTGAGGGA CTAAAGGGTT GTAGAGTCGG CAAACTAGTA   
  
  
- GTGTTGGGTG GGGGTTATTT GGAGGGTAGT AGTCTAAAGT TGGAGACACT AAAGAGGATG TTAGTGTTGG   
  
  
- GGTTAAAACC CGGCAAGCTA GAAGTAGTGT TGGGCGTAGT CTTGTTGGGC TTGTTGTTAT TGATGCCGAA   
  
  
- ACTAAAGTAT CTGCTGGAGT AGTTTCGTCG TGTCAGGGAG CTTAGGTTGC TGAGAGTAGT TCAGGTGGAC   
  
  
- TATAACCGCG CCGAGTTAGT TGCCGAGTCT AGTGGGTGGC CGTTTGGCGA GGTCGCCCGG CGGATGATGA   
  
  
- AGTTCCTTCG GGAGTAGCGG CGGCGCCGGC CGGGCGGGGC GGGGCGAGCC GAAAGTAGCA TACTCCACCA   
  
  
- CGTCTGGTAG GCTCGGATGT TCCGCAAAAG TCCTTAAAGC GGTCAGAGAG AGAAGAGGTG AAAGCGGCGG   
  
  
- TTGGTCCGCT AAGAACTCCG CCACCTGTGC CGCCGGAAGT AGGTGTAGTA GCTAAAGCTG TAGCCTAAGC   
  
  
- CGCCGGTGAC CCGGAGCAAG GAGGCGCTTG ACCAGCTGTT TCGACTAGGA CGCTTTGAAT CAAGTCACCA   
  
  
- CAATGCTTAG TGGCGGTAGC AAGGGCTTCT TAAGCCCTAG CTTTCGTTCG ATCACTCTCT TTTAGACAGC   
  
  
- GTTAAACGGG CTCTAGAGTT GTAGTTGAAG GTGTAGCTAA TACAGAATAA AGTCTCGAAA CTTTAGAATA   
  
  
- GGAAGTTTAG ACAGTTTAAG TAGCTTTCCC TCTTTAACCG CGACGTAGAA AGCGGCCGGC ACAAGGTGGC   
  
  
- CGATCCCTCG CCTTAAAGCT TCAAATAGCG ACTAGAGGCT AGTTAGAGCG GCTCGAGGCA GCACCACCAC   
  
  
- CTGTCTCTAC AGCCCTAACT GTAGCCTTGC AGCAGCAAAT CGTACTTAAA GCAGCGGCCT TAACTTAAGA   
  
  
- TGTGGCCCTA CGACCTCAGC GAACTGCGTC GGTGGCGGCC CCCCGAGTAG CCGCCACACC TGACGCACTC   
  
  
- TGCCTAACTC TGCAAACAAG AGGTGGGTTC CTAGTACCGC CGCCATCTCC GCCGCCGCAG CCGCAGCCGG   
  
  
- CCCGCCTCCT GCCGCACCTC CCTCCGTAAG CGCCGCCGCC CCTAATCCCG CCACCCCAAA TCGGTCAAAC   
  
  
- GACTAAAGGT CCGGCTTACA AACAACTCCT CTCGAGTCCA ACCACCGAAG GTGCACCGTT TCGCGGTACC   
  
  
- TCTCTACTAC GAAATGACCG TACTGGCGGG TGAGCAGCGG TGTAGTCGAA CCTCCACAAT

+     E2Fb

| Site Name | Organism | Position | Strand | Matrix score. | sequence | function |
| --- | --- | --- | --- | --- | --- | --- |
| E2Fb | Nicotiana tabacum | 2196 | + | 8 | TTTGCCGC |  |

>HU05G02245.1   
+ -Up\_Stream \_Len000ATAATT TATCTTTCTT TAATTTCGTT TCATGTAAAA AATTTCAGTC TCATCTGCGA   
  
  
+ TTGAATCTCT TTTCAATTCG CATCAATAAT TCATCCAATT CAAATGTGCC TTTCTTAATT TCATTACATT   
  
  
+ GGGTTGGTAT AAATGTTAGC ACGATAGATT GGAATAGCAA GGCTCTAATT CAATTATGAC TTGAATGTAT   
  
  
+ GCATGCTTTT ATCATTAGTT AATTAACATT TTCTGTATTG AAATCAGCTC GAAAGAGAAC CATATGTAGT   
  
  
+ TGTACTTGCG ACGATATATA TGTTTAGTTT AGTTAGTTCT TACCTGTTTT GGAATTGTTG GGGAGATCTG   
  
  
+ AGGAAGGAGC CCGCTTCAAT CAAAGTTCAT GGCAATAAGC CAAAGTGAGG TTCCATCCTA AAACCAATTG   
  
  
+ GCAATAGGAA GAGTAGCCCA CTTAACTTGT ATACTATGAC TCTTCTTTTC AAACTCCCAA TGTGGGACTT   
  
  
+ CTTTACTCAT GTGTGATATT CCCAACAGGA ATGATCAGTT CCACATGTTT TCATCTCTTT GGACTCGAAC   
  
  
+ TTCGTGTAAT TGTATATGCC ATGGCACAAA TAGTTTTGAT GGTGCCACCA AGTTGCTCAA TCAATGTAAT   
  
  
+ ACTGCTACTA TCTAACTTGC CTTGGTGGAC ATCAATAAGA TCACGAGATG AGGAGAACTG AATCTTGGCC   
  
  
+ AACCACGTCG TTTGACTTCT CTTACCAATA AAGATTGAAT AGACAGTGTT TCTCTCCCCC TTCCATTCTT   
  
  
+ CTTTTCTAGT GATGTCGAAA AAGACTTTAT ACATGTCCAG CAACGCTAGC CACAAAATAT TCCATTTCTG   
  
  
+ TAATCCACTC ATGTCAATTA AACTTGTATC ATCAAATGGT AGTTTCCGTA CTAATCACTA CATCTAGATA   
  
  
+ TTCCTTGATA AATTTAATTT GACTTTAAAG GCTCGATCTT TTTAAGAAAA GTTTTGTTGC TTACAACCTG   
  
  
+ TAAAGCCTAG AGAACAAAGC CAAAGAAGAA ATTTAAGTTC TATATATAGC ATAATCTCTC AAAAAATATA   
  
  
+ TTTTTTAAAT ATAGTGCGAT ATTGACTGAC CTAATATGGG TTATTACAGG AGATGGGGAG ATATCTACAT   
  
  
+ TAACTACTTG AACAACTTAA ATTTTCATTT TAACTCGGTC ATTAAGAACA CTAAGAACTT TATATATTAG   
  
  
+ TGATATCTCA GGCGTAAAAG AAACTACTTT GAATTAATCC ATGCAATCCT ACTCTTTATT TCCATCCGTT   
  
  
+ AAAATTTTCC TTCAAAACTC CCTTCTGTTC ATTATTAATT CAATATCGTA TTACACTTGT TGGATTATTT   
  
  
+ CTGTGATTTG ATCGTCATTT TCTATATATG AATAACTACA AAAATCATAG TCCGAGTACA TTAATATTAA   
  
  
+ TAAAATAGGA TGAGTGATAA CCTCATATAG TGCGTTTATC CCTACCACTA GGCTGGATTT CTGCATAAAG   
  
  
+ AAGAAATTGA ACATATGGAA AAAGAAATAA AGAGAAGCAA CTTCCTTGAG TGGGAGAAGA ATCGTAGAAA   
  
  
+ GTTAAACTTA TGAAAAAGAG CATTAACAAA AAGGAAATTA TAAGGCTTAC CGTTGAAGCC TCATATAATT   
  
  
+ TTGCAAGAGA CAGACAAAGA AAGTGTCTTT CAAATGTCAT ATTTCTCGAG CATGCATGAA AAATGTACTT   
  
  
+ CGGCTACAAT AGTAATAGTA TATATGTTGG CTTCATACAC TTGACCCCAA TGTATGTCAA CGTGACTAGT   
  
  
+ GACCACACAC ATCGTTGTCC CTGCCCGCCC CACTACCCAA ACTCCCCGCA CGCAAAGATA AAAAGCCCCC   
  
  
+ CAATTTTTAT TTGCCTCCCA AAAAGCCCAC CCGCGCTTTT TTATTTGCCT TCTTTTTTCC TACTTAAACC   
  
  
+ ACCTCCTCAA CGAACTTTGC AACATACATA CTAGTCTATA AGCAATTTAG AGGGAAGGGA AGCAACCTAA   
  
  
+ ACAGAGCGAG ATTGGCGGGG TGTGTTTGAG GGCTTGCGTT TTGGTGCAAT AGGAATGAAA GTGCCCATTT   
  
  
+ CAACCACCAT CCAAACTTGC AACTCCGGCA CCGGCAAACC ACTTTCCTTT GAGAATACTG CCCTACATAC   
  
  
+ CGCCTTTCAA GTTCCCAATA CCACCTACGA ACCCACCTCG GTTCTTGACC TCCAGCGCAG CCCTAGCCCC   
  
  
+ ACCTCCACCC AAAAACCGCC CTTTGCCGCC TCCAACCACC AGCCTGCCTC GGCTGATCTG GACAACTTGG   
  
  
+ ACGGGTGGGA TTCAATCCTA TCAGAGCTGG GGCTTAATGA CGAATTTACC CCTAATTCCA AACTCTGGTC   
  
  
+ CCAAATTAGT CCGTCTGATC CTCACCTCCC CCAACTCCCT GATTTCCCAA CATCTCAGCC GTTTGATCAT   
  
  
+ CACAACCCAC CCCCAATAAA CCTCCCATCA TCAGATTTCA ACCTCTGTGA TTTCTCCTAC AATCACAACC   
  
  
+ CCAATTTTGG GCCGTTCGAT CTTCATCACA ACCCGCATCA GAACAACCCG AACAACAATA ACTACGGCTT   
  
  
+ TGATTTCATA GACGACCTCA TCAAAGCAGC ACAGTCCCTC GAATCCAACG ACTCTCATCA AGTCCACCTG   
  
  
+ ATATTGGCGC GGCTCAATCA ACGGCTCAGA TCACCCACCG GCAAACCGCT CCAGCGGGCC GCCTACTACT   
  
  
+ TCAAGGAAGC CCTCATCGCC GCCGCGGCCG GCCCGCCCCG CCCCGCTCGG CTTTCATCGT ATGAGGTGGT   
  
  
+ GCAGACCATC CGAGCCTACA AGGCGTTTTC AGGAATTTCG CCAGTCTCTC TCTTCTCCAC TTTCGCCGCC   
  
  
+ AACCAGGCGA TTCTTGAGGC GGTGGACACG GCGGCCTTCA TCCACATCAT CGATTTCGAC ATCGGATTCG   
  
  
+ GCGGCCACTG GGCCTCGTTC CTCCGCGAAC TGGTCGACAA AGCTGATCCT GCGAAACTTA GTTCAGTGGT   
  
  
+ GTTACGAATC ACCGCCATCG TTCCCGAAGA ATTCGGGATC GAAAGCAAGC TAGTGAGAGA AAATCTGTCG   
  
  
+ CAATTTGCCC GAGATCTCAA CATCAACTTC CACATCGATT ATGTCTTATT TCAGAGCTTT GAAATCTTAT   
  
  
+ CCTTCAAATC TGTCAAATTC ATCGAAAGGG AGAAATTGGC GCTGCATCTT TCGCCGGCCG TGTTCCACCG   
  
  
+ GCTAGGGAGC GGAATTTCGA AGTTTATCGC TGATCTCCGA TCAATCTCGC CGAGCTCCGT CGTGGTGGTG   
  
  
+ GACAGAGATG TCGGGATTGA CATCGGAACG TCGTCGTTTA GCATGAATTT CGTCGCCGGA ATTGAATTCT   
  
  
+ ACACCGGGAT GCTGGAGTCG CTTGACGCAG CCACCGCCGG GGGGCTCATC GGCGGTGTGG ACTGCGTGAG   
  
  
+ ACGGATTGAG ACGTTTGTTC TCCACCCAAG GATCATGGCG GCGGTAGAGG CGGCGGCGTC GGCGTCGGCC   
  
  
+ GGGCGGAGGA CGGCGTGGAG GGAGGCATTC GCGGCGGCGG GGATTAGGGC GGTGGGGTTT AGCCAGTTTG   
  
  
+ CTGATTTCCA GGCCGAATGT TTGTTGAGGA GAGCTCAGGT TGGTGGCTTC CACGTGGCAA AGCGCCATGG   
  
  
+ AGAGATGATG CTTTACTGGC ATGACCGCCC ACTCGTCGCC ACATCAGCTT GGAGGTGTTA   

- -Up\_Stream \_Len000TATTAA ATAGAAAGAA ATTAAAGCAA AGTACATTTT TTAAAGTCAG AGTAGACGCT   
  
  
- AACTTAGAGA AAAGTTAAGC GTAGTTATTA AGTAGGTTAA GTTTACACGG AAAGAATTAA AGTAATGTAA   
  
  
- CCCAACCATA TTTACAATCG TGCTATCTAA CCTTATCGTT CCGAGATTAA GTTAATACTG AACTTACATA   
  
  
- CGTACGAAAA TAGTAATCAA TTAATTGTAA AAGACATAAC TTTAGTCGAG CTTTCTCTTG GTATACATCA   
  
  
- ACATGAACGC TGCTATATAT ACAAATCAAA TCAATCAAGA ATGGACAAAA CCTTAACAAC CCCTCTAGAC   
  
  
- TCCTTCCTCG GGCGAAGTTA GTTTCAAGTA CCGTTATTCG GTTTCACTCC AAGGTAGGAT TTTGGTTAAC   
  
  
- CGTTATCCTT CTCATCGGGT GAATTGAACA TATGATACTG AGAAGAAAAG TTTGAGGGTT ACACCCTGAA   
  
  
- GAAATGAGTA CACACTATAA GGGTTGTCCT TACTAGTCAA GGTGTACAAA AGTAGAGAAA CCTGAGCTTG   
  
  
- AAGCACATTA ACATATACGG TACCGTGTTT ATCAAAACTA CCACGGTGGT TCAACGAGTT AGTTACATTA   
  
  
- TGACGATGAT AGATTGAACG GAACCACCTG TAGTTATTCT AGTGCTCTAC TCCTCTTGAC TTAGAACCGG   
  
  
- TTGGTGCAGC AAACTGAAGA GAATGGTTAT TTCTAACTTA TCTGTCACAA AGAGAGGGGG AAGGTAAGAA   
  
  
- GAAAAGATCA CTACAGCTTT TTCTGAAATA TGTACAGGTC GTTGCGATCG GTGTTTTATA AGGTAAAGAC   
  
  
- ATTAGGTGAG TACAGTTAAT TTGAACATAG TAGTTTACCA TCAAAGGCAT GATTAGTGAT GTAGATCTAT   
  
  
- AAGGAACTAT TTAAATTAAA CTGAAATTTC CGAGCTAGAA AAATTCTTTT CAAAACAACG AATGTTGGAC   
  
  
- ATTTCGGATC TCTTGTTTCG GTTTCTTCTT TAAATTCAAG ATATATATCG TATTAGAGAG TTTTTTATAT   
  
  
- AAAAAATTTA TATCACGCTA TAACTGACTG GATTATACCC AATAATGTCC TCTACCCCTC TATAGATGTA   
  
  
- ATTGATGAAC TTGTTGAATT TAAAAGTAAA ATTGAGCCAG TAATTCTTGT GATTCTTGAA ATATATAATC   
  
  
- ACTATAGAGT CCGCATTTTC TTTGATGAAA CTTAATTAGG TACGTTAGGA TGAGAAATAA AGGTAGGCAA   
  
  
- TTTTAAAAGG AAGTTTTGAG GGAAGACAAG TAATAATTAA GTTATAGCAT AATGTGAACA ACCTAATAAA   
  
  
- GACACTAAAC TAGCAGTAAA AGATATATAC TTATTGATGT TTTTAGTATC AGGCTCATGT AATTATAATT   
  
  
- ATTTTATCCT ACTCACTATT GGAGTATATC ACGCAAATAG GGATGGTGAT CCGACCTAAA GACGTATTTC   
  
  
- TTCTTTAACT TGTATACCTT TTTCTTTATT TCTCTTCGTT GAAGGAACTC ACCCTCTTCT TAGCATCTTT   
  
  
- CAATTTGAAT ACTTTTTCTC GTAATTGTTT TTCCTTTAAT ATTCCGAATG GCAACTTCGG AGTATATTAA   
  
  
- AACGTTCTCT GTCTGTTTCT TTCACAGAAA GTTTACAGTA TAAAGAGCTC GTACGTACTT TTTACATGAA   
  
  
- GCCGATGTTA TCATTATCAT ATATACAACC GAAGTATGTG AACTGGGGTT ACATACAGTT GCACTGATCA   
  
  
- CTGGTGTGTG TAGCAACAGG GACGGGCGGG GTGATGGGTT TGAGGGGCGT GCGTTTCTAT TTTTCGGGGG   
  
  
- GTTAAAAATA AACGGAGGGT TTTTCGGGTG GGCGCGAAAA AATAAACGGA AGAAAAAAGG ATGAATTTGG   
  
  
- TGGAGGAGTT GCTTGAAACG TTGTATGTAT GATCAGATAT TCGTTAAATC TCCCTTCCCT TCGTTGGATT   
  
  
- TGTCTCGCTC TAACCGCCCC ACACAAACTC CCGAACGCAA AACCACGTTA TCCTTACTTT CACGGGTAAA   
  
  
- GTTGGTGGTA GGTTTGAACG TTGAGGCCGT GGCCGTTTGG TGAAAGGAAA CTCTTATGAC GGGATGTATG   
  
  
- GCGGAAAGTT CAAGGGTTAT GGTGGATGCT TGGGTGGAGC CAAGAACTGG AGGTCGCGTC GGGATCGGGG   
  
  
- TGGAGGTGGG TTTTTGGCGG GAAACGGCGG AGGTTGGTGG TCGGACGGAG CCGACTAGAC CTGTTGAACC   
  
  
- TGCCCACCCT AAGTTAGGAT AGTCTCGACC CCGAATTACT GCTTAAATGG GGATTAAGGT TTGAGACCAG   
  
  
- GGTTTAATCA GGCAGACTAG GAGTGGAGGG GGTTGAGGGA CTAAAGGGTT GTAGAGTCGG CAAACTAGTA   
  
  
- GTGTTGGGTG GGGGTTATTT GGAGGGTAGT AGTCTAAAGT TGGAGACACT AAAGAGGATG TTAGTGTTGG   
  
  
- GGTTAAAACC CGGCAAGCTA GAAGTAGTGT TGGGCGTAGT CTTGTTGGGC TTGTTGTTAT TGATGCCGAA   
  
  
- ACTAAAGTAT CTGCTGGAGT AGTTTCGTCG TGTCAGGGAG CTTAGGTTGC TGAGAGTAGT TCAGGTGGAC   
  
  
- TATAACCGCG CCGAGTTAGT TGCCGAGTCT AGTGGGTGGC CGTTTGGCGA GGTCGCCCGG CGGATGATGA   
  
  
- AGTTCCTTCG GGAGTAGCGG CGGCGCCGGC CGGGCGGGGC GGGGCGAGCC GAAAGTAGCA TACTCCACCA   
  
  
- CGTCTGGTAG GCTCGGATGT TCCGCAAAAG TCCTTAAAGC GGTCAGAGAG AGAAGAGGTG AAAGCGGCGG   
  
  
- TTGGTCCGCT AAGAACTCCG CCACCTGTGC CGCCGGAAGT AGGTGTAGTA GCTAAAGCTG TAGCCTAAGC   
  
  
- CGCCGGTGAC CCGGAGCAAG GAGGCGCTTG ACCAGCTGTT TCGACTAGGA CGCTTTGAAT CAAGTCACCA   
  
  
- CAATGCTTAG TGGCGGTAGC AAGGGCTTCT TAAGCCCTAG CTTTCGTTCG ATCACTCTCT TTTAGACAGC   
  
  
- GTTAAACGGG CTCTAGAGTT GTAGTTGAAG GTGTAGCTAA TACAGAATAA AGTCTCGAAA CTTTAGAATA   
  
  
- GGAAGTTTAG ACAGTTTAAG TAGCTTTCCC TCTTTAACCG CGACGTAGAA AGCGGCCGGC ACAAGGTGGC   
  
  
- CGATCCCTCG CCTTAAAGCT TCAAATAGCG ACTAGAGGCT AGTTAGAGCG GCTCGAGGCA GCACCACCAC   
  
  
- CTGTCTCTAC AGCCCTAACT GTAGCCTTGC AGCAGCAAAT CGTACTTAAA GCAGCGGCCT TAACTTAAGA   
  
  
- TGTGGCCCTA CGACCTCAGC GAACTGCGTC GGTGGCGGCC CCCCGAGTAG CCGCCACACC TGACGCACTC   
  
  
- TGCCTAACTC TGCAAACAAG AGGTGGGTTC CTAGTACCGC CGCCATCTCC GCCGCCGCAG CCGCAGCCGG   
  
  
- CCCGCCTCCT GCCGCACCTC CCTCCGTAAG CGCCGCCGCC CCTAATCCCG CCACCCCAAA TCGGTCAAAC   
  
  
- GACTAAAGGT CCGGCTTACA AACAACTCCT CTCGAGTCCA ACCACCGAAG GTGCACCGTT TCGCGGTACC   
  
  
- TCTCTACTAC GAAATGACCG TACTGGCGGG TGAGCAGCGG TGTAGTCGAA CCTCCACAAT

+     ERE

| Site Name | Organism | Position | Strand | Matrix score. | sequence | function |
| --- | --- | --- | --- | --- | --- | --- |
| ERE | Nicotiana glutinos | 2527 | + | 8 | ATTTCATA |  |

>HU05G02245.1   
+ -Up\_Stream \_Len000ATAATT TATCTTTCTT TAATTTCGTT TCATGTAAAA AATTTCAGTC TCATCTGCGA   
  
  
+ TTGAATCTCT TTTCAATTCG CATCAATAAT TCATCCAATT CAAATGTGCC TTTCTTAATT TCATTACATT   
  
  
+ GGGTTGGTAT AAATGTTAGC ACGATAGATT GGAATAGCAA GGCTCTAATT CAATTATGAC TTGAATGTAT   
  
  
+ GCATGCTTTT ATCATTAGTT AATTAACATT TTCTGTATTG AAATCAGCTC GAAAGAGAAC CATATGTAGT   
  
  
+ TGTACTTGCG ACGATATATA TGTTTAGTTT AGTTAGTTCT TACCTGTTTT GGAATTGTTG GGGAGATCTG   
  
  
+ AGGAAGGAGC CCGCTTCAAT CAAAGTTCAT GGCAATAAGC CAAAGTGAGG TTCCATCCTA AAACCAATTG   
  
  
+ GCAATAGGAA GAGTAGCCCA CTTAACTTGT ATACTATGAC TCTTCTTTTC AAACTCCCAA TGTGGGACTT   
  
  
+ CTTTACTCAT GTGTGATATT CCCAACAGGA ATGATCAGTT CCACATGTTT TCATCTCTTT GGACTCGAAC   
  
  
+ TTCGTGTAAT TGTATATGCC ATGGCACAAA TAGTTTTGAT GGTGCCACCA AGTTGCTCAA TCAATGTAAT   
  
  
+ ACTGCTACTA TCTAACTTGC CTTGGTGGAC ATCAATAAGA TCACGAGATG AGGAGAACTG AATCTTGGCC   
  
  
+ AACCACGTCG TTTGACTTCT CTTACCAATA AAGATTGAAT AGACAGTGTT TCTCTCCCCC TTCCATTCTT   
  
  
+ CTTTTCTAGT GATGTCGAAA AAGACTTTAT ACATGTCCAG CAACGCTAGC CACAAAATAT TCCATTTCTG   
  
  
+ TAATCCACTC ATGTCAATTA AACTTGTATC ATCAAATGGT AGTTTCCGTA CTAATCACTA CATCTAGATA   
  
  
+ TTCCTTGATA AATTTAATTT GACTTTAAAG GCTCGATCTT TTTAAGAAAA GTTTTGTTGC TTACAACCTG   
  
  
+ TAAAGCCTAG AGAACAAAGC CAAAGAAGAA ATTTAAGTTC TATATATAGC ATAATCTCTC AAAAAATATA   
  
  
+ TTTTTTAAAT ATAGTGCGAT ATTGACTGAC CTAATATGGG TTATTACAGG AGATGGGGAG ATATCTACAT   
  
  
+ TAACTACTTG AACAACTTAA ATTTTCATTT TAACTCGGTC ATTAAGAACA CTAAGAACTT TATATATTAG   
  
  
+ TGATATCTCA GGCGTAAAAG AAACTACTTT GAATTAATCC ATGCAATCCT ACTCTTTATT TCCATCCGTT   
  
  
+ AAAATTTTCC TTCAAAACTC CCTTCTGTTC ATTATTAATT CAATATCGTA TTACACTTGT TGGATTATTT   
  
  
+ CTGTGATTTG ATCGTCATTT TCTATATATG AATAACTACA AAAATCATAG TCCGAGTACA TTAATATTAA   
  
  
+ TAAAATAGGA TGAGTGATAA CCTCATATAG TGCGTTTATC CCTACCACTA GGCTGGATTT CTGCATAAAG   
  
  
+ AAGAAATTGA ACATATGGAA AAAGAAATAA AGAGAAGCAA CTTCCTTGAG TGGGAGAAGA ATCGTAGAAA   
  
  
+ GTTAAACTTA TGAAAAAGAG CATTAACAAA AAGGAAATTA TAAGGCTTAC CGTTGAAGCC TCATATAATT   
  
  
+ TTGCAAGAGA CAGACAAAGA AAGTGTCTTT CAAATGTCAT ATTTCTCGAG CATGCATGAA AAATGTACTT   
  
  
+ CGGCTACAAT AGTAATAGTA TATATGTTGG CTTCATACAC TTGACCCCAA TGTATGTCAA CGTGACTAGT   
  
  
+ GACCACACAC ATCGTTGTCC CTGCCCGCCC CACTACCCAA ACTCCCCGCA CGCAAAGATA AAAAGCCCCC   
  
  
+ CAATTTTTAT TTGCCTCCCA AAAAGCCCAC CCGCGCTTTT TTATTTGCCT TCTTTTTTCC TACTTAAACC   
  
  
+ ACCTCCTCAA CGAACTTTGC AACATACATA CTAGTCTATA AGCAATTTAG AGGGAAGGGA AGCAACCTAA   
  
  
+ ACAGAGCGAG ATTGGCGGGG TGTGTTTGAG GGCTTGCGTT TTGGTGCAAT AGGAATGAAA GTGCCCATTT   
  
  
+ CAACCACCAT CCAAACTTGC AACTCCGGCA CCGGCAAACC ACTTTCCTTT GAGAATACTG CCCTACATAC   
  
  
+ CGCCTTTCAA GTTCCCAATA CCACCTACGA ACCCACCTCG GTTCTTGACC TCCAGCGCAG CCCTAGCCCC   
  
  
+ ACCTCCACCC AAAAACCGCC CTTTGCCGCC TCCAACCACC AGCCTGCCTC GGCTGATCTG GACAACTTGG   
  
  
+ ACGGGTGGGA TTCAATCCTA TCAGAGCTGG GGCTTAATGA CGAATTTACC CCTAATTCCA AACTCTGGTC   
  
  
+ CCAAATTAGT CCGTCTGATC CTCACCTCCC CCAACTCCCT GATTTCCCAA CATCTCAGCC GTTTGATCAT   
  
  
+ CACAACCCAC CCCCAATAAA CCTCCCATCA TCAGATTTCA ACCTCTGTGA TTTCTCCTAC AATCACAACC   
  
  
+ CCAATTTTGG GCCGTTCGAT CTTCATCACA ACCCGCATCA GAACAACCCG AACAACAATA ACTACGGCTT   
  
  
+ TGATTTCATA GACGACCTCA TCAAAGCAGC ACAGTCCCTC GAATCCAACG ACTCTCATCA AGTCCACCTG   
  
  
+ ATATTGGCGC GGCTCAATCA ACGGCTCAGA TCACCCACCG GCAAACCGCT CCAGCGGGCC GCCTACTACT   
  
  
+ TCAAGGAAGC CCTCATCGCC GCCGCGGCCG GCCCGCCCCG CCCCGCTCGG CTTTCATCGT ATGAGGTGGT   
  
  
+ GCAGACCATC CGAGCCTACA AGGCGTTTTC AGGAATTTCG CCAGTCTCTC TCTTCTCCAC TTTCGCCGCC   
  
  
+ AACCAGGCGA TTCTTGAGGC GGTGGACACG GCGGCCTTCA TCCACATCAT CGATTTCGAC ATCGGATTCG   
  
  
+ GCGGCCACTG GGCCTCGTTC CTCCGCGAAC TGGTCGACAA AGCTGATCCT GCGAAACTTA GTTCAGTGGT   
  
  
+ GTTACGAATC ACCGCCATCG TTCCCGAAGA ATTCGGGATC GAAAGCAAGC TAGTGAGAGA AAATCTGTCG   
  
  
+ CAATTTGCCC GAGATCTCAA CATCAACTTC CACATCGATT ATGTCTTATT TCAGAGCTTT GAAATCTTAT   
  
  
+ CCTTCAAATC TGTCAAATTC ATCGAAAGGG AGAAATTGGC GCTGCATCTT TCGCCGGCCG TGTTCCACCG   
  
  
+ GCTAGGGAGC GGAATTTCGA AGTTTATCGC TGATCTCCGA TCAATCTCGC CGAGCTCCGT CGTGGTGGTG   
  
  
+ GACAGAGATG TCGGGATTGA CATCGGAACG TCGTCGTTTA GCATGAATTT CGTCGCCGGA ATTGAATTCT   
  
  
+ ACACCGGGAT GCTGGAGTCG CTTGACGCAG CCACCGCCGG GGGGCTCATC GGCGGTGTGG ACTGCGTGAG   
  
  
+ ACGGATTGAG ACGTTTGTTC TCCACCCAAG GATCATGGCG GCGGTAGAGG CGGCGGCGTC GGCGTCGGCC   
  
  
+ GGGCGGAGGA CGGCGTGGAG GGAGGCATTC GCGGCGGCGG GGATTAGGGC GGTGGGGTTT AGCCAGTTTG   
  
  
+ CTGATTTCCA GGCCGAATGT TTGTTGAGGA GAGCTCAGGT TGGTGGCTTC CACGTGGCAA AGCGCCATGG   
  
  
+ AGAGATGATG CTTTACTGGC ATGACCGCCC ACTCGTCGCC ACATCAGCTT GGAGGTGTTA   

- -Up\_Stream \_Len000TATTAA ATAGAAAGAA ATTAAAGCAA AGTACATTTT TTAAAGTCAG AGTAGACGCT   
  
  
- AACTTAGAGA AAAGTTAAGC GTAGTTATTA AGTAGGTTAA GTTTACACGG AAAGAATTAA AGTAATGTAA   
  
  
- CCCAACCATA TTTACAATCG TGCTATCTAA CCTTATCGTT CCGAGATTAA GTTAATACTG AACTTACATA   
  
  
- CGTACGAAAA TAGTAATCAA TTAATTGTAA AAGACATAAC TTTAGTCGAG CTTTCTCTTG GTATACATCA   
  
  
- ACATGAACGC TGCTATATAT ACAAATCAAA TCAATCAAGA ATGGACAAAA CCTTAACAAC CCCTCTAGAC   
  
  
- TCCTTCCTCG GGCGAAGTTA GTTTCAAGTA CCGTTATTCG GTTTCACTCC AAGGTAGGAT TTTGGTTAAC   
  
  
- CGTTATCCTT CTCATCGGGT GAATTGAACA TATGATACTG AGAAGAAAAG TTTGAGGGTT ACACCCTGAA   
  
  
- GAAATGAGTA CACACTATAA GGGTTGTCCT TACTAGTCAA GGTGTACAAA AGTAGAGAAA CCTGAGCTTG   
  
  
- AAGCACATTA ACATATACGG TACCGTGTTT ATCAAAACTA CCACGGTGGT TCAACGAGTT AGTTACATTA   
  
  
- TGACGATGAT AGATTGAACG GAACCACCTG TAGTTATTCT AGTGCTCTAC TCCTCTTGAC TTAGAACCGG   
  
  
- TTGGTGCAGC AAACTGAAGA GAATGGTTAT TTCTAACTTA TCTGTCACAA AGAGAGGGGG AAGGTAAGAA   
  
  
- GAAAAGATCA CTACAGCTTT TTCTGAAATA TGTACAGGTC GTTGCGATCG GTGTTTTATA AGGTAAAGAC   
  
  
- ATTAGGTGAG TACAGTTAAT TTGAACATAG TAGTTTACCA TCAAAGGCAT GATTAGTGAT GTAGATCTAT   
  
  
- AAGGAACTAT TTAAATTAAA CTGAAATTTC CGAGCTAGAA AAATTCTTTT CAAAACAACG AATGTTGGAC   
  
  
- ATTTCGGATC TCTTGTTTCG GTTTCTTCTT TAAATTCAAG ATATATATCG TATTAGAGAG TTTTTTATAT   
  
  
- AAAAAATTTA TATCACGCTA TAACTGACTG GATTATACCC AATAATGTCC TCTACCCCTC TATAGATGTA   
  
  
- ATTGATGAAC TTGTTGAATT TAAAAGTAAA ATTGAGCCAG TAATTCTTGT GATTCTTGAA ATATATAATC   
  
  
- ACTATAGAGT CCGCATTTTC TTTGATGAAA CTTAATTAGG TACGTTAGGA TGAGAAATAA AGGTAGGCAA   
  
  
- TTTTAAAAGG AAGTTTTGAG GGAAGACAAG TAATAATTAA GTTATAGCAT AATGTGAACA ACCTAATAAA   
  
  
- GACACTAAAC TAGCAGTAAA AGATATATAC TTATTGATGT TTTTAGTATC AGGCTCATGT AATTATAATT   
  
  
- ATTTTATCCT ACTCACTATT GGAGTATATC ACGCAAATAG GGATGGTGAT CCGACCTAAA GACGTATTTC   
  
  
- TTCTTTAACT TGTATACCTT TTTCTTTATT TCTCTTCGTT GAAGGAACTC ACCCTCTTCT TAGCATCTTT   
  
  
- CAATTTGAAT ACTTTTTCTC GTAATTGTTT TTCCTTTAAT ATTCCGAATG GCAACTTCGG AGTATATTAA   
  
  
- AACGTTCTCT GTCTGTTTCT TTCACAGAAA GTTTACAGTA TAAAGAGCTC GTACGTACTT TTTACATGAA   
  
  
- GCCGATGTTA TCATTATCAT ATATACAACC GAAGTATGTG AACTGGGGTT ACATACAGTT GCACTGATCA   
  
  
- CTGGTGTGTG TAGCAACAGG GACGGGCGGG GTGATGGGTT TGAGGGGCGT GCGTTTCTAT TTTTCGGGGG   
  
  
- GTTAAAAATA AACGGAGGGT TTTTCGGGTG GGCGCGAAAA AATAAACGGA AGAAAAAAGG ATGAATTTGG   
  
  
- TGGAGGAGTT GCTTGAAACG TTGTATGTAT GATCAGATAT TCGTTAAATC TCCCTTCCCT TCGTTGGATT   
  
  
- TGTCTCGCTC TAACCGCCCC ACACAAACTC CCGAACGCAA AACCACGTTA TCCTTACTTT CACGGGTAAA   
  
  
- GTTGGTGGTA GGTTTGAACG TTGAGGCCGT GGCCGTTTGG TGAAAGGAAA CTCTTATGAC GGGATGTATG   
  
  
- GCGGAAAGTT CAAGGGTTAT GGTGGATGCT TGGGTGGAGC CAAGAACTGG AGGTCGCGTC GGGATCGGGG   
  
  
- TGGAGGTGGG TTTTTGGCGG GAAACGGCGG AGGTTGGTGG TCGGACGGAG CCGACTAGAC CTGTTGAACC   
  
  
- TGCCCACCCT AAGTTAGGAT AGTCTCGACC CCGAATTACT GCTTAAATGG GGATTAAGGT TTGAGACCAG   
  
  
- GGTTTAATCA GGCAGACTAG GAGTGGAGGG GGTTGAGGGA CTAAAGGGTT GTAGAGTCGG CAAACTAGTA   
  
  
- GTGTTGGGTG GGGGTTATTT GGAGGGTAGT AGTCTAAAGT TGGAGACACT AAAGAGGATG TTAGTGTTGG   
  
  
- GGTTAAAACC CGGCAAGCTA GAAGTAGTGT TGGGCGTAGT CTTGTTGGGC TTGTTGTTAT TGATGCCGAA   
  
  
- ACTAAAGTAT CTGCTGGAGT AGTTTCGTCG TGTCAGGGAG CTTAGGTTGC TGAGAGTAGT TCAGGTGGAC   
  
  
- TATAACCGCG CCGAGTTAGT TGCCGAGTCT AGTGGGTGGC CGTTTGGCGA GGTCGCCCGG CGGATGATGA   
  
  
- AGTTCCTTCG GGAGTAGCGG CGGCGCCGGC CGGGCGGGGC GGGGCGAGCC GAAAGTAGCA TACTCCACCA   
  
  
- CGTCTGGTAG GCTCGGATGT TCCGCAAAAG TCCTTAAAGC GGTCAGAGAG AGAAGAGGTG AAAGCGGCGG   
  
  
- TTGGTCCGCT AAGAACTCCG CCACCTGTGC CGCCGGAAGT AGGTGTAGTA GCTAAAGCTG TAGCCTAAGC   
  
  
- CGCCGGTGAC CCGGAGCAAG GAGGCGCTTG ACCAGCTGTT TCGACTAGGA CGCTTTGAAT CAAGTCACCA   
  
  
- CAATGCTTAG TGGCGGTAGC AAGGGCTTCT TAAGCCCTAG CTTTCGTTCG ATCACTCTCT TTTAGACAGC   
  
  
- GTTAAACGGG CTCTAGAGTT GTAGTTGAAG GTGTAGCTAA TACAGAATAA AGTCTCGAAA CTTTAGAATA   
  
  
- GGAAGTTTAG ACAGTTTAAG TAGCTTTCCC TCTTTAACCG CGACGTAGAA AGCGGCCGGC ACAAGGTGGC   
  
  
- CGATCCCTCG CCTTAAAGCT TCAAATAGCG ACTAGAGGCT AGTTAGAGCG GCTCGAGGCA GCACCACCAC   
  
  
- CTGTCTCTAC AGCCCTAACT GTAGCCTTGC AGCAGCAAAT CGTACTTAAA GCAGCGGCCT TAACTTAAGA   
  
  
- TGTGGCCCTA CGACCTCAGC GAACTGCGTC GGTGGCGGCC CCCCGAGTAG CCGCCACACC TGACGCACTC   
  
  
- TGCCTAACTC TGCAAACAAG AGGTGGGTTC CTAGTACCGC CGCCATCTCC GCCGCCGCAG CCGCAGCCGG   
  
  
- CCCGCCTCCT GCCGCACCTC CCTCCGTAAG CGCCGCCGCC CCTAATCCCG CCACCCCAAA TCGGTCAAAC   
  
  
- GACTAAAGGT CCGGCTTACA AACAACTCCT CTCGAGTCCA ACCACCGAAG GTGCACCGTT TCGCGGTACC   
  
  
- TCTCTACTAC GAAATGACCG TACTGGCGGG TGAGCAGCGG TGTAGTCGAA CCTCCACAAT

+     G-Box

| Site Name | Organism | Position | Strand | Matrix score. | sequence | function |
| --- | --- | --- | --- | --- | --- | --- |
| G-Box | Triticum aestivum | 3553 | + | 10 | TCCACATGGCA | cis-acting regulatory element involved in light responsiveness |
| G-Box | Pisum sativum | 3555 | - | 6 | CACGTG | cis-acting regulatory element involved in light responsiveness |
| G-Box | Pisum sativum | 1743 | - | 6 | CACGTT | cis-acting regulatory element involved in light responsiveness |

>HU05G02245.1   
+ -Up\_Stream \_Len000ATAATT TATCTTTCTT TAATTTCGTT TCATGTAAAA AATTTCAGTC TCATCTGCGA   
  
  
+ TTGAATCTCT TTTCAATTCG CATCAATAAT TCATCCAATT CAAATGTGCC TTTCTTAATT TCATTACATT   
  
  
+ GGGTTGGTAT AAATGTTAGC ACGATAGATT GGAATAGCAA GGCTCTAATT CAATTATGAC TTGAATGTAT   
  
  
+ GCATGCTTTT ATCATTAGTT AATTAACATT TTCTGTATTG AAATCAGCTC GAAAGAGAAC CATATGTAGT   
  
  
+ TGTACTTGCG ACGATATATA TGTTTAGTTT AGTTAGTTCT TACCTGTTTT GGAATTGTTG GGGAGATCTG   
  
  
+ AGGAAGGAGC CCGCTTCAAT CAAAGTTCAT GGCAATAAGC CAAAGTGAGG TTCCATCCTA AAACCAATTG   
  
  
+ GCAATAGGAA GAGTAGCCCA CTTAACTTGT ATACTATGAC TCTTCTTTTC AAACTCCCAA TGTGGGACTT   
  
  
+ CTTTACTCAT GTGTGATATT CCCAACAGGA ATGATCAGTT CCACATGTTT TCATCTCTTT GGACTCGAAC   
  
  
+ TTCGTGTAAT TGTATATGCC ATGGCACAAA TAGTTTTGAT GGTGCCACCA AGTTGCTCAA TCAATGTAAT   
  
  
+ ACTGCTACTA TCTAACTTGC CTTGGTGGAC ATCAATAAGA TCACGAGATG AGGAGAACTG AATCTTGGCC   
  
  
+ AACCACGTCG TTTGACTTCT CTTACCAATA AAGATTGAAT AGACAGTGTT TCTCTCCCCC TTCCATTCTT   
  
  
+ CTTTTCTAGT GATGTCGAAA AAGACTTTAT ACATGTCCAG CAACGCTAGC CACAAAATAT TCCATTTCTG   
  
  
+ TAATCCACTC ATGTCAATTA AACTTGTATC ATCAAATGGT AGTTTCCGTA CTAATCACTA CATCTAGATA   
  
  
+ TTCCTTGATA AATTTAATTT GACTTTAAAG GCTCGATCTT TTTAAGAAAA GTTTTGTTGC TTACAACCTG   
  
  
+ TAAAGCCTAG AGAACAAAGC CAAAGAAGAA ATTTAAGTTC TATATATAGC ATAATCTCTC AAAAAATATA   
  
  
+ TTTTTTAAAT ATAGTGCGAT ATTGACTGAC CTAATATGGG TTATTACAGG AGATGGGGAG ATATCTACAT   
  
  
+ TAACTACTTG AACAACTTAA ATTTTCATTT TAACTCGGTC ATTAAGAACA CTAAGAACTT TATATATTAG   
  
  
+ TGATATCTCA GGCGTAAAAG AAACTACTTT GAATTAATCC ATGCAATCCT ACTCTTTATT TCCATCCGTT   
  
  
+ AAAATTTTCC TTCAAAACTC CCTTCTGTTC ATTATTAATT CAATATCGTA TTACACTTGT TGGATTATTT   
  
  
+ CTGTGATTTG ATCGTCATTT TCTATATATG AATAACTACA AAAATCATAG TCCGAGTACA TTAATATTAA   
  
  
+ TAAAATAGGA TGAGTGATAA CCTCATATAG TGCGTTTATC CCTACCACTA GGCTGGATTT CTGCATAAAG   
  
  
+ AAGAAATTGA ACATATGGAA AAAGAAATAA AGAGAAGCAA CTTCCTTGAG TGGGAGAAGA ATCGTAGAAA   
  
  
+ GTTAAACTTA TGAAAAAGAG CATTAACAAA AAGGAAATTA TAAGGCTTAC CGTTGAAGCC TCATATAATT   
  
  
+ TTGCAAGAGA CAGACAAAGA AAGTGTCTTT CAAATGTCAT ATTTCTCGAG CATGCATGAA AAATGTACTT   
  
  
+ CGGCTACAAT AGTAATAGTA TATATGTTGG CTTCATACAC TTGACCCCAA TGTATGTCAA CGTGACTAGT   
  
  
+ GACCACACAC ATCGTTGTCC CTGCCCGCCC CACTACCCAA ACTCCCCGCA CGCAAAGATA AAAAGCCCCC   
  
  
+ CAATTTTTAT TTGCCTCCCA AAAAGCCCAC CCGCGCTTTT TTATTTGCCT TCTTTTTTCC TACTTAAACC   
  
  
+ ACCTCCTCAA CGAACTTTGC AACATACATA CTAGTCTATA AGCAATTTAG AGGGAAGGGA AGCAACCTAA   
  
  
+ ACAGAGCGAG ATTGGCGGGG TGTGTTTGAG GGCTTGCGTT TTGGTGCAAT AGGAATGAAA GTGCCCATTT   
  
  
+ CAACCACCAT CCAAACTTGC AACTCCGGCA CCGGCAAACC ACTTTCCTTT GAGAATACTG CCCTACATAC   
  
  
+ CGCCTTTCAA GTTCCCAATA CCACCTACGA ACCCACCTCG GTTCTTGACC TCCAGCGCAG CCCTAGCCCC   
  
  
+ ACCTCCACCC AAAAACCGCC CTTTGCCGCC TCCAACCACC AGCCTGCCTC GGCTGATCTG GACAACTTGG   
  
  
+ ACGGGTGGGA TTCAATCCTA TCAGAGCTGG GGCTTAATGA CGAATTTACC CCTAATTCCA AACTCTGGTC   
  
  
+ CCAAATTAGT CCGTCTGATC CTCACCTCCC CCAACTCCCT GATTTCCCAA CATCTCAGCC GTTTGATCAT   
  
  
+ CACAACCCAC CCCCAATAAA CCTCCCATCA TCAGATTTCA ACCTCTGTGA TTTCTCCTAC AATCACAACC   
  
  
+ CCAATTTTGG GCCGTTCGAT CTTCATCACA ACCCGCATCA GAACAACCCG AACAACAATA ACTACGGCTT   
  
  
+ TGATTTCATA GACGACCTCA TCAAAGCAGC ACAGTCCCTC GAATCCAACG ACTCTCATCA AGTCCACCTG   
  
  
+ ATATTGGCGC GGCTCAATCA ACGGCTCAGA TCACCCACCG GCAAACCGCT CCAGCGGGCC GCCTACTACT   
  
  
+ TCAAGGAAGC CCTCATCGCC GCCGCGGCCG GCCCGCCCCG CCCCGCTCGG CTTTCATCGT ATGAGGTGGT   
  
  
+ GCAGACCATC CGAGCCTACA AGGCGTTTTC AGGAATTTCG CCAGTCTCTC TCTTCTCCAC TTTCGCCGCC   
  
  
+ AACCAGGCGA TTCTTGAGGC GGTGGACACG GCGGCCTTCA TCCACATCAT CGATTTCGAC ATCGGATTCG   
  
  
+ GCGGCCACTG GGCCTCGTTC CTCCGCGAAC TGGTCGACAA AGCTGATCCT GCGAAACTTA GTTCAGTGGT   
  
  
+ GTTACGAATC ACCGCCATCG TTCCCGAAGA ATTCGGGATC GAAAGCAAGC TAGTGAGAGA AAATCTGTCG   
  
  
+ CAATTTGCCC GAGATCTCAA CATCAACTTC CACATCGATT ATGTCTTATT TCAGAGCTTT GAAATCTTAT   
  
  
+ CCTTCAAATC TGTCAAATTC ATCGAAAGGG AGAAATTGGC GCTGCATCTT TCGCCGGCCG TGTTCCACCG   
  
  
+ GCTAGGGAGC GGAATTTCGA AGTTTATCGC TGATCTCCGA TCAATCTCGC CGAGCTCCGT CGTGGTGGTG   
  
  
+ GACAGAGATG TCGGGATTGA CATCGGAACG TCGTCGTTTA GCATGAATTT CGTCGCCGGA ATTGAATTCT   
  
  
+ ACACCGGGAT GCTGGAGTCG CTTGACGCAG CCACCGCCGG GGGGCTCATC GGCGGTGTGG ACTGCGTGAG   
  
  
+ ACGGATTGAG ACGTTTGTTC TCCACCCAAG GATCATGGCG GCGGTAGAGG CGGCGGCGTC GGCGTCGGCC   
  
  
+ GGGCGGAGGA CGGCGTGGAG GGAGGCATTC GCGGCGGCGG GGATTAGGGC GGTGGGGTTT AGCCAGTTTG   
  
  
+ CTGATTTCCA GGCCGAATGT TTGTTGAGGA GAGCTCAGGT TGGTGGCTTC CACGTGGCAA AGCGCCATGG   
  
  
+ AGAGATGATG CTTTACTGGC ATGACCGCCC ACTCGTCGCC ACATCAGCTT GGAGGTGTTA   

- -Up\_Stream \_Len000TATTAA ATAGAAAGAA ATTAAAGCAA AGTACATTTT TTAAAGTCAG AGTAGACGCT   
  
  
- AACTTAGAGA AAAGTTAAGC GTAGTTATTA AGTAGGTTAA GTTTACACGG AAAGAATTAA AGTAATGTAA   
  
  
- CCCAACCATA TTTACAATCG TGCTATCTAA CCTTATCGTT CCGAGATTAA GTTAATACTG AACTTACATA   
  
  
- CGTACGAAAA TAGTAATCAA TTAATTGTAA AAGACATAAC TTTAGTCGAG CTTTCTCTTG GTATACATCA   
  
  
- ACATGAACGC TGCTATATAT ACAAATCAAA TCAATCAAGA ATGGACAAAA CCTTAACAAC CCCTCTAGAC   
  
  
- TCCTTCCTCG GGCGAAGTTA GTTTCAAGTA CCGTTATTCG GTTTCACTCC AAGGTAGGAT TTTGGTTAAC   
  
  
- CGTTATCCTT CTCATCGGGT GAATTGAACA TATGATACTG AGAAGAAAAG TTTGAGGGTT ACACCCTGAA   
  
  
- GAAATGAGTA CACACTATAA GGGTTGTCCT TACTAGTCAA GGTGTACAAA AGTAGAGAAA CCTGAGCTTG   
  
  
- AAGCACATTA ACATATACGG TACCGTGTTT ATCAAAACTA CCACGGTGGT TCAACGAGTT AGTTACATTA   
  
  
- TGACGATGAT AGATTGAACG GAACCACCTG TAGTTATTCT AGTGCTCTAC TCCTCTTGAC TTAGAACCGG   
  
  
- TTGGTGCAGC AAACTGAAGA GAATGGTTAT TTCTAACTTA TCTGTCACAA AGAGAGGGGG AAGGTAAGAA   
  
  
- GAAAAGATCA CTACAGCTTT TTCTGAAATA TGTACAGGTC GTTGCGATCG GTGTTTTATA AGGTAAAGAC   
  
  
- ATTAGGTGAG TACAGTTAAT TTGAACATAG TAGTTTACCA TCAAAGGCAT GATTAGTGAT GTAGATCTAT   
  
  
- AAGGAACTAT TTAAATTAAA CTGAAATTTC CGAGCTAGAA AAATTCTTTT CAAAACAACG AATGTTGGAC   
  
  
- ATTTCGGATC TCTTGTTTCG GTTTCTTCTT TAAATTCAAG ATATATATCG TATTAGAGAG TTTTTTATAT   
  
  
- AAAAAATTTA TATCACGCTA TAACTGACTG GATTATACCC AATAATGTCC TCTACCCCTC TATAGATGTA   
  
  
- ATTGATGAAC TTGTTGAATT TAAAAGTAAA ATTGAGCCAG TAATTCTTGT GATTCTTGAA ATATATAATC   
  
  
- ACTATAGAGT CCGCATTTTC TTTGATGAAA CTTAATTAGG TACGTTAGGA TGAGAAATAA AGGTAGGCAA   
  
  
- TTTTAAAAGG AAGTTTTGAG GGAAGACAAG TAATAATTAA GTTATAGCAT AATGTGAACA ACCTAATAAA   
  
  
- GACACTAAAC TAGCAGTAAA AGATATATAC TTATTGATGT TTTTAGTATC AGGCTCATGT AATTATAATT   
  
  
- ATTTTATCCT ACTCACTATT GGAGTATATC ACGCAAATAG GGATGGTGAT CCGACCTAAA GACGTATTTC   
  
  
- TTCTTTAACT TGTATACCTT TTTCTTTATT TCTCTTCGTT GAAGGAACTC ACCCTCTTCT TAGCATCTTT   
  
  
- CAATTTGAAT ACTTTTTCTC GTAATTGTTT TTCCTTTAAT ATTCCGAATG GCAACTTCGG AGTATATTAA   
  
  
- AACGTTCTCT GTCTGTTTCT TTCACAGAAA GTTTACAGTA TAAAGAGCTC GTACGTACTT TTTACATGAA   
  
  
- GCCGATGTTA TCATTATCAT ATATACAACC GAAGTATGTG AACTGGGGTT ACATACAGTT GCACTGATCA   
  
  
- CTGGTGTGTG TAGCAACAGG GACGGGCGGG GTGATGGGTT TGAGGGGCGT GCGTTTCTAT TTTTCGGGGG   
  
  
- GTTAAAAATA AACGGAGGGT TTTTCGGGTG GGCGCGAAAA AATAAACGGA AGAAAAAAGG ATGAATTTGG   
  
  
- TGGAGGAGTT GCTTGAAACG TTGTATGTAT GATCAGATAT TCGTTAAATC TCCCTTCCCT TCGTTGGATT   
  
  
- TGTCTCGCTC TAACCGCCCC ACACAAACTC CCGAACGCAA AACCACGTTA TCCTTACTTT CACGGGTAAA   
  
  
- GTTGGTGGTA GGTTTGAACG TTGAGGCCGT GGCCGTTTGG TGAAAGGAAA CTCTTATGAC GGGATGTATG   
  
  
- GCGGAAAGTT CAAGGGTTAT GGTGGATGCT TGGGTGGAGC CAAGAACTGG AGGTCGCGTC GGGATCGGGG   
  
  
- TGGAGGTGGG TTTTTGGCGG GAAACGGCGG AGGTTGGTGG TCGGACGGAG CCGACTAGAC CTGTTGAACC   
  
  
- TGCCCACCCT AAGTTAGGAT AGTCTCGACC CCGAATTACT GCTTAAATGG GGATTAAGGT TTGAGACCAG   
  
  
- GGTTTAATCA GGCAGACTAG GAGTGGAGGG GGTTGAGGGA CTAAAGGGTT GTAGAGTCGG CAAACTAGTA   
  
  
- GTGTTGGGTG GGGGTTATTT GGAGGGTAGT AGTCTAAAGT TGGAGACACT AAAGAGGATG TTAGTGTTGG   
  
  
- GGTTAAAACC CGGCAAGCTA GAAGTAGTGT TGGGCGTAGT CTTGTTGGGC TTGTTGTTAT TGATGCCGAA   
  
  
- ACTAAAGTAT CTGCTGGAGT AGTTTCGTCG TGTCAGGGAG CTTAGGTTGC TGAGAGTAGT TCAGGTGGAC   
  
  
- TATAACCGCG CCGAGTTAGT TGCCGAGTCT AGTGGGTGGC CGTTTGGCGA GGTCGCCCGG CGGATGATGA   
  
  
- AGTTCCTTCG GGAGTAGCGG CGGCGCCGGC CGGGCGGGGC GGGGCGAGCC GAAAGTAGCA TACTCCACCA   
  
  
- CGTCTGGTAG GCTCGGATGT TCCGCAAAAG TCCTTAAAGC GGTCAGAGAG AGAAGAGGTG AAAGCGGCGG   
  
  
- TTGGTCCGCT AAGAACTCCG CCACCTGTGC CGCCGGAAGT AGGTGTAGTA GCTAAAGCTG TAGCCTAAGC   
  
  
- CGCCGGTGAC CCGGAGCAAG GAGGCGCTTG ACCAGCTGTT TCGACTAGGA CGCTTTGAAT CAAGTCACCA   
  
  
- CAATGCTTAG TGGCGGTAGC AAGGGCTTCT TAAGCCCTAG CTTTCGTTCG ATCACTCTCT TTTAGACAGC   
  
  
- GTTAAACGGG CTCTAGAGTT GTAGTTGAAG GTGTAGCTAA TACAGAATAA AGTCTCGAAA CTTTAGAATA   
  
  
- GGAAGTTTAG ACAGTTTAAG TAGCTTTCCC TCTTTAACCG CGACGTAGAA AGCGGCCGGC ACAAGGTGGC   
  
  
- CGATCCCTCG CCTTAAAGCT TCAAATAGCG ACTAGAGGCT AGTTAGAGCG GCTCGAGGCA GCACCACCAC   
  
  
- CTGTCTCTAC AGCCCTAACT GTAGCCTTGC AGCAGCAAAT CGTACTTAAA GCAGCGGCCT TAACTTAAGA   
  
  
- TGTGGCCCTA CGACCTCAGC GAACTGCGTC GGTGGCGGCC CCCCGAGTAG CCGCCACACC TGACGCACTC   
  
  
- TGCCTAACTC TGCAAACAAG AGGTGGGTTC CTAGTACCGC CGCCATCTCC GCCGCCGCAG CCGCAGCCGG   
  
  
- CCCGCCTCCT GCCGCACCTC CCTCCGTAAG CGCCGCCGCC CCTAATCCCG CCACCCCAAA TCGGTCAAAC   
  
  
- GACTAAAGGT CCGGCTTACA AACAACTCCT CTCGAGTCCA ACCACCGAAG GTGCACCGTT TCGCGGTACC   
  
  
- TCTCTACTAC GAAATGACCG TACTGGCGGG TGAGCAGCGG TGTAGTCGAA CCTCCACAAT

+     G-box

| Site Name | Organism | Position | Strand | Matrix score. | sequence | function |
| --- | --- | --- | --- | --- | --- | --- |
| G-box | Arabidopsis thaliana | 3553 | - | 10 | GCCACGTGGA | cis-acting regulatory element involved in light responsiveness |
| G-box | Arabidopsis thaliana | 3555 | - | 6 | CACGTG | cis-acting regulatory element involved in light responsiveness |
| G-box | Arabidopsis thaliana | 3551 | + | 13 | CTTCCACGTGGCA | cis-acting regulatory element involved in light responsiveness |
| G-box | Zea mays | 708 | + | 6 | CACGTC | cis-acting regulatory element involved in light responsiveness |
| G-box | Zea mays | 3213 | - | 6 | CACGAC | cis-acting regulatory element involved in light responsiveness |

>HU05G02245.1   
+ -Up\_Stream \_Len000ATAATT TATCTTTCTT TAATTTCGTT TCATGTAAAA AATTTCAGTC TCATCTGCGA   
  
  
+ TTGAATCTCT TTTCAATTCG CATCAATAAT TCATCCAATT CAAATGTGCC TTTCTTAATT TCATTACATT   
  
  
+ GGGTTGGTAT AAATGTTAGC ACGATAGATT GGAATAGCAA GGCTCTAATT CAATTATGAC TTGAATGTAT   
  
  
+ GCATGCTTTT ATCATTAGTT AATTAACATT TTCTGTATTG AAATCAGCTC GAAAGAGAAC CATATGTAGT   
  
  
+ TGTACTTGCG ACGATATATA TGTTTAGTTT AGTTAGTTCT TACCTGTTTT GGAATTGTTG GGGAGATCTG   
  
  
+ AGGAAGGAGC CCGCTTCAAT CAAAGTTCAT GGCAATAAGC CAAAGTGAGG TTCCATCCTA AAACCAATTG   
  
  
+ GCAATAGGAA GAGTAGCCCA CTTAACTTGT ATACTATGAC TCTTCTTTTC AAACTCCCAA TGTGGGACTT   
  
  
+ CTTTACTCAT GTGTGATATT CCCAACAGGA ATGATCAGTT CCACATGTTT TCATCTCTTT GGACTCGAAC   
  
  
+ TTCGTGTAAT TGTATATGCC ATGGCACAAA TAGTTTTGAT GGTGCCACCA AGTTGCTCAA TCAATGTAAT   
  
  
+ ACTGCTACTA TCTAACTTGC CTTGGTGGAC ATCAATAAGA TCACGAGATG AGGAGAACTG AATCTTGGCC   
  
  
+ AACCACGTCG TTTGACTTCT CTTACCAATA AAGATTGAAT AGACAGTGTT TCTCTCCCCC TTCCATTCTT   
  
  
+ CTTTTCTAGT GATGTCGAAA AAGACTTTAT ACATGTCCAG CAACGCTAGC CACAAAATAT TCCATTTCTG   
  
  
+ TAATCCACTC ATGTCAATTA AACTTGTATC ATCAAATGGT AGTTTCCGTA CTAATCACTA CATCTAGATA   
  
  
+ TTCCTTGATA AATTTAATTT GACTTTAAAG GCTCGATCTT TTTAAGAAAA GTTTTGTTGC TTACAACCTG   
  
  
+ TAAAGCCTAG AGAACAAAGC CAAAGAAGAA ATTTAAGTTC TATATATAGC ATAATCTCTC AAAAAATATA   
  
  
+ TTTTTTAAAT ATAGTGCGAT ATTGACTGAC CTAATATGGG TTATTACAGG AGATGGGGAG ATATCTACAT   
  
  
+ TAACTACTTG AACAACTTAA ATTTTCATTT TAACTCGGTC ATTAAGAACA CTAAGAACTT TATATATTAG   
  
  
+ TGATATCTCA GGCGTAAAAG AAACTACTTT GAATTAATCC ATGCAATCCT ACTCTTTATT TCCATCCGTT   
  
  
+ AAAATTTTCC TTCAAAACTC CCTTCTGTTC ATTATTAATT CAATATCGTA TTACACTTGT TGGATTATTT   
  
  
+ CTGTGATTTG ATCGTCATTT TCTATATATG AATAACTACA AAAATCATAG TCCGAGTACA TTAATATTAA   
  
  
+ TAAAATAGGA TGAGTGATAA CCTCATATAG TGCGTTTATC CCTACCACTA GGCTGGATTT CTGCATAAAG   
  
  
+ AAGAAATTGA ACATATGGAA AAAGAAATAA AGAGAAGCAA CTTCCTTGAG TGGGAGAAGA ATCGTAGAAA   
  
  
+ GTTAAACTTA TGAAAAAGAG CATTAACAAA AAGGAAATTA TAAGGCTTAC CGTTGAAGCC TCATATAATT   
  
  
+ TTGCAAGAGA CAGACAAAGA AAGTGTCTTT CAAATGTCAT ATTTCTCGAG CATGCATGAA AAATGTACTT   
  
  
+ CGGCTACAAT AGTAATAGTA TATATGTTGG CTTCATACAC TTGACCCCAA TGTATGTCAA CGTGACTAGT   
  
  
+ GACCACACAC ATCGTTGTCC CTGCCCGCCC CACTACCCAA ACTCCCCGCA CGCAAAGATA AAAAGCCCCC   
  
  
+ CAATTTTTAT TTGCCTCCCA AAAAGCCCAC CCGCGCTTTT TTATTTGCCT TCTTTTTTCC TACTTAAACC   
  
  
+ ACCTCCTCAA CGAACTTTGC AACATACATA CTAGTCTATA AGCAATTTAG AGGGAAGGGA AGCAACCTAA   
  
  
+ ACAGAGCGAG ATTGGCGGGG TGTGTTTGAG GGCTTGCGTT TTGGTGCAAT AGGAATGAAA GTGCCCATTT   
  
  
+ CAACCACCAT CCAAACTTGC AACTCCGGCA CCGGCAAACC ACTTTCCTTT GAGAATACTG CCCTACATAC   
  
  
+ CGCCTTTCAA GTTCCCAATA CCACCTACGA ACCCACCTCG GTTCTTGACC TCCAGCGCAG CCCTAGCCCC   
  
  
+ ACCTCCACCC AAAAACCGCC CTTTGCCGCC TCCAACCACC AGCCTGCCTC GGCTGATCTG GACAACTTGG   
  
  
+ ACGGGTGGGA TTCAATCCTA TCAGAGCTGG GGCTTAATGA CGAATTTACC CCTAATTCCA AACTCTGGTC   
  
  
+ CCAAATTAGT CCGTCTGATC CTCACCTCCC CCAACTCCCT GATTTCCCAA CATCTCAGCC GTTTGATCAT   
  
  
+ CACAACCCAC CCCCAATAAA CCTCCCATCA TCAGATTTCA ACCTCTGTGA TTTCTCCTAC AATCACAACC   
  
  
+ CCAATTTTGG GCCGTTCGAT CTTCATCACA ACCCGCATCA GAACAACCCG AACAACAATA ACTACGGCTT   
  
  
+ TGATTTCATA GACGACCTCA TCAAAGCAGC ACAGTCCCTC GAATCCAACG ACTCTCATCA AGTCCACCTG   
  
  
+ ATATTGGCGC GGCTCAATCA ACGGCTCAGA TCACCCACCG GCAAACCGCT CCAGCGGGCC GCCTACTACT   
  
  
+ TCAAGGAAGC CCTCATCGCC GCCGCGGCCG GCCCGCCCCG CCCCGCTCGG CTTTCATCGT ATGAGGTGGT   
  
  
+ GCAGACCATC CGAGCCTACA AGGCGTTTTC AGGAATTTCG CCAGTCTCTC TCTTCTCCAC TTTCGCCGCC   
  
  
+ AACCAGGCGA TTCTTGAGGC GGTGGACACG GCGGCCTTCA TCCACATCAT CGATTTCGAC ATCGGATTCG   
  
  
+ GCGGCCACTG GGCCTCGTTC CTCCGCGAAC TGGTCGACAA AGCTGATCCT GCGAAACTTA GTTCAGTGGT   
  
  
+ GTTACGAATC ACCGCCATCG TTCCCGAAGA ATTCGGGATC GAAAGCAAGC TAGTGAGAGA AAATCTGTCG   
  
  
+ CAATTTGCCC GAGATCTCAA CATCAACTTC CACATCGATT ATGTCTTATT TCAGAGCTTT GAAATCTTAT   
  
  
+ CCTTCAAATC TGTCAAATTC ATCGAAAGGG AGAAATTGGC GCTGCATCTT TCGCCGGCCG TGTTCCACCG   
  
  
+ GCTAGGGAGC GGAATTTCGA AGTTTATCGC TGATCTCCGA TCAATCTCGC CGAGCTCCGT CGTGGTGGTG   
  
  
+ GACAGAGATG TCGGGATTGA CATCGGAACG TCGTCGTTTA GCATGAATTT CGTCGCCGGA ATTGAATTCT   
  
  
+ ACACCGGGAT GCTGGAGTCG CTTGACGCAG CCACCGCCGG GGGGCTCATC GGCGGTGTGG ACTGCGTGAG   
  
  
+ ACGGATTGAG ACGTTTGTTC TCCACCCAAG GATCATGGCG GCGGTAGAGG CGGCGGCGTC GGCGTCGGCC   
  
  
+ GGGCGGAGGA CGGCGTGGAG GGAGGCATTC GCGGCGGCGG GGATTAGGGC GGTGGGGTTT AGCCAGTTTG   
  
  
+ CTGATTTCCA GGCCGAATGT TTGTTGAGGA GAGCTCAGGT TGGTGGCTTC CACGTGGCAA AGCGCCATGG   
  
  
+ AGAGATGATG CTTTACTGGC ATGACCGCCC ACTCGTCGCC ACATCAGCTT GGAGGTGTTA   

- -Up\_Stream \_Len000TATTAA ATAGAAAGAA ATTAAAGCAA AGTACATTTT TTAAAGTCAG AGTAGACGCT   
  
  
- AACTTAGAGA AAAGTTAAGC GTAGTTATTA AGTAGGTTAA GTTTACACGG AAAGAATTAA AGTAATGTAA   
  
  
- CCCAACCATA TTTACAATCG TGCTATCTAA CCTTATCGTT CCGAGATTAA GTTAATACTG AACTTACATA   
  
  
- CGTACGAAAA TAGTAATCAA TTAATTGTAA AAGACATAAC TTTAGTCGAG CTTTCTCTTG GTATACATCA   
  
  
- ACATGAACGC TGCTATATAT ACAAATCAAA TCAATCAAGA ATGGACAAAA CCTTAACAAC CCCTCTAGAC   
  
  
- TCCTTCCTCG GGCGAAGTTA GTTTCAAGTA CCGTTATTCG GTTTCACTCC AAGGTAGGAT TTTGGTTAAC   
  
  
- CGTTATCCTT CTCATCGGGT GAATTGAACA TATGATACTG AGAAGAAAAG TTTGAGGGTT ACACCCTGAA   
  
  
- GAAATGAGTA CACACTATAA GGGTTGTCCT TACTAGTCAA GGTGTACAAA AGTAGAGAAA CCTGAGCTTG   
  
  
- AAGCACATTA ACATATACGG TACCGTGTTT ATCAAAACTA CCACGGTGGT TCAACGAGTT AGTTACATTA   
  
  
- TGACGATGAT AGATTGAACG GAACCACCTG TAGTTATTCT AGTGCTCTAC TCCTCTTGAC TTAGAACCGG   
  
  
- TTGGTGCAGC AAACTGAAGA GAATGGTTAT TTCTAACTTA TCTGTCACAA AGAGAGGGGG AAGGTAAGAA   
  
  
- GAAAAGATCA CTACAGCTTT TTCTGAAATA TGTACAGGTC GTTGCGATCG GTGTTTTATA AGGTAAAGAC   
  
  
- ATTAGGTGAG TACAGTTAAT TTGAACATAG TAGTTTACCA TCAAAGGCAT GATTAGTGAT GTAGATCTAT   
  
  
- AAGGAACTAT TTAAATTAAA CTGAAATTTC CGAGCTAGAA AAATTCTTTT CAAAACAACG AATGTTGGAC   
  
  
- ATTTCGGATC TCTTGTTTCG GTTTCTTCTT TAAATTCAAG ATATATATCG TATTAGAGAG TTTTTTATAT   
  
  
- AAAAAATTTA TATCACGCTA TAACTGACTG GATTATACCC AATAATGTCC TCTACCCCTC TATAGATGTA   
  
  
- ATTGATGAAC TTGTTGAATT TAAAAGTAAA ATTGAGCCAG TAATTCTTGT GATTCTTGAA ATATATAATC   
  
  
- ACTATAGAGT CCGCATTTTC TTTGATGAAA CTTAATTAGG TACGTTAGGA TGAGAAATAA AGGTAGGCAA   
  
  
- TTTTAAAAGG AAGTTTTGAG GGAAGACAAG TAATAATTAA GTTATAGCAT AATGTGAACA ACCTAATAAA   
  
  
- GACACTAAAC TAGCAGTAAA AGATATATAC TTATTGATGT TTTTAGTATC AGGCTCATGT AATTATAATT   
  
  
- ATTTTATCCT ACTCACTATT GGAGTATATC ACGCAAATAG GGATGGTGAT CCGACCTAAA GACGTATTTC   
  
  
- TTCTTTAACT TGTATACCTT TTTCTTTATT TCTCTTCGTT GAAGGAACTC ACCCTCTTCT TAGCATCTTT   
  
  
- CAATTTGAAT ACTTTTTCTC GTAATTGTTT TTCCTTTAAT ATTCCGAATG GCAACTTCGG AGTATATTAA   
  
  
- AACGTTCTCT GTCTGTTTCT TTCACAGAAA GTTTACAGTA TAAAGAGCTC GTACGTACTT TTTACATGAA   
  
  
- GCCGATGTTA TCATTATCAT ATATACAACC GAAGTATGTG AACTGGGGTT ACATACAGTT GCACTGATCA   
  
  
- CTGGTGTGTG TAGCAACAGG GACGGGCGGG GTGATGGGTT TGAGGGGCGT GCGTTTCTAT TTTTCGGGGG   
  
  
- GTTAAAAATA AACGGAGGGT TTTTCGGGTG GGCGCGAAAA AATAAACGGA AGAAAAAAGG ATGAATTTGG   
  
  
- TGGAGGAGTT GCTTGAAACG TTGTATGTAT GATCAGATAT TCGTTAAATC TCCCTTCCCT TCGTTGGATT   
  
  
- TGTCTCGCTC TAACCGCCCC ACACAAACTC CCGAACGCAA AACCACGTTA TCCTTACTTT CACGGGTAAA   
  
  
- GTTGGTGGTA GGTTTGAACG TTGAGGCCGT GGCCGTTTGG TGAAAGGAAA CTCTTATGAC GGGATGTATG   
  
  
- GCGGAAAGTT CAAGGGTTAT GGTGGATGCT TGGGTGGAGC CAAGAACTGG AGGTCGCGTC GGGATCGGGG   
  
  
- TGGAGGTGGG TTTTTGGCGG GAAACGGCGG AGGTTGGTGG TCGGACGGAG CCGACTAGAC CTGTTGAACC   
  
  
- TGCCCACCCT AAGTTAGGAT AGTCTCGACC CCGAATTACT GCTTAAATGG GGATTAAGGT TTGAGACCAG   
  
  
- GGTTTAATCA GGCAGACTAG GAGTGGAGGG GGTTGAGGGA CTAAAGGGTT GTAGAGTCGG CAAACTAGTA   
  
  
- GTGTTGGGTG GGGGTTATTT GGAGGGTAGT AGTCTAAAGT TGGAGACACT AAAGAGGATG TTAGTGTTGG   
  
  
- GGTTAAAACC CGGCAAGCTA GAAGTAGTGT TGGGCGTAGT CTTGTTGGGC TTGTTGTTAT TGATGCCGAA   
  
  
- ACTAAAGTAT CTGCTGGAGT AGTTTCGTCG TGTCAGGGAG CTTAGGTTGC TGAGAGTAGT TCAGGTGGAC   
  
  
- TATAACCGCG CCGAGTTAGT TGCCGAGTCT AGTGGGTGGC CGTTTGGCGA GGTCGCCCGG CGGATGATGA   
  
  
- AGTTCCTTCG GGAGTAGCGG CGGCGCCGGC CGGGCGGGGC GGGGCGAGCC GAAAGTAGCA TACTCCACCA   
  
  
- CGTCTGGTAG GCTCGGATGT TCCGCAAAAG TCCTTAAAGC GGTCAGAGAG AGAAGAGGTG AAAGCGGCGG   
  
  
- TTGGTCCGCT AAGAACTCCG CCACCTGTGC CGCCGGAAGT AGGTGTAGTA GCTAAAGCTG TAGCCTAAGC   
  
  
- CGCCGGTGAC CCGGAGCAAG GAGGCGCTTG ACCAGCTGTT TCGACTAGGA CGCTTTGAAT CAAGTCACCA   
  
  
- CAATGCTTAG TGGCGGTAGC AAGGGCTTCT TAAGCCCTAG CTTTCGTTCG ATCACTCTCT TTTAGACAGC   
  
  
- GTTAAACGGG CTCTAGAGTT GTAGTTGAAG GTGTAGCTAA TACAGAATAA AGTCTCGAAA CTTTAGAATA   
  
  
- GGAAGTTTAG ACAGTTTAAG TAGCTTTCCC TCTTTAACCG CGACGTAGAA AGCGGCCGGC ACAAGGTGGC   
  
  
- CGATCCCTCG CCTTAAAGCT TCAAATAGCG ACTAGAGGCT AGTTAGAGCG GCTCGAGGCA GCACCACCAC   
  
  
- CTGTCTCTAC AGCCCTAACT GTAGCCTTGC AGCAGCAAAT CGTACTTAAA GCAGCGGCCT TAACTTAAGA   
  
  
- TGTGGCCCTA CGACCTCAGC GAACTGCGTC GGTGGCGGCC CCCCGAGTAG CCGCCACACC TGACGCACTC   
  
  
- TGCCTAACTC TGCAAACAAG AGGTGGGTTC CTAGTACCGC CGCCATCTCC GCCGCCGCAG CCGCAGCCGG   
  
  
- CCCGCCTCCT GCCGCACCTC CCTCCGTAAG CGCCGCCGCC CCTAATCCCG CCACCCCAAA TCGGTCAAAC   
  
  
- GACTAAAGGT CCGGCTTACA AACAACTCCT CTCGAGTCCA ACCACCGAAG GTGCACCGTT TCGCGGTACC   
  
  
- TCTCTACTAC GAAATGACCG TACTGGCGGG TGAGCAGCGG TGTAGTCGAA CCTCCACAAT

+     GATA-motif

| Site Name | Organism | Position | Strand | Matrix score. | sequence | function |
| --- | --- | --- | --- | --- | --- | --- |
| GATA-motif | Arabidopsis thaliana | 2260 | - | 7 | GATAGGA | part of a light responsive element |
| GATA-motif | Solanum tuberosum | 3079 | - | 9 | AAGGATAAGG | part of a light responsive element |
| GATA-motif | Arabidopsis thaliana | 3077 | - | 10 | AAGATAAGATT | part of a light responsive element |

>HU05G02245.1   
+ -Up\_Stream \_Len000ATAATT TATCTTTCTT TAATTTCGTT TCATGTAAAA AATTTCAGTC TCATCTGCGA   
  
  
+ TTGAATCTCT TTTCAATTCG CATCAATAAT TCATCCAATT CAAATGTGCC TTTCTTAATT TCATTACATT   
  
  
+ GGGTTGGTAT AAATGTTAGC ACGATAGATT GGAATAGCAA GGCTCTAATT CAATTATGAC TTGAATGTAT   
  
  
+ GCATGCTTTT ATCATTAGTT AATTAACATT TTCTGTATTG AAATCAGCTC GAAAGAGAAC CATATGTAGT   
  
  
+ TGTACTTGCG ACGATATATA TGTTTAGTTT AGTTAGTTCT TACCTGTTTT GGAATTGTTG GGGAGATCTG   
  
  
+ AGGAAGGAGC CCGCTTCAAT CAAAGTTCAT GGCAATAAGC CAAAGTGAGG TTCCATCCTA AAACCAATTG   
  
  
+ GCAATAGGAA GAGTAGCCCA CTTAACTTGT ATACTATGAC TCTTCTTTTC AAACTCCCAA TGTGGGACTT   
  
  
+ CTTTACTCAT GTGTGATATT CCCAACAGGA ATGATCAGTT CCACATGTTT TCATCTCTTT GGACTCGAAC   
  
  
+ TTCGTGTAAT TGTATATGCC ATGGCACAAA TAGTTTTGAT GGTGCCACCA AGTTGCTCAA TCAATGTAAT   
  
  
+ ACTGCTACTA TCTAACTTGC CTTGGTGGAC ATCAATAAGA TCACGAGATG AGGAGAACTG AATCTTGGCC   
  
  
+ AACCACGTCG TTTGACTTCT CTTACCAATA AAGATTGAAT AGACAGTGTT TCTCTCCCCC TTCCATTCTT   
  
  
+ CTTTTCTAGT GATGTCGAAA AAGACTTTAT ACATGTCCAG CAACGCTAGC CACAAAATAT TCCATTTCTG   
  
  
+ TAATCCACTC ATGTCAATTA AACTTGTATC ATCAAATGGT AGTTTCCGTA CTAATCACTA CATCTAGATA   
  
  
+ TTCCTTGATA AATTTAATTT GACTTTAAAG GCTCGATCTT TTTAAGAAAA GTTTTGTTGC TTACAACCTG   
  
  
+ TAAAGCCTAG AGAACAAAGC CAAAGAAGAA ATTTAAGTTC TATATATAGC ATAATCTCTC AAAAAATATA   
  
  
+ TTTTTTAAAT ATAGTGCGAT ATTGACTGAC CTAATATGGG TTATTACAGG AGATGGGGAG ATATCTACAT   
  
  
+ TAACTACTTG AACAACTTAA ATTTTCATTT TAACTCGGTC ATTAAGAACA CTAAGAACTT TATATATTAG   
  
  
+ TGATATCTCA GGCGTAAAAG AAACTACTTT GAATTAATCC ATGCAATCCT ACTCTTTATT TCCATCCGTT   
  
  
+ AAAATTTTCC TTCAAAACTC CCTTCTGTTC ATTATTAATT CAATATCGTA TTACACTTGT TGGATTATTT   
  
  
+ CTGTGATTTG ATCGTCATTT TCTATATATG AATAACTACA AAAATCATAG TCCGAGTACA TTAATATTAA   
  
  
+ TAAAATAGGA TGAGTGATAA CCTCATATAG TGCGTTTATC CCTACCACTA GGCTGGATTT CTGCATAAAG   
  
  
+ AAGAAATTGA ACATATGGAA AAAGAAATAA AGAGAAGCAA CTTCCTTGAG TGGGAGAAGA ATCGTAGAAA   
  
  
+ GTTAAACTTA TGAAAAAGAG CATTAACAAA AAGGAAATTA TAAGGCTTAC CGTTGAAGCC TCATATAATT   
  
  
+ TTGCAAGAGA CAGACAAAGA AAGTGTCTTT CAAATGTCAT ATTTCTCGAG CATGCATGAA AAATGTACTT   
  
  
+ CGGCTACAAT AGTAATAGTA TATATGTTGG CTTCATACAC TTGACCCCAA TGTATGTCAA CGTGACTAGT   
  
  
+ GACCACACAC ATCGTTGTCC CTGCCCGCCC CACTACCCAA ACTCCCCGCA CGCAAAGATA AAAAGCCCCC   
  
  
+ CAATTTTTAT TTGCCTCCCA AAAAGCCCAC CCGCGCTTTT TTATTTGCCT TCTTTTTTCC TACTTAAACC   
  
  
+ ACCTCCTCAA CGAACTTTGC AACATACATA CTAGTCTATA AGCAATTTAG AGGGAAGGGA AGCAACCTAA   
  
  
+ ACAGAGCGAG ATTGGCGGGG TGTGTTTGAG GGCTTGCGTT TTGGTGCAAT AGGAATGAAA GTGCCCATTT   
  
  
+ CAACCACCAT CCAAACTTGC AACTCCGGCA CCGGCAAACC ACTTTCCTTT GAGAATACTG CCCTACATAC   
  
  
+ CGCCTTTCAA GTTCCCAATA CCACCTACGA ACCCACCTCG GTTCTTGACC TCCAGCGCAG CCCTAGCCCC   
  
  
+ ACCTCCACCC AAAAACCGCC CTTTGCCGCC TCCAACCACC AGCCTGCCTC GGCTGATCTG GACAACTTGG   
  
  
+ ACGGGTGGGA TTCAATCCTA TCAGAGCTGG GGCTTAATGA CGAATTTACC CCTAATTCCA AACTCTGGTC   
  
  
+ CCAAATTAGT CCGTCTGATC CTCACCTCCC CCAACTCCCT GATTTCCCAA CATCTCAGCC GTTTGATCAT   
  
  
+ CACAACCCAC CCCCAATAAA CCTCCCATCA TCAGATTTCA ACCTCTGTGA TTTCTCCTAC AATCACAACC   
  
  
+ CCAATTTTGG GCCGTTCGAT CTTCATCACA ACCCGCATCA GAACAACCCG AACAACAATA ACTACGGCTT   
  
  
+ TGATTTCATA GACGACCTCA TCAAAGCAGC ACAGTCCCTC GAATCCAACG ACTCTCATCA AGTCCACCTG   
  
  
+ ATATTGGCGC GGCTCAATCA ACGGCTCAGA TCACCCACCG GCAAACCGCT CCAGCGGGCC GCCTACTACT   
  
  
+ TCAAGGAAGC CCTCATCGCC GCCGCGGCCG GCCCGCCCCG CCCCGCTCGG CTTTCATCGT ATGAGGTGGT   
  
  
+ GCAGACCATC CGAGCCTACA AGGCGTTTTC AGGAATTTCG CCAGTCTCTC TCTTCTCCAC TTTCGCCGCC   
  
  
+ AACCAGGCGA TTCTTGAGGC GGTGGACACG GCGGCCTTCA TCCACATCAT CGATTTCGAC ATCGGATTCG   
  
  
+ GCGGCCACTG GGCCTCGTTC CTCCGCGAAC TGGTCGACAA AGCTGATCCT GCGAAACTTA GTTCAGTGGT   
  
  
+ GTTACGAATC ACCGCCATCG TTCCCGAAGA ATTCGGGATC GAAAGCAAGC TAGTGAGAGA AAATCTGTCG   
  
  
+ CAATTTGCCC GAGATCTCAA CATCAACTTC CACATCGATT ATGTCTTATT TCAGAGCTTT GAAATCTTAT   
  
  
+ CCTTCAAATC TGTCAAATTC ATCGAAAGGG AGAAATTGGC GCTGCATCTT TCGCCGGCCG TGTTCCACCG   
  
  
+ GCTAGGGAGC GGAATTTCGA AGTTTATCGC TGATCTCCGA TCAATCTCGC CGAGCTCCGT CGTGGTGGTG   
  
  
+ GACAGAGATG TCGGGATTGA CATCGGAACG TCGTCGTTTA GCATGAATTT CGTCGCCGGA ATTGAATTCT   
  
  
+ ACACCGGGAT GCTGGAGTCG CTTGACGCAG CCACCGCCGG GGGGCTCATC GGCGGTGTGG ACTGCGTGAG   
  
  
+ ACGGATTGAG ACGTTTGTTC TCCACCCAAG GATCATGGCG GCGGTAGAGG CGGCGGCGTC GGCGTCGGCC   
  
  
+ GGGCGGAGGA CGGCGTGGAG GGAGGCATTC GCGGCGGCGG GGATTAGGGC GGTGGGGTTT AGCCAGTTTG   
  
  
+ CTGATTTCCA GGCCGAATGT TTGTTGAGGA GAGCTCAGGT TGGTGGCTTC CACGTGGCAA AGCGCCATGG   
  
  
+ AGAGATGATG CTTTACTGGC ATGACCGCCC ACTCGTCGCC ACATCAGCTT GGAGGTGTTA   

- -Up\_Stream \_Len000TATTAA ATAGAAAGAA ATTAAAGCAA AGTACATTTT TTAAAGTCAG AGTAGACGCT   
  
  
- AACTTAGAGA AAAGTTAAGC GTAGTTATTA AGTAGGTTAA GTTTACACGG AAAGAATTAA AGTAATGTAA   
  
  
- CCCAACCATA TTTACAATCG TGCTATCTAA CCTTATCGTT CCGAGATTAA GTTAATACTG AACTTACATA   
  
  
- CGTACGAAAA TAGTAATCAA TTAATTGTAA AAGACATAAC TTTAGTCGAG CTTTCTCTTG GTATACATCA   
  
  
- ACATGAACGC TGCTATATAT ACAAATCAAA TCAATCAAGA ATGGACAAAA CCTTAACAAC CCCTCTAGAC   
  
  
- TCCTTCCTCG GGCGAAGTTA GTTTCAAGTA CCGTTATTCG GTTTCACTCC AAGGTAGGAT TTTGGTTAAC   
  
  
- CGTTATCCTT CTCATCGGGT GAATTGAACA TATGATACTG AGAAGAAAAG TTTGAGGGTT ACACCCTGAA   
  
  
- GAAATGAGTA CACACTATAA GGGTTGTCCT TACTAGTCAA GGTGTACAAA AGTAGAGAAA CCTGAGCTTG   
  
  
- AAGCACATTA ACATATACGG TACCGTGTTT ATCAAAACTA CCACGGTGGT TCAACGAGTT AGTTACATTA   
  
  
- TGACGATGAT AGATTGAACG GAACCACCTG TAGTTATTCT AGTGCTCTAC TCCTCTTGAC TTAGAACCGG   
  
  
- TTGGTGCAGC AAACTGAAGA GAATGGTTAT TTCTAACTTA TCTGTCACAA AGAGAGGGGG AAGGTAAGAA   
  
  
- GAAAAGATCA CTACAGCTTT TTCTGAAATA TGTACAGGTC GTTGCGATCG GTGTTTTATA AGGTAAAGAC   
  
  
- ATTAGGTGAG TACAGTTAAT TTGAACATAG TAGTTTACCA TCAAAGGCAT GATTAGTGAT GTAGATCTAT   
  
  
- AAGGAACTAT TTAAATTAAA CTGAAATTTC CGAGCTAGAA AAATTCTTTT CAAAACAACG AATGTTGGAC   
  
  
- ATTTCGGATC TCTTGTTTCG GTTTCTTCTT TAAATTCAAG ATATATATCG TATTAGAGAG TTTTTTATAT   
  
  
- AAAAAATTTA TATCACGCTA TAACTGACTG GATTATACCC AATAATGTCC TCTACCCCTC TATAGATGTA   
  
  
- ATTGATGAAC TTGTTGAATT TAAAAGTAAA ATTGAGCCAG TAATTCTTGT GATTCTTGAA ATATATAATC   
  
  
- ACTATAGAGT CCGCATTTTC TTTGATGAAA CTTAATTAGG TACGTTAGGA TGAGAAATAA AGGTAGGCAA   
  
  
- TTTTAAAAGG AAGTTTTGAG GGAAGACAAG TAATAATTAA GTTATAGCAT AATGTGAACA ACCTAATAAA   
  
  
- GACACTAAAC TAGCAGTAAA AGATATATAC TTATTGATGT TTTTAGTATC AGGCTCATGT AATTATAATT   
  
  
- ATTTTATCCT ACTCACTATT GGAGTATATC ACGCAAATAG GGATGGTGAT CCGACCTAAA GACGTATTTC   
  
  
- TTCTTTAACT TGTATACCTT TTTCTTTATT TCTCTTCGTT GAAGGAACTC ACCCTCTTCT TAGCATCTTT   
  
  
- CAATTTGAAT ACTTTTTCTC GTAATTGTTT TTCCTTTAAT ATTCCGAATG GCAACTTCGG AGTATATTAA   
  
  
- AACGTTCTCT GTCTGTTTCT TTCACAGAAA GTTTACAGTA TAAAGAGCTC GTACGTACTT TTTACATGAA   
  
  
- GCCGATGTTA TCATTATCAT ATATACAACC GAAGTATGTG AACTGGGGTT ACATACAGTT GCACTGATCA   
  
  
- CTGGTGTGTG TAGCAACAGG GACGGGCGGG GTGATGGGTT TGAGGGGCGT GCGTTTCTAT TTTTCGGGGG   
  
  
- GTTAAAAATA AACGGAGGGT TTTTCGGGTG GGCGCGAAAA AATAAACGGA AGAAAAAAGG ATGAATTTGG   
  
  
- TGGAGGAGTT GCTTGAAACG TTGTATGTAT GATCAGATAT TCGTTAAATC TCCCTTCCCT TCGTTGGATT   
  
  
- TGTCTCGCTC TAACCGCCCC ACACAAACTC CCGAACGCAA AACCACGTTA TCCTTACTTT CACGGGTAAA   
  
  
- GTTGGTGGTA GGTTTGAACG TTGAGGCCGT GGCCGTTTGG TGAAAGGAAA CTCTTATGAC GGGATGTATG   
  
  
- GCGGAAAGTT CAAGGGTTAT GGTGGATGCT TGGGTGGAGC CAAGAACTGG AGGTCGCGTC GGGATCGGGG   
  
  
- TGGAGGTGGG TTTTTGGCGG GAAACGGCGG AGGTTGGTGG TCGGACGGAG CCGACTAGAC CTGTTGAACC   
  
  
- TGCCCACCCT AAGTTAGGAT AGTCTCGACC CCGAATTACT GCTTAAATGG GGATTAAGGT TTGAGACCAG   
  
  
- GGTTTAATCA GGCAGACTAG GAGTGGAGGG GGTTGAGGGA CTAAAGGGTT GTAGAGTCGG CAAACTAGTA   
  
  
- GTGTTGGGTG GGGGTTATTT GGAGGGTAGT AGTCTAAAGT TGGAGACACT AAAGAGGATG TTAGTGTTGG   
  
  
- GGTTAAAACC CGGCAAGCTA GAAGTAGTGT TGGGCGTAGT CTTGTTGGGC TTGTTGTTAT TGATGCCGAA   
  
  
- ACTAAAGTAT CTGCTGGAGT AGTTTCGTCG TGTCAGGGAG CTTAGGTTGC TGAGAGTAGT TCAGGTGGAC   
  
  
- TATAACCGCG CCGAGTTAGT TGCCGAGTCT AGTGGGTGGC CGTTTGGCGA GGTCGCCCGG CGGATGATGA   
  
  
- AGTTCCTTCG GGAGTAGCGG CGGCGCCGGC CGGGCGGGGC GGGGCGAGCC GAAAGTAGCA TACTCCACCA   
  
  
- CGTCTGGTAG GCTCGGATGT TCCGCAAAAG TCCTTAAAGC GGTCAGAGAG AGAAGAGGTG AAAGCGGCGG   
  
  
- TTGGTCCGCT AAGAACTCCG CCACCTGTGC CGCCGGAAGT AGGTGTAGTA GCTAAAGCTG TAGCCTAAGC   
  
  
- CGCCGGTGAC CCGGAGCAAG GAGGCGCTTG ACCAGCTGTT TCGACTAGGA CGCTTTGAAT CAAGTCACCA   
  
  
- CAATGCTTAG TGGCGGTAGC AAGGGCTTCT TAAGCCCTAG CTTTCGTTCG ATCACTCTCT TTTAGACAGC   
  
  
- GTTAAACGGG CTCTAGAGTT GTAGTTGAAG GTGTAGCTAA TACAGAATAA AGTCTCGAAA CTTTAGAATA   
  
  
- GGAAGTTTAG ACAGTTTAAG TAGCTTTCCC TCTTTAACCG CGACGTAGAA AGCGGCCGGC ACAAGGTGGC   
  
  
- CGATCCCTCG CCTTAAAGCT TCAAATAGCG ACTAGAGGCT AGTTAGAGCG GCTCGAGGCA GCACCACCAC   
  
  
- CTGTCTCTAC AGCCCTAACT GTAGCCTTGC AGCAGCAAAT CGTACTTAAA GCAGCGGCCT TAACTTAAGA   
  
  
- TGTGGCCCTA CGACCTCAGC GAACTGCGTC GGTGGCGGCC CCCCGAGTAG CCGCCACACC TGACGCACTC   
  
  
- TGCCTAACTC TGCAAACAAG AGGTGGGTTC CTAGTACCGC CGCCATCTCC GCCGCCGCAG CCGCAGCCGG   
  
  
- CCCGCCTCCT GCCGCACCTC CCTCCGTAAG CGCCGCCGCC CCTAATCCCG CCACCCCAAA TCGGTCAAAC   
  
  
- GACTAAAGGT CCGGCTTACA AACAACTCCT CTCGAGTCCA ACCACCGAAG GTGCACCGTT TCGCGGTACC   
  
  
- TCTCTACTAC GAAATGACCG TACTGGCGGG TGAGCAGCGG TGTAGTCGAA CCTCCACAAT

+     GC-motif

| Site Name | Organism | Position | Strand | Matrix score. | sequence | function |
| --- | --- | --- | --- | --- | --- | --- |
| GC-motif | Zea mays | 3332 | - | 6 | CCCCCG | enhancer-like element involved in anoxic specific inducibility |

>HU05G02245.1   
+ -Up\_Stream \_Len000ATAATT TATCTTTCTT TAATTTCGTT TCATGTAAAA AATTTCAGTC TCATCTGCGA   
  
  
+ TTGAATCTCT TTTCAATTCG CATCAATAAT TCATCCAATT CAAATGTGCC TTTCTTAATT TCATTACATT   
  
  
+ GGGTTGGTAT AAATGTTAGC ACGATAGATT GGAATAGCAA GGCTCTAATT CAATTATGAC TTGAATGTAT   
  
  
+ GCATGCTTTT ATCATTAGTT AATTAACATT TTCTGTATTG AAATCAGCTC GAAAGAGAAC CATATGTAGT   
  
  
+ TGTACTTGCG ACGATATATA TGTTTAGTTT AGTTAGTTCT TACCTGTTTT GGAATTGTTG GGGAGATCTG   
  
  
+ AGGAAGGAGC CCGCTTCAAT CAAAGTTCAT GGCAATAAGC CAAAGTGAGG TTCCATCCTA AAACCAATTG   
  
  
+ GCAATAGGAA GAGTAGCCCA CTTAACTTGT ATACTATGAC TCTTCTTTTC AAACTCCCAA TGTGGGACTT   
  
  
+ CTTTACTCAT GTGTGATATT CCCAACAGGA ATGATCAGTT CCACATGTTT TCATCTCTTT GGACTCGAAC   
  
  
+ TTCGTGTAAT TGTATATGCC ATGGCACAAA TAGTTTTGAT GGTGCCACCA AGTTGCTCAA TCAATGTAAT   
  
  
+ ACTGCTACTA TCTAACTTGC CTTGGTGGAC ATCAATAAGA TCACGAGATG AGGAGAACTG AATCTTGGCC   
  
  
+ AACCACGTCG TTTGACTTCT CTTACCAATA AAGATTGAAT AGACAGTGTT TCTCTCCCCC TTCCATTCTT   
  
  
+ CTTTTCTAGT GATGTCGAAA AAGACTTTAT ACATGTCCAG CAACGCTAGC CACAAAATAT TCCATTTCTG   
  
  
+ TAATCCACTC ATGTCAATTA AACTTGTATC ATCAAATGGT AGTTTCCGTA CTAATCACTA CATCTAGATA   
  
  
+ TTCCTTGATA AATTTAATTT GACTTTAAAG GCTCGATCTT TTTAAGAAAA GTTTTGTTGC TTACAACCTG   
  
  
+ TAAAGCCTAG AGAACAAAGC CAAAGAAGAA ATTTAAGTTC TATATATAGC ATAATCTCTC AAAAAATATA   
  
  
+ TTTTTTAAAT ATAGTGCGAT ATTGACTGAC CTAATATGGG TTATTACAGG AGATGGGGAG ATATCTACAT   
  
  
+ TAACTACTTG AACAACTTAA ATTTTCATTT TAACTCGGTC ATTAAGAACA CTAAGAACTT TATATATTAG   
  
  
+ TGATATCTCA GGCGTAAAAG AAACTACTTT GAATTAATCC ATGCAATCCT ACTCTTTATT TCCATCCGTT   
  
  
+ AAAATTTTCC TTCAAAACTC CCTTCTGTTC ATTATTAATT CAATATCGTA TTACACTTGT TGGATTATTT   
  
  
+ CTGTGATTTG ATCGTCATTT TCTATATATG AATAACTACA AAAATCATAG TCCGAGTACA TTAATATTAA   
  
  
+ TAAAATAGGA TGAGTGATAA CCTCATATAG TGCGTTTATC CCTACCACTA GGCTGGATTT CTGCATAAAG   
  
  
+ AAGAAATTGA ACATATGGAA AAAGAAATAA AGAGAAGCAA CTTCCTTGAG TGGGAGAAGA ATCGTAGAAA   
  
  
+ GTTAAACTTA TGAAAAAGAG CATTAACAAA AAGGAAATTA TAAGGCTTAC CGTTGAAGCC TCATATAATT   
  
  
+ TTGCAAGAGA CAGACAAAGA AAGTGTCTTT CAAATGTCAT ATTTCTCGAG CATGCATGAA AAATGTACTT   
  
  
+ CGGCTACAAT AGTAATAGTA TATATGTTGG CTTCATACAC TTGACCCCAA TGTATGTCAA CGTGACTAGT   
  
  
+ GACCACACAC ATCGTTGTCC CTGCCCGCCC CACTACCCAA ACTCCCCGCA CGCAAAGATA AAAAGCCCCC   
  
  
+ CAATTTTTAT TTGCCTCCCA AAAAGCCCAC CCGCGCTTTT TTATTTGCCT TCTTTTTTCC TACTTAAACC   
  
  
+ ACCTCCTCAA CGAACTTTGC AACATACATA CTAGTCTATA AGCAATTTAG AGGGAAGGGA AGCAACCTAA   
  
  
+ ACAGAGCGAG ATTGGCGGGG TGTGTTTGAG GGCTTGCGTT TTGGTGCAAT AGGAATGAAA GTGCCCATTT   
  
  
+ CAACCACCAT CCAAACTTGC AACTCCGGCA CCGGCAAACC ACTTTCCTTT GAGAATACTG CCCTACATAC   
  
  
+ CGCCTTTCAA GTTCCCAATA CCACCTACGA ACCCACCTCG GTTCTTGACC TCCAGCGCAG CCCTAGCCCC   
  
  
+ ACCTCCACCC AAAAACCGCC CTTTGCCGCC TCCAACCACC AGCCTGCCTC GGCTGATCTG GACAACTTGG   
  
  
+ ACGGGTGGGA TTCAATCCTA TCAGAGCTGG GGCTTAATGA CGAATTTACC CCTAATTCCA AACTCTGGTC   
  
  
+ CCAAATTAGT CCGTCTGATC CTCACCTCCC CCAACTCCCT GATTTCCCAA CATCTCAGCC GTTTGATCAT   
  
  
+ CACAACCCAC CCCCAATAAA CCTCCCATCA TCAGATTTCA ACCTCTGTGA TTTCTCCTAC AATCACAACC   
  
  
+ CCAATTTTGG GCCGTTCGAT CTTCATCACA ACCCGCATCA GAACAACCCG AACAACAATA ACTACGGCTT   
  
  
+ TGATTTCATA GACGACCTCA TCAAAGCAGC ACAGTCCCTC GAATCCAACG ACTCTCATCA AGTCCACCTG   
  
  
+ ATATTGGCGC GGCTCAATCA ACGGCTCAGA TCACCCACCG GCAAACCGCT CCAGCGGGCC GCCTACTACT   
  
  
+ TCAAGGAAGC CCTCATCGCC GCCGCGGCCG GCCCGCCCCG CCCCGCTCGG CTTTCATCGT ATGAGGTGGT   
  
  
+ GCAGACCATC CGAGCCTACA AGGCGTTTTC AGGAATTTCG CCAGTCTCTC TCTTCTCCAC TTTCGCCGCC   
  
  
+ AACCAGGCGA TTCTTGAGGC GGTGGACACG GCGGCCTTCA TCCACATCAT CGATTTCGAC ATCGGATTCG   
  
  
+ GCGGCCACTG GGCCTCGTTC CTCCGCGAAC TGGTCGACAA AGCTGATCCT GCGAAACTTA GTTCAGTGGT   
  
  
+ GTTACGAATC ACCGCCATCG TTCCCGAAGA ATTCGGGATC GAAAGCAAGC TAGTGAGAGA AAATCTGTCG   
  
  
+ CAATTTGCCC GAGATCTCAA CATCAACTTC CACATCGATT ATGTCTTATT TCAGAGCTTT GAAATCTTAT   
  
  
+ CCTTCAAATC TGTCAAATTC ATCGAAAGGG AGAAATTGGC GCTGCATCTT TCGCCGGCCG TGTTCCACCG   
  
  
+ GCTAGGGAGC GGAATTTCGA AGTTTATCGC TGATCTCCGA TCAATCTCGC CGAGCTCCGT CGTGGTGGTG   
  
  
+ GACAGAGATG TCGGGATTGA CATCGGAACG TCGTCGTTTA GCATGAATTT CGTCGCCGGA ATTGAATTCT   
  
  
+ ACACCGGGAT GCTGGAGTCG CTTGACGCAG CCACCGCCGG GGGGCTCATC GGCGGTGTGG ACTGCGTGAG   
  
  
+ ACGGATTGAG ACGTTTGTTC TCCACCCAAG GATCATGGCG GCGGTAGAGG CGGCGGCGTC GGCGTCGGCC   
  
  
+ GGGCGGAGGA CGGCGTGGAG GGAGGCATTC GCGGCGGCGG GGATTAGGGC GGTGGGGTTT AGCCAGTTTG   
  
  
+ CTGATTTCCA GGCCGAATGT TTGTTGAGGA GAGCTCAGGT TGGTGGCTTC CACGTGGCAA AGCGCCATGG   
  
  
+ AGAGATGATG CTTTACTGGC ATGACCGCCC ACTCGTCGCC ACATCAGCTT GGAGGTGTTA   

- -Up\_Stream \_Len000TATTAA ATAGAAAGAA ATTAAAGCAA AGTACATTTT TTAAAGTCAG AGTAGACGCT   
  
  
- AACTTAGAGA AAAGTTAAGC GTAGTTATTA AGTAGGTTAA GTTTACACGG AAAGAATTAA AGTAATGTAA   
  
  
- CCCAACCATA TTTACAATCG TGCTATCTAA CCTTATCGTT CCGAGATTAA GTTAATACTG AACTTACATA   
  
  
- CGTACGAAAA TAGTAATCAA TTAATTGTAA AAGACATAAC TTTAGTCGAG CTTTCTCTTG GTATACATCA   
  
  
- ACATGAACGC TGCTATATAT ACAAATCAAA TCAATCAAGA ATGGACAAAA CCTTAACAAC CCCTCTAGAC   
  
  
- TCCTTCCTCG GGCGAAGTTA GTTTCAAGTA CCGTTATTCG GTTTCACTCC AAGGTAGGAT TTTGGTTAAC   
  
  
- CGTTATCCTT CTCATCGGGT GAATTGAACA TATGATACTG AGAAGAAAAG TTTGAGGGTT ACACCCTGAA   
  
  
- GAAATGAGTA CACACTATAA GGGTTGTCCT TACTAGTCAA GGTGTACAAA AGTAGAGAAA CCTGAGCTTG   
  
  
- AAGCACATTA ACATATACGG TACCGTGTTT ATCAAAACTA CCACGGTGGT TCAACGAGTT AGTTACATTA   
  
  
- TGACGATGAT AGATTGAACG GAACCACCTG TAGTTATTCT AGTGCTCTAC TCCTCTTGAC TTAGAACCGG   
  
  
- TTGGTGCAGC AAACTGAAGA GAATGGTTAT TTCTAACTTA TCTGTCACAA AGAGAGGGGG AAGGTAAGAA   
  
  
- GAAAAGATCA CTACAGCTTT TTCTGAAATA TGTACAGGTC GTTGCGATCG GTGTTTTATA AGGTAAAGAC   
  
  
- ATTAGGTGAG TACAGTTAAT TTGAACATAG TAGTTTACCA TCAAAGGCAT GATTAGTGAT GTAGATCTAT   
  
  
- AAGGAACTAT TTAAATTAAA CTGAAATTTC CGAGCTAGAA AAATTCTTTT CAAAACAACG AATGTTGGAC   
  
  
- ATTTCGGATC TCTTGTTTCG GTTTCTTCTT TAAATTCAAG ATATATATCG TATTAGAGAG TTTTTTATAT   
  
  
- AAAAAATTTA TATCACGCTA TAACTGACTG GATTATACCC AATAATGTCC TCTACCCCTC TATAGATGTA   
  
  
- ATTGATGAAC TTGTTGAATT TAAAAGTAAA ATTGAGCCAG TAATTCTTGT GATTCTTGAA ATATATAATC   
  
  
- ACTATAGAGT CCGCATTTTC TTTGATGAAA CTTAATTAGG TACGTTAGGA TGAGAAATAA AGGTAGGCAA   
  
  
- TTTTAAAAGG AAGTTTTGAG GGAAGACAAG TAATAATTAA GTTATAGCAT AATGTGAACA ACCTAATAAA   
  
  
- GACACTAAAC TAGCAGTAAA AGATATATAC TTATTGATGT TTTTAGTATC AGGCTCATGT AATTATAATT   
  
  
- ATTTTATCCT ACTCACTATT GGAGTATATC ACGCAAATAG GGATGGTGAT CCGACCTAAA GACGTATTTC   
  
  
- TTCTTTAACT TGTATACCTT TTTCTTTATT TCTCTTCGTT GAAGGAACTC ACCCTCTTCT TAGCATCTTT   
  
  
- CAATTTGAAT ACTTTTTCTC GTAATTGTTT TTCCTTTAAT ATTCCGAATG GCAACTTCGG AGTATATTAA   
  
  
- AACGTTCTCT GTCTGTTTCT TTCACAGAAA GTTTACAGTA TAAAGAGCTC GTACGTACTT TTTACATGAA   
  
  
- GCCGATGTTA TCATTATCAT ATATACAACC GAAGTATGTG AACTGGGGTT ACATACAGTT GCACTGATCA   
  
  
- CTGGTGTGTG TAGCAACAGG GACGGGCGGG GTGATGGGTT TGAGGGGCGT GCGTTTCTAT TTTTCGGGGG   
  
  
- GTTAAAAATA AACGGAGGGT TTTTCGGGTG GGCGCGAAAA AATAAACGGA AGAAAAAAGG ATGAATTTGG   
  
  
- TGGAGGAGTT GCTTGAAACG TTGTATGTAT GATCAGATAT TCGTTAAATC TCCCTTCCCT TCGTTGGATT   
  
  
- TGTCTCGCTC TAACCGCCCC ACACAAACTC CCGAACGCAA AACCACGTTA TCCTTACTTT CACGGGTAAA   
  
  
- GTTGGTGGTA GGTTTGAACG TTGAGGCCGT GGCCGTTTGG TGAAAGGAAA CTCTTATGAC GGGATGTATG   
  
  
- GCGGAAAGTT CAAGGGTTAT GGTGGATGCT TGGGTGGAGC CAAGAACTGG AGGTCGCGTC GGGATCGGGG   
  
  
- TGGAGGTGGG TTTTTGGCGG GAAACGGCGG AGGTTGGTGG TCGGACGGAG CCGACTAGAC CTGTTGAACC   
  
  
- TGCCCACCCT AAGTTAGGAT AGTCTCGACC CCGAATTACT GCTTAAATGG GGATTAAGGT TTGAGACCAG   
  
  
- GGTTTAATCA GGCAGACTAG GAGTGGAGGG GGTTGAGGGA CTAAAGGGTT GTAGAGTCGG CAAACTAGTA   
  
  
- GTGTTGGGTG GGGGTTATTT GGAGGGTAGT AGTCTAAAGT TGGAGACACT AAAGAGGATG TTAGTGTTGG   
  
  
- GGTTAAAACC CGGCAAGCTA GAAGTAGTGT TGGGCGTAGT CTTGTTGGGC TTGTTGTTAT TGATGCCGAA   
  
  
- ACTAAAGTAT CTGCTGGAGT AGTTTCGTCG TGTCAGGGAG CTTAGGTTGC TGAGAGTAGT TCAGGTGGAC   
  
  
- TATAACCGCG CCGAGTTAGT TGCCGAGTCT AGTGGGTGGC CGTTTGGCGA GGTCGCCCGG CGGATGATGA   
  
  
- AGTTCCTTCG GGAGTAGCGG CGGCGCCGGC CGGGCGGGGC GGGGCGAGCC GAAAGTAGCA TACTCCACCA   
  
  
- CGTCTGGTAG GCTCGGATGT TCCGCAAAAG TCCTTAAAGC GGTCAGAGAG AGAAGAGGTG AAAGCGGCGG   
  
  
- TTGGTCCGCT AAGAACTCCG CCACCTGTGC CGCCGGAAGT AGGTGTAGTA GCTAAAGCTG TAGCCTAAGC   
  
  
- CGCCGGTGAC CCGGAGCAAG GAGGCGCTTG ACCAGCTGTT TCGACTAGGA CGCTTTGAAT CAAGTCACCA   
  
  
- CAATGCTTAG TGGCGGTAGC AAGGGCTTCT TAAGCCCTAG CTTTCGTTCG ATCACTCTCT TTTAGACAGC   
  
  
- GTTAAACGGG CTCTAGAGTT GTAGTTGAAG GTGTAGCTAA TACAGAATAA AGTCTCGAAA CTTTAGAATA   
  
  
- GGAAGTTTAG ACAGTTTAAG TAGCTTTCCC TCTTTAACCG CGACGTAGAA AGCGGCCGGC ACAAGGTGGC   
  
  
- CGATCCCTCG CCTTAAAGCT TCAAATAGCG ACTAGAGGCT AGTTAGAGCG GCTCGAGGCA GCACCACCAC   
  
  
- CTGTCTCTAC AGCCCTAACT GTAGCCTTGC AGCAGCAAAT CGTACTTAAA GCAGCGGCCT TAACTTAAGA   
  
  
- TGTGGCCCTA CGACCTCAGC GAACTGCGTC GGTGGCGGCC CCCCGAGTAG CCGCCACACC TGACGCACTC   
  
  
- TGCCTAACTC TGCAAACAAG AGGTGGGTTC CTAGTACCGC CGCCATCTCC GCCGCCGCAG CCGCAGCCGG   
  
  
- CCCGCCTCCT GCCGCACCTC CCTCCGTAAG CGCCGCCGCC CCTAATCCCG CCACCCCAAA TCGGTCAAAC   
  
  
- GACTAAAGGT CCGGCTTACA AACAACTCCT CTCGAGTCCA ACCACCGAAG GTGCACCGTT TCGCGGTACC   
  
  
- TCTCTACTAC GAAATGACCG TACTGGCGGG TGAGCAGCGG TGTAGTCGAA CCTCCACAAT

+     L-box

| Site Name | Organism | Position | Strand | Matrix score. | sequence | function |
| --- | --- | --- | --- | --- | --- | --- |
| L-box | Petroselinum crispum | 2122 | + | 10 | ATCCCACCTAC | part of a light responsive element |

>HU05G02245.1   
+ -Up\_Stream \_Len000ATAATT TATCTTTCTT TAATTTCGTT TCATGTAAAA AATTTCAGTC TCATCTGCGA   
  
  
+ TTGAATCTCT TTTCAATTCG CATCAATAAT TCATCCAATT CAAATGTGCC TTTCTTAATT TCATTACATT   
  
  
+ GGGTTGGTAT AAATGTTAGC ACGATAGATT GGAATAGCAA GGCTCTAATT CAATTATGAC TTGAATGTAT   
  
  
+ GCATGCTTTT ATCATTAGTT AATTAACATT TTCTGTATTG AAATCAGCTC GAAAGAGAAC CATATGTAGT   
  
  
+ TGTACTTGCG ACGATATATA TGTTTAGTTT AGTTAGTTCT TACCTGTTTT GGAATTGTTG GGGAGATCTG   
  
  
+ AGGAAGGAGC CCGCTTCAAT CAAAGTTCAT GGCAATAAGC CAAAGTGAGG TTCCATCCTA AAACCAATTG   
  
  
+ GCAATAGGAA GAGTAGCCCA CTTAACTTGT ATACTATGAC TCTTCTTTTC AAACTCCCAA TGTGGGACTT   
  
  
+ CTTTACTCAT GTGTGATATT CCCAACAGGA ATGATCAGTT CCACATGTTT TCATCTCTTT GGACTCGAAC   
  
  
+ TTCGTGTAAT TGTATATGCC ATGGCACAAA TAGTTTTGAT GGTGCCACCA AGTTGCTCAA TCAATGTAAT   
  
  
+ ACTGCTACTA TCTAACTTGC CTTGGTGGAC ATCAATAAGA TCACGAGATG AGGAGAACTG AATCTTGGCC   
  
  
+ AACCACGTCG TTTGACTTCT CTTACCAATA AAGATTGAAT AGACAGTGTT TCTCTCCCCC TTCCATTCTT   
  
  
+ CTTTTCTAGT GATGTCGAAA AAGACTTTAT ACATGTCCAG CAACGCTAGC CACAAAATAT TCCATTTCTG   
  
  
+ TAATCCACTC ATGTCAATTA AACTTGTATC ATCAAATGGT AGTTTCCGTA CTAATCACTA CATCTAGATA   
  
  
+ TTCCTTGATA AATTTAATTT GACTTTAAAG GCTCGATCTT TTTAAGAAAA GTTTTGTTGC TTACAACCTG   
  
  
+ TAAAGCCTAG AGAACAAAGC CAAAGAAGAA ATTTAAGTTC TATATATAGC ATAATCTCTC AAAAAATATA   
  
  
+ TTTTTTAAAT ATAGTGCGAT ATTGACTGAC CTAATATGGG TTATTACAGG AGATGGGGAG ATATCTACAT   
  
  
+ TAACTACTTG AACAACTTAA ATTTTCATTT TAACTCGGTC ATTAAGAACA CTAAGAACTT TATATATTAG   
  
  
+ TGATATCTCA GGCGTAAAAG AAACTACTTT GAATTAATCC ATGCAATCCT ACTCTTTATT TCCATCCGTT   
  
  
+ AAAATTTTCC TTCAAAACTC CCTTCTGTTC ATTATTAATT CAATATCGTA TTACACTTGT TGGATTATTT   
  
  
+ CTGTGATTTG ATCGTCATTT TCTATATATG AATAACTACA AAAATCATAG TCCGAGTACA TTAATATTAA   
  
  
+ TAAAATAGGA TGAGTGATAA CCTCATATAG TGCGTTTATC CCTACCACTA GGCTGGATTT CTGCATAAAG   
  
  
+ AAGAAATTGA ACATATGGAA AAAGAAATAA AGAGAAGCAA CTTCCTTGAG TGGGAGAAGA ATCGTAGAAA   
  
  
+ GTTAAACTTA TGAAAAAGAG CATTAACAAA AAGGAAATTA TAAGGCTTAC CGTTGAAGCC TCATATAATT   
  
  
+ TTGCAAGAGA CAGACAAAGA AAGTGTCTTT CAAATGTCAT ATTTCTCGAG CATGCATGAA AAATGTACTT   
  
  
+ CGGCTACAAT AGTAATAGTA TATATGTTGG CTTCATACAC TTGACCCCAA TGTATGTCAA CGTGACTAGT   
  
  
+ GACCACACAC ATCGTTGTCC CTGCCCGCCC CACTACCCAA ACTCCCCGCA CGCAAAGATA AAAAGCCCCC   
  
  
+ CAATTTTTAT TTGCCTCCCA AAAAGCCCAC CCGCGCTTTT TTATTTGCCT TCTTTTTTCC TACTTAAACC   
  
  
+ ACCTCCTCAA CGAACTTTGC AACATACATA CTAGTCTATA AGCAATTTAG AGGGAAGGGA AGCAACCTAA   
  
  
+ ACAGAGCGAG ATTGGCGGGG TGTGTTTGAG GGCTTGCGTT TTGGTGCAAT AGGAATGAAA GTGCCCATTT   
  
  
+ CAACCACCAT CCAAACTTGC AACTCCGGCA CCGGCAAACC ACTTTCCTTT GAGAATACTG CCCTACATAC   
  
  
+ CGCCTTTCAA GTTCCCAATA CCACCTACGA ACCCACCTCG GTTCTTGACC TCCAGCGCAG CCCTAGCCCC   
  
  
+ ACCTCCACCC AAAAACCGCC CTTTGCCGCC TCCAACCACC AGCCTGCCTC GGCTGATCTG GACAACTTGG   
  
  
+ ACGGGTGGGA TTCAATCCTA TCAGAGCTGG GGCTTAATGA CGAATTTACC CCTAATTCCA AACTCTGGTC   
  
  
+ CCAAATTAGT CCGTCTGATC CTCACCTCCC CCAACTCCCT GATTTCCCAA CATCTCAGCC GTTTGATCAT   
  
  
+ CACAACCCAC CCCCAATAAA CCTCCCATCA TCAGATTTCA ACCTCTGTGA TTTCTCCTAC AATCACAACC   
  
  
+ CCAATTTTGG GCCGTTCGAT CTTCATCACA ACCCGCATCA GAACAACCCG AACAACAATA ACTACGGCTT   
  
  
+ TGATTTCATA GACGACCTCA TCAAAGCAGC ACAGTCCCTC GAATCCAACG ACTCTCATCA AGTCCACCTG   
  
  
+ ATATTGGCGC GGCTCAATCA ACGGCTCAGA TCACCCACCG GCAAACCGCT CCAGCGGGCC GCCTACTACT   
  
  
+ TCAAGGAAGC CCTCATCGCC GCCGCGGCCG GCCCGCCCCG CCCCGCTCGG CTTTCATCGT ATGAGGTGGT   
  
  
+ GCAGACCATC CGAGCCTACA AGGCGTTTTC AGGAATTTCG CCAGTCTCTC TCTTCTCCAC TTTCGCCGCC   
  
  
+ AACCAGGCGA TTCTTGAGGC GGTGGACACG GCGGCCTTCA TCCACATCAT CGATTTCGAC ATCGGATTCG   
  
  
+ GCGGCCACTG GGCCTCGTTC CTCCGCGAAC TGGTCGACAA AGCTGATCCT GCGAAACTTA GTTCAGTGGT   
  
  
+ GTTACGAATC ACCGCCATCG TTCCCGAAGA ATTCGGGATC GAAAGCAAGC TAGTGAGAGA AAATCTGTCG   
  
  
+ CAATTTGCCC GAGATCTCAA CATCAACTTC CACATCGATT ATGTCTTATT TCAGAGCTTT GAAATCTTAT   
  
  
+ CCTTCAAATC TGTCAAATTC ATCGAAAGGG AGAAATTGGC GCTGCATCTT TCGCCGGCCG TGTTCCACCG   
  
  
+ GCTAGGGAGC GGAATTTCGA AGTTTATCGC TGATCTCCGA TCAATCTCGC CGAGCTCCGT CGTGGTGGTG   
  
  
+ GACAGAGATG TCGGGATTGA CATCGGAACG TCGTCGTTTA GCATGAATTT CGTCGCCGGA ATTGAATTCT   
  
  
+ ACACCGGGAT GCTGGAGTCG CTTGACGCAG CCACCGCCGG GGGGCTCATC GGCGGTGTGG ACTGCGTGAG   
  
  
+ ACGGATTGAG ACGTTTGTTC TCCACCCAAG GATCATGGCG GCGGTAGAGG CGGCGGCGTC GGCGTCGGCC   
  
  
+ GGGCGGAGGA CGGCGTGGAG GGAGGCATTC GCGGCGGCGG GGATTAGGGC GGTGGGGTTT AGCCAGTTTG   
  
  
+ CTGATTTCCA GGCCGAATGT TTGTTGAGGA GAGCTCAGGT TGGTGGCTTC CACGTGGCAA AGCGCCATGG   
  
  
+ AGAGATGATG CTTTACTGGC ATGACCGCCC ACTCGTCGCC ACATCAGCTT GGAGGTGTTA   

- -Up\_Stream \_Len000TATTAA ATAGAAAGAA ATTAAAGCAA AGTACATTTT TTAAAGTCAG AGTAGACGCT   
  
  
- AACTTAGAGA AAAGTTAAGC GTAGTTATTA AGTAGGTTAA GTTTACACGG AAAGAATTAA AGTAATGTAA   
  
  
- CCCAACCATA TTTACAATCG TGCTATCTAA CCTTATCGTT CCGAGATTAA GTTAATACTG AACTTACATA   
  
  
- CGTACGAAAA TAGTAATCAA TTAATTGTAA AAGACATAAC TTTAGTCGAG CTTTCTCTTG GTATACATCA   
  
  
- ACATGAACGC TGCTATATAT ACAAATCAAA TCAATCAAGA ATGGACAAAA CCTTAACAAC CCCTCTAGAC   
  
  
- TCCTTCCTCG GGCGAAGTTA GTTTCAAGTA CCGTTATTCG GTTTCACTCC AAGGTAGGAT TTTGGTTAAC   
  
  
- CGTTATCCTT CTCATCGGGT GAATTGAACA TATGATACTG AGAAGAAAAG TTTGAGGGTT ACACCCTGAA   
  
  
- GAAATGAGTA CACACTATAA GGGTTGTCCT TACTAGTCAA GGTGTACAAA AGTAGAGAAA CCTGAGCTTG   
  
  
- AAGCACATTA ACATATACGG TACCGTGTTT ATCAAAACTA CCACGGTGGT TCAACGAGTT AGTTACATTA   
  
  
- TGACGATGAT AGATTGAACG GAACCACCTG TAGTTATTCT AGTGCTCTAC TCCTCTTGAC TTAGAACCGG   
  
  
- TTGGTGCAGC AAACTGAAGA GAATGGTTAT TTCTAACTTA TCTGTCACAA AGAGAGGGGG AAGGTAAGAA   
  
  
- GAAAAGATCA CTACAGCTTT TTCTGAAATA TGTACAGGTC GTTGCGATCG GTGTTTTATA AGGTAAAGAC   
  
  
- ATTAGGTGAG TACAGTTAAT TTGAACATAG TAGTTTACCA TCAAAGGCAT GATTAGTGAT GTAGATCTAT   
  
  
- AAGGAACTAT TTAAATTAAA CTGAAATTTC CGAGCTAGAA AAATTCTTTT CAAAACAACG AATGTTGGAC   
  
  
- ATTTCGGATC TCTTGTTTCG GTTTCTTCTT TAAATTCAAG ATATATATCG TATTAGAGAG TTTTTTATAT   
  
  
- AAAAAATTTA TATCACGCTA TAACTGACTG GATTATACCC AATAATGTCC TCTACCCCTC TATAGATGTA   
  
  
- ATTGATGAAC TTGTTGAATT TAAAAGTAAA ATTGAGCCAG TAATTCTTGT GATTCTTGAA ATATATAATC   
  
  
- ACTATAGAGT CCGCATTTTC TTTGATGAAA CTTAATTAGG TACGTTAGGA TGAGAAATAA AGGTAGGCAA   
  
  
- TTTTAAAAGG AAGTTTTGAG GGAAGACAAG TAATAATTAA GTTATAGCAT AATGTGAACA ACCTAATAAA   
  
  
- GACACTAAAC TAGCAGTAAA AGATATATAC TTATTGATGT TTTTAGTATC AGGCTCATGT AATTATAATT   
  
  
- ATTTTATCCT ACTCACTATT GGAGTATATC ACGCAAATAG GGATGGTGAT CCGACCTAAA GACGTATTTC   
  
  
- TTCTTTAACT TGTATACCTT TTTCTTTATT TCTCTTCGTT GAAGGAACTC ACCCTCTTCT TAGCATCTTT   
  
  
- CAATTTGAAT ACTTTTTCTC GTAATTGTTT TTCCTTTAAT ATTCCGAATG GCAACTTCGG AGTATATTAA   
  
  
- AACGTTCTCT GTCTGTTTCT TTCACAGAAA GTTTACAGTA TAAAGAGCTC GTACGTACTT TTTACATGAA   
  
  
- GCCGATGTTA TCATTATCAT ATATACAACC GAAGTATGTG AACTGGGGTT ACATACAGTT GCACTGATCA   
  
  
- CTGGTGTGTG TAGCAACAGG GACGGGCGGG GTGATGGGTT TGAGGGGCGT GCGTTTCTAT TTTTCGGGGG   
  
  
- GTTAAAAATA AACGGAGGGT TTTTCGGGTG GGCGCGAAAA AATAAACGGA AGAAAAAAGG ATGAATTTGG   
  
  
- TGGAGGAGTT GCTTGAAACG TTGTATGTAT GATCAGATAT TCGTTAAATC TCCCTTCCCT TCGTTGGATT   
  
  
- TGTCTCGCTC TAACCGCCCC ACACAAACTC CCGAACGCAA AACCACGTTA TCCTTACTTT CACGGGTAAA   
  
  
- GTTGGTGGTA GGTTTGAACG TTGAGGCCGT GGCCGTTTGG TGAAAGGAAA CTCTTATGAC GGGATGTATG   
  
  
- GCGGAAAGTT CAAGGGTTAT GGTGGATGCT TGGGTGGAGC CAAGAACTGG AGGTCGCGTC GGGATCGGGG   
  
  
- TGGAGGTGGG TTTTTGGCGG GAAACGGCGG AGGTTGGTGG TCGGACGGAG CCGACTAGAC CTGTTGAACC   
  
  
- TGCCCACCCT AAGTTAGGAT AGTCTCGACC CCGAATTACT GCTTAAATGG GGATTAAGGT TTGAGACCAG   
  
  
- GGTTTAATCA GGCAGACTAG GAGTGGAGGG GGTTGAGGGA CTAAAGGGTT GTAGAGTCGG CAAACTAGTA   
  
  
- GTGTTGGGTG GGGGTTATTT GGAGGGTAGT AGTCTAAAGT TGGAGACACT AAAGAGGATG TTAGTGTTGG   
  
  
- GGTTAAAACC CGGCAAGCTA GAAGTAGTGT TGGGCGTAGT CTTGTTGGGC TTGTTGTTAT TGATGCCGAA   
  
  
- ACTAAAGTAT CTGCTGGAGT AGTTTCGTCG TGTCAGGGAG CTTAGGTTGC TGAGAGTAGT TCAGGTGGAC   
  
  
- TATAACCGCG CCGAGTTAGT TGCCGAGTCT AGTGGGTGGC CGTTTGGCGA GGTCGCCCGG CGGATGATGA   
  
  
- AGTTCCTTCG GGAGTAGCGG CGGCGCCGGC CGGGCGGGGC GGGGCGAGCC GAAAGTAGCA TACTCCACCA   
  
  
- CGTCTGGTAG GCTCGGATGT TCCGCAAAAG TCCTTAAAGC GGTCAGAGAG AGAAGAGGTG AAAGCGGCGG   
  
  
- TTGGTCCGCT AAGAACTCCG CCACCTGTGC CGCCGGAAGT AGGTGTAGTA GCTAAAGCTG TAGCCTAAGC   
  
  
- CGCCGGTGAC CCGGAGCAAG GAGGCGCTTG ACCAGCTGTT TCGACTAGGA CGCTTTGAAT CAAGTCACCA   
  
  
- CAATGCTTAG TGGCGGTAGC AAGGGCTTCT TAAGCCCTAG CTTTCGTTCG ATCACTCTCT TTTAGACAGC   
  
  
- GTTAAACGGG CTCTAGAGTT GTAGTTGAAG GTGTAGCTAA TACAGAATAA AGTCTCGAAA CTTTAGAATA   
  
  
- GGAAGTTTAG ACAGTTTAAG TAGCTTTCCC TCTTTAACCG CGACGTAGAA AGCGGCCGGC ACAAGGTGGC   
  
  
- CGATCCCTCG CCTTAAAGCT TCAAATAGCG ACTAGAGGCT AGTTAGAGCG GCTCGAGGCA GCACCACCAC   
  
  
- CTGTCTCTAC AGCCCTAACT GTAGCCTTGC AGCAGCAAAT CGTACTTAAA GCAGCGGCCT TAACTTAAGA   
  
  
- TGTGGCCCTA CGACCTCAGC GAACTGCGTC GGTGGCGGCC CCCCGAGTAG CCGCCACACC TGACGCACTC   
  
  
- TGCCTAACTC TGCAAACAAG AGGTGGGTTC CTAGTACCGC CGCCATCTCC GCCGCCGCAG CCGCAGCCGG   
  
  
- CCCGCCTCCT GCCGCACCTC CCTCCGTAAG CGCCGCCGCC CCTAATCCCG CCACCCCAAA TCGGTCAAAC   
  
  
- GACTAAAGGT CCGGCTTACA AACAACTCCT CTCGAGTCCA ACCACCGAAG GTGCACCGTT TCGCGGTACC   
  
  
- TCTCTACTAC GAAATGACCG TACTGGCGGG TGAGCAGCGG TGTAGTCGAA CCTCCACAAT

+     MRE

| Site Name | Organism | Position | Strand | Matrix score. | sequence | function |
| --- | --- | --- | --- | --- | --- | --- |
| MRE | Petroselinum crispum | 1958 | + | 7 | AACCTAA | MYB binding site involved in light responsiveness |

>HU05G02245.1   
+ -Up\_Stream \_Len000ATAATT TATCTTTCTT TAATTTCGTT TCATGTAAAA AATTTCAGTC TCATCTGCGA   
  
  
+ TTGAATCTCT TTTCAATTCG CATCAATAAT TCATCCAATT CAAATGTGCC TTTCTTAATT TCATTACATT   
  
  
+ GGGTTGGTAT AAATGTTAGC ACGATAGATT GGAATAGCAA GGCTCTAATT CAATTATGAC TTGAATGTAT   
  
  
+ GCATGCTTTT ATCATTAGTT AATTAACATT TTCTGTATTG AAATCAGCTC GAAAGAGAAC CATATGTAGT   
  
  
+ TGTACTTGCG ACGATATATA TGTTTAGTTT AGTTAGTTCT TACCTGTTTT GGAATTGTTG GGGAGATCTG   
  
  
+ AGGAAGGAGC CCGCTTCAAT CAAAGTTCAT GGCAATAAGC CAAAGTGAGG TTCCATCCTA AAACCAATTG   
  
  
+ GCAATAGGAA GAGTAGCCCA CTTAACTTGT ATACTATGAC TCTTCTTTTC AAACTCCCAA TGTGGGACTT   
  
  
+ CTTTACTCAT GTGTGATATT CCCAACAGGA ATGATCAGTT CCACATGTTT TCATCTCTTT GGACTCGAAC   
  
  
+ TTCGTGTAAT TGTATATGCC ATGGCACAAA TAGTTTTGAT GGTGCCACCA AGTTGCTCAA TCAATGTAAT   
  
  
+ ACTGCTACTA TCTAACTTGC CTTGGTGGAC ATCAATAAGA TCACGAGATG AGGAGAACTG AATCTTGGCC   
  
  
+ AACCACGTCG TTTGACTTCT CTTACCAATA AAGATTGAAT AGACAGTGTT TCTCTCCCCC TTCCATTCTT   
  
  
+ CTTTTCTAGT GATGTCGAAA AAGACTTTAT ACATGTCCAG CAACGCTAGC CACAAAATAT TCCATTTCTG   
  
  
+ TAATCCACTC ATGTCAATTA AACTTGTATC ATCAAATGGT AGTTTCCGTA CTAATCACTA CATCTAGATA   
  
  
+ TTCCTTGATA AATTTAATTT GACTTTAAAG GCTCGATCTT TTTAAGAAAA GTTTTGTTGC TTACAACCTG   
  
  
+ TAAAGCCTAG AGAACAAAGC CAAAGAAGAA ATTTAAGTTC TATATATAGC ATAATCTCTC AAAAAATATA   
  
  
+ TTTTTTAAAT ATAGTGCGAT ATTGACTGAC CTAATATGGG TTATTACAGG AGATGGGGAG ATATCTACAT   
  
  
+ TAACTACTTG AACAACTTAA ATTTTCATTT TAACTCGGTC ATTAAGAACA CTAAGAACTT TATATATTAG   
  
  
+ TGATATCTCA GGCGTAAAAG AAACTACTTT GAATTAATCC ATGCAATCCT ACTCTTTATT TCCATCCGTT   
  
  
+ AAAATTTTCC TTCAAAACTC CCTTCTGTTC ATTATTAATT CAATATCGTA TTACACTTGT TGGATTATTT   
  
  
+ CTGTGATTTG ATCGTCATTT TCTATATATG AATAACTACA AAAATCATAG TCCGAGTACA TTAATATTAA   
  
  
+ TAAAATAGGA TGAGTGATAA CCTCATATAG TGCGTTTATC CCTACCACTA GGCTGGATTT CTGCATAAAG   
  
  
+ AAGAAATTGA ACATATGGAA AAAGAAATAA AGAGAAGCAA CTTCCTTGAG TGGGAGAAGA ATCGTAGAAA   
  
  
+ GTTAAACTTA TGAAAAAGAG CATTAACAAA AAGGAAATTA TAAGGCTTAC CGTTGAAGCC TCATATAATT   
  
  
+ TTGCAAGAGA CAGACAAAGA AAGTGTCTTT CAAATGTCAT ATTTCTCGAG CATGCATGAA AAATGTACTT   
  
  
+ CGGCTACAAT AGTAATAGTA TATATGTTGG CTTCATACAC TTGACCCCAA TGTATGTCAA CGTGACTAGT   
  
  
+ GACCACACAC ATCGTTGTCC CTGCCCGCCC CACTACCCAA ACTCCCCGCA CGCAAAGATA AAAAGCCCCC   
  
  
+ CAATTTTTAT TTGCCTCCCA AAAAGCCCAC CCGCGCTTTT TTATTTGCCT TCTTTTTTCC TACTTAAACC   
  
  
+ ACCTCCTCAA CGAACTTTGC AACATACATA CTAGTCTATA AGCAATTTAG AGGGAAGGGA AGCAACCTAA   
  
  
+ ACAGAGCGAG ATTGGCGGGG TGTGTTTGAG GGCTTGCGTT TTGGTGCAAT AGGAATGAAA GTGCCCATTT   
  
  
+ CAACCACCAT CCAAACTTGC AACTCCGGCA CCGGCAAACC ACTTTCCTTT GAGAATACTG CCCTACATAC   
  
  
+ CGCCTTTCAA GTTCCCAATA CCACCTACGA ACCCACCTCG GTTCTTGACC TCCAGCGCAG CCCTAGCCCC   
  
  
+ ACCTCCACCC AAAAACCGCC CTTTGCCGCC TCCAACCACC AGCCTGCCTC GGCTGATCTG GACAACTTGG   
  
  
+ ACGGGTGGGA TTCAATCCTA TCAGAGCTGG GGCTTAATGA CGAATTTACC CCTAATTCCA AACTCTGGTC   
  
  
+ CCAAATTAGT CCGTCTGATC CTCACCTCCC CCAACTCCCT GATTTCCCAA CATCTCAGCC GTTTGATCAT   
  
  
+ CACAACCCAC CCCCAATAAA CCTCCCATCA TCAGATTTCA ACCTCTGTGA TTTCTCCTAC AATCACAACC   
  
  
+ CCAATTTTGG GCCGTTCGAT CTTCATCACA ACCCGCATCA GAACAACCCG AACAACAATA ACTACGGCTT   
  
  
+ TGATTTCATA GACGACCTCA TCAAAGCAGC ACAGTCCCTC GAATCCAACG ACTCTCATCA AGTCCACCTG   
  
  
+ ATATTGGCGC GGCTCAATCA ACGGCTCAGA TCACCCACCG GCAAACCGCT CCAGCGGGCC GCCTACTACT   
  
  
+ TCAAGGAAGC CCTCATCGCC GCCGCGGCCG GCCCGCCCCG CCCCGCTCGG CTTTCATCGT ATGAGGTGGT   
  
  
+ GCAGACCATC CGAGCCTACA AGGCGTTTTC AGGAATTTCG CCAGTCTCTC TCTTCTCCAC TTTCGCCGCC   
  
  
+ AACCAGGCGA TTCTTGAGGC GGTGGACACG GCGGCCTTCA TCCACATCAT CGATTTCGAC ATCGGATTCG   
  
  
+ GCGGCCACTG GGCCTCGTTC CTCCGCGAAC TGGTCGACAA AGCTGATCCT GCGAAACTTA GTTCAGTGGT   
  
  
+ GTTACGAATC ACCGCCATCG TTCCCGAAGA ATTCGGGATC GAAAGCAAGC TAGTGAGAGA AAATCTGTCG   
  
  
+ CAATTTGCCC GAGATCTCAA CATCAACTTC CACATCGATT ATGTCTTATT TCAGAGCTTT GAAATCTTAT   
  
  
+ CCTTCAAATC TGTCAAATTC ATCGAAAGGG AGAAATTGGC GCTGCATCTT TCGCCGGCCG TGTTCCACCG   
  
  
+ GCTAGGGAGC GGAATTTCGA AGTTTATCGC TGATCTCCGA TCAATCTCGC CGAGCTCCGT CGTGGTGGTG   
  
  
+ GACAGAGATG TCGGGATTGA CATCGGAACG TCGTCGTTTA GCATGAATTT CGTCGCCGGA ATTGAATTCT   
  
  
+ ACACCGGGAT GCTGGAGTCG CTTGACGCAG CCACCGCCGG GGGGCTCATC GGCGGTGTGG ACTGCGTGAG   
  
  
+ ACGGATTGAG ACGTTTGTTC TCCACCCAAG GATCATGGCG GCGGTAGAGG CGGCGGCGTC GGCGTCGGCC   
  
  
+ GGGCGGAGGA CGGCGTGGAG GGAGGCATTC GCGGCGGCGG GGATTAGGGC GGTGGGGTTT AGCCAGTTTG   
  
  
+ CTGATTTCCA GGCCGAATGT TTGTTGAGGA GAGCTCAGGT TGGTGGCTTC CACGTGGCAA AGCGCCATGG   
  
  
+ AGAGATGATG CTTTACTGGC ATGACCGCCC ACTCGTCGCC ACATCAGCTT GGAGGTGTTA   

- -Up\_Stream \_Len000TATTAA ATAGAAAGAA ATTAAAGCAA AGTACATTTT TTAAAGTCAG AGTAGACGCT   
  
  
- AACTTAGAGA AAAGTTAAGC GTAGTTATTA AGTAGGTTAA GTTTACACGG AAAGAATTAA AGTAATGTAA   
  
  
- CCCAACCATA TTTACAATCG TGCTATCTAA CCTTATCGTT CCGAGATTAA GTTAATACTG AACTTACATA   
  
  
- CGTACGAAAA TAGTAATCAA TTAATTGTAA AAGACATAAC TTTAGTCGAG CTTTCTCTTG GTATACATCA   
  
  
- ACATGAACGC TGCTATATAT ACAAATCAAA TCAATCAAGA ATGGACAAAA CCTTAACAAC CCCTCTAGAC   
  
  
- TCCTTCCTCG GGCGAAGTTA GTTTCAAGTA CCGTTATTCG GTTTCACTCC AAGGTAGGAT TTTGGTTAAC   
  
  
- CGTTATCCTT CTCATCGGGT GAATTGAACA TATGATACTG AGAAGAAAAG TTTGAGGGTT ACACCCTGAA   
  
  
- GAAATGAGTA CACACTATAA GGGTTGTCCT TACTAGTCAA GGTGTACAAA AGTAGAGAAA CCTGAGCTTG   
  
  
- AAGCACATTA ACATATACGG TACCGTGTTT ATCAAAACTA CCACGGTGGT TCAACGAGTT AGTTACATTA   
  
  
- TGACGATGAT AGATTGAACG GAACCACCTG TAGTTATTCT AGTGCTCTAC TCCTCTTGAC TTAGAACCGG   
  
  
- TTGGTGCAGC AAACTGAAGA GAATGGTTAT TTCTAACTTA TCTGTCACAA AGAGAGGGGG AAGGTAAGAA   
  
  
- GAAAAGATCA CTACAGCTTT TTCTGAAATA TGTACAGGTC GTTGCGATCG GTGTTTTATA AGGTAAAGAC   
  
  
- ATTAGGTGAG TACAGTTAAT TTGAACATAG TAGTTTACCA TCAAAGGCAT GATTAGTGAT GTAGATCTAT   
  
  
- AAGGAACTAT TTAAATTAAA CTGAAATTTC CGAGCTAGAA AAATTCTTTT CAAAACAACG AATGTTGGAC   
  
  
- ATTTCGGATC TCTTGTTTCG GTTTCTTCTT TAAATTCAAG ATATATATCG TATTAGAGAG TTTTTTATAT   
  
  
- AAAAAATTTA TATCACGCTA TAACTGACTG GATTATACCC AATAATGTCC TCTACCCCTC TATAGATGTA   
  
  
- ATTGATGAAC TTGTTGAATT TAAAAGTAAA ATTGAGCCAG TAATTCTTGT GATTCTTGAA ATATATAATC   
  
  
- ACTATAGAGT CCGCATTTTC TTTGATGAAA CTTAATTAGG TACGTTAGGA TGAGAAATAA AGGTAGGCAA   
  
  
- TTTTAAAAGG AAGTTTTGAG GGAAGACAAG TAATAATTAA GTTATAGCAT AATGTGAACA ACCTAATAAA   
  
  
- GACACTAAAC TAGCAGTAAA AGATATATAC TTATTGATGT TTTTAGTATC AGGCTCATGT AATTATAATT   
  
  
- ATTTTATCCT ACTCACTATT GGAGTATATC ACGCAAATAG GGATGGTGAT CCGACCTAAA GACGTATTTC   
  
  
- TTCTTTAACT TGTATACCTT TTTCTTTATT TCTCTTCGTT GAAGGAACTC ACCCTCTTCT TAGCATCTTT   
  
  
- CAATTTGAAT ACTTTTTCTC GTAATTGTTT TTCCTTTAAT ATTCCGAATG GCAACTTCGG AGTATATTAA   
  
  
- AACGTTCTCT GTCTGTTTCT TTCACAGAAA GTTTACAGTA TAAAGAGCTC GTACGTACTT TTTACATGAA   
  
  
- GCCGATGTTA TCATTATCAT ATATACAACC GAAGTATGTG AACTGGGGTT ACATACAGTT GCACTGATCA   
  
  
- CTGGTGTGTG TAGCAACAGG GACGGGCGGG GTGATGGGTT TGAGGGGCGT GCGTTTCTAT TTTTCGGGGG   
  
  
- GTTAAAAATA AACGGAGGGT TTTTCGGGTG GGCGCGAAAA AATAAACGGA AGAAAAAAGG ATGAATTTGG   
  
  
- TGGAGGAGTT GCTTGAAACG TTGTATGTAT GATCAGATAT TCGTTAAATC TCCCTTCCCT TCGTTGGATT   
  
  
- TGTCTCGCTC TAACCGCCCC ACACAAACTC CCGAACGCAA AACCACGTTA TCCTTACTTT CACGGGTAAA   
  
  
- GTTGGTGGTA GGTTTGAACG TTGAGGCCGT GGCCGTTTGG TGAAAGGAAA CTCTTATGAC GGGATGTATG   
  
  
- GCGGAAAGTT CAAGGGTTAT GGTGGATGCT TGGGTGGAGC CAAGAACTGG AGGTCGCGTC GGGATCGGGG   
  
  
- TGGAGGTGGG TTTTTGGCGG GAAACGGCGG AGGTTGGTGG TCGGACGGAG CCGACTAGAC CTGTTGAACC   
  
  
- TGCCCACCCT AAGTTAGGAT AGTCTCGACC CCGAATTACT GCTTAAATGG GGATTAAGGT TTGAGACCAG   
  
  
- GGTTTAATCA GGCAGACTAG GAGTGGAGGG GGTTGAGGGA CTAAAGGGTT GTAGAGTCGG CAAACTAGTA   
  
  
- GTGTTGGGTG GGGGTTATTT GGAGGGTAGT AGTCTAAAGT TGGAGACACT AAAGAGGATG TTAGTGTTGG   
  
  
- GGTTAAAACC CGGCAAGCTA GAAGTAGTGT TGGGCGTAGT CTTGTTGGGC TTGTTGTTAT TGATGCCGAA   
  
  
- ACTAAAGTAT CTGCTGGAGT AGTTTCGTCG TGTCAGGGAG CTTAGGTTGC TGAGAGTAGT TCAGGTGGAC   
  
  
- TATAACCGCG CCGAGTTAGT TGCCGAGTCT AGTGGGTGGC CGTTTGGCGA GGTCGCCCGG CGGATGATGA   
  
  
- AGTTCCTTCG GGAGTAGCGG CGGCGCCGGC CGGGCGGGGC GGGGCGAGCC GAAAGTAGCA TACTCCACCA   
  
  
- CGTCTGGTAG GCTCGGATGT TCCGCAAAAG TCCTTAAAGC GGTCAGAGAG AGAAGAGGTG AAAGCGGCGG   
  
  
- TTGGTCCGCT AAGAACTCCG CCACCTGTGC CGCCGGAAGT AGGTGTAGTA GCTAAAGCTG TAGCCTAAGC   
  
  
- CGCCGGTGAC CCGGAGCAAG GAGGCGCTTG ACCAGCTGTT TCGACTAGGA CGCTTTGAAT CAAGTCACCA   
  
  
- CAATGCTTAG TGGCGGTAGC AAGGGCTTCT TAAGCCCTAG CTTTCGTTCG ATCACTCTCT TTTAGACAGC   
  
  
- GTTAAACGGG CTCTAGAGTT GTAGTTGAAG GTGTAGCTAA TACAGAATAA AGTCTCGAAA CTTTAGAATA   
  
  
- GGAAGTTTAG ACAGTTTAAG TAGCTTTCCC TCTTTAACCG CGACGTAGAA AGCGGCCGGC ACAAGGTGGC   
  
  
- CGATCCCTCG CCTTAAAGCT TCAAATAGCG ACTAGAGGCT AGTTAGAGCG GCTCGAGGCA GCACCACCAC   
  
  
- CTGTCTCTAC AGCCCTAACT GTAGCCTTGC AGCAGCAAAT CGTACTTAAA GCAGCGGCCT TAACTTAAGA   
  
  
- TGTGGCCCTA CGACCTCAGC GAACTGCGTC GGTGGCGGCC CCCCGAGTAG CCGCCACACC TGACGCACTC   
  
  
- TGCCTAACTC TGCAAACAAG AGGTGGGTTC CTAGTACCGC CGCCATCTCC GCCGCCGCAG CCGCAGCCGG   
  
  
- CCCGCCTCCT GCCGCACCTC CCTCCGTAAG CGCCGCCGCC CCTAATCCCG CCACCCCAAA TCGGTCAAAC   
  
  
- GACTAAAGGT CCGGCTTACA AACAACTCCT CTCGAGTCCA ACCACCGAAG GTGCACCGTT TCGCGGTACC   
  
  
- TCTCTACTAC GAAATGACCG TACTGGCGGG TGAGCAGCGG TGTAGTCGAA CCTCCACAAT

+     MYB

| Site Name | Organism | Position | Strand | Matrix score. | sequence | function |
| --- | --- | --- | --- | --- | --- | --- |
| MYB | Arabidopsis thaliana | 2804 | + | 6 | CAACCA |  |
| MYB | Arabidopsis thaliana | 2207 | + | 6 | CAACCA |  |
| MYB | Arabidopsis thaliana | 704 | + | 6 | CAACCA |  |
| MYB | Arabidopsis thaliana | 2035 | + | 6 | CAACCA |  |
| MYB | Arabidopsis thaliana | 517 | + | 6 | CAACAG |  |

>HU05G02245.1   
+ -Up\_Stream \_Len000ATAATT TATCTTTCTT TAATTTCGTT TCATGTAAAA AATTTCAGTC TCATCTGCGA   
  
  
+ TTGAATCTCT TTTCAATTCG CATCAATAAT TCATCCAATT CAAATGTGCC TTTCTTAATT TCATTACATT   
  
  
+ GGGTTGGTAT AAATGTTAGC ACGATAGATT GGAATAGCAA GGCTCTAATT CAATTATGAC TTGAATGTAT   
  
  
+ GCATGCTTTT ATCATTAGTT AATTAACATT TTCTGTATTG AAATCAGCTC GAAAGAGAAC CATATGTAGT   
  
  
+ TGTACTTGCG ACGATATATA TGTTTAGTTT AGTTAGTTCT TACCTGTTTT GGAATTGTTG GGGAGATCTG   
  
  
+ AGGAAGGAGC CCGCTTCAAT CAAAGTTCAT GGCAATAAGC CAAAGTGAGG TTCCATCCTA AAACCAATTG   
  
  
+ GCAATAGGAA GAGTAGCCCA CTTAACTTGT ATACTATGAC TCTTCTTTTC AAACTCCCAA TGTGGGACTT   
  
  
+ CTTTACTCAT GTGTGATATT CCCAACAGGA ATGATCAGTT CCACATGTTT TCATCTCTTT GGACTCGAAC   
  
  
+ TTCGTGTAAT TGTATATGCC ATGGCACAAA TAGTTTTGAT GGTGCCACCA AGTTGCTCAA TCAATGTAAT   
  
  
+ ACTGCTACTA TCTAACTTGC CTTGGTGGAC ATCAATAAGA TCACGAGATG AGGAGAACTG AATCTTGGCC   
  
  
+ AACCACGTCG TTTGACTTCT CTTACCAATA AAGATTGAAT AGACAGTGTT TCTCTCCCCC TTCCATTCTT   
  
  
+ CTTTTCTAGT GATGTCGAAA AAGACTTTAT ACATGTCCAG CAACGCTAGC CACAAAATAT TCCATTTCTG   
  
  
+ TAATCCACTC ATGTCAATTA AACTTGTATC ATCAAATGGT AGTTTCCGTA CTAATCACTA CATCTAGATA   
  
  
+ TTCCTTGATA AATTTAATTT GACTTTAAAG GCTCGATCTT TTTAAGAAAA GTTTTGTTGC TTACAACCTG   
  
  
+ TAAAGCCTAG AGAACAAAGC CAAAGAAGAA ATTTAAGTTC TATATATAGC ATAATCTCTC AAAAAATATA   
  
  
+ TTTTTTAAAT ATAGTGCGAT ATTGACTGAC CTAATATGGG TTATTACAGG AGATGGGGAG ATATCTACAT   
  
  
+ TAACTACTTG AACAACTTAA ATTTTCATTT TAACTCGGTC ATTAAGAACA CTAAGAACTT TATATATTAG   
  
  
+ TGATATCTCA GGCGTAAAAG AAACTACTTT GAATTAATCC ATGCAATCCT ACTCTTTATT TCCATCCGTT   
  
  
+ AAAATTTTCC TTCAAAACTC CCTTCTGTTC ATTATTAATT CAATATCGTA TTACACTTGT TGGATTATTT   
  
  
+ CTGTGATTTG ATCGTCATTT TCTATATATG AATAACTACA AAAATCATAG TCCGAGTACA TTAATATTAA   
  
  
+ TAAAATAGGA TGAGTGATAA CCTCATATAG TGCGTTTATC CCTACCACTA GGCTGGATTT CTGCATAAAG   
  
  
+ AAGAAATTGA ACATATGGAA AAAGAAATAA AGAGAAGCAA CTTCCTTGAG TGGGAGAAGA ATCGTAGAAA   
  
  
+ GTTAAACTTA TGAAAAAGAG CATTAACAAA AAGGAAATTA TAAGGCTTAC CGTTGAAGCC TCATATAATT   
  
  
+ TTGCAAGAGA CAGACAAAGA AAGTGTCTTT CAAATGTCAT ATTTCTCGAG CATGCATGAA AAATGTACTT   
  
  
+ CGGCTACAAT AGTAATAGTA TATATGTTGG CTTCATACAC TTGACCCCAA TGTATGTCAA CGTGACTAGT   
  
  
+ GACCACACAC ATCGTTGTCC CTGCCCGCCC CACTACCCAA ACTCCCCGCA CGCAAAGATA AAAAGCCCCC   
  
  
+ CAATTTTTAT TTGCCTCCCA AAAAGCCCAC CCGCGCTTTT TTATTTGCCT TCTTTTTTCC TACTTAAACC   
  
  
+ ACCTCCTCAA CGAACTTTGC AACATACATA CTAGTCTATA AGCAATTTAG AGGGAAGGGA AGCAACCTAA   
  
  
+ ACAGAGCGAG ATTGGCGGGG TGTGTTTGAG GGCTTGCGTT TTGGTGCAAT AGGAATGAAA GTGCCCATTT   
  
  
+ CAACCACCAT CCAAACTTGC AACTCCGGCA CCGGCAAACC ACTTTCCTTT GAGAATACTG CCCTACATAC   
  
  
+ CGCCTTTCAA GTTCCCAATA CCACCTACGA ACCCACCTCG GTTCTTGACC TCCAGCGCAG CCCTAGCCCC   
  
  
+ ACCTCCACCC AAAAACCGCC CTTTGCCGCC TCCAACCACC AGCCTGCCTC GGCTGATCTG GACAACTTGG   
  
  
+ ACGGGTGGGA TTCAATCCTA TCAGAGCTGG GGCTTAATGA CGAATTTACC CCTAATTCCA AACTCTGGTC   
  
  
+ CCAAATTAGT CCGTCTGATC CTCACCTCCC CCAACTCCCT GATTTCCCAA CATCTCAGCC GTTTGATCAT   
  
  
+ CACAACCCAC CCCCAATAAA CCTCCCATCA TCAGATTTCA ACCTCTGTGA TTTCTCCTAC AATCACAACC   
  
  
+ CCAATTTTGG GCCGTTCGAT CTTCATCACA ACCCGCATCA GAACAACCCG AACAACAATA ACTACGGCTT   
  
  
+ TGATTTCATA GACGACCTCA TCAAAGCAGC ACAGTCCCTC GAATCCAACG ACTCTCATCA AGTCCACCTG   
  
  
+ ATATTGGCGC GGCTCAATCA ACGGCTCAGA TCACCCACCG GCAAACCGCT CCAGCGGGCC GCCTACTACT   
  
  
+ TCAAGGAAGC CCTCATCGCC GCCGCGGCCG GCCCGCCCCG CCCCGCTCGG CTTTCATCGT ATGAGGTGGT   
  
  
+ GCAGACCATC CGAGCCTACA AGGCGTTTTC AGGAATTTCG CCAGTCTCTC TCTTCTCCAC TTTCGCCGCC   
  
  
+ AACCAGGCGA TTCTTGAGGC GGTGGACACG GCGGCCTTCA TCCACATCAT CGATTTCGAC ATCGGATTCG   
  
  
+ GCGGCCACTG GGCCTCGTTC CTCCGCGAAC TGGTCGACAA AGCTGATCCT GCGAAACTTA GTTCAGTGGT   
  
  
+ GTTACGAATC ACCGCCATCG TTCCCGAAGA ATTCGGGATC GAAAGCAAGC TAGTGAGAGA AAATCTGTCG   
  
  
+ CAATTTGCCC GAGATCTCAA CATCAACTTC CACATCGATT ATGTCTTATT TCAGAGCTTT GAAATCTTAT   
  
  
+ CCTTCAAATC TGTCAAATTC ATCGAAAGGG AGAAATTGGC GCTGCATCTT TCGCCGGCCG TGTTCCACCG   
  
  
+ GCTAGGGAGC GGAATTTCGA AGTTTATCGC TGATCTCCGA TCAATCTCGC CGAGCTCCGT CGTGGTGGTG   
  
  
+ GACAGAGATG TCGGGATTGA CATCGGAACG TCGTCGTTTA GCATGAATTT CGTCGCCGGA ATTGAATTCT   
  
  
+ ACACCGGGAT GCTGGAGTCG CTTGACGCAG CCACCGCCGG GGGGCTCATC GGCGGTGTGG ACTGCGTGAG   
  
  
+ ACGGATTGAG ACGTTTGTTC TCCACCCAAG GATCATGGCG GCGGTAGAGG CGGCGGCGTC GGCGTCGGCC   
  
  
+ GGGCGGAGGA CGGCGTGGAG GGAGGCATTC GCGGCGGCGG GGATTAGGGC GGTGGGGTTT AGCCAGTTTG   
  
  
+ CTGATTTCCA GGCCGAATGT TTGTTGAGGA GAGCTCAGGT TGGTGGCTTC CACGTGGCAA AGCGCCATGG   
  
  
+ AGAGATGATG CTTTACTGGC ATGACCGCCC ACTCGTCGCC ACATCAGCTT GGAGGTGTTA   

- -Up\_Stream \_Len000TATTAA ATAGAAAGAA ATTAAAGCAA AGTACATTTT TTAAAGTCAG AGTAGACGCT   
  
  
- AACTTAGAGA AAAGTTAAGC GTAGTTATTA AGTAGGTTAA GTTTACACGG AAAGAATTAA AGTAATGTAA   
  
  
- CCCAACCATA TTTACAATCG TGCTATCTAA CCTTATCGTT CCGAGATTAA GTTAATACTG AACTTACATA   
  
  
- CGTACGAAAA TAGTAATCAA TTAATTGTAA AAGACATAAC TTTAGTCGAG CTTTCTCTTG GTATACATCA   
  
  
- ACATGAACGC TGCTATATAT ACAAATCAAA TCAATCAAGA ATGGACAAAA CCTTAACAAC CCCTCTAGAC   
  
  
- TCCTTCCTCG GGCGAAGTTA GTTTCAAGTA CCGTTATTCG GTTTCACTCC AAGGTAGGAT TTTGGTTAAC   
  
  
- CGTTATCCTT CTCATCGGGT GAATTGAACA TATGATACTG AGAAGAAAAG TTTGAGGGTT ACACCCTGAA   
  
  
- GAAATGAGTA CACACTATAA GGGTTGTCCT TACTAGTCAA GGTGTACAAA AGTAGAGAAA CCTGAGCTTG   
  
  
- AAGCACATTA ACATATACGG TACCGTGTTT ATCAAAACTA CCACGGTGGT TCAACGAGTT AGTTACATTA   
  
  
- TGACGATGAT AGATTGAACG GAACCACCTG TAGTTATTCT AGTGCTCTAC TCCTCTTGAC TTAGAACCGG   
  
  
- TTGGTGCAGC AAACTGAAGA GAATGGTTAT TTCTAACTTA TCTGTCACAA AGAGAGGGGG AAGGTAAGAA   
  
  
- GAAAAGATCA CTACAGCTTT TTCTGAAATA TGTACAGGTC GTTGCGATCG GTGTTTTATA AGGTAAAGAC   
  
  
- ATTAGGTGAG TACAGTTAAT TTGAACATAG TAGTTTACCA TCAAAGGCAT GATTAGTGAT GTAGATCTAT   
  
  
- AAGGAACTAT TTAAATTAAA CTGAAATTTC CGAGCTAGAA AAATTCTTTT CAAAACAACG AATGTTGGAC   
  
  
- ATTTCGGATC TCTTGTTTCG GTTTCTTCTT TAAATTCAAG ATATATATCG TATTAGAGAG TTTTTTATAT   
  
  
- AAAAAATTTA TATCACGCTA TAACTGACTG GATTATACCC AATAATGTCC TCTACCCCTC TATAGATGTA   
  
  
- ATTGATGAAC TTGTTGAATT TAAAAGTAAA ATTGAGCCAG TAATTCTTGT GATTCTTGAA ATATATAATC   
  
  
- ACTATAGAGT CCGCATTTTC TTTGATGAAA CTTAATTAGG TACGTTAGGA TGAGAAATAA AGGTAGGCAA   
  
  
- TTTTAAAAGG AAGTTTTGAG GGAAGACAAG TAATAATTAA GTTATAGCAT AATGTGAACA ACCTAATAAA   
  
  
- GACACTAAAC TAGCAGTAAA AGATATATAC TTATTGATGT TTTTAGTATC AGGCTCATGT AATTATAATT   
  
  
- ATTTTATCCT ACTCACTATT GGAGTATATC ACGCAAATAG GGATGGTGAT CCGACCTAAA GACGTATTTC   
  
  
- TTCTTTAACT TGTATACCTT TTTCTTTATT TCTCTTCGTT GAAGGAACTC ACCCTCTTCT TAGCATCTTT   
  
  
- CAATTTGAAT ACTTTTTCTC GTAATTGTTT TTCCTTTAAT ATTCCGAATG GCAACTTCGG AGTATATTAA   
  
  
- AACGTTCTCT GTCTGTTTCT TTCACAGAAA GTTTACAGTA TAAAGAGCTC GTACGTACTT TTTACATGAA   
  
  
- GCCGATGTTA TCATTATCAT ATATACAACC GAAGTATGTG AACTGGGGTT ACATACAGTT GCACTGATCA   
  
  
- CTGGTGTGTG TAGCAACAGG GACGGGCGGG GTGATGGGTT TGAGGGGCGT GCGTTTCTAT TTTTCGGGGG   
  
  
- GTTAAAAATA AACGGAGGGT TTTTCGGGTG GGCGCGAAAA AATAAACGGA AGAAAAAAGG ATGAATTTGG   
  
  
- TGGAGGAGTT GCTTGAAACG TTGTATGTAT GATCAGATAT TCGTTAAATC TCCCTTCCCT TCGTTGGATT   
  
  
- TGTCTCGCTC TAACCGCCCC ACACAAACTC CCGAACGCAA AACCACGTTA TCCTTACTTT CACGGGTAAA   
  
  
- GTTGGTGGTA GGTTTGAACG TTGAGGCCGT GGCCGTTTGG TGAAAGGAAA CTCTTATGAC GGGATGTATG   
  
  
- GCGGAAAGTT CAAGGGTTAT GGTGGATGCT TGGGTGGAGC CAAGAACTGG AGGTCGCGTC GGGATCGGGG   
  
  
- TGGAGGTGGG TTTTTGGCGG GAAACGGCGG AGGTTGGTGG TCGGACGGAG CCGACTAGAC CTGTTGAACC   
  
  
- TGCCCACCCT AAGTTAGGAT AGTCTCGACC CCGAATTACT GCTTAAATGG GGATTAAGGT TTGAGACCAG   
  
  
- GGTTTAATCA GGCAGACTAG GAGTGGAGGG GGTTGAGGGA CTAAAGGGTT GTAGAGTCGG CAAACTAGTA   
  
  
- GTGTTGGGTG GGGGTTATTT GGAGGGTAGT AGTCTAAAGT TGGAGACACT AAAGAGGATG TTAGTGTTGG   
  
  
- GGTTAAAACC CGGCAAGCTA GAAGTAGTGT TGGGCGTAGT CTTGTTGGGC TTGTTGTTAT TGATGCCGAA   
  
  
- ACTAAAGTAT CTGCTGGAGT AGTTTCGTCG TGTCAGGGAG CTTAGGTTGC TGAGAGTAGT TCAGGTGGAC   
  
  
- TATAACCGCG CCGAGTTAGT TGCCGAGTCT AGTGGGTGGC CGTTTGGCGA GGTCGCCCGG CGGATGATGA   
  
  
- AGTTCCTTCG GGAGTAGCGG CGGCGCCGGC CGGGCGGGGC GGGGCGAGCC GAAAGTAGCA TACTCCACCA   
  
  
- CGTCTGGTAG GCTCGGATGT TCCGCAAAAG TCCTTAAAGC GGTCAGAGAG AGAAGAGGTG AAAGCGGCGG   
  
  
- TTGGTCCGCT AAGAACTCCG CCACCTGTGC CGCCGGAAGT AGGTGTAGTA GCTAAAGCTG TAGCCTAAGC   
  
  
- CGCCGGTGAC CCGGAGCAAG GAGGCGCTTG ACCAGCTGTT TCGACTAGGA CGCTTTGAAT CAAGTCACCA   
  
  
- CAATGCTTAG TGGCGGTAGC AAGGGCTTCT TAAGCCCTAG CTTTCGTTCG ATCACTCTCT TTTAGACAGC   
  
  
- GTTAAACGGG CTCTAGAGTT GTAGTTGAAG GTGTAGCTAA TACAGAATAA AGTCTCGAAA CTTTAGAATA   
  
  
- GGAAGTTTAG ACAGTTTAAG TAGCTTTCCC TCTTTAACCG CGACGTAGAA AGCGGCCGGC ACAAGGTGGC   
  
  
- CGATCCCTCG CCTTAAAGCT TCAAATAGCG ACTAGAGGCT AGTTAGAGCG GCTCGAGGCA GCACCACCAC   
  
  
- CTGTCTCTAC AGCCCTAACT GTAGCCTTGC AGCAGCAAAT CGTACTTAAA GCAGCGGCCT TAACTTAAGA   
  
  
- TGTGGCCCTA CGACCTCAGC GAACTGCGTC GGTGGCGGCC CCCCGAGTAG CCGCCACACC TGACGCACTC   
  
  
- TGCCTAACTC TGCAAACAAG AGGTGGGTTC CTAGTACCGC CGCCATCTCC GCCGCCGCAG CCGCAGCCGG   
  
  
- CCCGCCTCCT GCCGCACCTC CCTCCGTAAG CGCCGCCGCC CCTAATCCCG CCACCCCAAA TCGGTCAAAC   
  
  
- GACTAAAGGT CCGGCTTACA AACAACTCCT CTCGAGTCCA ACCACCGAAG GTGCACCGTT TCGCGGTACC   
  
  
- TCTCTACTAC GAAATGACCG TACTGGCGGG TGAGCAGCGG TGTAGTCGAA CCTCCACAAT

+     MYB recognition site

| Site Name | Organism | Position | Strand | Matrix score. | sequence | function |
| --- | --- | --- | --- | --- | --- | --- |
| MYB recognition site | Arabidopsis thaliana | 2613 | - | 6 | CCGTTG |  |
| MYB recognition site | Arabidopsis thaliana | 1594 | + | 6 | CCGTTG |  |

>HU05G02245.1   
+ -Up\_Stream \_Len000ATAATT TATCTTTCTT TAATTTCGTT TCATGTAAAA AATTTCAGTC TCATCTGCGA   
  
  
+ TTGAATCTCT TTTCAATTCG CATCAATAAT TCATCCAATT CAAATGTGCC TTTCTTAATT TCATTACATT   
  
  
+ GGGTTGGTAT AAATGTTAGC ACGATAGATT GGAATAGCAA GGCTCTAATT CAATTATGAC TTGAATGTAT   
  
  
+ GCATGCTTTT ATCATTAGTT AATTAACATT TTCTGTATTG AAATCAGCTC GAAAGAGAAC CATATGTAGT   
  
  
+ TGTACTTGCG ACGATATATA TGTTTAGTTT AGTTAGTTCT TACCTGTTTT GGAATTGTTG GGGAGATCTG   
  
  
+ AGGAAGGAGC CCGCTTCAAT CAAAGTTCAT GGCAATAAGC CAAAGTGAGG TTCCATCCTA AAACCAATTG   
  
  
+ GCAATAGGAA GAGTAGCCCA CTTAACTTGT ATACTATGAC TCTTCTTTTC AAACTCCCAA TGTGGGACTT   
  
  
+ CTTTACTCAT GTGTGATATT CCCAACAGGA ATGATCAGTT CCACATGTTT TCATCTCTTT GGACTCGAAC   
  
  
+ TTCGTGTAAT TGTATATGCC ATGGCACAAA TAGTTTTGAT GGTGCCACCA AGTTGCTCAA TCAATGTAAT   
  
  
+ ACTGCTACTA TCTAACTTGC CTTGGTGGAC ATCAATAAGA TCACGAGATG AGGAGAACTG AATCTTGGCC   
  
  
+ AACCACGTCG TTTGACTTCT CTTACCAATA AAGATTGAAT AGACAGTGTT TCTCTCCCCC TTCCATTCTT   
  
  
+ CTTTTCTAGT GATGTCGAAA AAGACTTTAT ACATGTCCAG CAACGCTAGC CACAAAATAT TCCATTTCTG   
  
  
+ TAATCCACTC ATGTCAATTA AACTTGTATC ATCAAATGGT AGTTTCCGTA CTAATCACTA CATCTAGATA   
  
  
+ TTCCTTGATA AATTTAATTT GACTTTAAAG GCTCGATCTT TTTAAGAAAA GTTTTGTTGC TTACAACCTG   
  
  
+ TAAAGCCTAG AGAACAAAGC CAAAGAAGAA ATTTAAGTTC TATATATAGC ATAATCTCTC AAAAAATATA   
  
  
+ TTTTTTAAAT ATAGTGCGAT ATTGACTGAC CTAATATGGG TTATTACAGG AGATGGGGAG ATATCTACAT   
  
  
+ TAACTACTTG AACAACTTAA ATTTTCATTT TAACTCGGTC ATTAAGAACA CTAAGAACTT TATATATTAG   
  
  
+ TGATATCTCA GGCGTAAAAG AAACTACTTT GAATTAATCC ATGCAATCCT ACTCTTTATT TCCATCCGTT   
  
  
+ AAAATTTTCC TTCAAAACTC CCTTCTGTTC ATTATTAATT CAATATCGTA TTACACTTGT TGGATTATTT   
  
  
+ CTGTGATTTG ATCGTCATTT TCTATATATG AATAACTACA AAAATCATAG TCCGAGTACA TTAATATTAA   
  
  
+ TAAAATAGGA TGAGTGATAA CCTCATATAG TGCGTTTATC CCTACCACTA GGCTGGATTT CTGCATAAAG   
  
  
+ AAGAAATTGA ACATATGGAA AAAGAAATAA AGAGAAGCAA CTTCCTTGAG TGGGAGAAGA ATCGTAGAAA   
  
  
+ GTTAAACTTA TGAAAAAGAG CATTAACAAA AAGGAAATTA TAAGGCTTAC CGTTGAAGCC TCATATAATT   
  
  
+ TTGCAAGAGA CAGACAAAGA AAGTGTCTTT CAAATGTCAT ATTTCTCGAG CATGCATGAA AAATGTACTT   
  
  
+ CGGCTACAAT AGTAATAGTA TATATGTTGG CTTCATACAC TTGACCCCAA TGTATGTCAA CGTGACTAGT   
  
  
+ GACCACACAC ATCGTTGTCC CTGCCCGCCC CACTACCCAA ACTCCCCGCA CGCAAAGATA AAAAGCCCCC   
  
  
+ CAATTTTTAT TTGCCTCCCA AAAAGCCCAC CCGCGCTTTT TTATTTGCCT TCTTTTTTCC TACTTAAACC   
  
  
+ ACCTCCTCAA CGAACTTTGC AACATACATA CTAGTCTATA AGCAATTTAG AGGGAAGGGA AGCAACCTAA   
  
  
+ ACAGAGCGAG ATTGGCGGGG TGTGTTTGAG GGCTTGCGTT TTGGTGCAAT AGGAATGAAA GTGCCCATTT   
  
  
+ CAACCACCAT CCAAACTTGC AACTCCGGCA CCGGCAAACC ACTTTCCTTT GAGAATACTG CCCTACATAC   
  
  
+ CGCCTTTCAA GTTCCCAATA CCACCTACGA ACCCACCTCG GTTCTTGACC TCCAGCGCAG CCCTAGCCCC   
  
  
+ ACCTCCACCC AAAAACCGCC CTTTGCCGCC TCCAACCACC AGCCTGCCTC GGCTGATCTG GACAACTTGG   
  
  
+ ACGGGTGGGA TTCAATCCTA TCAGAGCTGG GGCTTAATGA CGAATTTACC CCTAATTCCA AACTCTGGTC   
  
  
+ CCAAATTAGT CCGTCTGATC CTCACCTCCC CCAACTCCCT GATTTCCCAA CATCTCAGCC GTTTGATCAT   
  
  
+ CACAACCCAC CCCCAATAAA CCTCCCATCA TCAGATTTCA ACCTCTGTGA TTTCTCCTAC AATCACAACC   
  
  
+ CCAATTTTGG GCCGTTCGAT CTTCATCACA ACCCGCATCA GAACAACCCG AACAACAATA ACTACGGCTT   
  
  
+ TGATTTCATA GACGACCTCA TCAAAGCAGC ACAGTCCCTC GAATCCAACG ACTCTCATCA AGTCCACCTG   
  
  
+ ATATTGGCGC GGCTCAATCA ACGGCTCAGA TCACCCACCG GCAAACCGCT CCAGCGGGCC GCCTACTACT   
  
  
+ TCAAGGAAGC CCTCATCGCC GCCGCGGCCG GCCCGCCCCG CCCCGCTCGG CTTTCATCGT ATGAGGTGGT   
  
  
+ GCAGACCATC CGAGCCTACA AGGCGTTTTC AGGAATTTCG CCAGTCTCTC TCTTCTCCAC TTTCGCCGCC   
  
  
+ AACCAGGCGA TTCTTGAGGC GGTGGACACG GCGGCCTTCA TCCACATCAT CGATTTCGAC ATCGGATTCG   
  
  
+ GCGGCCACTG GGCCTCGTTC CTCCGCGAAC TGGTCGACAA AGCTGATCCT GCGAAACTTA GTTCAGTGGT   
  
  
+ GTTACGAATC ACCGCCATCG TTCCCGAAGA ATTCGGGATC GAAAGCAAGC TAGTGAGAGA AAATCTGTCG   
  
  
+ CAATTTGCCC GAGATCTCAA CATCAACTTC CACATCGATT ATGTCTTATT TCAGAGCTTT GAAATCTTAT   
  
  
+ CCTTCAAATC TGTCAAATTC ATCGAAAGGG AGAAATTGGC GCTGCATCTT TCGCCGGCCG TGTTCCACCG   
  
  
+ GCTAGGGAGC GGAATTTCGA AGTTTATCGC TGATCTCCGA TCAATCTCGC CGAGCTCCGT CGTGGTGGTG   
  
  
+ GACAGAGATG TCGGGATTGA CATCGGAACG TCGTCGTTTA GCATGAATTT CGTCGCCGGA ATTGAATTCT   
  
  
+ ACACCGGGAT GCTGGAGTCG CTTGACGCAG CCACCGCCGG GGGGCTCATC GGCGGTGTGG ACTGCGTGAG   
  
  
+ ACGGATTGAG ACGTTTGTTC TCCACCCAAG GATCATGGCG GCGGTAGAGG CGGCGGCGTC GGCGTCGGCC   
  
  
+ GGGCGGAGGA CGGCGTGGAG GGAGGCATTC GCGGCGGCGG GGATTAGGGC GGTGGGGTTT AGCCAGTTTG   
  
  
+ CTGATTTCCA GGCCGAATGT TTGTTGAGGA GAGCTCAGGT TGGTGGCTTC CACGTGGCAA AGCGCCATGG   
  
  
+ AGAGATGATG CTTTACTGGC ATGACCGCCC ACTCGTCGCC ACATCAGCTT GGAGGTGTTA   

- -Up\_Stream \_Len000TATTAA ATAGAAAGAA ATTAAAGCAA AGTACATTTT TTAAAGTCAG AGTAGACGCT   
  
  
- AACTTAGAGA AAAGTTAAGC GTAGTTATTA AGTAGGTTAA GTTTACACGG AAAGAATTAA AGTAATGTAA   
  
  
- CCCAACCATA TTTACAATCG TGCTATCTAA CCTTATCGTT CCGAGATTAA GTTAATACTG AACTTACATA   
  
  
- CGTACGAAAA TAGTAATCAA TTAATTGTAA AAGACATAAC TTTAGTCGAG CTTTCTCTTG GTATACATCA   
  
  
- ACATGAACGC TGCTATATAT ACAAATCAAA TCAATCAAGA ATGGACAAAA CCTTAACAAC CCCTCTAGAC   
  
  
- TCCTTCCTCG GGCGAAGTTA GTTTCAAGTA CCGTTATTCG GTTTCACTCC AAGGTAGGAT TTTGGTTAAC   
  
  
- CGTTATCCTT CTCATCGGGT GAATTGAACA TATGATACTG AGAAGAAAAG TTTGAGGGTT ACACCCTGAA   
  
  
- GAAATGAGTA CACACTATAA GGGTTGTCCT TACTAGTCAA GGTGTACAAA AGTAGAGAAA CCTGAGCTTG   
  
  
- AAGCACATTA ACATATACGG TACCGTGTTT ATCAAAACTA CCACGGTGGT TCAACGAGTT AGTTACATTA   
  
  
- TGACGATGAT AGATTGAACG GAACCACCTG TAGTTATTCT AGTGCTCTAC TCCTCTTGAC TTAGAACCGG   
  
  
- TTGGTGCAGC AAACTGAAGA GAATGGTTAT TTCTAACTTA TCTGTCACAA AGAGAGGGGG AAGGTAAGAA   
  
  
- GAAAAGATCA CTACAGCTTT TTCTGAAATA TGTACAGGTC GTTGCGATCG GTGTTTTATA AGGTAAAGAC   
  
  
- ATTAGGTGAG TACAGTTAAT TTGAACATAG TAGTTTACCA TCAAAGGCAT GATTAGTGAT GTAGATCTAT   
  
  
- AAGGAACTAT TTAAATTAAA CTGAAATTTC CGAGCTAGAA AAATTCTTTT CAAAACAACG AATGTTGGAC   
  
  
- ATTTCGGATC TCTTGTTTCG GTTTCTTCTT TAAATTCAAG ATATATATCG TATTAGAGAG TTTTTTATAT   
  
  
- AAAAAATTTA TATCACGCTA TAACTGACTG GATTATACCC AATAATGTCC TCTACCCCTC TATAGATGTA   
  
  
- ATTGATGAAC TTGTTGAATT TAAAAGTAAA ATTGAGCCAG TAATTCTTGT GATTCTTGAA ATATATAATC   
  
  
- ACTATAGAGT CCGCATTTTC TTTGATGAAA CTTAATTAGG TACGTTAGGA TGAGAAATAA AGGTAGGCAA   
  
  
- TTTTAAAAGG AAGTTTTGAG GGAAGACAAG TAATAATTAA GTTATAGCAT AATGTGAACA ACCTAATAAA   
  
  
- GACACTAAAC TAGCAGTAAA AGATATATAC TTATTGATGT TTTTAGTATC AGGCTCATGT AATTATAATT   
  
  
- ATTTTATCCT ACTCACTATT GGAGTATATC ACGCAAATAG GGATGGTGAT CCGACCTAAA GACGTATTTC   
  
  
- TTCTTTAACT TGTATACCTT TTTCTTTATT TCTCTTCGTT GAAGGAACTC ACCCTCTTCT TAGCATCTTT   
  
  
- CAATTTGAAT ACTTTTTCTC GTAATTGTTT TTCCTTTAAT ATTCCGAATG GCAACTTCGG AGTATATTAA   
  
  
- AACGTTCTCT GTCTGTTTCT TTCACAGAAA GTTTACAGTA TAAAGAGCTC GTACGTACTT TTTACATGAA   
  
  
- GCCGATGTTA TCATTATCAT ATATACAACC GAAGTATGTG AACTGGGGTT ACATACAGTT GCACTGATCA   
  
  
- CTGGTGTGTG TAGCAACAGG GACGGGCGGG GTGATGGGTT TGAGGGGCGT GCGTTTCTAT TTTTCGGGGG   
  
  
- GTTAAAAATA AACGGAGGGT TTTTCGGGTG GGCGCGAAAA AATAAACGGA AGAAAAAAGG ATGAATTTGG   
  
  
- TGGAGGAGTT GCTTGAAACG TTGTATGTAT GATCAGATAT TCGTTAAATC TCCCTTCCCT TCGTTGGATT   
  
  
- TGTCTCGCTC TAACCGCCCC ACACAAACTC CCGAACGCAA AACCACGTTA TCCTTACTTT CACGGGTAAA   
  
  
- GTTGGTGGTA GGTTTGAACG TTGAGGCCGT GGCCGTTTGG TGAAAGGAAA CTCTTATGAC GGGATGTATG   
  
  
- GCGGAAAGTT CAAGGGTTAT GGTGGATGCT TGGGTGGAGC CAAGAACTGG AGGTCGCGTC GGGATCGGGG   
  
  
- TGGAGGTGGG TTTTTGGCGG GAAACGGCGG AGGTTGGTGG TCGGACGGAG CCGACTAGAC CTGTTGAACC   
  
  
- TGCCCACCCT AAGTTAGGAT AGTCTCGACC CCGAATTACT GCTTAAATGG GGATTAAGGT TTGAGACCAG   
  
  
- GGTTTAATCA GGCAGACTAG GAGTGGAGGG GGTTGAGGGA CTAAAGGGTT GTAGAGTCGG CAAACTAGTA   
  
  
- GTGTTGGGTG GGGGTTATTT GGAGGGTAGT AGTCTAAAGT TGGAGACACT AAAGAGGATG TTAGTGTTGG   
  
  
- GGTTAAAACC CGGCAAGCTA GAAGTAGTGT TGGGCGTAGT CTTGTTGGGC TTGTTGTTAT TGATGCCGAA   
  
  
- ACTAAAGTAT CTGCTGGAGT AGTTTCGTCG TGTCAGGGAG CTTAGGTTGC TGAGAGTAGT TCAGGTGGAC   
  
  
- TATAACCGCG CCGAGTTAGT TGCCGAGTCT AGTGGGTGGC CGTTTGGCGA GGTCGCCCGG CGGATGATGA   
  
  
- AGTTCCTTCG GGAGTAGCGG CGGCGCCGGC CGGGCGGGGC GGGGCGAGCC GAAAGTAGCA TACTCCACCA   
  
  
- CGTCTGGTAG GCTCGGATGT TCCGCAAAAG TCCTTAAAGC GGTCAGAGAG AGAAGAGGTG AAAGCGGCGG   
  
  
- TTGGTCCGCT AAGAACTCCG CCACCTGTGC CGCCGGAAGT AGGTGTAGTA GCTAAAGCTG TAGCCTAAGC   
  
  
- CGCCGGTGAC CCGGAGCAAG GAGGCGCTTG ACCAGCTGTT TCGACTAGGA CGCTTTGAAT CAAGTCACCA   
  
  
- CAATGCTTAG TGGCGGTAGC AAGGGCTTCT TAAGCCCTAG CTTTCGTTCG ATCACTCTCT TTTAGACAGC   
  
  
- GTTAAACGGG CTCTAGAGTT GTAGTTGAAG GTGTAGCTAA TACAGAATAA AGTCTCGAAA CTTTAGAATA   
  
  
- GGAAGTTTAG ACAGTTTAAG TAGCTTTCCC TCTTTAACCG CGACGTAGAA AGCGGCCGGC ACAAGGTGGC   
  
  
- CGATCCCTCG CCTTAAAGCT TCAAATAGCG ACTAGAGGCT AGTTAGAGCG GCTCGAGGCA GCACCACCAC   
  
  
- CTGTCTCTAC AGCCCTAACT GTAGCCTTGC AGCAGCAAAT CGTACTTAAA GCAGCGGCCT TAACTTAAGA   
  
  
- TGTGGCCCTA CGACCTCAGC GAACTGCGTC GGTGGCGGCC CCCCGAGTAG CCGCCACACC TGACGCACTC   
  
  
- TGCCTAACTC TGCAAACAAG AGGTGGGTTC CTAGTACCGC CGCCATCTCC GCCGCCGCAG CCGCAGCCGG   
  
  
- CCCGCCTCCT GCCGCACCTC CCTCCGTAAG CGCCGCCGCC CCTAATCCCG CCACCCCAAA TCGGTCAAAC   
  
  
- GACTAAAGGT CCGGCTTACA AACAACTCCT CTCGAGTCCA ACCACCGAAG GTGCACCGTT TCGCGGTACC   
  
  
- TCTCTACTAC GAAATGACCG TACTGGCGGG TGAGCAGCGG TGTAGTCGAA CCTCCACAAT

+     MYC

| Site Name | Organism | Position | Strand | Matrix score. | sequence | function |
| --- | --- | --- | --- | --- | --- | --- |
| MYC | Arabidopsis thaliana | 1645 | - | 6 | CATTTG |  |
| MYC | Arabidopsis thaliana | 877 | - | 6 | CATTTG |  |
| MYC | Arabidopsis thaliana | 536 | - | 6 | CATGTG |  |
| MYC | Arabidopsis thaliana | 419 | + | 6 | CAATTG |  |
| MYC | Arabidopsis thaliana | 502 | + | 6 | CATGTG |  |
| MYC | Arabidopsis thaliana | 115 | - | 6 | CATTTG |  |

>HU05G02245.1   
+ -Up\_Stream \_Len000ATAATT TATCTTTCTT TAATTTCGTT TCATGTAAAA AATTTCAGTC TCATCTGCGA   
  
  
+ TTGAATCTCT TTTCAATTCG CATCAATAAT TCATCCAATT CAAATGTGCC TTTCTTAATT TCATTACATT   
  
  
+ GGGTTGGTAT AAATGTTAGC ACGATAGATT GGAATAGCAA GGCTCTAATT CAATTATGAC TTGAATGTAT   
  
  
+ GCATGCTTTT ATCATTAGTT AATTAACATT TTCTGTATTG AAATCAGCTC GAAAGAGAAC CATATGTAGT   
  
  
+ TGTACTTGCG ACGATATATA TGTTTAGTTT AGTTAGTTCT TACCTGTTTT GGAATTGTTG GGGAGATCTG   
  
  
+ AGGAAGGAGC CCGCTTCAAT CAAAGTTCAT GGCAATAAGC CAAAGTGAGG TTCCATCCTA AAACCAATTG   
  
  
+ GCAATAGGAA GAGTAGCCCA CTTAACTTGT ATACTATGAC TCTTCTTTTC AAACTCCCAA TGTGGGACTT   
  
  
+ CTTTACTCAT GTGTGATATT CCCAACAGGA ATGATCAGTT CCACATGTTT TCATCTCTTT GGACTCGAAC   
  
  
+ TTCGTGTAAT TGTATATGCC ATGGCACAAA TAGTTTTGAT GGTGCCACCA AGTTGCTCAA TCAATGTAAT   
  
  
+ ACTGCTACTA TCTAACTTGC CTTGGTGGAC ATCAATAAGA TCACGAGATG AGGAGAACTG AATCTTGGCC   
  
  
+ AACCACGTCG TTTGACTTCT CTTACCAATA AAGATTGAAT AGACAGTGTT TCTCTCCCCC TTCCATTCTT   
  
  
+ CTTTTCTAGT GATGTCGAAA AAGACTTTAT ACATGTCCAG CAACGCTAGC CACAAAATAT TCCATTTCTG   
  
  
+ TAATCCACTC ATGTCAATTA AACTTGTATC ATCAAATGGT AGTTTCCGTA CTAATCACTA CATCTAGATA   
  
  
+ TTCCTTGATA AATTTAATTT GACTTTAAAG GCTCGATCTT TTTAAGAAAA GTTTTGTTGC TTACAACCTG   
  
  
+ TAAAGCCTAG AGAACAAAGC CAAAGAAGAA ATTTAAGTTC TATATATAGC ATAATCTCTC AAAAAATATA   
  
  
+ TTTTTTAAAT ATAGTGCGAT ATTGACTGAC CTAATATGGG TTATTACAGG AGATGGGGAG ATATCTACAT   
  
  
+ TAACTACTTG AACAACTTAA ATTTTCATTT TAACTCGGTC ATTAAGAACA CTAAGAACTT TATATATTAG   
  
  
+ TGATATCTCA GGCGTAAAAG AAACTACTTT GAATTAATCC ATGCAATCCT ACTCTTTATT TCCATCCGTT   
  
  
+ AAAATTTTCC TTCAAAACTC CCTTCTGTTC ATTATTAATT CAATATCGTA TTACACTTGT TGGATTATTT   
  
  
+ CTGTGATTTG ATCGTCATTT TCTATATATG AATAACTACA AAAATCATAG TCCGAGTACA TTAATATTAA   
  
  
+ TAAAATAGGA TGAGTGATAA CCTCATATAG TGCGTTTATC CCTACCACTA GGCTGGATTT CTGCATAAAG   
  
  
+ AAGAAATTGA ACATATGGAA AAAGAAATAA AGAGAAGCAA CTTCCTTGAG TGGGAGAAGA ATCGTAGAAA   
  
  
+ GTTAAACTTA TGAAAAAGAG CATTAACAAA AAGGAAATTA TAAGGCTTAC CGTTGAAGCC TCATATAATT   
  
  
+ TTGCAAGAGA CAGACAAAGA AAGTGTCTTT CAAATGTCAT ATTTCTCGAG CATGCATGAA AAATGTACTT   
  
  
+ CGGCTACAAT AGTAATAGTA TATATGTTGG CTTCATACAC TTGACCCCAA TGTATGTCAA CGTGACTAGT   
  
  
+ GACCACACAC ATCGTTGTCC CTGCCCGCCC CACTACCCAA ACTCCCCGCA CGCAAAGATA AAAAGCCCCC   
  
  
+ CAATTTTTAT TTGCCTCCCA AAAAGCCCAC CCGCGCTTTT TTATTTGCCT TCTTTTTTCC TACTTAAACC   
  
  
+ ACCTCCTCAA CGAACTTTGC AACATACATA CTAGTCTATA AGCAATTTAG AGGGAAGGGA AGCAACCTAA   
  
  
+ ACAGAGCGAG ATTGGCGGGG TGTGTTTGAG GGCTTGCGTT TTGGTGCAAT AGGAATGAAA GTGCCCATTT   
  
  
+ CAACCACCAT CCAAACTTGC AACTCCGGCA CCGGCAAACC ACTTTCCTTT GAGAATACTG CCCTACATAC   
  
  
+ CGCCTTTCAA GTTCCCAATA CCACCTACGA ACCCACCTCG GTTCTTGACC TCCAGCGCAG CCCTAGCCCC   
  
  
+ ACCTCCACCC AAAAACCGCC CTTTGCCGCC TCCAACCACC AGCCTGCCTC GGCTGATCTG GACAACTTGG   
  
  
+ ACGGGTGGGA TTCAATCCTA TCAGAGCTGG GGCTTAATGA CGAATTTACC CCTAATTCCA AACTCTGGTC   
  
  
+ CCAAATTAGT CCGTCTGATC CTCACCTCCC CCAACTCCCT GATTTCCCAA CATCTCAGCC GTTTGATCAT   
  
  
+ CACAACCCAC CCCCAATAAA CCTCCCATCA TCAGATTTCA ACCTCTGTGA TTTCTCCTAC AATCACAACC   
  
  
+ CCAATTTTGG GCCGTTCGAT CTTCATCACA ACCCGCATCA GAACAACCCG AACAACAATA ACTACGGCTT   
  
  
+ TGATTTCATA GACGACCTCA TCAAAGCAGC ACAGTCCCTC GAATCCAACG ACTCTCATCA AGTCCACCTG   
  
  
+ ATATTGGCGC GGCTCAATCA ACGGCTCAGA TCACCCACCG GCAAACCGCT CCAGCGGGCC GCCTACTACT   
  
  
+ TCAAGGAAGC CCTCATCGCC GCCGCGGCCG GCCCGCCCCG CCCCGCTCGG CTTTCATCGT ATGAGGTGGT   
  
  
+ GCAGACCATC CGAGCCTACA AGGCGTTTTC AGGAATTTCG CCAGTCTCTC TCTTCTCCAC TTTCGCCGCC   
  
  
+ AACCAGGCGA TTCTTGAGGC GGTGGACACG GCGGCCTTCA TCCACATCAT CGATTTCGAC ATCGGATTCG   
  
  
+ GCGGCCACTG GGCCTCGTTC CTCCGCGAAC TGGTCGACAA AGCTGATCCT GCGAAACTTA GTTCAGTGGT   
  
  
+ GTTACGAATC ACCGCCATCG TTCCCGAAGA ATTCGGGATC GAAAGCAAGC TAGTGAGAGA AAATCTGTCG   
  
  
+ CAATTTGCCC GAGATCTCAA CATCAACTTC CACATCGATT ATGTCTTATT TCAGAGCTTT GAAATCTTAT   
  
  
+ CCTTCAAATC TGTCAAATTC ATCGAAAGGG AGAAATTGGC GCTGCATCTT TCGCCGGCCG TGTTCCACCG   
  
  
+ GCTAGGGAGC GGAATTTCGA AGTTTATCGC TGATCTCCGA TCAATCTCGC CGAGCTCCGT CGTGGTGGTG   
  
  
+ GACAGAGATG TCGGGATTGA CATCGGAACG TCGTCGTTTA GCATGAATTT CGTCGCCGGA ATTGAATTCT   
  
  
+ ACACCGGGAT GCTGGAGTCG CTTGACGCAG CCACCGCCGG GGGGCTCATC GGCGGTGTGG ACTGCGTGAG   
  
  
+ ACGGATTGAG ACGTTTGTTC TCCACCCAAG GATCATGGCG GCGGTAGAGG CGGCGGCGTC GGCGTCGGCC   
  
  
+ GGGCGGAGGA CGGCGTGGAG GGAGGCATTC GCGGCGGCGG GGATTAGGGC GGTGGGGTTT AGCCAGTTTG   
  
  
+ CTGATTTCCA GGCCGAATGT TTGTTGAGGA GAGCTCAGGT TGGTGGCTTC CACGTGGCAA AGCGCCATGG   
  
  
+ AGAGATGATG CTTTACTGGC ATGACCGCCC ACTCGTCGCC ACATCAGCTT GGAGGTGTTA   

- -Up\_Stream \_Len000TATTAA ATAGAAAGAA ATTAAAGCAA AGTACATTTT TTAAAGTCAG AGTAGACGCT   
  
  
- AACTTAGAGA AAAGTTAAGC GTAGTTATTA AGTAGGTTAA GTTTACACGG AAAGAATTAA AGTAATGTAA   
  
  
- CCCAACCATA TTTACAATCG TGCTATCTAA CCTTATCGTT CCGAGATTAA GTTAATACTG AACTTACATA   
  
  
- CGTACGAAAA TAGTAATCAA TTAATTGTAA AAGACATAAC TTTAGTCGAG CTTTCTCTTG GTATACATCA   
  
  
- ACATGAACGC TGCTATATAT ACAAATCAAA TCAATCAAGA ATGGACAAAA CCTTAACAAC CCCTCTAGAC   
  
  
- TCCTTCCTCG GGCGAAGTTA GTTTCAAGTA CCGTTATTCG GTTTCACTCC AAGGTAGGAT TTTGGTTAAC   
  
  
- CGTTATCCTT CTCATCGGGT GAATTGAACA TATGATACTG AGAAGAAAAG TTTGAGGGTT ACACCCTGAA   
  
  
- GAAATGAGTA CACACTATAA GGGTTGTCCT TACTAGTCAA GGTGTACAAA AGTAGAGAAA CCTGAGCTTG   
  
  
- AAGCACATTA ACATATACGG TACCGTGTTT ATCAAAACTA CCACGGTGGT TCAACGAGTT AGTTACATTA   
  
  
- TGACGATGAT AGATTGAACG GAACCACCTG TAGTTATTCT AGTGCTCTAC TCCTCTTGAC TTAGAACCGG   
  
  
- TTGGTGCAGC AAACTGAAGA GAATGGTTAT TTCTAACTTA TCTGTCACAA AGAGAGGGGG AAGGTAAGAA   
  
  
- GAAAAGATCA CTACAGCTTT TTCTGAAATA TGTACAGGTC GTTGCGATCG GTGTTTTATA AGGTAAAGAC   
  
  
- ATTAGGTGAG TACAGTTAAT TTGAACATAG TAGTTTACCA TCAAAGGCAT GATTAGTGAT GTAGATCTAT   
  
  
- AAGGAACTAT TTAAATTAAA CTGAAATTTC CGAGCTAGAA AAATTCTTTT CAAAACAACG AATGTTGGAC   
  
  
- ATTTCGGATC TCTTGTTTCG GTTTCTTCTT TAAATTCAAG ATATATATCG TATTAGAGAG TTTTTTATAT   
  
  
- AAAAAATTTA TATCACGCTA TAACTGACTG GATTATACCC AATAATGTCC TCTACCCCTC TATAGATGTA   
  
  
- ATTGATGAAC TTGTTGAATT TAAAAGTAAA ATTGAGCCAG TAATTCTTGT GATTCTTGAA ATATATAATC   
  
  
- ACTATAGAGT CCGCATTTTC TTTGATGAAA CTTAATTAGG TACGTTAGGA TGAGAAATAA AGGTAGGCAA   
  
  
- TTTTAAAAGG AAGTTTTGAG GGAAGACAAG TAATAATTAA GTTATAGCAT AATGTGAACA ACCTAATAAA   
  
  
- GACACTAAAC TAGCAGTAAA AGATATATAC TTATTGATGT TTTTAGTATC AGGCTCATGT AATTATAATT   
  
  
- ATTTTATCCT ACTCACTATT GGAGTATATC ACGCAAATAG GGATGGTGAT CCGACCTAAA GACGTATTTC   
  
  
- TTCTTTAACT TGTATACCTT TTTCTTTATT TCTCTTCGTT GAAGGAACTC ACCCTCTTCT TAGCATCTTT   
  
  
- CAATTTGAAT ACTTTTTCTC GTAATTGTTT TTCCTTTAAT ATTCCGAATG GCAACTTCGG AGTATATTAA   
  
  
- AACGTTCTCT GTCTGTTTCT TTCACAGAAA GTTTACAGTA TAAAGAGCTC GTACGTACTT TTTACATGAA   
  
  
- GCCGATGTTA TCATTATCAT ATATACAACC GAAGTATGTG AACTGGGGTT ACATACAGTT GCACTGATCA   
  
  
- CTGGTGTGTG TAGCAACAGG GACGGGCGGG GTGATGGGTT TGAGGGGCGT GCGTTTCTAT TTTTCGGGGG   
  
  
- GTTAAAAATA AACGGAGGGT TTTTCGGGTG GGCGCGAAAA AATAAACGGA AGAAAAAAGG ATGAATTTGG   
  
  
- TGGAGGAGTT GCTTGAAACG TTGTATGTAT GATCAGATAT TCGTTAAATC TCCCTTCCCT TCGTTGGATT   
  
  
- TGTCTCGCTC TAACCGCCCC ACACAAACTC CCGAACGCAA AACCACGTTA TCCTTACTTT CACGGGTAAA   
  
  
- GTTGGTGGTA GGTTTGAACG TTGAGGCCGT GGCCGTTTGG TGAAAGGAAA CTCTTATGAC GGGATGTATG   
  
  
- GCGGAAAGTT CAAGGGTTAT GGTGGATGCT TGGGTGGAGC CAAGAACTGG AGGTCGCGTC GGGATCGGGG   
  
  
- TGGAGGTGGG TTTTTGGCGG GAAACGGCGG AGGTTGGTGG TCGGACGGAG CCGACTAGAC CTGTTGAACC   
  
  
- TGCCCACCCT AAGTTAGGAT AGTCTCGACC CCGAATTACT GCTTAAATGG GGATTAAGGT TTGAGACCAG   
  
  
- GGTTTAATCA GGCAGACTAG GAGTGGAGGG GGTTGAGGGA CTAAAGGGTT GTAGAGTCGG CAAACTAGTA   
  
  
- GTGTTGGGTG GGGGTTATTT GGAGGGTAGT AGTCTAAAGT TGGAGACACT AAAGAGGATG TTAGTGTTGG   
  
  
- GGTTAAAACC CGGCAAGCTA GAAGTAGTGT TGGGCGTAGT CTTGTTGGGC TTGTTGTTAT TGATGCCGAA   
  
  
- ACTAAAGTAT CTGCTGGAGT AGTTTCGTCG TGTCAGGGAG CTTAGGTTGC TGAGAGTAGT TCAGGTGGAC   
  
  
- TATAACCGCG CCGAGTTAGT TGCCGAGTCT AGTGGGTGGC CGTTTGGCGA GGTCGCCCGG CGGATGATGA   
  
  
- AGTTCCTTCG GGAGTAGCGG CGGCGCCGGC CGGGCGGGGC GGGGCGAGCC GAAAGTAGCA TACTCCACCA   
  
  
- CGTCTGGTAG GCTCGGATGT TCCGCAAAAG TCCTTAAAGC GGTCAGAGAG AGAAGAGGTG AAAGCGGCGG   
  
  
- TTGGTCCGCT AAGAACTCCG CCACCTGTGC CGCCGGAAGT AGGTGTAGTA GCTAAAGCTG TAGCCTAAGC   
  
  
- CGCCGGTGAC CCGGAGCAAG GAGGCGCTTG ACCAGCTGTT TCGACTAGGA CGCTTTGAAT CAAGTCACCA   
  
  
- CAATGCTTAG TGGCGGTAGC AAGGGCTTCT TAAGCCCTAG CTTTCGTTCG ATCACTCTCT TTTAGACAGC   
  
  
- GTTAAACGGG CTCTAGAGTT GTAGTTGAAG GTGTAGCTAA TACAGAATAA AGTCTCGAAA CTTTAGAATA   
  
  
- GGAAGTTTAG ACAGTTTAAG TAGCTTTCCC TCTTTAACCG CGACGTAGAA AGCGGCCGGC ACAAGGTGGC   
  
  
- CGATCCCTCG CCTTAAAGCT TCAAATAGCG ACTAGAGGCT AGTTAGAGCG GCTCGAGGCA GCACCACCAC   
  
  
- CTGTCTCTAC AGCCCTAACT GTAGCCTTGC AGCAGCAAAT CGTACTTAAA GCAGCGGCCT TAACTTAAGA   
  
  
- TGTGGCCCTA CGACCTCAGC GAACTGCGTC GGTGGCGGCC CCCCGAGTAG CCGCCACACC TGACGCACTC   
  
  
- TGCCTAACTC TGCAAACAAG AGGTGGGTTC CTAGTACCGC CGCCATCTCC GCCGCCGCAG CCGCAGCCGG   
  
  
- CCCGCCTCCT GCCGCACCTC CCTCCGTAAG CGCCGCCGCC CCTAATCCCG CCACCCCAAA TCGGTCAAAC   
  
  
- GACTAAAGGT CCGGCTTACA AACAACTCCT CTCGAGTCCA ACCACCGAAG GTGCACCGTT TCGCGGTACC   
  
  
- TCTCTACTAC GAAATGACCG TACTGGCGGG TGAGCAGCGG TGTAGTCGAA CCTCCACAAT

+     Myb-binding site

| Site Name | Organism | Position | Strand | Matrix score. | sequence | function |
| --- | --- | --- | --- | --- | --- | --- |
| Myb-binding site | Nicotiana tabacum | 517 | + | 6 | CAACAG |  |

>HU05G02245.1   
+ -Up\_Stream \_Len000ATAATT TATCTTTCTT TAATTTCGTT TCATGTAAAA AATTTCAGTC TCATCTGCGA   
  
  
+ TTGAATCTCT TTTCAATTCG CATCAATAAT TCATCCAATT CAAATGTGCC TTTCTTAATT TCATTACATT   
  
  
+ GGGTTGGTAT AAATGTTAGC ACGATAGATT GGAATAGCAA GGCTCTAATT CAATTATGAC TTGAATGTAT   
  
  
+ GCATGCTTTT ATCATTAGTT AATTAACATT TTCTGTATTG AAATCAGCTC GAAAGAGAAC CATATGTAGT   
  
  
+ TGTACTTGCG ACGATATATA TGTTTAGTTT AGTTAGTTCT TACCTGTTTT GGAATTGTTG GGGAGATCTG   
  
  
+ AGGAAGGAGC CCGCTTCAAT CAAAGTTCAT GGCAATAAGC CAAAGTGAGG TTCCATCCTA AAACCAATTG   
  
  
+ GCAATAGGAA GAGTAGCCCA CTTAACTTGT ATACTATGAC TCTTCTTTTC AAACTCCCAA TGTGGGACTT   
  
  
+ CTTTACTCAT GTGTGATATT CCCAACAGGA ATGATCAGTT CCACATGTTT TCATCTCTTT GGACTCGAAC   
  
  
+ TTCGTGTAAT TGTATATGCC ATGGCACAAA TAGTTTTGAT GGTGCCACCA AGTTGCTCAA TCAATGTAAT   
  
  
+ ACTGCTACTA TCTAACTTGC CTTGGTGGAC ATCAATAAGA TCACGAGATG AGGAGAACTG AATCTTGGCC   
  
  
+ AACCACGTCG TTTGACTTCT CTTACCAATA AAGATTGAAT AGACAGTGTT TCTCTCCCCC TTCCATTCTT   
  
  
+ CTTTTCTAGT GATGTCGAAA AAGACTTTAT ACATGTCCAG CAACGCTAGC CACAAAATAT TCCATTTCTG   
  
  
+ TAATCCACTC ATGTCAATTA AACTTGTATC ATCAAATGGT AGTTTCCGTA CTAATCACTA CATCTAGATA   
  
  
+ TTCCTTGATA AATTTAATTT GACTTTAAAG GCTCGATCTT TTTAAGAAAA GTTTTGTTGC TTACAACCTG   
  
  
+ TAAAGCCTAG AGAACAAAGC CAAAGAAGAA ATTTAAGTTC TATATATAGC ATAATCTCTC AAAAAATATA   
  
  
+ TTTTTTAAAT ATAGTGCGAT ATTGACTGAC CTAATATGGG TTATTACAGG AGATGGGGAG ATATCTACAT   
  
  
+ TAACTACTTG AACAACTTAA ATTTTCATTT TAACTCGGTC ATTAAGAACA CTAAGAACTT TATATATTAG   
  
  
+ TGATATCTCA GGCGTAAAAG AAACTACTTT GAATTAATCC ATGCAATCCT ACTCTTTATT TCCATCCGTT   
  
  
+ AAAATTTTCC TTCAAAACTC CCTTCTGTTC ATTATTAATT CAATATCGTA TTACACTTGT TGGATTATTT   
  
  
+ CTGTGATTTG ATCGTCATTT TCTATATATG AATAACTACA AAAATCATAG TCCGAGTACA TTAATATTAA   
  
  
+ TAAAATAGGA TGAGTGATAA CCTCATATAG TGCGTTTATC CCTACCACTA GGCTGGATTT CTGCATAAAG   
  
  
+ AAGAAATTGA ACATATGGAA AAAGAAATAA AGAGAAGCAA CTTCCTTGAG TGGGAGAAGA ATCGTAGAAA   
  
  
+ GTTAAACTTA TGAAAAAGAG CATTAACAAA AAGGAAATTA TAAGGCTTAC CGTTGAAGCC TCATATAATT   
  
  
+ TTGCAAGAGA CAGACAAAGA AAGTGTCTTT CAAATGTCAT ATTTCTCGAG CATGCATGAA AAATGTACTT   
  
  
+ CGGCTACAAT AGTAATAGTA TATATGTTGG CTTCATACAC TTGACCCCAA TGTATGTCAA CGTGACTAGT   
  
  
+ GACCACACAC ATCGTTGTCC CTGCCCGCCC CACTACCCAA ACTCCCCGCA CGCAAAGATA AAAAGCCCCC   
  
  
+ CAATTTTTAT TTGCCTCCCA AAAAGCCCAC CCGCGCTTTT TTATTTGCCT TCTTTTTTCC TACTTAAACC   
  
  
+ ACCTCCTCAA CGAACTTTGC AACATACATA CTAGTCTATA AGCAATTTAG AGGGAAGGGA AGCAACCTAA   
  
  
+ ACAGAGCGAG ATTGGCGGGG TGTGTTTGAG GGCTTGCGTT TTGGTGCAAT AGGAATGAAA GTGCCCATTT   
  
  
+ CAACCACCAT CCAAACTTGC AACTCCGGCA CCGGCAAACC ACTTTCCTTT GAGAATACTG CCCTACATAC   
  
  
+ CGCCTTTCAA GTTCCCAATA CCACCTACGA ACCCACCTCG GTTCTTGACC TCCAGCGCAG CCCTAGCCCC   
  
  
+ ACCTCCACCC AAAAACCGCC CTTTGCCGCC TCCAACCACC AGCCTGCCTC GGCTGATCTG GACAACTTGG   
  
  
+ ACGGGTGGGA TTCAATCCTA TCAGAGCTGG GGCTTAATGA CGAATTTACC CCTAATTCCA AACTCTGGTC   
  
  
+ CCAAATTAGT CCGTCTGATC CTCACCTCCC CCAACTCCCT GATTTCCCAA CATCTCAGCC GTTTGATCAT   
  
  
+ CACAACCCAC CCCCAATAAA CCTCCCATCA TCAGATTTCA ACCTCTGTGA TTTCTCCTAC AATCACAACC   
  
  
+ CCAATTTTGG GCCGTTCGAT CTTCATCACA ACCCGCATCA GAACAACCCG AACAACAATA ACTACGGCTT   
  
  
+ TGATTTCATA GACGACCTCA TCAAAGCAGC ACAGTCCCTC GAATCCAACG ACTCTCATCA AGTCCACCTG   
  
  
+ ATATTGGCGC GGCTCAATCA ACGGCTCAGA TCACCCACCG GCAAACCGCT CCAGCGGGCC GCCTACTACT   
  
  
+ TCAAGGAAGC CCTCATCGCC GCCGCGGCCG GCCCGCCCCG CCCCGCTCGG CTTTCATCGT ATGAGGTGGT   
  
  
+ GCAGACCATC CGAGCCTACA AGGCGTTTTC AGGAATTTCG CCAGTCTCTC TCTTCTCCAC TTTCGCCGCC   
  
  
+ AACCAGGCGA TTCTTGAGGC GGTGGACACG GCGGCCTTCA TCCACATCAT CGATTTCGAC ATCGGATTCG   
  
  
+ GCGGCCACTG GGCCTCGTTC CTCCGCGAAC TGGTCGACAA AGCTGATCCT GCGAAACTTA GTTCAGTGGT   
  
  
+ GTTACGAATC ACCGCCATCG TTCCCGAAGA ATTCGGGATC GAAAGCAAGC TAGTGAGAGA AAATCTGTCG   
  
  
+ CAATTTGCCC GAGATCTCAA CATCAACTTC CACATCGATT ATGTCTTATT TCAGAGCTTT GAAATCTTAT   
  
  
+ CCTTCAAATC TGTCAAATTC ATCGAAAGGG AGAAATTGGC GCTGCATCTT TCGCCGGCCG TGTTCCACCG   
  
  
+ GCTAGGGAGC GGAATTTCGA AGTTTATCGC TGATCTCCGA TCAATCTCGC CGAGCTCCGT CGTGGTGGTG   
  
  
+ GACAGAGATG TCGGGATTGA CATCGGAACG TCGTCGTTTA GCATGAATTT CGTCGCCGGA ATTGAATTCT   
  
  
+ ACACCGGGAT GCTGGAGTCG CTTGACGCAG CCACCGCCGG GGGGCTCATC GGCGGTGTGG ACTGCGTGAG   
  
  
+ ACGGATTGAG ACGTTTGTTC TCCACCCAAG GATCATGGCG GCGGTAGAGG CGGCGGCGTC GGCGTCGGCC   
  
  
+ GGGCGGAGGA CGGCGTGGAG GGAGGCATTC GCGGCGGCGG GGATTAGGGC GGTGGGGTTT AGCCAGTTTG   
  
  
+ CTGATTTCCA GGCCGAATGT TTGTTGAGGA GAGCTCAGGT TGGTGGCTTC CACGTGGCAA AGCGCCATGG   
  
  
+ AGAGATGATG CTTTACTGGC ATGACCGCCC ACTCGTCGCC ACATCAGCTT GGAGGTGTTA   

- -Up\_Stream \_Len000TATTAA ATAGAAAGAA ATTAAAGCAA AGTACATTTT TTAAAGTCAG AGTAGACGCT   
  
  
- AACTTAGAGA AAAGTTAAGC GTAGTTATTA AGTAGGTTAA GTTTACACGG AAAGAATTAA AGTAATGTAA   
  
  
- CCCAACCATA TTTACAATCG TGCTATCTAA CCTTATCGTT CCGAGATTAA GTTAATACTG AACTTACATA   
  
  
- CGTACGAAAA TAGTAATCAA TTAATTGTAA AAGACATAAC TTTAGTCGAG CTTTCTCTTG GTATACATCA   
  
  
- ACATGAACGC TGCTATATAT ACAAATCAAA TCAATCAAGA ATGGACAAAA CCTTAACAAC CCCTCTAGAC   
  
  
- TCCTTCCTCG GGCGAAGTTA GTTTCAAGTA CCGTTATTCG GTTTCACTCC AAGGTAGGAT TTTGGTTAAC   
  
  
- CGTTATCCTT CTCATCGGGT GAATTGAACA TATGATACTG AGAAGAAAAG TTTGAGGGTT ACACCCTGAA   
  
  
- GAAATGAGTA CACACTATAA GGGTTGTCCT TACTAGTCAA GGTGTACAAA AGTAGAGAAA CCTGAGCTTG   
  
  
- AAGCACATTA ACATATACGG TACCGTGTTT ATCAAAACTA CCACGGTGGT TCAACGAGTT AGTTACATTA   
  
  
- TGACGATGAT AGATTGAACG GAACCACCTG TAGTTATTCT AGTGCTCTAC TCCTCTTGAC TTAGAACCGG   
  
  
- TTGGTGCAGC AAACTGAAGA GAATGGTTAT TTCTAACTTA TCTGTCACAA AGAGAGGGGG AAGGTAAGAA   
  
  
- GAAAAGATCA CTACAGCTTT TTCTGAAATA TGTACAGGTC GTTGCGATCG GTGTTTTATA AGGTAAAGAC   
  
  
- ATTAGGTGAG TACAGTTAAT TTGAACATAG TAGTTTACCA TCAAAGGCAT GATTAGTGAT GTAGATCTAT   
  
  
- AAGGAACTAT TTAAATTAAA CTGAAATTTC CGAGCTAGAA AAATTCTTTT CAAAACAACG AATGTTGGAC   
  
  
- ATTTCGGATC TCTTGTTTCG GTTTCTTCTT TAAATTCAAG ATATATATCG TATTAGAGAG TTTTTTATAT   
  
  
- AAAAAATTTA TATCACGCTA TAACTGACTG GATTATACCC AATAATGTCC TCTACCCCTC TATAGATGTA   
  
  
- ATTGATGAAC TTGTTGAATT TAAAAGTAAA ATTGAGCCAG TAATTCTTGT GATTCTTGAA ATATATAATC   
  
  
- ACTATAGAGT CCGCATTTTC TTTGATGAAA CTTAATTAGG TACGTTAGGA TGAGAAATAA AGGTAGGCAA   
  
  
- TTTTAAAAGG AAGTTTTGAG GGAAGACAAG TAATAATTAA GTTATAGCAT AATGTGAACA ACCTAATAAA   
  
  
- GACACTAAAC TAGCAGTAAA AGATATATAC TTATTGATGT TTTTAGTATC AGGCTCATGT AATTATAATT   
  
  
- ATTTTATCCT ACTCACTATT GGAGTATATC ACGCAAATAG GGATGGTGAT CCGACCTAAA GACGTATTTC   
  
  
- TTCTTTAACT TGTATACCTT TTTCTTTATT TCTCTTCGTT GAAGGAACTC ACCCTCTTCT TAGCATCTTT   
  
  
- CAATTTGAAT ACTTTTTCTC GTAATTGTTT TTCCTTTAAT ATTCCGAATG GCAACTTCGG AGTATATTAA   
  
  
- AACGTTCTCT GTCTGTTTCT TTCACAGAAA GTTTACAGTA TAAAGAGCTC GTACGTACTT TTTACATGAA   
  
  
- GCCGATGTTA TCATTATCAT ATATACAACC GAAGTATGTG AACTGGGGTT ACATACAGTT GCACTGATCA   
  
  
- CTGGTGTGTG TAGCAACAGG GACGGGCGGG GTGATGGGTT TGAGGGGCGT GCGTTTCTAT TTTTCGGGGG   
  
  
- GTTAAAAATA AACGGAGGGT TTTTCGGGTG GGCGCGAAAA AATAAACGGA AGAAAAAAGG ATGAATTTGG   
  
  
- TGGAGGAGTT GCTTGAAACG TTGTATGTAT GATCAGATAT TCGTTAAATC TCCCTTCCCT TCGTTGGATT   
  
  
- TGTCTCGCTC TAACCGCCCC ACACAAACTC CCGAACGCAA AACCACGTTA TCCTTACTTT CACGGGTAAA   
  
  
- GTTGGTGGTA GGTTTGAACG TTGAGGCCGT GGCCGTTTGG TGAAAGGAAA CTCTTATGAC GGGATGTATG   
  
  
- GCGGAAAGTT CAAGGGTTAT GGTGGATGCT TGGGTGGAGC CAAGAACTGG AGGTCGCGTC GGGATCGGGG   
  
  
- TGGAGGTGGG TTTTTGGCGG GAAACGGCGG AGGTTGGTGG TCGGACGGAG CCGACTAGAC CTGTTGAACC   
  
  
- TGCCCACCCT AAGTTAGGAT AGTCTCGACC CCGAATTACT GCTTAAATGG GGATTAAGGT TTGAGACCAG   
  
  
- GGTTTAATCA GGCAGACTAG GAGTGGAGGG GGTTGAGGGA CTAAAGGGTT GTAGAGTCGG CAAACTAGTA   
  
  
- GTGTTGGGTG GGGGTTATTT GGAGGGTAGT AGTCTAAAGT TGGAGACACT AAAGAGGATG TTAGTGTTGG   
  
  
- GGTTAAAACC CGGCAAGCTA GAAGTAGTGT TGGGCGTAGT CTTGTTGGGC TTGTTGTTAT TGATGCCGAA   
  
  
- ACTAAAGTAT CTGCTGGAGT AGTTTCGTCG TGTCAGGGAG CTTAGGTTGC TGAGAGTAGT TCAGGTGGAC   
  
  
- TATAACCGCG CCGAGTTAGT TGCCGAGTCT AGTGGGTGGC CGTTTGGCGA GGTCGCCCGG CGGATGATGA   
  
  
- AGTTCCTTCG GGAGTAGCGG CGGCGCCGGC CGGGCGGGGC GGGGCGAGCC GAAAGTAGCA TACTCCACCA   
  
  
- CGTCTGGTAG GCTCGGATGT TCCGCAAAAG TCCTTAAAGC GGTCAGAGAG AGAAGAGGTG AAAGCGGCGG   
  
  
- TTGGTCCGCT AAGAACTCCG CCACCTGTGC CGCCGGAAGT AGGTGTAGTA GCTAAAGCTG TAGCCTAAGC   
  
  
- CGCCGGTGAC CCGGAGCAAG GAGGCGCTTG ACCAGCTGTT TCGACTAGGA CGCTTTGAAT CAAGTCACCA   
  
  
- CAATGCTTAG TGGCGGTAGC AAGGGCTTCT TAAGCCCTAG CTTTCGTTCG ATCACTCTCT TTTAGACAGC   
  
  
- GTTAAACGGG CTCTAGAGTT GTAGTTGAAG GTGTAGCTAA TACAGAATAA AGTCTCGAAA CTTTAGAATA   
  
  
- GGAAGTTTAG ACAGTTTAAG TAGCTTTCCC TCTTTAACCG CGACGTAGAA AGCGGCCGGC ACAAGGTGGC   
  
  
- CGATCCCTCG CCTTAAAGCT TCAAATAGCG ACTAGAGGCT AGTTAGAGCG GCTCGAGGCA GCACCACCAC   
  
  
- CTGTCTCTAC AGCCCTAACT GTAGCCTTGC AGCAGCAAAT CGTACTTAAA GCAGCGGCCT TAACTTAAGA   
  
  
- TGTGGCCCTA CGACCTCAGC GAACTGCGTC GGTGGCGGCC CCCCGAGTAG CCGCCACACC TGACGCACTC   
  
  
- TGCCTAACTC TGCAAACAAG AGGTGGGTTC CTAGTACCGC CGCCATCTCC GCCGCCGCAG CCGCAGCCGG   
  
  
- CCCGCCTCCT GCCGCACCTC CCTCCGTAAG CGCCGCCGCC CCTAATCCCG CCACCCCAAA TCGGTCAAAC   
  
  
- GACTAAAGGT CCGGCTTACA AACAACTCCT CTCGAGTCCA ACCACCGAAG GTGCACCGTT TCGCGGTACC   
  
  
- TCTCTACTAC GAAATGACCG TACTGGCGGG TGAGCAGCGG TGTAGTCGAA CCTCCACAAT

+     Myc

| Site Name | Organism | Position | Strand | Matrix score. | sequence | function |
| --- | --- | --- | --- | --- | --- | --- |
| Myc | Arabidopsis thaliana | 722 | + | 7 | TCTCTTA |  |

>HU05G02245.1   
+ -Up\_Stream \_Len000ATAATT TATCTTTCTT TAATTTCGTT TCATGTAAAA AATTTCAGTC TCATCTGCGA   
  
  
+ TTGAATCTCT TTTCAATTCG CATCAATAAT TCATCCAATT CAAATGTGCC TTTCTTAATT TCATTACATT   
  
  
+ GGGTTGGTAT AAATGTTAGC ACGATAGATT GGAATAGCAA GGCTCTAATT CAATTATGAC TTGAATGTAT   
  
  
+ GCATGCTTTT ATCATTAGTT AATTAACATT TTCTGTATTG AAATCAGCTC GAAAGAGAAC CATATGTAGT   
  
  
+ TGTACTTGCG ACGATATATA TGTTTAGTTT AGTTAGTTCT TACCTGTTTT GGAATTGTTG GGGAGATCTG   
  
  
+ AGGAAGGAGC CCGCTTCAAT CAAAGTTCAT GGCAATAAGC CAAAGTGAGG TTCCATCCTA AAACCAATTG   
  
  
+ GCAATAGGAA GAGTAGCCCA CTTAACTTGT ATACTATGAC TCTTCTTTTC AAACTCCCAA TGTGGGACTT   
  
  
+ CTTTACTCAT GTGTGATATT CCCAACAGGA ATGATCAGTT CCACATGTTT TCATCTCTTT GGACTCGAAC   
  
  
+ TTCGTGTAAT TGTATATGCC ATGGCACAAA TAGTTTTGAT GGTGCCACCA AGTTGCTCAA TCAATGTAAT   
  
  
+ ACTGCTACTA TCTAACTTGC CTTGGTGGAC ATCAATAAGA TCACGAGATG AGGAGAACTG AATCTTGGCC   
  
  
+ AACCACGTCG TTTGACTTCT CTTACCAATA AAGATTGAAT AGACAGTGTT TCTCTCCCCC TTCCATTCTT   
  
  
+ CTTTTCTAGT GATGTCGAAA AAGACTTTAT ACATGTCCAG CAACGCTAGC CACAAAATAT TCCATTTCTG   
  
  
+ TAATCCACTC ATGTCAATTA AACTTGTATC ATCAAATGGT AGTTTCCGTA CTAATCACTA CATCTAGATA   
  
  
+ TTCCTTGATA AATTTAATTT GACTTTAAAG GCTCGATCTT TTTAAGAAAA GTTTTGTTGC TTACAACCTG   
  
  
+ TAAAGCCTAG AGAACAAAGC CAAAGAAGAA ATTTAAGTTC TATATATAGC ATAATCTCTC AAAAAATATA   
  
  
+ TTTTTTAAAT ATAGTGCGAT ATTGACTGAC CTAATATGGG TTATTACAGG AGATGGGGAG ATATCTACAT   
  
  
+ TAACTACTTG AACAACTTAA ATTTTCATTT TAACTCGGTC ATTAAGAACA CTAAGAACTT TATATATTAG   
  
  
+ TGATATCTCA GGCGTAAAAG AAACTACTTT GAATTAATCC ATGCAATCCT ACTCTTTATT TCCATCCGTT   
  
  
+ AAAATTTTCC TTCAAAACTC CCTTCTGTTC ATTATTAATT CAATATCGTA TTACACTTGT TGGATTATTT   
  
  
+ CTGTGATTTG ATCGTCATTT TCTATATATG AATAACTACA AAAATCATAG TCCGAGTACA TTAATATTAA   
  
  
+ TAAAATAGGA TGAGTGATAA CCTCATATAG TGCGTTTATC CCTACCACTA GGCTGGATTT CTGCATAAAG   
  
  
+ AAGAAATTGA ACATATGGAA AAAGAAATAA AGAGAAGCAA CTTCCTTGAG TGGGAGAAGA ATCGTAGAAA   
  
  
+ GTTAAACTTA TGAAAAAGAG CATTAACAAA AAGGAAATTA TAAGGCTTAC CGTTGAAGCC TCATATAATT   
  
  
+ TTGCAAGAGA CAGACAAAGA AAGTGTCTTT CAAATGTCAT ATTTCTCGAG CATGCATGAA AAATGTACTT   
  
  
+ CGGCTACAAT AGTAATAGTA TATATGTTGG CTTCATACAC TTGACCCCAA TGTATGTCAA CGTGACTAGT   
  
  
+ GACCACACAC ATCGTTGTCC CTGCCCGCCC CACTACCCAA ACTCCCCGCA CGCAAAGATA AAAAGCCCCC   
  
  
+ CAATTTTTAT TTGCCTCCCA AAAAGCCCAC CCGCGCTTTT TTATTTGCCT TCTTTTTTCC TACTTAAACC   
  
  
+ ACCTCCTCAA CGAACTTTGC AACATACATA CTAGTCTATA AGCAATTTAG AGGGAAGGGA AGCAACCTAA   
  
  
+ ACAGAGCGAG ATTGGCGGGG TGTGTTTGAG GGCTTGCGTT TTGGTGCAAT AGGAATGAAA GTGCCCATTT   
  
  
+ CAACCACCAT CCAAACTTGC AACTCCGGCA CCGGCAAACC ACTTTCCTTT GAGAATACTG CCCTACATAC   
  
  
+ CGCCTTTCAA GTTCCCAATA CCACCTACGA ACCCACCTCG GTTCTTGACC TCCAGCGCAG CCCTAGCCCC   
  
  
+ ACCTCCACCC AAAAACCGCC CTTTGCCGCC TCCAACCACC AGCCTGCCTC GGCTGATCTG GACAACTTGG   
  
  
+ ACGGGTGGGA TTCAATCCTA TCAGAGCTGG GGCTTAATGA CGAATTTACC CCTAATTCCA AACTCTGGTC   
  
  
+ CCAAATTAGT CCGTCTGATC CTCACCTCCC CCAACTCCCT GATTTCCCAA CATCTCAGCC GTTTGATCAT   
  
  
+ CACAACCCAC CCCCAATAAA CCTCCCATCA TCAGATTTCA ACCTCTGTGA TTTCTCCTAC AATCACAACC   
  
  
+ CCAATTTTGG GCCGTTCGAT CTTCATCACA ACCCGCATCA GAACAACCCG AACAACAATA ACTACGGCTT   
  
  
+ TGATTTCATA GACGACCTCA TCAAAGCAGC ACAGTCCCTC GAATCCAACG ACTCTCATCA AGTCCACCTG   
  
  
+ ATATTGGCGC GGCTCAATCA ACGGCTCAGA TCACCCACCG GCAAACCGCT CCAGCGGGCC GCCTACTACT   
  
  
+ TCAAGGAAGC CCTCATCGCC GCCGCGGCCG GCCCGCCCCG CCCCGCTCGG CTTTCATCGT ATGAGGTGGT   
  
  
+ GCAGACCATC CGAGCCTACA AGGCGTTTTC AGGAATTTCG CCAGTCTCTC TCTTCTCCAC TTTCGCCGCC   
  
  
+ AACCAGGCGA TTCTTGAGGC GGTGGACACG GCGGCCTTCA TCCACATCAT CGATTTCGAC ATCGGATTCG   
  
  
+ GCGGCCACTG GGCCTCGTTC CTCCGCGAAC TGGTCGACAA AGCTGATCCT GCGAAACTTA GTTCAGTGGT   
  
  
+ GTTACGAATC ACCGCCATCG TTCCCGAAGA ATTCGGGATC GAAAGCAAGC TAGTGAGAGA AAATCTGTCG   
  
  
+ CAATTTGCCC GAGATCTCAA CATCAACTTC CACATCGATT ATGTCTTATT TCAGAGCTTT GAAATCTTAT   
  
  
+ CCTTCAAATC TGTCAAATTC ATCGAAAGGG AGAAATTGGC GCTGCATCTT TCGCCGGCCG TGTTCCACCG   
  
  
+ GCTAGGGAGC GGAATTTCGA AGTTTATCGC TGATCTCCGA TCAATCTCGC CGAGCTCCGT CGTGGTGGTG   
  
  
+ GACAGAGATG TCGGGATTGA CATCGGAACG TCGTCGTTTA GCATGAATTT CGTCGCCGGA ATTGAATTCT   
  
  
+ ACACCGGGAT GCTGGAGTCG CTTGACGCAG CCACCGCCGG GGGGCTCATC GGCGGTGTGG ACTGCGTGAG   
  
  
+ ACGGATTGAG ACGTTTGTTC TCCACCCAAG GATCATGGCG GCGGTAGAGG CGGCGGCGTC GGCGTCGGCC   
  
  
+ GGGCGGAGGA CGGCGTGGAG GGAGGCATTC GCGGCGGCGG GGATTAGGGC GGTGGGGTTT AGCCAGTTTG   
  
  
+ CTGATTTCCA GGCCGAATGT TTGTTGAGGA GAGCTCAGGT TGGTGGCTTC CACGTGGCAA AGCGCCATGG   
  
  
+ AGAGATGATG CTTTACTGGC ATGACCGCCC ACTCGTCGCC ACATCAGCTT GGAGGTGTTA   

- -Up\_Stream \_Len000TATTAA ATAGAAAGAA ATTAAAGCAA AGTACATTTT TTAAAGTCAG AGTAGACGCT   
  
  
- AACTTAGAGA AAAGTTAAGC GTAGTTATTA AGTAGGTTAA GTTTACACGG AAAGAATTAA AGTAATGTAA   
  
  
- CCCAACCATA TTTACAATCG TGCTATCTAA CCTTATCGTT CCGAGATTAA GTTAATACTG AACTTACATA   
  
  
- CGTACGAAAA TAGTAATCAA TTAATTGTAA AAGACATAAC TTTAGTCGAG CTTTCTCTTG GTATACATCA   
  
  
- ACATGAACGC TGCTATATAT ACAAATCAAA TCAATCAAGA ATGGACAAAA CCTTAACAAC CCCTCTAGAC   
  
  
- TCCTTCCTCG GGCGAAGTTA GTTTCAAGTA CCGTTATTCG GTTTCACTCC AAGGTAGGAT TTTGGTTAAC   
  
  
- CGTTATCCTT CTCATCGGGT GAATTGAACA TATGATACTG AGAAGAAAAG TTTGAGGGTT ACACCCTGAA   
  
  
- GAAATGAGTA CACACTATAA GGGTTGTCCT TACTAGTCAA GGTGTACAAA AGTAGAGAAA CCTGAGCTTG   
  
  
- AAGCACATTA ACATATACGG TACCGTGTTT ATCAAAACTA CCACGGTGGT TCAACGAGTT AGTTACATTA   
  
  
- TGACGATGAT AGATTGAACG GAACCACCTG TAGTTATTCT AGTGCTCTAC TCCTCTTGAC TTAGAACCGG   
  
  
- TTGGTGCAGC AAACTGAAGA GAATGGTTAT TTCTAACTTA TCTGTCACAA AGAGAGGGGG AAGGTAAGAA   
  
  
- GAAAAGATCA CTACAGCTTT TTCTGAAATA TGTACAGGTC GTTGCGATCG GTGTTTTATA AGGTAAAGAC   
  
  
- ATTAGGTGAG TACAGTTAAT TTGAACATAG TAGTTTACCA TCAAAGGCAT GATTAGTGAT GTAGATCTAT   
  
  
- AAGGAACTAT TTAAATTAAA CTGAAATTTC CGAGCTAGAA AAATTCTTTT CAAAACAACG AATGTTGGAC   
  
  
- ATTTCGGATC TCTTGTTTCG GTTTCTTCTT TAAATTCAAG ATATATATCG TATTAGAGAG TTTTTTATAT   
  
  
- AAAAAATTTA TATCACGCTA TAACTGACTG GATTATACCC AATAATGTCC TCTACCCCTC TATAGATGTA   
  
  
- ATTGATGAAC TTGTTGAATT TAAAAGTAAA ATTGAGCCAG TAATTCTTGT GATTCTTGAA ATATATAATC   
  
  
- ACTATAGAGT CCGCATTTTC TTTGATGAAA CTTAATTAGG TACGTTAGGA TGAGAAATAA AGGTAGGCAA   
  
  
- TTTTAAAAGG AAGTTTTGAG GGAAGACAAG TAATAATTAA GTTATAGCAT AATGTGAACA ACCTAATAAA   
  
  
- GACACTAAAC TAGCAGTAAA AGATATATAC TTATTGATGT TTTTAGTATC AGGCTCATGT AATTATAATT   
  
  
- ATTTTATCCT ACTCACTATT GGAGTATATC ACGCAAATAG GGATGGTGAT CCGACCTAAA GACGTATTTC   
  
  
- TTCTTTAACT TGTATACCTT TTTCTTTATT TCTCTTCGTT GAAGGAACTC ACCCTCTTCT TAGCATCTTT   
  
  
- CAATTTGAAT ACTTTTTCTC GTAATTGTTT TTCCTTTAAT ATTCCGAATG GCAACTTCGG AGTATATTAA   
  
  
- AACGTTCTCT GTCTGTTTCT TTCACAGAAA GTTTACAGTA TAAAGAGCTC GTACGTACTT TTTACATGAA   
  
  
- GCCGATGTTA TCATTATCAT ATATACAACC GAAGTATGTG AACTGGGGTT ACATACAGTT GCACTGATCA   
  
  
- CTGGTGTGTG TAGCAACAGG GACGGGCGGG GTGATGGGTT TGAGGGGCGT GCGTTTCTAT TTTTCGGGGG   
  
  
- GTTAAAAATA AACGGAGGGT TTTTCGGGTG GGCGCGAAAA AATAAACGGA AGAAAAAAGG ATGAATTTGG   
  
  
- TGGAGGAGTT GCTTGAAACG TTGTATGTAT GATCAGATAT TCGTTAAATC TCCCTTCCCT TCGTTGGATT   
  
  
- TGTCTCGCTC TAACCGCCCC ACACAAACTC CCGAACGCAA AACCACGTTA TCCTTACTTT CACGGGTAAA   
  
  
- GTTGGTGGTA GGTTTGAACG TTGAGGCCGT GGCCGTTTGG TGAAAGGAAA CTCTTATGAC GGGATGTATG   
  
  
- GCGGAAAGTT CAAGGGTTAT GGTGGATGCT TGGGTGGAGC CAAGAACTGG AGGTCGCGTC GGGATCGGGG   
  
  
- TGGAGGTGGG TTTTTGGCGG GAAACGGCGG AGGTTGGTGG TCGGACGGAG CCGACTAGAC CTGTTGAACC   
  
  
- TGCCCACCCT AAGTTAGGAT AGTCTCGACC CCGAATTACT GCTTAAATGG GGATTAAGGT TTGAGACCAG   
  
  
- GGTTTAATCA GGCAGACTAG GAGTGGAGGG GGTTGAGGGA CTAAAGGGTT GTAGAGTCGG CAAACTAGTA   
  
  
- GTGTTGGGTG GGGGTTATTT GGAGGGTAGT AGTCTAAAGT TGGAGACACT AAAGAGGATG TTAGTGTTGG   
  
  
- GGTTAAAACC CGGCAAGCTA GAAGTAGTGT TGGGCGTAGT CTTGTTGGGC TTGTTGTTAT TGATGCCGAA   
  
  
- ACTAAAGTAT CTGCTGGAGT AGTTTCGTCG TGTCAGGGAG CTTAGGTTGC TGAGAGTAGT TCAGGTGGAC   
  
  
- TATAACCGCG CCGAGTTAGT TGCCGAGTCT AGTGGGTGGC CGTTTGGCGA GGTCGCCCGG CGGATGATGA   
  
  
- AGTTCCTTCG GGAGTAGCGG CGGCGCCGGC CGGGCGGGGC GGGGCGAGCC GAAAGTAGCA TACTCCACCA   
  
  
- CGTCTGGTAG GCTCGGATGT TCCGCAAAAG TCCTTAAAGC GGTCAGAGAG AGAAGAGGTG AAAGCGGCGG   
  
  
- TTGGTCCGCT AAGAACTCCG CCACCTGTGC CGCCGGAAGT AGGTGTAGTA GCTAAAGCTG TAGCCTAAGC   
  
  
- CGCCGGTGAC CCGGAGCAAG GAGGCGCTTG ACCAGCTGTT TCGACTAGGA CGCTTTGAAT CAAGTCACCA   
  
  
- CAATGCTTAG TGGCGGTAGC AAGGGCTTCT TAAGCCCTAG CTTTCGTTCG ATCACTCTCT TTTAGACAGC   
  
  
- GTTAAACGGG CTCTAGAGTT GTAGTTGAAG GTGTAGCTAA TACAGAATAA AGTCTCGAAA CTTTAGAATA   
  
  
- GGAAGTTTAG ACAGTTTAAG TAGCTTTCCC TCTTTAACCG CGACGTAGAA AGCGGCCGGC ACAAGGTGGC   
  
  
- CGATCCCTCG CCTTAAAGCT TCAAATAGCG ACTAGAGGCT AGTTAGAGCG GCTCGAGGCA GCACCACCAC   
  
  
- CTGTCTCTAC AGCCCTAACT GTAGCCTTGC AGCAGCAAAT CGTACTTAAA GCAGCGGCCT TAACTTAAGA   
  
  
- TGTGGCCCTA CGACCTCAGC GAACTGCGTC GGTGGCGGCC CCCCGAGTAG CCGCCACACC TGACGCACTC   
  
  
- TGCCTAACTC TGCAAACAAG AGGTGGGTTC CTAGTACCGC CGCCATCTCC GCCGCCGCAG CCGCAGCCGG   
  
  
- CCCGCCTCCT GCCGCACCTC CCTCCGTAAG CGCCGCCGCC CCTAATCCCG CCACCCCAAA TCGGTCAAAC   
  
  
- GACTAAAGGT CCGGCTTACA AACAACTCCT CTCGAGTCCA ACCACCGAAG GTGCACCGTT TCGCGGTACC   
  
  
- TCTCTACTAC GAAATGACCG TACTGGCGGG TGAGCAGCGG TGTAGTCGAA CCTCCACAAT

+     O2-site

| Site Name | Organism | Position | Strand | Matrix score. | sequence | function |
| --- | --- | --- | --- | --- | --- | --- |
| O2-site | Zea mays | 2846 | - | 10 | GATGATGTGG | cis-acting regulatory element involved in zein metabolism regulation |
| O2-site | Zea mays | 3613 | - | 9 | GATGATGTGG | cis-acting regulatory element involved in zein metabolism regulation |

>HU05G02245.1   
+ -Up\_Stream \_Len000ATAATT TATCTTTCTT TAATTTCGTT TCATGTAAAA AATTTCAGTC TCATCTGCGA   
  
  
+ TTGAATCTCT TTTCAATTCG CATCAATAAT TCATCCAATT CAAATGTGCC TTTCTTAATT TCATTACATT   
  
  
+ GGGTTGGTAT AAATGTTAGC ACGATAGATT GGAATAGCAA GGCTCTAATT CAATTATGAC TTGAATGTAT   
  
  
+ GCATGCTTTT ATCATTAGTT AATTAACATT TTCTGTATTG AAATCAGCTC GAAAGAGAAC CATATGTAGT   
  
  
+ TGTACTTGCG ACGATATATA TGTTTAGTTT AGTTAGTTCT TACCTGTTTT GGAATTGTTG GGGAGATCTG   
  
  
+ AGGAAGGAGC CCGCTTCAAT CAAAGTTCAT GGCAATAAGC CAAAGTGAGG TTCCATCCTA AAACCAATTG   
  
  
+ GCAATAGGAA GAGTAGCCCA CTTAACTTGT ATACTATGAC TCTTCTTTTC AAACTCCCAA TGTGGGACTT   
  
  
+ CTTTACTCAT GTGTGATATT CCCAACAGGA ATGATCAGTT CCACATGTTT TCATCTCTTT GGACTCGAAC   
  
  
+ TTCGTGTAAT TGTATATGCC ATGGCACAAA TAGTTTTGAT GGTGCCACCA AGTTGCTCAA TCAATGTAAT   
  
  
+ ACTGCTACTA TCTAACTTGC CTTGGTGGAC ATCAATAAGA TCACGAGATG AGGAGAACTG AATCTTGGCC   
  
  
+ AACCACGTCG TTTGACTTCT CTTACCAATA AAGATTGAAT AGACAGTGTT TCTCTCCCCC TTCCATTCTT   
  
  
+ CTTTTCTAGT GATGTCGAAA AAGACTTTAT ACATGTCCAG CAACGCTAGC CACAAAATAT TCCATTTCTG   
  
  
+ TAATCCACTC ATGTCAATTA AACTTGTATC ATCAAATGGT AGTTTCCGTA CTAATCACTA CATCTAGATA   
  
  
+ TTCCTTGATA AATTTAATTT GACTTTAAAG GCTCGATCTT TTTAAGAAAA GTTTTGTTGC TTACAACCTG   
  
  
+ TAAAGCCTAG AGAACAAAGC CAAAGAAGAA ATTTAAGTTC TATATATAGC ATAATCTCTC AAAAAATATA   
  
  
+ TTTTTTAAAT ATAGTGCGAT ATTGACTGAC CTAATATGGG TTATTACAGG AGATGGGGAG ATATCTACAT   
  
  
+ TAACTACTTG AACAACTTAA ATTTTCATTT TAACTCGGTC ATTAAGAACA CTAAGAACTT TATATATTAG   
  
  
+ TGATATCTCA GGCGTAAAAG AAACTACTTT GAATTAATCC ATGCAATCCT ACTCTTTATT TCCATCCGTT   
  
  
+ AAAATTTTCC TTCAAAACTC CCTTCTGTTC ATTATTAATT CAATATCGTA TTACACTTGT TGGATTATTT   
  
  
+ CTGTGATTTG ATCGTCATTT TCTATATATG AATAACTACA AAAATCATAG TCCGAGTACA TTAATATTAA   
  
  
+ TAAAATAGGA TGAGTGATAA CCTCATATAG TGCGTTTATC CCTACCACTA GGCTGGATTT CTGCATAAAG   
  
  
+ AAGAAATTGA ACATATGGAA AAAGAAATAA AGAGAAGCAA CTTCCTTGAG TGGGAGAAGA ATCGTAGAAA   
  
  
+ GTTAAACTTA TGAAAAAGAG CATTAACAAA AAGGAAATTA TAAGGCTTAC CGTTGAAGCC TCATATAATT   
  
  
+ TTGCAAGAGA CAGACAAAGA AAGTGTCTTT CAAATGTCAT ATTTCTCGAG CATGCATGAA AAATGTACTT   
  
  
+ CGGCTACAAT AGTAATAGTA TATATGTTGG CTTCATACAC TTGACCCCAA TGTATGTCAA CGTGACTAGT   
  
  
+ GACCACACAC ATCGTTGTCC CTGCCCGCCC CACTACCCAA ACTCCCCGCA CGCAAAGATA AAAAGCCCCC   
  
  
+ CAATTTTTAT TTGCCTCCCA AAAAGCCCAC CCGCGCTTTT TTATTTGCCT TCTTTTTTCC TACTTAAACC   
  
  
+ ACCTCCTCAA CGAACTTTGC AACATACATA CTAGTCTATA AGCAATTTAG AGGGAAGGGA AGCAACCTAA   
  
  
+ ACAGAGCGAG ATTGGCGGGG TGTGTTTGAG GGCTTGCGTT TTGGTGCAAT AGGAATGAAA GTGCCCATTT   
  
  
+ CAACCACCAT CCAAACTTGC AACTCCGGCA CCGGCAAACC ACTTTCCTTT GAGAATACTG CCCTACATAC   
  
  
+ CGCCTTTCAA GTTCCCAATA CCACCTACGA ACCCACCTCG GTTCTTGACC TCCAGCGCAG CCCTAGCCCC   
  
  
+ ACCTCCACCC AAAAACCGCC CTTTGCCGCC TCCAACCACC AGCCTGCCTC GGCTGATCTG GACAACTTGG   
  
  
+ ACGGGTGGGA TTCAATCCTA TCAGAGCTGG GGCTTAATGA CGAATTTACC CCTAATTCCA AACTCTGGTC   
  
  
+ CCAAATTAGT CCGTCTGATC CTCACCTCCC CCAACTCCCT GATTTCCCAA CATCTCAGCC GTTTGATCAT   
  
  
+ CACAACCCAC CCCCAATAAA CCTCCCATCA TCAGATTTCA ACCTCTGTGA TTTCTCCTAC AATCACAACC   
  
  
+ CCAATTTTGG GCCGTTCGAT CTTCATCACA ACCCGCATCA GAACAACCCG AACAACAATA ACTACGGCTT   
  
  
+ TGATTTCATA GACGACCTCA TCAAAGCAGC ACAGTCCCTC GAATCCAACG ACTCTCATCA AGTCCACCTG   
  
  
+ ATATTGGCGC GGCTCAATCA ACGGCTCAGA TCACCCACCG GCAAACCGCT CCAGCGGGCC GCCTACTACT   
  
  
+ TCAAGGAAGC CCTCATCGCC GCCGCGGCCG GCCCGCCCCG CCCCGCTCGG CTTTCATCGT ATGAGGTGGT   
  
  
+ GCAGACCATC CGAGCCTACA AGGCGTTTTC AGGAATTTCG CCAGTCTCTC TCTTCTCCAC TTTCGCCGCC   
  
  
+ AACCAGGCGA TTCTTGAGGC GGTGGACACG GCGGCCTTCA TCCACATCAT CGATTTCGAC ATCGGATTCG   
  
  
+ GCGGCCACTG GGCCTCGTTC CTCCGCGAAC TGGTCGACAA AGCTGATCCT GCGAAACTTA GTTCAGTGGT   
  
  
+ GTTACGAATC ACCGCCATCG TTCCCGAAGA ATTCGGGATC GAAAGCAAGC TAGTGAGAGA AAATCTGTCG   
  
  
+ CAATTTGCCC GAGATCTCAA CATCAACTTC CACATCGATT ATGTCTTATT TCAGAGCTTT GAAATCTTAT   
  
  
+ CCTTCAAATC TGTCAAATTC ATCGAAAGGG AGAAATTGGC GCTGCATCTT TCGCCGGCCG TGTTCCACCG   
  
  
+ GCTAGGGAGC GGAATTTCGA AGTTTATCGC TGATCTCCGA TCAATCTCGC CGAGCTCCGT CGTGGTGGTG   
  
  
+ GACAGAGATG TCGGGATTGA CATCGGAACG TCGTCGTTTA GCATGAATTT CGTCGCCGGA ATTGAATTCT   
  
  
+ ACACCGGGAT GCTGGAGTCG CTTGACGCAG CCACCGCCGG GGGGCTCATC GGCGGTGTGG ACTGCGTGAG   
  
  
+ ACGGATTGAG ACGTTTGTTC TCCACCCAAG GATCATGGCG GCGGTAGAGG CGGCGGCGTC GGCGTCGGCC   
  
  
+ GGGCGGAGGA CGGCGTGGAG GGAGGCATTC GCGGCGGCGG GGATTAGGGC GGTGGGGTTT AGCCAGTTTG   
  
  
+ CTGATTTCCA GGCCGAATGT TTGTTGAGGA GAGCTCAGGT TGGTGGCTTC CACGTGGCAA AGCGCCATGG   
  
  
+ AGAGATGATG CTTTACTGGC ATGACCGCCC ACTCGTCGCC ACATCAGCTT GGAGGTGTTA   

- -Up\_Stream \_Len000TATTAA ATAGAAAGAA ATTAAAGCAA AGTACATTTT TTAAAGTCAG AGTAGACGCT   
  
  
- AACTTAGAGA AAAGTTAAGC GTAGTTATTA AGTAGGTTAA GTTTACACGG AAAGAATTAA AGTAATGTAA   
  
  
- CCCAACCATA TTTACAATCG TGCTATCTAA CCTTATCGTT CCGAGATTAA GTTAATACTG AACTTACATA   
  
  
- CGTACGAAAA TAGTAATCAA TTAATTGTAA AAGACATAAC TTTAGTCGAG CTTTCTCTTG GTATACATCA   
  
  
- ACATGAACGC TGCTATATAT ACAAATCAAA TCAATCAAGA ATGGACAAAA CCTTAACAAC CCCTCTAGAC   
  
  
- TCCTTCCTCG GGCGAAGTTA GTTTCAAGTA CCGTTATTCG GTTTCACTCC AAGGTAGGAT TTTGGTTAAC   
  
  
- CGTTATCCTT CTCATCGGGT GAATTGAACA TATGATACTG AGAAGAAAAG TTTGAGGGTT ACACCCTGAA   
  
  
- GAAATGAGTA CACACTATAA GGGTTGTCCT TACTAGTCAA GGTGTACAAA AGTAGAGAAA CCTGAGCTTG   
  
  
- AAGCACATTA ACATATACGG TACCGTGTTT ATCAAAACTA CCACGGTGGT TCAACGAGTT AGTTACATTA   
  
  
- TGACGATGAT AGATTGAACG GAACCACCTG TAGTTATTCT AGTGCTCTAC TCCTCTTGAC TTAGAACCGG   
  
  
- TTGGTGCAGC AAACTGAAGA GAATGGTTAT TTCTAACTTA TCTGTCACAA AGAGAGGGGG AAGGTAAGAA   
  
  
- GAAAAGATCA CTACAGCTTT TTCTGAAATA TGTACAGGTC GTTGCGATCG GTGTTTTATA AGGTAAAGAC   
  
  
- ATTAGGTGAG TACAGTTAAT TTGAACATAG TAGTTTACCA TCAAAGGCAT GATTAGTGAT GTAGATCTAT   
  
  
- AAGGAACTAT TTAAATTAAA CTGAAATTTC CGAGCTAGAA AAATTCTTTT CAAAACAACG AATGTTGGAC   
  
  
- ATTTCGGATC TCTTGTTTCG GTTTCTTCTT TAAATTCAAG ATATATATCG TATTAGAGAG TTTTTTATAT   
  
  
- AAAAAATTTA TATCACGCTA TAACTGACTG GATTATACCC AATAATGTCC TCTACCCCTC TATAGATGTA   
  
  
- ATTGATGAAC TTGTTGAATT TAAAAGTAAA ATTGAGCCAG TAATTCTTGT GATTCTTGAA ATATATAATC   
  
  
- ACTATAGAGT CCGCATTTTC TTTGATGAAA CTTAATTAGG TACGTTAGGA TGAGAAATAA AGGTAGGCAA   
  
  
- TTTTAAAAGG AAGTTTTGAG GGAAGACAAG TAATAATTAA GTTATAGCAT AATGTGAACA ACCTAATAAA   
  
  
- GACACTAAAC TAGCAGTAAA AGATATATAC TTATTGATGT TTTTAGTATC AGGCTCATGT AATTATAATT   
  
  
- ATTTTATCCT ACTCACTATT GGAGTATATC ACGCAAATAG GGATGGTGAT CCGACCTAAA GACGTATTTC   
  
  
- TTCTTTAACT TGTATACCTT TTTCTTTATT TCTCTTCGTT GAAGGAACTC ACCCTCTTCT TAGCATCTTT   
  
  
- CAATTTGAAT ACTTTTTCTC GTAATTGTTT TTCCTTTAAT ATTCCGAATG GCAACTTCGG AGTATATTAA   
  
  
- AACGTTCTCT GTCTGTTTCT TTCACAGAAA GTTTACAGTA TAAAGAGCTC GTACGTACTT TTTACATGAA   
  
  
- GCCGATGTTA TCATTATCAT ATATACAACC GAAGTATGTG AACTGGGGTT ACATACAGTT GCACTGATCA   
  
  
- CTGGTGTGTG TAGCAACAGG GACGGGCGGG GTGATGGGTT TGAGGGGCGT GCGTTTCTAT TTTTCGGGGG   
  
  
- GTTAAAAATA AACGGAGGGT TTTTCGGGTG GGCGCGAAAA AATAAACGGA AGAAAAAAGG ATGAATTTGG   
  
  
- TGGAGGAGTT GCTTGAAACG TTGTATGTAT GATCAGATAT TCGTTAAATC TCCCTTCCCT TCGTTGGATT   
  
  
- TGTCTCGCTC TAACCGCCCC ACACAAACTC CCGAACGCAA AACCACGTTA TCCTTACTTT CACGGGTAAA   
  
  
- GTTGGTGGTA GGTTTGAACG TTGAGGCCGT GGCCGTTTGG TGAAAGGAAA CTCTTATGAC GGGATGTATG   
  
  
- GCGGAAAGTT CAAGGGTTAT GGTGGATGCT TGGGTGGAGC CAAGAACTGG AGGTCGCGTC GGGATCGGGG   
  
  
- TGGAGGTGGG TTTTTGGCGG GAAACGGCGG AGGTTGGTGG TCGGACGGAG CCGACTAGAC CTGTTGAACC   
  
  
- TGCCCACCCT AAGTTAGGAT AGTCTCGACC CCGAATTACT GCTTAAATGG GGATTAAGGT TTGAGACCAG   
  
  
- GGTTTAATCA GGCAGACTAG GAGTGGAGGG GGTTGAGGGA CTAAAGGGTT GTAGAGTCGG CAAACTAGTA   
  
  
- GTGTTGGGTG GGGGTTATTT GGAGGGTAGT AGTCTAAAGT TGGAGACACT AAAGAGGATG TTAGTGTTGG   
  
  
- GGTTAAAACC CGGCAAGCTA GAAGTAGTGT TGGGCGTAGT CTTGTTGGGC TTGTTGTTAT TGATGCCGAA   
  
  
- ACTAAAGTAT CTGCTGGAGT AGTTTCGTCG TGTCAGGGAG CTTAGGTTGC TGAGAGTAGT TCAGGTGGAC   
  
  
- TATAACCGCG CCGAGTTAGT TGCCGAGTCT AGTGGGTGGC CGTTTGGCGA GGTCGCCCGG CGGATGATGA   
  
  
- AGTTCCTTCG GGAGTAGCGG CGGCGCCGGC CGGGCGGGGC GGGGCGAGCC GAAAGTAGCA TACTCCACCA   
  
  
- CGTCTGGTAG GCTCGGATGT TCCGCAAAAG TCCTTAAAGC GGTCAGAGAG AGAAGAGGTG AAAGCGGCGG   
  
  
- TTGGTCCGCT AAGAACTCCG CCACCTGTGC CGCCGGAAGT AGGTGTAGTA GCTAAAGCTG TAGCCTAAGC   
  
  
- CGCCGGTGAC CCGGAGCAAG GAGGCGCTTG ACCAGCTGTT TCGACTAGGA CGCTTTGAAT CAAGTCACCA   
  
  
- CAATGCTTAG TGGCGGTAGC AAGGGCTTCT TAAGCCCTAG CTTTCGTTCG ATCACTCTCT TTTAGACAGC   
  
  
- GTTAAACGGG CTCTAGAGTT GTAGTTGAAG GTGTAGCTAA TACAGAATAA AGTCTCGAAA CTTTAGAATA   
  
  
- GGAAGTTTAG ACAGTTTAAG TAGCTTTCCC TCTTTAACCG CGACGTAGAA AGCGGCCGGC ACAAGGTGGC   
  
  
- CGATCCCTCG CCTTAAAGCT TCAAATAGCG ACTAGAGGCT AGTTAGAGCG GCTCGAGGCA GCACCACCAC   
  
  
- CTGTCTCTAC AGCCCTAACT GTAGCCTTGC AGCAGCAAAT CGTACTTAAA GCAGCGGCCT TAACTTAAGA   
  
  
- TGTGGCCCTA CGACCTCAGC GAACTGCGTC GGTGGCGGCC CCCCGAGTAG CCGCCACACC TGACGCACTC   
  
  
- TGCCTAACTC TGCAAACAAG AGGTGGGTTC CTAGTACCGC CGCCATCTCC GCCGCCGCAG CCGCAGCCGG   
  
  
- CCCGCCTCCT GCCGCACCTC CCTCCGTAAG CGCCGCCGCC CCTAATCCCG CCACCCCAAA TCGGTCAAAC   
  
  
- GACTAAAGGT CCGGCTTACA AACAACTCCT CTCGAGTCCA ACCACCGAAG GTGCACCGTT TCGCGGTACC   
  
  
- TCTCTACTAC GAAATGACCG TACTGGCGGG TGAGCAGCGG TGTAGTCGAA CCTCCACAAT

+     RY-element

| Site Name | Organism | Position | Strand | Matrix score. | sequence | function |
| --- | --- | --- | --- | --- | --- | --- |
| RY-element | Helianthus annuus | 1665 | + | 8 | CATGCATG | cis-acting regulatory element involved in seed-specific regulation |

>HU05G02245.1   
+ -Up\_Stream \_Len000ATAATT TATCTTTCTT TAATTTCGTT TCATGTAAAA AATTTCAGTC TCATCTGCGA   
  
  
+ TTGAATCTCT TTTCAATTCG CATCAATAAT TCATCCAATT CAAATGTGCC TTTCTTAATT TCATTACATT   
  
  
+ GGGTTGGTAT AAATGTTAGC ACGATAGATT GGAATAGCAA GGCTCTAATT CAATTATGAC TTGAATGTAT   
  
  
+ GCATGCTTTT ATCATTAGTT AATTAACATT TTCTGTATTG AAATCAGCTC GAAAGAGAAC CATATGTAGT   
  
  
+ TGTACTTGCG ACGATATATA TGTTTAGTTT AGTTAGTTCT TACCTGTTTT GGAATTGTTG GGGAGATCTG   
  
  
+ AGGAAGGAGC CCGCTTCAAT CAAAGTTCAT GGCAATAAGC CAAAGTGAGG TTCCATCCTA AAACCAATTG   
  
  
+ GCAATAGGAA GAGTAGCCCA CTTAACTTGT ATACTATGAC TCTTCTTTTC AAACTCCCAA TGTGGGACTT   
  
  
+ CTTTACTCAT GTGTGATATT CCCAACAGGA ATGATCAGTT CCACATGTTT TCATCTCTTT GGACTCGAAC   
  
  
+ TTCGTGTAAT TGTATATGCC ATGGCACAAA TAGTTTTGAT GGTGCCACCA AGTTGCTCAA TCAATGTAAT   
  
  
+ ACTGCTACTA TCTAACTTGC CTTGGTGGAC ATCAATAAGA TCACGAGATG AGGAGAACTG AATCTTGGCC   
  
  
+ AACCACGTCG TTTGACTTCT CTTACCAATA AAGATTGAAT AGACAGTGTT TCTCTCCCCC TTCCATTCTT   
  
  
+ CTTTTCTAGT GATGTCGAAA AAGACTTTAT ACATGTCCAG CAACGCTAGC CACAAAATAT TCCATTTCTG   
  
  
+ TAATCCACTC ATGTCAATTA AACTTGTATC ATCAAATGGT AGTTTCCGTA CTAATCACTA CATCTAGATA   
  
  
+ TTCCTTGATA AATTTAATTT GACTTTAAAG GCTCGATCTT TTTAAGAAAA GTTTTGTTGC TTACAACCTG   
  
  
+ TAAAGCCTAG AGAACAAAGC CAAAGAAGAA ATTTAAGTTC TATATATAGC ATAATCTCTC AAAAAATATA   
  
  
+ TTTTTTAAAT ATAGTGCGAT ATTGACTGAC CTAATATGGG TTATTACAGG AGATGGGGAG ATATCTACAT   
  
  
+ TAACTACTTG AACAACTTAA ATTTTCATTT TAACTCGGTC ATTAAGAACA CTAAGAACTT TATATATTAG   
  
  
+ TGATATCTCA GGCGTAAAAG AAACTACTTT GAATTAATCC ATGCAATCCT ACTCTTTATT TCCATCCGTT   
  
  
+ AAAATTTTCC TTCAAAACTC CCTTCTGTTC ATTATTAATT CAATATCGTA TTACACTTGT TGGATTATTT   
  
  
+ CTGTGATTTG ATCGTCATTT TCTATATATG AATAACTACA AAAATCATAG TCCGAGTACA TTAATATTAA   
  
  
+ TAAAATAGGA TGAGTGATAA CCTCATATAG TGCGTTTATC CCTACCACTA GGCTGGATTT CTGCATAAAG   
  
  
+ AAGAAATTGA ACATATGGAA AAAGAAATAA AGAGAAGCAA CTTCCTTGAG TGGGAGAAGA ATCGTAGAAA   
  
  
+ GTTAAACTTA TGAAAAAGAG CATTAACAAA AAGGAAATTA TAAGGCTTAC CGTTGAAGCC TCATATAATT   
  
  
+ TTGCAAGAGA CAGACAAAGA AAGTGTCTTT CAAATGTCAT ATTTCTCGAG CATGCATGAA AAATGTACTT   
  
  
+ CGGCTACAAT AGTAATAGTA TATATGTTGG CTTCATACAC TTGACCCCAA TGTATGTCAA CGTGACTAGT   
  
  
+ GACCACACAC ATCGTTGTCC CTGCCCGCCC CACTACCCAA ACTCCCCGCA CGCAAAGATA AAAAGCCCCC   
  
  
+ CAATTTTTAT TTGCCTCCCA AAAAGCCCAC CCGCGCTTTT TTATTTGCCT TCTTTTTTCC TACTTAAACC   
  
  
+ ACCTCCTCAA CGAACTTTGC AACATACATA CTAGTCTATA AGCAATTTAG AGGGAAGGGA AGCAACCTAA   
  
  
+ ACAGAGCGAG ATTGGCGGGG TGTGTTTGAG GGCTTGCGTT TTGGTGCAAT AGGAATGAAA GTGCCCATTT   
  
  
+ CAACCACCAT CCAAACTTGC AACTCCGGCA CCGGCAAACC ACTTTCCTTT GAGAATACTG CCCTACATAC   
  
  
+ CGCCTTTCAA GTTCCCAATA CCACCTACGA ACCCACCTCG GTTCTTGACC TCCAGCGCAG CCCTAGCCCC   
  
  
+ ACCTCCACCC AAAAACCGCC CTTTGCCGCC TCCAACCACC AGCCTGCCTC GGCTGATCTG GACAACTTGG   
  
  
+ ACGGGTGGGA TTCAATCCTA TCAGAGCTGG GGCTTAATGA CGAATTTACC CCTAATTCCA AACTCTGGTC   
  
  
+ CCAAATTAGT CCGTCTGATC CTCACCTCCC CCAACTCCCT GATTTCCCAA CATCTCAGCC GTTTGATCAT   
  
  
+ CACAACCCAC CCCCAATAAA CCTCCCATCA TCAGATTTCA ACCTCTGTGA TTTCTCCTAC AATCACAACC   
  
  
+ CCAATTTTGG GCCGTTCGAT CTTCATCACA ACCCGCATCA GAACAACCCG AACAACAATA ACTACGGCTT   
  
  
+ TGATTTCATA GACGACCTCA TCAAAGCAGC ACAGTCCCTC GAATCCAACG ACTCTCATCA AGTCCACCTG   
  
  
+ ATATTGGCGC GGCTCAATCA ACGGCTCAGA TCACCCACCG GCAAACCGCT CCAGCGGGCC GCCTACTACT   
  
  
+ TCAAGGAAGC CCTCATCGCC GCCGCGGCCG GCCCGCCCCG CCCCGCTCGG CTTTCATCGT ATGAGGTGGT   
  
  
+ GCAGACCATC CGAGCCTACA AGGCGTTTTC AGGAATTTCG CCAGTCTCTC TCTTCTCCAC TTTCGCCGCC   
  
  
+ AACCAGGCGA TTCTTGAGGC GGTGGACACG GCGGCCTTCA TCCACATCAT CGATTTCGAC ATCGGATTCG   
  
  
+ GCGGCCACTG GGCCTCGTTC CTCCGCGAAC TGGTCGACAA AGCTGATCCT GCGAAACTTA GTTCAGTGGT   
  
  
+ GTTACGAATC ACCGCCATCG TTCCCGAAGA ATTCGGGATC GAAAGCAAGC TAGTGAGAGA AAATCTGTCG   
  
  
+ CAATTTGCCC GAGATCTCAA CATCAACTTC CACATCGATT ATGTCTTATT TCAGAGCTTT GAAATCTTAT   
  
  
+ CCTTCAAATC TGTCAAATTC ATCGAAAGGG AGAAATTGGC GCTGCATCTT TCGCCGGCCG TGTTCCACCG   
  
  
+ GCTAGGGAGC GGAATTTCGA AGTTTATCGC TGATCTCCGA TCAATCTCGC CGAGCTCCGT CGTGGTGGTG   
  
  
+ GACAGAGATG TCGGGATTGA CATCGGAACG TCGTCGTTTA GCATGAATTT CGTCGCCGGA ATTGAATTCT   
  
  
+ ACACCGGGAT GCTGGAGTCG CTTGACGCAG CCACCGCCGG GGGGCTCATC GGCGGTGTGG ACTGCGTGAG   
  
  
+ ACGGATTGAG ACGTTTGTTC TCCACCCAAG GATCATGGCG GCGGTAGAGG CGGCGGCGTC GGCGTCGGCC   
  
  
+ GGGCGGAGGA CGGCGTGGAG GGAGGCATTC GCGGCGGCGG GGATTAGGGC GGTGGGGTTT AGCCAGTTTG   
  
  
+ CTGATTTCCA GGCCGAATGT TTGTTGAGGA GAGCTCAGGT TGGTGGCTTC CACGTGGCAA AGCGCCATGG   
  
  
+ AGAGATGATG CTTTACTGGC ATGACCGCCC ACTCGTCGCC ACATCAGCTT GGAGGTGTTA   

- -Up\_Stream \_Len000TATTAA ATAGAAAGAA ATTAAAGCAA AGTACATTTT TTAAAGTCAG AGTAGACGCT   
  
  
- AACTTAGAGA AAAGTTAAGC GTAGTTATTA AGTAGGTTAA GTTTACACGG AAAGAATTAA AGTAATGTAA   
  
  
- CCCAACCATA TTTACAATCG TGCTATCTAA CCTTATCGTT CCGAGATTAA GTTAATACTG AACTTACATA   
  
  
- CGTACGAAAA TAGTAATCAA TTAATTGTAA AAGACATAAC TTTAGTCGAG CTTTCTCTTG GTATACATCA   
  
  
- ACATGAACGC TGCTATATAT ACAAATCAAA TCAATCAAGA ATGGACAAAA CCTTAACAAC CCCTCTAGAC   
  
  
- TCCTTCCTCG GGCGAAGTTA GTTTCAAGTA CCGTTATTCG GTTTCACTCC AAGGTAGGAT TTTGGTTAAC   
  
  
- CGTTATCCTT CTCATCGGGT GAATTGAACA TATGATACTG AGAAGAAAAG TTTGAGGGTT ACACCCTGAA   
  
  
- GAAATGAGTA CACACTATAA GGGTTGTCCT TACTAGTCAA GGTGTACAAA AGTAGAGAAA CCTGAGCTTG   
  
  
- AAGCACATTA ACATATACGG TACCGTGTTT ATCAAAACTA CCACGGTGGT TCAACGAGTT AGTTACATTA   
  
  
- TGACGATGAT AGATTGAACG GAACCACCTG TAGTTATTCT AGTGCTCTAC TCCTCTTGAC TTAGAACCGG   
  
  
- TTGGTGCAGC AAACTGAAGA GAATGGTTAT TTCTAACTTA TCTGTCACAA AGAGAGGGGG AAGGTAAGAA   
  
  
- GAAAAGATCA CTACAGCTTT TTCTGAAATA TGTACAGGTC GTTGCGATCG GTGTTTTATA AGGTAAAGAC   
  
  
- ATTAGGTGAG TACAGTTAAT TTGAACATAG TAGTTTACCA TCAAAGGCAT GATTAGTGAT GTAGATCTAT   
  
  
- AAGGAACTAT TTAAATTAAA CTGAAATTTC CGAGCTAGAA AAATTCTTTT CAAAACAACG AATGTTGGAC   
  
  
- ATTTCGGATC TCTTGTTTCG GTTTCTTCTT TAAATTCAAG ATATATATCG TATTAGAGAG TTTTTTATAT   
  
  
- AAAAAATTTA TATCACGCTA TAACTGACTG GATTATACCC AATAATGTCC TCTACCCCTC TATAGATGTA   
  
  
- ATTGATGAAC TTGTTGAATT TAAAAGTAAA ATTGAGCCAG TAATTCTTGT GATTCTTGAA ATATATAATC   
  
  
- ACTATAGAGT CCGCATTTTC TTTGATGAAA CTTAATTAGG TACGTTAGGA TGAGAAATAA AGGTAGGCAA   
  
  
- TTTTAAAAGG AAGTTTTGAG GGAAGACAAG TAATAATTAA GTTATAGCAT AATGTGAACA ACCTAATAAA   
  
  
- GACACTAAAC TAGCAGTAAA AGATATATAC TTATTGATGT TTTTAGTATC AGGCTCATGT AATTATAATT   
  
  
- ATTTTATCCT ACTCACTATT GGAGTATATC ACGCAAATAG GGATGGTGAT CCGACCTAAA GACGTATTTC   
  
  
- TTCTTTAACT TGTATACCTT TTTCTTTATT TCTCTTCGTT GAAGGAACTC ACCCTCTTCT TAGCATCTTT   
  
  
- CAATTTGAAT ACTTTTTCTC GTAATTGTTT TTCCTTTAAT ATTCCGAATG GCAACTTCGG AGTATATTAA   
  
  
- AACGTTCTCT GTCTGTTTCT TTCACAGAAA GTTTACAGTA TAAAGAGCTC GTACGTACTT TTTACATGAA   
  
  
- GCCGATGTTA TCATTATCAT ATATACAACC GAAGTATGTG AACTGGGGTT ACATACAGTT GCACTGATCA   
  
  
- CTGGTGTGTG TAGCAACAGG GACGGGCGGG GTGATGGGTT TGAGGGGCGT GCGTTTCTAT TTTTCGGGGG   
  
  
- GTTAAAAATA AACGGAGGGT TTTTCGGGTG GGCGCGAAAA AATAAACGGA AGAAAAAAGG ATGAATTTGG   
  
  
- TGGAGGAGTT GCTTGAAACG TTGTATGTAT GATCAGATAT TCGTTAAATC TCCCTTCCCT TCGTTGGATT   
  
  
- TGTCTCGCTC TAACCGCCCC ACACAAACTC CCGAACGCAA AACCACGTTA TCCTTACTTT CACGGGTAAA   
  
  
- GTTGGTGGTA GGTTTGAACG TTGAGGCCGT GGCCGTTTGG TGAAAGGAAA CTCTTATGAC GGGATGTATG   
  
  
- GCGGAAAGTT CAAGGGTTAT GGTGGATGCT TGGGTGGAGC CAAGAACTGG AGGTCGCGTC GGGATCGGGG   
  
  
- TGGAGGTGGG TTTTTGGCGG GAAACGGCGG AGGTTGGTGG TCGGACGGAG CCGACTAGAC CTGTTGAACC   
  
  
- TGCCCACCCT AAGTTAGGAT AGTCTCGACC CCGAATTACT GCTTAAATGG GGATTAAGGT TTGAGACCAG   
  
  
- GGTTTAATCA GGCAGACTAG GAGTGGAGGG GGTTGAGGGA CTAAAGGGTT GTAGAGTCGG CAAACTAGTA   
  
  
- GTGTTGGGTG GGGGTTATTT GGAGGGTAGT AGTCTAAAGT TGGAGACACT AAAGAGGATG TTAGTGTTGG   
  
  
- GGTTAAAACC CGGCAAGCTA GAAGTAGTGT TGGGCGTAGT CTTGTTGGGC TTGTTGTTAT TGATGCCGAA   
  
  
- ACTAAAGTAT CTGCTGGAGT AGTTTCGTCG TGTCAGGGAG CTTAGGTTGC TGAGAGTAGT TCAGGTGGAC   
  
  
- TATAACCGCG CCGAGTTAGT TGCCGAGTCT AGTGGGTGGC CGTTTGGCGA GGTCGCCCGG CGGATGATGA   
  
  
- AGTTCCTTCG GGAGTAGCGG CGGCGCCGGC CGGGCGGGGC GGGGCGAGCC GAAAGTAGCA TACTCCACCA   
  
  
- CGTCTGGTAG GCTCGGATGT TCCGCAAAAG TCCTTAAAGC GGTCAGAGAG AGAAGAGGTG AAAGCGGCGG   
  
  
- TTGGTCCGCT AAGAACTCCG CCACCTGTGC CGCCGGAAGT AGGTGTAGTA GCTAAAGCTG TAGCCTAAGC   
  
  
- CGCCGGTGAC CCGGAGCAAG GAGGCGCTTG ACCAGCTGTT TCGACTAGGA CGCTTTGAAT CAAGTCACCA   
  
  
- CAATGCTTAG TGGCGGTAGC AAGGGCTTCT TAAGCCCTAG CTTTCGTTCG ATCACTCTCT TTTAGACAGC   
  
  
- GTTAAACGGG CTCTAGAGTT GTAGTTGAAG GTGTAGCTAA TACAGAATAA AGTCTCGAAA CTTTAGAATA   
  
  
- GGAAGTTTAG ACAGTTTAAG TAGCTTTCCC TCTTTAACCG CGACGTAGAA AGCGGCCGGC ACAAGGTGGC   
  
  
- CGATCCCTCG CCTTAAAGCT TCAAATAGCG ACTAGAGGCT AGTTAGAGCG GCTCGAGGCA GCACCACCAC   
  
  
- CTGTCTCTAC AGCCCTAACT GTAGCCTTGC AGCAGCAAAT CGTACTTAAA GCAGCGGCCT TAACTTAAGA   
  
  
- TGTGGCCCTA CGACCTCAGC GAACTGCGTC GGTGGCGGCC CCCCGAGTAG CCGCCACACC TGACGCACTC   
  
  
- TGCCTAACTC TGCAAACAAG AGGTGGGTTC CTAGTACCGC CGCCATCTCC GCCGCCGCAG CCGCAGCCGG   
  
  
- CCCGCCTCCT GCCGCACCTC CCTCCGTAAG CGCCGCCGCC CCTAATCCCG CCACCCCAAA TCGGTCAAAC   
  
  
- GACTAAAGGT CCGGCTTACA AACAACTCCT CTCGAGTCCA ACCACCGAAG GTGCACCGTT TCGCGGTACC   
  
  
- TCTCTACTAC GAAATGACCG TACTGGCGGG TGAGCAGCGG TGTAGTCGAA CCTCCACAAT

+     STRE

| Site Name | Organism | Position | Strand | Matrix score. | sequence | function |
| --- | --- | --- | --- | --- | --- | --- |
| STRE | Arabidopsis thaliana | 761 | - | 5 | AGGGG |  |
| STRE | Arabidopsis thaliana | 2293 | - | 5 | AGGGG |  |

>HU05G02245.1   
+ -Up\_Stream \_Len000ATAATT TATCTTTCTT TAATTTCGTT TCATGTAAAA AATTTCAGTC TCATCTGCGA   
  
  
+ TTGAATCTCT TTTCAATTCG CATCAATAAT TCATCCAATT CAAATGTGCC TTTCTTAATT TCATTACATT   
  
  
+ GGGTTGGTAT AAATGTTAGC ACGATAGATT GGAATAGCAA GGCTCTAATT CAATTATGAC TTGAATGTAT   
  
  
+ GCATGCTTTT ATCATTAGTT AATTAACATT TTCTGTATTG AAATCAGCTC GAAAGAGAAC CATATGTAGT   
  
  
+ TGTACTTGCG ACGATATATA TGTTTAGTTT AGTTAGTTCT TACCTGTTTT GGAATTGTTG GGGAGATCTG   
  
  
+ AGGAAGGAGC CCGCTTCAAT CAAAGTTCAT GGCAATAAGC CAAAGTGAGG TTCCATCCTA AAACCAATTG   
  
  
+ GCAATAGGAA GAGTAGCCCA CTTAACTTGT ATACTATGAC TCTTCTTTTC AAACTCCCAA TGTGGGACTT   
  
  
+ CTTTACTCAT GTGTGATATT CCCAACAGGA ATGATCAGTT CCACATGTTT TCATCTCTTT GGACTCGAAC   
  
  
+ TTCGTGTAAT TGTATATGCC ATGGCACAAA TAGTTTTGAT GGTGCCACCA AGTTGCTCAA TCAATGTAAT   
  
  
+ ACTGCTACTA TCTAACTTGC CTTGGTGGAC ATCAATAAGA TCACGAGATG AGGAGAACTG AATCTTGGCC   
  
  
+ AACCACGTCG TTTGACTTCT CTTACCAATA AAGATTGAAT AGACAGTGTT TCTCTCCCCC TTCCATTCTT   
  
  
+ CTTTTCTAGT GATGTCGAAA AAGACTTTAT ACATGTCCAG CAACGCTAGC CACAAAATAT TCCATTTCTG   
  
  
+ TAATCCACTC ATGTCAATTA AACTTGTATC ATCAAATGGT AGTTTCCGTA CTAATCACTA CATCTAGATA   
  
  
+ TTCCTTGATA AATTTAATTT GACTTTAAAG GCTCGATCTT TTTAAGAAAA GTTTTGTTGC TTACAACCTG   
  
  
+ TAAAGCCTAG AGAACAAAGC CAAAGAAGAA ATTTAAGTTC TATATATAGC ATAATCTCTC AAAAAATATA   
  
  
+ TTTTTTAAAT ATAGTGCGAT ATTGACTGAC CTAATATGGG TTATTACAGG AGATGGGGAG ATATCTACAT   
  
  
+ TAACTACTTG AACAACTTAA ATTTTCATTT TAACTCGGTC ATTAAGAACA CTAAGAACTT TATATATTAG   
  
  
+ TGATATCTCA GGCGTAAAAG AAACTACTTT GAATTAATCC ATGCAATCCT ACTCTTTATT TCCATCCGTT   
  
  
+ AAAATTTTCC TTCAAAACTC CCTTCTGTTC ATTATTAATT CAATATCGTA TTACACTTGT TGGATTATTT   
  
  
+ CTGTGATTTG ATCGTCATTT TCTATATATG AATAACTACA AAAATCATAG TCCGAGTACA TTAATATTAA   
  
  
+ TAAAATAGGA TGAGTGATAA CCTCATATAG TGCGTTTATC CCTACCACTA GGCTGGATTT CTGCATAAAG   
  
  
+ AAGAAATTGA ACATATGGAA AAAGAAATAA AGAGAAGCAA CTTCCTTGAG TGGGAGAAGA ATCGTAGAAA   
  
  
+ GTTAAACTTA TGAAAAAGAG CATTAACAAA AAGGAAATTA TAAGGCTTAC CGTTGAAGCC TCATATAATT   
  
  
+ TTGCAAGAGA CAGACAAAGA AAGTGTCTTT CAAATGTCAT ATTTCTCGAG CATGCATGAA AAATGTACTT   
  
  
+ CGGCTACAAT AGTAATAGTA TATATGTTGG CTTCATACAC TTGACCCCAA TGTATGTCAA CGTGACTAGT   
  
  
+ GACCACACAC ATCGTTGTCC CTGCCCGCCC CACTACCCAA ACTCCCCGCA CGCAAAGATA AAAAGCCCCC   
  
  
+ CAATTTTTAT TTGCCTCCCA AAAAGCCCAC CCGCGCTTTT TTATTTGCCT TCTTTTTTCC TACTTAAACC   
  
  
+ ACCTCCTCAA CGAACTTTGC AACATACATA CTAGTCTATA AGCAATTTAG AGGGAAGGGA AGCAACCTAA   
  
  
+ ACAGAGCGAG ATTGGCGGGG TGTGTTTGAG GGCTTGCGTT TTGGTGCAAT AGGAATGAAA GTGCCCATTT   
  
  
+ CAACCACCAT CCAAACTTGC AACTCCGGCA CCGGCAAACC ACTTTCCTTT GAGAATACTG CCCTACATAC   
  
  
+ CGCCTTTCAA GTTCCCAATA CCACCTACGA ACCCACCTCG GTTCTTGACC TCCAGCGCAG CCCTAGCCCC   
  
  
+ ACCTCCACCC AAAAACCGCC CTTTGCCGCC TCCAACCACC AGCCTGCCTC GGCTGATCTG GACAACTTGG   
  
  
+ ACGGGTGGGA TTCAATCCTA TCAGAGCTGG GGCTTAATGA CGAATTTACC CCTAATTCCA AACTCTGGTC   
  
  
+ CCAAATTAGT CCGTCTGATC CTCACCTCCC CCAACTCCCT GATTTCCCAA CATCTCAGCC GTTTGATCAT   
  
  
+ CACAACCCAC CCCCAATAAA CCTCCCATCA TCAGATTTCA ACCTCTGTGA TTTCTCCTAC AATCACAACC   
  
  
+ CCAATTTTGG GCCGTTCGAT CTTCATCACA ACCCGCATCA GAACAACCCG AACAACAATA ACTACGGCTT   
  
  
+ TGATTTCATA GACGACCTCA TCAAAGCAGC ACAGTCCCTC GAATCCAACG ACTCTCATCA AGTCCACCTG   
  
  
+ ATATTGGCGC GGCTCAATCA ACGGCTCAGA TCACCCACCG GCAAACCGCT CCAGCGGGCC GCCTACTACT   
  
  
+ TCAAGGAAGC CCTCATCGCC GCCGCGGCCG GCCCGCCCCG CCCCGCTCGG CTTTCATCGT ATGAGGTGGT   
  
  
+ GCAGACCATC CGAGCCTACA AGGCGTTTTC AGGAATTTCG CCAGTCTCTC TCTTCTCCAC TTTCGCCGCC   
  
  
+ AACCAGGCGA TTCTTGAGGC GGTGGACACG GCGGCCTTCA TCCACATCAT CGATTTCGAC ATCGGATTCG   
  
  
+ GCGGCCACTG GGCCTCGTTC CTCCGCGAAC TGGTCGACAA AGCTGATCCT GCGAAACTTA GTTCAGTGGT   
  
  
+ GTTACGAATC ACCGCCATCG TTCCCGAAGA ATTCGGGATC GAAAGCAAGC TAGTGAGAGA AAATCTGTCG   
  
  
+ CAATTTGCCC GAGATCTCAA CATCAACTTC CACATCGATT ATGTCTTATT TCAGAGCTTT GAAATCTTAT   
  
  
+ CCTTCAAATC TGTCAAATTC ATCGAAAGGG AGAAATTGGC GCTGCATCTT TCGCCGGCCG TGTTCCACCG   
  
  
+ GCTAGGGAGC GGAATTTCGA AGTTTATCGC TGATCTCCGA TCAATCTCGC CGAGCTCCGT CGTGGTGGTG   
  
  
+ GACAGAGATG TCGGGATTGA CATCGGAACG TCGTCGTTTA GCATGAATTT CGTCGCCGGA ATTGAATTCT   
  
  
+ ACACCGGGAT GCTGGAGTCG CTTGACGCAG CCACCGCCGG GGGGCTCATC GGCGGTGTGG ACTGCGTGAG   
  
  
+ ACGGATTGAG ACGTTTGTTC TCCACCCAAG GATCATGGCG GCGGTAGAGG CGGCGGCGTC GGCGTCGGCC   
  
  
+ GGGCGGAGGA CGGCGTGGAG GGAGGCATTC GCGGCGGCGG GGATTAGGGC GGTGGGGTTT AGCCAGTTTG   
  
  
+ CTGATTTCCA GGCCGAATGT TTGTTGAGGA GAGCTCAGGT TGGTGGCTTC CACGTGGCAA AGCGCCATGG   
  
  
+ AGAGATGATG CTTTACTGGC ATGACCGCCC ACTCGTCGCC ACATCAGCTT GGAGGTGTTA   

- -Up\_Stream \_Len000TATTAA ATAGAAAGAA ATTAAAGCAA AGTACATTTT TTAAAGTCAG AGTAGACGCT   
  
  
- AACTTAGAGA AAAGTTAAGC GTAGTTATTA AGTAGGTTAA GTTTACACGG AAAGAATTAA AGTAATGTAA   
  
  
- CCCAACCATA TTTACAATCG TGCTATCTAA CCTTATCGTT CCGAGATTAA GTTAATACTG AACTTACATA   
  
  
- CGTACGAAAA TAGTAATCAA TTAATTGTAA AAGACATAAC TTTAGTCGAG CTTTCTCTTG GTATACATCA   
  
  
- ACATGAACGC TGCTATATAT ACAAATCAAA TCAATCAAGA ATGGACAAAA CCTTAACAAC CCCTCTAGAC   
  
  
- TCCTTCCTCG GGCGAAGTTA GTTTCAAGTA CCGTTATTCG GTTTCACTCC AAGGTAGGAT TTTGGTTAAC   
  
  
- CGTTATCCTT CTCATCGGGT GAATTGAACA TATGATACTG AGAAGAAAAG TTTGAGGGTT ACACCCTGAA   
  
  
- GAAATGAGTA CACACTATAA GGGTTGTCCT TACTAGTCAA GGTGTACAAA AGTAGAGAAA CCTGAGCTTG   
  
  
- AAGCACATTA ACATATACGG TACCGTGTTT ATCAAAACTA CCACGGTGGT TCAACGAGTT AGTTACATTA   
  
  
- TGACGATGAT AGATTGAACG GAACCACCTG TAGTTATTCT AGTGCTCTAC TCCTCTTGAC TTAGAACCGG   
  
  
- TTGGTGCAGC AAACTGAAGA GAATGGTTAT TTCTAACTTA TCTGTCACAA AGAGAGGGGG AAGGTAAGAA   
  
  
- GAAAAGATCA CTACAGCTTT TTCTGAAATA TGTACAGGTC GTTGCGATCG GTGTTTTATA AGGTAAAGAC   
  
  
- ATTAGGTGAG TACAGTTAAT TTGAACATAG TAGTTTACCA TCAAAGGCAT GATTAGTGAT GTAGATCTAT   
  
  
- AAGGAACTAT TTAAATTAAA CTGAAATTTC CGAGCTAGAA AAATTCTTTT CAAAACAACG AATGTTGGAC   
  
  
- ATTTCGGATC TCTTGTTTCG GTTTCTTCTT TAAATTCAAG ATATATATCG TATTAGAGAG TTTTTTATAT   
  
  
- AAAAAATTTA TATCACGCTA TAACTGACTG GATTATACCC AATAATGTCC TCTACCCCTC TATAGATGTA   
  
  
- ATTGATGAAC TTGTTGAATT TAAAAGTAAA ATTGAGCCAG TAATTCTTGT GATTCTTGAA ATATATAATC   
  
  
- ACTATAGAGT CCGCATTTTC TTTGATGAAA CTTAATTAGG TACGTTAGGA TGAGAAATAA AGGTAGGCAA   
  
  
- TTTTAAAAGG AAGTTTTGAG GGAAGACAAG TAATAATTAA GTTATAGCAT AATGTGAACA ACCTAATAAA   
  
  
- GACACTAAAC TAGCAGTAAA AGATATATAC TTATTGATGT TTTTAGTATC AGGCTCATGT AATTATAATT   
  
  
- ATTTTATCCT ACTCACTATT GGAGTATATC ACGCAAATAG GGATGGTGAT CCGACCTAAA GACGTATTTC   
  
  
- TTCTTTAACT TGTATACCTT TTTCTTTATT TCTCTTCGTT GAAGGAACTC ACCCTCTTCT TAGCATCTTT   
  
  
- CAATTTGAAT ACTTTTTCTC GTAATTGTTT TTCCTTTAAT ATTCCGAATG GCAACTTCGG AGTATATTAA   
  
  
- AACGTTCTCT GTCTGTTTCT TTCACAGAAA GTTTACAGTA TAAAGAGCTC GTACGTACTT TTTACATGAA   
  
  
- GCCGATGTTA TCATTATCAT ATATACAACC GAAGTATGTG AACTGGGGTT ACATACAGTT GCACTGATCA   
  
  
- CTGGTGTGTG TAGCAACAGG GACGGGCGGG GTGATGGGTT TGAGGGGCGT GCGTTTCTAT TTTTCGGGGG   
  
  
- GTTAAAAATA AACGGAGGGT TTTTCGGGTG GGCGCGAAAA AATAAACGGA AGAAAAAAGG ATGAATTTGG   
  
  
- TGGAGGAGTT GCTTGAAACG TTGTATGTAT GATCAGATAT TCGTTAAATC TCCCTTCCCT TCGTTGGATT   
  
  
- TGTCTCGCTC TAACCGCCCC ACACAAACTC CCGAACGCAA AACCACGTTA TCCTTACTTT CACGGGTAAA   
  
  
- GTTGGTGGTA GGTTTGAACG TTGAGGCCGT GGCCGTTTGG TGAAAGGAAA CTCTTATGAC GGGATGTATG   
  
  
- GCGGAAAGTT CAAGGGTTAT GGTGGATGCT TGGGTGGAGC CAAGAACTGG AGGTCGCGTC GGGATCGGGG   
  
  
- TGGAGGTGGG TTTTTGGCGG GAAACGGCGG AGGTTGGTGG TCGGACGGAG CCGACTAGAC CTGTTGAACC   
  
  
- TGCCCACCCT AAGTTAGGAT AGTCTCGACC CCGAATTACT GCTTAAATGG GGATTAAGGT TTGAGACCAG   
  
  
- GGTTTAATCA GGCAGACTAG GAGTGGAGGG GGTTGAGGGA CTAAAGGGTT GTAGAGTCGG CAAACTAGTA   
  
  
- GTGTTGGGTG GGGGTTATTT GGAGGGTAGT AGTCTAAAGT TGGAGACACT AAAGAGGATG TTAGTGTTGG   
  
  
- GGTTAAAACC CGGCAAGCTA GAAGTAGTGT TGGGCGTAGT CTTGTTGGGC TTGTTGTTAT TGATGCCGAA   
  
  
- ACTAAAGTAT CTGCTGGAGT AGTTTCGTCG TGTCAGGGAG CTTAGGTTGC TGAGAGTAGT TCAGGTGGAC   
  
  
- TATAACCGCG CCGAGTTAGT TGCCGAGTCT AGTGGGTGGC CGTTTGGCGA GGTCGCCCGG CGGATGATGA   
  
  
- AGTTCCTTCG GGAGTAGCGG CGGCGCCGGC CGGGCGGGGC GGGGCGAGCC GAAAGTAGCA TACTCCACCA   
  
  
- CGTCTGGTAG GCTCGGATGT TCCGCAAAAG TCCTTAAAGC GGTCAGAGAG AGAAGAGGTG AAAGCGGCGG   
  
  
- TTGGTCCGCT AAGAACTCCG CCACCTGTGC CGCCGGAAGT AGGTGTAGTA GCTAAAGCTG TAGCCTAAGC   
  
  
- CGCCGGTGAC CCGGAGCAAG GAGGCGCTTG ACCAGCTGTT TCGACTAGGA CGCTTTGAAT CAAGTCACCA   
  
  
- CAATGCTTAG TGGCGGTAGC AAGGGCTTCT TAAGCCCTAG CTTTCGTTCG ATCACTCTCT TTTAGACAGC   
  
  
- GTTAAACGGG CTCTAGAGTT GTAGTTGAAG GTGTAGCTAA TACAGAATAA AGTCTCGAAA CTTTAGAATA   
  
  
- GGAAGTTTAG ACAGTTTAAG TAGCTTTCCC TCTTTAACCG CGACGTAGAA AGCGGCCGGC ACAAGGTGGC   
  
  
- CGATCCCTCG CCTTAAAGCT TCAAATAGCG ACTAGAGGCT AGTTAGAGCG GCTCGAGGCA GCACCACCAC   
  
  
- CTGTCTCTAC AGCCCTAACT GTAGCCTTGC AGCAGCAAAT CGTACTTAAA GCAGCGGCCT TAACTTAAGA   
  
  
- TGTGGCCCTA CGACCTCAGC GAACTGCGTC GGTGGCGGCC CCCCGAGTAG CCGCCACACC TGACGCACTC   
  
  
- TGCCTAACTC TGCAAACAAG AGGTGGGTTC CTAGTACCGC CGCCATCTCC GCCGCCGCAG CCGCAGCCGG   
  
  
- CCCGCCTCCT GCCGCACCTC CCTCCGTAAG CGCCGCCGCC CCTAATCCCG CCACCCCAAA TCGGTCAAAC   
  
  
- GACTAAAGGT CCGGCTTACA AACAACTCCT CTCGAGTCCA ACCACCGAAG GTGCACCGTT TCGCGGTACC   
  
  
- TCTCTACTAC GAAATGACCG TACTGGCGGG TGAGCAGCGG TGTAGTCGAA CCTCCACAAT

+     Sp1

| Site Name | Organism | Position | Strand | Matrix score. | sequence | function |
| --- | --- | --- | --- | --- | --- | --- |
| Sp1 | Oryza sativa | 3599 | - | 6 | GGGCGG | light responsive element |
| Sp1 | Oryza sativa | 3481 | + | 6 | GGGCGG | light responsive element |
| Sp1 | Oryza sativa | 2702 | - | 6 | GGGCGG | light responsive element |
| Sp1 | Oryza sativa | 3435 | + | 6 | GGGCGG | light responsive element |
| Sp1 | Oryza sativa | 2697 | - | 6 | GGGCGG | light responsive element |
| Sp1 | Oryza sativa | 2190 | - | 6 | GGGCGG | light responsive element |
| Sp1 | Oryza sativa | 1779 | - | 6 | GGGCGG | light responsive element |

>HU05G02245.1   
+ -Up\_Stream \_Len000ATAATT TATCTTTCTT TAATTTCGTT TCATGTAAAA AATTTCAGTC TCATCTGCGA   
  
  
+ TTGAATCTCT TTTCAATTCG CATCAATAAT TCATCCAATT CAAATGTGCC TTTCTTAATT TCATTACATT   
  
  
+ GGGTTGGTAT AAATGTTAGC ACGATAGATT GGAATAGCAA GGCTCTAATT CAATTATGAC TTGAATGTAT   
  
  
+ GCATGCTTTT ATCATTAGTT AATTAACATT TTCTGTATTG AAATCAGCTC GAAAGAGAAC CATATGTAGT   
  
  
+ TGTACTTGCG ACGATATATA TGTTTAGTTT AGTTAGTTCT TACCTGTTTT GGAATTGTTG GGGAGATCTG   
  
  
+ AGGAAGGAGC CCGCTTCAAT CAAAGTTCAT GGCAATAAGC CAAAGTGAGG TTCCATCCTA AAACCAATTG   
  
  
+ GCAATAGGAA GAGTAGCCCA CTTAACTTGT ATACTATGAC TCTTCTTTTC AAACTCCCAA TGTGGGACTT   
  
  
+ CTTTACTCAT GTGTGATATT CCCAACAGGA ATGATCAGTT CCACATGTTT TCATCTCTTT GGACTCGAAC   
  
  
+ TTCGTGTAAT TGTATATGCC ATGGCACAAA TAGTTTTGAT GGTGCCACCA AGTTGCTCAA TCAATGTAAT   
  
  
+ ACTGCTACTA TCTAACTTGC CTTGGTGGAC ATCAATAAGA TCACGAGATG AGGAGAACTG AATCTTGGCC   
  
  
+ AACCACGTCG TTTGACTTCT CTTACCAATA AAGATTGAAT AGACAGTGTT TCTCTCCCCC TTCCATTCTT   
  
  
+ CTTTTCTAGT GATGTCGAAA AAGACTTTAT ACATGTCCAG CAACGCTAGC CACAAAATAT TCCATTTCTG   
  
  
+ TAATCCACTC ATGTCAATTA AACTTGTATC ATCAAATGGT AGTTTCCGTA CTAATCACTA CATCTAGATA   
  
  
+ TTCCTTGATA AATTTAATTT GACTTTAAAG GCTCGATCTT TTTAAGAAAA GTTTTGTTGC TTACAACCTG   
  
  
+ TAAAGCCTAG AGAACAAAGC CAAAGAAGAA ATTTAAGTTC TATATATAGC ATAATCTCTC AAAAAATATA   
  
  
+ TTTTTTAAAT ATAGTGCGAT ATTGACTGAC CTAATATGGG TTATTACAGG AGATGGGGAG ATATCTACAT   
  
  
+ TAACTACTTG AACAACTTAA ATTTTCATTT TAACTCGGTC ATTAAGAACA CTAAGAACTT TATATATTAG   
  
  
+ TGATATCTCA GGCGTAAAAG AAACTACTTT GAATTAATCC ATGCAATCCT ACTCTTTATT TCCATCCGTT   
  
  
+ AAAATTTTCC TTCAAAACTC CCTTCTGTTC ATTATTAATT CAATATCGTA TTACACTTGT TGGATTATTT   
  
  
+ CTGTGATTTG ATCGTCATTT TCTATATATG AATAACTACA AAAATCATAG TCCGAGTACA TTAATATTAA   
  
  
+ TAAAATAGGA TGAGTGATAA CCTCATATAG TGCGTTTATC CCTACCACTA GGCTGGATTT CTGCATAAAG   
  
  
+ AAGAAATTGA ACATATGGAA AAAGAAATAA AGAGAAGCAA CTTCCTTGAG TGGGAGAAGA ATCGTAGAAA   
  
  
+ GTTAAACTTA TGAAAAAGAG CATTAACAAA AAGGAAATTA TAAGGCTTAC CGTTGAAGCC TCATATAATT   
  
  
+ TTGCAAGAGA CAGACAAAGA AAGTGTCTTT CAAATGTCAT ATTTCTCGAG CATGCATGAA AAATGTACTT   
  
  
+ CGGCTACAAT AGTAATAGTA TATATGTTGG CTTCATACAC TTGACCCCAA TGTATGTCAA CGTGACTAGT   
  
  
+ GACCACACAC ATCGTTGTCC CTGCCCGCCC CACTACCCAA ACTCCCCGCA CGCAAAGATA AAAAGCCCCC   
  
  
+ CAATTTTTAT TTGCCTCCCA AAAAGCCCAC CCGCGCTTTT TTATTTGCCT TCTTTTTTCC TACTTAAACC   
  
  
+ ACCTCCTCAA CGAACTTTGC AACATACATA CTAGTCTATA AGCAATTTAG AGGGAAGGGA AGCAACCTAA   
  
  
+ ACAGAGCGAG ATTGGCGGGG TGTGTTTGAG GGCTTGCGTT TTGGTGCAAT AGGAATGAAA GTGCCCATTT   
  
  
+ CAACCACCAT CCAAACTTGC AACTCCGGCA CCGGCAAACC ACTTTCCTTT GAGAATACTG CCCTACATAC   
  
  
+ CGCCTTTCAA GTTCCCAATA CCACCTACGA ACCCACCTCG GTTCTTGACC TCCAGCGCAG CCCTAGCCCC   
  
  
+ ACCTCCACCC AAAAACCGCC CTTTGCCGCC TCCAACCACC AGCCTGCCTC GGCTGATCTG GACAACTTGG   
  
  
+ ACGGGTGGGA TTCAATCCTA TCAGAGCTGG GGCTTAATGA CGAATTTACC CCTAATTCCA AACTCTGGTC   
  
  
+ CCAAATTAGT CCGTCTGATC CTCACCTCCC CCAACTCCCT GATTTCCCAA CATCTCAGCC GTTTGATCAT   
  
  
+ CACAACCCAC CCCCAATAAA CCTCCCATCA TCAGATTTCA ACCTCTGTGA TTTCTCCTAC AATCACAACC   
  
  
+ CCAATTTTGG GCCGTTCGAT CTTCATCACA ACCCGCATCA GAACAACCCG AACAACAATA ACTACGGCTT   
  
  
+ TGATTTCATA GACGACCTCA TCAAAGCAGC ACAGTCCCTC GAATCCAACG ACTCTCATCA AGTCCACCTG   
  
  
+ ATATTGGCGC GGCTCAATCA ACGGCTCAGA TCACCCACCG GCAAACCGCT CCAGCGGGCC GCCTACTACT   
  
  
+ TCAAGGAAGC CCTCATCGCC GCCGCGGCCG GCCCGCCCCG CCCCGCTCGG CTTTCATCGT ATGAGGTGGT   
  
  
+ GCAGACCATC CGAGCCTACA AGGCGTTTTC AGGAATTTCG CCAGTCTCTC TCTTCTCCAC TTTCGCCGCC   
  
  
+ AACCAGGCGA TTCTTGAGGC GGTGGACACG GCGGCCTTCA TCCACATCAT CGATTTCGAC ATCGGATTCG   
  
  
+ GCGGCCACTG GGCCTCGTTC CTCCGCGAAC TGGTCGACAA AGCTGATCCT GCGAAACTTA GTTCAGTGGT   
  
  
+ GTTACGAATC ACCGCCATCG TTCCCGAAGA ATTCGGGATC GAAAGCAAGC TAGTGAGAGA AAATCTGTCG   
  
  
+ CAATTTGCCC GAGATCTCAA CATCAACTTC CACATCGATT ATGTCTTATT TCAGAGCTTT GAAATCTTAT   
  
  
+ CCTTCAAATC TGTCAAATTC ATCGAAAGGG AGAAATTGGC GCTGCATCTT TCGCCGGCCG TGTTCCACCG   
  
  
+ GCTAGGGAGC GGAATTTCGA AGTTTATCGC TGATCTCCGA TCAATCTCGC CGAGCTCCGT CGTGGTGGTG   
  
  
+ GACAGAGATG TCGGGATTGA CATCGGAACG TCGTCGTTTA GCATGAATTT CGTCGCCGGA ATTGAATTCT   
  
  
+ ACACCGGGAT GCTGGAGTCG CTTGACGCAG CCACCGCCGG GGGGCTCATC GGCGGTGTGG ACTGCGTGAG   
  
  
+ ACGGATTGAG ACGTTTGTTC TCCACCCAAG GATCATGGCG GCGGTAGAGG CGGCGGCGTC GGCGTCGGCC   
  
  
+ GGGCGGAGGA CGGCGTGGAG GGAGGCATTC GCGGCGGCGG GGATTAGGGC GGTGGGGTTT AGCCAGTTTG   
  
  
+ CTGATTTCCA GGCCGAATGT TTGTTGAGGA GAGCTCAGGT TGGTGGCTTC CACGTGGCAA AGCGCCATGG   
  
  
+ AGAGATGATG CTTTACTGGC ATGACCGCCC ACTCGTCGCC ACATCAGCTT GGAGGTGTTA   

- -Up\_Stream \_Len000TATTAA ATAGAAAGAA ATTAAAGCAA AGTACATTTT TTAAAGTCAG AGTAGACGCT   
  
  
- AACTTAGAGA AAAGTTAAGC GTAGTTATTA AGTAGGTTAA GTTTACACGG AAAGAATTAA AGTAATGTAA   
  
  
- CCCAACCATA TTTACAATCG TGCTATCTAA CCTTATCGTT CCGAGATTAA GTTAATACTG AACTTACATA   
  
  
- CGTACGAAAA TAGTAATCAA TTAATTGTAA AAGACATAAC TTTAGTCGAG CTTTCTCTTG GTATACATCA   
  
  
- ACATGAACGC TGCTATATAT ACAAATCAAA TCAATCAAGA ATGGACAAAA CCTTAACAAC CCCTCTAGAC   
  
  
- TCCTTCCTCG GGCGAAGTTA GTTTCAAGTA CCGTTATTCG GTTTCACTCC AAGGTAGGAT TTTGGTTAAC   
  
  
- CGTTATCCTT CTCATCGGGT GAATTGAACA TATGATACTG AGAAGAAAAG TTTGAGGGTT ACACCCTGAA   
  
  
- GAAATGAGTA CACACTATAA GGGTTGTCCT TACTAGTCAA GGTGTACAAA AGTAGAGAAA CCTGAGCTTG   
  
  
- AAGCACATTA ACATATACGG TACCGTGTTT ATCAAAACTA CCACGGTGGT TCAACGAGTT AGTTACATTA   
  
  
- TGACGATGAT AGATTGAACG GAACCACCTG TAGTTATTCT AGTGCTCTAC TCCTCTTGAC TTAGAACCGG   
  
  
- TTGGTGCAGC AAACTGAAGA GAATGGTTAT TTCTAACTTA TCTGTCACAA AGAGAGGGGG AAGGTAAGAA   
  
  
- GAAAAGATCA CTACAGCTTT TTCTGAAATA TGTACAGGTC GTTGCGATCG GTGTTTTATA AGGTAAAGAC   
  
  
- ATTAGGTGAG TACAGTTAAT TTGAACATAG TAGTTTACCA TCAAAGGCAT GATTAGTGAT GTAGATCTAT   
  
  
- AAGGAACTAT TTAAATTAAA CTGAAATTTC CGAGCTAGAA AAATTCTTTT CAAAACAACG AATGTTGGAC   
  
  
- ATTTCGGATC TCTTGTTTCG GTTTCTTCTT TAAATTCAAG ATATATATCG TATTAGAGAG TTTTTTATAT   
  
  
- AAAAAATTTA TATCACGCTA TAACTGACTG GATTATACCC AATAATGTCC TCTACCCCTC TATAGATGTA   
  
  
- ATTGATGAAC TTGTTGAATT TAAAAGTAAA ATTGAGCCAG TAATTCTTGT GATTCTTGAA ATATATAATC   
  
  
- ACTATAGAGT CCGCATTTTC TTTGATGAAA CTTAATTAGG TACGTTAGGA TGAGAAATAA AGGTAGGCAA   
  
  
- TTTTAAAAGG AAGTTTTGAG GGAAGACAAG TAATAATTAA GTTATAGCAT AATGTGAACA ACCTAATAAA   
  
  
- GACACTAAAC TAGCAGTAAA AGATATATAC TTATTGATGT TTTTAGTATC AGGCTCATGT AATTATAATT   
  
  
- ATTTTATCCT ACTCACTATT GGAGTATATC ACGCAAATAG GGATGGTGAT CCGACCTAAA GACGTATTTC   
  
  
- TTCTTTAACT TGTATACCTT TTTCTTTATT TCTCTTCGTT GAAGGAACTC ACCCTCTTCT TAGCATCTTT   
  
  
- CAATTTGAAT ACTTTTTCTC GTAATTGTTT TTCCTTTAAT ATTCCGAATG GCAACTTCGG AGTATATTAA   
  
  
- AACGTTCTCT GTCTGTTTCT TTCACAGAAA GTTTACAGTA TAAAGAGCTC GTACGTACTT TTTACATGAA   
  
  
- GCCGATGTTA TCATTATCAT ATATACAACC GAAGTATGTG AACTGGGGTT ACATACAGTT GCACTGATCA   
  
  
- CTGGTGTGTG TAGCAACAGG GACGGGCGGG GTGATGGGTT TGAGGGGCGT GCGTTTCTAT TTTTCGGGGG   
  
  
- GTTAAAAATA AACGGAGGGT TTTTCGGGTG GGCGCGAAAA AATAAACGGA AGAAAAAAGG ATGAATTTGG   
  
  
- TGGAGGAGTT GCTTGAAACG TTGTATGTAT GATCAGATAT TCGTTAAATC TCCCTTCCCT TCGTTGGATT   
  
  
- TGTCTCGCTC TAACCGCCCC ACACAAACTC CCGAACGCAA AACCACGTTA TCCTTACTTT CACGGGTAAA   
  
  
- GTTGGTGGTA GGTTTGAACG TTGAGGCCGT GGCCGTTTGG TGAAAGGAAA CTCTTATGAC GGGATGTATG   
  
  
- GCGGAAAGTT CAAGGGTTAT GGTGGATGCT TGGGTGGAGC CAAGAACTGG AGGTCGCGTC GGGATCGGGG   
  
  
- TGGAGGTGGG TTTTTGGCGG GAAACGGCGG AGGTTGGTGG TCGGACGGAG CCGACTAGAC CTGTTGAACC   
  
  
- TGCCCACCCT AAGTTAGGAT AGTCTCGACC CCGAATTACT GCTTAAATGG GGATTAAGGT TTGAGACCAG   
  
  
- GGTTTAATCA GGCAGACTAG GAGTGGAGGG GGTTGAGGGA CTAAAGGGTT GTAGAGTCGG CAAACTAGTA   
  
  
- GTGTTGGGTG GGGGTTATTT GGAGGGTAGT AGTCTAAAGT TGGAGACACT AAAGAGGATG TTAGTGTTGG   
  
  
- GGTTAAAACC CGGCAAGCTA GAAGTAGTGT TGGGCGTAGT CTTGTTGGGC TTGTTGTTAT TGATGCCGAA   
  
  
- ACTAAAGTAT CTGCTGGAGT AGTTTCGTCG TGTCAGGGAG CTTAGGTTGC TGAGAGTAGT TCAGGTGGAC   
  
  
- TATAACCGCG CCGAGTTAGT TGCCGAGTCT AGTGGGTGGC CGTTTGGCGA GGTCGCCCGG CGGATGATGA   
  
  
- AGTTCCTTCG GGAGTAGCGG CGGCGCCGGC CGGGCGGGGC GGGGCGAGCC GAAAGTAGCA TACTCCACCA   
  
  
- CGTCTGGTAG GCTCGGATGT TCCGCAAAAG TCCTTAAAGC GGTCAGAGAG AGAAGAGGTG AAAGCGGCGG   
  
  
- TTGGTCCGCT AAGAACTCCG CCACCTGTGC CGCCGGAAGT AGGTGTAGTA GCTAAAGCTG TAGCCTAAGC   
  
  
- CGCCGGTGAC CCGGAGCAAG GAGGCGCTTG ACCAGCTGTT TCGACTAGGA CGCTTTGAAT CAAGTCACCA   
  
  
- CAATGCTTAG TGGCGGTAGC AAGGGCTTCT TAAGCCCTAG CTTTCGTTCG ATCACTCTCT TTTAGACAGC   
  
  
- GTTAAACGGG CTCTAGAGTT GTAGTTGAAG GTGTAGCTAA TACAGAATAA AGTCTCGAAA CTTTAGAATA   
  
  
- GGAAGTTTAG ACAGTTTAAG TAGCTTTCCC TCTTTAACCG CGACGTAGAA AGCGGCCGGC ACAAGGTGGC   
  
  
- CGATCCCTCG CCTTAAAGCT TCAAATAGCG ACTAGAGGCT AGTTAGAGCG GCTCGAGGCA GCACCACCAC   
  
  
- CTGTCTCTAC AGCCCTAACT GTAGCCTTGC AGCAGCAAAT CGTACTTAAA GCAGCGGCCT TAACTTAAGA   
  
  
- TGTGGCCCTA CGACCTCAGC GAACTGCGTC GGTGGCGGCC CCCCGAGTAG CCGCCACACC TGACGCACTC   
  
  
- TGCCTAACTC TGCAAACAAG AGGTGGGTTC CTAGTACCGC CGCCATCTCC GCCGCCGCAG CCGCAGCCGG   
  
  
- CCCGCCTCCT GCCGCACCTC CCTCCGTAAG CGCCGCCGCC CCTAATCCCG CCACCCCAAA TCGGTCAAAC   
  
  
- GACTAAAGGT CCGGCTTACA AACAACTCCT CTCGAGTCCA ACCACCGAAG GTGCACCGTT TCGCGGTACC   
  
  
- TCTCTACTAC GAAATGACCG TACTGGCGGG TGAGCAGCGG TGTAGTCGAA CCTCCACAAT

+     TATA-box

| Site Name | Organism | Position | Strand | Matrix score. | sequence | function |
| --- | --- | --- | --- | --- | --- | --- |
| TATA-box | Brassica napus | 1050 | + | 6 | ATATAT | core promoter element around -30 of transcription start |
| TATA-box | Arabidopsis thaliana | 1029 | + | 4 | TATA | core promoter element around -30 of transcription start |
| TATA-box | Helianthus annuus | 452 | - | 6 | TATACA | core promoter element around -30 of transcription start |
| TATA-box | Arabidopsis thaliana | 301 | + | 4 | TATA | core promoter element around -30 of transcription start |
| TATA-box | Brassica napus | 300 | + | 6 | ATATAT | core promoter element around -30 of transcription start |
| TATA-box | Arabidopsis thaliana | 299 | + | 6 | TATATA | core promoter element around -30 of transcription start |
| TATA-box | Brassica napus | 298 | + | 6 | ATATAT | core promoter element around -30 of transcription start |
| TATA-box | Helianthus annuus | 800 | - | 6 | TATAAA | core promoter element around -30 of transcription start |
| TATA-box | Arabidopsis thaliana | 1705 | + | 4 | TATA | core promoter element around -30 of transcription start |
| TATA-box | Arabidopsis thaliana | 1703 | + | 6 | TATATA | core promoter element around -30 of transcription start |
| TATA-box | Arabidopsis thaliana | 1058 | - | 8 | TATTTAAA | core promoter element around -30 of transcription start |
| TATA-box | Arabidopsis thaliana | 152 | + | 4 | TATA | core promoter element around -30 of transcription start |
| TATA-box | Lycopersicon esculentum | 1182 | - | 9 | taTATAAAg | core promoter element around -30 of transcription start |
| TATA-box | Arabidopsis thaliana | 801 | - | 5 | TATAA | core promoter element around -30 of transcription start |
| TATA-box | Arabidopsis thaliana | 1608 | + | 4 | TATA | core promoter element around -30 of transcription start |
| TATA-box | Brassica napus | 1026 | + | 6 | ATATAT | core promoter element around -30 of transcription start |
| TATA-box | Brassica napus | 1704 | + | 6 | ATATAT | core promoter element around -30 of transcription start |
| TATA-box | Arabidopsis thaliana | 1064 | + | 4 | TATA | core promoter element around -30 of transcription start |
| TATA-box | Arabidopsis thaliana | 1051 | + | 4 | TATA | core promoter element around -30 of transcription start |
| TATA-box | Arabidopsis thaliana | 1359 | + | 4 | TATA | core promoter element around -30 of transcription start |
| TATA-box | Arabidopsis thaliana | 1357 | + | 6 | TATATA | core promoter element around -30 of transcription start |
| TATA-box | Arabidopsis thaliana | 1027 | + | 6 | TATATA | core promoter element around -30 of transcription start |
| TATA-box | Brassica napus | 1358 | + | 6 | ATATAT | core promoter element around -30 of transcription start |
| TATA-box | Oryza sativa | 1371 | + | 7 | TACAAAA | core promoter element around -30 of transcription start |
| TATA-box | Arabidopsis thaliana | 1187 | + | 4 | TATA | core promoter element around -30 of transcription start |
| TATA-box | Arabidopsis thaliana | 1430 | + | 4 | TATA | core promoter element around -30 of transcription start |
| TATA-box | Arabidopsis thaliana | 802 | + | 4 | TATA | core promoter element around -30 of transcription start |
| TATA-box | Arabidopsis thaliana | 577 | + | 4 | TATA | core promoter element around -30 of transcription start |
| TATA-box | Arabidopsis thaliana | 1582 | - | 5 | TATAA | core promoter element around -30 of transcription start |
| TATA-box | Arabidopsis thaliana | 1025 | + | 6 | TATATA | core promoter element around -30 of transcription start |
| TATA-box | Brassica napus | 1186 | + | 6 | ATATAT | core promoter element around -30 of transcription start |
| TATA-box | Arabidopsis thaliana | 1931 | - | 4 | TATA | core promoter element around -30 of transcription start |
| TATA-box | Arabidopsis thaliana | 733 | + | 8 | TAAAGATT | core promoter element around -30 of transcription start |
| TATA-box | Brassica napus | 1581 | + | 6 | ATTATA | core promoter element around -30 of transcription start |
| TATA-box | Arabidopsis thaliana | 1185 | + | 6 | TATATA | core promoter element around -30 of transcription start |
| TATA-box | Arabidopsis thaliana | 454 | + | 4 | TATA | core promoter element around -30 of transcription start |
| TATA-box | Helianthus annuus | 575 | - | 6 | TATACA | core promoter element around -30 of transcription start |
| TATA-box | Arabidopsis thaliana | 1583 | + | 4 | TATA | core promoter element around -30 of transcription start |
| TATA-box | Arabidopsis thaliana | 1184 | - | 7 | TATATAA | core promoter element around -30 of transcription start |
| TATA-box | Brassica oleracea | 1607 | + | 6 | ATATAA | core promoter element around -30 of transcription start |
| TATA-box | Helianthus annuus | 1183 | - | 6 | TATAAA | core promoter element around -30 of transcription start |

>HU05G02245.1   
+ -Up\_Stream \_Len000ATAATT TATCTTTCTT TAATTTCGTT TCATGTAAAA AATTTCAGTC TCATCTGCGA   
  
  
+ TTGAATCTCT TTTCAATTCG CATCAATAAT TCATCCAATT CAAATGTGCC TTTCTTAATT TCATTACATT   
  
  
+ GGGTTGGTAT AAATGTTAGC ACGATAGATT GGAATAGCAA GGCTCTAATT CAATTATGAC TTGAATGTAT   
  
  
+ GCATGCTTTT ATCATTAGTT AATTAACATT TTCTGTATTG AAATCAGCTC GAAAGAGAAC CATATGTAGT   
  
  
+ TGTACTTGCG ACGATATATA TGTTTAGTTT AGTTAGTTCT TACCTGTTTT GGAATTGTTG GGGAGATCTG   
  
  
+ AGGAAGGAGC CCGCTTCAAT CAAAGTTCAT GGCAATAAGC CAAAGTGAGG TTCCATCCTA AAACCAATTG   
  
  
+ GCAATAGGAA GAGTAGCCCA CTTAACTTGT ATACTATGAC TCTTCTTTTC AAACTCCCAA TGTGGGACTT   
  
  
+ CTTTACTCAT GTGTGATATT CCCAACAGGA ATGATCAGTT CCACATGTTT TCATCTCTTT GGACTCGAAC   
  
  
+ TTCGTGTAAT TGTATATGCC ATGGCACAAA TAGTTTTGAT GGTGCCACCA AGTTGCTCAA TCAATGTAAT   
  
  
+ ACTGCTACTA TCTAACTTGC CTTGGTGGAC ATCAATAAGA TCACGAGATG AGGAGAACTG AATCTTGGCC   
  
  
+ AACCACGTCG TTTGACTTCT CTTACCAATA AAGATTGAAT AGACAGTGTT TCTCTCCCCC TTCCATTCTT   
  
  
+ CTTTTCTAGT GATGTCGAAA AAGACTTTAT ACATGTCCAG CAACGCTAGC CACAAAATAT TCCATTTCTG   
  
  
+ TAATCCACTC ATGTCAATTA AACTTGTATC ATCAAATGGT AGTTTCCGTA CTAATCACTA CATCTAGATA   
  
  
+ TTCCTTGATA AATTTAATTT GACTTTAAAG GCTCGATCTT TTTAAGAAAA GTTTTGTTGC TTACAACCTG   
  
  
+ TAAAGCCTAG AGAACAAAGC CAAAGAAGAA ATTTAAGTTC TATATATAGC ATAATCTCTC AAAAAATATA   
  
  
+ TTTTTTAAAT ATAGTGCGAT ATTGACTGAC CTAATATGGG TTATTACAGG AGATGGGGAG ATATCTACAT   
  
  
+ TAACTACTTG AACAACTTAA ATTTTCATTT TAACTCGGTC ATTAAGAACA CTAAGAACTT TATATATTAG   
  
  
+ TGATATCTCA GGCGTAAAAG AAACTACTTT GAATTAATCC ATGCAATCCT ACTCTTTATT TCCATCCGTT   
  
  
+ AAAATTTTCC TTCAAAACTC CCTTCTGTTC ATTATTAATT CAATATCGTA TTACACTTGT TGGATTATTT   
  
  
+ CTGTGATTTG ATCGTCATTT TCTATATATG AATAACTACA AAAATCATAG TCCGAGTACA TTAATATTAA   
  
  
+ TAAAATAGGA TGAGTGATAA CCTCATATAG TGCGTTTATC CCTACCACTA GGCTGGATTT CTGCATAAAG   
  
  
+ AAGAAATTGA ACATATGGAA AAAGAAATAA AGAGAAGCAA CTTCCTTGAG TGGGAGAAGA ATCGTAGAAA   
  
  
+ GTTAAACTTA TGAAAAAGAG CATTAACAAA AAGGAAATTA TAAGGCTTAC CGTTGAAGCC TCATATAATT   
  
  
+ TTGCAAGAGA CAGACAAAGA AAGTGTCTTT CAAATGTCAT ATTTCTCGAG CATGCATGAA AAATGTACTT   
  
  
+ CGGCTACAAT AGTAATAGTA TATATGTTGG CTTCATACAC TTGACCCCAA TGTATGTCAA CGTGACTAGT   
  
  
+ GACCACACAC ATCGTTGTCC CTGCCCGCCC CACTACCCAA ACTCCCCGCA CGCAAAGATA AAAAGCCCCC   
  
  
+ CAATTTTTAT TTGCCTCCCA AAAAGCCCAC CCGCGCTTTT TTATTTGCCT TCTTTTTTCC TACTTAAACC   
  
  
+ ACCTCCTCAA CGAACTTTGC AACATACATA CTAGTCTATA AGCAATTTAG AGGGAAGGGA AGCAACCTAA   
  
  
+ ACAGAGCGAG ATTGGCGGGG TGTGTTTGAG GGCTTGCGTT TTGGTGCAAT AGGAATGAAA GTGCCCATTT   
  
  
+ CAACCACCAT CCAAACTTGC AACTCCGGCA CCGGCAAACC ACTTTCCTTT GAGAATACTG CCCTACATAC   
  
  
+ CGCCTTTCAA GTTCCCAATA CCACCTACGA ACCCACCTCG GTTCTTGACC TCCAGCGCAG CCCTAGCCCC   
  
  
+ ACCTCCACCC AAAAACCGCC CTTTGCCGCC TCCAACCACC AGCCTGCCTC GGCTGATCTG GACAACTTGG   
  
  
+ ACGGGTGGGA TTCAATCCTA TCAGAGCTGG GGCTTAATGA CGAATTTACC CCTAATTCCA AACTCTGGTC   
  
  
+ CCAAATTAGT CCGTCTGATC CTCACCTCCC CCAACTCCCT GATTTCCCAA CATCTCAGCC GTTTGATCAT   
  
  
+ CACAACCCAC CCCCAATAAA CCTCCCATCA TCAGATTTCA ACCTCTGTGA TTTCTCCTAC AATCACAACC   
  
  
+ CCAATTTTGG GCCGTTCGAT CTTCATCACA ACCCGCATCA GAACAACCCG AACAACAATA ACTACGGCTT   
  
  
+ TGATTTCATA GACGACCTCA TCAAAGCAGC ACAGTCCCTC GAATCCAACG ACTCTCATCA AGTCCACCTG   
  
  
+ ATATTGGCGC GGCTCAATCA ACGGCTCAGA TCACCCACCG GCAAACCGCT CCAGCGGGCC GCCTACTACT   
  
  
+ TCAAGGAAGC CCTCATCGCC GCCGCGGCCG GCCCGCCCCG CCCCGCTCGG CTTTCATCGT ATGAGGTGGT   
  
  
+ GCAGACCATC CGAGCCTACA AGGCGTTTTC AGGAATTTCG CCAGTCTCTC TCTTCTCCAC TTTCGCCGCC   
  
  
+ AACCAGGCGA TTCTTGAGGC GGTGGACACG GCGGCCTTCA TCCACATCAT CGATTTCGAC ATCGGATTCG   
  
  
+ GCGGCCACTG GGCCTCGTTC CTCCGCGAAC TGGTCGACAA AGCTGATCCT GCGAAACTTA GTTCAGTGGT   
  
  
+ GTTACGAATC ACCGCCATCG TTCCCGAAGA ATTCGGGATC GAAAGCAAGC TAGTGAGAGA AAATCTGTCG   
  
  
+ CAATTTGCCC GAGATCTCAA CATCAACTTC CACATCGATT ATGTCTTATT TCAGAGCTTT GAAATCTTAT   
  
  
+ CCTTCAAATC TGTCAAATTC ATCGAAAGGG AGAAATTGGC GCTGCATCTT TCGCCGGCCG TGTTCCACCG   
  
  
+ GCTAGGGAGC GGAATTTCGA AGTTTATCGC TGATCTCCGA TCAATCTCGC CGAGCTCCGT CGTGGTGGTG   
  
  
+ GACAGAGATG TCGGGATTGA CATCGGAACG TCGTCGTTTA GCATGAATTT CGTCGCCGGA ATTGAATTCT   
  
  
+ ACACCGGGAT GCTGGAGTCG CTTGACGCAG CCACCGCCGG GGGGCTCATC GGCGGTGTGG ACTGCGTGAG   
  
  
+ ACGGATTGAG ACGTTTGTTC TCCACCCAAG GATCATGGCG GCGGTAGAGG CGGCGGCGTC GGCGTCGGCC   
  
  
+ GGGCGGAGGA CGGCGTGGAG GGAGGCATTC GCGGCGGCGG GGATTAGGGC GGTGGGGTTT AGCCAGTTTG   
  
  
+ CTGATTTCCA GGCCGAATGT TTGTTGAGGA GAGCTCAGGT TGGTGGCTTC CACGTGGCAA AGCGCCATGG   
  
  
+ AGAGATGATG CTTTACTGGC ATGACCGCCC ACTCGTCGCC ACATCAGCTT GGAGGTGTTA   

- -Up\_Stream \_Len000TATTAA ATAGAAAGAA ATTAAAGCAA AGTACATTTT TTAAAGTCAG AGTAGACGCT   
  
  
- AACTTAGAGA AAAGTTAAGC GTAGTTATTA AGTAGGTTAA GTTTACACGG AAAGAATTAA AGTAATGTAA   
  
  
- CCCAACCATA TTTACAATCG TGCTATCTAA CCTTATCGTT CCGAGATTAA GTTAATACTG AACTTACATA   
  
  
- CGTACGAAAA TAGTAATCAA TTAATTGTAA AAGACATAAC TTTAGTCGAG CTTTCTCTTG GTATACATCA   
  
  
- ACATGAACGC TGCTATATAT ACAAATCAAA TCAATCAAGA ATGGACAAAA CCTTAACAAC CCCTCTAGAC   
  
  
- TCCTTCCTCG GGCGAAGTTA GTTTCAAGTA CCGTTATTCG GTTTCACTCC AAGGTAGGAT TTTGGTTAAC   
  
  
- CGTTATCCTT CTCATCGGGT GAATTGAACA TATGATACTG AGAAGAAAAG TTTGAGGGTT ACACCCTGAA   
  
  
- GAAATGAGTA CACACTATAA GGGTTGTCCT TACTAGTCAA GGTGTACAAA AGTAGAGAAA CCTGAGCTTG   
  
  
- AAGCACATTA ACATATACGG TACCGTGTTT ATCAAAACTA CCACGGTGGT TCAACGAGTT AGTTACATTA   
  
  
- TGACGATGAT AGATTGAACG GAACCACCTG TAGTTATTCT AGTGCTCTAC TCCTCTTGAC TTAGAACCGG   
  
  
- TTGGTGCAGC AAACTGAAGA GAATGGTTAT TTCTAACTTA TCTGTCACAA AGAGAGGGGG AAGGTAAGAA   
  
  
- GAAAAGATCA CTACAGCTTT TTCTGAAATA TGTACAGGTC GTTGCGATCG GTGTTTTATA AGGTAAAGAC   
  
  
- ATTAGGTGAG TACAGTTAAT TTGAACATAG TAGTTTACCA TCAAAGGCAT GATTAGTGAT GTAGATCTAT   
  
  
- AAGGAACTAT TTAAATTAAA CTGAAATTTC CGAGCTAGAA AAATTCTTTT CAAAACAACG AATGTTGGAC   
  
  
- ATTTCGGATC TCTTGTTTCG GTTTCTTCTT TAAATTCAAG ATATATATCG TATTAGAGAG TTTTTTATAT   
  
  
- AAAAAATTTA TATCACGCTA TAACTGACTG GATTATACCC AATAATGTCC TCTACCCCTC TATAGATGTA   
  
  
- ATTGATGAAC TTGTTGAATT TAAAAGTAAA ATTGAGCCAG TAATTCTTGT GATTCTTGAA ATATATAATC   
  
  
- ACTATAGAGT CCGCATTTTC TTTGATGAAA CTTAATTAGG TACGTTAGGA TGAGAAATAA AGGTAGGCAA   
  
  
- TTTTAAAAGG AAGTTTTGAG GGAAGACAAG TAATAATTAA GTTATAGCAT AATGTGAACA ACCTAATAAA   
  
  
- GACACTAAAC TAGCAGTAAA AGATATATAC TTATTGATGT TTTTAGTATC AGGCTCATGT AATTATAATT   
  
  
- ATTTTATCCT ACTCACTATT GGAGTATATC ACGCAAATAG GGATGGTGAT CCGACCTAAA GACGTATTTC   
  
  
- TTCTTTAACT TGTATACCTT TTTCTTTATT TCTCTTCGTT GAAGGAACTC ACCCTCTTCT TAGCATCTTT   
  
  
- CAATTTGAAT ACTTTTTCTC GTAATTGTTT TTCCTTTAAT ATTCCGAATG GCAACTTCGG AGTATATTAA   
  
  
- AACGTTCTCT GTCTGTTTCT TTCACAGAAA GTTTACAGTA TAAAGAGCTC GTACGTACTT TTTACATGAA   
  
  
- GCCGATGTTA TCATTATCAT ATATACAACC GAAGTATGTG AACTGGGGTT ACATACAGTT GCACTGATCA   
  
  
- CTGGTGTGTG TAGCAACAGG GACGGGCGGG GTGATGGGTT TGAGGGGCGT GCGTTTCTAT TTTTCGGGGG   
  
  
- GTTAAAAATA AACGGAGGGT TTTTCGGGTG GGCGCGAAAA AATAAACGGA AGAAAAAAGG ATGAATTTGG   
  
  
- TGGAGGAGTT GCTTGAAACG TTGTATGTAT GATCAGATAT TCGTTAAATC TCCCTTCCCT TCGTTGGATT   
  
  
- TGTCTCGCTC TAACCGCCCC ACACAAACTC CCGAACGCAA AACCACGTTA TCCTTACTTT CACGGGTAAA   
  
  
- GTTGGTGGTA GGTTTGAACG TTGAGGCCGT GGCCGTTTGG TGAAAGGAAA CTCTTATGAC GGGATGTATG   
  
  
- GCGGAAAGTT CAAGGGTTAT GGTGGATGCT TGGGTGGAGC CAAGAACTGG AGGTCGCGTC GGGATCGGGG   
  
  
- TGGAGGTGGG TTTTTGGCGG GAAACGGCGG AGGTTGGTGG TCGGACGGAG CCGACTAGAC CTGTTGAACC   
  
  
- TGCCCACCCT AAGTTAGGAT AGTCTCGACC CCGAATTACT GCTTAAATGG GGATTAAGGT TTGAGACCAG   
  
  
- GGTTTAATCA GGCAGACTAG GAGTGGAGGG GGTTGAGGGA CTAAAGGGTT GTAGAGTCGG CAAACTAGTA   
  
  
- GTGTTGGGTG GGGGTTATTT GGAGGGTAGT AGTCTAAAGT TGGAGACACT AAAGAGGATG TTAGTGTTGG   
  
  
- GGTTAAAACC CGGCAAGCTA GAAGTAGTGT TGGGCGTAGT CTTGTTGGGC TTGTTGTTAT TGATGCCGAA   
  
  
- ACTAAAGTAT CTGCTGGAGT AGTTTCGTCG TGTCAGGGAG CTTAGGTTGC TGAGAGTAGT TCAGGTGGAC   
  
  
- TATAACCGCG CCGAGTTAGT TGCCGAGTCT AGTGGGTGGC CGTTTGGCGA GGTCGCCCGG CGGATGATGA   
  
  
- AGTTCCTTCG GGAGTAGCGG CGGCGCCGGC CGGGCGGGGC GGGGCGAGCC GAAAGTAGCA TACTCCACCA   
  
  
- CGTCTGGTAG GCTCGGATGT TCCGCAAAAG TCCTTAAAGC GGTCAGAGAG AGAAGAGGTG AAAGCGGCGG   
  
  
- TTGGTCCGCT AAGAACTCCG CCACCTGTGC CGCCGGAAGT AGGTGTAGTA GCTAAAGCTG TAGCCTAAGC   
  
  
- CGCCGGTGAC CCGGAGCAAG GAGGCGCTTG ACCAGCTGTT TCGACTAGGA CGCTTTGAAT CAAGTCACCA   
  
  
- CAATGCTTAG TGGCGGTAGC AAGGGCTTCT TAAGCCCTAG CTTTCGTTCG ATCACTCTCT TTTAGACAGC   
  
  
- GTTAAACGGG CTCTAGAGTT GTAGTTGAAG GTGTAGCTAA TACAGAATAA AGTCTCGAAA CTTTAGAATA   
  
  
- GGAAGTTTAG ACAGTTTAAG TAGCTTTCCC TCTTTAACCG CGACGTAGAA AGCGGCCGGC ACAAGGTGGC   
  
  
- CGATCCCTCG CCTTAAAGCT TCAAATAGCG ACTAGAGGCT AGTTAGAGCG GCTCGAGGCA GCACCACCAC   
  
  
- CTGTCTCTAC AGCCCTAACT GTAGCCTTGC AGCAGCAAAT CGTACTTAAA GCAGCGGCCT TAACTTAAGA   
  
  
- TGTGGCCCTA CGACCTCAGC GAACTGCGTC GGTGGCGGCC CCCCGAGTAG CCGCCACACC TGACGCACTC   
  
  
- TGCCTAACTC TGCAAACAAG AGGTGGGTTC CTAGTACCGC CGCCATCTCC GCCGCCGCAG CCGCAGCCGG   
  
  
- CCCGCCTCCT GCCGCACCTC CCTCCGTAAG CGCCGCCGCC CCTAATCCCG CCACCCCAAA TCGGTCAAAC   
  
  
- GACTAAAGGT CCGGCTTACA AACAACTCCT CTCGAGTCCA ACCACCGAAG GTGCACCGTT TCGCGGTACC   
  
  
- TCTCTACTAC GAAATGACCG TACTGGCGGG TGAGCAGCGG TGTAGTCGAA CCTCCACAAT

+     TCA-element

| Site Name | Organism | Position | Strand | Matrix score. | sequence | function |
| --- | --- | --- | --- | --- | --- | --- |
| TCA-element | Nicotiana tabacum | 948 | + | 9 | CCATCTTTTT | cis-acting element involved in salicylic acid responsiveness |
| TCA-element | Nicotiana tabacum | 1872 | + | 9 | CCATCTTTTT | cis-acting element involved in salicylic acid responsiveness |

>HU05G02245.1   
+ -Up\_Stream \_Len000ATAATT TATCTTTCTT TAATTTCGTT TCATGTAAAA AATTTCAGTC TCATCTGCGA   
  
  
+ TTGAATCTCT TTTCAATTCG CATCAATAAT TCATCCAATT CAAATGTGCC TTTCTTAATT TCATTACATT   
  
  
+ GGGTTGGTAT AAATGTTAGC ACGATAGATT GGAATAGCAA GGCTCTAATT CAATTATGAC TTGAATGTAT   
  
  
+ GCATGCTTTT ATCATTAGTT AATTAACATT TTCTGTATTG AAATCAGCTC GAAAGAGAAC CATATGTAGT   
  
  
+ TGTACTTGCG ACGATATATA TGTTTAGTTT AGTTAGTTCT TACCTGTTTT GGAATTGTTG GGGAGATCTG   
  
  
+ AGGAAGGAGC CCGCTTCAAT CAAAGTTCAT GGCAATAAGC CAAAGTGAGG TTCCATCCTA AAACCAATTG   
  
  
+ GCAATAGGAA GAGTAGCCCA CTTAACTTGT ATACTATGAC TCTTCTTTTC AAACTCCCAA TGTGGGACTT   
  
  
+ CTTTACTCAT GTGTGATATT CCCAACAGGA ATGATCAGTT CCACATGTTT TCATCTCTTT GGACTCGAAC   
  
  
+ TTCGTGTAAT TGTATATGCC ATGGCACAAA TAGTTTTGAT GGTGCCACCA AGTTGCTCAA TCAATGTAAT   
  
  
+ ACTGCTACTA TCTAACTTGC CTTGGTGGAC ATCAATAAGA TCACGAGATG AGGAGAACTG AATCTTGGCC   
  
  
+ AACCACGTCG TTTGACTTCT CTTACCAATA AAGATTGAAT AGACAGTGTT TCTCTCCCCC TTCCATTCTT   
  
  
+ CTTTTCTAGT GATGTCGAAA AAGACTTTAT ACATGTCCAG CAACGCTAGC CACAAAATAT TCCATTTCTG   
  
  
+ TAATCCACTC ATGTCAATTA AACTTGTATC ATCAAATGGT AGTTTCCGTA CTAATCACTA CATCTAGATA   
  
  
+ TTCCTTGATA AATTTAATTT GACTTTAAAG GCTCGATCTT TTTAAGAAAA GTTTTGTTGC TTACAACCTG   
  
  
+ TAAAGCCTAG AGAACAAAGC CAAAGAAGAA ATTTAAGTTC TATATATAGC ATAATCTCTC AAAAAATATA   
  
  
+ TTTTTTAAAT ATAGTGCGAT ATTGACTGAC CTAATATGGG TTATTACAGG AGATGGGGAG ATATCTACAT   
  
  
+ TAACTACTTG AACAACTTAA ATTTTCATTT TAACTCGGTC ATTAAGAACA CTAAGAACTT TATATATTAG   
  
  
+ TGATATCTCA GGCGTAAAAG AAACTACTTT GAATTAATCC ATGCAATCCT ACTCTTTATT TCCATCCGTT   
  
  
+ AAAATTTTCC TTCAAAACTC CCTTCTGTTC ATTATTAATT CAATATCGTA TTACACTTGT TGGATTATTT   
  
  
+ CTGTGATTTG ATCGTCATTT TCTATATATG AATAACTACA AAAATCATAG TCCGAGTACA TTAATATTAA   
  
  
+ TAAAATAGGA TGAGTGATAA CCTCATATAG TGCGTTTATC CCTACCACTA GGCTGGATTT CTGCATAAAG   
  
  
+ AAGAAATTGA ACATATGGAA AAAGAAATAA AGAGAAGCAA CTTCCTTGAG TGGGAGAAGA ATCGTAGAAA   
  
  
+ GTTAAACTTA TGAAAAAGAG CATTAACAAA AAGGAAATTA TAAGGCTTAC CGTTGAAGCC TCATATAATT   
  
  
+ TTGCAAGAGA CAGACAAAGA AAGTGTCTTT CAAATGTCAT ATTTCTCGAG CATGCATGAA AAATGTACTT   
  
  
+ CGGCTACAAT AGTAATAGTA TATATGTTGG CTTCATACAC TTGACCCCAA TGTATGTCAA CGTGACTAGT   
  
  
+ GACCACACAC ATCGTTGTCC CTGCCCGCCC CACTACCCAA ACTCCCCGCA CGCAAAGATA AAAAGCCCCC   
  
  
+ CAATTTTTAT TTGCCTCCCA AAAAGCCCAC CCGCGCTTTT TTATTTGCCT TCTTTTTTCC TACTTAAACC   
  
  
+ ACCTCCTCAA CGAACTTTGC AACATACATA CTAGTCTATA AGCAATTTAG AGGGAAGGGA AGCAACCTAA   
  
  
+ ACAGAGCGAG ATTGGCGGGG TGTGTTTGAG GGCTTGCGTT TTGGTGCAAT AGGAATGAAA GTGCCCATTT   
  
  
+ CAACCACCAT CCAAACTTGC AACTCCGGCA CCGGCAAACC ACTTTCCTTT GAGAATACTG CCCTACATAC   
  
  
+ CGCCTTTCAA GTTCCCAATA CCACCTACGA ACCCACCTCG GTTCTTGACC TCCAGCGCAG CCCTAGCCCC   
  
  
+ ACCTCCACCC AAAAACCGCC CTTTGCCGCC TCCAACCACC AGCCTGCCTC GGCTGATCTG GACAACTTGG   
  
  
+ ACGGGTGGGA TTCAATCCTA TCAGAGCTGG GGCTTAATGA CGAATTTACC CCTAATTCCA AACTCTGGTC   
  
  
+ CCAAATTAGT CCGTCTGATC CTCACCTCCC CCAACTCCCT GATTTCCCAA CATCTCAGCC GTTTGATCAT   
  
  
+ CACAACCCAC CCCCAATAAA CCTCCCATCA TCAGATTTCA ACCTCTGTGA TTTCTCCTAC AATCACAACC   
  
  
+ CCAATTTTGG GCCGTTCGAT CTTCATCACA ACCCGCATCA GAACAACCCG AACAACAATA ACTACGGCTT   
  
  
+ TGATTTCATA GACGACCTCA TCAAAGCAGC ACAGTCCCTC GAATCCAACG ACTCTCATCA AGTCCACCTG   
  
  
+ ATATTGGCGC GGCTCAATCA ACGGCTCAGA TCACCCACCG GCAAACCGCT CCAGCGGGCC GCCTACTACT   
  
  
+ TCAAGGAAGC CCTCATCGCC GCCGCGGCCG GCCCGCCCCG CCCCGCTCGG CTTTCATCGT ATGAGGTGGT   
  
  
+ GCAGACCATC CGAGCCTACA AGGCGTTTTC AGGAATTTCG CCAGTCTCTC TCTTCTCCAC TTTCGCCGCC   
  
  
+ AACCAGGCGA TTCTTGAGGC GGTGGACACG GCGGCCTTCA TCCACATCAT CGATTTCGAC ATCGGATTCG   
  
  
+ GCGGCCACTG GGCCTCGTTC CTCCGCGAAC TGGTCGACAA AGCTGATCCT GCGAAACTTA GTTCAGTGGT   
  
  
+ GTTACGAATC ACCGCCATCG TTCCCGAAGA ATTCGGGATC GAAAGCAAGC TAGTGAGAGA AAATCTGTCG   
  
  
+ CAATTTGCCC GAGATCTCAA CATCAACTTC CACATCGATT ATGTCTTATT TCAGAGCTTT GAAATCTTAT   
  
  
+ CCTTCAAATC TGTCAAATTC ATCGAAAGGG AGAAATTGGC GCTGCATCTT TCGCCGGCCG TGTTCCACCG   
  
  
+ GCTAGGGAGC GGAATTTCGA AGTTTATCGC TGATCTCCGA TCAATCTCGC CGAGCTCCGT CGTGGTGGTG   
  
  
+ GACAGAGATG TCGGGATTGA CATCGGAACG TCGTCGTTTA GCATGAATTT CGTCGCCGGA ATTGAATTCT   
  
  
+ ACACCGGGAT GCTGGAGTCG CTTGACGCAG CCACCGCCGG GGGGCTCATC GGCGGTGTGG ACTGCGTGAG   
  
  
+ ACGGATTGAG ACGTTTGTTC TCCACCCAAG GATCATGGCG GCGGTAGAGG CGGCGGCGTC GGCGTCGGCC   
  
  
+ GGGCGGAGGA CGGCGTGGAG GGAGGCATTC GCGGCGGCGG GGATTAGGGC GGTGGGGTTT AGCCAGTTTG   
  
  
+ CTGATTTCCA GGCCGAATGT TTGTTGAGGA GAGCTCAGGT TGGTGGCTTC CACGTGGCAA AGCGCCATGG   
  
  
+ AGAGATGATG CTTTACTGGC ATGACCGCCC ACTCGTCGCC ACATCAGCTT GGAGGTGTTA   

- -Up\_Stream \_Len000TATTAA ATAGAAAGAA ATTAAAGCAA AGTACATTTT TTAAAGTCAG AGTAGACGCT   
  
  
- AACTTAGAGA AAAGTTAAGC GTAGTTATTA AGTAGGTTAA GTTTACACGG AAAGAATTAA AGTAATGTAA   
  
  
- CCCAACCATA TTTACAATCG TGCTATCTAA CCTTATCGTT CCGAGATTAA GTTAATACTG AACTTACATA   
  
  
- CGTACGAAAA TAGTAATCAA TTAATTGTAA AAGACATAAC TTTAGTCGAG CTTTCTCTTG GTATACATCA   
  
  
- ACATGAACGC TGCTATATAT ACAAATCAAA TCAATCAAGA ATGGACAAAA CCTTAACAAC CCCTCTAGAC   
  
  
- TCCTTCCTCG GGCGAAGTTA GTTTCAAGTA CCGTTATTCG GTTTCACTCC AAGGTAGGAT TTTGGTTAAC   
  
  
- CGTTATCCTT CTCATCGGGT GAATTGAACA TATGATACTG AGAAGAAAAG TTTGAGGGTT ACACCCTGAA   
  
  
- GAAATGAGTA CACACTATAA GGGTTGTCCT TACTAGTCAA GGTGTACAAA AGTAGAGAAA CCTGAGCTTG   
  
  
- AAGCACATTA ACATATACGG TACCGTGTTT ATCAAAACTA CCACGGTGGT TCAACGAGTT AGTTACATTA   
  
  
- TGACGATGAT AGATTGAACG GAACCACCTG TAGTTATTCT AGTGCTCTAC TCCTCTTGAC TTAGAACCGG   
  
  
- TTGGTGCAGC AAACTGAAGA GAATGGTTAT TTCTAACTTA TCTGTCACAA AGAGAGGGGG AAGGTAAGAA   
  
  
- GAAAAGATCA CTACAGCTTT TTCTGAAATA TGTACAGGTC GTTGCGATCG GTGTTTTATA AGGTAAAGAC   
  
  
- ATTAGGTGAG TACAGTTAAT TTGAACATAG TAGTTTACCA TCAAAGGCAT GATTAGTGAT GTAGATCTAT   
  
  
- AAGGAACTAT TTAAATTAAA CTGAAATTTC CGAGCTAGAA AAATTCTTTT CAAAACAACG AATGTTGGAC   
  
  
- ATTTCGGATC TCTTGTTTCG GTTTCTTCTT TAAATTCAAG ATATATATCG TATTAGAGAG TTTTTTATAT   
  
  
- AAAAAATTTA TATCACGCTA TAACTGACTG GATTATACCC AATAATGTCC TCTACCCCTC TATAGATGTA   
  
  
- ATTGATGAAC TTGTTGAATT TAAAAGTAAA ATTGAGCCAG TAATTCTTGT GATTCTTGAA ATATATAATC   
  
  
- ACTATAGAGT CCGCATTTTC TTTGATGAAA CTTAATTAGG TACGTTAGGA TGAGAAATAA AGGTAGGCAA   
  
  
- TTTTAAAAGG AAGTTTTGAG GGAAGACAAG TAATAATTAA GTTATAGCAT AATGTGAACA ACCTAATAAA   
  
  
- GACACTAAAC TAGCAGTAAA AGATATATAC TTATTGATGT TTTTAGTATC AGGCTCATGT AATTATAATT   
  
  
- ATTTTATCCT ACTCACTATT GGAGTATATC ACGCAAATAG GGATGGTGAT CCGACCTAAA GACGTATTTC   
  
  
- TTCTTTAACT TGTATACCTT TTTCTTTATT TCTCTTCGTT GAAGGAACTC ACCCTCTTCT TAGCATCTTT   
  
  
- CAATTTGAAT ACTTTTTCTC GTAATTGTTT TTCCTTTAAT ATTCCGAATG GCAACTTCGG AGTATATTAA   
  
  
- AACGTTCTCT GTCTGTTTCT TTCACAGAAA GTTTACAGTA TAAAGAGCTC GTACGTACTT TTTACATGAA   
  
  
- GCCGATGTTA TCATTATCAT ATATACAACC GAAGTATGTG AACTGGGGTT ACATACAGTT GCACTGATCA   
  
  
- CTGGTGTGTG TAGCAACAGG GACGGGCGGG GTGATGGGTT TGAGGGGCGT GCGTTTCTAT TTTTCGGGGG   
  
  
- GTTAAAAATA AACGGAGGGT TTTTCGGGTG GGCGCGAAAA AATAAACGGA AGAAAAAAGG ATGAATTTGG   
  
  
- TGGAGGAGTT GCTTGAAACG TTGTATGTAT GATCAGATAT TCGTTAAATC TCCCTTCCCT TCGTTGGATT   
  
  
- TGTCTCGCTC TAACCGCCCC ACACAAACTC CCGAACGCAA AACCACGTTA TCCTTACTTT CACGGGTAAA   
  
  
- GTTGGTGGTA GGTTTGAACG TTGAGGCCGT GGCCGTTTGG TGAAAGGAAA CTCTTATGAC GGGATGTATG   
  
  
- GCGGAAAGTT CAAGGGTTAT GGTGGATGCT TGGGTGGAGC CAAGAACTGG AGGTCGCGTC GGGATCGGGG   
  
  
- TGGAGGTGGG TTTTTGGCGG GAAACGGCGG AGGTTGGTGG TCGGACGGAG CCGACTAGAC CTGTTGAACC   
  
  
- TGCCCACCCT AAGTTAGGAT AGTCTCGACC CCGAATTACT GCTTAAATGG GGATTAAGGT TTGAGACCAG   
  
  
- GGTTTAATCA GGCAGACTAG GAGTGGAGGG GGTTGAGGGA CTAAAGGGTT GTAGAGTCGG CAAACTAGTA   
  
  
- GTGTTGGGTG GGGGTTATTT GGAGGGTAGT AGTCTAAAGT TGGAGACACT AAAGAGGATG TTAGTGTTGG   
  
  
- GGTTAAAACC CGGCAAGCTA GAAGTAGTGT TGGGCGTAGT CTTGTTGGGC TTGTTGTTAT TGATGCCGAA   
  
  
- ACTAAAGTAT CTGCTGGAGT AGTTTCGTCG TGTCAGGGAG CTTAGGTTGC TGAGAGTAGT TCAGGTGGAC   
  
  
- TATAACCGCG CCGAGTTAGT TGCCGAGTCT AGTGGGTGGC CGTTTGGCGA GGTCGCCCGG CGGATGATGA   
  
  
- AGTTCCTTCG GGAGTAGCGG CGGCGCCGGC CGGGCGGGGC GGGGCGAGCC GAAAGTAGCA TACTCCACCA   
  
  
- CGTCTGGTAG GCTCGGATGT TCCGCAAAAG TCCTTAAAGC GGTCAGAGAG AGAAGAGGTG AAAGCGGCGG   
  
  
- TTGGTCCGCT AAGAACTCCG CCACCTGTGC CGCCGGAAGT AGGTGTAGTA GCTAAAGCTG TAGCCTAAGC   
  
  
- CGCCGGTGAC CCGGAGCAAG GAGGCGCTTG ACCAGCTGTT TCGACTAGGA CGCTTTGAAT CAAGTCACCA   
  
  
- CAATGCTTAG TGGCGGTAGC AAGGGCTTCT TAAGCCCTAG CTTTCGTTCG ATCACTCTCT TTTAGACAGC   
  
  
- GTTAAACGGG CTCTAGAGTT GTAGTTGAAG GTGTAGCTAA TACAGAATAA AGTCTCGAAA CTTTAGAATA   
  
  
- GGAAGTTTAG ACAGTTTAAG TAGCTTTCCC TCTTTAACCG CGACGTAGAA AGCGGCCGGC ACAAGGTGGC   
  
  
- CGATCCCTCG CCTTAAAGCT TCAAATAGCG ACTAGAGGCT AGTTAGAGCG GCTCGAGGCA GCACCACCAC   
  
  
- CTGTCTCTAC AGCCCTAACT GTAGCCTTGC AGCAGCAAAT CGTACTTAAA GCAGCGGCCT TAACTTAAGA   
  
  
- TGTGGCCCTA CGACCTCAGC GAACTGCGTC GGTGGCGGCC CCCCGAGTAG CCGCCACACC TGACGCACTC   
  
  
- TGCCTAACTC TGCAAACAAG AGGTGGGTTC CTAGTACCGC CGCCATCTCC GCCGCCGCAG CCGCAGCCGG   
  
  
- CCCGCCTCCT GCCGCACCTC CCTCCGTAAG CGCCGCCGCC CCTAATCCCG CCACCCCAAA TCGGTCAAAC   
  
  
- GACTAAAGGT CCGGCTTACA AACAACTCCT CTCGAGTCCA ACCACCGAAG GTGCACCGTT TCGCGGTACC   
  
  
- TCTCTACTAC GAAATGACCG TACTGGCGGG TGAGCAGCGG TGTAGTCGAA CCTCCACAAT

+     TCCC-motif

| Site Name | Organism | Position | Strand | Matrix score. | sequence | function |
| --- | --- | --- | --- | --- | --- | --- |
| TCCC-motif | Spinacia oleracea | 3111 | - | 7 | TCTCCCT | part of a light responsive element |

>HU05G02245.1   
+ -Up\_Stream \_Len000ATAATT TATCTTTCTT TAATTTCGTT TCATGTAAAA AATTTCAGTC TCATCTGCGA   
  
  
+ TTGAATCTCT TTTCAATTCG CATCAATAAT TCATCCAATT CAAATGTGCC TTTCTTAATT TCATTACATT   
  
  
+ GGGTTGGTAT AAATGTTAGC ACGATAGATT GGAATAGCAA GGCTCTAATT CAATTATGAC TTGAATGTAT   
  
  
+ GCATGCTTTT ATCATTAGTT AATTAACATT TTCTGTATTG AAATCAGCTC GAAAGAGAAC CATATGTAGT   
  
  
+ TGTACTTGCG ACGATATATA TGTTTAGTTT AGTTAGTTCT TACCTGTTTT GGAATTGTTG GGGAGATCTG   
  
  
+ AGGAAGGAGC CCGCTTCAAT CAAAGTTCAT GGCAATAAGC CAAAGTGAGG TTCCATCCTA AAACCAATTG   
  
  
+ GCAATAGGAA GAGTAGCCCA CTTAACTTGT ATACTATGAC TCTTCTTTTC AAACTCCCAA TGTGGGACTT   
  
  
+ CTTTACTCAT GTGTGATATT CCCAACAGGA ATGATCAGTT CCACATGTTT TCATCTCTTT GGACTCGAAC   
  
  
+ TTCGTGTAAT TGTATATGCC ATGGCACAAA TAGTTTTGAT GGTGCCACCA AGTTGCTCAA TCAATGTAAT   
  
  
+ ACTGCTACTA TCTAACTTGC CTTGGTGGAC ATCAATAAGA TCACGAGATG AGGAGAACTG AATCTTGGCC   
  
  
+ AACCACGTCG TTTGACTTCT CTTACCAATA AAGATTGAAT AGACAGTGTT TCTCTCCCCC TTCCATTCTT   
  
  
+ CTTTTCTAGT GATGTCGAAA AAGACTTTAT ACATGTCCAG CAACGCTAGC CACAAAATAT TCCATTTCTG   
  
  
+ TAATCCACTC ATGTCAATTA AACTTGTATC ATCAAATGGT AGTTTCCGTA CTAATCACTA CATCTAGATA   
  
  
+ TTCCTTGATA AATTTAATTT GACTTTAAAG GCTCGATCTT TTTAAGAAAA GTTTTGTTGC TTACAACCTG   
  
  
+ TAAAGCCTAG AGAACAAAGC CAAAGAAGAA ATTTAAGTTC TATATATAGC ATAATCTCTC AAAAAATATA   
  
  
+ TTTTTTAAAT ATAGTGCGAT ATTGACTGAC CTAATATGGG TTATTACAGG AGATGGGGAG ATATCTACAT   
  
  
+ TAACTACTTG AACAACTTAA ATTTTCATTT TAACTCGGTC ATTAAGAACA CTAAGAACTT TATATATTAG   
  
  
+ TGATATCTCA GGCGTAAAAG AAACTACTTT GAATTAATCC ATGCAATCCT ACTCTTTATT TCCATCCGTT   
  
  
+ AAAATTTTCC TTCAAAACTC CCTTCTGTTC ATTATTAATT CAATATCGTA TTACACTTGT TGGATTATTT   
  
  
+ CTGTGATTTG ATCGTCATTT TCTATATATG AATAACTACA AAAATCATAG TCCGAGTACA TTAATATTAA   
  
  
+ TAAAATAGGA TGAGTGATAA CCTCATATAG TGCGTTTATC CCTACCACTA GGCTGGATTT CTGCATAAAG   
  
  
+ AAGAAATTGA ACATATGGAA AAAGAAATAA AGAGAAGCAA CTTCCTTGAG TGGGAGAAGA ATCGTAGAAA   
  
  
+ GTTAAACTTA TGAAAAAGAG CATTAACAAA AAGGAAATTA TAAGGCTTAC CGTTGAAGCC TCATATAATT   
  
  
+ TTGCAAGAGA CAGACAAAGA AAGTGTCTTT CAAATGTCAT ATTTCTCGAG CATGCATGAA AAATGTACTT   
  
  
+ CGGCTACAAT AGTAATAGTA TATATGTTGG CTTCATACAC TTGACCCCAA TGTATGTCAA CGTGACTAGT   
  
  
+ GACCACACAC ATCGTTGTCC CTGCCCGCCC CACTACCCAA ACTCCCCGCA CGCAAAGATA AAAAGCCCCC   
  
  
+ CAATTTTTAT TTGCCTCCCA AAAAGCCCAC CCGCGCTTTT TTATTTGCCT TCTTTTTTCC TACTTAAACC   
  
  
+ ACCTCCTCAA CGAACTTTGC AACATACATA CTAGTCTATA AGCAATTTAG AGGGAAGGGA AGCAACCTAA   
  
  
+ ACAGAGCGAG ATTGGCGGGG TGTGTTTGAG GGCTTGCGTT TTGGTGCAAT AGGAATGAAA GTGCCCATTT   
  
  
+ CAACCACCAT CCAAACTTGC AACTCCGGCA CCGGCAAACC ACTTTCCTTT GAGAATACTG CCCTACATAC   
  
  
+ CGCCTTTCAA GTTCCCAATA CCACCTACGA ACCCACCTCG GTTCTTGACC TCCAGCGCAG CCCTAGCCCC   
  
  
+ ACCTCCACCC AAAAACCGCC CTTTGCCGCC TCCAACCACC AGCCTGCCTC GGCTGATCTG GACAACTTGG   
  
  
+ ACGGGTGGGA TTCAATCCTA TCAGAGCTGG GGCTTAATGA CGAATTTACC CCTAATTCCA AACTCTGGTC   
  
  
+ CCAAATTAGT CCGTCTGATC CTCACCTCCC CCAACTCCCT GATTTCCCAA CATCTCAGCC GTTTGATCAT   
  
  
+ CACAACCCAC CCCCAATAAA CCTCCCATCA TCAGATTTCA ACCTCTGTGA TTTCTCCTAC AATCACAACC   
  
  
+ CCAATTTTGG GCCGTTCGAT CTTCATCACA ACCCGCATCA GAACAACCCG AACAACAATA ACTACGGCTT   
  
  
+ TGATTTCATA GACGACCTCA TCAAAGCAGC ACAGTCCCTC GAATCCAACG ACTCTCATCA AGTCCACCTG   
  
  
+ ATATTGGCGC GGCTCAATCA ACGGCTCAGA TCACCCACCG GCAAACCGCT CCAGCGGGCC GCCTACTACT   
  
  
+ TCAAGGAAGC CCTCATCGCC GCCGCGGCCG GCCCGCCCCG CCCCGCTCGG CTTTCATCGT ATGAGGTGGT   
  
  
+ GCAGACCATC CGAGCCTACA AGGCGTTTTC AGGAATTTCG CCAGTCTCTC TCTTCTCCAC TTTCGCCGCC   
  
  
+ AACCAGGCGA TTCTTGAGGC GGTGGACACG GCGGCCTTCA TCCACATCAT CGATTTCGAC ATCGGATTCG   
  
  
+ GCGGCCACTG GGCCTCGTTC CTCCGCGAAC TGGTCGACAA AGCTGATCCT GCGAAACTTA GTTCAGTGGT   
  
  
+ GTTACGAATC ACCGCCATCG TTCCCGAAGA ATTCGGGATC GAAAGCAAGC TAGTGAGAGA AAATCTGTCG   
  
  
+ CAATTTGCCC GAGATCTCAA CATCAACTTC CACATCGATT ATGTCTTATT TCAGAGCTTT GAAATCTTAT   
  
  
+ CCTTCAAATC TGTCAAATTC ATCGAAAGGG AGAAATTGGC GCTGCATCTT TCGCCGGCCG TGTTCCACCG   
  
  
+ GCTAGGGAGC GGAATTTCGA AGTTTATCGC TGATCTCCGA TCAATCTCGC CGAGCTCCGT CGTGGTGGTG   
  
  
+ GACAGAGATG TCGGGATTGA CATCGGAACG TCGTCGTTTA GCATGAATTT CGTCGCCGGA ATTGAATTCT   
  
  
+ ACACCGGGAT GCTGGAGTCG CTTGACGCAG CCACCGCCGG GGGGCTCATC GGCGGTGTGG ACTGCGTGAG   
  
  
+ ACGGATTGAG ACGTTTGTTC TCCACCCAAG GATCATGGCG GCGGTAGAGG CGGCGGCGTC GGCGTCGGCC   
  
  
+ GGGCGGAGGA CGGCGTGGAG GGAGGCATTC GCGGCGGCGG GGATTAGGGC GGTGGGGTTT AGCCAGTTTG   
  
  
+ CTGATTTCCA GGCCGAATGT TTGTTGAGGA GAGCTCAGGT TGGTGGCTTC CACGTGGCAA AGCGCCATGG   
  
  
+ AGAGATGATG CTTTACTGGC ATGACCGCCC ACTCGTCGCC ACATCAGCTT GGAGGTGTTA   

- -Up\_Stream \_Len000TATTAA ATAGAAAGAA ATTAAAGCAA AGTACATTTT TTAAAGTCAG AGTAGACGCT   
  
  
- AACTTAGAGA AAAGTTAAGC GTAGTTATTA AGTAGGTTAA GTTTACACGG AAAGAATTAA AGTAATGTAA   
  
  
- CCCAACCATA TTTACAATCG TGCTATCTAA CCTTATCGTT CCGAGATTAA GTTAATACTG AACTTACATA   
  
  
- CGTACGAAAA TAGTAATCAA TTAATTGTAA AAGACATAAC TTTAGTCGAG CTTTCTCTTG GTATACATCA   
  
  
- ACATGAACGC TGCTATATAT ACAAATCAAA TCAATCAAGA ATGGACAAAA CCTTAACAAC CCCTCTAGAC   
  
  
- TCCTTCCTCG GGCGAAGTTA GTTTCAAGTA CCGTTATTCG GTTTCACTCC AAGGTAGGAT TTTGGTTAAC   
  
  
- CGTTATCCTT CTCATCGGGT GAATTGAACA TATGATACTG AGAAGAAAAG TTTGAGGGTT ACACCCTGAA   
  
  
- GAAATGAGTA CACACTATAA GGGTTGTCCT TACTAGTCAA GGTGTACAAA AGTAGAGAAA CCTGAGCTTG   
  
  
- AAGCACATTA ACATATACGG TACCGTGTTT ATCAAAACTA CCACGGTGGT TCAACGAGTT AGTTACATTA   
  
  
- TGACGATGAT AGATTGAACG GAACCACCTG TAGTTATTCT AGTGCTCTAC TCCTCTTGAC TTAGAACCGG   
  
  
- TTGGTGCAGC AAACTGAAGA GAATGGTTAT TTCTAACTTA TCTGTCACAA AGAGAGGGGG AAGGTAAGAA   
  
  
- GAAAAGATCA CTACAGCTTT TTCTGAAATA TGTACAGGTC GTTGCGATCG GTGTTTTATA AGGTAAAGAC   
  
  
- ATTAGGTGAG TACAGTTAAT TTGAACATAG TAGTTTACCA TCAAAGGCAT GATTAGTGAT GTAGATCTAT   
  
  
- AAGGAACTAT TTAAATTAAA CTGAAATTTC CGAGCTAGAA AAATTCTTTT CAAAACAACG AATGTTGGAC   
  
  
- ATTTCGGATC TCTTGTTTCG GTTTCTTCTT TAAATTCAAG ATATATATCG TATTAGAGAG TTTTTTATAT   
  
  
- AAAAAATTTA TATCACGCTA TAACTGACTG GATTATACCC AATAATGTCC TCTACCCCTC TATAGATGTA   
  
  
- ATTGATGAAC TTGTTGAATT TAAAAGTAAA ATTGAGCCAG TAATTCTTGT GATTCTTGAA ATATATAATC   
  
  
- ACTATAGAGT CCGCATTTTC TTTGATGAAA CTTAATTAGG TACGTTAGGA TGAGAAATAA AGGTAGGCAA   
  
  
- TTTTAAAAGG AAGTTTTGAG GGAAGACAAG TAATAATTAA GTTATAGCAT AATGTGAACA ACCTAATAAA   
  
  
- GACACTAAAC TAGCAGTAAA AGATATATAC TTATTGATGT TTTTAGTATC AGGCTCATGT AATTATAATT   
  
  
- ATTTTATCCT ACTCACTATT GGAGTATATC ACGCAAATAG GGATGGTGAT CCGACCTAAA GACGTATTTC   
  
  
- TTCTTTAACT TGTATACCTT TTTCTTTATT TCTCTTCGTT GAAGGAACTC ACCCTCTTCT TAGCATCTTT   
  
  
- CAATTTGAAT ACTTTTTCTC GTAATTGTTT TTCCTTTAAT ATTCCGAATG GCAACTTCGG AGTATATTAA   
  
  
- AACGTTCTCT GTCTGTTTCT TTCACAGAAA GTTTACAGTA TAAAGAGCTC GTACGTACTT TTTACATGAA   
  
  
- GCCGATGTTA TCATTATCAT ATATACAACC GAAGTATGTG AACTGGGGTT ACATACAGTT GCACTGATCA   
  
  
- CTGGTGTGTG TAGCAACAGG GACGGGCGGG GTGATGGGTT TGAGGGGCGT GCGTTTCTAT TTTTCGGGGG   
  
  
- GTTAAAAATA AACGGAGGGT TTTTCGGGTG GGCGCGAAAA AATAAACGGA AGAAAAAAGG ATGAATTTGG   
  
  
- TGGAGGAGTT GCTTGAAACG TTGTATGTAT GATCAGATAT TCGTTAAATC TCCCTTCCCT TCGTTGGATT   
  
  
- TGTCTCGCTC TAACCGCCCC ACACAAACTC CCGAACGCAA AACCACGTTA TCCTTACTTT CACGGGTAAA   
  
  
- GTTGGTGGTA GGTTTGAACG TTGAGGCCGT GGCCGTTTGG TGAAAGGAAA CTCTTATGAC GGGATGTATG   
  
  
- GCGGAAAGTT CAAGGGTTAT GGTGGATGCT TGGGTGGAGC CAAGAACTGG AGGTCGCGTC GGGATCGGGG   
  
  
- TGGAGGTGGG TTTTTGGCGG GAAACGGCGG AGGTTGGTGG TCGGACGGAG CCGACTAGAC CTGTTGAACC   
  
  
- TGCCCACCCT AAGTTAGGAT AGTCTCGACC CCGAATTACT GCTTAAATGG GGATTAAGGT TTGAGACCAG   
  
  
- GGTTTAATCA GGCAGACTAG GAGTGGAGGG GGTTGAGGGA CTAAAGGGTT GTAGAGTCGG CAAACTAGTA   
  
  
- GTGTTGGGTG GGGGTTATTT GGAGGGTAGT AGTCTAAAGT TGGAGACACT AAAGAGGATG TTAGTGTTGG   
  
  
- GGTTAAAACC CGGCAAGCTA GAAGTAGTGT TGGGCGTAGT CTTGTTGGGC TTGTTGTTAT TGATGCCGAA   
  
  
- ACTAAAGTAT CTGCTGGAGT AGTTTCGTCG TGTCAGGGAG CTTAGGTTGC TGAGAGTAGT TCAGGTGGAC   
  
  
- TATAACCGCG CCGAGTTAGT TGCCGAGTCT AGTGGGTGGC CGTTTGGCGA GGTCGCCCGG CGGATGATGA   
  
  
- AGTTCCTTCG GGAGTAGCGG CGGCGCCGGC CGGGCGGGGC GGGGCGAGCC GAAAGTAGCA TACTCCACCA   
  
  
- CGTCTGGTAG GCTCGGATGT TCCGCAAAAG TCCTTAAAGC GGTCAGAGAG AGAAGAGGTG AAAGCGGCGG   
  
  
- TTGGTCCGCT AAGAACTCCG CCACCTGTGC CGCCGGAAGT AGGTGTAGTA GCTAAAGCTG TAGCCTAAGC   
  
  
- CGCCGGTGAC CCGGAGCAAG GAGGCGCTTG ACCAGCTGTT TCGACTAGGA CGCTTTGAAT CAAGTCACCA   
  
  
- CAATGCTTAG TGGCGGTAGC AAGGGCTTCT TAAGCCCTAG CTTTCGTTCG ATCACTCTCT TTTAGACAGC   
  
  
- GTTAAACGGG CTCTAGAGTT GTAGTTGAAG GTGTAGCTAA TACAGAATAA AGTCTCGAAA CTTTAGAATA   
  
  
- GGAAGTTTAG ACAGTTTAAG TAGCTTTCCC TCTTTAACCG CGACGTAGAA AGCGGCCGGC ACAAGGTGGC   
  
  
- CGATCCCTCG CCTTAAAGCT TCAAATAGCG ACTAGAGGCT AGTTAGAGCG GCTCGAGGCA GCACCACCAC   
  
  
- CTGTCTCTAC AGCCCTAACT GTAGCCTTGC AGCAGCAAAT CGTACTTAAA GCAGCGGCCT TAACTTAAGA   
  
  
- TGTGGCCCTA CGACCTCAGC GAACTGCGTC GGTGGCGGCC CCCCGAGTAG CCGCCACACC TGACGCACTC   
  
  
- TGCCTAACTC TGCAAACAAG AGGTGGGTTC CTAGTACCGC CGCCATCTCC GCCGCCGCAG CCGCAGCCGG   
  
  
- CCCGCCTCCT GCCGCACCTC CCTCCGTAAG CGCCGCCGCC CCTAATCCCG CCACCCCAAA TCGGTCAAAC   
  
  
- GACTAAAGGT CCGGCTTACA AACAACTCCT CTCGAGTCCA ACCACCGAAG GTGCACCGTT TCGCGGTACC   
  
  
- TCTCTACTAC GAAATGACCG TACTGGCGGG TGAGCAGCGG TGTAGTCGAA CCTCCACAAT

+     TCT-motif

| Site Name | Organism | Position | Strand | Matrix score. | sequence | function |
| --- | --- | --- | --- | --- | --- | --- |
| TCT-motif | Arabidopsis thaliana | 724 | + | 6 | TCTTAC | part of a light responsive element |
| TCT-motif | Arabidopsis thaliana | 322 | + | 6 | TCTTAC | part of a light responsive element |

>HU05G02245.1   
+ -Up\_Stream \_Len000ATAATT TATCTTTCTT TAATTTCGTT TCATGTAAAA AATTTCAGTC TCATCTGCGA   
  
  
+ TTGAATCTCT TTTCAATTCG CATCAATAAT TCATCCAATT CAAATGTGCC TTTCTTAATT TCATTACATT   
  
  
+ GGGTTGGTAT AAATGTTAGC ACGATAGATT GGAATAGCAA GGCTCTAATT CAATTATGAC TTGAATGTAT   
  
  
+ GCATGCTTTT ATCATTAGTT AATTAACATT TTCTGTATTG AAATCAGCTC GAAAGAGAAC CATATGTAGT   
  
  
+ TGTACTTGCG ACGATATATA TGTTTAGTTT AGTTAGTTCT TACCTGTTTT GGAATTGTTG GGGAGATCTG   
  
  
+ AGGAAGGAGC CCGCTTCAAT CAAAGTTCAT GGCAATAAGC CAAAGTGAGG TTCCATCCTA AAACCAATTG   
  
  
+ GCAATAGGAA GAGTAGCCCA CTTAACTTGT ATACTATGAC TCTTCTTTTC AAACTCCCAA TGTGGGACTT   
  
  
+ CTTTACTCAT GTGTGATATT CCCAACAGGA ATGATCAGTT CCACATGTTT TCATCTCTTT GGACTCGAAC   
  
  
+ TTCGTGTAAT TGTATATGCC ATGGCACAAA TAGTTTTGAT GGTGCCACCA AGTTGCTCAA TCAATGTAAT   
  
  
+ ACTGCTACTA TCTAACTTGC CTTGGTGGAC ATCAATAAGA TCACGAGATG AGGAGAACTG AATCTTGGCC   
  
  
+ AACCACGTCG TTTGACTTCT CTTACCAATA AAGATTGAAT AGACAGTGTT TCTCTCCCCC TTCCATTCTT   
  
  
+ CTTTTCTAGT GATGTCGAAA AAGACTTTAT ACATGTCCAG CAACGCTAGC CACAAAATAT TCCATTTCTG   
  
  
+ TAATCCACTC ATGTCAATTA AACTTGTATC ATCAAATGGT AGTTTCCGTA CTAATCACTA CATCTAGATA   
  
  
+ TTCCTTGATA AATTTAATTT GACTTTAAAG GCTCGATCTT TTTAAGAAAA GTTTTGTTGC TTACAACCTG   
  
  
+ TAAAGCCTAG AGAACAAAGC CAAAGAAGAA ATTTAAGTTC TATATATAGC ATAATCTCTC AAAAAATATA   
  
  
+ TTTTTTAAAT ATAGTGCGAT ATTGACTGAC CTAATATGGG TTATTACAGG AGATGGGGAG ATATCTACAT   
  
  
+ TAACTACTTG AACAACTTAA ATTTTCATTT TAACTCGGTC ATTAAGAACA CTAAGAACTT TATATATTAG   
  
  
+ TGATATCTCA GGCGTAAAAG AAACTACTTT GAATTAATCC ATGCAATCCT ACTCTTTATT TCCATCCGTT   
  
  
+ AAAATTTTCC TTCAAAACTC CCTTCTGTTC ATTATTAATT CAATATCGTA TTACACTTGT TGGATTATTT   
  
  
+ CTGTGATTTG ATCGTCATTT TCTATATATG AATAACTACA AAAATCATAG TCCGAGTACA TTAATATTAA   
  
  
+ TAAAATAGGA TGAGTGATAA CCTCATATAG TGCGTTTATC CCTACCACTA GGCTGGATTT CTGCATAAAG   
  
  
+ AAGAAATTGA ACATATGGAA AAAGAAATAA AGAGAAGCAA CTTCCTTGAG TGGGAGAAGA ATCGTAGAAA   
  
  
+ GTTAAACTTA TGAAAAAGAG CATTAACAAA AAGGAAATTA TAAGGCTTAC CGTTGAAGCC TCATATAATT   
  
  
+ TTGCAAGAGA CAGACAAAGA AAGTGTCTTT CAAATGTCAT ATTTCTCGAG CATGCATGAA AAATGTACTT   
  
  
+ CGGCTACAAT AGTAATAGTA TATATGTTGG CTTCATACAC TTGACCCCAA TGTATGTCAA CGTGACTAGT   
  
  
+ GACCACACAC ATCGTTGTCC CTGCCCGCCC CACTACCCAA ACTCCCCGCA CGCAAAGATA AAAAGCCCCC   
  
  
+ CAATTTTTAT TTGCCTCCCA AAAAGCCCAC CCGCGCTTTT TTATTTGCCT TCTTTTTTCC TACTTAAACC   
  
  
+ ACCTCCTCAA CGAACTTTGC AACATACATA CTAGTCTATA AGCAATTTAG AGGGAAGGGA AGCAACCTAA   
  
  
+ ACAGAGCGAG ATTGGCGGGG TGTGTTTGAG GGCTTGCGTT TTGGTGCAAT AGGAATGAAA GTGCCCATTT   
  
  
+ CAACCACCAT CCAAACTTGC AACTCCGGCA CCGGCAAACC ACTTTCCTTT GAGAATACTG CCCTACATAC   
  
  
+ CGCCTTTCAA GTTCCCAATA CCACCTACGA ACCCACCTCG GTTCTTGACC TCCAGCGCAG CCCTAGCCCC   
  
  
+ ACCTCCACCC AAAAACCGCC CTTTGCCGCC TCCAACCACC AGCCTGCCTC GGCTGATCTG GACAACTTGG   
  
  
+ ACGGGTGGGA TTCAATCCTA TCAGAGCTGG GGCTTAATGA CGAATTTACC CCTAATTCCA AACTCTGGTC   
  
  
+ CCAAATTAGT CCGTCTGATC CTCACCTCCC CCAACTCCCT GATTTCCCAA CATCTCAGCC GTTTGATCAT   
  
  
+ CACAACCCAC CCCCAATAAA CCTCCCATCA TCAGATTTCA ACCTCTGTGA TTTCTCCTAC AATCACAACC   
  
  
+ CCAATTTTGG GCCGTTCGAT CTTCATCACA ACCCGCATCA GAACAACCCG AACAACAATA ACTACGGCTT   
  
  
+ TGATTTCATA GACGACCTCA TCAAAGCAGC ACAGTCCCTC GAATCCAACG ACTCTCATCA AGTCCACCTG   
  
  
+ ATATTGGCGC GGCTCAATCA ACGGCTCAGA TCACCCACCG GCAAACCGCT CCAGCGGGCC GCCTACTACT   
  
  
+ TCAAGGAAGC CCTCATCGCC GCCGCGGCCG GCCCGCCCCG CCCCGCTCGG CTTTCATCGT ATGAGGTGGT   
  
  
+ GCAGACCATC CGAGCCTACA AGGCGTTTTC AGGAATTTCG CCAGTCTCTC TCTTCTCCAC TTTCGCCGCC   
  
  
+ AACCAGGCGA TTCTTGAGGC GGTGGACACG GCGGCCTTCA TCCACATCAT CGATTTCGAC ATCGGATTCG   
  
  
+ GCGGCCACTG GGCCTCGTTC CTCCGCGAAC TGGTCGACAA AGCTGATCCT GCGAAACTTA GTTCAGTGGT   
  
  
+ GTTACGAATC ACCGCCATCG TTCCCGAAGA ATTCGGGATC GAAAGCAAGC TAGTGAGAGA AAATCTGTCG   
  
  
+ CAATTTGCCC GAGATCTCAA CATCAACTTC CACATCGATT ATGTCTTATT TCAGAGCTTT GAAATCTTAT   
  
  
+ CCTTCAAATC TGTCAAATTC ATCGAAAGGG AGAAATTGGC GCTGCATCTT TCGCCGGCCG TGTTCCACCG   
  
  
+ GCTAGGGAGC GGAATTTCGA AGTTTATCGC TGATCTCCGA TCAATCTCGC CGAGCTCCGT CGTGGTGGTG   
  
  
+ GACAGAGATG TCGGGATTGA CATCGGAACG TCGTCGTTTA GCATGAATTT CGTCGCCGGA ATTGAATTCT   
  
  
+ ACACCGGGAT GCTGGAGTCG CTTGACGCAG CCACCGCCGG GGGGCTCATC GGCGGTGTGG ACTGCGTGAG   
  
  
+ ACGGATTGAG ACGTTTGTTC TCCACCCAAG GATCATGGCG GCGGTAGAGG CGGCGGCGTC GGCGTCGGCC   
  
  
+ GGGCGGAGGA CGGCGTGGAG GGAGGCATTC GCGGCGGCGG GGATTAGGGC GGTGGGGTTT AGCCAGTTTG   
  
  
+ CTGATTTCCA GGCCGAATGT TTGTTGAGGA GAGCTCAGGT TGGTGGCTTC CACGTGGCAA AGCGCCATGG   
  
  
+ AGAGATGATG CTTTACTGGC ATGACCGCCC ACTCGTCGCC ACATCAGCTT GGAGGTGTTA   

- -Up\_Stream \_Len000TATTAA ATAGAAAGAA ATTAAAGCAA AGTACATTTT TTAAAGTCAG AGTAGACGCT   
  
  
- AACTTAGAGA AAAGTTAAGC GTAGTTATTA AGTAGGTTAA GTTTACACGG AAAGAATTAA AGTAATGTAA   
  
  
- CCCAACCATA TTTACAATCG TGCTATCTAA CCTTATCGTT CCGAGATTAA GTTAATACTG AACTTACATA   
  
  
- CGTACGAAAA TAGTAATCAA TTAATTGTAA AAGACATAAC TTTAGTCGAG CTTTCTCTTG GTATACATCA   
  
  
- ACATGAACGC TGCTATATAT ACAAATCAAA TCAATCAAGA ATGGACAAAA CCTTAACAAC CCCTCTAGAC   
  
  
- TCCTTCCTCG GGCGAAGTTA GTTTCAAGTA CCGTTATTCG GTTTCACTCC AAGGTAGGAT TTTGGTTAAC   
  
  
- CGTTATCCTT CTCATCGGGT GAATTGAACA TATGATACTG AGAAGAAAAG TTTGAGGGTT ACACCCTGAA   
  
  
- GAAATGAGTA CACACTATAA GGGTTGTCCT TACTAGTCAA GGTGTACAAA AGTAGAGAAA CCTGAGCTTG   
  
  
- AAGCACATTA ACATATACGG TACCGTGTTT ATCAAAACTA CCACGGTGGT TCAACGAGTT AGTTACATTA   
  
  
- TGACGATGAT AGATTGAACG GAACCACCTG TAGTTATTCT AGTGCTCTAC TCCTCTTGAC TTAGAACCGG   
  
  
- TTGGTGCAGC AAACTGAAGA GAATGGTTAT TTCTAACTTA TCTGTCACAA AGAGAGGGGG AAGGTAAGAA   
  
  
- GAAAAGATCA CTACAGCTTT TTCTGAAATA TGTACAGGTC GTTGCGATCG GTGTTTTATA AGGTAAAGAC   
  
  
- ATTAGGTGAG TACAGTTAAT TTGAACATAG TAGTTTACCA TCAAAGGCAT GATTAGTGAT GTAGATCTAT   
  
  
- AAGGAACTAT TTAAATTAAA CTGAAATTTC CGAGCTAGAA AAATTCTTTT CAAAACAACG AATGTTGGAC   
  
  
- ATTTCGGATC TCTTGTTTCG GTTTCTTCTT TAAATTCAAG ATATATATCG TATTAGAGAG TTTTTTATAT   
  
  
- AAAAAATTTA TATCACGCTA TAACTGACTG GATTATACCC AATAATGTCC TCTACCCCTC TATAGATGTA   
  
  
- ATTGATGAAC TTGTTGAATT TAAAAGTAAA ATTGAGCCAG TAATTCTTGT GATTCTTGAA ATATATAATC   
  
  
- ACTATAGAGT CCGCATTTTC TTTGATGAAA CTTAATTAGG TACGTTAGGA TGAGAAATAA AGGTAGGCAA   
  
  
- TTTTAAAAGG AAGTTTTGAG GGAAGACAAG TAATAATTAA GTTATAGCAT AATGTGAACA ACCTAATAAA   
  
  
- GACACTAAAC TAGCAGTAAA AGATATATAC TTATTGATGT TTTTAGTATC AGGCTCATGT AATTATAATT   
  
  
- ATTTTATCCT ACTCACTATT GGAGTATATC ACGCAAATAG GGATGGTGAT CCGACCTAAA GACGTATTTC   
  
  
- TTCTTTAACT TGTATACCTT TTTCTTTATT TCTCTTCGTT GAAGGAACTC ACCCTCTTCT TAGCATCTTT   
  
  
- CAATTTGAAT ACTTTTTCTC GTAATTGTTT TTCCTTTAAT ATTCCGAATG GCAACTTCGG AGTATATTAA   
  
  
- AACGTTCTCT GTCTGTTTCT TTCACAGAAA GTTTACAGTA TAAAGAGCTC GTACGTACTT TTTACATGAA   
  
  
- GCCGATGTTA TCATTATCAT ATATACAACC GAAGTATGTG AACTGGGGTT ACATACAGTT GCACTGATCA   
  
  
- CTGGTGTGTG TAGCAACAGG GACGGGCGGG GTGATGGGTT TGAGGGGCGT GCGTTTCTAT TTTTCGGGGG   
  
  
- GTTAAAAATA AACGGAGGGT TTTTCGGGTG GGCGCGAAAA AATAAACGGA AGAAAAAAGG ATGAATTTGG   
  
  
- TGGAGGAGTT GCTTGAAACG TTGTATGTAT GATCAGATAT TCGTTAAATC TCCCTTCCCT TCGTTGGATT   
  
  
- TGTCTCGCTC TAACCGCCCC ACACAAACTC CCGAACGCAA AACCACGTTA TCCTTACTTT CACGGGTAAA   
  
  
- GTTGGTGGTA GGTTTGAACG TTGAGGCCGT GGCCGTTTGG TGAAAGGAAA CTCTTATGAC GGGATGTATG   
  
  
- GCGGAAAGTT CAAGGGTTAT GGTGGATGCT TGGGTGGAGC CAAGAACTGG AGGTCGCGTC GGGATCGGGG   
  
  
- TGGAGGTGGG TTTTTGGCGG GAAACGGCGG AGGTTGGTGG TCGGACGGAG CCGACTAGAC CTGTTGAACC   
  
  
- TGCCCACCCT AAGTTAGGAT AGTCTCGACC CCGAATTACT GCTTAAATGG GGATTAAGGT TTGAGACCAG   
  
  
- GGTTTAATCA GGCAGACTAG GAGTGGAGGG GGTTGAGGGA CTAAAGGGTT GTAGAGTCGG CAAACTAGTA   
  
  
- GTGTTGGGTG GGGGTTATTT GGAGGGTAGT AGTCTAAAGT TGGAGACACT AAAGAGGATG TTAGTGTTGG   
  
  
- GGTTAAAACC CGGCAAGCTA GAAGTAGTGT TGGGCGTAGT CTTGTTGGGC TTGTTGTTAT TGATGCCGAA   
  
  
- ACTAAAGTAT CTGCTGGAGT AGTTTCGTCG TGTCAGGGAG CTTAGGTTGC TGAGAGTAGT TCAGGTGGAC   
  
  
- TATAACCGCG CCGAGTTAGT TGCCGAGTCT AGTGGGTGGC CGTTTGGCGA GGTCGCCCGG CGGATGATGA   
  
  
- AGTTCCTTCG GGAGTAGCGG CGGCGCCGGC CGGGCGGGGC GGGGCGAGCC GAAAGTAGCA TACTCCACCA   
  
  
- CGTCTGGTAG GCTCGGATGT TCCGCAAAAG TCCTTAAAGC GGTCAGAGAG AGAAGAGGTG AAAGCGGCGG   
  
  
- TTGGTCCGCT AAGAACTCCG CCACCTGTGC CGCCGGAAGT AGGTGTAGTA GCTAAAGCTG TAGCCTAAGC
[truncated: 98,019 more chars]
