# Supplementary material for: Genome-Wide Identification and Expression Pattern of the GRAS Gene Family in Pitaya (Selenicereus undatus L.)
Source: Biology (Basel). 2022 Dec 21;12(1):11. doi: 10.3390/biology12010011 (PMC9854919; doi:10.3390/biology12010011)
Supplement: Supplementary file 1 [file biology-12-00011-s001.zip › Supplementary file S5/HU06G00029.1_plantcare.html]

Content-Type: text/html; charset=ISO-8859-1


PlantCARE


Webmaster Firefox specific output  
To save the result:
click on the frame with the right mouse button and save the source code as a text file with extension .html  
REFERENCE:PlantCARE: a database of plant cis-acting regulatory elements and a portal to tools for in silico analysis of promoter sequences.  
Lescot, M., Déhais, P., Moreau, Y., De Moor, B., Rouzé ,P.,and Rombauts, S.  
Nucleic Acids Res., Database issue(2002), 30(1):325-327.   


---

>HU06G00029.1   
+ +Up\_Stream \_Len000TGATAA TTATTTTTAT TGATTTAGGA AAGGATATCA AAGTATATTT TGTTTTTAGG   
  
  
+ TAGTTAAGAG ATTCTGTTGC CAAAAAAAAA ATCAAGATAT TATATATTTT TAATTATGAC AGTAATTATT   
  
  
+ TTATTGAAAA TCCAACGGTT GTGATTATAT AATCAAATAA TCTAAAGGTT AAAAATATCA AATTTATTAG   
  
  
+ AAAATTCGAA AAAGTCACGT CATCACAACA TTGCTTTTAT ATATAGTATA GATTCCTTCG ACTTCTAAAC   
  
  
+ TAATCACTTG AAATTAGAAA TTTGAAACTT TCTAATATAA ATGCATGGTC TTCTTTCCAA GATTATAATC   
  
  
+ CGCAATTGCT TAAATGTAGG GTACAAATAT CAAATTTAGA AGTGACTATT GAAGCCATGA AAGATATTGT   
  
  
+ GATCGTATTG AGGGTGGTTG TGAGCAATAG GATGAAACAG GAAAAAAAAA CCCTAATGTT TTCAAATTTA   
  
  
+ TGGGTGGCTT TGGTAGTAGA TAAGTGAATA GTGTGCAAGG GAATGGCACC AATATGTTTG GATGGAACGA   
  
  
+ TGAGAGTAAG ACGTTAGGAA AGCAACAAGA GAAGGGAAAA GGCAGTAGGG AGAGAATTAT GCGTGTGAAT   
  
  
+ AATATTATAA CAGACGCTTT CGTATGATTA TTAAATACAA GACTTTGAGC ATACATATAA ACTTGTAACT   
  
  
+ TTCTTTACCT ATTGTCAATT AATTTTTGAA TTGAATCATC TGCGACTTAT GCATACAAGA TGATATGATG   
  
  
+ CATATTGGGC CAAGCATTTG ATCTCATGTC CCTTATGGAT CTAGATCCCA ATAGACAAAC TTGCATAAGT   
  
  
+ CAAGGGATGT GTTTCTAATA CAAAGGCTTT GTACTCAAGC AATTAGTTTT GAATTCATTT TCAGTAGTTT   
  
  
+ AATTTCCAAA TCTTATAATT TTTACAATGG CATAAATTTT TAGGAGTTTT GAGTAAATAA ATTATATACA   
  
  
+ ACGAAGATGT CATTTACGAA TTAATATGAC GCACAAAAAA AAAGAAAAAA AATTAATGGT AGCTTCTATA   
  
  
+ CTTGACTCCA TGTTGACAAT TCCCTCTCTC ACATAATGGT TAAAACTTGC AACATCATCC AGGACCCATA   
  
  
+ TGACATGGGT GGTGGTAGAC AGTGTGTTGT GGTAGCCTCA CTCCATGCTT GCCAATTTGT ATCATTGCGC   
  
  
+ TAGTCTTATT CGTCCTTAGA GATAATTGAC TAAGCTCTAA TGCTCTATCG TGGCTGCTGC AATATAAATG   
  
  
+ ATTTTTTGAC CTCAAAATGA AGACAGCTAG TTGTAAATTA AGAATCTTCG TCCCTTCATT TTTTGTGTAC   
  
  
+ GTGTGTATCT GCGTATGTGT TGAGAGACTA GCGGACTATT GATTGAGTTT CAATCAAAAA AGATGACCAT   
  
  
+ CTTAAGTATT TTTAATTACG CAAACTTCTT CATTTAAAAA AATGTTACTT TTATTTGAAA GAAATTGTGA   
  
  
+ ATAATACAAG GAATAGTTCA ATTAATATAT AATCCATAAC ATATTACCTC CTATAGGCAA AAAGAAGATT   
  
  
+ ATCGTATTAA TTTGGGATCA TCCCACCCTA AATTTAAAGA AACCAAAACA AATTACTAAG CAATATCTTT   
  
  
+ CTGTGTGAAA AAATAGTCAG AAACAAAGCG ATCCGACCGG CCAAATCGAG ATAAACACAG CCAAAGAACC   
  
  
+ GAAGTTGGAC CAAAGTGAGC CGAGTTGAAA GGCATTCATA TGGGGTTTAA TCATAACACA AACATAATTA   
  
  
+ GATTGATTGA CGTCTTTAAT CAGCGTTTTT GTCTTTGTTC GGTGCTCCCC TCACTCGCCC CTCCGCTCTT   
  
  
+ TAAAGCTTAC CTCACTCCCC CCATTCCCAC TCCTTTTCTC TCTCCTCTCT CGCTCTCACA CGCTCCGTCT   
  
  
+ GTCAGCCCCT TGTCATCTTC CCCCGTCTTC CTCGCAAAAA TCTCCAGCAA AATTTATAGC CGTTGTTGTG   
  
  
+ CCCATGGTGA TCTGATGTGA GAAGAGATTC AGCATTTAAG ATTATTGGGT CCAGATGGCT TATATGTGCG   
  
  
+ CCGACAGTGG TAATCTCATG GCTATTGCCC AACAAGTCAT CAAGCAAAAA CAACAGCAAG AACAGCAGCA   
  
  
+ ACACCAACAG CCTCATCCCC AGTTCGTCGG GTCGAATAAT AACCCCTTTT GCACCAGCCC ATGGCCCGGA   
  
  
+ CCCGTCCCTT CATCCGCCAT GTCTGCCAGC CCTCCGCCCC CTCTTGGGTT CTCCGCCGCC GCGTTTCCGG   
  
  
+ ACCCGTTTCA GGTGGGCCCG CCCTGCCCGG ATGGGGCTGA ACCGGGTTTC CAGTTTGCCC ATTTGGACCA   
  
  
+ CCACTCGAGT GGTTTCCGGT TTGCTGATTT TTGCGGTGGG GGTGGTGGTG AGTTTGACTC GGATGAGTGG   
  
  
+ ATGGAGAGTT TGATGGGTGG CGGCGGCGGT GACTCGCGGC GACTGAGAGT TCTAACCTCC AATCCCATTG   
  
  
+ CGACGCGTGG CAAGGGTCCG GTGATTTCGG TCTCTATGGT GCTGATCCGT TTGCGACGTG TTCGGAAGAC   
  
  
+ CGTTGCAGCC GCTATTCACG CTCAGCCACT GCCGCAACAG CAGCCGTCGT GGGTCCCGCC TTCTCCGCCG   
  
  
+ AAAGACACGA AGCAGTCGGC CCCACCTCAA AAGCAAAGCG ACGCCGCTGG ACCCTCGTTC TCATCCGAGG   
  
  
+ AGGAACCAGA GCCGTCATGG CCGCCGCTAA TTGCGGCGTT GTTGGAGTGC GCCAAACTCG CCGAGTCCGA   
  
  
+ CCCGGAACAC GCTGTCAAAT CGGTGATTCG ACTCAGAGAC TCAGTCTCAC AGCAGGGAGT TCCAATCCAG   
  
  
+ AGAGTGTCCT ATTACTTCTC CGAGGCACTC TACCACCGCC TTTCTCTCTC CTCCGCCCCT CAACCTCTCC   
  
  
+ CAGAAACGAC GTCGTCTGAG GAGTACACTC TCTCTTACAA GGCCCTGAAC GACGCGTGCC CGTACCATAA   
  
  
+ ATTCGCTCAC TTGACCGCAA ATCAGGCGAT TCTAGAAGCC ACTGATTCAG CCAAGAAGAT CCACATCGTT   
  
  
+ GATTTTGGGA TAATCCAGGG AGTCCAATGG GCTGCTCTGC TACAAGCCCT GGCAACCCGG CCAGCCGGGA   
  
  
+ AGCCCGAATT GATTCGAATA TCCGGAATTC CTGCTCCGGG TCTGGGGCAA TCTCCGGCGG CATCGCTGCT   
  
  
+ CGCAACTGGG AATCGCCTCC GTGAGTTCGC GAAGGTTCTA GATCTCAAGT TCGAGTTTGA GCCGGTACTC   
  
  
+ ACTCCAATCA GAGAGCTAAA CGGGTCGAAC TTCCGGGTCG ACCCAGATGA GATACTAGCT GTCAATTTCA   
  
  
+ TGCTTCAATT GTACAACCTA TTAGACGAAA CCCTGGTCGC TGTCGACGCC GCCCTGAAGC TGGCCAAGTC   
  
  
+ ATTGAACCCC AAAATCGTCA CTTTGGGTGA GTACGAGGCG GGTCTGAATC GGGCAGGTTT CTTGACCCGG   
  
  
+ TTCAAGACGG CCCTCAAGTA CTATTTGGCA ATGTTTGAAT CGGTTGAGCC CAACTTGGCC CGGGACTCGC   
  
  
+ CCGAGAGGGC TCAGGTTGAG AGGCTGTTAT TGGGCCGGCG GATCATGGGC TTAGTTGGGC CGGAGTTACC   
  
  
+ GGGAACGAGA AGGGAAAGGA TGGAGTGTAA GGAACAATGG AAGGGTTTAA TGGAAAGTGC CGGTTTCGAG   
  
  
+ CCGTGCCAAA GCAGAAACTA CGTCAGTGAA AAAGACAAGT TTTACCACTC CAGATTGAAT TACAGAGAGT   
  
  
+ G  

- +Up\_Stream \_Len000ACTATT AATAAAAATA ACTAAATCCT TTCCTATAGT TTCATATAAA ACAAAAATCC   
  
  
- ATCAATTCTC TAAGACAACG GTTTTTTTTT TAGTTCTATA ATATATAAAA ATTAATACTG TCATTAATAA   
  
  
- AATAACTTTT AGGTTGCCAA CACTAATATA TTAGTTTATT AGATTTCCAA TTTTTATAGT TTAAATAATC   
  
  
- TTTTAAGCTT TTTCAGTGCA GTAGTGTTGT AACGAAAATA TATATCATAT CTAAGGAAGC TGAAGATTTG   
  
  
- ATTAGTGAAC TTTAATCTTT AAACTTTGAA AGATTATATT TACGTACCAG AAGAAAGGTT CTAATATTAG   
  
  
- GCGTTAACGA ATTTACATCC CATGTTTATA GTTTAAATCT TCACTGATAA CTTCGGTACT TTCTATAACA   
  
  
- CTAGCATAAC TCCCACCAAC ACTCGTTATC CTACTTTGTC CTTTTTTTTT GGGATTACAA AAGTTTAAAT   
  
  
- ACCCACCGAA ACCATCATCT ATTCACTTAT CACACGTTCC CTTACCGTGG TTATACAAAC CTACCTTGCT   
  
  
- ACTCTCATTC TGCAATCCTT TCGTTGTTCT CTTCCCTTTT CCGTCATCCC TCTCTTAATA CGCACACTTA   
  
  
- TTATAATATT GTCTGCGAAA GCATACTAAT AATTTATGTT CTGAAACTCG TATGTATATT TGAACATTGA   
  
  
- AAGAAATGGA TAACAGTTAA TTAAAAACTT AACTTAGTAG ACGCTGAATA CGTATGTTCT ACTATACTAC   
  
  
- GTATAACCCG GTTCGTAAAC TAGAGTACAG GGAATACCTA GATCTAGGGT TATCTGTTTG AACGTATTCA   
  
  
- GTTCCCTACA CAAAGATTAT GTTTCCGAAA CATGAGTTCG TTAATCAAAA CTTAAGTAAA AGTCATCAAA   
  
  
- TTAAAGGTTT AGAATATTAA AAATGTTACC GTATTTAAAA ATCCTCAAAA CTCATTTATT TAATATATGT   
  
  
- TGCTTCTACA GTAAATGCTT AATTATACTG CGTGTTTTTT TTTCTTTTTT TTAATTACCA TCGAAGATAT   
  
  
- GAACTGAGGT ACAACTGTTA AGGGAGAGAG TGTATTACCA ATTTTGAACG TTGTAGTAGG TCCTGGGTAT   
  
  
- ACTGTACCCA CCACCATCTG TCACACAACA CCATCGGAGT GAGGTACGAA CGGTTAAACA TAGTAACGCG   
  
  
- ATCAGAATAA GCAGGAATCT CTATTAACTG ATTCGAGATT ACGAGATAGC ACCGACGACG TTATATTTAC   
  
  
- TAAAAAACTG GAGTTTTACT TCTGTCGATC AACATTTAAT TCTTAGAAGC AGGGAAGTAA AAAACACATG   
  
  
- CACACATAGA CGCATACACA ACTCTCTGAT CGCCTGATAA CTAACTCAAA GTTAGTTTTT TCTACTGGTA   
  
  
- GAATTCATAA AAATTAATGC GTTTGAAGAA GTAAATTTTT TTACAATGAA AATAAACTTT CTTTAACACT   
  
  
- TATTATGTTC CTTATCAAGT TAATTATATA TTAGGTATTG TATAATGGAG GATATCCGTT TTTCTTCTAA   
  
  
- TAGCATAATT AAACCCTAGT AGGGTGGGAT TTAAATTTCT TTGGTTTTGT TTAATGATTC GTTATAGAAA   
  
  
- GACACACTTT TTTATCAGTC TTTGTTTCGC TAGGCTGGCC GGTTTAGCTC TATTTGTGTC GGTTTCTTGG   
  
  
- CTTCAACCTG GTTTCACTCG GCTCAACTTT CCGTAAGTAT ACCCCAAATT AGTATTGTGT TTGTATTAAT   
  
  
- CTAACTAACT GCAGAAATTA GTCGCAAAAA CAGAAACAAG CCACGAGGGG AGTGAGCGGG GAGGCGAGAA   
  
  
- ATTTCGAATG GAGTGAGGGG GGTAAGGGTG AGGAAAAGAG AGAGGAGAGA GCGAGAGTGT GCGAGGCAGA   
  
  
- CAGTCGGGGA ACAGTAGAAG GGGGCAGAAG GAGCGTTTTT AGAGGTCGTT TTAAATATCG GCAACAACAC   
  
  
- GGGTACCACT AGACTACACT CTTCTCTAAG TCGTAAATTC TAATAACCCA GGTCTACCGA ATATACACGC   
  
  
- GGCTGTCACC ATTAGAGTAC CGATAACGGG TTGTTCAGTA GTTCGTTTTT GTTGTCGTTC TTGTCGTCGT   
  
  
- TGTGGTTGTC GGAGTAGGGG TCAAGCAGCC CAGCTTATTA TTGGGGAAAA CGTGGTCGGG TACCGGGCCT   
  
  
- GGGCAGGGAA GTAGGCGGTA CAGACGGTCG GGAGGCGGGG GAGAACCCAA GAGGCGGCGG CGCAAAGGCC   
  
  
- TGGGCAAAGT CCACCCGGGC GGGACGGGCC TACCCCGACT TGGCCCAAAG GTCAAACGGG TAAACCTGGT   
  
  
- GGTGAGCTCA CCAAAGGCCA AACGACTAAA AACGCCACCC CCACCACCAC TCAAACTGAG CCTACTCACC   
  
  
- TACCTCTCAA ACTACCCACC GCCGCCGCCA CTGAGCGCCG CTGACTCTCA AGATTGGAGG TTAGGGTAAC   
  
  
- GCTGCGCACC GTTCCCAGGC CACTAAAGCC AGAGATACCA CGACTAGGCA AACGCTGCAC AAGCCTTCTG   
  
  
- GCAACGTCGG CGATAAGTGC GAGTCGGTGA CGGCGTTGTC GTCGGCAGCA CCCAGGGCGG AAGAGGCGGC   
  
  
- TTTCTGTGCT TCGTCAGCCG GGGTGGAGTT TTCGTTTCGC TGCGGCGACC TGGGAGCAAG AGTAGGCTCC   
  
  
- TCCTTGGTCT CGGCAGTACC GGCGGCGATT AACGCCGCAA CAACCTCACG CGGTTTGAGC GGCTCAGGCT   
  
  
- GGGCCTTGTG CGACAGTTTA GCCACTAAGC TGAGTCTCTG AGTCAGAGTG TCGTCCCTCA AGGTTAGGTC   
  
  
- TCTCACAGGA TAATGAAGAG GCTCCGTGAG ATGGTGGCGG AAAGAGAGAG GAGGCGGGGA GTTGGAGAGG   
  
  
- GTCTTTGCTG CAGCAGACTC CTCATGTGAG AGAGAATGTT CCGGGACTTG CTGCGCACGG GCATGGTATT   
  
  
- TAAGCGAGTG AACTGGCGTT TAGTCCGCTA AGATCTTCGG TGACTAAGTC GGTTCTTCTA GGTGTAGCAA   
  
  
- CTAAAACCCT ATTAGGTCCC TCAGGTTACC CGACGAGACG ATGTTCGGGA CCGTTGGGCC GGTCGGCCCT   
  
  
- TCGGGCTTAA CTAAGCTTAT AGGCCTTAAG GACGAGGCCC AGACCCCGTT AGAGGCCGCC GTAGCGACGA   
  
  
- GCGTTGACCC TTAGCGGAGG CACTCAAGCG CTTCCAAGAT CTAGAGTTCA AGCTCAAACT CGGCCATGAG   
  
  
- TGAGGTTAGT CTCTCGATTT GCCCAGCTTG AAGGCCCAGC TGGGTCTACT CTATGATCGA CAGTTAAAGT   
  
  
- ACGAAGTTAA CATGTTGGAT AATCTGCTTT GGGACCAGCG ACAGCTGCGG CGGGACTTCG ACCGGTTCAG   
  
  
- TAACTTGGGG TTTTAGCAGT GAAACCCACT CATGCTCCGC CCAGACTTAG CCCGTCCAAA GAACTGGGCC   
  
  
- AAGTTCTGCC GGGAGTTCAT GATAAACCGT TACAAACTTA GCCAACTCGG GTTGAACCGG GCCCTGAGCG   
  
  
- GGCTCTCCCG AGTCCAACTC TCCGACAATA ACCCGGCCGC CTAGTACCCG AATCAACCCG GCCTCAATGG   
  
  
- CCCTTGCTCT TCCCTTTCCT ACCTCACATT CCTTGTTACC TTCCCAAATT ACCTTTCACG GCCAAAGCTC   
  
  
- GGCACGGTTT CGTCTTTGAT GCAGTCACTT TTTCTGTTCA AAATGGTGAG GTCTAACTTA ATGTCTCTCA   
  
  
- C

  
  
Motifs Found  

+   

| Site Name | Organism | Position | Strand | Matrix score. | sequence | function |
| --- | --- | --- | --- | --- | --- | --- |
|  | organism | 510 | - | 4 | motif\_sequence | short\_function |
|  | organism | 3134 | + | 4 | motif\_sequence | short\_function |
|  | organism | 3049 | + | 4 | motif\_sequence | short\_function |
|  | organism | 2999 | - | 4 | motif\_sequence | short\_function |
|  | organism | 2869 | + | 4 | motif\_sequence | short\_function |
|  | organism | 1985 | - | 4 | motif\_sequence | short\_function |
|  | organism | 1934 | + | 4 | motif\_sequence | short\_function |
|  | organism | 1909 | + | 4 | motif\_sequence | short\_function |
|  | organism | 1341 | + | 4 | motif\_sequence | short\_function |
|  | organism | 2850 | + | 4 | motif\_sequence | short\_function |
|  | organism | 2094 | - | 4 | motif\_sequence | short\_function |
|  | organism | 987 | - | 4 | motif\_sequence | short\_function |
|  | organism | 1308 | + | 4 | motif\_sequence | short\_function |
|  | organism | 1538 | - | 4 | motif\_sequence | short\_function |
|  | organism | 742 | + | 4 | motif\_sequence | short\_function |
|  | organism | 613 | - | 4 | motif\_sequence | short\_function |
|  | organism | 1864 | + | 4 | motif\_sequence | short\_function |
|  | organism | 2832 | + | 4 | motif\_sequence | short\_function |
|  | organism | 2387 | - | 4 | motif\_sequence | short\_function |
|  | organism | 2028 | + | 4 | motif\_sequence | short\_function |
|  | organism | 800 | + | 4 | motif\_sequence | short\_function |

>HU06G00029.1   
+ +Up\_Stream \_Len000TGATAA TTATTTTTAT TGATTTAGGA AAGGATATCA AAGTATATTT TGTTTTTAGG   
  
  
+ TAGTTAAGAG ATTCTGTTGC CAAAAAAAAA ATCAAGATAT TATATATTTT TAATTATGAC AGTAATTATT   
  
  
+ TTATTGAAAA TCCAACGGTT GTGATTATAT AATCAAATAA TCTAAAGGTT AAAAATATCA AATTTATTAG   
  
  
+ AAAATTCGAA AAAGTCACGT CATCACAACA TTGCTTTTAT ATATAGTATA GATTCCTTCG ACTTCTAAAC   
  
  
+ TAATCACTTG AAATTAGAAA TTTGAAACTT TCTAATATAA ATGCATGGTC TTCTTTCCAA GATTATAATC   
  
  
+ CGCAATTGCT TAAATGTAGG GTACAAATAT CAAATTTAGA AGTGACTATT GAAGCCATGA AAGATATTGT   
  
  
+ GATCGTATTG AGGGTGGTTG TGAGCAATAG GATGAAACAG GAAAAAAAAA CCCTAATGTT TTCAAATTTA   
  
  
+ TGGGTGGCTT TGGTAGTAGA TAAGTGAATA GTGTGCAAGG GAATGGCACC AATATGTTTG GATGGAACGA   
  
  
+ TGAGAGTAAG ACGTTAGGAA AGCAACAAGA GAAGGGAAAA GGCAGTAGGG AGAGAATTAT GCGTGTGAAT   
  
  
+ AATATTATAA CAGACGCTTT CGTATGATTA TTAAATACAA GACTTTGAGC ATACATATAA ACTTGTAACT   
  
  
+ TTCTTTACCT ATTGTCAATT AATTTTTGAA TTGAATCATC TGCGACTTAT GCATACAAGA TGATATGATG   
  
  
+ CATATTGGGC CAAGCATTTG ATCTCATGTC CCTTATGGAT CTAGATCCCA ATAGACAAAC TTGCATAAGT   
  
  
+ CAAGGGATGT GTTTCTAATA CAAAGGCTTT GTACTCAAGC AATTAGTTTT GAATTCATTT TCAGTAGTTT   
  
  
+ AATTTCCAAA TCTTATAATT TTTACAATGG CATAAATTTT TAGGAGTTTT GAGTAAATAA ATTATATACA   
  
  
+ ACGAAGATGT CATTTACGAA TTAATATGAC GCACAAAAAA AAAGAAAAAA AATTAATGGT AGCTTCTATA   
  
  
+ CTTGACTCCA TGTTGACAAT TCCCTCTCTC ACATAATGGT TAAAACTTGC AACATCATCC AGGACCCATA   
  
  
+ TGACATGGGT GGTGGTAGAC AGTGTGTTGT GGTAGCCTCA CTCCATGCTT GCCAATTTGT ATCATTGCGC   
  
  
+ TAGTCTTATT CGTCCTTAGA GATAATTGAC TAAGCTCTAA TGCTCTATCG TGGCTGCTGC AATATAAATG   
  
  
+ ATTTTTTGAC CTCAAAATGA AGACAGCTAG TTGTAAATTA AGAATCTTCG TCCCTTCATT TTTTGTGTAC   
  
  
+ GTGTGTATCT GCGTATGTGT TGAGAGACTA GCGGACTATT GATTGAGTTT CAATCAAAAA AGATGACCAT   
  
  
+ CTTAAGTATT TTTAATTACG CAAACTTCTT CATTTAAAAA AATGTTACTT TTATTTGAAA GAAATTGTGA   
  
  
+ ATAATACAAG GAATAGTTCA ATTAATATAT AATCCATAAC ATATTACCTC CTATAGGCAA AAAGAAGATT   
  
  
+ ATCGTATTAA TTTGGGATCA TCCCACCCTA AATTTAAAGA AACCAAAACA AATTACTAAG CAATATCTTT   
  
  
+ CTGTGTGAAA AAATAGTCAG AAACAAAGCG ATCCGACCGG CCAAATCGAG ATAAACACAG CCAAAGAACC   
  
  
+ GAAGTTGGAC CAAAGTGAGC CGAGTTGAAA GGCATTCATA TGGGGTTTAA TCATAACACA AACATAATTA   
  
  
+ GATTGATTGA CGTCTTTAAT CAGCGTTTTT GTCTTTGTTC GGTGCTCCCC TCACTCGCCC CTCCGCTCTT   
  
  
+ TAAAGCTTAC CTCACTCCCC CCATTCCCAC TCCTTTTCTC TCTCCTCTCT CGCTCTCACA CGCTCCGTCT   
  
  
+ GTCAGCCCCT TGTCATCTTC CCCCGTCTTC CTCGCAAAAA TCTCCAGCAA AATTTATAGC CGTTGTTGTG   
  
  
+ CCCATGGTGA TCTGATGTGA GAAGAGATTC AGCATTTAAG ATTATTGGGT CCAGATGGCT TATATGTGCG   
  
  
+ CCGACAGTGG TAATCTCATG GCTATTGCCC AACAAGTCAT CAAGCAAAAA CAACAGCAAG AACAGCAGCA   
  
  
+ ACACCAACAG CCTCATCCCC AGTTCGTCGG GTCGAATAAT AACCCCTTTT GCACCAGCCC ATGGCCCGGA   
  
  
+ CCCGTCCCTT CATCCGCCAT GTCTGCCAGC CCTCCGCCCC CTCTTGGGTT CTCCGCCGCC GCGTTTCCGG   
  
  
+ ACCCGTTTCA GGTGGGCCCG CCCTGCCCGG ATGGGGCTGA ACCGGGTTTC CAGTTTGCCC ATTTGGACCA   
  
  
+ CCACTCGAGT GGTTTCCGGT TTGCTGATTT TTGCGGTGGG GGTGGTGGTG AGTTTGACTC GGATGAGTGG   
  
  
+ ATGGAGAGTT TGATGGGTGG CGGCGGCGGT GACTCGCGGC GACTGAGAGT TCTAACCTCC AATCCCATTG   
  
  
+ CGACGCGTGG CAAGGGTCCG GTGATTTCGG TCTCTATGGT GCTGATCCGT TTGCGACGTG TTCGGAAGAC   
  
  
+ CGTTGCAGCC GCTATTCACG CTCAGCCACT GCCGCAACAG CAGCCGTCGT GGGTCCCGCC TTCTCCGCCG   
  
  
+ AAAGACACGA AGCAGTCGGC CCCACCTCAA AAGCAAAGCG ACGCCGCTGG ACCCTCGTTC TCATCCGAGG   
  
  
+ AGGAACCAGA GCCGTCATGG CCGCCGCTAA TTGCGGCGTT GTTGGAGTGC GCCAAACTCG CCGAGTCCGA   
  
  
+ CCCGGAACAC GCTGTCAAAT CGGTGATTCG ACTCAGAGAC TCAGTCTCAC AGCAGGGAGT TCCAATCCAG   
  
  
+ AGAGTGTCCT ATTACTTCTC CGAGGCACTC TACCACCGCC TTTCTCTCTC CTCCGCCCCT CAACCTCTCC   
  
  
+ CAGAAACGAC GTCGTCTGAG GAGTACACTC TCTCTTACAA GGCCCTGAAC GACGCGTGCC CGTACCATAA   
  
  
+ ATTCGCTCAC TTGACCGCAA ATCAGGCGAT TCTAGAAGCC ACTGATTCAG CCAAGAAGAT CCACATCGTT   
  
  
+ GATTTTGGGA TAATCCAGGG AGTCCAATGG GCTGCTCTGC TACAAGCCCT GGCAACCCGG CCAGCCGGGA   
  
  
+ AGCCCGAATT GATTCGAATA TCCGGAATTC CTGCTCCGGG TCTGGGGCAA TCTCCGGCGG CATCGCTGCT   
  
  
+ CGCAACTGGG AATCGCCTCC GTGAGTTCGC GAAGGTTCTA GATCTCAAGT TCGAGTTTGA GCCGGTACTC   
  
  
+ ACTCCAATCA GAGAGCTAAA CGGGTCGAAC TTCCGGGTCG ACCCAGATGA GATACTAGCT GTCAATTTCA   
  
  
+ TGCTTCAATT GTACAACCTA TTAGACGAAA CCCTGGTCGC TGTCGACGCC GCCCTGAAGC TGGCCAAGTC   
  
  
+ ATTGAACCCC AAAATCGTCA CTTTGGGTGA GTACGAGGCG GGTCTGAATC GGGCAGGTTT CTTGACCCGG   
  
  
+ TTCAAGACGG CCCTCAAGTA CTATTTGGCA ATGTTTGAAT CGGTTGAGCC CAACTTGGCC CGGGACTCGC   
  
  
+ CCGAGAGGGC TCAGGTTGAG AGGCTGTTAT TGGGCCGGCG GATCATGGGC TTAGTTGGGC CGGAGTTACC   
  
  
+ GGGAACGAGA AGGGAAAGGA TGGAGTGTAA GGAACAATGG AAGGGTTTAA TGGAAAGTGC CGGTTTCGAG   
  
  
+ CCGTGCCAAA GCAGAAACTA CGTCAGTGAA AAAGACAAGT TTTACCACTC CAGATTGAAT TACAGAGAGT   
  
  
+ G  

- +Up\_Stream \_Len000ACTATT AATAAAAATA ACTAAATCCT TTCCTATAGT TTCATATAAA ACAAAAATCC   
  
  
- ATCAATTCTC TAAGACAACG GTTTTTTTTT TAGTTCTATA ATATATAAAA ATTAATACTG TCATTAATAA   
  
  
- AATAACTTTT AGGTTGCCAA CACTAATATA TTAGTTTATT AGATTTCCAA TTTTTATAGT TTAAATAATC   
  
  
- TTTTAAGCTT TTTCAGTGCA GTAGTGTTGT AACGAAAATA TATATCATAT CTAAGGAAGC TGAAGATTTG   
  
  
- ATTAGTGAAC TTTAATCTTT AAACTTTGAA AGATTATATT TACGTACCAG AAGAAAGGTT CTAATATTAG   
  
  
- GCGTTAACGA ATTTACATCC CATGTTTATA GTTTAAATCT TCACTGATAA CTTCGGTACT TTCTATAACA   
  
  
- CTAGCATAAC TCCCACCAAC ACTCGTTATC CTACTTTGTC CTTTTTTTTT GGGATTACAA AAGTTTAAAT   
  
  
- ACCCACCGAA ACCATCATCT ATTCACTTAT CACACGTTCC CTTACCGTGG TTATACAAAC CTACCTTGCT   
  
  
- ACTCTCATTC TGCAATCCTT TCGTTGTTCT CTTCCCTTTT CCGTCATCCC TCTCTTAATA CGCACACTTA   
  
  
- TTATAATATT GTCTGCGAAA GCATACTAAT AATTTATGTT CTGAAACTCG TATGTATATT TGAACATTGA   
  
  
- AAGAAATGGA TAACAGTTAA TTAAAAACTT AACTTAGTAG ACGCTGAATA CGTATGTTCT ACTATACTAC   
  
  
- GTATAACCCG GTTCGTAAAC TAGAGTACAG GGAATACCTA GATCTAGGGT TATCTGTTTG AACGTATTCA   
  
  
- GTTCCCTACA CAAAGATTAT GTTTCCGAAA CATGAGTTCG TTAATCAAAA CTTAAGTAAA AGTCATCAAA   
  
  
- TTAAAGGTTT AGAATATTAA AAATGTTACC GTATTTAAAA ATCCTCAAAA CTCATTTATT TAATATATGT   
  
  
- TGCTTCTACA GTAAATGCTT AATTATACTG CGTGTTTTTT TTTCTTTTTT TTAATTACCA TCGAAGATAT   
  
  
- GAACTGAGGT ACAACTGTTA AGGGAGAGAG TGTATTACCA ATTTTGAACG TTGTAGTAGG TCCTGGGTAT   
  
  
- ACTGTACCCA CCACCATCTG TCACACAACA CCATCGGAGT GAGGTACGAA CGGTTAAACA TAGTAACGCG   
  
  
- ATCAGAATAA GCAGGAATCT CTATTAACTG ATTCGAGATT ACGAGATAGC ACCGACGACG TTATATTTAC   
  
  
- TAAAAAACTG GAGTTTTACT TCTGTCGATC AACATTTAAT TCTTAGAAGC AGGGAAGTAA AAAACACATG   
  
  
- CACACATAGA CGCATACACA ACTCTCTGAT CGCCTGATAA CTAACTCAAA GTTAGTTTTT TCTACTGGTA   
  
  
- GAATTCATAA AAATTAATGC GTTTGAAGAA GTAAATTTTT TTACAATGAA AATAAACTTT CTTTAACACT   
  
  
- TATTATGTTC CTTATCAAGT TAATTATATA TTAGGTATTG TATAATGGAG GATATCCGTT TTTCTTCTAA   
  
  
- TAGCATAATT AAACCCTAGT AGGGTGGGAT TTAAATTTCT TTGGTTTTGT TTAATGATTC GTTATAGAAA   
  
  
- GACACACTTT TTTATCAGTC TTTGTTTCGC TAGGCTGGCC GGTTTAGCTC TATTTGTGTC GGTTTCTTGG   
  
  
- CTTCAACCTG GTTTCACTCG GCTCAACTTT CCGTAAGTAT ACCCCAAATT AGTATTGTGT TTGTATTAAT   
  
  
- CTAACTAACT GCAGAAATTA GTCGCAAAAA CAGAAACAAG CCACGAGGGG AGTGAGCGGG GAGGCGAGAA   
  
  
- ATTTCGAATG GAGTGAGGGG GGTAAGGGTG AGGAAAAGAG AGAGGAGAGA GCGAGAGTGT GCGAGGCAGA   
  
  
- CAGTCGGGGA ACAGTAGAAG GGGGCAGAAG GAGCGTTTTT AGAGGTCGTT TTAAATATCG GCAACAACAC   
  
  
- GGGTACCACT AGACTACACT CTTCTCTAAG TCGTAAATTC TAATAACCCA GGTCTACCGA ATATACACGC   
  
  
- GGCTGTCACC ATTAGAGTAC CGATAACGGG TTGTTCAGTA GTTCGTTTTT GTTGTCGTTC TTGTCGTCGT   
  
  
- TGTGGTTGTC GGAGTAGGGG TCAAGCAGCC CAGCTTATTA TTGGGGAAAA CGTGGTCGGG TACCGGGCCT   
  
  
- GGGCAGGGAA GTAGGCGGTA CAGACGGTCG GGAGGCGGGG GAGAACCCAA GAGGCGGCGG CGCAAAGGCC   
  
  
- TGGGCAAAGT CCACCCGGGC GGGACGGGCC TACCCCGACT TGGCCCAAAG GTCAAACGGG TAAACCTGGT   
  
  
- GGTGAGCTCA CCAAAGGCCA AACGACTAAA AACGCCACCC CCACCACCAC TCAAACTGAG CCTACTCACC   
  
  
- TACCTCTCAA ACTACCCACC GCCGCCGCCA CTGAGCGCCG CTGACTCTCA AGATTGGAGG TTAGGGTAAC   
  
  
- GCTGCGCACC GTTCCCAGGC CACTAAAGCC AGAGATACCA CGACTAGGCA AACGCTGCAC AAGCCTTCTG   
  
  
- GCAACGTCGG CGATAAGTGC GAGTCGGTGA CGGCGTTGTC GTCGGCAGCA CCCAGGGCGG AAGAGGCGGC   
  
  
- TTTCTGTGCT TCGTCAGCCG GGGTGGAGTT TTCGTTTCGC TGCGGCGACC TGGGAGCAAG AGTAGGCTCC   
  
  
- TCCTTGGTCT CGGCAGTACC GGCGGCGATT AACGCCGCAA CAACCTCACG CGGTTTGAGC GGCTCAGGCT   
  
  
- GGGCCTTGTG CGACAGTTTA GCCACTAAGC TGAGTCTCTG AGTCAGAGTG TCGTCCCTCA AGGTTAGGTC   
  
  
- TCTCACAGGA TAATGAAGAG GCTCCGTGAG ATGGTGGCGG AAAGAGAGAG GAGGCGGGGA GTTGGAGAGG   
  
  
- GTCTTTGCTG CAGCAGACTC CTCATGTGAG AGAGAATGTT CCGGGACTTG CTGCGCACGG GCATGGTATT   
  
  
- TAAGCGAGTG AACTGGCGTT TAGTCCGCTA AGATCTTCGG TGACTAAGTC GGTTCTTCTA GGTGTAGCAA   
  
  
- CTAAAACCCT ATTAGGTCCC TCAGGTTACC CGACGAGACG ATGTTCGGGA CCGTTGGGCC GGTCGGCCCT   
  
  
- TCGGGCTTAA CTAAGCTTAT AGGCCTTAAG GACGAGGCCC AGACCCCGTT AGAGGCCGCC GTAGCGACGA   
  
  
- GCGTTGACCC TTAGCGGAGG CACTCAAGCG CTTCCAAGAT CTAGAGTTCA AGCTCAAACT CGGCCATGAG   
  
  
- TGAGGTTAGT CTCTCGATTT GCCCAGCTTG AAGGCCCAGC TGGGTCTACT CTATGATCGA CAGTTAAAGT   
  
  
- ACGAAGTTAA CATGTTGGAT AATCTGCTTT GGGACCAGCG ACAGCTGCGG CGGGACTTCG ACCGGTTCAG   
  
  
- TAACTTGGGG TTTTAGCAGT GAAACCCACT CATGCTCCGC CCAGACTTAG CCCGTCCAAA GAACTGGGCC   
  
  
- AAGTTCTGCC GGGAGTTCAT GATAAACCGT TACAAACTTA GCCAACTCGG GTTGAACCGG GCCCTGAGCG   
  
  
- GGCTCTCCCG AGTCCAACTC TCCGACAATA ACCCGGCCGC CTAGTACCCG AATCAACCCG GCCTCAATGG   
  
  
- CCCTTGCTCT TCCCTTTCCT ACCTCACATT CCTTGTTACC TTCCCAAATT ACCTTTCACG GCCAAAGCTC   
  
  
- GGCACGGTTT CGTCTTTGAT GCAGTCACTT TTTCTGTTCA AAATGGTGAG GTCTAACTTA ATGTCTCTCA   
  
  
- C

+     A-box

| Site Name | Organism | Position | Strand | Matrix score. | sequence | function |
| --- | --- | --- | --- | --- | --- | --- |
| A-box | Petroselinum crispum | 2176 | + | 6 | CCGTCC | cis-acting regulatory element |

>HU06G00029.1   
+ +Up\_Stream \_Len000TGATAA TTATTTTTAT TGATTTAGGA AAGGATATCA AAGTATATTT TGTTTTTAGG   
  
  
+ TAGTTAAGAG ATTCTGTTGC CAAAAAAAAA ATCAAGATAT TATATATTTT TAATTATGAC AGTAATTATT   
  
  
+ TTATTGAAAA TCCAACGGTT GTGATTATAT AATCAAATAA TCTAAAGGTT AAAAATATCA AATTTATTAG   
  
  
+ AAAATTCGAA AAAGTCACGT CATCACAACA TTGCTTTTAT ATATAGTATA GATTCCTTCG ACTTCTAAAC   
  
  
+ TAATCACTTG AAATTAGAAA TTTGAAACTT TCTAATATAA ATGCATGGTC TTCTTTCCAA GATTATAATC   
  
  
+ CGCAATTGCT TAAATGTAGG GTACAAATAT CAAATTTAGA AGTGACTATT GAAGCCATGA AAGATATTGT   
  
  
+ GATCGTATTG AGGGTGGTTG TGAGCAATAG GATGAAACAG GAAAAAAAAA CCCTAATGTT TTCAAATTTA   
  
  
+ TGGGTGGCTT TGGTAGTAGA TAAGTGAATA GTGTGCAAGG GAATGGCACC AATATGTTTG GATGGAACGA   
  
  
+ TGAGAGTAAG ACGTTAGGAA AGCAACAAGA GAAGGGAAAA GGCAGTAGGG AGAGAATTAT GCGTGTGAAT   
  
  
+ AATATTATAA CAGACGCTTT CGTATGATTA TTAAATACAA GACTTTGAGC ATACATATAA ACTTGTAACT   
  
  
+ TTCTTTACCT ATTGTCAATT AATTTTTGAA TTGAATCATC TGCGACTTAT GCATACAAGA TGATATGATG   
  
  
+ CATATTGGGC CAAGCATTTG ATCTCATGTC CCTTATGGAT CTAGATCCCA ATAGACAAAC TTGCATAAGT   
  
  
+ CAAGGGATGT GTTTCTAATA CAAAGGCTTT GTACTCAAGC AATTAGTTTT GAATTCATTT TCAGTAGTTT   
  
  
+ AATTTCCAAA TCTTATAATT TTTACAATGG CATAAATTTT TAGGAGTTTT GAGTAAATAA ATTATATACA   
  
  
+ ACGAAGATGT CATTTACGAA TTAATATGAC GCACAAAAAA AAAGAAAAAA AATTAATGGT AGCTTCTATA   
  
  
+ CTTGACTCCA TGTTGACAAT TCCCTCTCTC ACATAATGGT TAAAACTTGC AACATCATCC AGGACCCATA   
  
  
+ TGACATGGGT GGTGGTAGAC AGTGTGTTGT GGTAGCCTCA CTCCATGCTT GCCAATTTGT ATCATTGCGC   
  
  
+ TAGTCTTATT CGTCCTTAGA GATAATTGAC TAAGCTCTAA TGCTCTATCG TGGCTGCTGC AATATAAATG   
  
  
+ ATTTTTTGAC CTCAAAATGA AGACAGCTAG TTGTAAATTA AGAATCTTCG TCCCTTCATT TTTTGTGTAC   
  
  
+ GTGTGTATCT GCGTATGTGT TGAGAGACTA GCGGACTATT GATTGAGTTT CAATCAAAAA AGATGACCAT   
  
  
+ CTTAAGTATT TTTAATTACG CAAACTTCTT CATTTAAAAA AATGTTACTT TTATTTGAAA GAAATTGTGA   
  
  
+ ATAATACAAG GAATAGTTCA ATTAATATAT AATCCATAAC ATATTACCTC CTATAGGCAA AAAGAAGATT   
  
  
+ ATCGTATTAA TTTGGGATCA TCCCACCCTA AATTTAAAGA AACCAAAACA AATTACTAAG CAATATCTTT   
  
  
+ CTGTGTGAAA AAATAGTCAG AAACAAAGCG ATCCGACCGG CCAAATCGAG ATAAACACAG CCAAAGAACC   
  
  
+ GAAGTTGGAC CAAAGTGAGC CGAGTTGAAA GGCATTCATA TGGGGTTTAA TCATAACACA AACATAATTA   
  
  
+ GATTGATTGA CGTCTTTAAT CAGCGTTTTT GTCTTTGTTC GGTGCTCCCC TCACTCGCCC CTCCGCTCTT   
  
  
+ TAAAGCTTAC CTCACTCCCC CCATTCCCAC TCCTTTTCTC TCTCCTCTCT CGCTCTCACA CGCTCCGTCT   
  
  
+ GTCAGCCCCT TGTCATCTTC CCCCGTCTTC CTCGCAAAAA TCTCCAGCAA AATTTATAGC CGTTGTTGTG   
  
  
+ CCCATGGTGA TCTGATGTGA GAAGAGATTC AGCATTTAAG ATTATTGGGT CCAGATGGCT TATATGTGCG   
  
  
+ CCGACAGTGG TAATCTCATG GCTATTGCCC AACAAGTCAT CAAGCAAAAA CAACAGCAAG AACAGCAGCA   
  
  
+ ACACCAACAG CCTCATCCCC AGTTCGTCGG GTCGAATAAT AACCCCTTTT GCACCAGCCC ATGGCCCGGA   
  
  
+ CCCGTCCCTT CATCCGCCAT GTCTGCCAGC CCTCCGCCCC CTCTTGGGTT CTCCGCCGCC GCGTTTCCGG   
  
  
+ ACCCGTTTCA GGTGGGCCCG CCCTGCCCGG ATGGGGCTGA ACCGGGTTTC CAGTTTGCCC ATTTGGACCA   
  
  
+ CCACTCGAGT GGTTTCCGGT TTGCTGATTT TTGCGGTGGG GGTGGTGGTG AGTTTGACTC GGATGAGTGG   
  
  
+ ATGGAGAGTT TGATGGGTGG CGGCGGCGGT GACTCGCGGC GACTGAGAGT TCTAACCTCC AATCCCATTG   
  
  
+ CGACGCGTGG CAAGGGTCCG GTGATTTCGG TCTCTATGGT GCTGATCCGT TTGCGACGTG TTCGGAAGAC   
  
  
+ CGTTGCAGCC GCTATTCACG CTCAGCCACT GCCGCAACAG CAGCCGTCGT GGGTCCCGCC TTCTCCGCCG   
  
  
+ AAAGACACGA AGCAGTCGGC CCCACCTCAA AAGCAAAGCG ACGCCGCTGG ACCCTCGTTC TCATCCGAGG   
  
  
+ AGGAACCAGA GCCGTCATGG CCGCCGCTAA TTGCGGCGTT GTTGGAGTGC GCCAAACTCG CCGAGTCCGA   
  
  
+ CCCGGAACAC GCTGTCAAAT CGGTGATTCG ACTCAGAGAC TCAGTCTCAC AGCAGGGAGT TCCAATCCAG   
  
  
+ AGAGTGTCCT ATTACTTCTC CGAGGCACTC TACCACCGCC TTTCTCTCTC CTCCGCCCCT CAACCTCTCC   
  
  
+ CAGAAACGAC GTCGTCTGAG GAGTACACTC TCTCTTACAA GGCCCTGAAC GACGCGTGCC CGTACCATAA   
  
  
+ ATTCGCTCAC TTGACCGCAA ATCAGGCGAT TCTAGAAGCC ACTGATTCAG CCAAGAAGAT CCACATCGTT   
  
  
+ GATTTTGGGA TAATCCAGGG AGTCCAATGG GCTGCTCTGC TACAAGCCCT GGCAACCCGG CCAGCCGGGA   
  
  
+ AGCCCGAATT GATTCGAATA TCCGGAATTC CTGCTCCGGG TCTGGGGCAA TCTCCGGCGG CATCGCTGCT   
  
  
+ CGCAACTGGG AATCGCCTCC GTGAGTTCGC GAAGGTTCTA GATCTCAAGT TCGAGTTTGA GCCGGTACTC   
  
  
+ ACTCCAATCA GAGAGCTAAA CGGGTCGAAC TTCCGGGTCG ACCCAGATGA GATACTAGCT GTCAATTTCA   
  
  
+ TGCTTCAATT GTACAACCTA TTAGACGAAA CCCTGGTCGC TGTCGACGCC GCCCTGAAGC TGGCCAAGTC   
  
  
+ ATTGAACCCC AAAATCGTCA CTTTGGGTGA GTACGAGGCG GGTCTGAATC GGGCAGGTTT CTTGACCCGG   
  
  
+ TTCAAGACGG CCCTCAAGTA CTATTTGGCA ATGTTTGAAT CGGTTGAGCC CAACTTGGCC CGGGACTCGC   
  
  
+ CCGAGAGGGC TCAGGTTGAG AGGCTGTTAT TGGGCCGGCG GATCATGGGC TTAGTTGGGC CGGAGTTACC   
  
  
+ GGGAACGAGA AGGGAAAGGA TGGAGTGTAA GGAACAATGG AAGGGTTTAA TGGAAAGTGC CGGTTTCGAG   
  
  
+ CCGTGCCAAA GCAGAAACTA CGTCAGTGAA AAAGACAAGT TTTACCACTC CAGATTGAAT TACAGAGAGT   
  
  
+ G  

- +Up\_Stream \_Len000ACTATT AATAAAAATA ACTAAATCCT TTCCTATAGT TTCATATAAA ACAAAAATCC   
  
  
- ATCAATTCTC TAAGACAACG GTTTTTTTTT TAGTTCTATA ATATATAAAA ATTAATACTG TCATTAATAA   
  
  
- AATAACTTTT AGGTTGCCAA CACTAATATA TTAGTTTATT AGATTTCCAA TTTTTATAGT TTAAATAATC   
  
  
- TTTTAAGCTT TTTCAGTGCA GTAGTGTTGT AACGAAAATA TATATCATAT CTAAGGAAGC TGAAGATTTG   
  
  
- ATTAGTGAAC TTTAATCTTT AAACTTTGAA AGATTATATT TACGTACCAG AAGAAAGGTT CTAATATTAG   
  
  
- GCGTTAACGA ATTTACATCC CATGTTTATA GTTTAAATCT TCACTGATAA CTTCGGTACT TTCTATAACA   
  
  
- CTAGCATAAC TCCCACCAAC ACTCGTTATC CTACTTTGTC CTTTTTTTTT GGGATTACAA AAGTTTAAAT   
  
  
- ACCCACCGAA ACCATCATCT ATTCACTTAT CACACGTTCC CTTACCGTGG TTATACAAAC CTACCTTGCT   
  
  
- ACTCTCATTC TGCAATCCTT TCGTTGTTCT CTTCCCTTTT CCGTCATCCC TCTCTTAATA CGCACACTTA   
  
  
- TTATAATATT GTCTGCGAAA GCATACTAAT AATTTATGTT CTGAAACTCG TATGTATATT TGAACATTGA   
  
  
- AAGAAATGGA TAACAGTTAA TTAAAAACTT AACTTAGTAG ACGCTGAATA CGTATGTTCT ACTATACTAC   
  
  
- GTATAACCCG GTTCGTAAAC TAGAGTACAG GGAATACCTA GATCTAGGGT TATCTGTTTG AACGTATTCA   
  
  
- GTTCCCTACA CAAAGATTAT GTTTCCGAAA CATGAGTTCG TTAATCAAAA CTTAAGTAAA AGTCATCAAA   
  
  
- TTAAAGGTTT AGAATATTAA AAATGTTACC GTATTTAAAA ATCCTCAAAA CTCATTTATT TAATATATGT   
  
  
- TGCTTCTACA GTAAATGCTT AATTATACTG CGTGTTTTTT TTTCTTTTTT TTAATTACCA TCGAAGATAT   
  
  
- GAACTGAGGT ACAACTGTTA AGGGAGAGAG TGTATTACCA ATTTTGAACG TTGTAGTAGG TCCTGGGTAT   
  
  
- ACTGTACCCA CCACCATCTG TCACACAACA CCATCGGAGT GAGGTACGAA CGGTTAAACA TAGTAACGCG   
  
  
- ATCAGAATAA GCAGGAATCT CTATTAACTG ATTCGAGATT ACGAGATAGC ACCGACGACG TTATATTTAC   
  
  
- TAAAAAACTG GAGTTTTACT TCTGTCGATC AACATTTAAT TCTTAGAAGC AGGGAAGTAA AAAACACATG   
  
  
- CACACATAGA CGCATACACA ACTCTCTGAT CGCCTGATAA CTAACTCAAA GTTAGTTTTT TCTACTGGTA   
  
  
- GAATTCATAA AAATTAATGC GTTTGAAGAA GTAAATTTTT TTACAATGAA AATAAACTTT CTTTAACACT   
  
  
- TATTATGTTC CTTATCAAGT TAATTATATA TTAGGTATTG TATAATGGAG GATATCCGTT TTTCTTCTAA   
  
  
- TAGCATAATT AAACCCTAGT AGGGTGGGAT TTAAATTTCT TTGGTTTTGT TTAATGATTC GTTATAGAAA   
  
  
- GACACACTTT TTTATCAGTC TTTGTTTCGC TAGGCTGGCC GGTTTAGCTC TATTTGTGTC GGTTTCTTGG   
  
  
- CTTCAACCTG GTTTCACTCG GCTCAACTTT CCGTAAGTAT ACCCCAAATT AGTATTGTGT TTGTATTAAT   
  
  
- CTAACTAACT GCAGAAATTA GTCGCAAAAA CAGAAACAAG CCACGAGGGG AGTGAGCGGG GAGGCGAGAA   
  
  
- ATTTCGAATG GAGTGAGGGG GGTAAGGGTG AGGAAAAGAG AGAGGAGAGA GCGAGAGTGT GCGAGGCAGA   
  
  
- CAGTCGGGGA ACAGTAGAAG GGGGCAGAAG GAGCGTTTTT AGAGGTCGTT TTAAATATCG GCAACAACAC   
  
  
- GGGTACCACT AGACTACACT CTTCTCTAAG TCGTAAATTC TAATAACCCA GGTCTACCGA ATATACACGC   
  
  
- GGCTGTCACC ATTAGAGTAC CGATAACGGG TTGTTCAGTA GTTCGTTTTT GTTGTCGTTC TTGTCGTCGT   
  
  
- TGTGGTTGTC GGAGTAGGGG TCAAGCAGCC CAGCTTATTA TTGGGGAAAA CGTGGTCGGG TACCGGGCCT   
  
  
- GGGCAGGGAA GTAGGCGGTA CAGACGGTCG GGAGGCGGGG GAGAACCCAA GAGGCGGCGG CGCAAAGGCC   
  
  
- TGGGCAAAGT CCACCCGGGC GGGACGGGCC TACCCCGACT TGGCCCAAAG GTCAAACGGG TAAACCTGGT   
  
  
- GGTGAGCTCA CCAAAGGCCA AACGACTAAA AACGCCACCC CCACCACCAC TCAAACTGAG CCTACTCACC   
  
  
- TACCTCTCAA ACTACCCACC GCCGCCGCCA CTGAGCGCCG CTGACTCTCA AGATTGGAGG TTAGGGTAAC   
  
  
- GCTGCGCACC GTTCCCAGGC CACTAAAGCC AGAGATACCA CGACTAGGCA AACGCTGCAC AAGCCTTCTG   
  
  
- GCAACGTCGG CGATAAGTGC GAGTCGGTGA CGGCGTTGTC GTCGGCAGCA CCCAGGGCGG AAGAGGCGGC   
  
  
- TTTCTGTGCT TCGTCAGCCG GGGTGGAGTT TTCGTTTCGC TGCGGCGACC TGGGAGCAAG AGTAGGCTCC   
  
  
- TCCTTGGTCT CGGCAGTACC GGCGGCGATT AACGCCGCAA CAACCTCACG CGGTTTGAGC GGCTCAGGCT   
  
  
- GGGCCTTGTG CGACAGTTTA GCCACTAAGC TGAGTCTCTG AGTCAGAGTG TCGTCCCTCA AGGTTAGGTC   
  
  
- TCTCACAGGA TAATGAAGAG GCTCCGTGAG ATGGTGGCGG AAAGAGAGAG GAGGCGGGGA GTTGGAGAGG   
  
  
- GTCTTTGCTG CAGCAGACTC CTCATGTGAG AGAGAATGTT CCGGGACTTG CTGCGCACGG GCATGGTATT   
  
  
- TAAGCGAGTG AACTGGCGTT TAGTCCGCTA AGATCTTCGG TGACTAAGTC GGTTCTTCTA GGTGTAGCAA   
  
  
- CTAAAACCCT ATTAGGTCCC TCAGGTTACC CGACGAGACG ATGTTCGGGA CCGTTGGGCC GGTCGGCCCT   
  
  
- TCGGGCTTAA CTAAGCTTAT AGGCCTTAAG GACGAGGCCC AGACCCCGTT AGAGGCCGCC GTAGCGACGA   
  
  
- GCGTTGACCC TTAGCGGAGG CACTCAAGCG CTTCCAAGAT CTAGAGTTCA AGCTCAAACT CGGCCATGAG   
  
  
- TGAGGTTAGT CTCTCGATTT GCCCAGCTTG AAGGCCCAGC TGGGTCTACT CTATGATCGA CAGTTAAAGT   
  
  
- ACGAAGTTAA CATGTTGGAT AATCTGCTTT GGGACCAGCG ACAGCTGCGG CGGGACTTCG ACCGGTTCAG   
  
  
- TAACTTGGGG TTTTAGCAGT GAAACCCACT CATGCTCCGC CCAGACTTAG CCCGTCCAAA GAACTGGGCC   
  
  
- AAGTTCTGCC GGGAGTTCAT GATAAACCGT TACAAACTTA GCCAACTCGG GTTGAACCGG GCCCTGAGCG   
  
  
- GGCTCTCCCG AGTCCAACTC TCCGACAATA ACCCGGCCGC CTAGTACCCG AATCAACCCG GCCTCAATGG   
  
  
- CCCTTGCTCT TCCCTTTCCT ACCTCACATT CCTTGTTACC TTCCCAAATT ACCTTTCACG GCCAAAGCTC   
  
  
- GGCACGGTTT CGTCTTTGAT GCAGTCACTT TTTCTGTTCA AAATGGTGAG GTCTAACTTA ATGTCTCTCA   
  
  
- C

+     AAGAA-motif

| Site Name | Organism | Position | Strand | Matrix score. | sequence | function |
| --- | --- | --- | --- | --- | --- | --- |
| AAGAA-motif | Avena sativa | 704 | - | 10 | gGTAAAGAAA |  |
| AAGAA-motif | Avena sativa | 1461 | + | 7 | GAAAGAA |  |
| AAGAA-motif | Avena sativa | 335 | - | 7 | GAAAGAA |  |
| AAGAA-motif | Avena sativa | 3422 | - | 9 | gGTAAAGAAA |  |

>HU06G00029.1   
+ +Up\_Stream \_Len000TGATAA TTATTTTTAT TGATTTAGGA AAGGATATCA AAGTATATTT TGTTTTTAGG   
  
  
+ TAGTTAAGAG ATTCTGTTGC CAAAAAAAAA ATCAAGATAT TATATATTTT TAATTATGAC AGTAATTATT   
  
  
+ TTATTGAAAA TCCAACGGTT GTGATTATAT AATCAAATAA TCTAAAGGTT AAAAATATCA AATTTATTAG   
  
  
+ AAAATTCGAA AAAGTCACGT CATCACAACA TTGCTTTTAT ATATAGTATA GATTCCTTCG ACTTCTAAAC   
  
  
+ TAATCACTTG AAATTAGAAA TTTGAAACTT TCTAATATAA ATGCATGGTC TTCTTTCCAA GATTATAATC   
  
  
+ CGCAATTGCT TAAATGTAGG GTACAAATAT CAAATTTAGA AGTGACTATT GAAGCCATGA AAGATATTGT   
  
  
+ GATCGTATTG AGGGTGGTTG TGAGCAATAG GATGAAACAG GAAAAAAAAA CCCTAATGTT TTCAAATTTA   
  
  
+ TGGGTGGCTT TGGTAGTAGA TAAGTGAATA GTGTGCAAGG GAATGGCACC AATATGTTTG GATGGAACGA   
  
  
+ TGAGAGTAAG ACGTTAGGAA AGCAACAAGA GAAGGGAAAA GGCAGTAGGG AGAGAATTAT GCGTGTGAAT   
  
  
+ AATATTATAA CAGACGCTTT CGTATGATTA TTAAATACAA GACTTTGAGC ATACATATAA ACTTGTAACT   
  
  
+ TTCTTTACCT ATTGTCAATT AATTTTTGAA TTGAATCATC TGCGACTTAT GCATACAAGA TGATATGATG   
  
  
+ CATATTGGGC CAAGCATTTG ATCTCATGTC CCTTATGGAT CTAGATCCCA ATAGACAAAC TTGCATAAGT   
  
  
+ CAAGGGATGT GTTTCTAATA CAAAGGCTTT GTACTCAAGC AATTAGTTTT GAATTCATTT TCAGTAGTTT   
  
  
+ AATTTCCAAA TCTTATAATT TTTACAATGG CATAAATTTT TAGGAGTTTT GAGTAAATAA ATTATATACA   
  
  
+ ACGAAGATGT CATTTACGAA TTAATATGAC GCACAAAAAA AAAGAAAAAA AATTAATGGT AGCTTCTATA   
  
  
+ CTTGACTCCA TGTTGACAAT TCCCTCTCTC ACATAATGGT TAAAACTTGC AACATCATCC AGGACCCATA   
  
  
+ TGACATGGGT GGTGGTAGAC AGTGTGTTGT GGTAGCCTCA CTCCATGCTT GCCAATTTGT ATCATTGCGC   
  
  
+ TAGTCTTATT CGTCCTTAGA GATAATTGAC TAAGCTCTAA TGCTCTATCG TGGCTGCTGC AATATAAATG   
  
  
+ ATTTTTTGAC CTCAAAATGA AGACAGCTAG TTGTAAATTA AGAATCTTCG TCCCTTCATT TTTTGTGTAC   
  
  
+ GTGTGTATCT GCGTATGTGT TGAGAGACTA GCGGACTATT GATTGAGTTT CAATCAAAAA AGATGACCAT   
  
  
+ CTTAAGTATT TTTAATTACG CAAACTTCTT CATTTAAAAA AATGTTACTT TTATTTGAAA GAAATTGTGA   
  
  
+ ATAATACAAG GAATAGTTCA ATTAATATAT AATCCATAAC ATATTACCTC CTATAGGCAA AAAGAAGATT   
  
  
+ ATCGTATTAA TTTGGGATCA TCCCACCCTA AATTTAAAGA AACCAAAACA AATTACTAAG CAATATCTTT   
  
  
+ CTGTGTGAAA AAATAGTCAG AAACAAAGCG ATCCGACCGG CCAAATCGAG ATAAACACAG CCAAAGAACC   
  
  
+ GAAGTTGGAC CAAAGTGAGC CGAGTTGAAA GGCATTCATA TGGGGTTTAA TCATAACACA AACATAATTA   
  
  
+ GATTGATTGA CGTCTTTAAT CAGCGTTTTT GTCTTTGTTC GGTGCTCCCC TCACTCGCCC CTCCGCTCTT   
  
  
+ TAAAGCTTAC CTCACTCCCC CCATTCCCAC TCCTTTTCTC TCTCCTCTCT CGCTCTCACA CGCTCCGTCT   
  
  
+ GTCAGCCCCT TGTCATCTTC CCCCGTCTTC CTCGCAAAAA TCTCCAGCAA AATTTATAGC CGTTGTTGTG   
  
  
+ CCCATGGTGA TCTGATGTGA GAAGAGATTC AGCATTTAAG ATTATTGGGT CCAGATGGCT TATATGTGCG   
  
  
+ CCGACAGTGG TAATCTCATG GCTATTGCCC AACAAGTCAT CAAGCAAAAA CAACAGCAAG AACAGCAGCA   
  
  
+ ACACCAACAG CCTCATCCCC AGTTCGTCGG GTCGAATAAT AACCCCTTTT GCACCAGCCC ATGGCCCGGA   
  
  
+ CCCGTCCCTT CATCCGCCAT GTCTGCCAGC CCTCCGCCCC CTCTTGGGTT CTCCGCCGCC GCGTTTCCGG   
  
  
+ ACCCGTTTCA GGTGGGCCCG CCCTGCCCGG ATGGGGCTGA ACCGGGTTTC CAGTTTGCCC ATTTGGACCA   
  
  
+ CCACTCGAGT GGTTTCCGGT TTGCTGATTT TTGCGGTGGG GGTGGTGGTG AGTTTGACTC GGATGAGTGG   
  
  
+ ATGGAGAGTT TGATGGGTGG CGGCGGCGGT GACTCGCGGC GACTGAGAGT TCTAACCTCC AATCCCATTG   
  
  
+ CGACGCGTGG CAAGGGTCCG GTGATTTCGG TCTCTATGGT GCTGATCCGT TTGCGACGTG TTCGGAAGAC   
  
  
+ CGTTGCAGCC GCTATTCACG CTCAGCCACT GCCGCAACAG CAGCCGTCGT GGGTCCCGCC TTCTCCGCCG   
  
  
+ AAAGACACGA AGCAGTCGGC CCCACCTCAA AAGCAAAGCG ACGCCGCTGG ACCCTCGTTC TCATCCGAGG   
  
  
+ AGGAACCAGA GCCGTCATGG CCGCCGCTAA TTGCGGCGTT GTTGGAGTGC GCCAAACTCG CCGAGTCCGA   
  
  
+ CCCGGAACAC GCTGTCAAAT CGGTGATTCG ACTCAGAGAC TCAGTCTCAC AGCAGGGAGT TCCAATCCAG   
  
  
+ AGAGTGTCCT ATTACTTCTC CGAGGCACTC TACCACCGCC TTTCTCTCTC CTCCGCCCCT CAACCTCTCC   
  
  
+ CAGAAACGAC GTCGTCTGAG GAGTACACTC TCTCTTACAA GGCCCTGAAC GACGCGTGCC CGTACCATAA   
  
  
+ ATTCGCTCAC TTGACCGCAA ATCAGGCGAT TCTAGAAGCC ACTGATTCAG CCAAGAAGAT CCACATCGTT   
  
  
+ GATTTTGGGA TAATCCAGGG AGTCCAATGG GCTGCTCTGC TACAAGCCCT GGCAACCCGG CCAGCCGGGA   
  
  
+ AGCCCGAATT GATTCGAATA TCCGGAATTC CTGCTCCGGG TCTGGGGCAA TCTCCGGCGG CATCGCTGCT   
  
  
+ CGCAACTGGG AATCGCCTCC GTGAGTTCGC GAAGGTTCTA GATCTCAAGT TCGAGTTTGA GCCGGTACTC   
  
  
+ ACTCCAATCA GAGAGCTAAA CGGGTCGAAC TTCCGGGTCG ACCCAGATGA GATACTAGCT GTCAATTTCA   
  
  
+ TGCTTCAATT GTACAACCTA TTAGACGAAA CCCTGGTCGC TGTCGACGCC GCCCTGAAGC TGGCCAAGTC   
  
  
+ ATTGAACCCC AAAATCGTCA CTTTGGGTGA GTACGAGGCG GGTCTGAATC GGGCAGGTTT CTTGACCCGG   
  
  
+ TTCAAGACGG CCCTCAAGTA CTATTTGGCA ATGTTTGAAT CGGTTGAGCC CAACTTGGCC CGGGACTCGC   
  
  
+ CCGAGAGGGC TCAGGTTGAG AGGCTGTTAT TGGGCCGGCG GATCATGGGC TTAGTTGGGC CGGAGTTACC   
  
  
+ GGGAACGAGA AGGGAAAGGA TGGAGTGTAA GGAACAATGG AAGGGTTTAA TGGAAAGTGC CGGTTTCGAG   
  
  
+ CCGTGCCAAA GCAGAAACTA CGTCAGTGAA AAAGACAAGT TTTACCACTC CAGATTGAAT TACAGAGAGT   
  
  
+ G  

- +Up\_Stream \_Len000ACTATT AATAAAAATA ACTAAATCCT TTCCTATAGT TTCATATAAA ACAAAAATCC   
  
  
- ATCAATTCTC TAAGACAACG GTTTTTTTTT TAGTTCTATA ATATATAAAA ATTAATACTG TCATTAATAA   
  
  
- AATAACTTTT AGGTTGCCAA CACTAATATA TTAGTTTATT AGATTTCCAA TTTTTATAGT TTAAATAATC   
  
  
- TTTTAAGCTT TTTCAGTGCA GTAGTGTTGT AACGAAAATA TATATCATAT CTAAGGAAGC TGAAGATTTG   
  
  
- ATTAGTGAAC TTTAATCTTT AAACTTTGAA AGATTATATT TACGTACCAG AAGAAAGGTT CTAATATTAG   
  
  
- GCGTTAACGA ATTTACATCC CATGTTTATA GTTTAAATCT TCACTGATAA CTTCGGTACT TTCTATAACA   
  
  
- CTAGCATAAC TCCCACCAAC ACTCGTTATC CTACTTTGTC CTTTTTTTTT GGGATTACAA AAGTTTAAAT   
  
  
- ACCCACCGAA ACCATCATCT ATTCACTTAT CACACGTTCC CTTACCGTGG TTATACAAAC CTACCTTGCT   
  
  
- ACTCTCATTC TGCAATCCTT TCGTTGTTCT CTTCCCTTTT CCGTCATCCC TCTCTTAATA CGCACACTTA   
  
  
- TTATAATATT GTCTGCGAAA GCATACTAAT AATTTATGTT CTGAAACTCG TATGTATATT TGAACATTGA   
  
  
- AAGAAATGGA TAACAGTTAA TTAAAAACTT AACTTAGTAG ACGCTGAATA CGTATGTTCT ACTATACTAC   
  
  
- GTATAACCCG GTTCGTAAAC TAGAGTACAG GGAATACCTA GATCTAGGGT TATCTGTTTG AACGTATTCA   
  
  
- GTTCCCTACA CAAAGATTAT GTTTCCGAAA CATGAGTTCG TTAATCAAAA CTTAAGTAAA AGTCATCAAA   
  
  
- TTAAAGGTTT AGAATATTAA AAATGTTACC GTATTTAAAA ATCCTCAAAA CTCATTTATT TAATATATGT   
  
  
- TGCTTCTACA GTAAATGCTT AATTATACTG CGTGTTTTTT TTTCTTTTTT TTAATTACCA TCGAAGATAT   
  
  
- GAACTGAGGT ACAACTGTTA AGGGAGAGAG TGTATTACCA ATTTTGAACG TTGTAGTAGG TCCTGGGTAT   
  
  
- ACTGTACCCA CCACCATCTG TCACACAACA CCATCGGAGT GAGGTACGAA CGGTTAAACA TAGTAACGCG   
  
  
- ATCAGAATAA GCAGGAATCT CTATTAACTG ATTCGAGATT ACGAGATAGC ACCGACGACG TTATATTTAC   
  
  
- TAAAAAACTG GAGTTTTACT TCTGTCGATC AACATTTAAT TCTTAGAAGC AGGGAAGTAA AAAACACATG   
  
  
- CACACATAGA CGCATACACA ACTCTCTGAT CGCCTGATAA CTAACTCAAA GTTAGTTTTT TCTACTGGTA   
  
  
- GAATTCATAA AAATTAATGC GTTTGAAGAA GTAAATTTTT TTACAATGAA AATAAACTTT CTTTAACACT   
  
  
- TATTATGTTC CTTATCAAGT TAATTATATA TTAGGTATTG TATAATGGAG GATATCCGTT TTTCTTCTAA   
  
  
- TAGCATAATT AAACCCTAGT AGGGTGGGAT TTAAATTTCT TTGGTTTTGT TTAATGATTC GTTATAGAAA   
  
  
- GACACACTTT TTTATCAGTC TTTGTTTCGC TAGGCTGGCC GGTTTAGCTC TATTTGTGTC GGTTTCTTGG   
  
  
- CTTCAACCTG GTTTCACTCG GCTCAACTTT CCGTAAGTAT ACCCCAAATT AGTATTGTGT TTGTATTAAT   
  
  
- CTAACTAACT GCAGAAATTA GTCGCAAAAA CAGAAACAAG CCACGAGGGG AGTGAGCGGG GAGGCGAGAA   
  
  
- ATTTCGAATG GAGTGAGGGG GGTAAGGGTG AGGAAAAGAG AGAGGAGAGA GCGAGAGTGT GCGAGGCAGA   
  
  
- CAGTCGGGGA ACAGTAGAAG GGGGCAGAAG GAGCGTTTTT AGAGGTCGTT TTAAATATCG GCAACAACAC   
  
  
- GGGTACCACT AGACTACACT CTTCTCTAAG TCGTAAATTC TAATAACCCA GGTCTACCGA ATATACACGC   
  
  
- GGCTGTCACC ATTAGAGTAC CGATAACGGG TTGTTCAGTA GTTCGTTTTT GTTGTCGTTC TTGTCGTCGT   
  
  
- TGTGGTTGTC GGAGTAGGGG TCAAGCAGCC CAGCTTATTA TTGGGGAAAA CGTGGTCGGG TACCGGGCCT   
  
  
- GGGCAGGGAA GTAGGCGGTA CAGACGGTCG GGAGGCGGGG GAGAACCCAA GAGGCGGCGG CGCAAAGGCC   
  
  
- TGGGCAAAGT CCACCCGGGC GGGACGGGCC TACCCCGACT TGGCCCAAAG GTCAAACGGG TAAACCTGGT   
  
  
- GGTGAGCTCA CCAAAGGCCA AACGACTAAA AACGCCACCC CCACCACCAC TCAAACTGAG CCTACTCACC   
  
  
- TACCTCTCAA ACTACCCACC GCCGCCGCCA CTGAGCGCCG CTGACTCTCA AGATTGGAGG TTAGGGTAAC   
  
  
- GCTGCGCACC GTTCCCAGGC CACTAAAGCC AGAGATACCA CGACTAGGCA AACGCTGCAC AAGCCTTCTG   
  
  
- GCAACGTCGG CGATAAGTGC GAGTCGGTGA CGGCGTTGTC GTCGGCAGCA CCCAGGGCGG AAGAGGCGGC   
  
  
- TTTCTGTGCT TCGTCAGCCG GGGTGGAGTT TTCGTTTCGC TGCGGCGACC TGGGAGCAAG AGTAGGCTCC   
  
  
- TCCTTGGTCT CGGCAGTACC GGCGGCGATT AACGCCGCAA CAACCTCACG CGGTTTGAGC GGCTCAGGCT   
  
  
- GGGCCTTGTG CGACAGTTTA GCCACTAAGC TGAGTCTCTG AGTCAGAGTG TCGTCCCTCA AGGTTAGGTC   
  
  
- TCTCACAGGA TAATGAAGAG GCTCCGTGAG ATGGTGGCGG AAAGAGAGAG GAGGCGGGGA GTTGGAGAGG   
  
  
- GTCTTTGCTG CAGCAGACTC CTCATGTGAG AGAGAATGTT CCGGGACTTG CTGCGCACGG GCATGGTATT   
  
  
- TAAGCGAGTG AACTGGCGTT TAGTCCGCTA AGATCTTCGG TGACTAAGTC GGTTCTTCTA GGTGTAGCAA   
  
  
- CTAAAACCCT ATTAGGTCCC TCAGGTTACC CGACGAGACG ATGTTCGGGA CCGTTGGGCC GGTCGGCCCT   
  
  
- TCGGGCTTAA CTAAGCTTAT AGGCCTTAAG GACGAGGCCC AGACCCCGTT AGAGGCCGCC GTAGCGACGA   
  
  
- GCGTTGACCC TTAGCGGAGG CACTCAAGCG CTTCCAAGAT CTAGAGTTCA AGCTCAAACT CGGCCATGAG   
  
  
- TGAGGTTAGT CTCTCGATTT GCCCAGCTTG AAGGCCCAGC TGGGTCTACT CTATGATCGA CAGTTAAAGT   
  
  
- ACGAAGTTAA CATGTTGGAT AATCTGCTTT GGGACCAGCG ACAGCTGCGG CGGGACTTCG ACCGGTTCAG   
  
  
- TAACTTGGGG TTTTAGCAGT GAAACCCACT CATGCTCCGC CCAGACTTAG CCCGTCCAAA GAACTGGGCC   
  
  
- AAGTTCTGCC GGGAGTTCAT GATAAACCGT TACAAACTTA GCCAACTCGG GTTGAACCGG GCCCTGAGCG   
  
  
- GGCTCTCCCG AGTCCAACTC TCCGACAATA ACCCGGCCGC CTAGTACCCG AATCAACCCG GCCTCAATGG   
  
  
- CCCTTGCTCT TCCCTTTCCT ACCTCACATT CCTTGTTACC TTCCCAAATT ACCTTTCACG GCCAAAGCTC   
  
  
- GGCACGGTTT CGTCTTTGAT GCAGTCACTT TTTCTGTTCA AAATGGTGAG GTCTAACTTA ATGTCTCTCA   
  
  
- C

+     ABRE

| Site Name | Organism | Position | Strand | Matrix score. | sequence | function |
| --- | --- | --- | --- | --- | --- | --- |
| ABRE | Arabidopsis thaliana | 2510 | + | 5 | ACGTG | cis-acting element involved in the abscisic acid responsiveness |
| ABRE | Oryza sativa | 2456 | + | 9 | GCCGCGTGGC | cis-acting element involved in the abscisic acid responsiveness |
| ABRE | Arabidopsis thaliana | 1333 | + | 5 | ACGTG | cis-acting element involved in the abscisic acid responsiveness |
| ABRE | Arabidopsis thaliana | 230 | - | 5 | ACGTG | cis-acting element involved in the abscisic acid responsiveness |
| ABRE | Arabidopsis thaliana | 2286 | - | 7 | AACCCGG | cis-acting element involved in the abscisic acid responsiveness |
| ABRE | Arabidopsis thaliana | 3068 | + | 7 | AACCCGG | cis-acting element involved in the abscisic acid responsiveness |

>HU06G00029.1   
+ +Up\_Stream \_Len000TGATAA TTATTTTTAT TGATTTAGGA AAGGATATCA AAGTATATTT TGTTTTTAGG   
  
  
+ TAGTTAAGAG ATTCTGTTGC CAAAAAAAAA ATCAAGATAT TATATATTTT TAATTATGAC AGTAATTATT   
  
  
+ TTATTGAAAA TCCAACGGTT GTGATTATAT AATCAAATAA TCTAAAGGTT AAAAATATCA AATTTATTAG   
  
  
+ AAAATTCGAA AAAGTCACGT CATCACAACA TTGCTTTTAT ATATAGTATA GATTCCTTCG ACTTCTAAAC   
  
  
+ TAATCACTTG AAATTAGAAA TTTGAAACTT TCTAATATAA ATGCATGGTC TTCTTTCCAA GATTATAATC   
  
  
+ CGCAATTGCT TAAATGTAGG GTACAAATAT CAAATTTAGA AGTGACTATT GAAGCCATGA AAGATATTGT   
  
  
+ GATCGTATTG AGGGTGGTTG TGAGCAATAG GATGAAACAG GAAAAAAAAA CCCTAATGTT TTCAAATTTA   
  
  
+ TGGGTGGCTT TGGTAGTAGA TAAGTGAATA GTGTGCAAGG GAATGGCACC AATATGTTTG GATGGAACGA   
  
  
+ TGAGAGTAAG ACGTTAGGAA AGCAACAAGA GAAGGGAAAA GGCAGTAGGG AGAGAATTAT GCGTGTGAAT   
  
  
+ AATATTATAA CAGACGCTTT CGTATGATTA TTAAATACAA GACTTTGAGC ATACATATAA ACTTGTAACT   
  
  
+ TTCTTTACCT ATTGTCAATT AATTTTTGAA TTGAATCATC TGCGACTTAT GCATACAAGA TGATATGATG   
  
  
+ CATATTGGGC CAAGCATTTG ATCTCATGTC CCTTATGGAT CTAGATCCCA ATAGACAAAC TTGCATAAGT   
  
  
+ CAAGGGATGT GTTTCTAATA CAAAGGCTTT GTACTCAAGC AATTAGTTTT GAATTCATTT TCAGTAGTTT   
  
  
+ AATTTCCAAA TCTTATAATT TTTACAATGG CATAAATTTT TAGGAGTTTT GAGTAAATAA ATTATATACA   
  
  
+ ACGAAGATGT CATTTACGAA TTAATATGAC GCACAAAAAA AAAGAAAAAA AATTAATGGT AGCTTCTATA   
  
  
+ CTTGACTCCA TGTTGACAAT TCCCTCTCTC ACATAATGGT TAAAACTTGC AACATCATCC AGGACCCATA   
  
  
+ TGACATGGGT GGTGGTAGAC AGTGTGTTGT GGTAGCCTCA CTCCATGCTT GCCAATTTGT ATCATTGCGC   
  
  
+ TAGTCTTATT CGTCCTTAGA GATAATTGAC TAAGCTCTAA TGCTCTATCG TGGCTGCTGC AATATAAATG   
  
  
+ ATTTTTTGAC CTCAAAATGA AGACAGCTAG TTGTAAATTA AGAATCTTCG TCCCTTCATT TTTTGTGTAC   
  
  
+ GTGTGTATCT GCGTATGTGT TGAGAGACTA GCGGACTATT GATTGAGTTT CAATCAAAAA AGATGACCAT   
  
  
+ CTTAAGTATT TTTAATTACG CAAACTTCTT CATTTAAAAA AATGTTACTT TTATTTGAAA GAAATTGTGA   
  
  
+ ATAATACAAG GAATAGTTCA ATTAATATAT AATCCATAAC ATATTACCTC CTATAGGCAA AAAGAAGATT   
  
  
+ ATCGTATTAA TTTGGGATCA TCCCACCCTA AATTTAAAGA AACCAAAACA AATTACTAAG CAATATCTTT   
  
  
+ CTGTGTGAAA AAATAGTCAG AAACAAAGCG ATCCGACCGG CCAAATCGAG ATAAACACAG CCAAAGAACC   
  
  
+ GAAGTTGGAC CAAAGTGAGC CGAGTTGAAA GGCATTCATA TGGGGTTTAA TCATAACACA AACATAATTA   
  
  
+ GATTGATTGA CGTCTTTAAT CAGCGTTTTT GTCTTTGTTC GGTGCTCCCC TCACTCGCCC CTCCGCTCTT   
  
  
+ TAAAGCTTAC CTCACTCCCC CCATTCCCAC TCCTTTTCTC TCTCCTCTCT CGCTCTCACA CGCTCCGTCT   
  
  
+ GTCAGCCCCT TGTCATCTTC CCCCGTCTTC CTCGCAAAAA TCTCCAGCAA AATTTATAGC CGTTGTTGTG   
  
  
+ CCCATGGTGA TCTGATGTGA GAAGAGATTC AGCATTTAAG ATTATTGGGT CCAGATGGCT TATATGTGCG   
  
  
+ CCGACAGTGG TAATCTCATG GCTATTGCCC AACAAGTCAT CAAGCAAAAA CAACAGCAAG AACAGCAGCA   
  
  
+ ACACCAACAG CCTCATCCCC AGTTCGTCGG GTCGAATAAT AACCCCTTTT GCACCAGCCC ATGGCCCGGA   
  
  
+ CCCGTCCCTT CATCCGCCAT GTCTGCCAGC CCTCCGCCCC CTCTTGGGTT CTCCGCCGCC GCGTTTCCGG   
  
  
+ ACCCGTTTCA GGTGGGCCCG CCCTGCCCGG ATGGGGCTGA ACCGGGTTTC CAGTTTGCCC ATTTGGACCA   
  
  
+ CCACTCGAGT GGTTTCCGGT TTGCTGATTT TTGCGGTGGG GGTGGTGGTG AGTTTGACTC GGATGAGTGG   
  
  
+ ATGGAGAGTT TGATGGGTGG CGGCGGCGGT GACTCGCGGC GACTGAGAGT TCTAACCTCC AATCCCATTG   
  
  
+ CGACGCGTGG CAAGGGTCCG GTGATTTCGG TCTCTATGGT GCTGATCCGT TTGCGACGTG TTCGGAAGAC   
  
  
+ CGTTGCAGCC GCTATTCACG CTCAGCCACT GCCGCAACAG CAGCCGTCGT GGGTCCCGCC TTCTCCGCCG   
  
  
+ AAAGACACGA AGCAGTCGGC CCCACCTCAA AAGCAAAGCG ACGCCGCTGG ACCCTCGTTC TCATCCGAGG   
  
  
+ AGGAACCAGA GCCGTCATGG CCGCCGCTAA TTGCGGCGTT GTTGGAGTGC GCCAAACTCG CCGAGTCCGA   
  
  
+ CCCGGAACAC GCTGTCAAAT CGGTGATTCG ACTCAGAGAC TCAGTCTCAC AGCAGGGAGT TCCAATCCAG   
  
  
+ AGAGTGTCCT ATTACTTCTC CGAGGCACTC TACCACCGCC TTTCTCTCTC CTCCGCCCCT CAACCTCTCC   
  
  
+ CAGAAACGAC GTCGTCTGAG GAGTACACTC TCTCTTACAA GGCCCTGAAC GACGCGTGCC CGTACCATAA   
  
  
+ ATTCGCTCAC TTGACCGCAA ATCAGGCGAT TCTAGAAGCC ACTGATTCAG CCAAGAAGAT CCACATCGTT   
  
  
+ GATTTTGGGA TAATCCAGGG AGTCCAATGG GCTGCTCTGC TACAAGCCCT GGCAACCCGG CCAGCCGGGA   
  
  
+ AGCCCGAATT GATTCGAATA TCCGGAATTC CTGCTCCGGG TCTGGGGCAA TCTCCGGCGG CATCGCTGCT   
  
  
+ CGCAACTGGG AATCGCCTCC GTGAGTTCGC GAAGGTTCTA GATCTCAAGT TCGAGTTTGA GCCGGTACTC   
  
  
+ ACTCCAATCA GAGAGCTAAA CGGGTCGAAC TTCCGGGTCG ACCCAGATGA GATACTAGCT GTCAATTTCA   
  
  
+ TGCTTCAATT GTACAACCTA TTAGACGAAA CCCTGGTCGC TGTCGACGCC GCCCTGAAGC TGGCCAAGTC   
  
  
+ ATTGAACCCC AAAATCGTCA CTTTGGGTGA GTACGAGGCG GGTCTGAATC GGGCAGGTTT CTTGACCCGG   
  
  
+ TTCAAGACGG CCCTCAAGTA CTATTTGGCA ATGTTTGAAT CGGTTGAGCC CAACTTGGCC CGGGACTCGC   
  
  
+ CCGAGAGGGC TCAGGTTGAG AGGCTGTTAT TGGGCCGGCG GATCATGGGC TTAGTTGGGC CGGAGTTACC   
  
  
+ GGGAACGAGA AGGGAAAGGA TGGAGTGTAA GGAACAATGG AAGGGTTTAA TGGAAAGTGC CGGTTTCGAG   
  
  
+ CCGTGCCAAA GCAGAAACTA CGTCAGTGAA AAAGACAAGT TTTACCACTC CAGATTGAAT TACAGAGAGT   
  
  
+ G  

- +Up\_Stream \_Len000ACTATT AATAAAAATA ACTAAATCCT TTCCTATAGT TTCATATAAA ACAAAAATCC   
  
  
- ATCAATTCTC TAAGACAACG GTTTTTTTTT TAGTTCTATA ATATATAAAA ATTAATACTG TCATTAATAA   
  
  
- AATAACTTTT AGGTTGCCAA CACTAATATA TTAGTTTATT AGATTTCCAA TTTTTATAGT TTAAATAATC   
  
  
- TTTTAAGCTT TTTCAGTGCA GTAGTGTTGT AACGAAAATA TATATCATAT CTAAGGAAGC TGAAGATTTG   
  
  
- ATTAGTGAAC TTTAATCTTT AAACTTTGAA AGATTATATT TACGTACCAG AAGAAAGGTT CTAATATTAG   
  
  
- GCGTTAACGA ATTTACATCC CATGTTTATA GTTTAAATCT TCACTGATAA CTTCGGTACT TTCTATAACA   
  
  
- CTAGCATAAC TCCCACCAAC ACTCGTTATC CTACTTTGTC CTTTTTTTTT GGGATTACAA AAGTTTAAAT   
  
  
- ACCCACCGAA ACCATCATCT ATTCACTTAT CACACGTTCC CTTACCGTGG TTATACAAAC CTACCTTGCT   
  
  
- ACTCTCATTC TGCAATCCTT TCGTTGTTCT CTTCCCTTTT CCGTCATCCC TCTCTTAATA CGCACACTTA   
  
  
- TTATAATATT GTCTGCGAAA GCATACTAAT AATTTATGTT CTGAAACTCG TATGTATATT TGAACATTGA   
  
  
- AAGAAATGGA TAACAGTTAA TTAAAAACTT AACTTAGTAG ACGCTGAATA CGTATGTTCT ACTATACTAC   
  
  
- GTATAACCCG GTTCGTAAAC TAGAGTACAG GGAATACCTA GATCTAGGGT TATCTGTTTG AACGTATTCA   
  
  
- GTTCCCTACA CAAAGATTAT GTTTCCGAAA CATGAGTTCG TTAATCAAAA CTTAAGTAAA AGTCATCAAA   
  
  
- TTAAAGGTTT AGAATATTAA AAATGTTACC GTATTTAAAA ATCCTCAAAA CTCATTTATT TAATATATGT   
  
  
- TGCTTCTACA GTAAATGCTT AATTATACTG CGTGTTTTTT TTTCTTTTTT TTAATTACCA TCGAAGATAT   
  
  
- GAACTGAGGT ACAACTGTTA AGGGAGAGAG TGTATTACCA ATTTTGAACG TTGTAGTAGG TCCTGGGTAT   
  
  
- ACTGTACCCA CCACCATCTG TCACACAACA CCATCGGAGT GAGGTACGAA CGGTTAAACA TAGTAACGCG   
  
  
- ATCAGAATAA GCAGGAATCT CTATTAACTG ATTCGAGATT ACGAGATAGC ACCGACGACG TTATATTTAC   
  
  
- TAAAAAACTG GAGTTTTACT TCTGTCGATC AACATTTAAT TCTTAGAAGC AGGGAAGTAA AAAACACATG   
  
  
- CACACATAGA CGCATACACA ACTCTCTGAT CGCCTGATAA CTAACTCAAA GTTAGTTTTT TCTACTGGTA   
  
  
- GAATTCATAA AAATTAATGC GTTTGAAGAA GTAAATTTTT TTACAATGAA AATAAACTTT CTTTAACACT   
  
  
- TATTATGTTC CTTATCAAGT TAATTATATA TTAGGTATTG TATAATGGAG GATATCCGTT TTTCTTCTAA   
  
  
- TAGCATAATT AAACCCTAGT AGGGTGGGAT TTAAATTTCT TTGGTTTTGT TTAATGATTC GTTATAGAAA   
  
  
- GACACACTTT TTTATCAGTC TTTGTTTCGC TAGGCTGGCC GGTTTAGCTC TATTTGTGTC GGTTTCTTGG   
  
  
- CTTCAACCTG GTTTCACTCG GCTCAACTTT CCGTAAGTAT ACCCCAAATT AGTATTGTGT TTGTATTAAT   
  
  
- CTAACTAACT GCAGAAATTA GTCGCAAAAA CAGAAACAAG CCACGAGGGG AGTGAGCGGG GAGGCGAGAA   
  
  
- ATTTCGAATG GAGTGAGGGG GGTAAGGGTG AGGAAAAGAG AGAGGAGAGA GCGAGAGTGT GCGAGGCAGA   
  
  
- CAGTCGGGGA ACAGTAGAAG GGGGCAGAAG GAGCGTTTTT AGAGGTCGTT TTAAATATCG GCAACAACAC   
  
  
- GGGTACCACT AGACTACACT CTTCTCTAAG TCGTAAATTC TAATAACCCA GGTCTACCGA ATATACACGC   
  
  
- GGCTGTCACC ATTAGAGTAC CGATAACGGG TTGTTCAGTA GTTCGTTTTT GTTGTCGTTC TTGTCGTCGT   
  
  
- TGTGGTTGTC GGAGTAGGGG TCAAGCAGCC CAGCTTATTA TTGGGGAAAA CGTGGTCGGG TACCGGGCCT   
  
  
- GGGCAGGGAA GTAGGCGGTA CAGACGGTCG GGAGGCGGGG GAGAACCCAA GAGGCGGCGG CGCAAAGGCC   
  
  
- TGGGCAAAGT CCACCCGGGC GGGACGGGCC TACCCCGACT TGGCCCAAAG GTCAAACGGG TAAACCTGGT   
  
  
- GGTGAGCTCA CCAAAGGCCA AACGACTAAA AACGCCACCC CCACCACCAC TCAAACTGAG CCTACTCACC   
  
  
- TACCTCTCAA ACTACCCACC GCCGCCGCCA CTGAGCGCCG CTGACTCTCA AGATTGGAGG TTAGGGTAAC   
  
  
- GCTGCGCACC GTTCCCAGGC CACTAAAGCC AGAGATACCA CGACTAGGCA AACGCTGCAC AAGCCTTCTG   
  
  
- GCAACGTCGG CGATAAGTGC GAGTCGGTGA CGGCGTTGTC GTCGGCAGCA CCCAGGGCGG AAGAGGCGGC   
  
  
- TTTCTGTGCT TCGTCAGCCG GGGTGGAGTT TTCGTTTCGC TGCGGCGACC TGGGAGCAAG AGTAGGCTCC   
  
  
- TCCTTGGTCT CGGCAGTACC GGCGGCGATT AACGCCGCAA CAACCTCACG CGGTTTGAGC GGCTCAGGCT   
  
  
- GGGCCTTGTG CGACAGTTTA GCCACTAAGC TGAGTCTCTG AGTCAGAGTG TCGTCCCTCA AGGTTAGGTC   
  
  
- TCTCACAGGA TAATGAAGAG GCTCCGTGAG ATGGTGGCGG AAAGAGAGAG GAGGCGGGGA GTTGGAGAGG   
  
  
- GTCTTTGCTG CAGCAGACTC CTCATGTGAG AGAGAATGTT CCGGGACTTG CTGCGCACGG GCATGGTATT   
  
  
- TAAGCGAGTG AACTGGCGTT TAGTCCGCTA AGATCTTCGG TGACTAAGTC GGTTCTTCTA GGTGTAGCAA   
  
  
- CTAAAACCCT ATTAGGTCCC TCAGGTTACC CGACGAGACG ATGTTCGGGA CCGTTGGGCC GGTCGGCCCT   
  
  
- TCGGGCTTAA CTAAGCTTAT AGGCCTTAAG GACGAGGCCC AGACCCCGTT AGAGGCCGCC GTAGCGACGA   
  
  
- GCGTTGACCC TTAGCGGAGG CACTCAAGCG CTTCCAAGAT CTAGAGTTCA AGCTCAAACT CGGCCATGAG   
  
  
- TGAGGTTAGT CTCTCGATTT GCCCAGCTTG AAGGCCCAGC TGGGTCTACT CTATGATCGA CAGTTAAAGT   
  
  
- ACGAAGTTAA CATGTTGGAT AATCTGCTTT GGGACCAGCG ACAGCTGCGG CGGGACTTCG ACCGGTTCAG   
  
  
- TAACTTGGGG TTTTAGCAGT GAAACCCACT CATGCTCCGC CCAGACTTAG CCCGTCCAAA GAACTGGGCC   
  
  
- AAGTTCTGCC GGGAGTTCAT GATAAACCGT TACAAACTTA GCCAACTCGG GTTGAACCGG GCCCTGAGCG   
  
  
- GGCTCTCCCG AGTCCAACTC TCCGACAATA ACCCGGCCGC CTAGTACCCG AATCAACCCG GCCTCAATGG   
  
  
- CCCTTGCTCT TCCCTTTCCT ACCTCACATT CCTTGTTACC TTCCCAAATT ACCTTTCACG GCCAAAGCTC   
  
  
- GGCACGGTTT CGTCTTTGAT GCAGTCACTT TTTCTGTTCA AAATGGTGAG GTCTAACTTA ATGTCTCTCA   
  
  
- C

+     ABRE3a

| Site Name | Organism | Position | Strand | Matrix score. | sequence | function |
| --- | --- | --- | --- | --- | --- | --- |
| ABRE3a | Zea mays | 1332 | + | 6 | TACGTG |  |

>HU06G00029.1   
+ +Up\_Stream \_Len000TGATAA TTATTTTTAT TGATTTAGGA AAGGATATCA AAGTATATTT TGTTTTTAGG   
  
  
+ TAGTTAAGAG ATTCTGTTGC CAAAAAAAAA ATCAAGATAT TATATATTTT TAATTATGAC AGTAATTATT   
  
  
+ TTATTGAAAA TCCAACGGTT GTGATTATAT AATCAAATAA TCTAAAGGTT AAAAATATCA AATTTATTAG   
  
  
+ AAAATTCGAA AAAGTCACGT CATCACAACA TTGCTTTTAT ATATAGTATA GATTCCTTCG ACTTCTAAAC   
  
  
+ TAATCACTTG AAATTAGAAA TTTGAAACTT TCTAATATAA ATGCATGGTC TTCTTTCCAA GATTATAATC   
  
  
+ CGCAATTGCT TAAATGTAGG GTACAAATAT CAAATTTAGA AGTGACTATT GAAGCCATGA AAGATATTGT   
  
  
+ GATCGTATTG AGGGTGGTTG TGAGCAATAG GATGAAACAG GAAAAAAAAA CCCTAATGTT TTCAAATTTA   
  
  
+ TGGGTGGCTT TGGTAGTAGA TAAGTGAATA GTGTGCAAGG GAATGGCACC AATATGTTTG GATGGAACGA   
  
  
+ TGAGAGTAAG ACGTTAGGAA AGCAACAAGA GAAGGGAAAA GGCAGTAGGG AGAGAATTAT GCGTGTGAAT   
  
  
+ AATATTATAA CAGACGCTTT CGTATGATTA TTAAATACAA GACTTTGAGC ATACATATAA ACTTGTAACT   
  
  
+ TTCTTTACCT ATTGTCAATT AATTTTTGAA TTGAATCATC TGCGACTTAT GCATACAAGA TGATATGATG   
  
  
+ CATATTGGGC CAAGCATTTG ATCTCATGTC CCTTATGGAT CTAGATCCCA ATAGACAAAC TTGCATAAGT   
  
  
+ CAAGGGATGT GTTTCTAATA CAAAGGCTTT GTACTCAAGC AATTAGTTTT GAATTCATTT TCAGTAGTTT   
  
  
+ AATTTCCAAA TCTTATAATT TTTACAATGG CATAAATTTT TAGGAGTTTT GAGTAAATAA ATTATATACA   
  
  
+ ACGAAGATGT CATTTACGAA TTAATATGAC GCACAAAAAA AAAGAAAAAA AATTAATGGT AGCTTCTATA   
  
  
+ CTTGACTCCA TGTTGACAAT TCCCTCTCTC ACATAATGGT TAAAACTTGC AACATCATCC AGGACCCATA   
  
  
+ TGACATGGGT GGTGGTAGAC AGTGTGTTGT GGTAGCCTCA CTCCATGCTT GCCAATTTGT ATCATTGCGC   
  
  
+ TAGTCTTATT CGTCCTTAGA GATAATTGAC TAAGCTCTAA TGCTCTATCG TGGCTGCTGC AATATAAATG   
  
  
+ ATTTTTTGAC CTCAAAATGA AGACAGCTAG TTGTAAATTA AGAATCTTCG TCCCTTCATT TTTTGTGTAC   
  
  
+ GTGTGTATCT GCGTATGTGT TGAGAGACTA GCGGACTATT GATTGAGTTT CAATCAAAAA AGATGACCAT   
  
  
+ CTTAAGTATT TTTAATTACG CAAACTTCTT CATTTAAAAA AATGTTACTT TTATTTGAAA GAAATTGTGA   
  
  
+ ATAATACAAG GAATAGTTCA ATTAATATAT AATCCATAAC ATATTACCTC CTATAGGCAA AAAGAAGATT   
  
  
+ ATCGTATTAA TTTGGGATCA TCCCACCCTA AATTTAAAGA AACCAAAACA AATTACTAAG CAATATCTTT   
  
  
+ CTGTGTGAAA AAATAGTCAG AAACAAAGCG ATCCGACCGG CCAAATCGAG ATAAACACAG CCAAAGAACC   
  
  
+ GAAGTTGGAC CAAAGTGAGC CGAGTTGAAA GGCATTCATA TGGGGTTTAA TCATAACACA AACATAATTA   
  
  
+ GATTGATTGA CGTCTTTAAT CAGCGTTTTT GTCTTTGTTC GGTGCTCCCC TCACTCGCCC CTCCGCTCTT   
  
  
+ TAAAGCTTAC CTCACTCCCC CCATTCCCAC TCCTTTTCTC TCTCCTCTCT CGCTCTCACA CGCTCCGTCT   
  
  
+ GTCAGCCCCT TGTCATCTTC CCCCGTCTTC CTCGCAAAAA TCTCCAGCAA AATTTATAGC CGTTGTTGTG   
  
  
+ CCCATGGTGA TCTGATGTGA GAAGAGATTC AGCATTTAAG ATTATTGGGT CCAGATGGCT TATATGTGCG   
  
  
+ CCGACAGTGG TAATCTCATG GCTATTGCCC AACAAGTCAT CAAGCAAAAA CAACAGCAAG AACAGCAGCA   
  
  
+ ACACCAACAG CCTCATCCCC AGTTCGTCGG GTCGAATAAT AACCCCTTTT GCACCAGCCC ATGGCCCGGA   
  
  
+ CCCGTCCCTT CATCCGCCAT GTCTGCCAGC CCTCCGCCCC CTCTTGGGTT CTCCGCCGCC GCGTTTCCGG   
  
  
+ ACCCGTTTCA GGTGGGCCCG CCCTGCCCGG ATGGGGCTGA ACCGGGTTTC CAGTTTGCCC ATTTGGACCA   
  
  
+ CCACTCGAGT GGTTTCCGGT TTGCTGATTT TTGCGGTGGG GGTGGTGGTG AGTTTGACTC GGATGAGTGG   
  
  
+ ATGGAGAGTT TGATGGGTGG CGGCGGCGGT GACTCGCGGC GACTGAGAGT TCTAACCTCC AATCCCATTG   
  
  
+ CGACGCGTGG CAAGGGTCCG GTGATTTCGG TCTCTATGGT GCTGATCCGT TTGCGACGTG TTCGGAAGAC   
  
  
+ CGTTGCAGCC GCTATTCACG CTCAGCCACT GCCGCAACAG CAGCCGTCGT GGGTCCCGCC TTCTCCGCCG   
  
  
+ AAAGACACGA AGCAGTCGGC CCCACCTCAA AAGCAAAGCG ACGCCGCTGG ACCCTCGTTC TCATCCGAGG   
  
  
+ AGGAACCAGA GCCGTCATGG CCGCCGCTAA TTGCGGCGTT GTTGGAGTGC GCCAAACTCG CCGAGTCCGA   
  
  
+ CCCGGAACAC GCTGTCAAAT CGGTGATTCG ACTCAGAGAC TCAGTCTCAC AGCAGGGAGT TCCAATCCAG   
  
  
+ AGAGTGTCCT ATTACTTCTC CGAGGCACTC TACCACCGCC TTTCTCTCTC CTCCGCCCCT CAACCTCTCC   
  
  
+ CAGAAACGAC GTCGTCTGAG GAGTACACTC TCTCTTACAA GGCCCTGAAC GACGCGTGCC CGTACCATAA   
  
  
+ ATTCGCTCAC TTGACCGCAA ATCAGGCGAT TCTAGAAGCC ACTGATTCAG CCAAGAAGAT CCACATCGTT   
  
  
+ GATTTTGGGA TAATCCAGGG AGTCCAATGG GCTGCTCTGC TACAAGCCCT GGCAACCCGG CCAGCCGGGA   
  
  
+ AGCCCGAATT GATTCGAATA TCCGGAATTC CTGCTCCGGG TCTGGGGCAA TCTCCGGCGG CATCGCTGCT   
  
  
+ CGCAACTGGG AATCGCCTCC GTGAGTTCGC GAAGGTTCTA GATCTCAAGT TCGAGTTTGA GCCGGTACTC   
  
  
+ ACTCCAATCA GAGAGCTAAA CGGGTCGAAC TTCCGGGTCG ACCCAGATGA GATACTAGCT GTCAATTTCA   
  
  
+ TGCTTCAATT GTACAACCTA TTAGACGAAA CCCTGGTCGC TGTCGACGCC GCCCTGAAGC TGGCCAAGTC   
  
  
+ ATTGAACCCC AAAATCGTCA CTTTGGGTGA GTACGAGGCG GGTCTGAATC GGGCAGGTTT CTTGACCCGG   
  
  
+ TTCAAGACGG CCCTCAAGTA CTATTTGGCA ATGTTTGAAT CGGTTGAGCC CAACTTGGCC CGGGACTCGC   
  
  
+ CCGAGAGGGC TCAGGTTGAG AGGCTGTTAT TGGGCCGGCG GATCATGGGC TTAGTTGGGC CGGAGTTACC   
  
  
+ GGGAACGAGA AGGGAAAGGA TGGAGTGTAA GGAACAATGG AAGGGTTTAA TGGAAAGTGC CGGTTTCGAG   
  
  
+ CCGTGCCAAA GCAGAAACTA CGTCAGTGAA AAAGACAAGT TTTACCACTC CAGATTGAAT TACAGAGAGT   
  
  
+ G  

- +Up\_Stream \_Len000ACTATT AATAAAAATA ACTAAATCCT TTCCTATAGT TTCATATAAA ACAAAAATCC   
  
  
- ATCAATTCTC TAAGACAACG GTTTTTTTTT TAGTTCTATA ATATATAAAA ATTAATACTG TCATTAATAA   
  
  
- AATAACTTTT AGGTTGCCAA CACTAATATA TTAGTTTATT AGATTTCCAA TTTTTATAGT TTAAATAATC   
  
  
- TTTTAAGCTT TTTCAGTGCA GTAGTGTTGT AACGAAAATA TATATCATAT CTAAGGAAGC TGAAGATTTG   
  
  
- ATTAGTGAAC TTTAATCTTT AAACTTTGAA AGATTATATT TACGTACCAG AAGAAAGGTT CTAATATTAG   
  
  
- GCGTTAACGA ATTTACATCC CATGTTTATA GTTTAAATCT TCACTGATAA CTTCGGTACT TTCTATAACA   
  
  
- CTAGCATAAC TCCCACCAAC ACTCGTTATC CTACTTTGTC CTTTTTTTTT GGGATTACAA AAGTTTAAAT   
  
  
- ACCCACCGAA ACCATCATCT ATTCACTTAT CACACGTTCC CTTACCGTGG TTATACAAAC CTACCTTGCT   
  
  
- ACTCTCATTC TGCAATCCTT TCGTTGTTCT CTTCCCTTTT CCGTCATCCC TCTCTTAATA CGCACACTTA   
  
  
- TTATAATATT GTCTGCGAAA GCATACTAAT AATTTATGTT CTGAAACTCG TATGTATATT TGAACATTGA   
  
  
- AAGAAATGGA TAACAGTTAA TTAAAAACTT AACTTAGTAG ACGCTGAATA CGTATGTTCT ACTATACTAC   
  
  
- GTATAACCCG GTTCGTAAAC TAGAGTACAG GGAATACCTA GATCTAGGGT TATCTGTTTG AACGTATTCA   
  
  
- GTTCCCTACA CAAAGATTAT GTTTCCGAAA CATGAGTTCG TTAATCAAAA CTTAAGTAAA AGTCATCAAA   
  
  
- TTAAAGGTTT AGAATATTAA AAATGTTACC GTATTTAAAA ATCCTCAAAA CTCATTTATT TAATATATGT   
  
  
- TGCTTCTACA GTAAATGCTT AATTATACTG CGTGTTTTTT TTTCTTTTTT TTAATTACCA TCGAAGATAT   
  
  
- GAACTGAGGT ACAACTGTTA AGGGAGAGAG TGTATTACCA ATTTTGAACG TTGTAGTAGG TCCTGGGTAT   
  
  
- ACTGTACCCA CCACCATCTG TCACACAACA CCATCGGAGT GAGGTACGAA CGGTTAAACA TAGTAACGCG   
  
  
- ATCAGAATAA GCAGGAATCT CTATTAACTG ATTCGAGATT ACGAGATAGC ACCGACGACG TTATATTTAC   
  
  
- TAAAAAACTG GAGTTTTACT TCTGTCGATC AACATTTAAT TCTTAGAAGC AGGGAAGTAA AAAACACATG   
  
  
- CACACATAGA CGCATACACA ACTCTCTGAT CGCCTGATAA CTAACTCAAA GTTAGTTTTT TCTACTGGTA   
  
  
- GAATTCATAA AAATTAATGC GTTTGAAGAA GTAAATTTTT TTACAATGAA AATAAACTTT CTTTAACACT   
  
  
- TATTATGTTC CTTATCAAGT TAATTATATA TTAGGTATTG TATAATGGAG GATATCCGTT TTTCTTCTAA   
  
  
- TAGCATAATT AAACCCTAGT AGGGTGGGAT TTAAATTTCT TTGGTTTTGT TTAATGATTC GTTATAGAAA   
  
  
- GACACACTTT TTTATCAGTC TTTGTTTCGC TAGGCTGGCC GGTTTAGCTC TATTTGTGTC GGTTTCTTGG   
  
  
- CTTCAACCTG GTTTCACTCG GCTCAACTTT CCGTAAGTAT ACCCCAAATT AGTATTGTGT TTGTATTAAT   
  
  
- CTAACTAACT GCAGAAATTA GTCGCAAAAA CAGAAACAAG CCACGAGGGG AGTGAGCGGG GAGGCGAGAA   
  
  
- ATTTCGAATG GAGTGAGGGG GGTAAGGGTG AGGAAAAGAG AGAGGAGAGA GCGAGAGTGT GCGAGGCAGA   
  
  
- CAGTCGGGGA ACAGTAGAAG GGGGCAGAAG GAGCGTTTTT AGAGGTCGTT TTAAATATCG GCAACAACAC   
  
  
- GGGTACCACT AGACTACACT CTTCTCTAAG TCGTAAATTC TAATAACCCA GGTCTACCGA ATATACACGC   
  
  
- GGCTGTCACC ATTAGAGTAC CGATAACGGG TTGTTCAGTA GTTCGTTTTT GTTGTCGTTC TTGTCGTCGT   
  
  
- TGTGGTTGTC GGAGTAGGGG TCAAGCAGCC CAGCTTATTA TTGGGGAAAA CGTGGTCGGG TACCGGGCCT   
  
  
- GGGCAGGGAA GTAGGCGGTA CAGACGGTCG GGAGGCGGGG GAGAACCCAA GAGGCGGCGG CGCAAAGGCC   
  
  
- TGGGCAAAGT CCACCCGGGC GGGACGGGCC TACCCCGACT TGGCCCAAAG GTCAAACGGG TAAACCTGGT   
  
  
- GGTGAGCTCA CCAAAGGCCA AACGACTAAA AACGCCACCC CCACCACCAC TCAAACTGAG CCTACTCACC   
  
  
- TACCTCTCAA ACTACCCACC GCCGCCGCCA CTGAGCGCCG CTGACTCTCA AGATTGGAGG TTAGGGTAAC   
  
  
- GCTGCGCACC GTTCCCAGGC CACTAAAGCC AGAGATACCA CGACTAGGCA AACGCTGCAC AAGCCTTCTG   
  
  
- GCAACGTCGG CGATAAGTGC GAGTCGGTGA CGGCGTTGTC GTCGGCAGCA CCCAGGGCGG AAGAGGCGGC   
  
  
- TTTCTGTGCT TCGTCAGCCG GGGTGGAGTT TTCGTTTCGC TGCGGCGACC TGGGAGCAAG AGTAGGCTCC   
  
  
- TCCTTGGTCT CGGCAGTACC GGCGGCGATT AACGCCGCAA CAACCTCACG CGGTTTGAGC GGCTCAGGCT   
  
  
- GGGCCTTGTG CGACAGTTTA GCCACTAAGC TGAGTCTCTG AGTCAGAGTG TCGTCCCTCA AGGTTAGGTC   
  
  
- TCTCACAGGA TAATGAAGAG GCTCCGTGAG ATGGTGGCGG AAAGAGAGAG GAGGCGGGGA GTTGGAGAGG   
  
  
- GTCTTTGCTG CAGCAGACTC CTCATGTGAG AGAGAATGTT CCGGGACTTG CTGCGCACGG GCATGGTATT   
  
  
- TAAGCGAGTG AACTGGCGTT TAGTCCGCTA AGATCTTCGG TGACTAAGTC GGTTCTTCTA GGTGTAGCAA   
  
  
- CTAAAACCCT ATTAGGTCCC TCAGGTTACC CGACGAGACG ATGTTCGGGA CCGTTGGGCC GGTCGGCCCT   
  
  
- TCGGGCTTAA CTAAGCTTAT AGGCCTTAAG GACGAGGCCC AGACCCCGTT AGAGGCCGCC GTAGCGACGA   
  
  
- GCGTTGACCC TTAGCGGAGG CACTCAAGCG CTTCCAAGAT CTAGAGTTCA AGCTCAAACT CGGCCATGAG   
  
  
- TGAGGTTAGT CTCTCGATTT GCCCAGCTTG AAGGCCCAGC TGGGTCTACT CTATGATCGA CAGTTAAAGT   
  
  
- ACGAAGTTAA CATGTTGGAT AATCTGCTTT GGGACCAGCG ACAGCTGCGG CGGGACTTCG ACCGGTTCAG   
  
  
- TAACTTGGGG TTTTAGCAGT GAAACCCACT CATGCTCCGC CCAGACTTAG CCCGTCCAAA GAACTGGGCC   
  
  
- AAGTTCTGCC GGGAGTTCAT GATAAACCGT TACAAACTTA GCCAACTCGG GTTGAACCGG GCCCTGAGCG   
  
  
- GGCTCTCCCG AGTCCAACTC TCCGACAATA ACCCGGCCGC CTAGTACCCG AATCAACCCG GCCTCAATGG   
  
  
- CCCTTGCTCT TCCCTTTCCT ACCTCACATT CCTTGTTACC TTCCCAAATT ACCTTTCACG GCCAAAGCTC   
  
  
- GGCACGGTTT CGTCTTTGAT GCAGTCACTT TTTCTGTTCA AAATGGTGAG GTCTAACTTA ATGTCTCTCA   
  
  
- C

+     ABRE4

| Site Name | Organism | Position | Strand | Matrix score. | sequence | function |
| --- | --- | --- | --- | --- | --- | --- |
| ABRE4 | Zea mays | 1332 | - | 6 | CACGTA |  |

>HU06G00029.1   
+ +Up\_Stream \_Len000TGATAA TTATTTTTAT TGATTTAGGA AAGGATATCA AAGTATATTT TGTTTTTAGG   
  
  
+ TAGTTAAGAG ATTCTGTTGC CAAAAAAAAA ATCAAGATAT TATATATTTT TAATTATGAC AGTAATTATT   
  
  
+ TTATTGAAAA TCCAACGGTT GTGATTATAT AATCAAATAA TCTAAAGGTT AAAAATATCA AATTTATTAG   
  
  
+ AAAATTCGAA AAAGTCACGT CATCACAACA TTGCTTTTAT ATATAGTATA GATTCCTTCG ACTTCTAAAC   
  
  
+ TAATCACTTG AAATTAGAAA TTTGAAACTT TCTAATATAA ATGCATGGTC TTCTTTCCAA GATTATAATC   
  
  
+ CGCAATTGCT TAAATGTAGG GTACAAATAT CAAATTTAGA AGTGACTATT GAAGCCATGA AAGATATTGT   
  
  
+ GATCGTATTG AGGGTGGTTG TGAGCAATAG GATGAAACAG GAAAAAAAAA CCCTAATGTT TTCAAATTTA   
  
  
+ TGGGTGGCTT TGGTAGTAGA TAAGTGAATA GTGTGCAAGG GAATGGCACC AATATGTTTG GATGGAACGA   
  
  
+ TGAGAGTAAG ACGTTAGGAA AGCAACAAGA GAAGGGAAAA GGCAGTAGGG AGAGAATTAT GCGTGTGAAT   
  
  
+ AATATTATAA CAGACGCTTT CGTATGATTA TTAAATACAA GACTTTGAGC ATACATATAA ACTTGTAACT   
  
  
+ TTCTTTACCT ATTGTCAATT AATTTTTGAA TTGAATCATC TGCGACTTAT GCATACAAGA TGATATGATG   
  
  
+ CATATTGGGC CAAGCATTTG ATCTCATGTC CCTTATGGAT CTAGATCCCA ATAGACAAAC TTGCATAAGT   
  
  
+ CAAGGGATGT GTTTCTAATA CAAAGGCTTT GTACTCAAGC AATTAGTTTT GAATTCATTT TCAGTAGTTT   
  
  
+ AATTTCCAAA TCTTATAATT TTTACAATGG CATAAATTTT TAGGAGTTTT GAGTAAATAA ATTATATACA   
  
  
+ ACGAAGATGT CATTTACGAA TTAATATGAC GCACAAAAAA AAAGAAAAAA AATTAATGGT AGCTTCTATA   
  
  
+ CTTGACTCCA TGTTGACAAT TCCCTCTCTC ACATAATGGT TAAAACTTGC AACATCATCC AGGACCCATA   
  
  
+ TGACATGGGT GGTGGTAGAC AGTGTGTTGT GGTAGCCTCA CTCCATGCTT GCCAATTTGT ATCATTGCGC   
  
  
+ TAGTCTTATT CGTCCTTAGA GATAATTGAC TAAGCTCTAA TGCTCTATCG TGGCTGCTGC AATATAAATG   
  
  
+ ATTTTTTGAC CTCAAAATGA AGACAGCTAG TTGTAAATTA AGAATCTTCG TCCCTTCATT TTTTGTGTAC   
  
  
+ GTGTGTATCT GCGTATGTGT TGAGAGACTA GCGGACTATT GATTGAGTTT CAATCAAAAA AGATGACCAT   
  
  
+ CTTAAGTATT TTTAATTACG CAAACTTCTT CATTTAAAAA AATGTTACTT TTATTTGAAA GAAATTGTGA   
  
  
+ ATAATACAAG GAATAGTTCA ATTAATATAT AATCCATAAC ATATTACCTC CTATAGGCAA AAAGAAGATT   
  
  
+ ATCGTATTAA TTTGGGATCA TCCCACCCTA AATTTAAAGA AACCAAAACA AATTACTAAG CAATATCTTT   
  
  
+ CTGTGTGAAA AAATAGTCAG AAACAAAGCG ATCCGACCGG CCAAATCGAG ATAAACACAG CCAAAGAACC   
  
  
+ GAAGTTGGAC CAAAGTGAGC CGAGTTGAAA GGCATTCATA TGGGGTTTAA TCATAACACA AACATAATTA   
  
  
+ GATTGATTGA CGTCTTTAAT CAGCGTTTTT GTCTTTGTTC GGTGCTCCCC TCACTCGCCC CTCCGCTCTT   
  
  
+ TAAAGCTTAC CTCACTCCCC CCATTCCCAC TCCTTTTCTC TCTCCTCTCT CGCTCTCACA CGCTCCGTCT   
  
  
+ GTCAGCCCCT TGTCATCTTC CCCCGTCTTC CTCGCAAAAA TCTCCAGCAA AATTTATAGC CGTTGTTGTG   
  
  
+ CCCATGGTGA TCTGATGTGA GAAGAGATTC AGCATTTAAG ATTATTGGGT CCAGATGGCT TATATGTGCG   
  
  
+ CCGACAGTGG TAATCTCATG GCTATTGCCC AACAAGTCAT CAAGCAAAAA CAACAGCAAG AACAGCAGCA   
  
  
+ ACACCAACAG CCTCATCCCC AGTTCGTCGG GTCGAATAAT AACCCCTTTT GCACCAGCCC ATGGCCCGGA   
  
  
+ CCCGTCCCTT CATCCGCCAT GTCTGCCAGC CCTCCGCCCC CTCTTGGGTT CTCCGCCGCC GCGTTTCCGG   
  
  
+ ACCCGTTTCA GGTGGGCCCG CCCTGCCCGG ATGGGGCTGA ACCGGGTTTC CAGTTTGCCC ATTTGGACCA   
  
  
+ CCACTCGAGT GGTTTCCGGT TTGCTGATTT TTGCGGTGGG GGTGGTGGTG AGTTTGACTC GGATGAGTGG   
  
  
+ ATGGAGAGTT TGATGGGTGG CGGCGGCGGT GACTCGCGGC GACTGAGAGT TCTAACCTCC AATCCCATTG   
  
  
+ CGACGCGTGG CAAGGGTCCG GTGATTTCGG TCTCTATGGT GCTGATCCGT TTGCGACGTG TTCGGAAGAC   
  
  
+ CGTTGCAGCC GCTATTCACG CTCAGCCACT GCCGCAACAG CAGCCGTCGT GGGTCCCGCC TTCTCCGCCG   
  
  
+ AAAGACACGA AGCAGTCGGC CCCACCTCAA AAGCAAAGCG ACGCCGCTGG ACCCTCGTTC TCATCCGAGG   
  
  
+ AGGAACCAGA GCCGTCATGG CCGCCGCTAA TTGCGGCGTT GTTGGAGTGC GCCAAACTCG CCGAGTCCGA   
  
  
+ CCCGGAACAC GCTGTCAAAT CGGTGATTCG ACTCAGAGAC TCAGTCTCAC AGCAGGGAGT TCCAATCCAG   
  
  
+ AGAGTGTCCT ATTACTTCTC CGAGGCACTC TACCACCGCC TTTCTCTCTC CTCCGCCCCT CAACCTCTCC   
  
  
+ CAGAAACGAC GTCGTCTGAG GAGTACACTC TCTCTTACAA GGCCCTGAAC GACGCGTGCC CGTACCATAA   
  
  
+ ATTCGCTCAC TTGACCGCAA ATCAGGCGAT TCTAGAAGCC ACTGATTCAG CCAAGAAGAT CCACATCGTT   
  
  
+ GATTTTGGGA TAATCCAGGG AGTCCAATGG GCTGCTCTGC TACAAGCCCT GGCAACCCGG CCAGCCGGGA   
  
  
+ AGCCCGAATT GATTCGAATA TCCGGAATTC CTGCTCCGGG TCTGGGGCAA TCTCCGGCGG CATCGCTGCT   
  
  
+ CGCAACTGGG AATCGCCTCC GTGAGTTCGC GAAGGTTCTA GATCTCAAGT TCGAGTTTGA GCCGGTACTC   
  
  
+ ACTCCAATCA GAGAGCTAAA CGGGTCGAAC TTCCGGGTCG ACCCAGATGA GATACTAGCT GTCAATTTCA   
  
  
+ TGCTTCAATT GTACAACCTA TTAGACGAAA CCCTGGTCGC TGTCGACGCC GCCCTGAAGC TGGCCAAGTC   
  
  
+ ATTGAACCCC AAAATCGTCA CTTTGGGTGA GTACGAGGCG GGTCTGAATC GGGCAGGTTT CTTGACCCGG   
  
  
+ TTCAAGACGG CCCTCAAGTA CTATTTGGCA ATGTTTGAAT CGGTTGAGCC CAACTTGGCC CGGGACTCGC   
  
  
+ CCGAGAGGGC TCAGGTTGAG AGGCTGTTAT TGGGCCGGCG GATCATGGGC TTAGTTGGGC CGGAGTTACC   
  
  
+ GGGAACGAGA AGGGAAAGGA TGGAGTGTAA GGAACAATGG AAGGGTTTAA TGGAAAGTGC CGGTTTCGAG   
  
  
+ CCGTGCCAAA GCAGAAACTA CGTCAGTGAA AAAGACAAGT TTTACCACTC CAGATTGAAT TACAGAGAGT   
  
  
+ G  

- +Up\_Stream \_Len000ACTATT AATAAAAATA ACTAAATCCT TTCCTATAGT TTCATATAAA ACAAAAATCC   
  
  
- ATCAATTCTC TAAGACAACG GTTTTTTTTT TAGTTCTATA ATATATAAAA ATTAATACTG TCATTAATAA   
  
  
- AATAACTTTT AGGTTGCCAA CACTAATATA TTAGTTTATT AGATTTCCAA TTTTTATAGT TTAAATAATC   
  
  
- TTTTAAGCTT TTTCAGTGCA GTAGTGTTGT AACGAAAATA TATATCATAT CTAAGGAAGC TGAAGATTTG   
  
  
- ATTAGTGAAC TTTAATCTTT AAACTTTGAA AGATTATATT TACGTACCAG AAGAAAGGTT CTAATATTAG   
  
  
- GCGTTAACGA ATTTACATCC CATGTTTATA GTTTAAATCT TCACTGATAA CTTCGGTACT TTCTATAACA   
  
  
- CTAGCATAAC TCCCACCAAC ACTCGTTATC CTACTTTGTC CTTTTTTTTT GGGATTACAA AAGTTTAAAT   
  
  
- ACCCACCGAA ACCATCATCT ATTCACTTAT CACACGTTCC CTTACCGTGG TTATACAAAC CTACCTTGCT   
  
  
- ACTCTCATTC TGCAATCCTT TCGTTGTTCT CTTCCCTTTT CCGTCATCCC TCTCTTAATA CGCACACTTA   
  
  
- TTATAATATT GTCTGCGAAA GCATACTAAT AATTTATGTT CTGAAACTCG TATGTATATT TGAACATTGA   
  
  
- AAGAAATGGA TAACAGTTAA TTAAAAACTT AACTTAGTAG ACGCTGAATA CGTATGTTCT ACTATACTAC   
  
  
- GTATAACCCG GTTCGTAAAC TAGAGTACAG GGAATACCTA GATCTAGGGT TATCTGTTTG AACGTATTCA   
  
  
- GTTCCCTACA CAAAGATTAT GTTTCCGAAA CATGAGTTCG TTAATCAAAA CTTAAGTAAA AGTCATCAAA   
  
  
- TTAAAGGTTT AGAATATTAA AAATGTTACC GTATTTAAAA ATCCTCAAAA CTCATTTATT TAATATATGT   
  
  
- TGCTTCTACA GTAAATGCTT AATTATACTG CGTGTTTTTT TTTCTTTTTT TTAATTACCA TCGAAGATAT   
  
  
- GAACTGAGGT ACAACTGTTA AGGGAGAGAG TGTATTACCA ATTTTGAACG TTGTAGTAGG TCCTGGGTAT   
  
  
- ACTGTACCCA CCACCATCTG TCACACAACA CCATCGGAGT GAGGTACGAA CGGTTAAACA TAGTAACGCG   
  
  
- ATCAGAATAA GCAGGAATCT CTATTAACTG ATTCGAGATT ACGAGATAGC ACCGACGACG TTATATTTAC   
  
  
- TAAAAAACTG GAGTTTTACT TCTGTCGATC AACATTTAAT TCTTAGAAGC AGGGAAGTAA AAAACACATG   
  
  
- CACACATAGA CGCATACACA ACTCTCTGAT CGCCTGATAA CTAACTCAAA GTTAGTTTTT TCTACTGGTA   
  
  
- GAATTCATAA AAATTAATGC GTTTGAAGAA GTAAATTTTT TTACAATGAA AATAAACTTT CTTTAACACT   
  
  
- TATTATGTTC CTTATCAAGT TAATTATATA TTAGGTATTG TATAATGGAG GATATCCGTT TTTCTTCTAA   
  
  
- TAGCATAATT AAACCCTAGT AGGGTGGGAT TTAAATTTCT TTGGTTTTGT TTAATGATTC GTTATAGAAA   
  
  
- GACACACTTT TTTATCAGTC TTTGTTTCGC TAGGCTGGCC GGTTTAGCTC TATTTGTGTC GGTTTCTTGG   
  
  
- CTTCAACCTG GTTTCACTCG GCTCAACTTT CCGTAAGTAT ACCCCAAATT AGTATTGTGT TTGTATTAAT   
  
  
- CTAACTAACT GCAGAAATTA GTCGCAAAAA CAGAAACAAG CCACGAGGGG AGTGAGCGGG GAGGCGAGAA   
  
  
- ATTTCGAATG GAGTGAGGGG GGTAAGGGTG AGGAAAAGAG AGAGGAGAGA GCGAGAGTGT GCGAGGCAGA   
  
  
- CAGTCGGGGA ACAGTAGAAG GGGGCAGAAG GAGCGTTTTT AGAGGTCGTT TTAAATATCG GCAACAACAC   
  
  
- GGGTACCACT AGACTACACT CTTCTCTAAG TCGTAAATTC TAATAACCCA GGTCTACCGA ATATACACGC   
  
  
- GGCTGTCACC ATTAGAGTAC CGATAACGGG TTGTTCAGTA GTTCGTTTTT GTTGTCGTTC TTGTCGTCGT   
  
  
- TGTGGTTGTC GGAGTAGGGG TCAAGCAGCC CAGCTTATTA TTGGGGAAAA CGTGGTCGGG TACCGGGCCT   
  
  
- GGGCAGGGAA GTAGGCGGTA CAGACGGTCG GGAGGCGGGG GAGAACCCAA GAGGCGGCGG CGCAAAGGCC   
  
  
- TGGGCAAAGT CCACCCGGGC GGGACGGGCC TACCCCGACT TGGCCCAAAG GTCAAACGGG TAAACCTGGT   
  
  
- GGTGAGCTCA CCAAAGGCCA AACGACTAAA AACGCCACCC CCACCACCAC TCAAACTGAG CCTACTCACC   
  
  
- TACCTCTCAA ACTACCCACC GCCGCCGCCA CTGAGCGCCG CTGACTCTCA AGATTGGAGG TTAGGGTAAC   
  
  
- GCTGCGCACC GTTCCCAGGC CACTAAAGCC AGAGATACCA CGACTAGGCA AACGCTGCAC AAGCCTTCTG   
  
  
- GCAACGTCGG CGATAAGTGC GAGTCGGTGA CGGCGTTGTC GTCGGCAGCA CCCAGGGCGG AAGAGGCGGC   
  
  
- TTTCTGTGCT TCGTCAGCCG GGGTGGAGTT TTCGTTTCGC TGCGGCGACC TGGGAGCAAG AGTAGGCTCC   
  
  
- TCCTTGGTCT CGGCAGTACC GGCGGCGATT AACGCCGCAA CAACCTCACG CGGTTTGAGC GGCTCAGGCT   
  
  
- GGGCCTTGTG CGACAGTTTA GCCACTAAGC TGAGTCTCTG AGTCAGAGTG TCGTCCCTCA AGGTTAGGTC   
  
  
- TCTCACAGGA TAATGAAGAG GCTCCGTGAG ATGGTGGCGG AAAGAGAGAG GAGGCGGGGA GTTGGAGAGG   
  
  
- GTCTTTGCTG CAGCAGACTC CTCATGTGAG AGAGAATGTT CCGGGACTTG CTGCGCACGG GCATGGTATT   
  
  
- TAAGCGAGTG AACTGGCGTT TAGTCCGCTA AGATCTTCGG TGACTAAGTC GGTTCTTCTA GGTGTAGCAA   
  
  
- CTAAAACCCT ATTAGGTCCC TCAGGTTACC CGACGAGACG ATGTTCGGGA CCGTTGGGCC GGTCGGCCCT   
  
  
- TCGGGCTTAA CTAAGCTTAT AGGCCTTAAG GACGAGGCCC AGACCCCGTT AGAGGCCGCC GTAGCGACGA   
  
  
- GCGTTGACCC TTAGCGGAGG CACTCAAGCG CTTCCAAGAT CTAGAGTTCA AGCTCAAACT CGGCCATGAG   
  
  
- TGAGGTTAGT CTCTCGATTT GCCCAGCTTG AAGGCCCAGC TGGGTCTACT CTATGATCGA CAGTTAAAGT   
  
  
- ACGAAGTTAA CATGTTGGAT AATCTGCTTT GGGACCAGCG ACAGCTGCGG CGGGACTTCG ACCGGTTCAG   
  
  
- TAACTTGGGG TTTTAGCAGT GAAACCCACT CATGCTCCGC CCAGACTTAG CCCGTCCAAA GAACTGGGCC   
  
  
- AAGTTCTGCC GGGAGTTCAT GATAAACCGT TACAAACTTA GCCAACTCGG GTTGAACCGG GCCCTGAGCG   
  
  
- GGCTCTCCCG AGTCCAACTC TCCGACAATA ACCCGGCCGC CTAGTACCCG AATCAACCCG GCCTCAATGG   
  
  
- CCCTTGCTCT TCCCTTTCCT ACCTCACATT CCTTGTTACC TTCCCAAATT ACCTTTCACG GCCAAAGCTC   
  
  
- GGCACGGTTT CGTCTTTGAT GCAGTCACTT TTTCTGTTCA AAATGGTGAG GTCTAACTTA ATGTCTCTCA   
  
  
- C

+     ACE

| Site Name | Organism | Position | Strand | Matrix score. | sequence | function |
| --- | --- | --- | --- | --- | --- | --- |
| ACE | Petroselinum crispum | 572 | - | 9 | CTAACGTATT | cis-acting element involved in light responsiveness |

>HU06G00029.1   
+ +Up\_Stream \_Len000TGATAA TTATTTTTAT TGATTTAGGA AAGGATATCA AAGTATATTT TGTTTTTAGG   
  
  
+ TAGTTAAGAG ATTCTGTTGC CAAAAAAAAA ATCAAGATAT TATATATTTT TAATTATGAC AGTAATTATT   
  
  
+ TTATTGAAAA TCCAACGGTT GTGATTATAT AATCAAATAA TCTAAAGGTT AAAAATATCA AATTTATTAG   
  
  
+ AAAATTCGAA AAAGTCACGT CATCACAACA TTGCTTTTAT ATATAGTATA GATTCCTTCG ACTTCTAAAC   
  
  
+ TAATCACTTG AAATTAGAAA TTTGAAACTT TCTAATATAA ATGCATGGTC TTCTTTCCAA GATTATAATC   
  
  
+ CGCAATTGCT TAAATGTAGG GTACAAATAT CAAATTTAGA AGTGACTATT GAAGCCATGA AAGATATTGT   
  
  
+ GATCGTATTG AGGGTGGTTG TGAGCAATAG GATGAAACAG GAAAAAAAAA CCCTAATGTT TTCAAATTTA   
  
  
+ TGGGTGGCTT TGGTAGTAGA TAAGTGAATA GTGTGCAAGG GAATGGCACC AATATGTTTG GATGGAACGA   
  
  
+ TGAGAGTAAG ACGTTAGGAA AGCAACAAGA GAAGGGAAAA GGCAGTAGGG AGAGAATTAT GCGTGTGAAT   
  
  
+ AATATTATAA CAGACGCTTT CGTATGATTA TTAAATACAA GACTTTGAGC ATACATATAA ACTTGTAACT   
  
  
+ TTCTTTACCT ATTGTCAATT AATTTTTGAA TTGAATCATC TGCGACTTAT GCATACAAGA TGATATGATG   
  
  
+ CATATTGGGC CAAGCATTTG ATCTCATGTC CCTTATGGAT CTAGATCCCA ATAGACAAAC TTGCATAAGT   
  
  
+ CAAGGGATGT GTTTCTAATA CAAAGGCTTT GTACTCAAGC AATTAGTTTT GAATTCATTT TCAGTAGTTT   
  
  
+ AATTTCCAAA TCTTATAATT TTTACAATGG CATAAATTTT TAGGAGTTTT GAGTAAATAA ATTATATACA   
  
  
+ ACGAAGATGT CATTTACGAA TTAATATGAC GCACAAAAAA AAAGAAAAAA AATTAATGGT AGCTTCTATA   
  
  
+ CTTGACTCCA TGTTGACAAT TCCCTCTCTC ACATAATGGT TAAAACTTGC AACATCATCC AGGACCCATA   
  
  
+ TGACATGGGT GGTGGTAGAC AGTGTGTTGT GGTAGCCTCA CTCCATGCTT GCCAATTTGT ATCATTGCGC   
  
  
+ TAGTCTTATT CGTCCTTAGA GATAATTGAC TAAGCTCTAA TGCTCTATCG TGGCTGCTGC AATATAAATG   
  
  
+ ATTTTTTGAC CTCAAAATGA AGACAGCTAG TTGTAAATTA AGAATCTTCG TCCCTTCATT TTTTGTGTAC   
  
  
+ GTGTGTATCT GCGTATGTGT TGAGAGACTA GCGGACTATT GATTGAGTTT CAATCAAAAA AGATGACCAT   
  
  
+ CTTAAGTATT TTTAATTACG CAAACTTCTT CATTTAAAAA AATGTTACTT TTATTTGAAA GAAATTGTGA   
  
  
+ ATAATACAAG GAATAGTTCA ATTAATATAT AATCCATAAC ATATTACCTC CTATAGGCAA AAAGAAGATT   
  
  
+ ATCGTATTAA TTTGGGATCA TCCCACCCTA AATTTAAAGA AACCAAAACA AATTACTAAG CAATATCTTT   
  
  
+ CTGTGTGAAA AAATAGTCAG AAACAAAGCG ATCCGACCGG CCAAATCGAG ATAAACACAG CCAAAGAACC   
  
  
+ GAAGTTGGAC CAAAGTGAGC CGAGTTGAAA GGCATTCATA TGGGGTTTAA TCATAACACA AACATAATTA   
  
  
+ GATTGATTGA CGTCTTTAAT CAGCGTTTTT GTCTTTGTTC GGTGCTCCCC TCACTCGCCC CTCCGCTCTT   
  
  
+ TAAAGCTTAC CTCACTCCCC CCATTCCCAC TCCTTTTCTC TCTCCTCTCT CGCTCTCACA CGCTCCGTCT   
  
  
+ GTCAGCCCCT TGTCATCTTC CCCCGTCTTC CTCGCAAAAA TCTCCAGCAA AATTTATAGC CGTTGTTGTG   
  
  
+ CCCATGGTGA TCTGATGTGA GAAGAGATTC AGCATTTAAG ATTATTGGGT CCAGATGGCT TATATGTGCG   
  
  
+ CCGACAGTGG TAATCTCATG GCTATTGCCC AACAAGTCAT CAAGCAAAAA CAACAGCAAG AACAGCAGCA   
  
  
+ ACACCAACAG CCTCATCCCC AGTTCGTCGG GTCGAATAAT AACCCCTTTT GCACCAGCCC ATGGCCCGGA   
  
  
+ CCCGTCCCTT CATCCGCCAT GTCTGCCAGC CCTCCGCCCC CTCTTGGGTT CTCCGCCGCC GCGTTTCCGG   
  
  
+ ACCCGTTTCA GGTGGGCCCG CCCTGCCCGG ATGGGGCTGA ACCGGGTTTC CAGTTTGCCC ATTTGGACCA   
  
  
+ CCACTCGAGT GGTTTCCGGT TTGCTGATTT TTGCGGTGGG GGTGGTGGTG AGTTTGACTC GGATGAGTGG   
  
  
+ ATGGAGAGTT TGATGGGTGG CGGCGGCGGT GACTCGCGGC GACTGAGAGT TCTAACCTCC AATCCCATTG   
  
  
+ CGACGCGTGG CAAGGGTCCG GTGATTTCGG TCTCTATGGT GCTGATCCGT TTGCGACGTG TTCGGAAGAC   
  
  
+ CGTTGCAGCC GCTATTCACG CTCAGCCACT GCCGCAACAG CAGCCGTCGT GGGTCCCGCC TTCTCCGCCG   
  
  
+ AAAGACACGA AGCAGTCGGC CCCACCTCAA AAGCAAAGCG ACGCCGCTGG ACCCTCGTTC TCATCCGAGG   
  
  
+ AGGAACCAGA GCCGTCATGG CCGCCGCTAA TTGCGGCGTT GTTGGAGTGC GCCAAACTCG CCGAGTCCGA   
  
  
+ CCCGGAACAC GCTGTCAAAT CGGTGATTCG ACTCAGAGAC TCAGTCTCAC AGCAGGGAGT TCCAATCCAG   
  
  
+ AGAGTGTCCT ATTACTTCTC CGAGGCACTC TACCACCGCC TTTCTCTCTC CTCCGCCCCT CAACCTCTCC   
  
  
+ CAGAAACGAC GTCGTCTGAG GAGTACACTC TCTCTTACAA GGCCCTGAAC GACGCGTGCC CGTACCATAA   
  
  
+ ATTCGCTCAC TTGACCGCAA ATCAGGCGAT TCTAGAAGCC ACTGATTCAG CCAAGAAGAT CCACATCGTT   
  
  
+ GATTTTGGGA TAATCCAGGG AGTCCAATGG GCTGCTCTGC TACAAGCCCT GGCAACCCGG CCAGCCGGGA   
  
  
+ AGCCCGAATT GATTCGAATA TCCGGAATTC CTGCTCCGGG TCTGGGGCAA TCTCCGGCGG CATCGCTGCT   
  
  
+ CGCAACTGGG AATCGCCTCC GTGAGTTCGC GAAGGTTCTA GATCTCAAGT TCGAGTTTGA GCCGGTACTC   
  
  
+ ACTCCAATCA GAGAGCTAAA CGGGTCGAAC TTCCGGGTCG ACCCAGATGA GATACTAGCT GTCAATTTCA   
  
  
+ TGCTTCAATT GTACAACCTA TTAGACGAAA CCCTGGTCGC TGTCGACGCC GCCCTGAAGC TGGCCAAGTC   
  
  
+ ATTGAACCCC AAAATCGTCA CTTTGGGTGA GTACGAGGCG GGTCTGAATC GGGCAGGTTT CTTGACCCGG   
  
  
+ TTCAAGACGG CCCTCAAGTA CTATTTGGCA ATGTTTGAAT CGGTTGAGCC CAACTTGGCC CGGGACTCGC   
  
  
+ CCGAGAGGGC TCAGGTTGAG AGGCTGTTAT TGGGCCGGCG GATCATGGGC TTAGTTGGGC CGGAGTTACC   
  
  
+ GGGAACGAGA AGGGAAAGGA TGGAGTGTAA GGAACAATGG AAGGGTTTAA TGGAAAGTGC CGGTTTCGAG   
  
  
+ CCGTGCCAAA GCAGAAACTA CGTCAGTGAA AAAGACAAGT TTTACCACTC CAGATTGAAT TACAGAGAGT   
  
  
+ G  

- +Up\_Stream \_Len000ACTATT AATAAAAATA ACTAAATCCT TTCCTATAGT TTCATATAAA ACAAAAATCC   
  
  
- ATCAATTCTC TAAGACAACG GTTTTTTTTT TAGTTCTATA ATATATAAAA ATTAATACTG TCATTAATAA   
  
  
- AATAACTTTT AGGTTGCCAA CACTAATATA TTAGTTTATT AGATTTCCAA TTTTTATAGT TTAAATAATC   
  
  
- TTTTAAGCTT TTTCAGTGCA GTAGTGTTGT AACGAAAATA TATATCATAT CTAAGGAAGC TGAAGATTTG   
  
  
- ATTAGTGAAC TTTAATCTTT AAACTTTGAA AGATTATATT TACGTACCAG AAGAAAGGTT CTAATATTAG   
  
  
- GCGTTAACGA ATTTACATCC CATGTTTATA GTTTAAATCT TCACTGATAA CTTCGGTACT TTCTATAACA   
  
  
- CTAGCATAAC TCCCACCAAC ACTCGTTATC CTACTTTGTC CTTTTTTTTT GGGATTACAA AAGTTTAAAT   
  
  
- ACCCACCGAA ACCATCATCT ATTCACTTAT CACACGTTCC CTTACCGTGG TTATACAAAC CTACCTTGCT   
  
  
- ACTCTCATTC TGCAATCCTT TCGTTGTTCT CTTCCCTTTT CCGTCATCCC TCTCTTAATA CGCACACTTA   
  
  
- TTATAATATT GTCTGCGAAA GCATACTAAT AATTTATGTT CTGAAACTCG TATGTATATT TGAACATTGA   
  
  
- AAGAAATGGA TAACAGTTAA TTAAAAACTT AACTTAGTAG ACGCTGAATA CGTATGTTCT ACTATACTAC   
  
  
- GTATAACCCG GTTCGTAAAC TAGAGTACAG GGAATACCTA GATCTAGGGT TATCTGTTTG AACGTATTCA   
  
  
- GTTCCCTACA CAAAGATTAT GTTTCCGAAA CATGAGTTCG TTAATCAAAA CTTAAGTAAA AGTCATCAAA   
  
  
- TTAAAGGTTT AGAATATTAA AAATGTTACC GTATTTAAAA ATCCTCAAAA CTCATTTATT TAATATATGT   
  
  
- TGCTTCTACA GTAAATGCTT AATTATACTG CGTGTTTTTT TTTCTTTTTT TTAATTACCA TCGAAGATAT   
  
  
- GAACTGAGGT ACAACTGTTA AGGGAGAGAG TGTATTACCA ATTTTGAACG TTGTAGTAGG TCCTGGGTAT   
  
  
- ACTGTACCCA CCACCATCTG TCACACAACA CCATCGGAGT GAGGTACGAA CGGTTAAACA TAGTAACGCG   
  
  
- ATCAGAATAA GCAGGAATCT CTATTAACTG ATTCGAGATT ACGAGATAGC ACCGACGACG TTATATTTAC   
  
  
- TAAAAAACTG GAGTTTTACT TCTGTCGATC AACATTTAAT TCTTAGAAGC AGGGAAGTAA AAAACACATG   
  
  
- CACACATAGA CGCATACACA ACTCTCTGAT CGCCTGATAA CTAACTCAAA GTTAGTTTTT TCTACTGGTA   
  
  
- GAATTCATAA AAATTAATGC GTTTGAAGAA GTAAATTTTT TTACAATGAA AATAAACTTT CTTTAACACT   
  
  
- TATTATGTTC CTTATCAAGT TAATTATATA TTAGGTATTG TATAATGGAG GATATCCGTT TTTCTTCTAA   
  
  
- TAGCATAATT AAACCCTAGT AGGGTGGGAT TTAAATTTCT TTGGTTTTGT TTAATGATTC GTTATAGAAA   
  
  
- GACACACTTT TTTATCAGTC TTTGTTTCGC TAGGCTGGCC GGTTTAGCTC TATTTGTGTC GGTTTCTTGG   
  
  
- CTTCAACCTG GTTTCACTCG GCTCAACTTT CCGTAAGTAT ACCCCAAATT AGTATTGTGT TTGTATTAAT   
  
  
- CTAACTAACT GCAGAAATTA GTCGCAAAAA CAGAAACAAG CCACGAGGGG AGTGAGCGGG GAGGCGAGAA   
  
  
- ATTTCGAATG GAGTGAGGGG GGTAAGGGTG AGGAAAAGAG AGAGGAGAGA GCGAGAGTGT GCGAGGCAGA   
  
  
- CAGTCGGGGA ACAGTAGAAG GGGGCAGAAG GAGCGTTTTT AGAGGTCGTT TTAAATATCG GCAACAACAC   
  
  
- GGGTACCACT AGACTACACT CTTCTCTAAG TCGTAAATTC TAATAACCCA GGTCTACCGA ATATACACGC   
  
  
- GGCTGTCACC ATTAGAGTAC CGATAACGGG TTGTTCAGTA GTTCGTTTTT GTTGTCGTTC TTGTCGTCGT   
  
  
- TGTGGTTGTC GGAGTAGGGG TCAAGCAGCC CAGCTTATTA TTGGGGAAAA CGTGGTCGGG TACCGGGCCT   
  
  
- GGGCAGGGAA GTAGGCGGTA CAGACGGTCG GGAGGCGGGG GAGAACCCAA GAGGCGGCGG CGCAAAGGCC   
  
  
- TGGGCAAAGT CCACCCGGGC GGGACGGGCC TACCCCGACT TGGCCCAAAG GTCAAACGGG TAAACCTGGT   
  
  
- GGTGAGCTCA CCAAAGGCCA AACGACTAAA AACGCCACCC CCACCACCAC TCAAACTGAG CCTACTCACC   
  
  
- TACCTCTCAA ACTACCCACC GCCGCCGCCA CTGAGCGCCG CTGACTCTCA AGATTGGAGG TTAGGGTAAC   
  
  
- GCTGCGCACC GTTCCCAGGC CACTAAAGCC AGAGATACCA CGACTAGGCA AACGCTGCAC AAGCCTTCTG   
  
  
- GCAACGTCGG CGATAAGTGC GAGTCGGTGA CGGCGTTGTC GTCGGCAGCA CCCAGGGCGG AAGAGGCGGC   
  
  
- TTTCTGTGCT TCGTCAGCCG GGGTGGAGTT TTCGTTTCGC TGCGGCGACC TGGGAGCAAG AGTAGGCTCC   
  
  
- TCCTTGGTCT CGGCAGTACC GGCGGCGATT AACGCCGCAA CAACCTCACG CGGTTTGAGC GGCTCAGGCT   
  
  
- GGGCCTTGTG CGACAGTTTA GCCACTAAGC TGAGTCTCTG AGTCAGAGTG TCGTCCCTCA AGGTTAGGTC   
  
  
- TCTCACAGGA TAATGAAGAG GCTCCGTGAG ATGGTGGCGG AAAGAGAGAG GAGGCGGGGA GTTGGAGAGG   
  
  
- GTCTTTGCTG CAGCAGACTC CTCATGTGAG AGAGAATGTT CCGGGACTTG CTGCGCACGG GCATGGTATT   
  
  
- TAAGCGAGTG AACTGGCGTT TAGTCCGCTA AGATCTTCGG TGACTAAGTC GGTTCTTCTA GGTGTAGCAA   
  
  
- CTAAAACCCT ATTAGGTCCC TCAGGTTACC CGACGAGACG ATGTTCGGGA CCGTTGGGCC GGTCGGCCCT   
  
  
- TCGGGCTTAA CTAAGCTTAT AGGCCTTAAG GACGAGGCCC AGACCCCGTT AGAGGCCGCC GTAGCGACGA   
  
  
- GCGTTGACCC TTAGCGGAGG CACTCAAGCG CTTCCAAGAT CTAGAGTTCA AGCTCAAACT CGGCCATGAG   
  
  
- TGAGGTTAGT CTCTCGATTT GCCCAGCTTG AAGGCCCAGC TGGGTCTACT CTATGATCGA CAGTTAAAGT   
  
  
- ACGAAGTTAA CATGTTGGAT AATCTGCTTT GGGACCAGCG ACAGCTGCGG CGGGACTTCG ACCGGTTCAG   
  
  
- TAACTTGGGG TTTTAGCAGT GAAACCCACT CATGCTCCGC CCAGACTTAG CCCGTCCAAA GAACTGGGCC   
  
  
- AAGTTCTGCC GGGAGTTCAT GATAAACCGT TACAAACTTA GCCAACTCGG GTTGAACCGG GCCCTGAGCG   
  
  
- GGCTCTCCCG AGTCCAACTC TCCGACAATA ACCCGGCCGC CTAGTACCCG AATCAACCCG GCCTCAATGG   
  
  
- CCCTTGCTCT TCCCTTTCCT ACCTCACATT CCTTGTTACC TTCCCAAATT ACCTTTCACG GCCAAAGCTC   
  
  
- GGCACGGTTT CGTCTTTGAT GCAGTCACTT TTTCTGTTCA AAATGGTGAG GTCTAACTTA ATGTCTCTCA   
  
  
- C

+     AE-box

| Site Name | Organism | Position | Strand | Matrix score. | sequence | function |
| --- | --- | --- | --- | --- | --- | --- |
| AE-box | Arabidopsis thaliana | 1633 | + | 8 | AGAAACAA | part of a module for light response |

>HU06G00029.1   
+ +Up\_Stream \_Len000TGATAA TTATTTTTAT TGATTTAGGA AAGGATATCA AAGTATATTT TGTTTTTAGG   
  
  
+ TAGTTAAGAG ATTCTGTTGC CAAAAAAAAA ATCAAGATAT TATATATTTT TAATTATGAC AGTAATTATT   
  
  
+ TTATTGAAAA TCCAACGGTT GTGATTATAT AATCAAATAA TCTAAAGGTT AAAAATATCA AATTTATTAG   
  
  
+ AAAATTCGAA AAAGTCACGT CATCACAACA TTGCTTTTAT ATATAGTATA GATTCCTTCG ACTTCTAAAC   
  
  
+ TAATCACTTG AAATTAGAAA TTTGAAACTT TCTAATATAA ATGCATGGTC TTCTTTCCAA GATTATAATC   
  
  
+ CGCAATTGCT TAAATGTAGG GTACAAATAT CAAATTTAGA AGTGACTATT GAAGCCATGA AAGATATTGT   
  
  
+ GATCGTATTG AGGGTGGTTG TGAGCAATAG GATGAAACAG GAAAAAAAAA CCCTAATGTT TTCAAATTTA   
  
  
+ TGGGTGGCTT TGGTAGTAGA TAAGTGAATA GTGTGCAAGG GAATGGCACC AATATGTTTG GATGGAACGA   
  
  
+ TGAGAGTAAG ACGTTAGGAA AGCAACAAGA GAAGGGAAAA GGCAGTAGGG AGAGAATTAT GCGTGTGAAT   
  
  
+ AATATTATAA CAGACGCTTT CGTATGATTA TTAAATACAA GACTTTGAGC ATACATATAA ACTTGTAACT   
  
  
+ TTCTTTACCT ATTGTCAATT AATTTTTGAA TTGAATCATC TGCGACTTAT GCATACAAGA TGATATGATG   
  
  
+ CATATTGGGC CAAGCATTTG ATCTCATGTC CCTTATGGAT CTAGATCCCA ATAGACAAAC TTGCATAAGT   
  
  
+ CAAGGGATGT GTTTCTAATA CAAAGGCTTT GTACTCAAGC AATTAGTTTT GAATTCATTT TCAGTAGTTT   
  
  
+ AATTTCCAAA TCTTATAATT TTTACAATGG CATAAATTTT TAGGAGTTTT GAGTAAATAA ATTATATACA   
  
  
+ ACGAAGATGT CATTTACGAA TTAATATGAC GCACAAAAAA AAAGAAAAAA AATTAATGGT AGCTTCTATA   
  
  
+ CTTGACTCCA TGTTGACAAT TCCCTCTCTC ACATAATGGT TAAAACTTGC AACATCATCC AGGACCCATA   
  
  
+ TGACATGGGT GGTGGTAGAC AGTGTGTTGT GGTAGCCTCA CTCCATGCTT GCCAATTTGT ATCATTGCGC   
  
  
+ TAGTCTTATT CGTCCTTAGA GATAATTGAC TAAGCTCTAA TGCTCTATCG TGGCTGCTGC AATATAAATG   
  
  
+ ATTTTTTGAC CTCAAAATGA AGACAGCTAG TTGTAAATTA AGAATCTTCG TCCCTTCATT TTTTGTGTAC   
  
  
+ GTGTGTATCT GCGTATGTGT TGAGAGACTA GCGGACTATT GATTGAGTTT CAATCAAAAA AGATGACCAT   
  
  
+ CTTAAGTATT TTTAATTACG CAAACTTCTT CATTTAAAAA AATGTTACTT TTATTTGAAA GAAATTGTGA   
  
  
+ ATAATACAAG GAATAGTTCA ATTAATATAT AATCCATAAC ATATTACCTC CTATAGGCAA AAAGAAGATT   
  
  
+ ATCGTATTAA TTTGGGATCA TCCCACCCTA AATTTAAAGA AACCAAAACA AATTACTAAG CAATATCTTT   
  
  
+ CTGTGTGAAA AAATAGTCAG AAACAAAGCG ATCCGACCGG CCAAATCGAG ATAAACACAG CCAAAGAACC   
  
  
+ GAAGTTGGAC CAAAGTGAGC CGAGTTGAAA GGCATTCATA TGGGGTTTAA TCATAACACA AACATAATTA   
  
  
+ GATTGATTGA CGTCTTTAAT CAGCGTTTTT GTCTTTGTTC GGTGCTCCCC TCACTCGCCC CTCCGCTCTT   
  
  
+ TAAAGCTTAC CTCACTCCCC CCATTCCCAC TCCTTTTCTC TCTCCTCTCT CGCTCTCACA CGCTCCGTCT   
  
  
+ GTCAGCCCCT TGTCATCTTC CCCCGTCTTC CTCGCAAAAA TCTCCAGCAA AATTTATAGC CGTTGTTGTG   
  
  
+ CCCATGGTGA TCTGATGTGA GAAGAGATTC AGCATTTAAG ATTATTGGGT CCAGATGGCT TATATGTGCG   
  
  
+ CCGACAGTGG TAATCTCATG GCTATTGCCC AACAAGTCAT CAAGCAAAAA CAACAGCAAG AACAGCAGCA   
  
  
+ ACACCAACAG CCTCATCCCC AGTTCGTCGG GTCGAATAAT AACCCCTTTT GCACCAGCCC ATGGCCCGGA   
  
  
+ CCCGTCCCTT CATCCGCCAT GTCTGCCAGC CCTCCGCCCC CTCTTGGGTT CTCCGCCGCC GCGTTTCCGG   
  
  
+ ACCCGTTTCA GGTGGGCCCG CCCTGCCCGG ATGGGGCTGA ACCGGGTTTC CAGTTTGCCC ATTTGGACCA   
  
  
+ CCACTCGAGT GGTTTCCGGT TTGCTGATTT TTGCGGTGGG GGTGGTGGTG AGTTTGACTC GGATGAGTGG   
  
  
+ ATGGAGAGTT TGATGGGTGG CGGCGGCGGT GACTCGCGGC GACTGAGAGT TCTAACCTCC AATCCCATTG   
  
  
+ CGACGCGTGG CAAGGGTCCG GTGATTTCGG TCTCTATGGT GCTGATCCGT TTGCGACGTG TTCGGAAGAC   
  
  
+ CGTTGCAGCC GCTATTCACG CTCAGCCACT GCCGCAACAG CAGCCGTCGT GGGTCCCGCC TTCTCCGCCG   
  
  
+ AAAGACACGA AGCAGTCGGC CCCACCTCAA AAGCAAAGCG ACGCCGCTGG ACCCTCGTTC TCATCCGAGG   
  
  
+ AGGAACCAGA GCCGTCATGG CCGCCGCTAA TTGCGGCGTT GTTGGAGTGC GCCAAACTCG CCGAGTCCGA   
  
  
+ CCCGGAACAC GCTGTCAAAT CGGTGATTCG ACTCAGAGAC TCAGTCTCAC AGCAGGGAGT TCCAATCCAG   
  
  
+ AGAGTGTCCT ATTACTTCTC CGAGGCACTC TACCACCGCC TTTCTCTCTC CTCCGCCCCT CAACCTCTCC   
  
  
+ CAGAAACGAC GTCGTCTGAG GAGTACACTC TCTCTTACAA GGCCCTGAAC GACGCGTGCC CGTACCATAA   
  
  
+ ATTCGCTCAC TTGACCGCAA ATCAGGCGAT TCTAGAAGCC ACTGATTCAG CCAAGAAGAT CCACATCGTT   
  
  
+ GATTTTGGGA TAATCCAGGG AGTCCAATGG GCTGCTCTGC TACAAGCCCT GGCAACCCGG CCAGCCGGGA   
  
  
+ AGCCCGAATT GATTCGAATA TCCGGAATTC CTGCTCCGGG TCTGGGGCAA TCTCCGGCGG CATCGCTGCT   
  
  
+ CGCAACTGGG AATCGCCTCC GTGAGTTCGC GAAGGTTCTA GATCTCAAGT TCGAGTTTGA GCCGGTACTC   
  
  
+ ACTCCAATCA GAGAGCTAAA CGGGTCGAAC TTCCGGGTCG ACCCAGATGA GATACTAGCT GTCAATTTCA   
  
  
+ TGCTTCAATT GTACAACCTA TTAGACGAAA CCCTGGTCGC TGTCGACGCC GCCCTGAAGC TGGCCAAGTC   
  
  
+ ATTGAACCCC AAAATCGTCA CTTTGGGTGA GTACGAGGCG GGTCTGAATC GGGCAGGTTT CTTGACCCGG   
  
  
+ TTCAAGACGG CCCTCAAGTA CTATTTGGCA ATGTTTGAAT CGGTTGAGCC CAACTTGGCC CGGGACTCGC   
  
  
+ CCGAGAGGGC TCAGGTTGAG AGGCTGTTAT TGGGCCGGCG GATCATGGGC TTAGTTGGGC CGGAGTTACC   
  
  
+ GGGAACGAGA AGGGAAAGGA TGGAGTGTAA GGAACAATGG AAGGGTTTAA TGGAAAGTGC CGGTTTCGAG   
  
  
+ CCGTGCCAAA GCAGAAACTA CGTCAGTGAA AAAGACAAGT TTTACCACTC CAGATTGAAT TACAGAGAGT   
  
  
+ G  

- +Up\_Stream \_Len000ACTATT AATAAAAATA ACTAAATCCT TTCCTATAGT TTCATATAAA ACAAAAATCC   
  
  
- ATCAATTCTC TAAGACAACG GTTTTTTTTT TAGTTCTATA ATATATAAAA ATTAATACTG TCATTAATAA   
  
  
- AATAACTTTT AGGTTGCCAA CACTAATATA TTAGTTTATT AGATTTCCAA TTTTTATAGT TTAAATAATC   
  
  
- TTTTAAGCTT TTTCAGTGCA GTAGTGTTGT AACGAAAATA TATATCATAT CTAAGGAAGC TGAAGATTTG   
  
  
- ATTAGTGAAC TTTAATCTTT AAACTTTGAA AGATTATATT TACGTACCAG AAGAAAGGTT CTAATATTAG   
  
  
- GCGTTAACGA ATTTACATCC CATGTTTATA GTTTAAATCT TCACTGATAA CTTCGGTACT TTCTATAACA   
  
  
- CTAGCATAAC TCCCACCAAC ACTCGTTATC CTACTTTGTC CTTTTTTTTT GGGATTACAA AAGTTTAAAT   
  
  
- ACCCACCGAA ACCATCATCT ATTCACTTAT CACACGTTCC CTTACCGTGG TTATACAAAC CTACCTTGCT   
  
  
- ACTCTCATTC TGCAATCCTT TCGTTGTTCT CTTCCCTTTT CCGTCATCCC TCTCTTAATA CGCACACTTA   
  
  
- TTATAATATT GTCTGCGAAA GCATACTAAT AATTTATGTT CTGAAACTCG TATGTATATT TGAACATTGA   
  
  
- AAGAAATGGA TAACAGTTAA TTAAAAACTT AACTTAGTAG ACGCTGAATA CGTATGTTCT ACTATACTAC   
  
  
- GTATAACCCG GTTCGTAAAC TAGAGTACAG GGAATACCTA GATCTAGGGT TATCTGTTTG AACGTATTCA   
  
  
- GTTCCCTACA CAAAGATTAT GTTTCCGAAA CATGAGTTCG TTAATCAAAA CTTAAGTAAA AGTCATCAAA   
  
  
- TTAAAGGTTT AGAATATTAA AAATGTTACC GTATTTAAAA ATCCTCAAAA CTCATTTATT TAATATATGT   
  
  
- TGCTTCTACA GTAAATGCTT AATTATACTG CGTGTTTTTT TTTCTTTTTT TTAATTACCA TCGAAGATAT   
  
  
- GAACTGAGGT ACAACTGTTA AGGGAGAGAG TGTATTACCA ATTTTGAACG TTGTAGTAGG TCCTGGGTAT   
  
  
- ACTGTACCCA CCACCATCTG TCACACAACA CCATCGGAGT GAGGTACGAA CGGTTAAACA TAGTAACGCG   
  
  
- ATCAGAATAA GCAGGAATCT CTATTAACTG ATTCGAGATT ACGAGATAGC ACCGACGACG TTATATTTAC   
  
  
- TAAAAAACTG GAGTTTTACT TCTGTCGATC AACATTTAAT TCTTAGAAGC AGGGAAGTAA AAAACACATG   
  
  
- CACACATAGA CGCATACACA ACTCTCTGAT CGCCTGATAA CTAACTCAAA GTTAGTTTTT TCTACTGGTA   
  
  
- GAATTCATAA AAATTAATGC GTTTGAAGAA GTAAATTTTT TTACAATGAA AATAAACTTT CTTTAACACT   
  
  
- TATTATGTTC CTTATCAAGT TAATTATATA TTAGGTATTG TATAATGGAG GATATCCGTT TTTCTTCTAA   
  
  
- TAGCATAATT AAACCCTAGT AGGGTGGGAT TTAAATTTCT TTGGTTTTGT TTAATGATTC GTTATAGAAA   
  
  
- GACACACTTT TTTATCAGTC TTTGTTTCGC TAGGCTGGCC GGTTTAGCTC TATTTGTGTC GGTTTCTTGG   
  
  
- CTTCAACCTG GTTTCACTCG GCTCAACTTT CCGTAAGTAT ACCCCAAATT AGTATTGTGT TTGTATTAAT   
  
  
- CTAACTAACT GCAGAAATTA GTCGCAAAAA CAGAAACAAG CCACGAGGGG AGTGAGCGGG GAGGCGAGAA   
  
  
- ATTTCGAATG GAGTGAGGGG GGTAAGGGTG AGGAAAAGAG AGAGGAGAGA GCGAGAGTGT GCGAGGCAGA   
  
  
- CAGTCGGGGA ACAGTAGAAG GGGGCAGAAG GAGCGTTTTT AGAGGTCGTT TTAAATATCG GCAACAACAC   
  
  
- GGGTACCACT AGACTACACT CTTCTCTAAG TCGTAAATTC TAATAACCCA GGTCTACCGA ATATACACGC   
  
  
- GGCTGTCACC ATTAGAGTAC CGATAACGGG TTGTTCAGTA GTTCGTTTTT GTTGTCGTTC TTGTCGTCGT   
  
  
- TGTGGTTGTC GGAGTAGGGG TCAAGCAGCC CAGCTTATTA TTGGGGAAAA CGTGGTCGGG TACCGGGCCT   
  
  
- GGGCAGGGAA GTAGGCGGTA CAGACGGTCG GGAGGCGGGG GAGAACCCAA GAGGCGGCGG CGCAAAGGCC   
  
  
- TGGGCAAAGT CCACCCGGGC GGGACGGGCC TACCCCGACT TGGCCCAAAG GTCAAACGGG TAAACCTGGT   
  
  
- GGTGAGCTCA CCAAAGGCCA AACGACTAAA AACGCCACCC CCACCACCAC TCAAACTGAG CCTACTCACC   
  
  
- TACCTCTCAA ACTACCCACC GCCGCCGCCA CTGAGCGCCG CTGACTCTCA AGATTGGAGG TTAGGGTAAC   
  
  
- GCTGCGCACC GTTCCCAGGC CACTAAAGCC AGAGATACCA CGACTAGGCA AACGCTGCAC AAGCCTTCTG   
  
  
- GCAACGTCGG CGATAAGTGC GAGTCGGTGA CGGCGTTGTC GTCGGCAGCA CCCAGGGCGG AAGAGGCGGC   
  
  
- TTTCTGTGCT TCGTCAGCCG GGGTGGAGTT TTCGTTTCGC TGCGGCGACC TGGGAGCAAG AGTAGGCTCC   
  
  
- TCCTTGGTCT CGGCAGTACC GGCGGCGATT AACGCCGCAA CAACCTCACG CGGTTTGAGC GGCTCAGGCT   
  
  
- GGGCCTTGTG CGACAGTTTA GCCACTAAGC TGAGTCTCTG AGTCAGAGTG TCGTCCCTCA AGGTTAGGTC   
  
  
- TCTCACAGGA TAATGAAGAG GCTCCGTGAG ATGGTGGCGG AAAGAGAGAG GAGGCGGGGA GTTGGAGAGG   
  
  
- GTCTTTGCTG CAGCAGACTC CTCATGTGAG AGAGAATGTT CCGGGACTTG CTGCGCACGG GCATGGTATT   
  
  
- TAAGCGAGTG AACTGGCGTT TAGTCCGCTA AGATCTTCGG TGACTAAGTC GGTTCTTCTA GGTGTAGCAA   
  
  
- CTAAAACCCT ATTAGGTCCC TCAGGTTACC CGACGAGACG ATGTTCGGGA CCGTTGGGCC GGTCGGCCCT   
  
  
- TCGGGCTTAA CTAAGCTTAT AGGCCTTAAG GACGAGGCCC AGACCCCGTT AGAGGCCGCC GTAGCGACGA   
  
  
- GCGTTGACCC TTAGCGGAGG CACTCAAGCG CTTCCAAGAT CTAGAGTTCA AGCTCAAACT CGGCCATGAG   
  
  
- TGAGGTTAGT CTCTCGATTT GCCCAGCTTG AAGGCCCAGC TGGGTCTACT CTATGATCGA CAGTTAAAGT   
  
  
- ACGAAGTTAA CATGTTGGAT AATCTGCTTT GGGACCAGCG ACAGCTGCGG CGGGACTTCG ACCGGTTCAG   
  
  
- TAACTTGGGG TTTTAGCAGT GAAACCCACT CATGCTCCGC CCAGACTTAG CCCGTCCAAA GAACTGGGCC   
  
  
- AAGTTCTGCC GGGAGTTCAT GATAAACCGT TACAAACTTA GCCAACTCGG GTTGAACCGG GCCCTGAGCG   
  
  
- GGCTCTCCCG AGTCCAACTC TCCGACAATA ACCCGGCCGC CTAGTACCCG AATCAACCCG GCCTCAATGG   
  
  
- CCCTTGCTCT TCCCTTTCCT ACCTCACATT CCTTGTTACC TTCCCAAATT ACCTTTCACG GCCAAAGCTC   
  
  
- GGCACGGTTT CGTCTTTGAT GCAGTCACTT TTTCTGTTCA AAATGGTGAG GTCTAACTTA ATGTCTCTCA   
  
  
- C

+     ARE

| Site Name | Organism | Position | Strand | Matrix score. | sequence | function |
| --- | --- | --- | --- | --- | --- | --- |
| ARE | Zea mays | 1584 | + | 6 | AAACCA | cis-acting regulatory element essential for the anaerobic induction |
| ARE | Zea mays | 2324 | - | 6 | AAACCA | cis-acting regulatory element essential for the anaerobic induction |

>HU06G00029.1   
+ +Up\_Stream \_Len000TGATAA TTATTTTTAT TGATTTAGGA AAGGATATCA AAGTATATTT TGTTTTTAGG   
  
  
+ TAGTTAAGAG ATTCTGTTGC CAAAAAAAAA ATCAAGATAT TATATATTTT TAATTATGAC AGTAATTATT   
  
  
+ TTATTGAAAA TCCAACGGTT GTGATTATAT AATCAAATAA TCTAAAGGTT AAAAATATCA AATTTATTAG   
  
  
+ AAAATTCGAA AAAGTCACGT CATCACAACA TTGCTTTTAT ATATAGTATA GATTCCTTCG ACTTCTAAAC   
  
  
+ TAATCACTTG AAATTAGAAA TTTGAAACTT TCTAATATAA ATGCATGGTC TTCTTTCCAA GATTATAATC   
  
  
+ CGCAATTGCT TAAATGTAGG GTACAAATAT CAAATTTAGA AGTGACTATT GAAGCCATGA AAGATATTGT   
  
  
+ GATCGTATTG AGGGTGGTTG TGAGCAATAG GATGAAACAG GAAAAAAAAA CCCTAATGTT TTCAAATTTA   
  
  
+ TGGGTGGCTT TGGTAGTAGA TAAGTGAATA GTGTGCAAGG GAATGGCACC AATATGTTTG GATGGAACGA   
  
  
+ TGAGAGTAAG ACGTTAGGAA AGCAACAAGA GAAGGGAAAA GGCAGTAGGG AGAGAATTAT GCGTGTGAAT   
  
  
+ AATATTATAA CAGACGCTTT CGTATGATTA TTAAATACAA GACTTTGAGC ATACATATAA ACTTGTAACT   
  
  
+ TTCTTTACCT ATTGTCAATT AATTTTTGAA TTGAATCATC TGCGACTTAT GCATACAAGA TGATATGATG   
  
  
+ CATATTGGGC CAAGCATTTG ATCTCATGTC CCTTATGGAT CTAGATCCCA ATAGACAAAC TTGCATAAGT   
  
  
+ CAAGGGATGT GTTTCTAATA CAAAGGCTTT GTACTCAAGC AATTAGTTTT GAATTCATTT TCAGTAGTTT   
  
  
+ AATTTCCAAA TCTTATAATT TTTACAATGG CATAAATTTT TAGGAGTTTT GAGTAAATAA ATTATATACA   
  
  
+ ACGAAGATGT CATTTACGAA TTAATATGAC GCACAAAAAA AAAGAAAAAA AATTAATGGT AGCTTCTATA   
  
  
+ CTTGACTCCA TGTTGACAAT TCCCTCTCTC ACATAATGGT TAAAACTTGC AACATCATCC AGGACCCATA   
  
  
+ TGACATGGGT GGTGGTAGAC AGTGTGTTGT GGTAGCCTCA CTCCATGCTT GCCAATTTGT ATCATTGCGC   
  
  
+ TAGTCTTATT CGTCCTTAGA GATAATTGAC TAAGCTCTAA TGCTCTATCG TGGCTGCTGC AATATAAATG   
  
  
+ ATTTTTTGAC CTCAAAATGA AGACAGCTAG TTGTAAATTA AGAATCTTCG TCCCTTCATT TTTTGTGTAC   
  
  
+ GTGTGTATCT GCGTATGTGT TGAGAGACTA GCGGACTATT GATTGAGTTT CAATCAAAAA AGATGACCAT   
  
  
+ CTTAAGTATT TTTAATTACG CAAACTTCTT CATTTAAAAA AATGTTACTT TTATTTGAAA GAAATTGTGA   
  
  
+ ATAATACAAG GAATAGTTCA ATTAATATAT AATCCATAAC ATATTACCTC CTATAGGCAA AAAGAAGATT   
  
  
+ ATCGTATTAA TTTGGGATCA TCCCACCCTA AATTTAAAGA AACCAAAACA AATTACTAAG CAATATCTTT   
  
  
+ CTGTGTGAAA AAATAGTCAG AAACAAAGCG ATCCGACCGG CCAAATCGAG ATAAACACAG CCAAAGAACC   
  
  
+ GAAGTTGGAC CAAAGTGAGC CGAGTTGAAA GGCATTCATA TGGGGTTTAA TCATAACACA AACATAATTA   
  
  
+ GATTGATTGA CGTCTTTAAT CAGCGTTTTT GTCTTTGTTC GGTGCTCCCC TCACTCGCCC CTCCGCTCTT   
  
  
+ TAAAGCTTAC CTCACTCCCC CCATTCCCAC TCCTTTTCTC TCTCCTCTCT CGCTCTCACA CGCTCCGTCT   
  
  
+ GTCAGCCCCT TGTCATCTTC CCCCGTCTTC CTCGCAAAAA TCTCCAGCAA AATTTATAGC CGTTGTTGTG   
  
  
+ CCCATGGTGA TCTGATGTGA GAAGAGATTC AGCATTTAAG ATTATTGGGT CCAGATGGCT TATATGTGCG   
  
  
+ CCGACAGTGG TAATCTCATG GCTATTGCCC AACAAGTCAT CAAGCAAAAA CAACAGCAAG AACAGCAGCA   
  
  
+ ACACCAACAG CCTCATCCCC AGTTCGTCGG GTCGAATAAT AACCCCTTTT GCACCAGCCC ATGGCCCGGA   
  
  
+ CCCGTCCCTT CATCCGCCAT GTCTGCCAGC CCTCCGCCCC CTCTTGGGTT CTCCGCCGCC GCGTTTCCGG   
  
  
+ ACCCGTTTCA GGTGGGCCCG CCCTGCCCGG ATGGGGCTGA ACCGGGTTTC CAGTTTGCCC ATTTGGACCA   
  
  
+ CCACTCGAGT GGTTTCCGGT TTGCTGATTT TTGCGGTGGG GGTGGTGGTG AGTTTGACTC GGATGAGTGG   
  
  
+ ATGGAGAGTT TGATGGGTGG CGGCGGCGGT GACTCGCGGC GACTGAGAGT TCTAACCTCC AATCCCATTG   
  
  
+ CGACGCGTGG CAAGGGTCCG GTGATTTCGG TCTCTATGGT GCTGATCCGT TTGCGACGTG TTCGGAAGAC   
  
  
+ CGTTGCAGCC GCTATTCACG CTCAGCCACT GCCGCAACAG CAGCCGTCGT GGGTCCCGCC TTCTCCGCCG   
  
  
+ AAAGACACGA AGCAGTCGGC CCCACCTCAA AAGCAAAGCG ACGCCGCTGG ACCCTCGTTC TCATCCGAGG   
  
  
+ AGGAACCAGA GCCGTCATGG CCGCCGCTAA TTGCGGCGTT GTTGGAGTGC GCCAAACTCG CCGAGTCCGA   
  
  
+ CCCGGAACAC GCTGTCAAAT CGGTGATTCG ACTCAGAGAC TCAGTCTCAC AGCAGGGAGT TCCAATCCAG   
  
  
+ AGAGTGTCCT ATTACTTCTC CGAGGCACTC TACCACCGCC TTTCTCTCTC CTCCGCCCCT CAACCTCTCC   
  
  
+ CAGAAACGAC GTCGTCTGAG GAGTACACTC TCTCTTACAA GGCCCTGAAC GACGCGTGCC CGTACCATAA   
  
  
+ ATTCGCTCAC TTGACCGCAA ATCAGGCGAT TCTAGAAGCC ACTGATTCAG CCAAGAAGAT CCACATCGTT   
  
  
+ GATTTTGGGA TAATCCAGGG AGTCCAATGG GCTGCTCTGC TACAAGCCCT GGCAACCCGG CCAGCCGGGA   
  
  
+ AGCCCGAATT GATTCGAATA TCCGGAATTC CTGCTCCGGG TCTGGGGCAA TCTCCGGCGG CATCGCTGCT   
  
  
+ CGCAACTGGG AATCGCCTCC GTGAGTTCGC GAAGGTTCTA GATCTCAAGT TCGAGTTTGA GCCGGTACTC   
  
  
+ ACTCCAATCA GAGAGCTAAA CGGGTCGAAC TTCCGGGTCG ACCCAGATGA GATACTAGCT GTCAATTTCA   
  
  
+ TGCTTCAATT GTACAACCTA TTAGACGAAA CCCTGGTCGC TGTCGACGCC GCCCTGAAGC TGGCCAAGTC   
  
  
+ ATTGAACCCC AAAATCGTCA CTTTGGGTGA GTACGAGGCG GGTCTGAATC GGGCAGGTTT CTTGACCCGG   
  
  
+ TTCAAGACGG CCCTCAAGTA CTATTTGGCA ATGTTTGAAT CGGTTGAGCC CAACTTGGCC CGGGACTCGC   
  
  
+ CCGAGAGGGC TCAGGTTGAG AGGCTGTTAT TGGGCCGGCG GATCATGGGC TTAGTTGGGC CGGAGTTACC   
  
  
+ GGGAACGAGA AGGGAAAGGA TGGAGTGTAA GGAACAATGG AAGGGTTTAA TGGAAAGTGC CGGTTTCGAG   
  
  
+ CCGTGCCAAA GCAGAAACTA CGTCAGTGAA AAAGACAAGT TTTACCACTC CAGATTGAAT TACAGAGAGT   
  
  
+ G  

- +Up\_Stream \_Len000ACTATT AATAAAAATA ACTAAATCCT TTCCTATAGT TTCATATAAA ACAAAAATCC   
  
  
- ATCAATTCTC TAAGACAACG GTTTTTTTTT TAGTTCTATA ATATATAAAA ATTAATACTG TCATTAATAA   
  
  
- AATAACTTTT AGGTTGCCAA CACTAATATA TTAGTTTATT AGATTTCCAA TTTTTATAGT TTAAATAATC   
  
  
- TTTTAAGCTT TTTCAGTGCA GTAGTGTTGT AACGAAAATA TATATCATAT CTAAGGAAGC TGAAGATTTG   
  
  
- ATTAGTGAAC TTTAATCTTT AAACTTTGAA AGATTATATT TACGTACCAG AAGAAAGGTT CTAATATTAG   
  
  
- GCGTTAACGA ATTTACATCC CATGTTTATA GTTTAAATCT TCACTGATAA CTTCGGTACT TTCTATAACA   
  
  
- CTAGCATAAC TCCCACCAAC ACTCGTTATC CTACTTTGTC CTTTTTTTTT GGGATTACAA AAGTTTAAAT   
  
  
- ACCCACCGAA ACCATCATCT ATTCACTTAT CACACGTTCC CTTACCGTGG TTATACAAAC CTACCTTGCT   
  
  
- ACTCTCATTC TGCAATCCTT TCGTTGTTCT CTTCCCTTTT CCGTCATCCC TCTCTTAATA CGCACACTTA   
  
  
- TTATAATATT GTCTGCGAAA GCATACTAAT AATTTATGTT CTGAAACTCG TATGTATATT TGAACATTGA   
  
  
- AAGAAATGGA TAACAGTTAA TTAAAAACTT AACTTAGTAG ACGCTGAATA CGTATGTTCT ACTATACTAC   
  
  
- GTATAACCCG GTTCGTAAAC TAGAGTACAG GGAATACCTA GATCTAGGGT TATCTGTTTG AACGTATTCA   
  
  
- GTTCCCTACA CAAAGATTAT GTTTCCGAAA CATGAGTTCG TTAATCAAAA CTTAAGTAAA AGTCATCAAA   
  
  
- TTAAAGGTTT AGAATATTAA AAATGTTACC GTATTTAAAA ATCCTCAAAA CTCATTTATT TAATATATGT   
  
  
- TGCTTCTACA GTAAATGCTT AATTATACTG CGTGTTTTTT TTTCTTTTTT TTAATTACCA TCGAAGATAT   
  
  
- GAACTGAGGT ACAACTGTTA AGGGAGAGAG TGTATTACCA ATTTTGAACG TTGTAGTAGG TCCTGGGTAT   
  
  
- ACTGTACCCA CCACCATCTG TCACACAACA CCATCGGAGT GAGGTACGAA CGGTTAAACA TAGTAACGCG   
  
  
- ATCAGAATAA GCAGGAATCT CTATTAACTG ATTCGAGATT ACGAGATAGC ACCGACGACG TTATATTTAC   
  
  
- TAAAAAACTG GAGTTTTACT TCTGTCGATC AACATTTAAT TCTTAGAAGC AGGGAAGTAA AAAACACATG   
  
  
- CACACATAGA CGCATACACA ACTCTCTGAT CGCCTGATAA CTAACTCAAA GTTAGTTTTT TCTACTGGTA   
  
  
- GAATTCATAA AAATTAATGC GTTTGAAGAA GTAAATTTTT TTACAATGAA AATAAACTTT CTTTAACACT   
  
  
- TATTATGTTC CTTATCAAGT TAATTATATA TTAGGTATTG TATAATGGAG GATATCCGTT TTTCTTCTAA   
  
  
- TAGCATAATT AAACCCTAGT AGGGTGGGAT TTAAATTTCT TTGGTTTTGT TTAATGATTC GTTATAGAAA   
  
  
- GACACACTTT TTTATCAGTC TTTGTTTCGC TAGGCTGGCC GGTTTAGCTC TATTTGTGTC GGTTTCTTGG   
  
  
- CTTCAACCTG GTTTCACTCG GCTCAACTTT CCGTAAGTAT ACCCCAAATT AGTATTGTGT TTGTATTAAT   
  
  
- CTAACTAACT GCAGAAATTA GTCGCAAAAA CAGAAACAAG CCACGAGGGG AGTGAGCGGG GAGGCGAGAA   
  
  
- ATTTCGAATG GAGTGAGGGG GGTAAGGGTG AGGAAAAGAG AGAGGAGAGA GCGAGAGTGT GCGAGGCAGA   
  
  
- CAGTCGGGGA ACAGTAGAAG GGGGCAGAAG GAGCGTTTTT AGAGGTCGTT TTAAATATCG GCAACAACAC   
  
  
- GGGTACCACT AGACTACACT CTTCTCTAAG TCGTAAATTC TAATAACCCA GGTCTACCGA ATATACACGC   
  
  
- GGCTGTCACC ATTAGAGTAC CGATAACGGG TTGTTCAGTA GTTCGTTTTT GTTGTCGTTC TTGTCGTCGT   
  
  
- TGTGGTTGTC GGAGTAGGGG TCAAGCAGCC CAGCTTATTA TTGGGGAAAA CGTGGTCGGG TACCGGGCCT   
  
  
- GGGCAGGGAA GTAGGCGGTA CAGACGGTCG GGAGGCGGGG GAGAACCCAA GAGGCGGCGG CGCAAAGGCC   
  
  
- TGGGCAAAGT CCACCCGGGC GGGACGGGCC TACCCCGACT TGGCCCAAAG GTCAAACGGG TAAACCTGGT   
  
  
- GGTGAGCTCA CCAAAGGCCA AACGACTAAA AACGCCACCC CCACCACCAC TCAAACTGAG CCTACTCACC   
  
  
- TACCTCTCAA ACTACCCACC GCCGCCGCCA CTGAGCGCCG CTGACTCTCA AGATTGGAGG TTAGGGTAAC   
  
  
- GCTGCGCACC GTTCCCAGGC CACTAAAGCC AGAGATACCA CGACTAGGCA AACGCTGCAC AAGCCTTCTG   
  
  
- GCAACGTCGG CGATAAGTGC GAGTCGGTGA CGGCGTTGTC GTCGGCAGCA CCCAGGGCGG AAGAGGCGGC   
  
  
- TTTCTGTGCT TCGTCAGCCG GGGTGGAGTT TTCGTTTCGC TGCGGCGACC TGGGAGCAAG AGTAGGCTCC   
  
  
- TCCTTGGTCT CGGCAGTACC GGCGGCGATT AACGCCGCAA CAACCTCACG CGGTTTGAGC GGCTCAGGCT   
  
  
- GGGCCTTGTG CGACAGTTTA GCCACTAAGC TGAGTCTCTG AGTCAGAGTG TCGTCCCTCA AGGTTAGGTC   
  
  
- TCTCACAGGA TAATGAAGAG GCTCCGTGAG ATGGTGGCGG AAAGAGAGAG GAGGCGGGGA GTTGGAGAGG   
  
  
- GTCTTTGCTG CAGCAGACTC CTCATGTGAG AGAGAATGTT CCGGGACTTG CTGCGCACGG GCATGGTATT   
  
  
- TAAGCGAGTG AACTGGCGTT TAGTCCGCTA AGATCTTCGG TGACTAAGTC GGTTCTTCTA GGTGTAGCAA   
  
  
- CTAAAACCCT ATTAGGTCCC TCAGGTTACC CGACGAGACG ATGTTCGGGA CCGTTGGGCC GGTCGGCCCT   
  
  
- TCGGGCTTAA CTAAGCTTAT AGGCCTTAAG GACGAGGCCC AGACCCCGTT AGAGGCCGCC GTAGCGACGA   
  
  
- GCGTTGACCC TTAGCGGAGG CACTCAAGCG CTTCCAAGAT CTAGAGTTCA AGCTCAAACT CGGCCATGAG   
  
  
- TGAGGTTAGT CTCTCGATTT GCCCAGCTTG AAGGCCCAGC TGGGTCTACT CTATGATCGA CAGTTAAAGT   
  
  
- ACGAAGTTAA CATGTTGGAT AATCTGCTTT GGGACCAGCG ACAGCTGCGG CGGGACTTCG ACCGGTTCAG   
  
  
- TAACTTGGGG TTTTAGCAGT GAAACCCACT CATGCTCCGC CCAGACTTAG CCCGTCCAAA GAACTGGGCC   
  
  
- AAGTTCTGCC GGGAGTTCAT GATAAACCGT TACAAACTTA GCCAACTCGG GTTGAACCGG GCCCTGAGCG   
  
  
- GGCTCTCCCG AGTCCAACTC TCCGACAATA ACCCGGCCGC CTAGTACCCG AATCAACCCG GCCTCAATGG   
  
  
- CCCTTGCTCT TCCCTTTCCT ACCTCACATT CCTTGTTACC TTCCCAAATT ACCTTTCACG GCCAAAGCTC   
  
  
- GGCACGGTTT CGTCTTTGAT GCAGTCACTT TTTCTGTTCA AAATGGTGAG GTCTAACTTA ATGTCTCTCA   
  
  
- C

+     AT~TATA-box

| Site Name | Organism | Position | Strand | Matrix score. | sequence | function |
| --- | --- | --- | --- | --- | --- | --- |
| AT~TATA-box | Arabidopsis thaliana | 252 | + | 6 | TATATA |  |
| AT~TATA-box | Arabidopsis thaliana | 170 | + | 6 | TATATA |  |
| AT~TATA-box | Arabidopsis thaliana | 254 | + | 6 | TATATA |  |
| AT~TATA-box | Arabidopsis thaliana | 115 | + | 6 | TATATA |  |
| AT~TATA-box | Arabidopsis thaliana | 1500 | + | 6 | TATATA |  |
| AT~TATA-box | Arabidopsis thaliana | 250 | - | 8 | TATATAAA |  |
| AT~TATA-box | Arabidopsis thaliana | 977 | + | 6 | TATATA |  |

>HU06G00029.1   
+ +Up\_Stream \_Len000TGATAA TTATTTTTAT TGATTTAGGA AAGGATATCA AAGTATATTT TGTTTTTAGG   
  
  
+ TAGTTAAGAG ATTCTGTTGC CAAAAAAAAA ATCAAGATAT TATATATTTT TAATTATGAC AGTAATTATT   
  
  
+ TTATTGAAAA TCCAACGGTT GTGATTATAT AATCAAATAA TCTAAAGGTT AAAAATATCA AATTTATTAG   
  
  
+ AAAATTCGAA AAAGTCACGT CATCACAACA TTGCTTTTAT ATATAGTATA GATTCCTTCG ACTTCTAAAC   
  
  
+ TAATCACTTG AAATTAGAAA TTTGAAACTT TCTAATATAA ATGCATGGTC TTCTTTCCAA GATTATAATC   
  
  
+ CGCAATTGCT TAAATGTAGG GTACAAATAT CAAATTTAGA AGTGACTATT GAAGCCATGA AAGATATTGT   
  
  
+ GATCGTATTG AGGGTGGTTG TGAGCAATAG GATGAAACAG GAAAAAAAAA CCCTAATGTT TTCAAATTTA   
  
  
+ TGGGTGGCTT TGGTAGTAGA TAAGTGAATA GTGTGCAAGG GAATGGCACC AATATGTTTG GATGGAACGA   
  
  
+ TGAGAGTAAG ACGTTAGGAA AGCAACAAGA GAAGGGAAAA GGCAGTAGGG AGAGAATTAT GCGTGTGAAT   
  
  
+ AATATTATAA CAGACGCTTT CGTATGATTA TTAAATACAA GACTTTGAGC ATACATATAA ACTTGTAACT   
  
  
+ TTCTTTACCT ATTGTCAATT AATTTTTGAA TTGAATCATC TGCGACTTAT GCATACAAGA TGATATGATG   
  
  
+ CATATTGGGC CAAGCATTTG ATCTCATGTC CCTTATGGAT CTAGATCCCA ATAGACAAAC TTGCATAAGT   
  
  
+ CAAGGGATGT GTTTCTAATA CAAAGGCTTT GTACTCAAGC AATTAGTTTT GAATTCATTT TCAGTAGTTT   
  
  
+ AATTTCCAAA TCTTATAATT TTTACAATGG CATAAATTTT TAGGAGTTTT GAGTAAATAA ATTATATACA   
  
  
+ ACGAAGATGT CATTTACGAA TTAATATGAC GCACAAAAAA AAAGAAAAAA AATTAATGGT AGCTTCTATA   
  
  
+ CTTGACTCCA TGTTGACAAT TCCCTCTCTC ACATAATGGT TAAAACTTGC AACATCATCC AGGACCCATA   
  
  
+ TGACATGGGT GGTGGTAGAC AGTGTGTTGT GGTAGCCTCA CTCCATGCTT GCCAATTTGT ATCATTGCGC   
  
  
+ TAGTCTTATT CGTCCTTAGA GATAATTGAC TAAGCTCTAA TGCTCTATCG TGGCTGCTGC AATATAAATG   
  
  
+ ATTTTTTGAC CTCAAAATGA AGACAGCTAG TTGTAAATTA AGAATCTTCG TCCCTTCATT TTTTGTGTAC   
  
  
+ GTGTGTATCT GCGTATGTGT TGAGAGACTA GCGGACTATT GATTGAGTTT CAATCAAAAA AGATGACCAT   
  
  
+ CTTAAGTATT TTTAATTACG CAAACTTCTT CATTTAAAAA AATGTTACTT TTATTTGAAA GAAATTGTGA   
  
  
+ ATAATACAAG GAATAGTTCA ATTAATATAT AATCCATAAC ATATTACCTC CTATAGGCAA AAAGAAGATT   
  
  
+ ATCGTATTAA TTTGGGATCA TCCCACCCTA AATTTAAAGA AACCAAAACA AATTACTAAG CAATATCTTT   
  
  
+ CTGTGTGAAA AAATAGTCAG AAACAAAGCG ATCCGACCGG CCAAATCGAG ATAAACACAG CCAAAGAACC   
  
  
+ GAAGTTGGAC CAAAGTGAGC CGAGTTGAAA GGCATTCATA TGGGGTTTAA TCATAACACA AACATAATTA   
  
  
+ GATTGATTGA CGTCTTTAAT CAGCGTTTTT GTCTTTGTTC GGTGCTCCCC TCACTCGCCC CTCCGCTCTT   
  
  
+ TAAAGCTTAC CTCACTCCCC CCATTCCCAC TCCTTTTCTC TCTCCTCTCT CGCTCTCACA CGCTCCGTCT   
  
  
+ GTCAGCCCCT TGTCATCTTC CCCCGTCTTC CTCGCAAAAA TCTCCAGCAA AATTTATAGC CGTTGTTGTG   
  
  
+ CCCATGGTGA TCTGATGTGA GAAGAGATTC AGCATTTAAG ATTATTGGGT CCAGATGGCT TATATGTGCG   
  
  
+ CCGACAGTGG TAATCTCATG GCTATTGCCC AACAAGTCAT CAAGCAAAAA CAACAGCAAG AACAGCAGCA   
  
  
+ ACACCAACAG CCTCATCCCC AGTTCGTCGG GTCGAATAAT AACCCCTTTT GCACCAGCCC ATGGCCCGGA   
  
  
+ CCCGTCCCTT CATCCGCCAT GTCTGCCAGC CCTCCGCCCC CTCTTGGGTT CTCCGCCGCC GCGTTTCCGG   
  
  
+ ACCCGTTTCA GGTGGGCCCG CCCTGCCCGG ATGGGGCTGA ACCGGGTTTC CAGTTTGCCC ATTTGGACCA   
  
  
+ CCACTCGAGT GGTTTCCGGT TTGCTGATTT TTGCGGTGGG GGTGGTGGTG AGTTTGACTC GGATGAGTGG   
  
  
+ ATGGAGAGTT TGATGGGTGG CGGCGGCGGT GACTCGCGGC GACTGAGAGT TCTAACCTCC AATCCCATTG   
  
  
+ CGACGCGTGG CAAGGGTCCG GTGATTTCGG TCTCTATGGT GCTGATCCGT TTGCGACGTG TTCGGAAGAC   
  
  
+ CGTTGCAGCC GCTATTCACG CTCAGCCACT GCCGCAACAG CAGCCGTCGT GGGTCCCGCC TTCTCCGCCG   
  
  
+ AAAGACACGA AGCAGTCGGC CCCACCTCAA AAGCAAAGCG ACGCCGCTGG ACCCTCGTTC TCATCCGAGG   
  
  
+ AGGAACCAGA GCCGTCATGG CCGCCGCTAA TTGCGGCGTT GTTGGAGTGC GCCAAACTCG CCGAGTCCGA   
  
  
+ CCCGGAACAC GCTGTCAAAT CGGTGATTCG ACTCAGAGAC TCAGTCTCAC AGCAGGGAGT TCCAATCCAG   
  
  
+ AGAGTGTCCT ATTACTTCTC CGAGGCACTC TACCACCGCC TTTCTCTCTC CTCCGCCCCT CAACCTCTCC   
  
  
+ CAGAAACGAC GTCGTCTGAG GAGTACACTC TCTCTTACAA GGCCCTGAAC GACGCGTGCC CGTACCATAA   
  
  
+ ATTCGCTCAC TTGACCGCAA ATCAGGCGAT TCTAGAAGCC ACTGATTCAG CCAAGAAGAT CCACATCGTT   
  
  
+ GATTTTGGGA TAATCCAGGG AGTCCAATGG GCTGCTCTGC TACAAGCCCT GGCAACCCGG CCAGCCGGGA   
  
  
+ AGCCCGAATT GATTCGAATA TCCGGAATTC CTGCTCCGGG TCTGGGGCAA TCTCCGGCGG CATCGCTGCT   
  
  
+ CGCAACTGGG AATCGCCTCC GTGAGTTCGC GAAGGTTCTA GATCTCAAGT TCGAGTTTGA GCCGGTACTC   
  
  
+ ACTCCAATCA GAGAGCTAAA CGGGTCGAAC TTCCGGGTCG ACCCAGATGA GATACTAGCT GTCAATTTCA   
  
  
+ TGCTTCAATT GTACAACCTA TTAGACGAAA CCCTGGTCGC TGTCGACGCC GCCCTGAAGC TGGCCAAGTC   
  
  
+ ATTGAACCCC AAAATCGTCA CTTTGGGTGA GTACGAGGCG GGTCTGAATC GGGCAGGTTT CTTGACCCGG   
  
  
+ TTCAAGACGG CCCTCAAGTA CTATTTGGCA ATGTTTGAAT CGGTTGAGCC CAACTTGGCC CGGGACTCGC   
  
  
+ CCGAGAGGGC TCAGGTTGAG AGGCTGTTAT TGGGCCGGCG GATCATGGGC TTAGTTGGGC CGGAGTTACC   
  
  
+ GGGAACGAGA AGGGAAAGGA TGGAGTGTAA GGAACAATGG AAGGGTTTAA TGGAAAGTGC CGGTTTCGAG   
  
  
+ CCGTGCCAAA GCAGAAACTA CGTCAGTGAA AAAGACAAGT TTTACCACTC CAGATTGAAT TACAGAGAGT   
  
  
+ G  

- +Up\_Stream \_Len000ACTATT AATAAAAATA ACTAAATCCT TTCCTATAGT TTCATATAAA ACAAAAATCC   
  
  
- ATCAATTCTC TAAGACAACG GTTTTTTTTT TAGTTCTATA ATATATAAAA ATTAATACTG TCATTAATAA   
  
  
- AATAACTTTT AGGTTGCCAA CACTAATATA TTAGTTTATT AGATTTCCAA TTTTTATAGT TTAAATAATC   
  
  
- TTTTAAGCTT TTTCAGTGCA GTAGTGTTGT AACGAAAATA TATATCATAT CTAAGGAAGC TGAAGATTTG   
  
  
- ATTAGTGAAC TTTAATCTTT AAACTTTGAA AGATTATATT TACGTACCAG AAGAAAGGTT CTAATATTAG   
  
  
- GCGTTAACGA ATTTACATCC CATGTTTATA GTTTAAATCT TCACTGATAA CTTCGGTACT TTCTATAACA   
  
  
- CTAGCATAAC TCCCACCAAC ACTCGTTATC CTACTTTGTC CTTTTTTTTT GGGATTACAA AAGTTTAAAT   
  
  
- ACCCACCGAA ACCATCATCT ATTCACTTAT CACACGTTCC CTTACCGTGG TTATACAAAC CTACCTTGCT   
  
  
- ACTCTCATTC TGCAATCCTT TCGTTGTTCT CTTCCCTTTT CCGTCATCCC TCTCTTAATA CGCACACTTA   
  
  
- TTATAATATT GTCTGCGAAA GCATACTAAT AATTTATGTT CTGAAACTCG TATGTATATT TGAACATTGA   
  
  
- AAGAAATGGA TAACAGTTAA TTAAAAACTT AACTTAGTAG ACGCTGAATA CGTATGTTCT ACTATACTAC   
  
  
- GTATAACCCG GTTCGTAAAC TAGAGTACAG GGAATACCTA GATCTAGGGT TATCTGTTTG AACGTATTCA   
  
  
- GTTCCCTACA CAAAGATTAT GTTTCCGAAA CATGAGTTCG TTAATCAAAA CTTAAGTAAA AGTCATCAAA   
  
  
- TTAAAGGTTT AGAATATTAA AAATGTTACC GTATTTAAAA ATCCTCAAAA CTCATTTATT TAATATATGT   
  
  
- TGCTTCTACA GTAAATGCTT AATTATACTG CGTGTTTTTT TTTCTTTTTT TTAATTACCA TCGAAGATAT   
  
  
- GAACTGAGGT ACAACTGTTA AGGGAGAGAG TGTATTACCA ATTTTGAACG TTGTAGTAGG TCCTGGGTAT   
  
  
- ACTGTACCCA CCACCATCTG TCACACAACA CCATCGGAGT GAGGTACGAA CGGTTAAACA TAGTAACGCG   
  
  
- ATCAGAATAA GCAGGAATCT CTATTAACTG ATTCGAGATT ACGAGATAGC ACCGACGACG TTATATTTAC   
  
  
- TAAAAAACTG GAGTTTTACT TCTGTCGATC AACATTTAAT TCTTAGAAGC AGGGAAGTAA AAAACACATG   
  
  
- CACACATAGA CGCATACACA ACTCTCTGAT CGCCTGATAA CTAACTCAAA GTTAGTTTTT TCTACTGGTA   
  
  
- GAATTCATAA AAATTAATGC GTTTGAAGAA GTAAATTTTT TTACAATGAA AATAAACTTT CTTTAACACT   
  
  
- TATTATGTTC CTTATCAAGT TAATTATATA TTAGGTATTG TATAATGGAG GATATCCGTT TTTCTTCTAA   
  
  
- TAGCATAATT AAACCCTAGT AGGGTGGGAT TTAAATTTCT TTGGTTTTGT TTAATGATTC GTTATAGAAA   
  
  
- GACACACTTT TTTATCAGTC TTTGTTTCGC TAGGCTGGCC GGTTTAGCTC TATTTGTGTC GGTTTCTTGG   
  
  
- CTTCAACCTG GTTTCACTCG GCTCAACTTT CCGTAAGTAT ACCCCAAATT AGTATTGTGT TTGTATTAAT   
  
  
- CTAACTAACT GCAGAAATTA GTCGCAAAAA CAGAAACAAG CCACGAGGGG AGTGAGCGGG GAGGCGAGAA   
  
  
- ATTTCGAATG GAGTGAGGGG GGTAAGGGTG AGGAAAAGAG AGAGGAGAGA GCGAGAGTGT GCGAGGCAGA   
  
  
- CAGTCGGGGA ACAGTAGAAG GGGGCAGAAG GAGCGTTTTT AGAGGTCGTT TTAAATATCG GCAACAACAC   
  
  
- GGGTACCACT AGACTACACT CTTCTCTAAG TCGTAAATTC TAATAACCCA GGTCTACCGA ATATACACGC   
  
  
- GGCTGTCACC ATTAGAGTAC CGATAACGGG TTGTTCAGTA GTTCGTTTTT GTTGTCGTTC TTGTCGTCGT   
  
  
- TGTGGTTGTC GGAGTAGGGG TCAAGCAGCC CAGCTTATTA TTGGGGAAAA CGTGGTCGGG TACCGGGCCT   
  
  
- GGGCAGGGAA GTAGGCGGTA CAGACGGTCG GGAGGCGGGG GAGAACCCAA GAGGCGGCGG CGCAAAGGCC   
  
  
- TGGGCAAAGT CCACCCGGGC GGGACGGGCC TACCCCGACT TGGCCCAAAG GTCAAACGGG TAAACCTGGT   
  
  
- GGTGAGCTCA CCAAAGGCCA AACGACTAAA AACGCCACCC CCACCACCAC TCAAACTGAG CCTACTCACC   
  
  
- TACCTCTCAA ACTACCCACC GCCGCCGCCA CTGAGCGCCG CTGACTCTCA AGATTGGAGG TTAGGGTAAC   
  
  
- GCTGCGCACC GTTCCCAGGC CACTAAAGCC AGAGATACCA CGACTAGGCA AACGCTGCAC AAGCCTTCTG   
  
  
- GCAACGTCGG CGATAAGTGC GAGTCGGTGA CGGCGTTGTC GTCGGCAGCA CCCAGGGCGG AAGAGGCGGC   
  
  
- TTTCTGTGCT TCGTCAGCCG GGGTGGAGTT TTCGTTTCGC TGCGGCGACC TGGGAGCAAG AGTAGGCTCC   
  
  
- TCCTTGGTCT CGGCAGTACC GGCGGCGATT AACGCCGCAA CAACCTCACG CGGTTTGAGC GGCTCAGGCT   
  
  
- GGGCCTTGTG CGACAGTTTA GCCACTAAGC TGAGTCTCTG AGTCAGAGTG TCGTCCCTCA AGGTTAGGTC   
  
  
- TCTCACAGGA TAATGAAGAG GCTCCGTGAG ATGGTGGCGG AAAGAGAGAG GAGGCGGGGA GTTGGAGAGG   
  
  
- GTCTTTGCTG CAGCAGACTC CTCATGTGAG AGAGAATGTT CCGGGACTTG CTGCGCACGG GCATGGTATT   
  
  
- TAAGCGAGTG AACTGGCGTT TAGTCCGCTA AGATCTTCGG TGACTAAGTC GGTTCTTCTA GGTGTAGCAA   
  
  
- CTAAAACCCT ATTAGGTCCC TCAGGTTACC CGACGAGACG ATGTTCGGGA CCGTTGGGCC GGTCGGCCCT   
  
  
- TCGGGCTTAA CTAAGCTTAT AGGCCTTAAG GACGAGGCCC AGACCCCGTT AGAGGCCGCC GTAGCGACGA   
  
  
- GCGTTGACCC TTAGCGGAGG CACTCAAGCG CTTCCAAGAT CTAGAGTTCA AGCTCAAACT CGGCCATGAG   
  
  
- TGAGGTTAGT CTCTCGATTT GCCCAGCTTG AAGGCCCAGC TGGGTCTACT CTATGATCGA CAGTTAAAGT   
  
  
- ACGAAGTTAA CATGTTGGAT AATCTGCTTT GGGACCAGCG ACAGCTGCGG CGGGACTTCG ACCGGTTCAG   
  
  
- TAACTTGGGG TTTTAGCAGT GAAACCCACT CATGCTCCGC CCAGACTTAG CCCGTCCAAA GAACTGGGCC   
  
  
- AAGTTCTGCC GGGAGTTCAT GATAAACCGT TACAAACTTA GCCAACTCGG GTTGAACCGG GCCCTGAGCG   
  
  
- GGCTCTCCCG AGTCCAACTC TCCGACAATA ACCCGGCCGC CTAGTACCCG AATCAACCCG GCCTCAATGG   
  
  
- CCCTTGCTCT TCCCTTTCCT ACCTCACATT CCTTGTTACC TTCCCAAATT ACCTTTCACG GCCAAAGCTC   
  
  
- GGCACGGTTT CGTCTTTGAT GCAGTCACTT TTTCTGTTCA AAATGGTGAG GTCTAACTTA ATGTCTCTCA   
  
  
- C

+     Box 4

| Site Name | Organism | Position | Strand | Matrix score. | sequence | function |
| --- | --- | --- | --- | --- | --- | --- |
| Box 4 | Petroselinum crispum | 1036 | + | 6 | ATTAAT | part of a conserved DNA module involved in light responsiveness |
| Box 4 | Petroselinum crispum | 1550 | + | 6 | ATTAAT | part of a conserved DNA module involved in light responsiveness |
| Box 4 | Petroselinum crispum | 1004 | + | 6 | ATTAAT | part of a conserved DNA module involved in light responsiveness |
| Box 4 | Petroselinum crispum | 1495 | + | 6 | ATTAAT | part of a conserved DNA module involved in light responsiveness |
| Box 4 | Petroselinum crispum | 722 | + | 6 | ATTAAT | part of a conserved DNA module involved in light responsiveness |

>HU06G00029.1   
+ +Up\_Stream \_Len000TGATAA TTATTTTTAT TGATTTAGGA AAGGATATCA AAGTATATTT TGTTTTTAGG   
  
  
+ TAGTTAAGAG ATTCTGTTGC CAAAAAAAAA ATCAAGATAT TATATATTTT TAATTATGAC AGTAATTATT   
  
  
+ TTATTGAAAA TCCAACGGTT GTGATTATAT AATCAAATAA TCTAAAGGTT AAAAATATCA AATTTATTAG   
  
  
+ AAAATTCGAA AAAGTCACGT CATCACAACA TTGCTTTTAT ATATAGTATA GATTCCTTCG ACTTCTAAAC   
  
  
+ TAATCACTTG AAATTAGAAA TTTGAAACTT TCTAATATAA ATGCATGGTC TTCTTTCCAA GATTATAATC   
  
  
+ CGCAATTGCT TAAATGTAGG GTACAAATAT CAAATTTAGA AGTGACTATT GAAGCCATGA AAGATATTGT   
  
  
+ GATCGTATTG AGGGTGGTTG TGAGCAATAG GATGAAACAG GAAAAAAAAA CCCTAATGTT TTCAAATTTA   
  
  
+ TGGGTGGCTT TGGTAGTAGA TAAGTGAATA GTGTGCAAGG GAATGGCACC AATATGTTTG GATGGAACGA   
  
  
+ TGAGAGTAAG ACGTTAGGAA AGCAACAAGA GAAGGGAAAA GGCAGTAGGG AGAGAATTAT GCGTGTGAAT   
  
  
+ AATATTATAA CAGACGCTTT CGTATGATTA TTAAATACAA GACTTTGAGC ATACATATAA ACTTGTAACT   
  
  
+ TTCTTTACCT ATTGTCAATT AATTTTTGAA TTGAATCATC TGCGACTTAT GCATACAAGA TGATATGATG   
  
  
+ CATATTGGGC CAAGCATTTG ATCTCATGTC CCTTATGGAT CTAGATCCCA ATAGACAAAC TTGCATAAGT   
  
  
+ CAAGGGATGT GTTTCTAATA CAAAGGCTTT GTACTCAAGC AATTAGTTTT GAATTCATTT TCAGTAGTTT   
  
  
+ AATTTCCAAA TCTTATAATT TTTACAATGG CATAAATTTT TAGGAGTTTT GAGTAAATAA ATTATATACA   
  
  
+ ACGAAGATGT CATTTACGAA TTAATATGAC GCACAAAAAA AAAGAAAAAA AATTAATGGT AGCTTCTATA   
  
  
+ CTTGACTCCA TGTTGACAAT TCCCTCTCTC ACATAATGGT TAAAACTTGC AACATCATCC AGGACCCATA   
  
  
+ TGACATGGGT GGTGGTAGAC AGTGTGTTGT GGTAGCCTCA CTCCATGCTT GCCAATTTGT ATCATTGCGC   
  
  
+ TAGTCTTATT CGTCCTTAGA GATAATTGAC TAAGCTCTAA TGCTCTATCG TGGCTGCTGC AATATAAATG   
  
  
+ ATTTTTTGAC CTCAAAATGA AGACAGCTAG TTGTAAATTA AGAATCTTCG TCCCTTCATT TTTTGTGTAC   
  
  
+ GTGTGTATCT GCGTATGTGT TGAGAGACTA GCGGACTATT GATTGAGTTT CAATCAAAAA AGATGACCAT   
  
  
+ CTTAAGTATT TTTAATTACG CAAACTTCTT CATTTAAAAA AATGTTACTT TTATTTGAAA GAAATTGTGA   
  
  
+ ATAATACAAG GAATAGTTCA ATTAATATAT AATCCATAAC ATATTACCTC CTATAGGCAA AAAGAAGATT   
  
  
+ ATCGTATTAA TTTGGGATCA TCCCACCCTA AATTTAAAGA AACCAAAACA AATTACTAAG CAATATCTTT   
  
  
+ CTGTGTGAAA AAATAGTCAG AAACAAAGCG ATCCGACCGG CCAAATCGAG ATAAACACAG CCAAAGAACC   
  
  
+ GAAGTTGGAC CAAAGTGAGC CGAGTTGAAA GGCATTCATA TGGGGTTTAA TCATAACACA AACATAATTA   
  
  
+ GATTGATTGA CGTCTTTAAT CAGCGTTTTT GTCTTTGTTC GGTGCTCCCC TCACTCGCCC CTCCGCTCTT   
  
  
+ TAAAGCTTAC CTCACTCCCC CCATTCCCAC TCCTTTTCTC TCTCCTCTCT CGCTCTCACA CGCTCCGTCT   
  
  
+ GTCAGCCCCT TGTCATCTTC CCCCGTCTTC CTCGCAAAAA TCTCCAGCAA AATTTATAGC CGTTGTTGTG   
  
  
+ CCCATGGTGA TCTGATGTGA GAAGAGATTC AGCATTTAAG ATTATTGGGT CCAGATGGCT TATATGTGCG   
  
  
+ CCGACAGTGG TAATCTCATG GCTATTGCCC AACAAGTCAT CAAGCAAAAA CAACAGCAAG AACAGCAGCA   
  
  
+ ACACCAACAG CCTCATCCCC AGTTCGTCGG GTCGAATAAT AACCCCTTTT GCACCAGCCC ATGGCCCGGA   
  
  
+ CCCGTCCCTT CATCCGCCAT GTCTGCCAGC CCTCCGCCCC CTCTTGGGTT CTCCGCCGCC GCGTTTCCGG   
  
  
+ ACCCGTTTCA GGTGGGCCCG CCCTGCCCGG ATGGGGCTGA ACCGGGTTTC CAGTTTGCCC ATTTGGACCA   
  
  
+ CCACTCGAGT GGTTTCCGGT TTGCTGATTT TTGCGGTGGG GGTGGTGGTG AGTTTGACTC GGATGAGTGG   
  
  
+ ATGGAGAGTT TGATGGGTGG CGGCGGCGGT GACTCGCGGC GACTGAGAGT TCTAACCTCC AATCCCATTG   
  
  
+ CGACGCGTGG CAAGGGTCCG GTGATTTCGG TCTCTATGGT GCTGATCCGT TTGCGACGTG TTCGGAAGAC   
  
  
+ CGTTGCAGCC GCTATTCACG CTCAGCCACT GCCGCAACAG CAGCCGTCGT GGGTCCCGCC TTCTCCGCCG   
  
  
+ AAAGACACGA AGCAGTCGGC CCCACCTCAA AAGCAAAGCG ACGCCGCTGG ACCCTCGTTC TCATCCGAGG   
  
  
+ AGGAACCAGA GCCGTCATGG CCGCCGCTAA TTGCGGCGTT GTTGGAGTGC GCCAAACTCG CCGAGTCCGA   
  
  
+ CCCGGAACAC GCTGTCAAAT CGGTGATTCG ACTCAGAGAC TCAGTCTCAC AGCAGGGAGT TCCAATCCAG   
  
  
+ AGAGTGTCCT ATTACTTCTC CGAGGCACTC TACCACCGCC TTTCTCTCTC CTCCGCCCCT CAACCTCTCC   
  
  
+ CAGAAACGAC GTCGTCTGAG GAGTACACTC TCTCTTACAA GGCCCTGAAC GACGCGTGCC CGTACCATAA   
  
  
+ ATTCGCTCAC TTGACCGCAA ATCAGGCGAT TCTAGAAGCC ACTGATTCAG CCAAGAAGAT CCACATCGTT   
  
  
+ GATTTTGGGA TAATCCAGGG AGTCCAATGG GCTGCTCTGC TACAAGCCCT GGCAACCCGG CCAGCCGGGA   
  
  
+ AGCCCGAATT GATTCGAATA TCCGGAATTC CTGCTCCGGG TCTGGGGCAA TCTCCGGCGG CATCGCTGCT   
  
  
+ CGCAACTGGG AATCGCCTCC GTGAGTTCGC GAAGGTTCTA GATCTCAAGT TCGAGTTTGA GCCGGTACTC   
  
  
+ ACTCCAATCA GAGAGCTAAA CGGGTCGAAC TTCCGGGTCG ACCCAGATGA GATACTAGCT GTCAATTTCA   
  
  
+ TGCTTCAATT GTACAACCTA TTAGACGAAA CCCTGGTCGC TGTCGACGCC GCCCTGAAGC TGGCCAAGTC   
  
  
+ ATTGAACCCC AAAATCGTCA CTTTGGGTGA GTACGAGGCG GGTCTGAATC GGGCAGGTTT CTTGACCCGG   
  
  
+ TTCAAGACGG CCCTCAAGTA CTATTTGGCA ATGTTTGAAT CGGTTGAGCC CAACTTGGCC CGGGACTCGC   
  
  
+ CCGAGAGGGC TCAGGTTGAG AGGCTGTTAT TGGGCCGGCG GATCATGGGC TTAGTTGGGC CGGAGTTACC   
  
  
+ GGGAACGAGA AGGGAAAGGA TGGAGTGTAA GGAACAATGG AAGGGTTTAA TGGAAAGTGC CGGTTTCGAG   
  
  
+ CCGTGCCAAA GCAGAAACTA CGTCAGTGAA AAAGACAAGT TTTACCACTC CAGATTGAAT TACAGAGAGT   
  
  
+ G  

- +Up\_Stream \_Len000ACTATT AATAAAAATA ACTAAATCCT TTCCTATAGT TTCATATAAA ACAAAAATCC   
  
  
- ATCAATTCTC TAAGACAACG GTTTTTTTTT TAGTTCTATA ATATATAAAA ATTAATACTG TCATTAATAA   
  
  
- AATAACTTTT AGGTTGCCAA CACTAATATA TTAGTTTATT AGATTTCCAA TTTTTATAGT TTAAATAATC   
  
  
- TTTTAAGCTT TTTCAGTGCA GTAGTGTTGT AACGAAAATA TATATCATAT CTAAGGAAGC TGAAGATTTG   
  
  
- ATTAGTGAAC TTTAATCTTT AAACTTTGAA AGATTATATT TACGTACCAG AAGAAAGGTT CTAATATTAG   
  
  
- GCGTTAACGA ATTTACATCC CATGTTTATA GTTTAAATCT TCACTGATAA CTTCGGTACT TTCTATAACA   
  
  
- CTAGCATAAC TCCCACCAAC ACTCGTTATC CTACTTTGTC CTTTTTTTTT GGGATTACAA AAGTTTAAAT   
  
  
- ACCCACCGAA ACCATCATCT ATTCACTTAT CACACGTTCC CTTACCGTGG TTATACAAAC CTACCTTGCT   
  
  
- ACTCTCATTC TGCAATCCTT TCGTTGTTCT CTTCCCTTTT CCGTCATCCC TCTCTTAATA CGCACACTTA   
  
  
- TTATAATATT GTCTGCGAAA GCATACTAAT AATTTATGTT CTGAAACTCG TATGTATATT TGAACATTGA   
  
  
- AAGAAATGGA TAACAGTTAA TTAAAAACTT AACTTAGTAG ACGCTGAATA CGTATGTTCT ACTATACTAC   
  
  
- GTATAACCCG GTTCGTAAAC TAGAGTACAG GGAATACCTA GATCTAGGGT TATCTGTTTG AACGTATTCA   
  
  
- GTTCCCTACA CAAAGATTAT GTTTCCGAAA CATGAGTTCG TTAATCAAAA CTTAAGTAAA AGTCATCAAA   
  
  
- TTAAAGGTTT AGAATATTAA AAATGTTACC GTATTTAAAA ATCCTCAAAA CTCATTTATT TAATATATGT   
  
  
- TGCTTCTACA GTAAATGCTT AATTATACTG CGTGTTTTTT TTTCTTTTTT TTAATTACCA TCGAAGATAT   
  
  
- GAACTGAGGT ACAACTGTTA AGGGAGAGAG TGTATTACCA ATTTTGAACG TTGTAGTAGG TCCTGGGTAT   
  
  
- ACTGTACCCA CCACCATCTG TCACACAACA CCATCGGAGT GAGGTACGAA CGGTTAAACA TAGTAACGCG   
  
  
- ATCAGAATAA GCAGGAATCT CTATTAACTG ATTCGAGATT ACGAGATAGC ACCGACGACG TTATATTTAC   
  
  
- TAAAAAACTG GAGTTTTACT TCTGTCGATC AACATTTAAT TCTTAGAAGC AGGGAAGTAA AAAACACATG   
  
  
- CACACATAGA CGCATACACA ACTCTCTGAT CGCCTGATAA CTAACTCAAA GTTAGTTTTT TCTACTGGTA   
  
  
- GAATTCATAA AAATTAATGC GTTTGAAGAA GTAAATTTTT TTACAATGAA AATAAACTTT CTTTAACACT   
  
  
- TATTATGTTC CTTATCAAGT TAATTATATA TTAGGTATTG TATAATGGAG GATATCCGTT TTTCTTCTAA   
  
  
- TAGCATAATT AAACCCTAGT AGGGTGGGAT TTAAATTTCT TTGGTTTTGT TTAATGATTC GTTATAGAAA   
  
  
- GACACACTTT TTTATCAGTC TTTGTTTCGC TAGGCTGGCC GGTTTAGCTC TATTTGTGTC GGTTTCTTGG   
  
  
- CTTCAACCTG GTTTCACTCG GCTCAACTTT CCGTAAGTAT ACCCCAAATT AGTATTGTGT TTGTATTAAT   
  
  
- CTAACTAACT GCAGAAATTA GTCGCAAAAA CAGAAACAAG CCACGAGGGG AGTGAGCGGG GAGGCGAGAA   
  
  
- ATTTCGAATG GAGTGAGGGG GGTAAGGGTG AGGAAAAGAG AGAGGAGAGA GCGAGAGTGT GCGAGGCAGA   
  
  
- CAGTCGGGGA ACAGTAGAAG GGGGCAGAAG GAGCGTTTTT AGAGGTCGTT TTAAATATCG GCAACAACAC   
  
  
- GGGTACCACT AGACTACACT CTTCTCTAAG TCGTAAATTC TAATAACCCA GGTCTACCGA ATATACACGC   
  
  
- GGCTGTCACC ATTAGAGTAC CGATAACGGG TTGTTCAGTA GTTCGTTTTT GTTGTCGTTC TTGTCGTCGT   
  
  
- TGTGGTTGTC GGAGTAGGGG TCAAGCAGCC CAGCTTATTA TTGGGGAAAA CGTGGTCGGG TACCGGGCCT   
  
  
- GGGCAGGGAA GTAGGCGGTA CAGACGGTCG GGAGGCGGGG GAGAACCCAA GAGGCGGCGG CGCAAAGGCC   
  
  
- TGGGCAAAGT CCACCCGGGC GGGACGGGCC TACCCCGACT TGGCCCAAAG GTCAAACGGG TAAACCTGGT   
  
  
- GGTGAGCTCA CCAAAGGCCA AACGACTAAA AACGCCACCC CCACCACCAC TCAAACTGAG CCTACTCACC   
  
  
- TACCTCTCAA ACTACCCACC GCCGCCGCCA CTGAGCGCCG CTGACTCTCA AGATTGGAGG TTAGGGTAAC   
  
  
- GCTGCGCACC GTTCCCAGGC CACTAAAGCC AGAGATACCA CGACTAGGCA AACGCTGCAC AAGCCTTCTG   
  
  
- GCAACGTCGG CGATAAGTGC GAGTCGGTGA CGGCGTTGTC GTCGGCAGCA CCCAGGGCGG AAGAGGCGGC   
  
  
- TTTCTGTGCT TCGTCAGCCG GGGTGGAGTT TTCGTTTCGC TGCGGCGACC TGGGAGCAAG AGTAGGCTCC   
  
  
- TCCTTGGTCT CGGCAGTACC GGCGGCGATT AACGCCGCAA CAACCTCACG CGGTTTGAGC GGCTCAGGCT   
  
  
- GGGCCTTGTG CGACAGTTTA GCCACTAAGC TGAGTCTCTG AGTCAGAGTG TCGTCCCTCA AGGTTAGGTC   
  
  
- TCTCACAGGA TAATGAAGAG GCTCCGTGAG ATGGTGGCGG AAAGAGAGAG GAGGCGGGGA GTTGGAGAGG   
  
  
- GTCTTTGCTG CAGCAGACTC CTCATGTGAG AGAGAATGTT CCGGGACTTG CTGCGCACGG GCATGGTATT   
  
  
- TAAGCGAGTG AACTGGCGTT TAGTCCGCTA AGATCTTCGG TGACTAAGTC GGTTCTTCTA GGTGTAGCAA   
  
  
- CTAAAACCCT ATTAGGTCCC TCAGGTTACC CGACGAGACG ATGTTCGGGA CCGTTGGGCC GGTCGGCCCT   
  
  
- TCGGGCTTAA CTAAGCTTAT AGGCCTTAAG GACGAGGCCC AGACCCCGTT AGAGGCCGCC GTAGCGACGA   
  
  
- GCGTTGACCC TTAGCGGAGG CACTCAAGCG CTTCCAAGAT CTAGAGTTCA AGCTCAAACT CGGCCATGAG   
  
  
- TGAGGTTAGT CTCTCGATTT GCCCAGCTTG AAGGCCCAGC TGGGTCTACT CTATGATCGA CAGTTAAAGT   
  
  
- ACGAAGTTAA CATGTTGGAT AATCTGCTTT GGGACCAGCG ACAGCTGCGG CGGGACTTCG ACCGGTTCAG   
  
  
- TAACTTGGGG TTTTAGCAGT GAAACCCACT CATGCTCCGC CCAGACTTAG CCCGTCCAAA GAACTGGGCC   
  
  
- AAGTTCTGCC GGGAGTTCAT GATAAACCGT TACAAACTTA GCCAACTCGG GTTGAACCGG GCCCTGAGCG   
  
  
- GGCTCTCCCG AGTCCAACTC TCCGACAATA ACCCGGCCGC CTAGTACCCG AATCAACCCG GCCTCAATGG   
  
  
- CCCTTGCTCT TCCCTTTCCT ACCTCACATT CCTTGTTACC TTCCCAAATT ACCTTTCACG GCCAAAGCTC   
  
  
- GGCACGGTTT CGTCTTTGAT GCAGTCACTT TTTCTGTTCA AAATGGTGAG GTCTAACTTA ATGTCTCTCA   
  
  
- C

+     CAAT-box

| Site Name | Organism | Position | Strand | Matrix score. | sequence | function |
| --- | --- | --- | --- | --- | --- | --- |
| CAAT-box | Nicotiana glutinosa | 449 | + | 4 | CAAT |  |
| CAAT-box | Nicotiana glutinosa | 420 | - | 4 | CAAT |  |
| CAAT-box | Nicotiana glutinosa | 244 | - | 4 | CAAT |  |
| CAAT-box | Nicotiana glutinosa | 402 | - | 4 | CAAT |  |
| CAAT-box | Nicotiana glutinosa | 359 | - | 4 | CAAT |  |
| CAAT-box | Nicotiana glutinosa | 3609 | + | 4 | CAAT |  |
| CAAT-box | Nicotiana glutinosa | 2451 | - | 4 | CAAT |  |
| CAAT-box | Nicotiana glutinosa | 2444 | + | 4 | CAAT |  |
| CAAT-box | Nicotiana glutinosa | 1605 | + | 4 | CAAT |  |
| CAAT-box | Pisum sativum | 1593 | + | 5 | CAAAT | common cis-acting element in promoter and enhancer regions |
| CAAT-box | Nicotiana glutinosa | 1493 | + | 4 | CAAT |  |
| CAAT-box | Pisum sativum | 304 | - | 5 | CAAAT | common cis-acting element in promoter and enhancer regions |
| CAAT-box | Pisum sativum | 1457 | - | 5 | CAAAT | common cis-acting element in promoter and enhancer regions |
| CAAT-box | Pisum sativum | 378 | + | 5 | CAAAT | common cis-acting element in promoter and enhancer regions |
| CAAT-box | Pisum sativum | 487 | + | 5 | CAAAT | common cis-acting element in promoter and enhancer regions |
| CAAT-box | Nicotiana glutinosa | 1071 | + | 4 | CAAT |  |
| CAAT-box | Nicotiana glutinosa | 3463 | + | 4 | CAAT |  |
| CAAT-box | Nicotiana glutinosa | 3287 | + | 4 | CAAT |  |
| CAAT-box | Nicotiana glutinosa | 1376 | - | 4 | CAAT |  |
| CAAT-box | Arabidopsis thaliana | 3533 | - | 5 | CCAAT | common cis-acting element in promoter and enhancer regions |
| CAAT-box | Nicotiana glutinosa | 3229 | + | 4 | CAAT |  |
| CAAT-box | Arabidopsis thaliana | 3228 | + | 5 | CCAAT | common cis-acting element in promoter and enhancer regions |
| CAAT-box | Nicotiana glutinosa | 3092 | - | 4 | CAAT |  |
| CAAT-box | Pisum sativum | 178 | + | 5 | CAAAT | common cis-acting element in promoter and enhancer regions |
| CAAT-box | Nicotiana glutinosa | 147 | - | 4 | CAAT |  |
| CAAT-box | Nicotiana glutinosa | 33 | - | 4 | CAAT |  |
| CAAT-box | Pisum sativum | 2305 | - | 5 | CAAAT | common cis-acting element in promoter and enhancer regions |
| CAAT-box | Nicotiana glutinosa | 884 | + | 4 | CAAT |  |
| CAAT-box | Nicotiana glutinosa | 1372 | - | 4 | CAAT |  |
| CAAT-box | Nicotiana glutinosa | 1219 | - | 4 | CAAT |  |
| CAAT-box | Nicotiana glutinosa | 3132 | + | 4 | CAAT |  |
| CAAT-box | Arabidopsis thaliana | 2443 | + | 5 | CCAAT | common cis-acting element in promoter and enhancer regions |
| CAAT-box | Nicotiana glutinosa | 1385 | + | 4 | CAAT |  |
| CAAT-box | Nicotiana glutinosa | 3365 | - | 4 | CAAT |  |
| CAAT-box | Nicotiana glutinosa | 3302 | - | 4 | CAAT |  |
| CAAT-box | Nicotiana glutinosa | 3039 | + | 4 | CAAT |  |
| CAAT-box | Arabidopsis thaliana | 3038 | + | 5 | CCAAT | common cis-acting element in promoter and enhancer regions |
| CAAT-box | Nicotiana glutinosa | 431 | - | 4 | CAAT |  |
| CAAT-box | Nicotiana glutinosa | 2694 | - | 4 | CAAT |  |
| CAAT-box | Nicotiana glutinosa | 357 | + | 4 | CAAT |  |
| CAAT-box | Pisum sativum | 921 | + | 5 | CAAAT | common cis-acting element in promoter and enhancer regions |
| CAAT-box | Pisum sativum | 1179 | - | 5 | CAAAT | common cis-acting element in promoter and enhancer regions |
| CAAT-box | Arabidopsis thaliana | 822 | + | 5 | CCAAT | common cis-acting element in promoter and enhancer regions |
| CAAT-box | Pisum sativum | 1656 | + | 5 | CAAAT | common cis-acting element in promoter and enhancer regions |
| CAAT-box | Nicotiana glutinosa | 544 | + | 4 | CAAT |  |
| CAAT-box | Nicotiana glutinosa | 2058 | - | 4 | CAAT |  |
| CAAT-box | Nicotiana glutinosa | 1177 | + | 4 | CAAT |  |
| CAAT-box | Pisum sativum | 1554 | - | 5 | CAAAT | common cis-acting element in promoter and enhancer regions |
| CAAT-box | Arabidopsis thaliana | 1176 | + | 5 | CCAAT | common cis-acting element in promoter and enhancer regions |
| CAAT-box | Nicotiana glutinosa | 3698 | - | 4 | CAAT |  |
| CAAT-box | Nicotiana glutinosa | 1188 | - | 4 | CAAT |  |
| CAAT-box | Nicotiana glutinosa | 715 | - | 4 | CAAT |  |
| CAAT-box | Arabidopsis thaliana | 2796 | + | 5 | CCAAT | common cis-acting element in promoter and enhancer regions |
| CAAT-box | Nicotiana glutinosa | 2797 | + | 4 | CAAT |  |
| CAAT-box | Nicotiana glutinosa | 1468 | - | 4 | CAAT |  |
| CAAT-box | Pisum sativum | 3457 | - | 5 | CAAAT | common cis-acting element in promoter and enhancer regions |
| CAAT-box | Pisum sativum | 385 | + | 5 | CAAAT | common cis-acting element in promoter and enhancer regions |
| CAAT-box | Nicotiana glutinosa | 1756 | - | 4 | CAAT |  |
| CAAT-box | Nicotiana glutinosa | 1254 | + | 4 | CAAT |  |
| CAAT-box | Nicotiana glutinosa | 3300 | + | 4 | CAAT |  |
| CAAT-box | Pisum sativum | 2962 | + | 5 | CAAAT | common cis-acting element in promoter and enhancer regions |
| CAAT-box | Pisum sativum | 2750 | + | 5 | CAAAT | common cis-acting element in promoter and enhancer regions |
| CAAT-box | Nicotiana glutinosa | 1760 | - | 4 | CAAT |  |
| CAAT-box | Arabidopsis thaliana | 2008 | - | 5 | CCAAT | common cis-acting element in promoter and enhancer regions |
| CAAT-box | Pisum sativum | 203 | + | 5 | CAAAT | common cis-acting element in promoter and enhancer regions |
| CAAT-box | Nicotiana glutinosa | 823 | + | 4 | CAAT |  |
| CAAT-box | Nicotiana glutinosa | 939 | + | 4 | CAAT |  |
| CAAT-box | Arabidopsis thaliana | 778 | - | 5 | CCAAT | common cis-acting element in promoter and enhancer regions |
| CAAT-box | Pisum sativum | 790 | - | 5 | CAAAT | common cis-acting element in promoter and enhancer regions |
| CAAT-box | Nicotiana glutinosa | 720 | + | 4 | CAAT |  |
| CAAT-box | Nicotiana glutinosa | 734 | - | 4 | CAAT |  |
| CAAT-box | Arabidopsis thaliana | 543 | + | 5 | CCAAT | common cis-acting element in promoter and enhancer regions |

>HU06G00029.1   
+ +Up\_Stream \_Len000TGATAA TTATTTTTAT TGATTTAGGA AAGGATATCA AAGTATATTT TGTTTTTAGG   
  
  
+ TAGTTAAGAG ATTCTGTTGC CAAAAAAAAA ATCAAGATAT TATATATTTT TAATTATGAC AGTAATTATT   
  
  
+ TTATTGAAAA TCCAACGGTT GTGATTATAT AATCAAATAA TCTAAAGGTT AAAAATATCA AATTTATTAG   
  
  
+ AAAATTCGAA AAAGTCACGT CATCACAACA TTGCTTTTAT ATATAGTATA GATTCCTTCG ACTTCTAAAC   
  
  
+ TAATCACTTG AAATTAGAAA TTTGAAACTT TCTAATATAA ATGCATGGTC TTCTTTCCAA GATTATAATC   
  
  
+ CGCAATTGCT TAAATGTAGG GTACAAATAT CAAATTTAGA AGTGACTATT GAAGCCATGA AAGATATTGT   
  
  
+ GATCGTATTG AGGGTGGTTG TGAGCAATAG GATGAAACAG GAAAAAAAAA CCCTAATGTT TTCAAATTTA   
  
  
+ TGGGTGGCTT TGGTAGTAGA TAAGTGAATA GTGTGCAAGG GAATGGCACC AATATGTTTG GATGGAACGA   
  
  
+ TGAGAGTAAG ACGTTAGGAA AGCAACAAGA GAAGGGAAAA GGCAGTAGGG AGAGAATTAT GCGTGTGAAT   
  
  
+ AATATTATAA CAGACGCTTT CGTATGATTA TTAAATACAA GACTTTGAGC ATACATATAA ACTTGTAACT   
  
  
+ TTCTTTACCT ATTGTCAATT AATTTTTGAA TTGAATCATC TGCGACTTAT GCATACAAGA TGATATGATG   
  
  
+ CATATTGGGC CAAGCATTTG ATCTCATGTC CCTTATGGAT CTAGATCCCA ATAGACAAAC TTGCATAAGT   
  
  
+ CAAGGGATGT GTTTCTAATA CAAAGGCTTT GTACTCAAGC AATTAGTTTT GAATTCATTT TCAGTAGTTT   
  
  
+ AATTTCCAAA TCTTATAATT TTTACAATGG CATAAATTTT TAGGAGTTTT GAGTAAATAA ATTATATACA   
  
  
+ ACGAAGATGT CATTTACGAA TTAATATGAC GCACAAAAAA AAAGAAAAAA AATTAATGGT AGCTTCTATA   
  
  
+ CTTGACTCCA TGTTGACAAT TCCCTCTCTC ACATAATGGT TAAAACTTGC AACATCATCC AGGACCCATA   
  
  
+ TGACATGGGT GGTGGTAGAC AGTGTGTTGT GGTAGCCTCA CTCCATGCTT GCCAATTTGT ATCATTGCGC   
  
  
+ TAGTCTTATT CGTCCTTAGA GATAATTGAC TAAGCTCTAA TGCTCTATCG TGGCTGCTGC AATATAAATG   
  
  
+ ATTTTTTGAC CTCAAAATGA AGACAGCTAG TTGTAAATTA AGAATCTTCG TCCCTTCATT TTTTGTGTAC   
  
  
+ GTGTGTATCT GCGTATGTGT TGAGAGACTA GCGGACTATT GATTGAGTTT CAATCAAAAA AGATGACCAT   
  
  
+ CTTAAGTATT TTTAATTACG CAAACTTCTT CATTTAAAAA AATGTTACTT TTATTTGAAA GAAATTGTGA   
  
  
+ ATAATACAAG GAATAGTTCA ATTAATATAT AATCCATAAC ATATTACCTC CTATAGGCAA AAAGAAGATT   
  
  
+ ATCGTATTAA TTTGGGATCA TCCCACCCTA AATTTAAAGA AACCAAAACA AATTACTAAG CAATATCTTT   
  
  
+ CTGTGTGAAA AAATAGTCAG AAACAAAGCG ATCCGACCGG CCAAATCGAG ATAAACACAG CCAAAGAACC   
  
  
+ GAAGTTGGAC CAAAGTGAGC CGAGTTGAAA GGCATTCATA TGGGGTTTAA TCATAACACA AACATAATTA   
  
  
+ GATTGATTGA CGTCTTTAAT CAGCGTTTTT GTCTTTGTTC GGTGCTCCCC TCACTCGCCC CTCCGCTCTT   
  
  
+ TAAAGCTTAC CTCACTCCCC CCATTCCCAC TCCTTTTCTC TCTCCTCTCT CGCTCTCACA CGCTCCGTCT   
  
  
+ GTCAGCCCCT TGTCATCTTC CCCCGTCTTC CTCGCAAAAA TCTCCAGCAA AATTTATAGC CGTTGTTGTG   
  
  
+ CCCATGGTGA TCTGATGTGA GAAGAGATTC AGCATTTAAG ATTATTGGGT CCAGATGGCT TATATGTGCG   
  
  
+ CCGACAGTGG TAATCTCATG GCTATTGCCC AACAAGTCAT CAAGCAAAAA CAACAGCAAG AACAGCAGCA   
  
  
+ ACACCAACAG CCTCATCCCC AGTTCGTCGG GTCGAATAAT AACCCCTTTT GCACCAGCCC ATGGCCCGGA   
  
  
+ CCCGTCCCTT CATCCGCCAT GTCTGCCAGC CCTCCGCCCC CTCTTGGGTT CTCCGCCGCC GCGTTTCCGG   
  
  
+ ACCCGTTTCA GGTGGGCCCG CCCTGCCCGG ATGGGGCTGA ACCGGGTTTC CAGTTTGCCC ATTTGGACCA   
  
  
+ CCACTCGAGT GGTTTCCGGT TTGCTGATTT TTGCGGTGGG GGTGGTGGTG AGTTTGACTC GGATGAGTGG   
  
  
+ ATGGAGAGTT TGATGGGTGG CGGCGGCGGT GACTCGCGGC GACTGAGAGT TCTAACCTCC AATCCCATTG   
  
  
+ CGACGCGTGG CAAGGGTCCG GTGATTTCGG TCTCTATGGT GCTGATCCGT TTGCGACGTG TTCGGAAGAC   
  
  
+ CGTTGCAGCC GCTATTCACG CTCAGCCACT GCCGCAACAG CAGCCGTCGT GGGTCCCGCC TTCTCCGCCG   
  
  
+ AAAGACACGA AGCAGTCGGC CCCACCTCAA AAGCAAAGCG ACGCCGCTGG ACCCTCGTTC TCATCCGAGG   
  
  
+ AGGAACCAGA GCCGTCATGG CCGCCGCTAA TTGCGGCGTT GTTGGAGTGC GCCAAACTCG CCGAGTCCGA   
  
  
+ CCCGGAACAC GCTGTCAAAT CGGTGATTCG ACTCAGAGAC TCAGTCTCAC AGCAGGGAGT TCCAATCCAG   
  
  
+ AGAGTGTCCT ATTACTTCTC CGAGGCACTC TACCACCGCC TTTCTCTCTC CTCCGCCCCT CAACCTCTCC   
  
  
+ CAGAAACGAC GTCGTCTGAG GAGTACACTC TCTCTTACAA GGCCCTGAAC GACGCGTGCC CGTACCATAA   
  
  
+ ATTCGCTCAC TTGACCGCAA ATCAGGCGAT TCTAGAAGCC ACTGATTCAG CCAAGAAGAT CCACATCGTT   
  
  
+ GATTTTGGGA TAATCCAGGG AGTCCAATGG GCTGCTCTGC TACAAGCCCT GGCAACCCGG CCAGCCGGGA   
  
  
+ AGCCCGAATT GATTCGAATA TCCGGAATTC CTGCTCCGGG TCTGGGGCAA TCTCCGGCGG CATCGCTGCT   
  
  
+ CGCAACTGGG AATCGCCTCC GTGAGTTCGC GAAGGTTCTA GATCTCAAGT TCGAGTTTGA GCCGGTACTC   
  
  
+ ACTCCAATCA GAGAGCTAAA CGGGTCGAAC TTCCGGGTCG ACCCAGATGA GATACTAGCT GTCAATTTCA   
  
  
+ TGCTTCAATT GTACAACCTA TTAGACGAAA CCCTGGTCGC TGTCGACGCC GCCCTGAAGC TGGCCAAGTC   
  
  
+ ATTGAACCCC AAAATCGTCA CTTTGGGTGA GTACGAGGCG GGTCTGAATC GGGCAGGTTT CTTGACCCGG   
  
  
+ TTCAAGACGG CCCTCAAGTA CTATTTGGCA ATGTTTGAAT CGGTTGAGCC CAACTTGGCC CGGGACTCGC   
  
  
+ CCGAGAGGGC TCAGGTTGAG AGGCTGTTAT TGGGCCGGCG GATCATGGGC TTAGTTGGGC CGGAGTTACC   
  
  
+ GGGAACGAGA AGGGAAAGGA TGGAGTGTAA GGAACAATGG AAGGGTTTAA TGGAAAGTGC CGGTTTCGAG   
  
  
+ CCGTGCCAAA GCAGAAACTA CGTCAGTGAA AAAGACAAGT TTTACCACTC CAGATTGAAT TACAGAGAGT   
  
  
+ G  

- +Up\_Stream \_Len000ACTATT AATAAAAATA ACTAAATCCT TTCCTATAGT TTCATATAAA ACAAAAATCC   
  
  
- ATCAATTCTC TAAGACAACG GTTTTTTTTT TAGTTCTATA ATATATAAAA ATTAATACTG TCATTAATAA   
  
  
- AATAACTTTT AGGTTGCCAA CACTAATATA TTAGTTTATT AGATTTCCAA TTTTTATAGT TTAAATAATC   
  
  
- TTTTAAGCTT TTTCAGTGCA GTAGTGTTGT AACGAAAATA TATATCATAT CTAAGGAAGC TGAAGATTTG   
  
  
- ATTAGTGAAC TTTAATCTTT AAACTTTGAA AGATTATATT TACGTACCAG AAGAAAGGTT CTAATATTAG   
  
  
- GCGTTAACGA ATTTACATCC CATGTTTATA GTTTAAATCT TCACTGATAA CTTCGGTACT TTCTATAACA   
  
  
- CTAGCATAAC TCCCACCAAC ACTCGTTATC CTACTTTGTC CTTTTTTTTT GGGATTACAA AAGTTTAAAT   
  
  
- ACCCACCGAA ACCATCATCT ATTCACTTAT CACACGTTCC CTTACCGTGG TTATACAAAC CTACCTTGCT   
  
  
- ACTCTCATTC TGCAATCCTT TCGTTGTTCT CTTCCCTTTT CCGTCATCCC TCTCTTAATA CGCACACTTA   
  
  
- TTATAATATT GTCTGCGAAA GCATACTAAT AATTTATGTT CTGAAACTCG TATGTATATT TGAACATTGA   
  
  
- AAGAAATGGA TAACAGTTAA TTAAAAACTT AACTTAGTAG ACGCTGAATA CGTATGTTCT ACTATACTAC   
  
  
- GTATAACCCG GTTCGTAAAC TAGAGTACAG GGAATACCTA GATCTAGGGT TATCTGTTTG AACGTATTCA   
  
  
- GTTCCCTACA CAAAGATTAT GTTTCCGAAA CATGAGTTCG TTAATCAAAA CTTAAGTAAA AGTCATCAAA   
  
  
- TTAAAGGTTT AGAATATTAA AAATGTTACC GTATTTAAAA ATCCTCAAAA CTCATTTATT TAATATATGT   
  
  
- TGCTTCTACA GTAAATGCTT AATTATACTG CGTGTTTTTT TTTCTTTTTT TTAATTACCA TCGAAGATAT   
  
  
- GAACTGAGGT ACAACTGTTA AGGGAGAGAG TGTATTACCA ATTTTGAACG TTGTAGTAGG TCCTGGGTAT   
  
  
- ACTGTACCCA CCACCATCTG TCACACAACA CCATCGGAGT GAGGTACGAA CGGTTAAACA TAGTAACGCG   
  
  
- ATCAGAATAA GCAGGAATCT CTATTAACTG ATTCGAGATT ACGAGATAGC ACCGACGACG TTATATTTAC   
  
  
- TAAAAAACTG GAGTTTTACT TCTGTCGATC AACATTTAAT TCTTAGAAGC AGGGAAGTAA AAAACACATG   
  
  
- CACACATAGA CGCATACACA ACTCTCTGAT CGCCTGATAA CTAACTCAAA GTTAGTTTTT TCTACTGGTA   
  
  
- GAATTCATAA AAATTAATGC GTTTGAAGAA GTAAATTTTT TTACAATGAA AATAAACTTT CTTTAACACT   
  
  
- TATTATGTTC CTTATCAAGT TAATTATATA TTAGGTATTG TATAATGGAG GATATCCGTT TTTCTTCTAA   
  
  
- TAGCATAATT AAACCCTAGT AGGGTGGGAT TTAAATTTCT TTGGTTTTGT TTAATGATTC GTTATAGAAA   
  
  
- GACACACTTT TTTATCAGTC TTTGTTTCGC TAGGCTGGCC GGTTTAGCTC TATTTGTGTC GGTTTCTTGG   
  
  
- CTTCAACCTG GTTTCACTCG GCTCAACTTT CCGTAAGTAT ACCCCAAATT AGTATTGTGT TTGTATTAAT   
  
  
- CTAACTAACT GCAGAAATTA GTCGCAAAAA CAGAAACAAG CCACGAGGGG AGTGAGCGGG GAGGCGAGAA   
  
  
- ATTTCGAATG GAGTGAGGGG GGTAAGGGTG AGGAAAAGAG AGAGGAGAGA GCGAGAGTGT GCGAGGCAGA   
  
  
- CAGTCGGGGA ACAGTAGAAG GGGGCAGAAG GAGCGTTTTT AGAGGTCGTT TTAAATATCG GCAACAACAC   
  
  
- GGGTACCACT AGACTACACT CTTCTCTAAG TCGTAAATTC TAATAACCCA GGTCTACCGA ATATACACGC   
  
  
- GGCTGTCACC ATTAGAGTAC CGATAACGGG TTGTTCAGTA GTTCGTTTTT GTTGTCGTTC TTGTCGTCGT   
  
  
- TGTGGTTGTC GGAGTAGGGG TCAAGCAGCC CAGCTTATTA TTGGGGAAAA CGTGGTCGGG TACCGGGCCT   
  
  
- GGGCAGGGAA GTAGGCGGTA CAGACGGTCG GGAGGCGGGG GAGAACCCAA GAGGCGGCGG CGCAAAGGCC   
  
  
- TGGGCAAAGT CCACCCGGGC GGGACGGGCC TACCCCGACT TGGCCCAAAG GTCAAACGGG TAAACCTGGT   
  
  
- GGTGAGCTCA CCAAAGGCCA AACGACTAAA AACGCCACCC CCACCACCAC TCAAACTGAG CCTACTCACC   
  
  
- TACCTCTCAA ACTACCCACC GCCGCCGCCA CTGAGCGCCG CTGACTCTCA AGATTGGAGG TTAGGGTAAC   
  
  
- GCTGCGCACC GTTCCCAGGC CACTAAAGCC AGAGATACCA CGACTAGGCA AACGCTGCAC AAGCCTTCTG   
  
  
- GCAACGTCGG CGATAAGTGC GAGTCGGTGA CGGCGTTGTC GTCGGCAGCA CCCAGGGCGG AAGAGGCGGC   
  
  
- TTTCTGTGCT TCGTCAGCCG GGGTGGAGTT TTCGTTTCGC TGCGGCGACC TGGGAGCAAG AGTAGGCTCC   
  
  
- TCCTTGGTCT CGGCAGTACC GGCGGCGATT AACGCCGCAA CAACCTCACG CGGTTTGAGC GGCTCAGGCT   
  
  
- GGGCCTTGTG CGACAGTTTA GCCACTAAGC TGAGTCTCTG AGTCAGAGTG TCGTCCCTCA AGGTTAGGTC   
  
  
- TCTCACAGGA TAATGAAGAG GCTCCGTGAG ATGGTGGCGG AAAGAGAGAG GAGGCGGGGA GTTGGAGAGG   
  
  
- GTCTTTGCTG CAGCAGACTC CTCATGTGAG AGAGAATGTT CCGGGACTTG CTGCGCACGG GCATGGTATT   
  
  
- TAAGCGAGTG AACTGGCGTT TAGTCCGCTA AGATCTTCGG TGACTAAGTC GGTTCTTCTA GGTGTAGCAA   
  
  
- CTAAAACCCT ATTAGGTCCC TCAGGTTACC CGACGAGACG ATGTTCGGGA CCGTTGGGCC GGTCGGCCCT   
  
  
- TCGGGCTTAA CTAAGCTTAT AGGCCTTAAG GACGAGGCCC AGACCCCGTT AGAGGCCGCC GTAGCGACGA   
  
  
- GCGTTGACCC TTAGCGGAGG CACTCAAGCG CTTCCAAGAT CTAGAGTTCA AGCTCAAACT CGGCCATGAG   
  
  
- TGAGGTTAGT CTCTCGATTT GCCCAGCTTG AAGGCCCAGC TGGGTCTACT CTATGATCGA CAGTTAAAGT   
  
  
- ACGAAGTTAA CATGTTGGAT AATCTGCTTT GGGACCAGCG ACAGCTGCGG CGGGACTTCG ACCGGTTCAG   
  
  
- TAACTTGGGG TTTTAGCAGT GAAACCCACT CATGCTCCGC CCAGACTTAG CCCGTCCAAA GAACTGGGCC   
  
  
- AAGTTCTGCC GGGAGTTCAT GATAAACCGT TACAAACTTA GCCAACTCGG GTTGAACCGG GCCCTGAGCG   
  
  
- GGCTCTCCCG AGTCCAACTC TCCGACAATA ACCCGGCCGC CTAGTACCCG AATCAACCCG GCCTCAATGG   
  
  
- CCCTTGCTCT TCCCTTTCCT ACCTCACATT CCTTGTTACC TTCCCAAATT ACCTTTCACG GCCAAAGCTC   
  
  
- GGCACGGTTT CGTCTTTGAT GCAGTCACTT TTTCTGTTCA AAATGGTGAG GTCTAACTTA ATGTCTCTCA   
  
  
- C

+     CAT-box

| Site Name | Organism | Position | Strand | Matrix score. | sequence | function |
| --- | --- | --- | --- | --- | --- | --- |
| CAT-box | Arabidopsis thaliana | 2982 | + | 6 | GCCACT | cis-acting regulatory element related to meristem expression |
| CAT-box | Arabidopsis thaliana | 2549 | + | 6 | GCCACT | cis-acting regulatory element related to meristem expression |

>HU06G00029.1   
+ +Up\_Stream \_Len000TGATAA TTATTTTTAT TGATTTAGGA AAGGATATCA AAGTATATTT TGTTTTTAGG   
  
  
+ TAGTTAAGAG ATTCTGTTGC CAAAAAAAAA ATCAAGATAT TATATATTTT TAATTATGAC AGTAATTATT   
  
  
+ TTATTGAAAA TCCAACGGTT GTGATTATAT AATCAAATAA TCTAAAGGTT AAAAATATCA AATTTATTAG   
  
  
+ AAAATTCGAA AAAGTCACGT CATCACAACA TTGCTTTTAT ATATAGTATA GATTCCTTCG ACTTCTAAAC   
  
  
+ TAATCACTTG AAATTAGAAA TTTGAAACTT TCTAATATAA ATGCATGGTC TTCTTTCCAA GATTATAATC   
  
  
+ CGCAATTGCT TAAATGTAGG GTACAAATAT CAAATTTAGA AGTGACTATT GAAGCCATGA AAGATATTGT   
  
  
+ GATCGTATTG AGGGTGGTTG TGAGCAATAG GATGAAACAG GAAAAAAAAA CCCTAATGTT TTCAAATTTA   
  
  
+ TGGGTGGCTT TGGTAGTAGA TAAGTGAATA GTGTGCAAGG GAATGGCACC AATATGTTTG GATGGAACGA   
  
  
+ TGAGAGTAAG ACGTTAGGAA AGCAACAAGA GAAGGGAAAA GGCAGTAGGG AGAGAATTAT GCGTGTGAAT   
  
  
+ AATATTATAA CAGACGCTTT CGTATGATTA TTAAATACAA GACTTTGAGC ATACATATAA ACTTGTAACT   
  
  
+ TTCTTTACCT ATTGTCAATT AATTTTTGAA TTGAATCATC TGCGACTTAT GCATACAAGA TGATATGATG   
  
  
+ CATATTGGGC CAAGCATTTG ATCTCATGTC CCTTATGGAT CTAGATCCCA ATAGACAAAC TTGCATAAGT   
  
  
+ CAAGGGATGT GTTTCTAATA CAAAGGCTTT GTACTCAAGC AATTAGTTTT GAATTCATTT TCAGTAGTTT   
  
  
+ AATTTCCAAA TCTTATAATT TTTACAATGG CATAAATTTT TAGGAGTTTT GAGTAAATAA ATTATATACA   
  
  
+ ACGAAGATGT CATTTACGAA TTAATATGAC GCACAAAAAA AAAGAAAAAA AATTAATGGT AGCTTCTATA   
  
  
+ CTTGACTCCA TGTTGACAAT TCCCTCTCTC ACATAATGGT TAAAACTTGC AACATCATCC AGGACCCATA   
  
  
+ TGACATGGGT GGTGGTAGAC AGTGTGTTGT GGTAGCCTCA CTCCATGCTT GCCAATTTGT ATCATTGCGC   
  
  
+ TAGTCTTATT CGTCCTTAGA GATAATTGAC TAAGCTCTAA TGCTCTATCG TGGCTGCTGC AATATAAATG   
  
  
+ ATTTTTTGAC CTCAAAATGA AGACAGCTAG TTGTAAATTA AGAATCTTCG TCCCTTCATT TTTTGTGTAC   
  
  
+ GTGTGTATCT GCGTATGTGT TGAGAGACTA GCGGACTATT GATTGAGTTT CAATCAAAAA AGATGACCAT   
  
  
+ CTTAAGTATT TTTAATTACG CAAACTTCTT CATTTAAAAA AATGTTACTT TTATTTGAAA GAAATTGTGA   
  
  
+ ATAATACAAG GAATAGTTCA ATTAATATAT AATCCATAAC ATATTACCTC CTATAGGCAA AAAGAAGATT   
  
  
+ ATCGTATTAA TTTGGGATCA TCCCACCCTA AATTTAAAGA AACCAAAACA AATTACTAAG CAATATCTTT   
  
  
+ CTGTGTGAAA AAATAGTCAG AAACAAAGCG ATCCGACCGG CCAAATCGAG ATAAACACAG CCAAAGAACC   
  
  
+ GAAGTTGGAC CAAAGTGAGC CGAGTTGAAA GGCATTCATA TGGGGTTTAA TCATAACACA AACATAATTA   
  
  
+ GATTGATTGA CGTCTTTAAT CAGCGTTTTT GTCTTTGTTC GGTGCTCCCC TCACTCGCCC CTCCGCTCTT   
  
  
+ TAAAGCTTAC CTCACTCCCC CCATTCCCAC TCCTTTTCTC TCTCCTCTCT CGCTCTCACA CGCTCCGTCT   
  
  
+ GTCAGCCCCT TGTCATCTTC CCCCGTCTTC CTCGCAAAAA TCTCCAGCAA AATTTATAGC CGTTGTTGTG   
  
  
+ CCCATGGTGA TCTGATGTGA GAAGAGATTC AGCATTTAAG ATTATTGGGT CCAGATGGCT TATATGTGCG   
  
  
+ CCGACAGTGG TAATCTCATG GCTATTGCCC AACAAGTCAT CAAGCAAAAA CAACAGCAAG AACAGCAGCA   
  
  
+ ACACCAACAG CCTCATCCCC AGTTCGTCGG GTCGAATAAT AACCCCTTTT GCACCAGCCC ATGGCCCGGA   
  
  
+ CCCGTCCCTT CATCCGCCAT GTCTGCCAGC CCTCCGCCCC CTCTTGGGTT CTCCGCCGCC GCGTTTCCGG   
  
  
+ ACCCGTTTCA GGTGGGCCCG CCCTGCCCGG ATGGGGCTGA ACCGGGTTTC CAGTTTGCCC ATTTGGACCA   
  
  
+ CCACTCGAGT GGTTTCCGGT TTGCTGATTT TTGCGGTGGG GGTGGTGGTG AGTTTGACTC GGATGAGTGG   
  
  
+ ATGGAGAGTT TGATGGGTGG CGGCGGCGGT GACTCGCGGC GACTGAGAGT TCTAACCTCC AATCCCATTG   
  
  
+ CGACGCGTGG CAAGGGTCCG GTGATTTCGG TCTCTATGGT GCTGATCCGT TTGCGACGTG TTCGGAAGAC   
  
  
+ CGTTGCAGCC GCTATTCACG CTCAGCCACT GCCGCAACAG CAGCCGTCGT GGGTCCCGCC TTCTCCGCCG   
  
  
+ AAAGACACGA AGCAGTCGGC CCCACCTCAA AAGCAAAGCG ACGCCGCTGG ACCCTCGTTC TCATCCGAGG   
  
  
+ AGGAACCAGA GCCGTCATGG CCGCCGCTAA TTGCGGCGTT GTTGGAGTGC GCCAAACTCG CCGAGTCCGA   
  
  
+ CCCGGAACAC GCTGTCAAAT CGGTGATTCG ACTCAGAGAC TCAGTCTCAC AGCAGGGAGT TCCAATCCAG   
  
  
+ AGAGTGTCCT ATTACTTCTC CGAGGCACTC TACCACCGCC TTTCTCTCTC CTCCGCCCCT CAACCTCTCC   
  
  
+ CAGAAACGAC GTCGTCTGAG GAGTACACTC TCTCTTACAA GGCCCTGAAC GACGCGTGCC CGTACCATAA   
  
  
+ ATTCGCTCAC TTGACCGCAA ATCAGGCGAT TCTAGAAGCC ACTGATTCAG CCAAGAAGAT CCACATCGTT   
  
  
+ GATTTTGGGA TAATCCAGGG AGTCCAATGG GCTGCTCTGC TACAAGCCCT GGCAACCCGG CCAGCCGGGA   
  
  
+ AGCCCGAATT GATTCGAATA TCCGGAATTC CTGCTCCGGG TCTGGGGCAA TCTCCGGCGG CATCGCTGCT   
  
  
+ CGCAACTGGG AATCGCCTCC GTGAGTTCGC GAAGGTTCTA GATCTCAAGT TCGAGTTTGA GCCGGTACTC   
  
  
+ ACTCCAATCA GAGAGCTAAA CGGGTCGAAC TTCCGGGTCG ACCCAGATGA GATACTAGCT GTCAATTTCA   
  
  
+ TGCTTCAATT GTACAACCTA TTAGACGAAA CCCTGGTCGC TGTCGACGCC GCCCTGAAGC TGGCCAAGTC   
  
  
+ ATTGAACCCC AAAATCGTCA CTTTGGGTGA GTACGAGGCG GGTCTGAATC GGGCAGGTTT CTTGACCCGG   
  
  
+ TTCAAGACGG CCCTCAAGTA CTATTTGGCA ATGTTTGAAT CGGTTGAGCC CAACTTGGCC CGGGACTCGC   
  
  
+ CCGAGAGGGC TCAGGTTGAG AGGCTGTTAT TGGGCCGGCG GATCATGGGC TTAGTTGGGC CGGAGTTACC   
  
  
+ GGGAACGAGA AGGGAAAGGA TGGAGTGTAA GGAACAATGG AAGGGTTTAA TGGAAAGTGC CGGTTTCGAG   
  
  
+ CCGTGCCAAA GCAGAAACTA CGTCAGTGAA AAAGACAAGT TTTACCACTC CAGATTGAAT TACAGAGAGT   
  
  
+ G  

- +Up\_Stream \_Len000ACTATT AATAAAAATA ACTAAATCCT TTCCTATAGT TTCATATAAA ACAAAAATCC   
  
  
- ATCAATTCTC TAAGACAACG GTTTTTTTTT TAGTTCTATA ATATATAAAA ATTAATACTG TCATTAATAA   
  
  
- AATAACTTTT AGGTTGCCAA CACTAATATA TTAGTTTATT AGATTTCCAA TTTTTATAGT TTAAATAATC   
  
  
- TTTTAAGCTT TTTCAGTGCA GTAGTGTTGT AACGAAAATA TATATCATAT CTAAGGAAGC TGAAGATTTG   
  
  
- ATTAGTGAAC TTTAATCTTT AAACTTTGAA AGATTATATT TACGTACCAG AAGAAAGGTT CTAATATTAG   
  
  
- GCGTTAACGA ATTTACATCC CATGTTTATA GTTTAAATCT TCACTGATAA CTTCGGTACT TTCTATAACA   
  
  
- CTAGCATAAC TCCCACCAAC ACTCGTTATC CTACTTTGTC CTTTTTTTTT GGGATTACAA AAGTTTAAAT   
  
  
- ACCCACCGAA ACCATCATCT ATTCACTTAT CACACGTTCC CTTACCGTGG TTATACAAAC CTACCTTGCT   
  
  
- ACTCTCATTC TGCAATCCTT TCGTTGTTCT CTTCCCTTTT CCGTCATCCC TCTCTTAATA CGCACACTTA   
  
  
- TTATAATATT GTCTGCGAAA GCATACTAAT AATTTATGTT CTGAAACTCG TATGTATATT TGAACATTGA   
  
  
- AAGAAATGGA TAACAGTTAA TTAAAAACTT AACTTAGTAG ACGCTGAATA CGTATGTTCT ACTATACTAC   
  
  
- GTATAACCCG GTTCGTAAAC TAGAGTACAG GGAATACCTA GATCTAGGGT TATCTGTTTG AACGTATTCA   
  
  
- GTTCCCTACA CAAAGATTAT GTTTCCGAAA CATGAGTTCG TTAATCAAAA CTTAAGTAAA AGTCATCAAA   
  
  
- TTAAAGGTTT AGAATATTAA AAATGTTACC GTATTTAAAA ATCCTCAAAA CTCATTTATT TAATATATGT   
  
  
- TGCTTCTACA GTAAATGCTT AATTATACTG CGTGTTTTTT TTTCTTTTTT TTAATTACCA TCGAAGATAT   
  
  
- GAACTGAGGT ACAACTGTTA AGGGAGAGAG TGTATTACCA ATTTTGAACG TTGTAGTAGG TCCTGGGTAT   
  
  
- ACTGTACCCA CCACCATCTG TCACACAACA CCATCGGAGT GAGGTACGAA CGGTTAAACA TAGTAACGCG   
  
  
- ATCAGAATAA GCAGGAATCT CTATTAACTG ATTCGAGATT ACGAGATAGC ACCGACGACG TTATATTTAC   
  
  
- TAAAAAACTG GAGTTTTACT TCTGTCGATC AACATTTAAT TCTTAGAAGC AGGGAAGTAA AAAACACATG   
  
  
- CACACATAGA CGCATACACA ACTCTCTGAT CGCCTGATAA CTAACTCAAA GTTAGTTTTT TCTACTGGTA   
  
  
- GAATTCATAA AAATTAATGC GTTTGAAGAA GTAAATTTTT TTACAATGAA AATAAACTTT CTTTAACACT   
  
  
- TATTATGTTC CTTATCAAGT TAATTATATA TTAGGTATTG TATAATGGAG GATATCCGTT TTTCTTCTAA   
  
  
- TAGCATAATT AAACCCTAGT AGGGTGGGAT TTAAATTTCT TTGGTTTTGT TTAATGATTC GTTATAGAAA   
  
  
- GACACACTTT TTTATCAGTC TTTGTTTCGC TAGGCTGGCC GGTTTAGCTC TATTTGTGTC GGTTTCTTGG   
  
  
- CTTCAACCTG GTTTCACTCG GCTCAACTTT CCGTAAGTAT ACCCCAAATT AGTATTGTGT TTGTATTAAT   
  
  
- CTAACTAACT GCAGAAATTA GTCGCAAAAA CAGAAACAAG CCACGAGGGG AGTGAGCGGG GAGGCGAGAA   
  
  
- ATTTCGAATG GAGTGAGGGG GGTAAGGGTG AGGAAAAGAG AGAGGAGAGA GCGAGAGTGT GCGAGGCAGA   
  
  
- CAGTCGGGGA ACAGTAGAAG GGGGCAGAAG GAGCGTTTTT AGAGGTCGTT TTAAATATCG GCAACAACAC   
  
  
- GGGTACCACT AGACTACACT CTTCTCTAAG TCGTAAATTC TAATAACCCA GGTCTACCGA ATATACACGC   
  
  
- GGCTGTCACC ATTAGAGTAC CGATAACGGG TTGTTCAGTA GTTCGTTTTT GTTGTCGTTC TTGTCGTCGT   
  
  
- TGTGGTTGTC GGAGTAGGGG TCAAGCAGCC CAGCTTATTA TTGGGGAAAA CGTGGTCGGG TACCGGGCCT   
  
  
- GGGCAGGGAA GTAGGCGGTA CAGACGGTCG GGAGGCGGGG GAGAACCCAA GAGGCGGCGG CGCAAAGGCC   
  
  
- TGGGCAAAGT CCACCCGGGC GGGACGGGCC TACCCCGACT TGGCCCAAAG GTCAAACGGG TAAACCTGGT   
  
  
- GGTGAGCTCA CCAAAGGCCA AACGACTAAA AACGCCACCC CCACCACCAC TCAAACTGAG CCTACTCACC   
  
  
- TACCTCTCAA ACTACCCACC GCCGCCGCCA CTGAGCGCCG CTGACTCTCA AGATTGGAGG TTAGGGTAAC   
  
  
- GCTGCGCACC GTTCCCAGGC CACTAAAGCC AGAGATACCA CGACTAGGCA AACGCTGCAC AAGCCTTCTG   
  
  
- GCAACGTCGG CGATAAGTGC GAGTCGGTGA CGGCGTTGTC GTCGGCAGCA CCCAGGGCGG AAGAGGCGGC   
  
  
- TTTCTGTGCT TCGTCAGCCG GGGTGGAGTT TTCGTTTCGC TGCGGCGACC TGGGAGCAAG AGTAGGCTCC   
  
  
- TCCTTGGTCT CGGCAGTACC GGCGGCGATT AACGCCGCAA CAACCTCACG CGGTTTGAGC GGCTCAGGCT   
  
  
- GGGCCTTGTG CGACAGTTTA GCCACTAAGC TGAGTCTCTG AGTCAGAGTG TCGTCCCTCA AGGTTAGGTC   
  
  
- TCTCACAGGA TAATGAAGAG GCTCCGTGAG ATGGTGGCGG AAAGAGAGAG GAGGCGGGGA GTTGGAGAGG   
  
  
- GTCTTTGCTG CAGCAGACTC CTCATGTGAG AGAGAATGTT CCGGGACTTG CTGCGCACGG GCATGGTATT   
  
  
- TAAGCGAGTG AACTGGCGTT TAGTCCGCTA AGATCTTCGG TGACTAAGTC GGTTCTTCTA GGTGTAGCAA   
  
  
- CTAAAACCCT ATTAGGTCCC TCAGGTTACC CGACGAGACG ATGTTCGGGA CCGTTGGGCC GGTCGGCCCT   
  
  
- TCGGGCTTAA CTAAGCTTAT AGGCCTTAAG GACGAGGCCC AGACCCCGTT AGAGGCCGCC GTAGCGACGA   
  
  
- GCGTTGACCC TTAGCGGAGG CACTCAAGCG CTTCCAAGAT CTAGAGTTCA AGCTCAAACT CGGCCATGAG   
  
  
- TGAGGTTAGT CTCTCGATTT GCCCAGCTTG AAGGCCCAGC TGGGTCTACT CTATGATCGA CAGTTAAAGT   
  
  
- ACGAAGTTAA CATGTTGGAT AATCTGCTTT GGGACCAGCG ACAGCTGCGG CGGGACTTCG ACCGGTTCAG   
  
  
- TAACTTGGGG TTTTAGCAGT GAAACCCACT CATGCTCCGC CCAGACTTAG CCCGTCCAAA GAACTGGGCC   
  
  
- AAGTTCTGCC GGGAGTTCAT GATAAACCGT TACAAACTTA GCCAACTCGG GTTGAACCGG GCCCTGAGCG   
  
  
- GGCTCTCCCG AGTCCAACTC TCCGACAATA ACCCGGCCGC CTAGTACCCG AATCAACCCG GCCTCAATGG   
  
  
- CCCTTGCTCT TCCCTTTCCT ACCTCACATT CCTTGTTACC TTCCCAAATT ACCTTTCACG GCCAAAGCTC   
  
  
- GGCACGGTTT CGTCTTTGAT GCAGTCACTT TTTCTGTTCA AAATGGTGAG GTCTAACTTA ATGTCTCTCA   
  
  
- C

+     CCAAT-box

| Site Name | Organism | Position | Strand | Matrix score. | sequence | function |
| --- | --- | --- | --- | --- | --- | --- |
| CCAAT-box | Hordeum vulgare | 1954 | - | 6 | CAACGG | MYBHv1 binding site |
| CCAAT-box | Hordeum vulgare | 2524 | - | 6 | CAACGG | MYBHv1 binding site |
| CCAAT-box | Hordeum vulgare | 157 | + | 6 | CAACGG | MYBHv1 binding site |

>HU06G00029.1   
+ +Up\_Stream \_Len000TGATAA TTATTTTTAT TGATTTAGGA AAGGATATCA AAGTATATTT TGTTTTTAGG   
  
  
+ TAGTTAAGAG ATTCTGTTGC CAAAAAAAAA ATCAAGATAT TATATATTTT TAATTATGAC AGTAATTATT   
  
  
+ TTATTGAAAA TCCAACGGTT GTGATTATAT AATCAAATAA TCTAAAGGTT AAAAATATCA AATTTATTAG   
  
  
+ AAAATTCGAA AAAGTCACGT CATCACAACA TTGCTTTTAT ATATAGTATA GATTCCTTCG ACTTCTAAAC   
  
  
+ TAATCACTTG AAATTAGAAA TTTGAAACTT TCTAATATAA ATGCATGGTC TTCTTTCCAA GATTATAATC   
  
  
+ CGCAATTGCT TAAATGTAGG GTACAAATAT CAAATTTAGA AGTGACTATT GAAGCCATGA AAGATATTGT   
  
  
+ GATCGTATTG AGGGTGGTTG TGAGCAATAG GATGAAACAG GAAAAAAAAA CCCTAATGTT TTCAAATTTA   
  
  
+ TGGGTGGCTT TGGTAGTAGA TAAGTGAATA GTGTGCAAGG GAATGGCACC AATATGTTTG GATGGAACGA   
  
  
+ TGAGAGTAAG ACGTTAGGAA AGCAACAAGA GAAGGGAAAA GGCAGTAGGG AGAGAATTAT GCGTGTGAAT   
  
  
+ AATATTATAA CAGACGCTTT CGTATGATTA TTAAATACAA GACTTTGAGC ATACATATAA ACTTGTAACT   
  
  
+ TTCTTTACCT ATTGTCAATT AATTTTTGAA TTGAATCATC TGCGACTTAT GCATACAAGA TGATATGATG   
  
  
+ CATATTGGGC CAAGCATTTG ATCTCATGTC CCTTATGGAT CTAGATCCCA ATAGACAAAC TTGCATAAGT   
  
  
+ CAAGGGATGT GTTTCTAATA CAAAGGCTTT GTACTCAAGC AATTAGTTTT GAATTCATTT TCAGTAGTTT   
  
  
+ AATTTCCAAA TCTTATAATT TTTACAATGG CATAAATTTT TAGGAGTTTT GAGTAAATAA ATTATATACA   
  
  
+ ACGAAGATGT CATTTACGAA TTAATATGAC GCACAAAAAA AAAGAAAAAA AATTAATGGT AGCTTCTATA   
  
  
+ CTTGACTCCA TGTTGACAAT TCCCTCTCTC ACATAATGGT TAAAACTTGC AACATCATCC AGGACCCATA   
  
  
+ TGACATGGGT GGTGGTAGAC AGTGTGTTGT GGTAGCCTCA CTCCATGCTT GCCAATTTGT ATCATTGCGC   
  
  
+ TAGTCTTATT CGTCCTTAGA GATAATTGAC TAAGCTCTAA TGCTCTATCG TGGCTGCTGC AATATAAATG   
  
  
+ ATTTTTTGAC CTCAAAATGA AGACAGCTAG TTGTAAATTA AGAATCTTCG TCCCTTCATT TTTTGTGTAC   
  
  
+ GTGTGTATCT GCGTATGTGT TGAGAGACTA GCGGACTATT GATTGAGTTT CAATCAAAAA AGATGACCAT   
  
  
+ CTTAAGTATT TTTAATTACG CAAACTTCTT CATTTAAAAA AATGTTACTT TTATTTGAAA GAAATTGTGA   
  
  
+ ATAATACAAG GAATAGTTCA ATTAATATAT AATCCATAAC ATATTACCTC CTATAGGCAA AAAGAAGATT   
  
  
+ ATCGTATTAA TTTGGGATCA TCCCACCCTA AATTTAAAGA AACCAAAACA AATTACTAAG CAATATCTTT   
  
  
+ CTGTGTGAAA AAATAGTCAG AAACAAAGCG ATCCGACCGG CCAAATCGAG ATAAACACAG CCAAAGAACC   
  
  
+ GAAGTTGGAC CAAAGTGAGC CGAGTTGAAA GGCATTCATA TGGGGTTTAA TCATAACACA AACATAATTA   
  
  
+ GATTGATTGA CGTCTTTAAT CAGCGTTTTT GTCTTTGTTC GGTGCTCCCC TCACTCGCCC CTCCGCTCTT   
  
  
+ TAAAGCTTAC CTCACTCCCC CCATTCCCAC TCCTTTTCTC TCTCCTCTCT CGCTCTCACA CGCTCCGTCT   
  
  
+ GTCAGCCCCT TGTCATCTTC CCCCGTCTTC CTCGCAAAAA TCTCCAGCAA AATTTATAGC CGTTGTTGTG   
  
  
+ CCCATGGTGA TCTGATGTGA GAAGAGATTC AGCATTTAAG ATTATTGGGT CCAGATGGCT TATATGTGCG   
  
  
+ CCGACAGTGG TAATCTCATG GCTATTGCCC AACAAGTCAT CAAGCAAAAA CAACAGCAAG AACAGCAGCA   
  
  
+ ACACCAACAG CCTCATCCCC AGTTCGTCGG GTCGAATAAT AACCCCTTTT GCACCAGCCC ATGGCCCGGA   
  
  
+ CCCGTCCCTT CATCCGCCAT GTCTGCCAGC CCTCCGCCCC CTCTTGGGTT CTCCGCCGCC GCGTTTCCGG   
  
  
+ ACCCGTTTCA GGTGGGCCCG CCCTGCCCGG ATGGGGCTGA ACCGGGTTTC CAGTTTGCCC ATTTGGACCA   
  
  
+ CCACTCGAGT GGTTTCCGGT TTGCTGATTT TTGCGGTGGG GGTGGTGGTG AGTTTGACTC GGATGAGTGG   
  
  
+ ATGGAGAGTT TGATGGGTGG CGGCGGCGGT GACTCGCGGC GACTGAGAGT TCTAACCTCC AATCCCATTG   
  
  
+ CGACGCGTGG CAAGGGTCCG GTGATTTCGG TCTCTATGGT GCTGATCCGT TTGCGACGTG TTCGGAAGAC   
  
  
+ CGTTGCAGCC GCTATTCACG CTCAGCCACT GCCGCAACAG CAGCCGTCGT GGGTCCCGCC TTCTCCGCCG   
  
  
+ AAAGACACGA AGCAGTCGGC CCCACCTCAA AAGCAAAGCG ACGCCGCTGG ACCCTCGTTC TCATCCGAGG   
  
  
+ AGGAACCAGA GCCGTCATGG CCGCCGCTAA TTGCGGCGTT GTTGGAGTGC GCCAAACTCG CCGAGTCCGA   
  
  
+ CCCGGAACAC GCTGTCAAAT CGGTGATTCG ACTCAGAGAC TCAGTCTCAC AGCAGGGAGT TCCAATCCAG   
  
  
+ AGAGTGTCCT ATTACTTCTC CGAGGCACTC TACCACCGCC TTTCTCTCTC CTCCGCCCCT CAACCTCTCC   
  
  
+ CAGAAACGAC GTCGTCTGAG GAGTACACTC TCTCTTACAA GGCCCTGAAC GACGCGTGCC CGTACCATAA   
  
  
+ ATTCGCTCAC TTGACCGCAA ATCAGGCGAT TCTAGAAGCC ACTGATTCAG CCAAGAAGAT CCACATCGTT   
  
  
+ GATTTTGGGA TAATCCAGGG AGTCCAATGG GCTGCTCTGC TACAAGCCCT GGCAACCCGG CCAGCCGGGA   
  
  
+ AGCCCGAATT GATTCGAATA TCCGGAATTC CTGCTCCGGG TCTGGGGCAA TCTCCGGCGG CATCGCTGCT   
  
  
+ CGCAACTGGG AATCGCCTCC GTGAGTTCGC GAAGGTTCTA GATCTCAAGT TCGAGTTTGA GCCGGTACTC   
  
  
+ ACTCCAATCA GAGAGCTAAA CGGGTCGAAC TTCCGGGTCG ACCCAGATGA GATACTAGCT GTCAATTTCA   
  
  
+ TGCTTCAATT GTACAACCTA TTAGACGAAA CCCTGGTCGC TGTCGACGCC GCCCTGAAGC TGGCCAAGTC   
  
  
+ ATTGAACCCC AAAATCGTCA CTTTGGGTGA GTACGAGGCG GGTCTGAATC GGGCAGGTTT CTTGACCCGG   
  
  
+ TTCAAGACGG CCCTCAAGTA CTATTTGGCA ATGTTTGAAT CGGTTGAGCC CAACTTGGCC CGGGACTCGC   
  
  
+ CCGAGAGGGC TCAGGTTGAG AGGCTGTTAT TGGGCCGGCG GATCATGGGC TTAGTTGGGC CGGAGTTACC   
  
  
+ GGGAACGAGA AGGGAAAGGA TGGAGTGTAA GGAACAATGG AAGGGTTTAA TGGAAAGTGC CGGTTTCGAG   
  
  
+ CCGTGCCAAA GCAGAAACTA CGTCAGTGAA AAAGACAAGT TTTACCACTC CAGATTGAAT TACAGAGAGT   
  
  
+ G  

- +Up\_Stream \_Len000ACTATT AATAAAAATA ACTAAATCCT TTCCTATAGT TTCATATAAA ACAAAAATCC   
  
  
- ATCAATTCTC TAAGACAACG GTTTTTTTTT TAGTTCTATA ATATATAAAA ATTAATACTG TCATTAATAA   
  
  
- AATAACTTTT AGGTTGCCAA CACTAATATA TTAGTTTATT AGATTTCCAA TTTTTATAGT TTAAATAATC   
  
  
- TTTTAAGCTT TTTCAGTGCA GTAGTGTTGT AACGAAAATA TATATCATAT CTAAGGAAGC TGAAGATTTG   
  
  
- ATTAGTGAAC TTTAATCTTT AAACTTTGAA AGATTATATT TACGTACCAG AAGAAAGGTT CTAATATTAG   
  
  
- GCGTTAACGA ATTTACATCC CATGTTTATA GTTTAAATCT TCACTGATAA CTTCGGTACT TTCTATAACA   
  
  
- CTAGCATAAC TCCCACCAAC ACTCGTTATC CTACTTTGTC CTTTTTTTTT GGGATTACAA AAGTTTAAAT   
  
  
- ACCCACCGAA ACCATCATCT ATTCACTTAT CACACGTTCC CTTACCGTGG TTATACAAAC CTACCTTGCT   
  
  
- ACTCTCATTC TGCAATCCTT TCGTTGTTCT CTTCCCTTTT CCGTCATCCC TCTCTTAATA CGCACACTTA   
  
  
- TTATAATATT GTCTGCGAAA GCATACTAAT AATTTATGTT CTGAAACTCG TATGTATATT TGAACATTGA   
  
  
- AAGAAATGGA TAACAGTTAA TTAAAAACTT AACTTAGTAG ACGCTGAATA CGTATGTTCT ACTATACTAC   
  
  
- GTATAACCCG GTTCGTAAAC TAGAGTACAG GGAATACCTA GATCTAGGGT TATCTGTTTG AACGTATTCA   
  
  
- GTTCCCTACA CAAAGATTAT GTTTCCGAAA CATGAGTTCG TTAATCAAAA CTTAAGTAAA AGTCATCAAA   
  
  
- TTAAAGGTTT AGAATATTAA AAATGTTACC GTATTTAAAA ATCCTCAAAA CTCATTTATT TAATATATGT   
  
  
- TGCTTCTACA GTAAATGCTT AATTATACTG CGTGTTTTTT TTTCTTTTTT TTAATTACCA TCGAAGATAT   
  
  
- GAACTGAGGT ACAACTGTTA AGGGAGAGAG TGTATTACCA ATTTTGAACG TTGTAGTAGG TCCTGGGTAT   
  
  
- ACTGTACCCA CCACCATCTG TCACACAACA CCATCGGAGT GAGGTACGAA CGGTTAAACA TAGTAACGCG   
  
  
- ATCAGAATAA GCAGGAATCT CTATTAACTG ATTCGAGATT ACGAGATAGC ACCGACGACG TTATATTTAC   
  
  
- TAAAAAACTG GAGTTTTACT TCTGTCGATC AACATTTAAT TCTTAGAAGC AGGGAAGTAA AAAACACATG   
  
  
- CACACATAGA CGCATACACA ACTCTCTGAT CGCCTGATAA CTAACTCAAA GTTAGTTTTT TCTACTGGTA   
  
  
- GAATTCATAA AAATTAATGC GTTTGAAGAA GTAAATTTTT TTACAATGAA AATAAACTTT CTTTAACACT   
  
  
- TATTATGTTC CTTATCAAGT TAATTATATA TTAGGTATTG TATAATGGAG GATATCCGTT TTTCTTCTAA   
  
  
- TAGCATAATT AAACCCTAGT AGGGTGGGAT TTAAATTTCT TTGGTTTTGT TTAATGATTC GTTATAGAAA   
  
  
- GACACACTTT TTTATCAGTC TTTGTTTCGC TAGGCTGGCC GGTTTAGCTC TATTTGTGTC GGTTTCTTGG   
  
  
- CTTCAACCTG GTTTCACTCG GCTCAACTTT CCGTAAGTAT ACCCCAAATT AGTATTGTGT TTGTATTAAT   
  
  
- CTAACTAACT GCAGAAATTA GTCGCAAAAA CAGAAACAAG CCACGAGGGG AGTGAGCGGG GAGGCGAGAA   
  
  
- ATTTCGAATG GAGTGAGGGG GGTAAGGGTG AGGAAAAGAG AGAGGAGAGA GCGAGAGTGT GCGAGGCAGA   
  
  
- CAGTCGGGGA ACAGTAGAAG GGGGCAGAAG GAGCGTTTTT AGAGGTCGTT TTAAATATCG GCAACAACAC   
  
  
- GGGTACCACT AGACTACACT CTTCTCTAAG TCGTAAATTC TAATAACCCA GGTCTACCGA ATATACACGC   
  
  
- GGCTGTCACC ATTAGAGTAC CGATAACGGG TTGTTCAGTA GTTCGTTTTT GTTGTCGTTC TTGTCGTCGT   
  
  
- TGTGGTTGTC GGAGTAGGGG TCAAGCAGCC CAGCTTATTA TTGGGGAAAA CGTGGTCGGG TACCGGGCCT   
  
  
- GGGCAGGGAA GTAGGCGGTA CAGACGGTCG GGAGGCGGGG GAGAACCCAA GAGGCGGCGG CGCAAAGGCC   
  
  
- TGGGCAAAGT CCACCCGGGC GGGACGGGCC TACCCCGACT TGGCCCAAAG GTCAAACGGG TAAACCTGGT   
  
  
- GGTGAGCTCA CCAAAGGCCA AACGACTAAA AACGCCACCC CCACCACCAC TCAAACTGAG CCTACTCACC   
  
  
- TACCTCTCAA ACTACCCACC GCCGCCGCCA CTGAGCGCCG CTGACTCTCA AGATTGGAGG TTAGGGTAAC   
  
  
- GCTGCGCACC GTTCCCAGGC CACTAAAGCC AGAGATACCA CGACTAGGCA AACGCTGCAC AAGCCTTCTG   
  
  
- GCAACGTCGG CGATAAGTGC GAGTCGGTGA CGGCGTTGTC GTCGGCAGCA CCCAGGGCGG AAGAGGCGGC   
  
  
- TTTCTGTGCT TCGTCAGCCG GGGTGGAGTT TTCGTTTCGC TGCGGCGACC TGGGAGCAAG AGTAGGCTCC   
  
  
- TCCTTGGTCT CGGCAGTACC GGCGGCGATT AACGCCGCAA CAACCTCACG CGGTTTGAGC GGCTCAGGCT   
  
  
- GGGCCTTGTG CGACAGTTTA GCCACTAAGC TGAGTCTCTG AGTCAGAGTG TCGTCCCTCA AGGTTAGGTC   
  
  
- TCTCACAGGA TAATGAAGAG GCTCCGTGAG ATGGTGGCGG AAAGAGAGAG GAGGCGGGGA GTTGGAGAGG   
  
  
- GTCTTTGCTG CAGCAGACTC CTCATGTGAG AGAGAATGTT CCGGGACTTG CTGCGCACGG GCATGGTATT   
  
  
- TAAGCGAGTG AACTGGCGTT TAGTCCGCTA AGATCTTCGG TGACTAAGTC GGTTCTTCTA GGTGTAGCAA   
  
  
- CTAAAACCCT ATTAGGTCCC TCAGGTTACC CGACGAGACG ATGTTCGGGA CCGTTGGGCC GGTCGGCCCT   
  
  
- TCGGGCTTAA CTAAGCTTAT AGGCCTTAAG GACGAGGCCC AGACCCCGTT AGAGGCCGCC GTAGCGACGA   
  
  
- GCGTTGACCC TTAGCGGAGG CACTCAAGCG CTTCCAAGAT CTAGAGTTCA AGCTCAAACT CGGCCATGAG   
  
  
- TGAGGTTAGT CTCTCGATTT GCCCAGCTTG AAGGCCCAGC TGGGTCTACT CTATGATCGA CAGTTAAAGT   
  
  
- ACGAAGTTAA CATGTTGGAT AATCTGCTTT GGGACCAGCG ACAGCTGCGG CGGGACTTCG ACCGGTTCAG   
  
  
- TAACTTGGGG TTTTAGCAGT GAAACCCACT CATGCTCCGC CCAGACTTAG CCCGTCCAAA GAACTGGGCC   
  
  
- AAGTTCTGCC GGGAGTTCAT GATAAACCGT TACAAACTTA GCCAACTCGG GTTGAACCGG GCCCTGAGCG   
  
  
- GGCTCTCCCG AGTCCAACTC TCCGACAATA ACCCGGCCGC CTAGTACCCG AATCAACCCG GCCTCAATGG   
  
  
- CCCTTGCTCT TCCCTTTCCT ACCTCACATT CCTTGTTACC TTCCCAAATT ACCTTTCACG GCCAAAGCTC   
  
  
- GGCACGGTTT CGTCTTTGAT GCAGTCACTT TTTCTGTTCA AAATGGTGAG GTCTAACTTA ATGTCTCTCA   
  
  
- C

+     CCGTCC motif

| Site Name | Organism | Position | Strand | Matrix score. | sequence | function |
| --- | --- | --- | --- | --- | --- | --- |
| CCGTCC motif | Nicotiana tabacum | 2176 | + | 6 | CCGTCC |  |

>HU06G00029.1   
+ +Up\_Stream \_Len000TGATAA TTATTTTTAT TGATTTAGGA AAGGATATCA AAGTATATTT TGTTTTTAGG   
  
  
+ TAGTTAAGAG ATTCTGTTGC CAAAAAAAAA ATCAAGATAT TATATATTTT TAATTATGAC AGTAATTATT   
  
  
+ TTATTGAAAA TCCAACGGTT GTGATTATAT AATCAAATAA TCTAAAGGTT AAAAATATCA AATTTATTAG   
  
  
+ AAAATTCGAA AAAGTCACGT CATCACAACA TTGCTTTTAT ATATAGTATA GATTCCTTCG ACTTCTAAAC   
  
  
+ TAATCACTTG AAATTAGAAA TTTGAAACTT TCTAATATAA ATGCATGGTC TTCTTTCCAA GATTATAATC   
  
  
+ CGCAATTGCT TAAATGTAGG GTACAAATAT CAAATTTAGA AGTGACTATT GAAGCCATGA AAGATATTGT   
  
  
+ GATCGTATTG AGGGTGGTTG TGAGCAATAG GATGAAACAG GAAAAAAAAA CCCTAATGTT TTCAAATTTA   
  
  
+ TGGGTGGCTT TGGTAGTAGA TAAGTGAATA GTGTGCAAGG GAATGGCACC AATATGTTTG GATGGAACGA   
  
  
+ TGAGAGTAAG ACGTTAGGAA AGCAACAAGA GAAGGGAAAA GGCAGTAGGG AGAGAATTAT GCGTGTGAAT   
  
  
+ AATATTATAA CAGACGCTTT CGTATGATTA TTAAATACAA GACTTTGAGC ATACATATAA ACTTGTAACT   
  
  
+ TTCTTTACCT ATTGTCAATT AATTTTTGAA TTGAATCATC TGCGACTTAT GCATACAAGA TGATATGATG   
  
  
+ CATATTGGGC CAAGCATTTG ATCTCATGTC CCTTATGGAT CTAGATCCCA ATAGACAAAC TTGCATAAGT   
  
  
+ CAAGGGATGT GTTTCTAATA CAAAGGCTTT GTACTCAAGC AATTAGTTTT GAATTCATTT TCAGTAGTTT   
  
  
+ AATTTCCAAA TCTTATAATT TTTACAATGG CATAAATTTT TAGGAGTTTT GAGTAAATAA ATTATATACA   
  
  
+ ACGAAGATGT CATTTACGAA TTAATATGAC GCACAAAAAA AAAGAAAAAA AATTAATGGT AGCTTCTATA   
  
  
+ CTTGACTCCA TGTTGACAAT TCCCTCTCTC ACATAATGGT TAAAACTTGC AACATCATCC AGGACCCATA   
  
  
+ TGACATGGGT GGTGGTAGAC AGTGTGTTGT GGTAGCCTCA CTCCATGCTT GCCAATTTGT ATCATTGCGC   
  
  
+ TAGTCTTATT CGTCCTTAGA GATAATTGAC TAAGCTCTAA TGCTCTATCG TGGCTGCTGC AATATAAATG   
  
  
+ ATTTTTTGAC CTCAAAATGA AGACAGCTAG TTGTAAATTA AGAATCTTCG TCCCTTCATT TTTTGTGTAC   
  
  
+ GTGTGTATCT GCGTATGTGT TGAGAGACTA GCGGACTATT GATTGAGTTT CAATCAAAAA AGATGACCAT   
  
  
+ CTTAAGTATT TTTAATTACG CAAACTTCTT CATTTAAAAA AATGTTACTT TTATTTGAAA GAAATTGTGA   
  
  
+ ATAATACAAG GAATAGTTCA ATTAATATAT AATCCATAAC ATATTACCTC CTATAGGCAA AAAGAAGATT   
  
  
+ ATCGTATTAA TTTGGGATCA TCCCACCCTA AATTTAAAGA AACCAAAACA AATTACTAAG CAATATCTTT   
  
  
+ CTGTGTGAAA AAATAGTCAG AAACAAAGCG ATCCGACCGG CCAAATCGAG ATAAACACAG CCAAAGAACC   
  
  
+ GAAGTTGGAC CAAAGTGAGC CGAGTTGAAA GGCATTCATA TGGGGTTTAA TCATAACACA AACATAATTA   
  
  
+ GATTGATTGA CGTCTTTAAT CAGCGTTTTT GTCTTTGTTC GGTGCTCCCC TCACTCGCCC CTCCGCTCTT   
  
  
+ TAAAGCTTAC CTCACTCCCC CCATTCCCAC TCCTTTTCTC TCTCCTCTCT CGCTCTCACA CGCTCCGTCT   
  
  
+ GTCAGCCCCT TGTCATCTTC CCCCGTCTTC CTCGCAAAAA TCTCCAGCAA AATTTATAGC CGTTGTTGTG   
  
  
+ CCCATGGTGA TCTGATGTGA GAAGAGATTC AGCATTTAAG ATTATTGGGT CCAGATGGCT TATATGTGCG   
  
  
+ CCGACAGTGG TAATCTCATG GCTATTGCCC AACAAGTCAT CAAGCAAAAA CAACAGCAAG AACAGCAGCA   
  
  
+ ACACCAACAG CCTCATCCCC AGTTCGTCGG GTCGAATAAT AACCCCTTTT GCACCAGCCC ATGGCCCGGA   
  
  
+ CCCGTCCCTT CATCCGCCAT GTCTGCCAGC CCTCCGCCCC CTCTTGGGTT CTCCGCCGCC GCGTTTCCGG   
  
  
+ ACCCGTTTCA GGTGGGCCCG CCCTGCCCGG ATGGGGCTGA ACCGGGTTTC CAGTTTGCCC ATTTGGACCA   
  
  
+ CCACTCGAGT GGTTTCCGGT TTGCTGATTT TTGCGGTGGG GGTGGTGGTG AGTTTGACTC GGATGAGTGG   
  
  
+ ATGGAGAGTT TGATGGGTGG CGGCGGCGGT GACTCGCGGC GACTGAGAGT TCTAACCTCC AATCCCATTG   
  
  
+ CGACGCGTGG CAAGGGTCCG GTGATTTCGG TCTCTATGGT GCTGATCCGT TTGCGACGTG TTCGGAAGAC   
  
  
+ CGTTGCAGCC GCTATTCACG CTCAGCCACT GCCGCAACAG CAGCCGTCGT GGGTCCCGCC TTCTCCGCCG   
  
  
+ AAAGACACGA AGCAGTCGGC CCCACCTCAA AAGCAAAGCG ACGCCGCTGG ACCCTCGTTC TCATCCGAGG   
  
  
+ AGGAACCAGA GCCGTCATGG CCGCCGCTAA TTGCGGCGTT GTTGGAGTGC GCCAAACTCG CCGAGTCCGA   
  
  
+ CCCGGAACAC GCTGTCAAAT CGGTGATTCG ACTCAGAGAC TCAGTCTCAC AGCAGGGAGT TCCAATCCAG   
  
  
+ AGAGTGTCCT ATTACTTCTC CGAGGCACTC TACCACCGCC TTTCTCTCTC CTCCGCCCCT CAACCTCTCC   
  
  
+ CAGAAACGAC GTCGTCTGAG GAGTACACTC TCTCTTACAA GGCCCTGAAC GACGCGTGCC CGTACCATAA   
  
  
+ ATTCGCTCAC TTGACCGCAA ATCAGGCGAT TCTAGAAGCC ACTGATTCAG CCAAGAAGAT CCACATCGTT   
  
  
+ GATTTTGGGA TAATCCAGGG AGTCCAATGG GCTGCTCTGC TACAAGCCCT GGCAACCCGG CCAGCCGGGA   
  
  
+ AGCCCGAATT GATTCGAATA TCCGGAATTC CTGCTCCGGG TCTGGGGCAA TCTCCGGCGG CATCGCTGCT   
  
  
+ CGCAACTGGG AATCGCCTCC GTGAGTTCGC GAAGGTTCTA GATCTCAAGT TCGAGTTTGA GCCGGTACTC   
  
  
+ ACTCCAATCA GAGAGCTAAA CGGGTCGAAC TTCCGGGTCG ACCCAGATGA GATACTAGCT GTCAATTTCA   
  
  
+ TGCTTCAATT GTACAACCTA TTAGACGAAA CCCTGGTCGC TGTCGACGCC GCCCTGAAGC TGGCCAAGTC   
  
  
+ ATTGAACCCC AAAATCGTCA CTTTGGGTGA GTACGAGGCG GGTCTGAATC GGGCAGGTTT CTTGACCCGG   
  
  
+ TTCAAGACGG CCCTCAAGTA CTATTTGGCA ATGTTTGAAT CGGTTGAGCC CAACTTGGCC CGGGACTCGC   
  
  
+ CCGAGAGGGC TCAGGTTGAG AGGCTGTTAT TGGGCCGGCG GATCATGGGC TTAGTTGGGC CGGAGTTACC   
  
  
+ GGGAACGAGA AGGGAAAGGA TGGAGTGTAA GGAACAATGG AAGGGTTTAA TGGAAAGTGC CGGTTTCGAG   
  
  
+ CCGTGCCAAA GCAGAAACTA CGTCAGTGAA AAAGACAAGT TTTACCACTC CAGATTGAAT TACAGAGAGT   
  
  
+ G  

- +Up\_Stream \_Len000ACTATT AATAAAAATA ACTAAATCCT TTCCTATAGT TTCATATAAA ACAAAAATCC   
  
  
- ATCAATTCTC TAAGACAACG GTTTTTTTTT TAGTTCTATA ATATATAAAA ATTAATACTG TCATTAATAA   
  
  
- AATAACTTTT AGGTTGCCAA CACTAATATA TTAGTTTATT AGATTTCCAA TTTTTATAGT TTAAATAATC   
  
  
- TTTTAAGCTT TTTCAGTGCA GTAGTGTTGT AACGAAAATA TATATCATAT CTAAGGAAGC TGAAGATTTG   
  
  
- ATTAGTGAAC TTTAATCTTT AAACTTTGAA AGATTATATT TACGTACCAG AAGAAAGGTT CTAATATTAG   
  
  
- GCGTTAACGA ATTTACATCC CATGTTTATA GTTTAAATCT TCACTGATAA CTTCGGTACT TTCTATAACA   
  
  
- CTAGCATAAC TCCCACCAAC ACTCGTTATC CTACTTTGTC CTTTTTTTTT GGGATTACAA AAGTTTAAAT   
  
  
- ACCCACCGAA ACCATCATCT ATTCACTTAT CACACGTTCC CTTACCGTGG TTATACAAAC CTACCTTGCT   
  
  
- ACTCTCATTC TGCAATCCTT TCGTTGTTCT CTTCCCTTTT CCGTCATCCC TCTCTTAATA CGCACACTTA   
  
  
- TTATAATATT GTCTGCGAAA GCATACTAAT AATTTATGTT CTGAAACTCG TATGTATATT TGAACATTGA   
  
  
- AAGAAATGGA TAACAGTTAA TTAAAAACTT AACTTAGTAG ACGCTGAATA CGTATGTTCT ACTATACTAC   
  
  
- GTATAACCCG GTTCGTAAAC TAGAGTACAG GGAATACCTA GATCTAGGGT TATCTGTTTG AACGTATTCA   
  
  
- GTTCCCTACA CAAAGATTAT GTTTCCGAAA CATGAGTTCG TTAATCAAAA CTTAAGTAAA AGTCATCAAA   
  
  
- TTAAAGGTTT AGAATATTAA AAATGTTACC GTATTTAAAA ATCCTCAAAA CTCATTTATT TAATATATGT   
  
  
- TGCTTCTACA GTAAATGCTT AATTATACTG CGTGTTTTTT TTTCTTTTTT TTAATTACCA TCGAAGATAT   
  
  
- GAACTGAGGT ACAACTGTTA AGGGAGAGAG TGTATTACCA ATTTTGAACG TTGTAGTAGG TCCTGGGTAT   
  
  
- ACTGTACCCA CCACCATCTG TCACACAACA CCATCGGAGT GAGGTACGAA CGGTTAAACA TAGTAACGCG   
  
  
- ATCAGAATAA GCAGGAATCT CTATTAACTG ATTCGAGATT ACGAGATAGC ACCGACGACG TTATATTTAC   
  
  
- TAAAAAACTG GAGTTTTACT TCTGTCGATC AACATTTAAT TCTTAGAAGC AGGGAAGTAA AAAACACATG   
  
  
- CACACATAGA CGCATACACA ACTCTCTGAT CGCCTGATAA CTAACTCAAA GTTAGTTTTT TCTACTGGTA   
  
  
- GAATTCATAA AAATTAATGC GTTTGAAGAA GTAAATTTTT TTACAATGAA AATAAACTTT CTTTAACACT   
  
  
- TATTATGTTC CTTATCAAGT TAATTATATA TTAGGTATTG TATAATGGAG GATATCCGTT TTTCTTCTAA   
  
  
- TAGCATAATT AAACCCTAGT AGGGTGGGAT TTAAATTTCT TTGGTTTTGT TTAATGATTC GTTATAGAAA   
  
  
- GACACACTTT TTTATCAGTC TTTGTTTCGC TAGGCTGGCC GGTTTAGCTC TATTTGTGTC GGTTTCTTGG   
  
  
- CTTCAACCTG GTTTCACTCG GCTCAACTTT CCGTAAGTAT ACCCCAAATT AGTATTGTGT TTGTATTAAT   
  
  
- CTAACTAACT GCAGAAATTA GTCGCAAAAA CAGAAACAAG CCACGAGGGG AGTGAGCGGG GAGGCGAGAA   
  
  
- ATTTCGAATG GAGTGAGGGG GGTAAGGGTG AGGAAAAGAG AGAGGAGAGA GCGAGAGTGT GCGAGGCAGA   
  
  
- CAGTCGGGGA ACAGTAGAAG GGGGCAGAAG GAGCGTTTTT AGAGGTCGTT TTAAATATCG GCAACAACAC   
  
  
- GGGTACCACT AGACTACACT CTTCTCTAAG TCGTAAATTC TAATAACCCA GGTCTACCGA ATATACACGC   
  
  
- GGCTGTCACC ATTAGAGTAC CGATAACGGG TTGTTCAGTA GTTCGTTTTT GTTGTCGTTC TTGTCGTCGT   
  
  
- TGTGGTTGTC GGAGTAGGGG TCAAGCAGCC CAGCTTATTA TTGGGGAAAA CGTGGTCGGG TACCGGGCCT   
  
  
- GGGCAGGGAA GTAGGCGGTA CAGACGGTCG GGAGGCGGGG GAGAACCCAA GAGGCGGCGG CGCAAAGGCC   
  
  
- TGGGCAAAGT CCACCCGGGC GGGACGGGCC TACCCCGACT TGGCCCAAAG GTCAAACGGG TAAACCTGGT   
  
  
- GGTGAGCTCA CCAAAGGCCA AACGACTAAA AACGCCACCC CCACCACCAC TCAAACTGAG CCTACTCACC   
  
  
- TACCTCTCAA ACTACCCACC GCCGCCGCCA CTGAGCGCCG CTGACTCTCA AGATTGGAGG TTAGGGTAAC   
  
  
- GCTGCGCACC GTTCCCAGGC CACTAAAGCC AGAGATACCA CGACTAGGCA AACGCTGCAC AAGCCTTCTG   
  
  
- GCAACGTCGG CGATAAGTGC GAGTCGGTGA CGGCGTTGTC GTCGGCAGCA CCCAGGGCGG AAGAGGCGGC   
  
  
- TTTCTGTGCT TCGTCAGCCG GGGTGGAGTT TTCGTTTCGC TGCGGCGACC TGGGAGCAAG AGTAGGCTCC   
  
  
- TCCTTGGTCT CGGCAGTACC GGCGGCGATT AACGCCGCAA CAACCTCACG CGGTTTGAGC GGCTCAGGCT   
  
  
- GGGCCTTGTG CGACAGTTTA GCCACTAAGC TGAGTCTCTG AGTCAGAGTG TCGTCCCTCA AGGTTAGGTC   
  
  
- TCTCACAGGA TAATGAAGAG GCTCCGTGAG ATGGTGGCGG AAAGAGAGAG GAGGCGGGGA GTTGGAGAGG   
  
  
- GTCTTTGCTG CAGCAGACTC CTCATGTGAG AGAGAATGTT CCGGGACTTG CTGCGCACGG GCATGGTATT   
  
  
- TAAGCGAGTG AACTGGCGTT TAGTCCGCTA AGATCTTCGG TGACTAAGTC GGTTCTTCTA GGTGTAGCAA   
  
  
- CTAAAACCCT ATTAGGTCCC TCAGGTTACC CGACGAGACG ATGTTCGGGA CCGTTGGGCC GGTCGGCCCT   
  
  
- TCGGGCTTAA CTAAGCTTAT AGGCCTTAAG GACGAGGCCC AGACCCCGTT AGAGGCCGCC GTAGCGACGA   
  
  
- GCGTTGACCC TTAGCGGAGG CACTCAAGCG CTTCCAAGAT CTAGAGTTCA AGCTCAAACT CGGCCATGAG   
  
  
- TGAGGTTAGT CTCTCGATTT GCCCAGCTTG AAGGCCCAGC TGGGTCTACT CTATGATCGA CAGTTAAAGT   
  
  
- ACGAAGTTAA CATGTTGGAT AATCTGCTTT GGGACCAGCG ACAGCTGCGG CGGGACTTCG ACCGGTTCAG   
  
  
- TAACTTGGGG TTTTAGCAGT GAAACCCACT CATGCTCCGC CCAGACTTAG CCCGTCCAAA GAACTGGGCC   
  
  
- AAGTTCTGCC GGGAGTTCAT GATAAACCGT TACAAACTTA GCCAACTCGG GTTGAACCGG GCCCTGAGCG   
  
  
- GGCTCTCCCG AGTCCAACTC TCCGACAATA ACCCGGCCGC CTAGTACCCG AATCAACCCG GCCTCAATGG   
  
  
- CCCTTGCTCT TCCCTTTCCT ACCTCACATT CCTTGTTACC TTCCCAAATT ACCTTTCACG GCCAAAGCTC   
  
  
- GGCACGGTTT CGTCTTTGAT GCAGTCACTT TTTCTGTTCA AAATGGTGAG GTCTAACTTA ATGTCTCTCA   
  
  
- C

+     CCGTCC-box

| Site Name | Organism | Position | Strand | Matrix score. | sequence | function |
| --- | --- | --- | --- | --- | --- | --- |
| CCGTCC-box | Petroselinum hortense | 2176 | + | 6 | CCGTCC |  |

>HU06G00029.1   
+ +Up\_Stream \_Len000TGATAA TTATTTTTAT TGATTTAGGA AAGGATATCA AAGTATATTT TGTTTTTAGG   
  
  
+ TAGTTAAGAG ATTCTGTTGC CAAAAAAAAA ATCAAGATAT TATATATTTT TAATTATGAC AGTAATTATT   
  
  
+ TTATTGAAAA TCCAACGGTT GTGATTATAT AATCAAATAA TCTAAAGGTT AAAAATATCA AATTTATTAG   
  
  
+ AAAATTCGAA AAAGTCACGT CATCACAACA TTGCTTTTAT ATATAGTATA GATTCCTTCG ACTTCTAAAC   
  
  
+ TAATCACTTG AAATTAGAAA TTTGAAACTT TCTAATATAA ATGCATGGTC TTCTTTCCAA GATTATAATC   
  
  
+ CGCAATTGCT TAAATGTAGG GTACAAATAT CAAATTTAGA AGTGACTATT GAAGCCATGA AAGATATTGT   
  
  
+ GATCGTATTG AGGGTGGTTG TGAGCAATAG GATGAAACAG GAAAAAAAAA CCCTAATGTT TTCAAATTTA   
  
  
+ TGGGTGGCTT TGGTAGTAGA TAAGTGAATA GTGTGCAAGG GAATGGCACC AATATGTTTG GATGGAACGA   
  
  
+ TGAGAGTAAG ACGTTAGGAA AGCAACAAGA GAAGGGAAAA GGCAGTAGGG AGAGAATTAT GCGTGTGAAT   
  
  
+ AATATTATAA CAGACGCTTT CGTATGATTA TTAAATACAA GACTTTGAGC ATACATATAA ACTTGTAACT   
  
  
+ TTCTTTACCT ATTGTCAATT AATTTTTGAA TTGAATCATC TGCGACTTAT GCATACAAGA TGATATGATG   
  
  
+ CATATTGGGC CAAGCATTTG ATCTCATGTC CCTTATGGAT CTAGATCCCA ATAGACAAAC TTGCATAAGT   
  
  
+ CAAGGGATGT GTTTCTAATA CAAAGGCTTT GTACTCAAGC AATTAGTTTT GAATTCATTT TCAGTAGTTT   
  
  
+ AATTTCCAAA TCTTATAATT TTTACAATGG CATAAATTTT TAGGAGTTTT GAGTAAATAA ATTATATACA   
  
  
+ ACGAAGATGT CATTTACGAA TTAATATGAC GCACAAAAAA AAAGAAAAAA AATTAATGGT AGCTTCTATA   
  
  
+ CTTGACTCCA TGTTGACAAT TCCCTCTCTC ACATAATGGT TAAAACTTGC AACATCATCC AGGACCCATA   
  
  
+ TGACATGGGT GGTGGTAGAC AGTGTGTTGT GGTAGCCTCA CTCCATGCTT GCCAATTTGT ATCATTGCGC   
  
  
+ TAGTCTTATT CGTCCTTAGA GATAATTGAC TAAGCTCTAA TGCTCTATCG TGGCTGCTGC AATATAAATG   
  
  
+ ATTTTTTGAC CTCAAAATGA AGACAGCTAG TTGTAAATTA AGAATCTTCG TCCCTTCATT TTTTGTGTAC   
  
  
+ GTGTGTATCT GCGTATGTGT TGAGAGACTA GCGGACTATT GATTGAGTTT CAATCAAAAA AGATGACCAT   
  
  
+ CTTAAGTATT TTTAATTACG CAAACTTCTT CATTTAAAAA AATGTTACTT TTATTTGAAA GAAATTGTGA   
  
  
+ ATAATACAAG GAATAGTTCA ATTAATATAT AATCCATAAC ATATTACCTC CTATAGGCAA AAAGAAGATT   
  
  
+ ATCGTATTAA TTTGGGATCA TCCCACCCTA AATTTAAAGA AACCAAAACA AATTACTAAG CAATATCTTT   
  
  
+ CTGTGTGAAA AAATAGTCAG AAACAAAGCG ATCCGACCGG CCAAATCGAG ATAAACACAG CCAAAGAACC   
  
  
+ GAAGTTGGAC CAAAGTGAGC CGAGTTGAAA GGCATTCATA TGGGGTTTAA TCATAACACA AACATAATTA   
  
  
+ GATTGATTGA CGTCTTTAAT CAGCGTTTTT GTCTTTGTTC GGTGCTCCCC TCACTCGCCC CTCCGCTCTT   
  
  
+ TAAAGCTTAC CTCACTCCCC CCATTCCCAC TCCTTTTCTC TCTCCTCTCT CGCTCTCACA CGCTCCGTCT   
  
  
+ GTCAGCCCCT TGTCATCTTC CCCCGTCTTC CTCGCAAAAA TCTCCAGCAA AATTTATAGC CGTTGTTGTG   
  
  
+ CCCATGGTGA TCTGATGTGA GAAGAGATTC AGCATTTAAG ATTATTGGGT CCAGATGGCT TATATGTGCG   
  
  
+ CCGACAGTGG TAATCTCATG GCTATTGCCC AACAAGTCAT CAAGCAAAAA CAACAGCAAG AACAGCAGCA   
  
  
+ ACACCAACAG CCTCATCCCC AGTTCGTCGG GTCGAATAAT AACCCCTTTT GCACCAGCCC ATGGCCCGGA   
  
  
+ CCCGTCCCTT CATCCGCCAT GTCTGCCAGC CCTCCGCCCC CTCTTGGGTT CTCCGCCGCC GCGTTTCCGG   
  
  
+ ACCCGTTTCA GGTGGGCCCG CCCTGCCCGG ATGGGGCTGA ACCGGGTTTC CAGTTTGCCC ATTTGGACCA   
  
  
+ CCACTCGAGT GGTTTCCGGT TTGCTGATTT TTGCGGTGGG GGTGGTGGTG AGTTTGACTC GGATGAGTGG   
  
  
+ ATGGAGAGTT TGATGGGTGG CGGCGGCGGT GACTCGCGGC GACTGAGAGT TCTAACCTCC AATCCCATTG   
  
  
+ CGACGCGTGG CAAGGGTCCG GTGATTTCGG TCTCTATGGT GCTGATCCGT TTGCGACGTG TTCGGAAGAC   
  
  
+ CGTTGCAGCC GCTATTCACG CTCAGCCACT GCCGCAACAG CAGCCGTCGT GGGTCCCGCC TTCTCCGCCG   
  
  
+ AAAGACACGA AGCAGTCGGC CCCACCTCAA AAGCAAAGCG ACGCCGCTGG ACCCTCGTTC TCATCCGAGG   
  
  
+ AGGAACCAGA GCCGTCATGG CCGCCGCTAA TTGCGGCGTT GTTGGAGTGC GCCAAACTCG CCGAGTCCGA   
  
  
+ CCCGGAACAC GCTGTCAAAT CGGTGATTCG ACTCAGAGAC TCAGTCTCAC AGCAGGGAGT TCCAATCCAG   
  
  
+ AGAGTGTCCT ATTACTTCTC CGAGGCACTC TACCACCGCC TTTCTCTCTC CTCCGCCCCT CAACCTCTCC   
  
  
+ CAGAAACGAC GTCGTCTGAG GAGTACACTC TCTCTTACAA GGCCCTGAAC GACGCGTGCC CGTACCATAA   
  
  
+ ATTCGCTCAC TTGACCGCAA ATCAGGCGAT TCTAGAAGCC ACTGATTCAG CCAAGAAGAT CCACATCGTT   
  
  
+ GATTTTGGGA TAATCCAGGG AGTCCAATGG GCTGCTCTGC TACAAGCCCT GGCAACCCGG CCAGCCGGGA   
  
  
+ AGCCCGAATT GATTCGAATA TCCGGAATTC CTGCTCCGGG TCTGGGGCAA TCTCCGGCGG CATCGCTGCT   
  
  
+ CGCAACTGGG AATCGCCTCC GTGAGTTCGC GAAGGTTCTA GATCTCAAGT TCGAGTTTGA GCCGGTACTC   
  
  
+ ACTCCAATCA GAGAGCTAAA CGGGTCGAAC TTCCGGGTCG ACCCAGATGA GATACTAGCT GTCAATTTCA   
  
  
+ TGCTTCAATT GTACAACCTA TTAGACGAAA CCCTGGTCGC TGTCGACGCC GCCCTGAAGC TGGCCAAGTC   
  
  
+ ATTGAACCCC AAAATCGTCA CTTTGGGTGA GTACGAGGCG GGTCTGAATC GGGCAGGTTT CTTGACCCGG   
  
  
+ TTCAAGACGG CCCTCAAGTA CTATTTGGCA ATGTTTGAAT CGGTTGAGCC CAACTTGGCC CGGGACTCGC   
  
  
+ CCGAGAGGGC TCAGGTTGAG AGGCTGTTAT TGGGCCGGCG GATCATGGGC TTAGTTGGGC CGGAGTTACC   
  
  
+ GGGAACGAGA AGGGAAAGGA TGGAGTGTAA GGAACAATGG AAGGGTTTAA TGGAAAGTGC CGGTTTCGAG   
  
  
+ CCGTGCCAAA GCAGAAACTA CGTCAGTGAA AAAGACAAGT TTTACCACTC CAGATTGAAT TACAGAGAGT   
  
  
+ G  

- +Up\_Stream \_Len000ACTATT AATAAAAATA ACTAAATCCT TTCCTATAGT TTCATATAAA ACAAAAATCC   
  
  
- ATCAATTCTC TAAGACAACG GTTTTTTTTT TAGTTCTATA ATATATAAAA ATTAATACTG TCATTAATAA   
  
  
- AATAACTTTT AGGTTGCCAA CACTAATATA TTAGTTTATT AGATTTCCAA TTTTTATAGT TTAAATAATC   
  
  
- TTTTAAGCTT TTTCAGTGCA GTAGTGTTGT AACGAAAATA TATATCATAT CTAAGGAAGC TGAAGATTTG   
  
  
- ATTAGTGAAC TTTAATCTTT AAACTTTGAA AGATTATATT TACGTACCAG AAGAAAGGTT CTAATATTAG   
  
  
- GCGTTAACGA ATTTACATCC CATGTTTATA GTTTAAATCT TCACTGATAA CTTCGGTACT TTCTATAACA   
  
  
- CTAGCATAAC TCCCACCAAC ACTCGTTATC CTACTTTGTC CTTTTTTTTT GGGATTACAA AAGTTTAAAT   
  
  
- ACCCACCGAA ACCATCATCT ATTCACTTAT CACACGTTCC CTTACCGTGG TTATACAAAC CTACCTTGCT   
  
  
- ACTCTCATTC TGCAATCCTT TCGTTGTTCT CTTCCCTTTT CCGTCATCCC TCTCTTAATA CGCACACTTA   
  
  
- TTATAATATT GTCTGCGAAA GCATACTAAT AATTTATGTT CTGAAACTCG TATGTATATT TGAACATTGA   
  
  
- AAGAAATGGA TAACAGTTAA TTAAAAACTT AACTTAGTAG ACGCTGAATA CGTATGTTCT ACTATACTAC   
  
  
- GTATAACCCG GTTCGTAAAC TAGAGTACAG GGAATACCTA GATCTAGGGT TATCTGTTTG AACGTATTCA   
  
  
- GTTCCCTACA CAAAGATTAT GTTTCCGAAA CATGAGTTCG TTAATCAAAA CTTAAGTAAA AGTCATCAAA   
  
  
- TTAAAGGTTT AGAATATTAA AAATGTTACC GTATTTAAAA ATCCTCAAAA CTCATTTATT TAATATATGT   
  
  
- TGCTTCTACA GTAAATGCTT AATTATACTG CGTGTTTTTT TTTCTTTTTT TTAATTACCA TCGAAGATAT   
  
  
- GAACTGAGGT ACAACTGTTA AGGGAGAGAG TGTATTACCA ATTTTGAACG TTGTAGTAGG TCCTGGGTAT   
  
  
- ACTGTACCCA CCACCATCTG TCACACAACA CCATCGGAGT GAGGTACGAA CGGTTAAACA TAGTAACGCG   
  
  
- ATCAGAATAA GCAGGAATCT CTATTAACTG ATTCGAGATT ACGAGATAGC ACCGACGACG TTATATTTAC   
  
  
- TAAAAAACTG GAGTTTTACT TCTGTCGATC AACATTTAAT TCTTAGAAGC AGGGAAGTAA AAAACACATG   
  
  
- CACACATAGA CGCATACACA ACTCTCTGAT CGCCTGATAA CTAACTCAAA GTTAGTTTTT TCTACTGGTA   
  
  
- GAATTCATAA AAATTAATGC GTTTGAAGAA GTAAATTTTT TTACAATGAA AATAAACTTT CTTTAACACT   
  
  
- TATTATGTTC CTTATCAAGT TAATTATATA TTAGGTATTG TATAATGGAG GATATCCGTT TTTCTTCTAA   
  
  
- TAGCATAATT AAACCCTAGT AGGGTGGGAT TTAAATTTCT TTGGTTTTGT TTAATGATTC GTTATAGAAA   
  
  
- GACACACTTT TTTATCAGTC TTTGTTTCGC TAGGCTGGCC GGTTTAGCTC TATTTGTGTC GGTTTCTTGG   
  
  
- CTTCAACCTG GTTTCACTCG GCTCAACTTT CCGTAAGTAT ACCCCAAATT AGTATTGTGT TTGTATTAAT   
  
  
- CTAACTAACT GCAGAAATTA GTCGCAAAAA CAGAAACAAG CCACGAGGGG AGTGAGCGGG GAGGCGAGAA   
  
  
- ATTTCGAATG GAGTGAGGGG GGTAAGGGTG AGGAAAAGAG AGAGGAGAGA GCGAGAGTGT GCGAGGCAGA   
  
  
- CAGTCGGGGA ACAGTAGAAG GGGGCAGAAG GAGCGTTTTT AGAGGTCGTT TTAAATATCG GCAACAACAC   
  
  
- GGGTACCACT AGACTACACT CTTCTCTAAG TCGTAAATTC TAATAACCCA GGTCTACCGA ATATACACGC   
  
  
- GGCTGTCACC ATTAGAGTAC CGATAACGGG TTGTTCAGTA GTTCGTTTTT GTTGTCGTTC TTGTCGTCGT   
  
  
- TGTGGTTGTC GGAGTAGGGG TCAAGCAGCC CAGCTTATTA TTGGGGAAAA CGTGGTCGGG TACCGGGCCT   
  
  
- GGGCAGGGAA GTAGGCGGTA CAGACGGTCG GGAGGCGGGG GAGAACCCAA GAGGCGGCGG CGCAAAGGCC   
  
  
- TGGGCAAAGT CCACCCGGGC GGGACGGGCC TACCCCGACT TGGCCCAAAG GTCAAACGGG TAAACCTGGT   
  
  
- GGTGAGCTCA CCAAAGGCCA AACGACTAAA AACGCCACCC CCACCACCAC TCAAACTGAG CCTACTCACC   
  
  
- TACCTCTCAA ACTACCCACC GCCGCCGCCA CTGAGCGCCG CTGACTCTCA AGATTGGAGG TTAGGGTAAC   
  
  
- GCTGCGCACC GTTCCCAGGC CACTAAAGCC AGAGATACCA CGACTAGGCA AACGCTGCAC AAGCCTTCTG   
  
  
- GCAACGTCGG CGATAAGTGC GAGTCGGTGA CGGCGTTGTC GTCGGCAGCA CCCAGGGCGG AAGAGGCGGC   
  
  
- TTTCTGTGCT TCGTCAGCCG GGGTGGAGTT TTCGTTTCGC TGCGGCGACC TGGGAGCAAG AGTAGGCTCC   
  
  
- TCCTTGGTCT CGGCAGTACC GGCGGCGATT AACGCCGCAA CAACCTCACG CGGTTTGAGC GGCTCAGGCT   
  
  
- GGGCCTTGTG CGACAGTTTA GCCACTAAGC TGAGTCTCTG AGTCAGAGTG TCGTCCCTCA AGGTTAGGTC   
  
  
- TCTCACAGGA TAATGAAGAG GCTCCGTGAG ATGGTGGCGG AAAGAGAGAG GAGGCGGGGA GTTGGAGAGG   
  
  
- GTCTTTGCTG CAGCAGACTC CTCATGTGAG AGAGAATGTT CCGGGACTTG CTGCGCACGG GCATGGTATT   
  
  
- TAAGCGAGTG AACTGGCGTT TAGTCCGCTA AGATCTTCGG TGACTAAGTC GGTTCTTCTA GGTGTAGCAA   
  
  
- CTAAAACCCT ATTAGGTCCC TCAGGTTACC CGACGAGACG ATGTTCGGGA CCGTTGGGCC GGTCGGCCCT   
  
  
- TCGGGCTTAA CTAAGCTTAT AGGCCTTAAG GACGAGGCCC AGACCCCGTT AGAGGCCGCC GTAGCGACGA   
  
  
- GCGTTGACCC TTAGCGGAGG CACTCAAGCG CTTCCAAGAT CTAGAGTTCA AGCTCAAACT CGGCCATGAG   
  
  
- TGAGGTTAGT CTCTCGATTT GCCCAGCTTG AAGGCCCAGC TGGGTCTACT CTATGATCGA CAGTTAAAGT   
  
  
- ACGAAGTTAA CATGTTGGAT AATCTGCTTT GGGACCAGCG ACAGCTGCGG CGGGACTTCG ACCGGTTCAG   
  
  
- TAACTTGGGG TTTTAGCAGT GAAACCCACT CATGCTCCGC CCAGACTTAG CCCGTCCAAA GAACTGGGCC   
  
  
- AAGTTCTGCC GGGAGTTCAT GATAAACCGT TACAAACTTA GCCAACTCGG GTTGAACCGG GCCCTGAGCG   
  
  
- GGCTCTCCCG AGTCCAACTC TCCGACAATA ACCCGGCCGC CTAGTACCCG AATCAACCCG GCCTCAATGG   
  
  
- CCCTTGCTCT TCCCTTTCCT ACCTCACATT CCTTGTTACC TTCCCAAATT ACCTTTCACG GCCAAAGCTC   
  
  
- GGCACGGTTT CGTCTTTGAT GCAGTCACTT TTTCTGTTCA AAATGGTGAG GTCTAACTTA ATGTCTCTCA   
  
  
- C

+     CGTCA-motif

| Site Name | Organism | Position | Strand | Matrix score. | sequence | function |
| --- | --- | --- | --- | --- | --- | --- |
| CGTCA-motif | Hordeum vulgare | 3380 | + | 5 | CGTCA | cis-acting regulatory element involved in the MeJA-responsiveness |
| CGTCA-motif | Hordeum vulgare | 3665 | + | 5 | CGTCA | cis-acting regulatory element involved in the MeJA-responsiveness |
| CGTCA-motif | Hordeum vulgare | 1011 | - | 5 | CGTCA | cis-acting regulatory element involved in the MeJA-responsiveness |
| CGTCA-motif | Hordeum vulgare | 2677 | + | 5 | CGTCA | cis-acting regulatory element involved in the MeJA-responsiveness |
| CGTCA-motif | Hordeum vulgare | 1762 | - | 5 | CGTCA | cis-acting regulatory element involved in the MeJA-responsiveness |
| CGTCA-motif | Hordeum vulgare | 232 | + | 5 | CGTCA | cis-acting regulatory element involved in the MeJA-responsiveness |

>HU06G00029.1   
+ +Up\_Stream \_Len000TGATAA TTATTTTTAT TGATTTAGGA AAGGATATCA AAGTATATTT TGTTTTTAGG   
  
  
+ TAGTTAAGAG ATTCTGTTGC CAAAAAAAAA ATCAAGATAT TATATATTTT TAATTATGAC AGTAATTATT   
  
  
+ TTATTGAAAA TCCAACGGTT GTGATTATAT AATCAAATAA TCTAAAGGTT AAAAATATCA AATTTATTAG   
  
  
+ AAAATTCGAA AAAGTCACGT CATCACAACA TTGCTTTTAT ATATAGTATA GATTCCTTCG ACTTCTAAAC   
  
  
+ TAATCACTTG AAATTAGAAA TTTGAAACTT TCTAATATAA ATGCATGGTC TTCTTTCCAA GATTATAATC   
  
  
+ CGCAATTGCT TAAATGTAGG GTACAAATAT CAAATTTAGA AGTGACTATT GAAGCCATGA AAGATATTGT   
  
  
+ GATCGTATTG AGGGTGGTTG TGAGCAATAG GATGAAACAG GAAAAAAAAA CCCTAATGTT TTCAAATTTA   
  
  
+ TGGGTGGCTT TGGTAGTAGA TAAGTGAATA GTGTGCAAGG GAATGGCACC AATATGTTTG GATGGAACGA   
  
  
+ TGAGAGTAAG ACGTTAGGAA AGCAACAAGA GAAGGGAAAA GGCAGTAGGG AGAGAATTAT GCGTGTGAAT   
  
  
+ AATATTATAA CAGACGCTTT CGTATGATTA TTAAATACAA GACTTTGAGC ATACATATAA ACTTGTAACT   
  
  
+ TTCTTTACCT ATTGTCAATT AATTTTTGAA TTGAATCATC TGCGACTTAT GCATACAAGA TGATATGATG   
  
  
+ CATATTGGGC CAAGCATTTG ATCTCATGTC CCTTATGGAT CTAGATCCCA ATAGACAAAC TTGCATAAGT   
  
  
+ CAAGGGATGT GTTTCTAATA CAAAGGCTTT GTACTCAAGC AATTAGTTTT GAATTCATTT TCAGTAGTTT   
  
  
+ AATTTCCAAA TCTTATAATT TTTACAATGG CATAAATTTT TAGGAGTTTT GAGTAAATAA ATTATATACA   
  
  
+ ACGAAGATGT CATTTACGAA TTAATATGAC GCACAAAAAA AAAGAAAAAA AATTAATGGT AGCTTCTATA   
  
  
+ CTTGACTCCA TGTTGACAAT TCCCTCTCTC ACATAATGGT TAAAACTTGC AACATCATCC AGGACCCATA   
  
  
+ TGACATGGGT GGTGGTAGAC AGTGTGTTGT GGTAGCCTCA CTCCATGCTT GCCAATTTGT ATCATTGCGC   
  
  
+ TAGTCTTATT CGTCCTTAGA GATAATTGAC TAAGCTCTAA TGCTCTATCG TGGCTGCTGC AATATAAATG   
  
  
+ ATTTTTTGAC CTCAAAATGA AGACAGCTAG TTGTAAATTA AGAATCTTCG TCCCTTCATT TTTTGTGTAC   
  
  
+ GTGTGTATCT GCGTATGTGT TGAGAGACTA GCGGACTATT GATTGAGTTT CAATCAAAAA AGATGACCAT   
  
  
+ CTTAAGTATT TTTAATTACG CAAACTTCTT CATTTAAAAA AATGTTACTT TTATTTGAAA GAAATTGTGA   
  
  
+ ATAATACAAG GAATAGTTCA ATTAATATAT AATCCATAAC ATATTACCTC CTATAGGCAA AAAGAAGATT   
  
  
+ ATCGTATTAA TTTGGGATCA TCCCACCCTA AATTTAAAGA AACCAAAACA AATTACTAAG CAATATCTTT   
  
  
+ CTGTGTGAAA AAATAGTCAG AAACAAAGCG ATCCGACCGG CCAAATCGAG ATAAACACAG CCAAAGAACC   
  
  
+ GAAGTTGGAC CAAAGTGAGC CGAGTTGAAA GGCATTCATA TGGGGTTTAA TCATAACACA AACATAATTA   
  
  
+ GATTGATTGA CGTCTTTAAT CAGCGTTTTT GTCTTTGTTC GGTGCTCCCC TCACTCGCCC CTCCGCTCTT   
  
  
+ TAAAGCTTAC CTCACTCCCC CCATTCCCAC TCCTTTTCTC TCTCCTCTCT CGCTCTCACA CGCTCCGTCT   
  
  
+ GTCAGCCCCT TGTCATCTTC CCCCGTCTTC CTCGCAAAAA TCTCCAGCAA AATTTATAGC CGTTGTTGTG   
  
  
+ CCCATGGTGA TCTGATGTGA GAAGAGATTC AGCATTTAAG ATTATTGGGT CCAGATGGCT TATATGTGCG   
  
  
+ CCGACAGTGG TAATCTCATG GCTATTGCCC AACAAGTCAT CAAGCAAAAA CAACAGCAAG AACAGCAGCA   
  
  
+ ACACCAACAG CCTCATCCCC AGTTCGTCGG GTCGAATAAT AACCCCTTTT GCACCAGCCC ATGGCCCGGA   
  
  
+ CCCGTCCCTT CATCCGCCAT GTCTGCCAGC CCTCCGCCCC CTCTTGGGTT CTCCGCCGCC GCGTTTCCGG   
  
  
+ ACCCGTTTCA GGTGGGCCCG CCCTGCCCGG ATGGGGCTGA ACCGGGTTTC CAGTTTGCCC ATTTGGACCA   
  
  
+ CCACTCGAGT GGTTTCCGGT TTGCTGATTT TTGCGGTGGG GGTGGTGGTG AGTTTGACTC GGATGAGTGG   
  
  
+ ATGGAGAGTT TGATGGGTGG CGGCGGCGGT GACTCGCGGC GACTGAGAGT TCTAACCTCC AATCCCATTG   
  
  
+ CGACGCGTGG CAAGGGTCCG GTGATTTCGG TCTCTATGGT GCTGATCCGT TTGCGACGTG TTCGGAAGAC   
  
  
+ CGTTGCAGCC GCTATTCACG CTCAGCCACT GCCGCAACAG CAGCCGTCGT GGGTCCCGCC TTCTCCGCCG   
  
  
+ AAAGACACGA AGCAGTCGGC CCCACCTCAA AAGCAAAGCG ACGCCGCTGG ACCCTCGTTC TCATCCGAGG   
  
  
+ AGGAACCAGA GCCGTCATGG CCGCCGCTAA TTGCGGCGTT GTTGGAGTGC GCCAAACTCG CCGAGTCCGA   
  
  
+ CCCGGAACAC GCTGTCAAAT CGGTGATTCG ACTCAGAGAC TCAGTCTCAC AGCAGGGAGT TCCAATCCAG   
  
  
+ AGAGTGTCCT ATTACTTCTC CGAGGCACTC TACCACCGCC TTTCTCTCTC CTCCGCCCCT CAACCTCTCC   
  
  
+ CAGAAACGAC GTCGTCTGAG GAGTACACTC TCTCTTACAA GGCCCTGAAC GACGCGTGCC CGTACCATAA   
  
  
+ ATTCGCTCAC TTGACCGCAA ATCAGGCGAT TCTAGAAGCC ACTGATTCAG CCAAGAAGAT CCACATCGTT   
  
  
+ GATTTTGGGA TAATCCAGGG AGTCCAATGG GCTGCTCTGC TACAAGCCCT GGCAACCCGG CCAGCCGGGA   
  
  
+ AGCCCGAATT GATTCGAATA TCCGGAATTC CTGCTCCGGG TCTGGGGCAA TCTCCGGCGG CATCGCTGCT   
  
  
+ CGCAACTGGG AATCGCCTCC GTGAGTTCGC GAAGGTTCTA GATCTCAAGT TCGAGTTTGA GCCGGTACTC   
  
  
+ ACTCCAATCA GAGAGCTAAA CGGGTCGAAC TTCCGGGTCG ACCCAGATGA GATACTAGCT GTCAATTTCA   
  
  
+ TGCTTCAATT GTACAACCTA TTAGACGAAA CCCTGGTCGC TGTCGACGCC GCCCTGAAGC TGGCCAAGTC   
  
  
+ ATTGAACCCC AAAATCGTCA CTTTGGGTGA GTACGAGGCG GGTCTGAATC GGGCAGGTTT CTTGACCCGG   
  
  
+ TTCAAGACGG CCCTCAAGTA CTATTTGGCA ATGTTTGAAT CGGTTGAGCC CAACTTGGCC CGGGACTCGC   
  
  
+ CCGAGAGGGC TCAGGTTGAG AGGCTGTTAT TGGGCCGGCG GATCATGGGC TTAGTTGGGC CGGAGTTACC   
  
  
+ GGGAACGAGA AGGGAAAGGA TGGAGTGTAA GGAACAATGG AAGGGTTTAA TGGAAAGTGC CGGTTTCGAG   
  
  
+ CCGTGCCAAA GCAGAAACTA CGTCAGTGAA AAAGACAAGT TTTACCACTC CAGATTGAAT TACAGAGAGT   
  
  
+ G  

- +Up\_Stream \_Len000ACTATT AATAAAAATA ACTAAATCCT TTCCTATAGT TTCATATAAA ACAAAAATCC   
  
  
- ATCAATTCTC TAAGACAACG GTTTTTTTTT TAGTTCTATA ATATATAAAA ATTAATACTG TCATTAATAA   
  
  
- AATAACTTTT AGGTTGCCAA CACTAATATA TTAGTTTATT AGATTTCCAA TTTTTATAGT TTAAATAATC   
  
  
- TTTTAAGCTT TTTCAGTGCA GTAGTGTTGT AACGAAAATA TATATCATAT CTAAGGAAGC TGAAGATTTG   
  
  
- ATTAGTGAAC TTTAATCTTT AAACTTTGAA AGATTATATT TACGTACCAG AAGAAAGGTT CTAATATTAG   
  
  
- GCGTTAACGA ATTTACATCC CATGTTTATA GTTTAAATCT TCACTGATAA CTTCGGTACT TTCTATAACA   
  
  
- CTAGCATAAC TCCCACCAAC ACTCGTTATC CTACTTTGTC CTTTTTTTTT GGGATTACAA AAGTTTAAAT   
  
  
- ACCCACCGAA ACCATCATCT ATTCACTTAT CACACGTTCC CTTACCGTGG TTATACAAAC CTACCTTGCT   
  
  
- ACTCTCATTC TGCAATCCTT TCGTTGTTCT CTTCCCTTTT CCGTCATCCC TCTCTTAATA CGCACACTTA   
  
  
- TTATAATATT GTCTGCGAAA GCATACTAAT AATTTATGTT CTGAAACTCG TATGTATATT TGAACATTGA   
  
  
- AAGAAATGGA TAACAGTTAA TTAAAAACTT AACTTAGTAG ACGCTGAATA CGTATGTTCT ACTATACTAC   
  
  
- GTATAACCCG GTTCGTAAAC TAGAGTACAG GGAATACCTA GATCTAGGGT TATCTGTTTG AACGTATTCA   
  
  
- GTTCCCTACA CAAAGATTAT GTTTCCGAAA CATGAGTTCG TTAATCAAAA CTTAAGTAAA AGTCATCAAA   
  
  
- TTAAAGGTTT AGAATATTAA AAATGTTACC GTATTTAAAA ATCCTCAAAA CTCATTTATT TAATATATGT   
  
  
- TGCTTCTACA GTAAATGCTT AATTATACTG CGTGTTTTTT TTTCTTTTTT TTAATTACCA TCGAAGATAT   
  
  
- GAACTGAGGT ACAACTGTTA AGGGAGAGAG TGTATTACCA ATTTTGAACG TTGTAGTAGG TCCTGGGTAT   
  
  
- ACTGTACCCA CCACCATCTG TCACACAACA CCATCGGAGT GAGGTACGAA CGGTTAAACA TAGTAACGCG   
  
  
- ATCAGAATAA GCAGGAATCT CTATTAACTG ATTCGAGATT ACGAGATAGC ACCGACGACG TTATATTTAC   
  
  
- TAAAAAACTG GAGTTTTACT TCTGTCGATC AACATTTAAT TCTTAGAAGC AGGGAAGTAA AAAACACATG   
  
  
- CACACATAGA CGCATACACA ACTCTCTGAT CGCCTGATAA CTAACTCAAA GTTAGTTTTT TCTACTGGTA   
  
  
- GAATTCATAA AAATTAATGC GTTTGAAGAA GTAAATTTTT TTACAATGAA AATAAACTTT CTTTAACACT   
  
  
- TATTATGTTC CTTATCAAGT TAATTATATA TTAGGTATTG TATAATGGAG GATATCCGTT TTTCTTCTAA   
  
  
- TAGCATAATT AAACCCTAGT AGGGTGGGAT TTAAATTTCT TTGGTTTTGT TTAATGATTC GTTATAGAAA   
  
  
- GACACACTTT TTTATCAGTC TTTGTTTCGC TAGGCTGGCC GGTTTAGCTC TATTTGTGTC GGTTTCTTGG   
  
  
- CTTCAACCTG GTTTCACTCG GCTCAACTTT CCGTAAGTAT ACCCCAAATT AGTATTGTGT TTGTATTAAT   
  
  
- CTAACTAACT GCAGAAATTA GTCGCAAAAA CAGAAACAAG CCACGAGGGG AGTGAGCGGG GAGGCGAGAA   
  
  
- ATTTCGAATG GAGTGAGGGG GGTAAGGGTG AGGAAAAGAG AGAGGAGAGA GCGAGAGTGT GCGAGGCAGA   
  
  
- CAGTCGGGGA ACAGTAGAAG GGGGCAGAAG GAGCGTTTTT AGAGGTCGTT TTAAATATCG GCAACAACAC   
  
  
- GGGTACCACT AGACTACACT CTTCTCTAAG TCGTAAATTC TAATAACCCA GGTCTACCGA ATATACACGC   
  
  
- GGCTGTCACC ATTAGAGTAC CGATAACGGG TTGTTCAGTA GTTCGTTTTT GTTGTCGTTC TTGTCGTCGT   
  
  
- TGTGGTTGTC GGAGTAGGGG TCAAGCAGCC CAGCTTATTA TTGGGGAAAA CGTGGTCGGG TACCGGGCCT   
  
  
- GGGCAGGGAA GTAGGCGGTA CAGACGGTCG GGAGGCGGGG GAGAACCCAA GAGGCGGCGG CGCAAAGGCC   
  
  
- TGGGCAAAGT CCACCCGGGC GGGACGGGCC TACCCCGACT TGGCCCAAAG GTCAAACGGG TAAACCTGGT   
  
  
- GGTGAGCTCA CCAAAGGCCA AACGACTAAA AACGCCACCC CCACCACCAC TCAAACTGAG CCTACTCACC   
  
  
- TACCTCTCAA ACTACCCACC GCCGCCGCCA CTGAGCGCCG CTGACTCTCA AGATTGGAGG TTAGGGTAAC   
  
  
- GCTGCGCACC GTTCCCAGGC CACTAAAGCC AGAGATACCA CGACTAGGCA AACGCTGCAC AAGCCTTCTG   
  
  
- GCAACGTCGG CGATAAGTGC GAGTCGGTGA CGGCGTTGTC GTCGGCAGCA CCCAGGGCGG AAGAGGCGGC   
  
  
- TTTCTGTGCT TCGTCAGCCG GGGTGGAGTT TTCGTTTCGC TGCGGCGACC TGGGAGCAAG AGTAGGCTCC   
  
  
- TCCTTGGTCT CGGCAGTACC GGCGGCGATT AACGCCGCAA CAACCTCACG CGGTTTGAGC GGCTCAGGCT   
  
  
- GGGCCTTGTG CGACAGTTTA GCCACTAAGC TGAGTCTCTG AGTCAGAGTG TCGTCCCTCA AGGTTAGGTC   
  
  
- TCTCACAGGA TAATGAAGAG GCTCCGTGAG ATGGTGGCGG AAAGAGAGAG GAGGCGGGGA GTTGGAGAGG   
  
  
- GTCTTTGCTG CAGCAGACTC CTCATGTGAG AGAGAATGTT CCGGGACTTG CTGCGCACGG GCATGGTATT   
  
  
- TAAGCGAGTG AACTGGCGTT TAGTCCGCTA AGATCTTCGG TGACTAAGTC GGTTCTTCTA GGTGTAGCAA   
  
  
- CTAAAACCCT ATTAGGTCCC TCAGGTTACC CGACGAGACG ATGTTCGGGA CCGTTGGGCC GGTCGGCCCT   
  
  
- TCGGGCTTAA CTAAGCTTAT AGGCCTTAAG GACGAGGCCC AGACCCCGTT AGAGGCCGCC GTAGCGACGA   
  
  
- GCGTTGACCC TTAGCGGAGG CACTCAAGCG CTTCCAAGAT CTAGAGTTCA AGCTCAAACT CGGCCATGAG   
  
  
- TGAGGTTAGT CTCTCGATTT GCCCAGCTTG AAGGCCCAGC TGGGTCTACT CTATGATCGA CAGTTAAAGT   
  
  
- ACGAAGTTAA CATGTTGGAT AATCTGCTTT GGGACCAGCG ACAGCTGCGG CGGGACTTCG ACCGGTTCAG   
  
  
- TAACTTGGGG TTTTAGCAGT GAAACCCACT CATGCTCCGC CCAGACTTAG CCCGTCCAAA GAACTGGGCC   
  
  
- AAGTTCTGCC GGGAGTTCAT GATAAACCGT TACAAACTTA GCCAACTCGG GTTGAACCGG GCCCTGAGCG   
  
  
- GGCTCTCCCG AGTCCAACTC TCCGACAATA ACCCGGCCGC CTAGTACCCG AATCAACCCG GCCTCAATGG   
  
  
- CCCTTGCTCT TCCCTTTCCT ACCTCACATT CCTTGTTACC TTCCCAAATT ACCTTTCACG GCCAAAGCTC   
  
  
- GGCACGGTTT CGTCTTTGAT GCAGTCACTT TTTCTGTTCA AAATGGTGAG GTCTAACTTA ATGTCTCTCA   
  
  
- C

+     DRE core

| Site Name | Organism | Position | Strand | Matrix score. | sequence | function |
| --- | --- | --- | --- | --- | --- | --- |
| DRE core | Arabidopsis thaliana | 2034 | + | 6 | GCCGAC |  |
| DRE core | Arabidopsis thaliana | 2609 | - | 6 | GCCGAC |  |

>HU06G00029.1   
+ +Up\_Stream \_Len000TGATAA TTATTTTTAT TGATTTAGGA AAGGATATCA AAGTATATTT TGTTTTTAGG   
  
  
+ TAGTTAAGAG ATTCTGTTGC CAAAAAAAAA ATCAAGATAT TATATATTTT TAATTATGAC AGTAATTATT   
  
  
+ TTATTGAAAA TCCAACGGTT GTGATTATAT AATCAAATAA TCTAAAGGTT AAAAATATCA AATTTATTAG   
  
  
+ AAAATTCGAA AAAGTCACGT CATCACAACA TTGCTTTTAT ATATAGTATA GATTCCTTCG ACTTCTAAAC   
  
  
+ TAATCACTTG AAATTAGAAA TTTGAAACTT TCTAATATAA ATGCATGGTC TTCTTTCCAA GATTATAATC   
  
  
+ CGCAATTGCT TAAATGTAGG GTACAAATAT CAAATTTAGA AGTGACTATT GAAGCCATGA AAGATATTGT   
  
  
+ GATCGTATTG AGGGTGGTTG TGAGCAATAG GATGAAACAG GAAAAAAAAA CCCTAATGTT TTCAAATTTA   
  
  
+ TGGGTGGCTT TGGTAGTAGA TAAGTGAATA GTGTGCAAGG GAATGGCACC AATATGTTTG GATGGAACGA   
  
  
+ TGAGAGTAAG ACGTTAGGAA AGCAACAAGA GAAGGGAAAA GGCAGTAGGG AGAGAATTAT GCGTGTGAAT   
  
  
+ AATATTATAA CAGACGCTTT CGTATGATTA TTAAATACAA GACTTTGAGC ATACATATAA ACTTGTAACT   
  
  
+ TTCTTTACCT ATTGTCAATT AATTTTTGAA TTGAATCATC TGCGACTTAT GCATACAAGA TGATATGATG   
  
  
+ CATATTGGGC CAAGCATTTG ATCTCATGTC CCTTATGGAT CTAGATCCCA ATAGACAAAC TTGCATAAGT   
  
  
+ CAAGGGATGT GTTTCTAATA CAAAGGCTTT GTACTCAAGC AATTAGTTTT GAATTCATTT TCAGTAGTTT   
  
  
+ AATTTCCAAA TCTTATAATT TTTACAATGG CATAAATTTT TAGGAGTTTT GAGTAAATAA ATTATATACA   
  
  
+ ACGAAGATGT CATTTACGAA TTAATATGAC GCACAAAAAA AAAGAAAAAA AATTAATGGT AGCTTCTATA   
  
  
+ CTTGACTCCA TGTTGACAAT TCCCTCTCTC ACATAATGGT TAAAACTTGC AACATCATCC AGGACCCATA   
  
  
+ TGACATGGGT GGTGGTAGAC AGTGTGTTGT GGTAGCCTCA CTCCATGCTT GCCAATTTGT ATCATTGCGC   
  
  
+ TAGTCTTATT CGTCCTTAGA GATAATTGAC TAAGCTCTAA TGCTCTATCG TGGCTGCTGC AATATAAATG   
  
  
+ ATTTTTTGAC CTCAAAATGA AGACAGCTAG TTGTAAATTA AGAATCTTCG TCCCTTCATT TTTTGTGTAC   
  
  
+ GTGTGTATCT GCGTATGTGT TGAGAGACTA GCGGACTATT GATTGAGTTT CAATCAAAAA AGATGACCAT   
  
  
+ CTTAAGTATT TTTAATTACG CAAACTTCTT CATTTAAAAA AATGTTACTT TTATTTGAAA GAAATTGTGA   
  
  
+ ATAATACAAG GAATAGTTCA ATTAATATAT AATCCATAAC ATATTACCTC CTATAGGCAA AAAGAAGATT   
  
  
+ ATCGTATTAA TTTGGGATCA TCCCACCCTA AATTTAAAGA AACCAAAACA AATTACTAAG CAATATCTTT   
  
  
+ CTGTGTGAAA AAATAGTCAG AAACAAAGCG ATCCGACCGG CCAAATCGAG ATAAACACAG CCAAAGAACC   
  
  
+ GAAGTTGGAC CAAAGTGAGC CGAGTTGAAA GGCATTCATA TGGGGTTTAA TCATAACACA AACATAATTA   
  
  
+ GATTGATTGA CGTCTTTAAT CAGCGTTTTT GTCTTTGTTC GGTGCTCCCC TCACTCGCCC CTCCGCTCTT   
  
  
+ TAAAGCTTAC CTCACTCCCC CCATTCCCAC TCCTTTTCTC TCTCCTCTCT CGCTCTCACA CGCTCCGTCT   
  
  
+ GTCAGCCCCT TGTCATCTTC CCCCGTCTTC CTCGCAAAAA TCTCCAGCAA AATTTATAGC CGTTGTTGTG   
  
  
+ CCCATGGTGA TCTGATGTGA GAAGAGATTC AGCATTTAAG ATTATTGGGT CCAGATGGCT TATATGTGCG   
  
  
+ CCGACAGTGG TAATCTCATG GCTATTGCCC AACAAGTCAT CAAGCAAAAA CAACAGCAAG AACAGCAGCA   
  
  
+ ACACCAACAG CCTCATCCCC AGTTCGTCGG GTCGAATAAT AACCCCTTTT GCACCAGCCC ATGGCCCGGA   
  
  
+ CCCGTCCCTT CATCCGCCAT GTCTGCCAGC CCTCCGCCCC CTCTTGGGTT CTCCGCCGCC GCGTTTCCGG   
  
  
+ ACCCGTTTCA GGTGGGCCCG CCCTGCCCGG ATGGGGCTGA ACCGGGTTTC CAGTTTGCCC ATTTGGACCA   
  
  
+ CCACTCGAGT GGTTTCCGGT TTGCTGATTT TTGCGGTGGG GGTGGTGGTG AGTTTGACTC GGATGAGTGG   
  
  
+ ATGGAGAGTT TGATGGGTGG CGGCGGCGGT GACTCGCGGC GACTGAGAGT TCTAACCTCC AATCCCATTG   
  
  
+ CGACGCGTGG CAAGGGTCCG GTGATTTCGG TCTCTATGGT GCTGATCCGT TTGCGACGTG TTCGGAAGAC   
  
  
+ CGTTGCAGCC GCTATTCACG CTCAGCCACT GCCGCAACAG CAGCCGTCGT GGGTCCCGCC TTCTCCGCCG   
  
  
+ AAAGACACGA AGCAGTCGGC CCCACCTCAA AAGCAAAGCG ACGCCGCTGG ACCCTCGTTC TCATCCGAGG   
  
  
+ AGGAACCAGA GCCGTCATGG CCGCCGCTAA TTGCGGCGTT GTTGGAGTGC GCCAAACTCG CCGAGTCCGA   
  
  
+ CCCGGAACAC GCTGTCAAAT CGGTGATTCG ACTCAGAGAC TCAGTCTCAC AGCAGGGAGT TCCAATCCAG   
  
  
+ AGAGTGTCCT ATTACTTCTC CGAGGCACTC TACCACCGCC TTTCTCTCTC CTCCGCCCCT CAACCTCTCC   
  
  
+ CAGAAACGAC GTCGTCTGAG GAGTACACTC TCTCTTACAA GGCCCTGAAC GACGCGTGCC CGTACCATAA   
  
  
+ ATTCGCTCAC TTGACCGCAA ATCAGGCGAT TCTAGAAGCC ACTGATTCAG CCAAGAAGAT CCACATCGTT   
  
  
+ GATTTTGGGA TAATCCAGGG AGTCCAATGG GCTGCTCTGC TACAAGCCCT GGCAACCCGG CCAGCCGGGA   
  
  
+ AGCCCGAATT GATTCGAATA TCCGGAATTC CTGCTCCGGG TCTGGGGCAA TCTCCGGCGG CATCGCTGCT   
  
  
+ CGCAACTGGG AATCGCCTCC GTGAGTTCGC GAAGGTTCTA GATCTCAAGT TCGAGTTTGA GCCGGTACTC   
  
  
+ ACTCCAATCA GAGAGCTAAA CGGGTCGAAC TTCCGGGTCG ACCCAGATGA GATACTAGCT GTCAATTTCA   
  
  
+ TGCTTCAATT GTACAACCTA TTAGACGAAA CCCTGGTCGC TGTCGACGCC GCCCTGAAGC TGGCCAAGTC   
  
  
+ ATTGAACCCC AAAATCGTCA CTTTGGGTGA GTACGAGGCG GGTCTGAATC GGGCAGGTTT CTTGACCCGG   
  
  
+ TTCAAGACGG CCCTCAAGTA CTATTTGGCA ATGTTTGAAT CGGTTGAGCC CAACTTGGCC CGGGACTCGC   
  
  
+ CCGAGAGGGC TCAGGTTGAG AGGCTGTTAT TGGGCCGGCG GATCATGGGC TTAGTTGGGC CGGAGTTACC   
  
  
+ GGGAACGAGA AGGGAAAGGA TGGAGTGTAA GGAACAATGG AAGGGTTTAA TGGAAAGTGC CGGTTTCGAG   
  
  
+ CCGTGCCAAA GCAGAAACTA CGTCAGTGAA AAAGACAAGT TTTACCACTC CAGATTGAAT TACAGAGAGT   
  
  
+ G  

- +Up\_Stream \_Len000ACTATT AATAAAAATA ACTAAATCCT TTCCTATAGT TTCATATAAA ACAAAAATCC   
  
  
- ATCAATTCTC TAAGACAACG GTTTTTTTTT TAGTTCTATA ATATATAAAA ATTAATACTG TCATTAATAA   
  
  
- AATAACTTTT AGGTTGCCAA CACTAATATA TTAGTTTATT AGATTTCCAA TTTTTATAGT TTAAATAATC   
  
  
- TTTTAAGCTT TTTCAGTGCA GTAGTGTTGT AACGAAAATA TATATCATAT CTAAGGAAGC TGAAGATTTG   
  
  
- ATTAGTGAAC TTTAATCTTT AAACTTTGAA AGATTATATT TACGTACCAG AAGAAAGGTT CTAATATTAG   
  
  
- GCGTTAACGA ATTTACATCC CATGTTTATA GTTTAAATCT TCACTGATAA CTTCGGTACT TTCTATAACA   
  
  
- CTAGCATAAC TCCCACCAAC ACTCGTTATC CTACTTTGTC CTTTTTTTTT GGGATTACAA AAGTTTAAAT   
  
  
- ACCCACCGAA ACCATCATCT ATTCACTTAT CACACGTTCC CTTACCGTGG TTATACAAAC CTACCTTGCT   
  
  
- ACTCTCATTC TGCAATCCTT TCGTTGTTCT CTTCCCTTTT CCGTCATCCC TCTCTTAATA CGCACACTTA   
  
  
- TTATAATATT GTCTGCGAAA GCATACTAAT AATTTATGTT CTGAAACTCG TATGTATATT TGAACATTGA   
  
  
- AAGAAATGGA TAACAGTTAA TTAAAAACTT AACTTAGTAG ACGCTGAATA CGTATGTTCT ACTATACTAC   
  
  
- GTATAACCCG GTTCGTAAAC TAGAGTACAG GGAATACCTA GATCTAGGGT TATCTGTTTG AACGTATTCA   
  
  
- GTTCCCTACA CAAAGATTAT GTTTCCGAAA CATGAGTTCG TTAATCAAAA CTTAAGTAAA AGTCATCAAA   
  
  
- TTAAAGGTTT AGAATATTAA AAATGTTACC GTATTTAAAA ATCCTCAAAA CTCATTTATT TAATATATGT   
  
  
- TGCTTCTACA GTAAATGCTT AATTATACTG CGTGTTTTTT TTTCTTTTTT TTAATTACCA TCGAAGATAT   
  
  
- GAACTGAGGT ACAACTGTTA AGGGAGAGAG TGTATTACCA ATTTTGAACG TTGTAGTAGG TCCTGGGTAT   
  
  
- ACTGTACCCA CCACCATCTG TCACACAACA CCATCGGAGT GAGGTACGAA CGGTTAAACA TAGTAACGCG   
  
  
- ATCAGAATAA GCAGGAATCT CTATTAACTG ATTCGAGATT ACGAGATAGC ACCGACGACG TTATATTTAC   
  
  
- TAAAAAACTG GAGTTTTACT TCTGTCGATC AACATTTAAT TCTTAGAAGC AGGGAAGTAA AAAACACATG   
  
  
- CACACATAGA CGCATACACA ACTCTCTGAT CGCCTGATAA CTAACTCAAA GTTAGTTTTT TCTACTGGTA   
  
  
- GAATTCATAA AAATTAATGC GTTTGAAGAA GTAAATTTTT TTACAATGAA AATAAACTTT CTTTAACACT   
  
  
- TATTATGTTC CTTATCAAGT TAATTATATA TTAGGTATTG TATAATGGAG GATATCCGTT TTTCTTCTAA   
  
  
- TAGCATAATT AAACCCTAGT AGGGTGGGAT TTAAATTTCT TTGGTTTTGT TTAATGATTC GTTATAGAAA   
  
  
- GACACACTTT TTTATCAGTC TTTGTTTCGC TAGGCTGGCC GGTTTAGCTC TATTTGTGTC GGTTTCTTGG   
  
  
- CTTCAACCTG GTTTCACTCG GCTCAACTTT CCGTAAGTAT ACCCCAAATT AGTATTGTGT TTGTATTAAT   
  
  
- CTAACTAACT GCAGAAATTA GTCGCAAAAA CAGAAACAAG CCACGAGGGG AGTGAGCGGG GAGGCGAGAA   
  
  
- ATTTCGAATG GAGTGAGGGG GGTAAGGGTG AGGAAAAGAG AGAGGAGAGA GCGAGAGTGT GCGAGGCAGA   
  
  
- CAGTCGGGGA ACAGTAGAAG GGGGCAGAAG GAGCGTTTTT AGAGGTCGTT TTAAATATCG GCAACAACAC   
  
  
- GGGTACCACT AGACTACACT CTTCTCTAAG TCGTAAATTC TAATAACCCA GGTCTACCGA ATATACACGC   
  
  
- GGCTGTCACC ATTAGAGTAC CGATAACGGG TTGTTCAGTA GTTCGTTTTT GTTGTCGTTC TTGTCGTCGT   
  
  
- TGTGGTTGTC GGAGTAGGGG TCAAGCAGCC CAGCTTATTA TTGGGGAAAA CGTGGTCGGG TACCGGGCCT   
  
  
- GGGCAGGGAA GTAGGCGGTA CAGACGGTCG GGAGGCGGGG GAGAACCCAA GAGGCGGCGG CGCAAAGGCC   
  
  
- TGGGCAAAGT CCACCCGGGC GGGACGGGCC TACCCCGACT TGGCCCAAAG GTCAAACGGG TAAACCTGGT   
  
  
- GGTGAGCTCA CCAAAGGCCA AACGACTAAA AACGCCACCC CCACCACCAC TCAAACTGAG CCTACTCACC   
  
  
- TACCTCTCAA ACTACCCACC GCCGCCGCCA CTGAGCGCCG CTGACTCTCA AGATTGGAGG TTAGGGTAAC   
  
  
- GCTGCGCACC GTTCCCAGGC CACTAAAGCC AGAGATACCA CGACTAGGCA AACGCTGCAC AAGCCTTCTG   
  
  
- GCAACGTCGG CGATAAGTGC GAGTCGGTGA CGGCGTTGTC GTCGGCAGCA CCCAGGGCGG AAGAGGCGGC   
  
  
- TTTCTGTGCT TCGTCAGCCG GGGTGGAGTT TTCGTTTCGC TGCGGCGACC TGGGAGCAAG AGTAGGCTCC   
  
  
- TCCTTGGTCT CGGCAGTACC GGCGGCGATT AACGCCGCAA CAACCTCACG CGGTTTGAGC GGCTCAGGCT   
  
  
- GGGCCTTGTG CGACAGTTTA GCCACTAAGC TGAGTCTCTG AGTCAGAGTG TCGTCCCTCA AGGTTAGGTC   
  
  
- TCTCACAGGA TAATGAAGAG GCTCCGTGAG ATGGTGGCGG AAAGAGAGAG GAGGCGGGGA GTTGGAGAGG   
  
  
- GTCTTTGCTG CAGCAGACTC CTCATGTGAG AGAGAATGTT CCGGGACTTG CTGCGCACGG GCATGGTATT   
  
  
- TAAGCGAGTG AACTGGCGTT TAGTCCGCTA AGATCTTCGG TGACTAAGTC GGTTCTTCTA GGTGTAGCAA   
  
  
- CTAAAACCCT ATTAGGTCCC TCAGGTTACC CGACGAGACG ATGTTCGGGA CCGTTGGGCC GGTCGGCCCT   
  
  
- TCGGGCTTAA CTAAGCTTAT AGGCCTTAAG GACGAGGCCC AGACCCCGTT AGAGGCCGCC GTAGCGACGA   
  
  
- GCGTTGACCC TTAGCGGAGG CACTCAAGCG CTTCCAAGAT CTAGAGTTCA AGCTCAAACT CGGCCATGAG   
  
  
- TGAGGTTAGT CTCTCGATTT GCCCAGCTTG AAGGCCCAGC TGGGTCTACT CTATGATCGA CAGTTAAAGT   
  
  
- ACGAAGTTAA CATGTTGGAT AATCTGCTTT GGGACCAGCG ACAGCTGCGG CGGGACTTCG ACCGGTTCAG   
  
  
- TAACTTGGGG TTTTAGCAGT GAAACCCACT CATGCTCCGC CCAGACTTAG CCCGTCCAAA GAACTGGGCC   
  
  
- AAGTTCTGCC GGGAGTTCAT GATAAACCGT TACAAACTTA GCCAACTCGG GTTGAACCGG GCCCTGAGCG   
  
  
- GGCTCTCCCG AGTCCAACTC TCCGACAATA ACCCGGCCGC CTAGTACCCG AATCAACCCG GCCTCAATGG   
  
  
- CCCTTGCTCT TCCCTTTCCT ACCTCACATT CCTTGTTACC TTCCCAAATT ACCTTTCACG GCCAAAGCTC   
  
  
- GGCACGGTTT CGTCTTTGAT GCAGTCACTT TTTCTGTTCA AAATGGTGAG GTCTAACTTA ATGTCTCTCA   
  
  
- C

+     G-box

| Site Name | Organism | Position | Strand | Matrix score. | sequence | function |
| --- | --- | --- | --- | --- | --- | --- |
| G-box | Zea mays | 230 | + | 6 | CACGTC | cis-acting regulatory element involved in light responsiveness |
| G-box | Zea mays | 2509 | - | 6 | CACGTC | cis-acting regulatory element involved in light responsiveness |
| G-box | Arabidopsis thaliana | 1332 | + | 6 | TACGTG | cis-acting regulatory element involved in light responsiveness |
| G-box | Zea mays | 2570 | - | 6 | CACGAC | cis-acting regulatory element involved in light responsiveness |

>HU06G00029.1   
+ +Up\_Stream \_Len000TGATAA TTATTTTTAT TGATTTAGGA AAGGATATCA AAGTATATTT TGTTTTTAGG   
  
  
+ TAGTTAAGAG ATTCTGTTGC CAAAAAAAAA ATCAAGATAT TATATATTTT TAATTATGAC AGTAATTATT   
  
  
+ TTATTGAAAA TCCAACGGTT GTGATTATAT AATCAAATAA TCTAAAGGTT AAAAATATCA AATTTATTAG   
  
  
+ AAAATTCGAA AAAGTCACGT CATCACAACA TTGCTTTTAT ATATAGTATA GATTCCTTCG ACTTCTAAAC   
  
  
+ TAATCACTTG AAATTAGAAA TTTGAAACTT TCTAATATAA ATGCATGGTC TTCTTTCCAA GATTATAATC   
  
  
+ CGCAATTGCT TAAATGTAGG GTACAAATAT CAAATTTAGA AGTGACTATT GAAGCCATGA AAGATATTGT   
  
  
+ GATCGTATTG AGGGTGGTTG TGAGCAATAG GATGAAACAG GAAAAAAAAA CCCTAATGTT TTCAAATTTA   
  
  
+ TGGGTGGCTT TGGTAGTAGA TAAGTGAATA GTGTGCAAGG GAATGGCACC AATATGTTTG GATGGAACGA   
  
  
+ TGAGAGTAAG ACGTTAGGAA AGCAACAAGA GAAGGGAAAA GGCAGTAGGG AGAGAATTAT GCGTGTGAAT   
  
  
+ AATATTATAA CAGACGCTTT CGTATGATTA TTAAATACAA GACTTTGAGC ATACATATAA ACTTGTAACT   
  
  
+ TTCTTTACCT ATTGTCAATT AATTTTTGAA TTGAATCATC TGCGACTTAT GCATACAAGA TGATATGATG   
  
  
+ CATATTGGGC CAAGCATTTG ATCTCATGTC CCTTATGGAT CTAGATCCCA ATAGACAAAC TTGCATAAGT   
  
  
+ CAAGGGATGT GTTTCTAATA CAAAGGCTTT GTACTCAAGC AATTAGTTTT GAATTCATTT TCAGTAGTTT   
  
  
+ AATTTCCAAA TCTTATAATT TTTACAATGG CATAAATTTT TAGGAGTTTT GAGTAAATAA ATTATATACA   
  
  
+ ACGAAGATGT CATTTACGAA TTAATATGAC GCACAAAAAA AAAGAAAAAA AATTAATGGT AGCTTCTATA   
  
  
+ CTTGACTCCA TGTTGACAAT TCCCTCTCTC ACATAATGGT TAAAACTTGC AACATCATCC AGGACCCATA   
  
  
+ TGACATGGGT GGTGGTAGAC AGTGTGTTGT GGTAGCCTCA CTCCATGCTT GCCAATTTGT ATCATTGCGC   
  
  
+ TAGTCTTATT CGTCCTTAGA GATAATTGAC TAAGCTCTAA TGCTCTATCG TGGCTGCTGC AATATAAATG   
  
  
+ ATTTTTTGAC CTCAAAATGA AGACAGCTAG TTGTAAATTA AGAATCTTCG TCCCTTCATT TTTTGTGTAC   
  
  
+ GTGTGTATCT GCGTATGTGT TGAGAGACTA GCGGACTATT GATTGAGTTT CAATCAAAAA AGATGACCAT   
  
  
+ CTTAAGTATT TTTAATTACG CAAACTTCTT CATTTAAAAA AATGTTACTT TTATTTGAAA GAAATTGTGA   
  
  
+ ATAATACAAG GAATAGTTCA ATTAATATAT AATCCATAAC ATATTACCTC CTATAGGCAA AAAGAAGATT   
  
  
+ ATCGTATTAA TTTGGGATCA TCCCACCCTA AATTTAAAGA AACCAAAACA AATTACTAAG CAATATCTTT   
  
  
+ CTGTGTGAAA AAATAGTCAG AAACAAAGCG ATCCGACCGG CCAAATCGAG ATAAACACAG CCAAAGAACC   
  
  
+ GAAGTTGGAC CAAAGTGAGC CGAGTTGAAA GGCATTCATA TGGGGTTTAA TCATAACACA AACATAATTA   
  
  
+ GATTGATTGA CGTCTTTAAT CAGCGTTTTT GTCTTTGTTC GGTGCTCCCC TCACTCGCCC CTCCGCTCTT   
  
  
+ TAAAGCTTAC CTCACTCCCC CCATTCCCAC TCCTTTTCTC TCTCCTCTCT CGCTCTCACA CGCTCCGTCT   
  
  
+ GTCAGCCCCT TGTCATCTTC CCCCGTCTTC CTCGCAAAAA TCTCCAGCAA AATTTATAGC CGTTGTTGTG   
  
  
+ CCCATGGTGA TCTGATGTGA GAAGAGATTC AGCATTTAAG ATTATTGGGT CCAGATGGCT TATATGTGCG   
  
  
+ CCGACAGTGG TAATCTCATG GCTATTGCCC AACAAGTCAT CAAGCAAAAA CAACAGCAAG AACAGCAGCA   
  
  
+ ACACCAACAG CCTCATCCCC AGTTCGTCGG GTCGAATAAT AACCCCTTTT GCACCAGCCC ATGGCCCGGA   
  
  
+ CCCGTCCCTT CATCCGCCAT GTCTGCCAGC CCTCCGCCCC CTCTTGGGTT CTCCGCCGCC GCGTTTCCGG   
  
  
+ ACCCGTTTCA GGTGGGCCCG CCCTGCCCGG ATGGGGCTGA ACCGGGTTTC CAGTTTGCCC ATTTGGACCA   
  
  
+ CCACTCGAGT GGTTTCCGGT TTGCTGATTT TTGCGGTGGG GGTGGTGGTG AGTTTGACTC GGATGAGTGG   
  
  
+ ATGGAGAGTT TGATGGGTGG CGGCGGCGGT GACTCGCGGC GACTGAGAGT TCTAACCTCC AATCCCATTG   
  
  
+ CGACGCGTGG CAAGGGTCCG GTGATTTCGG TCTCTATGGT GCTGATCCGT TTGCGACGTG TTCGGAAGAC   
  
  
+ CGTTGCAGCC GCTATTCACG CTCAGCCACT GCCGCAACAG CAGCCGTCGT GGGTCCCGCC TTCTCCGCCG   
  
  
+ AAAGACACGA AGCAGTCGGC CCCACCTCAA AAGCAAAGCG ACGCCGCTGG ACCCTCGTTC TCATCCGAGG   
  
  
+ AGGAACCAGA GCCGTCATGG CCGCCGCTAA TTGCGGCGTT GTTGGAGTGC GCCAAACTCG CCGAGTCCGA   
  
  
+ CCCGGAACAC GCTGTCAAAT CGGTGATTCG ACTCAGAGAC TCAGTCTCAC AGCAGGGAGT TCCAATCCAG   
  
  
+ AGAGTGTCCT ATTACTTCTC CGAGGCACTC TACCACCGCC TTTCTCTCTC CTCCGCCCCT CAACCTCTCC   
  
  
+ CAGAAACGAC GTCGTCTGAG GAGTACACTC TCTCTTACAA GGCCCTGAAC GACGCGTGCC CGTACCATAA   
  
  
+ ATTCGCTCAC TTGACCGCAA ATCAGGCGAT TCTAGAAGCC ACTGATTCAG CCAAGAAGAT CCACATCGTT   
  
  
+ GATTTTGGGA TAATCCAGGG AGTCCAATGG GCTGCTCTGC TACAAGCCCT GGCAACCCGG CCAGCCGGGA   
  
  
+ AGCCCGAATT GATTCGAATA TCCGGAATTC CTGCTCCGGG TCTGGGGCAA TCTCCGGCGG CATCGCTGCT   
  
  
+ CGCAACTGGG AATCGCCTCC GTGAGTTCGC GAAGGTTCTA GATCTCAAGT TCGAGTTTGA GCCGGTACTC   
  
  
+ ACTCCAATCA GAGAGCTAAA CGGGTCGAAC TTCCGGGTCG ACCCAGATGA GATACTAGCT GTCAATTTCA   
  
  
+ TGCTTCAATT GTACAACCTA TTAGACGAAA CCCTGGTCGC TGTCGACGCC GCCCTGAAGC TGGCCAAGTC   
  
  
+ ATTGAACCCC AAAATCGTCA CTTTGGGTGA GTACGAGGCG GGTCTGAATC GGGCAGGTTT CTTGACCCGG   
  
  
+ TTCAAGACGG CCCTCAAGTA CTATTTGGCA ATGTTTGAAT CGGTTGAGCC CAACTTGGCC CGGGACTCGC   
  
  
+ CCGAGAGGGC TCAGGTTGAG AGGCTGTTAT TGGGCCGGCG GATCATGGGC TTAGTTGGGC CGGAGTTACC   
  
  
+ GGGAACGAGA AGGGAAAGGA TGGAGTGTAA GGAACAATGG AAGGGTTTAA TGGAAAGTGC CGGTTTCGAG   
  
  
+ CCGTGCCAAA GCAGAAACTA CGTCAGTGAA AAAGACAAGT TTTACCACTC CAGATTGAAT TACAGAGAGT   
  
  
+ G  

- +Up\_Stream \_Len000ACTATT AATAAAAATA ACTAAATCCT TTCCTATAGT TTCATATAAA ACAAAAATCC   
  
  
- ATCAATTCTC TAAGACAACG GTTTTTTTTT TAGTTCTATA ATATATAAAA ATTAATACTG TCATTAATAA   
  
  
- AATAACTTTT AGGTTGCCAA CACTAATATA TTAGTTTATT AGATTTCCAA TTTTTATAGT TTAAATAATC   
  
  
- TTTTAAGCTT TTTCAGTGCA GTAGTGTTGT AACGAAAATA TATATCATAT CTAAGGAAGC TGAAGATTTG   
  
  
- ATTAGTGAAC TTTAATCTTT AAACTTTGAA AGATTATATT TACGTACCAG AAGAAAGGTT CTAATATTAG   
  
  
- GCGTTAACGA ATTTACATCC CATGTTTATA GTTTAAATCT TCACTGATAA CTTCGGTACT TTCTATAACA   
  
  
- CTAGCATAAC TCCCACCAAC ACTCGTTATC CTACTTTGTC CTTTTTTTTT GGGATTACAA AAGTTTAAAT   
  
  
- ACCCACCGAA ACCATCATCT ATTCACTTAT CACACGTTCC CTTACCGTGG TTATACAAAC CTACCTTGCT   
  
  
- ACTCTCATTC TGCAATCCTT TCGTTGTTCT CTTCCCTTTT CCGTCATCCC TCTCTTAATA CGCACACTTA   
  
  
- TTATAATATT GTCTGCGAAA GCATACTAAT AATTTATGTT CTGAAACTCG TATGTATATT TGAACATTGA   
  
  
- AAGAAATGGA TAACAGTTAA TTAAAAACTT AACTTAGTAG ACGCTGAATA CGTATGTTCT ACTATACTAC   
  
  
- GTATAACCCG GTTCGTAAAC TAGAGTACAG GGAATACCTA GATCTAGGGT TATCTGTTTG AACGTATTCA   
  
  
- GTTCCCTACA CAAAGATTAT GTTTCCGAAA CATGAGTTCG TTAATCAAAA CTTAAGTAAA AGTCATCAAA   
  
  
- TTAAAGGTTT AGAATATTAA AAATGTTACC GTATTTAAAA ATCCTCAAAA CTCATTTATT TAATATATGT   
  
  
- TGCTTCTACA GTAAATGCTT AATTATACTG CGTGTTTTTT TTTCTTTTTT TTAATTACCA TCGAAGATAT   
  
  
- GAACTGAGGT ACAACTGTTA AGGGAGAGAG TGTATTACCA ATTTTGAACG TTGTAGTAGG TCCTGGGTAT   
  
  
- ACTGTACCCA CCACCATCTG TCACACAACA CCATCGGAGT GAGGTACGAA CGGTTAAACA TAGTAACGCG   
  
  
- ATCAGAATAA GCAGGAATCT CTATTAACTG ATTCGAGATT ACGAGATAGC ACCGACGACG TTATATTTAC   
  
  
- TAAAAAACTG GAGTTTTACT TCTGTCGATC AACATTTAAT TCTTAGAAGC AGGGAAGTAA AAAACACATG   
  
  
- CACACATAGA CGCATACACA ACTCTCTGAT CGCCTGATAA CTAACTCAAA GTTAGTTTTT TCTACTGGTA   
  
  
- GAATTCATAA AAATTAATGC GTTTGAAGAA GTAAATTTTT TTACAATGAA AATAAACTTT CTTTAACACT   
  
  
- TATTATGTTC CTTATCAAGT TAATTATATA TTAGGTATTG TATAATGGAG GATATCCGTT TTTCTTCTAA   
  
  
- TAGCATAATT AAACCCTAGT AGGGTGGGAT TTAAATTTCT TTGGTTTTGT TTAATGATTC GTTATAGAAA   
  
  
- GACACACTTT TTTATCAGTC TTTGTTTCGC TAGGCTGGCC GGTTTAGCTC TATTTGTGTC GGTTTCTTGG   
  
  
- CTTCAACCTG GTTTCACTCG GCTCAACTTT CCGTAAGTAT ACCCCAAATT AGTATTGTGT TTGTATTAAT   
  
  
- CTAACTAACT GCAGAAATTA GTCGCAAAAA CAGAAACAAG CCACGAGGGG AGTGAGCGGG GAGGCGAGAA   
  
  
- ATTTCGAATG GAGTGAGGGG GGTAAGGGTG AGGAAAAGAG AGAGGAGAGA GCGAGAGTGT GCGAGGCAGA   
  
  
- CAGTCGGGGA ACAGTAGAAG GGGGCAGAAG GAGCGTTTTT AGAGGTCGTT TTAAATATCG GCAACAACAC   
  
  
- GGGTACCACT AGACTACACT CTTCTCTAAG TCGTAAATTC TAATAACCCA GGTCTACCGA ATATACACGC   
  
  
- GGCTGTCACC ATTAGAGTAC CGATAACGGG TTGTTCAGTA GTTCGTTTTT GTTGTCGTTC TTGTCGTCGT   
  
  
- TGTGGTTGTC GGAGTAGGGG TCAAGCAGCC CAGCTTATTA TTGGGGAAAA CGTGGTCGGG TACCGGGCCT   
  
  
- GGGCAGGGAA GTAGGCGGTA CAGACGGTCG GGAGGCGGGG GAGAACCCAA GAGGCGGCGG CGCAAAGGCC   
  
  
- TGGGCAAAGT CCACCCGGGC GGGACGGGCC TACCCCGACT TGGCCCAAAG GTCAAACGGG TAAACCTGGT   
  
  
- GGTGAGCTCA CCAAAGGCCA AACGACTAAA AACGCCACCC CCACCACCAC TCAAACTGAG CCTACTCACC   
  
  
- TACCTCTCAA ACTACCCACC GCCGCCGCCA CTGAGCGCCG CTGACTCTCA AGATTGGAGG TTAGGGTAAC   
  
  
- GCTGCGCACC GTTCCCAGGC CACTAAAGCC AGAGATACCA CGACTAGGCA AACGCTGCAC AAGCCTTCTG   
  
  
- GCAACGTCGG CGATAAGTGC GAGTCGGTGA CGGCGTTGTC GTCGGCAGCA CCCAGGGCGG AAGAGGCGGC   
  
  
- TTTCTGTGCT TCGTCAGCCG GGGTGGAGTT TTCGTTTCGC TGCGGCGACC TGGGAGCAAG AGTAGGCTCC   
  
  
- TCCTTGGTCT CGGCAGTACC GGCGGCGATT AACGCCGCAA CAACCTCACG CGGTTTGAGC GGCTCAGGCT   
  
  
- GGGCCTTGTG CGACAGTTTA GCCACTAAGC TGAGTCTCTG AGTCAGAGTG TCGTCCCTCA AGGTTAGGTC   
  
  
- TCTCACAGGA TAATGAAGAG GCTCCGTGAG ATGGTGGCGG AAAGAGAGAG GAGGCGGGGA GTTGGAGAGG   
  
  
- GTCTTTGCTG CAGCAGACTC CTCATGTGAG AGAGAATGTT CCGGGACTTG CTGCGCACGG GCATGGTATT   
  
  
- TAAGCGAGTG AACTGGCGTT TAGTCCGCTA AGATCTTCGG TGACTAAGTC GGTTCTTCTA GGTGTAGCAA   
  
  
- CTAAAACCCT ATTAGGTCCC TCAGGTTACC CGACGAGACG ATGTTCGGGA CCGTTGGGCC GGTCGGCCCT   
  
  
- TCGGGCTTAA CTAAGCTTAT AGGCCTTAAG GACGAGGCCC AGACCCCGTT AGAGGCCGCC GTAGCGACGA   
  
  
- GCGTTGACCC TTAGCGGAGG CACTCAAGCG CTTCCAAGAT CTAGAGTTCA AGCTCAAACT CGGCCATGAG   
  
  
- TGAGGTTAGT CTCTCGATTT GCCCAGCTTG AAGGCCCAGC TGGGTCTACT CTATGATCGA CAGTTAAAGT   
  
  
- ACGAAGTTAA CATGTTGGAT AATCTGCTTT GGGACCAGCG ACAGCTGCGG CGGGACTTCG ACCGGTTCAG   
  
  
- TAACTTGGGG TTTTAGCAGT GAAACCCACT CATGCTCCGC CCAGACTTAG CCCGTCCAAA GAACTGGGCC   
  
  
- AAGTTCTGCC GGGAGTTCAT GATAAACCGT TACAAACTTA GCCAACTCGG GTTGAACCGG GCCCTGAGCG   
  
  
- GGCTCTCCCG AGTCCAACTC TCCGACAATA ACCCGGCCGC CTAGTACCCG AATCAACCCG GCCTCAATGG   
  
  
- CCCTTGCTCT TCCCTTTCCT ACCTCACATT CCTTGTTACC TTCCCAAATT ACCTTTCACG GCCAAAGCTC   
  
  
- GGCACGGTTT CGTCTTTGAT GCAGTCACTT TTTCTGTTCA AAATGGTGAG GTCTAACTTA ATGTCTCTCA   
  
  
- C

+     GARE-motif

| Site Name | Organism | Position | Strand | Matrix score. | sequence | function |
| --- | --- | --- | --- | --- | --- | --- |
| GARE-motif | Brassica oleracea | 87 | + | 7 | TCTGTTG | gibberellin-responsive element |

>HU06G00029.1   
+ +Up\_Stream \_Len000TGATAA TTATTTTTAT TGATTTAGGA AAGGATATCA AAGTATATTT TGTTTTTAGG   
  
  
+ TAGTTAAGAG ATTCTGTTGC CAAAAAAAAA ATCAAGATAT TATATATTTT TAATTATGAC AGTAATTATT   
  
  
+ TTATTGAAAA TCCAACGGTT GTGATTATAT AATCAAATAA TCTAAAGGTT AAAAATATCA AATTTATTAG   
  
  
+ AAAATTCGAA AAAGTCACGT CATCACAACA TTGCTTTTAT ATATAGTATA GATTCCTTCG ACTTCTAAAC   
  
  
+ TAATCACTTG AAATTAGAAA TTTGAAACTT TCTAATATAA ATGCATGGTC TTCTTTCCAA GATTATAATC   
  
  
+ CGCAATTGCT TAAATGTAGG GTACAAATAT CAAATTTAGA AGTGACTATT GAAGCCATGA AAGATATTGT   
  
  
+ GATCGTATTG AGGGTGGTTG TGAGCAATAG GATGAAACAG GAAAAAAAAA CCCTAATGTT TTCAAATTTA   
  
  
+ TGGGTGGCTT TGGTAGTAGA TAAGTGAATA GTGTGCAAGG GAATGGCACC AATATGTTTG GATGGAACGA   
  
  
+ TGAGAGTAAG ACGTTAGGAA AGCAACAAGA GAAGGGAAAA GGCAGTAGGG AGAGAATTAT GCGTGTGAAT   
  
  
+ AATATTATAA CAGACGCTTT CGTATGATTA TTAAATACAA GACTTTGAGC ATACATATAA ACTTGTAACT   
  
  
+ TTCTTTACCT ATTGTCAATT AATTTTTGAA TTGAATCATC TGCGACTTAT GCATACAAGA TGATATGATG   
  
  
+ CATATTGGGC CAAGCATTTG ATCTCATGTC CCTTATGGAT CTAGATCCCA ATAGACAAAC TTGCATAAGT   
  
  
+ CAAGGGATGT GTTTCTAATA CAAAGGCTTT GTACTCAAGC AATTAGTTTT GAATTCATTT TCAGTAGTTT   
  
  
+ AATTTCCAAA TCTTATAATT TTTACAATGG CATAAATTTT TAGGAGTTTT GAGTAAATAA ATTATATACA   
  
  
+ ACGAAGATGT CATTTACGAA TTAATATGAC GCACAAAAAA AAAGAAAAAA AATTAATGGT AGCTTCTATA   
  
  
+ CTTGACTCCA TGTTGACAAT TCCCTCTCTC ACATAATGGT TAAAACTTGC AACATCATCC AGGACCCATA   
  
  
+ TGACATGGGT GGTGGTAGAC AGTGTGTTGT GGTAGCCTCA CTCCATGCTT GCCAATTTGT ATCATTGCGC   
  
  
+ TAGTCTTATT CGTCCTTAGA GATAATTGAC TAAGCTCTAA TGCTCTATCG TGGCTGCTGC AATATAAATG   
  
  
+ ATTTTTTGAC CTCAAAATGA AGACAGCTAG TTGTAAATTA AGAATCTTCG TCCCTTCATT TTTTGTGTAC   
  
  
+ GTGTGTATCT GCGTATGTGT TGAGAGACTA GCGGACTATT GATTGAGTTT CAATCAAAAA AGATGACCAT   
  
  
+ CTTAAGTATT TTTAATTACG CAAACTTCTT CATTTAAAAA AATGTTACTT TTATTTGAAA GAAATTGTGA   
  
  
+ ATAATACAAG GAATAGTTCA ATTAATATAT AATCCATAAC ATATTACCTC CTATAGGCAA AAAGAAGATT   
  
  
+ ATCGTATTAA TTTGGGATCA TCCCACCCTA AATTTAAAGA AACCAAAACA AATTACTAAG CAATATCTTT   
  
  
+ CTGTGTGAAA AAATAGTCAG AAACAAAGCG ATCCGACCGG CCAAATCGAG ATAAACACAG CCAAAGAACC   
  
  
+ GAAGTTGGAC CAAAGTGAGC CGAGTTGAAA GGCATTCATA TGGGGTTTAA TCATAACACA AACATAATTA   
  
  
+ GATTGATTGA CGTCTTTAAT CAGCGTTTTT GTCTTTGTTC GGTGCTCCCC TCACTCGCCC CTCCGCTCTT   
  
  
+ TAAAGCTTAC CTCACTCCCC CCATTCCCAC TCCTTTTCTC TCTCCTCTCT CGCTCTCACA CGCTCCGTCT   
  
  
+ GTCAGCCCCT TGTCATCTTC CCCCGTCTTC CTCGCAAAAA TCTCCAGCAA AATTTATAGC CGTTGTTGTG   
  
  
+ CCCATGGTGA TCTGATGTGA GAAGAGATTC AGCATTTAAG ATTATTGGGT CCAGATGGCT TATATGTGCG   
  
  
+ CCGACAGTGG TAATCTCATG GCTATTGCCC AACAAGTCAT CAAGCAAAAA CAACAGCAAG AACAGCAGCA   
  
  
+ ACACCAACAG CCTCATCCCC AGTTCGTCGG GTCGAATAAT AACCCCTTTT GCACCAGCCC ATGGCCCGGA   
  
  
+ CCCGTCCCTT CATCCGCCAT GTCTGCCAGC CCTCCGCCCC CTCTTGGGTT CTCCGCCGCC GCGTTTCCGG   
  
  
+ ACCCGTTTCA GGTGGGCCCG CCCTGCCCGG ATGGGGCTGA ACCGGGTTTC CAGTTTGCCC ATTTGGACCA   
  
  
+ CCACTCGAGT GGTTTCCGGT TTGCTGATTT TTGCGGTGGG GGTGGTGGTG AGTTTGACTC GGATGAGTGG   
  
  
+ ATGGAGAGTT TGATGGGTGG CGGCGGCGGT GACTCGCGGC GACTGAGAGT TCTAACCTCC AATCCCATTG   
  
  
+ CGACGCGTGG CAAGGGTCCG GTGATTTCGG TCTCTATGGT GCTGATCCGT TTGCGACGTG TTCGGAAGAC   
  
  
+ CGTTGCAGCC GCTATTCACG CTCAGCCACT GCCGCAACAG CAGCCGTCGT GGGTCCCGCC TTCTCCGCCG   
  
  
+ AAAGACACGA AGCAGTCGGC CCCACCTCAA AAGCAAAGCG ACGCCGCTGG ACCCTCGTTC TCATCCGAGG   
  
  
+ AGGAACCAGA GCCGTCATGG CCGCCGCTAA TTGCGGCGTT GTTGGAGTGC GCCAAACTCG CCGAGTCCGA   
  
  
+ CCCGGAACAC GCTGTCAAAT CGGTGATTCG ACTCAGAGAC TCAGTCTCAC AGCAGGGAGT TCCAATCCAG   
  
  
+ AGAGTGTCCT ATTACTTCTC CGAGGCACTC TACCACCGCC TTTCTCTCTC CTCCGCCCCT CAACCTCTCC   
  
  
+ CAGAAACGAC GTCGTCTGAG GAGTACACTC TCTCTTACAA GGCCCTGAAC GACGCGTGCC CGTACCATAA   
  
  
+ ATTCGCTCAC TTGACCGCAA ATCAGGCGAT TCTAGAAGCC ACTGATTCAG CCAAGAAGAT CCACATCGTT   
  
  
+ GATTTTGGGA TAATCCAGGG AGTCCAATGG GCTGCTCTGC TACAAGCCCT GGCAACCCGG CCAGCCGGGA   
  
  
+ AGCCCGAATT GATTCGAATA TCCGGAATTC CTGCTCCGGG TCTGGGGCAA TCTCCGGCGG CATCGCTGCT   
  
  
+ CGCAACTGGG AATCGCCTCC GTGAGTTCGC GAAGGTTCTA GATCTCAAGT TCGAGTTTGA GCCGGTACTC   
  
  
+ ACTCCAATCA GAGAGCTAAA CGGGTCGAAC TTCCGGGTCG ACCCAGATGA GATACTAGCT GTCAATTTCA   
  
  
+ TGCTTCAATT GTACAACCTA TTAGACGAAA CCCTGGTCGC TGTCGACGCC GCCCTGAAGC TGGCCAAGTC   
  
  
+ ATTGAACCCC AAAATCGTCA CTTTGGGTGA GTACGAGGCG GGTCTGAATC GGGCAGGTTT CTTGACCCGG   
  
  
+ TTCAAGACGG CCCTCAAGTA CTATTTGGCA ATGTTTGAAT CGGTTGAGCC CAACTTGGCC CGGGACTCGC   
  
  
+ CCGAGAGGGC TCAGGTTGAG AGGCTGTTAT TGGGCCGGCG GATCATGGGC TTAGTTGGGC CGGAGTTACC   
  
  
+ GGGAACGAGA AGGGAAAGGA TGGAGTGTAA GGAACAATGG AAGGGTTTAA TGGAAAGTGC CGGTTTCGAG   
  
  
+ CCGTGCCAAA GCAGAAACTA CGTCAGTGAA AAAGACAAGT TTTACCACTC CAGATTGAAT TACAGAGAGT   
  
  
+ G  

- +Up\_Stream \_Len000ACTATT AATAAAAATA ACTAAATCCT TTCCTATAGT TTCATATAAA ACAAAAATCC   
  
  
- ATCAATTCTC TAAGACAACG GTTTTTTTTT TAGTTCTATA ATATATAAAA ATTAATACTG TCATTAATAA   
  
  
- AATAACTTTT AGGTTGCCAA CACTAATATA TTAGTTTATT AGATTTCCAA TTTTTATAGT TTAAATAATC   
  
  
- TTTTAAGCTT TTTCAGTGCA GTAGTGTTGT AACGAAAATA TATATCATAT CTAAGGAAGC TGAAGATTTG   
  
  
- ATTAGTGAAC TTTAATCTTT AAACTTTGAA AGATTATATT TACGTACCAG AAGAAAGGTT CTAATATTAG   
  
  
- GCGTTAACGA ATTTACATCC CATGTTTATA GTTTAAATCT TCACTGATAA CTTCGGTACT TTCTATAACA   
  
  
- CTAGCATAAC TCCCACCAAC ACTCGTTATC CTACTTTGTC CTTTTTTTTT GGGATTACAA AAGTTTAAAT   
  
  
- ACCCACCGAA ACCATCATCT ATTCACTTAT CACACGTTCC CTTACCGTGG TTATACAAAC CTACCTTGCT   
  
  
- ACTCTCATTC TGCAATCCTT TCGTTGTTCT CTTCCCTTTT CCGTCATCCC TCTCTTAATA CGCACACTTA   
  
  
- TTATAATATT GTCTGCGAAA GCATACTAAT AATTTATGTT CTGAAACTCG TATGTATATT TGAACATTGA   
  
  
- AAGAAATGGA TAACAGTTAA TTAAAAACTT AACTTAGTAG ACGCTGAATA CGTATGTTCT ACTATACTAC   
  
  
- GTATAACCCG GTTCGTAAAC TAGAGTACAG GGAATACCTA GATCTAGGGT TATCTGTTTG AACGTATTCA   
  
  
- GTTCCCTACA CAAAGATTAT GTTTCCGAAA CATGAGTTCG TTAATCAAAA CTTAAGTAAA AGTCATCAAA   
  
  
- TTAAAGGTTT AGAATATTAA AAATGTTACC GTATTTAAAA ATCCTCAAAA CTCATTTATT TAATATATGT   
  
  
- TGCTTCTACA GTAAATGCTT AATTATACTG CGTGTTTTTT TTTCTTTTTT TTAATTACCA TCGAAGATAT   
  
  
- GAACTGAGGT ACAACTGTTA AGGGAGAGAG TGTATTACCA ATTTTGAACG TTGTAGTAGG TCCTGGGTAT   
  
  
- ACTGTACCCA CCACCATCTG TCACACAACA CCATCGGAGT GAGGTACGAA CGGTTAAACA TAGTAACGCG   
  
  
- ATCAGAATAA GCAGGAATCT CTATTAACTG ATTCGAGATT ACGAGATAGC ACCGACGACG TTATATTTAC   
  
  
- TAAAAAACTG GAGTTTTACT TCTGTCGATC AACATTTAAT TCTTAGAAGC AGGGAAGTAA AAAACACATG   
  
  
- CACACATAGA CGCATACACA ACTCTCTGAT CGCCTGATAA CTAACTCAAA GTTAGTTTTT TCTACTGGTA   
  
  
- GAATTCATAA AAATTAATGC GTTTGAAGAA GTAAATTTTT TTACAATGAA AATAAACTTT CTTTAACACT   
  
  
- TATTATGTTC CTTATCAAGT TAATTATATA TTAGGTATTG TATAATGGAG GATATCCGTT TTTCTTCTAA   
  
  
- TAGCATAATT AAACCCTAGT AGGGTGGGAT TTAAATTTCT TTGGTTTTGT TTAATGATTC GTTATAGAAA   
  
  
- GACACACTTT TTTATCAGTC TTTGTTTCGC TAGGCTGGCC GGTTTAGCTC TATTTGTGTC GGTTTCTTGG   
  
  
- CTTCAACCTG GTTTCACTCG GCTCAACTTT CCGTAAGTAT ACCCCAAATT AGTATTGTGT TTGTATTAAT   
  
  
- CTAACTAACT GCAGAAATTA GTCGCAAAAA CAGAAACAAG CCACGAGGGG AGTGAGCGGG GAGGCGAGAA   
  
  
- ATTTCGAATG GAGTGAGGGG GGTAAGGGTG AGGAAAAGAG AGAGGAGAGA GCGAGAGTGT GCGAGGCAGA   
  
  
- CAGTCGGGGA ACAGTAGAAG GGGGCAGAAG GAGCGTTTTT AGAGGTCGTT TTAAATATCG GCAACAACAC   
  
  
- GGGTACCACT AGACTACACT CTTCTCTAAG TCGTAAATTC TAATAACCCA GGTCTACCGA ATATACACGC   
  
  
- GGCTGTCACC ATTAGAGTAC CGATAACGGG TTGTTCAGTA GTTCGTTTTT GTTGTCGTTC TTGTCGTCGT   
  
  
- TGTGGTTGTC GGAGTAGGGG TCAAGCAGCC CAGCTTATTA TTGGGGAAAA CGTGGTCGGG TACCGGGCCT   
  
  
- GGGCAGGGAA GTAGGCGGTA CAGACGGTCG GGAGGCGGGG GAGAACCCAA GAGGCGGCGG CGCAAAGGCC   
  
  
- TGGGCAAAGT CCACCCGGGC GGGACGGGCC TACCCCGACT TGGCCCAAAG GTCAAACGGG TAAACCTGGT   
  
  
- GGTGAGCTCA CCAAAGGCCA AACGACTAAA AACGCCACCC CCACCACCAC TCAAACTGAG CCTACTCACC   
  
  
- TACCTCTCAA ACTACCCACC GCCGCCGCCA CTGAGCGCCG CTGACTCTCA AGATTGGAGG TTAGGGTAAC   
  
  
- GCTGCGCACC GTTCCCAGGC CACTAAAGCC AGAGATACCA CGACTAGGCA AACGCTGCAC AAGCCTTCTG   
  
  
- GCAACGTCGG CGATAAGTGC GAGTCGGTGA CGGCGTTGTC GTCGGCAGCA CCCAGGGCGG AAGAGGCGGC   
  
  
- TTTCTGTGCT TCGTCAGCCG GGGTGGAGTT TTCGTTTCGC TGCGGCGACC TGGGAGCAAG AGTAGGCTCC   
  
  
- TCCTTGGTCT CGGCAGTACC GGCGGCGATT AACGCCGCAA CAACCTCACG CGGTTTGAGC GGCTCAGGCT   
  
  
- GGGCCTTGTG CGACAGTTTA GCCACTAAGC TGAGTCTCTG AGTCAGAGTG TCGTCCCTCA AGGTTAGGTC   
  
  
- TCTCACAGGA TAATGAAGAG GCTCCGTGAG ATGGTGGCGG AAAGAGAGAG GAGGCGGGGA GTTGGAGAGG   
  
  
- GTCTTTGCTG CAGCAGACTC CTCATGTGAG AGAGAATGTT CCGGGACTTG CTGCGCACGG GCATGGTATT   
  
  
- TAAGCGAGTG AACTGGCGTT TAGTCCGCTA AGATCTTCGG TGACTAAGTC GGTTCTTCTA GGTGTAGCAA   
  
  
- CTAAAACCCT ATTAGGTCCC TCAGGTTACC CGACGAGACG ATGTTCGGGA CCGTTGGGCC GGTCGGCCCT   
  
  
- TCGGGCTTAA CTAAGCTTAT AGGCCTTAAG GACGAGGCCC AGACCCCGTT AGAGGCCGCC GTAGCGACGA   
  
  
- GCGTTGACCC TTAGCGGAGG CACTCAAGCG CTTCCAAGAT CTAGAGTTCA AGCTCAAACT CGGCCATGAG   
  
  
- TGAGGTTAGT CTCTCGATTT GCCCAGCTTG AAGGCCCAGC TGGGTCTACT CTATGATCGA CAGTTAAAGT   
  
  
- ACGAAGTTAA CATGTTGGAT AATCTGCTTT GGGACCAGCG ACAGCTGCGG CGGGACTTCG ACCGGTTCAG   
  
  
- TAACTTGGGG TTTTAGCAGT GAAACCCACT CATGCTCCGC CCAGACTTAG CCCGTCCAAA GAACTGGGCC   
  
  
- AAGTTCTGCC GGGAGTTCAT GATAAACCGT TACAAACTTA GCCAACTCGG GTTGAACCGG GCCCTGAGCG   
  
  
- GGCTCTCCCG AGTCCAACTC TCCGACAATA ACCCGGCCGC CTAGTACCCG AATCAACCCG GCCTCAATGG   
  
  
- CCCTTGCTCT TCCCTTTCCT ACCTCACATT CCTTGTTACC TTCCCAAATT ACCTTTCACG GCCAAAGCTC   
  
  
- GGCACGGTTT CGTCTTTGAT GCAGTCACTT TTTCTGTTCA AAATGGTGAG GTCTAACTTA ATGTCTCTCA   
  
  
- C

+     GC-motif

| Site Name | Organism | Position | Strand | Matrix score. | sequence | function |
| --- | --- | --- | --- | --- | --- | --- |
| GC-motif | Zea mays | 1914 | + | 6 | CCCCCG | enhancer-like element involved in anoxic specific inducibility |

>HU06G00029.1   
+ +Up\_Stream \_Len000TGATAA TTATTTTTAT TGATTTAGGA AAGGATATCA AAGTATATTT TGTTTTTAGG   
  
  
+ TAGTTAAGAG ATTCTGTTGC CAAAAAAAAA ATCAAGATAT TATATATTTT TAATTATGAC AGTAATTATT   
  
  
+ TTATTGAAAA TCCAACGGTT GTGATTATAT AATCAAATAA TCTAAAGGTT AAAAATATCA AATTTATTAG   
  
  
+ AAAATTCGAA AAAGTCACGT CATCACAACA TTGCTTTTAT ATATAGTATA GATTCCTTCG ACTTCTAAAC   
  
  
+ TAATCACTTG AAATTAGAAA TTTGAAACTT TCTAATATAA ATGCATGGTC TTCTTTCCAA GATTATAATC   
  
  
+ CGCAATTGCT TAAATGTAGG GTACAAATAT CAAATTTAGA AGTGACTATT GAAGCCATGA AAGATATTGT   
  
  
+ GATCGTATTG AGGGTGGTTG TGAGCAATAG GATGAAACAG GAAAAAAAAA CCCTAATGTT TTCAAATTTA   
  
  
+ TGGGTGGCTT TGGTAGTAGA TAAGTGAATA GTGTGCAAGG GAATGGCACC AATATGTTTG GATGGAACGA   
  
  
+ TGAGAGTAAG ACGTTAGGAA AGCAACAAGA GAAGGGAAAA GGCAGTAGGG AGAGAATTAT GCGTGTGAAT   
  
  
+ AATATTATAA CAGACGCTTT CGTATGATTA TTAAATACAA GACTTTGAGC ATACATATAA ACTTGTAACT   
  
  
+ TTCTTTACCT ATTGTCAATT AATTTTTGAA TTGAATCATC TGCGACTTAT GCATACAAGA TGATATGATG   
  
  
+ CATATTGGGC CAAGCATTTG ATCTCATGTC CCTTATGGAT CTAGATCCCA ATAGACAAAC TTGCATAAGT   
  
  
+ CAAGGGATGT GTTTCTAATA CAAAGGCTTT GTACTCAAGC AATTAGTTTT GAATTCATTT TCAGTAGTTT   
  
  
+ AATTTCCAAA TCTTATAATT TTTACAATGG CATAAATTTT TAGGAGTTTT GAGTAAATAA ATTATATACA   
  
  
+ ACGAAGATGT CATTTACGAA TTAATATGAC GCACAAAAAA AAAGAAAAAA AATTAATGGT AGCTTCTATA   
  
  
+ CTTGACTCCA TGTTGACAAT TCCCTCTCTC ACATAATGGT TAAAACTTGC AACATCATCC AGGACCCATA   
  
  
+ TGACATGGGT GGTGGTAGAC AGTGTGTTGT GGTAGCCTCA CTCCATGCTT GCCAATTTGT ATCATTGCGC   
  
  
+ TAGTCTTATT CGTCCTTAGA GATAATTGAC TAAGCTCTAA TGCTCTATCG TGGCTGCTGC AATATAAATG   
  
  
+ ATTTTTTGAC CTCAAAATGA AGACAGCTAG TTGTAAATTA AGAATCTTCG TCCCTTCATT TTTTGTGTAC   
  
  
+ GTGTGTATCT GCGTATGTGT TGAGAGACTA GCGGACTATT GATTGAGTTT CAATCAAAAA AGATGACCAT   
  
  
+ CTTAAGTATT TTTAATTACG CAAACTTCTT CATTTAAAAA AATGTTACTT TTATTTGAAA GAAATTGTGA   
  
  
+ ATAATACAAG GAATAGTTCA ATTAATATAT AATCCATAAC ATATTACCTC CTATAGGCAA AAAGAAGATT   
  
  
+ ATCGTATTAA TTTGGGATCA TCCCACCCTA AATTTAAAGA AACCAAAACA AATTACTAAG CAATATCTTT   
  
  
+ CTGTGTGAAA AAATAGTCAG AAACAAAGCG ATCCGACCGG CCAAATCGAG ATAAACACAG CCAAAGAACC   
  
  
+ GAAGTTGGAC CAAAGTGAGC CGAGTTGAAA GGCATTCATA TGGGGTTTAA TCATAACACA AACATAATTA   
  
  
+ GATTGATTGA CGTCTTTAAT CAGCGTTTTT GTCTTTGTTC GGTGCTCCCC TCACTCGCCC CTCCGCTCTT   
  
  
+ TAAAGCTTAC CTCACTCCCC CCATTCCCAC TCCTTTTCTC TCTCCTCTCT CGCTCTCACA CGCTCCGTCT   
  
  
+ GTCAGCCCCT TGTCATCTTC CCCCGTCTTC CTCGCAAAAA TCTCCAGCAA AATTTATAGC CGTTGTTGTG   
  
  
+ CCCATGGTGA TCTGATGTGA GAAGAGATTC AGCATTTAAG ATTATTGGGT CCAGATGGCT TATATGTGCG   
  
  
+ CCGACAGTGG TAATCTCATG GCTATTGCCC AACAAGTCAT CAAGCAAAAA CAACAGCAAG AACAGCAGCA   
  
  
+ ACACCAACAG CCTCATCCCC AGTTCGTCGG GTCGAATAAT AACCCCTTTT GCACCAGCCC ATGGCCCGGA   
  
  
+ CCCGTCCCTT CATCCGCCAT GTCTGCCAGC CCTCCGCCCC CTCTTGGGTT CTCCGCCGCC GCGTTTCCGG   
  
  
+ ACCCGTTTCA GGTGGGCCCG CCCTGCCCGG ATGGGGCTGA ACCGGGTTTC CAGTTTGCCC ATTTGGACCA   
  
  
+ CCACTCGAGT GGTTTCCGGT TTGCTGATTT TTGCGGTGGG GGTGGTGGTG AGTTTGACTC GGATGAGTGG   
  
  
+ ATGGAGAGTT TGATGGGTGG CGGCGGCGGT GACTCGCGGC GACTGAGAGT TCTAACCTCC AATCCCATTG   
  
  
+ CGACGCGTGG CAAGGGTCCG GTGATTTCGG TCTCTATGGT GCTGATCCGT TTGCGACGTG TTCGGAAGAC   
  
  
+ CGTTGCAGCC GCTATTCACG CTCAGCCACT GCCGCAACAG CAGCCGTCGT GGGTCCCGCC TTCTCCGCCG   
  
  
+ AAAGACACGA AGCAGTCGGC CCCACCTCAA AAGCAAAGCG ACGCCGCTGG ACCCTCGTTC TCATCCGAGG   
  
  
+ AGGAACCAGA GCCGTCATGG CCGCCGCTAA TTGCGGCGTT GTTGGAGTGC GCCAAACTCG CCGAGTCCGA   
  
  
+ CCCGGAACAC GCTGTCAAAT CGGTGATTCG ACTCAGAGAC TCAGTCTCAC AGCAGGGAGT TCCAATCCAG   
  
  
+ AGAGTGTCCT ATTACTTCTC CGAGGCACTC TACCACCGCC TTTCTCTCTC CTCCGCCCCT CAACCTCTCC   
  
  
+ CAGAAACGAC GTCGTCTGAG GAGTACACTC TCTCTTACAA GGCCCTGAAC GACGCGTGCC CGTACCATAA   
  
  
+ ATTCGCTCAC TTGACCGCAA ATCAGGCGAT TCTAGAAGCC ACTGATTCAG CCAAGAAGAT CCACATCGTT   
  
  
+ GATTTTGGGA TAATCCAGGG AGTCCAATGG GCTGCTCTGC TACAAGCCCT GGCAACCCGG CCAGCCGGGA   
  
  
+ AGCCCGAATT GATTCGAATA TCCGGAATTC CTGCTCCGGG TCTGGGGCAA TCTCCGGCGG CATCGCTGCT   
  
  
+ CGCAACTGGG AATCGCCTCC GTGAGTTCGC GAAGGTTCTA GATCTCAAGT TCGAGTTTGA GCCGGTACTC   
  
  
+ ACTCCAATCA GAGAGCTAAA CGGGTCGAAC TTCCGGGTCG ACCCAGATGA GATACTAGCT GTCAATTTCA   
  
  
+ TGCTTCAATT GTACAACCTA TTAGACGAAA CCCTGGTCGC TGTCGACGCC GCCCTGAAGC TGGCCAAGTC   
  
  
+ ATTGAACCCC AAAATCGTCA CTTTGGGTGA GTACGAGGCG GGTCTGAATC GGGCAGGTTT CTTGACCCGG   
  
  
+ TTCAAGACGG CCCTCAAGTA CTATTTGGCA ATGTTTGAAT CGGTTGAGCC CAACTTGGCC CGGGACTCGC   
  
  
+ CCGAGAGGGC TCAGGTTGAG AGGCTGTTAT TGGGCCGGCG GATCATGGGC TTAGTTGGGC CGGAGTTACC   
  
  
+ GGGAACGAGA AGGGAAAGGA TGGAGTGTAA GGAACAATGG AAGGGTTTAA TGGAAAGTGC CGGTTTCGAG   
  
  
+ CCGTGCCAAA GCAGAAACTA CGTCAGTGAA AAAGACAAGT TTTACCACTC CAGATTGAAT TACAGAGAGT   
  
  
+ G  

- +Up\_Stream \_Len000ACTATT AATAAAAATA ACTAAATCCT TTCCTATAGT TTCATATAAA ACAAAAATCC   
  
  
- ATCAATTCTC TAAGACAACG GTTTTTTTTT TAGTTCTATA ATATATAAAA ATTAATACTG TCATTAATAA   
  
  
- AATAACTTTT AGGTTGCCAA CACTAATATA TTAGTTTATT AGATTTCCAA TTTTTATAGT TTAAATAATC   
  
  
- TTTTAAGCTT TTTCAGTGCA GTAGTGTTGT AACGAAAATA TATATCATAT CTAAGGAAGC TGAAGATTTG   
  
  
- ATTAGTGAAC TTTAATCTTT AAACTTTGAA AGATTATATT TACGTACCAG AAGAAAGGTT CTAATATTAG   
  
  
- GCGTTAACGA ATTTACATCC CATGTTTATA GTTTAAATCT TCACTGATAA CTTCGGTACT TTCTATAACA   
  
  
- CTAGCATAAC TCCCACCAAC ACTCGTTATC CTACTTTGTC CTTTTTTTTT GGGATTACAA AAGTTTAAAT   
  
  
- ACCCACCGAA ACCATCATCT ATTCACTTAT CACACGTTCC CTTACCGTGG TTATACAAAC CTACCTTGCT   
  
  
- ACTCTCATTC TGCAATCCTT TCGTTGTTCT CTTCCCTTTT CCGTCATCCC TCTCTTAATA CGCACACTTA   
  
  
- TTATAATATT GTCTGCGAAA GCATACTAAT AATTTATGTT CTGAAACTCG TATGTATATT TGAACATTGA   
  
  
- AAGAAATGGA TAACAGTTAA TTAAAAACTT AACTTAGTAG ACGCTGAATA CGTATGTTCT ACTATACTAC   
  
  
- GTATAACCCG GTTCGTAAAC TAGAGTACAG GGAATACCTA GATCTAGGGT TATCTGTTTG AACGTATTCA   
  
  
- GTTCCCTACA CAAAGATTAT GTTTCCGAAA CATGAGTTCG TTAATCAAAA CTTAAGTAAA AGTCATCAAA   
  
  
- TTAAAGGTTT AGAATATTAA AAATGTTACC GTATTTAAAA ATCCTCAAAA CTCATTTATT TAATATATGT   
  
  
- TGCTTCTACA GTAAATGCTT AATTATACTG CGTGTTTTTT TTTCTTTTTT TTAATTACCA TCGAAGATAT   
  
  
- GAACTGAGGT ACAACTGTTA AGGGAGAGAG TGTATTACCA ATTTTGAACG TTGTAGTAGG TCCTGGGTAT   
  
  
- ACTGTACCCA CCACCATCTG TCACACAACA CCATCGGAGT GAGGTACGAA CGGTTAAACA TAGTAACGCG   
  
  
- ATCAGAATAA GCAGGAATCT CTATTAACTG ATTCGAGATT ACGAGATAGC ACCGACGACG TTATATTTAC   
  
  
- TAAAAAACTG GAGTTTTACT TCTGTCGATC AACATTTAAT TCTTAGAAGC AGGGAAGTAA AAAACACATG   
  
  
- CACACATAGA CGCATACACA ACTCTCTGAT CGCCTGATAA CTAACTCAAA GTTAGTTTTT TCTACTGGTA   
  
  
- GAATTCATAA AAATTAATGC GTTTGAAGAA GTAAATTTTT TTACAATGAA AATAAACTTT CTTTAACACT   
  
  
- TATTATGTTC CTTATCAAGT TAATTATATA TTAGGTATTG TATAATGGAG GATATCCGTT TTTCTTCTAA   
  
  
- TAGCATAATT AAACCCTAGT AGGGTGGGAT TTAAATTTCT TTGGTTTTGT TTAATGATTC GTTATAGAAA   
  
  
- GACACACTTT TTTATCAGTC TTTGTTTCGC TAGGCTGGCC GGTTTAGCTC TATTTGTGTC GGTTTCTTGG   
  
  
- CTTCAACCTG GTTTCACTCG GCTCAACTTT CCGTAAGTAT ACCCCAAATT AGTATTGTGT TTGTATTAAT   
  
  
- CTAACTAACT GCAGAAATTA GTCGCAAAAA CAGAAACAAG CCACGAGGGG AGTGAGCGGG GAGGCGAGAA   
  
  
- ATTTCGAATG GAGTGAGGGG GGTAAGGGTG AGGAAAAGAG AGAGGAGAGA GCGAGAGTGT GCGAGGCAGA   
  
  
- CAGTCGGGGA ACAGTAGAAG GGGGCAGAAG GAGCGTTTTT AGAGGTCGTT TTAAATATCG GCAACAACAC   
  
  
- GGGTACCACT AGACTACACT CTTCTCTAAG TCGTAAATTC TAATAACCCA GGTCTACCGA ATATACACGC   
  
  
- GGCTGTCACC ATTAGAGTAC CGATAACGGG TTGTTCAGTA GTTCGTTTTT GTTGTCGTTC TTGTCGTCGT   
  
  
- TGTGGTTGTC GGAGTAGGGG TCAAGCAGCC CAGCTTATTA TTGGGGAAAA CGTGGTCGGG TACCGGGCCT   
  
  
- GGGCAGGGAA GTAGGCGGTA CAGACGGTCG GGAGGCGGGG GAGAACCCAA GAGGCGGCGG CGCAAAGGCC   
  
  
- TGGGCAAAGT CCACCCGGGC GGGACGGGCC TACCCCGACT TGGCCCAAAG GTCAAACGGG TAAACCTGGT   
  
  
- GGTGAGCTCA CCAAAGGCCA AACGACTAAA AACGCCACCC CCACCACCAC TCAAACTGAG CCTACTCACC   
  
  
- TACCTCTCAA ACTACCCACC GCCGCCGCCA CTGAGCGCCG CTGACTCTCA AGATTGGAGG TTAGGGTAAC   
  
  
- GCTGCGCACC GTTCCCAGGC CACTAAAGCC AGAGATACCA CGACTAGGCA AACGCTGCAC AAGCCTTCTG   
  
  
- GCAACGTCGG CGATAAGTGC GAGTCGGTGA CGGCGTTGTC GTCGGCAGCA CCCAGGGCGG AAGAGGCGGC   
  
  
- TTTCTGTGCT TCGTCAGCCG GGGTGGAGTT TTCGTTTCGC TGCGGCGACC TGGGAGCAAG AGTAGGCTCC   
  
  
- TCCTTGGTCT CGGCAGTACC GGCGGCGATT AACGCCGCAA CAACCTCACG CGGTTTGAGC GGCTCAGGCT   
  
  
- GGGCCTTGTG CGACAGTTTA GCCACTAAGC TGAGTCTCTG AGTCAGAGTG TCGTCCCTCA AGGTTAGGTC   
  
  
- TCTCACAGGA TAATGAAGAG GCTCCGTGAG ATGGTGGCGG AAAGAGAGAG GAGGCGGGGA GTTGGAGAGG   
  
  
- GTCTTTGCTG CAGCAGACTC CTCATGTGAG AGAGAATGTT CCGGGACTTG CTGCGCACGG GCATGGTATT   
  
  
- TAAGCGAGTG AACTGGCGTT TAGTCCGCTA AGATCTTCGG TGACTAAGTC GGTTCTTCTA GGTGTAGCAA   
  
  
- CTAAAACCCT ATTAGGTCCC TCAGGTTACC CGACGAGACG ATGTTCGGGA CCGTTGGGCC GGTCGGCCCT   
  
  
- TCGGGCTTAA CTAAGCTTAT AGGCCTTAAG GACGAGGCCC AGACCCCGTT AGAGGCCGCC GTAGCGACGA   
  
  
- GCGTTGACCC TTAGCGGAGG CACTCAAGCG CTTCCAAGAT CTAGAGTTCA AGCTCAAACT CGGCCATGAG   
  
  
- TGAGGTTAGT CTCTCGATTT GCCCAGCTTG AAGGCCCAGC TGGGTCTACT CTATGATCGA CAGTTAAAGT   
  
  
- ACGAAGTTAA CATGTTGGAT AATCTGCTTT GGGACCAGCG ACAGCTGCGG CGGGACTTCG ACCGGTTCAG   
  
  
- TAACTTGGGG TTTTAGCAGT GAAACCCACT CATGCTCCGC CCAGACTTAG CCCGTCCAAA GAACTGGGCC   
  
  
- AAGTTCTGCC GGGAGTTCAT GATAAACCGT TACAAACTTA GCCAACTCGG GTTGAACCGG GCCCTGAGCG   
  
  
- GGCTCTCCCG AGTCCAACTC TCCGACAATA ACCCGGCCGC CTAGTACCCG AATCAACCCG GCCTCAATGG   
  
  
- CCCTTGCTCT TCCCTTTCCT ACCTCACATT CCTTGTTACC TTCCCAAATT ACCTTTCACG GCCAAAGCTC   
  
  
- GGCACGGTTT CGTCTTTGAT GCAGTCACTT TTTCTGTTCA AAATGGTGAG GTCTAACTTA ATGTCTCTCA   
  
  
- C

+     GT1-motif

| Site Name | Organism | Position | Strand | Matrix score. | sequence | function |
| --- | --- | --- | --- | --- | --- | --- |
| GT1-motif | Arabidopsis thaliana | 1092 | + | 6 | GGTTAA | light responsive element |
| GT1-motif | Arabidopsis thaliana | 191 | + | 6 | GGTTAA | light responsive element |

>HU06G00029.1   
+ +Up\_Stream \_Len000TGATAA TTATTTTTAT TGATTTAGGA AAGGATATCA AAGTATATTT TGTTTTTAGG   
  
  
+ TAGTTAAGAG ATTCTGTTGC CAAAAAAAAA ATCAAGATAT TATATATTTT TAATTATGAC AGTAATTATT   
  
  
+ TTATTGAAAA TCCAACGGTT GTGATTATAT AATCAAATAA TCTAAAGGTT AAAAATATCA AATTTATTAG   
  
  
+ AAAATTCGAA AAAGTCACGT CATCACAACA TTGCTTTTAT ATATAGTATA GATTCCTTCG ACTTCTAAAC   
  
  
+ TAATCACTTG AAATTAGAAA TTTGAAACTT TCTAATATAA ATGCATGGTC TTCTTTCCAA GATTATAATC   
  
  
+ CGCAATTGCT TAAATGTAGG GTACAAATAT CAAATTTAGA AGTGACTATT GAAGCCATGA AAGATATTGT   
  
  
+ GATCGTATTG AGGGTGGTTG TGAGCAATAG GATGAAACAG GAAAAAAAAA CCCTAATGTT TTCAAATTTA   
  
  
+ TGGGTGGCTT TGGTAGTAGA TAAGTGAATA GTGTGCAAGG GAATGGCACC AATATGTTTG GATGGAACGA   
  
  
+ TGAGAGTAAG ACGTTAGGAA AGCAACAAGA GAAGGGAAAA GGCAGTAGGG AGAGAATTAT GCGTGTGAAT   
  
  
+ AATATTATAA CAGACGCTTT CGTATGATTA TTAAATACAA GACTTTGAGC ATACATATAA ACTTGTAACT   
  
  
+ TTCTTTACCT ATTGTCAATT AATTTTTGAA TTGAATCATC TGCGACTTAT GCATACAAGA TGATATGATG   
  
  
+ CATATTGGGC CAAGCATTTG ATCTCATGTC CCTTATGGAT CTAGATCCCA ATAGACAAAC TTGCATAAGT   
  
  
+ CAAGGGATGT GTTTCTAATA CAAAGGCTTT GTACTCAAGC AATTAGTTTT GAATTCATTT TCAGTAGTTT   
  
  
+ AATTTCCAAA TCTTATAATT TTTACAATGG CATAAATTTT TAGGAGTTTT GAGTAAATAA ATTATATACA   
  
  
+ ACGAAGATGT CATTTACGAA TTAATATGAC GCACAAAAAA AAAGAAAAAA AATTAATGGT AGCTTCTATA   
  
  
+ CTTGACTCCA TGTTGACAAT TCCCTCTCTC ACATAATGGT TAAAACTTGC AACATCATCC AGGACCCATA   
  
  
+ TGACATGGGT GGTGGTAGAC AGTGTGTTGT GGTAGCCTCA CTCCATGCTT GCCAATTTGT ATCATTGCGC   
  
  
+ TAGTCTTATT CGTCCTTAGA GATAATTGAC TAAGCTCTAA TGCTCTATCG TGGCTGCTGC AATATAAATG   
  
  
+ ATTTTTTGAC CTCAAAATGA AGACAGCTAG TTGTAAATTA AGAATCTTCG TCCCTTCATT TTTTGTGTAC   
  
  
+ GTGTGTATCT GCGTATGTGT TGAGAGACTA GCGGACTATT GATTGAGTTT CAATCAAAAA AGATGACCAT   
  
  
+ CTTAAGTATT TTTAATTACG CAAACTTCTT CATTTAAAAA AATGTTACTT TTATTTGAAA GAAATTGTGA   
  
  
+ ATAATACAAG GAATAGTTCA ATTAATATAT AATCCATAAC ATATTACCTC CTATAGGCAA AAAGAAGATT   
  
  
+ ATCGTATTAA TTTGGGATCA TCCCACCCTA AATTTAAAGA AACCAAAACA AATTACTAAG CAATATCTTT   
  
  
+ CTGTGTGAAA AAATAGTCAG AAACAAAGCG ATCCGACCGG CCAAATCGAG ATAAACACAG CCAAAGAACC   
  
  
+ GAAGTTGGAC CAAAGTGAGC CGAGTTGAAA GGCATTCATA TGGGGTTTAA TCATAACACA AACATAATTA   
  
  
+ GATTGATTGA CGTCTTTAAT CAGCGTTTTT GTCTTTGTTC GGTGCTCCCC TCACTCGCCC CTCCGCTCTT   
  
  
+ TAAAGCTTAC CTCACTCCCC CCATTCCCAC TCCTTTTCTC TCTCCTCTCT CGCTCTCACA CGCTCCGTCT   
  
  
+ GTCAGCCCCT TGTCATCTTC CCCCGTCTTC CTCGCAAAAA TCTCCAGCAA AATTTATAGC CGTTGTTGTG   
  
  
+ CCCATGGTGA TCTGATGTGA GAAGAGATTC AGCATTTAAG ATTATTGGGT CCAGATGGCT TATATGTGCG   
  
  
+ CCGACAGTGG TAATCTCATG GCTATTGCCC AACAAGTCAT CAAGCAAAAA CAACAGCAAG AACAGCAGCA   
  
  
+ ACACCAACAG CCTCATCCCC AGTTCGTCGG GTCGAATAAT AACCCCTTTT GCACCAGCCC ATGGCCCGGA   
  
  
+ CCCGTCCCTT CATCCGCCAT GTCTGCCAGC CCTCCGCCCC CTCTTGGGTT CTCCGCCGCC GCGTTTCCGG   
  
  
+ ACCCGTTTCA GGTGGGCCCG CCCTGCCCGG ATGGGGCTGA ACCGGGTTTC CAGTTTGCCC ATTTGGACCA   
  
  
+ CCACTCGAGT GGTTTCCGGT TTGCTGATTT TTGCGGTGGG GGTGGTGGTG AGTTTGACTC GGATGAGTGG   
  
  
+ ATGGAGAGTT TGATGGGTGG CGGCGGCGGT GACTCGCGGC GACTGAGAGT TCTAACCTCC AATCCCATTG   
  
  
+ CGACGCGTGG CAAGGGTCCG GTGATTTCGG TCTCTATGGT GCTGATCCGT TTGCGACGTG TTCGGAAGAC   
  
  
+ CGTTGCAGCC GCTATTCACG CTCAGCCACT GCCGCAACAG CAGCCGTCGT GGGTCCCGCC TTCTCCGCCG   
  
  
+ AAAGACACGA AGCAGTCGGC CCCACCTCAA AAGCAAAGCG ACGCCGCTGG ACCCTCGTTC TCATCCGAGG   
  
  
+ AGGAACCAGA GCCGTCATGG CCGCCGCTAA TTGCGGCGTT GTTGGAGTGC GCCAAACTCG CCGAGTCCGA   
  
  
+ CCCGGAACAC GCTGTCAAAT CGGTGATTCG ACTCAGAGAC TCAGTCTCAC AGCAGGGAGT TCCAATCCAG   
  
  
+ AGAGTGTCCT ATTACTTCTC CGAGGCACTC TACCACCGCC TTTCTCTCTC CTCCGCCCCT CAACCTCTCC   
  
  
+ CAGAAACGAC GTCGTCTGAG GAGTACACTC TCTCTTACAA GGCCCTGAAC GACGCGTGCC CGTACCATAA   
  
  
+ ATTCGCTCAC TTGACCGCAA ATCAGGCGAT TCTAGAAGCC ACTGATTCAG CCAAGAAGAT CCACATCGTT   
  
  
+ GATTTTGGGA TAATCCAGGG AGTCCAATGG GCTGCTCTGC TACAAGCCCT GGCAACCCGG CCAGCCGGGA   
  
  
+ AGCCCGAATT GATTCGAATA TCCGGAATTC CTGCTCCGGG TCTGGGGCAA TCTCCGGCGG CATCGCTGCT   
  
  
+ CGCAACTGGG AATCGCCTCC GTGAGTTCGC GAAGGTTCTA GATCTCAAGT TCGAGTTTGA GCCGGTACTC   
  
  
+ ACTCCAATCA GAGAGCTAAA CGGGTCGAAC TTCCGGGTCG ACCCAGATGA GATACTAGCT GTCAATTTCA   
  
  
+ TGCTTCAATT GTACAACCTA TTAGACGAAA CCCTGGTCGC TGTCGACGCC GCCCTGAAGC TGGCCAAGTC   
  
  
+ ATTGAACCCC AAAATCGTCA CTTTGGGTGA GTACGAGGCG GGTCTGAATC GGGCAGGTTT CTTGACCCGG   
  
  
+ TTCAAGACGG CCCTCAAGTA CTATTTGGCA ATGTTTGAAT CGGTTGAGCC CAACTTGGCC CGGGACTCGC   
  
  
+ CCGAGAGGGC TCAGGTTGAG AGGCTGTTAT TGGGCCGGCG GATCATGGGC TTAGTTGGGC CGGAGTTACC   
  
  
+ GGGAACGAGA AGGGAAAGGA TGGAGTGTAA GGAACAATGG AAGGGTTTAA TGGAAAGTGC CGGTTTCGAG   
  
  
+ CCGTGCCAAA GCAGAAACTA CGTCAGTGAA AAAGACAAGT TTTACCACTC CAGATTGAAT TACAGAGAGT   
  
  
+ G  

- +Up\_Stream \_Len000ACTATT AATAAAAATA ACTAAATCCT TTCCTATAGT TTCATATAAA ACAAAAATCC   
  
  
- ATCAATTCTC TAAGACAACG GTTTTTTTTT TAGTTCTATA ATATATAAAA ATTAATACTG TCATTAATAA   
  
  
- AATAACTTTT AGGTTGCCAA CACTAATATA TTAGTTTATT AGATTTCCAA TTTTTATAGT TTAAATAATC   
  
  
- TTTTAAGCTT TTTCAGTGCA GTAGTGTTGT AACGAAAATA TATATCATAT CTAAGGAAGC TGAAGATTTG   
  
  
- ATTAGTGAAC TTTAATCTTT AAACTTTGAA AGATTATATT TACGTACCAG AAGAAAGGTT CTAATATTAG   
  
  
- GCGTTAACGA ATTTACATCC CATGTTTATA GTTTAAATCT TCACTGATAA CTTCGGTACT TTCTATAACA   
  
  
- CTAGCATAAC TCCCACCAAC ACTCGTTATC CTACTTTGTC CTTTTTTTTT GGGATTACAA AAGTTTAAAT   
  
  
- ACCCACCGAA ACCATCATCT ATTCACTTAT CACACGTTCC CTTACCGTGG TTATACAAAC CTACCTTGCT   
  
  
- ACTCTCATTC TGCAATCCTT TCGTTGTTCT CTTCCCTTTT CCGTCATCCC TCTCTTAATA CGCACACTTA   
  
  
- TTATAATATT GTCTGCGAAA GCATACTAAT AATTTATGTT CTGAAACTCG TATGTATATT TGAACATTGA   
  
  
- AAGAAATGGA TAACAGTTAA TTAAAAACTT AACTTAGTAG ACGCTGAATA CGTATGTTCT ACTATACTAC   
  
  
- GTATAACCCG GTTCGTAAAC TAGAGTACAG GGAATACCTA GATCTAGGGT TATCTGTTTG AACGTATTCA   
  
  
- GTTCCCTACA CAAAGATTAT GTTTCCGAAA CATGAGTTCG TTAATCAAAA CTTAAGTAAA AGTCATCAAA   
  
  
- TTAAAGGTTT AGAATATTAA AAATGTTACC GTATTTAAAA ATCCTCAAAA CTCATTTATT TAATATATGT   
  
  
- TGCTTCTACA GTAAATGCTT AATTATACTG CGTGTTTTTT TTTCTTTTTT TTAATTACCA TCGAAGATAT   
  
  
- GAACTGAGGT ACAACTGTTA AGGGAGAGAG TGTATTACCA ATTTTGAACG TTGTAGTAGG TCCTGGGTAT   
  
  
- ACTGTACCCA CCACCATCTG TCACACAACA CCATCGGAGT GAGGTACGAA CGGTTAAACA TAGTAACGCG   
  
  
- ATCAGAATAA GCAGGAATCT CTATTAACTG ATTCGAGATT ACGAGATAGC ACCGACGACG TTATATTTAC   
  
  
- TAAAAAACTG GAGTTTTACT TCTGTCGATC AACATTTAAT TCTTAGAAGC AGGGAAGTAA AAAACACATG   
  
  
- CACACATAGA CGCATACACA ACTCTCTGAT CGCCTGATAA CTAACTCAAA GTTAGTTTTT TCTACTGGTA   
  
  
- GAATTCATAA AAATTAATGC GTTTGAAGAA GTAAATTTTT TTACAATGAA AATAAACTTT CTTTAACACT   
  
  
- TATTATGTTC CTTATCAAGT TAATTATATA TTAGGTATTG TATAATGGAG GATATCCGTT TTTCTTCTAA   
  
  
- TAGCATAATT AAACCCTAGT AGGGTGGGAT TTAAATTTCT TTGGTTTTGT TTAATGATTC GTTATAGAAA   
  
  
- GACACACTTT TTTATCAGTC TTTGTTTCGC TAGGCTGGCC GGTTTAGCTC TATTTGTGTC GGTTTCTTGG   
  
  
- CTTCAACCTG GTTTCACTCG GCTCAACTTT CCGTAAGTAT ACCCCAAATT AGTATTGTGT TTGTATTAAT   
  
  
- CTAACTAACT GCAGAAATTA GTCGCAAAAA CAGAAACAAG CCACGAGGGG AGTGAGCGGG GAGGCGAGAA   
  
  
- ATTTCGAATG GAGTGAGGGG GGTAAGGGTG AGGAAAAGAG AGAGGAGAGA GCGAGAGTGT GCGAGGCAGA   
  
  
- CAGTCGGGGA ACAGTAGAAG GGGGCAGAAG GAGCGTTTTT AGAGGTCGTT TTAAATATCG GCAACAACAC   
  
  
- GGGTACCACT AGACTACACT CTTCTCTAAG TCGTAAATTC TAATAACCCA GGTCTACCGA ATATACACGC   
  
  
- GGCTGTCACC ATTAGAGTAC CGATAACGGG TTGTTCAGTA GTTCGTTTTT GTTGTCGTTC TTGTCGTCGT   
  
  
- TGTGGTTGTC GGAGTAGGGG TCAAGCAGCC CAGCTTATTA TTGGGGAAAA CGTGGTCGGG TACCGGGCCT   
  
  
- GGGCAGGGAA GTAGGCGGTA CAGACGGTCG GGAGGCGGGG GAGAACCCAA GAGGCGGCGG CGCAAAGGCC   
  
  
- TGGGCAAAGT CCACCCGGGC GGGACGGGCC TACCCCGACT TGGCCCAAAG GTCAAACGGG TAAACCTGGT   
  
  
- GGTGAGCTCA CCAAAGGCCA AACGACTAAA AACGCCACCC CCACCACCAC TCAAACTGAG CCTACTCACC   
  
  
- TACCTCTCAA ACTACCCACC GCCGCCGCCA CTGAGCGCCG CTGACTCTCA AGATTGGAGG TTAGGGTAAC   
  
  
- GCTGCGCACC GTTCCCAGGC CACTAAAGCC AGAGATACCA CGACTAGGCA AACGCTGCAC AAGCCTTCTG   
  
  
- GCAACGTCGG CGATAAGTGC GAGTCGGTGA CGGCGTTGTC GTCGGCAGCA CCCAGGGCGG AAGAGGCGGC   
  
  
- TTTCTGTGCT TCGTCAGCCG GGGTGGAGTT TTCGTTTCGC TGCGGCGACC TGGGAGCAAG AGTAGGCTCC   
  
  
- TCCTTGGTCT CGGCAGTACC GGCGGCGATT AACGCCGCAA CAACCTCACG CGGTTTGAGC GGCTCAGGCT   
  
  
- GGGCCTTGTG CGACAGTTTA GCCACTAAGC TGAGTCTCTG AGTCAGAGTG TCGTCCCTCA AGGTTAGGTC   
  
  
- TCTCACAGGA TAATGAAGAG GCTCCGTGAG ATGGTGGCGG AAAGAGAGAG GAGGCGGGGA GTTGGAGAGG   
  
  
- GTCTTTGCTG CAGCAGACTC CTCATGTGAG AGAGAATGTT CCGGGACTTG CTGCGCACGG GCATGGTATT   
  
  
- TAAGCGAGTG AACTGGCGTT TAGTCCGCTA AGATCTTCGG TGACTAAGTC GGTTCTTCTA GGTGTAGCAA   
  
  
- CTAAAACCCT ATTAGGTCCC TCAGGTTACC CGACGAGACG ATGTTCGGGA CCGTTGGGCC GGTCGGCCCT   
  
  
- TCGGGCTTAA CTAAGCTTAT AGGCCTTAAG GACGAGGCCC AGACCCCGTT AGAGGCCGCC GTAGCGACGA   
  
  
- GCGTTGACCC TTAGCGGAGG CACTCAAGCG CTTCCAAGAT CTAGAGTTCA AGCTCAAACT CGGCCATGAG   
  
  
- TGAGGTTAGT CTCTCGATTT GCCCAGCTTG AAGGCCCAGC TGGGTCTACT CTATGATCGA CAGTTAAAGT   
  
  
- ACGAAGTTAA CATGTTGGAT AATCTGCTTT GGGACCAGCG ACAGCTGCGG CGGGACTTCG ACCGGTTCAG   
  
  
- TAACTTGGGG TTTTAGCAGT GAAACCCACT CATGCTCCGC CCAGACTTAG CCCGTCCAAA GAACTGGGCC   
  
  
- AAGTTCTGCC GGGAGTTCAT GATAAACCGT TACAAACTTA GCCAACTCGG GTTGAACCGG GCCCTGAGCG   
  
  
- GGCTCTCCCG AGTCCAACTC TCCGACAATA ACCCGGCCGC CTAGTACCCG AATCAACCCG GCCTCAATGG   
  
  
- CCCTTGCTCT TCCCTTTCCT ACCTCACATT CCTTGTTACC TTCCCAAATT ACCTTTCACG GCCAAAGCTC   
  
  
- GGCACGGTTT CGTCTTTGAT GCAGTCACTT TTTCTGTTCA AAATGGTGAG GTCTAACTTA ATGTCTCTCA   
  
  
- C

+     GTGGC-motif

| Site Name | Organism | Position | Strand | Matrix score. | sequence | function |
| --- | --- | --- | --- | --- | --- | --- |
| GTGGC-motif | Spinacia oleracea | 84 | + | 10 | GATTCTGTGGC | part of a light responsive element |

>HU06G00029.1   
+ +Up\_Stream \_Len000TGATAA TTATTTTTAT TGATTTAGGA AAGGATATCA AAGTATATTT TGTTTTTAGG   
  
  
+ TAGTTAAGAG ATTCTGTTGC CAAAAAAAAA ATCAAGATAT TATATATTTT TAATTATGAC AGTAATTATT   
  
  
+ TTATTGAAAA TCCAACGGTT GTGATTATAT AATCAAATAA TCTAAAGGTT AAAAATATCA AATTTATTAG   
  
  
+ AAAATTCGAA AAAGTCACGT CATCACAACA TTGCTTTTAT ATATAGTATA GATTCCTTCG ACTTCTAAAC   
  
  
+ TAATCACTTG AAATTAGAAA TTTGAAACTT TCTAATATAA ATGCATGGTC TTCTTTCCAA GATTATAATC   
  
  
+ CGCAATTGCT TAAATGTAGG GTACAAATAT CAAATTTAGA AGTGACTATT GAAGCCATGA AAGATATTGT   
  
  
+ GATCGTATTG AGGGTGGTTG TGAGCAATAG GATGAAACAG GAAAAAAAAA CCCTAATGTT TTCAAATTTA   
  
  
+ TGGGTGGCTT TGGTAGTAGA TAAGTGAATA GTGTGCAAGG GAATGGCACC AATATGTTTG GATGGAACGA   
  
  
+ TGAGAGTAAG ACGTTAGGAA AGCAACAAGA GAAGGGAAAA GGCAGTAGGG AGAGAATTAT GCGTGTGAAT   
  
  
+ AATATTATAA CAGACGCTTT CGTATGATTA TTAAATACAA GACTTTGAGC ATACATATAA ACTTGTAACT   
  
  
+ TTCTTTACCT ATTGTCAATT AATTTTTGAA TTGAATCATC TGCGACTTAT GCATACAAGA TGATATGATG   
  
  
+ CATATTGGGC CAAGCATTTG ATCTCATGTC CCTTATGGAT CTAGATCCCA ATAGACAAAC TTGCATAAGT   
  
  
+ CAAGGGATGT GTTTCTAATA CAAAGGCTTT GTACTCAAGC AATTAGTTTT GAATTCATTT TCAGTAGTTT   
  
  
+ AATTTCCAAA TCTTATAATT TTTACAATGG CATAAATTTT TAGGAGTTTT GAGTAAATAA ATTATATACA   
  
  
+ ACGAAGATGT CATTTACGAA TTAATATGAC GCACAAAAAA AAAGAAAAAA AATTAATGGT AGCTTCTATA   
  
  
+ CTTGACTCCA TGTTGACAAT TCCCTCTCTC ACATAATGGT TAAAACTTGC AACATCATCC AGGACCCATA   
  
  
+ TGACATGGGT GGTGGTAGAC AGTGTGTTGT GGTAGCCTCA CTCCATGCTT GCCAATTTGT ATCATTGCGC   
  
  
+ TAGTCTTATT CGTCCTTAGA GATAATTGAC TAAGCTCTAA TGCTCTATCG TGGCTGCTGC AATATAAATG   
  
  
+ ATTTTTTGAC CTCAAAATGA AGACAGCTAG TTGTAAATTA AGAATCTTCG TCCCTTCATT TTTTGTGTAC   
  
  
+ GTGTGTATCT GCGTATGTGT TGAGAGACTA GCGGACTATT GATTGAGTTT CAATCAAAAA AGATGACCAT   
  
  
+ CTTAAGTATT TTTAATTACG CAAACTTCTT CATTTAAAAA AATGTTACTT TTATTTGAAA GAAATTGTGA   
  
  
+ ATAATACAAG GAATAGTTCA ATTAATATAT AATCCATAAC ATATTACCTC CTATAGGCAA AAAGAAGATT   
  
  
+ ATCGTATTAA TTTGGGATCA TCCCACCCTA AATTTAAAGA AACCAAAACA AATTACTAAG CAATATCTTT   
  
  
+ CTGTGTGAAA AAATAGTCAG AAACAAAGCG ATCCGACCGG CCAAATCGAG ATAAACACAG CCAAAGAACC   
  
  
+ GAAGTTGGAC CAAAGTGAGC CGAGTTGAAA GGCATTCATA TGGGGTTTAA TCATAACACA AACATAATTA   
  
  
+ GATTGATTGA CGTCTTTAAT CAGCGTTTTT GTCTTTGTTC GGTGCTCCCC TCACTCGCCC CTCCGCTCTT   
  
  
+ TAAAGCTTAC CTCACTCCCC CCATTCCCAC TCCTTTTCTC TCTCCTCTCT CGCTCTCACA CGCTCCGTCT   
  
  
+ GTCAGCCCCT TGTCATCTTC CCCCGTCTTC CTCGCAAAAA TCTCCAGCAA AATTTATAGC CGTTGTTGTG   
  
  
+ CCCATGGTGA TCTGATGTGA GAAGAGATTC AGCATTTAAG ATTATTGGGT CCAGATGGCT TATATGTGCG   
  
  
+ CCGACAGTGG TAATCTCATG GCTATTGCCC AACAAGTCAT CAAGCAAAAA CAACAGCAAG AACAGCAGCA   
  
  
+ ACACCAACAG CCTCATCCCC AGTTCGTCGG GTCGAATAAT AACCCCTTTT GCACCAGCCC ATGGCCCGGA   
  
  
+ CCCGTCCCTT CATCCGCCAT GTCTGCCAGC CCTCCGCCCC CTCTTGGGTT CTCCGCCGCC GCGTTTCCGG   
  
  
+ ACCCGTTTCA GGTGGGCCCG CCCTGCCCGG ATGGGGCTGA ACCGGGTTTC CAGTTTGCCC ATTTGGACCA   
  
  
+ CCACTCGAGT GGTTTCCGGT TTGCTGATTT TTGCGGTGGG GGTGGTGGTG AGTTTGACTC GGATGAGTGG   
  
  
+ ATGGAGAGTT TGATGGGTGG CGGCGGCGGT GACTCGCGGC GACTGAGAGT TCTAACCTCC AATCCCATTG   
  
  
+ CGACGCGTGG CAAGGGTCCG GTGATTTCGG TCTCTATGGT GCTGATCCGT TTGCGACGTG TTCGGAAGAC   
  
  
+ CGTTGCAGCC GCTATTCACG CTCAGCCACT GCCGCAACAG CAGCCGTCGT GGGTCCCGCC TTCTCCGCCG   
  
  
+ AAAGACACGA AGCAGTCGGC CCCACCTCAA AAGCAAAGCG ACGCCGCTGG ACCCTCGTTC TCATCCGAGG   
  
  
+ AGGAACCAGA GCCGTCATGG CCGCCGCTAA TTGCGGCGTT GTTGGAGTGC GCCAAACTCG CCGAGTCCGA   
  
  
+ CCCGGAACAC GCTGTCAAAT CGGTGATTCG ACTCAGAGAC TCAGTCTCAC AGCAGGGAGT TCCAATCCAG   
  
  
+ AGAGTGTCCT ATTACTTCTC CGAGGCACTC TACCACCGCC TTTCTCTCTC CTCCGCCCCT CAACCTCTCC   
  
  
+ CAGAAACGAC GTCGTCTGAG GAGTACACTC TCTCTTACAA GGCCCTGAAC GACGCGTGCC CGTACCATAA   
  
  
+ ATTCGCTCAC TTGACCGCAA ATCAGGCGAT TCTAGAAGCC ACTGATTCAG CCAAGAAGAT CCACATCGTT   
  
  
+ GATTTTGGGA TAATCCAGGG AGTCCAATGG GCTGCTCTGC TACAAGCCCT GGCAACCCGG CCAGCCGGGA   
  
  
+ AGCCCGAATT GATTCGAATA TCCGGAATTC CTGCTCCGGG TCTGGGGCAA TCTCCGGCGG CATCGCTGCT   
  
  
+ CGCAACTGGG AATCGCCTCC GTGAGTTCGC GAAGGTTCTA GATCTCAAGT TCGAGTTTGA GCCGGTACTC   
  
  
+ ACTCCAATCA GAGAGCTAAA CGGGTCGAAC TTCCGGGTCG ACCCAGATGA GATACTAGCT GTCAATTTCA   
  
  
+ TGCTTCAATT GTACAACCTA TTAGACGAAA CCCTGGTCGC TGTCGACGCC GCCCTGAAGC TGGCCAAGTC   
  
  
+ ATTGAACCCC AAAATCGTCA CTTTGGGTGA GTACGAGGCG GGTCTGAATC GGGCAGGTTT CTTGACCCGG   
  
  
+ TTCAAGACGG CCCTCAAGTA CTATTTGGCA ATGTTTGAAT CGGTTGAGCC CAACTTGGCC CGGGACTCGC   
  
  
+ CCGAGAGGGC TCAGGTTGAG AGGCTGTTAT TGGGCCGGCG GATCATGGGC TTAGTTGGGC CGGAGTTACC   
  
  
+ GGGAACGAGA AGGGAAAGGA TGGAGTGTAA GGAACAATGG AAGGGTTTAA TGGAAAGTGC CGGTTTCGAG   
  
  
+ CCGTGCCAAA GCAGAAACTA CGTCAGTGAA AAAGACAAGT TTTACCACTC CAGATTGAAT TACAGAGAGT   
  
  
+ G  

- +Up\_Stream \_Len000ACTATT AATAAAAATA ACTAAATCCT TTCCTATAGT TTCATATAAA ACAAAAATCC   
  
  
- ATCAATTCTC TAAGACAACG GTTTTTTTTT TAGTTCTATA ATATATAAAA ATTAATACTG TCATTAATAA   
  
  
- AATAACTTTT AGGTTGCCAA CACTAATATA TTAGTTTATT AGATTTCCAA TTTTTATAGT TTAAATAATC   
  
  
- TTTTAAGCTT TTTCAGTGCA GTAGTGTTGT AACGAAAATA TATATCATAT CTAAGGAAGC TGAAGATTTG   
  
  
- ATTAGTGAAC TTTAATCTTT AAACTTTGAA AGATTATATT TACGTACCAG AAGAAAGGTT CTAATATTAG   
  
  
- GCGTTAACGA ATTTACATCC CATGTTTATA GTTTAAATCT TCACTGATAA CTTCGGTACT TTCTATAACA   
  
  
- CTAGCATAAC TCCCACCAAC ACTCGTTATC CTACTTTGTC CTTTTTTTTT GGGATTACAA AAGTTTAAAT   
  
  
- ACCCACCGAA ACCATCATCT ATTCACTTAT CACACGTTCC CTTACCGTGG TTATACAAAC CTACCTTGCT   
  
  
- ACTCTCATTC TGCAATCCTT TCGTTGTTCT CTTCCCTTTT CCGTCATCCC TCTCTTAATA CGCACACTTA   
  
  
- TTATAATATT GTCTGCGAAA GCATACTAAT AATTTATGTT CTGAAACTCG TATGTATATT TGAACATTGA   
  
  
- AAGAAATGGA TAACAGTTAA TTAAAAACTT AACTTAGTAG ACGCTGAATA CGTATGTTCT ACTATACTAC   
  
  
- GTATAACCCG GTTCGTAAAC TAGAGTACAG GGAATACCTA GATCTAGGGT TATCTGTTTG AACGTATTCA   
  
  
- GTTCCCTACA CAAAGATTAT GTTTCCGAAA CATGAGTTCG TTAATCAAAA CTTAAGTAAA AGTCATCAAA   
  
  
- TTAAAGGTTT AGAATATTAA AAATGTTACC GTATTTAAAA ATCCTCAAAA CTCATTTATT TAATATATGT   
  
  
- TGCTTCTACA GTAAATGCTT AATTATACTG CGTGTTTTTT TTTCTTTTTT TTAATTACCA TCGAAGATAT   
  
  
- GAACTGAGGT ACAACTGTTA AGGGAGAGAG TGTATTACCA ATTTTGAACG TTGTAGTAGG TCCTGGGTAT   
  
  
- ACTGTACCCA CCACCATCTG TCACACAACA CCATCGGAGT GAGGTACGAA CGGTTAAACA TAGTAACGCG   
  
  
- ATCAGAATAA GCAGGAATCT CTATTAACTG ATTCGAGATT ACGAGATAGC ACCGACGACG TTATATTTAC   
  
  
- TAAAAAACTG GAGTTTTACT TCTGTCGATC AACATTTAAT TCTTAGAAGC AGGGAAGTAA AAAACACATG   
  
  
- CACACATAGA CGCATACACA ACTCTCTGAT CGCCTGATAA CTAACTCAAA GTTAGTTTTT TCTACTGGTA   
  
  
- GAATTCATAA AAATTAATGC GTTTGAAGAA GTAAATTTTT TTACAATGAA AATAAACTTT CTTTAACACT   
  
  
- TATTATGTTC CTTATCAAGT TAATTATATA TTAGGTATTG TATAATGGAG GATATCCGTT TTTCTTCTAA   
  
  
- TAGCATAATT AAACCCTAGT AGGGTGGGAT TTAAATTTCT TTGGTTTTGT TTAATGATTC GTTATAGAAA   
  
  
- GACACACTTT TTTATCAGTC TTTGTTTCGC TAGGCTGGCC GGTTTAGCTC TATTTGTGTC GGTTTCTTGG   
  
  
- CTTCAACCTG GTTTCACTCG GCTCAACTTT CCGTAAGTAT ACCCCAAATT AGTATTGTGT TTGTATTAAT   
  
  
- CTAACTAACT GCAGAAATTA GTCGCAAAAA CAGAAACAAG CCACGAGGGG AGTGAGCGGG GAGGCGAGAA   
  
  
- ATTTCGAATG GAGTGAGGGG GGTAAGGGTG AGGAAAAGAG AGAGGAGAGA GCGAGAGTGT GCGAGGCAGA   
  
  
- CAGTCGGGGA ACAGTAGAAG GGGGCAGAAG GAGCGTTTTT AGAGGTCGTT TTAAATATCG GCAACAACAC   
  
  
- GGGTACCACT AGACTACACT CTTCTCTAAG TCGTAAATTC TAATAACCCA GGTCTACCGA ATATACACGC   
  
  
- GGCTGTCACC ATTAGAGTAC CGATAACGGG TTGTTCAGTA GTTCGTTTTT GTTGTCGTTC TTGTCGTCGT   
  
  
- TGTGGTTGTC GGAGTAGGGG TCAAGCAGCC CAGCTTATTA TTGGGGAAAA CGTGGTCGGG TACCGGGCCT   
  
  
- GGGCAGGGAA GTAGGCGGTA CAGACGGTCG GGAGGCGGGG GAGAACCCAA GAGGCGGCGG CGCAAAGGCC   
  
  
- TGGGCAAAGT CCACCCGGGC GGGACGGGCC TACCCCGACT TGGCCCAAAG GTCAAACGGG TAAACCTGGT   
  
  
- GGTGAGCTCA CCAAAGGCCA AACGACTAAA AACGCCACCC CCACCACCAC TCAAACTGAG CCTACTCACC   
  
  
- TACCTCTCAA ACTACCCACC GCCGCCGCCA CTGAGCGCCG CTGACTCTCA AGATTGGAGG TTAGGGTAAC   
  
  
- GCTGCGCACC GTTCCCAGGC CACTAAAGCC AGAGATACCA CGACTAGGCA AACGCTGCAC AAGCCTTCTG   
  
  
- GCAACGTCGG CGATAAGTGC GAGTCGGTGA CGGCGTTGTC GTCGGCAGCA CCCAGGGCGG AAGAGGCGGC   
  
  
- TTTCTGTGCT TCGTCAGCCG GGGTGGAGTT TTCGTTTCGC TGCGGCGACC TGGGAGCAAG AGTAGGCTCC   
  
  
- TCCTTGGTCT CGGCAGTACC GGCGGCGATT AACGCCGCAA CAACCTCACG CGGTTTGAGC GGCTCAGGCT   
  
  
- GGGCCTTGTG CGACAGTTTA GCCACTAAGC TGAGTCTCTG AGTCAGAGTG TCGTCCCTCA AGGTTAGGTC   
  
  
- TCTCACAGGA TAATGAAGAG GCTCCGTGAG ATGGTGGCGG AAAGAGAGAG GAGGCGGGGA GTTGGAGAGG   
  
  
- GTCTTTGCTG CAGCAGACTC CTCATGTGAG AGAGAATGTT CCGGGACTTG CTGCGCACGG GCATGGTATT   
  
  
- TAAGCGAGTG AACTGGCGTT TAGTCCGCTA AGATCTTCGG TGACTAAGTC GGTTCTTCTA GGTGTAGCAA   
  
  
- CTAAAACCCT ATTAGGTCCC TCAGGTTACC CGACGAGACG ATGTTCGGGA CCGTTGGGCC GGTCGGCCCT   
  
  
- TCGGGCTTAA CTAAGCTTAT AGGCCTTAAG GACGAGGCCC AGACCCCGTT AGAGGCCGCC GTAGCGACGA   
  
  
- GCGTTGACCC TTAGCGGAGG CACTCAAGCG CTTCCAAGAT CTAGAGTTCA AGCTCAAACT CGGCCATGAG   
  
  
- TGAGGTTAGT CTCTCGATTT GCCCAGCTTG AAGGCCCAGC TGGGTCTACT CTATGATCGA CAGTTAAAGT   
  
  
- ACGAAGTTAA CATGTTGGAT AATCTGCTTT GGGACCAGCG ACAGCTGCGG CGGGACTTCG ACCGGTTCAG   
  
  
- TAACTTGGGG TTTTAGCAGT GAAACCCACT CATGCTCCGC CCAGACTTAG CCCGTCCAAA GAACTGGGCC   
  
  
- AAGTTCTGCC GGGAGTTCAT GATAAACCGT TACAAACTTA GCCAACTCGG GTTGAACCGG GCCCTGAGCG   
  
  
- GGCTCTCCCG AGTCCAACTC TCCGACAATA ACCCGGCCGC CTAGTACCCG AATCAACCCG GCCTCAATGG   
  
  
- CCCTTGCTCT TCCCTTTCCT ACCTCACATT CCTTGTTACC TTCCCAAATT ACCTTTCACG GCCAAAGCTC   
  
  
- GGCACGGTTT CGTCTTTGAT GCAGTCACTT TTTCTGTTCA AAATGGTGAG GTCTAACTTA ATGTCTCTCA   
  
  
- C

+     LTR

| Site Name | Organism | Position | Strand | Matrix score. | sequence | function |
| --- | --- | --- | --- | --- | --- | --- |
| LTR | Hordeum vulgare | 2592 | + | 6 | CCGAAA | cis-acting element involved in low-temperature responsiveness |
| LTR | Hordeum vulgare | 2479 | - | 6 | CCGAAA | cis-acting element involved in low-temperature responsiveness |

>HU06G00029.1   
+ +Up\_Stream \_Len000TGATAA TTATTTTTAT TGATTTAGGA AAGGATATCA AAGTATATTT TGTTTTTAGG   
  
  
+ TAGTTAAGAG ATTCTGTTGC CAAAAAAAAA ATCAAGATAT TATATATTTT TAATTATGAC AGTAATTATT   
  
  
+ TTATTGAAAA TCCAACGGTT GTGATTATAT AATCAAATAA TCTAAAGGTT AAAAATATCA AATTTATTAG   
  
  
+ AAAATTCGAA AAAGTCACGT CATCACAACA TTGCTTTTAT ATATAGTATA GATTCCTTCG ACTTCTAAAC   
  
  
+ TAATCACTTG AAATTAGAAA TTTGAAACTT TCTAATATAA ATGCATGGTC TTCTTTCCAA GATTATAATC   
  
  
+ CGCAATTGCT TAAATGTAGG GTACAAATAT CAAATTTAGA AGTGACTATT GAAGCCATGA AAGATATTGT   
  
  
+ GATCGTATTG AGGGTGGTTG TGAGCAATAG GATGAAACAG GAAAAAAAAA CCCTAATGTT TTCAAATTTA   
  
  
+ TGGGTGGCTT TGGTAGTAGA TAAGTGAATA GTGTGCAAGG GAATGGCACC AATATGTTTG GATGGAACGA   
  
  
+ TGAGAGTAAG ACGTTAGGAA AGCAACAAGA GAAGGGAAAA GGCAGTAGGG AGAGAATTAT GCGTGTGAAT   
  
  
+ AATATTATAA CAGACGCTTT CGTATGATTA TTAAATACAA GACTTTGAGC ATACATATAA ACTTGTAACT   
  
  
+ TTCTTTACCT ATTGTCAATT AATTTTTGAA TTGAATCATC TGCGACTTAT GCATACAAGA TGATATGATG   
  
  
+ CATATTGGGC CAAGCATTTG ATCTCATGTC CCTTATGGAT CTAGATCCCA ATAGACAAAC TTGCATAAGT   
  
  
+ CAAGGGATGT GTTTCTAATA CAAAGGCTTT GTACTCAAGC AATTAGTTTT GAATTCATTT TCAGTAGTTT   
  
  
+ AATTTCCAAA TCTTATAATT TTTACAATGG CATAAATTTT TAGGAGTTTT GAGTAAATAA ATTATATACA   
  
  
+ ACGAAGATGT CATTTACGAA TTAATATGAC GCACAAAAAA AAAGAAAAAA AATTAATGGT AGCTTCTATA   
  
  
+ CTTGACTCCA TGTTGACAAT TCCCTCTCTC ACATAATGGT TAAAACTTGC AACATCATCC AGGACCCATA   
  
  
+ TGACATGGGT GGTGGTAGAC AGTGTGTTGT GGTAGCCTCA CTCCATGCTT GCCAATTTGT ATCATTGCGC   
  
  
+ TAGTCTTATT CGTCCTTAGA GATAATTGAC TAAGCTCTAA TGCTCTATCG TGGCTGCTGC AATATAAATG   
  
  
+ ATTTTTTGAC CTCAAAATGA AGACAGCTAG TTGTAAATTA AGAATCTTCG TCCCTTCATT TTTTGTGTAC   
  
  
+ GTGTGTATCT GCGTATGTGT TGAGAGACTA GCGGACTATT GATTGAGTTT CAATCAAAAA AGATGACCAT   
  
  
+ CTTAAGTATT TTTAATTACG CAAACTTCTT CATTTAAAAA AATGTTACTT TTATTTGAAA GAAATTGTGA   
  
  
+ ATAATACAAG GAATAGTTCA ATTAATATAT AATCCATAAC ATATTACCTC CTATAGGCAA AAAGAAGATT   
  
  
+ ATCGTATTAA TTTGGGATCA TCCCACCCTA AATTTAAAGA AACCAAAACA AATTACTAAG CAATATCTTT   
  
  
+ CTGTGTGAAA AAATAGTCAG AAACAAAGCG ATCCGACCGG CCAAATCGAG ATAAACACAG CCAAAGAACC   
  
  
+ GAAGTTGGAC CAAAGTGAGC CGAGTTGAAA GGCATTCATA TGGGGTTTAA TCATAACACA AACATAATTA   
  
  
+ GATTGATTGA CGTCTTTAAT CAGCGTTTTT GTCTTTGTTC GGTGCTCCCC TCACTCGCCC CTCCGCTCTT   
  
  
+ TAAAGCTTAC CTCACTCCCC CCATTCCCAC TCCTTTTCTC TCTCCTCTCT CGCTCTCACA CGCTCCGTCT   
  
  
+ GTCAGCCCCT TGTCATCTTC CCCCGTCTTC CTCGCAAAAA TCTCCAGCAA AATTTATAGC CGTTGTTGTG   
  
  
+ CCCATGGTGA TCTGATGTGA GAAGAGATTC AGCATTTAAG ATTATTGGGT CCAGATGGCT TATATGTGCG   
  
  
+ CCGACAGTGG TAATCTCATG GCTATTGCCC AACAAGTCAT CAAGCAAAAA CAACAGCAAG AACAGCAGCA   
  
  
+ ACACCAACAG CCTCATCCCC AGTTCGTCGG GTCGAATAAT AACCCCTTTT GCACCAGCCC ATGGCCCGGA   
  
  
+ CCCGTCCCTT CATCCGCCAT GTCTGCCAGC CCTCCGCCCC CTCTTGGGTT CTCCGCCGCC GCGTTTCCGG   
  
  
+ ACCCGTTTCA GGTGGGCCCG CCCTGCCCGG ATGGGGCTGA ACCGGGTTTC CAGTTTGCCC ATTTGGACCA   
  
  
+ CCACTCGAGT GGTTTCCGGT TTGCTGATTT TTGCGGTGGG GGTGGTGGTG AGTTTGACTC GGATGAGTGG   
  
  
+ ATGGAGAGTT TGATGGGTGG CGGCGGCGGT GACTCGCGGC GACTGAGAGT TCTAACCTCC AATCCCATTG   
  
  
+ CGACGCGTGG CAAGGGTCCG GTGATTTCGG TCTCTATGGT GCTGATCCGT TTGCGACGTG TTCGGAAGAC   
  
  
+ CGTTGCAGCC GCTATTCACG CTCAGCCACT GCCGCAACAG CAGCCGTCGT GGGTCCCGCC TTCTCCGCCG   
  
  
+ AAAGACACGA AGCAGTCGGC CCCACCTCAA AAGCAAAGCG ACGCCGCTGG ACCCTCGTTC TCATCCGAGG   
  
  
+ AGGAACCAGA GCCGTCATGG CCGCCGCTAA TTGCGGCGTT GTTGGAGTGC GCCAAACTCG CCGAGTCCGA   
  
  
+ CCCGGAACAC GCTGTCAAAT CGGTGATTCG ACTCAGAGAC TCAGTCTCAC AGCAGGGAGT TCCAATCCAG   
  
  
+ AGAGTGTCCT ATTACTTCTC CGAGGCACTC TACCACCGCC TTTCTCTCTC CTCCGCCCCT CAACCTCTCC   
  
  
+ CAGAAACGAC GTCGTCTGAG GAGTACACTC TCTCTTACAA GGCCCTGAAC GACGCGTGCC CGTACCATAA   
  
  
+ ATTCGCTCAC TTGACCGCAA ATCAGGCGAT TCTAGAAGCC ACTGATTCAG CCAAGAAGAT CCACATCGTT   
  
  
+ GATTTTGGGA TAATCCAGGG AGTCCAATGG GCTGCTCTGC TACAAGCCCT GGCAACCCGG CCAGCCGGGA   
  
  
+ AGCCCGAATT GATTCGAATA TCCGGAATTC CTGCTCCGGG TCTGGGGCAA TCTCCGGCGG CATCGCTGCT   
  
  
+ CGCAACTGGG AATCGCCTCC GTGAGTTCGC GAAGGTTCTA GATCTCAAGT TCGAGTTTGA GCCGGTACTC   
  
  
+ ACTCCAATCA GAGAGCTAAA CGGGTCGAAC TTCCGGGTCG ACCCAGATGA GATACTAGCT GTCAATTTCA   
  
  
+ TGCTTCAATT GTACAACCTA TTAGACGAAA CCCTGGTCGC TGTCGACGCC GCCCTGAAGC TGGCCAAGTC   
  
  
+ ATTGAACCCC AAAATCGTCA CTTTGGGTGA GTACGAGGCG GGTCTGAATC GGGCAGGTTT CTTGACCCGG   
  
  
+ TTCAAGACGG CCCTCAAGTA CTATTTGGCA ATGTTTGAAT CGGTTGAGCC CAACTTGGCC CGGGACTCGC   
  
  
+ CCGAGAGGGC TCAGGTTGAG AGGCTGTTAT TGGGCCGGCG GATCATGGGC TTAGTTGGGC CGGAGTTACC   
  
  
+ GGGAACGAGA AGGGAAAGGA TGGAGTGTAA GGAACAATGG AAGGGTTTAA TGGAAAGTGC CGGTTTCGAG   
  
  
+ CCGTGCCAAA GCAGAAACTA CGTCAGTGAA AAAGACAAGT TTTACCACTC CAGATTGAAT TACAGAGAGT   
  
  
+ G  

- +Up\_Stream \_Len000ACTATT AATAAAAATA ACTAAATCCT TTCCTATAGT TTCATATAAA ACAAAAATCC   
  
  
- ATCAATTCTC TAAGACAACG GTTTTTTTTT TAGTTCTATA ATATATAAAA ATTAATACTG TCATTAATAA   
  
  
- AATAACTTTT AGGTTGCCAA CACTAATATA TTAGTTTATT AGATTTCCAA TTTTTATAGT TTAAATAATC   
  
  
- TTTTAAGCTT TTTCAGTGCA GTAGTGTTGT AACGAAAATA TATATCATAT CTAAGGAAGC TGAAGATTTG   
  
  
- ATTAGTGAAC TTTAATCTTT AAACTTTGAA AGATTATATT TACGTACCAG AAGAAAGGTT CTAATATTAG   
  
  
- GCGTTAACGA ATTTACATCC CATGTTTATA GTTTAAATCT TCACTGATAA CTTCGGTACT TTCTATAACA   
  
  
- CTAGCATAAC TCCCACCAAC ACTCGTTATC CTACTTTGTC CTTTTTTTTT GGGATTACAA AAGTTTAAAT   
  
  
- ACCCACCGAA ACCATCATCT ATTCACTTAT CACACGTTCC CTTACCGTGG TTATACAAAC CTACCTTGCT   
  
  
- ACTCTCATTC TGCAATCCTT TCGTTGTTCT CTTCCCTTTT CCGTCATCCC TCTCTTAATA CGCACACTTA   
  
  
- TTATAATATT GTCTGCGAAA GCATACTAAT AATTTATGTT CTGAAACTCG TATGTATATT TGAACATTGA   
  
  
- AAGAAATGGA TAACAGTTAA TTAAAAACTT AACTTAGTAG ACGCTGAATA CGTATGTTCT ACTATACTAC   
  
  
- GTATAACCCG GTTCGTAAAC TAGAGTACAG GGAATACCTA GATCTAGGGT TATCTGTTTG AACGTATTCA   
  
  
- GTTCCCTACA CAAAGATTAT GTTTCCGAAA CATGAGTTCG TTAATCAAAA CTTAAGTAAA AGTCATCAAA   
  
  
- TTAAAGGTTT AGAATATTAA AAATGTTACC GTATTTAAAA ATCCTCAAAA CTCATTTATT TAATATATGT   
  
  
- TGCTTCTACA GTAAATGCTT AATTATACTG CGTGTTTTTT TTTCTTTTTT TTAATTACCA TCGAAGATAT   
  
  
- GAACTGAGGT ACAACTGTTA AGGGAGAGAG TGTATTACCA ATTTTGAACG TTGTAGTAGG TCCTGGGTAT   
  
  
- ACTGTACCCA CCACCATCTG TCACACAACA CCATCGGAGT GAGGTACGAA CGGTTAAACA TAGTAACGCG   
  
  
- ATCAGAATAA GCAGGAATCT CTATTAACTG ATTCGAGATT ACGAGATAGC ACCGACGACG TTATATTTAC   
  
  
- TAAAAAACTG GAGTTTTACT TCTGTCGATC AACATTTAAT TCTTAGAAGC AGGGAAGTAA AAAACACATG   
  
  
- CACACATAGA CGCATACACA ACTCTCTGAT CGCCTGATAA CTAACTCAAA GTTAGTTTTT TCTACTGGTA   
  
  
- GAATTCATAA AAATTAATGC GTTTGAAGAA GTAAATTTTT TTACAATGAA AATAAACTTT CTTTAACACT   
  
  
- TATTATGTTC CTTATCAAGT TAATTATATA TTAGGTATTG TATAATGGAG GATATCCGTT TTTCTTCTAA   
  
  
- TAGCATAATT AAACCCTAGT AGGGTGGGAT TTAAATTTCT TTGGTTTTGT TTAATGATTC GTTATAGAAA   
  
  
- GACACACTTT TTTATCAGTC TTTGTTTCGC TAGGCTGGCC GGTTTAGCTC TATTTGTGTC GGTTTCTTGG   
  
  
- CTTCAACCTG GTTTCACTCG GCTCAACTTT CCGTAAGTAT ACCCCAAATT AGTATTGTGT TTGTATTAAT   
  
  
- CTAACTAACT GCAGAAATTA GTCGCAAAAA CAGAAACAAG CCACGAGGGG AGTGAGCGGG GAGGCGAGAA   
  
  
- ATTTCGAATG GAGTGAGGGG GGTAAGGGTG AGGAAAAGAG AGAGGAGAGA GCGAGAGTGT GCGAGGCAGA   
  
  
- CAGTCGGGGA ACAGTAGAAG GGGGCAGAAG GAGCGTTTTT AGAGGTCGTT TTAAATATCG GCAACAACAC   
  
  
- GGGTACCACT AGACTACACT CTTCTCTAAG TCGTAAATTC TAATAACCCA GGTCTACCGA ATATACACGC   
  
  
- GGCTGTCACC ATTAGAGTAC CGATAACGGG TTGTTCAGTA GTTCGTTTTT GTTGTCGTTC TTGTCGTCGT   
  
  
- TGTGGTTGTC GGAGTAGGGG TCAAGCAGCC CAGCTTATTA TTGGGGAAAA CGTGGTCGGG TACCGGGCCT   
  
  
- GGGCAGGGAA GTAGGCGGTA CAGACGGTCG GGAGGCGGGG GAGAACCCAA GAGGCGGCGG CGCAAAGGCC   
  
  
- TGGGCAAAGT CCACCCGGGC GGGACGGGCC TACCCCGACT TGGCCCAAAG GTCAAACGGG TAAACCTGGT   
  
  
- GGTGAGCTCA CCAAAGGCCA AACGACTAAA AACGCCACCC CCACCACCAC TCAAACTGAG CCTACTCACC   
  
  
- TACCTCTCAA ACTACCCACC GCCGCCGCCA CTGAGCGCCG CTGACTCTCA AGATTGGAGG TTAGGGTAAC   
  
  
- GCTGCGCACC GTTCCCAGGC CACTAAAGCC AGAGATACCA CGACTAGGCA AACGCTGCAC AAGCCTTCTG   
  
  
- GCAACGTCGG CGATAAGTGC GAGTCGGTGA CGGCGTTGTC GTCGGCAGCA CCCAGGGCGG AAGAGGCGGC   
  
  
- TTTCTGTGCT TCGTCAGCCG GGGTGGAGTT TTCGTTTCGC TGCGGCGACC TGGGAGCAAG AGTAGGCTCC   
  
  
- TCCTTGGTCT CGGCAGTACC GGCGGCGATT AACGCCGCAA CAACCTCACG CGGTTTGAGC GGCTCAGGCT   
  
  
- GGGCCTTGTG CGACAGTTTA GCCACTAAGC TGAGTCTCTG AGTCAGAGTG TCGTCCCTCA AGGTTAGGTC   
  
  
- TCTCACAGGA TAATGAAGAG GCTCCGTGAG ATGGTGGCGG AAAGAGAGAG GAGGCGGGGA GTTGGAGAGG   
  
  
- GTCTTTGCTG CAGCAGACTC CTCATGTGAG AGAGAATGTT CCGGGACTTG CTGCGCACGG GCATGGTATT   
  
  
- TAAGCGAGTG AACTGGCGTT TAGTCCGCTA AGATCTTCGG TGACTAAGTC GGTTCTTCTA GGTGTAGCAA   
  
  
- CTAAAACCCT ATTAGGTCCC TCAGGTTACC CGACGAGACG ATGTTCGGGA CCGTTGGGCC GGTCGGCCCT   
  
  
- TCGGGCTTAA CTAAGCTTAT AGGCCTTAAG GACGAGGCCC AGACCCCGTT AGAGGCCGCC GTAGCGACGA   
  
  
- GCGTTGACCC TTAGCGGAGG CACTCAAGCG CTTCCAAGAT CTAGAGTTCA AGCTCAAACT CGGCCATGAG   
  
  
- TGAGGTTAGT CTCTCGATTT GCCCAGCTTG AAGGCCCAGC TGGGTCTACT CTATGATCGA CAGTTAAAGT   
  
  
- ACGAAGTTAA CATGTTGGAT AATCTGCTTT GGGACCAGCG ACAGCTGCGG CGGGACTTCG ACCGGTTCAG   
  
  
- TAACTTGGGG TTTTAGCAGT GAAACCCACT CATGCTCCGC CCAGACTTAG CCCGTCCAAA GAACTGGGCC   
  
  
- AAGTTCTGCC GGGAGTTCAT GATAAACCGT TACAAACTTA GCCAACTCGG GTTGAACCGG GCCCTGAGCG   
  
  
- GGCTCTCCCG AGTCCAACTC TCCGACAATA ACCCGGCCGC CTAGTACCCG AATCAACCCG GCCTCAATGG   
  
  
- CCCTTGCTCT TCCCTTTCCT ACCTCACATT CCTTGTTACC TTCCCAAATT ACCTTTCACG GCCAAAGCTC   
  
  
- GGCACGGTTT CGTCTTTGAT GCAGTCACTT TTTCTGTTCA AAATGGTGAG GTCTAACTTA ATGTCTCTCA   
  
  
- C

+     MBS

| Site Name | Organism | Position | Strand | Matrix score. | sequence | function |
| --- | --- | --- | --- | --- | --- | --- |
| MBS | Arabidopsis thaliana | 3157 | + | 6 | CAACTG | MYB binding site involved in drought-inducibility |

>HU06G00029.1   
+ +Up\_Stream \_Len000TGATAA TTATTTTTAT TGATTTAGGA AAGGATATCA AAGTATATTT TGTTTTTAGG   
  
  
+ TAGTTAAGAG ATTCTGTTGC CAAAAAAAAA ATCAAGATAT TATATATTTT TAATTATGAC AGTAATTATT   
  
  
+ TTATTGAAAA TCCAACGGTT GTGATTATAT AATCAAATAA TCTAAAGGTT AAAAATATCA AATTTATTAG   
  
  
+ AAAATTCGAA AAAGTCACGT CATCACAACA TTGCTTTTAT ATATAGTATA GATTCCTTCG ACTTCTAAAC   
  
  
+ TAATCACTTG AAATTAGAAA TTTGAAACTT TCTAATATAA ATGCATGGTC TTCTTTCCAA GATTATAATC   
  
  
+ CGCAATTGCT TAAATGTAGG GTACAAATAT CAAATTTAGA AGTGACTATT GAAGCCATGA AAGATATTGT   
  
  
+ GATCGTATTG AGGGTGGTTG TGAGCAATAG GATGAAACAG GAAAAAAAAA CCCTAATGTT TTCAAATTTA   
  
  
+ TGGGTGGCTT TGGTAGTAGA TAAGTGAATA GTGTGCAAGG GAATGGCACC AATATGTTTG GATGGAACGA   
  
  
+ TGAGAGTAAG ACGTTAGGAA AGCAACAAGA GAAGGGAAAA GGCAGTAGGG AGAGAATTAT GCGTGTGAAT   
  
  
+ AATATTATAA CAGACGCTTT CGTATGATTA TTAAATACAA GACTTTGAGC ATACATATAA ACTTGTAACT   
  
  
+ TTCTTTACCT ATTGTCAATT AATTTTTGAA TTGAATCATC TGCGACTTAT GCATACAAGA TGATATGATG   
  
  
+ CATATTGGGC CAAGCATTTG ATCTCATGTC CCTTATGGAT CTAGATCCCA ATAGACAAAC TTGCATAAGT   
  
  
+ CAAGGGATGT GTTTCTAATA CAAAGGCTTT GTACTCAAGC AATTAGTTTT GAATTCATTT TCAGTAGTTT   
  
  
+ AATTTCCAAA TCTTATAATT TTTACAATGG CATAAATTTT TAGGAGTTTT GAGTAAATAA ATTATATACA   
  
  
+ ACGAAGATGT CATTTACGAA TTAATATGAC GCACAAAAAA AAAGAAAAAA AATTAATGGT AGCTTCTATA   
  
  
+ CTTGACTCCA TGTTGACAAT TCCCTCTCTC ACATAATGGT TAAAACTTGC AACATCATCC AGGACCCATA   
  
  
+ TGACATGGGT GGTGGTAGAC AGTGTGTTGT GGTAGCCTCA CTCCATGCTT GCCAATTTGT ATCATTGCGC   
  
  
+ TAGTCTTATT CGTCCTTAGA GATAATTGAC TAAGCTCTAA TGCTCTATCG TGGCTGCTGC AATATAAATG   
  
  
+ ATTTTTTGAC CTCAAAATGA AGACAGCTAG TTGTAAATTA AGAATCTTCG TCCCTTCATT TTTTGTGTAC   
  
  
+ GTGTGTATCT GCGTATGTGT TGAGAGACTA GCGGACTATT GATTGAGTTT CAATCAAAAA AGATGACCAT   
  
  
+ CTTAAGTATT TTTAATTACG CAAACTTCTT CATTTAAAAA AATGTTACTT TTATTTGAAA GAAATTGTGA   
  
  
+ ATAATACAAG GAATAGTTCA ATTAATATAT AATCCATAAC ATATTACCTC CTATAGGCAA AAAGAAGATT   
  
  
+ ATCGTATTAA TTTGGGATCA TCCCACCCTA AATTTAAAGA AACCAAAACA AATTACTAAG CAATATCTTT   
  
  
+ CTGTGTGAAA AAATAGTCAG AAACAAAGCG ATCCGACCGG CCAAATCGAG ATAAACACAG CCAAAGAACC   
  
  
+ GAAGTTGGAC CAAAGTGAGC CGAGTTGAAA GGCATTCATA TGGGGTTTAA TCATAACACA AACATAATTA   
  
  
+ GATTGATTGA CGTCTTTAAT CAGCGTTTTT GTCTTTGTTC GGTGCTCCCC TCACTCGCCC CTCCGCTCTT   
  
  
+ TAAAGCTTAC CTCACTCCCC CCATTCCCAC TCCTTTTCTC TCTCCTCTCT CGCTCTCACA CGCTCCGTCT   
  
  
+ GTCAGCCCCT TGTCATCTTC CCCCGTCTTC CTCGCAAAAA TCTCCAGCAA AATTTATAGC CGTTGTTGTG   
  
  
+ CCCATGGTGA TCTGATGTGA GAAGAGATTC AGCATTTAAG ATTATTGGGT CCAGATGGCT TATATGTGCG   
  
  
+ CCGACAGTGG TAATCTCATG GCTATTGCCC AACAAGTCAT CAAGCAAAAA CAACAGCAAG AACAGCAGCA   
  
  
+ ACACCAACAG CCTCATCCCC AGTTCGTCGG GTCGAATAAT AACCCCTTTT GCACCAGCCC ATGGCCCGGA   
  
  
+ CCCGTCCCTT CATCCGCCAT GTCTGCCAGC CCTCCGCCCC CTCTTGGGTT CTCCGCCGCC GCGTTTCCGG   
  
  
+ ACCCGTTTCA GGTGGGCCCG CCCTGCCCGG ATGGGGCTGA ACCGGGTTTC CAGTTTGCCC ATTTGGACCA   
  
  
+ CCACTCGAGT GGTTTCCGGT TTGCTGATTT TTGCGGTGGG GGTGGTGGTG AGTTTGACTC GGATGAGTGG   
  
  
+ ATGGAGAGTT TGATGGGTGG CGGCGGCGGT GACTCGCGGC GACTGAGAGT TCTAACCTCC AATCCCATTG   
  
  
+ CGACGCGTGG CAAGGGTCCG GTGATTTCGG TCTCTATGGT GCTGATCCGT TTGCGACGTG TTCGGAAGAC   
  
  
+ CGTTGCAGCC GCTATTCACG CTCAGCCACT GCCGCAACAG CAGCCGTCGT GGGTCCCGCC TTCTCCGCCG   
  
  
+ AAAGACACGA AGCAGTCGGC CCCACCTCAA AAGCAAAGCG ACGCCGCTGG ACCCTCGTTC TCATCCGAGG   
  
  
+ AGGAACCAGA GCCGTCATGG CCGCCGCTAA TTGCGGCGTT GTTGGAGTGC GCCAAACTCG CCGAGTCCGA   
  
  
+ CCCGGAACAC GCTGTCAAAT CGGTGATTCG ACTCAGAGAC TCAGTCTCAC AGCAGGGAGT TCCAATCCAG   
  
  
+ AGAGTGTCCT ATTACTTCTC CGAGGCACTC TACCACCGCC TTTCTCTCTC CTCCGCCCCT CAACCTCTCC   
  
  
+ CAGAAACGAC GTCGTCTGAG GAGTACACTC TCTCTTACAA GGCCCTGAAC GACGCGTGCC CGTACCATAA   
  
  
+ ATTCGCTCAC TTGACCGCAA ATCAGGCGAT TCTAGAAGCC ACTGATTCAG CCAAGAAGAT CCACATCGTT   
  
  
+ GATTTTGGGA TAATCCAGGG AGTCCAATGG GCTGCTCTGC TACAAGCCCT GGCAACCCGG CCAGCCGGGA   
  
  
+ AGCCCGAATT GATTCGAATA TCCGGAATTC CTGCTCCGGG TCTGGGGCAA TCTCCGGCGG CATCGCTGCT   
  
  
+ CGCAACTGGG AATCGCCTCC GTGAGTTCGC GAAGGTTCTA GATCTCAAGT TCGAGTTTGA GCCGGTACTC   
  
  
+ ACTCCAATCA GAGAGCTAAA CGGGTCGAAC TTCCGGGTCG ACCCAGATGA GATACTAGCT GTCAATTTCA   
  
  
+ TGCTTCAATT GTACAACCTA TTAGACGAAA CCCTGGTCGC TGTCGACGCC GCCCTGAAGC TGGCCAAGTC   
  
  
+ ATTGAACCCC AAAATCGTCA CTTTGGGTGA GTACGAGGCG GGTCTGAATC GGGCAGGTTT CTTGACCCGG   
  
  
+ TTCAAGACGG CCCTCAAGTA CTATTTGGCA ATGTTTGAAT CGGTTGAGCC CAACTTGGCC CGGGACTCGC   
  
  
+ CCGAGAGGGC TCAGGTTGAG AGGCTGTTAT TGGGCCGGCG GATCATGGGC TTAGTTGGGC CGGAGTTACC   
  
  
+ GGGAACGAGA AGGGAAAGGA TGGAGTGTAA GGAACAATGG AAGGGTTTAA TGGAAAGTGC CGGTTTCGAG   
  
  
+ CCGTGCCAAA GCAGAAACTA CGTCAGTGAA AAAGACAAGT TTTACCACTC CAGATTGAAT TACAGAGAGT   
  
  
+ G  

- +Up\_Stream \_Len000ACTATT AATAAAAATA ACTAAATCCT TTCCTATAGT TTCATATAAA ACAAAAATCC   
  
  
- ATCAATTCTC TAAGACAACG GTTTTTTTTT TAGTTCTATA ATATATAAAA ATTAATACTG TCATTAATAA   
  
  
- AATAACTTTT AGGTTGCCAA CACTAATATA TTAGTTTATT AGATTTCCAA TTTTTATAGT TTAAATAATC   
  
  
- TTTTAAGCTT TTTCAGTGCA GTAGTGTTGT AACGAAAATA TATATCATAT CTAAGGAAGC TGAAGATTTG   
  
  
- ATTAGTGAAC TTTAATCTTT AAACTTTGAA AGATTATATT TACGTACCAG AAGAAAGGTT CTAATATTAG   
  
  
- GCGTTAACGA ATTTACATCC CATGTTTATA GTTTAAATCT TCACTGATAA CTTCGGTACT TTCTATAACA   
  
  
- CTAGCATAAC TCCCACCAAC ACTCGTTATC CTACTTTGTC CTTTTTTTTT GGGATTACAA AAGTTTAAAT   
  
  
- ACCCACCGAA ACCATCATCT ATTCACTTAT CACACGTTCC CTTACCGTGG TTATACAAAC CTACCTTGCT   
  
  
- ACTCTCATTC TGCAATCCTT TCGTTGTTCT CTTCCCTTTT CCGTCATCCC TCTCTTAATA CGCACACTTA   
  
  
- TTATAATATT GTCTGCGAAA GCATACTAAT AATTTATGTT CTGAAACTCG TATGTATATT TGAACATTGA   
  
  
- AAGAAATGGA TAACAGTTAA TTAAAAACTT AACTTAGTAG ACGCTGAATA CGTATGTTCT ACTATACTAC   
  
  
- GTATAACCCG GTTCGTAAAC TAGAGTACAG GGAATACCTA GATCTAGGGT TATCTGTTTG AACGTATTCA   
  
  
- GTTCCCTACA CAAAGATTAT GTTTCCGAAA CATGAGTTCG TTAATCAAAA CTTAAGTAAA AGTCATCAAA   
  
  
- TTAAAGGTTT AGAATATTAA AAATGTTACC GTATTTAAAA ATCCTCAAAA CTCATTTATT TAATATATGT   
  
  
- TGCTTCTACA GTAAATGCTT AATTATACTG CGTGTTTTTT TTTCTTTTTT TTAATTACCA TCGAAGATAT   
  
  
- GAACTGAGGT ACAACTGTTA AGGGAGAGAG TGTATTACCA ATTTTGAACG TTGTAGTAGG TCCTGGGTAT   
  
  
- ACTGTACCCA CCACCATCTG TCACACAACA CCATCGGAGT GAGGTACGAA CGGTTAAACA TAGTAACGCG   
  
  
- ATCAGAATAA GCAGGAATCT CTATTAACTG ATTCGAGATT ACGAGATAGC ACCGACGACG TTATATTTAC   
  
  
- TAAAAAACTG GAGTTTTACT TCTGTCGATC AACATTTAAT TCTTAGAAGC AGGGAAGTAA AAAACACATG   
  
  
- CACACATAGA CGCATACACA ACTCTCTGAT CGCCTGATAA CTAACTCAAA GTTAGTTTTT TCTACTGGTA   
  
  
- GAATTCATAA AAATTAATGC GTTTGAAGAA GTAAATTTTT TTACAATGAA AATAAACTTT CTTTAACACT   
  
  
- TATTATGTTC CTTATCAAGT TAATTATATA TTAGGTATTG TATAATGGAG GATATCCGTT TTTCTTCTAA   
  
  
- TAGCATAATT AAACCCTAGT AGGGTGGGAT TTAAATTTCT TTGGTTTTGT TTAATGATTC GTTATAGAAA   
  
  
- GACACACTTT TTTATCAGTC TTTGTTTCGC TAGGCTGGCC GGTTTAGCTC TATTTGTGTC GGTTTCTTGG   
  
  
- CTTCAACCTG GTTTCACTCG GCTCAACTTT CCGTAAGTAT ACCCCAAATT AGTATTGTGT TTGTATTAAT   
  
  
- CTAACTAACT GCAGAAATTA GTCGCAAAAA CAGAAACAAG CCACGAGGGG AGTGAGCGGG GAGGCGAGAA   
  
  
- ATTTCGAATG GAGTGAGGGG GGTAAGGGTG AGGAAAAGAG AGAGGAGAGA GCGAGAGTGT GCGAGGCAGA   
  
  
- CAGTCGGGGA ACAGTAGAAG GGGGCAGAAG GAGCGTTTTT AGAGGTCGTT TTAAATATCG GCAACAACAC   
  
  
- GGGTACCACT AGACTACACT CTTCTCTAAG TCGTAAATTC TAATAACCCA GGTCTACCGA ATATACACGC   
  
  
- GGCTGTCACC ATTAGAGTAC CGATAACGGG TTGTTCAGTA GTTCGTTTTT GTTGTCGTTC TTGTCGTCGT   
  
  
- TGTGGTTGTC GGAGTAGGGG TCAAGCAGCC CAGCTTATTA TTGGGGAAAA CGTGGTCGGG TACCGGGCCT   
  
  
- GGGCAGGGAA GTAGGCGGTA CAGACGGTCG GGAGGCGGGG GAGAACCCAA GAGGCGGCGG CGCAAAGGCC   
  
  
- TGGGCAAAGT CCACCCGGGC GGGACGGGCC TACCCCGACT TGGCCCAAAG GTCAAACGGG TAAACCTGGT   
  
  
- GGTGAGCTCA CCAAAGGCCA AACGACTAAA AACGCCACCC CCACCACCAC TCAAACTGAG CCTACTCACC   
  
  
- TACCTCTCAA ACTACCCACC GCCGCCGCCA CTGAGCGCCG CTGACTCTCA AGATTGGAGG TTAGGGTAAC   
  
  
- GCTGCGCACC GTTCCCAGGC CACTAAAGCC AGAGATACCA CGACTAGGCA AACGCTGCAC AAGCCTTCTG   
  
  
- GCAACGTCGG CGATAAGTGC GAGTCGGTGA CGGCGTTGTC GTCGGCAGCA CCCAGGGCGG AAGAGGCGGC   
  
  
- TTTCTGTGCT TCGTCAGCCG GGGTGGAGTT TTCGTTTCGC TGCGGCGACC TGGGAGCAAG AGTAGGCTCC   
  
  
- TCCTTGGTCT CGGCAGTACC GGCGGCGATT AACGCCGCAA CAACCTCACG CGGTTTGAGC GGCTCAGGCT   
  
  
- GGGCCTTGTG CGACAGTTTA GCCACTAAGC TGAGTCTCTG AGTCAGAGTG TCGTCCCTCA AGGTTAGGTC   
  
  
- TCTCACAGGA TAATGAAGAG GCTCCGTGAG ATGGTGGCGG AAAGAGAGAG GAGGCGGGGA GTTGGAGAGG   
  
  
- GTCTTTGCTG CAGCAGACTC CTCATGTGAG AGAGAATGTT CCGGGACTTG CTGCGCACGG GCATGGTATT   
  
  
- TAAGCGAGTG AACTGGCGTT TAGTCCGCTA AGATCTTCGG TGACTAAGTC GGTTCTTCTA GGTGTAGCAA   
  
  
- CTAAAACCCT ATTAGGTCCC TCAGGTTACC CGACGAGACG ATGTTCGGGA CCGTTGGGCC GGTCGGCCCT   
  
  
- TCGGGCTTAA CTAAGCTTAT AGGCCTTAAG GACGAGGCCC AGACCCCGTT AGAGGCCGCC GTAGCGACGA   
  
  
- GCGTTGACCC TTAGCGGAGG CACTCAAGCG CTTCCAAGAT CTAGAGTTCA AGCTCAAACT CGGCCATGAG   
  
  
- TGAGGTTAGT CTCTCGATTT GCCCAGCTTG AAGGCCCAGC TGGGTCTACT CTATGATCGA CAGTTAAAGT   
  
  
- ACGAAGTTAA CATGTTGGAT AATCTGCTTT GGGACCAGCG ACAGCTGCGG CGGGACTTCG ACCGGTTCAG   
  
  
- TAACTTGGGG TTTTAGCAGT GAAACCCACT CATGCTCCGC CCAGACTTAG CCCGTCCAAA GAACTGGGCC   
  
  
- AAGTTCTGCC GGGAGTTCAT GATAAACCGT TACAAACTTA GCCAACTCGG GTTGAACCGG GCCCTGAGCG   
  
  
- GGCTCTCCCG AGTCCAACTC TCCGACAATA ACCCGGCCGC CTAGTACCCG AATCAACCCG GCCTCAATGG   
  
  
- CCCTTGCTCT TCCCTTTCCT ACCTCACATT CCTTGTTACC TTCCCAAATT ACCTTTCACG GCCAAAGCTC   
  
  
- GGCACGGTTT CGTCTTTGAT GCAGTCACTT TTTCTGTTCA AAATGGTGAG GTCTAACTTA ATGTCTCTCA   
  
  
- C

+     MSA-like

| Site Name | Organism | Position | Strand | Matrix score. | sequence | function |
| --- | --- | --- | --- | --- | --- | --- |
| MSA-like | Catharanthus roseus | 155 | + | 9 | TCCAACGGT | cis-acting element involved in cell cycle regulation |

>HU06G00029.1   
+ +Up\_Stream \_Len000TGATAA TTATTTTTAT TGATTTAGGA AAGGATATCA AAGTATATTT TGTTTTTAGG   
  
  
+ TAGTTAAGAG ATTCTGTTGC CAAAAAAAAA ATCAAGATAT TATATATTTT TAATTATGAC AGTAATTATT   
  
  
+ TTATTGAAAA TCCAACGGTT GTGATTATAT AATCAAATAA TCTAAAGGTT AAAAATATCA AATTTATTAG   
  
  
+ AAAATTCGAA AAAGTCACGT CATCACAACA TTGCTTTTAT ATATAGTATA GATTCCTTCG ACTTCTAAAC   
  
  
+ TAATCACTTG AAATTAGAAA TTTGAAACTT TCTAATATAA ATGCATGGTC TTCTTTCCAA GATTATAATC   
  
  
+ CGCAATTGCT TAAATGTAGG GTACAAATAT CAAATTTAGA AGTGACTATT GAAGCCATGA AAGATATTGT   
  
  
+ GATCGTATTG AGGGTGGTTG TGAGCAATAG GATGAAACAG GAAAAAAAAA CCCTAATGTT TTCAAATTTA   
  
  
+ TGGGTGGCTT TGGTAGTAGA TAAGTGAATA GTGTGCAAGG GAATGGCACC AATATGTTTG GATGGAACGA   
  
  
+ TGAGAGTAAG ACGTTAGGAA AGCAACAAGA GAAGGGAAAA GGCAGTAGGG AGAGAATTAT GCGTGTGAAT   
  
  
+ AATATTATAA CAGACGCTTT CGTATGATTA TTAAATACAA GACTTTGAGC ATACATATAA ACTTGTAACT   
  
  
+ TTCTTTACCT ATTGTCAATT AATTTTTGAA TTGAATCATC TGCGACTTAT GCATACAAGA TGATATGATG   
  
  
+ CATATTGGGC CAAGCATTTG ATCTCATGTC CCTTATGGAT CTAGATCCCA ATAGACAAAC TTGCATAAGT   
  
  
+ CAAGGGATGT GTTTCTAATA CAAAGGCTTT GTACTCAAGC AATTAGTTTT GAATTCATTT TCAGTAGTTT   
  
  
+ AATTTCCAAA TCTTATAATT TTTACAATGG CATAAATTTT TAGGAGTTTT GAGTAAATAA ATTATATACA   
  
  
+ ACGAAGATGT CATTTACGAA TTAATATGAC GCACAAAAAA AAAGAAAAAA AATTAATGGT AGCTTCTATA   
  
  
+ CTTGACTCCA TGTTGACAAT TCCCTCTCTC ACATAATGGT TAAAACTTGC AACATCATCC AGGACCCATA   
  
  
+ TGACATGGGT GGTGGTAGAC AGTGTGTTGT GGTAGCCTCA CTCCATGCTT GCCAATTTGT ATCATTGCGC   
  
  
+ TAGTCTTATT CGTCCTTAGA GATAATTGAC TAAGCTCTAA TGCTCTATCG TGGCTGCTGC AATATAAATG   
  
  
+ ATTTTTTGAC CTCAAAATGA AGACAGCTAG TTGTAAATTA AGAATCTTCG TCCCTTCATT TTTTGTGTAC   
  
  
+ GTGTGTATCT GCGTATGTGT TGAGAGACTA GCGGACTATT GATTGAGTTT CAATCAAAAA AGATGACCAT   
  
  
+ CTTAAGTATT TTTAATTACG CAAACTTCTT CATTTAAAAA AATGTTACTT TTATTTGAAA GAAATTGTGA   
  
  
+ ATAATACAAG GAATAGTTCA ATTAATATAT AATCCATAAC ATATTACCTC CTATAGGCAA AAAGAAGATT   
  
  
+ ATCGTATTAA TTTGGGATCA TCCCACCCTA AATTTAAAGA AACCAAAACA AATTACTAAG CAATATCTTT   
  
  
+ CTGTGTGAAA AAATAGTCAG AAACAAAGCG ATCCGACCGG CCAAATCGAG ATAAACACAG CCAAAGAACC   
  
  
+ GAAGTTGGAC CAAAGTGAGC CGAGTTGAAA GGCATTCATA TGGGGTTTAA TCATAACACA AACATAATTA   
  
  
+ GATTGATTGA CGTCTTTAAT CAGCGTTTTT GTCTTTGTTC GGTGCTCCCC TCACTCGCCC CTCCGCTCTT   
  
  
+ TAAAGCTTAC CTCACTCCCC CCATTCCCAC TCCTTTTCTC TCTCCTCTCT CGCTCTCACA CGCTCCGTCT   
  
  
+ GTCAGCCCCT TGTCATCTTC CCCCGTCTTC CTCGCAAAAA TCTCCAGCAA AATTTATAGC CGTTGTTGTG   
  
  
+ CCCATGGTGA TCTGATGTGA GAAGAGATTC AGCATTTAAG ATTATTGGGT CCAGATGGCT TATATGTGCG   
  
  
+ CCGACAGTGG TAATCTCATG GCTATTGCCC AACAAGTCAT CAAGCAAAAA CAACAGCAAG AACAGCAGCA   
  
  
+ ACACCAACAG CCTCATCCCC AGTTCGTCGG GTCGAATAAT AACCCCTTTT GCACCAGCCC ATGGCCCGGA   
  
  
+ CCCGTCCCTT CATCCGCCAT GTCTGCCAGC CCTCCGCCCC CTCTTGGGTT CTCCGCCGCC GCGTTTCCGG   
  
  
+ ACCCGTTTCA GGTGGGCCCG CCCTGCCCGG ATGGGGCTGA ACCGGGTTTC CAGTTTGCCC ATTTGGACCA   
  
  
+ CCACTCGAGT GGTTTCCGGT TTGCTGATTT TTGCGGTGGG GGTGGTGGTG AGTTTGACTC GGATGAGTGG   
  
  
+ ATGGAGAGTT TGATGGGTGG CGGCGGCGGT GACTCGCGGC GACTGAGAGT TCTAACCTCC AATCCCATTG   
  
  
+ CGACGCGTGG CAAGGGTCCG GTGATTTCGG TCTCTATGGT GCTGATCCGT TTGCGACGTG TTCGGAAGAC   
  
  
+ CGTTGCAGCC GCTATTCACG CTCAGCCACT GCCGCAACAG CAGCCGTCGT GGGTCCCGCC TTCTCCGCCG   
  
  
+ AAAGACACGA AGCAGTCGGC CCCACCTCAA AAGCAAAGCG ACGCCGCTGG ACCCTCGTTC TCATCCGAGG   
  
  
+ AGGAACCAGA GCCGTCATGG CCGCCGCTAA TTGCGGCGTT GTTGGAGTGC GCCAAACTCG CCGAGTCCGA   
  
  
+ CCCGGAACAC GCTGTCAAAT CGGTGATTCG ACTCAGAGAC TCAGTCTCAC AGCAGGGAGT TCCAATCCAG   
  
  
+ AGAGTGTCCT ATTACTTCTC CGAGGCACTC TACCACCGCC TTTCTCTCTC CTCCGCCCCT CAACCTCTCC   
  
  
+ CAGAAACGAC GTCGTCTGAG GAGTACACTC TCTCTTACAA GGCCCTGAAC GACGCGTGCC CGTACCATAA   
  
  
+ ATTCGCTCAC TTGACCGCAA ATCAGGCGAT TCTAGAAGCC ACTGATTCAG CCAAGAAGAT CCACATCGTT   
  
  
+ GATTTTGGGA TAATCCAGGG AGTCCAATGG GCTGCTCTGC TACAAGCCCT GGCAACCCGG CCAGCCGGGA   
  
  
+ AGCCCGAATT GATTCGAATA TCCGGAATTC CTGCTCCGGG TCTGGGGCAA TCTCCGGCGG CATCGCTGCT   
  
  
+ CGCAACTGGG AATCGCCTCC GTGAGTTCGC GAAGGTTCTA GATCTCAAGT TCGAGTTTGA GCCGGTACTC   
  
  
+ ACTCCAATCA GAGAGCTAAA CGGGTCGAAC TTCCGGGTCG ACCCAGATGA GATACTAGCT GTCAATTTCA   
  
  
+ TGCTTCAATT GTACAACCTA TTAGACGAAA CCCTGGTCGC TGTCGACGCC GCCCTGAAGC TGGCCAAGTC   
  
  
+ ATTGAACCCC AAAATCGTCA CTTTGGGTGA GTACGAGGCG GGTCTGAATC GGGCAGGTTT CTTGACCCGG   
  
  
+ TTCAAGACGG CCCTCAAGTA CTATTTGGCA ATGTTTGAAT CGGTTGAGCC CAACTTGGCC CGGGACTCGC   
  
  
+ CCGAGAGGGC TCAGGTTGAG AGGCTGTTAT TGGGCCGGCG GATCATGGGC TTAGTTGGGC CGGAGTTACC   
  
  
+ GGGAACGAGA AGGGAAAGGA TGGAGTGTAA GGAACAATGG AAGGGTTTAA TGGAAAGTGC CGGTTTCGAG   
  
  
+ CCGTGCCAAA GCAGAAACTA CGTCAGTGAA AAAGACAAGT TTTACCACTC CAGATTGAAT TACAGAGAGT   
  
  
+ G  

- +Up\_Stream \_Len000ACTATT AATAAAAATA ACTAAATCCT TTCCTATAGT TTCATATAAA ACAAAAATCC   
  
  
- ATCAATTCTC TAAGACAACG GTTTTTTTTT TAGTTCTATA ATATATAAAA ATTAATACTG TCATTAATAA   
  
  
- AATAACTTTT AGGTTGCCAA CACTAATATA TTAGTTTATT AGATTTCCAA TTTTTATAGT TTAAATAATC   
  
  
- TTTTAAGCTT TTTCAGTGCA GTAGTGTTGT AACGAAAATA TATATCATAT CTAAGGAAGC TGAAGATTTG   
  
  
- ATTAGTGAAC TTTAATCTTT AAACTTTGAA AGATTATATT TACGTACCAG AAGAAAGGTT CTAATATTAG   
  
  
- GCGTTAACGA ATTTACATCC CATGTTTATA GTTTAAATCT TCACTGATAA CTTCGGTACT TTCTATAACA   
  
  
- CTAGCATAAC TCCCACCAAC ACTCGTTATC CTACTTTGTC CTTTTTTTTT GGGATTACAA AAGTTTAAAT   
  
  
- ACCCACCGAA ACCATCATCT ATTCACTTAT CACACGTTCC CTTACCGTGG TTATACAAAC CTACCTTGCT   
  
  
- ACTCTCATTC TGCAATCCTT TCGTTGTTCT CTTCCCTTTT CCGTCATCCC TCTCTTAATA CGCACACTTA   
  
  
- TTATAATATT GTCTGCGAAA GCATACTAAT AATTTATGTT CTGAAACTCG TATGTATATT TGAACATTGA   
  
  
- AAGAAATGGA TAACAGTTAA TTAAAAACTT AACTTAGTAG ACGCTGAATA CGTATGTTCT ACTATACTAC   
  
  
- GTATAACCCG GTTCGTAAAC TAGAGTACAG GGAATACCTA GATCTAGGGT TATCTGTTTG AACGTATTCA   
  
  
- GTTCCCTACA CAAAGATTAT GTTTCCGAAA CATGAGTTCG TTAATCAAAA CTTAAGTAAA AGTCATCAAA   
  
  
- TTAAAGGTTT AGAATATTAA AAATGTTACC GTATTTAAAA ATCCTCAAAA CTCATTTATT TAATATATGT   
  
  
- TGCTTCTACA GTAAATGCTT AATTATACTG CGTGTTTTTT TTTCTTTTTT TTAATTACCA TCGAAGATAT   
  
  
- GAACTGAGGT ACAACTGTTA AGGGAGAGAG TGTATTACCA ATTTTGAACG TTGTAGTAGG TCCTGGGTAT   
  
  
- ACTGTACCCA CCACCATCTG TCACACAACA CCATCGGAGT GAGGTACGAA CGGTTAAACA TAGTAACGCG   
  
  
- ATCAGAATAA GCAGGAATCT CTATTAACTG ATTCGAGATT ACGAGATAGC ACCGACGACG TTATATTTAC   
  
  
- TAAAAAACTG GAGTTTTACT TCTGTCGATC AACATTTAAT TCTTAGAAGC AGGGAAGTAA AAAACACATG   
  
  
- CACACATAGA CGCATACACA ACTCTCTGAT CGCCTGATAA CTAACTCAAA GTTAGTTTTT TCTACTGGTA   
  
  
- GAATTCATAA AAATTAATGC GTTTGAAGAA GTAAATTTTT TTACAATGAA AATAAACTTT CTTTAACACT   
  
  
- TATTATGTTC CTTATCAAGT TAATTATATA TTAGGTATTG TATAATGGAG GATATCCGTT TTTCTTCTAA   
  
  
- TAGCATAATT AAACCCTAGT AGGGTGGGAT TTAAATTTCT TTGGTTTTGT TTAATGATTC GTTATAGAAA   
  
  
- GACACACTTT TTTATCAGTC TTTGTTTCGC TAGGCTGGCC GGTTTAGCTC TATTTGTGTC GGTTTCTTGG   
  
  
- CTTCAACCTG GTTTCACTCG GCTCAACTTT CCGTAAGTAT ACCCCAAATT AGTATTGTGT TTGTATTAAT   
  
  
- CTAACTAACT GCAGAAATTA GTCGCAAAAA CAGAAACAAG CCACGAGGGG AGTGAGCGGG GAGGCGAGAA   
  
  
- ATTTCGAATG GAGTGAGGGG GGTAAGGGTG AGGAAAAGAG AGAGGAGAGA GCGAGAGTGT GCGAGGCAGA   
  
  
- CAGTCGGGGA ACAGTAGAAG GGGGCAGAAG GAGCGTTTTT AGAGGTCGTT TTAAATATCG GCAACAACAC   
  
  
- GGGTACCACT AGACTACACT CTTCTCTAAG TCGTAAATTC TAATAACCCA GGTCTACCGA ATATACACGC   
  
  
- GGCTGTCACC ATTAGAGTAC CGATAACGGG TTGTTCAGTA GTTCGTTTTT GTTGTCGTTC TTGTCGTCGT   
  
  
- TGTGGTTGTC GGAGTAGGGG TCAAGCAGCC CAGCTTATTA TTGGGGAAAA CGTGGTCGGG TACCGGGCCT   
  
  
- GGGCAGGGAA GTAGGCGGTA CAGACGGTCG GGAGGCGGGG GAGAACCCAA GAGGCGGCGG CGCAAAGGCC   
  
  
- TGGGCAAAGT CCACCCGGGC GGGACGGGCC TACCCCGACT TGGCCCAAAG GTCAAACGGG TAAACCTGGT   
  
  
- GGTGAGCTCA CCAAAGGCCA AACGACTAAA AACGCCACCC CCACCACCAC TCAAACTGAG CCTACTCACC   
  
  
- TACCTCTCAA ACTACCCACC GCCGCCGCCA CTGAGCGCCG CTGACTCTCA AGATTGGAGG TTAGGGTAAC   
  
  
- GCTGCGCACC GTTCCCAGGC CACTAAAGCC AGAGATACCA CGACTAGGCA AACGCTGCAC AAGCCTTCTG   
  
  
- GCAACGTCGG CGATAAGTGC GAGTCGGTGA CGGCGTTGTC GTCGGCAGCA CCCAGGGCGG AAGAGGCGGC   
  
  
- TTTCTGTGCT TCGTCAGCCG GGGTGGAGTT TTCGTTTCGC TGCGGCGACC TGGGAGCAAG AGTAGGCTCC   
  
  
- TCCTTGGTCT CGGCAGTACC GGCGGCGATT AACGCCGCAA CAACCTCACG CGGTTTGAGC GGCTCAGGCT   
  
  
- GGGCCTTGTG CGACAGTTTA GCCACTAAGC TGAGTCTCTG AGTCAGAGTG TCGTCCCTCA AGGTTAGGTC   
  
  
- TCTCACAGGA TAATGAAGAG GCTCCGTGAG ATGGTGGCGG AAAGAGAGAG GAGGCGGGGA GTTGGAGAGG   
  
  
- GTCTTTGCTG CAGCAGACTC CTCATGTGAG AGAGAATGTT CCGGGACTTG CTGCGCACGG GCATGGTATT   
  
  
- TAAGCGAGTG AACTGGCGTT TAGTCCGCTA AGATCTTCGG TGACTAAGTC GGTTCTTCTA GGTGTAGCAA   
  
  
- CTAAAACCCT ATTAGGTCCC TCAGGTTACC CGACGAGACG ATGTTCGGGA CCGTTGGGCC GGTCGGCCCT   
  
  
- TCGGGCTTAA CTAAGCTTAT AGGCCTTAAG GACGAGGCCC AGACCCCGTT AGAGGCCGCC GTAGCGACGA   
  
  
- GCGTTGACCC TTAGCGGAGG CACTCAAGCG CTTCCAAGAT CTAGAGTTCA AGCTCAAACT CGGCCATGAG   
  
  
- TGAGGTTAGT CTCTCGATTT GCCCAGCTTG AAGGCCCAGC TGGGTCTACT CTATGATCGA CAGTTAAAGT   
  
  
- ACGAAGTTAA CATGTTGGAT AATCTGCTTT GGGACCAGCG ACAGCTGCGG CGGGACTTCG ACCGGTTCAG   
  
  
- TAACTTGGGG TTTTAGCAGT GAAACCCACT CATGCTCCGC CCAGACTTAG CCCGTCCAAA GAACTGGGCC   
  
  
- AAGTTCTGCC GGGAGTTCAT GATAAACCGT TACAAACTTA GCCAACTCGG GTTGAACCGG GCCCTGAGCG   
  
  
- GGCTCTCCCG AGTCCAACTC TCCGACAATA ACCCGGCCGC CTAGTACCCG AATCAACCCG GCCTCAATGG   
  
  
- CCCTTGCTCT TCCCTTTCCT ACCTCACATT CCTTGTTACC TTCCCAAATT ACCTTTCACG GCCAAAGCTC   
  
  
- GGCACGGTTT CGTCTTTGAT GCAGTCACTT TTTCTGTTCA AAATGGTGAG GTCTAACTTA ATGTCTCTCA   
  
  
- C

+     MYB

| Site Name | Organism | Position | Strand | Matrix score. | sequence | function |
| --- | --- | --- | --- | --- | --- | --- |
| MYB | Arabidopsis thaliana | 2559 | + | 6 | CAACAG |  |
| MYB | Arabidopsis thaliana | 2109 | + | 6 | CAACAG |  |
| MYB | Arabidopsis thaliana | 2085 | + | 6 | CAACAG |  |
| MYB | Arabidopsis thaliana | 1091 | - | 6 | TAACCA |  |
| MYB | Arabidopsis thaliana | 88 | - | 6 | CAACAG |  |
| MYB | Arabidopsis thaliana | 439 | - | 6 | CAACCA |  |

>HU06G00029.1   
+ +Up\_Stream \_Len000TGATAA TTATTTTTAT TGATTTAGGA AAGGATATCA AAGTATATTT TGTTTTTAGG   
  
  
+ TAGTTAAGAG ATTCTGTTGC CAAAAAAAAA ATCAAGATAT TATATATTTT TAATTATGAC AGTAATTATT   
  
  
+ TTATTGAAAA TCCAACGGTT GTGATTATAT AATCAAATAA TCTAAAGGTT AAAAATATCA AATTTATTAG   
  
  
+ AAAATTCGAA AAAGTCACGT CATCACAACA TTGCTTTTAT ATATAGTATA GATTCCTTCG ACTTCTAAAC   
  
  
+ TAATCACTTG AAATTAGAAA TTTGAAACTT TCTAATATAA ATGCATGGTC TTCTTTCCAA GATTATAATC   
  
  
+ CGCAATTGCT TAAATGTAGG GTACAAATAT CAAATTTAGA AGTGACTATT GAAGCCATGA AAGATATTGT   
  
  
+ GATCGTATTG AGGGTGGTTG TGAGCAATAG GATGAAACAG GAAAAAAAAA CCCTAATGTT TTCAAATTTA   
  
  
+ TGGGTGGCTT TGGTAGTAGA TAAGTGAATA GTGTGCAAGG GAATGGCACC AATATGTTTG GATGGAACGA   
  
  
+ TGAGAGTAAG ACGTTAGGAA AGCAACAAGA GAAGGGAAAA GGCAGTAGGG AGAGAATTAT GCGTGTGAAT   
  
  
+ AATATTATAA CAGACGCTTT CGTATGATTA TTAAATACAA GACTTTGAGC ATACATATAA ACTTGTAACT   
  
  
+ TTCTTTACCT ATTGTCAATT AATTTTTGAA TTGAATCATC TGCGACTTAT GCATACAAGA TGATATGATG   
  
  
+ CATATTGGGC CAAGCATTTG ATCTCATGTC CCTTATGGAT CTAGATCCCA ATAGACAAAC TTGCATAAGT   
  
  
+ CAAGGGATGT GTTTCTAATA CAAAGGCTTT GTACTCAAGC AATTAGTTTT GAATTCATTT TCAGTAGTTT   
  
  
+ AATTTCCAAA TCTTATAATT TTTACAATGG CATAAATTTT TAGGAGTTTT GAGTAAATAA ATTATATACA   
  
  
+ ACGAAGATGT CATTTACGAA TTAATATGAC GCACAAAAAA AAAGAAAAAA AATTAATGGT AGCTTCTATA   
  
  
+ CTTGACTCCA TGTTGACAAT TCCCTCTCTC ACATAATGGT TAAAACTTGC AACATCATCC AGGACCCATA   
  
  
+ TGACATGGGT GGTGGTAGAC AGTGTGTTGT GGTAGCCTCA CTCCATGCTT GCCAATTTGT ATCATTGCGC   
  
  
+ TAGTCTTATT CGTCCTTAGA GATAATTGAC TAAGCTCTAA TGCTCTATCG TGGCTGCTGC AATATAAATG   
  
  
+ ATTTTTTGAC CTCAAAATGA AGACAGCTAG TTGTAAATTA AGAATCTTCG TCCCTTCATT TTTTGTGTAC   
  
  
+ GTGTGTATCT GCGTATGTGT TGAGAGACTA GCGGACTATT GATTGAGTTT CAATCAAAAA AGATGACCAT   
  
  
+ CTTAAGTATT TTTAATTACG CAAACTTCTT CATTTAAAAA AATGTTACTT TTATTTGAAA GAAATTGTGA   
  
  
+ ATAATACAAG GAATAGTTCA ATTAATATAT AATCCATAAC ATATTACCTC CTATAGGCAA AAAGAAGATT   
  
  
+ ATCGTATTAA TTTGGGATCA TCCCACCCTA AATTTAAAGA AACCAAAACA AATTACTAAG CAATATCTTT   
  
  
+ CTGTGTGAAA AAATAGTCAG AAACAAAGCG ATCCGACCGG CCAAATCGAG ATAAACACAG CCAAAGAACC   
  
  
+ GAAGTTGGAC CAAAGTGAGC CGAGTTGAAA GGCATTCATA TGGGGTTTAA TCATAACACA AACATAATTA   
  
  
+ GATTGATTGA CGTCTTTAAT CAGCGTTTTT GTCTTTGTTC GGTGCTCCCC TCACTCGCCC CTCCGCTCTT   
  
  
+ TAAAGCTTAC CTCACTCCCC CCATTCCCAC TCCTTTTCTC TCTCCTCTCT CGCTCTCACA CGCTCCGTCT   
  
  
+ GTCAGCCCCT TGTCATCTTC CCCCGTCTTC CTCGCAAAAA TCTCCAGCAA AATTTATAGC CGTTGTTGTG   
  
  
+ CCCATGGTGA TCTGATGTGA GAAGAGATTC AGCATTTAAG ATTATTGGGT CCAGATGGCT TATATGTGCG   
  
  
+ CCGACAGTGG TAATCTCATG GCTATTGCCC AACAAGTCAT CAAGCAAAAA CAACAGCAAG AACAGCAGCA   
  
  
+ ACACCAACAG CCTCATCCCC AGTTCGTCGG GTCGAATAAT AACCCCTTTT GCACCAGCCC ATGGCCCGGA   
  
  
+ CCCGTCCCTT CATCCGCCAT GTCTGCCAGC CCTCCGCCCC CTCTTGGGTT CTCCGCCGCC GCGTTTCCGG   
  
  
+ ACCCGTTTCA GGTGGGCCCG CCCTGCCCGG ATGGGGCTGA ACCGGGTTTC CAGTTTGCCC ATTTGGACCA   
  
  
+ CCACTCGAGT GGTTTCCGGT TTGCTGATTT TTGCGGTGGG GGTGGTGGTG AGTTTGACTC GGATGAGTGG   
  
  
+ ATGGAGAGTT TGATGGGTGG CGGCGGCGGT GACTCGCGGC GACTGAGAGT TCTAACCTCC AATCCCATTG   
  
  
+ CGACGCGTGG CAAGGGTCCG GTGATTTCGG TCTCTATGGT GCTGATCCGT TTGCGACGTG TTCGGAAGAC   
  
  
+ CGTTGCAGCC GCTATTCACG CTCAGCCACT GCCGCAACAG CAGCCGTCGT GGGTCCCGCC TTCTCCGCCG   
  
  
+ AAAGACACGA AGCAGTCGGC CCCACCTCAA AAGCAAAGCG ACGCCGCTGG ACCCTCGTTC TCATCCGAGG   
  
  
+ AGGAACCAGA GCCGTCATGG CCGCCGCTAA TTGCGGCGTT GTTGGAGTGC GCCAAACTCG CCGAGTCCGA   
  
  
+ CCCGGAACAC GCTGTCAAAT CGGTGATTCG ACTCAGAGAC TCAGTCTCAC AGCAGGGAGT TCCAATCCAG   
  
  
+ AGAGTGTCCT ATTACTTCTC CGAGGCACTC TACCACCGCC TTTCTCTCTC CTCCGCCCCT CAACCTCTCC   
  
  
+ CAGAAACGAC GTCGTCTGAG GAGTACACTC TCTCTTACAA GGCCCTGAAC GACGCGTGCC CGTACCATAA   
  
  
+ ATTCGCTCAC TTGACCGCAA ATCAGGCGAT TCTAGAAGCC ACTGATTCAG CCAAGAAGAT CCACATCGTT   
  
  
+ GATTTTGGGA TAATCCAGGG AGTCCAATGG GCTGCTCTGC TACAAGCCCT GGCAACCCGG CCAGCCGGGA   
  
  
+ AGCCCGAATT GATTCGAATA TCCGGAATTC CTGCTCCGGG TCTGGGGCAA TCTCCGGCGG CATCGCTGCT   
  
  
+ CGCAACTGGG AATCGCCTCC GTGAGTTCGC GAAGGTTCTA GATCTCAAGT TCGAGTTTGA GCCGGTACTC   
  
  
+ ACTCCAATCA GAGAGCTAAA CGGGTCGAAC TTCCGGGTCG ACCCAGATGA GATACTAGCT GTCAATTTCA   
  
  
+ TGCTTCAATT GTACAACCTA TTAGACGAAA CCCTGGTCGC TGTCGACGCC GCCCTGAAGC TGGCCAAGTC   
  
  
+ ATTGAACCCC AAAATCGTCA CTTTGGGTGA GTACGAGGCG GGTCTGAATC GGGCAGGTTT CTTGACCCGG   
  
  
+ TTCAAGACGG CCCTCAAGTA CTATTTGGCA ATGTTTGAAT CGGTTGAGCC CAACTTGGCC CGGGACTCGC   
  
  
+ CCGAGAGGGC TCAGGTTGAG AGGCTGTTAT TGGGCCGGCG GATCATGGGC TTAGTTGGGC CGGAGTTACC   
  
  
+ GGGAACGAGA AGGGAAAGGA TGGAGTGTAA GGAACAATGG AAGGGTTTAA TGGAAAGTGC CGGTTTCGAG   
  
  
+ CCGTGCCAAA GCAGAAACTA CGTCAGTGAA AAAGACAAGT TTTACCACTC CAGATTGAAT TACAGAGAGT   
  
  
+ G  

- +Up\_Stream \_Len000ACTATT AATAAAAATA ACTAAATCCT TTCCTATAGT TTCATATAAA ACAAAAATCC   
  
  
- ATCAATTCTC TAAGACAACG GTTTTTTTTT TAGTTCTATA ATATATAAAA ATTAATACTG TCATTAATAA   
  
  
- AATAACTTTT AGGTTGCCAA CACTAATATA TTAGTTTATT AGATTTCCAA TTTTTATAGT TTAAATAATC   
  
  
- TTTTAAGCTT TTTCAGTGCA GTAGTGTTGT AACGAAAATA TATATCATAT CTAAGGAAGC TGAAGATTTG   
  
  
- ATTAGTGAAC TTTAATCTTT AAACTTTGAA AGATTATATT TACGTACCAG AAGAAAGGTT CTAATATTAG   
  
  
- GCGTTAACGA ATTTACATCC CATGTTTATA GTTTAAATCT TCACTGATAA CTTCGGTACT TTCTATAACA   
  
  
- CTAGCATAAC TCCCACCAAC ACTCGTTATC CTACTTTGTC CTTTTTTTTT GGGATTACAA AAGTTTAAAT   
  
  
- ACCCACCGAA ACCATCATCT ATTCACTTAT CACACGTTCC CTTACCGTGG TTATACAAAC CTACCTTGCT   
  
  
- ACTCTCATTC TGCAATCCTT TCGTTGTTCT CTTCCCTTTT CCGTCATCCC TCTCTTAATA CGCACACTTA   
  
  
- TTATAATATT GTCTGCGAAA GCATACTAAT AATTTATGTT CTGAAACTCG TATGTATATT TGAACATTGA   
  
  
- AAGAAATGGA TAACAGTTAA TTAAAAACTT AACTTAGTAG ACGCTGAATA CGTATGTTCT ACTATACTAC   
  
  
- GTATAACCCG GTTCGTAAAC TAGAGTACAG GGAATACCTA GATCTAGGGT TATCTGTTTG AACGTATTCA   
  
  
- GTTCCCTACA CAAAGATTAT GTTTCCGAAA CATGAGTTCG TTAATCAAAA CTTAAGTAAA AGTCATCAAA   
  
  
- TTAAAGGTTT AGAATATTAA AAATGTTACC GTATTTAAAA ATCCTCAAAA CTCATTTATT TAATATATGT   
  
  
- TGCTTCTACA GTAAATGCTT AATTATACTG CGTGTTTTTT TTTCTTTTTT TTAATTACCA TCGAAGATAT   
  
  
- GAACTGAGGT ACAACTGTTA AGGGAGAGAG TGTATTACCA ATTTTGAACG TTGTAGTAGG TCCTGGGTAT   
  
  
- ACTGTACCCA CCACCATCTG TCACACAACA CCATCGGAGT GAGGTACGAA CGGTTAAACA TAGTAACGCG   
  
  
- ATCAGAATAA GCAGGAATCT CTATTAACTG ATTCGAGATT ACGAGATAGC ACCGACGACG TTATATTTAC   
  
  
- TAAAAAACTG GAGTTTTACT TCTGTCGATC AACATTTAAT TCTTAGAAGC AGGGAAGTAA AAAACACATG   
  
  
- CACACATAGA CGCATACACA ACTCTCTGAT CGCCTGATAA CTAACTCAAA GTTAGTTTTT TCTACTGGTA   
  
  
- GAATTCATAA AAATTAATGC GTTTGAAGAA GTAAATTTTT TTACAATGAA AATAAACTTT CTTTAACACT   
  
  
- TATTATGTTC CTTATCAAGT TAATTATATA TTAGGTATTG TATAATGGAG GATATCCGTT TTTCTTCTAA   
  
  
- TAGCATAATT AAACCCTAGT AGGGTGGGAT TTAAATTTCT TTGGTTTTGT TTAATGATTC GTTATAGAAA   
  
  
- GACACACTTT TTTATCAGTC TTTGTTTCGC TAGGCTGGCC GGTTTAGCTC TATTTGTGTC GGTTTCTTGG   
  
  
- CTTCAACCTG GTTTCACTCG GCTCAACTTT CCGTAAGTAT ACCCCAAATT AGTATTGTGT TTGTATTAAT   
  
  
- CTAACTAACT GCAGAAATTA GTCGCAAAAA CAGAAACAAG CCACGAGGGG AGTGAGCGGG GAGGCGAGAA   
  
  
- ATTTCGAATG GAGTGAGGGG GGTAAGGGTG AGGAAAAGAG AGAGGAGAGA GCGAGAGTGT GCGAGGCAGA   
  
  
- CAGTCGGGGA ACAGTAGAAG GGGGCAGAAG GAGCGTTTTT AGAGGTCGTT TTAAATATCG GCAACAACAC   
  
  
- GGGTACCACT AGACTACACT CTTCTCTAAG TCGTAAATTC TAATAACCCA GGTCTACCGA ATATACACGC   
  
  
- GGCTGTCACC ATTAGAGTAC CGATAACGGG TTGTTCAGTA GTTCGTTTTT GTTGTCGTTC TTGTCGTCGT   
  
  
- TGTGGTTGTC GGAGTAGGGG TCAAGCAGCC CAGCTTATTA TTGGGGAAAA CGTGGTCGGG TACCGGGCCT   
  
  
- GGGCAGGGAA GTAGGCGGTA CAGACGGTCG GGAGGCGGGG GAGAACCCAA GAGGCGGCGG CGCAAAGGCC   
  
  
- TGGGCAAAGT CCACCCGGGC GGGACGGGCC TACCCCGACT TGGCCCAAAG GTCAAACGGG TAAACCTGGT   
  
  
- GGTGAGCTCA CCAAAGGCCA AACGACTAAA AACGCCACCC CCACCACCAC TCAAACTGAG CCTACTCACC   
  
  
- TACCTCTCAA ACTACCCACC GCCGCCGCCA CTGAGCGCCG CTGACTCTCA AGATTGGAGG TTAGGGTAAC   
  
  
- GCTGCGCACC GTTCCCAGGC CACTAAAGCC AGAGATACCA CGACTAGGCA AACGCTGCAC AAGCCTTCTG   
  
  
- GCAACGTCGG CGATAAGTGC GAGTCGGTGA CGGCGTTGTC GTCGGCAGCA CCCAGGGCGG AAGAGGCGGC   
  
  
- TTTCTGTGCT TCGTCAGCCG GGGTGGAGTT TTCGTTTCGC TGCGGCGACC TGGGAGCAAG AGTAGGCTCC   
  
  
- TCCTTGGTCT CGGCAGTACC GGCGGCGATT AACGCCGCAA CAACCTCACG CGGTTTGAGC GGCTCAGGCT   
  
  
- GGGCCTTGTG CGACAGTTTA GCCACTAAGC TGAGTCTCTG AGTCAGAGTG TCGTCCCTCA AGGTTAGGTC   
  
  
- TCTCACAGGA TAATGAAGAG GCTCCGTGAG ATGGTGGCGG AAAGAGAGAG GAGGCGGGGA GTTGGAGAGG   
  
  
- GTCTTTGCTG CAGCAGACTC CTCATGTGAG AGAGAATGTT CCGGGACTTG CTGCGCACGG GCATGGTATT   
  
  
- TAAGCGAGTG AACTGGCGTT TAGTCCGCTA AGATCTTCGG TGACTAAGTC GGTTCTTCTA GGTGTAGCAA   
  
  
- CTAAAACCCT ATTAGGTCCC TCAGGTTACC CGACGAGACG ATGTTCGGGA CCGTTGGGCC GGTCGGCCCT   
  
  
- TCGGGCTTAA CTAAGCTTAT AGGCCTTAAG GACGAGGCCC AGACCCCGTT AGAGGCCGCC GTAGCGACGA   
  
  
- GCGTTGACCC TTAGCGGAGG CACTCAAGCG CTTCCAAGAT CTAGAGTTCA AGCTCAAACT CGGCCATGAG   
  
  
- TGAGGTTAGT CTCTCGATTT GCCCAGCTTG AAGGCCCAGC TGGGTCTACT CTATGATCGA CAGTTAAAGT   
  
  
- ACGAAGTTAA CATGTTGGAT AATCTGCTTT GGGACCAGCG ACAGCTGCGG CGGGACTTCG ACCGGTTCAG   
  
  
- TAACTTGGGG TTTTAGCAGT GAAACCCACT CATGCTCCGC CCAGACTTAG CCCGTCCAAA GAACTGGGCC   
  
  
- AAGTTCTGCC GGGAGTTCAT GATAAACCGT TACAAACTTA GCCAACTCGG GTTGAACCGG GCCCTGAGCG   
  
  
- GGCTCTCCCG AGTCCAACTC TCCGACAATA ACCCGGCCGC CTAGTACCCG AATCAACCCG GCCTCAATGG   
  
  
- CCCTTGCTCT TCCCTTTCCT ACCTCACATT CCTTGTTACC TTCCCAAATT ACCTTTCACG GCCAAAGCTC   
  
  
- GGCACGGTTT CGTCTTTGAT GCAGTCACTT TTTCTGTTCA AAATGGTGAG GTCTAACTTA ATGTCTCTCA   
  
  
- C

+     MYB recognition site

| Site Name | Organism | Position | Strand | Matrix score. | sequence | function |
| --- | --- | --- | --- | --- | --- | --- |
| MYB recognition site | Arabidopsis thaliana | 1954 | + | 6 | CCGTTG |  |
| MYB recognition site | Arabidopsis thaliana | 2524 | + | 6 | CCGTTG |  |
| MYB recognition site | Arabidopsis thaliana | 157 | - | 6 | CCGTTG |  |

>HU06G00029.1   
+ +Up\_Stream \_Len000TGATAA TTATTTTTAT TGATTTAGGA AAGGATATCA AAGTATATTT TGTTTTTAGG   
  
  
+ TAGTTAAGAG ATTCTGTTGC CAAAAAAAAA ATCAAGATAT TATATATTTT TAATTATGAC AGTAATTATT   
  
  
+ TTATTGAAAA TCCAACGGTT GTGATTATAT AATCAAATAA TCTAAAGGTT AAAAATATCA AATTTATTAG   
  
  
+ AAAATTCGAA AAAGTCACGT CATCACAACA TTGCTTTTAT ATATAGTATA GATTCCTTCG ACTTCTAAAC   
  
  
+ TAATCACTTG AAATTAGAAA TTTGAAACTT TCTAATATAA ATGCATGGTC TTCTTTCCAA GATTATAATC   
  
  
+ CGCAATTGCT TAAATGTAGG GTACAAATAT CAAATTTAGA AGTGACTATT GAAGCCATGA AAGATATTGT   
  
  
+ GATCGTATTG AGGGTGGTTG TGAGCAATAG GATGAAACAG GAAAAAAAAA CCCTAATGTT TTCAAATTTA   
  
  
+ TGGGTGGCTT TGGTAGTAGA TAAGTGAATA GTGTGCAAGG GAATGGCACC AATATGTTTG GATGGAACGA   
  
  
+ TGAGAGTAAG ACGTTAGGAA AGCAACAAGA GAAGGGAAAA GGCAGTAGGG AGAGAATTAT GCGTGTGAAT   
  
  
+ AATATTATAA CAGACGCTTT CGTATGATTA TTAAATACAA GACTTTGAGC ATACATATAA ACTTGTAACT   
  
  
+ TTCTTTACCT ATTGTCAATT AATTTTTGAA TTGAATCATC TGCGACTTAT GCATACAAGA TGATATGATG   
  
  
+ CATATTGGGC CAAGCATTTG ATCTCATGTC CCTTATGGAT CTAGATCCCA ATAGACAAAC TTGCATAAGT   
  
  
+ CAAGGGATGT GTTTCTAATA CAAAGGCTTT GTACTCAAGC AATTAGTTTT GAATTCATTT TCAGTAGTTT   
  
  
+ AATTTCCAAA TCTTATAATT TTTACAATGG CATAAATTTT TAGGAGTTTT GAGTAAATAA ATTATATACA   
  
  
+ ACGAAGATGT CATTTACGAA TTAATATGAC GCACAAAAAA AAAGAAAAAA AATTAATGGT AGCTTCTATA   
  
  
+ CTTGACTCCA TGTTGACAAT TCCCTCTCTC ACATAATGGT TAAAACTTGC AACATCATCC AGGACCCATA   
  
  
+ TGACATGGGT GGTGGTAGAC AGTGTGTTGT GGTAGCCTCA CTCCATGCTT GCCAATTTGT ATCATTGCGC   
  
  
+ TAGTCTTATT CGTCCTTAGA GATAATTGAC TAAGCTCTAA TGCTCTATCG TGGCTGCTGC AATATAAATG   
  
  
+ ATTTTTTGAC CTCAAAATGA AGACAGCTAG TTGTAAATTA AGAATCTTCG TCCCTTCATT TTTTGTGTAC   
  
  
+ GTGTGTATCT GCGTATGTGT TGAGAGACTA GCGGACTATT GATTGAGTTT CAATCAAAAA AGATGACCAT   
  
  
+ CTTAAGTATT TTTAATTACG CAAACTTCTT CATTTAAAAA AATGTTACTT TTATTTGAAA GAAATTGTGA   
  
  
+ ATAATACAAG GAATAGTTCA ATTAATATAT AATCCATAAC ATATTACCTC CTATAGGCAA AAAGAAGATT   
  
  
+ ATCGTATTAA TTTGGGATCA TCCCACCCTA AATTTAAAGA AACCAAAACA AATTACTAAG CAATATCTTT   
  
  
+ CTGTGTGAAA AAATAGTCAG AAACAAAGCG ATCCGACCGG CCAAATCGAG ATAAACACAG CCAAAGAACC   
  
  
+ GAAGTTGGAC CAAAGTGAGC CGAGTTGAAA GGCATTCATA TGGGGTTTAA TCATAACACA AACATAATTA   
  
  
+ GATTGATTGA CGTCTTTAAT CAGCGTTTTT GTCTTTGTTC GGTGCTCCCC TCACTCGCCC CTCCGCTCTT   
  
  
+ TAAAGCTTAC CTCACTCCCC CCATTCCCAC TCCTTTTCTC TCTCCTCTCT CGCTCTCACA CGCTCCGTCT   
  
  
+ GTCAGCCCCT TGTCATCTTC CCCCGTCTTC CTCGCAAAAA TCTCCAGCAA AATTTATAGC CGTTGTTGTG   
  
  
+ CCCATGGTGA TCTGATGTGA GAAGAGATTC AGCATTTAAG ATTATTGGGT CCAGATGGCT TATATGTGCG   
  
  
+ CCGACAGTGG TAATCTCATG GCTATTGCCC AACAAGTCAT CAAGCAAAAA CAACAGCAAG AACAGCAGCA   
  
  
+ ACACCAACAG CCTCATCCCC AGTTCGTCGG GTCGAATAAT AACCCCTTTT GCACCAGCCC ATGGCCCGGA   
  
  
+ CCCGTCCCTT CATCCGCCAT GTCTGCCAGC CCTCCGCCCC CTCTTGGGTT CTCCGCCGCC GCGTTTCCGG   
  
  
+ ACCCGTTTCA GGTGGGCCCG CCCTGCCCGG ATGGGGCTGA ACCGGGTTTC CAGTTTGCCC ATTTGGACCA   
  
  
+ CCACTCGAGT GGTTTCCGGT TTGCTGATTT TTGCGGTGGG GGTGGTGGTG AGTTTGACTC GGATGAGTGG   
  
  
+ ATGGAGAGTT TGATGGGTGG CGGCGGCGGT GACTCGCGGC GACTGAGAGT TCTAACCTCC AATCCCATTG   
  
  
+ CGACGCGTGG CAAGGGTCCG GTGATTTCGG TCTCTATGGT GCTGATCCGT TTGCGACGTG TTCGGAAGAC   
  
  
+ CGTTGCAGCC GCTATTCACG CTCAGCCACT GCCGCAACAG CAGCCGTCGT GGGTCCCGCC TTCTCCGCCG   
  
  
+ AAAGACACGA AGCAGTCGGC CCCACCTCAA AAGCAAAGCG ACGCCGCTGG ACCCTCGTTC TCATCCGAGG   
  
  
+ AGGAACCAGA GCCGTCATGG CCGCCGCTAA TTGCGGCGTT GTTGGAGTGC GCCAAACTCG CCGAGTCCGA   
  
  
+ CCCGGAACAC GCTGTCAAAT CGGTGATTCG ACTCAGAGAC TCAGTCTCAC AGCAGGGAGT TCCAATCCAG   
  
  
+ AGAGTGTCCT ATTACTTCTC CGAGGCACTC TACCACCGCC TTTCTCTCTC CTCCGCCCCT CAACCTCTCC   
  
  
+ CAGAAACGAC GTCGTCTGAG GAGTACACTC TCTCTTACAA GGCCCTGAAC GACGCGTGCC CGTACCATAA   
  
  
+ ATTCGCTCAC TTGACCGCAA ATCAGGCGAT TCTAGAAGCC ACTGATTCAG CCAAGAAGAT CCACATCGTT   
  
  
+ GATTTTGGGA TAATCCAGGG AGTCCAATGG GCTGCTCTGC TACAAGCCCT GGCAACCCGG CCAGCCGGGA   
  
  
+ AGCCCGAATT GATTCGAATA TCCGGAATTC CTGCTCCGGG TCTGGGGCAA TCTCCGGCGG CATCGCTGCT   
  
  
+ CGCAACTGGG AATCGCCTCC GTGAGTTCGC GAAGGTTCTA GATCTCAAGT TCGAGTTTGA GCCGGTACTC   
  
  
+ ACTCCAATCA GAGAGCTAAA CGGGTCGAAC TTCCGGGTCG ACCCAGATGA GATACTAGCT GTCAATTTCA   
  
  
+ TGCTTCAATT GTACAACCTA TTAGACGAAA CCCTGGTCGC TGTCGACGCC GCCCTGAAGC TGGCCAAGTC   
  
  
+ ATTGAACCCC AAAATCGTCA CTTTGGGTGA GTACGAGGCG GGTCTGAATC GGGCAGGTTT CTTGACCCGG   
  
  
+ TTCAAGACGG CCCTCAAGTA CTATTTGGCA ATGTTTGAAT CGGTTGAGCC CAACTTGGCC CGGGACTCGC   
  
  
+ CCGAGAGGGC TCAGGTTGAG AGGCTGTTAT TGGGCCGGCG GATCATGGGC TTAGTTGGGC CGGAGTTACC   
  
  
+ GGGAACGAGA AGGGAAAGGA TGGAGTGTAA GGAACAATGG AAGGGTTTAA TGGAAAGTGC CGGTTTCGAG   
  
  
+ CCGTGCCAAA GCAGAAACTA CGTCAGTGAA AAAGACAAGT TTTACCACTC CAGATTGAAT TACAGAGAGT   
  
  
+ G  

- +Up\_Stream \_Len000ACTATT AATAAAAATA ACTAAATCCT TTCCTATAGT TTCATATAAA ACAAAAATCC   
  
  
- ATCAATTCTC TAAGACAACG GTTTTTTTTT TAGTTCTATA ATATATAAAA ATTAATACTG TCATTAATAA   
  
  
- AATAACTTTT AGGTTGCCAA CACTAATATA TTAGTTTATT AGATTTCCAA TTTTTATAGT TTAAATAATC   
  
  
- TTTTAAGCTT TTTCAGTGCA GTAGTGTTGT AACGAAAATA TATATCATAT CTAAGGAAGC TGAAGATTTG   
  
  
- ATTAGTGAAC TTTAATCTTT AAACTTTGAA AGATTATATT TACGTACCAG AAGAAAGGTT CTAATATTAG   
  
  
- GCGTTAACGA ATTTACATCC CATGTTTATA GTTTAAATCT TCACTGATAA CTTCGGTACT TTCTATAACA   
  
  
- CTAGCATAAC TCCCACCAAC ACTCGTTATC CTACTTTGTC CTTTTTTTTT GGGATTACAA AAGTTTAAAT   
  
  
- ACCCACCGAA ACCATCATCT ATTCACTTAT CACACGTTCC CTTACCGTGG TTATACAAAC CTACCTTGCT   
  
  
- ACTCTCATTC TGCAATCCTT TCGTTGTTCT CTTCCCTTTT CCGTCATCCC TCTCTTAATA CGCACACTTA   
  
  
- TTATAATATT GTCTGCGAAA GCATACTAAT AATTTATGTT CTGAAACTCG TATGTATATT TGAACATTGA   
  
  
- AAGAAATGGA TAACAGTTAA TTAAAAACTT AACTTAGTAG ACGCTGAATA CGTATGTTCT ACTATACTAC   
  
  
- GTATAACCCG GTTCGTAAAC TAGAGTACAG GGAATACCTA GATCTAGGGT TATCTGTTTG AACGTATTCA   
  
  
- GTTCCCTACA CAAAGATTAT GTTTCCGAAA CATGAGTTCG TTAATCAAAA CTTAAGTAAA AGTCATCAAA   
  
  
- TTAAAGGTTT AGAATATTAA AAATGTTACC GTATTTAAAA ATCCTCAAAA CTCATTTATT TAATATATGT   
  
  
- TGCTTCTACA GTAAATGCTT AATTATACTG CGTGTTTTTT TTTCTTTTTT TTAATTACCA TCGAAGATAT   
  
  
- GAACTGAGGT ACAACTGTTA AGGGAGAGAG TGTATTACCA ATTTTGAACG TTGTAGTAGG TCCTGGGTAT   
  
  
- ACTGTACCCA CCACCATCTG TCACACAACA CCATCGGAGT GAGGTACGAA CGGTTAAACA TAGTAACGCG   
  
  
- ATCAGAATAA GCAGGAATCT CTATTAACTG ATTCGAGATT ACGAGATAGC ACCGACGACG TTATATTTAC   
  
  
- TAAAAAACTG GAGTTTTACT TCTGTCGATC AACATTTAAT TCTTAGAAGC AGGGAAGTAA AAAACACATG   
  
  
- CACACATAGA CGCATACACA ACTCTCTGAT CGCCTGATAA CTAACTCAAA GTTAGTTTTT TCTACTGGTA   
  
  
- GAATTCATAA AAATTAATGC GTTTGAAGAA GTAAATTTTT TTACAATGAA AATAAACTTT CTTTAACACT   
  
  
- TATTATGTTC CTTATCAAGT TAATTATATA TTAGGTATTG TATAATGGAG GATATCCGTT TTTCTTCTAA   
  
  
- TAGCATAATT AAACCCTAGT AGGGTGGGAT TTAAATTTCT TTGGTTTTGT TTAATGATTC GTTATAGAAA   
  
  
- GACACACTTT TTTATCAGTC TTTGTTTCGC TAGGCTGGCC GGTTTAGCTC TATTTGTGTC GGTTTCTTGG   
  
  
- CTTCAACCTG GTTTCACTCG GCTCAACTTT CCGTAAGTAT ACCCCAAATT AGTATTGTGT TTGTATTAAT   
  
  
- CTAACTAACT GCAGAAATTA GTCGCAAAAA CAGAAACAAG CCACGAGGGG AGTGAGCGGG GAGGCGAGAA   
  
  
- ATTTCGAATG GAGTGAGGGG GGTAAGGGTG AGGAAAAGAG AGAGGAGAGA GCGAGAGTGT GCGAGGCAGA   
  
  
- CAGTCGGGGA ACAGTAGAAG GGGGCAGAAG GAGCGTTTTT AGAGGTCGTT TTAAATATCG GCAACAACAC   
  
  
- GGGTACCACT AGACTACACT CTTCTCTAAG TCGTAAATTC TAATAACCCA GGTCTACCGA ATATACACGC   
  
  
- GGCTGTCACC ATTAGAGTAC CGATAACGGG TTGTTCAGTA GTTCGTTTTT GTTGTCGTTC TTGTCGTCGT   
  
  
- TGTGGTTGTC GGAGTAGGGG TCAAGCAGCC CAGCTTATTA TTGGGGAAAA CGTGGTCGGG TACCGGGCCT   
  
  
- GGGCAGGGAA GTAGGCGGTA CAGACGGTCG GGAGGCGGGG GAGAACCCAA GAGGCGGCGG CGCAAAGGCC   
  
  
- TGGGCAAAGT CCACCCGGGC GGGACGGGCC TACCCCGACT TGGCCCAAAG GTCAAACGGG TAAACCTGGT   
  
  
- GGTGAGCTCA CCAAAGGCCA AACGACTAAA AACGCCACCC CCACCACCAC TCAAACTGAG CCTACTCACC   
  
  
- TACCTCTCAA ACTACCCACC GCCGCCGCCA CTGAGCGCCG CTGACTCTCA AGATTGGAGG TTAGGGTAAC   
  
  
- GCTGCGCACC GTTCCCAGGC CACTAAAGCC AGAGATACCA CGACTAGGCA AACGCTGCAC AAGCCTTCTG   
  
  
- GCAACGTCGG CGATAAGTGC GAGTCGGTGA CGGCGTTGTC GTCGGCAGCA CCCAGGGCGG AAGAGGCGGC   
  
  
- TTTCTGTGCT TCGTCAGCCG GGGTGGAGTT TTCGTTTCGC TGCGGCGACC TGGGAGCAAG AGTAGGCTCC   
  
  
- TCCTTGGTCT CGGCAGTACC GGCGGCGATT AACGCCGCAA CAACCTCACG CGGTTTGAGC GGCTCAGGCT   
  
  
- GGGCCTTGTG CGACAGTTTA GCCACTAAGC TGAGTCTCTG AGTCAGAGTG TCGTCCCTCA AGGTTAGGTC   
  
  
- TCTCACAGGA TAATGAAGAG GCTCCGTGAG ATGGTGGCGG AAAGAGAGAG GAGGCGGGGA GTTGGAGAGG   
  
  
- GTCTTTGCTG CAGCAGACTC CTCATGTGAG AGAGAATGTT CCGGGACTTG CTGCGCACGG GCATGGTATT   
  
  
- TAAGCGAGTG AACTGGCGTT TAGTCCGCTA AGATCTTCGG TGACTAAGTC GGTTCTTCTA GGTGTAGCAA   
  
  
- CTAAAACCCT ATTAGGTCCC TCAGGTTACC CGACGAGACG ATGTTCGGGA CCGTTGGGCC GGTCGGCCCT   
  
  
- TCGGGCTTAA CTAAGCTTAT AGGCCTTAAG GACGAGGCCC AGACCCCGTT AGAGGCCGCC GTAGCGACGA   
  
  
- GCGTTGACCC TTAGCGGAGG CACTCAAGCG CTTCCAAGAT CTAGAGTTCA AGCTCAAACT CGGCCATGAG   
  
  
- TGAGGTTAGT CTCTCGATTT GCCCAGCTTG AAGGCCCAGC TGGGTCTACT CTATGATCGA CAGTTAAAGT   
  
  
- ACGAAGTTAA CATGTTGGAT AATCTGCTTT GGGACCAGCG ACAGCTGCGG CGGGACTTCG ACCGGTTCAG   
  
  
- TAACTTGGGG TTTTAGCAGT GAAACCCACT CATGCTCCGC CCAGACTTAG CCCGTCCAAA GAACTGGGCC   
  
  
- AAGTTCTGCC GGGAGTTCAT GATAAACCGT TACAAACTTA GCCAACTCGG GTTGAACCGG GCCCTGAGCG   
  
  
- GGCTCTCCCG AGTCCAACTC TCCGACAATA ACCCGGCCGC CTAGTACCCG AATCAACCCG GCCTCAATGG   
  
  
- CCCTTGCTCT TCCCTTTCCT ACCTCACATT CCTTGTTACC TTCCCAAATT ACCTTTCACG GCCAAAGCTC   
  
  
- GGCACGGTTT CGTCTTTGAT GCAGTCACTT TTTCTGTTCA AAATGGTGAG GTCTAACTTA ATGTCTCTCA   
  
  
- C

+     MYB-like sequence

| Site Name | Organism | Position | Strand | Matrix score. | sequence | function |
| --- | --- | --- | --- | --- | --- | --- |
| MYB-like sequence | Arabidopsis thaliana | 1091 | - | 6 | TAACCA |  |

>HU06G00029.1   
+ +Up\_Stream \_Len000TGATAA TTATTTTTAT TGATTTAGGA AAGGATATCA AAGTATATTT TGTTTTTAGG   
  
  
+ TAGTTAAGAG ATTCTGTTGC CAAAAAAAAA ATCAAGATAT TATATATTTT TAATTATGAC AGTAATTATT   
  
  
+ TTATTGAAAA TCCAACGGTT GTGATTATAT AATCAAATAA TCTAAAGGTT AAAAATATCA AATTTATTAG   
  
  
+ AAAATTCGAA AAAGTCACGT CATCACAACA TTGCTTTTAT ATATAGTATA GATTCCTTCG ACTTCTAAAC   
  
  
+ TAATCACTTG AAATTAGAAA TTTGAAACTT TCTAATATAA ATGCATGGTC TTCTTTCCAA GATTATAATC   
  
  
+ CGCAATTGCT TAAATGTAGG GTACAAATAT CAAATTTAGA AGTGACTATT GAAGCCATGA AAGATATTGT   
  
  
+ GATCGTATTG AGGGTGGTTG TGAGCAATAG GATGAAACAG GAAAAAAAAA CCCTAATGTT TTCAAATTTA   
  
  
+ TGGGTGGCTT TGGTAGTAGA TAAGTGAATA GTGTGCAAGG GAATGGCACC AATATGTTTG GATGGAACGA   
  
  
+ TGAGAGTAAG ACGTTAGGAA AGCAACAAGA GAAGGGAAAA GGCAGTAGGG AGAGAATTAT GCGTGTGAAT   
  
  
+ AATATTATAA CAGACGCTTT CGTATGATTA TTAAATACAA GACTTTGAGC ATACATATAA ACTTGTAACT   
  
  
+ TTCTTTACCT ATTGTCAATT AATTTTTGAA TTGAATCATC TGCGACTTAT GCATACAAGA TGATATGATG   
  
  
+ CATATTGGGC CAAGCATTTG ATCTCATGTC CCTTATGGAT CTAGATCCCA ATAGACAAAC TTGCATAAGT   
  
  
+ CAAGGGATGT GTTTCTAATA CAAAGGCTTT GTACTCAAGC AATTAGTTTT GAATTCATTT TCAGTAGTTT   
  
  
+ AATTTCCAAA TCTTATAATT TTTACAATGG CATAAATTTT TAGGAGTTTT GAGTAAATAA ATTATATACA   
  
  
+ ACGAAGATGT CATTTACGAA TTAATATGAC GCACAAAAAA AAAGAAAAAA AATTAATGGT AGCTTCTATA   
  
  
+ CTTGACTCCA TGTTGACAAT TCCCTCTCTC ACATAATGGT TAAAACTTGC AACATCATCC AGGACCCATA   
  
  
+ TGACATGGGT GGTGGTAGAC AGTGTGTTGT GGTAGCCTCA CTCCATGCTT GCCAATTTGT ATCATTGCGC   
  
  
+ TAGTCTTATT CGTCCTTAGA GATAATTGAC TAAGCTCTAA TGCTCTATCG TGGCTGCTGC AATATAAATG   
  
  
+ ATTTTTTGAC CTCAAAATGA AGACAGCTAG TTGTAAATTA AGAATCTTCG TCCCTTCATT TTTTGTGTAC   
  
  
+ GTGTGTATCT GCGTATGTGT TGAGAGACTA GCGGACTATT GATTGAGTTT CAATCAAAAA AGATGACCAT   
  
  
+ CTTAAGTATT TTTAATTACG CAAACTTCTT CATTTAAAAA AATGTTACTT TTATTTGAAA GAAATTGTGA   
  
  
+ ATAATACAAG GAATAGTTCA ATTAATATAT AATCCATAAC ATATTACCTC CTATAGGCAA AAAGAAGATT   
  
  
+ ATCGTATTAA TTTGGGATCA TCCCACCCTA AATTTAAAGA AACCAAAACA AATTACTAAG CAATATCTTT   
  
  
+ CTGTGTGAAA AAATAGTCAG AAACAAAGCG ATCCGACCGG CCAAATCGAG ATAAACACAG CCAAAGAACC   
  
  
+ GAAGTTGGAC CAAAGTGAGC CGAGTTGAAA GGCATTCATA TGGGGTTTAA TCATAACACA AACATAATTA   
  
  
+ GATTGATTGA CGTCTTTAAT CAGCGTTTTT GTCTTTGTTC GGTGCTCCCC TCACTCGCCC CTCCGCTCTT   
  
  
+ TAAAGCTTAC CTCACTCCCC CCATTCCCAC TCCTTTTCTC TCTCCTCTCT CGCTCTCACA CGCTCCGTCT   
  
  
+ GTCAGCCCCT TGTCATCTTC CCCCGTCTTC CTCGCAAAAA TCTCCAGCAA AATTTATAGC CGTTGTTGTG   
  
  
+ CCCATGGTGA TCTGATGTGA GAAGAGATTC AGCATTTAAG ATTATTGGGT CCAGATGGCT TATATGTGCG   
  
  
+ CCGACAGTGG TAATCTCATG GCTATTGCCC AACAAGTCAT CAAGCAAAAA CAACAGCAAG AACAGCAGCA   
  
  
+ ACACCAACAG CCTCATCCCC AGTTCGTCGG GTCGAATAAT AACCCCTTTT GCACCAGCCC ATGGCCCGGA   
  
  
+ CCCGTCCCTT CATCCGCCAT GTCTGCCAGC CCTCCGCCCC CTCTTGGGTT CTCCGCCGCC GCGTTTCCGG   
  
  
+ ACCCGTTTCA GGTGGGCCCG CCCTGCCCGG ATGGGGCTGA ACCGGGTTTC CAGTTTGCCC ATTTGGACCA   
  
  
+ CCACTCGAGT GGTTTCCGGT TTGCTGATTT TTGCGGTGGG GGTGGTGGTG AGTTTGACTC GGATGAGTGG   
  
  
+ ATGGAGAGTT TGATGGGTGG CGGCGGCGGT GACTCGCGGC GACTGAGAGT TCTAACCTCC AATCCCATTG   
  
  
+ CGACGCGTGG CAAGGGTCCG GTGATTTCGG TCTCTATGGT GCTGATCCGT TTGCGACGTG TTCGGAAGAC   
  
  
+ CGTTGCAGCC GCTATTCACG CTCAGCCACT GCCGCAACAG CAGCCGTCGT GGGTCCCGCC TTCTCCGCCG   
  
  
+ AAAGACACGA AGCAGTCGGC CCCACCTCAA AAGCAAAGCG ACGCCGCTGG ACCCTCGTTC TCATCCGAGG   
  
  
+ AGGAACCAGA GCCGTCATGG CCGCCGCTAA TTGCGGCGTT GTTGGAGTGC GCCAAACTCG CCGAGTCCGA   
  
  
+ CCCGGAACAC GCTGTCAAAT CGGTGATTCG ACTCAGAGAC TCAGTCTCAC AGCAGGGAGT TCCAATCCAG   
  
  
+ AGAGTGTCCT ATTACTTCTC CGAGGCACTC TACCACCGCC TTTCTCTCTC CTCCGCCCCT CAACCTCTCC   
  
  
+ CAGAAACGAC GTCGTCTGAG GAGTACACTC TCTCTTACAA GGCCCTGAAC GACGCGTGCC CGTACCATAA   
  
  
+ ATTCGCTCAC TTGACCGCAA ATCAGGCGAT TCTAGAAGCC ACTGATTCAG CCAAGAAGAT CCACATCGTT   
  
  
+ GATTTTGGGA TAATCCAGGG AGTCCAATGG GCTGCTCTGC TACAAGCCCT GGCAACCCGG CCAGCCGGGA   
  
  
+ AGCCCGAATT GATTCGAATA TCCGGAATTC CTGCTCCGGG TCTGGGGCAA TCTCCGGCGG CATCGCTGCT   
  
  
+ CGCAACTGGG AATCGCCTCC GTGAGTTCGC GAAGGTTCTA GATCTCAAGT TCGAGTTTGA GCCGGTACTC   
  
  
+ ACTCCAATCA GAGAGCTAAA CGGGTCGAAC TTCCGGGTCG ACCCAGATGA GATACTAGCT GTCAATTTCA   
  
  
+ TGCTTCAATT GTACAACCTA TTAGACGAAA CCCTGGTCGC TGTCGACGCC GCCCTGAAGC TGGCCAAGTC   
  
  
+ ATTGAACCCC AAAATCGTCA CTTTGGGTGA GTACGAGGCG GGTCTGAATC GGGCAGGTTT CTTGACCCGG   
  
  
+ TTCAAGACGG CCCTCAAGTA CTATTTGGCA ATGTTTGAAT CGGTTGAGCC CAACTTGGCC CGGGACTCGC   
  
  
+ CCGAGAGGGC TCAGGTTGAG AGGCTGTTAT TGGGCCGGCG GATCATGGGC TTAGTTGGGC CGGAGTTACC   
  
  
+ GGGAACGAGA AGGGAAAGGA TGGAGTGTAA GGAACAATGG AAGGGTTTAA TGGAAAGTGC CGGTTTCGAG   
  
  
+ CCGTGCCAAA GCAGAAACTA CGTCAGTGAA AAAGACAAGT TTTACCACTC CAGATTGAAT TACAGAGAGT   
  
  
+ G  

- +Up\_Stream \_Len000ACTATT AATAAAAATA ACTAAATCCT TTCCTATAGT TTCATATAAA ACAAAAATCC   
  
  
- ATCAATTCTC TAAGACAACG GTTTTTTTTT TAGTTCTATA ATATATAAAA ATTAATACTG TCATTAATAA   
  
  
- AATAACTTTT AGGTTGCCAA CACTAATATA TTAGTTTATT AGATTTCCAA TTTTTATAGT TTAAATAATC   
  
  
- TTTTAAGCTT TTTCAGTGCA GTAGTGTTGT AACGAAAATA TATATCATAT CTAAGGAAGC TGAAGATTTG   
  
  
- ATTAGTGAAC TTTAATCTTT AAACTTTGAA AGATTATATT TACGTACCAG AAGAAAGGTT CTAATATTAG   
  
  
- GCGTTAACGA ATTTACATCC CATGTTTATA GTTTAAATCT TCACTGATAA CTTCGGTACT TTCTATAACA   
  
  
- CTAGCATAAC TCCCACCAAC ACTCGTTATC CTACTTTGTC CTTTTTTTTT GGGATTACAA AAGTTTAAAT   
  
  
- ACCCACCGAA ACCATCATCT ATTCACTTAT CACACGTTCC CTTACCGTGG TTATACAAAC CTACCTTGCT   
  
  
- ACTCTCATTC TGCAATCCTT TCGTTGTTCT CTTCCCTTTT CCGTCATCCC TCTCTTAATA CGCACACTTA   
  
  
- TTATAATATT GTCTGCGAAA GCATACTAAT AATTTATGTT CTGAAACTCG TATGTATATT TGAACATTGA   
  
  
- AAGAAATGGA TAACAGTTAA TTAAAAACTT AACTTAGTAG ACGCTGAATA CGTATGTTCT ACTATACTAC   
  
  
- GTATAACCCG GTTCGTAAAC TAGAGTACAG GGAATACCTA GATCTAGGGT TATCTGTTTG AACGTATTCA   
  
  
- GTTCCCTACA CAAAGATTAT GTTTCCGAAA CATGAGTTCG TTAATCAAAA CTTAAGTAAA AGTCATCAAA   
  
  
- TTAAAGGTTT AGAATATTAA AAATGTTACC GTATTTAAAA ATCCTCAAAA CTCATTTATT TAATATATGT   
  
  
- TGCTTCTACA GTAAATGCTT AATTATACTG CGTGTTTTTT TTTCTTTTTT TTAATTACCA TCGAAGATAT   
  
  
- GAACTGAGGT ACAACTGTTA AGGGAGAGAG TGTATTACCA ATTTTGAACG TTGTAGTAGG TCCTGGGTAT   
  
  
- ACTGTACCCA CCACCATCTG TCACACAACA CCATCGGAGT GAGGTACGAA CGGTTAAACA TAGTAACGCG   
  
  
- ATCAGAATAA GCAGGAATCT CTATTAACTG ATTCGAGATT ACGAGATAGC ACCGACGACG TTATATTTAC   
  
  
- TAAAAAACTG GAGTTTTACT TCTGTCGATC AACATTTAAT TCTTAGAAGC AGGGAAGTAA AAAACACATG   
  
  
- CACACATAGA CGCATACACA ACTCTCTGAT CGCCTGATAA CTAACTCAAA GTTAGTTTTT TCTACTGGTA   
  
  
- GAATTCATAA AAATTAATGC GTTTGAAGAA GTAAATTTTT TTACAATGAA AATAAACTTT CTTTAACACT   
  
  
- TATTATGTTC CTTATCAAGT TAATTATATA TTAGGTATTG TATAATGGAG GATATCCGTT TTTCTTCTAA   
  
  
- TAGCATAATT AAACCCTAGT AGGGTGGGAT TTAAATTTCT TTGGTTTTGT TTAATGATTC GTTATAGAAA   
  
  
- GACACACTTT TTTATCAGTC TTTGTTTCGC TAGGCTGGCC GGTTTAGCTC TATTTGTGTC GGTTTCTTGG   
  
  
- CTTCAACCTG GTTTCACTCG GCTCAACTTT CCGTAAGTAT ACCCCAAATT AGTATTGTGT TTGTATTAAT   
  
  
- CTAACTAACT GCAGAAATTA GTCGCAAAAA CAGAAACAAG CCACGAGGGG AGTGAGCGGG GAGGCGAGAA   
  
  
- ATTTCGAATG GAGTGAGGGG GGTAAGGGTG AGGAAAAGAG AGAGGAGAGA GCGAGAGTGT GCGAGGCAGA   
  
  
- CAGTCGGGGA ACAGTAGAAG GGGGCAGAAG GAGCGTTTTT AGAGGTCGTT TTAAATATCG GCAACAACAC   
  
  
- GGGTACCACT AGACTACACT CTTCTCTAAG TCGTAAATTC TAATAACCCA GGTCTACCGA ATATACACGC   
  
  
- GGCTGTCACC ATTAGAGTAC CGATAACGGG TTGTTCAGTA GTTCGTTTTT GTTGTCGTTC TTGTCGTCGT   
  
  
- TGTGGTTGTC GGAGTAGGGG TCAAGCAGCC CAGCTTATTA TTGGGGAAAA CGTGGTCGGG TACCGGGCCT   
  
  
- GGGCAGGGAA GTAGGCGGTA CAGACGGTCG GGAGGCGGGG GAGAACCCAA GAGGCGGCGG CGCAAAGGCC   
  
  
- TGGGCAAAGT CCACCCGGGC GGGACGGGCC TACCCCGACT TGGCCCAAAG GTCAAACGGG TAAACCTGGT   
  
  
- GGTGAGCTCA CCAAAGGCCA AACGACTAAA AACGCCACCC CCACCACCAC TCAAACTGAG CCTACTCACC   
  
  
- TACCTCTCAA ACTACCCACC GCCGCCGCCA CTGAGCGCCG CTGACTCTCA AGATTGGAGG TTAGGGTAAC   
  
  
- GCTGCGCACC GTTCCCAGGC CACTAAAGCC AGAGATACCA CGACTAGGCA AACGCTGCAC AAGCCTTCTG   
  
  
- GCAACGTCGG CGATAAGTGC GAGTCGGTGA CGGCGTTGTC GTCGGCAGCA CCCAGGGCGG AAGAGGCGGC   
  
  
- TTTCTGTGCT TCGTCAGCCG GGGTGGAGTT TTCGTTTCGC TGCGGCGACC TGGGAGCAAG AGTAGGCTCC   
  
  
- TCCTTGGTCT CGGCAGTACC GGCGGCGATT AACGCCGCAA CAACCTCACG CGGTTTGAGC GGCTCAGGCT   
  
  
- GGGCCTTGTG CGACAGTTTA GCCACTAAGC TGAGTCTCTG AGTCAGAGTG TCGTCCCTCA AGGTTAGGTC   
  
  
- TCTCACAGGA TAATGAAGAG GCTCCGTGAG ATGGTGGCGG AAAGAGAGAG GAGGCGGGGA GTTGGAGAGG   
  
  
- GTCTTTGCTG CAGCAGACTC CTCATGTGAG AGAGAATGTT CCGGGACTTG CTGCGCACGG GCATGGTATT   
  
  
- TAAGCGAGTG AACTGGCGTT TAGTCCGCTA AGATCTTCGG TGACTAAGTC GGTTCTTCTA GGTGTAGCAA   
  
  
- CTAAAACCCT ATTAGGTCCC TCAGGTTACC CGACGAGACG ATGTTCGGGA CCGTTGGGCC GGTCGGCCCT   
  
  
- TCGGGCTTAA CTAAGCTTAT AGGCCTTAAG GACGAGGCCC AGACCCCGTT AGAGGCCGCC GTAGCGACGA   
  
  
- GCGTTGACCC TTAGCGGAGG CACTCAAGCG CTTCCAAGAT CTAGAGTTCA AGCTCAAACT CGGCCATGAG   
  
  
- TGAGGTTAGT CTCTCGATTT GCCCAGCTTG AAGGCCCAGC TGGGTCTACT CTATGATCGA CAGTTAAAGT   
  
  
- ACGAAGTTAA CATGTTGGAT AATCTGCTTT GGGACCAGCG ACAGCTGCGG CGGGACTTCG ACCGGTTCAG   
  
  
- TAACTTGGGG TTTTAGCAGT GAAACCCACT CATGCTCCGC CCAGACTTAG CCCGTCCAAA GAACTGGGCC   
  
  
- AAGTTCTGCC GGGAGTTCAT GATAAACCGT TACAAACTTA GCCAACTCGG GTTGAACCGG GCCCTGAGCG   
  
  
- GGCTCTCCCG AGTCCAACTC TCCGACAATA ACCCGGCCGC CTAGTACCCG AATCAACCCG GCCTCAATGG   
  
  
- CCCTTGCTCT TCCCTTTCCT ACCTCACATT CCTTGTTACC TTCCCAAATT ACCTTTCACG GCCAAAGCTC   
  
  
- GGCACGGTTT CGTCTTTGAT GCAGTCACTT TTTCTGTTCA AAATGGTGAG GTCTAACTTA ATGTCTCTCA   
  
  
- C

+     MYC

| Site Name | Organism | Position | Strand | Matrix score. | sequence | function |
| --- | --- | --- | --- | --- | --- | --- |
| MYC | Arabidopsis thaliana | 789 | + | 6 | CATTTG |  |
| MYC | Arabidopsis thaliana | 357 | + | 6 | CAATTG |  |
| MYC | Arabidopsis thaliana | 3300 | - | 6 | CAATTG |  |
| MYC | Arabidopsis thaliana | 2304 | + | 6 | CATTTG |  |

>HU06G00029.1   
+ +Up\_Stream \_Len000TGATAA TTATTTTTAT TGATTTAGGA AAGGATATCA AAGTATATTT TGTTTTTAGG   
  
  
+ TAGTTAAGAG ATTCTGTTGC CAAAAAAAAA ATCAAGATAT TATATATTTT TAATTATGAC AGTAATTATT   
  
  
+ TTATTGAAAA TCCAACGGTT GTGATTATAT AATCAAATAA TCTAAAGGTT AAAAATATCA AATTTATTAG   
  
  
+ AAAATTCGAA AAAGTCACGT CATCACAACA TTGCTTTTAT ATATAGTATA GATTCCTTCG ACTTCTAAAC   
  
  
+ TAATCACTTG AAATTAGAAA TTTGAAACTT TCTAATATAA ATGCATGGTC TTCTTTCCAA GATTATAATC   
  
  
+ CGCAATTGCT TAAATGTAGG GTACAAATAT CAAATTTAGA AGTGACTATT GAAGCCATGA AAGATATTGT   
  
  
+ GATCGTATTG AGGGTGGTTG TGAGCAATAG GATGAAACAG GAAAAAAAAA CCCTAATGTT TTCAAATTTA   
  
  
+ TGGGTGGCTT TGGTAGTAGA TAAGTGAATA GTGTGCAAGG GAATGGCACC AATATGTTTG GATGGAACGA   
  
  
+ TGAGAGTAAG ACGTTAGGAA AGCAACAAGA GAAGGGAAAA GGCAGTAGGG AGAGAATTAT GCGTGTGAAT   
  
  
+ AATATTATAA CAGACGCTTT CGTATGATTA TTAAATACAA GACTTTGAGC ATACATATAA ACTTGTAACT   
  
  
+ TTCTTTACCT ATTGTCAATT AATTTTTGAA TTGAATCATC TGCGACTTAT GCATACAAGA TGATATGATG   
  
  
+ CATATTGGGC CAAGCATTTG ATCTCATGTC CCTTATGGAT CTAGATCCCA ATAGACAAAC TTGCATAAGT   
  
  
+ CAAGGGATGT GTTTCTAATA CAAAGGCTTT GTACTCAAGC AATTAGTTTT GAATTCATTT TCAGTAGTTT   
  
  
+ AATTTCCAAA TCTTATAATT TTTACAATGG CATAAATTTT TAGGAGTTTT GAGTAAATAA ATTATATACA   
  
  
+ ACGAAGATGT CATTTACGAA TTAATATGAC GCACAAAAAA AAAGAAAAAA AATTAATGGT AGCTTCTATA   
  
  
+ CTTGACTCCA TGTTGACAAT TCCCTCTCTC ACATAATGGT TAAAACTTGC AACATCATCC AGGACCCATA   
  
  
+ TGACATGGGT GGTGGTAGAC AGTGTGTTGT GGTAGCCTCA CTCCATGCTT GCCAATTTGT ATCATTGCGC   
  
  
+ TAGTCTTATT CGTCCTTAGA GATAATTGAC TAAGCTCTAA TGCTCTATCG TGGCTGCTGC AATATAAATG   
  
  
+ ATTTTTTGAC CTCAAAATGA AGACAGCTAG TTGTAAATTA AGAATCTTCG TCCCTTCATT TTTTGTGTAC   
  
  
+ GTGTGTATCT GCGTATGTGT TGAGAGACTA GCGGACTATT GATTGAGTTT CAATCAAAAA AGATGACCAT   
  
  
+ CTTAAGTATT TTTAATTACG CAAACTTCTT CATTTAAAAA AATGTTACTT TTATTTGAAA GAAATTGTGA   
  
  
+ ATAATACAAG GAATAGTTCA ATTAATATAT AATCCATAAC ATATTACCTC CTATAGGCAA AAAGAAGATT   
  
  
+ ATCGTATTAA TTTGGGATCA TCCCACCCTA AATTTAAAGA AACCAAAACA AATTACTAAG CAATATCTTT   
  
  
+ CTGTGTGAAA AAATAGTCAG AAACAAAGCG ATCCGACCGG CCAAATCGAG ATAAACACAG CCAAAGAACC   
  
  
+ GAAGTTGGAC CAAAGTGAGC CGAGTTGAAA GGCATTCATA TGGGGTTTAA TCATAACACA AACATAATTA   
  
  
+ GATTGATTGA CGTCTTTAAT CAGCGTTTTT GTCTTTGTTC GGTGCTCCCC TCACTCGCCC CTCCGCTCTT   
  
  
+ TAAAGCTTAC CTCACTCCCC CCATTCCCAC TCCTTTTCTC TCTCCTCTCT CGCTCTCACA CGCTCCGTCT   
  
  
+ GTCAGCCCCT TGTCATCTTC CCCCGTCTTC CTCGCAAAAA TCTCCAGCAA AATTTATAGC CGTTGTTGTG   
  
  
+ CCCATGGTGA TCTGATGTGA GAAGAGATTC AGCATTTAAG ATTATTGGGT CCAGATGGCT TATATGTGCG   
  
  
+ CCGACAGTGG TAATCTCATG GCTATTGCCC AACAAGTCAT CAAGCAAAAA CAACAGCAAG AACAGCAGCA   
  
  
+ ACACCAACAG CCTCATCCCC AGTTCGTCGG GTCGAATAAT AACCCCTTTT GCACCAGCCC ATGGCCCGGA   
  
  
+ CCCGTCCCTT CATCCGCCAT GTCTGCCAGC CCTCCGCCCC CTCTTGGGTT CTCCGCCGCC GCGTTTCCGG   
  
  
+ ACCCGTTTCA GGTGGGCCCG CCCTGCCCGG ATGGGGCTGA ACCGGGTTTC CAGTTTGCCC ATTTGGACCA   
  
  
+ CCACTCGAGT GGTTTCCGGT TTGCTGATTT TTGCGGTGGG GGTGGTGGTG AGTTTGACTC GGATGAGTGG   
  
  
+ ATGGAGAGTT TGATGGGTGG CGGCGGCGGT GACTCGCGGC GACTGAGAGT TCTAACCTCC AATCCCATTG   
  
  
+ CGACGCGTGG CAAGGGTCCG GTGATTTCGG TCTCTATGGT GCTGATCCGT TTGCGACGTG TTCGGAAGAC   
  
  
+ CGTTGCAGCC GCTATTCACG CTCAGCCACT GCCGCAACAG CAGCCGTCGT GGGTCCCGCC TTCTCCGCCG   
  
  
+ AAAGACACGA AGCAGTCGGC CCCACCTCAA AAGCAAAGCG ACGCCGCTGG ACCCTCGTTC TCATCCGAGG   
  
  
+ AGGAACCAGA GCCGTCATGG CCGCCGCTAA TTGCGGCGTT GTTGGAGTGC GCCAAACTCG CCGAGTCCGA   
  
  
+ CCCGGAACAC GCTGTCAAAT CGGTGATTCG ACTCAGAGAC TCAGTCTCAC AGCAGGGAGT TCCAATCCAG   
  
  
+ AGAGTGTCCT ATTACTTCTC CGAGGCACTC TACCACCGCC TTTCTCTCTC CTCCGCCCCT CAACCTCTCC   
  
  
+ CAGAAACGAC GTCGTCTGAG GAGTACACTC TCTCTTACAA GGCCCTGAAC GACGCGTGCC CGTACCATAA   
  
  
+ ATTCGCTCAC TTGACCGCAA ATCAGGCGAT TCTAGAAGCC ACTGATTCAG CCAAGAAGAT CCACATCGTT   
  
  
+ GATTTTGGGA TAATCCAGGG AGTCCAATGG GCTGCTCTGC TACAAGCCCT GGCAACCCGG CCAGCCGGGA   
  
  
+ AGCCCGAATT GATTCGAATA TCCGGAATTC CTGCTCCGGG TCTGGGGCAA TCTCCGGCGG CATCGCTGCT   
  
  
+ CGCAACTGGG AATCGCCTCC GTGAGTTCGC GAAGGTTCTA GATCTCAAGT TCGAGTTTGA GCCGGTACTC   
  
  
+ ACTCCAATCA GAGAGCTAAA CGGGTCGAAC TTCCGGGTCG ACCCAGATGA GATACTAGCT GTCAATTTCA   
  
  
+ TGCTTCAATT GTACAACCTA TTAGACGAAA CCCTGGTCGC TGTCGACGCC GCCCTGAAGC TGGCCAAGTC   
  
  
+ ATTGAACCCC AAAATCGTCA CTTTGGGTGA GTACGAGGCG GGTCTGAATC GGGCAGGTTT CTTGACCCGG   
  
  
+ TTCAAGACGG CCCTCAAGTA CTATTTGGCA ATGTTTGAAT CGGTTGAGCC CAACTTGGCC CGGGACTCGC   
  
  
+ CCGAGAGGGC TCAGGTTGAG AGGCTGTTAT TGGGCCGGCG GATCATGGGC TTAGTTGGGC CGGAGTTACC   
  
  
+ GGGAACGAGA AGGGAAAGGA TGGAGTGTAA GGAACAATGG AAGGGTTTAA TGGAAAGTGC CGGTTTCGAG   
  
  
+ CCGTGCCAAA GCAGAAACTA CGTCAGTGAA AAAGACAAGT TTTACCACTC CAGATTGAAT TACAGAGAGT   
  
  
+ G  

- +Up\_Stream \_Len000ACTATT AATAAAAATA ACTAAATCCT TTCCTATAGT TTCATATAAA ACAAAAATCC   
  
  
- ATCAATTCTC TAAGACAACG GTTTTTTTTT TAGTTCTATA ATATATAAAA ATTAATACTG TCATTAATAA   
  
  
- AATAACTTTT AGGTTGCCAA CACTAATATA TTAGTTTATT AGATTTCCAA TTTTTATAGT TTAAATAATC   
  
  
- TTTTAAGCTT TTTCAGTGCA GTAGTGTTGT AACGAAAATA TATATCATAT CTAAGGAAGC TGAAGATTTG   
  
  
- ATTAGTGAAC TTTAATCTTT AAACTTTGAA AGATTATATT TACGTACCAG AAGAAAGGTT CTAATATTAG   
  
  
- GCGTTAACGA ATTTACATCC CATGTTTATA GTTTAAATCT TCACTGATAA CTTCGGTACT TTCTATAACA   
  
  
- CTAGCATAAC TCCCACCAAC ACTCGTTATC CTACTTTGTC CTTTTTTTTT GGGATTACAA AAGTTTAAAT   
  
  
- ACCCACCGAA ACCATCATCT ATTCACTTAT CACACGTTCC CTTACCGTGG TTATACAAAC CTACCTTGCT   
  
  
- ACTCTCATTC TGCAATCCTT TCGTTGTTCT CTTCCCTTTT CCGTCATCCC TCTCTTAATA CGCACACTTA   
  
  
- TTATAATATT GTCTGCGAAA GCATACTAAT AATTTATGTT CTGAAACTCG TATGTATATT TGAACATTGA   
  
  
- AAGAAATGGA TAACAGTTAA TTAAAAACTT AACTTAGTAG ACGCTGAATA CGTATGTTCT ACTATACTAC   
  
  
- GTATAACCCG GTTCGTAAAC TAGAGTACAG GGAATACCTA GATCTAGGGT TATCTGTTTG AACGTATTCA   
  
  
- GTTCCCTACA CAAAGATTAT GTTTCCGAAA CATGAGTTCG TTAATCAAAA CTTAAGTAAA AGTCATCAAA   
  
  
- TTAAAGGTTT AGAATATTAA AAATGTTACC GTATTTAAAA ATCCTCAAAA CTCATTTATT TAATATATGT   
  
  
- TGCTTCTACA GTAAATGCTT AATTATACTG CGTGTTTTTT TTTCTTTTTT TTAATTACCA TCGAAGATAT   
  
  
- GAACTGAGGT ACAACTGTTA AGGGAGAGAG TGTATTACCA ATTTTGAACG TTGTAGTAGG TCCTGGGTAT   
  
  
- ACTGTACCCA CCACCATCTG TCACACAACA CCATCGGAGT GAGGTACGAA CGGTTAAACA TAGTAACGCG   
  
  
- ATCAGAATAA GCAGGAATCT CTATTAACTG ATTCGAGATT ACGAGATAGC ACCGACGACG TTATATTTAC   
  
  
- TAAAAAACTG GAGTTTTACT TCTGTCGATC AACATTTAAT TCTTAGAAGC AGGGAAGTAA AAAACACATG   
  
  
- CACACATAGA CGCATACACA ACTCTCTGAT CGCCTGATAA CTAACTCAAA GTTAGTTTTT TCTACTGGTA   
  
  
- GAATTCATAA AAATTAATGC GTTTGAAGAA GTAAATTTTT TTACAATGAA AATAAACTTT CTTTAACACT   
  
  
- TATTATGTTC CTTATCAAGT TAATTATATA TTAGGTATTG TATAATGGAG GATATCCGTT TTTCTTCTAA   
  
  
- TAGCATAATT AAACCCTAGT AGGGTGGGAT TTAAATTTCT TTGGTTTTGT TTAATGATTC GTTATAGAAA   
  
  
- GACACACTTT TTTATCAGTC TTTGTTTCGC TAGGCTGGCC GGTTTAGCTC TATTTGTGTC GGTTTCTTGG   
  
  
- CTTCAACCTG GTTTCACTCG GCTCAACTTT CCGTAAGTAT ACCCCAAATT AGTATTGTGT TTGTATTAAT   
  
  
- CTAACTAACT GCAGAAATTA GTCGCAAAAA CAGAAACAAG CCACGAGGGG AGTGAGCGGG GAGGCGAGAA   
  
  
- ATTTCGAATG GAGTGAGGGG GGTAAGGGTG AGGAAAAGAG AGAGGAGAGA GCGAGAGTGT GCGAGGCAGA   
  
  
- CAGTCGGGGA ACAGTAGAAG GGGGCAGAAG GAGCGTTTTT AGAGGTCGTT TTAAATATCG GCAACAACAC   
  
  
- GGGTACCACT AGACTACACT CTTCTCTAAG TCGTAAATTC TAATAACCCA GGTCTACCGA ATATACACGC   
  
  
- GGCTGTCACC ATTAGAGTAC CGATAACGGG TTGTTCAGTA GTTCGTTTTT GTTGTCGTTC TTGTCGTCGT   
  
  
- TGTGGTTGTC GGAGTAGGGG TCAAGCAGCC CAGCTTATTA TTGGGGAAAA CGTGGTCGGG TACCGGGCCT   
  
  
- GGGCAGGGAA GTAGGCGGTA CAGACGGTCG GGAGGCGGGG GAGAACCCAA GAGGCGGCGG CGCAAAGGCC   
  
  
- TGGGCAAAGT CCACCCGGGC GGGACGGGCC TACCCCGACT TGGCCCAAAG GTCAAACGGG TAAACCTGGT   
  
  
- GGTGAGCTCA CCAAAGGCCA AACGACTAAA AACGCCACCC CCACCACCAC TCAAACTGAG CCTACTCACC   
  
  
- TACCTCTCAA ACTACCCACC GCCGCCGCCA CTGAGCGCCG CTGACTCTCA AGATTGGAGG TTAGGGTAAC   
  
  
- GCTGCGCACC GTTCCCAGGC CACTAAAGCC AGAGATACCA CGACTAGGCA AACGCTGCAC AAGCCTTCTG   
  
  
- GCAACGTCGG CGATAAGTGC GAGTCGGTGA CGGCGTTGTC GTCGGCAGCA CCCAGGGCGG AAGAGGCGGC   
  
  
- TTTCTGTGCT TCGTCAGCCG GGGTGGAGTT TTCGTTTCGC TGCGGCGACC TGGGAGCAAG AGTAGGCTCC   
  
  
- TCCTTGGTCT CGGCAGTACC GGCGGCGATT AACGCCGCAA CAACCTCACG CGGTTTGAGC GGCTCAGGCT   
  
  
- GGGCCTTGTG CGACAGTTTA GCCACTAAGC TGAGTCTCTG AGTCAGAGTG TCGTCCCTCA AGGTTAGGTC   
  
  
- TCTCACAGGA TAATGAAGAG GCTCCGTGAG ATGGTGGCGG AAAGAGAGAG GAGGCGGGGA GTTGGAGAGG   
  
  
- GTCTTTGCTG CAGCAGACTC CTCATGTGAG AGAGAATGTT CCGGGACTTG CTGCGCACGG GCATGGTATT   
  
  
- TAAGCGAGTG AACTGGCGTT TAGTCCGCTA AGATCTTCGG TGACTAAGTC GGTTCTTCTA GGTGTAGCAA   
  
  
- CTAAAACCCT ATTAGGTCCC TCAGGTTACC CGACGAGACG ATGTTCGGGA CCGTTGGGCC GGTCGGCCCT   
  
  
- TCGGGCTTAA CTAAGCTTAT AGGCCTTAAG GACGAGGCCC AGACCCCGTT AGAGGCCGCC GTAGCGACGA   
  
  
- GCGTTGACCC TTAGCGGAGG CACTCAAGCG CTTCCAAGAT CTAGAGTTCA AGCTCAAACT CGGCCATGAG   
  
  
- TGAGGTTAGT CTCTCGATTT GCCCAGCTTG AAGGCCCAGC TGGGTCTACT CTATGATCGA CAGTTAAAGT   
  
  
- ACGAAGTTAA CATGTTGGAT AATCTGCTTT GGGACCAGCG ACAGCTGCGG CGGGACTTCG ACCGGTTCAG   
  
  
- TAACTTGGGG TTTTAGCAGT GAAACCCACT CATGCTCCGC CCAGACTTAG CCCGTCCAAA GAACTGGGCC   
  
  
- AAGTTCTGCC GGGAGTTCAT GATAAACCGT TACAAACTTA GCCAACTCGG GTTGAACCGG GCCCTGAGCG   
  
  
- GGCTCTCCCG AGTCCAACTC TCCGACAATA ACCCGGCCGC CTAGTACCCG AATCAACCCG GCCTCAATGG   
  
  
- CCCTTGCTCT TCCCTTTCCT ACCTCACATT CCTTGTTACC TTCCCAAATT ACCTTTCACG GCCAAAGCTC   
  
  
- GGCACGGTTT CGTCTTTGAT GCAGTCACTT TTTCTGTTCA AAATGGTGAG GTCTAACTTA ATGTCTCTCA   
  
  
- C

+     Myb

| Site Name | Organism | Position | Strand | Matrix score. | sequence | function |
| --- | --- | --- | --- | --- | --- | --- |
| Myb | Arabidopsis thaliana | 3157 | + | 6 | CAACTG |  |

>HU06G00029.1   
+ +Up\_Stream \_Len000TGATAA TTATTTTTAT TGATTTAGGA AAGGATATCA AAGTATATTT TGTTTTTAGG   
  
  
+ TAGTTAAGAG ATTCTGTTGC CAAAAAAAAA ATCAAGATAT TATATATTTT TAATTATGAC AGTAATTATT   
  
  
+ TTATTGAAAA TCCAACGGTT GTGATTATAT AATCAAATAA TCTAAAGGTT AAAAATATCA AATTTATTAG   
  
  
+ AAAATTCGAA AAAGTCACGT CATCACAACA TTGCTTTTAT ATATAGTATA GATTCCTTCG ACTTCTAAAC   
  
  
+ TAATCACTTG AAATTAGAAA TTTGAAACTT TCTAATATAA ATGCATGGTC TTCTTTCCAA GATTATAATC   
  
  
+ CGCAATTGCT TAAATGTAGG GTACAAATAT CAAATTTAGA AGTGACTATT GAAGCCATGA AAGATATTGT   
  
  
+ GATCGTATTG AGGGTGGTTG TGAGCAATAG GATGAAACAG GAAAAAAAAA CCCTAATGTT TTCAAATTTA   
  
  
+ TGGGTGGCTT TGGTAGTAGA TAAGTGAATA GTGTGCAAGG GAATGGCACC AATATGTTTG GATGGAACGA   
  
  
+ TGAGAGTAAG ACGTTAGGAA AGCAACAAGA GAAGGGAAAA GGCAGTAGGG AGAGAATTAT GCGTGTGAAT   
  
  
+ AATATTATAA CAGACGCTTT CGTATGATTA TTAAATACAA GACTTTGAGC ATACATATAA ACTTGTAACT   
  
  
+ TTCTTTACCT ATTGTCAATT AATTTTTGAA TTGAATCATC TGCGACTTAT GCATACAAGA TGATATGATG   
  
  
+ CATATTGGGC CAAGCATTTG ATCTCATGTC CCTTATGGAT CTAGATCCCA ATAGACAAAC TTGCATAAGT   
  
  
+ CAAGGGATGT GTTTCTAATA CAAAGGCTTT GTACTCAAGC AATTAGTTTT GAATTCATTT TCAGTAGTTT   
  
  
+ AATTTCCAAA TCTTATAATT TTTACAATGG CATAAATTTT TAGGAGTTTT GAGTAAATAA ATTATATACA   
  
  
+ ACGAAGATGT CATTTACGAA TTAATATGAC GCACAAAAAA AAAGAAAAAA AATTAATGGT AGCTTCTATA   
  
  
+ CTTGACTCCA TGTTGACAAT TCCCTCTCTC ACATAATGGT TAAAACTTGC AACATCATCC AGGACCCATA   
  
  
+ TGACATGGGT GGTGGTAGAC AGTGTGTTGT GGTAGCCTCA CTCCATGCTT GCCAATTTGT ATCATTGCGC   
  
  
+ TAGTCTTATT CGTCCTTAGA GATAATTGAC TAAGCTCTAA TGCTCTATCG TGGCTGCTGC AATATAAATG   
  
  
+ ATTTTTTGAC CTCAAAATGA AGACAGCTAG TTGTAAATTA AGAATCTTCG TCCCTTCATT TTTTGTGTAC   
  
  
+ GTGTGTATCT GCGTATGTGT TGAGAGACTA GCGGACTATT GATTGAGTTT CAATCAAAAA AGATGACCAT   
  
  
+ CTTAAGTATT TTTAATTACG CAAACTTCTT CATTTAAAAA AATGTTACTT TTATTTGAAA GAAATTGTGA   
  
  
+ ATAATACAAG GAATAGTTCA ATTAATATAT AATCCATAAC ATATTACCTC CTATAGGCAA AAAGAAGATT   
  
  
+ ATCGTATTAA TTTGGGATCA TCCCACCCTA AATTTAAAGA AACCAAAACA AATTACTAAG CAATATCTTT   
  
  
+ CTGTGTGAAA AAATAGTCAG AAACAAAGCG ATCCGACCGG CCAAATCGAG ATAAACACAG CCAAAGAACC   
  
  
+ GAAGTTGGAC CAAAGTGAGC CGAGTTGAAA GGCATTCATA TGGGGTTTAA TCATAACACA AACATAATTA   
  
  
+ GATTGATTGA CGTCTTTAAT CAGCGTTTTT GTCTTTGTTC GGTGCTCCCC TCACTCGCCC CTCCGCTCTT   
  
  
+ TAAAGCTTAC CTCACTCCCC CCATTCCCAC TCCTTTTCTC TCTCCTCTCT CGCTCTCACA CGCTCCGTCT   
  
  
+ GTCAGCCCCT TGTCATCTTC CCCCGTCTTC CTCGCAAAAA TCTCCAGCAA AATTTATAGC CGTTGTTGTG   
  
  
+ CCCATGGTGA TCTGATGTGA GAAGAGATTC AGCATTTAAG ATTATTGGGT CCAGATGGCT TATATGTGCG   
  
  
+ CCGACAGTGG TAATCTCATG GCTATTGCCC AACAAGTCAT CAAGCAAAAA CAACAGCAAG AACAGCAGCA   
  
  
+ ACACCAACAG CCTCATCCCC AGTTCGTCGG GTCGAATAAT AACCCCTTTT GCACCAGCCC ATGGCCCGGA   
  
  
+ CCCGTCCCTT CATCCGCCAT GTCTGCCAGC CCTCCGCCCC CTCTTGGGTT CTCCGCCGCC GCGTTTCCGG   
  
  
+ ACCCGTTTCA GGTGGGCCCG CCCTGCCCGG ATGGGGCTGA ACCGGGTTTC CAGTTTGCCC ATTTGGACCA   
  
  
+ CCACTCGAGT GGTTTCCGGT TTGCTGATTT TTGCGGTGGG GGTGGTGGTG AGTTTGACTC GGATGAGTGG   
  
  
+ ATGGAGAGTT TGATGGGTGG CGGCGGCGGT GACTCGCGGC GACTGAGAGT TCTAACCTCC AATCCCATTG   
  
  
+ CGACGCGTGG CAAGGGTCCG GTGATTTCGG TCTCTATGGT GCTGATCCGT TTGCGACGTG TTCGGAAGAC   
  
  
+ CGTTGCAGCC GCTATTCACG CTCAGCCACT GCCGCAACAG CAGCCGTCGT GGGTCCCGCC TTCTCCGCCG   
  
  
+ AAAGACACGA AGCAGTCGGC CCCACCTCAA AAGCAAAGCG ACGCCGCTGG ACCCTCGTTC TCATCCGAGG   
  
  
+ AGGAACCAGA GCCGTCATGG CCGCCGCTAA TTGCGGCGTT GTTGGAGTGC GCCAAACTCG CCGAGTCCGA   
  
  
+ CCCGGAACAC GCTGTCAAAT CGGTGATTCG ACTCAGAGAC TCAGTCTCAC AGCAGGGAGT TCCAATCCAG   
  
  
+ AGAGTGTCCT ATTACTTCTC CGAGGCACTC TACCACCGCC TTTCTCTCTC CTCCGCCCCT CAACCTCTCC   
  
  
+ CAGAAACGAC GTCGTCTGAG GAGTACACTC TCTCTTACAA GGCCCTGAAC GACGCGTGCC CGTACCATAA   
  
  
+ ATTCGCTCAC TTGACCGCAA ATCAGGCGAT TCTAGAAGCC ACTGATTCAG CCAAGAAGAT CCACATCGTT   
  
  
+ GATTTTGGGA TAATCCAGGG AGTCCAATGG GCTGCTCTGC TACAAGCCCT GGCAACCCGG CCAGCCGGGA   
  
  
+ AGCCCGAATT GATTCGAATA TCCGGAATTC CTGCTCCGGG TCTGGGGCAA TCTCCGGCGG CATCGCTGCT   
  
  
+ CGCAACTGGG AATCGCCTCC GTGAGTTCGC GAAGGTTCTA GATCTCAAGT TCGAGTTTGA GCCGGTACTC   
  
  
+ ACTCCAATCA GAGAGCTAAA CGGGTCGAAC TTCCGGGTCG ACCCAGATGA GATACTAGCT GTCAATTTCA   
  
  
+ TGCTTCAATT GTACAACCTA TTAGACGAAA CCCTGGTCGC TGTCGACGCC GCCCTGAAGC TGGCCAAGTC   
  
  
+ ATTGAACCCC AAAATCGTCA CTTTGGGTGA GTACGAGGCG GGTCTGAATC GGGCAGGTTT CTTGACCCGG   
  
  
+ TTCAAGACGG CCCTCAAGTA CTATTTGGCA ATGTTTGAAT CGGTTGAGCC CAACTTGGCC CGGGACTCGC   
  
  
+ CCGAGAGGGC TCAGGTTGAG AGGCTGTTAT TGGGCCGGCG GATCATGGGC TTAGTTGGGC CGGAGTTACC   
  
  
+ GGGAACGAGA AGGGAAAGGA TGGAGTGTAA GGAACAATGG AAGGGTTTAA TGGAAAGTGC CGGTTTCGAG   
  
  
+ CCGTGCCAAA GCAGAAACTA CGTCAGTGAA AAAGACAAGT TTTACCACTC CAGATTGAAT TACAGAGAGT   
  
  
+ G  

- +Up\_Stream \_Len000ACTATT AATAAAAATA ACTAAATCCT TTCCTATAGT TTCATATAAA ACAAAAATCC   
  
  
- ATCAATTCTC TAAGACAACG GTTTTTTTTT TAGTTCTATA ATATATAAAA ATTAATACTG TCATTAATAA   
  
  
- AATAACTTTT AGGTTGCCAA CACTAATATA TTAGTTTATT AGATTTCCAA TTTTTATAGT TTAAATAATC   
  
  
- TTTTAAGCTT TTTCAGTGCA GTAGTGTTGT AACGAAAATA TATATCATAT CTAAGGAAGC TGAAGATTTG   
  
  
- ATTAGTGAAC TTTAATCTTT AAACTTTGAA AGATTATATT TACGTACCAG AAGAAAGGTT CTAATATTAG   
  
  
- GCGTTAACGA ATTTACATCC CATGTTTATA GTTTAAATCT TCACTGATAA CTTCGGTACT TTCTATAACA   
  
  
- CTAGCATAAC TCCCACCAAC ACTCGTTATC CTACTTTGTC CTTTTTTTTT GGGATTACAA AAGTTTAAAT   
  
  
- ACCCACCGAA ACCATCATCT ATTCACTTAT CACACGTTCC CTTACCGTGG TTATACAAAC CTACCTTGCT   
  
  
- ACTCTCATTC TGCAATCCTT TCGTTGTTCT CTTCCCTTTT CCGTCATCCC TCTCTTAATA CGCACACTTA   
  
  
- TTATAATATT GTCTGCGAAA GCATACTAAT AATTTATGTT CTGAAACTCG TATGTATATT TGAACATTGA   
  
  
- AAGAAATGGA TAACAGTTAA TTAAAAACTT AACTTAGTAG ACGCTGAATA CGTATGTTCT ACTATACTAC   
  
  
- GTATAACCCG GTTCGTAAAC TAGAGTACAG GGAATACCTA GATCTAGGGT TATCTGTTTG AACGTATTCA   
  
  
- GTTCCCTACA CAAAGATTAT GTTTCCGAAA CATGAGTTCG TTAATCAAAA CTTAAGTAAA AGTCATCAAA   
  
  
- TTAAAGGTTT AGAATATTAA AAATGTTACC GTATTTAAAA ATCCTCAAAA CTCATTTATT TAATATATGT   
  
  
- TGCTTCTACA GTAAATGCTT AATTATACTG CGTGTTTTTT TTTCTTTTTT TTAATTACCA TCGAAGATAT   
  
  
- GAACTGAGGT ACAACTGTTA AGGGAGAGAG TGTATTACCA ATTTTGAACG TTGTAGTAGG TCCTGGGTAT   
  
  
- ACTGTACCCA CCACCATCTG TCACACAACA CCATCGGAGT GAGGTACGAA CGGTTAAACA TAGTAACGCG   
  
  
- ATCAGAATAA GCAGGAATCT CTATTAACTG ATTCGAGATT ACGAGATAGC ACCGACGACG TTATATTTAC   
  
  
- TAAAAAACTG GAGTTTTACT TCTGTCGATC AACATTTAAT TCTTAGAAGC AGGGAAGTAA AAAACACATG   
  
  
- CACACATAGA CGCATACACA ACTCTCTGAT CGCCTGATAA CTAACTCAAA GTTAGTTTTT TCTACTGGTA   
  
  
- GAATTCATAA AAATTAATGC GTTTGAAGAA GTAAATTTTT TTACAATGAA AATAAACTTT CTTTAACACT   
  
  
- TATTATGTTC CTTATCAAGT TAATTATATA TTAGGTATTG TATAATGGAG GATATCCGTT TTTCTTCTAA   
  
  
- TAGCATAATT AAACCCTAGT AGGGTGGGAT TTAAATTTCT TTGGTTTTGT TTAATGATTC GTTATAGAAA   
  
  
- GACACACTTT TTTATCAGTC TTTGTTTCGC TAGGCTGGCC GGTTTAGCTC TATTTGTGTC GGTTTCTTGG   
  
  
- CTTCAACCTG GTTTCACTCG GCTCAACTTT CCGTAAGTAT ACCCCAAATT AGTATTGTGT TTGTATTAAT   
  
  
- CTAACTAACT GCAGAAATTA GTCGCAAAAA CAGAAACAAG CCACGAGGGG AGTGAGCGGG GAGGCGAGAA   
  
  
- ATTTCGAATG GAGTGAGGGG GGTAAGGGTG AGGAAAAGAG AGAGGAGAGA GCGAGAGTGT GCGAGGCAGA   
  
  
- CAGTCGGGGA ACAGTAGAAG GGGGCAGAAG GAGCGTTTTT AGAGGTCGTT TTAAATATCG GCAACAACAC   
  
  
- GGGTACCACT AGACTACACT CTTCTCTAAG TCGTAAATTC TAATAACCCA GGTCTACCGA ATATACACGC   
  
  
- GGCTGTCACC ATTAGAGTAC CGATAACGGG TTGTTCAGTA GTTCGTTTTT GTTGTCGTTC TTGTCGTCGT   
  
  
- TGTGGTTGTC GGAGTAGGGG TCAAGCAGCC CAGCTTATTA TTGGGGAAAA CGTGGTCGGG TACCGGGCCT   
  
  
- GGGCAGGGAA GTAGGCGGTA CAGACGGTCG GGAGGCGGGG GAGAACCCAA GAGGCGGCGG CGCAAAGGCC   
  
  
- TGGGCAAAGT CCACCCGGGC GGGACGGGCC TACCCCGACT TGGCCCAAAG GTCAAACGGG TAAACCTGGT   
  
  
- GGTGAGCTCA CCAAAGGCCA AACGACTAAA AACGCCACCC CCACCACCAC TCAAACTGAG CCTACTCACC   
  
  
- TACCTCTCAA ACTACCCACC GCCGCCGCCA CTGAGCGCCG CTGACTCTCA AGATTGGAGG TTAGGGTAAC   
  
  
- GCTGCGCACC GTTCCCAGGC CACTAAAGCC AGAGATACCA CGACTAGGCA AACGCTGCAC AAGCCTTCTG   
  
  
- GCAACGTCGG CGATAAGTGC GAGTCGGTGA CGGCGTTGTC GTCGGCAGCA CCCAGGGCGG AAGAGGCGGC   
  
  
- TTTCTGTGCT TCGTCAGCCG GGGTGGAGTT TTCGTTTCGC TGCGGCGACC TGGGAGCAAG AGTAGGCTCC   
  
  
- TCCTTGGTCT CGGCAGTACC GGCGGCGATT AACGCCGCAA CAACCTCACG CGGTTTGAGC GGCTCAGGCT   
  
  
- GGGCCTTGTG CGACAGTTTA GCCACTAAGC TGAGTCTCTG AGTCAGAGTG TCGTCCCTCA AGGTTAGGTC   
  
  
- TCTCACAGGA TAATGAAGAG GCTCCGTGAG ATGGTGGCGG AAAGAGAGAG GAGGCGGGGA GTTGGAGAGG   
  
  
- GTCTTTGCTG CAGCAGACTC CTCATGTGAG AGAGAATGTT CCGGGACTTG CTGCGCACGG GCATGGTATT   
  
  
- TAAGCGAGTG AACTGGCGTT TAGTCCGCTA AGATCTTCGG TGACTAAGTC GGTTCTTCTA GGTGTAGCAA   
  
  
- CTAAAACCCT ATTAGGTCCC TCAGGTTACC CGACGAGACG ATGTTCGGGA CCGTTGGGCC GGTCGGCCCT   
  
  
- TCGGGCTTAA CTAAGCTTAT AGGCCTTAAG GACGAGGCCC AGACCCCGTT AGAGGCCGCC GTAGCGACGA   
  
  
- GCGTTGACCC TTAGCGGAGG CACTCAAGCG CTTCCAAGAT CTAGAGTTCA AGCTCAAACT CGGCCATGAG   
  
  
- TGAGGTTAGT CTCTCGATTT GCCCAGCTTG AAGGCCCAGC TGGGTCTACT CTATGATCGA CAGTTAAAGT   
  
  
- ACGAAGTTAA CATGTTGGAT AATCTGCTTT GGGACCAGCG ACAGCTGCGG CGGGACTTCG ACCGGTTCAG   
  
  
- TAACTTGGGG TTTTAGCAGT GAAACCCACT CATGCTCCGC CCAGACTTAG CCCGTCCAAA GAACTGGGCC   
  
  
- AAGTTCTGCC GGGAGTTCAT GATAAACCGT TACAAACTTA GCCAACTCGG GTTGAACCGG GCCCTGAGCG   
  
  
- GGCTCTCCCG AGTCCAACTC TCCGACAATA ACCCGGCCGC CTAGTACCCG AATCAACCCG GCCTCAATGG   
  
  
- CCCTTGCTCT TCCCTTTCCT ACCTCACATT CCTTGTTACC TTCCCAAATT ACCTTTCACG GCCAAAGCTC   
  
  
- GGCACGGTTT CGTCTTTGAT GCAGTCACTT TTTCTGTTCA AAATGGTGAG GTCTAACTTA ATGTCTCTCA   
  
  
- C

+     Myb-binding site

| Site Name | Organism | Position | Strand | Matrix score. | sequence | function |
| --- | --- | --- | --- | --- | --- | --- |
| Myb-binding site | Nicotiana tabacum | 2109 | + | 6 | CAACAG |  |
| Myb-binding site | Nicotiana tabacum | 2559 | + | 6 | CAACAG |  |
| Myb-binding site | Nicotiana tabacum | 2085 | + | 6 | CAACAG |  |
| Myb-binding site | Nicotiana tabacum | 88 | - | 6 | CAACAG |  |

>HU06G00029.1   
+ +Up\_Stream \_Len000TGATAA TTATTTTTAT TGATTTAGGA AAGGATATCA AAGTATATTT TGTTTTTAGG   
  
  
+ TAGTTAAGAG ATTCTGTTGC CAAAAAAAAA ATCAAGATAT TATATATTTT TAATTATGAC AGTAATTATT   
  
  
+ TTATTGAAAA TCCAACGGTT GTGATTATAT AATCAAATAA TCTAAAGGTT AAAAATATCA AATTTATTAG   
  
  
+ AAAATTCGAA AAAGTCACGT CATCACAACA TTGCTTTTAT ATATAGTATA GATTCCTTCG ACTTCTAAAC   
  
  
+ TAATCACTTG AAATTAGAAA TTTGAAACTT TCTAATATAA ATGCATGGTC TTCTTTCCAA GATTATAATC   
  
  
+ CGCAATTGCT TAAATGTAGG GTACAAATAT CAAATTTAGA AGTGACTATT GAAGCCATGA AAGATATTGT   
  
  
+ GATCGTATTG AGGGTGGTTG TGAGCAATAG GATGAAACAG GAAAAAAAAA CCCTAATGTT TTCAAATTTA   
  
  
+ TGGGTGGCTT TGGTAGTAGA TAAGTGAATA GTGTGCAAGG GAATGGCACC AATATGTTTG GATGGAACGA   
  
  
+ TGAGAGTAAG ACGTTAGGAA AGCAACAAGA GAAGGGAAAA GGCAGTAGGG AGAGAATTAT GCGTGTGAAT   
  
  
+ AATATTATAA CAGACGCTTT CGTATGATTA TTAAATACAA GACTTTGAGC ATACATATAA ACTTGTAACT   
  
  
+ TTCTTTACCT ATTGTCAATT AATTTTTGAA TTGAATCATC TGCGACTTAT GCATACAAGA TGATATGATG   
  
  
+ CATATTGGGC CAAGCATTTG ATCTCATGTC CCTTATGGAT CTAGATCCCA ATAGACAAAC TTGCATAAGT   
  
  
+ CAAGGGATGT GTTTCTAATA CAAAGGCTTT GTACTCAAGC AATTAGTTTT GAATTCATTT TCAGTAGTTT   
  
  
+ AATTTCCAAA TCTTATAATT TTTACAATGG CATAAATTTT TAGGAGTTTT GAGTAAATAA ATTATATACA   
  
  
+ ACGAAGATGT CATTTACGAA TTAATATGAC GCACAAAAAA AAAGAAAAAA AATTAATGGT AGCTTCTATA   
  
  
+ CTTGACTCCA TGTTGACAAT TCCCTCTCTC ACATAATGGT TAAAACTTGC AACATCATCC AGGACCCATA   
  
  
+ TGACATGGGT GGTGGTAGAC AGTGTGTTGT GGTAGCCTCA CTCCATGCTT GCCAATTTGT ATCATTGCGC   
  
  
+ TAGTCTTATT CGTCCTTAGA GATAATTGAC TAAGCTCTAA TGCTCTATCG TGGCTGCTGC AATATAAATG   
  
  
+ ATTTTTTGAC CTCAAAATGA AGACAGCTAG TTGTAAATTA AGAATCTTCG TCCCTTCATT TTTTGTGTAC   
  
  
+ GTGTGTATCT GCGTATGTGT TGAGAGACTA GCGGACTATT GATTGAGTTT CAATCAAAAA AGATGACCAT   
  
  
+ CTTAAGTATT TTTAATTACG CAAACTTCTT CATTTAAAAA AATGTTACTT TTATTTGAAA GAAATTGTGA   
  
  
+ ATAATACAAG GAATAGTTCA ATTAATATAT AATCCATAAC ATATTACCTC CTATAGGCAA AAAGAAGATT   
  
  
+ ATCGTATTAA TTTGGGATCA TCCCACCCTA AATTTAAAGA AACCAAAACA AATTACTAAG CAATATCTTT   
  
  
+ CTGTGTGAAA AAATAGTCAG AAACAAAGCG ATCCGACCGG CCAAATCGAG ATAAACACAG CCAAAGAACC   
  
  
+ GAAGTTGGAC CAAAGTGAGC CGAGTTGAAA GGCATTCATA TGGGGTTTAA TCATAACACA AACATAATTA   
  
  
+ GATTGATTGA CGTCTTTAAT CAGCGTTTTT GTCTTTGTTC GGTGCTCCCC TCACTCGCCC CTCCGCTCTT   
  
  
+ TAAAGCTTAC CTCACTCCCC CCATTCCCAC TCCTTTTCTC TCTCCTCTCT CGCTCTCACA CGCTCCGTCT   
  
  
+ GTCAGCCCCT TGTCATCTTC CCCCGTCTTC CTCGCAAAAA TCTCCAGCAA AATTTATAGC CGTTGTTGTG   
  
  
+ CCCATGGTGA TCTGATGTGA GAAGAGATTC AGCATTTAAG ATTATTGGGT CCAGATGGCT TATATGTGCG   
  
  
+ CCGACAGTGG TAATCTCATG GCTATTGCCC AACAAGTCAT CAAGCAAAAA CAACAGCAAG AACAGCAGCA   
  
  
+ ACACCAACAG CCTCATCCCC AGTTCGTCGG GTCGAATAAT AACCCCTTTT GCACCAGCCC ATGGCCCGGA   
  
  
+ CCCGTCCCTT CATCCGCCAT GTCTGCCAGC CCTCCGCCCC CTCTTGGGTT CTCCGCCGCC GCGTTTCCGG   
  
  
+ ACCCGTTTCA GGTGGGCCCG CCCTGCCCGG ATGGGGCTGA ACCGGGTTTC CAGTTTGCCC ATTTGGACCA   
  
  
+ CCACTCGAGT GGTTTCCGGT TTGCTGATTT TTGCGGTGGG GGTGGTGGTG AGTTTGACTC GGATGAGTGG   
  
  
+ ATGGAGAGTT TGATGGGTGG CGGCGGCGGT GACTCGCGGC GACTGAGAGT TCTAACCTCC AATCCCATTG   
  
  
+ CGACGCGTGG CAAGGGTCCG GTGATTTCGG TCTCTATGGT GCTGATCCGT TTGCGACGTG TTCGGAAGAC   
  
  
+ CGTTGCAGCC GCTATTCACG CTCAGCCACT GCCGCAACAG CAGCCGTCGT GGGTCCCGCC TTCTCCGCCG   
  
  
+ AAAGACACGA AGCAGTCGGC CCCACCTCAA AAGCAAAGCG ACGCCGCTGG ACCCTCGTTC TCATCCGAGG   
  
  
+ AGGAACCAGA GCCGTCATGG CCGCCGCTAA TTGCGGCGTT GTTGGAGTGC GCCAAACTCG CCGAGTCCGA   
  
  
+ CCCGGAACAC GCTGTCAAAT CGGTGATTCG ACTCAGAGAC TCAGTCTCAC AGCAGGGAGT TCCAATCCAG   
  
  
+ AGAGTGTCCT ATTACTTCTC CGAGGCACTC TACCACCGCC TTTCTCTCTC CTCCGCCCCT CAACCTCTCC   
  
  
+ CAGAAACGAC GTCGTCTGAG GAGTACACTC TCTCTTACAA GGCCCTGAAC GACGCGTGCC CGTACCATAA   
  
  
+ ATTCGCTCAC TTGACCGCAA ATCAGGCGAT TCTAGAAGCC ACTGATTCAG CCAAGAAGAT CCACATCGTT   
  
  
+ GATTTTGGGA TAATCCAGGG AGTCCAATGG GCTGCTCTGC TACAAGCCCT GGCAACCCGG CCAGCCGGGA   
  
  
+ AGCCCGAATT GATTCGAATA TCCGGAATTC CTGCTCCGGG TCTGGGGCAA TCTCCGGCGG CATCGCTGCT   
  
  
+ CGCAACTGGG AATCGCCTCC GTGAGTTCGC GAAGGTTCTA GATCTCAAGT TCGAGTTTGA GCCGGTACTC   
  
  
+ ACTCCAATCA GAGAGCTAAA CGGGTCGAAC TTCCGGGTCG ACCCAGATGA GATACTAGCT GTCAATTTCA   
  
  
+ TGCTTCAATT GTACAACCTA TTAGACGAAA CCCTGGTCGC TGTCGACGCC GCCCTGAAGC TGGCCAAGTC   
  
  
+ ATTGAACCCC AAAATCGTCA CTTTGGGTGA GTACGAGGCG GGTCTGAATC GGGCAGGTTT CTTGACCCGG   
  
  
+ TTCAAGACGG CCCTCAAGTA CTATTTGGCA ATGTTTGAAT CGGTTGAGCC CAACTTGGCC CGGGACTCGC   
  
  
+ CCGAGAGGGC TCAGGTTGAG AGGCTGTTAT TGGGCCGGCG GATCATGGGC TTAGTTGGGC CGGAGTTACC   
  
  
+ GGGAACGAGA AGGGAAAGGA TGGAGTGTAA GGAACAATGG AAGGGTTTAA TGGAAAGTGC CGGTTTCGAG   
  
  
+ CCGTGCCAAA GCAGAAACTA CGTCAGTGAA AAAGACAAGT TTTACCACTC CAGATTGAAT TACAGAGAGT   
  
  
+ G  

- +Up\_Stream \_Len000ACTATT AATAAAAATA ACTAAATCCT TTCCTATAGT TTCATATAAA ACAAAAATCC   
  
  
- ATCAATTCTC TAAGACAACG GTTTTTTTTT TAGTTCTATA ATATATAAAA ATTAATACTG TCATTAATAA   
  
  
- AATAACTTTT AGGTTGCCAA CACTAATATA TTAGTTTATT AGATTTCCAA TTTTTATAGT TTAAATAATC   
  
  
- TTTTAAGCTT TTTCAGTGCA GTAGTGTTGT AACGAAAATA TATATCATAT CTAAGGAAGC TGAAGATTTG   
  
  
- ATTAGTGAAC TTTAATCTTT AAACTTTGAA AGATTATATT TACGTACCAG AAGAAAGGTT CTAATATTAG   
  
  
- GCGTTAACGA ATTTACATCC CATGTTTATA GTTTAAATCT TCACTGATAA CTTCGGTACT TTCTATAACA   
  
  
- CTAGCATAAC TCCCACCAAC ACTCGTTATC CTACTTTGTC CTTTTTTTTT GGGATTACAA AAGTTTAAAT   
  
  
- ACCCACCGAA ACCATCATCT ATTCACTTAT CACACGTTCC CTTACCGTGG TTATACAAAC CTACCTTGCT   
  
  
- ACTCTCATTC TGCAATCCTT TCGTTGTTCT CTTCCCTTTT CCGTCATCCC TCTCTTAATA CGCACACTTA   
  
  
- TTATAATATT GTCTGCGAAA GCATACTAAT AATTTATGTT CTGAAACTCG TATGTATATT TGAACATTGA   
  
  
- AAGAAATGGA TAACAGTTAA TTAAAAACTT AACTTAGTAG ACGCTGAATA CGTATGTTCT ACTATACTAC   
  
  
- GTATAACCCG GTTCGTAAAC TAGAGTACAG GGAATACCTA GATCTAGGGT TATCTGTTTG AACGTATTCA   
  
  
- GTTCCCTACA CAAAGATTAT GTTTCCGAAA CATGAGTTCG TTAATCAAAA CTTAAGTAAA AGTCATCAAA   
  
  
- TTAAAGGTTT AGAATATTAA AAATGTTACC GTATTTAAAA ATCCTCAAAA CTCATTTATT TAATATATGT   
  
  
- TGCTTCTACA GTAAATGCTT AATTATACTG CGTGTTTTTT TTTCTTTTTT TTAATTACCA TCGAAGATAT   
  
  
- GAACTGAGGT ACAACTGTTA AGGGAGAGAG TGTATTACCA ATTTTGAACG TTGTAGTAGG TCCTGGGTAT   
  
  
- ACTGTACCCA CCACCATCTG TCACACAACA CCATCGGAGT GAGGTACGAA CGGTTAAACA TAGTAACGCG   
  
  
- ATCAGAATAA GCAGGAATCT CTATTAACTG ATTCGAGATT ACGAGATAGC ACCGACGACG TTATATTTAC   
  
  
- TAAAAAACTG GAGTTTTACT TCTGTCGATC AACATTTAAT TCTTAGAAGC AGGGAAGTAA AAAACACATG   
  
  
- CACACATAGA CGCATACACA ACTCTCTGAT CGCCTGATAA CTAACTCAAA GTTAGTTTTT TCTACTGGTA   
  
  
- GAATTCATAA AAATTAATGC GTTTGAAGAA GTAAATTTTT TTACAATGAA AATAAACTTT CTTTAACACT   
  
  
- TATTATGTTC CTTATCAAGT TAATTATATA TTAGGTATTG TATAATGGAG GATATCCGTT TTTCTTCTAA   
  
  
- TAGCATAATT AAACCCTAGT AGGGTGGGAT TTAAATTTCT TTGGTTTTGT TTAATGATTC GTTATAGAAA   
  
  
- GACACACTTT TTTATCAGTC TTTGTTTCGC TAGGCTGGCC GGTTTAGCTC TATTTGTGTC GGTTTCTTGG   
  
  
- CTTCAACCTG GTTTCACTCG GCTCAACTTT CCGTAAGTAT ACCCCAAATT AGTATTGTGT TTGTATTAAT   
  
  
- CTAACTAACT GCAGAAATTA GTCGCAAAAA CAGAAACAAG CCACGAGGGG AGTGAGCGGG GAGGCGAGAA   
  
  
- ATTTCGAATG GAGTGAGGGG GGTAAGGGTG AGGAAAAGAG AGAGGAGAGA GCGAGAGTGT GCGAGGCAGA   
  
  
- CAGTCGGGGA ACAGTAGAAG GGGGCAGAAG GAGCGTTTTT AGAGGTCGTT TTAAATATCG GCAACAACAC   
  
  
- GGGTACCACT AGACTACACT CTTCTCTAAG TCGTAAATTC TAATAACCCA GGTCTACCGA ATATACACGC   
  
  
- GGCTGTCACC ATTAGAGTAC CGATAACGGG TTGTTCAGTA GTTCGTTTTT GTTGTCGTTC TTGTCGTCGT   
  
  
- TGTGGTTGTC GGAGTAGGGG TCAAGCAGCC CAGCTTATTA TTGGGGAAAA CGTGGTCGGG TACCGGGCCT   
  
  
- GGGCAGGGAA GTAGGCGGTA CAGACGGTCG GGAGGCGGGG GAGAACCCAA GAGGCGGCGG CGCAAAGGCC   
  
  
- TGGGCAAAGT CCACCCGGGC GGGACGGGCC TACCCCGACT TGGCCCAAAG GTCAAACGGG TAAACCTGGT   
  
  
- GGTGAGCTCA CCAAAGGCCA AACGACTAAA AACGCCACCC CCACCACCAC TCAAACTGAG CCTACTCACC   
  
  
- TACCTCTCAA ACTACCCACC GCCGCCGCCA CTGAGCGCCG CTGACTCTCA AGATTGGAGG TTAGGGTAAC   
  
  
- GCTGCGCACC GTTCCCAGGC CACTAAAGCC AGAGATACCA CGACTAGGCA AACGCTGCAC AAGCCTTCTG   
  
  
- GCAACGTCGG CGATAAGTGC GAGTCGGTGA CGGCGTTGTC GTCGGCAGCA CCCAGGGCGG AAGAGGCGGC   
  
  
- TTTCTGTGCT TCGTCAGCCG GGGTGGAGTT TTCGTTTCGC TGCGGCGACC TGGGAGCAAG AGTAGGCTCC   
  
  
- TCCTTGGTCT CGGCAGTACC GGCGGCGATT AACGCCGCAA CAACCTCACG CGGTTTGAGC GGCTCAGGCT   
  
  
- GGGCCTTGTG CGACAGTTTA GCCACTAAGC TGAGTCTCTG AGTCAGAGTG TCGTCCCTCA AGGTTAGGTC   
  
  
- TCTCACAGGA TAATGAAGAG GCTCCGTGAG ATGGTGGCGG AAAGAGAGAG GAGGCGGGGA GTTGGAGAGG   
  
  
- GTCTTTGCTG CAGCAGACTC CTCATGTGAG AGAGAATGTT CCGGGACTTG CTGCGCACGG GCATGGTATT   
  
  
- TAAGCGAGTG AACTGGCGTT TAGTCCGCTA AGATCTTCGG TGACTAAGTC GGTTCTTCTA GGTGTAGCAA   
  
  
- CTAAAACCCT ATTAGGTCCC TCAGGTTACC CGACGAGACG ATGTTCGGGA CCGTTGGGCC GGTCGGCCCT   
  
  
- TCGGGCTTAA CTAAGCTTAT AGGCCTTAAG GACGAGGCCC AGACCCCGTT AGAGGCCGCC GTAGCGACGA   
  
  
- GCGTTGACCC TTAGCGGAGG CACTCAAGCG CTTCCAAGAT CTAGAGTTCA AGCTCAAACT CGGCCATGAG   
  
  
- TGAGGTTAGT CTCTCGATTT GCCCAGCTTG AAGGCCCAGC TGGGTCTACT CTATGATCGA CAGTTAAAGT   
  
  
- ACGAAGTTAA CATGTTGGAT AATCTGCTTT GGGACCAGCG ACAGCTGCGG CGGGACTTCG ACCGGTTCAG   
  
  
- TAACTTGGGG TTTTAGCAGT GAAACCCACT CATGCTCCGC CCAGACTTAG CCCGTCCAAA GAACTGGGCC   
  
  
- AAGTTCTGCC GGGAGTTCAT GATAAACCGT TACAAACTTA GCCAACTCGG GTTGAACCGG GCCCTGAGCG   
  
  
- GGCTCTCCCG AGTCCAACTC TCCGACAATA ACCCGGCCGC CTAGTACCCG AATCAACCCG GCCTCAATGG   
  
  
- CCCTTGCTCT TCCCTTTCCT ACCTCACATT CCTTGTTACC TTCCCAAATT ACCTTTCACG GCCAAAGCTC   
  
  
- GGCACGGTTT CGTCTTTGAT GCAGTCACTT TTTCTGTTCA AAATGGTGAG GTCTAACTTA ATGTCTCTCA   
  
  
- C

+     Myc

| Site Name | Organism | Position | Strand | Matrix score. | sequence | function |
| --- | --- | --- | --- | --- | --- | --- |
| Myc | Arabidopsis thaliana | 2905 | + | 7 | TCTCTTA |  |
| Myc | Arabidopsis thaliana | 79 | - | 7 | TCTCTTA |  |

>HU06G00029.1   
+ +Up\_Stream \_Len000TGATAA TTATTTTTAT TGATTTAGGA AAGGATATCA AAGTATATTT TGTTTTTAGG   
  
  
+ TAGTTAAGAG ATTCTGTTGC CAAAAAAAAA ATCAAGATAT TATATATTTT TAATTATGAC AGTAATTATT   
  
  
+ TTATTGAAAA TCCAACGGTT GTGATTATAT AATCAAATAA TCTAAAGGTT AAAAATATCA AATTTATTAG   
  
  
+ AAAATTCGAA AAAGTCACGT CATCACAACA TTGCTTTTAT ATATAGTATA GATTCCTTCG ACTTCTAAAC   
  
  
+ TAATCACTTG AAATTAGAAA TTTGAAACTT TCTAATATAA ATGCATGGTC TTCTTTCCAA GATTATAATC   
  
  
+ CGCAATTGCT TAAATGTAGG GTACAAATAT CAAATTTAGA AGTGACTATT GAAGCCATGA AAGATATTGT   
  
  
+ GATCGTATTG AGGGTGGTTG TGAGCAATAG GATGAAACAG GAAAAAAAAA CCCTAATGTT TTCAAATTTA   
  
  
+ TGGGTGGCTT TGGTAGTAGA TAAGTGAATA GTGTGCAAGG GAATGGCACC AATATGTTTG GATGGAACGA   
  
  
+ TGAGAGTAAG ACGTTAGGAA AGCAACAAGA GAAGGGAAAA GGCAGTAGGG AGAGAATTAT GCGTGTGAAT   
  
  
+ AATATTATAA CAGACGCTTT CGTATGATTA TTAAATACAA GACTTTGAGC ATACATATAA ACTTGTAACT   
  
  
+ TTCTTTACCT ATTGTCAATT AATTTTTGAA TTGAATCATC TGCGACTTAT GCATACAAGA TGATATGATG   
  
  
+ CATATTGGGC CAAGCATTTG ATCTCATGTC CCTTATGGAT CTAGATCCCA ATAGACAAAC TTGCATAAGT   
  
  
+ CAAGGGATGT GTTTCTAATA CAAAGGCTTT GTACTCAAGC AATTAGTTTT GAATTCATTT TCAGTAGTTT   
  
  
+ AATTTCCAAA TCTTATAATT TTTACAATGG CATAAATTTT TAGGAGTTTT GAGTAAATAA ATTATATACA   
  
  
+ ACGAAGATGT CATTTACGAA TTAATATGAC GCACAAAAAA AAAGAAAAAA AATTAATGGT AGCTTCTATA   
  
  
+ CTTGACTCCA TGTTGACAAT TCCCTCTCTC ACATAATGGT TAAAACTTGC AACATCATCC AGGACCCATA   
  
  
+ TGACATGGGT GGTGGTAGAC AGTGTGTTGT GGTAGCCTCA CTCCATGCTT GCCAATTTGT ATCATTGCGC   
  
  
+ TAGTCTTATT CGTCCTTAGA GATAATTGAC TAAGCTCTAA TGCTCTATCG TGGCTGCTGC AATATAAATG   
  
  
+ ATTTTTTGAC CTCAAAATGA AGACAGCTAG TTGTAAATTA AGAATCTTCG TCCCTTCATT TTTTGTGTAC   
  
  
+ GTGTGTATCT GCGTATGTGT TGAGAGACTA GCGGACTATT GATTGAGTTT CAATCAAAAA AGATGACCAT   
  
  
+ CTTAAGTATT TTTAATTACG CAAACTTCTT CATTTAAAAA AATGTTACTT TTATTTGAAA GAAATTGTGA   
  
  
+ ATAATACAAG GAATAGTTCA ATTAATATAT AATCCATAAC ATATTACCTC CTATAGGCAA AAAGAAGATT   
  
  
+ ATCGTATTAA TTTGGGATCA TCCCACCCTA AATTTAAAGA AACCAAAACA AATTACTAAG CAATATCTTT   
  
  
+ CTGTGTGAAA AAATAGTCAG AAACAAAGCG ATCCGACCGG CCAAATCGAG ATAAACACAG CCAAAGAACC   
  
  
+ GAAGTTGGAC CAAAGTGAGC CGAGTTGAAA GGCATTCATA TGGGGTTTAA TCATAACACA AACATAATTA   
  
  
+ GATTGATTGA CGTCTTTAAT CAGCGTTTTT GTCTTTGTTC GGTGCTCCCC TCACTCGCCC CTCCGCTCTT   
  
  
+ TAAAGCTTAC CTCACTCCCC CCATTCCCAC TCCTTTTCTC TCTCCTCTCT CGCTCTCACA CGCTCCGTCT   
  
  
+ GTCAGCCCCT TGTCATCTTC CCCCGTCTTC CTCGCAAAAA TCTCCAGCAA AATTTATAGC CGTTGTTGTG   
  
  
+ CCCATGGTGA TCTGATGTGA GAAGAGATTC AGCATTTAAG ATTATTGGGT CCAGATGGCT TATATGTGCG   
  
  
+ CCGACAGTGG TAATCTCATG GCTATTGCCC AACAAGTCAT CAAGCAAAAA CAACAGCAAG AACAGCAGCA   
  
  
+ ACACCAACAG CCTCATCCCC AGTTCGTCGG GTCGAATAAT AACCCCTTTT GCACCAGCCC ATGGCCCGGA   
  
  
+ CCCGTCCCTT CATCCGCCAT GTCTGCCAGC CCTCCGCCCC CTCTTGGGTT CTCCGCCGCC GCGTTTCCGG   
  
  
+ ACCCGTTTCA GGTGGGCCCG CCCTGCCCGG ATGGGGCTGA ACCGGGTTTC CAGTTTGCCC ATTTGGACCA   
  
  
+ CCACTCGAGT GGTTTCCGGT TTGCTGATTT TTGCGGTGGG GGTGGTGGTG AGTTTGACTC GGATGAGTGG   
  
  
+ ATGGAGAGTT TGATGGGTGG CGGCGGCGGT GACTCGCGGC GACTGAGAGT TCTAACCTCC AATCCCATTG   
  
  
+ CGACGCGTGG CAAGGGTCCG GTGATTTCGG TCTCTATGGT GCTGATCCGT TTGCGACGTG TTCGGAAGAC   
  
  
+ CGTTGCAGCC GCTATTCACG CTCAGCCACT GCCGCAACAG CAGCCGTCGT GGGTCCCGCC TTCTCCGCCG   
  
  
+ AAAGACACGA AGCAGTCGGC CCCACCTCAA AAGCAAAGCG ACGCCGCTGG ACCCTCGTTC TCATCCGAGG   
  
  
+ AGGAACCAGA GCCGTCATGG CCGCCGCTAA TTGCGGCGTT GTTGGAGTGC GCCAAACTCG CCGAGTCCGA   
  
  
+ CCCGGAACAC GCTGTCAAAT CGGTGATTCG ACTCAGAGAC TCAGTCTCAC AGCAGGGAGT TCCAATCCAG   
  
  
+ AGAGTGTCCT ATTACTTCTC CGAGGCACTC TACCACCGCC TTTCTCTCTC CTCCGCCCCT CAACCTCTCC   
  
  
+ CAGAAACGAC GTCGTCTGAG GAGTACACTC TCTCTTACAA GGCCCTGAAC GACGCGTGCC CGTACCATAA   
  
  
+ ATTCGCTCAC TTGACCGCAA ATCAGGCGAT TCTAGAAGCC ACTGATTCAG CCAAGAAGAT CCACATCGTT   
  
  
+ GATTTTGGGA TAATCCAGGG AGTCCAATGG GCTGCTCTGC TACAAGCCCT GGCAACCCGG CCAGCCGGGA   
  
  
+ AGCCCGAATT GATTCGAATA TCCGGAATTC CTGCTCCGGG TCTGGGGCAA TCTCCGGCGG CATCGCTGCT   
  
  
+ CGCAACTGGG AATCGCCTCC GTGAGTTCGC GAAGGTTCTA GATCTCAAGT TCGAGTTTGA GCCGGTACTC   
  
  
+ ACTCCAATCA GAGAGCTAAA CGGGTCGAAC TTCCGGGTCG ACCCAGATGA GATACTAGCT GTCAATTTCA   
  
  
+ TGCTTCAATT GTACAACCTA TTAGACGAAA CCCTGGTCGC TGTCGACGCC GCCCTGAAGC TGGCCAAGTC   
  
  
+ ATTGAACCCC AAAATCGTCA CTTTGGGTGA GTACGAGGCG GGTCTGAATC GGGCAGGTTT CTTGACCCGG   
  
  
+ TTCAAGACGG CCCTCAAGTA CTATTTGGCA ATGTTTGAAT CGGTTGAGCC CAACTTGGCC CGGGACTCGC   
  
  
+ CCGAGAGGGC TCAGGTTGAG AGGCTGTTAT TGGGCCGGCG GATCATGGGC TTAGTTGGGC CGGAGTTACC   
  
  
+ GGGAACGAGA AGGGAAAGGA TGGAGTGTAA GGAACAATGG AAGGGTTTAA TGGAAAGTGC CGGTTTCGAG   
  
  
+ CCGTGCCAAA GCAGAAACTA CGTCAGTGAA AAAGACAAGT TTTACCACTC CAGATTGAAT TACAGAGAGT   
  
  
+ G  

- +Up\_Stream \_Len000ACTATT AATAAAAATA ACTAAATCCT TTCCTATAGT TTCATATAAA ACAAAAATCC   
  
  
- ATCAATTCTC TAAGACAACG GTTTTTTTTT TAGTTCTATA ATATATAAAA ATTAATACTG TCATTAATAA   
  
  
- AATAACTTTT AGGTTGCCAA CACTAATATA TTAGTTTATT AGATTTCCAA TTTTTATAGT TTAAATAATC   
  
  
- TTTTAAGCTT TTTCAGTGCA GTAGTGTTGT AACGAAAATA TATATCATAT CTAAGGAAGC TGAAGATTTG   
  
  
- ATTAGTGAAC TTTAATCTTT AAACTTTGAA AGATTATATT TACGTACCAG AAGAAAGGTT CTAATATTAG   
  
  
- GCGTTAACGA ATTTACATCC CATGTTTATA GTTTAAATCT TCACTGATAA CTTCGGTACT TTCTATAACA   
  
  
- CTAGCATAAC TCCCACCAAC ACTCGTTATC CTACTTTGTC CTTTTTTTTT GGGATTACAA AAGTTTAAAT   
  
  
- ACCCACCGAA ACCATCATCT ATTCACTTAT CACACGTTCC CTTACCGTGG TTATACAAAC CTACCTTGCT   
  
  
- ACTCTCATTC TGCAATCCTT TCGTTGTTCT CTTCCCTTTT CCGTCATCCC TCTCTTAATA CGCACACTTA   
  
  
- TTATAATATT GTCTGCGAAA GCATACTAAT AATTTATGTT CTGAAACTCG TATGTATATT TGAACATTGA   
  
  
- AAGAAATGGA TAACAGTTAA TTAAAAACTT AACTTAGTAG ACGCTGAATA CGTATGTTCT ACTATACTAC   
  
  
- GTATAACCCG GTTCGTAAAC TAGAGTACAG GGAATACCTA GATCTAGGGT TATCTGTTTG AACGTATTCA   
  
  
- GTTCCCTACA CAAAGATTAT GTTTCCGAAA CATGAGTTCG TTAATCAAAA CTTAAGTAAA AGTCATCAAA   
  
  
- TTAAAGGTTT AGAATATTAA AAATGTTACC GTATTTAAAA ATCCTCAAAA CTCATTTATT TAATATATGT   
  
  
- TGCTTCTACA GTAAATGCTT AATTATACTG CGTGTTTTTT TTTCTTTTTT TTAATTACCA TCGAAGATAT   
  
  
- GAACTGAGGT ACAACTGTTA AGGGAGAGAG TGTATTACCA ATTTTGAACG TTGTAGTAGG TCCTGGGTAT   
  
  
- ACTGTACCCA CCACCATCTG TCACACAACA CCATCGGAGT GAGGTACGAA CGGTTAAACA TAGTAACGCG   
  
  
- ATCAGAATAA GCAGGAATCT CTATTAACTG ATTCGAGATT ACGAGATAGC ACCGACGACG TTATATTTAC   
  
  
- TAAAAAACTG GAGTTTTACT TCTGTCGATC AACATTTAAT TCTTAGAAGC AGGGAAGTAA AAAACACATG   
  
  
- CACACATAGA CGCATACACA ACTCTCTGAT CGCCTGATAA CTAACTCAAA GTTAGTTTTT TCTACTGGTA   
  
  
- GAATTCATAA AAATTAATGC GTTTGAAGAA GTAAATTTTT TTACAATGAA AATAAACTTT CTTTAACACT   
  
  
- TATTATGTTC CTTATCAAGT TAATTATATA TTAGGTATTG TATAATGGAG GATATCCGTT TTTCTTCTAA   
  
  
- TAGCATAATT AAACCCTAGT AGGGTGGGAT TTAAATTTCT TTGGTTTTGT TTAATGATTC GTTATAGAAA   
  
  
- GACACACTTT TTTATCAGTC TTTGTTTCGC TAGGCTGGCC GGTTTAGCTC TATTTGTGTC GGTTTCTTGG   
  
  
- CTTCAACCTG GTTTCACTCG GCTCAACTTT CCGTAAGTAT ACCCCAAATT AGTATTGTGT TTGTATTAAT   
  
  
- CTAACTAACT GCAGAAATTA GTCGCAAAAA CAGAAACAAG CCACGAGGGG AGTGAGCGGG GAGGCGAGAA   
  
  
- ATTTCGAATG GAGTGAGGGG GGTAAGGGTG AGGAAAAGAG AGAGGAGAGA GCGAGAGTGT GCGAGGCAGA   
  
  
- CAGTCGGGGA ACAGTAGAAG GGGGCAGAAG GAGCGTTTTT AGAGGTCGTT TTAAATATCG GCAACAACAC   
  
  
- GGGTACCACT AGACTACACT CTTCTCTAAG TCGTAAATTC TAATAACCCA GGTCTACCGA ATATACACGC   
  
  
- GGCTGTCACC ATTAGAGTAC CGATAACGGG TTGTTCAGTA GTTCGTTTTT GTTGTCGTTC TTGTCGTCGT   
  
  
- TGTGGTTGTC GGAGTAGGGG TCAAGCAGCC CAGCTTATTA TTGGGGAAAA CGTGGTCGGG TACCGGGCCT   
  
  
- GGGCAGGGAA GTAGGCGGTA CAGACGGTCG GGAGGCGGGG GAGAACCCAA GAGGCGGCGG CGCAAAGGCC   
  
  
- TGGGCAAAGT CCACCCGGGC GGGACGGGCC TACCCCGACT TGGCCCAAAG GTCAAACGGG TAAACCTGGT   
  
  
- GGTGAGCTCA CCAAAGGCCA AACGACTAAA AACGCCACCC CCACCACCAC TCAAACTGAG CCTACTCACC   
  
  
- TACCTCTCAA ACTACCCACC GCCGCCGCCA CTGAGCGCCG CTGACTCTCA AGATTGGAGG TTAGGGTAAC   
  
  
- GCTGCGCACC GTTCCCAGGC CACTAAAGCC AGAGATACCA CGACTAGGCA AACGCTGCAC AAGCCTTCTG   
  
  
- GCAACGTCGG CGATAAGTGC GAGTCGGTGA CGGCGTTGTC GTCGGCAGCA CCCAGGGCGG AAGAGGCGGC   
  
  
- TTTCTGTGCT TCGTCAGCCG GGGTGGAGTT TTCGTTTCGC TGCGGCGACC TGGGAGCAAG AGTAGGCTCC   
  
  
- TCCTTGGTCT CGGCAGTACC GGCGGCGATT AACGCCGCAA CAACCTCACG CGGTTTGAGC GGCTCAGGCT   
  
  
- GGGCCTTGTG CGACAGTTTA GCCACTAAGC TGAGTCTCTG AGTCAGAGTG TCGTCCCTCA AGGTTAGGTC   
  
  
- TCTCACAGGA TAATGAAGAG GCTCCGTGAG ATGGTGGCGG AAAGAGAGAG GAGGCGGGGA GTTGGAGAGG   
  
  
- GTCTTTGCTG CAGCAGACTC CTCATGTGAG AGAGAATGTT CCGGGACTTG CTGCGCACGG GCATGGTATT   
  
  
- TAAGCGAGTG AACTGGCGTT TAGTCCGCTA AGATCTTCGG TGACTAAGTC GGTTCTTCTA GGTGTAGCAA   
  
  
- CTAAAACCCT ATTAGGTCCC TCAGGTTACC CGACGAGACG ATGTTCGGGA CCGTTGGGCC GGTCGGCCCT   
  
  
- TCGGGCTTAA CTAAGCTTAT AGGCCTTAAG GACGAGGCCC AGACCCCGTT AGAGGCCGCC GTAGCGACGA   
  
  
- GCGTTGACCC TTAGCGGAGG CACTCAAGCG CTTCCAAGAT CTAGAGTTCA AGCTCAAACT CGGCCATGAG   
  
  
- TGAGGTTAGT CTCTCGATTT GCCCAGCTTG AAGGCCCAGC TGGGTCTACT CTATGATCGA CAGTTAAAGT   
  
  
- ACGAAGTTAA CATGTTGGAT AATCTGCTTT GGGACCAGCG ACAGCTGCGG CGGGACTTCG ACCGGTTCAG   
  
  
- TAACTTGGGG TTTTAGCAGT GAAACCCACT CATGCTCCGC CCAGACTTAG CCCGTCCAAA GAACTGGGCC   
  
  
- AAGTTCTGCC GGGAGTTCAT GATAAACCGT TACAAACTTA GCCAACTCGG GTTGAACCGG GCCCTGAGCG   
  
  
- GGCTCTCCCG AGTCCAACTC TCCGACAATA ACCCGGCCGC CTAGTACCCG AATCAACCCG GCCTCAATGG   
  
  
- CCCTTGCTCT TCCCTTTCCT ACCTCACATT CCTTGTTACC TTCCCAAATT ACCTTTCACG GCCAAAGCTC   
  
  
- GGCACGGTTT CGTCTTTGAT GCAGTCACTT TTTCTGTTCA AAATGGTGAG GTCTAACTTA ATGTCTCTCA   
  
  
- C

+     O2-site

| Site Name | Organism | Position | Strand | Matrix score. | sequence | function |
| --- | --- | --- | --- | --- | --- | --- |
| O2-site | Zea mays | 1902 | - | 9 | GATGACATGG | cis-acting regulatory element involved in zein metabolism regulation |
| O2-site | Zea mays | 229 | - | 9 | GTTGACGTGA | cis-acting regulatory element involved in zein metabolism regulation |
| O2-site | Zea mays | 763 | + | 8.5 | GATGA(C/T)(A/G)TG(A/G) | cis-acting regulatory element involved in zein metabolism regulation |
| O2-site | Zea mays | 1123 | + | 9 | GATGACATGG | cis-acting regulatory element involved in zein metabolism regulation |
| O2-site | Zea mays | 1104 | - | 9 | GATGATGTGG | cis-acting regulatory element involved in zein metabolism regulation |

>HU06G00029.1   
+ +Up\_Stream \_Len000TGATAA TTATTTTTAT TGATTTAGGA AAGGATATCA AAGTATATTT TGTTTTTAGG   
  
  
+ TAGTTAAGAG ATTCTGTTGC CAAAAAAAAA ATCAAGATAT TATATATTTT TAATTATGAC AGTAATTATT   
  
  
+ TTATTGAAAA TCCAACGGTT GTGATTATAT AATCAAATAA TCTAAAGGTT AAAAATATCA AATTTATTAG   
  
  
+ AAAATTCGAA AAAGTCACGT CATCACAACA TTGCTTTTAT ATATAGTATA GATTCCTTCG ACTTCTAAAC   
  
  
+ TAATCACTTG AAATTAGAAA TTTGAAACTT TCTAATATAA ATGCATGGTC TTCTTTCCAA GATTATAATC   
  
  
+ CGCAATTGCT TAAATGTAGG GTACAAATAT CAAATTTAGA AGTGACTATT GAAGCCATGA AAGATATTGT   
  
  
+ GATCGTATTG AGGGTGGTTG TGAGCAATAG GATGAAACAG GAAAAAAAAA CCCTAATGTT TTCAAATTTA   
  
  
+ TGGGTGGCTT TGGTAGTAGA TAAGTGAATA GTGTGCAAGG GAATGGCACC AATATGTTTG GATGGAACGA   
  
  
+ TGAGAGTAAG ACGTTAGGAA AGCAACAAGA GAAGGGAAAA GGCAGTAGGG AGAGAATTAT GCGTGTGAAT   
  
  
+ AATATTATAA CAGACGCTTT CGTATGATTA TTAAATACAA GACTTTGAGC ATACATATAA ACTTGTAACT   
  
  
+ TTCTTTACCT ATTGTCAATT AATTTTTGAA TTGAATCATC TGCGACTTAT GCATACAAGA TGATATGATG   
  
  
+ CATATTGGGC CAAGCATTTG ATCTCATGTC CCTTATGGAT CTAGATCCCA ATAGACAAAC TTGCATAAGT   
  
  
+ CAAGGGATGT GTTTCTAATA CAAAGGCTTT GTACTCAAGC AATTAGTTTT GAATTCATTT TCAGTAGTTT   
  
  
+ AATTTCCAAA TCTTATAATT TTTACAATGG CATAAATTTT TAGGAGTTTT GAGTAAATAA ATTATATACA   
  
  
+ ACGAAGATGT CATTTACGAA TTAATATGAC GCACAAAAAA AAAGAAAAAA AATTAATGGT AGCTTCTATA   
  
  
+ CTTGACTCCA TGTTGACAAT TCCCTCTCTC ACATAATGGT TAAAACTTGC AACATCATCC AGGACCCATA   
  
  
+ TGACATGGGT GGTGGTAGAC AGTGTGTTGT GGTAGCCTCA CTCCATGCTT GCCAATTTGT ATCATTGCGC   
  
  
+ TAGTCTTATT CGTCCTTAGA GATAATTGAC TAAGCTCTAA TGCTCTATCG TGGCTGCTGC AATATAAATG   
  
  
+ ATTTTTTGAC CTCAAAATGA AGACAGCTAG TTGTAAATTA AGAATCTTCG TCCCTTCATT TTTTGTGTAC   
  
  
+ GTGTGTATCT GCGTATGTGT TGAGAGACTA GCGGACTATT GATTGAGTTT CAATCAAAAA AGATGACCAT   
  
  
+ CTTAAGTATT TTTAATTACG CAAACTTCTT CATTTAAAAA AATGTTACTT TTATTTGAAA GAAATTGTGA   
  
  
+ ATAATACAAG GAATAGTTCA ATTAATATAT AATCCATAAC ATATTACCTC CTATAGGCAA AAAGAAGATT   
  
  
+ ATCGTATTAA TTTGGGATCA TCCCACCCTA AATTTAAAGA AACCAAAACA AATTACTAAG CAATATCTTT   
  
  
+ CTGTGTGAAA AAATAGTCAG AAACAAAGCG ATCCGACCGG CCAAATCGAG ATAAACACAG CCAAAGAACC   
  
  
+ GAAGTTGGAC CAAAGTGAGC CGAGTTGAAA GGCATTCATA TGGGGTTTAA TCATAACACA AACATAATTA   
  
  
+ GATTGATTGA CGTCTTTAAT CAGCGTTTTT GTCTTTGTTC GGTGCTCCCC TCACTCGCCC CTCCGCTCTT   
  
  
+ TAAAGCTTAC CTCACTCCCC CCATTCCCAC TCCTTTTCTC TCTCCTCTCT CGCTCTCACA CGCTCCGTCT   
  
  
+ GTCAGCCCCT TGTCATCTTC CCCCGTCTTC CTCGCAAAAA TCTCCAGCAA AATTTATAGC CGTTGTTGTG   
  
  
+ CCCATGGTGA TCTGATGTGA GAAGAGATTC AGCATTTAAG ATTATTGGGT CCAGATGGCT TATATGTGCG   
  
  
+ CCGACAGTGG TAATCTCATG GCTATTGCCC AACAAGTCAT CAAGCAAAAA CAACAGCAAG AACAGCAGCA   
  
  
+ ACACCAACAG CCTCATCCCC AGTTCGTCGG GTCGAATAAT AACCCCTTTT GCACCAGCCC ATGGCCCGGA   
  
  
+ CCCGTCCCTT CATCCGCCAT GTCTGCCAGC CCTCCGCCCC CTCTTGGGTT CTCCGCCGCC GCGTTTCCGG   
  
  
+ ACCCGTTTCA GGTGGGCCCG CCCTGCCCGG ATGGGGCTGA ACCGGGTTTC CAGTTTGCCC ATTTGGACCA   
  
  
+ CCACTCGAGT GGTTTCCGGT TTGCTGATTT TTGCGGTGGG GGTGGTGGTG AGTTTGACTC GGATGAGTGG   
  
  
+ ATGGAGAGTT TGATGGGTGG CGGCGGCGGT GACTCGCGGC GACTGAGAGT TCTAACCTCC AATCCCATTG   
  
  
+ CGACGCGTGG CAAGGGTCCG GTGATTTCGG TCTCTATGGT GCTGATCCGT TTGCGACGTG TTCGGAAGAC   
  
  
+ CGTTGCAGCC GCTATTCACG CTCAGCCACT GCCGCAACAG CAGCCGTCGT GGGTCCCGCC TTCTCCGCCG   
  
  
+ AAAGACACGA AGCAGTCGGC CCCACCTCAA AAGCAAAGCG ACGCCGCTGG ACCCTCGTTC TCATCCGAGG   
  
  
+ AGGAACCAGA GCCGTCATGG CCGCCGCTAA TTGCGGCGTT GTTGGAGTGC GCCAAACTCG CCGAGTCCGA   
  
  
+ CCCGGAACAC GCTGTCAAAT CGGTGATTCG ACTCAGAGAC TCAGTCTCAC AGCAGGGAGT TCCAATCCAG   
  
  
+ AGAGTGTCCT ATTACTTCTC CGAGGCACTC TACCACCGCC TTTCTCTCTC CTCCGCCCCT CAACCTCTCC   
  
  
+ CAGAAACGAC GTCGTCTGAG GAGTACACTC TCTCTTACAA GGCCCTGAAC GACGCGTGCC CGTACCATAA   
  
  
+ ATTCGCTCAC TTGACCGCAA ATCAGGCGAT TCTAGAAGCC ACTGATTCAG CCAAGAAGAT CCACATCGTT   
  
  
+ GATTTTGGGA TAATCCAGGG AGTCCAATGG GCTGCTCTGC TACAAGCCCT GGCAACCCGG CCAGCCGGGA   
  
  
+ AGCCCGAATT GATTCGAATA TCCGGAATTC CTGCTCCGGG TCTGGGGCAA TCTCCGGCGG CATCGCTGCT   
  
  
+ CGCAACTGGG AATCGCCTCC GTGAGTTCGC GAAGGTTCTA GATCTCAAGT TCGAGTTTGA GCCGGTACTC   
  
  
+ ACTCCAATCA GAGAGCTAAA CGGGTCGAAC TTCCGGGTCG ACCCAGATGA GATACTAGCT GTCAATTTCA   
  
  
+ TGCTTCAATT GTACAACCTA TTAGACGAAA CCCTGGTCGC TGTCGACGCC GCCCTGAAGC TGGCCAAGTC   
  
  
+ ATTGAACCCC AAAATCGTCA CTTTGGGTGA GTACGAGGCG GGTCTGAATC GGGCAGGTTT CTTGACCCGG   
  
  
+ TTCAAGACGG CCCTCAAGTA CTATTTGGCA ATGTTTGAAT CGGTTGAGCC CAACTTGGCC CGGGACTCGC   
  
  
+ CCGAGAGGGC TCAGGTTGAG AGGCTGTTAT TGGGCCGGCG GATCATGGGC TTAGTTGGGC CGGAGTTACC   
  
  
+ GGGAACGAGA AGGGAAAGGA TGGAGTGTAA GGAACAATGG AAGGGTTTAA TGGAAAGTGC CGGTTTCGAG   
  
  
+ CCGTGCCAAA GCAGAAACTA CGTCAGTGAA AAAGACAAGT TTTACCACTC CAGATTGAAT TACAGAGAGT   
  
  
+ G  

- +Up\_Stream \_Len000ACTATT AATAAAAATA ACTAAATCCT TTCCTATAGT TTCATATAAA ACAAAAATCC   
  
  
- ATCAATTCTC TAAGACAACG GTTTTTTTTT TAGTTCTATA ATATATAAAA ATTAATACTG TCATTAATAA   
  
  
- AATAACTTTT AGGTTGCCAA CACTAATATA TTAGTTTATT AGATTTCCAA TTTTTATAGT TTAAATAATC   
  
  
- TTTTAAGCTT TTTCAGTGCA GTAGTGTTGT AACGAAAATA TATATCATAT CTAAGGAAGC TGAAGATTTG   
  
  
- ATTAGTGAAC TTTAATCTTT AAACTTTGAA AGATTATATT TACGTACCAG AAGAAAGGTT CTAATATTAG   
  
  
- GCGTTAACGA ATTTACATCC CATGTTTATA GTTTAAATCT TCACTGATAA CTTCGGTACT TTCTATAACA   
  
  
- CTAGCATAAC TCCCACCAAC ACTCGTTATC CTACTTTGTC CTTTTTTTTT GGGATTACAA AAGTTTAAAT   
  
  
- ACCCACCGAA ACCATCATCT ATTCACTTAT CACACGTTCC CTTACCGTGG TTATACAAAC CTACCTTGCT   
  
  
- ACTCTCATTC TGCAATCCTT TCGTTGTTCT CTTCCCTTTT CCGTCATCCC TCTCTTAATA CGCACACTTA   
  
  
- TTATAATATT GTCTGCGAAA GCATACTAAT AATTTATGTT CTGAAACTCG TATGTATATT TGAACATTGA   
  
  
- AAGAAATGGA TAACAGTTAA TTAAAAACTT AACTTAGTAG ACGCTGAATA CGTATGTTCT ACTATACTAC   
  
  
- GTATAACCCG GTTCGTAAAC TAGAGTACAG GGAATACCTA GATCTAGGGT TATCTGTTTG AACGTATTCA   
  
  
- GTTCCCTACA CAAAGATTAT GTTTCCGAAA CATGAGTTCG TTAATCAAAA CTTAAGTAAA AGTCATCAAA   
  
  
- TTAAAGGTTT AGAATATTAA AAATGTTACC GTATTTAAAA ATCCTCAAAA CTCATTTATT TAATATATGT   
  
  
- TGCTTCTACA GTAAATGCTT AATTATACTG CGTGTTTTTT TTTCTTTTTT TTAATTACCA TCGAAGATAT   
  
  
- GAACTGAGGT ACAACTGTTA AGGGAGAGAG TGTATTACCA ATTTTGAACG TTGTAGTAGG TCCTGGGTAT   
  
  
- ACTGTACCCA CCACCATCTG TCACACAACA CCATCGGAGT GAGGTACGAA CGGTTAAACA TAGTAACGCG   
  
  
- ATCAGAATAA GCAGGAATCT CTATTAACTG ATTCGAGATT ACGAGATAGC ACCGACGACG TTATATTTAC   
  
  
- TAAAAAACTG GAGTTTTACT TCTGTCGATC AACATTTAAT TCTTAGAAGC AGGGAAGTAA AAAACACATG   
  
  
- CACACATAGA CGCATACACA ACTCTCTGAT CGCCTGATAA CTAACTCAAA GTTAGTTTTT TCTACTGGTA   
  
  
- GAATTCATAA AAATTAATGC GTTTGAAGAA GTAAATTTTT TTACAATGAA AATAAACTTT CTTTAACACT   
  
  
- TATTATGTTC CTTATCAAGT TAATTATATA TTAGGTATTG TATAATGGAG GATATCCGTT TTTCTTCTAA   
  
  
- TAGCATAATT AAACCCTAGT AGGGTGGGAT TTAAATTTCT TTGGTTTTGT TTAATGATTC GTTATAGAAA   
  
  
- GACACACTTT TTTATCAGTC TTTGTTTCGC TAGGCTGGCC GGTTTAGCTC TATTTGTGTC GGTTTCTTGG   
  
  
- CTTCAACCTG GTTTCACTCG GCTCAACTTT CCGTAAGTAT ACCCCAAATT AGTATTGTGT TTGTATTAAT   
  
  
- CTAACTAACT GCAGAAATTA GTCGCAAAAA CAGAAACAAG CCACGAGGGG AGTGAGCGGG GAGGCGAGAA   
  
  
- ATTTCGAATG GAGTGAGGGG GGTAAGGGTG AGGAAAAGAG AGAGGAGAGA GCGAGAGTGT GCGAGGCAGA   
  
  
- CAGTCGGGGA ACAGTAGAAG GGGGCAGAAG GAGCGTTTTT AGAGGTCGTT TTAAATATCG GCAACAACAC   
  
  
- GGGTACCACT AGACTACACT CTTCTCTAAG TCGTAAATTC TAATAACCCA GGTCTACCGA ATATACACGC   
  
  
- GGCTGTCACC ATTAGAGTAC CGATAACGGG TTGTTCAGTA GTTCGTTTTT GTTGTCGTTC TTGTCGTCGT   
  
  
- TGTGGTTGTC GGAGTAGGGG TCAAGCAGCC CAGCTTATTA TTGGGGAAAA CGTGGTCGGG TACCGGGCCT   
  
  
- GGGCAGGGAA GTAGGCGGTA CAGACGGTCG GGAGGCGGGG GAGAACCCAA GAGGCGGCGG CGCAAAGGCC   
  
  
- TGGGCAAAGT CCACCCGGGC GGGACGGGCC TACCCCGACT TGGCCCAAAG GTCAAACGGG TAAACCTGGT   
  
  
- GGTGAGCTCA CCAAAGGCCA AACGACTAAA AACGCCACCC CCACCACCAC TCAAACTGAG CCTACTCACC   
  
  
- TACCTCTCAA ACTACCCACC GCCGCCGCCA CTGAGCGCCG CTGACTCTCA AGATTGGAGG TTAGGGTAAC   
  
  
- GCTGCGCACC GTTCCCAGGC CACTAAAGCC AGAGATACCA CGACTAGGCA AACGCTGCAC AAGCCTTCTG   
  
  
- GCAACGTCGG CGATAAGTGC GAGTCGGTGA CGGCGTTGTC GTCGGCAGCA CCCAGGGCGG AAGAGGCGGC   
  
  
- TTTCTGTGCT TCGTCAGCCG GGGTGGAGTT TTCGTTTCGC TGCGGCGACC TGGGAGCAAG AGTAGGCTCC   
  
  
- TCCTTGGTCT CGGCAGTACC GGCGGCGATT AACGCCGCAA CAACCTCACG CGGTTTGAGC GGCTCAGGCT   
  
  
- GGGCCTTGTG CGACAGTTTA GCCACTAAGC TGAGTCTCTG AGTCAGAGTG TCGTCCCTCA AGGTTAGGTC   
  
  
- TCTCACAGGA TAATGAAGAG GCTCCGTGAG ATGGTGGCGG AAAGAGAGAG GAGGCGGGGA GTTGGAGAGG   
  
  
- GTCTTTGCTG CAGCAGACTC CTCATGTGAG AGAGAATGTT CCGGGACTTG CTGCGCACGG GCATGGTATT   
  
  
- TAAGCGAGTG AACTGGCGTT TAGTCCGCTA AGATCTTCGG TGACTAAGTC GGTTCTTCTA GGTGTAGCAA   
  
  
- CTAAAACCCT ATTAGGTCCC TCAGGTTACC CGACGAGACG ATGTTCGGGA CCGTTGGGCC GGTCGGCCCT   
  
  
- TCGGGCTTAA CTAAGCTTAT AGGCCTTAAG GACGAGGCCC AGACCCCGTT AGAGGCCGCC GTAGCGACGA   
  
  
- GCGTTGACCC TTAGCGGAGG CACTCAAGCG CTTCCAAGAT CTAGAGTTCA AGCTCAAACT CGGCCATGAG   
  
  
- TGAGGTTAGT CTCTCGATTT GCCCAGCTTG AAGGCCCAGC TGGGTCTACT CTATGATCGA CAGTTAAAGT   
  
  
- ACGAAGTTAA CATGTTGGAT AATCTGCTTT GGGACCAGCG ACAGCTGCGG CGGGACTTCG ACCGGTTCAG   
  
  
- TAACTTGGGG TTTTAGCAGT GAAACCCACT CATGCTCCGC CCAGACTTAG CCCGTCCAAA GAACTGGGCC   
  
  
- AAGTTCTGCC GGGAGTTCAT GATAAACCGT TACAAACTTA GCCAACTCGG GTTGAACCGG GCCCTGAGCG   
  
  
- GGCTCTCCCG AGTCCAACTC TCCGACAATA ACCCGGCCGC CTAGTACCCG AATCAACCCG GCCTCAATGG   
  
  
- CCCTTGCTCT TCCCTTTCCT ACCTCACATT CCTTGTTACC TTCCCAAATT ACCTTTCACG GCCAAAGCTC   
  
  
- GGCACGGTTT CGTCTTTGAT GCAGTCACTT TTTCTGTTCA AAATGGTGAG GTCTAACTTA ATGTCTCTCA   
  
  
- C

+     P-box

| Site Name | Organism | Position | Strand | Matrix score. | sequence | function |
| --- | --- | --- | --- | --- | --- | --- |
| P-box | Oryza sativa | 2149 | + | 7 | CCTTTTG | gibberellin-responsive element |

>HU06G00029.1   
+ +Up\_Stream \_Len000TGATAA TTATTTTTAT TGATTTAGGA AAGGATATCA AAGTATATTT TGTTTTTAGG   
  
  
+ TAGTTAAGAG ATTCTGTTGC CAAAAAAAAA ATCAAGATAT TATATATTTT TAATTATGAC AGTAATTATT   
  
  
+ TTATTGAAAA TCCAACGGTT GTGATTATAT AATCAAATAA TCTAAAGGTT AAAAATATCA AATTTATTAG   
  
  
+ AAAATTCGAA AAAGTCACGT CATCACAACA TTGCTTTTAT ATATAGTATA GATTCCTTCG ACTTCTAAAC   
  
  
+ TAATCACTTG AAATTAGAAA TTTGAAACTT TCTAATATAA ATGCATGGTC TTCTTTCCAA GATTATAATC   
  
  
+ CGCAATTGCT TAAATGTAGG GTACAAATAT CAAATTTAGA AGTGACTATT GAAGCCATGA AAGATATTGT   
  
  
+ GATCGTATTG AGGGTGGTTG TGAGCAATAG GATGAAACAG GAAAAAAAAA CCCTAATGTT TTCAAATTTA   
  
  
+ TGGGTGGCTT TGGTAGTAGA TAAGTGAATA GTGTGCAAGG GAATGGCACC AATATGTTTG GATGGAACGA   
  
  
+ TGAGAGTAAG ACGTTAGGAA AGCAACAAGA GAAGGGAAAA GGCAGTAGGG AGAGAATTAT GCGTGTGAAT   
  
  
+ AATATTATAA CAGACGCTTT CGTATGATTA TTAAATACAA GACTTTGAGC ATACATATAA ACTTGTAACT   
  
  
+ TTCTTTACCT ATTGTCAATT AATTTTTGAA TTGAATCATC TGCGACTTAT GCATACAAGA TGATATGATG   
  
  
+ CATATTGGGC CAAGCATTTG ATCTCATGTC CCTTATGGAT CTAGATCCCA ATAGACAAAC TTGCATAAGT   
  
  
+ CAAGGGATGT GTTTCTAATA CAAAGGCTTT GTACTCAAGC AATTAGTTTT GAATTCATTT TCAGTAGTTT   
  
  
+ AATTTCCAAA TCTTATAATT TTTACAATGG CATAAATTTT TAGGAGTTTT GAGTAAATAA ATTATATACA   
  
  
+ ACGAAGATGT CATTTACGAA TTAATATGAC GCACAAAAAA AAAGAAAAAA AATTAATGGT AGCTTCTATA   
  
  
+ CTTGACTCCA TGTTGACAAT TCCCTCTCTC ACATAATGGT TAAAACTTGC AACATCATCC AGGACCCATA   
  
  
+ TGACATGGGT GGTGGTAGAC AGTGTGTTGT GGTAGCCTCA CTCCATGCTT GCCAATTTGT ATCATTGCGC   
  
  
+ TAGTCTTATT CGTCCTTAGA GATAATTGAC TAAGCTCTAA TGCTCTATCG TGGCTGCTGC AATATAAATG   
  
  
+ ATTTTTTGAC CTCAAAATGA AGACAGCTAG TTGTAAATTA AGAATCTTCG TCCCTTCATT TTTTGTGTAC   
  
  
+ GTGTGTATCT GCGTATGTGT TGAGAGACTA GCGGACTATT GATTGAGTTT CAATCAAAAA AGATGACCAT   
  
  
+ CTTAAGTATT TTTAATTACG CAAACTTCTT CATTTAAAAA AATGTTACTT TTATTTGAAA GAAATTGTGA   
  
  
+ ATAATACAAG GAATAGTTCA ATTAATATAT AATCCATAAC ATATTACCTC CTATAGGCAA AAAGAAGATT   
  
  
+ ATCGTATTAA TTTGGGATCA TCCCACCCTA AATTTAAAGA AACCAAAACA AATTACTAAG CAATATCTTT   
  
  
+ CTGTGTGAAA AAATAGTCAG AAACAAAGCG ATCCGACCGG CCAAATCGAG ATAAACACAG CCAAAGAACC   
  
  
+ GAAGTTGGAC CAAAGTGAGC CGAGTTGAAA GGCATTCATA TGGGGTTTAA TCATAACACA AACATAATTA   
  
  
+ GATTGATTGA CGTCTTTAAT CAGCGTTTTT GTCTTTGTTC GGTGCTCCCC TCACTCGCCC CTCCGCTCTT   
  
  
+ TAAAGCTTAC CTCACTCCCC CCATTCCCAC TCCTTTTCTC TCTCCTCTCT CGCTCTCACA CGCTCCGTCT   
  
  
+ GTCAGCCCCT TGTCATCTTC CCCCGTCTTC CTCGCAAAAA TCTCCAGCAA AATTTATAGC CGTTGTTGTG   
  
  
+ CCCATGGTGA TCTGATGTGA GAAGAGATTC AGCATTTAAG ATTATTGGGT CCAGATGGCT TATATGTGCG   
  
  
+ CCGACAGTGG TAATCTCATG GCTATTGCCC AACAAGTCAT CAAGCAAAAA CAACAGCAAG AACAGCAGCA   
  
  
+ ACACCAACAG CCTCATCCCC AGTTCGTCGG GTCGAATAAT AACCCCTTTT GCACCAGCCC ATGGCCCGGA   
  
  
+ CCCGTCCCTT CATCCGCCAT GTCTGCCAGC CCTCCGCCCC CTCTTGGGTT CTCCGCCGCC GCGTTTCCGG   
  
  
+ ACCCGTTTCA GGTGGGCCCG CCCTGCCCGG ATGGGGCTGA ACCGGGTTTC CAGTTTGCCC ATTTGGACCA   
  
  
+ CCACTCGAGT GGTTTCCGGT TTGCTGATTT TTGCGGTGGG GGTGGTGGTG AGTTTGACTC GGATGAGTGG   
  
  
+ ATGGAGAGTT TGATGGGTGG CGGCGGCGGT GACTCGCGGC GACTGAGAGT TCTAACCTCC AATCCCATTG   
  
  
+ CGACGCGTGG CAAGGGTCCG GTGATTTCGG TCTCTATGGT GCTGATCCGT TTGCGACGTG TTCGGAAGAC   
  
  
+ CGTTGCAGCC GCTATTCACG CTCAGCCACT GCCGCAACAG CAGCCGTCGT GGGTCCCGCC TTCTCCGCCG   
  
  
+ AAAGACACGA AGCAGTCGGC CCCACCTCAA AAGCAAAGCG ACGCCGCTGG ACCCTCGTTC TCATCCGAGG   
  
  
+ AGGAACCAGA GCCGTCATGG CCGCCGCTAA TTGCGGCGTT GTTGGAGTGC GCCAAACTCG CCGAGTCCGA   
  
  
+ CCCGGAACAC GCTGTCAAAT CGGTGATTCG ACTCAGAGAC TCAGTCTCAC AGCAGGGAGT TCCAATCCAG   
  
  
+ AGAGTGTCCT ATTACTTCTC CGAGGCACTC TACCACCGCC TTTCTCTCTC CTCCGCCCCT CAACCTCTCC   
  
  
+ CAGAAACGAC GTCGTCTGAG GAGTACACTC TCTCTTACAA GGCCCTGAAC GACGCGTGCC CGTACCATAA   
  
  
+ ATTCGCTCAC TTGACCGCAA ATCAGGCGAT TCTAGAAGCC ACTGATTCAG CCAAGAAGAT CCACATCGTT   
  
  
+ GATTTTGGGA TAATCCAGGG AGTCCAATGG GCTGCTCTGC TACAAGCCCT GGCAACCCGG CCAGCCGGGA   
  
  
+ AGCCCGAATT GATTCGAATA TCCGGAATTC CTGCTCCGGG TCTGGGGCAA TCTCCGGCGG CATCGCTGCT   
  
  
+ CGCAACTGGG AATCGCCTCC GTGAGTTCGC GAAGGTTCTA GATCTCAAGT TCGAGTTTGA GCCGGTACTC   
  
  
+ ACTCCAATCA GAGAGCTAAA CGGGTCGAAC TTCCGGGTCG ACCCAGATGA GATACTAGCT GTCAATTTCA   
  
  
+ TGCTTCAATT GTACAACCTA TTAGACGAAA CCCTGGTCGC TGTCGACGCC GCCCTGAAGC TGGCCAAGTC   
  
  
+ ATTGAACCCC AAAATCGTCA CTTTGGGTGA GTACGAGGCG GGTCTGAATC GGGCAGGTTT CTTGACCCGG   
  
  
+ TTCAAGACGG CCCTCAAGTA CTATTTGGCA ATGTTTGAAT CGGTTGAGCC CAACTTGGCC CGGGACTCGC   
  
  
+ CCGAGAGGGC TCAGGTTGAG AGGCTGTTAT TGGGCCGGCG GATCATGGGC TTAGTTGGGC CGGAGTTACC   
  
  
+ GGGAACGAGA AGGGAAAGGA TGGAGTGTAA GGAACAATGG AAGGGTTTAA TGGAAAGTGC CGGTTTCGAG   
  
  
+ CCGTGCCAAA GCAGAAACTA CGTCAGTGAA AAAGACAAGT TTTACCACTC CAGATTGAAT TACAGAGAGT   
  
  
+ G  

- +Up\_Stream \_Len000ACTATT AATAAAAATA ACTAAATCCT TTCCTATAGT TTCATATAAA ACAAAAATCC   
  
  
- ATCAATTCTC TAAGACAACG GTTTTTTTTT TAGTTCTATA ATATATAAAA ATTAATACTG TCATTAATAA   
  
  
- AATAACTTTT AGGTTGCCAA CACTAATATA TTAGTTTATT AGATTTCCAA TTTTTATAGT TTAAATAATC   
  
  
- TTTTAAGCTT TTTCAGTGCA GTAGTGTTGT AACGAAAATA TATATCATAT CTAAGGAAGC TGAAGATTTG   
  
  
- ATTAGTGAAC TTTAATCTTT AAACTTTGAA AGATTATATT TACGTACCAG AAGAAAGGTT CTAATATTAG   
  
  
- GCGTTAACGA ATTTACATCC CATGTTTATA GTTTAAATCT TCACTGATAA CTTCGGTACT TTCTATAACA   
  
  
- CTAGCATAAC TCCCACCAAC ACTCGTTATC CTACTTTGTC CTTTTTTTTT GGGATTACAA AAGTTTAAAT   
  
  
- ACCCACCGAA ACCATCATCT ATTCACTTAT CACACGTTCC CTTACCGTGG TTATACAAAC CTACCTTGCT   
  
  
- ACTCTCATTC TGCAATCCTT TCGTTGTTCT CTTCCCTTTT CCGTCATCCC TCTCTTAATA CGCACACTTA   
  
  
- TTATAATATT GTCTGCGAAA GCATACTAAT AATTTATGTT CTGAAACTCG TATGTATATT TGAACATTGA   
  
  
- AAGAAATGGA TAACAGTTAA TTAAAAACTT AACTTAGTAG ACGCTGAATA CGTATGTTCT ACTATACTAC   
  
  
- GTATAACCCG GTTCGTAAAC TAGAGTACAG GGAATACCTA GATCTAGGGT TATCTGTTTG AACGTATTCA   
  
  
- GTTCCCTACA CAAAGATTAT GTTTCCGAAA CATGAGTTCG TTAATCAAAA CTTAAGTAAA AGTCATCAAA   
  
  
- TTAAAGGTTT AGAATATTAA AAATGTTACC GTATTTAAAA ATCCTCAAAA CTCATTTATT TAATATATGT   
  
  
- TGCTTCTACA GTAAATGCTT AATTATACTG CGTGTTTTTT TTTCTTTTTT TTAATTACCA TCGAAGATAT   
  
  
- GAACTGAGGT ACAACTGTTA AGGGAGAGAG TGTATTACCA ATTTTGAACG TTGTAGTAGG TCCTGGGTAT   
  
  
- ACTGTACCCA CCACCATCTG TCACACAACA CCATCGGAGT GAGGTACGAA CGGTTAAACA TAGTAACGCG   
  
  
- ATCAGAATAA GCAGGAATCT CTATTAACTG ATTCGAGATT ACGAGATAGC ACCGACGACG TTATATTTAC   
  
  
- TAAAAAACTG GAGTTTTACT TCTGTCGATC AACATTTAAT TCTTAGAAGC AGGGAAGTAA AAAACACATG   
  
  
- CACACATAGA CGCATACACA ACTCTCTGAT CGCCTGATAA CTAACTCAAA GTTAGTTTTT TCTACTGGTA   
  
  
- GAATTCATAA AAATTAATGC GTTTGAAGAA GTAAATTTTT TTACAATGAA AATAAACTTT CTTTAACACT   
  
  
- TATTATGTTC CTTATCAAGT TAATTATATA TTAGGTATTG TATAATGGAG GATATCCGTT TTTCTTCTAA   
  
  
- TAGCATAATT AAACCCTAGT AGGGTGGGAT TTAAATTTCT TTGGTTTTGT TTAATGATTC GTTATAGAAA   
  
  
- GACACACTTT TTTATCAGTC TTTGTTTCGC TAGGCTGGCC GGTTTAGCTC TATTTGTGTC GGTTTCTTGG   
  
  
- CTTCAACCTG GTTTCACTCG GCTCAACTTT CCGTAAGTAT ACCCCAAATT AGTATTGTGT TTGTATTAAT   
  
  
- CTAACTAACT GCAGAAATTA GTCGCAAAAA CAGAAACAAG CCACGAGGGG AGTGAGCGGG GAGGCGAGAA   
  
  
- ATTTCGAATG GAGTGAGGGG GGTAAGGGTG AGGAAAAGAG AGAGGAGAGA GCGAGAGTGT GCGAGGCAGA   
  
  
- CAGTCGGGGA ACAGTAGAAG GGGGCAGAAG GAGCGTTTTT AGAGGTCGTT TTAAATATCG GCAACAACAC   
  
  
- GGGTACCACT AGACTACACT CTTCTCTAAG TCGTAAATTC TAATAACCCA GGTCTACCGA ATATACACGC   
  
  
- GGCTGTCACC ATTAGAGTAC CGATAACGGG TTGTTCAGTA GTTCGTTTTT GTTGTCGTTC TTGTCGTCGT   
  
  
- TGTGGTTGTC GGAGTAGGGG TCAAGCAGCC CAGCTTATTA TTGGGGAAAA CGTGGTCGGG TACCGGGCCT   
  
  
- GGGCAGGGAA GTAGGCGGTA CAGACGGTCG GGAGGCGGGG GAGAACCCAA GAGGCGGCGG CGCAAAGGCC   
  
  
- TGGGCAAAGT CCACCCGGGC GGGACGGGCC TACCCCGACT TGGCCCAAAG GTCAAACGGG TAAACCTGGT   
  
  
- GGTGAGCTCA CCAAAGGCCA AACGACTAAA AACGCCACCC CCACCACCAC TCAAACTGAG CCTACTCACC   
  
  
- TACCTCTCAA ACTACCCACC GCCGCCGCCA CTGAGCGCCG CTGACTCTCA AGATTGGAGG TTAGGGTAAC   
  
  
- GCTGCGCACC GTTCCCAGGC CACTAAAGCC AGAGATACCA CGACTAGGCA AACGCTGCAC AAGCCTTCTG   
  
  
- GCAACGTCGG CGATAAGTGC GAGTCGGTGA CGGCGTTGTC GTCGGCAGCA CCCAGGGCGG AAGAGGCGGC   
  
  
- TTTCTGTGCT TCGTCAGCCG GGGTGGAGTT TTCGTTTCGC TGCGGCGACC TGGGAGCAAG AGTAGGCTCC   
  
  
- TCCTTGGTCT CGGCAGTACC GGCGGCGATT AACGCCGCAA CAACCTCACG CGGTTTGAGC GGCTCAGGCT   
  
  
- GGGCCTTGTG CGACAGTTTA GCCACTAAGC TGAGTCTCTG AGTCAGAGTG TCGTCCCTCA AGGTTAGGTC   
  
  
- TCTCACAGGA TAATGAAGAG GCTCCGTGAG ATGGTGGCGG AAAGAGAGAG GAGGCGGGGA GTTGGAGAGG   
  
  
- GTCTTTGCTG CAGCAGACTC CTCATGTGAG AGAGAATGTT CCGGGACTTG CTGCGCACGG GCATGGTATT   
  
  
- TAAGCGAGTG AACTGGCGTT TAGTCCGCTA AGATCTTCGG TGACTAAGTC GGTTCTTCTA GGTGTAGCAA   
  
  
- CTAAAACCCT ATTAGGTCCC TCAGGTTACC CGACGAGACG ATGTTCGGGA CCGTTGGGCC GGTCGGCCCT   
  
  
- TCGGGCTTAA CTAAGCTTAT AGGCCTTAAG GACGAGGCCC AGACCCCGTT AGAGGCCGCC GTAGCGACGA   
  
  
- GCGTTGACCC TTAGCGGAGG CACTCAAGCG CTTCCAAGAT CTAGAGTTCA AGCTCAAACT CGGCCATGAG   
  
  
- TGAGGTTAGT CTCTCGATTT GCCCAGCTTG AAGGCCCAGC TGGGTCTACT CTATGATCGA CAGTTAAAGT   
  
  
- ACGAAGTTAA CATGTTGGAT AATCTGCTTT GGGACCAGCG ACAGCTGCGG CGGGACTTCG ACCGGTTCAG   
  
  
- TAACTTGGGG TTTTAGCAGT GAAACCCACT CATGCTCCGC CCAGACTTAG CCCGTCCAAA GAACTGGGCC   
  
  
- AAGTTCTGCC GGGAGTTCAT GATAAACCGT TACAAACTTA GCCAACTCGG GTTGAACCGG GCCCTGAGCG   
  
  
- GGCTCTCCCG AGTCCAACTC TCCGACAATA ACCCGGCCGC CTAGTACCCG AATCAACCCG GCCTCAATGG   
  
  
- CCCTTGCTCT TCCCTTTCCT ACCTCACATT CCTTGTTACC TTCCCAAATT ACCTTTCACG GCCAAAGCTC   
  
  
- GGCACGGTTT CGTCTTTGAT GCAGTCACTT TTTCTGTTCA AAATGGTGAG GTCTAACTTA ATGTCTCTCA   
  
  
- C

+     STRE

| Site Name | Organism | Position | Strand | Matrix score. | sequence | function |
| --- | --- | --- | --- | --- | --- | --- |
| STRE | Arabidopsis thaliana | 1801 | - | 5 | AGGGG |  |
| STRE | Arabidopsis thaliana | 1900 | - | 5 | AGGGG |  |
| STRE | Arabidopsis thaliana | 2860 | - | 5 | AGGGG |  |
| STRE | Arabidopsis thaliana | 2212 | - | 5 | AGGGG |  |
| STRE | Arabidopsis thaliana | 1812 | - | 5 | AGGGG |  |
| STRE | Arabidopsis thaliana | 2147 | - | 5 | AGGGG |  |

>HU06G00029.1   
+ +Up\_Stream \_Len000TGATAA TTATTTTTAT TGATTTAGGA AAGGATATCA AAGTATATTT TGTTTTTAGG   
  
  
+ TAGTTAAGAG ATTCTGTTGC CAAAAAAAAA ATCAAGATAT TATATATTTT TAATTATGAC AGTAATTATT   
  
  
+ TTATTGAAAA TCCAACGGTT GTGATTATAT AATCAAATAA TCTAAAGGTT AAAAATATCA AATTTATTAG   
  
  
+ AAAATTCGAA AAAGTCACGT CATCACAACA TTGCTTTTAT ATATAGTATA GATTCCTTCG ACTTCTAAAC   
  
  
+ TAATCACTTG AAATTAGAAA TTTGAAACTT TCTAATATAA ATGCATGGTC TTCTTTCCAA GATTATAATC   
  
  
+ CGCAATTGCT TAAATGTAGG GTACAAATAT CAAATTTAGA AGTGACTATT GAAGCCATGA AAGATATTGT   
  
  
+ GATCGTATTG AGGGTGGTTG TGAGCAATAG GATGAAACAG GAAAAAAAAA CCCTAATGTT TTCAAATTTA   
  
  
+ TGGGTGGCTT TGGTAGTAGA TAAGTGAATA GTGTGCAAGG GAATGGCACC AATATGTTTG GATGGAACGA   
  
  
+ TGAGAGTAAG ACGTTAGGAA AGCAACAAGA GAAGGGAAAA GGCAGTAGGG AGAGAATTAT GCGTGTGAAT   
  
  
+ AATATTATAA CAGACGCTTT CGTATGATTA TTAAATACAA GACTTTGAGC ATACATATAA ACTTGTAACT   
  
  
+ TTCTTTACCT ATTGTCAATT AATTTTTGAA TTGAATCATC TGCGACTTAT GCATACAAGA TGATATGATG   
  
  
+ CATATTGGGC CAAGCATTTG ATCTCATGTC CCTTATGGAT CTAGATCCCA ATAGACAAAC TTGCATAAGT   
  
  
+ CAAGGGATGT GTTTCTAATA CAAAGGCTTT GTACTCAAGC AATTAGTTTT GAATTCATTT TCAGTAGTTT   
  
  
+ AATTTCCAAA TCTTATAATT TTTACAATGG CATAAATTTT TAGGAGTTTT GAGTAAATAA ATTATATACA   
  
  
+ ACGAAGATGT CATTTACGAA TTAATATGAC GCACAAAAAA AAAGAAAAAA AATTAATGGT AGCTTCTATA   
  
  
+ CTTGACTCCA TGTTGACAAT TCCCTCTCTC ACATAATGGT TAAAACTTGC AACATCATCC AGGACCCATA   
  
  
+ TGACATGGGT GGTGGTAGAC AGTGTGTTGT GGTAGCCTCA CTCCATGCTT GCCAATTTGT ATCATTGCGC   
  
  
+ TAGTCTTATT CGTCCTTAGA GATAATTGAC TAAGCTCTAA TGCTCTATCG TGGCTGCTGC AATATAAATG   
  
  
+ ATTTTTTGAC CTCAAAATGA AGACAGCTAG TTGTAAATTA AGAATCTTCG TCCCTTCATT TTTTGTGTAC   
  
  
+ GTGTGTATCT GCGTATGTGT TGAGAGACTA GCGGACTATT GATTGAGTTT CAATCAAAAA AGATGACCAT   
  
  
+ CTTAAGTATT TTTAATTACG CAAACTTCTT CATTTAAAAA AATGTTACTT TTATTTGAAA GAAATTGTGA   
  
  
+ ATAATACAAG GAATAGTTCA ATTAATATAT AATCCATAAC ATATTACCTC CTATAGGCAA AAAGAAGATT   
  
  
+ ATCGTATTAA TTTGGGATCA TCCCACCCTA AATTTAAAGA AACCAAAACA AATTACTAAG CAATATCTTT   
  
  
+ CTGTGTGAAA AAATAGTCAG AAACAAAGCG ATCCGACCGG CCAAATCGAG ATAAACACAG CCAAAGAACC   
  
  
+ GAAGTTGGAC CAAAGTGAGC CGAGTTGAAA GGCATTCATA TGGGGTTTAA TCATAACACA AACATAATTA   
  
  
+ GATTGATTGA CGTCTTTAAT CAGCGTTTTT GTCTTTGTTC GGTGCTCCCC TCACTCGCCC CTCCGCTCTT   
  
  
+ TAAAGCTTAC CTCACTCCCC CCATTCCCAC TCCTTTTCTC TCTCCTCTCT CGCTCTCACA CGCTCCGTCT   
  
  
+ GTCAGCCCCT TGTCATCTTC CCCCGTCTTC CTCGCAAAAA TCTCCAGCAA AATTTATAGC CGTTGTTGTG   
  
  
+ CCCATGGTGA TCTGATGTGA GAAGAGATTC AGCATTTAAG ATTATTGGGT CCAGATGGCT TATATGTGCG   
  
  
+ CCGACAGTGG TAATCTCATG GCTATTGCCC AACAAGTCAT CAAGCAAAAA CAACAGCAAG AACAGCAGCA   
  
  
+ ACACCAACAG CCTCATCCCC AGTTCGTCGG GTCGAATAAT AACCCCTTTT GCACCAGCCC ATGGCCCGGA   
  
  
+ CCCGTCCCTT CATCCGCCAT GTCTGCCAGC CCTCCGCCCC CTCTTGGGTT CTCCGCCGCC GCGTTTCCGG   
  
  
+ ACCCGTTTCA GGTGGGCCCG CCCTGCCCGG ATGGGGCTGA ACCGGGTTTC CAGTTTGCCC ATTTGGACCA   
  
  
+ CCACTCGAGT GGTTTCCGGT TTGCTGATTT TTGCGGTGGG GGTGGTGGTG AGTTTGACTC GGATGAGTGG   
  
  
+ ATGGAGAGTT TGATGGGTGG CGGCGGCGGT GACTCGCGGC GACTGAGAGT TCTAACCTCC AATCCCATTG   
  
  
+ CGACGCGTGG CAAGGGTCCG GTGATTTCGG TCTCTATGGT GCTGATCCGT TTGCGACGTG TTCGGAAGAC   
  
  
+ CGTTGCAGCC GCTATTCACG CTCAGCCACT GCCGCAACAG CAGCCGTCGT GGGTCCCGCC TTCTCCGCCG   
  
  
+ AAAGACACGA AGCAGTCGGC CCCACCTCAA AAGCAAAGCG ACGCCGCTGG ACCCTCGTTC TCATCCGAGG   
  
  
+ AGGAACCAGA GCCGTCATGG CCGCCGCTAA TTGCGGCGTT GTTGGAGTGC GCCAAACTCG CCGAGTCCGA   
  
  
+ CCCGGAACAC GCTGTCAAAT CGGTGATTCG ACTCAGAGAC TCAGTCTCAC AGCAGGGAGT TCCAATCCAG   
  
  
+ AGAGTGTCCT ATTACTTCTC CGAGGCACTC TACCACCGCC TTTCTCTCTC CTCCGCCCCT CAACCTCTCC   
  
  
+ CAGAAACGAC GTCGTCTGAG GAGTACACTC TCTCTTACAA GGCCCTGAAC GACGCGTGCC CGTACCATAA   
  
  
+ ATTCGCTCAC TTGACCGCAA ATCAGGCGAT TCTAGAAGCC ACTGATTCAG CCAAGAAGAT CCACATCGTT   
  
  
+ GATTTTGGGA TAATCCAGGG AGTCCAATGG GCTGCTCTGC TACAAGCCCT GGCAACCCGG CCAGCCGGGA   
  
  
+ AGCCCGAATT GATTCGAATA TCCGGAATTC CTGCTCCGGG TCTGGGGCAA TCTCCGGCGG CATCGCTGCT   
  
  
+ CGCAACTGGG AATCGCCTCC GTGAGTTCGC GAAGGTTCTA GATCTCAAGT TCGAGTTTGA GCCGGTACTC   
  
  
+ ACTCCAATCA GAGAGCTAAA CGGGTCGAAC TTCCGGGTCG ACCCAGATGA GATACTAGCT GTCAATTTCA   
  
  
+ TGCTTCAATT GTACAACCTA TTAGACGAAA CCCTGGTCGC TGTCGACGCC GCCCTGAAGC TGGCCAAGTC   
  
  
+ ATTGAACCCC AAAATCGTCA CTTTGGGTGA GTACGAGGCG GGTCTGAATC GGGCAGGTTT CTTGACCCGG   
  
  
+ TTCAAGACGG CCCTCAAGTA CTATTTGGCA ATGTTTGAAT CGGTTGAGCC CAACTTGGCC CGGGACTCGC   
  
  
+ CCGAGAGGGC TCAGGTTGAG AGGCTGTTAT TGGGCCGGCG GATCATGGGC TTAGTTGGGC CGGAGTTACC   
  
  
+ GGGAACGAGA AGGGAAAGGA TGGAGTGTAA GGAACAATGG AAGGGTTTAA TGGAAAGTGC CGGTTTCGAG   
  
  
+ CCGTGCCAAA GCAGAAACTA CGTCAGTGAA AAAGACAAGT TTTACCACTC CAGATTGAAT TACAGAGAGT   
  
  
+ G  

- +Up\_Stream \_Len000ACTATT AATAAAAATA ACTAAATCCT TTCCTATAGT TTCATATAAA ACAAAAATCC   
  
  
- ATCAATTCTC TAAGACAACG GTTTTTTTTT TAGTTCTATA ATATATAAAA ATTAATACTG TCATTAATAA   
  
  
- AATAACTTTT AGGTTGCCAA CACTAATATA TTAGTTTATT AGATTTCCAA TTTTTATAGT TTAAATAATC   
  
  
- TTTTAAGCTT TTTCAGTGCA GTAGTGTTGT AACGAAAATA TATATCATAT CTAAGGAAGC TGAAGATTTG   
  
  
- ATTAGTGAAC TTTAATCTTT AAACTTTGAA AGATTATATT TACGTACCAG AAGAAAGGTT CTAATATTAG   
  
  
- GCGTTAACGA ATTTACATCC CATGTTTATA GTTTAAATCT TCACTGATAA CTTCGGTACT TTCTATAACA   
  
  
- CTAGCATAAC TCCCACCAAC ACTCGTTATC CTACTTTGTC CTTTTTTTTT GGGATTACAA AAGTTTAAAT   
  
  
- ACCCACCGAA ACCATCATCT ATTCACTTAT CACACGTTCC CTTACCGTGG TTATACAAAC CTACCTTGCT   
  
  
- ACTCTCATTC TGCAATCCTT TCGTTGTTCT CTTCCCTTTT CCGTCATCCC TCTCTTAATA CGCACACTTA   
  
  
- TTATAATATT GTCTGCGAAA GCATACTAAT AATTTATGTT CTGAAACTCG TATGTATATT TGAACATTGA   
  
  
- AAGAAATGGA TAACAGTTAA TTAAAAACTT AACTTAGTAG ACGCTGAATA CGTATGTTCT ACTATACTAC   
  
  
- GTATAACCCG GTTCGTAAAC TAGAGTACAG GGAATACCTA GATCTAGGGT TATCTGTTTG AACGTATTCA   
  
  
- GTTCCCTACA CAAAGATTAT GTTTCCGAAA CATGAGTTCG TTAATCAAAA CTTAAGTAAA AGTCATCAAA   
  
  
- TTAAAGGTTT AGAATATTAA AAATGTTACC GTATTTAAAA ATCCTCAAAA CTCATTTATT TAATATATGT   
  
  
- TGCTTCTACA GTAAATGCTT AATTATACTG CGTGTTTTTT TTTCTTTTTT TTAATTACCA TCGAAGATAT   
  
  
- GAACTGAGGT ACAACTGTTA AGGGAGAGAG TGTATTACCA ATTTTGAACG TTGTAGTAGG TCCTGGGTAT   
  
  
- ACTGTACCCA CCACCATCTG TCACACAACA CCATCGGAGT GAGGTACGAA CGGTTAAACA TAGTAACGCG   
  
  
- ATCAGAATAA GCAGGAATCT CTATTAACTG ATTCGAGATT ACGAGATAGC ACCGACGACG TTATATTTAC   
  
  
- TAAAAAACTG GAGTTTTACT TCTGTCGATC AACATTTAAT TCTTAGAAGC AGGGAAGTAA AAAACACATG   
  
  
- CACACATAGA CGCATACACA ACTCTCTGAT CGCCTGATAA CTAACTCAAA GTTAGTTTTT TCTACTGGTA   
  
  
- GAATTCATAA AAATTAATGC GTTTGAAGAA GTAAATTTTT TTACAATGAA AATAAACTTT CTTTAACACT   
  
  
- TATTATGTTC CTTATCAAGT TAATTATATA TTAGGTATTG TATAATGGAG GATATCCGTT TTTCTTCTAA   
  
  
- TAGCATAATT AAACCCTAGT AGGGTGGGAT TTAAATTTCT TTGGTTTTGT TTAATGATTC GTTATAGAAA   
  
  
- GACACACTTT TTTATCAGTC TTTGTTTCGC TAGGCTGGCC GGTTTAGCTC TATTTGTGTC GGTTTCTTGG   
  
  
- CTTCAACCTG GTTTCACTCG GCTCAACTTT CCGTAAGTAT ACCCCAAATT AGTATTGTGT TTGTATTAAT   
  
  
- CTAACTAACT GCAGAAATTA GTCGCAAAAA CAGAAACAAG CCACGAGGGG AGTGAGCGGG GAGGCGAGAA   
  
  
- ATTTCGAATG GAGTGAGGGG GGTAAGGGTG AGGAAAAGAG AGAGGAGAGA GCGAGAGTGT GCGAGGCAGA   
  
  
- CAGTCGGGGA ACAGTAGAAG GGGGCAGAAG GAGCGTTTTT AGAGGTCGTT TTAAATATCG GCAACAACAC   
  
  
- GGGTACCACT AGACTACACT CTTCTCTAAG TCGTAAATTC TAATAACCCA GGTCTACCGA ATATACACGC   
  
  
- GGCTGTCACC ATTAGAGTAC CGATAACGGG TTGTTCAGTA GTTCGTTTTT GTTGTCGTTC TTGTCGTCGT   
  
  
- TGTGGTTGTC GGAGTAGGGG TCAAGCAGCC CAGCTTATTA TTGGGGAAAA CGTGGTCGGG TACCGGGCCT   
  
  
- GGGCAGGGAA GTAGGCGGTA CAGACGGTCG GGAGGCGGGG GAGAACCCAA GAGGCGGCGG CGCAAAGGCC   
  
  
- TGGGCAAAGT CCACCCGGGC GGGACGGGCC TACCCCGACT TGGCCCAAAG GTCAAACGGG TAAACCTGGT   
  
  
- GGTGAGCTCA CCAAAGGCCA AACGACTAAA AACGCCACCC CCACCACCAC TCAAACTGAG CCTACTCACC   
  
  
- TACCTCTCAA ACTACCCACC GCCGCCGCCA CTGAGCGCCG CTGACTCTCA AGATTGGAGG TTAGGGTAAC   
  
  
- GCTGCGCACC GTTCCCAGGC CACTAAAGCC AGAGATACCA CGACTAGGCA AACGCTGCAC AAGCCTTCTG   
  
  
- GCAACGTCGG CGATAAGTGC GAGTCGGTGA CGGCGTTGTC GTCGGCAGCA CCCAGGGCGG AAGAGGCGGC   
  
  
- TTTCTGTGCT TCGTCAGCCG GGGTGGAGTT TTCGTTTCGC TGCGGCGACC TGGGAGCAAG AGTAGGCTCC   
  
  
- TCCTTGGTCT CGGCAGTACC GGCGGCGATT AACGCCGCAA CAACCTCACG CGGTTTGAGC GGCTCAGGCT   
  
  
- GGGCCTTGTG CGACAGTTTA GCCACTAAGC TGAGTCTCTG AGTCAGAGTG TCGTCCCTCA AGGTTAGGTC   
  
  
- TCTCACAGGA TAATGAAGAG GCTCCGTGAG ATGGTGGCGG AAAGAGAGAG GAGGCGGGGA GTTGGAGAGG   
  
  
- GTCTTTGCTG CAGCAGACTC CTCATGTGAG AGAGAATGTT CCGGGACTTG CTGCGCACGG GCATGGTATT   
  
  
- TAAGCGAGTG AACTGGCGTT TAGTCCGCTA AGATCTTCGG TGACTAAGTC GGTTCTTCTA GGTGTAGCAA   
  
  
- CTAAAACCCT ATTAGGTCCC TCAGGTTACC CGACGAGACG ATGTTCGGGA CCGTTGGGCC GGTCGGCCCT   
  
  
- TCGGGCTTAA CTAAGCTTAT AGGCCTTAAG GACGAGGCCC AGACCCCGTT AGAGGCCGCC GTAGCGACGA   
  
  
- GCGTTGACCC TTAGCGGAGG CACTCAAGCG CTTCCAAGAT CTAGAGTTCA AGCTCAAACT CGGCCATGAG   
  
  
- TGAGGTTAGT CTCTCGATTT GCCCAGCTTG AAGGCCCAGC TGGGTCTACT CTATGATCGA CAGTTAAAGT   
  
  
- ACGAAGTTAA CATGTTGGAT AATCTGCTTT GGGACCAGCG ACAGCTGCGG CGGGACTTCG ACCGGTTCAG   
  
  
- TAACTTGGGG TTTTAGCAGT GAAACCCACT CATGCTCCGC CCAGACTTAG CCCGTCCAAA GAACTGGGCC   
  
  
- AAGTTCTGCC GGGAGTTCAT GATAAACCGT TACAAACTTA GCCAACTCGG GTTGAACCGG GCCCTGAGCG   
  
  
- GGCTCTCCCG AGTCCAACTC TCCGACAATA ACCCGGCCGC CTAGTACCCG AATCAACCCG GCCTCAATGG   
  
  
- CCCTTGCTCT TCCCTTTCCT ACCTCACATT CCTTGTTACC TTCCCAAATT ACCTTTCACG GCCAAAGCTC   
  
  
- GGCACGGTTT CGTCTTTGAT GCAGTCACTT TTTCTGTTCA AAATGGTGAG GTCTAACTTA ATGTCTCTCA   
  
  
- C

+     Sp1

| Site Name | Organism | Position | Strand | Matrix score. | sequence | function |
| --- | --- | --- | --- | --- | --- | --- |
| Sp1 | Oryza sativa | 3343 | - | 6 | GGGCGG | light responsive element |
| Sp1 | Oryza sativa | 2857 | - | 6 | GGGCGG | light responsive element |
| Sp1 | Oryza sativa | 2208 | - | 6 | GGGCGG | light responsive element |
| Sp1 | Oryza sativa | 2262 | - | 6 | GGGCGG | light responsive element |

>HU06G00029.1   
+ +Up\_Stream \_Len000TGATAA TTATTTTTAT TGATTTAGGA AAGGATATCA AAGTATATTT TGTTTTTAGG   
  
  
+ TAGTTAAGAG ATTCTGTTGC CAAAAAAAAA ATCAAGATAT TATATATTTT TAATTATGAC AGTAATTATT   
  
  
+ TTATTGAAAA TCCAACGGTT GTGATTATAT AATCAAATAA TCTAAAGGTT AAAAATATCA AATTTATTAG   
  
  
+ AAAATTCGAA AAAGTCACGT CATCACAACA TTGCTTTTAT ATATAGTATA GATTCCTTCG ACTTCTAAAC   
  
  
+ TAATCACTTG AAATTAGAAA TTTGAAACTT TCTAATATAA ATGCATGGTC TTCTTTCCAA GATTATAATC   
  
  
+ CGCAATTGCT TAAATGTAGG GTACAAATAT CAAATTTAGA AGTGACTATT GAAGCCATGA AAGATATTGT   
  
  
+ GATCGTATTG AGGGTGGTTG TGAGCAATAG GATGAAACAG GAAAAAAAAA CCCTAATGTT TTCAAATTTA   
  
  
+ TGGGTGGCTT TGGTAGTAGA TAAGTGAATA GTGTGCAAGG GAATGGCACC AATATGTTTG GATGGAACGA   
  
  
+ TGAGAGTAAG ACGTTAGGAA AGCAACAAGA GAAGGGAAAA GGCAGTAGGG AGAGAATTAT GCGTGTGAAT   
  
  
+ AATATTATAA CAGACGCTTT CGTATGATTA TTAAATACAA GACTTTGAGC ATACATATAA ACTTGTAACT   
  
  
+ TTCTTTACCT ATTGTCAATT AATTTTTGAA TTGAATCATC TGCGACTTAT GCATACAAGA TGATATGATG   
  
  
+ CATATTGGGC CAAGCATTTG ATCTCATGTC CCTTATGGAT CTAGATCCCA ATAGACAAAC TTGCATAAGT   
  
  
+ CAAGGGATGT GTTTCTAATA CAAAGGCTTT GTACTCAAGC AATTAGTTTT GAATTCATTT TCAGTAGTTT   
  
  
+ AATTTCCAAA TCTTATAATT TTTACAATGG CATAAATTTT TAGGAGTTTT GAGTAAATAA ATTATATACA   
  
  
+ ACGAAGATGT CATTTACGAA TTAATATGAC GCACAAAAAA AAAGAAAAAA AATTAATGGT AGCTTCTATA   
  
  
+ CTTGACTCCA TGTTGACAAT TCCCTCTCTC ACATAATGGT TAAAACTTGC AACATCATCC AGGACCCATA   
  
  
+ TGACATGGGT GGTGGTAGAC AGTGTGTTGT GGTAGCCTCA CTCCATGCTT GCCAATTTGT ATCATTGCGC   
  
  
+ TAGTCTTATT CGTCCTTAGA GATAATTGAC TAAGCTCTAA TGCTCTATCG TGGCTGCTGC AATATAAATG   
  
  
+ ATTTTTTGAC CTCAAAATGA AGACAGCTAG TTGTAAATTA AGAATCTTCG TCCCTTCATT TTTTGTGTAC   
  
  
+ GTGTGTATCT GCGTATGTGT TGAGAGACTA GCGGACTATT GATTGAGTTT CAATCAAAAA AGATGACCAT   
  
  
+ CTTAAGTATT TTTAATTACG CAAACTTCTT CATTTAAAAA AATGTTACTT TTATTTGAAA GAAATTGTGA   
  
  
+ ATAATACAAG GAATAGTTCA ATTAATATAT AATCCATAAC ATATTACCTC CTATAGGCAA AAAGAAGATT   
  
  
+ ATCGTATTAA TTTGGGATCA TCCCACCCTA AATTTAAAGA AACCAAAACA AATTACTAAG CAATATCTTT   
  
  
+ CTGTGTGAAA AAATAGTCAG AAACAAAGCG ATCCGACCGG CCAAATCGAG ATAAACACAG CCAAAGAACC   
  
  
+ GAAGTTGGAC CAAAGTGAGC CGAGTTGAAA GGCATTCATA TGGGGTTTAA TCATAACACA AACATAATTA   
  
  
+ GATTGATTGA CGTCTTTAAT CAGCGTTTTT GTCTTTGTTC GGTGCTCCCC TCACTCGCCC CTCCGCTCTT   
  
  
+ TAAAGCTTAC CTCACTCCCC CCATTCCCAC TCCTTTTCTC TCTCCTCTCT CGCTCTCACA CGCTCCGTCT   
  
  
+ GTCAGCCCCT TGTCATCTTC CCCCGTCTTC CTCGCAAAAA TCTCCAGCAA AATTTATAGC CGTTGTTGTG   
  
  
+ CCCATGGTGA TCTGATGTGA GAAGAGATTC AGCATTTAAG ATTATTGGGT CCAGATGGCT TATATGTGCG   
  
  
+ CCGACAGTGG TAATCTCATG GCTATTGCCC AACAAGTCAT CAAGCAAAAA CAACAGCAAG AACAGCAGCA   
  
  
+ ACACCAACAG CCTCATCCCC AGTTCGTCGG GTCGAATAAT AACCCCTTTT GCACCAGCCC ATGGCCCGGA   
  
  
+ CCCGTCCCTT CATCCGCCAT GTCTGCCAGC CCTCCGCCCC CTCTTGGGTT CTCCGCCGCC GCGTTTCCGG   
  
  
+ ACCCGTTTCA GGTGGGCCCG CCCTGCCCGG ATGGGGCTGA ACCGGGTTTC CAGTTTGCCC ATTTGGACCA   
  
  
+ CCACTCGAGT GGTTTCCGGT TTGCTGATTT TTGCGGTGGG GGTGGTGGTG AGTTTGACTC GGATGAGTGG   
  
  
+ ATGGAGAGTT TGATGGGTGG CGGCGGCGGT GACTCGCGGC GACTGAGAGT TCTAACCTCC AATCCCATTG   
  
  
+ CGACGCGTGG CAAGGGTCCG GTGATTTCGG TCTCTATGGT GCTGATCCGT TTGCGACGTG TTCGGAAGAC   
  
  
+ CGTTGCAGCC GCTATTCACG CTCAGCCACT GCCGCAACAG CAGCCGTCGT GGGTCCCGCC TTCTCCGCCG   
  
  
+ AAAGACACGA AGCAGTCGGC CCCACCTCAA AAGCAAAGCG ACGCCGCTGG ACCCTCGTTC TCATCCGAGG   
  
  
+ AGGAACCAGA GCCGTCATGG CCGCCGCTAA TTGCGGCGTT GTTGGAGTGC GCCAAACTCG CCGAGTCCGA   
  
  
+ CCCGGAACAC GCTGTCAAAT CGGTGATTCG ACTCAGAGAC TCAGTCTCAC AGCAGGGAGT TCCAATCCAG   
  
  
+ AGAGTGTCCT ATTACTTCTC CGAGGCACTC TACCACCGCC TTTCTCTCTC CTCCGCCCCT CAACCTCTCC   
  
  
+ CAGAAACGAC GTCGTCTGAG GAGTACACTC TCTCTTACAA GGCCCTGAAC GACGCGTGCC CGTACCATAA   
  
  
+ ATTCGCTCAC TTGACCGCAA ATCAGGCGAT TCTAGAAGCC ACTGATTCAG CCAAGAAGAT CCACATCGTT   
  
  
+ GATTTTGGGA TAATCCAGGG AGTCCAATGG GCTGCTCTGC TACAAGCCCT GGCAACCCGG CCAGCCGGGA   
  
  
+ AGCCCGAATT GATTCGAATA TCCGGAATTC CTGCTCCGGG TCTGGGGCAA TCTCCGGCGG CATCGCTGCT   
  
  
+ CGCAACTGGG AATCGCCTCC GTGAGTTCGC GAAGGTTCTA GATCTCAAGT TCGAGTTTGA GCCGGTACTC   
  
  
+ ACTCCAATCA GAGAGCTAAA CGGGTCGAAC TTCCGGGTCG ACCCAGATGA GATACTAGCT GTCAATTTCA   
  
  
+ TGCTTCAATT GTACAACCTA TTAGACGAAA CCCTGGTCGC TGTCGACGCC GCCCTGAAGC TGGCCAAGTC   
  
  
+ ATTGAACCCC AAAATCGTCA CTTTGGGTGA GTACGAGGCG GGTCTGAATC GGGCAGGTTT CTTGACCCGG   
  
  
+ TTCAAGACGG CCCTCAAGTA CTATTTGGCA ATGTTTGAAT CGGTTGAGCC CAACTTGGCC CGGGACTCGC   
  
  
+ CCGAGAGGGC TCAGGTTGAG AGGCTGTTAT TGGGCCGGCG GATCATGGGC TTAGTTGGGC CGGAGTTACC   
  
  
+ GGGAACGAGA AGGGAAAGGA TGGAGTGTAA GGAACAATGG AAGGGTTTAA TGGAAAGTGC CGGTTTCGAG   
  
  
+ CCGTGCCAAA GCAGAAACTA CGTCAGTGAA AAAGACAAGT TTTACCACTC CAGATTGAAT TACAGAGAGT   
  
  
+ G  

- +Up\_Stream \_Len000ACTATT AATAAAAATA ACTAAATCCT TTCCTATAGT TTCATATAAA ACAAAAATCC   
  
  
- ATCAATTCTC TAAGACAACG GTTTTTTTTT TAGTTCTATA ATATATAAAA ATTAATACTG TCATTAATAA   
  
  
- AATAACTTTT AGGTTGCCAA CACTAATATA TTAGTTTATT AGATTTCCAA TTTTTATAGT TTAAATAATC   
  
  
- TTTTAAGCTT TTTCAGTGCA GTAGTGTTGT AACGAAAATA TATATCATAT CTAAGGAAGC TGAAGATTTG   
  
  
- ATTAGTGAAC TTTAATCTTT AAACTTTGAA AGATTATATT TACGTACCAG AAGAAAGGTT CTAATATTAG   
  
  
- GCGTTAACGA ATTTACATCC CATGTTTATA GTTTAAATCT TCACTGATAA CTTCGGTACT TTCTATAACA   
  
  
- CTAGCATAAC TCCCACCAAC ACTCGTTATC CTACTTTGTC CTTTTTTTTT GGGATTACAA AAGTTTAAAT   
  
  
- ACCCACCGAA ACCATCATCT ATTCACTTAT CACACGTTCC CTTACCGTGG TTATACAAAC CTACCTTGCT   
  
  
- ACTCTCATTC TGCAATCCTT TCGTTGTTCT CTTCCCTTTT CCGTCATCCC TCTCTTAATA CGCACACTTA   
  
  
- TTATAATATT GTCTGCGAAA GCATACTAAT AATTTATGTT CTGAAACTCG TATGTATATT TGAACATTGA   
  
  
- AAGAAATGGA TAACAGTTAA TTAAAAACTT AACTTAGTAG ACGCTGAATA CGTATGTTCT ACTATACTAC   
  
  
- GTATAACCCG GTTCGTAAAC TAGAGTACAG GGAATACCTA GATCTAGGGT TATCTGTTTG AACGTATTCA   
  
  
- GTTCCCTACA CAAAGATTAT GTTTCCGAAA CATGAGTTCG TTAATCAAAA CTTAAGTAAA AGTCATCAAA   
  
  
- TTAAAGGTTT AGAATATTAA AAATGTTACC GTATTTAAAA ATCCTCAAAA CTCATTTATT TAATATATGT   
  
  
- TGCTTCTACA GTAAATGCTT AATTATACTG CGTGTTTTTT TTTCTTTTTT TTAATTACCA TCGAAGATAT   
  
  
- GAACTGAGGT ACAACTGTTA AGGGAGAGAG TGTATTACCA ATTTTGAACG TTGTAGTAGG TCCTGGGTAT   
  
  
- ACTGTACCCA CCACCATCTG TCACACAACA CCATCGGAGT GAGGTACGAA CGGTTAAACA TAGTAACGCG   
  
  
- ATCAGAATAA GCAGGAATCT CTATTAACTG ATTCGAGATT ACGAGATAGC ACCGACGACG TTATATTTAC   
  
  
- TAAAAAACTG GAGTTTTACT TCTGTCGATC AACATTTAAT TCTTAGAAGC AGGGAAGTAA AAAACACATG   
  
  
- CACACATAGA CGCATACACA ACTCTCTGAT CGCCTGATAA CTAACTCAAA GTTAGTTTTT TCTACTGGTA   
  
  
- GAATTCATAA AAATTAATGC GTTTGAAGAA GTAAATTTTT TTACAATGAA AATAAACTTT CTTTAACACT   
  
  
- TATTATGTTC CTTATCAAGT TAATTATATA TTAGGTATTG TATAATGGAG GATATCCGTT TTTCTTCTAA   
  
  
- TAGCATAATT AAACCCTAGT AGGGTGGGAT TTAAATTTCT TTGGTTTTGT TTAATGATTC GTTATAGAAA   
  
  
- GACACACTTT TTTATCAGTC TTTGTTTCGC TAGGCTGGCC GGTTTAGCTC TATTTGTGTC GGTTTCTTGG   
  
  
- CTTCAACCTG GTTTCACTCG GCTCAACTTT CCGTAAGTAT ACCCCAAATT AGTATTGTGT TTGTATTAAT   
  
  
- CTAACTAACT GCAGAAATTA GTCGCAAAAA CAGAAACAAG CCACGAGGGG AGTGAGCGGG GAGGCGAGAA   
  
  
- ATTTCGAATG GAGTGAGGGG GGTAAGGGTG AGGAAAAGAG AGAGGAGAGA GCGAGAGTGT GCGAGGCAGA   
  
  
- CAGTCGGGGA ACAGTAGAAG GGGGCAGAAG GAGCGTTTTT AGAGGTCGTT TTAAATATCG GCAACAACAC   
  
  
- GGGTACCACT AGACTACACT CTTCTCTAAG TCGTAAATTC TAATAACCCA GGTCTACCGA ATATACACGC   
  
  
- GGCTGTCACC ATTAGAGTAC CGATAACGGG TTGTTCAGTA GTTCGTTTTT GTTGTCGTTC TTGTCGTCGT   
  
  
- TGTGGTTGTC GGAGTAGGGG TCAAGCAGCC CAGCTTATTA TTGGGGAAAA CGTGGTCGGG TACCGGGCCT   
  
  
- GGGCAGGGAA GTAGGCGGTA CAGACGGTCG GGAGGCGGGG GAGAACCCAA GAGGCGGCGG CGCAAAGGCC   
  
  
- TGGGCAAAGT CCACCCGGGC GGGACGGGCC TACCCCGACT TGGCCCAAAG GTCAAACGGG TAAACCTGGT   
  
  
- GGTGAGCTCA CCAAAGGCCA AACGACTAAA AACGCCACCC CCACCACCAC TCAAACTGAG CCTACTCACC   
  
  
- TACCTCTCAA ACTACCCACC GCCGCCGCCA CTGAGCGCCG CTGACTCTCA AGATTGGAGG TTAGGGTAAC   
  
  
- GCTGCGCACC GTTCCCAGGC CACTAAAGCC AGAGATACCA CGACTAGGCA AACGCTGCAC AAGCCTTCTG   
  
  
- GCAACGTCGG CGATAAGTGC GAGTCGGTGA CGGCGTTGTC GTCGGCAGCA CCCAGGGCGG AAGAGGCGGC   
  
  
- TTTCTGTGCT TCGTCAGCCG GGGTGGAGTT TTCGTTTCGC TGCGGCGACC TGGGAGCAAG AGTAGGCTCC   
  
  
- TCCTTGGTCT CGGCAGTACC GGCGGCGATT AACGCCGCAA CAACCTCACG CGGTTTGAGC GGCTCAGGCT   
  
  
- GGGCCTTGTG CGACAGTTTA GCCACTAAGC TGAGTCTCTG AGTCAGAGTG TCGTCCCTCA AGGTTAGGTC   
  
  
- TCTCACAGGA TAATGAAGAG GCTCCGTGAG ATGGTGGCGG AAAGAGAGAG GAGGCGGGGA GTTGGAGAGG   
  
  
- GTCTTTGCTG CAGCAGACTC CTCATGTGAG AGAGAATGTT CCGGGACTTG CTGCGCACGG GCATGGTATT   
  
  
- TAAGCGAGTG AACTGGCGTT TAGTCCGCTA AGATCTTCGG TGACTAAGTC GGTTCTTCTA GGTGTAGCAA   
  
  
- CTAAAACCCT ATTAGGTCCC TCAGGTTACC CGACGAGACG ATGTTCGGGA CCGTTGGGCC GGTCGGCCCT   
  
  
- TCGGGCTTAA CTAAGCTTAT AGGCCTTAAG GACGAGGCCC AGACCCCGTT AGAGGCCGCC GTAGCGACGA   
  
  
- GCGTTGACCC TTAGCGGAGG CACTCAAGCG CTTCCAAGAT CTAGAGTTCA AGCTCAAACT CGGCCATGAG   
  
  
- TGAGGTTAGT CTCTCGATTT GCCCAGCTTG AAGGCCCAGC TGGGTCTACT CTATGATCGA CAGTTAAAGT   
  
  
- ACGAAGTTAA CATGTTGGAT AATCTGCTTT GGGACCAGCG ACAGCTGCGG CGGGACTTCG ACCGGTTCAG   
  
  
- TAACTTGGGG TTTTAGCAGT GAAACCCACT CATGCTCCGC CCAGACTTAG CCCGTCCAAA GAACTGGGCC   
  
  
- AAGTTCTGCC GGGAGTTCAT GATAAACCGT TACAAACTTA GCCAACTCGG GTTGAACCGG GCCCTGAGCG   
  
  
- GGCTCTCCCG AGTCCAACTC TCCGACAATA ACCCGGCCGC CTAGTACCCG AATCAACCCG GCCTCAATGG   
  
  
- CCCTTGCTCT TCCCTTTCCT ACCTCACATT CCTTGTTACC TTCCCAAATT ACCTTTCACG GCCAAAGCTC   
  
  
- GGCACGGTTT CGTCTTTGAT GCAGTCACTT TTTCTGTTCA AAATGGTGAG GTCTAACTTA ATGTCTCTCA   
  
  
- C

+     TATA-box

| Site Name | Organism | Position | Strand | Matrix score. | sequence | function |
| --- | --- | --- | --- | --- | --- | --- |
| TATA-box | Arabidopsis thaliana | 2025 | - | 4 | TATA | core promoter element around -30 of transcription start |
| TATA-box | Arabidopsis thaliana | 1948 | - | 5 | TATAA | core promoter element around -30 of transcription start |
| TATA-box | Brassica napus | 638 | + | 6 | ATTATA | core promoter element around -30 of transcription start |
| TATA-box | Arabidopsis thaliana | 1526 | + | 4 | TATA | core promoter element around -30 of transcription start |
| TATA-box | Arabidopsis thaliana | 348 | + | 4 | TATA | core promoter element around -30 of transcription start |
| TATA-box | Brassica oleracea | 319 | + | 6 | ATATAA | core promoter element around -30 of transcription start |
| TATA-box | Pisum sativum | 249 | - | 7 | TATAAAA | core promoter element around -30 of transcription start |
| TATA-box | Brassica oleracea | 1501 | + | 6 | ATATAA | core promoter element around -30 of transcription start |
| TATA-box | Helianthus annuus | 250 | - | 6 | TATAAA | core promoter element around -30 of transcription start |
| TATA-box | Brassica napus | 113 | + | 6 | ATTATA | core promoter element around -30 of transcription start |
| TATA-box | Arabidopsis thaliana | 169 | - | 7 | TATATAA | core promoter element around -30 of transcription start |
| TATA-box | Arabidopsis thaliana | 251 | - | 7 | TATATAA | core promoter element around -30 of transcription start |
| TATA-box | Arabidopsis thaliana | 1949 | - | 4 | TATA | core promoter element around -30 of transcription start |
| TATA-box | Arabidopsis thaliana | 928 | + | 4 | TATA | core promoter element around -30 of transcription start |
| TATA-box | Arabidopsis thaliana | 117 | + | 4 | TATA | core promoter element around -30 of transcription start |
| TATA-box | Brassica napus | 116 | + | 6 | ATATAT | core promoter element around -30 of transcription start |
| TATA-box | Arabidopsis thaliana | 261 | + | 4 | TATA | core promoter element around -30 of transcription start |
| TATA-box | Brassica juncea | 1946 | - | 7 | TATAAAT | core promoter element around -30 of transcription start |
| TATA-box | Brassica oleracea | 171 | + | 6 | ATATAA | core promoter element around -30 of transcription start |
| TATA-box | Arabidopsis thaliana | 254 | + | 6 | TATATA | core promoter element around -30 of transcription start |
| TATA-box | Brassica napus | 346 | + | 6 | ATTATA | core promoter element around -30 of transcription start |
| TATA-box | Arabidopsis thaliana | 347 | - | 5 | TATAA | core promoter element around -30 of transcription start |
| TATA-box | Arabidopsis thaliana | 256 | + | 4 | TATA | core promoter element around -30 of transcription start |
| TATA-box | Arabidopsis thaliana | 172 | + | 4 | TATA | core promoter element around -30 of transcription start |
| TATA-box | Arabidopsis thaliana | 252 | + | 6 | TATATA | core promoter element around -30 of transcription start |
| TATA-box | Arabidopsis thaliana | 58 | + | 4 | TATA | core promoter element around -30 of transcription start |
| TATA-box | Arabidopsis thaliana | 1500 | + | 6 | TATATA | core promoter element around -30 of transcription start |
| TATA-box | Arabidopsis thaliana | 114 | - | 7 | TATATAA | core promoter element around -30 of transcription start |
| TATA-box | Arabidopsis thaliana | 1257 | + | 4 | TATA | core promoter element around -30 of transcription start |
| TATA-box | Arabidopsis thaliana | 1502 | + | 4 | TATA | core promoter element around -30 of transcription start |
| TATA-box | Arabidopsis thaliana | 115 | + | 6 | TATATA | core promoter element around -30 of transcription start |
| TATA-box | Brassica oleracea | 1256 | + | 6 | ATATAA | core promoter element around -30 of transcription start |
| TATA-box | Arabidopsis thaliana | 1051 | + | 4 | TATA | core promoter element around -30 of transcription start |
| TATA-box | Arabidopsis thaliana | 979 | + | 4 | TATA | core promoter element around -30 of transcription start |
| TATA-box | Arabidopsis thaliana | 170 | + | 6 | TATATA | core promoter element around -30 of transcription start |
| TATA-box | Arabidopsis thaliana | 639 | - | 5 | TATAA | core promoter element around -30 of transcription start |
| TATA-box | Arabidopsis thaliana | 2024 | - | 5 | TATAA | core promoter element around -30 of transcription start |
| TATA-box | Arabidopsis thaliana | 320 | + | 4 | TATA | core promoter element around -30 of transcription start |
| TATA-box | Brassica napus | 168 | + | 6 | ATTATA | core promoter element around -30 of transcription start |
| TATA-box | Brassica napus | 975 | + | 6 | ATTATA | core promoter element around -30 of transcription start |
| TATA-box | Brassica napus | 253 | + | 6 | ATATAT | core promoter element around -30 of transcription start |
| TATA-box | Helianthus annuus | 1947 | - | 6 | TATAAA | core promoter element around -30 of transcription start |
| TATA-box | Brassica oleracea | 689 | + | 6 | ATATAA | core promoter element around -30 of transcription start |
| TATA-box | Brassica napus | 1499 | + | 6 | ATATAT | core promoter element around -30 of transcription start |
| TATA-box | Arabidopsis thaliana | 976 | - | 7 | TATATAA | core promoter element around -30 of transcription start |
| TATA-box | Arabidopsis thaliana | 690 | + | 4 | TATA | core promoter element around -30 of transcription start |
| TATA-box | Arabidopsis thaliana | 977 | + | 6 | TATATA | core promoter element around -30 of transcription start |
| TATA-box | Arabidopsis thaliana | 640 | + | 4 | TATA | core promoter element around -30 of transcription start |
| TATA-box | Arabidopsis thaliana | 927 | - | 5 | TATAA | core promoter element around -30 of transcription start |

>HU06G00029.1   
+ +Up\_Stream \_Len000TGATAA TTATTTTTAT TGATTTAGGA AAGGATATCA AAGTATATTT TGTTTTTAGG   
  
  
+ TAGTTAAGAG ATTCTGTTGC CAAAAAAAAA ATCAAGATAT TATATATTTT TAATTATGAC AGTAATTATT   
  
  
+ TTATTGAAAA TCCAACGGTT GTGATTATAT AATCAAATAA TCTAAAGGTT AAAAATATCA AATTTATTAG   
  
  
+ AAAATTCGAA AAAGTCACGT CATCACAACA TTGCTTTTAT ATATAGTATA GATTCCTTCG ACTTCTAAAC   
  
  
+ TAATCACTTG AAATTAGAAA TTTGAAACTT TCTAATATAA ATGCATGGTC TTCTTTCCAA GATTATAATC   
  
  
+ CGCAATTGCT TAAATGTAGG GTACAAATAT CAAATTTAGA AGTGACTATT GAAGCCATGA AAGATATTGT   
  
  
+ GATCGTATTG AGGGTGGTTG TGAGCAATAG GATGAAACAG GAAAAAAAAA CCCTAATGTT TTCAAATTTA   
  
  
+ TGGGTGGCTT TGGTAGTAGA TAAGTGAATA GTGTGCAAGG GAATGGCACC AATATGTTTG GATGGAACGA   
  
  
+ TGAGAGTAAG ACGTTAGGAA AGCAACAAGA GAAGGGAAAA GGCAGTAGGG AGAGAATTAT GCGTGTGAAT   
  
  
+ AATATTATAA CAGACGCTTT CGTATGATTA TTAAATACAA GACTTTGAGC ATACATATAA ACTTGTAACT   
  
  
+ TTCTTTACCT ATTGTCAATT AATTTTTGAA TTGAATCATC TGCGACTTAT GCATACAAGA TGATATGATG   
  
  
+ CATATTGGGC CAAGCATTTG ATCTCATGTC CCTTATGGAT CTAGATCCCA ATAGACAAAC TTGCATAAGT   
  
  
+ CAAGGGATGT GTTTCTAATA CAAAGGCTTT GTACTCAAGC AATTAGTTTT GAATTCATTT TCAGTAGTTT   
  
  
+ AATTTCCAAA TCTTATAATT TTTACAATGG CATAAATTTT TAGGAGTTTT GAGTAAATAA ATTATATACA   
  
  
+ ACGAAGATGT CATTTACGAA TTAATATGAC GCACAAAAAA AAAGAAAAAA AATTAATGGT AGCTTCTATA   
  
  
+ CTTGACTCCA TGTTGACAAT TCCCTCTCTC ACATAATGGT TAAAACTTGC AACATCATCC AGGACCCATA   
  
  
+ TGACATGGGT GGTGGTAGAC AGTGTGTTGT GGTAGCCTCA CTCCATGCTT GCCAATTTGT ATCATTGCGC   
  
  
+ TAGTCTTATT CGTCCTTAGA GATAATTGAC TAAGCTCTAA TGCTCTATCG TGGCTGCTGC AATATAAATG   
  
  
+ ATTTTTTGAC CTCAAAATGA AGACAGCTAG TTGTAAATTA AGAATCTTCG TCCCTTCATT TTTTGTGTAC   
  
  
+ GTGTGTATCT GCGTATGTGT TGAGAGACTA GCGGACTATT GATTGAGTTT CAATCAAAAA AGATGACCAT   
  
  
+ CTTAAGTATT TTTAATTACG CAAACTTCTT CATTTAAAAA AATGTTACTT TTATTTGAAA GAAATTGTGA   
  
  
+ ATAATACAAG GAATAGTTCA ATTAATATAT AATCCATAAC ATATTACCTC CTATAGGCAA AAAGAAGATT   
  
  
+ ATCGTATTAA TTTGGGATCA TCCCACCCTA AATTTAAAGA AACCAAAACA AATTACTAAG CAATATCTTT   
  
  
+ CTGTGTGAAA AAATAGTCAG AAACAAAGCG ATCCGACCGG CCAAATCGAG ATAAACACAG CCAAAGAACC   
  
  
+ GAAGTTGGAC CAAAGTGAGC CGAGTTGAAA GGCATTCATA TGGGGTTTAA TCATAACACA AACATAATTA   
  
  
+ GATTGATTGA CGTCTTTAAT CAGCGTTTTT GTCTTTGTTC GGTGCTCCCC TCACTCGCCC CTCCGCTCTT   
  
  
+ TAAAGCTTAC CTCACTCCCC CCATTCCCAC TCCTTTTCTC TCTCCTCTCT CGCTCTCACA CGCTCCGTCT   
  
  
+ GTCAGCCCCT TGTCATCTTC CCCCGTCTTC CTCGCAAAAA TCTCCAGCAA AATTTATAGC CGTTGTTGTG   
  
  
+ CCCATGGTGA TCTGATGTGA GAAGAGATTC AGCATTTAAG ATTATTGGGT CCAGATGGCT TATATGTGCG   
  
  
+ CCGACAGTGG TAATCTCATG GCTATTGCCC AACAAGTCAT CAAGCAAAAA CAACAGCAAG AACAGCAGCA   
  
  
+ ACACCAACAG CCTCATCCCC AGTTCGTCGG GTCGAATAAT AACCCCTTTT GCACCAGCCC ATGGCCCGGA   
  
  
+ CCCGTCCCTT CATCCGCCAT GTCTGCCAGC CCTCCGCCCC CTCTTGGGTT CTCCGCCGCC GCGTTTCCGG   
  
  
+ ACCCGTTTCA GGTGGGCCCG CCCTGCCCGG ATGGGGCTGA ACCGGGTTTC CAGTTTGCCC ATTTGGACCA   
  
  
+ CCACTCGAGT GGTTTCCGGT TTGCTGATTT TTGCGGTGGG GGTGGTGGTG AGTTTGACTC GGATGAGTGG   
  
  
+ ATGGAGAGTT TGATGGGTGG CGGCGGCGGT GACTCGCGGC GACTGAGAGT TCTAACCTCC AATCCCATTG   
  
  
+ CGACGCGTGG CAAGGGTCCG GTGATTTCGG TCTCTATGGT GCTGATCCGT TTGCGACGTG TTCGGAAGAC   
  
  
+ CGTTGCAGCC GCTATTCACG CTCAGCCACT GCCGCAACAG CAGCCGTCGT GGGTCCCGCC TTCTCCGCCG   
  
  
+ AAAGACACGA AGCAGTCGGC CCCACCTCAA AAGCAAAGCG ACGCCGCTGG ACCCTCGTTC TCATCCGAGG   
  
  
+ AGGAACCAGA GCCGTCATGG CCGCCGCTAA TTGCGGCGTT GTTGGAGTGC GCCAAACTCG CCGAGTCCGA   
  
  
+ CCCGGAACAC GCTGTCAAAT CGGTGATTCG ACTCAGAGAC TCAGTCTCAC AGCAGGGAGT TCCAATCCAG   
  
  
+ AGAGTGTCCT ATTACTTCTC CGAGGCACTC TACCACCGCC TTTCTCTCTC CTCCGCCCCT CAACCTCTCC   
  
  
+ CAGAAACGAC GTCGTCTGAG GAGTACACTC TCTCTTACAA GGCCCTGAAC GACGCGTGCC CGTACCATAA   
  
  
+ ATTCGCTCAC TTGACCGCAA ATCAGGCGAT TCTAGAAGCC ACTGATTCAG CCAAGAAGAT CCACATCGTT   
  
  
+ GATTTTGGGA TAATCCAGGG AGTCCAATGG GCTGCTCTGC TACAAGCCCT GGCAACCCGG CCAGCCGGGA   
  
  
+ AGCCCGAATT GATTCGAATA TCCGGAATTC CTGCTCCGGG TCTGGGGCAA TCTCCGGCGG CATCGCTGCT   
  
  
+ CGCAACTGGG AATCGCCTCC GTGAGTTCGC GAAGGTTCTA GATCTCAAGT TCGAGTTTGA GCCGGTACTC   
  
  
+ ACTCCAATCA GAGAGCTAAA CGGGTCGAAC TTCCGGGTCG ACCCAGATGA GATACTAGCT GTCAATTTCA   
  
  
+ TGCTTCAATT GTACAACCTA TTAGACGAAA CCCTGGTCGC TGTCGACGCC GCCCTGAAGC TGGCCAAGTC   
  
  
+ ATTGAACCCC AAAATCGTCA CTTTGGGTGA GTACGAGGCG GGTCTGAATC GGGCAGGTTT CTTGACCCGG   
  
  
+ TTCAAGACGG CCCTCAAGTA CTATTTGGCA ATGTTTGAAT CGGTTGAGCC CAACTTGGCC CGGGACTCGC   
  
  
+ CCGAGAGGGC TCAGGTTGAG AGGCTGTTAT TGGGCCGGCG GATCATGGGC TTAGTTGGGC CGGAGTTACC   
  
  
+ GGGAACGAGA AGGGAAAGGA TGGAGTGTAA GGAACAATGG AAGGGTTTAA TGGAAAGTGC CGGTTTCGAG   
  
  
+ CCGTGCCAAA GCAGAAACTA CGTCAGTGAA AAAGACAAGT TTTACCACTC CAGATTGAAT TACAGAGAGT   
  
  
+ G  

- +Up\_Stream \_Len000ACTATT AATAAAAATA ACTAAATCCT TTCCTATAGT TTCATATAAA ACAAAAATCC   
  
  
- ATCAATTCTC TAAGACAACG GTTTTTTTTT TAGTTCTATA ATATATAAAA ATTAATACTG TCATTAATAA   
  
  
- AATAACTTTT AGGTTGCCAA CACTAATATA TTAGTTTATT AGATTTCCAA TTTTTATAGT TTAAATAATC   
  
  
- TTTTAAGCTT TTTCAGTGCA GTAGTGTTGT AACGAAAATA TATATCATAT CTAAGGAAGC TGAAGATTTG   
  
  
- ATTAGTGAAC TTTAATCTTT AAACTTTGAA AGATTATATT TACGTACCAG AAGAAAGGTT CTAATATTAG   
  
  
- GCGTTAACGA ATTTACATCC CATGTTTATA GTTTAAATCT TCACTGATAA CTTCGGTACT TTCTATAACA   
  
  
- CTAGCATAAC TCCCACCAAC ACTCGTTATC CTACTTTGTC CTTTTTTTTT GGGATTACAA AAGTTTAAAT   
  
  
- ACCCACCGAA ACCATCATCT ATTCACTTAT CACACGTTCC CTTACCGTGG TTATACAAAC CTACCTTGCT   
  
  
- ACTCTCATTC TGCAATCCTT TCGTTGTTCT CTTCCCTTTT CCGTCATCCC TCTCTTAATA CGCACACTTA   
  
  
- TTATAATATT GTCTGCGAAA GCATACTAAT AATTTATGTT CTGAAACTCG TATGTATATT TGAACATTGA   
  
  
- AAGAAATGGA TAACAGTTAA TTAAAAACTT AACTTAGTAG ACGCTGAATA CGTATGTTCT ACTATACTAC   
  
  
- GTATAACCCG GTTCGTAAAC TAGAGTACAG GGAATACCTA GATCTAGGGT TATCTGTTTG AACGTATTCA   
  
  
- GTTCCCTACA CAAAGATTAT GTTTCCGAAA CATGAGTTCG TTAATCAAAA CTTAAGTAAA AGTCATCAAA   
  
  
- TTAAAGGTTT AGAATATTAA AAATGTTACC GTATTTAAAA ATCCTCAAAA CTCATTTATT TAATATATGT   
  
  
- TGCTTCTACA GTAAATGCTT AATTATACTG CGTGTTTTTT TTTCTTTTTT TTAATTACCA TCGAAGATAT   
  
  
- GAACTGAGGT ACAACTGTTA AGGGAGAGAG TGTATTACCA ATTTTGAACG TTGTAGTAGG TCCTGGGTAT   
  
  
- ACTGTACCCA CCACCATCTG TCACACAACA CCATCGGAGT GAGGTACGAA CGGTTAAACA TAGTAACGCG   
  
  
- ATCAGAATAA GCAGGAATCT CTATTAACTG ATTCGAGATT ACGAGATAGC ACCGACGACG TTATATTTAC   
  
  
- TAAAAAACTG GAGTTTTACT TCTGTCGATC AACATTTAAT TCTTAGAAGC AGGGAAGTAA AAAACACATG   
  
  
- CACACATAGA CGCATACACA ACTCTCTGAT CGCCTGATAA CTAACTCAAA GTTAGTTTTT TCTACTGGTA   
  
  
- GAATTCATAA AAATTAATGC GTTTGAAGAA GTAAATTTTT TTACAATGAA AATAAACTTT CTTTAACACT   
  
  
- TATTATGTTC CTTATCAAGT TAATTATATA TTAGGTATTG TATAATGGAG GATATCCGTT TTTCTTCTAA   
  
  
- TAGCATAATT AAACCCTAGT AGGGTGGGAT TTAAATTTCT TTGGTTTTGT TTAATGATTC GTTATAGAAA   
  
  
- GACACACTTT TTTATCAGTC TTTGTTTCGC TAGGCTGGCC GGTTTAGCTC TATTTGTGTC GGTTTCTTGG   
  
  
- CTTCAACCTG GTTTCACTCG GCTCAACTTT CCGTAAGTAT ACCCCAAATT AGTATTGTGT TTGTATTAAT   
  
  
- CTAACTAACT GCAGAAATTA GTCGCAAAAA CAGAAACAAG CCACGAGGGG AGTGAGCGGG GAGGCGAGAA   
  
  
- ATTTCGAATG GAGTGAGGGG GGTAAGGGTG AGGAAAAGAG AGAGGAGAGA GCGAGAGTGT GCGAGGCAGA   
  
  
- CAGTCGGGGA ACAGTAGAAG GGGGCAGAAG GAGCGTTTTT AGAGGTCGTT TTAAATATCG GCAACAACAC   
  
  
- GGGTACCACT AGACTACACT CTTCTCTAAG TCGTAAATTC TAATAACCCA GGTCTACCGA ATATACACGC   
  
  
- GGCTGTCACC ATTAGAGTAC CGATAACGGG TTGTTCAGTA GTTCGTTTTT GTTGTCGTTC TTGTCGTCGT   
  
  
- TGTGGTTGTC GGAGTAGGGG TCAAGCAGCC CAGCTTATTA TTGGGGAAAA CGTGGTCGGG TACCGGGCCT   
  
  
- GGGCAGGGAA GTAGGCGGTA CAGACGGTCG GGAGGCGGGG GAGAACCCAA GAGGCGGCGG CGCAAAGGCC   
  
  
- TGGGCAAAGT CCACCCGGGC GGGACGGGCC TACCCCGACT TGGCCCAAAG GTCAAACGGG TAAACCTGGT   
  
  
- GGTGAGCTCA CCAAAGGCCA AACGACTAAA AACGCCACCC CCACCACCAC TCAAACTGAG CCTACTCACC   
  
  
- TACCTCTCAA ACTACCCACC GCCGCCGCCA CTGAGCGCCG CTGACTCTCA AGATTGGAGG TTAGGGTAAC   
  
  
- GCTGCGCACC GTTCCCAGGC CACTAAAGCC AGAGATACCA CGACTAGGCA AACGCTGCAC AAGCCTTCTG   
  
  
- GCAACGTCGG CGATAAGTGC GAGTCGGTGA CGGCGTTGTC GTCGGCAGCA CCCAGGGCGG AAGAGGCGGC   
  
  
- TTTCTGTGCT TCGTCAGCCG GGGTGGAGTT TTCGTTTCGC TGCGGCGACC TGGGAGCAAG AGTAGGCTCC   
  
  
- TCCTTGGTCT CGGCAGTACC GGCGGCGATT AACGCCGCAA CAACCTCACG CGGTTTGAGC GGCTCAGGCT   
  
  
- GGGCCTTGTG CGACAGTTTA GCCACTAAGC TGAGTCTCTG AGTCAGAGTG TCGTCCCTCA AGGTTAGGTC   
  
  
- TCTCACAGGA TAATGAAGAG GCTCCGTGAG ATGGTGGCGG AAAGAGAGAG GAGGCGGGGA GTTGGAGAGG   
  
  
- GTCTTTGCTG CAGCAGACTC CTCATGTGAG AGAGAATGTT CCGGGACTTG CTGCGCACGG GCATGGTATT   
  
  
- TAAGCGAGTG AACTGGCGTT TAGTCCGCTA AGATCTTCGG TGACTAAGTC GGTTCTTCTA GGTGTAGCAA   
  
  
- CTAAAACCCT ATTAGGTCCC TCAGGTTACC CGACGAGACG ATGTTCGGGA CCGTTGGGCC GGTCGGCCCT   
  
  
- TCGGGCTTAA CTAAGCTTAT AGGCCTTAAG GACGAGGCCC AGACCCCGTT AGAGGCCGCC GTAGCGACGA   
  
  
- GCGTTGACCC TTAGCGGAGG CACTCAAGCG CTTCCAAGAT CTAGAGTTCA AGCTCAAACT CGGCCATGAG   
  
  
- TGAGGTTAGT CTCTCGATTT GCCCAGCTTG AAGGCCCAGC TGGGTCTACT CTATGATCGA CAGTTAAAGT   
  
  
- ACGAAGTTAA CATGTTGGAT AATCTGCTTT GGGACCAGCG ACAGCTGCGG CGGGACTTCG ACCGGTTCAG   
  
  
- TAACTTGGGG TTTTAGCAGT GAAACCCACT CATGCTCCGC CCAGACTTAG CCCGTCCAAA GAACTGGGCC   
  
  
- AAGTTCTGCC GGGAGTTCAT GATAAACCGT TACAAACTTA GCCAACTCGG GTTGAACCGG GCCCTGAGCG   
  
  
- GGCTCTCCCG AGTCCAACTC TCCGACAATA ACCCGGCCGC CTAGTACCCG AATCAACCCG GCCTCAATGG   
  
  
- CCCTTGCTCT TCCCTTTCCT ACCTCACATT CCTTGTTACC TTCCCAAATT ACCTTTCACG GCCAAAGCTC   
  
  
- GGCACGGTTT CGTCTTTGAT GCAGTCACTT TTTCTGTTCA AAATGGTGAG GTCTAACTTA ATGTCTCTCA   
  
  
- C

+     TATC-box

| Site Name | Organism | Position | Strand | Matrix score. | sequence | function |
| --- | --- | --- | --- | --- | --- | --- |
| TATC-box | Oryza sativa | 3020 | - | 7 | TATCCCA | cis-acting element involved in gibberellin-responsiveness |

>HU06G00029.1   
+ +Up\_Stream \_Len000TGATAA TTATTTTTAT TGATTTAGGA AAGGATATCA AAGTATATTT TGTTTTTAGG   
  
  
+ TAGTTAAGAG ATTCTGTTGC CAAAAAAAAA ATCAAGATAT TATATATTTT TAATTATGAC AGTAATTATT   
  
  
+ TTATTGAAAA TCCAACGGTT GTGATTATAT AATCAAATAA TCTAAAGGTT AAAAATATCA AATTTATTAG   
  
  
+ AAAATTCGAA AAAGTCACGT CATCACAACA TTGCTTTTAT ATATAGTATA GATTCCTTCG ACTTCTAAAC   
  
  
+ TAATCACTTG AAATTAGAAA TTTGAAACTT TCTAATATAA ATGCATGGTC TTCTTTCCAA GATTATAATC   
  
  
+ CGCAATTGCT TAAATGTAGG GTACAAATAT CAAATTTAGA AGTGACTATT GAAGCCATGA AAGATATTGT   
  
  
+ GATCGTATTG AGGGTGGTTG TGAGCAATAG GATGAAACAG GAAAAAAAAA CCCTAATGTT TTCAAATTTA   
  
  
+ TGGGTGGCTT TGGTAGTAGA TAAGTGAATA GTGTGCAAGG GAATGGCACC AATATGTTTG GATGGAACGA   
  
  
+ TGAGAGTAAG ACGTTAGGAA AGCAACAAGA GAAGGGAAAA GGCAGTAGGG AGAGAATTAT GCGTGTGAAT   
  
  
+ AATATTATAA CAGACGCTTT CGTATGATTA TTAAATACAA GACTTTGAGC ATACATATAA ACTTGTAACT   
  
  
+ TTCTTTACCT ATTGTCAATT AATTTTTGAA TTGAATCATC TGCGACTTAT GCATACAAGA TGATATGATG   
  
  
+ CATATTGGGC CAAGCATTTG ATCTCATGTC CCTTATGGAT CTAGATCCCA ATAGACAAAC TTGCATAAGT   
  
  
+ CAAGGGATGT GTTTCTAATA CAAAGGCTTT GTACTCAAGC AATTAGTTTT GAATTCATTT TCAGTAGTTT   
  
  
+ AATTTCCAAA TCTTATAATT TTTACAATGG CATAAATTTT TAGGAGTTTT GAGTAAATAA ATTATATACA   
  
  
+ ACGAAGATGT CATTTACGAA TTAATATGAC GCACAAAAAA AAAGAAAAAA AATTAATGGT AGCTTCTATA   
  
  
+ CTTGACTCCA TGTTGACAAT TCCCTCTCTC ACATAATGGT TAAAACTTGC AACATCATCC AGGACCCATA   
  
  
+ TGACATGGGT GGTGGTAGAC AGTGTGTTGT GGTAGCCTCA CTCCATGCTT GCCAATTTGT ATCATTGCGC   
  
  
+ TAGTCTTATT CGTCCTTAGA GATAATTGAC TAAGCTCTAA TGCTCTATCG TGGCTGCTGC AATATAAATG   
  
  
+ ATTTTTTGAC CTCAAAATGA AGACAGCTAG TTGTAAATTA AGAATCTTCG TCCCTTCATT TTTTGTGTAC   
  
  
+ GTGTGTATCT GCGTATGTGT TGAGAGACTA GCGGACTATT GATTGAGTTT CAATCAAAAA AGATGACCAT   
  
  
+ CTTAAGTATT TTTAATTACG CAAACTTCTT CATTTAAAAA AATGTTACTT TTATTTGAAA GAAATTGTGA   
  
  
+ ATAATACAAG GAATAGTTCA ATTAATATAT AATCCATAAC ATATTACCTC CTATAGGCAA AAAGAAGATT   
  
  
+ ATCGTATTAA TTTGGGATCA TCCCACCCTA AATTTAAAGA AACCAAAACA AATTACTAAG CAATATCTTT   
  
  
+ CTGTGTGAAA AAATAGTCAG AAACAAAGCG ATCCGACCGG CCAAATCGAG ATAAACACAG CCAAAGAACC   
  
  
+ GAAGTTGGAC CAAAGTGAGC CGAGTTGAAA GGCATTCATA TGGGGTTTAA TCATAACACA AACATAATTA   
  
  
+ GATTGATTGA CGTCTTTAAT CAGCGTTTTT GTCTTTGTTC GGTGCTCCCC TCACTCGCCC CTCCGCTCTT   
  
  
+ TAAAGCTTAC CTCACTCCCC CCATTCCCAC TCCTTTTCTC TCTCCTCTCT CGCTCTCACA CGCTCCGTCT   
  
  
+ GTCAGCCCCT TGTCATCTTC CCCCGTCTTC CTCGCAAAAA TCTCCAGCAA AATTTATAGC CGTTGTTGTG   
  
  
+ CCCATGGTGA TCTGATGTGA GAAGAGATTC AGCATTTAAG ATTATTGGGT CCAGATGGCT TATATGTGCG   
  
  
+ CCGACAGTGG TAATCTCATG GCTATTGCCC AACAAGTCAT CAAGCAAAAA CAACAGCAAG AACAGCAGCA   
  
  
+ ACACCAACAG CCTCATCCCC AGTTCGTCGG GTCGAATAAT AACCCCTTTT GCACCAGCCC ATGGCCCGGA   
  
  
+ CCCGTCCCTT CATCCGCCAT GTCTGCCAGC CCTCCGCCCC CTCTTGGGTT CTCCGCCGCC GCGTTTCCGG   
  
  
+ ACCCGTTTCA GGTGGGCCCG CCCTGCCCGG ATGGGGCTGA ACCGGGTTTC CAGTTTGCCC ATTTGGACCA   
  
  
+ CCACTCGAGT GGTTTCCGGT TTGCTGATTT TTGCGGTGGG GGTGGTGGTG AGTTTGACTC GGATGAGTGG   
  
  
+ ATGGAGAGTT TGATGGGTGG CGGCGGCGGT GACTCGCGGC GACTGAGAGT TCTAACCTCC AATCCCATTG   
  
  
+ CGACGCGTGG CAAGGGTCCG GTGATTTCGG TCTCTATGGT GCTGATCCGT TTGCGACGTG TTCGGAAGAC   
  
  
+ CGTTGCAGCC GCTATTCACG CTCAGCCACT GCCGCAACAG CAGCCGTCGT GGGTCCCGCC TTCTCCGCCG   
  
  
+ AAAGACACGA AGCAGTCGGC CCCACCTCAA AAGCAAAGCG ACGCCGCTGG ACCCTCGTTC TCATCCGAGG   
  
  
+ AGGAACCAGA GCCGTCATGG CCGCCGCTAA TTGCGGCGTT GTTGGAGTGC GCCAAACTCG CCGAGTCCGA   
  
  
+ CCCGGAACAC GCTGTCAAAT CGGTGATTCG ACTCAGAGAC TCAGTCTCAC AGCAGGGAGT TCCAATCCAG   
  
  
+ AGAGTGTCCT ATTACTTCTC CGAGGCACTC TACCACCGCC TTTCTCTCTC CTCCGCCCCT CAACCTCTCC   
  
  
+ CAGAAACGAC GTCGTCTGAG GAGTACACTC TCTCTTACAA GGCCCTGAAC GACGCGTGCC CGTACCATAA   
  
  
+ ATTCGCTCAC TTGACCGCAA ATCAGGCGAT TCTAGAAGCC ACTGATTCAG CCAAGAAGAT CCACATCGTT   
  
  
+ GATTTTGGGA TAATCCAGGG AGTCCAATGG GCTGCTCTGC TACAAGCCCT GGCAACCCGG CCAGCCGGGA   
  
  
+ AGCCCGAATT GATTCGAATA TCCGGAATTC CTGCTCCGGG TCTGGGGCAA TCTCCGGCGG CATCGCTGCT   
  
  
+ CGCAACTGGG AATCGCCTCC GTGAGTTCGC GAAGGTTCTA GATCTCAAGT TCGAGTTTGA GCCGGTACTC   
  
  
+ ACTCCAATCA GAGAGCTAAA CGGGTCGAAC TTCCGGGTCG ACCCAGATGA GATACTAGCT GTCAATTTCA   
  
  
+ TGCTTCAATT GTACAACCTA TTAGACGAAA CCCTGGTCGC TGTCGACGCC GCCCTGAAGC TGGCCAAGTC   
  
  
+ ATTGAACCCC AAAATCGTCA CTTTGGGTGA GTACGAGGCG GGTCTGAATC GGGCAGGTTT CTTGACCCGG   
  
  
+ TTCAAGACGG CCCTCAAGTA CTATTTGGCA ATGTTTGAAT CGGTTGAGCC CAACTTGGCC CGGGACTCGC   
  
  
+ CCGAGAGGGC TCAGGTTGAG AGGCTGTTAT TGGGCCGGCG GATCATGGGC TTAGTTGGGC CGGAGTTACC   
  
  
+ GGGAACGAGA AGGGAAAGGA TGGAGTGTAA GGAACAATGG AAGGGTTTAA TGGAAAGTGC CGGTTTCGAG   
  
  
+ CCGTGCCAAA GCAGAAACTA CGTCAGTGAA AAAGACAAGT TTTACCACTC CAGATTGAAT TACAGAGAGT   
  
  
+ G  

- +Up\_Stream \_Len000ACTATT AATAAAAATA ACTAAATCCT TTCCTATAGT TTCATATAAA ACAAAAATCC   
  
  
- ATCAATTCTC TAAGACAACG GTTTTTTTTT TAGTTCTATA ATATATAAAA ATTAATACTG TCATTAATAA   
  
  
- AATAACTTTT AGGTTGCCAA CACTAATATA TTAGTTTATT AGATTTCCAA TTTTTATAGT TTAAATAATC   
  
  
- TTTTAAGCTT TTTCAGTGCA GTAGTGTTGT AACGAAAATA TATATCATAT CTAAGGAAGC TGAAGATTTG   
  
  
- ATTAGTGAAC TTTAATCTTT AAACTTTGAA AGATTATATT TACGTACCAG AAGAAAGGTT CTAATATTAG   
  
  
- GCGTTAACGA ATTTACATCC CATGTTTATA GTTTAAATCT TCACTGATAA CTTCGGTACT TTCTATAACA   
  
  
- CTAGCATAAC TCCCACCAAC ACTCGTTATC CTACTTTGTC CTTTTTTTTT GGGATTACAA AAGTTTAAAT   
  
  
- ACCCACCGAA ACCATCATCT ATTCACTTAT CACACGTTCC CTTACCGTGG TTATACAAAC CTACCTTGCT   
  
  
- ACTCTCATTC TGCAATCCTT TCGTTGTTCT CTTCCCTTTT CCGTCATCCC TCTCTTAATA CGCACACTTA   
  
  
- TTATAATATT GTCTGCGAAA GCATACTAAT AATTTATGTT CTGAAACTCG TATGTATATT TGAACATTGA   
  
  
- AAGAAATGGA TAACAGTTAA TTAAAAACTT AACTTAGTAG ACGCTGAATA CGTATGTTCT ACTATACTAC   
  
  
- GTATAACCCG GTTCGTAAAC TAGAGTACAG GGAATACCTA GATCTAGGGT TATCTGTTTG AACGTATTCA   
  
  
- GTTCCCTACA CAAAGATTAT GTTTCCGAAA CATGAGTTCG TTAATCAAAA CTTAAGTAAA AGTCATCAAA   
  
  
- TTAAAGGTTT AGAATATTAA AAATGTTACC GTATTTAAAA ATCCTCAAAA CTCATTTATT TAATATATGT   
  
  
- TGCTTCTACA GTAAATGCTT AATTATACTG CGTGTTTTTT TTTCTTTTTT TTAATTACCA TCGAAGATAT   
  
  
- GAACTGAGGT ACAACTGTTA AGGGAGAGAG TGTATTACCA ATTTTGAACG TTGTAGTAGG TCCTGGGTAT   
  
  
- ACTGTACCCA CCACCATCTG TCACACAACA CCATCGGAGT GAGGTACGAA CGGTTAAACA TAGTAACGCG   
  
  
- ATCAGAATAA GCAGGAATCT CTATTAACTG ATTCGAGATT ACGAGATAGC ACCGACGACG TTATATTTAC   
  
  
- TAAAAAACTG GAGTTTTACT TCTGTCGATC AACATTTAAT TCTTAGAAGC AGGGAAGTAA AAAACACATG   
  
  
- CACACATAGA CGCATACACA ACTCTCTGAT CGCCTGATAA CTAACTCAAA GTTAGTTTTT TCTACTGGTA   
  
  
- GAATTCATAA AAATTAATGC GTTTGAAGAA GTAAATTTTT TTACAATGAA AATAAACTTT CTTTAACACT   
  
  
- TATTATGTTC CTTATCAAGT TAATTATATA TTAGGTATTG TATAATGGAG GATATCCGTT TTTCTTCTAA   
  
  
- TAGCATAATT AAACCCTAGT AGGGTGGGAT TTAAATTTCT TTGGTTTTGT TTAATGATTC GTTATAGAAA   
  
  
- GACACACTTT TTTATCAGTC TTTGTTTCGC TAGGCTGGCC GGTTTAGCTC TATTTGTGTC GGTTTCTTGG   
  
  
- CTTCAACCTG GTTTCACTCG GCTCAACTTT CCGTAAGTAT ACCCCAAATT AGTATTGTGT TTGTATTAAT   
  
  
- CTAACTAACT GCAGAAATTA GTCGCAAAAA CAGAAACAAG CCACGAGGGG AGTGAGCGGG GAGGCGAGAA   
  
  
- ATTTCGAATG GAGTGAGGGG GGTAAGGGTG AGGAAAAGAG AGAGGAGAGA GCGAGAGTGT GCGAGGCAGA   
  
  
- CAGTCGGGGA ACAGTAGAAG GGGGCAGAAG GAGCGTTTTT AGAGGTCGTT TTAAATATCG GCAACAACAC   
  
  
- GGGTACCACT AGACTACACT CTTCTCTAAG TCGTAAATTC TAATAACCCA GGTCTACCGA ATATACACGC   
  
  
- GGCTGTCACC ATTAGAGTAC CGATAACGGG TTGTTCAGTA GTTCGTTTTT GTTGTCGTTC TTGTCGTCGT   
  
  
- TGTGGTTGTC GGAGTAGGGG TCAAGCAGCC CAGCTTATTA TTGGGGAAAA CGTGGTCGGG TACCGGGCCT
[truncated: 139,811 more chars]
