# Supplementary material for: Genome-Wide Identification and Expression Pattern of the GRAS Gene Family in Pitaya (Selenicereus undatus L.)
Source: Biology (Basel). 2022 Dec 21;12(1):11. doi: 10.3390/biology12010011 (PMC9854919; doi:10.3390/biology12010011)
Supplement: Supplementary file 1 [file biology-12-00011-s001.zip › Supplementary file S5/HU06G00283.1_plantcare.html]

Content-Type: text/html; charset=ISO-8859-1


PlantCARE


Webmaster Firefox specific output  
To save the result:
click on the frame with the right mouse button and save the source code as a text file with extension .html  
REFERENCE:PlantCARE: a database of plant cis-acting regulatory elements and a portal to tools for in silico analysis of promoter sequences.  
Lescot, M., Déhais, P., Moreau, Y., De Moor, B., Rouzé ,P.,and Rombauts, S.  
Nucleic Acids Res., Database issue(2002), 30(1):325-327.   


---

>HU06G00283.1   
+ -Up\_Stream \_Len000GATTTT ACTTCCAATT TTCTACTTAA ATTTGGTTAT CACGTTCGAA TTTTTCAGGT   
  
  
+ AAATACAAAC TGTTAGTGGC CATCCTTTTT TTTTGGGTCT AATCCATCCA GATGTGCTAA TACTTTCTGT   
  
  
+ TTTAGATGAG AAGTTTTAAT GCTTTTTTAG ATGTGTAATT TTTTTAAACA TAAATAGTCT TGTCGAATAA   
  
  
+ GATTTGCTAG TAATTTTTGT AAGAGAAATT ATGTGTATTT TTTATTATAT CAGAATATTA GATCGATCTC   
  
  
+ AATTTAATTA CTTATGAAAA AAAAAGTGGA TATCTTTAAT CAATTTGGCA AGCTCTCATG CATTAATTGT   
  
  
+ AGTCGTACGA ACTTATTTAC ATCACTCATC TGAATTTGCT ACCATTTACA CCTCAAATTT TATTTTAATG   
  
  
+ CCATAAAGAG AATAGAATAT ACTGTCTATT TGTAGTAGTT TTACAGATAA AAACAAATAA GCAAATGAAA   
  
  
+ ATAGATAGAG GATTTTTTTG CTATTATCCA CGTCGATCCA TTGAATTATT TTGTCGCTAT TTGGTTAAGT   
  
  
+ GAAATAAAAT ACTGAAGTAG AATGGAGGAT GTTGATAATG ACCTATGTCC CTCAAAGTTT AATCATGTAG   
  
  
+ TTTCTACGAA TTTCAAGCCA TTATAAGACA TACAGATTTT TATGATGTTG AAAATACTCT TACCTCCTTG   
  
  
+ TCACAATATT AACCTGAAGC CAATGAAATG AAACAGAACT AATTAAATTG AATACATAAT CAATCCATAT   
  
  
+ TAATTTGTTT TGACAACTAT TTACAATCAT CGATCTCTTT AATTAGGTCT ATAAGCTATA GAGTAATTAC   
  
  
+ TCAAAACTGT TTTGTAAGAC AACTTACTCC ATATTGGCTT ATTAGTATAT TGCCTCCATT CACATGAATA   
  
  
+ CTTCATGAGA GATTGCCTAA AACTCATATT GTCATCACCT TAAGTCAAAC ATATTTTGTT ATGAAATGAC   
  
  
+ ATCAAGAATT ACCCTCTGAC AAGAAAAATA TACCTGACTG ACATAATCGT ACCACATCTG TTAGTCCTCT   
  
  
+ TTCTACCACT TTGTAGAACA TAATGTGACA GTAATACATC CATCCCAAAA GAAAAGACGA AAAGAAGAGT   
  
  
+ GGAGAGTCAC GTATACGCGT TGAATTCAGG AAAATTAAAG GAACTCATGA AGACCCCATA TAGTGCTCCA   
  
  
+ ACCTGAACCT GCAGCCCACT ACTACATGCC TAAATTAAAG AGCAAAGTTA TGAGAGAAAC CAAAAAAGCT   
  
  
+ CTTTGAGAGA GTGGAGGAAG AATCATGGAA AGCTAGCTAG GCTTAGCAAG AAAGAGCAAC AGCTAGCTAG   
  
  
+ CAAAAGGCTT CTCTTTGAAG CCTTAGAAAT CTTTAGAGAT AAAAAGTTGG TGGCTTTTTA AGGGCTGTGA   
  
  
+ TATGTCCCCC ATGTAAAAAT GCAGCAATAA TGTTAATGTC ATGCTTTCTT TCATGGTCCC TAATGCGAAA   
  
  
+ TCAATCATGC ATGTCAACGT GACTCAGTGG CTCACCATAT ATGATCTGAT TTCCCCGCAC ACAGAGATAA   
  
  
+ AAAGCCCCCA ATTATAACCT CCCAAAAGCC CCCCCTTTTC CTCCTACCCA ACCCACACCT CCCCATTTCA   
  
  
+ TCAGGGCTCG CTTCTTAAGT TATCGATTAA AAAAAGAGTG AGTAAACGTA CCCGAATTGA AGAGGGGTGT   
  
  
+ TCGAAGCTTA TACTTGCTTG ATTAGGAATG AAAGTCCCCA TTTCATCCGC CCCCCAAACC GCCGCCACCA   
  
  
+ CCAGTAAATC GCTATCTTGC AACAATACTG CCTTGAATAC TGCCTTTCAA GCCCCCAATT CAATTTCAAC   
  
  
+ CCTTGTTAAC AACAACAAAC CCTCCTACGA GCCTACCTCG GTTCTAGACC TCCGGCGCAG CCCAAGCCCC   
  
  
+ GCCACCAACG CCGCCCTGCC ACTGCCCACT GACGCGGGCA TCAATAAACC CAAGCCTGCC TCTGCTGACC   
  
  
+ TGGACAATTT GGAGGGTGAG GGTTGGGATT CAATCCTATC AGAGCTGGGG CTTAATGATG ATTCTAATAA   
  
  
+ TCCTAACTCC AAACTTTGGT CATCCCAAAT TAGCTCATCA TGTGATTCCC ACCTCACACA GCTCCCTGAG   
  
  
+ TTCCCCTCCT CTCAGCCGTT GGATCACACA CGCACCCCCA ATAATTAACT TCTCCCCCTC TGATTACCCC   
  
  
+ ATCTCTGATT TATCCTACGG TCAGAATCTC AGCCCCAATT TTGGGCAGTT TGAGCTTCAT CTTCATCAGC   
  
  
+ CCAACGTCAA TAACAATGGG CTTGACTTCA TAGAGGACCT AATTAGAGCA GCTCAAGCCT ACGAATCCAA   
  
  
+ CGACTCCCAC CAGGTCCAGC TGGTATTGGC GCGGCTCAAT CAACGGCTCA GATCACCCGC CGGAAAACCC   
  
  
+ CTCCACCGCG CCGCCTTCTA CTTCAAGGAG GCCCTCCAAC ACCTCGCTGT CGGCGGAGGC CCGGCCCGGC   
  
  
+ CTGTCCGGCT GTCATCCTAC GAGGTGGTGC AGACCATCCG GGCCCACAAG AACTTTTCCG GCATATCACT   
  
  
+ GATATCCCTC TTCTCCACCT TCGCCGCCAA TCAGGCGATT CTGGAGGCGG TGGATGGGGC TGCGTTCATC   
  
  
+ CACATCATCG ATTTCGACAT CGGATTTGGC GGCCACTGGG CGTCGTTTAT GCGTGAGCTT GTCGACAAGG   
  
  
+ CCGACCCGAA TAAGATCAGC TCCTTGGTGT TCCGAATCAC GGCGATCGTA CCCGAAGAGT TCGGGATCGA   
  
  
+ AAGCAAGCTG GTGAGAGAGA ATCTTACGCA ATTCGCTCGT GATCTCAACA TCAATGTCCA TATGGAGTAT   
  
  
+ GTCCTTATTC AAAGCTTCGA GTTCTTATCC TTCAAATCGG TCAAATTCAT CGACGGTGAG AAATTGGCGG   
  
  
+ TCCATCTATC TCCGGCGATG TTTCACCGGC TTCAAGGCGG AATCTCGAAA TTCCTAGCCG ATCTCCGGGG   
  
  
+ AATTTCGCCA AGCACCGTCG TGGTGGTGGA CAGTGAGGTA GGAATCGACA CGGGAACTTC GTCCTTCAGC   
  
  
+ GTCAACTTCG TCGCCGGTAT CGAATTCTAC ACCGGAATGC TCGAATCGCT CGACGTCACC TCCGCCAGCG   
  
  
+ GTTTTGCAGG CGGCGGCGGC GGCGACGCTG TGAGAAGGAT CGAGACGTAC GTGCTCCGGC CGAGGATTAC   
  
  
+ GGCGGCGGTG GAGGCGGCAT CTGCGGCGGC GGTAGCGCGG CGGAGCGGTT GGAGAGAGTC GTTCGCGGTG   
  
  
+ GCTAGGATGA GGGCGGTGGG GTTCAGCCAG TTTGCTGATT TCCAGGCGGA GTGTTTGCTG AGGAAGGTTC   
  
  
+ AGGTTGGCGG GTTCCACGTG GCGAAACGTC ATGGGGAGAT GATGCTTTGC TGGCATGATA GGCCCCTTGT   
  
  
+ GTCCACGTCG GCTTGGAGGT GTTA  

- -Up\_Stream \_Len000CTAAAA TGAAGGTTAA AAGATGAATT TAAACCAATA GTGCAAGCTT AAAAAGTCCA   
  
  
- TTTATGTTTG ACAATCACCG GTAGGAAAAA AAAACCCAGA TTAGGTAGGT CTACACGATT ATGAAAGACA   
  
  
- AAATCTACTC TTCAAAATTA CGAAAAAATC TACACATTAA AAAAATTTGT ATTTATCAGA ACAGCTTATT   
  
  
- CTAAACGATC ATTAAAAACA TTCTCTTTAA TACACATAAA AAATAATATA GTCTTATAAT CTAGCTAGAG   
  
  
- TTAAATTAAT GAATACTTTT TTTTTCACCT ATAGAAATTA GTTAAACCGT TCGAGAGTAC GTAATTAACA   
  
  
- TCAGCATGCT TGAATAAATG TAGTGAGTAG ACTTAAACGA TGGTAAATGT GGAGTTTAAA ATAAAATTAC   
  
  
- GGTATTTCTC TTATCTTATA TGACAGATAA ACATCATCAA AATGTCTATT TTTGTTTATT CGTTTACTTT   
  
  
- TATCTATCTC CTAAAAAAAC GATAATAGGT GCAGCTAGGT AACTTAATAA AACAGCGATA AACCAATTCA   
  
  
- CTTTATTTTA TGACTTCATC TTACCTCCTA CAACTATTAC TGGATACAGG GAGTTTCAAA TTAGTACATC   
  
  
- AAAGATGCTT AAAGTTCGGT AATATTCTGT ATGTCTAAAA ATACTACAAC TTTTATGAGA ATGGAGGAAC   
  
  
- AGTGTTATAA TTGGACTTCG GTTACTTTAC TTTGTCTTGA TTAATTTAAC TTATGTATTA GTTAGGTATA   
  
  
- ATTAAACAAA ACTGTTGATA AATGTTAGTA GCTAGAGAAA TTAATCCAGA TATTCGATAT CTCATTAATG   
  
  
- AGTTTTGACA AAACATTCTG TTGAATGAGG TATAACCGAA TAATCATATA ACGGAGGTAA GTGTACTTAT   
  
  
- GAAGTACTCT CTAACGGATT TTGAGTATAA CAGTAGTGGA ATTCAGTTTG TATAAAACAA TACTTTACTG   
  
  
- TAGTTCTTAA TGGGAGACTG TTCTTTTTAT ATGGACTGAC TGTATTAGCA TGGTGTAGAC AATCAGGAGA   
  
  
- AAGATGGTGA AACATCTTGT ATTACACTGT CATTATGTAG GTAGGGTTTT CTTTTCTGCT TTTCTTCTCA   
  
  
- CCTCTCAGTG CATATGCGCA ACTTAAGTCC TTTTAATTTC CTTGAGTACT TCTGGGGTAT ATCACGAGGT   
  
  
- TGGACTTGGA CGTCGGGTGA TGATGTACGG ATTTAATTTC TCGTTTCAAT ACTCTCTTTG GTTTTTTCGA   
  
  
- GAAACTCTCT CACCTCCTTC TTAGTACCTT TCGATCGATC CGAATCGTTC TTTCTCGTTG TCGATCGATC   
  
  
- GTTTTCCGAA GAGAAACTTC GGAATCTTTA GAAATCTCTA TTTTTCAACC ACCGAAAAAT TCCCGACACT   
  
  
- ATACAGGGGG TACATTTTTA CGTCGTTATT ACAATTACAG TACGAAAGAA AGTACCAGGG ATTACGCTTT   
  
  
- AGTTAGTACG TACAGTTGCA CTGAGTCACC GAGTGGTATA TACTAGACTA AAGGGGCGTG TGTCTCTATT   
  
  
- TTTCGGGGGT TAATATTGGA GGGTTTTCGG GGGGGAAAAG GAGGATGGGT TGGGTGTGGA GGGGTAAAGT   
  
  
- AGTCCCGAGC GAAGAATTCA ATAGCTAATT TTTTTCTCAC TCATTTGCAT GGGCTTAACT TCTCCCCACA   
  
  
- AGCTTCGAAT ATGAACGAAC TAATCCTTAC TTTCAGGGGT AAAGTAGGCG GGGGGTTTGG CGGCGGTGGT   
  
  
- GGTCATTTAG CGATAGAACG TTGTTATGAC GGAACTTATG ACGGAAAGTT CGGGGGTTAA GTTAAAGTTG   
  
  
- GGAACAATTG TTGTTGTTTG GGAGGATGCT CGGATGGAGC CAAGATCTGG AGGCCGCGTC GGGTTCGGGG   
  
  
- CGGTGGTTGC GGCGGGACGG TGACGGGTGA CTGCGCCCGT AGTTATTTGG GTTCGGACGG AGACGACTGG   
  
  
- ACCTGTTAAA CCTCCCACTC CCAACCCTAA GTTAGGATAG TCTCGACCCC GAATTACTAC TAAGATTATT   
  
  
- AGGATTGAGG TTTGAAACCA GTAGGGTTTA ATCGAGTAGT ACACTAAGGG TGGAGTGTGT CGAGGGACTC   
  
  
- AAGGGGAGGA GAGTCGGCAA CCTAGTGTGT GCGTGGGGGT TATTAATTGA AGAGGGGGAG ACTAATGGGG   
  
  
- TAGAGACTAA ATAGGATGCC AGTCTTAGAG TCGGGGTTAA AACCCGTCAA ACTCGAAGTA GAAGTAGTCG   
  
  
- GGTTGCAGTT ATTGTTACCC GAACTGAAGT ATCTCCTGGA TTAATCTCGT CGAGTTCGGA TGCTTAGGTT   
  
  
- GCTGAGGGTG GTCCAGGTCG ACCATAACCG CGCCGAGTTA GTTGCCGAGT CTAGTGGGCG GCCTTTTGGG   
  
  
- GAGGTGGCGC GGCGGAAGAT GAAGTTCCTC CGGGAGGTTG TGGAGCGACA GCCGCCTCCG GGCCGGGCCG   
  
  
- GACAGGCCGA CAGTAGGATG CTCCACCACG TCTGGTAGGC CCGGGTGTTC TTGAAAAGGC CGTATAGTGA   
  
  
- CTATAGGGAG AAGAGGTGGA AGCGGCGGTT AGTCCGCTAA GACCTCCGCC ACCTACCCCG ACGCAAGTAG   
  
  
- GTGTAGTAGC TAAAGCTGTA GCCTAAACCG CCGGTGACCC GCAGCAAATA CGCACTCGAA CAGCTGTTCC   
  
  
- GGCTGGGCTT ATTCTAGTCG AGGAACCACA AGGCTTAGTG CCGCTAGCAT GGGCTTCTCA AGCCCTAGCT   
  
  
- TTCGTTCGAC CACTCTCTCT TAGAATGCGT TAAGCGAGCA CTAGAGTTGT AGTTACAGGT ATACCTCATA   
  
  
- CAGGAATAAG TTTCGAAGCT CAAGAATAGG AAGTTTAGCC AGTTTAAGTA GCTGCCACTC TTTAACCGCC   
  
  
- AGGTAGATAG AGGCCGCTAC AAAGTGGCCG AAGTTCCGCC TTAGAGCTTT AAGGATCGGC TAGAGGCCCC   
  
  
- TTAAAGCGGT TCGTGGCAGC ACCACCACCT GTCACTCCAT CCTTAGCTGT GCCCTTGAAG CAGGAAGTCG   
  
  
- CAGTTGAAGC AGCGGCCATA GCTTAAGATG TGGCCTTACG AGCTTAGCGA GCTGCAGTGG AGGCGGTCGC   
  
  
- CAAAACGTCC GCCGCCGCCG CCGCTGCGAC ACTCTTCCTA GCTCTGCATG CACGAGGCCG GCTCCTAATG   
  
  
- CCGCCGCCAC CTCCGCCGTA GACGCCGCCG CCATCGCGCC GCCTCGCCAA CCTCTCTCAG CAAGCGCCAC   
  
  
- CGATCCTACT CCCGCCACCC CAAGTCGGTC AAACGACTAA AGGTCCGCCT CACAAACGAC TCCTTCCAAG   
  
  
- TCCAACCGCC CAAGGTGCAC CGCTTTGCAG TACCCCTCTA CTACGAAACG ACCGTACTAT CCGGGGAACA   
  
  
- CAGGTGCAGC CGAACCTCCA CAAT

  
  
Motifs Found  

+   

| Site Name | Organism | Position | Strand | Matrix score. | sequence | function |
| --- | --- | --- | --- | --- | --- | --- |
|  | organism | 3329 | - | 4 | motif\_sequence | short\_function |
|  | organism | 3173 | + | 4 | motif\_sequence | short\_function |
|  | organism | 1406 | + | 4 | motif\_sequence | short\_function |
|  | organism | 2455 | + | 4 | motif\_sequence | short\_function |
|  | organism | 2233 | + | 4 | motif\_sequence | short\_function |
|  | organism | 2972 | - | 4 | motif\_sequence | short\_function |
|  | organism | 2718 | - | 4 | motif\_sequence | short\_function |
|  | organism | 1125 | - | 4 | motif\_sequence | short\_function |
|  | organism | 2532 | + | 4 | motif\_sequence | short\_function |
|  | organism | 1118 | - | 4 | motif\_sequence | short\_function |
|  | organism | 3205 | - | 4 | motif\_sequence | short\_function |
|  | organism | 2882 | + | 4 | motif\_sequence | short\_function |
|  | organism | 2788 | + | 4 | motif\_sequence | short\_function |
|  | organism | 609 | + | 4 | motif\_sequence | short\_function |
|  | organism | 2935 | + | 4 | motif\_sequence | short\_function |
|  | organism | 1673 | - | 4 | motif\_sequence | short\_function |
|  | organism | 1954 | + | 4 | motif\_sequence | short\_function |
|  | organism | 2803 | + | 4 | motif\_sequence | short\_function |
|  | organism | 126 | + | 4 | motif\_sequence | short\_function |
|  | organism | 1070 | - | 4 | motif\_sequence | short\_function |

>HU06G00283.1   
+ -Up\_Stream \_Len000GATTTT ACTTCCAATT TTCTACTTAA ATTTGGTTAT CACGTTCGAA TTTTTCAGGT   
  
  
+ AAATACAAAC TGTTAGTGGC CATCCTTTTT TTTTGGGTCT AATCCATCCA GATGTGCTAA TACTTTCTGT   
  
  
+ TTTAGATGAG AAGTTTTAAT GCTTTTTTAG ATGTGTAATT TTTTTAAACA TAAATAGTCT TGTCGAATAA   
  
  
+ GATTTGCTAG TAATTTTTGT AAGAGAAATT ATGTGTATTT TTTATTATAT CAGAATATTA GATCGATCTC   
  
  
+ AATTTAATTA CTTATGAAAA AAAAAGTGGA TATCTTTAAT CAATTTGGCA AGCTCTCATG CATTAATTGT   
  
  
+ AGTCGTACGA ACTTATTTAC ATCACTCATC TGAATTTGCT ACCATTTACA CCTCAAATTT TATTTTAATG   
  
  
+ CCATAAAGAG AATAGAATAT ACTGTCTATT TGTAGTAGTT TTACAGATAA AAACAAATAA GCAAATGAAA   
  
  
+ ATAGATAGAG GATTTTTTTG CTATTATCCA CGTCGATCCA TTGAATTATT TTGTCGCTAT TTGGTTAAGT   
  
  
+ GAAATAAAAT ACTGAAGTAG AATGGAGGAT GTTGATAATG ACCTATGTCC CTCAAAGTTT AATCATGTAG   
  
  
+ TTTCTACGAA TTTCAAGCCA TTATAAGACA TACAGATTTT TATGATGTTG AAAATACTCT TACCTCCTTG   
  
  
+ TCACAATATT AACCTGAAGC CAATGAAATG AAACAGAACT AATTAAATTG AATACATAAT CAATCCATAT   
  
  
+ TAATTTGTTT TGACAACTAT TTACAATCAT CGATCTCTTT AATTAGGTCT ATAAGCTATA GAGTAATTAC   
  
  
+ TCAAAACTGT TTTGTAAGAC AACTTACTCC ATATTGGCTT ATTAGTATAT TGCCTCCATT CACATGAATA   
  
  
+ CTTCATGAGA GATTGCCTAA AACTCATATT GTCATCACCT TAAGTCAAAC ATATTTTGTT ATGAAATGAC   
  
  
+ ATCAAGAATT ACCCTCTGAC AAGAAAAATA TACCTGACTG ACATAATCGT ACCACATCTG TTAGTCCTCT   
  
  
+ TTCTACCACT TTGTAGAACA TAATGTGACA GTAATACATC CATCCCAAAA GAAAAGACGA AAAGAAGAGT   
  
  
+ GGAGAGTCAC GTATACGCGT TGAATTCAGG AAAATTAAAG GAACTCATGA AGACCCCATA TAGTGCTCCA   
  
  
+ ACCTGAACCT GCAGCCCACT ACTACATGCC TAAATTAAAG AGCAAAGTTA TGAGAGAAAC CAAAAAAGCT   
  
  
+ CTTTGAGAGA GTGGAGGAAG AATCATGGAA AGCTAGCTAG GCTTAGCAAG AAAGAGCAAC AGCTAGCTAG   
  
  
+ CAAAAGGCTT CTCTTTGAAG CCTTAGAAAT CTTTAGAGAT AAAAAGTTGG TGGCTTTTTA AGGGCTGTGA   
  
  
+ TATGTCCCCC ATGTAAAAAT GCAGCAATAA TGTTAATGTC ATGCTTTCTT TCATGGTCCC TAATGCGAAA   
  
  
+ TCAATCATGC ATGTCAACGT GACTCAGTGG CTCACCATAT ATGATCTGAT TTCCCCGCAC ACAGAGATAA   
  
  
+ AAAGCCCCCA ATTATAACCT CCCAAAAGCC CCCCCTTTTC CTCCTACCCA ACCCACACCT CCCCATTTCA   
  
  
+ TCAGGGCTCG CTTCTTAAGT TATCGATTAA AAAAAGAGTG AGTAAACGTA CCCGAATTGA AGAGGGGTGT   
  
  
+ TCGAAGCTTA TACTTGCTTG ATTAGGAATG AAAGTCCCCA TTTCATCCGC CCCCCAAACC GCCGCCACCA   
  
  
+ CCAGTAAATC GCTATCTTGC AACAATACTG CCTTGAATAC TGCCTTTCAA GCCCCCAATT CAATTTCAAC   
  
  
+ CCTTGTTAAC AACAACAAAC CCTCCTACGA GCCTACCTCG GTTCTAGACC TCCGGCGCAG CCCAAGCCCC   
  
  
+ GCCACCAACG CCGCCCTGCC ACTGCCCACT GACGCGGGCA TCAATAAACC CAAGCCTGCC TCTGCTGACC   
  
  
+ TGGACAATTT GGAGGGTGAG GGTTGGGATT CAATCCTATC AGAGCTGGGG CTTAATGATG ATTCTAATAA   
  
  
+ TCCTAACTCC AAACTTTGGT CATCCCAAAT TAGCTCATCA TGTGATTCCC ACCTCACACA GCTCCCTGAG   
  
  
+ TTCCCCTCCT CTCAGCCGTT GGATCACACA CGCACCCCCA ATAATTAACT TCTCCCCCTC TGATTACCCC   
  
  
+ ATCTCTGATT TATCCTACGG TCAGAATCTC AGCCCCAATT TTGGGCAGTT TGAGCTTCAT CTTCATCAGC   
  
  
+ CCAACGTCAA TAACAATGGG CTTGACTTCA TAGAGGACCT AATTAGAGCA GCTCAAGCCT ACGAATCCAA   
  
  
+ CGACTCCCAC CAGGTCCAGC TGGTATTGGC GCGGCTCAAT CAACGGCTCA GATCACCCGC CGGAAAACCC   
  
  
+ CTCCACCGCG CCGCCTTCTA CTTCAAGGAG GCCCTCCAAC ACCTCGCTGT CGGCGGAGGC CCGGCCCGGC   
  
  
+ CTGTCCGGCT GTCATCCTAC GAGGTGGTGC AGACCATCCG GGCCCACAAG AACTTTTCCG GCATATCACT   
  
  
+ GATATCCCTC TTCTCCACCT TCGCCGCCAA TCAGGCGATT CTGGAGGCGG TGGATGGGGC TGCGTTCATC   
  
  
+ CACATCATCG ATTTCGACAT CGGATTTGGC GGCCACTGGG CGTCGTTTAT GCGTGAGCTT GTCGACAAGG   
  
  
+ CCGACCCGAA TAAGATCAGC TCCTTGGTGT TCCGAATCAC GGCGATCGTA CCCGAAGAGT TCGGGATCGA   
  
  
+ AAGCAAGCTG GTGAGAGAGA ATCTTACGCA ATTCGCTCGT GATCTCAACA TCAATGTCCA TATGGAGTAT   
  
  
+ GTCCTTATTC AAAGCTTCGA GTTCTTATCC TTCAAATCGG TCAAATTCAT CGACGGTGAG AAATTGGCGG   
  
  
+ TCCATCTATC TCCGGCGATG TTTCACCGGC TTCAAGGCGG AATCTCGAAA TTCCTAGCCG ATCTCCGGGG   
  
  
+ AATTTCGCCA AGCACCGTCG TGGTGGTGGA CAGTGAGGTA GGAATCGACA CGGGAACTTC GTCCTTCAGC   
  
  
+ GTCAACTTCG TCGCCGGTAT CGAATTCTAC ACCGGAATGC TCGAATCGCT CGACGTCACC TCCGCCAGCG   
  
  
+ GTTTTGCAGG CGGCGGCGGC GGCGACGCTG TGAGAAGGAT CGAGACGTAC GTGCTCCGGC CGAGGATTAC   
  
  
+ GGCGGCGGTG GAGGCGGCAT CTGCGGCGGC GGTAGCGCGG CGGAGCGGTT GGAGAGAGTC GTTCGCGGTG   
  
  
+ GCTAGGATGA GGGCGGTGGG GTTCAGCCAG TTTGCTGATT TCCAGGCGGA GTGTTTGCTG AGGAAGGTTC   
  
  
+ AGGTTGGCGG GTTCCACGTG GCGAAACGTC ATGGGGAGAT GATGCTTTGC TGGCATGATA GGCCCCTTGT   
  
  
+ GTCCACGTCG GCTTGGAGGT GTTA  

- -Up\_Stream \_Len000CTAAAA TGAAGGTTAA AAGATGAATT TAAACCAATA GTGCAAGCTT AAAAAGTCCA   
  
  
- TTTATGTTTG ACAATCACCG GTAGGAAAAA AAAACCCAGA TTAGGTAGGT CTACACGATT ATGAAAGACA   
  
  
- AAATCTACTC TTCAAAATTA CGAAAAAATC TACACATTAA AAAAATTTGT ATTTATCAGA ACAGCTTATT   
  
  
- CTAAACGATC ATTAAAAACA TTCTCTTTAA TACACATAAA AAATAATATA GTCTTATAAT CTAGCTAGAG   
  
  
- TTAAATTAAT GAATACTTTT TTTTTCACCT ATAGAAATTA GTTAAACCGT TCGAGAGTAC GTAATTAACA   
  
  
- TCAGCATGCT TGAATAAATG TAGTGAGTAG ACTTAAACGA TGGTAAATGT GGAGTTTAAA ATAAAATTAC   
  
  
- GGTATTTCTC TTATCTTATA TGACAGATAA ACATCATCAA AATGTCTATT TTTGTTTATT CGTTTACTTT   
  
  
- TATCTATCTC CTAAAAAAAC GATAATAGGT GCAGCTAGGT AACTTAATAA AACAGCGATA AACCAATTCA   
  
  
- CTTTATTTTA TGACTTCATC TTACCTCCTA CAACTATTAC TGGATACAGG GAGTTTCAAA TTAGTACATC   
  
  
- AAAGATGCTT AAAGTTCGGT AATATTCTGT ATGTCTAAAA ATACTACAAC TTTTATGAGA ATGGAGGAAC   
  
  
- AGTGTTATAA TTGGACTTCG GTTACTTTAC TTTGTCTTGA TTAATTTAAC TTATGTATTA GTTAGGTATA   
  
  
- ATTAAACAAA ACTGTTGATA AATGTTAGTA GCTAGAGAAA TTAATCCAGA TATTCGATAT CTCATTAATG   
  
  
- AGTTTTGACA AAACATTCTG TTGAATGAGG TATAACCGAA TAATCATATA ACGGAGGTAA GTGTACTTAT   
  
  
- GAAGTACTCT CTAACGGATT TTGAGTATAA CAGTAGTGGA ATTCAGTTTG TATAAAACAA TACTTTACTG   
  
  
- TAGTTCTTAA TGGGAGACTG TTCTTTTTAT ATGGACTGAC TGTATTAGCA TGGTGTAGAC AATCAGGAGA   
  
  
- AAGATGGTGA AACATCTTGT ATTACACTGT CATTATGTAG GTAGGGTTTT CTTTTCTGCT TTTCTTCTCA   
  
  
- CCTCTCAGTG CATATGCGCA ACTTAAGTCC TTTTAATTTC CTTGAGTACT TCTGGGGTAT ATCACGAGGT   
  
  
- TGGACTTGGA CGTCGGGTGA TGATGTACGG ATTTAATTTC TCGTTTCAAT ACTCTCTTTG GTTTTTTCGA   
  
  
- GAAACTCTCT CACCTCCTTC TTAGTACCTT TCGATCGATC CGAATCGTTC TTTCTCGTTG TCGATCGATC   
  
  
- GTTTTCCGAA GAGAAACTTC GGAATCTTTA GAAATCTCTA TTTTTCAACC ACCGAAAAAT TCCCGACACT   
  
  
- ATACAGGGGG TACATTTTTA CGTCGTTATT ACAATTACAG TACGAAAGAA AGTACCAGGG ATTACGCTTT   
  
  
- AGTTAGTACG TACAGTTGCA CTGAGTCACC GAGTGGTATA TACTAGACTA AAGGGGCGTG TGTCTCTATT   
  
  
- TTTCGGGGGT TAATATTGGA GGGTTTTCGG GGGGGAAAAG GAGGATGGGT TGGGTGTGGA GGGGTAAAGT   
  
  
- AGTCCCGAGC GAAGAATTCA ATAGCTAATT TTTTTCTCAC TCATTTGCAT GGGCTTAACT TCTCCCCACA   
  
  
- AGCTTCGAAT ATGAACGAAC TAATCCTTAC TTTCAGGGGT AAAGTAGGCG GGGGGTTTGG CGGCGGTGGT   
  
  
- GGTCATTTAG CGATAGAACG TTGTTATGAC GGAACTTATG ACGGAAAGTT CGGGGGTTAA GTTAAAGTTG   
  
  
- GGAACAATTG TTGTTGTTTG GGAGGATGCT CGGATGGAGC CAAGATCTGG AGGCCGCGTC GGGTTCGGGG   
  
  
- CGGTGGTTGC GGCGGGACGG TGACGGGTGA CTGCGCCCGT AGTTATTTGG GTTCGGACGG AGACGACTGG   
  
  
- ACCTGTTAAA CCTCCCACTC CCAACCCTAA GTTAGGATAG TCTCGACCCC GAATTACTAC TAAGATTATT   
  
  
- AGGATTGAGG TTTGAAACCA GTAGGGTTTA ATCGAGTAGT ACACTAAGGG TGGAGTGTGT CGAGGGACTC   
  
  
- AAGGGGAGGA GAGTCGGCAA CCTAGTGTGT GCGTGGGGGT TATTAATTGA AGAGGGGGAG ACTAATGGGG   
  
  
- TAGAGACTAA ATAGGATGCC AGTCTTAGAG TCGGGGTTAA AACCCGTCAA ACTCGAAGTA GAAGTAGTCG   
  
  
- GGTTGCAGTT ATTGTTACCC GAACTGAAGT ATCTCCTGGA TTAATCTCGT CGAGTTCGGA TGCTTAGGTT   
  
  
- GCTGAGGGTG GTCCAGGTCG ACCATAACCG CGCCGAGTTA GTTGCCGAGT CTAGTGGGCG GCCTTTTGGG   
  
  
- GAGGTGGCGC GGCGGAAGAT GAAGTTCCTC CGGGAGGTTG TGGAGCGACA GCCGCCTCCG GGCCGGGCCG   
  
  
- GACAGGCCGA CAGTAGGATG CTCCACCACG TCTGGTAGGC CCGGGTGTTC TTGAAAAGGC CGTATAGTGA   
  
  
- CTATAGGGAG AAGAGGTGGA AGCGGCGGTT AGTCCGCTAA GACCTCCGCC ACCTACCCCG ACGCAAGTAG   
  
  
- GTGTAGTAGC TAAAGCTGTA GCCTAAACCG CCGGTGACCC GCAGCAAATA CGCACTCGAA CAGCTGTTCC   
  
  
- GGCTGGGCTT ATTCTAGTCG AGGAACCACA AGGCTTAGTG CCGCTAGCAT GGGCTTCTCA AGCCCTAGCT   
  
  
- TTCGTTCGAC CACTCTCTCT TAGAATGCGT TAAGCGAGCA CTAGAGTTGT AGTTACAGGT ATACCTCATA   
  
  
- CAGGAATAAG TTTCGAAGCT CAAGAATAGG AAGTTTAGCC AGTTTAAGTA GCTGCCACTC TTTAACCGCC   
  
  
- AGGTAGATAG AGGCCGCTAC AAAGTGGCCG AAGTTCCGCC TTAGAGCTTT AAGGATCGGC TAGAGGCCCC   
  
  
- TTAAAGCGGT TCGTGGCAGC ACCACCACCT GTCACTCCAT CCTTAGCTGT GCCCTTGAAG CAGGAAGTCG   
  
  
- CAGTTGAAGC AGCGGCCATA GCTTAAGATG TGGCCTTACG AGCTTAGCGA GCTGCAGTGG AGGCGGTCGC   
  
  
- CAAAACGTCC GCCGCCGCCG CCGCTGCGAC ACTCTTCCTA GCTCTGCATG CACGAGGCCG GCTCCTAATG   
  
  
- CCGCCGCCAC CTCCGCCGTA GACGCCGCCG CCATCGCGCC GCCTCGCCAA CCTCTCTCAG CAAGCGCCAC   
  
  
- CGATCCTACT CCCGCCACCC CAAGTCGGTC AAACGACTAA AGGTCCGCCT CACAAACGAC TCCTTCCAAG   
  
  
- TCCAACCGCC CAAGGTGCAC CGCTTTGCAG TACCCCTCTA CTACGAAACG ACCGTACTAT CCGGGGAACA   
  
  
- CAGGTGCAGC CGAACCTCCA CAAT

+     AAGAA-motif

| Site Name | Organism | Position | Strand | Matrix score. | sequence | function |
| --- | --- | --- | --- | --- | --- | --- |
| AAGAA-motif | Avena sativa | 1450 | - | 7 | GAAAGAA |  |

>HU06G00283.1   
+ -Up\_Stream \_Len000GATTTT ACTTCCAATT TTCTACTTAA ATTTGGTTAT CACGTTCGAA TTTTTCAGGT   
  
  
+ AAATACAAAC TGTTAGTGGC CATCCTTTTT TTTTGGGTCT AATCCATCCA GATGTGCTAA TACTTTCTGT   
  
  
+ TTTAGATGAG AAGTTTTAAT GCTTTTTTAG ATGTGTAATT TTTTTAAACA TAAATAGTCT TGTCGAATAA   
  
  
+ GATTTGCTAG TAATTTTTGT AAGAGAAATT ATGTGTATTT TTTATTATAT CAGAATATTA GATCGATCTC   
  
  
+ AATTTAATTA CTTATGAAAA AAAAAGTGGA TATCTTTAAT CAATTTGGCA AGCTCTCATG CATTAATTGT   
  
  
+ AGTCGTACGA ACTTATTTAC ATCACTCATC TGAATTTGCT ACCATTTACA CCTCAAATTT TATTTTAATG   
  
  
+ CCATAAAGAG AATAGAATAT ACTGTCTATT TGTAGTAGTT TTACAGATAA AAACAAATAA GCAAATGAAA   
  
  
+ ATAGATAGAG GATTTTTTTG CTATTATCCA CGTCGATCCA TTGAATTATT TTGTCGCTAT TTGGTTAAGT   
  
  
+ GAAATAAAAT ACTGAAGTAG AATGGAGGAT GTTGATAATG ACCTATGTCC CTCAAAGTTT AATCATGTAG   
  
  
+ TTTCTACGAA TTTCAAGCCA TTATAAGACA TACAGATTTT TATGATGTTG AAAATACTCT TACCTCCTTG   
  
  
+ TCACAATATT AACCTGAAGC CAATGAAATG AAACAGAACT AATTAAATTG AATACATAAT CAATCCATAT   
  
  
+ TAATTTGTTT TGACAACTAT TTACAATCAT CGATCTCTTT AATTAGGTCT ATAAGCTATA GAGTAATTAC   
  
  
+ TCAAAACTGT TTTGTAAGAC AACTTACTCC ATATTGGCTT ATTAGTATAT TGCCTCCATT CACATGAATA   
  
  
+ CTTCATGAGA GATTGCCTAA AACTCATATT GTCATCACCT TAAGTCAAAC ATATTTTGTT ATGAAATGAC   
  
  
+ ATCAAGAATT ACCCTCTGAC AAGAAAAATA TACCTGACTG ACATAATCGT ACCACATCTG TTAGTCCTCT   
  
  
+ TTCTACCACT TTGTAGAACA TAATGTGACA GTAATACATC CATCCCAAAA GAAAAGACGA AAAGAAGAGT   
  
  
+ GGAGAGTCAC GTATACGCGT TGAATTCAGG AAAATTAAAG GAACTCATGA AGACCCCATA TAGTGCTCCA   
  
  
+ ACCTGAACCT GCAGCCCACT ACTACATGCC TAAATTAAAG AGCAAAGTTA TGAGAGAAAC CAAAAAAGCT   
  
  
+ CTTTGAGAGA GTGGAGGAAG AATCATGGAA AGCTAGCTAG GCTTAGCAAG AAAGAGCAAC AGCTAGCTAG   
  
  
+ CAAAAGGCTT CTCTTTGAAG CCTTAGAAAT CTTTAGAGAT AAAAAGTTGG TGGCTTTTTA AGGGCTGTGA   
  
  
+ TATGTCCCCC ATGTAAAAAT GCAGCAATAA TGTTAATGTC ATGCTTTCTT TCATGGTCCC TAATGCGAAA   
  
  
+ TCAATCATGC ATGTCAACGT GACTCAGTGG CTCACCATAT ATGATCTGAT TTCCCCGCAC ACAGAGATAA   
  
  
+ AAAGCCCCCA ATTATAACCT CCCAAAAGCC CCCCCTTTTC CTCCTACCCA ACCCACACCT CCCCATTTCA   
  
  
+ TCAGGGCTCG CTTCTTAAGT TATCGATTAA AAAAAGAGTG AGTAAACGTA CCCGAATTGA AGAGGGGTGT   
  
  
+ TCGAAGCTTA TACTTGCTTG ATTAGGAATG AAAGTCCCCA TTTCATCCGC CCCCCAAACC GCCGCCACCA   
  
  
+ CCAGTAAATC GCTATCTTGC AACAATACTG CCTTGAATAC TGCCTTTCAA GCCCCCAATT CAATTTCAAC   
  
  
+ CCTTGTTAAC AACAACAAAC CCTCCTACGA GCCTACCTCG GTTCTAGACC TCCGGCGCAG CCCAAGCCCC   
  
  
+ GCCACCAACG CCGCCCTGCC ACTGCCCACT GACGCGGGCA TCAATAAACC CAAGCCTGCC TCTGCTGACC   
  
  
+ TGGACAATTT GGAGGGTGAG GGTTGGGATT CAATCCTATC AGAGCTGGGG CTTAATGATG ATTCTAATAA   
  
  
+ TCCTAACTCC AAACTTTGGT CATCCCAAAT TAGCTCATCA TGTGATTCCC ACCTCACACA GCTCCCTGAG   
  
  
+ TTCCCCTCCT CTCAGCCGTT GGATCACACA CGCACCCCCA ATAATTAACT TCTCCCCCTC TGATTACCCC   
  
  
+ ATCTCTGATT TATCCTACGG TCAGAATCTC AGCCCCAATT TTGGGCAGTT TGAGCTTCAT CTTCATCAGC   
  
  
+ CCAACGTCAA TAACAATGGG CTTGACTTCA TAGAGGACCT AATTAGAGCA GCTCAAGCCT ACGAATCCAA   
  
  
+ CGACTCCCAC CAGGTCCAGC TGGTATTGGC GCGGCTCAAT CAACGGCTCA GATCACCCGC CGGAAAACCC   
  
  
+ CTCCACCGCG CCGCCTTCTA CTTCAAGGAG GCCCTCCAAC ACCTCGCTGT CGGCGGAGGC CCGGCCCGGC   
  
  
+ CTGTCCGGCT GTCATCCTAC GAGGTGGTGC AGACCATCCG GGCCCACAAG AACTTTTCCG GCATATCACT   
  
  
+ GATATCCCTC TTCTCCACCT TCGCCGCCAA TCAGGCGATT CTGGAGGCGG TGGATGGGGC TGCGTTCATC   
  
  
+ CACATCATCG ATTTCGACAT CGGATTTGGC GGCCACTGGG CGTCGTTTAT GCGTGAGCTT GTCGACAAGG   
  
  
+ CCGACCCGAA TAAGATCAGC TCCTTGGTGT TCCGAATCAC GGCGATCGTA CCCGAAGAGT TCGGGATCGA   
  
  
+ AAGCAAGCTG GTGAGAGAGA ATCTTACGCA ATTCGCTCGT GATCTCAACA TCAATGTCCA TATGGAGTAT   
  
  
+ GTCCTTATTC AAAGCTTCGA GTTCTTATCC TTCAAATCGG TCAAATTCAT CGACGGTGAG AAATTGGCGG   
  
  
+ TCCATCTATC TCCGGCGATG TTTCACCGGC TTCAAGGCGG AATCTCGAAA TTCCTAGCCG ATCTCCGGGG   
  
  
+ AATTTCGCCA AGCACCGTCG TGGTGGTGGA CAGTGAGGTA GGAATCGACA CGGGAACTTC GTCCTTCAGC   
  
  
+ GTCAACTTCG TCGCCGGTAT CGAATTCTAC ACCGGAATGC TCGAATCGCT CGACGTCACC TCCGCCAGCG   
  
  
+ GTTTTGCAGG CGGCGGCGGC GGCGACGCTG TGAGAAGGAT CGAGACGTAC GTGCTCCGGC CGAGGATTAC   
  
  
+ GGCGGCGGTG GAGGCGGCAT CTGCGGCGGC GGTAGCGCGG CGGAGCGGTT GGAGAGAGTC GTTCGCGGTG   
  
  
+ GCTAGGATGA GGGCGGTGGG GTTCAGCCAG TTTGCTGATT TCCAGGCGGA GTGTTTGCTG AGGAAGGTTC   
  
  
+ AGGTTGGCGG GTTCCACGTG GCGAAACGTC ATGGGGAGAT GATGCTTTGC TGGCATGATA GGCCCCTTGT   
  
  
+ GTCCACGTCG GCTTGGAGGT GTTA  

- -Up\_Stream \_Len000CTAAAA TGAAGGTTAA AAGATGAATT TAAACCAATA GTGCAAGCTT AAAAAGTCCA   
  
  
- TTTATGTTTG ACAATCACCG GTAGGAAAAA AAAACCCAGA TTAGGTAGGT CTACACGATT ATGAAAGACA   
  
  
- AAATCTACTC TTCAAAATTA CGAAAAAATC TACACATTAA AAAAATTTGT ATTTATCAGA ACAGCTTATT   
  
  
- CTAAACGATC ATTAAAAACA TTCTCTTTAA TACACATAAA AAATAATATA GTCTTATAAT CTAGCTAGAG   
  
  
- TTAAATTAAT GAATACTTTT TTTTTCACCT ATAGAAATTA GTTAAACCGT TCGAGAGTAC GTAATTAACA   
  
  
- TCAGCATGCT TGAATAAATG TAGTGAGTAG ACTTAAACGA TGGTAAATGT GGAGTTTAAA ATAAAATTAC   
  
  
- GGTATTTCTC TTATCTTATA TGACAGATAA ACATCATCAA AATGTCTATT TTTGTTTATT CGTTTACTTT   
  
  
- TATCTATCTC CTAAAAAAAC GATAATAGGT GCAGCTAGGT AACTTAATAA AACAGCGATA AACCAATTCA   
  
  
- CTTTATTTTA TGACTTCATC TTACCTCCTA CAACTATTAC TGGATACAGG GAGTTTCAAA TTAGTACATC   
  
  
- AAAGATGCTT AAAGTTCGGT AATATTCTGT ATGTCTAAAA ATACTACAAC TTTTATGAGA ATGGAGGAAC   
  
  
- AGTGTTATAA TTGGACTTCG GTTACTTTAC TTTGTCTTGA TTAATTTAAC TTATGTATTA GTTAGGTATA   
  
  
- ATTAAACAAA ACTGTTGATA AATGTTAGTA GCTAGAGAAA TTAATCCAGA TATTCGATAT CTCATTAATG   
  
  
- AGTTTTGACA AAACATTCTG TTGAATGAGG TATAACCGAA TAATCATATA ACGGAGGTAA GTGTACTTAT   
  
  
- GAAGTACTCT CTAACGGATT TTGAGTATAA CAGTAGTGGA ATTCAGTTTG TATAAAACAA TACTTTACTG   
  
  
- TAGTTCTTAA TGGGAGACTG TTCTTTTTAT ATGGACTGAC TGTATTAGCA TGGTGTAGAC AATCAGGAGA   
  
  
- AAGATGGTGA AACATCTTGT ATTACACTGT CATTATGTAG GTAGGGTTTT CTTTTCTGCT TTTCTTCTCA   
  
  
- CCTCTCAGTG CATATGCGCA ACTTAAGTCC TTTTAATTTC CTTGAGTACT TCTGGGGTAT ATCACGAGGT   
  
  
- TGGACTTGGA CGTCGGGTGA TGATGTACGG ATTTAATTTC TCGTTTCAAT ACTCTCTTTG GTTTTTTCGA   
  
  
- GAAACTCTCT CACCTCCTTC TTAGTACCTT TCGATCGATC CGAATCGTTC TTTCTCGTTG TCGATCGATC   
  
  
- GTTTTCCGAA GAGAAACTTC GGAATCTTTA GAAATCTCTA TTTTTCAACC ACCGAAAAAT TCCCGACACT   
  
  
- ATACAGGGGG TACATTTTTA CGTCGTTATT ACAATTACAG TACGAAAGAA AGTACCAGGG ATTACGCTTT   
  
  
- AGTTAGTACG TACAGTTGCA CTGAGTCACC GAGTGGTATA TACTAGACTA AAGGGGCGTG TGTCTCTATT   
  
  
- TTTCGGGGGT TAATATTGGA GGGTTTTCGG GGGGGAAAAG GAGGATGGGT TGGGTGTGGA GGGGTAAAGT   
  
  
- AGTCCCGAGC GAAGAATTCA ATAGCTAATT TTTTTCTCAC TCATTTGCAT GGGCTTAACT TCTCCCCACA   
  
  
- AGCTTCGAAT ATGAACGAAC TAATCCTTAC TTTCAGGGGT AAAGTAGGCG GGGGGTTTGG CGGCGGTGGT   
  
  
- GGTCATTTAG CGATAGAACG TTGTTATGAC GGAACTTATG ACGGAAAGTT CGGGGGTTAA GTTAAAGTTG   
  
  
- GGAACAATTG TTGTTGTTTG GGAGGATGCT CGGATGGAGC CAAGATCTGG AGGCCGCGTC GGGTTCGGGG   
  
  
- CGGTGGTTGC GGCGGGACGG TGACGGGTGA CTGCGCCCGT AGTTATTTGG GTTCGGACGG AGACGACTGG   
  
  
- ACCTGTTAAA CCTCCCACTC CCAACCCTAA GTTAGGATAG TCTCGACCCC GAATTACTAC TAAGATTATT   
  
  
- AGGATTGAGG TTTGAAACCA GTAGGGTTTA ATCGAGTAGT ACACTAAGGG TGGAGTGTGT CGAGGGACTC   
  
  
- AAGGGGAGGA GAGTCGGCAA CCTAGTGTGT GCGTGGGGGT TATTAATTGA AGAGGGGGAG ACTAATGGGG   
  
  
- TAGAGACTAA ATAGGATGCC AGTCTTAGAG TCGGGGTTAA AACCCGTCAA ACTCGAAGTA GAAGTAGTCG   
  
  
- GGTTGCAGTT ATTGTTACCC GAACTGAAGT ATCTCCTGGA TTAATCTCGT CGAGTTCGGA TGCTTAGGTT   
  
  
- GCTGAGGGTG GTCCAGGTCG ACCATAACCG CGCCGAGTTA GTTGCCGAGT CTAGTGGGCG GCCTTTTGGG   
  
  
- GAGGTGGCGC GGCGGAAGAT GAAGTTCCTC CGGGAGGTTG TGGAGCGACA GCCGCCTCCG GGCCGGGCCG   
  
  
- GACAGGCCGA CAGTAGGATG CTCCACCACG TCTGGTAGGC CCGGGTGTTC TTGAAAAGGC CGTATAGTGA   
  
  
- CTATAGGGAG AAGAGGTGGA AGCGGCGGTT AGTCCGCTAA GACCTCCGCC ACCTACCCCG ACGCAAGTAG   
  
  
- GTGTAGTAGC TAAAGCTGTA GCCTAAACCG CCGGTGACCC GCAGCAAATA CGCACTCGAA CAGCTGTTCC   
  
  
- GGCTGGGCTT ATTCTAGTCG AGGAACCACA AGGCTTAGTG CCGCTAGCAT GGGCTTCTCA AGCCCTAGCT   
  
  
- TTCGTTCGAC CACTCTCTCT TAGAATGCGT TAAGCGAGCA CTAGAGTTGT AGTTACAGGT ATACCTCATA   
  
  
- CAGGAATAAG TTTCGAAGCT CAAGAATAGG AAGTTTAGCC AGTTTAAGTA GCTGCCACTC TTTAACCGCC   
  
  
- AGGTAGATAG AGGCCGCTAC AAAGTGGCCG AAGTTCCGCC TTAGAGCTTT AAGGATCGGC TAGAGGCCCC   
  
  
- TTAAAGCGGT TCGTGGCAGC ACCACCACCT GTCACTCCAT CCTTAGCTGT GCCCTTGAAG CAGGAAGTCG   
  
  
- CAGTTGAAGC AGCGGCCATA GCTTAAGATG TGGCCTTACG AGCTTAGCGA GCTGCAGTGG AGGCGGTCGC   
  
  
- CAAAACGTCC GCCGCCGCCG CCGCTGCGAC ACTCTTCCTA GCTCTGCATG CACGAGGCCG GCTCCTAATG   
  
  
- CCGCCGCCAC CTCCGCCGTA GACGCCGCCG CCATCGCGCC GCCTCGCCAA CCTCTCTCAG CAAGCGCCAC   
  
  
- CGATCCTACT CCCGCCACCC CAAGTCGGTC AAACGACTAA AGGTCCGCCT CACAAACGAC TCCTTCCAAG   
  
  
- TCCAACCGCC CAAGGTGCAC CGCTTTGCAG TACCCCTCTA CTACGAAACG ACCGTACTAT CCGGGGAACA   
  
  
- CAGGTGCAGC CGAACCTCCA CAAT

+     ABRE

| Site Name | Organism | Position | Strand | Matrix score. | sequence | function |
| --- | --- | --- | --- | --- | --- | --- |
| ABRE | Arabidopsis thaliana | 3368 | - | 5 | ACGTG | cis-acting element involved in the abscisic acid responsiveness |
| ABRE | Arabidopsis thaliana | 3310 | + | 5 | ACGTG | cis-acting element involved in the abscisic acid responsiveness |
| ABRE | Oryza sativa | 3126 | + | 10 | GACACGTACGT | cis-acting element involved in the abscisic acid responsiveness |
| ABRE | Arabidopsis thaliana | 2190 | + | 7 | TACGGTC | cis-acting element involved in the abscisic acid responsiveness |
| ABRE | Arabidopsis thaliana | 3309 | - | 6 | CACGTG | cis-acting element involved in the abscisic acid responsiveness |
| ABRE | Arabidopsis thaliana | 55 | - | 5 | ACGTG | cis-acting element involved in the abscisic acid responsiveness |
| ABRE | Hordeum vulgare | 3130 | + | 9 | CGTACGTGCA | cis-acting element involved in the abscisic acid responsiveness |
| ABRE | Arabidopsis thaliana | 1491 | + | 5 | ACGTG | cis-acting element involved in the abscisic acid responsiveness |
| ABRE | Arabidopsis thaliana | 3133 | + | 5 | ACGTG | cis-acting element involved in the abscisic acid responsiveness |
| ABRE | Arabidopsis thaliana | 523 | - | 5 | ACGTG | cis-acting element involved in the abscisic acid responsiveness |
| ABRE | Arabidopsis thaliana | 1132 | - | 5 | ACGTG | cis-acting element involved in the abscisic acid responsiveness |

>HU06G00283.1   
+ -Up\_Stream \_Len000GATTTT ACTTCCAATT TTCTACTTAA ATTTGGTTAT CACGTTCGAA TTTTTCAGGT   
  
  
+ AAATACAAAC TGTTAGTGGC CATCCTTTTT TTTTGGGTCT AATCCATCCA GATGTGCTAA TACTTTCTGT   
  
  
+ TTTAGATGAG AAGTTTTAAT GCTTTTTTAG ATGTGTAATT TTTTTAAACA TAAATAGTCT TGTCGAATAA   
  
  
+ GATTTGCTAG TAATTTTTGT AAGAGAAATT ATGTGTATTT TTTATTATAT CAGAATATTA GATCGATCTC   
  
  
+ AATTTAATTA CTTATGAAAA AAAAAGTGGA TATCTTTAAT CAATTTGGCA AGCTCTCATG CATTAATTGT   
  
  
+ AGTCGTACGA ACTTATTTAC ATCACTCATC TGAATTTGCT ACCATTTACA CCTCAAATTT TATTTTAATG   
  
  
+ CCATAAAGAG AATAGAATAT ACTGTCTATT TGTAGTAGTT TTACAGATAA AAACAAATAA GCAAATGAAA   
  
  
+ ATAGATAGAG GATTTTTTTG CTATTATCCA CGTCGATCCA TTGAATTATT TTGTCGCTAT TTGGTTAAGT   
  
  
+ GAAATAAAAT ACTGAAGTAG AATGGAGGAT GTTGATAATG ACCTATGTCC CTCAAAGTTT AATCATGTAG   
  
  
+ TTTCTACGAA TTTCAAGCCA TTATAAGACA TACAGATTTT TATGATGTTG AAAATACTCT TACCTCCTTG   
  
  
+ TCACAATATT AACCTGAAGC CAATGAAATG AAACAGAACT AATTAAATTG AATACATAAT CAATCCATAT   
  
  
+ TAATTTGTTT TGACAACTAT TTACAATCAT CGATCTCTTT AATTAGGTCT ATAAGCTATA GAGTAATTAC   
  
  
+ TCAAAACTGT TTTGTAAGAC AACTTACTCC ATATTGGCTT ATTAGTATAT TGCCTCCATT CACATGAATA   
  
  
+ CTTCATGAGA GATTGCCTAA AACTCATATT GTCATCACCT TAAGTCAAAC ATATTTTGTT ATGAAATGAC   
  
  
+ ATCAAGAATT ACCCTCTGAC AAGAAAAATA TACCTGACTG ACATAATCGT ACCACATCTG TTAGTCCTCT   
  
  
+ TTCTACCACT TTGTAGAACA TAATGTGACA GTAATACATC CATCCCAAAA GAAAAGACGA AAAGAAGAGT   
  
  
+ GGAGAGTCAC GTATACGCGT TGAATTCAGG AAAATTAAAG GAACTCATGA AGACCCCATA TAGTGCTCCA   
  
  
+ ACCTGAACCT GCAGCCCACT ACTACATGCC TAAATTAAAG AGCAAAGTTA TGAGAGAAAC CAAAAAAGCT   
  
  
+ CTTTGAGAGA GTGGAGGAAG AATCATGGAA AGCTAGCTAG GCTTAGCAAG AAAGAGCAAC AGCTAGCTAG   
  
  
+ CAAAAGGCTT CTCTTTGAAG CCTTAGAAAT CTTTAGAGAT AAAAAGTTGG TGGCTTTTTA AGGGCTGTGA   
  
  
+ TATGTCCCCC ATGTAAAAAT GCAGCAATAA TGTTAATGTC ATGCTTTCTT TCATGGTCCC TAATGCGAAA   
  
  
+ TCAATCATGC ATGTCAACGT GACTCAGTGG CTCACCATAT ATGATCTGAT TTCCCCGCAC ACAGAGATAA   
  
  
+ AAAGCCCCCA ATTATAACCT CCCAAAAGCC CCCCCTTTTC CTCCTACCCA ACCCACACCT CCCCATTTCA   
  
  
+ TCAGGGCTCG CTTCTTAAGT TATCGATTAA AAAAAGAGTG AGTAAACGTA CCCGAATTGA AGAGGGGTGT   
  
  
+ TCGAAGCTTA TACTTGCTTG ATTAGGAATG AAAGTCCCCA TTTCATCCGC CCCCCAAACC GCCGCCACCA   
  
  
+ CCAGTAAATC GCTATCTTGC AACAATACTG CCTTGAATAC TGCCTTTCAA GCCCCCAATT CAATTTCAAC   
  
  
+ CCTTGTTAAC AACAACAAAC CCTCCTACGA GCCTACCTCG GTTCTAGACC TCCGGCGCAG CCCAAGCCCC   
  
  
+ GCCACCAACG CCGCCCTGCC ACTGCCCACT GACGCGGGCA TCAATAAACC CAAGCCTGCC TCTGCTGACC   
  
  
+ TGGACAATTT GGAGGGTGAG GGTTGGGATT CAATCCTATC AGAGCTGGGG CTTAATGATG ATTCTAATAA   
  
  
+ TCCTAACTCC AAACTTTGGT CATCCCAAAT TAGCTCATCA TGTGATTCCC ACCTCACACA GCTCCCTGAG   
  
  
+ TTCCCCTCCT CTCAGCCGTT GGATCACACA CGCACCCCCA ATAATTAACT TCTCCCCCTC TGATTACCCC   
  
  
+ ATCTCTGATT TATCCTACGG TCAGAATCTC AGCCCCAATT TTGGGCAGTT TGAGCTTCAT CTTCATCAGC   
  
  
+ CCAACGTCAA TAACAATGGG CTTGACTTCA TAGAGGACCT AATTAGAGCA GCTCAAGCCT ACGAATCCAA   
  
  
+ CGACTCCCAC CAGGTCCAGC TGGTATTGGC GCGGCTCAAT CAACGGCTCA GATCACCCGC CGGAAAACCC   
  
  
+ CTCCACCGCG CCGCCTTCTA CTTCAAGGAG GCCCTCCAAC ACCTCGCTGT CGGCGGAGGC CCGGCCCGGC   
  
  
+ CTGTCCGGCT GTCATCCTAC GAGGTGGTGC AGACCATCCG GGCCCACAAG AACTTTTCCG GCATATCACT   
  
  
+ GATATCCCTC TTCTCCACCT TCGCCGCCAA TCAGGCGATT CTGGAGGCGG TGGATGGGGC TGCGTTCATC   
  
  
+ CACATCATCG ATTTCGACAT CGGATTTGGC GGCCACTGGG CGTCGTTTAT GCGTGAGCTT GTCGACAAGG   
  
  
+ CCGACCCGAA TAAGATCAGC TCCTTGGTGT TCCGAATCAC GGCGATCGTA CCCGAAGAGT TCGGGATCGA   
  
  
+ AAGCAAGCTG GTGAGAGAGA ATCTTACGCA ATTCGCTCGT GATCTCAACA TCAATGTCCA TATGGAGTAT   
  
  
+ GTCCTTATTC AAAGCTTCGA GTTCTTATCC TTCAAATCGG TCAAATTCAT CGACGGTGAG AAATTGGCGG   
  
  
+ TCCATCTATC TCCGGCGATG TTTCACCGGC TTCAAGGCGG AATCTCGAAA TTCCTAGCCG ATCTCCGGGG   
  
  
+ AATTTCGCCA AGCACCGTCG TGGTGGTGGA CAGTGAGGTA GGAATCGACA CGGGAACTTC GTCCTTCAGC   
  
  
+ GTCAACTTCG TCGCCGGTAT CGAATTCTAC ACCGGAATGC TCGAATCGCT CGACGTCACC TCCGCCAGCG   
  
  
+ GTTTTGCAGG CGGCGGCGGC GGCGACGCTG TGAGAAGGAT CGAGACGTAC GTGCTCCGGC CGAGGATTAC   
  
  
+ GGCGGCGGTG GAGGCGGCAT CTGCGGCGGC GGTAGCGCGG CGGAGCGGTT GGAGAGAGTC GTTCGCGGTG   
  
  
+ GCTAGGATGA GGGCGGTGGG GTTCAGCCAG TTTGCTGATT TCCAGGCGGA GTGTTTGCTG AGGAAGGTTC   
  
  
+ AGGTTGGCGG GTTCCACGTG GCGAAACGTC ATGGGGAGAT GATGCTTTGC TGGCATGATA GGCCCCTTGT   
  
  
+ GTCCACGTCG GCTTGGAGGT GTTA  

- -Up\_Stream \_Len000CTAAAA TGAAGGTTAA AAGATGAATT TAAACCAATA GTGCAAGCTT AAAAAGTCCA   
  
  
- TTTATGTTTG ACAATCACCG GTAGGAAAAA AAAACCCAGA TTAGGTAGGT CTACACGATT ATGAAAGACA   
  
  
- AAATCTACTC TTCAAAATTA CGAAAAAATC TACACATTAA AAAAATTTGT ATTTATCAGA ACAGCTTATT   
  
  
- CTAAACGATC ATTAAAAACA TTCTCTTTAA TACACATAAA AAATAATATA GTCTTATAAT CTAGCTAGAG   
  
  
- TTAAATTAAT GAATACTTTT TTTTTCACCT ATAGAAATTA GTTAAACCGT TCGAGAGTAC GTAATTAACA   
  
  
- TCAGCATGCT TGAATAAATG TAGTGAGTAG ACTTAAACGA TGGTAAATGT GGAGTTTAAA ATAAAATTAC   
  
  
- GGTATTTCTC TTATCTTATA TGACAGATAA ACATCATCAA AATGTCTATT TTTGTTTATT CGTTTACTTT   
  
  
- TATCTATCTC CTAAAAAAAC GATAATAGGT GCAGCTAGGT AACTTAATAA AACAGCGATA AACCAATTCA   
  
  
- CTTTATTTTA TGACTTCATC TTACCTCCTA CAACTATTAC TGGATACAGG GAGTTTCAAA TTAGTACATC   
  
  
- AAAGATGCTT AAAGTTCGGT AATATTCTGT ATGTCTAAAA ATACTACAAC TTTTATGAGA ATGGAGGAAC   
  
  
- AGTGTTATAA TTGGACTTCG GTTACTTTAC TTTGTCTTGA TTAATTTAAC TTATGTATTA GTTAGGTATA   
  
  
- ATTAAACAAA ACTGTTGATA AATGTTAGTA GCTAGAGAAA TTAATCCAGA TATTCGATAT CTCATTAATG   
  
  
- AGTTTTGACA AAACATTCTG TTGAATGAGG TATAACCGAA TAATCATATA ACGGAGGTAA GTGTACTTAT   
  
  
- GAAGTACTCT CTAACGGATT TTGAGTATAA CAGTAGTGGA ATTCAGTTTG TATAAAACAA TACTTTACTG   
  
  
- TAGTTCTTAA TGGGAGACTG TTCTTTTTAT ATGGACTGAC TGTATTAGCA TGGTGTAGAC AATCAGGAGA   
  
  
- AAGATGGTGA AACATCTTGT ATTACACTGT CATTATGTAG GTAGGGTTTT CTTTTCTGCT TTTCTTCTCA   
  
  
- CCTCTCAGTG CATATGCGCA ACTTAAGTCC TTTTAATTTC CTTGAGTACT TCTGGGGTAT ATCACGAGGT   
  
  
- TGGACTTGGA CGTCGGGTGA TGATGTACGG ATTTAATTTC TCGTTTCAAT ACTCTCTTTG GTTTTTTCGA   
  
  
- GAAACTCTCT CACCTCCTTC TTAGTACCTT TCGATCGATC CGAATCGTTC TTTCTCGTTG TCGATCGATC   
  
  
- GTTTTCCGAA GAGAAACTTC GGAATCTTTA GAAATCTCTA TTTTTCAACC ACCGAAAAAT TCCCGACACT   
  
  
- ATACAGGGGG TACATTTTTA CGTCGTTATT ACAATTACAG TACGAAAGAA AGTACCAGGG ATTACGCTTT   
  
  
- AGTTAGTACG TACAGTTGCA CTGAGTCACC GAGTGGTATA TACTAGACTA AAGGGGCGTG TGTCTCTATT   
  
  
- TTTCGGGGGT TAATATTGGA GGGTTTTCGG GGGGGAAAAG GAGGATGGGT TGGGTGTGGA GGGGTAAAGT   
  
  
- AGTCCCGAGC GAAGAATTCA ATAGCTAATT TTTTTCTCAC TCATTTGCAT GGGCTTAACT TCTCCCCACA   
  
  
- AGCTTCGAAT ATGAACGAAC TAATCCTTAC TTTCAGGGGT AAAGTAGGCG GGGGGTTTGG CGGCGGTGGT   
  
  
- GGTCATTTAG CGATAGAACG TTGTTATGAC GGAACTTATG ACGGAAAGTT CGGGGGTTAA GTTAAAGTTG   
  
  
- GGAACAATTG TTGTTGTTTG GGAGGATGCT CGGATGGAGC CAAGATCTGG AGGCCGCGTC GGGTTCGGGG   
  
  
- CGGTGGTTGC GGCGGGACGG TGACGGGTGA CTGCGCCCGT AGTTATTTGG GTTCGGACGG AGACGACTGG   
  
  
- ACCTGTTAAA CCTCCCACTC CCAACCCTAA GTTAGGATAG TCTCGACCCC GAATTACTAC TAAGATTATT   
  
  
- AGGATTGAGG TTTGAAACCA GTAGGGTTTA ATCGAGTAGT ACACTAAGGG TGGAGTGTGT CGAGGGACTC   
  
  
- AAGGGGAGGA GAGTCGGCAA CCTAGTGTGT GCGTGGGGGT TATTAATTGA AGAGGGGGAG ACTAATGGGG   
  
  
- TAGAGACTAA ATAGGATGCC AGTCTTAGAG TCGGGGTTAA AACCCGTCAA ACTCGAAGTA GAAGTAGTCG   
  
  
- GGTTGCAGTT ATTGTTACCC GAACTGAAGT ATCTCCTGGA TTAATCTCGT CGAGTTCGGA TGCTTAGGTT   
  
  
- GCTGAGGGTG GTCCAGGTCG ACCATAACCG CGCCGAGTTA GTTGCCGAGT CTAGTGGGCG GCCTTTTGGG   
  
  
- GAGGTGGCGC GGCGGAAGAT GAAGTTCCTC CGGGAGGTTG TGGAGCGACA GCCGCCTCCG GGCCGGGCCG   
  
  
- GACAGGCCGA CAGTAGGATG CTCCACCACG TCTGGTAGGC CCGGGTGTTC TTGAAAAGGC CGTATAGTGA   
  
  
- CTATAGGGAG AAGAGGTGGA AGCGGCGGTT AGTCCGCTAA GACCTCCGCC ACCTACCCCG ACGCAAGTAG   
  
  
- GTGTAGTAGC TAAAGCTGTA GCCTAAACCG CCGGTGACCC GCAGCAAATA CGCACTCGAA CAGCTGTTCC   
  
  
- GGCTGGGCTT ATTCTAGTCG AGGAACCACA AGGCTTAGTG CCGCTAGCAT GGGCTTCTCA AGCCCTAGCT   
  
  
- TTCGTTCGAC CACTCTCTCT TAGAATGCGT TAAGCGAGCA CTAGAGTTGT AGTTACAGGT ATACCTCATA   
  
  
- CAGGAATAAG TTTCGAAGCT CAAGAATAGG AAGTTTAGCC AGTTTAAGTA GCTGCCACTC TTTAACCGCC   
  
  
- AGGTAGATAG AGGCCGCTAC AAAGTGGCCG AAGTTCCGCC TTAGAGCTTT AAGGATCGGC TAGAGGCCCC   
  
  
- TTAAAGCGGT TCGTGGCAGC ACCACCACCT GTCACTCCAT CCTTAGCTGT GCCCTTGAAG CAGGAAGTCG   
  
  
- CAGTTGAAGC AGCGGCCATA GCTTAAGATG TGGCCTTACG AGCTTAGCGA GCTGCAGTGG AGGCGGTCGC   
  
  
- CAAAACGTCC GCCGCCGCCG CCGCTGCGAC ACTCTTCCTA GCTCTGCATG CACGAGGCCG GCTCCTAATG   
  
  
- CCGCCGCCAC CTCCGCCGTA GACGCCGCCG CCATCGCGCC GCCTCGCCAA CCTCTCTCAG CAAGCGCCAC   
  
  
- CGATCCTACT CCCGCCACCC CAAGTCGGTC AAACGACTAA AGGTCCGCCT CACAAACGAC TCCTTCCAAG   
  
  
- TCCAACCGCC CAAGGTGCAC CGCTTTGCAG TACCCCTCTA CTACGAAACG ACCGTACTAT CCGGGGAACA   
  
  
- CAGGTGCAGC CGAACCTCCA CAAT

+     ABRE2

| Site Name | Organism | Position | Strand | Matrix score. | sequence | function |
| --- | --- | --- | --- | --- | --- | --- |
| ABRE2 | Zea mays | 3308 | - | 8 | CCACGTGG |  |

>HU06G00283.1   
+ -Up\_Stream \_Len000GATTTT ACTTCCAATT TTCTACTTAA ATTTGGTTAT CACGTTCGAA TTTTTCAGGT   
  
  
+ AAATACAAAC TGTTAGTGGC CATCCTTTTT TTTTGGGTCT AATCCATCCA GATGTGCTAA TACTTTCTGT   
  
  
+ TTTAGATGAG AAGTTTTAAT GCTTTTTTAG ATGTGTAATT TTTTTAAACA TAAATAGTCT TGTCGAATAA   
  
  
+ GATTTGCTAG TAATTTTTGT AAGAGAAATT ATGTGTATTT TTTATTATAT CAGAATATTA GATCGATCTC   
  
  
+ AATTTAATTA CTTATGAAAA AAAAAGTGGA TATCTTTAAT CAATTTGGCA AGCTCTCATG CATTAATTGT   
  
  
+ AGTCGTACGA ACTTATTTAC ATCACTCATC TGAATTTGCT ACCATTTACA CCTCAAATTT TATTTTAATG   
  
  
+ CCATAAAGAG AATAGAATAT ACTGTCTATT TGTAGTAGTT TTACAGATAA AAACAAATAA GCAAATGAAA   
  
  
+ ATAGATAGAG GATTTTTTTG CTATTATCCA CGTCGATCCA TTGAATTATT TTGTCGCTAT TTGGTTAAGT   
  
  
+ GAAATAAAAT ACTGAAGTAG AATGGAGGAT GTTGATAATG ACCTATGTCC CTCAAAGTTT AATCATGTAG   
  
  
+ TTTCTACGAA TTTCAAGCCA TTATAAGACA TACAGATTTT TATGATGTTG AAAATACTCT TACCTCCTTG   
  
  
+ TCACAATATT AACCTGAAGC CAATGAAATG AAACAGAACT AATTAAATTG AATACATAAT CAATCCATAT   
  
  
+ TAATTTGTTT TGACAACTAT TTACAATCAT CGATCTCTTT AATTAGGTCT ATAAGCTATA GAGTAATTAC   
  
  
+ TCAAAACTGT TTTGTAAGAC AACTTACTCC ATATTGGCTT ATTAGTATAT TGCCTCCATT CACATGAATA   
  
  
+ CTTCATGAGA GATTGCCTAA AACTCATATT GTCATCACCT TAAGTCAAAC ATATTTTGTT ATGAAATGAC   
  
  
+ ATCAAGAATT ACCCTCTGAC AAGAAAAATA TACCTGACTG ACATAATCGT ACCACATCTG TTAGTCCTCT   
  
  
+ TTCTACCACT TTGTAGAACA TAATGTGACA GTAATACATC CATCCCAAAA GAAAAGACGA AAAGAAGAGT   
  
  
+ GGAGAGTCAC GTATACGCGT TGAATTCAGG AAAATTAAAG GAACTCATGA AGACCCCATA TAGTGCTCCA   
  
  
+ ACCTGAACCT GCAGCCCACT ACTACATGCC TAAATTAAAG AGCAAAGTTA TGAGAGAAAC CAAAAAAGCT   
  
  
+ CTTTGAGAGA GTGGAGGAAG AATCATGGAA AGCTAGCTAG GCTTAGCAAG AAAGAGCAAC AGCTAGCTAG   
  
  
+ CAAAAGGCTT CTCTTTGAAG CCTTAGAAAT CTTTAGAGAT AAAAAGTTGG TGGCTTTTTA AGGGCTGTGA   
  
  
+ TATGTCCCCC ATGTAAAAAT GCAGCAATAA TGTTAATGTC ATGCTTTCTT TCATGGTCCC TAATGCGAAA   
  
  
+ TCAATCATGC ATGTCAACGT GACTCAGTGG CTCACCATAT ATGATCTGAT TTCCCCGCAC ACAGAGATAA   
  
  
+ AAAGCCCCCA ATTATAACCT CCCAAAAGCC CCCCCTTTTC CTCCTACCCA ACCCACACCT CCCCATTTCA   
  
  
+ TCAGGGCTCG CTTCTTAAGT TATCGATTAA AAAAAGAGTG AGTAAACGTA CCCGAATTGA AGAGGGGTGT   
  
  
+ TCGAAGCTTA TACTTGCTTG ATTAGGAATG AAAGTCCCCA TTTCATCCGC CCCCCAAACC GCCGCCACCA   
  
  
+ CCAGTAAATC GCTATCTTGC AACAATACTG CCTTGAATAC TGCCTTTCAA GCCCCCAATT CAATTTCAAC   
  
  
+ CCTTGTTAAC AACAACAAAC CCTCCTACGA GCCTACCTCG GTTCTAGACC TCCGGCGCAG CCCAAGCCCC   
  
  
+ GCCACCAACG CCGCCCTGCC ACTGCCCACT GACGCGGGCA TCAATAAACC CAAGCCTGCC TCTGCTGACC   
  
  
+ TGGACAATTT GGAGGGTGAG GGTTGGGATT CAATCCTATC AGAGCTGGGG CTTAATGATG ATTCTAATAA   
  
  
+ TCCTAACTCC AAACTTTGGT CATCCCAAAT TAGCTCATCA TGTGATTCCC ACCTCACACA GCTCCCTGAG   
  
  
+ TTCCCCTCCT CTCAGCCGTT GGATCACACA CGCACCCCCA ATAATTAACT TCTCCCCCTC TGATTACCCC   
  
  
+ ATCTCTGATT TATCCTACGG TCAGAATCTC AGCCCCAATT TTGGGCAGTT TGAGCTTCAT CTTCATCAGC   
  
  
+ CCAACGTCAA TAACAATGGG CTTGACTTCA TAGAGGACCT AATTAGAGCA GCTCAAGCCT ACGAATCCAA   
  
  
+ CGACTCCCAC CAGGTCCAGC TGGTATTGGC GCGGCTCAAT CAACGGCTCA GATCACCCGC CGGAAAACCC   
  
  
+ CTCCACCGCG CCGCCTTCTA CTTCAAGGAG GCCCTCCAAC ACCTCGCTGT CGGCGGAGGC CCGGCCCGGC   
  
  
+ CTGTCCGGCT GTCATCCTAC GAGGTGGTGC AGACCATCCG GGCCCACAAG AACTTTTCCG GCATATCACT   
  
  
+ GATATCCCTC TTCTCCACCT TCGCCGCCAA TCAGGCGATT CTGGAGGCGG TGGATGGGGC TGCGTTCATC   
  
  
+ CACATCATCG ATTTCGACAT CGGATTTGGC GGCCACTGGG CGTCGTTTAT GCGTGAGCTT GTCGACAAGG   
  
  
+ CCGACCCGAA TAAGATCAGC TCCTTGGTGT TCCGAATCAC GGCGATCGTA CCCGAAGAGT TCGGGATCGA   
  
  
+ AAGCAAGCTG GTGAGAGAGA ATCTTACGCA ATTCGCTCGT GATCTCAACA TCAATGTCCA TATGGAGTAT   
  
  
+ GTCCTTATTC AAAGCTTCGA GTTCTTATCC TTCAAATCGG TCAAATTCAT CGACGGTGAG AAATTGGCGG   
  
  
+ TCCATCTATC TCCGGCGATG TTTCACCGGC TTCAAGGCGG AATCTCGAAA TTCCTAGCCG ATCTCCGGGG   
  
  
+ AATTTCGCCA AGCACCGTCG TGGTGGTGGA CAGTGAGGTA GGAATCGACA CGGGAACTTC GTCCTTCAGC   
  
  
+ GTCAACTTCG TCGCCGGTAT CGAATTCTAC ACCGGAATGC TCGAATCGCT CGACGTCACC TCCGCCAGCG   
  
  
+ GTTTTGCAGG CGGCGGCGGC GGCGACGCTG TGAGAAGGAT CGAGACGTAC GTGCTCCGGC CGAGGATTAC   
  
  
+ GGCGGCGGTG GAGGCGGCAT CTGCGGCGGC GGTAGCGCGG CGGAGCGGTT GGAGAGAGTC GTTCGCGGTG   
  
  
+ GCTAGGATGA GGGCGGTGGG GTTCAGCCAG TTTGCTGATT TCCAGGCGGA GTGTTTGCTG AGGAAGGTTC   
  
  
+ AGGTTGGCGG GTTCCACGTG GCGAAACGTC ATGGGGAGAT GATGCTTTGC TGGCATGATA GGCCCCTTGT   
  
  
+ GTCCACGTCG GCTTGGAGGT GTTA  

- -Up\_Stream \_Len000CTAAAA TGAAGGTTAA AAGATGAATT TAAACCAATA GTGCAAGCTT AAAAAGTCCA   
  
  
- TTTATGTTTG ACAATCACCG GTAGGAAAAA AAAACCCAGA TTAGGTAGGT CTACACGATT ATGAAAGACA   
  
  
- AAATCTACTC TTCAAAATTA CGAAAAAATC TACACATTAA AAAAATTTGT ATTTATCAGA ACAGCTTATT   
  
  
- CTAAACGATC ATTAAAAACA TTCTCTTTAA TACACATAAA AAATAATATA GTCTTATAAT CTAGCTAGAG   
  
  
- TTAAATTAAT GAATACTTTT TTTTTCACCT ATAGAAATTA GTTAAACCGT TCGAGAGTAC GTAATTAACA   
  
  
- TCAGCATGCT TGAATAAATG TAGTGAGTAG ACTTAAACGA TGGTAAATGT GGAGTTTAAA ATAAAATTAC   
  
  
- GGTATTTCTC TTATCTTATA TGACAGATAA ACATCATCAA AATGTCTATT TTTGTTTATT CGTTTACTTT   
  
  
- TATCTATCTC CTAAAAAAAC GATAATAGGT GCAGCTAGGT AACTTAATAA AACAGCGATA AACCAATTCA   
  
  
- CTTTATTTTA TGACTTCATC TTACCTCCTA CAACTATTAC TGGATACAGG GAGTTTCAAA TTAGTACATC   
  
  
- AAAGATGCTT AAAGTTCGGT AATATTCTGT ATGTCTAAAA ATACTACAAC TTTTATGAGA ATGGAGGAAC   
  
  
- AGTGTTATAA TTGGACTTCG GTTACTTTAC TTTGTCTTGA TTAATTTAAC TTATGTATTA GTTAGGTATA   
  
  
- ATTAAACAAA ACTGTTGATA AATGTTAGTA GCTAGAGAAA TTAATCCAGA TATTCGATAT CTCATTAATG   
  
  
- AGTTTTGACA AAACATTCTG TTGAATGAGG TATAACCGAA TAATCATATA ACGGAGGTAA GTGTACTTAT   
  
  
- GAAGTACTCT CTAACGGATT TTGAGTATAA CAGTAGTGGA ATTCAGTTTG TATAAAACAA TACTTTACTG   
  
  
- TAGTTCTTAA TGGGAGACTG TTCTTTTTAT ATGGACTGAC TGTATTAGCA TGGTGTAGAC AATCAGGAGA   
  
  
- AAGATGGTGA AACATCTTGT ATTACACTGT CATTATGTAG GTAGGGTTTT CTTTTCTGCT TTTCTTCTCA   
  
  
- CCTCTCAGTG CATATGCGCA ACTTAAGTCC TTTTAATTTC CTTGAGTACT TCTGGGGTAT ATCACGAGGT   
  
  
- TGGACTTGGA CGTCGGGTGA TGATGTACGG ATTTAATTTC TCGTTTCAAT ACTCTCTTTG GTTTTTTCGA   
  
  
- GAAACTCTCT CACCTCCTTC TTAGTACCTT TCGATCGATC CGAATCGTTC TTTCTCGTTG TCGATCGATC   
  
  
- GTTTTCCGAA GAGAAACTTC GGAATCTTTA GAAATCTCTA TTTTTCAACC ACCGAAAAAT TCCCGACACT   
  
  
- ATACAGGGGG TACATTTTTA CGTCGTTATT ACAATTACAG TACGAAAGAA AGTACCAGGG ATTACGCTTT   
  
  
- AGTTAGTACG TACAGTTGCA CTGAGTCACC GAGTGGTATA TACTAGACTA AAGGGGCGTG TGTCTCTATT   
  
  
- TTTCGGGGGT TAATATTGGA GGGTTTTCGG GGGGGAAAAG GAGGATGGGT TGGGTGTGGA GGGGTAAAGT   
  
  
- AGTCCCGAGC GAAGAATTCA ATAGCTAATT TTTTTCTCAC TCATTTGCAT GGGCTTAACT TCTCCCCACA   
  
  
- AGCTTCGAAT ATGAACGAAC TAATCCTTAC TTTCAGGGGT AAAGTAGGCG GGGGGTTTGG CGGCGGTGGT   
  
  
- GGTCATTTAG CGATAGAACG TTGTTATGAC GGAACTTATG ACGGAAAGTT CGGGGGTTAA GTTAAAGTTG   
  
  
- GGAACAATTG TTGTTGTTTG GGAGGATGCT CGGATGGAGC CAAGATCTGG AGGCCGCGTC GGGTTCGGGG   
  
  
- CGGTGGTTGC GGCGGGACGG TGACGGGTGA CTGCGCCCGT AGTTATTTGG GTTCGGACGG AGACGACTGG   
  
  
- ACCTGTTAAA CCTCCCACTC CCAACCCTAA GTTAGGATAG TCTCGACCCC GAATTACTAC TAAGATTATT   
  
  
- AGGATTGAGG TTTGAAACCA GTAGGGTTTA ATCGAGTAGT ACACTAAGGG TGGAGTGTGT CGAGGGACTC   
  
  
- AAGGGGAGGA GAGTCGGCAA CCTAGTGTGT GCGTGGGGGT TATTAATTGA AGAGGGGGAG ACTAATGGGG   
  
  
- TAGAGACTAA ATAGGATGCC AGTCTTAGAG TCGGGGTTAA AACCCGTCAA ACTCGAAGTA GAAGTAGTCG   
  
  
- GGTTGCAGTT ATTGTTACCC GAACTGAAGT ATCTCCTGGA TTAATCTCGT CGAGTTCGGA TGCTTAGGTT   
  
  
- GCTGAGGGTG GTCCAGGTCG ACCATAACCG CGCCGAGTTA GTTGCCGAGT CTAGTGGGCG GCCTTTTGGG   
  
  
- GAGGTGGCGC GGCGGAAGAT GAAGTTCCTC CGGGAGGTTG TGGAGCGACA GCCGCCTCCG GGCCGGGCCG   
  
  
- GACAGGCCGA CAGTAGGATG CTCCACCACG TCTGGTAGGC CCGGGTGTTC TTGAAAAGGC CGTATAGTGA   
  
  
- CTATAGGGAG AAGAGGTGGA AGCGGCGGTT AGTCCGCTAA GACCTCCGCC ACCTACCCCG ACGCAAGTAG   
  
  
- GTGTAGTAGC TAAAGCTGTA GCCTAAACCG CCGGTGACCC GCAGCAAATA CGCACTCGAA CAGCTGTTCC   
  
  
- GGCTGGGCTT ATTCTAGTCG AGGAACCACA AGGCTTAGTG CCGCTAGCAT GGGCTTCTCA AGCCCTAGCT   
  
  
- TTCGTTCGAC CACTCTCTCT TAGAATGCGT TAAGCGAGCA CTAGAGTTGT AGTTACAGGT ATACCTCATA   
  
  
- CAGGAATAAG TTTCGAAGCT CAAGAATAGG AAGTTTAGCC AGTTTAAGTA GCTGCCACTC TTTAACCGCC   
  
  
- AGGTAGATAG AGGCCGCTAC AAAGTGGCCG AAGTTCCGCC TTAGAGCTTT AAGGATCGGC TAGAGGCCCC   
  
  
- TTAAAGCGGT TCGTGGCAGC ACCACCACCT GTCACTCCAT CCTTAGCTGT GCCCTTGAAG CAGGAAGTCG   
  
  
- CAGTTGAAGC AGCGGCCATA GCTTAAGATG TGGCCTTACG AGCTTAGCGA GCTGCAGTGG AGGCGGTCGC   
  
  
- CAAAACGTCC GCCGCCGCCG CCGCTGCGAC ACTCTTCCTA GCTCTGCATG CACGAGGCCG GCTCCTAATG   
  
  
- CCGCCGCCAC CTCCGCCGTA GACGCCGCCG CCATCGCGCC GCCTCGCCAA CCTCTCTCAG CAAGCGCCAC   
  
  
- CGATCCTACT CCCGCCACCC CAAGTCGGTC AAACGACTAA AGGTCCGCCT CACAAACGAC TCCTTCCAAG   
  
  
- TCCAACCGCC CAAGGTGCAC CGCTTTGCAG TACCCCTCTA CTACGAAACG ACCGTACTAT CCGGGGAACA   
  
  
- CAGGTGCAGC CGAACCTCCA CAAT

+     ABRE3a

| Site Name | Organism | Position | Strand | Matrix score. | sequence | function |
| --- | --- | --- | --- | --- | --- | --- |
| ABRE3a | Zea mays | 1132 | - | 6 | TACGTG |  |
| ABRE3a | Zea mays | 3132 | + | 6 | TACGTG |  |

>HU06G00283.1   
+ -Up\_Stream \_Len000GATTTT ACTTCCAATT TTCTACTTAA ATTTGGTTAT CACGTTCGAA TTTTTCAGGT   
  
  
+ AAATACAAAC TGTTAGTGGC CATCCTTTTT TTTTGGGTCT AATCCATCCA GATGTGCTAA TACTTTCTGT   
  
  
+ TTTAGATGAG AAGTTTTAAT GCTTTTTTAG ATGTGTAATT TTTTTAAACA TAAATAGTCT TGTCGAATAA   
  
  
+ GATTTGCTAG TAATTTTTGT AAGAGAAATT ATGTGTATTT TTTATTATAT CAGAATATTA GATCGATCTC   
  
  
+ AATTTAATTA CTTATGAAAA AAAAAGTGGA TATCTTTAAT CAATTTGGCA AGCTCTCATG CATTAATTGT   
  
  
+ AGTCGTACGA ACTTATTTAC ATCACTCATC TGAATTTGCT ACCATTTACA CCTCAAATTT TATTTTAATG   
  
  
+ CCATAAAGAG AATAGAATAT ACTGTCTATT TGTAGTAGTT TTACAGATAA AAACAAATAA GCAAATGAAA   
  
  
+ ATAGATAGAG GATTTTTTTG CTATTATCCA CGTCGATCCA TTGAATTATT TTGTCGCTAT TTGGTTAAGT   
  
  
+ GAAATAAAAT ACTGAAGTAG AATGGAGGAT GTTGATAATG ACCTATGTCC CTCAAAGTTT AATCATGTAG   
  
  
+ TTTCTACGAA TTTCAAGCCA TTATAAGACA TACAGATTTT TATGATGTTG AAAATACTCT TACCTCCTTG   
  
  
+ TCACAATATT AACCTGAAGC CAATGAAATG AAACAGAACT AATTAAATTG AATACATAAT CAATCCATAT   
  
  
+ TAATTTGTTT TGACAACTAT TTACAATCAT CGATCTCTTT AATTAGGTCT ATAAGCTATA GAGTAATTAC   
  
  
+ TCAAAACTGT TTTGTAAGAC AACTTACTCC ATATTGGCTT ATTAGTATAT TGCCTCCATT CACATGAATA   
  
  
+ CTTCATGAGA GATTGCCTAA AACTCATATT GTCATCACCT TAAGTCAAAC ATATTTTGTT ATGAAATGAC   
  
  
+ ATCAAGAATT ACCCTCTGAC AAGAAAAATA TACCTGACTG ACATAATCGT ACCACATCTG TTAGTCCTCT   
  
  
+ TTCTACCACT TTGTAGAACA TAATGTGACA GTAATACATC CATCCCAAAA GAAAAGACGA AAAGAAGAGT   
  
  
+ GGAGAGTCAC GTATACGCGT TGAATTCAGG AAAATTAAAG GAACTCATGA AGACCCCATA TAGTGCTCCA   
  
  
+ ACCTGAACCT GCAGCCCACT ACTACATGCC TAAATTAAAG AGCAAAGTTA TGAGAGAAAC CAAAAAAGCT   
  
  
+ CTTTGAGAGA GTGGAGGAAG AATCATGGAA AGCTAGCTAG GCTTAGCAAG AAAGAGCAAC AGCTAGCTAG   
  
  
+ CAAAAGGCTT CTCTTTGAAG CCTTAGAAAT CTTTAGAGAT AAAAAGTTGG TGGCTTTTTA AGGGCTGTGA   
  
  
+ TATGTCCCCC ATGTAAAAAT GCAGCAATAA TGTTAATGTC ATGCTTTCTT TCATGGTCCC TAATGCGAAA   
  
  
+ TCAATCATGC ATGTCAACGT GACTCAGTGG CTCACCATAT ATGATCTGAT TTCCCCGCAC ACAGAGATAA   
  
  
+ AAAGCCCCCA ATTATAACCT CCCAAAAGCC CCCCCTTTTC CTCCTACCCA ACCCACACCT CCCCATTTCA   
  
  
+ TCAGGGCTCG CTTCTTAAGT TATCGATTAA AAAAAGAGTG AGTAAACGTA CCCGAATTGA AGAGGGGTGT   
  
  
+ TCGAAGCTTA TACTTGCTTG ATTAGGAATG AAAGTCCCCA TTTCATCCGC CCCCCAAACC GCCGCCACCA   
  
  
+ CCAGTAAATC GCTATCTTGC AACAATACTG CCTTGAATAC TGCCTTTCAA GCCCCCAATT CAATTTCAAC   
  
  
+ CCTTGTTAAC AACAACAAAC CCTCCTACGA GCCTACCTCG GTTCTAGACC TCCGGCGCAG CCCAAGCCCC   
  
  
+ GCCACCAACG CCGCCCTGCC ACTGCCCACT GACGCGGGCA TCAATAAACC CAAGCCTGCC TCTGCTGACC   
  
  
+ TGGACAATTT GGAGGGTGAG GGTTGGGATT CAATCCTATC AGAGCTGGGG CTTAATGATG ATTCTAATAA   
  
  
+ TCCTAACTCC AAACTTTGGT CATCCCAAAT TAGCTCATCA TGTGATTCCC ACCTCACACA GCTCCCTGAG   
  
  
+ TTCCCCTCCT CTCAGCCGTT GGATCACACA CGCACCCCCA ATAATTAACT TCTCCCCCTC TGATTACCCC   
  
  
+ ATCTCTGATT TATCCTACGG TCAGAATCTC AGCCCCAATT TTGGGCAGTT TGAGCTTCAT CTTCATCAGC   
  
  
+ CCAACGTCAA TAACAATGGG CTTGACTTCA TAGAGGACCT AATTAGAGCA GCTCAAGCCT ACGAATCCAA   
  
  
+ CGACTCCCAC CAGGTCCAGC TGGTATTGGC GCGGCTCAAT CAACGGCTCA GATCACCCGC CGGAAAACCC   
  
  
+ CTCCACCGCG CCGCCTTCTA CTTCAAGGAG GCCCTCCAAC ACCTCGCTGT CGGCGGAGGC CCGGCCCGGC   
  
  
+ CTGTCCGGCT GTCATCCTAC GAGGTGGTGC AGACCATCCG GGCCCACAAG AACTTTTCCG GCATATCACT   
  
  
+ GATATCCCTC TTCTCCACCT TCGCCGCCAA TCAGGCGATT CTGGAGGCGG TGGATGGGGC TGCGTTCATC   
  
  
+ CACATCATCG ATTTCGACAT CGGATTTGGC GGCCACTGGG CGTCGTTTAT GCGTGAGCTT GTCGACAAGG   
  
  
+ CCGACCCGAA TAAGATCAGC TCCTTGGTGT TCCGAATCAC GGCGATCGTA CCCGAAGAGT TCGGGATCGA   
  
  
+ AAGCAAGCTG GTGAGAGAGA ATCTTACGCA ATTCGCTCGT GATCTCAACA TCAATGTCCA TATGGAGTAT   
  
  
+ GTCCTTATTC AAAGCTTCGA GTTCTTATCC TTCAAATCGG TCAAATTCAT CGACGGTGAG AAATTGGCGG   
  
  
+ TCCATCTATC TCCGGCGATG TTTCACCGGC TTCAAGGCGG AATCTCGAAA TTCCTAGCCG ATCTCCGGGG   
  
  
+ AATTTCGCCA AGCACCGTCG TGGTGGTGGA CAGTGAGGTA GGAATCGACA CGGGAACTTC GTCCTTCAGC   
  
  
+ GTCAACTTCG TCGCCGGTAT CGAATTCTAC ACCGGAATGC TCGAATCGCT CGACGTCACC TCCGCCAGCG   
  
  
+ GTTTTGCAGG CGGCGGCGGC GGCGACGCTG TGAGAAGGAT CGAGACGTAC GTGCTCCGGC CGAGGATTAC   
  
  
+ GGCGGCGGTG GAGGCGGCAT CTGCGGCGGC GGTAGCGCGG CGGAGCGGTT GGAGAGAGTC GTTCGCGGTG   
  
  
+ GCTAGGATGA GGGCGGTGGG GTTCAGCCAG TTTGCTGATT TCCAGGCGGA GTGTTTGCTG AGGAAGGTTC   
  
  
+ AGGTTGGCGG GTTCCACGTG GCGAAACGTC ATGGGGAGAT GATGCTTTGC TGGCATGATA GGCCCCTTGT   
  
  
+ GTCCACGTCG GCTTGGAGGT GTTA  

- -Up\_Stream \_Len000CTAAAA TGAAGGTTAA AAGATGAATT TAAACCAATA GTGCAAGCTT AAAAAGTCCA   
  
  
- TTTATGTTTG ACAATCACCG GTAGGAAAAA AAAACCCAGA TTAGGTAGGT CTACACGATT ATGAAAGACA   
  
  
- AAATCTACTC TTCAAAATTA CGAAAAAATC TACACATTAA AAAAATTTGT ATTTATCAGA ACAGCTTATT   
  
  
- CTAAACGATC ATTAAAAACA TTCTCTTTAA TACACATAAA AAATAATATA GTCTTATAAT CTAGCTAGAG   
  
  
- TTAAATTAAT GAATACTTTT TTTTTCACCT ATAGAAATTA GTTAAACCGT TCGAGAGTAC GTAATTAACA   
  
  
- TCAGCATGCT TGAATAAATG TAGTGAGTAG ACTTAAACGA TGGTAAATGT GGAGTTTAAA ATAAAATTAC   
  
  
- GGTATTTCTC TTATCTTATA TGACAGATAA ACATCATCAA AATGTCTATT TTTGTTTATT CGTTTACTTT   
  
  
- TATCTATCTC CTAAAAAAAC GATAATAGGT GCAGCTAGGT AACTTAATAA AACAGCGATA AACCAATTCA   
  
  
- CTTTATTTTA TGACTTCATC TTACCTCCTA CAACTATTAC TGGATACAGG GAGTTTCAAA TTAGTACATC   
  
  
- AAAGATGCTT AAAGTTCGGT AATATTCTGT ATGTCTAAAA ATACTACAAC TTTTATGAGA ATGGAGGAAC   
  
  
- AGTGTTATAA TTGGACTTCG GTTACTTTAC TTTGTCTTGA TTAATTTAAC TTATGTATTA GTTAGGTATA   
  
  
- ATTAAACAAA ACTGTTGATA AATGTTAGTA GCTAGAGAAA TTAATCCAGA TATTCGATAT CTCATTAATG   
  
  
- AGTTTTGACA AAACATTCTG TTGAATGAGG TATAACCGAA TAATCATATA ACGGAGGTAA GTGTACTTAT   
  
  
- GAAGTACTCT CTAACGGATT TTGAGTATAA CAGTAGTGGA ATTCAGTTTG TATAAAACAA TACTTTACTG   
  
  
- TAGTTCTTAA TGGGAGACTG TTCTTTTTAT ATGGACTGAC TGTATTAGCA TGGTGTAGAC AATCAGGAGA   
  
  
- AAGATGGTGA AACATCTTGT ATTACACTGT CATTATGTAG GTAGGGTTTT CTTTTCTGCT TTTCTTCTCA   
  
  
- CCTCTCAGTG CATATGCGCA ACTTAAGTCC TTTTAATTTC CTTGAGTACT TCTGGGGTAT ATCACGAGGT   
  
  
- TGGACTTGGA CGTCGGGTGA TGATGTACGG ATTTAATTTC TCGTTTCAAT ACTCTCTTTG GTTTTTTCGA   
  
  
- GAAACTCTCT CACCTCCTTC TTAGTACCTT TCGATCGATC CGAATCGTTC TTTCTCGTTG TCGATCGATC   
  
  
- GTTTTCCGAA GAGAAACTTC GGAATCTTTA GAAATCTCTA TTTTTCAACC ACCGAAAAAT TCCCGACACT   
  
  
- ATACAGGGGG TACATTTTTA CGTCGTTATT ACAATTACAG TACGAAAGAA AGTACCAGGG ATTACGCTTT   
  
  
- AGTTAGTACG TACAGTTGCA CTGAGTCACC GAGTGGTATA TACTAGACTA AAGGGGCGTG TGTCTCTATT   
  
  
- TTTCGGGGGT TAATATTGGA GGGTTTTCGG GGGGGAAAAG GAGGATGGGT TGGGTGTGGA GGGGTAAAGT   
  
  
- AGTCCCGAGC GAAGAATTCA ATAGCTAATT TTTTTCTCAC TCATTTGCAT GGGCTTAACT TCTCCCCACA   
  
  
- AGCTTCGAAT ATGAACGAAC TAATCCTTAC TTTCAGGGGT AAAGTAGGCG GGGGGTTTGG CGGCGGTGGT   
  
  
- GGTCATTTAG CGATAGAACG TTGTTATGAC GGAACTTATG ACGGAAAGTT CGGGGGTTAA GTTAAAGTTG   
  
  
- GGAACAATTG TTGTTGTTTG GGAGGATGCT CGGATGGAGC CAAGATCTGG AGGCCGCGTC GGGTTCGGGG   
  
  
- CGGTGGTTGC GGCGGGACGG TGACGGGTGA CTGCGCCCGT AGTTATTTGG GTTCGGACGG AGACGACTGG   
  
  
- ACCTGTTAAA CCTCCCACTC CCAACCCTAA GTTAGGATAG TCTCGACCCC GAATTACTAC TAAGATTATT   
  
  
- AGGATTGAGG TTTGAAACCA GTAGGGTTTA ATCGAGTAGT ACACTAAGGG TGGAGTGTGT CGAGGGACTC   
  
  
- AAGGGGAGGA GAGTCGGCAA CCTAGTGTGT GCGTGGGGGT TATTAATTGA AGAGGGGGAG ACTAATGGGG   
  
  
- TAGAGACTAA ATAGGATGCC AGTCTTAGAG TCGGGGTTAA AACCCGTCAA ACTCGAAGTA GAAGTAGTCG   
  
  
- GGTTGCAGTT ATTGTTACCC GAACTGAAGT ATCTCCTGGA TTAATCTCGT CGAGTTCGGA TGCTTAGGTT   
  
  
- GCTGAGGGTG GTCCAGGTCG ACCATAACCG CGCCGAGTTA GTTGCCGAGT CTAGTGGGCG GCCTTTTGGG   
  
  
- GAGGTGGCGC GGCGGAAGAT GAAGTTCCTC CGGGAGGTTG TGGAGCGACA GCCGCCTCCG GGCCGGGCCG   
  
  
- GACAGGCCGA CAGTAGGATG CTCCACCACG TCTGGTAGGC CCGGGTGTTC TTGAAAAGGC CGTATAGTGA   
  
  
- CTATAGGGAG AAGAGGTGGA AGCGGCGGTT AGTCCGCTAA GACCTCCGCC ACCTACCCCG ACGCAAGTAG   
  
  
- GTGTAGTAGC TAAAGCTGTA GCCTAAACCG CCGGTGACCC GCAGCAAATA CGCACTCGAA CAGCTGTTCC   
  
  
- GGCTGGGCTT ATTCTAGTCG AGGAACCACA AGGCTTAGTG CCGCTAGCAT GGGCTTCTCA AGCCCTAGCT   
  
  
- TTCGTTCGAC CACTCTCTCT TAGAATGCGT TAAGCGAGCA CTAGAGTTGT AGTTACAGGT ATACCTCATA   
  
  
- CAGGAATAAG TTTCGAAGCT CAAGAATAGG AAGTTTAGCC AGTTTAAGTA GCTGCCACTC TTTAACCGCC   
  
  
- AGGTAGATAG AGGCCGCTAC AAAGTGGCCG AAGTTCCGCC TTAGAGCTTT AAGGATCGGC TAGAGGCCCC   
  
  
- TTAAAGCGGT TCGTGGCAGC ACCACCACCT GTCACTCCAT CCTTAGCTGT GCCCTTGAAG CAGGAAGTCG   
  
  
- CAGTTGAAGC AGCGGCCATA GCTTAAGATG TGGCCTTACG AGCTTAGCGA GCTGCAGTGG AGGCGGTCGC   
  
  
- CAAAACGTCC GCCGCCGCCG CCGCTGCGAC ACTCTTCCTA GCTCTGCATG CACGAGGCCG GCTCCTAATG   
  
  
- CCGCCGCCAC CTCCGCCGTA GACGCCGCCG CCATCGCGCC GCCTCGCCAA CCTCTCTCAG CAAGCGCCAC   
  
  
- CGATCCTACT CCCGCCACCC CAAGTCGGTC AAACGACTAA AGGTCCGCCT CACAAACGAC TCCTTCCAAG   
  
  
- TCCAACCGCC CAAGGTGCAC CGCTTTGCAG TACCCCTCTA CTACGAAACG ACCGTACTAT CCGGGGAACA   
  
  
- CAGGTGCAGC CGAACCTCCA CAAT

+     ABRE4

| Site Name | Organism | Position | Strand | Matrix score. | sequence | function |
| --- | --- | --- | --- | --- | --- | --- |
| ABRE4 | Zea mays | 1132 | + | 6 | CACGTA |  |
| ABRE4 | Zea mays | 3132 | - | 6 | CACGTA |  |

>HU06G00283.1   
+ -Up\_Stream \_Len000GATTTT ACTTCCAATT TTCTACTTAA ATTTGGTTAT CACGTTCGAA TTTTTCAGGT   
  
  
+ AAATACAAAC TGTTAGTGGC CATCCTTTTT TTTTGGGTCT AATCCATCCA GATGTGCTAA TACTTTCTGT   
  
  
+ TTTAGATGAG AAGTTTTAAT GCTTTTTTAG ATGTGTAATT TTTTTAAACA TAAATAGTCT TGTCGAATAA   
  
  
+ GATTTGCTAG TAATTTTTGT AAGAGAAATT ATGTGTATTT TTTATTATAT CAGAATATTA GATCGATCTC   
  
  
+ AATTTAATTA CTTATGAAAA AAAAAGTGGA TATCTTTAAT CAATTTGGCA AGCTCTCATG CATTAATTGT   
  
  
+ AGTCGTACGA ACTTATTTAC ATCACTCATC TGAATTTGCT ACCATTTACA CCTCAAATTT TATTTTAATG   
  
  
+ CCATAAAGAG AATAGAATAT ACTGTCTATT TGTAGTAGTT TTACAGATAA AAACAAATAA GCAAATGAAA   
  
  
+ ATAGATAGAG GATTTTTTTG CTATTATCCA CGTCGATCCA TTGAATTATT TTGTCGCTAT TTGGTTAAGT   
  
  
+ GAAATAAAAT ACTGAAGTAG AATGGAGGAT GTTGATAATG ACCTATGTCC CTCAAAGTTT AATCATGTAG   
  
  
+ TTTCTACGAA TTTCAAGCCA TTATAAGACA TACAGATTTT TATGATGTTG AAAATACTCT TACCTCCTTG   
  
  
+ TCACAATATT AACCTGAAGC CAATGAAATG AAACAGAACT AATTAAATTG AATACATAAT CAATCCATAT   
  
  
+ TAATTTGTTT TGACAACTAT TTACAATCAT CGATCTCTTT AATTAGGTCT ATAAGCTATA GAGTAATTAC   
  
  
+ TCAAAACTGT TTTGTAAGAC AACTTACTCC ATATTGGCTT ATTAGTATAT TGCCTCCATT CACATGAATA   
  
  
+ CTTCATGAGA GATTGCCTAA AACTCATATT GTCATCACCT TAAGTCAAAC ATATTTTGTT ATGAAATGAC   
  
  
+ ATCAAGAATT ACCCTCTGAC AAGAAAAATA TACCTGACTG ACATAATCGT ACCACATCTG TTAGTCCTCT   
  
  
+ TTCTACCACT TTGTAGAACA TAATGTGACA GTAATACATC CATCCCAAAA GAAAAGACGA AAAGAAGAGT   
  
  
+ GGAGAGTCAC GTATACGCGT TGAATTCAGG AAAATTAAAG GAACTCATGA AGACCCCATA TAGTGCTCCA   
  
  
+ ACCTGAACCT GCAGCCCACT ACTACATGCC TAAATTAAAG AGCAAAGTTA TGAGAGAAAC CAAAAAAGCT   
  
  
+ CTTTGAGAGA GTGGAGGAAG AATCATGGAA AGCTAGCTAG GCTTAGCAAG AAAGAGCAAC AGCTAGCTAG   
  
  
+ CAAAAGGCTT CTCTTTGAAG CCTTAGAAAT CTTTAGAGAT AAAAAGTTGG TGGCTTTTTA AGGGCTGTGA   
  
  
+ TATGTCCCCC ATGTAAAAAT GCAGCAATAA TGTTAATGTC ATGCTTTCTT TCATGGTCCC TAATGCGAAA   
  
  
+ TCAATCATGC ATGTCAACGT GACTCAGTGG CTCACCATAT ATGATCTGAT TTCCCCGCAC ACAGAGATAA   
  
  
+ AAAGCCCCCA ATTATAACCT CCCAAAAGCC CCCCCTTTTC CTCCTACCCA ACCCACACCT CCCCATTTCA   
  
  
+ TCAGGGCTCG CTTCTTAAGT TATCGATTAA AAAAAGAGTG AGTAAACGTA CCCGAATTGA AGAGGGGTGT   
  
  
+ TCGAAGCTTA TACTTGCTTG ATTAGGAATG AAAGTCCCCA TTTCATCCGC CCCCCAAACC GCCGCCACCA   
  
  
+ CCAGTAAATC GCTATCTTGC AACAATACTG CCTTGAATAC TGCCTTTCAA GCCCCCAATT CAATTTCAAC   
  
  
+ CCTTGTTAAC AACAACAAAC CCTCCTACGA GCCTACCTCG GTTCTAGACC TCCGGCGCAG CCCAAGCCCC   
  
  
+ GCCACCAACG CCGCCCTGCC ACTGCCCACT GACGCGGGCA TCAATAAACC CAAGCCTGCC TCTGCTGACC   
  
  
+ TGGACAATTT GGAGGGTGAG GGTTGGGATT CAATCCTATC AGAGCTGGGG CTTAATGATG ATTCTAATAA   
  
  
+ TCCTAACTCC AAACTTTGGT CATCCCAAAT TAGCTCATCA TGTGATTCCC ACCTCACACA GCTCCCTGAG   
  
  
+ TTCCCCTCCT CTCAGCCGTT GGATCACACA CGCACCCCCA ATAATTAACT TCTCCCCCTC TGATTACCCC   
  
  
+ ATCTCTGATT TATCCTACGG TCAGAATCTC AGCCCCAATT TTGGGCAGTT TGAGCTTCAT CTTCATCAGC   
  
  
+ CCAACGTCAA TAACAATGGG CTTGACTTCA TAGAGGACCT AATTAGAGCA GCTCAAGCCT ACGAATCCAA   
  
  
+ CGACTCCCAC CAGGTCCAGC TGGTATTGGC GCGGCTCAAT CAACGGCTCA GATCACCCGC CGGAAAACCC   
  
  
+ CTCCACCGCG CCGCCTTCTA CTTCAAGGAG GCCCTCCAAC ACCTCGCTGT CGGCGGAGGC CCGGCCCGGC   
  
  
+ CTGTCCGGCT GTCATCCTAC GAGGTGGTGC AGACCATCCG GGCCCACAAG AACTTTTCCG GCATATCACT   
  
  
+ GATATCCCTC TTCTCCACCT TCGCCGCCAA TCAGGCGATT CTGGAGGCGG TGGATGGGGC TGCGTTCATC   
  
  
+ CACATCATCG ATTTCGACAT CGGATTTGGC GGCCACTGGG CGTCGTTTAT GCGTGAGCTT GTCGACAAGG   
  
  
+ CCGACCCGAA TAAGATCAGC TCCTTGGTGT TCCGAATCAC GGCGATCGTA CCCGAAGAGT TCGGGATCGA   
  
  
+ AAGCAAGCTG GTGAGAGAGA ATCTTACGCA ATTCGCTCGT GATCTCAACA TCAATGTCCA TATGGAGTAT   
  
  
+ GTCCTTATTC AAAGCTTCGA GTTCTTATCC TTCAAATCGG TCAAATTCAT CGACGGTGAG AAATTGGCGG   
  
  
+ TCCATCTATC TCCGGCGATG TTTCACCGGC TTCAAGGCGG AATCTCGAAA TTCCTAGCCG ATCTCCGGGG   
  
  
+ AATTTCGCCA AGCACCGTCG TGGTGGTGGA CAGTGAGGTA GGAATCGACA CGGGAACTTC GTCCTTCAGC   
  
  
+ GTCAACTTCG TCGCCGGTAT CGAATTCTAC ACCGGAATGC TCGAATCGCT CGACGTCACC TCCGCCAGCG   
  
  
+ GTTTTGCAGG CGGCGGCGGC GGCGACGCTG TGAGAAGGAT CGAGACGTAC GTGCTCCGGC CGAGGATTAC   
  
  
+ GGCGGCGGTG GAGGCGGCAT CTGCGGCGGC GGTAGCGCGG CGGAGCGGTT GGAGAGAGTC GTTCGCGGTG   
  
  
+ GCTAGGATGA GGGCGGTGGG GTTCAGCCAG TTTGCTGATT TCCAGGCGGA GTGTTTGCTG AGGAAGGTTC   
  
  
+ AGGTTGGCGG GTTCCACGTG GCGAAACGTC ATGGGGAGAT GATGCTTTGC TGGCATGATA GGCCCCTTGT   
  
  
+ GTCCACGTCG GCTTGGAGGT GTTA  

- -Up\_Stream \_Len000CTAAAA TGAAGGTTAA AAGATGAATT TAAACCAATA GTGCAAGCTT AAAAAGTCCA   
  
  
- TTTATGTTTG ACAATCACCG GTAGGAAAAA AAAACCCAGA TTAGGTAGGT CTACACGATT ATGAAAGACA   
  
  
- AAATCTACTC TTCAAAATTA CGAAAAAATC TACACATTAA AAAAATTTGT ATTTATCAGA ACAGCTTATT   
  
  
- CTAAACGATC ATTAAAAACA TTCTCTTTAA TACACATAAA AAATAATATA GTCTTATAAT CTAGCTAGAG   
  
  
- TTAAATTAAT GAATACTTTT TTTTTCACCT ATAGAAATTA GTTAAACCGT TCGAGAGTAC GTAATTAACA   
  
  
- TCAGCATGCT TGAATAAATG TAGTGAGTAG ACTTAAACGA TGGTAAATGT GGAGTTTAAA ATAAAATTAC   
  
  
- GGTATTTCTC TTATCTTATA TGACAGATAA ACATCATCAA AATGTCTATT TTTGTTTATT CGTTTACTTT   
  
  
- TATCTATCTC CTAAAAAAAC GATAATAGGT GCAGCTAGGT AACTTAATAA AACAGCGATA AACCAATTCA   
  
  
- CTTTATTTTA TGACTTCATC TTACCTCCTA CAACTATTAC TGGATACAGG GAGTTTCAAA TTAGTACATC   
  
  
- AAAGATGCTT AAAGTTCGGT AATATTCTGT ATGTCTAAAA ATACTACAAC TTTTATGAGA ATGGAGGAAC   
  
  
- AGTGTTATAA TTGGACTTCG GTTACTTTAC TTTGTCTTGA TTAATTTAAC TTATGTATTA GTTAGGTATA   
  
  
- ATTAAACAAA ACTGTTGATA AATGTTAGTA GCTAGAGAAA TTAATCCAGA TATTCGATAT CTCATTAATG   
  
  
- AGTTTTGACA AAACATTCTG TTGAATGAGG TATAACCGAA TAATCATATA ACGGAGGTAA GTGTACTTAT   
  
  
- GAAGTACTCT CTAACGGATT TTGAGTATAA CAGTAGTGGA ATTCAGTTTG TATAAAACAA TACTTTACTG   
  
  
- TAGTTCTTAA TGGGAGACTG TTCTTTTTAT ATGGACTGAC TGTATTAGCA TGGTGTAGAC AATCAGGAGA   
  
  
- AAGATGGTGA AACATCTTGT ATTACACTGT CATTATGTAG GTAGGGTTTT CTTTTCTGCT TTTCTTCTCA   
  
  
- CCTCTCAGTG CATATGCGCA ACTTAAGTCC TTTTAATTTC CTTGAGTACT TCTGGGGTAT ATCACGAGGT   
  
  
- TGGACTTGGA CGTCGGGTGA TGATGTACGG ATTTAATTTC TCGTTTCAAT ACTCTCTTTG GTTTTTTCGA   
  
  
- GAAACTCTCT CACCTCCTTC TTAGTACCTT TCGATCGATC CGAATCGTTC TTTCTCGTTG TCGATCGATC   
  
  
- GTTTTCCGAA GAGAAACTTC GGAATCTTTA GAAATCTCTA TTTTTCAACC ACCGAAAAAT TCCCGACACT   
  
  
- ATACAGGGGG TACATTTTTA CGTCGTTATT ACAATTACAG TACGAAAGAA AGTACCAGGG ATTACGCTTT   
  
  
- AGTTAGTACG TACAGTTGCA CTGAGTCACC GAGTGGTATA TACTAGACTA AAGGGGCGTG TGTCTCTATT   
  
  
- TTTCGGGGGT TAATATTGGA GGGTTTTCGG GGGGGAAAAG GAGGATGGGT TGGGTGTGGA GGGGTAAAGT   
  
  
- AGTCCCGAGC GAAGAATTCA ATAGCTAATT TTTTTCTCAC TCATTTGCAT GGGCTTAACT TCTCCCCACA   
  
  
- AGCTTCGAAT ATGAACGAAC TAATCCTTAC TTTCAGGGGT AAAGTAGGCG GGGGGTTTGG CGGCGGTGGT   
  
  
- GGTCATTTAG CGATAGAACG TTGTTATGAC GGAACTTATG ACGGAAAGTT CGGGGGTTAA GTTAAAGTTG   
  
  
- GGAACAATTG TTGTTGTTTG GGAGGATGCT CGGATGGAGC CAAGATCTGG AGGCCGCGTC GGGTTCGGGG   
  
  
- CGGTGGTTGC GGCGGGACGG TGACGGGTGA CTGCGCCCGT AGTTATTTGG GTTCGGACGG AGACGACTGG   
  
  
- ACCTGTTAAA CCTCCCACTC CCAACCCTAA GTTAGGATAG TCTCGACCCC GAATTACTAC TAAGATTATT   
  
  
- AGGATTGAGG TTTGAAACCA GTAGGGTTTA ATCGAGTAGT ACACTAAGGG TGGAGTGTGT CGAGGGACTC   
  
  
- AAGGGGAGGA GAGTCGGCAA CCTAGTGTGT GCGTGGGGGT TATTAATTGA AGAGGGGGAG ACTAATGGGG   
  
  
- TAGAGACTAA ATAGGATGCC AGTCTTAGAG TCGGGGTTAA AACCCGTCAA ACTCGAAGTA GAAGTAGTCG   
  
  
- GGTTGCAGTT ATTGTTACCC GAACTGAAGT ATCTCCTGGA TTAATCTCGT CGAGTTCGGA TGCTTAGGTT   
  
  
- GCTGAGGGTG GTCCAGGTCG ACCATAACCG CGCCGAGTTA GTTGCCGAGT CTAGTGGGCG GCCTTTTGGG   
  
  
- GAGGTGGCGC GGCGGAAGAT GAAGTTCCTC CGGGAGGTTG TGGAGCGACA GCCGCCTCCG GGCCGGGCCG   
  
  
- GACAGGCCGA CAGTAGGATG CTCCACCACG TCTGGTAGGC CCGGGTGTTC TTGAAAAGGC CGTATAGTGA   
  
  
- CTATAGGGAG AAGAGGTGGA AGCGGCGGTT AGTCCGCTAA GACCTCCGCC ACCTACCCCG ACGCAAGTAG   
  
  
- GTGTAGTAGC TAAAGCTGTA GCCTAAACCG CCGGTGACCC GCAGCAAATA CGCACTCGAA CAGCTGTTCC   
  
  
- GGCTGGGCTT ATTCTAGTCG AGGAACCACA AGGCTTAGTG CCGCTAGCAT GGGCTTCTCA AGCCCTAGCT   
  
  
- TTCGTTCGAC CACTCTCTCT TAGAATGCGT TAAGCGAGCA CTAGAGTTGT AGTTACAGGT ATACCTCATA   
  
  
- CAGGAATAAG TTTCGAAGCT CAAGAATAGG AAGTTTAGCC AGTTTAAGTA GCTGCCACTC TTTAACCGCC   
  
  
- AGGTAGATAG AGGCCGCTAC AAAGTGGCCG AAGTTCCGCC TTAGAGCTTT AAGGATCGGC TAGAGGCCCC   
  
  
- TTAAAGCGGT TCGTGGCAGC ACCACCACCT GTCACTCCAT CCTTAGCTGT GCCCTTGAAG CAGGAAGTCG   
  
  
- CAGTTGAAGC AGCGGCCATA GCTTAAGATG TGGCCTTACG AGCTTAGCGA GCTGCAGTGG AGGCGGTCGC   
  
  
- CAAAACGTCC GCCGCCGCCG CCGCTGCGAC ACTCTTCCTA GCTCTGCATG CACGAGGCCG GCTCCTAATG   
  
  
- CCGCCGCCAC CTCCGCCGTA GACGCCGCCG CCATCGCGCC GCCTCGCCAA CCTCTCTCAG CAAGCGCCAC   
  
  
- CGATCCTACT CCCGCCACCC CAAGTCGGTC AAACGACTAA AGGTCCGCCT CACAAACGAC TCCTTCCAAG   
  
  
- TCCAACCGCC CAAGGTGCAC CGCTTTGCAG TACCCCTCTA CTACGAAACG ACCGTACTAT CCGGGGAACA   
  
  
- CAGGTGCAGC CGAACCTCCA CAAT

+     ARE

| Site Name | Organism | Position | Strand | Matrix score. | sequence | function |
| --- | --- | --- | --- | --- | --- | --- |
| ARE | Zea mays | 1251 | + | 6 | AAACCA | cis-acting regulatory element essential for the anaerobic induction |

>HU06G00283.1   
+ -Up\_Stream \_Len000GATTTT ACTTCCAATT TTCTACTTAA ATTTGGTTAT CACGTTCGAA TTTTTCAGGT   
  
  
+ AAATACAAAC TGTTAGTGGC CATCCTTTTT TTTTGGGTCT AATCCATCCA GATGTGCTAA TACTTTCTGT   
  
  
+ TTTAGATGAG AAGTTTTAAT GCTTTTTTAG ATGTGTAATT TTTTTAAACA TAAATAGTCT TGTCGAATAA   
  
  
+ GATTTGCTAG TAATTTTTGT AAGAGAAATT ATGTGTATTT TTTATTATAT CAGAATATTA GATCGATCTC   
  
  
+ AATTTAATTA CTTATGAAAA AAAAAGTGGA TATCTTTAAT CAATTTGGCA AGCTCTCATG CATTAATTGT   
  
  
+ AGTCGTACGA ACTTATTTAC ATCACTCATC TGAATTTGCT ACCATTTACA CCTCAAATTT TATTTTAATG   
  
  
+ CCATAAAGAG AATAGAATAT ACTGTCTATT TGTAGTAGTT TTACAGATAA AAACAAATAA GCAAATGAAA   
  
  
+ ATAGATAGAG GATTTTTTTG CTATTATCCA CGTCGATCCA TTGAATTATT TTGTCGCTAT TTGGTTAAGT   
  
  
+ GAAATAAAAT ACTGAAGTAG AATGGAGGAT GTTGATAATG ACCTATGTCC CTCAAAGTTT AATCATGTAG   
  
  
+ TTTCTACGAA TTTCAAGCCA TTATAAGACA TACAGATTTT TATGATGTTG AAAATACTCT TACCTCCTTG   
  
  
+ TCACAATATT AACCTGAAGC CAATGAAATG AAACAGAACT AATTAAATTG AATACATAAT CAATCCATAT   
  
  
+ TAATTTGTTT TGACAACTAT TTACAATCAT CGATCTCTTT AATTAGGTCT ATAAGCTATA GAGTAATTAC   
  
  
+ TCAAAACTGT TTTGTAAGAC AACTTACTCC ATATTGGCTT ATTAGTATAT TGCCTCCATT CACATGAATA   
  
  
+ CTTCATGAGA GATTGCCTAA AACTCATATT GTCATCACCT TAAGTCAAAC ATATTTTGTT ATGAAATGAC   
  
  
+ ATCAAGAATT ACCCTCTGAC AAGAAAAATA TACCTGACTG ACATAATCGT ACCACATCTG TTAGTCCTCT   
  
  
+ TTCTACCACT TTGTAGAACA TAATGTGACA GTAATACATC CATCCCAAAA GAAAAGACGA AAAGAAGAGT   
  
  
+ GGAGAGTCAC GTATACGCGT TGAATTCAGG AAAATTAAAG GAACTCATGA AGACCCCATA TAGTGCTCCA   
  
  
+ ACCTGAACCT GCAGCCCACT ACTACATGCC TAAATTAAAG AGCAAAGTTA TGAGAGAAAC CAAAAAAGCT   
  
  
+ CTTTGAGAGA GTGGAGGAAG AATCATGGAA AGCTAGCTAG GCTTAGCAAG AAAGAGCAAC AGCTAGCTAG   
  
  
+ CAAAAGGCTT CTCTTTGAAG CCTTAGAAAT CTTTAGAGAT AAAAAGTTGG TGGCTTTTTA AGGGCTGTGA   
  
  
+ TATGTCCCCC ATGTAAAAAT GCAGCAATAA TGTTAATGTC ATGCTTTCTT TCATGGTCCC TAATGCGAAA   
  
  
+ TCAATCATGC ATGTCAACGT GACTCAGTGG CTCACCATAT ATGATCTGAT TTCCCCGCAC ACAGAGATAA   
  
  
+ AAAGCCCCCA ATTATAACCT CCCAAAAGCC CCCCCTTTTC CTCCTACCCA ACCCACACCT CCCCATTTCA   
  
  
+ TCAGGGCTCG CTTCTTAAGT TATCGATTAA AAAAAGAGTG AGTAAACGTA CCCGAATTGA AGAGGGGTGT   
  
  
+ TCGAAGCTTA TACTTGCTTG ATTAGGAATG AAAGTCCCCA TTTCATCCGC CCCCCAAACC GCCGCCACCA   
  
  
+ CCAGTAAATC GCTATCTTGC AACAATACTG CCTTGAATAC TGCCTTTCAA GCCCCCAATT CAATTTCAAC   
  
  
+ CCTTGTTAAC AACAACAAAC CCTCCTACGA GCCTACCTCG GTTCTAGACC TCCGGCGCAG CCCAAGCCCC   
  
  
+ GCCACCAACG CCGCCCTGCC ACTGCCCACT GACGCGGGCA TCAATAAACC CAAGCCTGCC TCTGCTGACC   
  
  
+ TGGACAATTT GGAGGGTGAG GGTTGGGATT CAATCCTATC AGAGCTGGGG CTTAATGATG ATTCTAATAA   
  
  
+ TCCTAACTCC AAACTTTGGT CATCCCAAAT TAGCTCATCA TGTGATTCCC ACCTCACACA GCTCCCTGAG   
  
  
+ TTCCCCTCCT CTCAGCCGTT GGATCACACA CGCACCCCCA ATAATTAACT TCTCCCCCTC TGATTACCCC   
  
  
+ ATCTCTGATT TATCCTACGG TCAGAATCTC AGCCCCAATT TTGGGCAGTT TGAGCTTCAT CTTCATCAGC   
  
  
+ CCAACGTCAA TAACAATGGG CTTGACTTCA TAGAGGACCT AATTAGAGCA GCTCAAGCCT ACGAATCCAA   
  
  
+ CGACTCCCAC CAGGTCCAGC TGGTATTGGC GCGGCTCAAT CAACGGCTCA GATCACCCGC CGGAAAACCC   
  
  
+ CTCCACCGCG CCGCCTTCTA CTTCAAGGAG GCCCTCCAAC ACCTCGCTGT CGGCGGAGGC CCGGCCCGGC   
  
  
+ CTGTCCGGCT GTCATCCTAC GAGGTGGTGC AGACCATCCG GGCCCACAAG AACTTTTCCG GCATATCACT   
  
  
+ GATATCCCTC TTCTCCACCT TCGCCGCCAA TCAGGCGATT CTGGAGGCGG TGGATGGGGC TGCGTTCATC   
  
  
+ CACATCATCG ATTTCGACAT CGGATTTGGC GGCCACTGGG CGTCGTTTAT GCGTGAGCTT GTCGACAAGG   
  
  
+ CCGACCCGAA TAAGATCAGC TCCTTGGTGT TCCGAATCAC GGCGATCGTA CCCGAAGAGT TCGGGATCGA   
  
  
+ AAGCAAGCTG GTGAGAGAGA ATCTTACGCA ATTCGCTCGT GATCTCAACA TCAATGTCCA TATGGAGTAT   
  
  
+ GTCCTTATTC AAAGCTTCGA GTTCTTATCC TTCAAATCGG TCAAATTCAT CGACGGTGAG AAATTGGCGG   
  
  
+ TCCATCTATC TCCGGCGATG TTTCACCGGC TTCAAGGCGG AATCTCGAAA TTCCTAGCCG ATCTCCGGGG   
  
  
+ AATTTCGCCA AGCACCGTCG TGGTGGTGGA CAGTGAGGTA GGAATCGACA CGGGAACTTC GTCCTTCAGC   
  
  
+ GTCAACTTCG TCGCCGGTAT CGAATTCTAC ACCGGAATGC TCGAATCGCT CGACGTCACC TCCGCCAGCG   
  
  
+ GTTTTGCAGG CGGCGGCGGC GGCGACGCTG TGAGAAGGAT CGAGACGTAC GTGCTCCGGC CGAGGATTAC   
  
  
+ GGCGGCGGTG GAGGCGGCAT CTGCGGCGGC GGTAGCGCGG CGGAGCGGTT GGAGAGAGTC GTTCGCGGTG   
  
  
+ GCTAGGATGA GGGCGGTGGG GTTCAGCCAG TTTGCTGATT TCCAGGCGGA GTGTTTGCTG AGGAAGGTTC   
  
  
+ AGGTTGGCGG GTTCCACGTG GCGAAACGTC ATGGGGAGAT GATGCTTTGC TGGCATGATA GGCCCCTTGT   
  
  
+ GTCCACGTCG GCTTGGAGGT GTTA  

- -Up\_Stream \_Len000CTAAAA TGAAGGTTAA AAGATGAATT TAAACCAATA GTGCAAGCTT AAAAAGTCCA   
  
  
- TTTATGTTTG ACAATCACCG GTAGGAAAAA AAAACCCAGA TTAGGTAGGT CTACACGATT ATGAAAGACA   
  
  
- AAATCTACTC TTCAAAATTA CGAAAAAATC TACACATTAA AAAAATTTGT ATTTATCAGA ACAGCTTATT   
  
  
- CTAAACGATC ATTAAAAACA TTCTCTTTAA TACACATAAA AAATAATATA GTCTTATAAT CTAGCTAGAG   
  
  
- TTAAATTAAT GAATACTTTT TTTTTCACCT ATAGAAATTA GTTAAACCGT TCGAGAGTAC GTAATTAACA   
  
  
- TCAGCATGCT TGAATAAATG TAGTGAGTAG ACTTAAACGA TGGTAAATGT GGAGTTTAAA ATAAAATTAC   
  
  
- GGTATTTCTC TTATCTTATA TGACAGATAA ACATCATCAA AATGTCTATT TTTGTTTATT CGTTTACTTT   
  
  
- TATCTATCTC CTAAAAAAAC GATAATAGGT GCAGCTAGGT AACTTAATAA AACAGCGATA AACCAATTCA   
  
  
- CTTTATTTTA TGACTTCATC TTACCTCCTA CAACTATTAC TGGATACAGG GAGTTTCAAA TTAGTACATC   
  
  
- AAAGATGCTT AAAGTTCGGT AATATTCTGT ATGTCTAAAA ATACTACAAC TTTTATGAGA ATGGAGGAAC   
  
  
- AGTGTTATAA TTGGACTTCG GTTACTTTAC TTTGTCTTGA TTAATTTAAC TTATGTATTA GTTAGGTATA   
  
  
- ATTAAACAAA ACTGTTGATA AATGTTAGTA GCTAGAGAAA TTAATCCAGA TATTCGATAT CTCATTAATG   
  
  
- AGTTTTGACA AAACATTCTG TTGAATGAGG TATAACCGAA TAATCATATA ACGGAGGTAA GTGTACTTAT   
  
  
- GAAGTACTCT CTAACGGATT TTGAGTATAA CAGTAGTGGA ATTCAGTTTG TATAAAACAA TACTTTACTG   
  
  
- TAGTTCTTAA TGGGAGACTG TTCTTTTTAT ATGGACTGAC TGTATTAGCA TGGTGTAGAC AATCAGGAGA   
  
  
- AAGATGGTGA AACATCTTGT ATTACACTGT CATTATGTAG GTAGGGTTTT CTTTTCTGCT TTTCTTCTCA   
  
  
- CCTCTCAGTG CATATGCGCA ACTTAAGTCC TTTTAATTTC CTTGAGTACT TCTGGGGTAT ATCACGAGGT   
  
  
- TGGACTTGGA CGTCGGGTGA TGATGTACGG ATTTAATTTC TCGTTTCAAT ACTCTCTTTG GTTTTTTCGA   
  
  
- GAAACTCTCT CACCTCCTTC TTAGTACCTT TCGATCGATC CGAATCGTTC TTTCTCGTTG TCGATCGATC   
  
  
- GTTTTCCGAA GAGAAACTTC GGAATCTTTA GAAATCTCTA TTTTTCAACC ACCGAAAAAT TCCCGACACT   
  
  
- ATACAGGGGG TACATTTTTA CGTCGTTATT ACAATTACAG TACGAAAGAA AGTACCAGGG ATTACGCTTT   
  
  
- AGTTAGTACG TACAGTTGCA CTGAGTCACC GAGTGGTATA TACTAGACTA AAGGGGCGTG TGTCTCTATT   
  
  
- TTTCGGGGGT TAATATTGGA GGGTTTTCGG GGGGGAAAAG GAGGATGGGT TGGGTGTGGA GGGGTAAAGT   
  
  
- AGTCCCGAGC GAAGAATTCA ATAGCTAATT TTTTTCTCAC TCATTTGCAT GGGCTTAACT TCTCCCCACA   
  
  
- AGCTTCGAAT ATGAACGAAC TAATCCTTAC TTTCAGGGGT AAAGTAGGCG GGGGGTTTGG CGGCGGTGGT   
  
  
- GGTCATTTAG CGATAGAACG TTGTTATGAC GGAACTTATG ACGGAAAGTT CGGGGGTTAA GTTAAAGTTG   
  
  
- GGAACAATTG TTGTTGTTTG GGAGGATGCT CGGATGGAGC CAAGATCTGG AGGCCGCGTC GGGTTCGGGG   
  
  
- CGGTGGTTGC GGCGGGACGG TGACGGGTGA CTGCGCCCGT AGTTATTTGG GTTCGGACGG AGACGACTGG   
  
  
- ACCTGTTAAA CCTCCCACTC CCAACCCTAA GTTAGGATAG TCTCGACCCC GAATTACTAC TAAGATTATT   
  
  
- AGGATTGAGG TTTGAAACCA GTAGGGTTTA ATCGAGTAGT ACACTAAGGG TGGAGTGTGT CGAGGGACTC   
  
  
- AAGGGGAGGA GAGTCGGCAA CCTAGTGTGT GCGTGGGGGT TATTAATTGA AGAGGGGGAG ACTAATGGGG   
  
  
- TAGAGACTAA ATAGGATGCC AGTCTTAGAG TCGGGGTTAA AACCCGTCAA ACTCGAAGTA GAAGTAGTCG   
  
  
- GGTTGCAGTT ATTGTTACCC GAACTGAAGT ATCTCCTGGA TTAATCTCGT CGAGTTCGGA TGCTTAGGTT   
  
  
- GCTGAGGGTG GTCCAGGTCG ACCATAACCG CGCCGAGTTA GTTGCCGAGT CTAGTGGGCG GCCTTTTGGG   
  
  
- GAGGTGGCGC GGCGGAAGAT GAAGTTCCTC CGGGAGGTTG TGGAGCGACA GCCGCCTCCG GGCCGGGCCG   
  
  
- GACAGGCCGA CAGTAGGATG CTCCACCACG TCTGGTAGGC CCGGGTGTTC TTGAAAAGGC CGTATAGTGA   
  
  
- CTATAGGGAG AAGAGGTGGA AGCGGCGGTT AGTCCGCTAA GACCTCCGCC ACCTACCCCG ACGCAAGTAG   
  
  
- GTGTAGTAGC TAAAGCTGTA GCCTAAACCG CCGGTGACCC GCAGCAAATA CGCACTCGAA CAGCTGTTCC   
  
  
- GGCTGGGCTT ATTCTAGTCG AGGAACCACA AGGCTTAGTG CCGCTAGCAT GGGCTTCTCA AGCCCTAGCT   
  
  
- TTCGTTCGAC CACTCTCTCT TAGAATGCGT TAAGCGAGCA CTAGAGTTGT AGTTACAGGT ATACCTCATA   
  
  
- CAGGAATAAG TTTCGAAGCT CAAGAATAGG AAGTTTAGCC AGTTTAAGTA GCTGCCACTC TTTAACCGCC   
  
  
- AGGTAGATAG AGGCCGCTAC AAAGTGGCCG AAGTTCCGCC TTAGAGCTTT AAGGATCGGC TAGAGGCCCC   
  
  
- TTAAAGCGGT TCGTGGCAGC ACCACCACCT GTCACTCCAT CCTTAGCTGT GCCCTTGAAG CAGGAAGTCG   
  
  
- CAGTTGAAGC AGCGGCCATA GCTTAAGATG TGGCCTTACG AGCTTAGCGA GCTGCAGTGG AGGCGGTCGC   
  
  
- CAAAACGTCC GCCGCCGCCG CCGCTGCGAC ACTCTTCCTA GCTCTGCATG CACGAGGCCG GCTCCTAATG   
  
  
- CCGCCGCCAC CTCCGCCGTA GACGCCGCCG CCATCGCGCC GCCTCGCCAA CCTCTCTCAG CAAGCGCCAC   
  
  
- CGATCCTACT CCCGCCACCC CAAGTCGGTC AAACGACTAA AGGTCCGCCT CACAAACGAC TCCTTCCAAG   
  
  
- TCCAACCGCC CAAGGTGCAC CGCTTTGCAG TACCCCTCTA CTACGAAACG ACCGTACTAT CCGGGGAACA   
  
  
- CAGGTGCAGC CGAACCTCCA CAAT

+     AT-rich sequence

| Site Name | Organism | Position | Strand | Matrix score. | sequence | function |
| --- | --- | --- | --- | --- | --- | --- |
| AT-rich sequence | Pisum sativum | 569 | + | 9 | TAAAATACT | element for maximal elicitor-mediated activation (2copies) |

>HU06G00283.1   
+ -Up\_Stream \_Len000GATTTT ACTTCCAATT TTCTACTTAA ATTTGGTTAT CACGTTCGAA TTTTTCAGGT   
  
  
+ AAATACAAAC TGTTAGTGGC CATCCTTTTT TTTTGGGTCT AATCCATCCA GATGTGCTAA TACTTTCTGT   
  
  
+ TTTAGATGAG AAGTTTTAAT GCTTTTTTAG ATGTGTAATT TTTTTAAACA TAAATAGTCT TGTCGAATAA   
  
  
+ GATTTGCTAG TAATTTTTGT AAGAGAAATT ATGTGTATTT TTTATTATAT CAGAATATTA GATCGATCTC   
  
  
+ AATTTAATTA CTTATGAAAA AAAAAGTGGA TATCTTTAAT CAATTTGGCA AGCTCTCATG CATTAATTGT   
  
  
+ AGTCGTACGA ACTTATTTAC ATCACTCATC TGAATTTGCT ACCATTTACA CCTCAAATTT TATTTTAATG   
  
  
+ CCATAAAGAG AATAGAATAT ACTGTCTATT TGTAGTAGTT TTACAGATAA AAACAAATAA GCAAATGAAA   
  
  
+ ATAGATAGAG GATTTTTTTG CTATTATCCA CGTCGATCCA TTGAATTATT TTGTCGCTAT TTGGTTAAGT   
  
  
+ GAAATAAAAT ACTGAAGTAG AATGGAGGAT GTTGATAATG ACCTATGTCC CTCAAAGTTT AATCATGTAG   
  
  
+ TTTCTACGAA TTTCAAGCCA TTATAAGACA TACAGATTTT TATGATGTTG AAAATACTCT TACCTCCTTG   
  
  
+ TCACAATATT AACCTGAAGC CAATGAAATG AAACAGAACT AATTAAATTG AATACATAAT CAATCCATAT   
  
  
+ TAATTTGTTT TGACAACTAT TTACAATCAT CGATCTCTTT AATTAGGTCT ATAAGCTATA GAGTAATTAC   
  
  
+ TCAAAACTGT TTTGTAAGAC AACTTACTCC ATATTGGCTT ATTAGTATAT TGCCTCCATT CACATGAATA   
  
  
+ CTTCATGAGA GATTGCCTAA AACTCATATT GTCATCACCT TAAGTCAAAC ATATTTTGTT ATGAAATGAC   
  
  
+ ATCAAGAATT ACCCTCTGAC AAGAAAAATA TACCTGACTG ACATAATCGT ACCACATCTG TTAGTCCTCT   
  
  
+ TTCTACCACT TTGTAGAACA TAATGTGACA GTAATACATC CATCCCAAAA GAAAAGACGA AAAGAAGAGT   
  
  
+ GGAGAGTCAC GTATACGCGT TGAATTCAGG AAAATTAAAG GAACTCATGA AGACCCCATA TAGTGCTCCA   
  
  
+ ACCTGAACCT GCAGCCCACT ACTACATGCC TAAATTAAAG AGCAAAGTTA TGAGAGAAAC CAAAAAAGCT   
  
  
+ CTTTGAGAGA GTGGAGGAAG AATCATGGAA AGCTAGCTAG GCTTAGCAAG AAAGAGCAAC AGCTAGCTAG   
  
  
+ CAAAAGGCTT CTCTTTGAAG CCTTAGAAAT CTTTAGAGAT AAAAAGTTGG TGGCTTTTTA AGGGCTGTGA   
  
  
+ TATGTCCCCC ATGTAAAAAT GCAGCAATAA TGTTAATGTC ATGCTTTCTT TCATGGTCCC TAATGCGAAA   
  
  
+ TCAATCATGC ATGTCAACGT GACTCAGTGG CTCACCATAT ATGATCTGAT TTCCCCGCAC ACAGAGATAA   
  
  
+ AAAGCCCCCA ATTATAACCT CCCAAAAGCC CCCCCTTTTC CTCCTACCCA ACCCACACCT CCCCATTTCA   
  
  
+ TCAGGGCTCG CTTCTTAAGT TATCGATTAA AAAAAGAGTG AGTAAACGTA CCCGAATTGA AGAGGGGTGT   
  
  
+ TCGAAGCTTA TACTTGCTTG ATTAGGAATG AAAGTCCCCA TTTCATCCGC CCCCCAAACC GCCGCCACCA   
  
  
+ CCAGTAAATC GCTATCTTGC AACAATACTG CCTTGAATAC TGCCTTTCAA GCCCCCAATT CAATTTCAAC   
  
  
+ CCTTGTTAAC AACAACAAAC CCTCCTACGA GCCTACCTCG GTTCTAGACC TCCGGCGCAG CCCAAGCCCC   
  
  
+ GCCACCAACG CCGCCCTGCC ACTGCCCACT GACGCGGGCA TCAATAAACC CAAGCCTGCC TCTGCTGACC   
  
  
+ TGGACAATTT GGAGGGTGAG GGTTGGGATT CAATCCTATC AGAGCTGGGG CTTAATGATG ATTCTAATAA   
  
  
+ TCCTAACTCC AAACTTTGGT CATCCCAAAT TAGCTCATCA TGTGATTCCC ACCTCACACA GCTCCCTGAG   
  
  
+ TTCCCCTCCT CTCAGCCGTT GGATCACACA CGCACCCCCA ATAATTAACT TCTCCCCCTC TGATTACCCC   
  
  
+ ATCTCTGATT TATCCTACGG TCAGAATCTC AGCCCCAATT TTGGGCAGTT TGAGCTTCAT CTTCATCAGC   
  
  
+ CCAACGTCAA TAACAATGGG CTTGACTTCA TAGAGGACCT AATTAGAGCA GCTCAAGCCT ACGAATCCAA   
  
  
+ CGACTCCCAC CAGGTCCAGC TGGTATTGGC GCGGCTCAAT CAACGGCTCA GATCACCCGC CGGAAAACCC   
  
  
+ CTCCACCGCG CCGCCTTCTA CTTCAAGGAG GCCCTCCAAC ACCTCGCTGT CGGCGGAGGC CCGGCCCGGC   
  
  
+ CTGTCCGGCT GTCATCCTAC GAGGTGGTGC AGACCATCCG GGCCCACAAG AACTTTTCCG GCATATCACT   
  
  
+ GATATCCCTC TTCTCCACCT TCGCCGCCAA TCAGGCGATT CTGGAGGCGG TGGATGGGGC TGCGTTCATC   
  
  
+ CACATCATCG ATTTCGACAT CGGATTTGGC GGCCACTGGG CGTCGTTTAT GCGTGAGCTT GTCGACAAGG   
  
  
+ CCGACCCGAA TAAGATCAGC TCCTTGGTGT TCCGAATCAC GGCGATCGTA CCCGAAGAGT TCGGGATCGA   
  
  
+ AAGCAAGCTG GTGAGAGAGA ATCTTACGCA ATTCGCTCGT GATCTCAACA TCAATGTCCA TATGGAGTAT   
  
  
+ GTCCTTATTC AAAGCTTCGA GTTCTTATCC TTCAAATCGG TCAAATTCAT CGACGGTGAG AAATTGGCGG   
  
  
+ TCCATCTATC TCCGGCGATG TTTCACCGGC TTCAAGGCGG AATCTCGAAA TTCCTAGCCG ATCTCCGGGG   
  
  
+ AATTTCGCCA AGCACCGTCG TGGTGGTGGA CAGTGAGGTA GGAATCGACA CGGGAACTTC GTCCTTCAGC   
  
  
+ GTCAACTTCG TCGCCGGTAT CGAATTCTAC ACCGGAATGC TCGAATCGCT CGACGTCACC TCCGCCAGCG   
  
  
+ GTTTTGCAGG CGGCGGCGGC GGCGACGCTG TGAGAAGGAT CGAGACGTAC GTGCTCCGGC CGAGGATTAC   
  
  
+ GGCGGCGGTG GAGGCGGCAT CTGCGGCGGC GGTAGCGCGG CGGAGCGGTT GGAGAGAGTC GTTCGCGGTG   
  
  
+ GCTAGGATGA GGGCGGTGGG GTTCAGCCAG TTTGCTGATT TCCAGGCGGA GTGTTTGCTG AGGAAGGTTC   
  
  
+ AGGTTGGCGG GTTCCACGTG GCGAAACGTC ATGGGGAGAT GATGCTTTGC TGGCATGATA GGCCCCTTGT   
  
  
+ GTCCACGTCG GCTTGGAGGT GTTA  

- -Up\_Stream \_Len000CTAAAA TGAAGGTTAA AAGATGAATT TAAACCAATA GTGCAAGCTT AAAAAGTCCA   
  
  
- TTTATGTTTG ACAATCACCG GTAGGAAAAA AAAACCCAGA TTAGGTAGGT CTACACGATT ATGAAAGACA   
  
  
- AAATCTACTC TTCAAAATTA CGAAAAAATC TACACATTAA AAAAATTTGT ATTTATCAGA ACAGCTTATT   
  
  
- CTAAACGATC ATTAAAAACA TTCTCTTTAA TACACATAAA AAATAATATA GTCTTATAAT CTAGCTAGAG   
  
  
- TTAAATTAAT GAATACTTTT TTTTTCACCT ATAGAAATTA GTTAAACCGT TCGAGAGTAC GTAATTAACA   
  
  
- TCAGCATGCT TGAATAAATG TAGTGAGTAG ACTTAAACGA TGGTAAATGT GGAGTTTAAA ATAAAATTAC   
  
  
- GGTATTTCTC TTATCTTATA TGACAGATAA ACATCATCAA AATGTCTATT TTTGTTTATT CGTTTACTTT   
  
  
- TATCTATCTC CTAAAAAAAC GATAATAGGT GCAGCTAGGT AACTTAATAA AACAGCGATA AACCAATTCA   
  
  
- CTTTATTTTA TGACTTCATC TTACCTCCTA CAACTATTAC TGGATACAGG GAGTTTCAAA TTAGTACATC   
  
  
- AAAGATGCTT AAAGTTCGGT AATATTCTGT ATGTCTAAAA ATACTACAAC TTTTATGAGA ATGGAGGAAC   
  
  
- AGTGTTATAA TTGGACTTCG GTTACTTTAC TTTGTCTTGA TTAATTTAAC TTATGTATTA GTTAGGTATA   
  
  
- ATTAAACAAA ACTGTTGATA AATGTTAGTA GCTAGAGAAA TTAATCCAGA TATTCGATAT CTCATTAATG   
  
  
- AGTTTTGACA AAACATTCTG TTGAATGAGG TATAACCGAA TAATCATATA ACGGAGGTAA GTGTACTTAT   
  
  
- GAAGTACTCT CTAACGGATT TTGAGTATAA CAGTAGTGGA ATTCAGTTTG TATAAAACAA TACTTTACTG   
  
  
- TAGTTCTTAA TGGGAGACTG TTCTTTTTAT ATGGACTGAC TGTATTAGCA TGGTGTAGAC AATCAGGAGA   
  
  
- AAGATGGTGA AACATCTTGT ATTACACTGT CATTATGTAG GTAGGGTTTT CTTTTCTGCT TTTCTTCTCA   
  
  
- CCTCTCAGTG CATATGCGCA ACTTAAGTCC TTTTAATTTC CTTGAGTACT TCTGGGGTAT ATCACGAGGT   
  
  
- TGGACTTGGA CGTCGGGTGA TGATGTACGG ATTTAATTTC TCGTTTCAAT ACTCTCTTTG GTTTTTTCGA   
  
  
- GAAACTCTCT CACCTCCTTC TTAGTACCTT TCGATCGATC CGAATCGTTC TTTCTCGTTG TCGATCGATC   
  
  
- GTTTTCCGAA GAGAAACTTC GGAATCTTTA GAAATCTCTA TTTTTCAACC ACCGAAAAAT TCCCGACACT   
  
  
- ATACAGGGGG TACATTTTTA CGTCGTTATT ACAATTACAG TACGAAAGAA AGTACCAGGG ATTACGCTTT   
  
  
- AGTTAGTACG TACAGTTGCA CTGAGTCACC GAGTGGTATA TACTAGACTA AAGGGGCGTG TGTCTCTATT   
  
  
- TTTCGGGGGT TAATATTGGA GGGTTTTCGG GGGGGAAAAG GAGGATGGGT TGGGTGTGGA GGGGTAAAGT   
  
  
- AGTCCCGAGC GAAGAATTCA ATAGCTAATT TTTTTCTCAC TCATTTGCAT GGGCTTAACT TCTCCCCACA   
  
  
- AGCTTCGAAT ATGAACGAAC TAATCCTTAC TTTCAGGGGT AAAGTAGGCG GGGGGTTTGG CGGCGGTGGT   
  
  
- GGTCATTTAG CGATAGAACG TTGTTATGAC GGAACTTATG ACGGAAAGTT CGGGGGTTAA GTTAAAGTTG   
  
  
- GGAACAATTG TTGTTGTTTG GGAGGATGCT CGGATGGAGC CAAGATCTGG AGGCCGCGTC GGGTTCGGGG   
  
  
- CGGTGGTTGC GGCGGGACGG TGACGGGTGA CTGCGCCCGT AGTTATTTGG GTTCGGACGG AGACGACTGG   
  
  
- ACCTGTTAAA CCTCCCACTC CCAACCCTAA GTTAGGATAG TCTCGACCCC GAATTACTAC TAAGATTATT   
  
  
- AGGATTGAGG TTTGAAACCA GTAGGGTTTA ATCGAGTAGT ACACTAAGGG TGGAGTGTGT CGAGGGACTC   
  
  
- AAGGGGAGGA GAGTCGGCAA CCTAGTGTGT GCGTGGGGGT TATTAATTGA AGAGGGGGAG ACTAATGGGG   
  
  
- TAGAGACTAA ATAGGATGCC AGTCTTAGAG TCGGGGTTAA AACCCGTCAA ACTCGAAGTA GAAGTAGTCG   
  
  
- GGTTGCAGTT ATTGTTACCC GAACTGAAGT ATCTCCTGGA TTAATCTCGT CGAGTTCGGA TGCTTAGGTT   
  
  
- GCTGAGGGTG GTCCAGGTCG ACCATAACCG CGCCGAGTTA GTTGCCGAGT CTAGTGGGCG GCCTTTTGGG   
  
  
- GAGGTGGCGC GGCGGAAGAT GAAGTTCCTC CGGGAGGTTG TGGAGCGACA GCCGCCTCCG GGCCGGGCCG   
  
  
- GACAGGCCGA CAGTAGGATG CTCCACCACG TCTGGTAGGC CCGGGTGTTC TTGAAAAGGC CGTATAGTGA   
  
  
- CTATAGGGAG AAGAGGTGGA AGCGGCGGTT AGTCCGCTAA GACCTCCGCC ACCTACCCCG ACGCAAGTAG   
  
  
- GTGTAGTAGC TAAAGCTGTA GCCTAAACCG CCGGTGACCC GCAGCAAATA CGCACTCGAA CAGCTGTTCC   
  
  
- GGCTGGGCTT ATTCTAGTCG AGGAACCACA AGGCTTAGTG CCGCTAGCAT GGGCTTCTCA AGCCCTAGCT   
  
  
- TTCGTTCGAC CACTCTCTCT TAGAATGCGT TAAGCGAGCA CTAGAGTTGT AGTTACAGGT ATACCTCATA   
  
  
- CAGGAATAAG TTTCGAAGCT CAAGAATAGG AAGTTTAGCC AGTTTAAGTA GCTGCCACTC TTTAACCGCC   
  
  
- AGGTAGATAG AGGCCGCTAC AAAGTGGCCG AAGTTCCGCC TTAGAGCTTT AAGGATCGGC TAGAGGCCCC   
  
  
- TTAAAGCGGT TCGTGGCAGC ACCACCACCT GTCACTCCAT CCTTAGCTGT GCCCTTGAAG CAGGAAGTCG   
  
  
- CAGTTGAAGC AGCGGCCATA GCTTAAGATG TGGCCTTACG AGCTTAGCGA GCTGCAGTGG AGGCGGTCGC   
  
  
- CAAAACGTCC GCCGCCGCCG CCGCTGCGAC ACTCTTCCTA GCTCTGCATG CACGAGGCCG GCTCCTAATG   
  
  
- CCGCCGCCAC CTCCGCCGTA GACGCCGCCG CCATCGCGCC GCCTCGCCAA CCTCTCTCAG CAAGCGCCAC   
  
  
- CGATCCTACT CCCGCCACCC CAAGTCGGTC AAACGACTAA AGGTCCGCCT CACAAACGAC TCCTTCCAAG   
  
  
- TCCAACCGCC CAAGGTGCAC CGCTTTGCAG TACCCCTCTA CTACGAAACG ACCGTACTAT CCGGGGAACA   
  
  
- CAGGTGCAGC CGAACCTCCA CAAT

+     AuxRR-core

| Site Name | Organism | Position | Strand | Matrix score. | sequence | function |
| --- | --- | --- | --- | --- | --- | --- |
| AuxRR-core | Nicotiana tabacum | 2873 | + | 7 | GGTCCAT | cis-acting regulatory element involved in auxin responsiveness |

>HU06G00283.1   
+ -Up\_Stream \_Len000GATTTT ACTTCCAATT TTCTACTTAA ATTTGGTTAT CACGTTCGAA TTTTTCAGGT   
  
  
+ AAATACAAAC TGTTAGTGGC CATCCTTTTT TTTTGGGTCT AATCCATCCA GATGTGCTAA TACTTTCTGT   
  
  
+ TTTAGATGAG AAGTTTTAAT GCTTTTTTAG ATGTGTAATT TTTTTAAACA TAAATAGTCT TGTCGAATAA   
  
  
+ GATTTGCTAG TAATTTTTGT AAGAGAAATT ATGTGTATTT TTTATTATAT CAGAATATTA GATCGATCTC   
  
  
+ AATTTAATTA CTTATGAAAA AAAAAGTGGA TATCTTTAAT CAATTTGGCA AGCTCTCATG CATTAATTGT   
  
  
+ AGTCGTACGA ACTTATTTAC ATCACTCATC TGAATTTGCT ACCATTTACA CCTCAAATTT TATTTTAATG   
  
  
+ CCATAAAGAG AATAGAATAT ACTGTCTATT TGTAGTAGTT TTACAGATAA AAACAAATAA GCAAATGAAA   
  
  
+ ATAGATAGAG GATTTTTTTG CTATTATCCA CGTCGATCCA TTGAATTATT TTGTCGCTAT TTGGTTAAGT   
  
  
+ GAAATAAAAT ACTGAAGTAG AATGGAGGAT GTTGATAATG ACCTATGTCC CTCAAAGTTT AATCATGTAG   
  
  
+ TTTCTACGAA TTTCAAGCCA TTATAAGACA TACAGATTTT TATGATGTTG AAAATACTCT TACCTCCTTG   
  
  
+ TCACAATATT AACCTGAAGC CAATGAAATG AAACAGAACT AATTAAATTG AATACATAAT CAATCCATAT   
  
  
+ TAATTTGTTT TGACAACTAT TTACAATCAT CGATCTCTTT AATTAGGTCT ATAAGCTATA GAGTAATTAC   
  
  
+ TCAAAACTGT TTTGTAAGAC AACTTACTCC ATATTGGCTT ATTAGTATAT TGCCTCCATT CACATGAATA   
  
  
+ CTTCATGAGA GATTGCCTAA AACTCATATT GTCATCACCT TAAGTCAAAC ATATTTTGTT ATGAAATGAC   
  
  
+ ATCAAGAATT ACCCTCTGAC AAGAAAAATA TACCTGACTG ACATAATCGT ACCACATCTG TTAGTCCTCT   
  
  
+ TTCTACCACT TTGTAGAACA TAATGTGACA GTAATACATC CATCCCAAAA GAAAAGACGA AAAGAAGAGT   
  
  
+ GGAGAGTCAC GTATACGCGT TGAATTCAGG AAAATTAAAG GAACTCATGA AGACCCCATA TAGTGCTCCA   
  
  
+ ACCTGAACCT GCAGCCCACT ACTACATGCC TAAATTAAAG AGCAAAGTTA TGAGAGAAAC CAAAAAAGCT   
  
  
+ CTTTGAGAGA GTGGAGGAAG AATCATGGAA AGCTAGCTAG GCTTAGCAAG AAAGAGCAAC AGCTAGCTAG   
  
  
+ CAAAAGGCTT CTCTTTGAAG CCTTAGAAAT CTTTAGAGAT AAAAAGTTGG TGGCTTTTTA AGGGCTGTGA   
  
  
+ TATGTCCCCC ATGTAAAAAT GCAGCAATAA TGTTAATGTC ATGCTTTCTT TCATGGTCCC TAATGCGAAA   
  
  
+ TCAATCATGC ATGTCAACGT GACTCAGTGG CTCACCATAT ATGATCTGAT TTCCCCGCAC ACAGAGATAA   
  
  
+ AAAGCCCCCA ATTATAACCT CCCAAAAGCC CCCCCTTTTC CTCCTACCCA ACCCACACCT CCCCATTTCA   
  
  
+ TCAGGGCTCG CTTCTTAAGT TATCGATTAA AAAAAGAGTG AGTAAACGTA CCCGAATTGA AGAGGGGTGT   
  
  
+ TCGAAGCTTA TACTTGCTTG ATTAGGAATG AAAGTCCCCA TTTCATCCGC CCCCCAAACC GCCGCCACCA   
  
  
+ CCAGTAAATC GCTATCTTGC AACAATACTG CCTTGAATAC TGCCTTTCAA GCCCCCAATT CAATTTCAAC   
  
  
+ CCTTGTTAAC AACAACAAAC CCTCCTACGA GCCTACCTCG GTTCTAGACC TCCGGCGCAG CCCAAGCCCC   
  
  
+ GCCACCAACG CCGCCCTGCC ACTGCCCACT GACGCGGGCA TCAATAAACC CAAGCCTGCC TCTGCTGACC   
  
  
+ TGGACAATTT GGAGGGTGAG GGTTGGGATT CAATCCTATC AGAGCTGGGG CTTAATGATG ATTCTAATAA   
  
  
+ TCCTAACTCC AAACTTTGGT CATCCCAAAT TAGCTCATCA TGTGATTCCC ACCTCACACA GCTCCCTGAG   
  
  
+ TTCCCCTCCT CTCAGCCGTT GGATCACACA CGCACCCCCA ATAATTAACT TCTCCCCCTC TGATTACCCC   
  
  
+ ATCTCTGATT TATCCTACGG TCAGAATCTC AGCCCCAATT TTGGGCAGTT TGAGCTTCAT CTTCATCAGC   
  
  
+ CCAACGTCAA TAACAATGGG CTTGACTTCA TAGAGGACCT AATTAGAGCA GCTCAAGCCT ACGAATCCAA   
  
  
+ CGACTCCCAC CAGGTCCAGC TGGTATTGGC GCGGCTCAAT CAACGGCTCA GATCACCCGC CGGAAAACCC   
  
  
+ CTCCACCGCG CCGCCTTCTA CTTCAAGGAG GCCCTCCAAC ACCTCGCTGT CGGCGGAGGC CCGGCCCGGC   
  
  
+ CTGTCCGGCT GTCATCCTAC GAGGTGGTGC AGACCATCCG GGCCCACAAG AACTTTTCCG GCATATCACT   
  
  
+ GATATCCCTC TTCTCCACCT TCGCCGCCAA TCAGGCGATT CTGGAGGCGG TGGATGGGGC TGCGTTCATC   
  
  
+ CACATCATCG ATTTCGACAT CGGATTTGGC GGCCACTGGG CGTCGTTTAT GCGTGAGCTT GTCGACAAGG   
  
  
+ CCGACCCGAA TAAGATCAGC TCCTTGGTGT TCCGAATCAC GGCGATCGTA CCCGAAGAGT TCGGGATCGA   
  
  
+ AAGCAAGCTG GTGAGAGAGA ATCTTACGCA ATTCGCTCGT GATCTCAACA TCAATGTCCA TATGGAGTAT   
  
  
+ GTCCTTATTC AAAGCTTCGA GTTCTTATCC TTCAAATCGG TCAAATTCAT CGACGGTGAG AAATTGGCGG   
  
  
+ TCCATCTATC TCCGGCGATG TTTCACCGGC TTCAAGGCGG AATCTCGAAA TTCCTAGCCG ATCTCCGGGG   
  
  
+ AATTTCGCCA AGCACCGTCG TGGTGGTGGA CAGTGAGGTA GGAATCGACA CGGGAACTTC GTCCTTCAGC   
  
  
+ GTCAACTTCG TCGCCGGTAT CGAATTCTAC ACCGGAATGC TCGAATCGCT CGACGTCACC TCCGCCAGCG   
  
  
+ GTTTTGCAGG CGGCGGCGGC GGCGACGCTG TGAGAAGGAT CGAGACGTAC GTGCTCCGGC CGAGGATTAC   
  
  
+ GGCGGCGGTG GAGGCGGCAT CTGCGGCGGC GGTAGCGCGG CGGAGCGGTT GGAGAGAGTC GTTCGCGGTG   
  
  
+ GCTAGGATGA GGGCGGTGGG GTTCAGCCAG TTTGCTGATT TCCAGGCGGA GTGTTTGCTG AGGAAGGTTC   
  
  
+ AGGTTGGCGG GTTCCACGTG GCGAAACGTC ATGGGGAGAT GATGCTTTGC TGGCATGATA GGCCCCTTGT   
  
  
+ GTCCACGTCG GCTTGGAGGT GTTA  

- -Up\_Stream \_Len000CTAAAA TGAAGGTTAA AAGATGAATT TAAACCAATA GTGCAAGCTT AAAAAGTCCA   
  
  
- TTTATGTTTG ACAATCACCG GTAGGAAAAA AAAACCCAGA TTAGGTAGGT CTACACGATT ATGAAAGACA   
  
  
- AAATCTACTC TTCAAAATTA CGAAAAAATC TACACATTAA AAAAATTTGT ATTTATCAGA ACAGCTTATT   
  
  
- CTAAACGATC ATTAAAAACA TTCTCTTTAA TACACATAAA AAATAATATA GTCTTATAAT CTAGCTAGAG   
  
  
- TTAAATTAAT GAATACTTTT TTTTTCACCT ATAGAAATTA GTTAAACCGT TCGAGAGTAC GTAATTAACA   
  
  
- TCAGCATGCT TGAATAAATG TAGTGAGTAG ACTTAAACGA TGGTAAATGT GGAGTTTAAA ATAAAATTAC   
  
  
- GGTATTTCTC TTATCTTATA TGACAGATAA ACATCATCAA AATGTCTATT TTTGTTTATT CGTTTACTTT   
  
  
- TATCTATCTC CTAAAAAAAC GATAATAGGT GCAGCTAGGT AACTTAATAA AACAGCGATA AACCAATTCA   
  
  
- CTTTATTTTA TGACTTCATC TTACCTCCTA CAACTATTAC TGGATACAGG GAGTTTCAAA TTAGTACATC   
  
  
- AAAGATGCTT AAAGTTCGGT AATATTCTGT ATGTCTAAAA ATACTACAAC TTTTATGAGA ATGGAGGAAC   
  
  
- AGTGTTATAA TTGGACTTCG GTTACTTTAC TTTGTCTTGA TTAATTTAAC TTATGTATTA GTTAGGTATA   
  
  
- ATTAAACAAA ACTGTTGATA AATGTTAGTA GCTAGAGAAA TTAATCCAGA TATTCGATAT CTCATTAATG   
  
  
- AGTTTTGACA AAACATTCTG TTGAATGAGG TATAACCGAA TAATCATATA ACGGAGGTAA GTGTACTTAT   
  
  
- GAAGTACTCT CTAACGGATT TTGAGTATAA CAGTAGTGGA ATTCAGTTTG TATAAAACAA TACTTTACTG   
  
  
- TAGTTCTTAA TGGGAGACTG TTCTTTTTAT ATGGACTGAC TGTATTAGCA TGGTGTAGAC AATCAGGAGA   
  
  
- AAGATGGTGA AACATCTTGT ATTACACTGT CATTATGTAG GTAGGGTTTT CTTTTCTGCT TTTCTTCTCA   
  
  
- CCTCTCAGTG CATATGCGCA ACTTAAGTCC TTTTAATTTC CTTGAGTACT TCTGGGGTAT ATCACGAGGT   
  
  
- TGGACTTGGA CGTCGGGTGA TGATGTACGG ATTTAATTTC TCGTTTCAAT ACTCTCTTTG GTTTTTTCGA   
  
  
- GAAACTCTCT CACCTCCTTC TTAGTACCTT TCGATCGATC CGAATCGTTC TTTCTCGTTG TCGATCGATC   
  
  
- GTTTTCCGAA GAGAAACTTC GGAATCTTTA GAAATCTCTA TTTTTCAACC ACCGAAAAAT TCCCGACACT   
  
  
- ATACAGGGGG TACATTTTTA CGTCGTTATT ACAATTACAG TACGAAAGAA AGTACCAGGG ATTACGCTTT   
  
  
- AGTTAGTACG TACAGTTGCA CTGAGTCACC GAGTGGTATA TACTAGACTA AAGGGGCGTG TGTCTCTATT   
  
  
- TTTCGGGGGT TAATATTGGA GGGTTTTCGG GGGGGAAAAG GAGGATGGGT TGGGTGTGGA GGGGTAAAGT   
  
  
- AGTCCCGAGC GAAGAATTCA ATAGCTAATT TTTTTCTCAC TCATTTGCAT GGGCTTAACT TCTCCCCACA   
  
  
- AGCTTCGAAT ATGAACGAAC TAATCCTTAC TTTCAGGGGT AAAGTAGGCG GGGGGTTTGG CGGCGGTGGT   
  
  
- GGTCATTTAG CGATAGAACG TTGTTATGAC GGAACTTATG ACGGAAAGTT CGGGGGTTAA GTTAAAGTTG   
  
  
- GGAACAATTG TTGTTGTTTG GGAGGATGCT CGGATGGAGC CAAGATCTGG AGGCCGCGTC GGGTTCGGGG   
  
  
- CGGTGGTTGC GGCGGGACGG TGACGGGTGA CTGCGCCCGT AGTTATTTGG GTTCGGACGG AGACGACTGG   
  
  
- ACCTGTTAAA CCTCCCACTC CCAACCCTAA GTTAGGATAG TCTCGACCCC GAATTACTAC TAAGATTATT   
  
  
- AGGATTGAGG TTTGAAACCA GTAGGGTTTA ATCGAGTAGT ACACTAAGGG TGGAGTGTGT CGAGGGACTC   
  
  
- AAGGGGAGGA GAGTCGGCAA CCTAGTGTGT GCGTGGGGGT TATTAATTGA AGAGGGGGAG ACTAATGGGG   
  
  
- TAGAGACTAA ATAGGATGCC AGTCTTAGAG TCGGGGTTAA AACCCGTCAA ACTCGAAGTA GAAGTAGTCG   
  
  
- GGTTGCAGTT ATTGTTACCC GAACTGAAGT ATCTCCTGGA TTAATCTCGT CGAGTTCGGA TGCTTAGGTT   
  
  
- GCTGAGGGTG GTCCAGGTCG ACCATAACCG CGCCGAGTTA GTTGCCGAGT CTAGTGGGCG GCCTTTTGGG   
  
  
- GAGGTGGCGC GGCGGAAGAT GAAGTTCCTC CGGGAGGTTG TGGAGCGACA GCCGCCTCCG GGCCGGGCCG   
  
  
- GACAGGCCGA CAGTAGGATG CTCCACCACG TCTGGTAGGC CCGGGTGTTC TTGAAAAGGC CGTATAGTGA   
  
  
- CTATAGGGAG AAGAGGTGGA AGCGGCGGTT AGTCCGCTAA GACCTCCGCC ACCTACCCCG ACGCAAGTAG   
  
  
- GTGTAGTAGC TAAAGCTGTA GCCTAAACCG CCGGTGACCC GCAGCAAATA CGCACTCGAA CAGCTGTTCC   
  
  
- GGCTGGGCTT ATTCTAGTCG AGGAACCACA AGGCTTAGTG CCGCTAGCAT GGGCTTCTCA AGCCCTAGCT   
  
  
- TTCGTTCGAC CACTCTCTCT TAGAATGCGT TAAGCGAGCA CTAGAGTTGT AGTTACAGGT ATACCTCATA   
  
  
- CAGGAATAAG TTTCGAAGCT CAAGAATAGG AAGTTTAGCC AGTTTAAGTA GCTGCCACTC TTTAACCGCC   
  
  
- AGGTAGATAG AGGCCGCTAC AAAGTGGCCG AAGTTCCGCC TTAGAGCTTT AAGGATCGGC TAGAGGCCCC   
  
  
- TTAAAGCGGT TCGTGGCAGC ACCACCACCT GTCACTCCAT CCTTAGCTGT GCCCTTGAAG CAGGAAGTCG   
  
  
- CAGTTGAAGC AGCGGCCATA GCTTAAGATG TGGCCTTACG AGCTTAGCGA GCTGCAGTGG AGGCGGTCGC   
  
  
- CAAAACGTCC GCCGCCGCCG CCGCTGCGAC ACTCTTCCTA GCTCTGCATG CACGAGGCCG GCTCCTAATG   
  
  
- CCGCCGCCAC CTCCGCCGTA GACGCCGCCG CCATCGCGCC GCCTCGCCAA CCTCTCTCAG CAAGCGCCAC   
  
  
- CGATCCTACT CCCGCCACCC CAAGTCGGTC AAACGACTAA AGGTCCGCCT CACAAACGAC TCCTTCCAAG   
  
  
- TCCAACCGCC CAAGGTGCAC CGCTTTGCAG TACCCCTCTA CTACGAAACG ACCGTACTAT CCGGGGAACA   
  
  
- CAGGTGCAGC CGAACCTCCA CAAT

+     Box 4

| Site Name | Organism | Position | Strand | Matrix score. | sequence | function |
| --- | --- | --- | --- | --- | --- | --- |
| Box 4 | Petroselinum crispum | 346 | + | 6 | ATTAAT | part of a conserved DNA module involved in light responsiveness |
| Box 4 | Petroselinum crispum | 773 | + | 6 | ATTAAT | part of a conserved DNA module involved in light responsiveness |

>HU06G00283.1   
+ -Up\_Stream \_Len000GATTTT ACTTCCAATT TTCTACTTAA ATTTGGTTAT CACGTTCGAA TTTTTCAGGT   
  
  
+ AAATACAAAC TGTTAGTGGC CATCCTTTTT TTTTGGGTCT AATCCATCCA GATGTGCTAA TACTTTCTGT   
  
  
+ TTTAGATGAG AAGTTTTAAT GCTTTTTTAG ATGTGTAATT TTTTTAAACA TAAATAGTCT TGTCGAATAA   
  
  
+ GATTTGCTAG TAATTTTTGT AAGAGAAATT ATGTGTATTT TTTATTATAT CAGAATATTA GATCGATCTC   
  
  
+ AATTTAATTA CTTATGAAAA AAAAAGTGGA TATCTTTAAT CAATTTGGCA AGCTCTCATG CATTAATTGT   
  
  
+ AGTCGTACGA ACTTATTTAC ATCACTCATC TGAATTTGCT ACCATTTACA CCTCAAATTT TATTTTAATG   
  
  
+ CCATAAAGAG AATAGAATAT ACTGTCTATT TGTAGTAGTT TTACAGATAA AAACAAATAA GCAAATGAAA   
  
  
+ ATAGATAGAG GATTTTTTTG CTATTATCCA CGTCGATCCA TTGAATTATT TTGTCGCTAT TTGGTTAAGT   
  
  
+ GAAATAAAAT ACTGAAGTAG AATGGAGGAT GTTGATAATG ACCTATGTCC CTCAAAGTTT AATCATGTAG   
  
  
+ TTTCTACGAA TTTCAAGCCA TTATAAGACA TACAGATTTT TATGATGTTG AAAATACTCT TACCTCCTTG   
  
  
+ TCACAATATT AACCTGAAGC CAATGAAATG AAACAGAACT AATTAAATTG AATACATAAT CAATCCATAT   
  
  
+ TAATTTGTTT TGACAACTAT TTACAATCAT CGATCTCTTT AATTAGGTCT ATAAGCTATA GAGTAATTAC   
  
  
+ TCAAAACTGT TTTGTAAGAC AACTTACTCC ATATTGGCTT ATTAGTATAT TGCCTCCATT CACATGAATA   
  
  
+ CTTCATGAGA GATTGCCTAA AACTCATATT GTCATCACCT TAAGTCAAAC ATATTTTGTT ATGAAATGAC   
  
  
+ ATCAAGAATT ACCCTCTGAC AAGAAAAATA TACCTGACTG ACATAATCGT ACCACATCTG TTAGTCCTCT   
  
  
+ TTCTACCACT TTGTAGAACA TAATGTGACA GTAATACATC CATCCCAAAA GAAAAGACGA AAAGAAGAGT   
  
  
+ GGAGAGTCAC GTATACGCGT TGAATTCAGG AAAATTAAAG GAACTCATGA AGACCCCATA TAGTGCTCCA   
  
  
+ ACCTGAACCT GCAGCCCACT ACTACATGCC TAAATTAAAG AGCAAAGTTA TGAGAGAAAC CAAAAAAGCT   
  
  
+ CTTTGAGAGA GTGGAGGAAG AATCATGGAA AGCTAGCTAG GCTTAGCAAG AAAGAGCAAC AGCTAGCTAG   
  
  
+ CAAAAGGCTT CTCTTTGAAG CCTTAGAAAT CTTTAGAGAT AAAAAGTTGG TGGCTTTTTA AGGGCTGTGA   
  
  
+ TATGTCCCCC ATGTAAAAAT GCAGCAATAA TGTTAATGTC ATGCTTTCTT TCATGGTCCC TAATGCGAAA   
  
  
+ TCAATCATGC ATGTCAACGT GACTCAGTGG CTCACCATAT ATGATCTGAT TTCCCCGCAC ACAGAGATAA   
  
  
+ AAAGCCCCCA ATTATAACCT CCCAAAAGCC CCCCCTTTTC CTCCTACCCA ACCCACACCT CCCCATTTCA   
  
  
+ TCAGGGCTCG CTTCTTAAGT TATCGATTAA AAAAAGAGTG AGTAAACGTA CCCGAATTGA AGAGGGGTGT   
  
  
+ TCGAAGCTTA TACTTGCTTG ATTAGGAATG AAAGTCCCCA TTTCATCCGC CCCCCAAACC GCCGCCACCA   
  
  
+ CCAGTAAATC GCTATCTTGC AACAATACTG CCTTGAATAC TGCCTTTCAA GCCCCCAATT CAATTTCAAC   
  
  
+ CCTTGTTAAC AACAACAAAC CCTCCTACGA GCCTACCTCG GTTCTAGACC TCCGGCGCAG CCCAAGCCCC   
  
  
+ GCCACCAACG CCGCCCTGCC ACTGCCCACT GACGCGGGCA TCAATAAACC CAAGCCTGCC TCTGCTGACC   
  
  
+ TGGACAATTT GGAGGGTGAG GGTTGGGATT CAATCCTATC AGAGCTGGGG CTTAATGATG ATTCTAATAA   
  
  
+ TCCTAACTCC AAACTTTGGT CATCCCAAAT TAGCTCATCA TGTGATTCCC ACCTCACACA GCTCCCTGAG   
  
  
+ TTCCCCTCCT CTCAGCCGTT GGATCACACA CGCACCCCCA ATAATTAACT TCTCCCCCTC TGATTACCCC   
  
  
+ ATCTCTGATT TATCCTACGG TCAGAATCTC AGCCCCAATT TTGGGCAGTT TGAGCTTCAT CTTCATCAGC   
  
  
+ CCAACGTCAA TAACAATGGG CTTGACTTCA TAGAGGACCT AATTAGAGCA GCTCAAGCCT ACGAATCCAA   
  
  
+ CGACTCCCAC CAGGTCCAGC TGGTATTGGC GCGGCTCAAT CAACGGCTCA GATCACCCGC CGGAAAACCC   
  
  
+ CTCCACCGCG CCGCCTTCTA CTTCAAGGAG GCCCTCCAAC ACCTCGCTGT CGGCGGAGGC CCGGCCCGGC   
  
  
+ CTGTCCGGCT GTCATCCTAC GAGGTGGTGC AGACCATCCG GGCCCACAAG AACTTTTCCG GCATATCACT   
  
  
+ GATATCCCTC TTCTCCACCT TCGCCGCCAA TCAGGCGATT CTGGAGGCGG TGGATGGGGC TGCGTTCATC   
  
  
+ CACATCATCG ATTTCGACAT CGGATTTGGC GGCCACTGGG CGTCGTTTAT GCGTGAGCTT GTCGACAAGG   
  
  
+ CCGACCCGAA TAAGATCAGC TCCTTGGTGT TCCGAATCAC GGCGATCGTA CCCGAAGAGT TCGGGATCGA   
  
  
+ AAGCAAGCTG GTGAGAGAGA ATCTTACGCA ATTCGCTCGT GATCTCAACA TCAATGTCCA TATGGAGTAT   
  
  
+ GTCCTTATTC AAAGCTTCGA GTTCTTATCC TTCAAATCGG TCAAATTCAT CGACGGTGAG AAATTGGCGG   
  
  
+ TCCATCTATC TCCGGCGATG TTTCACCGGC TTCAAGGCGG AATCTCGAAA TTCCTAGCCG ATCTCCGGGG   
  
  
+ AATTTCGCCA AGCACCGTCG TGGTGGTGGA CAGTGAGGTA GGAATCGACA CGGGAACTTC GTCCTTCAGC   
  
  
+ GTCAACTTCG TCGCCGGTAT CGAATTCTAC ACCGGAATGC TCGAATCGCT CGACGTCACC TCCGCCAGCG   
  
  
+ GTTTTGCAGG CGGCGGCGGC GGCGACGCTG TGAGAAGGAT CGAGACGTAC GTGCTCCGGC CGAGGATTAC   
  
  
+ GGCGGCGGTG GAGGCGGCAT CTGCGGCGGC GGTAGCGCGG CGGAGCGGTT GGAGAGAGTC GTTCGCGGTG   
  
  
+ GCTAGGATGA GGGCGGTGGG GTTCAGCCAG TTTGCTGATT TCCAGGCGGA GTGTTTGCTG AGGAAGGTTC   
  
  
+ AGGTTGGCGG GTTCCACGTG GCGAAACGTC ATGGGGAGAT GATGCTTTGC TGGCATGATA GGCCCCTTGT   
  
  
+ GTCCACGTCG GCTTGGAGGT GTTA  

- -Up\_Stream \_Len000CTAAAA TGAAGGTTAA AAGATGAATT TAAACCAATA GTGCAAGCTT AAAAAGTCCA   
  
  
- TTTATGTTTG ACAATCACCG GTAGGAAAAA AAAACCCAGA TTAGGTAGGT CTACACGATT ATGAAAGACA   
  
  
- AAATCTACTC TTCAAAATTA CGAAAAAATC TACACATTAA AAAAATTTGT ATTTATCAGA ACAGCTTATT   
  
  
- CTAAACGATC ATTAAAAACA TTCTCTTTAA TACACATAAA AAATAATATA GTCTTATAAT CTAGCTAGAG   
  
  
- TTAAATTAAT GAATACTTTT TTTTTCACCT ATAGAAATTA GTTAAACCGT TCGAGAGTAC GTAATTAACA   
  
  
- TCAGCATGCT TGAATAAATG TAGTGAGTAG ACTTAAACGA TGGTAAATGT GGAGTTTAAA ATAAAATTAC   
  
  
- GGTATTTCTC TTATCTTATA TGACAGATAA ACATCATCAA AATGTCTATT TTTGTTTATT CGTTTACTTT   
  
  
- TATCTATCTC CTAAAAAAAC GATAATAGGT GCAGCTAGGT AACTTAATAA AACAGCGATA AACCAATTCA   
  
  
- CTTTATTTTA TGACTTCATC TTACCTCCTA CAACTATTAC TGGATACAGG GAGTTTCAAA TTAGTACATC   
  
  
- AAAGATGCTT AAAGTTCGGT AATATTCTGT ATGTCTAAAA ATACTACAAC TTTTATGAGA ATGGAGGAAC   
  
  
- AGTGTTATAA TTGGACTTCG GTTACTTTAC TTTGTCTTGA TTAATTTAAC TTATGTATTA GTTAGGTATA   
  
  
- ATTAAACAAA ACTGTTGATA AATGTTAGTA GCTAGAGAAA TTAATCCAGA TATTCGATAT CTCATTAATG   
  
  
- AGTTTTGACA AAACATTCTG TTGAATGAGG TATAACCGAA TAATCATATA ACGGAGGTAA GTGTACTTAT   
  
  
- GAAGTACTCT CTAACGGATT TTGAGTATAA CAGTAGTGGA ATTCAGTTTG TATAAAACAA TACTTTACTG   
  
  
- TAGTTCTTAA TGGGAGACTG TTCTTTTTAT ATGGACTGAC TGTATTAGCA TGGTGTAGAC AATCAGGAGA   
  
  
- AAGATGGTGA AACATCTTGT ATTACACTGT CATTATGTAG GTAGGGTTTT CTTTTCTGCT TTTCTTCTCA   
  
  
- CCTCTCAGTG CATATGCGCA ACTTAAGTCC TTTTAATTTC CTTGAGTACT TCTGGGGTAT ATCACGAGGT   
  
  
- TGGACTTGGA CGTCGGGTGA TGATGTACGG ATTTAATTTC TCGTTTCAAT ACTCTCTTTG GTTTTTTCGA   
  
  
- GAAACTCTCT CACCTCCTTC TTAGTACCTT TCGATCGATC CGAATCGTTC TTTCTCGTTG TCGATCGATC   
  
  
- GTTTTCCGAA GAGAAACTTC GGAATCTTTA GAAATCTCTA TTTTTCAACC ACCGAAAAAT TCCCGACACT   
  
  
- ATACAGGGGG TACATTTTTA CGTCGTTATT ACAATTACAG TACGAAAGAA AGTACCAGGG ATTACGCTTT   
  
  
- AGTTAGTACG TACAGTTGCA CTGAGTCACC GAGTGGTATA TACTAGACTA AAGGGGCGTG TGTCTCTATT   
  
  
- TTTCGGGGGT TAATATTGGA GGGTTTTCGG GGGGGAAAAG GAGGATGGGT TGGGTGTGGA GGGGTAAAGT   
  
  
- AGTCCCGAGC GAAGAATTCA ATAGCTAATT TTTTTCTCAC TCATTTGCAT GGGCTTAACT TCTCCCCACA   
  
  
- AGCTTCGAAT ATGAACGAAC TAATCCTTAC TTTCAGGGGT AAAGTAGGCG GGGGGTTTGG CGGCGGTGGT   
  
  
- GGTCATTTAG CGATAGAACG TTGTTATGAC GGAACTTATG ACGGAAAGTT CGGGGGTTAA GTTAAAGTTG   
  
  
- GGAACAATTG TTGTTGTTTG GGAGGATGCT CGGATGGAGC CAAGATCTGG AGGCCGCGTC GGGTTCGGGG   
  
  
- CGGTGGTTGC GGCGGGACGG TGACGGGTGA CTGCGCCCGT AGTTATTTGG GTTCGGACGG AGACGACTGG   
  
  
- ACCTGTTAAA CCTCCCACTC CCAACCCTAA GTTAGGATAG TCTCGACCCC GAATTACTAC TAAGATTATT   
  
  
- AGGATTGAGG TTTGAAACCA GTAGGGTTTA ATCGAGTAGT ACACTAAGGG TGGAGTGTGT CGAGGGACTC   
  
  
- AAGGGGAGGA GAGTCGGCAA CCTAGTGTGT GCGTGGGGGT TATTAATTGA AGAGGGGGAG ACTAATGGGG   
  
  
- TAGAGACTAA ATAGGATGCC AGTCTTAGAG TCGGGGTTAA AACCCGTCAA ACTCGAAGTA GAAGTAGTCG   
  
  
- GGTTGCAGTT ATTGTTACCC GAACTGAAGT ATCTCCTGGA TTAATCTCGT CGAGTTCGGA TGCTTAGGTT   
  
  
- GCTGAGGGTG GTCCAGGTCG ACCATAACCG CGCCGAGTTA GTTGCCGAGT CTAGTGGGCG GCCTTTTGGG   
  
  
- GAGGTGGCGC GGCGGAAGAT GAAGTTCCTC CGGGAGGTTG TGGAGCGACA GCCGCCTCCG GGCCGGGCCG   
  
  
- GACAGGCCGA CAGTAGGATG CTCCACCACG TCTGGTAGGC CCGGGTGTTC TTGAAAAGGC CGTATAGTGA   
  
  
- CTATAGGGAG AAGAGGTGGA AGCGGCGGTT AGTCCGCTAA GACCTCCGCC ACCTACCCCG ACGCAAGTAG   
  
  
- GTGTAGTAGC TAAAGCTGTA GCCTAAACCG CCGGTGACCC GCAGCAAATA CGCACTCGAA CAGCTGTTCC   
  
  
- GGCTGGGCTT ATTCTAGTCG AGGAACCACA AGGCTTAGTG CCGCTAGCAT GGGCTTCTCA AGCCCTAGCT   
  
  
- TTCGTTCGAC CACTCTCTCT TAGAATGCGT TAAGCGAGCA CTAGAGTTGT AGTTACAGGT ATACCTCATA   
  
  
- CAGGAATAAG TTTCGAAGCT CAAGAATAGG AAGTTTAGCC AGTTTAAGTA GCTGCCACTC TTTAACCGCC   
  
  
- AGGTAGATAG AGGCCGCTAC AAAGTGGCCG AAGTTCCGCC TTAGAGCTTT AAGGATCGGC TAGAGGCCCC   
  
  
- TTAAAGCGGT TCGTGGCAGC ACCACCACCT GTCACTCCAT CCTTAGCTGT GCCCTTGAAG CAGGAAGTCG   
  
  
- CAGTTGAAGC AGCGGCCATA GCTTAAGATG TGGCCTTACG AGCTTAGCGA GCTGCAGTGG AGGCGGTCGC   
  
  
- CAAAACGTCC GCCGCCGCCG CCGCTGCGAC ACTCTTCCTA GCTCTGCATG CACGAGGCCG GCTCCTAATG   
  
  
- CCGCCGCCAC CTCCGCCGTA GACGCCGCCG CCATCGCGCC GCCTCGCCAA CCTCTCTCAG CAAGCGCCAC   
  
  
- CGATCCTACT CCCGCCACCC CAAGTCGGTC AAACGACTAA AGGTCCGCCT CACAAACGAC TCCTTCCAAG   
  
  
- TCCAACCGCC CAAGGTGCAC CGCTTTGCAG TACCCCTCTA CTACGAAACG ACCGTACTAT CCGGGGAACA   
  
  
- CAGGTGCAGC CGAACCTCCA CAAT

+     Box II

| Site Name | Organism | Position | Strand | Matrix score. | sequence | function |
| --- | --- | --- | --- | --- | --- | --- |
| Box II | Petroselinum crispum | 3308 | + | 9 | CCACGTGGC | part of a light responsive element |

>HU06G00283.1   
+ -Up\_Stream \_Len000GATTTT ACTTCCAATT TTCTACTTAA ATTTGGTTAT CACGTTCGAA TTTTTCAGGT   
  
  
+ AAATACAAAC TGTTAGTGGC CATCCTTTTT TTTTGGGTCT AATCCATCCA GATGTGCTAA TACTTTCTGT   
  
  
+ TTTAGATGAG AAGTTTTAAT GCTTTTTTAG ATGTGTAATT TTTTTAAACA TAAATAGTCT TGTCGAATAA   
  
  
+ GATTTGCTAG TAATTTTTGT AAGAGAAATT ATGTGTATTT TTTATTATAT CAGAATATTA GATCGATCTC   
  
  
+ AATTTAATTA CTTATGAAAA AAAAAGTGGA TATCTTTAAT CAATTTGGCA AGCTCTCATG CATTAATTGT   
  
  
+ AGTCGTACGA ACTTATTTAC ATCACTCATC TGAATTTGCT ACCATTTACA CCTCAAATTT TATTTTAATG   
  
  
+ CCATAAAGAG AATAGAATAT ACTGTCTATT TGTAGTAGTT TTACAGATAA AAACAAATAA GCAAATGAAA   
  
  
+ ATAGATAGAG GATTTTTTTG CTATTATCCA CGTCGATCCA TTGAATTATT TTGTCGCTAT TTGGTTAAGT   
  
  
+ GAAATAAAAT ACTGAAGTAG AATGGAGGAT GTTGATAATG ACCTATGTCC CTCAAAGTTT AATCATGTAG   
  
  
+ TTTCTACGAA TTTCAAGCCA TTATAAGACA TACAGATTTT TATGATGTTG AAAATACTCT TACCTCCTTG   
  
  
+ TCACAATATT AACCTGAAGC CAATGAAATG AAACAGAACT AATTAAATTG AATACATAAT CAATCCATAT   
  
  
+ TAATTTGTTT TGACAACTAT TTACAATCAT CGATCTCTTT AATTAGGTCT ATAAGCTATA GAGTAATTAC   
  
  
+ TCAAAACTGT TTTGTAAGAC AACTTACTCC ATATTGGCTT ATTAGTATAT TGCCTCCATT CACATGAATA   
  
  
+ CTTCATGAGA GATTGCCTAA AACTCATATT GTCATCACCT TAAGTCAAAC ATATTTTGTT ATGAAATGAC   
  
  
+ ATCAAGAATT ACCCTCTGAC AAGAAAAATA TACCTGACTG ACATAATCGT ACCACATCTG TTAGTCCTCT   
  
  
+ TTCTACCACT TTGTAGAACA TAATGTGACA GTAATACATC CATCCCAAAA GAAAAGACGA AAAGAAGAGT   
  
  
+ GGAGAGTCAC GTATACGCGT TGAATTCAGG AAAATTAAAG GAACTCATGA AGACCCCATA TAGTGCTCCA   
  
  
+ ACCTGAACCT GCAGCCCACT ACTACATGCC TAAATTAAAG AGCAAAGTTA TGAGAGAAAC CAAAAAAGCT   
  
  
+ CTTTGAGAGA GTGGAGGAAG AATCATGGAA AGCTAGCTAG GCTTAGCAAG AAAGAGCAAC AGCTAGCTAG   
  
  
+ CAAAAGGCTT CTCTTTGAAG CCTTAGAAAT CTTTAGAGAT AAAAAGTTGG TGGCTTTTTA AGGGCTGTGA   
  
  
+ TATGTCCCCC ATGTAAAAAT GCAGCAATAA TGTTAATGTC ATGCTTTCTT TCATGGTCCC TAATGCGAAA   
  
  
+ TCAATCATGC ATGTCAACGT GACTCAGTGG CTCACCATAT ATGATCTGAT TTCCCCGCAC ACAGAGATAA   
  
  
+ AAAGCCCCCA ATTATAACCT CCCAAAAGCC CCCCCTTTTC CTCCTACCCA ACCCACACCT CCCCATTTCA   
  
  
+ TCAGGGCTCG CTTCTTAAGT TATCGATTAA AAAAAGAGTG AGTAAACGTA CCCGAATTGA AGAGGGGTGT   
  
  
+ TCGAAGCTTA TACTTGCTTG ATTAGGAATG AAAGTCCCCA TTTCATCCGC CCCCCAAACC GCCGCCACCA   
  
  
+ CCAGTAAATC GCTATCTTGC AACAATACTG CCTTGAATAC TGCCTTTCAA GCCCCCAATT CAATTTCAAC   
  
  
+ CCTTGTTAAC AACAACAAAC CCTCCTACGA GCCTACCTCG GTTCTAGACC TCCGGCGCAG CCCAAGCCCC   
  
  
+ GCCACCAACG CCGCCCTGCC ACTGCCCACT GACGCGGGCA TCAATAAACC CAAGCCTGCC TCTGCTGACC   
  
  
+ TGGACAATTT GGAGGGTGAG GGTTGGGATT CAATCCTATC AGAGCTGGGG CTTAATGATG ATTCTAATAA   
  
  
+ TCCTAACTCC AAACTTTGGT CATCCCAAAT TAGCTCATCA TGTGATTCCC ACCTCACACA GCTCCCTGAG   
  
  
+ TTCCCCTCCT CTCAGCCGTT GGATCACACA CGCACCCCCA ATAATTAACT TCTCCCCCTC TGATTACCCC   
  
  
+ ATCTCTGATT TATCCTACGG TCAGAATCTC AGCCCCAATT TTGGGCAGTT TGAGCTTCAT CTTCATCAGC   
  
  
+ CCAACGTCAA TAACAATGGG CTTGACTTCA TAGAGGACCT AATTAGAGCA GCTCAAGCCT ACGAATCCAA   
  
  
+ CGACTCCCAC CAGGTCCAGC TGGTATTGGC GCGGCTCAAT CAACGGCTCA GATCACCCGC CGGAAAACCC   
  
  
+ CTCCACCGCG CCGCCTTCTA CTTCAAGGAG GCCCTCCAAC ACCTCGCTGT CGGCGGAGGC CCGGCCCGGC   
  
  
+ CTGTCCGGCT GTCATCCTAC GAGGTGGTGC AGACCATCCG GGCCCACAAG AACTTTTCCG GCATATCACT   
  
  
+ GATATCCCTC TTCTCCACCT TCGCCGCCAA TCAGGCGATT CTGGAGGCGG TGGATGGGGC TGCGTTCATC   
  
  
+ CACATCATCG ATTTCGACAT CGGATTTGGC GGCCACTGGG CGTCGTTTAT GCGTGAGCTT GTCGACAAGG   
  
  
+ CCGACCCGAA TAAGATCAGC TCCTTGGTGT TCCGAATCAC GGCGATCGTA CCCGAAGAGT TCGGGATCGA   
  
  
+ AAGCAAGCTG GTGAGAGAGA ATCTTACGCA ATTCGCTCGT GATCTCAACA TCAATGTCCA TATGGAGTAT   
  
  
+ GTCCTTATTC AAAGCTTCGA GTTCTTATCC TTCAAATCGG TCAAATTCAT CGACGGTGAG AAATTGGCGG   
  
  
+ TCCATCTATC TCCGGCGATG TTTCACCGGC TTCAAGGCGG AATCTCGAAA TTCCTAGCCG ATCTCCGGGG   
  
  
+ AATTTCGCCA AGCACCGTCG TGGTGGTGGA CAGTGAGGTA GGAATCGACA CGGGAACTTC GTCCTTCAGC   
  
  
+ GTCAACTTCG TCGCCGGTAT CGAATTCTAC ACCGGAATGC TCGAATCGCT CGACGTCACC TCCGCCAGCG   
  
  
+ GTTTTGCAGG CGGCGGCGGC GGCGACGCTG TGAGAAGGAT CGAGACGTAC GTGCTCCGGC CGAGGATTAC   
  
  
+ GGCGGCGGTG GAGGCGGCAT CTGCGGCGGC GGTAGCGCGG CGGAGCGGTT GGAGAGAGTC GTTCGCGGTG   
  
  
+ GCTAGGATGA GGGCGGTGGG GTTCAGCCAG TTTGCTGATT TCCAGGCGGA GTGTTTGCTG AGGAAGGTTC   
  
  
+ AGGTTGGCGG GTTCCACGTG GCGAAACGTC ATGGGGAGAT GATGCTTTGC TGGCATGATA GGCCCCTTGT   
  
  
+ GTCCACGTCG GCTTGGAGGT GTTA  

- -Up\_Stream \_Len000CTAAAA TGAAGGTTAA AAGATGAATT TAAACCAATA GTGCAAGCTT AAAAAGTCCA   
  
  
- TTTATGTTTG ACAATCACCG GTAGGAAAAA AAAACCCAGA TTAGGTAGGT CTACACGATT ATGAAAGACA   
  
  
- AAATCTACTC TTCAAAATTA CGAAAAAATC TACACATTAA AAAAATTTGT ATTTATCAGA ACAGCTTATT   
  
  
- CTAAACGATC ATTAAAAACA TTCTCTTTAA TACACATAAA AAATAATATA GTCTTATAAT CTAGCTAGAG   
  
  
- TTAAATTAAT GAATACTTTT TTTTTCACCT ATAGAAATTA GTTAAACCGT TCGAGAGTAC GTAATTAACA   
  
  
- TCAGCATGCT TGAATAAATG TAGTGAGTAG ACTTAAACGA TGGTAAATGT GGAGTTTAAA ATAAAATTAC   
  
  
- GGTATTTCTC TTATCTTATA TGACAGATAA ACATCATCAA AATGTCTATT TTTGTTTATT CGTTTACTTT   
  
  
- TATCTATCTC CTAAAAAAAC GATAATAGGT GCAGCTAGGT AACTTAATAA AACAGCGATA AACCAATTCA   
  
  
- CTTTATTTTA TGACTTCATC TTACCTCCTA CAACTATTAC TGGATACAGG GAGTTTCAAA TTAGTACATC   
  
  
- AAAGATGCTT AAAGTTCGGT AATATTCTGT ATGTCTAAAA ATACTACAAC TTTTATGAGA ATGGAGGAAC   
  
  
- AGTGTTATAA TTGGACTTCG GTTACTTTAC TTTGTCTTGA TTAATTTAAC TTATGTATTA GTTAGGTATA   
  
  
- ATTAAACAAA ACTGTTGATA AATGTTAGTA GCTAGAGAAA TTAATCCAGA TATTCGATAT CTCATTAATG   
  
  
- AGTTTTGACA AAACATTCTG TTGAATGAGG TATAACCGAA TAATCATATA ACGGAGGTAA GTGTACTTAT   
  
  
- GAAGTACTCT CTAACGGATT TTGAGTATAA CAGTAGTGGA ATTCAGTTTG TATAAAACAA TACTTTACTG   
  
  
- TAGTTCTTAA TGGGAGACTG TTCTTTTTAT ATGGACTGAC TGTATTAGCA TGGTGTAGAC AATCAGGAGA   
  
  
- AAGATGGTGA AACATCTTGT ATTACACTGT CATTATGTAG GTAGGGTTTT CTTTTCTGCT TTTCTTCTCA   
  
  
- CCTCTCAGTG CATATGCGCA ACTTAAGTCC TTTTAATTTC CTTGAGTACT TCTGGGGTAT ATCACGAGGT   
  
  
- TGGACTTGGA CGTCGGGTGA TGATGTACGG ATTTAATTTC TCGTTTCAAT ACTCTCTTTG GTTTTTTCGA   
  
  
- GAAACTCTCT CACCTCCTTC TTAGTACCTT TCGATCGATC CGAATCGTTC TTTCTCGTTG TCGATCGATC   
  
  
- GTTTTCCGAA GAGAAACTTC GGAATCTTTA GAAATCTCTA TTTTTCAACC ACCGAAAAAT TCCCGACACT   
  
  
- ATACAGGGGG TACATTTTTA CGTCGTTATT ACAATTACAG TACGAAAGAA AGTACCAGGG ATTACGCTTT   
  
  
- AGTTAGTACG TACAGTTGCA CTGAGTCACC GAGTGGTATA TACTAGACTA AAGGGGCGTG TGTCTCTATT   
  
  
- TTTCGGGGGT TAATATTGGA GGGTTTTCGG GGGGGAAAAG GAGGATGGGT TGGGTGTGGA GGGGTAAAGT   
  
  
- AGTCCCGAGC GAAGAATTCA ATAGCTAATT TTTTTCTCAC TCATTTGCAT GGGCTTAACT TCTCCCCACA   
  
  
- AGCTTCGAAT ATGAACGAAC TAATCCTTAC TTTCAGGGGT AAAGTAGGCG GGGGGTTTGG CGGCGGTGGT   
  
  
- GGTCATTTAG CGATAGAACG TTGTTATGAC GGAACTTATG ACGGAAAGTT CGGGGGTTAA GTTAAAGTTG   
  
  
- GGAACAATTG TTGTTGTTTG GGAGGATGCT CGGATGGAGC CAAGATCTGG AGGCCGCGTC GGGTTCGGGG   
  
  
- CGGTGGTTGC GGCGGGACGG TGACGGGTGA CTGCGCCCGT AGTTATTTGG GTTCGGACGG AGACGACTGG   
  
  
- ACCTGTTAAA CCTCCCACTC CCAACCCTAA GTTAGGATAG TCTCGACCCC GAATTACTAC TAAGATTATT   
  
  
- AGGATTGAGG TTTGAAACCA GTAGGGTTTA ATCGAGTAGT ACACTAAGGG TGGAGTGTGT CGAGGGACTC   
  
  
- AAGGGGAGGA GAGTCGGCAA CCTAGTGTGT GCGTGGGGGT TATTAATTGA AGAGGGGGAG ACTAATGGGG   
  
  
- TAGAGACTAA ATAGGATGCC AGTCTTAGAG TCGGGGTTAA AACCCGTCAA ACTCGAAGTA GAAGTAGTCG   
  
  
- GGTTGCAGTT ATTGTTACCC GAACTGAAGT ATCTCCTGGA TTAATCTCGT CGAGTTCGGA TGCTTAGGTT   
  
  
- GCTGAGGGTG GTCCAGGTCG ACCATAACCG CGCCGAGTTA GTTGCCGAGT CTAGTGGGCG GCCTTTTGGG   
  
  
- GAGGTGGCGC GGCGGAAGAT GAAGTTCCTC CGGGAGGTTG TGGAGCGACA GCCGCCTCCG GGCCGGGCCG   
  
  
- GACAGGCCGA CAGTAGGATG CTCCACCACG TCTGGTAGGC CCGGGTGTTC TTGAAAAGGC CGTATAGTGA   
  
  
- CTATAGGGAG AAGAGGTGGA AGCGGCGGTT AGTCCGCTAA GACCTCCGCC ACCTACCCCG ACGCAAGTAG   
  
  
- GTGTAGTAGC TAAAGCTGTA GCCTAAACCG CCGGTGACCC GCAGCAAATA CGCACTCGAA CAGCTGTTCC   
  
  
- GGCTGGGCTT ATTCTAGTCG AGGAACCACA AGGCTTAGTG CCGCTAGCAT GGGCTTCTCA AGCCCTAGCT   
  
  
- TTCGTTCGAC CACTCTCTCT TAGAATGCGT TAAGCGAGCA CTAGAGTTGT AGTTACAGGT ATACCTCATA   
  
  
- CAGGAATAAG TTTCGAAGCT CAAGAATAGG AAGTTTAGCC AGTTTAAGTA GCTGCCACTC TTTAACCGCC   
  
  
- AGGTAGATAG AGGCCGCTAC AAAGTGGCCG AAGTTCCGCC TTAGAGCTTT AAGGATCGGC TAGAGGCCCC   
  
  
- TTAAAGCGGT TCGTGGCAGC ACCACCACCT GTCACTCCAT CCTTAGCTGT GCCCTTGAAG CAGGAAGTCG   
  
  
- CAGTTGAAGC AGCGGCCATA GCTTAAGATG TGGCCTTACG AGCTTAGCGA GCTGCAGTGG AGGCGGTCGC   
  
  
- CAAAACGTCC GCCGCCGCCG CCGCTGCGAC ACTCTTCCTA GCTCTGCATG CACGAGGCCG GCTCCTAATG   
  
  
- CCGCCGCCAC CTCCGCCGTA GACGCCGCCG CCATCGCGCC GCCTCGCCAA CCTCTCTCAG CAAGCGCCAC   
  
  
- CGATCCTACT CCCGCCACCC CAAGTCGGTC AAACGACTAA AGGTCCGCCT CACAAACGAC TCCTTCCAAG   
  
  
- TCCAACCGCC CAAGGTGCAC CGCTTTGCAG TACCCCTCTA CTACGAAACG ACCGTACTAT CCGGGGAACA   
  
  
- CAGGTGCAGC CGAACCTCCA CAAT

+     CAAT-box

| Site Name | Organism | Position | Strand | Matrix score. | sequence | function |
| --- | --- | --- | --- | --- | --- | --- |
| CAAT-box | Arabidopsis thaliana | 2867 | - | 5 | CCAAT | common cis-acting element in promoter and enhancer regions |
| CAAT-box | Nicotiana glutinosa | 2786 | + | 4 | CAAT |  |
| CAAT-box | Arabidopsis thaliana | 2209 | + | 5 | CCAAT | common cis-acting element in promoter and enhancer regions |
| CAAT-box | Nicotiana glutinosa | 893 | - | 4 | CAAT |  |
| CAAT-box | Nicotiana glutinosa | 765 | + | 4 | CAAT |  |
| CAAT-box | Nicotiana glutinosa | 751 | - | 4 | CAAT |  |
| CAAT-box | Arabidopsis thaliana | 724 | + | 5 | CCAAT | common cis-acting element in promoter and enhancer regions |
| CAAT-box | Pisum sativum | 553 | - | 5 | CAAAT | common cis-acting element in promoter and enhancer regions |
| CAAT-box | Arabidopsis thaliana | 877 | - | 5 | CCAAT | common cis-acting element in promoter and enhancer regions |
| CAAT-box | Nicotiana glutinosa | 708 | + | 4 | CAAT |  |
| CAAT-box | Pisum sativum | 486 | + | 5 | CAAAT | common cis-acting element in promoter and enhancer regions |
| CAAT-box | Nicotiana glutinosa | 798 | + | 4 | CAAT |  |
| CAAT-box | Arabidopsis thaliana | 2208 | + | 8 | CCCAATTT | common cis-acting element in promoter and enhancer regions |
| CAAT-box | Nicotiana glutinosa | 534 | - | 4 | CAAT |  |
| CAAT-box | Nicotiana glutinosa | 325 | + | 4 | CAAT |  |
| CAAT-box | Nicotiana glutinosa | 1553 | + | 4 | CAAT |  |
| CAAT-box | Arabidopsis thaliana | 2142 | + | 5 | CCAAT | common cis-acting element in promoter and enhancer regions |
| CAAT-box | Pisum sativum | 2060 | + | 5 | CAAAT | common cis-acting element in promoter and enhancer regions |
| CAAT-box | Nicotiana glutinosa | 1777 | + | 4 | CAAT |  |
| CAAT-box | Pisum sativum | 2837 | + | 5 | CAAAT | common cis-acting element in promoter and enhancer regions |
| CAAT-box | Pisum sativum | 388 | - | 5 | CAAAT | common cis-acting element in promoter and enhancer regions |
| CAAT-box | Arabidopsis thaliana | 1552 | + | 5 | CCAAT | common cis-acting element in promoter and enhancer regions |
| CAAT-box | Nicotiana glutinosa | 1995 | + | 4 | CAAT |  |
| CAAT-box | Nicotiana glutinosa | 2763 | + | 4 | CAAT |  |
| CAAT-box | Pisum sativum | 452 | - | 5 | CAAAT | common cis-acting element in promoter and enhancer regions |
| CAAT-box | Pisum sativum | 2846 | + | 5 | CAAAT | common cis-acting element in promoter and enhancer regions |
| CAAT-box | Arabidopsis thaliana | 2551 | + | 5 | CCAAT | common cis-acting element in promoter and enhancer regions |
| CAAT-box | Nicotiana glutinosa | 2351 | + | 4 | CAAT |  |
| CAAT-box | Arabidopsis thaliana | 2339 | - | 5 | CCAAT | common cis-acting element in promoter and enhancer regions |
| CAAT-box | Nicotiana glutinosa | 2258 | + | 4 | CAAT |  |
| CAAT-box | Nicotiana glutinosa | 2252 | + | 4 | CAAT |  |
| CAAT-box | Nicotiana glutinosa | 725 | + | 4 | CAAT |  |
| CAAT-box | Pisum sativum | 777 | - | 5 | CAAAT | common cis-acting element in promoter and enhancer regions |
| CAAT-box | Nicotiana glutinosa | 2143 | + | 4 | CAAT |  |
| CAAT-box | Pisum sativum | 1971 | - | 5 | CAAAT | common cis-acting element in promoter and enhancer regions |
| CAAT-box | Pisum sativum | 478 | + | 5 | CAAAT | common cis-acting element in promoter and enhancer regions |
| CAAT-box | Nicotiana glutinosa | 1969 | + | 4 | CAAT |  |
| CAAT-box | Arabidopsis thaliana | 1809 | + | 5 | CCAAT | common cis-acting element in promoter and enhancer regions |
| CAAT-box | Nicotiana glutinosa | 1810 | + | 4 | CAAT |  |
| CAAT-box | Nicotiana glutinosa | 350 | - | 4 | CAAT |  |
| CAAT-box | Nicotiana glutinosa | 1670 | - | 4 | CAAT |  |
| CAAT-box | Nicotiana glutinosa | 2552 | + | 4 | CAAT |  |
| CAAT-box | Pisum sativum | 216 | - | 5 | CAAAT | common cis-acting element in promoter and enhancer regions |
| CAAT-box | Nicotiana glutinosa | 2210 | + | 4 | CAAT |  |
| CAAT-box | Pisum sativum | 2618 | - | 5 | CAAAT | common cis-acting element in promoter and enhancer regions |
| CAAT-box | Nicotiana glutinosa | 1476 | + | 4 | CAAT |  |
| CAAT-box | Nicotiana glutinosa | 1815 | + | 4 | CAAT |  |
| CAAT-box | Nicotiana glutinosa | 1429 | + | 4 | CAAT |  |
| CAAT-box | Pisum sativum | 408 | + | 5 | CAAAT | common cis-acting element in promoter and enhancer regions |
| CAAT-box | Nicotiana glutinosa | 1936 | + | 4 | CAAT |  |
| CAAT-box | Nicotiana glutinosa | 926 | - | 4 | CAAT |  |
| CAAT-box | Pisum sativum | 45 | - | 5 | CAAAT | common cis-acting element in promoter and enhancer regions |
| CAAT-box | Arabidopsis thaliana | 29 | + | 5 | CCAAT | common cis-acting element in promoter and enhancer regions |
| CAAT-box | Nicotiana glutinosa | 30 | + | 4 | CAAT |  |
| CAAT-box | Nicotiana glutinosa | 942 | - | 4 | CAAT |  |
| CAAT-box | Nicotiana glutinosa | 284 | + | 4 | CAAT |  |
| CAAT-box | Pisum sativum | 327 | - | 5 | CAAAT | common cis-acting element in promoter and enhancer regions |

>HU06G00283.1   
+ -Up\_Stream \_Len000GATTTT ACTTCCAATT TTCTACTTAA ATTTGGTTAT CACGTTCGAA TTTTTCAGGT   
  
  
+ AAATACAAAC TGTTAGTGGC CATCCTTTTT TTTTGGGTCT AATCCATCCA GATGTGCTAA TACTTTCTGT   
  
  
+ TTTAGATGAG AAGTTTTAAT GCTTTTTTAG ATGTGTAATT TTTTTAAACA TAAATAGTCT TGTCGAATAA   
  
  
+ GATTTGCTAG TAATTTTTGT AAGAGAAATT ATGTGTATTT TTTATTATAT CAGAATATTA GATCGATCTC   
  
  
+ AATTTAATTA CTTATGAAAA AAAAAGTGGA TATCTTTAAT CAATTTGGCA AGCTCTCATG CATTAATTGT   
  
  
+ AGTCGTACGA ACTTATTTAC ATCACTCATC TGAATTTGCT ACCATTTACA CCTCAAATTT TATTTTAATG   
  
  
+ CCATAAAGAG AATAGAATAT ACTGTCTATT TGTAGTAGTT TTACAGATAA AAACAAATAA GCAAATGAAA   
  
  
+ ATAGATAGAG GATTTTTTTG CTATTATCCA CGTCGATCCA TTGAATTATT TTGTCGCTAT TTGGTTAAGT   
  
  
+ GAAATAAAAT ACTGAAGTAG AATGGAGGAT GTTGATAATG ACCTATGTCC CTCAAAGTTT AATCATGTAG   
  
  
+ TTTCTACGAA TTTCAAGCCA TTATAAGACA TACAGATTTT TATGATGTTG AAAATACTCT TACCTCCTTG   
  
  
+ TCACAATATT AACCTGAAGC CAATGAAATG AAACAGAACT AATTAAATTG AATACATAAT CAATCCATAT   
  
  
+ TAATTTGTTT TGACAACTAT TTACAATCAT CGATCTCTTT AATTAGGTCT ATAAGCTATA GAGTAATTAC   
  
  
+ TCAAAACTGT TTTGTAAGAC AACTTACTCC ATATTGGCTT ATTAGTATAT TGCCTCCATT CACATGAATA   
  
  
+ CTTCATGAGA GATTGCCTAA AACTCATATT GTCATCACCT TAAGTCAAAC ATATTTTGTT ATGAAATGAC   
  
  
+ ATCAAGAATT ACCCTCTGAC AAGAAAAATA TACCTGACTG ACATAATCGT ACCACATCTG TTAGTCCTCT   
  
  
+ TTCTACCACT TTGTAGAACA TAATGTGACA GTAATACATC CATCCCAAAA GAAAAGACGA AAAGAAGAGT   
  
  
+ GGAGAGTCAC GTATACGCGT TGAATTCAGG AAAATTAAAG GAACTCATGA AGACCCCATA TAGTGCTCCA   
  
  
+ ACCTGAACCT GCAGCCCACT ACTACATGCC TAAATTAAAG AGCAAAGTTA TGAGAGAAAC CAAAAAAGCT   
  
  
+ CTTTGAGAGA GTGGAGGAAG AATCATGGAA AGCTAGCTAG GCTTAGCAAG AAAGAGCAAC AGCTAGCTAG   
  
  
+ CAAAAGGCTT CTCTTTGAAG CCTTAGAAAT CTTTAGAGAT AAAAAGTTGG TGGCTTTTTA AGGGCTGTGA   
  
  
+ TATGTCCCCC ATGTAAAAAT GCAGCAATAA TGTTAATGTC ATGCTTTCTT TCATGGTCCC TAATGCGAAA   
  
  
+ TCAATCATGC ATGTCAACGT GACTCAGTGG CTCACCATAT ATGATCTGAT TTCCCCGCAC ACAGAGATAA   
  
  
+ AAAGCCCCCA ATTATAACCT CCCAAAAGCC CCCCCTTTTC CTCCTACCCA ACCCACACCT CCCCATTTCA   
  
  
+ TCAGGGCTCG CTTCTTAAGT TATCGATTAA AAAAAGAGTG AGTAAACGTA CCCGAATTGA AGAGGGGTGT   
  
  
+ TCGAAGCTTA TACTTGCTTG ATTAGGAATG AAAGTCCCCA TTTCATCCGC CCCCCAAACC GCCGCCACCA   
  
  
+ CCAGTAAATC GCTATCTTGC AACAATACTG CCTTGAATAC TGCCTTTCAA GCCCCCAATT CAATTTCAAC   
  
  
+ CCTTGTTAAC AACAACAAAC CCTCCTACGA GCCTACCTCG GTTCTAGACC TCCGGCGCAG CCCAAGCCCC   
  
  
+ GCCACCAACG CCGCCCTGCC ACTGCCCACT GACGCGGGCA TCAATAAACC CAAGCCTGCC TCTGCTGACC   
  
  
+ TGGACAATTT GGAGGGTGAG GGTTGGGATT CAATCCTATC AGAGCTGGGG CTTAATGATG ATTCTAATAA   
  
  
+ TCCTAACTCC AAACTTTGGT CATCCCAAAT TAGCTCATCA TGTGATTCCC ACCTCACACA GCTCCCTGAG   
  
  
+ TTCCCCTCCT CTCAGCCGTT GGATCACACA CGCACCCCCA ATAATTAACT TCTCCCCCTC TGATTACCCC   
  
  
+ ATCTCTGATT TATCCTACGG TCAGAATCTC AGCCCCAATT TTGGGCAGTT TGAGCTTCAT CTTCATCAGC   
  
  
+ CCAACGTCAA TAACAATGGG CTTGACTTCA TAGAGGACCT AATTAGAGCA GCTCAAGCCT ACGAATCCAA   
  
  
+ CGACTCCCAC CAGGTCCAGC TGGTATTGGC GCGGCTCAAT CAACGGCTCA GATCACCCGC CGGAAAACCC   
  
  
+ CTCCACCGCG CCGCCTTCTA CTTCAAGGAG GCCCTCCAAC ACCTCGCTGT CGGCGGAGGC CCGGCCCGGC   
  
  
+ CTGTCCGGCT GTCATCCTAC GAGGTGGTGC AGACCATCCG GGCCCACAAG AACTTTTCCG GCATATCACT   
  
  
+ GATATCCCTC TTCTCCACCT TCGCCGCCAA TCAGGCGATT CTGGAGGCGG TGGATGGGGC TGCGTTCATC   
  
  
+ CACATCATCG ATTTCGACAT CGGATTTGGC GGCCACTGGG CGTCGTTTAT GCGTGAGCTT GTCGACAAGG   
  
  
+ CCGACCCGAA TAAGATCAGC TCCTTGGTGT TCCGAATCAC GGCGATCGTA CCCGAAGAGT TCGGGATCGA   
  
  
+ AAGCAAGCTG GTGAGAGAGA ATCTTACGCA ATTCGCTCGT GATCTCAACA TCAATGTCCA TATGGAGTAT   
  
  
+ GTCCTTATTC AAAGCTTCGA GTTCTTATCC TTCAAATCGG TCAAATTCAT CGACGGTGAG AAATTGGCGG   
  
  
+ TCCATCTATC TCCGGCGATG TTTCACCGGC TTCAAGGCGG AATCTCGAAA TTCCTAGCCG ATCTCCGGGG   
  
  
+ AATTTCGCCA AGCACCGTCG TGGTGGTGGA CAGTGAGGTA GGAATCGACA CGGGAACTTC GTCCTTCAGC   
  
  
+ GTCAACTTCG TCGCCGGTAT CGAATTCTAC ACCGGAATGC TCGAATCGCT CGACGTCACC TCCGCCAGCG   
  
  
+ GTTTTGCAGG CGGCGGCGGC GGCGACGCTG TGAGAAGGAT CGAGACGTAC GTGCTCCGGC CGAGGATTAC   
  
  
+ GGCGGCGGTG GAGGCGGCAT CTGCGGCGGC GGTAGCGCGG CGGAGCGGTT GGAGAGAGTC GTTCGCGGTG   
  
  
+ GCTAGGATGA GGGCGGTGGG GTTCAGCCAG TTTGCTGATT TCCAGGCGGA GTGTTTGCTG AGGAAGGTTC   
  
  
+ AGGTTGGCGG GTTCCACGTG GCGAAACGTC ATGGGGAGAT GATGCTTTGC TGGCATGATA GGCCCCTTGT   
  
  
+ GTCCACGTCG GCTTGGAGGT GTTA  

- -Up\_Stream \_Len000CTAAAA TGAAGGTTAA AAGATGAATT TAAACCAATA GTGCAAGCTT AAAAAGTCCA   
  
  
- TTTATGTTTG ACAATCACCG GTAGGAAAAA AAAACCCAGA TTAGGTAGGT CTACACGATT ATGAAAGACA   
  
  
- AAATCTACTC TTCAAAATTA CGAAAAAATC TACACATTAA AAAAATTTGT ATTTATCAGA ACAGCTTATT   
  
  
- CTAAACGATC ATTAAAAACA TTCTCTTTAA TACACATAAA AAATAATATA GTCTTATAAT CTAGCTAGAG   
  
  
- TTAAATTAAT GAATACTTTT TTTTTCACCT ATAGAAATTA GTTAAACCGT TCGAGAGTAC GTAATTAACA   
  
  
- TCAGCATGCT TGAATAAATG TAGTGAGTAG ACTTAAACGA TGGTAAATGT GGAGTTTAAA ATAAAATTAC   
  
  
- GGTATTTCTC TTATCTTATA TGACAGATAA ACATCATCAA AATGTCTATT TTTGTTTATT CGTTTACTTT   
  
  
- TATCTATCTC CTAAAAAAAC GATAATAGGT GCAGCTAGGT AACTTAATAA AACAGCGATA AACCAATTCA   
  
  
- CTTTATTTTA TGACTTCATC TTACCTCCTA CAACTATTAC TGGATACAGG GAGTTTCAAA TTAGTACATC   
  
  
- AAAGATGCTT AAAGTTCGGT AATATTCTGT ATGTCTAAAA ATACTACAAC TTTTATGAGA ATGGAGGAAC   
  
  
- AGTGTTATAA TTGGACTTCG GTTACTTTAC TTTGTCTTGA TTAATTTAAC TTATGTATTA GTTAGGTATA   
  
  
- ATTAAACAAA ACTGTTGATA AATGTTAGTA GCTAGAGAAA TTAATCCAGA TATTCGATAT CTCATTAATG   
  
  
- AGTTTTGACA AAACATTCTG TTGAATGAGG TATAACCGAA TAATCATATA ACGGAGGTAA GTGTACTTAT   
  
  
- GAAGTACTCT CTAACGGATT TTGAGTATAA CAGTAGTGGA ATTCAGTTTG TATAAAACAA TACTTTACTG   
  
  
- TAGTTCTTAA TGGGAGACTG TTCTTTTTAT ATGGACTGAC TGTATTAGCA TGGTGTAGAC AATCAGGAGA   
  
  
- AAGATGGTGA AACATCTTGT ATTACACTGT CATTATGTAG GTAGGGTTTT CTTTTCTGCT TTTCTTCTCA   
  
  
- CCTCTCAGTG CATATGCGCA ACTTAAGTCC TTTTAATTTC CTTGAGTACT TCTGGGGTAT ATCACGAGGT   
  
  
- TGGACTTGGA CGTCGGGTGA TGATGTACGG ATTTAATTTC TCGTTTCAAT ACTCTCTTTG GTTTTTTCGA   
  
  
- GAAACTCTCT CACCTCCTTC TTAGTACCTT TCGATCGATC CGAATCGTTC TTTCTCGTTG TCGATCGATC   
  
  
- GTTTTCCGAA GAGAAACTTC GGAATCTTTA GAAATCTCTA TTTTTCAACC ACCGAAAAAT TCCCGACACT   
  
  
- ATACAGGGGG TACATTTTTA CGTCGTTATT ACAATTACAG TACGAAAGAA AGTACCAGGG ATTACGCTTT   
  
  
- AGTTAGTACG TACAGTTGCA CTGAGTCACC GAGTGGTATA TACTAGACTA AAGGGGCGTG TGTCTCTATT   
  
  
- TTTCGGGGGT TAATATTGGA GGGTTTTCGG GGGGGAAAAG GAGGATGGGT TGGGTGTGGA GGGGTAAAGT   
  
  
- AGTCCCGAGC GAAGAATTCA ATAGCTAATT TTTTTCTCAC TCATTTGCAT GGGCTTAACT TCTCCCCACA   
  
  
- AGCTTCGAAT ATGAACGAAC TAATCCTTAC TTTCAGGGGT AAAGTAGGCG GGGGGTTTGG CGGCGGTGGT   
  
  
- GGTCATTTAG CGATAGAACG TTGTTATGAC GGAACTTATG ACGGAAAGTT CGGGGGTTAA GTTAAAGTTG   
  
  
- GGAACAATTG TTGTTGTTTG GGAGGATGCT CGGATGGAGC CAAGATCTGG AGGCCGCGTC GGGTTCGGGG   
  
  
- CGGTGGTTGC GGCGGGACGG TGACGGGTGA CTGCGCCCGT AGTTATTTGG GTTCGGACGG AGACGACTGG   
  
  
- ACCTGTTAAA CCTCCCACTC CCAACCCTAA GTTAGGATAG TCTCGACCCC GAATTACTAC TAAGATTATT   
  
  
- AGGATTGAGG TTTGAAACCA GTAGGGTTTA ATCGAGTAGT ACACTAAGGG TGGAGTGTGT CGAGGGACTC   
  
  
- AAGGGGAGGA GAGTCGGCAA CCTAGTGTGT GCGTGGGGGT TATTAATTGA AGAGGGGGAG ACTAATGGGG   
  
  
- TAGAGACTAA ATAGGATGCC AGTCTTAGAG TCGGGGTTAA AACCCGTCAA ACTCGAAGTA GAAGTAGTCG   
  
  
- GGTTGCAGTT ATTGTTACCC GAACTGAAGT ATCTCCTGGA TTAATCTCGT CGAGTTCGGA TGCTTAGGTT   
  
  
- GCTGAGGGTG GTCCAGGTCG ACCATAACCG CGCCGAGTTA GTTGCCGAGT CTAGTGGGCG GCCTTTTGGG   
  
  
- GAGGTGGCGC GGCGGAAGAT GAAGTTCCTC CGGGAGGTTG TGGAGCGACA GCCGCCTCCG GGCCGGGCCG   
  
  
- GACAGGCCGA CAGTAGGATG CTCCACCACG TCTGGTAGGC CCGGGTGTTC TTGAAAAGGC CGTATAGTGA   
  
  
- CTATAGGGAG AAGAGGTGGA AGCGGCGGTT AGTCCGCTAA GACCTCCGCC ACCTACCCCG ACGCAAGTAG   
  
  
- GTGTAGTAGC TAAAGCTGTA GCCTAAACCG CCGGTGACCC GCAGCAAATA CGCACTCGAA CAGCTGTTCC   
  
  
- GGCTGGGCTT ATTCTAGTCG AGGAACCACA AGGCTTAGTG CCGCTAGCAT GGGCTTCTCA AGCCCTAGCT   
  
  
- TTCGTTCGAC CACTCTCTCT TAGAATGCGT TAAGCGAGCA CTAGAGTTGT AGTTACAGGT ATACCTCATA   
  
  
- CAGGAATAAG TTTCGAAGCT CAAGAATAGG AAGTTTAGCC AGTTTAAGTA GCTGCCACTC TTTAACCGCC   
  
  
- AGGTAGATAG AGGCCGCTAC AAAGTGGCCG AAGTTCCGCC TTAGAGCTTT AAGGATCGGC TAGAGGCCCC   
  
  
- TTAAAGCGGT TCGTGGCAGC ACCACCACCT GTCACTCCAT CCTTAGCTGT GCCCTTGAAG CAGGAAGTCG   
  
  
- CAGTTGAAGC AGCGGCCATA GCTTAAGATG TGGCCTTACG AGCTTAGCGA GCTGCAGTGG AGGCGGTCGC   
  
  
- CAAAACGTCC GCCGCCGCCG CCGCTGCGAC ACTCTTCCTA GCTCTGCATG CACGAGGCCG GCTCCTAATG   
  
  
- CCGCCGCCAC CTCCGCCGTA GACGCCGCCG CCATCGCGCC GCCTCGCCAA CCTCTCTCAG CAAGCGCCAC   
  
  
- CGATCCTACT CCCGCCACCC CAAGTCGGTC AAACGACTAA AGGTCCGCCT CACAAACGAC TCCTTCCAAG   
  
  
- TCCAACCGCC CAAGGTGCAC CGCTTTGCAG TACCCCTCTA CTACGAAACG ACCGTACTAT CCGGGGAACA   
  
  
- CAGGTGCAGC CGAACCTCCA CAAT

+     CAT-box

| Site Name | Organism | Position | Strand | Matrix score. | sequence | function |
| --- | --- | --- | --- | --- | --- | --- |
| CAT-box | Arabidopsis thaliana | 1500 | - | 6 | GCCACT | cis-acting regulatory element related to meristem expression |
| CAT-box | Arabidopsis thaliana | 89 | - | 6 | GCCACT | cis-acting regulatory element related to meristem expression |
| CAT-box | Arabidopsis thaliana | 2626 | + | 6 | GCCACT | cis-acting regulatory element related to meristem expression |
| CAT-box | Arabidopsis thaliana | 1912 | + | 6 | GCCACT | cis-acting regulatory element related to meristem expression |

>HU06G00283.1   
+ -Up\_Stream \_Len000GATTTT ACTTCCAATT TTCTACTTAA ATTTGGTTAT CACGTTCGAA TTTTTCAGGT   
  
  
+ AAATACAAAC TGTTAGTGGC CATCCTTTTT TTTTGGGTCT AATCCATCCA GATGTGCTAA TACTTTCTGT   
  
  
+ TTTAGATGAG AAGTTTTAAT GCTTTTTTAG ATGTGTAATT TTTTTAAACA TAAATAGTCT TGTCGAATAA   
  
  
+ GATTTGCTAG TAATTTTTGT AAGAGAAATT ATGTGTATTT TTTATTATAT CAGAATATTA GATCGATCTC   
  
  
+ AATTTAATTA CTTATGAAAA AAAAAGTGGA TATCTTTAAT CAATTTGGCA AGCTCTCATG CATTAATTGT   
  
  
+ AGTCGTACGA ACTTATTTAC ATCACTCATC TGAATTTGCT ACCATTTACA CCTCAAATTT TATTTTAATG   
  
  
+ CCATAAAGAG AATAGAATAT ACTGTCTATT TGTAGTAGTT TTACAGATAA AAACAAATAA GCAAATGAAA   
  
  
+ ATAGATAGAG GATTTTTTTG CTATTATCCA CGTCGATCCA TTGAATTATT TTGTCGCTAT TTGGTTAAGT   
  
  
+ GAAATAAAAT ACTGAAGTAG AATGGAGGAT GTTGATAATG ACCTATGTCC CTCAAAGTTT AATCATGTAG   
  
  
+ TTTCTACGAA TTTCAAGCCA TTATAAGACA TACAGATTTT TATGATGTTG AAAATACTCT TACCTCCTTG   
  
  
+ TCACAATATT AACCTGAAGC CAATGAAATG AAACAGAACT AATTAAATTG AATACATAAT CAATCCATAT   
  
  
+ TAATTTGTTT TGACAACTAT TTACAATCAT CGATCTCTTT AATTAGGTCT ATAAGCTATA GAGTAATTAC   
  
  
+ TCAAAACTGT TTTGTAAGAC AACTTACTCC ATATTGGCTT ATTAGTATAT TGCCTCCATT CACATGAATA   
  
  
+ CTTCATGAGA GATTGCCTAA AACTCATATT GTCATCACCT TAAGTCAAAC ATATTTTGTT ATGAAATGAC   
  
  
+ ATCAAGAATT ACCCTCTGAC AAGAAAAATA TACCTGACTG ACATAATCGT ACCACATCTG TTAGTCCTCT   
  
  
+ TTCTACCACT TTGTAGAACA TAATGTGACA GTAATACATC CATCCCAAAA GAAAAGACGA AAAGAAGAGT   
  
  
+ GGAGAGTCAC GTATACGCGT TGAATTCAGG AAAATTAAAG GAACTCATGA AGACCCCATA TAGTGCTCCA   
  
  
+ ACCTGAACCT GCAGCCCACT ACTACATGCC TAAATTAAAG AGCAAAGTTA TGAGAGAAAC CAAAAAAGCT   
  
  
+ CTTTGAGAGA GTGGAGGAAG AATCATGGAA AGCTAGCTAG GCTTAGCAAG AAAGAGCAAC AGCTAGCTAG   
  
  
+ CAAAAGGCTT CTCTTTGAAG CCTTAGAAAT CTTTAGAGAT AAAAAGTTGG TGGCTTTTTA AGGGCTGTGA   
  
  
+ TATGTCCCCC ATGTAAAAAT GCAGCAATAA TGTTAATGTC ATGCTTTCTT TCATGGTCCC TAATGCGAAA   
  
  
+ TCAATCATGC ATGTCAACGT GACTCAGTGG CTCACCATAT ATGATCTGAT TTCCCCGCAC ACAGAGATAA   
  
  
+ AAAGCCCCCA ATTATAACCT CCCAAAAGCC CCCCCTTTTC CTCCTACCCA ACCCACACCT CCCCATTTCA   
  
  
+ TCAGGGCTCG CTTCTTAAGT TATCGATTAA AAAAAGAGTG AGTAAACGTA CCCGAATTGA AGAGGGGTGT   
  
  
+ TCGAAGCTTA TACTTGCTTG ATTAGGAATG AAAGTCCCCA TTTCATCCGC CCCCCAAACC GCCGCCACCA   
  
  
+ CCAGTAAATC GCTATCTTGC AACAATACTG CCTTGAATAC TGCCTTTCAA GCCCCCAATT CAATTTCAAC   
  
  
+ CCTTGTTAAC AACAACAAAC CCTCCTACGA GCCTACCTCG GTTCTAGACC TCCGGCGCAG CCCAAGCCCC   
  
  
+ GCCACCAACG CCGCCCTGCC ACTGCCCACT GACGCGGGCA TCAATAAACC CAAGCCTGCC TCTGCTGACC   
  
  
+ TGGACAATTT GGAGGGTGAG GGTTGGGATT CAATCCTATC AGAGCTGGGG CTTAATGATG ATTCTAATAA   
  
  
+ TCCTAACTCC AAACTTTGGT CATCCCAAAT TAGCTCATCA TGTGATTCCC ACCTCACACA GCTCCCTGAG   
  
  
+ TTCCCCTCCT CTCAGCCGTT GGATCACACA CGCACCCCCA ATAATTAACT TCTCCCCCTC TGATTACCCC   
  
  
+ ATCTCTGATT TATCCTACGG TCAGAATCTC AGCCCCAATT TTGGGCAGTT TGAGCTTCAT CTTCATCAGC   
  
  
+ CCAACGTCAA TAACAATGGG CTTGACTTCA TAGAGGACCT AATTAGAGCA GCTCAAGCCT ACGAATCCAA   
  
  
+ CGACTCCCAC CAGGTCCAGC TGGTATTGGC GCGGCTCAAT CAACGGCTCA GATCACCCGC CGGAAAACCC   
  
  
+ CTCCACCGCG CCGCCTTCTA CTTCAAGGAG GCCCTCCAAC ACCTCGCTGT CGGCGGAGGC CCGGCCCGGC   
  
  
+ CTGTCCGGCT GTCATCCTAC GAGGTGGTGC AGACCATCCG GGCCCACAAG AACTTTTCCG GCATATCACT   
  
  
+ GATATCCCTC TTCTCCACCT TCGCCGCCAA TCAGGCGATT CTGGAGGCGG TGGATGGGGC TGCGTTCATC   
  
  
+ CACATCATCG ATTTCGACAT CGGATTTGGC GGCCACTGGG CGTCGTTTAT GCGTGAGCTT GTCGACAAGG   
  
  
+ CCGACCCGAA TAAGATCAGC TCCTTGGTGT TCCGAATCAC GGCGATCGTA CCCGAAGAGT TCGGGATCGA   
  
  
+ AAGCAAGCTG GTGAGAGAGA ATCTTACGCA ATTCGCTCGT GATCTCAACA TCAATGTCCA TATGGAGTAT   
  
  
+ GTCCTTATTC AAAGCTTCGA GTTCTTATCC TTCAAATCGG TCAAATTCAT CGACGGTGAG AAATTGGCGG   
  
  
+ TCCATCTATC TCCGGCGATG TTTCACCGGC TTCAAGGCGG AATCTCGAAA TTCCTAGCCG ATCTCCGGGG   
  
  
+ AATTTCGCCA AGCACCGTCG TGGTGGTGGA CAGTGAGGTA GGAATCGACA CGGGAACTTC GTCCTTCAGC   
  
  
+ GTCAACTTCG TCGCCGGTAT CGAATTCTAC ACCGGAATGC TCGAATCGCT CGACGTCACC TCCGCCAGCG   
  
  
+ GTTTTGCAGG CGGCGGCGGC GGCGACGCTG TGAGAAGGAT CGAGACGTAC GTGCTCCGGC CGAGGATTAC   
  
  
+ GGCGGCGGTG GAGGCGGCAT CTGCGGCGGC GGTAGCGCGG CGGAGCGGTT GGAGAGAGTC GTTCGCGGTG   
  
  
+ GCTAGGATGA GGGCGGTGGG GTTCAGCCAG TTTGCTGATT TCCAGGCGGA GTGTTTGCTG AGGAAGGTTC   
  
  
+ AGGTTGGCGG GTTCCACGTG GCGAAACGTC ATGGGGAGAT GATGCTTTGC TGGCATGATA GGCCCCTTGT   
  
  
+ GTCCACGTCG GCTTGGAGGT GTTA  

- -Up\_Stream \_Len000CTAAAA TGAAGGTTAA AAGATGAATT TAAACCAATA GTGCAAGCTT AAAAAGTCCA   
  
  
- TTTATGTTTG ACAATCACCG GTAGGAAAAA AAAACCCAGA TTAGGTAGGT CTACACGATT ATGAAAGACA   
  
  
- AAATCTACTC TTCAAAATTA CGAAAAAATC TACACATTAA AAAAATTTGT ATTTATCAGA ACAGCTTATT   
  
  
- CTAAACGATC ATTAAAAACA TTCTCTTTAA TACACATAAA AAATAATATA GTCTTATAAT CTAGCTAGAG   
  
  
- TTAAATTAAT GAATACTTTT TTTTTCACCT ATAGAAATTA GTTAAACCGT TCGAGAGTAC GTAATTAACA   
  
  
- TCAGCATGCT TGAATAAATG TAGTGAGTAG ACTTAAACGA TGGTAAATGT GGAGTTTAAA ATAAAATTAC   
  
  
- GGTATTTCTC TTATCTTATA TGACAGATAA ACATCATCAA AATGTCTATT TTTGTTTATT CGTTTACTTT   
  
  
- TATCTATCTC CTAAAAAAAC GATAATAGGT GCAGCTAGGT AACTTAATAA AACAGCGATA AACCAATTCA   
  
  
- CTTTATTTTA TGACTTCATC TTACCTCCTA CAACTATTAC TGGATACAGG GAGTTTCAAA TTAGTACATC   
  
  
- AAAGATGCTT AAAGTTCGGT AATATTCTGT ATGTCTAAAA ATACTACAAC TTTTATGAGA ATGGAGGAAC   
  
  
- AGTGTTATAA TTGGACTTCG GTTACTTTAC TTTGTCTTGA TTAATTTAAC TTATGTATTA GTTAGGTATA   
  
  
- ATTAAACAAA ACTGTTGATA AATGTTAGTA GCTAGAGAAA TTAATCCAGA TATTCGATAT CTCATTAATG   
  
  
- AGTTTTGACA AAACATTCTG TTGAATGAGG TATAACCGAA TAATCATATA ACGGAGGTAA GTGTACTTAT   
  
  
- GAAGTACTCT CTAACGGATT TTGAGTATAA CAGTAGTGGA ATTCAGTTTG TATAAAACAA TACTTTACTG   
  
  
- TAGTTCTTAA TGGGAGACTG TTCTTTTTAT ATGGACTGAC TGTATTAGCA TGGTGTAGAC AATCAGGAGA   
  
  
- AAGATGGTGA AACATCTTGT ATTACACTGT CATTATGTAG GTAGGGTTTT CTTTTCTGCT TTTCTTCTCA   
  
  
- CCTCTCAGTG CATATGCGCA ACTTAAGTCC TTTTAATTTC CTTGAGTACT TCTGGGGTAT ATCACGAGGT   
  
  
- TGGACTTGGA CGTCGGGTGA TGATGTACGG ATTTAATTTC TCGTTTCAAT ACTCTCTTTG GTTTTTTCGA   
  
  
- GAAACTCTCT CACCTCCTTC TTAGTACCTT TCGATCGATC CGAATCGTTC TTTCTCGTTG TCGATCGATC   
  
  
- GTTTTCCGAA GAGAAACTTC GGAATCTTTA GAAATCTCTA TTTTTCAACC ACCGAAAAAT TCCCGACACT   
  
  
- ATACAGGGGG TACATTTTTA CGTCGTTATT ACAATTACAG TACGAAAGAA AGTACCAGGG ATTACGCTTT   
  
  
- AGTTAGTACG TACAGTTGCA CTGAGTCACC GAGTGGTATA TACTAGACTA AAGGGGCGTG TGTCTCTATT   
  
  
- TTTCGGGGGT TAATATTGGA GGGTTTTCGG GGGGGAAAAG GAGGATGGGT TGGGTGTGGA GGGGTAAAGT   
  
  
- AGTCCCGAGC GAAGAATTCA ATAGCTAATT TTTTTCTCAC TCATTTGCAT GGGCTTAACT TCTCCCCACA   
  
  
- AGCTTCGAAT ATGAACGAAC TAATCCTTAC TTTCAGGGGT AAAGTAGGCG GGGGGTTTGG CGGCGGTGGT   
  
  
- GGTCATTTAG CGATAGAACG TTGTTATGAC GGAACTTATG ACGGAAAGTT CGGGGGTTAA GTTAAAGTTG   
  
  
- GGAACAATTG TTGTTGTTTG GGAGGATGCT CGGATGGAGC CAAGATCTGG AGGCCGCGTC GGGTTCGGGG   
  
  
- CGGTGGTTGC GGCGGGACGG TGACGGGTGA CTGCGCCCGT AGTTATTTGG GTTCGGACGG AGACGACTGG   
  
  
- ACCTGTTAAA CCTCCCACTC CCAACCCTAA GTTAGGATAG TCTCGACCCC GAATTACTAC TAAGATTATT   
  
  
- AGGATTGAGG TTTGAAACCA GTAGGGTTTA ATCGAGTAGT ACACTAAGGG TGGAGTGTGT CGAGGGACTC   
  
  
- AAGGGGAGGA GAGTCGGCAA CCTAGTGTGT GCGTGGGGGT TATTAATTGA AGAGGGGGAG ACTAATGGGG   
  
  
- TAGAGACTAA ATAGGATGCC AGTCTTAGAG TCGGGGTTAA AACCCGTCAA ACTCGAAGTA GAAGTAGTCG   
  
  
- GGTTGCAGTT ATTGTTACCC GAACTGAAGT ATCTCCTGGA TTAATCTCGT CGAGTTCGGA TGCTTAGGTT   
  
  
- GCTGAGGGTG GTCCAGGTCG ACCATAACCG CGCCGAGTTA GTTGCCGAGT CTAGTGGGCG GCCTTTTGGG   
  
  
- GAGGTGGCGC GGCGGAAGAT GAAGTTCCTC CGGGAGGTTG TGGAGCGACA GCCGCCTCCG GGCCGGGCCG   
  
  
- GACAGGCCGA CAGTAGGATG CTCCACCACG TCTGGTAGGC CCGGGTGTTC TTGAAAAGGC CGTATAGTGA   
  
  
- CTATAGGGAG AAGAGGTGGA AGCGGCGGTT AGTCCGCTAA GACCTCCGCC ACCTACCCCG ACGCAAGTAG   
  
  
- GTGTAGTAGC TAAAGCTGTA GCCTAAACCG CCGGTGACCC GCAGCAAATA CGCACTCGAA CAGCTGTTCC   
  
  
- GGCTGGGCTT ATTCTAGTCG AGGAACCACA AGGCTTAGTG CCGCTAGCAT GGGCTTCTCA AGCCCTAGCT   
  
  
- TTCGTTCGAC CACTCTCTCT TAGAATGCGT TAAGCGAGCA CTAGAGTTGT AGTTACAGGT ATACCTCATA   
  
  
- CAGGAATAAG TTTCGAAGCT CAAGAATAGG AAGTTTAGCC AGTTTAAGTA GCTGCCACTC TTTAACCGCC   
  
  
- AGGTAGATAG AGGCCGCTAC AAAGTGGCCG AAGTTCCGCC TTAGAGCTTT AAGGATCGGC TAGAGGCCCC   
  
  
- TTAAAGCGGT TCGTGGCAGC ACCACCACCT GTCACTCCAT CCTTAGCTGT GCCCTTGAAG CAGGAAGTCG   
  
  
- CAGTTGAAGC AGCGGCCATA GCTTAAGATG TGGCCTTACG AGCTTAGCGA GCTGCAGTGG AGGCGGTCGC   
  
  
- CAAAACGTCC GCCGCCGCCG CCGCTGCGAC ACTCTTCCTA GCTCTGCATG CACGAGGCCG GCTCCTAATG   
  
  
- CCGCCGCCAC CTCCGCCGTA GACGCCGCCG CCATCGCGCC GCCTCGCCAA CCTCTCTCAG CAAGCGCCAC   
  
  
- CGATCCTACT CCCGCCACCC CAAGTCGGTC AAACGACTAA AGGTCCGCCT CACAAACGAC TCCTTCCAAG   
  
  
- TCCAACCGCC CAAGGTGCAC CGCTTTGCAG TACCCCTCTA CTACGAAACG ACCGTACTAT CCGGGGAACA   
  
  
- CAGGTGCAGC CGAACCTCCA CAAT

+     CCAAT-box

| Site Name | Organism | Position | Strand | Matrix score. | sequence | function |
| --- | --- | --- | --- | --- | --- | --- |
| CCAAT-box | Hordeum vulgare | 2355 | + | 6 | CAACGG | MYBHv1 binding site |
| CCAAT-box | Hordeum vulgare | 2120 | - | 6 | CAACGG | MYBHv1 binding site |

>HU06G00283.1   
+ -Up\_Stream \_Len000GATTTT ACTTCCAATT TTCTACTTAA ATTTGGTTAT CACGTTCGAA TTTTTCAGGT   
  
  
+ AAATACAAAC TGTTAGTGGC CATCCTTTTT TTTTGGGTCT AATCCATCCA GATGTGCTAA TACTTTCTGT   
  
  
+ TTTAGATGAG AAGTTTTAAT GCTTTTTTAG ATGTGTAATT TTTTTAAACA TAAATAGTCT TGTCGAATAA   
  
  
+ GATTTGCTAG TAATTTTTGT AAGAGAAATT ATGTGTATTT TTTATTATAT CAGAATATTA GATCGATCTC   
  
  
+ AATTTAATTA CTTATGAAAA AAAAAGTGGA TATCTTTAAT CAATTTGGCA AGCTCTCATG CATTAATTGT   
  
  
+ AGTCGTACGA ACTTATTTAC ATCACTCATC TGAATTTGCT ACCATTTACA CCTCAAATTT TATTTTAATG   
  
  
+ CCATAAAGAG AATAGAATAT ACTGTCTATT TGTAGTAGTT TTACAGATAA AAACAAATAA GCAAATGAAA   
  
  
+ ATAGATAGAG GATTTTTTTG CTATTATCCA CGTCGATCCA TTGAATTATT TTGTCGCTAT TTGGTTAAGT   
  
  
+ GAAATAAAAT ACTGAAGTAG AATGGAGGAT GTTGATAATG ACCTATGTCC CTCAAAGTTT AATCATGTAG   
  
  
+ TTTCTACGAA TTTCAAGCCA TTATAAGACA TACAGATTTT TATGATGTTG AAAATACTCT TACCTCCTTG   
  
  
+ TCACAATATT AACCTGAAGC CAATGAAATG AAACAGAACT AATTAAATTG AATACATAAT CAATCCATAT   
  
  
+ TAATTTGTTT TGACAACTAT TTACAATCAT CGATCTCTTT AATTAGGTCT ATAAGCTATA GAGTAATTAC   
  
  
+ TCAAAACTGT TTTGTAAGAC AACTTACTCC ATATTGGCTT ATTAGTATAT TGCCTCCATT CACATGAATA   
  
  
+ CTTCATGAGA GATTGCCTAA AACTCATATT GTCATCACCT TAAGTCAAAC ATATTTTGTT ATGAAATGAC   
  
  
+ ATCAAGAATT ACCCTCTGAC AAGAAAAATA TACCTGACTG ACATAATCGT ACCACATCTG TTAGTCCTCT   
  
  
+ TTCTACCACT TTGTAGAACA TAATGTGACA GTAATACATC CATCCCAAAA GAAAAGACGA AAAGAAGAGT   
  
  
+ GGAGAGTCAC GTATACGCGT TGAATTCAGG AAAATTAAAG GAACTCATGA AGACCCCATA TAGTGCTCCA   
  
  
+ ACCTGAACCT GCAGCCCACT ACTACATGCC TAAATTAAAG AGCAAAGTTA TGAGAGAAAC CAAAAAAGCT   
  
  
+ CTTTGAGAGA GTGGAGGAAG AATCATGGAA AGCTAGCTAG GCTTAGCAAG AAAGAGCAAC AGCTAGCTAG   
  
  
+ CAAAAGGCTT CTCTTTGAAG CCTTAGAAAT CTTTAGAGAT AAAAAGTTGG TGGCTTTTTA AGGGCTGTGA   
  
  
+ TATGTCCCCC ATGTAAAAAT GCAGCAATAA TGTTAATGTC ATGCTTTCTT TCATGGTCCC TAATGCGAAA   
  
  
+ TCAATCATGC ATGTCAACGT GACTCAGTGG CTCACCATAT ATGATCTGAT TTCCCCGCAC ACAGAGATAA   
  
  
+ AAAGCCCCCA ATTATAACCT CCCAAAAGCC CCCCCTTTTC CTCCTACCCA ACCCACACCT CCCCATTTCA   
  
  
+ TCAGGGCTCG CTTCTTAAGT TATCGATTAA AAAAAGAGTG AGTAAACGTA CCCGAATTGA AGAGGGGTGT   
  
  
+ TCGAAGCTTA TACTTGCTTG ATTAGGAATG AAAGTCCCCA TTTCATCCGC CCCCCAAACC GCCGCCACCA   
  
  
+ CCAGTAAATC GCTATCTTGC AACAATACTG CCTTGAATAC TGCCTTTCAA GCCCCCAATT CAATTTCAAC   
  
  
+ CCTTGTTAAC AACAACAAAC CCTCCTACGA GCCTACCTCG GTTCTAGACC TCCGGCGCAG CCCAAGCCCC   
  
  
+ GCCACCAACG CCGCCCTGCC ACTGCCCACT GACGCGGGCA TCAATAAACC CAAGCCTGCC TCTGCTGACC   
  
  
+ TGGACAATTT GGAGGGTGAG GGTTGGGATT CAATCCTATC AGAGCTGGGG CTTAATGATG ATTCTAATAA   
  
  
+ TCCTAACTCC AAACTTTGGT CATCCCAAAT TAGCTCATCA TGTGATTCCC ACCTCACACA GCTCCCTGAG   
  
  
+ TTCCCCTCCT CTCAGCCGTT GGATCACACA CGCACCCCCA ATAATTAACT TCTCCCCCTC TGATTACCCC   
  
  
+ ATCTCTGATT TATCCTACGG TCAGAATCTC AGCCCCAATT TTGGGCAGTT TGAGCTTCAT CTTCATCAGC   
  
  
+ CCAACGTCAA TAACAATGGG CTTGACTTCA TAGAGGACCT AATTAGAGCA GCTCAAGCCT ACGAATCCAA   
  
  
+ CGACTCCCAC CAGGTCCAGC TGGTATTGGC GCGGCTCAAT CAACGGCTCA GATCACCCGC CGGAAAACCC   
  
  
+ CTCCACCGCG CCGCCTTCTA CTTCAAGGAG GCCCTCCAAC ACCTCGCTGT CGGCGGAGGC CCGGCCCGGC   
  
  
+ CTGTCCGGCT GTCATCCTAC GAGGTGGTGC AGACCATCCG GGCCCACAAG AACTTTTCCG GCATATCACT   
  
  
+ GATATCCCTC TTCTCCACCT TCGCCGCCAA TCAGGCGATT CTGGAGGCGG TGGATGGGGC TGCGTTCATC   
  
  
+ CACATCATCG ATTTCGACAT CGGATTTGGC GGCCACTGGG CGTCGTTTAT GCGTGAGCTT GTCGACAAGG   
  
  
+ CCGACCCGAA TAAGATCAGC TCCTTGGTGT TCCGAATCAC GGCGATCGTA CCCGAAGAGT TCGGGATCGA   
  
  
+ AAGCAAGCTG GTGAGAGAGA ATCTTACGCA ATTCGCTCGT GATCTCAACA TCAATGTCCA TATGGAGTAT   
  
  
+ GTCCTTATTC AAAGCTTCGA GTTCTTATCC TTCAAATCGG TCAAATTCAT CGACGGTGAG AAATTGGCGG   
  
  
+ TCCATCTATC TCCGGCGATG TTTCACCGGC TTCAAGGCGG AATCTCGAAA TTCCTAGCCG ATCTCCGGGG   
  
  
+ AATTTCGCCA AGCACCGTCG TGGTGGTGGA CAGTGAGGTA GGAATCGACA CGGGAACTTC GTCCTTCAGC   
  
  
+ GTCAACTTCG TCGCCGGTAT CGAATTCTAC ACCGGAATGC TCGAATCGCT CGACGTCACC TCCGCCAGCG   
  
  
+ GTTTTGCAGG CGGCGGCGGC GGCGACGCTG TGAGAAGGAT CGAGACGTAC GTGCTCCGGC CGAGGATTAC   
  
  
+ GGCGGCGGTG GAGGCGGCAT CTGCGGCGGC GGTAGCGCGG CGGAGCGGTT GGAGAGAGTC GTTCGCGGTG   
  
  
+ GCTAGGATGA GGGCGGTGGG GTTCAGCCAG TTTGCTGATT TCCAGGCGGA GTGTTTGCTG AGGAAGGTTC   
  
  
+ AGGTTGGCGG GTTCCACGTG GCGAAACGTC ATGGGGAGAT GATGCTTTGC TGGCATGATA GGCCCCTTGT   
  
  
+ GTCCACGTCG GCTTGGAGGT GTTA  

- -Up\_Stream \_Len000CTAAAA TGAAGGTTAA AAGATGAATT TAAACCAATA GTGCAAGCTT AAAAAGTCCA   
  
  
- TTTATGTTTG ACAATCACCG GTAGGAAAAA AAAACCCAGA TTAGGTAGGT CTACACGATT ATGAAAGACA   
  
  
- AAATCTACTC TTCAAAATTA CGAAAAAATC TACACATTAA AAAAATTTGT ATTTATCAGA ACAGCTTATT   
  
  
- CTAAACGATC ATTAAAAACA TTCTCTTTAA TACACATAAA AAATAATATA GTCTTATAAT CTAGCTAGAG   
  
  
- TTAAATTAAT GAATACTTTT TTTTTCACCT ATAGAAATTA GTTAAACCGT TCGAGAGTAC GTAATTAACA   
  
  
- TCAGCATGCT TGAATAAATG TAGTGAGTAG ACTTAAACGA TGGTAAATGT GGAGTTTAAA ATAAAATTAC   
  
  
- GGTATTTCTC TTATCTTATA TGACAGATAA ACATCATCAA AATGTCTATT TTTGTTTATT CGTTTACTTT   
  
  
- TATCTATCTC CTAAAAAAAC GATAATAGGT GCAGCTAGGT AACTTAATAA AACAGCGATA AACCAATTCA   
  
  
- CTTTATTTTA TGACTTCATC TTACCTCCTA CAACTATTAC TGGATACAGG GAGTTTCAAA TTAGTACATC   
  
  
- AAAGATGCTT AAAGTTCGGT AATATTCTGT ATGTCTAAAA ATACTACAAC TTTTATGAGA ATGGAGGAAC   
  
  
- AGTGTTATAA TTGGACTTCG GTTACTTTAC TTTGTCTTGA TTAATTTAAC TTATGTATTA GTTAGGTATA   
  
  
- ATTAAACAAA ACTGTTGATA AATGTTAGTA GCTAGAGAAA TTAATCCAGA TATTCGATAT CTCATTAATG   
  
  
- AGTTTTGACA AAACATTCTG TTGAATGAGG TATAACCGAA TAATCATATA ACGGAGGTAA GTGTACTTAT   
  
  
- GAAGTACTCT CTAACGGATT TTGAGTATAA CAGTAGTGGA ATTCAGTTTG TATAAAACAA TACTTTACTG   
  
  
- TAGTTCTTAA TGGGAGACTG TTCTTTTTAT ATGGACTGAC TGTATTAGCA TGGTGTAGAC AATCAGGAGA   
  
  
- AAGATGGTGA AACATCTTGT ATTACACTGT CATTATGTAG GTAGGGTTTT CTTTTCTGCT TTTCTTCTCA   
  
  
- CCTCTCAGTG CATATGCGCA ACTTAAGTCC TTTTAATTTC CTTGAGTACT TCTGGGGTAT ATCACGAGGT   
  
  
- TGGACTTGGA CGTCGGGTGA TGATGTACGG ATTTAATTTC TCGTTTCAAT ACTCTCTTTG GTTTTTTCGA   
  
  
- GAAACTCTCT CACCTCCTTC TTAGTACCTT TCGATCGATC CGAATCGTTC TTTCTCGTTG TCGATCGATC   
  
  
- GTTTTCCGAA GAGAAACTTC GGAATCTTTA GAAATCTCTA TTTTTCAACC ACCGAAAAAT TCCCGACACT   
  
  
- ATACAGGGGG TACATTTTTA CGTCGTTATT ACAATTACAG TACGAAAGAA AGTACCAGGG ATTACGCTTT   
  
  
- AGTTAGTACG TACAGTTGCA CTGAGTCACC GAGTGGTATA TACTAGACTA AAGGGGCGTG TGTCTCTATT   
  
  
- TTTCGGGGGT TAATATTGGA GGGTTTTCGG GGGGGAAAAG GAGGATGGGT TGGGTGTGGA GGGGTAAAGT   
  
  
- AGTCCCGAGC GAAGAATTCA ATAGCTAATT TTTTTCTCAC TCATTTGCAT GGGCTTAACT TCTCCCCACA   
  
  
- AGCTTCGAAT ATGAACGAAC TAATCCTTAC TTTCAGGGGT AAAGTAGGCG GGGGGTTTGG CGGCGGTGGT   
  
  
- GGTCATTTAG CGATAGAACG TTGTTATGAC GGAACTTATG ACGGAAAGTT CGGGGGTTAA GTTAAAGTTG   
  
  
- GGAACAATTG TTGTTGTTTG GGAGGATGCT CGGATGGAGC CAAGATCTGG AGGCCGCGTC GGGTTCGGGG   
  
  
- CGGTGGTTGC GGCGGGACGG TGACGGGTGA CTGCGCCCGT AGTTATTTGG GTTCGGACGG AGACGACTGG   
  
  
- ACCTGTTAAA CCTCCCACTC CCAACCCTAA GTTAGGATAG TCTCGACCCC GAATTACTAC TAAGATTATT   
  
  
- AGGATTGAGG TTTGAAACCA GTAGGGTTTA ATCGAGTAGT ACACTAAGGG TGGAGTGTGT CGAGGGACTC   
  
  
- AAGGGGAGGA GAGTCGGCAA CCTAGTGTGT GCGTGGGGGT TATTAATTGA AGAGGGGGAG ACTAATGGGG   
  
  
- TAGAGACTAA ATAGGATGCC AGTCTTAGAG TCGGGGTTAA AACCCGTCAA ACTCGAAGTA GAAGTAGTCG   
  
  
- GGTTGCAGTT ATTGTTACCC GAACTGAAGT ATCTCCTGGA TTAATCTCGT CGAGTTCGGA TGCTTAGGTT   
  
  
- GCTGAGGGTG GTCCAGGTCG ACCATAACCG CGCCGAGTTA GTTGCCGAGT CTAGTGGGCG GCCTTTTGGG   
  
  
- GAGGTGGCGC GGCGGAAGAT GAAGTTCCTC CGGGAGGTTG TGGAGCGACA GCCGCCTCCG GGCCGGGCCG   
  
  
- GACAGGCCGA CAGTAGGATG CTCCACCACG TCTGGTAGGC CCGGGTGTTC TTGAAAAGGC CGTATAGTGA   
  
  
- CTATAGGGAG AAGAGGTGGA AGCGGCGGTT AGTCCGCTAA GACCTCCGCC ACCTACCCCG ACGCAAGTAG   
  
  
- GTGTAGTAGC TAAAGCTGTA GCCTAAACCG CCGGTGACCC GCAGCAAATA CGCACTCGAA CAGCTGTTCC   
  
  
- GGCTGGGCTT ATTCTAGTCG AGGAACCACA AGGCTTAGTG CCGCTAGCAT GGGCTTCTCA AGCCCTAGCT   
  
  
- TTCGTTCGAC CACTCTCTCT TAGAATGCGT TAAGCGAGCA CTAGAGTTGT AGTTACAGGT ATACCTCATA   
  
  
- CAGGAATAAG TTTCGAAGCT CAAGAATAGG AAGTTTAGCC AGTTTAAGTA GCTGCCACTC TTTAACCGCC   
  
  
- AGGTAGATAG AGGCCGCTAC AAAGTGGCCG AAGTTCCGCC TTAGAGCTTT AAGGATCGGC TAGAGGCCCC   
  
  
- TTAAAGCGGT TCGTGGCAGC ACCACCACCT GTCACTCCAT CCTTAGCTGT GCCCTTGAAG CAGGAAGTCG   
  
  
- CAGTTGAAGC AGCGGCCATA GCTTAAGATG TGGCCTTACG AGCTTAGCGA GCTGCAGTGG AGGCGGTCGC   
  
  
- CAAAACGTCC GCCGCCGCCG CCGCTGCGAC ACTCTTCCTA GCTCTGCATG CACGAGGCCG GCTCCTAATG   
  
  
- CCGCCGCCAC CTCCGCCGTA GACGCCGCCG CCATCGCGCC GCCTCGCCAA CCTCTCTCAG CAAGCGCCAC   
  
  
- CGATCCTACT CCCGCCACCC CAAGTCGGTC AAACGACTAA AGGTCCGCCT CACAAACGAC TCCTTCCAAG   
  
  
- TCCAACCGCC CAAGGTGCAC CGCTTTGCAG TACCCCTCTA CTACGAAACG ACCGTACTAT CCGGGGAACA   
  
  
- CAGGTGCAGC CGAACCTCCA CAAT

+     CGTCA-motif

| Site Name | Organism | Position | Strand | Matrix score. | sequence | function |
| --- | --- | --- | --- | --- | --- | --- |
| CGTCA-motif | Hordeum vulgare | 3321 | + | 5 | CGTCA | cis-acting regulatory element involved in the MeJA-responsiveness |
| CGTCA-motif | Hordeum vulgare | 3068 | + | 5 | CGTCA | cis-acting regulatory element involved in the MeJA-responsiveness |
| CGTCA-motif | Hordeum vulgare | 1924 | - | 5 | CGTCA | cis-acting regulatory element involved in the MeJA-responsiveness |
| CGTCA-motif | Hordeum vulgare | 2249 | + | 5 | CGTCA | cis-acting regulatory element involved in the MeJA-responsiveness |
| CGTCA-motif | Hordeum vulgare | 3014 | + | 5 | CGTCA | cis-acting regulatory element involved in the MeJA-responsiveness |

>HU06G00283.1   
+ -Up\_Stream \_Len000GATTTT ACTTCCAATT TTCTACTTAA ATTTGGTTAT CACGTTCGAA TTTTTCAGGT   
  
  
+ AAATACAAAC TGTTAGTGGC CATCCTTTTT TTTTGGGTCT AATCCATCCA GATGTGCTAA TACTTTCTGT   
  
  
+ TTTAGATGAG AAGTTTTAAT GCTTTTTTAG ATGTGTAATT TTTTTAAACA TAAATAGTCT TGTCGAATAA   
  
  
+ GATTTGCTAG TAATTTTTGT AAGAGAAATT ATGTGTATTT TTTATTATAT CAGAATATTA GATCGATCTC   
  
  
+ AATTTAATTA CTTATGAAAA AAAAAGTGGA TATCTTTAAT CAATTTGGCA AGCTCTCATG CATTAATTGT   
  
  
+ AGTCGTACGA ACTTATTTAC ATCACTCATC TGAATTTGCT ACCATTTACA CCTCAAATTT TATTTTAATG   
  
  
+ CCATAAAGAG AATAGAATAT ACTGTCTATT TGTAGTAGTT TTACAGATAA AAACAAATAA GCAAATGAAA   
  
  
+ ATAGATAGAG GATTTTTTTG CTATTATCCA CGTCGATCCA TTGAATTATT TTGTCGCTAT TTGGTTAAGT   
  
  
+ GAAATAAAAT ACTGAAGTAG AATGGAGGAT GTTGATAATG ACCTATGTCC CTCAAAGTTT AATCATGTAG   
  
  
+ TTTCTACGAA TTTCAAGCCA TTATAAGACA TACAGATTTT TATGATGTTG AAAATACTCT TACCTCCTTG   
  
  
+ TCACAATATT AACCTGAAGC CAATGAAATG AAACAGAACT AATTAAATTG AATACATAAT CAATCCATAT   
  
  
+ TAATTTGTTT TGACAACTAT TTACAATCAT CGATCTCTTT AATTAGGTCT ATAAGCTATA GAGTAATTAC   
  
  
+ TCAAAACTGT TTTGTAAGAC AACTTACTCC ATATTGGCTT ATTAGTATAT TGCCTCCATT CACATGAATA   
  
  
+ CTTCATGAGA GATTGCCTAA AACTCATATT GTCATCACCT TAAGTCAAAC ATATTTTGTT ATGAAATGAC   
  
  
+ ATCAAGAATT ACCCTCTGAC AAGAAAAATA TACCTGACTG ACATAATCGT ACCACATCTG TTAGTCCTCT   
  
  
+ TTCTACCACT TTGTAGAACA TAATGTGACA GTAATACATC CATCCCAAAA GAAAAGACGA AAAGAAGAGT   
  
  
+ GGAGAGTCAC GTATACGCGT TGAATTCAGG AAAATTAAAG GAACTCATGA AGACCCCATA TAGTGCTCCA   
  
  
+ ACCTGAACCT GCAGCCCACT ACTACATGCC TAAATTAAAG AGCAAAGTTA TGAGAGAAAC CAAAAAAGCT   
  
  
+ CTTTGAGAGA GTGGAGGAAG AATCATGGAA AGCTAGCTAG GCTTAGCAAG AAAGAGCAAC AGCTAGCTAG   
  
  
+ CAAAAGGCTT CTCTTTGAAG CCTTAGAAAT CTTTAGAGAT AAAAAGTTGG TGGCTTTTTA AGGGCTGTGA   
  
  
+ TATGTCCCCC ATGTAAAAAT GCAGCAATAA TGTTAATGTC ATGCTTTCTT TCATGGTCCC TAATGCGAAA   
  
  
+ TCAATCATGC ATGTCAACGT GACTCAGTGG CTCACCATAT ATGATCTGAT TTCCCCGCAC ACAGAGATAA   
  
  
+ AAAGCCCCCA ATTATAACCT CCCAAAAGCC CCCCCTTTTC CTCCTACCCA ACCCACACCT CCCCATTTCA   
  
  
+ TCAGGGCTCG CTTCTTAAGT TATCGATTAA AAAAAGAGTG AGTAAACGTA CCCGAATTGA AGAGGGGTGT   
  
  
+ TCGAAGCTTA TACTTGCTTG ATTAGGAATG AAAGTCCCCA TTTCATCCGC CCCCCAAACC GCCGCCACCA   
  
  
+ CCAGTAAATC GCTATCTTGC AACAATACTG CCTTGAATAC TGCCTTTCAA GCCCCCAATT CAATTTCAAC   
  
  
+ CCTTGTTAAC AACAACAAAC CCTCCTACGA GCCTACCTCG GTTCTAGACC TCCGGCGCAG CCCAAGCCCC   
  
  
+ GCCACCAACG CCGCCCTGCC ACTGCCCACT GACGCGGGCA TCAATAAACC CAAGCCTGCC TCTGCTGACC   
  
  
+ TGGACAATTT GGAGGGTGAG GGTTGGGATT CAATCCTATC AGAGCTGGGG CTTAATGATG ATTCTAATAA   
  
  
+ TCCTAACTCC AAACTTTGGT CATCCCAAAT TAGCTCATCA TGTGATTCCC ACCTCACACA GCTCCCTGAG   
  
  
+ TTCCCCTCCT CTCAGCCGTT GGATCACACA CGCACCCCCA ATAATTAACT TCTCCCCCTC TGATTACCCC   
  
  
+ ATCTCTGATT TATCCTACGG TCAGAATCTC AGCCCCAATT TTGGGCAGTT TGAGCTTCAT CTTCATCAGC   
  
  
+ CCAACGTCAA TAACAATGGG CTTGACTTCA TAGAGGACCT AATTAGAGCA GCTCAAGCCT ACGAATCCAA   
  
  
+ CGACTCCCAC CAGGTCCAGC TGGTATTGGC GCGGCTCAAT CAACGGCTCA GATCACCCGC CGGAAAACCC   
  
  
+ CTCCACCGCG CCGCCTTCTA CTTCAAGGAG GCCCTCCAAC ACCTCGCTGT CGGCGGAGGC CCGGCCCGGC   
  
  
+ CTGTCCGGCT GTCATCCTAC GAGGTGGTGC AGACCATCCG GGCCCACAAG AACTTTTCCG GCATATCACT   
  
  
+ GATATCCCTC TTCTCCACCT TCGCCGCCAA TCAGGCGATT CTGGAGGCGG TGGATGGGGC TGCGTTCATC   
  
  
+ CACATCATCG ATTTCGACAT CGGATTTGGC GGCCACTGGG CGTCGTTTAT GCGTGAGCTT GTCGACAAGG   
  
  
+ CCGACCCGAA TAAGATCAGC TCCTTGGTGT TCCGAATCAC GGCGATCGTA CCCGAAGAGT TCGGGATCGA   
  
  
+ AAGCAAGCTG GTGAGAGAGA ATCTTACGCA ATTCGCTCGT GATCTCAACA TCAATGTCCA TATGGAGTAT   
  
  
+ GTCCTTATTC AAAGCTTCGA GTTCTTATCC TTCAAATCGG TCAAATTCAT CGACGGTGAG AAATTGGCGG   
  
  
+ TCCATCTATC TCCGGCGATG TTTCACCGGC TTCAAGGCGG AATCTCGAAA TTCCTAGCCG ATCTCCGGGG   
  
  
+ AATTTCGCCA AGCACCGTCG TGGTGGTGGA CAGTGAGGTA GGAATCGACA CGGGAACTTC GTCCTTCAGC   
  
  
+ GTCAACTTCG TCGCCGGTAT CGAATTCTAC ACCGGAATGC TCGAATCGCT CGACGTCACC TCCGCCAGCG   
  
  
+ GTTTTGCAGG CGGCGGCGGC GGCGACGCTG TGAGAAGGAT CGAGACGTAC GTGCTCCGGC CGAGGATTAC   
  
  
+ GGCGGCGGTG GAGGCGGCAT CTGCGGCGGC GGTAGCGCGG CGGAGCGGTT GGAGAGAGTC GTTCGCGGTG   
  
  
+ GCTAGGATGA GGGCGGTGGG GTTCAGCCAG TTTGCTGATT TCCAGGCGGA GTGTTTGCTG AGGAAGGTTC   
  
  
+ AGGTTGGCGG GTTCCACGTG GCGAAACGTC ATGGGGAGAT GATGCTTTGC TGGCATGATA GGCCCCTTGT   
  
  
+ GTCCACGTCG GCTTGGAGGT GTTA  

- -Up\_Stream \_Len000CTAAAA TGAAGGTTAA AAGATGAATT TAAACCAATA GTGCAAGCTT AAAAAGTCCA   
  
  
- TTTATGTTTG ACAATCACCG GTAGGAAAAA AAAACCCAGA TTAGGTAGGT CTACACGATT ATGAAAGACA   
  
  
- AAATCTACTC TTCAAAATTA CGAAAAAATC TACACATTAA AAAAATTTGT ATTTATCAGA ACAGCTTATT   
  
  
- CTAAACGATC ATTAAAAACA TTCTCTTTAA TACACATAAA AAATAATATA GTCTTATAAT CTAGCTAGAG   
  
  
- TTAAATTAAT GAATACTTTT TTTTTCACCT ATAGAAATTA GTTAAACCGT TCGAGAGTAC GTAATTAACA   
  
  
- TCAGCATGCT TGAATAAATG TAGTGAGTAG ACTTAAACGA TGGTAAATGT GGAGTTTAAA ATAAAATTAC   
  
  
- GGTATTTCTC TTATCTTATA TGACAGATAA ACATCATCAA AATGTCTATT TTTGTTTATT CGTTTACTTT   
  
  
- TATCTATCTC CTAAAAAAAC GATAATAGGT GCAGCTAGGT AACTTAATAA AACAGCGATA AACCAATTCA   
  
  
- CTTTATTTTA TGACTTCATC TTACCTCCTA CAACTATTAC TGGATACAGG GAGTTTCAAA TTAGTACATC   
  
  
- AAAGATGCTT AAAGTTCGGT AATATTCTGT ATGTCTAAAA ATACTACAAC TTTTATGAGA ATGGAGGAAC   
  
  
- AGTGTTATAA TTGGACTTCG GTTACTTTAC TTTGTCTTGA TTAATTTAAC TTATGTATTA GTTAGGTATA   
  
  
- ATTAAACAAA ACTGTTGATA AATGTTAGTA GCTAGAGAAA TTAATCCAGA TATTCGATAT CTCATTAATG   
  
  
- AGTTTTGACA AAACATTCTG TTGAATGAGG TATAACCGAA TAATCATATA ACGGAGGTAA GTGTACTTAT   
  
  
- GAAGTACTCT CTAACGGATT TTGAGTATAA CAGTAGTGGA ATTCAGTTTG TATAAAACAA TACTTTACTG   
  
  
- TAGTTCTTAA TGGGAGACTG TTCTTTTTAT ATGGACTGAC TGTATTAGCA TGGTGTAGAC AATCAGGAGA   
  
  
- AAGATGGTGA AACATCTTGT ATTACACTGT CATTATGTAG GTAGGGTTTT CTTTTCTGCT TTTCTTCTCA   
  
  
- CCTCTCAGTG CATATGCGCA ACTTAAGTCC TTTTAATTTC CTTGAGTACT TCTGGGGTAT ATCACGAGGT   
  
  
- TGGACTTGGA CGTCGGGTGA TGATGTACGG ATTTAATTTC TCGTTTCAAT ACTCTCTTTG GTTTTTTCGA   
  
  
- GAAACTCTCT CACCTCCTTC TTAGTACCTT TCGATCGATC CGAATCGTTC TTTCTCGTTG TCGATCGATC   
  
  
- GTTTTCCGAA GAGAAACTTC GGAATCTTTA GAAATCTCTA TTTTTCAACC ACCGAAAAAT TCCCGACACT   
  
  
- ATACAGGGGG TACATTTTTA CGTCGTTATT ACAATTACAG TACGAAAGAA AGTACCAGGG ATTACGCTTT   
  
  
- AGTTAGTACG TACAGTTGCA CTGAGTCACC GAGTGGTATA TACTAGACTA AAGGGGCGTG TGTCTCTATT   
  
  
- TTTCGGGGGT TAATATTGGA GGGTTTTCGG GGGGGAAAAG GAGGATGGGT TGGGTGTGGA GGGGTAAAGT   
  
  
- AGTCCCGAGC GAAGAATTCA ATAGCTAATT TTTTTCTCAC TCATTTGCAT GGGCTTAACT TCTCCCCACA   
  
  
- AGCTTCGAAT ATGAACGAAC TAATCCTTAC TTTCAGGGGT AAAGTAGGCG GGGGGTTTGG CGGCGGTGGT   
  
  
- GGTCATTTAG CGATAGAACG TTGTTATGAC GGAACTTATG ACGGAAAGTT CGGGGGTTAA GTTAAAGTTG   
  
  
- GGAACAATTG TTGTTGTTTG GGAGGATGCT CGGATGGAGC CAAGATCTGG AGGCCGCGTC GGGTTCGGGG   
  
  
- CGGTGGTTGC GGCGGGACGG TGACGGGTGA CTGCGCCCGT AGTTATTTGG GTTCGGACGG AGACGACTGG   
  
  
- ACCTGTTAAA CCTCCCACTC CCAACCCTAA GTTAGGATAG TCTCGACCCC GAATTACTAC TAAGATTATT   
  
  
- AGGATTGAGG TTTGAAACCA GTAGGGTTTA ATCGAGTAGT ACACTAAGGG TGGAGTGTGT CGAGGGACTC   
  
  
- AAGGGGAGGA GAGTCGGCAA CCTAGTGTGT GCGTGGGGGT TATTAATTGA AGAGGGGGAG ACTAATGGGG   
  
  
- TAGAGACTAA ATAGGATGCC AGTCTTAGAG TCGGGGTTAA AACCCGTCAA ACTCGAAGTA GAAGTAGTCG   
  
  
- GGTTGCAGTT ATTGTTACCC GAACTGAAGT ATCTCCTGGA TTAATCTCGT CGAGTTCGGA TGCTTAGGTT   
  
  
- GCTGAGGGTG GTCCAGGTCG ACCATAACCG CGCCGAGTTA GTTGCCGAGT CTAGTGGGCG GCCTTTTGGG   
  
  
- GAGGTGGCGC GGCGGAAGAT GAAGTTCCTC CGGGAGGTTG TGGAGCGACA GCCGCCTCCG GGCCGGGCCG   
  
  
- GACAGGCCGA CAGTAGGATG CTCCACCACG TCTGGTAGGC CCGGGTGTTC TTGAAAAGGC CGTATAGTGA   
  
  
- CTATAGGGAG AAGAGGTGGA AGCGGCGGTT AGTCCGCTAA GACCTCCGCC ACCTACCCCG ACGCAAGTAG   
  
  
- GTGTAGTAGC TAAAGCTGTA GCCTAAACCG CCGGTGACCC GCAGCAAATA CGCACTCGAA CAGCTGTTCC   
  
  
- GGCTGGGCTT ATTCTAGTCG AGGAACCACA AGGCTTAGTG CCGCTAGCAT GGGCTTCTCA AGCCCTAGCT   
  
  
- TTCGTTCGAC CACTCTCTCT TAGAATGCGT TAAGCGAGCA CTAGAGTTGT AGTTACAGGT ATACCTCATA   
  
  
- CAGGAATAAG TTTCGAAGCT CAAGAATAGG AAGTTTAGCC AGTTTAAGTA GCTGCCACTC TTTAACCGCC   
  
  
- AGGTAGATAG AGGCCGCTAC AAAGTGGCCG AAGTTCCGCC TTAGAGCTTT AAGGATCGGC TAGAGGCCCC   
  
  
- TTAAAGCGGT TCGTGGCAGC ACCACCACCT GTCACTCCAT CCTTAGCTGT GCCCTTGAAG CAGGAAGTCG   
  
  
- CAGTTGAAGC AGCGGCCATA GCTTAAGATG TGGCCTTACG AGCTTAGCGA GCTGCAGTGG AGGCGGTCGC   
  
  
- CAAAACGTCC GCCGCCGCCG CCGCTGCGAC ACTCTTCCTA GCTCTGCATG CACGAGGCCG GCTCCTAATG   
  
  
- CCGCCGCCAC CTCCGCCGTA GACGCCGCCG CCATCGCGCC GCCTCGCCAA CCTCTCTCAG CAAGCGCCAC   
  
  
- CGATCCTACT CCCGCCACCC CAAGTCGGTC AAACGACTAA AGGTCCGCCT CACAAACGAC TCCTTCCAAG   
  
  
- TCCAACCGCC CAAGGTGCAC CGCTTTGCAG TACCCCTCTA CTACGAAACG ACCGTACTAT CCGGGGAACA   
  
  
- CAGGTGCAGC CGAACCTCCA CAAT

+     DRE core

| Site Name | Organism | Position | Strand | Matrix score. | sequence | function |
| --- | --- | --- | --- | --- | --- | --- |
| DRE core | Arabidopsis thaliana | 2664 | + | 6 | GCCGAC |  |
| DRE core | Arabidopsis thaliana | 3371 | - | 6 | GCCGAC |  |
| DRE core | Arabidopsis thaliana | 2433 | - | 6 | GCCGAC |  |

>HU06G00283.1   
+ -Up\_Stream \_Len000GATTTT ACTTCCAATT TTCTACTTAA ATTTGGTTAT CACGTTCGAA TTTTTCAGGT   
  
  
+ AAATACAAAC TGTTAGTGGC CATCCTTTTT TTTTGGGTCT AATCCATCCA GATGTGCTAA TACTTTCTGT   
  
  
+ TTTAGATGAG AAGTTTTAAT GCTTTTTTAG ATGTGTAATT TTTTTAAACA TAAATAGTCT TGTCGAATAA   
  
  
+ GATTTGCTAG TAATTTTTGT AAGAGAAATT ATGTGTATTT TTTATTATAT CAGAATATTA GATCGATCTC   
  
  
+ AATTTAATTA CTTATGAAAA AAAAAGTGGA TATCTTTAAT CAATTTGGCA AGCTCTCATG CATTAATTGT   
  
  
+ AGTCGTACGA ACTTATTTAC ATCACTCATC TGAATTTGCT ACCATTTACA CCTCAAATTT TATTTTAATG   
  
  
+ CCATAAAGAG AATAGAATAT ACTGTCTATT TGTAGTAGTT TTACAGATAA AAACAAATAA GCAAATGAAA   
  
  
+ ATAGATAGAG GATTTTTTTG CTATTATCCA CGTCGATCCA TTGAATTATT TTGTCGCTAT TTGGTTAAGT   
  
  
+ GAAATAAAAT ACTGAAGTAG AATGGAGGAT GTTGATAATG ACCTATGTCC CTCAAAGTTT AATCATGTAG   
  
  
+ TTTCTACGAA TTTCAAGCCA TTATAAGACA TACAGATTTT TATGATGTTG AAAATACTCT TACCTCCTTG   
  
  
+ TCACAATATT AACCTGAAGC CAATGAAATG AAACAGAACT AATTAAATTG AATACATAAT CAATCCATAT   
  
  
+ TAATTTGTTT TGACAACTAT TTACAATCAT CGATCTCTTT AATTAGGTCT ATAAGCTATA GAGTAATTAC   
  
  
+ TCAAAACTGT TTTGTAAGAC AACTTACTCC ATATTGGCTT ATTAGTATAT TGCCTCCATT CACATGAATA   
  
  
+ CTTCATGAGA GATTGCCTAA AACTCATATT GTCATCACCT TAAGTCAAAC ATATTTTGTT ATGAAATGAC   
  
  
+ ATCAAGAATT ACCCTCTGAC AAGAAAAATA TACCTGACTG ACATAATCGT ACCACATCTG TTAGTCCTCT   
  
  
+ TTCTACCACT TTGTAGAACA TAATGTGACA GTAATACATC CATCCCAAAA GAAAAGACGA AAAGAAGAGT   
  
  
+ GGAGAGTCAC GTATACGCGT TGAATTCAGG AAAATTAAAG GAACTCATGA AGACCCCATA TAGTGCTCCA   
  
  
+ ACCTGAACCT GCAGCCCACT ACTACATGCC TAAATTAAAG AGCAAAGTTA TGAGAGAAAC CAAAAAAGCT   
  
  
+ CTTTGAGAGA GTGGAGGAAG AATCATGGAA AGCTAGCTAG GCTTAGCAAG AAAGAGCAAC AGCTAGCTAG   
  
  
+ CAAAAGGCTT CTCTTTGAAG CCTTAGAAAT CTTTAGAGAT AAAAAGTTGG TGGCTTTTTA AGGGCTGTGA   
  
  
+ TATGTCCCCC ATGTAAAAAT GCAGCAATAA TGTTAATGTC ATGCTTTCTT TCATGGTCCC TAATGCGAAA   
  
  
+ TCAATCATGC ATGTCAACGT GACTCAGTGG CTCACCATAT ATGATCTGAT TTCCCCGCAC ACAGAGATAA   
  
  
+ AAAGCCCCCA ATTATAACCT CCCAAAAGCC CCCCCTTTTC CTCCTACCCA ACCCACACCT CCCCATTTCA   
  
  
+ TCAGGGCTCG CTTCTTAAGT TATCGATTAA AAAAAGAGTG AGTAAACGTA CCCGAATTGA AGAGGGGTGT   
  
  
+ TCGAAGCTTA TACTTGCTTG ATTAGGAATG AAAGTCCCCA TTTCATCCGC CCCCCAAACC GCCGCCACCA   
  
  
+ CCAGTAAATC GCTATCTTGC AACAATACTG CCTTGAATAC TGCCTTTCAA GCCCCCAATT CAATTTCAAC   
  
  
+ CCTTGTTAAC AACAACAAAC CCTCCTACGA GCCTACCTCG GTTCTAGACC TCCGGCGCAG CCCAAGCCCC   
  
  
+ GCCACCAACG CCGCCCTGCC ACTGCCCACT GACGCGGGCA TCAATAAACC CAAGCCTGCC TCTGCTGACC   
  
  
+ TGGACAATTT GGAGGGTGAG GGTTGGGATT CAATCCTATC AGAGCTGGGG CTTAATGATG ATTCTAATAA   
  
  
+ TCCTAACTCC AAACTTTGGT CATCCCAAAT TAGCTCATCA TGTGATTCCC ACCTCACACA GCTCCCTGAG   
  
  
+ TTCCCCTCCT CTCAGCCGTT GGATCACACA CGCACCCCCA ATAATTAACT TCTCCCCCTC TGATTACCCC   
  
  
+ ATCTCTGATT TATCCTACGG TCAGAATCTC AGCCCCAATT TTGGGCAGTT TGAGCTTCAT CTTCATCAGC   
  
  
+ CCAACGTCAA TAACAATGGG CTTGACTTCA TAGAGGACCT AATTAGAGCA GCTCAAGCCT ACGAATCCAA   
  
  
+ CGACTCCCAC CAGGTCCAGC TGGTATTGGC GCGGCTCAAT CAACGGCTCA GATCACCCGC CGGAAAACCC   
  
  
+ CTCCACCGCG CCGCCTTCTA CTTCAAGGAG GCCCTCCAAC ACCTCGCTGT CGGCGGAGGC CCGGCCCGGC   
  
  
+ CTGTCCGGCT GTCATCCTAC GAGGTGGTGC AGACCATCCG GGCCCACAAG AACTTTTCCG GCATATCACT   
  
  
+ GATATCCCTC TTCTCCACCT TCGCCGCCAA TCAGGCGATT CTGGAGGCGG TGGATGGGGC TGCGTTCATC   
  
  
+ CACATCATCG ATTTCGACAT CGGATTTGGC GGCCACTGGG CGTCGTTTAT GCGTGAGCTT GTCGACAAGG   
  
  
+ CCGACCCGAA TAAGATCAGC TCCTTGGTGT TCCGAATCAC GGCGATCGTA CCCGAAGAGT TCGGGATCGA   
  
  
+ AAGCAAGCTG GTGAGAGAGA ATCTTACGCA ATTCGCTCGT GATCTCAACA TCAATGTCCA TATGGAGTAT   
  
  
+ GTCCTTATTC AAAGCTTCGA GTTCTTATCC TTCAAATCGG TCAAATTCAT CGACGGTGAG AAATTGGCGG   
  
  
+ TCCATCTATC TCCGGCGATG TTTCACCGGC TTCAAGGCGG AATCTCGAAA TTCCTAGCCG ATCTCCGGGG   
  
  
+ AATTTCGCCA AGCACCGTCG TGGTGGTGGA CAGTGAGGTA GGAATCGACA CGGGAACTTC GTCCTTCAGC   
  
  
+ GTCAACTTCG TCGCCGGTAT CGAATTCTAC ACCGGAATGC TCGAATCGCT CGACGTCACC TCCGCCAGCG   
  
  
+ GTTTTGCAGG CGGCGGCGGC GGCGACGCTG TGAGAAGGAT CGAGACGTAC GTGCTCCGGC CGAGGATTAC   
  
  
+ GGCGGCGGTG GAGGCGGCAT CTGCGGCGGC GGTAGCGCGG CGGAGCGGTT GGAGAGAGTC GTTCGCGGTG   
  
  
+ GCTAGGATGA GGGCGGTGGG GTTCAGCCAG TTTGCTGATT TCCAGGCGGA GTGTTTGCTG AGGAAGGTTC   
  
  
+ AGGTTGGCGG GTTCCACGTG GCGAAACGTC ATGGGGAGAT GATGCTTTGC TGGCATGATA GGCCCCTTGT   
  
  
+ GTCCACGTCG GCTTGGAGGT GTTA  

- -Up\_Stream \_Len000CTAAAA TGAAGGTTAA AAGATGAATT TAAACCAATA GTGCAAGCTT AAAAAGTCCA   
  
  
- TTTATGTTTG ACAATCACCG GTAGGAAAAA AAAACCCAGA TTAGGTAGGT CTACACGATT ATGAAAGACA   
  
  
- AAATCTACTC TTCAAAATTA CGAAAAAATC TACACATTAA AAAAATTTGT ATTTATCAGA ACAGCTTATT   
  
  
- CTAAACGATC ATTAAAAACA TTCTCTTTAA TACACATAAA AAATAATATA GTCTTATAAT CTAGCTAGAG   
  
  
- TTAAATTAAT GAATACTTTT TTTTTCACCT ATAGAAATTA GTTAAACCGT TCGAGAGTAC GTAATTAACA   
  
  
- TCAGCATGCT TGAATAAATG TAGTGAGTAG ACTTAAACGA TGGTAAATGT GGAGTTTAAA ATAAAATTAC   
  
  
- GGTATTTCTC TTATCTTATA TGACAGATAA ACATCATCAA AATGTCTATT TTTGTTTATT CGTTTACTTT   
  
  
- TATCTATCTC CTAAAAAAAC GATAATAGGT GCAGCTAGGT AACTTAATAA AACAGCGATA AACCAATTCA   
  
  
- CTTTATTTTA TGACTTCATC TTACCTCCTA CAACTATTAC TGGATACAGG GAGTTTCAAA TTAGTACATC   
  
  
- AAAGATGCTT AAAGTTCGGT AATATTCTGT ATGTCTAAAA ATACTACAAC TTTTATGAGA ATGGAGGAAC   
  
  
- AGTGTTATAA TTGGACTTCG GTTACTTTAC TTTGTCTTGA TTAATTTAAC TTATGTATTA GTTAGGTATA   
  
  
- ATTAAACAAA ACTGTTGATA AATGTTAGTA GCTAGAGAAA TTAATCCAGA TATTCGATAT CTCATTAATG   
  
  
- AGTTTTGACA AAACATTCTG TTGAATGAGG TATAACCGAA TAATCATATA ACGGAGGTAA GTGTACTTAT   
  
  
- GAAGTACTCT CTAACGGATT TTGAGTATAA CAGTAGTGGA ATTCAGTTTG TATAAAACAA TACTTTACTG   
  
  
- TAGTTCTTAA TGGGAGACTG TTCTTTTTAT ATGGACTGAC TGTATTAGCA TGGTGTAGAC AATCAGGAGA   
  
  
- AAGATGGTGA AACATCTTGT ATTACACTGT CATTATGTAG GTAGGGTTTT CTTTTCTGCT TTTCTTCTCA   
  
  
- CCTCTCAGTG CATATGCGCA ACTTAAGTCC TTTTAATTTC CTTGAGTACT TCTGGGGTAT ATCACGAGGT   
  
  
- TGGACTTGGA CGTCGGGTGA TGATGTACGG ATTTAATTTC TCGTTTCAAT ACTCTCTTTG GTTTTTTCGA   
  
  
- GAAACTCTCT CACCTCCTTC TTAGTACCTT TCGATCGATC CGAATCGTTC TTTCTCGTTG TCGATCGATC   
  
  
- GTTTTCCGAA GAGAAACTTC GGAATCTTTA GAAATCTCTA TTTTTCAACC ACCGAAAAAT TCCCGACACT   
  
  
- ATACAGGGGG TACATTTTTA CGTCGTTATT ACAATTACAG TACGAAAGAA AGTACCAGGG ATTACGCTTT   
  
  
- AGTTAGTACG TACAGTTGCA CTGAGTCACC GAGTGGTATA TACTAGACTA AAGGGGCGTG TGTCTCTATT   
  
  
- TTTCGGGGGT TAATATTGGA GGGTTTTCGG GGGGGAAAAG GAGGATGGGT TGGGTGTGGA GGGGTAAAGT   
  
  
- AGTCCCGAGC GAAGAATTCA ATAGCTAATT TTTTTCTCAC TCATTTGCAT GGGCTTAACT TCTCCCCACA   
  
  
- AGCTTCGAAT ATGAACGAAC TAATCCTTAC TTTCAGGGGT AAAGTAGGCG GGGGGTTTGG CGGCGGTGGT   
  
  
- GGTCATTTAG CGATAGAACG TTGTTATGAC GGAACTTATG ACGGAAAGTT CGGGGGTTAA GTTAAAGTTG   
  
  
- GGAACAATTG TTGTTGTTTG GGAGGATGCT CGGATGGAGC CAAGATCTGG AGGCCGCGTC GGGTTCGGGG   
  
  
- CGGTGGTTGC GGCGGGACGG TGACGGGTGA CTGCGCCCGT AGTTATTTGG GTTCGGACGG AGACGACTGG   
  
  
- ACCTGTTAAA CCTCCCACTC CCAACCCTAA GTTAGGATAG TCTCGACCCC GAATTACTAC TAAGATTATT   
  
  
- AGGATTGAGG TTTGAAACCA GTAGGGTTTA ATCGAGTAGT ACACTAAGGG TGGAGTGTGT CGAGGGACTC   
  
  
- AAGGGGAGGA GAGTCGGCAA CCTAGTGTGT GCGTGGGGGT TATTAATTGA AGAGGGGGAG ACTAATGGGG   
  
  
- TAGAGACTAA ATAGGATGCC AGTCTTAGAG TCGGGGTTAA AACCCGTCAA ACTCGAAGTA GAAGTAGTCG   
  
  
- GGTTGCAGTT ATTGTTACCC GAACTGAAGT ATCTCCTGGA TTAATCTCGT CGAGTTCGGA TGCTTAGGTT   
  
  
- GCTGAGGGTG GTCCAGGTCG ACCATAACCG CGCCGAGTTA GTTGCCGAGT CTAGTGGGCG GCCTTTTGGG   
  
  
- GAGGTGGCGC GGCGGAAGAT GAAGTTCCTC CGGGAGGTTG TGGAGCGACA GCCGCCTCCG GGCCGGGCCG   
  
  
- GACAGGCCGA CAGTAGGATG CTCCACCACG TCTGGTAGGC CCGGGTGTTC TTGAAAAGGC CGTATAGTGA   
  
  
- CTATAGGGAG AAGAGGTGGA AGCGGCGGTT AGTCCGCTAA GACCTCCGCC ACCTACCCCG ACGCAAGTAG   
  
  
- GTGTAGTAGC TAAAGCTGTA GCCTAAACCG CCGGTGACCC GCAGCAAATA CGCACTCGAA CAGCTGTTCC   
  
  
- GGCTGGGCTT ATTCTAGTCG AGGAACCACA AGGCTTAGTG CCGCTAGCAT GGGCTTCTCA AGCCCTAGCT   
  
  
- TTCGTTCGAC CACTCTCTCT TAGAATGCGT TAAGCGAGCA CTAGAGTTGT AGTTACAGGT ATACCTCATA   
  
  
- CAGGAATAAG TTTCGAAGCT CAAGAATAGG AAGTTTAGCC AGTTTAAGTA GCTGCCACTC TTTAACCGCC   
  
  
- AGGTAGATAG AGGCCGCTAC AAAGTGGCCG AAGTTCCGCC TTAGAGCTTT AAGGATCGGC TAGAGGCCCC   
  
  
- TTAAAGCGGT TCGTGGCAGC ACCACCACCT GTCACTCCAT CCTTAGCTGT GCCCTTGAAG CAGGAAGTCG   
  
  
- CAGTTGAAGC AGCGGCCATA GCTTAAGATG TGGCCTTACG AGCTTAGCGA GCTGCAGTGG AGGCGGTCGC   
  
  
- CAAAACGTCC GCCGCCGCCG CCGCTGCGAC ACTCTTCCTA GCTCTGCATG CACGAGGCCG GCTCCTAATG   
  
  
- CCGCCGCCAC CTCCGCCGTA GACGCCGCCG CCATCGCGCC GCCTCGCCAA CCTCTCTCAG CAAGCGCCAC   
  
  
- CGATCCTACT CCCGCCACCC CAAGTCGGTC AAACGACTAA AGGTCCGCCT CACAAACGAC TCCTTCCAAG   
  
  
- TCCAACCGCC CAAGGTGCAC CGCTTTGCAG TACCCCTCTA CTACGAAACG ACCGTACTAT CCGGGGAACA   
  
  
- CAGGTGCAGC CGAACCTCCA CAAT

+     ERE

| Site Name | Organism | Position | Strand | Matrix score. | sequence | function |
| --- | --- | --- | --- | --- | --- | --- |
| ERE | Nicotiana glutinos | 974 | - | 8 | ATTTCATA |  |

>HU06G00283.1   
+ -Up\_Stream \_Len000GATTTT ACTTCCAATT TTCTACTTAA ATTTGGTTAT CACGTTCGAA TTTTTCAGGT   
  
  
+ AAATACAAAC TGTTAGTGGC CATCCTTTTT TTTTGGGTCT AATCCATCCA GATGTGCTAA TACTTTCTGT   
  
  
+ TTTAGATGAG AAGTTTTAAT GCTTTTTTAG ATGTGTAATT TTTTTAAACA TAAATAGTCT TGTCGAATAA   
  
  
+ GATTTGCTAG TAATTTTTGT AAGAGAAATT ATGTGTATTT TTTATTATAT CAGAATATTA GATCGATCTC   
  
  
+ AATTTAATTA CTTATGAAAA AAAAAGTGGA TATCTTTAAT CAATTTGGCA AGCTCTCATG CATTAATTGT   
  
  
+ AGTCGTACGA ACTTATTTAC ATCACTCATC TGAATTTGCT ACCATTTACA CCTCAAATTT TATTTTAATG   
  
  
+ CCATAAAGAG AATAGAATAT ACTGTCTATT TGTAGTAGTT TTACAGATAA AAACAAATAA GCAAATGAAA   
  
  
+ ATAGATAGAG GATTTTTTTG CTATTATCCA CGTCGATCCA TTGAATTATT TTGTCGCTAT TTGGTTAAGT   
  
  
+ GAAATAAAAT ACTGAAGTAG AATGGAGGAT GTTGATAATG ACCTATGTCC CTCAAAGTTT AATCATGTAG   
  
  
+ TTTCTACGAA TTTCAAGCCA TTATAAGACA TACAGATTTT TATGATGTTG AAAATACTCT TACCTCCTTG   
  
  
+ TCACAATATT AACCTGAAGC CAATGAAATG AAACAGAACT AATTAAATTG AATACATAAT CAATCCATAT   
  
  
+ TAATTTGTTT TGACAACTAT TTACAATCAT CGATCTCTTT AATTAGGTCT ATAAGCTATA GAGTAATTAC   
  
  
+ TCAAAACTGT TTTGTAAGAC AACTTACTCC ATATTGGCTT ATTAGTATAT TGCCTCCATT CACATGAATA   
  
  
+ CTTCATGAGA GATTGCCTAA AACTCATATT GTCATCACCT TAAGTCAAAC ATATTTTGTT ATGAAATGAC   
  
  
+ ATCAAGAATT ACCCTCTGAC AAGAAAAATA TACCTGACTG ACATAATCGT ACCACATCTG TTAGTCCTCT   
  
  
+ TTCTACCACT TTGTAGAACA TAATGTGACA GTAATACATC CATCCCAAAA GAAAAGACGA AAAGAAGAGT   
  
  
+ GGAGAGTCAC GTATACGCGT TGAATTCAGG AAAATTAAAG GAACTCATGA AGACCCCATA TAGTGCTCCA   
  
  
+ ACCTGAACCT GCAGCCCACT ACTACATGCC TAAATTAAAG AGCAAAGTTA TGAGAGAAAC CAAAAAAGCT   
  
  
+ CTTTGAGAGA GTGGAGGAAG AATCATGGAA AGCTAGCTAG GCTTAGCAAG AAAGAGCAAC AGCTAGCTAG   
  
  
+ CAAAAGGCTT CTCTTTGAAG CCTTAGAAAT CTTTAGAGAT AAAAAGTTGG TGGCTTTTTA AGGGCTGTGA   
  
  
+ TATGTCCCCC ATGTAAAAAT GCAGCAATAA TGTTAATGTC ATGCTTTCTT TCATGGTCCC TAATGCGAAA   
  
  
+ TCAATCATGC ATGTCAACGT GACTCAGTGG CTCACCATAT ATGATCTGAT TTCCCCGCAC ACAGAGATAA   
  
  
+ AAAGCCCCCA ATTATAACCT CCCAAAAGCC CCCCCTTTTC CTCCTACCCA ACCCACACCT CCCCATTTCA   
  
  
+ TCAGGGCTCG CTTCTTAAGT TATCGATTAA AAAAAGAGTG AGTAAACGTA CCCGAATTGA AGAGGGGTGT   
  
  
+ TCGAAGCTTA TACTTGCTTG ATTAGGAATG AAAGTCCCCA TTTCATCCGC CCCCCAAACC GCCGCCACCA   
  
  
+ CCAGTAAATC GCTATCTTGC AACAATACTG CCTTGAATAC TGCCTTTCAA GCCCCCAATT CAATTTCAAC   
  
  
+ CCTTGTTAAC AACAACAAAC CCTCCTACGA GCCTACCTCG GTTCTAGACC TCCGGCGCAG CCCAAGCCCC   
  
  
+ GCCACCAACG CCGCCCTGCC ACTGCCCACT GACGCGGGCA TCAATAAACC CAAGCCTGCC TCTGCTGACC   
  
  
+ TGGACAATTT GGAGGGTGAG GGTTGGGATT CAATCCTATC AGAGCTGGGG CTTAATGATG ATTCTAATAA   
  
  
+ TCCTAACTCC AAACTTTGGT CATCCCAAAT TAGCTCATCA TGTGATTCCC ACCTCACACA GCTCCCTGAG   
  
  
+ TTCCCCTCCT CTCAGCCGTT GGATCACACA CGCACCCCCA ATAATTAACT TCTCCCCCTC TGATTACCCC   
  
  
+ ATCTCTGATT TATCCTACGG TCAGAATCTC AGCCCCAATT TTGGGCAGTT TGAGCTTCAT CTTCATCAGC   
  
  
+ CCAACGTCAA TAACAATGGG CTTGACTTCA TAGAGGACCT AATTAGAGCA GCTCAAGCCT ACGAATCCAA   
  
  
+ CGACTCCCAC CAGGTCCAGC TGGTATTGGC GCGGCTCAAT CAACGGCTCA GATCACCCGC CGGAAAACCC   
  
  
+ CTCCACCGCG CCGCCTTCTA CTTCAAGGAG GCCCTCCAAC ACCTCGCTGT CGGCGGAGGC CCGGCCCGGC   
  
  
+ CTGTCCGGCT GTCATCCTAC GAGGTGGTGC AGACCATCCG GGCCCACAAG AACTTTTCCG GCATATCACT   
  
  
+ GATATCCCTC TTCTCCACCT TCGCCGCCAA TCAGGCGATT CTGGAGGCGG TGGATGGGGC TGCGTTCATC   
  
  
+ CACATCATCG ATTTCGACAT CGGATTTGGC GGCCACTGGG CGTCGTTTAT GCGTGAGCTT GTCGACAAGG   
  
  
+ CCGACCCGAA TAAGATCAGC TCCTTGGTGT TCCGAATCAC GGCGATCGTA CCCGAAGAGT TCGGGATCGA   
  
  
+ AAGCAAGCTG GTGAGAGAGA ATCTTACGCA ATTCGCTCGT GATCTCAACA TCAATGTCCA TATGGAGTAT   
  
  
+ GTCCTTATTC AAAGCTTCGA GTTCTTATCC TTCAAATCGG TCAAATTCAT CGACGGTGAG AAATTGGCGG   
  
  
+ TCCATCTATC TCCGGCGATG TTTCACCGGC TTCAAGGCGG AATCTCGAAA TTCCTAGCCG ATCTCCGGGG   
  
  
+ AATTTCGCCA AGCACCGTCG TGGTGGTGGA CAGTGAGGTA GGAATCGACA CGGGAACTTC GTCCTTCAGC   
  
  
+ GTCAACTTCG TCGCCGGTAT CGAATTCTAC ACCGGAATGC TCGAATCGCT CGACGTCACC TCCGCCAGCG   
  
  
+ GTTTTGCAGG CGGCGGCGGC GGCGACGCTG TGAGAAGGAT CGAGACGTAC GTGCTCCGGC CGAGGATTAC   
  
  
+ GGCGGCGGTG GAGGCGGCAT CTGCGGCGGC GGTAGCGCGG CGGAGCGGTT GGAGAGAGTC GTTCGCGGTG   
  
  
+ GCTAGGATGA GGGCGGTGGG GTTCAGCCAG TTTGCTGATT TCCAGGCGGA GTGTTTGCTG AGGAAGGTTC   
  
  
+ AGGTTGGCGG GTTCCACGTG GCGAAACGTC ATGGGGAGAT GATGCTTTGC TGGCATGATA GGCCCCTTGT   
  
  
+ GTCCACGTCG GCTTGGAGGT GTTA  

- -Up\_Stream \_Len000CTAAAA TGAAGGTTAA AAGATGAATT TAAACCAATA GTGCAAGCTT AAAAAGTCCA   
  
  
- TTTATGTTTG ACAATCACCG GTAGGAAAAA AAAACCCAGA TTAGGTAGGT CTACACGATT ATGAAAGACA   
  
  
- AAATCTACTC TTCAAAATTA CGAAAAAATC TACACATTAA AAAAATTTGT ATTTATCAGA ACAGCTTATT   
  
  
- CTAAACGATC ATTAAAAACA TTCTCTTTAA TACACATAAA AAATAATATA GTCTTATAAT CTAGCTAGAG   
  
  
- TTAAATTAAT GAATACTTTT TTTTTCACCT ATAGAAATTA GTTAAACCGT TCGAGAGTAC GTAATTAACA   
  
  
- TCAGCATGCT TGAATAAATG TAGTGAGTAG ACTTAAACGA TGGTAAATGT GGAGTTTAAA ATAAAATTAC   
  
  
- GGTATTTCTC TTATCTTATA TGACAGATAA ACATCATCAA AATGTCTATT TTTGTTTATT CGTTTACTTT   
  
  
- TATCTATCTC CTAAAAAAAC GATAATAGGT GCAGCTAGGT AACTTAATAA AACAGCGATA AACCAATTCA   
  
  
- CTTTATTTTA TGACTTCATC TTACCTCCTA CAACTATTAC TGGATACAGG GAGTTTCAAA TTAGTACATC   
  
  
- AAAGATGCTT AAAGTTCGGT AATATTCTGT ATGTCTAAAA ATACTACAAC TTTTATGAGA ATGGAGGAAC   
  
  
- AGTGTTATAA TTGGACTTCG GTTACTTTAC TTTGTCTTGA TTAATTTAAC TTATGTATTA GTTAGGTATA   
  
  
- ATTAAACAAA ACTGTTGATA AATGTTAGTA GCTAGAGAAA TTAATCCAGA TATTCGATAT CTCATTAATG   
  
  
- AGTTTTGACA AAACATTCTG TTGAATGAGG TATAACCGAA TAATCATATA ACGGAGGTAA GTGTACTTAT   
  
  
- GAAGTACTCT CTAACGGATT TTGAGTATAA CAGTAGTGGA ATTCAGTTTG TATAAAACAA TACTTTACTG   
  
  
- TAGTTCTTAA TGGGAGACTG TTCTTTTTAT ATGGACTGAC TGTATTAGCA TGGTGTAGAC AATCAGGAGA   
  
  
- AAGATGGTGA AACATCTTGT ATTACACTGT CATTATGTAG GTAGGGTTTT CTTTTCTGCT TTTCTTCTCA   
  
  
- CCTCTCAGTG CATATGCGCA ACTTAAGTCC TTTTAATTTC CTTGAGTACT TCTGGGGTAT ATCACGAGGT   
  
  
- TGGACTTGGA CGTCGGGTGA TGATGTACGG ATTTAATTTC TCGTTTCAAT ACTCTCTTTG GTTTTTTCGA   
  
  
- GAAACTCTCT CACCTCCTTC TTAGTACCTT TCGATCGATC CGAATCGTTC TTTCTCGTTG TCGATCGATC   
  
  
- GTTTTCCGAA GAGAAACTTC GGAATCTTTA GAAATCTCTA TTTTTCAACC ACCGAAAAAT TCCCGACACT   
  
  
- ATACAGGGGG TACATTTTTA CGTCGTTATT ACAATTACAG TACGAAAGAA AGTACCAGGG ATTACGCTTT   
  
  
- AGTTAGTACG TACAGTTGCA CTGAGTCACC GAGTGGTATA TACTAGACTA AAGGGGCGTG TGTCTCTATT   
  
  
- TTTCGGGGGT TAATATTGGA GGGTTTTCGG GGGGGAAAAG GAGGATGGGT TGGGTGTGGA GGGGTAAAGT   
  
  
- AGTCCCGAGC GAAGAATTCA ATAGCTAATT TTTTTCTCAC TCATTTGCAT GGGCTTAACT TCTCCCCACA   
  
  
- AGCTTCGAAT ATGAACGAAC TAATCCTTAC TTTCAGGGGT AAAGTAGGCG GGGGGTTTGG CGGCGGTGGT   
  
  
- GGTCATTTAG CGATAGAACG TTGTTATGAC GGAACTTATG ACGGAAAGTT CGGGGGTTAA GTTAAAGTTG   
  
  
- GGAACAATTG TTGTTGTTTG GGAGGATGCT CGGATGGAGC CAAGATCTGG AGGCCGCGTC GGGTTCGGGG   
  
  
- CGGTGGTTGC GGCGGGACGG TGACGGGTGA CTGCGCCCGT AGTTATTTGG GTTCGGACGG AGACGACTGG   
  
  
- ACCTGTTAAA CCTCCCACTC CCAACCCTAA GTTAGGATAG TCTCGACCCC GAATTACTAC TAAGATTATT   
  
  
- AGGATTGAGG TTTGAAACCA GTAGGGTTTA ATCGAGTAGT ACACTAAGGG TGGAGTGTGT CGAGGGACTC   
  
  
- AAGGGGAGGA GAGTCGGCAA CCTAGTGTGT GCGTGGGGGT TATTAATTGA AGAGGGGGAG ACTAATGGGG   
  
  
- TAGAGACTAA ATAGGATGCC AGTCTTAGAG TCGGGGTTAA AACCCGTCAA ACTCGAAGTA GAAGTAGTCG   
  
  
- GGTTGCAGTT ATTGTTACCC GAACTGAAGT ATCTCCTGGA TTAATCTCGT CGAGTTCGGA TGCTTAGGTT   
  
  
- GCTGAGGGTG GTCCAGGTCG ACCATAACCG CGCCGAGTTA GTTGCCGAGT CTAGTGGGCG GCCTTTTGGG   
  
  
- GAGGTGGCGC GGCGGAAGAT GAAGTTCCTC CGGGAGGTTG TGGAGCGACA GCCGCCTCCG GGCCGGGCCG   
  
  
- GACAGGCCGA CAGTAGGATG CTCCACCACG TCTGGTAGGC CCGGGTGTTC TTGAAAAGGC CGTATAGTGA   
  
  
- CTATAGGGAG AAGAGGTGGA AGCGGCGGTT AGTCCGCTAA GACCTCCGCC ACCTACCCCG ACGCAAGTAG   
  
  
- GTGTAGTAGC TAAAGCTGTA GCCTAAACCG CCGGTGACCC GCAGCAAATA CGCACTCGAA CAGCTGTTCC   
  
  
- GGCTGGGCTT ATTCTAGTCG AGGAACCACA AGGCTTAGTG CCGCTAGCAT GGGCTTCTCA AGCCCTAGCT   
  
  
- TTCGTTCGAC CACTCTCTCT TAGAATGCGT TAAGCGAGCA CTAGAGTTGT AGTTACAGGT ATACCTCATA   
  
  
- CAGGAATAAG TTTCGAAGCT CAAGAATAGG AAGTTTAGCC AGTTTAAGTA GCTGCCACTC TTTAACCGCC   
  
  
- AGGTAGATAG AGGCCGCTAC AAAGTGGCCG AAGTTCCGCC TTAGAGCTTT AAGGATCGGC TAGAGGCCCC   
  
  
- TTAAAGCGGT TCGTGGCAGC ACCACCACCT GTCACTCCAT CCTTAGCTGT GCCCTTGAAG CAGGAAGTCG   
  
  
- CAGTTGAAGC AGCGGCCATA GCTTAAGATG TGGCCTTACG AGCTTAGCGA GCTGCAGTGG AGGCGGTCGC   
  
  
- CAAAACGTCC GCCGCCGCCG CCGCTGCGAC ACTCTTCCTA GCTCTGCATG CACGAGGCCG GCTCCTAATG   
  
  
- CCGCCGCCAC CTCCGCCGTA GACGCCGCCG CCATCGCGCC GCCTCGCCAA CCTCTCTCAG CAAGCGCCAC   
  
  
- CGATCCTACT CCCGCCACCC CAAGTCGGTC AAACGACTAA AGGTCCGCCT CACAAACGAC TCCTTCCAAG   
  
  
- TCCAACCGCC CAAGGTGCAC CGCTTTGCAG TACCCCTCTA CTACGAAACG ACCGTACTAT CCGGGGAACA   
  
  
- CAGGTGCAGC CGAACCTCCA CAAT

+     F-box

| Site Name | Organism | Position | Strand | Matrix score. | sequence | function |
| --- | --- | --- | --- | --- | --- | --- |
| F-box | Lycopersicon esculentum | 488 | - | 10 | CTATTCTCATT |  |
| F-box | Lycopersicon esculentum | 429 | - | 10 | CTATTCTCATT |  |

>HU06G00283.1   
+ -Up\_Stream \_Len000GATTTT ACTTCCAATT TTCTACTTAA ATTTGGTTAT CACGTTCGAA TTTTTCAGGT   
  
  
+ AAATACAAAC TGTTAGTGGC CATCCTTTTT TTTTGGGTCT AATCCATCCA GATGTGCTAA TACTTTCTGT   
  
  
+ TTTAGATGAG AAGTTTTAAT GCTTTTTTAG ATGTGTAATT TTTTTAAACA TAAATAGTCT TGTCGAATAA   
  
  
+ GATTTGCTAG TAATTTTTGT AAGAGAAATT ATGTGTATTT TTTATTATAT CAGAATATTA GATCGATCTC   
  
  
+ AATTTAATTA CTTATGAAAA AAAAAGTGGA TATCTTTAAT CAATTTGGCA AGCTCTCATG CATTAATTGT   
  
  
+ AGTCGTACGA ACTTATTTAC ATCACTCATC TGAATTTGCT ACCATTTACA CCTCAAATTT TATTTTAATG   
  
  
+ CCATAAAGAG AATAGAATAT ACTGTCTATT TGTAGTAGTT TTACAGATAA AAACAAATAA GCAAATGAAA   
  
  
+ ATAGATAGAG GATTTTTTTG CTATTATCCA CGTCGATCCA TTGAATTATT TTGTCGCTAT TTGGTTAAGT   
  
  
+ GAAATAAAAT ACTGAAGTAG AATGGAGGAT GTTGATAATG ACCTATGTCC CTCAAAGTTT AATCATGTAG   
  
  
+ TTTCTACGAA TTTCAAGCCA TTATAAGACA TACAGATTTT TATGATGTTG AAAATACTCT TACCTCCTTG   
  
  
+ TCACAATATT AACCTGAAGC CAATGAAATG AAACAGAACT AATTAAATTG AATACATAAT CAATCCATAT   
  
  
+ TAATTTGTTT TGACAACTAT TTACAATCAT CGATCTCTTT AATTAGGTCT ATAAGCTATA GAGTAATTAC   
  
  
+ TCAAAACTGT TTTGTAAGAC AACTTACTCC ATATTGGCTT ATTAGTATAT TGCCTCCATT CACATGAATA   
  
  
+ CTTCATGAGA GATTGCCTAA AACTCATATT GTCATCACCT TAAGTCAAAC ATATTTTGTT ATGAAATGAC   
  
  
+ ATCAAGAATT ACCCTCTGAC AAGAAAAATA TACCTGACTG ACATAATCGT ACCACATCTG TTAGTCCTCT   
  
  
+ TTCTACCACT TTGTAGAACA TAATGTGACA GTAATACATC CATCCCAAAA GAAAAGACGA AAAGAAGAGT   
  
  
+ GGAGAGTCAC GTATACGCGT TGAATTCAGG AAAATTAAAG GAACTCATGA AGACCCCATA TAGTGCTCCA   
  
  
+ ACCTGAACCT GCAGCCCACT ACTACATGCC TAAATTAAAG AGCAAAGTTA TGAGAGAAAC CAAAAAAGCT   
  
  
+ CTTTGAGAGA GTGGAGGAAG AATCATGGAA AGCTAGCTAG GCTTAGCAAG AAAGAGCAAC AGCTAGCTAG   
  
  
+ CAAAAGGCTT CTCTTTGAAG CCTTAGAAAT CTTTAGAGAT AAAAAGTTGG TGGCTTTTTA AGGGCTGTGA   
  
  
+ TATGTCCCCC ATGTAAAAAT GCAGCAATAA TGTTAATGTC ATGCTTTCTT TCATGGTCCC TAATGCGAAA   
  
  
+ TCAATCATGC ATGTCAACGT GACTCAGTGG CTCACCATAT ATGATCTGAT TTCCCCGCAC ACAGAGATAA   
  
  
+ AAAGCCCCCA ATTATAACCT CCCAAAAGCC CCCCCTTTTC CTCCTACCCA ACCCACACCT CCCCATTTCA   
  
  
+ TCAGGGCTCG CTTCTTAAGT TATCGATTAA AAAAAGAGTG AGTAAACGTA CCCGAATTGA AGAGGGGTGT   
  
  
+ TCGAAGCTTA TACTTGCTTG ATTAGGAATG AAAGTCCCCA TTTCATCCGC CCCCCAAACC GCCGCCACCA   
  
  
+ CCAGTAAATC GCTATCTTGC AACAATACTG CCTTGAATAC TGCCTTTCAA GCCCCCAATT CAATTTCAAC   
  
  
+ CCTTGTTAAC AACAACAAAC CCTCCTACGA GCCTACCTCG GTTCTAGACC TCCGGCGCAG CCCAAGCCCC   
  
  
+ GCCACCAACG CCGCCCTGCC ACTGCCCACT GACGCGGGCA TCAATAAACC CAAGCCTGCC TCTGCTGACC   
  
  
+ TGGACAATTT GGAGGGTGAG GGTTGGGATT CAATCCTATC AGAGCTGGGG CTTAATGATG ATTCTAATAA   
  
  
+ TCCTAACTCC AAACTTTGGT CATCCCAAAT TAGCTCATCA TGTGATTCCC ACCTCACACA GCTCCCTGAG   
  
  
+ TTCCCCTCCT CTCAGCCGTT GGATCACACA CGCACCCCCA ATAATTAACT TCTCCCCCTC TGATTACCCC   
  
  
+ ATCTCTGATT TATCCTACGG TCAGAATCTC AGCCCCAATT TTGGGCAGTT TGAGCTTCAT CTTCATCAGC   
  
  
+ CCAACGTCAA TAACAATGGG CTTGACTTCA TAGAGGACCT AATTAGAGCA GCTCAAGCCT ACGAATCCAA   
  
  
+ CGACTCCCAC CAGGTCCAGC TGGTATTGGC GCGGCTCAAT CAACGGCTCA GATCACCCGC CGGAAAACCC   
  
  
+ CTCCACCGCG CCGCCTTCTA CTTCAAGGAG GCCCTCCAAC ACCTCGCTGT CGGCGGAGGC CCGGCCCGGC   
  
  
+ CTGTCCGGCT GTCATCCTAC GAGGTGGTGC AGACCATCCG GGCCCACAAG AACTTTTCCG GCATATCACT   
  
  
+ GATATCCCTC TTCTCCACCT TCGCCGCCAA TCAGGCGATT CTGGAGGCGG TGGATGGGGC TGCGTTCATC   
  
  
+ CACATCATCG ATTTCGACAT CGGATTTGGC GGCCACTGGG CGTCGTTTAT GCGTGAGCTT GTCGACAAGG   
  
  
+ CCGACCCGAA TAAGATCAGC TCCTTGGTGT TCCGAATCAC GGCGATCGTA CCCGAAGAGT TCGGGATCGA   
  
  
+ AAGCAAGCTG GTGAGAGAGA ATCTTACGCA ATTCGCTCGT GATCTCAACA TCAATGTCCA TATGGAGTAT   
  
  
+ GTCCTTATTC AAAGCTTCGA GTTCTTATCC TTCAAATCGG TCAAATTCAT CGACGGTGAG AAATTGGCGG   
  
  
+ TCCATCTATC TCCGGCGATG TTTCACCGGC TTCAAGGCGG AATCTCGAAA TTCCTAGCCG ATCTCCGGGG   
  
  
+ AATTTCGCCA AGCACCGTCG TGGTGGTGGA CAGTGAGGTA GGAATCGACA CGGGAACTTC GTCCTTCAGC   
  
  
+ GTCAACTTCG TCGCCGGTAT CGAATTCTAC ACCGGAATGC TCGAATCGCT CGACGTCACC TCCGCCAGCG   
  
  
+ GTTTTGCAGG CGGCGGCGGC GGCGACGCTG TGAGAAGGAT CGAGACGTAC GTGCTCCGGC CGAGGATTAC   
  
  
+ GGCGGCGGTG GAGGCGGCAT CTGCGGCGGC GGTAGCGCGG CGGAGCGGTT GGAGAGAGTC GTTCGCGGTG   
  
  
+ GCTAGGATGA GGGCGGTGGG GTTCAGCCAG TTTGCTGATT TCCAGGCGGA GTGTTTGCTG AGGAAGGTTC   
  
  
+ AGGTTGGCGG GTTCCACGTG GCGAAACGTC ATGGGGAGAT GATGCTTTGC TGGCATGATA GGCCCCTTGT   
  
  
+ GTCCACGTCG GCTTGGAGGT GTTA  

- -Up\_Stream \_Len000CTAAAA TGAAGGTTAA AAGATGAATT TAAACCAATA GTGCAAGCTT AAAAAGTCCA   
  
  
- TTTATGTTTG ACAATCACCG GTAGGAAAAA AAAACCCAGA TTAGGTAGGT CTACACGATT ATGAAAGACA   
  
  
- AAATCTACTC TTCAAAATTA CGAAAAAATC TACACATTAA AAAAATTTGT ATTTATCAGA ACAGCTTATT   
  
  
- CTAAACGATC ATTAAAAACA TTCTCTTTAA TACACATAAA AAATAATATA GTCTTATAAT CTAGCTAGAG   
  
  
- TTAAATTAAT GAATACTTTT TTTTTCACCT ATAGAAATTA GTTAAACCGT TCGAGAGTAC GTAATTAACA   
  
  
- TCAGCATGCT TGAATAAATG TAGTGAGTAG ACTTAAACGA TGGTAAATGT GGAGTTTAAA ATAAAATTAC   
  
  
- GGTATTTCTC TTATCTTATA TGACAGATAA ACATCATCAA AATGTCTATT TTTGTTTATT CGTTTACTTT   
  
  
- TATCTATCTC CTAAAAAAAC GATAATAGGT GCAGCTAGGT AACTTAATAA AACAGCGATA AACCAATTCA   
  
  
- CTTTATTTTA TGACTTCATC TTACCTCCTA CAACTATTAC TGGATACAGG GAGTTTCAAA TTAGTACATC   
  
  
- AAAGATGCTT AAAGTTCGGT AATATTCTGT ATGTCTAAAA ATACTACAAC TTTTATGAGA ATGGAGGAAC   
  
  
- AGTGTTATAA TTGGACTTCG GTTACTTTAC TTTGTCTTGA TTAATTTAAC TTATGTATTA GTTAGGTATA   
  
  
- ATTAAACAAA ACTGTTGATA AATGTTAGTA GCTAGAGAAA TTAATCCAGA TATTCGATAT CTCATTAATG   
  
  
- AGTTTTGACA AAACATTCTG TTGAATGAGG TATAACCGAA TAATCATATA ACGGAGGTAA GTGTACTTAT   
  
  
- GAAGTACTCT CTAACGGATT TTGAGTATAA CAGTAGTGGA ATTCAGTTTG TATAAAACAA TACTTTACTG   
  
  
- TAGTTCTTAA TGGGAGACTG TTCTTTTTAT ATGGACTGAC TGTATTAGCA TGGTGTAGAC AATCAGGAGA   
  
  
- AAGATGGTGA AACATCTTGT ATTACACTGT CATTATGTAG GTAGGGTTTT CTTTTCTGCT TTTCTTCTCA   
  
  
- CCTCTCAGTG CATATGCGCA ACTTAAGTCC TTTTAATTTC CTTGAGTACT TCTGGGGTAT ATCACGAGGT   
  
  
- TGGACTTGGA CGTCGGGTGA TGATGTACGG ATTTAATTTC TCGTTTCAAT ACTCTCTTTG GTTTTTTCGA   
  
  
- GAAACTCTCT CACCTCCTTC TTAGTACCTT TCGATCGATC CGAATCGTTC TTTCTCGTTG TCGATCGATC   
  
  
- GTTTTCCGAA GAGAAACTTC GGAATCTTTA GAAATCTCTA TTTTTCAACC ACCGAAAAAT TCCCGACACT   
  
  
- ATACAGGGGG TACATTTTTA CGTCGTTATT ACAATTACAG TACGAAAGAA AGTACCAGGG ATTACGCTTT   
  
  
- AGTTAGTACG TACAGTTGCA CTGAGTCACC GAGTGGTATA TACTAGACTA AAGGGGCGTG TGTCTCTATT   
  
  
- TTTCGGGGGT TAATATTGGA GGGTTTTCGG GGGGGAAAAG GAGGATGGGT TGGGTGTGGA GGGGTAAAGT   
  
  
- AGTCCCGAGC GAAGAATTCA ATAGCTAATT TTTTTCTCAC TCATTTGCAT GGGCTTAACT TCTCCCCACA   
  
  
- AGCTTCGAAT ATGAACGAAC TAATCCTTAC TTTCAGGGGT AAAGTAGGCG GGGGGTTTGG CGGCGGTGGT   
  
  
- GGTCATTTAG CGATAGAACG TTGTTATGAC GGAACTTATG ACGGAAAGTT CGGGGGTTAA GTTAAAGTTG   
  
  
- GGAACAATTG TTGTTGTTTG GGAGGATGCT CGGATGGAGC CAAGATCTGG AGGCCGCGTC GGGTTCGGGG   
  
  
- CGGTGGTTGC GGCGGGACGG TGACGGGTGA CTGCGCCCGT AGTTATTTGG GTTCGGACGG AGACGACTGG   
  
  
- ACCTGTTAAA CCTCCCACTC CCAACCCTAA GTTAGGATAG TCTCGACCCC GAATTACTAC TAAGATTATT   
  
  
- AGGATTGAGG TTTGAAACCA GTAGGGTTTA ATCGAGTAGT ACACTAAGGG TGGAGTGTGT CGAGGGACTC   
  
  
- AAGGGGAGGA GAGTCGGCAA CCTAGTGTGT GCGTGGGGGT TATTAATTGA AGAGGGGGAG ACTAATGGGG   
  
  
- TAGAGACTAA ATAGGATGCC AGTCTTAGAG TCGGGGTTAA AACCCGTCAA ACTCGAAGTA GAAGTAGTCG   
  
  
- GGTTGCAGTT ATTGTTACCC GAACTGAAGT ATCTCCTGGA TTAATCTCGT CGAGTTCGGA TGCTTAGGTT   
  
  
- GCTGAGGGTG GTCCAGGTCG ACCATAACCG CGCCGAGTTA GTTGCCGAGT CTAGTGGGCG GCCTTTTGGG   
  
  
- GAGGTGGCGC GGCGGAAGAT GAAGTTCCTC CGGGAGGTTG TGGAGCGACA GCCGCCTCCG GGCCGGGCCG   
  
  
- GACAGGCCGA CAGTAGGATG CTCCACCACG TCTGGTAGGC CCGGGTGTTC TTGAAAAGGC CGTATAGTGA   
  
  
- CTATAGGGAG AAGAGGTGGA AGCGGCGGTT AGTCCGCTAA GACCTCCGCC ACCTACCCCG ACGCAAGTAG   
  
  
- GTGTAGTAGC TAAAGCTGTA GCCTAAACCG CCGGTGACCC GCAGCAAATA CGCACTCGAA CAGCTGTTCC   
  
  
- GGCTGGGCTT ATTCTAGTCG AGGAACCACA AGGCTTAGTG CCGCTAGCAT GGGCTTCTCA AGCCCTAGCT   
  
  
- TTCGTTCGAC CACTCTCTCT TAGAATGCGT TAAGCGAGCA CTAGAGTTGT AGTTACAGGT ATACCTCATA   
  
  
- CAGGAATAAG TTTCGAAGCT CAAGAATAGG AAGTTTAGCC AGTTTAAGTA GCTGCCACTC TTTAACCGCC   
  
  
- AGGTAGATAG AGGCCGCTAC AAAGTGGCCG AAGTTCCGCC TTAGAGCTTT AAGGATCGGC TAGAGGCCCC   
  
  
- TTAAAGCGGT TCGTGGCAGC ACCACCACCT GTCACTCCAT CCTTAGCTGT GCCCTTGAAG CAGGAAGTCG   
  
  
- CAGTTGAAGC AGCGGCCATA GCTTAAGATG TGGCCTTACG AGCTTAGCGA GCTGCAGTGG AGGCGGTCGC   
  
  
- CAAAACGTCC GCCGCCGCCG CCGCTGCGAC ACTCTTCCTA GCTCTGCATG CACGAGGCCG GCTCCTAATG   
  
  
- CCGCCGCCAC CTCCGCCGTA GACGCCGCCG CCATCGCGCC GCCTCGCCAA CCTCTCTCAG CAAGCGCCAC   
  
  
- CGATCCTACT CCCGCCACCC CAAGTCGGTC AAACGACTAA AGGTCCGCCT CACAAACGAC TCCTTCCAAG   
  
  
- TCCAACCGCC CAAGGTGCAC CGCTTTGCAG TACCCCTCTA CTACGAAACG ACCGTACTAT CCGGGGAACA   
  
  
- CAGGTGCAGC CGAACCTCCA CAAT

+     G-Box

| Site Name | Organism | Position | Strand | Matrix score. | sequence | function |
| --- | --- | --- | --- | --- | --- | --- |
| G-Box | Pisum sativum | 1490 | - | 6 | CACGTT | cis-acting regulatory element involved in light responsiveness |
| G-Box | Pisum sativum | 3309 | - | 6 | CACGTG | cis-acting regulatory element involved in light responsiveness |
| G-Box | Pisum sativum | 55 | + | 6 | CACGTT | cis-acting regulatory element involved in light responsiveness |

>HU06G00283.1   
+ -Up\_Stream \_Len000GATTTT ACTTCCAATT TTCTACTTAA ATTTGGTTAT CACGTTCGAA TTTTTCAGGT   
  
  
+ AAATACAAAC TGTTAGTGGC CATCCTTTTT TTTTGGGTCT AATCCATCCA GATGTGCTAA TACTTTCTGT   
  
  
+ TTTAGATGAG AAGTTTTAAT GCTTTTTTAG ATGTGTAATT TTTTTAAACA TAAATAGTCT TGTCGAATAA   
  
  
+ GATTTGCTAG TAATTTTTGT AAGAGAAATT ATGTGTATTT TTTATTATAT CAGAATATTA GATCGATCTC   
  
  
+ AATTTAATTA CTTATGAAAA AAAAAGTGGA TATCTTTAAT CAATTTGGCA AGCTCTCATG CATTAATTGT   
  
  
+ AGTCGTACGA ACTTATTTAC ATCACTCATC TGAATTTGCT ACCATTTACA CCTCAAATTT TATTTTAATG   
  
  
+ CCATAAAGAG AATAGAATAT ACTGTCTATT TGTAGTAGTT TTACAGATAA AAACAAATAA GCAAATGAAA   
  
  
+ ATAGATAGAG GATTTTTTTG CTATTATCCA CGTCGATCCA TTGAATTATT TTGTCGCTAT TTGGTTAAGT   
  
  
+ GAAATAAAAT ACTGAAGTAG AATGGAGGAT GTTGATAATG ACCTATGTCC CTCAAAGTTT AATCATGTAG   
  
  
+ TTTCTACGAA TTTCAAGCCA TTATAAGACA TACAGATTTT TATGATGTTG AAAATACTCT TACCTCCTTG   
  
  
+ TCACAATATT AACCTGAAGC CAATGAAATG AAACAGAACT AATTAAATTG AATACATAAT CAATCCATAT   
  
  
+ TAATTTGTTT TGACAACTAT TTACAATCAT CGATCTCTTT AATTAGGTCT ATAAGCTATA GAGTAATTAC   
  
  
+ TCAAAACTGT TTTGTAAGAC AACTTACTCC ATATTGGCTT ATTAGTATAT TGCCTCCATT CACATGAATA   
  
  
+ CTTCATGAGA GATTGCCTAA AACTCATATT GTCATCACCT TAAGTCAAAC ATATTTTGTT ATGAAATGAC   
  
  
+ ATCAAGAATT ACCCTCTGAC AAGAAAAATA TACCTGACTG ACATAATCGT ACCACATCTG TTAGTCCTCT   
  
  
+ TTCTACCACT TTGTAGAACA TAATGTGACA GTAATACATC CATCCCAAAA GAAAAGACGA AAAGAAGAGT   
  
  
+ GGAGAGTCAC GTATACGCGT TGAATTCAGG AAAATTAAAG GAACTCATGA AGACCCCATA TAGTGCTCCA   
  
  
+ ACCTGAACCT GCAGCCCACT ACTACATGCC TAAATTAAAG AGCAAAGTTA TGAGAGAAAC CAAAAAAGCT   
  
  
+ CTTTGAGAGA GTGGAGGAAG AATCATGGAA AGCTAGCTAG GCTTAGCAAG AAAGAGCAAC AGCTAGCTAG   
  
  
+ CAAAAGGCTT CTCTTTGAAG CCTTAGAAAT CTTTAGAGAT AAAAAGTTGG TGGCTTTTTA AGGGCTGTGA   
  
  
+ TATGTCCCCC ATGTAAAAAT GCAGCAATAA TGTTAATGTC ATGCTTTCTT TCATGGTCCC TAATGCGAAA   
  
  
+ TCAATCATGC ATGTCAACGT GACTCAGTGG CTCACCATAT ATGATCTGAT TTCCCCGCAC ACAGAGATAA   
  
  
+ AAAGCCCCCA ATTATAACCT CCCAAAAGCC CCCCCTTTTC CTCCTACCCA ACCCACACCT CCCCATTTCA   
  
  
+ TCAGGGCTCG CTTCTTAAGT TATCGATTAA AAAAAGAGTG AGTAAACGTA CCCGAATTGA AGAGGGGTGT   
  
  
+ TCGAAGCTTA TACTTGCTTG ATTAGGAATG AAAGTCCCCA TTTCATCCGC CCCCCAAACC GCCGCCACCA   
  
  
+ CCAGTAAATC GCTATCTTGC AACAATACTG CCTTGAATAC TGCCTTTCAA GCCCCCAATT CAATTTCAAC   
  
  
+ CCTTGTTAAC AACAACAAAC CCTCCTACGA GCCTACCTCG GTTCTAGACC TCCGGCGCAG CCCAAGCCCC   
  
  
+ GCCACCAACG CCGCCCTGCC ACTGCCCACT GACGCGGGCA TCAATAAACC CAAGCCTGCC TCTGCTGACC   
  
  
+ TGGACAATTT GGAGGGTGAG GGTTGGGATT CAATCCTATC AGAGCTGGGG CTTAATGATG ATTCTAATAA   
  
  
+ TCCTAACTCC AAACTTTGGT CATCCCAAAT TAGCTCATCA TGTGATTCCC ACCTCACACA GCTCCCTGAG   
  
  
+ TTCCCCTCCT CTCAGCCGTT GGATCACACA CGCACCCCCA ATAATTAACT TCTCCCCCTC TGATTACCCC   
  
  
+ ATCTCTGATT TATCCTACGG TCAGAATCTC AGCCCCAATT TTGGGCAGTT TGAGCTTCAT CTTCATCAGC   
  
  
+ CCAACGTCAA TAACAATGGG CTTGACTTCA TAGAGGACCT AATTAGAGCA GCTCAAGCCT ACGAATCCAA   
  
  
+ CGACTCCCAC CAGGTCCAGC TGGTATTGGC GCGGCTCAAT CAACGGCTCA GATCACCCGC CGGAAAACCC   
  
  
+ CTCCACCGCG CCGCCTTCTA CTTCAAGGAG GCCCTCCAAC ACCTCGCTGT CGGCGGAGGC CCGGCCCGGC   
  
  
+ CTGTCCGGCT GTCATCCTAC GAGGTGGTGC AGACCATCCG GGCCCACAAG AACTTTTCCG GCATATCACT   
  
  
+ GATATCCCTC TTCTCCACCT TCGCCGCCAA TCAGGCGATT CTGGAGGCGG TGGATGGGGC TGCGTTCATC   
  
  
+ CACATCATCG ATTTCGACAT CGGATTTGGC GGCCACTGGG CGTCGTTTAT GCGTGAGCTT GTCGACAAGG   
  
  
+ CCGACCCGAA TAAGATCAGC TCCTTGGTGT TCCGAATCAC GGCGATCGTA CCCGAAGAGT TCGGGATCGA   
  
  
+ AAGCAAGCTG GTGAGAGAGA ATCTTACGCA ATTCGCTCGT GATCTCAACA TCAATGTCCA TATGGAGTAT   
  
  
+ GTCCTTATTC AAAGCTTCGA GTTCTTATCC TTCAAATCGG TCAAATTCAT CGACGGTGAG AAATTGGCGG   
  
  
+ TCCATCTATC TCCGGCGATG TTTCACCGGC TTCAAGGCGG AATCTCGAAA TTCCTAGCCG ATCTCCGGGG   
  
  
+ AATTTCGCCA AGCACCGTCG TGGTGGTGGA CAGTGAGGTA GGAATCGACA CGGGAACTTC GTCCTTCAGC   
  
  
+ GTCAACTTCG TCGCCGGTAT CGAATTCTAC ACCGGAATGC TCGAATCGCT CGACGTCACC TCCGCCAGCG   
  
  
+ GTTTTGCAGG CGGCGGCGGC GGCGACGCTG TGAGAAGGAT CGAGACGTAC GTGCTCCGGC CGAGGATTAC   
  
  
+ GGCGGCGGTG GAGGCGGCAT CTGCGGCGGC GGTAGCGCGG CGGAGCGGTT GGAGAGAGTC GTTCGCGGTG   
  
  
+ GCTAGGATGA GGGCGGTGGG GTTCAGCCAG TTTGCTGATT TCCAGGCGGA GTGTTTGCTG AGGAAGGTTC   
  
  
+ AGGTTGGCGG GTTCCACGTG GCGAAACGTC ATGGGGAGAT GATGCTTTGC TGGCATGATA GGCCCCTTGT   
  
  
+ GTCCACGTCG GCTTGGAGGT GTTA  

- -Up\_Stream \_Len000CTAAAA TGAAGGTTAA AAGATGAATT TAAACCAATA GTGCAAGCTT AAAAAGTCCA   
  
  
- TTTATGTTTG ACAATCACCG GTAGGAAAAA AAAACCCAGA TTAGGTAGGT CTACACGATT ATGAAAGACA   
  
  
- AAATCTACTC TTCAAAATTA CGAAAAAATC TACACATTAA AAAAATTTGT ATTTATCAGA ACAGCTTATT   
  
  
- CTAAACGATC ATTAAAAACA TTCTCTTTAA TACACATAAA AAATAATATA GTCTTATAAT CTAGCTAGAG   
  
  
- TTAAATTAAT GAATACTTTT TTTTTCACCT ATAGAAATTA GTTAAACCGT TCGAGAGTAC GTAATTAACA   
  
  
- TCAGCATGCT TGAATAAATG TAGTGAGTAG ACTTAAACGA TGGTAAATGT GGAGTTTAAA ATAAAATTAC   
  
  
- GGTATTTCTC TTATCTTATA TGACAGATAA ACATCATCAA AATGTCTATT TTTGTTTATT CGTTTACTTT   
  
  
- TATCTATCTC CTAAAAAAAC GATAATAGGT GCAGCTAGGT AACTTAATAA AACAGCGATA AACCAATTCA   
  
  
- CTTTATTTTA TGACTTCATC TTACCTCCTA CAACTATTAC TGGATACAGG GAGTTTCAAA TTAGTACATC   
  
  
- AAAGATGCTT AAAGTTCGGT AATATTCTGT ATGTCTAAAA ATACTACAAC TTTTATGAGA ATGGAGGAAC   
  
  
- AGTGTTATAA TTGGACTTCG GTTACTTTAC TTTGTCTTGA TTAATTTAAC TTATGTATTA GTTAGGTATA   
  
  
- ATTAAACAAA ACTGTTGATA AATGTTAGTA GCTAGAGAAA TTAATCCAGA TATTCGATAT CTCATTAATG   
  
  
- AGTTTTGACA AAACATTCTG TTGAATGAGG TATAACCGAA TAATCATATA ACGGAGGTAA GTGTACTTAT   
  
  
- GAAGTACTCT CTAACGGATT TTGAGTATAA CAGTAGTGGA ATTCAGTTTG TATAAAACAA TACTTTACTG   
  
  
- TAGTTCTTAA TGGGAGACTG TTCTTTTTAT ATGGACTGAC TGTATTAGCA TGGTGTAGAC AATCAGGAGA   
  
  
- AAGATGGTGA AACATCTTGT ATTACACTGT CATTATGTAG GTAGGGTTTT CTTTTCTGCT TTTCTTCTCA   
  
  
- CCTCTCAGTG CATATGCGCA ACTTAAGTCC TTTTAATTTC CTTGAGTACT TCTGGGGTAT ATCACGAGGT   
  
  
- TGGACTTGGA CGTCGGGTGA TGATGTACGG ATTTAATTTC TCGTTTCAAT ACTCTCTTTG GTTTTTTCGA   
  
  
- GAAACTCTCT CACCTCCTTC TTAGTACCTT TCGATCGATC CGAATCGTTC TTTCTCGTTG TCGATCGATC   
  
  
- GTTTTCCGAA GAGAAACTTC GGAATCTTTA GAAATCTCTA TTTTTCAACC ACCGAAAAAT TCCCGACACT   
  
  
- ATACAGGGGG TACATTTTTA CGTCGTTATT ACAATTACAG TACGAAAGAA AGTACCAGGG ATTACGCTTT   
  
  
- AGTTAGTACG TACAGTTGCA CTGAGTCACC GAGTGGTATA TACTAGACTA AAGGGGCGTG TGTCTCTATT   
  
  
- TTTCGGGGGT TAATATTGGA GGGTTTTCGG GGGGGAAAAG GAGGATGGGT TGGGTGTGGA GGGGTAAAGT   
  
  
- AGTCCCGAGC GAAGAATTCA ATAGCTAATT TTTTTCTCAC TCATTTGCAT GGGCTTAACT TCTCCCCACA   
  
  
- AGCTTCGAAT ATGAACGAAC TAATCCTTAC TTTCAGGGGT AAAGTAGGCG GGGGGTTTGG CGGCGGTGGT   
  
  
- GGTCATTTAG CGATAGAACG TTGTTATGAC GGAACTTATG ACGGAAAGTT CGGGGGTTAA GTTAAAGTTG   
  
  
- GGAACAATTG TTGTTGTTTG GGAGGATGCT CGGATGGAGC CAAGATCTGG AGGCCGCGTC GGGTTCGGGG   
  
  
- CGGTGGTTGC GGCGGGACGG TGACGGGTGA CTGCGCCCGT AGTTATTTGG GTTCGGACGG AGACGACTGG   
  
  
- ACCTGTTAAA CCTCCCACTC CCAACCCTAA GTTAGGATAG TCTCGACCCC GAATTACTAC TAAGATTATT   
  
  
- AGGATTGAGG TTTGAAACCA GTAGGGTTTA ATCGAGTAGT ACACTAAGGG TGGAGTGTGT CGAGGGACTC   
  
  
- AAGGGGAGGA GAGTCGGCAA CCTAGTGTGT GCGTGGGGGT TATTAATTGA AGAGGGGGAG ACTAATGGGG   
  
  
- TAGAGACTAA ATAGGATGCC AGTCTTAGAG TCGGGGTTAA AACCCGTCAA ACTCGAAGTA GAAGTAGTCG   
  
  
- GGTTGCAGTT ATTGTTACCC GAACTGAAGT ATCTCCTGGA TTAATCTCGT CGAGTTCGGA TGCTTAGGTT   
  
  
- GCTGAGGGTG GTCCAGGTCG ACCATAACCG CGCCGAGTTA GTTGCCGAGT CTAGTGGGCG GCCTTTTGGG   
  
  
- GAGGTGGCGC GGCGGAAGAT GAAGTTCCTC CGGGAGGTTG TGGAGCGACA GCCGCCTCCG GGCCGGGCCG   
  
  
- GACAGGCCGA CAGTAGGATG CTCCACCACG TCTGGTAGGC CCGGGTGTTC TTGAAAAGGC CGTATAGTGA   
  
  
- CTATAGGGAG AAGAGGTGGA AGCGGCGGTT AGTCCGCTAA GACCTCCGCC ACCTACCCCG ACGCAAGTAG   
  
  
- GTGTAGTAGC TAAAGCTGTA GCCTAAACCG CCGGTGACCC GCAGCAAATA CGCACTCGAA CAGCTGTTCC   
  
  
- GGCTGGGCTT ATTCTAGTCG AGGAACCACA AGGCTTAGTG CCGCTAGCAT GGGCTTCTCA AGCCCTAGCT   
  
  
- TTCGTTCGAC CACTCTCTCT TAGAATGCGT TAAGCGAGCA CTAGAGTTGT AGTTACAGGT ATACCTCATA   
  
  
- CAGGAATAAG TTTCGAAGCT CAAGAATAGG AAGTTTAGCC AGTTTAAGTA GCTGCCACTC TTTAACCGCC   
  
  
- AGGTAGATAG AGGCCGCTAC AAAGTGGCCG AAGTTCCGCC TTAGAGCTTT AAGGATCGGC TAGAGGCCCC   
  
  
- TTAAAGCGGT TCGTGGCAGC ACCACCACCT GTCACTCCAT CCTTAGCTGT GCCCTTGAAG CAGGAAGTCG   
  
  
- CAGTTGAAGC AGCGGCCATA GCTTAAGATG TGGCCTTACG AGCTTAGCGA GCTGCAGTGG AGGCGGTCGC   
  
  
- CAAAACGTCC GCCGCCGCCG CCGCTGCGAC ACTCTTCCTA GCTCTGCATG CACGAGGCCG GCTCCTAATG   
  
  
- CCGCCGCCAC CTCCGCCGTA GACGCCGCCG CCATCGCGCC GCCTCGCCAA CCTCTCTCAG CAAGCGCCAC   
  
  
- CGATCCTACT CCCGCCACCC CAAGTCGGTC AAACGACTAA AGGTCCGCCT CACAAACGAC TCCTTCCAAG   
  
  
- TCCAACCGCC CAAGGTGCAC CGCTTTGCAG TACCCCTCTA CTACGAAACG ACCGTACTAT CCGGGGAACA   
  
  
- CAGGTGCAGC CGAACCTCCA CAAT

+     G-box

| Site Name | Organism | Position | Strand | Matrix score. | sequence | function |
| --- | --- | --- | --- | --- | --- | --- |
| G-box | Arabidopsis thaliana | 3132 | + | 6 | TACGTG | cis-acting regulatory element involved in light responsiveness |
| G-box | Arabidopsis thaliana | 3309 | - | 6 | CACGTG | cis-acting regulatory element involved in light responsiveness |
| G-box | Zea mays | 3368 | + | 6 | CACGTC | cis-acting regulatory element involved in light responsiveness |
| G-box | Arabidopsis thaliana | 3307 | - | 10 | GCCACGTGGA | cis-acting regulatory element involved in light responsiveness |
| G-box | Zea mays | 523 | + | 6 | CACGTC | cis-acting regulatory element involved in light responsiveness |
| G-box | Arabidopsis thaliana | 1132 | - | 6 | TACGTG | cis-acting regulatory element involved in light responsiveness |
| G-box | Zea mays | 2961 | - | 6 | CACGAC | cis-acting regulatory element involved in light responsiveness |

>HU06G00283.1   
+ -Up\_Stream \_Len000GATTTT ACTTCCAATT TTCTACTTAA ATTTGGTTAT CACGTTCGAA TTTTTCAGGT   
  
  
+ AAATACAAAC TGTTAGTGGC CATCCTTTTT TTTTGGGTCT AATCCATCCA GATGTGCTAA TACTTTCTGT   
  
  
+ TTTAGATGAG AAGTTTTAAT GCTTTTTTAG ATGTGTAATT TTTTTAAACA TAAATAGTCT TGTCGAATAA   
  
  
+ GATTTGCTAG TAATTTTTGT AAGAGAAATT ATGTGTATTT TTTATTATAT CAGAATATTA GATCGATCTC   
  
  
+ AATTTAATTA CTTATGAAAA AAAAAGTGGA TATCTTTAAT CAATTTGGCA AGCTCTCATG CATTAATTGT   
  
  
+ AGTCGTACGA ACTTATTTAC ATCACTCATC TGAATTTGCT ACCATTTACA CCTCAAATTT TATTTTAATG   
  
  
+ CCATAAAGAG AATAGAATAT ACTGTCTATT TGTAGTAGTT TTACAGATAA AAACAAATAA GCAAATGAAA   
  
  
+ ATAGATAGAG GATTTTTTTG CTATTATCCA CGTCGATCCA TTGAATTATT TTGTCGCTAT TTGGTTAAGT   
  
  
+ GAAATAAAAT ACTGAAGTAG AATGGAGGAT GTTGATAATG ACCTATGTCC CTCAAAGTTT AATCATGTAG   
  
  
+ TTTCTACGAA TTTCAAGCCA TTATAAGACA TACAGATTTT TATGATGTTG AAAATACTCT TACCTCCTTG   
  
  
+ TCACAATATT AACCTGAAGC CAATGAAATG AAACAGAACT AATTAAATTG AATACATAAT CAATCCATAT   
  
  
+ TAATTTGTTT TGACAACTAT TTACAATCAT CGATCTCTTT AATTAGGTCT ATAAGCTATA GAGTAATTAC   
  
  
+ TCAAAACTGT TTTGTAAGAC AACTTACTCC ATATTGGCTT ATTAGTATAT TGCCTCCATT CACATGAATA   
  
  
+ CTTCATGAGA GATTGCCTAA AACTCATATT GTCATCACCT TAAGTCAAAC ATATTTTGTT ATGAAATGAC   
  
  
+ ATCAAGAATT ACCCTCTGAC AAGAAAAATA TACCTGACTG ACATAATCGT ACCACATCTG TTAGTCCTCT   
  
  
+ TTCTACCACT TTGTAGAACA TAATGTGACA GTAATACATC CATCCCAAAA GAAAAGACGA AAAGAAGAGT   
  
  
+ GGAGAGTCAC GTATACGCGT TGAATTCAGG AAAATTAAAG GAACTCATGA AGACCCCATA TAGTGCTCCA   
  
  
+ ACCTGAACCT GCAGCCCACT ACTACATGCC TAAATTAAAG AGCAAAGTTA TGAGAGAAAC CAAAAAAGCT   
  
  
+ CTTTGAGAGA GTGGAGGAAG AATCATGGAA AGCTAGCTAG GCTTAGCAAG AAAGAGCAAC AGCTAGCTAG   
  
  
+ CAAAAGGCTT CTCTTTGAAG CCTTAGAAAT CTTTAGAGAT AAAAAGTTGG TGGCTTTTTA AGGGCTGTGA   
  
  
+ TATGTCCCCC ATGTAAAAAT GCAGCAATAA TGTTAATGTC ATGCTTTCTT TCATGGTCCC TAATGCGAAA   
  
  
+ TCAATCATGC ATGTCAACGT GACTCAGTGG CTCACCATAT ATGATCTGAT TTCCCCGCAC ACAGAGATAA   
  
  
+ AAAGCCCCCA ATTATAACCT CCCAAAAGCC CCCCCTTTTC CTCCTACCCA ACCCACACCT CCCCATTTCA   
  
  
+ TCAGGGCTCG CTTCTTAAGT TATCGATTAA AAAAAGAGTG AGTAAACGTA CCCGAATTGA AGAGGGGTGT   
  
  
+ TCGAAGCTTA TACTTGCTTG ATTAGGAATG AAAGTCCCCA TTTCATCCGC CCCCCAAACC GCCGCCACCA   
  
  
+ CCAGTAAATC GCTATCTTGC AACAATACTG CCTTGAATAC TGCCTTTCAA GCCCCCAATT CAATTTCAAC   
  
  
+ CCTTGTTAAC AACAACAAAC CCTCCTACGA GCCTACCTCG GTTCTAGACC TCCGGCGCAG CCCAAGCCCC   
  
  
+ GCCACCAACG CCGCCCTGCC ACTGCCCACT GACGCGGGCA TCAATAAACC CAAGCCTGCC TCTGCTGACC   
  
  
+ TGGACAATTT GGAGGGTGAG GGTTGGGATT CAATCCTATC AGAGCTGGGG CTTAATGATG ATTCTAATAA   
  
  
+ TCCTAACTCC AAACTTTGGT CATCCCAAAT TAGCTCATCA TGTGATTCCC ACCTCACACA GCTCCCTGAG   
  
  
+ TTCCCCTCCT CTCAGCCGTT GGATCACACA CGCACCCCCA ATAATTAACT TCTCCCCCTC TGATTACCCC   
  
  
+ ATCTCTGATT TATCCTACGG TCAGAATCTC AGCCCCAATT TTGGGCAGTT TGAGCTTCAT CTTCATCAGC   
  
  
+ CCAACGTCAA TAACAATGGG CTTGACTTCA TAGAGGACCT AATTAGAGCA GCTCAAGCCT ACGAATCCAA   
  
  
+ CGACTCCCAC CAGGTCCAGC TGGTATTGGC GCGGCTCAAT CAACGGCTCA GATCACCCGC CGGAAAACCC   
  
  
+ CTCCACCGCG CCGCCTTCTA CTTCAAGGAG GCCCTCCAAC ACCTCGCTGT CGGCGGAGGC CCGGCCCGGC   
  
  
+ CTGTCCGGCT GTCATCCTAC GAGGTGGTGC AGACCATCCG GGCCCACAAG AACTTTTCCG GCATATCACT   
  
  
+ GATATCCCTC TTCTCCACCT TCGCCGCCAA TCAGGCGATT CTGGAGGCGG TGGATGGGGC TGCGTTCATC   
  
  
+ CACATCATCG ATTTCGACAT CGGATTTGGC GGCCACTGGG CGTCGTTTAT GCGTGAGCTT GTCGACAAGG   
  
  
+ CCGACCCGAA TAAGATCAGC TCCTTGGTGT TCCGAATCAC GGCGATCGTA CCCGAAGAGT TCGGGATCGA   
  
  
+ AAGCAAGCTG GTGAGAGAGA ATCTTACGCA ATTCGCTCGT GATCTCAACA TCAATGTCCA TATGGAGTAT   
  
  
+ GTCCTTATTC AAAGCTTCGA GTTCTTATCC TTCAAATCGG TCAAATTCAT CGACGGTGAG AAATTGGCGG   
  
  
+ TCCATCTATC TCCGGCGATG TTTCACCGGC TTCAAGGCGG AATCTCGAAA TTCCTAGCCG ATCTCCGGGG   
  
  
+ AATTTCGCCA AGCACCGTCG TGGTGGTGGA CAGTGAGGTA GGAATCGACA CGGGAACTTC GTCCTTCAGC   
  
  
+ GTCAACTTCG TCGCCGGTAT CGAATTCTAC ACCGGAATGC TCGAATCGCT CGACGTCACC TCCGCCAGCG   
  
  
+ GTTTTGCAGG CGGCGGCGGC GGCGACGCTG TGAGAAGGAT CGAGACGTAC GTGCTCCGGC CGAGGATTAC   
  
  
+ GGCGGCGGTG GAGGCGGCAT CTGCGGCGGC GGTAGCGCGG CGGAGCGGTT GGAGAGAGTC GTTCGCGGTG   
  
  
+ GCTAGGATGA GGGCGGTGGG GTTCAGCCAG TTTGCTGATT TCCAGGCGGA GTGTTTGCTG AGGAAGGTTC   
  
  
+ AGGTTGGCGG GTTCCACGTG GCGAAACGTC ATGGGGAGAT GATGCTTTGC TGGCATGATA GGCCCCTTGT   
  
  
+ GTCCACGTCG GCTTGGAGGT GTTA  

- -Up\_Stream \_Len000CTAAAA TGAAGGTTAA AAGATGAATT TAAACCAATA GTGCAAGCTT AAAAAGTCCA   
  
  
- TTTATGTTTG ACAATCACCG GTAGGAAAAA AAAACCCAGA TTAGGTAGGT CTACACGATT ATGAAAGACA   
  
  
- AAATCTACTC TTCAAAATTA CGAAAAAATC TACACATTAA AAAAATTTGT ATTTATCAGA ACAGCTTATT   
  
  
- CTAAACGATC ATTAAAAACA TTCTCTTTAA TACACATAAA AAATAATATA GTCTTATAAT CTAGCTAGAG   
  
  
- TTAAATTAAT GAATACTTTT TTTTTCACCT ATAGAAATTA GTTAAACCGT TCGAGAGTAC GTAATTAACA   
  
  
- TCAGCATGCT TGAATAAATG TAGTGAGTAG ACTTAAACGA TGGTAAATGT GGAGTTTAAA ATAAAATTAC   
  
  
- GGTATTTCTC TTATCTTATA TGACAGATAA ACATCATCAA AATGTCTATT TTTGTTTATT CGTTTACTTT   
  
  
- TATCTATCTC CTAAAAAAAC GATAATAGGT GCAGCTAGGT AACTTAATAA AACAGCGATA AACCAATTCA   
  
  
- CTTTATTTTA TGACTTCATC TTACCTCCTA CAACTATTAC TGGATACAGG GAGTTTCAAA TTAGTACATC   
  
  
- AAAGATGCTT AAAGTTCGGT AATATTCTGT ATGTCTAAAA ATACTACAAC TTTTATGAGA ATGGAGGAAC   
  
  
- AGTGTTATAA TTGGACTTCG GTTACTTTAC TTTGTCTTGA TTAATTTAAC TTATGTATTA GTTAGGTATA   
  
  
- ATTAAACAAA ACTGTTGATA AATGTTAGTA GCTAGAGAAA TTAATCCAGA TATTCGATAT CTCATTAATG   
  
  
- AGTTTTGACA AAACATTCTG TTGAATGAGG TATAACCGAA TAATCATATA ACGGAGGTAA GTGTACTTAT   
  
  
- GAAGTACTCT CTAACGGATT TTGAGTATAA CAGTAGTGGA ATTCAGTTTG TATAAAACAA TACTTTACTG   
  
  
- TAGTTCTTAA TGGGAGACTG TTCTTTTTAT ATGGACTGAC TGTATTAGCA TGGTGTAGAC AATCAGGAGA   
  
  
- AAGATGGTGA AACATCTTGT ATTACACTGT CATTATGTAG GTAGGGTTTT CTTTTCTGCT TTTCTTCTCA   
  
  
- CCTCTCAGTG CATATGCGCA ACTTAAGTCC TTTTAATTTC CTTGAGTACT TCTGGGGTAT ATCACGAGGT   
  
  
- TGGACTTGGA CGTCGGGTGA TGATGTACGG ATTTAATTTC TCGTTTCAAT ACTCTCTTTG GTTTTTTCGA   
  
  
- GAAACTCTCT CACCTCCTTC TTAGTACCTT TCGATCGATC CGAATCGTTC TTTCTCGTTG TCGATCGATC   
  
  
- GTTTTCCGAA GAGAAACTTC GGAATCTTTA GAAATCTCTA TTTTTCAACC ACCGAAAAAT TCCCGACACT   
  
  
- ATACAGGGGG TACATTTTTA CGTCGTTATT ACAATTACAG TACGAAAGAA AGTACCAGGG ATTACGCTTT   
  
  
- AGTTAGTACG TACAGTTGCA CTGAGTCACC GAGTGGTATA TACTAGACTA AAGGGGCGTG TGTCTCTATT   
  
  
- TTTCGGGGGT TAATATTGGA GGGTTTTCGG GGGGGAAAAG GAGGATGGGT TGGGTGTGGA GGGGTAAAGT   
  
  
- AGTCCCGAGC GAAGAATTCA ATAGCTAATT TTTTTCTCAC TCATTTGCAT GGGCTTAACT TCTCCCCACA   
  
  
- AGCTTCGAAT ATGAACGAAC TAATCCTTAC TTTCAGGGGT AAAGTAGGCG GGGGGTTTGG CGGCGGTGGT   
  
  
- GGTCATTTAG CGATAGAACG TTGTTATGAC GGAACTTATG ACGGAAAGTT CGGGGGTTAA GTTAAAGTTG   
  
  
- GGAACAATTG TTGTTGTTTG GGAGGATGCT CGGATGGAGC CAAGATCTGG AGGCCGCGTC GGGTTCGGGG   
  
  
- CGGTGGTTGC GGCGGGACGG TGACGGGTGA CTGCGCCCGT AGTTATTTGG GTTCGGACGG AGACGACTGG   
  
  
- ACCTGTTAAA CCTCCCACTC CCAACCCTAA GTTAGGATAG TCTCGACCCC GAATTACTAC TAAGATTATT   
  
  
- AGGATTGAGG TTTGAAACCA GTAGGGTTTA ATCGAGTAGT ACACTAAGGG TGGAGTGTGT CGAGGGACTC   
  
  
- AAGGGGAGGA GAGTCGGCAA CCTAGTGTGT GCGTGGGGGT TATTAATTGA AGAGGGGGAG ACTAATGGGG   
  
  
- TAGAGACTAA ATAGGATGCC AGTCTTAGAG TCGGGGTTAA AACCCGTCAA ACTCGAAGTA GAAGTAGTCG   
  
  
- GGTTGCAGTT ATTGTTACCC GAACTGAAGT ATCTCCTGGA TTAATCTCGT CGAGTTCGGA TGCTTAGGTT   
  
  
- GCTGAGGGTG GTCCAGGTCG ACCATAACCG CGCCGAGTTA GTTGCCGAGT CTAGTGGGCG GCCTTTTGGG   
  
  
- GAGGTGGCGC GGCGGAAGAT GAAGTTCCTC CGGGAGGTTG TGGAGCGACA GCCGCCTCCG GGCCGGGCCG   
  
  
- GACAGGCCGA CAGTAGGATG CTCCACCACG TCTGGTAGGC CCGGGTGTTC TTGAAAAGGC CGTATAGTGA   
  
  
- CTATAGGGAG AAGAGGTGGA AGCGGCGGTT AGTCCGCTAA GACCTCCGCC ACCTACCCCG ACGCAAGTAG   
  
  
- GTGTAGTAGC TAAAGCTGTA GCCTAAACCG CCGGTGACCC GCAGCAAATA CGCACTCGAA CAGCTGTTCC   
  
  
- GGCTGGGCTT ATTCTAGTCG AGGAACCACA AGGCTTAGTG CCGCTAGCAT GGGCTTCTCA AGCCCTAGCT   
  
  
- TTCGTTCGAC CACTCTCTCT TAGAATGCGT TAAGCGAGCA CTAGAGTTGT AGTTACAGGT ATACCTCATA   
  
  
- CAGGAATAAG TTTCGAAGCT CAAGAATAGG AAGTTTAGCC AGTTTAAGTA GCTGCCACTC TTTAACCGCC   
  
  
- AGGTAGATAG AGGCCGCTAC AAAGTGGCCG AAGTTCCGCC TTAGAGCTTT AAGGATCGGC TAGAGGCCCC   
  
  
- TTAAAGCGGT TCGTGGCAGC ACCACCACCT GTCACTCCAT CCTTAGCTGT GCCCTTGAAG CAGGAAGTCG   
  
  
- CAGTTGAAGC AGCGGCCATA GCTTAAGATG TGGCCTTACG AGCTTAGCGA GCTGCAGTGG AGGCGGTCGC   
  
  
- CAAAACGTCC GCCGCCGCCG CCGCTGCGAC ACTCTTCCTA GCTCTGCATG CACGAGGCCG GCTCCTAATG   
  
  
- CCGCCGCCAC CTCCGCCGTA GACGCCGCCG CCATCGCGCC GCCTCGCCAA CCTCTCTCAG CAAGCGCCAC   
  
  
- CGATCCTACT CCCGCCACCC CAAGTCGGTC AAACGACTAA AGGTCCGCCT CACAAACGAC TCCTTCCAAG   
  
  
- TCCAACCGCC CAAGGTGCAC CGCTTTGCAG TACCCCTCTA CTACGAAACG ACCGTACTAT CCGGGGAACA   
  
  
- CAGGTGCAGC CGAACCTCCA CAAT

+     GATA-motif

| Site Name | Organism | Position | Strand | Matrix score. | sequence | function |
| --- | --- | --- | --- | --- | --- | --- |
| GATA-motif | Solanum tuberosum | 2827 | - | 9 | AAGGATAAGG | part of a light responsive element |
| GATA-motif | Arabidopsis thaliana | 1998 | - | 7 | GATAGGA | part of a light responsive element |

>HU06G00283.1   
+ -Up\_Stream \_Len000GATTTT ACTTCCAATT TTCTACTTAA ATTTGGTTAT CACGTTCGAA TTTTTCAGGT   
  
  
+ AAATACAAAC TGTTAGTGGC CATCCTTTTT TTTTGGGTCT AATCCATCCA GATGTGCTAA TACTTTCTGT   
  
  
+ TTTAGATGAG AAGTTTTAAT GCTTTTTTAG ATGTGTAATT TTTTTAAACA TAAATAGTCT TGTCGAATAA   
  
  
+ GATTTGCTAG TAATTTTTGT AAGAGAAATT ATGTGTATTT TTTATTATAT CAGAATATTA GATCGATCTC   
  
  
+ AATTTAATTA CTTATGAAAA AAAAAGTGGA TATCTTTAAT CAATTTGGCA AGCTCTCATG CATTAATTGT   
  
  
+ AGTCGTACGA ACTTATTTAC ATCACTCATC TGAATTTGCT ACCATTTACA CCTCAAATTT TATTTTAATG   
  
  
+ CCATAAAGAG AATAGAATAT ACTGTCTATT TGTAGTAGTT TTACAGATAA AAACAAATAA GCAAATGAAA   
  
  
+ ATAGATAGAG GATTTTTTTG CTATTATCCA CGTCGATCCA TTGAATTATT TTGTCGCTAT TTGGTTAAGT   
  
  
+ GAAATAAAAT ACTGAAGTAG AATGGAGGAT GTTGATAATG ACCTATGTCC CTCAAAGTTT AATCATGTAG   
  
  
+ TTTCTACGAA TTTCAAGCCA TTATAAGACA TACAGATTTT TATGATGTTG AAAATACTCT TACCTCCTTG   
  
  
+ TCACAATATT AACCTGAAGC CAATGAAATG AAACAGAACT AATTAAATTG AATACATAAT CAATCCATAT   
  
  
+ TAATTTGTTT TGACAACTAT TTACAATCAT CGATCTCTTT AATTAGGTCT ATAAGCTATA GAGTAATTAC   
  
  
+ TCAAAACTGT TTTGTAAGAC AACTTACTCC ATATTGGCTT ATTAGTATAT TGCCTCCATT CACATGAATA   
  
  
+ CTTCATGAGA GATTGCCTAA AACTCATATT GTCATCACCT TAAGTCAAAC ATATTTTGTT ATGAAATGAC   
  
  
+ ATCAAGAATT ACCCTCTGAC AAGAAAAATA TACCTGACTG ACATAATCGT ACCACATCTG TTAGTCCTCT   
  
  
+ TTCTACCACT TTGTAGAACA TAATGTGACA GTAATACATC CATCCCAAAA GAAAAGACGA AAAGAAGAGT   
  
  
+ GGAGAGTCAC GTATACGCGT TGAATTCAGG AAAATTAAAG GAACTCATGA AGACCCCATA TAGTGCTCCA   
  
  
+ ACCTGAACCT GCAGCCCACT ACTACATGCC TAAATTAAAG AGCAAAGTTA TGAGAGAAAC CAAAAAAGCT   
  
  
+ CTTTGAGAGA GTGGAGGAAG AATCATGGAA AGCTAGCTAG GCTTAGCAAG AAAGAGCAAC AGCTAGCTAG   
  
  
+ CAAAAGGCTT CTCTTTGAAG CCTTAGAAAT CTTTAGAGAT AAAAAGTTGG TGGCTTTTTA AGGGCTGTGA   
  
  
+ TATGTCCCCC ATGTAAAAAT GCAGCAATAA TGTTAATGTC ATGCTTTCTT TCATGGTCCC TAATGCGAAA   
  
  
+ TCAATCATGC ATGTCAACGT GACTCAGTGG CTCACCATAT ATGATCTGAT TTCCCCGCAC ACAGAGATAA   
  
  
+ AAAGCCCCCA ATTATAACCT CCCAAAAGCC CCCCCTTTTC CTCCTACCCA ACCCACACCT CCCCATTTCA   
  
  
+ TCAGGGCTCG CTTCTTAAGT TATCGATTAA AAAAAGAGTG AGTAAACGTA CCCGAATTGA AGAGGGGTGT   
  
  
+ TCGAAGCTTA TACTTGCTTG ATTAGGAATG AAAGTCCCCA TTTCATCCGC CCCCCAAACC GCCGCCACCA   
  
  
+ CCAGTAAATC GCTATCTTGC AACAATACTG CCTTGAATAC TGCCTTTCAA GCCCCCAATT CAATTTCAAC   
  
  
+ CCTTGTTAAC AACAACAAAC CCTCCTACGA GCCTACCTCG GTTCTAGACC TCCGGCGCAG CCCAAGCCCC   
  
  
+ GCCACCAACG CCGCCCTGCC ACTGCCCACT GACGCGGGCA TCAATAAACC CAAGCCTGCC TCTGCTGACC   
  
  
+ TGGACAATTT GGAGGGTGAG GGTTGGGATT CAATCCTATC AGAGCTGGGG CTTAATGATG ATTCTAATAA   
  
  
+ TCCTAACTCC AAACTTTGGT CATCCCAAAT TAGCTCATCA TGTGATTCCC ACCTCACACA GCTCCCTGAG   
  
  
+ TTCCCCTCCT CTCAGCCGTT GGATCACACA CGCACCCCCA ATAATTAACT TCTCCCCCTC TGATTACCCC   
  
  
+ ATCTCTGATT TATCCTACGG TCAGAATCTC AGCCCCAATT TTGGGCAGTT TGAGCTTCAT CTTCATCAGC   
  
  
+ CCAACGTCAA TAACAATGGG CTTGACTTCA TAGAGGACCT AATTAGAGCA GCTCAAGCCT ACGAATCCAA   
  
  
+ CGACTCCCAC CAGGTCCAGC TGGTATTGGC GCGGCTCAAT CAACGGCTCA GATCACCCGC CGGAAAACCC   
  
  
+ CTCCACCGCG CCGCCTTCTA CTTCAAGGAG GCCCTCCAAC ACCTCGCTGT CGGCGGAGGC CCGGCCCGGC   
  
  
+ CTGTCCGGCT GTCATCCTAC GAGGTGGTGC AGACCATCCG GGCCCACAAG AACTTTTCCG GCATATCACT   
  
  
+ GATATCCCTC TTCTCCACCT TCGCCGCCAA TCAGGCGATT CTGGAGGCGG TGGATGGGGC TGCGTTCATC   
  
  
+ CACATCATCG ATTTCGACAT CGGATTTGGC GGCCACTGGG CGTCGTTTAT GCGTGAGCTT GTCGACAAGG   
  
  
+ CCGACCCGAA TAAGATCAGC TCCTTGGTGT TCCGAATCAC GGCGATCGTA CCCGAAGAGT TCGGGATCGA   
  
  
+ AAGCAAGCTG GTGAGAGAGA ATCTTACGCA ATTCGCTCGT GATCTCAACA TCAATGTCCA TATGGAGTAT   
  
  
+ GTCCTTATTC AAAGCTTCGA GTTCTTATCC TTCAAATCGG TCAAATTCAT CGACGGTGAG AAATTGGCGG   
  
  
+ TCCATCTATC TCCGGCGATG TTTCACCGGC TTCAAGGCGG AATCTCGAAA TTCCTAGCCG ATCTCCGGGG   
  
  
+ AATTTCGCCA AGCACCGTCG TGGTGGTGGA CAGTGAGGTA GGAATCGACA CGGGAACTTC GTCCTTCAGC   
  
  
+ GTCAACTTCG TCGCCGGTAT CGAATTCTAC ACCGGAATGC TCGAATCGCT CGACGTCACC TCCGCCAGCG   
  
  
+ GTTTTGCAGG CGGCGGCGGC GGCGACGCTG TGAGAAGGAT CGAGACGTAC GTGCTCCGGC CGAGGATTAC   
  
  
+ GGCGGCGGTG GAGGCGGCAT CTGCGGCGGC GGTAGCGCGG CGGAGCGGTT GGAGAGAGTC GTTCGCGGTG   
  
  
+ GCTAGGATGA GGGCGGTGGG GTTCAGCCAG TTTGCTGATT TCCAGGCGGA GTGTTTGCTG AGGAAGGTTC   
  
  
+ AGGTTGGCGG GTTCCACGTG GCGAAACGTC ATGGGGAGAT GATGCTTTGC TGGCATGATA GGCCCCTTGT   
  
  
+ GTCCACGTCG GCTTGGAGGT GTTA  

- -Up\_Stream \_Len000CTAAAA TGAAGGTTAA AAGATGAATT TAAACCAATA GTGCAAGCTT AAAAAGTCCA   
  
  
- TTTATGTTTG ACAATCACCG GTAGGAAAAA AAAACCCAGA TTAGGTAGGT CTACACGATT ATGAAAGACA   
  
  
- AAATCTACTC TTCAAAATTA CGAAAAAATC TACACATTAA AAAAATTTGT ATTTATCAGA ACAGCTTATT   
  
  
- CTAAACGATC ATTAAAAACA TTCTCTTTAA TACACATAAA AAATAATATA GTCTTATAAT CTAGCTAGAG   
  
  
- TTAAATTAAT GAATACTTTT TTTTTCACCT ATAGAAATTA GTTAAACCGT TCGAGAGTAC GTAATTAACA   
  
  
- TCAGCATGCT TGAATAAATG TAGTGAGTAG ACTTAAACGA TGGTAAATGT GGAGTTTAAA ATAAAATTAC   
  
  
- GGTATTTCTC TTATCTTATA TGACAGATAA ACATCATCAA AATGTCTATT TTTGTTTATT CGTTTACTTT   
  
  
- TATCTATCTC CTAAAAAAAC GATAATAGGT GCAGCTAGGT AACTTAATAA AACAGCGATA AACCAATTCA   
  
  
- CTTTATTTTA TGACTTCATC TTACCTCCTA CAACTATTAC TGGATACAGG GAGTTTCAAA TTAGTACATC   
  
  
- AAAGATGCTT AAAGTTCGGT AATATTCTGT ATGTCTAAAA ATACTACAAC TTTTATGAGA ATGGAGGAAC   
  
  
- AGTGTTATAA TTGGACTTCG GTTACTTTAC TTTGTCTTGA TTAATTTAAC TTATGTATTA GTTAGGTATA   
  
  
- ATTAAACAAA ACTGTTGATA AATGTTAGTA GCTAGAGAAA TTAATCCAGA TATTCGATAT CTCATTAATG   
  
  
- AGTTTTGACA AAACATTCTG TTGAATGAGG TATAACCGAA TAATCATATA ACGGAGGTAA GTGTACTTAT   
  
  
- GAAGTACTCT CTAACGGATT TTGAGTATAA CAGTAGTGGA ATTCAGTTTG TATAAAACAA TACTTTACTG   
  
  
- TAGTTCTTAA TGGGAGACTG TTCTTTTTAT ATGGACTGAC TGTATTAGCA TGGTGTAGAC AATCAGGAGA   
  
  
- AAGATGGTGA AACATCTTGT ATTACACTGT CATTATGTAG GTAGGGTTTT CTTTTCTGCT TTTCTTCTCA   
  
  
- CCTCTCAGTG CATATGCGCA ACTTAAGTCC TTTTAATTTC CTTGAGTACT TCTGGGGTAT ATCACGAGGT   
  
  
- TGGACTTGGA CGTCGGGTGA TGATGTACGG ATTTAATTTC TCGTTTCAAT ACTCTCTTTG GTTTTTTCGA   
  
  
- GAAACTCTCT CACCTCCTTC TTAGTACCTT TCGATCGATC CGAATCGTTC TTTCTCGTTG TCGATCGATC   
  
  
- GTTTTCCGAA GAGAAACTTC GGAATCTTTA GAAATCTCTA TTTTTCAACC ACCGAAAAAT TCCCGACACT   
  
  
- ATACAGGGGG TACATTTTTA CGTCGTTATT ACAATTACAG TACGAAAGAA AGTACCAGGG ATTACGCTTT   
  
  
- AGTTAGTACG TACAGTTGCA CTGAGTCACC GAGTGGTATA TACTAGACTA AAGGGGCGTG TGTCTCTATT   
  
  
- TTTCGGGGGT TAATATTGGA GGGTTTTCGG GGGGGAAAAG GAGGATGGGT TGGGTGTGGA GGGGTAAAGT   
  
  
- AGTCCCGAGC GAAGAATTCA ATAGCTAATT TTTTTCTCAC TCATTTGCAT GGGCTTAACT TCTCCCCACA   
  
  
- AGCTTCGAAT ATGAACGAAC TAATCCTTAC TTTCAGGGGT AAAGTAGGCG GGGGGTTTGG CGGCGGTGGT   
  
  
- GGTCATTTAG CGATAGAACG TTGTTATGAC GGAACTTATG ACGGAAAGTT CGGGGGTTAA GTTAAAGTTG   
  
  
- GGAACAATTG TTGTTGTTTG GGAGGATGCT CGGATGGAGC CAAGATCTGG AGGCCGCGTC GGGTTCGGGG   
  
  
- CGGTGGTTGC GGCGGGACGG TGACGGGTGA CTGCGCCCGT AGTTATTTGG GTTCGGACGG AGACGACTGG   
  
  
- ACCTGTTAAA CCTCCCACTC CCAACCCTAA GTTAGGATAG TCTCGACCCC GAATTACTAC TAAGATTATT   
  
  
- AGGATTGAGG TTTGAAACCA GTAGGGTTTA ATCGAGTAGT ACACTAAGGG TGGAGTGTGT CGAGGGACTC   
  
  
- AAGGGGAGGA GAGTCGGCAA CCTAGTGTGT GCGTGGGGGT TATTAATTGA AGAGGGGGAG ACTAATGGGG   
  
  
- TAGAGACTAA ATAGGATGCC AGTCTTAGAG TCGGGGTTAA AACCCGTCAA ACTCGAAGTA GAAGTAGTCG   
  
  
- GGTTGCAGTT ATTGTTACCC GAACTGAAGT ATCTCCTGGA TTAATCTCGT CGAGTTCGGA TGCTTAGGTT   
  
  
- GCTGAGGGTG GTCCAGGTCG ACCATAACCG CGCCGAGTTA GTTGCCGAGT CTAGTGGGCG GCCTTTTGGG   
  
  
- GAGGTGGCGC GGCGGAAGAT GAAGTTCCTC CGGGAGGTTG TGGAGCGACA GCCGCCTCCG GGCCGGGCCG   
  
  
- GACAGGCCGA CAGTAGGATG CTCCACCACG TCTGGTAGGC CCGGGTGTTC TTGAAAAGGC CGTATAGTGA   
  
  
- CTATAGGGAG AAGAGGTGGA AGCGGCGGTT AGTCCGCTAA GACCTCCGCC ACCTACCCCG ACGCAAGTAG   
  
  
- GTGTAGTAGC TAAAGCTGTA GCCTAAACCG CCGGTGACCC GCAGCAAATA CGCACTCGAA CAGCTGTTCC   
  
  
- GGCTGGGCTT ATTCTAGTCG AGGAACCACA AGGCTTAGTG CCGCTAGCAT GGGCTTCTCA AGCCCTAGCT   
  
  
- TTCGTTCGAC CACTCTCTCT TAGAATGCGT TAAGCGAGCA CTAGAGTTGT AGTTACAGGT ATACCTCATA   
  
  
- CAGGAATAAG TTTCGAAGCT CAAGAATAGG AAGTTTAGCC AGTTTAAGTA GCTGCCACTC TTTAACCGCC   
  
  
- AGGTAGATAG AGGCCGCTAC AAAGTGGCCG AAGTTCCGCC TTAGAGCTTT AAGGATCGGC TAGAGGCCCC   
  
  
- TTAAAGCGGT TCGTGGCAGC ACCACCACCT GTCACTCCAT CCTTAGCTGT GCCCTTGAAG CAGGAAGTCG   
  
  
- CAGTTGAAGC AGCGGCCATA GCTTAAGATG TGGCCTTACG AGCTTAGCGA GCTGCAGTGG AGGCGGTCGC   
  
  
- CAAAACGTCC GCCGCCGCCG CCGCTGCGAC ACTCTTCCTA GCTCTGCATG CACGAGGCCG GCTCCTAATG   
  
  
- CCGCCGCCAC CTCCGCCGTA GACGCCGCCG CCATCGCGCC GCCTCGCCAA CCTCTCTCAG CAAGCGCCAC   
  
  
- CGATCCTACT CCCGCCACCC CAAGTCGGTC AAACGACTAA AGGTCCGCCT CACAAACGAC TCCTTCCAAG   
  
  
- TCCAACCGCC CAAGGTGCAC CGCTTTGCAG TACCCCTCTA CTACGAAACG ACCGTACTAT CCGGGGAACA   
  
  
- CAGGTGCAGC CGAACCTCCA CAAT

+     GCN4\_motif

| Site Name | Organism | Position | Strand | Matrix score. | sequence | function |
| --- | --- | --- | --- | --- | --- | --- |
| GCN4\_motif | Oryza sativa | 1494 | - | 7 | TGAGTCA | cis-regulatory element involved in endosperm expression |

>HU06G00283.1   
+ -Up\_Stream \_Len000GATTTT ACTTCCAATT TTCTACTTAA ATTTGGTTAT CACGTTCGAA TTTTTCAGGT   
  
  
+ AAATACAAAC TGTTAGTGGC CATCCTTTTT TTTTGGGTCT AATCCATCCA GATGTGCTAA TACTTTCTGT   
  
  
+ TTTAGATGAG AAGTTTTAAT GCTTTTTTAG ATGTGTAATT TTTTTAAACA TAAATAGTCT TGTCGAATAA   
  
  
+ GATTTGCTAG TAATTTTTGT AAGAGAAATT ATGTGTATTT TTTATTATAT CAGAATATTA GATCGATCTC   
  
  
+ AATTTAATTA CTTATGAAAA AAAAAGTGGA TATCTTTAAT CAATTTGGCA AGCTCTCATG CATTAATTGT   
  
  
+ AGTCGTACGA ACTTATTTAC ATCACTCATC TGAATTTGCT ACCATTTACA CCTCAAATTT TATTTTAATG   
  
  
+ CCATAAAGAG AATAGAATAT ACTGTCTATT TGTAGTAGTT TTACAGATAA AAACAAATAA GCAAATGAAA   
  
  
+ ATAGATAGAG GATTTTTTTG CTATTATCCA CGTCGATCCA TTGAATTATT TTGTCGCTAT TTGGTTAAGT   
  
  
+ GAAATAAAAT ACTGAAGTAG AATGGAGGAT GTTGATAATG ACCTATGTCC CTCAAAGTTT AATCATGTAG   
  
  
+ TTTCTACGAA TTTCAAGCCA TTATAAGACA TACAGATTTT TATGATGTTG AAAATACTCT TACCTCCTTG   
  
  
+ TCACAATATT AACCTGAAGC CAATGAAATG AAACAGAACT AATTAAATTG AATACATAAT CAATCCATAT   
  
  
+ TAATTTGTTT TGACAACTAT TTACAATCAT CGATCTCTTT AATTAGGTCT ATAAGCTATA GAGTAATTAC   
  
  
+ TCAAAACTGT TTTGTAAGAC AACTTACTCC ATATTGGCTT ATTAGTATAT TGCCTCCATT CACATGAATA   
  
  
+ CTTCATGAGA GATTGCCTAA AACTCATATT GTCATCACCT TAAGTCAAAC ATATTTTGTT ATGAAATGAC   
  
  
+ ATCAAGAATT ACCCTCTGAC AAGAAAAATA TACCTGACTG ACATAATCGT ACCACATCTG TTAGTCCTCT   
  
  
+ TTCTACCACT TTGTAGAACA TAATGTGACA GTAATACATC CATCCCAAAA GAAAAGACGA AAAGAAGAGT   
  
  
+ GGAGAGTCAC GTATACGCGT TGAATTCAGG AAAATTAAAG GAACTCATGA AGACCCCATA TAGTGCTCCA   
  
  
+ ACCTGAACCT GCAGCCCACT ACTACATGCC TAAATTAAAG AGCAAAGTTA TGAGAGAAAC CAAAAAAGCT   
  
  
+ CTTTGAGAGA GTGGAGGAAG AATCATGGAA AGCTAGCTAG GCTTAGCAAG AAAGAGCAAC AGCTAGCTAG   
  
  
+ CAAAAGGCTT CTCTTTGAAG CCTTAGAAAT CTTTAGAGAT AAAAAGTTGG TGGCTTTTTA AGGGCTGTGA   
  
  
+ TATGTCCCCC ATGTAAAAAT GCAGCAATAA TGTTAATGTC ATGCTTTCTT TCATGGTCCC TAATGCGAAA   
  
  
+ TCAATCATGC ATGTCAACGT GACTCAGTGG CTCACCATAT ATGATCTGAT TTCCCCGCAC ACAGAGATAA   
  
  
+ AAAGCCCCCA ATTATAACCT CCCAAAAGCC CCCCCTTTTC CTCCTACCCA ACCCACACCT CCCCATTTCA   
  
  
+ TCAGGGCTCG CTTCTTAAGT TATCGATTAA AAAAAGAGTG AGTAAACGTA CCCGAATTGA AGAGGGGTGT   
  
  
+ TCGAAGCTTA TACTTGCTTG ATTAGGAATG AAAGTCCCCA TTTCATCCGC CCCCCAAACC GCCGCCACCA   
  
  
+ CCAGTAAATC GCTATCTTGC AACAATACTG CCTTGAATAC TGCCTTTCAA GCCCCCAATT CAATTTCAAC   
  
  
+ CCTTGTTAAC AACAACAAAC CCTCCTACGA GCCTACCTCG GTTCTAGACC TCCGGCGCAG CCCAAGCCCC   
  
  
+ GCCACCAACG CCGCCCTGCC ACTGCCCACT GACGCGGGCA TCAATAAACC CAAGCCTGCC TCTGCTGACC   
  
  
+ TGGACAATTT GGAGGGTGAG GGTTGGGATT CAATCCTATC AGAGCTGGGG CTTAATGATG ATTCTAATAA   
  
  
+ TCCTAACTCC AAACTTTGGT CATCCCAAAT TAGCTCATCA TGTGATTCCC ACCTCACACA GCTCCCTGAG   
  
  
+ TTCCCCTCCT CTCAGCCGTT GGATCACACA CGCACCCCCA ATAATTAACT TCTCCCCCTC TGATTACCCC   
  
  
+ ATCTCTGATT TATCCTACGG TCAGAATCTC AGCCCCAATT TTGGGCAGTT TGAGCTTCAT CTTCATCAGC   
  
  
+ CCAACGTCAA TAACAATGGG CTTGACTTCA TAGAGGACCT AATTAGAGCA GCTCAAGCCT ACGAATCCAA   
  
  
+ CGACTCCCAC CAGGTCCAGC TGGTATTGGC GCGGCTCAAT CAACGGCTCA GATCACCCGC CGGAAAACCC   
  
  
+ CTCCACCGCG CCGCCTTCTA CTTCAAGGAG GCCCTCCAAC ACCTCGCTGT CGGCGGAGGC CCGGCCCGGC   
  
  
+ CTGTCCGGCT GTCATCCTAC GAGGTGGTGC AGACCATCCG GGCCCACAAG AACTTTTCCG GCATATCACT   
  
  
+ GATATCCCTC TTCTCCACCT TCGCCGCCAA TCAGGCGATT CTGGAGGCGG TGGATGGGGC TGCGTTCATC   
  
  
+ CACATCATCG ATTTCGACAT CGGATTTGGC GGCCACTGGG CGTCGTTTAT GCGTGAGCTT GTCGACAAGG   
  
  
+ CCGACCCGAA TAAGATCAGC TCCTTGGTGT TCCGAATCAC GGCGATCGTA CCCGAAGAGT TCGGGATCGA   
  
  
+ AAGCAAGCTG GTGAGAGAGA ATCTTACGCA ATTCGCTCGT GATCTCAACA TCAATGTCCA TATGGAGTAT   
  
  
+ GTCCTTATTC AAAGCTTCGA GTTCTTATCC TTCAAATCGG TCAAATTCAT CGACGGTGAG AAATTGGCGG   
  
  
+ TCCATCTATC TCCGGCGATG TTTCACCGGC TTCAAGGCGG AATCTCGAAA TTCCTAGCCG ATCTCCGGGG   
  
  
+ AATTTCGCCA AGCACCGTCG TGGTGGTGGA CAGTGAGGTA GGAATCGACA CGGGAACTTC GTCCTTCAGC   
  
  
+ GTCAACTTCG TCGCCGGTAT CGAATTCTAC ACCGGAATGC TCGAATCGCT CGACGTCACC TCCGCCAGCG   
  
  
+ GTTTTGCAGG CGGCGGCGGC GGCGACGCTG TGAGAAGGAT CGAGACGTAC GTGCTCCGGC CGAGGATTAC   
  
  
+ GGCGGCGGTG GAGGCGGCAT CTGCGGCGGC GGTAGCGCGG CGGAGCGGTT GGAGAGAGTC GTTCGCGGTG   
  
  
+ GCTAGGATGA GGGCGGTGGG GTTCAGCCAG TTTGCTGATT TCCAGGCGGA GTGTTTGCTG AGGAAGGTTC   
  
  
+ AGGTTGGCGG GTTCCACGTG GCGAAACGTC ATGGGGAGAT GATGCTTTGC TGGCATGATA GGCCCCTTGT   
  
  
+ GTCCACGTCG GCTTGGAGGT GTTA  

- -Up\_Stream \_Len000CTAAAA TGAAGGTTAA AAGATGAATT TAAACCAATA GTGCAAGCTT AAAAAGTCCA   
  
  
- TTTATGTTTG ACAATCACCG GTAGGAAAAA AAAACCCAGA TTAGGTAGGT CTACACGATT ATGAAAGACA   
  
  
- AAATCTACTC TTCAAAATTA CGAAAAAATC TACACATTAA AAAAATTTGT ATTTATCAGA ACAGCTTATT   
  
  
- CTAAACGATC ATTAAAAACA TTCTCTTTAA TACACATAAA AAATAATATA GTCTTATAAT CTAGCTAGAG   
  
  
- TTAAATTAAT GAATACTTTT TTTTTCACCT ATAGAAATTA GTTAAACCGT TCGAGAGTAC GTAATTAACA   
  
  
- TCAGCATGCT TGAATAAATG TAGTGAGTAG ACTTAAACGA TGGTAAATGT GGAGTTTAAA ATAAAATTAC   
  
  
- GGTATTTCTC TTATCTTATA TGACAGATAA ACATCATCAA AATGTCTATT TTTGTTTATT CGTTTACTTT   
  
  
- TATCTATCTC CTAAAAAAAC GATAATAGGT GCAGCTAGGT AACTTAATAA AACAGCGATA AACCAATTCA   
  
  
- CTTTATTTTA TGACTTCATC TTACCTCCTA CAACTATTAC TGGATACAGG GAGTTTCAAA TTAGTACATC   
  
  
- AAAGATGCTT AAAGTTCGGT AATATTCTGT ATGTCTAAAA ATACTACAAC TTTTATGAGA ATGGAGGAAC   
  
  
- AGTGTTATAA TTGGACTTCG GTTACTTTAC TTTGTCTTGA TTAATTTAAC TTATGTATTA GTTAGGTATA   
  
  
- ATTAAACAAA ACTGTTGATA AATGTTAGTA GCTAGAGAAA TTAATCCAGA TATTCGATAT CTCATTAATG   
  
  
- AGTTTTGACA AAACATTCTG TTGAATGAGG TATAACCGAA TAATCATATA ACGGAGGTAA GTGTACTTAT   
  
  
- GAAGTACTCT CTAACGGATT TTGAGTATAA CAGTAGTGGA ATTCAGTTTG TATAAAACAA TACTTTACTG   
  
  
- TAGTTCTTAA TGGGAGACTG TTCTTTTTAT ATGGACTGAC TGTATTAGCA TGGTGTAGAC AATCAGGAGA   
  
  
- AAGATGGTGA AACATCTTGT ATTACACTGT CATTATGTAG GTAGGGTTTT CTTTTCTGCT TTTCTTCTCA   
  
  
- CCTCTCAGTG CATATGCGCA ACTTAAGTCC TTTTAATTTC CTTGAGTACT TCTGGGGTAT ATCACGAGGT   
  
  
- TGGACTTGGA CGTCGGGTGA TGATGTACGG ATTTAATTTC TCGTTTCAAT ACTCTCTTTG GTTTTTTCGA   
  
  
- GAAACTCTCT CACCTCCTTC TTAGTACCTT TCGATCGATC CGAATCGTTC TTTCTCGTTG TCGATCGATC   
  
  
- GTTTTCCGAA GAGAAACTTC GGAATCTTTA GAAATCTCTA TTTTTCAACC ACCGAAAAAT TCCCGACACT   
  
  
- ATACAGGGGG TACATTTTTA CGTCGTTATT ACAATTACAG TACGAAAGAA AGTACCAGGG ATTACGCTTT   
  
  
- AGTTAGTACG TACAGTTGCA CTGAGTCACC GAGTGGTATA TACTAGACTA AAGGGGCGTG TGTCTCTATT   
  
  
- TTTCGGGGGT TAATATTGGA GGGTTTTCGG GGGGGAAAAG GAGGATGGGT TGGGTGTGGA GGGGTAAAGT   
  
  
- AGTCCCGAGC GAAGAATTCA ATAGCTAATT TTTTTCTCAC TCATTTGCAT GGGCTTAACT TCTCCCCACA   
  
  
- AGCTTCGAAT ATGAACGAAC TAATCCTTAC TTTCAGGGGT AAAGTAGGCG GGGGGTTTGG CGGCGGTGGT   
  
  
- GGTCATTTAG CGATAGAACG TTGTTATGAC GGAACTTATG ACGGAAAGTT CGGGGGTTAA GTTAAAGTTG   
  
  
- GGAACAATTG TTGTTGTTTG GGAGGATGCT CGGATGGAGC CAAGATCTGG AGGCCGCGTC GGGTTCGGGG   
  
  
- CGGTGGTTGC GGCGGGACGG TGACGGGTGA CTGCGCCCGT AGTTATTTGG GTTCGGACGG AGACGACTGG   
  
  
- ACCTGTTAAA CCTCCCACTC CCAACCCTAA GTTAGGATAG TCTCGACCCC GAATTACTAC TAAGATTATT   
  
  
- AGGATTGAGG TTTGAAACCA GTAGGGTTTA ATCGAGTAGT ACACTAAGGG TGGAGTGTGT CGAGGGACTC   
  
  
- AAGGGGAGGA GAGTCGGCAA CCTAGTGTGT GCGTGGGGGT TATTAATTGA AGAGGGGGAG ACTAATGGGG   
  
  
- TAGAGACTAA ATAGGATGCC AGTCTTAGAG TCGGGGTTAA AACCCGTCAA ACTCGAAGTA GAAGTAGTCG   
  
  
- GGTTGCAGTT ATTGTTACCC GAACTGAAGT ATCTCCTGGA TTAATCTCGT CGAGTTCGGA TGCTTAGGTT   
  
  
- GCTGAGGGTG GTCCAGGTCG ACCATAACCG CGCCGAGTTA GTTGCCGAGT CTAGTGGGCG GCCTTTTGGG   
  
  
- GAGGTGGCGC GGCGGAAGAT GAAGTTCCTC CGGGAGGTTG TGGAGCGACA GCCGCCTCCG GGCCGGGCCG   
  
  
- GACAGGCCGA CAGTAGGATG CTCCACCACG TCTGGTAGGC CCGGGTGTTC TTGAAAAGGC CGTATAGTGA   
  
  
- CTATAGGGAG AAGAGGTGGA AGCGGCGGTT AGTCCGCTAA GACCTCCGCC ACCTACCCCG ACGCAAGTAG   
  
  
- GTGTAGTAGC TAAAGCTGTA GCCTAAACCG CCGGTGACCC GCAGCAAATA CGCACTCGAA CAGCTGTTCC   
  
  
- GGCTGGGCTT ATTCTAGTCG AGGAACCACA AGGCTTAGTG CCGCTAGCAT GGGCTTCTCA AGCCCTAGCT   
  
  
- TTCGTTCGAC CACTCTCTCT TAGAATGCGT TAAGCGAGCA CTAGAGTTGT AGTTACAGGT ATACCTCATA   
  
  
- CAGGAATAAG TTTCGAAGCT CAAGAATAGG AAGTTTAGCC AGTTTAAGTA GCTGCCACTC TTTAACCGCC   
  
  
- AGGTAGATAG AGGCCGCTAC AAAGTGGCCG AAGTTCCGCC TTAGAGCTTT AAGGATCGGC TAGAGGCCCC   
  
  
- TTAAAGCGGT TCGTGGCAGC ACCACCACCT GTCACTCCAT CCTTAGCTGT GCCCTTGAAG CAGGAAGTCG   
  
  
- CAGTTGAAGC AGCGGCCATA GCTTAAGATG TGGCCTTACG AGCTTAGCGA GCTGCAGTGG AGGCGGTCGC   
  
  
- CAAAACGTCC GCCGCCGCCG CCGCTGCGAC ACTCTTCCTA GCTCTGCATG CACGAGGCCG GCTCCTAATG   
  
  
- CCGCCGCCAC CTCCGCCGTA GACGCCGCCG CCATCGCGCC GCCTCGCCAA CCTCTCTCAG CAAGCGCCAC   
  
  
- CGATCCTACT CCCGCCACCC CAAGTCGGTC AAACGACTAA AGGTCCGCCT CACAAACGAC TCCTTCCAAG   
  
  
- TCCAACCGCC CAAGGTGCAC CGCTTTGCAG TACCCCTCTA CTACGAAACG ACCGTACTAT CCGGGGAACA   
  
  
- CAGGTGCAGC CGAACCTCCA CAAT

+     GT1-motif

| Site Name | Organism | Position | Strand | Matrix score. | sequence | function |
| --- | --- | --- | --- | --- | --- | --- |
| GT1-motif | Arabidopsis thaliana | 557 | + | 6 | GGTTAA | light responsive element |
| GT1-motif | Arabidopsis thaliana | 713 | - | 6 | GGTTAA | light responsive element |
| GT1-motif | Avena sativa | 712 | - | 7 | GGTTAAT | light responsive element |

>HU06G00283.1   
+ -Up\_Stream \_Len000GATTTT ACTTCCAATT TTCTACTTAA ATTTGGTTAT CACGTTCGAA TTTTTCAGGT   
  
  
+ AAATACAAAC TGTTAGTGGC CATCCTTTTT TTTTGGGTCT AATCCATCCA GATGTGCTAA TACTTTCTGT   
  
  
+ TTTAGATGAG AAGTTTTAAT GCTTTTTTAG ATGTGTAATT TTTTTAAACA TAAATAGTCT TGTCGAATAA   
  
  
+ GATTTGCTAG TAATTTTTGT AAGAGAAATT ATGTGTATTT TTTATTATAT CAGAATATTA GATCGATCTC   
  
  
+ AATTTAATTA CTTATGAAAA AAAAAGTGGA TATCTTTAAT CAATTTGGCA AGCTCTCATG CATTAATTGT   
  
  
+ AGTCGTACGA ACTTATTTAC ATCACTCATC TGAATTTGCT ACCATTTACA CCTCAAATTT TATTTTAATG   
  
  
+ CCATAAAGAG AATAGAATAT ACTGTCTATT TGTAGTAGTT TTACAGATAA AAACAAATAA GCAAATGAAA   
  
  
+ ATAGATAGAG GATTTTTTTG CTATTATCCA CGTCGATCCA TTGAATTATT TTGTCGCTAT TTGGTTAAGT   
  
  
+ GAAATAAAAT ACTGAAGTAG AATGGAGGAT GTTGATAATG ACCTATGTCC CTCAAAGTTT AATCATGTAG   
  
  
+ TTTCTACGAA TTTCAAGCCA TTATAAGACA TACAGATTTT TATGATGTTG AAAATACTCT TACCTCCTTG   
  
  
+ TCACAATATT AACCTGAAGC CAATGAAATG AAACAGAACT AATTAAATTG AATACATAAT CAATCCATAT   
  
  
+ TAATTTGTTT TGACAACTAT TTACAATCAT CGATCTCTTT AATTAGGTCT ATAAGCTATA GAGTAATTAC   
  
  
+ TCAAAACTGT TTTGTAAGAC AACTTACTCC ATATTGGCTT ATTAGTATAT TGCCTCCATT CACATGAATA   
  
  
+ CTTCATGAGA GATTGCCTAA AACTCATATT GTCATCACCT TAAGTCAAAC ATATTTTGTT ATGAAATGAC   
  
  
+ ATCAAGAATT ACCCTCTGAC AAGAAAAATA TACCTGACTG ACATAATCGT ACCACATCTG TTAGTCCTCT   
  
  
+ TTCTACCACT TTGTAGAACA TAATGTGACA GTAATACATC CATCCCAAAA GAAAAGACGA AAAGAAGAGT   
  
  
+ GGAGAGTCAC GTATACGCGT TGAATTCAGG AAAATTAAAG GAACTCATGA AGACCCCATA TAGTGCTCCA   
  
  
+ ACCTGAACCT GCAGCCCACT ACTACATGCC TAAATTAAAG AGCAAAGTTA TGAGAGAAAC CAAAAAAGCT   
  
  
+ CTTTGAGAGA GTGGAGGAAG AATCATGGAA AGCTAGCTAG GCTTAGCAAG AAAGAGCAAC AGCTAGCTAG   
  
  
+ CAAAAGGCTT CTCTTTGAAG CCTTAGAAAT CTTTAGAGAT AAAAAGTTGG TGGCTTTTTA AGGGCTGTGA   
  
  
+ TATGTCCCCC ATGTAAAAAT GCAGCAATAA TGTTAATGTC ATGCTTTCTT TCATGGTCCC TAATGCGAAA   
  
  
+ TCAATCATGC ATGTCAACGT GACTCAGTGG CTCACCATAT ATGATCTGAT TTCCCCGCAC ACAGAGATAA   
  
  
+ AAAGCCCCCA ATTATAACCT CCCAAAAGCC CCCCCTTTTC CTCCTACCCA ACCCACACCT CCCCATTTCA   
  
  
+ TCAGGGCTCG CTTCTTAAGT TATCGATTAA AAAAAGAGTG AGTAAACGTA CCCGAATTGA AGAGGGGTGT   
  
  
+ TCGAAGCTTA TACTTGCTTG ATTAGGAATG AAAGTCCCCA TTTCATCCGC CCCCCAAACC GCCGCCACCA   
  
  
+ CCAGTAAATC GCTATCTTGC AACAATACTG CCTTGAATAC TGCCTTTCAA GCCCCCAATT CAATTTCAAC   
  
  
+ CCTTGTTAAC AACAACAAAC CCTCCTACGA GCCTACCTCG GTTCTAGACC TCCGGCGCAG CCCAAGCCCC   
  
  
+ GCCACCAACG CCGCCCTGCC ACTGCCCACT GACGCGGGCA TCAATAAACC CAAGCCTGCC TCTGCTGACC   
  
  
+ TGGACAATTT GGAGGGTGAG GGTTGGGATT CAATCCTATC AGAGCTGGGG CTTAATGATG ATTCTAATAA   
  
  
+ TCCTAACTCC AAACTTTGGT CATCCCAAAT TAGCTCATCA TGTGATTCCC ACCTCACACA GCTCCCTGAG   
  
  
+ TTCCCCTCCT CTCAGCCGTT GGATCACACA CGCACCCCCA ATAATTAACT TCTCCCCCTC TGATTACCCC   
  
  
+ ATCTCTGATT TATCCTACGG TCAGAATCTC AGCCCCAATT TTGGGCAGTT TGAGCTTCAT CTTCATCAGC   
  
  
+ CCAACGTCAA TAACAATGGG CTTGACTTCA TAGAGGACCT AATTAGAGCA GCTCAAGCCT ACGAATCCAA   
  
  
+ CGACTCCCAC CAGGTCCAGC TGGTATTGGC GCGGCTCAAT CAACGGCTCA GATCACCCGC CGGAAAACCC   
  
  
+ CTCCACCGCG CCGCCTTCTA CTTCAAGGAG GCCCTCCAAC ACCTCGCTGT CGGCGGAGGC CCGGCCCGGC   
  
  
+ CTGTCCGGCT GTCATCCTAC GAGGTGGTGC AGACCATCCG GGCCCACAAG AACTTTTCCG GCATATCACT   
  
  
+ GATATCCCTC TTCTCCACCT TCGCCGCCAA TCAGGCGATT CTGGAGGCGG TGGATGGGGC TGCGTTCATC   
  
  
+ CACATCATCG ATTTCGACAT CGGATTTGGC GGCCACTGGG CGTCGTTTAT GCGTGAGCTT GTCGACAAGG   
  
  
+ CCGACCCGAA TAAGATCAGC TCCTTGGTGT TCCGAATCAC GGCGATCGTA CCCGAAGAGT TCGGGATCGA   
  
  
+ AAGCAAGCTG GTGAGAGAGA ATCTTACGCA ATTCGCTCGT GATCTCAACA TCAATGTCCA TATGGAGTAT   
  
  
+ GTCCTTATTC AAAGCTTCGA GTTCTTATCC TTCAAATCGG TCAAATTCAT CGACGGTGAG AAATTGGCGG   
  
  
+ TCCATCTATC TCCGGCGATG TTTCACCGGC TTCAAGGCGG AATCTCGAAA TTCCTAGCCG ATCTCCGGGG   
  
  
+ AATTTCGCCA AGCACCGTCG TGGTGGTGGA CAGTGAGGTA GGAATCGACA CGGGAACTTC GTCCTTCAGC   
  
  
+ GTCAACTTCG TCGCCGGTAT CGAATTCTAC ACCGGAATGC TCGAATCGCT CGACGTCACC TCCGCCAGCG   
  
  
+ GTTTTGCAGG CGGCGGCGGC GGCGACGCTG TGAGAAGGAT CGAGACGTAC GTGCTCCGGC CGAGGATTAC   
  
  
+ GGCGGCGGTG GAGGCGGCAT CTGCGGCGGC GGTAGCGCGG CGGAGCGGTT GGAGAGAGTC GTTCGCGGTG   
  
  
+ GCTAGGATGA GGGCGGTGGG GTTCAGCCAG TTTGCTGATT TCCAGGCGGA GTGTTTGCTG AGGAAGGTTC   
  
  
+ AGGTTGGCGG GTTCCACGTG GCGAAACGTC ATGGGGAGAT GATGCTTTGC TGGCATGATA GGCCCCTTGT   
  
  
+ GTCCACGTCG GCTTGGAGGT GTTA  

- -Up\_Stream \_Len000CTAAAA TGAAGGTTAA AAGATGAATT TAAACCAATA GTGCAAGCTT AAAAAGTCCA   
  
  
- TTTATGTTTG ACAATCACCG GTAGGAAAAA AAAACCCAGA TTAGGTAGGT CTACACGATT ATGAAAGACA   
  
  
- AAATCTACTC TTCAAAATTA CGAAAAAATC TACACATTAA AAAAATTTGT ATTTATCAGA ACAGCTTATT   
  
  
- CTAAACGATC ATTAAAAACA TTCTCTTTAA TACACATAAA AAATAATATA GTCTTATAAT CTAGCTAGAG   
  
  
- TTAAATTAAT GAATACTTTT TTTTTCACCT ATAGAAATTA GTTAAACCGT TCGAGAGTAC GTAATTAACA   
  
  
- TCAGCATGCT TGAATAAATG TAGTGAGTAG ACTTAAACGA TGGTAAATGT GGAGTTTAAA ATAAAATTAC   
  
  
- GGTATTTCTC TTATCTTATA TGACAGATAA ACATCATCAA AATGTCTATT TTTGTTTATT CGTTTACTTT   
  
  
- TATCTATCTC CTAAAAAAAC GATAATAGGT GCAGCTAGGT AACTTAATAA AACAGCGATA AACCAATTCA   
  
  
- CTTTATTTTA TGACTTCATC TTACCTCCTA CAACTATTAC TGGATACAGG GAGTTTCAAA TTAGTACATC   
  
  
- AAAGATGCTT AAAGTTCGGT AATATTCTGT ATGTCTAAAA ATACTACAAC TTTTATGAGA ATGGAGGAAC   
  
  
- AGTGTTATAA TTGGACTTCG GTTACTTTAC TTTGTCTTGA TTAATTTAAC TTATGTATTA GTTAGGTATA   
  
  
- ATTAAACAAA ACTGTTGATA AATGTTAGTA GCTAGAGAAA TTAATCCAGA TATTCGATAT CTCATTAATG   
  
  
- AGTTTTGACA AAACATTCTG TTGAATGAGG TATAACCGAA TAATCATATA ACGGAGGTAA GTGTACTTAT   
  
  
- GAAGTACTCT CTAACGGATT TTGAGTATAA CAGTAGTGGA ATTCAGTTTG TATAAAACAA TACTTTACTG   
  
  
- TAGTTCTTAA TGGGAGACTG TTCTTTTTAT ATGGACTGAC TGTATTAGCA TGGTGTAGAC AATCAGGAGA   
  
  
- AAGATGGTGA AACATCTTGT ATTACACTGT CATTATGTAG GTAGGGTTTT CTTTTCTGCT TTTCTTCTCA   
  
  
- CCTCTCAGTG CATATGCGCA ACTTAAGTCC TTTTAATTTC CTTGAGTACT TCTGGGGTAT ATCACGAGGT   
  
  
- TGGACTTGGA CGTCGGGTGA TGATGTACGG ATTTAATTTC TCGTTTCAAT ACTCTCTTTG GTTTTTTCGA   
  
  
- GAAACTCTCT CACCTCCTTC TTAGTACCTT TCGATCGATC CGAATCGTTC TTTCTCGTTG TCGATCGATC   
  
  
- GTTTTCCGAA GAGAAACTTC GGAATCTTTA GAAATCTCTA TTTTTCAACC ACCGAAAAAT TCCCGACACT   
  
  
- ATACAGGGGG TACATTTTTA CGTCGTTATT ACAATTACAG TACGAAAGAA AGTACCAGGG ATTACGCTTT   
  
  
- AGTTAGTACG TACAGTTGCA CTGAGTCACC GAGTGGTATA TACTAGACTA AAGGGGCGTG TGTCTCTATT   
  
  
- TTTCGGGGGT TAATATTGGA GGGTTTTCGG GGGGGAAAAG GAGGATGGGT TGGGTGTGGA GGGGTAAAGT   
  
  
- AGTCCCGAGC GAAGAATTCA ATAGCTAATT TTTTTCTCAC TCATTTGCAT GGGCTTAACT TCTCCCCACA   
  
  
- AGCTTCGAAT ATGAACGAAC TAATCCTTAC TTTCAGGGGT AAAGTAGGCG GGGGGTTTGG CGGCGGTGGT   
  
  
- GGTCATTTAG CGATAGAACG TTGTTATGAC GGAACTTATG ACGGAAAGTT CGGGGGTTAA GTTAAAGTTG   
  
  
- GGAACAATTG TTGTTGTTTG GGAGGATGCT CGGATGGAGC CAAGATCTGG AGGCCGCGTC GGGTTCGGGG   
  
  
- CGGTGGTTGC GGCGGGACGG TGACGGGTGA CTGCGCCCGT AGTTATTTGG GTTCGGACGG AGACGACTGG   
  
  
- ACCTGTTAAA CCTCCCACTC CCAACCCTAA GTTAGGATAG TCTCGACCCC GAATTACTAC TAAGATTATT   
  
  
- AGGATTGAGG TTTGAAACCA GTAGGGTTTA ATCGAGTAGT ACACTAAGGG TGGAGTGTGT CGAGGGACTC   
  
  
- AAGGGGAGGA GAGTCGGCAA CCTAGTGTGT GCGTGGGGGT TATTAATTGA AGAGGGGGAG ACTAATGGGG   
  
  
- TAGAGACTAA ATAGGATGCC AGTCTTAGAG TCGGGGTTAA AACCCGTCAA ACTCGAAGTA GAAGTAGTCG   
  
  
- GGTTGCAGTT ATTGTTACCC GAACTGAAGT ATCTCCTGGA TTAATCTCGT CGAGTTCGGA TGCTTAGGTT   
  
  
- GCTGAGGGTG GTCCAGGTCG ACCATAACCG CGCCGAGTTA GTTGCCGAGT CTAGTGGGCG GCCTTTTGGG   
  
  
- GAGGTGGCGC GGCGGAAGAT GAAGTTCCTC CGGGAGGTTG TGGAGCGACA GCCGCCTCCG GGCCGGGCCG   
  
  
- GACAGGCCGA CAGTAGGATG CTCCACCACG TCTGGTAGGC CCGGGTGTTC TTGAAAAGGC CGTATAGTGA   
  
  
- CTATAGGGAG AAGAGGTGGA AGCGGCGGTT AGTCCGCTAA GACCTCCGCC ACCTACCCCG ACGCAAGTAG   
  
  
- GTGTAGTAGC TAAAGCTGTA GCCTAAACCG CCGGTGACCC GCAGCAAATA CGCACTCGAA CAGCTGTTCC   
  
  
- GGCTGGGCTT ATTCTAGTCG AGGAACCACA AGGCTTAGTG CCGCTAGCAT GGGCTTCTCA AGCCCTAGCT   
  
  
- TTCGTTCGAC CACTCTCTCT TAGAATGCGT TAAGCGAGCA CTAGAGTTGT AGTTACAGGT ATACCTCATA   
  
  
- CAGGAATAAG TTTCGAAGCT CAAGAATAGG AAGTTTAGCC AGTTTAAGTA GCTGCCACTC TTTAACCGCC   
  
  
- AGGTAGATAG AGGCCGCTAC AAAGTGGCCG AAGTTCCGCC TTAGAGCTTT AAGGATCGGC TAGAGGCCCC   
  
  
- TTAAAGCGGT TCGTGGCAGC ACCACCACCT GTCACTCCAT CCTTAGCTGT GCCCTTGAAG CAGGAAGTCG   
  
  
- CAGTTGAAGC AGCGGCCATA GCTTAAGATG TGGCCTTACG AGCTTAGCGA GCTGCAGTGG AGGCGGTCGC   
  
  
- CAAAACGTCC GCCGCCGCCG CCGCTGCGAC ACTCTTCCTA GCTCTGCATG CACGAGGCCG GCTCCTAATG   
  
  
- CCGCCGCCAC CTCCGCCGTA GACGCCGCCG CCATCGCGCC GCCTCGCCAA CCTCTCTCAG CAAGCGCCAC   
  
  
- CGATCCTACT CCCGCCACCC CAAGTCGGTC AAACGACTAA AGGTCCGCCT CACAAACGAC TCCTTCCAAG   
  
  
- TCCAACCGCC CAAGGTGCAC CGCTTTGCAG TACCCCTCTA CTACGAAACG ACCGTACTAT CCGGGGAACA   
  
  
- CAGGTGCAGC CGAACCTCCA CAAT

+     Gap-box

| Site Name | Organism | Position | Strand | Matrix score. | sequence | function |
| --- | --- | --- | --- | --- | --- | --- |
| Gap-box | Arabidopsis thaliana | 486 | + | 9.5 | CAAATGAA(A/G)A | part of a light responsive element |

>HU06G00283.1   
+ -Up\_Stream \_Len000GATTTT ACTTCCAATT TTCTACTTAA ATTTGGTTAT CACGTTCGAA TTTTTCAGGT   
  
  
+ AAATACAAAC TGTTAGTGGC CATCCTTTTT TTTTGGGTCT AATCCATCCA GATGTGCTAA TACTTTCTGT   
  
  
+ TTTAGATGAG AAGTTTTAAT GCTTTTTTAG ATGTGTAATT TTTTTAAACA TAAATAGTCT TGTCGAATAA   
  
  
+ GATTTGCTAG TAATTTTTGT AAGAGAAATT ATGTGTATTT TTTATTATAT CAGAATATTA GATCGATCTC   
  
  
+ AATTTAATTA CTTATGAAAA AAAAAGTGGA TATCTTTAAT CAATTTGGCA AGCTCTCATG CATTAATTGT   
  
  
+ AGTCGTACGA ACTTATTTAC ATCACTCATC TGAATTTGCT ACCATTTACA CCTCAAATTT TATTTTAATG   
  
  
+ CCATAAAGAG AATAGAATAT ACTGTCTATT TGTAGTAGTT TTACAGATAA AAACAAATAA GCAAATGAAA   
  
  
+ ATAGATAGAG GATTTTTTTG CTATTATCCA CGTCGATCCA TTGAATTATT TTGTCGCTAT TTGGTTAAGT   
  
  
+ GAAATAAAAT ACTGAAGTAG AATGGAGGAT GTTGATAATG ACCTATGTCC CTCAAAGTTT AATCATGTAG   
  
  
+ TTTCTACGAA TTTCAAGCCA TTATAAGACA TACAGATTTT TATGATGTTG AAAATACTCT TACCTCCTTG   
  
  
+ TCACAATATT AACCTGAAGC CAATGAAATG AAACAGAACT AATTAAATTG AATACATAAT CAATCCATAT   
  
  
+ TAATTTGTTT TGACAACTAT TTACAATCAT CGATCTCTTT AATTAGGTCT ATAAGCTATA GAGTAATTAC   
  
  
+ TCAAAACTGT TTTGTAAGAC AACTTACTCC ATATTGGCTT ATTAGTATAT TGCCTCCATT CACATGAATA   
  
  
+ CTTCATGAGA GATTGCCTAA AACTCATATT GTCATCACCT TAAGTCAAAC ATATTTTGTT ATGAAATGAC   
  
  
+ ATCAAGAATT ACCCTCTGAC AAGAAAAATA TACCTGACTG ACATAATCGT ACCACATCTG TTAGTCCTCT   
  
  
+ TTCTACCACT TTGTAGAACA TAATGTGACA GTAATACATC CATCCCAAAA GAAAAGACGA AAAGAAGAGT   
  
  
+ GGAGAGTCAC GTATACGCGT TGAATTCAGG AAAATTAAAG GAACTCATGA AGACCCCATA TAGTGCTCCA   
  
  
+ ACCTGAACCT GCAGCCCACT ACTACATGCC TAAATTAAAG AGCAAAGTTA TGAGAGAAAC CAAAAAAGCT   
  
  
+ CTTTGAGAGA GTGGAGGAAG AATCATGGAA AGCTAGCTAG GCTTAGCAAG AAAGAGCAAC AGCTAGCTAG   
  
  
+ CAAAAGGCTT CTCTTTGAAG CCTTAGAAAT CTTTAGAGAT AAAAAGTTGG TGGCTTTTTA AGGGCTGTGA   
  
  
+ TATGTCCCCC ATGTAAAAAT GCAGCAATAA TGTTAATGTC ATGCTTTCTT TCATGGTCCC TAATGCGAAA   
  
  
+ TCAATCATGC ATGTCAACGT GACTCAGTGG CTCACCATAT ATGATCTGAT TTCCCCGCAC ACAGAGATAA   
  
  
+ AAAGCCCCCA ATTATAACCT CCCAAAAGCC CCCCCTTTTC CTCCTACCCA ACCCACACCT CCCCATTTCA   
  
  
+ TCAGGGCTCG CTTCTTAAGT TATCGATTAA AAAAAGAGTG AGTAAACGTA CCCGAATTGA AGAGGGGTGT   
  
  
+ TCGAAGCTTA TACTTGCTTG ATTAGGAATG AAAGTCCCCA TTTCATCCGC CCCCCAAACC GCCGCCACCA   
  
  
+ CCAGTAAATC GCTATCTTGC AACAATACTG CCTTGAATAC TGCCTTTCAA GCCCCCAATT CAATTTCAAC   
  
  
+ CCTTGTTAAC AACAACAAAC CCTCCTACGA GCCTACCTCG GTTCTAGACC TCCGGCGCAG CCCAAGCCCC   
  
  
+ GCCACCAACG CCGCCCTGCC ACTGCCCACT GACGCGGGCA TCAATAAACC CAAGCCTGCC TCTGCTGACC   
  
  
+ TGGACAATTT GGAGGGTGAG GGTTGGGATT CAATCCTATC AGAGCTGGGG CTTAATGATG ATTCTAATAA   
  
  
+ TCCTAACTCC AAACTTTGGT CATCCCAAAT TAGCTCATCA TGTGATTCCC ACCTCACACA GCTCCCTGAG   
  
  
+ TTCCCCTCCT CTCAGCCGTT GGATCACACA CGCACCCCCA ATAATTAACT TCTCCCCCTC TGATTACCCC   
  
  
+ ATCTCTGATT TATCCTACGG TCAGAATCTC AGCCCCAATT TTGGGCAGTT TGAGCTTCAT CTTCATCAGC   
  
  
+ CCAACGTCAA TAACAATGGG CTTGACTTCA TAGAGGACCT AATTAGAGCA GCTCAAGCCT ACGAATCCAA   
  
  
+ CGACTCCCAC CAGGTCCAGC TGGTATTGGC GCGGCTCAAT CAACGGCTCA GATCACCCGC CGGAAAACCC   
  
  
+ CTCCACCGCG CCGCCTTCTA CTTCAAGGAG GCCCTCCAAC ACCTCGCTGT CGGCGGAGGC CCGGCCCGGC   
  
  
+ CTGTCCGGCT GTCATCCTAC GAGGTGGTGC AGACCATCCG GGCCCACAAG AACTTTTCCG GCATATCACT   
  
  
+ GATATCCCTC TTCTCCACCT TCGCCGCCAA TCAGGCGATT CTGGAGGCGG TGGATGGGGC TGCGTTCATC   
  
  
+ CACATCATCG ATTTCGACAT CGGATTTGGC GGCCACTGGG CGTCGTTTAT GCGTGAGCTT GTCGACAAGG   
  
  
+ CCGACCCGAA TAAGATCAGC TCCTTGGTGT TCCGAATCAC GGCGATCGTA CCCGAAGAGT TCGGGATCGA   
  
  
+ AAGCAAGCTG GTGAGAGAGA ATCTTACGCA ATTCGCTCGT GATCTCAACA TCAATGTCCA TATGGAGTAT   
  
  
+ GTCCTTATTC AAAGCTTCGA GTTCTTATCC TTCAAATCGG TCAAATTCAT CGACGGTGAG AAATTGGCGG   
  
  
+ TCCATCTATC TCCGGCGATG TTTCACCGGC TTCAAGGCGG AATCTCGAAA TTCCTAGCCG ATCTCCGGGG   
  
  
+ AATTTCGCCA AGCACCGTCG TGGTGGTGGA CAGTGAGGTA GGAATCGACA CGGGAACTTC GTCCTTCAGC   
  
  
+ GTCAACTTCG TCGCCGGTAT CGAATTCTAC ACCGGAATGC TCGAATCGCT CGACGTCACC TCCGCCAGCG   
  
  
+ GTTTTGCAGG CGGCGGCGGC GGCGACGCTG TGAGAAGGAT CGAGACGTAC GTGCTCCGGC CGAGGATTAC   
  
  
+ GGCGGCGGTG GAGGCGGCAT CTGCGGCGGC GGTAGCGCGG CGGAGCGGTT GGAGAGAGTC GTTCGCGGTG   
  
  
+ GCTAGGATGA GGGCGGTGGG GTTCAGCCAG TTTGCTGATT TCCAGGCGGA GTGTTTGCTG AGGAAGGTTC   
  
  
+ AGGTTGGCGG GTTCCACGTG GCGAAACGTC ATGGGGAGAT GATGCTTTGC TGGCATGATA GGCCCCTTGT   
  
  
+ GTCCACGTCG GCTTGGAGGT GTTA  

- -Up\_Stream \_Len000CTAAAA TGAAGGTTAA AAGATGAATT TAAACCAATA GTGCAAGCTT AAAAAGTCCA   
  
  
- TTTATGTTTG ACAATCACCG GTAGGAAAAA AAAACCCAGA TTAGGTAGGT CTACACGATT ATGAAAGACA   
  
  
- AAATCTACTC TTCAAAATTA CGAAAAAATC TACACATTAA AAAAATTTGT ATTTATCAGA ACAGCTTATT   
  
  
- CTAAACGATC ATTAAAAACA TTCTCTTTAA TACACATAAA AAATAATATA GTCTTATAAT CTAGCTAGAG   
  
  
- TTAAATTAAT GAATACTTTT TTTTTCACCT ATAGAAATTA GTTAAACCGT TCGAGAGTAC GTAATTAACA   
  
  
- TCAGCATGCT TGAATAAATG TAGTGAGTAG ACTTAAACGA TGGTAAATGT GGAGTTTAAA ATAAAATTAC   
  
  
- GGTATTTCTC TTATCTTATA TGACAGATAA ACATCATCAA AATGTCTATT TTTGTTTATT CGTTTACTTT   
  
  
- TATCTATCTC CTAAAAAAAC GATAATAGGT GCAGCTAGGT AACTTAATAA AACAGCGATA AACCAATTCA   
  
  
- CTTTATTTTA TGACTTCATC TTACCTCCTA CAACTATTAC TGGATACAGG GAGTTTCAAA TTAGTACATC   
  
  
- AAAGATGCTT AAAGTTCGGT AATATTCTGT ATGTCTAAAA ATACTACAAC TTTTATGAGA ATGGAGGAAC   
  
  
- AGTGTTATAA TTGGACTTCG GTTACTTTAC TTTGTCTTGA TTAATTTAAC TTATGTATTA GTTAGGTATA   
  
  
- ATTAAACAAA ACTGTTGATA AATGTTAGTA GCTAGAGAAA TTAATCCAGA TATTCGATAT CTCATTAATG   
  
  
- AGTTTTGACA AAACATTCTG TTGAATGAGG TATAACCGAA TAATCATATA ACGGAGGTAA GTGTACTTAT   
  
  
- GAAGTACTCT CTAACGGATT TTGAGTATAA CAGTAGTGGA ATTCAGTTTG TATAAAACAA TACTTTACTG   
  
  
- TAGTTCTTAA TGGGAGACTG TTCTTTTTAT ATGGACTGAC TGTATTAGCA TGGTGTAGAC AATCAGGAGA   
  
  
- AAGATGGTGA AACATCTTGT ATTACACTGT CATTATGTAG GTAGGGTTTT CTTTTCTGCT TTTCTTCTCA   
  
  
- CCTCTCAGTG CATATGCGCA ACTTAAGTCC TTTTAATTTC CTTGAGTACT TCTGGGGTAT ATCACGAGGT   
  
  
- TGGACTTGGA CGTCGGGTGA TGATGTACGG ATTTAATTTC TCGTTTCAAT ACTCTCTTTG GTTTTTTCGA   
  
  
- GAAACTCTCT CACCTCCTTC TTAGTACCTT TCGATCGATC CGAATCGTTC TTTCTCGTTG TCGATCGATC   
  
  
- GTTTTCCGAA GAGAAACTTC GGAATCTTTA GAAATCTCTA TTTTTCAACC ACCGAAAAAT TCCCGACACT   
  
  
- ATACAGGGGG TACATTTTTA CGTCGTTATT ACAATTACAG TACGAAAGAA AGTACCAGGG ATTACGCTTT   
  
  
- AGTTAGTACG TACAGTTGCA CTGAGTCACC GAGTGGTATA TACTAGACTA AAGGGGCGTG TGTCTCTATT   
  
  
- TTTCGGGGGT TAATATTGGA GGGTTTTCGG GGGGGAAAAG GAGGATGGGT TGGGTGTGGA GGGGTAAAGT   
  
  
- AGTCCCGAGC GAAGAATTCA ATAGCTAATT TTTTTCTCAC TCATTTGCAT GGGCTTAACT TCTCCCCACA   
  
  
- AGCTTCGAAT ATGAACGAAC TAATCCTTAC TTTCAGGGGT AAAGTAGGCG GGGGGTTTGG CGGCGGTGGT   
  
  
- GGTCATTTAG CGATAGAACG TTGTTATGAC GGAACTTATG ACGGAAAGTT CGGGGGTTAA GTTAAAGTTG   
  
  
- GGAACAATTG TTGTTGTTTG GGAGGATGCT CGGATGGAGC CAAGATCTGG AGGCCGCGTC GGGTTCGGGG   
  
  
- CGGTGGTTGC GGCGGGACGG TGACGGGTGA CTGCGCCCGT AGTTATTTGG GTTCGGACGG AGACGACTGG   
  
  
- ACCTGTTAAA CCTCCCACTC CCAACCCTAA GTTAGGATAG TCTCGACCCC GAATTACTAC TAAGATTATT   
  
  
- AGGATTGAGG TTTGAAACCA GTAGGGTTTA ATCGAGTAGT ACACTAAGGG TGGAGTGTGT CGAGGGACTC   
  
  
- AAGGGGAGGA GAGTCGGCAA CCTAGTGTGT GCGTGGGGGT TATTAATTGA AGAGGGGGAG ACTAATGGGG   
  
  
- TAGAGACTAA ATAGGATGCC AGTCTTAGAG TCGGGGTTAA AACCCGTCAA ACTCGAAGTA GAAGTAGTCG   
  
  
- GGTTGCAGTT ATTGTTACCC GAACTGAAGT ATCTCCTGGA TTAATCTCGT CGAGTTCGGA TGCTTAGGTT   
  
  
- GCTGAGGGTG GTCCAGGTCG ACCATAACCG CGCCGAGTTA GTTGCCGAGT CTAGTGGGCG GCCTTTTGGG   
  
  
- GAGGTGGCGC GGCGGAAGAT GAAGTTCCTC CGGGAGGTTG TGGAGCGACA GCCGCCTCCG GGCCGGGCCG   
  
  
- GACAGGCCGA CAGTAGGATG CTCCACCACG TCTGGTAGGC CCGGGTGTTC TTGAAAAGGC CGTATAGTGA   
  
  
- CTATAGGGAG AAGAGGTGGA AGCGGCGGTT AGTCCGCTAA GACCTCCGCC ACCTACCCCG ACGCAAGTAG   
  
  
- GTGTAGTAGC TAAAGCTGTA GCCTAAACCG CCGGTGACCC GCAGCAAATA CGCACTCGAA CAGCTGTTCC   
  
  
- GGCTGGGCTT ATTCTAGTCG AGGAACCACA AGGCTTAGTG CCGCTAGCAT GGGCTTCTCA AGCCCTAGCT   
  
  
- TTCGTTCGAC CACTCTCTCT TAGAATGCGT TAAGCGAGCA CTAGAGTTGT AGTTACAGGT ATACCTCATA   
  
  
- CAGGAATAAG TTTCGAAGCT CAAGAATAGG AAGTTTAGCC AGTTTAAGTA GCTGCCACTC TTTAACCGCC   
  
  
- AGGTAGATAG AGGCCGCTAC AAAGTGGCCG AAGTTCCGCC TTAGAGCTTT AAGGATCGGC TAGAGGCCCC   
  
  
- TTAAAGCGGT TCGTGGCAGC ACCACCACCT GTCACTCCAT CCTTAGCTGT GCCCTTGAAG CAGGAAGTCG   
  
  
- CAGTTGAAGC AGCGGCCATA GCTTAAGATG TGGCCTTACG AGCTTAGCGA GCTGCAGTGG AGGCGGTCGC   
  
  
- CAAAACGTCC GCCGCCGCCG CCGCTGCGAC ACTCTTCCTA GCTCTGCATG CACGAGGCCG GCTCCTAATG   
  
  
- CCGCCGCCAC CTCCGCCGTA GACGCCGCCG CCATCGCGCC GCCTCGCCAA CCTCTCTCAG CAAGCGCCAC   
  
  
- CGATCCTACT CCCGCCACCC CAAGTCGGTC AAACGACTAA AGGTCCGCCT CACAAACGAC TCCTTCCAAG   
  
  
- TCCAACCGCC CAAGGTGCAC CGCTTTGCAG TACCCCTCTA CTACGAAACG ACCGTACTAT CCGGGGAACA   
  
  
- CAGGTGCAGC CGAACCTCCA CAAT

+     MYB

| Site Name | Organism | Position | Strand | Matrix score. | sequence | function |
| --- | --- | --- | --- | --- | --- | --- |
| MYB | Arabidopsis thaliana | 1321 | + | 6 | CAACAG |  |
| MYB | Arabidopsis thaliana | 556 | - | 6 | TAACCA |  |
| MYB | Arabidopsis thaliana | 48 | - | 6 | TAACCA |  |

>HU06G00283.1   
+ -Up\_Stream \_Len000GATTTT ACTTCCAATT TTCTACTTAA ATTTGGTTAT CACGTTCGAA TTTTTCAGGT   
  
  
+ AAATACAAAC TGTTAGTGGC CATCCTTTTT TTTTGGGTCT AATCCATCCA GATGTGCTAA TACTTTCTGT   
  
  
+ TTTAGATGAG AAGTTTTAAT GCTTTTTTAG ATGTGTAATT TTTTTAAACA TAAATAGTCT TGTCGAATAA   
  
  
+ GATTTGCTAG TAATTTTTGT AAGAGAAATT ATGTGTATTT TTTATTATAT CAGAATATTA GATCGATCTC   
  
  
+ AATTTAATTA CTTATGAAAA AAAAAGTGGA TATCTTTAAT CAATTTGGCA AGCTCTCATG CATTAATTGT   
  
  
+ AGTCGTACGA ACTTATTTAC ATCACTCATC TGAATTTGCT ACCATTTACA CCTCAAATTT TATTTTAATG   
  
  
+ CCATAAAGAG AATAGAATAT ACTGTCTATT TGTAGTAGTT TTACAGATAA AAACAAATAA GCAAATGAAA   
  
  
+ ATAGATAGAG GATTTTTTTG CTATTATCCA CGTCGATCCA TTGAATTATT TTGTCGCTAT TTGGTTAAGT   
  
  
+ GAAATAAAAT ACTGAAGTAG AATGGAGGAT GTTGATAATG ACCTATGTCC CTCAAAGTTT AATCATGTAG   
  
  
+ TTTCTACGAA TTTCAAGCCA TTATAAGACA TACAGATTTT TATGATGTTG AAAATACTCT TACCTCCTTG   
  
  
+ TCACAATATT AACCTGAAGC CAATGAAATG AAACAGAACT AATTAAATTG AATACATAAT CAATCCATAT   
  
  
+ TAATTTGTTT TGACAACTAT TTACAATCAT CGATCTCTTT AATTAGGTCT ATAAGCTATA GAGTAATTAC   
  
  
+ TCAAAACTGT TTTGTAAGAC AACTTACTCC ATATTGGCTT ATTAGTATAT TGCCTCCATT CACATGAATA   
  
  
+ CTTCATGAGA GATTGCCTAA AACTCATATT GTCATCACCT TAAGTCAAAC ATATTTTGTT ATGAAATGAC   
  
  
+ ATCAAGAATT ACCCTCTGAC AAGAAAAATA TACCTGACTG ACATAATCGT ACCACATCTG TTAGTCCTCT   
  
  
+ TTCTACCACT TTGTAGAACA TAATGTGACA GTAATACATC CATCCCAAAA GAAAAGACGA AAAGAAGAGT   
  
  
+ GGAGAGTCAC GTATACGCGT TGAATTCAGG AAAATTAAAG GAACTCATGA AGACCCCATA TAGTGCTCCA   
  
  
+ ACCTGAACCT GCAGCCCACT ACTACATGCC TAAATTAAAG AGCAAAGTTA TGAGAGAAAC CAAAAAAGCT   
  
  
+ CTTTGAGAGA GTGGAGGAAG AATCATGGAA AGCTAGCTAG GCTTAGCAAG AAAGAGCAAC AGCTAGCTAG   
  
  
+ CAAAAGGCTT CTCTTTGAAG CCTTAGAAAT CTTTAGAGAT AAAAAGTTGG TGGCTTTTTA AGGGCTGTGA   
  
  
+ TATGTCCCCC ATGTAAAAAT GCAGCAATAA TGTTAATGTC ATGCTTTCTT TCATGGTCCC TAATGCGAAA   
  
  
+ TCAATCATGC ATGTCAACGT GACTCAGTGG CTCACCATAT ATGATCTGAT TTCCCCGCAC ACAGAGATAA   
  
  
+ AAAGCCCCCA ATTATAACCT CCCAAAAGCC CCCCCTTTTC CTCCTACCCA ACCCACACCT CCCCATTTCA   
  
  
+ TCAGGGCTCG CTTCTTAAGT TATCGATTAA AAAAAGAGTG AGTAAACGTA CCCGAATTGA AGAGGGGTGT   
  
  
+ TCGAAGCTTA TACTTGCTTG ATTAGGAATG AAAGTCCCCA TTTCATCCGC CCCCCAAACC GCCGCCACCA   
  
  
+ CCAGTAAATC GCTATCTTGC AACAATACTG CCTTGAATAC TGCCTTTCAA GCCCCCAATT CAATTTCAAC   
  
  
+ CCTTGTTAAC AACAACAAAC CCTCCTACGA GCCTACCTCG GTTCTAGACC TCCGGCGCAG CCCAAGCCCC   
  
  
+ GCCACCAACG CCGCCCTGCC ACTGCCCACT GACGCGGGCA TCAATAAACC CAAGCCTGCC TCTGCTGACC   
  
  
+ TGGACAATTT GGAGGGTGAG GGTTGGGATT CAATCCTATC AGAGCTGGGG CTTAATGATG ATTCTAATAA   
  
  
+ TCCTAACTCC AAACTTTGGT CATCCCAAAT TAGCTCATCA TGTGATTCCC ACCTCACACA GCTCCCTGAG   
  
  
+ TTCCCCTCCT CTCAGCCGTT GGATCACACA CGCACCCCCA ATAATTAACT TCTCCCCCTC TGATTACCCC   
  
  
+ ATCTCTGATT TATCCTACGG TCAGAATCTC AGCCCCAATT TTGGGCAGTT TGAGCTTCAT CTTCATCAGC   
  
  
+ CCAACGTCAA TAACAATGGG CTTGACTTCA TAGAGGACCT AATTAGAGCA GCTCAAGCCT ACGAATCCAA   
  
  
+ CGACTCCCAC CAGGTCCAGC TGGTATTGGC GCGGCTCAAT CAACGGCTCA GATCACCCGC CGGAAAACCC   
  
  
+ CTCCACCGCG CCGCCTTCTA CTTCAAGGAG GCCCTCCAAC ACCTCGCTGT CGGCGGAGGC CCGGCCCGGC   
  
  
+ CTGTCCGGCT GTCATCCTAC GAGGTGGTGC AGACCATCCG GGCCCACAAG AACTTTTCCG GCATATCACT   
  
  
+ GATATCCCTC TTCTCCACCT TCGCCGCCAA TCAGGCGATT CTGGAGGCGG TGGATGGGGC TGCGTTCATC   
  
  
+ CACATCATCG ATTTCGACAT CGGATTTGGC GGCCACTGGG CGTCGTTTAT GCGTGAGCTT GTCGACAAGG   
  
  
+ CCGACCCGAA TAAGATCAGC TCCTTGGTGT TCCGAATCAC GGCGATCGTA CCCGAAGAGT TCGGGATCGA   
  
  
+ AAGCAAGCTG GTGAGAGAGA ATCTTACGCA ATTCGCTCGT GATCTCAACA TCAATGTCCA TATGGAGTAT   
  
  
+ GTCCTTATTC AAAGCTTCGA GTTCTTATCC TTCAAATCGG TCAAATTCAT CGACGGTGAG AAATTGGCGG   
  
  
+ TCCATCTATC TCCGGCGATG TTTCACCGGC TTCAAGGCGG AATCTCGAAA TTCCTAGCCG ATCTCCGGGG   
  
  
+ AATTTCGCCA AGCACCGTCG TGGTGGTGGA CAGTGAGGTA GGAATCGACA CGGGAACTTC GTCCTTCAGC   
  
  
+ GTCAACTTCG TCGCCGGTAT CGAATTCTAC ACCGGAATGC TCGAATCGCT CGACGTCACC TCCGCCAGCG   
  
  
+ GTTTTGCAGG CGGCGGCGGC GGCGACGCTG TGAGAAGGAT CGAGACGTAC GTGCTCCGGC CGAGGATTAC   
  
  
+ GGCGGCGGTG GAGGCGGCAT CTGCGGCGGC GGTAGCGCGG CGGAGCGGTT GGAGAGAGTC GTTCGCGGTG   
  
  
+ GCTAGGATGA GGGCGGTGGG GTTCAGCCAG TTTGCTGATT TCCAGGCGGA GTGTTTGCTG AGGAAGGTTC   
  
  
+ AGGTTGGCGG GTTCCACGTG GCGAAACGTC ATGGGGAGAT GATGCTTTGC TGGCATGATA GGCCCCTTGT   
  
  
+ GTCCACGTCG GCTTGGAGGT GTTA  

- -Up\_Stream \_Len000CTAAAA TGAAGGTTAA AAGATGAATT TAAACCAATA GTGCAAGCTT AAAAAGTCCA   
  
  
- TTTATGTTTG ACAATCACCG GTAGGAAAAA AAAACCCAGA TTAGGTAGGT CTACACGATT ATGAAAGACA   
  
  
- AAATCTACTC TTCAAAATTA CGAAAAAATC TACACATTAA AAAAATTTGT ATTTATCAGA ACAGCTTATT   
  
  
- CTAAACGATC ATTAAAAACA TTCTCTTTAA TACACATAAA AAATAATATA GTCTTATAAT CTAGCTAGAG   
  
  
- TTAAATTAAT GAATACTTTT TTTTTCACCT ATAGAAATTA GTTAAACCGT TCGAGAGTAC GTAATTAACA   
  
  
- TCAGCATGCT TGAATAAATG TAGTGAGTAG ACTTAAACGA TGGTAAATGT GGAGTTTAAA ATAAAATTAC   
  
  
- GGTATTTCTC TTATCTTATA TGACAGATAA ACATCATCAA AATGTCTATT TTTGTTTATT CGTTTACTTT   
  
  
- TATCTATCTC CTAAAAAAAC GATAATAGGT GCAGCTAGGT AACTTAATAA AACAGCGATA AACCAATTCA   
  
  
- CTTTATTTTA TGACTTCATC TTACCTCCTA CAACTATTAC TGGATACAGG GAGTTTCAAA TTAGTACATC   
  
  
- AAAGATGCTT AAAGTTCGGT AATATTCTGT ATGTCTAAAA ATACTACAAC TTTTATGAGA ATGGAGGAAC   
  
  
- AGTGTTATAA TTGGACTTCG GTTACTTTAC TTTGTCTTGA TTAATTTAAC TTATGTATTA GTTAGGTATA   
  
  
- ATTAAACAAA ACTGTTGATA AATGTTAGTA GCTAGAGAAA TTAATCCAGA TATTCGATAT CTCATTAATG   
  
  
- AGTTTTGACA AAACATTCTG TTGAATGAGG TATAACCGAA TAATCATATA ACGGAGGTAA GTGTACTTAT   
  
  
- GAAGTACTCT CTAACGGATT TTGAGTATAA CAGTAGTGGA ATTCAGTTTG TATAAAACAA TACTTTACTG   
  
  
- TAGTTCTTAA TGGGAGACTG TTCTTTTTAT ATGGACTGAC TGTATTAGCA TGGTGTAGAC AATCAGGAGA   
  
  
- AAGATGGTGA AACATCTTGT ATTACACTGT CATTATGTAG GTAGGGTTTT CTTTTCTGCT TTTCTTCTCA   
  
  
- CCTCTCAGTG CATATGCGCA ACTTAAGTCC TTTTAATTTC CTTGAGTACT TCTGGGGTAT ATCACGAGGT   
  
  
- TGGACTTGGA CGTCGGGTGA TGATGTACGG ATTTAATTTC TCGTTTCAAT ACTCTCTTTG GTTTTTTCGA   
  
  
- GAAACTCTCT CACCTCCTTC TTAGTACCTT TCGATCGATC CGAATCGTTC TTTCTCGTTG TCGATCGATC   
  
  
- GTTTTCCGAA GAGAAACTTC GGAATCTTTA GAAATCTCTA TTTTTCAACC ACCGAAAAAT TCCCGACACT   
  
  
- ATACAGGGGG TACATTTTTA CGTCGTTATT ACAATTACAG TACGAAAGAA AGTACCAGGG ATTACGCTTT   
  
  
- AGTTAGTACG TACAGTTGCA CTGAGTCACC GAGTGGTATA TACTAGACTA AAGGGGCGTG TGTCTCTATT   
  
  
- TTTCGGGGGT TAATATTGGA GGGTTTTCGG GGGGGAAAAG GAGGATGGGT TGGGTGTGGA GGGGTAAAGT   
  
  
- AGTCCCGAGC GAAGAATTCA ATAGCTAATT TTTTTCTCAC TCATTTGCAT GGGCTTAACT TCTCCCCACA   
  
  
- AGCTTCGAAT ATGAACGAAC TAATCCTTAC TTTCAGGGGT AAAGTAGGCG GGGGGTTTGG CGGCGGTGGT   
  
  
- GGTCATTTAG CGATAGAACG TTGTTATGAC GGAACTTATG ACGGAAAGTT CGGGGGTTAA GTTAAAGTTG   
  
  
- GGAACAATTG TTGTTGTTTG GGAGGATGCT CGGATGGAGC CAAGATCTGG AGGCCGCGTC GGGTTCGGGG   
  
  
- CGGTGGTTGC GGCGGGACGG TGACGGGTGA CTGCGCCCGT AGTTATTTGG GTTCGGACGG AGACGACTGG   
  
  
- ACCTGTTAAA CCTCCCACTC CCAACCCTAA GTTAGGATAG TCTCGACCCC GAATTACTAC TAAGATTATT   
  
  
- AGGATTGAGG TTTGAAACCA GTAGGGTTTA ATCGAGTAGT ACACTAAGGG TGGAGTGTGT CGAGGGACTC   
  
  
- AAGGGGAGGA GAGTCGGCAA CCTAGTGTGT GCGTGGGGGT TATTAATTGA AGAGGGGGAG ACTAATGGGG   
  
  
- TAGAGACTAA ATAGGATGCC AGTCTTAGAG TCGGGGTTAA AACCCGTCAA ACTCGAAGTA GAAGTAGTCG   
  
  
- GGTTGCAGTT ATTGTTACCC GAACTGAAGT ATCTCCTGGA TTAATCTCGT CGAGTTCGGA TGCTTAGGTT   
  
  
- GCTGAGGGTG GTCCAGGTCG ACCATAACCG CGCCGAGTTA GTTGCCGAGT CTAGTGGGCG GCCTTTTGGG   
  
  
- GAGGTGGCGC GGCGGAAGAT GAAGTTCCTC CGGGAGGTTG TGGAGCGACA GCCGCCTCCG GGCCGGGCCG   
  
  
- GACAGGCCGA CAGTAGGATG CTCCACCACG TCTGGTAGGC CCGGGTGTTC TTGAAAAGGC CGTATAGTGA   
  
  
- CTATAGGGAG AAGAGGTGGA AGCGGCGGTT AGTCCGCTAA GACCTCCGCC ACCTACCCCG ACGCAAGTAG   
  
  
- GTGTAGTAGC TAAAGCTGTA GCCTAAACCG CCGGTGACCC GCAGCAAATA CGCACTCGAA CAGCTGTTCC   
  
  
- GGCTGGGCTT ATTCTAGTCG AGGAACCACA AGGCTTAGTG CCGCTAGCAT GGGCTTCTCA AGCCCTAGCT   
  
  
- TTCGTTCGAC CACTCTCTCT TAGAATGCGT TAAGCGAGCA CTAGAGTTGT AGTTACAGGT ATACCTCATA   
  
  
- CAGGAATAAG TTTCGAAGCT CAAGAATAGG AAGTTTAGCC AGTTTAAGTA GCTGCCACTC TTTAACCGCC   
  
  
- AGGTAGATAG AGGCCGCTAC AAAGTGGCCG AAGTTCCGCC TTAGAGCTTT AAGGATCGGC TAGAGGCCCC   
  
  
- TTAAAGCGGT TCGTGGCAGC ACCACCACCT GTCACTCCAT CCTTAGCTGT GCCCTTGAAG CAGGAAGTCG   
  
  
- CAGTTGAAGC AGCGGCCATA GCTTAAGATG TGGCCTTACG AGCTTAGCGA GCTGCAGTGG AGGCGGTCGC   
  
  
- CAAAACGTCC GCCGCCGCCG CCGCTGCGAC ACTCTTCCTA GCTCTGCATG CACGAGGCCG GCTCCTAATG   
  
  
- CCGCCGCCAC CTCCGCCGTA GACGCCGCCG CCATCGCGCC GCCTCGCCAA CCTCTCTCAG CAAGCGCCAC   
  
  
- CGATCCTACT CCCGCCACCC CAAGTCGGTC AAACGACTAA AGGTCCGCCT CACAAACGAC TCCTTCCAAG   
  
  
- TCCAACCGCC CAAGGTGCAC CGCTTTGCAG TACCCCTCTA CTACGAAACG ACCGTACTAT CCGGGGAACA   
  
  
- CAGGTGCAGC CGAACCTCCA CAAT

+     MYB recognition site

| Site Name | Organism | Position | Strand | Matrix score. | sequence | function |
| --- | --- | --- | --- | --- | --- | --- |
| MYB recognition site | Arabidopsis thaliana | 2120 | + | 6 | CCGTTG |  |
| MYB recognition site | Arabidopsis thaliana | 2355 | - | 6 | CCGTTG |  |

>HU06G00283.1   
+ -Up\_Stream \_Len000GATTTT ACTTCCAATT TTCTACTTAA ATTTGGTTAT CACGTTCGAA TTTTTCAGGT   
  
  
+ AAATACAAAC TGTTAGTGGC CATCCTTTTT TTTTGGGTCT AATCCATCCA GATGTGCTAA TACTTTCTGT   
  
  
+ TTTAGATGAG AAGTTTTAAT GCTTTTTTAG ATGTGTAATT TTTTTAAACA TAAATAGTCT TGTCGAATAA   
  
  
+ GATTTGCTAG TAATTTTTGT AAGAGAAATT ATGTGTATTT TTTATTATAT CAGAATATTA GATCGATCTC   
  
  
+ AATTTAATTA CTTATGAAAA AAAAAGTGGA TATCTTTAAT CAATTTGGCA AGCTCTCATG CATTAATTGT   
  
  
+ AGTCGTACGA ACTTATTTAC ATCACTCATC TGAATTTGCT ACCATTTACA CCTCAAATTT TATTTTAATG   
  
  
+ CCATAAAGAG AATAGAATAT ACTGTCTATT TGTAGTAGTT TTACAGATAA AAACAAATAA GCAAATGAAA   
  
  
+ ATAGATAGAG GATTTTTTTG CTATTATCCA CGTCGATCCA TTGAATTATT TTGTCGCTAT TTGGTTAAGT   
  
  
+ GAAATAAAAT ACTGAAGTAG AATGGAGGAT GTTGATAATG ACCTATGTCC CTCAAAGTTT AATCATGTAG   
  
  
+ TTTCTACGAA TTTCAAGCCA TTATAAGACA TACAGATTTT TATGATGTTG AAAATACTCT TACCTCCTTG   
  
  
+ TCACAATATT AACCTGAAGC CAATGAAATG AAACAGAACT AATTAAATTG AATACATAAT CAATCCATAT   
  
  
+ TAATTTGTTT TGACAACTAT TTACAATCAT CGATCTCTTT AATTAGGTCT ATAAGCTATA GAGTAATTAC   
  
  
+ TCAAAACTGT TTTGTAAGAC AACTTACTCC ATATTGGCTT ATTAGTATAT TGCCTCCATT CACATGAATA   
  
  
+ CTTCATGAGA GATTGCCTAA AACTCATATT GTCATCACCT TAAGTCAAAC ATATTTTGTT ATGAAATGAC   
  
  
+ ATCAAGAATT ACCCTCTGAC AAGAAAAATA TACCTGACTG ACATAATCGT ACCACATCTG TTAGTCCTCT   
  
  
+ TTCTACCACT TTGTAGAACA TAATGTGACA GTAATACATC CATCCCAAAA GAAAAGACGA AAAGAAGAGT   
  
  
+ GGAGAGTCAC GTATACGCGT TGAATTCAGG AAAATTAAAG GAACTCATGA AGACCCCATA TAGTGCTCCA   
  
  
+ ACCTGAACCT GCAGCCCACT ACTACATGCC TAAATTAAAG AGCAAAGTTA TGAGAGAAAC CAAAAAAGCT   
  
  
+ CTTTGAGAGA GTGGAGGAAG AATCATGGAA AGCTAGCTAG GCTTAGCAAG AAAGAGCAAC AGCTAGCTAG   
  
  
+ CAAAAGGCTT CTCTTTGAAG CCTTAGAAAT CTTTAGAGAT AAAAAGTTGG TGGCTTTTTA AGGGCTGTGA   
  
  
+ TATGTCCCCC ATGTAAAAAT GCAGCAATAA TGTTAATGTC ATGCTTTCTT TCATGGTCCC TAATGCGAAA   
  
  
+ TCAATCATGC ATGTCAACGT GACTCAGTGG CTCACCATAT ATGATCTGAT TTCCCCGCAC ACAGAGATAA   
  
  
+ AAAGCCCCCA ATTATAACCT CCCAAAAGCC CCCCCTTTTC CTCCTACCCA ACCCACACCT CCCCATTTCA   
  
  
+ TCAGGGCTCG CTTCTTAAGT TATCGATTAA AAAAAGAGTG AGTAAACGTA CCCGAATTGA AGAGGGGTGT   
  
  
+ TCGAAGCTTA TACTTGCTTG ATTAGGAATG AAAGTCCCCA TTTCATCCGC CCCCCAAACC GCCGCCACCA   
  
  
+ CCAGTAAATC GCTATCTTGC AACAATACTG CCTTGAATAC TGCCTTTCAA GCCCCCAATT CAATTTCAAC   
  
  
+ CCTTGTTAAC AACAACAAAC CCTCCTACGA GCCTACCTCG GTTCTAGACC TCCGGCGCAG CCCAAGCCCC   
  
  
+ GCCACCAACG CCGCCCTGCC ACTGCCCACT GACGCGGGCA TCAATAAACC CAAGCCTGCC TCTGCTGACC   
  
  
+ TGGACAATTT GGAGGGTGAG GGTTGGGATT CAATCCTATC AGAGCTGGGG CTTAATGATG ATTCTAATAA   
  
  
+ TCCTAACTCC AAACTTTGGT CATCCCAAAT TAGCTCATCA TGTGATTCCC ACCTCACACA GCTCCCTGAG   
  
  
+ TTCCCCTCCT CTCAGCCGTT GGATCACACA CGCACCCCCA ATAATTAACT TCTCCCCCTC TGATTACCCC   
  
  
+ ATCTCTGATT TATCCTACGG TCAGAATCTC AGCCCCAATT TTGGGCAGTT TGAGCTTCAT CTTCATCAGC   
  
  
+ CCAACGTCAA TAACAATGGG CTTGACTTCA TAGAGGACCT AATTAGAGCA GCTCAAGCCT ACGAATCCAA   
  
  
+ CGACTCCCAC CAGGTCCAGC TGGTATTGGC GCGGCTCAAT CAACGGCTCA GATCACCCGC CGGAAAACCC   
  
  
+ CTCCACCGCG CCGCCTTCTA CTTCAAGGAG GCCCTCCAAC ACCTCGCTGT CGGCGGAGGC CCGGCCCGGC   
  
  
+ CTGTCCGGCT GTCATCCTAC GAGGTGGTGC AGACCATCCG GGCCCACAAG AACTTTTCCG GCATATCACT   
  
  
+ GATATCCCTC TTCTCCACCT TCGCCGCCAA TCAGGCGATT CTGGAGGCGG TGGATGGGGC TGCGTTCATC   
  
  
+ CACATCATCG ATTTCGACAT CGGATTTGGC GGCCACTGGG CGTCGTTTAT GCGTGAGCTT GTCGACAAGG   
  
  
+ CCGACCCGAA TAAGATCAGC TCCTTGGTGT TCCGAATCAC GGCGATCGTA CCCGAAGAGT TCGGGATCGA   
  
  
+ AAGCAAGCTG GTGAGAGAGA ATCTTACGCA ATTCGCTCGT GATCTCAACA TCAATGTCCA TATGGAGTAT   
  
  
+ GTCCTTATTC AAAGCTTCGA GTTCTTATCC TTCAAATCGG TCAAATTCAT CGACGGTGAG AAATTGGCGG   
  
  
+ TCCATCTATC TCCGGCGATG TTTCACCGGC TTCAAGGCGG AATCTCGAAA TTCCTAGCCG ATCTCCGGGG   
  
  
+ AATTTCGCCA AGCACCGTCG TGGTGGTGGA CAGTGAGGTA GGAATCGACA CGGGAACTTC GTCCTTCAGC   
  
  
+ GTCAACTTCG TCGCCGGTAT CGAATTCTAC ACCGGAATGC TCGAATCGCT CGACGTCACC TCCGCCAGCG   
  
  
+ GTTTTGCAGG CGGCGGCGGC GGCGACGCTG TGAGAAGGAT CGAGACGTAC GTGCTCCGGC CGAGGATTAC   
  
  
+ GGCGGCGGTG GAGGCGGCAT CTGCGGCGGC GGTAGCGCGG CGGAGCGGTT GGAGAGAGTC GTTCGCGGTG   
  
  
+ GCTAGGATGA GGGCGGTGGG GTTCAGCCAG TTTGCTGATT TCCAGGCGGA GTGTTTGCTG AGGAAGGTTC   
  
  
+ AGGTTGGCGG GTTCCACGTG GCGAAACGTC ATGGGGAGAT GATGCTTTGC TGGCATGATA GGCCCCTTGT   
  
  
+ GTCCACGTCG GCTTGGAGGT GTTA  

- -Up\_Stream \_Len000CTAAAA TGAAGGTTAA AAGATGAATT TAAACCAATA GTGCAAGCTT AAAAAGTCCA   
  
  
- TTTATGTTTG ACAATCACCG GTAGGAAAAA AAAACCCAGA TTAGGTAGGT CTACACGATT ATGAAAGACA   
  
  
- AAATCTACTC TTCAAAATTA CGAAAAAATC TACACATTAA AAAAATTTGT ATTTATCAGA ACAGCTTATT   
  
  
- CTAAACGATC ATTAAAAACA TTCTCTTTAA TACACATAAA AAATAATATA GTCTTATAAT CTAGCTAGAG   
  
  
- TTAAATTAAT GAATACTTTT TTTTTCACCT ATAGAAATTA GTTAAACCGT TCGAGAGTAC GTAATTAACA   
  
  
- TCAGCATGCT TGAATAAATG TAGTGAGTAG ACTTAAACGA TGGTAAATGT GGAGTTTAAA ATAAAATTAC   
  
  
- GGTATTTCTC TTATCTTATA TGACAGATAA ACATCATCAA AATGTCTATT TTTGTTTATT CGTTTACTTT   
  
  
- TATCTATCTC CTAAAAAAAC GATAATAGGT GCAGCTAGGT AACTTAATAA AACAGCGATA AACCAATTCA   
  
  
- CTTTATTTTA TGACTTCATC TTACCTCCTA CAACTATTAC TGGATACAGG GAGTTTCAAA TTAGTACATC   
  
  
- AAAGATGCTT AAAGTTCGGT AATATTCTGT ATGTCTAAAA ATACTACAAC TTTTATGAGA ATGGAGGAAC   
  
  
- AGTGTTATAA TTGGACTTCG GTTACTTTAC TTTGTCTTGA TTAATTTAAC TTATGTATTA GTTAGGTATA   
  
  
- ATTAAACAAA ACTGTTGATA AATGTTAGTA GCTAGAGAAA TTAATCCAGA TATTCGATAT CTCATTAATG   
  
  
- AGTTTTGACA AAACATTCTG TTGAATGAGG TATAACCGAA TAATCATATA ACGGAGGTAA GTGTACTTAT   
  
  
- GAAGTACTCT CTAACGGATT TTGAGTATAA CAGTAGTGGA ATTCAGTTTG TATAAAACAA TACTTTACTG   
  
  
- TAGTTCTTAA TGGGAGACTG TTCTTTTTAT ATGGACTGAC TGTATTAGCA TGGTGTAGAC AATCAGGAGA   
  
  
- AAGATGGTGA AACATCTTGT ATTACACTGT CATTATGTAG GTAGGGTTTT CTTTTCTGCT TTTCTTCTCA   
  
  
- CCTCTCAGTG CATATGCGCA ACTTAAGTCC TTTTAATTTC CTTGAGTACT TCTGGGGTAT ATCACGAGGT   
  
  
- TGGACTTGGA CGTCGGGTGA TGATGTACGG ATTTAATTTC TCGTTTCAAT ACTCTCTTTG GTTTTTTCGA   
  
  
- GAAACTCTCT CACCTCCTTC TTAGTACCTT TCGATCGATC CGAATCGTTC TTTCTCGTTG TCGATCGATC   
  
  
- GTTTTCCGAA GAGAAACTTC GGAATCTTTA GAAATCTCTA TTTTTCAACC ACCGAAAAAT TCCCGACACT   
  
  
- ATACAGGGGG TACATTTTTA CGTCGTTATT ACAATTACAG TACGAAAGAA AGTACCAGGG ATTACGCTTT   
  
  
- AGTTAGTACG TACAGTTGCA CTGAGTCACC GAGTGGTATA TACTAGACTA AAGGGGCGTG TGTCTCTATT   
  
  
- TTTCGGGGGT TAATATTGGA GGGTTTTCGG GGGGGAAAAG GAGGATGGGT TGGGTGTGGA GGGGTAAAGT   
  
  
- AGTCCCGAGC GAAGAATTCA ATAGCTAATT TTTTTCTCAC TCATTTGCAT GGGCTTAACT TCTCCCCACA   
  
  
- AGCTTCGAAT ATGAACGAAC TAATCCTTAC TTTCAGGGGT AAAGTAGGCG GGGGGTTTGG CGGCGGTGGT   
  
  
- GGTCATTTAG CGATAGAACG TTGTTATGAC GGAACTTATG ACGGAAAGTT CGGGGGTTAA GTTAAAGTTG   
  
  
- GGAACAATTG TTGTTGTTTG GGAGGATGCT CGGATGGAGC CAAGATCTGG AGGCCGCGTC GGGTTCGGGG   
  
  
- CGGTGGTTGC GGCGGGACGG TGACGGGTGA CTGCGCCCGT AGTTATTTGG GTTCGGACGG AGACGACTGG   
  
  
- ACCTGTTAAA CCTCCCACTC CCAACCCTAA GTTAGGATAG TCTCGACCCC GAATTACTAC TAAGATTATT   
  
  
- AGGATTGAGG TTTGAAACCA GTAGGGTTTA ATCGAGTAGT ACACTAAGGG TGGAGTGTGT CGAGGGACTC   
  
  
- AAGGGGAGGA GAGTCGGCAA CCTAGTGTGT GCGTGGGGGT TATTAATTGA AGAGGGGGAG ACTAATGGGG   
  
  
- TAGAGACTAA ATAGGATGCC AGTCTTAGAG TCGGGGTTAA AACCCGTCAA ACTCGAAGTA GAAGTAGTCG   
  
  
- GGTTGCAGTT ATTGTTACCC GAACTGAAGT ATCTCCTGGA TTAATCTCGT CGAGTTCGGA TGCTTAGGTT   
  
  
- GCTGAGGGTG GTCCAGGTCG ACCATAACCG CGCCGAGTTA GTTGCCGAGT CTAGTGGGCG GCCTTTTGGG   
  
  
- GAGGTGGCGC GGCGGAAGAT GAAGTTCCTC CGGGAGGTTG TGGAGCGACA GCCGCCTCCG GGCCGGGCCG   
  
  
- GACAGGCCGA CAGTAGGATG CTCCACCACG TCTGGTAGGC CCGGGTGTTC TTGAAAAGGC CGTATAGTGA   
  
  
- CTATAGGGAG AAGAGGTGGA AGCGGCGGTT AGTCCGCTAA GACCTCCGCC ACCTACCCCG ACGCAAGTAG   
  
  
- GTGTAGTAGC TAAAGCTGTA GCCTAAACCG CCGGTGACCC GCAGCAAATA CGCACTCGAA CAGCTGTTCC   
  
  
- GGCTGGGCTT ATTCTAGTCG AGGAACCACA AGGCTTAGTG CCGCTAGCAT GGGCTTCTCA AGCCCTAGCT   
  
  
- TTCGTTCGAC CACTCTCTCT TAGAATGCGT TAAGCGAGCA CTAGAGTTGT AGTTACAGGT ATACCTCATA   
  
  
- CAGGAATAAG TTTCGAAGCT CAAGAATAGG AAGTTTAGCC AGTTTAAGTA GCTGCCACTC TTTAACCGCC   
  
  
- AGGTAGATAG AGGCCGCTAC AAAGTGGCCG AAGTTCCGCC TTAGAGCTTT AAGGATCGGC TAGAGGCCCC   
  
  
- TTAAAGCGGT TCGTGGCAGC ACCACCACCT GTCACTCCAT CCTTAGCTGT GCCCTTGAAG CAGGAAGTCG   
  
  
- CAGTTGAAGC AGCGGCCATA GCTTAAGATG TGGCCTTACG AGCTTAGCGA GCTGCAGTGG AGGCGGTCGC   
  
  
- CAAAACGTCC GCCGCCGCCG CCGCTGCGAC ACTCTTCCTA GCTCTGCATG CACGAGGCCG GCTCCTAATG   
  
  
- CCGCCGCCAC CTCCGCCGTA GACGCCGCCG CCATCGCGCC GCCTCGCCAA CCTCTCTCAG CAAGCGCCAC   
  
  
- CGATCCTACT CCCGCCACCC CAAGTCGGTC AAACGACTAA AGGTCCGCCT CACAAACGAC TCCTTCCAAG   
  
  
- TCCAACCGCC CAAGGTGCAC CGCTTTGCAG TACCCCTCTA CTACGAAACG ACCGTACTAT CCGGGGAACA   
  
  
- CAGGTGCAGC CGAACCTCCA CAAT

+     MYB-like sequence

| Site Name | Organism | Position | Strand | Matrix score. | sequence | function |
| --- | --- | --- | --- | --- | --- | --- |
| MYB-like sequence | Arabidopsis thaliana | 48 | - | 6 | TAACCA |  |
| MYB-like sequence | Arabidopsis thaliana | 556 | - | 6 | TAACCA |  |

>HU06G00283.1   
+ -Up\_Stream \_Len000GATTTT ACTTCCAATT TTCTACTTAA ATTTGGTTAT CACGTTCGAA TTTTTCAGGT   
  
  
+ AAATACAAAC TGTTAGTGGC CATCCTTTTT TTTTGGGTCT AATCCATCCA GATGTGCTAA TACTTTCTGT   
  
  
+ TTTAGATGAG AAGTTTTAAT GCTTTTTTAG ATGTGTAATT TTTTTAAACA TAAATAGTCT TGTCGAATAA   
  
  
+ GATTTGCTAG TAATTTTTGT AAGAGAAATT ATGTGTATTT TTTATTATAT CAGAATATTA GATCGATCTC   
  
  
+ AATTTAATTA CTTATGAAAA AAAAAGTGGA TATCTTTAAT CAATTTGGCA AGCTCTCATG CATTAATTGT   
  
  
+ AGTCGTACGA ACTTATTTAC ATCACTCATC TGAATTTGCT ACCATTTACA CCTCAAATTT TATTTTAATG   
  
  
+ CCATAAAGAG AATAGAATAT ACTGTCTATT TGTAGTAGTT TTACAGATAA AAACAAATAA GCAAATGAAA   
  
  
+ ATAGATAGAG GATTTTTTTG CTATTATCCA CGTCGATCCA TTGAATTATT TTGTCGCTAT TTGGTTAAGT   
  
  
+ GAAATAAAAT ACTGAAGTAG AATGGAGGAT GTTGATAATG ACCTATGTCC CTCAAAGTTT AATCATGTAG   
  
  
+ TTTCTACGAA TTTCAAGCCA TTATAAGACA TACAGATTTT TATGATGTTG AAAATACTCT TACCTCCTTG   
  
  
+ TCACAATATT AACCTGAAGC CAATGAAATG AAACAGAACT AATTAAATTG AATACATAAT CAATCCATAT   
  
  
+ TAATTTGTTT TGACAACTAT TTACAATCAT CGATCTCTTT AATTAGGTCT ATAAGCTATA GAGTAATTAC   
  
  
+ TCAAAACTGT TTTGTAAGAC AACTTACTCC ATATTGGCTT ATTAGTATAT TGCCTCCATT CACATGAATA   
  
  
+ CTTCATGAGA GATTGCCTAA AACTCATATT GTCATCACCT TAAGTCAAAC ATATTTTGTT ATGAAATGAC   
  
  
+ ATCAAGAATT ACCCTCTGAC AAGAAAAATA TACCTGACTG ACATAATCGT ACCACATCTG TTAGTCCTCT   
  
  
+ TTCTACCACT TTGTAGAACA TAATGTGACA GTAATACATC CATCCCAAAA GAAAAGACGA AAAGAAGAGT   
  
  
+ GGAGAGTCAC GTATACGCGT TGAATTCAGG AAAATTAAAG GAACTCATGA AGACCCCATA TAGTGCTCCA   
  
  
+ ACCTGAACCT GCAGCCCACT ACTACATGCC TAAATTAAAG AGCAAAGTTA TGAGAGAAAC CAAAAAAGCT   
  
  
+ CTTTGAGAGA GTGGAGGAAG AATCATGGAA AGCTAGCTAG GCTTAGCAAG AAAGAGCAAC AGCTAGCTAG   
  
  
+ CAAAAGGCTT CTCTTTGAAG CCTTAGAAAT CTTTAGAGAT AAAAAGTTGG TGGCTTTTTA AGGGCTGTGA   
  
  
+ TATGTCCCCC ATGTAAAAAT GCAGCAATAA TGTTAATGTC ATGCTTTCTT TCATGGTCCC TAATGCGAAA   
  
  
+ TCAATCATGC ATGTCAACGT GACTCAGTGG CTCACCATAT ATGATCTGAT TTCCCCGCAC ACAGAGATAA   
  
  
+ AAAGCCCCCA ATTATAACCT CCCAAAAGCC CCCCCTTTTC CTCCTACCCA ACCCACACCT CCCCATTTCA   
  
  
+ TCAGGGCTCG CTTCTTAAGT TATCGATTAA AAAAAGAGTG AGTAAACGTA CCCGAATTGA AGAGGGGTGT   
  
  
+ TCGAAGCTTA TACTTGCTTG ATTAGGAATG AAAGTCCCCA TTTCATCCGC CCCCCAAACC GCCGCCACCA   
  
  
+ CCAGTAAATC GCTATCTTGC AACAATACTG CCTTGAATAC TGCCTTTCAA GCCCCCAATT CAATTTCAAC   
  
  
+ CCTTGTTAAC AACAACAAAC CCTCCTACGA GCCTACCTCG GTTCTAGACC TCCGGCGCAG CCCAAGCCCC   
  
  
+ GCCACCAACG CCGCCCTGCC ACTGCCCACT GACGCGGGCA TCAATAAACC CAAGCCTGCC TCTGCTGACC   
  
  
+ TGGACAATTT GGAGGGTGAG GGTTGGGATT CAATCCTATC AGAGCTGGGG CTTAATGATG ATTCTAATAA   
  
  
+ TCCTAACTCC AAACTTTGGT CATCCCAAAT TAGCTCATCA TGTGATTCCC ACCTCACACA GCTCCCTGAG   
  
  
+ TTCCCCTCCT CTCAGCCGTT GGATCACACA CGCACCCCCA ATAATTAACT TCTCCCCCTC TGATTACCCC   
  
  
+ ATCTCTGATT TATCCTACGG TCAGAATCTC AGCCCCAATT TTGGGCAGTT TGAGCTTCAT CTTCATCAGC   
  
  
+ CCAACGTCAA TAACAATGGG CTTGACTTCA TAGAGGACCT AATTAGAGCA GCTCAAGCCT ACGAATCCAA   
  
  
+ CGACTCCCAC CAGGTCCAGC TGGTATTGGC GCGGCTCAAT CAACGGCTCA GATCACCCGC CGGAAAACCC   
  
  
+ CTCCACCGCG CCGCCTTCTA CTTCAAGGAG GCCCTCCAAC ACCTCGCTGT CGGCGGAGGC CCGGCCCGGC   
  
  
+ CTGTCCGGCT GTCATCCTAC GAGGTGGTGC AGACCATCCG GGCCCACAAG AACTTTTCCG GCATATCACT   
  
  
+ GATATCCCTC TTCTCCACCT TCGCCGCCAA TCAGGCGATT CTGGAGGCGG TGGATGGGGC TGCGTTCATC   
  
  
+ CACATCATCG ATTTCGACAT CGGATTTGGC GGCCACTGGG CGTCGTTTAT GCGTGAGCTT GTCGACAAGG   
  
  
+ CCGACCCGAA TAAGATCAGC TCCTTGGTGT TCCGAATCAC GGCGATCGTA CCCGAAGAGT TCGGGATCGA   
  
  
+ AAGCAAGCTG GTGAGAGAGA ATCTTACGCA ATTCGCTCGT GATCTCAACA TCAATGTCCA TATGGAGTAT   
  
  
+ GTCCTTATTC AAAGCTTCGA GTTCTTATCC TTCAAATCGG TCAAATTCAT CGACGGTGAG AAATTGGCGG   
  
  
+ TCCATCTATC TCCGGCGATG TTTCACCGGC TTCAAGGCGG AATCTCGAAA TTCCTAGCCG ATCTCCGGGG   
  
  
+ AATTTCGCCA AGCACCGTCG TGGTGGTGGA CAGTGAGGTA GGAATCGACA CGGGAACTTC GTCCTTCAGC   
  
  
+ GTCAACTTCG TCGCCGGTAT CGAATTCTAC ACCGGAATGC TCGAATCGCT CGACGTCACC TCCGCCAGCG   
  
  
+ GTTTTGCAGG CGGCGGCGGC GGCGACGCTG TGAGAAGGAT CGAGACGTAC GTGCTCCGGC CGAGGATTAC   
  
  
+ GGCGGCGGTG GAGGCGGCAT CTGCGGCGGC GGTAGCGCGG CGGAGCGGTT GGAGAGAGTC GTTCGCGGTG   
  
  
+ GCTAGGATGA GGGCGGTGGG GTTCAGCCAG TTTGCTGATT TCCAGGCGGA GTGTTTGCTG AGGAAGGTTC   
  
  
+ AGGTTGGCGG GTTCCACGTG GCGAAACGTC ATGGGGAGAT GATGCTTTGC TGGCATGATA GGCCCCTTGT   
  
  
+ GTCCACGTCG GCTTGGAGGT GTTA  

- -Up\_Stream \_Len000CTAAAA TGAAGGTTAA AAGATGAATT TAAACCAATA GTGCAAGCTT AAAAAGTCCA   
  
  
- TTTATGTTTG ACAATCACCG GTAGGAAAAA AAAACCCAGA TTAGGTAGGT CTACACGATT ATGAAAGACA   
  
  
- AAATCTACTC TTCAAAATTA CGAAAAAATC TACACATTAA AAAAATTTGT ATTTATCAGA ACAGCTTATT   
  
  
- CTAAACGATC ATTAAAAACA TTCTCTTTAA TACACATAAA AAATAATATA GTCTTATAAT CTAGCTAGAG   
  
  
- TTAAATTAAT GAATACTTTT TTTTTCACCT ATAGAAATTA GTTAAACCGT TCGAGAGTAC GTAATTAACA   
  
  
- TCAGCATGCT TGAATAAATG TAGTGAGTAG ACTTAAACGA TGGTAAATGT GGAGTTTAAA ATAAAATTAC   
  
  
- GGTATTTCTC TTATCTTATA TGACAGATAA ACATCATCAA AATGTCTATT TTTGTTTATT CGTTTACTTT   
  
  
- TATCTATCTC CTAAAAAAAC GATAATAGGT GCAGCTAGGT AACTTAATAA AACAGCGATA AACCAATTCA   
  
  
- CTTTATTTTA TGACTTCATC TTACCTCCTA CAACTATTAC TGGATACAGG GAGTTTCAAA TTAGTACATC   
  
  
- AAAGATGCTT AAAGTTCGGT AATATTCTGT ATGTCTAAAA ATACTACAAC TTTTATGAGA ATGGAGGAAC   
  
  
- AGTGTTATAA TTGGACTTCG GTTACTTTAC TTTGTCTTGA TTAATTTAAC TTATGTATTA GTTAGGTATA   
  
  
- ATTAAACAAA ACTGTTGATA AATGTTAGTA GCTAGAGAAA TTAATCCAGA TATTCGATAT CTCATTAATG   
  
  
- AGTTTTGACA AAACATTCTG TTGAATGAGG TATAACCGAA TAATCATATA ACGGAGGTAA GTGTACTTAT   
  
  
- GAAGTACTCT CTAACGGATT TTGAGTATAA CAGTAGTGGA ATTCAGTTTG TATAAAACAA TACTTTACTG   
  
  
- TAGTTCTTAA TGGGAGACTG TTCTTTTTAT ATGGACTGAC TGTATTAGCA TGGTGTAGAC AATCAGGAGA   
  
  
- AAGATGGTGA AACATCTTGT ATTACACTGT CATTATGTAG GTAGGGTTTT CTTTTCTGCT TTTCTTCTCA   
  
  
- CCTCTCAGTG CATATGCGCA ACTTAAGTCC TTTTAATTTC CTTGAGTACT TCTGGGGTAT ATCACGAGGT   
  
  
- TGGACTTGGA CGTCGGGTGA TGATGTACGG ATTTAATTTC TCGTTTCAAT ACTCTCTTTG GTTTTTTCGA   
  
  
- GAAACTCTCT CACCTCCTTC TTAGTACCTT TCGATCGATC CGAATCGTTC TTTCTCGTTG TCGATCGATC   
  
  
- GTTTTCCGAA GAGAAACTTC GGAATCTTTA GAAATCTCTA TTTTTCAACC ACCGAAAAAT TCCCGACACT   
  
  
- ATACAGGGGG TACATTTTTA CGTCGTTATT ACAATTACAG TACGAAAGAA AGTACCAGGG ATTACGCTTT   
  
  
- AGTTAGTACG TACAGTTGCA CTGAGTCACC GAGTGGTATA TACTAGACTA AAGGGGCGTG TGTCTCTATT   
  
  
- TTTCGGGGGT TAATATTGGA GGGTTTTCGG GGGGGAAAAG GAGGATGGGT TGGGTGTGGA GGGGTAAAGT   
  
  
- AGTCCCGAGC GAAGAATTCA ATAGCTAATT TTTTTCTCAC TCATTTGCAT GGGCTTAACT TCTCCCCACA   
  
  
- AGCTTCGAAT ATGAACGAAC TAATCCTTAC TTTCAGGGGT AAAGTAGGCG GGGGGTTTGG CGGCGGTGGT   
  
  
- GGTCATTTAG CGATAGAACG TTGTTATGAC GGAACTTATG ACGGAAAGTT CGGGGGTTAA GTTAAAGTTG   
  
  
- GGAACAATTG TTGTTGTTTG GGAGGATGCT CGGATGGAGC CAAGATCTGG AGGCCGCGTC GGGTTCGGGG   
  
  
- CGGTGGTTGC GGCGGGACGG TGACGGGTGA CTGCGCCCGT AGTTATTTGG GTTCGGACGG AGACGACTGG   
  
  
- ACCTGTTAAA CCTCCCACTC CCAACCCTAA GTTAGGATAG TCTCGACCCC GAATTACTAC TAAGATTATT   
  
  
- AGGATTGAGG TTTGAAACCA GTAGGGTTTA ATCGAGTAGT ACACTAAGGG TGGAGTGTGT CGAGGGACTC   
  
  
- AAGGGGAGGA GAGTCGGCAA CCTAGTGTGT GCGTGGGGGT TATTAATTGA AGAGGGGGAG ACTAATGGGG   
  
  
- TAGAGACTAA ATAGGATGCC AGTCTTAGAG TCGGGGTTAA AACCCGTCAA ACTCGAAGTA GAAGTAGTCG   
  
  
- GGTTGCAGTT ATTGTTACCC GAACTGAAGT ATCTCCTGGA TTAATCTCGT CGAGTTCGGA TGCTTAGGTT   
  
  
- GCTGAGGGTG GTCCAGGTCG ACCATAACCG CGCCGAGTTA GTTGCCGAGT CTAGTGGGCG GCCTTTTGGG   
  
  
- GAGGTGGCGC GGCGGAAGAT GAAGTTCCTC CGGGAGGTTG TGGAGCGACA GCCGCCTCCG GGCCGGGCCG   
  
  
- GACAGGCCGA CAGTAGGATG CTCCACCACG TCTGGTAGGC CCGGGTGTTC TTGAAAAGGC CGTATAGTGA   
  
  
- CTATAGGGAG AAGAGGTGGA AGCGGCGGTT AGTCCGCTAA GACCTCCGCC ACCTACCCCG ACGCAAGTAG   
  
  
- GTGTAGTAGC TAAAGCTGTA GCCTAAACCG CCGGTGACCC GCAGCAAATA CGCACTCGAA CAGCTGTTCC   
  
  
- GGCTGGGCTT ATTCTAGTCG AGGAACCACA AGGCTTAGTG CCGCTAGCAT GGGCTTCTCA AGCCCTAGCT   
  
  
- TTCGTTCGAC CACTCTCTCT TAGAATGCGT TAAGCGAGCA CTAGAGTTGT AGTTACAGGT ATACCTCATA   
  
  
- CAGGAATAAG TTTCGAAGCT CAAGAATAGG AAGTTTAGCC AGTTTAAGTA GCTGCCACTC TTTAACCGCC   
  
  
- AGGTAGATAG AGGCCGCTAC AAAGTGGCCG AAGTTCCGCC TTAGAGCTTT AAGGATCGGC TAGAGGCCCC   
  
  
- TTAAAGCGGT TCGTGGCAGC ACCACCACCT GTCACTCCAT CCTTAGCTGT GCCCTTGAAG CAGGAAGTCG   
  
  
- CAGTTGAAGC AGCGGCCATA GCTTAAGATG TGGCCTTACG AGCTTAGCGA GCTGCAGTGG AGGCGGTCGC   
  
  
- CAAAACGTCC GCCGCCGCCG CCGCTGCGAC ACTCTTCCTA GCTCTGCATG CACGAGGCCG GCTCCTAATG   
  
  
- CCGCCGCCAC CTCCGCCGTA GACGCCGCCG CCATCGCGCC GCCTCGCCAA CCTCTCTCAG CAAGCGCCAC   
  
  
- CGATCCTACT CCCGCCACCC CAAGTCGGTC AAACGACTAA AGGTCCGCCT CACAAACGAC TCCTTCCAAG   
  
  
- TCCAACCGCC CAAGGTGCAC CGCTTTGCAG TACCCCTCTA CTACGAAACG ACCGTACTAT CCGGGGAACA   
  
  
- CAGGTGCAGC CGAACCTCCA CAAT

+     MYC

| Site Name | Organism | Position | Strand | Matrix score. | sequence | function |
| --- | --- | --- | --- | --- | --- | --- |
| MYC | Arabidopsis thaliana | 2073 | + | 6 | CATGTG |  |
| MYC | Arabidopsis thaliana | 905 | - | 6 | CATGTG |  |
| MYC | Arabidopsis thaliana | 486 | - | 6 | CATTTG |  |

>HU06G00283.1   
+ -Up\_Stream \_Len000GATTTT ACTTCCAATT TTCTACTTAA ATTTGGTTAT CACGTTCGAA TTTTTCAGGT   
  
  
+ AAATACAAAC TGTTAGTGGC CATCCTTTTT TTTTGGGTCT AATCCATCCA GATGTGCTAA TACTTTCTGT   
  
  
+ TTTAGATGAG AAGTTTTAAT GCTTTTTTAG ATGTGTAATT TTTTTAAACA TAAATAGTCT TGTCGAATAA   
  
  
+ GATTTGCTAG TAATTTTTGT AAGAGAAATT ATGTGTATTT TTTATTATAT CAGAATATTA GATCGATCTC   
  
  
+ AATTTAATTA CTTATGAAAA AAAAAGTGGA TATCTTTAAT CAATTTGGCA AGCTCTCATG CATTAATTGT   
  
  
+ AGTCGTACGA ACTTATTTAC ATCACTCATC TGAATTTGCT ACCATTTACA CCTCAAATTT TATTTTAATG   
  
  
+ CCATAAAGAG AATAGAATAT ACTGTCTATT TGTAGTAGTT TTACAGATAA AAACAAATAA GCAAATGAAA   
  
  
+ ATAGATAGAG GATTTTTTTG CTATTATCCA CGTCGATCCA TTGAATTATT TTGTCGCTAT TTGGTTAAGT   
  
  
+ GAAATAAAAT ACTGAAGTAG AATGGAGGAT GTTGATAATG ACCTATGTCC CTCAAAGTTT AATCATGTAG   
  
  
+ TTTCTACGAA TTTCAAGCCA TTATAAGACA TACAGATTTT TATGATGTTG AAAATACTCT TACCTCCTTG   
  
  
+ TCACAATATT AACCTGAAGC CAATGAAATG AAACAGAACT AATTAAATTG AATACATAAT CAATCCATAT   
  
  
+ TAATTTGTTT TGACAACTAT TTACAATCAT CGATCTCTTT AATTAGGTCT ATAAGCTATA GAGTAATTAC   
  
  
+ TCAAAACTGT TTTGTAAGAC AACTTACTCC ATATTGGCTT ATTAGTATAT TGCCTCCATT CACATGAATA   
  
  
+ CTTCATGAGA GATTGCCTAA AACTCATATT GTCATCACCT TAAGTCAAAC ATATTTTGTT ATGAAATGAC   
  
  
+ ATCAAGAATT ACCCTCTGAC AAGAAAAATA TACCTGACTG ACATAATCGT ACCACATCTG TTAGTCCTCT   
  
  
+ TTCTACCACT TTGTAGAACA TAATGTGACA GTAATACATC CATCCCAAAA GAAAAGACGA AAAGAAGAGT   
  
  
+ GGAGAGTCAC GTATACGCGT TGAATTCAGG AAAATTAAAG GAACTCATGA AGACCCCATA TAGTGCTCCA   
  
  
+ ACCTGAACCT GCAGCCCACT ACTACATGCC TAAATTAAAG AGCAAAGTTA TGAGAGAAAC CAAAAAAGCT   
  
  
+ CTTTGAGAGA GTGGAGGAAG AATCATGGAA AGCTAGCTAG GCTTAGCAAG AAAGAGCAAC AGCTAGCTAG   
  
  
+ CAAAAGGCTT CTCTTTGAAG CCTTAGAAAT CTTTAGAGAT AAAAAGTTGG TGGCTTTTTA AGGGCTGTGA   
  
  
+ TATGTCCCCC ATGTAAAAAT GCAGCAATAA TGTTAATGTC ATGCTTTCTT TCATGGTCCC TAATGCGAAA   
  
  
+ TCAATCATGC ATGTCAACGT GACTCAGTGG CTCACCATAT ATGATCTGAT TTCCCCGCAC ACAGAGATAA   
  
  
+ AAAGCCCCCA ATTATAACCT CCCAAAAGCC CCCCCTTTTC CTCCTACCCA ACCCACACCT CCCCATTTCA   
  
  
+ TCAGGGCTCG CTTCTTAAGT TATCGATTAA AAAAAGAGTG AGTAAACGTA CCCGAATTGA AGAGGGGTGT   
  
  
+ TCGAAGCTTA TACTTGCTTG ATTAGGAATG AAAGTCCCCA TTTCATCCGC CCCCCAAACC GCCGCCACCA   
  
  
+ CCAGTAAATC GCTATCTTGC AACAATACTG CCTTGAATAC TGCCTTTCAA GCCCCCAATT CAATTTCAAC   
  
  
+ CCTTGTTAAC AACAACAAAC CCTCCTACGA GCCTACCTCG GTTCTAGACC TCCGGCGCAG CCCAAGCCCC   
  
  
+ GCCACCAACG CCGCCCTGCC ACTGCCCACT GACGCGGGCA TCAATAAACC CAAGCCTGCC TCTGCTGACC   
  
  
+ TGGACAATTT GGAGGGTGAG GGTTGGGATT CAATCCTATC AGAGCTGGGG CTTAATGATG ATTCTAATAA   
  
  
+ TCCTAACTCC AAACTTTGGT CATCCCAAAT TAGCTCATCA TGTGATTCCC ACCTCACACA GCTCCCTGAG   
  
  
+ TTCCCCTCCT CTCAGCCGTT GGATCACACA CGCACCCCCA ATAATTAACT TCTCCCCCTC TGATTACCCC   
  
  
+ ATCTCTGATT TATCCTACGG TCAGAATCTC AGCCCCAATT TTGGGCAGTT TGAGCTTCAT CTTCATCAGC   
  
  
+ CCAACGTCAA TAACAATGGG CTTGACTTCA TAGAGGACCT AATTAGAGCA GCTCAAGCCT ACGAATCCAA   
  
  
+ CGACTCCCAC CAGGTCCAGC TGGTATTGGC GCGGCTCAAT CAACGGCTCA GATCACCCGC CGGAAAACCC   
  
  
+ CTCCACCGCG CCGCCTTCTA CTTCAAGGAG GCCCTCCAAC ACCTCGCTGT CGGCGGAGGC CCGGCCCGGC   
  
  
+ CTGTCCGGCT GTCATCCTAC GAGGTGGTGC AGACCATCCG GGCCCACAAG AACTTTTCCG GCATATCACT   
  
  
+ GATATCCCTC TTCTCCACCT TCGCCGCCAA TCAGGCGATT CTGGAGGCGG TGGATGGGGC TGCGTTCATC   
  
  
+ CACATCATCG ATTTCGACAT CGGATTTGGC GGCCACTGGG CGTCGTTTAT GCGTGAGCTT GTCGACAAGG   
  
  
+ CCGACCCGAA TAAGATCAGC TCCTTGGTGT TCCGAATCAC GGCGATCGTA CCCGAAGAGT TCGGGATCGA   
  
  
+ AAGCAAGCTG GTGAGAGAGA ATCTTACGCA ATTCGCTCGT GATCTCAACA TCAATGTCCA TATGGAGTAT   
  
  
+ GTCCTTATTC AAAGCTTCGA GTTCTTATCC TTCAAATCGG TCAAATTCAT CGACGGTGAG AAATTGGCGG   
  
  
+ TCCATCTATC TCCGGCGATG TTTCACCGGC TTCAAGGCGG AATCTCGAAA TTCCTAGCCG ATCTCCGGGG   
  
  
+ AATTTCGCCA AGCACCGTCG TGGTGGTGGA CAGTGAGGTA GGAATCGACA CGGGAACTTC GTCCTTCAGC   
  
  
+ GTCAACTTCG TCGCCGGTAT CGAATTCTAC ACCGGAATGC TCGAATCGCT CGACGTCACC TCCGCCAGCG   
  
  
+ GTTTTGCAGG CGGCGGCGGC GGCGACGCTG TGAGAAGGAT CGAGACGTAC GTGCTCCGGC CGAGGATTAC   
  
  
+ GGCGGCGGTG GAGGCGGCAT CTGCGGCGGC GGTAGCGCGG CGGAGCGGTT GGAGAGAGTC GTTCGCGGTG   
  
  
+ GCTAGGATGA GGGCGGTGGG GTTCAGCCAG TTTGCTGATT TCCAGGCGGA GTGTTTGCTG AGGAAGGTTC   
  
  
+ AGGTTGGCGG GTTCCACGTG GCGAAACGTC ATGGGGAGAT GATGCTTTGC TGGCATGATA GGCCCCTTGT   
  
  
+ GTCCACGTCG GCTTGGAGGT GTTA  

- -Up\_Stream \_Len000CTAAAA TGAAGGTTAA AAGATGAATT TAAACCAATA GTGCAAGCTT AAAAAGTCCA   
  
  
- TTTATGTTTG ACAATCACCG GTAGGAAAAA AAAACCCAGA TTAGGTAGGT CTACACGATT ATGAAAGACA   
  
  
- AAATCTACTC TTCAAAATTA CGAAAAAATC TACACATTAA AAAAATTTGT ATTTATCAGA ACAGCTTATT   
  
  
- CTAAACGATC ATTAAAAACA TTCTCTTTAA TACACATAAA AAATAATATA GTCTTATAAT CTAGCTAGAG   
  
  
- TTAAATTAAT GAATACTTTT TTTTTCACCT ATAGAAATTA GTTAAACCGT TCGAGAGTAC GTAATTAACA   
  
  
- TCAGCATGCT TGAATAAATG TAGTGAGTAG ACTTAAACGA TGGTAAATGT GGAGTTTAAA ATAAAATTAC   
  
  
- GGTATTTCTC TTATCTTATA TGACAGATAA ACATCATCAA AATGTCTATT TTTGTTTATT CGTTTACTTT   
  
  
- TATCTATCTC CTAAAAAAAC GATAATAGGT GCAGCTAGGT AACTTAATAA AACAGCGATA AACCAATTCA   
  
  
- CTTTATTTTA TGACTTCATC TTACCTCCTA CAACTATTAC TGGATACAGG GAGTTTCAAA TTAGTACATC   
  
  
- AAAGATGCTT AAAGTTCGGT AATATTCTGT ATGTCTAAAA ATACTACAAC TTTTATGAGA ATGGAGGAAC   
  
  
- AGTGTTATAA TTGGACTTCG GTTACTTTAC TTTGTCTTGA TTAATTTAAC TTATGTATTA GTTAGGTATA   
  
  
- ATTAAACAAA ACTGTTGATA AATGTTAGTA GCTAGAGAAA TTAATCCAGA TATTCGATAT CTCATTAATG   
  
  
- AGTTTTGACA AAACATTCTG TTGAATGAGG TATAACCGAA TAATCATATA ACGGAGGTAA GTGTACTTAT   
  
  
- GAAGTACTCT CTAACGGATT TTGAGTATAA CAGTAGTGGA ATTCAGTTTG TATAAAACAA TACTTTACTG   
  
  
- TAGTTCTTAA TGGGAGACTG TTCTTTTTAT ATGGACTGAC TGTATTAGCA TGGTGTAGAC AATCAGGAGA   
  
  
- AAGATGGTGA AACATCTTGT ATTACACTGT CATTATGTAG GTAGGGTTTT CTTTTCTGCT TTTCTTCTCA   
  
  
- CCTCTCAGTG CATATGCGCA ACTTAAGTCC TTTTAATTTC CTTGAGTACT TCTGGGGTAT ATCACGAGGT   
  
  
- TGGACTTGGA CGTCGGGTGA TGATGTACGG ATTTAATTTC TCGTTTCAAT ACTCTCTTTG GTTTTTTCGA   
  
  
- GAAACTCTCT CACCTCCTTC TTAGTACCTT TCGATCGATC CGAATCGTTC TTTCTCGTTG TCGATCGATC   
  
  
- GTTTTCCGAA GAGAAACTTC GGAATCTTTA GAAATCTCTA TTTTTCAACC ACCGAAAAAT TCCCGACACT   
  
  
- ATACAGGGGG TACATTTTTA CGTCGTTATT ACAATTACAG TACGAAAGAA AGTACCAGGG ATTACGCTTT   
  
  
- AGTTAGTACG TACAGTTGCA CTGAGTCACC GAGTGGTATA TACTAGACTA AAGGGGCGTG TGTCTCTATT   
  
  
- TTTCGGGGGT TAATATTGGA GGGTTTTCGG GGGGGAAAAG GAGGATGGGT TGGGTGTGGA GGGGTAAAGT   
  
  
- AGTCCCGAGC GAAGAATTCA ATAGCTAATT TTTTTCTCAC TCATTTGCAT GGGCTTAACT TCTCCCCACA   
  
  
- AGCTTCGAAT ATGAACGAAC TAATCCTTAC TTTCAGGGGT AAAGTAGGCG GGGGGTTTGG CGGCGGTGGT   
  
  
- GGTCATTTAG CGATAGAACG TTGTTATGAC GGAACTTATG ACGGAAAGTT CGGGGGTTAA GTTAAAGTTG   
  
  
- GGAACAATTG TTGTTGTTTG GGAGGATGCT CGGATGGAGC CAAGATCTGG AGGCCGCGTC GGGTTCGGGG   
  
  
- CGGTGGTTGC GGCGGGACGG TGACGGGTGA CTGCGCCCGT AGTTATTTGG GTTCGGACGG AGACGACTGG   
  
  
- ACCTGTTAAA CCTCCCACTC CCAACCCTAA GTTAGGATAG TCTCGACCCC GAATTACTAC TAAGATTATT   
  
  
- AGGATTGAGG TTTGAAACCA GTAGGGTTTA ATCGAGTAGT ACACTAAGGG TGGAGTGTGT CGAGGGACTC   
  
  
- AAGGGGAGGA GAGTCGGCAA CCTAGTGTGT GCGTGGGGGT TATTAATTGA AGAGGGGGAG ACTAATGGGG   
  
  
- TAGAGACTAA ATAGGATGCC AGTCTTAGAG TCGGGGTTAA AACCCGTCAA ACTCGAAGTA GAAGTAGTCG   
  
  
- GGTTGCAGTT ATTGTTACCC GAACTGAAGT ATCTCCTGGA TTAATCTCGT CGAGTTCGGA TGCTTAGGTT   
  
  
- GCTGAGGGTG GTCCAGGTCG ACCATAACCG CGCCGAGTTA GTTGCCGAGT CTAGTGGGCG GCCTTTTGGG   
  
  
- GAGGTGGCGC GGCGGAAGAT GAAGTTCCTC CGGGAGGTTG TGGAGCGACA GCCGCCTCCG GGCCGGGCCG   
  
  
- GACAGGCCGA CAGTAGGATG CTCCACCACG TCTGGTAGGC CCGGGTGTTC TTGAAAAGGC CGTATAGTGA   
  
  
- CTATAGGGAG AAGAGGTGGA AGCGGCGGTT AGTCCGCTAA GACCTCCGCC ACCTACCCCG ACGCAAGTAG   
  
  
- GTGTAGTAGC TAAAGCTGTA GCCTAAACCG CCGGTGACCC GCAGCAAATA CGCACTCGAA CAGCTGTTCC   
  
  
- GGCTGGGCTT ATTCTAGTCG AGGAACCACA AGGCTTAGTG CCGCTAGCAT GGGCTTCTCA AGCCCTAGCT   
  
  
- TTCGTTCGAC CACTCTCTCT TAGAATGCGT TAAGCGAGCA CTAGAGTTGT AGTTACAGGT ATACCTCATA   
  
  
- CAGGAATAAG TTTCGAAGCT CAAGAATAGG AAGTTTAGCC AGTTTAAGTA GCTGCCACTC TTTAACCGCC   
  
  
- AGGTAGATAG AGGCCGCTAC AAAGTGGCCG AAGTTCCGCC TTAGAGCTTT AAGGATCGGC TAGAGGCCCC   
  
  
- TTAAAGCGGT TCGTGGCAGC ACCACCACCT GTCACTCCAT CCTTAGCTGT GCCCTTGAAG CAGGAAGTCG   
  
  
- CAGTTGAAGC AGCGGCCATA GCTTAAGATG TGGCCTTACG AGCTTAGCGA GCTGCAGTGG AGGCGGTCGC   
  
  
- CAAAACGTCC GCCGCCGCCG CCGCTGCGAC ACTCTTCCTA GCTCTGCATG CACGAGGCCG GCTCCTAATG   
  
  
- CCGCCGCCAC CTCCGCCGTA GACGCCGCCG CCATCGCGCC GCCTCGCCAA CCTCTCTCAG CAAGCGCCAC   
  
  
- CGATCCTACT CCCGCCACCC CAAGTCGGTC AAACGACTAA AGGTCCGCCT CACAAACGAC TCCTTCCAAG   
  
  
- TCCAACCGCC CAAGGTGCAC CGCTTTGCAG TACCCCTCTA CTACGAAACG ACCGTACTAT CCGGGGAACA   
  
  
- CAGGTGCAGC CGAACCTCCA CAAT

+     Myb-binding site

| Site Name | Organism | Position | Strand | Matrix score. | sequence | function |
| --- | --- | --- | --- | --- | --- | --- |
| Myb-binding site | Nicotiana tabacum | 1321 | + | 6 | CAACAG |  |

>HU06G00283.1   
+ -Up\_Stream \_Len000GATTTT ACTTCCAATT TTCTACTTAA ATTTGGTTAT CACGTTCGAA TTTTTCAGGT   
  
  
+ AAATACAAAC TGTTAGTGGC CATCCTTTTT TTTTGGGTCT AATCCATCCA GATGTGCTAA TACTTTCTGT   
  
  
+ TTTAGATGAG AAGTTTTAAT GCTTTTTTAG ATGTGTAATT TTTTTAAACA TAAATAGTCT TGTCGAATAA   
  
  
+ GATTTGCTAG TAATTTTTGT AAGAGAAATT ATGTGTATTT TTTATTATAT CAGAATATTA GATCGATCTC   
  
  
+ AATTTAATTA CTTATGAAAA AAAAAGTGGA TATCTTTAAT CAATTTGGCA AGCTCTCATG CATTAATTGT   
  
  
+ AGTCGTACGA ACTTATTTAC ATCACTCATC TGAATTTGCT ACCATTTACA CCTCAAATTT TATTTTAATG   
  
  
+ CCATAAAGAG AATAGAATAT ACTGTCTATT TGTAGTAGTT TTACAGATAA AAACAAATAA GCAAATGAAA   
  
  
+ ATAGATAGAG GATTTTTTTG CTATTATCCA CGTCGATCCA TTGAATTATT TTGTCGCTAT TTGGTTAAGT   
  
  
+ GAAATAAAAT ACTGAAGTAG AATGGAGGAT GTTGATAATG ACCTATGTCC CTCAAAGTTT AATCATGTAG   
  
  
+ TTTCTACGAA TTTCAAGCCA TTATAAGACA TACAGATTTT TATGATGTTG AAAATACTCT TACCTCCTTG   
  
  
+ TCACAATATT AACCTGAAGC CAATGAAATG AAACAGAACT AATTAAATTG AATACATAAT CAATCCATAT   
  
  
+ TAATTTGTTT TGACAACTAT TTACAATCAT CGATCTCTTT AATTAGGTCT ATAAGCTATA GAGTAATTAC   
  
  
+ TCAAAACTGT TTTGTAAGAC AACTTACTCC ATATTGGCTT ATTAGTATAT TGCCTCCATT CACATGAATA   
  
  
+ CTTCATGAGA GATTGCCTAA AACTCATATT GTCATCACCT TAAGTCAAAC ATATTTTGTT ATGAAATGAC   
  
  
+ ATCAAGAATT ACCCTCTGAC AAGAAAAATA TACCTGACTG ACATAATCGT ACCACATCTG TTAGTCCTCT   
  
  
+ TTCTACCACT TTGTAGAACA TAATGTGACA GTAATACATC CATCCCAAAA GAAAAGACGA AAAGAAGAGT   
  
  
+ GGAGAGTCAC GTATACGCGT TGAATTCAGG AAAATTAAAG GAACTCATGA AGACCCCATA TAGTGCTCCA   
  
  
+ ACCTGAACCT GCAGCCCACT ACTACATGCC TAAATTAAAG AGCAAAGTTA TGAGAGAAAC CAAAAAAGCT   
  
  
+ CTTTGAGAGA GTGGAGGAAG AATCATGGAA AGCTAGCTAG GCTTAGCAAG AAAGAGCAAC AGCTAGCTAG   
  
  
+ CAAAAGGCTT CTCTTTGAAG CCTTAGAAAT CTTTAGAGAT AAAAAGTTGG TGGCTTTTTA AGGGCTGTGA   
  
  
+ TATGTCCCCC ATGTAAAAAT GCAGCAATAA TGTTAATGTC ATGCTTTCTT TCATGGTCCC TAATGCGAAA   
  
  
+ TCAATCATGC ATGTCAACGT GACTCAGTGG CTCACCATAT ATGATCTGAT TTCCCCGCAC ACAGAGATAA   
  
  
+ AAAGCCCCCA ATTATAACCT CCCAAAAGCC CCCCCTTTTC CTCCTACCCA ACCCACACCT CCCCATTTCA   
  
  
+ TCAGGGCTCG CTTCTTAAGT TATCGATTAA AAAAAGAGTG AGTAAACGTA CCCGAATTGA AGAGGGGTGT   
  
  
+ TCGAAGCTTA TACTTGCTTG ATTAGGAATG AAAGTCCCCA TTTCATCCGC CCCCCAAACC GCCGCCACCA   
  
  
+ CCAGTAAATC GCTATCTTGC AACAATACTG CCTTGAATAC TGCCTTTCAA GCCCCCAATT CAATTTCAAC   
  
  
+ CCTTGTTAAC AACAACAAAC CCTCCTACGA GCCTACCTCG GTTCTAGACC TCCGGCGCAG CCCAAGCCCC   
  
  
+ GCCACCAACG CCGCCCTGCC ACTGCCCACT GACGCGGGCA TCAATAAACC CAAGCCTGCC TCTGCTGACC   
  
  
+ TGGACAATTT GGAGGGTGAG GGTTGGGATT CAATCCTATC AGAGCTGGGG CTTAATGATG ATTCTAATAA   
  
  
+ TCCTAACTCC AAACTTTGGT CATCCCAAAT TAGCTCATCA TGTGATTCCC ACCTCACACA GCTCCCTGAG   
  
  
+ TTCCCCTCCT CTCAGCCGTT GGATCACACA CGCACCCCCA ATAATTAACT TCTCCCCCTC TGATTACCCC   
  
  
+ ATCTCTGATT TATCCTACGG TCAGAATCTC AGCCCCAATT TTGGGCAGTT TGAGCTTCAT CTTCATCAGC   
  
  
+ CCAACGTCAA TAACAATGGG CTTGACTTCA TAGAGGACCT AATTAGAGCA GCTCAAGCCT ACGAATCCAA   
  
  
+ CGACTCCCAC CAGGTCCAGC TGGTATTGGC GCGGCTCAAT CAACGGCTCA GATCACCCGC CGGAAAACCC   
  
  
+ CTCCACCGCG CCGCCTTCTA CTTCAAGGAG GCCCTCCAAC ACCTCGCTGT CGGCGGAGGC CCGGCCCGGC   
  
  
+ CTGTCCGGCT GTCATCCTAC GAGGTGGTGC AGACCATCCG GGCCCACAAG AACTTTTCCG GCATATCACT   
  
  
+ GATATCCCTC TTCTCCACCT TCGCCGCCAA TCAGGCGATT CTGGAGGCGG TGGATGGGGC TGCGTTCATC   
  
  
+ CACATCATCG ATTTCGACAT CGGATTTGGC GGCCACTGGG CGTCGTTTAT GCGTGAGCTT GTCGACAAGG   
  
  
+ CCGACCCGAA TAAGATCAGC TCCTTGGTGT TCCGAATCAC GGCGATCGTA CCCGAAGAGT TCGGGATCGA   
  
  
+ AAGCAAGCTG GTGAGAGAGA ATCTTACGCA ATTCGCTCGT GATCTCAACA TCAATGTCCA TATGGAGTAT   
  
  
+ GTCCTTATTC AAAGCTTCGA GTTCTTATCC TTCAAATCGG TCAAATTCAT CGACGGTGAG AAATTGGCGG   
  
  
+ TCCATCTATC TCCGGCGATG TTTCACCGGC TTCAAGGCGG AATCTCGAAA TTCCTAGCCG ATCTCCGGGG   
  
  
+ AATTTCGCCA AGCACCGTCG TGGTGGTGGA CAGTGAGGTA GGAATCGACA CGGGAACTTC GTCCTTCAGC   
  
  
+ GTCAACTTCG TCGCCGGTAT CGAATTCTAC ACCGGAATGC TCGAATCGCT CGACGTCACC TCCGCCAGCG   
  
  
+ GTTTTGCAGG CGGCGGCGGC GGCGACGCTG TGAGAAGGAT CGAGACGTAC GTGCTCCGGC CGAGGATTAC   
  
  
+ GGCGGCGGTG GAGGCGGCAT CTGCGGCGGC GGTAGCGCGG CGGAGCGGTT GGAGAGAGTC GTTCGCGGTG   
  
  
+ GCTAGGATGA GGGCGGTGGG GTTCAGCCAG TTTGCTGATT TCCAGGCGGA GTGTTTGCTG AGGAAGGTTC   
  
  
+ AGGTTGGCGG GTTCCACGTG GCGAAACGTC ATGGGGAGAT GATGCTTTGC TGGCATGATA GGCCCCTTGT   
  
  
+ GTCCACGTCG GCTTGGAGGT GTTA  

- -Up\_Stream \_Len000CTAAAA TGAAGGTTAA AAGATGAATT TAAACCAATA GTGCAAGCTT AAAAAGTCCA   
  
  
- TTTATGTTTG ACAATCACCG GTAGGAAAAA AAAACCCAGA TTAGGTAGGT CTACACGATT ATGAAAGACA   
  
  
- AAATCTACTC TTCAAAATTA CGAAAAAATC TACACATTAA AAAAATTTGT ATTTATCAGA ACAGCTTATT   
  
  
- CTAAACGATC ATTAAAAACA TTCTCTTTAA TACACATAAA AAATAATATA GTCTTATAAT CTAGCTAGAG   
  
  
- TTAAATTAAT GAATACTTTT TTTTTCACCT ATAGAAATTA GTTAAACCGT TCGAGAGTAC GTAATTAACA   
  
  
- TCAGCATGCT TGAATAAATG TAGTGAGTAG ACTTAAACGA TGGTAAATGT GGAGTTTAAA ATAAAATTAC   
  
  
- GGTATTTCTC TTATCTTATA TGACAGATAA ACATCATCAA AATGTCTATT TTTGTTTATT CGTTTACTTT   
  
  
- TATCTATCTC CTAAAAAAAC GATAATAGGT GCAGCTAGGT AACTTAATAA AACAGCGATA AACCAATTCA   
  
  
- CTTTATTTTA TGACTTCATC TTACCTCCTA CAACTATTAC TGGATACAGG GAGTTTCAAA TTAGTACATC   
  
  
- AAAGATGCTT AAAGTTCGGT AATATTCTGT ATGTCTAAAA ATACTACAAC TTTTATGAGA ATGGAGGAAC   
  
  
- AGTGTTATAA TTGGACTTCG GTTACTTTAC TTTGTCTTGA TTAATTTAAC TTATGTATTA GTTAGGTATA   
  
  
- ATTAAACAAA ACTGTTGATA AATGTTAGTA GCTAGAGAAA TTAATCCAGA TATTCGATAT CTCATTAATG   
  
  
- AGTTTTGACA AAACATTCTG TTGAATGAGG TATAACCGAA TAATCATATA ACGGAGGTAA GTGTACTTAT   
  
  
- GAAGTACTCT CTAACGGATT TTGAGTATAA CAGTAGTGGA ATTCAGTTTG TATAAAACAA TACTTTACTG   
  
  
- TAGTTCTTAA TGGGAGACTG TTCTTTTTAT ATGGACTGAC TGTATTAGCA TGGTGTAGAC AATCAGGAGA   
  
  
- AAGATGGTGA AACATCTTGT ATTACACTGT CATTATGTAG GTAGGGTTTT CTTTTCTGCT TTTCTTCTCA   
  
  
- CCTCTCAGTG CATATGCGCA ACTTAAGTCC TTTTAATTTC CTTGAGTACT TCTGGGGTAT ATCACGAGGT   
  
  
- TGGACTTGGA CGTCGGGTGA TGATGTACGG ATTTAATTTC TCGTTTCAAT ACTCTCTTTG GTTTTTTCGA   
  
  
- GAAACTCTCT CACCTCCTTC TTAGTACCTT TCGATCGATC CGAATCGTTC TTTCTCGTTG TCGATCGATC   
  
  
- GTTTTCCGAA GAGAAACTTC GGAATCTTTA GAAATCTCTA TTTTTCAACC ACCGAAAAAT TCCCGACACT   
  
  
- ATACAGGGGG TACATTTTTA CGTCGTTATT ACAATTACAG TACGAAAGAA AGTACCAGGG ATTACGCTTT   
  
  
- AGTTAGTACG TACAGTTGCA CTGAGTCACC GAGTGGTATA TACTAGACTA AAGGGGCGTG TGTCTCTATT   
  
  
- TTTCGGGGGT TAATATTGGA GGGTTTTCGG GGGGGAAAAG GAGGATGGGT TGGGTGTGGA GGGGTAAAGT   
  
  
- AGTCCCGAGC GAAGAATTCA ATAGCTAATT TTTTTCTCAC TCATTTGCAT GGGCTTAACT TCTCCCCACA   
  
  
- AGCTTCGAAT ATGAACGAAC TAATCCTTAC TTTCAGGGGT AAAGTAGGCG GGGGGTTTGG CGGCGGTGGT   
  
  
- GGTCATTTAG CGATAGAACG TTGTTATGAC GGAACTTATG ACGGAAAGTT CGGGGGTTAA GTTAAAGTTG   
  
  
- GGAACAATTG TTGTTGTTTG GGAGGATGCT CGGATGGAGC CAAGATCTGG AGGCCGCGTC GGGTTCGGGG   
  
  
- CGGTGGTTGC GGCGGGACGG TGACGGGTGA CTGCGCCCGT AGTTATTTGG GTTCGGACGG AGACGACTGG   
  
  
- ACCTGTTAAA CCTCCCACTC CCAACCCTAA GTTAGGATAG TCTCGACCCC GAATTACTAC TAAGATTATT   
  
  
- AGGATTGAGG TTTGAAACCA GTAGGGTTTA ATCGAGTAGT ACACTAAGGG TGGAGTGTGT CGAGGGACTC   
  
  
- AAGGGGAGGA GAGTCGGCAA CCTAGTGTGT GCGTGGGGGT TATTAATTGA AGAGGGGGAG ACTAATGGGG   
  
  
- TAGAGACTAA ATAGGATGCC AGTCTTAGAG TCGGGGTTAA AACCCGTCAA ACTCGAAGTA GAAGTAGTCG   
  
  
- GGTTGCAGTT ATTGTTACCC GAACTGAAGT ATCTCCTGGA TTAATCTCGT CGAGTTCGGA TGCTTAGGTT   
  
  
- GCTGAGGGTG GTCCAGGTCG ACCATAACCG CGCCGAGTTA GTTGCCGAGT CTAGTGGGCG GCCTTTTGGG   
  
  
- GAGGTGGCGC GGCGGAAGAT GAAGTTCCTC CGGGAGGTTG TGGAGCGACA GCCGCCTCCG GGCCGGGCCG   
  
  
- GACAGGCCGA CAGTAGGATG CTCCACCACG TCTGGTAGGC CCGGGTGTTC TTGAAAAGGC CGTATAGTGA   
  
  
- CTATAGGGAG AAGAGGTGGA AGCGGCGGTT AGTCCGCTAA GACCTCCGCC ACCTACCCCG ACGCAAGTAG   
  
  
- GTGTAGTAGC TAAAGCTGTA GCCTAAACCG CCGGTGACCC GCAGCAAATA CGCACTCGAA CAGCTGTTCC   
  
  
- GGCTGGGCTT ATTCTAGTCG AGGAACCACA AGGCTTAGTG CCGCTAGCAT GGGCTTCTCA AGCCCTAGCT   
  
  
- TTCGTTCGAC CACTCTCTCT TAGAATGCGT TAAGCGAGCA CTAGAGTTGT AGTTACAGGT ATACCTCATA   
  
  
- CAGGAATAAG TTTCGAAGCT CAAGAATAGG AAGTTTAGCC AGTTTAAGTA GCTGCCACTC TTTAACCGCC   
  
  
- AGGTAGATAG AGGCCGCTAC AAAGTGGCCG AAGTTCCGCC TTAGAGCTTT AAGGATCGGC TAGAGGCCCC   
  
  
- TTAAAGCGGT TCGTGGCAGC ACCACCACCT GTCACTCCAT CCTTAGCTGT GCCCTTGAAG CAGGAAGTCG   
  
  
- CAGTTGAAGC AGCGGCCATA GCTTAAGATG TGGCCTTACG AGCTTAGCGA GCTGCAGTGG AGGCGGTCGC   
  
  
- CAAAACGTCC GCCGCCGCCG CCGCTGCGAC ACTCTTCCTA GCTCTGCATG CACGAGGCCG GCTCCTAATG   
  
  
- CCGCCGCCAC CTCCGCCGTA GACGCCGCCG CCATCGCGCC GCCTCGCCAA CCTCTCTCAG CAAGCGCCAC   
  
  
- CGATCCTACT CCCGCCACCC CAAGTCGGTC AAACGACTAA AGGTCCGCCT CACAAACGAC TCCTTCCAAG   
  
  
- TCCAACCGCC CAAGGTGCAC CGCTTTGCAG TACCCCTCTA CTACGAAACG ACCGTACTAT CCGGGGAACA   
  
  
- CAGGTGCAGC CGAACCTCCA CAAT

+     Myc

| Site Name | Organism | Position | Strand | Matrix score. | sequence | function |
| --- | --- | --- | --- | --- | --- | --- |
| Myc | Arabidopsis thaliana | 234 | - | 7 | TCTCTTA |  |

>HU06G00283.1   
+ -Up\_Stream \_Len000GATTTT ACTTCCAATT TTCTACTTAA ATTTGGTTAT CACGTTCGAA TTTTTCAGGT   
  
  
+ AAATACAAAC TGTTAGTGGC CATCCTTTTT TTTTGGGTCT AATCCATCCA GATGTGCTAA TACTTTCTGT   
  
  
+ TTTAGATGAG AAGTTTTAAT GCTTTTTTAG ATGTGTAATT TTTTTAAACA TAAATAGTCT TGTCGAATAA   
  
  
+ GATTTGCTAG TAATTTTTGT AAGAGAAATT ATGTGTATTT TTTATTATAT CAGAATATTA GATCGATCTC   
  
  
+ AATTTAATTA CTTATGAAAA AAAAAGTGGA TATCTTTAAT CAATTTGGCA AGCTCTCATG CATTAATTGT   
  
  
+ AGTCGTACGA ACTTATTTAC ATCACTCATC TGAATTTGCT ACCATTTACA CCTCAAATTT TATTTTAATG   
  
  
+ CCATAAAGAG AATAGAATAT ACTGTCTATT TGTAGTAGTT TTACAGATAA AAACAAATAA GCAAATGAAA   
  
  
+ ATAGATAGAG GATTTTTTTG CTATTATCCA CGTCGATCCA TTGAATTATT TTGTCGCTAT TTGGTTAAGT   
  
  
+ GAAATAAAAT ACTGAAGTAG AATGGAGGAT GTTGATAATG ACCTATGTCC CTCAAAGTTT AATCATGTAG   
  
  
+ TTTCTACGAA TTTCAAGCCA TTATAAGACA TACAGATTTT TATGATGTTG AAAATACTCT TACCTCCTTG   
  
  
+ TCACAATATT AACCTGAAGC CAATGAAATG AAACAGAACT AATTAAATTG AATACATAAT CAATCCATAT   
  
  
+ TAATTTGTTT TGACAACTAT TTACAATCAT CGATCTCTTT AATTAGGTCT ATAAGCTATA GAGTAATTAC   
  
  
+ TCAAAACTGT TTTGTAAGAC AACTTACTCC ATATTGGCTT ATTAGTATAT TGCCTCCATT CACATGAATA   
  
  
+ CTTCATGAGA GATTGCCTAA AACTCATATT GTCATCACCT TAAGTCAAAC ATATTTTGTT ATGAAATGAC   
  
  
+ ATCAAGAATT ACCCTCTGAC AAGAAAAATA TACCTGACTG ACATAATCGT ACCACATCTG TTAGTCCTCT   
  
  
+ TTCTACCACT TTGTAGAACA TAATGTGACA GTAATACATC CATCCCAAAA GAAAAGACGA AAAGAAGAGT   
  
  
+ GGAGAGTCAC GTATACGCGT TGAATTCAGG AAAATTAAAG GAACTCATGA AGACCCCATA TAGTGCTCCA   
  
  
+ ACCTGAACCT GCAGCCCACT ACTACATGCC TAAATTAAAG AGCAAAGTTA TGAGAGAAAC CAAAAAAGCT   
  
  
+ CTTTGAGAGA GTGGAGGAAG AATCATGGAA AGCTAGCTAG GCTTAGCAAG AAAGAGCAAC AGCTAGCTAG   
  
  
+ CAAAAGGCTT CTCTTTGAAG CCTTAGAAAT CTTTAGAGAT AAAAAGTTGG TGGCTTTTTA AGGGCTGTGA   
  
  
+ TATGTCCCCC ATGTAAAAAT GCAGCAATAA TGTTAATGTC ATGCTTTCTT TCATGGTCCC TAATGCGAAA   
  
  
+ TCAATCATGC ATGTCAACGT GACTCAGTGG CTCACCATAT ATGATCTGAT TTCCCCGCAC ACAGAGATAA   
  
  
+ AAAGCCCCCA ATTATAACCT CCCAAAAGCC CCCCCTTTTC CTCCTACCCA ACCCACACCT CCCCATTTCA   
  
  
+ TCAGGGCTCG CTTCTTAAGT TATCGATTAA AAAAAGAGTG AGTAAACGTA CCCGAATTGA AGAGGGGTGT   
  
  
+ TCGAAGCTTA TACTTGCTTG ATTAGGAATG AAAGTCCCCA TTTCATCCGC CCCCCAAACC GCCGCCACCA   
  
  
+ CCAGTAAATC GCTATCTTGC AACAATACTG CCTTGAATAC TGCCTTTCAA GCCCCCAATT CAATTTCAAC   
  
  
+ CCTTGTTAAC AACAACAAAC CCTCCTACGA GCCTACCTCG GTTCTAGACC TCCGGCGCAG CCCAAGCCCC   
  
  
+ GCCACCAACG CCGCCCTGCC ACTGCCCACT GACGCGGGCA TCAATAAACC CAAGCCTGCC TCTGCTGACC   
  
  
+ TGGACAATTT GGAGGGTGAG GGTTGGGATT CAATCCTATC AGAGCTGGGG CTTAATGATG ATTCTAATAA   
  
  
+ TCCTAACTCC AAACTTTGGT CATCCCAAAT TAGCTCATCA TGTGATTCCC ACCTCACACA GCTCCCTGAG   
  
  
+ TTCCCCTCCT CTCAGCCGTT GGATCACACA CGCACCCCCA ATAATTAACT TCTCCCCCTC TGATTACCCC   
  
  
+ ATCTCTGATT TATCCTACGG TCAGAATCTC AGCCCCAATT TTGGGCAGTT TGAGCTTCAT CTTCATCAGC   
  
  
+ CCAACGTCAA TAACAATGGG CTTGACTTCA TAGAGGACCT AATTAGAGCA GCTCAAGCCT ACGAATCCAA   
  
  
+ CGACTCCCAC CAGGTCCAGC TGGTATTGGC GCGGCTCAAT CAACGGCTCA GATCACCCGC CGGAAAACCC   
  
  
+ CTCCACCGCG CCGCCTTCTA CTTCAAGGAG GCCCTCCAAC ACCTCGCTGT CGGCGGAGGC CCGGCCCGGC   
  
  
+ CTGTCCGGCT GTCATCCTAC GAGGTGGTGC AGACCATCCG GGCCCACAAG AACTTTTCCG GCATATCACT   
  
  
+ GATATCCCTC TTCTCCACCT TCGCCGCCAA TCAGGCGATT CTGGAGGCGG TGGATGGGGC TGCGTTCATC   
  
  
+ CACATCATCG ATTTCGACAT CGGATTTGGC GGCCACTGGG CGTCGTTTAT GCGTGAGCTT GTCGACAAGG   
  
  
+ CCGACCCGAA TAAGATCAGC TCCTTGGTGT TCCGAATCAC GGCGATCGTA CCCGAAGAGT TCGGGATCGA   
  
  
+ AAGCAAGCTG GTGAGAGAGA ATCTTACGCA ATTCGCTCGT GATCTCAACA TCAATGTCCA TATGGAGTAT   
  
  
+ GTCCTTATTC AAAGCTTCGA GTTCTTATCC TTCAAATCGG TCAAATTCAT CGACGGTGAG AAATTGGCGG   
  
  
+ TCCATCTATC TCCGGCGATG TTTCACCGGC TTCAAGGCGG AATCTCGAAA TTCCTAGCCG ATCTCCGGGG   
  
  
+ AATTTCGCCA AGCACCGTCG TGGTGGTGGA CAGTGAGGTA GGAATCGACA CGGGAACTTC GTCCTTCAGC   
  
  
+ GTCAACTTCG TCGCCGGTAT CGAATTCTAC ACCGGAATGC TCGAATCGCT CGACGTCACC TCCGCCAGCG   
  
  
+ GTTTTGCAGG CGGCGGCGGC GGCGACGCTG TGAGAAGGAT CGAGACGTAC GTGCTCCGGC CGAGGATTAC   
  
  
+ GGCGGCGGTG GAGGCGGCAT CTGCGGCGGC GGTAGCGCGG CGGAGCGGTT GGAGAGAGTC GTTCGCGGTG   
  
  
+ GCTAGGATGA GGGCGGTGGG GTTCAGCCAG TTTGCTGATT TCCAGGCGGA GTGTTTGCTG AGGAAGGTTC   
  
  
+ AGGTTGGCGG GTTCCACGTG GCGAAACGTC ATGGGGAGAT GATGCTTTGC TGGCATGATA GGCCCCTTGT   
  
  
+ GTCCACGTCG GCTTGGAGGT GTTA  

- -Up\_Stream \_Len000CTAAAA TGAAGGTTAA AAGATGAATT TAAACCAATA GTGCAAGCTT AAAAAGTCCA   
  
  
- TTTATGTTTG ACAATCACCG GTAGGAAAAA AAAACCCAGA TTAGGTAGGT CTACACGATT ATGAAAGACA   
  
  
- AAATCTACTC TTCAAAATTA CGAAAAAATC TACACATTAA AAAAATTTGT ATTTATCAGA ACAGCTTATT   
  
  
- CTAAACGATC ATTAAAAACA TTCTCTTTAA TACACATAAA AAATAATATA GTCTTATAAT CTAGCTAGAG   
  
  
- TTAAATTAAT GAATACTTTT TTTTTCACCT ATAGAAATTA GTTAAACCGT TCGAGAGTAC GTAATTAACA   
  
  
- TCAGCATGCT TGAATAAATG TAGTGAGTAG ACTTAAACGA TGGTAAATGT GGAGTTTAAA ATAAAATTAC   
  
  
- GGTATTTCTC TTATCTTATA TGACAGATAA ACATCATCAA AATGTCTATT TTTGTTTATT CGTTTACTTT   
  
  
- TATCTATCTC CTAAAAAAAC GATAATAGGT GCAGCTAGGT AACTTAATAA AACAGCGATA AACCAATTCA   
  
  
- CTTTATTTTA TGACTTCATC TTACCTCCTA CAACTATTAC TGGATACAGG GAGTTTCAAA TTAGTACATC   
  
  
- AAAGATGCTT AAAGTTCGGT AATATTCTGT ATGTCTAAAA ATACTACAAC TTTTATGAGA ATGGAGGAAC   
  
  
- AGTGTTATAA TTGGACTTCG GTTACTTTAC TTTGTCTTGA TTAATTTAAC TTATGTATTA GTTAGGTATA   
  
  
- ATTAAACAAA ACTGTTGATA AATGTTAGTA GCTAGAGAAA TTAATCCAGA TATTCGATAT CTCATTAATG   
  
  
- AGTTTTGACA AAACATTCTG TTGAATGAGG TATAACCGAA TAATCATATA ACGGAGGTAA GTGTACTTAT   
  
  
- GAAGTACTCT CTAACGGATT TTGAGTATAA CAGTAGTGGA ATTCAGTTTG TATAAAACAA TACTTTACTG   
  
  
- TAGTTCTTAA TGGGAGACTG TTCTTTTTAT ATGGACTGAC TGTATTAGCA TGGTGTAGAC AATCAGGAGA   
  
  
- AAGATGGTGA AACATCTTGT ATTACACTGT CATTATGTAG GTAGGGTTTT CTTTTCTGCT TTTCTTCTCA   
  
  
- CCTCTCAGTG CATATGCGCA ACTTAAGTCC TTTTAATTTC CTTGAGTACT TCTGGGGTAT ATCACGAGGT   
  
  
- TGGACTTGGA CGTCGGGTGA TGATGTACGG ATTTAATTTC TCGTTTCAAT ACTCTCTTTG GTTTTTTCGA   
  
  
- GAAACTCTCT CACCTCCTTC TTAGTACCTT TCGATCGATC CGAATCGTTC TTTCTCGTTG TCGATCGATC   
  
  
- GTTTTCCGAA GAGAAACTTC GGAATCTTTA GAAATCTCTA TTTTTCAACC ACCGAAAAAT TCCCGACACT   
  
  
- ATACAGGGGG TACATTTTTA CGTCGTTATT ACAATTACAG TACGAAAGAA AGTACCAGGG ATTACGCTTT   
  
  
- AGTTAGTACG TACAGTTGCA CTGAGTCACC GAGTGGTATA TACTAGACTA AAGGGGCGTG TGTCTCTATT   
  
  
- TTTCGGGGGT TAATATTGGA GGGTTTTCGG GGGGGAAAAG GAGGATGGGT TGGGTGTGGA GGGGTAAAGT   
  
  
- AGTCCCGAGC GAAGAATTCA ATAGCTAATT TTTTTCTCAC TCATTTGCAT GGGCTTAACT TCTCCCCACA   
  
  
- AGCTTCGAAT ATGAACGAAC TAATCCTTAC TTTCAGGGGT AAAGTAGGCG GGGGGTTTGG CGGCGGTGGT   
  
  
- GGTCATTTAG CGATAGAACG TTGTTATGAC GGAACTTATG ACGGAAAGTT CGGGGGTTAA GTTAAAGTTG   
  
  
- GGAACAATTG TTGTTGTTTG GGAGGATGCT CGGATGGAGC CAAGATCTGG AGGCCGCGTC GGGTTCGGGG   
  
  
- CGGTGGTTGC GGCGGGACGG TGACGGGTGA CTGCGCCCGT AGTTATTTGG GTTCGGACGG AGACGACTGG   
  
  
- ACCTGTTAAA CCTCCCACTC CCAACCCTAA GTTAGGATAG TCTCGACCCC GAATTACTAC TAAGATTATT   
  
  
- AGGATTGAGG TTTGAAACCA GTAGGGTTTA ATCGAGTAGT ACACTAAGGG TGGAGTGTGT CGAGGGACTC   
  
  
- AAGGGGAGGA GAGTCGGCAA CCTAGTGTGT GCGTGGGGGT TATTAATTGA AGAGGGGGAG ACTAATGGGG   
  
  
- TAGAGACTAA ATAGGATGCC AGTCTTAGAG TCGGGGTTAA AACCCGTCAA ACTCGAAGTA GAAGTAGTCG   
  
  
- GGTTGCAGTT ATTGTTACCC GAACTGAAGT ATCTCCTGGA TTAATCTCGT CGAGTTCGGA TGCTTAGGTT   
  
  
- GCTGAGGGTG GTCCAGGTCG ACCATAACCG CGCCGAGTTA GTTGCCGAGT CTAGTGGGCG GCCTTTTGGG   
  
  
- GAGGTGGCGC GGCGGAAGAT GAAGTTCCTC CGGGAGGTTG TGGAGCGACA GCCGCCTCCG GGCCGGGCCG   
  
  
- GACAGGCCGA CAGTAGGATG CTCCACCACG TCTGGTAGGC CCGGGTGTTC TTGAAAAGGC CGTATAGTGA   
  
  
- CTATAGGGAG AAGAGGTGGA AGCGGCGGTT AGTCCGCTAA GACCTCCGCC ACCTACCCCG ACGCAAGTAG   
  
  
- GTGTAGTAGC TAAAGCTGTA GCCTAAACCG CCGGTGACCC GCAGCAAATA CGCACTCGAA CAGCTGTTCC   
  
  
- GGCTGGGCTT ATTCTAGTCG AGGAACCACA AGGCTTAGTG CCGCTAGCAT GGGCTTCTCA AGCCCTAGCT   
  
  
- TTCGTTCGAC CACTCTCTCT TAGAATGCGT TAAGCGAGCA CTAGAGTTGT AGTTACAGGT ATACCTCATA   
  
  
- CAGGAATAAG TTTCGAAGCT CAAGAATAGG AAGTTTAGCC AGTTTAAGTA GCTGCCACTC TTTAACCGCC   
  
  
- AGGTAGATAG AGGCCGCTAC AAAGTGGCCG AAGTTCCGCC TTAGAGCTTT AAGGATCGGC TAGAGGCCCC   
  
  
- TTAAAGCGGT TCGTGGCAGC ACCACCACCT GTCACTCCAT CCTTAGCTGT GCCCTTGAAG CAGGAAGTCG   
  
  
- CAGTTGAAGC AGCGGCCATA GCTTAAGATG TGGCCTTACG AGCTTAGCGA GCTGCAGTGG AGGCGGTCGC   
  
  
- CAAAACGTCC GCCGCCGCCG CCGCTGCGAC ACTCTTCCTA GCTCTGCATG CACGAGGCCG GCTCCTAATG   
  
  
- CCGCCGCCAC CTCCGCCGTA GACGCCGCCG CCATCGCGCC GCCTCGCCAA CCTCTCTCAG CAAGCGCCAC   
  
  
- CGATCCTACT CCCGCCACCC CAAGTCGGTC AAACGACTAA AGGTCCGCCT CACAAACGAC TCCTTCCAAG   
  
  
- TCCAACCGCC CAAGGTGCAC CGCTTTGCAG TACCCCTCTA CTACGAAACG ACCGTACTAT CCGGGGAACA   
  
  
- CAGGTGCAGC CGAACCTCCA CAAT

+     O2-site

| Site Name | Organism | Position | Strand | Matrix score. | sequence | function |
| --- | --- | --- | --- | --- | --- | --- |
| O2-site | Zea mays | 1607 | - | 9 | GATGACATGG | cis-acting regulatory element involved in zein metabolism regulation |
| O2-site | Zea mays | 1722 | - | 9 | GATGACATGG | cis-acting regulatory element involved in zein metabolism regulation |
| O2-site | Zea mays | 2594 | - | 10 | GATGATGTGG | cis-acting regulatory element involved in zein metabolism regulation |

>HU06G00283.1   
+ -Up\_Stream \_Len000GATTTT ACTTCCAATT TTCTACTTAA ATTTGGTTAT CACGTTCGAA TTTTTCAGGT   
  
  
+ AAATACAAAC TGTTAGTGGC CATCCTTTTT TTTTGGGTCT AATCCATCCA GATGTGCTAA TACTTTCTGT   
  
  
+ TTTAGATGAG AAGTTTTAAT GCTTTTTTAG ATGTGTAATT TTTTTAAACA TAAATAGTCT TGTCGAATAA   
  
  
+ GATTTGCTAG TAATTTTTGT AAGAGAAATT ATGTGTATTT TTTATTATAT CAGAATATTA GATCGATCTC   
  
  
+ AATTTAATTA CTTATGAAAA AAAAAGTGGA TATCTTTAAT CAATTTGGCA AGCTCTCATG CATTAATTGT   
  
  
+ AGTCGTACGA ACTTATTTAC ATCACTCATC TGAATTTGCT ACCATTTACA CCTCAAATTT TATTTTAATG   
  
  
+ CCATAAAGAG AATAGAATAT ACTGTCTATT TGTAGTAGTT TTACAGATAA AAACAAATAA GCAAATGAAA   
  
  
+ ATAGATAGAG GATTTTTTTG CTATTATCCA CGTCGATCCA TTGAATTATT TTGTCGCTAT TTGGTTAAGT   
  
  
+ GAAATAAAAT ACTGAAGTAG AATGGAGGAT GTTGATAATG ACCTATGTCC CTCAAAGTTT AATCATGTAG   
  
  
+ TTTCTACGAA TTTCAAGCCA TTATAAGACA TACAGATTTT TATGATGTTG AAAATACTCT TACCTCCTTG   
  
  
+ TCACAATATT AACCTGAAGC CAATGAAATG AAACAGAACT AATTAAATTG AATACATAAT CAATCCATAT   
  
  
+ TAATTTGTTT TGACAACTAT TTACAATCAT CGATCTCTTT AATTAGGTCT ATAAGCTATA GAGTAATTAC   
  
  
+ TCAAAACTGT TTTGTAAGAC AACTTACTCC ATATTGGCTT ATTAGTATAT TGCCTCCATT CACATGAATA   
  
  
+ CTTCATGAGA GATTGCCTAA AACTCATATT GTCATCACCT TAAGTCAAAC ATATTTTGTT ATGAAATGAC   
  
  
+ ATCAAGAATT ACCCTCTGAC AAGAAAAATA TACCTGACTG ACATAATCGT ACCACATCTG TTAGTCCTCT   
  
  
+ TTCTACCACT TTGTAGAACA TAATGTGACA GTAATACATC CATCCCAAAA GAAAAGACGA AAAGAAGAGT   
  
  
+ GGAGAGTCAC GTATACGCGT TGAATTCAGG AAAATTAAAG GAACTCATGA AGACCCCATA TAGTGCTCCA   
  
  
+ ACCTGAACCT GCAGCCCACT ACTACATGCC TAAATTAAAG AGCAAAGTTA TGAGAGAAAC CAAAAAAGCT   
  
  
+ CTTTGAGAGA GTGGAGGAAG AATCATGGAA AGCTAGCTAG GCTTAGCAAG AAAGAGCAAC AGCTAGCTAG   
  
  
+ CAAAAGGCTT CTCTTTGAAG CCTTAGAAAT CTTTAGAGAT AAAAAGTTGG TGGCTTTTTA AGGGCTGTGA   
  
  
+ TATGTCCCCC ATGTAAAAAT GCAGCAATAA TGTTAATGTC ATGCTTTCTT TCATGGTCCC TAATGCGAAA   
  
  
+ TCAATCATGC ATGTCAACGT GACTCAGTGG CTCACCATAT ATGATCTGAT TTCCCCGCAC ACAGAGATAA   
  
  
+ AAAGCCCCCA ATTATAACCT CCCAAAAGCC CCCCCTTTTC CTCCTACCCA ACCCACACCT CCCCATTTCA   
  
  
+ TCAGGGCTCG CTTCTTAAGT TATCGATTAA AAAAAGAGTG AGTAAACGTA CCCGAATTGA AGAGGGGTGT   
  
  
+ TCGAAGCTTA TACTTGCTTG ATTAGGAATG AAAGTCCCCA TTTCATCCGC CCCCCAAACC GCCGCCACCA   
  
  
+ CCAGTAAATC GCTATCTTGC AACAATACTG CCTTGAATAC TGCCTTTCAA GCCCCCAATT CAATTTCAAC   
  
  
+ CCTTGTTAAC AACAACAAAC CCTCCTACGA GCCTACCTCG GTTCTAGACC TCCGGCGCAG CCCAAGCCCC   
  
  
+ GCCACCAACG CCGCCCTGCC ACTGCCCACT GACGCGGGCA TCAATAAACC CAAGCCTGCC TCTGCTGACC   
  
  
+ TGGACAATTT GGAGGGTGAG GGTTGGGATT CAATCCTATC AGAGCTGGGG CTTAATGATG ATTCTAATAA   
  
  
+ TCCTAACTCC AAACTTTGGT CATCCCAAAT TAGCTCATCA TGTGATTCCC ACCTCACACA GCTCCCTGAG   
  
  
+ TTCCCCTCCT CTCAGCCGTT GGATCACACA CGCACCCCCA ATAATTAACT TCTCCCCCTC TGATTACCCC   
  
  
+ ATCTCTGATT TATCCTACGG TCAGAATCTC AGCCCCAATT TTGGGCAGTT TGAGCTTCAT CTTCATCAGC   
  
  
+ CCAACGTCAA TAACAATGGG CTTGACTTCA TAGAGGACCT AATTAGAGCA GCTCAAGCCT ACGAATCCAA   
  
  
+ CGACTCCCAC CAGGTCCAGC TGGTATTGGC GCGGCTCAAT CAACGGCTCA GATCACCCGC CGGAAAACCC   
  
  
+ CTCCACCGCG CCGCCTTCTA CTTCAAGGAG GCCCTCCAAC ACCTCGCTGT CGGCGGAGGC CCGGCCCGGC   
  
  
+ CTGTCCGGCT GTCATCCTAC GAGGTGGTGC AGACCATCCG GGCCCACAAG AACTTTTCCG GCATATCACT   
  
  
+ GATATCCCTC TTCTCCACCT TCGCCGCCAA TCAGGCGATT CTGGAGGCGG TGGATGGGGC TGCGTTCATC   
  
  
+ CACATCATCG ATTTCGACAT CGGATTTGGC GGCCACTGGG CGTCGTTTAT GCGTGAGCTT GTCGACAAGG   
  
  
+ CCGACCCGAA TAAGATCAGC TCCTTGGTGT TCCGAATCAC GGCGATCGTA CCCGAAGAGT TCGGGATCGA   
  
  
+ AAGCAAGCTG GTGAGAGAGA ATCTTACGCA ATTCGCTCGT GATCTCAACA TCAATGTCCA TATGGAGTAT   
  
  
+ GTCCTTATTC AAAGCTTCGA GTTCTTATCC TTCAAATCGG TCAAATTCAT CGACGGTGAG AAATTGGCGG   
  
  
+ TCCATCTATC TCCGGCGATG TTTCACCGGC TTCAAGGCGG AATCTCGAAA TTCCTAGCCG ATCTCCGGGG   
  
  
+ AATTTCGCCA AGCACCGTCG TGGTGGTGGA CAGTGAGGTA GGAATCGACA CGGGAACTTC GTCCTTCAGC   
  
  
+ GTCAACTTCG TCGCCGGTAT CGAATTCTAC ACCGGAATGC TCGAATCGCT CGACGTCACC TCCGCCAGCG   
  
  
+ GTTTTGCAGG CGGCGGCGGC GGCGACGCTG TGAGAAGGAT CGAGACGTAC GTGCTCCGGC CGAGGATTAC   
  
  
+ GGCGGCGGTG GAGGCGGCAT CTGCGGCGGC GGTAGCGCGG CGGAGCGGTT GGAGAGAGTC GTTCGCGGTG   
  
  
+ GCTAGGATGA GGGCGGTGGG GTTCAGCCAG TTTGCTGATT TCCAGGCGGA GTGTTTGCTG AGGAAGGTTC   
  
  
+ AGGTTGGCGG GTTCCACGTG GCGAAACGTC ATGGGGAGAT GATGCTTTGC TGGCATGATA GGCCCCTTGT   
  
  
+ GTCCACGTCG GCTTGGAGGT GTTA  

- -Up\_Stream \_Len000CTAAAA TGAAGGTTAA AAGATGAATT TAAACCAATA GTGCAAGCTT AAAAAGTCCA   
  
  
- TTTATGTTTG ACAATCACCG GTAGGAAAAA AAAACCCAGA TTAGGTAGGT CTACACGATT ATGAAAGACA   
  
  
- AAATCTACTC TTCAAAATTA CGAAAAAATC TACACATTAA AAAAATTTGT ATTTATCAGA ACAGCTTATT   
  
  
- CTAAACGATC ATTAAAAACA TTCTCTTTAA TACACATAAA AAATAATATA GTCTTATAAT CTAGCTAGAG   
  
  
- TTAAATTAAT GAATACTTTT TTTTTCACCT ATAGAAATTA GTTAAACCGT TCGAGAGTAC GTAATTAACA   
  
  
- TCAGCATGCT TGAATAAATG TAGTGAGTAG ACTTAAACGA TGGTAAATGT GGAGTTTAAA ATAAAATTAC   
  
  
- GGTATTTCTC TTATCTTATA TGACAGATAA ACATCATCAA AATGTCTATT TTTGTTTATT CGTTTACTTT   
  
  
- TATCTATCTC CTAAAAAAAC GATAATAGGT GCAGCTAGGT AACTTAATAA AACAGCGATA AACCAATTCA   
  
  
- CTTTATTTTA TGACTTCATC TTACCTCCTA CAACTATTAC TGGATACAGG GAGTTTCAAA TTAGTACATC   
  
  
- AAAGATGCTT AAAGTTCGGT AATATTCTGT ATGTCTAAAA ATACTACAAC TTTTATGAGA ATGGAGGAAC   
  
  
- AGTGTTATAA TTGGACTTCG GTTACTTTAC TTTGTCTTGA TTAATTTAAC TTATGTATTA GTTAGGTATA   
  
  
- ATTAAACAAA ACTGTTGATA AATGTTAGTA GCTAGAGAAA TTAATCCAGA TATTCGATAT CTCATTAATG   
  
  
- AGTTTTGACA AAACATTCTG TTGAATGAGG TATAACCGAA TAATCATATA ACGGAGGTAA GTGTACTTAT   
  
  
- GAAGTACTCT CTAACGGATT TTGAGTATAA CAGTAGTGGA ATTCAGTTTG TATAAAACAA TACTTTACTG   
  
  
- TAGTTCTTAA TGGGAGACTG TTCTTTTTAT ATGGACTGAC TGTATTAGCA TGGTGTAGAC AATCAGGAGA   
  
  
- AAGATGGTGA AACATCTTGT ATTACACTGT CATTATGTAG GTAGGGTTTT CTTTTCTGCT TTTCTTCTCA   
  
  
- CCTCTCAGTG CATATGCGCA ACTTAAGTCC TTTTAATTTC CTTGAGTACT TCTGGGGTAT ATCACGAGGT   
  
  
- TGGACTTGGA CGTCGGGTGA TGATGTACGG ATTTAATTTC TCGTTTCAAT ACTCTCTTTG GTTTTTTCGA   
  
  
- GAAACTCTCT CACCTCCTTC TTAGTACCTT TCGATCGATC CGAATCGTTC TTTCTCGTTG TCGATCGATC   
  
  
- GTTTTCCGAA GAGAAACTTC GGAATCTTTA GAAATCTCTA TTTTTCAACC ACCGAAAAAT TCCCGACACT   
  
  
- ATACAGGGGG TACATTTTTA CGTCGTTATT ACAATTACAG TACGAAAGAA AGTACCAGGG ATTACGCTTT   
  
  
- AGTTAGTACG TACAGTTGCA CTGAGTCACC GAGTGGTATA TACTAGACTA AAGGGGCGTG TGTCTCTATT   
  
  
- TTTCGGGGGT TAATATTGGA GGGTTTTCGG GGGGGAAAAG GAGGATGGGT TGGGTGTGGA GGGGTAAAGT   
  
  
- AGTCCCGAGC GAAGAATTCA ATAGCTAATT TTTTTCTCAC TCATTTGCAT GGGCTTAACT TCTCCCCACA   
  
  
- AGCTTCGAAT ATGAACGAAC TAATCCTTAC TTTCAGGGGT AAAGTAGGCG GGGGGTTTGG CGGCGGTGGT   
  
  
- GGTCATTTAG CGATAGAACG TTGTTATGAC GGAACTTATG ACGGAAAGTT CGGGGGTTAA GTTAAAGTTG   
  
  
- GGAACAATTG TTGTTGTTTG GGAGGATGCT CGGATGGAGC CAAGATCTGG AGGCCGCGTC GGGTTCGGGG   
  
  
- CGGTGGTTGC GGCGGGACGG TGACGGGTGA CTGCGCCCGT AGTTATTTGG GTTCGGACGG AGACGACTGG   
  
  
- ACCTGTTAAA CCTCCCACTC CCAACCCTAA GTTAGGATAG TCTCGACCCC GAATTACTAC TAAGATTATT   
  
  
- AGGATTGAGG TTTGAAACCA GTAGGGTTTA ATCGAGTAGT ACACTAAGGG TGGAGTGTGT CGAGGGACTC   
  
  
- AAGGGGAGGA GAGTCGGCAA CCTAGTGTGT GCGTGGGGGT TATTAATTGA AGAGGGGGAG ACTAATGGGG   
  
  
- TAGAGACTAA ATAGGATGCC AGTCTTAGAG TCGGGGTTAA AACCCGTCAA ACTCGAAGTA GAAGTAGTCG   
  
  
- GGTTGCAGTT ATTGTTACCC GAACTGAAGT ATCTCCTGGA TTAATCTCGT CGAGTTCGGA TGCTTAGGTT   
  
  
- GCTGAGGGTG GTCCAGGTCG ACCATAACCG CGCCGAGTTA GTTGCCGAGT CTAGTGGGCG GCCTTTTGGG   
  
  
- GAGGTGGCGC GGCGGAAGAT GAAGTTCCTC CGGGAGGTTG TGGAGCGACA GCCGCCTCCG GGCCGGGCCG   
  
  
- GACAGGCCGA CAGTAGGATG CTCCACCACG TCTGGTAGGC CCGGGTGTTC TTGAAAAGGC CGTATAGTGA   
  
  
- CTATAGGGAG AAGAGGTGGA AGCGGCGGTT AGTCCGCTAA GACCTCCGCC ACCTACCCCG ACGCAAGTAG   
  
  
- GTGTAGTAGC TAAAGCTGTA GCCTAAACCG CCGGTGACCC GCAGCAAATA CGCACTCGAA CAGCTGTTCC   
  
  
- GGCTGGGCTT ATTCTAGTCG AGGAACCACA AGGCTTAGTG CCGCTAGCAT GGGCTTCTCA AGCCCTAGCT   
  
  
- TTCGTTCGAC CACTCTCTCT TAGAATGCGT TAAGCGAGCA CTAGAGTTGT AGTTACAGGT ATACCTCATA   
  
  
- CAGGAATAAG TTTCGAAGCT CAAGAATAGG AAGTTTAGCC AGTTTAAGTA GCTGCCACTC TTTAACCGCC   
  
  
- AGGTAGATAG AGGCCGCTAC AAAGTGGCCG AAGTTCCGCC TTAGAGCTTT AAGGATCGGC TAGAGGCCCC   
  
  
- TTAAAGCGGT TCGTGGCAGC ACCACCACCT GTCACTCCAT CCTTAGCTGT GCCCTTGAAG CAGGAAGTCG   
  
  
- CAGTTGAAGC AGCGGCCATA GCTTAAGATG TGGCCTTACG AGCTTAGCGA GCTGCAGTGG AGGCGGTCGC   
  
  
- CAAAACGTCC GCCGCCGCCG CCGCTGCGAC ACTCTTCCTA GCTCTGCATG CACGAGGCCG GCTCCTAATG   
  
  
- CCGCCGCCAC CTCCGCCGTA GACGCCGCCG CCATCGCGCC GCCTCGCCAA CCTCTCTCAG CAAGCGCCAC   
  
  
- CGATCCTACT CCCGCCACCC CAAGTCGGTC AAACGACTAA AGGTCCGCCT CACAAACGAC TCCTTCCAAG   
  
  
- TCCAACCGCC CAAGGTGCAC CGCTTTGCAG TACCCCTCTA CTACGAAACG ACCGTACTAT CCGGGGAACA   
  
  
- CAGGTGCAGC CGAACCTCCA CAAT

+     P-box

| Site Name | Organism | Position | Strand | Matrix score. | sequence | function |
| --- | --- | --- | --- | --- | --- | --- |
| P-box | Oryza sativa | 1335 | - | 7 | CCTTTTG | gibberellin-responsive element |

>HU06G00283.1   
+ -Up\_Stream \_Len000GATTTT ACTTCCAATT TTCTACTTAA ATTTGGTTAT CACGTTCGAA TTTTTCAGGT   
  
  
+ AAATACAAAC TGTTAGTGGC CATCCTTTTT TTTTGGGTCT AATCCATCCA GATGTGCTAA TACTTTCTGT   
  
  
+ TTTAGATGAG AAGTTTTAAT GCTTTTTTAG ATGTGTAATT TTTTTAAACA TAAATAGTCT TGTCGAATAA   
  
  
+ GATTTGCTAG TAATTTTTGT AAGAGAAATT ATGTGTATTT TTTATTATAT CAGAATATTA GATCGATCTC   
  
  
+ AATTTAATTA CTTATGAAAA AAAAAGTGGA TATCTTTAAT CAATTTGGCA AGCTCTCATG CATTAATTGT   
  
  
+ AGTCGTACGA ACTTATTTAC ATCACTCATC TGAATTTGCT ACCATTTACA CCTCAAATTT TATTTTAATG   
  
  
+ CCATAAAGAG AATAGAATAT ACTGTCTATT TGTAGTAGTT TTACAGATAA AAACAAATAA GCAAATGAAA   
  
  
+ ATAGATAGAG GATTTTTTTG CTATTATCCA CGTCGATCCA TTGAATTATT TTGTCGCTAT TTGGTTAAGT   
  
  
+ GAAATAAAAT ACTGAAGTAG AATGGAGGAT GTTGATAATG ACCTATGTCC CTCAAAGTTT AATCATGTAG   
  
  
+ TTTCTACGAA TTTCAAGCCA TTATAAGACA TACAGATTTT TATGATGTTG AAAATACTCT TACCTCCTTG   
  
  
+ TCACAATATT AACCTGAAGC CAATGAAATG AAACAGAACT AATTAAATTG AATACATAAT CAATCCATAT   
  
  
+ TAATTTGTTT TGACAACTAT TTACAATCAT CGATCTCTTT AATTAGGTCT ATAAGCTATA GAGTAATTAC   
  
  
+ TCAAAACTGT TTTGTAAGAC AACTTACTCC ATATTGGCTT ATTAGTATAT TGCCTCCATT CACATGAATA   
  
  
+ CTTCATGAGA GATTGCCTAA AACTCATATT GTCATCACCT TAAGTCAAAC ATATTTTGTT ATGAAATGAC   
  
  
+ ATCAAGAATT ACCCTCTGAC AAGAAAAATA TACCTGACTG ACATAATCGT ACCACATCTG TTAGTCCTCT   
  
  
+ TTCTACCACT TTGTAGAACA TAATGTGACA GTAATACATC CATCCCAAAA GAAAAGACGA AAAGAAGAGT   
  
  
+ GGAGAGTCAC GTATACGCGT TGAATTCAGG AAAATTAAAG GAACTCATGA AGACCCCATA TAGTGCTCCA   
  
  
+ ACCTGAACCT GCAGCCCACT ACTACATGCC TAAATTAAAG AGCAAAGTTA TGAGAGAAAC CAAAAAAGCT   
  
  
+ CTTTGAGAGA GTGGAGGAAG AATCATGGAA AGCTAGCTAG GCTTAGCAAG AAAGAGCAAC AGCTAGCTAG   
  
  
+ CAAAAGGCTT CTCTTTGAAG CCTTAGAAAT CTTTAGAGAT AAAAAGTTGG TGGCTTTTTA AGGGCTGTGA   
  
  
+ TATGTCCCCC ATGTAAAAAT GCAGCAATAA TGTTAATGTC ATGCTTTCTT TCATGGTCCC TAATGCGAAA   
  
  
+ TCAATCATGC ATGTCAACGT GACTCAGTGG CTCACCATAT ATGATCTGAT TTCCCCGCAC ACAGAGATAA   
  
  
+ AAAGCCCCCA ATTATAACCT CCCAAAAGCC CCCCCTTTTC CTCCTACCCA ACCCACACCT CCCCATTTCA   
  
  
+ TCAGGGCTCG CTTCTTAAGT TATCGATTAA AAAAAGAGTG AGTAAACGTA CCCGAATTGA AGAGGGGTGT   
  
  
+ TCGAAGCTTA TACTTGCTTG ATTAGGAATG AAAGTCCCCA TTTCATCCGC CCCCCAAACC GCCGCCACCA   
  
  
+ CCAGTAAATC GCTATCTTGC AACAATACTG CCTTGAATAC TGCCTTTCAA GCCCCCAATT CAATTTCAAC   
  
  
+ CCTTGTTAAC AACAACAAAC CCTCCTACGA GCCTACCTCG GTTCTAGACC TCCGGCGCAG CCCAAGCCCC   
  
  
+ GCCACCAACG CCGCCCTGCC ACTGCCCACT GACGCGGGCA TCAATAAACC CAAGCCTGCC TCTGCTGACC   
  
  
+ TGGACAATTT GGAGGGTGAG GGTTGGGATT CAATCCTATC AGAGCTGGGG CTTAATGATG ATTCTAATAA   
  
  
+ TCCTAACTCC AAACTTTGGT CATCCCAAAT TAGCTCATCA TGTGATTCCC ACCTCACACA GCTCCCTGAG   
  
  
+ TTCCCCTCCT CTCAGCCGTT GGATCACACA CGCACCCCCA ATAATTAACT TCTCCCCCTC TGATTACCCC   
  
  
+ ATCTCTGATT TATCCTACGG TCAGAATCTC AGCCCCAATT TTGGGCAGTT TGAGCTTCAT CTTCATCAGC   
  
  
+ CCAACGTCAA TAACAATGGG CTTGACTTCA TAGAGGACCT AATTAGAGCA GCTCAAGCCT ACGAATCCAA   
  
  
+ CGACTCCCAC CAGGTCCAGC TGGTATTGGC GCGGCTCAAT CAACGGCTCA GATCACCCGC CGGAAAACCC   
  
  
+ CTCCACCGCG CCGCCTTCTA CTTCAAGGAG GCCCTCCAAC ACCTCGCTGT CGGCGGAGGC CCGGCCCGGC   
  
  
+ CTGTCCGGCT GTCATCCTAC GAGGTGGTGC AGACCATCCG GGCCCACAAG AACTTTTCCG GCATATCACT   
  
  
+ GATATCCCTC TTCTCCACCT TCGCCGCCAA TCAGGCGATT CTGGAGGCGG TGGATGGGGC TGCGTTCATC   
  
  
+ CACATCATCG ATTTCGACAT CGGATTTGGC GGCCACTGGG CGTCGTTTAT GCGTGAGCTT GTCGACAAGG   
  
  
+ CCGACCCGAA TAAGATCAGC TCCTTGGTGT TCCGAATCAC GGCGATCGTA CCCGAAGAGT TCGGGATCGA   
  
  
+ AAGCAAGCTG GTGAGAGAGA ATCTTACGCA ATTCGCTCGT GATCTCAACA TCAATGTCCA TATGGAGTAT   
  
  
+ GTCCTTATTC AAAGCTTCGA GTTCTTATCC TTCAAATCGG TCAAATTCAT CGACGGTGAG AAATTGGCGG   
  
  
+ TCCATCTATC TCCGGCGATG TTTCACCGGC TTCAAGGCGG AATCTCGAAA TTCCTAGCCG ATCTCCGGGG   
  
  
+ AATTTCGCCA AGCACCGTCG TGGTGGTGGA CAGTGAGGTA GGAATCGACA CGGGAACTTC GTCCTTCAGC   
  
  
+ GTCAACTTCG TCGCCGGTAT CGAATTCTAC ACCGGAATGC TCGAATCGCT CGACGTCACC TCCGCCAGCG   
  
  
+ GTTTTGCAGG CGGCGGCGGC GGCGACGCTG TGAGAAGGAT CGAGACGTAC GTGCTCCGGC CGAGGATTAC   
  
  
+ GGCGGCGGTG GAGGCGGCAT CTGCGGCGGC GGTAGCGCGG CGGAGCGGTT GGAGAGAGTC GTTCGCGGTG   
  
  
+ GCTAGGATGA GGGCGGTGGG GTTCAGCCAG TTTGCTGATT TCCAGGCGGA GTGTTTGCTG AGGAAGGTTC   
  
  
+ AGGTTGGCGG GTTCCACGTG GCGAAACGTC ATGGGGAGAT GATGCTTTGC TGGCATGATA GGCCCCTTGT   
  
  
+ GTCCACGTCG GCTTGGAGGT GTTA  

- -Up\_Stream \_Len000CTAAAA TGAAGGTTAA AAGATGAATT TAAACCAATA GTGCAAGCTT AAAAAGTCCA   
  
  
- TTTATGTTTG ACAATCACCG GTAGGAAAAA AAAACCCAGA TTAGGTAGGT CTACACGATT ATGAAAGACA   
  
  
- AAATCTACTC TTCAAAATTA CGAAAAAATC TACACATTAA AAAAATTTGT ATTTATCAGA ACAGCTTATT   
  
  
- CTAAACGATC ATTAAAAACA TTCTCTTTAA TACACATAAA AAATAATATA GTCTTATAAT CTAGCTAGAG   
  
  
- TTAAATTAAT GAATACTTTT TTTTTCACCT ATAGAAATTA GTTAAACCGT TCGAGAGTAC GTAATTAACA   
  
  
- TCAGCATGCT TGAATAAATG TAGTGAGTAG ACTTAAACGA TGGTAAATGT GGAGTTTAAA ATAAAATTAC   
  
  
- GGTATTTCTC TTATCTTATA TGACAGATAA ACATCATCAA AATGTCTATT TTTGTTTATT CGTTTACTTT   
  
  
- TATCTATCTC CTAAAAAAAC GATAATAGGT GCAGCTAGGT AACTTAATAA AACAGCGATA AACCAATTCA   
  
  
- CTTTATTTTA TGACTTCATC TTACCTCCTA CAACTATTAC TGGATACAGG GAGTTTCAAA TTAGTACATC   
  
  
- AAAGATGCTT AAAGTTCGGT AATATTCTGT ATGTCTAAAA ATACTACAAC TTTTATGAGA ATGGAGGAAC   
  
  
- AGTGTTATAA TTGGACTTCG GTTACTTTAC TTTGTCTTGA TTAATTTAAC TTATGTATTA GTTAGGTATA   
  
  
- ATTAAACAAA ACTGTTGATA AATGTTAGTA GCTAGAGAAA TTAATCCAGA TATTCGATAT CTCATTAATG   
  
  
- AGTTTTGACA AAACATTCTG TTGAATGAGG TATAACCGAA TAATCATATA ACGGAGGTAA GTGTACTTAT   
  
  
- GAAGTACTCT CTAACGGATT TTGAGTATAA CAGTAGTGGA ATTCAGTTTG TATAAAACAA TACTTTACTG   
  
  
- TAGTTCTTAA TGGGAGACTG TTCTTTTTAT ATGGACTGAC TGTATTAGCA TGGTGTAGAC AATCAGGAGA   
  
  
- AAGATGGTGA AACATCTTGT ATTACACTGT CATTATGTAG GTAGGGTTTT CTTTTCTGCT TTTCTTCTCA   
  
  
- CCTCTCAGTG CATATGCGCA ACTTAAGTCC TTTTAATTTC CTTGAGTACT TCTGGGGTAT ATCACGAGGT   
  
  
- TGGACTTGGA CGTCGGGTGA TGATGTACGG ATTTAATTTC TCGTTTCAAT ACTCTCTTTG GTTTTTTCGA   
  
  
- GAAACTCTCT CACCTCCTTC TTAGTACCTT TCGATCGATC CGAATCGTTC TTTCTCGTTG TCGATCGATC   
  
  
- GTTTTCCGAA GAGAAACTTC GGAATCTTTA GAAATCTCTA TTTTTCAACC ACCGAAAAAT TCCCGACACT   
  
  
- ATACAGGGGG TACATTTTTA CGTCGTTATT ACAATTACAG TACGAAAGAA AGTACCAGGG ATTACGCTTT   
  
  
- AGTTAGTACG TACAGTTGCA CTGAGTCACC GAGTGGTATA TACTAGACTA AAGGGGCGTG TGTCTCTATT   
  
  
- TTTCGGGGGT TAATATTGGA GGGTTTTCGG GGGGGAAAAG GAGGATGGGT TGGGTGTGGA GGGGTAAAGT   
  
  
- AGTCCCGAGC GAAGAATTCA ATAGCTAATT TTTTTCTCAC TCATTTGCAT GGGCTTAACT TCTCCCCACA   
  
  
- AGCTTCGAAT ATGAACGAAC TAATCCTTAC TTTCAGGGGT AAAGTAGGCG GGGGGTTTGG CGGCGGTGGT   
  
  
- GGTCATTTAG CGATAGAACG TTGTTATGAC GGAACTTATG ACGGAAAGTT CGGGGGTTAA GTTAAAGTTG   
  
  
- GGAACAATTG TTGTTGTTTG GGAGGATGCT CGGATGGAGC CAAGATCTGG AGGCCGCGTC GGGTTCGGGG   
  
  
- CGGTGGTTGC GGCGGGACGG TGACGGGTGA CTGCGCCCGT AGTTATTTGG GTTCGGACGG AGACGACTGG   
  
  
- ACCTGTTAAA CCTCCCACTC CCAACCCTAA GTTAGGATAG TCTCGACCCC GAATTACTAC TAAGATTATT   
  
  
- AGGATTGAGG TTTGAAACCA GTAGGGTTTA ATCGAGTAGT ACACTAAGGG TGGAGTGTGT CGAGGGACTC   
  
  
- AAGGGGAGGA GAGTCGGCAA CCTAGTGTGT GCGTGGGGGT TATTAATTGA AGAGGGGGAG ACTAATGGGG   
  
  
- TAGAGACTAA ATAGGATGCC AGTCTTAGAG TCGGGGTTAA AACCCGTCAA ACTCGAAGTA GAAGTAGTCG   
  
  
- GGTTGCAGTT ATTGTTACCC GAACTGAAGT ATCTCCTGGA TTAATCTCGT CGAGTTCGGA TGCTTAGGTT   
  
  
- GCTGAGGGTG GTCCAGGTCG ACCATAACCG CGCCGAGTTA GTTGCCGAGT CTAGTGGGCG GCCTTTTGGG   
  
  
- GAGGTGGCGC GGCGGAAGAT GAAGTTCCTC CGGGAGGTTG TGGAGCGACA GCCGCCTCCG GGCCGGGCCG   
  
  
- GACAGGCCGA CAGTAGGATG CTCCACCACG TCTGGTAGGC CCGGGTGTTC TTGAAAAGGC CGTATAGTGA   
  
  
- CTATAGGGAG AAGAGGTGGA AGCGGCGGTT AGTCCGCTAA GACCTCCGCC ACCTACCCCG ACGCAAGTAG   
  
  
- GTGTAGTAGC TAAAGCTGTA GCCTAAACCG CCGGTGACCC GCAGCAAATA CGCACTCGAA CAGCTGTTCC   
  
  
- GGCTGGGCTT ATTCTAGTCG AGGAACCACA AGGCTTAGTG CCGCTAGCAT GGGCTTCTCA AGCCCTAGCT   
  
  
- TTCGTTCGAC CACTCTCTCT TAGAATGCGT TAAGCGAGCA CTAGAGTTGT AGTTACAGGT ATACCTCATA   
  
  
- CAGGAATAAG TTTCGAAGCT CAAGAATAGG AAGTTTAGCC AGTTTAAGTA GCTGCCACTC TTTAACCGCC   
  
  
- AGGTAGATAG AGGCCGCTAC AAAGTGGCCG AAGTTCCGCC TTAGAGCTTT AAGGATCGGC TAGAGGCCCC   
  
  
- TTAAAGCGGT TCGTGGCAGC ACCACCACCT GTCACTCCAT CCTTAGCTGT GCCCTTGAAG CAGGAAGTCG   
  
  
- CAGTTGAAGC AGCGGCCATA GCTTAAGATG TGGCCTTACG AGCTTAGCGA GCTGCAGTGG AGGCGGTCGC   
  
  
- CAAAACGTCC GCCGCCGCCG CCGCTGCGAC ACTCTTCCTA GCTCTGCATG CACGAGGCCG GCTCCTAATG   
  
  
- CCGCCGCCAC CTCCGCCGTA GACGCCGCCG CCATCGCGCC GCCTCGCCAA CCTCTCTCAG CAAGCGCCAC   
  
  
- CGATCCTACT CCCGCCACCC CAAGTCGGTC AAACGACTAA AGGTCCGCCT CACAAACGAC TCCTTCCAAG   
  
  
- TCCAACCGCC CAAGGTGCAC CGCTTTGCAG TACCCCTCTA CTACGAAACG ACCGTACTAT CCGGGGAACA   
  
  
- CAGGTGCAGC CGAACCTCCA CAAT

+     RY-element

| Site Name | Organism | Position | Strand | Matrix score. | sequence | function |
| --- | --- | --- | --- | --- | --- | --- |
| RY-element | Helianthus annuus | 1480 | + | 8 | CATGCATG | cis-acting regulatory element involved in seed-specific regulation |

>HU06G00283.1   
+ -Up\_Stream \_Len000GATTTT ACTTCCAATT TTCTACTTAA ATTTGGTTAT CACGTTCGAA TTTTTCAGGT   
  
  
+ AAATACAAAC TGTTAGTGGC CATCCTTTTT TTTTGGGTCT AATCCATCCA GATGTGCTAA TACTTTCTGT   
  
  
+ TTTAGATGAG AAGTTTTAAT GCTTTTTTAG ATGTGTAATT TTTTTAAACA TAAATAGTCT TGTCGAATAA   
  
  
+ GATTTGCTAG TAATTTTTGT AAGAGAAATT ATGTGTATTT TTTATTATAT CAGAATATTA GATCGATCTC   
  
  
+ AATTTAATTA CTTATGAAAA AAAAAGTGGA TATCTTTAAT CAATTTGGCA AGCTCTCATG CATTAATTGT   
  
  
+ AGTCGTACGA ACTTATTTAC ATCACTCATC TGAATTTGCT ACCATTTACA CCTCAAATTT TATTTTAATG   
  
  
+ CCATAAAGAG AATAGAATAT ACTGTCTATT TGTAGTAGTT TTACAGATAA AAACAAATAA GCAAATGAAA   
  
  
+ ATAGATAGAG GATTTTTTTG CTATTATCCA CGTCGATCCA TTGAATTATT TTGTCGCTAT TTGGTTAAGT   
  
  
+ GAAATAAAAT ACTGAAGTAG AATGGAGGAT GTTGATAATG ACCTATGTCC CTCAAAGTTT AATCATGTAG   
  
  
+ TTTCTACGAA TTTCAAGCCA TTATAAGACA TACAGATTTT TATGATGTTG AAAATACTCT TACCTCCTTG   
  
  
+ TCACAATATT AACCTGAAGC CAATGAAATG AAACAGAACT AATTAAATTG AATACATAAT CAATCCATAT   
  
  
+ TAATTTGTTT TGACAACTAT TTACAATCAT CGATCTCTTT AATTAGGTCT ATAAGCTATA GAGTAATTAC   
  
  
+ TCAAAACTGT TTTGTAAGAC AACTTACTCC ATATTGGCTT ATTAGTATAT TGCCTCCATT CACATGAATA   
  
  
+ CTTCATGAGA GATTGCCTAA AACTCATATT GTCATCACCT TAAGTCAAAC ATATTTTGTT ATGAAATGAC   
  
  
+ ATCAAGAATT ACCCTCTGAC AAGAAAAATA TACCTGACTG ACATAATCGT ACCACATCTG TTAGTCCTCT   
  
  
+ TTCTACCACT TTGTAGAACA TAATGTGACA GTAATACATC CATCCCAAAA GAAAAGACGA AAAGAAGAGT   
  
  
+ GGAGAGTCAC GTATACGCGT TGAATTCAGG AAAATTAAAG GAACTCATGA AGACCCCATA TAGTGCTCCA   
  
  
+ ACCTGAACCT GCAGCCCACT ACTACATGCC TAAATTAAAG AGCAAAGTTA TGAGAGAAAC CAAAAAAGCT   
  
  
+ CTTTGAGAGA GTGGAGGAAG AATCATGGAA AGCTAGCTAG GCTTAGCAAG AAAGAGCAAC AGCTAGCTAG   
  
  
+ CAAAAGGCTT CTCTTTGAAG CCTTAGAAAT CTTTAGAGAT AAAAAGTTGG TGGCTTTTTA AGGGCTGTGA   
  
  
+ TATGTCCCCC ATGTAAAAAT GCAGCAATAA TGTTAATGTC ATGCTTTCTT TCATGGTCCC TAATGCGAAA   
  
  
+ TCAATCATGC ATGTCAACGT GACTCAGTGG CTCACCATAT ATGATCTGAT TTCCCCGCAC ACAGAGATAA   
  
  
+ AAAGCCCCCA ATTATAACCT CCCAAAAGCC CCCCCTTTTC CTCCTACCCA ACCCACACCT CCCCATTTCA   
  
  
+ TCAGGGCTCG CTTCTTAAGT TATCGATTAA AAAAAGAGTG AGTAAACGTA CCCGAATTGA AGAGGGGTGT   
  
  
+ TCGAAGCTTA TACTTGCTTG ATTAGGAATG AAAGTCCCCA TTTCATCCGC CCCCCAAACC GCCGCCACCA   
  
  
+ CCAGTAAATC GCTATCTTGC AACAATACTG CCTTGAATAC TGCCTTTCAA GCCCCCAATT CAATTTCAAC   
  
  
+ CCTTGTTAAC AACAACAAAC CCTCCTACGA GCCTACCTCG GTTCTAGACC TCCGGCGCAG CCCAAGCCCC   
  
  
+ GCCACCAACG CCGCCCTGCC ACTGCCCACT GACGCGGGCA TCAATAAACC CAAGCCTGCC TCTGCTGACC   
  
  
+ TGGACAATTT GGAGGGTGAG GGTTGGGATT CAATCCTATC AGAGCTGGGG CTTAATGATG ATTCTAATAA   
  
  
+ TCCTAACTCC AAACTTTGGT CATCCCAAAT TAGCTCATCA TGTGATTCCC ACCTCACACA GCTCCCTGAG   
  
  
+ TTCCCCTCCT CTCAGCCGTT GGATCACACA CGCACCCCCA ATAATTAACT TCTCCCCCTC TGATTACCCC   
  
  
+ ATCTCTGATT TATCCTACGG TCAGAATCTC AGCCCCAATT TTGGGCAGTT TGAGCTTCAT CTTCATCAGC   
  
  
+ CCAACGTCAA TAACAATGGG CTTGACTTCA TAGAGGACCT AATTAGAGCA GCTCAAGCCT ACGAATCCAA   
  
  
+ CGACTCCCAC CAGGTCCAGC TGGTATTGGC GCGGCTCAAT CAACGGCTCA GATCACCCGC CGGAAAACCC   
  
  
+ CTCCACCGCG CCGCCTTCTA CTTCAAGGAG GCCCTCCAAC ACCTCGCTGT CGGCGGAGGC CCGGCCCGGC   
  
  
+ CTGTCCGGCT GTCATCCTAC GAGGTGGTGC AGACCATCCG GGCCCACAAG AACTTTTCCG GCATATCACT   
  
  
+ GATATCCCTC TTCTCCACCT TCGCCGCCAA TCAGGCGATT CTGGAGGCGG TGGATGGGGC TGCGTTCATC   
  
  
+ CACATCATCG ATTTCGACAT CGGATTTGGC GGCCACTGGG CGTCGTTTAT GCGTGAGCTT GTCGACAAGG   
  
  
+ CCGACCCGAA TAAGATCAGC TCCTTGGTGT TCCGAATCAC GGCGATCGTA CCCGAAGAGT TCGGGATCGA   
  
  
+ AAGCAAGCTG GTGAGAGAGA ATCTTACGCA ATTCGCTCGT GATCTCAACA TCAATGTCCA TATGGAGTAT   
  
  
+ GTCCTTATTC AAAGCTTCGA GTTCTTATCC TTCAAATCGG TCAAATTCAT CGACGGTGAG AAATTGGCGG   
  
  
+ TCCATCTATC TCCGGCGATG TTTCACCGGC TTCAAGGCGG AATCTCGAAA TTCCTAGCCG ATCTCCGGGG   
  
  
+ AATTTCGCCA AGCACCGTCG TGGTGGTGGA CAGTGAGGTA GGAATCGACA CGGGAACTTC GTCCTTCAGC   
  
  
+ GTCAACTTCG TCGCCGGTAT CGAATTCTAC ACCGGAATGC TCGAATCGCT CGACGTCACC TCCGCCAGCG   
  
  
+ GTTTTGCAGG CGGCGGCGGC GGCGACGCTG TGAGAAGGAT CGAGACGTAC GTGCTCCGGC CGAGGATTAC   
  
  
+ GGCGGCGGTG GAGGCGGCAT CTGCGGCGGC GGTAGCGCGG CGGAGCGGTT GGAGAGAGTC GTTCGCGGTG   
  
  
+ GCTAGGATGA GGGCGGTGGG GTTCAGCCAG TTTGCTGATT TCCAGGCGGA GTGTTTGCTG AGGAAGGTTC   
  
  
+ AGGTTGGCGG GTTCCACGTG GCGAAACGTC ATGGGGAGAT GATGCTTTGC TGGCATGATA GGCCCCTTGT   
  
  
+ GTCCACGTCG GCTTGGAGGT GTTA  

- -Up\_Stream \_Len000CTAAAA TGAAGGTTAA AAGATGAATT TAAACCAATA GTGCAAGCTT AAAAAGTCCA   
  
  
- TTTATGTTTG ACAATCACCG GTAGGAAAAA AAAACCCAGA TTAGGTAGGT CTACACGATT ATGAAAGACA   
  
  
- AAATCTACTC TTCAAAATTA CGAAAAAATC TACACATTAA AAAAATTTGT ATTTATCAGA ACAGCTTATT   
  
  
- CTAAACGATC ATTAAAAACA TTCTCTTTAA TACACATAAA AAATAATATA GTCTTATAAT CTAGCTAGAG   
  
  
- TTAAATTAAT GAATACTTTT TTTTTCACCT ATAGAAATTA GTTAAACCGT TCGAGAGTAC GTAATTAACA   
  
  
- TCAGCATGCT TGAATAAATG TAGTGAGTAG ACTTAAACGA TGGTAAATGT GGAGTTTAAA ATAAAATTAC   
  
  
- GGTATTTCTC TTATCTTATA TGACAGATAA ACATCATCAA AATGTCTATT TTTGTTTATT CGTTTACTTT   
  
  
- TATCTATCTC CTAAAAAAAC GATAATAGGT GCAGCTAGGT AACTTAATAA AACAGCGATA AACCAATTCA   
  
  
- CTTTATTTTA TGACTTCATC TTACCTCCTA CAACTATTAC TGGATACAGG GAGTTTCAAA TTAGTACATC   
  
  
- AAAGATGCTT AAAGTTCGGT AATATTCTGT ATGTCTAAAA ATACTACAAC TTTTATGAGA ATGGAGGAAC   
  
  
- AGTGTTATAA TTGGACTTCG GTTACTTTAC TTTGTCTTGA TTAATTTAAC TTATGTATTA GTTAGGTATA   
  
  
- ATTAAACAAA ACTGTTGATA AATGTTAGTA GCTAGAGAAA TTAATCCAGA TATTCGATAT CTCATTAATG   
  
  
- AGTTTTGACA AAACATTCTG TTGAATGAGG TATAACCGAA TAATCATATA ACGGAGGTAA GTGTACTTAT   
  
  
- GAAGTACTCT CTAACGGATT TTGAGTATAA CAGTAGTGGA ATTCAGTTTG TATAAAACAA TACTTTACTG   
  
  
- TAGTTCTTAA TGGGAGACTG TTCTTTTTAT ATGGACTGAC TGTATTAGCA TGGTGTAGAC AATCAGGAGA   
  
  
- AAGATGGTGA AACATCTTGT ATTACACTGT CATTATGTAG GTAGGGTTTT CTTTTCTGCT TTTCTTCTCA   
  
  
- CCTCTCAGTG CATATGCGCA ACTTAAGTCC TTTTAATTTC CTTGAGTACT TCTGGGGTAT ATCACGAGGT   
  
  
- TGGACTTGGA CGTCGGGTGA TGATGTACGG ATTTAATTTC TCGTTTCAAT ACTCTCTTTG GTTTTTTCGA   
  
  
- GAAACTCTCT CACCTCCTTC TTAGTACCTT TCGATCGATC CGAATCGTTC TTTCTCGTTG TCGATCGATC   
  
  
- GTTTTCCGAA GAGAAACTTC GGAATCTTTA GAAATCTCTA TTTTTCAACC ACCGAAAAAT TCCCGACACT   
  
  
- ATACAGGGGG TACATTTTTA CGTCGTTATT ACAATTACAG TACGAAAGAA AGTACCAGGG ATTACGCTTT   
  
  
- AGTTAGTACG TACAGTTGCA CTGAGTCACC GAGTGGTATA TACTAGACTA AAGGGGCGTG TGTCTCTATT   
  
  
- TTTCGGGGGT TAATATTGGA GGGTTTTCGG GGGGGAAAAG GAGGATGGGT TGGGTGTGGA GGGGTAAAGT   
  
  
- AGTCCCGAGC GAAGAATTCA ATAGCTAATT TTTTTCTCAC TCATTTGCAT GGGCTTAACT TCTCCCCACA   
  
  
- AGCTTCGAAT ATGAACGAAC TAATCCTTAC TTTCAGGGGT AAAGTAGGCG GGGGGTTTGG CGGCGGTGGT   
  
  
- GGTCATTTAG CGATAGAACG TTGTTATGAC GGAACTTATG ACGGAAAGTT CGGGGGTTAA GTTAAAGTTG   
  
  
- GGAACAATTG TTGTTGTTTG GGAGGATGCT CGGATGGAGC CAAGATCTGG AGGCCGCGTC GGGTTCGGGG   
  
  
- CGGTGGTTGC GGCGGGACGG TGACGGGTGA CTGCGCCCGT AGTTATTTGG GTTCGGACGG AGACGACTGG   
  
  
- ACCTGTTAAA CCTCCCACTC CCAACCCTAA GTTAGGATAG TCTCGACCCC GAATTACTAC TAAGATTATT   
  
  
- AGGATTGAGG TTTGAAACCA GTAGGGTTTA ATCGAGTAGT ACACTAAGGG TGGAGTGTGT CGAGGGACTC   
  
  
- AAGGGGAGGA GAGTCGGCAA CCTAGTGTGT GCGTGGGGGT TATTAATTGA AGAGGGGGAG ACTAATGGGG   
  
  
- TAGAGACTAA ATAGGATGCC AGTCTTAGAG TCGGGGTTAA AACCCGTCAA ACTCGAAGTA GAAGTAGTCG   
  
  
- GGTTGCAGTT ATTGTTACCC GAACTGAAGT ATCTCCTGGA TTAATCTCGT CGAGTTCGGA TGCTTAGGTT   
  
  
- GCTGAGGGTG GTCCAGGTCG ACCATAACCG CGCCGAGTTA GTTGCCGAGT CTAGTGGGCG GCCTTTTGGG   
  
  
- GAGGTGGCGC GGCGGAAGAT GAAGTTCCTC CGGGAGGTTG TGGAGCGACA GCCGCCTCCG GGCCGGGCCG   
  
  
- GACAGGCCGA CAGTAGGATG CTCCACCACG TCTGGTAGGC CCGGGTGTTC TTGAAAAGGC CGTATAGTGA   
  
  
- CTATAGGGAG AAGAGGTGGA AGCGGCGGTT AGTCCGCTAA GACCTCCGCC ACCTACCCCG ACGCAAGTAG   
  
  
- GTGTAGTAGC TAAAGCTGTA GCCTAAACCG CCGGTGACCC GCAGCAAATA CGCACTCGAA CAGCTGTTCC   
  
  
- GGCTGGGCTT ATTCTAGTCG AGGAACCACA AGGCTTAGTG CCGCTAGCAT GGGCTTCTCA AGCCCTAGCT   
  
  
- TTCGTTCGAC CACTCTCTCT TAGAATGCGT TAAGCGAGCA CTAGAGTTGT AGTTACAGGT ATACCTCATA   
  
  
- CAGGAATAAG TTTCGAAGCT CAAGAATAGG AAGTTTAGCC AGTTTAAGTA GCTGCCACTC TTTAACCGCC   
  
  
- AGGTAGATAG AGGCCGCTAC AAAGTGGCCG AAGTTCCGCC TTAGAGCTTT AAGGATCGGC TAGAGGCCCC   
  
  
- TTAAAGCGGT TCGTGGCAGC ACCACCACCT GTCACTCCAT CCTTAGCTGT GCCCTTGAAG CAGGAAGTCG   
  
  
- CAGTTGAAGC AGCGGCCATA GCTTAAGATG TGGCCTTACG AGCTTAGCGA GCTGCAGTGG AGGCGGTCGC   
  
  
- CAAAACGTCC GCCGCCGCCG CCGCTGCGAC ACTCTTCCTA GCTCTGCATG CACGAGGCCG GCTCCTAATG   
  
  
- CCGCCGCCAC CTCCGCCGTA GACGCCGCCG CCATCGCGCC GCCTCGCCAA CCTCTCTCAG CAAGCGCCAC   
  
  
- CGATCCTACT CCCGCCACCC CAAGTCGGTC AAACGACTAA AGGTCCGCCT CACAAACGAC TCCTTCCAAG   
  
  
- TCCAACCGCC CAAGGTGCAC CGCTTTGCAG TACCCCTCTA CTACGAAACG ACCGTACTAT CCGGGGAACA   
  
  
- CAGGTGCAGC CGAACCTCCA CAAT

+     STRE

| Site Name | Organism | Position | Strand | Matrix score. | sequence | function |
| --- | --- | --- | --- | --- | --- | --- |
| STRE | Arabidopsis thaliana | 2382 | - | 5 | AGGGG |  |
| STRE | Arabidopsis thaliana | 1677 | + | 5 | AGGGG |  |
| STRE | Arabidopsis thaliana | 1576 | - | 5 | AGGGG |  |
| STRE | Arabidopsis thaliana | 3357 | - | 5 | AGGGG |  |
| STRE | Arabidopsis thaliana | 2107 | - | 5 | AGGGG |  |
| STRE | Arabidopsis thaliana | 2159 | - | 5 | AGGGG |  |

>HU06G00283.1   
+ -Up\_Stream \_Len000GATTTT ACTTCCAATT TTCTACTTAA ATTTGGTTAT CACGTTCGAA TTTTTCAGGT   
  
  
+ AAATACAAAC TGTTAGTGGC CATCCTTTTT TTTTGGGTCT AATCCATCCA GATGTGCTAA TACTTTCTGT   
  
  
+ TTTAGATGAG AAGTTTTAAT GCTTTTTTAG ATGTGTAATT TTTTTAAACA TAAATAGTCT TGTCGAATAA   
  
  
+ GATTTGCTAG TAATTTTTGT AAGAGAAATT ATGTGTATTT TTTATTATAT CAGAATATTA GATCGATCTC   
  
  
+ AATTTAATTA CTTATGAAAA AAAAAGTGGA TATCTTTAAT CAATTTGGCA AGCTCTCATG CATTAATTGT   
  
  
+ AGTCGTACGA ACTTATTTAC ATCACTCATC TGAATTTGCT ACCATTTACA CCTCAAATTT TATTTTAATG   
  
  
+ CCATAAAGAG AATAGAATAT ACTGTCTATT TGTAGTAGTT TTACAGATAA AAACAAATAA GCAAATGAAA   
  
  
+ ATAGATAGAG GATTTTTTTG CTATTATCCA CGTCGATCCA TTGAATTATT TTGTCGCTAT TTGGTTAAGT   
  
  
+ GAAATAAAAT ACTGAAGTAG AATGGAGGAT GTTGATAATG ACCTATGTCC CTCAAAGTTT AATCATGTAG   
  
  
+ TTTCTACGAA TTTCAAGCCA TTATAAGACA TACAGATTTT TATGATGTTG AAAATACTCT TACCTCCTTG   
  
  
+ TCACAATATT AACCTGAAGC CAATGAAATG AAACAGAACT AATTAAATTG AATACATAAT CAATCCATAT   
  
  
+ TAATTTGTTT TGACAACTAT TTACAATCAT CGATCTCTTT AATTAGGTCT ATAAGCTATA GAGTAATTAC   
  
  
+ TCAAAACTGT TTTGTAAGAC AACTTACTCC ATATTGGCTT ATTAGTATAT TGCCTCCATT CACATGAATA   
  
  
+ CTTCATGAGA GATTGCCTAA AACTCATATT GTCATCACCT TAAGTCAAAC ATATTTTGTT ATGAAATGAC   
  
  
+ ATCAAGAATT ACCCTCTGAC AAGAAAAATA TACCTGACTG ACATAATCGT ACCACATCTG TTAGTCCTCT   
  
  
+ TTCTACCACT TTGTAGAACA TAATGTGACA GTAATACATC CATCCCAAAA GAAAAGACGA AAAGAAGAGT   
  
  
+ GGAGAGTCAC GTATACGCGT TGAATTCAGG AAAATTAAAG GAACTCATGA AGACCCCATA TAGTGCTCCA   
  
  
+ ACCTGAACCT GCAGCCCACT ACTACATGCC TAAATTAAAG AGCAAAGTTA TGAGAGAAAC CAAAAAAGCT   
  
  
+ CTTTGAGAGA GTGGAGGAAG AATCATGGAA AGCTAGCTAG GCTTAGCAAG AAAGAGCAAC AGCTAGCTAG   
  
  
+ CAAAAGGCTT CTCTTTGAAG CCTTAGAAAT CTTTAGAGAT AAAAAGTTGG TGGCTTTTTA AGGGCTGTGA   
  
  
+ TATGTCCCCC ATGTAAAAAT GCAGCAATAA TGTTAATGTC ATGCTTTCTT TCATGGTCCC TAATGCGAAA   
  
  
+ TCAATCATGC ATGTCAACGT GACTCAGTGG CTCACCATAT ATGATCTGAT TTCCCCGCAC ACAGAGATAA   
  
  
+ AAAGCCCCCA ATTATAACCT CCCAAAAGCC CCCCCTTTTC CTCCTACCCA ACCCACACCT CCCCATTTCA   
  
  
+ TCAGGGCTCG CTTCTTAAGT TATCGATTAA AAAAAGAGTG AGTAAACGTA CCCGAATTGA AGAGGGGTGT   
  
  
+ TCGAAGCTTA TACTTGCTTG ATTAGGAATG AAAGTCCCCA TTTCATCCGC CCCCCAAACC GCCGCCACCA   
  
  
+ CCAGTAAATC GCTATCTTGC AACAATACTG CCTTGAATAC TGCCTTTCAA GCCCCCAATT CAATTTCAAC   
  
  
+ CCTTGTTAAC AACAACAAAC CCTCCTACGA GCCTACCTCG GTTCTAGACC TCCGGCGCAG CCCAAGCCCC   
  
  
+ GCCACCAACG CCGCCCTGCC ACTGCCCACT GACGCGGGCA TCAATAAACC CAAGCCTGCC TCTGCTGACC   
  
  
+ TGGACAATTT GGAGGGTGAG GGTTGGGATT CAATCCTATC AGAGCTGGGG CTTAATGATG ATTCTAATAA   
  
  
+ TCCTAACTCC AAACTTTGGT CATCCCAAAT TAGCTCATCA TGTGATTCCC ACCTCACACA GCTCCCTGAG   
  
  
+ TTCCCCTCCT CTCAGCCGTT GGATCACACA CGCACCCCCA ATAATTAACT TCTCCCCCTC TGATTACCCC   
  
  
+ ATCTCTGATT TATCCTACGG TCAGAATCTC AGCCCCAATT TTGGGCAGTT TGAGCTTCAT CTTCATCAGC   
  
  
+ CCAACGTCAA TAACAATGGG CTTGACTTCA TAGAGGACCT AATTAGAGCA GCTCAAGCCT ACGAATCCAA   
  
  
+ CGACTCCCAC CAGGTCCAGC TGGTATTGGC GCGGCTCAAT CAACGGCTCA GATCACCCGC CGGAAAACCC   
  
  
+ CTCCACCGCG CCGCCTTCTA CTTCAAGGAG GCCCTCCAAC ACCTCGCTGT CGGCGGAGGC CCGGCCCGGC   
  
  
+ CTGTCCGGCT GTCATCCTAC GAGGTGGTGC AGACCATCCG GGCCCACAAG AACTTTTCCG GCATATCACT   
  
  
+ GATATCCCTC TTCTCCACCT TCGCCGCCAA TCAGGCGATT CTGGAGGCGG TGGATGGGGC TGCGTTCATC   
  
  
+ CACATCATCG ATTTCGACAT CGGATTTGGC GGCCACTGGG CGTCGTTTAT GCGTGAGCTT GTCGACAAGG   
  
  
+ CCGACCCGAA TAAGATCAGC TCCTTGGTGT TCCGAATCAC GGCGATCGTA CCCGAAGAGT TCGGGATCGA   
  
  
+ AAGCAAGCTG GTGAGAGAGA ATCTTACGCA ATTCGCTCGT GATCTCAACA TCAATGTCCA TATGGAGTAT   
  
  
+ GTCCTTATTC AAAGCTTCGA GTTCTTATCC TTCAAATCGG TCAAATTCAT CGACGGTGAG AAATTGGCGG   
  
  
+ TCCATCTATC TCCGGCGATG TTTCACCGGC TTCAAGGCGG AATCTCGAAA TTCCTAGCCG ATCTCCGGGG   
  
  
+ AATTTCGCCA AGCACCGTCG TGGTGGTGGA CAGTGAGGTA GGAATCGACA CGGGAACTTC GTCCTTCAGC   
  
  
+ GTCAACTTCG TCGCCGGTAT CGAATTCTAC ACCGGAATGC TCGAATCGCT CGACGTCACC TCCGCCAGCG   
  
  
+ GTTTTGCAGG CGGCGGCGGC GGCGACGCTG TGAGAAGGAT CGAGACGTAC GTGCTCCGGC CGAGGATTAC   
  
  
+ GGCGGCGGTG GAGGCGGCAT CTGCGGCGGC GGTAGCGCGG CGGAGCGGTT GGAGAGAGTC GTTCGCGGTG   
  
  
+ GCTAGGATGA GGGCGGTGGG GTTCAGCCAG TTTGCTGATT TCCAGGCGGA GTGTTTGCTG AGGAAGGTTC   
  
  
+ AGGTTGGCGG GTTCCACGTG GCGAAACGTC ATGGGGAGAT GATGCTTTGC TGGCATGATA GGCCCCTTGT   
  
  
+ GTCCACGTCG GCTTGGAGGT GTTA  

- -Up\_Stream \_Len000CTAAAA TGAAGGTTAA AAGATGAATT TAAACCAATA GTGCAAGCTT AAAAAGTCCA   
  
  
- TTTATGTTTG ACAATCACCG GTAGGAAAAA AAAACCCAGA TTAGGTAGGT CTACACGATT ATGAAAGACA   
  
  
- AAATCTACTC TTCAAAATTA CGAAAAAATC TACACATTAA AAAAATTTGT ATTTATCAGA ACAGCTTATT   
  
  
- CTAAACGATC ATTAAAAACA TTCTCTTTAA TACACATAAA AAATAATATA GTCTTATAAT CTAGCTAGAG   
  
  
- TTAAATTAAT GAATACTTTT TTTTTCACCT ATAGAAATTA GTTAAACCGT TCGAGAGTAC GTAATTAACA   
  
  
- TCAGCATGCT TGAATAAATG TAGTGAGTAG ACTTAAACGA TGGTAAATGT GGAGTTTAAA ATAAAATTAC   
  
  
- GGTATTTCTC TTATCTTATA TGACAGATAA ACATCATCAA AATGTCTATT TTTGTTTATT CGTTTACTTT   
  
  
- TATCTATCTC CTAAAAAAAC GATAATAGGT GCAGCTAGGT AACTTAATAA AACAGCGATA AACCAATTCA   
  
  
- CTTTATTTTA TGACTTCATC TTACCTCCTA CAACTATTAC TGGATACAGG GAGTTTCAAA TTAGTACATC   
  
  
- AAAGATGCTT AAAGTTCGGT AATATTCTGT ATGTCTAAAA ATACTACAAC TTTTATGAGA ATGGAGGAAC   
  
  
- AGTGTTATAA TTGGACTTCG GTTACTTTAC TTTGTCTTGA TTAATTTAAC TTATGTATTA GTTAGGTATA   
  
  
- ATTAAACAAA ACTGTTGATA AATGTTAGTA GCTAGAGAAA TTAATCCAGA TATTCGATAT CTCATTAATG   
  
  
- AGTTTTGACA AAACATTCTG TTGAATGAGG TATAACCGAA TAATCATATA ACGGAGGTAA GTGTACTTAT   
  
  
- GAAGTACTCT CTAACGGATT TTGAGTATAA CAGTAGTGGA ATTCAGTTTG TATAAAACAA TACTTTACTG   
  
  
- TAGTTCTTAA TGGGAGACTG TTCTTTTTAT ATGGACTGAC TGTATTAGCA TGGTGTAGAC AATCAGGAGA   
  
  
- AAGATGGTGA AACATCTTGT ATTACACTGT CATTATGTAG GTAGGGTTTT CTTTTCTGCT TTTCTTCTCA   
  
  
- CCTCTCAGTG CATATGCGCA ACTTAAGTCC TTTTAATTTC CTTGAGTACT TCTGGGGTAT ATCACGAGGT   
  
  
- TGGACTTGGA CGTCGGGTGA TGATGTACGG ATTTAATTTC TCGTTTCAAT ACTCTCTTTG GTTTTTTCGA   
  
  
- GAAACTCTCT CACCTCCTTC TTAGTACCTT TCGATCGATC CGAATCGTTC TTTCTCGTTG TCGATCGATC   
  
  
- GTTTTCCGAA GAGAAACTTC GGAATCTTTA GAAATCTCTA TTTTTCAACC ACCGAAAAAT TCCCGACACT   
  
  
- ATACAGGGGG TACATTTTTA CGTCGTTATT ACAATTACAG TACGAAAGAA AGTACCAGGG ATTACGCTTT   
  
  
- AGTTAGTACG TACAGTTGCA CTGAGTCACC GAGTGGTATA TACTAGACTA AAGGGGCGTG TGTCTCTATT   
  
  
- TTTCGGGGGT TAATATTGGA GGGTTTTCGG GGGGGAAAAG GAGGATGGGT TGGGTGTGGA GGGGTAAAGT   
  
  
- AGTCCCGAGC GAAGAATTCA ATAGCTAATT TTTTTCTCAC TCATTTGCAT GGGCTTAACT TCTCCCCACA   
  
  
- AGCTTCGAAT ATGAACGAAC TAATCCTTAC TTTCAGGGGT AAAGTAGGCG GGGGGTTTGG CGGCGGTGGT   
  
  
- GGTCATTTAG CGATAGAACG TTGTTATGAC GGAACTTATG ACGGAAAGTT CGGGGGTTAA GTTAAAGTTG   
  
  
- GGAACAATTG TTGTTGTTTG GGAGGATGCT CGGATGGAGC CAAGATCTGG AGGCCGCGTC GGGTTCGGGG   
  
  
- CGGTGGTTGC GGCGGGACGG TGACGGGTGA CTGCGCCCGT AGTTATTTGG GTTCGGACGG AGACGACTGG   
  
  
- ACCTGTTAAA CCTCCCACTC CCAACCCTAA GTTAGGATAG TCTCGACCCC GAATTACTAC TAAGATTATT   
  
  
- AGGATTGAGG TTTGAAACCA GTAGGGTTTA ATCGAGTAGT ACACTAAGGG TGGAGTGTGT CGAGGGACTC   
  
  
- AAGGGGAGGA GAGTCGGCAA CCTAGTGTGT GCGTGGGGGT TATTAATTGA AGAGGGGGAG ACTAATGGGG   
  
  
- TAGAGACTAA ATAGGATGCC AGTCTTAGAG TCGGGGTTAA AACCCGTCAA ACTCGAAGTA GAAGTAGTCG   
  
  
- GGTTGCAGTT ATTGTTACCC GAACTGAAGT ATCTCCTGGA TTAATCTCGT CGAGTTCGGA TGCTTAGGTT   
  
  
- GCTGAGGGTG GTCCAGGTCG ACCATAACCG CGCCGAGTTA GTTGCCGAGT CTAGTGGGCG GCCTTTTGGG   
  
  
- GAGGTGGCGC GGCGGAAGAT GAAGTTCCTC CGGGAGGTTG TGGAGCGACA GCCGCCTCCG GGCCGGGCCG   
  
  
- GACAGGCCGA CAGTAGGATG CTCCACCACG TCTGGTAGGC CCGGGTGTTC TTGAAAAGGC CGTATAGTGA   
  
  
- CTATAGGGAG AAGAGGTGGA AGCGGCGGTT AGTCCGCTAA GACCTCCGCC ACCTACCCCG ACGCAAGTAG   
  
  
- GTGTAGTAGC TAAAGCTGTA GCCTAAACCG CCGGTGACCC GCAGCAAATA CGCACTCGAA CAGCTGTTCC   
  
  
- GGCTGGGCTT ATTCTAGTCG AGGAACCACA AGGCTTAGTG CCGCTAGCAT GGGCTTCTCA AGCCCTAGCT   
  
  
- TTCGTTCGAC CACTCTCTCT TAGAATGCGT TAAGCGAGCA CTAGAGTTGT AGTTACAGGT ATACCTCATA   
  
  
- CAGGAATAAG TTTCGAAGCT CAAGAATAGG AAGTTTAGCC AGTTTAAGTA GCTGCCACTC TTTAACCGCC   
  
  
- AGGTAGATAG AGGCCGCTAC AAAGTGGCCG AAGTTCCGCC TTAGAGCTTT AAGGATCGGC TAGAGGCCCC   
  
  
- TTAAAGCGGT TCGTGGCAGC ACCACCACCT GTCACTCCAT CCTTAGCTGT GCCCTTGAAG CAGGAAGTCG   
  
  
- CAGTTGAAGC AGCGGCCATA GCTTAAGATG TGGCCTTACG AGCTTAGCGA GCTGCAGTGG AGGCGGTCGC   
  
  
- CAAAACGTCC GCCGCCGCCG CCGCTGCGAC ACTCTTCCTA GCTCTGCATG CACGAGGCCG GCTCCTAATG   
  
  
- CCGCCGCCAC CTCCGCCGTA GACGCCGCCG CCATCGCGCC GCCTCGCCAA CCTCTCTCAG CAAGCGCCAC   
  
  
- CGATCCTACT CCCGCCACCC CAAGTCGGTC AAACGACTAA AGGTCCGCCT CACAAACGAC TCCTTCCAAG   
  
  
- TCCAACCGCC CAAGGTGCAC CGCTTTGCAG TACCCCTCTA CTACGAAACG ACCGTACTAT CCGGGGAACA   
  
  
- CAGGTGCAGC CGAACCTCCA CAAT

+     Sp1

| Site Name | Organism | Position | Strand | Matrix score. | sequence | function |
| --- | --- | --- | --- | --- | --- | --- |
| Sp1 | Oryza sativa | 3235 | + | 6 | GGGCGG | light responsive element |
| Sp1 | Oryza sativa | 1905 | - | 6 | GGGCGG | light responsive element |
| Sp1 | Oryza sativa | 1731 | - | 6 | GGGCGG | light responsive element |

>HU06G00283.1   
+ -Up\_Stream \_Len000GATTTT ACTTCCAATT TTCTACTTAA ATTTGGTTAT CACGTTCGAA TTTTTCAGGT   
  
  
+ AAATACAAAC TGTTAGTGGC CATCCTTTTT TTTTGGGTCT AATCCATCCA GATGTGCTAA TACTTTCTGT   
  
  
+ TTTAGATGAG AAGTTTTAAT GCTTTTTTAG ATGTGTAATT TTTTTAAACA TAAATAGTCT TGTCGAATAA   
  
  
+ GATTTGCTAG TAATTTTTGT AAGAGAAATT ATGTGTATTT TTTATTATAT CAGAATATTA GATCGATCTC   
  
  
+ AATTTAATTA CTTATGAAAA AAAAAGTGGA TATCTTTAAT CAATTTGGCA AGCTCTCATG CATTAATTGT   
  
  
+ AGTCGTACGA ACTTATTTAC ATCACTCATC TGAATTTGCT ACCATTTACA CCTCAAATTT TATTTTAATG   
  
  
+ CCATAAAGAG AATAGAATAT ACTGTCTATT TGTAGTAGTT TTACAGATAA AAACAAATAA GCAAATGAAA   
  
  
+ ATAGATAGAG GATTTTTTTG CTATTATCCA CGTCGATCCA TTGAATTATT TTGTCGCTAT TTGGTTAAGT   
  
  
+ GAAATAAAAT ACTGAAGTAG AATGGAGGAT GTTGATAATG ACCTATGTCC CTCAAAGTTT AATCATGTAG   
  
  
+ TTTCTACGAA TTTCAAGCCA TTATAAGACA TACAGATTTT TATGATGTTG AAAATACTCT TACCTCCTTG   
  
  
+ TCACAATATT AACCTGAAGC CAATGAAATG AAACAGAACT AATTAAATTG AATACATAAT CAATCCATAT   
  
  
+ TAATTTGTTT TGACAACTAT TTACAATCAT CGATCTCTTT AATTAGGTCT ATAAGCTATA GAGTAATTAC   
  
  
+ TCAAAACTGT TTTGTAAGAC AACTTACTCC ATATTGGCTT ATTAGTATAT TGCCTCCATT CACATGAATA   
  
  
+ CTTCATGAGA GATTGCCTAA AACTCATATT GTCATCACCT TAAGTCAAAC ATATTTTGTT ATGAAATGAC   
  
  
+ ATCAAGAATT ACCCTCTGAC AAGAAAAATA TACCTGACTG ACATAATCGT ACCACATCTG TTAGTCCTCT   
  
  
+ TTCTACCACT TTGTAGAACA TAATGTGACA GTAATACATC CATCCCAAAA GAAAAGACGA AAAGAAGAGT   
  
  
+ GGAGAGTCAC GTATACGCGT TGAATTCAGG AAAATTAAAG GAACTCATGA AGACCCCATA TAGTGCTCCA   
  
  
+ ACCTGAACCT GCAGCCCACT ACTACATGCC TAAATTAAAG AGCAAAGTTA TGAGAGAAAC CAAAAAAGCT   
  
  
+ CTTTGAGAGA GTGGAGGAAG AATCATGGAA AGCTAGCTAG GCTTAGCAAG AAAGAGCAAC AGCTAGCTAG   
  
  
+ CAAAAGGCTT CTCTTTGAAG CCTTAGAAAT CTTTAGAGAT AAAAAGTTGG TGGCTTTTTA AGGGCTGTGA   
  
  
+ TATGTCCCCC ATGTAAAAAT GCAGCAATAA TGTTAATGTC ATGCTTTCTT TCATGGTCCC TAATGCGAAA   
  
  
+ TCAATCATGC ATGTCAACGT GACTCAGTGG CTCACCATAT ATGATCTGAT TTCCCCGCAC ACAGAGATAA   
  
  
+ AAAGCCCCCA ATTATAACCT CCCAAAAGCC CCCCCTTTTC CTCCTACCCA ACCCACACCT CCCCATTTCA   
  
  
+ TCAGGGCTCG CTTCTTAAGT TATCGATTAA AAAAAGAGTG AGTAAACGTA CCCGAATTGA AGAGGGGTGT   
  
  
+ TCGAAGCTTA TACTTGCTTG ATTAGGAATG AAAGTCCCCA TTTCATCCGC CCCCCAAACC GCCGCCACCA   
  
  
+ CCAGTAAATC GCTATCTTGC AACAATACTG CCTTGAATAC TGCCTTTCAA GCCCCCAATT CAATTTCAAC   
  
  
+ CCTTGTTAAC AACAACAAAC CCTCCTACGA GCCTACCTCG GTTCTAGACC TCCGGCGCAG CCCAAGCCCC   
  
  
+ GCCACCAACG CCGCCCTGCC ACTGCCCACT GACGCGGGCA TCAATAAACC CAAGCCTGCC TCTGCTGACC   
  
  
+ TGGACAATTT GGAGGGTGAG GGTTGGGATT CAATCCTATC AGAGCTGGGG CTTAATGATG ATTCTAATAA   
  
  
+ TCCTAACTCC AAACTTTGGT CATCCCAAAT TAGCTCATCA TGTGATTCCC ACCTCACACA GCTCCCTGAG   
  
  
+ TTCCCCTCCT CTCAGCCGTT GGATCACACA CGCACCCCCA ATAATTAACT TCTCCCCCTC TGATTACCCC   
  
  
+ ATCTCTGATT TATCCTACGG TCAGAATCTC AGCCCCAATT TTGGGCAGTT TGAGCTTCAT CTTCATCAGC   
  
  
+ CCAACGTCAA TAACAATGGG CTTGACTTCA TAGAGGACCT AATTAGAGCA GCTCAAGCCT ACGAATCCAA   
  
  
+ CGACTCCCAC CAGGTCCAGC TGGTATTGGC GCGGCTCAAT CAACGGCTCA GATCACCCGC CGGAAAACCC   
  
  
+ CTCCACCGCG CCGCCTTCTA CTTCAAGGAG GCCCTCCAAC ACCTCGCTGT CGGCGGAGGC CCGGCCCGGC   
  
  
+ CTGTCCGGCT GTCATCCTAC GAGGTGGTGC AGACCATCCG GGCCCACAAG AACTTTTCCG GCATATCACT   
  
  
+ GATATCCCTC TTCTCCACCT TCGCCGCCAA TCAGGCGATT CTGGAGGCGG TGGATGGGGC TGCGTTCATC   
  
  
+ CACATCATCG ATTTCGACAT CGGATTTGGC GGCCACTGGG CGTCGTTTAT GCGTGAGCTT GTCGACAAGG   
  
  
+ CCGACCCGAA TAAGATCAGC TCCTTGGTGT TCCGAATCAC GGCGATCGTA CCCGAAGAGT TCGGGATCGA   
  
  
+ AAGCAAGCTG GTGAGAGAGA ATCTTACGCA ATTCGCTCGT GATCTCAACA TCAATGTCCA TATGGAGTAT   
  
  
+ GTCCTTATTC AAAGCTTCGA GTTCTTATCC TTCAAATCGG TCAAATTCAT CGACGGTGAG AAATTGGCGG   
  
  
+ TCCATCTATC TCCGGCGATG TTTCACCGGC TTCAAGGCGG AATCTCGAAA TTCCTAGCCG ATCTCCGGGG   
  
  
+ AATTTCGCCA AGCACCGTCG TGGTGGTGGA CAGTGAGGTA GGAATCGACA CGGGAACTTC GTCCTTCAGC   
  
  
+ GTCAACTTCG TCGCCGGTAT CGAATTCTAC ACCGGAATGC TCGAATCGCT CGACGTCACC TCCGCCAGCG   
  
  
+ GTTTTGCAGG CGGCGGCGGC GGCGACGCTG TGAGAAGGAT CGAGACGTAC GTGCTCCGGC CGAGGATTAC   
  
  
+ GGCGGCGGTG GAGGCGGCAT CTGCGGCGGC GGTAGCGCGG CGGAGCGGTT GGAGAGAGTC GTTCGCGGTG   
  
  
+ GCTAGGATGA GGGCGGTGGG GTTCAGCCAG TTTGCTGATT TCCAGGCGGA GTGTTTGCTG AGGAAGGTTC   
  
  
+ AGGTTGGCGG GTTCCACGTG GCGAAACGTC ATGGGGAGAT GATGCTTTGC TGGCATGATA GGCCCCTTGT   
  
  
+ GTCCACGTCG GCTTGGAGGT GTTA  

- -Up\_Stream \_Len000CTAAAA TGAAGGTTAA AAGATGAATT TAAACCAATA GTGCAAGCTT AAAAAGTCCA   
  
  
- TTTATGTTTG ACAATCACCG GTAGGAAAAA AAAACCCAGA TTAGGTAGGT CTACACGATT ATGAAAGACA   
  
  
- AAATCTACTC TTCAAAATTA CGAAAAAATC TACACATTAA AAAAATTTGT ATTTATCAGA ACAGCTTATT   
  
  
- CTAAACGATC ATTAAAAACA TTCTCTTTAA TACACATAAA AAATAATATA GTCTTATAAT CTAGCTAGAG   
  
  
- TTAAATTAAT GAATACTTTT TTTTTCACCT ATAGAAATTA GTTAAACCGT TCGAGAGTAC GTAATTAACA   
  
  
- TCAGCATGCT TGAATAAATG TAGTGAGTAG ACTTAAACGA TGGTAAATGT GGAGTTTAAA ATAAAATTAC   
  
  
- GGTATTTCTC TTATCTTATA TGACAGATAA ACATCATCAA AATGTCTATT TTTGTTTATT CGTTTACTTT   
  
  
- TATCTATCTC CTAAAAAAAC GATAATAGGT GCAGCTAGGT AACTTAATAA AACAGCGATA AACCAATTCA   
  
  
- CTTTATTTTA TGACTTCATC TTACCTCCTA CAACTATTAC TGGATACAGG GAGTTTCAAA TTAGTACATC   
  
  
- AAAGATGCTT AAAGTTCGGT AATATTCTGT ATGTCTAAAA ATACTACAAC TTTTATGAGA ATGGAGGAAC   
  
  
- AGTGTTATAA TTGGACTTCG GTTACTTTAC TTTGTCTTGA TTAATTTAAC TTATGTATTA GTTAGGTATA   
  
  
- ATTAAACAAA ACTGTTGATA AATGTTAGTA GCTAGAGAAA TTAATCCAGA TATTCGATAT CTCATTAATG   
  
  
- AGTTTTGACA AAACATTCTG TTGAATGAGG TATAACCGAA TAATCATATA ACGGAGGTAA GTGTACTTAT   
  
  
- GAAGTACTCT CTAACGGATT TTGAGTATAA CAGTAGTGGA ATTCAGTTTG TATAAAACAA TACTTTACTG   
  
  
- TAGTTCTTAA TGGGAGACTG TTCTTTTTAT ATGGACTGAC TGTATTAGCA TGGTGTAGAC AATCAGGAGA   
  
  
- AAGATGGTGA AACATCTTGT ATTACACTGT CATTATGTAG GTAGGGTTTT CTTTTCTGCT TTTCTTCTCA   
  
  
- CCTCTCAGTG CATATGCGCA ACTTAAGTCC TTTTAATTTC CTTGAGTACT TCTGGGGTAT ATCACGAGGT   
  
  
- TGGACTTGGA CGTCGGGTGA TGATGTACGG ATTTAATTTC TCGTTTCAAT ACTCTCTTTG GTTTTTTCGA   
  
  
- GAAACTCTCT CACCTCCTTC TTAGTACCTT TCGATCGATC CGAATCGTTC TTTCTCGTTG TCGATCGATC   
  
  
- GTTTTCCGAA GAGAAACTTC GGAATCTTTA GAAATCTCTA TTTTTCAACC ACCGAAAAAT TCCCGACACT   
  
  
- ATACAGGGGG TACATTTTTA CGTCGTTATT ACAATTACAG TACGAAAGAA AGTACCAGGG ATTACGCTTT   
  
  
- AGTTAGTACG TACAGTTGCA CTGAGTCACC GAGTGGTATA TACTAGACTA AAGGGGCGTG TGTCTCTATT   
  
  
- TTTCGGGGGT TAATATTGGA GGGTTTTCGG GGGGGAAAAG GAGGATGGGT TGGGTGTGGA GGGGTAAAGT   
  
  
- AGTCCCGAGC GAAGAATTCA ATAGCTAATT TTTTTCTCAC TCATTTGCAT GGGCTTAACT TCTCCCCACA   
  
  
- AGCTTCGAAT ATGAACGAAC TAATCCTTAC TTTCAGGGGT AAAGTAGGCG GGGGGTTTGG CGGCGGTGGT   
  
  
- GGTCATTTAG CGATAGAACG TTGTTATGAC GGAACTTATG ACGGAAAGTT CGGGGGTTAA GTTAAAGTTG   
  
  
- GGAACAATTG TTGTTGTTTG GGAGGATGCT CGGATGGAGC CAAGATCTGG AGGCCGCGTC GGGTTCGGGG   
  
  
- CGGTGGTTGC GGCGGGACGG TGACGGGTGA CTGCGCCCGT AGTTATTTGG GTTCGGACGG AGACGACTGG   
  
  
- ACCTGTTAAA CCTCCCACTC CCAACCCTAA GTTAGGATAG TCTCGACCCC GAATTACTAC TAAGATTATT   
  
  
- AGGATTGAGG TTTGAAACCA GTAGGGTTTA ATCGAGTAGT ACACTAAGGG TGGAGTGTGT CGAGGGACTC   
  
  
- AAGGGGAGGA GAGTCGGCAA CCTAGTGTGT GCGTGGGGGT TATTAATTGA AGAGGGGGAG ACTAATGGGG   
  
  
- TAGAGACTAA ATAGGATGCC AGTCTTAGAG TCGGGGTTAA AACCCGTCAA ACTCGAAGTA GAAGTAGTCG   
  
  
- GGTTGCAGTT ATTGTTACCC GAACTGAAGT ATCTCCTGGA TTAATCTCGT CGAGTTCGGA TGCTTAGGTT   
  
  
- GCTGAGGGTG GTCCAGGTCG ACCATAACCG CGCCGAGTTA GTTGCCGAGT CTAGTGGGCG GCCTTTTGGG   
  
  
- GAGGTGGCGC GGCGGAAGAT GAAGTTCCTC CGGGAGGTTG TGGAGCGACA GCCGCCTCCG GGCCGGGCCG   
  
  
- GACAGGCCGA CAGTAGGATG CTCCACCACG TCTGGTAGGC CCGGGTGTTC TTGAAAAGGC CGTATAGTGA   
  
  
- CTATAGGGAG AAGAGGTGGA AGCGGCGGTT AGTCCGCTAA GACCTCCGCC ACCTACCCCG ACGCAAGTAG   
  
  
- GTGTAGTAGC TAAAGCTGTA GCCTAAACCG CCGGTGACCC GCAGCAAATA CGCACTCGAA CAGCTGTTCC   
  
  
- GGCTGGGCTT ATTCTAGTCG AGGAACCACA AGGCTTAGTG CCGCTAGCAT GGGCTTCTCA AGCCCTAGCT   
  
  
- TTCGTTCGAC CACTCTCTCT TAGAATGCGT TAAGCGAGCA CTAGAGTTGT AGTTACAGGT ATACCTCATA   
  
  
- CAGGAATAAG TTTCGAAGCT CAAGAATAGG AAGTTTAGCC AGTTTAAGTA GCTGCCACTC TTTAACCGCC   
  
  
- AGGTAGATAG AGGCCGCTAC AAAGTGGCCG AAGTTCCGCC TTAGAGCTTT AAGGATCGGC TAGAGGCCCC   
  
  
- TTAAAGCGGT TCGTGGCAGC ACCACCACCT GTCACTCCAT CCTTAGCTGT GCCCTTGAAG CAGGAAGTCG   
  
  
- CAGTTGAAGC AGCGGCCATA GCTTAAGATG TGGCCTTACG AGCTTAGCGA GCTGCAGTGG AGGCGGTCGC   
  
  
- CAAAACGTCC GCCGCCGCCG CCGCTGCGAC ACTCTTCCTA GCTCTGCATG CACGAGGCCG GCTCCTAATG   
  
  
- CCGCCGCCAC CTCCGCCGTA GACGCCGCCG CCATCGCGCC GCCTCGCCAA CCTCTCTCAG CAAGCGCCAC   
  
  
- CGATCCTACT CCCGCCACCC CAAGTCGGTC AAACGACTAA AGGTCCGCCT CACAAACGAC TCCTTCCAAG   
  
  
- TCCAACCGCC CAAGGTGCAC CGCTTTGCAG TACCCCTCTA CTACGAAACG ACCGTACTAT CCGGGGAACA   
  
  
- CAGGTGCAGC CGAACCTCCA CAAT

+     TATA-box

| Site Name | Organism | Position | Strand | Matrix score. | sequence | function |
| --- | --- | --- | --- | --- | --- | --- |
| TATA-box | Brassica napus | 1511 | + | 6 | ATATAT | core promoter element around -30 of transcription start |
| TATA-box | Brassica napus | 654 | + | 6 | ATTATA | core promoter element around -30 of transcription start |
| TATA-box | Brassica napus | 1555 | + | 6 | ATTATA | core promoter element around -30 of transcription start |
| TATA-box | Oryza sativa | 854 | - | 7 | TACAAAA | core promoter element around -30 of transcription start |
| TATA-box | Arabidopsis thaliana | 824 | + | 4 | TATA | core promoter element around -30 of transcription start |
| TATA-box | Arabidopsis thaliana | 655 | - | 5 | TATAA | core promoter element around -30 of transcription start |
| TATA-box | Arabidopsis thaliana | 1557 | + | 4 | TATA | core promoter element around -30 of transcription start |
| TATA-box | Arabidopsis thaliana | 656 | + | 4 | TATA | core promoter element around -30 of transcription start |
| TATA-box | Arabidopsis thaliana | 259 | - | 5 | TATAA | core promoter element around -30 of transcription start |
| TATA-box | Arabidopsis thaliana | 1013 | + | 4 | TATA | core promoter element around -30 of transcription start |
| TATA-box | Arabidopsis thaliana | 442 | + | 4 | TATA | core promoter element around -30 of transcription start |
| TATA-box | Arabidopsis thaliana | 260 | + | 4 | TATA | core promoter element around -30 of transcription start |
| TATA-box | Brassica napus | 258 | + | 6 | ATTATA | core promoter element around -30 of transcription start |
| TATA-box | Arabidopsis thaliana | 1183 | + | 4 | TATA | core promoter element around -30 of transcription start |
| TATA-box | Arabidopsis thaliana | 1692 | - | 5 | TATAA | core promoter element around -30 of transcription start |
| TATA-box | Oryza sativa | 229 | - | 7 | TACAAAA | core promoter element around -30 of transcription start |
| TATA-box | Arabidopsis thaliana | 890 | + | 4 | TATA | core promoter element around -30 of transcription start |
| TATA-box | Arabidopsis thaliana | 1512 | + | 4 | TATA | core promoter element around -30 of transcription start |
| TATA-box | Arabidopsis thaliana | 1693 | + | 4 | TATA | core promoter element around -30 of transcription start |
| TATA-box | Arabidopsis thaliana | 831 | + | 4 | TATA | core promoter element around -30 of transcription start |
| TATA-box | Arabidopsis thaliana | 1136 | + | 4 | TATA | core promoter element around -30 of transcription start |
| TATA-box | Arabidopsis thaliana | 1556 | - | 5 | TATAA | core promoter element around -30 of transcription start |
| TATA-box | Arabidopsis thaliana | 1362 | - | 8 | TAAAGATT | core promoter element around -30 of transcription start |

>HU06G00283.1   
+ -Up\_Stream \_Len000GATTTT ACTTCCAATT TTCTACTTAA ATTTGGTTAT CACGTTCGAA TTTTTCAGGT   
  
  
+ AAATACAAAC TGTTAGTGGC CATCCTTTTT TTTTGGGTCT AATCCATCCA GATGTGCTAA TACTTTCTGT   
  
  
+ TTTAGATGAG AAGTTTTAAT GCTTTTTTAG ATGTGTAATT TTTTTAAACA TAAATAGTCT TGTCGAATAA   
  
  
+ GATTTGCTAG TAATTTTTGT AAGAGAAATT ATGTGTATTT TTTATTATAT CAGAATATTA GATCGATCTC   
  
  
+ AATTTAATTA CTTATGAAAA AAAAAGTGGA TATCTTTAAT CAATTTGGCA AGCTCTCATG CATTAATTGT   
  
  
+ AGTCGTACGA ACTTATTTAC ATCACTCATC TGAATTTGCT ACCATTTACA CCTCAAATTT TATTTTAATG   
  
  
+ CCATAAAGAG AATAGAATAT ACTGTCTATT TGTAGTAGTT TTACAGATAA AAACAAATAA GCAAATGAAA   
  
  
+ ATAGATAGAG GATTTTTTTG CTATTATCCA CGTCGATCCA TTGAATTATT TTGTCGCTAT TTGGTTAAGT   
  
  
+ GAAATAAAAT ACTGAAGTAG AATGGAGGAT GTTGATAATG ACCTATGTCC CTCAAAGTTT AATCATGTAG   
  
  
+ TTTCTACGAA TTTCAAGCCA TTATAAGACA TACAGATTTT TATGATGTTG AAAATACTCT TACCTCCTTG   
  
  
+ TCACAATATT AACCTGAAGC CAATGAAATG AAACAGAACT AATTAAATTG AATACATAAT CAATCCATAT   
  
  
+ TAATTTGTTT TGACAACTAT TTACAATCAT CGATCTCTTT AATTAGGTCT ATAAGCTATA GAGTAATTAC   
  
  
+ TCAAAACTGT TTTGTAAGAC AACTTACTCC ATATTGGCTT ATTAGTATAT TGCCTCCATT CACATGAATA   
  
  
+ CTTCATGAGA GATTGCCTAA AACTCATATT GTCATCACCT TAAGTCAAAC ATATTTTGTT ATGAAATGAC   
  
  
+ ATCAAGAATT ACCCTCTGAC AAGAAAAATA TACCTGACTG ACATAATCGT ACCACATCTG TTAGTCCTCT   
  
  
+ TTCTACCACT TTGTAGAACA TAATGTGACA GTAATACATC CATCCCAAAA GAAAAGACGA AAAGAAGAGT   
  
  
+ GGAGAGTCAC GTATACGCGT TGAATTCAGG AAAATTAAAG GAACTCATGA AGACCCCATA TAGTGCTCCA   
  
  
+ ACCTGAACCT GCAGCCCACT ACTACATGCC TAAATTAAAG AGCAAAGTTA TGAGAGAAAC CAAAAAAGCT   
  
  
+ CTTTGAGAGA GTGGAGGAAG AATCATGGAA AGCTAGCTAG GCTTAGCAAG AAAGAGCAAC AGCTAGCTAG   
  
  
+ CAAAAGGCTT CTCTTTGAAG CCTTAGAAAT CTTTAGAGAT AAAAAGTTGG TGGCTTTTTA AGGGCTGTGA   
  
  
+ TATGTCCCCC ATGTAAAAAT GCAGCAATAA TGTTAATGTC ATGCTTTCTT TCATGGTCCC TAATGCGAAA   
  
  
+ TCAATCATGC ATGTCAACGT GACTCAGTGG CTCACCATAT ATGATCTGAT TTCCCCGCAC ACAGAGATAA   
  
  
+ AAAGCCCCCA ATTATAACCT CCCAAAAGCC CCCCCTTTTC CTCCTACCCA ACCCACACCT CCCCATTTCA   
  
  
+ TCAGGGCTCG CTTCTTAAGT TATCGATTAA AAAAAGAGTG AGTAAACGTA CCCGAATTGA AGAGGGGTGT   
  
  
+ TCGAAGCTTA TACTTGCTTG ATTAGGAATG AAAGTCCCCA TTTCATCCGC CCCCCAAACC GCCGCCACCA   
  
  
+ CCAGTAAATC GCTATCTTGC AACAATACTG CCTTGAATAC TGCCTTTCAA GCCCCCAATT CAATTTCAAC   
  
  
+ CCTTGTTAAC AACAACAAAC CCTCCTACGA GCCTACCTCG GTTCTAGACC TCCGGCGCAG CCCAAGCCCC   
  
  
+ GCCACCAACG CCGCCCTGCC ACTGCCCACT GACGCGGGCA TCAATAAACC CAAGCCTGCC TCTGCTGACC   
  
  
+ TGGACAATTT GGAGGGTGAG GGTTGGGATT CAATCCTATC AGAGCTGGGG CTTAATGATG ATTCTAATAA   
  
  
+ TCCTAACTCC AAACTTTGGT CATCCCAAAT TAGCTCATCA TGTGATTCCC ACCTCACACA GCTCCCTGAG   
  
  
+ TTCCCCTCCT CTCAGCCGTT GGATCACACA CGCACCCCCA ATAATTAACT TCTCCCCCTC TGATTACCCC   
  
  
+ ATCTCTGATT TATCCTACGG TCAGAATCTC AGCCCCAATT TTGGGCAGTT TGAGCTTCAT CTTCATCAGC   
  
  
+ CCAACGTCAA TAACAATGGG CTTGACTTCA TAGAGGACCT AATTAGAGCA GCTCAAGCCT ACGAATCCAA   
  
  
+ CGACTCCCAC CAGGTCCAGC TGGTATTGGC GCGGCTCAAT CAACGGCTCA GATCACCCGC CGGAAAACCC   
  
  
+ CTCCACCGCG CCGCCTTCTA CTTCAAGGAG GCCCTCCAAC ACCTCGCTGT CGGCGGAGGC CCGGCCCGGC   
  
  
+ CTGTCCGGCT GTCATCCTAC GAGGTGGTGC AGACCATCCG GGCCCACAAG AACTTTTCCG GCATATCACT   
  
  
+ GATATCCCTC TTCTCCACCT TCGCCGCCAA TCAGGCGATT CTGGAGGCGG TGGATGGGGC TGCGTTCATC   
  
  
+ CACATCATCG ATTTCGACAT CGGATTTGGC GGCCACTGGG CGTCGTTTAT GCGTGAGCTT GTCGACAAGG   
  
  
+ CCGACCCGAA TAAGATCAGC TCCTTGGTGT TCCGAATCAC GGCGATCGTA CCCGAAGAGT TCGGGATCGA   
  
  
+ AAGCAAGCTG GTGAGAGAGA ATCTTACGCA ATTCGCTCGT GATCTCAACA TCAATGTCCA TATGGAGTAT   
  
  
+ GTCCTTATTC AAAGCTTCGA GTTCTTATCC TTCAAATCGG TCAAATTCAT CGACGGTGAG AAATTGGCGG   
  
  
+ TCCATCTATC TCCGGCGATG TTTCACCGGC TTCAAGGCGG AATCTCGAAA TTCCTAGCCG ATCTCCGGGG   
  
  
+ AATTTCGCCA AGCACCGTCG TGGTGGTGGA CAGTGAGGTA GGAATCGACA CGGGAACTTC GTCCTTCAGC   
  
  
+ GTCAACTTCG TCGCCGGTAT CGAATTCTAC ACCGGAATGC TCGAATCGCT CGACGTCACC TCCGCCAGCG   
  
  
+ GTTTTGCAGG CGGCGGCGGC GGCGACGCTG TGAGAAGGAT CGAGACGTAC GTGCTCCGGC CGAGGATTAC   
  
  
+ GGCGGCGGTG GAGGCGGCAT CTGCGGCGGC GGTAGCGCGG CGGAGCGGTT GGAGAGAGTC GTTCGCGGTG   
  
  
+ GCTAGGATGA GGGCGGTGGG GTTCAGCCAG TTTGCTGATT TCCAGGCGGA GTGTTTGCTG AGGAAGGTTC   
  
  
+ AGGTTGGCGG GTTCCACGTG GCGAAACGTC ATGGGGAGAT GATGCTTTGC TGGCATGATA GGCCCCTTGT   
  
  
+ GTCCACGTCG GCTTGGAGGT GTTA  

- -Up\_Stream \_Len000CTAAAA TGAAGGTTAA AAGATGAATT TAAACCAATA GTGCAAGCTT AAAAAGTCCA   
  
  
- TTTATGTTTG ACAATCACCG GTAGGAAAAA AAAACCCAGA TTAGGTAGGT CTACACGATT ATGAAAGACA   
  
  
- AAATCTACTC TTCAAAATTA CGAAAAAATC TACACATTAA AAAAATTTGT ATTTATCAGA ACAGCTTATT   
  
  
- CTAAACGATC ATTAAAAACA TTCTCTTTAA TACACATAAA AAATAATATA GTCTTATAAT CTAGCTAGAG   
  
  
- TTAAATTAAT GAATACTTTT TTTTTCACCT ATAGAAATTA GTTAAACCGT TCGAGAGTAC GTAATTAACA   
  
  
- TCAGCATGCT TGAATAAATG TAGTGAGTAG ACTTAAACGA TGGTAAATGT GGAGTTTAAA ATAAAATTAC   
  
  
- GGTATTTCTC TTATCTTATA TGACAGATAA ACATCATCAA AATGTCTATT TTTGTTTATT CGTTTACTTT   
  
  
- TATCTATCTC CTAAAAAAAC GATAATAGGT GCAGCTAGGT AACTTAATAA AACAGCGATA AACCAATTCA   
  
  
- CTTTATTTTA TGACTTCATC TTACCTCCTA CAACTATTAC TGGATACAGG GAGTTTCAAA TTAGTACATC   
  
  
- AAAGATGCTT AAAGTTCGGT AATATTCTGT ATGTCTAAAA ATACTACAAC TTTTATGAGA ATGGAGGAAC   
  
  
- AGTGTTATAA TTGGACTTCG GTTACTTTAC TTTGTCTTGA TTAATTTAAC TTATGTATTA GTTAGGTATA   
  
  
- ATTAAACAAA ACTGTTGATA AATGTTAGTA GCTAGAGAAA TTAATCCAGA TATTCGATAT CTCATTAATG   
  
  
- AGTTTTGACA AAACATTCTG TTGAATGAGG TATAACCGAA TAATCATATA ACGGAGGTAA GTGTACTTAT   
  
  
- GAAGTACTCT CTAACGGATT TTGAGTATAA CAGTAGTGGA ATTCAGTTTG TATAAAACAA TACTTTACTG   
  
  
- TAGTTCTTAA TGGGAGACTG TTCTTTTTAT ATGGACTGAC TGTATTAGCA TGGTGTAGAC AATCAGGAGA   
  
  
- AAGATGGTGA AACATCTTGT ATTACACTGT CATTATGTAG GTAGGGTTTT CTTTTCTGCT TTTCTTCTCA   
  
  
- CCTCTCAGTG CATATGCGCA ACTTAAGTCC TTTTAATTTC CTTGAGTACT TCTGGGGTAT ATCACGAGGT   
  
  
- TGGACTTGGA CGTCGGGTGA TGATGTACGG ATTTAATTTC TCGTTTCAAT ACTCTCTTTG GTTTTTTCGA   
  
  
- GAAACTCTCT CACCTCCTTC TTAGTACCTT TCGATCGATC CGAATCGTTC TTTCTCGTTG TCGATCGATC   
  
  
- GTTTTCCGAA GAGAAACTTC GGAATCTTTA GAAATCTCTA TTTTTCAACC ACCGAAAAAT TCCCGACACT   
  
  
- ATACAGGGGG TACATTTTTA CGTCGTTATT ACAATTACAG TACGAAAGAA AGTACCAGGG ATTACGCTTT   
  
  
- AGTTAGTACG TACAGTTGCA CTGAGTCACC GAGTGGTATA TACTAGACTA AAGGGGCGTG TGTCTCTATT   
  
  
- TTTCGGGGGT TAATATTGGA GGGTTTTCGG GGGGGAAAAG GAGGATGGGT TGGGTGTGGA GGGGTAAAGT   
  
  
- AGTCCCGAGC GAAGAATTCA ATAGCTAATT TTTTTCTCAC TCATTTGCAT GGGCTTAACT TCTCCCCACA   
  
  
- AGCTTCGAAT ATGAACGAAC TAATCCTTAC TTTCAGGGGT AAAGTAGGCG GGGGGTTTGG CGGCGGTGGT   
  
  
- GGTCATTTAG CGATAGAACG TTGTTATGAC GGAACTTATG ACGGAAAGTT CGGGGGTTAA GTTAAAGTTG   
  
  
- GGAACAATTG TTGTTGTTTG GGAGGATGCT CGGATGGAGC CAAGATCTGG AGGCCGCGTC GGGTTCGGGG   
  
  
- CGGTGGTTGC GGCGGGACGG TGACGGGTGA CTGCGCCCGT AGTTATTTGG GTTCGGACGG AGACGACTGG   
  
  
- ACCTGTTAAA CCTCCCACTC CCAACCCTAA GTTAGGATAG TCTCGACCCC GAATTACTAC TAAGATTATT   
  
  
- AGGATTGAGG TTTGAAACCA GTAGGGTTTA ATCGAGTAGT ACACTAAGGG TGGAGTGTGT CGAGGGACTC   
  
  
- AAGGGGAGGA GAGTCGGCAA CCTAGTGTGT GCGTGGGGGT TATTAATTGA AGAGGGGGAG ACTAATGGGG   
  
  
- TAGAGACTAA ATAGGATGCC AGTCTTAGAG TCGGGGTTAA AACCCGTCAA ACTCGAAGTA GAAGTAGTCG   
  
  
- GGTTGCAGTT ATTGTTACCC GAACTGAAGT ATCTCCTGGA TTAATCTCGT CGAGTTCGGA TGCTTAGGTT   
  
  
- GCTGAGGGTG GTCCAGGTCG ACCATAACCG CGCCGAGTTA GTTGCCGAGT CTAGTGGGCG GCCTTTTGGG   
  
  
- GAGGTGGCGC GGCGGAAGAT GAAGTTCCTC CGGGAGGTTG TGGAGCGACA GCCGCCTCCG GGCCGGGCCG   
  
  
- GACAGGCCGA CAGTAGGATG CTCCACCACG TCTGGTAGGC CCGGGTGTTC TTGAAAAGGC CGTATAGTGA   
  
  
- CTATAGGGAG AAGAGGTGGA AGCGGCGGTT AGTCCGCTAA GACCTCCGCC ACCTACCCCG ACGCAAGTAG   
  
  
- GTGTAGTAGC TAAAGCTGTA GCCTAAACCG CCGGTGACCC GCAGCAAATA CGCACTCGAA CAGCTGTTCC   
  
  
- GGCTGGGCTT ATTCTAGTCG AGGAACCACA AGGCTTAGTG CCGCTAGCAT GGGCTTCTCA AGCCCTAGCT   
  
  
- TTCGTTCGAC CACTCTCTCT TAGAATGCGT TAAGCGAGCA CTAGAGTTGT AGTTACAGGT ATACCTCATA   
  
  
- CAGGAATAAG TTTCGAAGCT CAAGAATAGG AAGTTTAGCC AGTTTAAGTA GCTGCCACTC TTTAACCGCC   
  
  
- AGGTAGATAG AGGCCGCTAC AAAGTGGCCG AAGTTCCGCC TTAGAGCTTT AAGGATCGGC TAGAGGCCCC   
  
  
- TTAAAGCGGT TCGTGGCAGC ACCACCACCT GTCACTCCAT CCTTAGCTGT GCCCTTGAAG CAGGAAGTCG   
  
  
- CAGTTGAAGC AGCGGCCATA GCTTAAGATG TGGCCTTACG AGCTTAGCGA GCTGCAGTGG AGGCGGTCGC   
  
  
- CAAAACGTCC GCCGCCGCCG CCGCTGCGAC ACTCTTCCTA GCTCTGCATG CACGAGGCCG GCTCCTAATG   
  
  
- CCGCCGCCAC CTCCGCCGTA GACGCCGCCG CCATCGCGCC GCCTCGCCAA CCTCTCTCAG CAAGCGCCAC   
  
  
- CGATCCTACT CCCGCCACCC CAAGTCGGTC AAACGACTAA AGGTCCGCCT CACAAACGAC TCCTTCCAAG   
  
  
- TCCAACCGCC CAAGGTGCAC CGCTTTGCAG TACCCCTCTA CTACGAAACG ACCGTACTAT CCGGGGAACA   
  
  
- CAGGTGCAGC CGAACCTCCA CAAT

+     TC-rich repeats

| Site Name | Organism | Position | Strand | Matrix score. | sequence | function |
| --- | --- | --- | --- | --- | --- | --- |
| TC-rich repeats | Nicotiana tabacum | 858 | - | 9 | GTTTTCTTAC | cis-acting element involved in defense and stress responsiveness |

>HU06G00283.1   
+ -Up\_Stream \_Len000GATTTT ACTTCCAATT TTCTACTTAA ATTTGGTTAT CACGTTCGAA TTTTTCAGGT   
  
  
+ AAATACAAAC TGTTAGTGGC CATCCTTTTT TTTTGGGTCT AATCCATCCA GATGTGCTAA TACTTTCTGT   
  
  
+ TTTAGATGAG AAGTTTTAAT GCTTTTTTAG ATGTGTAATT TTTTTAAACA TAAATAGTCT TGTCGAATAA   
  
  
+ GATTTGCTAG TAATTTTTGT AAGAGAAATT ATGTGTATTT TTTATTATAT CAGAATATTA GATCGATCTC   
  
  
+ AATTTAATTA CTTATGAAAA AAAAAGTGGA TATCTTTAAT CAATTTGGCA AGCTCTCATG CATTAATTGT   
  
  
+ AGTCGTACGA ACTTATTTAC ATCACTCATC TGAATTTGCT ACCATTTACA CCTCAAATTT TATTTTAATG   
  
  
+ CCATAAAGAG AATAGAATAT ACTGTCTATT TGTAGTAGTT TTACAGATAA AAACAAATAA GCAAATGAAA   
  
  
+ ATAGATAGAG GATTTTTTTG CTATTATCCA CGTCGATCCA TTGAATTATT TTGTCGCTAT TTGGTTAAGT   
  
  
+ GAAATAAAAT ACTGAAGTAG AATGGAGGAT GTTGATAATG ACCTATGTCC CTCAAAGTTT AATCATGTAG   
  
  
+ TTTCTACGAA TTTCAAGCCA TTATAAGACA TACAGATTTT TATGATGTTG AAAATACTCT TACCTCCTTG   
  
  
+ TCACAATATT AACCTGAAGC CAATGAAATG AAACAGAACT AATTAAATTG AATACATAAT CAATCCATAT   
  
  
+ TAATTTGTTT TGACAACTAT TTACAATCAT CGATCTCTTT AATTAGGTCT ATAAGCTATA GAGTAATTAC   
  
  
+ TCAAAACTGT TTTGTAAGAC AACTTACTCC ATATTGGCTT ATTAGTATAT TGCCTCCATT CACATGAATA   
  
  
+ CTTCATGAGA GATTGCCTAA AACTCATATT GTCATCACCT TAAGTCAAAC ATATTTTGTT ATGAAATGAC   
  
  
+ ATCAAGAATT ACCCTCTGAC AAGAAAAATA TACCTGACTG ACATAATCGT ACCACATCTG TTAGTCCTCT   
  
  
+ TTCTACCACT TTGTAGAACA TAATGTGACA GTAATACATC CATCCCAAAA GAAAAGACGA AAAGAAGAGT   
  
  
+ GGAGAGTCAC GTATACGCGT TGAATTCAGG AAAATTAAAG GAACTCATGA AGACCCCATA TAGTGCTCCA   
  
  
+ ACCTGAACCT GCAGCCCACT ACTACATGCC TAAATTAAAG AGCAAAGTTA TGAGAGAAAC CAAAAAAGCT   
  
  
+ CTTTGAGAGA GTGGAGGAAG AATCATGGAA AGCTAGCTAG GCTTAGCAAG AAAGAGCAAC AGCTAGCTAG   
  
  
+ CAAAAGGCTT CTCTTTGAAG CCTTAGAAAT CTTTAGAGAT AAAAAGTTGG TGGCTTTTTA AGGGCTGTGA   
  
  
+ TATGTCCCCC ATGTAAAAAT GCAGCAATAA TGTTAATGTC ATGCTTTCTT TCATGGTCCC TAATGCGAAA   
  
  
+ TCAATCATGC ATGTCAACGT GACTCAGTGG CTCACCATAT ATGATCTGAT TTCCCCGCAC ACAGAGATAA   
  
  
+ AAAGCCCCCA ATTATAACCT CCCAAAAGCC CCCCCTTTTC CTCCTACCCA ACCCACACCT CCCCATTTCA   
  
  
+ TCAGGGCTCG CTTCTTAAGT TATCGATTAA AAAAAGAGTG AGTAAACGTA CCCGAATTGA AGAGGGGTGT   
  
  
+ TCGAAGCTTA TACTTGCTTG ATTAGGAATG AAAGTCCCCA TTTCATCCGC CCCCCAAACC GCCGCCACCA   
  
  
+ CCAGTAAATC GCTATCTTGC AACAATACTG CCTTGAATAC TGCCTTTCAA GCCCCCAATT CAATTTCAAC   
  
  
+ CCTTGTTAAC AACAACAAAC CCTCCTACGA GCCTACCTCG GTTCTAGACC TCCGGCGCAG CCCAAGCCCC   
  
  
+ GCCACCAACG CCGCCCTGCC ACTGCCCACT GACGCGGGCA TCAATAAACC CAAGCCTGCC TCTGCTGACC   
  
  
+ TGGACAATTT GGAGGGTGAG GGTTGGGATT CAATCCTATC AGAGCTGGGG CTTAATGATG ATTCTAATAA   
  
  
+ TCCTAACTCC AAACTTTGGT CATCCCAAAT TAGCTCATCA TGTGATTCCC ACCTCACACA GCTCCCTGAG   
  
  
+ TTCCCCTCCT CTCAGCCGTT GGATCACACA CGCACCCCCA ATAATTAACT TCTCCCCCTC TGATTACCCC   
  
  
+ ATCTCTGATT TATCCTACGG TCAGAATCTC AGCCCCAATT TTGGGCAGTT TGAGCTTCAT CTTCATCAGC   
  
  
+ CCAACGTCAA TAACAATGGG CTTGACTTCA TAGAGGACCT AATTAGAGCA GCTCAAGCCT ACGAATCCAA   
  
  
+ CGACTCCCAC CAGGTCCAGC TGGTATTGGC GCGGCTCAAT CAACGGCTCA GATCACCCGC CGGAAAACCC   
  
  
+ CTCCACCGCG CCGCCTTCTA CTTCAAGGAG GCCCTCCAAC ACCTCGCTGT CGGCGGAGGC CCGGCCCGGC   
  
  
+ CTGTCCGGCT GTCATCCTAC GAGGTGGTGC AGACCATCCG GGCCCACAAG AACTTTTCCG GCATATCACT   
  
  
+ GATATCCCTC TTCTCCACCT TCGCCGCCAA TCAGGCGATT CTGGAGGCGG TGGATGGGGC TGCGTTCATC   
  
  
+ CACATCATCG ATTTCGACAT CGGATTTGGC GGCCACTGGG CGTCGTTTAT GCGTGAGCTT GTCGACAAGG   
  
  
+ CCGACCCGAA TAAGATCAGC TCCTTGGTGT TCCGAATCAC GGCGATCGTA CCCGAAGAGT TCGGGATCGA   
  
  
+ AAGCAAGCTG GTGAGAGAGA ATCTTACGCA ATTCGCTCGT GATCTCAACA TCAATGTCCA TATGGAGTAT   
  
  
+ GTCCTTATTC AAAGCTTCGA GTTCTTATCC TTCAAATCGG TCAAATTCAT CGACGGTGAG AAATTGGCGG   
  
  
+ TCCATCTATC TCCGGCGATG TTTCACCGGC TTCAAGGCGG AATCTCGAAA TTCCTAGCCG ATCTCCGGGG   
  
  
+ AATTTCGCCA AGCACCGTCG TGGTGGTGGA CAGTGAGGTA GGAATCGACA CGGGAACTTC GTCCTTCAGC   
  
  
+ GTCAACTTCG TCGCCGGTAT CGAATTCTAC ACCGGAATGC TCGAATCGCT CGACGTCACC TCCGCCAGCG   
  
  
+ GTTTTGCAGG CGGCGGCGGC GGCGACGCTG TGAGAAGGAT CGAGACGTAC GTGCTCCGGC CGAGGATTAC   
  
  
+ GGCGGCGGTG GAGGCGGCAT CTGCGGCGGC GGTAGCGCGG CGGAGCGGTT GGAGAGAGTC GTTCGCGGTG   
  
  
+ GCTAGGATGA GGGCGGTGGG GTTCAGCCAG TTTGCTGATT TCCAGGCGGA GTGTTTGCTG AGGAAGGTTC   
  
  
+ AGGTTGGCGG GTTCCACGTG GCGAAACGTC ATGGGGAGAT GATGCTTTGC TGGCATGATA GGCCCCTTGT   
  
  
+ GTCCACGTCG GCTTGGAGGT GTTA  

- -Up\_Stream \_Len000CTAAAA TGAAGGTTAA AAGATGAATT TAAACCAATA GTGCAAGCTT AAAAAGTCCA   
  
  
- TTTATGTTTG ACAATCACCG GTAGGAAAAA AAAACCCAGA TTAGGTAGGT CTACACGATT ATGAAAGACA   
  
  
- AAATCTACTC TTCAAAATTA CGAAAAAATC TACACATTAA AAAAATTTGT ATTTATCAGA ACAGCTTATT   
  
  
- CTAAACGATC ATTAAAAACA TTCTCTTTAA TACACATAAA AAATAATATA GTCTTATAAT CTAGCTAGAG   
  
  
- TTAAATTAAT GAATACTTTT TTTTTCACCT ATAGAAATTA GTTAAACCGT TCGAGAGTAC GTAATTAACA   
  
  
- TCAGCATGCT TGAATAAATG TAGTGAGTAG ACTTAAACGA TGGTAAATGT GGAGTTTAAA ATAAAATTAC   
  
  
- GGTATTTCTC TTATCTTATA TGACAGATAA ACATCATCAA AATGTCTATT TTTGTTTATT CGTTTACTTT   
  
  
- TATCTATCTC CTAAAAAAAC GATAATAGGT GCAGCTAGGT AACTTAATAA AACAGCGATA AACCAATTCA   
  
  
- CTTTATTTTA TGACTTCATC TTACCTCCTA CAACTATTAC TGGATACAGG GAGTTTCAAA TTAGTACATC   
  
  
- AAAGATGCTT AAAGTTCGGT AATATTCTGT ATGTCTAAAA ATACTACAAC TTTTATGAGA ATGGAGGAAC   
  
  
- AGTGTTATAA TTGGACTTCG GTTACTTTAC TTTGTCTTGA TTAATTTAAC TTATGTATTA GTTAGGTATA   
  
  
- ATTAAACAAA ACTGTTGATA AATGTTAGTA GCTAGAGAAA TTAATCCAGA TATTCGATAT CTCATTAATG   
  
  
- AGTTTTGACA AAACATTCTG TTGAATGAGG TATAACCGAA TAATCATATA ACGGAGGTAA GTGTACTTAT   
  
  
- GAAGTACTCT CTAACGGATT TTGAGTATAA CAGTAGTGGA ATTCAGTTTG TATAAAACAA TACTTTACTG   
  
  
- TAGTTCTTAA TGGGAGACTG TTCTTTTTAT ATGGACTGAC TGTATTAGCA TGGTGTAGAC AATCAGGAGA   
  
  
- AAGATGGTGA AACATCTTGT ATTACACTGT CATTATGTAG GTAGGGTTTT CTTTTCTGCT TTTCTTCTCA   
  
  
- CCTCTCAGTG CATATGCGCA ACTTAAGTCC TTTTAATTTC CTTGAGTACT TCTGGGGTAT ATCACGAGGT   
  
  
- TGGACTTGGA CGTCGGGTGA TGATGTACGG ATTTAATTTC TCGTTTCAAT ACTCTCTTTG GTTTTTTCGA   
  
  
- GAAACTCTCT CACCTCCTTC TTAGTACCTT TCGATCGATC CGAATCGTTC TTTCTCGTTG TCGATCGATC   
  
  
- GTTTTCCGAA GAGAAACTTC GGAATCTTTA GAAATCTCTA TTTTTCAACC ACCGAAAAAT TCCCGACACT   
  
  
- ATACAGGGGG TACATTTTTA CGTCGTTATT ACAATTACAG TACGAAAGAA AGTACCAGGG ATTACGCTTT   
  
  
- AGTTAGTACG TACAGTTGCA CTGAGTCACC GAGTGGTATA TACTAGACTA AAGGGGCGTG TGTCTCTATT   
  
  
- TTTCGGGGGT TAATATTGGA GGGTTTTCGG GGGGGAAAAG GAGGATGGGT TGGGTGTGGA GGGGTAAAGT   
  
  
- AGTCCCGAGC GAAGAATTCA ATAGCTAATT TTTTTCTCAC TCATTTGCAT GGGCTTAACT TCTCCCCACA   
  
  
- AGCTTCGAAT ATGAACGAAC TAATCCTTAC TTTCAGGGGT AAAGTAGGCG GGGGGTTTGG CGGCGGTGGT   
  
  
- GGTCATTTAG CGATAGAACG TTGTTATGAC GGAACTTATG ACGGAAAGTT CGGGGGTTAA GTTAAAGTTG   
  
  
- GGAACAATTG TTGTTGTTTG GGAGGATGCT CGGATGGAGC CAAGATCTGG AGGCCGCGTC GGGTTCGGGG   
  
  
- CGGTGGTTGC GGCGGGACGG TGACGGGTGA CTGCGCCCGT AGTTATTTGG GTTCGGACGG AGACGACTGG   
  
  
- ACCTGTTAAA CCTCCCACTC CCAACCCTAA GTTAGGATAG TCTCGACCCC GAATTACTAC TAAGATTATT   
  
  
- AGGATTGAGG TTTGAAACCA GTAGGGTTTA ATCGAGTAGT ACACTAAGGG TGGAGTGTGT CGAGGGACTC   
  
  
- AAGGGGAGGA GAGTCGGCAA CCTAGTGTGT GCGTGGGGGT TATTAATTGA AGAGGGGGAG ACTAATGGGG   
  
  
- TAGAGACTAA ATAGGATGCC AGTCTTAGAG TCGGGGTTAA AACCCGTCAA ACTCGAAGTA GAAGTAGTCG   
  
  
- GGTTGCAGTT ATTGTTACCC GAACTGAAGT ATCTCCTGGA TTAATCTCGT CGAGTTCGGA TGCTTAGGTT   
  
  
- GCTGAGGGTG GTCCAGGTCG ACCATAACCG CGCCGAGTTA GTTGCCGAGT CTAGTGGGCG GCCTTTTGGG   
  
  
- GAGGTGGCGC GGCGGAAGAT GAAGTTCCTC CGGGAGGTTG TGGAGCGACA GCCGCCTCCG GGCCGGGCCG   
  
  
- GACAGGCCGA CAGTAGGATG CTCCACCACG TCTGGTAGGC CCGGGTGTTC TTGAAAAGGC CGTATAGTGA   
  
  
- CTATAGGGAG AAGAGGTGGA AGCGGCGGTT AGTCCGCTAA GACCTCCGCC ACCTACCCCG ACGCAAGTAG   
  
  
- GTGTAGTAGC TAAAGCTGTA GCCTAAACCG CCGGTGACCC GCAGCAAATA CGCACTCGAA CAGCTGTTCC   
  
  
- GGCTGGGCTT ATTCTAGTCG AGGAACCACA AGGCTTAGTG CCGCTAGCAT GGGCTTCTCA AGCCCTAGCT   
  
  
- TTCGTTCGAC CACTCTCTCT TAGAATGCGT TAAGCGAGCA CTAGAGTTGT AGTTACAGGT ATACCTCATA   
  
  
- CAGGAATAAG TTTCGAAGCT CAAGAATAGG AAGTTTAGCC AGTTTAAGTA GCTGCCACTC TTTAACCGCC   
  
  
- AGGTAGATAG AGGCCGCTAC AAAGTGGCCG AAGTTCCGCC TTAGAGCTTT AAGGATCGGC TAGAGGCCCC   
  
  
- TTAAAGCGGT TCGTGGCAGC ACCACCACCT GTCACTCCAT CCTTAGCTGT GCCCTTGAAG CAGGAAGTCG   
  
  
- CAGTTGAAGC AGCGGCCATA GCTTAAGATG TGGCCTTACG AGCTTAGCGA GCTGCAGTGG AGGCGGTCGC   
  
  
- CAAAACGTCC GCCGCCGCCG CCGCTGCGAC ACTCTTCCTA GCTCTGCATG CACGAGGCCG GCTCCTAATG   
  
  
- CCGCCGCCAC CTCCGCCGTA GACGCCGCCG CCATCGCGCC GCCTCGCCAA CCTCTCTCAG CAAGCGCCAC   
  
  
- CGATCCTACT CCCGCCACCC CAAGTCGGTC AAACGACTAA AGGTCCGCCT CACAAACGAC TCCTTCCAAG   
  
  
- TCCAACCGCC CAAGGTGCAC CGCTTTGCAG TACCCCTCTA CTACGAAACG ACCGTACTAT CCGGGGAACA   
  
  
- CAGGTGCAGC CGAACCTCCA CAAT

+     TCA

| Site Name | Organism | Position | Strand | Matrix score. | sequence | function |
| --- | --- | --- | --- | --- | --- | --- |
| TCA | Pisum sativum | 2231 | + | 10 | TCATCTTCAT |  |

>HU06G00283.1   
+ -Up\_Stream \_Len000GATTTT ACTTCCAATT TTCTACTTAA ATTTGGTTAT CACGTTCGAA TTTTTCAGGT   
  
  
+ AAATACAAAC TGTTAGTGGC CATCCTTTTT TTTTGGGTCT AATCCATCCA GATGTGCTAA TACTTTCTGT   
  
  
+ TTTAGATGAG AAGTTTTAAT GCTTTTTTAG ATGTGTAATT TTTTTAAACA TAAATAGTCT TGTCGAATAA   
  
  
+ GATTTGCTAG TAATTTTTGT AAGAGAAATT ATGTGTATTT TTTATTATAT CAGAATATTA GATCGATCTC   
  
  
+ AATTTAATTA CTTATGAAAA AAAAAGTGGA TATCTTTAAT CAATTTGGCA AGCTCTCATG CATTAATTGT   
  
  
+ AGTCGTACGA ACTTATTTAC ATCACTCATC TGAATTTGCT ACCATTTACA CCTCAAATTT TATTTTAATG   
  
  
+ CCATAAAGAG AATAGAATAT ACTGTCTATT TGTAGTAGTT TTACAGATAA AAACAAATAA GCAAATGAAA   
  
  
+ ATAGATAGAG GATTTTTTTG CTATTATCCA CGTCGATCCA TTGAATTATT TTGTCGCTAT TTGGTTAAGT   
  
  
+ GAAATAAAAT ACTGAAGTAG AATGGAGGAT GTTGATAATG ACCTATGTCC CTCAAAGTTT AATCATGTAG   
  
  
+ TTTCTACGAA TTTCAAGCCA TTATAAGACA TACAGATTTT TATGATGTTG AAAATACTCT TACCTCCTTG   
  
  
+ TCACAATATT AACCTGAAGC CAATGAAATG AAACAGAACT AATTAAATTG AATACATAAT CAATCCATAT   
  
  
+ TAATTTGTTT TGACAACTAT TTACAATCAT CGATCTCTTT AATTAGGTCT ATAAGCTATA GAGTAATTAC   
  
  
+ TCAAAACTGT TTTGTAAGAC AACTTACTCC ATATTGGCTT ATTAGTATAT TGCCTCCATT CACATGAATA   
  
  
+ CTTCATGAGA GATTGCCTAA AACTCATATT GTCATCACCT TAAGTCAAAC ATATTTTGTT ATGAAATGAC   
  
  
+ ATCAAGAATT ACCCTCTGAC AAGAAAAATA TACCTGACTG ACATAATCGT ACCACATCTG TTAGTCCTCT   
  
  
+ TTCTACCACT TTGTAGAACA TAATGTGACA GTAATACATC CATCCCAAAA GAAAAGACGA AAAGAAGAGT   
  
  
+ GGAGAGTCAC GTATACGCGT TGAATTCAGG AAAATTAAAG GAACTCATGA AGACCCCATA TAGTGCTCCA   
  
  
+ ACCTGAACCT GCAGCCCACT ACTACATGCC TAAATTAAAG AGCAAAGTTA TGAGAGAAAC CAAAAAAGCT   
  
  
+ CTTTGAGAGA GTGGAGGAAG AATCATGGAA AGCTAGCTAG GCTTAGCAAG AAAGAGCAAC AGCTAGCTAG   
  
  
+ CAAAAGGCTT CTCTTTGAAG CCTTAGAAAT CTTTAGAGAT AAAAAGTTGG TGGCTTTTTA AGGGCTGTGA   
  
  
+ TATGTCCCCC ATGTAAAAAT GCAGCAATAA TGTTAATGTC ATGCTTTCTT TCATGGTCCC TAATGCGAAA   
  
  
+ TCAATCATGC ATGTCAACGT GACTCAGTGG CTCACCATAT ATGATCTGAT TTCCCCGCAC ACAGAGATAA   
  
  
+ AAAGCCCCCA ATTATAACCT CCCAAAAGCC CCCCCTTTTC CTCCTACCCA ACCCACACCT CCCCATTTCA   
  
  
+ TCAGGGCTCG CTTCTTAAGT TATCGATTAA AAAAAGAGTG AGTAAACGTA CCCGAATTGA AGAGGGGTGT   
  
  
+ TCGAAGCTTA TACTTGCTTG ATTAGGAATG AAAGTCCCCA TTTCATCCGC CCCCCAAACC GCCGCCACCA   
  
  
+ CCAGTAAATC GCTATCTTGC AACAATACTG CCTTGAATAC TGCCTTTCAA GCCCCCAATT CAATTTCAAC   
  
  
+ CCTTGTTAAC AACAACAAAC CCTCCTACGA GCCTACCTCG GTTCTAGACC TCCGGCGCAG CCCAAGCCCC   
  
  
+ GCCACCAACG CCGCCCTGCC ACTGCCCACT GACGCGGGCA TCAATAAACC CAAGCCTGCC TCTGCTGACC   
  
  
+ TGGACAATTT GGAGGGTGAG GGTTGGGATT CAATCCTATC AGAGCTGGGG CTTAATGATG ATTCTAATAA   
  
  
+ TCCTAACTCC AAACTTTGGT CATCCCAAAT TAGCTCATCA TGTGATTCCC ACCTCACACA GCTCCCTGAG   
  
  
+ TTCCCCTCCT CTCAGCCGTT GGATCACACA CGCACCCCCA ATAATTAACT TCTCCCCCTC TGATTACCCC   
  
  
+ ATCTCTGATT TATCCTACGG TCAGAATCTC AGCCCCAATT TTGGGCAGTT TGAGCTTCAT CTTCATCAGC   
  
  
+ CCAACGTCAA TAACAATGGG CTTGACTTCA TAGAGGACCT AATTAGAGCA GCTCAAGCCT ACGAATCCAA   
  
  
+ CGACTCCCAC CAGGTCCAGC TGGTATTGGC GCGGCTCAAT CAACGGCTCA GATCACCCGC CGGAAAACCC   
  
  
+ CTCCACCGCG CCGCCTTCTA CTTCAAGGAG GCCCTCCAAC ACCTCGCTGT CGGCGGAGGC CCGGCCCGGC   
  
  
+ CTGTCCGGCT GTCATCCTAC GAGGTGGTGC AGACCATCCG GGCCCACAAG AACTTTTCCG GCATATCACT   
  
  
+ GATATCCCTC TTCTCCACCT TCGCCGCCAA TCAGGCGATT CTGGAGGCGG TGGATGGGGC TGCGTTCATC   
  
  
+ CACATCATCG ATTTCGACAT CGGATTTGGC GGCCACTGGG CGTCGTTTAT GCGTGAGCTT GTCGACAAGG   
  
  
+ CCGACCCGAA TAAGATCAGC TCCTTGGTGT TCCGAATCAC GGCGATCGTA CCCGAAGAGT TCGGGATCGA   
  
  
+ AAGCAAGCTG GTGAGAGAGA ATCTTACGCA ATTCGCTCGT GATCTCAACA TCAATGTCCA TATGGAGTAT   
  
  
+ GTCCTTATTC AAAGCTTCGA GTTCTTATCC TTCAAATCGG TCAAATTCAT CGACGGTGAG AAATTGGCGG   
  
  
+ TCCATCTATC TCCGGCGATG TTTCACCGGC TTCAAGGCGG AATCTCGAAA TTCCTAGCCG ATCTCCGGGG   
  
  
+ AATTTCGCCA AGCACCGTCG TGGTGGTGGA CAGTGAGGTA GGAATCGACA CGGGAACTTC GTCCTTCAGC   
  
  
+ GTCAACTTCG TCGCCGGTAT CGAATTCTAC ACCGGAATGC TCGAATCGCT CGACGTCACC TCCGCCAGCG   
  
  
+ GTTTTGCAGG CGGCGGCGGC GGCGACGCTG TGAGAAGGAT CGAGACGTAC GTGCTCCGGC CGAGGATTAC   
  
  
+ GGCGGCGGTG GAGGCGGCAT CTGCGGCGGC GGTAGCGCGG CGGAGCGGTT GGAGAGAGTC GTTCGCGGTG   
  
  
+ GCTAGGATGA GGGCGGTGGG GTTCAGCCAG TTTGCTGATT TCCAGGCGGA GTGTTTGCTG AGGAAGGTTC   
  
  
+ AGGTTGGCGG GTTCCACGTG GCGAAACGTC ATGGGGAGAT GATGCTTTGC TGGCATGATA GGCCCCTTGT   
  
  
+ GTCCACGTCG GCTTGGAGGT GTTA  

- -Up\_Stream \_Len000CTAAAA TGAAGGTTAA AAGATGAATT TAAACCAATA GTGCAAGCTT AAAAAGTCCA   
  
  
- TTTATGTTTG ACAATCACCG GTAGGAAAAA AAAACCCAGA TTAGGTAGGT CTACACGATT ATGAAAGACA   
  
  
- AAATCTACTC TTCAAAATTA CGAAAAAATC TACACATTAA AAAAATTTGT ATTTATCAGA ACAGCTTATT   
  
  
- CTAAACGATC ATTAAAAACA TTCTCTTTAA TACACATAAA AAATAATATA GTCTTATAAT CTAGCTAGAG   
  
  
- TTAAATTAAT GAATACTTTT TTTTTCACCT ATAGAAATTA GTTAAACCGT TCGAGAGTAC GTAATTAACA   
  
  
- TCAGCATGCT TGAATAAATG TAGTGAGTAG ACTTAAACGA TGGTAAATGT GGAGTTTAAA ATAAAATTAC   
  
  
- GGTATTTCTC TTATCTTATA TGACAGATAA ACATCATCAA AATGTCTATT TTTGTTTATT CGTTTACTTT   
  
  
- TATCTATCTC CTAAAAAAAC GATAATAGGT GCAGCTAGGT AACTTAATAA AACAGCGATA AACCAATTCA   
  
  
- CTTTATTTTA TGACTTCATC TTACCTCCTA CAACTATTAC TGGATACAGG GAGTTTCAAA TTAGTACATC   
  
  
- AAAGATGCTT AAAGTTCGGT AATATTCTGT ATGTCTAAAA ATACTACAAC TTTTATGAGA ATGGAGGAAC   
  
  
- AGTGTTATAA TTGGACTTCG GTTACTTTAC TTTGTCTTGA TTAATTTAAC TTATGTATTA GTTAGGTATA   
  
  
- ATTAAACAAA ACTGTTGATA AATGTTAGTA GCTAGAGAAA TTAATCCAGA TATTCGATAT CTCATTAATG   
  
  
- AGTTTTGACA AAACATTCTG TTGAATGAGG TATAACCGAA TAATCATATA ACGGAGGTAA GTGTACTTAT   
  
  
- GAAGTACTCT CTAACGGATT TTGAGTATAA CAGTAGTGGA ATTCAGTTTG TATAAAACAA TACTTTACTG   
  
  
- TAGTTCTTAA TGGGAGACTG TTCTTTTTAT ATGGACTGAC TGTATTAGCA TGGTGTAGAC AATCAGGAGA   
  
  
- AAGATGGTGA AACATCTTGT ATTACACTGT CATTATGTAG GTAGGGTTTT CTTTTCTGCT TTTCTTCTCA   
  
  
- CCTCTCAGTG CATATGCGCA ACTTAAGTCC TTTTAATTTC CTTGAGTACT TCTGGGGTAT ATCACGAGGT   
  
  
- TGGACTTGGA CGTCGGGTGA TGATGTACGG ATTTAATTTC TCGTTTCAAT ACTCTCTTTG GTTTTTTCGA   
  
  
- GAAACTCTCT CACCTCCTTC TTAGTACCTT TCGATCGATC CGAATCGTTC TTTCTCGTTG TCGATCGATC   
  
  
- GTTTTCCGAA GAGAAACTTC GGAATCTTTA GAAATCTCTA TTTTTCAACC ACCGAAAAAT TCCCGACACT   
  
  
- ATACAGGGGG TACATTTTTA CGTCGTTATT ACAATTACAG TACGAAAGAA AGTACCAGGG ATTACGCTTT   
  
  
- AGTTAGTACG TACAGTTGCA CTGAGTCACC GAGTGGTATA TACTAGACTA AAGGGGCGTG TGTCTCTATT   
  
  
- TTTCGGGGGT TAATATTGGA GGGTTTTCGG GGGGGAAAAG GAGGATGGGT TGGGTGTGGA GGGGTAAAGT   
  
  
- AGTCCCGAGC GAAGAATTCA ATAGCTAATT TTTTTCTCAC TCATTTGCAT GGGCTTAACT TCTCCCCACA   
  
  
- AGCTTCGAAT ATGAACGAAC TAATCCTTAC TTTCAGGGGT AAAGTAGGCG GGGGGTTTGG CGGCGGTGGT   
  
  
- GGTCATTTAG CGATAGAACG TTGTTATGAC GGAACTTATG ACGGAAAGTT CGGGGGTTAA GTTAAAGTTG   
  
  
- GGAACAATTG TTGTTGTTTG GGAGGATGCT CGGATGGAGC CAAGATCTGG AGGCCGCGTC GGGTTCGGGG   
  
  
- CGGTGGTTGC GGCGGGACGG TGACGGGTGA CTGCGCCCGT AGTTATTTGG GTTCGGACGG AGACGACTGG   
  
  
- ACCTGTTAAA CCTCCCACTC CCAACCCTAA GTTAGGATAG TCTCGACCCC GAATTACTAC TAAGATTATT   
  
  
- AGGATTGAGG TTTGAAACCA GTAGGGTTTA ATCGAGTAGT ACACTAAGGG TGGAGTGTGT CGAGGGACTC   
  
  
- AAGGGGAGGA GAGTCGGCAA CCTAGTGTGT GCGTGGGGGT TATTAATTGA AGAGGGGGAG ACTAATGGGG   
  
  
- TAGAGACTAA ATAGGATGCC AGTCTTAGAG TCGGGGTTAA AACCCGTCAA ACTCGAAGTA GAAGTAGTCG   
  
  
- GGTTGCAGTT ATTGTTACCC GAACTGAAGT ATCTCCTGGA TTAATCTCGT CGAGTTCGGA TGCTTAGGTT   
  
  
- GCTGAGGGTG GTCCAGGTCG ACCATAACCG CGCCGAGTTA GTTGCCGAGT CTAGTGGGCG GCCTTTTGGG   
  
  
- GAGGTGGCGC GGCGGAAGAT GAAGTTCCTC CGGGAGGTTG TGGAGCGACA GCCGCCTCCG GGCCGGGCCG   
  
  
- GACAGGCCGA CAGTAGGATG CTCCACCACG TCTGGTAGGC CCGGGTGTTC TTGAAAAGGC CGTATAGTGA   
  
  
- CTATAGGGAG AAGAGGTGGA AGCGGCGGTT AGTCCGCTAA GACCTCCGCC ACCTACCCCG ACGCAAGTAG   
  
  
- GTGTAGTAGC TAAAGCTGTA GCCTAAACCG CCGGTGACCC GCAGCAAATA CGCACTCGAA CAGCTGTTCC   
  
  
- GGCTGGGCTT ATTCTAGTCG AGGAACCACA AGGCTTAGTG CCGCTAGCAT GGGCTTCTCA AGCCCTAGCT   
  
  
- TTCGTTCGAC CACTCTCTCT TAGAATGCGT TAAGCGAGCA CTAGAGTTGT AGTTACAGGT ATACCTCATA   
  
  
- CAGGAATAAG TTTCGAAGCT CAAGAATAGG AAGTTTAGCC AGTTTAAGTA GCTGCCACTC TTTAACCGCC   
  
  
- AGGTAGATAG AGGCCGCTAC AAAGTGGCCG AAGTTCCGCC TTAGAGCTTT AAGGATCGGC TAGAGGCCCC   
  
  
- TTAAAGCGGT TCGTGGCAGC ACCACCACCT GTCACTCCAT CCTTAGCTGT GCCCTTGAAG CAGGAAGTCG   
  
  
- CAGTTGAAGC AGCGGCCATA GCTTAAGATG TGGCCTTACG AGCTTAGCGA GCTGCAGTGG AGGCGGTCGC   
  
  
- CAAAACGTCC GCCGCCGCCG CCGCTGCGAC ACTCTTCCTA GCTCTGCATG CACGAGGCCG GCTCCTAATG   
  
  
- CCGCCGCCAC CTCCGCCGTA GACGCCGCCG CCATCGCGCC GCCTCGCCAA CCTCTCTCAG CAAGCGCCAC   
  
  
- CGATCCTACT CCCGCCACCC CAAGTCGGTC AAACGACTAA AGGTCCGCCT CACAAACGAC TCCTTCCAAG   
  
  
- TCCAACCGCC CAAGGTGCAC CGCTTTGCAG TACCCCTCTA CTACGAAACG ACCGTACTAT CCGGGGAACA   
  
  
- CAGGTGCAGC CGAACCTCCA CAAT

+     TCA-element

| Site Name | Organism | Position | Strand | Matrix score. | sequence | function |
| --- | --- | --- | --- | --- | --- | --- |
| TCA-element | Brassica oleracea | 1953 | - | 9 | TCAGAAGAGG | cis-acting element involved in salicylic acid responsiveness |
| TCA-element | Nicotiana tabacum | 1375 | - | 9 | CCATCTTTTT | cis-acting element involved in salicylic acid responsiveness |
| TCA-element | Nicotiana tabacum | 94 | + | 9 | CCATCTTTTT | cis-acting element involved in salicylic acid responsiveness |

>HU06G00283.1   
+ -Up\_Stream \_Len000GATTTT ACTTCCAATT TTCTACTTAA ATTTGGTTAT CACGTTCGAA TTTTTCAGGT   
  
  
+ AAATACAAAC TGTTAGTGGC CATCCTTTTT TTTTGGGTCT AATCCATCCA GATGTGCTAA TACTTTCTGT   
  
  
+ TTTAGATGAG AAGTTTTAAT GCTTTTTTAG ATGTGTAATT TTTTTAAACA TAAATAGTCT TGTCGAATAA   
  
  
+ GATTTGCTAG TAATTTTTGT AAGAGAAATT ATGTGTATTT TTTATTATAT CAGAATATTA GATCGATCTC   
  
  
+ AATTTAATTA CTTATGAAAA AAAAAGTGGA TATCTTTAAT CAATTTGGCA AGCTCTCATG CATTAATTGT   
  
  
+ AGTCGTACGA ACTTATTTAC ATCACTCATC TGAATTTGCT ACCATTTACA CCTCAAATTT TATTTTAATG   
  
  
+ CCATAAAGAG AATAGAATAT ACTGTCTATT TGTAGTAGTT TTACAGATAA AAACAAATAA GCAAATGAAA   
  
  
+ ATAGATAGAG GATTTTTTTG CTATTATCCA CGTCGATCCA TTGAATTATT TTGTCGCTAT TTGGTTAAGT   
  
  
+ GAAATAAAAT ACTGAAGTAG AATGGAGGAT GTTGATAATG ACCTATGTCC CTCAAAGTTT AATCATGTAG   
  
  
+ TTTCTACGAA TTTCAAGCCA TTATAAGACA TACAGATTTT TATGATGTTG AAAATACTCT TACCTCCTTG   
  
  
+ TCACAATATT AACCTGAAGC CAATGAAATG AAACAGAACT AATTAAATTG AATACATAAT CAATCCATAT   
  
  
+ TAATTTGTTT TGACAACTAT TTACAATCAT CGATCTCTTT AATTAGGTCT ATAAGCTATA GAGTAATTAC   
  
  
+ TCAAAACTGT TTTGTAAGAC AACTTACTCC ATATTGGCTT ATTAGTATAT TGCCTCCATT CACATGAATA   
  
  
+ CTTCATGAGA GATTGCCTAA AACTCATATT GTCATCACCT TAAGTCAAAC ATATTTTGTT ATGAAATGAC   
  
  
+ ATCAAGAATT ACCCTCTGAC AAGAAAAATA TACCTGACTG ACATAATCGT ACCACATCTG TTAGTCCTCT   
  
  
+ TTCTACCACT TTGTAGAACA TAATGTGACA GTAATACATC CATCCCAAAA GAAAAGACGA AAAGAAGAGT   
  
  
+ GGAGAGTCAC GTATACGCGT TGAATTCAGG AAAATTAAAG GAACTCATGA AGACCCCATA TAGTGCTCCA   
  
  
+ ACCTGAACCT GCAGCCCACT ACTACATGCC TAAATTAAAG AGCAAAGTTA TGAGAGAAAC CAAAAAAGCT   
  
  
+ CTTTGAGAGA GTGGAGGAAG AATCATGGAA AGCTAGCTAG GCTTAGCAAG AAAGAGCAAC AGCTAGCTAG   
  
  
+ CAAAAGGCTT CTCTTTGAAG CCTTAGAAAT CTTTAGAGAT AAAAAGTTGG TGGCTTTTTA AGGGCTGTGA   
  
  
+ TATGTCCCCC ATGTAAAAAT GCAGCAATAA TGTTAATGTC ATGCTTTCTT TCATGGTCCC TAATGCGAAA   
  
  
+ TCAATCATGC ATGTCAACGT GACTCAGTGG CTCACCATAT ATGATCTGAT TTCCCCGCAC ACAGAGATAA   
  
  
+ AAAGCCCCCA ATTATAACCT CCCAAAAGCC CCCCCTTTTC CTCCTACCCA ACCCACACCT CCCCATTTCA   
  
  
+ TCAGGGCTCG CTTCTTAAGT TATCGATTAA AAAAAGAGTG AGTAAACGTA CCCGAATTGA AGAGGGGTGT   
  
  
+ TCGAAGCTTA TACTTGCTTG ATTAGGAATG AAAGTCCCCA TTTCATCCGC CCCCCAAACC GCCGCCACCA   
  
  
+ CCAGTAAATC GCTATCTTGC AACAATACTG CCTTGAATAC TGCCTTTCAA GCCCCCAATT CAATTTCAAC   
  
  
+ CCTTGTTAAC AACAACAAAC CCTCCTACGA GCCTACCTCG GTTCTAGACC TCCGGCGCAG CCCAAGCCCC   
  
  
+ GCCACCAACG CCGCCCTGCC ACTGCCCACT GACGCGGGCA TCAATAAACC CAAGCCTGCC TCTGCTGACC   
  
  
+ TGGACAATTT GGAGGGTGAG GGTTGGGATT CAATCCTATC AGAGCTGGGG CTTAATGATG ATTCTAATAA   
  
  
+ TCCTAACTCC AAACTTTGGT CATCCCAAAT TAGCTCATCA TGTGATTCCC ACCTCACACA GCTCCCTGAG   
  
  
+ TTCCCCTCCT CTCAGCCGTT GGATCACACA CGCACCCCCA ATAATTAACT TCTCCCCCTC TGATTACCCC   
  
  
+ ATCTCTGATT TATCCTACGG TCAGAATCTC AGCCCCAATT TTGGGCAGTT TGAGCTTCAT CTTCATCAGC   
  
  
+ CCAACGTCAA TAACAATGGG CTTGACTTCA TAGAGGACCT AATTAGAGCA GCTCAAGCCT ACGAATCCAA   
  
  
+ CGACTCCCAC CAGGTCCAGC TGGTATTGGC GCGGCTCAAT CAACGGCTCA GATCACCCGC CGGAAAACCC   
  
  
+ CTCCACCGCG CCGCCTTCTA CTTCAAGGAG GCCCTCCAAC ACCTCGCTGT CGGCGGAGGC CCGGCCCGGC   
  
  
+ CTGTCCGGCT GTCATCCTAC GAGGTGGTGC AGACCATCCG GGCCCACAAG AACTTTTCCG GCATATCACT   
  
  
+ GATATCCCTC TTCTCCACCT TCGCCGCCAA TCAGGCGATT CTGGAGGCGG TGGATGGGGC TGCGTTCATC   
  
  
+ CACATCATCG ATTTCGACAT CGGATTTGGC GGCCACTGGG CGTCGTTTAT GCGTGAGCTT GTCGACAAGG   
  
  
+ CCGACCCGAA TAAGATCAGC TCCTTGGTGT TCCGAATCAC GGCGATCGTA CCCGAAGAGT TCGGGATCGA   
  
  
+ AAGCAAGCTG GTGAGAGAGA ATCTTACGCA ATTCGCTCGT GATCTCAACA TCAATGTCCA TATGGAGTAT   
  
  
+ GTCCTTATTC AAAGCTTCGA GTTCTTATCC TTCAAATCGG TCAAATTCAT CGACGGTGAG AAATTGGCGG   
  
  
+ TCCATCTATC TCCGGCGATG TTTCACCGGC TTCAAGGCGG AATCTCGAAA TTCCTAGCCG ATCTCCGGGG   
  
  
+ AATTTCGCCA AGCACCGTCG TGGTGGTGGA CAGTGAGGTA GGAATCGACA CGGGAACTTC GTCCTTCAGC   
  
  
+ GTCAACTTCG TCGCCGGTAT CGAATTCTAC ACCGGAATGC TCGAATCGCT CGACGTCACC TCCGCCAGCG   
  
  
+ GTTTTGCAGG CGGCGGCGGC GGCGACGCTG TGAGAAGGAT CGAGACGTAC GTGCTCCGGC CGAGGATTAC   
  
  
+ GGCGGCGGTG GAGGCGGCAT CTGCGGCGGC GGTAGCGCGG CGGAGCGGTT GGAGAGAGTC GTTCGCGGTG   
  
  
+ GCTAGGATGA GGGCGGTGGG GTTCAGCCAG TTTGCTGATT TCCAGGCGGA GTGTTTGCTG AGGAAGGTTC   
  
  
+ AGGTTGGCGG GTTCCACGTG GCGAAACGTC ATGGGGAGAT GATGCTTTGC TGGCATGATA GGCCCCTTGT   
  
  
+ GTCCACGTCG GCTTGGAGGT GTTA  

- -Up\_Stream \_Len000CTAAAA TGAAGGTTAA AAGATGAATT TAAACCAATA GTGCAAGCTT AAAAAGTCCA   
  
  
- TTTATGTTTG ACAATCACCG GTAGGAAAAA AAAACCCAGA TTAGGTAGGT CTACACGATT ATGAAAGACA   
  
  
- AAATCTACTC TTCAAAATTA CGAAAAAATC TACACATTAA AAAAATTTGT ATTTATCAGA ACAGCTTATT   
  
  
- CTAAACGATC ATTAAAAACA TTCTCTTTAA TACACATAAA AAATAATATA GTCTTATAAT CTAGCTAGAG   
  
  
- TTAAATTAAT GAATACTTTT TTTTTCACCT ATAGAAATTA GTTAAACCGT TCGAGAGTAC GTAATTAACA   
  
  
- TCAGCATGCT TGAATAAATG TAGTGAGTAG ACTTAAACGA TGGTAAATGT GGAGTTTAAA ATAAAATTAC   
  
  
- GGTATTTCTC TTATCTTATA TGACAGATAA ACATCATCAA AATGTCTATT TTTGTTTATT CGTTTACTTT   
  
  
- TATCTATCTC CTAAAAAAAC GATAATAGGT GCAGCTAGGT AACTTAATAA AACAGCGATA AACCAATTCA   
  
  
- CTTTATTTTA TGACTTCATC TTACCTCCTA CAACTATTAC TGGATACAGG GAGTTTCAAA TTAGTACATC   
  
  
- AAAGATGCTT AAAGTTCGGT AATATTCTGT ATGTCTAAAA ATACTACAAC TTTTATGAGA ATGGAGGAAC   
  
  
- AGTGTTATAA TTGGACTTCG GTTACTTTAC TTTGTCTTGA TTAATTTAAC TTATGTATTA GTTAGGTATA   
  
  
- ATTAAACAAA ACTGTTGATA AATGTTAGTA GCTAGAGAAA TTAATCCAGA TATTCGATAT CTCATTAATG   
  
  
- AGTTTTGACA AAACATTCTG TTGAATGAGG TATAACCGAA TAATCATATA ACGGAGGTAA GTGTACTTAT   
  
  
- GAAGTACTCT CTAACGGATT TTGAGTATAA CAGTAGTGGA ATTCAGTTTG TATAAAACAA TACTTTACTG   
  
  
- TAGTTCTTAA TGGGAGACTG TTCTTTTTAT ATGGACTGAC TGTATTAGCA TGGTGTAGAC AATCAGGAGA   
  
  
- AAGATGGTGA AACATCTTGT ATTACACTGT CATTATGTAG GTAGGGTTTT CTTTTCTGCT TTTCTTCTCA   
  
  
- CCTCTCAGTG CATATGCGCA ACTTAAGTCC TTTTAATTTC CTTGAGTACT TCTGGGGTAT ATCACGAGGT   
  
  
- TGGACTTGGA CGTCGGGTGA TGATGTACGG ATTTAATTTC TCGTTTCAAT ACTCTCTTTG GTTTTTTCGA   
  
  
- GAAACTCTCT CACCTCCTTC TTAGTACCTT TCGATCGATC CGAATCGTTC TTTCTCGTTG TCGATCGATC   
  
  
- GTTTTCCGAA GAGAAACTTC GGAATCTTTA GAAATCTCTA TTTTTCAACC ACCGAAAAAT TCCCGACACT   
  
  
- ATACAGGGGG TACATTTTTA CGTCGTTATT ACAATTACAG TACGAAAGAA AGTACCAGGG ATTACGCTTT   
  
  
- AGTTAGTACG TACAGTTGCA CTGAGTCACC GAGTGGTATA TACTAGACTA AAGGGGCGTG TGTCTCTATT   
  
  
- TTTCGGGGGT TAATATTGGA GGGTTTTCGG GGGGGAAAAG GAGGATGGGT TGGGTGTGGA GGGGTAAAGT   
  
  
- AGTCCCGAGC GAAGAATTCA ATAGCTAATT TTTTTCTCAC TCATTTGCAT GGGCTTAACT TCTCCCCACA   
  
  
- AGCTTCGAAT ATGAACGAAC TAATCCTTAC TTTCAGGGGT AAAGTAGGCG GGGGGTTTGG CGGCGGTGGT   
  
  
- GGTCATTTAG CGATAGAACG TTGTTATGAC GGAACTTATG ACGGAAAGTT CGGGGGTTAA GTTAAAGTTG   
  
  
- GGAACAATTG TTGTTGTTTG GGAGGATGCT CGGATGGAGC CAAGATCTGG AGGCCGCGTC GGGTTCGGGG   
  
  
- CGGTGGTTGC GGCGGGACGG TGACGGGTGA CTGCGCCCGT AGTTATTTGG GTTCGGACGG AGACGACTGG   
  
  
- ACCTGTTAAA CCTCCCACTC CCAACCCTAA GTTAGGATAG TCTCGACCCC GAATTACTAC TAAGATTATT   
  
  
- AGGATTGAGG TTTGAAACCA GTAGGGTTTA ATCGAGTAGT ACACTAAGGG TGGAGTGTGT CGAGGGACTC   
  
  
- AAGGGGAGGA GAGTCGGCAA CCTAGTGTGT GCGTGGGGGT TATTAATTGA AGAGGGGGAG ACTAATGGGG   
  
  
- TAGAGACTAA ATAGGATGCC AGTCTTAGAG TCGGGGTTAA AACCCGTCAA ACTCGAAGTA GAAGTAGTCG   
  
  
- GGTTGCAGTT ATTGTTACCC GAACTGAAGT ATCTCCTGGA TTAATCTCGT CGAGTTCGGA TGCTTAGGTT   
  
  
- GCTGAGGGTG GTCCAGGTCG ACCATAACCG CGCCGAGTTA GTTGCCGAGT CTAGTGGGCG GCCTTTTGGG   
  
  
- GAGGTGGCGC GGCGGAAGAT GAAGTTCCTC CGGGAGGTTG TGGAGCGACA GCCGCCTCCG GGCCGGGCCG   
  
  
- GACAGGCCGA CAGTAGGATG CTCCACCACG TCTGGTAGGC CCGGGTGTTC TTGAAAAGGC CGTATAGTGA   
  
  
- CTATAGGGAG AAGAGGTGGA AGCGGCGGTT AGTCCGCTAA GACCTCCGCC ACCTACCCCG ACGCAAGTAG   
  
  
- GTGTAGTAGC TAAAGCTGTA GCCTAAACCG CCGGTGACCC GCAGCAAATA CGCACTCGAA CAGCTGTTCC   
  
  
- GGCTGGGCTT ATTCTAGTCG AGGAACCACA AGGCTTAGTG CCGCTAGCAT GGGCTTCTCA AGCCCTAGCT   
  
  
- TTCGTTCGAC CACTCTCTCT TAGAATGCGT TAAGCGAGCA CTAGAGTTGT AGTTACAGGT ATACCTCATA   
  
  
- CAGGAATAAG TTTCGAAGCT CAAGAATAGG AAGTTTAGCC AGTTTAAGTA GCTGCCACTC TTTAACCGCC   
  
  
- AGGTAGATAG AGGCCGCTAC AAAGTGGCCG AAGTTCCGCC TTAGAGCTTT AAGGATCGGC TAGAGGCCCC   
  
  
- TTAAAGCGGT TCGTGGCAGC ACCACCACCT GTCACTCCAT CCTTAGCTGT GCCCTTGAAG CAGGAAGTCG   
  
  
- CAGTTGAAGC AGCGGCCATA GCTTAAGATG TGGCCTTACG AGCTTAGCGA GCTGCAGTGG AGGCGGTCGC   
  
  
- CAAAACGTCC GCCGCCGCCG CCGCTGCGAC ACTCTTCCTA GCTCTGCATG CACGAGGCCG GCTCCTAATG   
  
  
- CCGCCGCCAC CTCCGCCGTA GACGCCGCCG CCATCGCGCC GCCTCGCCAA CCTCTCTCAG CAAGCGCCAC   
  
  
- CGATCCTACT CCCGCCACCC CAAGTCGGTC AAACGACTAA AGGTCCGCCT CACAAACGAC TCCTTCCAAG   
  
  
- TCCAACCGCC CAAGGTGCAC CGCTTTGCAG TACCCCTCTA CTACGAAACG ACCGTACTAT CCGGGGAACA   
  
  
- CAGGTGCAGC CGAACCTCCA CAAT

+     TCT-motif

| Site Name | Organism | Position | Strand | Matrix score. | sequence | function |
| --- | --- | --- | --- | --- | --- | --- |
| TCT-motif | Arabidopsis thaliana | 692 | + | 6 | TCTTAC | part of a light responsive element |
| TCT-motif | Arabidopsis thaliana | 233 | - | 6 | TCTTAC | part of a light responsive element |
| TCT-motif | Arabidopsis thaliana | 2756 | + | 6 | TCTTAC | part of a light responsive element |
| TCT-motif | Arabidopsis thaliana | 858 | - | 6 | TCTTAC | part of a light responsive element |

>HU06G00283.1   
+ -Up\_Stream \_Len000GATTTT ACTTCCAATT TTCTACTTAA ATTTGGTTAT CACGTTCGAA TTTTTCAGGT   
  
  
+ AAATACAAAC TGTTAGTGGC CATCCTTTTT TTTTGGGTCT AATCCATCCA GATGTGCTAA TACTTTCTGT   
  
  
+ TTTAGATGAG AAGTTTTAAT GCTTTTTTAG ATGTGTAATT TTTTTAAACA TAAATAGTCT TGTCGAATAA   
  
  
+ GATTTGCTAG TAATTTTTGT AAGAGAAATT ATGTGTATTT TTTATTATAT CAGAATATTA GATCGATCTC   
  
  
+ AATTTAATTA CTTATGAAAA AAAAAGTGGA TATCTTTAAT CAATTTGGCA AGCTCTCATG CATTAATTGT   
  
  
+ AGTCGTACGA ACTTATTTAC ATCACTCATC TGAATTTGCT ACCATTTACA CCTCAAATTT TATTTTAATG   
  
  
+ CCATAAAGAG AATAGAATAT ACTGTCTATT TGTAGTAGTT TTACAGATAA AAACAAATAA GCAAATGAAA   
  
  
+ ATAGATAGAG GATTTTTTTG CTATTATCCA CGTCGATCCA TTGAATTATT TTGTCGCTAT TTGGTTAAGT   
  
  
+ GAAATAAAAT ACTGAAGTAG AATGGAGGAT GTTGATAATG ACCTATGTCC CTCAAAGTTT AATCATGTAG   
  
  
+ TTTCTACGAA TTTCAAGCCA TTATAAGACA TACAGATTTT TATGATGTTG AAAATACTCT TACCTCCTTG   
  
  
+ TCACAATATT AACCTGAAGC CAATGAAATG AAACAGAACT AATTAAATTG AATACATAAT CAATCCATAT   
  
  
+ TAATTTGTTT TGACAACTAT TTACAATCAT CGATCTCTTT AATTAGGTCT ATAAGCTATA GAGTAATTAC   
  
  
+ TCAAAACTGT TTTGTAAGAC AACTTACTCC ATATTGGCTT ATTAGTATAT TGCCTCCATT CACATGAATA   
  
  
+ CTTCATGAGA GATTGCCTAA AACTCATATT GTCATCACCT TAAGTCAAAC ATATTTTGTT ATGAAATGAC   
  
  
+ ATCAAGAATT ACCCTCTGAC AAGAAAAATA TACCTGACTG ACATAATCGT ACCACATCTG TTAGTCCTCT   
  
  
+ TTCTACCACT TTGTAGAACA TAATGTGACA GTAATACATC CATCCCAAAA GAAAAGACGA AAAGAAGAGT   
  
  
+ GGAGAGTCAC GTATACGCGT TGAATTCAGG AAAATTAAAG GAACTCATGA AGACCCCATA TAGTGCTCCA   
  
  
+ ACCTGAACCT GCAGCCCACT ACTACATGCC TAAATTAAAG AGCAAAGTTA TGAGAGAAAC CAAAAAAGCT   
  
  
+ CTTTGAGAGA GTGGAGGAAG AATCATGGAA AGCTAGCTAG GCTTAGCAAG AAAGAGCAAC AGCTAGCTAG   
  
  
+ CAAAAGGCTT CTCTTTGAAG CCTTAGAAAT CTTTAGAGAT AAAAAGTTGG TGGCTTTTTA AGGGCTGTGA   
  
  
+ TATGTCCCCC ATGTAAAAAT GCAGCAATAA TGTTAATGTC ATGCTTTCTT TCATGGTCCC TAATGCGAAA   
  
  
+ TCAATCATGC ATGTCAACGT GACTCAGTGG CTCACCATAT ATGATCTGAT TTCCCCGCAC ACAGAGATAA   
  
  
+ AAAGCCCCCA ATTATAACCT CCCAAAAGCC CCCCCTTTTC CTCCTACCCA ACCCACACCT CCCCATTTCA   
  
  
+ TCAGGGCTCG CTTCTTAAGT TATCGATTAA AAAAAGAGTG AGTAAACGTA CCCGAATTGA AGAGGGGTGT   
  
  
+ TCGAAGCTTA TACTTGCTTG ATTAGGAATG AAAGTCCCCA TTTCATCCGC CCCCCAAACC GCCGCCACCA   
  
  
+ CCAGTAAATC GCTATCTTGC AACAATACTG CCTTGAATAC TGCCTTTCAA GCCCCCAATT CAATTTCAAC   
  
  
+ CCTTGTTAAC AACAACAAAC CCTCCTACGA GCCTACCTCG GTTCTAGACC TCCGGCGCAG CCCAAGCCCC   
  
  
+ GCCACCAACG CCGCCCTGCC ACTGCCCACT GACGCGGGCA TCAATAAACC CAAGCCTGCC TCTGCTGACC   
  
  
+ TGGACAATTT GGAGGGTGAG GGTTGGGATT CAATCCTATC AGAGCTGGGG CTTAATGATG ATTCTAATAA   
  
  
+ TCCTAACTCC AAACTTTGGT CATCCCAAAT TAGCTCATCA TGTGATTCCC ACCTCACACA GCTCCCTGAG   
  
  
+ TTCCCCTCCT CTCAGCCGTT GGATCACACA CGCACCCCCA ATAATTAACT TCTCCCCCTC TGATTACCCC   
  
  
+ ATCTCTGATT TATCCTACGG TCAGAATCTC AGCCCCAATT TTGGGCAGTT TGAGCTTCAT CTTCATCAGC   
  
  
+ CCAACGTCAA TAACAATGGG CTTGACTTCA TAGAGGACCT AATTAGAGCA GCTCAAGCCT ACGAATCCAA   
  
  
+ CGACTCCCAC CAGGTCCAGC TGGTATTGGC GCGGCTCAAT CAACGGCTCA GATCACCCGC CGGAAAACCC   
  
  
+ CTCCACCGCG CCGCCTTCTA CTTCAAGGAG GCCCTCCAAC ACCTCGCTGT CGGCGGAGGC CCGGCCCGGC   
  
  
+ CTGTCCGGCT GTCATCCTAC GAGGTGGTGC AGACCATCCG GGCCCACAAG AACTTTTCCG GCATATCACT   
  
  
+ GATATCCCTC TTCTCCACCT TCGCCGCCAA TCAGGCGATT CTGGAGGCGG TGGATGGGGC TGCGTTCATC   
  
  
+ CACATCATCG ATTTCGACAT CGGATTTGGC GGCCACTGGG CGTCGTTTAT GCGTGAGCTT GTCGACAAGG   
  
  
+ CCGACCCGAA TAAGATCAGC TCCTTGGTGT TCCGAATCAC GGCGATCGTA CCCGAAGAGT TCGGGATCGA   
  
  
+ AAGCAAGCTG GTGAGAGAGA ATCTTACGCA ATTCGCTCGT GATCTCAACA TCAATGTCCA TATGGAGTAT   
  
  
+ GTCCTTATTC AAAGCTTCGA GTTCTTATCC TTCAAATCGG TCAAATTCAT CGACGGTGAG AAATTGGCGG   
  
  
+ TCCATCTATC TCCGGCGATG TTTCACCGGC TTCAAGGCGG AATCTCGAAA TTCCTAGCCG ATCTCCGGGG   
  
  
+ AATTTCGCCA AGCACCGTCG TGGTGGTGGA CAGTGAGGTA GGAATCGACA CGGGAACTTC GTCCTTCAGC   
  
  
+ GTCAACTTCG TCGCCGGTAT CGAATTCTAC ACCGGAATGC TCGAATCGCT CGACGTCACC TCCGCCAGCG   
  
  
+ GTTTTGCAGG CGGCGGCGGC GGCGACGCTG TGAGAAGGAT CGAGACGTAC GTGCTCCGGC CGAGGATTAC   
  
  
+ GGCGGCGGTG GAGGCGGCAT CTGCGGCGGC GGTAGCGCGG CGGAGCGGTT GGAGAGAGTC GTTCGCGGTG   
  
  
+ GCTAGGATGA GGGCGGTGGG GTTCAGCCAG TTTGCTGATT TCCAGGCGGA GTGTTTGCTG AGGAAGGTTC   
  
  
+ AGGTTGGCGG GTTCCACGTG GCGAAACGTC ATGGGGAGAT GATGCTTTGC TGGCATGATA GGCCCCTTGT   
  
  
+ GTCCACGTCG GCTTGGAGGT GTTA  

- -Up\_Stream \_Len000CTAAAA TGAAGGTTAA AAGATGAATT TAAACCAATA GTGCAAGCTT AAAAAGTCCA   
  
  
- TTTATGTTTG ACAATCACCG GTAGGAAAAA AAAACCCAGA TTAGGTAGGT CTACACGATT ATGAAAGACA   
  
  
- AAATCTACTC TTCAAAATTA CGAAAAAATC TACACATTAA AAAAATTTGT ATTTATCAGA ACAGCTTATT   
  
  
- CTAAACGATC ATTAAAAACA TTCTCTTTAA TACACATAAA AAATAATATA GTCTTATAAT CTAGCTAGAG   
  
  
- TTAAATTAAT GAATACTTTT TTTTTCACCT ATAGAAATTA GTTAAACCGT TCGAGAGTAC GTAATTAACA   
  
  
- TCAGCATGCT TGAATAAATG TAGTGAGTAG ACTTAAACGA TGGTAAATGT GGAGTTTAAA ATAAAATTAC   
  
  
- GGTATTTCTC TTATCTTATA TGACAGATAA ACATCATCAA AATGTCTATT TTTGTTTATT CGTTTACTTT   
  
  
- TATCTATCTC CTAAAAAAAC GATAATAGGT GCAGCTAGGT AACTTAATAA AACAGCGATA AACCAATTCA   
  
  
- CTTTATTTTA TGACTTCATC TTACCTCCTA CAACTATTAC TGGATACAGG GAGTTTCAAA TTAGTACATC   
  
  
- AAAGATGCTT AAAGTTCGGT AATATTCTGT ATGTCTAAAA ATACTACAAC TTTTATGAGA ATGGAGGAAC   
  
  
- AGTGTTATAA TTGGACTTCG GTTACTTTAC TTTGTCTTGA TTAATTTAAC TTATGTATTA GTTAGGTATA   
  
  
- ATTAAACAAA ACTGTTGATA AATGTTAGTA GCTAGAGAAA TTAATCCAGA TATTCGATAT CTCATTAATG   
  
  
- AGTTTTGACA AAACATTCTG TTGAATGAGG TATAACCGAA TAATCATATA ACGGAGGTAA GTGTACTTAT   
  
  
- GAAGTACTCT CTAACGGATT TTGAGTATAA CAGTAGTGGA ATTCAGTTTG TATAAAACAA TACTTTACTG   
  
  
- TAGTTCTTAA TGGGAGACTG TTCTTTTTAT ATGGACTGAC TGTATTAGCA TGGTGTAGAC AATCAGGAGA   
  
  
- AAGATGGTGA AACATCTTGT ATTACACTGT CATTATGTAG GTAGGGTTTT CTTTTCTGCT TTTCTTCTCA   
  
  
- CCTCTCAGTG CATATGCGCA ACTTAAGTCC TTTTAATTTC CTTGAGTACT TCTGGGGTAT ATCACGAGGT   
  
  
- TGGACTTGGA CGTCGGGTGA TGATGTACGG ATTTAATTTC TCGTTTCAAT ACTCTCTTTG GTTTTTTCGA   
  
  
- GAAACTCTCT CACCTCCTTC TTAGTACCTT TCGATCGATC CGAATCGTTC TTTCTCGTTG TCGATCGATC   
  
  
- GTTTTCCGAA GAGAAACTTC GGAATCTTTA GAAATCTCTA TTTTTCAACC ACCGAAAAAT TCCCGACACT   
  
  
- ATACAGGGGG TACATTTTTA CGTCGTTATT ACAATTACAG TACGAAAGAA AGTACCAGGG ATTACGCTTT   
  
  
- AGTTAGTACG TACAGTTGCA CTGAGTCACC GAGTGGTATA TACTAGACTA AAGGGGCGTG TGTCTCTATT   
  
  
- TTTCGGGGGT TAATATTGGA GGGTTTTCGG GGGGGAAAAG GAGGATGGGT TGGGTGTGGA GGGGTAAAGT   
  
  
- AGTCCCGAGC GAAGAATTCA ATAGCTAATT TTTTTCTCAC TCATTTGCAT GGGCTTAACT TCTCCCCACA   
  
  
- AGCTTCGAAT ATGAACGAAC TAATCCTTAC TTTCAGGGGT AAAGTAGGCG GGGGGTTTGG CGGCGGTGGT   
  
  
- GGTCATTTAG CGATAGAACG TTGTTATGAC GGAACTTATG ACGGAAAGTT CGGGGGTTAA GTTAAAGTTG   
  
  
- GGAACAATTG TTGTTGTTTG GGAGGATGCT CGGATGGAGC CAAGATCTGG AGGCCGCGTC GGGTTCGGGG   
  
  
- CGGTGGTTGC GGCGGGACGG TGACGGGTGA CTGCGCCCGT AGTTATTTGG GTTCGGACGG AGACGACTGG   
  
  
- ACCTGTTAAA CCTCCCACTC CCAACCCTAA GTTAGGATAG TCTCGACCCC GAATTACTAC TAAGATTATT   
  
  
- AGGATTGAGG TTTGAAACCA GTAGGGTTTA ATCGAGTAGT ACACTAAGGG TGGAGTGTGT CGAGGGACTC   
  
  
- AAGGGGAGGA GAGTCGGCAA CCTAGTGTGT GCGTGGGGGT TATTAATTGA AGAGGGGGAG ACTAATGGGG   
  
  
- TAGAGACTAA ATAGGATGCC AGTCTTAGAG TCGGGGTTAA AACCCGTCAA ACTCGAAGTA GAAGTAGTCG   
  
  
- GGTTGCAGTT ATTGTTACCC GAACTGAAGT ATCTCCTGGA TTAATCTCGT CGAGTTCGGA TGCTTAGGTT   
  
  
- GCTGAGGGTG GTCCAGGTCG ACCATAACCG CGCCGAGTTA GTTGCCGAGT CTAGTGGGCG GCCTTTTGGG   
  
  
- GAGGTGGCGC GGCGGAAGAT GAAGTTCCTC CGGGAGGTTG TGGAGCGACA GCCGCCTCCG GGCCGGGCCG   
  
  
- GACAGGCCGA CAGTAGGATG CTCCACCACG TCTGGTAGGC CCGGGTGTTC TTGAAAAGGC CGTATAGTGA   
  
  
- CTATAGGGAG AAGAGGTGGA AGCGGCGGTT AGTCCGCTAA GACCTCCGCC ACCTACCCCG ACGCAAGTAG   
  
  
- GTGTAGTAGC TAAAGCTGTA GCCTAAACCG CCGGTGACCC GCAGCAAATA CGCACTCGAA CAGCTGTTCC   
  
  
- GGCTGGGCTT ATTCTAGTCG AGGAACCACA AGGCTTAGTG CCGCTAGCAT GGGCTTCTCA AGCCCTAGCT   
  
  
- TTCGTTCGAC CACTCTCTCT TAGAATGCGT TAAGCGAGCA CTAGAGTTGT AGTTACAGGT ATACCTCATA   
  
  
- CAGGAATAAG TTTCGAAGCT CAAGAATAGG AAGTTTAGCC AGTTTAAGTA GCTGCCACTC TTTAACCGCC   
  
  
- AGGTAGATAG AGGCCGCTAC AAAGTGGCCG AAGTTCCGCC TTAGAGCTTT AAGGATCGGC TAGAGGCCCC   
  
  
- TTAAAGCGGT TCGTGGCAGC ACCACCACCT GTCACTCCAT CCTTAGCTGT GCCCTTGAAG CAGGAAGTCG   
  
  
- CAGTTGAAGC AGCGGCCATA GCTTAAGATG TGGCCTTACG AGCTTAGCGA GCTGCAGTGG AGGCGGTCGC   
  
  
- CAAAACGTCC GCCGCCGCCG CCGCTGCGAC ACTCTTCCTA GCTCTGCATG CACGAGGCCG GCTCCTAATG   
  
  
- CCGCCGCCAC CTCCGCCGTA GACGCCGCCG CCATCGCGCC GCCTCGCCAA CCTCTCTCAG CAAGCGCCAC   
  
  
- CGATCCTACT CCCGCCACCC CAAGTCGGTC AAACGACTAA AGGTCCGCCT CACAAACGAC TCCTTCCAAG   
  
  
- TCCAACCGCC CAAGGTGCAC CGCTTTGCAG TACCCCTCTA CTACGAAACG ACCGTACTAT CCGGGGAACA   
  
  
- CAGGTGCAGC CGAACCTCCA CAAT

+     TGA-element

| Site Name | Organism | Position | Strand | Matrix score. | sequence | function |
| --- | --- | --- | --- | --- | --- | --- |
| TGA-element | Brassica oleracea | 3212 | - | 6 | AACGAC | auxin-responsive element |
| TGA-element | Brassica oleracea | 2313 | + | 6 | AACGAC | auxin-responsive element |
| TGA-element | Brassica oleracea | 2636 | - | 6 | AACGAC | auxin-responsive element |

>HU06G00283.1   
+ -Up\_Stream \_Len000GATTTT ACTTCCAATT TTCTACTTAA ATTTGGTTAT CACGTTCGAA TTTTTCAGGT   
  
  
+ AAATACAAAC TGTTAGTGGC CATCCTTTTT TTTTGGGTCT AATCCATCCA GATGTGCTAA TACTTTCTGT   
  
  
+ TTTAGATGAG AAGTTTTAAT GCTTTTTTAG ATGTGTAATT TTTTTAAACA TAAATAGTCT TGTCGAATAA   
  
  
+ GATTTGCTAG TAATTTTTGT AAGAGAAATT ATGTGTATTT TTTATTATAT CAGAATATTA GATCGATCTC   
  
  
+ AATTTAATTA CTTATGAAAA AAAAAGTGGA TATCTTTAAT CAATTTGGCA AGCTCTCATG CATTAATTGT   
  
  
+ AGTCGTACGA ACTTATTTAC ATCACTCATC TGAATTTGCT ACCATTTACA CCTCAAATTT TATTTTAATG   
  
  
+ CCATAAAGAG AATAGAATAT ACTGTCTATT TGTAGTAGTT TTACAGATAA AAACAAATAA GCAAATGAAA   
  
  
+ ATAGATAGAG GATTTTTTTG CTATTATCCA CGTCGATCCA TTGAATTATT TTGTCGCTAT TTGGTTAAGT   
  
  
+ GAAATAAAAT ACTGAAGTAG AATGGAGGAT GTTGATAATG ACCTATGTCC CTCAAAGTTT AATCATGTAG   
  
  
+ TTTCTACGAA TTTCAAGCCA TTATAAGACA TACAGATTTT TATGATGTTG AAAATACTCT TACCTCCTTG   
  
  
+ TCACAATATT AACCTGAAGC CAATGAAATG AAACAGAACT AATTAAATTG AATACATAAT CAATCCATAT   
  
  
+ TAATTTGTTT TGACAACTAT TTACAATCAT CGATCTCTTT AATTAGGTCT ATAAGCTATA GAGTAATTAC   
  
  
+ TCAAAACTGT TTTGTAAGAC AACTTACTCC ATATTGGCTT ATTAGTATAT TGCCTCCATT CACATGAATA   
  
  
+ CTTCATGAGA GATTGCCTAA AACTCATATT GTCATCACCT TAAGTCAAAC ATATTTTGTT ATGAAATGAC   
  
  
+ ATCAAGAATT ACCCTCTGAC AAGAAAAATA TACCTGACTG ACATAATCGT ACCACATCTG TTAGTCCTCT   
  
  
+ TTCTACCACT TTGTAGAACA TAATGTGACA GTAATACATC CATCCCAAAA GAAAAGACGA AAAGAAGAGT   
  
  
+ GGAGAGTCAC GTATACGCGT TGAATTCAGG AAAATTAAAG GAACTCATGA AGACCCCATA TAGTGCTCCA   
  
  
+ ACCTGAACCT GCAGCCCACT ACTACATGCC TAAATTAAAG AGCAAAGTTA TGAGAGAAAC CAAAAAAGCT   
  
  
+ CTTTGAGAGA GTGGAGGAAG AATCATGGAA AGCTAGCTAG GCTTAGCAAG AAAGAGCAAC AGCTAGCTAG   
  
  
+ CAAAAGGCTT CTCTTTGAAG CCTTAGAAAT CTTTAGAGAT AAAAAGTTGG TGGCTTTTTA AGGGCTGTGA   
  
  
+ TATGTCCCCC ATGTAAAAAT GCAGCAATAA TGTTAATGTC ATGCTTTCTT TCATGGTCCC TAATGCGAAA   
  
  
+ TCAATCATGC ATGTCAACGT GACTCAGTGG CTCACCATAT ATGATCTGAT TTCCCCGCAC ACAGAGATAA   
  
  
+ AAAGCCCCCA ATTATAACCT CCCAAAAGCC CCCCCTTTTC CTCCTACCCA ACCCACACCT CCCCATTTCA   
  
  
+ TCAGGGCTCG CTTCTTAAGT TATCGATTAA AAAAAGAGTG AGTAAACGTA CCCGAATTGA AGAGGGGTGT   
  
  
+ TCGAAGCTTA TACTTGCTTG ATTAGGAATG AAAGTCCCCA TTTCATCCGC CCCCCAAACC GCCGCCACCA   
  
  
+ CCAGTAAATC GCTATCTTGC AACAATACTG CCTTGAATAC TGCCTTTCAA GCCCCCAATT CAATTTCAAC   
  
  
+ CCTTGTTAAC AACAACAAAC CCTCCTACGA GCCTACCTCG GTTCTAGACC TCCGGCGCAG CCCAAGCCCC   
  
  
+ GCCACCAACG CCGCCCTGCC ACTGCCCACT GACGCGGGCA TCAATAAACC CAAGCCTGCC TCTGCTGACC   
  
  
+ TGGACAATTT GGAGGGTGAG GGTTGGGATT CAATCCTATC AGAGCTGGGG CTTAATGATG ATTCTAATAA   
  
  
+ TCCTAACTCC AAACTTTGGT CATCCCAAAT TAGCTCATCA TGTGATTCCC ACCTCACACA GCTCCCTGAG   
  
  
+ TTCCCCTCCT CTCAGCCGTT GGATCACACA CGCACCCCCA ATAATTAACT TCTCCCCCTC TGATTACCCC   
  
  
+ ATCTCTGATT TATCCTACGG TCAGAATCTC AGCCCCAATT TTGGGCAGTT TGAGCTTCAT CTTCATCAGC   
  
  
+ CCAACGTCAA TAACAATGGG CTTGACTTCA TAGAGGACCT AATTAGAGCA GCTCAAGCCT ACGAATCCAA   
  
  
+ CGACTCCCAC CAGGTCCAGC TGGTATTGGC GCGGCTCAAT CAACGGCTCA GATCACCCGC CGGAAAACCC   
  
  
+ CTCCACCGCG CCGCCTTCTA CTTCAAGGAG GCCCTCCAAC ACCTCGCTGT CGGCGGAGGC CCGGCCCGGC   
  
  
+ CTGTCCGGCT GTCATCCTAC GAGGTGGTGC AGACCATCCG GGCCCACAAG AACTTTTCCG GCATATCACT   
  
  
+ GATATCCCTC TTCTCCACCT TCGCCGCCAA TCAGGCGATT CTGGAGGCGG TGGATGGGGC TGCGTTCATC   
  
  
+ CACATCATCG ATTTCGACAT CGGATTTGGC GGCCACTGGG CGTCGTTTAT GCGTGAGCTT GTCGACAAGG   
  
  
+ CCGACCCGAA TAAGATCAGC TCCTTGGTGT TCCGAATCAC GGCGATCGTA CCCGAAGAGT TCGGGATCGA   
  
  
+ AAGCAAGCTG GTGAGAGAGA ATCTTACGCA ATTCGCTCGT GATCTCAACA TCAATGTCCA TATGGAGTAT   
  
  
+ GTCCTTATTC AAAGCTTCGA GTTCTTATCC TTCAAATCGG TCAAATTCAT CGACGGTGAG AAATTGGCGG   
  
  
+ TCCATCTATC TCCGGCGATG TTTCACCGGC TTCAAGGCGG AATCTCGAAA TTCCTAGCCG ATCTCCGGGG   
  
  
+ AATTTCGCCA AGCACCGTCG TGGTGGTGGA CAGTGAGGTA GGAATCGACA CGGGAACTTC GTCCTTCAGC   
  
  
+ GTCAACTTCG TCGCCGGTAT CGAATTCTAC ACCGGAATGC TCGAATCGCT CGACGTCACC TCCGCCAGCG   
  
  
+ GTTTTGCAGG CGGCGGCGGC GGCGACGCTG TGAGAAGGAT CGAGACGTAC GTGCTCCGGC CGAGGATTAC   
  
  
+ GGCGGCGGTG GAGGCGGCAT CTGCGGCGGC GGTAGCGCGG CGGAGCGGTT GGAGAGAGTC GTTCGCGGTG   
  
  
+ GCTAGGATGA GGGCGGTGGG GTTCAGCCAG TTTGCTGATT TCCAGGCGGA GTGTTTGCTG AGGAAGGTTC   
  
  
+ AGGTTGGCGG GTTCCACGTG GCGAAACGTC ATGGGGAGAT GATGCTTTGC TGGCATGATA GGCCCCTTGT   
  
  
+ GTCCACGTCG GCTTGGAGGT GTTA  

- -Up\_Stream \_Len000CTAAAA TGAAGGTTAA AAGATGAATT TAAACCAATA GTGCAAGCTT AAAAAGTCCA   
  
  
- TTTATGTTTG ACAATCACCG GTAGGAAAAA AAAACCCAGA TTAGGTAGGT CTACACGATT ATGAAAGACA   
  
  
- AAATCTACTC TTCAAAATTA CGAAAAAATC TACACATTAA AAAAATTTGT ATTTATCAGA ACAGCTTATT   
  
  
- CTAAACGATC ATTAAAAACA TTCTCTTTAA TACACATAAA AAATAATATA GTCTTATAAT CTAGCTAGAG   
  
  
- TTAAATTAAT GAATACTTTT TTTTTCACCT ATAGAAATTA GTTAAACCGT TCGAGAGTAC GTAATTAACA   
  
  
- TCAGCATGCT TGAATAAATG TAGTGAGTAG ACTTAAACGA TGGTAAATGT GGAGTTTAAA ATAAAATTAC   
  
  
- GGTATTTCTC TTATCTTATA TGACAGATAA ACATCATCAA AATGTCTATT TTTGTTTATT CGTTTACTTT   
  
  
- TATCTATCTC CTAAAAAAAC GATAATAGGT GCAGCTAGGT AACTTAATAA AACAGCGATA AACCAATTCA   
  
  
- CTTTATTTTA TGACTTCATC TTACCTCCTA CAACTATTAC TGGATACAGG GAGTTTCAAA TTAGTACATC   
  
  
- AAAGATGCTT AAAGTTCGGT AATATTCTGT ATGTCTAAAA ATACTACAAC TTTTATGAGA ATGGAGGAAC   
  
  
- AGTGTTATAA TTGGACTTCG GTTACTTTAC TTTGTCTTGA TTAATTTAAC TTATGTATTA GTTAGGTATA   
  
  
- ATTAAACAAA ACTGTTGATA AATGTTAGTA GCTAGAGAAA TTAATCCAGA TATTCGATAT CTCATTAATG   
  
  
- AGTTTTGACA AAACATTCTG TTGAATGAGG TATAACCGAA TAATCATATA ACGGAGGTAA GTGTACTTAT   
  
  
- GAAGTACTCT CTAACGGATT TTGAGTATAA CAGTAGTGGA ATTCAGTTTG TATAAAACAA TACTTTACTG   
  
  
- TAGTTCTTAA TGGGAGACTG TTCTTTTTAT ATGGACTGAC TGTATTAGCA TGGTGTAGAC AATCAGGAGA   
  
  
- AAGATGGTGA AACATCTTGT ATTACACTGT CATTATGTAG GTAGGGTTTT CTTTTCTGCT TTTCTTCTCA   
  
  
- CCTCTCAGTG CATATGCGCA ACTTAAGTCC TTTTAATTTC CTTGAGTACT TCTGGGGTAT ATCACGAGGT   
  
  
- TGGACTTGGA CGTCGGGTGA TGATGTACGG ATTTAATTTC TCGTTTCAAT ACTCTCTTTG GTTTTTTCGA   
  
  
- GAAACTCTCT CACCTCCTTC TTAGTACCTT TCGATCGATC CGAATCGTTC TTTCTCGTTG TCGATCGATC   
  
  
- GTTTTCCGAA GAGAAACTTC GGAATCTTTA GAAATCTCTA TTTTTCAACC ACCGAAAAAT TCCCGACACT   
  
  
- ATACAGGGGG TACATTTTTA CGTCGTTATT ACAATTACAG TACGAAAGAA AGTACCAGGG ATTACGCTTT   
  
  
- AGTTAGTACG TACAGTTGCA CTGAGTCACC GAGTGGTATA TACTAGACTA AAGGGGCGTG TGTCTCTATT   
  
  
- TTTCGGGGGT TAATATTGGA GGGTTTTCGG GGGGGAAAAG GAGGATGGGT TGGGTGTGGA GGGGTAAAGT   
  
  
- AGTCCCGAGC GAAGAATTCA ATAGCTAATT TTTTTCTCAC TCATTTGCAT GGGCTTAACT TCTCCCCACA   
  
  
- AGCTTCGAAT ATGAACGAAC TAATCCTTAC TTTCAGGGGT AAAGTAGGCG GGGGGTTTGG CGGCGGTGGT   
  
  
- GGTCATTTAG CGATAGAACG TTGTTATGAC GGAACTTATG ACGGAAAGTT CGGGGGTTAA GTTAAAGTTG   
  
  
- GGAACAATTG TTGTTGTTTG GGAGGATGCT CGGATGGAGC CAAGATCTGG AGGCCGCGTC GGGTTCGGGG   
  
  
- CGGTGGTTGC GGCGGGACGG TGACGGGTGA CTGCGCCCGT AGTTATTTGG GTTCGGACGG AGACGACTGG   
  
  
- ACCTGTTAAA CCTCCCACTC CCAACCCTAA GTTAGGATAG TCTCGACCCC GAATTACTAC TAAGATTATT   
  
  
- AGGATTGAGG TTTGAAACCA GTAGGGTTTA ATCGAGTAGT ACACTAAGGG TGGAGTGTGT CGAGGGACTC   
  
  
- AAGGGGAGGA GAGTCGGCAA CCTAGTGTGT GCGTGGGGGT TATTAATTGA AGAGGGGGAG ACTAATGGGG   
  
  
- TAGAGACTAA ATAGGATGCC AGTCTTAGAG TCGGGGTTAA AACCCGTCAA ACTCGAAGTA GAAGTAGTCG   
  
  
- GGTTGCAGTT ATTGTTACCC GAACTGAAGT ATCTCCTGGA TTAATCTCGT CGAGTTCGGA TGCTTAGGTT   
  
  
- GCTGAGGGTG GTCCAGGTCG ACCATAACCG CGCCGAGTTA GTTGCCGAGT CTAGTGGGCG GCCTTTTGGG   
  
  
- GAGGTGGCGC GGCGGAAGAT GAAGTTCCTC CGGGAGGTTG TGGAGCGACA GCCGCCTCCG GGCCGGGCCG   
  
  
- GACAGGCCGA CAGTAGGATG CTCCACCACG TCTGGTAGGC CCGGGTGTTC TTGAAAAGGC CGTATAGTGA   
  
  
- CTATAGGGAG AAGAGGTGGA AGCGGCGGTT AGTCCGCTAA GACCTCCGCC ACCTACCCCG ACGCAAGTAG   
  
  
- GTGTAGTAGC TAAAGCTGTA GCCTAAACCG CCGGTGACCC GCAGCAAATA CGCACTCGAA CAGCTGTTCC   
  
  
- GGCTGGGCTT ATTCTAGTCG AGGAACCACA AGGCTTAGTG CCGCTAGCAT GGGCTTCTCA AGCCCTAGCT   
  
  
- TTCGTTCGAC CACTCTCTCT TAGAATGCGT TAAGCGAGCA CTAGAGTTGT AGTTACAGGT ATACCTCATA   
  
  
- CAGGAATAAG TTTCGAAGCT CAAGAATAGG AAGTTTAGCC AGTTTAAGTA GCTGCCACTC TTTAACCGCC   
  
  
- AGGTAGATAG AGGCCGCTAC AAAGTGGCCG AAGTTCCGCC TTAGAGCTTT AAGGATCGGC TAGAGGCCCC   
  
  
- TTAAAGCGGT TCGTGGCAGC ACCACCACCT GTCACTCCAT CCTTAGCTGT GCCCTTGAAG CAGGAAGTCG   
  
  
- CAGTTGAAGC AGCGGCCATA GCTTAAGATG TGGCCTTACG AGCTTAGCGA GCTGCAGTGG AGGCGGTCGC   
  
  
- CAAAACGTCC GCCGCCGCCG CCGCTGCGAC ACTCTTCCTA GCTCTGCATG CACGAGGCCG GCTCCTAATG   
  
  
- CCGCCGCCAC CTCCGCCGTA GACGCCGCCG CCATCGCGCC GCCTCGCCAA CCTCTCTCAG CAAGCGCCAC   
  
  
- CGATCCTACT CCCGCCACCC CAAGTCGGTC AAACGACTAA AGGTCCGCCT CACAAACGAC TCCTTCCAAG   
  
  
- TCCAACCGCC CAAGGTGCAC CGCTTTGCAG TACCCCTCTA CTACGAAACG ACCGTACTAT CCGGGGAACA   
  
  
- CAGGTGCAGC CGAACCTCCA CAAT

+     TGACG-motif

| Site Name | Organism | Position | Strand | Matrix score. | sequence | function |
| --- | --- | --- | --- | --- | --- | --- |
| TGACG-motif | Hordeum vulgare | 3321 | - | 5 | TGACG | cis-acting regulatory element involved in the MeJA-responsiveness |
| TGACG-motif | Hordeum vulgare | 2249 | - | 5 | TGACG | cis-acting regulatory element involved in the MeJA-responsiveness |
| TGACG-motif | Hordeum vulgare | 3014 | - | 5 | TGACG | cis-acting regulatory element involved in the MeJA-responsiveness |
| TGACG-motif | Hordeum vulgare | 1924 | + | 5 | TGACG | cis-acting regulatory element involved in the MeJA-responsiveness |
| TGACG-motif | Hordeum vulgare | 3068 | - | 5 | TGACG | cis-acting regulatory element involved in the MeJA-responsiveness |

>HU06G00283.1   
+ -Up\_Stream \_Len000GATTTT ACTTCCAATT TTCTACTTAA ATTTGGTTAT CACGTTCGAA TTTTTCAGGT   
  
  
+ AAATACAAAC TGTTAGTGGC CATCCTTTTT TTTTGGGTCT AATCCATCCA GATGTGCTAA TACTTTCTGT   
  
  
+ TTTAGATGAG AAGTTTTAAT GCTTTTTTAG ATGTGTAATT TTTTTAAACA TAAATAGTCT TGTCGAATAA   
  
  
+ GATTTGCTAG TAATTTTTGT AAGAGAAATT ATGTGTATTT TTTATTATAT CAGAATATTA GATCGATCTC   
  
  
+ AATTTAATTA CTTATGAAAA AAAAAGTGGA TATCTTTAAT CAATTTGGCA AGCTCTCATG CATTAATTGT   
  
  
+ AGTCGTACGA ACTTATTTAC ATCACTCATC TGAATTTGCT ACCATTTACA CCTCAAATTT TATTTTAATG   
  
  
+ CCATAAAGAG AATAGAATAT ACTGTCTATT TGTAGTAGTT TTACAGATAA AAACAAATAA GCAAATGAAA   
  
  
+ ATAGATAGAG GATTTTTTTG CTATTATCCA CGTCGATCCA TTGAATTATT TTGTCGCTAT TTGGTTAAGT   
  
  
+ GAAATAAAAT ACTGAAGTAG AATGGAGGAT GTTGATAATG ACCTATGTCC CTCAAAGTTT AATCATGTAG   
  
  
+ TTTCTACGAA TTTCAAGCCA TTATAAGACA TACAGATTTT TATGATGTTG AAAATACTCT TACCTCCTTG   
  
  
+ TCACAATATT AACCTGAAGC CAATGAAATG AAACAGAACT AATTAAATTG AATACATAAT CAATCCATAT   
  
  
+ TAATTTGTTT TGACAACTAT TTACAATCAT CGATCTCTTT AATTAGGTCT ATAAGCTATA GAGTAATTAC   
  
  
+ TCAAAACTGT TTTGTAAGAC AACTTACTCC ATATTGGCTT ATTAGTATAT TGCCTCCATT CACATGAATA   
  
  
+ CTTCATGAGA GATTGCCTAA AACTCATATT GTCATCACCT TAAGTCAAAC ATATTTTGTT ATGAAATGAC   
  
  
+ ATCAAGAATT ACCCTCTGAC AAGAAAAATA TACCTGACTG ACATAATCGT ACCACATCTG TTAGTCCTCT   
  
  
+ TTCTACCACT TTGTAGAACA TAATGTGACA GTAATACATC CATCCCAAAA GAAAAGACGA AAAGAAGAGT   
  
  
+ GGAGAGTCAC GTATACGCGT TGAATTCAGG AAAATTAAAG GAACTCATGA AGACCCCATA TAGTGCTCCA   
  
  
+ ACCTGAACCT GCAGCCCACT ACTACATGCC TAAATTAAAG AGCAAAGTTA TGAGAGAAAC CAAAAAAGCT   
  
  
+ CTTTGAGAGA GTGGAGGAAG AATCATGGAA AGCTAGCTAG GCTTAGCAAG AAAGAGCAAC AGCTAGCTAG   
  
  
+ CAAAAGGCTT CTCTTTGAAG CCTTAGAAAT CTTTAGAGAT AAAAAGTTGG TGGCTTTTTA AGGGCTGTGA   
  
  
+ TATGTCCCCC ATGTAAAAAT GCAGCAATAA TGTTAATGTC ATGCTTTCTT TCATGGTCCC TAATGCGAAA   
  
  
+ TCAATCATGC ATGTCAACGT GACTCAGTGG CTCACCATAT ATGATCTGAT TTCCCCGCAC ACAGAGATAA   
  
  
+ AAAGCCCCCA ATTATAACCT CCCAAAAGCC CCCCCTTTTC CTCCTACCCA ACCCACACCT CCCCATTTCA   
  
  
+ TCAGGGCTCG CTTCTTAAGT TATCGATTAA AAAAAGAGTG AGTAAACGTA CCCGAATTGA AGAGGGGTGT   
  
  
+ TCGAAGCTTA TACTTGCTTG ATTAGGAATG AAAGTCCCCA TTTCATCCGC CCCCCAAACC GCCGCCACCA   
  
  
+ CCAGTAAATC GCTATCTTGC AACAATACTG CCTTGAATAC TGCCTTTCAA GCCCCCAATT CAATTTCAAC   
  
  
+ CCTTGTTAAC AACAACAAAC CCTCCTACGA GCCTACCTCG GTTCTAGACC TCCGGCGCAG CCCAAGCCCC   
  
  
+ GCCACCAACG CCGCCCTGCC ACTGCCCACT GACGCGGGCA TCAATAAACC CAAGCCTGCC TCTGCTGACC   
  
  
+ TGGACAATTT GGAGGGTGAG GGTTGGGATT CAATCCTATC AGAGCTGGGG CTTAATGATG ATTCTAATAA   
  
  
+ TCCTAACTCC AAACTTTGGT CATCCCAAAT TAGCTCATCA TGTGATTCCC ACCTCACACA GCTCCCTGAG   
  
  
+ TTCCCCTCCT CTCAGCCGTT GGATCACACA CGCACCCCCA ATAATTAACT TCTCCCCCTC TGATTACCCC   
  
  
+ ATCTCTGATT TATCCTACGG TCAGAATCTC AGCCCCAATT TTGGGCAGTT TGAGCTTCAT CTTCATCAGC   
  
  
+ CCAACGTCAA TAACAATGGG CTTGACTTCA TAGAGGACCT AATTAGAGCA GCTCAAGCCT ACGAATCCAA   
  
  
+ CGACTCCCAC CAGGTCCAGC TGGTATTGGC GCGGCTCAAT CAACGGCTCA GATCACCCGC CGGAAAACCC   
  
  
+ CTCCACCGCG CCGCCTTCTA CTTCAAGGAG GCCCTCCAAC ACCTCGCTGT CGGCGGAGGC CCGGCCCGGC   
  
  
+ CTGTCCGGCT GTCATCCTAC GAGGTGGTGC AGACCATCCG GGCCCACAAG AACTTTTCCG GCATATCACT   
  
  
+ GATATCCCTC TTCTCCACCT TCGCCGCCAA TCAGGCGATT CTGGAGGCGG TGGATGGGGC TGCGTTCATC   
  
  
+ CACATCATCG ATTTCGACAT CGGATTTGGC GGCCACTGGG CGTCGTTTAT GCGTGAGCTT GTCGACAAGG   
  
  
+ CCGACCCGAA TAAGATCAGC TCCTTGGTGT TCCGAATCAC GGCGATCGTA CCCGAAGAGT TCGGGATCGA   
  
  
+ AAGCAAGCTG GTGAGAGAGA ATCTTACGCA ATTCGCTCGT GATCTCAACA TCAATGTCCA TATGGAGTAT   
  
  
+ GTCCTTATTC AAAGCTTCGA GTTCTTATCC TTCAAATCGG TCAAATTCAT CGACGGTGAG AAATTGGCGG   
  
  
+ TCCATCTATC TCCGGCGATG TTTCACCGGC TTCAAGGCGG AATCTCGAAA TTCCTAGCCG ATCTCCGGGG   
  
  
+ AATTTCGCCA AGCACCGTCG TGGTGGTGGA CAGTGAGGTA GGAATCGACA CGGGAACTTC GTCCTTCAGC   
  
  
+ GTCAACTTCG TCGCCGGTAT CGAATTCTAC ACCGGAATGC TCGAATCGCT CGACGTCACC TCCGCCAGCG   
  
  
+ GTTTTGCAGG CGGCGGCGGC GGCGACGCTG TGAGAAGGAT CGAGACGTAC GTGCTCCGGC CGAGGATTAC   
  
  
+ GGCGGCGGTG GAGGCGGCAT CTGCGGCGGC GGTAGCGCGG CGGAGCGGTT GGAGAGAGTC GTTCGCGGTG   
  
  
+ GCTAGGATGA GGGCGGTGGG GTTCAGCCAG TTTGCTGATT TCCAGGCGGA GTGTTTGCTG AGGAAGGTTC   
  
  
+ AGGTTGGCGG GTTCCACGTG GCGAAACGTC ATGGGGAGAT GATGCTTTGC TGGCATGATA GGCCCCTTGT   
  
  
+ GTCCACGTCG GCTTGGAGGT GTTA  

- -Up\_Stream \_Len000CTAAAA TGAAGGTTAA AAGATGAATT TAAACCAATA GTGCAAGCTT AAAAAGTCCA   
  
  
- TTTATGTTTG ACAATCACCG GTAGGAAAAA AAAACCCAGA TTAGGTAGGT CTACACGATT ATGAAAGACA   
  
  
- AAATCTACTC TTCAAAATTA CGAAAAAATC TACACATTAA AAAAATTTGT ATTTATCAGA ACAGCTTATT   
  
  
- CTAAACGATC ATTAAAAACA TTCTCTTTAA TACACATAAA AAATAATATA GTCTTATAAT CTAGCTAGAG   
  
  
- TTAAATTAAT GAATACTTTT TTTTTCACCT ATAGAAATTA GTTAAACCGT TCGAGAGTAC GTAATTAACA   
  
  
- TCAGCATGCT TGAATAAATG TAGTGAGTAG ACTTAAACGA TGGTAAATGT GGAGTTTAAA ATAAAATTAC   
  
  
- GGTATTTCTC TTATCTTATA TGACAGATAA ACATCATCAA AATGTCTATT TTTGTTTATT CGTTTACTTT   
  
  
- TATCTATCTC CTAAAAAAAC GATAATAGGT GCAGCTAGGT AACTTAATAA AACAGCGATA AACCAATTCA   
  
  
- CTTTATTTTA TGACTTCATC TTACCTCCTA CAACTATTAC TGGATACAGG GAGTTTCAAA TTAGTACATC   
  
  
- AAAGATGCTT AAAGTTCGGT AATATTCTGT ATGTCTAAAA ATACTACAAC TTTTATGAGA ATGGAGGAAC   
  
  
- AGTGTTATAA TTGGACTTCG GTTACTTTAC TTTGTCTTGA TTAATTTAAC TTATGTATTA GTTAGGTATA   
  
  
- ATTAAACAAA ACTGTTGATA AATGTTAGTA GCTAGAGAAA TTAATCCAGA TATTCGATAT CTCATTAATG   
  
  
- AGTTTTGACA AAACATTCTG TTGAATGAGG TATAACCGAA TAATCATATA ACGGAGGTAA GTGTACTTAT   
  
  
- GAAGTACTCT CTAACGGATT TTGAGTATAA CAGTAGTGGA ATTCAGTTTG TATAAAACAA TACTTTACTG   
  
  
- TAGTTCTTAA TGGGAGACTG TTCTTTTTAT ATGGACTGAC TGTATTAGCA TGGTGTAGAC AATCAGGAGA   
  
  
- AAGATGGTGA AACATCTTGT ATTACACTGT CATTATGTAG GTAGGGTTTT CTTTTCTGCT TTTCTTCTCA   
  
  
- CCTCTCAGTG CATATGCGCA ACTTAAGTCC TTTTAATTTC CTTGAGTACT TCTGGGGTAT ATCACGAGGT   
  
  
- TGGACTTGGA CGTCGGGTGA TGATGTACGG ATTTAATTTC TCGTTTCAAT ACTCTCTTTG GTTTTTTCGA   
  
  
- GAAACTCTCT CACCTCCTTC TTAGTACCTT TCGATCGATC CGAATCGTTC TTTCTCGTTG TCGATCGATC   
  
  
- GTTTTCCGAA GAGAAACTTC GGAATCTTTA GAAATCTCTA TTTTTCAACC ACCGAAAAAT TCCCGACACT   
  
  
- ATACAGGGGG TACATTTTTA CGTCGTTATT ACAATTACAG TACGAAAGAA AGTACCAGGG ATTACGCTTT   
  
  
- AGTTAGTACG TACAGTTGCA CTGAGTCACC GAGTGGTATA TACTAGACTA AAGGGGCGTG TGTCTCTATT   
  
  
- TTTCGGGGGT TAATATTGGA GGGTTTTCGG GGGGGAAAAG GAGGATGGGT TGGGTGTGGA GGGGTAAAGT   
  
  
- AGTCCCGAGC GAAGAATTCA ATAGCTAATT TTTTTCTCAC TCATTTGCAT GGGCTTAACT TCTCCCCACA   
  
  
- AGCTTCGAAT ATGAACGAAC TAATCCTTAC TTTCAGGGGT AAAGTAGGCG GGGGGTTTGG CGGCGGTGGT   
  
  
- GGTCATTTAG CGATAGAACG TTGTTATGAC GGAACTTATG ACGGAAAGTT CGGGGGTTAA GTTAAAGTTG   
  
  
- GGAACAATTG TTGTTGTTTG GGAGGATGCT CGGATGGAGC CAAGATCTGG AGGCCGCGTC GGGTTCGGGG   
  
  
- CGGTGGTTGC GGCGGGACGG TGACGGGTGA CTGCGCCCGT AGTTATTTGG GTTCGGACGG AGACGACTGG   
  
  
- ACCTGTTAAA CCTCCCACTC CCAACCCTAA GTTAGGATAG TCTCGACCCC GAATTACTAC TAAGATTATT   
  
  
- AGGATTGAGG TTTGAAACCA GTAGGGTTTA ATCGAGTAGT ACACTAAGGG TGGAGTGTGT CGAGGGACTC   
  
  
- AAGGGGAGGA GAGTCGGCAA CCTAGTGTGT GCGTGGGGGT TATTAATTGA AGAGGGGGAG ACTAATGGGG   
  
  
- TAGAGACTAA ATAGGATGCC AGTCTTAGAG TCGGGGTTAA AACCCGTCAA ACTCGAAGTA GAAGTAGTCG   
  
  
- GGTTGCAGTT ATTGTTACCC GAACTGAAGT ATCTCCTGGA TTAATCTCGT CGAGTTCGGA TGCTTAGGTT   
  
  
- GCTGAGGGTG GTCCAGGTCG ACCATAACCG CGCCGAGTTA GTTGCCGAGT CTAGTGGGCG GCCTTTTGGG   
  
  
- GAGGTGGCGC GGCGGAAGAT GAAGTTCCTC CGGGAGGTTG TGGAGCGACA GCCGCCTCCG GGCCGGGCCG   
  
  
- GACAGGCCGA CAGTAGGATG CTCCACCACG TCTGGTAGGC CCGGGTGTTC TTGAAAAGGC CGTATAGTGA   
  
  
- CTATAGGGAG AAGAGGTGGA AGCGGCGGTT AGTCCGCTAA GACCTCCGCC ACCTACCCCG ACGCAAGTAG   
  
  
- GTGTAGTAGC TAAAGCTGTA GCCTAAACCG CCGGTGACCC GCAGCAAATA CGCACTCGAA CAGCTGTTCC   
  
  
- GGCTGGGCTT ATTCTAGTCG AGGAACCACA AGGCTTAGTG CCGCTAGCAT GGGCTTCTCA AGCCCTAGCT   
  
  
- TTCGTTCGAC CACTCTCTCT TAGAATGCGT TAAGCGAGCA CTAGAGTTGT AGTTACAGGT ATACCTCATA   
  
  
- CAGGAATAAG TTTCGAAGCT CAAGAATAGG AAGTTTAGCC AGTTTAAGTA GCTGCCACTC TTTAACCGCC   
  
  
- AGGTAGATAG AGGCCGCTAC AAAGTGGCCG AAGTTCCGCC TTAGAGCTTT AAGGATCGGC TAGAGGCCCC   
  
  
- TTAAAGCGGT TCGTGGCAGC ACCACCACCT GTCACTCCAT CCTTAGCTGT GCCCTTGAAG CAGGAAGTCG   
  
  
- CAGTTGAAGC AGCGGCCATA GCTTAAGATG TGGCCTTACG AGCTTAGCGA GCTGCAGTGG AGGCGGTCGC   
  
  
- CAAAACGTCC GCCGCCGCCG CCGCTGCGAC ACTCTTCCTA GCTCTGCATG CACGAGGCCG GCTCCTAATG   
  
  
- CCGCCGCCAC CTCCGCCGTA GACGCCGCCG CCATCGCGCC GCCTCGCCAA CCTCTCTCAG CAAGCGCCAC   
  
  
- CGATCCTACT CCCGCCACCC CAAGTCGGTC AAACGACTAA AGGTCCGCCT CACAAACGAC TCCTTCCAAG   
  
  
- TCCAACCGCC CAAGGTGCAC CGCTTTGCAG TACCCCTCTA CTACGAAACG ACCGTACTAT CCGGGGAACA   
  
  
- CAGGTGCAGC CGAACCTCCA CAAT

+     Unnamed\_\_1

| Site Name | Organism | Position | Strand | Matrix score. | sequence | function |
| --- | --- | --- | --- | --- | --- | --- |
| Unnamed\_\_1 | Zea mays | 3367 | - | 5 | CGTGG |  |
| Unnamed\_\_1 | Zea mays | 3311 | + | 5 | CGTGG |  |
| Unnamed\_\_1 | Zea mays | 3308 | - | 5 | CGTGG |  |
| Unnamed\_\_1 | Zea mays | 2963 | + | 5 | CGTGG |  |
| Unnamed\_\_1 | Zea mays | 522 | - | 5 | CGTGG |  |
| Unnamed\_\_1 | Petunia sp. | 3307 | - | 9 | GCCACGTGGC |  |

>HU06G00283.1   
+ -Up\_Stream \_Len000GATTTT ACTTCCAATT TTCTACTTAA ATTTGGTTAT CACGTTCGAA TTTTTCAGGT   
  
  
+ AAATACAAAC TGTTAGTGGC CATCCTTTTT TTTTGGGTCT AATCCATCCA GATGTGCTAA TACTTTCTGT   
  
  
+ TTTAGATGAG AAGTTTTAAT GCTTTTTTAG ATGTGTAATT TTTTTAAACA TAAATAGTCT TGTCGAATAA   
  
  
+ GATTTGCTAG TAATTTTTGT AAGAGAAATT ATGTGTATTT TTTATTATAT CAGAATATTA GATCGATCTC   
  
  
+ AATTTAATTA CTTATGAAAA AAAAAGTGGA TATCTTTAAT CAATTTGGCA AGCTCTCATG CATTAATTGT   
  
  
+ AGTCGTACGA ACTTATTTAC ATCACTCATC TGAATTTGCT ACCATTTACA CCTCAAATTT TATTTTAATG   
  
  
+ CCATAAAGAG AATAGAATAT ACTGTCTATT TGTAGTAGTT TTACAGATAA AAACAAATAA GCAAATGAAA   
  
  
+ ATAGATAGAG GATTTTTTTG CTATTATCCA CGTCGATCCA TTGAATTATT TTGTCGCTAT TTGGTTAAGT   
  
  
+ GAAATAAAAT ACTGAAGTAG AATGGAGGAT GTTGATAATG ACCTATGTCC CTCAAAGTTT AATCATGTAG   
  
  
+ TTTCTACGAA TTTCAAGCCA TTATAAGACA TACAGATTTT TATGATGTTG AAAATACTCT TACCTCCTTG   
  
  
+ TCACAATATT AACCTGAAGC CAATGAAATG AAACAGAACT AATTAAATTG AATACATAAT CAATCCATAT   
  
  
+ TAATTTGTTT TGACAACTAT TTACAATCAT CGATCTCTTT AATTAGGTCT ATAAGCTATA GAGTAATTAC   
  
  
+ TCAAAACTGT TTTGTAAGAC AACTTACTCC ATATTGGCTT ATTAGTATAT TGCCTCCATT CACATGAATA   
  
  
+ CTTCATGAGA GATTGCCTAA AACTCATATT GTCATCACCT TAAGTCAAAC ATATTTTGTT ATGAAATGAC   
  
  
+ ATCAAGAATT ACCCTCTGAC AAGAAAAATA TACCTGACTG ACATAATCGT ACCACATCTG TTAGTCCTCT   
  
  
+ TTCTACCACT TTGTAGAACA TAATGTGACA GTAATACATC CATCCCAAAA GAAAAGACGA AAAGAAGAGT   
  
  
+ GGAGAGTCAC GTATACGCGT TGAATTCAGG AAAATTAAAG GAACTCATGA AGACCCCATA TAGTGCTCCA   
  
  
+ ACCTGAACCT GCAGCCCACT ACTACATGCC TAAATTAAAG AGCAAAGTTA TGAGAGAAAC CAAAAAAGCT   
  
  
+ CTTTGAGAGA GTGGAGGAAG AATCATGGAA AGCTAGCTAG GCTTAGCAAG AAAGAGCAAC AGCTAGCTAG   
  
  
+ CAAAAGGCTT CTCTTTGAAG CCTTAGAAAT CTTTAGAGAT AAAAAGTTGG TGGCTTTTTA AGGGCTGTGA   
  
  
+ TATGTCCCCC ATGTAAAAAT GCAGCAATAA TGTTAATGTC ATGCTTTCTT TCATGGTCCC TAATGCGAAA   
  
  
+ TCAATCATGC ATGTCAACGT GACTCAGTGG CTCACCATAT ATGATCTGAT TTCCCCGCAC ACAGAGATAA   
  
  
+ AAAGCCCCCA ATTATAACCT CCCAAAAGCC CCCCCTTTTC CTCCTACCCA ACCCACACCT CCCCATTTCA   
  
  
+ TCAGGGCTCG CTTCTTAAGT TATCGATTAA AAAAAGAGTG AGTAAACGTA CCCGAATTGA AGAGGGGTGT   
  
  
+ TCGAAGCTTA TACTTGCTTG ATTAGGAATG AAAGTCCCCA TTTCATCCGC CCCCCAAACC GCCGCCACCA   
  
  
+ CCAGTAAATC GCTATCTTGC AACAATACTG CCTTGAATAC TGCCTTTCAA GCCCCCAATT CAATTTCAAC   
  
  
+ CCTTGTTAAC AACAACAAAC CCTCCTACGA GCCTACCTCG GTTCTAGACC TCCGGCGCAG CCCAAGCCCC   
  
  
+ GCCACCAACG CCGCCCTGCC ACTGCCCACT GACGCGGGCA TCAATAAACC CAAGCCTGCC TCTGCTGACC   
  
  
+ TGGACAATTT GGAGGGTGAG GGTTGGGATT CAATCCTATC AGAGCTGGGG CTTAATGATG ATTCTAATAA   
  
  
+ TCCTAACTCC AAACTTTGGT CATCCCAAAT TAGCTCATCA TGTGATTCCC ACCTCACACA GCTCCCTGAG   
  
  
+ TTCCCCTCCT CTCAGCCGTT GGATCACACA CGCACCCCCA ATAATTAACT TCTCCCCCTC TGATTACCCC   
  
  
+ ATCTCTGATT TATCCTACGG TCAGAATCTC AGCCCCAATT TTGGGCAGTT TGAGCTTCAT CTTCATCAGC   
  
  
+ CCAACGTCAA TAACAATGGG CTTGACTTCA TAGAGGACCT AATTAGAGCA GCTCAAGCCT ACGAATCCAA   
  
  
+ CGACTCCCAC CAGGTCCAGC TGGTATTGGC GCGGCTCAAT CAACGGCTCA GATCACCCGC CGGAAAACCC   
  
  
+ CTCCACCGCG CCGCCTTCTA CTTCAAGGAG GCCCTCCAAC ACCTCGCTGT CGGCGGAGGC CCGGCCCGGC   
  
  
+ CTGTCCGGCT GTCATCCTAC GAGGTGGTGC AGACCATCCG GGCCCACAAG AACTTTTCCG GCATATCACT   
  
  
+ GATATCCCTC TTCTCCACCT TCGCCGCCAA TCAGGCGATT CTGGAGGCGG TGGATGGGGC TGCGTTCATC   
  
  
+ CACATCATCG ATTTCGACAT CGGATTTGGC GGCCACTGGG CGTCGTTTAT GCGTGAGCTT GTCGACAAGG   
  
  
+ CCGACCCGAA TAAGATCAGC TCCTTGGTGT TCCGAATCAC GGCGATCGTA CCCGAAGAGT TCGGGATCGA   
  
  
+ AAGCAAGCTG GTGAGAGAGA ATCTTACGCA ATTCGCTCGT GATCTCAACA TCAATGTCCA TATGGAGTAT   
  
  
+ GTCCTTATTC AAAGCTTCGA GTTCTTATCC TTCAAATCGG TCAAATTCAT CGACGGTGAG AAATTGGCGG   
  
  
+ TCCATCTATC TCCGGCGATG TTTCACCGGC TTCAAGGCGG AATCTCGAAA TTCCTAGCCG ATCTCCGGGG   
  
  
+ AATTTCGCCA AGCACCGTCG TGGTGGTGGA CAGTGAGGTA GGAATCGACA CGGGAACTTC GTCCTTCAGC   
  
  
+ GTCAACTTCG TCGCCGGTAT CGAATTCTAC ACCGGAATGC TCGAATCGCT CGACGTCACC TCCGCCAGCG   
  
  
+ GTTTTGCAGG CGGCGGCGGC GGCGACGCTG TGAGAAGGAT CGAGACGTAC GTGCTCCGGC CGAGGATTAC   
  
  
+ GGCGGCGGTG GAGGCGGCAT CTGCGGCGGC GGTAGCGCGG CGGAGCGGTT GGAGAGAGTC GTTCGCGGTG   
  
  
+ GCTAGGATGA GGGCGGTGGG GTTCAGCCAG TTTGCTGATT TCCAGGCGGA GTGTTTGCTG AGGAAGGTTC   
  
  
+ AGGTTGGCGG GTTCCACGTG GCGAAACGTC ATGGGGAGAT GATGCTTTGC TGGCATGATA GGCCCCTTGT   
  
  
+ GTCCACGTCG GCTTGGAGGT GTTA  

- -Up\_Stream \_Len000CTAAAA TGAAGGTTAA AAGATGAATT TAAACCAATA GTGCAAGCTT AAAAAGTCCA   
  
  
- TTTATGTTTG ACAATCACCG GTAGGAAAAA AAAACCCAGA TTAGGTAGGT CTACACGATT ATGAAAGACA   
  
  
- AAATCTACTC TTCAAAATTA CGAAAAAATC TACACATTAA AAAAATTTGT ATTTATCAGA ACAGCTTATT   
  
  
- CTAAACGATC ATTAAAAACA TTCTCTTTAA TACACATAAA AAATAATATA GTCTTATAAT CTAGCTAGAG   
  
  
- TTAAATTAAT GAATACTTTT TTTTTCACCT ATAGAAATTA GTTAAACCGT TCGAGAGTAC GTAATTAACA   
  
  
- TCAGCATGCT TGAATAAATG TAGTGAGTAG ACTTAAACGA TGGTAAATGT GGAGTTTAAA ATAAAATTAC   
  
  
- GGTATTTCTC TTATCTTATA TGACAGATAA ACATCATCAA AATGTCTATT TTTGTTTATT CGTTTACTTT   
  
  
- TATCTATCTC CTAAAAAAAC GATAATAGGT GCAGCTAGGT AACTTAATAA AACAGCGATA AACCAATTCA   
  
  
- CTTTATTTTA TGACTTCATC TTACCTCCTA CAACTATTAC TGGATACAGG GAGTTTCAAA TTAGTACATC   
  
  
- AAAGATGCTT AAAGTTCGGT AATATTCTGT ATGTCTAAAA ATACTACAAC TTTTATGAGA ATGGAGGAAC   
  
  
- AGTGTTATAA TTGGACTTCG GTTACTTTAC TTTGTCTTGA TTAATTTAAC TTATGTATTA GTTAGGTATA   
  
  
- ATTAAACAAA ACTGTTGATA AATGTTAGTA GCTAGAGAAA TTAATCCAGA TATTCGATAT CTCATTAATG   
  
  
- AGTTTTGACA AAACATTCTG TTGAATGAGG TATAACCGAA TAATCATATA ACGGAGGTAA GTGTACTTAT   
  
  
- GAAGTACTCT CTAACGGATT TTGAGTATAA CAGTAGTGGA ATTCAGTTTG TATAAAACAA TACTTTACTG   
  
  
- TAGTTCTTAA TGGGAGACTG TTCTTTTTAT ATGGACTGAC TGTATTAGCA TGGTGTAGAC AATCAGGAGA   
  
  
- AAGATGGTGA AACATCTTGT ATTACACTGT CATTATGTAG GTAGGGTTTT CTTTTCTGCT TTTCTTCTCA   
  
  
- CCTCTCAGTG CATATGCGCA ACTTAAGTCC TTTTAATTTC CTTGAGTACT TCTGGGGTAT ATCACGAGGT   
  
  
- TGGACTTGGA CGTCGGGTGA TGATGTACGG ATTTAATTTC TCGTTTCAAT ACTCTCTTTG GTTTTTTCGA   
  
  
- GAAACTCTCT CACCTCCTTC TTAGTACCTT TCGATCGATC CGAATCGTTC TTTCTCGTTG TCGATCGATC   
  
  
- GTTTTCCGAA GAGAAACTTC GGAATCTTTA GAAATCTCTA TTTTTCAACC ACCGAAAAAT TCCCGACACT   
  
  
- ATACAGGGGG TACATTTTTA CGTCGTTATT ACAATTACAG TACGAAAGAA AGTACCAGGG ATTACGCTTT   
  
  
- AGTTAGTACG TACAGTTGCA CTGAGTCACC GAGTGGTATA TACTAGACTA AAGGGGCGTG TGTCTCTATT   
  
  
- TTTCGGGGGT TAATATTGGA GGGTTTTCGG GGGGGAAAAG GAGGATGGGT TGGGTGTGGA GGGGTAAAGT   
  
  
- AGTCCCGAGC GAAGAATTCA ATAGCTAATT TTTTTCTCAC TCATTTGCAT GGGCTTAACT TCTCCCCACA   
  
  
- AGCTTCGAAT ATGAACGAAC TAATCCTTAC TTTCAGGGGT AAAGTAGGCG GGGGGTTTGG CGGCGGTGGT   
  
  
- GGTCATTTAG CGATAGAACG TTGTTATGAC GGAACTTATG ACGGAAAGTT CGGGGGTTAA GTTAAAGTTG   
  
  
- GGAACAATTG TTGTTGTTTG GGAGGATGCT CGGATGGAGC CAAGATCTGG AGGCCGCGTC GGGTTCGGGG   
  
  
- CGGTGGTTGC GGCGGGACGG TGACGGGTGA CTGCGCCCGT AGTTATTTGG GTTCGGACGG AGACGACTGG   
  
  
- ACCTGTTAAA CCTCCCACTC CCAACCCTAA GTTAGGATAG TCTCGACCCC GAATTACTAC TAAGATTATT   
  
  
- AGGATTGAGG TTTGAAACCA GTAGGGTTTA ATCGAGTAGT ACACTAAGGG TGGAGTGTGT CGAGGGACTC   
  
  
- AAGGGGAGGA GAGTCGGCAA CCTAGTGTGT GCGTGGGGGT TATTAATTGA AGAGGGGGAG ACTAATGGGG   
  
  
- TAGAGACTAA ATAGGATGCC AGTCTTAGAG TCGGGGTTAA AACCCGTCAA ACTCGAAGTA GAAGTAGTCG   
  
  
- GGTTGCAGTT ATTGTTACCC GAACTGAAGT ATCTCCTGGA TTAATCTCGT CGAGTTCGGA TGCTTAGGTT   
  
  
- GCTGAGGGTG GTCCAGGTCG ACCATAACCG CGCCGAGTTA GTTGCCGAGT CTAGTGGGCG GCCTTTTGGG   
  
  
- GAGGTGGCGC GGCGGAAGAT GAAGTTCCTC CGGGAGGTTG TGGAGCGACA GCCGCCTCCG GGCCGGGCCG   
  
  
- GACAGGCCGA CAGTAGGATG CTCCACCACG TCTGGTAGGC CCGGGTGTTC TTGAAAAGGC CGTATAGTGA
[truncated: 108,950 more chars]
