# Supplementary material for: Genome-Wide Identification and Expression Pattern of the GRAS Gene Family in Pitaya (Selenicereus undatus L.)
Source: Biology (Basel). 2022 Dec 21;12(1):11. doi: 10.3390/biology12010011 (PMC9854919; doi:10.3390/biology12010011)
Supplement: Supplementary file 1 [file biology-12-00011-s001.zip › Supplementary file S5/HU06G00358.1_plantcare.html]

Content-Type: text/html; charset=ISO-8859-1


PlantCARE


Webmaster Firefox specific output  
To save the result:
click on the frame with the right mouse button and save the source code as a text file with extension .html  
REFERENCE:PlantCARE: a database of plant cis-acting regulatory elements and a portal to tools for in silico analysis of promoter sequences.  
Lescot, M., Déhais, P., Moreau, Y., De Moor, B., Rouzé ,P.,and Rombauts, S.  
Nucleic Acids Res., Database issue(2002), 30(1):325-327.   


---

>HU06G00358.1   
+ -Up\_Stream \_Len000GGGTTT TTTTAAACTC AAAATTAGTT ATTGTATCAA TTGAATTTGG AAAACTATTA   
  
  
+ TTTTCGACTT ATTATAGAGA ATTTGTTGTT AATGAATGCT GATAAATACT GATAATTAGT GTTGTGAGTG   
  
  
+ TGAATTTGTG ATGATGAGCA ATGATTAGCG TTAAAATTAT AACTAGGATA AATAATTAAG TGTTGATGAA   
  
  
+ TGTTGATTGA TGATGATGAG TACTGATTGG TGCGGTGCTG GTAATAAATA TTTATGATAA GTTAAAAAAA   
  
  
+ AAAAACACAT CCTAAACAAT CACTATGTAA TATACTAAAT GAAAAAAATT GGTAATGTGA TTTGACAATC   
  
  
+ TAAGGGTAAG GGTGTTTTAC CCACACATGT TATGAAATTG GCTTACTATT TACAAACTTT TTACAGTTAT   
  
  
+ ATGATTCTAC CTATGTGTTA TCATCTTTCC GTCGATGTAA TTGATTATAT TCAACACAAT TTAATTCATT   
  
  
+ ACAATGTAGC TAATTGATAC TCAAAATAAT GAACTATTAA TAAATAATTA ATTATAACTT TTAAAAGTTT   
  
  
+ GTTAATTTAA AATTATATAA TATATAAATT TAGGCTACAT TAATCAAAAA ATTAGGCGTG TTCATCGGTC   
  
  
+ CAAACTTGAA CCGGACCAGA CCGGACCGAA GATCGATTAA GAGAAAAAGT ACAGACTGAG GACCGGATCG   
  
  
+ AATAAGAGTC GCACCGGACC AGGATCGGAC CGGAAAAATT TGGACCAGAT CGGGACCGAT CGAGGTAGAC   
  
  
+ CGAATTTATT TTCAAATACG ATAGGAATAA AACTTATTGA TTCAATTGAT TTTGGCCAAA ATCATGCTAT   
  
  
+ TATTGATTTT ATTTGCTAAC TCTCAAGAGT ATTAGTTTGC ACTTCGCATT ATCTTTGGCC AAAAAAATCA   
  
  
+ AAAAGAAAGT CTCAAACTCT TAATAGTTTT TTATTTTATT TGGTTTATTT TTAAAATAGT CGGCCTTCGG   
  
  
+ TCCGGACTAG TGGACCGAAT AAGGTAATTT TTCGAATCGA GGACCAGACC AAATGCACTC AGTTCGAACC   
  
  
+ GGTTCAGTTC GGTCTGACTC GATTTTTGGT CCGGTCCAAG TTTGAACAGA ATAATGTACA CACCTAGAAA   
  
  
+ AAAAGAATTA TGCATATATA CTTAAGGCCC CCAACCCTTT TTCCCTTTCC GCCAATACTT TTTACCAAGT   
  
  
+ CCAATTCTTT TCTTCTTAGC TATGGCTAGT AGTTATCCCT CCCTAGCTTC CACCCTAAGT TTTGAGACAA   
  
  
+ AATCCCATCT ATGTTAATAT TTATTTCTTG CACATGTCAC CCAATACAAG GGTTTCATTG AAAAGAAAAG   
  
  
+ GTATTAGATG TAAGATATGT ATGTATATAA ATAGTTTTGC AATAGACTAG AAGGACTAAA AAGGAAAAAG   
  
  
+ GAAGTTTAAA AGCAAAAAAC TTTCCCCTCT AGAAAAAAGG AAACCAGATT CCCTTTTCTA CCCCAATTCC   
  
  
+ CATAGTCTTT ACTCTGTCTC TCTCTCTCTG TCTCTCTTAC TCATGATGTT GAGGGAGGAA GGAATGAATT   
  
  
+ AAGGACCCTA AGCTAAGATC CAACCGTAAG ATATTGCCCC TTTAATCACC CATTTCATAT CTCTATCCAC   
  
  
+ AGTTAAAAAC CCACCTCCTC CATCCACATA ACTGATATAT CTCTTCTACA CCATACTATA AGGAAGAAGA   
  
  
+ GAAAAATAGG AGTGTATTGT ATTTTTATTT GAGTAATTTA TTTTAAGGAG AGAAATTTAC ATGGTTGGGT   
  
  
+ TAAATGGATA CCTTGTTTAG ACTAGTGAGT TTCCAACAAC CACACCACCA CCATCACCAA CACCAACACC   
  
  
+ AACACCACCA ACAGCAATCT TCTGATCAAT CCTCTTTGAA TAATACTACT ACTTATTCCA CAAATTCAAG   
  
  
+ CAGTTCCAGA TCTTCCCAAC AAACCCATCA TAATCATAAT CATACTCAGT ATTATCACCA ACATCATCCT   
  
  
+ ACTTACCCCC ACCGCCACCA TCAGGAGGAA GAAGAAGAAT GCTTCAACTT TTACATGGAT GAAGAAAACT   
  
  
+ TCTCTTCTTC TTCCTCCCAC AACAAACATC ATCCTCAACC TCCTTCCTCC GCCACCACCT CCTCGTATTA   
  
  
+ CCCTTACTAC TCCACCCCCT TCGATGTTCC CTCTACCACC ACCACCTCCA CCTCCACCCC CACGCCCACC   
  
  
+ AACACCACCA CCACCACCCC CCCAACCCAG TATGGCGGCG GCGGCGGTGG GTTTGACTCC AGCTGCTTCT   
  
  
+ CCTACTCCCC TGCCCGAGAC CTCAACCCGG AGCTCTTCTC CTCGTCCGGG TTCTCGGGCA AGTGGGCTTC   
  
  
+ CAACATCCTC TTGGAAGCCG CTCGTGCCAT CTCTGAGAGG AACACGAGCC GCCTCCACCA GCTCCTATGG   
  
  
+ ATGGTCAACG AGCTCAGCTC CCCGTACGGT GACGTGGAGC AGAAGATAGC TTCATACTTC GGCCAAGCCC   
  
  
+ TCTTCACCCG CATGACCGAG TCAGGGGACC GCACCTACCG CACCATGCGG TCCGCCGTGG ACAAGACCTT   
  
  
+ CTCCTTTGAG TCTACGAGAA AGATGGTACT CAAGTTCCAG GAGGTTAGCC CTTGGACCAC CTTTGGACAC   
  
  
+ GTGGCAGCTA ATGGAGCCCT CATTGATGCC TTTGACGGTG AATCCAAAAT CCACATCATC GACATCAGCA   
  
  
+ CCACATTCTG CACCCAGTGG CCCACCCTCC TGGAGGCGCT GGCCACCCGA ATGGATGACG CGCCCTACCT   
  
  
+ GAGGCTGACC ACTGTGGTGG TCAACAAGTC CGGGAATGAA GGTCCCACCG GAGGCGGGTC CCACAGGGTG   
  
  
+ ATGAAAGAGA TTGGGACCCG CCTCGAGAAG TTTGCTAGGC TAATGGGAGT GCCTTTCAAA TTTAATGTGG   
  
  
+ TCCACCACGG GGCTGATTTA TCCGACTTGG ATTTCTCTCA GCTGGATATT AACGATGACG AGGCATTAGC   
  
  
+ CATCAACTGT GTCAACTCGC TCCACTCGGT CAATATCCAC CGCCGTGACT CGGTCATCTC GGCCTTCCGG   
  
  
+ CGGCTCCACC CTAGGGTGGT GACCGTGGTG GAGGAGGAGG CTGACCTTGC TGACGTGGGG GCGGAGGGGT   
  
  
+ ACGAGTTCTA CAGGGGGTTT AATGAGTGTT TAAGGTGGGT TAGGGTTTAC TTTGAGGCCC TGGAAGAGTG   
  
  
+ CTTTCCTAGG ACAAGCAATG AGAAACTCAT GCTCGAGCGG GCCGCAGGGC GGGCTCTGGT GGACCTCCTG   
  
  
+ GCCTGCCCCA AGCCCGCATC ATCCGAGCGG AGGGAGGCAG CTGCGCGGTG GTCCGGGAGG ATGCAGGGGG   
  
  
+ CCGGGTTCGG CCACGTGGGG TTCAGTGATG AGGTCTGTGA TGACGTCAAG GCCCTCCTAA GGAGGTACAA   
  
  
+ GGAGGGGTGG TCCATGATAC AATGTTCCGC CGCGGATGGC GGCGCCTCCG CCGGAATATT CTTGTCGTGG   
  
  
+ AGGGATCAGC CGGTGGTGTG GGCCAGTGCA TGGAAGCCTT A  

- -Up\_Stream \_Len000CCCAAA AAAATTTGAG TTTTAATCAA TAACATAGTT AACTTAAACC TTTTGATAAT   
  
  
- AAAAGCTGAA TAATATCTCT TAAACAACAA TTACTTACGA CTATTTATGA CTATTAATCA CAACACTCAC   
  
  
- ACTTAAACAC TACTACTCGT TACTAATCGC AATTTTAATA TTGATCCTAT TTATTAATTC ACAACTACTT   
  
  
- ACAACTAACT ACTACTACTC ATGACTAACC ACGCCACGAC CATTATTTAT AAATACTATT CAATTTTTTT   
  
  
- TTTTTGTGTA GGATTTGTTA GTGATACATT ATATGATTTA CTTTTTTTAA CCATTACACT AAACTGTTAG   
  
  
- ATTCCCATTC CCACAAAATG GGTGTGTACA ATACTTTAAC CGAATGATAA ATGTTTGAAA AATGTCAATA   
  
  
- TACTAAGATG GATACACAAT AGTAGAAAGG CAGCTACATT AACTAATATA AGTTGTGTTA AATTAAGTAA   
  
  
- TGTTACATCG ATTAACTATG AGTTTTATTA CTTGATAATT ATTTATTAAT TAATATTGAA AATTTTCAAA   
  
  
- CAATTAAATT TTAATATATT ATATATTTAA ATCCGATGTA ATTAGTTTTT TAATCCGCAC AAGTAGCCAG   
  
  
- GTTTGAACTT GGCCTGGTCT GGCCTGGCTT CTAGCTAATT CTCTTTTTCA TGTCTGACTC CTGGCCTAGC   
  
  
- TTATTCTCAG CGTGGCCTGG TCCTAGCCTG GCCTTTTTAA ACCTGGTCTA GCCCTGGCTA GCTCCATCTG   
  
  
- GCTTAAATAA AAGTTTATGC TATCCTTATT TTGAATAACT AAGTTAACTA AAACCGGTTT TAGTACGATA   
  
  
- ATAACTAAAA TAAACGATTG AGAGTTCTCA TAATCAAACG TGAAGCGTAA TAGAAACCGG TTTTTTTAGT   
  
  
- TTTTCTTTCA GAGTTTGAGA ATTATCAAAA AATAAAATAA ACCAAATAAA AATTTTATCA GCCGGAAGCC   
  
  
- AGGCCTGATC ACCTGGCTTA TTCCATTAAA AAGCTTAGCT CCTGGTCTGG TTTACGTGAG TCAAGCTTGG   
  
  
- CCAAGTCAAG CCAGACTGAG CTAAAAACCA GGCCAGGTTC AAACTTGTCT TATTACATGT GTGGATCTTT   
  
  
- TTTTCTTAAT ACGTATATAT GAATTCCGGG GGTTGGGAAA AAGGGAAAGG CGGTTATGAA AAATGGTTCA   
  
  
- GGTTAAGAAA AGAAGAATCG ATACCGATCA TCAATAGGGA GGGATCGAAG GTGGGATTCA AAACTCTGTT   
  
  
- TTAGGGTAGA TACAATTATA AATAAAGAAC GTGTACAGTG GGTTATGTTC CCAAAGTAAC TTTTCTTTTC   
  
  
- CATAATCTAC ATTCTATACA TACATATATT TATCAAAACG TTATCTGATC TTCCTGATTT TTCCTTTTTC   
  
  
- CTTCAAATTT TCGTTTTTTG AAAGGGGAGA TCTTTTTTCC TTTGGTCTAA GGGAAAAGAT GGGGTTAAGG   
  
  
- GTATCAGAAA TGAGACAGAG AGAGAGAGAC AGAGAGAATG AGTACTACAA CTCCCTCCTT CCTTACTTAA   
  
  
- TTCCTGGGAT TCGATTCTAG GTTGGCATTC TATAACGGGG AAATTAGTGG GTAAAGTATA GAGATAGGTG   
  
  
- TCAATTTTTG GGTGGAGGAG GTAGGTGTAT TGACTATATA GAGAAGATGT GGTATGATAT TCCTTCTTCT   
  
  
- CTTTTTATCC TCACATAACA TAAAAATAAA CTCATTAAAT AAAATTCCTC TCTTTAAATG TACCAACCCA   
  
  
- ATTTACCTAT GGAACAAATC TGATCACTCA AAGGTTGTTG GTGTGGTGGT GGTAGTGGTT GTGGTTGTGG   
  
  
- TTGTGGTGGT TGTCGTTAGA AGACTAGTTA GGAGAAACTT ATTATGATGA TGAATAAGGT GTTTAAGTTC   
  
  
- GTCAAGGTCT AGAAGGGTTG TTTGGGTAGT ATTAGTATTA GTATGAGTCA TAATAGTGGT TGTAGTAGGA   
  
  
- TGAATGGGGG TGGCGGTGGT AGTCCTCCTT CTTCTTCTTA CGAAGTTGAA AATGTACCTA CTTCTTTTGA   
  
  
- AGAGAAGAAG AAGGAGGGTG TTGTTTGTAG TAGGAGTTGG AGGAAGGAGG CGGTGGTGGA GGAGCATAAT   
  
  
- GGGAATGATG AGGTGGGGGA AGCTACAAGG GAGATGGTGG TGGTGGAGGT GGAGGTGGGG GTGCGGGTGG   
  
  
- TTGTGGTGGT GGTGGTGGGG GGGTTGGGTC ATACCGCCGC CGCCGCCACC CAAACTGAGG TCGACGAAGA   
  
  
- GGATGAGGGG ACGGGCTCTG GAGTTGGGCC TCGAGAAGAG GAGCAGGCCC AAGAGCCCGT TCACCCGAAG   
  
  
- GTTGTAGGAG AACCTTCGGC GAGCACGGTA GAGACTCTCC TTGTGCTCGG CGGAGGTGGT CGAGGATACC   
  
  
- TACCAGTTGC TCGAGTCGAG GGGCATGCCA CTGCACCTCG TCTTCTATCG AAGTATGAAG CCGGTTCGGG   
  
  
- AGAAGTGGGC GTACTGGCTC AGTCCCCTGG CGTGGATGGC GTGGTACGCC AGGCGGCACC TGTTCTGGAA   
  
  
- GAGGAAACTC AGATGCTCTT TCTACCATGA GTTCAAGGTC CTCCAATCGG GAACCTGGTG GAAACCTGTG   
  
  
- CACCGTCGAT TACCTCGGGA GTAACTACGG AAACTGCCAC TTAGGTTTTA GGTGTAGTAG CTGTAGTCGT   
  
  
- GGTGTAAGAC GTGGGTCACC GGGTGGGAGG ACCTCCGCGA CCGGTGGGCT TACCTACTGC GCGGGATGGA   
  
  
- CTCCGACTGG TGACACCACC AGTTGTTCAG GCCCTTACTT CCAGGGTGGC CTCCGCCCAG GGTGTCCCAC   
  
  
- TACTTTCTCT AACCCTGGGC GGAGCTCTTC AAACGATCCG ATTACCCTCA CGGAAAGTTT AAATTACACC   
  
  
- AGGTGGTGCC CCGACTAAAT AGGCTGAACC TAAAGAGAGT CGACCTATAA TTGCTACTGC TCCGTAATCG   
  
  
- GTAGTTGACA CAGTTGAGCG AGGTGAGCCA GTTATAGGTG GCGGCACTGA GCCAGTAGAG CCGGAAGGCC   
  
  
- GCCGAGGTGG GATCCCACCA CTGGCACCAC CTCCTCCTCC GACTGGAACG ACTGCACCCC CGCCTCCCCA   
  
  
- TGCTCAAGAT GTCCCCCAAA TTACTCACAA ATTCCACCCA ATCCCAAATG AAACTCCGGG ACCTTCTCAC   
  
  
- GAAAGGATCC TGTTCGTTAC TCTTTGAGTA CGAGCTCGCC CGGCGTCCCG CCCGAGACCA CCTGGAGGAC   
  
  
- CGGACGGGGT TCGGGCGTAG TAGGCTCGCC TCCCTCCGTC GACGCGCCAC CAGGCCCTCC TACGTCCCCC   
  
  
- GGCCCAAGCC GGTGCACCCC AAGTCACTAC TCCAGACACT ACTGCAGTTC CGGGAGGATT CCTCCATGTT   
  
  
- CCTCCCCACC AGGTACTATG TTACAAGGCG GCGCCTACCG CCGCGGAGGC GGCCTTATAA GAACAGCACC   
  
  
- TCCCTAGTCG GCCACCACAC CCGGTCACGT ACCTTCGGAA T

  
  
Motifs Found  

+   

| Site Name | Organism | Position | Strand | Matrix score. | sequence | function |
| --- | --- | --- | --- | --- | --- | --- |
|  | organism | 1731 | - | 4 | motif\_sequence | short\_function |
|  | organism | 1108 | + | 4 | motif\_sequence | short\_function |
|  | organism | 683 | - | 4 | motif\_sequence | short\_function |
|  | organism | 1294 | - | 4 | motif\_sequence | short\_function |
|  | organism | 2277 | + | 4 | motif\_sequence | short\_function |
|  | organism | 662 | - | 4 | motif\_sequence | short\_function |
|  | organism | 3386 | + | 4 | motif\_sequence | short\_function |
|  | organism | 1680 | - | 4 | motif\_sequence | short\_function |
|  | organism | 1655 | + | 4 | motif\_sequence | short\_function |
|  | organism | 1841 | + | 4 | motif\_sequence | short\_function |
|  | organism | 2454 | + | 4 | motif\_sequence | short\_function |
|  | organism | 1098 | - | 4 | motif\_sequence | short\_function |
|  | organism | 3147 | - | 4 | motif\_sequence | short\_function |
|  | organism | 1904 | + | 4 | motif\_sequence | short\_function |
|  | organism | 2135 | + | 4 | motif\_sequence | short\_function |
|  | organism | 2426 | - | 4 | motif\_sequence | short\_function |
|  | organism | 2128 | + | 4 | motif\_sequence | short\_function |
|  | organism | 2036 | + | 4 | motif\_sequence | short\_function |

>HU06G00358.1   
+ -Up\_Stream \_Len000GGGTTT TTTTAAACTC AAAATTAGTT ATTGTATCAA TTGAATTTGG AAAACTATTA   
  
  
+ TTTTCGACTT ATTATAGAGA ATTTGTTGTT AATGAATGCT GATAAATACT GATAATTAGT GTTGTGAGTG   
  
  
+ TGAATTTGTG ATGATGAGCA ATGATTAGCG TTAAAATTAT AACTAGGATA AATAATTAAG TGTTGATGAA   
  
  
+ TGTTGATTGA TGATGATGAG TACTGATTGG TGCGGTGCTG GTAATAAATA TTTATGATAA GTTAAAAAAA   
  
  
+ AAAAACACAT CCTAAACAAT CACTATGTAA TATACTAAAT GAAAAAAATT GGTAATGTGA TTTGACAATC   
  
  
+ TAAGGGTAAG GGTGTTTTAC CCACACATGT TATGAAATTG GCTTACTATT TACAAACTTT TTACAGTTAT   
  
  
+ ATGATTCTAC CTATGTGTTA TCATCTTTCC GTCGATGTAA TTGATTATAT TCAACACAAT TTAATTCATT   
  
  
+ ACAATGTAGC TAATTGATAC TCAAAATAAT GAACTATTAA TAAATAATTA ATTATAACTT TTAAAAGTTT   
  
  
+ GTTAATTTAA AATTATATAA TATATAAATT TAGGCTACAT TAATCAAAAA ATTAGGCGTG TTCATCGGTC   
  
  
+ CAAACTTGAA CCGGACCAGA CCGGACCGAA GATCGATTAA GAGAAAAAGT ACAGACTGAG GACCGGATCG   
  
  
+ AATAAGAGTC GCACCGGACC AGGATCGGAC CGGAAAAATT TGGACCAGAT CGGGACCGAT CGAGGTAGAC   
  
  
+ CGAATTTATT TTCAAATACG ATAGGAATAA AACTTATTGA TTCAATTGAT TTTGGCCAAA ATCATGCTAT   
  
  
+ TATTGATTTT ATTTGCTAAC TCTCAAGAGT ATTAGTTTGC ACTTCGCATT ATCTTTGGCC AAAAAAATCA   
  
  
+ AAAAGAAAGT CTCAAACTCT TAATAGTTTT TTATTTTATT TGGTTTATTT TTAAAATAGT CGGCCTTCGG   
  
  
+ TCCGGACTAG TGGACCGAAT AAGGTAATTT TTCGAATCGA GGACCAGACC AAATGCACTC AGTTCGAACC   
  
  
+ GGTTCAGTTC GGTCTGACTC GATTTTTGGT CCGGTCCAAG TTTGAACAGA ATAATGTACA CACCTAGAAA   
  
  
+ AAAAGAATTA TGCATATATA CTTAAGGCCC CCAACCCTTT TTCCCTTTCC GCCAATACTT TTTACCAAGT   
  
  
+ CCAATTCTTT TCTTCTTAGC TATGGCTAGT AGTTATCCCT CCCTAGCTTC CACCCTAAGT TTTGAGACAA   
  
  
+ AATCCCATCT ATGTTAATAT TTATTTCTTG CACATGTCAC CCAATACAAG GGTTTCATTG AAAAGAAAAG   
  
  
+ GTATTAGATG TAAGATATGT ATGTATATAA ATAGTTTTGC AATAGACTAG AAGGACTAAA AAGGAAAAAG   
  
  
+ GAAGTTTAAA AGCAAAAAAC TTTCCCCTCT AGAAAAAAGG AAACCAGATT CCCTTTTCTA CCCCAATTCC   
  
  
+ CATAGTCTTT ACTCTGTCTC TCTCTCTCTG TCTCTCTTAC TCATGATGTT GAGGGAGGAA GGAATGAATT   
  
  
+ AAGGACCCTA AGCTAAGATC CAACCGTAAG ATATTGCCCC TTTAATCACC CATTTCATAT CTCTATCCAC   
  
  
+ AGTTAAAAAC CCACCTCCTC CATCCACATA ACTGATATAT CTCTTCTACA CCATACTATA AGGAAGAAGA   
  
  
+ GAAAAATAGG AGTGTATTGT ATTTTTATTT GAGTAATTTA TTTTAAGGAG AGAAATTTAC ATGGTTGGGT   
  
  
+ TAAATGGATA CCTTGTTTAG ACTAGTGAGT TTCCAACAAC CACACCACCA CCATCACCAA CACCAACACC   
  
  
+ AACACCACCA ACAGCAATCT TCTGATCAAT CCTCTTTGAA TAATACTACT ACTTATTCCA CAAATTCAAG   
  
  
+ CAGTTCCAGA TCTTCCCAAC AAACCCATCA TAATCATAAT CATACTCAGT ATTATCACCA ACATCATCCT   
  
  
+ ACTTACCCCC ACCGCCACCA TCAGGAGGAA GAAGAAGAAT GCTTCAACTT TTACATGGAT GAAGAAAACT   
  
  
+ TCTCTTCTTC TTCCTCCCAC AACAAACATC ATCCTCAACC TCCTTCCTCC GCCACCACCT CCTCGTATTA   
  
  
+ CCCTTACTAC TCCACCCCCT TCGATGTTCC CTCTACCACC ACCACCTCCA CCTCCACCCC CACGCCCACC   
  
  
+ AACACCACCA CCACCACCCC CCCAACCCAG TATGGCGGCG GCGGCGGTGG GTTTGACTCC AGCTGCTTCT   
  
  
+ CCTACTCCCC TGCCCGAGAC CTCAACCCGG AGCTCTTCTC CTCGTCCGGG TTCTCGGGCA AGTGGGCTTC   
  
  
+ CAACATCCTC TTGGAAGCCG CTCGTGCCAT CTCTGAGAGG AACACGAGCC GCCTCCACCA GCTCCTATGG   
  
  
+ ATGGTCAACG AGCTCAGCTC CCCGTACGGT GACGTGGAGC AGAAGATAGC TTCATACTTC GGCCAAGCCC   
  
  
+ TCTTCACCCG CATGACCGAG TCAGGGGACC GCACCTACCG CACCATGCGG TCCGCCGTGG ACAAGACCTT   
  
  
+ CTCCTTTGAG TCTACGAGAA AGATGGTACT CAAGTTCCAG GAGGTTAGCC CTTGGACCAC CTTTGGACAC   
  
  
+ GTGGCAGCTA ATGGAGCCCT CATTGATGCC TTTGACGGTG AATCCAAAAT CCACATCATC GACATCAGCA   
  
  
+ CCACATTCTG CACCCAGTGG CCCACCCTCC TGGAGGCGCT GGCCACCCGA ATGGATGACG CGCCCTACCT   
  
  
+ GAGGCTGACC ACTGTGGTGG TCAACAAGTC CGGGAATGAA GGTCCCACCG GAGGCGGGTC CCACAGGGTG   
  
  
+ ATGAAAGAGA TTGGGACCCG CCTCGAGAAG TTTGCTAGGC TAATGGGAGT GCCTTTCAAA TTTAATGTGG   
  
  
+ TCCACCACGG GGCTGATTTA TCCGACTTGG ATTTCTCTCA GCTGGATATT AACGATGACG AGGCATTAGC   
  
  
+ CATCAACTGT GTCAACTCGC TCCACTCGGT CAATATCCAC CGCCGTGACT CGGTCATCTC GGCCTTCCGG   
  
  
+ CGGCTCCACC CTAGGGTGGT GACCGTGGTG GAGGAGGAGG CTGACCTTGC TGACGTGGGG GCGGAGGGGT   
  
  
+ ACGAGTTCTA CAGGGGGTTT AATGAGTGTT TAAGGTGGGT TAGGGTTTAC TTTGAGGCCC TGGAAGAGTG   
  
  
+ CTTTCCTAGG ACAAGCAATG AGAAACTCAT GCTCGAGCGG GCCGCAGGGC GGGCTCTGGT GGACCTCCTG   
  
  
+ GCCTGCCCCA AGCCCGCATC ATCCGAGCGG AGGGAGGCAG CTGCGCGGTG GTCCGGGAGG ATGCAGGGGG   
  
  
+ CCGGGTTCGG CCACGTGGGG TTCAGTGATG AGGTCTGTGA TGACGTCAAG GCCCTCCTAA GGAGGTACAA   
  
  
+ GGAGGGGTGG TCCATGATAC AATGTTCCGC CGCGGATGGC GGCGCCTCCG CCGGAATATT CTTGTCGTGG   
  
  
+ AGGGATCAGC CGGTGGTGTG GGCCAGTGCA TGGAAGCCTT A  

- -Up\_Stream \_Len000CCCAAA AAAATTTGAG TTTTAATCAA TAACATAGTT AACTTAAACC TTTTGATAAT   
  
  
- AAAAGCTGAA TAATATCTCT TAAACAACAA TTACTTACGA CTATTTATGA CTATTAATCA CAACACTCAC   
  
  
- ACTTAAACAC TACTACTCGT TACTAATCGC AATTTTAATA TTGATCCTAT TTATTAATTC ACAACTACTT   
  
  
- ACAACTAACT ACTACTACTC ATGACTAACC ACGCCACGAC CATTATTTAT AAATACTATT CAATTTTTTT   
  
  
- TTTTTGTGTA GGATTTGTTA GTGATACATT ATATGATTTA CTTTTTTTAA CCATTACACT AAACTGTTAG   
  
  
- ATTCCCATTC CCACAAAATG GGTGTGTACA ATACTTTAAC CGAATGATAA ATGTTTGAAA AATGTCAATA   
  
  
- TACTAAGATG GATACACAAT AGTAGAAAGG CAGCTACATT AACTAATATA AGTTGTGTTA AATTAAGTAA   
  
  
- TGTTACATCG ATTAACTATG AGTTTTATTA CTTGATAATT ATTTATTAAT TAATATTGAA AATTTTCAAA   
  
  
- CAATTAAATT TTAATATATT ATATATTTAA ATCCGATGTA ATTAGTTTTT TAATCCGCAC AAGTAGCCAG   
  
  
- GTTTGAACTT GGCCTGGTCT GGCCTGGCTT CTAGCTAATT CTCTTTTTCA TGTCTGACTC CTGGCCTAGC   
  
  
- TTATTCTCAG CGTGGCCTGG TCCTAGCCTG GCCTTTTTAA ACCTGGTCTA GCCCTGGCTA GCTCCATCTG   
  
  
- GCTTAAATAA AAGTTTATGC TATCCTTATT TTGAATAACT AAGTTAACTA AAACCGGTTT TAGTACGATA   
  
  
- ATAACTAAAA TAAACGATTG AGAGTTCTCA TAATCAAACG TGAAGCGTAA TAGAAACCGG TTTTTTTAGT   
  
  
- TTTTCTTTCA GAGTTTGAGA ATTATCAAAA AATAAAATAA ACCAAATAAA AATTTTATCA GCCGGAAGCC   
  
  
- AGGCCTGATC ACCTGGCTTA TTCCATTAAA AAGCTTAGCT CCTGGTCTGG TTTACGTGAG TCAAGCTTGG   
  
  
- CCAAGTCAAG CCAGACTGAG CTAAAAACCA GGCCAGGTTC AAACTTGTCT TATTACATGT GTGGATCTTT   
  
  
- TTTTCTTAAT ACGTATATAT GAATTCCGGG GGTTGGGAAA AAGGGAAAGG CGGTTATGAA AAATGGTTCA   
  
  
- GGTTAAGAAA AGAAGAATCG ATACCGATCA TCAATAGGGA GGGATCGAAG GTGGGATTCA AAACTCTGTT   
  
  
- TTAGGGTAGA TACAATTATA AATAAAGAAC GTGTACAGTG GGTTATGTTC CCAAAGTAAC TTTTCTTTTC   
  
  
- CATAATCTAC ATTCTATACA TACATATATT TATCAAAACG TTATCTGATC TTCCTGATTT TTCCTTTTTC   
  
  
- CTTCAAATTT TCGTTTTTTG AAAGGGGAGA TCTTTTTTCC TTTGGTCTAA GGGAAAAGAT GGGGTTAAGG   
  
  
- GTATCAGAAA TGAGACAGAG AGAGAGAGAC AGAGAGAATG AGTACTACAA CTCCCTCCTT CCTTACTTAA   
  
  
- TTCCTGGGAT TCGATTCTAG GTTGGCATTC TATAACGGGG AAATTAGTGG GTAAAGTATA GAGATAGGTG   
  
  
- TCAATTTTTG GGTGGAGGAG GTAGGTGTAT TGACTATATA GAGAAGATGT GGTATGATAT TCCTTCTTCT   
  
  
- CTTTTTATCC TCACATAACA TAAAAATAAA CTCATTAAAT AAAATTCCTC TCTTTAAATG TACCAACCCA   
  
  
- ATTTACCTAT GGAACAAATC TGATCACTCA AAGGTTGTTG GTGTGGTGGT GGTAGTGGTT GTGGTTGTGG   
  
  
- TTGTGGTGGT TGTCGTTAGA AGACTAGTTA GGAGAAACTT ATTATGATGA TGAATAAGGT GTTTAAGTTC   
  
  
- GTCAAGGTCT AGAAGGGTTG TTTGGGTAGT ATTAGTATTA GTATGAGTCA TAATAGTGGT TGTAGTAGGA   
  
  
- TGAATGGGGG TGGCGGTGGT AGTCCTCCTT CTTCTTCTTA CGAAGTTGAA AATGTACCTA CTTCTTTTGA   
  
  
- AGAGAAGAAG AAGGAGGGTG TTGTTTGTAG TAGGAGTTGG AGGAAGGAGG CGGTGGTGGA GGAGCATAAT   
  
  
- GGGAATGATG AGGTGGGGGA AGCTACAAGG GAGATGGTGG TGGTGGAGGT GGAGGTGGGG GTGCGGGTGG   
  
  
- TTGTGGTGGT GGTGGTGGGG GGGTTGGGTC ATACCGCCGC CGCCGCCACC CAAACTGAGG TCGACGAAGA   
  
  
- GGATGAGGGG ACGGGCTCTG GAGTTGGGCC TCGAGAAGAG GAGCAGGCCC AAGAGCCCGT TCACCCGAAG   
  
  
- GTTGTAGGAG AACCTTCGGC GAGCACGGTA GAGACTCTCC TTGTGCTCGG CGGAGGTGGT CGAGGATACC   
  
  
- TACCAGTTGC TCGAGTCGAG GGGCATGCCA CTGCACCTCG TCTTCTATCG AAGTATGAAG CCGGTTCGGG   
  
  
- AGAAGTGGGC GTACTGGCTC AGTCCCCTGG CGTGGATGGC GTGGTACGCC AGGCGGCACC TGTTCTGGAA   
  
  
- GAGGAAACTC AGATGCTCTT TCTACCATGA GTTCAAGGTC CTCCAATCGG GAACCTGGTG GAAACCTGTG   
  
  
- CACCGTCGAT TACCTCGGGA GTAACTACGG AAACTGCCAC TTAGGTTTTA GGTGTAGTAG CTGTAGTCGT   
  
  
- GGTGTAAGAC GTGGGTCACC GGGTGGGAGG ACCTCCGCGA CCGGTGGGCT TACCTACTGC GCGGGATGGA   
  
  
- CTCCGACTGG TGACACCACC AGTTGTTCAG GCCCTTACTT CCAGGGTGGC CTCCGCCCAG GGTGTCCCAC   
  
  
- TACTTTCTCT AACCCTGGGC GGAGCTCTTC AAACGATCCG ATTACCCTCA CGGAAAGTTT AAATTACACC   
  
  
- AGGTGGTGCC CCGACTAAAT AGGCTGAACC TAAAGAGAGT CGACCTATAA TTGCTACTGC TCCGTAATCG   
  
  
- GTAGTTGACA CAGTTGAGCG AGGTGAGCCA GTTATAGGTG GCGGCACTGA GCCAGTAGAG CCGGAAGGCC   
  
  
- GCCGAGGTGG GATCCCACCA CTGGCACCAC CTCCTCCTCC GACTGGAACG ACTGCACCCC CGCCTCCCCA   
  
  
- TGCTCAAGAT GTCCCCCAAA TTACTCACAA ATTCCACCCA ATCCCAAATG AAACTCCGGG ACCTTCTCAC   
  
  
- GAAAGGATCC TGTTCGTTAC TCTTTGAGTA CGAGCTCGCC CGGCGTCCCG CCCGAGACCA CCTGGAGGAC   
  
  
- CGGACGGGGT TCGGGCGTAG TAGGCTCGCC TCCCTCCGTC GACGCGCCAC CAGGCCCTCC TACGTCCCCC   
  
  
- GGCCCAAGCC GGTGCACCCC AAGTCACTAC TCCAGACACT ACTGCAGTTC CGGGAGGATT CCTCCATGTT   
  
  
- CCTCCCCACC AGGTACTATG TTACAAGGCG GCGCCTACCG CCGCGGAGGC GGCCTTATAA GAACAGCACC   
  
  
- TCCCTAGTCG GCCACCACAC CCGGTCACGT ACCTTCGGAA T

+     ABRE

| Site Name | Organism | Position | Strand | Matrix score. | sequence | function |
| --- | --- | --- | --- | --- | --- | --- |
| ABRE | Arabidopsis thaliana | 3307 | + | 5 | ACGTG | cis-acting element involved in the abscisic acid responsiveness |
| ABRE | Arabidopsis thaliana | 2592 | - | 6 | CACGTG | cis-acting element involved in the abscisic acid responsiveness |
| ABRE | Arabidopsis thaliana | 3306 | - | 6 | CACGTG | cis-acting element involved in the abscisic acid responsiveness |
| ABRE | Arabidopsis thaliana | 2593 | + | 5 | ACGTG | cis-acting element involved in the abscisic acid responsiveness |
| ABRE | Arabidopsis thaliana | 2290 | - | 7 | AACCCGG | cis-acting element involved in the abscisic acid responsiveness |
| ABRE | Arabidopsis thaliana | 3067 | + | 5 | ACGTG | cis-acting element involved in the abscisic acid responsiveness |
| ABRE | Arabidopsis thaliana | 2416 | + | 5 | ACGTG | cis-acting element involved in the abscisic acid responsiveness |
| ABRE | Arabidopsis thaliana | 3295 | - | 7 | AACCCGG | cis-acting element involved in the abscisic acid responsiveness |
| ABRE | Arabidopsis thaliana | 2268 | + | 7 | AACCCGG | cis-acting element involved in the abscisic acid responsiveness |
| ABRE | Hordeum vulgare | 2590 | - | 9 | GCAACGTGTC | cis-acting element involved in the abscisic acid responsiveness |

>HU06G00358.1   
+ -Up\_Stream \_Len000GGGTTT TTTTAAACTC AAAATTAGTT ATTGTATCAA TTGAATTTGG AAAACTATTA   
  
  
+ TTTTCGACTT ATTATAGAGA ATTTGTTGTT AATGAATGCT GATAAATACT GATAATTAGT GTTGTGAGTG   
  
  
+ TGAATTTGTG ATGATGAGCA ATGATTAGCG TTAAAATTAT AACTAGGATA AATAATTAAG TGTTGATGAA   
  
  
+ TGTTGATTGA TGATGATGAG TACTGATTGG TGCGGTGCTG GTAATAAATA TTTATGATAA GTTAAAAAAA   
  
  
+ AAAAACACAT CCTAAACAAT CACTATGTAA TATACTAAAT GAAAAAAATT GGTAATGTGA TTTGACAATC   
  
  
+ TAAGGGTAAG GGTGTTTTAC CCACACATGT TATGAAATTG GCTTACTATT TACAAACTTT TTACAGTTAT   
  
  
+ ATGATTCTAC CTATGTGTTA TCATCTTTCC GTCGATGTAA TTGATTATAT TCAACACAAT TTAATTCATT   
  
  
+ ACAATGTAGC TAATTGATAC TCAAAATAAT GAACTATTAA TAAATAATTA ATTATAACTT TTAAAAGTTT   
  
  
+ GTTAATTTAA AATTATATAA TATATAAATT TAGGCTACAT TAATCAAAAA ATTAGGCGTG TTCATCGGTC   
  
  
+ CAAACTTGAA CCGGACCAGA CCGGACCGAA GATCGATTAA GAGAAAAAGT ACAGACTGAG GACCGGATCG   
  
  
+ AATAAGAGTC GCACCGGACC AGGATCGGAC CGGAAAAATT TGGACCAGAT CGGGACCGAT CGAGGTAGAC   
  
  
+ CGAATTTATT TTCAAATACG ATAGGAATAA AACTTATTGA TTCAATTGAT TTTGGCCAAA ATCATGCTAT   
  
  
+ TATTGATTTT ATTTGCTAAC TCTCAAGAGT ATTAGTTTGC ACTTCGCATT ATCTTTGGCC AAAAAAATCA   
  
  
+ AAAAGAAAGT CTCAAACTCT TAATAGTTTT TTATTTTATT TGGTTTATTT TTAAAATAGT CGGCCTTCGG   
  
  
+ TCCGGACTAG TGGACCGAAT AAGGTAATTT TTCGAATCGA GGACCAGACC AAATGCACTC AGTTCGAACC   
  
  
+ GGTTCAGTTC GGTCTGACTC GATTTTTGGT CCGGTCCAAG TTTGAACAGA ATAATGTACA CACCTAGAAA   
  
  
+ AAAAGAATTA TGCATATATA CTTAAGGCCC CCAACCCTTT TTCCCTTTCC GCCAATACTT TTTACCAAGT   
  
  
+ CCAATTCTTT TCTTCTTAGC TATGGCTAGT AGTTATCCCT CCCTAGCTTC CACCCTAAGT TTTGAGACAA   
  
  
+ AATCCCATCT ATGTTAATAT TTATTTCTTG CACATGTCAC CCAATACAAG GGTTTCATTG AAAAGAAAAG   
  
  
+ GTATTAGATG TAAGATATGT ATGTATATAA ATAGTTTTGC AATAGACTAG AAGGACTAAA AAGGAAAAAG   
  
  
+ GAAGTTTAAA AGCAAAAAAC TTTCCCCTCT AGAAAAAAGG AAACCAGATT CCCTTTTCTA CCCCAATTCC   
  
  
+ CATAGTCTTT ACTCTGTCTC TCTCTCTCTG TCTCTCTTAC TCATGATGTT GAGGGAGGAA GGAATGAATT   
  
  
+ AAGGACCCTA AGCTAAGATC CAACCGTAAG ATATTGCCCC TTTAATCACC CATTTCATAT CTCTATCCAC   
  
  
+ AGTTAAAAAC CCACCTCCTC CATCCACATA ACTGATATAT CTCTTCTACA CCATACTATA AGGAAGAAGA   
  
  
+ GAAAAATAGG AGTGTATTGT ATTTTTATTT GAGTAATTTA TTTTAAGGAG AGAAATTTAC ATGGTTGGGT   
  
  
+ TAAATGGATA CCTTGTTTAG ACTAGTGAGT TTCCAACAAC CACACCACCA CCATCACCAA CACCAACACC   
  
  
+ AACACCACCA ACAGCAATCT TCTGATCAAT CCTCTTTGAA TAATACTACT ACTTATTCCA CAAATTCAAG   
  
  
+ CAGTTCCAGA TCTTCCCAAC AAACCCATCA TAATCATAAT CATACTCAGT ATTATCACCA ACATCATCCT   
  
  
+ ACTTACCCCC ACCGCCACCA TCAGGAGGAA GAAGAAGAAT GCTTCAACTT TTACATGGAT GAAGAAAACT   
  
  
+ TCTCTTCTTC TTCCTCCCAC AACAAACATC ATCCTCAACC TCCTTCCTCC GCCACCACCT CCTCGTATTA   
  
  
+ CCCTTACTAC TCCACCCCCT TCGATGTTCC CTCTACCACC ACCACCTCCA CCTCCACCCC CACGCCCACC   
  
  
+ AACACCACCA CCACCACCCC CCCAACCCAG TATGGCGGCG GCGGCGGTGG GTTTGACTCC AGCTGCTTCT   
  
  
+ CCTACTCCCC TGCCCGAGAC CTCAACCCGG AGCTCTTCTC CTCGTCCGGG TTCTCGGGCA AGTGGGCTTC   
  
  
+ CAACATCCTC TTGGAAGCCG CTCGTGCCAT CTCTGAGAGG AACACGAGCC GCCTCCACCA GCTCCTATGG   
  
  
+ ATGGTCAACG AGCTCAGCTC CCCGTACGGT GACGTGGAGC AGAAGATAGC TTCATACTTC GGCCAAGCCC   
  
  
+ TCTTCACCCG CATGACCGAG TCAGGGGACC GCACCTACCG CACCATGCGG TCCGCCGTGG ACAAGACCTT   
  
  
+ CTCCTTTGAG TCTACGAGAA AGATGGTACT CAAGTTCCAG GAGGTTAGCC CTTGGACCAC CTTTGGACAC   
  
  
+ GTGGCAGCTA ATGGAGCCCT CATTGATGCC TTTGACGGTG AATCCAAAAT CCACATCATC GACATCAGCA   
  
  
+ CCACATTCTG CACCCAGTGG CCCACCCTCC TGGAGGCGCT GGCCACCCGA ATGGATGACG CGCCCTACCT   
  
  
+ GAGGCTGACC ACTGTGGTGG TCAACAAGTC CGGGAATGAA GGTCCCACCG GAGGCGGGTC CCACAGGGTG   
  
  
+ ATGAAAGAGA TTGGGACCCG CCTCGAGAAG TTTGCTAGGC TAATGGGAGT GCCTTTCAAA TTTAATGTGG   
  
  
+ TCCACCACGG GGCTGATTTA TCCGACTTGG ATTTCTCTCA GCTGGATATT AACGATGACG AGGCATTAGC   
  
  
+ CATCAACTGT GTCAACTCGC TCCACTCGGT CAATATCCAC CGCCGTGACT CGGTCATCTC GGCCTTCCGG   
  
  
+ CGGCTCCACC CTAGGGTGGT GACCGTGGTG GAGGAGGAGG CTGACCTTGC TGACGTGGGG GCGGAGGGGT   
  
  
+ ACGAGTTCTA CAGGGGGTTT AATGAGTGTT TAAGGTGGGT TAGGGTTTAC TTTGAGGCCC TGGAAGAGTG   
  
  
+ CTTTCCTAGG ACAAGCAATG AGAAACTCAT GCTCGAGCGG GCCGCAGGGC GGGCTCTGGT GGACCTCCTG   
  
  
+ GCCTGCCCCA AGCCCGCATC ATCCGAGCGG AGGGAGGCAG CTGCGCGGTG GTCCGGGAGG ATGCAGGGGG   
  
  
+ CCGGGTTCGG CCACGTGGGG TTCAGTGATG AGGTCTGTGA TGACGTCAAG GCCCTCCTAA GGAGGTACAA   
  
  
+ GGAGGGGTGG TCCATGATAC AATGTTCCGC CGCGGATGGC GGCGCCTCCG CCGGAATATT CTTGTCGTGG   
  
  
+ AGGGATCAGC CGGTGGTGTG GGCCAGTGCA TGGAAGCCTT A  

- -Up\_Stream \_Len000CCCAAA AAAATTTGAG TTTTAATCAA TAACATAGTT AACTTAAACC TTTTGATAAT   
  
  
- AAAAGCTGAA TAATATCTCT TAAACAACAA TTACTTACGA CTATTTATGA CTATTAATCA CAACACTCAC   
  
  
- ACTTAAACAC TACTACTCGT TACTAATCGC AATTTTAATA TTGATCCTAT TTATTAATTC ACAACTACTT   
  
  
- ACAACTAACT ACTACTACTC ATGACTAACC ACGCCACGAC CATTATTTAT AAATACTATT CAATTTTTTT   
  
  
- TTTTTGTGTA GGATTTGTTA GTGATACATT ATATGATTTA CTTTTTTTAA CCATTACACT AAACTGTTAG   
  
  
- ATTCCCATTC CCACAAAATG GGTGTGTACA ATACTTTAAC CGAATGATAA ATGTTTGAAA AATGTCAATA   
  
  
- TACTAAGATG GATACACAAT AGTAGAAAGG CAGCTACATT AACTAATATA AGTTGTGTTA AATTAAGTAA   
  
  
- TGTTACATCG ATTAACTATG AGTTTTATTA CTTGATAATT ATTTATTAAT TAATATTGAA AATTTTCAAA   
  
  
- CAATTAAATT TTAATATATT ATATATTTAA ATCCGATGTA ATTAGTTTTT TAATCCGCAC AAGTAGCCAG   
  
  
- GTTTGAACTT GGCCTGGTCT GGCCTGGCTT CTAGCTAATT CTCTTTTTCA TGTCTGACTC CTGGCCTAGC   
  
  
- TTATTCTCAG CGTGGCCTGG TCCTAGCCTG GCCTTTTTAA ACCTGGTCTA GCCCTGGCTA GCTCCATCTG   
  
  
- GCTTAAATAA AAGTTTATGC TATCCTTATT TTGAATAACT AAGTTAACTA AAACCGGTTT TAGTACGATA   
  
  
- ATAACTAAAA TAAACGATTG AGAGTTCTCA TAATCAAACG TGAAGCGTAA TAGAAACCGG TTTTTTTAGT   
  
  
- TTTTCTTTCA GAGTTTGAGA ATTATCAAAA AATAAAATAA ACCAAATAAA AATTTTATCA GCCGGAAGCC   
  
  
- AGGCCTGATC ACCTGGCTTA TTCCATTAAA AAGCTTAGCT CCTGGTCTGG TTTACGTGAG TCAAGCTTGG   
  
  
- CCAAGTCAAG CCAGACTGAG CTAAAAACCA GGCCAGGTTC AAACTTGTCT TATTACATGT GTGGATCTTT   
  
  
- TTTTCTTAAT ACGTATATAT GAATTCCGGG GGTTGGGAAA AAGGGAAAGG CGGTTATGAA AAATGGTTCA   
  
  
- GGTTAAGAAA AGAAGAATCG ATACCGATCA TCAATAGGGA GGGATCGAAG GTGGGATTCA AAACTCTGTT   
  
  
- TTAGGGTAGA TACAATTATA AATAAAGAAC GTGTACAGTG GGTTATGTTC CCAAAGTAAC TTTTCTTTTC   
  
  
- CATAATCTAC ATTCTATACA TACATATATT TATCAAAACG TTATCTGATC TTCCTGATTT TTCCTTTTTC   
  
  
- CTTCAAATTT TCGTTTTTTG AAAGGGGAGA TCTTTTTTCC TTTGGTCTAA GGGAAAAGAT GGGGTTAAGG   
  
  
- GTATCAGAAA TGAGACAGAG AGAGAGAGAC AGAGAGAATG AGTACTACAA CTCCCTCCTT CCTTACTTAA   
  
  
- TTCCTGGGAT TCGATTCTAG GTTGGCATTC TATAACGGGG AAATTAGTGG GTAAAGTATA GAGATAGGTG   
  
  
- TCAATTTTTG GGTGGAGGAG GTAGGTGTAT TGACTATATA GAGAAGATGT GGTATGATAT TCCTTCTTCT   
  
  
- CTTTTTATCC TCACATAACA TAAAAATAAA CTCATTAAAT AAAATTCCTC TCTTTAAATG TACCAACCCA   
  
  
- ATTTACCTAT GGAACAAATC TGATCACTCA AAGGTTGTTG GTGTGGTGGT GGTAGTGGTT GTGGTTGTGG   
  
  
- TTGTGGTGGT TGTCGTTAGA AGACTAGTTA GGAGAAACTT ATTATGATGA TGAATAAGGT GTTTAAGTTC   
  
  
- GTCAAGGTCT AGAAGGGTTG TTTGGGTAGT ATTAGTATTA GTATGAGTCA TAATAGTGGT TGTAGTAGGA   
  
  
- TGAATGGGGG TGGCGGTGGT AGTCCTCCTT CTTCTTCTTA CGAAGTTGAA AATGTACCTA CTTCTTTTGA   
  
  
- AGAGAAGAAG AAGGAGGGTG TTGTTTGTAG TAGGAGTTGG AGGAAGGAGG CGGTGGTGGA GGAGCATAAT   
  
  
- GGGAATGATG AGGTGGGGGA AGCTACAAGG GAGATGGTGG TGGTGGAGGT GGAGGTGGGG GTGCGGGTGG   
  
  
- TTGTGGTGGT GGTGGTGGGG GGGTTGGGTC ATACCGCCGC CGCCGCCACC CAAACTGAGG TCGACGAAGA   
  
  
- GGATGAGGGG ACGGGCTCTG GAGTTGGGCC TCGAGAAGAG GAGCAGGCCC AAGAGCCCGT TCACCCGAAG   
  
  
- GTTGTAGGAG AACCTTCGGC GAGCACGGTA GAGACTCTCC TTGTGCTCGG CGGAGGTGGT CGAGGATACC   
  
  
- TACCAGTTGC TCGAGTCGAG GGGCATGCCA CTGCACCTCG TCTTCTATCG AAGTATGAAG CCGGTTCGGG   
  
  
- AGAAGTGGGC GTACTGGCTC AGTCCCCTGG CGTGGATGGC GTGGTACGCC AGGCGGCACC TGTTCTGGAA   
  
  
- GAGGAAACTC AGATGCTCTT TCTACCATGA GTTCAAGGTC CTCCAATCGG GAACCTGGTG GAAACCTGTG   
  
  
- CACCGTCGAT TACCTCGGGA GTAACTACGG AAACTGCCAC TTAGGTTTTA GGTGTAGTAG CTGTAGTCGT   
  
  
- GGTGTAAGAC GTGGGTCACC GGGTGGGAGG ACCTCCGCGA CCGGTGGGCT TACCTACTGC GCGGGATGGA   
  
  
- CTCCGACTGG TGACACCACC AGTTGTTCAG GCCCTTACTT CCAGGGTGGC CTCCGCCCAG GGTGTCCCAC   
  
  
- TACTTTCTCT AACCCTGGGC GGAGCTCTTC AAACGATCCG ATTACCCTCA CGGAAAGTTT AAATTACACC   
  
  
- AGGTGGTGCC CCGACTAAAT AGGCTGAACC TAAAGAGAGT CGACCTATAA TTGCTACTGC TCCGTAATCG   
  
  
- GTAGTTGACA CAGTTGAGCG AGGTGAGCCA GTTATAGGTG GCGGCACTGA GCCAGTAGAG CCGGAAGGCC   
  
  
- GCCGAGGTGG GATCCCACCA CTGGCACCAC CTCCTCCTCC GACTGGAACG ACTGCACCCC CGCCTCCCCA   
  
  
- TGCTCAAGAT GTCCCCCAAA TTACTCACAA ATTCCACCCA ATCCCAAATG AAACTCCGGG ACCTTCTCAC   
  
  
- GAAAGGATCC TGTTCGTTAC TCTTTGAGTA CGAGCTCGCC CGGCGTCCCG CCCGAGACCA CCTGGAGGAC   
  
  
- CGGACGGGGT TCGGGCGTAG TAGGCTCGCC TCCCTCCGTC GACGCGCCAC CAGGCCCTCC TACGTCCCCC   
  
  
- GGCCCAAGCC GGTGCACCCC AAGTCACTAC TCCAGACACT ACTGCAGTTC CGGGAGGATT CCTCCATGTT   
  
  
- CCTCCCCACC AGGTACTATG TTACAAGGCG GCGCCTACCG CCGCGGAGGC GGCCTTATAA GAACAGCACC   
  
  
- TCCCTAGTCG GCCACCACAC CCGGTCACGT ACCTTCGGAA T

+     ABRE2

| Site Name | Organism | Position | Strand | Matrix score. | sequence | function |
| --- | --- | --- | --- | --- | --- | --- |
| ABRE2 | Zea mays | 3305 | - | 8 | CCACGTGG |  |

>HU06G00358.1   
+ -Up\_Stream \_Len000GGGTTT TTTTAAACTC AAAATTAGTT ATTGTATCAA TTGAATTTGG AAAACTATTA   
  
  
+ TTTTCGACTT ATTATAGAGA ATTTGTTGTT AATGAATGCT GATAAATACT GATAATTAGT GTTGTGAGTG   
  
  
+ TGAATTTGTG ATGATGAGCA ATGATTAGCG TTAAAATTAT AACTAGGATA AATAATTAAG TGTTGATGAA   
  
  
+ TGTTGATTGA TGATGATGAG TACTGATTGG TGCGGTGCTG GTAATAAATA TTTATGATAA GTTAAAAAAA   
  
  
+ AAAAACACAT CCTAAACAAT CACTATGTAA TATACTAAAT GAAAAAAATT GGTAATGTGA TTTGACAATC   
  
  
+ TAAGGGTAAG GGTGTTTTAC CCACACATGT TATGAAATTG GCTTACTATT TACAAACTTT TTACAGTTAT   
  
  
+ ATGATTCTAC CTATGTGTTA TCATCTTTCC GTCGATGTAA TTGATTATAT TCAACACAAT TTAATTCATT   
  
  
+ ACAATGTAGC TAATTGATAC TCAAAATAAT GAACTATTAA TAAATAATTA ATTATAACTT TTAAAAGTTT   
  
  
+ GTTAATTTAA AATTATATAA TATATAAATT TAGGCTACAT TAATCAAAAA ATTAGGCGTG TTCATCGGTC   
  
  
+ CAAACTTGAA CCGGACCAGA CCGGACCGAA GATCGATTAA GAGAAAAAGT ACAGACTGAG GACCGGATCG   
  
  
+ AATAAGAGTC GCACCGGACC AGGATCGGAC CGGAAAAATT TGGACCAGAT CGGGACCGAT CGAGGTAGAC   
  
  
+ CGAATTTATT TTCAAATACG ATAGGAATAA AACTTATTGA TTCAATTGAT TTTGGCCAAA ATCATGCTAT   
  
  
+ TATTGATTTT ATTTGCTAAC TCTCAAGAGT ATTAGTTTGC ACTTCGCATT ATCTTTGGCC AAAAAAATCA   
  
  
+ AAAAGAAAGT CTCAAACTCT TAATAGTTTT TTATTTTATT TGGTTTATTT TTAAAATAGT CGGCCTTCGG   
  
  
+ TCCGGACTAG TGGACCGAAT AAGGTAATTT TTCGAATCGA GGACCAGACC AAATGCACTC AGTTCGAACC   
  
  
+ GGTTCAGTTC GGTCTGACTC GATTTTTGGT CCGGTCCAAG TTTGAACAGA ATAATGTACA CACCTAGAAA   
  
  
+ AAAAGAATTA TGCATATATA CTTAAGGCCC CCAACCCTTT TTCCCTTTCC GCCAATACTT TTTACCAAGT   
  
  
+ CCAATTCTTT TCTTCTTAGC TATGGCTAGT AGTTATCCCT CCCTAGCTTC CACCCTAAGT TTTGAGACAA   
  
  
+ AATCCCATCT ATGTTAATAT TTATTTCTTG CACATGTCAC CCAATACAAG GGTTTCATTG AAAAGAAAAG   
  
  
+ GTATTAGATG TAAGATATGT ATGTATATAA ATAGTTTTGC AATAGACTAG AAGGACTAAA AAGGAAAAAG   
  
  
+ GAAGTTTAAA AGCAAAAAAC TTTCCCCTCT AGAAAAAAGG AAACCAGATT CCCTTTTCTA CCCCAATTCC   
  
  
+ CATAGTCTTT ACTCTGTCTC TCTCTCTCTG TCTCTCTTAC TCATGATGTT GAGGGAGGAA GGAATGAATT   
  
  
+ AAGGACCCTA AGCTAAGATC CAACCGTAAG ATATTGCCCC TTTAATCACC CATTTCATAT CTCTATCCAC   
  
  
+ AGTTAAAAAC CCACCTCCTC CATCCACATA ACTGATATAT CTCTTCTACA CCATACTATA AGGAAGAAGA   
  
  
+ GAAAAATAGG AGTGTATTGT ATTTTTATTT GAGTAATTTA TTTTAAGGAG AGAAATTTAC ATGGTTGGGT   
  
  
+ TAAATGGATA CCTTGTTTAG ACTAGTGAGT TTCCAACAAC CACACCACCA CCATCACCAA CACCAACACC   
  
  
+ AACACCACCA ACAGCAATCT TCTGATCAAT CCTCTTTGAA TAATACTACT ACTTATTCCA CAAATTCAAG   
  
  
+ CAGTTCCAGA TCTTCCCAAC AAACCCATCA TAATCATAAT CATACTCAGT ATTATCACCA ACATCATCCT   
  
  
+ ACTTACCCCC ACCGCCACCA TCAGGAGGAA GAAGAAGAAT GCTTCAACTT TTACATGGAT GAAGAAAACT   
  
  
+ TCTCTTCTTC TTCCTCCCAC AACAAACATC ATCCTCAACC TCCTTCCTCC GCCACCACCT CCTCGTATTA   
  
  
+ CCCTTACTAC TCCACCCCCT TCGATGTTCC CTCTACCACC ACCACCTCCA CCTCCACCCC CACGCCCACC   
  
  
+ AACACCACCA CCACCACCCC CCCAACCCAG TATGGCGGCG GCGGCGGTGG GTTTGACTCC AGCTGCTTCT   
  
  
+ CCTACTCCCC TGCCCGAGAC CTCAACCCGG AGCTCTTCTC CTCGTCCGGG TTCTCGGGCA AGTGGGCTTC   
  
  
+ CAACATCCTC TTGGAAGCCG CTCGTGCCAT CTCTGAGAGG AACACGAGCC GCCTCCACCA GCTCCTATGG   
  
  
+ ATGGTCAACG AGCTCAGCTC CCCGTACGGT GACGTGGAGC AGAAGATAGC TTCATACTTC GGCCAAGCCC   
  
  
+ TCTTCACCCG CATGACCGAG TCAGGGGACC GCACCTACCG CACCATGCGG TCCGCCGTGG ACAAGACCTT   
  
  
+ CTCCTTTGAG TCTACGAGAA AGATGGTACT CAAGTTCCAG GAGGTTAGCC CTTGGACCAC CTTTGGACAC   
  
  
+ GTGGCAGCTA ATGGAGCCCT CATTGATGCC TTTGACGGTG AATCCAAAAT CCACATCATC GACATCAGCA   
  
  
+ CCACATTCTG CACCCAGTGG CCCACCCTCC TGGAGGCGCT GGCCACCCGA ATGGATGACG CGCCCTACCT   
  
  
+ GAGGCTGACC ACTGTGGTGG TCAACAAGTC CGGGAATGAA GGTCCCACCG GAGGCGGGTC CCACAGGGTG   
  
  
+ ATGAAAGAGA TTGGGACCCG CCTCGAGAAG TTTGCTAGGC TAATGGGAGT GCCTTTCAAA TTTAATGTGG   
  
  
+ TCCACCACGG GGCTGATTTA TCCGACTTGG ATTTCTCTCA GCTGGATATT AACGATGACG AGGCATTAGC   
  
  
+ CATCAACTGT GTCAACTCGC TCCACTCGGT CAATATCCAC CGCCGTGACT CGGTCATCTC GGCCTTCCGG   
  
  
+ CGGCTCCACC CTAGGGTGGT GACCGTGGTG GAGGAGGAGG CTGACCTTGC TGACGTGGGG GCGGAGGGGT   
  
  
+ ACGAGTTCTA CAGGGGGTTT AATGAGTGTT TAAGGTGGGT TAGGGTTTAC TTTGAGGCCC TGGAAGAGTG   
  
  
+ CTTTCCTAGG ACAAGCAATG AGAAACTCAT GCTCGAGCGG GCCGCAGGGC GGGCTCTGGT GGACCTCCTG   
  
  
+ GCCTGCCCCA AGCCCGCATC ATCCGAGCGG AGGGAGGCAG CTGCGCGGTG GTCCGGGAGG ATGCAGGGGG   
  
  
+ CCGGGTTCGG CCACGTGGGG TTCAGTGATG AGGTCTGTGA TGACGTCAAG GCCCTCCTAA GGAGGTACAA   
  
  
+ GGAGGGGTGG TCCATGATAC AATGTTCCGC CGCGGATGGC GGCGCCTCCG CCGGAATATT CTTGTCGTGG   
  
  
+ AGGGATCAGC CGGTGGTGTG GGCCAGTGCA TGGAAGCCTT A  

- -Up\_Stream \_Len000CCCAAA AAAATTTGAG TTTTAATCAA TAACATAGTT AACTTAAACC TTTTGATAAT   
  
  
- AAAAGCTGAA TAATATCTCT TAAACAACAA TTACTTACGA CTATTTATGA CTATTAATCA CAACACTCAC   
  
  
- ACTTAAACAC TACTACTCGT TACTAATCGC AATTTTAATA TTGATCCTAT TTATTAATTC ACAACTACTT   
  
  
- ACAACTAACT ACTACTACTC ATGACTAACC ACGCCACGAC CATTATTTAT AAATACTATT CAATTTTTTT   
  
  
- TTTTTGTGTA GGATTTGTTA GTGATACATT ATATGATTTA CTTTTTTTAA CCATTACACT AAACTGTTAG   
  
  
- ATTCCCATTC CCACAAAATG GGTGTGTACA ATACTTTAAC CGAATGATAA ATGTTTGAAA AATGTCAATA   
  
  
- TACTAAGATG GATACACAAT AGTAGAAAGG CAGCTACATT AACTAATATA AGTTGTGTTA AATTAAGTAA   
  
  
- TGTTACATCG ATTAACTATG AGTTTTATTA CTTGATAATT ATTTATTAAT TAATATTGAA AATTTTCAAA   
  
  
- CAATTAAATT TTAATATATT ATATATTTAA ATCCGATGTA ATTAGTTTTT TAATCCGCAC AAGTAGCCAG   
  
  
- GTTTGAACTT GGCCTGGTCT GGCCTGGCTT CTAGCTAATT CTCTTTTTCA TGTCTGACTC CTGGCCTAGC   
  
  
- TTATTCTCAG CGTGGCCTGG TCCTAGCCTG GCCTTTTTAA ACCTGGTCTA GCCCTGGCTA GCTCCATCTG   
  
  
- GCTTAAATAA AAGTTTATGC TATCCTTATT TTGAATAACT AAGTTAACTA AAACCGGTTT TAGTACGATA   
  
  
- ATAACTAAAA TAAACGATTG AGAGTTCTCA TAATCAAACG TGAAGCGTAA TAGAAACCGG TTTTTTTAGT   
  
  
- TTTTCTTTCA GAGTTTGAGA ATTATCAAAA AATAAAATAA ACCAAATAAA AATTTTATCA GCCGGAAGCC   
  
  
- AGGCCTGATC ACCTGGCTTA TTCCATTAAA AAGCTTAGCT CCTGGTCTGG TTTACGTGAG TCAAGCTTGG   
  
  
- CCAAGTCAAG CCAGACTGAG CTAAAAACCA GGCCAGGTTC AAACTTGTCT TATTACATGT GTGGATCTTT   
  
  
- TTTTCTTAAT ACGTATATAT GAATTCCGGG GGTTGGGAAA AAGGGAAAGG CGGTTATGAA AAATGGTTCA   
  
  
- GGTTAAGAAA AGAAGAATCG ATACCGATCA TCAATAGGGA GGGATCGAAG GTGGGATTCA AAACTCTGTT   
  
  
- TTAGGGTAGA TACAATTATA AATAAAGAAC GTGTACAGTG GGTTATGTTC CCAAAGTAAC TTTTCTTTTC   
  
  
- CATAATCTAC ATTCTATACA TACATATATT TATCAAAACG TTATCTGATC TTCCTGATTT TTCCTTTTTC   
  
  
- CTTCAAATTT TCGTTTTTTG AAAGGGGAGA TCTTTTTTCC TTTGGTCTAA GGGAAAAGAT GGGGTTAAGG   
  
  
- GTATCAGAAA TGAGACAGAG AGAGAGAGAC AGAGAGAATG AGTACTACAA CTCCCTCCTT CCTTACTTAA   
  
  
- TTCCTGGGAT TCGATTCTAG GTTGGCATTC TATAACGGGG AAATTAGTGG GTAAAGTATA GAGATAGGTG   
  
  
- TCAATTTTTG GGTGGAGGAG GTAGGTGTAT TGACTATATA GAGAAGATGT GGTATGATAT TCCTTCTTCT   
  
  
- CTTTTTATCC TCACATAACA TAAAAATAAA CTCATTAAAT AAAATTCCTC TCTTTAAATG TACCAACCCA   
  
  
- ATTTACCTAT GGAACAAATC TGATCACTCA AAGGTTGTTG GTGTGGTGGT GGTAGTGGTT GTGGTTGTGG   
  
  
- TTGTGGTGGT TGTCGTTAGA AGACTAGTTA GGAGAAACTT ATTATGATGA TGAATAAGGT GTTTAAGTTC   
  
  
- GTCAAGGTCT AGAAGGGTTG TTTGGGTAGT ATTAGTATTA GTATGAGTCA TAATAGTGGT TGTAGTAGGA   
  
  
- TGAATGGGGG TGGCGGTGGT AGTCCTCCTT CTTCTTCTTA CGAAGTTGAA AATGTACCTA CTTCTTTTGA   
  
  
- AGAGAAGAAG AAGGAGGGTG TTGTTTGTAG TAGGAGTTGG AGGAAGGAGG CGGTGGTGGA GGAGCATAAT   
  
  
- GGGAATGATG AGGTGGGGGA AGCTACAAGG GAGATGGTGG TGGTGGAGGT GGAGGTGGGG GTGCGGGTGG   
  
  
- TTGTGGTGGT GGTGGTGGGG GGGTTGGGTC ATACCGCCGC CGCCGCCACC CAAACTGAGG TCGACGAAGA   
  
  
- GGATGAGGGG ACGGGCTCTG GAGTTGGGCC TCGAGAAGAG GAGCAGGCCC AAGAGCCCGT TCACCCGAAG   
  
  
- GTTGTAGGAG AACCTTCGGC GAGCACGGTA GAGACTCTCC TTGTGCTCGG CGGAGGTGGT CGAGGATACC   
  
  
- TACCAGTTGC TCGAGTCGAG GGGCATGCCA CTGCACCTCG TCTTCTATCG AAGTATGAAG CCGGTTCGGG   
  
  
- AGAAGTGGGC GTACTGGCTC AGTCCCCTGG CGTGGATGGC GTGGTACGCC AGGCGGCACC TGTTCTGGAA   
  
  
- GAGGAAACTC AGATGCTCTT TCTACCATGA GTTCAAGGTC CTCCAATCGG GAACCTGGTG GAAACCTGTG   
  
  
- CACCGTCGAT TACCTCGGGA GTAACTACGG AAACTGCCAC TTAGGTTTTA GGTGTAGTAG CTGTAGTCGT   
  
  
- GGTGTAAGAC GTGGGTCACC GGGTGGGAGG ACCTCCGCGA CCGGTGGGCT TACCTACTGC GCGGGATGGA   
  
  
- CTCCGACTGG TGACACCACC AGTTGTTCAG GCCCTTACTT CCAGGGTGGC CTCCGCCCAG GGTGTCCCAC   
  
  
- TACTTTCTCT AACCCTGGGC GGAGCTCTTC AAACGATCCG ATTACCCTCA CGGAAAGTTT AAATTACACC   
  
  
- AGGTGGTGCC CCGACTAAAT AGGCTGAACC TAAAGAGAGT CGACCTATAA TTGCTACTGC TCCGTAATCG   
  
  
- GTAGTTGACA CAGTTGAGCG AGGTGAGCCA GTTATAGGTG GCGGCACTGA GCCAGTAGAG CCGGAAGGCC   
  
  
- GCCGAGGTGG GATCCCACCA CTGGCACCAC CTCCTCCTCC GACTGGAACG ACTGCACCCC CGCCTCCCCA   
  
  
- TGCTCAAGAT GTCCCCCAAA TTACTCACAA ATTCCACCCA ATCCCAAATG AAACTCCGGG ACCTTCTCAC   
  
  
- GAAAGGATCC TGTTCGTTAC TCTTTGAGTA CGAGCTCGCC CGGCGTCCCG CCCGAGACCA CCTGGAGGAC   
  
  
- CGGACGGGGT TCGGGCGTAG TAGGCTCGCC TCCCTCCGTC GACGCGCCAC CAGGCCCTCC TACGTCCCCC   
  
  
- GGCCCAAGCC GGTGCACCCC AAGTCACTAC TCCAGACACT ACTGCAGTTC CGGGAGGATT CCTCCATGTT   
  
  
- CCTCCCCACC AGGTACTATG TTACAAGGCG GCGCCTACCG CCGCGGAGGC GGCCTTATAA GAACAGCACC   
  
  
- TCCCTAGTCG GCCACCACAC CCGGTCACGT ACCTTCGGAA T

+     AC-I

| Site Name | Organism | Position | Strand | Matrix score. | sequence | function |
| --- | --- | --- | --- | --- | --- | --- |
| AC-I | Phaseolus vulgaris | 2483 | + | 8.5 | (T/C)C(T/C)(C/T)ACC(T/C)ACC |  |
| AC-I | Phaseolus vulgaris | 3118 | - | 8.5 | (T/C)C(T/C)(C/T)ACC(T/C)ACC |  |

>HU06G00358.1   
+ -Up\_Stream \_Len000GGGTTT TTTTAAACTC AAAATTAGTT ATTGTATCAA TTGAATTTGG AAAACTATTA   
  
  
+ TTTTCGACTT ATTATAGAGA ATTTGTTGTT AATGAATGCT GATAAATACT GATAATTAGT GTTGTGAGTG   
  
  
+ TGAATTTGTG ATGATGAGCA ATGATTAGCG TTAAAATTAT AACTAGGATA AATAATTAAG TGTTGATGAA   
  
  
+ TGTTGATTGA TGATGATGAG TACTGATTGG TGCGGTGCTG GTAATAAATA TTTATGATAA GTTAAAAAAA   
  
  
+ AAAAACACAT CCTAAACAAT CACTATGTAA TATACTAAAT GAAAAAAATT GGTAATGTGA TTTGACAATC   
  
  
+ TAAGGGTAAG GGTGTTTTAC CCACACATGT TATGAAATTG GCTTACTATT TACAAACTTT TTACAGTTAT   
  
  
+ ATGATTCTAC CTATGTGTTA TCATCTTTCC GTCGATGTAA TTGATTATAT TCAACACAAT TTAATTCATT   
  
  
+ ACAATGTAGC TAATTGATAC TCAAAATAAT GAACTATTAA TAAATAATTA ATTATAACTT TTAAAAGTTT   
  
  
+ GTTAATTTAA AATTATATAA TATATAAATT TAGGCTACAT TAATCAAAAA ATTAGGCGTG TTCATCGGTC   
  
  
+ CAAACTTGAA CCGGACCAGA CCGGACCGAA GATCGATTAA GAGAAAAAGT ACAGACTGAG GACCGGATCG   
  
  
+ AATAAGAGTC GCACCGGACC AGGATCGGAC CGGAAAAATT TGGACCAGAT CGGGACCGAT CGAGGTAGAC   
  
  
+ CGAATTTATT TTCAAATACG ATAGGAATAA AACTTATTGA TTCAATTGAT TTTGGCCAAA ATCATGCTAT   
  
  
+ TATTGATTTT ATTTGCTAAC TCTCAAGAGT ATTAGTTTGC ACTTCGCATT ATCTTTGGCC AAAAAAATCA   
  
  
+ AAAAGAAAGT CTCAAACTCT TAATAGTTTT TTATTTTATT TGGTTTATTT TTAAAATAGT CGGCCTTCGG   
  
  
+ TCCGGACTAG TGGACCGAAT AAGGTAATTT TTCGAATCGA GGACCAGACC AAATGCACTC AGTTCGAACC   
  
  
+ GGTTCAGTTC GGTCTGACTC GATTTTTGGT CCGGTCCAAG TTTGAACAGA ATAATGTACA CACCTAGAAA   
  
  
+ AAAAGAATTA TGCATATATA CTTAAGGCCC CCAACCCTTT TTCCCTTTCC GCCAATACTT TTTACCAAGT   
  
  
+ CCAATTCTTT TCTTCTTAGC TATGGCTAGT AGTTATCCCT CCCTAGCTTC CACCCTAAGT TTTGAGACAA   
  
  
+ AATCCCATCT ATGTTAATAT TTATTTCTTG CACATGTCAC CCAATACAAG GGTTTCATTG AAAAGAAAAG   
  
  
+ GTATTAGATG TAAGATATGT ATGTATATAA ATAGTTTTGC AATAGACTAG AAGGACTAAA AAGGAAAAAG   
  
  
+ GAAGTTTAAA AGCAAAAAAC TTTCCCCTCT AGAAAAAAGG AAACCAGATT CCCTTTTCTA CCCCAATTCC   
  
  
+ CATAGTCTTT ACTCTGTCTC TCTCTCTCTG TCTCTCTTAC TCATGATGTT GAGGGAGGAA GGAATGAATT   
  
  
+ AAGGACCCTA AGCTAAGATC CAACCGTAAG ATATTGCCCC TTTAATCACC CATTTCATAT CTCTATCCAC   
  
  
+ AGTTAAAAAC CCACCTCCTC CATCCACATA ACTGATATAT CTCTTCTACA CCATACTATA AGGAAGAAGA   
  
  
+ GAAAAATAGG AGTGTATTGT ATTTTTATTT GAGTAATTTA TTTTAAGGAG AGAAATTTAC ATGGTTGGGT   
  
  
+ TAAATGGATA CCTTGTTTAG ACTAGTGAGT TTCCAACAAC CACACCACCA CCATCACCAA CACCAACACC   
  
  
+ AACACCACCA ACAGCAATCT TCTGATCAAT CCTCTTTGAA TAATACTACT ACTTATTCCA CAAATTCAAG   
  
  
+ CAGTTCCAGA TCTTCCCAAC AAACCCATCA TAATCATAAT CATACTCAGT ATTATCACCA ACATCATCCT   
  
  
+ ACTTACCCCC ACCGCCACCA TCAGGAGGAA GAAGAAGAAT GCTTCAACTT TTACATGGAT GAAGAAAACT   
  
  
+ TCTCTTCTTC TTCCTCCCAC AACAAACATC ATCCTCAACC TCCTTCCTCC GCCACCACCT CCTCGTATTA   
  
  
+ CCCTTACTAC TCCACCCCCT TCGATGTTCC CTCTACCACC ACCACCTCCA CCTCCACCCC CACGCCCACC   
  
  
+ AACACCACCA CCACCACCCC CCCAACCCAG TATGGCGGCG GCGGCGGTGG GTTTGACTCC AGCTGCTTCT   
  
  
+ CCTACTCCCC TGCCCGAGAC CTCAACCCGG AGCTCTTCTC CTCGTCCGGG TTCTCGGGCA AGTGGGCTTC   
  
  
+ CAACATCCTC TTGGAAGCCG CTCGTGCCAT CTCTGAGAGG AACACGAGCC GCCTCCACCA GCTCCTATGG   
  
  
+ ATGGTCAACG AGCTCAGCTC CCCGTACGGT GACGTGGAGC AGAAGATAGC TTCATACTTC GGCCAAGCCC   
  
  
+ TCTTCACCCG CATGACCGAG TCAGGGGACC GCACCTACCG CACCATGCGG TCCGCCGTGG ACAAGACCTT   
  
  
+ CTCCTTTGAG TCTACGAGAA AGATGGTACT CAAGTTCCAG GAGGTTAGCC CTTGGACCAC CTTTGGACAC   
  
  
+ GTGGCAGCTA ATGGAGCCCT CATTGATGCC TTTGACGGTG AATCCAAAAT CCACATCATC GACATCAGCA   
  
  
+ CCACATTCTG CACCCAGTGG CCCACCCTCC TGGAGGCGCT GGCCACCCGA ATGGATGACG CGCCCTACCT   
  
  
+ GAGGCTGACC ACTGTGGTGG TCAACAAGTC CGGGAATGAA GGTCCCACCG GAGGCGGGTC CCACAGGGTG   
  
  
+ ATGAAAGAGA TTGGGACCCG CCTCGAGAAG TTTGCTAGGC TAATGGGAGT GCCTTTCAAA TTTAATGTGG   
  
  
+ TCCACCACGG GGCTGATTTA TCCGACTTGG ATTTCTCTCA GCTGGATATT AACGATGACG AGGCATTAGC   
  
  
+ CATCAACTGT GTCAACTCGC TCCACTCGGT CAATATCCAC CGCCGTGACT CGGTCATCTC GGCCTTCCGG   
  
  
+ CGGCTCCACC CTAGGGTGGT GACCGTGGTG GAGGAGGAGG CTGACCTTGC TGACGTGGGG GCGGAGGGGT   
  
  
+ ACGAGTTCTA CAGGGGGTTT AATGAGTGTT TAAGGTGGGT TAGGGTTTAC TTTGAGGCCC TGGAAGAGTG   
  
  
+ CTTTCCTAGG ACAAGCAATG AGAAACTCAT GCTCGAGCGG GCCGCAGGGC GGGCTCTGGT GGACCTCCTG   
  
  
+ GCCTGCCCCA AGCCCGCATC ATCCGAGCGG AGGGAGGCAG CTGCGCGGTG GTCCGGGAGG ATGCAGGGGG   
  
  
+ CCGGGTTCGG CCACGTGGGG TTCAGTGATG AGGTCTGTGA TGACGTCAAG GCCCTCCTAA GGAGGTACAA   
  
  
+ GGAGGGGTGG TCCATGATAC AATGTTCCGC CGCGGATGGC GGCGCCTCCG CCGGAATATT CTTGTCGTGG   
  
  
+ AGGGATCAGC CGGTGGTGTG GGCCAGTGCA TGGAAGCCTT A  

- -Up\_Stream \_Len000CCCAAA AAAATTTGAG TTTTAATCAA TAACATAGTT AACTTAAACC TTTTGATAAT   
  
  
- AAAAGCTGAA TAATATCTCT TAAACAACAA TTACTTACGA CTATTTATGA CTATTAATCA CAACACTCAC   
  
  
- ACTTAAACAC TACTACTCGT TACTAATCGC AATTTTAATA TTGATCCTAT TTATTAATTC ACAACTACTT   
  
  
- ACAACTAACT ACTACTACTC ATGACTAACC ACGCCACGAC CATTATTTAT AAATACTATT CAATTTTTTT   
  
  
- TTTTTGTGTA GGATTTGTTA GTGATACATT ATATGATTTA CTTTTTTTAA CCATTACACT AAACTGTTAG   
  
  
- ATTCCCATTC CCACAAAATG GGTGTGTACA ATACTTTAAC CGAATGATAA ATGTTTGAAA AATGTCAATA   
  
  
- TACTAAGATG GATACACAAT AGTAGAAAGG CAGCTACATT AACTAATATA AGTTGTGTTA AATTAAGTAA   
  
  
- TGTTACATCG ATTAACTATG AGTTTTATTA CTTGATAATT ATTTATTAAT TAATATTGAA AATTTTCAAA   
  
  
- CAATTAAATT TTAATATATT ATATATTTAA ATCCGATGTA ATTAGTTTTT TAATCCGCAC AAGTAGCCAG   
  
  
- GTTTGAACTT GGCCTGGTCT GGCCTGGCTT CTAGCTAATT CTCTTTTTCA TGTCTGACTC CTGGCCTAGC   
  
  
- TTATTCTCAG CGTGGCCTGG TCCTAGCCTG GCCTTTTTAA ACCTGGTCTA GCCCTGGCTA GCTCCATCTG   
  
  
- GCTTAAATAA AAGTTTATGC TATCCTTATT TTGAATAACT AAGTTAACTA AAACCGGTTT TAGTACGATA   
  
  
- ATAACTAAAA TAAACGATTG AGAGTTCTCA TAATCAAACG TGAAGCGTAA TAGAAACCGG TTTTTTTAGT   
  
  
- TTTTCTTTCA GAGTTTGAGA ATTATCAAAA AATAAAATAA ACCAAATAAA AATTTTATCA GCCGGAAGCC   
  
  
- AGGCCTGATC ACCTGGCTTA TTCCATTAAA AAGCTTAGCT CCTGGTCTGG TTTACGTGAG TCAAGCTTGG   
  
  
- CCAAGTCAAG CCAGACTGAG CTAAAAACCA GGCCAGGTTC AAACTTGTCT TATTACATGT GTGGATCTTT   
  
  
- TTTTCTTAAT ACGTATATAT GAATTCCGGG GGTTGGGAAA AAGGGAAAGG CGGTTATGAA AAATGGTTCA   
  
  
- GGTTAAGAAA AGAAGAATCG ATACCGATCA TCAATAGGGA GGGATCGAAG GTGGGATTCA AAACTCTGTT   
  
  
- TTAGGGTAGA TACAATTATA AATAAAGAAC GTGTACAGTG GGTTATGTTC CCAAAGTAAC TTTTCTTTTC   
  
  
- CATAATCTAC ATTCTATACA TACATATATT TATCAAAACG TTATCTGATC TTCCTGATTT TTCCTTTTTC   
  
  
- CTTCAAATTT TCGTTTTTTG AAAGGGGAGA TCTTTTTTCC TTTGGTCTAA GGGAAAAGAT GGGGTTAAGG   
  
  
- GTATCAGAAA TGAGACAGAG AGAGAGAGAC AGAGAGAATG AGTACTACAA CTCCCTCCTT CCTTACTTAA   
  
  
- TTCCTGGGAT TCGATTCTAG GTTGGCATTC TATAACGGGG AAATTAGTGG GTAAAGTATA GAGATAGGTG   
  
  
- TCAATTTTTG GGTGGAGGAG GTAGGTGTAT TGACTATATA GAGAAGATGT GGTATGATAT TCCTTCTTCT   
  
  
- CTTTTTATCC TCACATAACA TAAAAATAAA CTCATTAAAT AAAATTCCTC TCTTTAAATG TACCAACCCA   
  
  
- ATTTACCTAT GGAACAAATC TGATCACTCA AAGGTTGTTG GTGTGGTGGT GGTAGTGGTT GTGGTTGTGG   
  
  
- TTGTGGTGGT TGTCGTTAGA AGACTAGTTA GGAGAAACTT ATTATGATGA TGAATAAGGT GTTTAAGTTC   
  
  
- GTCAAGGTCT AGAAGGGTTG TTTGGGTAGT ATTAGTATTA GTATGAGTCA TAATAGTGGT TGTAGTAGGA   
  
  
- TGAATGGGGG TGGCGGTGGT AGTCCTCCTT CTTCTTCTTA CGAAGTTGAA AATGTACCTA CTTCTTTTGA   
  
  
- AGAGAAGAAG AAGGAGGGTG TTGTTTGTAG TAGGAGTTGG AGGAAGGAGG CGGTGGTGGA GGAGCATAAT   
  
  
- GGGAATGATG AGGTGGGGGA AGCTACAAGG GAGATGGTGG TGGTGGAGGT GGAGGTGGGG GTGCGGGTGG   
  
  
- TTGTGGTGGT GGTGGTGGGG GGGTTGGGTC ATACCGCCGC CGCCGCCACC CAAACTGAGG TCGACGAAGA   
  
  
- GGATGAGGGG ACGGGCTCTG GAGTTGGGCC TCGAGAAGAG GAGCAGGCCC AAGAGCCCGT TCACCCGAAG   
  
  
- GTTGTAGGAG AACCTTCGGC GAGCACGGTA GAGACTCTCC TTGTGCTCGG CGGAGGTGGT CGAGGATACC   
  
  
- TACCAGTTGC TCGAGTCGAG GGGCATGCCA CTGCACCTCG TCTTCTATCG AAGTATGAAG CCGGTTCGGG   
  
  
- AGAAGTGGGC GTACTGGCTC AGTCCCCTGG CGTGGATGGC GTGGTACGCC AGGCGGCACC TGTTCTGGAA   
  
  
- GAGGAAACTC AGATGCTCTT TCTACCATGA GTTCAAGGTC CTCCAATCGG GAACCTGGTG GAAACCTGTG   
  
  
- CACCGTCGAT TACCTCGGGA GTAACTACGG AAACTGCCAC TTAGGTTTTA GGTGTAGTAG CTGTAGTCGT   
  
  
- GGTGTAAGAC GTGGGTCACC GGGTGGGAGG ACCTCCGCGA CCGGTGGGCT TACCTACTGC GCGGGATGGA   
  
  
- CTCCGACTGG TGACACCACC AGTTGTTCAG GCCCTTACTT CCAGGGTGGC CTCCGCCCAG GGTGTCCCAC   
  
  
- TACTTTCTCT AACCCTGGGC GGAGCTCTTC AAACGATCCG ATTACCCTCA CGGAAAGTTT AAATTACACC   
  
  
- AGGTGGTGCC CCGACTAAAT AGGCTGAACC TAAAGAGAGT CGACCTATAA TTGCTACTGC TCCGTAATCG   
  
  
- GTAGTTGACA CAGTTGAGCG AGGTGAGCCA GTTATAGGTG GCGGCACTGA GCCAGTAGAG CCGGAAGGCC   
  
  
- GCCGAGGTGG GATCCCACCA CTGGCACCAC CTCCTCCTCC GACTGGAACG ACTGCACCCC CGCCTCCCCA   
  
  
- TGCTCAAGAT GTCCCCCAAA TTACTCACAA ATTCCACCCA ATCCCAAATG AAACTCCGGG ACCTTCTCAC   
  
  
- GAAAGGATCC TGTTCGTTAC TCTTTGAGTA CGAGCTCGCC CGGCGTCCCG CCCGAGACCA CCTGGAGGAC   
  
  
- CGGACGGGGT TCGGGCGTAG TAGGCTCGCC TCCCTCCGTC GACGCGCCAC CAGGCCCTCC TACGTCCCCC   
  
  
- GGCCCAAGCC GGTGCACCCC AAGTCACTAC TCCAGACACT ACTGCAGTTC CGGGAGGATT CCTCCATGTT   
  
  
- CCTCCCCACC AGGTACTATG TTACAAGGCG GCGCCTACCG CCGCGGAGGC GGCCTTATAA GAACAGCACC   
  
  
- TCCCTAGTCG GCCACCACAC CCGGTCACGT ACCTTCGGAA T

+     AC-II

| Site Name | Organism | Position | Strand | Matrix score. | sequence | function |
| --- | --- | --- | --- | --- | --- | --- |
| AC-II | Phaseolus vulgaris | 2185 | + | 11 | CCACCAACCCCC |  |

>HU06G00358.1   
+ -Up\_Stream \_Len000GGGTTT TTTTAAACTC AAAATTAGTT ATTGTATCAA TTGAATTTGG AAAACTATTA   
  
  
+ TTTTCGACTT ATTATAGAGA ATTTGTTGTT AATGAATGCT GATAAATACT GATAATTAGT GTTGTGAGTG   
  
  
+ TGAATTTGTG ATGATGAGCA ATGATTAGCG TTAAAATTAT AACTAGGATA AATAATTAAG TGTTGATGAA   
  
  
+ TGTTGATTGA TGATGATGAG TACTGATTGG TGCGGTGCTG GTAATAAATA TTTATGATAA GTTAAAAAAA   
  
  
+ AAAAACACAT CCTAAACAAT CACTATGTAA TATACTAAAT GAAAAAAATT GGTAATGTGA TTTGACAATC   
  
  
+ TAAGGGTAAG GGTGTTTTAC CCACACATGT TATGAAATTG GCTTACTATT TACAAACTTT TTACAGTTAT   
  
  
+ ATGATTCTAC CTATGTGTTA TCATCTTTCC GTCGATGTAA TTGATTATAT TCAACACAAT TTAATTCATT   
  
  
+ ACAATGTAGC TAATTGATAC TCAAAATAAT GAACTATTAA TAAATAATTA ATTATAACTT TTAAAAGTTT   
  
  
+ GTTAATTTAA AATTATATAA TATATAAATT TAGGCTACAT TAATCAAAAA ATTAGGCGTG TTCATCGGTC   
  
  
+ CAAACTTGAA CCGGACCAGA CCGGACCGAA GATCGATTAA GAGAAAAAGT ACAGACTGAG GACCGGATCG   
  
  
+ AATAAGAGTC GCACCGGACC AGGATCGGAC CGGAAAAATT TGGACCAGAT CGGGACCGAT CGAGGTAGAC   
  
  
+ CGAATTTATT TTCAAATACG ATAGGAATAA AACTTATTGA TTCAATTGAT TTTGGCCAAA ATCATGCTAT   
  
  
+ TATTGATTTT ATTTGCTAAC TCTCAAGAGT ATTAGTTTGC ACTTCGCATT ATCTTTGGCC AAAAAAATCA   
  
  
+ AAAAGAAAGT CTCAAACTCT TAATAGTTTT TTATTTTATT TGGTTTATTT TTAAAATAGT CGGCCTTCGG   
  
  
+ TCCGGACTAG TGGACCGAAT AAGGTAATTT TTCGAATCGA GGACCAGACC AAATGCACTC AGTTCGAACC   
  
  
+ GGTTCAGTTC GGTCTGACTC GATTTTTGGT CCGGTCCAAG TTTGAACAGA ATAATGTACA CACCTAGAAA   
  
  
+ AAAAGAATTA TGCATATATA CTTAAGGCCC CCAACCCTTT TTCCCTTTCC GCCAATACTT TTTACCAAGT   
  
  
+ CCAATTCTTT TCTTCTTAGC TATGGCTAGT AGTTATCCCT CCCTAGCTTC CACCCTAAGT TTTGAGACAA   
  
  
+ AATCCCATCT ATGTTAATAT TTATTTCTTG CACATGTCAC CCAATACAAG GGTTTCATTG AAAAGAAAAG   
  
  
+ GTATTAGATG TAAGATATGT ATGTATATAA ATAGTTTTGC AATAGACTAG AAGGACTAAA AAGGAAAAAG   
  
  
+ GAAGTTTAAA AGCAAAAAAC TTTCCCCTCT AGAAAAAAGG AAACCAGATT CCCTTTTCTA CCCCAATTCC   
  
  
+ CATAGTCTTT ACTCTGTCTC TCTCTCTCTG TCTCTCTTAC TCATGATGTT GAGGGAGGAA GGAATGAATT   
  
  
+ AAGGACCCTA AGCTAAGATC CAACCGTAAG ATATTGCCCC TTTAATCACC CATTTCATAT CTCTATCCAC   
  
  
+ AGTTAAAAAC CCACCTCCTC CATCCACATA ACTGATATAT CTCTTCTACA CCATACTATA AGGAAGAAGA   
  
  
+ GAAAAATAGG AGTGTATTGT ATTTTTATTT GAGTAATTTA TTTTAAGGAG AGAAATTTAC ATGGTTGGGT   
  
  
+ TAAATGGATA CCTTGTTTAG ACTAGTGAGT TTCCAACAAC CACACCACCA CCATCACCAA CACCAACACC   
  
  
+ AACACCACCA ACAGCAATCT TCTGATCAAT CCTCTTTGAA TAATACTACT ACTTATTCCA CAAATTCAAG   
  
  
+ CAGTTCCAGA TCTTCCCAAC AAACCCATCA TAATCATAAT CATACTCAGT ATTATCACCA ACATCATCCT   
  
  
+ ACTTACCCCC ACCGCCACCA TCAGGAGGAA GAAGAAGAAT GCTTCAACTT TTACATGGAT GAAGAAAACT   
  
  
+ TCTCTTCTTC TTCCTCCCAC AACAAACATC ATCCTCAACC TCCTTCCTCC GCCACCACCT CCTCGTATTA   
  
  
+ CCCTTACTAC TCCACCCCCT TCGATGTTCC CTCTACCACC ACCACCTCCA CCTCCACCCC CACGCCCACC   
  
  
+ AACACCACCA CCACCACCCC CCCAACCCAG TATGGCGGCG GCGGCGGTGG GTTTGACTCC AGCTGCTTCT   
  
  
+ CCTACTCCCC TGCCCGAGAC CTCAACCCGG AGCTCTTCTC CTCGTCCGGG TTCTCGGGCA AGTGGGCTTC   
  
  
+ CAACATCCTC TTGGAAGCCG CTCGTGCCAT CTCTGAGAGG AACACGAGCC GCCTCCACCA GCTCCTATGG   
  
  
+ ATGGTCAACG AGCTCAGCTC CCCGTACGGT GACGTGGAGC AGAAGATAGC TTCATACTTC GGCCAAGCCC   
  
  
+ TCTTCACCCG CATGACCGAG TCAGGGGACC GCACCTACCG CACCATGCGG TCCGCCGTGG ACAAGACCTT   
  
  
+ CTCCTTTGAG TCTACGAGAA AGATGGTACT CAAGTTCCAG GAGGTTAGCC CTTGGACCAC CTTTGGACAC   
  
  
+ GTGGCAGCTA ATGGAGCCCT CATTGATGCC TTTGACGGTG AATCCAAAAT CCACATCATC GACATCAGCA   
  
  
+ CCACATTCTG CACCCAGTGG CCCACCCTCC TGGAGGCGCT GGCCACCCGA ATGGATGACG CGCCCTACCT   
  
  
+ GAGGCTGACC ACTGTGGTGG TCAACAAGTC CGGGAATGAA GGTCCCACCG GAGGCGGGTC CCACAGGGTG   
  
  
+ ATGAAAGAGA TTGGGACCCG CCTCGAGAAG TTTGCTAGGC TAATGGGAGT GCCTTTCAAA TTTAATGTGG   
  
  
+ TCCACCACGG GGCTGATTTA TCCGACTTGG ATTTCTCTCA GCTGGATATT AACGATGACG AGGCATTAGC   
  
  
+ CATCAACTGT GTCAACTCGC TCCACTCGGT CAATATCCAC CGCCGTGACT CGGTCATCTC GGCCTTCCGG   
  
  
+ CGGCTCCACC CTAGGGTGGT GACCGTGGTG GAGGAGGAGG CTGACCTTGC TGACGTGGGG GCGGAGGGGT   
  
  
+ ACGAGTTCTA CAGGGGGTTT AATGAGTGTT TAAGGTGGGT TAGGGTTTAC TTTGAGGCCC TGGAAGAGTG   
  
  
+ CTTTCCTAGG ACAAGCAATG AGAAACTCAT GCTCGAGCGG GCCGCAGGGC GGGCTCTGGT GGACCTCCTG   
  
  
+ GCCTGCCCCA AGCCCGCATC ATCCGAGCGG AGGGAGGCAG CTGCGCGGTG GTCCGGGAGG ATGCAGGGGG   
  
  
+ CCGGGTTCGG CCACGTGGGG TTCAGTGATG AGGTCTGTGA TGACGTCAAG GCCCTCCTAA GGAGGTACAA   
  
  
+ GGAGGGGTGG TCCATGATAC AATGTTCCGC CGCGGATGGC GGCGCCTCCG CCGGAATATT CTTGTCGTGG   
  
  
+ AGGGATCAGC CGGTGGTGTG GGCCAGTGCA TGGAAGCCTT A  

- -Up\_Stream \_Len000CCCAAA AAAATTTGAG TTTTAATCAA TAACATAGTT AACTTAAACC TTTTGATAAT   
  
  
- AAAAGCTGAA TAATATCTCT TAAACAACAA TTACTTACGA CTATTTATGA CTATTAATCA CAACACTCAC   
  
  
- ACTTAAACAC TACTACTCGT TACTAATCGC AATTTTAATA TTGATCCTAT TTATTAATTC ACAACTACTT   
  
  
- ACAACTAACT ACTACTACTC ATGACTAACC ACGCCACGAC CATTATTTAT AAATACTATT CAATTTTTTT   
  
  
- TTTTTGTGTA GGATTTGTTA GTGATACATT ATATGATTTA CTTTTTTTAA CCATTACACT AAACTGTTAG   
  
  
- ATTCCCATTC CCACAAAATG GGTGTGTACA ATACTTTAAC CGAATGATAA ATGTTTGAAA AATGTCAATA   
  
  
- TACTAAGATG GATACACAAT AGTAGAAAGG CAGCTACATT AACTAATATA AGTTGTGTTA AATTAAGTAA   
  
  
- TGTTACATCG ATTAACTATG AGTTTTATTA CTTGATAATT ATTTATTAAT TAATATTGAA AATTTTCAAA   
  
  
- CAATTAAATT TTAATATATT ATATATTTAA ATCCGATGTA ATTAGTTTTT TAATCCGCAC AAGTAGCCAG   
  
  
- GTTTGAACTT GGCCTGGTCT GGCCTGGCTT CTAGCTAATT CTCTTTTTCA TGTCTGACTC CTGGCCTAGC   
  
  
- TTATTCTCAG CGTGGCCTGG TCCTAGCCTG GCCTTTTTAA ACCTGGTCTA GCCCTGGCTA GCTCCATCTG   
  
  
- GCTTAAATAA AAGTTTATGC TATCCTTATT TTGAATAACT AAGTTAACTA AAACCGGTTT TAGTACGATA   
  
  
- ATAACTAAAA TAAACGATTG AGAGTTCTCA TAATCAAACG TGAAGCGTAA TAGAAACCGG TTTTTTTAGT   
  
  
- TTTTCTTTCA GAGTTTGAGA ATTATCAAAA AATAAAATAA ACCAAATAAA AATTTTATCA GCCGGAAGCC   
  
  
- AGGCCTGATC ACCTGGCTTA TTCCATTAAA AAGCTTAGCT CCTGGTCTGG TTTACGTGAG TCAAGCTTGG   
  
  
- CCAAGTCAAG CCAGACTGAG CTAAAAACCA GGCCAGGTTC AAACTTGTCT TATTACATGT GTGGATCTTT   
  
  
- TTTTCTTAAT ACGTATATAT GAATTCCGGG GGTTGGGAAA AAGGGAAAGG CGGTTATGAA AAATGGTTCA   
  
  
- GGTTAAGAAA AGAAGAATCG ATACCGATCA TCAATAGGGA GGGATCGAAG GTGGGATTCA AAACTCTGTT   
  
  
- TTAGGGTAGA TACAATTATA AATAAAGAAC GTGTACAGTG GGTTATGTTC CCAAAGTAAC TTTTCTTTTC   
  
  
- CATAATCTAC ATTCTATACA TACATATATT TATCAAAACG TTATCTGATC TTCCTGATTT TTCCTTTTTC   
  
  
- CTTCAAATTT TCGTTTTTTG AAAGGGGAGA TCTTTTTTCC TTTGGTCTAA GGGAAAAGAT GGGGTTAAGG   
  
  
- GTATCAGAAA TGAGACAGAG AGAGAGAGAC AGAGAGAATG AGTACTACAA CTCCCTCCTT CCTTACTTAA   
  
  
- TTCCTGGGAT TCGATTCTAG GTTGGCATTC TATAACGGGG AAATTAGTGG GTAAAGTATA GAGATAGGTG   
  
  
- TCAATTTTTG GGTGGAGGAG GTAGGTGTAT TGACTATATA GAGAAGATGT GGTATGATAT TCCTTCTTCT   
  
  
- CTTTTTATCC TCACATAACA TAAAAATAAA CTCATTAAAT AAAATTCCTC TCTTTAAATG TACCAACCCA   
  
  
- ATTTACCTAT GGAACAAATC TGATCACTCA AAGGTTGTTG GTGTGGTGGT GGTAGTGGTT GTGGTTGTGG   
  
  
- TTGTGGTGGT TGTCGTTAGA AGACTAGTTA GGAGAAACTT ATTATGATGA TGAATAAGGT GTTTAAGTTC   
  
  
- GTCAAGGTCT AGAAGGGTTG TTTGGGTAGT ATTAGTATTA GTATGAGTCA TAATAGTGGT TGTAGTAGGA   
  
  
- TGAATGGGGG TGGCGGTGGT AGTCCTCCTT CTTCTTCTTA CGAAGTTGAA AATGTACCTA CTTCTTTTGA   
  
  
- AGAGAAGAAG AAGGAGGGTG TTGTTTGTAG TAGGAGTTGG AGGAAGGAGG CGGTGGTGGA GGAGCATAAT   
  
  
- GGGAATGATG AGGTGGGGGA AGCTACAAGG GAGATGGTGG TGGTGGAGGT GGAGGTGGGG GTGCGGGTGG   
  
  
- TTGTGGTGGT GGTGGTGGGG GGGTTGGGTC ATACCGCCGC CGCCGCCACC CAAACTGAGG TCGACGAAGA   
  
  
- GGATGAGGGG ACGGGCTCTG GAGTTGGGCC TCGAGAAGAG GAGCAGGCCC AAGAGCCCGT TCACCCGAAG   
  
  
- GTTGTAGGAG AACCTTCGGC GAGCACGGTA GAGACTCTCC TTGTGCTCGG CGGAGGTGGT CGAGGATACC   
  
  
- TACCAGTTGC TCGAGTCGAG GGGCATGCCA CTGCACCTCG TCTTCTATCG AAGTATGAAG CCGGTTCGGG   
  
  
- AGAAGTGGGC GTACTGGCTC AGTCCCCTGG CGTGGATGGC GTGGTACGCC AGGCGGCACC TGTTCTGGAA   
  
  
- GAGGAAACTC AGATGCTCTT TCTACCATGA GTTCAAGGTC CTCCAATCGG GAACCTGGTG GAAACCTGTG   
  
  
- CACCGTCGAT TACCTCGGGA GTAACTACGG AAACTGCCAC TTAGGTTTTA GGTGTAGTAG CTGTAGTCGT   
  
  
- GGTGTAAGAC GTGGGTCACC GGGTGGGAGG ACCTCCGCGA CCGGTGGGCT TACCTACTGC GCGGGATGGA   
  
  
- CTCCGACTGG TGACACCACC AGTTGTTCAG GCCCTTACTT CCAGGGTGGC CTCCGCCCAG GGTGTCCCAC   
  
  
- TACTTTCTCT AACCCTGGGC GGAGCTCTTC AAACGATCCG ATTACCCTCA CGGAAAGTTT AAATTACACC   
  
  
- AGGTGGTGCC CCGACTAAAT AGGCTGAACC TAAAGAGAGT CGACCTATAA TTGCTACTGC TCCGTAATCG   
  
  
- GTAGTTGACA CAGTTGAGCG AGGTGAGCCA GTTATAGGTG GCGGCACTGA GCCAGTAGAG CCGGAAGGCC   
  
  
- GCCGAGGTGG GATCCCACCA CTGGCACCAC CTCCTCCTCC GACTGGAACG ACTGCACCCC CGCCTCCCCA   
  
  
- TGCTCAAGAT GTCCCCCAAA TTACTCACAA ATTCCACCCA ATCCCAAATG AAACTCCGGG ACCTTCTCAC   
  
  
- GAAAGGATCC TGTTCGTTAC TCTTTGAGTA CGAGCTCGCC CGGCGTCCCG CCCGAGACCA CCTGGAGGAC   
  
  
- CGGACGGGGT TCGGGCGTAG TAGGCTCGCC TCCCTCCGTC GACGCGCCAC CAGGCCCTCC TACGTCCCCC   
  
  
- GGCCCAAGCC GGTGCACCCC AAGTCACTAC TCCAGACACT ACTGCAGTTC CGGGAGGATT CCTCCATGTT   
  
  
- CCTCCCCACC AGGTACTATG TTACAAGGCG GCGCCTACCG CCGCGGAGGC GGCCTTATAA GAACAGCACC   
  
  
- TCCCTAGTCG GCCACCACAC CCGGTCACGT ACCTTCGGAA T

+     ACE

| Site Name | Organism | Position | Strand | Matrix score. | sequence | function |
| --- | --- | --- | --- | --- | --- | --- |
| ACE | Petroselinum crispum | 789 | - | 9 | CTAACGTATT | cis-acting element involved in light responsiveness |

>HU06G00358.1   
+ -Up\_Stream \_Len000GGGTTT TTTTAAACTC AAAATTAGTT ATTGTATCAA TTGAATTTGG AAAACTATTA   
  
  
+ TTTTCGACTT ATTATAGAGA ATTTGTTGTT AATGAATGCT GATAAATACT GATAATTAGT GTTGTGAGTG   
  
  
+ TGAATTTGTG ATGATGAGCA ATGATTAGCG TTAAAATTAT AACTAGGATA AATAATTAAG TGTTGATGAA   
  
  
+ TGTTGATTGA TGATGATGAG TACTGATTGG TGCGGTGCTG GTAATAAATA TTTATGATAA GTTAAAAAAA   
  
  
+ AAAAACACAT CCTAAACAAT CACTATGTAA TATACTAAAT GAAAAAAATT GGTAATGTGA TTTGACAATC   
  
  
+ TAAGGGTAAG GGTGTTTTAC CCACACATGT TATGAAATTG GCTTACTATT TACAAACTTT TTACAGTTAT   
  
  
+ ATGATTCTAC CTATGTGTTA TCATCTTTCC GTCGATGTAA TTGATTATAT TCAACACAAT TTAATTCATT   
  
  
+ ACAATGTAGC TAATTGATAC TCAAAATAAT GAACTATTAA TAAATAATTA ATTATAACTT TTAAAAGTTT   
  
  
+ GTTAATTTAA AATTATATAA TATATAAATT TAGGCTACAT TAATCAAAAA ATTAGGCGTG TTCATCGGTC   
  
  
+ CAAACTTGAA CCGGACCAGA CCGGACCGAA GATCGATTAA GAGAAAAAGT ACAGACTGAG GACCGGATCG   
  
  
+ AATAAGAGTC GCACCGGACC AGGATCGGAC CGGAAAAATT TGGACCAGAT CGGGACCGAT CGAGGTAGAC   
  
  
+ CGAATTTATT TTCAAATACG ATAGGAATAA AACTTATTGA TTCAATTGAT TTTGGCCAAA ATCATGCTAT   
  
  
+ TATTGATTTT ATTTGCTAAC TCTCAAGAGT ATTAGTTTGC ACTTCGCATT ATCTTTGGCC AAAAAAATCA   
  
  
+ AAAAGAAAGT CTCAAACTCT TAATAGTTTT TTATTTTATT TGGTTTATTT TTAAAATAGT CGGCCTTCGG   
  
  
+ TCCGGACTAG TGGACCGAAT AAGGTAATTT TTCGAATCGA GGACCAGACC AAATGCACTC AGTTCGAACC   
  
  
+ GGTTCAGTTC GGTCTGACTC GATTTTTGGT CCGGTCCAAG TTTGAACAGA ATAATGTACA CACCTAGAAA   
  
  
+ AAAAGAATTA TGCATATATA CTTAAGGCCC CCAACCCTTT TTCCCTTTCC GCCAATACTT TTTACCAAGT   
  
  
+ CCAATTCTTT TCTTCTTAGC TATGGCTAGT AGTTATCCCT CCCTAGCTTC CACCCTAAGT TTTGAGACAA   
  
  
+ AATCCCATCT ATGTTAATAT TTATTTCTTG CACATGTCAC CCAATACAAG GGTTTCATTG AAAAGAAAAG   
  
  
+ GTATTAGATG TAAGATATGT ATGTATATAA ATAGTTTTGC AATAGACTAG AAGGACTAAA AAGGAAAAAG   
  
  
+ GAAGTTTAAA AGCAAAAAAC TTTCCCCTCT AGAAAAAAGG AAACCAGATT CCCTTTTCTA CCCCAATTCC   
  
  
+ CATAGTCTTT ACTCTGTCTC TCTCTCTCTG TCTCTCTTAC TCATGATGTT GAGGGAGGAA GGAATGAATT   
  
  
+ AAGGACCCTA AGCTAAGATC CAACCGTAAG ATATTGCCCC TTTAATCACC CATTTCATAT CTCTATCCAC   
  
  
+ AGTTAAAAAC CCACCTCCTC CATCCACATA ACTGATATAT CTCTTCTACA CCATACTATA AGGAAGAAGA   
  
  
+ GAAAAATAGG AGTGTATTGT ATTTTTATTT GAGTAATTTA TTTTAAGGAG AGAAATTTAC ATGGTTGGGT   
  
  
+ TAAATGGATA CCTTGTTTAG ACTAGTGAGT TTCCAACAAC CACACCACCA CCATCACCAA CACCAACACC   
  
  
+ AACACCACCA ACAGCAATCT TCTGATCAAT CCTCTTTGAA TAATACTACT ACTTATTCCA CAAATTCAAG   
  
  
+ CAGTTCCAGA TCTTCCCAAC AAACCCATCA TAATCATAAT CATACTCAGT ATTATCACCA ACATCATCCT   
  
  
+ ACTTACCCCC ACCGCCACCA TCAGGAGGAA GAAGAAGAAT GCTTCAACTT TTACATGGAT GAAGAAAACT   
  
  
+ TCTCTTCTTC TTCCTCCCAC AACAAACATC ATCCTCAACC TCCTTCCTCC GCCACCACCT CCTCGTATTA   
  
  
+ CCCTTACTAC TCCACCCCCT TCGATGTTCC CTCTACCACC ACCACCTCCA CCTCCACCCC CACGCCCACC   
  
  
+ AACACCACCA CCACCACCCC CCCAACCCAG TATGGCGGCG GCGGCGGTGG GTTTGACTCC AGCTGCTTCT   
  
  
+ CCTACTCCCC TGCCCGAGAC CTCAACCCGG AGCTCTTCTC CTCGTCCGGG TTCTCGGGCA AGTGGGCTTC   
  
  
+ CAACATCCTC TTGGAAGCCG CTCGTGCCAT CTCTGAGAGG AACACGAGCC GCCTCCACCA GCTCCTATGG   
  
  
+ ATGGTCAACG AGCTCAGCTC CCCGTACGGT GACGTGGAGC AGAAGATAGC TTCATACTTC GGCCAAGCCC   
  
  
+ TCTTCACCCG CATGACCGAG TCAGGGGACC GCACCTACCG CACCATGCGG TCCGCCGTGG ACAAGACCTT   
  
  
+ CTCCTTTGAG TCTACGAGAA AGATGGTACT CAAGTTCCAG GAGGTTAGCC CTTGGACCAC CTTTGGACAC   
  
  
+ GTGGCAGCTA ATGGAGCCCT CATTGATGCC TTTGACGGTG AATCCAAAAT CCACATCATC GACATCAGCA   
  
  
+ CCACATTCTG CACCCAGTGG CCCACCCTCC TGGAGGCGCT GGCCACCCGA ATGGATGACG CGCCCTACCT   
  
  
+ GAGGCTGACC ACTGTGGTGG TCAACAAGTC CGGGAATGAA GGTCCCACCG GAGGCGGGTC CCACAGGGTG   
  
  
+ ATGAAAGAGA TTGGGACCCG CCTCGAGAAG TTTGCTAGGC TAATGGGAGT GCCTTTCAAA TTTAATGTGG   
  
  
+ TCCACCACGG GGCTGATTTA TCCGACTTGG ATTTCTCTCA GCTGGATATT AACGATGACG AGGCATTAGC   
  
  
+ CATCAACTGT GTCAACTCGC TCCACTCGGT CAATATCCAC CGCCGTGACT CGGTCATCTC GGCCTTCCGG   
  
  
+ CGGCTCCACC CTAGGGTGGT GACCGTGGTG GAGGAGGAGG CTGACCTTGC TGACGTGGGG GCGGAGGGGT   
  
  
+ ACGAGTTCTA CAGGGGGTTT AATGAGTGTT TAAGGTGGGT TAGGGTTTAC TTTGAGGCCC TGGAAGAGTG   
  
  
+ CTTTCCTAGG ACAAGCAATG AGAAACTCAT GCTCGAGCGG GCCGCAGGGC GGGCTCTGGT GGACCTCCTG   
  
  
+ GCCTGCCCCA AGCCCGCATC ATCCGAGCGG AGGGAGGCAG CTGCGCGGTG GTCCGGGAGG ATGCAGGGGG   
  
  
+ CCGGGTTCGG CCACGTGGGG TTCAGTGATG AGGTCTGTGA TGACGTCAAG GCCCTCCTAA GGAGGTACAA   
  
  
+ GGAGGGGTGG TCCATGATAC AATGTTCCGC CGCGGATGGC GGCGCCTCCG CCGGAATATT CTTGTCGTGG   
  
  
+ AGGGATCAGC CGGTGGTGTG GGCCAGTGCA TGGAAGCCTT A  

- -Up\_Stream \_Len000CCCAAA AAAATTTGAG TTTTAATCAA TAACATAGTT AACTTAAACC TTTTGATAAT   
  
  
- AAAAGCTGAA TAATATCTCT TAAACAACAA TTACTTACGA CTATTTATGA CTATTAATCA CAACACTCAC   
  
  
- ACTTAAACAC TACTACTCGT TACTAATCGC AATTTTAATA TTGATCCTAT TTATTAATTC ACAACTACTT   
  
  
- ACAACTAACT ACTACTACTC ATGACTAACC ACGCCACGAC CATTATTTAT AAATACTATT CAATTTTTTT   
  
  
- TTTTTGTGTA GGATTTGTTA GTGATACATT ATATGATTTA CTTTTTTTAA CCATTACACT AAACTGTTAG   
  
  
- ATTCCCATTC CCACAAAATG GGTGTGTACA ATACTTTAAC CGAATGATAA ATGTTTGAAA AATGTCAATA   
  
  
- TACTAAGATG GATACACAAT AGTAGAAAGG CAGCTACATT AACTAATATA AGTTGTGTTA AATTAAGTAA   
  
  
- TGTTACATCG ATTAACTATG AGTTTTATTA CTTGATAATT ATTTATTAAT TAATATTGAA AATTTTCAAA   
  
  
- CAATTAAATT TTAATATATT ATATATTTAA ATCCGATGTA ATTAGTTTTT TAATCCGCAC AAGTAGCCAG   
  
  
- GTTTGAACTT GGCCTGGTCT GGCCTGGCTT CTAGCTAATT CTCTTTTTCA TGTCTGACTC CTGGCCTAGC   
  
  
- TTATTCTCAG CGTGGCCTGG TCCTAGCCTG GCCTTTTTAA ACCTGGTCTA GCCCTGGCTA GCTCCATCTG   
  
  
- GCTTAAATAA AAGTTTATGC TATCCTTATT TTGAATAACT AAGTTAACTA AAACCGGTTT TAGTACGATA   
  
  
- ATAACTAAAA TAAACGATTG AGAGTTCTCA TAATCAAACG TGAAGCGTAA TAGAAACCGG TTTTTTTAGT   
  
  
- TTTTCTTTCA GAGTTTGAGA ATTATCAAAA AATAAAATAA ACCAAATAAA AATTTTATCA GCCGGAAGCC   
  
  
- AGGCCTGATC ACCTGGCTTA TTCCATTAAA AAGCTTAGCT CCTGGTCTGG TTTACGTGAG TCAAGCTTGG   
  
  
- CCAAGTCAAG CCAGACTGAG CTAAAAACCA GGCCAGGTTC AAACTTGTCT TATTACATGT GTGGATCTTT   
  
  
- TTTTCTTAAT ACGTATATAT GAATTCCGGG GGTTGGGAAA AAGGGAAAGG CGGTTATGAA AAATGGTTCA   
  
  
- GGTTAAGAAA AGAAGAATCG ATACCGATCA TCAATAGGGA GGGATCGAAG GTGGGATTCA AAACTCTGTT   
  
  
- TTAGGGTAGA TACAATTATA AATAAAGAAC GTGTACAGTG GGTTATGTTC CCAAAGTAAC TTTTCTTTTC   
  
  
- CATAATCTAC ATTCTATACA TACATATATT TATCAAAACG TTATCTGATC TTCCTGATTT TTCCTTTTTC   
  
  
- CTTCAAATTT TCGTTTTTTG AAAGGGGAGA TCTTTTTTCC TTTGGTCTAA GGGAAAAGAT GGGGTTAAGG   
  
  
- GTATCAGAAA TGAGACAGAG AGAGAGAGAC AGAGAGAATG AGTACTACAA CTCCCTCCTT CCTTACTTAA   
  
  
- TTCCTGGGAT TCGATTCTAG GTTGGCATTC TATAACGGGG AAATTAGTGG GTAAAGTATA GAGATAGGTG   
  
  
- TCAATTTTTG GGTGGAGGAG GTAGGTGTAT TGACTATATA GAGAAGATGT GGTATGATAT TCCTTCTTCT   
  
  
- CTTTTTATCC TCACATAACA TAAAAATAAA CTCATTAAAT AAAATTCCTC TCTTTAAATG TACCAACCCA   
  
  
- ATTTACCTAT GGAACAAATC TGATCACTCA AAGGTTGTTG GTGTGGTGGT GGTAGTGGTT GTGGTTGTGG   
  
  
- TTGTGGTGGT TGTCGTTAGA AGACTAGTTA GGAGAAACTT ATTATGATGA TGAATAAGGT GTTTAAGTTC   
  
  
- GTCAAGGTCT AGAAGGGTTG TTTGGGTAGT ATTAGTATTA GTATGAGTCA TAATAGTGGT TGTAGTAGGA   
  
  
- TGAATGGGGG TGGCGGTGGT AGTCCTCCTT CTTCTTCTTA CGAAGTTGAA AATGTACCTA CTTCTTTTGA   
  
  
- AGAGAAGAAG AAGGAGGGTG TTGTTTGTAG TAGGAGTTGG AGGAAGGAGG CGGTGGTGGA GGAGCATAAT   
  
  
- GGGAATGATG AGGTGGGGGA AGCTACAAGG GAGATGGTGG TGGTGGAGGT GGAGGTGGGG GTGCGGGTGG   
  
  
- TTGTGGTGGT GGTGGTGGGG GGGTTGGGTC ATACCGCCGC CGCCGCCACC CAAACTGAGG TCGACGAAGA   
  
  
- GGATGAGGGG ACGGGCTCTG GAGTTGGGCC TCGAGAAGAG GAGCAGGCCC AAGAGCCCGT TCACCCGAAG   
  
  
- GTTGTAGGAG AACCTTCGGC GAGCACGGTA GAGACTCTCC TTGTGCTCGG CGGAGGTGGT CGAGGATACC   
  
  
- TACCAGTTGC TCGAGTCGAG GGGCATGCCA CTGCACCTCG TCTTCTATCG AAGTATGAAG CCGGTTCGGG   
  
  
- AGAAGTGGGC GTACTGGCTC AGTCCCCTGG CGTGGATGGC GTGGTACGCC AGGCGGCACC TGTTCTGGAA   
  
  
- GAGGAAACTC AGATGCTCTT TCTACCATGA GTTCAAGGTC CTCCAATCGG GAACCTGGTG GAAACCTGTG   
  
  
- CACCGTCGAT TACCTCGGGA GTAACTACGG AAACTGCCAC TTAGGTTTTA GGTGTAGTAG CTGTAGTCGT   
  
  
- GGTGTAAGAC GTGGGTCACC GGGTGGGAGG ACCTCCGCGA CCGGTGGGCT TACCTACTGC GCGGGATGGA   
  
  
- CTCCGACTGG TGACACCACC AGTTGTTCAG GCCCTTACTT CCAGGGTGGC CTCCGCCCAG GGTGTCCCAC   
  
  
- TACTTTCTCT AACCCTGGGC GGAGCTCTTC AAACGATCCG ATTACCCTCA CGGAAAGTTT AAATTACACC   
  
  
- AGGTGGTGCC CCGACTAAAT AGGCTGAACC TAAAGAGAGT CGACCTATAA TTGCTACTGC TCCGTAATCG   
  
  
- GTAGTTGACA CAGTTGAGCG AGGTGAGCCA GTTATAGGTG GCGGCACTGA GCCAGTAGAG CCGGAAGGCC   
  
  
- GCCGAGGTGG GATCCCACCA CTGGCACCAC CTCCTCCTCC GACTGGAACG ACTGCACCCC CGCCTCCCCA   
  
  
- TGCTCAAGAT GTCCCCCAAA TTACTCACAA ATTCCACCCA ATCCCAAATG AAACTCCGGG ACCTTCTCAC   
  
  
- GAAAGGATCC TGTTCGTTAC TCTTTGAGTA CGAGCTCGCC CGGCGTCCCG CCCGAGACCA CCTGGAGGAC   
  
  
- CGGACGGGGT TCGGGCGTAG TAGGCTCGCC TCCCTCCGTC GACGCGCCAC CAGGCCCTCC TACGTCCCCC   
  
  
- GGCCCAAGCC GGTGCACCCC AAGTCACTAC TCCAGACACT ACTGCAGTTC CGGGAGGATT CCTCCATGTT   
  
  
- CCTCCCCACC AGGTACTATG TTACAAGGCG GCGCCTACCG CCGCGGAGGC GGCCTTATAA GAACAGCACC   
  
  
- TCCCTAGTCG GCCACCACAC CCGGTCACGT ACCTTCGGAA T

+     ARE

| Site Name | Organism | Position | Strand | Matrix score. | sequence | function |
| --- | --- | --- | --- | --- | --- | --- |
| ARE | Zea mays | 955 | - | 6 | AAACCA | cis-acting regulatory element essential for the anaerobic induction |
| ARE | Zea mays | 1445 | + | 6 | AAACCA | cis-acting regulatory element essential for the anaerobic induction |

>HU06G00358.1   
+ -Up\_Stream \_Len000GGGTTT TTTTAAACTC AAAATTAGTT ATTGTATCAA TTGAATTTGG AAAACTATTA   
  
  
+ TTTTCGACTT ATTATAGAGA ATTTGTTGTT AATGAATGCT GATAAATACT GATAATTAGT GTTGTGAGTG   
  
  
+ TGAATTTGTG ATGATGAGCA ATGATTAGCG TTAAAATTAT AACTAGGATA AATAATTAAG TGTTGATGAA   
  
  
+ TGTTGATTGA TGATGATGAG TACTGATTGG TGCGGTGCTG GTAATAAATA TTTATGATAA GTTAAAAAAA   
  
  
+ AAAAACACAT CCTAAACAAT CACTATGTAA TATACTAAAT GAAAAAAATT GGTAATGTGA TTTGACAATC   
  
  
+ TAAGGGTAAG GGTGTTTTAC CCACACATGT TATGAAATTG GCTTACTATT TACAAACTTT TTACAGTTAT   
  
  
+ ATGATTCTAC CTATGTGTTA TCATCTTTCC GTCGATGTAA TTGATTATAT TCAACACAAT TTAATTCATT   
  
  
+ ACAATGTAGC TAATTGATAC TCAAAATAAT GAACTATTAA TAAATAATTA ATTATAACTT TTAAAAGTTT   
  
  
+ GTTAATTTAA AATTATATAA TATATAAATT TAGGCTACAT TAATCAAAAA ATTAGGCGTG TTCATCGGTC   
  
  
+ CAAACTTGAA CCGGACCAGA CCGGACCGAA GATCGATTAA GAGAAAAAGT ACAGACTGAG GACCGGATCG   
  
  
+ AATAAGAGTC GCACCGGACC AGGATCGGAC CGGAAAAATT TGGACCAGAT CGGGACCGAT CGAGGTAGAC   
  
  
+ CGAATTTATT TTCAAATACG ATAGGAATAA AACTTATTGA TTCAATTGAT TTTGGCCAAA ATCATGCTAT   
  
  
+ TATTGATTTT ATTTGCTAAC TCTCAAGAGT ATTAGTTTGC ACTTCGCATT ATCTTTGGCC AAAAAAATCA   
  
  
+ AAAAGAAAGT CTCAAACTCT TAATAGTTTT TTATTTTATT TGGTTTATTT TTAAAATAGT CGGCCTTCGG   
  
  
+ TCCGGACTAG TGGACCGAAT AAGGTAATTT TTCGAATCGA GGACCAGACC AAATGCACTC AGTTCGAACC   
  
  
+ GGTTCAGTTC GGTCTGACTC GATTTTTGGT CCGGTCCAAG TTTGAACAGA ATAATGTACA CACCTAGAAA   
  
  
+ AAAAGAATTA TGCATATATA CTTAAGGCCC CCAACCCTTT TTCCCTTTCC GCCAATACTT TTTACCAAGT   
  
  
+ CCAATTCTTT TCTTCTTAGC TATGGCTAGT AGTTATCCCT CCCTAGCTTC CACCCTAAGT TTTGAGACAA   
  
  
+ AATCCCATCT ATGTTAATAT TTATTTCTTG CACATGTCAC CCAATACAAG GGTTTCATTG AAAAGAAAAG   
  
  
+ GTATTAGATG TAAGATATGT ATGTATATAA ATAGTTTTGC AATAGACTAG AAGGACTAAA AAGGAAAAAG   
  
  
+ GAAGTTTAAA AGCAAAAAAC TTTCCCCTCT AGAAAAAAGG AAACCAGATT CCCTTTTCTA CCCCAATTCC   
  
  
+ CATAGTCTTT ACTCTGTCTC TCTCTCTCTG TCTCTCTTAC TCATGATGTT GAGGGAGGAA GGAATGAATT   
  
  
+ AAGGACCCTA AGCTAAGATC CAACCGTAAG ATATTGCCCC TTTAATCACC CATTTCATAT CTCTATCCAC   
  
  
+ AGTTAAAAAC CCACCTCCTC CATCCACATA ACTGATATAT CTCTTCTACA CCATACTATA AGGAAGAAGA   
  
  
+ GAAAAATAGG AGTGTATTGT ATTTTTATTT GAGTAATTTA TTTTAAGGAG AGAAATTTAC ATGGTTGGGT   
  
  
+ TAAATGGATA CCTTGTTTAG ACTAGTGAGT TTCCAACAAC CACACCACCA CCATCACCAA CACCAACACC   
  
  
+ AACACCACCA ACAGCAATCT TCTGATCAAT CCTCTTTGAA TAATACTACT ACTTATTCCA CAAATTCAAG   
  
  
+ CAGTTCCAGA TCTTCCCAAC AAACCCATCA TAATCATAAT CATACTCAGT ATTATCACCA ACATCATCCT   
  
  
+ ACTTACCCCC ACCGCCACCA TCAGGAGGAA GAAGAAGAAT GCTTCAACTT TTACATGGAT GAAGAAAACT   
  
  
+ TCTCTTCTTC TTCCTCCCAC AACAAACATC ATCCTCAACC TCCTTCCTCC GCCACCACCT CCTCGTATTA   
  
  
+ CCCTTACTAC TCCACCCCCT TCGATGTTCC CTCTACCACC ACCACCTCCA CCTCCACCCC CACGCCCACC   
  
  
+ AACACCACCA CCACCACCCC CCCAACCCAG TATGGCGGCG GCGGCGGTGG GTTTGACTCC AGCTGCTTCT   
  
  
+ CCTACTCCCC TGCCCGAGAC CTCAACCCGG AGCTCTTCTC CTCGTCCGGG TTCTCGGGCA AGTGGGCTTC   
  
  
+ CAACATCCTC TTGGAAGCCG CTCGTGCCAT CTCTGAGAGG AACACGAGCC GCCTCCACCA GCTCCTATGG   
  
  
+ ATGGTCAACG AGCTCAGCTC CCCGTACGGT GACGTGGAGC AGAAGATAGC TTCATACTTC GGCCAAGCCC   
  
  
+ TCTTCACCCG CATGACCGAG TCAGGGGACC GCACCTACCG CACCATGCGG TCCGCCGTGG ACAAGACCTT   
  
  
+ CTCCTTTGAG TCTACGAGAA AGATGGTACT CAAGTTCCAG GAGGTTAGCC CTTGGACCAC CTTTGGACAC   
  
  
+ GTGGCAGCTA ATGGAGCCCT CATTGATGCC TTTGACGGTG AATCCAAAAT CCACATCATC GACATCAGCA   
  
  
+ CCACATTCTG CACCCAGTGG CCCACCCTCC TGGAGGCGCT GGCCACCCGA ATGGATGACG CGCCCTACCT   
  
  
+ GAGGCTGACC ACTGTGGTGG TCAACAAGTC CGGGAATGAA GGTCCCACCG GAGGCGGGTC CCACAGGGTG   
  
  
+ ATGAAAGAGA TTGGGACCCG CCTCGAGAAG TTTGCTAGGC TAATGGGAGT GCCTTTCAAA TTTAATGTGG   
  
  
+ TCCACCACGG GGCTGATTTA TCCGACTTGG ATTTCTCTCA GCTGGATATT AACGATGACG AGGCATTAGC   
  
  
+ CATCAACTGT GTCAACTCGC TCCACTCGGT CAATATCCAC CGCCGTGACT CGGTCATCTC GGCCTTCCGG   
  
  
+ CGGCTCCACC CTAGGGTGGT GACCGTGGTG GAGGAGGAGG CTGACCTTGC TGACGTGGGG GCGGAGGGGT   
  
  
+ ACGAGTTCTA CAGGGGGTTT AATGAGTGTT TAAGGTGGGT TAGGGTTTAC TTTGAGGCCC TGGAAGAGTG   
  
  
+ CTTTCCTAGG ACAAGCAATG AGAAACTCAT GCTCGAGCGG GCCGCAGGGC GGGCTCTGGT GGACCTCCTG   
  
  
+ GCCTGCCCCA AGCCCGCATC ATCCGAGCGG AGGGAGGCAG CTGCGCGGTG GTCCGGGAGG ATGCAGGGGG   
  
  
+ CCGGGTTCGG CCACGTGGGG TTCAGTGATG AGGTCTGTGA TGACGTCAAG GCCCTCCTAA GGAGGTACAA   
  
  
+ GGAGGGGTGG TCCATGATAC AATGTTCCGC CGCGGATGGC GGCGCCTCCG CCGGAATATT CTTGTCGTGG   
  
  
+ AGGGATCAGC CGGTGGTGTG GGCCAGTGCA TGGAAGCCTT A  

- -Up\_Stream \_Len000CCCAAA AAAATTTGAG TTTTAATCAA TAACATAGTT AACTTAAACC TTTTGATAAT   
  
  
- AAAAGCTGAA TAATATCTCT TAAACAACAA TTACTTACGA CTATTTATGA CTATTAATCA CAACACTCAC   
  
  
- ACTTAAACAC TACTACTCGT TACTAATCGC AATTTTAATA TTGATCCTAT TTATTAATTC ACAACTACTT   
  
  
- ACAACTAACT ACTACTACTC ATGACTAACC ACGCCACGAC CATTATTTAT AAATACTATT CAATTTTTTT   
  
  
- TTTTTGTGTA GGATTTGTTA GTGATACATT ATATGATTTA CTTTTTTTAA CCATTACACT AAACTGTTAG   
  
  
- ATTCCCATTC CCACAAAATG GGTGTGTACA ATACTTTAAC CGAATGATAA ATGTTTGAAA AATGTCAATA   
  
  
- TACTAAGATG GATACACAAT AGTAGAAAGG CAGCTACATT AACTAATATA AGTTGTGTTA AATTAAGTAA   
  
  
- TGTTACATCG ATTAACTATG AGTTTTATTA CTTGATAATT ATTTATTAAT TAATATTGAA AATTTTCAAA   
  
  
- CAATTAAATT TTAATATATT ATATATTTAA ATCCGATGTA ATTAGTTTTT TAATCCGCAC AAGTAGCCAG   
  
  
- GTTTGAACTT GGCCTGGTCT GGCCTGGCTT CTAGCTAATT CTCTTTTTCA TGTCTGACTC CTGGCCTAGC   
  
  
- TTATTCTCAG CGTGGCCTGG TCCTAGCCTG GCCTTTTTAA ACCTGGTCTA GCCCTGGCTA GCTCCATCTG   
  
  
- GCTTAAATAA AAGTTTATGC TATCCTTATT TTGAATAACT AAGTTAACTA AAACCGGTTT TAGTACGATA   
  
  
- ATAACTAAAA TAAACGATTG AGAGTTCTCA TAATCAAACG TGAAGCGTAA TAGAAACCGG TTTTTTTAGT   
  
  
- TTTTCTTTCA GAGTTTGAGA ATTATCAAAA AATAAAATAA ACCAAATAAA AATTTTATCA GCCGGAAGCC   
  
  
- AGGCCTGATC ACCTGGCTTA TTCCATTAAA AAGCTTAGCT CCTGGTCTGG TTTACGTGAG TCAAGCTTGG   
  
  
- CCAAGTCAAG CCAGACTGAG CTAAAAACCA GGCCAGGTTC AAACTTGTCT TATTACATGT GTGGATCTTT   
  
  
- TTTTCTTAAT ACGTATATAT GAATTCCGGG GGTTGGGAAA AAGGGAAAGG CGGTTATGAA AAATGGTTCA   
  
  
- GGTTAAGAAA AGAAGAATCG ATACCGATCA TCAATAGGGA GGGATCGAAG GTGGGATTCA AAACTCTGTT   
  
  
- TTAGGGTAGA TACAATTATA AATAAAGAAC GTGTACAGTG GGTTATGTTC CCAAAGTAAC TTTTCTTTTC   
  
  
- CATAATCTAC ATTCTATACA TACATATATT TATCAAAACG TTATCTGATC TTCCTGATTT TTCCTTTTTC   
  
  
- CTTCAAATTT TCGTTTTTTG AAAGGGGAGA TCTTTTTTCC TTTGGTCTAA GGGAAAAGAT GGGGTTAAGG   
  
  
- GTATCAGAAA TGAGACAGAG AGAGAGAGAC AGAGAGAATG AGTACTACAA CTCCCTCCTT CCTTACTTAA   
  
  
- TTCCTGGGAT TCGATTCTAG GTTGGCATTC TATAACGGGG AAATTAGTGG GTAAAGTATA GAGATAGGTG   
  
  
- TCAATTTTTG GGTGGAGGAG GTAGGTGTAT TGACTATATA GAGAAGATGT GGTATGATAT TCCTTCTTCT   
  
  
- CTTTTTATCC TCACATAACA TAAAAATAAA CTCATTAAAT AAAATTCCTC TCTTTAAATG TACCAACCCA   
  
  
- ATTTACCTAT GGAACAAATC TGATCACTCA AAGGTTGTTG GTGTGGTGGT GGTAGTGGTT GTGGTTGTGG   
  
  
- TTGTGGTGGT TGTCGTTAGA AGACTAGTTA GGAGAAACTT ATTATGATGA TGAATAAGGT GTTTAAGTTC   
  
  
- GTCAAGGTCT AGAAGGGTTG TTTGGGTAGT ATTAGTATTA GTATGAGTCA TAATAGTGGT TGTAGTAGGA   
  
  
- TGAATGGGGG TGGCGGTGGT AGTCCTCCTT CTTCTTCTTA CGAAGTTGAA AATGTACCTA CTTCTTTTGA   
  
  
- AGAGAAGAAG AAGGAGGGTG TTGTTTGTAG TAGGAGTTGG AGGAAGGAGG CGGTGGTGGA GGAGCATAAT   
  
  
- GGGAATGATG AGGTGGGGGA AGCTACAAGG GAGATGGTGG TGGTGGAGGT GGAGGTGGGG GTGCGGGTGG   
  
  
- TTGTGGTGGT GGTGGTGGGG GGGTTGGGTC ATACCGCCGC CGCCGCCACC CAAACTGAGG TCGACGAAGA   
  
  
- GGATGAGGGG ACGGGCTCTG GAGTTGGGCC TCGAGAAGAG GAGCAGGCCC AAGAGCCCGT TCACCCGAAG   
  
  
- GTTGTAGGAG AACCTTCGGC GAGCACGGTA GAGACTCTCC TTGTGCTCGG CGGAGGTGGT CGAGGATACC   
  
  
- TACCAGTTGC TCGAGTCGAG GGGCATGCCA CTGCACCTCG TCTTCTATCG AAGTATGAAG CCGGTTCGGG   
  
  
- AGAAGTGGGC GTACTGGCTC AGTCCCCTGG CGTGGATGGC GTGGTACGCC AGGCGGCACC TGTTCTGGAA   
  
  
- GAGGAAACTC AGATGCTCTT TCTACCATGA GTTCAAGGTC CTCCAATCGG GAACCTGGTG GAAACCTGTG   
  
  
- CACCGTCGAT TACCTCGGGA GTAACTACGG AAACTGCCAC TTAGGTTTTA GGTGTAGTAG CTGTAGTCGT   
  
  
- GGTGTAAGAC GTGGGTCACC GGGTGGGAGG ACCTCCGCGA CCGGTGGGCT TACCTACTGC GCGGGATGGA   
  
  
- CTCCGACTGG TGACACCACC AGTTGTTCAG GCCCTTACTT CCAGGGTGGC CTCCGCCCAG GGTGTCCCAC   
  
  
- TACTTTCTCT AACCCTGGGC GGAGCTCTTC AAACGATCCG ATTACCCTCA CGGAAAGTTT AAATTACACC   
  
  
- AGGTGGTGCC CCGACTAAAT AGGCTGAACC TAAAGAGAGT CGACCTATAA TTGCTACTGC TCCGTAATCG   
  
  
- GTAGTTGACA CAGTTGAGCG AGGTGAGCCA GTTATAGGTG GCGGCACTGA GCCAGTAGAG CCGGAAGGCC   
  
  
- GCCGAGGTGG GATCCCACCA CTGGCACCAC CTCCTCCTCC GACTGGAACG ACTGCACCCC CGCCTCCCCA   
  
  
- TGCTCAAGAT GTCCCCCAAA TTACTCACAA ATTCCACCCA ATCCCAAATG AAACTCCGGG ACCTTCTCAC   
  
  
- GAAAGGATCC TGTTCGTTAC TCTTTGAGTA CGAGCTCGCC CGGCGTCCCG CCCGAGACCA CCTGGAGGAC   
  
  
- CGGACGGGGT TCGGGCGTAG TAGGCTCGCC TCCCTCCGTC GACGCGCCAC CAGGCCCTCC TACGTCCCCC   
  
  
- GGCCCAAGCC GGTGCACCCC AAGTCACTAC TCCAGACACT ACTGCAGTTC CGGGAGGATT CCTCCATGTT   
  
  
- CCTCCCCACC AGGTACTATG TTACAAGGCG GCGCCTACCG CCGCGGAGGC GGCCTTATAA GAACAGCACC   
  
  
- TCCCTAGTCG GCCACCACAC CCGGTCACGT ACCTTCGGAA T

+     AT~TATA-box

| Site Name | Organism | Position | Strand | Matrix score. | sequence | function |
| --- | --- | --- | --- | --- | --- | --- |
| AT~TATA-box | Arabidopsis thaliana | 1139 | + | 6 | TATATA |  |
| AT~TATA-box | Arabidopsis thaliana | 585 | + | 6 | TATATA |  |
| AT~TATA-box | Arabidopsis thaliana | 578 | + | 6 | TATATA |  |
| AT~TATA-box | Arabidopsis thaliana | 1358 | + | 6 | TATATA |  |

>HU06G00358.1   
+ -Up\_Stream \_Len000GGGTTT TTTTAAACTC AAAATTAGTT ATTGTATCAA TTGAATTTGG AAAACTATTA   
  
  
+ TTTTCGACTT ATTATAGAGA ATTTGTTGTT AATGAATGCT GATAAATACT GATAATTAGT GTTGTGAGTG   
  
  
+ TGAATTTGTG ATGATGAGCA ATGATTAGCG TTAAAATTAT AACTAGGATA AATAATTAAG TGTTGATGAA   
  
  
+ TGTTGATTGA TGATGATGAG TACTGATTGG TGCGGTGCTG GTAATAAATA TTTATGATAA GTTAAAAAAA   
  
  
+ AAAAACACAT CCTAAACAAT CACTATGTAA TATACTAAAT GAAAAAAATT GGTAATGTGA TTTGACAATC   
  
  
+ TAAGGGTAAG GGTGTTTTAC CCACACATGT TATGAAATTG GCTTACTATT TACAAACTTT TTACAGTTAT   
  
  
+ ATGATTCTAC CTATGTGTTA TCATCTTTCC GTCGATGTAA TTGATTATAT TCAACACAAT TTAATTCATT   
  
  
+ ACAATGTAGC TAATTGATAC TCAAAATAAT GAACTATTAA TAAATAATTA ATTATAACTT TTAAAAGTTT   
  
  
+ GTTAATTTAA AATTATATAA TATATAAATT TAGGCTACAT TAATCAAAAA ATTAGGCGTG TTCATCGGTC   
  
  
+ CAAACTTGAA CCGGACCAGA CCGGACCGAA GATCGATTAA GAGAAAAAGT ACAGACTGAG GACCGGATCG   
  
  
+ AATAAGAGTC GCACCGGACC AGGATCGGAC CGGAAAAATT TGGACCAGAT CGGGACCGAT CGAGGTAGAC   
  
  
+ CGAATTTATT TTCAAATACG ATAGGAATAA AACTTATTGA TTCAATTGAT TTTGGCCAAA ATCATGCTAT   
  
  
+ TATTGATTTT ATTTGCTAAC TCTCAAGAGT ATTAGTTTGC ACTTCGCATT ATCTTTGGCC AAAAAAATCA   
  
  
+ AAAAGAAAGT CTCAAACTCT TAATAGTTTT TTATTTTATT TGGTTTATTT TTAAAATAGT CGGCCTTCGG   
  
  
+ TCCGGACTAG TGGACCGAAT AAGGTAATTT TTCGAATCGA GGACCAGACC AAATGCACTC AGTTCGAACC   
  
  
+ GGTTCAGTTC GGTCTGACTC GATTTTTGGT CCGGTCCAAG TTTGAACAGA ATAATGTACA CACCTAGAAA   
  
  
+ AAAAGAATTA TGCATATATA CTTAAGGCCC CCAACCCTTT TTCCCTTTCC GCCAATACTT TTTACCAAGT   
  
  
+ CCAATTCTTT TCTTCTTAGC TATGGCTAGT AGTTATCCCT CCCTAGCTTC CACCCTAAGT TTTGAGACAA   
  
  
+ AATCCCATCT ATGTTAATAT TTATTTCTTG CACATGTCAC CCAATACAAG GGTTTCATTG AAAAGAAAAG   
  
  
+ GTATTAGATG TAAGATATGT ATGTATATAA ATAGTTTTGC AATAGACTAG AAGGACTAAA AAGGAAAAAG   
  
  
+ GAAGTTTAAA AGCAAAAAAC TTTCCCCTCT AGAAAAAAGG AAACCAGATT CCCTTTTCTA CCCCAATTCC   
  
  
+ CATAGTCTTT ACTCTGTCTC TCTCTCTCTG TCTCTCTTAC TCATGATGTT GAGGGAGGAA GGAATGAATT   
  
  
+ AAGGACCCTA AGCTAAGATC CAACCGTAAG ATATTGCCCC TTTAATCACC CATTTCATAT CTCTATCCAC   
  
  
+ AGTTAAAAAC CCACCTCCTC CATCCACATA ACTGATATAT CTCTTCTACA CCATACTATA AGGAAGAAGA   
  
  
+ GAAAAATAGG AGTGTATTGT ATTTTTATTT GAGTAATTTA TTTTAAGGAG AGAAATTTAC ATGGTTGGGT   
  
  
+ TAAATGGATA CCTTGTTTAG ACTAGTGAGT TTCCAACAAC CACACCACCA CCATCACCAA CACCAACACC   
  
  
+ AACACCACCA ACAGCAATCT TCTGATCAAT CCTCTTTGAA TAATACTACT ACTTATTCCA CAAATTCAAG   
  
  
+ CAGTTCCAGA TCTTCCCAAC AAACCCATCA TAATCATAAT CATACTCAGT ATTATCACCA ACATCATCCT   
  
  
+ ACTTACCCCC ACCGCCACCA TCAGGAGGAA GAAGAAGAAT GCTTCAACTT TTACATGGAT GAAGAAAACT   
  
  
+ TCTCTTCTTC TTCCTCCCAC AACAAACATC ATCCTCAACC TCCTTCCTCC GCCACCACCT CCTCGTATTA   
  
  
+ CCCTTACTAC TCCACCCCCT TCGATGTTCC CTCTACCACC ACCACCTCCA CCTCCACCCC CACGCCCACC   
  
  
+ AACACCACCA CCACCACCCC CCCAACCCAG TATGGCGGCG GCGGCGGTGG GTTTGACTCC AGCTGCTTCT   
  
  
+ CCTACTCCCC TGCCCGAGAC CTCAACCCGG AGCTCTTCTC CTCGTCCGGG TTCTCGGGCA AGTGGGCTTC   
  
  
+ CAACATCCTC TTGGAAGCCG CTCGTGCCAT CTCTGAGAGG AACACGAGCC GCCTCCACCA GCTCCTATGG   
  
  
+ ATGGTCAACG AGCTCAGCTC CCCGTACGGT GACGTGGAGC AGAAGATAGC TTCATACTTC GGCCAAGCCC   
  
  
+ TCTTCACCCG CATGACCGAG TCAGGGGACC GCACCTACCG CACCATGCGG TCCGCCGTGG ACAAGACCTT   
  
  
+ CTCCTTTGAG TCTACGAGAA AGATGGTACT CAAGTTCCAG GAGGTTAGCC CTTGGACCAC CTTTGGACAC   
  
  
+ GTGGCAGCTA ATGGAGCCCT CATTGATGCC TTTGACGGTG AATCCAAAAT CCACATCATC GACATCAGCA   
  
  
+ CCACATTCTG CACCCAGTGG CCCACCCTCC TGGAGGCGCT GGCCACCCGA ATGGATGACG CGCCCTACCT   
  
  
+ GAGGCTGACC ACTGTGGTGG TCAACAAGTC CGGGAATGAA GGTCCCACCG GAGGCGGGTC CCACAGGGTG   
  
  
+ ATGAAAGAGA TTGGGACCCG CCTCGAGAAG TTTGCTAGGC TAATGGGAGT GCCTTTCAAA TTTAATGTGG   
  
  
+ TCCACCACGG GGCTGATTTA TCCGACTTGG ATTTCTCTCA GCTGGATATT AACGATGACG AGGCATTAGC   
  
  
+ CATCAACTGT GTCAACTCGC TCCACTCGGT CAATATCCAC CGCCGTGACT CGGTCATCTC GGCCTTCCGG   
  
  
+ CGGCTCCACC CTAGGGTGGT GACCGTGGTG GAGGAGGAGG CTGACCTTGC TGACGTGGGG GCGGAGGGGT   
  
  
+ ACGAGTTCTA CAGGGGGTTT AATGAGTGTT TAAGGTGGGT TAGGGTTTAC TTTGAGGCCC TGGAAGAGTG   
  
  
+ CTTTCCTAGG ACAAGCAATG AGAAACTCAT GCTCGAGCGG GCCGCAGGGC GGGCTCTGGT GGACCTCCTG   
  
  
+ GCCTGCCCCA AGCCCGCATC ATCCGAGCGG AGGGAGGCAG CTGCGCGGTG GTCCGGGAGG ATGCAGGGGG   
  
  
+ CCGGGTTCGG CCACGTGGGG TTCAGTGATG AGGTCTGTGA TGACGTCAAG GCCCTCCTAA GGAGGTACAA   
  
  
+ GGAGGGGTGG TCCATGATAC AATGTTCCGC CGCGGATGGC GGCGCCTCCG CCGGAATATT CTTGTCGTGG   
  
  
+ AGGGATCAGC CGGTGGTGTG GGCCAGTGCA TGGAAGCCTT A  

- -Up\_Stream \_Len000CCCAAA AAAATTTGAG TTTTAATCAA TAACATAGTT AACTTAAACC TTTTGATAAT   
  
  
- AAAAGCTGAA TAATATCTCT TAAACAACAA TTACTTACGA CTATTTATGA CTATTAATCA CAACACTCAC   
  
  
- ACTTAAACAC TACTACTCGT TACTAATCGC AATTTTAATA TTGATCCTAT TTATTAATTC ACAACTACTT   
  
  
- ACAACTAACT ACTACTACTC ATGACTAACC ACGCCACGAC CATTATTTAT AAATACTATT CAATTTTTTT   
  
  
- TTTTTGTGTA GGATTTGTTA GTGATACATT ATATGATTTA CTTTTTTTAA CCATTACACT AAACTGTTAG   
  
  
- ATTCCCATTC CCACAAAATG GGTGTGTACA ATACTTTAAC CGAATGATAA ATGTTTGAAA AATGTCAATA   
  
  
- TACTAAGATG GATACACAAT AGTAGAAAGG CAGCTACATT AACTAATATA AGTTGTGTTA AATTAAGTAA   
  
  
- TGTTACATCG ATTAACTATG AGTTTTATTA CTTGATAATT ATTTATTAAT TAATATTGAA AATTTTCAAA   
  
  
- CAATTAAATT TTAATATATT ATATATTTAA ATCCGATGTA ATTAGTTTTT TAATCCGCAC AAGTAGCCAG   
  
  
- GTTTGAACTT GGCCTGGTCT GGCCTGGCTT CTAGCTAATT CTCTTTTTCA TGTCTGACTC CTGGCCTAGC   
  
  
- TTATTCTCAG CGTGGCCTGG TCCTAGCCTG GCCTTTTTAA ACCTGGTCTA GCCCTGGCTA GCTCCATCTG   
  
  
- GCTTAAATAA AAGTTTATGC TATCCTTATT TTGAATAACT AAGTTAACTA AAACCGGTTT TAGTACGATA   
  
  
- ATAACTAAAA TAAACGATTG AGAGTTCTCA TAATCAAACG TGAAGCGTAA TAGAAACCGG TTTTTTTAGT   
  
  
- TTTTCTTTCA GAGTTTGAGA ATTATCAAAA AATAAAATAA ACCAAATAAA AATTTTATCA GCCGGAAGCC   
  
  
- AGGCCTGATC ACCTGGCTTA TTCCATTAAA AAGCTTAGCT CCTGGTCTGG TTTACGTGAG TCAAGCTTGG   
  
  
- CCAAGTCAAG CCAGACTGAG CTAAAAACCA GGCCAGGTTC AAACTTGTCT TATTACATGT GTGGATCTTT   
  
  
- TTTTCTTAAT ACGTATATAT GAATTCCGGG GGTTGGGAAA AAGGGAAAGG CGGTTATGAA AAATGGTTCA   
  
  
- GGTTAAGAAA AGAAGAATCG ATACCGATCA TCAATAGGGA GGGATCGAAG GTGGGATTCA AAACTCTGTT   
  
  
- TTAGGGTAGA TACAATTATA AATAAAGAAC GTGTACAGTG GGTTATGTTC CCAAAGTAAC TTTTCTTTTC   
  
  
- CATAATCTAC ATTCTATACA TACATATATT TATCAAAACG TTATCTGATC TTCCTGATTT TTCCTTTTTC   
  
  
- CTTCAAATTT TCGTTTTTTG AAAGGGGAGA TCTTTTTTCC TTTGGTCTAA GGGAAAAGAT GGGGTTAAGG   
  
  
- GTATCAGAAA TGAGACAGAG AGAGAGAGAC AGAGAGAATG AGTACTACAA CTCCCTCCTT CCTTACTTAA   
  
  
- TTCCTGGGAT TCGATTCTAG GTTGGCATTC TATAACGGGG AAATTAGTGG GTAAAGTATA GAGATAGGTG   
  
  
- TCAATTTTTG GGTGGAGGAG GTAGGTGTAT TGACTATATA GAGAAGATGT GGTATGATAT TCCTTCTTCT   
  
  
- CTTTTTATCC TCACATAACA TAAAAATAAA CTCATTAAAT AAAATTCCTC TCTTTAAATG TACCAACCCA   
  
  
- ATTTACCTAT GGAACAAATC TGATCACTCA AAGGTTGTTG GTGTGGTGGT GGTAGTGGTT GTGGTTGTGG   
  
  
- TTGTGGTGGT TGTCGTTAGA AGACTAGTTA GGAGAAACTT ATTATGATGA TGAATAAGGT GTTTAAGTTC   
  
  
- GTCAAGGTCT AGAAGGGTTG TTTGGGTAGT ATTAGTATTA GTATGAGTCA TAATAGTGGT TGTAGTAGGA   
  
  
- TGAATGGGGG TGGCGGTGGT AGTCCTCCTT CTTCTTCTTA CGAAGTTGAA AATGTACCTA CTTCTTTTGA   
  
  
- AGAGAAGAAG AAGGAGGGTG TTGTTTGTAG TAGGAGTTGG AGGAAGGAGG CGGTGGTGGA GGAGCATAAT   
  
  
- GGGAATGATG AGGTGGGGGA AGCTACAAGG GAGATGGTGG TGGTGGAGGT GGAGGTGGGG GTGCGGGTGG   
  
  
- TTGTGGTGGT GGTGGTGGGG GGGTTGGGTC ATACCGCCGC CGCCGCCACC CAAACTGAGG TCGACGAAGA   
  
  
- GGATGAGGGG ACGGGCTCTG GAGTTGGGCC TCGAGAAGAG GAGCAGGCCC AAGAGCCCGT TCACCCGAAG   
  
  
- GTTGTAGGAG AACCTTCGGC GAGCACGGTA GAGACTCTCC TTGTGCTCGG CGGAGGTGGT CGAGGATACC   
  
  
- TACCAGTTGC TCGAGTCGAG GGGCATGCCA CTGCACCTCG TCTTCTATCG AAGTATGAAG CCGGTTCGGG   
  
  
- AGAAGTGGGC GTACTGGCTC AGTCCCCTGG CGTGGATGGC GTGGTACGCC AGGCGGCACC TGTTCTGGAA   
  
  
- GAGGAAACTC AGATGCTCTT TCTACCATGA GTTCAAGGTC CTCCAATCGG GAACCTGGTG GAAACCTGTG   
  
  
- CACCGTCGAT TACCTCGGGA GTAACTACGG AAACTGCCAC TTAGGTTTTA GGTGTAGTAG CTGTAGTCGT   
  
  
- GGTGTAAGAC GTGGGTCACC GGGTGGGAGG ACCTCCGCGA CCGGTGGGCT TACCTACTGC GCGGGATGGA   
  
  
- CTCCGACTGG TGACACCACC AGTTGTTCAG GCCCTTACTT CCAGGGTGGC CTCCGCCCAG GGTGTCCCAC   
  
  
- TACTTTCTCT AACCCTGGGC GGAGCTCTTC AAACGATCCG ATTACCCTCA CGGAAAGTTT AAATTACACC   
  
  
- AGGTGGTGCC CCGACTAAAT AGGCTGAACC TAAAGAGAGT CGACCTATAA TTGCTACTGC TCCGTAATCG   
  
  
- GTAGTTGACA CAGTTGAGCG AGGTGAGCCA GTTATAGGTG GCGGCACTGA GCCAGTAGAG CCGGAAGGCC   
  
  
- GCCGAGGTGG GATCCCACCA CTGGCACCAC CTCCTCCTCC GACTGGAACG ACTGCACCCC CGCCTCCCCA   
  
  
- TGCTCAAGAT GTCCCCCAAA TTACTCACAA ATTCCACCCA ATCCCAAATG AAACTCCGGG ACCTTCTCAC   
  
  
- GAAAGGATCC TGTTCGTTAC TCTTTGAGTA CGAGCTCGCC CGGCGTCCCG CCCGAGACCA CCTGGAGGAC   
  
  
- CGGACGGGGT TCGGGCGTAG TAGGCTCGCC TCCCTCCGTC GACGCGCCAC CAGGCCCTCC TACGTCCCCC   
  
  
- GGCCCAAGCC GGTGCACCCC AAGTCACTAC TCCAGACACT ACTGCAGTTC CGGGAGGATT CCTCCATGTT   
  
  
- CCTCCCCACC AGGTACTATG TTACAAGGCG GCGCCTACCG CCGCGGAGGC GGCCTTATAA GAACAGCACC   
  
  
- TCCCTAGTCG GCCACCACAC CCGGTCACGT ACCTTCGGAA T

+     AuxRR-core

| Site Name | Organism | Position | Strand | Matrix score. | sequence | function |
| --- | --- | --- | --- | --- | --- | --- |
| AuxRR-core | Nicotiana tabacum | 3373 | + | 7 | GGTCCAT | cis-acting regulatory element involved in auxin responsiveness |

>HU06G00358.1   
+ -Up\_Stream \_Len000GGGTTT TTTTAAACTC AAAATTAGTT ATTGTATCAA TTGAATTTGG AAAACTATTA   
  
  
+ TTTTCGACTT ATTATAGAGA ATTTGTTGTT AATGAATGCT GATAAATACT GATAATTAGT GTTGTGAGTG   
  
  
+ TGAATTTGTG ATGATGAGCA ATGATTAGCG TTAAAATTAT AACTAGGATA AATAATTAAG TGTTGATGAA   
  
  
+ TGTTGATTGA TGATGATGAG TACTGATTGG TGCGGTGCTG GTAATAAATA TTTATGATAA GTTAAAAAAA   
  
  
+ AAAAACACAT CCTAAACAAT CACTATGTAA TATACTAAAT GAAAAAAATT GGTAATGTGA TTTGACAATC   
  
  
+ TAAGGGTAAG GGTGTTTTAC CCACACATGT TATGAAATTG GCTTACTATT TACAAACTTT TTACAGTTAT   
  
  
+ ATGATTCTAC CTATGTGTTA TCATCTTTCC GTCGATGTAA TTGATTATAT TCAACACAAT TTAATTCATT   
  
  
+ ACAATGTAGC TAATTGATAC TCAAAATAAT GAACTATTAA TAAATAATTA ATTATAACTT TTAAAAGTTT   
  
  
+ GTTAATTTAA AATTATATAA TATATAAATT TAGGCTACAT TAATCAAAAA ATTAGGCGTG TTCATCGGTC   
  
  
+ CAAACTTGAA CCGGACCAGA CCGGACCGAA GATCGATTAA GAGAAAAAGT ACAGACTGAG GACCGGATCG   
  
  
+ AATAAGAGTC GCACCGGACC AGGATCGGAC CGGAAAAATT TGGACCAGAT CGGGACCGAT CGAGGTAGAC   
  
  
+ CGAATTTATT TTCAAATACG ATAGGAATAA AACTTATTGA TTCAATTGAT TTTGGCCAAA ATCATGCTAT   
  
  
+ TATTGATTTT ATTTGCTAAC TCTCAAGAGT ATTAGTTTGC ACTTCGCATT ATCTTTGGCC AAAAAAATCA   
  
  
+ AAAAGAAAGT CTCAAACTCT TAATAGTTTT TTATTTTATT TGGTTTATTT TTAAAATAGT CGGCCTTCGG   
  
  
+ TCCGGACTAG TGGACCGAAT AAGGTAATTT TTCGAATCGA GGACCAGACC AAATGCACTC AGTTCGAACC   
  
  
+ GGTTCAGTTC GGTCTGACTC GATTTTTGGT CCGGTCCAAG TTTGAACAGA ATAATGTACA CACCTAGAAA   
  
  
+ AAAAGAATTA TGCATATATA CTTAAGGCCC CCAACCCTTT TTCCCTTTCC GCCAATACTT TTTACCAAGT   
  
  
+ CCAATTCTTT TCTTCTTAGC TATGGCTAGT AGTTATCCCT CCCTAGCTTC CACCCTAAGT TTTGAGACAA   
  
  
+ AATCCCATCT ATGTTAATAT TTATTTCTTG CACATGTCAC CCAATACAAG GGTTTCATTG AAAAGAAAAG   
  
  
+ GTATTAGATG TAAGATATGT ATGTATATAA ATAGTTTTGC AATAGACTAG AAGGACTAAA AAGGAAAAAG   
  
  
+ GAAGTTTAAA AGCAAAAAAC TTTCCCCTCT AGAAAAAAGG AAACCAGATT CCCTTTTCTA CCCCAATTCC   
  
  
+ CATAGTCTTT ACTCTGTCTC TCTCTCTCTG TCTCTCTTAC TCATGATGTT GAGGGAGGAA GGAATGAATT   
  
  
+ AAGGACCCTA AGCTAAGATC CAACCGTAAG ATATTGCCCC TTTAATCACC CATTTCATAT CTCTATCCAC   
  
  
+ AGTTAAAAAC CCACCTCCTC CATCCACATA ACTGATATAT CTCTTCTACA CCATACTATA AGGAAGAAGA   
  
  
+ GAAAAATAGG AGTGTATTGT ATTTTTATTT GAGTAATTTA TTTTAAGGAG AGAAATTTAC ATGGTTGGGT   
  
  
+ TAAATGGATA CCTTGTTTAG ACTAGTGAGT TTCCAACAAC CACACCACCA CCATCACCAA CACCAACACC   
  
  
+ AACACCACCA ACAGCAATCT TCTGATCAAT CCTCTTTGAA TAATACTACT ACTTATTCCA CAAATTCAAG   
  
  
+ CAGTTCCAGA TCTTCCCAAC AAACCCATCA TAATCATAAT CATACTCAGT ATTATCACCA ACATCATCCT   
  
  
+ ACTTACCCCC ACCGCCACCA TCAGGAGGAA GAAGAAGAAT GCTTCAACTT TTACATGGAT GAAGAAAACT   
  
  
+ TCTCTTCTTC TTCCTCCCAC AACAAACATC ATCCTCAACC TCCTTCCTCC GCCACCACCT CCTCGTATTA   
  
  
+ CCCTTACTAC TCCACCCCCT TCGATGTTCC CTCTACCACC ACCACCTCCA CCTCCACCCC CACGCCCACC   
  
  
+ AACACCACCA CCACCACCCC CCCAACCCAG TATGGCGGCG GCGGCGGTGG GTTTGACTCC AGCTGCTTCT   
  
  
+ CCTACTCCCC TGCCCGAGAC CTCAACCCGG AGCTCTTCTC CTCGTCCGGG TTCTCGGGCA AGTGGGCTTC   
  
  
+ CAACATCCTC TTGGAAGCCG CTCGTGCCAT CTCTGAGAGG AACACGAGCC GCCTCCACCA GCTCCTATGG   
  
  
+ ATGGTCAACG AGCTCAGCTC CCCGTACGGT GACGTGGAGC AGAAGATAGC TTCATACTTC GGCCAAGCCC   
  
  
+ TCTTCACCCG CATGACCGAG TCAGGGGACC GCACCTACCG CACCATGCGG TCCGCCGTGG ACAAGACCTT   
  
  
+ CTCCTTTGAG TCTACGAGAA AGATGGTACT CAAGTTCCAG GAGGTTAGCC CTTGGACCAC CTTTGGACAC   
  
  
+ GTGGCAGCTA ATGGAGCCCT CATTGATGCC TTTGACGGTG AATCCAAAAT CCACATCATC GACATCAGCA   
  
  
+ CCACATTCTG CACCCAGTGG CCCACCCTCC TGGAGGCGCT GGCCACCCGA ATGGATGACG CGCCCTACCT   
  
  
+ GAGGCTGACC ACTGTGGTGG TCAACAAGTC CGGGAATGAA GGTCCCACCG GAGGCGGGTC CCACAGGGTG   
  
  
+ ATGAAAGAGA TTGGGACCCG CCTCGAGAAG TTTGCTAGGC TAATGGGAGT GCCTTTCAAA TTTAATGTGG   
  
  
+ TCCACCACGG GGCTGATTTA TCCGACTTGG ATTTCTCTCA GCTGGATATT AACGATGACG AGGCATTAGC   
  
  
+ CATCAACTGT GTCAACTCGC TCCACTCGGT CAATATCCAC CGCCGTGACT CGGTCATCTC GGCCTTCCGG   
  
  
+ CGGCTCCACC CTAGGGTGGT GACCGTGGTG GAGGAGGAGG CTGACCTTGC TGACGTGGGG GCGGAGGGGT   
  
  
+ ACGAGTTCTA CAGGGGGTTT AATGAGTGTT TAAGGTGGGT TAGGGTTTAC TTTGAGGCCC TGGAAGAGTG   
  
  
+ CTTTCCTAGG ACAAGCAATG AGAAACTCAT GCTCGAGCGG GCCGCAGGGC GGGCTCTGGT GGACCTCCTG   
  
  
+ GCCTGCCCCA AGCCCGCATC ATCCGAGCGG AGGGAGGCAG CTGCGCGGTG GTCCGGGAGG ATGCAGGGGG   
  
  
+ CCGGGTTCGG CCACGTGGGG TTCAGTGATG AGGTCTGTGA TGACGTCAAG GCCCTCCTAA GGAGGTACAA   
  
  
+ GGAGGGGTGG TCCATGATAC AATGTTCCGC CGCGGATGGC GGCGCCTCCG CCGGAATATT CTTGTCGTGG   
  
  
+ AGGGATCAGC CGGTGGTGTG GGCCAGTGCA TGGAAGCCTT A  

- -Up\_Stream \_Len000CCCAAA AAAATTTGAG TTTTAATCAA TAACATAGTT AACTTAAACC TTTTGATAAT   
  
  
- AAAAGCTGAA TAATATCTCT TAAACAACAA TTACTTACGA CTATTTATGA CTATTAATCA CAACACTCAC   
  
  
- ACTTAAACAC TACTACTCGT TACTAATCGC AATTTTAATA TTGATCCTAT TTATTAATTC ACAACTACTT   
  
  
- ACAACTAACT ACTACTACTC ATGACTAACC ACGCCACGAC CATTATTTAT AAATACTATT CAATTTTTTT   
  
  
- TTTTTGTGTA GGATTTGTTA GTGATACATT ATATGATTTA CTTTTTTTAA CCATTACACT AAACTGTTAG   
  
  
- ATTCCCATTC CCACAAAATG GGTGTGTACA ATACTTTAAC CGAATGATAA ATGTTTGAAA AATGTCAATA   
  
  
- TACTAAGATG GATACACAAT AGTAGAAAGG CAGCTACATT AACTAATATA AGTTGTGTTA AATTAAGTAA   
  
  
- TGTTACATCG ATTAACTATG AGTTTTATTA CTTGATAATT ATTTATTAAT TAATATTGAA AATTTTCAAA   
  
  
- CAATTAAATT TTAATATATT ATATATTTAA ATCCGATGTA ATTAGTTTTT TAATCCGCAC AAGTAGCCAG   
  
  
- GTTTGAACTT GGCCTGGTCT GGCCTGGCTT CTAGCTAATT CTCTTTTTCA TGTCTGACTC CTGGCCTAGC   
  
  
- TTATTCTCAG CGTGGCCTGG TCCTAGCCTG GCCTTTTTAA ACCTGGTCTA GCCCTGGCTA GCTCCATCTG   
  
  
- GCTTAAATAA AAGTTTATGC TATCCTTATT TTGAATAACT AAGTTAACTA AAACCGGTTT TAGTACGATA   
  
  
- ATAACTAAAA TAAACGATTG AGAGTTCTCA TAATCAAACG TGAAGCGTAA TAGAAACCGG TTTTTTTAGT   
  
  
- TTTTCTTTCA GAGTTTGAGA ATTATCAAAA AATAAAATAA ACCAAATAAA AATTTTATCA GCCGGAAGCC   
  
  
- AGGCCTGATC ACCTGGCTTA TTCCATTAAA AAGCTTAGCT CCTGGTCTGG TTTACGTGAG TCAAGCTTGG   
  
  
- CCAAGTCAAG CCAGACTGAG CTAAAAACCA GGCCAGGTTC AAACTTGTCT TATTACATGT GTGGATCTTT   
  
  
- TTTTCTTAAT ACGTATATAT GAATTCCGGG GGTTGGGAAA AAGGGAAAGG CGGTTATGAA AAATGGTTCA   
  
  
- GGTTAAGAAA AGAAGAATCG ATACCGATCA TCAATAGGGA GGGATCGAAG GTGGGATTCA AAACTCTGTT   
  
  
- TTAGGGTAGA TACAATTATA AATAAAGAAC GTGTACAGTG GGTTATGTTC CCAAAGTAAC TTTTCTTTTC   
  
  
- CATAATCTAC ATTCTATACA TACATATATT TATCAAAACG TTATCTGATC TTCCTGATTT TTCCTTTTTC   
  
  
- CTTCAAATTT TCGTTTTTTG AAAGGGGAGA TCTTTTTTCC TTTGGTCTAA GGGAAAAGAT GGGGTTAAGG   
  
  
- GTATCAGAAA TGAGACAGAG AGAGAGAGAC AGAGAGAATG AGTACTACAA CTCCCTCCTT CCTTACTTAA   
  
  
- TTCCTGGGAT TCGATTCTAG GTTGGCATTC TATAACGGGG AAATTAGTGG GTAAAGTATA GAGATAGGTG   
  
  
- TCAATTTTTG GGTGGAGGAG GTAGGTGTAT TGACTATATA GAGAAGATGT GGTATGATAT TCCTTCTTCT   
  
  
- CTTTTTATCC TCACATAACA TAAAAATAAA CTCATTAAAT AAAATTCCTC TCTTTAAATG TACCAACCCA   
  
  
- ATTTACCTAT GGAACAAATC TGATCACTCA AAGGTTGTTG GTGTGGTGGT GGTAGTGGTT GTGGTTGTGG   
  
  
- TTGTGGTGGT TGTCGTTAGA AGACTAGTTA GGAGAAACTT ATTATGATGA TGAATAAGGT GTTTAAGTTC   
  
  
- GTCAAGGTCT AGAAGGGTTG TTTGGGTAGT ATTAGTATTA GTATGAGTCA TAATAGTGGT TGTAGTAGGA   
  
  
- TGAATGGGGG TGGCGGTGGT AGTCCTCCTT CTTCTTCTTA CGAAGTTGAA AATGTACCTA CTTCTTTTGA   
  
  
- AGAGAAGAAG AAGGAGGGTG TTGTTTGTAG TAGGAGTTGG AGGAAGGAGG CGGTGGTGGA GGAGCATAAT   
  
  
- GGGAATGATG AGGTGGGGGA AGCTACAAGG GAGATGGTGG TGGTGGAGGT GGAGGTGGGG GTGCGGGTGG   
  
  
- TTGTGGTGGT GGTGGTGGGG GGGTTGGGTC ATACCGCCGC CGCCGCCACC CAAACTGAGG TCGACGAAGA   
  
  
- GGATGAGGGG ACGGGCTCTG GAGTTGGGCC TCGAGAAGAG GAGCAGGCCC AAGAGCCCGT TCACCCGAAG   
  
  
- GTTGTAGGAG AACCTTCGGC GAGCACGGTA GAGACTCTCC TTGTGCTCGG CGGAGGTGGT CGAGGATACC   
  
  
- TACCAGTTGC TCGAGTCGAG GGGCATGCCA CTGCACCTCG TCTTCTATCG AAGTATGAAG CCGGTTCGGG   
  
  
- AGAAGTGGGC GTACTGGCTC AGTCCCCTGG CGTGGATGGC GTGGTACGCC AGGCGGCACC TGTTCTGGAA   
  
  
- GAGGAAACTC AGATGCTCTT TCTACCATGA GTTCAAGGTC CTCCAATCGG GAACCTGGTG GAAACCTGTG   
  
  
- CACCGTCGAT TACCTCGGGA GTAACTACGG AAACTGCCAC TTAGGTTTTA GGTGTAGTAG CTGTAGTCGT   
  
  
- GGTGTAAGAC GTGGGTCACC GGGTGGGAGG ACCTCCGCGA CCGGTGGGCT TACCTACTGC GCGGGATGGA   
  
  
- CTCCGACTGG TGACACCACC AGTTGTTCAG GCCCTTACTT CCAGGGTGGC CTCCGCCCAG GGTGTCCCAC   
  
  
- TACTTTCTCT AACCCTGGGC GGAGCTCTTC AAACGATCCG ATTACCCTCA CGGAAAGTTT AAATTACACC   
  
  
- AGGTGGTGCC CCGACTAAAT AGGCTGAACC TAAAGAGAGT CGACCTATAA TTGCTACTGC TCCGTAATCG   
  
  
- GTAGTTGACA CAGTTGAGCG AGGTGAGCCA GTTATAGGTG GCGGCACTGA GCCAGTAGAG CCGGAAGGCC   
  
  
- GCCGAGGTGG GATCCCACCA CTGGCACCAC CTCCTCCTCC GACTGGAACG ACTGCACCCC CGCCTCCCCA   
  
  
- TGCTCAAGAT GTCCCCCAAA TTACTCACAA ATTCCACCCA ATCCCAAATG AAACTCCGGG ACCTTCTCAC   
  
  
- GAAAGGATCC TGTTCGTTAC TCTTTGAGTA CGAGCTCGCC CGGCGTCCCG CCCGAGACCA CCTGGAGGAC   
  
  
- CGGACGGGGT TCGGGCGTAG TAGGCTCGCC TCCCTCCGTC GACGCGCCAC CAGGCCCTCC TACGTCCCCC   
  
  
- GGCCCAAGCC GGTGCACCCC AAGTCACTAC TCCAGACACT ACTGCAGTTC CGGGAGGATT CCTCCATGTT   
  
  
- CCTCCCCACC AGGTACTATG TTACAAGGCG GCGCCTACCG CCGCGGAGGC GGCCTTATAA GAACAGCACC   
  
  
- TCCCTAGTCG GCCACCACAC CCGGTCACGT ACCTTCGGAA T

+     Box 4

| Site Name | Organism | Position | Strand | Matrix score. | sequence | function |
| --- | --- | --- | --- | --- | --- | --- |
| Box 4 | Petroselinum crispum | 603 | + | 6 | ATTAAT | part of a conserved DNA module involved in light responsiveness |
| Box 4 | Petroselinum crispum | 541 | + | 6 | ATTAAT | part of a conserved DNA module involved in light responsiveness |
| Box 4 | Petroselinum crispum | 530 | + | 6 | ATTAAT | part of a conserved DNA module involved in light responsiveness |

>HU06G00358.1   
+ -Up\_Stream \_Len000GGGTTT TTTTAAACTC AAAATTAGTT ATTGTATCAA TTGAATTTGG AAAACTATTA   
  
  
+ TTTTCGACTT ATTATAGAGA ATTTGTTGTT AATGAATGCT GATAAATACT GATAATTAGT GTTGTGAGTG   
  
  
+ TGAATTTGTG ATGATGAGCA ATGATTAGCG TTAAAATTAT AACTAGGATA AATAATTAAG TGTTGATGAA   
  
  
+ TGTTGATTGA TGATGATGAG TACTGATTGG TGCGGTGCTG GTAATAAATA TTTATGATAA GTTAAAAAAA   
  
  
+ AAAAACACAT CCTAAACAAT CACTATGTAA TATACTAAAT GAAAAAAATT GGTAATGTGA TTTGACAATC   
  
  
+ TAAGGGTAAG GGTGTTTTAC CCACACATGT TATGAAATTG GCTTACTATT TACAAACTTT TTACAGTTAT   
  
  
+ ATGATTCTAC CTATGTGTTA TCATCTTTCC GTCGATGTAA TTGATTATAT TCAACACAAT TTAATTCATT   
  
  
+ ACAATGTAGC TAATTGATAC TCAAAATAAT GAACTATTAA TAAATAATTA ATTATAACTT TTAAAAGTTT   
  
  
+ GTTAATTTAA AATTATATAA TATATAAATT TAGGCTACAT TAATCAAAAA ATTAGGCGTG TTCATCGGTC   
  
  
+ CAAACTTGAA CCGGACCAGA CCGGACCGAA GATCGATTAA GAGAAAAAGT ACAGACTGAG GACCGGATCG   
  
  
+ AATAAGAGTC GCACCGGACC AGGATCGGAC CGGAAAAATT TGGACCAGAT CGGGACCGAT CGAGGTAGAC   
  
  
+ CGAATTTATT TTCAAATACG ATAGGAATAA AACTTATTGA TTCAATTGAT TTTGGCCAAA ATCATGCTAT   
  
  
+ TATTGATTTT ATTTGCTAAC TCTCAAGAGT ATTAGTTTGC ACTTCGCATT ATCTTTGGCC AAAAAAATCA   
  
  
+ AAAAGAAAGT CTCAAACTCT TAATAGTTTT TTATTTTATT TGGTTTATTT TTAAAATAGT CGGCCTTCGG   
  
  
+ TCCGGACTAG TGGACCGAAT AAGGTAATTT TTCGAATCGA GGACCAGACC AAATGCACTC AGTTCGAACC   
  
  
+ GGTTCAGTTC GGTCTGACTC GATTTTTGGT CCGGTCCAAG TTTGAACAGA ATAATGTACA CACCTAGAAA   
  
  
+ AAAAGAATTA TGCATATATA CTTAAGGCCC CCAACCCTTT TTCCCTTTCC GCCAATACTT TTTACCAAGT   
  
  
+ CCAATTCTTT TCTTCTTAGC TATGGCTAGT AGTTATCCCT CCCTAGCTTC CACCCTAAGT TTTGAGACAA   
  
  
+ AATCCCATCT ATGTTAATAT TTATTTCTTG CACATGTCAC CCAATACAAG GGTTTCATTG AAAAGAAAAG   
  
  
+ GTATTAGATG TAAGATATGT ATGTATATAA ATAGTTTTGC AATAGACTAG AAGGACTAAA AAGGAAAAAG   
  
  
+ GAAGTTTAAA AGCAAAAAAC TTTCCCCTCT AGAAAAAAGG AAACCAGATT CCCTTTTCTA CCCCAATTCC   
  
  
+ CATAGTCTTT ACTCTGTCTC TCTCTCTCTG TCTCTCTTAC TCATGATGTT GAGGGAGGAA GGAATGAATT   
  
  
+ AAGGACCCTA AGCTAAGATC CAACCGTAAG ATATTGCCCC TTTAATCACC CATTTCATAT CTCTATCCAC   
  
  
+ AGTTAAAAAC CCACCTCCTC CATCCACATA ACTGATATAT CTCTTCTACA CCATACTATA AGGAAGAAGA   
  
  
+ GAAAAATAGG AGTGTATTGT ATTTTTATTT GAGTAATTTA TTTTAAGGAG AGAAATTTAC ATGGTTGGGT   
  
  
+ TAAATGGATA CCTTGTTTAG ACTAGTGAGT TTCCAACAAC CACACCACCA CCATCACCAA CACCAACACC   
  
  
+ AACACCACCA ACAGCAATCT TCTGATCAAT CCTCTTTGAA TAATACTACT ACTTATTCCA CAAATTCAAG   
  
  
+ CAGTTCCAGA TCTTCCCAAC AAACCCATCA TAATCATAAT CATACTCAGT ATTATCACCA ACATCATCCT   
  
  
+ ACTTACCCCC ACCGCCACCA TCAGGAGGAA GAAGAAGAAT GCTTCAACTT TTACATGGAT GAAGAAAACT   
  
  
+ TCTCTTCTTC TTCCTCCCAC AACAAACATC ATCCTCAACC TCCTTCCTCC GCCACCACCT CCTCGTATTA   
  
  
+ CCCTTACTAC TCCACCCCCT TCGATGTTCC CTCTACCACC ACCACCTCCA CCTCCACCCC CACGCCCACC   
  
  
+ AACACCACCA CCACCACCCC CCCAACCCAG TATGGCGGCG GCGGCGGTGG GTTTGACTCC AGCTGCTTCT   
  
  
+ CCTACTCCCC TGCCCGAGAC CTCAACCCGG AGCTCTTCTC CTCGTCCGGG TTCTCGGGCA AGTGGGCTTC   
  
  
+ CAACATCCTC TTGGAAGCCG CTCGTGCCAT CTCTGAGAGG AACACGAGCC GCCTCCACCA GCTCCTATGG   
  
  
+ ATGGTCAACG AGCTCAGCTC CCCGTACGGT GACGTGGAGC AGAAGATAGC TTCATACTTC GGCCAAGCCC   
  
  
+ TCTTCACCCG CATGACCGAG TCAGGGGACC GCACCTACCG CACCATGCGG TCCGCCGTGG ACAAGACCTT   
  
  
+ CTCCTTTGAG TCTACGAGAA AGATGGTACT CAAGTTCCAG GAGGTTAGCC CTTGGACCAC CTTTGGACAC   
  
  
+ GTGGCAGCTA ATGGAGCCCT CATTGATGCC TTTGACGGTG AATCCAAAAT CCACATCATC GACATCAGCA   
  
  
+ CCACATTCTG CACCCAGTGG CCCACCCTCC TGGAGGCGCT GGCCACCCGA ATGGATGACG CGCCCTACCT   
  
  
+ GAGGCTGACC ACTGTGGTGG TCAACAAGTC CGGGAATGAA GGTCCCACCG GAGGCGGGTC CCACAGGGTG   
  
  
+ ATGAAAGAGA TTGGGACCCG CCTCGAGAAG TTTGCTAGGC TAATGGGAGT GCCTTTCAAA TTTAATGTGG   
  
  
+ TCCACCACGG GGCTGATTTA TCCGACTTGG ATTTCTCTCA GCTGGATATT AACGATGACG AGGCATTAGC   
  
  
+ CATCAACTGT GTCAACTCGC TCCACTCGGT CAATATCCAC CGCCGTGACT CGGTCATCTC GGCCTTCCGG   
  
  
+ CGGCTCCACC CTAGGGTGGT GACCGTGGTG GAGGAGGAGG CTGACCTTGC TGACGTGGGG GCGGAGGGGT   
  
  
+ ACGAGTTCTA CAGGGGGTTT AATGAGTGTT TAAGGTGGGT TAGGGTTTAC TTTGAGGCCC TGGAAGAGTG   
  
  
+ CTTTCCTAGG ACAAGCAATG AGAAACTCAT GCTCGAGCGG GCCGCAGGGC GGGCTCTGGT GGACCTCCTG   
  
  
+ GCCTGCCCCA AGCCCGCATC ATCCGAGCGG AGGGAGGCAG CTGCGCGGTG GTCCGGGAGG ATGCAGGGGG   
  
  
+ CCGGGTTCGG CCACGTGGGG TTCAGTGATG AGGTCTGTGA TGACGTCAAG GCCCTCCTAA GGAGGTACAA   
  
  
+ GGAGGGGTGG TCCATGATAC AATGTTCCGC CGCGGATGGC GGCGCCTCCG CCGGAATATT CTTGTCGTGG   
  
  
+ AGGGATCAGC CGGTGGTGTG GGCCAGTGCA TGGAAGCCTT A  

- -Up\_Stream \_Len000CCCAAA AAAATTTGAG TTTTAATCAA TAACATAGTT AACTTAAACC TTTTGATAAT   
  
  
- AAAAGCTGAA TAATATCTCT TAAACAACAA TTACTTACGA CTATTTATGA CTATTAATCA CAACACTCAC   
  
  
- ACTTAAACAC TACTACTCGT TACTAATCGC AATTTTAATA TTGATCCTAT TTATTAATTC ACAACTACTT   
  
  
- ACAACTAACT ACTACTACTC ATGACTAACC ACGCCACGAC CATTATTTAT AAATACTATT CAATTTTTTT   
  
  
- TTTTTGTGTA GGATTTGTTA GTGATACATT ATATGATTTA CTTTTTTTAA CCATTACACT AAACTGTTAG   
  
  
- ATTCCCATTC CCACAAAATG GGTGTGTACA ATACTTTAAC CGAATGATAA ATGTTTGAAA AATGTCAATA   
  
  
- TACTAAGATG GATACACAAT AGTAGAAAGG CAGCTACATT AACTAATATA AGTTGTGTTA AATTAAGTAA   
  
  
- TGTTACATCG ATTAACTATG AGTTTTATTA CTTGATAATT ATTTATTAAT TAATATTGAA AATTTTCAAA   
  
  
- CAATTAAATT TTAATATATT ATATATTTAA ATCCGATGTA ATTAGTTTTT TAATCCGCAC AAGTAGCCAG   
  
  
- GTTTGAACTT GGCCTGGTCT GGCCTGGCTT CTAGCTAATT CTCTTTTTCA TGTCTGACTC CTGGCCTAGC   
  
  
- TTATTCTCAG CGTGGCCTGG TCCTAGCCTG GCCTTTTTAA ACCTGGTCTA GCCCTGGCTA GCTCCATCTG   
  
  
- GCTTAAATAA AAGTTTATGC TATCCTTATT TTGAATAACT AAGTTAACTA AAACCGGTTT TAGTACGATA   
  
  
- ATAACTAAAA TAAACGATTG AGAGTTCTCA TAATCAAACG TGAAGCGTAA TAGAAACCGG TTTTTTTAGT   
  
  
- TTTTCTTTCA GAGTTTGAGA ATTATCAAAA AATAAAATAA ACCAAATAAA AATTTTATCA GCCGGAAGCC   
  
  
- AGGCCTGATC ACCTGGCTTA TTCCATTAAA AAGCTTAGCT CCTGGTCTGG TTTACGTGAG TCAAGCTTGG   
  
  
- CCAAGTCAAG CCAGACTGAG CTAAAAACCA GGCCAGGTTC AAACTTGTCT TATTACATGT GTGGATCTTT   
  
  
- TTTTCTTAAT ACGTATATAT GAATTCCGGG GGTTGGGAAA AAGGGAAAGG CGGTTATGAA AAATGGTTCA   
  
  
- GGTTAAGAAA AGAAGAATCG ATACCGATCA TCAATAGGGA GGGATCGAAG GTGGGATTCA AAACTCTGTT   
  
  
- TTAGGGTAGA TACAATTATA AATAAAGAAC GTGTACAGTG GGTTATGTTC CCAAAGTAAC TTTTCTTTTC   
  
  
- CATAATCTAC ATTCTATACA TACATATATT TATCAAAACG TTATCTGATC TTCCTGATTT TTCCTTTTTC   
  
  
- CTTCAAATTT TCGTTTTTTG AAAGGGGAGA TCTTTTTTCC TTTGGTCTAA GGGAAAAGAT GGGGTTAAGG   
  
  
- GTATCAGAAA TGAGACAGAG AGAGAGAGAC AGAGAGAATG AGTACTACAA CTCCCTCCTT CCTTACTTAA   
  
  
- TTCCTGGGAT TCGATTCTAG GTTGGCATTC TATAACGGGG AAATTAGTGG GTAAAGTATA GAGATAGGTG   
  
  
- TCAATTTTTG GGTGGAGGAG GTAGGTGTAT TGACTATATA GAGAAGATGT GGTATGATAT TCCTTCTTCT   
  
  
- CTTTTTATCC TCACATAACA TAAAAATAAA CTCATTAAAT AAAATTCCTC TCTTTAAATG TACCAACCCA   
  
  
- ATTTACCTAT GGAACAAATC TGATCACTCA AAGGTTGTTG GTGTGGTGGT GGTAGTGGTT GTGGTTGTGG   
  
  
- TTGTGGTGGT TGTCGTTAGA AGACTAGTTA GGAGAAACTT ATTATGATGA TGAATAAGGT GTTTAAGTTC   
  
  
- GTCAAGGTCT AGAAGGGTTG TTTGGGTAGT ATTAGTATTA GTATGAGTCA TAATAGTGGT TGTAGTAGGA   
  
  
- TGAATGGGGG TGGCGGTGGT AGTCCTCCTT CTTCTTCTTA CGAAGTTGAA AATGTACCTA CTTCTTTTGA   
  
  
- AGAGAAGAAG AAGGAGGGTG TTGTTTGTAG TAGGAGTTGG AGGAAGGAGG CGGTGGTGGA GGAGCATAAT   
  
  
- GGGAATGATG AGGTGGGGGA AGCTACAAGG GAGATGGTGG TGGTGGAGGT GGAGGTGGGG GTGCGGGTGG   
  
  
- TTGTGGTGGT GGTGGTGGGG GGGTTGGGTC ATACCGCCGC CGCCGCCACC CAAACTGAGG TCGACGAAGA   
  
  
- GGATGAGGGG ACGGGCTCTG GAGTTGGGCC TCGAGAAGAG GAGCAGGCCC AAGAGCCCGT TCACCCGAAG   
  
  
- GTTGTAGGAG AACCTTCGGC GAGCACGGTA GAGACTCTCC TTGTGCTCGG CGGAGGTGGT CGAGGATACC   
  
  
- TACCAGTTGC TCGAGTCGAG GGGCATGCCA CTGCACCTCG TCTTCTATCG AAGTATGAAG CCGGTTCGGG   
  
  
- AGAAGTGGGC GTACTGGCTC AGTCCCCTGG CGTGGATGGC GTGGTACGCC AGGCGGCACC TGTTCTGGAA   
  
  
- GAGGAAACTC AGATGCTCTT TCTACCATGA GTTCAAGGTC CTCCAATCGG GAACCTGGTG GAAACCTGTG   
  
  
- CACCGTCGAT TACCTCGGGA GTAACTACGG AAACTGCCAC TTAGGTTTTA GGTGTAGTAG CTGTAGTCGT   
  
  
- GGTGTAAGAC GTGGGTCACC GGGTGGGAGG ACCTCCGCGA CCGGTGGGCT TACCTACTGC GCGGGATGGA   
  
  
- CTCCGACTGG TGACACCACC AGTTGTTCAG GCCCTTACTT CCAGGGTGGC CTCCGCCCAG GGTGTCCCAC   
  
  
- TACTTTCTCT AACCCTGGGC GGAGCTCTTC AAACGATCCG ATTACCCTCA CGGAAAGTTT AAATTACACC   
  
  
- AGGTGGTGCC CCGACTAAAT AGGCTGAACC TAAAGAGAGT CGACCTATAA TTGCTACTGC TCCGTAATCG   
  
  
- GTAGTTGACA CAGTTGAGCG AGGTGAGCCA GTTATAGGTG GCGGCACTGA GCCAGTAGAG CCGGAAGGCC   
  
  
- GCCGAGGTGG GATCCCACCA CTGGCACCAC CTCCTCCTCC GACTGGAACG ACTGCACCCC CGCCTCCCCA   
  
  
- TGCTCAAGAT GTCCCCCAAA TTACTCACAA ATTCCACCCA ATCCCAAATG AAACTCCGGG ACCTTCTCAC   
  
  
- GAAAGGATCC TGTTCGTTAC TCTTTGAGTA CGAGCTCGCC CGGCGTCCCG CCCGAGACCA CCTGGAGGAC   
  
  
- CGGACGGGGT TCGGGCGTAG TAGGCTCGCC TCCCTCCGTC GACGCGCCAC CAGGCCCTCC TACGTCCCCC   
  
  
- GGCCCAAGCC GGTGCACCCC AAGTCACTAC TCCAGACACT ACTGCAGTTC CGGGAGGATT CCTCCATGTT   
  
  
- CCTCCCCACC AGGTACTATG TTACAAGGCG GCGCCTACCG CCGCGGAGGC GGCCTTATAA GAACAGCACC   
  
  
- TCCCTAGTCG GCCACCACAC CCGGTCACGT ACCTTCGGAA T

+     Box II

| Site Name | Organism | Position | Strand | Matrix score. | sequence | function |
| --- | --- | --- | --- | --- | --- | --- |
| Box II | Petroselinum crispum | 3304 | - | 9 | CCACGTGGC | part of a light responsive element |
| Box II | Solanum tuberosum | 253 | + | 9 | TGGTAATAA | part of a light responsive element |

>HU06G00358.1   
+ -Up\_Stream \_Len000GGGTTT TTTTAAACTC AAAATTAGTT ATTGTATCAA TTGAATTTGG AAAACTATTA   
  
  
+ TTTTCGACTT ATTATAGAGA ATTTGTTGTT AATGAATGCT GATAAATACT GATAATTAGT GTTGTGAGTG   
  
  
+ TGAATTTGTG ATGATGAGCA ATGATTAGCG TTAAAATTAT AACTAGGATA AATAATTAAG TGTTGATGAA   
  
  
+ TGTTGATTGA TGATGATGAG TACTGATTGG TGCGGTGCTG GTAATAAATA TTTATGATAA GTTAAAAAAA   
  
  
+ AAAAACACAT CCTAAACAAT CACTATGTAA TATACTAAAT GAAAAAAATT GGTAATGTGA TTTGACAATC   
  
  
+ TAAGGGTAAG GGTGTTTTAC CCACACATGT TATGAAATTG GCTTACTATT TACAAACTTT TTACAGTTAT   
  
  
+ ATGATTCTAC CTATGTGTTA TCATCTTTCC GTCGATGTAA TTGATTATAT TCAACACAAT TTAATTCATT   
  
  
+ ACAATGTAGC TAATTGATAC TCAAAATAAT GAACTATTAA TAAATAATTA ATTATAACTT TTAAAAGTTT   
  
  
+ GTTAATTTAA AATTATATAA TATATAAATT TAGGCTACAT TAATCAAAAA ATTAGGCGTG TTCATCGGTC   
  
  
+ CAAACTTGAA CCGGACCAGA CCGGACCGAA GATCGATTAA GAGAAAAAGT ACAGACTGAG GACCGGATCG   
  
  
+ AATAAGAGTC GCACCGGACC AGGATCGGAC CGGAAAAATT TGGACCAGAT CGGGACCGAT CGAGGTAGAC   
  
  
+ CGAATTTATT TTCAAATACG ATAGGAATAA AACTTATTGA TTCAATTGAT TTTGGCCAAA ATCATGCTAT   
  
  
+ TATTGATTTT ATTTGCTAAC TCTCAAGAGT ATTAGTTTGC ACTTCGCATT ATCTTTGGCC AAAAAAATCA   
  
  
+ AAAAGAAAGT CTCAAACTCT TAATAGTTTT TTATTTTATT TGGTTTATTT TTAAAATAGT CGGCCTTCGG   
  
  
+ TCCGGACTAG TGGACCGAAT AAGGTAATTT TTCGAATCGA GGACCAGACC AAATGCACTC AGTTCGAACC   
  
  
+ GGTTCAGTTC GGTCTGACTC GATTTTTGGT CCGGTCCAAG TTTGAACAGA ATAATGTACA CACCTAGAAA   
  
  
+ AAAAGAATTA TGCATATATA CTTAAGGCCC CCAACCCTTT TTCCCTTTCC GCCAATACTT TTTACCAAGT   
  
  
+ CCAATTCTTT TCTTCTTAGC TATGGCTAGT AGTTATCCCT CCCTAGCTTC CACCCTAAGT TTTGAGACAA   
  
  
+ AATCCCATCT ATGTTAATAT TTATTTCTTG CACATGTCAC CCAATACAAG GGTTTCATTG AAAAGAAAAG   
  
  
+ GTATTAGATG TAAGATATGT ATGTATATAA ATAGTTTTGC AATAGACTAG AAGGACTAAA AAGGAAAAAG   
  
  
+ GAAGTTTAAA AGCAAAAAAC TTTCCCCTCT AGAAAAAAGG AAACCAGATT CCCTTTTCTA CCCCAATTCC   
  
  
+ CATAGTCTTT ACTCTGTCTC TCTCTCTCTG TCTCTCTTAC TCATGATGTT GAGGGAGGAA GGAATGAATT   
  
  
+ AAGGACCCTA AGCTAAGATC CAACCGTAAG ATATTGCCCC TTTAATCACC CATTTCATAT CTCTATCCAC   
  
  
+ AGTTAAAAAC CCACCTCCTC CATCCACATA ACTGATATAT CTCTTCTACA CCATACTATA AGGAAGAAGA   
  
  
+ GAAAAATAGG AGTGTATTGT ATTTTTATTT GAGTAATTTA TTTTAAGGAG AGAAATTTAC ATGGTTGGGT   
  
  
+ TAAATGGATA CCTTGTTTAG ACTAGTGAGT TTCCAACAAC CACACCACCA CCATCACCAA CACCAACACC   
  
  
+ AACACCACCA ACAGCAATCT TCTGATCAAT CCTCTTTGAA TAATACTACT ACTTATTCCA CAAATTCAAG   
  
  
+ CAGTTCCAGA TCTTCCCAAC AAACCCATCA TAATCATAAT CATACTCAGT ATTATCACCA ACATCATCCT   
  
  
+ ACTTACCCCC ACCGCCACCA TCAGGAGGAA GAAGAAGAAT GCTTCAACTT TTACATGGAT GAAGAAAACT   
  
  
+ TCTCTTCTTC TTCCTCCCAC AACAAACATC ATCCTCAACC TCCTTCCTCC GCCACCACCT CCTCGTATTA   
  
  
+ CCCTTACTAC TCCACCCCCT TCGATGTTCC CTCTACCACC ACCACCTCCA CCTCCACCCC CACGCCCACC   
  
  
+ AACACCACCA CCACCACCCC CCCAACCCAG TATGGCGGCG GCGGCGGTGG GTTTGACTCC AGCTGCTTCT   
  
  
+ CCTACTCCCC TGCCCGAGAC CTCAACCCGG AGCTCTTCTC CTCGTCCGGG TTCTCGGGCA AGTGGGCTTC   
  
  
+ CAACATCCTC TTGGAAGCCG CTCGTGCCAT CTCTGAGAGG AACACGAGCC GCCTCCACCA GCTCCTATGG   
  
  
+ ATGGTCAACG AGCTCAGCTC CCCGTACGGT GACGTGGAGC AGAAGATAGC TTCATACTTC GGCCAAGCCC   
  
  
+ TCTTCACCCG CATGACCGAG TCAGGGGACC GCACCTACCG CACCATGCGG TCCGCCGTGG ACAAGACCTT   
  
  
+ CTCCTTTGAG TCTACGAGAA AGATGGTACT CAAGTTCCAG GAGGTTAGCC CTTGGACCAC CTTTGGACAC   
  
  
+ GTGGCAGCTA ATGGAGCCCT CATTGATGCC TTTGACGGTG AATCCAAAAT CCACATCATC GACATCAGCA   
  
  
+ CCACATTCTG CACCCAGTGG CCCACCCTCC TGGAGGCGCT GGCCACCCGA ATGGATGACG CGCCCTACCT   
  
  
+ GAGGCTGACC ACTGTGGTGG TCAACAAGTC CGGGAATGAA GGTCCCACCG GAGGCGGGTC CCACAGGGTG   
  
  
+ ATGAAAGAGA TTGGGACCCG CCTCGAGAAG TTTGCTAGGC TAATGGGAGT GCCTTTCAAA TTTAATGTGG   
  
  
+ TCCACCACGG GGCTGATTTA TCCGACTTGG ATTTCTCTCA GCTGGATATT AACGATGACG AGGCATTAGC   
  
  
+ CATCAACTGT GTCAACTCGC TCCACTCGGT CAATATCCAC CGCCGTGACT CGGTCATCTC GGCCTTCCGG   
  
  
+ CGGCTCCACC CTAGGGTGGT GACCGTGGTG GAGGAGGAGG CTGACCTTGC TGACGTGGGG GCGGAGGGGT   
  
  
+ ACGAGTTCTA CAGGGGGTTT AATGAGTGTT TAAGGTGGGT TAGGGTTTAC TTTGAGGCCC TGGAAGAGTG   
  
  
+ CTTTCCTAGG ACAAGCAATG AGAAACTCAT GCTCGAGCGG GCCGCAGGGC GGGCTCTGGT GGACCTCCTG   
  
  
+ GCCTGCCCCA AGCCCGCATC ATCCGAGCGG AGGGAGGCAG CTGCGCGGTG GTCCGGGAGG ATGCAGGGGG   
  
  
+ CCGGGTTCGG CCACGTGGGG TTCAGTGATG AGGTCTGTGA TGACGTCAAG GCCCTCCTAA GGAGGTACAA   
  
  
+ GGAGGGGTGG TCCATGATAC AATGTTCCGC CGCGGATGGC GGCGCCTCCG CCGGAATATT CTTGTCGTGG   
  
  
+ AGGGATCAGC CGGTGGTGTG GGCCAGTGCA TGGAAGCCTT A  

- -Up\_Stream \_Len000CCCAAA AAAATTTGAG TTTTAATCAA TAACATAGTT AACTTAAACC TTTTGATAAT   
  
  
- AAAAGCTGAA TAATATCTCT TAAACAACAA TTACTTACGA CTATTTATGA CTATTAATCA CAACACTCAC   
  
  
- ACTTAAACAC TACTACTCGT TACTAATCGC AATTTTAATA TTGATCCTAT TTATTAATTC ACAACTACTT   
  
  
- ACAACTAACT ACTACTACTC ATGACTAACC ACGCCACGAC CATTATTTAT AAATACTATT CAATTTTTTT   
  
  
- TTTTTGTGTA GGATTTGTTA GTGATACATT ATATGATTTA CTTTTTTTAA CCATTACACT AAACTGTTAG   
  
  
- ATTCCCATTC CCACAAAATG GGTGTGTACA ATACTTTAAC CGAATGATAA ATGTTTGAAA AATGTCAATA   
  
  
- TACTAAGATG GATACACAAT AGTAGAAAGG CAGCTACATT AACTAATATA AGTTGTGTTA AATTAAGTAA   
  
  
- TGTTACATCG ATTAACTATG AGTTTTATTA CTTGATAATT ATTTATTAAT TAATATTGAA AATTTTCAAA   
  
  
- CAATTAAATT TTAATATATT ATATATTTAA ATCCGATGTA ATTAGTTTTT TAATCCGCAC AAGTAGCCAG   
  
  
- GTTTGAACTT GGCCTGGTCT GGCCTGGCTT CTAGCTAATT CTCTTTTTCA TGTCTGACTC CTGGCCTAGC   
  
  
- TTATTCTCAG CGTGGCCTGG TCCTAGCCTG GCCTTTTTAA ACCTGGTCTA GCCCTGGCTA GCTCCATCTG   
  
  
- GCTTAAATAA AAGTTTATGC TATCCTTATT TTGAATAACT AAGTTAACTA AAACCGGTTT TAGTACGATA   
  
  
- ATAACTAAAA TAAACGATTG AGAGTTCTCA TAATCAAACG TGAAGCGTAA TAGAAACCGG TTTTTTTAGT   
  
  
- TTTTCTTTCA GAGTTTGAGA ATTATCAAAA AATAAAATAA ACCAAATAAA AATTTTATCA GCCGGAAGCC   
  
  
- AGGCCTGATC ACCTGGCTTA TTCCATTAAA AAGCTTAGCT CCTGGTCTGG TTTACGTGAG TCAAGCTTGG   
  
  
- CCAAGTCAAG CCAGACTGAG CTAAAAACCA GGCCAGGTTC AAACTTGTCT TATTACATGT GTGGATCTTT   
  
  
- TTTTCTTAAT ACGTATATAT GAATTCCGGG GGTTGGGAAA AAGGGAAAGG CGGTTATGAA AAATGGTTCA   
  
  
- GGTTAAGAAA AGAAGAATCG ATACCGATCA TCAATAGGGA GGGATCGAAG GTGGGATTCA AAACTCTGTT   
  
  
- TTAGGGTAGA TACAATTATA AATAAAGAAC GTGTACAGTG GGTTATGTTC CCAAAGTAAC TTTTCTTTTC   
  
  
- CATAATCTAC ATTCTATACA TACATATATT TATCAAAACG TTATCTGATC TTCCTGATTT TTCCTTTTTC   
  
  
- CTTCAAATTT TCGTTTTTTG AAAGGGGAGA TCTTTTTTCC TTTGGTCTAA GGGAAAAGAT GGGGTTAAGG   
  
  
- GTATCAGAAA TGAGACAGAG AGAGAGAGAC AGAGAGAATG AGTACTACAA CTCCCTCCTT CCTTACTTAA   
  
  
- TTCCTGGGAT TCGATTCTAG GTTGGCATTC TATAACGGGG AAATTAGTGG GTAAAGTATA GAGATAGGTG   
  
  
- TCAATTTTTG GGTGGAGGAG GTAGGTGTAT TGACTATATA GAGAAGATGT GGTATGATAT TCCTTCTTCT   
  
  
- CTTTTTATCC TCACATAACA TAAAAATAAA CTCATTAAAT AAAATTCCTC TCTTTAAATG TACCAACCCA   
  
  
- ATTTACCTAT GGAACAAATC TGATCACTCA AAGGTTGTTG GTGTGGTGGT GGTAGTGGTT GTGGTTGTGG   
  
  
- TTGTGGTGGT TGTCGTTAGA AGACTAGTTA GGAGAAACTT ATTATGATGA TGAATAAGGT GTTTAAGTTC   
  
  
- GTCAAGGTCT AGAAGGGTTG TTTGGGTAGT ATTAGTATTA GTATGAGTCA TAATAGTGGT TGTAGTAGGA   
  
  
- TGAATGGGGG TGGCGGTGGT AGTCCTCCTT CTTCTTCTTA CGAAGTTGAA AATGTACCTA CTTCTTTTGA   
  
  
- AGAGAAGAAG AAGGAGGGTG TTGTTTGTAG TAGGAGTTGG AGGAAGGAGG CGGTGGTGGA GGAGCATAAT   
  
  
- GGGAATGATG AGGTGGGGGA AGCTACAAGG GAGATGGTGG TGGTGGAGGT GGAGGTGGGG GTGCGGGTGG   
  
  
- TTGTGGTGGT GGTGGTGGGG GGGTTGGGTC ATACCGCCGC CGCCGCCACC CAAACTGAGG TCGACGAAGA   
  
  
- GGATGAGGGG ACGGGCTCTG GAGTTGGGCC TCGAGAAGAG GAGCAGGCCC AAGAGCCCGT TCACCCGAAG   
  
  
- GTTGTAGGAG AACCTTCGGC GAGCACGGTA GAGACTCTCC TTGTGCTCGG CGGAGGTGGT CGAGGATACC   
  
  
- TACCAGTTGC TCGAGTCGAG GGGCATGCCA CTGCACCTCG TCTTCTATCG AAGTATGAAG CCGGTTCGGG   
  
  
- AGAAGTGGGC GTACTGGCTC AGTCCCCTGG CGTGGATGGC GTGGTACGCC AGGCGGCACC TGTTCTGGAA   
  
  
- GAGGAAACTC AGATGCTCTT TCTACCATGA GTTCAAGGTC CTCCAATCGG GAACCTGGTG GAAACCTGTG   
  
  
- CACCGTCGAT TACCTCGGGA GTAACTACGG AAACTGCCAC TTAGGTTTTA GGTGTAGTAG CTGTAGTCGT   
  
  
- GGTGTAAGAC GTGGGTCACC GGGTGGGAGG ACCTCCGCGA CCGGTGGGCT TACCTACTGC GCGGGATGGA   
  
  
- CTCCGACTGG TGACACCACC AGTTGTTCAG GCCCTTACTT CCAGGGTGGC CTCCGCCCAG GGTGTCCCAC   
  
  
- TACTTTCTCT AACCCTGGGC GGAGCTCTTC AAACGATCCG ATTACCCTCA CGGAAAGTTT AAATTACACC   
  
  
- AGGTGGTGCC CCGACTAAAT AGGCTGAACC TAAAGAGAGT CGACCTATAA TTGCTACTGC TCCGTAATCG   
  
  
- GTAGTTGACA CAGTTGAGCG AGGTGAGCCA GTTATAGGTG GCGGCACTGA GCCAGTAGAG CCGGAAGGCC   
  
  
- GCCGAGGTGG GATCCCACCA CTGGCACCAC CTCCTCCTCC GACTGGAACG ACTGCACCCC CGCCTCCCCA   
  
  
- TGCTCAAGAT GTCCCCCAAA TTACTCACAA ATTCCACCCA ATCCCAAATG AAACTCCGGG ACCTTCTCAC   
  
  
- GAAAGGATCC TGTTCGTTAC TCTTTGAGTA CGAGCTCGCC CGGCGTCCCG CCCGAGACCA CCTGGAGGAC   
  
  
- CGGACGGGGT TCGGGCGTAG TAGGCTCGCC TCCCTCCGTC GACGCGCCAC CAGGCCCTCC TACGTCCCCC   
  
  
- GGCCCAAGCC GGTGCACCCC AAGTCACTAC TCCAGACACT ACTGCAGTTC CGGGAGGATT CCTCCATGTT   
  
  
- CCTCCCCACC AGGTACTATG TTACAAGGCG GCGCCTACCG CCGCGGAGGC GGCCTTATAA GAACAGCACC   
  
  
- TCCCTAGTCG GCCACCACAC CCGGTCACGT ACCTTCGGAA T

+     CAAT-box

| Site Name | Organism | Position | Strand | Matrix score. | sequence | function |
| --- | --- | --- | --- | --- | --- | --- |
| CAAT-box | Nicotiana glutinosa | 3384 | + | 4 | CAAT |  |
| CAAT-box | Pisum sativum | 2861 | + | 5 | CAAAT | common cis-acting element in promoter and enhancer regions |
| CAAT-box | Nicotiana glutinosa | 846 | - | 4 | CAAT |  |
| CAAT-box | Nicotiana glutinosa | 464 | - | 4 | CAAT |  |
| CAAT-box | Nicotiana glutinosa | 350 | + | 4 | CAAT |  |
| CAAT-box | Nicotiana glutinosa | 819 | - | 4 | CAAT |  |
| CAAT-box | Pisum sativum | 344 | - | 5 | CAAAT | common cis-acting element in promoter and enhancer regions |
| CAAT-box | Arabidopsis thaliana | 1195 | + | 5 | CCAAT | common cis-acting element in promoter and enhancer regions |
| CAAT-box | Nicotiana glutinosa | 1700 | - | 4 | CAAT |  |
| CAAT-box | Nicotiana glutinosa | 2616 | - | 4 | CAAT |  |
| CAAT-box | Pisum sativum | 1885 | + | 5 | CAAAT | common cis-acting element in promoter and enhancer regions |
| CAAT-box | Nicotiana glutinosa | 1177 | + | 4 | CAAT |  |
| CAAT-box | Nicotiana glutinosa | 163 | + | 4 | CAAT |  |
| CAAT-box | Pisum sativum | 952 | - | 5 | CAAAT | common cis-acting element in promoter and enhancer regions |
| CAAT-box | Pisum sativum | 855 | - | 5 | CAAAT | common cis-acting element in promoter and enhancer regions |
| CAAT-box | Nicotiana glutinosa | 301 | + | 4 | CAAT |  |
| CAAT-box | Nicotiana glutinosa | 220 | - | 4 | CAAT |  |
| CAAT-box | Nicotiana glutinosa | 54 | - | 4 | CAAT |  |
| CAAT-box | Nicotiana glutinosa | 2975 | + | 4 | CAAT |  |
| CAAT-box | Arabidopsis thaliana | 1176 | + | 5 | CCAAT | common cis-acting element in promoter and enhancer regions |
| CAAT-box | Pisum sativum | 1034 | + | 5 | CAAAT | common cis-acting element in promoter and enhancer regions |
| CAAT-box | Pisum sativum | 787 | + | 5 | CAAAT | common cis-acting element in promoter and enhancer regions |
| CAAT-box | Nicotiana glutinosa | 3170 | + | 4 | CAAT |  |
| CAAT-box | Arabidopsis thaliana | 391 | - | 5 | CCAAT | common cis-acting element in promoter and enhancer regions |
| CAAT-box | Nicotiana glutinosa | 481 | + | 4 | CAAT |  |
| CAAT-box | Pisum sativum | 95 | - | 5 | CAAAT | common cis-acting element in promoter and enhancer regions |
| CAAT-box | Nicotiana glutinosa | 1196 | + | 4 | CAAT |  |
| CAAT-box | Arabidopsis thaliana | 240 | - | 5 | CCAAT | common cis-acting element in promoter and enhancer regions |
| CAAT-box | Arabidopsis thaliana | 332 | - | 5 | CCAAT | common cis-acting element in promoter and enhancer regions |
| CAAT-box | Nicotiana glutinosa | 1468 | + | 4 | CAAT |  |
| CAAT-box | Nicotiana glutinosa | 1839 | + | 4 | CAAT |  |
| CAAT-box | Pisum sativum | 148 | - | 5 | CAAAT | common cis-acting element in promoter and enhancer regions |
| CAAT-box | Pisum sativum | 59 | - | 5 | CAAAT | common cis-acting element in promoter and enhancer regions |
| CAAT-box | Arabidopsis thaliana | 1305 | + | 5 | CCAAT | common cis-acting element in promoter and enhancer regions |
| CAAT-box | Nicotiana glutinosa | 810 | - | 4 | CAAT |  |
| CAAT-box | Nicotiana glutinosa | 52 | + | 4 | CAAT |  |
| CAAT-box | Nicotiana glutinosa | 45 | - | 4 | CAAT |  |
| CAAT-box | Pisum sativum | 742 | - | 5 | CAAAT | common cis-acting element in promoter and enhancer regions |
| CAAT-box | Pisum sativum | 1711 | - | 5 | CAAAT | common cis-acting element in promoter and enhancer regions |
| CAAT-box | Nicotiana glutinosa | 1577 | - | 4 | CAAT |  |
| CAAT-box | Nicotiana glutinosa | 1851 | + | 4 | CAAT |  |
| CAAT-box | Nicotiana glutinosa | 817 | + | 4 | CAAT |  |
| CAAT-box | Nicotiana glutinosa | 507 | - | 4 | CAAT |  |
| CAAT-box | Nicotiana glutinosa | 1306 | + | 4 | CAAT |  |
| CAAT-box | Nicotiana glutinosa | 496 | + | 4 | CAAT |  |
| CAAT-box | Arabidopsis thaliana | 2814 | - | 5 | CCAAT | common cis-acting element in promoter and enhancer regions |
| CAAT-box | Nicotiana glutinosa | 1374 | + | 4 | CAAT |  |
| CAAT-box | Nicotiana glutinosa | 1321 | - | 4 | CAAT |  |
| CAAT-box | Arabidopsis thaliana | 1467 | + | 5 | CCAAT | common cis-acting element in promoter and enhancer regions |

>HU06G00358.1   
+ -Up\_Stream \_Len000GGGTTT TTTTAAACTC AAAATTAGTT ATTGTATCAA TTGAATTTGG AAAACTATTA   
  
  
+ TTTTCGACTT ATTATAGAGA ATTTGTTGTT AATGAATGCT GATAAATACT GATAATTAGT GTTGTGAGTG   
  
  
+ TGAATTTGTG ATGATGAGCA ATGATTAGCG TTAAAATTAT AACTAGGATA AATAATTAAG TGTTGATGAA   
  
  
+ TGTTGATTGA TGATGATGAG TACTGATTGG TGCGGTGCTG GTAATAAATA TTTATGATAA GTTAAAAAAA   
  
  
+ AAAAACACAT CCTAAACAAT CACTATGTAA TATACTAAAT GAAAAAAATT GGTAATGTGA TTTGACAATC   
  
  
+ TAAGGGTAAG GGTGTTTTAC CCACACATGT TATGAAATTG GCTTACTATT TACAAACTTT TTACAGTTAT   
  
  
+ ATGATTCTAC CTATGTGTTA TCATCTTTCC GTCGATGTAA TTGATTATAT TCAACACAAT TTAATTCATT   
  
  
+ ACAATGTAGC TAATTGATAC TCAAAATAAT GAACTATTAA TAAATAATTA ATTATAACTT TTAAAAGTTT   
  
  
+ GTTAATTTAA AATTATATAA TATATAAATT TAGGCTACAT TAATCAAAAA ATTAGGCGTG TTCATCGGTC   
  
  
+ CAAACTTGAA CCGGACCAGA CCGGACCGAA GATCGATTAA GAGAAAAAGT ACAGACTGAG GACCGGATCG   
  
  
+ AATAAGAGTC GCACCGGACC AGGATCGGAC CGGAAAAATT TGGACCAGAT CGGGACCGAT CGAGGTAGAC   
  
  
+ CGAATTTATT TTCAAATACG ATAGGAATAA AACTTATTGA TTCAATTGAT TTTGGCCAAA ATCATGCTAT   
  
  
+ TATTGATTTT ATTTGCTAAC TCTCAAGAGT ATTAGTTTGC ACTTCGCATT ATCTTTGGCC AAAAAAATCA   
  
  
+ AAAAGAAAGT CTCAAACTCT TAATAGTTTT TTATTTTATT TGGTTTATTT TTAAAATAGT CGGCCTTCGG   
  
  
+ TCCGGACTAG TGGACCGAAT AAGGTAATTT TTCGAATCGA GGACCAGACC AAATGCACTC AGTTCGAACC   
  
  
+ GGTTCAGTTC GGTCTGACTC GATTTTTGGT CCGGTCCAAG TTTGAACAGA ATAATGTACA CACCTAGAAA   
  
  
+ AAAAGAATTA TGCATATATA CTTAAGGCCC CCAACCCTTT TTCCCTTTCC GCCAATACTT TTTACCAAGT   
  
  
+ CCAATTCTTT TCTTCTTAGC TATGGCTAGT AGTTATCCCT CCCTAGCTTC CACCCTAAGT TTTGAGACAA   
  
  
+ AATCCCATCT ATGTTAATAT TTATTTCTTG CACATGTCAC CCAATACAAG GGTTTCATTG AAAAGAAAAG   
  
  
+ GTATTAGATG TAAGATATGT ATGTATATAA ATAGTTTTGC AATAGACTAG AAGGACTAAA AAGGAAAAAG   
  
  
+ GAAGTTTAAA AGCAAAAAAC TTTCCCCTCT AGAAAAAAGG AAACCAGATT CCCTTTTCTA CCCCAATTCC   
  
  
+ CATAGTCTTT ACTCTGTCTC TCTCTCTCTG TCTCTCTTAC TCATGATGTT GAGGGAGGAA GGAATGAATT   
  
  
+ AAGGACCCTA AGCTAAGATC CAACCGTAAG ATATTGCCCC TTTAATCACC CATTTCATAT CTCTATCCAC   
  
  
+ AGTTAAAAAC CCACCTCCTC CATCCACATA ACTGATATAT CTCTTCTACA CCATACTATA AGGAAGAAGA   
  
  
+ GAAAAATAGG AGTGTATTGT ATTTTTATTT GAGTAATTTA TTTTAAGGAG AGAAATTTAC ATGGTTGGGT   
  
  
+ TAAATGGATA CCTTGTTTAG ACTAGTGAGT TTCCAACAAC CACACCACCA CCATCACCAA CACCAACACC   
  
  
+ AACACCACCA ACAGCAATCT TCTGATCAAT CCTCTTTGAA TAATACTACT ACTTATTCCA CAAATTCAAG   
  
  
+ CAGTTCCAGA TCTTCCCAAC AAACCCATCA TAATCATAAT CATACTCAGT ATTATCACCA ACATCATCCT   
  
  
+ ACTTACCCCC ACCGCCACCA TCAGGAGGAA GAAGAAGAAT GCTTCAACTT TTACATGGAT GAAGAAAACT   
  
  
+ TCTCTTCTTC TTCCTCCCAC AACAAACATC ATCCTCAACC TCCTTCCTCC GCCACCACCT CCTCGTATTA   
  
  
+ CCCTTACTAC TCCACCCCCT TCGATGTTCC CTCTACCACC ACCACCTCCA CCTCCACCCC CACGCCCACC   
  
  
+ AACACCACCA CCACCACCCC CCCAACCCAG TATGGCGGCG GCGGCGGTGG GTTTGACTCC AGCTGCTTCT   
  
  
+ CCTACTCCCC TGCCCGAGAC CTCAACCCGG AGCTCTTCTC CTCGTCCGGG TTCTCGGGCA AGTGGGCTTC   
  
  
+ CAACATCCTC TTGGAAGCCG CTCGTGCCAT CTCTGAGAGG AACACGAGCC GCCTCCACCA GCTCCTATGG   
  
  
+ ATGGTCAACG AGCTCAGCTC CCCGTACGGT GACGTGGAGC AGAAGATAGC TTCATACTTC GGCCAAGCCC   
  
  
+ TCTTCACCCG CATGACCGAG TCAGGGGACC GCACCTACCG CACCATGCGG TCCGCCGTGG ACAAGACCTT   
  
  
+ CTCCTTTGAG TCTACGAGAA AGATGGTACT CAAGTTCCAG GAGGTTAGCC CTTGGACCAC CTTTGGACAC   
  
  
+ GTGGCAGCTA ATGGAGCCCT CATTGATGCC TTTGACGGTG AATCCAAAAT CCACATCATC GACATCAGCA   
  
  
+ CCACATTCTG CACCCAGTGG CCCACCCTCC TGGAGGCGCT GGCCACCCGA ATGGATGACG CGCCCTACCT   
  
  
+ GAGGCTGACC ACTGTGGTGG TCAACAAGTC CGGGAATGAA GGTCCCACCG GAGGCGGGTC CCACAGGGTG   
  
  
+ ATGAAAGAGA TTGGGACCCG CCTCGAGAAG TTTGCTAGGC TAATGGGAGT GCCTTTCAAA TTTAATGTGG   
  
  
+ TCCACCACGG GGCTGATTTA TCCGACTTGG ATTTCTCTCA GCTGGATATT AACGATGACG AGGCATTAGC   
  
  
+ CATCAACTGT GTCAACTCGC TCCACTCGGT CAATATCCAC CGCCGTGACT CGGTCATCTC GGCCTTCCGG   
  
  
+ CGGCTCCACC CTAGGGTGGT GACCGTGGTG GAGGAGGAGG CTGACCTTGC TGACGTGGGG GCGGAGGGGT   
  
  
+ ACGAGTTCTA CAGGGGGTTT AATGAGTGTT TAAGGTGGGT TAGGGTTTAC TTTGAGGCCC TGGAAGAGTG   
  
  
+ CTTTCCTAGG ACAAGCAATG AGAAACTCAT GCTCGAGCGG GCCGCAGGGC GGGCTCTGGT GGACCTCCTG   
  
  
+ GCCTGCCCCA AGCCCGCATC ATCCGAGCGG AGGGAGGCAG CTGCGCGGTG GTCCGGGAGG ATGCAGGGGG   
  
  
+ CCGGGTTCGG CCACGTGGGG TTCAGTGATG AGGTCTGTGA TGACGTCAAG GCCCTCCTAA GGAGGTACAA   
  
  
+ GGAGGGGTGG TCCATGATAC AATGTTCCGC CGCGGATGGC GGCGCCTCCG CCGGAATATT CTTGTCGTGG   
  
  
+ AGGGATCAGC CGGTGGTGTG GGCCAGTGCA TGGAAGCCTT A  

- -Up\_Stream \_Len000CCCAAA AAAATTTGAG TTTTAATCAA TAACATAGTT AACTTAAACC TTTTGATAAT   
  
  
- AAAAGCTGAA TAATATCTCT TAAACAACAA TTACTTACGA CTATTTATGA CTATTAATCA CAACACTCAC   
  
  
- ACTTAAACAC TACTACTCGT TACTAATCGC AATTTTAATA TTGATCCTAT TTATTAATTC ACAACTACTT   
  
  
- ACAACTAACT ACTACTACTC ATGACTAACC ACGCCACGAC CATTATTTAT AAATACTATT CAATTTTTTT   
  
  
- TTTTTGTGTA GGATTTGTTA GTGATACATT ATATGATTTA CTTTTTTTAA CCATTACACT AAACTGTTAG   
  
  
- ATTCCCATTC CCACAAAATG GGTGTGTACA ATACTTTAAC CGAATGATAA ATGTTTGAAA AATGTCAATA   
  
  
- TACTAAGATG GATACACAAT AGTAGAAAGG CAGCTACATT AACTAATATA AGTTGTGTTA AATTAAGTAA   
  
  
- TGTTACATCG ATTAACTATG AGTTTTATTA CTTGATAATT ATTTATTAAT TAATATTGAA AATTTTCAAA   
  
  
- CAATTAAATT TTAATATATT ATATATTTAA ATCCGATGTA ATTAGTTTTT TAATCCGCAC AAGTAGCCAG   
  
  
- GTTTGAACTT GGCCTGGTCT GGCCTGGCTT CTAGCTAATT CTCTTTTTCA TGTCTGACTC CTGGCCTAGC   
  
  
- TTATTCTCAG CGTGGCCTGG TCCTAGCCTG GCCTTTTTAA ACCTGGTCTA GCCCTGGCTA GCTCCATCTG   
  
  
- GCTTAAATAA AAGTTTATGC TATCCTTATT TTGAATAACT AAGTTAACTA AAACCGGTTT TAGTACGATA   
  
  
- ATAACTAAAA TAAACGATTG AGAGTTCTCA TAATCAAACG TGAAGCGTAA TAGAAACCGG TTTTTTTAGT   
  
  
- TTTTCTTTCA GAGTTTGAGA ATTATCAAAA AATAAAATAA ACCAAATAAA AATTTTATCA GCCGGAAGCC   
  
  
- AGGCCTGATC ACCTGGCTTA TTCCATTAAA AAGCTTAGCT CCTGGTCTGG TTTACGTGAG TCAAGCTTGG   
  
  
- CCAAGTCAAG CCAGACTGAG CTAAAAACCA GGCCAGGTTC AAACTTGTCT TATTACATGT GTGGATCTTT   
  
  
- TTTTCTTAAT ACGTATATAT GAATTCCGGG GGTTGGGAAA AAGGGAAAGG CGGTTATGAA AAATGGTTCA   
  
  
- GGTTAAGAAA AGAAGAATCG ATACCGATCA TCAATAGGGA GGGATCGAAG GTGGGATTCA AAACTCTGTT   
  
  
- TTAGGGTAGA TACAATTATA AATAAAGAAC GTGTACAGTG GGTTATGTTC CCAAAGTAAC TTTTCTTTTC   
  
  
- CATAATCTAC ATTCTATACA TACATATATT TATCAAAACG TTATCTGATC TTCCTGATTT TTCCTTTTTC   
  
  
- CTTCAAATTT TCGTTTTTTG AAAGGGGAGA TCTTTTTTCC TTTGGTCTAA GGGAAAAGAT GGGGTTAAGG   
  
  
- GTATCAGAAA TGAGACAGAG AGAGAGAGAC AGAGAGAATG AGTACTACAA CTCCCTCCTT CCTTACTTAA   
  
  
- TTCCTGGGAT TCGATTCTAG GTTGGCATTC TATAACGGGG AAATTAGTGG GTAAAGTATA GAGATAGGTG   
  
  
- TCAATTTTTG GGTGGAGGAG GTAGGTGTAT TGACTATATA GAGAAGATGT GGTATGATAT TCCTTCTTCT   
  
  
- CTTTTTATCC TCACATAACA TAAAAATAAA CTCATTAAAT AAAATTCCTC TCTTTAAATG TACCAACCCA   
  
  
- ATTTACCTAT GGAACAAATC TGATCACTCA AAGGTTGTTG GTGTGGTGGT GGTAGTGGTT GTGGTTGTGG   
  
  
- TTGTGGTGGT TGTCGTTAGA AGACTAGTTA GGAGAAACTT ATTATGATGA TGAATAAGGT GTTTAAGTTC   
  
  
- GTCAAGGTCT AGAAGGGTTG TTTGGGTAGT ATTAGTATTA GTATGAGTCA TAATAGTGGT TGTAGTAGGA   
  
  
- TGAATGGGGG TGGCGGTGGT AGTCCTCCTT CTTCTTCTTA CGAAGTTGAA AATGTACCTA CTTCTTTTGA   
  
  
- AGAGAAGAAG AAGGAGGGTG TTGTTTGTAG TAGGAGTTGG AGGAAGGAGG CGGTGGTGGA GGAGCATAAT   
  
  
- GGGAATGATG AGGTGGGGGA AGCTACAAGG GAGATGGTGG TGGTGGAGGT GGAGGTGGGG GTGCGGGTGG   
  
  
- TTGTGGTGGT GGTGGTGGGG GGGTTGGGTC ATACCGCCGC CGCCGCCACC CAAACTGAGG TCGACGAAGA   
  
  
- GGATGAGGGG ACGGGCTCTG GAGTTGGGCC TCGAGAAGAG GAGCAGGCCC AAGAGCCCGT TCACCCGAAG   
  
  
- GTTGTAGGAG AACCTTCGGC GAGCACGGTA GAGACTCTCC TTGTGCTCGG CGGAGGTGGT CGAGGATACC   
  
  
- TACCAGTTGC TCGAGTCGAG GGGCATGCCA CTGCACCTCG TCTTCTATCG AAGTATGAAG CCGGTTCGGG   
  
  
- AGAAGTGGGC GTACTGGCTC AGTCCCCTGG CGTGGATGGC GTGGTACGCC AGGCGGCACC TGTTCTGGAA   
  
  
- GAGGAAACTC AGATGCTCTT TCTACCATGA GTTCAAGGTC CTCCAATCGG GAACCTGGTG GAAACCTGTG   
  
  
- CACCGTCGAT TACCTCGGGA GTAACTACGG AAACTGCCAC TTAGGTTTTA GGTGTAGTAG CTGTAGTCGT   
  
  
- GGTGTAAGAC GTGGGTCACC GGGTGGGAGG ACCTCCGCGA CCGGTGGGCT TACCTACTGC GCGGGATGGA   
  
  
- CTCCGACTGG TGACACCACC AGTTGTTCAG GCCCTTACTT CCAGGGTGGC CTCCGCCCAG GGTGTCCCAC   
  
  
- TACTTTCTCT AACCCTGGGC GGAGCTCTTC AAACGATCCG ATTACCCTCA CGGAAAGTTT AAATTACACC   
  
  
- AGGTGGTGCC CCGACTAAAT AGGCTGAACC TAAAGAGAGT CGACCTATAA TTGCTACTGC TCCGTAATCG   
  
  
- GTAGTTGACA CAGTTGAGCG AGGTGAGCCA GTTATAGGTG GCGGCACTGA GCCAGTAGAG CCGGAAGGCC   
  
  
- GCCGAGGTGG GATCCCACCA CTGGCACCAC CTCCTCCTCC GACTGGAACG ACTGCACCCC CGCCTCCCCA   
  
  
- TGCTCAAGAT GTCCCCCAAA TTACTCACAA ATTCCACCCA ATCCCAAATG AAACTCCGGG ACCTTCTCAC   
  
  
- GAAAGGATCC TGTTCGTTAC TCTTTGAGTA CGAGCTCGCC CGGCGTCCCG CCCGAGACCA CCTGGAGGAC   
  
  
- CGGACGGGGT TCGGGCGTAG TAGGCTCGCC TCCCTCCGTC GACGCGCCAC CAGGCCCTCC TACGTCCCCC   
  
  
- GGCCCAAGCC GGTGCACCCC AAGTCACTAC TCCAGACACT ACTGCAGTTC CGGGAGGATT CCTCCATGTT   
  
  
- CCTCCCCACC AGGTACTATG TTACAAGGCG GCGCCTACCG CCGCGGAGGC GGCCTTATAA GAACAGCACC   
  
  
- TCCCTAGTCG GCCACCACAC CCGGTCACGT ACCTTCGGAA T

+     CAT-box

| Site Name | Organism | Position | Strand | Matrix score. | sequence | function |
| --- | --- | --- | --- | --- | --- | --- |
| CAT-box | Arabidopsis thaliana | 2680 | - | 6 | GCCACT | cis-acting regulatory element related to meristem expression |

>HU06G00358.1   
+ -Up\_Stream \_Len000GGGTTT TTTTAAACTC AAAATTAGTT ATTGTATCAA TTGAATTTGG AAAACTATTA   
  
  
+ TTTTCGACTT ATTATAGAGA ATTTGTTGTT AATGAATGCT GATAAATACT GATAATTAGT GTTGTGAGTG   
  
  
+ TGAATTTGTG ATGATGAGCA ATGATTAGCG TTAAAATTAT AACTAGGATA AATAATTAAG TGTTGATGAA   
  
  
+ TGTTGATTGA TGATGATGAG TACTGATTGG TGCGGTGCTG GTAATAAATA TTTATGATAA GTTAAAAAAA   
  
  
+ AAAAACACAT CCTAAACAAT CACTATGTAA TATACTAAAT GAAAAAAATT GGTAATGTGA TTTGACAATC   
  
  
+ TAAGGGTAAG GGTGTTTTAC CCACACATGT TATGAAATTG GCTTACTATT TACAAACTTT TTACAGTTAT   
  
  
+ ATGATTCTAC CTATGTGTTA TCATCTTTCC GTCGATGTAA TTGATTATAT TCAACACAAT TTAATTCATT   
  
  
+ ACAATGTAGC TAATTGATAC TCAAAATAAT GAACTATTAA TAAATAATTA ATTATAACTT TTAAAAGTTT   
  
  
+ GTTAATTTAA AATTATATAA TATATAAATT TAGGCTACAT TAATCAAAAA ATTAGGCGTG TTCATCGGTC   
  
  
+ CAAACTTGAA CCGGACCAGA CCGGACCGAA GATCGATTAA GAGAAAAAGT ACAGACTGAG GACCGGATCG   
  
  
+ AATAAGAGTC GCACCGGACC AGGATCGGAC CGGAAAAATT TGGACCAGAT CGGGACCGAT CGAGGTAGAC   
  
  
+ CGAATTTATT TTCAAATACG ATAGGAATAA AACTTATTGA TTCAATTGAT TTTGGCCAAA ATCATGCTAT   
  
  
+ TATTGATTTT ATTTGCTAAC TCTCAAGAGT ATTAGTTTGC ACTTCGCATT ATCTTTGGCC AAAAAAATCA   
  
  
+ AAAAGAAAGT CTCAAACTCT TAATAGTTTT TTATTTTATT TGGTTTATTT TTAAAATAGT CGGCCTTCGG   
  
  
+ TCCGGACTAG TGGACCGAAT AAGGTAATTT TTCGAATCGA GGACCAGACC AAATGCACTC AGTTCGAACC   
  
  
+ GGTTCAGTTC GGTCTGACTC GATTTTTGGT CCGGTCCAAG TTTGAACAGA ATAATGTACA CACCTAGAAA   
  
  
+ AAAAGAATTA TGCATATATA CTTAAGGCCC CCAACCCTTT TTCCCTTTCC GCCAATACTT TTTACCAAGT   
  
  
+ CCAATTCTTT TCTTCTTAGC TATGGCTAGT AGTTATCCCT CCCTAGCTTC CACCCTAAGT TTTGAGACAA   
  
  
+ AATCCCATCT ATGTTAATAT TTATTTCTTG CACATGTCAC CCAATACAAG GGTTTCATTG AAAAGAAAAG   
  
  
+ GTATTAGATG TAAGATATGT ATGTATATAA ATAGTTTTGC AATAGACTAG AAGGACTAAA AAGGAAAAAG   
  
  
+ GAAGTTTAAA AGCAAAAAAC TTTCCCCTCT AGAAAAAAGG AAACCAGATT CCCTTTTCTA CCCCAATTCC   
  
  
+ CATAGTCTTT ACTCTGTCTC TCTCTCTCTG TCTCTCTTAC TCATGATGTT GAGGGAGGAA GGAATGAATT   
  
  
+ AAGGACCCTA AGCTAAGATC CAACCGTAAG ATATTGCCCC TTTAATCACC CATTTCATAT CTCTATCCAC   
  
  
+ AGTTAAAAAC CCACCTCCTC CATCCACATA ACTGATATAT CTCTTCTACA CCATACTATA AGGAAGAAGA   
  
  
+ GAAAAATAGG AGTGTATTGT ATTTTTATTT GAGTAATTTA TTTTAAGGAG AGAAATTTAC ATGGTTGGGT   
  
  
+ TAAATGGATA CCTTGTTTAG ACTAGTGAGT TTCCAACAAC CACACCACCA CCATCACCAA CACCAACACC   
  
  
+ AACACCACCA ACAGCAATCT TCTGATCAAT CCTCTTTGAA TAATACTACT ACTTATTCCA CAAATTCAAG   
  
  
+ CAGTTCCAGA TCTTCCCAAC AAACCCATCA TAATCATAAT CATACTCAGT ATTATCACCA ACATCATCCT   
  
  
+ ACTTACCCCC ACCGCCACCA TCAGGAGGAA GAAGAAGAAT GCTTCAACTT TTACATGGAT GAAGAAAACT   
  
  
+ TCTCTTCTTC TTCCTCCCAC AACAAACATC ATCCTCAACC TCCTTCCTCC GCCACCACCT CCTCGTATTA   
  
  
+ CCCTTACTAC TCCACCCCCT TCGATGTTCC CTCTACCACC ACCACCTCCA CCTCCACCCC CACGCCCACC   
  
  
+ AACACCACCA CCACCACCCC CCCAACCCAG TATGGCGGCG GCGGCGGTGG GTTTGACTCC AGCTGCTTCT   
  
  
+ CCTACTCCCC TGCCCGAGAC CTCAACCCGG AGCTCTTCTC CTCGTCCGGG TTCTCGGGCA AGTGGGCTTC   
  
  
+ CAACATCCTC TTGGAAGCCG CTCGTGCCAT CTCTGAGAGG AACACGAGCC GCCTCCACCA GCTCCTATGG   
  
  
+ ATGGTCAACG AGCTCAGCTC CCCGTACGGT GACGTGGAGC AGAAGATAGC TTCATACTTC GGCCAAGCCC   
  
  
+ TCTTCACCCG CATGACCGAG TCAGGGGACC GCACCTACCG CACCATGCGG TCCGCCGTGG ACAAGACCTT   
  
  
+ CTCCTTTGAG TCTACGAGAA AGATGGTACT CAAGTTCCAG GAGGTTAGCC CTTGGACCAC CTTTGGACAC   
  
  
+ GTGGCAGCTA ATGGAGCCCT CATTGATGCC TTTGACGGTG AATCCAAAAT CCACATCATC GACATCAGCA   
  
  
+ CCACATTCTG CACCCAGTGG CCCACCCTCC TGGAGGCGCT GGCCACCCGA ATGGATGACG CGCCCTACCT   
  
  
+ GAGGCTGACC ACTGTGGTGG TCAACAAGTC CGGGAATGAA GGTCCCACCG GAGGCGGGTC CCACAGGGTG   
  
  
+ ATGAAAGAGA TTGGGACCCG CCTCGAGAAG TTTGCTAGGC TAATGGGAGT GCCTTTCAAA TTTAATGTGG   
  
  
+ TCCACCACGG GGCTGATTTA TCCGACTTGG ATTTCTCTCA GCTGGATATT AACGATGACG AGGCATTAGC   
  
  
+ CATCAACTGT GTCAACTCGC TCCACTCGGT CAATATCCAC CGCCGTGACT CGGTCATCTC GGCCTTCCGG   
  
  
+ CGGCTCCACC CTAGGGTGGT GACCGTGGTG GAGGAGGAGG CTGACCTTGC TGACGTGGGG GCGGAGGGGT   
  
  
+ ACGAGTTCTA CAGGGGGTTT AATGAGTGTT TAAGGTGGGT TAGGGTTTAC TTTGAGGCCC TGGAAGAGTG   
  
  
+ CTTTCCTAGG ACAAGCAATG AGAAACTCAT GCTCGAGCGG GCCGCAGGGC GGGCTCTGGT GGACCTCCTG   
  
  
+ GCCTGCCCCA AGCCCGCATC ATCCGAGCGG AGGGAGGCAG CTGCGCGGTG GTCCGGGAGG ATGCAGGGGG   
  
  
+ CCGGGTTCGG CCACGTGGGG TTCAGTGATG AGGTCTGTGA TGACGTCAAG GCCCTCCTAA GGAGGTACAA   
  
  
+ GGAGGGGTGG TCCATGATAC AATGTTCCGC CGCGGATGGC GGCGCCTCCG CCGGAATATT CTTGTCGTGG   
  
  
+ AGGGATCAGC CGGTGGTGTG GGCCAGTGCA TGGAAGCCTT A  

- -Up\_Stream \_Len000CCCAAA AAAATTTGAG TTTTAATCAA TAACATAGTT AACTTAAACC TTTTGATAAT   
  
  
- AAAAGCTGAA TAATATCTCT TAAACAACAA TTACTTACGA CTATTTATGA CTATTAATCA CAACACTCAC   
  
  
- ACTTAAACAC TACTACTCGT TACTAATCGC AATTTTAATA TTGATCCTAT TTATTAATTC ACAACTACTT   
  
  
- ACAACTAACT ACTACTACTC ATGACTAACC ACGCCACGAC CATTATTTAT AAATACTATT CAATTTTTTT   
  
  
- TTTTTGTGTA GGATTTGTTA GTGATACATT ATATGATTTA CTTTTTTTAA CCATTACACT AAACTGTTAG   
  
  
- ATTCCCATTC CCACAAAATG GGTGTGTACA ATACTTTAAC CGAATGATAA ATGTTTGAAA AATGTCAATA   
  
  
- TACTAAGATG GATACACAAT AGTAGAAAGG CAGCTACATT AACTAATATA AGTTGTGTTA AATTAAGTAA   
  
  
- TGTTACATCG ATTAACTATG AGTTTTATTA CTTGATAATT ATTTATTAAT TAATATTGAA AATTTTCAAA   
  
  
- CAATTAAATT TTAATATATT ATATATTTAA ATCCGATGTA ATTAGTTTTT TAATCCGCAC AAGTAGCCAG   
  
  
- GTTTGAACTT GGCCTGGTCT GGCCTGGCTT CTAGCTAATT CTCTTTTTCA TGTCTGACTC CTGGCCTAGC   
  
  
- TTATTCTCAG CGTGGCCTGG TCCTAGCCTG GCCTTTTTAA ACCTGGTCTA GCCCTGGCTA GCTCCATCTG   
  
  
- GCTTAAATAA AAGTTTATGC TATCCTTATT TTGAATAACT AAGTTAACTA AAACCGGTTT TAGTACGATA   
  
  
- ATAACTAAAA TAAACGATTG AGAGTTCTCA TAATCAAACG TGAAGCGTAA TAGAAACCGG TTTTTTTAGT   
  
  
- TTTTCTTTCA GAGTTTGAGA ATTATCAAAA AATAAAATAA ACCAAATAAA AATTTTATCA GCCGGAAGCC   
  
  
- AGGCCTGATC ACCTGGCTTA TTCCATTAAA AAGCTTAGCT CCTGGTCTGG TTTACGTGAG TCAAGCTTGG   
  
  
- CCAAGTCAAG CCAGACTGAG CTAAAAACCA GGCCAGGTTC AAACTTGTCT TATTACATGT GTGGATCTTT   
  
  
- TTTTCTTAAT ACGTATATAT GAATTCCGGG GGTTGGGAAA AAGGGAAAGG CGGTTATGAA AAATGGTTCA   
  
  
- GGTTAAGAAA AGAAGAATCG ATACCGATCA TCAATAGGGA GGGATCGAAG GTGGGATTCA AAACTCTGTT   
  
  
- TTAGGGTAGA TACAATTATA AATAAAGAAC GTGTACAGTG GGTTATGTTC CCAAAGTAAC TTTTCTTTTC   
  
  
- CATAATCTAC ATTCTATACA TACATATATT TATCAAAACG TTATCTGATC TTCCTGATTT TTCCTTTTTC   
  
  
- CTTCAAATTT TCGTTTTTTG AAAGGGGAGA TCTTTTTTCC TTTGGTCTAA GGGAAAAGAT GGGGTTAAGG   
  
  
- GTATCAGAAA TGAGACAGAG AGAGAGAGAC AGAGAGAATG AGTACTACAA CTCCCTCCTT CCTTACTTAA   
  
  
- TTCCTGGGAT TCGATTCTAG GTTGGCATTC TATAACGGGG AAATTAGTGG GTAAAGTATA GAGATAGGTG   
  
  
- TCAATTTTTG GGTGGAGGAG GTAGGTGTAT TGACTATATA GAGAAGATGT GGTATGATAT TCCTTCTTCT   
  
  
- CTTTTTATCC TCACATAACA TAAAAATAAA CTCATTAAAT AAAATTCCTC TCTTTAAATG TACCAACCCA   
  
  
- ATTTACCTAT GGAACAAATC TGATCACTCA AAGGTTGTTG GTGTGGTGGT GGTAGTGGTT GTGGTTGTGG   
  
  
- TTGTGGTGGT TGTCGTTAGA AGACTAGTTA GGAGAAACTT ATTATGATGA TGAATAAGGT GTTTAAGTTC   
  
  
- GTCAAGGTCT AGAAGGGTTG TTTGGGTAGT ATTAGTATTA GTATGAGTCA TAATAGTGGT TGTAGTAGGA   
  
  
- TGAATGGGGG TGGCGGTGGT AGTCCTCCTT CTTCTTCTTA CGAAGTTGAA AATGTACCTA CTTCTTTTGA   
  
  
- AGAGAAGAAG AAGGAGGGTG TTGTTTGTAG TAGGAGTTGG AGGAAGGAGG CGGTGGTGGA GGAGCATAAT   
  
  
- GGGAATGATG AGGTGGGGGA AGCTACAAGG GAGATGGTGG TGGTGGAGGT GGAGGTGGGG GTGCGGGTGG   
  
  
- TTGTGGTGGT GGTGGTGGGG GGGTTGGGTC ATACCGCCGC CGCCGCCACC CAAACTGAGG TCGACGAAGA   
  
  
- GGATGAGGGG ACGGGCTCTG GAGTTGGGCC TCGAGAAGAG GAGCAGGCCC AAGAGCCCGT TCACCCGAAG   
  
  
- GTTGTAGGAG AACCTTCGGC GAGCACGGTA GAGACTCTCC TTGTGCTCGG CGGAGGTGGT CGAGGATACC   
  
  
- TACCAGTTGC TCGAGTCGAG GGGCATGCCA CTGCACCTCG TCTTCTATCG AAGTATGAAG CCGGTTCGGG   
  
  
- AGAAGTGGGC GTACTGGCTC AGTCCCCTGG CGTGGATGGC GTGGTACGCC AGGCGGCACC TGTTCTGGAA   
  
  
- GAGGAAACTC AGATGCTCTT TCTACCATGA GTTCAAGGTC CTCCAATCGG GAACCTGGTG GAAACCTGTG   
  
  
- CACCGTCGAT TACCTCGGGA GTAACTACGG AAACTGCCAC TTAGGTTTTA GGTGTAGTAG CTGTAGTCGT   
  
  
- GGTGTAAGAC GTGGGTCACC GGGTGGGAGG ACCTCCGCGA CCGGTGGGCT TACCTACTGC GCGGGATGGA   
  
  
- CTCCGACTGG TGACACCACC AGTTGTTCAG GCCCTTACTT CCAGGGTGGC CTCCGCCCAG GGTGTCCCAC   
  
  
- TACTTTCTCT AACCCTGGGC GGAGCTCTTC AAACGATCCG ATTACCCTCA CGGAAAGTTT AAATTACACC   
  
  
- AGGTGGTGCC CCGACTAAAT AGGCTGAACC TAAAGAGAGT CGACCTATAA TTGCTACTGC TCCGTAATCG   
  
  
- GTAGTTGACA CAGTTGAGCG AGGTGAGCCA GTTATAGGTG GCGGCACTGA GCCAGTAGAG CCGGAAGGCC   
  
  
- GCCGAGGTGG GATCCCACCA CTGGCACCAC CTCCTCCTCC GACTGGAACG ACTGCACCCC CGCCTCCCCA   
  
  
- TGCTCAAGAT GTCCCCCAAA TTACTCACAA ATTCCACCCA ATCCCAAATG AAACTCCGGG ACCTTCTCAC   
  
  
- GAAAGGATCC TGTTCGTTAC TCTTTGAGTA CGAGCTCGCC CGGCGTCCCG CCCGAGACCA CCTGGAGGAC   
  
  
- CGGACGGGGT TCGGGCGTAG TAGGCTCGCC TCCCTCCGTC GACGCGCCAC CAGGCCCTCC TACGTCCCCC   
  
  
- GGCCCAAGCC GGTGCACCCC AAGTCACTAC TCCAGACACT ACTGCAGTTC CGGGAGGATT CCTCCATGTT   
  
  
- CCTCCCCACC AGGTACTATG TTACAAGGCG GCGCCTACCG CCGCGGAGGC GGCCTTATAA GAACAGCACC   
  
  
- TCCCTAGTCG GCCACCACAC CCGGTCACGT ACCTTCGGAA T

+     CGTCA-motif

| Site Name | Organism | Position | Strand | Matrix score. | sequence | function |
| --- | --- | --- | --- | --- | --- | --- |
| CGTCA-motif | Hordeum vulgare | 2720 | - | 5 | CGTCA | cis-acting regulatory element involved in the MeJA-responsiveness |
| CGTCA-motif | Hordeum vulgare | 3338 | + | 5 | CGTCA | cis-acting regulatory element involved in the MeJA-responsiveness |
| CGTCA-motif | Hordeum vulgare | 3065 | - | 5 | CGTCA | cis-acting regulatory element involved in the MeJA-responsiveness |
| CGTCA-motif | Hordeum vulgare | 3335 | - | 5 | CGTCA | cis-acting regulatory element involved in the MeJA-responsiveness |
| CGTCA-motif | Hordeum vulgare | 2414 | - | 5 | CGTCA | cis-acting regulatory element involved in the MeJA-responsiveness |
| CGTCA-motif | Hordeum vulgare | 2627 | - | 5 | CGTCA | cis-acting regulatory element involved in the MeJA-responsiveness |
| CGTCA-motif | Hordeum vulgare | 2930 | - | 5 | CGTCA | cis-acting regulatory element involved in the MeJA-responsiveness |

>HU06G00358.1   
+ -Up\_Stream \_Len000GGGTTT TTTTAAACTC AAAATTAGTT ATTGTATCAA TTGAATTTGG AAAACTATTA   
  
  
+ TTTTCGACTT ATTATAGAGA ATTTGTTGTT AATGAATGCT GATAAATACT GATAATTAGT GTTGTGAGTG   
  
  
+ TGAATTTGTG ATGATGAGCA ATGATTAGCG TTAAAATTAT AACTAGGATA AATAATTAAG TGTTGATGAA   
  
  
+ TGTTGATTGA TGATGATGAG TACTGATTGG TGCGGTGCTG GTAATAAATA TTTATGATAA GTTAAAAAAA   
  
  
+ AAAAACACAT CCTAAACAAT CACTATGTAA TATACTAAAT GAAAAAAATT GGTAATGTGA TTTGACAATC   
  
  
+ TAAGGGTAAG GGTGTTTTAC CCACACATGT TATGAAATTG GCTTACTATT TACAAACTTT TTACAGTTAT   
  
  
+ ATGATTCTAC CTATGTGTTA TCATCTTTCC GTCGATGTAA TTGATTATAT TCAACACAAT TTAATTCATT   
  
  
+ ACAATGTAGC TAATTGATAC TCAAAATAAT GAACTATTAA TAAATAATTA ATTATAACTT TTAAAAGTTT   
  
  
+ GTTAATTTAA AATTATATAA TATATAAATT TAGGCTACAT TAATCAAAAA ATTAGGCGTG TTCATCGGTC   
  
  
+ CAAACTTGAA CCGGACCAGA CCGGACCGAA GATCGATTAA GAGAAAAAGT ACAGACTGAG GACCGGATCG   
  
  
+ AATAAGAGTC GCACCGGACC AGGATCGGAC CGGAAAAATT TGGACCAGAT CGGGACCGAT CGAGGTAGAC   
  
  
+ CGAATTTATT TTCAAATACG ATAGGAATAA AACTTATTGA TTCAATTGAT TTTGGCCAAA ATCATGCTAT   
  
  
+ TATTGATTTT ATTTGCTAAC TCTCAAGAGT ATTAGTTTGC ACTTCGCATT ATCTTTGGCC AAAAAAATCA   
  
  
+ AAAAGAAAGT CTCAAACTCT TAATAGTTTT TTATTTTATT TGGTTTATTT TTAAAATAGT CGGCCTTCGG   
  
  
+ TCCGGACTAG TGGACCGAAT AAGGTAATTT TTCGAATCGA GGACCAGACC AAATGCACTC AGTTCGAACC   
  
  
+ GGTTCAGTTC GGTCTGACTC GATTTTTGGT CCGGTCCAAG TTTGAACAGA ATAATGTACA CACCTAGAAA   
  
  
+ AAAAGAATTA TGCATATATA CTTAAGGCCC CCAACCCTTT TTCCCTTTCC GCCAATACTT TTTACCAAGT   
  
  
+ CCAATTCTTT TCTTCTTAGC TATGGCTAGT AGTTATCCCT CCCTAGCTTC CACCCTAAGT TTTGAGACAA   
  
  
+ AATCCCATCT ATGTTAATAT TTATTTCTTG CACATGTCAC CCAATACAAG GGTTTCATTG AAAAGAAAAG   
  
  
+ GTATTAGATG TAAGATATGT ATGTATATAA ATAGTTTTGC AATAGACTAG AAGGACTAAA AAGGAAAAAG   
  
  
+ GAAGTTTAAA AGCAAAAAAC TTTCCCCTCT AGAAAAAAGG AAACCAGATT CCCTTTTCTA CCCCAATTCC   
  
  
+ CATAGTCTTT ACTCTGTCTC TCTCTCTCTG TCTCTCTTAC TCATGATGTT GAGGGAGGAA GGAATGAATT   
  
  
+ AAGGACCCTA AGCTAAGATC CAACCGTAAG ATATTGCCCC TTTAATCACC CATTTCATAT CTCTATCCAC   
  
  
+ AGTTAAAAAC CCACCTCCTC CATCCACATA ACTGATATAT CTCTTCTACA CCATACTATA AGGAAGAAGA   
  
  
+ GAAAAATAGG AGTGTATTGT ATTTTTATTT GAGTAATTTA TTTTAAGGAG AGAAATTTAC ATGGTTGGGT   
  
  
+ TAAATGGATA CCTTGTTTAG ACTAGTGAGT TTCCAACAAC CACACCACCA CCATCACCAA CACCAACACC   
  
  
+ AACACCACCA ACAGCAATCT TCTGATCAAT CCTCTTTGAA TAATACTACT ACTTATTCCA CAAATTCAAG   
  
  
+ CAGTTCCAGA TCTTCCCAAC AAACCCATCA TAATCATAAT CATACTCAGT ATTATCACCA ACATCATCCT   
  
  
+ ACTTACCCCC ACCGCCACCA TCAGGAGGAA GAAGAAGAAT GCTTCAACTT TTACATGGAT GAAGAAAACT   
  
  
+ TCTCTTCTTC TTCCTCCCAC AACAAACATC ATCCTCAACC TCCTTCCTCC GCCACCACCT CCTCGTATTA   
  
  
+ CCCTTACTAC TCCACCCCCT TCGATGTTCC CTCTACCACC ACCACCTCCA CCTCCACCCC CACGCCCACC   
  
  
+ AACACCACCA CCACCACCCC CCCAACCCAG TATGGCGGCG GCGGCGGTGG GTTTGACTCC AGCTGCTTCT   
  
  
+ CCTACTCCCC TGCCCGAGAC CTCAACCCGG AGCTCTTCTC CTCGTCCGGG TTCTCGGGCA AGTGGGCTTC   
  
  
+ CAACATCCTC TTGGAAGCCG CTCGTGCCAT CTCTGAGAGG AACACGAGCC GCCTCCACCA GCTCCTATGG   
  
  
+ ATGGTCAACG AGCTCAGCTC CCCGTACGGT GACGTGGAGC AGAAGATAGC TTCATACTTC GGCCAAGCCC   
  
  
+ TCTTCACCCG CATGACCGAG TCAGGGGACC GCACCTACCG CACCATGCGG TCCGCCGTGG ACAAGACCTT   
  
  
+ CTCCTTTGAG TCTACGAGAA AGATGGTACT CAAGTTCCAG GAGGTTAGCC CTTGGACCAC CTTTGGACAC   
  
  
+ GTGGCAGCTA ATGGAGCCCT CATTGATGCC TTTGACGGTG AATCCAAAAT CCACATCATC GACATCAGCA   
  
  
+ CCACATTCTG CACCCAGTGG CCCACCCTCC TGGAGGCGCT GGCCACCCGA ATGGATGACG CGCCCTACCT   
  
  
+ GAGGCTGACC ACTGTGGTGG TCAACAAGTC CGGGAATGAA GGTCCCACCG GAGGCGGGTC CCACAGGGTG   
  
  
+ ATGAAAGAGA TTGGGACCCG CCTCGAGAAG TTTGCTAGGC TAATGGGAGT GCCTTTCAAA TTTAATGTGG   
  
  
+ TCCACCACGG GGCTGATTTA TCCGACTTGG ATTTCTCTCA GCTGGATATT AACGATGACG AGGCATTAGC   
  
  
+ CATCAACTGT GTCAACTCGC TCCACTCGGT CAATATCCAC CGCCGTGACT CGGTCATCTC GGCCTTCCGG   
  
  
+ CGGCTCCACC CTAGGGTGGT GACCGTGGTG GAGGAGGAGG CTGACCTTGC TGACGTGGGG GCGGAGGGGT   
  
  
+ ACGAGTTCTA CAGGGGGTTT AATGAGTGTT TAAGGTGGGT TAGGGTTTAC TTTGAGGCCC TGGAAGAGTG   
  
  
+ CTTTCCTAGG ACAAGCAATG AGAAACTCAT GCTCGAGCGG GCCGCAGGGC GGGCTCTGGT GGACCTCCTG   
  
  
+ GCCTGCCCCA AGCCCGCATC ATCCGAGCGG AGGGAGGCAG CTGCGCGGTG GTCCGGGAGG ATGCAGGGGG   
  
  
+ CCGGGTTCGG CCACGTGGGG TTCAGTGATG AGGTCTGTGA TGACGTCAAG GCCCTCCTAA GGAGGTACAA   
  
  
+ GGAGGGGTGG TCCATGATAC AATGTTCCGC CGCGGATGGC GGCGCCTCCG CCGGAATATT CTTGTCGTGG   
  
  
+ AGGGATCAGC CGGTGGTGTG GGCCAGTGCA TGGAAGCCTT A  

- -Up\_Stream \_Len000CCCAAA AAAATTTGAG TTTTAATCAA TAACATAGTT AACTTAAACC TTTTGATAAT   
  
  
- AAAAGCTGAA TAATATCTCT TAAACAACAA TTACTTACGA CTATTTATGA CTATTAATCA CAACACTCAC   
  
  
- ACTTAAACAC TACTACTCGT TACTAATCGC AATTTTAATA TTGATCCTAT TTATTAATTC ACAACTACTT   
  
  
- ACAACTAACT ACTACTACTC ATGACTAACC ACGCCACGAC CATTATTTAT AAATACTATT CAATTTTTTT   
  
  
- TTTTTGTGTA GGATTTGTTA GTGATACATT ATATGATTTA CTTTTTTTAA CCATTACACT AAACTGTTAG   
  
  
- ATTCCCATTC CCACAAAATG GGTGTGTACA ATACTTTAAC CGAATGATAA ATGTTTGAAA AATGTCAATA   
  
  
- TACTAAGATG GATACACAAT AGTAGAAAGG CAGCTACATT AACTAATATA AGTTGTGTTA AATTAAGTAA   
  
  
- TGTTACATCG ATTAACTATG AGTTTTATTA CTTGATAATT ATTTATTAAT TAATATTGAA AATTTTCAAA   
  
  
- CAATTAAATT TTAATATATT ATATATTTAA ATCCGATGTA ATTAGTTTTT TAATCCGCAC AAGTAGCCAG   
  
  
- GTTTGAACTT GGCCTGGTCT GGCCTGGCTT CTAGCTAATT CTCTTTTTCA TGTCTGACTC CTGGCCTAGC   
  
  
- TTATTCTCAG CGTGGCCTGG TCCTAGCCTG GCCTTTTTAA ACCTGGTCTA GCCCTGGCTA GCTCCATCTG   
  
  
- GCTTAAATAA AAGTTTATGC TATCCTTATT TTGAATAACT AAGTTAACTA AAACCGGTTT TAGTACGATA   
  
  
- ATAACTAAAA TAAACGATTG AGAGTTCTCA TAATCAAACG TGAAGCGTAA TAGAAACCGG TTTTTTTAGT   
  
  
- TTTTCTTTCA GAGTTTGAGA ATTATCAAAA AATAAAATAA ACCAAATAAA AATTTTATCA GCCGGAAGCC   
  
  
- AGGCCTGATC ACCTGGCTTA TTCCATTAAA AAGCTTAGCT CCTGGTCTGG TTTACGTGAG TCAAGCTTGG   
  
  
- CCAAGTCAAG CCAGACTGAG CTAAAAACCA GGCCAGGTTC AAACTTGTCT TATTACATGT GTGGATCTTT   
  
  
- TTTTCTTAAT ACGTATATAT GAATTCCGGG GGTTGGGAAA AAGGGAAAGG CGGTTATGAA AAATGGTTCA   
  
  
- GGTTAAGAAA AGAAGAATCG ATACCGATCA TCAATAGGGA GGGATCGAAG GTGGGATTCA AAACTCTGTT   
  
  
- TTAGGGTAGA TACAATTATA AATAAAGAAC GTGTACAGTG GGTTATGTTC CCAAAGTAAC TTTTCTTTTC   
  
  
- CATAATCTAC ATTCTATACA TACATATATT TATCAAAACG TTATCTGATC TTCCTGATTT TTCCTTTTTC   
  
  
- CTTCAAATTT TCGTTTTTTG AAAGGGGAGA TCTTTTTTCC TTTGGTCTAA GGGAAAAGAT GGGGTTAAGG   
  
  
- GTATCAGAAA TGAGACAGAG AGAGAGAGAC AGAGAGAATG AGTACTACAA CTCCCTCCTT CCTTACTTAA   
  
  
- TTCCTGGGAT TCGATTCTAG GTTGGCATTC TATAACGGGG AAATTAGTGG GTAAAGTATA GAGATAGGTG   
  
  
- TCAATTTTTG GGTGGAGGAG GTAGGTGTAT TGACTATATA GAGAAGATGT GGTATGATAT TCCTTCTTCT   
  
  
- CTTTTTATCC TCACATAACA TAAAAATAAA CTCATTAAAT AAAATTCCTC TCTTTAAATG TACCAACCCA   
  
  
- ATTTACCTAT GGAACAAATC TGATCACTCA AAGGTTGTTG GTGTGGTGGT GGTAGTGGTT GTGGTTGTGG   
  
  
- TTGTGGTGGT TGTCGTTAGA AGACTAGTTA GGAGAAACTT ATTATGATGA TGAATAAGGT GTTTAAGTTC   
  
  
- GTCAAGGTCT AGAAGGGTTG TTTGGGTAGT ATTAGTATTA GTATGAGTCA TAATAGTGGT TGTAGTAGGA   
  
  
- TGAATGGGGG TGGCGGTGGT AGTCCTCCTT CTTCTTCTTA CGAAGTTGAA AATGTACCTA CTTCTTTTGA   
  
  
- AGAGAAGAAG AAGGAGGGTG TTGTTTGTAG TAGGAGTTGG AGGAAGGAGG CGGTGGTGGA GGAGCATAAT   
  
  
- GGGAATGATG AGGTGGGGGA AGCTACAAGG GAGATGGTGG TGGTGGAGGT GGAGGTGGGG GTGCGGGTGG   
  
  
- TTGTGGTGGT GGTGGTGGGG GGGTTGGGTC ATACCGCCGC CGCCGCCACC CAAACTGAGG TCGACGAAGA   
  
  
- GGATGAGGGG ACGGGCTCTG GAGTTGGGCC TCGAGAAGAG GAGCAGGCCC AAGAGCCCGT TCACCCGAAG   
  
  
- GTTGTAGGAG AACCTTCGGC GAGCACGGTA GAGACTCTCC TTGTGCTCGG CGGAGGTGGT CGAGGATACC   
  
  
- TACCAGTTGC TCGAGTCGAG GGGCATGCCA CTGCACCTCG TCTTCTATCG AAGTATGAAG CCGGTTCGGG   
  
  
- AGAAGTGGGC GTACTGGCTC AGTCCCCTGG CGTGGATGGC GTGGTACGCC AGGCGGCACC TGTTCTGGAA   
  
  
- GAGGAAACTC AGATGCTCTT TCTACCATGA GTTCAAGGTC CTCCAATCGG GAACCTGGTG GAAACCTGTG   
  
  
- CACCGTCGAT TACCTCGGGA GTAACTACGG AAACTGCCAC TTAGGTTTTA GGTGTAGTAG CTGTAGTCGT   
  
  
- GGTGTAAGAC GTGGGTCACC GGGTGGGAGG ACCTCCGCGA CCGGTGGGCT TACCTACTGC GCGGGATGGA   
  
  
- CTCCGACTGG TGACACCACC AGTTGTTCAG GCCCTTACTT CCAGGGTGGC CTCCGCCCAG GGTGTCCCAC   
  
  
- TACTTTCTCT AACCCTGGGC GGAGCTCTTC AAACGATCCG ATTACCCTCA CGGAAAGTTT AAATTACACC   
  
  
- AGGTGGTGCC CCGACTAAAT AGGCTGAACC TAAAGAGAGT CGACCTATAA TTGCTACTGC TCCGTAATCG   
  
  
- GTAGTTGACA CAGTTGAGCG AGGTGAGCCA GTTATAGGTG GCGGCACTGA GCCAGTAGAG CCGGAAGGCC   
  
  
- GCCGAGGTGG GATCCCACCA CTGGCACCAC CTCCTCCTCC GACTGGAACG ACTGCACCCC CGCCTCCCCA   
  
  
- TGCTCAAGAT GTCCCCCAAA TTACTCACAA ATTCCACCCA ATCCCAAATG AAACTCCGGG ACCTTCTCAC   
  
  
- GAAAGGATCC TGTTCGTTAC TCTTTGAGTA CGAGCTCGCC CGGCGTCCCG CCCGAGACCA CCTGGAGGAC   
  
  
- CGGACGGGGT TCGGGCGTAG TAGGCTCGCC TCCCTCCGTC GACGCGCCAC CAGGCCCTCC TACGTCCCCC   
  
  
- GGCCCAAGCC GGTGCACCCC AAGTCACTAC TCCAGACACT ACTGCAGTTC CGGGAGGATT CCTCCATGTT   
  
  
- CCTCCCCACC AGGTACTATG TTACAAGGCG GCGCCTACCG CCGCGGAGGC GGCCTTATAA GAACAGCACC   
  
  
- TCCCTAGTCG GCCACCACAC CCGGTCACGT ACCTTCGGAA T

+     DRE core

| Site Name | Organism | Position | Strand | Matrix score. | sequence | function |
| --- | --- | --- | --- | --- | --- | --- |
| DRE core | Arabidopsis thaliana | 973 | - | 6 | GCCGAC |  |

>HU06G00358.1   
+ -Up\_Stream \_Len000GGGTTT TTTTAAACTC AAAATTAGTT ATTGTATCAA TTGAATTTGG AAAACTATTA   
  
  
+ TTTTCGACTT ATTATAGAGA ATTTGTTGTT AATGAATGCT GATAAATACT GATAATTAGT GTTGTGAGTG   
  
  
+ TGAATTTGTG ATGATGAGCA ATGATTAGCG TTAAAATTAT AACTAGGATA AATAATTAAG TGTTGATGAA   
  
  
+ TGTTGATTGA TGATGATGAG TACTGATTGG TGCGGTGCTG GTAATAAATA TTTATGATAA GTTAAAAAAA   
  
  
+ AAAAACACAT CCTAAACAAT CACTATGTAA TATACTAAAT GAAAAAAATT GGTAATGTGA TTTGACAATC   
  
  
+ TAAGGGTAAG GGTGTTTTAC CCACACATGT TATGAAATTG GCTTACTATT TACAAACTTT TTACAGTTAT   
  
  
+ ATGATTCTAC CTATGTGTTA TCATCTTTCC GTCGATGTAA TTGATTATAT TCAACACAAT TTAATTCATT   
  
  
+ ACAATGTAGC TAATTGATAC TCAAAATAAT GAACTATTAA TAAATAATTA ATTATAACTT TTAAAAGTTT   
  
  
+ GTTAATTTAA AATTATATAA TATATAAATT TAGGCTACAT TAATCAAAAA ATTAGGCGTG TTCATCGGTC   
  
  
+ CAAACTTGAA CCGGACCAGA CCGGACCGAA GATCGATTAA GAGAAAAAGT ACAGACTGAG GACCGGATCG   
  
  
+ AATAAGAGTC GCACCGGACC AGGATCGGAC CGGAAAAATT TGGACCAGAT CGGGACCGAT CGAGGTAGAC   
  
  
+ CGAATTTATT TTCAAATACG ATAGGAATAA AACTTATTGA TTCAATTGAT TTTGGCCAAA ATCATGCTAT   
  
  
+ TATTGATTTT ATTTGCTAAC TCTCAAGAGT ATTAGTTTGC ACTTCGCATT ATCTTTGGCC AAAAAAATCA   
  
  
+ AAAAGAAAGT CTCAAACTCT TAATAGTTTT TTATTTTATT TGGTTTATTT TTAAAATAGT CGGCCTTCGG   
  
  
+ TCCGGACTAG TGGACCGAAT AAGGTAATTT TTCGAATCGA GGACCAGACC AAATGCACTC AGTTCGAACC   
  
  
+ GGTTCAGTTC GGTCTGACTC GATTTTTGGT CCGGTCCAAG TTTGAACAGA ATAATGTACA CACCTAGAAA   
  
  
+ AAAAGAATTA TGCATATATA CTTAAGGCCC CCAACCCTTT TTCCCTTTCC GCCAATACTT TTTACCAAGT   
  
  
+ CCAATTCTTT TCTTCTTAGC TATGGCTAGT AGTTATCCCT CCCTAGCTTC CACCCTAAGT TTTGAGACAA   
  
  
+ AATCCCATCT ATGTTAATAT TTATTTCTTG CACATGTCAC CCAATACAAG GGTTTCATTG AAAAGAAAAG   
  
  
+ GTATTAGATG TAAGATATGT ATGTATATAA ATAGTTTTGC AATAGACTAG AAGGACTAAA AAGGAAAAAG   
  
  
+ GAAGTTTAAA AGCAAAAAAC TTTCCCCTCT AGAAAAAAGG AAACCAGATT CCCTTTTCTA CCCCAATTCC   
  
  
+ CATAGTCTTT ACTCTGTCTC TCTCTCTCTG TCTCTCTTAC TCATGATGTT GAGGGAGGAA GGAATGAATT   
  
  
+ AAGGACCCTA AGCTAAGATC CAACCGTAAG ATATTGCCCC TTTAATCACC CATTTCATAT CTCTATCCAC   
  
  
+ AGTTAAAAAC CCACCTCCTC CATCCACATA ACTGATATAT CTCTTCTACA CCATACTATA AGGAAGAAGA   
  
  
+ GAAAAATAGG AGTGTATTGT ATTTTTATTT GAGTAATTTA TTTTAAGGAG AGAAATTTAC ATGGTTGGGT   
  
  
+ TAAATGGATA CCTTGTTTAG ACTAGTGAGT TTCCAACAAC CACACCACCA CCATCACCAA CACCAACACC   
  
  
+ AACACCACCA ACAGCAATCT TCTGATCAAT CCTCTTTGAA TAATACTACT ACTTATTCCA CAAATTCAAG   
  
  
+ CAGTTCCAGA TCTTCCCAAC AAACCCATCA TAATCATAAT CATACTCAGT ATTATCACCA ACATCATCCT   
  
  
+ ACTTACCCCC ACCGCCACCA TCAGGAGGAA GAAGAAGAAT GCTTCAACTT TTACATGGAT GAAGAAAACT   
  
  
+ TCTCTTCTTC TTCCTCCCAC AACAAACATC ATCCTCAACC TCCTTCCTCC GCCACCACCT CCTCGTATTA   
  
  
+ CCCTTACTAC TCCACCCCCT TCGATGTTCC CTCTACCACC ACCACCTCCA CCTCCACCCC CACGCCCACC   
  
  
+ AACACCACCA CCACCACCCC CCCAACCCAG TATGGCGGCG GCGGCGGTGG GTTTGACTCC AGCTGCTTCT   
  
  
+ CCTACTCCCC TGCCCGAGAC CTCAACCCGG AGCTCTTCTC CTCGTCCGGG TTCTCGGGCA AGTGGGCTTC   
  
  
+ CAACATCCTC TTGGAAGCCG CTCGTGCCAT CTCTGAGAGG AACACGAGCC GCCTCCACCA GCTCCTATGG   
  
  
+ ATGGTCAACG AGCTCAGCTC CCCGTACGGT GACGTGGAGC AGAAGATAGC TTCATACTTC GGCCAAGCCC   
  
  
+ TCTTCACCCG CATGACCGAG TCAGGGGACC GCACCTACCG CACCATGCGG TCCGCCGTGG ACAAGACCTT   
  
  
+ CTCCTTTGAG TCTACGAGAA AGATGGTACT CAAGTTCCAG GAGGTTAGCC CTTGGACCAC CTTTGGACAC   
  
  
+ GTGGCAGCTA ATGGAGCCCT CATTGATGCC TTTGACGGTG AATCCAAAAT CCACATCATC GACATCAGCA   
  
  
+ CCACATTCTG CACCCAGTGG CCCACCCTCC TGGAGGCGCT GGCCACCCGA ATGGATGACG CGCCCTACCT   
  
  
+ GAGGCTGACC ACTGTGGTGG TCAACAAGTC CGGGAATGAA GGTCCCACCG GAGGCGGGTC CCACAGGGTG   
  
  
+ ATGAAAGAGA TTGGGACCCG CCTCGAGAAG TTTGCTAGGC TAATGGGAGT GCCTTTCAAA TTTAATGTGG   
  
  
+ TCCACCACGG GGCTGATTTA TCCGACTTGG ATTTCTCTCA GCTGGATATT AACGATGACG AGGCATTAGC   
  
  
+ CATCAACTGT GTCAACTCGC TCCACTCGGT CAATATCCAC CGCCGTGACT CGGTCATCTC GGCCTTCCGG   
  
  
+ CGGCTCCACC CTAGGGTGGT GACCGTGGTG GAGGAGGAGG CTGACCTTGC TGACGTGGGG GCGGAGGGGT   
  
  
+ ACGAGTTCTA CAGGGGGTTT AATGAGTGTT TAAGGTGGGT TAGGGTTTAC TTTGAGGCCC TGGAAGAGTG   
  
  
+ CTTTCCTAGG ACAAGCAATG AGAAACTCAT GCTCGAGCGG GCCGCAGGGC GGGCTCTGGT GGACCTCCTG   
  
  
+ GCCTGCCCCA AGCCCGCATC ATCCGAGCGG AGGGAGGCAG CTGCGCGGTG GTCCGGGAGG ATGCAGGGGG   
  
  
+ CCGGGTTCGG CCACGTGGGG TTCAGTGATG AGGTCTGTGA TGACGTCAAG GCCCTCCTAA GGAGGTACAA   
  
  
+ GGAGGGGTGG TCCATGATAC AATGTTCCGC CGCGGATGGC GGCGCCTCCG CCGGAATATT CTTGTCGTGG   
  
  
+ AGGGATCAGC CGGTGGTGTG GGCCAGTGCA TGGAAGCCTT A  

- -Up\_Stream \_Len000CCCAAA AAAATTTGAG TTTTAATCAA TAACATAGTT AACTTAAACC TTTTGATAAT   
  
  
- AAAAGCTGAA TAATATCTCT TAAACAACAA TTACTTACGA CTATTTATGA CTATTAATCA CAACACTCAC   
  
  
- ACTTAAACAC TACTACTCGT TACTAATCGC AATTTTAATA TTGATCCTAT TTATTAATTC ACAACTACTT   
  
  
- ACAACTAACT ACTACTACTC ATGACTAACC ACGCCACGAC CATTATTTAT AAATACTATT CAATTTTTTT   
  
  
- TTTTTGTGTA GGATTTGTTA GTGATACATT ATATGATTTA CTTTTTTTAA CCATTACACT AAACTGTTAG   
  
  
- ATTCCCATTC CCACAAAATG GGTGTGTACA ATACTTTAAC CGAATGATAA ATGTTTGAAA AATGTCAATA   
  
  
- TACTAAGATG GATACACAAT AGTAGAAAGG CAGCTACATT AACTAATATA AGTTGTGTTA AATTAAGTAA   
  
  
- TGTTACATCG ATTAACTATG AGTTTTATTA CTTGATAATT ATTTATTAAT TAATATTGAA AATTTTCAAA   
  
  
- CAATTAAATT TTAATATATT ATATATTTAA ATCCGATGTA ATTAGTTTTT TAATCCGCAC AAGTAGCCAG   
  
  
- GTTTGAACTT GGCCTGGTCT GGCCTGGCTT CTAGCTAATT CTCTTTTTCA TGTCTGACTC CTGGCCTAGC   
  
  
- TTATTCTCAG CGTGGCCTGG TCCTAGCCTG GCCTTTTTAA ACCTGGTCTA GCCCTGGCTA GCTCCATCTG   
  
  
- GCTTAAATAA AAGTTTATGC TATCCTTATT TTGAATAACT AAGTTAACTA AAACCGGTTT TAGTACGATA   
  
  
- ATAACTAAAA TAAACGATTG AGAGTTCTCA TAATCAAACG TGAAGCGTAA TAGAAACCGG TTTTTTTAGT   
  
  
- TTTTCTTTCA GAGTTTGAGA ATTATCAAAA AATAAAATAA ACCAAATAAA AATTTTATCA GCCGGAAGCC   
  
  
- AGGCCTGATC ACCTGGCTTA TTCCATTAAA AAGCTTAGCT CCTGGTCTGG TTTACGTGAG TCAAGCTTGG   
  
  
- CCAAGTCAAG CCAGACTGAG CTAAAAACCA GGCCAGGTTC AAACTTGTCT TATTACATGT GTGGATCTTT   
  
  
- TTTTCTTAAT ACGTATATAT GAATTCCGGG GGTTGGGAAA AAGGGAAAGG CGGTTATGAA AAATGGTTCA   
  
  
- GGTTAAGAAA AGAAGAATCG ATACCGATCA TCAATAGGGA GGGATCGAAG GTGGGATTCA AAACTCTGTT   
  
  
- TTAGGGTAGA TACAATTATA AATAAAGAAC GTGTACAGTG GGTTATGTTC CCAAAGTAAC TTTTCTTTTC   
  
  
- CATAATCTAC ATTCTATACA TACATATATT TATCAAAACG TTATCTGATC TTCCTGATTT TTCCTTTTTC   
  
  
- CTTCAAATTT TCGTTTTTTG AAAGGGGAGA TCTTTTTTCC TTTGGTCTAA GGGAAAAGAT GGGGTTAAGG   
  
  
- GTATCAGAAA TGAGACAGAG AGAGAGAGAC AGAGAGAATG AGTACTACAA CTCCCTCCTT CCTTACTTAA   
  
  
- TTCCTGGGAT TCGATTCTAG GTTGGCATTC TATAACGGGG AAATTAGTGG GTAAAGTATA GAGATAGGTG   
  
  
- TCAATTTTTG GGTGGAGGAG GTAGGTGTAT TGACTATATA GAGAAGATGT GGTATGATAT TCCTTCTTCT   
  
  
- CTTTTTATCC TCACATAACA TAAAAATAAA CTCATTAAAT AAAATTCCTC TCTTTAAATG TACCAACCCA   
  
  
- ATTTACCTAT GGAACAAATC TGATCACTCA AAGGTTGTTG GTGTGGTGGT GGTAGTGGTT GTGGTTGTGG   
  
  
- TTGTGGTGGT TGTCGTTAGA AGACTAGTTA GGAGAAACTT ATTATGATGA TGAATAAGGT GTTTAAGTTC   
  
  
- GTCAAGGTCT AGAAGGGTTG TTTGGGTAGT ATTAGTATTA GTATGAGTCA TAATAGTGGT TGTAGTAGGA   
  
  
- TGAATGGGGG TGGCGGTGGT AGTCCTCCTT CTTCTTCTTA CGAAGTTGAA AATGTACCTA CTTCTTTTGA   
  
  
- AGAGAAGAAG AAGGAGGGTG TTGTTTGTAG TAGGAGTTGG AGGAAGGAGG CGGTGGTGGA GGAGCATAAT   
  
  
- GGGAATGATG AGGTGGGGGA AGCTACAAGG GAGATGGTGG TGGTGGAGGT GGAGGTGGGG GTGCGGGTGG   
  
  
- TTGTGGTGGT GGTGGTGGGG GGGTTGGGTC ATACCGCCGC CGCCGCCACC CAAACTGAGG TCGACGAAGA   
  
  
- GGATGAGGGG ACGGGCTCTG GAGTTGGGCC TCGAGAAGAG GAGCAGGCCC AAGAGCCCGT TCACCCGAAG   
  
  
- GTTGTAGGAG AACCTTCGGC GAGCACGGTA GAGACTCTCC TTGTGCTCGG CGGAGGTGGT CGAGGATACC   
  
  
- TACCAGTTGC TCGAGTCGAG GGGCATGCCA CTGCACCTCG TCTTCTATCG AAGTATGAAG CCGGTTCGGG   
  
  
- AGAAGTGGGC GTACTGGCTC AGTCCCCTGG CGTGGATGGC GTGGTACGCC AGGCGGCACC TGTTCTGGAA   
  
  
- GAGGAAACTC AGATGCTCTT TCTACCATGA GTTCAAGGTC CTCCAATCGG GAACCTGGTG GAAACCTGTG   
  
  
- CACCGTCGAT TACCTCGGGA GTAACTACGG AAACTGCCAC TTAGGTTTTA GGTGTAGTAG CTGTAGTCGT   
  
  
- GGTGTAAGAC GTGGGTCACC GGGTGGGAGG ACCTCCGCGA CCGGTGGGCT TACCTACTGC GCGGGATGGA   
  
  
- CTCCGACTGG TGACACCACC AGTTGTTCAG GCCCTTACTT CCAGGGTGGC CTCCGCCCAG GGTGTCCCAC   
  
  
- TACTTTCTCT AACCCTGGGC GGAGCTCTTC AAACGATCCG ATTACCCTCA CGGAAAGTTT AAATTACACC   
  
  
- AGGTGGTGCC CCGACTAAAT AGGCTGAACC TAAAGAGAGT CGACCTATAA TTGCTACTGC TCCGTAATCG   
  
  
- GTAGTTGACA CAGTTGAGCG AGGTGAGCCA GTTATAGGTG GCGGCACTGA GCCAGTAGAG CCGGAAGGCC   
  
  
- GCCGAGGTGG GATCCCACCA CTGGCACCAC CTCCTCCTCC GACTGGAACG ACTGCACCCC CGCCTCCCCA   
  
  
- TGCTCAAGAT GTCCCCCAAA TTACTCACAA ATTCCACCCA ATCCCAAATG AAACTCCGGG ACCTTCTCAC   
  
  
- GAAAGGATCC TGTTCGTTAC TCTTTGAGTA CGAGCTCGCC CGGCGTCCCG CCCGAGACCA CCTGGAGGAC   
  
  
- CGGACGGGGT TCGGGCGTAG TAGGCTCGCC TCCCTCCGTC GACGCGCCAC CAGGCCCTCC TACGTCCCCC   
  
  
- GGCCCAAGCC GGTGCACCCC AAGTCACTAC TCCAGACACT ACTGCAGTTC CGGGAGGATT CCTCCATGTT   
  
  
- CCTCCCCACC AGGTACTATG TTACAAGGCG GCGCCTACCG CCGCGGAGGC GGCCTTATAA GAACAGCACC   
  
  
- TCCCTAGTCG GCCACCACAC CCGGTCACGT ACCTTCGGAA T

+     ERE

| Site Name | Organism | Position | Strand | Matrix score. | sequence | function |
| --- | --- | --- | --- | --- | --- | --- |
| ERE | Nicotiana glutinos | 1596 | + | 8 | ATTTCATA |  |
| ERE | Nicotiana glutinos | 964 | - | 8 | ATTTTAAA |  |
| ERE | Nicotiana glutinos | 385 | - | 8 | ATTTCATA |  |
| ERE | Nicotiana glutinos | 570 | - | 8 | ATTTTAAA |  |

>HU06G00358.1   
+ -Up\_Stream \_Len000GGGTTT TTTTAAACTC AAAATTAGTT ATTGTATCAA TTGAATTTGG AAAACTATTA   
  
  
+ TTTTCGACTT ATTATAGAGA ATTTGTTGTT AATGAATGCT GATAAATACT GATAATTAGT GTTGTGAGTG   
  
  
+ TGAATTTGTG ATGATGAGCA ATGATTAGCG TTAAAATTAT AACTAGGATA AATAATTAAG TGTTGATGAA   
  
  
+ TGTTGATTGA TGATGATGAG TACTGATTGG TGCGGTGCTG GTAATAAATA TTTATGATAA GTTAAAAAAA   
  
  
+ AAAAACACAT CCTAAACAAT CACTATGTAA TATACTAAAT GAAAAAAATT GGTAATGTGA TTTGACAATC   
  
  
+ TAAGGGTAAG GGTGTTTTAC CCACACATGT TATGAAATTG GCTTACTATT TACAAACTTT TTACAGTTAT   
  
  
+ ATGATTCTAC CTATGTGTTA TCATCTTTCC GTCGATGTAA TTGATTATAT TCAACACAAT TTAATTCATT   
  
  
+ ACAATGTAGC TAATTGATAC TCAAAATAAT GAACTATTAA TAAATAATTA ATTATAACTT TTAAAAGTTT   
  
  
+ GTTAATTTAA AATTATATAA TATATAAATT TAGGCTACAT TAATCAAAAA ATTAGGCGTG TTCATCGGTC   
  
  
+ CAAACTTGAA CCGGACCAGA CCGGACCGAA GATCGATTAA GAGAAAAAGT ACAGACTGAG GACCGGATCG   
  
  
+ AATAAGAGTC GCACCGGACC AGGATCGGAC CGGAAAAATT TGGACCAGAT CGGGACCGAT CGAGGTAGAC   
  
  
+ CGAATTTATT TTCAAATACG ATAGGAATAA AACTTATTGA TTCAATTGAT TTTGGCCAAA ATCATGCTAT   
  
  
+ TATTGATTTT ATTTGCTAAC TCTCAAGAGT ATTAGTTTGC ACTTCGCATT ATCTTTGGCC AAAAAAATCA   
  
  
+ AAAAGAAAGT CTCAAACTCT TAATAGTTTT TTATTTTATT TGGTTTATTT TTAAAATAGT CGGCCTTCGG   
  
  
+ TCCGGACTAG TGGACCGAAT AAGGTAATTT TTCGAATCGA GGACCAGACC AAATGCACTC AGTTCGAACC   
  
  
+ GGTTCAGTTC GGTCTGACTC GATTTTTGGT CCGGTCCAAG TTTGAACAGA ATAATGTACA CACCTAGAAA   
  
  
+ AAAAGAATTA TGCATATATA CTTAAGGCCC CCAACCCTTT TTCCCTTTCC GCCAATACTT TTTACCAAGT   
  
  
+ CCAATTCTTT TCTTCTTAGC TATGGCTAGT AGTTATCCCT CCCTAGCTTC CACCCTAAGT TTTGAGACAA   
  
  
+ AATCCCATCT ATGTTAATAT TTATTTCTTG CACATGTCAC CCAATACAAG GGTTTCATTG AAAAGAAAAG   
  
  
+ GTATTAGATG TAAGATATGT ATGTATATAA ATAGTTTTGC AATAGACTAG AAGGACTAAA AAGGAAAAAG   
  
  
+ GAAGTTTAAA AGCAAAAAAC TTTCCCCTCT AGAAAAAAGG AAACCAGATT CCCTTTTCTA CCCCAATTCC   
  
  
+ CATAGTCTTT ACTCTGTCTC TCTCTCTCTG TCTCTCTTAC TCATGATGTT GAGGGAGGAA GGAATGAATT   
  
  
+ AAGGACCCTA AGCTAAGATC CAACCGTAAG ATATTGCCCC TTTAATCACC CATTTCATAT CTCTATCCAC   
  
  
+ AGTTAAAAAC CCACCTCCTC CATCCACATA ACTGATATAT CTCTTCTACA CCATACTATA AGGAAGAAGA   
  
  
+ GAAAAATAGG AGTGTATTGT ATTTTTATTT GAGTAATTTA TTTTAAGGAG AGAAATTTAC ATGGTTGGGT   
  
  
+ TAAATGGATA CCTTGTTTAG ACTAGTGAGT TTCCAACAAC CACACCACCA CCATCACCAA CACCAACACC   
  
  
+ AACACCACCA ACAGCAATCT TCTGATCAAT CCTCTTTGAA TAATACTACT ACTTATTCCA CAAATTCAAG   
  
  
+ CAGTTCCAGA TCTTCCCAAC AAACCCATCA TAATCATAAT CATACTCAGT ATTATCACCA ACATCATCCT   
  
  
+ ACTTACCCCC ACCGCCACCA TCAGGAGGAA GAAGAAGAAT GCTTCAACTT TTACATGGAT GAAGAAAACT   
  
  
+ TCTCTTCTTC TTCCTCCCAC AACAAACATC ATCCTCAACC TCCTTCCTCC GCCACCACCT CCTCGTATTA   
  
  
+ CCCTTACTAC TCCACCCCCT TCGATGTTCC CTCTACCACC ACCACCTCCA CCTCCACCCC CACGCCCACC   
  
  
+ AACACCACCA CCACCACCCC CCCAACCCAG TATGGCGGCG GCGGCGGTGG GTTTGACTCC AGCTGCTTCT   
  
  
+ CCTACTCCCC TGCCCGAGAC CTCAACCCGG AGCTCTTCTC CTCGTCCGGG TTCTCGGGCA AGTGGGCTTC   
  
  
+ CAACATCCTC TTGGAAGCCG CTCGTGCCAT CTCTGAGAGG AACACGAGCC GCCTCCACCA GCTCCTATGG   
  
  
+ ATGGTCAACG AGCTCAGCTC CCCGTACGGT GACGTGGAGC AGAAGATAGC TTCATACTTC GGCCAAGCCC   
  
  
+ TCTTCACCCG CATGACCGAG TCAGGGGACC GCACCTACCG CACCATGCGG TCCGCCGTGG ACAAGACCTT   
  
  
+ CTCCTTTGAG TCTACGAGAA AGATGGTACT CAAGTTCCAG GAGGTTAGCC CTTGGACCAC CTTTGGACAC   
  
  
+ GTGGCAGCTA ATGGAGCCCT CATTGATGCC TTTGACGGTG AATCCAAAAT CCACATCATC GACATCAGCA   
  
  
+ CCACATTCTG CACCCAGTGG CCCACCCTCC TGGAGGCGCT GGCCACCCGA ATGGATGACG CGCCCTACCT   
  
  
+ GAGGCTGACC ACTGTGGTGG TCAACAAGTC CGGGAATGAA GGTCCCACCG GAGGCGGGTC CCACAGGGTG   
  
  
+ ATGAAAGAGA TTGGGACCCG CCTCGAGAAG TTTGCTAGGC TAATGGGAGT GCCTTTCAAA TTTAATGTGG   
  
  
+ TCCACCACGG GGCTGATTTA TCCGACTTGG ATTTCTCTCA GCTGGATATT AACGATGACG AGGCATTAGC   
  
  
+ CATCAACTGT GTCAACTCGC TCCACTCGGT CAATATCCAC CGCCGTGACT CGGTCATCTC GGCCTTCCGG   
  
  
+ CGGCTCCACC CTAGGGTGGT GACCGTGGTG GAGGAGGAGG CTGACCTTGC TGACGTGGGG GCGGAGGGGT   
  
  
+ ACGAGTTCTA CAGGGGGTTT AATGAGTGTT TAAGGTGGGT TAGGGTTTAC TTTGAGGCCC TGGAAGAGTG   
  
  
+ CTTTCCTAGG ACAAGCAATG AGAAACTCAT GCTCGAGCGG GCCGCAGGGC GGGCTCTGGT GGACCTCCTG   
  
  
+ GCCTGCCCCA AGCCCGCATC ATCCGAGCGG AGGGAGGCAG CTGCGCGGTG GTCCGGGAGG ATGCAGGGGG   
  
  
+ CCGGGTTCGG CCACGTGGGG TTCAGTGATG AGGTCTGTGA TGACGTCAAG GCCCTCCTAA GGAGGTACAA   
  
  
+ GGAGGGGTGG TCCATGATAC AATGTTCCGC CGCGGATGGC GGCGCCTCCG CCGGAATATT CTTGTCGTGG   
  
  
+ AGGGATCAGC CGGTGGTGTG GGCCAGTGCA TGGAAGCCTT A  

- -Up\_Stream \_Len000CCCAAA AAAATTTGAG TTTTAATCAA TAACATAGTT AACTTAAACC TTTTGATAAT   
  
  
- AAAAGCTGAA TAATATCTCT TAAACAACAA TTACTTACGA CTATTTATGA CTATTAATCA CAACACTCAC   
  
  
- ACTTAAACAC TACTACTCGT TACTAATCGC AATTTTAATA TTGATCCTAT TTATTAATTC ACAACTACTT   
  
  
- ACAACTAACT ACTACTACTC ATGACTAACC ACGCCACGAC CATTATTTAT AAATACTATT CAATTTTTTT   
  
  
- TTTTTGTGTA GGATTTGTTA GTGATACATT ATATGATTTA CTTTTTTTAA CCATTACACT AAACTGTTAG   
  
  
- ATTCCCATTC CCACAAAATG GGTGTGTACA ATACTTTAAC CGAATGATAA ATGTTTGAAA AATGTCAATA   
  
  
- TACTAAGATG GATACACAAT AGTAGAAAGG CAGCTACATT AACTAATATA AGTTGTGTTA AATTAAGTAA   
  
  
- TGTTACATCG ATTAACTATG AGTTTTATTA CTTGATAATT ATTTATTAAT TAATATTGAA AATTTTCAAA   
  
  
- CAATTAAATT TTAATATATT ATATATTTAA ATCCGATGTA ATTAGTTTTT TAATCCGCAC AAGTAGCCAG   
  
  
- GTTTGAACTT GGCCTGGTCT GGCCTGGCTT CTAGCTAATT CTCTTTTTCA TGTCTGACTC CTGGCCTAGC   
  
  
- TTATTCTCAG CGTGGCCTGG TCCTAGCCTG GCCTTTTTAA ACCTGGTCTA GCCCTGGCTA GCTCCATCTG   
  
  
- GCTTAAATAA AAGTTTATGC TATCCTTATT TTGAATAACT AAGTTAACTA AAACCGGTTT TAGTACGATA   
  
  
- ATAACTAAAA TAAACGATTG AGAGTTCTCA TAATCAAACG TGAAGCGTAA TAGAAACCGG TTTTTTTAGT   
  
  
- TTTTCTTTCA GAGTTTGAGA ATTATCAAAA AATAAAATAA ACCAAATAAA AATTTTATCA GCCGGAAGCC   
  
  
- AGGCCTGATC ACCTGGCTTA TTCCATTAAA AAGCTTAGCT CCTGGTCTGG TTTACGTGAG TCAAGCTTGG   
  
  
- CCAAGTCAAG CCAGACTGAG CTAAAAACCA GGCCAGGTTC AAACTTGTCT TATTACATGT GTGGATCTTT   
  
  
- TTTTCTTAAT ACGTATATAT GAATTCCGGG GGTTGGGAAA AAGGGAAAGG CGGTTATGAA AAATGGTTCA   
  
  
- GGTTAAGAAA AGAAGAATCG ATACCGATCA TCAATAGGGA GGGATCGAAG GTGGGATTCA AAACTCTGTT   
  
  
- TTAGGGTAGA TACAATTATA AATAAAGAAC GTGTACAGTG GGTTATGTTC CCAAAGTAAC TTTTCTTTTC   
  
  
- CATAATCTAC ATTCTATACA TACATATATT TATCAAAACG TTATCTGATC TTCCTGATTT TTCCTTTTTC   
  
  
- CTTCAAATTT TCGTTTTTTG AAAGGGGAGA TCTTTTTTCC TTTGGTCTAA GGGAAAAGAT GGGGTTAAGG   
  
  
- GTATCAGAAA TGAGACAGAG AGAGAGAGAC AGAGAGAATG AGTACTACAA CTCCCTCCTT CCTTACTTAA   
  
  
- TTCCTGGGAT TCGATTCTAG GTTGGCATTC TATAACGGGG AAATTAGTGG GTAAAGTATA GAGATAGGTG   
  
  
- TCAATTTTTG GGTGGAGGAG GTAGGTGTAT TGACTATATA GAGAAGATGT GGTATGATAT TCCTTCTTCT   
  
  
- CTTTTTATCC TCACATAACA TAAAAATAAA CTCATTAAAT AAAATTCCTC TCTTTAAATG TACCAACCCA   
  
  
- ATTTACCTAT GGAACAAATC TGATCACTCA AAGGTTGTTG GTGTGGTGGT GGTAGTGGTT GTGGTTGTGG   
  
  
- TTGTGGTGGT TGTCGTTAGA AGACTAGTTA GGAGAAACTT ATTATGATGA TGAATAAGGT GTTTAAGTTC   
  
  
- GTCAAGGTCT AGAAGGGTTG TTTGGGTAGT ATTAGTATTA GTATGAGTCA TAATAGTGGT TGTAGTAGGA   
  
  
- TGAATGGGGG TGGCGGTGGT AGTCCTCCTT CTTCTTCTTA CGAAGTTGAA AATGTACCTA CTTCTTTTGA   
  
  
- AGAGAAGAAG AAGGAGGGTG TTGTTTGTAG TAGGAGTTGG AGGAAGGAGG CGGTGGTGGA GGAGCATAAT   
  
  
- GGGAATGATG AGGTGGGGGA AGCTACAAGG GAGATGGTGG TGGTGGAGGT GGAGGTGGGG GTGCGGGTGG   
  
  
- TTGTGGTGGT GGTGGTGGGG GGGTTGGGTC ATACCGCCGC CGCCGCCACC CAAACTGAGG TCGACGAAGA   
  
  
- GGATGAGGGG ACGGGCTCTG GAGTTGGGCC TCGAGAAGAG GAGCAGGCCC AAGAGCCCGT TCACCCGAAG   
  
  
- GTTGTAGGAG AACCTTCGGC GAGCACGGTA GAGACTCTCC TTGTGCTCGG CGGAGGTGGT CGAGGATACC   
  
  
- TACCAGTTGC TCGAGTCGAG GGGCATGCCA CTGCACCTCG TCTTCTATCG AAGTATGAAG CCGGTTCGGG   
  
  
- AGAAGTGGGC GTACTGGCTC AGTCCCCTGG CGTGGATGGC GTGGTACGCC AGGCGGCACC TGTTCTGGAA   
  
  
- GAGGAAACTC AGATGCTCTT TCTACCATGA GTTCAAGGTC CTCCAATCGG GAACCTGGTG GAAACCTGTG   
  
  
- CACCGTCGAT TACCTCGGGA GTAACTACGG AAACTGCCAC TTAGGTTTTA GGTGTAGTAG CTGTAGTCGT   
  
  
- GGTGTAAGAC GTGGGTCACC GGGTGGGAGG ACCTCCGCGA CCGGTGGGCT TACCTACTGC GCGGGATGGA   
  
  
- CTCCGACTGG TGACACCACC AGTTGTTCAG GCCCTTACTT CCAGGGTGGC CTCCGCCCAG GGTGTCCCAC   
  
  
- TACTTTCTCT AACCCTGGGC GGAGCTCTTC AAACGATCCG ATTACCCTCA CGGAAAGTTT AAATTACACC   
  
  
- AGGTGGTGCC CCGACTAAAT AGGCTGAACC TAAAGAGAGT CGACCTATAA TTGCTACTGC TCCGTAATCG   
  
  
- GTAGTTGACA CAGTTGAGCG AGGTGAGCCA GTTATAGGTG GCGGCACTGA GCCAGTAGAG CCGGAAGGCC   
  
  
- GCCGAGGTGG GATCCCACCA CTGGCACCAC CTCCTCCTCC GACTGGAACG ACTGCACCCC CGCCTCCCCA   
  
  
- TGCTCAAGAT GTCCCCCAAA TTACTCACAA ATTCCACCCA ATCCCAAATG AAACTCCGGG ACCTTCTCAC   
  
  
- GAAAGGATCC TGTTCGTTAC TCTTTGAGTA CGAGCTCGCC CGGCGTCCCG CCCGAGACCA CCTGGAGGAC   
  
  
- CGGACGGGGT TCGGGCGTAG TAGGCTCGCC TCCCTCCGTC GACGCGCCAC CAGGCCCTCC TACGTCCCCC   
  
  
- GGCCCAAGCC GGTGCACCCC AAGTCACTAC TCCAGACACT ACTGCAGTTC CGGGAGGATT CCTCCATGTT   
  
  
- CCTCCCCACC AGGTACTATG TTACAAGGCG GCGCCTACCG CCGCGGAGGC GGCCTTATAA GAACAGCACC   
  
  
- TCCCTAGTCG GCCACCACAC CCGGTCACGT ACCTTCGGAA T

+     G-Box

| Site Name | Organism | Position | Strand | Matrix score. | sequence | function |
| --- | --- | --- | --- | --- | --- | --- |
| G-Box | Pisum sativum | 2592 | - | 6 | CACGTG | cis-acting regulatory element involved in light responsiveness |
| G-Box | Pisum sativum | 3306 | - | 6 | CACGTG | cis-acting regulatory element involved in light responsiveness |

>HU06G00358.1   
+ -Up\_Stream \_Len000GGGTTT TTTTAAACTC AAAATTAGTT ATTGTATCAA TTGAATTTGG AAAACTATTA   
  
  
+ TTTTCGACTT ATTATAGAGA ATTTGTTGTT AATGAATGCT GATAAATACT GATAATTAGT GTTGTGAGTG   
  
  
+ TGAATTTGTG ATGATGAGCA ATGATTAGCG TTAAAATTAT AACTAGGATA AATAATTAAG TGTTGATGAA   
  
  
+ TGTTGATTGA TGATGATGAG TACTGATTGG TGCGGTGCTG GTAATAAATA TTTATGATAA GTTAAAAAAA   
  
  
+ AAAAACACAT CCTAAACAAT CACTATGTAA TATACTAAAT GAAAAAAATT GGTAATGTGA TTTGACAATC   
  
  
+ TAAGGGTAAG GGTGTTTTAC CCACACATGT TATGAAATTG GCTTACTATT TACAAACTTT TTACAGTTAT   
  
  
+ ATGATTCTAC CTATGTGTTA TCATCTTTCC GTCGATGTAA TTGATTATAT TCAACACAAT TTAATTCATT   
  
  
+ ACAATGTAGC TAATTGATAC TCAAAATAAT GAACTATTAA TAAATAATTA ATTATAACTT TTAAAAGTTT   
  
  
+ GTTAATTTAA AATTATATAA TATATAAATT TAGGCTACAT TAATCAAAAA ATTAGGCGTG TTCATCGGTC   
  
  
+ CAAACTTGAA CCGGACCAGA CCGGACCGAA GATCGATTAA GAGAAAAAGT ACAGACTGAG GACCGGATCG   
  
  
+ AATAAGAGTC GCACCGGACC AGGATCGGAC CGGAAAAATT TGGACCAGAT CGGGACCGAT CGAGGTAGAC   
  
  
+ CGAATTTATT TTCAAATACG ATAGGAATAA AACTTATTGA TTCAATTGAT TTTGGCCAAA ATCATGCTAT   
  
  
+ TATTGATTTT ATTTGCTAAC TCTCAAGAGT ATTAGTTTGC ACTTCGCATT ATCTTTGGCC AAAAAAATCA   
  
  
+ AAAAGAAAGT CTCAAACTCT TAATAGTTTT TTATTTTATT TGGTTTATTT TTAAAATAGT CGGCCTTCGG   
  
  
+ TCCGGACTAG TGGACCGAAT AAGGTAATTT TTCGAATCGA GGACCAGACC AAATGCACTC AGTTCGAACC   
  
  
+ GGTTCAGTTC GGTCTGACTC GATTTTTGGT CCGGTCCAAG TTTGAACAGA ATAATGTACA CACCTAGAAA   
  
  
+ AAAAGAATTA TGCATATATA CTTAAGGCCC CCAACCCTTT TTCCCTTTCC GCCAATACTT TTTACCAAGT   
  
  
+ CCAATTCTTT TCTTCTTAGC TATGGCTAGT AGTTATCCCT CCCTAGCTTC CACCCTAAGT TTTGAGACAA   
  
  
+ AATCCCATCT ATGTTAATAT TTATTTCTTG CACATGTCAC CCAATACAAG GGTTTCATTG AAAAGAAAAG   
  
  
+ GTATTAGATG TAAGATATGT ATGTATATAA ATAGTTTTGC AATAGACTAG AAGGACTAAA AAGGAAAAAG   
  
  
+ GAAGTTTAAA AGCAAAAAAC TTTCCCCTCT AGAAAAAAGG AAACCAGATT CCCTTTTCTA CCCCAATTCC   
  
  
+ CATAGTCTTT ACTCTGTCTC TCTCTCTCTG TCTCTCTTAC TCATGATGTT GAGGGAGGAA GGAATGAATT   
  
  
+ AAGGACCCTA AGCTAAGATC CAACCGTAAG ATATTGCCCC TTTAATCACC CATTTCATAT CTCTATCCAC   
  
  
+ AGTTAAAAAC CCACCTCCTC CATCCACATA ACTGATATAT CTCTTCTACA CCATACTATA AGGAAGAAGA   
  
  
+ GAAAAATAGG AGTGTATTGT ATTTTTATTT GAGTAATTTA TTTTAAGGAG AGAAATTTAC ATGGTTGGGT   
  
  
+ TAAATGGATA CCTTGTTTAG ACTAGTGAGT TTCCAACAAC CACACCACCA CCATCACCAA CACCAACACC   
  
  
+ AACACCACCA ACAGCAATCT TCTGATCAAT CCTCTTTGAA TAATACTACT ACTTATTCCA CAAATTCAAG   
  
  
+ CAGTTCCAGA TCTTCCCAAC AAACCCATCA TAATCATAAT CATACTCAGT ATTATCACCA ACATCATCCT   
  
  
+ ACTTACCCCC ACCGCCACCA TCAGGAGGAA GAAGAAGAAT GCTTCAACTT TTACATGGAT GAAGAAAACT   
  
  
+ TCTCTTCTTC TTCCTCCCAC AACAAACATC ATCCTCAACC TCCTTCCTCC GCCACCACCT CCTCGTATTA   
  
  
+ CCCTTACTAC TCCACCCCCT TCGATGTTCC CTCTACCACC ACCACCTCCA CCTCCACCCC CACGCCCACC   
  
  
+ AACACCACCA CCACCACCCC CCCAACCCAG TATGGCGGCG GCGGCGGTGG GTTTGACTCC AGCTGCTTCT   
  
  
+ CCTACTCCCC TGCCCGAGAC CTCAACCCGG AGCTCTTCTC CTCGTCCGGG TTCTCGGGCA AGTGGGCTTC   
  
  
+ CAACATCCTC TTGGAAGCCG CTCGTGCCAT CTCTGAGAGG AACACGAGCC GCCTCCACCA GCTCCTATGG   
  
  
+ ATGGTCAACG AGCTCAGCTC CCCGTACGGT GACGTGGAGC AGAAGATAGC TTCATACTTC GGCCAAGCCC   
  
  
+ TCTTCACCCG CATGACCGAG TCAGGGGACC GCACCTACCG CACCATGCGG TCCGCCGTGG ACAAGACCTT   
  
  
+ CTCCTTTGAG TCTACGAGAA AGATGGTACT CAAGTTCCAG GAGGTTAGCC CTTGGACCAC CTTTGGACAC   
  
  
+ GTGGCAGCTA ATGGAGCCCT CATTGATGCC TTTGACGGTG AATCCAAAAT CCACATCATC GACATCAGCA   
  
  
+ CCACATTCTG CACCCAGTGG CCCACCCTCC TGGAGGCGCT GGCCACCCGA ATGGATGACG CGCCCTACCT   
  
  
+ GAGGCTGACC ACTGTGGTGG TCAACAAGTC CGGGAATGAA GGTCCCACCG GAGGCGGGTC CCACAGGGTG   
  
  
+ ATGAAAGAGA TTGGGACCCG CCTCGAGAAG TTTGCTAGGC TAATGGGAGT GCCTTTCAAA TTTAATGTGG   
  
  
+ TCCACCACGG GGCTGATTTA TCCGACTTGG ATTTCTCTCA GCTGGATATT AACGATGACG AGGCATTAGC   
  
  
+ CATCAACTGT GTCAACTCGC TCCACTCGGT CAATATCCAC CGCCGTGACT CGGTCATCTC GGCCTTCCGG   
  
  
+ CGGCTCCACC CTAGGGTGGT GACCGTGGTG GAGGAGGAGG CTGACCTTGC TGACGTGGGG GCGGAGGGGT   
  
  
+ ACGAGTTCTA CAGGGGGTTT AATGAGTGTT TAAGGTGGGT TAGGGTTTAC TTTGAGGCCC TGGAAGAGTG   
  
  
+ CTTTCCTAGG ACAAGCAATG AGAAACTCAT GCTCGAGCGG GCCGCAGGGC GGGCTCTGGT GGACCTCCTG   
  
  
+ GCCTGCCCCA AGCCCGCATC ATCCGAGCGG AGGGAGGCAG CTGCGCGGTG GTCCGGGAGG ATGCAGGGGG   
  
  
+ CCGGGTTCGG CCACGTGGGG TTCAGTGATG AGGTCTGTGA TGACGTCAAG GCCCTCCTAA GGAGGTACAA   
  
  
+ GGAGGGGTGG TCCATGATAC AATGTTCCGC CGCGGATGGC GGCGCCTCCG CCGGAATATT CTTGTCGTGG   
  
  
+ AGGGATCAGC CGGTGGTGTG GGCCAGTGCA TGGAAGCCTT A  

- -Up\_Stream \_Len000CCCAAA AAAATTTGAG TTTTAATCAA TAACATAGTT AACTTAAACC TTTTGATAAT   
  
  
- AAAAGCTGAA TAATATCTCT TAAACAACAA TTACTTACGA CTATTTATGA CTATTAATCA CAACACTCAC   
  
  
- ACTTAAACAC TACTACTCGT TACTAATCGC AATTTTAATA TTGATCCTAT TTATTAATTC ACAACTACTT   
  
  
- ACAACTAACT ACTACTACTC ATGACTAACC ACGCCACGAC CATTATTTAT AAATACTATT CAATTTTTTT   
  
  
- TTTTTGTGTA GGATTTGTTA GTGATACATT ATATGATTTA CTTTTTTTAA CCATTACACT AAACTGTTAG   
  
  
- ATTCCCATTC CCACAAAATG GGTGTGTACA ATACTTTAAC CGAATGATAA ATGTTTGAAA AATGTCAATA   
  
  
- TACTAAGATG GATACACAAT AGTAGAAAGG CAGCTACATT AACTAATATA AGTTGTGTTA AATTAAGTAA   
  
  
- TGTTACATCG ATTAACTATG AGTTTTATTA CTTGATAATT ATTTATTAAT TAATATTGAA AATTTTCAAA   
  
  
- CAATTAAATT TTAATATATT ATATATTTAA ATCCGATGTA ATTAGTTTTT TAATCCGCAC AAGTAGCCAG   
  
  
- GTTTGAACTT GGCCTGGTCT GGCCTGGCTT CTAGCTAATT CTCTTTTTCA TGTCTGACTC CTGGCCTAGC   
  
  
- TTATTCTCAG CGTGGCCTGG TCCTAGCCTG GCCTTTTTAA ACCTGGTCTA GCCCTGGCTA GCTCCATCTG   
  
  
- GCTTAAATAA AAGTTTATGC TATCCTTATT TTGAATAACT AAGTTAACTA AAACCGGTTT TAGTACGATA   
  
  
- ATAACTAAAA TAAACGATTG AGAGTTCTCA TAATCAAACG TGAAGCGTAA TAGAAACCGG TTTTTTTAGT   
  
  
- TTTTCTTTCA GAGTTTGAGA ATTATCAAAA AATAAAATAA ACCAAATAAA AATTTTATCA GCCGGAAGCC   
  
  
- AGGCCTGATC ACCTGGCTTA TTCCATTAAA AAGCTTAGCT CCTGGTCTGG TTTACGTGAG TCAAGCTTGG   
  
  
- CCAAGTCAAG CCAGACTGAG CTAAAAACCA GGCCAGGTTC AAACTTGTCT TATTACATGT GTGGATCTTT   
  
  
- TTTTCTTAAT ACGTATATAT GAATTCCGGG GGTTGGGAAA AAGGGAAAGG CGGTTATGAA AAATGGTTCA   
  
  
- GGTTAAGAAA AGAAGAATCG ATACCGATCA TCAATAGGGA GGGATCGAAG GTGGGATTCA AAACTCTGTT   
  
  
- TTAGGGTAGA TACAATTATA AATAAAGAAC GTGTACAGTG GGTTATGTTC CCAAAGTAAC TTTTCTTTTC   
  
  
- CATAATCTAC ATTCTATACA TACATATATT TATCAAAACG TTATCTGATC TTCCTGATTT TTCCTTTTTC   
  
  
- CTTCAAATTT TCGTTTTTTG AAAGGGGAGA TCTTTTTTCC TTTGGTCTAA GGGAAAAGAT GGGGTTAAGG   
  
  
- GTATCAGAAA TGAGACAGAG AGAGAGAGAC AGAGAGAATG AGTACTACAA CTCCCTCCTT CCTTACTTAA   
  
  
- TTCCTGGGAT TCGATTCTAG GTTGGCATTC TATAACGGGG AAATTAGTGG GTAAAGTATA GAGATAGGTG   
  
  
- TCAATTTTTG GGTGGAGGAG GTAGGTGTAT TGACTATATA GAGAAGATGT GGTATGATAT TCCTTCTTCT   
  
  
- CTTTTTATCC TCACATAACA TAAAAATAAA CTCATTAAAT AAAATTCCTC TCTTTAAATG TACCAACCCA   
  
  
- ATTTACCTAT GGAACAAATC TGATCACTCA AAGGTTGTTG GTGTGGTGGT GGTAGTGGTT GTGGTTGTGG   
  
  
- TTGTGGTGGT TGTCGTTAGA AGACTAGTTA GGAGAAACTT ATTATGATGA TGAATAAGGT GTTTAAGTTC   
  
  
- GTCAAGGTCT AGAAGGGTTG TTTGGGTAGT ATTAGTATTA GTATGAGTCA TAATAGTGGT TGTAGTAGGA   
  
  
- TGAATGGGGG TGGCGGTGGT AGTCCTCCTT CTTCTTCTTA CGAAGTTGAA AATGTACCTA CTTCTTTTGA   
  
  
- AGAGAAGAAG AAGGAGGGTG TTGTTTGTAG TAGGAGTTGG AGGAAGGAGG CGGTGGTGGA GGAGCATAAT   
  
  
- GGGAATGATG AGGTGGGGGA AGCTACAAGG GAGATGGTGG TGGTGGAGGT GGAGGTGGGG GTGCGGGTGG   
  
  
- TTGTGGTGGT GGTGGTGGGG GGGTTGGGTC ATACCGCCGC CGCCGCCACC CAAACTGAGG TCGACGAAGA   
  
  
- GGATGAGGGG ACGGGCTCTG GAGTTGGGCC TCGAGAAGAG GAGCAGGCCC AAGAGCCCGT TCACCCGAAG   
  
  
- GTTGTAGGAG AACCTTCGGC GAGCACGGTA GAGACTCTCC TTGTGCTCGG CGGAGGTGGT CGAGGATACC   
  
  
- TACCAGTTGC TCGAGTCGAG GGGCATGCCA CTGCACCTCG TCTTCTATCG AAGTATGAAG CCGGTTCGGG   
  
  
- AGAAGTGGGC GTACTGGCTC AGTCCCCTGG CGTGGATGGC GTGGTACGCC AGGCGGCACC TGTTCTGGAA   
  
  
- GAGGAAACTC AGATGCTCTT TCTACCATGA GTTCAAGGTC CTCCAATCGG GAACCTGGTG GAAACCTGTG   
  
  
- CACCGTCGAT TACCTCGGGA GTAACTACGG AAACTGCCAC TTAGGTTTTA GGTGTAGTAG CTGTAGTCGT   
  
  
- GGTGTAAGAC GTGGGTCACC GGGTGGGAGG ACCTCCGCGA CCGGTGGGCT TACCTACTGC GCGGGATGGA   
  
  
- CTCCGACTGG TGACACCACC AGTTGTTCAG GCCCTTACTT CCAGGGTGGC CTCCGCCCAG GGTGTCCCAC   
  
  
- TACTTTCTCT AACCCTGGGC GGAGCTCTTC AAACGATCCG ATTACCCTCA CGGAAAGTTT AAATTACACC   
  
  
- AGGTGGTGCC CCGACTAAAT AGGCTGAACC TAAAGAGAGT CGACCTATAA TTGCTACTGC TCCGTAATCG   
  
  
- GTAGTTGACA CAGTTGAGCG AGGTGAGCCA GTTATAGGTG GCGGCACTGA GCCAGTAGAG CCGGAAGGCC   
  
  
- GCCGAGGTGG GATCCCACCA CTGGCACCAC CTCCTCCTCC GACTGGAACG ACTGCACCCC CGCCTCCCCA   
  
  
- TGCTCAAGAT GTCCCCCAAA TTACTCACAA ATTCCACCCA ATCCCAAATG AAACTCCGGG ACCTTCTCAC   
  
  
- GAAAGGATCC TGTTCGTTAC TCTTTGAGTA CGAGCTCGCC CGGCGTCCCG CCCGAGACCA CCTGGAGGAC   
  
  
- CGGACGGGGT TCGGGCGTAG TAGGCTCGCC TCCCTCCGTC GACGCGCCAC CAGGCCCTCC TACGTCCCCC   
  
  
- GGCCCAAGCC GGTGCACCCC AAGTCACTAC TCCAGACACT ACTGCAGTTC CGGGAGGATT CCTCCATGTT   
  
  
- CCTCCCCACC AGGTACTATG TTACAAGGCG GCGCCTACCG CCGCGGAGGC GGCCTTATAA GAACAGCACC   
  
  
- TCCCTAGTCG GCCACCACAC CCGGTCACGT ACCTTCGGAA T

+     G-box

| Site Name | Organism | Position | Strand | Matrix score. | sequence | function |
| --- | --- | --- | --- | --- | --- | --- |
| G-box | Zea mays | 3428 | - | 6 | CACGAC | cis-acting regulatory element involved in light responsiveness |
| G-box | Arabidopsis thaliana | 3306 | - | 6 | CACGTG | cis-acting regulatory element involved in light responsiveness |
| G-box | Zea mays | 3066 | - | 6 | CACGTC | cis-acting regulatory element involved in light responsiveness |
| G-box | Brassica napus | 2591 | + | 9 | ACACGTGGC | cis-acting regulatory element involved in light responsiveness |
| G-box | Lycopersicon esculentum | 2589 | + | 11 | tgACACGTGGCA | cis-acting regulatory element involved in light responsiveness |
| G-box | Arabidopsis thaliana | 3304 | + | 9 | GCCACGTGGA | cis-acting regulatory element involved in light responsiveness |
| G-box | Arabidopsis thaliana | 2592 | - | 6 | CACGTG | cis-acting regulatory element involved in light responsiveness |
| G-box | Brassica oleracea | 435 | - | 9 | TAACACGTAG | cis-acting regulatory element involved in light responsiveness |
| G-box | Zea mays | 2415 | - | 6 | CACGTC | cis-acting regulatory element involved in light responsiveness |

>HU06G00358.1   
+ -Up\_Stream \_Len000GGGTTT TTTTAAACTC AAAATTAGTT ATTGTATCAA TTGAATTTGG AAAACTATTA   
  
  
+ TTTTCGACTT ATTATAGAGA ATTTGTTGTT AATGAATGCT GATAAATACT GATAATTAGT GTTGTGAGTG   
  
  
+ TGAATTTGTG ATGATGAGCA ATGATTAGCG TTAAAATTAT AACTAGGATA AATAATTAAG TGTTGATGAA   
  
  
+ TGTTGATTGA TGATGATGAG TACTGATTGG TGCGGTGCTG GTAATAAATA TTTATGATAA GTTAAAAAAA   
  
  
+ AAAAACACAT CCTAAACAAT CACTATGTAA TATACTAAAT GAAAAAAATT GGTAATGTGA TTTGACAATC   
  
  
+ TAAGGGTAAG GGTGTTTTAC CCACACATGT TATGAAATTG GCTTACTATT TACAAACTTT TTACAGTTAT   
  
  
+ ATGATTCTAC CTATGTGTTA TCATCTTTCC GTCGATGTAA TTGATTATAT TCAACACAAT TTAATTCATT   
  
  
+ ACAATGTAGC TAATTGATAC TCAAAATAAT GAACTATTAA TAAATAATTA ATTATAACTT TTAAAAGTTT   
  
  
+ GTTAATTTAA AATTATATAA TATATAAATT TAGGCTACAT TAATCAAAAA ATTAGGCGTG TTCATCGGTC   
  
  
+ CAAACTTGAA CCGGACCAGA CCGGACCGAA GATCGATTAA GAGAAAAAGT ACAGACTGAG GACCGGATCG   
  
  
+ AATAAGAGTC GCACCGGACC AGGATCGGAC CGGAAAAATT TGGACCAGAT CGGGACCGAT CGAGGTAGAC   
  
  
+ CGAATTTATT TTCAAATACG ATAGGAATAA AACTTATTGA TTCAATTGAT TTTGGCCAAA ATCATGCTAT   
  
  
+ TATTGATTTT ATTTGCTAAC TCTCAAGAGT ATTAGTTTGC ACTTCGCATT ATCTTTGGCC AAAAAAATCA   
  
  
+ AAAAGAAAGT CTCAAACTCT TAATAGTTTT TTATTTTATT TGGTTTATTT TTAAAATAGT CGGCCTTCGG   
  
  
+ TCCGGACTAG TGGACCGAAT AAGGTAATTT TTCGAATCGA GGACCAGACC AAATGCACTC AGTTCGAACC   
  
  
+ GGTTCAGTTC GGTCTGACTC GATTTTTGGT CCGGTCCAAG TTTGAACAGA ATAATGTACA CACCTAGAAA   
  
  
+ AAAAGAATTA TGCATATATA CTTAAGGCCC CCAACCCTTT TTCCCTTTCC GCCAATACTT TTTACCAAGT   
  
  
+ CCAATTCTTT TCTTCTTAGC TATGGCTAGT AGTTATCCCT CCCTAGCTTC CACCCTAAGT TTTGAGACAA   
  
  
+ AATCCCATCT ATGTTAATAT TTATTTCTTG CACATGTCAC CCAATACAAG GGTTTCATTG AAAAGAAAAG   
  
  
+ GTATTAGATG TAAGATATGT ATGTATATAA ATAGTTTTGC AATAGACTAG AAGGACTAAA AAGGAAAAAG   
  
  
+ GAAGTTTAAA AGCAAAAAAC TTTCCCCTCT AGAAAAAAGG AAACCAGATT CCCTTTTCTA CCCCAATTCC   
  
  
+ CATAGTCTTT ACTCTGTCTC TCTCTCTCTG TCTCTCTTAC TCATGATGTT GAGGGAGGAA GGAATGAATT   
  
  
+ AAGGACCCTA AGCTAAGATC CAACCGTAAG ATATTGCCCC TTTAATCACC CATTTCATAT CTCTATCCAC   
  
  
+ AGTTAAAAAC CCACCTCCTC CATCCACATA ACTGATATAT CTCTTCTACA CCATACTATA AGGAAGAAGA   
  
  
+ GAAAAATAGG AGTGTATTGT ATTTTTATTT GAGTAATTTA TTTTAAGGAG AGAAATTTAC ATGGTTGGGT   
  
  
+ TAAATGGATA CCTTGTTTAG ACTAGTGAGT TTCCAACAAC CACACCACCA CCATCACCAA CACCAACACC   
  
  
+ AACACCACCA ACAGCAATCT TCTGATCAAT CCTCTTTGAA TAATACTACT ACTTATTCCA CAAATTCAAG   
  
  
+ CAGTTCCAGA TCTTCCCAAC AAACCCATCA TAATCATAAT CATACTCAGT ATTATCACCA ACATCATCCT   
  
  
+ ACTTACCCCC ACCGCCACCA TCAGGAGGAA GAAGAAGAAT GCTTCAACTT TTACATGGAT GAAGAAAACT   
  
  
+ TCTCTTCTTC TTCCTCCCAC AACAAACATC ATCCTCAACC TCCTTCCTCC GCCACCACCT CCTCGTATTA   
  
  
+ CCCTTACTAC TCCACCCCCT TCGATGTTCC CTCTACCACC ACCACCTCCA CCTCCACCCC CACGCCCACC   
  
  
+ AACACCACCA CCACCACCCC CCCAACCCAG TATGGCGGCG GCGGCGGTGG GTTTGACTCC AGCTGCTTCT   
  
  
+ CCTACTCCCC TGCCCGAGAC CTCAACCCGG AGCTCTTCTC CTCGTCCGGG TTCTCGGGCA AGTGGGCTTC   
  
  
+ CAACATCCTC TTGGAAGCCG CTCGTGCCAT CTCTGAGAGG AACACGAGCC GCCTCCACCA GCTCCTATGG   
  
  
+ ATGGTCAACG AGCTCAGCTC CCCGTACGGT GACGTGGAGC AGAAGATAGC TTCATACTTC GGCCAAGCCC   
  
  
+ TCTTCACCCG CATGACCGAG TCAGGGGACC GCACCTACCG CACCATGCGG TCCGCCGTGG ACAAGACCTT   
  
  
+ CTCCTTTGAG TCTACGAGAA AGATGGTACT CAAGTTCCAG GAGGTTAGCC CTTGGACCAC CTTTGGACAC   
  
  
+ GTGGCAGCTA ATGGAGCCCT CATTGATGCC TTTGACGGTG AATCCAAAAT CCACATCATC GACATCAGCA   
  
  
+ CCACATTCTG CACCCAGTGG CCCACCCTCC TGGAGGCGCT GGCCACCCGA ATGGATGACG CGCCCTACCT   
  
  
+ GAGGCTGACC ACTGTGGTGG TCAACAAGTC CGGGAATGAA GGTCCCACCG GAGGCGGGTC CCACAGGGTG   
  
  
+ ATGAAAGAGA TTGGGACCCG CCTCGAGAAG TTTGCTAGGC TAATGGGAGT GCCTTTCAAA TTTAATGTGG   
  
  
+ TCCACCACGG GGCTGATTTA TCCGACTTGG ATTTCTCTCA GCTGGATATT AACGATGACG AGGCATTAGC   
  
  
+ CATCAACTGT GTCAACTCGC TCCACTCGGT CAATATCCAC CGCCGTGACT CGGTCATCTC GGCCTTCCGG   
  
  
+ CGGCTCCACC CTAGGGTGGT GACCGTGGTG GAGGAGGAGG CTGACCTTGC TGACGTGGGG GCGGAGGGGT   
  
  
+ ACGAGTTCTA CAGGGGGTTT AATGAGTGTT TAAGGTGGGT TAGGGTTTAC TTTGAGGCCC TGGAAGAGTG   
  
  
+ CTTTCCTAGG ACAAGCAATG AGAAACTCAT GCTCGAGCGG GCCGCAGGGC GGGCTCTGGT GGACCTCCTG   
  
  
+ GCCTGCCCCA AGCCCGCATC ATCCGAGCGG AGGGAGGCAG CTGCGCGGTG GTCCGGGAGG ATGCAGGGGG   
  
  
+ CCGGGTTCGG CCACGTGGGG TTCAGTGATG AGGTCTGTGA TGACGTCAAG GCCCTCCTAA GGAGGTACAA   
  
  
+ GGAGGGGTGG TCCATGATAC AATGTTCCGC CGCGGATGGC GGCGCCTCCG CCGGAATATT CTTGTCGTGG   
  
  
+ AGGGATCAGC CGGTGGTGTG GGCCAGTGCA TGGAAGCCTT A  

- -Up\_Stream \_Len000CCCAAA AAAATTTGAG TTTTAATCAA TAACATAGTT AACTTAAACC TTTTGATAAT   
  
  
- AAAAGCTGAA TAATATCTCT TAAACAACAA TTACTTACGA CTATTTATGA CTATTAATCA CAACACTCAC   
  
  
- ACTTAAACAC TACTACTCGT TACTAATCGC AATTTTAATA TTGATCCTAT TTATTAATTC ACAACTACTT   
  
  
- ACAACTAACT ACTACTACTC ATGACTAACC ACGCCACGAC CATTATTTAT AAATACTATT CAATTTTTTT   
  
  
- TTTTTGTGTA GGATTTGTTA GTGATACATT ATATGATTTA CTTTTTTTAA CCATTACACT AAACTGTTAG   
  
  
- ATTCCCATTC CCACAAAATG GGTGTGTACA ATACTTTAAC CGAATGATAA ATGTTTGAAA AATGTCAATA   
  
  
- TACTAAGATG GATACACAAT AGTAGAAAGG CAGCTACATT AACTAATATA AGTTGTGTTA AATTAAGTAA   
  
  
- TGTTACATCG ATTAACTATG AGTTTTATTA CTTGATAATT ATTTATTAAT TAATATTGAA AATTTTCAAA   
  
  
- CAATTAAATT TTAATATATT ATATATTTAA ATCCGATGTA ATTAGTTTTT TAATCCGCAC AAGTAGCCAG   
  
  
- GTTTGAACTT GGCCTGGTCT GGCCTGGCTT CTAGCTAATT CTCTTTTTCA TGTCTGACTC CTGGCCTAGC   
  
  
- TTATTCTCAG CGTGGCCTGG TCCTAGCCTG GCCTTTTTAA ACCTGGTCTA GCCCTGGCTA GCTCCATCTG   
  
  
- GCTTAAATAA AAGTTTATGC TATCCTTATT TTGAATAACT AAGTTAACTA AAACCGGTTT TAGTACGATA   
  
  
- ATAACTAAAA TAAACGATTG AGAGTTCTCA TAATCAAACG TGAAGCGTAA TAGAAACCGG TTTTTTTAGT   
  
  
- TTTTCTTTCA GAGTTTGAGA ATTATCAAAA AATAAAATAA ACCAAATAAA AATTTTATCA GCCGGAAGCC   
  
  
- AGGCCTGATC ACCTGGCTTA TTCCATTAAA AAGCTTAGCT CCTGGTCTGG TTTACGTGAG TCAAGCTTGG   
  
  
- CCAAGTCAAG CCAGACTGAG CTAAAAACCA GGCCAGGTTC AAACTTGTCT TATTACATGT GTGGATCTTT   
  
  
- TTTTCTTAAT ACGTATATAT GAATTCCGGG GGTTGGGAAA AAGGGAAAGG CGGTTATGAA AAATGGTTCA   
  
  
- GGTTAAGAAA AGAAGAATCG ATACCGATCA TCAATAGGGA GGGATCGAAG GTGGGATTCA AAACTCTGTT   
  
  
- TTAGGGTAGA TACAATTATA AATAAAGAAC GTGTACAGTG GGTTATGTTC CCAAAGTAAC TTTTCTTTTC   
  
  
- CATAATCTAC ATTCTATACA TACATATATT TATCAAAACG TTATCTGATC TTCCTGATTT TTCCTTTTTC   
  
  
- CTTCAAATTT TCGTTTTTTG AAAGGGGAGA TCTTTTTTCC TTTGGTCTAA GGGAAAAGAT GGGGTTAAGG   
  
  
- GTATCAGAAA TGAGACAGAG AGAGAGAGAC AGAGAGAATG AGTACTACAA CTCCCTCCTT CCTTACTTAA   
  
  
- TTCCTGGGAT TCGATTCTAG GTTGGCATTC TATAACGGGG AAATTAGTGG GTAAAGTATA GAGATAGGTG   
  
  
- TCAATTTTTG GGTGGAGGAG GTAGGTGTAT TGACTATATA GAGAAGATGT GGTATGATAT TCCTTCTTCT   
  
  
- CTTTTTATCC TCACATAACA TAAAAATAAA CTCATTAAAT AAAATTCCTC TCTTTAAATG TACCAACCCA   
  
  
- ATTTACCTAT GGAACAAATC TGATCACTCA AAGGTTGTTG GTGTGGTGGT GGTAGTGGTT GTGGTTGTGG   
  
  
- TTGTGGTGGT TGTCGTTAGA AGACTAGTTA GGAGAAACTT ATTATGATGA TGAATAAGGT GTTTAAGTTC   
  
  
- GTCAAGGTCT AGAAGGGTTG TTTGGGTAGT ATTAGTATTA GTATGAGTCA TAATAGTGGT TGTAGTAGGA   
  
  
- TGAATGGGGG TGGCGGTGGT AGTCCTCCTT CTTCTTCTTA CGAAGTTGAA AATGTACCTA CTTCTTTTGA   
  
  
- AGAGAAGAAG AAGGAGGGTG TTGTTTGTAG TAGGAGTTGG AGGAAGGAGG CGGTGGTGGA GGAGCATAAT   
  
  
- GGGAATGATG AGGTGGGGGA AGCTACAAGG GAGATGGTGG TGGTGGAGGT GGAGGTGGGG GTGCGGGTGG   
  
  
- TTGTGGTGGT GGTGGTGGGG GGGTTGGGTC ATACCGCCGC CGCCGCCACC CAAACTGAGG TCGACGAAGA   
  
  
- GGATGAGGGG ACGGGCTCTG GAGTTGGGCC TCGAGAAGAG GAGCAGGCCC AAGAGCCCGT TCACCCGAAG   
  
  
- GTTGTAGGAG AACCTTCGGC GAGCACGGTA GAGACTCTCC TTGTGCTCGG CGGAGGTGGT CGAGGATACC   
  
  
- TACCAGTTGC TCGAGTCGAG GGGCATGCCA CTGCACCTCG TCTTCTATCG AAGTATGAAG CCGGTTCGGG   
  
  
- AGAAGTGGGC GTACTGGCTC AGTCCCCTGG CGTGGATGGC GTGGTACGCC AGGCGGCACC TGTTCTGGAA   
  
  
- GAGGAAACTC AGATGCTCTT TCTACCATGA GTTCAAGGTC CTCCAATCGG GAACCTGGTG GAAACCTGTG   
  
  
- CACCGTCGAT TACCTCGGGA GTAACTACGG AAACTGCCAC TTAGGTTTTA GGTGTAGTAG CTGTAGTCGT   
  
  
- GGTGTAAGAC GTGGGTCACC GGGTGGGAGG ACCTCCGCGA CCGGTGGGCT TACCTACTGC GCGGGATGGA   
  
  
- CTCCGACTGG TGACACCACC AGTTGTTCAG GCCCTTACTT CCAGGGTGGC CTCCGCCCAG GGTGTCCCAC   
  
  
- TACTTTCTCT AACCCTGGGC GGAGCTCTTC AAACGATCCG ATTACCCTCA CGGAAAGTTT AAATTACACC   
  
  
- AGGTGGTGCC CCGACTAAAT AGGCTGAACC TAAAGAGAGT CGACCTATAA TTGCTACTGC TCCGTAATCG   
  
  
- GTAGTTGACA CAGTTGAGCG AGGTGAGCCA GTTATAGGTG GCGGCACTGA GCCAGTAGAG CCGGAAGGCC   
  
  
- GCCGAGGTGG GATCCCACCA CTGGCACCAC CTCCTCCTCC GACTGGAACG ACTGCACCCC CGCCTCCCCA   
  
  
- TGCTCAAGAT GTCCCCCAAA TTACTCACAA ATTCCACCCA ATCCCAAATG AAACTCCGGG ACCTTCTCAC   
  
  
- GAAAGGATCC TGTTCGTTAC TCTTTGAGTA CGAGCTCGCC CGGCGTCCCG CCCGAGACCA CCTGGAGGAC   
  
  
- CGGACGGGGT TCGGGCGTAG TAGGCTCGCC TCCCTCCGTC GACGCGCCAC CAGGCCCTCC TACGTCCCCC   
  
  
- GGCCCAAGCC GGTGCACCCC AAGTCACTAC TCCAGACACT ACTGCAGTTC CGGGAGGATT CCTCCATGTT   
  
  
- CCTCCCCACC AGGTACTATG TTACAAGGCG GCGCCTACCG CCGCGGAGGC GGCCTTATAA GAACAGCACC   
  
  
- TCCCTAGTCG GCCACCACAC CCGGTCACGT ACCTTCGGAA T

+     GATA-motif

| Site Name | Organism | Position | Strand | Matrix score. | sequence | function |
| --- | --- | --- | --- | --- | --- | --- |
| GATA-motif | Solanum tuberosum | 356 | + | 9 | AAGGATAAGG | part of a light responsive element |
| GATA-motif | Solanum tuberosum | 2521 | - | 9 | AAGGATAAGG | part of a light responsive element |
| GATA-motif | Arabidopsis thaliana | 794 | + | 7 | GATAGGA | part of a light responsive element |

>HU06G00358.1   
+ -Up\_Stream \_Len000GGGTTT TTTTAAACTC AAAATTAGTT ATTGTATCAA TTGAATTTGG AAAACTATTA   
  
  
+ TTTTCGACTT ATTATAGAGA ATTTGTTGTT AATGAATGCT GATAAATACT GATAATTAGT GTTGTGAGTG   
  
  
+ TGAATTTGTG ATGATGAGCA ATGATTAGCG TTAAAATTAT AACTAGGATA AATAATTAAG TGTTGATGAA   
  
  
+ TGTTGATTGA TGATGATGAG TACTGATTGG TGCGGTGCTG GTAATAAATA TTTATGATAA GTTAAAAAAA   
  
  
+ AAAAACACAT CCTAAACAAT CACTATGTAA TATACTAAAT GAAAAAAATT GGTAATGTGA TTTGACAATC   
  
  
+ TAAGGGTAAG GGTGTTTTAC CCACACATGT TATGAAATTG GCTTACTATT TACAAACTTT TTACAGTTAT   
  
  
+ ATGATTCTAC CTATGTGTTA TCATCTTTCC GTCGATGTAA TTGATTATAT TCAACACAAT TTAATTCATT   
  
  
+ ACAATGTAGC TAATTGATAC TCAAAATAAT GAACTATTAA TAAATAATTA ATTATAACTT TTAAAAGTTT   
  
  
+ GTTAATTTAA AATTATATAA TATATAAATT TAGGCTACAT TAATCAAAAA ATTAGGCGTG TTCATCGGTC   
  
  
+ CAAACTTGAA CCGGACCAGA CCGGACCGAA GATCGATTAA GAGAAAAAGT ACAGACTGAG GACCGGATCG   
  
  
+ AATAAGAGTC GCACCGGACC AGGATCGGAC CGGAAAAATT TGGACCAGAT CGGGACCGAT CGAGGTAGAC   
  
  
+ CGAATTTATT TTCAAATACG ATAGGAATAA AACTTATTGA TTCAATTGAT TTTGGCCAAA ATCATGCTAT   
  
  
+ TATTGATTTT ATTTGCTAAC TCTCAAGAGT ATTAGTTTGC ACTTCGCATT ATCTTTGGCC AAAAAAATCA   
  
  
+ AAAAGAAAGT CTCAAACTCT TAATAGTTTT TTATTTTATT TGGTTTATTT TTAAAATAGT CGGCCTTCGG   
  
  
+ TCCGGACTAG TGGACCGAAT AAGGTAATTT TTCGAATCGA GGACCAGACC AAATGCACTC AGTTCGAACC   
  
  
+ GGTTCAGTTC GGTCTGACTC GATTTTTGGT CCGGTCCAAG TTTGAACAGA ATAATGTACA CACCTAGAAA   
  
  
+ AAAAGAATTA TGCATATATA CTTAAGGCCC CCAACCCTTT TTCCCTTTCC GCCAATACTT TTTACCAAGT   
  
  
+ CCAATTCTTT TCTTCTTAGC TATGGCTAGT AGTTATCCCT CCCTAGCTTC CACCCTAAGT TTTGAGACAA   
  
  
+ AATCCCATCT ATGTTAATAT TTATTTCTTG CACATGTCAC CCAATACAAG GGTTTCATTG AAAAGAAAAG   
  
  
+ GTATTAGATG TAAGATATGT ATGTATATAA ATAGTTTTGC AATAGACTAG AAGGACTAAA AAGGAAAAAG   
  
  
+ GAAGTTTAAA AGCAAAAAAC TTTCCCCTCT AGAAAAAAGG AAACCAGATT CCCTTTTCTA CCCCAATTCC   
  
  
+ CATAGTCTTT ACTCTGTCTC TCTCTCTCTG TCTCTCTTAC TCATGATGTT GAGGGAGGAA GGAATGAATT   
  
  
+ AAGGACCCTA AGCTAAGATC CAACCGTAAG ATATTGCCCC TTTAATCACC CATTTCATAT CTCTATCCAC   
  
  
+ AGTTAAAAAC CCACCTCCTC CATCCACATA ACTGATATAT CTCTTCTACA CCATACTATA AGGAAGAAGA   
  
  
+ GAAAAATAGG AGTGTATTGT ATTTTTATTT GAGTAATTTA TTTTAAGGAG AGAAATTTAC ATGGTTGGGT   
  
  
+ TAAATGGATA CCTTGTTTAG ACTAGTGAGT TTCCAACAAC CACACCACCA CCATCACCAA CACCAACACC   
  
  
+ AACACCACCA ACAGCAATCT TCTGATCAAT CCTCTTTGAA TAATACTACT ACTTATTCCA CAAATTCAAG   
  
  
+ CAGTTCCAGA TCTTCCCAAC AAACCCATCA TAATCATAAT CATACTCAGT ATTATCACCA ACATCATCCT   
  
  
+ ACTTACCCCC ACCGCCACCA TCAGGAGGAA GAAGAAGAAT GCTTCAACTT TTACATGGAT GAAGAAAACT   
  
  
+ TCTCTTCTTC TTCCTCCCAC AACAAACATC ATCCTCAACC TCCTTCCTCC GCCACCACCT CCTCGTATTA   
  
  
+ CCCTTACTAC TCCACCCCCT TCGATGTTCC CTCTACCACC ACCACCTCCA CCTCCACCCC CACGCCCACC   
  
  
+ AACACCACCA CCACCACCCC CCCAACCCAG TATGGCGGCG GCGGCGGTGG GTTTGACTCC AGCTGCTTCT   
  
  
+ CCTACTCCCC TGCCCGAGAC CTCAACCCGG AGCTCTTCTC CTCGTCCGGG TTCTCGGGCA AGTGGGCTTC   
  
  
+ CAACATCCTC TTGGAAGCCG CTCGTGCCAT CTCTGAGAGG AACACGAGCC GCCTCCACCA GCTCCTATGG   
  
  
+ ATGGTCAACG AGCTCAGCTC CCCGTACGGT GACGTGGAGC AGAAGATAGC TTCATACTTC GGCCAAGCCC   
  
  
+ TCTTCACCCG CATGACCGAG TCAGGGGACC GCACCTACCG CACCATGCGG TCCGCCGTGG ACAAGACCTT   
  
  
+ CTCCTTTGAG TCTACGAGAA AGATGGTACT CAAGTTCCAG GAGGTTAGCC CTTGGACCAC CTTTGGACAC   
  
  
+ GTGGCAGCTA ATGGAGCCCT CATTGATGCC TTTGACGGTG AATCCAAAAT CCACATCATC GACATCAGCA   
  
  
+ CCACATTCTG CACCCAGTGG CCCACCCTCC TGGAGGCGCT GGCCACCCGA ATGGATGACG CGCCCTACCT   
  
  
+ GAGGCTGACC ACTGTGGTGG TCAACAAGTC CGGGAATGAA GGTCCCACCG GAGGCGGGTC CCACAGGGTG   
  
  
+ ATGAAAGAGA TTGGGACCCG CCTCGAGAAG TTTGCTAGGC TAATGGGAGT GCCTTTCAAA TTTAATGTGG   
  
  
+ TCCACCACGG GGCTGATTTA TCCGACTTGG ATTTCTCTCA GCTGGATATT AACGATGACG AGGCATTAGC   
  
  
+ CATCAACTGT GTCAACTCGC TCCACTCGGT CAATATCCAC CGCCGTGACT CGGTCATCTC GGCCTTCCGG   
  
  
+ CGGCTCCACC CTAGGGTGGT GACCGTGGTG GAGGAGGAGG CTGACCTTGC TGACGTGGGG GCGGAGGGGT   
  
  
+ ACGAGTTCTA CAGGGGGTTT AATGAGTGTT TAAGGTGGGT TAGGGTTTAC TTTGAGGCCC TGGAAGAGTG   
  
  
+ CTTTCCTAGG ACAAGCAATG AGAAACTCAT GCTCGAGCGG GCCGCAGGGC GGGCTCTGGT GGACCTCCTG   
  
  
+ GCCTGCCCCA AGCCCGCATC ATCCGAGCGG AGGGAGGCAG CTGCGCGGTG GTCCGGGAGG ATGCAGGGGG   
  
  
+ CCGGGTTCGG CCACGTGGGG TTCAGTGATG AGGTCTGTGA TGACGTCAAG GCCCTCCTAA GGAGGTACAA   
  
  
+ GGAGGGGTGG TCCATGATAC AATGTTCCGC CGCGGATGGC GGCGCCTCCG CCGGAATATT CTTGTCGTGG   
  
  
+ AGGGATCAGC CGGTGGTGTG GGCCAGTGCA TGGAAGCCTT A  

- -Up\_Stream \_Len000CCCAAA AAAATTTGAG TTTTAATCAA TAACATAGTT AACTTAAACC TTTTGATAAT   
  
  
- AAAAGCTGAA TAATATCTCT TAAACAACAA TTACTTACGA CTATTTATGA CTATTAATCA CAACACTCAC   
  
  
- ACTTAAACAC TACTACTCGT TACTAATCGC AATTTTAATA TTGATCCTAT TTATTAATTC ACAACTACTT   
  
  
- ACAACTAACT ACTACTACTC ATGACTAACC ACGCCACGAC CATTATTTAT AAATACTATT CAATTTTTTT   
  
  
- TTTTTGTGTA GGATTTGTTA GTGATACATT ATATGATTTA CTTTTTTTAA CCATTACACT AAACTGTTAG   
  
  
- ATTCCCATTC CCACAAAATG GGTGTGTACA ATACTTTAAC CGAATGATAA ATGTTTGAAA AATGTCAATA   
  
  
- TACTAAGATG GATACACAAT AGTAGAAAGG CAGCTACATT AACTAATATA AGTTGTGTTA AATTAAGTAA   
  
  
- TGTTACATCG ATTAACTATG AGTTTTATTA CTTGATAATT ATTTATTAAT TAATATTGAA AATTTTCAAA   
  
  
- CAATTAAATT TTAATATATT ATATATTTAA ATCCGATGTA ATTAGTTTTT TAATCCGCAC AAGTAGCCAG   
  
  
- GTTTGAACTT GGCCTGGTCT GGCCTGGCTT CTAGCTAATT CTCTTTTTCA TGTCTGACTC CTGGCCTAGC   
  
  
- TTATTCTCAG CGTGGCCTGG TCCTAGCCTG GCCTTTTTAA ACCTGGTCTA GCCCTGGCTA GCTCCATCTG   
  
  
- GCTTAAATAA AAGTTTATGC TATCCTTATT TTGAATAACT AAGTTAACTA AAACCGGTTT TAGTACGATA   
  
  
- ATAACTAAAA TAAACGATTG AGAGTTCTCA TAATCAAACG TGAAGCGTAA TAGAAACCGG TTTTTTTAGT   
  
  
- TTTTCTTTCA GAGTTTGAGA ATTATCAAAA AATAAAATAA ACCAAATAAA AATTTTATCA GCCGGAAGCC   
  
  
- AGGCCTGATC ACCTGGCTTA TTCCATTAAA AAGCTTAGCT CCTGGTCTGG TTTACGTGAG TCAAGCTTGG   
  
  
- CCAAGTCAAG CCAGACTGAG CTAAAAACCA GGCCAGGTTC AAACTTGTCT TATTACATGT GTGGATCTTT   
  
  
- TTTTCTTAAT ACGTATATAT GAATTCCGGG GGTTGGGAAA AAGGGAAAGG CGGTTATGAA AAATGGTTCA   
  
  
- GGTTAAGAAA AGAAGAATCG ATACCGATCA TCAATAGGGA GGGATCGAAG GTGGGATTCA AAACTCTGTT   
  
  
- TTAGGGTAGA TACAATTATA AATAAAGAAC GTGTACAGTG GGTTATGTTC CCAAAGTAAC TTTTCTTTTC   
  
  
- CATAATCTAC ATTCTATACA TACATATATT TATCAAAACG TTATCTGATC TTCCTGATTT TTCCTTTTTC   
  
  
- CTTCAAATTT TCGTTTTTTG AAAGGGGAGA TCTTTTTTCC TTTGGTCTAA GGGAAAAGAT GGGGTTAAGG   
  
  
- GTATCAGAAA TGAGACAGAG AGAGAGAGAC AGAGAGAATG AGTACTACAA CTCCCTCCTT CCTTACTTAA   
  
  
- TTCCTGGGAT TCGATTCTAG GTTGGCATTC TATAACGGGG AAATTAGTGG GTAAAGTATA GAGATAGGTG   
  
  
- TCAATTTTTG GGTGGAGGAG GTAGGTGTAT TGACTATATA GAGAAGATGT GGTATGATAT TCCTTCTTCT   
  
  
- CTTTTTATCC TCACATAACA TAAAAATAAA CTCATTAAAT AAAATTCCTC TCTTTAAATG TACCAACCCA   
  
  
- ATTTACCTAT GGAACAAATC TGATCACTCA AAGGTTGTTG GTGTGGTGGT GGTAGTGGTT GTGGTTGTGG   
  
  
- TTGTGGTGGT TGTCGTTAGA AGACTAGTTA GGAGAAACTT ATTATGATGA TGAATAAGGT GTTTAAGTTC   
  
  
- GTCAAGGTCT AGAAGGGTTG TTTGGGTAGT ATTAGTATTA GTATGAGTCA TAATAGTGGT TGTAGTAGGA   
  
  
- TGAATGGGGG TGGCGGTGGT AGTCCTCCTT CTTCTTCTTA CGAAGTTGAA AATGTACCTA CTTCTTTTGA   
  
  
- AGAGAAGAAG AAGGAGGGTG TTGTTTGTAG TAGGAGTTGG AGGAAGGAGG CGGTGGTGGA GGAGCATAAT   
  
  
- GGGAATGATG AGGTGGGGGA AGCTACAAGG GAGATGGTGG TGGTGGAGGT GGAGGTGGGG GTGCGGGTGG   
  
  
- TTGTGGTGGT GGTGGTGGGG GGGTTGGGTC ATACCGCCGC CGCCGCCACC CAAACTGAGG TCGACGAAGA   
  
  
- GGATGAGGGG ACGGGCTCTG GAGTTGGGCC TCGAGAAGAG GAGCAGGCCC AAGAGCCCGT TCACCCGAAG   
  
  
- GTTGTAGGAG AACCTTCGGC GAGCACGGTA GAGACTCTCC TTGTGCTCGG CGGAGGTGGT CGAGGATACC   
  
  
- TACCAGTTGC TCGAGTCGAG GGGCATGCCA CTGCACCTCG TCTTCTATCG AAGTATGAAG CCGGTTCGGG   
  
  
- AGAAGTGGGC GTACTGGCTC AGTCCCCTGG CGTGGATGGC GTGGTACGCC AGGCGGCACC TGTTCTGGAA   
  
  
- GAGGAAACTC AGATGCTCTT TCTACCATGA GTTCAAGGTC CTCCAATCGG GAACCTGGTG GAAACCTGTG   
  
  
- CACCGTCGAT TACCTCGGGA GTAACTACGG AAACTGCCAC TTAGGTTTTA GGTGTAGTAG CTGTAGTCGT   
  
  
- GGTGTAAGAC GTGGGTCACC GGGTGGGAGG ACCTCCGCGA CCGGTGGGCT TACCTACTGC GCGGGATGGA   
  
  
- CTCCGACTGG TGACACCACC AGTTGTTCAG GCCCTTACTT CCAGGGTGGC CTCCGCCCAG GGTGTCCCAC   
  
  
- TACTTTCTCT AACCCTGGGC GGAGCTCTTC AAACGATCCG ATTACCCTCA CGGAAAGTTT AAATTACACC   
  
  
- AGGTGGTGCC CCGACTAAAT AGGCTGAACC TAAAGAGAGT CGACCTATAA TTGCTACTGC TCCGTAATCG   
  
  
- GTAGTTGACA CAGTTGAGCG AGGTGAGCCA GTTATAGGTG GCGGCACTGA GCCAGTAGAG CCGGAAGGCC   
  
  
- GCCGAGGTGG GATCCCACCA CTGGCACCAC CTCCTCCTCC GACTGGAACG ACTGCACCCC CGCCTCCCCA   
  
  
- TGCTCAAGAT GTCCCCCAAA TTACTCACAA ATTCCACCCA ATCCCAAATG AAACTCCGGG ACCTTCTCAC   
  
  
- GAAAGGATCC TGTTCGTTAC TCTTTGAGTA CGAGCTCGCC CGGCGTCCCG CCCGAGACCA CCTGGAGGAC   
  
  
- CGGACGGGGT TCGGGCGTAG TAGGCTCGCC TCCCTCCGTC GACGCGCCAC CAGGCCCTCC TACGTCCCCC   
  
  
- GGCCCAAGCC GGTGCACCCC AAGTCACTAC TCCAGACACT ACTGCAGTTC CGGGAGGATT CCTCCATGTT   
  
  
- CCTCCCCACC AGGTACTATG TTACAAGGCG GCGCCTACCG CCGCGGAGGC GGCCTTATAA GAACAGCACC   
  
  
- TCCCTAGTCG GCCACCACAC CCGGTCACGT ACCTTCGGAA T

+     GT1-motif

| Site Name | Organism | Position | Strand | Matrix score. | sequence | function |
| --- | --- | --- | --- | --- | --- | --- |
| GT1-motif | Arabidopsis thaliana | 1752 | + | 6 | GGTTAA | light responsive element |

>HU06G00358.1   
+ -Up\_Stream \_Len000GGGTTT TTTTAAACTC AAAATTAGTT ATTGTATCAA TTGAATTTGG AAAACTATTA   
  
  
+ TTTTCGACTT ATTATAGAGA ATTTGTTGTT AATGAATGCT GATAAATACT GATAATTAGT GTTGTGAGTG   
  
  
+ TGAATTTGTG ATGATGAGCA ATGATTAGCG TTAAAATTAT AACTAGGATA AATAATTAAG TGTTGATGAA   
  
  
+ TGTTGATTGA TGATGATGAG TACTGATTGG TGCGGTGCTG GTAATAAATA TTTATGATAA GTTAAAAAAA   
  
  
+ AAAAACACAT CCTAAACAAT CACTATGTAA TATACTAAAT GAAAAAAATT GGTAATGTGA TTTGACAATC   
  
  
+ TAAGGGTAAG GGTGTTTTAC CCACACATGT TATGAAATTG GCTTACTATT TACAAACTTT TTACAGTTAT   
  
  
+ ATGATTCTAC CTATGTGTTA TCATCTTTCC GTCGATGTAA TTGATTATAT TCAACACAAT TTAATTCATT   
  
  
+ ACAATGTAGC TAATTGATAC TCAAAATAAT GAACTATTAA TAAATAATTA ATTATAACTT TTAAAAGTTT   
  
  
+ GTTAATTTAA AATTATATAA TATATAAATT TAGGCTACAT TAATCAAAAA ATTAGGCGTG TTCATCGGTC   
  
  
+ CAAACTTGAA CCGGACCAGA CCGGACCGAA GATCGATTAA GAGAAAAAGT ACAGACTGAG GACCGGATCG   
  
  
+ AATAAGAGTC GCACCGGACC AGGATCGGAC CGGAAAAATT TGGACCAGAT CGGGACCGAT CGAGGTAGAC   
  
  
+ CGAATTTATT TTCAAATACG ATAGGAATAA AACTTATTGA TTCAATTGAT TTTGGCCAAA ATCATGCTAT   
  
  
+ TATTGATTTT ATTTGCTAAC TCTCAAGAGT ATTAGTTTGC ACTTCGCATT ATCTTTGGCC AAAAAAATCA   
  
  
+ AAAAGAAAGT CTCAAACTCT TAATAGTTTT TTATTTTATT TGGTTTATTT TTAAAATAGT CGGCCTTCGG   
  
  
+ TCCGGACTAG TGGACCGAAT AAGGTAATTT TTCGAATCGA GGACCAGACC AAATGCACTC AGTTCGAACC   
  
  
+ GGTTCAGTTC GGTCTGACTC GATTTTTGGT CCGGTCCAAG TTTGAACAGA ATAATGTACA CACCTAGAAA   
  
  
+ AAAAGAATTA TGCATATATA CTTAAGGCCC CCAACCCTTT TTCCCTTTCC GCCAATACTT TTTACCAAGT   
  
  
+ CCAATTCTTT TCTTCTTAGC TATGGCTAGT AGTTATCCCT CCCTAGCTTC CACCCTAAGT TTTGAGACAA   
  
  
+ AATCCCATCT ATGTTAATAT TTATTTCTTG CACATGTCAC CCAATACAAG GGTTTCATTG AAAAGAAAAG   
  
  
+ GTATTAGATG TAAGATATGT ATGTATATAA ATAGTTTTGC AATAGACTAG AAGGACTAAA AAGGAAAAAG   
  
  
+ GAAGTTTAAA AGCAAAAAAC TTTCCCCTCT AGAAAAAAGG AAACCAGATT CCCTTTTCTA CCCCAATTCC   
  
  
+ CATAGTCTTT ACTCTGTCTC TCTCTCTCTG TCTCTCTTAC TCATGATGTT GAGGGAGGAA GGAATGAATT   
  
  
+ AAGGACCCTA AGCTAAGATC CAACCGTAAG ATATTGCCCC TTTAATCACC CATTTCATAT CTCTATCCAC   
  
  
+ AGTTAAAAAC CCACCTCCTC CATCCACATA ACTGATATAT CTCTTCTACA CCATACTATA AGGAAGAAGA   
  
  
+ GAAAAATAGG AGTGTATTGT ATTTTTATTT GAGTAATTTA TTTTAAGGAG AGAAATTTAC ATGGTTGGGT   
  
  
+ TAAATGGATA CCTTGTTTAG ACTAGTGAGT TTCCAACAAC CACACCACCA CCATCACCAA CACCAACACC   
  
  
+ AACACCACCA ACAGCAATCT TCTGATCAAT CCTCTTTGAA TAATACTACT ACTTATTCCA CAAATTCAAG   
  
  
+ CAGTTCCAGA TCTTCCCAAC AAACCCATCA TAATCATAAT CATACTCAGT ATTATCACCA ACATCATCCT   
  
  
+ ACTTACCCCC ACCGCCACCA TCAGGAGGAA GAAGAAGAAT GCTTCAACTT TTACATGGAT GAAGAAAACT   
  
  
+ TCTCTTCTTC TTCCTCCCAC AACAAACATC ATCCTCAACC TCCTTCCTCC GCCACCACCT CCTCGTATTA   
  
  
+ CCCTTACTAC TCCACCCCCT TCGATGTTCC CTCTACCACC ACCACCTCCA CCTCCACCCC CACGCCCACC   
  
  
+ AACACCACCA CCACCACCCC CCCAACCCAG TATGGCGGCG GCGGCGGTGG GTTTGACTCC AGCTGCTTCT   
  
  
+ CCTACTCCCC TGCCCGAGAC CTCAACCCGG AGCTCTTCTC CTCGTCCGGG TTCTCGGGCA AGTGGGCTTC   
  
  
+ CAACATCCTC TTGGAAGCCG CTCGTGCCAT CTCTGAGAGG AACACGAGCC GCCTCCACCA GCTCCTATGG   
  
  
+ ATGGTCAACG AGCTCAGCTC CCCGTACGGT GACGTGGAGC AGAAGATAGC TTCATACTTC GGCCAAGCCC   
  
  
+ TCTTCACCCG CATGACCGAG TCAGGGGACC GCACCTACCG CACCATGCGG TCCGCCGTGG ACAAGACCTT   
  
  
+ CTCCTTTGAG TCTACGAGAA AGATGGTACT CAAGTTCCAG GAGGTTAGCC CTTGGACCAC CTTTGGACAC   
  
  
+ GTGGCAGCTA ATGGAGCCCT CATTGATGCC TTTGACGGTG AATCCAAAAT CCACATCATC GACATCAGCA   
  
  
+ CCACATTCTG CACCCAGTGG CCCACCCTCC TGGAGGCGCT GGCCACCCGA ATGGATGACG CGCCCTACCT   
  
  
+ GAGGCTGACC ACTGTGGTGG TCAACAAGTC CGGGAATGAA GGTCCCACCG GAGGCGGGTC CCACAGGGTG   
  
  
+ ATGAAAGAGA TTGGGACCCG CCTCGAGAAG TTTGCTAGGC TAATGGGAGT GCCTTTCAAA TTTAATGTGG   
  
  
+ TCCACCACGG GGCTGATTTA TCCGACTTGG ATTTCTCTCA GCTGGATATT AACGATGACG AGGCATTAGC   
  
  
+ CATCAACTGT GTCAACTCGC TCCACTCGGT CAATATCCAC CGCCGTGACT CGGTCATCTC GGCCTTCCGG   
  
  
+ CGGCTCCACC CTAGGGTGGT GACCGTGGTG GAGGAGGAGG CTGACCTTGC TGACGTGGGG GCGGAGGGGT   
  
  
+ ACGAGTTCTA CAGGGGGTTT AATGAGTGTT TAAGGTGGGT TAGGGTTTAC TTTGAGGCCC TGGAAGAGTG   
  
  
+ CTTTCCTAGG ACAAGCAATG AGAAACTCAT GCTCGAGCGG GCCGCAGGGC GGGCTCTGGT GGACCTCCTG   
  
  
+ GCCTGCCCCA AGCCCGCATC ATCCGAGCGG AGGGAGGCAG CTGCGCGGTG GTCCGGGAGG ATGCAGGGGG   
  
  
+ CCGGGTTCGG CCACGTGGGG TTCAGTGATG AGGTCTGTGA TGACGTCAAG GCCCTCCTAA GGAGGTACAA   
  
  
+ GGAGGGGTGG TCCATGATAC AATGTTCCGC CGCGGATGGC GGCGCCTCCG CCGGAATATT CTTGTCGTGG   
  
  
+ AGGGATCAGC CGGTGGTGTG GGCCAGTGCA TGGAAGCCTT A  

- -Up\_Stream \_Len000CCCAAA AAAATTTGAG TTTTAATCAA TAACATAGTT AACTTAAACC TTTTGATAAT   
  
  
- AAAAGCTGAA TAATATCTCT TAAACAACAA TTACTTACGA CTATTTATGA CTATTAATCA CAACACTCAC   
  
  
- ACTTAAACAC TACTACTCGT TACTAATCGC AATTTTAATA TTGATCCTAT TTATTAATTC ACAACTACTT   
  
  
- ACAACTAACT ACTACTACTC ATGACTAACC ACGCCACGAC CATTATTTAT AAATACTATT CAATTTTTTT   
  
  
- TTTTTGTGTA GGATTTGTTA GTGATACATT ATATGATTTA CTTTTTTTAA CCATTACACT AAACTGTTAG   
  
  
- ATTCCCATTC CCACAAAATG GGTGTGTACA ATACTTTAAC CGAATGATAA ATGTTTGAAA AATGTCAATA   
  
  
- TACTAAGATG GATACACAAT AGTAGAAAGG CAGCTACATT AACTAATATA AGTTGTGTTA AATTAAGTAA   
  
  
- TGTTACATCG ATTAACTATG AGTTTTATTA CTTGATAATT ATTTATTAAT TAATATTGAA AATTTTCAAA   
  
  
- CAATTAAATT TTAATATATT ATATATTTAA ATCCGATGTA ATTAGTTTTT TAATCCGCAC AAGTAGCCAG   
  
  
- GTTTGAACTT GGCCTGGTCT GGCCTGGCTT CTAGCTAATT CTCTTTTTCA TGTCTGACTC CTGGCCTAGC   
  
  
- TTATTCTCAG CGTGGCCTGG TCCTAGCCTG GCCTTTTTAA ACCTGGTCTA GCCCTGGCTA GCTCCATCTG   
  
  
- GCTTAAATAA AAGTTTATGC TATCCTTATT TTGAATAACT AAGTTAACTA AAACCGGTTT TAGTACGATA   
  
  
- ATAACTAAAA TAAACGATTG AGAGTTCTCA TAATCAAACG TGAAGCGTAA TAGAAACCGG TTTTTTTAGT   
  
  
- TTTTCTTTCA GAGTTTGAGA ATTATCAAAA AATAAAATAA ACCAAATAAA AATTTTATCA GCCGGAAGCC   
  
  
- AGGCCTGATC ACCTGGCTTA TTCCATTAAA AAGCTTAGCT CCTGGTCTGG TTTACGTGAG TCAAGCTTGG   
  
  
- CCAAGTCAAG CCAGACTGAG CTAAAAACCA GGCCAGGTTC AAACTTGTCT TATTACATGT GTGGATCTTT   
  
  
- TTTTCTTAAT ACGTATATAT GAATTCCGGG GGTTGGGAAA AAGGGAAAGG CGGTTATGAA AAATGGTTCA   
  
  
- GGTTAAGAAA AGAAGAATCG ATACCGATCA TCAATAGGGA GGGATCGAAG GTGGGATTCA AAACTCTGTT   
  
  
- TTAGGGTAGA TACAATTATA AATAAAGAAC GTGTACAGTG GGTTATGTTC CCAAAGTAAC TTTTCTTTTC   
  
  
- CATAATCTAC ATTCTATACA TACATATATT TATCAAAACG TTATCTGATC TTCCTGATTT TTCCTTTTTC   
  
  
- CTTCAAATTT TCGTTTTTTG AAAGGGGAGA TCTTTTTTCC TTTGGTCTAA GGGAAAAGAT GGGGTTAAGG   
  
  
- GTATCAGAAA TGAGACAGAG AGAGAGAGAC AGAGAGAATG AGTACTACAA CTCCCTCCTT CCTTACTTAA   
  
  
- TTCCTGGGAT TCGATTCTAG GTTGGCATTC TATAACGGGG AAATTAGTGG GTAAAGTATA GAGATAGGTG   
  
  
- TCAATTTTTG GGTGGAGGAG GTAGGTGTAT TGACTATATA GAGAAGATGT GGTATGATAT TCCTTCTTCT   
  
  
- CTTTTTATCC TCACATAACA TAAAAATAAA CTCATTAAAT AAAATTCCTC TCTTTAAATG TACCAACCCA   
  
  
- ATTTACCTAT GGAACAAATC TGATCACTCA AAGGTTGTTG GTGTGGTGGT GGTAGTGGTT GTGGTTGTGG   
  
  
- TTGTGGTGGT TGTCGTTAGA AGACTAGTTA GGAGAAACTT ATTATGATGA TGAATAAGGT GTTTAAGTTC   
  
  
- GTCAAGGTCT AGAAGGGTTG TTTGGGTAGT ATTAGTATTA GTATGAGTCA TAATAGTGGT TGTAGTAGGA   
  
  
- TGAATGGGGG TGGCGGTGGT AGTCCTCCTT CTTCTTCTTA CGAAGTTGAA AATGTACCTA CTTCTTTTGA   
  
  
- AGAGAAGAAG AAGGAGGGTG TTGTTTGTAG TAGGAGTTGG AGGAAGGAGG CGGTGGTGGA GGAGCATAAT   
  
  
- GGGAATGATG AGGTGGGGGA AGCTACAAGG GAGATGGTGG TGGTGGAGGT GGAGGTGGGG GTGCGGGTGG   
  
  
- TTGTGGTGGT GGTGGTGGGG GGGTTGGGTC ATACCGCCGC CGCCGCCACC CAAACTGAGG TCGACGAAGA   
  
  
- GGATGAGGGG ACGGGCTCTG GAGTTGGGCC TCGAGAAGAG GAGCAGGCCC AAGAGCCCGT TCACCCGAAG   
  
  
- GTTGTAGGAG AACCTTCGGC GAGCACGGTA GAGACTCTCC TTGTGCTCGG CGGAGGTGGT CGAGGATACC   
  
  
- TACCAGTTGC TCGAGTCGAG GGGCATGCCA CTGCACCTCG TCTTCTATCG AAGTATGAAG CCGGTTCGGG   
  
  
- AGAAGTGGGC GTACTGGCTC AGTCCCCTGG CGTGGATGGC GTGGTACGCC AGGCGGCACC TGTTCTGGAA   
  
  
- GAGGAAACTC AGATGCTCTT TCTACCATGA GTTCAAGGTC CTCCAATCGG GAACCTGGTG GAAACCTGTG   
  
  
- CACCGTCGAT TACCTCGGGA GTAACTACGG AAACTGCCAC TTAGGTTTTA GGTGTAGTAG CTGTAGTCGT   
  
  
- GGTGTAAGAC GTGGGTCACC GGGTGGGAGG ACCTCCGCGA CCGGTGGGCT TACCTACTGC GCGGGATGGA   
  
  
- CTCCGACTGG TGACACCACC AGTTGTTCAG GCCCTTACTT CCAGGGTGGC CTCCGCCCAG GGTGTCCCAC   
  
  
- TACTTTCTCT AACCCTGGGC GGAGCTCTTC AAACGATCCG ATTACCCTCA CGGAAAGTTT AAATTACACC   
  
  
- AGGTGGTGCC CCGACTAAAT AGGCTGAACC TAAAGAGAGT CGACCTATAA TTGCTACTGC TCCGTAATCG   
  
  
- GTAGTTGACA CAGTTGAGCG AGGTGAGCCA GTTATAGGTG GCGGCACTGA GCCAGTAGAG CCGGAAGGCC   
  
  
- GCCGAGGTGG GATCCCACCA CTGGCACCAC CTCCTCCTCC GACTGGAACG ACTGCACCCC CGCCTCCCCA   
  
  
- TGCTCAAGAT GTCCCCCAAA TTACTCACAA ATTCCACCCA ATCCCAAATG AAACTCCGGG ACCTTCTCAC   
  
  
- GAAAGGATCC TGTTCGTTAC TCTTTGAGTA CGAGCTCGCC CGGCGTCCCG CCCGAGACCA CCTGGAGGAC   
  
  
- CGGACGGGGT TCGGGCGTAG TAGGCTCGCC TCCCTCCGTC GACGCGCCAC CAGGCCCTCC TACGTCCCCC   
  
  
- GGCCCAAGCC GGTGCACCCC AAGTCACTAC TCCAGACACT ACTGCAGTTC CGGGAGGATT CCTCCATGTT   
  
  
- CCTCCCCACC AGGTACTATG TTACAAGGCG GCGCCTACCG CCGCGGAGGC GGCCTTATAA GAACAGCACC   
  
  
- TCCCTAGTCG GCCACCACAC CCGGTCACGT ACCTTCGGAA T

+     MBS

| Site Name | Organism | Position | Strand | Matrix score. | sequence | function |
| --- | --- | --- | --- | --- | --- | --- |
| MBS | Arabidopsis thaliana | 2948 | + | 6 | CAACTG | MYB binding site involved in drought-inducibility |

>HU06G00358.1   
+ -Up\_Stream \_Len000GGGTTT TTTTAAACTC AAAATTAGTT ATTGTATCAA TTGAATTTGG AAAACTATTA   
  
  
+ TTTTCGACTT ATTATAGAGA ATTTGTTGTT AATGAATGCT GATAAATACT GATAATTAGT GTTGTGAGTG   
  
  
+ TGAATTTGTG ATGATGAGCA ATGATTAGCG TTAAAATTAT AACTAGGATA AATAATTAAG TGTTGATGAA   
  
  
+ TGTTGATTGA TGATGATGAG TACTGATTGG TGCGGTGCTG GTAATAAATA TTTATGATAA GTTAAAAAAA   
  
  
+ AAAAACACAT CCTAAACAAT CACTATGTAA TATACTAAAT GAAAAAAATT GGTAATGTGA TTTGACAATC   
  
  
+ TAAGGGTAAG GGTGTTTTAC CCACACATGT TATGAAATTG GCTTACTATT TACAAACTTT TTACAGTTAT   
  
  
+ ATGATTCTAC CTATGTGTTA TCATCTTTCC GTCGATGTAA TTGATTATAT TCAACACAAT TTAATTCATT   
  
  
+ ACAATGTAGC TAATTGATAC TCAAAATAAT GAACTATTAA TAAATAATTA ATTATAACTT TTAAAAGTTT   
  
  
+ GTTAATTTAA AATTATATAA TATATAAATT TAGGCTACAT TAATCAAAAA ATTAGGCGTG TTCATCGGTC   
  
  
+ CAAACTTGAA CCGGACCAGA CCGGACCGAA GATCGATTAA GAGAAAAAGT ACAGACTGAG GACCGGATCG   
  
  
+ AATAAGAGTC GCACCGGACC AGGATCGGAC CGGAAAAATT TGGACCAGAT CGGGACCGAT CGAGGTAGAC   
  
  
+ CGAATTTATT TTCAAATACG ATAGGAATAA AACTTATTGA TTCAATTGAT TTTGGCCAAA ATCATGCTAT   
  
  
+ TATTGATTTT ATTTGCTAAC TCTCAAGAGT ATTAGTTTGC ACTTCGCATT ATCTTTGGCC AAAAAAATCA   
  
  
+ AAAAGAAAGT CTCAAACTCT TAATAGTTTT TTATTTTATT TGGTTTATTT TTAAAATAGT CGGCCTTCGG   
  
  
+ TCCGGACTAG TGGACCGAAT AAGGTAATTT TTCGAATCGA GGACCAGACC AAATGCACTC AGTTCGAACC   
  
  
+ GGTTCAGTTC GGTCTGACTC GATTTTTGGT CCGGTCCAAG TTTGAACAGA ATAATGTACA CACCTAGAAA   
  
  
+ AAAAGAATTA TGCATATATA CTTAAGGCCC CCAACCCTTT TTCCCTTTCC GCCAATACTT TTTACCAAGT   
  
  
+ CCAATTCTTT TCTTCTTAGC TATGGCTAGT AGTTATCCCT CCCTAGCTTC CACCCTAAGT TTTGAGACAA   
  
  
+ AATCCCATCT ATGTTAATAT TTATTTCTTG CACATGTCAC CCAATACAAG GGTTTCATTG AAAAGAAAAG   
  
  
+ GTATTAGATG TAAGATATGT ATGTATATAA ATAGTTTTGC AATAGACTAG AAGGACTAAA AAGGAAAAAG   
  
  
+ GAAGTTTAAA AGCAAAAAAC TTTCCCCTCT AGAAAAAAGG AAACCAGATT CCCTTTTCTA CCCCAATTCC   
  
  
+ CATAGTCTTT ACTCTGTCTC TCTCTCTCTG TCTCTCTTAC TCATGATGTT GAGGGAGGAA GGAATGAATT   
  
  
+ AAGGACCCTA AGCTAAGATC CAACCGTAAG ATATTGCCCC TTTAATCACC CATTTCATAT CTCTATCCAC   
  
  
+ AGTTAAAAAC CCACCTCCTC CATCCACATA ACTGATATAT CTCTTCTACA CCATACTATA AGGAAGAAGA   
  
  
+ GAAAAATAGG AGTGTATTGT ATTTTTATTT GAGTAATTTA TTTTAAGGAG AGAAATTTAC ATGGTTGGGT   
  
  
+ TAAATGGATA CCTTGTTTAG ACTAGTGAGT TTCCAACAAC CACACCACCA CCATCACCAA CACCAACACC   
  
  
+ AACACCACCA ACAGCAATCT TCTGATCAAT CCTCTTTGAA TAATACTACT ACTTATTCCA CAAATTCAAG   
  
  
+ CAGTTCCAGA TCTTCCCAAC AAACCCATCA TAATCATAAT CATACTCAGT ATTATCACCA ACATCATCCT   
  
  
+ ACTTACCCCC ACCGCCACCA TCAGGAGGAA GAAGAAGAAT GCTTCAACTT TTACATGGAT GAAGAAAACT   
  
  
+ TCTCTTCTTC TTCCTCCCAC AACAAACATC ATCCTCAACC TCCTTCCTCC GCCACCACCT CCTCGTATTA   
  
  
+ CCCTTACTAC TCCACCCCCT TCGATGTTCC CTCTACCACC ACCACCTCCA CCTCCACCCC CACGCCCACC   
  
  
+ AACACCACCA CCACCACCCC CCCAACCCAG TATGGCGGCG GCGGCGGTGG GTTTGACTCC AGCTGCTTCT   
  
  
+ CCTACTCCCC TGCCCGAGAC CTCAACCCGG AGCTCTTCTC CTCGTCCGGG TTCTCGGGCA AGTGGGCTTC   
  
  
+ CAACATCCTC TTGGAAGCCG CTCGTGCCAT CTCTGAGAGG AACACGAGCC GCCTCCACCA GCTCCTATGG   
  
  
+ ATGGTCAACG AGCTCAGCTC CCCGTACGGT GACGTGGAGC AGAAGATAGC TTCATACTTC GGCCAAGCCC   
  
  
+ TCTTCACCCG CATGACCGAG TCAGGGGACC GCACCTACCG CACCATGCGG TCCGCCGTGG ACAAGACCTT   
  
  
+ CTCCTTTGAG TCTACGAGAA AGATGGTACT CAAGTTCCAG GAGGTTAGCC CTTGGACCAC CTTTGGACAC   
  
  
+ GTGGCAGCTA ATGGAGCCCT CATTGATGCC TTTGACGGTG AATCCAAAAT CCACATCATC GACATCAGCA   
  
  
+ CCACATTCTG CACCCAGTGG CCCACCCTCC TGGAGGCGCT GGCCACCCGA ATGGATGACG CGCCCTACCT   
  
  
+ GAGGCTGACC ACTGTGGTGG TCAACAAGTC CGGGAATGAA GGTCCCACCG GAGGCGGGTC CCACAGGGTG   
  
  
+ ATGAAAGAGA TTGGGACCCG CCTCGAGAAG TTTGCTAGGC TAATGGGAGT GCCTTTCAAA TTTAATGTGG   
  
  
+ TCCACCACGG GGCTGATTTA TCCGACTTGG ATTTCTCTCA GCTGGATATT AACGATGACG AGGCATTAGC   
  
  
+ CATCAACTGT GTCAACTCGC TCCACTCGGT CAATATCCAC CGCCGTGACT CGGTCATCTC GGCCTTCCGG   
  
  
+ CGGCTCCACC CTAGGGTGGT GACCGTGGTG GAGGAGGAGG CTGACCTTGC TGACGTGGGG GCGGAGGGGT   
  
  
+ ACGAGTTCTA CAGGGGGTTT AATGAGTGTT TAAGGTGGGT TAGGGTTTAC TTTGAGGCCC TGGAAGAGTG   
  
  
+ CTTTCCTAGG ACAAGCAATG AGAAACTCAT GCTCGAGCGG GCCGCAGGGC GGGCTCTGGT GGACCTCCTG   
  
  
+ GCCTGCCCCA AGCCCGCATC ATCCGAGCGG AGGGAGGCAG CTGCGCGGTG GTCCGGGAGG ATGCAGGGGG   
  
  
+ CCGGGTTCGG CCACGTGGGG TTCAGTGATG AGGTCTGTGA TGACGTCAAG GCCCTCCTAA GGAGGTACAA   
  
  
+ GGAGGGGTGG TCCATGATAC AATGTTCCGC CGCGGATGGC GGCGCCTCCG CCGGAATATT CTTGTCGTGG   
  
  
+ AGGGATCAGC CGGTGGTGTG GGCCAGTGCA TGGAAGCCTT A  

- -Up\_Stream \_Len000CCCAAA AAAATTTGAG TTTTAATCAA TAACATAGTT AACTTAAACC TTTTGATAAT   
  
  
- AAAAGCTGAA TAATATCTCT TAAACAACAA TTACTTACGA CTATTTATGA CTATTAATCA CAACACTCAC   
  
  
- ACTTAAACAC TACTACTCGT TACTAATCGC AATTTTAATA TTGATCCTAT TTATTAATTC ACAACTACTT   
  
  
- ACAACTAACT ACTACTACTC ATGACTAACC ACGCCACGAC CATTATTTAT AAATACTATT CAATTTTTTT   
  
  
- TTTTTGTGTA GGATTTGTTA GTGATACATT ATATGATTTA CTTTTTTTAA CCATTACACT AAACTGTTAG   
  
  
- ATTCCCATTC CCACAAAATG GGTGTGTACA ATACTTTAAC CGAATGATAA ATGTTTGAAA AATGTCAATA   
  
  
- TACTAAGATG GATACACAAT AGTAGAAAGG CAGCTACATT AACTAATATA AGTTGTGTTA AATTAAGTAA   
  
  
- TGTTACATCG ATTAACTATG AGTTTTATTA CTTGATAATT ATTTATTAAT TAATATTGAA AATTTTCAAA   
  
  
- CAATTAAATT TTAATATATT ATATATTTAA ATCCGATGTA ATTAGTTTTT TAATCCGCAC AAGTAGCCAG   
  
  
- GTTTGAACTT GGCCTGGTCT GGCCTGGCTT CTAGCTAATT CTCTTTTTCA TGTCTGACTC CTGGCCTAGC   
  
  
- TTATTCTCAG CGTGGCCTGG TCCTAGCCTG GCCTTTTTAA ACCTGGTCTA GCCCTGGCTA GCTCCATCTG   
  
  
- GCTTAAATAA AAGTTTATGC TATCCTTATT TTGAATAACT AAGTTAACTA AAACCGGTTT TAGTACGATA   
  
  
- ATAACTAAAA TAAACGATTG AGAGTTCTCA TAATCAAACG TGAAGCGTAA TAGAAACCGG TTTTTTTAGT   
  
  
- TTTTCTTTCA GAGTTTGAGA ATTATCAAAA AATAAAATAA ACCAAATAAA AATTTTATCA GCCGGAAGCC   
  
  
- AGGCCTGATC ACCTGGCTTA TTCCATTAAA AAGCTTAGCT CCTGGTCTGG TTTACGTGAG TCAAGCTTGG   
  
  
- CCAAGTCAAG CCAGACTGAG CTAAAAACCA GGCCAGGTTC AAACTTGTCT TATTACATGT GTGGATCTTT   
  
  
- TTTTCTTAAT ACGTATATAT GAATTCCGGG GGTTGGGAAA AAGGGAAAGG CGGTTATGAA AAATGGTTCA   
  
  
- GGTTAAGAAA AGAAGAATCG ATACCGATCA TCAATAGGGA GGGATCGAAG GTGGGATTCA AAACTCTGTT   
  
  
- TTAGGGTAGA TACAATTATA AATAAAGAAC GTGTACAGTG GGTTATGTTC CCAAAGTAAC TTTTCTTTTC   
  
  
- CATAATCTAC ATTCTATACA TACATATATT TATCAAAACG TTATCTGATC TTCCTGATTT TTCCTTTTTC   
  
  
- CTTCAAATTT TCGTTTTTTG AAAGGGGAGA TCTTTTTTCC TTTGGTCTAA GGGAAAAGAT GGGGTTAAGG   
  
  
- GTATCAGAAA TGAGACAGAG AGAGAGAGAC AGAGAGAATG AGTACTACAA CTCCCTCCTT CCTTACTTAA   
  
  
- TTCCTGGGAT TCGATTCTAG GTTGGCATTC TATAACGGGG AAATTAGTGG GTAAAGTATA GAGATAGGTG   
  
  
- TCAATTTTTG GGTGGAGGAG GTAGGTGTAT TGACTATATA GAGAAGATGT GGTATGATAT TCCTTCTTCT   
  
  
- CTTTTTATCC TCACATAACA TAAAAATAAA CTCATTAAAT AAAATTCCTC TCTTTAAATG TACCAACCCA   
  
  
- ATTTACCTAT GGAACAAATC TGATCACTCA AAGGTTGTTG GTGTGGTGGT GGTAGTGGTT GTGGTTGTGG   
  
  
- TTGTGGTGGT TGTCGTTAGA AGACTAGTTA GGAGAAACTT ATTATGATGA TGAATAAGGT GTTTAAGTTC   
  
  
- GTCAAGGTCT AGAAGGGTTG TTTGGGTAGT ATTAGTATTA GTATGAGTCA TAATAGTGGT TGTAGTAGGA   
  
  
- TGAATGGGGG TGGCGGTGGT AGTCCTCCTT CTTCTTCTTA CGAAGTTGAA AATGTACCTA CTTCTTTTGA   
  
  
- AGAGAAGAAG AAGGAGGGTG TTGTTTGTAG TAGGAGTTGG AGGAAGGAGG CGGTGGTGGA GGAGCATAAT   
  
  
- GGGAATGATG AGGTGGGGGA AGCTACAAGG GAGATGGTGG TGGTGGAGGT GGAGGTGGGG GTGCGGGTGG   
  
  
- TTGTGGTGGT GGTGGTGGGG GGGTTGGGTC ATACCGCCGC CGCCGCCACC CAAACTGAGG TCGACGAAGA   
  
  
- GGATGAGGGG ACGGGCTCTG GAGTTGGGCC TCGAGAAGAG GAGCAGGCCC AAGAGCCCGT TCACCCGAAG   
  
  
- GTTGTAGGAG AACCTTCGGC GAGCACGGTA GAGACTCTCC TTGTGCTCGG CGGAGGTGGT CGAGGATACC   
  
  
- TACCAGTTGC TCGAGTCGAG GGGCATGCCA CTGCACCTCG TCTTCTATCG AAGTATGAAG CCGGTTCGGG   
  
  
- AGAAGTGGGC GTACTGGCTC AGTCCCCTGG CGTGGATGGC GTGGTACGCC AGGCGGCACC TGTTCTGGAA   
  
  
- GAGGAAACTC AGATGCTCTT TCTACCATGA GTTCAAGGTC CTCCAATCGG GAACCTGGTG GAAACCTGTG   
  
  
- CACCGTCGAT TACCTCGGGA GTAACTACGG AAACTGCCAC TTAGGTTTTA GGTGTAGTAG CTGTAGTCGT   
  
  
- GGTGTAAGAC GTGGGTCACC GGGTGGGAGG ACCTCCGCGA CCGGTGGGCT TACCTACTGC GCGGGATGGA   
  
  
- CTCCGACTGG TGACACCACC AGTTGTTCAG GCCCTTACTT CCAGGGTGGC CTCCGCCCAG GGTGTCCCAC   
  
  
- TACTTTCTCT AACCCTGGGC GGAGCTCTTC AAACGATCCG ATTACCCTCA CGGAAAGTTT AAATTACACC   
  
  
- AGGTGGTGCC CCGACTAAAT AGGCTGAACC TAAAGAGAGT CGACCTATAA TTGCTACTGC TCCGTAATCG   
  
  
- GTAGTTGACA CAGTTGAGCG AGGTGAGCCA GTTATAGGTG GCGGCACTGA GCCAGTAGAG CCGGAAGGCC   
  
  
- GCCGAGGTGG GATCCCACCA CTGGCACCAC CTCCTCCTCC GACTGGAACG ACTGCACCCC CGCCTCCCCA   
  
  
- TGCTCAAGAT GTCCCCCAAA TTACTCACAA ATTCCACCCA ATCCCAAATG AAACTCCGGG ACCTTCTCAC   
  
  
- GAAAGGATCC TGTTCGTTAC TCTTTGAGTA CGAGCTCGCC CGGCGTCCCG CCCGAGACCA CCTGGAGGAC   
  
  
- CGGACGGGGT TCGGGCGTAG TAGGCTCGCC TCCCTCCGTC GACGCGCCAC CAGGCCCTCC TACGTCCCCC   
  
  
- GGCCCAAGCC GGTGCACCCC AAGTCACTAC TCCAGACACT ACTGCAGTTC CGGGAGGATT CCTCCATGTT   
  
  
- CCTCCCCACC AGGTACTATG TTACAAGGCG GCGCCTACCG CCGCGGAGGC GGCCTTATAA GAACAGCACC   
  
  
- TCCCTAGTCG GCCACCACAC CCGGTCACGT ACCTTCGGAA T

+     MBSI

| Site Name | Organism | Position | Strand | Matrix score. | sequence | function |
| --- | --- | --- | --- | --- | --- | --- |
| MBSI | Petunia hybrida | 412 | + | 11 | TTTTTACGGTTA | MYB binding site involved in flavonoid biosynthetic genes regulation |

>HU06G00358.1   
+ -Up\_Stream \_Len000GGGTTT TTTTAAACTC AAAATTAGTT ATTGTATCAA TTGAATTTGG AAAACTATTA   
  
  
+ TTTTCGACTT ATTATAGAGA ATTTGTTGTT AATGAATGCT GATAAATACT GATAATTAGT GTTGTGAGTG   
  
  
+ TGAATTTGTG ATGATGAGCA ATGATTAGCG TTAAAATTAT AACTAGGATA AATAATTAAG TGTTGATGAA   
  
  
+ TGTTGATTGA TGATGATGAG TACTGATTGG TGCGGTGCTG GTAATAAATA TTTATGATAA GTTAAAAAAA   
  
  
+ AAAAACACAT CCTAAACAAT CACTATGTAA TATACTAAAT GAAAAAAATT GGTAATGTGA TTTGACAATC   
  
  
+ TAAGGGTAAG GGTGTTTTAC CCACACATGT TATGAAATTG GCTTACTATT TACAAACTTT TTACAGTTAT   
  
  
+ ATGATTCTAC CTATGTGTTA TCATCTTTCC GTCGATGTAA TTGATTATAT TCAACACAAT TTAATTCATT   
  
  
+ ACAATGTAGC TAATTGATAC TCAAAATAAT GAACTATTAA TAAATAATTA ATTATAACTT TTAAAAGTTT   
  
  
+ GTTAATTTAA AATTATATAA TATATAAATT TAGGCTACAT TAATCAAAAA ATTAGGCGTG TTCATCGGTC   
  
  
+ CAAACTTGAA CCGGACCAGA CCGGACCGAA GATCGATTAA GAGAAAAAGT ACAGACTGAG GACCGGATCG   
  
  
+ AATAAGAGTC GCACCGGACC AGGATCGGAC CGGAAAAATT TGGACCAGAT CGGGACCGAT CGAGGTAGAC   
  
  
+ CGAATTTATT TTCAAATACG ATAGGAATAA AACTTATTGA TTCAATTGAT TTTGGCCAAA ATCATGCTAT   
  
  
+ TATTGATTTT ATTTGCTAAC TCTCAAGAGT ATTAGTTTGC ACTTCGCATT ATCTTTGGCC AAAAAAATCA   
  
  
+ AAAAGAAAGT CTCAAACTCT TAATAGTTTT TTATTTTATT TGGTTTATTT TTAAAATAGT CGGCCTTCGG   
  
  
+ TCCGGACTAG TGGACCGAAT AAGGTAATTT TTCGAATCGA GGACCAGACC AAATGCACTC AGTTCGAACC   
  
  
+ GGTTCAGTTC GGTCTGACTC GATTTTTGGT CCGGTCCAAG TTTGAACAGA ATAATGTACA CACCTAGAAA   
  
  
+ AAAAGAATTA TGCATATATA CTTAAGGCCC CCAACCCTTT TTCCCTTTCC GCCAATACTT TTTACCAAGT   
  
  
+ CCAATTCTTT TCTTCTTAGC TATGGCTAGT AGTTATCCCT CCCTAGCTTC CACCCTAAGT TTTGAGACAA   
  
  
+ AATCCCATCT ATGTTAATAT TTATTTCTTG CACATGTCAC CCAATACAAG GGTTTCATTG AAAAGAAAAG   
  
  
+ GTATTAGATG TAAGATATGT ATGTATATAA ATAGTTTTGC AATAGACTAG AAGGACTAAA AAGGAAAAAG   
  
  
+ GAAGTTTAAA AGCAAAAAAC TTTCCCCTCT AGAAAAAAGG AAACCAGATT CCCTTTTCTA CCCCAATTCC   
  
  
+ CATAGTCTTT ACTCTGTCTC TCTCTCTCTG TCTCTCTTAC TCATGATGTT GAGGGAGGAA GGAATGAATT   
  
  
+ AAGGACCCTA AGCTAAGATC CAACCGTAAG ATATTGCCCC TTTAATCACC CATTTCATAT CTCTATCCAC   
  
  
+ AGTTAAAAAC CCACCTCCTC CATCCACATA ACTGATATAT CTCTTCTACA CCATACTATA AGGAAGAAGA   
  
  
+ GAAAAATAGG AGTGTATTGT ATTTTTATTT GAGTAATTTA TTTTAAGGAG AGAAATTTAC ATGGTTGGGT   
  
  
+ TAAATGGATA CCTTGTTTAG ACTAGTGAGT TTCCAACAAC CACACCACCA CCATCACCAA CACCAACACC   
  
  
+ AACACCACCA ACAGCAATCT TCTGATCAAT CCTCTTTGAA TAATACTACT ACTTATTCCA CAAATTCAAG   
  
  
+ CAGTTCCAGA TCTTCCCAAC AAACCCATCA TAATCATAAT CATACTCAGT ATTATCACCA ACATCATCCT   
  
  
+ ACTTACCCCC ACCGCCACCA TCAGGAGGAA GAAGAAGAAT GCTTCAACTT TTACATGGAT GAAGAAAACT   
  
  
+ TCTCTTCTTC TTCCTCCCAC AACAAACATC ATCCTCAACC TCCTTCCTCC GCCACCACCT CCTCGTATTA   
  
  
+ CCCTTACTAC TCCACCCCCT TCGATGTTCC CTCTACCACC ACCACCTCCA CCTCCACCCC CACGCCCACC   
  
  
+ AACACCACCA CCACCACCCC CCCAACCCAG TATGGCGGCG GCGGCGGTGG GTTTGACTCC AGCTGCTTCT   
  
  
+ CCTACTCCCC TGCCCGAGAC CTCAACCCGG AGCTCTTCTC CTCGTCCGGG TTCTCGGGCA AGTGGGCTTC   
  
  
+ CAACATCCTC TTGGAAGCCG CTCGTGCCAT CTCTGAGAGG AACACGAGCC GCCTCCACCA GCTCCTATGG   
  
  
+ ATGGTCAACG AGCTCAGCTC CCCGTACGGT GACGTGGAGC AGAAGATAGC TTCATACTTC GGCCAAGCCC   
  
  
+ TCTTCACCCG CATGACCGAG TCAGGGGACC GCACCTACCG CACCATGCGG TCCGCCGTGG ACAAGACCTT   
  
  
+ CTCCTTTGAG TCTACGAGAA AGATGGTACT CAAGTTCCAG GAGGTTAGCC CTTGGACCAC CTTTGGACAC   
  
  
+ GTGGCAGCTA ATGGAGCCCT CATTGATGCC TTTGACGGTG AATCCAAAAT CCACATCATC GACATCAGCA   
  
  
+ CCACATTCTG CACCCAGTGG CCCACCCTCC TGGAGGCGCT GGCCACCCGA ATGGATGACG CGCCCTACCT   
  
  
+ GAGGCTGACC ACTGTGGTGG TCAACAAGTC CGGGAATGAA GGTCCCACCG GAGGCGGGTC CCACAGGGTG   
  
  
+ ATGAAAGAGA TTGGGACCCG CCTCGAGAAG TTTGCTAGGC TAATGGGAGT GCCTTTCAAA TTTAATGTGG   
  
  
+ TCCACCACGG GGCTGATTTA TCCGACTTGG ATTTCTCTCA GCTGGATATT AACGATGACG AGGCATTAGC   
  
  
+ CATCAACTGT GTCAACTCGC TCCACTCGGT CAATATCCAC CGCCGTGACT CGGTCATCTC GGCCTTCCGG   
  
  
+ CGGCTCCACC CTAGGGTGGT GACCGTGGTG GAGGAGGAGG CTGACCTTGC TGACGTGGGG GCGGAGGGGT   
  
  
+ ACGAGTTCTA CAGGGGGTTT AATGAGTGTT TAAGGTGGGT TAGGGTTTAC TTTGAGGCCC TGGAAGAGTG   
  
  
+ CTTTCCTAGG ACAAGCAATG AGAAACTCAT GCTCGAGCGG GCCGCAGGGC GGGCTCTGGT GGACCTCCTG   
  
  
+ GCCTGCCCCA AGCCCGCATC ATCCGAGCGG AGGGAGGCAG CTGCGCGGTG GTCCGGGAGG ATGCAGGGGG   
  
  
+ CCGGGTTCGG CCACGTGGGG TTCAGTGATG AGGTCTGTGA TGACGTCAAG GCCCTCCTAA GGAGGTACAA   
  
  
+ GGAGGGGTGG TCCATGATAC AATGTTCCGC CGCGGATGGC GGCGCCTCCG CCGGAATATT CTTGTCGTGG   
  
  
+ AGGGATCAGC CGGTGGTGTG GGCCAGTGCA TGGAAGCCTT A  

- -Up\_Stream \_Len000CCCAAA AAAATTTGAG TTTTAATCAA TAACATAGTT AACTTAAACC TTTTGATAAT   
  
  
- AAAAGCTGAA TAATATCTCT TAAACAACAA TTACTTACGA CTATTTATGA CTATTAATCA CAACACTCAC   
  
  
- ACTTAAACAC TACTACTCGT TACTAATCGC AATTTTAATA TTGATCCTAT TTATTAATTC ACAACTACTT   
  
  
- ACAACTAACT ACTACTACTC ATGACTAACC ACGCCACGAC CATTATTTAT AAATACTATT CAATTTTTTT   
  
  
- TTTTTGTGTA GGATTTGTTA GTGATACATT ATATGATTTA CTTTTTTTAA CCATTACACT AAACTGTTAG   
  
  
- ATTCCCATTC CCACAAAATG GGTGTGTACA ATACTTTAAC CGAATGATAA ATGTTTGAAA AATGTCAATA   
  
  
- TACTAAGATG GATACACAAT AGTAGAAAGG CAGCTACATT AACTAATATA AGTTGTGTTA AATTAAGTAA   
  
  
- TGTTACATCG ATTAACTATG AGTTTTATTA CTTGATAATT ATTTATTAAT TAATATTGAA AATTTTCAAA   
  
  
- CAATTAAATT TTAATATATT ATATATTTAA ATCCGATGTA ATTAGTTTTT TAATCCGCAC AAGTAGCCAG   
  
  
- GTTTGAACTT GGCCTGGTCT GGCCTGGCTT CTAGCTAATT CTCTTTTTCA TGTCTGACTC CTGGCCTAGC   
  
  
- TTATTCTCAG CGTGGCCTGG TCCTAGCCTG GCCTTTTTAA ACCTGGTCTA GCCCTGGCTA GCTCCATCTG   
  
  
- GCTTAAATAA AAGTTTATGC TATCCTTATT TTGAATAACT AAGTTAACTA AAACCGGTTT TAGTACGATA   
  
  
- ATAACTAAAA TAAACGATTG AGAGTTCTCA TAATCAAACG TGAAGCGTAA TAGAAACCGG TTTTTTTAGT   
  
  
- TTTTCTTTCA GAGTTTGAGA ATTATCAAAA AATAAAATAA ACCAAATAAA AATTTTATCA GCCGGAAGCC   
  
  
- AGGCCTGATC ACCTGGCTTA TTCCATTAAA AAGCTTAGCT CCTGGTCTGG TTTACGTGAG TCAAGCTTGG   
  
  
- CCAAGTCAAG CCAGACTGAG CTAAAAACCA GGCCAGGTTC AAACTTGTCT TATTACATGT GTGGATCTTT   
  
  
- TTTTCTTAAT ACGTATATAT GAATTCCGGG GGTTGGGAAA AAGGGAAAGG CGGTTATGAA AAATGGTTCA   
  
  
- GGTTAAGAAA AGAAGAATCG ATACCGATCA TCAATAGGGA GGGATCGAAG GTGGGATTCA AAACTCTGTT   
  
  
- TTAGGGTAGA TACAATTATA AATAAAGAAC GTGTACAGTG GGTTATGTTC CCAAAGTAAC TTTTCTTTTC   
  
  
- CATAATCTAC ATTCTATACA TACATATATT TATCAAAACG TTATCTGATC TTCCTGATTT TTCCTTTTTC   
  
  
- CTTCAAATTT TCGTTTTTTG AAAGGGGAGA TCTTTTTTCC TTTGGTCTAA GGGAAAAGAT GGGGTTAAGG   
  
  
- GTATCAGAAA TGAGACAGAG AGAGAGAGAC AGAGAGAATG AGTACTACAA CTCCCTCCTT CCTTACTTAA   
  
  
- TTCCTGGGAT TCGATTCTAG GTTGGCATTC TATAACGGGG AAATTAGTGG GTAAAGTATA GAGATAGGTG   
  
  
- TCAATTTTTG GGTGGAGGAG GTAGGTGTAT TGACTATATA GAGAAGATGT GGTATGATAT TCCTTCTTCT   
  
  
- CTTTTTATCC TCACATAACA TAAAAATAAA CTCATTAAAT AAAATTCCTC TCTTTAAATG TACCAACCCA   
  
  
- ATTTACCTAT GGAACAAATC TGATCACTCA AAGGTTGTTG GTGTGGTGGT GGTAGTGGTT GTGGTTGTGG   
  
  
- TTGTGGTGGT TGTCGTTAGA AGACTAGTTA GGAGAAACTT ATTATGATGA TGAATAAGGT GTTTAAGTTC   
  
  
- GTCAAGGTCT AGAAGGGTTG TTTGGGTAGT ATTAGTATTA GTATGAGTCA TAATAGTGGT TGTAGTAGGA   
  
  
- TGAATGGGGG TGGCGGTGGT AGTCCTCCTT CTTCTTCTTA CGAAGTTGAA AATGTACCTA CTTCTTTTGA   
  
  
- AGAGAAGAAG AAGGAGGGTG TTGTTTGTAG TAGGAGTTGG AGGAAGGAGG CGGTGGTGGA GGAGCATAAT   
  
  
- GGGAATGATG AGGTGGGGGA AGCTACAAGG GAGATGGTGG TGGTGGAGGT GGAGGTGGGG GTGCGGGTGG   
  
  
- TTGTGGTGGT GGTGGTGGGG GGGTTGGGTC ATACCGCCGC CGCCGCCACC CAAACTGAGG TCGACGAAGA   
  
  
- GGATGAGGGG ACGGGCTCTG GAGTTGGGCC TCGAGAAGAG GAGCAGGCCC AAGAGCCCGT TCACCCGAAG   
  
  
- GTTGTAGGAG AACCTTCGGC GAGCACGGTA GAGACTCTCC TTGTGCTCGG CGGAGGTGGT CGAGGATACC   
  
  
- TACCAGTTGC TCGAGTCGAG GGGCATGCCA CTGCACCTCG TCTTCTATCG AAGTATGAAG CCGGTTCGGG   
  
  
- AGAAGTGGGC GTACTGGCTC AGTCCCCTGG CGTGGATGGC GTGGTACGCC AGGCGGCACC TGTTCTGGAA   
  
  
- GAGGAAACTC AGATGCTCTT TCTACCATGA GTTCAAGGTC CTCCAATCGG GAACCTGGTG GAAACCTGTG   
  
  
- CACCGTCGAT TACCTCGGGA GTAACTACGG AAACTGCCAC TTAGGTTTTA GGTGTAGTAG CTGTAGTCGT   
  
  
- GGTGTAAGAC GTGGGTCACC GGGTGGGAGG ACCTCCGCGA CCGGTGGGCT TACCTACTGC GCGGGATGGA   
  
  
- CTCCGACTGG TGACACCACC AGTTGTTCAG GCCCTTACTT CCAGGGTGGC CTCCGCCCAG GGTGTCCCAC   
  
  
- TACTTTCTCT AACCCTGGGC GGAGCTCTTC AAACGATCCG ATTACCCTCA CGGAAAGTTT AAATTACACC   
  
  
- AGGTGGTGCC CCGACTAAAT AGGCTGAACC TAAAGAGAGT CGACCTATAA TTGCTACTGC TCCGTAATCG   
  
  
- GTAGTTGACA CAGTTGAGCG AGGTGAGCCA GTTATAGGTG GCGGCACTGA GCCAGTAGAG CCGGAAGGCC   
  
  
- GCCGAGGTGG GATCCCACCA CTGGCACCAC CTCCTCCTCC GACTGGAACG ACTGCACCCC CGCCTCCCCA   
  
  
- TGCTCAAGAT GTCCCCCAAA TTACTCACAA ATTCCACCCA ATCCCAAATG AAACTCCGGG ACCTTCTCAC   
  
  
- GAAAGGATCC TGTTCGTTAC TCTTTGAGTA CGAGCTCGCC CGGCGTCCCG CCCGAGACCA CCTGGAGGAC   
  
  
- CGGACGGGGT TCGGGCGTAG TAGGCTCGCC TCCCTCCGTC GACGCGCCAC CAGGCCCTCC TACGTCCCCC   
  
  
- GGCCCAAGCC GGTGCACCCC AAGTCACTAC TCCAGACACT ACTGCAGTTC CGGGAGGATT CCTCCATGTT   
  
  
- CCTCCCCACC AGGTACTATG TTACAAGGCG GCGCCTACCG CCGCGGAGGC GGCCTTATAA GAACAGCACC   
  
  
- TCCCTAGTCG GCCACCACAC CCGGTCACGT ACCTTCGGAA T

+     MYB

| Site Name | Organism | Position | Strand | Matrix score. | sequence | function |
| --- | --- | --- | --- | --- | --- | --- |
| MYB | Arabidopsis thaliana | 1833 | + | 6 | CAACAG |  |
| MYB | Arabidopsis thaliana | 1791 | + | 6 | CAACCA |  |
| MYB | Arabidopsis thaliana | 1746 | - | 6 | CAACCA |  |

>HU06G00358.1   
+ -Up\_Stream \_Len000GGGTTT TTTTAAACTC AAAATTAGTT ATTGTATCAA TTGAATTTGG AAAACTATTA   
  
  
+ TTTTCGACTT ATTATAGAGA ATTTGTTGTT AATGAATGCT GATAAATACT GATAATTAGT GTTGTGAGTG   
  
  
+ TGAATTTGTG ATGATGAGCA ATGATTAGCG TTAAAATTAT AACTAGGATA AATAATTAAG TGTTGATGAA   
  
  
+ TGTTGATTGA TGATGATGAG TACTGATTGG TGCGGTGCTG GTAATAAATA TTTATGATAA GTTAAAAAAA   
  
  
+ AAAAACACAT CCTAAACAAT CACTATGTAA TATACTAAAT GAAAAAAATT GGTAATGTGA TTTGACAATC   
  
  
+ TAAGGGTAAG GGTGTTTTAC CCACACATGT TATGAAATTG GCTTACTATT TACAAACTTT TTACAGTTAT   
  
  
+ ATGATTCTAC CTATGTGTTA TCATCTTTCC GTCGATGTAA TTGATTATAT TCAACACAAT TTAATTCATT   
  
  
+ ACAATGTAGC TAATTGATAC TCAAAATAAT GAACTATTAA TAAATAATTA ATTATAACTT TTAAAAGTTT   
  
  
+ GTTAATTTAA AATTATATAA TATATAAATT TAGGCTACAT TAATCAAAAA ATTAGGCGTG TTCATCGGTC   
  
  
+ CAAACTTGAA CCGGACCAGA CCGGACCGAA GATCGATTAA GAGAAAAAGT ACAGACTGAG GACCGGATCG   
  
  
+ AATAAGAGTC GCACCGGACC AGGATCGGAC CGGAAAAATT TGGACCAGAT CGGGACCGAT CGAGGTAGAC   
  
  
+ CGAATTTATT TTCAAATACG ATAGGAATAA AACTTATTGA TTCAATTGAT TTTGGCCAAA ATCATGCTAT   
  
  
+ TATTGATTTT ATTTGCTAAC TCTCAAGAGT ATTAGTTTGC ACTTCGCATT ATCTTTGGCC AAAAAAATCA   
  
  
+ AAAAGAAAGT CTCAAACTCT TAATAGTTTT TTATTTTATT TGGTTTATTT TTAAAATAGT CGGCCTTCGG   
  
  
+ TCCGGACTAG TGGACCGAAT AAGGTAATTT TTCGAATCGA GGACCAGACC AAATGCACTC AGTTCGAACC   
  
  
+ GGTTCAGTTC GGTCTGACTC GATTTTTGGT CCGGTCCAAG TTTGAACAGA ATAATGTACA CACCTAGAAA   
  
  
+ AAAAGAATTA TGCATATATA CTTAAGGCCC CCAACCCTTT TTCCCTTTCC GCCAATACTT TTTACCAAGT   
  
  
+ CCAATTCTTT TCTTCTTAGC TATGGCTAGT AGTTATCCCT CCCTAGCTTC CACCCTAAGT TTTGAGACAA   
  
  
+ AATCCCATCT ATGTTAATAT TTATTTCTTG CACATGTCAC CCAATACAAG GGTTTCATTG AAAAGAAAAG   
  
  
+ GTATTAGATG TAAGATATGT ATGTATATAA ATAGTTTTGC AATAGACTAG AAGGACTAAA AAGGAAAAAG   
  
  
+ GAAGTTTAAA AGCAAAAAAC TTTCCCCTCT AGAAAAAAGG AAACCAGATT CCCTTTTCTA CCCCAATTCC   
  
  
+ CATAGTCTTT ACTCTGTCTC TCTCTCTCTG TCTCTCTTAC TCATGATGTT GAGGGAGGAA GGAATGAATT   
  
  
+ AAGGACCCTA AGCTAAGATC CAACCGTAAG ATATTGCCCC TTTAATCACC CATTTCATAT CTCTATCCAC   
  
  
+ AGTTAAAAAC CCACCTCCTC CATCCACATA ACTGATATAT CTCTTCTACA CCATACTATA AGGAAGAAGA   
  
  
+ GAAAAATAGG AGTGTATTGT ATTTTTATTT GAGTAATTTA TTTTAAGGAG AGAAATTTAC ATGGTTGGGT   
  
  
+ TAAATGGATA CCTTGTTTAG ACTAGTGAGT TTCCAACAAC CACACCACCA CCATCACCAA CACCAACACC   
  
  
+ AACACCACCA ACAGCAATCT TCTGATCAAT CCTCTTTGAA TAATACTACT ACTTATTCCA CAAATTCAAG   
  
  
+ CAGTTCCAGA TCTTCCCAAC AAACCCATCA TAATCATAAT CATACTCAGT ATTATCACCA ACATCATCCT   
  
  
+ ACTTACCCCC ACCGCCACCA TCAGGAGGAA GAAGAAGAAT GCTTCAACTT TTACATGGAT GAAGAAAACT   
  
  
+ TCTCTTCTTC TTCCTCCCAC AACAAACATC ATCCTCAACC TCCTTCCTCC GCCACCACCT CCTCGTATTA   
  
  
+ CCCTTACTAC TCCACCCCCT TCGATGTTCC CTCTACCACC ACCACCTCCA CCTCCACCCC CACGCCCACC   
  
  
+ AACACCACCA CCACCACCCC CCCAACCCAG TATGGCGGCG GCGGCGGTGG GTTTGACTCC AGCTGCTTCT   
  
  
+ CCTACTCCCC TGCCCGAGAC CTCAACCCGG AGCTCTTCTC CTCGTCCGGG TTCTCGGGCA AGTGGGCTTC   
  
  
+ CAACATCCTC TTGGAAGCCG CTCGTGCCAT CTCTGAGAGG AACACGAGCC GCCTCCACCA GCTCCTATGG   
  
  
+ ATGGTCAACG AGCTCAGCTC CCCGTACGGT GACGTGGAGC AGAAGATAGC TTCATACTTC GGCCAAGCCC   
  
  
+ TCTTCACCCG CATGACCGAG TCAGGGGACC GCACCTACCG CACCATGCGG TCCGCCGTGG ACAAGACCTT   
  
  
+ CTCCTTTGAG TCTACGAGAA AGATGGTACT CAAGTTCCAG GAGGTTAGCC CTTGGACCAC CTTTGGACAC   
  
  
+ GTGGCAGCTA ATGGAGCCCT CATTGATGCC TTTGACGGTG AATCCAAAAT CCACATCATC GACATCAGCA   
  
  
+ CCACATTCTG CACCCAGTGG CCCACCCTCC TGGAGGCGCT GGCCACCCGA ATGGATGACG CGCCCTACCT   
  
  
+ GAGGCTGACC ACTGTGGTGG TCAACAAGTC CGGGAATGAA GGTCCCACCG GAGGCGGGTC CCACAGGGTG   
  
  
+ ATGAAAGAGA TTGGGACCCG CCTCGAGAAG TTTGCTAGGC TAATGGGAGT GCCTTTCAAA TTTAATGTGG   
  
  
+ TCCACCACGG GGCTGATTTA TCCGACTTGG ATTTCTCTCA GCTGGATATT AACGATGACG AGGCATTAGC   
  
  
+ CATCAACTGT GTCAACTCGC TCCACTCGGT CAATATCCAC CGCCGTGACT CGGTCATCTC GGCCTTCCGG   
  
  
+ CGGCTCCACC CTAGGGTGGT GACCGTGGTG GAGGAGGAGG CTGACCTTGC TGACGTGGGG GCGGAGGGGT   
  
  
+ ACGAGTTCTA CAGGGGGTTT AATGAGTGTT TAAGGTGGGT TAGGGTTTAC TTTGAGGCCC TGGAAGAGTG   
  
  
+ CTTTCCTAGG ACAAGCAATG AGAAACTCAT GCTCGAGCGG GCCGCAGGGC GGGCTCTGGT GGACCTCCTG   
  
  
+ GCCTGCCCCA AGCCCGCATC ATCCGAGCGG AGGGAGGCAG CTGCGCGGTG GTCCGGGAGG ATGCAGGGGG   
  
  
+ CCGGGTTCGG CCACGTGGGG TTCAGTGATG AGGTCTGTGA TGACGTCAAG GCCCTCCTAA GGAGGTACAA   
  
  
+ GGAGGGGTGG TCCATGATAC AATGTTCCGC CGCGGATGGC GGCGCCTCCG CCGGAATATT CTTGTCGTGG   
  
  
+ AGGGATCAGC CGGTGGTGTG GGCCAGTGCA TGGAAGCCTT A  

- -Up\_Stream \_Len000CCCAAA AAAATTTGAG TTTTAATCAA TAACATAGTT AACTTAAACC TTTTGATAAT   
  
  
- AAAAGCTGAA TAATATCTCT TAAACAACAA TTACTTACGA CTATTTATGA CTATTAATCA CAACACTCAC   
  
  
- ACTTAAACAC TACTACTCGT TACTAATCGC AATTTTAATA TTGATCCTAT TTATTAATTC ACAACTACTT   
  
  
- ACAACTAACT ACTACTACTC ATGACTAACC ACGCCACGAC CATTATTTAT AAATACTATT CAATTTTTTT   
  
  
- TTTTTGTGTA GGATTTGTTA GTGATACATT ATATGATTTA CTTTTTTTAA CCATTACACT AAACTGTTAG   
  
  
- ATTCCCATTC CCACAAAATG GGTGTGTACA ATACTTTAAC CGAATGATAA ATGTTTGAAA AATGTCAATA   
  
  
- TACTAAGATG GATACACAAT AGTAGAAAGG CAGCTACATT AACTAATATA AGTTGTGTTA AATTAAGTAA   
  
  
- TGTTACATCG ATTAACTATG AGTTTTATTA CTTGATAATT ATTTATTAAT TAATATTGAA AATTTTCAAA   
  
  
- CAATTAAATT TTAATATATT ATATATTTAA ATCCGATGTA ATTAGTTTTT TAATCCGCAC AAGTAGCCAG   
  
  
- GTTTGAACTT GGCCTGGTCT GGCCTGGCTT CTAGCTAATT CTCTTTTTCA TGTCTGACTC CTGGCCTAGC   
  
  
- TTATTCTCAG CGTGGCCTGG TCCTAGCCTG GCCTTTTTAA ACCTGGTCTA GCCCTGGCTA GCTCCATCTG   
  
  
- GCTTAAATAA AAGTTTATGC TATCCTTATT TTGAATAACT AAGTTAACTA AAACCGGTTT TAGTACGATA   
  
  
- ATAACTAAAA TAAACGATTG AGAGTTCTCA TAATCAAACG TGAAGCGTAA TAGAAACCGG TTTTTTTAGT   
  
  
- TTTTCTTTCA GAGTTTGAGA ATTATCAAAA AATAAAATAA ACCAAATAAA AATTTTATCA GCCGGAAGCC   
  
  
- AGGCCTGATC ACCTGGCTTA TTCCATTAAA AAGCTTAGCT CCTGGTCTGG TTTACGTGAG TCAAGCTTGG   
  
  
- CCAAGTCAAG CCAGACTGAG CTAAAAACCA GGCCAGGTTC AAACTTGTCT TATTACATGT GTGGATCTTT   
  
  
- TTTTCTTAAT ACGTATATAT GAATTCCGGG GGTTGGGAAA AAGGGAAAGG CGGTTATGAA AAATGGTTCA   
  
  
- GGTTAAGAAA AGAAGAATCG ATACCGATCA TCAATAGGGA GGGATCGAAG GTGGGATTCA AAACTCTGTT   
  
  
- TTAGGGTAGA TACAATTATA AATAAAGAAC GTGTACAGTG GGTTATGTTC CCAAAGTAAC TTTTCTTTTC   
  
  
- CATAATCTAC ATTCTATACA TACATATATT TATCAAAACG TTATCTGATC TTCCTGATTT TTCCTTTTTC   
  
  
- CTTCAAATTT TCGTTTTTTG AAAGGGGAGA TCTTTTTTCC TTTGGTCTAA GGGAAAAGAT GGGGTTAAGG   
  
  
- GTATCAGAAA TGAGACAGAG AGAGAGAGAC AGAGAGAATG AGTACTACAA CTCCCTCCTT CCTTACTTAA   
  
  
- TTCCTGGGAT TCGATTCTAG GTTGGCATTC TATAACGGGG AAATTAGTGG GTAAAGTATA GAGATAGGTG   
  
  
- TCAATTTTTG GGTGGAGGAG GTAGGTGTAT TGACTATATA GAGAAGATGT GGTATGATAT TCCTTCTTCT   
  
  
- CTTTTTATCC TCACATAACA TAAAAATAAA CTCATTAAAT AAAATTCCTC TCTTTAAATG TACCAACCCA   
  
  
- ATTTACCTAT GGAACAAATC TGATCACTCA AAGGTTGTTG GTGTGGTGGT GGTAGTGGTT GTGGTTGTGG   
  
  
- TTGTGGTGGT TGTCGTTAGA AGACTAGTTA GGAGAAACTT ATTATGATGA TGAATAAGGT GTTTAAGTTC   
  
  
- GTCAAGGTCT AGAAGGGTTG TTTGGGTAGT ATTAGTATTA GTATGAGTCA TAATAGTGGT TGTAGTAGGA   
  
  
- TGAATGGGGG TGGCGGTGGT AGTCCTCCTT CTTCTTCTTA CGAAGTTGAA AATGTACCTA CTTCTTTTGA   
  
  
- AGAGAAGAAG AAGGAGGGTG TTGTTTGTAG TAGGAGTTGG AGGAAGGAGG CGGTGGTGGA GGAGCATAAT   
  
  
- GGGAATGATG AGGTGGGGGA AGCTACAAGG GAGATGGTGG TGGTGGAGGT GGAGGTGGGG GTGCGGGTGG   
  
  
- TTGTGGTGGT GGTGGTGGGG GGGTTGGGTC ATACCGCCGC CGCCGCCACC CAAACTGAGG TCGACGAAGA   
  
  
- GGATGAGGGG ACGGGCTCTG GAGTTGGGCC TCGAGAAGAG GAGCAGGCCC AAGAGCCCGT TCACCCGAAG   
  
  
- GTTGTAGGAG AACCTTCGGC GAGCACGGTA GAGACTCTCC TTGTGCTCGG CGGAGGTGGT CGAGGATACC   
  
  
- TACCAGTTGC TCGAGTCGAG GGGCATGCCA CTGCACCTCG TCTTCTATCG AAGTATGAAG CCGGTTCGGG   
  
  
- AGAAGTGGGC GTACTGGCTC AGTCCCCTGG CGTGGATGGC GTGGTACGCC AGGCGGCACC TGTTCTGGAA   
  
  
- GAGGAAACTC AGATGCTCTT TCTACCATGA GTTCAAGGTC CTCCAATCGG GAACCTGGTG GAAACCTGTG   
  
  
- CACCGTCGAT TACCTCGGGA GTAACTACGG AAACTGCCAC TTAGGTTTTA GGTGTAGTAG CTGTAGTCGT   
  
  
- GGTGTAAGAC GTGGGTCACC GGGTGGGAGG ACCTCCGCGA CCGGTGGGCT TACCTACTGC GCGGGATGGA   
  
  
- CTCCGACTGG TGACACCACC AGTTGTTCAG GCCCTTACTT CCAGGGTGGC CTCCGCCCAG GGTGTCCCAC   
  
  
- TACTTTCTCT AACCCTGGGC GGAGCTCTTC AAACGATCCG ATTACCCTCA CGGAAAGTTT AAATTACACC   
  
  
- AGGTGGTGCC CCGACTAAAT AGGCTGAACC TAAAGAGAGT CGACCTATAA TTGCTACTGC TCCGTAATCG   
  
  
- GTAGTTGACA CAGTTGAGCG AGGTGAGCCA GTTATAGGTG GCGGCACTGA GCCAGTAGAG CCGGAAGGCC   
  
  
- GCCGAGGTGG GATCCCACCA CTGGCACCAC CTCCTCCTCC GACTGGAACG ACTGCACCCC CGCCTCCCCA   
  
  
- TGCTCAAGAT GTCCCCCAAA TTACTCACAA ATTCCACCCA ATCCCAAATG AAACTCCGGG ACCTTCTCAC   
  
  
- GAAAGGATCC TGTTCGTTAC TCTTTGAGTA CGAGCTCGCC CGGCGTCCCG CCCGAGACCA CCTGGAGGAC   
  
  
- CGGACGGGGT TCGGGCGTAG TAGGCTCGCC TCCCTCCGTC GACGCGCCAC CAGGCCCTCC TACGTCCCCC   
  
  
- GGCCCAAGCC GGTGCACCCC AAGTCACTAC TCCAGACACT ACTGCAGTTC CGGGAGGATT CCTCCATGTT   
  
  
- CCTCCCCACC AGGTACTATG TTACAAGGCG GCGCCTACCG CCGCGGAGGC GGCCTTATAA GAACAGCACC   
  
  
- TCCCTAGTCG GCCACCACAC CCGGTCACGT ACCTTCGGAA T

+     MYC

| Site Name | Organism | Position | Strand | Matrix score. | sequence | function |
| --- | --- | --- | --- | --- | --- | --- |
| MYC | Arabidopsis thaliana | 1295 | - | 6 | CATGTG |  |
| MYC | Arabidopsis thaliana | 817 | + | 6 | CAATTG |  |
| MYC | Arabidopsis thaliana | 1034 | - | 6 | CATTTG |  |
| MYC | Arabidopsis thaliana | 378 | - | 6 | CATGTG |  |
| MYC | Arabidopsis thaliana | 52 | + | 6 | CAATTG |  |

>HU06G00358.1   
+ -Up\_Stream \_Len000GGGTTT TTTTAAACTC AAAATTAGTT ATTGTATCAA TTGAATTTGG AAAACTATTA   
  
  
+ TTTTCGACTT ATTATAGAGA ATTTGTTGTT AATGAATGCT GATAAATACT GATAATTAGT GTTGTGAGTG   
  
  
+ TGAATTTGTG ATGATGAGCA ATGATTAGCG TTAAAATTAT AACTAGGATA AATAATTAAG TGTTGATGAA   
  
  
+ TGTTGATTGA TGATGATGAG TACTGATTGG TGCGGTGCTG GTAATAAATA TTTATGATAA GTTAAAAAAA   
  
  
+ AAAAACACAT CCTAAACAAT CACTATGTAA TATACTAAAT GAAAAAAATT GGTAATGTGA TTTGACAATC   
  
  
+ TAAGGGTAAG GGTGTTTTAC CCACACATGT TATGAAATTG GCTTACTATT TACAAACTTT TTACAGTTAT   
  
  
+ ATGATTCTAC CTATGTGTTA TCATCTTTCC GTCGATGTAA TTGATTATAT TCAACACAAT TTAATTCATT   
  
  
+ ACAATGTAGC TAATTGATAC TCAAAATAAT GAACTATTAA TAAATAATTA ATTATAACTT TTAAAAGTTT   
  
  
+ GTTAATTTAA AATTATATAA TATATAAATT TAGGCTACAT TAATCAAAAA ATTAGGCGTG TTCATCGGTC   
  
  
+ CAAACTTGAA CCGGACCAGA CCGGACCGAA GATCGATTAA GAGAAAAAGT ACAGACTGAG GACCGGATCG   
  
  
+ AATAAGAGTC GCACCGGACC AGGATCGGAC CGGAAAAATT TGGACCAGAT CGGGACCGAT CGAGGTAGAC   
  
  
+ CGAATTTATT TTCAAATACG ATAGGAATAA AACTTATTGA TTCAATTGAT TTTGGCCAAA ATCATGCTAT   
  
  
+ TATTGATTTT ATTTGCTAAC TCTCAAGAGT ATTAGTTTGC ACTTCGCATT ATCTTTGGCC AAAAAAATCA   
  
  
+ AAAAGAAAGT CTCAAACTCT TAATAGTTTT TTATTTTATT TGGTTTATTT TTAAAATAGT CGGCCTTCGG   
  
  
+ TCCGGACTAG TGGACCGAAT AAGGTAATTT TTCGAATCGA GGACCAGACC AAATGCACTC AGTTCGAACC   
  
  
+ GGTTCAGTTC GGTCTGACTC GATTTTTGGT CCGGTCCAAG TTTGAACAGA ATAATGTACA CACCTAGAAA   
  
  
+ AAAAGAATTA TGCATATATA CTTAAGGCCC CCAACCCTTT TTCCCTTTCC GCCAATACTT TTTACCAAGT   
  
  
+ CCAATTCTTT TCTTCTTAGC TATGGCTAGT AGTTATCCCT CCCTAGCTTC CACCCTAAGT TTTGAGACAA   
  
  
+ AATCCCATCT ATGTTAATAT TTATTTCTTG CACATGTCAC CCAATACAAG GGTTTCATTG AAAAGAAAAG   
  
  
+ GTATTAGATG TAAGATATGT ATGTATATAA ATAGTTTTGC AATAGACTAG AAGGACTAAA AAGGAAAAAG   
  
  
+ GAAGTTTAAA AGCAAAAAAC TTTCCCCTCT AGAAAAAAGG AAACCAGATT CCCTTTTCTA CCCCAATTCC   
  
  
+ CATAGTCTTT ACTCTGTCTC TCTCTCTCTG TCTCTCTTAC TCATGATGTT GAGGGAGGAA GGAATGAATT   
  
  
+ AAGGACCCTA AGCTAAGATC CAACCGTAAG ATATTGCCCC TTTAATCACC CATTTCATAT CTCTATCCAC   
  
  
+ AGTTAAAAAC CCACCTCCTC CATCCACATA ACTGATATAT CTCTTCTACA CCATACTATA AGGAAGAAGA   
  
  
+ GAAAAATAGG AGTGTATTGT ATTTTTATTT GAGTAATTTA TTTTAAGGAG AGAAATTTAC ATGGTTGGGT   
  
  
+ TAAATGGATA CCTTGTTTAG ACTAGTGAGT TTCCAACAAC CACACCACCA CCATCACCAA CACCAACACC   
  
  
+ AACACCACCA ACAGCAATCT TCTGATCAAT CCTCTTTGAA TAATACTACT ACTTATTCCA CAAATTCAAG   
  
  
+ CAGTTCCAGA TCTTCCCAAC AAACCCATCA TAATCATAAT CATACTCAGT ATTATCACCA ACATCATCCT   
  
  
+ ACTTACCCCC ACCGCCACCA TCAGGAGGAA GAAGAAGAAT GCTTCAACTT TTACATGGAT GAAGAAAACT   
  
  
+ TCTCTTCTTC TTCCTCCCAC AACAAACATC ATCCTCAACC TCCTTCCTCC GCCACCACCT CCTCGTATTA   
  
  
+ CCCTTACTAC TCCACCCCCT TCGATGTTCC CTCTACCACC ACCACCTCCA CCTCCACCCC CACGCCCACC   
  
  
+ AACACCACCA CCACCACCCC CCCAACCCAG TATGGCGGCG GCGGCGGTGG GTTTGACTCC AGCTGCTTCT   
  
  
+ CCTACTCCCC TGCCCGAGAC CTCAACCCGG AGCTCTTCTC CTCGTCCGGG TTCTCGGGCA AGTGGGCTTC   
  
  
+ CAACATCCTC TTGGAAGCCG CTCGTGCCAT CTCTGAGAGG AACACGAGCC GCCTCCACCA GCTCCTATGG   
  
  
+ ATGGTCAACG AGCTCAGCTC CCCGTACGGT GACGTGGAGC AGAAGATAGC TTCATACTTC GGCCAAGCCC   
  
  
+ TCTTCACCCG CATGACCGAG TCAGGGGACC GCACCTACCG CACCATGCGG TCCGCCGTGG ACAAGACCTT   
  
  
+ CTCCTTTGAG TCTACGAGAA AGATGGTACT CAAGTTCCAG GAGGTTAGCC CTTGGACCAC CTTTGGACAC   
  
  
+ GTGGCAGCTA ATGGAGCCCT CATTGATGCC TTTGACGGTG AATCCAAAAT CCACATCATC GACATCAGCA   
  
  
+ CCACATTCTG CACCCAGTGG CCCACCCTCC TGGAGGCGCT GGCCACCCGA ATGGATGACG CGCCCTACCT   
  
  
+ GAGGCTGACC ACTGTGGTGG TCAACAAGTC CGGGAATGAA GGTCCCACCG GAGGCGGGTC CCACAGGGTG   
  
  
+ ATGAAAGAGA TTGGGACCCG CCTCGAGAAG TTTGCTAGGC TAATGGGAGT GCCTTTCAAA TTTAATGTGG   
  
  
+ TCCACCACGG GGCTGATTTA TCCGACTTGG ATTTCTCTCA GCTGGATATT AACGATGACG AGGCATTAGC   
  
  
+ CATCAACTGT GTCAACTCGC TCCACTCGGT CAATATCCAC CGCCGTGACT CGGTCATCTC GGCCTTCCGG   
  
  
+ CGGCTCCACC CTAGGGTGGT GACCGTGGTG GAGGAGGAGG CTGACCTTGC TGACGTGGGG GCGGAGGGGT   
  
  
+ ACGAGTTCTA CAGGGGGTTT AATGAGTGTT TAAGGTGGGT TAGGGTTTAC TTTGAGGCCC TGGAAGAGTG   
  
  
+ CTTTCCTAGG ACAAGCAATG AGAAACTCAT GCTCGAGCGG GCCGCAGGGC GGGCTCTGGT GGACCTCCTG   
  
  
+ GCCTGCCCCA AGCCCGCATC ATCCGAGCGG AGGGAGGCAG CTGCGCGGTG GTCCGGGAGG ATGCAGGGGG   
  
  
+ CCGGGTTCGG CCACGTGGGG TTCAGTGATG AGGTCTGTGA TGACGTCAAG GCCCTCCTAA GGAGGTACAA   
  
  
+ GGAGGGGTGG TCCATGATAC AATGTTCCGC CGCGGATGGC GGCGCCTCCG CCGGAATATT CTTGTCGTGG   
  
  
+ AGGGATCAGC CGGTGGTGTG GGCCAGTGCA TGGAAGCCTT A  

- -Up\_Stream \_Len000CCCAAA AAAATTTGAG TTTTAATCAA TAACATAGTT AACTTAAACC TTTTGATAAT   
  
  
- AAAAGCTGAA TAATATCTCT TAAACAACAA TTACTTACGA CTATTTATGA CTATTAATCA CAACACTCAC   
  
  
- ACTTAAACAC TACTACTCGT TACTAATCGC AATTTTAATA TTGATCCTAT TTATTAATTC ACAACTACTT   
  
  
- ACAACTAACT ACTACTACTC ATGACTAACC ACGCCACGAC CATTATTTAT AAATACTATT CAATTTTTTT   
  
  
- TTTTTGTGTA GGATTTGTTA GTGATACATT ATATGATTTA CTTTTTTTAA CCATTACACT AAACTGTTAG   
  
  
- ATTCCCATTC CCACAAAATG GGTGTGTACA ATACTTTAAC CGAATGATAA ATGTTTGAAA AATGTCAATA   
  
  
- TACTAAGATG GATACACAAT AGTAGAAAGG CAGCTACATT AACTAATATA AGTTGTGTTA AATTAAGTAA   
  
  
- TGTTACATCG ATTAACTATG AGTTTTATTA CTTGATAATT ATTTATTAAT TAATATTGAA AATTTTCAAA   
  
  
- CAATTAAATT TTAATATATT ATATATTTAA ATCCGATGTA ATTAGTTTTT TAATCCGCAC AAGTAGCCAG   
  
  
- GTTTGAACTT GGCCTGGTCT GGCCTGGCTT CTAGCTAATT CTCTTTTTCA TGTCTGACTC CTGGCCTAGC   
  
  
- TTATTCTCAG CGTGGCCTGG TCCTAGCCTG GCCTTTTTAA ACCTGGTCTA GCCCTGGCTA GCTCCATCTG   
  
  
- GCTTAAATAA AAGTTTATGC TATCCTTATT TTGAATAACT AAGTTAACTA AAACCGGTTT TAGTACGATA   
  
  
- ATAACTAAAA TAAACGATTG AGAGTTCTCA TAATCAAACG TGAAGCGTAA TAGAAACCGG TTTTTTTAGT   
  
  
- TTTTCTTTCA GAGTTTGAGA ATTATCAAAA AATAAAATAA ACCAAATAAA AATTTTATCA GCCGGAAGCC   
  
  
- AGGCCTGATC ACCTGGCTTA TTCCATTAAA AAGCTTAGCT CCTGGTCTGG TTTACGTGAG TCAAGCTTGG   
  
  
- CCAAGTCAAG CCAGACTGAG CTAAAAACCA GGCCAGGTTC AAACTTGTCT TATTACATGT GTGGATCTTT   
  
  
- TTTTCTTAAT ACGTATATAT GAATTCCGGG GGTTGGGAAA AAGGGAAAGG CGGTTATGAA AAATGGTTCA   
  
  
- GGTTAAGAAA AGAAGAATCG ATACCGATCA TCAATAGGGA GGGATCGAAG GTGGGATTCA AAACTCTGTT   
  
  
- TTAGGGTAGA TACAATTATA AATAAAGAAC GTGTACAGTG GGTTATGTTC CCAAAGTAAC TTTTCTTTTC   
  
  
- CATAATCTAC ATTCTATACA TACATATATT TATCAAAACG TTATCTGATC TTCCTGATTT TTCCTTTTTC   
  
  
- CTTCAAATTT TCGTTTTTTG AAAGGGGAGA TCTTTTTTCC TTTGGTCTAA GGGAAAAGAT GGGGTTAAGG   
  
  
- GTATCAGAAA TGAGACAGAG AGAGAGAGAC AGAGAGAATG AGTACTACAA CTCCCTCCTT CCTTACTTAA   
  
  
- TTCCTGGGAT TCGATTCTAG GTTGGCATTC TATAACGGGG AAATTAGTGG GTAAAGTATA GAGATAGGTG   
  
  
- TCAATTTTTG GGTGGAGGAG GTAGGTGTAT TGACTATATA GAGAAGATGT GGTATGATAT TCCTTCTTCT   
  
  
- CTTTTTATCC TCACATAACA TAAAAATAAA CTCATTAAAT AAAATTCCTC TCTTTAAATG TACCAACCCA   
  
  
- ATTTACCTAT GGAACAAATC TGATCACTCA AAGGTTGTTG GTGTGGTGGT GGTAGTGGTT GTGGTTGTGG   
  
  
- TTGTGGTGGT TGTCGTTAGA AGACTAGTTA GGAGAAACTT ATTATGATGA TGAATAAGGT GTTTAAGTTC   
  
  
- GTCAAGGTCT AGAAGGGTTG TTTGGGTAGT ATTAGTATTA GTATGAGTCA TAATAGTGGT TGTAGTAGGA   
  
  
- TGAATGGGGG TGGCGGTGGT AGTCCTCCTT CTTCTTCTTA CGAAGTTGAA AATGTACCTA CTTCTTTTGA   
  
  
- AGAGAAGAAG AAGGAGGGTG TTGTTTGTAG TAGGAGTTGG AGGAAGGAGG CGGTGGTGGA GGAGCATAAT   
  
  
- GGGAATGATG AGGTGGGGGA AGCTACAAGG GAGATGGTGG TGGTGGAGGT GGAGGTGGGG GTGCGGGTGG   
  
  
- TTGTGGTGGT GGTGGTGGGG GGGTTGGGTC ATACCGCCGC CGCCGCCACC CAAACTGAGG TCGACGAAGA   
  
  
- GGATGAGGGG ACGGGCTCTG GAGTTGGGCC TCGAGAAGAG GAGCAGGCCC AAGAGCCCGT TCACCCGAAG   
  
  
- GTTGTAGGAG AACCTTCGGC GAGCACGGTA GAGACTCTCC TTGTGCTCGG CGGAGGTGGT CGAGGATACC   
  
  
- TACCAGTTGC TCGAGTCGAG GGGCATGCCA CTGCACCTCG TCTTCTATCG AAGTATGAAG CCGGTTCGGG   
  
  
- AGAAGTGGGC GTACTGGCTC AGTCCCCTGG CGTGGATGGC GTGGTACGCC AGGCGGCACC TGTTCTGGAA   
  
  
- GAGGAAACTC AGATGCTCTT TCTACCATGA GTTCAAGGTC CTCCAATCGG GAACCTGGTG GAAACCTGTG   
  
  
- CACCGTCGAT TACCTCGGGA GTAACTACGG AAACTGCCAC TTAGGTTTTA GGTGTAGTAG CTGTAGTCGT   
  
  
- GGTGTAAGAC GTGGGTCACC GGGTGGGAGG ACCTCCGCGA CCGGTGGGCT TACCTACTGC GCGGGATGGA   
  
  
- CTCCGACTGG TGACACCACC AGTTGTTCAG GCCCTTACTT CCAGGGTGGC CTCCGCCCAG GGTGTCCCAC   
  
  
- TACTTTCTCT AACCCTGGGC GGAGCTCTTC AAACGATCCG ATTACCCTCA CGGAAAGTTT AAATTACACC   
  
  
- AGGTGGTGCC CCGACTAAAT AGGCTGAACC TAAAGAGAGT CGACCTATAA TTGCTACTGC TCCGTAATCG   
  
  
- GTAGTTGACA CAGTTGAGCG AGGTGAGCCA GTTATAGGTG GCGGCACTGA GCCAGTAGAG CCGGAAGGCC   
  
  
- GCCGAGGTGG GATCCCACCA CTGGCACCAC CTCCTCCTCC GACTGGAACG ACTGCACCCC CGCCTCCCCA   
  
  
- TGCTCAAGAT GTCCCCCAAA TTACTCACAA ATTCCACCCA ATCCCAAATG AAACTCCGGG ACCTTCTCAC   
  
  
- GAAAGGATCC TGTTCGTTAC TCTTTGAGTA CGAGCTCGCC CGGCGTCCCG CCCGAGACCA CCTGGAGGAC   
  
  
- CGGACGGGGT TCGGGCGTAG TAGGCTCGCC TCCCTCCGTC GACGCGCCAC CAGGCCCTCC TACGTCCCCC   
  
  
- GGCCCAAGCC GGTGCACCCC AAGTCACTAC TCCAGACACT ACTGCAGTTC CGGGAGGATT CCTCCATGTT   
  
  
- CCTCCCCACC AGGTACTATG TTACAAGGCG GCGCCTACCG CCGCGGAGGC GGCCTTATAA GAACAGCACC   
  
  
- TCCCTAGTCG GCCACCACAC CCGGTCACGT ACCTTCGGAA T

+     Myb

| Site Name | Organism | Position | Strand | Matrix score. | sequence | function |
| --- | --- | --- | --- | --- | --- | --- |
| Myb | Arabidopsis thaliana | 418 | - | 6 | TAACTG |  |
| Myb | Arabidopsis thaliana | 1643 | + | 6 | TAACTG |  |
| Myb | Arabidopsis thaliana | 2948 | + | 6 | CAACTG |  |
| Myb | Arabidopsis thaliana | 1614 | - | 6 | TAACTG |  |

>HU06G00358.1   
+ -Up\_Stream \_Len000GGGTTT TTTTAAACTC AAAATTAGTT ATTGTATCAA TTGAATTTGG AAAACTATTA   
  
  
+ TTTTCGACTT ATTATAGAGA ATTTGTTGTT AATGAATGCT GATAAATACT GATAATTAGT GTTGTGAGTG   
  
  
+ TGAATTTGTG ATGATGAGCA ATGATTAGCG TTAAAATTAT AACTAGGATA AATAATTAAG TGTTGATGAA   
  
  
+ TGTTGATTGA TGATGATGAG TACTGATTGG TGCGGTGCTG GTAATAAATA TTTATGATAA GTTAAAAAAA   
  
  
+ AAAAACACAT CCTAAACAAT CACTATGTAA TATACTAAAT GAAAAAAATT GGTAATGTGA TTTGACAATC   
  
  
+ TAAGGGTAAG GGTGTTTTAC CCACACATGT TATGAAATTG GCTTACTATT TACAAACTTT TTACAGTTAT   
  
  
+ ATGATTCTAC CTATGTGTTA TCATCTTTCC GTCGATGTAA TTGATTATAT TCAACACAAT TTAATTCATT   
  
  
+ ACAATGTAGC TAATTGATAC TCAAAATAAT GAACTATTAA TAAATAATTA ATTATAACTT TTAAAAGTTT   
  
  
+ GTTAATTTAA AATTATATAA TATATAAATT TAGGCTACAT TAATCAAAAA ATTAGGCGTG TTCATCGGTC   
  
  
+ CAAACTTGAA CCGGACCAGA CCGGACCGAA GATCGATTAA GAGAAAAAGT ACAGACTGAG GACCGGATCG   
  
  
+ AATAAGAGTC GCACCGGACC AGGATCGGAC CGGAAAAATT TGGACCAGAT CGGGACCGAT CGAGGTAGAC   
  
  
+ CGAATTTATT TTCAAATACG ATAGGAATAA AACTTATTGA TTCAATTGAT TTTGGCCAAA ATCATGCTAT   
  
  
+ TATTGATTTT ATTTGCTAAC TCTCAAGAGT ATTAGTTTGC ACTTCGCATT ATCTTTGGCC AAAAAAATCA   
  
  
+ AAAAGAAAGT CTCAAACTCT TAATAGTTTT TTATTTTATT TGGTTTATTT TTAAAATAGT CGGCCTTCGG   
  
  
+ TCCGGACTAG TGGACCGAAT AAGGTAATTT TTCGAATCGA GGACCAGACC AAATGCACTC AGTTCGAACC   
  
  
+ GGTTCAGTTC GGTCTGACTC GATTTTTGGT CCGGTCCAAG TTTGAACAGA ATAATGTACA CACCTAGAAA   
  
  
+ AAAAGAATTA TGCATATATA CTTAAGGCCC CCAACCCTTT TTCCCTTTCC GCCAATACTT TTTACCAAGT   
  
  
+ CCAATTCTTT TCTTCTTAGC TATGGCTAGT AGTTATCCCT CCCTAGCTTC CACCCTAAGT TTTGAGACAA   
  
  
+ AATCCCATCT ATGTTAATAT TTATTTCTTG CACATGTCAC CCAATACAAG GGTTTCATTG AAAAGAAAAG   
  
  
+ GTATTAGATG TAAGATATGT ATGTATATAA ATAGTTTTGC AATAGACTAG AAGGACTAAA AAGGAAAAAG   
  
  
+ GAAGTTTAAA AGCAAAAAAC TTTCCCCTCT AGAAAAAAGG AAACCAGATT CCCTTTTCTA CCCCAATTCC   
  
  
+ CATAGTCTTT ACTCTGTCTC TCTCTCTCTG TCTCTCTTAC TCATGATGTT GAGGGAGGAA GGAATGAATT   
  
  
+ AAGGACCCTA AGCTAAGATC CAACCGTAAG ATATTGCCCC TTTAATCACC CATTTCATAT CTCTATCCAC   
  
  
+ AGTTAAAAAC CCACCTCCTC CATCCACATA ACTGATATAT CTCTTCTACA CCATACTATA AGGAAGAAGA   
  
  
+ GAAAAATAGG AGTGTATTGT ATTTTTATTT GAGTAATTTA TTTTAAGGAG AGAAATTTAC ATGGTTGGGT   
  
  
+ TAAATGGATA CCTTGTTTAG ACTAGTGAGT TTCCAACAAC CACACCACCA CCATCACCAA CACCAACACC   
  
  
+ AACACCACCA ACAGCAATCT TCTGATCAAT CCTCTTTGAA TAATACTACT ACTTATTCCA CAAATTCAAG   
  
  
+ CAGTTCCAGA TCTTCCCAAC AAACCCATCA TAATCATAAT CATACTCAGT ATTATCACCA ACATCATCCT   
  
  
+ ACTTACCCCC ACCGCCACCA TCAGGAGGAA GAAGAAGAAT GCTTCAACTT TTACATGGAT GAAGAAAACT   
  
  
+ TCTCTTCTTC TTCCTCCCAC AACAAACATC ATCCTCAACC TCCTTCCTCC GCCACCACCT CCTCGTATTA   
  
  
+ CCCTTACTAC TCCACCCCCT TCGATGTTCC CTCTACCACC ACCACCTCCA CCTCCACCCC CACGCCCACC   
  
  
+ AACACCACCA CCACCACCCC CCCAACCCAG TATGGCGGCG GCGGCGGTGG GTTTGACTCC AGCTGCTTCT   
  
  
+ CCTACTCCCC TGCCCGAGAC CTCAACCCGG AGCTCTTCTC CTCGTCCGGG TTCTCGGGCA AGTGGGCTTC   
  
  
+ CAACATCCTC TTGGAAGCCG CTCGTGCCAT CTCTGAGAGG AACACGAGCC GCCTCCACCA GCTCCTATGG   
  
  
+ ATGGTCAACG AGCTCAGCTC CCCGTACGGT GACGTGGAGC AGAAGATAGC TTCATACTTC GGCCAAGCCC   
  
  
+ TCTTCACCCG CATGACCGAG TCAGGGGACC GCACCTACCG CACCATGCGG TCCGCCGTGG ACAAGACCTT   
  
  
+ CTCCTTTGAG TCTACGAGAA AGATGGTACT CAAGTTCCAG GAGGTTAGCC CTTGGACCAC CTTTGGACAC   
  
  
+ GTGGCAGCTA ATGGAGCCCT CATTGATGCC TTTGACGGTG AATCCAAAAT CCACATCATC GACATCAGCA   
  
  
+ CCACATTCTG CACCCAGTGG CCCACCCTCC TGGAGGCGCT GGCCACCCGA ATGGATGACG CGCCCTACCT   
  
  
+ GAGGCTGACC ACTGTGGTGG TCAACAAGTC CGGGAATGAA GGTCCCACCG GAGGCGGGTC CCACAGGGTG   
  
  
+ ATGAAAGAGA TTGGGACCCG CCTCGAGAAG TTTGCTAGGC TAATGGGAGT GCCTTTCAAA TTTAATGTGG   
  
  
+ TCCACCACGG GGCTGATTTA TCCGACTTGG ATTTCTCTCA GCTGGATATT AACGATGACG AGGCATTAGC   
  
  
+ CATCAACTGT GTCAACTCGC TCCACTCGGT CAATATCCAC CGCCGTGACT CGGTCATCTC GGCCTTCCGG   
  
  
+ CGGCTCCACC CTAGGGTGGT GACCGTGGTG GAGGAGGAGG CTGACCTTGC TGACGTGGGG GCGGAGGGGT   
  
  
+ ACGAGTTCTA CAGGGGGTTT AATGAGTGTT TAAGGTGGGT TAGGGTTTAC TTTGAGGCCC TGGAAGAGTG   
  
  
+ CTTTCCTAGG ACAAGCAATG AGAAACTCAT GCTCGAGCGG GCCGCAGGGC GGGCTCTGGT GGACCTCCTG   
  
  
+ GCCTGCCCCA AGCCCGCATC ATCCGAGCGG AGGGAGGCAG CTGCGCGGTG GTCCGGGAGG ATGCAGGGGG   
  
  
+ CCGGGTTCGG CCACGTGGGG TTCAGTGATG AGGTCTGTGA TGACGTCAAG GCCCTCCTAA GGAGGTACAA   
  
  
+ GGAGGGGTGG TCCATGATAC AATGTTCCGC CGCGGATGGC GGCGCCTCCG CCGGAATATT CTTGTCGTGG   
  
  
+ AGGGATCAGC CGGTGGTGTG GGCCAGTGCA TGGAAGCCTT A  

- -Up\_Stream \_Len000CCCAAA AAAATTTGAG TTTTAATCAA TAACATAGTT AACTTAAACC TTTTGATAAT   
  
  
- AAAAGCTGAA TAATATCTCT TAAACAACAA TTACTTACGA CTATTTATGA CTATTAATCA CAACACTCAC   
  
  
- ACTTAAACAC TACTACTCGT TACTAATCGC AATTTTAATA TTGATCCTAT TTATTAATTC ACAACTACTT   
  
  
- ACAACTAACT ACTACTACTC ATGACTAACC ACGCCACGAC CATTATTTAT AAATACTATT CAATTTTTTT   
  
  
- TTTTTGTGTA GGATTTGTTA GTGATACATT ATATGATTTA CTTTTTTTAA CCATTACACT AAACTGTTAG   
  
  
- ATTCCCATTC CCACAAAATG GGTGTGTACA ATACTTTAAC CGAATGATAA ATGTTTGAAA AATGTCAATA   
  
  
- TACTAAGATG GATACACAAT AGTAGAAAGG CAGCTACATT AACTAATATA AGTTGTGTTA AATTAAGTAA   
  
  
- TGTTACATCG ATTAACTATG AGTTTTATTA CTTGATAATT ATTTATTAAT TAATATTGAA AATTTTCAAA   
  
  
- CAATTAAATT TTAATATATT ATATATTTAA ATCCGATGTA ATTAGTTTTT TAATCCGCAC AAGTAGCCAG   
  
  
- GTTTGAACTT GGCCTGGTCT GGCCTGGCTT CTAGCTAATT CTCTTTTTCA TGTCTGACTC CTGGCCTAGC   
  
  
- TTATTCTCAG CGTGGCCTGG TCCTAGCCTG GCCTTTTTAA ACCTGGTCTA GCCCTGGCTA GCTCCATCTG   
  
  
- GCTTAAATAA AAGTTTATGC TATCCTTATT TTGAATAACT AAGTTAACTA AAACCGGTTT TAGTACGATA   
  
  
- ATAACTAAAA TAAACGATTG AGAGTTCTCA TAATCAAACG TGAAGCGTAA TAGAAACCGG TTTTTTTAGT   
  
  
- TTTTCTTTCA GAGTTTGAGA ATTATCAAAA AATAAAATAA ACCAAATAAA AATTTTATCA GCCGGAAGCC   
  
  
- AGGCCTGATC ACCTGGCTTA TTCCATTAAA AAGCTTAGCT CCTGGTCTGG TTTACGTGAG TCAAGCTTGG   
  
  
- CCAAGTCAAG CCAGACTGAG CTAAAAACCA GGCCAGGTTC AAACTTGTCT TATTACATGT GTGGATCTTT   
  
  
- TTTTCTTAAT ACGTATATAT GAATTCCGGG GGTTGGGAAA AAGGGAAAGG CGGTTATGAA AAATGGTTCA   
  
  
- GGTTAAGAAA AGAAGAATCG ATACCGATCA TCAATAGGGA GGGATCGAAG GTGGGATTCA AAACTCTGTT   
  
  
- TTAGGGTAGA TACAATTATA AATAAAGAAC GTGTACAGTG GGTTATGTTC CCAAAGTAAC TTTTCTTTTC   
  
  
- CATAATCTAC ATTCTATACA TACATATATT TATCAAAACG TTATCTGATC TTCCTGATTT TTCCTTTTTC   
  
  
- CTTCAAATTT TCGTTTTTTG AAAGGGGAGA TCTTTTTTCC TTTGGTCTAA GGGAAAAGAT GGGGTTAAGG   
  
  
- GTATCAGAAA TGAGACAGAG AGAGAGAGAC AGAGAGAATG AGTACTACAA CTCCCTCCTT CCTTACTTAA   
  
  
- TTCCTGGGAT TCGATTCTAG GTTGGCATTC TATAACGGGG AAATTAGTGG GTAAAGTATA GAGATAGGTG   
  
  
- TCAATTTTTG GGTGGAGGAG GTAGGTGTAT TGACTATATA GAGAAGATGT GGTATGATAT TCCTTCTTCT   
  
  
- CTTTTTATCC TCACATAACA TAAAAATAAA CTCATTAAAT AAAATTCCTC TCTTTAAATG TACCAACCCA   
  
  
- ATTTACCTAT GGAACAAATC TGATCACTCA AAGGTTGTTG GTGTGGTGGT GGTAGTGGTT GTGGTTGTGG   
  
  
- TTGTGGTGGT TGTCGTTAGA AGACTAGTTA GGAGAAACTT ATTATGATGA TGAATAAGGT GTTTAAGTTC   
  
  
- GTCAAGGTCT AGAAGGGTTG TTTGGGTAGT ATTAGTATTA GTATGAGTCA TAATAGTGGT TGTAGTAGGA   
  
  
- TGAATGGGGG TGGCGGTGGT AGTCCTCCTT CTTCTTCTTA CGAAGTTGAA AATGTACCTA CTTCTTTTGA   
  
  
- AGAGAAGAAG AAGGAGGGTG TTGTTTGTAG TAGGAGTTGG AGGAAGGAGG CGGTGGTGGA GGAGCATAAT   
  
  
- GGGAATGATG AGGTGGGGGA AGCTACAAGG GAGATGGTGG TGGTGGAGGT GGAGGTGGGG GTGCGGGTGG   
  
  
- TTGTGGTGGT GGTGGTGGGG GGGTTGGGTC ATACCGCCGC CGCCGCCACC CAAACTGAGG TCGACGAAGA   
  
  
- GGATGAGGGG ACGGGCTCTG GAGTTGGGCC TCGAGAAGAG GAGCAGGCCC AAGAGCCCGT TCACCCGAAG   
  
  
- GTTGTAGGAG AACCTTCGGC GAGCACGGTA GAGACTCTCC TTGTGCTCGG CGGAGGTGGT CGAGGATACC   
  
  
- TACCAGTTGC TCGAGTCGAG GGGCATGCCA CTGCACCTCG TCTTCTATCG AAGTATGAAG CCGGTTCGGG   
  
  
- AGAAGTGGGC GTACTGGCTC AGTCCCCTGG CGTGGATGGC GTGGTACGCC AGGCGGCACC TGTTCTGGAA   
  
  
- GAGGAAACTC AGATGCTCTT TCTACCATGA GTTCAAGGTC CTCCAATCGG GAACCTGGTG GAAACCTGTG   
  
  
- CACCGTCGAT TACCTCGGGA GTAACTACGG AAACTGCCAC TTAGGTTTTA GGTGTAGTAG CTGTAGTCGT   
  
  
- GGTGTAAGAC GTGGGTCACC GGGTGGGAGG ACCTCCGCGA CCGGTGGGCT TACCTACTGC GCGGGATGGA   
  
  
- CTCCGACTGG TGACACCACC AGTTGTTCAG GCCCTTACTT CCAGGGTGGC CTCCGCCCAG GGTGTCCCAC   
  
  
- TACTTTCTCT AACCCTGGGC GGAGCTCTTC AAACGATCCG ATTACCCTCA CGGAAAGTTT AAATTACACC   
  
  
- AGGTGGTGCC CCGACTAAAT AGGCTGAACC TAAAGAGAGT CGACCTATAA TTGCTACTGC TCCGTAATCG   
  
  
- GTAGTTGACA CAGTTGAGCG AGGTGAGCCA GTTATAGGTG GCGGCACTGA GCCAGTAGAG CCGGAAGGCC   
  
  
- GCCGAGGTGG GATCCCACCA CTGGCACCAC CTCCTCCTCC GACTGGAACG ACTGCACCCC CGCCTCCCCA   
  
  
- TGCTCAAGAT GTCCCCCAAA TTACTCACAA ATTCCACCCA ATCCCAAATG AAACTCCGGG ACCTTCTCAC   
  
  
- GAAAGGATCC TGTTCGTTAC TCTTTGAGTA CGAGCTCGCC CGGCGTCCCG CCCGAGACCA CCTGGAGGAC   
  
  
- CGGACGGGGT TCGGGCGTAG TAGGCTCGCC TCCCTCCGTC GACGCGCCAC CAGGCCCTCC TACGTCCCCC   
  
  
- GGCCCAAGCC GGTGCACCCC AAGTCACTAC TCCAGACACT ACTGCAGTTC CGGGAGGATT CCTCCATGTT   
  
  
- CCTCCCCACC AGGTACTATG TTACAAGGCG GCGCCTACCG CCGCGGAGGC GGCCTTATAA GAACAGCACC   
  
  
- TCCCTAGTCG GCCACCACAC CCGGTCACGT ACCTTCGGAA T

+     Myb-binding site

| Site Name | Organism | Position | Strand | Matrix score. | sequence | function |
| --- | --- | --- | --- | --- | --- | --- |
| Myb-binding site | Nicotiana tabacum | 1833 | + | 6 | CAACAG |  |

>HU06G00358.1   
+ -Up\_Stream \_Len000GGGTTT TTTTAAACTC AAAATTAGTT ATTGTATCAA TTGAATTTGG AAAACTATTA   
  
  
+ TTTTCGACTT ATTATAGAGA ATTTGTTGTT AATGAATGCT GATAAATACT GATAATTAGT GTTGTGAGTG   
  
  
+ TGAATTTGTG ATGATGAGCA ATGATTAGCG TTAAAATTAT AACTAGGATA AATAATTAAG TGTTGATGAA   
  
  
+ TGTTGATTGA TGATGATGAG TACTGATTGG TGCGGTGCTG GTAATAAATA TTTATGATAA GTTAAAAAAA   
  
  
+ AAAAACACAT CCTAAACAAT CACTATGTAA TATACTAAAT GAAAAAAATT GGTAATGTGA TTTGACAATC   
  
  
+ TAAGGGTAAG GGTGTTTTAC CCACACATGT TATGAAATTG GCTTACTATT TACAAACTTT TTACAGTTAT   
  
  
+ ATGATTCTAC CTATGTGTTA TCATCTTTCC GTCGATGTAA TTGATTATAT TCAACACAAT TTAATTCATT   
  
  
+ ACAATGTAGC TAATTGATAC TCAAAATAAT GAACTATTAA TAAATAATTA ATTATAACTT TTAAAAGTTT   
  
  
+ GTTAATTTAA AATTATATAA TATATAAATT TAGGCTACAT TAATCAAAAA ATTAGGCGTG TTCATCGGTC   
  
  
+ CAAACTTGAA CCGGACCAGA CCGGACCGAA GATCGATTAA GAGAAAAAGT ACAGACTGAG GACCGGATCG   
  
  
+ AATAAGAGTC GCACCGGACC AGGATCGGAC CGGAAAAATT TGGACCAGAT CGGGACCGAT CGAGGTAGAC   
  
  
+ CGAATTTATT TTCAAATACG ATAGGAATAA AACTTATTGA TTCAATTGAT TTTGGCCAAA ATCATGCTAT   
  
  
+ TATTGATTTT ATTTGCTAAC TCTCAAGAGT ATTAGTTTGC ACTTCGCATT ATCTTTGGCC AAAAAAATCA   
  
  
+ AAAAGAAAGT CTCAAACTCT TAATAGTTTT TTATTTTATT TGGTTTATTT TTAAAATAGT CGGCCTTCGG   
  
  
+ TCCGGACTAG TGGACCGAAT AAGGTAATTT TTCGAATCGA GGACCAGACC AAATGCACTC AGTTCGAACC   
  
  
+ GGTTCAGTTC GGTCTGACTC GATTTTTGGT CCGGTCCAAG TTTGAACAGA ATAATGTACA CACCTAGAAA   
  
  
+ AAAAGAATTA TGCATATATA CTTAAGGCCC CCAACCCTTT TTCCCTTTCC GCCAATACTT TTTACCAAGT   
  
  
+ CCAATTCTTT TCTTCTTAGC TATGGCTAGT AGTTATCCCT CCCTAGCTTC CACCCTAAGT TTTGAGACAA   
  
  
+ AATCCCATCT ATGTTAATAT TTATTTCTTG CACATGTCAC CCAATACAAG GGTTTCATTG AAAAGAAAAG   
  
  
+ GTATTAGATG TAAGATATGT ATGTATATAA ATAGTTTTGC AATAGACTAG AAGGACTAAA AAGGAAAAAG   
  
  
+ GAAGTTTAAA AGCAAAAAAC TTTCCCCTCT AGAAAAAAGG AAACCAGATT CCCTTTTCTA CCCCAATTCC   
  
  
+ CATAGTCTTT ACTCTGTCTC TCTCTCTCTG TCTCTCTTAC TCATGATGTT GAGGGAGGAA GGAATGAATT   
  
  
+ AAGGACCCTA AGCTAAGATC CAACCGTAAG ATATTGCCCC TTTAATCACC CATTTCATAT CTCTATCCAC   
  
  
+ AGTTAAAAAC CCACCTCCTC CATCCACATA ACTGATATAT CTCTTCTACA CCATACTATA AGGAAGAAGA   
  
  
+ GAAAAATAGG AGTGTATTGT ATTTTTATTT GAGTAATTTA TTTTAAGGAG AGAAATTTAC ATGGTTGGGT   
  
  
+ TAAATGGATA CCTTGTTTAG ACTAGTGAGT TTCCAACAAC CACACCACCA CCATCACCAA CACCAACACC   
  
  
+ AACACCACCA ACAGCAATCT TCTGATCAAT CCTCTTTGAA TAATACTACT ACTTATTCCA CAAATTCAAG   
  
  
+ CAGTTCCAGA TCTTCCCAAC AAACCCATCA TAATCATAAT CATACTCAGT ATTATCACCA ACATCATCCT   
  
  
+ ACTTACCCCC ACCGCCACCA TCAGGAGGAA GAAGAAGAAT GCTTCAACTT TTACATGGAT GAAGAAAACT   
  
  
+ TCTCTTCTTC TTCCTCCCAC AACAAACATC ATCCTCAACC TCCTTCCTCC GCCACCACCT CCTCGTATTA   
  
  
+ CCCTTACTAC TCCACCCCCT TCGATGTTCC CTCTACCACC ACCACCTCCA CCTCCACCCC CACGCCCACC   
  
  
+ AACACCACCA CCACCACCCC CCCAACCCAG TATGGCGGCG GCGGCGGTGG GTTTGACTCC AGCTGCTTCT   
  
  
+ CCTACTCCCC TGCCCGAGAC CTCAACCCGG AGCTCTTCTC CTCGTCCGGG TTCTCGGGCA AGTGGGCTTC   
  
  
+ CAACATCCTC TTGGAAGCCG CTCGTGCCAT CTCTGAGAGG AACACGAGCC GCCTCCACCA GCTCCTATGG   
  
  
+ ATGGTCAACG AGCTCAGCTC CCCGTACGGT GACGTGGAGC AGAAGATAGC TTCATACTTC GGCCAAGCCC   
  
  
+ TCTTCACCCG CATGACCGAG TCAGGGGACC GCACCTACCG CACCATGCGG TCCGCCGTGG ACAAGACCTT   
  
  
+ CTCCTTTGAG TCTACGAGAA AGATGGTACT CAAGTTCCAG GAGGTTAGCC CTTGGACCAC CTTTGGACAC   
  
  
+ GTGGCAGCTA ATGGAGCCCT CATTGATGCC TTTGACGGTG AATCCAAAAT CCACATCATC GACATCAGCA   
  
  
+ CCACATTCTG CACCCAGTGG CCCACCCTCC TGGAGGCGCT GGCCACCCGA ATGGATGACG CGCCCTACCT   
  
  
+ GAGGCTGACC ACTGTGGTGG TCAACAAGTC CGGGAATGAA GGTCCCACCG GAGGCGGGTC CCACAGGGTG   
  
  
+ ATGAAAGAGA TTGGGACCCG CCTCGAGAAG TTTGCTAGGC TAATGGGAGT GCCTTTCAAA TTTAATGTGG   
  
  
+ TCCACCACGG GGCTGATTTA TCCGACTTGG ATTTCTCTCA GCTGGATATT AACGATGACG AGGCATTAGC   
  
  
+ CATCAACTGT GTCAACTCGC TCCACTCGGT CAATATCCAC CGCCGTGACT CGGTCATCTC GGCCTTCCGG   
  
  
+ CGGCTCCACC CTAGGGTGGT GACCGTGGTG GAGGAGGAGG CTGACCTTGC TGACGTGGGG GCGGAGGGGT   
  
  
+ ACGAGTTCTA CAGGGGGTTT AATGAGTGTT TAAGGTGGGT TAGGGTTTAC TTTGAGGCCC TGGAAGAGTG   
  
  
+ CTTTCCTAGG ACAAGCAATG AGAAACTCAT GCTCGAGCGG GCCGCAGGGC GGGCTCTGGT GGACCTCCTG   
  
  
+ GCCTGCCCCA AGCCCGCATC ATCCGAGCGG AGGGAGGCAG CTGCGCGGTG GTCCGGGAGG ATGCAGGGGG   
  
  
+ CCGGGTTCGG CCACGTGGGG TTCAGTGATG AGGTCTGTGA TGACGTCAAG GCCCTCCTAA GGAGGTACAA   
  
  
+ GGAGGGGTGG TCCATGATAC AATGTTCCGC CGCGGATGGC GGCGCCTCCG CCGGAATATT CTTGTCGTGG   
  
  
+ AGGGATCAGC CGGTGGTGTG GGCCAGTGCA TGGAAGCCTT A  

- -Up\_Stream \_Len000CCCAAA AAAATTTGAG TTTTAATCAA TAACATAGTT AACTTAAACC TTTTGATAAT   
  
  
- AAAAGCTGAA TAATATCTCT TAAACAACAA TTACTTACGA CTATTTATGA CTATTAATCA CAACACTCAC   
  
  
- ACTTAAACAC TACTACTCGT TACTAATCGC AATTTTAATA TTGATCCTAT TTATTAATTC ACAACTACTT   
  
  
- ACAACTAACT ACTACTACTC ATGACTAACC ACGCCACGAC CATTATTTAT AAATACTATT CAATTTTTTT   
  
  
- TTTTTGTGTA GGATTTGTTA GTGATACATT ATATGATTTA CTTTTTTTAA CCATTACACT AAACTGTTAG   
  
  
- ATTCCCATTC CCACAAAATG GGTGTGTACA ATACTTTAAC CGAATGATAA ATGTTTGAAA AATGTCAATA   
  
  
- TACTAAGATG GATACACAAT AGTAGAAAGG CAGCTACATT AACTAATATA AGTTGTGTTA AATTAAGTAA   
  
  
- TGTTACATCG ATTAACTATG AGTTTTATTA CTTGATAATT ATTTATTAAT TAATATTGAA AATTTTCAAA   
  
  
- CAATTAAATT TTAATATATT ATATATTTAA ATCCGATGTA ATTAGTTTTT TAATCCGCAC AAGTAGCCAG   
  
  
- GTTTGAACTT GGCCTGGTCT GGCCTGGCTT CTAGCTAATT CTCTTTTTCA TGTCTGACTC CTGGCCTAGC   
  
  
- TTATTCTCAG CGTGGCCTGG TCCTAGCCTG GCCTTTTTAA ACCTGGTCTA GCCCTGGCTA GCTCCATCTG   
  
  
- GCTTAAATAA AAGTTTATGC TATCCTTATT TTGAATAACT AAGTTAACTA AAACCGGTTT TAGTACGATA   
  
  
- ATAACTAAAA TAAACGATTG AGAGTTCTCA TAATCAAACG TGAAGCGTAA TAGAAACCGG TTTTTTTAGT   
  
  
- TTTTCTTTCA GAGTTTGAGA ATTATCAAAA AATAAAATAA ACCAAATAAA AATTTTATCA GCCGGAAGCC   
  
  
- AGGCCTGATC ACCTGGCTTA TTCCATTAAA AAGCTTAGCT CCTGGTCTGG TTTACGTGAG TCAAGCTTGG   
  
  
- CCAAGTCAAG CCAGACTGAG CTAAAAACCA GGCCAGGTTC AAACTTGTCT TATTACATGT GTGGATCTTT   
  
  
- TTTTCTTAAT ACGTATATAT GAATTCCGGG GGTTGGGAAA AAGGGAAAGG CGGTTATGAA AAATGGTTCA   
  
  
- GGTTAAGAAA AGAAGAATCG ATACCGATCA TCAATAGGGA GGGATCGAAG GTGGGATTCA AAACTCTGTT   
  
  
- TTAGGGTAGA TACAATTATA AATAAAGAAC GTGTACAGTG GGTTATGTTC CCAAAGTAAC TTTTCTTTTC   
  
  
- CATAATCTAC ATTCTATACA TACATATATT TATCAAAACG TTATCTGATC TTCCTGATTT TTCCTTTTTC   
  
  
- CTTCAAATTT TCGTTTTTTG AAAGGGGAGA TCTTTTTTCC TTTGGTCTAA GGGAAAAGAT GGGGTTAAGG   
  
  
- GTATCAGAAA TGAGACAGAG AGAGAGAGAC AGAGAGAATG AGTACTACAA CTCCCTCCTT CCTTACTTAA   
  
  
- TTCCTGGGAT TCGATTCTAG GTTGGCATTC TATAACGGGG AAATTAGTGG GTAAAGTATA GAGATAGGTG   
  
  
- TCAATTTTTG GGTGGAGGAG GTAGGTGTAT TGACTATATA GAGAAGATGT GGTATGATAT TCCTTCTTCT   
  
  
- CTTTTTATCC TCACATAACA TAAAAATAAA CTCATTAAAT AAAATTCCTC TCTTTAAATG TACCAACCCA   
  
  
- ATTTACCTAT GGAACAAATC TGATCACTCA AAGGTTGTTG GTGTGGTGGT GGTAGTGGTT GTGGTTGTGG   
  
  
- TTGTGGTGGT TGTCGTTAGA AGACTAGTTA GGAGAAACTT ATTATGATGA TGAATAAGGT GTTTAAGTTC   
  
  
- GTCAAGGTCT AGAAGGGTTG TTTGGGTAGT ATTAGTATTA GTATGAGTCA TAATAGTGGT TGTAGTAGGA   
  
  
- TGAATGGGGG TGGCGGTGGT AGTCCTCCTT CTTCTTCTTA CGAAGTTGAA AATGTACCTA CTTCTTTTGA   
  
  
- AGAGAAGAAG AAGGAGGGTG TTGTTTGTAG TAGGAGTTGG AGGAAGGAGG CGGTGGTGGA GGAGCATAAT   
  
  
- GGGAATGATG AGGTGGGGGA AGCTACAAGG GAGATGGTGG TGGTGGAGGT GGAGGTGGGG GTGCGGGTGG   
  
  
- TTGTGGTGGT GGTGGTGGGG GGGTTGGGTC ATACCGCCGC CGCCGCCACC CAAACTGAGG TCGACGAAGA   
  
  
- GGATGAGGGG ACGGGCTCTG GAGTTGGGCC TCGAGAAGAG GAGCAGGCCC AAGAGCCCGT TCACCCGAAG   
  
  
- GTTGTAGGAG AACCTTCGGC GAGCACGGTA GAGACTCTCC TTGTGCTCGG CGGAGGTGGT CGAGGATACC   
  
  
- TACCAGTTGC TCGAGTCGAG GGGCATGCCA CTGCACCTCG TCTTCTATCG AAGTATGAAG CCGGTTCGGG   
  
  
- AGAAGTGGGC GTACTGGCTC AGTCCCCTGG CGTGGATGGC GTGGTACGCC AGGCGGCACC TGTTCTGGAA   
  
  
- GAGGAAACTC AGATGCTCTT TCTACCATGA GTTCAAGGTC CTCCAATCGG GAACCTGGTG GAAACCTGTG   
  
  
- CACCGTCGAT TACCTCGGGA GTAACTACGG AAACTGCCAC TTAGGTTTTA GGTGTAGTAG CTGTAGTCGT   
  
  
- GGTGTAAGAC GTGGGTCACC GGGTGGGAGG ACCTCCGCGA CCGGTGGGCT TACCTACTGC GCGGGATGGA   
  
  
- CTCCGACTGG TGACACCACC AGTTGTTCAG GCCCTTACTT CCAGGGTGGC CTCCGCCCAG GGTGTCCCAC   
  
  
- TACTTTCTCT AACCCTGGGC GGAGCTCTTC AAACGATCCG ATTACCCTCA CGGAAAGTTT AAATTACACC   
  
  
- AGGTGGTGCC CCGACTAAAT AGGCTGAACC TAAAGAGAGT CGACCTATAA TTGCTACTGC TCCGTAATCG   
  
  
- GTAGTTGACA CAGTTGAGCG AGGTGAGCCA GTTATAGGTG GCGGCACTGA GCCAGTAGAG CCGGAAGGCC   
  
  
- GCCGAGGTGG GATCCCACCA CTGGCACCAC CTCCTCCTCC GACTGGAACG ACTGCACCCC CGCCTCCCCA   
  
  
- TGCTCAAGAT GTCCCCCAAA TTACTCACAA ATTCCACCCA ATCCCAAATG AAACTCCGGG ACCTTCTCAC   
  
  
- GAAAGGATCC TGTTCGTTAC TCTTTGAGTA CGAGCTCGCC CGGCGTCCCG CCCGAGACCA CCTGGAGGAC   
  
  
- CGGACGGGGT TCGGGCGTAG TAGGCTCGCC TCCCTCCGTC GACGCGCCAC CAGGCCCTCC TACGTCCCCC   
  
  
- GGCCCAAGCC GGTGCACCCC AAGTCACTAC TCCAGACACT ACTGCAGTTC CGGGAGGATT CCTCCATGTT   
  
  
- CCTCCCCACC AGGTACTATG TTACAAGGCG GCGCCTACCG CCGCGGAGGC GGCCTTATAA GAACAGCACC   
  
  
- TCCCTAGTCG GCCACCACAC CCGGTCACGT ACCTTCGGAA T

+     Myc

| Site Name | Organism | Position | Strand | Matrix score. | sequence | function |
| --- | --- | --- | --- | --- | --- | --- |
| Myc | Arabidopsis thaliana | 672 | - | 7 | TCTCTTA |  |
| Myc | Arabidopsis thaliana | 1507 | + | 7 | TCTCTTA |  |

>HU06G00358.1   
+ -Up\_Stream \_Len000GGGTTT TTTTAAACTC AAAATTAGTT ATTGTATCAA TTGAATTTGG AAAACTATTA   
  
  
+ TTTTCGACTT ATTATAGAGA ATTTGTTGTT AATGAATGCT GATAAATACT GATAATTAGT GTTGTGAGTG   
  
  
+ TGAATTTGTG ATGATGAGCA ATGATTAGCG TTAAAATTAT AACTAGGATA AATAATTAAG TGTTGATGAA   
  
  
+ TGTTGATTGA TGATGATGAG TACTGATTGG TGCGGTGCTG GTAATAAATA TTTATGATAA GTTAAAAAAA   
  
  
+ AAAAACACAT CCTAAACAAT CACTATGTAA TATACTAAAT GAAAAAAATT GGTAATGTGA TTTGACAATC   
  
  
+ TAAGGGTAAG GGTGTTTTAC CCACACATGT TATGAAATTG GCTTACTATT TACAAACTTT TTACAGTTAT   
  
  
+ ATGATTCTAC CTATGTGTTA TCATCTTTCC GTCGATGTAA TTGATTATAT TCAACACAAT TTAATTCATT   
  
  
+ ACAATGTAGC TAATTGATAC TCAAAATAAT GAACTATTAA TAAATAATTA ATTATAACTT TTAAAAGTTT   
  
  
+ GTTAATTTAA AATTATATAA TATATAAATT TAGGCTACAT TAATCAAAAA ATTAGGCGTG TTCATCGGTC   
  
  
+ CAAACTTGAA CCGGACCAGA CCGGACCGAA GATCGATTAA GAGAAAAAGT ACAGACTGAG GACCGGATCG   
  
  
+ AATAAGAGTC GCACCGGACC AGGATCGGAC CGGAAAAATT TGGACCAGAT CGGGACCGAT CGAGGTAGAC   
  
  
+ CGAATTTATT TTCAAATACG ATAGGAATAA AACTTATTGA TTCAATTGAT TTTGGCCAAA ATCATGCTAT   
  
  
+ TATTGATTTT ATTTGCTAAC TCTCAAGAGT ATTAGTTTGC ACTTCGCATT ATCTTTGGCC AAAAAAATCA   
  
  
+ AAAAGAAAGT CTCAAACTCT TAATAGTTTT TTATTTTATT TGGTTTATTT TTAAAATAGT CGGCCTTCGG   
  
  
+ TCCGGACTAG TGGACCGAAT AAGGTAATTT TTCGAATCGA GGACCAGACC AAATGCACTC AGTTCGAACC   
  
  
+ GGTTCAGTTC GGTCTGACTC GATTTTTGGT CCGGTCCAAG TTTGAACAGA ATAATGTACA CACCTAGAAA   
  
  
+ AAAAGAATTA TGCATATATA CTTAAGGCCC CCAACCCTTT TTCCCTTTCC GCCAATACTT TTTACCAAGT   
  
  
+ CCAATTCTTT TCTTCTTAGC TATGGCTAGT AGTTATCCCT CCCTAGCTTC CACCCTAAGT TTTGAGACAA   
  
  
+ AATCCCATCT ATGTTAATAT TTATTTCTTG CACATGTCAC CCAATACAAG GGTTTCATTG AAAAGAAAAG   
  
  
+ GTATTAGATG TAAGATATGT ATGTATATAA ATAGTTTTGC AATAGACTAG AAGGACTAAA AAGGAAAAAG   
  
  
+ GAAGTTTAAA AGCAAAAAAC TTTCCCCTCT AGAAAAAAGG AAACCAGATT CCCTTTTCTA CCCCAATTCC   
  
  
+ CATAGTCTTT ACTCTGTCTC TCTCTCTCTG TCTCTCTTAC TCATGATGTT GAGGGAGGAA GGAATGAATT   
  
  
+ AAGGACCCTA AGCTAAGATC CAACCGTAAG ATATTGCCCC TTTAATCACC CATTTCATAT CTCTATCCAC   
  
  
+ AGTTAAAAAC CCACCTCCTC CATCCACATA ACTGATATAT CTCTTCTACA CCATACTATA AGGAAGAAGA   
  
  
+ GAAAAATAGG AGTGTATTGT ATTTTTATTT GAGTAATTTA TTTTAAGGAG AGAAATTTAC ATGGTTGGGT   
  
  
+ TAAATGGATA CCTTGTTTAG ACTAGTGAGT TTCCAACAAC CACACCACCA CCATCACCAA CACCAACACC   
  
  
+ AACACCACCA ACAGCAATCT TCTGATCAAT CCTCTTTGAA TAATACTACT ACTTATTCCA CAAATTCAAG   
  
  
+ CAGTTCCAGA TCTTCCCAAC AAACCCATCA TAATCATAAT CATACTCAGT ATTATCACCA ACATCATCCT   
  
  
+ ACTTACCCCC ACCGCCACCA TCAGGAGGAA GAAGAAGAAT GCTTCAACTT TTACATGGAT GAAGAAAACT   
  
  
+ TCTCTTCTTC TTCCTCCCAC AACAAACATC ATCCTCAACC TCCTTCCTCC GCCACCACCT CCTCGTATTA   
  
  
+ CCCTTACTAC TCCACCCCCT TCGATGTTCC CTCTACCACC ACCACCTCCA CCTCCACCCC CACGCCCACC   
  
  
+ AACACCACCA CCACCACCCC CCCAACCCAG TATGGCGGCG GCGGCGGTGG GTTTGACTCC AGCTGCTTCT   
  
  
+ CCTACTCCCC TGCCCGAGAC CTCAACCCGG AGCTCTTCTC CTCGTCCGGG TTCTCGGGCA AGTGGGCTTC   
  
  
+ CAACATCCTC TTGGAAGCCG CTCGTGCCAT CTCTGAGAGG AACACGAGCC GCCTCCACCA GCTCCTATGG   
  
  
+ ATGGTCAACG AGCTCAGCTC CCCGTACGGT GACGTGGAGC AGAAGATAGC TTCATACTTC GGCCAAGCCC   
  
  
+ TCTTCACCCG CATGACCGAG TCAGGGGACC GCACCTACCG CACCATGCGG TCCGCCGTGG ACAAGACCTT   
  
  
+ CTCCTTTGAG TCTACGAGAA AGATGGTACT CAAGTTCCAG GAGGTTAGCC CTTGGACCAC CTTTGGACAC   
  
  
+ GTGGCAGCTA ATGGAGCCCT CATTGATGCC TTTGACGGTG AATCCAAAAT CCACATCATC GACATCAGCA   
  
  
+ CCACATTCTG CACCCAGTGG CCCACCCTCC TGGAGGCGCT GGCCACCCGA ATGGATGACG CGCCCTACCT   
  
  
+ GAGGCTGACC ACTGTGGTGG TCAACAAGTC CGGGAATGAA GGTCCCACCG GAGGCGGGTC CCACAGGGTG   
  
  
+ ATGAAAGAGA TTGGGACCCG CCTCGAGAAG TTTGCTAGGC TAATGGGAGT GCCTTTCAAA TTTAATGTGG   
  
  
+ TCCACCACGG GGCTGATTTA TCCGACTTGG ATTTCTCTCA GCTGGATATT AACGATGACG AGGCATTAGC   
  
  
+ CATCAACTGT GTCAACTCGC TCCACTCGGT CAATATCCAC CGCCGTGACT CGGTCATCTC GGCCTTCCGG   
  
  
+ CGGCTCCACC CTAGGGTGGT GACCGTGGTG GAGGAGGAGG CTGACCTTGC TGACGTGGGG GCGGAGGGGT   
  
  
+ ACGAGTTCTA CAGGGGGTTT AATGAGTGTT TAAGGTGGGT TAGGGTTTAC TTTGAGGCCC TGGAAGAGTG   
  
  
+ CTTTCCTAGG ACAAGCAATG AGAAACTCAT GCTCGAGCGG GCCGCAGGGC GGGCTCTGGT GGACCTCCTG   
  
  
+ GCCTGCCCCA AGCCCGCATC ATCCGAGCGG AGGGAGGCAG CTGCGCGGTG GTCCGGGAGG ATGCAGGGGG   
  
  
+ CCGGGTTCGG CCACGTGGGG TTCAGTGATG AGGTCTGTGA TGACGTCAAG GCCCTCCTAA GGAGGTACAA   
  
  
+ GGAGGGGTGG TCCATGATAC AATGTTCCGC CGCGGATGGC GGCGCCTCCG CCGGAATATT CTTGTCGTGG   
  
  
+ AGGGATCAGC CGGTGGTGTG GGCCAGTGCA TGGAAGCCTT A  

- -Up\_Stream \_Len000CCCAAA AAAATTTGAG TTTTAATCAA TAACATAGTT AACTTAAACC TTTTGATAAT   
  
  
- AAAAGCTGAA TAATATCTCT TAAACAACAA TTACTTACGA CTATTTATGA CTATTAATCA CAACACTCAC   
  
  
- ACTTAAACAC TACTACTCGT TACTAATCGC AATTTTAATA TTGATCCTAT TTATTAATTC ACAACTACTT   
  
  
- ACAACTAACT ACTACTACTC ATGACTAACC ACGCCACGAC CATTATTTAT AAATACTATT CAATTTTTTT   
  
  
- TTTTTGTGTA GGATTTGTTA GTGATACATT ATATGATTTA CTTTTTTTAA CCATTACACT AAACTGTTAG   
  
  
- ATTCCCATTC CCACAAAATG GGTGTGTACA ATACTTTAAC CGAATGATAA ATGTTTGAAA AATGTCAATA   
  
  
- TACTAAGATG GATACACAAT AGTAGAAAGG CAGCTACATT AACTAATATA AGTTGTGTTA AATTAAGTAA   
  
  
- TGTTACATCG ATTAACTATG AGTTTTATTA CTTGATAATT ATTTATTAAT TAATATTGAA AATTTTCAAA   
  
  
- CAATTAAATT TTAATATATT ATATATTTAA ATCCGATGTA ATTAGTTTTT TAATCCGCAC AAGTAGCCAG   
  
  
- GTTTGAACTT GGCCTGGTCT GGCCTGGCTT CTAGCTAATT CTCTTTTTCA TGTCTGACTC CTGGCCTAGC   
  
  
- TTATTCTCAG CGTGGCCTGG TCCTAGCCTG GCCTTTTTAA ACCTGGTCTA GCCCTGGCTA GCTCCATCTG   
  
  
- GCTTAAATAA AAGTTTATGC TATCCTTATT TTGAATAACT AAGTTAACTA AAACCGGTTT TAGTACGATA   
  
  
- ATAACTAAAA TAAACGATTG AGAGTTCTCA TAATCAAACG TGAAGCGTAA TAGAAACCGG TTTTTTTAGT   
  
  
- TTTTCTTTCA GAGTTTGAGA ATTATCAAAA AATAAAATAA ACCAAATAAA AATTTTATCA GCCGGAAGCC   
  
  
- AGGCCTGATC ACCTGGCTTA TTCCATTAAA AAGCTTAGCT CCTGGTCTGG TTTACGTGAG TCAAGCTTGG   
  
  
- CCAAGTCAAG CCAGACTGAG CTAAAAACCA GGCCAGGTTC AAACTTGTCT TATTACATGT GTGGATCTTT   
  
  
- TTTTCTTAAT ACGTATATAT GAATTCCGGG GGTTGGGAAA AAGGGAAAGG CGGTTATGAA AAATGGTTCA   
  
  
- GGTTAAGAAA AGAAGAATCG ATACCGATCA TCAATAGGGA GGGATCGAAG GTGGGATTCA AAACTCTGTT   
  
  
- TTAGGGTAGA TACAATTATA AATAAAGAAC GTGTACAGTG GGTTATGTTC CCAAAGTAAC TTTTCTTTTC   
  
  
- CATAATCTAC ATTCTATACA TACATATATT TATCAAAACG TTATCTGATC TTCCTGATTT TTCCTTTTTC   
  
  
- CTTCAAATTT TCGTTTTTTG AAAGGGGAGA TCTTTTTTCC TTTGGTCTAA GGGAAAAGAT GGGGTTAAGG   
  
  
- GTATCAGAAA TGAGACAGAG AGAGAGAGAC AGAGAGAATG AGTACTACAA CTCCCTCCTT CCTTACTTAA   
  
  
- TTCCTGGGAT TCGATTCTAG GTTGGCATTC TATAACGGGG AAATTAGTGG GTAAAGTATA GAGATAGGTG   
  
  
- TCAATTTTTG GGTGGAGGAG GTAGGTGTAT TGACTATATA GAGAAGATGT GGTATGATAT TCCTTCTTCT   
  
  
- CTTTTTATCC TCACATAACA TAAAAATAAA CTCATTAAAT AAAATTCCTC TCTTTAAATG TACCAACCCA   
  
  
- ATTTACCTAT GGAACAAATC TGATCACTCA AAGGTTGTTG GTGTGGTGGT GGTAGTGGTT GTGGTTGTGG   
  
  
- TTGTGGTGGT TGTCGTTAGA AGACTAGTTA GGAGAAACTT ATTATGATGA TGAATAAGGT GTTTAAGTTC   
  
  
- GTCAAGGTCT AGAAGGGTTG TTTGGGTAGT ATTAGTATTA GTATGAGTCA TAATAGTGGT TGTAGTAGGA   
  
  
- TGAATGGGGG TGGCGGTGGT AGTCCTCCTT CTTCTTCTTA CGAAGTTGAA AATGTACCTA CTTCTTTTGA   
  
  
- AGAGAAGAAG AAGGAGGGTG TTGTTTGTAG TAGGAGTTGG AGGAAGGAGG CGGTGGTGGA GGAGCATAAT   
  
  
- GGGAATGATG AGGTGGGGGA AGCTACAAGG GAGATGGTGG TGGTGGAGGT GGAGGTGGGG GTGCGGGTGG   
  
  
- TTGTGGTGGT GGTGGTGGGG GGGTTGGGTC ATACCGCCGC CGCCGCCACC CAAACTGAGG TCGACGAAGA   
  
  
- GGATGAGGGG ACGGGCTCTG GAGTTGGGCC TCGAGAAGAG GAGCAGGCCC AAGAGCCCGT TCACCCGAAG   
  
  
- GTTGTAGGAG AACCTTCGGC GAGCACGGTA GAGACTCTCC TTGTGCTCGG CGGAGGTGGT CGAGGATACC   
  
  
- TACCAGTTGC TCGAGTCGAG GGGCATGCCA CTGCACCTCG TCTTCTATCG AAGTATGAAG CCGGTTCGGG   
  
  
- AGAAGTGGGC GTACTGGCTC AGTCCCCTGG CGTGGATGGC GTGGTACGCC AGGCGGCACC TGTTCTGGAA   
  
  
- GAGGAAACTC AGATGCTCTT TCTACCATGA GTTCAAGGTC CTCCAATCGG GAACCTGGTG GAAACCTGTG   
  
  
- CACCGTCGAT TACCTCGGGA GTAACTACGG AAACTGCCAC TTAGGTTTTA GGTGTAGTAG CTGTAGTCGT   
  
  
- GGTGTAAGAC GTGGGTCACC GGGTGGGAGG ACCTCCGCGA CCGGTGGGCT TACCTACTGC GCGGGATGGA   
  
  
- CTCCGACTGG TGACACCACC AGTTGTTCAG GCCCTTACTT CCAGGGTGGC CTCCGCCCAG GGTGTCCCAC   
  
  
- TACTTTCTCT AACCCTGGGC GGAGCTCTTC AAACGATCCG ATTACCCTCA CGGAAAGTTT AAATTACACC   
  
  
- AGGTGGTGCC CCGACTAAAT AGGCTGAACC TAAAGAGAGT CGACCTATAA TTGCTACTGC TCCGTAATCG   
  
  
- GTAGTTGACA CAGTTGAGCG AGGTGAGCCA GTTATAGGTG GCGGCACTGA GCCAGTAGAG CCGGAAGGCC   
  
  
- GCCGAGGTGG GATCCCACCA CTGGCACCAC CTCCTCCTCC GACTGGAACG ACTGCACCCC CGCCTCCCCA   
  
  
- TGCTCAAGAT GTCCCCCAAA TTACTCACAA ATTCCACCCA ATCCCAAATG AAACTCCGGG ACCTTCTCAC   
  
  
- GAAAGGATCC TGTTCGTTAC TCTTTGAGTA CGAGCTCGCC CGGCGTCCCG CCCGAGACCA CCTGGAGGAC   
  
  
- CGGACGGGGT TCGGGCGTAG TAGGCTCGCC TCCCTCCGTC GACGCGCCAC CAGGCCCTCC TACGTCCCCC   
  
  
- GGCCCAAGCC GGTGCACCCC AAGTCACTAC TCCAGACACT ACTGCAGTTC CGGGAGGATT CCTCCATGTT   
  
  
- CCTCCCCACC AGGTACTATG TTACAAGGCG GCGCCTACCG CCGCGGAGGC GGCCTTATAA GAACAGCACC   
  
  
- TCCCTAGTCG GCCACCACAC CCGGTCACGT ACCTTCGGAA T

+     O2-site

| Site Name | Organism | Position | Strand | Matrix score. | sequence | function |
| --- | --- | --- | --- | --- | --- | --- |
| O2-site | Zea mays | 1953 | - | 9 | GATGATGTGG | cis-acting regulatory element involved in zein metabolism regulation |
| O2-site | Zea mays | 2645 | - | 10 | GATGATGTGG | cis-acting regulatory element involved in zein metabolism regulation |
| O2-site | Zea mays | 3238 | - | 9 | GATGATGTGG | cis-acting regulatory element involved in zein metabolism regulation |

>HU06G00358.1   
+ -Up\_Stream \_Len000GGGTTT TTTTAAACTC AAAATTAGTT ATTGTATCAA TTGAATTTGG AAAACTATTA   
  
  
+ TTTTCGACTT ATTATAGAGA ATTTGTTGTT AATGAATGCT GATAAATACT GATAATTAGT GTTGTGAGTG   
  
  
+ TGAATTTGTG ATGATGAGCA ATGATTAGCG TTAAAATTAT AACTAGGATA AATAATTAAG TGTTGATGAA   
  
  
+ TGTTGATTGA TGATGATGAG TACTGATTGG TGCGGTGCTG GTAATAAATA TTTATGATAA GTTAAAAAAA   
  
  
+ AAAAACACAT CCTAAACAAT CACTATGTAA TATACTAAAT GAAAAAAATT GGTAATGTGA TTTGACAATC   
  
  
+ TAAGGGTAAG GGTGTTTTAC CCACACATGT TATGAAATTG GCTTACTATT TACAAACTTT TTACAGTTAT   
  
  
+ ATGATTCTAC CTATGTGTTA TCATCTTTCC GTCGATGTAA TTGATTATAT TCAACACAAT TTAATTCATT   
  
  
+ ACAATGTAGC TAATTGATAC TCAAAATAAT GAACTATTAA TAAATAATTA ATTATAACTT TTAAAAGTTT   
  
  
+ GTTAATTTAA AATTATATAA TATATAAATT TAGGCTACAT TAATCAAAAA ATTAGGCGTG TTCATCGGTC   
  
  
+ CAAACTTGAA CCGGACCAGA CCGGACCGAA GATCGATTAA GAGAAAAAGT ACAGACTGAG GACCGGATCG   
  
  
+ AATAAGAGTC GCACCGGACC AGGATCGGAC CGGAAAAATT TGGACCAGAT CGGGACCGAT CGAGGTAGAC   
  
  
+ CGAATTTATT TTCAAATACG ATAGGAATAA AACTTATTGA TTCAATTGAT TTTGGCCAAA ATCATGCTAT   
  
  
+ TATTGATTTT ATTTGCTAAC TCTCAAGAGT ATTAGTTTGC ACTTCGCATT ATCTTTGGCC AAAAAAATCA   
  
  
+ AAAAGAAAGT CTCAAACTCT TAATAGTTTT TTATTTTATT TGGTTTATTT TTAAAATAGT CGGCCTTCGG   
  
  
+ TCCGGACTAG TGGACCGAAT AAGGTAATTT TTCGAATCGA GGACCAGACC AAATGCACTC AGTTCGAACC   
  
  
+ GGTTCAGTTC GGTCTGACTC GATTTTTGGT CCGGTCCAAG TTTGAACAGA ATAATGTACA CACCTAGAAA   
  
  
+ AAAAGAATTA TGCATATATA CTTAAGGCCC CCAACCCTTT TTCCCTTTCC GCCAATACTT TTTACCAAGT   
  
  
+ CCAATTCTTT TCTTCTTAGC TATGGCTAGT AGTTATCCCT CCCTAGCTTC CACCCTAAGT TTTGAGACAA   
  
  
+ AATCCCATCT ATGTTAATAT TTATTTCTTG CACATGTCAC CCAATACAAG GGTTTCATTG AAAAGAAAAG   
  
  
+ GTATTAGATG TAAGATATGT ATGTATATAA ATAGTTTTGC AATAGACTAG AAGGACTAAA AAGGAAAAAG   
  
  
+ GAAGTTTAAA AGCAAAAAAC TTTCCCCTCT AGAAAAAAGG AAACCAGATT CCCTTTTCTA CCCCAATTCC   
  
  
+ CATAGTCTTT ACTCTGTCTC TCTCTCTCTG TCTCTCTTAC TCATGATGTT GAGGGAGGAA GGAATGAATT   
  
  
+ AAGGACCCTA AGCTAAGATC CAACCGTAAG ATATTGCCCC TTTAATCACC CATTTCATAT CTCTATCCAC   
  
  
+ AGTTAAAAAC CCACCTCCTC CATCCACATA ACTGATATAT CTCTTCTACA CCATACTATA AGGAAGAAGA   
  
  
+ GAAAAATAGG AGTGTATTGT ATTTTTATTT GAGTAATTTA TTTTAAGGAG AGAAATTTAC ATGGTTGGGT   
  
  
+ TAAATGGATA CCTTGTTTAG ACTAGTGAGT TTCCAACAAC CACACCACCA CCATCACCAA CACCAACACC   
  
  
+ AACACCACCA ACAGCAATCT TCTGATCAAT CCTCTTTGAA TAATACTACT ACTTATTCCA CAAATTCAAG   
  
  
+ CAGTTCCAGA TCTTCCCAAC AAACCCATCA TAATCATAAT CATACTCAGT ATTATCACCA ACATCATCCT   
  
  
+ ACTTACCCCC ACCGCCACCA TCAGGAGGAA GAAGAAGAAT GCTTCAACTT TTACATGGAT GAAGAAAACT   
  
  
+ TCTCTTCTTC TTCCTCCCAC AACAAACATC ATCCTCAACC TCCTTCCTCC GCCACCACCT CCTCGTATTA   
  
  
+ CCCTTACTAC TCCACCCCCT TCGATGTTCC CTCTACCACC ACCACCTCCA CCTCCACCCC CACGCCCACC   
  
  
+ AACACCACCA CCACCACCCC CCCAACCCAG TATGGCGGCG GCGGCGGTGG GTTTGACTCC AGCTGCTTCT   
  
  
+ CCTACTCCCC TGCCCGAGAC CTCAACCCGG AGCTCTTCTC CTCGTCCGGG TTCTCGGGCA AGTGGGCTTC   
  
  
+ CAACATCCTC TTGGAAGCCG CTCGTGCCAT CTCTGAGAGG AACACGAGCC GCCTCCACCA GCTCCTATGG   
  
  
+ ATGGTCAACG AGCTCAGCTC CCCGTACGGT GACGTGGAGC AGAAGATAGC TTCATACTTC GGCCAAGCCC   
  
  
+ TCTTCACCCG CATGACCGAG TCAGGGGACC GCACCTACCG CACCATGCGG TCCGCCGTGG ACAAGACCTT   
  
  
+ CTCCTTTGAG TCTACGAGAA AGATGGTACT CAAGTTCCAG GAGGTTAGCC CTTGGACCAC CTTTGGACAC   
  
  
+ GTGGCAGCTA ATGGAGCCCT CATTGATGCC TTTGACGGTG AATCCAAAAT CCACATCATC GACATCAGCA   
  
  
+ CCACATTCTG CACCCAGTGG CCCACCCTCC TGGAGGCGCT GGCCACCCGA ATGGATGACG CGCCCTACCT   
  
  
+ GAGGCTGACC ACTGTGGTGG TCAACAAGTC CGGGAATGAA GGTCCCACCG GAGGCGGGTC CCACAGGGTG   
  
  
+ ATGAAAGAGA TTGGGACCCG CCTCGAGAAG TTTGCTAGGC TAATGGGAGT GCCTTTCAAA TTTAATGTGG   
  
  
+ TCCACCACGG GGCTGATTTA TCCGACTTGG ATTTCTCTCA GCTGGATATT AACGATGACG AGGCATTAGC   
  
  
+ CATCAACTGT GTCAACTCGC TCCACTCGGT CAATATCCAC CGCCGTGACT CGGTCATCTC GGCCTTCCGG   
  
  
+ CGGCTCCACC CTAGGGTGGT GACCGTGGTG GAGGAGGAGG CTGACCTTGC TGACGTGGGG GCGGAGGGGT   
  
  
+ ACGAGTTCTA CAGGGGGTTT AATGAGTGTT TAAGGTGGGT TAGGGTTTAC TTTGAGGCCC TGGAAGAGTG   
  
  
+ CTTTCCTAGG ACAAGCAATG AGAAACTCAT GCTCGAGCGG GCCGCAGGGC GGGCTCTGGT GGACCTCCTG   
  
  
+ GCCTGCCCCA AGCCCGCATC ATCCGAGCGG AGGGAGGCAG CTGCGCGGTG GTCCGGGAGG ATGCAGGGGG   
  
  
+ CCGGGTTCGG CCACGTGGGG TTCAGTGATG AGGTCTGTGA TGACGTCAAG GCCCTCCTAA GGAGGTACAA   
  
  
+ GGAGGGGTGG TCCATGATAC AATGTTCCGC CGCGGATGGC GGCGCCTCCG CCGGAATATT CTTGTCGTGG   
  
  
+ AGGGATCAGC CGGTGGTGTG GGCCAGTGCA TGGAAGCCTT A  

- -Up\_Stream \_Len000CCCAAA AAAATTTGAG TTTTAATCAA TAACATAGTT AACTTAAACC TTTTGATAAT   
  
  
- AAAAGCTGAA TAATATCTCT TAAACAACAA TTACTTACGA CTATTTATGA CTATTAATCA CAACACTCAC   
  
  
- ACTTAAACAC TACTACTCGT TACTAATCGC AATTTTAATA TTGATCCTAT TTATTAATTC ACAACTACTT   
  
  
- ACAACTAACT ACTACTACTC ATGACTAACC ACGCCACGAC CATTATTTAT AAATACTATT CAATTTTTTT   
  
  
- TTTTTGTGTA GGATTTGTTA GTGATACATT ATATGATTTA CTTTTTTTAA CCATTACACT AAACTGTTAG   
  
  
- ATTCCCATTC CCACAAAATG GGTGTGTACA ATACTTTAAC CGAATGATAA ATGTTTGAAA AATGTCAATA   
  
  
- TACTAAGATG GATACACAAT AGTAGAAAGG CAGCTACATT AACTAATATA AGTTGTGTTA AATTAAGTAA   
  
  
- TGTTACATCG ATTAACTATG AGTTTTATTA CTTGATAATT ATTTATTAAT TAATATTGAA AATTTTCAAA   
  
  
- CAATTAAATT TTAATATATT ATATATTTAA ATCCGATGTA ATTAGTTTTT TAATCCGCAC AAGTAGCCAG   
  
  
- GTTTGAACTT GGCCTGGTCT GGCCTGGCTT CTAGCTAATT CTCTTTTTCA TGTCTGACTC CTGGCCTAGC   
  
  
- TTATTCTCAG CGTGGCCTGG TCCTAGCCTG GCCTTTTTAA ACCTGGTCTA GCCCTGGCTA GCTCCATCTG   
  
  
- GCTTAAATAA AAGTTTATGC TATCCTTATT TTGAATAACT AAGTTAACTA AAACCGGTTT TAGTACGATA   
  
  
- ATAACTAAAA TAAACGATTG AGAGTTCTCA TAATCAAACG TGAAGCGTAA TAGAAACCGG TTTTTTTAGT   
  
  
- TTTTCTTTCA GAGTTTGAGA ATTATCAAAA AATAAAATAA ACCAAATAAA AATTTTATCA GCCGGAAGCC   
  
  
- AGGCCTGATC ACCTGGCTTA TTCCATTAAA AAGCTTAGCT CCTGGTCTGG TTTACGTGAG TCAAGCTTGG   
  
  
- CCAAGTCAAG CCAGACTGAG CTAAAAACCA GGCCAGGTTC AAACTTGTCT TATTACATGT GTGGATCTTT   
  
  
- TTTTCTTAAT ACGTATATAT GAATTCCGGG GGTTGGGAAA AAGGGAAAGG CGGTTATGAA AAATGGTTCA   
  
  
- GGTTAAGAAA AGAAGAATCG ATACCGATCA TCAATAGGGA GGGATCGAAG GTGGGATTCA AAACTCTGTT   
  
  
- TTAGGGTAGA TACAATTATA AATAAAGAAC GTGTACAGTG GGTTATGTTC CCAAAGTAAC TTTTCTTTTC   
  
  
- CATAATCTAC ATTCTATACA TACATATATT TATCAAAACG TTATCTGATC TTCCTGATTT TTCCTTTTTC   
  
  
- CTTCAAATTT TCGTTTTTTG AAAGGGGAGA TCTTTTTTCC TTTGGTCTAA GGGAAAAGAT GGGGTTAAGG   
  
  
- GTATCAGAAA TGAGACAGAG AGAGAGAGAC AGAGAGAATG AGTACTACAA CTCCCTCCTT CCTTACTTAA   
  
  
- TTCCTGGGAT TCGATTCTAG GTTGGCATTC TATAACGGGG AAATTAGTGG GTAAAGTATA GAGATAGGTG   
  
  
- TCAATTTTTG GGTGGAGGAG GTAGGTGTAT TGACTATATA GAGAAGATGT GGTATGATAT TCCTTCTTCT   
  
  
- CTTTTTATCC TCACATAACA TAAAAATAAA CTCATTAAAT AAAATTCCTC TCTTTAAATG TACCAACCCA   
  
  
- ATTTACCTAT GGAACAAATC TGATCACTCA AAGGTTGTTG GTGTGGTGGT GGTAGTGGTT GTGGTTGTGG   
  
  
- TTGTGGTGGT TGTCGTTAGA AGACTAGTTA GGAGAAACTT ATTATGATGA TGAATAAGGT GTTTAAGTTC   
  
  
- GTCAAGGTCT AGAAGGGTTG TTTGGGTAGT ATTAGTATTA GTATGAGTCA TAATAGTGGT TGTAGTAGGA   
  
  
- TGAATGGGGG TGGCGGTGGT AGTCCTCCTT CTTCTTCTTA CGAAGTTGAA AATGTACCTA CTTCTTTTGA   
  
  
- AGAGAAGAAG AAGGAGGGTG TTGTTTGTAG TAGGAGTTGG AGGAAGGAGG CGGTGGTGGA GGAGCATAAT   
  
  
- GGGAATGATG AGGTGGGGGA AGCTACAAGG GAGATGGTGG TGGTGGAGGT GGAGGTGGGG GTGCGGGTGG   
  
  
- TTGTGGTGGT GGTGGTGGGG GGGTTGGGTC ATACCGCCGC CGCCGCCACC CAAACTGAGG TCGACGAAGA   
  
  
- GGATGAGGGG ACGGGCTCTG GAGTTGGGCC TCGAGAAGAG GAGCAGGCCC AAGAGCCCGT TCACCCGAAG   
  
  
- GTTGTAGGAG AACCTTCGGC GAGCACGGTA GAGACTCTCC TTGTGCTCGG CGGAGGTGGT CGAGGATACC   
  
  
- TACCAGTTGC TCGAGTCGAG GGGCATGCCA CTGCACCTCG TCTTCTATCG AAGTATGAAG CCGGTTCGGG   
  
  
- AGAAGTGGGC GTACTGGCTC AGTCCCCTGG CGTGGATGGC GTGGTACGCC AGGCGGCACC TGTTCTGGAA   
  
  
- GAGGAAACTC AGATGCTCTT TCTACCATGA GTTCAAGGTC CTCCAATCGG GAACCTGGTG GAAACCTGTG   
  
  
- CACCGTCGAT TACCTCGGGA GTAACTACGG AAACTGCCAC TTAGGTTTTA GGTGTAGTAG CTGTAGTCGT   
  
  
- GGTGTAAGAC GTGGGTCACC GGGTGGGAGG ACCTCCGCGA CCGGTGGGCT TACCTACTGC GCGGGATGGA   
  
  
- CTCCGACTGG TGACACCACC AGTTGTTCAG GCCCTTACTT CCAGGGTGGC CTCCGCCCAG GGTGTCCCAC   
  
  
- TACTTTCTCT AACCCTGGGC GGAGCTCTTC AAACGATCCG ATTACCCTCA CGGAAAGTTT AAATTACACC   
  
  
- AGGTGGTGCC CCGACTAAAT AGGCTGAACC TAAAGAGAGT CGACCTATAA TTGCTACTGC TCCGTAATCG   
  
  
- GTAGTTGACA CAGTTGAGCG AGGTGAGCCA GTTATAGGTG GCGGCACTGA GCCAGTAGAG CCGGAAGGCC   
  
  
- GCCGAGGTGG GATCCCACCA CTGGCACCAC CTCCTCCTCC GACTGGAACG ACTGCACCCC CGCCTCCCCA   
  
  
- TGCTCAAGAT GTCCCCCAAA TTACTCACAA ATTCCACCCA ATCCCAAATG AAACTCCGGG ACCTTCTCAC   
  
  
- GAAAGGATCC TGTTCGTTAC TCTTTGAGTA CGAGCTCGCC CGGCGTCCCG CCCGAGACCA CCTGGAGGAC   
  
  
- CGGACGGGGT TCGGGCGTAG TAGGCTCGCC TCCCTCCGTC GACGCGCCAC CAGGCCCTCC TACGTCCCCC   
  
  
- GGCCCAAGCC GGTGCACCCC AAGTCACTAC TCCAGACACT ACTGCAGTTC CGGGAGGATT CCTCCATGTT   
  
  
- CCTCCCCACC AGGTACTATG TTACAAGGCG GCGCCTACCG CCGCGGAGGC GGCCTTATAA GAACAGCACC   
  
  
- TCCCTAGTCG GCCACCACAC CCGGTCACGT ACCTTCGGAA T

+     STRE

| Site Name | Organism | Position | Strand | Matrix score. | sequence | function |
| --- | --- | --- | --- | --- | --- | --- |
| STRE | Arabidopsis thaliana | 3079 | + | 5 | AGGGG |  |
| STRE | Arabidopsis thaliana | 2477 | + | 5 | AGGGG |  |
| STRE | Arabidopsis thaliana | 3367 | + | 5 | AGGGG |  |
| STRE | Arabidopsis thaliana | 3289 | + | 5 | AGGGG |  |
| STRE | Arabidopsis thaliana | 3096 | + | 5 | AGGGG |  |
| STRE | Arabidopsis thaliana | 1581 | - | 5 | AGGGG |  |
| STRE | Arabidopsis thaliana | 1428 | - | 5 | AGGGG |  |
| STRE | Arabidopsis thaliana | 2251 | - | 5 | AGGGG |  |
| STRE | Arabidopsis thaliana | 2120 | - | 5 | AGGGG |  |

>HU06G00358.1   
+ -Up\_Stream \_Len000GGGTTT TTTTAAACTC AAAATTAGTT ATTGTATCAA TTGAATTTGG AAAACTATTA   
  
  
+ TTTTCGACTT ATTATAGAGA ATTTGTTGTT AATGAATGCT GATAAATACT GATAATTAGT GTTGTGAGTG   
  
  
+ TGAATTTGTG ATGATGAGCA ATGATTAGCG TTAAAATTAT AACTAGGATA AATAATTAAG TGTTGATGAA   
  
  
+ TGTTGATTGA TGATGATGAG TACTGATTGG TGCGGTGCTG GTAATAAATA TTTATGATAA GTTAAAAAAA   
  
  
+ AAAAACACAT CCTAAACAAT CACTATGTAA TATACTAAAT GAAAAAAATT GGTAATGTGA TTTGACAATC   
  
  
+ TAAGGGTAAG GGTGTTTTAC CCACACATGT TATGAAATTG GCTTACTATT TACAAACTTT TTACAGTTAT   
  
  
+ ATGATTCTAC CTATGTGTTA TCATCTTTCC GTCGATGTAA TTGATTATAT TCAACACAAT TTAATTCATT   
  
  
+ ACAATGTAGC TAATTGATAC TCAAAATAAT GAACTATTAA TAAATAATTA ATTATAACTT TTAAAAGTTT   
  
  
+ GTTAATTTAA AATTATATAA TATATAAATT TAGGCTACAT TAATCAAAAA ATTAGGCGTG TTCATCGGTC   
  
  
+ CAAACTTGAA CCGGACCAGA CCGGACCGAA GATCGATTAA GAGAAAAAGT ACAGACTGAG GACCGGATCG   
  
  
+ AATAAGAGTC GCACCGGACC AGGATCGGAC CGGAAAAATT TGGACCAGAT CGGGACCGAT CGAGGTAGAC   
  
  
+ CGAATTTATT TTCAAATACG ATAGGAATAA AACTTATTGA TTCAATTGAT TTTGGCCAAA ATCATGCTAT   
  
  
+ TATTGATTTT ATTTGCTAAC TCTCAAGAGT ATTAGTTTGC ACTTCGCATT ATCTTTGGCC AAAAAAATCA   
  
  
+ AAAAGAAAGT CTCAAACTCT TAATAGTTTT TTATTTTATT TGGTTTATTT TTAAAATAGT CGGCCTTCGG   
  
  
+ TCCGGACTAG TGGACCGAAT AAGGTAATTT TTCGAATCGA GGACCAGACC AAATGCACTC AGTTCGAACC   
  
  
+ GGTTCAGTTC GGTCTGACTC GATTTTTGGT CCGGTCCAAG TTTGAACAGA ATAATGTACA CACCTAGAAA   
  
  
+ AAAAGAATTA TGCATATATA CTTAAGGCCC CCAACCCTTT TTCCCTTTCC GCCAATACTT TTTACCAAGT   
  
  
+ CCAATTCTTT TCTTCTTAGC TATGGCTAGT AGTTATCCCT CCCTAGCTTC CACCCTAAGT TTTGAGACAA   
  
  
+ AATCCCATCT ATGTTAATAT TTATTTCTTG CACATGTCAC CCAATACAAG GGTTTCATTG AAAAGAAAAG   
  
  
+ GTATTAGATG TAAGATATGT ATGTATATAA ATAGTTTTGC AATAGACTAG AAGGACTAAA AAGGAAAAAG   
  
  
+ GAAGTTTAAA AGCAAAAAAC TTTCCCCTCT AGAAAAAAGG AAACCAGATT CCCTTTTCTA CCCCAATTCC   
  
  
+ CATAGTCTTT ACTCTGTCTC TCTCTCTCTG TCTCTCTTAC TCATGATGTT GAGGGAGGAA GGAATGAATT   
  
  
+ AAGGACCCTA AGCTAAGATC CAACCGTAAG ATATTGCCCC TTTAATCACC CATTTCATAT CTCTATCCAC   
  
  
+ AGTTAAAAAC CCACCTCCTC CATCCACATA ACTGATATAT CTCTTCTACA CCATACTATA AGGAAGAAGA   
  
  
+ GAAAAATAGG AGTGTATTGT ATTTTTATTT GAGTAATTTA TTTTAAGGAG AGAAATTTAC ATGGTTGGGT   
  
  
+ TAAATGGATA CCTTGTTTAG ACTAGTGAGT TTCCAACAAC CACACCACCA CCATCACCAA CACCAACACC   
  
  
+ AACACCACCA ACAGCAATCT TCTGATCAAT CCTCTTTGAA TAATACTACT ACTTATTCCA CAAATTCAAG   
  
  
+ CAGTTCCAGA TCTTCCCAAC AAACCCATCA TAATCATAAT CATACTCAGT ATTATCACCA ACATCATCCT   
  
  
+ ACTTACCCCC ACCGCCACCA TCAGGAGGAA GAAGAAGAAT GCTTCAACTT TTACATGGAT GAAGAAAACT   
  
  
+ TCTCTTCTTC TTCCTCCCAC AACAAACATC ATCCTCAACC TCCTTCCTCC GCCACCACCT CCTCGTATTA   
  
  
+ CCCTTACTAC TCCACCCCCT TCGATGTTCC CTCTACCACC ACCACCTCCA CCTCCACCCC CACGCCCACC   
  
  
+ AACACCACCA CCACCACCCC CCCAACCCAG TATGGCGGCG GCGGCGGTGG GTTTGACTCC AGCTGCTTCT   
  
  
+ CCTACTCCCC TGCCCGAGAC CTCAACCCGG AGCTCTTCTC CTCGTCCGGG TTCTCGGGCA AGTGGGCTTC   
  
  
+ CAACATCCTC TTGGAAGCCG CTCGTGCCAT CTCTGAGAGG AACACGAGCC GCCTCCACCA GCTCCTATGG   
  
  
+ ATGGTCAACG AGCTCAGCTC CCCGTACGGT GACGTGGAGC AGAAGATAGC TTCATACTTC GGCCAAGCCC   
  
  
+ TCTTCACCCG CATGACCGAG TCAGGGGACC GCACCTACCG CACCATGCGG TCCGCCGTGG ACAAGACCTT   
  
  
+ CTCCTTTGAG TCTACGAGAA AGATGGTACT CAAGTTCCAG GAGGTTAGCC CTTGGACCAC CTTTGGACAC   
  
  
+ GTGGCAGCTA ATGGAGCCCT CATTGATGCC TTTGACGGTG AATCCAAAAT CCACATCATC GACATCAGCA   
  
  
+ CCACATTCTG CACCCAGTGG CCCACCCTCC TGGAGGCGCT GGCCACCCGA ATGGATGACG CGCCCTACCT   
  
  
+ GAGGCTGACC ACTGTGGTGG TCAACAAGTC CGGGAATGAA GGTCCCACCG GAGGCGGGTC CCACAGGGTG   
  
  
+ ATGAAAGAGA TTGGGACCCG CCTCGAGAAG TTTGCTAGGC TAATGGGAGT GCCTTTCAAA TTTAATGTGG   
  
  
+ TCCACCACGG GGCTGATTTA TCCGACTTGG ATTTCTCTCA GCTGGATATT AACGATGACG AGGCATTAGC   
  
  
+ CATCAACTGT GTCAACTCGC TCCACTCGGT CAATATCCAC CGCCGTGACT CGGTCATCTC GGCCTTCCGG   
  
  
+ CGGCTCCACC CTAGGGTGGT GACCGTGGTG GAGGAGGAGG CTGACCTTGC TGACGTGGGG GCGGAGGGGT   
  
  
+ ACGAGTTCTA CAGGGGGTTT AATGAGTGTT TAAGGTGGGT TAGGGTTTAC TTTGAGGCCC TGGAAGAGTG   
  
  
+ CTTTCCTAGG ACAAGCAATG AGAAACTCAT GCTCGAGCGG GCCGCAGGGC GGGCTCTGGT GGACCTCCTG   
  
  
+ GCCTGCCCCA AGCCCGCATC ATCCGAGCGG AGGGAGGCAG CTGCGCGGTG GTCCGGGAGG ATGCAGGGGG   
  
  
+ CCGGGTTCGG CCACGTGGGG TTCAGTGATG AGGTCTGTGA TGACGTCAAG GCCCTCCTAA GGAGGTACAA   
  
  
+ GGAGGGGTGG TCCATGATAC AATGTTCCGC CGCGGATGGC GGCGCCTCCG CCGGAATATT CTTGTCGTGG   
  
  
+ AGGGATCAGC CGGTGGTGTG GGCCAGTGCA TGGAAGCCTT A  

- -Up\_Stream \_Len000CCCAAA AAAATTTGAG TTTTAATCAA TAACATAGTT AACTTAAACC TTTTGATAAT   
  
  
- AAAAGCTGAA TAATATCTCT TAAACAACAA TTACTTACGA CTATTTATGA CTATTAATCA CAACACTCAC   
  
  
- ACTTAAACAC TACTACTCGT TACTAATCGC AATTTTAATA TTGATCCTAT TTATTAATTC ACAACTACTT   
  
  
- ACAACTAACT ACTACTACTC ATGACTAACC ACGCCACGAC CATTATTTAT AAATACTATT CAATTTTTTT   
  
  
- TTTTTGTGTA GGATTTGTTA GTGATACATT ATATGATTTA CTTTTTTTAA CCATTACACT AAACTGTTAG   
  
  
- ATTCCCATTC CCACAAAATG GGTGTGTACA ATACTTTAAC CGAATGATAA ATGTTTGAAA AATGTCAATA   
  
  
- TACTAAGATG GATACACAAT AGTAGAAAGG CAGCTACATT AACTAATATA AGTTGTGTTA AATTAAGTAA   
  
  
- TGTTACATCG ATTAACTATG AGTTTTATTA CTTGATAATT ATTTATTAAT TAATATTGAA AATTTTCAAA   
  
  
- CAATTAAATT TTAATATATT ATATATTTAA ATCCGATGTA ATTAGTTTTT TAATCCGCAC AAGTAGCCAG   
  
  
- GTTTGAACTT GGCCTGGTCT GGCCTGGCTT CTAGCTAATT CTCTTTTTCA TGTCTGACTC CTGGCCTAGC   
  
  
- TTATTCTCAG CGTGGCCTGG TCCTAGCCTG GCCTTTTTAA ACCTGGTCTA GCCCTGGCTA GCTCCATCTG   
  
  
- GCTTAAATAA AAGTTTATGC TATCCTTATT TTGAATAACT AAGTTAACTA AAACCGGTTT TAGTACGATA   
  
  
- ATAACTAAAA TAAACGATTG AGAGTTCTCA TAATCAAACG TGAAGCGTAA TAGAAACCGG TTTTTTTAGT   
  
  
- TTTTCTTTCA GAGTTTGAGA ATTATCAAAA AATAAAATAA ACCAAATAAA AATTTTATCA GCCGGAAGCC   
  
  
- AGGCCTGATC ACCTGGCTTA TTCCATTAAA AAGCTTAGCT CCTGGTCTGG TTTACGTGAG TCAAGCTTGG   
  
  
- CCAAGTCAAG CCAGACTGAG CTAAAAACCA GGCCAGGTTC AAACTTGTCT TATTACATGT GTGGATCTTT   
  
  
- TTTTCTTAAT ACGTATATAT GAATTCCGGG GGTTGGGAAA AAGGGAAAGG CGGTTATGAA AAATGGTTCA   
  
  
- GGTTAAGAAA AGAAGAATCG ATACCGATCA TCAATAGGGA GGGATCGAAG GTGGGATTCA AAACTCTGTT   
  
  
- TTAGGGTAGA TACAATTATA AATAAAGAAC GTGTACAGTG GGTTATGTTC CCAAAGTAAC TTTTCTTTTC   
  
  
- CATAATCTAC ATTCTATACA TACATATATT TATCAAAACG TTATCTGATC TTCCTGATTT TTCCTTTTTC   
  
  
- CTTCAAATTT TCGTTTTTTG AAAGGGGAGA TCTTTTTTCC TTTGGTCTAA GGGAAAAGAT GGGGTTAAGG   
  
  
- GTATCAGAAA TGAGACAGAG AGAGAGAGAC AGAGAGAATG AGTACTACAA CTCCCTCCTT CCTTACTTAA   
  
  
- TTCCTGGGAT TCGATTCTAG GTTGGCATTC TATAACGGGG AAATTAGTGG GTAAAGTATA GAGATAGGTG   
  
  
- TCAATTTTTG GGTGGAGGAG GTAGGTGTAT TGACTATATA GAGAAGATGT GGTATGATAT TCCTTCTTCT   
  
  
- CTTTTTATCC TCACATAACA TAAAAATAAA CTCATTAAAT AAAATTCCTC TCTTTAAATG TACCAACCCA   
  
  
- ATTTACCTAT GGAACAAATC TGATCACTCA AAGGTTGTTG GTGTGGTGGT GGTAGTGGTT GTGGTTGTGG   
  
  
- TTGTGGTGGT TGTCGTTAGA AGACTAGTTA GGAGAAACTT ATTATGATGA TGAATAAGGT GTTTAAGTTC   
  
  
- GTCAAGGTCT AGAAGGGTTG TTTGGGTAGT ATTAGTATTA GTATGAGTCA TAATAGTGGT TGTAGTAGGA   
  
  
- TGAATGGGGG TGGCGGTGGT AGTCCTCCTT CTTCTTCTTA CGAAGTTGAA AATGTACCTA CTTCTTTTGA   
  
  
- AGAGAAGAAG AAGGAGGGTG TTGTTTGTAG TAGGAGTTGG AGGAAGGAGG CGGTGGTGGA GGAGCATAAT   
  
  
- GGGAATGATG AGGTGGGGGA AGCTACAAGG GAGATGGTGG TGGTGGAGGT GGAGGTGGGG GTGCGGGTGG   
  
  
- TTGTGGTGGT GGTGGTGGGG GGGTTGGGTC ATACCGCCGC CGCCGCCACC CAAACTGAGG TCGACGAAGA   
  
  
- GGATGAGGGG ACGGGCTCTG GAGTTGGGCC TCGAGAAGAG GAGCAGGCCC AAGAGCCCGT TCACCCGAAG   
  
  
- GTTGTAGGAG AACCTTCGGC GAGCACGGTA GAGACTCTCC TTGTGCTCGG CGGAGGTGGT CGAGGATACC   
  
  
- TACCAGTTGC TCGAGTCGAG GGGCATGCCA CTGCACCTCG TCTTCTATCG AAGTATGAAG CCGGTTCGGG   
  
  
- AGAAGTGGGC GTACTGGCTC AGTCCCCTGG CGTGGATGGC GTGGTACGCC AGGCGGCACC TGTTCTGGAA   
  
  
- GAGGAAACTC AGATGCTCTT TCTACCATGA GTTCAAGGTC CTCCAATCGG GAACCTGGTG GAAACCTGTG   
  
  
- CACCGTCGAT TACCTCGGGA GTAACTACGG AAACTGCCAC TTAGGTTTTA GGTGTAGTAG CTGTAGTCGT   
  
  
- GGTGTAAGAC GTGGGTCACC GGGTGGGAGG ACCTCCGCGA CCGGTGGGCT TACCTACTGC GCGGGATGGA   
  
  
- CTCCGACTGG TGACACCACC AGTTGTTCAG GCCCTTACTT CCAGGGTGGC CTCCGCCCAG GGTGTCCCAC   
  
  
- TACTTTCTCT AACCCTGGGC GGAGCTCTTC AAACGATCCG ATTACCCTCA CGGAAAGTTT AAATTACACC   
  
  
- AGGTGGTGCC CCGACTAAAT AGGCTGAACC TAAAGAGAGT CGACCTATAA TTGCTACTGC TCCGTAATCG   
  
  
- GTAGTTGACA CAGTTGAGCG AGGTGAGCCA GTTATAGGTG GCGGCACTGA GCCAGTAGAG CCGGAAGGCC   
  
  
- GCCGAGGTGG GATCCCACCA CTGGCACCAC CTCCTCCTCC GACTGGAACG ACTGCACCCC CGCCTCCCCA   
  
  
- TGCTCAAGAT GTCCCCCAAA TTACTCACAA ATTCCACCCA ATCCCAAATG AAACTCCGGG ACCTTCTCAC   
  
  
- GAAAGGATCC TGTTCGTTAC TCTTTGAGTA CGAGCTCGCC CGGCGTCCCG CCCGAGACCA CCTGGAGGAC   
  
  
- CGGACGGGGT TCGGGCGTAG TAGGCTCGCC TCCCTCCGTC GACGCGCCAC CAGGCCCTCC TACGTCCCCC   
  
  
- GGCCCAAGCC GGTGCACCCC AAGTCACTAC TCCAGACACT ACTGCAGTTC CGGGAGGATT CCTCCATGTT   
  
  
- CCTCCCCACC AGGTACTATG TTACAAGGCG GCGCCTACCG CCGCGGAGGC GGCCTTATAA GAACAGCACC   
  
  
- TCCCTAGTCG GCCACCACAC CCGGTCACGT ACCTTCGGAA T

+     Sp1

| Site Name | Organism | Position | Strand | Matrix score. | sequence | function |
| --- | --- | --- | --- | --- | --- | --- |
| Sp1 | Oryza sativa | 3201 | + | 6 | GGGCGG | light responsive element |
| Sp1 | Oryza sativa | 3073 | + | 6 | GGGCGG | light responsive element |

>HU06G00358.1   
+ -Up\_Stream \_Len000GGGTTT TTTTAAACTC AAAATTAGTT ATTGTATCAA TTGAATTTGG AAAACTATTA   
  
  
+ TTTTCGACTT ATTATAGAGA ATTTGTTGTT AATGAATGCT GATAAATACT GATAATTAGT GTTGTGAGTG   
  
  
+ TGAATTTGTG ATGATGAGCA ATGATTAGCG TTAAAATTAT AACTAGGATA AATAATTAAG TGTTGATGAA   
  
  
+ TGTTGATTGA TGATGATGAG TACTGATTGG TGCGGTGCTG GTAATAAATA TTTATGATAA GTTAAAAAAA   
  
  
+ AAAAACACAT CCTAAACAAT CACTATGTAA TATACTAAAT GAAAAAAATT GGTAATGTGA TTTGACAATC   
  
  
+ TAAGGGTAAG GGTGTTTTAC CCACACATGT TATGAAATTG GCTTACTATT TACAAACTTT TTACAGTTAT   
  
  
+ ATGATTCTAC CTATGTGTTA TCATCTTTCC GTCGATGTAA TTGATTATAT TCAACACAAT TTAATTCATT   
  
  
+ ACAATGTAGC TAATTGATAC TCAAAATAAT GAACTATTAA TAAATAATTA ATTATAACTT TTAAAAGTTT   
  
  
+ GTTAATTTAA AATTATATAA TATATAAATT TAGGCTACAT TAATCAAAAA ATTAGGCGTG TTCATCGGTC   
  
  
+ CAAACTTGAA CCGGACCAGA CCGGACCGAA GATCGATTAA GAGAAAAAGT ACAGACTGAG GACCGGATCG   
  
  
+ AATAAGAGTC GCACCGGACC AGGATCGGAC CGGAAAAATT TGGACCAGAT CGGGACCGAT CGAGGTAGAC   
  
  
+ CGAATTTATT TTCAAATACG ATAGGAATAA AACTTATTGA TTCAATTGAT TTTGGCCAAA ATCATGCTAT   
  
  
+ TATTGATTTT ATTTGCTAAC TCTCAAGAGT ATTAGTTTGC ACTTCGCATT ATCTTTGGCC AAAAAAATCA   
  
  
+ AAAAGAAAGT CTCAAACTCT TAATAGTTTT TTATTTTATT TGGTTTATTT TTAAAATAGT CGGCCTTCGG   
  
  
+ TCCGGACTAG TGGACCGAAT AAGGTAATTT TTCGAATCGA GGACCAGACC AAATGCACTC AGTTCGAACC   
  
  
+ GGTTCAGTTC GGTCTGACTC GATTTTTGGT CCGGTCCAAG TTTGAACAGA ATAATGTACA CACCTAGAAA   
  
  
+ AAAAGAATTA TGCATATATA CTTAAGGCCC CCAACCCTTT TTCCCTTTCC GCCAATACTT TTTACCAAGT   
  
  
+ CCAATTCTTT TCTTCTTAGC TATGGCTAGT AGTTATCCCT CCCTAGCTTC CACCCTAAGT TTTGAGACAA   
  
  
+ AATCCCATCT ATGTTAATAT TTATTTCTTG CACATGTCAC CCAATACAAG GGTTTCATTG AAAAGAAAAG   
  
  
+ GTATTAGATG TAAGATATGT ATGTATATAA ATAGTTTTGC AATAGACTAG AAGGACTAAA AAGGAAAAAG   
  
  
+ GAAGTTTAAA AGCAAAAAAC TTTCCCCTCT AGAAAAAAGG AAACCAGATT CCCTTTTCTA CCCCAATTCC   
  
  
+ CATAGTCTTT ACTCTGTCTC TCTCTCTCTG TCTCTCTTAC TCATGATGTT GAGGGAGGAA GGAATGAATT   
  
  
+ AAGGACCCTA AGCTAAGATC CAACCGTAAG ATATTGCCCC TTTAATCACC CATTTCATAT CTCTATCCAC   
  
  
+ AGTTAAAAAC CCACCTCCTC CATCCACATA ACTGATATAT CTCTTCTACA CCATACTATA AGGAAGAAGA   
  
  
+ GAAAAATAGG AGTGTATTGT ATTTTTATTT GAGTAATTTA TTTTAAGGAG AGAAATTTAC ATGGTTGGGT   
  
  
+ TAAATGGATA CCTTGTTTAG ACTAGTGAGT TTCCAACAAC CACACCACCA CCATCACCAA CACCAACACC   
  
  
+ AACACCACCA ACAGCAATCT TCTGATCAAT CCTCTTTGAA TAATACTACT ACTTATTCCA CAAATTCAAG   
  
  
+ CAGTTCCAGA TCTTCCCAAC AAACCCATCA TAATCATAAT CATACTCAGT ATTATCACCA ACATCATCCT   
  
  
+ ACTTACCCCC ACCGCCACCA TCAGGAGGAA GAAGAAGAAT GCTTCAACTT TTACATGGAT GAAGAAAACT   
  
  
+ TCTCTTCTTC TTCCTCCCAC AACAAACATC ATCCTCAACC TCCTTCCTCC GCCACCACCT CCTCGTATTA   
  
  
+ CCCTTACTAC TCCACCCCCT TCGATGTTCC CTCTACCACC ACCACCTCCA CCTCCACCCC CACGCCCACC   
  
  
+ AACACCACCA CCACCACCCC CCCAACCCAG TATGGCGGCG GCGGCGGTGG GTTTGACTCC AGCTGCTTCT   
  
  
+ CCTACTCCCC TGCCCGAGAC CTCAACCCGG AGCTCTTCTC CTCGTCCGGG TTCTCGGGCA AGTGGGCTTC   
  
  
+ CAACATCCTC TTGGAAGCCG CTCGTGCCAT CTCTGAGAGG AACACGAGCC GCCTCCACCA GCTCCTATGG   
  
  
+ ATGGTCAACG AGCTCAGCTC CCCGTACGGT GACGTGGAGC AGAAGATAGC TTCATACTTC GGCCAAGCCC   
  
  
+ TCTTCACCCG CATGACCGAG TCAGGGGACC GCACCTACCG CACCATGCGG TCCGCCGTGG ACAAGACCTT   
  
  
+ CTCCTTTGAG TCTACGAGAA AGATGGTACT CAAGTTCCAG GAGGTTAGCC CTTGGACCAC CTTTGGACAC   
  
  
+ GTGGCAGCTA ATGGAGCCCT CATTGATGCC TTTGACGGTG AATCCAAAAT CCACATCATC GACATCAGCA   
  
  
+ CCACATTCTG CACCCAGTGG CCCACCCTCC TGGAGGCGCT GGCCACCCGA ATGGATGACG CGCCCTACCT   
  
  
+ GAGGCTGACC ACTGTGGTGG TCAACAAGTC CGGGAATGAA GGTCCCACCG GAGGCGGGTC CCACAGGGTG   
  
  
+ ATGAAAGAGA TTGGGACCCG CCTCGAGAAG TTTGCTAGGC TAATGGGAGT GCCTTTCAAA TTTAATGTGG   
  
  
+ TCCACCACGG GGCTGATTTA TCCGACTTGG ATTTCTCTCA GCTGGATATT AACGATGACG AGGCATTAGC   
  
  
+ CATCAACTGT GTCAACTCGC TCCACTCGGT CAATATCCAC CGCCGTGACT CGGTCATCTC GGCCTTCCGG   
  
  
+ CGGCTCCACC CTAGGGTGGT GACCGTGGTG GAGGAGGAGG CTGACCTTGC TGACGTGGGG GCGGAGGGGT   
  
  
+ ACGAGTTCTA CAGGGGGTTT AATGAGTGTT TAAGGTGGGT TAGGGTTTAC TTTGAGGCCC TGGAAGAGTG   
  
  
+ CTTTCCTAGG ACAAGCAATG AGAAACTCAT GCTCGAGCGG GCCGCAGGGC GGGCTCTGGT GGACCTCCTG   
  
  
+ GCCTGCCCCA AGCCCGCATC ATCCGAGCGG AGGGAGGCAG CTGCGCGGTG GTCCGGGAGG ATGCAGGGGG   
  
  
+ CCGGGTTCGG CCACGTGGGG TTCAGTGATG AGGTCTGTGA TGACGTCAAG GCCCTCCTAA GGAGGTACAA   
  
  
+ GGAGGGGTGG TCCATGATAC AATGTTCCGC CGCGGATGGC GGCGCCTCCG CCGGAATATT CTTGTCGTGG   
  
  
+ AGGGATCAGC CGGTGGTGTG GGCCAGTGCA TGGAAGCCTT A  

- -Up\_Stream \_Len000CCCAAA AAAATTTGAG TTTTAATCAA TAACATAGTT AACTTAAACC TTTTGATAAT   
  
  
- AAAAGCTGAA TAATATCTCT TAAACAACAA TTACTTACGA CTATTTATGA CTATTAATCA CAACACTCAC   
  
  
- ACTTAAACAC TACTACTCGT TACTAATCGC AATTTTAATA TTGATCCTAT TTATTAATTC ACAACTACTT   
  
  
- ACAACTAACT ACTACTACTC ATGACTAACC ACGCCACGAC CATTATTTAT AAATACTATT CAATTTTTTT   
  
  
- TTTTTGTGTA GGATTTGTTA GTGATACATT ATATGATTTA CTTTTTTTAA CCATTACACT AAACTGTTAG   
  
  
- ATTCCCATTC CCACAAAATG GGTGTGTACA ATACTTTAAC CGAATGATAA ATGTTTGAAA AATGTCAATA   
  
  
- TACTAAGATG GATACACAAT AGTAGAAAGG CAGCTACATT AACTAATATA AGTTGTGTTA AATTAAGTAA   
  
  
- TGTTACATCG ATTAACTATG AGTTTTATTA CTTGATAATT ATTTATTAAT TAATATTGAA AATTTTCAAA   
  
  
- CAATTAAATT TTAATATATT ATATATTTAA ATCCGATGTA ATTAGTTTTT TAATCCGCAC AAGTAGCCAG   
  
  
- GTTTGAACTT GGCCTGGTCT GGCCTGGCTT CTAGCTAATT CTCTTTTTCA TGTCTGACTC CTGGCCTAGC   
  
  
- TTATTCTCAG CGTGGCCTGG TCCTAGCCTG GCCTTTTTAA ACCTGGTCTA GCCCTGGCTA GCTCCATCTG   
  
  
- GCTTAAATAA AAGTTTATGC TATCCTTATT TTGAATAACT AAGTTAACTA AAACCGGTTT TAGTACGATA   
  
  
- ATAACTAAAA TAAACGATTG AGAGTTCTCA TAATCAAACG TGAAGCGTAA TAGAAACCGG TTTTTTTAGT   
  
  
- TTTTCTTTCA GAGTTTGAGA ATTATCAAAA AATAAAATAA ACCAAATAAA AATTTTATCA GCCGGAAGCC   
  
  
- AGGCCTGATC ACCTGGCTTA TTCCATTAAA AAGCTTAGCT CCTGGTCTGG TTTACGTGAG TCAAGCTTGG   
  
  
- CCAAGTCAAG CCAGACTGAG CTAAAAACCA GGCCAGGTTC AAACTTGTCT TATTACATGT GTGGATCTTT   
  
  
- TTTTCTTAAT ACGTATATAT GAATTCCGGG GGTTGGGAAA AAGGGAAAGG CGGTTATGAA AAATGGTTCA   
  
  
- GGTTAAGAAA AGAAGAATCG ATACCGATCA TCAATAGGGA GGGATCGAAG GTGGGATTCA AAACTCTGTT   
  
  
- TTAGGGTAGA TACAATTATA AATAAAGAAC GTGTACAGTG GGTTATGTTC CCAAAGTAAC TTTTCTTTTC   
  
  
- CATAATCTAC ATTCTATACA TACATATATT TATCAAAACG TTATCTGATC TTCCTGATTT TTCCTTTTTC   
  
  
- CTTCAAATTT TCGTTTTTTG AAAGGGGAGA TCTTTTTTCC TTTGGTCTAA GGGAAAAGAT GGGGTTAAGG   
  
  
- GTATCAGAAA TGAGACAGAG AGAGAGAGAC AGAGAGAATG AGTACTACAA CTCCCTCCTT CCTTACTTAA   
  
  
- TTCCTGGGAT TCGATTCTAG GTTGGCATTC TATAACGGGG AAATTAGTGG GTAAAGTATA GAGATAGGTG   
  
  
- TCAATTTTTG GGTGGAGGAG GTAGGTGTAT TGACTATATA GAGAAGATGT GGTATGATAT TCCTTCTTCT   
  
  
- CTTTTTATCC TCACATAACA TAAAAATAAA CTCATTAAAT AAAATTCCTC TCTTTAAATG TACCAACCCA   
  
  
- ATTTACCTAT GGAACAAATC TGATCACTCA AAGGTTGTTG GTGTGGTGGT GGTAGTGGTT GTGGTTGTGG   
  
  
- TTGTGGTGGT TGTCGTTAGA AGACTAGTTA GGAGAAACTT ATTATGATGA TGAATAAGGT GTTTAAGTTC   
  
  
- GTCAAGGTCT AGAAGGGTTG TTTGGGTAGT ATTAGTATTA GTATGAGTCA TAATAGTGGT TGTAGTAGGA   
  
  
- TGAATGGGGG TGGCGGTGGT AGTCCTCCTT CTTCTTCTTA CGAAGTTGAA AATGTACCTA CTTCTTTTGA   
  
  
- AGAGAAGAAG AAGGAGGGTG TTGTTTGTAG TAGGAGTTGG AGGAAGGAGG CGGTGGTGGA GGAGCATAAT   
  
  
- GGGAATGATG AGGTGGGGGA AGCTACAAGG GAGATGGTGG TGGTGGAGGT GGAGGTGGGG GTGCGGGTGG   
  
  
- TTGTGGTGGT GGTGGTGGGG GGGTTGGGTC ATACCGCCGC CGCCGCCACC CAAACTGAGG TCGACGAAGA   
  
  
- GGATGAGGGG ACGGGCTCTG GAGTTGGGCC TCGAGAAGAG GAGCAGGCCC AAGAGCCCGT TCACCCGAAG   
  
  
- GTTGTAGGAG AACCTTCGGC GAGCACGGTA GAGACTCTCC TTGTGCTCGG CGGAGGTGGT CGAGGATACC   
  
  
- TACCAGTTGC TCGAGTCGAG GGGCATGCCA CTGCACCTCG TCTTCTATCG AAGTATGAAG CCGGTTCGGG   
  
  
- AGAAGTGGGC GTACTGGCTC AGTCCCCTGG CGTGGATGGC GTGGTACGCC AGGCGGCACC TGTTCTGGAA   
  
  
- GAGGAAACTC AGATGCTCTT TCTACCATGA GTTCAAGGTC CTCCAATCGG GAACCTGGTG GAAACCTGTG   
  
  
- CACCGTCGAT TACCTCGGGA GTAACTACGG AAACTGCCAC TTAGGTTTTA GGTGTAGTAG CTGTAGTCGT   
  
  
- GGTGTAAGAC GTGGGTCACC GGGTGGGAGG ACCTCCGCGA CCGGTGGGCT TACCTACTGC GCGGGATGGA   
  
  
- CTCCGACTGG TGACACCACC AGTTGTTCAG GCCCTTACTT CCAGGGTGGC CTCCGCCCAG GGTGTCCCAC   
  
  
- TACTTTCTCT AACCCTGGGC GGAGCTCTTC AAACGATCCG ATTACCCTCA CGGAAAGTTT AAATTACACC   
  
  
- AGGTGGTGCC CCGACTAAAT AGGCTGAACC TAAAGAGAGT CGACCTATAA TTGCTACTGC TCCGTAATCG   
  
  
- GTAGTTGACA CAGTTGAGCG AGGTGAGCCA GTTATAGGTG GCGGCACTGA GCCAGTAGAG CCGGAAGGCC   
  
  
- GCCGAGGTGG GATCCCACCA CTGGCACCAC CTCCTCCTCC GACTGGAACG ACTGCACCCC CGCCTCCCCA   
  
  
- TGCTCAAGAT GTCCCCCAAA TTACTCACAA ATTCCACCCA ATCCCAAATG AAACTCCGGG ACCTTCTCAC   
  
  
- GAAAGGATCC TGTTCGTTAC TCTTTGAGTA CGAGCTCGCC CGGCGTCCCG CCCGAGACCA CCTGGAGGAC   
  
  
- CGGACGGGGT TCGGGCGTAG TAGGCTCGCC TCCCTCCGTC GACGCGCCAC CAGGCCCTCC TACGTCCCCC   
  
  
- GGCCCAAGCC GGTGCACCCC AAGTCACTAC TCCAGACACT ACTGCAGTTC CGGGAGGATT CCTCCATGTT   
  
  
- CCTCCCCACC AGGTACTATG TTACAAGGCG GCGCCTACCG CCGCGGAGGC GGCCTTATAA GAACAGCACC   
  
  
- TCCCTAGTCG GCCACCACAC CCGGTCACGT ACCTTCGGAA T

+     TATA-box

| Site Name | Organism | Position | Strand | Matrix score. | sequence | function |
| --- | --- | --- | --- | --- | --- | --- |
| TATA-box | Arabidopsis thaliana | 1141 | + | 4 | TATA | core promoter element around -30 of transcription start |
| TATA-box | Brassica napus | 1138 | + | 6 | ATATAT | core promoter element around -30 of transcription start |
| TATA-box | Arabidopsis thaliana | 182 | + | 4 | TATA | core promoter element around -30 of transcription start |
| TATA-box | Brassica napus | 85 | + | 6 | ATTATA | core promoter element around -30 of transcription start |
| TATA-box | Arabidopsis thaliana | 578 | + | 6 | TATATA | core promoter element around -30 of transcription start |
| TATA-box | Brassica napus | 576 | + | 6 | ATTATA | core promoter element around -30 of transcription start |
| TATA-box | Arabidopsis thaliana | 547 | + | 4 | TATA | core promoter element around -30 of transcription start |
| TATA-box | Arabidopsis thaliana | 86 | - | 5 | TATAA | core promoter element around -30 of transcription start |
| TATA-box | Brassica napus | 468 | + | 6 | ATTATA | core promoter element around -30 of transcription start |
| TATA-box | Brassica napus | 545 | + | 6 | ATTATA | core promoter element around -30 of transcription start |
| TATA-box | Arabidopsis thaliana | 87 | + | 4 | TATA | core promoter element around -30 of transcription start |
| TATA-box | Arabidopsis thaliana | 1650 | + | 4 | TATA | core promoter element around -30 of transcription start |
| TATA-box | Brassica oleracea | 586 | + | 6 | ATATAA | core promoter element around -30 of transcription start |
| TATA-box | Arabidopsis thaliana | 580 | + | 4 | TATA | core promoter element around -30 of transcription start |
| TATA-box | Arabidopsis thaliana | 1360 | + | 4 | TATA | core promoter element around -30 of transcription start |
| TATA-box | Brassica napus | 1649 | + | 6 | ATATAT | core promoter element around -30 of transcription start |
| TATA-box | Arabidopsis thaliana | 546 | - | 5 | TATAA | core promoter element around -30 of transcription start |
| TATA-box | Arabidopsis thaliana | 315 | + | 4 | TATA | core promoter element around -30 of transcription start |
| TATA-box | Arabidopsis thaliana | 1117 | + | 9 | ccTATAAAaa | core promoter element around -30 of transcription start |
| TATA-box | Arabidopsis thaliana | 421 | - | 5 | TATAA | core promoter element around -30 of transcription start |
| TATA-box | Arabidopsis thaliana | 577 | - | 7 | TATATAA | core promoter element around -30 of transcription start |
| TATA-box | Arabidopsis thaliana | 470 | + | 4 | TATA | core promoter element around -30 of transcription start |
| TATA-box | Arabidopsis thaliana | 469 | - | 5 | TATAA | core promoter element around -30 of transcription start |
| TATA-box | Arabidopsis thaliana | 1139 | + | 6 | TATATA | core promoter element around -30 of transcription start |
| TATA-box | Arabidopsis thaliana | 181 | - | 5 | TATAA | core promoter element around -30 of transcription start |
| TATA-box | Arabidopsis thaliana | 1358 | + | 6 | TATATA | core promoter element around -30 of transcription start |
| TATA-box | Brassica napus | 180 | + | 6 | ATTATA | core promoter element around -30 of transcription start |
| TATA-box | Arabidopsis thaliana | 1671 | + | 4 | TATA | core promoter element around -30 of transcription start |
| TATA-box | Arabidopsis thaliana | 422 | + | 4 | TATA | core promoter element around -30 of transcription start |
| TATA-box | Helianthus annuus | 1356 | - | 6 | TATACA | core promoter element around -30 of transcription start |
| TATA-box | Brassica oleracea | 1359 | + | 6 | ATATAA | core promoter element around -30 of transcription start |
| TATA-box | Arabidopsis thaliana | 587 | + | 4 | TATA | core promoter element around -30 of transcription start |
| TATA-box | Brassica oleracea | 579 | + | 6 | ATATAA | core promoter element around -30 of transcription start |
| TATA-box | Arabidopsis thaliana | 585 | + | 6 | TATATA | core promoter element around -30 of transcription start |
| TATA-box | Brassica napus | 584 | + | 6 | ATATAT | core promoter element around -30 of transcription start |

>HU06G00358.1   
+ -Up\_Stream \_Len000GGGTTT TTTTAAACTC AAAATTAGTT ATTGTATCAA TTGAATTTGG AAAACTATTA   
  
  
+ TTTTCGACTT ATTATAGAGA ATTTGTTGTT AATGAATGCT GATAAATACT GATAATTAGT GTTGTGAGTG   
  
  
+ TGAATTTGTG ATGATGAGCA ATGATTAGCG TTAAAATTAT AACTAGGATA AATAATTAAG TGTTGATGAA   
  
  
+ TGTTGATTGA TGATGATGAG TACTGATTGG TGCGGTGCTG GTAATAAATA TTTATGATAA GTTAAAAAAA   
  
  
+ AAAAACACAT CCTAAACAAT CACTATGTAA TATACTAAAT GAAAAAAATT GGTAATGTGA TTTGACAATC   
  
  
+ TAAGGGTAAG GGTGTTTTAC CCACACATGT TATGAAATTG GCTTACTATT TACAAACTTT TTACAGTTAT   
  
  
+ ATGATTCTAC CTATGTGTTA TCATCTTTCC GTCGATGTAA TTGATTATAT TCAACACAAT TTAATTCATT   
  
  
+ ACAATGTAGC TAATTGATAC TCAAAATAAT GAACTATTAA TAAATAATTA ATTATAACTT TTAAAAGTTT   
  
  
+ GTTAATTTAA AATTATATAA TATATAAATT TAGGCTACAT TAATCAAAAA ATTAGGCGTG TTCATCGGTC   
  
  
+ CAAACTTGAA CCGGACCAGA CCGGACCGAA GATCGATTAA GAGAAAAAGT ACAGACTGAG GACCGGATCG   
  
  
+ AATAAGAGTC GCACCGGACC AGGATCGGAC CGGAAAAATT TGGACCAGAT CGGGACCGAT CGAGGTAGAC   
  
  
+ CGAATTTATT TTCAAATACG ATAGGAATAA AACTTATTGA TTCAATTGAT TTTGGCCAAA ATCATGCTAT   
  
  
+ TATTGATTTT ATTTGCTAAC TCTCAAGAGT ATTAGTTTGC ACTTCGCATT ATCTTTGGCC AAAAAAATCA   
  
  
+ AAAAGAAAGT CTCAAACTCT TAATAGTTTT TTATTTTATT TGGTTTATTT TTAAAATAGT CGGCCTTCGG   
  
  
+ TCCGGACTAG TGGACCGAAT AAGGTAATTT TTCGAATCGA GGACCAGACC AAATGCACTC AGTTCGAACC   
  
  
+ GGTTCAGTTC GGTCTGACTC GATTTTTGGT CCGGTCCAAG TTTGAACAGA ATAATGTACA CACCTAGAAA   
  
  
+ AAAAGAATTA TGCATATATA CTTAAGGCCC CCAACCCTTT TTCCCTTTCC GCCAATACTT TTTACCAAGT   
  
  
+ CCAATTCTTT TCTTCTTAGC TATGGCTAGT AGTTATCCCT CCCTAGCTTC CACCCTAAGT TTTGAGACAA   
  
  
+ AATCCCATCT ATGTTAATAT TTATTTCTTG CACATGTCAC CCAATACAAG GGTTTCATTG AAAAGAAAAG   
  
  
+ GTATTAGATG TAAGATATGT ATGTATATAA ATAGTTTTGC AATAGACTAG AAGGACTAAA AAGGAAAAAG   
  
  
+ GAAGTTTAAA AGCAAAAAAC TTTCCCCTCT AGAAAAAAGG AAACCAGATT CCCTTTTCTA CCCCAATTCC   
  
  
+ CATAGTCTTT ACTCTGTCTC TCTCTCTCTG TCTCTCTTAC TCATGATGTT GAGGGAGGAA GGAATGAATT   
  
  
+ AAGGACCCTA AGCTAAGATC CAACCGTAAG ATATTGCCCC TTTAATCACC CATTTCATAT CTCTATCCAC   
  
  
+ AGTTAAAAAC CCACCTCCTC CATCCACATA ACTGATATAT CTCTTCTACA CCATACTATA AGGAAGAAGA   
  
  
+ GAAAAATAGG AGTGTATTGT ATTTTTATTT GAGTAATTTA TTTTAAGGAG AGAAATTTAC ATGGTTGGGT   
  
  
+ TAAATGGATA CCTTGTTTAG ACTAGTGAGT TTCCAACAAC CACACCACCA CCATCACCAA CACCAACACC   
  
  
+ AACACCACCA ACAGCAATCT TCTGATCAAT CCTCTTTGAA TAATACTACT ACTTATTCCA CAAATTCAAG   
  
  
+ CAGTTCCAGA TCTTCCCAAC AAACCCATCA TAATCATAAT CATACTCAGT ATTATCACCA ACATCATCCT   
  
  
+ ACTTACCCCC ACCGCCACCA TCAGGAGGAA GAAGAAGAAT GCTTCAACTT TTACATGGAT GAAGAAAACT   
  
  
+ TCTCTTCTTC TTCCTCCCAC AACAAACATC ATCCTCAACC TCCTTCCTCC GCCACCACCT CCTCGTATTA   
  
  
+ CCCTTACTAC TCCACCCCCT TCGATGTTCC CTCTACCACC ACCACCTCCA CCTCCACCCC CACGCCCACC   
  
  
+ AACACCACCA CCACCACCCC CCCAACCCAG TATGGCGGCG GCGGCGGTGG GTTTGACTCC AGCTGCTTCT   
  
  
+ CCTACTCCCC TGCCCGAGAC CTCAACCCGG AGCTCTTCTC CTCGTCCGGG TTCTCGGGCA AGTGGGCTTC   
  
  
+ CAACATCCTC TTGGAAGCCG CTCGTGCCAT CTCTGAGAGG AACACGAGCC GCCTCCACCA GCTCCTATGG   
  
  
+ ATGGTCAACG AGCTCAGCTC CCCGTACGGT GACGTGGAGC AGAAGATAGC TTCATACTTC GGCCAAGCCC   
  
  
+ TCTTCACCCG CATGACCGAG TCAGGGGACC GCACCTACCG CACCATGCGG TCCGCCGTGG ACAAGACCTT   
  
  
+ CTCCTTTGAG TCTACGAGAA AGATGGTACT CAAGTTCCAG GAGGTTAGCC CTTGGACCAC CTTTGGACAC   
  
  
+ GTGGCAGCTA ATGGAGCCCT CATTGATGCC TTTGACGGTG AATCCAAAAT CCACATCATC GACATCAGCA   
  
  
+ CCACATTCTG CACCCAGTGG CCCACCCTCC TGGAGGCGCT GGCCACCCGA ATGGATGACG CGCCCTACCT   
  
  
+ GAGGCTGACC ACTGTGGTGG TCAACAAGTC CGGGAATGAA GGTCCCACCG GAGGCGGGTC CCACAGGGTG   
  
  
+ ATGAAAGAGA TTGGGACCCG CCTCGAGAAG TTTGCTAGGC TAATGGGAGT GCCTTTCAAA TTTAATGTGG   
  
  
+ TCCACCACGG GGCTGATTTA TCCGACTTGG ATTTCTCTCA GCTGGATATT AACGATGACG AGGCATTAGC   
  
  
+ CATCAACTGT GTCAACTCGC TCCACTCGGT CAATATCCAC CGCCGTGACT CGGTCATCTC GGCCTTCCGG   
  
  
+ CGGCTCCACC CTAGGGTGGT GACCGTGGTG GAGGAGGAGG CTGACCTTGC TGACGTGGGG GCGGAGGGGT   
  
  
+ ACGAGTTCTA CAGGGGGTTT AATGAGTGTT TAAGGTGGGT TAGGGTTTAC TTTGAGGCCC TGGAAGAGTG   
  
  
+ CTTTCCTAGG ACAAGCAATG AGAAACTCAT GCTCGAGCGG GCCGCAGGGC GGGCTCTGGT GGACCTCCTG   
  
  
+ GCCTGCCCCA AGCCCGCATC ATCCGAGCGG AGGGAGGCAG CTGCGCGGTG GTCCGGGAGG ATGCAGGGGG   
  
  
+ CCGGGTTCGG CCACGTGGGG TTCAGTGATG AGGTCTGTGA TGACGTCAAG GCCCTCCTAA GGAGGTACAA   
  
  
+ GGAGGGGTGG TCCATGATAC AATGTTCCGC CGCGGATGGC GGCGCCTCCG CCGGAATATT CTTGTCGTGG   
  
  
+ AGGGATCAGC CGGTGGTGTG GGCCAGTGCA TGGAAGCCTT A  

- -Up\_Stream \_Len000CCCAAA AAAATTTGAG TTTTAATCAA TAACATAGTT AACTTAAACC TTTTGATAAT   
  
  
- AAAAGCTGAA TAATATCTCT TAAACAACAA TTACTTACGA CTATTTATGA CTATTAATCA CAACACTCAC   
  
  
- ACTTAAACAC TACTACTCGT TACTAATCGC AATTTTAATA TTGATCCTAT TTATTAATTC ACAACTACTT   
  
  
- ACAACTAACT ACTACTACTC ATGACTAACC ACGCCACGAC CATTATTTAT AAATACTATT CAATTTTTTT   
  
  
- TTTTTGTGTA GGATTTGTTA GTGATACATT ATATGATTTA CTTTTTTTAA CCATTACACT AAACTGTTAG   
  
  
- ATTCCCATTC CCACAAAATG GGTGTGTACA ATACTTTAAC CGAATGATAA ATGTTTGAAA AATGTCAATA   
  
  
- TACTAAGATG GATACACAAT AGTAGAAAGG CAGCTACATT AACTAATATA AGTTGTGTTA AATTAAGTAA   
  
  
- TGTTACATCG ATTAACTATG AGTTTTATTA CTTGATAATT ATTTATTAAT TAATATTGAA AATTTTCAAA   
  
  
- CAATTAAATT TTAATATATT ATATATTTAA ATCCGATGTA ATTAGTTTTT TAATCCGCAC AAGTAGCCAG   
  
  
- GTTTGAACTT GGCCTGGTCT GGCCTGGCTT CTAGCTAATT CTCTTTTTCA TGTCTGACTC CTGGCCTAGC   
  
  
- TTATTCTCAG CGTGGCCTGG TCCTAGCCTG GCCTTTTTAA ACCTGGTCTA GCCCTGGCTA GCTCCATCTG   
  
  
- GCTTAAATAA AAGTTTATGC TATCCTTATT TTGAATAACT AAGTTAACTA AAACCGGTTT TAGTACGATA   
  
  
- ATAACTAAAA TAAACGATTG AGAGTTCTCA TAATCAAACG TGAAGCGTAA TAGAAACCGG TTTTTTTAGT   
  
  
- TTTTCTTTCA GAGTTTGAGA ATTATCAAAA AATAAAATAA ACCAAATAAA AATTTTATCA GCCGGAAGCC   
  
  
- AGGCCTGATC ACCTGGCTTA TTCCATTAAA AAGCTTAGCT CCTGGTCTGG TTTACGTGAG TCAAGCTTGG   
  
  
- CCAAGTCAAG CCAGACTGAG CTAAAAACCA GGCCAGGTTC AAACTTGTCT TATTACATGT GTGGATCTTT   
  
  
- TTTTCTTAAT ACGTATATAT GAATTCCGGG GGTTGGGAAA AAGGGAAAGG CGGTTATGAA AAATGGTTCA   
  
  
- GGTTAAGAAA AGAAGAATCG ATACCGATCA TCAATAGGGA GGGATCGAAG GTGGGATTCA AAACTCTGTT   
  
  
- TTAGGGTAGA TACAATTATA AATAAAGAAC GTGTACAGTG GGTTATGTTC CCAAAGTAAC TTTTCTTTTC   
  
  
- CATAATCTAC ATTCTATACA TACATATATT TATCAAAACG TTATCTGATC TTCCTGATTT TTCCTTTTTC   
  
  
- CTTCAAATTT TCGTTTTTTG AAAGGGGAGA TCTTTTTTCC TTTGGTCTAA GGGAAAAGAT GGGGTTAAGG   
  
  
- GTATCAGAAA TGAGACAGAG AGAGAGAGAC AGAGAGAATG AGTACTACAA CTCCCTCCTT CCTTACTTAA   
  
  
- TTCCTGGGAT TCGATTCTAG GTTGGCATTC TATAACGGGG AAATTAGTGG GTAAAGTATA GAGATAGGTG   
  
  
- TCAATTTTTG GGTGGAGGAG GTAGGTGTAT TGACTATATA GAGAAGATGT GGTATGATAT TCCTTCTTCT   
  
  
- CTTTTTATCC TCACATAACA TAAAAATAAA CTCATTAAAT AAAATTCCTC TCTTTAAATG TACCAACCCA   
  
  
- ATTTACCTAT GGAACAAATC TGATCACTCA AAGGTTGTTG GTGTGGTGGT GGTAGTGGTT GTGGTTGTGG   
  
  
- TTGTGGTGGT TGTCGTTAGA AGACTAGTTA GGAGAAACTT ATTATGATGA TGAATAAGGT GTTTAAGTTC   
  
  
- GTCAAGGTCT AGAAGGGTTG TTTGGGTAGT ATTAGTATTA GTATGAGTCA TAATAGTGGT TGTAGTAGGA   
  
  
- TGAATGGGGG TGGCGGTGGT AGTCCTCCTT CTTCTTCTTA CGAAGTTGAA AATGTACCTA CTTCTTTTGA   
  
  
- AGAGAAGAAG AAGGAGGGTG TTGTTTGTAG TAGGAGTTGG AGGAAGGAGG CGGTGGTGGA GGAGCATAAT   
  
  
- GGGAATGATG AGGTGGGGGA AGCTACAAGG GAGATGGTGG TGGTGGAGGT GGAGGTGGGG GTGCGGGTGG   
  
  
- TTGTGGTGGT GGTGGTGGGG GGGTTGGGTC ATACCGCCGC CGCCGCCACC CAAACTGAGG TCGACGAAGA   
  
  
- GGATGAGGGG ACGGGCTCTG GAGTTGGGCC TCGAGAAGAG GAGCAGGCCC AAGAGCCCGT TCACCCGAAG   
  
  
- GTTGTAGGAG AACCTTCGGC GAGCACGGTA GAGACTCTCC TTGTGCTCGG CGGAGGTGGT CGAGGATACC   
  
  
- TACCAGTTGC TCGAGTCGAG GGGCATGCCA CTGCACCTCG TCTTCTATCG AAGTATGAAG CCGGTTCGGG   
  
  
- AGAAGTGGGC GTACTGGCTC AGTCCCCTGG CGTGGATGGC GTGGTACGCC AGGCGGCACC TGTTCTGGAA   
  
  
- GAGGAAACTC AGATGCTCTT TCTACCATGA GTTCAAGGTC CTCCAATCGG GAACCTGGTG GAAACCTGTG   
  
  
- CACCGTCGAT TACCTCGGGA GTAACTACGG AAACTGCCAC TTAGGTTTTA GGTGTAGTAG CTGTAGTCGT   
  
  
- GGTGTAAGAC GTGGGTCACC GGGTGGGAGG ACCTCCGCGA CCGGTGGGCT TACCTACTGC GCGGGATGGA   
  
  
- CTCCGACTGG TGACACCACC AGTTGTTCAG GCCCTTACTT CCAGGGTGGC CTCCGCCCAG GGTGTCCCAC   
  
  
- TACTTTCTCT AACCCTGGGC GGAGCTCTTC AAACGATCCG ATTACCCTCA CGGAAAGTTT AAATTACACC   
  
  
- AGGTGGTGCC CCGACTAAAT AGGCTGAACC TAAAGAGAGT CGACCTATAA TTGCTACTGC TCCGTAATCG   
  
  
- GTAGTTGACA CAGTTGAGCG AGGTGAGCCA GTTATAGGTG GCGGCACTGA GCCAGTAGAG CCGGAAGGCC   
  
  
- GCCGAGGTGG GATCCCACCA CTGGCACCAC CTCCTCCTCC GACTGGAACG ACTGCACCCC CGCCTCCCCA   
  
  
- TGCTCAAGAT GTCCCCCAAA TTACTCACAA ATTCCACCCA ATCCCAAATG AAACTCCGGG ACCTTCTCAC   
  
  
- GAAAGGATCC TGTTCGTTAC TCTTTGAGTA CGAGCTCGCC CGGCGTCCCG CCCGAGACCA CCTGGAGGAC   
  
  
- CGGACGGGGT TCGGGCGTAG TAGGCTCGCC TCCCTCCGTC GACGCGCCAC CAGGCCCTCC TACGTCCCCC   
  
  
- GGCCCAAGCC GGTGCACCCC AAGTCACTAC TCCAGACACT ACTGCAGTTC CGGGAGGATT CCTCCATGTT   
  
  
- CCTCCCCACC AGGTACTATG TTACAAGGCG GCGCCTACCG CCGCGGAGGC GGCCTTATAA GAACAGCACC   
  
  
- TCCCTAGTCG GCCACCACAC CCGGTCACGT ACCTTCGGAA T

+     TCA

| Site Name | Organism | Position | Strand | Matrix score. | sequence | function |
| --- | --- | --- | --- | --- | --- | --- |
| TCA | Pisum sativum | 224 | - | 9 | TCATCTTCAT |  |

>HU06G00358.1   
+ -Up\_Stream \_Len000GGGTTT TTTTAAACTC AAAATTAGTT ATTGTATCAA TTGAATTTGG AAAACTATTA   
  
  
+ TTTTCGACTT ATTATAGAGA ATTTGTTGTT AATGAATGCT GATAAATACT GATAATTAGT GTTGTGAGTG   
  
  
+ TGAATTTGTG ATGATGAGCA ATGATTAGCG TTAAAATTAT AACTAGGATA AATAATTAAG TGTTGATGAA   
  
  
+ TGTTGATTGA TGATGATGAG TACTGATTGG TGCGGTGCTG GTAATAAATA TTTATGATAA GTTAAAAAAA   
  
  
+ AAAAACACAT CCTAAACAAT CACTATGTAA TATACTAAAT GAAAAAAATT GGTAATGTGA TTTGACAATC   
  
  
+ TAAGGGTAAG GGTGTTTTAC CCACACATGT TATGAAATTG GCTTACTATT TACAAACTTT TTACAGTTAT   
  
  
+ ATGATTCTAC CTATGTGTTA TCATCTTTCC GTCGATGTAA TTGATTATAT TCAACACAAT TTAATTCATT   
  
  
+ ACAATGTAGC TAATTGATAC TCAAAATAAT GAACTATTAA TAAATAATTA ATTATAACTT TTAAAAGTTT   
  
  
+ GTTAATTTAA AATTATATAA TATATAAATT TAGGCTACAT TAATCAAAAA ATTAGGCGTG TTCATCGGTC   
  
  
+ CAAACTTGAA CCGGACCAGA CCGGACCGAA GATCGATTAA GAGAAAAAGT ACAGACTGAG GACCGGATCG   
  
  
+ AATAAGAGTC GCACCGGACC AGGATCGGAC CGGAAAAATT TGGACCAGAT CGGGACCGAT CGAGGTAGAC   
  
  
+ CGAATTTATT TTCAAATACG ATAGGAATAA AACTTATTGA TTCAATTGAT TTTGGCCAAA ATCATGCTAT   
  
  
+ TATTGATTTT ATTTGCTAAC TCTCAAGAGT ATTAGTTTGC ACTTCGCATT ATCTTTGGCC AAAAAAATCA   
  
  
+ AAAAGAAAGT CTCAAACTCT TAATAGTTTT TTATTTTATT TGGTTTATTT TTAAAATAGT CGGCCTTCGG   
  
  
+ TCCGGACTAG TGGACCGAAT AAGGTAATTT TTCGAATCGA GGACCAGACC AAATGCACTC AGTTCGAACC   
  
  
+ GGTTCAGTTC GGTCTGACTC GATTTTTGGT CCGGTCCAAG TTTGAACAGA ATAATGTACA CACCTAGAAA   
  
  
+ AAAAGAATTA TGCATATATA CTTAAGGCCC CCAACCCTTT TTCCCTTTCC GCCAATACTT TTTACCAAGT   
  
  
+ CCAATTCTTT TCTTCTTAGC TATGGCTAGT AGTTATCCCT CCCTAGCTTC CACCCTAAGT TTTGAGACAA   
  
  
+ AATCCCATCT ATGTTAATAT TTATTTCTTG CACATGTCAC CCAATACAAG GGTTTCATTG AAAAGAAAAG   
  
  
+ GTATTAGATG TAAGATATGT ATGTATATAA ATAGTTTTGC AATAGACTAG AAGGACTAAA AAGGAAAAAG   
  
  
+ GAAGTTTAAA AGCAAAAAAC TTTCCCCTCT AGAAAAAAGG AAACCAGATT CCCTTTTCTA CCCCAATTCC   
  
  
+ CATAGTCTTT ACTCTGTCTC TCTCTCTCTG TCTCTCTTAC TCATGATGTT GAGGGAGGAA GGAATGAATT   
  
  
+ AAGGACCCTA AGCTAAGATC CAACCGTAAG ATATTGCCCC TTTAATCACC CATTTCATAT CTCTATCCAC   
  
  
+ AGTTAAAAAC CCACCTCCTC CATCCACATA ACTGATATAT CTCTTCTACA CCATACTATA AGGAAGAAGA   
  
  
+ GAAAAATAGG AGTGTATTGT ATTTTTATTT GAGTAATTTA TTTTAAGGAG AGAAATTTAC ATGGTTGGGT   
  
  
+ TAAATGGATA CCTTGTTTAG ACTAGTGAGT TTCCAACAAC CACACCACCA CCATCACCAA CACCAACACC   
  
  
+ AACACCACCA ACAGCAATCT TCTGATCAAT CCTCTTTGAA TAATACTACT ACTTATTCCA CAAATTCAAG   
  
  
+ CAGTTCCAGA TCTTCCCAAC AAACCCATCA TAATCATAAT CATACTCAGT ATTATCACCA ACATCATCCT   
  
  
+ ACTTACCCCC ACCGCCACCA TCAGGAGGAA GAAGAAGAAT GCTTCAACTT TTACATGGAT GAAGAAAACT   
  
  
+ TCTCTTCTTC TTCCTCCCAC AACAAACATC ATCCTCAACC TCCTTCCTCC GCCACCACCT CCTCGTATTA   
  
  
+ CCCTTACTAC TCCACCCCCT TCGATGTTCC CTCTACCACC ACCACCTCCA CCTCCACCCC CACGCCCACC   
  
  
+ AACACCACCA CCACCACCCC CCCAACCCAG TATGGCGGCG GCGGCGGTGG GTTTGACTCC AGCTGCTTCT   
  
  
+ CCTACTCCCC TGCCCGAGAC CTCAACCCGG AGCTCTTCTC CTCGTCCGGG TTCTCGGGCA AGTGGGCTTC   
  
  
+ CAACATCCTC TTGGAAGCCG CTCGTGCCAT CTCTGAGAGG AACACGAGCC GCCTCCACCA GCTCCTATGG   
  
  
+ ATGGTCAACG AGCTCAGCTC CCCGTACGGT GACGTGGAGC AGAAGATAGC TTCATACTTC GGCCAAGCCC   
  
  
+ TCTTCACCCG CATGACCGAG TCAGGGGACC GCACCTACCG CACCATGCGG TCCGCCGTGG ACAAGACCTT   
  
  
+ CTCCTTTGAG TCTACGAGAA AGATGGTACT CAAGTTCCAG GAGGTTAGCC CTTGGACCAC CTTTGGACAC   
  
  
+ GTGGCAGCTA ATGGAGCCCT CATTGATGCC TTTGACGGTG AATCCAAAAT CCACATCATC GACATCAGCA   
  
  
+ CCACATTCTG CACCCAGTGG CCCACCCTCC TGGAGGCGCT GGCCACCCGA ATGGATGACG CGCCCTACCT   
  
  
+ GAGGCTGACC ACTGTGGTGG TCAACAAGTC CGGGAATGAA GGTCCCACCG GAGGCGGGTC CCACAGGGTG   
  
  
+ ATGAAAGAGA TTGGGACCCG CCTCGAGAAG TTTGCTAGGC TAATGGGAGT GCCTTTCAAA TTTAATGTGG   
  
  
+ TCCACCACGG GGCTGATTTA TCCGACTTGG ATTTCTCTCA GCTGGATATT AACGATGACG AGGCATTAGC   
  
  
+ CATCAACTGT GTCAACTCGC TCCACTCGGT CAATATCCAC CGCCGTGACT CGGTCATCTC GGCCTTCCGG   
  
  
+ CGGCTCCACC CTAGGGTGGT GACCGTGGTG GAGGAGGAGG CTGACCTTGC TGACGTGGGG GCGGAGGGGT   
  
  
+ ACGAGTTCTA CAGGGGGTTT AATGAGTGTT TAAGGTGGGT TAGGGTTTAC TTTGAGGCCC TGGAAGAGTG   
  
  
+ CTTTCCTAGG ACAAGCAATG AGAAACTCAT GCTCGAGCGG GCCGCAGGGC GGGCTCTGGT GGACCTCCTG   
  
  
+ GCCTGCCCCA AGCCCGCATC ATCCGAGCGG AGGGAGGCAG CTGCGCGGTG GTCCGGGAGG ATGCAGGGGG   
  
  
+ CCGGGTTCGG CCACGTGGGG TTCAGTGATG AGGTCTGTGA TGACGTCAAG GCCCTCCTAA GGAGGTACAA   
  
  
+ GGAGGGGTGG TCCATGATAC AATGTTCCGC CGCGGATGGC GGCGCCTCCG CCGGAATATT CTTGTCGTGG   
  
  
+ AGGGATCAGC CGGTGGTGTG GGCCAGTGCA TGGAAGCCTT A  

- -Up\_Stream \_Len000CCCAAA AAAATTTGAG TTTTAATCAA TAACATAGTT AACTTAAACC TTTTGATAAT   
  
  
- AAAAGCTGAA TAATATCTCT TAAACAACAA TTACTTACGA CTATTTATGA CTATTAATCA CAACACTCAC   
  
  
- ACTTAAACAC TACTACTCGT TACTAATCGC AATTTTAATA TTGATCCTAT TTATTAATTC ACAACTACTT   
  
  
- ACAACTAACT ACTACTACTC ATGACTAACC ACGCCACGAC CATTATTTAT AAATACTATT CAATTTTTTT   
  
  
- TTTTTGTGTA GGATTTGTTA GTGATACATT ATATGATTTA CTTTTTTTAA CCATTACACT AAACTGTTAG   
  
  
- ATTCCCATTC CCACAAAATG GGTGTGTACA ATACTTTAAC CGAATGATAA ATGTTTGAAA AATGTCAATA   
  
  
- TACTAAGATG GATACACAAT AGTAGAAAGG CAGCTACATT AACTAATATA AGTTGTGTTA AATTAAGTAA   
  
  
- TGTTACATCG ATTAACTATG AGTTTTATTA CTTGATAATT ATTTATTAAT TAATATTGAA AATTTTCAAA   
  
  
- CAATTAAATT TTAATATATT ATATATTTAA ATCCGATGTA ATTAGTTTTT TAATCCGCAC AAGTAGCCAG   
  
  
- GTTTGAACTT GGCCTGGTCT GGCCTGGCTT CTAGCTAATT CTCTTTTTCA TGTCTGACTC CTGGCCTAGC   
  
  
- TTATTCTCAG CGTGGCCTGG TCCTAGCCTG GCCTTTTTAA ACCTGGTCTA GCCCTGGCTA GCTCCATCTG   
  
  
- GCTTAAATAA AAGTTTATGC TATCCTTATT TTGAATAACT AAGTTAACTA AAACCGGTTT TAGTACGATA   
  
  
- ATAACTAAAA TAAACGATTG AGAGTTCTCA TAATCAAACG TGAAGCGTAA TAGAAACCGG TTTTTTTAGT   
  
  
- TTTTCTTTCA GAGTTTGAGA ATTATCAAAA AATAAAATAA ACCAAATAAA AATTTTATCA GCCGGAAGCC   
  
  
- AGGCCTGATC ACCTGGCTTA TTCCATTAAA AAGCTTAGCT CCTGGTCTGG TTTACGTGAG TCAAGCTTGG   
  
  
- CCAAGTCAAG CCAGACTGAG CTAAAAACCA GGCCAGGTTC AAACTTGTCT TATTACATGT GTGGATCTTT   
  
  
- TTTTCTTAAT ACGTATATAT GAATTCCGGG GGTTGGGAAA AAGGGAAAGG CGGTTATGAA AAATGGTTCA   
  
  
- GGTTAAGAAA AGAAGAATCG ATACCGATCA TCAATAGGGA GGGATCGAAG GTGGGATTCA AAACTCTGTT   
  
  
- TTAGGGTAGA TACAATTATA AATAAAGAAC GTGTACAGTG GGTTATGTTC CCAAAGTAAC TTTTCTTTTC   
  
  
- CATAATCTAC ATTCTATACA TACATATATT TATCAAAACG TTATCTGATC TTCCTGATTT TTCCTTTTTC   
  
  
- CTTCAAATTT TCGTTTTTTG AAAGGGGAGA TCTTTTTTCC TTTGGTCTAA GGGAAAAGAT GGGGTTAAGG   
  
  
- GTATCAGAAA TGAGACAGAG AGAGAGAGAC AGAGAGAATG AGTACTACAA CTCCCTCCTT CCTTACTTAA   
  
  
- TTCCTGGGAT TCGATTCTAG GTTGGCATTC TATAACGGGG AAATTAGTGG GTAAAGTATA GAGATAGGTG   
  
  
- TCAATTTTTG GGTGGAGGAG GTAGGTGTAT TGACTATATA GAGAAGATGT GGTATGATAT TCCTTCTTCT   
  
  
- CTTTTTATCC TCACATAACA TAAAAATAAA CTCATTAAAT AAAATTCCTC TCTTTAAATG TACCAACCCA   
  
  
- ATTTACCTAT GGAACAAATC TGATCACTCA AAGGTTGTTG GTGTGGTGGT GGTAGTGGTT GTGGTTGTGG   
  
  
- TTGTGGTGGT TGTCGTTAGA AGACTAGTTA GGAGAAACTT ATTATGATGA TGAATAAGGT GTTTAAGTTC   
  
  
- GTCAAGGTCT AGAAGGGTTG TTTGGGTAGT ATTAGTATTA GTATGAGTCA TAATAGTGGT TGTAGTAGGA   
  
  
- TGAATGGGGG TGGCGGTGGT AGTCCTCCTT CTTCTTCTTA CGAAGTTGAA AATGTACCTA CTTCTTTTGA   
  
  
- AGAGAAGAAG AAGGAGGGTG TTGTTTGTAG TAGGAGTTGG AGGAAGGAGG CGGTGGTGGA GGAGCATAAT   
  
  
- GGGAATGATG AGGTGGGGGA AGCTACAAGG GAGATGGTGG TGGTGGAGGT GGAGGTGGGG GTGCGGGTGG   
  
  
- TTGTGGTGGT GGTGGTGGGG GGGTTGGGTC ATACCGCCGC CGCCGCCACC CAAACTGAGG TCGACGAAGA   
  
  
- GGATGAGGGG ACGGGCTCTG GAGTTGGGCC TCGAGAAGAG GAGCAGGCCC AAGAGCCCGT TCACCCGAAG   
  
  
- GTTGTAGGAG AACCTTCGGC GAGCACGGTA GAGACTCTCC TTGTGCTCGG CGGAGGTGGT CGAGGATACC   
  
  
- TACCAGTTGC TCGAGTCGAG GGGCATGCCA CTGCACCTCG TCTTCTATCG AAGTATGAAG CCGGTTCGGG   
  
  
- AGAAGTGGGC GTACTGGCTC AGTCCCCTGG CGTGGATGGC GTGGTACGCC AGGCGGCACC TGTTCTGGAA   
  
  
- GAGGAAACTC AGATGCTCTT TCTACCATGA GTTCAAGGTC CTCCAATCGG GAACCTGGTG GAAACCTGTG   
  
  
- CACCGTCGAT TACCTCGGGA GTAACTACGG AAACTGCCAC TTAGGTTTTA GGTGTAGTAG CTGTAGTCGT   
  
  
- GGTGTAAGAC GTGGGTCACC GGGTGGGAGG ACCTCCGCGA CCGGTGGGCT TACCTACTGC GCGGGATGGA   
  
  
- CTCCGACTGG TGACACCACC AGTTGTTCAG GCCCTTACTT CCAGGGTGGC CTCCGCCCAG GGTGTCCCAC   
  
  
- TACTTTCTCT AACCCTGGGC GGAGCTCTTC AAACGATCCG ATTACCCTCA CGGAAAGTTT AAATTACACC   
  
  
- AGGTGGTGCC CCGACTAAAT AGGCTGAACC TAAAGAGAGT CGACCTATAA TTGCTACTGC TCCGTAATCG   
  
  
- GTAGTTGACA CAGTTGAGCG AGGTGAGCCA GTTATAGGTG GCGGCACTGA GCCAGTAGAG CCGGAAGGCC   
  
  
- GCCGAGGTGG GATCCCACCA CTGGCACCAC CTCCTCCTCC GACTGGAACG ACTGCACCCC CGCCTCCCCA   
  
  
- TGCTCAAGAT GTCCCCCAAA TTACTCACAA ATTCCACCCA ATCCCAAATG AAACTCCGGG ACCTTCTCAC   
  
  
- GAAAGGATCC TGTTCGTTAC TCTTTGAGTA CGAGCTCGCC CGGCGTCCCG CCCGAGACCA CCTGGAGGAC   
  
  
- CGGACGGGGT TCGGGCGTAG TAGGCTCGCC TCCCTCCGTC GACGCGCCAC CAGGCCCTCC TACGTCCCCC   
  
  
- GGCCCAAGCC GGTGCACCCC AAGTCACTAC TCCAGACACT ACTGCAGTTC CGGGAGGATT CCTCCATGTT   
  
  
- CCTCCCCACC AGGTACTATG TTACAAGGCG GCGCCTACCG CCGCGGAGGC GGCCTTATAA GAACAGCACC   
  
  
- TCCCTAGTCG GCCACCACAC CCGGTCACGT ACCTTCGGAA T

+     TCA-element

| Site Name | Organism | Position | Strand | Matrix score. | sequence | function |
| --- | --- | --- | --- | --- | --- | --- |
| TCA-element | Nicotiana tabacum | 2541 | - | 9 | CCATCTTTTT | cis-acting element involved in salicylic acid responsiveness |

>HU06G00358.1   
+ -Up\_Stream \_Len000GGGTTT TTTTAAACTC AAAATTAGTT ATTGTATCAA TTGAATTTGG AAAACTATTA   
  
  
+ TTTTCGACTT ATTATAGAGA ATTTGTTGTT AATGAATGCT GATAAATACT GATAATTAGT GTTGTGAGTG   
  
  
+ TGAATTTGTG ATGATGAGCA ATGATTAGCG TTAAAATTAT AACTAGGATA AATAATTAAG TGTTGATGAA   
  
  
+ TGTTGATTGA TGATGATGAG TACTGATTGG TGCGGTGCTG GTAATAAATA TTTATGATAA GTTAAAAAAA   
  
  
+ AAAAACACAT CCTAAACAAT CACTATGTAA TATACTAAAT GAAAAAAATT GGTAATGTGA TTTGACAATC   
  
  
+ TAAGGGTAAG GGTGTTTTAC CCACACATGT TATGAAATTG GCTTACTATT TACAAACTTT TTACAGTTAT   
  
  
+ ATGATTCTAC CTATGTGTTA TCATCTTTCC GTCGATGTAA TTGATTATAT TCAACACAAT TTAATTCATT   
  
  
+ ACAATGTAGC TAATTGATAC TCAAAATAAT GAACTATTAA TAAATAATTA ATTATAACTT TTAAAAGTTT   
  
  
+ GTTAATTTAA AATTATATAA TATATAAATT TAGGCTACAT TAATCAAAAA ATTAGGCGTG TTCATCGGTC   
  
  
+ CAAACTTGAA CCGGACCAGA CCGGACCGAA GATCGATTAA GAGAAAAAGT ACAGACTGAG GACCGGATCG   
  
  
+ AATAAGAGTC GCACCGGACC AGGATCGGAC CGGAAAAATT TGGACCAGAT CGGGACCGAT CGAGGTAGAC   
  
  
+ CGAATTTATT TTCAAATACG ATAGGAATAA AACTTATTGA TTCAATTGAT TTTGGCCAAA ATCATGCTAT   
  
  
+ TATTGATTTT ATTTGCTAAC TCTCAAGAGT ATTAGTTTGC ACTTCGCATT ATCTTTGGCC AAAAAAATCA   
  
  
+ AAAAGAAAGT CTCAAACTCT TAATAGTTTT TTATTTTATT TGGTTTATTT TTAAAATAGT CGGCCTTCGG   
  
  
+ TCCGGACTAG TGGACCGAAT AAGGTAATTT TTCGAATCGA GGACCAGACC AAATGCACTC AGTTCGAACC   
  
  
+ GGTTCAGTTC GGTCTGACTC GATTTTTGGT CCGGTCCAAG TTTGAACAGA ATAATGTACA CACCTAGAAA   
  
  
+ AAAAGAATTA TGCATATATA CTTAAGGCCC CCAACCCTTT TTCCCTTTCC GCCAATACTT TTTACCAAGT   
  
  
+ CCAATTCTTT TCTTCTTAGC TATGGCTAGT AGTTATCCCT CCCTAGCTTC CACCCTAAGT TTTGAGACAA   
  
  
+ AATCCCATCT ATGTTAATAT TTATTTCTTG CACATGTCAC CCAATACAAG GGTTTCATTG AAAAGAAAAG   
  
  
+ GTATTAGATG TAAGATATGT ATGTATATAA ATAGTTTTGC AATAGACTAG AAGGACTAAA AAGGAAAAAG   
  
  
+ GAAGTTTAAA AGCAAAAAAC TTTCCCCTCT AGAAAAAAGG AAACCAGATT CCCTTTTCTA CCCCAATTCC   
  
  
+ CATAGTCTTT ACTCTGTCTC TCTCTCTCTG TCTCTCTTAC TCATGATGTT GAGGGAGGAA GGAATGAATT   
  
  
+ AAGGACCCTA AGCTAAGATC CAACCGTAAG ATATTGCCCC TTTAATCACC CATTTCATAT CTCTATCCAC   
  
  
+ AGTTAAAAAC CCACCTCCTC CATCCACATA ACTGATATAT CTCTTCTACA CCATACTATA AGGAAGAAGA   
  
  
+ GAAAAATAGG AGTGTATTGT ATTTTTATTT GAGTAATTTA TTTTAAGGAG AGAAATTTAC ATGGTTGGGT   
  
  
+ TAAATGGATA CCTTGTTTAG ACTAGTGAGT TTCCAACAAC CACACCACCA CCATCACCAA CACCAACACC   
  
  
+ AACACCACCA ACAGCAATCT TCTGATCAAT CCTCTTTGAA TAATACTACT ACTTATTCCA CAAATTCAAG   
  
  
+ CAGTTCCAGA TCTTCCCAAC AAACCCATCA TAATCATAAT CATACTCAGT ATTATCACCA ACATCATCCT   
  
  
+ ACTTACCCCC ACCGCCACCA TCAGGAGGAA GAAGAAGAAT GCTTCAACTT TTACATGGAT GAAGAAAACT   
  
  
+ TCTCTTCTTC TTCCTCCCAC AACAAACATC ATCCTCAACC TCCTTCCTCC GCCACCACCT CCTCGTATTA   
  
  
+ CCCTTACTAC TCCACCCCCT TCGATGTTCC CTCTACCACC ACCACCTCCA CCTCCACCCC CACGCCCACC   
  
  
+ AACACCACCA CCACCACCCC CCCAACCCAG TATGGCGGCG GCGGCGGTGG GTTTGACTCC AGCTGCTTCT   
  
  
+ CCTACTCCCC TGCCCGAGAC CTCAACCCGG AGCTCTTCTC CTCGTCCGGG TTCTCGGGCA AGTGGGCTTC   
  
  
+ CAACATCCTC TTGGAAGCCG CTCGTGCCAT CTCTGAGAGG AACACGAGCC GCCTCCACCA GCTCCTATGG   
  
  
+ ATGGTCAACG AGCTCAGCTC CCCGTACGGT GACGTGGAGC AGAAGATAGC TTCATACTTC GGCCAAGCCC   
  
  
+ TCTTCACCCG CATGACCGAG TCAGGGGACC GCACCTACCG CACCATGCGG TCCGCCGTGG ACAAGACCTT   
  
  
+ CTCCTTTGAG TCTACGAGAA AGATGGTACT CAAGTTCCAG GAGGTTAGCC CTTGGACCAC CTTTGGACAC   
  
  
+ GTGGCAGCTA ATGGAGCCCT CATTGATGCC TTTGACGGTG AATCCAAAAT CCACATCATC GACATCAGCA   
  
  
+ CCACATTCTG CACCCAGTGG CCCACCCTCC TGGAGGCGCT GGCCACCCGA ATGGATGACG CGCCCTACCT   
  
  
+ GAGGCTGACC ACTGTGGTGG TCAACAAGTC CGGGAATGAA GGTCCCACCG GAGGCGGGTC CCACAGGGTG   
  
  
+ ATGAAAGAGA TTGGGACCCG CCTCGAGAAG TTTGCTAGGC TAATGGGAGT GCCTTTCAAA TTTAATGTGG   
  
  
+ TCCACCACGG GGCTGATTTA TCCGACTTGG ATTTCTCTCA GCTGGATATT AACGATGACG AGGCATTAGC   
  
  
+ CATCAACTGT GTCAACTCGC TCCACTCGGT CAATATCCAC CGCCGTGACT CGGTCATCTC GGCCTTCCGG   
  
  
+ CGGCTCCACC CTAGGGTGGT GACCGTGGTG GAGGAGGAGG CTGACCTTGC TGACGTGGGG GCGGAGGGGT   
  
  
+ ACGAGTTCTA CAGGGGGTTT AATGAGTGTT TAAGGTGGGT TAGGGTTTAC TTTGAGGCCC TGGAAGAGTG   
  
  
+ CTTTCCTAGG ACAAGCAATG AGAAACTCAT GCTCGAGCGG GCCGCAGGGC GGGCTCTGGT GGACCTCCTG   
  
  
+ GCCTGCCCCA AGCCCGCATC ATCCGAGCGG AGGGAGGCAG CTGCGCGGTG GTCCGGGAGG ATGCAGGGGG   
  
  
+ CCGGGTTCGG CCACGTGGGG TTCAGTGATG AGGTCTGTGA TGACGTCAAG GCCCTCCTAA GGAGGTACAA   
  
  
+ GGAGGGGTGG TCCATGATAC AATGTTCCGC CGCGGATGGC GGCGCCTCCG CCGGAATATT CTTGTCGTGG   
  
  
+ AGGGATCAGC CGGTGGTGTG GGCCAGTGCA TGGAAGCCTT A  

- -Up\_Stream \_Len000CCCAAA AAAATTTGAG TTTTAATCAA TAACATAGTT AACTTAAACC TTTTGATAAT   
  
  
- AAAAGCTGAA TAATATCTCT TAAACAACAA TTACTTACGA CTATTTATGA CTATTAATCA CAACACTCAC   
  
  
- ACTTAAACAC TACTACTCGT TACTAATCGC AATTTTAATA TTGATCCTAT TTATTAATTC ACAACTACTT   
  
  
- ACAACTAACT ACTACTACTC ATGACTAACC ACGCCACGAC CATTATTTAT AAATACTATT CAATTTTTTT   
  
  
- TTTTTGTGTA GGATTTGTTA GTGATACATT ATATGATTTA CTTTTTTTAA CCATTACACT AAACTGTTAG   
  
  
- ATTCCCATTC CCACAAAATG GGTGTGTACA ATACTTTAAC CGAATGATAA ATGTTTGAAA AATGTCAATA   
  
  
- TACTAAGATG GATACACAAT AGTAGAAAGG CAGCTACATT AACTAATATA AGTTGTGTTA AATTAAGTAA   
  
  
- TGTTACATCG ATTAACTATG AGTTTTATTA CTTGATAATT ATTTATTAAT TAATATTGAA AATTTTCAAA   
  
  
- CAATTAAATT TTAATATATT ATATATTTAA ATCCGATGTA ATTAGTTTTT TAATCCGCAC AAGTAGCCAG   
  
  
- GTTTGAACTT GGCCTGGTCT GGCCTGGCTT CTAGCTAATT CTCTTTTTCA TGTCTGACTC CTGGCCTAGC   
  
  
- TTATTCTCAG CGTGGCCTGG TCCTAGCCTG GCCTTTTTAA ACCTGGTCTA GCCCTGGCTA GCTCCATCTG   
  
  
- GCTTAAATAA AAGTTTATGC TATCCTTATT TTGAATAACT AAGTTAACTA AAACCGGTTT TAGTACGATA   
  
  
- ATAACTAAAA TAAACGATTG AGAGTTCTCA TAATCAAACG TGAAGCGTAA TAGAAACCGG TTTTTTTAGT   
  
  
- TTTTCTTTCA GAGTTTGAGA ATTATCAAAA AATAAAATAA ACCAAATAAA AATTTTATCA GCCGGAAGCC   
  
  
- AGGCCTGATC ACCTGGCTTA TTCCATTAAA AAGCTTAGCT CCTGGTCTGG TTTACGTGAG TCAAGCTTGG   
  
  
- CCAAGTCAAG CCAGACTGAG CTAAAAACCA GGCCAGGTTC AAACTTGTCT TATTACATGT GTGGATCTTT   
  
  
- TTTTCTTAAT ACGTATATAT GAATTCCGGG GGTTGGGAAA AAGGGAAAGG CGGTTATGAA AAATGGTTCA   
  
  
- GGTTAAGAAA AGAAGAATCG ATACCGATCA TCAATAGGGA GGGATCGAAG GTGGGATTCA AAACTCTGTT   
  
  
- TTAGGGTAGA TACAATTATA AATAAAGAAC GTGTACAGTG GGTTATGTTC CCAAAGTAAC TTTTCTTTTC   
  
  
- CATAATCTAC ATTCTATACA TACATATATT TATCAAAACG TTATCTGATC TTCCTGATTT TTCCTTTTTC   
  
  
- CTTCAAATTT TCGTTTTTTG AAAGGGGAGA TCTTTTTTCC TTTGGTCTAA GGGAAAAGAT GGGGTTAAGG   
  
  
- GTATCAGAAA TGAGACAGAG AGAGAGAGAC AGAGAGAATG AGTACTACAA CTCCCTCCTT CCTTACTTAA   
  
  
- TTCCTGGGAT TCGATTCTAG GTTGGCATTC TATAACGGGG AAATTAGTGG GTAAAGTATA GAGATAGGTG   
  
  
- TCAATTTTTG GGTGGAGGAG GTAGGTGTAT TGACTATATA GAGAAGATGT GGTATGATAT TCCTTCTTCT   
  
  
- CTTTTTATCC TCACATAACA TAAAAATAAA CTCATTAAAT AAAATTCCTC TCTTTAAATG TACCAACCCA   
  
  
- ATTTACCTAT GGAACAAATC TGATCACTCA AAGGTTGTTG GTGTGGTGGT GGTAGTGGTT GTGGTTGTGG   
  
  
- TTGTGGTGGT TGTCGTTAGA AGACTAGTTA GGAGAAACTT ATTATGATGA TGAATAAGGT GTTTAAGTTC   
  
  
- GTCAAGGTCT AGAAGGGTTG TTTGGGTAGT ATTAGTATTA GTATGAGTCA TAATAGTGGT TGTAGTAGGA   
  
  
- TGAATGGGGG TGGCGGTGGT AGTCCTCCTT CTTCTTCTTA CGAAGTTGAA AATGTACCTA CTTCTTTTGA   
  
  
- AGAGAAGAAG AAGGAGGGTG TTGTTTGTAG TAGGAGTTGG AGGAAGGAGG CGGTGGTGGA GGAGCATAAT   
  
  
- GGGAATGATG AGGTGGGGGA AGCTACAAGG GAGATGGTGG TGGTGGAGGT GGAGGTGGGG GTGCGGGTGG   
  
  
- TTGTGGTGGT GGTGGTGGGG GGGTTGGGTC ATACCGCCGC CGCCGCCACC CAAACTGAGG TCGACGAAGA   
  
  
- GGATGAGGGG ACGGGCTCTG GAGTTGGGCC TCGAGAAGAG GAGCAGGCCC AAGAGCCCGT TCACCCGAAG   
  
  
- GTTGTAGGAG AACCTTCGGC GAGCACGGTA GAGACTCTCC TTGTGCTCGG CGGAGGTGGT CGAGGATACC   
  
  
- TACCAGTTGC TCGAGTCGAG GGGCATGCCA CTGCACCTCG TCTTCTATCG AAGTATGAAG CCGGTTCGGG   
  
  
- AGAAGTGGGC GTACTGGCTC AGTCCCCTGG CGTGGATGGC GTGGTACGCC AGGCGGCACC TGTTCTGGAA   
  
  
- GAGGAAACTC AGATGCTCTT TCTACCATGA GTTCAAGGTC CTCCAATCGG GAACCTGGTG GAAACCTGTG   
  
  
- CACCGTCGAT TACCTCGGGA GTAACTACGG AAACTGCCAC TTAGGTTTTA GGTGTAGTAG CTGTAGTCGT   
  
  
- GGTGTAAGAC GTGGGTCACC GGGTGGGAGG ACCTCCGCGA CCGGTGGGCT TACCTACTGC GCGGGATGGA   
  
  
- CTCCGACTGG TGACACCACC AGTTGTTCAG GCCCTTACTT CCAGGGTGGC CTCCGCCCAG GGTGTCCCAC   
  
  
- TACTTTCTCT AACCCTGGGC GGAGCTCTTC AAACGATCCG ATTACCCTCA CGGAAAGTTT AAATTACACC   
  
  
- AGGTGGTGCC CCGACTAAAT AGGCTGAACC TAAAGAGAGT CGACCTATAA TTGCTACTGC TCCGTAATCG   
  
  
- GTAGTTGACA CAGTTGAGCG AGGTGAGCCA GTTATAGGTG GCGGCACTGA GCCAGTAGAG CCGGAAGGCC   
  
  
- GCCGAGGTGG GATCCCACCA CTGGCACCAC CTCCTCCTCC GACTGGAACG ACTGCACCCC CGCCTCCCCA   
  
  
- TGCTCAAGAT GTCCCCCAAA TTACTCACAA ATTCCACCCA ATCCCAAATG AAACTCCGGG ACCTTCTCAC   
  
  
- GAAAGGATCC TGTTCGTTAC TCTTTGAGTA CGAGCTCGCC CGGCGTCCCG CCCGAGACCA CCTGGAGGAC   
  
  
- CGGACGGGGT TCGGGCGTAG TAGGCTCGCC TCCCTCCGTC GACGCGCCAC CAGGCCCTCC TACGTCCCCC   
  
  
- GGCCCAAGCC GGTGCACCCC AAGTCACTAC TCCAGACACT ACTGCAGTTC CGGGAGGATT CCTCCATGTT   
  
  
- CCTCCCCACC AGGTACTATG TTACAAGGCG GCGCCTACCG CCGCGGAGGC GGCCTTATAA GAACAGCACC   
  
  
- TCCCTAGTCG GCCACCACAC CCGGTCACGT ACCTTCGGAA T

+     TCT-motif

| Site Name | Organism | Position | Strand | Matrix score. | sequence | function |
| --- | --- | --- | --- | --- | --- | --- |
| TCT-motif | Arabidopsis thaliana | 1570 | - | 6 | TCTTAC | part of a light responsive element |
| TCT-motif | Arabidopsis thaliana | 1509 | + | 6 | TCTTAC | part of a light responsive element |
| TCT-motif | Arabidopsis thaliana | 1344 | - | 6 | TCTTAC | part of a light responsive element |

>HU06G00358.1   
+ -Up\_Stream \_Len000GGGTTT TTTTAAACTC AAAATTAGTT ATTGTATCAA TTGAATTTGG AAAACTATTA   
  
  
+ TTTTCGACTT ATTATAGAGA ATTTGTTGTT AATGAATGCT GATAAATACT GATAATTAGT GTTGTGAGTG   
  
  
+ TGAATTTGTG ATGATGAGCA ATGATTAGCG TTAAAATTAT AACTAGGATA AATAATTAAG TGTTGATGAA   
  
  
+ TGTTGATTGA TGATGATGAG TACTGATTGG TGCGGTGCTG GTAATAAATA TTTATGATAA GTTAAAAAAA   
  
  
+ AAAAACACAT CCTAAACAAT CACTATGTAA TATACTAAAT GAAAAAAATT GGTAATGTGA TTTGACAATC   
  
  
+ TAAGGGTAAG GGTGTTTTAC CCACACATGT TATGAAATTG GCTTACTATT TACAAACTTT TTACAGTTAT   
  
  
+ ATGATTCTAC CTATGTGTTA TCATCTTTCC GTCGATGTAA TTGATTATAT TCAACACAAT TTAATTCATT   
  
  
+ ACAATGTAGC TAATTGATAC TCAAAATAAT GAACTATTAA TAAATAATTA ATTATAACTT TTAAAAGTTT   
  
  
+ GTTAATTTAA AATTATATAA TATATAAATT TAGGCTACAT TAATCAAAAA ATTAGGCGTG TTCATCGGTC   
  
  
+ CAAACTTGAA CCGGACCAGA CCGGACCGAA GATCGATTAA GAGAAAAAGT ACAGACTGAG GACCGGATCG   
  
  
+ AATAAGAGTC GCACCGGACC AGGATCGGAC CGGAAAAATT TGGACCAGAT CGGGACCGAT CGAGGTAGAC   
  
  
+ CGAATTTATT TTCAAATACG ATAGGAATAA AACTTATTGA TTCAATTGAT TTTGGCCAAA ATCATGCTAT   
  
  
+ TATTGATTTT ATTTGCTAAC TCTCAAGAGT ATTAGTTTGC ACTTCGCATT ATCTTTGGCC AAAAAAATCA   
  
  
+ AAAAGAAAGT CTCAAACTCT TAATAGTTTT TTATTTTATT TGGTTTATTT TTAAAATAGT CGGCCTTCGG   
  
  
+ TCCGGACTAG TGGACCGAAT AAGGTAATTT TTCGAATCGA GGACCAGACC AAATGCACTC AGTTCGAACC   
  
  
+ GGTTCAGTTC GGTCTGACTC GATTTTTGGT CCGGTCCAAG TTTGAACAGA ATAATGTACA CACCTAGAAA   
  
  
+ AAAAGAATTA TGCATATATA CTTAAGGCCC CCAACCCTTT TTCCCTTTCC GCCAATACTT TTTACCAAGT   
  
  
+ CCAATTCTTT TCTTCTTAGC TATGGCTAGT AGTTATCCCT CCCTAGCTTC CACCCTAAGT TTTGAGACAA   
  
  
+ AATCCCATCT ATGTTAATAT TTATTTCTTG CACATGTCAC CCAATACAAG GGTTTCATTG AAAAGAAAAG   
  
  
+ GTATTAGATG TAAGATATGT ATGTATATAA ATAGTTTTGC AATAGACTAG AAGGACTAAA AAGGAAAAAG   
  
  
+ GAAGTTTAAA AGCAAAAAAC TTTCCCCTCT AGAAAAAAGG AAACCAGATT CCCTTTTCTA CCCCAATTCC   
  
  
+ CATAGTCTTT ACTCTGTCTC TCTCTCTCTG TCTCTCTTAC TCATGATGTT GAGGGAGGAA GGAATGAATT   
  
  
+ AAGGACCCTA AGCTAAGATC CAACCGTAAG ATATTGCCCC TTTAATCACC CATTTCATAT CTCTATCCAC   
  
  
+ AGTTAAAAAC CCACCTCCTC CATCCACATA ACTGATATAT CTCTTCTACA CCATACTATA AGGAAGAAGA   
  
  
+ GAAAAATAGG AGTGTATTGT ATTTTTATTT GAGTAATTTA TTTTAAGGAG AGAAATTTAC ATGGTTGGGT   
  
  
+ TAAATGGATA CCTTGTTTAG ACTAGTGAGT TTCCAACAAC CACACCACCA CCATCACCAA CACCAACACC   
  
  
+ AACACCACCA ACAGCAATCT TCTGATCAAT CCTCTTTGAA TAATACTACT ACTTATTCCA CAAATTCAAG   
  
  
+ CAGTTCCAGA TCTTCCCAAC AAACCCATCA TAATCATAAT CATACTCAGT ATTATCACCA ACATCATCCT   
  
  
+ ACTTACCCCC ACCGCCACCA TCAGGAGGAA GAAGAAGAAT GCTTCAACTT TTACATGGAT GAAGAAAACT   
  
  
+ TCTCTTCTTC TTCCTCCCAC AACAAACATC ATCCTCAACC TCCTTCCTCC GCCACCACCT CCTCGTATTA   
  
  
+ CCCTTACTAC TCCACCCCCT TCGATGTTCC CTCTACCACC ACCACCTCCA CCTCCACCCC CACGCCCACC   
  
  
+ AACACCACCA CCACCACCCC CCCAACCCAG TATGGCGGCG GCGGCGGTGG GTTTGACTCC AGCTGCTTCT   
  
  
+ CCTACTCCCC TGCCCGAGAC CTCAACCCGG AGCTCTTCTC CTCGTCCGGG TTCTCGGGCA AGTGGGCTTC   
  
  
+ CAACATCCTC TTGGAAGCCG CTCGTGCCAT CTCTGAGAGG AACACGAGCC GCCTCCACCA GCTCCTATGG   
  
  
+ ATGGTCAACG AGCTCAGCTC CCCGTACGGT GACGTGGAGC AGAAGATAGC TTCATACTTC GGCCAAGCCC   
  
  
+ TCTTCACCCG CATGACCGAG TCAGGGGACC GCACCTACCG CACCATGCGG TCCGCCGTGG ACAAGACCTT   
  
  
+ CTCCTTTGAG TCTACGAGAA AGATGGTACT CAAGTTCCAG GAGGTTAGCC CTTGGACCAC CTTTGGACAC   
  
  
+ GTGGCAGCTA ATGGAGCCCT CATTGATGCC TTTGACGGTG AATCCAAAAT CCACATCATC GACATCAGCA   
  
  
+ CCACATTCTG CACCCAGTGG CCCACCCTCC TGGAGGCGCT GGCCACCCGA ATGGATGACG CGCCCTACCT   
  
  
+ GAGGCTGACC ACTGTGGTGG TCAACAAGTC CGGGAATGAA GGTCCCACCG GAGGCGGGTC CCACAGGGTG   
  
  
+ ATGAAAGAGA TTGGGACCCG CCTCGAGAAG TTTGCTAGGC TAATGGGAGT GCCTTTCAAA TTTAATGTGG   
  
  
+ TCCACCACGG GGCTGATTTA TCCGACTTGG ATTTCTCTCA GCTGGATATT AACGATGACG AGGCATTAGC   
  
  
+ CATCAACTGT GTCAACTCGC TCCACTCGGT CAATATCCAC CGCCGTGACT CGGTCATCTC GGCCTTCCGG   
  
  
+ CGGCTCCACC CTAGGGTGGT GACCGTGGTG GAGGAGGAGG CTGACCTTGC TGACGTGGGG GCGGAGGGGT   
  
  
+ ACGAGTTCTA CAGGGGGTTT AATGAGTGTT TAAGGTGGGT TAGGGTTTAC TTTGAGGCCC TGGAAGAGTG   
  
  
+ CTTTCCTAGG ACAAGCAATG AGAAACTCAT GCTCGAGCGG GCCGCAGGGC GGGCTCTGGT GGACCTCCTG   
  
  
+ GCCTGCCCCA AGCCCGCATC ATCCGAGCGG AGGGAGGCAG CTGCGCGGTG GTCCGGGAGG ATGCAGGGGG   
  
  
+ CCGGGTTCGG CCACGTGGGG TTCAGTGATG AGGTCTGTGA TGACGTCAAG GCCCTCCTAA GGAGGTACAA   
  
  
+ GGAGGGGTGG TCCATGATAC AATGTTCCGC CGCGGATGGC GGCGCCTCCG CCGGAATATT CTTGTCGTGG   
  
  
+ AGGGATCAGC CGGTGGTGTG GGCCAGTGCA TGGAAGCCTT A  

- -Up\_Stream \_Len000CCCAAA AAAATTTGAG TTTTAATCAA TAACATAGTT AACTTAAACC TTTTGATAAT   
  
  
- AAAAGCTGAA TAATATCTCT TAAACAACAA TTACTTACGA CTATTTATGA CTATTAATCA CAACACTCAC   
  
  
- ACTTAAACAC TACTACTCGT TACTAATCGC AATTTTAATA TTGATCCTAT TTATTAATTC ACAACTACTT   
  
  
- ACAACTAACT ACTACTACTC ATGACTAACC ACGCCACGAC CATTATTTAT AAATACTATT CAATTTTTTT   
  
  
- TTTTTGTGTA GGATTTGTTA GTGATACATT ATATGATTTA CTTTTTTTAA CCATTACACT AAACTGTTAG   
  
  
- ATTCCCATTC CCACAAAATG GGTGTGTACA ATACTTTAAC CGAATGATAA ATGTTTGAAA AATGTCAATA   
  
  
- TACTAAGATG GATACACAAT AGTAGAAAGG CAGCTACATT AACTAATATA AGTTGTGTTA AATTAAGTAA   
  
  
- TGTTACATCG ATTAACTATG AGTTTTATTA CTTGATAATT ATTTATTAAT TAATATTGAA AATTTTCAAA   
  
  
- CAATTAAATT TTAATATATT ATATATTTAA ATCCGATGTA ATTAGTTTTT TAATCCGCAC AAGTAGCCAG   
  
  
- GTTTGAACTT GGCCTGGTCT GGCCTGGCTT CTAGCTAATT CTCTTTTTCA TGTCTGACTC CTGGCCTAGC   
  
  
- TTATTCTCAG CGTGGCCTGG TCCTAGCCTG GCCTTTTTAA ACCTGGTCTA GCCCTGGCTA GCTCCATCTG   
  
  
- GCTTAAATAA AAGTTTATGC TATCCTTATT TTGAATAACT AAGTTAACTA AAACCGGTTT TAGTACGATA   
  
  
- ATAACTAAAA TAAACGATTG AGAGTTCTCA TAATCAAACG TGAAGCGTAA TAGAAACCGG TTTTTTTAGT   
  
  
- TTTTCTTTCA GAGTTTGAGA ATTATCAAAA AATAAAATAA ACCAAATAAA AATTTTATCA GCCGGAAGCC   
  
  
- AGGCCTGATC ACCTGGCTTA TTCCATTAAA AAGCTTAGCT CCTGGTCTGG TTTACGTGAG TCAAGCTTGG   
  
  
- CCAAGTCAAG CCAGACTGAG CTAAAAACCA GGCCAGGTTC AAACTTGTCT TATTACATGT GTGGATCTTT   
  
  
- TTTTCTTAAT ACGTATATAT GAATTCCGGG GGTTGGGAAA AAGGGAAAGG CGGTTATGAA AAATGGTTCA   
  
  
- GGTTAAGAAA AGAAGAATCG ATACCGATCA TCAATAGGGA GGGATCGAAG GTGGGATTCA AAACTCTGTT   
  
  
- TTAGGGTAGA TACAATTATA AATAAAGAAC GTGTACAGTG GGTTATGTTC CCAAAGTAAC TTTTCTTTTC   
  
  
- CATAATCTAC ATTCTATACA TACATATATT TATCAAAACG TTATCTGATC TTCCTGATTT TTCCTTTTTC   
  
  
- CTTCAAATTT TCGTTTTTTG AAAGGGGAGA TCTTTTTTCC TTTGGTCTAA GGGAAAAGAT GGGGTTAAGG   
  
  
- GTATCAGAAA TGAGACAGAG AGAGAGAGAC AGAGAGAATG AGTACTACAA CTCCCTCCTT CCTTACTTAA   
  
  
- TTCCTGGGAT TCGATTCTAG GTTGGCATTC TATAACGGGG AAATTAGTGG GTAAAGTATA GAGATAGGTG   
  
  
- TCAATTTTTG GGTGGAGGAG GTAGGTGTAT TGACTATATA GAGAAGATGT GGTATGATAT TCCTTCTTCT   
  
  
- CTTTTTATCC TCACATAACA TAAAAATAAA CTCATTAAAT AAAATTCCTC TCTTTAAATG TACCAACCCA   
  
  
- ATTTACCTAT GGAACAAATC TGATCACTCA AAGGTTGTTG GTGTGGTGGT GGTAGTGGTT GTGGTTGTGG   
  
  
- TTGTGGTGGT TGTCGTTAGA AGACTAGTTA GGAGAAACTT ATTATGATGA TGAATAAGGT GTTTAAGTTC   
  
  
- GTCAAGGTCT AGAAGGGTTG TTTGGGTAGT ATTAGTATTA GTATGAGTCA TAATAGTGGT TGTAGTAGGA   
  
  
- TGAATGGGGG TGGCGGTGGT AGTCCTCCTT CTTCTTCTTA CGAAGTTGAA AATGTACCTA CTTCTTTTGA   
  
  
- AGAGAAGAAG AAGGAGGGTG TTGTTTGTAG TAGGAGTTGG AGGAAGGAGG CGGTGGTGGA GGAGCATAAT   
  
  
- GGGAATGATG AGGTGGGGGA AGCTACAAGG GAGATGGTGG TGGTGGAGGT GGAGGTGGGG GTGCGGGTGG   
  
  
- TTGTGGTGGT GGTGGTGGGG GGGTTGGGTC ATACCGCCGC CGCCGCCACC CAAACTGAGG TCGACGAAGA   
  
  
- GGATGAGGGG ACGGGCTCTG GAGTTGGGCC TCGAGAAGAG GAGCAGGCCC AAGAGCCCGT TCACCCGAAG   
  
  
- GTTGTAGGAG AACCTTCGGC GAGCACGGTA GAGACTCTCC TTGTGCTCGG CGGAGGTGGT CGAGGATACC   
  
  
- TACCAGTTGC TCGAGTCGAG GGGCATGCCA CTGCACCTCG TCTTCTATCG AAGTATGAAG CCGGTTCGGG   
  
  
- AGAAGTGGGC GTACTGGCTC AGTCCCCTGG CGTGGATGGC GTGGTACGCC AGGCGGCACC TGTTCTGGAA   
  
  
- GAGGAAACTC AGATGCTCTT TCTACCATGA GTTCAAGGTC CTCCAATCGG GAACCTGGTG GAAACCTGTG   
  
  
- CACCGTCGAT TACCTCGGGA GTAACTACGG AAACTGCCAC TTAGGTTTTA GGTGTAGTAG CTGTAGTCGT   
  
  
- GGTGTAAGAC GTGGGTCACC GGGTGGGAGG ACCTCCGCGA CCGGTGGGCT TACCTACTGC GCGGGATGGA   
  
  
- CTCCGACTGG TGACACCACC AGTTGTTCAG GCCCTTACTT CCAGGGTGGC CTCCGCCCAG GGTGTCCCAC   
  
  
- TACTTTCTCT AACCCTGGGC GGAGCTCTTC AAACGATCCG ATTACCCTCA CGGAAAGTTT AAATTACACC   
  
  
- AGGTGGTGCC CCGACTAAAT AGGCTGAACC TAAAGAGAGT CGACCTATAA TTGCTACTGC TCCGTAATCG   
  
  
- GTAGTTGACA CAGTTGAGCG AGGTGAGCCA GTTATAGGTG GCGGCACTGA GCCAGTAGAG CCGGAAGGCC   
  
  
- GCCGAGGTGG GATCCCACCA CTGGCACCAC CTCCTCCTCC GACTGGAACG ACTGCACCCC CGCCTCCCCA   
  
  
- TGCTCAAGAT GTCCCCCAAA TTACTCACAA ATTCCACCCA ATCCCAAATG AAACTCCGGG ACCTTCTCAC   
  
  
- GAAAGGATCC TGTTCGTTAC TCTTTGAGTA CGAGCTCGCC CGGCGTCCCG CCCGAGACCA CCTGGAGGAC   
  
  
- CGGACGGGGT TCGGGCGTAG TAGGCTCGCC TCCCTCCGTC GACGCGCCAC CAGGCCCTCC TACGTCCCCC   
  
  
- GGCCCAAGCC GGTGCACCCC AAGTCACTAC TCCAGACACT ACTGCAGTTC CGGGAGGATT CCTCCATGTT   
  
  
- CCTCCCCACC AGGTACTATG TTACAAGGCG GCGCCTACCG CCGCGGAGGC GGCCTTATAA GAACAGCACC   
  
  
- TCCCTAGTCG GCCACCACAC CCGGTCACGT ACCTTCGGAA T

+     TGACG-motif

| Site Name | Organism | Position | Strand | Matrix score. | sequence | function |
| --- | --- | --- | --- | --- | --- | --- |
| TGACG-motif | Hordeum vulgare | 2627 | + | 5 | TGACG | cis-acting regulatory element involved in the MeJA-responsiveness |
| TGACG-motif | Hordeum vulgare | 3338 | - | 5 | TGACG | cis-acting regulatory element involved in the MeJA-responsiveness |
| TGACG-motif | Hordeum vulgare | 2414 | + | 5 | TGACG | cis-acting regulatory element involved in the MeJA-responsiveness |
| TGACG-motif | Hordeum vulgare | 2720 | + | 5 | TGACG | cis-acting regulatory element involved in the MeJA-responsiveness |
| TGACG-motif | Hordeum vulgare | 2930 | + | 5 | TGACG | cis-acting regulatory element involved in the MeJA-responsiveness |
| TGACG-motif | Hordeum vulgare | 3065 | + | 5 | TGACG | cis-acting regulatory element involved in the MeJA-responsiveness |
| TGACG-motif | Hordeum vulgare | 3335 | + | 5 | TGACG | cis-acting regulatory element involved in the MeJA-responsiveness |

>HU06G00358.1   
+ -Up\_Stream \_Len000GGGTTT TTTTAAACTC AAAATTAGTT ATTGTATCAA TTGAATTTGG AAAACTATTA   
  
  
+ TTTTCGACTT ATTATAGAGA ATTTGTTGTT AATGAATGCT GATAAATACT GATAATTAGT GTTGTGAGTG   
  
  
+ TGAATTTGTG ATGATGAGCA ATGATTAGCG TTAAAATTAT AACTAGGATA AATAATTAAG TGTTGATGAA   
  
  
+ TGTTGATTGA TGATGATGAG TACTGATTGG TGCGGTGCTG GTAATAAATA TTTATGATAA GTTAAAAAAA   
  
  
+ AAAAACACAT CCTAAACAAT CACTATGTAA TATACTAAAT GAAAAAAATT GGTAATGTGA TTTGACAATC   
  
  
+ TAAGGGTAAG GGTGTTTTAC CCACACATGT TATGAAATTG GCTTACTATT TACAAACTTT TTACAGTTAT   
  
  
+ ATGATTCTAC CTATGTGTTA TCATCTTTCC GTCGATGTAA TTGATTATAT TCAACACAAT TTAATTCATT   
  
  
+ ACAATGTAGC TAATTGATAC TCAAAATAAT GAACTATTAA TAAATAATTA ATTATAACTT TTAAAAGTTT   
  
  
+ GTTAATTTAA AATTATATAA TATATAAATT TAGGCTACAT TAATCAAAAA ATTAGGCGTG TTCATCGGTC   
  
  
+ CAAACTTGAA CCGGACCAGA CCGGACCGAA GATCGATTAA GAGAAAAAGT ACAGACTGAG GACCGGATCG   
  
  
+ AATAAGAGTC GCACCGGACC AGGATCGGAC CGGAAAAATT TGGACCAGAT CGGGACCGAT CGAGGTAGAC   
  
  
+ CGAATTTATT TTCAAATACG ATAGGAATAA AACTTATTGA TTCAATTGAT TTTGGCCAAA ATCATGCTAT   
  
  
+ TATTGATTTT ATTTGCTAAC TCTCAAGAGT ATTAGTTTGC ACTTCGCATT ATCTTTGGCC AAAAAAATCA   
  
  
+ AAAAGAAAGT CTCAAACTCT TAATAGTTTT TTATTTTATT TGGTTTATTT TTAAAATAGT CGGCCTTCGG   
  
  
+ TCCGGACTAG TGGACCGAAT AAGGTAATTT TTCGAATCGA GGACCAGACC AAATGCACTC AGTTCGAACC   
  
  
+ GGTTCAGTTC GGTCTGACTC GATTTTTGGT CCGGTCCAAG TTTGAACAGA ATAATGTACA CACCTAGAAA   
  
  
+ AAAAGAATTA TGCATATATA CTTAAGGCCC CCAACCCTTT TTCCCTTTCC GCCAATACTT TTTACCAAGT   
  
  
+ CCAATTCTTT TCTTCTTAGC TATGGCTAGT AGTTATCCCT CCCTAGCTTC CACCCTAAGT TTTGAGACAA   
  
  
+ AATCCCATCT ATGTTAATAT TTATTTCTTG CACATGTCAC CCAATACAAG GGTTTCATTG AAAAGAAAAG   
  
  
+ GTATTAGATG TAAGATATGT ATGTATATAA ATAGTTTTGC AATAGACTAG AAGGACTAAA AAGGAAAAAG   
  
  
+ GAAGTTTAAA AGCAAAAAAC TTTCCCCTCT AGAAAAAAGG AAACCAGATT CCCTTTTCTA CCCCAATTCC   
  
  
+ CATAGTCTTT ACTCTGTCTC TCTCTCTCTG TCTCTCTTAC TCATGATGTT GAGGGAGGAA GGAATGAATT   
  
  
+ AAGGACCCTA AGCTAAGATC CAACCGTAAG ATATTGCCCC TTTAATCACC CATTTCATAT CTCTATCCAC   
  
  
+ AGTTAAAAAC CCACCTCCTC CATCCACATA ACTGATATAT CTCTTCTACA CCATACTATA AGGAAGAAGA   
  
  
+ GAAAAATAGG AGTGTATTGT ATTTTTATTT GAGTAATTTA TTTTAAGGAG AGAAATTTAC ATGGTTGGGT   
  
  
+ TAAATGGATA CCTTGTTTAG ACTAGTGAGT TTCCAACAAC CACACCACCA CCATCACCAA CACCAACACC   
  
  
+ AACACCACCA ACAGCAATCT TCTGATCAAT CCTCTTTGAA TAATACTACT ACTTATTCCA CAAATTCAAG   
  
  
+ CAGTTCCAGA TCTTCCCAAC AAACCCATCA TAATCATAAT CATACTCAGT ATTATCACCA ACATCATCCT   
  
  
+ ACTTACCCCC ACCGCCACCA TCAGGAGGAA GAAGAAGAAT GCTTCAACTT TTACATGGAT GAAGAAAACT   
  
  
+ TCTCTTCTTC TTCCTCCCAC AACAAACATC ATCCTCAACC TCCTTCCTCC GCCACCACCT CCTCGTATTA   
  
  
+ CCCTTACTAC TCCACCCCCT TCGATGTTCC CTCTACCACC ACCACCTCCA CCTCCACCCC CACGCCCACC   
  
  
+ AACACCACCA CCACCACCCC CCCAACCCAG TATGGCGGCG GCGGCGGTGG GTTTGACTCC AGCTGCTTCT   
  
  
+ CCTACTCCCC TGCCCGAGAC CTCAACCCGG AGCTCTTCTC CTCGTCCGGG TTCTCGGGCA AGTGGGCTTC   
  
  
+ CAACATCCTC TTGGAAGCCG CTCGTGCCAT CTCTGAGAGG AACACGAGCC GCCTCCACCA GCTCCTATGG   
  
  
+ ATGGTCAACG AGCTCAGCTC CCCGTACGGT GACGTGGAGC AGAAGATAGC TTCATACTTC GGCCAAGCCC   
  
  
+ TCTTCACCCG CATGACCGAG TCAGGGGACC GCACCTACCG CACCATGCGG TCCGCCGTGG ACAAGACCTT   
  
  
+ CTCCTTTGAG TCTACGAGAA AGATGGTACT CAAGTTCCAG GAGGTTAGCC CTTGGACCAC CTTTGGACAC   
  
  
+ GTGGCAGCTA ATGGAGCCCT CATTGATGCC TTTGACGGTG AATCCAAAAT CCACATCATC GACATCAGCA   
  
  
+ CCACATTCTG CACCCAGTGG CCCACCCTCC TGGAGGCGCT GGCCACCCGA ATGGATGACG CGCCCTACCT   
  
  
+ GAGGCTGACC ACTGTGGTGG TCAACAAGTC CGGGAATGAA GGTCCCACCG GAGGCGGGTC CCACAGGGTG   
  
  
+ ATGAAAGAGA TTGGGACCCG CCTCGAGAAG TTTGCTAGGC TAATGGGAGT GCCTTTCAAA TTTAATGTGG   
  
  
+ TCCACCACGG GGCTGATTTA TCCGACTTGG ATTTCTCTCA GCTGGATATT AACGATGACG AGGCATTAGC   
  
  
+ CATCAACTGT GTCAACTCGC TCCACTCGGT CAATATCCAC CGCCGTGACT CGGTCATCTC GGCCTTCCGG   
  
  
+ CGGCTCCACC CTAGGGTGGT GACCGTGGTG GAGGAGGAGG CTGACCTTGC TGACGTGGGG GCGGAGGGGT   
  
  
+ ACGAGTTCTA CAGGGGGTTT AATGAGTGTT TAAGGTGGGT TAGGGTTTAC TTTGAGGCCC TGGAAGAGTG   
  
  
+ CTTTCCTAGG ACAAGCAATG AGAAACTCAT GCTCGAGCGG GCCGCAGGGC GGGCTCTGGT GGACCTCCTG   
  
  
+ GCCTGCCCCA AGCCCGCATC ATCCGAGCGG AGGGAGGCAG CTGCGCGGTG GTCCGGGAGG ATGCAGGGGG   
  
  
+ CCGGGTTCGG CCACGTGGGG TTCAGTGATG AGGTCTGTGA TGACGTCAAG GCCCTCCTAA GGAGGTACAA   
  
  
+ GGAGGGGTGG TCCATGATAC AATGTTCCGC CGCGGATGGC GGCGCCTCCG CCGGAATATT CTTGTCGTGG   
  
  
+ AGGGATCAGC CGGTGGTGTG GGCCAGTGCA TGGAAGCCTT A  

- -Up\_Stream \_Len000CCCAAA AAAATTTGAG TTTTAATCAA TAACATAGTT AACTTAAACC TTTTGATAAT   
  
  
- AAAAGCTGAA TAATATCTCT TAAACAACAA TTACTTACGA CTATTTATGA CTATTAATCA CAACACTCAC   
  
  
- ACTTAAACAC TACTACTCGT TACTAATCGC AATTTTAATA TTGATCCTAT TTATTAATTC ACAACTACTT   
  
  
- ACAACTAACT ACTACTACTC ATGACTAACC ACGCCACGAC CATTATTTAT AAATACTATT CAATTTTTTT   
  
  
- TTTTTGTGTA GGATTTGTTA GTGATACATT ATATGATTTA CTTTTTTTAA CCATTACACT AAACTGTTAG   
  
  
- ATTCCCATTC CCACAAAATG GGTGTGTACA ATACTTTAAC CGAATGATAA ATGTTTGAAA AATGTCAATA   
  
  
- TACTAAGATG GATACACAAT AGTAGAAAGG CAGCTACATT AACTAATATA AGTTGTGTTA AATTAAGTAA   
  
  
- TGTTACATCG ATTAACTATG AGTTTTATTA CTTGATAATT ATTTATTAAT TAATATTGAA AATTTTCAAA   
  
  
- CAATTAAATT TTAATATATT ATATATTTAA ATCCGATGTA ATTAGTTTTT TAATCCGCAC AAGTAGCCAG   
  
  
- GTTTGAACTT GGCCTGGTCT GGCCTGGCTT CTAGCTAATT CTCTTTTTCA TGTCTGACTC CTGGCCTAGC   
  
  
- TTATTCTCAG CGTGGCCTGG TCCTAGCCTG GCCTTTTTAA ACCTGGTCTA GCCCTGGCTA GCTCCATCTG   
  
  
- GCTTAAATAA AAGTTTATGC TATCCTTATT TTGAATAACT AAGTTAACTA AAACCGGTTT TAGTACGATA   
  
  
- ATAACTAAAA TAAACGATTG AGAGTTCTCA TAATCAAACG TGAAGCGTAA TAGAAACCGG TTTTTTTAGT   
  
  
- TTTTCTTTCA GAGTTTGAGA ATTATCAAAA AATAAAATAA ACCAAATAAA AATTTTATCA GCCGGAAGCC   
  
  
- AGGCCTGATC ACCTGGCTTA TTCCATTAAA AAGCTTAGCT CCTGGTCTGG TTTACGTGAG TCAAGCTTGG   
  
  
- CCAAGTCAAG CCAGACTGAG CTAAAAACCA GGCCAGGTTC AAACTTGTCT TATTACATGT GTGGATCTTT   
  
  
- TTTTCTTAAT ACGTATATAT GAATTCCGGG GGTTGGGAAA AAGGGAAAGG CGGTTATGAA AAATGGTTCA   
  
  
- GGTTAAGAAA AGAAGAATCG ATACCGATCA TCAATAGGGA GGGATCGAAG GTGGGATTCA AAACTCTGTT   
  
  
- TTAGGGTAGA TACAATTATA AATAAAGAAC GTGTACAGTG GGTTATGTTC CCAAAGTAAC TTTTCTTTTC   
  
  
- CATAATCTAC ATTCTATACA TACATATATT TATCAAAACG TTATCTGATC TTCCTGATTT TTCCTTTTTC   
  
  
- CTTCAAATTT TCGTTTTTTG AAAGGGGAGA TCTTTTTTCC TTTGGTCTAA GGGAAAAGAT GGGGTTAAGG   
  
  
- GTATCAGAAA TGAGACAGAG AGAGAGAGAC AGAGAGAATG AGTACTACAA CTCCCTCCTT CCTTACTTAA   
  
  
- TTCCTGGGAT TCGATTCTAG GTTGGCATTC TATAACGGGG AAATTAGTGG GTAAAGTATA GAGATAGGTG   
  
  
- TCAATTTTTG GGTGGAGGAG GTAGGTGTAT TGACTATATA GAGAAGATGT GGTATGATAT TCCTTCTTCT   
  
  
- CTTTTTATCC TCACATAACA TAAAAATAAA CTCATTAAAT AAAATTCCTC TCTTTAAATG TACCAACCCA   
  
  
- ATTTACCTAT GGAACAAATC TGATCACTCA AAGGTTGTTG GTGTGGTGGT GGTAGTGGTT GTGGTTGTGG   
  
  
- TTGTGGTGGT TGTCGTTAGA AGACTAGTTA GGAGAAACTT ATTATGATGA TGAATAAGGT GTTTAAGTTC   
  
  
- GTCAAGGTCT AGAAGGGTTG TTTGGGTAGT ATTAGTATTA GTATGAGTCA TAATAGTGGT TGTAGTAGGA   
  
  
- TGAATGGGGG TGGCGGTGGT AGTCCTCCTT CTTCTTCTTA CGAAGTTGAA AATGTACCTA CTTCTTTTGA   
  
  
- AGAGAAGAAG AAGGAGGGTG TTGTTTGTAG TAGGAGTTGG AGGAAGGAGG CGGTGGTGGA GGAGCATAAT   
  
  
- GGGAATGATG AGGTGGGGGA AGCTACAAGG GAGATGGTGG TGGTGGAGGT GGAGGTGGGG GTGCGGGTGG   
  
  
- TTGTGGTGGT GGTGGTGGGG GGGTTGGGTC ATACCGCCGC CGCCGCCACC CAAACTGAGG TCGACGAAGA   
  
  
- GGATGAGGGG ACGGGCTCTG GAGTTGGGCC TCGAGAAGAG GAGCAGGCCC AAGAGCCCGT TCACCCGAAG   
  
  
- GTTGTAGGAG AACCTTCGGC GAGCACGGTA GAGACTCTCC TTGTGCTCGG CGGAGGTGGT CGAGGATACC   
  
  
- TACCAGTTGC TCGAGTCGAG GGGCATGCCA CTGCACCTCG TCTTCTATCG AAGTATGAAG CCGGTTCGGG   
  
  
- AGAAGTGGGC GTACTGGCTC AGTCCCCTGG CGTGGATGGC GTGGTACGCC AGGCGGCACC TGTTCTGGAA   
  
  
- GAGGAAACTC AGATGCTCTT TCTACCATGA GTTCAAGGTC CTCCAATCGG GAACCTGGTG GAAACCTGTG   
  
  
- CACCGTCGAT TACCTCGGGA GTAACTACGG AAACTGCCAC TTAGGTTTTA GGTGTAGTAG CTGTAGTCGT   
  
  
- GGTGTAAGAC GTGGGTCACC GGGTGGGAGG ACCTCCGCGA CCGGTGGGCT TACCTACTGC GCGGGATGGA   
  
  
- CTCCGACTGG TGACACCACC AGTTGTTCAG GCCCTTACTT CCAGGGTGGC CTCCGCCCAG GGTGTCCCAC   
  
  
- TACTTTCTCT AACCCTGGGC GGAGCTCTTC AAACGATCCG ATTACCCTCA CGGAAAGTTT AAATTACACC   
  
  
- AGGTGGTGCC CCGACTAAAT AGGCTGAACC TAAAGAGAGT CGACCTATAA TTGCTACTGC TCCGTAATCG   
  
  
- GTAGTTGACA CAGTTGAGCG AGGTGAGCCA GTTATAGGTG GCGGCACTGA GCCAGTAGAG CCGGAAGGCC   
  
  
- GCCGAGGTGG GATCCCACCA CTGGCACCAC CTCCTCCTCC GACTGGAACG ACTGCACCCC CGCCTCCCCA   
  
  
- TGCTCAAGAT GTCCCCCAAA TTACTCACAA ATTCCACCCA ATCCCAAATG AAACTCCGGG ACCTTCTCAC   
  
  
- GAAAGGATCC TGTTCGTTAC TCTTTGAGTA CGAGCTCGCC CGGCGTCCCG CCCGAGACCA CCTGGAGGAC   
  
  
- CGGACGGGGT TCGGGCGTAG TAGGCTCGCC TCCCTCCGTC GACGCGCCAC CAGGCCCTCC TACGTCCCCC   
  
  
- GGCCCAAGCC GGTGCACCCC AAGTCACTAC TCCAGACACT ACTGCAGTTC CGGGAGGATT CCTCCATGTT   
  
  
- CCTCCCCACC AGGTACTATG TTACAAGGCG GCGCCTACCG CCGCGGAGGC GGCCTTATAA GAACAGCACC   
  
  
- TCCCTAGTCG GCCACCACAC CCGGTCACGT ACCTTCGGAA T

+     Unnamed\_\_1

| Site Name | Organism | Position | Strand | Matrix score. | sequence | function |
| --- | --- | --- | --- | --- | --- | --- |
| Unnamed\_\_1 | Zea mays | 3430 | + | 5 | CGTGG |  |
| Unnamed\_\_1 | Zea mays | 3305 | - | 5 | CGTGG |  |
| Unnamed\_\_1 | Petunia sp. | 3304 | - | 9 | GCCACGTGGC |  |
| Unnamed\_\_1 | Zea mays | 2164 | - | 5 | CGTGG |  |
| Unnamed\_\_1 | Zea mays | 2417 | + | 5 | CGTGG |  |
| Unnamed\_\_1 | Zea mays | 3308 | + | 5 | CGTGG |  |
| Unnamed\_\_1 | Zea mays | 2594 | + | 5 | CGTGG |  |
| Unnamed\_\_1 | Zea mays | 3038 | + | 5 | CGTGG |  |
| Unnamed\_\_1 | Zea mays | 3068 | + | 5 | CGTGG |  |
| Unnamed\_\_1 | Zea mays | 2510 | + | 5 | CGTGG |  |
| Unnamed\_\_1 | Zea mays | 2879 | - | 5 | CGTGG |  |
| Unnamed\_\_1 | Petunia sp. | 2590 | - | 9 | GCCACGTGGC |  |

>HU06G00358.1   
+ -Up\_Stream \_Len000GGGTTT TTTTAAACTC AAAATTAGTT ATTGTATCAA TTGAATTTGG AAAACTATTA   
  
  
+ TTTTCGACTT ATTATAGAGA ATTTGTTGTT AATGAATGCT GATAAATACT GATAATTAGT GTTGTGAGTG   
  
  
+ TGAATTTGTG ATGATGAGCA ATGATTAGCG TTAAAATTAT AACTAGGATA AATAATTAAG TGTTGATGAA   
  
  
+ TGTTGATTGA TGATGATGAG TACTGATTGG TGCGGTGCTG GTAATAAATA TTTATGATAA GTTAAAAAAA   
  
  
+ AAAAACACAT CCTAAACAAT CACTATGTAA TATACTAAAT GAAAAAAATT GGTAATGTGA TTTGACAATC   
  
  
+ TAAGGGTAAG GGTGTTTTAC CCACACATGT TATGAAATTG GCTTACTATT TACAAACTTT TTACAGTTAT   
  
  
+ ATGATTCTAC CTATGTGTTA TCATCTTTCC GTCGATGTAA TTGATTATAT TCAACACAAT TTAATTCATT   
  
  
+ ACAATGTAGC TAATTGATAC TCAAAATAAT GAACTATTAA TAAATAATTA ATTATAACTT TTAAAAGTTT   
  
  
+ GTTAATTTAA AATTATATAA TATATAAATT TAGGCTACAT TAATCAAAAA ATTAGGCGTG TTCATCGGTC   
  
  
+ CAAACTTGAA CCGGACCAGA CCGGACCGAA GATCGATTAA GAGAAAAAGT ACAGACTGAG GACCGGATCG   
  
  
+ AATAAGAGTC GCACCGGACC AGGATCGGAC CGGAAAAATT TGGACCAGAT CGGGACCGAT CGAGGTAGAC   
  
  
+ CGAATTTATT TTCAAATACG ATAGGAATAA AACTTATTGA TTCAATTGAT TTTGGCCAAA ATCATGCTAT   
  
  
+ TATTGATTTT ATTTGCTAAC TCTCAAGAGT ATTAGTTTGC ACTTCGCATT ATCTTTGGCC AAAAAAATCA   
  
  
+ AAAAGAAAGT CTCAAACTCT TAATAGTTTT TTATTTTATT TGGTTTATTT TTAAAATAGT CGGCCTTCGG   
  
  
+ TCCGGACTAG TGGACCGAAT AAGGTAATTT TTCGAATCGA GGACCAGACC AAATGCACTC AGTTCGAACC   
  
  
+ GGTTCAGTTC GGTCTGACTC GATTTTTGGT CCGGTCCAAG TTTGAACAGA ATAATGTACA CACCTAGAAA   
  
  
+ AAAAGAATTA TGCATATATA CTTAAGGCCC CCAACCCTTT TTCCCTTTCC GCCAATACTT TTTACCAAGT   
  
  
+ CCAATTCTTT TCTTCTTAGC TATGGCTAGT AGTTATCCCT CCCTAGCTTC CACCCTAAGT TTTGAGACAA   
  
  
+ AATCCCATCT ATGTTAATAT TTATTTCTTG CACATGTCAC CCAATACAAG GGTTTCATTG AAAAGAAAAG   
  
  
+ GTATTAGATG TAAGATATGT ATGTATATAA ATAGTTTTGC AATAGACTAG AAGGACTAAA AAGGAAAAAG   
  
  
+ GAAGTTTAAA AGCAAAAAAC TTTCCCCTCT AGAAAAAAGG AAACCAGATT CCCTTTTCTA CCCCAATTCC   
  
  
+ CATAGTCTTT ACTCTGTCTC TCTCTCTCTG TCTCTCTTAC TCATGATGTT GAGGGAGGAA GGAATGAATT   
  
  
+ AAGGACCCTA AGCTAAGATC CAACCGTAAG ATATTGCCCC TTTAATCACC CATTTCATAT CTCTATCCAC   
  
  
+ AGTTAAAAAC CCACCTCCTC CATCCACATA ACTGATATAT CTCTTCTACA CCATACTATA AGGAAGAAGA   
  
  
+ GAAAAATAGG AGTGTATTGT ATTTTTATTT GAGTAATTTA TTTTAAGGAG AGAAATTTAC ATGGTTGGGT   
  
  
+ TAAATGGATA CCTTGTTTAG ACTAGTGAGT TTCCAACAAC CACACCACCA CCATCACCAA CACCAACACC   
  
  
+ AACACCACCA ACAGCAATCT TCTGATCAAT CCTCTTTGAA TAATACTACT ACTTATTCCA CAAATTCAAG   
  
  
+ CAGTTCCAGA TCTTCCCAAC AAACCCATCA TAATCATAAT CATACTCAGT ATTATCACCA ACATCATCCT   
  
  
+ ACTTACCCCC ACCGCCACCA TCAGGAGGAA GAAGAAGAAT GCTTCAACTT TTACATGGAT GAAGAAAACT   
  
  
+ TCTCTTCTTC TTCCTCCCAC AACAAACATC ATCCTCAACC TCCTTCCTCC GCCACCACCT CCTCGTATTA   
  
  
+ CCCTTACTAC TCCACCCCCT TCGATGTTCC CTCTACCACC ACCACCTCCA CCTCCACCCC CACGCCCACC   
  
  
+ AACACCACCA CCACCACCCC CCCAACCCAG TATGGCGGCG GCGGCGGTGG GTTTGACTCC AGCTGCTTCT   
  
  
+ CCTACTCCCC TGCCCGAGAC CTCAACCCGG AGCTCTTCTC CTCGTCCGGG TTCTCGGGCA AGTGGGCTTC   
  
  
+ CAACATCCTC TTGGAAGCCG CTCGTGCCAT CTCTGAGAGG AACACGAGCC GCCTCCACCA GCTCCTATGG   
  
  
+ ATGGTCAACG AGCTCAGCTC CCCGTACGGT GACGTGGAGC AGAAGATAGC TTCATACTTC GGCCAAGCCC   
  
  
+ TCTTCACCCG CATGACCGAG TCAGGGGACC GCACCTACCG CACCATGCGG TCCGCCGTGG ACAAGACCTT   
  
  
+ CTCCTTTGAG TCTACGAGAA AGATGGTACT CAAGTTCCAG GAGGTTAGCC CTTGGACCAC CTTTGGACAC   
  
  
+ GTGGCAGCTA ATGGAGCCCT CATTGATGCC TTTGACGGTG AATCCAAAAT CCACATCATC GACATCAGCA   
  
  
+ CCACATTCTG CACCCAGTGG CCCACCCTCC TGGAGGCGCT GGCCACCCGA ATGGATGACG CGCCCTACCT   
  
  
+ GAGGCTGACC ACTGTGGTGG TCAACAAGTC CGGGAATGAA GGTCCCACCG GAGGCGGGTC CCACAGGGTG   
  
  
+ ATGAAAGAGA TTGGGACCCG CCTCGAGAAG TTTGCTAGGC TAATGGGAGT GCCTTTCAAA TTTAATGTGG   
  
  
+ TCCACCACGG GGCTGATTTA TCCGACTTGG ATTTCTCTCA GCTGGATATT AACGATGACG AGGCATTAGC   
  
  
+ CATCAACTGT GTCAACTCGC TCCACTCGGT CAATATCCAC CGCCGTGACT CGGTCATCTC GGCCTTCCGG   
  
  
+ CGGCTCCACC CTAGGGTGGT GACCGTGGTG GAGGAGGAGG CTGACCTTGC TGACGTGGGG GCGGAGGGGT   
  
  
+ ACGAGTTCTA CAGGGGGTTT AATGAGTGTT TAAGGTGGGT TAGGGTTTAC TTTGAGGCCC TGGAAGAGTG   
  
  
+ CTTTCCTAGG ACAAGCAATG AGAAACTCAT GCTCGAGCGG GCCGCAGGGC GGGCTCTGGT GGACCTCCTG   
  
  
+ GCCTGCCCCA AGCCCGCATC ATCCGAGCGG AGGGAGGCAG CTGCGCGGTG GTCCGGGAGG ATGCAGGGGG   
  
  
+ CCGGGTTCGG CCACGTGGGG TTCAGTGATG AGGTCTGTGA TGACGTCAAG GCCCTCCTAA GGAGGTACAA   
  
  
+ GGAGGGGTGG TCCATGATAC AATGTTCCGC CGCGGATGGC GGCGCCTCCG CCGGAATATT CTTGTCGTGG   
  
  
+ AGGGATCAGC CGGTGGTGTG GGCCAGTGCA TGGAAGCCTT A  

- -Up\_Stream \_Len000CCCAAA AAAATTTGAG TTTTAATCAA TAACATAGTT AACTTAAACC TTTTGATAAT   
  
  
- AAAAGCTGAA TAATATCTCT TAAACAACAA TTACTTACGA CTATTTATGA CTATTAATCA CAACACTCAC   
  
  
- ACTTAAACAC TACTACTCGT TACTAATCGC AATTTTAATA TTGATCCTAT TTATTAATTC ACAACTACTT   
  
  
- ACAACTAACT ACTACTACTC ATGACTAACC ACGCCACGAC CATTATTTAT AAATACTATT CAATTTTTTT   
  
  
- TTTTTGTGTA GGATTTGTTA GTGATACATT ATATGATTTA CTTTTTTTAA CCATTACACT AAACTGTTAG   
  
  
- ATTCCCATTC CCACAAAATG GGTGTGTACA ATACTTTAAC CGAATGATAA ATGTTTGAAA AATGTCAATA   
  
  
- TACTAAGATG GATACACAAT AGTAGAAAGG CAGCTACATT AACTAATATA AGTTGTGTTA AATTAAGTAA   
  
  
- TGTTACATCG ATTAACTATG AGTTTTATTA CTTGATAATT ATTTATTAAT TAATATTGAA AATTTTCAAA   
  
  
- CAATTAAATT TTAATATATT ATATATTTAA ATCCGATGTA ATTAGTTTTT TAATCCGCAC AAGTAGCCAG   
  
  
- GTTTGAACTT GGCCTGGTCT GGCCTGGCTT CTAGCTAATT CTCTTTTTCA TGTCTGACTC CTGGCCTAGC   
  
  
- TTATTCTCAG CGTGGCCTGG TCCTAGCCTG GCCTTTTTAA ACCTGGTCTA GCCCTGGCTA GCTCCATCTG   
  
  
- GCTTAAATAA AAGTTTATGC TATCCTTATT TTGAATAACT AAGTTAACTA AAACCGGTTT TAGTACGATA   
  
  
- ATAACTAAAA TAAACGATTG AGAGTTCTCA TAATCAAACG TGAAGCGTAA TAGAAACCGG TTTTTTTAGT   
  
  
- TTTTCTTTCA GAGTTTGAGA ATTATCAAAA AATAAAATAA ACCAAATAAA AATTTTATCA GCCGGAAGCC   
  
  
- AGGCCTGATC ACCTGGCTTA TTCCATTAAA AAGCTTAGCT CCTGGTCTGG TTTACGTGAG TCAAGCTTGG   
  
  
- CCAAGTCAAG CCAGACTGAG CTAAAAACCA GGCCAGGTTC AAACTTGTCT TATTACATGT GTGGATCTTT   
  
  
- TTTTCTTAAT ACGTATATAT GAATTCCGGG GGTTGGGAAA AAGGGAAAGG CGGTTATGAA AAATGGTTCA   
  
  
- GGTTAAGAAA AGAAGAATCG ATACCGATCA TCAATAGGGA GGGATCGAAG GTGGGATTCA AAACTCTGTT   
  
  
- TTAGGGTAGA TACAATTATA AATAAAGAAC GTGTACAGTG GGTTATGTTC CCAAAGTAAC TTTTCTTTTC   
  
  
- CATAATCTAC ATTCTATACA TACATATATT TATCAAAACG TTATCTGATC TTCCTGATTT TTCCTTTTTC   
  
  
- CTTCAAATTT TCGTTTTTTG AAAGGGGAGA TCTTTTTTCC TTTGGTCTAA GGGAAAAGAT GGGGTTAAGG   
  
  
- GTATCAGAAA TGAGACAGAG AGAGAGAGAC AGAGAGAATG AGTACTACAA CTCCCTCCTT CCTTACTTAA   
  
  
- TTCCTGGGAT TCGATTCTAG GTTGGCATTC TATAACGGGG AAATTAGTGG GTAAAGTATA GAGATAGGTG   
  
  
- TCAATTTTTG GGTGGAGGAG GTAGGTGTAT TGACTATATA GAGAAGATGT GGTATGATAT TCCTTCTTCT   
  
  
- CTTTTTATCC TCACATAACA TAAAAATAAA CTCATTAAAT AAAATTCCTC TCTTTAAATG TACCAACCCA   
  
  
- ATTTACCTAT GGAACAAATC TGATCACTCA AAGGTTGTTG GTGTGGTGGT GGTAGTGGTT GTGGTTGTGG   
  
  
- TTGTGGTGGT TGTCGTTAGA AGACTAGTTA GGAGAAACTT ATTATGATGA TGAATAAGGT GTTTAAGTTC   
  
  
- GTCAAGGTCT AGAAGGGTTG TTTGGGTAGT ATTAGTATTA GTATGAGTCA TAATAGTGGT TGTAGTAGGA   
  
  
- TGAATGGGGG TGGCGGTGGT AGTCCTCCTT CTTCTTCTTA CGAAGTTGAA AATGTACCTA CTTCTTTTGA   
  
  
- AGAGAAGAAG AAGGAGGGTG TTGTTTGTAG TAGGAGTTGG AGGAAGGAGG CGGTGGTGGA GGAGCATAAT   
  
  
- GGGAATGATG AGGTGGGGGA AGCTACAAGG GAGATGGTGG TGGTGGAGGT GGAGGTGGGG GTGCGGGTGG   
  
  
- TTGTGGTGGT GGTGGTGGGG GGGTTGGGTC ATACCGCCGC CGCCGCCACC CAAACTGAGG TCGACGAAGA   
  
  
- GGATGAGGGG ACGGGCTCTG GAGTTGGGCC TCGAGAAGAG GAGCAGGCCC AAGAGCCCGT TCACCCGAAG   
  
  
- GTTGTAGGAG AACCTTCGGC GAGCACGGTA GAGACTCTCC TTGTGCTCGG CGGAGGTGGT CGAGGATACC   
  
  
- TACCAGTTGC TCGAGTCGAG GGGCATGCCA CTGCACCTCG TCTTCTATCG AAGTATGAAG CCGGTTCGGG   
  
  
- AGAAGTGGGC GTACTGGCTC AGTCCCCTGG CGTGGATGGC GTGGTACGCC AGGCGGCACC TGTTCTGGAA   
  
  
- GAGGAAACTC AGATGCTCTT TCTACCATGA GTTCAAGGTC CTCCAATCGG GAACCTGGTG GAAACCTGTG   
  
  
- CACCGTCGAT TACCTCGGGA GTAACTACGG AAACTGCCAC TTAGGTTTTA GGTGTAGTAG CTGTAGTCGT   
  
  
- GGTGTAAGAC GTGGGTCACC GGGTGGGAGG ACCTCCGCGA CCGGTGGGCT TACCTACTGC GCGGGATGGA   
  
  
- CTCCGACTGG TGACACCACC AGTTGTTCAG GCCCTTACTT CCAGGGTGGC CTCCGCCCAG GGTGTCCCAC   
  
  
- TACTTTCTCT AACCCTGGGC GGAGCTCTTC AAACGATCCG ATTACCCTCA CGGAAAGTTT AAATTACACC   
  
  
- AGGTGGTGCC CCGACTAAAT AGGCTGAACC TAAAGAGAGT CGACCTATAA TTGCTACTGC TCCGTAATCG   
  
  
- GTAGTTGACA CAGTTGAGCG AGGTGAGCCA GTTATAGGTG GCGGCACTGA GCCAGTAGAG CCGGAAGGCC   
  
  
- GCCGAGGTGG GATCCCACCA CTGGCACCAC CTCCTCCTCC GACTGGAACG ACTGCACCCC CGCCTCCCCA   
  
  
- TGCTCAAGAT GTCCCCCAAA TTACTCACAA ATTCCACCCA ATCCCAAATG AAACTCCGGG ACCTTCTCAC   
  
  
- GAAAGGATCC TGTTCGTTAC TCTTTGAGTA CGAGCTCGCC CGGCGTCCCG CCCGAGACCA CCTGGAGGAC   
  
  
- CGGACGGGGT TCGGGCGTAG TAGGCTCGCC TCCCTCCGTC GACGCGCCAC CAGGCCCTCC TACGTCCCCC   
  
  
- GGCCCAAGCC GGTGCACCCC AAGTCACTAC TCCAGACACT ACTGCAGTTC CGGGAGGATT CCTCCATGTT   
  
  
- CCTCCCCACC AGGTACTATG TTACAAGGCG GCGCCTACCG CCGCGGAGGC GGCCTTATAA GAACAGCACC   
  
  
- TCCCTAGTCG GCCACCACAC CCGGTCACGT ACCTTCGGAA T

+     Unnamed\_\_4

| Site Name | Organism | Position | Strand | Matrix score. | sequence | function |
| --- | --- | --- | --- | --- | --- | --- |
| Unnamed\_\_4 | Petroselinum hortense | 3050 | - | 4 | CTCC |  |
| Unnamed\_\_4 | Petroselinum hortense | 3433 | - | 4 | CTCC |  |
| Unnamed\_\_4 | Petroselinum hortense | 3355 | - | 4 | CTCC |  |
| Unnamed\_\_4 | Petroselinum hortense | 3365 | - | 4 | CTCC |  |
| Unnamed\_\_4 | Petroselinum hortense | 3348 | + | 4 | CTCC |  |
| Unnamed\_\_4 | Petroselinum hortense | 2273 | - | 4 | CTCC |  |
| Unnamed\_\_4 | Petroselinum hortense | 2074 | + | 4 | CTCC |  |
| Unnamed\_\_4 | Petroselinum hortense | 2243 | + | 4 | CTCC |  |
| Unnamed\_\_4 | Petroselinum hortense | 2850 | - | 4 | CTCC |  |
| Unnamed\_\_4 | Petroselinum hortense | 3410 | + | 4 | CTCC |  |
| Unnamed\_\_4 | Petroselinum hortense | 2696 | - | 4 | CTCC |  |
| Unnamed\_\_4 | Petroselinum hortense | 1632 | + | 4 | CTCC |  |
| Unnamed\_\_4 | Petroselinum hortense | 1988 | - | 4 | CTCC |  |
| Unnamed\_\_4 | Petroselinum hortense | 2249 | + | 4 | CTCC |  |
| Unnamed\_\_4 | Petroselinum hortense | 2150 | + | 4 | CTCC |  |
| Unnamed\_\_4 | Petroselinum hortense | 3257 | - | 4 | CTCC |  |
| Unnamed\_\_4 | Petroselinum hortense | 2784 | - | 4 | CTCC |  |
| Unnamed\_\_4 | Petroselinum hortense | 2114 | + | 4 | CTCC |  |
| Unnamed\_\_4 | Petroselinum hortense | 3280 | - | 4 | CTCC |  |
| Unnamed\_\_4 | Petroselinum hortense | 2081 | + | 4 | CTCC |  |
| Unnamed\_\_4 | Petroselinum hortense | 3219 | + | 4 | CTCC |  |
| Unnamed\_\_4 | Petroselinum hortense | 3253 | - | 4 | CTCC |  |
| Unnamed\_\_4 | Petroselinum hortense | 2093 | + | 4 | CTCC |  |
| Unnamed\_\_4 | Petroselinum hortense | 2607 | - | 4 | CTCC |  |
| Unnamed\_\_4 | Petroselinum hortense | 2367 | + | 4 | CTCC |  |
| Unnamed\_\_4 | Petroselinum hortense | 3018 | + | 4 | CTCC |  |
| Unnamed\_\_4 | Petroselinum hortense | 2564 | - | 4 | CTCC |  |
| Unnamed\_\_4 | Petroselinum hortense | 1233 | + | 4 | CTCC |  |
| Unnamed\_\_4 | Petroselinum hortense | 1693 | - | 4 | CTCC |  |
| Unnamed\_\_4 | Petroselinum hortense | 2964 | + | 4 | CTCC |  |
| Unnamed\_\_4 | Petroselinum hortense | 3047 | - | 4 | CTCC |  |
| Unnamed\_\_4 | Petroselinum hortense | 2691 | + | 4 | CTCC |  |
| Unnamed\_\_4 | Petroselinum hortense | 3044 | - | 4 | CTCC |  |
| Unnamed\_\_4 | Petroselinum hortense | 2376 | + | 4 | CTCC |  |
| Unnamed\_\_4 | Petroselinum hortense | 2048 | + | 4 | CTCC |  |
| Unnamed\_\_4 | Petroselinum hortense | 1629 | + | 4 | CTCC |  |
| Unnamed\_\_4 | Petroselinum hortense | 2525 | + | 4 | CTCC |  |
| Unnamed\_\_4 | Petroselinum hortense | 2420 | - | 4 | CTCC |  |
| Unnamed\_\_4 | Petroselinum hortense | 3077 | - | 4 | CTCC |  |
| Unnamed\_\_4 | Petroselinum hortense | 1731 | - | 4 | CTCC |  |
| Unnamed\_\_4 | Petroselinum hortense | 1528 | - | 4 | CTCC |  |
| Unnamed\_\_4 | Petroselinum hortense | 2156 | + | 4 | CTCC |  |
| Unnamed\_\_4 | Petroselinum hortense | 2231 | + | 4 | CTCC |  |
| Unnamed\_\_4 | Petroselinum hortense | 2282 | + | 4 | CTCC |  |
| Unnamed\_\_4 | Petroselinum hortense | 2402 | + | 4 | CTCC |  |

>HU06G00358.1   
+ -Up\_Stream \_Len000GGGTTT TTTTAAACTC AAAATTAGTT ATTGTATCAA TTGAATTTGG AAAACTATTA   
  
  
+ TTTTCGACTT ATTATAGAGA ATTTGTTGTT AATGAATGCT GATAAATACT GATAATTAGT GTTGTGAGTG   
  
  
+ TGAATTTGTG ATGATGAGCA ATGATTAGCG TTAAAATTAT AACTAGGATA AATAATTAAG TGTTGATGAA   
  
  
+ TGTTGATTGA TGATGATGAG TACTGATTGG TGCGGTGCTG GTAATAAATA TTTATGATAA GTTAAAAAAA   
  
  
+ AAAAACACAT CCTAAACAAT CACTATGTAA TATACTAAAT GAAAAAAATT GGTAATGTGA TTTGACAATC   
  
  
+ TAAGGGTAAG GGTGTTTTAC CCACACATGT TATGAAATTG GCTTACTATT TACAAACTTT TTACAGTTAT   
  
  
+ ATGATTCTAC CTATGTGTTA TCATCTTTCC GTCGATGTAA TTGATTATAT TCAACACAAT TTAATTCATT   
  
  
+ ACAATGTAGC TAATTGATAC TCAAAATAAT GAACTATTAA TAAATAATTA ATTATAACTT TTAAAAGTTT   
  
  
+ GTTAATTTAA AATTATATAA TATATAAATT TAGGCTACAT TAATCAAAAA ATTAGGCGTG TTCATCGGTC   
  
  
+ CAAACTTGAA CCGGACCAGA CCGGACCGAA GATCGATTAA GAGAAAAAGT ACAGACTGAG GACCGGATCG   
  
  
+ AATAAGAGTC GCACCGGACC AGGATCGGAC CGGAAAAATT TGGACCAGAT CGGGACCGAT CGAGGTAGAC   
  
  
+ CGAATTTATT TTCAAATACG ATAGGAATAA AACTTATTGA TTCAATTGAT TTTGGCCAAA ATCATGCTAT   
  
  
+ TATTGATTTT ATTTGCTAAC TCTCAAGAGT ATTAGTTTGC ACTTCGCATT ATCTTTGGCC AAAAAAATCA   
  
  
+ AAAAGAAAGT CTCAAACTCT TAATAGTTTT TTATTTTATT TGGTTTATTT TTAAAATAGT CGGCCTTCGG   
  
  
+ TCCGGACTAG TGGACCGAAT AAGGTAATTT TTCGAATCGA GGACCAGACC AAATGCACTC AGTTCGAACC   
  
  
+ GGTTCAGTTC GGTCTGACTC GATTTTTGGT CCGGTCCAAG TTTGAACAGA ATAATGTACA CACCTAGAAA   
  
  
+ AAAAGAATTA TGCATATATA CTTAAGGCCC CCAACCCTTT TTCCCTTTCC GCCAATACTT TTTACCAAGT   
  
  
+ CCAATTCTTT TCTTCTTAGC TATGGCTAGT AGTTATCCCT CCCTAGCTTC CACCCTAAGT TTTGAGACAA   
  
  
+ AATCCCATCT ATGTTAATAT TTATTTCTTG CACATGTCAC CCAATACAAG GGTTTCATTG AAAAGAAAAG   
  
  
+ GTATTAGATG TAAGATATGT ATGTATATAA ATAGTTTTGC AATAGACTAG AAGGACTAAA AAGGAAAAAG   
  
  
+ GAAGTTTAAA AGCAAAAAAC TTTCCCCTCT AGAAAAAAGG AAACCAGATT CCCTTTTCTA CCCCAATTCC   
  
  
+ CATAGTCTTT ACTCTGTCTC TCTCTCTCTG TCTCTCTTAC TCATGATGTT GAGGGAGGAA GGAATGAATT   
  
  
+ AAGGACCCTA AGCTAAGATC CAACCGTAAG ATATTGCCCC TTTAATCACC CATTTCATAT CTCTATCCAC   
  
  
+ AGTTAAAAAC CCACCTCCTC CATCCACATA ACTGATATAT CTCTTCTACA CCATACTATA AGGAAGAAGA   
  
  
+ GAAAAATAGG AGTGTATTGT ATTTTTATTT GAGTAATTTA TTTTAAGGAG AGAAATTTAC ATGGTTGGGT   
  
  
+ TAAATGGATA CCTTGTTTAG ACTAGTGAGT TTCCAACAAC CACACCACCA CCATCACCAA CACCAACACC   
  
  
+ AACACCACCA ACAGCAATCT TCTGATCAAT CCTCTTTGAA TAATACTACT ACTTATTCCA CAAATTCAAG   
  
  
+ CAGTTCCAGA TCTTCCCAAC AAACCCATCA TAATCATAAT CATACTCAGT ATTATCACCA ACATCATCCT   
  
  
+ ACTTACCCCC ACCGCCACCA TCAGGAGGAA GAAGAAGAAT GCTTCAACTT TTACATGGAT GAAGAAAACT   
  
  
+ TCTCTTCTTC TTCCTCCCAC AACAAACATC ATCCTCAACC TCCTTCCTCC GCCACCACCT CCTCGTATTA   
  
  
+ CCCTTACTAC TCCACCCCCT TCGATGTTCC CTCTACCACC ACCACCTCCA CCTCCACCCC CACGCCCACC   
  
  
+ AACACCACCA CCACCACCCC CCCAACCCAG TATGGCGGCG GCGGCGGTGG GTTTGACTCC AGCTGCTTCT   
  
  
+ CCTACTCCCC TGCCCGAGAC CTCAACCCGG AGCTCTTCTC CTCGTCCGGG TTCTCGGGCA AGTGGGCTTC   
  
  
+ CAACATCCTC TTGGAAGCCG CTCGTGCCAT CTCTGAGAGG AACACGAGCC GCCTCCACCA GCTCCTATGG   
  
  
+ ATGGTCAACG AGCTCAGCTC CCCGTACGGT GACGTGGAGC AGAAGATAGC TTCATACTTC GGCCAAGCCC   
  
  
+ TCTTCACCCG CATGACCGAG TCAGGGGACC GCACCTACCG CACCATGCGG TCCGCCGTGG ACAAGACCTT   
  
  
+ CTCCTTTGAG TCTACGAGAA AGATGGTACT CAAGTTCCAG GAGGTTAGCC CTTGGACCAC CTTTGGACAC   
  
  
+ GTGGCAGCTA ATGGAGCCCT CATTGATGCC TTTGACGGTG AATCCAAAAT CCACATCATC GACATCAGCA   
  
  
+ CCACATTCTG CACCCAGTGG CCCACCCTCC TGGAGGCGCT GGCCACCCGA ATGGATGACG CGCCCTACCT   
  
  
+ GAGGCTGACC ACTGTGGTGG TCAACAAGTC CGGGAATGAA GGTCCCACCG GAGGCGGGTC CCACAGGGTG   
  
  
+ ATGAAAGAGA TTGGGACCCG CCTCGAGAAG TTTGCTAGGC TAATGGGAGT GCCTTTCAAA TTTAATGTGG   
  
  
+ TCCACCACGG GGCTGATTTA TCCGACTTGG ATTTCTCTCA GCTGGATATT AACGATGACG AGGCATTAGC   
  
  
+ CATCAACTGT GTCAACTCGC TCCACTCGGT CAATATCCAC CGCCGTGACT CGGTCATCTC GGCCTTCCGG   
  
  
+ CGGCTCCACC CTAGGGTGGT GACCGTGGTG GAGGAGGAGG CTGACCTTGC TGACGTGGGG GCGGAGGGGT   
  
  
+ ACGAGTTCTA CAGGGGGTTT AATGAGTGTT TAAGGTGGGT TAGGGTTTAC TTTGAGGCCC TGGAAGAGTG   
  
  
+ CTTTCCTAGG ACAAGCAATG AGAAACTCAT GCTCGAGCGG GCCGCAGGGC GGGCTCTGGT GGACCTCCTG   
  
  
+ GCCTGCCCCA AGCCCGCATC ATCCGAGCGG AGGGAGGCAG CTGCGCGGTG GTCCGGGAGG ATGCAGGGGG   
  
  
+ CCGGGTTCGG CCACGTGGGG TTCAGTGATG AGGTCTGTGA TGACGTCAAG GCCCTCCTAA GGAGGTACAA   
  
  
+ GGAGGGGTGG TCCATGATAC AATGTTCCGC CGCGGATGGC GGCGCCTCCG CCGGAATATT CTTGTCGTGG   
  
  
+ AGGGATCAGC CGGTGGTGTG GGCCAGTGCA TGGAAGCCTT A  

- -Up\_Stream \_Len000CCCAAA AAAATTTGAG TTTTAATCAA TAACATAGTT AACTTAAACC TTTTGATAAT   
  
  
- AAAAGCTGAA TAATATCTCT TAAACAACAA TTACTTACGA CTATTTATGA CTATTAATCA CAACACTCAC   
  
  
- ACTTAAACAC TACTACTCGT TACTAATCGC AATTTTAATA TTGATCCTAT TTATTAATTC ACAACTACTT   
  
  
- ACAACTAACT ACTACTACTC ATGACTAACC ACGCCACGAC CATTATTTAT AAATACTATT CAATTTTTTT   
  
  
- TTTTTGTGTA GGATTTGTTA GTGATACATT ATATGATTTA CTTTTTTTAA CCATTACACT AAACTGTTAG   
  
  
- ATTCCCATTC CCACAAAATG GGTGTGTACA ATACTTTAAC CGAATGATAA ATGTTTGAAA AATGTCAATA   
  
  
- TACTAAGATG GATACACAAT AGTAGAAAGG CAGCTACATT AACTAATATA AGTTGTGTTA AATTAAGTAA   
  
  
- TGTTACATCG ATTAACTATG AGTTTTATTA CTTGATAATT ATTTATTAAT TAATATTGAA AATTTTCAAA   
  
  
- CAATTAAATT TTAATATATT ATATATTTAA ATCCGATGTA ATTAGTTTTT TAATCCGCAC AAGTAGCCAG   
  
  
- GTTTGAACTT GGCCTGGTCT GGCCTGGCTT CTAGCTAATT CTCTTTTTCA TGTCTGACTC CTGGCCTAGC   
  
  
- TTATTCTCAG CGTGGCCTGG TCCTAGCCTG GCCTTTTTAA ACCTGGTCTA GCCCTGGCTA GCTCCATCTG   
  
  
- GCTTAAATAA AAGTTTATGC TATCCTTATT TTGAATAACT AAGTTAACTA AAACCGGTTT TAGTACGATA   
  
  
- ATAACTAAAA TAAACGATTG AGAGTTCTCA TAATCAAACG TGAAGCGTAA TAGAAACCGG TTTTTTTAGT   
  
  
- TTTTCTTTCA GAGTTTGAGA ATTATCAAAA AATAAAATAA ACCAAATAAA AATTTTATCA GCCGGAAGCC   
  
  
- AGGCCTGATC ACCTGGCTTA TTCCATTAAA AAGCTTAGCT CCTGGTCTGG TTTACGTGAG TCAAGCTTGG   
  
  
- CCAAGTCAAG CCAGACTGAG CTAAAAACCA GGCCAGGTTC AAACTTGTCT TATTACATGT GTGGATCTTT   
  
  
- TTTTCTTAAT ACGTATATAT GAATTCCGGG GGTTGGGAAA AAGGGAAAGG CGGTTATGAA AAATGGTTCA   
  
  
- GGTTAAGAAA AGAAGAATCG ATACCGATCA TCAATAGGGA GGGATCGAAG GTGGGATTCA AAACTCTGTT   
  
  
- TTAGGGTAGA TACAATTATA AATAAAGAAC GTGTACAGTG GGTTATGTTC CCAAAGTAAC TTTTCTTTTC   
  
  
- CATAATCTAC ATTCTATACA TACATATATT TATCAAAACG TTATCTGATC TTCCTGATTT TTCCTTTTTC   
  
  
- CTTCAAATTT TCGTTTTTTG AAAGGGGAGA TCTTTTTTCC TTTGGTCTAA GGGAAAAGAT GGGGTTAAGG   
  
  
- GTATCAGAAA TGAGACAGAG AGAGAGAGAC AGAGAGAATG AGTACTACAA CTCCCTCCTT CCTTACTTAA   
  
  
- TTCCTGGGAT TCGATTCTAG GTTGGCATTC TATAACGGGG AAATTAGTGG GTAAAGTATA GAGATAGGTG   
  
  
- TCAATTTTTG GGTGGAGGAG GTAGGTGTAT TGACTATATA GAGAAGATGT GGTATGATAT TCCTTCTTCT   
  
  
- CTTTTTATCC TCACATAACA TAAAAATAAA CTCATTAAAT AAAATTCCTC TCTTTAAATG TACCAACCCA   
  
  
- ATTTACCTAT GGAACAAATC TGATCACTCA AAGGTTGTTG GTGTGGTGGT GGTAGTGGTT GTGGTTGTGG   
  
  
- TTGTGGTGGT TGTCGTTAGA AGACTAGTTA GGAGAAACTT ATTATGATGA TGAATAAGGT GTTTAAGTTC   
  
  
- GTCAAGGTCT AGAAGGGTTG TTTGGGTAGT ATTAGTATTA GTATGAGTCA TAATAGTGGT TGTAGTAGGA   
  
  
- TGAATGGGGG TGGCGGTGGT AGTCCTCCTT CTTCTTCTTA CGAAGTTGAA AATGTACCTA CTTCTTTTGA   
  
  
- AGAGAAGAAG AAGGAGGGTG TTGTTTGTAG TAGGAGTTGG AGGAAGGAGG CGGTGGTGGA GGAGCATAAT   
  
  
- GGGAATGATG AGGTGGGGGA AGCTACAAGG GAGATGGTGG TGGTGGAGGT GGAGGTGGGG GTGCGGGTGG   
  
  
- TTGTGGTGGT GGTGGTGGGG GGGTTGGGTC ATACCGCCGC CGCCGCCACC CAAACTGAGG TCGACGAAGA   
  
  
- GGATGAGGGG ACGGGCTCTG GAGTTGGGCC TCGAGAAGAG GAGCAGGCCC AAGAGCCCGT TCACCCGAAG   
  
  
- GTTGTAGGAG AACCTTCGGC GAGCACGGTA GAGACTCTCC TTGTGCTCGG CGGAGGTGGT CGAGGATACC   
  
  
- TACCAGTTGC TCGAGTCGAG GGGCATGCCA CTGCACCTCG TCTTCTATCG AAGTATGAAG CCGGTTCGGG   
  
  
- AGAAGTGGGC GTACTGGCTC AGTCCCCTGG CGTGGATGGC GTGGTACGCC AGGCGGCACC TGTTCTGGAA   
  
  
- GAGGAAACTC AGATGCTCTT TCTACCATGA GTTCAAGGTC CTCCAATCGG GAACCTGGTG GAAACCTGTG   
  
  
- CACCGTCGAT TACCTCGGGA GTAACTACGG AAACTGCCAC TTAGGTTTTA GGTGTAGTAG CTGTAGTCGT   
  
  
- GGTGTAAGAC GTGGGTCACC GGGTGGGAGG ACCTCCGCGA CCGGTGGGCT TACCTACTGC GCGGGATGGA   
  
  
- CTCCGACTGG TGACACCACC AGTTGTTCAG GCCCTTACTT CCAGGGTGGC CTCCGCCCAG GGTGTCCCAC   
  
  
- TACTTTCTCT AACCCTGGGC GGAGCTCTTC AAACGATCCG ATTACCCTCA CGGAAAGTTT AAATTACACC   
  
  
- AGGTGGTGCC CCGACTAAAT AGGCTGAACC TAAAGAGAGT CGACCTATAA TTGCTACTGC TCCGTAATCG   
  
  
- GTAGTTGACA CAGTTGAGCG AGGTGAGCCA GTTATAGGTG GCGGCACTGA GCCAGTAGAG CCGGAAGGCC   
  
  
- GCCGAGGTGG GATCCCACCA CTGGCACCAC CTCCTCCTCC GACTGGAACG ACTGCACCCC CGCCTCCCCA   
  
  
- TGCTCAAGAT GTCCCCCAAA TTACTCACAA ATTCCACCCA ATCCCAAATG AAACTCCGGG ACCTTCTCAC   
  
  
- GAAAGGATCC TGTTCGTTAC TCTTTGAGTA CGAGCTCGCC CGGCGTCCCG CCCGAGACCA CCTGGAGGAC   
  
  
- CGGACGGGGT TCGGGCGTAG TAGGCTCGCC TCCCTCCGTC GACGCGCCAC CAGGCCCTCC TACGTCCCCC   
  
  
- GGCCCAAGCC GGTGCACCCC AAGTCACTAC TCCAGACACT ACTGCAGTTC CGGGAGGATT CCTCCATGTT   
  
  
- CCTCCCCACC AGGTACTATG TTACAAGGCG GCGCCTACCG CCGCGGAGGC GGCCTTATAA GAACAGCACC   
  
  
- TCCCTAGTCG GCCACCACAC CCGGTCACGT ACCTTCGGAA T

+     W box

| Site Name | Organism | Position | Strand | Matrix score. | sequence | function |
| --- | --- | --- | --- | --- | --- | --- |
| W box | Arabidopsis thaliana | 2753 | - | 6 | TTGACC |  |
| W box | Arabidopsis thaliana | 2972 | - | 6 | TTGACC |  |
| W box | Arabidopsis thaliana | 2387 | - | 6 | TTGACC |  |

>HU06G00358.1   
+ -Up\_Stream \_Len000GGGTTT TTTTAAACTC AAAATTAGTT ATTGTATCAA TTGAATTTGG AAAACTATTA   
  
  
+ TTTTCGACTT ATTATAGAGA ATTTGTTGTT AATGAATGCT GATAAATACT GATAATTAGT GTTGTGAGTG   
  
  
+ TGAATTTGTG ATGATGAGCA ATGATTAGCG TTAAAATTAT AACTAGGATA AATAATTAAG TGTTGATGAA   
  
  
+ TGTTGATTGA TGATGATGAG TACTGATTGG TGCGGTGCTG GTAATAAATA TTTATGATAA GTTAAAAAAA   
  
  
+ AAAAACACAT CCTAAACAAT CACTATGTAA TATACTAAAT GAAAAAAATT GGTAATGTGA TTTGACAATC   
  
  
+ TAAGGGTAAG GGTGTTTTAC CCACACATGT TATGAAATTG GCTTACTATT TACAAACTTT TTACAGTTAT   
  
  
+ ATGATTCTAC CTATGTGTTA TCATCTTTCC GTCGATGTAA TTGATTATAT TCAACACAAT TTAATTCATT   
  
  
+ ACAATGTAGC TAATTGATAC TCAAAATAAT GAACTATTAA TAAATAATTA ATTATAACTT TTAAAAGTTT   
  
  
+ GTTAATTTAA AATTATATAA TATATAAATT TAGGCTACAT TAATCAAAAA ATTAGGCGTG TTCATCGGTC   
  
  
+ CAAACTTGAA CCGGACCAGA CCGGACCGAA GATCGATTAA GAGAAAAAGT ACAGACTGAG GACCGGATCG   
  
  
+ AATAAGAGTC GCACCGGACC AGGATCGGAC CGGAAAAATT TGGACCAGAT CGGGACCGAT CGAGGTAGAC   
  
  
+ CGAATTTATT TTCAAATACG ATAGGAATAA AACTTATTGA TTCAATTGAT TTTGGCCAAA ATCATGCTAT   
  
  
+ TATTGATTTT ATTTGCTAAC TCTCAAGAGT ATTAGTTTGC ACTTCGCATT ATCTTTGGCC AAAAAAATCA   
  
  
+ AAAAGAAAGT CTCAAACTCT TAATAGTTTT TTATTTTATT TGGTTTATTT TTAAAATAGT CGGCCTTCGG   
  
  
+ TCCGGACTAG TGGACCGAAT AAGGTAATTT TTCGAATCGA GGACCAGACC AAATGCACTC AGTTCGAACC   
  
  
+ GGTTCAGTTC GGTCTGACTC GATTTTTGGT CCGGTCCAAG TTTGAACAGA ATAATGTACA CACCTAGAAA   
  
  
+ AAAAGAATTA TGCATATATA CTTAAGGCCC CCAACCCTTT TTCCCTTTCC GCCAATACTT TTTACCAAGT   
  
  
+ CCAATTCTTT TCTTCTTAGC TATGGCTAGT AGTTATCCCT CCCTAGCTTC CACCCTAAGT TTTGAGACAA   
  
  
+ AATCCCATCT ATGTTAATAT TTATTTCTTG CACATGTCAC CCAATACAAG GGTTTCATTG AAAAGAAAAG   
  
  
+ GTATTAGATG TAAGATATGT ATGTATATAA ATAGTTTTGC AATAGACTAG AAGGACTAAA AAGGAAAAAG   
  
  
+ GAAGTTTAAA AGCAAAAAAC TTTCCCCTCT AGAAAAAAGG AAACCAGATT CCCTTTTCTA CCCCAATTCC   
  
  
+ CATAGTCTTT ACTCTGTCTC TCTCTCTCTG TCTCTCTTAC TCATGATGTT GAGGGAGGAA GGAATGAATT   
  
  
+ AAGGACCCTA AGCTAAGATC CAACCGTAAG ATATTGCCCC TTTAATCACC CATTTCATAT CTCTATCCAC   
  
  
+ AGTTAAAAAC CCACCTCCTC CATCCACATA ACTGATATAT CTCTTCTACA CCATACTATA AGGAAGAAGA   
  
  
+ GAAAAATAGG AGTGTATTGT ATTTTTATTT GAGTAATTTA TTTTAAGGAG AGAAATTTAC ATGGTTGGGT   
  
  
+ TAAATGGATA CCTTGTTTAG ACTAGTGAGT TTCCAACAAC CACACCACCA CCATCACCAA CACCAACACC   
  
  
+ AACACCACCA ACAGCAATCT TCTGATCAAT CCTCTTTGAA TAATACTACT ACTTATTCCA CAAATTCAAG   
  
  
+ CAGTTCCAGA TCTTCCCAAC AAACCCATCA TAATCATAAT CATACTCAGT ATTATCACCA ACATCATCCT   
  
  
+ ACTTACCCCC ACCGCCACCA TCAGGAGGAA GAAGAAGAAT GCTTCAACTT TTACATGGAT GAAGAAAACT   
  
  
+ TCTCTTCTTC TTCCTCCCAC AACAAACATC ATCCTCAACC TCCTTCCTCC GCCACCACCT CCTCGTATTA   
  
  
+ CCCTTACTAC TCCACCCCCT TCGATGTTCC CTCTACCACC ACCACCTCCA CCTCCACCCC CACGCCCACC   
  
  
+ AACACCACCA CCACCACCCC CCCAACCCAG TATGGCGGCG GCGGCGGTGG GTTTGACTCC AGCTGCTTCT   
  
  
+ CCTACTCCCC TGCCCGAGAC CTCAACCCGG AGCTCTTCTC CTCGTCCGGG TTCTCGGGCA AGTGGGCTTC   
  
  
+ CAACATCCTC TTGGAAGCCG CTCGTGCCAT CTCTGAGAGG AACACGAGCC GCCTCCACCA GCTCCTATGG   
  
  
+ ATGGTCAACG AGCTCAGCTC CCCGTACGGT GACGTGGAGC AGAAGATAGC TTCATACTTC GGCCAAGCCC   
  
  
+ TCTTCACCCG CATGACCGAG TCAGGGGACC GCACCTACCG CACCATGCGG TCCGCCGTGG ACAAGACCTT   
  
  
+ CTCCTTTGAG TCTACGAGAA AGATGGTACT CAAGTTCCAG GAGGTTAGCC CTTGGACCAC CTTTGGACAC   
  
  
+ GTGGCAGCTA ATGGAGCCCT CATTGATGCC TTTGACGGTG AATCCAAAAT CCACATCATC GACATCAGCA   
  
  
+ CCACATTCTG CACCCAGTGG CCCACCCTCC TGGAGGCGCT GGCCACCCGA ATGGATGACG CGCCCTACCT   
  
  
+ GAGGCTGACC ACTGTGGTGG TCAACAAGTC CGGGAATGAA GGTCCCACCG GAGGCGGGTC CCACAGGGTG   
  
  
+ ATGAAAGAGA TTGGGACCCG CCTCGAGAAG TTTGCTAGGC TAATGGGAGT GCCTTTCAAA TTTAATGTGG   
  
  
+ TCCACCACGG GGCTGATTTA TCCGACTTGG ATTTCTCTCA GCTGGATATT AACGATGACG AGGCATTAGC   
  
  
+ CATCAACTGT GTCAACTCGC TCCACTCGGT CAATATCCAC CGCCGTGACT CGGTCATCTC GGCCTTCCGG   
  
  
+ CGGCTCCACC CTAGGGTGGT GACCGTGGTG GAGGAGGAGG CTGACCTTGC TGACGTGGGG GCGGAGGGGT   
  
  
+ ACGAGTTCTA CAGGGGGTTT AATGAGTGTT TAAGGTGGGT TAGGGTTTAC TTTGAGGCCC TGGAAGAGTG   
  
  
+ CTTTCCTAGG ACAAGCAATG AGAAACTCAT GCTCGAGCGG GCCGCAGGGC GGGCTCTGGT GGACCTCCTG   
  
  
+ GCCTGCCCCA AGCCCGCATC ATCCGAGCGG AGGGAGGCAG CTGCGCGGTG GTCCGGGAGG ATGCAGGGGG   
  
  
+ CCGGGTTCGG CCACGTGGGG TTCAGTGATG AGGTCTGTGA TGACGTCAAG GCCCTCCTAA GGAGGTACAA   
  
  
+ GGAGGGGTGG TCCATGATAC AATGTTCCGC CGCGGATGGC GGCGCCTCCG CCGGAATATT CTTGTCGTGG   
  
  
+ AGGGATCAGC CGGTGGTGTG GGCCAGTGCA TGGAAGCCTT A  

- -Up\_Stream \_Len000CCCAAA AAAATTTGAG TTTTAATCAA TAACATAGTT AACTTAAACC TTTTGATAAT   
  
  
- AAAAGCTGAA TAATATCTCT TAAACAACAA TTACTTACGA CTATTTATGA CTATTAATCA CAACACTCAC   
  
  
- ACTTAAACAC TACTACTCGT TACTAATCGC AATTTTAATA TTGATCCTAT TTATTAATTC ACAACTACTT   
  
  
- ACAACTAACT ACTACTACTC ATGACTAACC ACGCCACGAC CATTATTTAT AAATACTATT CAATTTTTTT   
  
  
- TTTTTGTGTA GGATTTGTTA GTGATACATT ATATGATTTA CTTTTTTTAA CCATTACACT AAACTGTTAG   
  
  
- ATTCCCATTC CCACAAAATG GGTGTGTACA ATACTTTAAC CGAATGATAA ATGTTTGAAA AATGTCAATA   
  
  
- TACTAAGATG GATACACAAT AGTAGAAAGG CAGCTACATT AACTAATATA AGTTGTGTTA AATTAAGTAA   
  
  
- TGTTACATCG ATTAACTATG AGTTTTATTA CTTGATAATT ATTTATTAAT TAATATTGAA AATTTTCAAA   
  
  
- CAATTAAATT TTAATATATT ATATATTTAA ATCCGATGTA ATTAGTTTTT TAATCCGCAC AAGTAGCCAG   
  
  
- GTTTGAACTT GGCCTGGTCT GGCCTGGCTT CTAGCTAATT CTCTTTTTCA TGTCTGACTC CTGGCCTAGC   
  
  
- TTATTCTCAG CGTGGCCTGG TCCTAGCCTG GCCTTTTTAA ACCTGGTCTA GCCCTGGCTA GCTCCATCTG   
  
  
- GCTTAAATAA AAGTTTATGC TATCCTTATT TTGAATAACT AAGTTAACTA AAACCGGTTT TAGTACGATA   
  
  
- ATAACTAAAA TAAACGATTG AGAGTTCTCA TAATCAAACG TGAAGCGTAA TAGAAACCGG TTTTTTTAGT   
  
  
- TTTTCTTTCA GAGTTTGAGA ATTATCAAAA AATAAAATAA ACCAAATAAA AATTTTATCA GCCGGAAGCC   
  
  
- AGGCCTGATC ACCTGGCTTA TTCCATTAAA AAGCTTAGCT CCTGGTCTGG TTTACGTGAG TCAAGCTTGG   
  
  
- CCAAGTCAAG CCAGACTGAG CTAAAAACCA GGCCAGGTTC AAACTTGTCT TATTACATGT GTGGATCTTT   
  
  
- TTTTCTTAAT ACGTATATAT GAATTCCGGG GGTTGGGAAA AAGGGAAAGG CGGTTATGAA AAATGGTTCA   
  
  
- GGTTAAGAAA AGAAGAATCG ATACCGATCA TCAATAGGGA GGGATCGAAG GTGGGATTCA AAACTCTGTT   
  
  
- TTAGGGTAGA TACAATTATA AATAAAGAAC GTGTACAGTG GGTTATGTTC CCAAAGTAAC TTTTCTTTTC   
  
  
- CATAATCTAC ATTCTATACA TACATATATT TATCAAAACG TTATCTGATC TTCCTGATTT TTCCTTTTTC   
  
  
- CTTCAAATTT TCGTTTTTTG AAAGGGGAGA TCTTTTTTCC TTTGGTCTAA GGGAAAAGAT GGGGTTAAGG   
  
  
- GTATCAGAAA TGAGACAGAG AGAGAGAGAC AGAGAGAATG AGTACTACAA CTCCCTCCTT CCTTACTTAA   
  
  
- TTCCTGGGAT TCGATTCTAG GTTGGCATTC TATAACGGGG AAATTAGTGG GTAAAGTATA GAGATAGGTG   
  
  
- TCAATTTTTG GGTGGAGGAG GTAGGTGTAT TGACTATATA GAGAAGATGT GGTATGATAT TCCTTCTTCT   
  
  
- CTTTTTATCC TCACATAACA TAAAAATAAA CTCATTAAAT AAAATTCCTC TCTTTAAATG TACCAACCCA   
  
  
- ATTTACCTAT GGAACAAATC TGATCACTCA AAGGTTGTTG GTGTGGTGGT GGTAGTGGTT GTGGTTGTGG   
  
  
- TTGTGGTGGT TGTCGTTAGA AGACTAGTTA GGAGAAACTT ATTATGATGA TGAATAAGGT GTTTAAGTTC   
  
  
- GTCAAGGTCT AGAAGGGTTG TTTGGGTAGT ATTAGTATTA GTATGAGTCA TAATAGTGGT TGTAGTAGGA   
  
  
- TGAATGGGGG TGGCGGTGGT AGTCCTCCTT CTTCTTCTTA CGAAGTTGAA AATGTACCTA CTTCTTTTGA   
  
  
- AGAGAAGAAG AAGGAGGGTG TTGTTTGTAG TAGGAGTTGG AGGAAGGAGG CGGTGGTGGA GGAGCATAAT   
  
  
- GGGAATGATG AGGTGGGGGA AGCTACAAGG GAGATGGTGG TGGTGGAGGT GGAGGTGGGG GTGCGGGTGG   
  
  
- TTGTGGTGGT GGTGGTGGGG GGGTTGGGTC ATACCGCCGC CGCCGCCACC CAAACTGAGG TCGACGAAGA   
  
  
- GGATGAGGGG ACGGGCTCTG GAGTTGGGCC TCGAGAAGAG GAGCAGGCCC AAGAGCCCGT TCACCCGAAG   
  
  
- GTTGTAGGAG AACCTTCGGC GAGCACGGTA GAGACTCTCC TTGTGCTCGG CGGAGGTGGT CGAGGATACC   
  
  
- TACCAGTTGC TCGAGTCGAG GGGCATGCCA CTGCACCTCG TCTTCTATCG AAGTATGAAG CCGGTTCGGG   
  
  
- AGAAGTGGGC GTACTGGCTC AGTCCCCTGG CGTGGATGGC GTGGTACGCC AGGCGGCACC TGTTCTGGAA   
  
  
- GAGGAAACTC AGATGCTCTT TCTACCATGA GTTCAAGGTC CTCCAATCGG GAACCTGGTG GAAACCTGTG   
  
  
- CACCGTCGAT TACCTCGGGA GTAACTACGG AAACTGCCAC TTAGGTTTTA GGTGTAGTAG CTGTAGTCGT   
  
  
- GGTGTAAGAC GTGGGTCACC GGGTGGGAGG ACCTCCGCGA CCGGTGGGCT TACCTACTGC GCGGGATGGA   
  
  
- CTCCGACTGG TGACACCACC AGTTGTTCAG GCCCTTACTT CCAGGGTGGC CTCCGCCCAG GGTGTCCCAC   
  
  
- TACTTTCTCT AACCCTGGGC GGAGCTCTTC AAACGATCCG ATTACCCTCA CGGAAAGTTT AAATTACACC   
  
  
- AGGTGGTGCC CCGACTAAAT AGGCTGAACC TAAAGAGAGT CGACCTATAA TTGCTACTGC TCCGTAATCG   
  
  
- GTAGTTGACA CAGTTGAGCG AGGTGAGCCA GTTATAGGTG GCGGCACTGA GCCAGTAGAG CCGGAAGGCC   
  
  
- GCCGAGGTGG GATCCCACCA CTGGCACCAC CTCCTCCTCC GACTGGAACG ACTGCACCCC CGCCTCCCCA   
  
  
- TGCTCAAGAT GTCCCCCAAA TTACTCACAA ATTCCACCCA ATCCCAAATG AAACTCCGGG ACCTTCTCAC   
  
  
- GAAAGGATCC TGTTCGTTAC TCTTTGAGTA CGAGCTCGCC CGGCGTCCCG CCCGAGACCA CCTGGAGGAC   
  
  
- CGGACGGGGT TCGGGCGTAG TAGGCTCGCC TCCCTCCGTC GACGCGCCAC CAGGCCCTCC TACGTCCCCC   
  
  
- GGCCCAAGCC GGTGCACCCC AAGTCACTAC TCCAGACACT ACTGCAGTTC CGGGAGGATT CCTCCATGTT   
  
  
- CCTCCCCACC AGGTACTATG TTACAAGGCG GCGCCTACCG CCGCGGAGGC GGCCTTATAA GAACAGCACC   
  
  
- TCCCTAGTCG GCCACCACAC CCGGTCACGT ACCTTCGGAA T

+     WRE3

| Site Name | Organism | Position | Strand | Matrix score. | sequence | function |
| --- | --- | --- | --- | --- | --- | --- |
| WRE3 | Pisum sativum | 3117 | - | 6 | CCACCT |  |
| WRE3 | Pisum sativum | 2581 | + | 6 | CCACCT |  |
| WRE3 | Pisum sativum | 2152 | + | 6 | CCACCT |  |
| WRE3 | Pisum sativum | 1625 | + | 6 | CCACCT |  |
| WRE3 | Pisum sativum | 2089 | + | 6 | CCACCT |  |
| WRE3 | Pisum sativum | 2146 | + | 6 | CCACCT |  |

>HU06G00358.1   
+ -Up\_Stream \_Len000GGGTTT TTTTAAACTC AAAATTAGTT ATTGTATCAA TTGAATTTGG AAAACTATTA   
  
  
+ TTTTCGACTT ATTATAGAGA ATTTGTTGTT AATGAATGCT GATAAATACT GATAATTAGT GTTGTGAGTG   
  
  
+ TGAATTTGTG ATGATGAGCA ATGATTAGCG TTAAAATTAT AACTAGGATA AATAATTAAG TGTTGATGAA   
  
  
+ TGTTGATTGA TGATGATGAG TACTGATTGG TGCGGTGCTG GTAATAAATA TTTATGATAA GTTAAAAAAA   
  
  
+ AAAAACACAT CCTAAACAAT CACTATGTAA TATACTAAAT GAAAAAAATT GGTAATGTGA TTTGACAATC   
  
  
+ TAAGGGTAAG GGTGTTTTAC CCACACATGT TATGAAATTG GCTTACTATT TACAAACTTT TTACAGTTAT   
  
  
+ ATGATTCTAC CTATGTGTTA TCATCTTTCC GTCGATGTAA TTGATTATAT TCAACACAAT TTAATTCATT   
  
  
+ ACAATGTAGC TAATTGATAC TCAAAATAAT GAACTATTAA TAAATAATTA ATTATAACTT TTAAAAGTTT   
  
  
+ GTTAATTTAA AATTATATAA TATATAAATT TAGGCTACAT TAATCAAAAA ATTAGGCGTG TTCATCGGTC   
  
  
+ CAAACTTGAA CCGGACCAGA CCGGACCGAA GATCGATTAA GAGAAAAAGT ACAGACTGAG GACCGGATCG   
  
  
+ AATAAGAGTC GCACCGGACC AGGATCGGAC CGGAAAAATT TGGACCAGAT CGGGACCGAT CGAGGTAGAC   
  
  
+ CGAATTTATT TTCAAATACG ATAGGAATAA AACTTATTGA TTCAATTGAT TTTGGCCAAA ATCATGCTAT   
  
  
+ TATTGATTTT ATTTGCTAAC TCTCAAGAGT ATTAGTTTGC ACTTCGCATT ATCTTTGGCC AAAAAAATCA   
  
  
+ AAAAGAAAGT CTCAAACTCT TAATAGTTTT TTATTTTATT TGGTTTATTT TTAAAATAGT CGGCCTTCGG   
  
  
+ TCCGGACTAG TGGACCGAAT AAGGTAATTT TTCGAATCGA GGACCAGACC AAATGCACTC AGTTCGAACC   
  
  
+ GGTTCAGTTC GGTCTGACTC GATTTTTGGT CCGGTCCAAG TTTGAACAGA ATAATGTACA CACCTAGAAA   
  
  
+ AAAAGAATTA TGCATATATA CTTAAGGCCC CCAACCCTTT TTCCCTTTCC GCCAATACTT TTTACCAAGT   
  
  
+ CCAATTCTTT TCTTCTTAGC TATGGCTAGT AGTTATCCCT CCCTAGCTTC CACCCTAAGT TTTGAGACAA   
  
  
+ AATCCCATCT ATGTTAATAT TTATTTCTTG CACATGTCAC CCAATACAAG GGTTTCATTG AAAAGAAAAG   
  
  
+ GTATTAGATG TAAGATATGT ATGTATATAA ATAGTTTTGC AATAGACTAG AAGGACTAAA AAGGAAAAAG   
  
  
+ GAAGTTTAAA AGCAAAAAAC TTTCCCCTCT AGAAAAAAGG AAACCAGATT CCCTTTTCTA CCCCAATTCC   
  
  
+ CATAGTCTTT ACTCTGTCTC TCTCTCTCTG TCTCTCTTAC TCATGATGTT GAGGGAGGAA GGAATGAATT   
  
  
+ AAGGACCCTA AGCTAAGATC CAACCGTAAG ATATTGCCCC TTTAATCACC CATTTCATAT CTCTATCCAC   
  
  
+ AGTTAAAAAC CCACCTCCTC CATCCACATA ACTGATATAT CTCTTCTACA CCATACTATA AGGAAGAAGA   
  
  
+ GAAAAATAGG AGTGTATTGT ATTTTTATTT GAGTAATTTA TTTTAAGGAG AGAAATTTAC ATGGTTGGGT   
  
  
+ TAAATGGATA CCTTGTTTAG ACTAGTGAGT TTCCAACAAC CACACCACCA CCATCACCAA CACCAACACC   
  
  
+ AACACCACCA ACAGCAATCT TCTGATCAAT CCTCTTTGAA TAATACTACT ACTTATTCCA CAAATTCAAG   
  
  
+ CAGTTCCAGA TCTTCCCAAC AAACCCATCA TAATCATAAT CATACTCAGT ATTATCACCA ACATCATCCT   
  
  
+ ACTTACCCCC ACCGCCACCA TCAGGAGGAA GAAGAAGAAT GCTTCAACTT TTACATGGAT GAAGAAAACT   
  
  
+ TCTCTTCTTC TTCCTCCCAC AACAAACATC ATCCTCAACC TCCTTCCTCC GCCACCACCT CCTCGTATTA   
  
  
+ CCCTTACTAC TCCACCCCCT TCGATGTTCC CTCTACCACC ACCACCTCCA CCTCCACCCC CACGCCCACC   
  
  
+ AACACCACCA CCACCACCCC CCCAACCCAG TATGGCGGCG GCGGCGGTGG GTTTGACTCC AGCTGCTTCT   
  
  
+ CCTACTCCCC TGCCCGAGAC CTCAACCCGG AGCTCTTCTC CTCGTCCGGG TTCTCGGGCA AGTGGGCTTC   
  
  
+ CAACATCCTC TTGGAAGCCG CTCGTGCCAT CTCTGAGAGG AACACGAGCC GCCTCCACCA GCTCCTATGG   
  
  
+ ATGGTCAACG AGCTCAGCTC CCCGTACGGT GACGTGGAGC AGAAGATAGC TTCATACTTC GGCCAAGCCC   
  
  
+ TCTTCACCCG CATGACCGAG TCAGGGGACC GCACCTACCG CACCATGCGG TCCGCCGTGG ACAAGACCTT   
  
  
+ CTCCTTTGAG TCTACGAGAA AGATGGTACT CAAGTTCCAG GAGGTTAGCC CTTGGACCAC CTTTGGACAC   
  
  
+ GTGGCAGCTA ATGGAGCCCT CATTGATGCC TTTGACGGTG AATCCAAAAT CCACATCATC GACATCAGCA   
  
  
+ CCACATTCTG CACCCAGTGG CCCACCCTCC TGGAGGCGCT GGCCACCCGA ATGGATGACG CGCCCTACCT   
  
  
+ GAGGCTGACC ACTGTGGTGG TCAACAAGTC CGGGAATGAA GGTCCCACCG GAGGCGGGTC CCACAGGGTG   
  
  
+ ATGAAAGAGA TTGGGACCCG CCTCGAGAAG TTTGCTAGGC TAATGGGAGT GCCTTTCAAA TTTAATGTGG   
  
  
+ TCCACCACGG GGCTGATTTA TCCGACTTGG ATTTCTCTCA GCTGGATATT AACGATGACG AGGCATTAGC   
  
  
+ CATCAACTGT GTCAACTCGC TCCACTCGGT CAATATCCAC CGCCGTGACT CGGTCATCTC GGCCTTCCGG   
  
  
+ CGGCTCCACC CTAGGGTGGT GACCGTGGTG GAGGAGGAGG CTGACCTTGC TGACGTGGGG GCGGAGGGGT   
  
  
+ ACGAGTTCTA CAGGGGGTTT AATGAGTGTT TAAGGTGGGT TAGGGTTTAC TTTGAGGCCC TGGAAGAGTG   
  
  
+ CTTTCCTAGG ACAAGCAATG AGAAACTCAT GCTCGAGCGG GCCGCAGGGC GGGCTCTGGT GGACCTCCTG   
  
  
+ GCCTGCCCCA AGCCCGCATC ATCCGAGCGG AGGGAGGCAG CTGCGCGGTG GTCCGGGAGG ATGCAGGGGG   
  
  
+ CCGGGTTCGG CCACGTGGGG TTCAGTGATG AGGTCTGTGA TGACGTCAAG GCCCTCCTAA GGAGGTACAA   
  
  
+ GGAGGGGTGG TCCATGATAC AATGTTCCGC CGCGGATGGC GGCGCCTCCG CCGGAATATT CTTGTCGTGG   
  
  
+ AGGGATCAGC CGGTGGTGTG GGCCAGTGCA TGGAAGCCTT A  

- -Up\_Stream \_Len000CCCAAA AAAATTTGAG TTTTAATCAA TAACATAGTT AACTTAAACC TTTTGATAAT   
  
  
- AAAAGCTGAA TAATATCTCT TAAACAACAA TTACTTACGA CTATTTATGA CTATTAATCA CAACACTCAC   
  
  
- ACTTAAACAC TACTACTCGT TACTAATCGC AATTTTAATA TTGATCCTAT TTATTAATTC ACAACTACTT   
  
  
- ACAACTAACT ACTACTACTC ATGACTAACC ACGCCACGAC CATTATTTAT AAATACTATT CAATTTTTTT   
  
  
- TTTTTGTGTA GGATTTGTTA GTGATACATT ATATGATTTA CTTTTTTTAA CCATTACACT AAACTGTTAG   
  
  
- ATTCCCATTC CCACAAAATG GGTGTGTACA ATACTTTAAC CGAATGATAA ATGTTTGAAA AATGTCAATA   
  
  
- TACTAAGATG GATACACAAT AGTAGAAAGG CAGCTACATT AACTAATATA AGTTGTGTTA AATTAAGTAA   
  
  
- TGTTACATCG ATTAACTATG AGTTTTATTA CTTGATAATT ATTTATTAAT TAATATTGAA AATTTTCAAA   
  
  
- CAATTAAATT TTAATATATT ATATATTTAA ATCCGATGTA ATTAGTTTTT TAATCCGCAC AAGTAGCCAG   
  
  
- GTTTGAACTT GGCCTGGTCT GGCCTGGCTT CTAGCTAATT CTCTTTTTCA TGTCTGACTC CTGGCCTAGC   
  
  
- TTATTCTCAG CGTGGCCTGG TCCTAGCCTG GCCTTTTTAA ACCTGGTCTA GCCCTGGCTA GCTCCATCTG   
  
  
- GCTTAAATAA AAGTTTATGC TATCCTTATT TTGAATAACT AAGTTAACTA AAACCGGTTT TAGTACGATA   
  
  
- ATAACTAAAA TAAACGATTG AGAGTTCTCA TAATCAAACG TGAAGCGTAA TAGAAACCGG TTTTTTTAGT   
  
  
- TTTTCTTTCA GAGTTTGAGA ATTATCAAAA AATAAAATAA ACCAAATAAA AATTTTATCA GCCGGAAGCC   
  
  
- AGGCCTGATC ACCTGGCTTA TTCCATTAAA AAGCTTAGCT CCTGGTCTGG TTTACGTGAG TCAAGCTTGG   
  
  
- CCAAGTCAAG CCAGACTGAG CTAAAAACCA GGCCAGGTTC AAACTTGTCT TATTACATGT GTGGATCTTT   
  
  
- TTTTCTTAAT ACGTATATAT GAATTCCGGG GGTTGGGAAA AAGGGAAAGG CGGTTATGAA AAATGGTTCA   
  
  
- GGTTAAGAAA AGAAGAATCG ATACCGATCA TCAATAGGGA GGGATCGAAG GTGGGATTCA AAACTCTGTT   
  
  
- TTAGGGTAGA TACAATTATA AATAAAGAAC GTGTACAGTG GGTTATGTTC CCAAAGTAAC TTTTCTTTTC   
  
  
- CATAATCTAC ATTCTATACA TACATATATT TATCAAAACG TTATCTGATC TTCCTGATTT TTCCTTTTTC   
  
  
- CTTCAAATTT TCGTTTTTTG AAAGGGGAGA TCTTTTTTCC TTTGGTCTAA GGGAAAAGAT GGGGTTAAGG   
  
  
- GTATCAGAAA TGAGACAGAG AGAGAGAGAC AGAGAGAATG AGTACTACAA CTCCCTCCTT CCTTACTTAA   
  
  
- TTCCTGGGAT TCGATTCTAG GTTGGCATTC TATAACGGGG AAATTAGTGG GTAAAGTATA GAGATAGGTG   
  
  
- TCAATTTTTG GGTGGAGGAG GTAGGTGTAT TGACTATATA GAGAAGATGT GGTATGATAT TCCTTCTTCT   
  
  
- CTTTTTATCC TCACATAACA TAAAAATAAA CTCATTAAAT AAAATTCCTC TCTTTAAATG TACCAACCCA   
  
  
- ATTTACCTAT GGAACAAATC TGATCACTCA AAGGTTGTTG GTGTGGTGGT GGTAGTGGTT GTGGTTGTGG   
  
  
- TTGTGGTGGT TGTCGTTAGA AGACTAGTTA GGAGAAACTT ATTATGATGA TGAATAAGGT GTTTAAGTTC   
  
  
- GTCAAGGTCT AGAAGGGTTG TTTGGGTAGT ATTAGTATTA GTATGAGTCA TAATAGTGGT TGTAGTAGGA   
  
  
- TGAATGGGGG TGGCGGTGGT AGTCCTCCTT CTTCTTCTTA CGAAGTTGAA AATGTACCTA CTTCTTTTGA   
  
  
- AGAGAAGAAG AAGGAGGGTG TTGTTTGTAG TAGGAGTTGG AGGAAGGAGG CGGTGGTGGA GGAGCATAAT   
  
  
- GGGAATGATG AGGTGGGGGA AGCTACAAGG GAGATGGTGG TGGTGGAGGT GGAGGTGGGG GTGCGGGTGG   
  
  
- TTGTGGTGGT GGTGGTGGGG GGGTTGGGTC ATACCGCCGC CGCCGCCACC CAAACTGAGG TCGACGAAGA   
  
  
- GGATGAGGGG ACGGGCTCTG GAGTTGGGCC TCGAGAAGAG GAGCAGGCCC AAGAGCCCGT TCACCCGAAG   
  
  
- GTTGTAGGAG AACCTTCGGC GAGCACGGTA GAGACTCTCC TTGTGCTCGG CGGAGGTGGT CGAGGATACC   
  
  
- TACCAGTTGC TCGAGTCGAG GGGCATGCCA CTGCACCTCG TCTTCTATCG AAGTATGAAG CCGGTTCGGG   
  
  
- AGAAGTGGGC GTACTGGCTC AGTCCCCTGG CGTGGATGGC GTGGTACGCC AGGCGGCACC TGTTCTGGAA   
  
  
- GAGGAAACTC AGATGCTCTT TCTACCATGA GTTCAAGGTC CTCCAATCGG GAACCTGGTG GAAACCTGTG   
  
  
- CACCGTCGAT TACCTCGGGA GTAACTACGG AAACTGCCAC TTAGGTTTTA GGTGTAGTAG CTGTAGTCGT   
  
  
- GGTGTAAGAC GTGGGTCACC GGGTGGGAGG ACCTCCGCGA CCGGTGGGCT TACCTACTGC GCGGGATGGA   
  
  
- CTCCGACTGG TGACACCACC AGTTGTTCAG GCCCTTACTT CCAGGGTGGC CTCCGCCCAG GGTGTCCCAC   
  
  
- TACTTTCTCT AACCCTGGGC GGAGCTCTTC AAACGATCCG ATTACCCTCA CGGAAAGTTT AAATTACACC   
  
  
- AGGTGGTGCC CCGACTAAAT AGGCTGAACC TAAAGAGAGT CGACCTATAA TTGCTACTGC TCCGTAATCG   
  
  
- GTAGTTGACA CAGTTGAGCG AGGTGAGCCA GTTATAGGTG GCGGCACTGA GCCAGTAGAG CCGGAAGGCC   
  
  
- GCCGAGGTGG GATCCCACCA CTGGCACCAC CTCCTCCTCC GACTGGAACG ACTGCACCCC CGCCTCCCCA   
  
  
- TGCTCAAGAT GTCCCCCAAA TTACTCACAA ATTCCACCCA ATCCCAAATG AAACTCCGGG ACCTTCTCAC   
  
  
- GAAAGGATCC TGTTCGTTAC TCTTTGAGTA CGAGCTCGCC CGGCGTCCCG CCCGAGACCA CCTGGAGGAC   
  
  
- CGGACGGGGT TCGGGCGTAG TAGGCTCGCC TCCCTCCGTC GACGCGCCAC CAGGCCCTCC TACGTCCCCC   
  
  
- GGCCCAAGCC GGTGCACCCC AAGTCACTAC TCCAGACACT ACTGCAGTTC CGGGAGGATT CCTCCATGTT   
  
  
- CCTCCCCACC AGGTACTATG TTACAAGGCG GCGCCTACCG CCGCGGAGGC GGCCTTATAA GAACAGCACC   
  
  
- TCCCTAGTCG GCCACCACAC CCGGTCACGT ACCTTCGGAA T

+     WUN-motif

| Site Name | Organism | Position | Strand | Matrix score. | sequence | function |
| --- | --- | --- | --- | --- | --- | --- |
| WUN-motif | Nicotiana glutinosa | 459 | - | 9 | CAATTACAT |  |
| WUN-motif | Nicotiana glutinosa | 1716 | - | 8 | AAATTACT |  |

>HU06G00358.1   
+ -Up\_Stream \_Len000GGGTTT TTTTAAACTC AAAATTAGTT ATTGTATCAA TTGAATTTGG AAAACTATTA   
  
  
+ TTTTCGACTT ATTATAGAGA ATTTGTTGTT AATGAATGCT GATAAATACT GATAATTAGT GTTGTGAGTG   
  
  
+ TGAATTTGTG ATGATGAGCA ATGATTAGCG TTAAAATTAT AACTAGGATA AATAATTAAG TGTTGATGAA   
  
  
+ TGTTGATTGA TGATGATGAG TACTGATTGG TGCGGTGCTG GTAATAAATA TTTATGATAA GTTAAAAAAA   
  
  
+ AAAAACACAT CCTAAACAAT CACTATGTAA TATACTAAAT GAAAAAAATT GGTAATGTGA TTTGACAATC   
  
  
+ TAAGGGTAAG GGTGTTTTAC CCACACATGT TATGAAATTG GCTTACTATT TACAAACTTT TTACAGTTAT   
  
  
+ ATGATTCTAC CTATGTGTTA TCATCTTTCC GTCGATGTAA TTGATTATAT TCAACACAAT TTAATTCATT   
  
  
+ ACAATGTAGC TAATTGATAC TCAAAATAAT GAACTATTAA TAAATAATTA ATTATAACTT TTAAAAGTTT   
  
  
+ GTTAATTTAA AATTATATAA TATATAAATT TAGGCTACAT TAATCAAAAA ATTAGGCGTG TTCATCGGTC   
  
  
+ CAAACTTGAA CCGGACCAGA CCGGACCGAA GATCGATTAA GAGAAAAAGT ACAGACTGAG GACCGGATCG   
  
  
+ AATAAGAGTC GCACCGGACC AGGATCGGAC CGGAAAAATT TGGACCAGAT CGGGACCGAT CGAGGTAGAC   
  
  
+ CGAATTTATT TTCAAATACG ATAGGAATAA AACTTATTGA TTCAATTGAT TTTGGCCAAA ATCATGCTAT   
  
  
+ TATTGATTTT ATTTGCTAAC TCTCAAGAGT ATTAGTTTGC ACTTCGCATT ATCTTTGGCC AAAAAAATCA   
  
  
+ AAAAGAAAGT CTCAAACTCT TAATAGTTTT TTATTTTATT TGGTTTATTT TTAAAATAGT CGGCCTTCGG   
  
  
+ TCCGGACTAG TGGACCGAAT AAGGTAATTT TTCGAATCGA GGACCAGACC AAATGCACTC AGTTCGAACC   
  
  
+ GGTTCAGTTC GGTCTGACTC GATTTTTGGT CCGGTCCAAG TTTGAACAGA ATAATGTACA CACCTAGAAA   
  
  
+ AAAAGAATTA TGCATATATA CTTAAGGCCC CCAACCCTTT TTCCCTTTCC GCCAATACTT TTTACCAAGT   
  
  
+ CCAATTCTTT TCTTCTTAGC TATGGCTAGT AGTTATCCCT CCCTAGCTTC CACCCTAAGT TTTGAGACAA   
  
  
+ AATCCCATCT ATGTTAATAT TTATTTCTTG CACATGTCAC CCAATACAAG GGTTTCATTG AAAAGAAAAG   
  
  
+ GTATTAGATG TAAGATATGT ATGTATATAA ATAGTTTTGC AATAGACTAG AAGGACTAAA AAGGAAAAAG   
  
  
+ GAAGTTTAAA AGCAAAAAAC TTTCCCCTCT AGAAAAAAGG AAACCAGATT CCCTTTTCTA CCCCAATTCC   
  
  
+ CATAGTCTTT ACTCTGTCTC TCTCTCTCTG TCTCTCTTAC TCATGATGTT GAGGGAGGAA GGAATGAATT   
  
  
+ AAGGACCCTA AGCTAAGATC CAACCGTAAG ATATTGCCCC TTTAATCACC CATTTCATAT CTCTATCCAC   
  
  
+ AGTTAAAAAC CCACCTCCTC CATCCACATA ACTGATATAT CTCTTCTACA CCATACTATA AGGAAGAAGA   
  
  
+ GAAAAATAGG AGTGTATTGT ATTTTTATTT GAGTAATTTA TTTTAAGGAG AGAAATTTAC ATGGTTGGGT   
  
  
+ TAAATGGATA CCTTGTTTAG ACTAGTGAGT TTCCAACAAC CACACCACCA CCATCACCAA CACCAACACC   
  
  
+ AACACCACCA ACAGCAATCT TCTGATCAAT CCTCTTTGAA TAATACTACT ACTTATTCCA CAAATTCAAG   
  
  
+ CAGTTCCAGA TCTTCCCAAC AAACCCATCA TAATCATAAT CATACTCAGT ATTATCACCA ACATCATCCT   
  
  
+ ACTTACCCCC ACCGCCACCA TCAGGAGGAA GAAGAAGAAT GCTTCAACTT TTACATGGAT GAAGAAAACT   
  
  
+ TCTCTTCTTC TTCCTCCCAC AACAAACATC ATCCTCAACC TCCTTCCTCC GCCACCACCT CCTCGTATTA   
  
  
+ CCCTTACTAC TCCACCCCCT TCGATGTTCC CTCTACCACC ACCACCTCCA CCTCCACCCC CACGCCCACC   
  
  
+ AACACCACCA CCACCACCCC CCCAACCCAG TATGGCGGCG GCGGCGGTGG GTTTGACTCC AGCTGCTTCT   
  
  
+ CCTACTCCCC TGCCCGAGAC CTCAACCCGG AGCTCTTCTC CTCGTCCGGG TTCTCGGGCA AGTGGGCTTC   
  
  
+ CAACATCCTC TTGGAAGCCG CTCGTGCCAT CTCTGAGAGG AACACGAGCC GCCTCCACCA GCTCCTATGG   
  
  
+ ATGGTCAACG AGCTCAGCTC CCCGTACGGT GACGTGGAGC AGAAGATAGC TTCATACTTC GGCCAAGCCC   
  
  
+ TCTTCACCCG CATGACCGAG TCAGGGGACC GCACCTACCG CACCATGCGG TCCGCCGTGG ACAAGACCTT   
  
  
+ CTCCTTTGAG TCTACGAGAA AGATGGTACT CAAGTTCCAG GAGGTTAGCC CTTGGACCAC CTTTGGACAC   
  
  
+ GTGGCAGCTA ATGGAGCCCT CATTGATGCC TTTGACGGTG AATCCAAAAT CCACATCATC GACATCAGCA   
  
  
+ CCACATTCTG CACCCAGTGG CCCACCCTCC TGGAGGCGCT GGCCACCCGA ATGGATGACG CGCCCTACCT   
  
  
+ GAGGCTGACC ACTGTGGTGG TCAACAAGTC CGGGAATGAA GGTCCCACCG GAGGCGGGTC CCACAGGGTG   
  
  
+ ATGAAAGAGA TTGGGACCCG CCTCGAGAAG TTTGCTAGGC TAATGGGAGT GCCTTTCAAA TTTAATGTGG   
  
  
+ TCCACCACGG GGCTGATTTA TCCGACTTGG ATTTCTCTCA GCTGGATATT AACGATGACG AGGCATTAGC   
  
  
+ CATCAACTGT GTCAACTCGC TCCACTCGGT CAATATCCAC CGCCGTGACT CGGTCATCTC GGCCTTCCGG   
  
  
+ CGGCTCCACC CTAGGGTGGT GACCGTGGTG GAGGAGGAGG CTGACCTTGC TGACGTGGGG GCGGAGGGGT   
  
  
+ ACGAGTTCTA CAGGGGGTTT AATGAGTGTT TAAGGTGGGT TAGGGTTTAC TTTGAGGCCC TGGAAGAGTG   
  
  
+ CTTTCCTAGG ACAAGCAATG AGAAACTCAT GCTCGAGCGG GCCGCAGGGC GGGCTCTGGT GGACCTCCTG   
  
  
+ GCCTGCCCCA AGCCCGCATC ATCCGAGCGG AGGGAGGCAG CTGCGCGGTG GTCCGGGAGG ATGCAGGGGG   
  
  
+ CCGGGTTCGG CCACGTGGGG TTCAGTGATG AGGTCTGTGA TGACGTCAAG GCCCTCCTAA GGAGGTACAA   
  
  
+ GGAGGGGTGG TCCATGATAC AATGTTCCGC CGCGGATGGC GGCGCCTCCG CCGGAATATT CTTGTCGTGG   
  
  
+ AGGGATCAGC CGGTGGTGTG GGCCAGTGCA TGGAAGCCTT A  

- -Up\_Stream \_Len000CCCAAA AAAATTTGAG TTTTAATCAA TAACATAGTT AACTTAAACC TTTTGATAAT   
  
  
- AAAAGCTGAA TAATATCTCT TAAACAACAA TTACTTACGA CTATTTATGA CTATTAATCA CAACACTCAC   
  
  
- ACTTAAACAC TACTACTCGT TACTAATCGC AATTTTAATA TTGATCCTAT TTATTAATTC ACAACTACTT   
  
  
- ACAACTAACT ACTACTACTC ATGACTAACC ACGCCACGAC CATTATTTAT AAATACTATT CAATTTTTTT   
  
  
- TTTTTGTGTA GGATTTGTTA GTGATACATT ATATGATTTA CTTTTTTTAA CCATTACACT AAACTGTTAG   
  
  
- ATTCCCATTC CCACAAAATG GGTGTGTACA ATACTTTAAC CGAATGATAA ATGTTTGAAA AATGTCAATA   
  
  
- TACTAAGATG GATACACAAT AGTAGAAAGG CAGCTACATT AACTAATATA AGTTGTGTTA AATTAAGTAA   
  
  
- TGTTACATCG ATTAACTATG AGTTTTATTA CTTGATAATT ATTTATTAAT TAATATTGAA AATTTTCAAA   
  
  
- CAATTAAATT TTAATATATT ATATATTTAA ATCCGATGTA ATTAGTTTTT TAATCCGCAC AAGTAGCCAG   
  
  
- GTTTGAACTT GGCCTGGTCT GGCCTGGCTT CTAGCTAATT CTCTTTTTCA TGTCTGACTC CTGGCCTAGC   
  
  
- TTATTCTCAG CGTGGCCTGG TCCTAGCCTG GCCTTTTTAA ACCTGGTCTA GCCCTGGCTA GCTCCATCTG   
  
  
- GCTTAAATAA AAGTTTATGC TATCCTTATT TTGAATAACT AAGTTAACTA AAACCGGTTT TAGTACGATA   
  
  
- ATAACTAAAA TAAACGATTG AGAGTTCTCA TAATCAAACG TGAAGCGTAA TAGAAACCGG TTTTTTTAGT   
  
  
- TTTTCTTTCA GAGTTTGAGA ATTATCAAAA AATAAAATAA ACCAAATAAA AATTTTATCA GCCGGAAGCC   
  
  
- AGGCCTGATC ACCTGGCTTA TTCCATTAAA AAGCTTAGCT CCTGGTCTGG TTTACGTGAG TCAAGCTTGG   
  
  
- CCAAGTCAAG CCAGACTGAG CTAAAAACCA GGCCAGGTTC AAACTTGTCT TATTACATGT GTGGATCTTT   
  
  
- TTTTCTTAAT ACGTATATAT GAATTCCGGG GGTTGGGAAA AAGGGAAAGG CGGTTATGAA AAATGGTTCA   
  
  
- GGTTAAGAAA AGAAGAATCG ATACCGATCA TCAATAGGGA GGGATCGAAG GTGGGATTCA AAACTCTGTT   
  
  
- TTAGGGTAGA TACAATTATA AATAAAGAAC GTGTACAGTG GGTTATGTTC CCAAAGTAAC TTTTCTTTTC   
  
  
- CATAATCTAC ATTCTATACA TACATATATT TATCAAAACG TTATCTGATC TTCCTGATTT TTCCTTTTTC   
  
  
- CTTCAAATTT TCGTTTTTTG AAAGGGGAGA TCTTTTTTCC TTTGGTCTAA GGGAAAAGAT GGGGTTAAGG   
  
  
- GTATCAGAAA TGAGACAGAG AGAGAGAGAC AGAGAGAATG AGTACTACAA CTCCCTCCTT CCTTACTTAA   
  
  
- TTCCTGGGAT TCGATTCTAG GTTGGCATTC TATAACGGGG AAATTAGTGG GTAAAGTATA GAGATAGGTG   
  
  
- TCAATTTTTG GGTGGAGGAG GTAGGTGTAT TGACTATATA GAGAAGATGT GGTATGATAT TCCTTCTTCT   
  
  
- CTTTTTATCC TCACATAACA TAAAAATAAA CTCATTAAAT AAAATTCCTC TCTTTAAATG TACCAACCCA   
  
  
- ATTTACCTAT GGAACAAATC TGATCACTCA AAGGTTGTTG GTGTGGTGGT GGTAGTGGTT GTGGTTGTGG   
  
  
- TTGTGGTGGT TGTCGTTAGA AGACTAGTTA GGAGAAACTT ATTATGATGA TGAATAAGGT GTTTAAGTTC   
  
  
- GTCAAGGTCT AGAAGGGTTG TTTGGGTAGT ATTAGTATTA GTATGAGTCA TAATAGTGGT TGTAGTAGGA   
  
  
- TGAATGGGGG TGGCGGTGGT AGTCCTCCTT CTTCTTCTTA CGAAGTTGAA AATGTACCTA CTTCTTTTGA   
  
  
- AGAGAAGAAG AAGGAGGGTG TTGTTTGTAG TAGGAGTTGG AGGAAGGAGG CGGTGGTGGA GGAGCATAAT   
  
  
- GGGAATGATG AGGTGGGGGA AGCTACAAGG GAGATGGTGG TGGTGGAGGT GGAGGTGGGG GTGCGGGTGG   
  
  
- TTGTGGTGGT GGTGGTGGGG GGGTTGGGTC ATACCGCCGC CGCCGCCACC CAAACTGAGG TCGACGAAGA   
  
  
- GGATGAGGGG ACGGGCTCTG GAGTTGGGCC TCGAGAAGAG GAGCAGGCCC AAGAGCCCGT TCACCCGAAG   
  
  
- GTTGTAGGAG AACCTTCGGC GAGCACGGTA GAGACTCTCC TTGTGCTCGG CGGAGGTGGT CGAGGATACC   
  
  
- TACCAGTTGC TCGAGTCGAG GGGCATGCCA CTGCACCTCG TCTTCTATCG AAGTATGAAG CCGGTTCGGG   
  
  
- AGAAGTGGGC GTACTGGCTC AGTCCCCTGG CGTGGATGGC GTGGTACGCC AGGCGGCACC TGTTCTGGAA   
  
  
- GAGGAAACTC AGATGCTCTT TCTACCATGA GTTCAAGGTC CTCCAATCGG GAACCTGGTG GAAACCTGTG   
  
  
- CACCGTCGAT TACCTCGGGA GTAACTACGG AAACTGCCAC TTAGGTTTTA GGTGTAGTAG CTGTAGTCGT   
  
  
- GGTGTAAGAC GTGGGTCACC GGGTGGGAGG ACCTCCGCGA CCGGTGGGCT TACCTACTGC GCGGGATGGA   
  
  
- CTCCGACTGG TGACACCACC AGTTGTTCAG GCCCTTACTT CCAGGGTGGC CTCCGCCCAG GGTGTCCCAC   
  
  
- TACTTTCTCT AACCCTGGGC GGAGCTCTTC AAACGATCCG ATTACCCTCA CGGAAAGTTT AAATTACACC   
  
  
- AGGTGGTGCC CCGACTAAAT AGGCTGAACC TAAAGAGAGT CGACCTATAA TTGCTACTGC TCCGTAATCG   
  
  
- GTAGTTGACA CAGTTGAGCG AGGTGAGCCA GTTATAGGTG GCGGCACTGA GCCAGTAGAG CCGGAAGGCC   
  
  
- GCCGAGGTGG GATCCCACCA CTGGCACCAC CTCCTCCTCC GACTGGAACG ACTGCACCCC CGCCTCCCCA   
  
  
- TGCTCAAGAT GTCCCCCAAA TTACTCACAA ATTCCACCCA ATCCCAAATG AAACTCCGGG ACCTTCTCAC   
  
  
- GAAAGGATCC TGTTCGTTAC TCTTTGAGTA CGAGCTCGCC CGGCGTCCCG CCCGAGACCA CCTGGAGGAC   
  
  
- CGGACGGGGT TCGGGCGTAG TAGGCTCGCC TCCCTCCGTC GACGCGCCAC CAGGCCCTCC TACGTCCCCC   
  
  
- GGCCCAAGCC GGTGCACCCC AAGTCACTAC TCCAGACACT ACTGCAGTTC CGGGAGGATT CCTCCATGTT   
  
  
- CCTCCCCACC AGGTACTATG TTACAAGGCG GCGCCTACCG CCGCGGAGGC GGCCTTATAA GAACAGCACC   
  
  
- TCCCTAGTCG GCCACCACAC CCGGTCACGT ACCTTCGGAA T

+     as-1

| Site Name | Organism | Position | Strand | Matrix score. | sequence | function |
| --- | --- | --- | --- | --- | --- | --- |
| as-1 | Arabidopsis thaliana | 3338 | - | 5 | TGACG |  |
| as-1 | Arabidopsis thaliana | 3335 | + | 5 | TGACG |  |
| as-1 | Arabidopsis thaliana | 2627 | + | 5 | TGACG |  |
| as-1 | Arabidopsis thaliana | 3065 | + | 5 | TGACG |  |
| as-1 | Arabidopsis thaliana | 2720 | + | 5 | TGACG |  |
| as-1 | Arabidopsis thaliana | 2414 | + | 5 | TGACG |  |
| as-1 | Arabidopsis thaliana | 2930 | + | 5 | TGACG |  |

>HU06G00358.1   
+ -Up\_Stream \_Len000GGGTTT TTTTAAACTC AAAATTAGTT ATTGTATCAA TTGAATTTGG AAAACTATTA   
  
  
+ TTTTCGACTT ATTATAGAGA ATTTGTTGTT AATGAATGCT GATAAATACT GATAATTAGT GTTGTGAGTG   
  
  
+ TGAATTTGTG ATGATGAGCA ATGATTAGCG TTAAAATTAT AACTAGGATA AATAATTAAG TGTTGATGAA   
  
  
+ TGTTGATTGA TGATGATGAG TACTGATTGG TGCGGTGCTG GTAATAAATA TTTATGATAA GTTAAAAAAA   
  
  
+ AAAAACACAT CCTAAACAAT CACTATGTAA TATACTAAAT GAAAAAAATT GGTAATGTGA TTTGACAATC   
  
  
+ TAAGGGTAAG GGTGTTTTAC CCACACATGT TATGAAATTG GCTTACTATT TACAAACTTT TTACAGTTAT   
  
  
+ ATGATTCTAC CTATGTGTTA TCATCTTTCC GTCGATGTAA TTGATTATAT TCAACACAAT TTAATTCATT   
  
  
+ ACAATGTAGC TAATTGATAC TCAAAATAAT GAACTATTAA TAAATAATTA ATTATAACTT TTAAAAGTTT   
  
  
+ GTTAATTTAA AATTATATAA TATATAAATT TAGGCTACAT TAATCAAAAA ATTAGGCGTG TTCATCGGTC   
  
  
+ CAAACTTGAA CCGGACCAGA CCGGACCGAA GATCGATTAA GAGAAAAAGT ACAGACTGAG GACCGGATCG   
  
  
+ AATAAGAGTC GCACCGGACC AGGATCGGAC CGGAAAAATT TGGACCAGAT CGGGACCGAT CGAGGTAGAC   
  
  
+ CGAATTTATT TTCAAATACG ATAGGAATAA AACTTATTGA TTCAATTGAT TTTGGCCAAA ATCATGCTAT   
  
  
+ TATTGATTTT ATTTGCTAAC TCTCAAGAGT ATTAGTTTGC ACTTCGCATT ATCTTTGGCC AAAAAAATCA   
  
  
+ AAAAGAAAGT CTCAAACTCT TAATAGTTTT TTATTTTATT TGGTTTATTT TTAAAATAGT CGGCCTTCGG   
  
  
+ TCCGGACTAG TGGACCGAAT AAGGTAATTT TTCGAATCGA GGACCAGACC AAATGCACTC AGTTCGAACC   
  
  
+ GGTTCAGTTC GGTCTGACTC GATTTTTGGT CCGGTCCAAG TTTGAACAGA ATAATGTACA CACCTAGAAA   
  
  
+ AAAAGAATTA TGCATATATA CTTAAGGCCC CCAACCCTTT TTCCCTTTCC GCCAATACTT TTTACCAAGT   
  
  
+ CCAATTCTTT TCTTCTTAGC TATGGCTAGT AGTTATCCCT CCCTAGCTTC CACCCTAAGT TTTGAGACAA   
  
  
+ AATCCCATCT ATGTTAATAT TTATTTCTTG CACATGTCAC CCAATACAAG GGTTTCATTG AAAAGAAAAG   
  
  
+ GTATTAGATG TAAGATATGT ATGTATATAA ATAGTTTTGC AATAGACTAG AAGGACTAAA AAGGAAAAAG   
  
  
+ GAAGTTTAAA AGCAAAAAAC TTTCCCCTCT AGAAAAAAGG AAACCAGATT CCCTTTTCTA CCCCAATTCC   
  
  
+ CATAGTCTTT ACTCTGTCTC TCTCTCTCTG TCTCTCTTAC TCATGATGTT GAGGGAGGAA GGAATGAATT   
  
  
+ AAGGACCCTA AGCTAAGATC CAACCGTAAG ATATTGCCCC TTTAATCACC CATTTCATAT CTCTATCCAC   
  
  
+ AGTTAAAAAC CCACCTCCTC CATCCACATA ACTGATATAT CTCTTCTACA CCATACTATA AGGAAGAAGA   
  
  
+ GAAAAATAGG AGTGTATTGT ATTTTTATTT GAGTAATTTA TTTTAAGGAG AGAAATTTAC ATGGTTGGGT   
  
  
+ TAAATGGATA CCTTGTTTAG ACTAGTGAGT TTCCAACAAC CACACCACCA CCATCACCAA CACCAACACC   
  
  
+ AACACCACCA ACAGCAATCT TCTGATCAAT CCTCTTTGAA TAATACTACT ACTTATTCCA CAAATTCAAG   
  
  
+ CAGTTCCAGA TCTTCCCAAC AAACCCATCA TAATCATAAT CATACTCAGT ATTATCACCA ACATCATCCT   
  
  
+ ACTTACCCCC ACCGCCACCA TCAGGAGGAA GAAGAAGAAT GCTTCAACTT TTACATGGAT GAAGAAAACT   
  
  
+ TCTCTTCTTC TTCCTCCCAC AACAAACATC ATCCTCAACC TCCTTCCTCC GCCACCACCT CCTCGTATTA   
  
  
+ CCCTTACTAC TCCACCCCCT TCGATGTTCC CTCTACCACC ACCACCTCCA CCTCCACCCC CACGCCCACC   
  
  
+ AACACCACCA CCACCACCCC CCCAACCCAG TATGGCGGCG GCGGCGGTGG GTTTGACTCC AGCTGCTTCT   
  
  
+ CCTACTCCCC TGCCCGAGAC CTCAACCCGG AGCTCTTCTC CTCGTCCGGG TTCTCGGGCA AGTGGGCTTC   
  
  
+ CAACATCCTC TTGGAAGCCG CTCGTGCCAT CTCTGAGAGG AACACGAGCC GCCTCCACCA GCTCCTATGG   
  
  
+ ATGGTCAACG AGCTCAGCTC CCCGTACGGT GACGTGGAGC AGAAGATAGC TTCATACTTC GGCCAAGCCC   
  
  
+ TCTTCACCCG CATGACCGAG TCAGGGGACC GCACCTACCG CACCATGCGG TCCGCCGTGG ACAAGACCTT   
  
  
+ CTCCTTTGAG TCTACGAGAA AGATGGTACT CAAGTTCCAG GAGGTTAGCC CTTGGACCAC CTTTGGACAC   
  
  
+ GTGGCAGCTA ATGGAGCCCT CATTGATGCC TTTGACGGTG AATCCAAAAT CCACATCATC GACATCAGCA   
  
  
+ CCACATTCTG CACCCAGTGG CCCACCCTCC TGGAGGCGCT GGCCACCCGA ATGGATGACG CGCCCTACCT   
  
  
+ GAGGCTGACC ACTGTGGTGG TCAACAAGTC CGGGAATGAA GGTCCCACCG GAGGCGGGTC CCACAGGGTG   
  
  
+ ATGAAAGAGA TTGGGACCCG CCTCGAGAAG TTTGCTAGGC TAATGGGAGT GCCTTTCAAA TTTAATGTGG   
  
  
+ TCCACCACGG GGCTGATTTA TCCGACTTGG ATTTCTCTCA GCTGGATATT AACGATGACG AGGCATTAGC   
  
  
+ CATCAACTGT GTCAACTCGC TCCACTCGGT CAATATCCAC CGCCGTGACT CGGTCATCTC GGCCTTCCGG   
  
  
+ CGGCTCCACC CTAGGGTGGT GACCGTGGTG GAGGAGGAGG CTGACCTTGC TGACGTGGGG GCGGAGGGGT   
  
  
+ ACGAGTTCTA CAGGGGGTTT AATGAGTGTT TAAGGTGGGT TAGGGTTTAC TTTGAGGCCC TGGAAGAGTG   
  
  
+ CTTTCCTAGG ACAAGCAATG AGAAACTCAT GCTCGAGCGG GCCGCAGGGC GGGCTCTGGT GGACCTCCTG   
  
  
+ GCCTGCCCCA AGCCCGCATC ATCCGAGCGG AGGGAGGCAG CTGCGCGGTG GTCCGGGAGG ATGCAGGGGG   
  
  
+ CCGGGTTCGG CCACGTGGGG TTCAGTGATG AGGTCTGTGA TGACGTCAAG GCCCTCCTAA GGAGGTACAA   
  
  
+ GGAGGGGTGG TCCATGATAC AATGTTCCGC CGCGGATGGC GGCGCCTCCG CCGGAATATT CTTGTCGTGG   
  
  
+ AGGGATCAGC CGGTGGTGTG GGCCAGTGCA TGGAAGCCTT A  

- -Up\_Stream \_Len000CCCAAA AAAATTTGAG TTTTAATCAA TAACATAGTT AACTTAAACC TTTTGATAAT   
  
  
- AAAAGCTGAA TAATATCTCT TAAACAACAA TTACTTACGA CTATTTATGA CTATTAATCA CAACACTCAC   
  
  
- ACTTAAACAC TACTACTCGT TACTAATCGC AATTTTAATA TTGATCCTAT TTATTAATTC ACAACTACTT   
  
  
- ACAACTAACT ACTACTACTC ATGACTAACC ACGCCACGAC CATTATTTAT AAATACTATT CAATTTTTTT   
  
  
- TTTTTGTGTA GGATTTGTTA GTGATACATT ATATGATTTA CTTTTTTTAA CCATTACACT AAACTGTTAG   
  
  
- ATTCCCATTC CCACAAAATG GGTGTGTACA ATACTTTAAC CGAATGATAA ATGTTTGAAA AATGTCAATA   
  
  
- TACTAAGATG GATACACAAT AGTAGAAAGG CAGCTACATT AACTAATATA AGTTGTGTTA AATTAAGTAA   
  
  
- TGTTACATCG ATTAACTATG AGTTTTATTA CTTGATAATT ATTTATTAAT TAATATTGAA AATTTTCAAA   
  
  
- CAATTAAATT TTAATATATT ATATATTTAA ATCCGATGTA ATTAGTTTTT TAATCCGCAC AAGTAGCCAG   
  
  
- GTTTGAACTT GGCCTGGTCT GGCCTGGCTT CTAGCTAATT CTCTTTTTCA TGTCTGACTC CTGGCCTAGC   
  
  
- TTATTCTCAG CGTGGCCTGG TCCTAGCCTG GCCTTTTTAA ACCTGGTCTA GCCCTGGCTA GCTCCATCTG   
  
  
- GCTTAAATAA AAGTTTATGC TATCCTTATT TTGAATAACT AAGTTAACTA AAACCGGTTT TAGTACGATA   
  
  
- ATAACTAAAA TAAACGATTG AGAGTTCTCA TAATCAAACG TGAAGCGTAA TAGAAACCGG TTTTTTTAGT   
  
  
- TTTTCTTTCA GAGTTTGAGA ATTATCAAAA AATAAAATAA ACCAAATAAA AATTTTATCA GCCGGAAGCC   
  
  
- AGGCCTGATC ACCTGGCTTA TTCCATTAAA AAGCTTAGCT CCTGGTCTGG TTTACGTGAG TCAAGCTTGG   
  
  
- CCAAGTCAAG CCAGACTGAG CTAAAAACCA GGCCAGGTTC AAACTTGTCT TATTACATGT GTGGATCTTT   
  
  
- TTTTCTTAAT ACGTATATAT GAATTCCGGG GGTTGGGAAA AAGGGAAAGG CGGTTATGAA AAATGGTTCA   
  
  
- GGTTAAGAAA AGAAGAATCG ATACCGATCA TCAATAGGGA GGGATCGAAG GTGGGATTCA AAACTCTGTT   
  
  
- TTAGGGTAGA TACAATTATA AATAAAGAAC GTGTACAGTG GGTTATGTTC CCAAAGTAAC TTTTCTTTTC   
  
  
- CATAATCTAC ATTCTATACA TACATATATT TATCAAAACG TTATCTGATC TTCCTGATTT TTCCTTTTTC   
  
  
- CTTCAAATTT TCGTTTTTTG AAAGGGGAGA TCTTTTTTCC TTTGGTCTAA GGGAAAAGAT GGGGTTAAGG   
  
  
- GTATCAGAAA TGAGACAGAG AGAGAGAGAC AGAGAGAATG AGTACTACAA CTCCCTCCTT CCTTACTTAA   
  
  
- TTCCTGGGAT TCGATTCTAG GTTGGCATTC TATAACGGGG AAATTAGTGG GTAAAGTATA GAGATAGGTG   
  
  
- TCAATTTTTG GGTGGAGGAG GTAGGTGTAT TGACTATATA GAGAAGATGT GGTATGATAT TCCTTCTTCT   
  
  
- CTTTTTATCC TCACATAACA TAAAAATAAA CTCATTAAAT AAAATTCCTC TCTTTAAATG TACCAACCCA   
  
  
- ATTTACCTAT GGAACAAATC TGATCACTCA AAGGTTGTTG GTGTGGTGGT GGTAGTGGTT GTGGTTGTGG   
  
  
- TTGTGGTGGT TGTCGTTAGA AGACTAGTTA GGAGAAACTT ATTATGATGA TGAATAAGGT GTTTAAGTTC   
  
  
- GTCAAGGTCT AGAAGGGTTG TTTGGGTAGT ATTAGTATTA GTATGAGTCA TAATAGTGGT TGTAGTAGGA   
  
  
- TGAATGGGGG TGGCGGTGGT AGTCCTCCTT CTTCTTCTTA CGAAGTTGAA AATGTACCTA CTTCTTTTGA   
  
  
- AGAGAAGAAG AAGGAGGGTG TTGTTTGTAG TAGGAGTTGG AGGAAGGAGG CGGTGGTGGA GGAGCATAAT   
  
  
- GGGAATGATG AGGTGGGGGA AGCTACAAGG GAGATGGTGG TGGTGGAGGT GGAGGTGGGG GTGCGGGTGG   
  
  
- TTGTGGTGGT GGTGGTGGGG GGGTTGGGTC ATACCGCCGC CGCCGCCACC CAAACTGAGG TCGACGAAGA   
  
  
- GGATGAGGGG ACGGGCTCTG GAGTTGGGCC TCGAGAAGAG GAGCAGGCCC AAGAGCCCGT TCACCCGAAG   
  
  
- GTTGTAGGAG AACCTTCGGC GAGCACGGTA GAGACTCTCC TTGTGCTCGG CGGAGGTGGT CGAGGATACC   
  
  
- TACCAGTTGC TCGAGTCGAG GGGCATGCCA CTGCACCTCG TCTTCTATCG AAGTATGAAG CCGGTTCGGG   
  
  
- AGAAGTGGGC GTACTGGCTC AGTCCCCTGG CGTGGATGGC GTGGTACGCC AGGCGGCACC TGTTCTGGAA   
  
  
- GAGGAAACTC AGATGCTCTT TCTACCATGA GTTCAAGGTC CTCCAATCGG GAACCTGGTG GAAACCTGTG   
  
  
- CACCGTCGAT TACCTCGGGA GTAACTACGG AAACTGCCAC TTAGGTTTTA GGTGTAGTAG CTGTAGTCGT   
  
  
- GGTGTAAGAC GTGGGTCACC GGGTGGGAGG ACCTCCGCGA CCGGTGGGCT TACCTACTGC GCGGGATGGA   
  
  
- CTCCGACTGG TGACACCACC AGTTGTTCAG GCCCTTACTT CCAGGGTGGC CTCCGCCCAG GGTGTCCCAC   
  
  
- TACTTTCTCT AACCCTGGGC GGAGCTCTTC AAACGATCCG ATTACCCTCA CGGAAAGTTT AAATTACACC   
  
  
- AGGTGGTGCC CCGACTAAAT AGGCTGAACC TAAAGAGAGT CGACCTATAA TTGCTACTGC TCCGTAATCG   
  
  
- GTAGTTGACA CAGTTGAGCG AGGTGAGCCA GTTATAGGTG GCGGCACTGA GCCAGTAGAG CCGGAAGGCC   
  
  
- GCCGAGGTGG GATCCCACCA CTGGCACCAC CTCCTCCTCC GACTGGAACG ACTGCACCCC CGCCTCCCCA   
  
  
- TGCTCAAGAT GTCCCCCAAA TTACTCACAA ATTCCACCCA ATCCCAAATG AAACTCCGGG ACCTTCTCAC   
  
  
- GAAAGGATCC TGTTCGTTAC TCTTTGAGTA CGAGCTCGCC CGGCGTCCCG CCCGAGACCA CCTGGAGGAC   
  
  
- CGGACGGGGT TCGGGCGTAG TAGGCTCGCC TCCCTCCGTC GACGCGCCAC CAGGCCCTCC TACGTCCCCC   
  
  
- GGCCCAAGCC GGTGCACCCC AAGTCACTAC TCCAGACACT ACTGCAGTTC CGGGAGGATT CCTCCATGTT   
  
  
- CCTCCCCACC AGGTACTATG TTACAAGGCG GCGCCTACCG CCGCGGAGGC GGCCTTATAA GAACAGCACC   
  
  
- TCCCTAGTCG GCCACCACAC CCGGTCACGT ACCTTCGGAA T
