# Supplementary material for: Genome-Wide Identification and Expression Pattern of the GRAS Gene Family in Pitaya (Selenicereus undatus L.)
Source: Biology (Basel). 2022 Dec 21;12(1):11. doi: 10.3390/biology12010011 (PMC9854919; doi:10.3390/biology12010011)
Supplement: Supplementary file 1 [file biology-12-00011-s001.zip › Supplementary file S5/HU06G00376.1_plantcare.html]

Content-Type: text/html; charset=ISO-8859-1


PlantCARE


Webmaster Firefox specific output  
To save the result:
click on the frame with the right mouse button and save the source code as a text file with extension .html  
REFERENCE:PlantCARE: a database of plant cis-acting regulatory elements and a portal to tools for in silico analysis of promoter sequences.  
Lescot, M., Déhais, P., Moreau, Y., De Moor, B., Rouzé ,P.,and Rombauts, S.  
Nucleic Acids Res., Database issue(2002), 30(1):325-327.   


---

>HU06G00376.1   
+ +Up\_Stream \_Len000AGAAGC TAATTAAACC AAACATGCAC GTATGTCCTA ATTAACATGA CTGTCCATCA   
  
  
+ TCTCCTCCCA TGTTGTTCTG TTGACAAGCC TGCACACCCA TGCTCCTCTC TCATGTCTAA CCTCGTCCCA   
  
  
+ ACGATCAAGA TCACTGTCTG TGAGGCCAGG GGAAGACTTG CTTTTATTTC CTTTTCTTTT GTTTACTCTC   
  
  
+ TGATTCCATT TAGCCATATA TACAAGGAGA ATGTCATGTG TATCTCATAT GTATATATAT AAGATTTTAT   
  
  
+ TTAGAAATAA AAATTTAAAC ACTATGTGAT TTATAGTTAT TATAATAAAT AGTATTTTTT AAACTATTTC   
  
  
+ ATTGATGAGA ACAGGAAAGT AAATTGCCGA CATGTTAATG TTATTATATC TAAAAATTAA ATCATAAAAT   
  
  
+ TAATTATAAT ATTTTAAAAA TATTTCAACT AAAATTTTAG AACAATAAGT GCGTAATACG GACTTAAAGG   
  
  
+ CTAGTTTATC GTAAAAATGT AAGTTCCTTT TGGATCTTAT GGTTGAATAT CTTGGTTGAT AAACTGTGAT   
  
  
+ TGGTTTTACA CTTTTACCAC ATATTCATAG AAGAATTATG TGCACGTTGA TGGTTCAAGA TGGGACAAAA   
  
  
+ GGAAACATGT ATTCTTCCCC CTTTTGCCTT TTCCCCTAAG CATTTGGATT GAGGTGTTGT CATCTGAATA   
  
  
+ TCAAAAATTC TTTTTGAAAA TTCAGTCTCA ATCAAAATTC AAAATTAAAA CAAAGACTTA AGAGTTTTAT   
  
  
+ TTGTTCTTAA CCTTCTTAAC TTTCTACTTT TCTTTTTTTT CTCATTCCCC CTCTTCAAAT CTCATTTATT   
  
  
+ CTTAACCCCC TCTTCCGTTC ATGCTCTCTC TGTCTATCAA CTAATATTAA TCTACCCCGA CTTTCAATTT   
  
  
+ GATGAGGTCT AATCCTTATC ATCGTATTGT GGTTGGGTTA ATTATGCAAG AAGGCAATAA ACCCCAGTGA   
  
  
+ CAAAGTCCAC GTTAAGTAGG CACCTCACCA TTAAGACATG CTCAGAAAAC ACCAAAACCA TTGAACACAA   
  
  
+ GTCCCCCAAG TCCCTTCCCT AGCTAGCCCA TTCTCTCTCC TCCCCTGTCT ATATCTACCA ACTCTTGCTC   
  
  
+ TTTGCTGAGC CTGAGTTGAA AGCGATACAC CCATAGCCTG TTCAGTTTTC AGTTTTCACA TTTGTCTTCC   
  
  
+ TCTGTTCAGT TGAAAGCGAT ACACTCTCAT TAGCTTTCAT TTTAAATACG TTCAATTCAC ACATAAATGG   
  
  
+ CTTTCAAAGC ATTGCCATTT TCGTTTAAAT GATGACAAAA TATATAGTAT CTGGCTATCT GCCCATATCT   
  
  
+ TGACCTTATT TACAAAGGCT GAGCCATCTT TTAATTTTTT TAGCCTCTTT TTTTAATCAA AAATAGAAAT   
  
  
+ TTCAAGAAAG CAGAAATCGT CAGATGACAG TGCAGACAGC GTTTTCGTTT CATACAAATC TCAAAAGCTG   
  
  
+ AAGTGTCAAT TTCTTCGATT ATTTTTGTCA GAAAATATTT ACGTCTCACC TTCACGTTGT TATTATTCTT   
  
  
+ TCTTTTTTAT TATTCTCATA ACTAGTAATA AAGTTAGCAA TGATAAAAAA TTATTCGATC CGAATATTTT   
  
  
+ AATTTATCTG ACCTAAAAAC ATAAGTAAAG GCACAAATTT TTCATCCAAA TTTTAATTTT TGATGTAATA   
  
  
+ TTTTTTATAT TTTTATTGTT CAAATCTGAT TTTAATCTTA TTTAAATTAT CTGACCTAAA AAAATCAAAT   
  
  
+ AATAATAAAC GTTAATTTTT TATTTAAATT TTGACATTAG TCAATCTGAC TTAAATTCGA ACCGAGCTTG   
  
  
+ AATTTTTTTG CCAGTGCTGG TAGTACTCCT GCTTTTGTAG CTCCCAATGC CATCGTACCA CCCTCTTCTA   
  
  
+ GTCTCGTCTC TTTCTCTGGT CTCCAATTAA TCACACCATC ATATCATACC GTATGATGAT ACAGTCCACT   
  
  
+ GAAATCCCAT TCTCACCGCC AATCTCTCTC CGCCACCATC CAACACTCTT AACGATGAAC AGGGCCGCCG   
  
  
+ CCTCGTCCTC CACACTCAGG CCGTGGCCGG GCAGCTTTCC CACCCAATCA AAATCTCTCT CCTCCGCCAA   
  
  
+ CTTCGGTAAC GCCAATTGCA TGGAGCAGCT CTTAGTCCAC TGCGCCGAAG CCATCGACAA CAATGACGCC   
  
  
+ ACCCCGGCCC AGCAAATCTT ATGGGTCCTG AATAACATAG CCCGACCCGA CGGCGACTCC ACCCAACGCC   
  
  
+ TCACGTGCGC ATTCCTACGT GCCTTAATCT CACGCGCCGT CCTCACTAGC ACCTGCAAGA TGGTAATCCC   
  
  
+ TCATTTCAAC CCCATCAATT CACCCCACAA ATTCTCGCTC CTCGAACTTG CCCACTTCGT CGATTTAACC   
  
  
+ CCTTGGCATC GATTCGGATT CACCGCCGCC AATTCGATCA TTCTGGAAGC TATTTCCGAC CTACCCGTTG   
  
  
+ TACACATTGT CGACCTCAGC ATCTCCCACT GTATGCAGAT CCCCACGTTG ATCGACTCCA TTGCGACCCG   
  
  
+ GTTGGAAGCC CCGGGTCGAG TCCCTCCTAT TGTCAAGCTC ACCGTCGGGG CTATTTCCGA CGAAATCCCG   
  
  
+ CCGGTGTTCG ATCTTCTGTC GTACGATGAG CTCGGAATGA GACTAATCAA CTTCGCTCGT TTTAGAAACA   
  
  
+ TCGTCCTCGA ATTCCAAGCA ATACCCACCT CCCCTTCCGA CGGATTTGCT TCGCTGTTGG AGGAGATTCG   
  
  
+ ACAAAGCAAG CTCTACTCCA ACGATGCGGC GGCGGTTATT GTGAATTGTC AGATGAGTTT GCATTTATTG   
  
  
+ CAGGAAGAGG AGGTGTCTTC ATCGTCGCCG TCGTCGATGA GGGGGATGTT TTTGCAGGCG GTGAGGAGCT   
  
  
+ TGGAGCCGAG CATGGTGGTG GTGGTGGAGG AGGACGTGGA TTTCACGGCG AGGAGTCTGG TGGGGCGGCT   
  
  
+ GAGATCGGCG TTTAATCACA TGTGGATACC CTTCGACACG GTGGACACGT TCTTGCCACG TGGGAGCCAG   
  
  
+ CAGAGAGAGT GGTTCGAGGC CGAGGTGTGC TGGAAGATTG AGAATGTGAT CGCTCATGAG GGACCCGCGA   
  
  
+ GGGTCGAGAG GCAGGAGCCC AGGGCCAAGT GGGCCCTCCG AATGAGGGAG GCCGAGTTTC AAGGGATCGA   
  
  
+ GTTCGGTGAT GAAGGTACGA CCGAGGTCAA GGCCATGCTG GAGGAGCATG CCGCTGGGTG GGGGTCTAAG   
  
  
+ AAGGAAGAGG ATGATCTTGT GCTCACTTGG AAGGGACATA GTGTTGTCTT TGCTTCTGCT TGGGTACCCA   
  
  
+ CTTA  

- +Up\_Stream \_Len000TCTTCG ATTAATTTGG TTTGTACGTG CATACAGGAT TAATTGTACT GACAGGTAGT   
  
  
- AGAGGAGGGT ACAACAAGAC AACTGTTCGG ACGTGTGGGT ACGAGGAGAG AGTACAGATT GGAGCAGGGT   
  
  
- TGCTAGTTCT AGTGACAGAC ACTCCGGTCC CCTTCTGAAC GAAAATAAAG GAAAAGAAAA CAAATGAGAG   
  
  
- ACTAAGGTAA ATCGGTATAT ATGTTCCTCT TACAGTACAC ATAGAGTATA CATATATATA TTCTAAAATA   
  
  
- AATCTTTATT TTTAAATTTG TGATACACTA AATATCAATA ATATTATTTA TCATAAAAAA TTTGATAAAG   
  
  
- TAACTACTCT TGTCCTTTCA TTTAACGGCT GTACAATTAC AATAATATAG ATTTTTAATT TAGTATTTTA   
  
  
- ATTAATATTA TAAAATTTTT ATAAAGTTGA TTTTAAAATC TTGTTATTCA CGCATTATGC CTGAATTTCC   
  
  
- GATCAAATAG CATTTTTACA TTCAAGGAAA ACCTAGAATA CCAACTTATA GAACCAACTA TTTGACACTA   
  
  
- ACCAAAATGT GAAAATGGTG TATAAGTATC TTCTTAATAC ACGTGCAACT ACCAAGTTCT ACCCTGTTTT   
  
  
- CCTTTGTACA TAAGAAGGGG GAAAACGGAA AAGGGGATTC GTAAACCTAA CTCCACAACA GTAGACTTAT   
  
  
- AGTTTTTAAG AAAAACTTTT AAGTCAGAGT TAGTTTTAAG TTTTAATTTT GTTTCTGAAT TCTCAAAATA   
  
  
- AACAAGAATT GGAAGAATTG AAAGATGAAA AGAAAAAAAA GAGTAAGGGG GAGAAGTTTA GAGTAAATAA   
  
  
- GAATTGGGGG AGAAGGCAAG TACGAGAGAG ACAGATAGTT GATTATAATT AGATGGGGCT GAAAGTTAAA   
  
  
- CTACTCCAGA TTAGGAATAG TAGCATAACA CCAACCCAAT TAATACGTTC TTCCGTTATT TGGGGTCACT   
  
  
- GTTTCAGGTG CAATTCATCC GTGGAGTGGT AATTCTGTAC GAGTCTTTTG TGGTTTTGGT AACTTGTGTT   
  
  
- CAGGGGGTTC AGGGAAGGGA TCGATCGGGT AAGAGAGAGG AGGGGACAGA TATAGATGGT TGAGAACGAG   
  
  
- AAACGACTCG GACTCAACTT TCGCTATGTG GGTATCGGAC AAGTCAAAAG TCAAAAGTGT AAACAGAAGG   
  
  
- AGACAAGTCA ACTTTCGCTA TGTGAGAGTA ATCGAAAGTA AAATTTATGC AAGTTAAGTG TGTATTTACC   
  
  
- GAAAGTTTCG TAACGGTAAA AGCAAATTTA CTACTGTTTT ATATATCATA GACCGATAGA CGGGTATAGA   
  
  
- ACTGGAATAA ATGTTTCCGA CTCGGTAGAA AATTAAAAAA ATCGGAGAAA AAAATTAGTT TTTATCTTTA   
  
  
- AAGTTCTTTC GTCTTTAGCA GTCTACTGTC ACGTCTGTCG CAAAAGCAAA GTATGTTTAG AGTTTTCGAC   
  
  
- TTCACAGTTA AAGAAGCTAA TAAAAACAGT CTTTTATAAA TGCAGAGTGG AAGTGCAACA ATAATAAGAA   
  
  
- AGAAAAAATA ATAAGAGTAT TGATCATTAT TTCAATCGTT ACTATTTTTT AATAAGCTAG GCTTATAAAA   
  
  
- TTAAATAGAC TGGATTTTTG TATTCATTTC CGTGTTTAAA AAGTAGGTTT AAAATTAAAA ACTACATTAT   
  
  
- AAAAAATATA AAAATAACAA GTTTAGACTA AAATTAGAAT AAATTTAATA GACTGGATTT TTTTAGTTTA   
  
  
- TTATTATTTG CAATTAAAAA ATAAATTTAA AACTGTAATC AGTTAGACTG AATTTAAGCT TGGCTCGAAC   
  
  
- TTAAAAAAAC GGTCACGACC ATCATGAGGA CGAAAACATC GAGGGTTACG GTAGCATGGT GGGAGAAGAT   
  
  
- CAGAGCAGAG AAAGAGACCA GAGGTTAATT AGTGTGGTAG TATAGTATGG CATACTACTA TGTCAGGTGA   
  
  
- CTTTAGGGTA AGAGTGGCGG TTAGAGAGAG GCGGTGGTAG GTTGTGAGAA TTGCTACTTG TCCCGGCGGC   
  
  
- GGAGCAGGAG GTGTGAGTCC GGCACCGGCC CGTCGAAAGG GTGGGTTAGT TTTAGAGAGA GGAGGCGGTT   
  
  
- GAAGCCATTG CGGTTAACGT ACCTCGTCGA GAATCAGGTG ACGCGGCTTC GGTAGCTGTT GTTACTGCGG   
  
  
- TGGGGCCGGG TCGTTTAGAA TACCCAGGAC TTATTGTATC GGGCTGGGCT GCCGCTGAGG TGGGTTGCGG   
  
  
- AGTGCACGCG TAAGGATGCA CGGAATTAGA GTGCGCGGCA GGAGTGATCG TGGACGTTCT ACCATTAGGG   
  
  
- AGTAAAGTTG GGGTAGTTAA GTGGGGTGTT TAAGAGCGAG GAGCTTGAAC GGGTGAAGCA GCTAAATTGG   
  
  
- GGAACCGTAG CTAAGCCTAA GTGGCGGCGG TTAAGCTAGT AAGACCTTCG ATAAAGGCTG GATGGGCAAC   
  
  
- ATGTGTAACA GCTGGAGTCG TAGAGGGTGA CATACGTCTA GGGGTGCAAC TAGCTGAGGT AACGCTGGGC   
  
  
- CAACCTTCGG GGCCCAGCTC AGGGAGGATA ACAGTTCGAG TGGCAGCCCC GATAAAGGCT GCTTTAGGGC   
  
  
- GGCCACAAGC TAGAAGACAG CATGCTACTC GAGCCTTACT CTGATTAGTT GAAGCGAGCA AAATCTTTGT   
  
  
- AGCAGGAGCT TAAGGTTCGT TATGGGTGGA GGGGAAGGCT GCCTAAACGA AGCGACAACC TCCTCTAAGC   
  
  
- TGTTTCGTTC GAGATGAGGT TGCTACGCCG CCGCCAATAA CACTTAACAG TCTACTCAAA CGTAAATAAC   
  
  
- GTCCTTCTCC TCCACAGAAG TAGCAGCGGC AGCAGCTACT CCCCCTACAA AAACGTCCGC CACTCCTCGA   
  
  
- ACCTCGGCTC GTACCACCAC CACCACCTCC TCCTGCACCT AAAGTGCCGC TCCTCAGACC ACCCCGCCGA   
  
  
- CTCTAGCCGC AAATTAGTGT ACACCTATGG GAAGCTGTGC CACCTGTGCA AGAACGGTGC ACCCTCGGTC   
  
  
- GTCTCTCTCA CCAAGCTCCG GCTCCACACG ACCTTCTAAC TCTTACACTA GCGAGTACTC CCTGGGCGCT   
  
  
- CCCAGCTCTC CGTCCTCGGG TCCCGGTTCA CCCGGGAGGC TTACTCCCTC CGGCTCAAAG TTCCCTAGCT   
  
  
- CAAGCCACTA CTTCCATGCT GGCTCCAGTT CCGGTACGAC CTCCTCGTAC GGCGACCCAC CCCCAGATTC   
  
  
- TTCCTTCTCC TACTAGAACA CGAGTGAACC TTCCCTGTAT CACAACAGAA ACGAAGACGA ACCCATGGGT   
  
  
- GAAT

  
  
Motifs Found  

+   

| Site Name | Organism | Position | Strand | Matrix score. | sequence | function |
| --- | --- | --- | --- | --- | --- | --- |
|  | organism | 3258 | - | 4 | motif\_sequence | short\_function |
|  | organism | 3014 | - | 4 | motif\_sequence | short\_function |
|  | organism | 1887 | + | 4 | motif\_sequence | short\_function |
|  | organism | 3047 | - | 4 | motif\_sequence | short\_function |
|  | organism | 363 | - | 4 | motif\_sequence | short\_function |
|  | organism | 65 | + | 4 | motif\_sequence | short\_function |
|  | organism | 2745 | + | 4 | motif\_sequence | short\_function |
|  | organism | 1089 | + | 4 | motif\_sequence | short\_function |
|  | organism | 74 | + | 4 | motif\_sequence | short\_function |
|  | organism | 1107 | + | 4 | motif\_sequence | short\_function |
|  | organism | 2808 | - | 4 | motif\_sequence | short\_function |
|  | organism | 3228 | - | 4 | motif\_sequence | short\_function |
|  | organism | 2091 | + | 4 | motif\_sequence | short\_function |
|  | organism | 2726 | - | 4 | motif\_sequence | short\_function |
|  | organism | 1321 | + | 4 | motif\_sequence | short\_function |
|  | organism | 1162 | + | 4 | motif\_sequence | short\_function |
|  | organism | 1196 | + | 4 | motif\_sequence | short\_function |
|  | organism | 47 | + | 4 | motif\_sequence | short\_function |
|  | organism | 2489 | - | 4 | motif\_sequence | short\_function |
|  | organism | 2605 | + | 4 | motif\_sequence | short\_function |
|  | organism | 2021 | - | 4 | motif\_sequence | short\_function |
|  | organism | 894 | + | 4 | motif\_sequence | short\_function |
|  | organism | 854 | + | 4 | motif\_sequence | short\_function |
|  | organism | 825 | + | 4 | motif\_sequence | short\_function |
|  | organism | 602 | + | 4 | motif\_sequence | short\_function |
|  | organism | 1990 | + | 4 | motif\_sequence | short\_function |
|  | organism | 2475 | + | 4 | motif\_sequence | short\_function |

>HU06G00376.1   
+ +Up\_Stream \_Len000AGAAGC TAATTAAACC AAACATGCAC GTATGTCCTA ATTAACATGA CTGTCCATCA   
  
  
+ TCTCCTCCCA TGTTGTTCTG TTGACAAGCC TGCACACCCA TGCTCCTCTC TCATGTCTAA CCTCGTCCCA   
  
  
+ ACGATCAAGA TCACTGTCTG TGAGGCCAGG GGAAGACTTG CTTTTATTTC CTTTTCTTTT GTTTACTCTC   
  
  
+ TGATTCCATT TAGCCATATA TACAAGGAGA ATGTCATGTG TATCTCATAT GTATATATAT AAGATTTTAT   
  
  
+ TTAGAAATAA AAATTTAAAC ACTATGTGAT TTATAGTTAT TATAATAAAT AGTATTTTTT AAACTATTTC   
  
  
+ ATTGATGAGA ACAGGAAAGT AAATTGCCGA CATGTTAATG TTATTATATC TAAAAATTAA ATCATAAAAT   
  
  
+ TAATTATAAT ATTTTAAAAA TATTTCAACT AAAATTTTAG AACAATAAGT GCGTAATACG GACTTAAAGG   
  
  
+ CTAGTTTATC GTAAAAATGT AAGTTCCTTT TGGATCTTAT GGTTGAATAT CTTGGTTGAT AAACTGTGAT   
  
  
+ TGGTTTTACA CTTTTACCAC ATATTCATAG AAGAATTATG TGCACGTTGA TGGTTCAAGA TGGGACAAAA   
  
  
+ GGAAACATGT ATTCTTCCCC CTTTTGCCTT TTCCCCTAAG CATTTGGATT GAGGTGTTGT CATCTGAATA   
  
  
+ TCAAAAATTC TTTTTGAAAA TTCAGTCTCA ATCAAAATTC AAAATTAAAA CAAAGACTTA AGAGTTTTAT   
  
  
+ TTGTTCTTAA CCTTCTTAAC TTTCTACTTT TCTTTTTTTT CTCATTCCCC CTCTTCAAAT CTCATTTATT   
  
  
+ CTTAACCCCC TCTTCCGTTC ATGCTCTCTC TGTCTATCAA CTAATATTAA TCTACCCCGA CTTTCAATTT   
  
  
+ GATGAGGTCT AATCCTTATC ATCGTATTGT GGTTGGGTTA ATTATGCAAG AAGGCAATAA ACCCCAGTGA   
  
  
+ CAAAGTCCAC GTTAAGTAGG CACCTCACCA TTAAGACATG CTCAGAAAAC ACCAAAACCA TTGAACACAA   
  
  
+ GTCCCCCAAG TCCCTTCCCT AGCTAGCCCA TTCTCTCTCC TCCCCTGTCT ATATCTACCA ACTCTTGCTC   
  
  
+ TTTGCTGAGC CTGAGTTGAA AGCGATACAC CCATAGCCTG TTCAGTTTTC AGTTTTCACA TTTGTCTTCC   
  
  
+ TCTGTTCAGT TGAAAGCGAT ACACTCTCAT TAGCTTTCAT TTTAAATACG TTCAATTCAC ACATAAATGG   
  
  
+ CTTTCAAAGC ATTGCCATTT TCGTTTAAAT GATGACAAAA TATATAGTAT CTGGCTATCT GCCCATATCT   
  
  
+ TGACCTTATT TACAAAGGCT GAGCCATCTT TTAATTTTTT TAGCCTCTTT TTTTAATCAA AAATAGAAAT   
  
  
+ TTCAAGAAAG CAGAAATCGT CAGATGACAG TGCAGACAGC GTTTTCGTTT CATACAAATC TCAAAAGCTG   
  
  
+ AAGTGTCAAT TTCTTCGATT ATTTTTGTCA GAAAATATTT ACGTCTCACC TTCACGTTGT TATTATTCTT   
  
  
+ TCTTTTTTAT TATTCTCATA ACTAGTAATA AAGTTAGCAA TGATAAAAAA TTATTCGATC CGAATATTTT   
  
  
+ AATTTATCTG ACCTAAAAAC ATAAGTAAAG GCACAAATTT TTCATCCAAA TTTTAATTTT TGATGTAATA   
  
  
+ TTTTTTATAT TTTTATTGTT CAAATCTGAT TTTAATCTTA TTTAAATTAT CTGACCTAAA AAAATCAAAT   
  
  
+ AATAATAAAC GTTAATTTTT TATTTAAATT TTGACATTAG TCAATCTGAC TTAAATTCGA ACCGAGCTTG   
  
  
+ AATTTTTTTG CCAGTGCTGG TAGTACTCCT GCTTTTGTAG CTCCCAATGC CATCGTACCA CCCTCTTCTA   
  
  
+ GTCTCGTCTC TTTCTCTGGT CTCCAATTAA TCACACCATC ATATCATACC GTATGATGAT ACAGTCCACT   
  
  
+ GAAATCCCAT TCTCACCGCC AATCTCTCTC CGCCACCATC CAACACTCTT AACGATGAAC AGGGCCGCCG   
  
  
+ CCTCGTCCTC CACACTCAGG CCGTGGCCGG GCAGCTTTCC CACCCAATCA AAATCTCTCT CCTCCGCCAA   
  
  
+ CTTCGGTAAC GCCAATTGCA TGGAGCAGCT CTTAGTCCAC TGCGCCGAAG CCATCGACAA CAATGACGCC   
  
  
+ ACCCCGGCCC AGCAAATCTT ATGGGTCCTG AATAACATAG CCCGACCCGA CGGCGACTCC ACCCAACGCC   
  
  
+ TCACGTGCGC ATTCCTACGT GCCTTAATCT CACGCGCCGT CCTCACTAGC ACCTGCAAGA TGGTAATCCC   
  
  
+ TCATTTCAAC CCCATCAATT CACCCCACAA ATTCTCGCTC CTCGAACTTG CCCACTTCGT CGATTTAACC   
  
  
+ CCTTGGCATC GATTCGGATT CACCGCCGCC AATTCGATCA TTCTGGAAGC TATTTCCGAC CTACCCGTTG   
  
  
+ TACACATTGT CGACCTCAGC ATCTCCCACT GTATGCAGAT CCCCACGTTG ATCGACTCCA TTGCGACCCG   
  
  
+ GTTGGAAGCC CCGGGTCGAG TCCCTCCTAT TGTCAAGCTC ACCGTCGGGG CTATTTCCGA CGAAATCCCG   
  
  
+ CCGGTGTTCG ATCTTCTGTC GTACGATGAG CTCGGAATGA GACTAATCAA CTTCGCTCGT TTTAGAAACA   
  
  
+ TCGTCCTCGA ATTCCAAGCA ATACCCACCT CCCCTTCCGA CGGATTTGCT TCGCTGTTGG AGGAGATTCG   
  
  
+ ACAAAGCAAG CTCTACTCCA ACGATGCGGC GGCGGTTATT GTGAATTGTC AGATGAGTTT GCATTTATTG   
  
  
+ CAGGAAGAGG AGGTGTCTTC ATCGTCGCCG TCGTCGATGA GGGGGATGTT TTTGCAGGCG GTGAGGAGCT   
  
  
+ TGGAGCCGAG CATGGTGGTG GTGGTGGAGG AGGACGTGGA TTTCACGGCG AGGAGTCTGG TGGGGCGGCT   
  
  
+ GAGATCGGCG TTTAATCACA TGTGGATACC CTTCGACACG GTGGACACGT TCTTGCCACG TGGGAGCCAG   
  
  
+ CAGAGAGAGT GGTTCGAGGC CGAGGTGTGC TGGAAGATTG AGAATGTGAT CGCTCATGAG GGACCCGCGA   
  
  
+ GGGTCGAGAG GCAGGAGCCC AGGGCCAAGT GGGCCCTCCG AATGAGGGAG GCCGAGTTTC AAGGGATCGA   
  
  
+ GTTCGGTGAT GAAGGTACGA CCGAGGTCAA GGCCATGCTG GAGGAGCATG CCGCTGGGTG GGGGTCTAAG   
  
  
+ AAGGAAGAGG ATGATCTTGT GCTCACTTGG AAGGGACATA GTGTTGTCTT TGCTTCTGCT TGGGTACCCA   
  
  
+ CTTA  

- +Up\_Stream \_Len000TCTTCG ATTAATTTGG TTTGTACGTG CATACAGGAT TAATTGTACT GACAGGTAGT   
  
  
- AGAGGAGGGT ACAACAAGAC AACTGTTCGG ACGTGTGGGT ACGAGGAGAG AGTACAGATT GGAGCAGGGT   
  
  
- TGCTAGTTCT AGTGACAGAC ACTCCGGTCC CCTTCTGAAC GAAAATAAAG GAAAAGAAAA CAAATGAGAG   
  
  
- ACTAAGGTAA ATCGGTATAT ATGTTCCTCT TACAGTACAC ATAGAGTATA CATATATATA TTCTAAAATA   
  
  
- AATCTTTATT TTTAAATTTG TGATACACTA AATATCAATA ATATTATTTA TCATAAAAAA TTTGATAAAG   
  
  
- TAACTACTCT TGTCCTTTCA TTTAACGGCT GTACAATTAC AATAATATAG ATTTTTAATT TAGTATTTTA   
  
  
- ATTAATATTA TAAAATTTTT ATAAAGTTGA TTTTAAAATC TTGTTATTCA CGCATTATGC CTGAATTTCC   
  
  
- GATCAAATAG CATTTTTACA TTCAAGGAAA ACCTAGAATA CCAACTTATA GAACCAACTA TTTGACACTA   
  
  
- ACCAAAATGT GAAAATGGTG TATAAGTATC TTCTTAATAC ACGTGCAACT ACCAAGTTCT ACCCTGTTTT   
  
  
- CCTTTGTACA TAAGAAGGGG GAAAACGGAA AAGGGGATTC GTAAACCTAA CTCCACAACA GTAGACTTAT   
  
  
- AGTTTTTAAG AAAAACTTTT AAGTCAGAGT TAGTTTTAAG TTTTAATTTT GTTTCTGAAT TCTCAAAATA   
  
  
- AACAAGAATT GGAAGAATTG AAAGATGAAA AGAAAAAAAA GAGTAAGGGG GAGAAGTTTA GAGTAAATAA   
  
  
- GAATTGGGGG AGAAGGCAAG TACGAGAGAG ACAGATAGTT GATTATAATT AGATGGGGCT GAAAGTTAAA   
  
  
- CTACTCCAGA TTAGGAATAG TAGCATAACA CCAACCCAAT TAATACGTTC TTCCGTTATT TGGGGTCACT   
  
  
- GTTTCAGGTG CAATTCATCC GTGGAGTGGT AATTCTGTAC GAGTCTTTTG TGGTTTTGGT AACTTGTGTT   
  
  
- CAGGGGGTTC AGGGAAGGGA TCGATCGGGT AAGAGAGAGG AGGGGACAGA TATAGATGGT TGAGAACGAG   
  
  
- AAACGACTCG GACTCAACTT TCGCTATGTG GGTATCGGAC AAGTCAAAAG TCAAAAGTGT AAACAGAAGG   
  
  
- AGACAAGTCA ACTTTCGCTA TGTGAGAGTA ATCGAAAGTA AAATTTATGC AAGTTAAGTG TGTATTTACC   
  
  
- GAAAGTTTCG TAACGGTAAA AGCAAATTTA CTACTGTTTT ATATATCATA GACCGATAGA CGGGTATAGA   
  
  
- ACTGGAATAA ATGTTTCCGA CTCGGTAGAA AATTAAAAAA ATCGGAGAAA AAAATTAGTT TTTATCTTTA   
  
  
- AAGTTCTTTC GTCTTTAGCA GTCTACTGTC ACGTCTGTCG CAAAAGCAAA GTATGTTTAG AGTTTTCGAC   
  
  
- TTCACAGTTA AAGAAGCTAA TAAAAACAGT CTTTTATAAA TGCAGAGTGG AAGTGCAACA ATAATAAGAA   
  
  
- AGAAAAAATA ATAAGAGTAT TGATCATTAT TTCAATCGTT ACTATTTTTT AATAAGCTAG GCTTATAAAA   
  
  
- TTAAATAGAC TGGATTTTTG TATTCATTTC CGTGTTTAAA AAGTAGGTTT AAAATTAAAA ACTACATTAT   
  
  
- AAAAAATATA AAAATAACAA GTTTAGACTA AAATTAGAAT AAATTTAATA GACTGGATTT TTTTAGTTTA   
  
  
- TTATTATTTG CAATTAAAAA ATAAATTTAA AACTGTAATC AGTTAGACTG AATTTAAGCT TGGCTCGAAC   
  
  
- TTAAAAAAAC GGTCACGACC ATCATGAGGA CGAAAACATC GAGGGTTACG GTAGCATGGT GGGAGAAGAT   
  
  
- CAGAGCAGAG AAAGAGACCA GAGGTTAATT AGTGTGGTAG TATAGTATGG CATACTACTA TGTCAGGTGA   
  
  
- CTTTAGGGTA AGAGTGGCGG TTAGAGAGAG GCGGTGGTAG GTTGTGAGAA TTGCTACTTG TCCCGGCGGC   
  
  
- GGAGCAGGAG GTGTGAGTCC GGCACCGGCC CGTCGAAAGG GTGGGTTAGT TTTAGAGAGA GGAGGCGGTT   
  
  
- GAAGCCATTG CGGTTAACGT ACCTCGTCGA GAATCAGGTG ACGCGGCTTC GGTAGCTGTT GTTACTGCGG   
  
  
- TGGGGCCGGG TCGTTTAGAA TACCCAGGAC TTATTGTATC GGGCTGGGCT GCCGCTGAGG TGGGTTGCGG   
  
  
- AGTGCACGCG TAAGGATGCA CGGAATTAGA GTGCGCGGCA GGAGTGATCG TGGACGTTCT ACCATTAGGG   
  
  
- AGTAAAGTTG GGGTAGTTAA GTGGGGTGTT TAAGAGCGAG GAGCTTGAAC GGGTGAAGCA GCTAAATTGG   
  
  
- GGAACCGTAG CTAAGCCTAA GTGGCGGCGG TTAAGCTAGT AAGACCTTCG ATAAAGGCTG GATGGGCAAC   
  
  
- ATGTGTAACA GCTGGAGTCG TAGAGGGTGA CATACGTCTA GGGGTGCAAC TAGCTGAGGT AACGCTGGGC   
  
  
- CAACCTTCGG GGCCCAGCTC AGGGAGGATA ACAGTTCGAG TGGCAGCCCC GATAAAGGCT GCTTTAGGGC   
  
  
- GGCCACAAGC TAGAAGACAG CATGCTACTC GAGCCTTACT CTGATTAGTT GAAGCGAGCA AAATCTTTGT   
  
  
- AGCAGGAGCT TAAGGTTCGT TATGGGTGGA GGGGAAGGCT GCCTAAACGA AGCGACAACC TCCTCTAAGC   
  
  
- TGTTTCGTTC GAGATGAGGT TGCTACGCCG CCGCCAATAA CACTTAACAG TCTACTCAAA CGTAAATAAC   
  
  
- GTCCTTCTCC TCCACAGAAG TAGCAGCGGC AGCAGCTACT CCCCCTACAA AAACGTCCGC CACTCCTCGA   
  
  
- ACCTCGGCTC GTACCACCAC CACCACCTCC TCCTGCACCT AAAGTGCCGC TCCTCAGACC ACCCCGCCGA   
  
  
- CTCTAGCCGC AAATTAGTGT ACACCTATGG GAAGCTGTGC CACCTGTGCA AGAACGGTGC ACCCTCGGTC   
  
  
- GTCTCTCTCA CCAAGCTCCG GCTCCACACG ACCTTCTAAC TCTTACACTA GCGAGTACTC CCTGGGCGCT   
  
  
- CCCAGCTCTC CGTCCTCGGG TCCCGGTTCA CCCGGGAGGC TTACTCCCTC CGGCTCAAAG TTCCCTAGCT   
  
  
- CAAGCCACTA CTTCCATGCT GGCTCCAGTT CCGGTACGAC CTCCTCGTAC GGCGACCCAC CCCCAGATTC   
  
  
- TTCCTTCTCC TACTAGAACA CGAGTGAACC TTCCCTGTAT CACAACAGAA ACGAAGACGA ACCCATGGGT   
  
  
- GAAT

+     A-box

| Site Name | Organism | Position | Strand | Matrix score. | sequence | function |
| --- | --- | --- | --- | --- | --- | --- |
| A-box | Petroselinum crispum | 2281 | + | 6 | CCGTCC | cis-acting regulatory element |

>HU06G00376.1   
+ +Up\_Stream \_Len000AGAAGC TAATTAAACC AAACATGCAC GTATGTCCTA ATTAACATGA CTGTCCATCA   
  
  
+ TCTCCTCCCA TGTTGTTCTG TTGACAAGCC TGCACACCCA TGCTCCTCTC TCATGTCTAA CCTCGTCCCA   
  
  
+ ACGATCAAGA TCACTGTCTG TGAGGCCAGG GGAAGACTTG CTTTTATTTC CTTTTCTTTT GTTTACTCTC   
  
  
+ TGATTCCATT TAGCCATATA TACAAGGAGA ATGTCATGTG TATCTCATAT GTATATATAT AAGATTTTAT   
  
  
+ TTAGAAATAA AAATTTAAAC ACTATGTGAT TTATAGTTAT TATAATAAAT AGTATTTTTT AAACTATTTC   
  
  
+ ATTGATGAGA ACAGGAAAGT AAATTGCCGA CATGTTAATG TTATTATATC TAAAAATTAA ATCATAAAAT   
  
  
+ TAATTATAAT ATTTTAAAAA TATTTCAACT AAAATTTTAG AACAATAAGT GCGTAATACG GACTTAAAGG   
  
  
+ CTAGTTTATC GTAAAAATGT AAGTTCCTTT TGGATCTTAT GGTTGAATAT CTTGGTTGAT AAACTGTGAT   
  
  
+ TGGTTTTACA CTTTTACCAC ATATTCATAG AAGAATTATG TGCACGTTGA TGGTTCAAGA TGGGACAAAA   
  
  
+ GGAAACATGT ATTCTTCCCC CTTTTGCCTT TTCCCCTAAG CATTTGGATT GAGGTGTTGT CATCTGAATA   
  
  
+ TCAAAAATTC TTTTTGAAAA TTCAGTCTCA ATCAAAATTC AAAATTAAAA CAAAGACTTA AGAGTTTTAT   
  
  
+ TTGTTCTTAA CCTTCTTAAC TTTCTACTTT TCTTTTTTTT CTCATTCCCC CTCTTCAAAT CTCATTTATT   
  
  
+ CTTAACCCCC TCTTCCGTTC ATGCTCTCTC TGTCTATCAA CTAATATTAA TCTACCCCGA CTTTCAATTT   
  
  
+ GATGAGGTCT AATCCTTATC ATCGTATTGT GGTTGGGTTA ATTATGCAAG AAGGCAATAA ACCCCAGTGA   
  
  
+ CAAAGTCCAC GTTAAGTAGG CACCTCACCA TTAAGACATG CTCAGAAAAC ACCAAAACCA TTGAACACAA   
  
  
+ GTCCCCCAAG TCCCTTCCCT AGCTAGCCCA TTCTCTCTCC TCCCCTGTCT ATATCTACCA ACTCTTGCTC   
  
  
+ TTTGCTGAGC CTGAGTTGAA AGCGATACAC CCATAGCCTG TTCAGTTTTC AGTTTTCACA TTTGTCTTCC   
  
  
+ TCTGTTCAGT TGAAAGCGAT ACACTCTCAT TAGCTTTCAT TTTAAATACG TTCAATTCAC ACATAAATGG   
  
  
+ CTTTCAAAGC ATTGCCATTT TCGTTTAAAT GATGACAAAA TATATAGTAT CTGGCTATCT GCCCATATCT   
  
  
+ TGACCTTATT TACAAAGGCT GAGCCATCTT TTAATTTTTT TAGCCTCTTT TTTTAATCAA AAATAGAAAT   
  
  
+ TTCAAGAAAG CAGAAATCGT CAGATGACAG TGCAGACAGC GTTTTCGTTT CATACAAATC TCAAAAGCTG   
  
  
+ AAGTGTCAAT TTCTTCGATT ATTTTTGTCA GAAAATATTT ACGTCTCACC TTCACGTTGT TATTATTCTT   
  
  
+ TCTTTTTTAT TATTCTCATA ACTAGTAATA AAGTTAGCAA TGATAAAAAA TTATTCGATC CGAATATTTT   
  
  
+ AATTTATCTG ACCTAAAAAC ATAAGTAAAG GCACAAATTT TTCATCCAAA TTTTAATTTT TGATGTAATA   
  
  
+ TTTTTTATAT TTTTATTGTT CAAATCTGAT TTTAATCTTA TTTAAATTAT CTGACCTAAA AAAATCAAAT   
  
  
+ AATAATAAAC GTTAATTTTT TATTTAAATT TTGACATTAG TCAATCTGAC TTAAATTCGA ACCGAGCTTG   
  
  
+ AATTTTTTTG CCAGTGCTGG TAGTACTCCT GCTTTTGTAG CTCCCAATGC CATCGTACCA CCCTCTTCTA   
  
  
+ GTCTCGTCTC TTTCTCTGGT CTCCAATTAA TCACACCATC ATATCATACC GTATGATGAT ACAGTCCACT   
  
  
+ GAAATCCCAT TCTCACCGCC AATCTCTCTC CGCCACCATC CAACACTCTT AACGATGAAC AGGGCCGCCG   
  
  
+ CCTCGTCCTC CACACTCAGG CCGTGGCCGG GCAGCTTTCC CACCCAATCA AAATCTCTCT CCTCCGCCAA   
  
  
+ CTTCGGTAAC GCCAATTGCA TGGAGCAGCT CTTAGTCCAC TGCGCCGAAG CCATCGACAA CAATGACGCC   
  
  
+ ACCCCGGCCC AGCAAATCTT ATGGGTCCTG AATAACATAG CCCGACCCGA CGGCGACTCC ACCCAACGCC   
  
  
+ TCACGTGCGC ATTCCTACGT GCCTTAATCT CACGCGCCGT CCTCACTAGC ACCTGCAAGA TGGTAATCCC   
  
  
+ TCATTTCAAC CCCATCAATT CACCCCACAA ATTCTCGCTC CTCGAACTTG CCCACTTCGT CGATTTAACC   
  
  
+ CCTTGGCATC GATTCGGATT CACCGCCGCC AATTCGATCA TTCTGGAAGC TATTTCCGAC CTACCCGTTG   
  
  
+ TACACATTGT CGACCTCAGC ATCTCCCACT GTATGCAGAT CCCCACGTTG ATCGACTCCA TTGCGACCCG   
  
  
+ GTTGGAAGCC CCGGGTCGAG TCCCTCCTAT TGTCAAGCTC ACCGTCGGGG CTATTTCCGA CGAAATCCCG   
  
  
+ CCGGTGTTCG ATCTTCTGTC GTACGATGAG CTCGGAATGA GACTAATCAA CTTCGCTCGT TTTAGAAACA   
  
  
+ TCGTCCTCGA ATTCCAAGCA ATACCCACCT CCCCTTCCGA CGGATTTGCT TCGCTGTTGG AGGAGATTCG   
  
  
+ ACAAAGCAAG CTCTACTCCA ACGATGCGGC GGCGGTTATT GTGAATTGTC AGATGAGTTT GCATTTATTG   
  
  
+ CAGGAAGAGG AGGTGTCTTC ATCGTCGCCG TCGTCGATGA GGGGGATGTT TTTGCAGGCG GTGAGGAGCT   
  
  
+ TGGAGCCGAG CATGGTGGTG GTGGTGGAGG AGGACGTGGA TTTCACGGCG AGGAGTCTGG TGGGGCGGCT   
  
  
+ GAGATCGGCG TTTAATCACA TGTGGATACC CTTCGACACG GTGGACACGT TCTTGCCACG TGGGAGCCAG   
  
  
+ CAGAGAGAGT GGTTCGAGGC CGAGGTGTGC TGGAAGATTG AGAATGTGAT CGCTCATGAG GGACCCGCGA   
  
  
+ GGGTCGAGAG GCAGGAGCCC AGGGCCAAGT GGGCCCTCCG AATGAGGGAG GCCGAGTTTC AAGGGATCGA   
  
  
+ GTTCGGTGAT GAAGGTACGA CCGAGGTCAA GGCCATGCTG GAGGAGCATG CCGCTGGGTG GGGGTCTAAG   
  
  
+ AAGGAAGAGG ATGATCTTGT GCTCACTTGG AAGGGACATA GTGTTGTCTT TGCTTCTGCT TGGGTACCCA   
  
  
+ CTTA  

- +Up\_Stream \_Len000TCTTCG ATTAATTTGG TTTGTACGTG CATACAGGAT TAATTGTACT GACAGGTAGT   
  
  
- AGAGGAGGGT ACAACAAGAC AACTGTTCGG ACGTGTGGGT ACGAGGAGAG AGTACAGATT GGAGCAGGGT   
  
  
- TGCTAGTTCT AGTGACAGAC ACTCCGGTCC CCTTCTGAAC GAAAATAAAG GAAAAGAAAA CAAATGAGAG   
  
  
- ACTAAGGTAA ATCGGTATAT ATGTTCCTCT TACAGTACAC ATAGAGTATA CATATATATA TTCTAAAATA   
  
  
- AATCTTTATT TTTAAATTTG TGATACACTA AATATCAATA ATATTATTTA TCATAAAAAA TTTGATAAAG   
  
  
- TAACTACTCT TGTCCTTTCA TTTAACGGCT GTACAATTAC AATAATATAG ATTTTTAATT TAGTATTTTA   
  
  
- ATTAATATTA TAAAATTTTT ATAAAGTTGA TTTTAAAATC TTGTTATTCA CGCATTATGC CTGAATTTCC   
  
  
- GATCAAATAG CATTTTTACA TTCAAGGAAA ACCTAGAATA CCAACTTATA GAACCAACTA TTTGACACTA   
  
  
- ACCAAAATGT GAAAATGGTG TATAAGTATC TTCTTAATAC ACGTGCAACT ACCAAGTTCT ACCCTGTTTT   
  
  
- CCTTTGTACA TAAGAAGGGG GAAAACGGAA AAGGGGATTC GTAAACCTAA CTCCACAACA GTAGACTTAT   
  
  
- AGTTTTTAAG AAAAACTTTT AAGTCAGAGT TAGTTTTAAG TTTTAATTTT GTTTCTGAAT TCTCAAAATA   
  
  
- AACAAGAATT GGAAGAATTG AAAGATGAAA AGAAAAAAAA GAGTAAGGGG GAGAAGTTTA GAGTAAATAA   
  
  
- GAATTGGGGG AGAAGGCAAG TACGAGAGAG ACAGATAGTT GATTATAATT AGATGGGGCT GAAAGTTAAA   
  
  
- CTACTCCAGA TTAGGAATAG TAGCATAACA CCAACCCAAT TAATACGTTC TTCCGTTATT TGGGGTCACT   
  
  
- GTTTCAGGTG CAATTCATCC GTGGAGTGGT AATTCTGTAC GAGTCTTTTG TGGTTTTGGT AACTTGTGTT   
  
  
- CAGGGGGTTC AGGGAAGGGA TCGATCGGGT AAGAGAGAGG AGGGGACAGA TATAGATGGT TGAGAACGAG   
  
  
- AAACGACTCG GACTCAACTT TCGCTATGTG GGTATCGGAC AAGTCAAAAG TCAAAAGTGT AAACAGAAGG   
  
  
- AGACAAGTCA ACTTTCGCTA TGTGAGAGTA ATCGAAAGTA AAATTTATGC AAGTTAAGTG TGTATTTACC   
  
  
- GAAAGTTTCG TAACGGTAAA AGCAAATTTA CTACTGTTTT ATATATCATA GACCGATAGA CGGGTATAGA   
  
  
- ACTGGAATAA ATGTTTCCGA CTCGGTAGAA AATTAAAAAA ATCGGAGAAA AAAATTAGTT TTTATCTTTA   
  
  
- AAGTTCTTTC GTCTTTAGCA GTCTACTGTC ACGTCTGTCG CAAAAGCAAA GTATGTTTAG AGTTTTCGAC   
  
  
- TTCACAGTTA AAGAAGCTAA TAAAAACAGT CTTTTATAAA TGCAGAGTGG AAGTGCAACA ATAATAAGAA   
  
  
- AGAAAAAATA ATAAGAGTAT TGATCATTAT TTCAATCGTT ACTATTTTTT AATAAGCTAG GCTTATAAAA   
  
  
- TTAAATAGAC TGGATTTTTG TATTCATTTC CGTGTTTAAA AAGTAGGTTT AAAATTAAAA ACTACATTAT   
  
  
- AAAAAATATA AAAATAACAA GTTTAGACTA AAATTAGAAT AAATTTAATA GACTGGATTT TTTTAGTTTA   
  
  
- TTATTATTTG CAATTAAAAA ATAAATTTAA AACTGTAATC AGTTAGACTG AATTTAAGCT TGGCTCGAAC   
  
  
- TTAAAAAAAC GGTCACGACC ATCATGAGGA CGAAAACATC GAGGGTTACG GTAGCATGGT GGGAGAAGAT   
  
  
- CAGAGCAGAG AAAGAGACCA GAGGTTAATT AGTGTGGTAG TATAGTATGG CATACTACTA TGTCAGGTGA   
  
  
- CTTTAGGGTA AGAGTGGCGG TTAGAGAGAG GCGGTGGTAG GTTGTGAGAA TTGCTACTTG TCCCGGCGGC   
  
  
- GGAGCAGGAG GTGTGAGTCC GGCACCGGCC CGTCGAAAGG GTGGGTTAGT TTTAGAGAGA GGAGGCGGTT   
  
  
- GAAGCCATTG CGGTTAACGT ACCTCGTCGA GAATCAGGTG ACGCGGCTTC GGTAGCTGTT GTTACTGCGG   
  
  
- TGGGGCCGGG TCGTTTAGAA TACCCAGGAC TTATTGTATC GGGCTGGGCT GCCGCTGAGG TGGGTTGCGG   
  
  
- AGTGCACGCG TAAGGATGCA CGGAATTAGA GTGCGCGGCA GGAGTGATCG TGGACGTTCT ACCATTAGGG   
  
  
- AGTAAAGTTG GGGTAGTTAA GTGGGGTGTT TAAGAGCGAG GAGCTTGAAC GGGTGAAGCA GCTAAATTGG   
  
  
- GGAACCGTAG CTAAGCCTAA GTGGCGGCGG TTAAGCTAGT AAGACCTTCG ATAAAGGCTG GATGGGCAAC   
  
  
- ATGTGTAACA GCTGGAGTCG TAGAGGGTGA CATACGTCTA GGGGTGCAAC TAGCTGAGGT AACGCTGGGC   
  
  
- CAACCTTCGG GGCCCAGCTC AGGGAGGATA ACAGTTCGAG TGGCAGCCCC GATAAAGGCT GCTTTAGGGC   
  
  
- GGCCACAAGC TAGAAGACAG CATGCTACTC GAGCCTTACT CTGATTAGTT GAAGCGAGCA AAATCTTTGT   
  
  
- AGCAGGAGCT TAAGGTTCGT TATGGGTGGA GGGGAAGGCT GCCTAAACGA AGCGACAACC TCCTCTAAGC   
  
  
- TGTTTCGTTC GAGATGAGGT TGCTACGCCG CCGCCAATAA CACTTAACAG TCTACTCAAA CGTAAATAAC   
  
  
- GTCCTTCTCC TCCACAGAAG TAGCAGCGGC AGCAGCTACT CCCCCTACAA AAACGTCCGC CACTCCTCGA   
  
  
- ACCTCGGCTC GTACCACCAC CACCACCTCC TCCTGCACCT AAAGTGCCGC TCCTCAGACC ACCCCGCCGA   
  
  
- CTCTAGCCGC AAATTAGTGT ACACCTATGG GAAGCTGTGC CACCTGTGCA AGAACGGTGC ACCCTCGGTC   
  
  
- GTCTCTCTCA CCAAGCTCCG GCTCCACACG ACCTTCTAAC TCTTACACTA GCGAGTACTC CCTGGGCGCT   
  
  
- CCCAGCTCTC CGTCCTCGGG TCCCGGTTCA CCCGGGAGGC TTACTCCCTC CGGCTCAAAG TTCCCTAGCT   
  
  
- CAAGCCACTA CTTCCATGCT GGCTCCAGTT CCGGTACGAC CTCCTCGTAC GGCGACCCAC CCCCAGATTC   
  
  
- TTCCTTCTCC TACTAGAACA CGAGTGAACC TTCCCTGTAT CACAACAGAA ACGAAGACGA ACCCATGGGT   
  
  
- GAAT

+     AAAC-motif

| Site Name | Organism | Position | Strand | Matrix score. | sequence | function |
| --- | --- | --- | --- | --- | --- | --- |
| AAAC-motif | Spinacia oleracea | 2079 | + | 11 | CAATCAAAACCT | light responsive element |

>HU06G00376.1   
+ +Up\_Stream \_Len000AGAAGC TAATTAAACC AAACATGCAC GTATGTCCTA ATTAACATGA CTGTCCATCA   
  
  
+ TCTCCTCCCA TGTTGTTCTG TTGACAAGCC TGCACACCCA TGCTCCTCTC TCATGTCTAA CCTCGTCCCA   
  
  
+ ACGATCAAGA TCACTGTCTG TGAGGCCAGG GGAAGACTTG CTTTTATTTC CTTTTCTTTT GTTTACTCTC   
  
  
+ TGATTCCATT TAGCCATATA TACAAGGAGA ATGTCATGTG TATCTCATAT GTATATATAT AAGATTTTAT   
  
  
+ TTAGAAATAA AAATTTAAAC ACTATGTGAT TTATAGTTAT TATAATAAAT AGTATTTTTT AAACTATTTC   
  
  
+ ATTGATGAGA ACAGGAAAGT AAATTGCCGA CATGTTAATG TTATTATATC TAAAAATTAA ATCATAAAAT   
  
  
+ TAATTATAAT ATTTTAAAAA TATTTCAACT AAAATTTTAG AACAATAAGT GCGTAATACG GACTTAAAGG   
  
  
+ CTAGTTTATC GTAAAAATGT AAGTTCCTTT TGGATCTTAT GGTTGAATAT CTTGGTTGAT AAACTGTGAT   
  
  
+ TGGTTTTACA CTTTTACCAC ATATTCATAG AAGAATTATG TGCACGTTGA TGGTTCAAGA TGGGACAAAA   
  
  
+ GGAAACATGT ATTCTTCCCC CTTTTGCCTT TTCCCCTAAG CATTTGGATT GAGGTGTTGT CATCTGAATA   
  
  
+ TCAAAAATTC TTTTTGAAAA TTCAGTCTCA ATCAAAATTC AAAATTAAAA CAAAGACTTA AGAGTTTTAT   
  
  
+ TTGTTCTTAA CCTTCTTAAC TTTCTACTTT TCTTTTTTTT CTCATTCCCC CTCTTCAAAT CTCATTTATT   
  
  
+ CTTAACCCCC TCTTCCGTTC ATGCTCTCTC TGTCTATCAA CTAATATTAA TCTACCCCGA CTTTCAATTT   
  
  
+ GATGAGGTCT AATCCTTATC ATCGTATTGT GGTTGGGTTA ATTATGCAAG AAGGCAATAA ACCCCAGTGA   
  
  
+ CAAAGTCCAC GTTAAGTAGG CACCTCACCA TTAAGACATG CTCAGAAAAC ACCAAAACCA TTGAACACAA   
  
  
+ GTCCCCCAAG TCCCTTCCCT AGCTAGCCCA TTCTCTCTCC TCCCCTGTCT ATATCTACCA ACTCTTGCTC   
  
  
+ TTTGCTGAGC CTGAGTTGAA AGCGATACAC CCATAGCCTG TTCAGTTTTC AGTTTTCACA TTTGTCTTCC   
  
  
+ TCTGTTCAGT TGAAAGCGAT ACACTCTCAT TAGCTTTCAT TTTAAATACG TTCAATTCAC ACATAAATGG   
  
  
+ CTTTCAAAGC ATTGCCATTT TCGTTTAAAT GATGACAAAA TATATAGTAT CTGGCTATCT GCCCATATCT   
  
  
+ TGACCTTATT TACAAAGGCT GAGCCATCTT TTAATTTTTT TAGCCTCTTT TTTTAATCAA AAATAGAAAT   
  
  
+ TTCAAGAAAG CAGAAATCGT CAGATGACAG TGCAGACAGC GTTTTCGTTT CATACAAATC TCAAAAGCTG   
  
  
+ AAGTGTCAAT TTCTTCGATT ATTTTTGTCA GAAAATATTT ACGTCTCACC TTCACGTTGT TATTATTCTT   
  
  
+ TCTTTTTTAT TATTCTCATA ACTAGTAATA AAGTTAGCAA TGATAAAAAA TTATTCGATC CGAATATTTT   
  
  
+ AATTTATCTG ACCTAAAAAC ATAAGTAAAG GCACAAATTT TTCATCCAAA TTTTAATTTT TGATGTAATA   
  
  
+ TTTTTTATAT TTTTATTGTT CAAATCTGAT TTTAATCTTA TTTAAATTAT CTGACCTAAA AAAATCAAAT   
  
  
+ AATAATAAAC GTTAATTTTT TATTTAAATT TTGACATTAG TCAATCTGAC TTAAATTCGA ACCGAGCTTG   
  
  
+ AATTTTTTTG CCAGTGCTGG TAGTACTCCT GCTTTTGTAG CTCCCAATGC CATCGTACCA CCCTCTTCTA   
  
  
+ GTCTCGTCTC TTTCTCTGGT CTCCAATTAA TCACACCATC ATATCATACC GTATGATGAT ACAGTCCACT   
  
  
+ GAAATCCCAT TCTCACCGCC AATCTCTCTC CGCCACCATC CAACACTCTT AACGATGAAC AGGGCCGCCG   
  
  
+ CCTCGTCCTC CACACTCAGG CCGTGGCCGG GCAGCTTTCC CACCCAATCA AAATCTCTCT CCTCCGCCAA   
  
  
+ CTTCGGTAAC GCCAATTGCA TGGAGCAGCT CTTAGTCCAC TGCGCCGAAG CCATCGACAA CAATGACGCC   
  
  
+ ACCCCGGCCC AGCAAATCTT ATGGGTCCTG AATAACATAG CCCGACCCGA CGGCGACTCC ACCCAACGCC   
  
  
+ TCACGTGCGC ATTCCTACGT GCCTTAATCT CACGCGCCGT CCTCACTAGC ACCTGCAAGA TGGTAATCCC   
  
  
+ TCATTTCAAC CCCATCAATT CACCCCACAA ATTCTCGCTC CTCGAACTTG CCCACTTCGT CGATTTAACC   
  
  
+ CCTTGGCATC GATTCGGATT CACCGCCGCC AATTCGATCA TTCTGGAAGC TATTTCCGAC CTACCCGTTG   
  
  
+ TACACATTGT CGACCTCAGC ATCTCCCACT GTATGCAGAT CCCCACGTTG ATCGACTCCA TTGCGACCCG   
  
  
+ GTTGGAAGCC CCGGGTCGAG TCCCTCCTAT TGTCAAGCTC ACCGTCGGGG CTATTTCCGA CGAAATCCCG   
  
  
+ CCGGTGTTCG ATCTTCTGTC GTACGATGAG CTCGGAATGA GACTAATCAA CTTCGCTCGT TTTAGAAACA   
  
  
+ TCGTCCTCGA ATTCCAAGCA ATACCCACCT CCCCTTCCGA CGGATTTGCT TCGCTGTTGG AGGAGATTCG   
  
  
+ ACAAAGCAAG CTCTACTCCA ACGATGCGGC GGCGGTTATT GTGAATTGTC AGATGAGTTT GCATTTATTG   
  
  
+ CAGGAAGAGG AGGTGTCTTC ATCGTCGCCG TCGTCGATGA GGGGGATGTT TTTGCAGGCG GTGAGGAGCT   
  
  
+ TGGAGCCGAG CATGGTGGTG GTGGTGGAGG AGGACGTGGA TTTCACGGCG AGGAGTCTGG TGGGGCGGCT   
  
  
+ GAGATCGGCG TTTAATCACA TGTGGATACC CTTCGACACG GTGGACACGT TCTTGCCACG TGGGAGCCAG   
  
  
+ CAGAGAGAGT GGTTCGAGGC CGAGGTGTGC TGGAAGATTG AGAATGTGAT CGCTCATGAG GGACCCGCGA   
  
  
+ GGGTCGAGAG GCAGGAGCCC AGGGCCAAGT GGGCCCTCCG AATGAGGGAG GCCGAGTTTC AAGGGATCGA   
  
  
+ GTTCGGTGAT GAAGGTACGA CCGAGGTCAA GGCCATGCTG GAGGAGCATG CCGCTGGGTG GGGGTCTAAG   
  
  
+ AAGGAAGAGG ATGATCTTGT GCTCACTTGG AAGGGACATA GTGTTGTCTT TGCTTCTGCT TGGGTACCCA   
  
  
+ CTTA  

- +Up\_Stream \_Len000TCTTCG ATTAATTTGG TTTGTACGTG CATACAGGAT TAATTGTACT GACAGGTAGT   
  
  
- AGAGGAGGGT ACAACAAGAC AACTGTTCGG ACGTGTGGGT ACGAGGAGAG AGTACAGATT GGAGCAGGGT   
  
  
- TGCTAGTTCT AGTGACAGAC ACTCCGGTCC CCTTCTGAAC GAAAATAAAG GAAAAGAAAA CAAATGAGAG   
  
  
- ACTAAGGTAA ATCGGTATAT ATGTTCCTCT TACAGTACAC ATAGAGTATA CATATATATA TTCTAAAATA   
  
  
- AATCTTTATT TTTAAATTTG TGATACACTA AATATCAATA ATATTATTTA TCATAAAAAA TTTGATAAAG   
  
  
- TAACTACTCT TGTCCTTTCA TTTAACGGCT GTACAATTAC AATAATATAG ATTTTTAATT TAGTATTTTA   
  
  
- ATTAATATTA TAAAATTTTT ATAAAGTTGA TTTTAAAATC TTGTTATTCA CGCATTATGC CTGAATTTCC   
  
  
- GATCAAATAG CATTTTTACA TTCAAGGAAA ACCTAGAATA CCAACTTATA GAACCAACTA TTTGACACTA   
  
  
- ACCAAAATGT GAAAATGGTG TATAAGTATC TTCTTAATAC ACGTGCAACT ACCAAGTTCT ACCCTGTTTT   
  
  
- CCTTTGTACA TAAGAAGGGG GAAAACGGAA AAGGGGATTC GTAAACCTAA CTCCACAACA GTAGACTTAT   
  
  
- AGTTTTTAAG AAAAACTTTT AAGTCAGAGT TAGTTTTAAG TTTTAATTTT GTTTCTGAAT TCTCAAAATA   
  
  
- AACAAGAATT GGAAGAATTG AAAGATGAAA AGAAAAAAAA GAGTAAGGGG GAGAAGTTTA GAGTAAATAA   
  
  
- GAATTGGGGG AGAAGGCAAG TACGAGAGAG ACAGATAGTT GATTATAATT AGATGGGGCT GAAAGTTAAA   
  
  
- CTACTCCAGA TTAGGAATAG TAGCATAACA CCAACCCAAT TAATACGTTC TTCCGTTATT TGGGGTCACT   
  
  
- GTTTCAGGTG CAATTCATCC GTGGAGTGGT AATTCTGTAC GAGTCTTTTG TGGTTTTGGT AACTTGTGTT   
  
  
- CAGGGGGTTC AGGGAAGGGA TCGATCGGGT AAGAGAGAGG AGGGGACAGA TATAGATGGT TGAGAACGAG   
  
  
- AAACGACTCG GACTCAACTT TCGCTATGTG GGTATCGGAC AAGTCAAAAG TCAAAAGTGT AAACAGAAGG   
  
  
- AGACAAGTCA ACTTTCGCTA TGTGAGAGTA ATCGAAAGTA AAATTTATGC AAGTTAAGTG TGTATTTACC   
  
  
- GAAAGTTTCG TAACGGTAAA AGCAAATTTA CTACTGTTTT ATATATCATA GACCGATAGA CGGGTATAGA   
  
  
- ACTGGAATAA ATGTTTCCGA CTCGGTAGAA AATTAAAAAA ATCGGAGAAA AAAATTAGTT TTTATCTTTA   
  
  
- AAGTTCTTTC GTCTTTAGCA GTCTACTGTC ACGTCTGTCG CAAAAGCAAA GTATGTTTAG AGTTTTCGAC   
  
  
- TTCACAGTTA AAGAAGCTAA TAAAAACAGT CTTTTATAAA TGCAGAGTGG AAGTGCAACA ATAATAAGAA   
  
  
- AGAAAAAATA ATAAGAGTAT TGATCATTAT TTCAATCGTT ACTATTTTTT AATAAGCTAG GCTTATAAAA   
  
  
- TTAAATAGAC TGGATTTTTG TATTCATTTC CGTGTTTAAA AAGTAGGTTT AAAATTAAAA ACTACATTAT   
  
  
- AAAAAATATA AAAATAACAA GTTTAGACTA AAATTAGAAT AAATTTAATA GACTGGATTT TTTTAGTTTA   
  
  
- TTATTATTTG CAATTAAAAA ATAAATTTAA AACTGTAATC AGTTAGACTG AATTTAAGCT TGGCTCGAAC   
  
  
- TTAAAAAAAC GGTCACGACC ATCATGAGGA CGAAAACATC GAGGGTTACG GTAGCATGGT GGGAGAAGAT   
  
  
- CAGAGCAGAG AAAGAGACCA GAGGTTAATT AGTGTGGTAG TATAGTATGG CATACTACTA TGTCAGGTGA   
  
  
- CTTTAGGGTA AGAGTGGCGG TTAGAGAGAG GCGGTGGTAG GTTGTGAGAA TTGCTACTTG TCCCGGCGGC   
  
  
- GGAGCAGGAG GTGTGAGTCC GGCACCGGCC CGTCGAAAGG GTGGGTTAGT TTTAGAGAGA GGAGGCGGTT   
  
  
- GAAGCCATTG CGGTTAACGT ACCTCGTCGA GAATCAGGTG ACGCGGCTTC GGTAGCTGTT GTTACTGCGG   
  
  
- TGGGGCCGGG TCGTTTAGAA TACCCAGGAC TTATTGTATC GGGCTGGGCT GCCGCTGAGG TGGGTTGCGG   
  
  
- AGTGCACGCG TAAGGATGCA CGGAATTAGA GTGCGCGGCA GGAGTGATCG TGGACGTTCT ACCATTAGGG   
  
  
- AGTAAAGTTG GGGTAGTTAA GTGGGGTGTT TAAGAGCGAG GAGCTTGAAC GGGTGAAGCA GCTAAATTGG   
  
  
- GGAACCGTAG CTAAGCCTAA GTGGCGGCGG TTAAGCTAGT AAGACCTTCG ATAAAGGCTG GATGGGCAAC   
  
  
- ATGTGTAACA GCTGGAGTCG TAGAGGGTGA CATACGTCTA GGGGTGCAAC TAGCTGAGGT AACGCTGGGC   
  
  
- CAACCTTCGG GGCCCAGCTC AGGGAGGATA ACAGTTCGAG TGGCAGCCCC GATAAAGGCT GCTTTAGGGC   
  
  
- GGCCACAAGC TAGAAGACAG CATGCTACTC GAGCCTTACT CTGATTAGTT GAAGCGAGCA AAATCTTTGT   
  
  
- AGCAGGAGCT TAAGGTTCGT TATGGGTGGA GGGGAAGGCT GCCTAAACGA AGCGACAACC TCCTCTAAGC   
  
  
- TGTTTCGTTC GAGATGAGGT TGCTACGCCG CCGCCAATAA CACTTAACAG TCTACTCAAA CGTAAATAAC   
  
  
- GTCCTTCTCC TCCACAGAAG TAGCAGCGGC AGCAGCTACT CCCCCTACAA AAACGTCCGC CACTCCTCGA   
  
  
- ACCTCGGCTC GTACCACCAC CACCACCTCC TCCTGCACCT AAAGTGCCGC TCCTCAGACC ACCCCGCCGA   
  
  
- CTCTAGCCGC AAATTAGTGT ACACCTATGG GAAGCTGTGC CACCTGTGCA AGAACGGTGC ACCCTCGGTC   
  
  
- GTCTCTCTCA CCAAGCTCCG GCTCCACACG ACCTTCTAAC TCTTACACTA GCGAGTACTC CCTGGGCGCT   
  
  
- CCCAGCTCTC CGTCCTCGGG TCCCGGTTCA CCCGGGAGGC TTACTCCCTC CGGCTCAAAG TTCCCTAGCT   
  
  
- CAAGCCACTA CTTCCATGCT GGCTCCAGTT CCGGTACGAC CTCCTCGTAC GGCGACCCAC CCCCAGATTC   
  
  
- TTCCTTCTCC TACTAGAACA CGAGTGAACC TTCCCTGTAT CACAACAGAA ACGAAGACGA ACCCATGGGT   
  
  
- GAAT

+     AAGAA-motif

| Site Name | Organism | Position | Strand | Matrix score. | sequence | function |
| --- | --- | --- | --- | --- | --- | --- |
| AAGAA-motif | Avena sativa | 1540 | - | 7 | GAAAGAA |  |

>HU06G00376.1   
+ +Up\_Stream \_Len000AGAAGC TAATTAAACC AAACATGCAC GTATGTCCTA ATTAACATGA CTGTCCATCA   
  
  
+ TCTCCTCCCA TGTTGTTCTG TTGACAAGCC TGCACACCCA TGCTCCTCTC TCATGTCTAA CCTCGTCCCA   
  
  
+ ACGATCAAGA TCACTGTCTG TGAGGCCAGG GGAAGACTTG CTTTTATTTC CTTTTCTTTT GTTTACTCTC   
  
  
+ TGATTCCATT TAGCCATATA TACAAGGAGA ATGTCATGTG TATCTCATAT GTATATATAT AAGATTTTAT   
  
  
+ TTAGAAATAA AAATTTAAAC ACTATGTGAT TTATAGTTAT TATAATAAAT AGTATTTTTT AAACTATTTC   
  
  
+ ATTGATGAGA ACAGGAAAGT AAATTGCCGA CATGTTAATG TTATTATATC TAAAAATTAA ATCATAAAAT   
  
  
+ TAATTATAAT ATTTTAAAAA TATTTCAACT AAAATTTTAG AACAATAAGT GCGTAATACG GACTTAAAGG   
  
  
+ CTAGTTTATC GTAAAAATGT AAGTTCCTTT TGGATCTTAT GGTTGAATAT CTTGGTTGAT AAACTGTGAT   
  
  
+ TGGTTTTACA CTTTTACCAC ATATTCATAG AAGAATTATG TGCACGTTGA TGGTTCAAGA TGGGACAAAA   
  
  
+ GGAAACATGT ATTCTTCCCC CTTTTGCCTT TTCCCCTAAG CATTTGGATT GAGGTGTTGT CATCTGAATA   
  
  
+ TCAAAAATTC TTTTTGAAAA TTCAGTCTCA ATCAAAATTC AAAATTAAAA CAAAGACTTA AGAGTTTTAT   
  
  
+ TTGTTCTTAA CCTTCTTAAC TTTCTACTTT TCTTTTTTTT CTCATTCCCC CTCTTCAAAT CTCATTTATT   
  
  
+ CTTAACCCCC TCTTCCGTTC ATGCTCTCTC TGTCTATCAA CTAATATTAA TCTACCCCGA CTTTCAATTT   
  
  
+ GATGAGGTCT AATCCTTATC ATCGTATTGT GGTTGGGTTA ATTATGCAAG AAGGCAATAA ACCCCAGTGA   
  
  
+ CAAAGTCCAC GTTAAGTAGG CACCTCACCA TTAAGACATG CTCAGAAAAC ACCAAAACCA TTGAACACAA   
  
  
+ GTCCCCCAAG TCCCTTCCCT AGCTAGCCCA TTCTCTCTCC TCCCCTGTCT ATATCTACCA ACTCTTGCTC   
  
  
+ TTTGCTGAGC CTGAGTTGAA AGCGATACAC CCATAGCCTG TTCAGTTTTC AGTTTTCACA TTTGTCTTCC   
  
  
+ TCTGTTCAGT TGAAAGCGAT ACACTCTCAT TAGCTTTCAT TTTAAATACG TTCAATTCAC ACATAAATGG   
  
  
+ CTTTCAAAGC ATTGCCATTT TCGTTTAAAT GATGACAAAA TATATAGTAT CTGGCTATCT GCCCATATCT   
  
  
+ TGACCTTATT TACAAAGGCT GAGCCATCTT TTAATTTTTT TAGCCTCTTT TTTTAATCAA AAATAGAAAT   
  
  
+ TTCAAGAAAG CAGAAATCGT CAGATGACAG TGCAGACAGC GTTTTCGTTT CATACAAATC TCAAAAGCTG   
  
  
+ AAGTGTCAAT TTCTTCGATT ATTTTTGTCA GAAAATATTT ACGTCTCACC TTCACGTTGT TATTATTCTT   
  
  
+ TCTTTTTTAT TATTCTCATA ACTAGTAATA AAGTTAGCAA TGATAAAAAA TTATTCGATC CGAATATTTT   
  
  
+ AATTTATCTG ACCTAAAAAC ATAAGTAAAG GCACAAATTT TTCATCCAAA TTTTAATTTT TGATGTAATA   
  
  
+ TTTTTTATAT TTTTATTGTT CAAATCTGAT TTTAATCTTA TTTAAATTAT CTGACCTAAA AAAATCAAAT   
  
  
+ AATAATAAAC GTTAATTTTT TATTTAAATT TTGACATTAG TCAATCTGAC TTAAATTCGA ACCGAGCTTG   
  
  
+ AATTTTTTTG CCAGTGCTGG TAGTACTCCT GCTTTTGTAG CTCCCAATGC CATCGTACCA CCCTCTTCTA   
  
  
+ GTCTCGTCTC TTTCTCTGGT CTCCAATTAA TCACACCATC ATATCATACC GTATGATGAT ACAGTCCACT   
  
  
+ GAAATCCCAT TCTCACCGCC AATCTCTCTC CGCCACCATC CAACACTCTT AACGATGAAC AGGGCCGCCG   
  
  
+ CCTCGTCCTC CACACTCAGG CCGTGGCCGG GCAGCTTTCC CACCCAATCA AAATCTCTCT CCTCCGCCAA   
  
  
+ CTTCGGTAAC GCCAATTGCA TGGAGCAGCT CTTAGTCCAC TGCGCCGAAG CCATCGACAA CAATGACGCC   
  
  
+ ACCCCGGCCC AGCAAATCTT ATGGGTCCTG AATAACATAG CCCGACCCGA CGGCGACTCC ACCCAACGCC   
  
  
+ TCACGTGCGC ATTCCTACGT GCCTTAATCT CACGCGCCGT CCTCACTAGC ACCTGCAAGA TGGTAATCCC   
  
  
+ TCATTTCAAC CCCATCAATT CACCCCACAA ATTCTCGCTC CTCGAACTTG CCCACTTCGT CGATTTAACC   
  
  
+ CCTTGGCATC GATTCGGATT CACCGCCGCC AATTCGATCA TTCTGGAAGC TATTTCCGAC CTACCCGTTG   
  
  
+ TACACATTGT CGACCTCAGC ATCTCCCACT GTATGCAGAT CCCCACGTTG ATCGACTCCA TTGCGACCCG   
  
  
+ GTTGGAAGCC CCGGGTCGAG TCCCTCCTAT TGTCAAGCTC ACCGTCGGGG CTATTTCCGA CGAAATCCCG   
  
  
+ CCGGTGTTCG ATCTTCTGTC GTACGATGAG CTCGGAATGA GACTAATCAA CTTCGCTCGT TTTAGAAACA   
  
  
+ TCGTCCTCGA ATTCCAAGCA ATACCCACCT CCCCTTCCGA CGGATTTGCT TCGCTGTTGG AGGAGATTCG   
  
  
+ ACAAAGCAAG CTCTACTCCA ACGATGCGGC GGCGGTTATT GTGAATTGTC AGATGAGTTT GCATTTATTG   
  
  
+ CAGGAAGAGG AGGTGTCTTC ATCGTCGCCG TCGTCGATGA GGGGGATGTT TTTGCAGGCG GTGAGGAGCT   
  
  
+ TGGAGCCGAG CATGGTGGTG GTGGTGGAGG AGGACGTGGA TTTCACGGCG AGGAGTCTGG TGGGGCGGCT   
  
  
+ GAGATCGGCG TTTAATCACA TGTGGATACC CTTCGACACG GTGGACACGT TCTTGCCACG TGGGAGCCAG   
  
  
+ CAGAGAGAGT GGTTCGAGGC CGAGGTGTGC TGGAAGATTG AGAATGTGAT CGCTCATGAG GGACCCGCGA   
  
  
+ GGGTCGAGAG GCAGGAGCCC AGGGCCAAGT GGGCCCTCCG AATGAGGGAG GCCGAGTTTC AAGGGATCGA   
  
  
+ GTTCGGTGAT GAAGGTACGA CCGAGGTCAA GGCCATGCTG GAGGAGCATG CCGCTGGGTG GGGGTCTAAG   
  
  
+ AAGGAAGAGG ATGATCTTGT GCTCACTTGG AAGGGACATA GTGTTGTCTT TGCTTCTGCT TGGGTACCCA   
  
  
+ CTTA  

- +Up\_Stream \_Len000TCTTCG ATTAATTTGG TTTGTACGTG CATACAGGAT TAATTGTACT GACAGGTAGT   
  
  
- AGAGGAGGGT ACAACAAGAC AACTGTTCGG ACGTGTGGGT ACGAGGAGAG AGTACAGATT GGAGCAGGGT   
  
  
- TGCTAGTTCT AGTGACAGAC ACTCCGGTCC CCTTCTGAAC GAAAATAAAG GAAAAGAAAA CAAATGAGAG   
  
  
- ACTAAGGTAA ATCGGTATAT ATGTTCCTCT TACAGTACAC ATAGAGTATA CATATATATA TTCTAAAATA   
  
  
- AATCTTTATT TTTAAATTTG TGATACACTA AATATCAATA ATATTATTTA TCATAAAAAA TTTGATAAAG   
  
  
- TAACTACTCT TGTCCTTTCA TTTAACGGCT GTACAATTAC AATAATATAG ATTTTTAATT TAGTATTTTA   
  
  
- ATTAATATTA TAAAATTTTT ATAAAGTTGA TTTTAAAATC TTGTTATTCA CGCATTATGC CTGAATTTCC   
  
  
- GATCAAATAG CATTTTTACA TTCAAGGAAA ACCTAGAATA CCAACTTATA GAACCAACTA TTTGACACTA   
  
  
- ACCAAAATGT GAAAATGGTG TATAAGTATC TTCTTAATAC ACGTGCAACT ACCAAGTTCT ACCCTGTTTT   
  
  
- CCTTTGTACA TAAGAAGGGG GAAAACGGAA AAGGGGATTC GTAAACCTAA CTCCACAACA GTAGACTTAT   
  
  
- AGTTTTTAAG AAAAACTTTT AAGTCAGAGT TAGTTTTAAG TTTTAATTTT GTTTCTGAAT TCTCAAAATA   
  
  
- AACAAGAATT GGAAGAATTG AAAGATGAAA AGAAAAAAAA GAGTAAGGGG GAGAAGTTTA GAGTAAATAA   
  
  
- GAATTGGGGG AGAAGGCAAG TACGAGAGAG ACAGATAGTT GATTATAATT AGATGGGGCT GAAAGTTAAA   
  
  
- CTACTCCAGA TTAGGAATAG TAGCATAACA CCAACCCAAT TAATACGTTC TTCCGTTATT TGGGGTCACT   
  
  
- GTTTCAGGTG CAATTCATCC GTGGAGTGGT AATTCTGTAC GAGTCTTTTG TGGTTTTGGT AACTTGTGTT   
  
  
- CAGGGGGTTC AGGGAAGGGA TCGATCGGGT AAGAGAGAGG AGGGGACAGA TATAGATGGT TGAGAACGAG   
  
  
- AAACGACTCG GACTCAACTT TCGCTATGTG GGTATCGGAC AAGTCAAAAG TCAAAAGTGT AAACAGAAGG   
  
  
- AGACAAGTCA ACTTTCGCTA TGTGAGAGTA ATCGAAAGTA AAATTTATGC AAGTTAAGTG TGTATTTACC   
  
  
- GAAAGTTTCG TAACGGTAAA AGCAAATTTA CTACTGTTTT ATATATCATA GACCGATAGA CGGGTATAGA   
  
  
- ACTGGAATAA ATGTTTCCGA CTCGGTAGAA AATTAAAAAA ATCGGAGAAA AAAATTAGTT TTTATCTTTA   
  
  
- AAGTTCTTTC GTCTTTAGCA GTCTACTGTC ACGTCTGTCG CAAAAGCAAA GTATGTTTAG AGTTTTCGAC   
  
  
- TTCACAGTTA AAGAAGCTAA TAAAAACAGT CTTTTATAAA TGCAGAGTGG AAGTGCAACA ATAATAAGAA   
  
  
- AGAAAAAATA ATAAGAGTAT TGATCATTAT TTCAATCGTT ACTATTTTTT AATAAGCTAG GCTTATAAAA   
  
  
- TTAAATAGAC TGGATTTTTG TATTCATTTC CGTGTTTAAA AAGTAGGTTT AAAATTAAAA ACTACATTAT   
  
  
- AAAAAATATA AAAATAACAA GTTTAGACTA AAATTAGAAT AAATTTAATA GACTGGATTT TTTTAGTTTA   
  
  
- TTATTATTTG CAATTAAAAA ATAAATTTAA AACTGTAATC AGTTAGACTG AATTTAAGCT TGGCTCGAAC   
  
  
- TTAAAAAAAC GGTCACGACC ATCATGAGGA CGAAAACATC GAGGGTTACG GTAGCATGGT GGGAGAAGAT   
  
  
- CAGAGCAGAG AAAGAGACCA GAGGTTAATT AGTGTGGTAG TATAGTATGG CATACTACTA TGTCAGGTGA   
  
  
- CTTTAGGGTA AGAGTGGCGG TTAGAGAGAG GCGGTGGTAG GTTGTGAGAA TTGCTACTTG TCCCGGCGGC   
  
  
- GGAGCAGGAG GTGTGAGTCC GGCACCGGCC CGTCGAAAGG GTGGGTTAGT TTTAGAGAGA GGAGGCGGTT   
  
  
- GAAGCCATTG CGGTTAACGT ACCTCGTCGA GAATCAGGTG ACGCGGCTTC GGTAGCTGTT GTTACTGCGG   
  
  
- TGGGGCCGGG TCGTTTAGAA TACCCAGGAC TTATTGTATC GGGCTGGGCT GCCGCTGAGG TGGGTTGCGG   
  
  
- AGTGCACGCG TAAGGATGCA CGGAATTAGA GTGCGCGGCA GGAGTGATCG TGGACGTTCT ACCATTAGGG   
  
  
- AGTAAAGTTG GGGTAGTTAA GTGGGGTGTT TAAGAGCGAG GAGCTTGAAC GGGTGAAGCA GCTAAATTGG   
  
  
- GGAACCGTAG CTAAGCCTAA GTGGCGGCGG TTAAGCTAGT AAGACCTTCG ATAAAGGCTG GATGGGCAAC   
  
  
- ATGTGTAACA GCTGGAGTCG TAGAGGGTGA CATACGTCTA GGGGTGCAAC TAGCTGAGGT AACGCTGGGC   
  
  
- CAACCTTCGG GGCCCAGCTC AGGGAGGATA ACAGTTCGAG TGGCAGCCCC GATAAAGGCT GCTTTAGGGC   
  
  
- GGCCACAAGC TAGAAGACAG CATGCTACTC GAGCCTTACT CTGATTAGTT GAAGCGAGCA AAATCTTTGT   
  
  
- AGCAGGAGCT TAAGGTTCGT TATGGGTGGA GGGGAAGGCT GCCTAAACGA AGCGACAACC TCCTCTAAGC   
  
  
- TGTTTCGTTC GAGATGAGGT TGCTACGCCG CCGCCAATAA CACTTAACAG TCTACTCAAA CGTAAATAAC   
  
  
- GTCCTTCTCC TCCACAGAAG TAGCAGCGGC AGCAGCTACT CCCCCTACAA AAACGTCCGC CACTCCTCGA   
  
  
- ACCTCGGCTC GTACCACCAC CACCACCTCC TCCTGCACCT AAAGTGCCGC TCCTCAGACC ACCCCGCCGA   
  
  
- CTCTAGCCGC AAATTAGTGT ACACCTATGG GAAGCTGTGC CACCTGTGCA AGAACGGTGC ACCCTCGGTC   
  
  
- GTCTCTCTCA CCAAGCTCCG GCTCCACACG ACCTTCTAAC TCTTACACTA GCGAGTACTC CCTGGGCGCT   
  
  
- CCCAGCTCTC CGTCCTCGGG TCCCGGTTCA CCCGGGAGGC TTACTCCCTC CGGCTCAAAG TTCCCTAGCT   
  
  
- CAAGCCACTA CTTCCATGCT GGCTCCAGTT CCGGTACGAC CTCCTCGTAC GGCGACCCAC CCCCAGATTC   
  
  
- TTCCTTCTCC TACTAGAACA CGAGTGAACC TTCCCTGTAT CACAACAGAA ACGAAGACGA ACCCATGGGT   
  
  
- GAAT

+     ABRE

| Site Name | Organism | Position | Strand | Matrix score. | sequence | function |
| --- | --- | --- | --- | --- | --- | --- |
| ABRE | Arabidopsis thaliana | 2990 | - | 5 | ACGTG | cis-acting element involved in the abscisic acid responsiveness |
| ABRE | Arabidopsis thaliana | 3001 | - | 6 | CACGTG | cis-acting element involved in the abscisic acid responsiveness |
| ABRE | Arabidopsis thaliana | 2261 | + | 5 | ACGTG | cis-acting element involved in the abscisic acid responsiveness |
| ABRE | Arabidopsis thaliana | 2246 | - | 6 | CACGTG | cis-acting element involved in the abscisic acid responsiveness |
| ABRE | Arabidopsis thaliana | 42 | - | 5 | ACGTG | cis-acting element involved in the abscisic acid responsiveness |
| ABRE | Hordeum vulgare | 40 | - | 9 | CGTACGTGCA | cis-acting element involved in the abscisic acid responsiveness |
| ABRE | Arabidopsis thaliana | 607 | - | 5 | ACGTG | cis-acting element involved in the abscisic acid responsiveness |
| ABRE | Arabidopsis thaliana | 3002 | + | 5 | ACGTG | cis-acting element involved in the abscisic acid responsiveness |
| ABRE | Arabidopsis thaliana | 992 | - | 5 | ACGTG | cis-acting element involved in the abscisic acid responsiveness |
| ABRE | Arabidopsis thaliana | 2498 | - | 5 | ACGTG | cis-acting element involved in the abscisic acid responsiveness |
| ABRE | Arabidopsis thaliana | 2908 | + | 5 | ACGTG | cis-acting element involved in the abscisic acid responsiveness |
| ABRE | Arabidopsis thaliana | 1527 | - | 5 | ACGTG | cis-acting element involved in the abscisic acid responsiveness |
| ABRE | Arabidopsis thaliana | 2247 | + | 5 | ACGTG | cis-acting element involved in the abscisic acid responsiveness |

>HU06G00376.1   
+ +Up\_Stream \_Len000AGAAGC TAATTAAACC AAACATGCAC GTATGTCCTA ATTAACATGA CTGTCCATCA   
  
  
+ TCTCCTCCCA TGTTGTTCTG TTGACAAGCC TGCACACCCA TGCTCCTCTC TCATGTCTAA CCTCGTCCCA   
  
  
+ ACGATCAAGA TCACTGTCTG TGAGGCCAGG GGAAGACTTG CTTTTATTTC CTTTTCTTTT GTTTACTCTC   
  
  
+ TGATTCCATT TAGCCATATA TACAAGGAGA ATGTCATGTG TATCTCATAT GTATATATAT AAGATTTTAT   
  
  
+ TTAGAAATAA AAATTTAAAC ACTATGTGAT TTATAGTTAT TATAATAAAT AGTATTTTTT AAACTATTTC   
  
  
+ ATTGATGAGA ACAGGAAAGT AAATTGCCGA CATGTTAATG TTATTATATC TAAAAATTAA ATCATAAAAT   
  
  
+ TAATTATAAT ATTTTAAAAA TATTTCAACT AAAATTTTAG AACAATAAGT GCGTAATACG GACTTAAAGG   
  
  
+ CTAGTTTATC GTAAAAATGT AAGTTCCTTT TGGATCTTAT GGTTGAATAT CTTGGTTGAT AAACTGTGAT   
  
  
+ TGGTTTTACA CTTTTACCAC ATATTCATAG AAGAATTATG TGCACGTTGA TGGTTCAAGA TGGGACAAAA   
  
  
+ GGAAACATGT ATTCTTCCCC CTTTTGCCTT TTCCCCTAAG CATTTGGATT GAGGTGTTGT CATCTGAATA   
  
  
+ TCAAAAATTC TTTTTGAAAA TTCAGTCTCA ATCAAAATTC AAAATTAAAA CAAAGACTTA AGAGTTTTAT   
  
  
+ TTGTTCTTAA CCTTCTTAAC TTTCTACTTT TCTTTTTTTT CTCATTCCCC CTCTTCAAAT CTCATTTATT   
  
  
+ CTTAACCCCC TCTTCCGTTC ATGCTCTCTC TGTCTATCAA CTAATATTAA TCTACCCCGA CTTTCAATTT   
  
  
+ GATGAGGTCT AATCCTTATC ATCGTATTGT GGTTGGGTTA ATTATGCAAG AAGGCAATAA ACCCCAGTGA   
  
  
+ CAAAGTCCAC GTTAAGTAGG CACCTCACCA TTAAGACATG CTCAGAAAAC ACCAAAACCA TTGAACACAA   
  
  
+ GTCCCCCAAG TCCCTTCCCT AGCTAGCCCA TTCTCTCTCC TCCCCTGTCT ATATCTACCA ACTCTTGCTC   
  
  
+ TTTGCTGAGC CTGAGTTGAA AGCGATACAC CCATAGCCTG TTCAGTTTTC AGTTTTCACA TTTGTCTTCC   
  
  
+ TCTGTTCAGT TGAAAGCGAT ACACTCTCAT TAGCTTTCAT TTTAAATACG TTCAATTCAC ACATAAATGG   
  
  
+ CTTTCAAAGC ATTGCCATTT TCGTTTAAAT GATGACAAAA TATATAGTAT CTGGCTATCT GCCCATATCT   
  
  
+ TGACCTTATT TACAAAGGCT GAGCCATCTT TTAATTTTTT TAGCCTCTTT TTTTAATCAA AAATAGAAAT   
  
  
+ TTCAAGAAAG CAGAAATCGT CAGATGACAG TGCAGACAGC GTTTTCGTTT CATACAAATC TCAAAAGCTG   
  
  
+ AAGTGTCAAT TTCTTCGATT ATTTTTGTCA GAAAATATTT ACGTCTCACC TTCACGTTGT TATTATTCTT   
  
  
+ TCTTTTTTAT TATTCTCATA ACTAGTAATA AAGTTAGCAA TGATAAAAAA TTATTCGATC CGAATATTTT   
  
  
+ AATTTATCTG ACCTAAAAAC ATAAGTAAAG GCACAAATTT TTCATCCAAA TTTTAATTTT TGATGTAATA   
  
  
+ TTTTTTATAT TTTTATTGTT CAAATCTGAT TTTAATCTTA TTTAAATTAT CTGACCTAAA AAAATCAAAT   
  
  
+ AATAATAAAC GTTAATTTTT TATTTAAATT TTGACATTAG TCAATCTGAC TTAAATTCGA ACCGAGCTTG   
  
  
+ AATTTTTTTG CCAGTGCTGG TAGTACTCCT GCTTTTGTAG CTCCCAATGC CATCGTACCA CCCTCTTCTA   
  
  
+ GTCTCGTCTC TTTCTCTGGT CTCCAATTAA TCACACCATC ATATCATACC GTATGATGAT ACAGTCCACT   
  
  
+ GAAATCCCAT TCTCACCGCC AATCTCTCTC CGCCACCATC CAACACTCTT AACGATGAAC AGGGCCGCCG   
  
  
+ CCTCGTCCTC CACACTCAGG CCGTGGCCGG GCAGCTTTCC CACCCAATCA AAATCTCTCT CCTCCGCCAA   
  
  
+ CTTCGGTAAC GCCAATTGCA TGGAGCAGCT CTTAGTCCAC TGCGCCGAAG CCATCGACAA CAATGACGCC   
  
  
+ ACCCCGGCCC AGCAAATCTT ATGGGTCCTG AATAACATAG CCCGACCCGA CGGCGACTCC ACCCAACGCC   
  
  
+ TCACGTGCGC ATTCCTACGT GCCTTAATCT CACGCGCCGT CCTCACTAGC ACCTGCAAGA TGGTAATCCC   
  
  
+ TCATTTCAAC CCCATCAATT CACCCCACAA ATTCTCGCTC CTCGAACTTG CCCACTTCGT CGATTTAACC   
  
  
+ CCTTGGCATC GATTCGGATT CACCGCCGCC AATTCGATCA TTCTGGAAGC TATTTCCGAC CTACCCGTTG   
  
  
+ TACACATTGT CGACCTCAGC ATCTCCCACT GTATGCAGAT CCCCACGTTG ATCGACTCCA TTGCGACCCG   
  
  
+ GTTGGAAGCC CCGGGTCGAG TCCCTCCTAT TGTCAAGCTC ACCGTCGGGG CTATTTCCGA CGAAATCCCG   
  
  
+ CCGGTGTTCG ATCTTCTGTC GTACGATGAG CTCGGAATGA GACTAATCAA CTTCGCTCGT TTTAGAAACA   
  
  
+ TCGTCCTCGA ATTCCAAGCA ATACCCACCT CCCCTTCCGA CGGATTTGCT TCGCTGTTGG AGGAGATTCG   
  
  
+ ACAAAGCAAG CTCTACTCCA ACGATGCGGC GGCGGTTATT GTGAATTGTC AGATGAGTTT GCATTTATTG   
  
  
+ CAGGAAGAGG AGGTGTCTTC ATCGTCGCCG TCGTCGATGA GGGGGATGTT TTTGCAGGCG GTGAGGAGCT   
  
  
+ TGGAGCCGAG CATGGTGGTG GTGGTGGAGG AGGACGTGGA TTTCACGGCG AGGAGTCTGG TGGGGCGGCT   
  
  
+ GAGATCGGCG TTTAATCACA TGTGGATACC CTTCGACACG GTGGACACGT TCTTGCCACG TGGGAGCCAG   
  
  
+ CAGAGAGAGT GGTTCGAGGC CGAGGTGTGC TGGAAGATTG AGAATGTGAT CGCTCATGAG GGACCCGCGA   
  
  
+ GGGTCGAGAG GCAGGAGCCC AGGGCCAAGT GGGCCCTCCG AATGAGGGAG GCCGAGTTTC AAGGGATCGA   
  
  
+ GTTCGGTGAT GAAGGTACGA CCGAGGTCAA GGCCATGCTG GAGGAGCATG CCGCTGGGTG GGGGTCTAAG   
  
  
+ AAGGAAGAGG ATGATCTTGT GCTCACTTGG AAGGGACATA GTGTTGTCTT TGCTTCTGCT TGGGTACCCA   
  
  
+ CTTA  

- +Up\_Stream \_Len000TCTTCG ATTAATTTGG TTTGTACGTG CATACAGGAT TAATTGTACT GACAGGTAGT   
  
  
- AGAGGAGGGT ACAACAAGAC AACTGTTCGG ACGTGTGGGT ACGAGGAGAG AGTACAGATT GGAGCAGGGT   
  
  
- TGCTAGTTCT AGTGACAGAC ACTCCGGTCC CCTTCTGAAC GAAAATAAAG GAAAAGAAAA CAAATGAGAG   
  
  
- ACTAAGGTAA ATCGGTATAT ATGTTCCTCT TACAGTACAC ATAGAGTATA CATATATATA TTCTAAAATA   
  
  
- AATCTTTATT TTTAAATTTG TGATACACTA AATATCAATA ATATTATTTA TCATAAAAAA TTTGATAAAG   
  
  
- TAACTACTCT TGTCCTTTCA TTTAACGGCT GTACAATTAC AATAATATAG ATTTTTAATT TAGTATTTTA   
  
  
- ATTAATATTA TAAAATTTTT ATAAAGTTGA TTTTAAAATC TTGTTATTCA CGCATTATGC CTGAATTTCC   
  
  
- GATCAAATAG CATTTTTACA TTCAAGGAAA ACCTAGAATA CCAACTTATA GAACCAACTA TTTGACACTA   
  
  
- ACCAAAATGT GAAAATGGTG TATAAGTATC TTCTTAATAC ACGTGCAACT ACCAAGTTCT ACCCTGTTTT   
  
  
- CCTTTGTACA TAAGAAGGGG GAAAACGGAA AAGGGGATTC GTAAACCTAA CTCCACAACA GTAGACTTAT   
  
  
- AGTTTTTAAG AAAAACTTTT AAGTCAGAGT TAGTTTTAAG TTTTAATTTT GTTTCTGAAT TCTCAAAATA   
  
  
- AACAAGAATT GGAAGAATTG AAAGATGAAA AGAAAAAAAA GAGTAAGGGG GAGAAGTTTA GAGTAAATAA   
  
  
- GAATTGGGGG AGAAGGCAAG TACGAGAGAG ACAGATAGTT GATTATAATT AGATGGGGCT GAAAGTTAAA   
  
  
- CTACTCCAGA TTAGGAATAG TAGCATAACA CCAACCCAAT TAATACGTTC TTCCGTTATT TGGGGTCACT   
  
  
- GTTTCAGGTG CAATTCATCC GTGGAGTGGT AATTCTGTAC GAGTCTTTTG TGGTTTTGGT AACTTGTGTT   
  
  
- CAGGGGGTTC AGGGAAGGGA TCGATCGGGT AAGAGAGAGG AGGGGACAGA TATAGATGGT TGAGAACGAG   
  
  
- AAACGACTCG GACTCAACTT TCGCTATGTG GGTATCGGAC AAGTCAAAAG TCAAAAGTGT AAACAGAAGG   
  
  
- AGACAAGTCA ACTTTCGCTA TGTGAGAGTA ATCGAAAGTA AAATTTATGC AAGTTAAGTG TGTATTTACC   
  
  
- GAAAGTTTCG TAACGGTAAA AGCAAATTTA CTACTGTTTT ATATATCATA GACCGATAGA CGGGTATAGA   
  
  
- ACTGGAATAA ATGTTTCCGA CTCGGTAGAA AATTAAAAAA ATCGGAGAAA AAAATTAGTT TTTATCTTTA   
  
  
- AAGTTCTTTC GTCTTTAGCA GTCTACTGTC ACGTCTGTCG CAAAAGCAAA GTATGTTTAG AGTTTTCGAC   
  
  
- TTCACAGTTA AAGAAGCTAA TAAAAACAGT CTTTTATAAA TGCAGAGTGG AAGTGCAACA ATAATAAGAA   
  
  
- AGAAAAAATA ATAAGAGTAT TGATCATTAT TTCAATCGTT ACTATTTTTT AATAAGCTAG GCTTATAAAA   
  
  
- TTAAATAGAC TGGATTTTTG TATTCATTTC CGTGTTTAAA AAGTAGGTTT AAAATTAAAA ACTACATTAT   
  
  
- AAAAAATATA AAAATAACAA GTTTAGACTA AAATTAGAAT AAATTTAATA GACTGGATTT TTTTAGTTTA   
  
  
- TTATTATTTG CAATTAAAAA ATAAATTTAA AACTGTAATC AGTTAGACTG AATTTAAGCT TGGCTCGAAC   
  
  
- TTAAAAAAAC GGTCACGACC ATCATGAGGA CGAAAACATC GAGGGTTACG GTAGCATGGT GGGAGAAGAT   
  
  
- CAGAGCAGAG AAAGAGACCA GAGGTTAATT AGTGTGGTAG TATAGTATGG CATACTACTA TGTCAGGTGA   
  
  
- CTTTAGGGTA AGAGTGGCGG TTAGAGAGAG GCGGTGGTAG GTTGTGAGAA TTGCTACTTG TCCCGGCGGC   
  
  
- GGAGCAGGAG GTGTGAGTCC GGCACCGGCC CGTCGAAAGG GTGGGTTAGT TTTAGAGAGA GGAGGCGGTT   
  
  
- GAAGCCATTG CGGTTAACGT ACCTCGTCGA GAATCAGGTG ACGCGGCTTC GGTAGCTGTT GTTACTGCGG   
  
  
- TGGGGCCGGG TCGTTTAGAA TACCCAGGAC TTATTGTATC GGGCTGGGCT GCCGCTGAGG TGGGTTGCGG   
  
  
- AGTGCACGCG TAAGGATGCA CGGAATTAGA GTGCGCGGCA GGAGTGATCG TGGACGTTCT ACCATTAGGG   
  
  
- AGTAAAGTTG GGGTAGTTAA GTGGGGTGTT TAAGAGCGAG GAGCTTGAAC GGGTGAAGCA GCTAAATTGG   
  
  
- GGAACCGTAG CTAAGCCTAA GTGGCGGCGG TTAAGCTAGT AAGACCTTCG ATAAAGGCTG GATGGGCAAC   
  
  
- ATGTGTAACA GCTGGAGTCG TAGAGGGTGA CATACGTCTA GGGGTGCAAC TAGCTGAGGT AACGCTGGGC   
  
  
- CAACCTTCGG GGCCCAGCTC AGGGAGGATA ACAGTTCGAG TGGCAGCCCC GATAAAGGCT GCTTTAGGGC   
  
  
- GGCCACAAGC TAGAAGACAG CATGCTACTC GAGCCTTACT CTGATTAGTT GAAGCGAGCA AAATCTTTGT   
  
  
- AGCAGGAGCT TAAGGTTCGT TATGGGTGGA GGGGAAGGCT GCCTAAACGA AGCGACAACC TCCTCTAAGC   
  
  
- TGTTTCGTTC GAGATGAGGT TGCTACGCCG CCGCCAATAA CACTTAACAG TCTACTCAAA CGTAAATAAC   
  
  
- GTCCTTCTCC TCCACAGAAG TAGCAGCGGC AGCAGCTACT CCCCCTACAA AAACGTCCGC CACTCCTCGA   
  
  
- ACCTCGGCTC GTACCACCAC CACCACCTCC TCCTGCACCT AAAGTGCCGC TCCTCAGACC ACCCCGCCGA   
  
  
- CTCTAGCCGC AAATTAGTGT ACACCTATGG GAAGCTGTGC CACCTGTGCA AGAACGGTGC ACCCTCGGTC   
  
  
- GTCTCTCTCA CCAAGCTCCG GCTCCACACG ACCTTCTAAC TCTTACACTA GCGAGTACTC CCTGGGCGCT   
  
  
- CCCAGCTCTC CGTCCTCGGG TCCCGGTTCA CCCGGGAGGC TTACTCCCTC CGGCTCAAAG TTCCCTAGCT   
  
  
- CAAGCCACTA CTTCCATGCT GGCTCCAGTT CCGGTACGAC CTCCTCGTAC GGCGACCCAC CCCCAGATTC   
  
  
- TTCCTTCTCC TACTAGAACA CGAGTGAACC TTCCCTGTAT CACAACAGAA ACGAAGACGA ACCCATGGGT   
  
  
- GAAT

+     ABRE2

| Site Name | Organism | Position | Strand | Matrix score. | sequence | function |
| --- | --- | --- | --- | --- | --- | --- |
| ABRE2 | Zea mays | 3000 | - | 8 | CCACGTGG |  |

>HU06G00376.1   
+ +Up\_Stream \_Len000AGAAGC TAATTAAACC AAACATGCAC GTATGTCCTA ATTAACATGA CTGTCCATCA   
  
  
+ TCTCCTCCCA TGTTGTTCTG TTGACAAGCC TGCACACCCA TGCTCCTCTC TCATGTCTAA CCTCGTCCCA   
  
  
+ ACGATCAAGA TCACTGTCTG TGAGGCCAGG GGAAGACTTG CTTTTATTTC CTTTTCTTTT GTTTACTCTC   
  
  
+ TGATTCCATT TAGCCATATA TACAAGGAGA ATGTCATGTG TATCTCATAT GTATATATAT AAGATTTTAT   
  
  
+ TTAGAAATAA AAATTTAAAC ACTATGTGAT TTATAGTTAT TATAATAAAT AGTATTTTTT AAACTATTTC   
  
  
+ ATTGATGAGA ACAGGAAAGT AAATTGCCGA CATGTTAATG TTATTATATC TAAAAATTAA ATCATAAAAT   
  
  
+ TAATTATAAT ATTTTAAAAA TATTTCAACT AAAATTTTAG AACAATAAGT GCGTAATACG GACTTAAAGG   
  
  
+ CTAGTTTATC GTAAAAATGT AAGTTCCTTT TGGATCTTAT GGTTGAATAT CTTGGTTGAT AAACTGTGAT   
  
  
+ TGGTTTTACA CTTTTACCAC ATATTCATAG AAGAATTATG TGCACGTTGA TGGTTCAAGA TGGGACAAAA   
  
  
+ GGAAACATGT ATTCTTCCCC CTTTTGCCTT TTCCCCTAAG CATTTGGATT GAGGTGTTGT CATCTGAATA   
  
  
+ TCAAAAATTC TTTTTGAAAA TTCAGTCTCA ATCAAAATTC AAAATTAAAA CAAAGACTTA AGAGTTTTAT   
  
  
+ TTGTTCTTAA CCTTCTTAAC TTTCTACTTT TCTTTTTTTT CTCATTCCCC CTCTTCAAAT CTCATTTATT   
  
  
+ CTTAACCCCC TCTTCCGTTC ATGCTCTCTC TGTCTATCAA CTAATATTAA TCTACCCCGA CTTTCAATTT   
  
  
+ GATGAGGTCT AATCCTTATC ATCGTATTGT GGTTGGGTTA ATTATGCAAG AAGGCAATAA ACCCCAGTGA   
  
  
+ CAAAGTCCAC GTTAAGTAGG CACCTCACCA TTAAGACATG CTCAGAAAAC ACCAAAACCA TTGAACACAA   
  
  
+ GTCCCCCAAG TCCCTTCCCT AGCTAGCCCA TTCTCTCTCC TCCCCTGTCT ATATCTACCA ACTCTTGCTC   
  
  
+ TTTGCTGAGC CTGAGTTGAA AGCGATACAC CCATAGCCTG TTCAGTTTTC AGTTTTCACA TTTGTCTTCC   
  
  
+ TCTGTTCAGT TGAAAGCGAT ACACTCTCAT TAGCTTTCAT TTTAAATACG TTCAATTCAC ACATAAATGG   
  
  
+ CTTTCAAAGC ATTGCCATTT TCGTTTAAAT GATGACAAAA TATATAGTAT CTGGCTATCT GCCCATATCT   
  
  
+ TGACCTTATT TACAAAGGCT GAGCCATCTT TTAATTTTTT TAGCCTCTTT TTTTAATCAA AAATAGAAAT   
  
  
+ TTCAAGAAAG CAGAAATCGT CAGATGACAG TGCAGACAGC GTTTTCGTTT CATACAAATC TCAAAAGCTG   
  
  
+ AAGTGTCAAT TTCTTCGATT ATTTTTGTCA GAAAATATTT ACGTCTCACC TTCACGTTGT TATTATTCTT   
  
  
+ TCTTTTTTAT TATTCTCATA ACTAGTAATA AAGTTAGCAA TGATAAAAAA TTATTCGATC CGAATATTTT   
  
  
+ AATTTATCTG ACCTAAAAAC ATAAGTAAAG GCACAAATTT TTCATCCAAA TTTTAATTTT TGATGTAATA   
  
  
+ TTTTTTATAT TTTTATTGTT CAAATCTGAT TTTAATCTTA TTTAAATTAT CTGACCTAAA AAAATCAAAT   
  
  
+ AATAATAAAC GTTAATTTTT TATTTAAATT TTGACATTAG TCAATCTGAC TTAAATTCGA ACCGAGCTTG   
  
  
+ AATTTTTTTG CCAGTGCTGG TAGTACTCCT GCTTTTGTAG CTCCCAATGC CATCGTACCA CCCTCTTCTA   
  
  
+ GTCTCGTCTC TTTCTCTGGT CTCCAATTAA TCACACCATC ATATCATACC GTATGATGAT ACAGTCCACT   
  
  
+ GAAATCCCAT TCTCACCGCC AATCTCTCTC CGCCACCATC CAACACTCTT AACGATGAAC AGGGCCGCCG   
  
  
+ CCTCGTCCTC CACACTCAGG CCGTGGCCGG GCAGCTTTCC CACCCAATCA AAATCTCTCT CCTCCGCCAA   
  
  
+ CTTCGGTAAC GCCAATTGCA TGGAGCAGCT CTTAGTCCAC TGCGCCGAAG CCATCGACAA CAATGACGCC   
  
  
+ ACCCCGGCCC AGCAAATCTT ATGGGTCCTG AATAACATAG CCCGACCCGA CGGCGACTCC ACCCAACGCC   
  
  
+ TCACGTGCGC ATTCCTACGT GCCTTAATCT CACGCGCCGT CCTCACTAGC ACCTGCAAGA TGGTAATCCC   
  
  
+ TCATTTCAAC CCCATCAATT CACCCCACAA ATTCTCGCTC CTCGAACTTG CCCACTTCGT CGATTTAACC   
  
  
+ CCTTGGCATC GATTCGGATT CACCGCCGCC AATTCGATCA TTCTGGAAGC TATTTCCGAC CTACCCGTTG   
  
  
+ TACACATTGT CGACCTCAGC ATCTCCCACT GTATGCAGAT CCCCACGTTG ATCGACTCCA TTGCGACCCG   
  
  
+ GTTGGAAGCC CCGGGTCGAG TCCCTCCTAT TGTCAAGCTC ACCGTCGGGG CTATTTCCGA CGAAATCCCG   
  
  
+ CCGGTGTTCG ATCTTCTGTC GTACGATGAG CTCGGAATGA GACTAATCAA CTTCGCTCGT TTTAGAAACA   
  
  
+ TCGTCCTCGA ATTCCAAGCA ATACCCACCT CCCCTTCCGA CGGATTTGCT TCGCTGTTGG AGGAGATTCG   
  
  
+ ACAAAGCAAG CTCTACTCCA ACGATGCGGC GGCGGTTATT GTGAATTGTC AGATGAGTTT GCATTTATTG   
  
  
+ CAGGAAGAGG AGGTGTCTTC ATCGTCGCCG TCGTCGATGA GGGGGATGTT TTTGCAGGCG GTGAGGAGCT   
  
  
+ TGGAGCCGAG CATGGTGGTG GTGGTGGAGG AGGACGTGGA TTTCACGGCG AGGAGTCTGG TGGGGCGGCT   
  
  
+ GAGATCGGCG TTTAATCACA TGTGGATACC CTTCGACACG GTGGACACGT TCTTGCCACG TGGGAGCCAG   
  
  
+ CAGAGAGAGT GGTTCGAGGC CGAGGTGTGC TGGAAGATTG AGAATGTGAT CGCTCATGAG GGACCCGCGA   
  
  
+ GGGTCGAGAG GCAGGAGCCC AGGGCCAAGT GGGCCCTCCG AATGAGGGAG GCCGAGTTTC AAGGGATCGA   
  
  
+ GTTCGGTGAT GAAGGTACGA CCGAGGTCAA GGCCATGCTG GAGGAGCATG CCGCTGGGTG GGGGTCTAAG   
  
  
+ AAGGAAGAGG ATGATCTTGT GCTCACTTGG AAGGGACATA GTGTTGTCTT TGCTTCTGCT TGGGTACCCA   
  
  
+ CTTA  

- +Up\_Stream \_Len000TCTTCG ATTAATTTGG TTTGTACGTG CATACAGGAT TAATTGTACT GACAGGTAGT   
  
  
- AGAGGAGGGT ACAACAAGAC AACTGTTCGG ACGTGTGGGT ACGAGGAGAG AGTACAGATT GGAGCAGGGT   
  
  
- TGCTAGTTCT AGTGACAGAC ACTCCGGTCC CCTTCTGAAC GAAAATAAAG GAAAAGAAAA CAAATGAGAG   
  
  
- ACTAAGGTAA ATCGGTATAT ATGTTCCTCT TACAGTACAC ATAGAGTATA CATATATATA TTCTAAAATA   
  
  
- AATCTTTATT TTTAAATTTG TGATACACTA AATATCAATA ATATTATTTA TCATAAAAAA TTTGATAAAG   
  
  
- TAACTACTCT TGTCCTTTCA TTTAACGGCT GTACAATTAC AATAATATAG ATTTTTAATT TAGTATTTTA   
  
  
- ATTAATATTA TAAAATTTTT ATAAAGTTGA TTTTAAAATC TTGTTATTCA CGCATTATGC CTGAATTTCC   
  
  
- GATCAAATAG CATTTTTACA TTCAAGGAAA ACCTAGAATA CCAACTTATA GAACCAACTA TTTGACACTA   
  
  
- ACCAAAATGT GAAAATGGTG TATAAGTATC TTCTTAATAC ACGTGCAACT ACCAAGTTCT ACCCTGTTTT   
  
  
- CCTTTGTACA TAAGAAGGGG GAAAACGGAA AAGGGGATTC GTAAACCTAA CTCCACAACA GTAGACTTAT   
  
  
- AGTTTTTAAG AAAAACTTTT AAGTCAGAGT TAGTTTTAAG TTTTAATTTT GTTTCTGAAT TCTCAAAATA   
  
  
- AACAAGAATT GGAAGAATTG AAAGATGAAA AGAAAAAAAA GAGTAAGGGG GAGAAGTTTA GAGTAAATAA   
  
  
- GAATTGGGGG AGAAGGCAAG TACGAGAGAG ACAGATAGTT GATTATAATT AGATGGGGCT GAAAGTTAAA   
  
  
- CTACTCCAGA TTAGGAATAG TAGCATAACA CCAACCCAAT TAATACGTTC TTCCGTTATT TGGGGTCACT   
  
  
- GTTTCAGGTG CAATTCATCC GTGGAGTGGT AATTCTGTAC GAGTCTTTTG TGGTTTTGGT AACTTGTGTT   
  
  
- CAGGGGGTTC AGGGAAGGGA TCGATCGGGT AAGAGAGAGG AGGGGACAGA TATAGATGGT TGAGAACGAG   
  
  
- AAACGACTCG GACTCAACTT TCGCTATGTG GGTATCGGAC AAGTCAAAAG TCAAAAGTGT AAACAGAAGG   
  
  
- AGACAAGTCA ACTTTCGCTA TGTGAGAGTA ATCGAAAGTA AAATTTATGC AAGTTAAGTG TGTATTTACC   
  
  
- GAAAGTTTCG TAACGGTAAA AGCAAATTTA CTACTGTTTT ATATATCATA GACCGATAGA CGGGTATAGA   
  
  
- ACTGGAATAA ATGTTTCCGA CTCGGTAGAA AATTAAAAAA ATCGGAGAAA AAAATTAGTT TTTATCTTTA   
  
  
- AAGTTCTTTC GTCTTTAGCA GTCTACTGTC ACGTCTGTCG CAAAAGCAAA GTATGTTTAG AGTTTTCGAC   
  
  
- TTCACAGTTA AAGAAGCTAA TAAAAACAGT CTTTTATAAA TGCAGAGTGG AAGTGCAACA ATAATAAGAA   
  
  
- AGAAAAAATA ATAAGAGTAT TGATCATTAT TTCAATCGTT ACTATTTTTT AATAAGCTAG GCTTATAAAA   
  
  
- TTAAATAGAC TGGATTTTTG TATTCATTTC CGTGTTTAAA AAGTAGGTTT AAAATTAAAA ACTACATTAT   
  
  
- AAAAAATATA AAAATAACAA GTTTAGACTA AAATTAGAAT AAATTTAATA GACTGGATTT TTTTAGTTTA   
  
  
- TTATTATTTG CAATTAAAAA ATAAATTTAA AACTGTAATC AGTTAGACTG AATTTAAGCT TGGCTCGAAC   
  
  
- TTAAAAAAAC GGTCACGACC ATCATGAGGA CGAAAACATC GAGGGTTACG GTAGCATGGT GGGAGAAGAT   
  
  
- CAGAGCAGAG AAAGAGACCA GAGGTTAATT AGTGTGGTAG TATAGTATGG CATACTACTA TGTCAGGTGA   
  
  
- CTTTAGGGTA AGAGTGGCGG TTAGAGAGAG GCGGTGGTAG GTTGTGAGAA TTGCTACTTG TCCCGGCGGC   
  
  
- GGAGCAGGAG GTGTGAGTCC GGCACCGGCC CGTCGAAAGG GTGGGTTAGT TTTAGAGAGA GGAGGCGGTT   
  
  
- GAAGCCATTG CGGTTAACGT ACCTCGTCGA GAATCAGGTG ACGCGGCTTC GGTAGCTGTT GTTACTGCGG   
  
  
- TGGGGCCGGG TCGTTTAGAA TACCCAGGAC TTATTGTATC GGGCTGGGCT GCCGCTGAGG TGGGTTGCGG   
  
  
- AGTGCACGCG TAAGGATGCA CGGAATTAGA GTGCGCGGCA GGAGTGATCG TGGACGTTCT ACCATTAGGG   
  
  
- AGTAAAGTTG GGGTAGTTAA GTGGGGTGTT TAAGAGCGAG GAGCTTGAAC GGGTGAAGCA GCTAAATTGG   
  
  
- GGAACCGTAG CTAAGCCTAA GTGGCGGCGG TTAAGCTAGT AAGACCTTCG ATAAAGGCTG GATGGGCAAC   
  
  
- ATGTGTAACA GCTGGAGTCG TAGAGGGTGA CATACGTCTA GGGGTGCAAC TAGCTGAGGT AACGCTGGGC   
  
  
- CAACCTTCGG GGCCCAGCTC AGGGAGGATA ACAGTTCGAG TGGCAGCCCC GATAAAGGCT GCTTTAGGGC   
  
  
- GGCCACAAGC TAGAAGACAG CATGCTACTC GAGCCTTACT CTGATTAGTT GAAGCGAGCA AAATCTTTGT   
  
  
- AGCAGGAGCT TAAGGTTCGT TATGGGTGGA GGGGAAGGCT GCCTAAACGA AGCGACAACC TCCTCTAAGC   
  
  
- TGTTTCGTTC GAGATGAGGT TGCTACGCCG CCGCCAATAA CACTTAACAG TCTACTCAAA CGTAAATAAC   
  
  
- GTCCTTCTCC TCCACAGAAG TAGCAGCGGC AGCAGCTACT CCCCCTACAA AAACGTCCGC CACTCCTCGA   
  
  
- ACCTCGGCTC GTACCACCAC CACCACCTCC TCCTGCACCT AAAGTGCCGC TCCTCAGACC ACCCCGCCGA   
  
  
- CTCTAGCCGC AAATTAGTGT ACACCTATGG GAAGCTGTGC CACCTGTGCA AGAACGGTGC ACCCTCGGTC   
  
  
- GTCTCTCTCA CCAAGCTCCG GCTCCACACG ACCTTCTAAC TCTTACACTA GCGAGTACTC CCTGGGCGCT   
  
  
- CCCAGCTCTC CGTCCTCGGG TCCCGGTTCA CCCGGGAGGC TTACTCCCTC CGGCTCAAAG TTCCCTAGCT   
  
  
- CAAGCCACTA CTTCCATGCT GGCTCCAGTT CCGGTACGAC CTCCTCGTAC GGCGACCCAC CCCCAGATTC   
  
  
- TTCCTTCTCC TACTAGAACA CGAGTGAACC TTCCCTGTAT CACAACAGAA ACGAAGACGA ACCCATGGGT   
  
  
- GAAT

+     ABRE3a

| Site Name | Organism | Position | Strand | Matrix score. | sequence | function |
| --- | --- | --- | --- | --- | --- | --- |
| ABRE3a | Zea mays | 42 | - | 6 | TACGTG |  |
| ABRE3a | Zea mays | 2260 | + | 6 | TACGTG |  |

>HU06G00376.1   
+ +Up\_Stream \_Len000AGAAGC TAATTAAACC AAACATGCAC GTATGTCCTA ATTAACATGA CTGTCCATCA   
  
  
+ TCTCCTCCCA TGTTGTTCTG TTGACAAGCC TGCACACCCA TGCTCCTCTC TCATGTCTAA CCTCGTCCCA   
  
  
+ ACGATCAAGA TCACTGTCTG TGAGGCCAGG GGAAGACTTG CTTTTATTTC CTTTTCTTTT GTTTACTCTC   
  
  
+ TGATTCCATT TAGCCATATA TACAAGGAGA ATGTCATGTG TATCTCATAT GTATATATAT AAGATTTTAT   
  
  
+ TTAGAAATAA AAATTTAAAC ACTATGTGAT TTATAGTTAT TATAATAAAT AGTATTTTTT AAACTATTTC   
  
  
+ ATTGATGAGA ACAGGAAAGT AAATTGCCGA CATGTTAATG TTATTATATC TAAAAATTAA ATCATAAAAT   
  
  
+ TAATTATAAT ATTTTAAAAA TATTTCAACT AAAATTTTAG AACAATAAGT GCGTAATACG GACTTAAAGG   
  
  
+ CTAGTTTATC GTAAAAATGT AAGTTCCTTT TGGATCTTAT GGTTGAATAT CTTGGTTGAT AAACTGTGAT   
  
  
+ TGGTTTTACA CTTTTACCAC ATATTCATAG AAGAATTATG TGCACGTTGA TGGTTCAAGA TGGGACAAAA   
  
  
+ GGAAACATGT ATTCTTCCCC CTTTTGCCTT TTCCCCTAAG CATTTGGATT GAGGTGTTGT CATCTGAATA   
  
  
+ TCAAAAATTC TTTTTGAAAA TTCAGTCTCA ATCAAAATTC AAAATTAAAA CAAAGACTTA AGAGTTTTAT   
  
  
+ TTGTTCTTAA CCTTCTTAAC TTTCTACTTT TCTTTTTTTT CTCATTCCCC CTCTTCAAAT CTCATTTATT   
  
  
+ CTTAACCCCC TCTTCCGTTC ATGCTCTCTC TGTCTATCAA CTAATATTAA TCTACCCCGA CTTTCAATTT   
  
  
+ GATGAGGTCT AATCCTTATC ATCGTATTGT GGTTGGGTTA ATTATGCAAG AAGGCAATAA ACCCCAGTGA   
  
  
+ CAAAGTCCAC GTTAAGTAGG CACCTCACCA TTAAGACATG CTCAGAAAAC ACCAAAACCA TTGAACACAA   
  
  
+ GTCCCCCAAG TCCCTTCCCT AGCTAGCCCA TTCTCTCTCC TCCCCTGTCT ATATCTACCA ACTCTTGCTC   
  
  
+ TTTGCTGAGC CTGAGTTGAA AGCGATACAC CCATAGCCTG TTCAGTTTTC AGTTTTCACA TTTGTCTTCC   
  
  
+ TCTGTTCAGT TGAAAGCGAT ACACTCTCAT TAGCTTTCAT TTTAAATACG TTCAATTCAC ACATAAATGG   
  
  
+ CTTTCAAAGC ATTGCCATTT TCGTTTAAAT GATGACAAAA TATATAGTAT CTGGCTATCT GCCCATATCT   
  
  
+ TGACCTTATT TACAAAGGCT GAGCCATCTT TTAATTTTTT TAGCCTCTTT TTTTAATCAA AAATAGAAAT   
  
  
+ TTCAAGAAAG CAGAAATCGT CAGATGACAG TGCAGACAGC GTTTTCGTTT CATACAAATC TCAAAAGCTG   
  
  
+ AAGTGTCAAT TTCTTCGATT ATTTTTGTCA GAAAATATTT ACGTCTCACC TTCACGTTGT TATTATTCTT   
  
  
+ TCTTTTTTAT TATTCTCATA ACTAGTAATA AAGTTAGCAA TGATAAAAAA TTATTCGATC CGAATATTTT   
  
  
+ AATTTATCTG ACCTAAAAAC ATAAGTAAAG GCACAAATTT TTCATCCAAA TTTTAATTTT TGATGTAATA   
  
  
+ TTTTTTATAT TTTTATTGTT CAAATCTGAT TTTAATCTTA TTTAAATTAT CTGACCTAAA AAAATCAAAT   
  
  
+ AATAATAAAC GTTAATTTTT TATTTAAATT TTGACATTAG TCAATCTGAC TTAAATTCGA ACCGAGCTTG   
  
  
+ AATTTTTTTG CCAGTGCTGG TAGTACTCCT GCTTTTGTAG CTCCCAATGC CATCGTACCA CCCTCTTCTA   
  
  
+ GTCTCGTCTC TTTCTCTGGT CTCCAATTAA TCACACCATC ATATCATACC GTATGATGAT ACAGTCCACT   
  
  
+ GAAATCCCAT TCTCACCGCC AATCTCTCTC CGCCACCATC CAACACTCTT AACGATGAAC AGGGCCGCCG   
  
  
+ CCTCGTCCTC CACACTCAGG CCGTGGCCGG GCAGCTTTCC CACCCAATCA AAATCTCTCT CCTCCGCCAA   
  
  
+ CTTCGGTAAC GCCAATTGCA TGGAGCAGCT CTTAGTCCAC TGCGCCGAAG CCATCGACAA CAATGACGCC   
  
  
+ ACCCCGGCCC AGCAAATCTT ATGGGTCCTG AATAACATAG CCCGACCCGA CGGCGACTCC ACCCAACGCC   
  
  
+ TCACGTGCGC ATTCCTACGT GCCTTAATCT CACGCGCCGT CCTCACTAGC ACCTGCAAGA TGGTAATCCC   
  
  
+ TCATTTCAAC CCCATCAATT CACCCCACAA ATTCTCGCTC CTCGAACTTG CCCACTTCGT CGATTTAACC   
  
  
+ CCTTGGCATC GATTCGGATT CACCGCCGCC AATTCGATCA TTCTGGAAGC TATTTCCGAC CTACCCGTTG   
  
  
+ TACACATTGT CGACCTCAGC ATCTCCCACT GTATGCAGAT CCCCACGTTG ATCGACTCCA TTGCGACCCG   
  
  
+ GTTGGAAGCC CCGGGTCGAG TCCCTCCTAT TGTCAAGCTC ACCGTCGGGG CTATTTCCGA CGAAATCCCG   
  
  
+ CCGGTGTTCG ATCTTCTGTC GTACGATGAG CTCGGAATGA GACTAATCAA CTTCGCTCGT TTTAGAAACA   
  
  
+ TCGTCCTCGA ATTCCAAGCA ATACCCACCT CCCCTTCCGA CGGATTTGCT TCGCTGTTGG AGGAGATTCG   
  
  
+ ACAAAGCAAG CTCTACTCCA ACGATGCGGC GGCGGTTATT GTGAATTGTC AGATGAGTTT GCATTTATTG   
  
  
+ CAGGAAGAGG AGGTGTCTTC ATCGTCGCCG TCGTCGATGA GGGGGATGTT TTTGCAGGCG GTGAGGAGCT   
  
  
+ TGGAGCCGAG CATGGTGGTG GTGGTGGAGG AGGACGTGGA TTTCACGGCG AGGAGTCTGG TGGGGCGGCT   
  
  
+ GAGATCGGCG TTTAATCACA TGTGGATACC CTTCGACACG GTGGACACGT TCTTGCCACG TGGGAGCCAG   
  
  
+ CAGAGAGAGT GGTTCGAGGC CGAGGTGTGC TGGAAGATTG AGAATGTGAT CGCTCATGAG GGACCCGCGA   
  
  
+ GGGTCGAGAG GCAGGAGCCC AGGGCCAAGT GGGCCCTCCG AATGAGGGAG GCCGAGTTTC AAGGGATCGA   
  
  
+ GTTCGGTGAT GAAGGTACGA CCGAGGTCAA GGCCATGCTG GAGGAGCATG CCGCTGGGTG GGGGTCTAAG   
  
  
+ AAGGAAGAGG ATGATCTTGT GCTCACTTGG AAGGGACATA GTGTTGTCTT TGCTTCTGCT TGGGTACCCA   
  
  
+ CTTA  

- +Up\_Stream \_Len000TCTTCG ATTAATTTGG TTTGTACGTG CATACAGGAT TAATTGTACT GACAGGTAGT   
  
  
- AGAGGAGGGT ACAACAAGAC AACTGTTCGG ACGTGTGGGT ACGAGGAGAG AGTACAGATT GGAGCAGGGT   
  
  
- TGCTAGTTCT AGTGACAGAC ACTCCGGTCC CCTTCTGAAC GAAAATAAAG GAAAAGAAAA CAAATGAGAG   
  
  
- ACTAAGGTAA ATCGGTATAT ATGTTCCTCT TACAGTACAC ATAGAGTATA CATATATATA TTCTAAAATA   
  
  
- AATCTTTATT TTTAAATTTG TGATACACTA AATATCAATA ATATTATTTA TCATAAAAAA TTTGATAAAG   
  
  
- TAACTACTCT TGTCCTTTCA TTTAACGGCT GTACAATTAC AATAATATAG ATTTTTAATT TAGTATTTTA   
  
  
- ATTAATATTA TAAAATTTTT ATAAAGTTGA TTTTAAAATC TTGTTATTCA CGCATTATGC CTGAATTTCC   
  
  
- GATCAAATAG CATTTTTACA TTCAAGGAAA ACCTAGAATA CCAACTTATA GAACCAACTA TTTGACACTA   
  
  
- ACCAAAATGT GAAAATGGTG TATAAGTATC TTCTTAATAC ACGTGCAACT ACCAAGTTCT ACCCTGTTTT   
  
  
- CCTTTGTACA TAAGAAGGGG GAAAACGGAA AAGGGGATTC GTAAACCTAA CTCCACAACA GTAGACTTAT   
  
  
- AGTTTTTAAG AAAAACTTTT AAGTCAGAGT TAGTTTTAAG TTTTAATTTT GTTTCTGAAT TCTCAAAATA   
  
  
- AACAAGAATT GGAAGAATTG AAAGATGAAA AGAAAAAAAA GAGTAAGGGG GAGAAGTTTA GAGTAAATAA   
  
  
- GAATTGGGGG AGAAGGCAAG TACGAGAGAG ACAGATAGTT GATTATAATT AGATGGGGCT GAAAGTTAAA   
  
  
- CTACTCCAGA TTAGGAATAG TAGCATAACA CCAACCCAAT TAATACGTTC TTCCGTTATT TGGGGTCACT   
  
  
- GTTTCAGGTG CAATTCATCC GTGGAGTGGT AATTCTGTAC GAGTCTTTTG TGGTTTTGGT AACTTGTGTT   
  
  
- CAGGGGGTTC AGGGAAGGGA TCGATCGGGT AAGAGAGAGG AGGGGACAGA TATAGATGGT TGAGAACGAG   
  
  
- AAACGACTCG GACTCAACTT TCGCTATGTG GGTATCGGAC AAGTCAAAAG TCAAAAGTGT AAACAGAAGG   
  
  
- AGACAAGTCA ACTTTCGCTA TGTGAGAGTA ATCGAAAGTA AAATTTATGC AAGTTAAGTG TGTATTTACC   
  
  
- GAAAGTTTCG TAACGGTAAA AGCAAATTTA CTACTGTTTT ATATATCATA GACCGATAGA CGGGTATAGA   
  
  
- ACTGGAATAA ATGTTTCCGA CTCGGTAGAA AATTAAAAAA ATCGGAGAAA AAAATTAGTT TTTATCTTTA   
  
  
- AAGTTCTTTC GTCTTTAGCA GTCTACTGTC ACGTCTGTCG CAAAAGCAAA GTATGTTTAG AGTTTTCGAC   
  
  
- TTCACAGTTA AAGAAGCTAA TAAAAACAGT CTTTTATAAA TGCAGAGTGG AAGTGCAACA ATAATAAGAA   
  
  
- AGAAAAAATA ATAAGAGTAT TGATCATTAT TTCAATCGTT ACTATTTTTT AATAAGCTAG GCTTATAAAA   
  
  
- TTAAATAGAC TGGATTTTTG TATTCATTTC CGTGTTTAAA AAGTAGGTTT AAAATTAAAA ACTACATTAT   
  
  
- AAAAAATATA AAAATAACAA GTTTAGACTA AAATTAGAAT AAATTTAATA GACTGGATTT TTTTAGTTTA   
  
  
- TTATTATTTG CAATTAAAAA ATAAATTTAA AACTGTAATC AGTTAGACTG AATTTAAGCT TGGCTCGAAC   
  
  
- TTAAAAAAAC GGTCACGACC ATCATGAGGA CGAAAACATC GAGGGTTACG GTAGCATGGT GGGAGAAGAT   
  
  
- CAGAGCAGAG AAAGAGACCA GAGGTTAATT AGTGTGGTAG TATAGTATGG CATACTACTA TGTCAGGTGA   
  
  
- CTTTAGGGTA AGAGTGGCGG TTAGAGAGAG GCGGTGGTAG GTTGTGAGAA TTGCTACTTG TCCCGGCGGC   
  
  
- GGAGCAGGAG GTGTGAGTCC GGCACCGGCC CGTCGAAAGG GTGGGTTAGT TTTAGAGAGA GGAGGCGGTT   
  
  
- GAAGCCATTG CGGTTAACGT ACCTCGTCGA GAATCAGGTG ACGCGGCTTC GGTAGCTGTT GTTACTGCGG   
  
  
- TGGGGCCGGG TCGTTTAGAA TACCCAGGAC TTATTGTATC GGGCTGGGCT GCCGCTGAGG TGGGTTGCGG   
  
  
- AGTGCACGCG TAAGGATGCA CGGAATTAGA GTGCGCGGCA GGAGTGATCG TGGACGTTCT ACCATTAGGG   
  
  
- AGTAAAGTTG GGGTAGTTAA GTGGGGTGTT TAAGAGCGAG GAGCTTGAAC GGGTGAAGCA GCTAAATTGG   
  
  
- GGAACCGTAG CTAAGCCTAA GTGGCGGCGG TTAAGCTAGT AAGACCTTCG ATAAAGGCTG GATGGGCAAC   
  
  
- ATGTGTAACA GCTGGAGTCG TAGAGGGTGA CATACGTCTA GGGGTGCAAC TAGCTGAGGT AACGCTGGGC   
  
  
- CAACCTTCGG GGCCCAGCTC AGGGAGGATA ACAGTTCGAG TGGCAGCCCC GATAAAGGCT GCTTTAGGGC   
  
  
- GGCCACAAGC TAGAAGACAG CATGCTACTC GAGCCTTACT CTGATTAGTT GAAGCGAGCA AAATCTTTGT   
  
  
- AGCAGGAGCT TAAGGTTCGT TATGGGTGGA GGGGAAGGCT GCCTAAACGA AGCGACAACC TCCTCTAAGC   
  
  
- TGTTTCGTTC GAGATGAGGT TGCTACGCCG CCGCCAATAA CACTTAACAG TCTACTCAAA CGTAAATAAC   
  
  
- GTCCTTCTCC TCCACAGAAG TAGCAGCGGC AGCAGCTACT CCCCCTACAA AAACGTCCGC CACTCCTCGA   
  
  
- ACCTCGGCTC GTACCACCAC CACCACCTCC TCCTGCACCT AAAGTGCCGC TCCTCAGACC ACCCCGCCGA   
  
  
- CTCTAGCCGC AAATTAGTGT ACACCTATGG GAAGCTGTGC CACCTGTGCA AGAACGGTGC ACCCTCGGTC   
  
  
- GTCTCTCTCA CCAAGCTCCG GCTCCACACG ACCTTCTAAC TCTTACACTA GCGAGTACTC CCTGGGCGCT   
  
  
- CCCAGCTCTC CGTCCTCGGG TCCCGGTTCA CCCGGGAGGC TTACTCCCTC CGGCTCAAAG TTCCCTAGCT   
  
  
- CAAGCCACTA CTTCCATGCT GGCTCCAGTT CCGGTACGAC CTCCTCGTAC GGCGACCCAC CCCCAGATTC   
  
  
- TTCCTTCTCC TACTAGAACA CGAGTGAACC TTCCCTGTAT CACAACAGAA ACGAAGACGA ACCCATGGGT   
  
  
- GAAT

+     ABRE4

| Site Name | Organism | Position | Strand | Matrix score. | sequence | function |
| --- | --- | --- | --- | --- | --- | --- |
| ABRE4 | Zea mays | 42 | + | 6 | CACGTA |  |
| ABRE4 | Zea mays | 2260 | - | 6 | CACGTA |  |

>HU06G00376.1   
+ +Up\_Stream \_Len000AGAAGC TAATTAAACC AAACATGCAC GTATGTCCTA ATTAACATGA CTGTCCATCA   
  
  
+ TCTCCTCCCA TGTTGTTCTG TTGACAAGCC TGCACACCCA TGCTCCTCTC TCATGTCTAA CCTCGTCCCA   
  
  
+ ACGATCAAGA TCACTGTCTG TGAGGCCAGG GGAAGACTTG CTTTTATTTC CTTTTCTTTT GTTTACTCTC   
  
  
+ TGATTCCATT TAGCCATATA TACAAGGAGA ATGTCATGTG TATCTCATAT GTATATATAT AAGATTTTAT   
  
  
+ TTAGAAATAA AAATTTAAAC ACTATGTGAT TTATAGTTAT TATAATAAAT AGTATTTTTT AAACTATTTC   
  
  
+ ATTGATGAGA ACAGGAAAGT AAATTGCCGA CATGTTAATG TTATTATATC TAAAAATTAA ATCATAAAAT   
  
  
+ TAATTATAAT ATTTTAAAAA TATTTCAACT AAAATTTTAG AACAATAAGT GCGTAATACG GACTTAAAGG   
  
  
+ CTAGTTTATC GTAAAAATGT AAGTTCCTTT TGGATCTTAT GGTTGAATAT CTTGGTTGAT AAACTGTGAT   
  
  
+ TGGTTTTACA CTTTTACCAC ATATTCATAG AAGAATTATG TGCACGTTGA TGGTTCAAGA TGGGACAAAA   
  
  
+ GGAAACATGT ATTCTTCCCC CTTTTGCCTT TTCCCCTAAG CATTTGGATT GAGGTGTTGT CATCTGAATA   
  
  
+ TCAAAAATTC TTTTTGAAAA TTCAGTCTCA ATCAAAATTC AAAATTAAAA CAAAGACTTA AGAGTTTTAT   
  
  
+ TTGTTCTTAA CCTTCTTAAC TTTCTACTTT TCTTTTTTTT CTCATTCCCC CTCTTCAAAT CTCATTTATT   
  
  
+ CTTAACCCCC TCTTCCGTTC ATGCTCTCTC TGTCTATCAA CTAATATTAA TCTACCCCGA CTTTCAATTT   
  
  
+ GATGAGGTCT AATCCTTATC ATCGTATTGT GGTTGGGTTA ATTATGCAAG AAGGCAATAA ACCCCAGTGA   
  
  
+ CAAAGTCCAC GTTAAGTAGG CACCTCACCA TTAAGACATG CTCAGAAAAC ACCAAAACCA TTGAACACAA   
  
  
+ GTCCCCCAAG TCCCTTCCCT AGCTAGCCCA TTCTCTCTCC TCCCCTGTCT ATATCTACCA ACTCTTGCTC   
  
  
+ TTTGCTGAGC CTGAGTTGAA AGCGATACAC CCATAGCCTG TTCAGTTTTC AGTTTTCACA TTTGTCTTCC   
  
  
+ TCTGTTCAGT TGAAAGCGAT ACACTCTCAT TAGCTTTCAT TTTAAATACG TTCAATTCAC ACATAAATGG   
  
  
+ CTTTCAAAGC ATTGCCATTT TCGTTTAAAT GATGACAAAA TATATAGTAT CTGGCTATCT GCCCATATCT   
  
  
+ TGACCTTATT TACAAAGGCT GAGCCATCTT TTAATTTTTT TAGCCTCTTT TTTTAATCAA AAATAGAAAT   
  
  
+ TTCAAGAAAG CAGAAATCGT CAGATGACAG TGCAGACAGC GTTTTCGTTT CATACAAATC TCAAAAGCTG   
  
  
+ AAGTGTCAAT TTCTTCGATT ATTTTTGTCA GAAAATATTT ACGTCTCACC TTCACGTTGT TATTATTCTT   
  
  
+ TCTTTTTTAT TATTCTCATA ACTAGTAATA AAGTTAGCAA TGATAAAAAA TTATTCGATC CGAATATTTT   
  
  
+ AATTTATCTG ACCTAAAAAC ATAAGTAAAG GCACAAATTT TTCATCCAAA TTTTAATTTT TGATGTAATA   
  
  
+ TTTTTTATAT TTTTATTGTT CAAATCTGAT TTTAATCTTA TTTAAATTAT CTGACCTAAA AAAATCAAAT   
  
  
+ AATAATAAAC GTTAATTTTT TATTTAAATT TTGACATTAG TCAATCTGAC TTAAATTCGA ACCGAGCTTG   
  
  
+ AATTTTTTTG CCAGTGCTGG TAGTACTCCT GCTTTTGTAG CTCCCAATGC CATCGTACCA CCCTCTTCTA   
  
  
+ GTCTCGTCTC TTTCTCTGGT CTCCAATTAA TCACACCATC ATATCATACC GTATGATGAT ACAGTCCACT   
  
  
+ GAAATCCCAT TCTCACCGCC AATCTCTCTC CGCCACCATC CAACACTCTT AACGATGAAC AGGGCCGCCG   
  
  
+ CCTCGTCCTC CACACTCAGG CCGTGGCCGG GCAGCTTTCC CACCCAATCA AAATCTCTCT CCTCCGCCAA   
  
  
+ CTTCGGTAAC GCCAATTGCA TGGAGCAGCT CTTAGTCCAC TGCGCCGAAG CCATCGACAA CAATGACGCC   
  
  
+ ACCCCGGCCC AGCAAATCTT ATGGGTCCTG AATAACATAG CCCGACCCGA CGGCGACTCC ACCCAACGCC   
  
  
+ TCACGTGCGC ATTCCTACGT GCCTTAATCT CACGCGCCGT CCTCACTAGC ACCTGCAAGA TGGTAATCCC   
  
  
+ TCATTTCAAC CCCATCAATT CACCCCACAA ATTCTCGCTC CTCGAACTTG CCCACTTCGT CGATTTAACC   
  
  
+ CCTTGGCATC GATTCGGATT CACCGCCGCC AATTCGATCA TTCTGGAAGC TATTTCCGAC CTACCCGTTG   
  
  
+ TACACATTGT CGACCTCAGC ATCTCCCACT GTATGCAGAT CCCCACGTTG ATCGACTCCA TTGCGACCCG   
  
  
+ GTTGGAAGCC CCGGGTCGAG TCCCTCCTAT TGTCAAGCTC ACCGTCGGGG CTATTTCCGA CGAAATCCCG   
  
  
+ CCGGTGTTCG ATCTTCTGTC GTACGATGAG CTCGGAATGA GACTAATCAA CTTCGCTCGT TTTAGAAACA   
  
  
+ TCGTCCTCGA ATTCCAAGCA ATACCCACCT CCCCTTCCGA CGGATTTGCT TCGCTGTTGG AGGAGATTCG   
  
  
+ ACAAAGCAAG CTCTACTCCA ACGATGCGGC GGCGGTTATT GTGAATTGTC AGATGAGTTT GCATTTATTG   
  
  
+ CAGGAAGAGG AGGTGTCTTC ATCGTCGCCG TCGTCGATGA GGGGGATGTT TTTGCAGGCG GTGAGGAGCT   
  
  
+ TGGAGCCGAG CATGGTGGTG GTGGTGGAGG AGGACGTGGA TTTCACGGCG AGGAGTCTGG TGGGGCGGCT   
  
  
+ GAGATCGGCG TTTAATCACA TGTGGATACC CTTCGACACG GTGGACACGT TCTTGCCACG TGGGAGCCAG   
  
  
+ CAGAGAGAGT GGTTCGAGGC CGAGGTGTGC TGGAAGATTG AGAATGTGAT CGCTCATGAG GGACCCGCGA   
  
  
+ GGGTCGAGAG GCAGGAGCCC AGGGCCAAGT GGGCCCTCCG AATGAGGGAG GCCGAGTTTC AAGGGATCGA   
  
  
+ GTTCGGTGAT GAAGGTACGA CCGAGGTCAA GGCCATGCTG GAGGAGCATG CCGCTGGGTG GGGGTCTAAG   
  
  
+ AAGGAAGAGG ATGATCTTGT GCTCACTTGG AAGGGACATA GTGTTGTCTT TGCTTCTGCT TGGGTACCCA   
  
  
+ CTTA  

- +Up\_Stream \_Len000TCTTCG ATTAATTTGG TTTGTACGTG CATACAGGAT TAATTGTACT GACAGGTAGT   
  
  
- AGAGGAGGGT ACAACAAGAC AACTGTTCGG ACGTGTGGGT ACGAGGAGAG AGTACAGATT GGAGCAGGGT   
  
  
- TGCTAGTTCT AGTGACAGAC ACTCCGGTCC CCTTCTGAAC GAAAATAAAG GAAAAGAAAA CAAATGAGAG   
  
  
- ACTAAGGTAA ATCGGTATAT ATGTTCCTCT TACAGTACAC ATAGAGTATA CATATATATA TTCTAAAATA   
  
  
- AATCTTTATT TTTAAATTTG TGATACACTA AATATCAATA ATATTATTTA TCATAAAAAA TTTGATAAAG   
  
  
- TAACTACTCT TGTCCTTTCA TTTAACGGCT GTACAATTAC AATAATATAG ATTTTTAATT TAGTATTTTA   
  
  
- ATTAATATTA TAAAATTTTT ATAAAGTTGA TTTTAAAATC TTGTTATTCA CGCATTATGC CTGAATTTCC   
  
  
- GATCAAATAG CATTTTTACA TTCAAGGAAA ACCTAGAATA CCAACTTATA GAACCAACTA TTTGACACTA   
  
  
- ACCAAAATGT GAAAATGGTG TATAAGTATC TTCTTAATAC ACGTGCAACT ACCAAGTTCT ACCCTGTTTT   
  
  
- CCTTTGTACA TAAGAAGGGG GAAAACGGAA AAGGGGATTC GTAAACCTAA CTCCACAACA GTAGACTTAT   
  
  
- AGTTTTTAAG AAAAACTTTT AAGTCAGAGT TAGTTTTAAG TTTTAATTTT GTTTCTGAAT TCTCAAAATA   
  
  
- AACAAGAATT GGAAGAATTG AAAGATGAAA AGAAAAAAAA GAGTAAGGGG GAGAAGTTTA GAGTAAATAA   
  
  
- GAATTGGGGG AGAAGGCAAG TACGAGAGAG ACAGATAGTT GATTATAATT AGATGGGGCT GAAAGTTAAA   
  
  
- CTACTCCAGA TTAGGAATAG TAGCATAACA CCAACCCAAT TAATACGTTC TTCCGTTATT TGGGGTCACT   
  
  
- GTTTCAGGTG CAATTCATCC GTGGAGTGGT AATTCTGTAC GAGTCTTTTG TGGTTTTGGT AACTTGTGTT   
  
  
- CAGGGGGTTC AGGGAAGGGA TCGATCGGGT AAGAGAGAGG AGGGGACAGA TATAGATGGT TGAGAACGAG   
  
  
- AAACGACTCG GACTCAACTT TCGCTATGTG GGTATCGGAC AAGTCAAAAG TCAAAAGTGT AAACAGAAGG   
  
  
- AGACAAGTCA ACTTTCGCTA TGTGAGAGTA ATCGAAAGTA AAATTTATGC AAGTTAAGTG TGTATTTACC   
  
  
- GAAAGTTTCG TAACGGTAAA AGCAAATTTA CTACTGTTTT ATATATCATA GACCGATAGA CGGGTATAGA   
  
  
- ACTGGAATAA ATGTTTCCGA CTCGGTAGAA AATTAAAAAA ATCGGAGAAA AAAATTAGTT TTTATCTTTA   
  
  
- AAGTTCTTTC GTCTTTAGCA GTCTACTGTC ACGTCTGTCG CAAAAGCAAA GTATGTTTAG AGTTTTCGAC   
  
  
- TTCACAGTTA AAGAAGCTAA TAAAAACAGT CTTTTATAAA TGCAGAGTGG AAGTGCAACA ATAATAAGAA   
  
  
- AGAAAAAATA ATAAGAGTAT TGATCATTAT TTCAATCGTT ACTATTTTTT AATAAGCTAG GCTTATAAAA   
  
  
- TTAAATAGAC TGGATTTTTG TATTCATTTC CGTGTTTAAA AAGTAGGTTT AAAATTAAAA ACTACATTAT   
  
  
- AAAAAATATA AAAATAACAA GTTTAGACTA AAATTAGAAT AAATTTAATA GACTGGATTT TTTTAGTTTA   
  
  
- TTATTATTTG CAATTAAAAA ATAAATTTAA AACTGTAATC AGTTAGACTG AATTTAAGCT TGGCTCGAAC   
  
  
- TTAAAAAAAC GGTCACGACC ATCATGAGGA CGAAAACATC GAGGGTTACG GTAGCATGGT GGGAGAAGAT   
  
  
- CAGAGCAGAG AAAGAGACCA GAGGTTAATT AGTGTGGTAG TATAGTATGG CATACTACTA TGTCAGGTGA   
  
  
- CTTTAGGGTA AGAGTGGCGG TTAGAGAGAG GCGGTGGTAG GTTGTGAGAA TTGCTACTTG TCCCGGCGGC   
  
  
- GGAGCAGGAG GTGTGAGTCC GGCACCGGCC CGTCGAAAGG GTGGGTTAGT TTTAGAGAGA GGAGGCGGTT   
  
  
- GAAGCCATTG CGGTTAACGT ACCTCGTCGA GAATCAGGTG ACGCGGCTTC GGTAGCTGTT GTTACTGCGG   
  
  
- TGGGGCCGGG TCGTTTAGAA TACCCAGGAC TTATTGTATC GGGCTGGGCT GCCGCTGAGG TGGGTTGCGG   
  
  
- AGTGCACGCG TAAGGATGCA CGGAATTAGA GTGCGCGGCA GGAGTGATCG TGGACGTTCT ACCATTAGGG   
  
  
- AGTAAAGTTG GGGTAGTTAA GTGGGGTGTT TAAGAGCGAG GAGCTTGAAC GGGTGAAGCA GCTAAATTGG   
  
  
- GGAACCGTAG CTAAGCCTAA GTGGCGGCGG TTAAGCTAGT AAGACCTTCG ATAAAGGCTG GATGGGCAAC   
  
  
- ATGTGTAACA GCTGGAGTCG TAGAGGGTGA CATACGTCTA GGGGTGCAAC TAGCTGAGGT AACGCTGGGC   
  
  
- CAACCTTCGG GGCCCAGCTC AGGGAGGATA ACAGTTCGAG TGGCAGCCCC GATAAAGGCT GCTTTAGGGC   
  
  
- GGCCACAAGC TAGAAGACAG CATGCTACTC GAGCCTTACT CTGATTAGTT GAAGCGAGCA AAATCTTTGT   
  
  
- AGCAGGAGCT TAAGGTTCGT TATGGGTGGA GGGGAAGGCT GCCTAAACGA AGCGACAACC TCCTCTAAGC   
  
  
- TGTTTCGTTC GAGATGAGGT TGCTACGCCG CCGCCAATAA CACTTAACAG TCTACTCAAA CGTAAATAAC   
  
  
- GTCCTTCTCC TCCACAGAAG TAGCAGCGGC AGCAGCTACT CCCCCTACAA AAACGTCCGC CACTCCTCGA   
  
  
- ACCTCGGCTC GTACCACCAC CACCACCTCC TCCTGCACCT AAAGTGCCGC TCCTCAGACC ACCCCGCCGA   
  
  
- CTCTAGCCGC AAATTAGTGT ACACCTATGG GAAGCTGTGC CACCTGTGCA AGAACGGTGC ACCCTCGGTC   
  
  
- GTCTCTCTCA CCAAGCTCCG GCTCCACACG ACCTTCTAAC TCTTACACTA GCGAGTACTC CCTGGGCGCT   
  
  
- CCCAGCTCTC CGTCCTCGGG TCCCGGTTCA CCCGGGAGGC TTACTCCCTC CGGCTCAAAG TTCCCTAGCT   
  
  
- CAAGCCACTA CTTCCATGCT GGCTCCAGTT CCGGTACGAC CTCCTCGTAC GGCGACCCAC CCCCAGATTC   
  
  
- TTCCTTCTCC TACTAGAACA CGAGTGAACC TTCCCTGTAT CACAACAGAA ACGAAGACGA ACCCATGGGT   
  
  
- GAAT

+     AC-I

| Site Name | Organism | Position | Strand | Matrix score. | sequence | function |
| --- | --- | --- | --- | --- | --- | --- |
| AC-I | Phaseolus vulgaris | 2439 | + | 8.5 | (T/C)C(T/C)(C/T)ACC(T/C)ACC |  |

>HU06G00376.1   
+ +Up\_Stream \_Len000AGAAGC TAATTAAACC AAACATGCAC GTATGTCCTA ATTAACATGA CTGTCCATCA   
  
  
+ TCTCCTCCCA TGTTGTTCTG TTGACAAGCC TGCACACCCA TGCTCCTCTC TCATGTCTAA CCTCGTCCCA   
  
  
+ ACGATCAAGA TCACTGTCTG TGAGGCCAGG GGAAGACTTG CTTTTATTTC CTTTTCTTTT GTTTACTCTC   
  
  
+ TGATTCCATT TAGCCATATA TACAAGGAGA ATGTCATGTG TATCTCATAT GTATATATAT AAGATTTTAT   
  
  
+ TTAGAAATAA AAATTTAAAC ACTATGTGAT TTATAGTTAT TATAATAAAT AGTATTTTTT AAACTATTTC   
  
  
+ ATTGATGAGA ACAGGAAAGT AAATTGCCGA CATGTTAATG TTATTATATC TAAAAATTAA ATCATAAAAT   
  
  
+ TAATTATAAT ATTTTAAAAA TATTTCAACT AAAATTTTAG AACAATAAGT GCGTAATACG GACTTAAAGG   
  
  
+ CTAGTTTATC GTAAAAATGT AAGTTCCTTT TGGATCTTAT GGTTGAATAT CTTGGTTGAT AAACTGTGAT   
  
  
+ TGGTTTTACA CTTTTACCAC ATATTCATAG AAGAATTATG TGCACGTTGA TGGTTCAAGA TGGGACAAAA   
  
  
+ GGAAACATGT ATTCTTCCCC CTTTTGCCTT TTCCCCTAAG CATTTGGATT GAGGTGTTGT CATCTGAATA   
  
  
+ TCAAAAATTC TTTTTGAAAA TTCAGTCTCA ATCAAAATTC AAAATTAAAA CAAAGACTTA AGAGTTTTAT   
  
  
+ TTGTTCTTAA CCTTCTTAAC TTTCTACTTT TCTTTTTTTT CTCATTCCCC CTCTTCAAAT CTCATTTATT   
  
  
+ CTTAACCCCC TCTTCCGTTC ATGCTCTCTC TGTCTATCAA CTAATATTAA TCTACCCCGA CTTTCAATTT   
  
  
+ GATGAGGTCT AATCCTTATC ATCGTATTGT GGTTGGGTTA ATTATGCAAG AAGGCAATAA ACCCCAGTGA   
  
  
+ CAAAGTCCAC GTTAAGTAGG CACCTCACCA TTAAGACATG CTCAGAAAAC ACCAAAACCA TTGAACACAA   
  
  
+ GTCCCCCAAG TCCCTTCCCT AGCTAGCCCA TTCTCTCTCC TCCCCTGTCT ATATCTACCA ACTCTTGCTC   
  
  
+ TTTGCTGAGC CTGAGTTGAA AGCGATACAC CCATAGCCTG TTCAGTTTTC AGTTTTCACA TTTGTCTTCC   
  
  
+ TCTGTTCAGT TGAAAGCGAT ACACTCTCAT TAGCTTTCAT TTTAAATACG TTCAATTCAC ACATAAATGG   
  
  
+ CTTTCAAAGC ATTGCCATTT TCGTTTAAAT GATGACAAAA TATATAGTAT CTGGCTATCT GCCCATATCT   
  
  
+ TGACCTTATT TACAAAGGCT GAGCCATCTT TTAATTTTTT TAGCCTCTTT TTTTAATCAA AAATAGAAAT   
  
  
+ TTCAAGAAAG CAGAAATCGT CAGATGACAG TGCAGACAGC GTTTTCGTTT CATACAAATC TCAAAAGCTG   
  
  
+ AAGTGTCAAT TTCTTCGATT ATTTTTGTCA GAAAATATTT ACGTCTCACC TTCACGTTGT TATTATTCTT   
  
  
+ TCTTTTTTAT TATTCTCATA ACTAGTAATA AAGTTAGCAA TGATAAAAAA TTATTCGATC CGAATATTTT   
  
  
+ AATTTATCTG ACCTAAAAAC ATAAGTAAAG GCACAAATTT TTCATCCAAA TTTTAATTTT TGATGTAATA   
  
  
+ TTTTTTATAT TTTTATTGTT CAAATCTGAT TTTAATCTTA TTTAAATTAT CTGACCTAAA AAAATCAAAT   
  
  
+ AATAATAAAC GTTAATTTTT TATTTAAATT TTGACATTAG TCAATCTGAC TTAAATTCGA ACCGAGCTTG   
  
  
+ AATTTTTTTG CCAGTGCTGG TAGTACTCCT GCTTTTGTAG CTCCCAATGC CATCGTACCA CCCTCTTCTA   
  
  
+ GTCTCGTCTC TTTCTCTGGT CTCCAATTAA TCACACCATC ATATCATACC GTATGATGAT ACAGTCCACT   
  
  
+ GAAATCCCAT TCTCACCGCC AATCTCTCTC CGCCACCATC CAACACTCTT AACGATGAAC AGGGCCGCCG   
  
  
+ CCTCGTCCTC CACACTCAGG CCGTGGCCGG GCAGCTTTCC CACCCAATCA AAATCTCTCT CCTCCGCCAA   
  
  
+ CTTCGGTAAC GCCAATTGCA TGGAGCAGCT CTTAGTCCAC TGCGCCGAAG CCATCGACAA CAATGACGCC   
  
  
+ ACCCCGGCCC AGCAAATCTT ATGGGTCCTG AATAACATAG CCCGACCCGA CGGCGACTCC ACCCAACGCC   
  
  
+ TCACGTGCGC ATTCCTACGT GCCTTAATCT CACGCGCCGT CCTCACTAGC ACCTGCAAGA TGGTAATCCC   
  
  
+ TCATTTCAAC CCCATCAATT CACCCCACAA ATTCTCGCTC CTCGAACTTG CCCACTTCGT CGATTTAACC   
  
  
+ CCTTGGCATC GATTCGGATT CACCGCCGCC AATTCGATCA TTCTGGAAGC TATTTCCGAC CTACCCGTTG   
  
  
+ TACACATTGT CGACCTCAGC ATCTCCCACT GTATGCAGAT CCCCACGTTG ATCGACTCCA TTGCGACCCG   
  
  
+ GTTGGAAGCC CCGGGTCGAG TCCCTCCTAT TGTCAAGCTC ACCGTCGGGG CTATTTCCGA CGAAATCCCG   
  
  
+ CCGGTGTTCG ATCTTCTGTC GTACGATGAG CTCGGAATGA GACTAATCAA CTTCGCTCGT TTTAGAAACA   
  
  
+ TCGTCCTCGA ATTCCAAGCA ATACCCACCT CCCCTTCCGA CGGATTTGCT TCGCTGTTGG AGGAGATTCG   
  
  
+ ACAAAGCAAG CTCTACTCCA ACGATGCGGC GGCGGTTATT GTGAATTGTC AGATGAGTTT GCATTTATTG   
  
  
+ CAGGAAGAGG AGGTGTCTTC ATCGTCGCCG TCGTCGATGA GGGGGATGTT TTTGCAGGCG GTGAGGAGCT   
  
  
+ TGGAGCCGAG CATGGTGGTG GTGGTGGAGG AGGACGTGGA TTTCACGGCG AGGAGTCTGG TGGGGCGGCT   
  
  
+ GAGATCGGCG TTTAATCACA TGTGGATACC CTTCGACACG GTGGACACGT TCTTGCCACG TGGGAGCCAG   
  
  
+ CAGAGAGAGT GGTTCGAGGC CGAGGTGTGC TGGAAGATTG AGAATGTGAT CGCTCATGAG GGACCCGCGA   
  
  
+ GGGTCGAGAG GCAGGAGCCC AGGGCCAAGT GGGCCCTCCG AATGAGGGAG GCCGAGTTTC AAGGGATCGA   
  
  
+ GTTCGGTGAT GAAGGTACGA CCGAGGTCAA GGCCATGCTG GAGGAGCATG CCGCTGGGTG GGGGTCTAAG   
  
  
+ AAGGAAGAGG ATGATCTTGT GCTCACTTGG AAGGGACATA GTGTTGTCTT TGCTTCTGCT TGGGTACCCA   
  
  
+ CTTA  

- +Up\_Stream \_Len000TCTTCG ATTAATTTGG TTTGTACGTG CATACAGGAT TAATTGTACT GACAGGTAGT   
  
  
- AGAGGAGGGT ACAACAAGAC AACTGTTCGG ACGTGTGGGT ACGAGGAGAG AGTACAGATT GGAGCAGGGT   
  
  
- TGCTAGTTCT AGTGACAGAC ACTCCGGTCC CCTTCTGAAC GAAAATAAAG GAAAAGAAAA CAAATGAGAG   
  
  
- ACTAAGGTAA ATCGGTATAT ATGTTCCTCT TACAGTACAC ATAGAGTATA CATATATATA TTCTAAAATA   
  
  
- AATCTTTATT TTTAAATTTG TGATACACTA AATATCAATA ATATTATTTA TCATAAAAAA TTTGATAAAG   
  
  
- TAACTACTCT TGTCCTTTCA TTTAACGGCT GTACAATTAC AATAATATAG ATTTTTAATT TAGTATTTTA   
  
  
- ATTAATATTA TAAAATTTTT ATAAAGTTGA TTTTAAAATC TTGTTATTCA CGCATTATGC CTGAATTTCC   
  
  
- GATCAAATAG CATTTTTACA TTCAAGGAAA ACCTAGAATA CCAACTTATA GAACCAACTA TTTGACACTA   
  
  
- ACCAAAATGT GAAAATGGTG TATAAGTATC TTCTTAATAC ACGTGCAACT ACCAAGTTCT ACCCTGTTTT   
  
  
- CCTTTGTACA TAAGAAGGGG GAAAACGGAA AAGGGGATTC GTAAACCTAA CTCCACAACA GTAGACTTAT   
  
  
- AGTTTTTAAG AAAAACTTTT AAGTCAGAGT TAGTTTTAAG TTTTAATTTT GTTTCTGAAT TCTCAAAATA   
  
  
- AACAAGAATT GGAAGAATTG AAAGATGAAA AGAAAAAAAA GAGTAAGGGG GAGAAGTTTA GAGTAAATAA   
  
  
- GAATTGGGGG AGAAGGCAAG TACGAGAGAG ACAGATAGTT GATTATAATT AGATGGGGCT GAAAGTTAAA   
  
  
- CTACTCCAGA TTAGGAATAG TAGCATAACA CCAACCCAAT TAATACGTTC TTCCGTTATT TGGGGTCACT   
  
  
- GTTTCAGGTG CAATTCATCC GTGGAGTGGT AATTCTGTAC GAGTCTTTTG TGGTTTTGGT AACTTGTGTT   
  
  
- CAGGGGGTTC AGGGAAGGGA TCGATCGGGT AAGAGAGAGG AGGGGACAGA TATAGATGGT TGAGAACGAG   
  
  
- AAACGACTCG GACTCAACTT TCGCTATGTG GGTATCGGAC AAGTCAAAAG TCAAAAGTGT AAACAGAAGG   
  
  
- AGACAAGTCA ACTTTCGCTA TGTGAGAGTA ATCGAAAGTA AAATTTATGC AAGTTAAGTG TGTATTTACC   
  
  
- GAAAGTTTCG TAACGGTAAA AGCAAATTTA CTACTGTTTT ATATATCATA GACCGATAGA CGGGTATAGA   
  
  
- ACTGGAATAA ATGTTTCCGA CTCGGTAGAA AATTAAAAAA ATCGGAGAAA AAAATTAGTT TTTATCTTTA   
  
  
- AAGTTCTTTC GTCTTTAGCA GTCTACTGTC ACGTCTGTCG CAAAAGCAAA GTATGTTTAG AGTTTTCGAC   
  
  
- TTCACAGTTA AAGAAGCTAA TAAAAACAGT CTTTTATAAA TGCAGAGTGG AAGTGCAACA ATAATAAGAA   
  
  
- AGAAAAAATA ATAAGAGTAT TGATCATTAT TTCAATCGTT ACTATTTTTT AATAAGCTAG GCTTATAAAA   
  
  
- TTAAATAGAC TGGATTTTTG TATTCATTTC CGTGTTTAAA AAGTAGGTTT AAAATTAAAA ACTACATTAT   
  
  
- AAAAAATATA AAAATAACAA GTTTAGACTA AAATTAGAAT AAATTTAATA GACTGGATTT TTTTAGTTTA   
  
  
- TTATTATTTG CAATTAAAAA ATAAATTTAA AACTGTAATC AGTTAGACTG AATTTAAGCT TGGCTCGAAC   
  
  
- TTAAAAAAAC GGTCACGACC ATCATGAGGA CGAAAACATC GAGGGTTACG GTAGCATGGT GGGAGAAGAT   
  
  
- CAGAGCAGAG AAAGAGACCA GAGGTTAATT AGTGTGGTAG TATAGTATGG CATACTACTA TGTCAGGTGA   
  
  
- CTTTAGGGTA AGAGTGGCGG TTAGAGAGAG GCGGTGGTAG GTTGTGAGAA TTGCTACTTG TCCCGGCGGC   
  
  
- GGAGCAGGAG GTGTGAGTCC GGCACCGGCC CGTCGAAAGG GTGGGTTAGT TTTAGAGAGA GGAGGCGGTT   
  
  
- GAAGCCATTG CGGTTAACGT ACCTCGTCGA GAATCAGGTG ACGCGGCTTC GGTAGCTGTT GTTACTGCGG   
  
  
- TGGGGCCGGG TCGTTTAGAA TACCCAGGAC TTATTGTATC GGGCTGGGCT GCCGCTGAGG TGGGTTGCGG   
  
  
- AGTGCACGCG TAAGGATGCA CGGAATTAGA GTGCGCGGCA GGAGTGATCG TGGACGTTCT ACCATTAGGG   
  
  
- AGTAAAGTTG GGGTAGTTAA GTGGGGTGTT TAAGAGCGAG GAGCTTGAAC GGGTGAAGCA GCTAAATTGG   
  
  
- GGAACCGTAG CTAAGCCTAA GTGGCGGCGG TTAAGCTAGT AAGACCTTCG ATAAAGGCTG GATGGGCAAC   
  
  
- ATGTGTAACA GCTGGAGTCG TAGAGGGTGA CATACGTCTA GGGGTGCAAC TAGCTGAGGT AACGCTGGGC   
  
  
- CAACCTTCGG GGCCCAGCTC AGGGAGGATA ACAGTTCGAG TGGCAGCCCC GATAAAGGCT GCTTTAGGGC   
  
  
- GGCCACAAGC TAGAAGACAG CATGCTACTC GAGCCTTACT CTGATTAGTT GAAGCGAGCA AAATCTTTGT   
  
  
- AGCAGGAGCT TAAGGTTCGT TATGGGTGGA GGGGAAGGCT GCCTAAACGA AGCGACAACC TCCTCTAAGC   
  
  
- TGTTTCGTTC GAGATGAGGT TGCTACGCCG CCGCCAATAA CACTTAACAG TCTACTCAAA CGTAAATAAC   
  
  
- GTCCTTCTCC TCCACAGAAG TAGCAGCGGC AGCAGCTACT CCCCCTACAA AAACGTCCGC CACTCCTCGA   
  
  
- ACCTCGGCTC GTACCACCAC CACCACCTCC TCCTGCACCT AAAGTGCCGC TCCTCAGACC ACCCCGCCGA   
  
  
- CTCTAGCCGC AAATTAGTGT ACACCTATGG GAAGCTGTGC CACCTGTGCA AGAACGGTGC ACCCTCGGTC   
  
  
- GTCTCTCTCA CCAAGCTCCG GCTCCACACG ACCTTCTAAC TCTTACACTA GCGAGTACTC CCTGGGCGCT   
  
  
- CCCAGCTCTC CGTCCTCGGG TCCCGGTTCA CCCGGGAGGC TTACTCCCTC CGGCTCAAAG TTCCCTAGCT   
  
  
- CAAGCCACTA CTTCCATGCT GGCTCCAGTT CCGGTACGAC CTCCTCGTAC GGCGACCCAC CCCCAGATTC   
  
  
- TTCCTTCTCC TACTAGAACA CGAGTGAACC TTCCCTGTAT CACAACAGAA ACGAAGACGA ACCCATGGGT   
  
  
- GAAT

+     ARE

| Site Name | Organism | Position | Strand | Matrix score. | sequence | function |
| --- | --- | --- | --- | --- | --- | --- |
| ARE | Zea mays | 1039 | + | 6 | AAACCA | cis-acting regulatory element essential for the anaerobic induction |
| ARE | Zea mays | 30 | + | 6 | AAACCA | cis-acting regulatory element essential for the anaerobic induction |
| ARE | Zea mays | 565 | - | 6 | AAACCA | cis-acting regulatory element essential for the anaerobic induction |

>HU06G00376.1   
+ +Up\_Stream \_Len000AGAAGC TAATTAAACC AAACATGCAC GTATGTCCTA ATTAACATGA CTGTCCATCA   
  
  
+ TCTCCTCCCA TGTTGTTCTG TTGACAAGCC TGCACACCCA TGCTCCTCTC TCATGTCTAA CCTCGTCCCA   
  
  
+ ACGATCAAGA TCACTGTCTG TGAGGCCAGG GGAAGACTTG CTTTTATTTC CTTTTCTTTT GTTTACTCTC   
  
  
+ TGATTCCATT TAGCCATATA TACAAGGAGA ATGTCATGTG TATCTCATAT GTATATATAT AAGATTTTAT   
  
  
+ TTAGAAATAA AAATTTAAAC ACTATGTGAT TTATAGTTAT TATAATAAAT AGTATTTTTT AAACTATTTC   
  
  
+ ATTGATGAGA ACAGGAAAGT AAATTGCCGA CATGTTAATG TTATTATATC TAAAAATTAA ATCATAAAAT   
  
  
+ TAATTATAAT ATTTTAAAAA TATTTCAACT AAAATTTTAG AACAATAAGT GCGTAATACG GACTTAAAGG   
  
  
+ CTAGTTTATC GTAAAAATGT AAGTTCCTTT TGGATCTTAT GGTTGAATAT CTTGGTTGAT AAACTGTGAT   
  
  
+ TGGTTTTACA CTTTTACCAC ATATTCATAG AAGAATTATG TGCACGTTGA TGGTTCAAGA TGGGACAAAA   
  
  
+ GGAAACATGT ATTCTTCCCC CTTTTGCCTT TTCCCCTAAG CATTTGGATT GAGGTGTTGT CATCTGAATA   
  
  
+ TCAAAAATTC TTTTTGAAAA TTCAGTCTCA ATCAAAATTC AAAATTAAAA CAAAGACTTA AGAGTTTTAT   
  
  
+ TTGTTCTTAA CCTTCTTAAC TTTCTACTTT TCTTTTTTTT CTCATTCCCC CTCTTCAAAT CTCATTTATT   
  
  
+ CTTAACCCCC TCTTCCGTTC ATGCTCTCTC TGTCTATCAA CTAATATTAA TCTACCCCGA CTTTCAATTT   
  
  
+ GATGAGGTCT AATCCTTATC ATCGTATTGT GGTTGGGTTA ATTATGCAAG AAGGCAATAA ACCCCAGTGA   
  
  
+ CAAAGTCCAC GTTAAGTAGG CACCTCACCA TTAAGACATG CTCAGAAAAC ACCAAAACCA TTGAACACAA   
  
  
+ GTCCCCCAAG TCCCTTCCCT AGCTAGCCCA TTCTCTCTCC TCCCCTGTCT ATATCTACCA ACTCTTGCTC   
  
  
+ TTTGCTGAGC CTGAGTTGAA AGCGATACAC CCATAGCCTG TTCAGTTTTC AGTTTTCACA TTTGTCTTCC   
  
  
+ TCTGTTCAGT TGAAAGCGAT ACACTCTCAT TAGCTTTCAT TTTAAATACG TTCAATTCAC ACATAAATGG   
  
  
+ CTTTCAAAGC ATTGCCATTT TCGTTTAAAT GATGACAAAA TATATAGTAT CTGGCTATCT GCCCATATCT   
  
  
+ TGACCTTATT TACAAAGGCT GAGCCATCTT TTAATTTTTT TAGCCTCTTT TTTTAATCAA AAATAGAAAT   
  
  
+ TTCAAGAAAG CAGAAATCGT CAGATGACAG TGCAGACAGC GTTTTCGTTT CATACAAATC TCAAAAGCTG   
  
  
+ AAGTGTCAAT TTCTTCGATT ATTTTTGTCA GAAAATATTT ACGTCTCACC TTCACGTTGT TATTATTCTT   
  
  
+ TCTTTTTTAT TATTCTCATA ACTAGTAATA AAGTTAGCAA TGATAAAAAA TTATTCGATC CGAATATTTT   
  
  
+ AATTTATCTG ACCTAAAAAC ATAAGTAAAG GCACAAATTT TTCATCCAAA TTTTAATTTT TGATGTAATA   
  
  
+ TTTTTTATAT TTTTATTGTT CAAATCTGAT TTTAATCTTA TTTAAATTAT CTGACCTAAA AAAATCAAAT   
  
  
+ AATAATAAAC GTTAATTTTT TATTTAAATT TTGACATTAG TCAATCTGAC TTAAATTCGA ACCGAGCTTG   
  
  
+ AATTTTTTTG CCAGTGCTGG TAGTACTCCT GCTTTTGTAG CTCCCAATGC CATCGTACCA CCCTCTTCTA   
  
  
+ GTCTCGTCTC TTTCTCTGGT CTCCAATTAA TCACACCATC ATATCATACC GTATGATGAT ACAGTCCACT   
  
  
+ GAAATCCCAT TCTCACCGCC AATCTCTCTC CGCCACCATC CAACACTCTT AACGATGAAC AGGGCCGCCG   
  
  
+ CCTCGTCCTC CACACTCAGG CCGTGGCCGG GCAGCTTTCC CACCCAATCA AAATCTCTCT CCTCCGCCAA   
  
  
+ CTTCGGTAAC GCCAATTGCA TGGAGCAGCT CTTAGTCCAC TGCGCCGAAG CCATCGACAA CAATGACGCC   
  
  
+ ACCCCGGCCC AGCAAATCTT ATGGGTCCTG AATAACATAG CCCGACCCGA CGGCGACTCC ACCCAACGCC   
  
  
+ TCACGTGCGC ATTCCTACGT GCCTTAATCT CACGCGCCGT CCTCACTAGC ACCTGCAAGA TGGTAATCCC   
  
  
+ TCATTTCAAC CCCATCAATT CACCCCACAA ATTCTCGCTC CTCGAACTTG CCCACTTCGT CGATTTAACC   
  
  
+ CCTTGGCATC GATTCGGATT CACCGCCGCC AATTCGATCA TTCTGGAAGC TATTTCCGAC CTACCCGTTG   
  
  
+ TACACATTGT CGACCTCAGC ATCTCCCACT GTATGCAGAT CCCCACGTTG ATCGACTCCA TTGCGACCCG   
  
  
+ GTTGGAAGCC CCGGGTCGAG TCCCTCCTAT TGTCAAGCTC ACCGTCGGGG CTATTTCCGA CGAAATCCCG   
  
  
+ CCGGTGTTCG ATCTTCTGTC GTACGATGAG CTCGGAATGA GACTAATCAA CTTCGCTCGT TTTAGAAACA   
  
  
+ TCGTCCTCGA ATTCCAAGCA ATACCCACCT CCCCTTCCGA CGGATTTGCT TCGCTGTTGG AGGAGATTCG   
  
  
+ ACAAAGCAAG CTCTACTCCA ACGATGCGGC GGCGGTTATT GTGAATTGTC AGATGAGTTT GCATTTATTG   
  
  
+ CAGGAAGAGG AGGTGTCTTC ATCGTCGCCG TCGTCGATGA GGGGGATGTT TTTGCAGGCG GTGAGGAGCT   
  
  
+ TGGAGCCGAG CATGGTGGTG GTGGTGGAGG AGGACGTGGA TTTCACGGCG AGGAGTCTGG TGGGGCGGCT   
  
  
+ GAGATCGGCG TTTAATCACA TGTGGATACC CTTCGACACG GTGGACACGT TCTTGCCACG TGGGAGCCAG   
  
  
+ CAGAGAGAGT GGTTCGAGGC CGAGGTGTGC TGGAAGATTG AGAATGTGAT CGCTCATGAG GGACCCGCGA   
  
  
+ GGGTCGAGAG GCAGGAGCCC AGGGCCAAGT GGGCCCTCCG AATGAGGGAG GCCGAGTTTC AAGGGATCGA   
  
  
+ GTTCGGTGAT GAAGGTACGA CCGAGGTCAA GGCCATGCTG GAGGAGCATG CCGCTGGGTG GGGGTCTAAG   
  
  
+ AAGGAAGAGG ATGATCTTGT GCTCACTTGG AAGGGACATA GTGTTGTCTT TGCTTCTGCT TGGGTACCCA   
  
  
+ CTTA  

- +Up\_Stream \_Len000TCTTCG ATTAATTTGG TTTGTACGTG CATACAGGAT TAATTGTACT GACAGGTAGT   
  
  
- AGAGGAGGGT ACAACAAGAC AACTGTTCGG ACGTGTGGGT ACGAGGAGAG AGTACAGATT GGAGCAGGGT   
  
  
- TGCTAGTTCT AGTGACAGAC ACTCCGGTCC CCTTCTGAAC GAAAATAAAG GAAAAGAAAA CAAATGAGAG   
  
  
- ACTAAGGTAA ATCGGTATAT ATGTTCCTCT TACAGTACAC ATAGAGTATA CATATATATA TTCTAAAATA   
  
  
- AATCTTTATT TTTAAATTTG TGATACACTA AATATCAATA ATATTATTTA TCATAAAAAA TTTGATAAAG   
  
  
- TAACTACTCT TGTCCTTTCA TTTAACGGCT GTACAATTAC AATAATATAG ATTTTTAATT TAGTATTTTA   
  
  
- ATTAATATTA TAAAATTTTT ATAAAGTTGA TTTTAAAATC TTGTTATTCA CGCATTATGC CTGAATTTCC   
  
  
- GATCAAATAG CATTTTTACA TTCAAGGAAA ACCTAGAATA CCAACTTATA GAACCAACTA TTTGACACTA   
  
  
- ACCAAAATGT GAAAATGGTG TATAAGTATC TTCTTAATAC ACGTGCAACT ACCAAGTTCT ACCCTGTTTT   
  
  
- CCTTTGTACA TAAGAAGGGG GAAAACGGAA AAGGGGATTC GTAAACCTAA CTCCACAACA GTAGACTTAT   
  
  
- AGTTTTTAAG AAAAACTTTT AAGTCAGAGT TAGTTTTAAG TTTTAATTTT GTTTCTGAAT TCTCAAAATA   
  
  
- AACAAGAATT GGAAGAATTG AAAGATGAAA AGAAAAAAAA GAGTAAGGGG GAGAAGTTTA GAGTAAATAA   
  
  
- GAATTGGGGG AGAAGGCAAG TACGAGAGAG ACAGATAGTT GATTATAATT AGATGGGGCT GAAAGTTAAA   
  
  
- CTACTCCAGA TTAGGAATAG TAGCATAACA CCAACCCAAT TAATACGTTC TTCCGTTATT TGGGGTCACT   
  
  
- GTTTCAGGTG CAATTCATCC GTGGAGTGGT AATTCTGTAC GAGTCTTTTG TGGTTTTGGT AACTTGTGTT   
  
  
- CAGGGGGTTC AGGGAAGGGA TCGATCGGGT AAGAGAGAGG AGGGGACAGA TATAGATGGT TGAGAACGAG   
  
  
- AAACGACTCG GACTCAACTT TCGCTATGTG GGTATCGGAC AAGTCAAAAG TCAAAAGTGT AAACAGAAGG   
  
  
- AGACAAGTCA ACTTTCGCTA TGTGAGAGTA ATCGAAAGTA AAATTTATGC AAGTTAAGTG TGTATTTACC   
  
  
- GAAAGTTTCG TAACGGTAAA AGCAAATTTA CTACTGTTTT ATATATCATA GACCGATAGA CGGGTATAGA   
  
  
- ACTGGAATAA ATGTTTCCGA CTCGGTAGAA AATTAAAAAA ATCGGAGAAA AAAATTAGTT TTTATCTTTA   
  
  
- AAGTTCTTTC GTCTTTAGCA GTCTACTGTC ACGTCTGTCG CAAAAGCAAA GTATGTTTAG AGTTTTCGAC   
  
  
- TTCACAGTTA AAGAAGCTAA TAAAAACAGT CTTTTATAAA TGCAGAGTGG AAGTGCAACA ATAATAAGAA   
  
  
- AGAAAAAATA ATAAGAGTAT TGATCATTAT TTCAATCGTT ACTATTTTTT AATAAGCTAG GCTTATAAAA   
  
  
- TTAAATAGAC TGGATTTTTG TATTCATTTC CGTGTTTAAA AAGTAGGTTT AAAATTAAAA ACTACATTAT   
  
  
- AAAAAATATA AAAATAACAA GTTTAGACTA AAATTAGAAT AAATTTAATA GACTGGATTT TTTTAGTTTA   
  
  
- TTATTATTTG CAATTAAAAA ATAAATTTAA AACTGTAATC AGTTAGACTG AATTTAAGCT TGGCTCGAAC   
  
  
- TTAAAAAAAC GGTCACGACC ATCATGAGGA CGAAAACATC GAGGGTTACG GTAGCATGGT GGGAGAAGAT   
  
  
- CAGAGCAGAG AAAGAGACCA GAGGTTAATT AGTGTGGTAG TATAGTATGG CATACTACTA TGTCAGGTGA   
  
  
- CTTTAGGGTA AGAGTGGCGG TTAGAGAGAG GCGGTGGTAG GTTGTGAGAA TTGCTACTTG TCCCGGCGGC   
  
  
- GGAGCAGGAG GTGTGAGTCC GGCACCGGCC CGTCGAAAGG GTGGGTTAGT TTTAGAGAGA GGAGGCGGTT   
  
  
- GAAGCCATTG CGGTTAACGT ACCTCGTCGA GAATCAGGTG ACGCGGCTTC GGTAGCTGTT GTTACTGCGG   
  
  
- TGGGGCCGGG TCGTTTAGAA TACCCAGGAC TTATTGTATC GGGCTGGGCT GCCGCTGAGG TGGGTTGCGG   
  
  
- AGTGCACGCG TAAGGATGCA CGGAATTAGA GTGCGCGGCA GGAGTGATCG TGGACGTTCT ACCATTAGGG   
  
  
- AGTAAAGTTG GGGTAGTTAA GTGGGGTGTT TAAGAGCGAG GAGCTTGAAC GGGTGAAGCA GCTAAATTGG   
  
  
- GGAACCGTAG CTAAGCCTAA GTGGCGGCGG TTAAGCTAGT AAGACCTTCG ATAAAGGCTG GATGGGCAAC   
  
  
- ATGTGTAACA GCTGGAGTCG TAGAGGGTGA CATACGTCTA GGGGTGCAAC TAGCTGAGGT AACGCTGGGC   
  
  
- CAACCTTCGG GGCCCAGCTC AGGGAGGATA ACAGTTCGAG TGGCAGCCCC GATAAAGGCT GCTTTAGGGC   
  
  
- GGCCACAAGC TAGAAGACAG CATGCTACTC GAGCCTTACT CTGATTAGTT GAAGCGAGCA AAATCTTTGT   
  
  
- AGCAGGAGCT TAAGGTTCGT TATGGGTGGA GGGGAAGGCT GCCTAAACGA AGCGACAACC TCCTCTAAGC   
  
  
- TGTTTCGTTC GAGATGAGGT TGCTACGCCG CCGCCAATAA CACTTAACAG TCTACTCAAA CGTAAATAAC   
  
  
- GTCCTTCTCC TCCACAGAAG TAGCAGCGGC AGCAGCTACT CCCCCTACAA AAACGTCCGC CACTCCTCGA   
  
  
- ACCTCGGCTC GTACCACCAC CACCACCTCC TCCTGCACCT AAAGTGCCGC TCCTCAGACC ACCCCGCCGA   
  
  
- CTCTAGCCGC AAATTAGTGT ACACCTATGG GAAGCTGTGC CACCTGTGCA AGAACGGTGC ACCCTCGGTC   
  
  
- GTCTCTCTCA CCAAGCTCCG GCTCCACACG ACCTTCTAAC TCTTACACTA GCGAGTACTC CCTGGGCGCT   
  
  
- CCCAGCTCTC CGTCCTCGGG TCCCGGTTCA CCCGGGAGGC TTACTCCCTC CGGCTCAAAG TTCCCTAGCT   
  
  
- CAAGCCACTA CTTCCATGCT GGCTCCAGTT CCGGTACGAC CTCCTCGTAC GGCGACCCAC CCCCAGATTC   
  
  
- TTCCTTCTCC TACTAGAACA CGAGTGAACC TTCCCTGTAT CACAACAGAA ACGAAGACGA ACCCATGGGT   
  
  
- GAAT

+     AT~TATA-box

| Site Name | Organism | Position | Strand | Matrix score. | sequence | function |
| --- | --- | --- | --- | --- | --- | --- |
| AT~TATA-box | Arabidopsis thaliana | 266 | + | 6 | TATATA |  |
| AT~TATA-box | Arabidopsis thaliana | 268 | + | 6 | TATATA |  |
| AT~TATA-box | Arabidopsis thaliana | 270 | + | 6 | TATATA |  |
| AT~TATA-box | Arabidopsis thaliana | 1305 | + | 6 | TATATA |  |
| AT~TATA-box | Arabidopsis thaliana | 231 | + | 6 | TATATA |  |

>HU06G00376.1   
+ +Up\_Stream \_Len000AGAAGC TAATTAAACC AAACATGCAC GTATGTCCTA ATTAACATGA CTGTCCATCA   
  
  
+ TCTCCTCCCA TGTTGTTCTG TTGACAAGCC TGCACACCCA TGCTCCTCTC TCATGTCTAA CCTCGTCCCA   
  
  
+ ACGATCAAGA TCACTGTCTG TGAGGCCAGG GGAAGACTTG CTTTTATTTC CTTTTCTTTT GTTTACTCTC   
  
  
+ TGATTCCATT TAGCCATATA TACAAGGAGA ATGTCATGTG TATCTCATAT GTATATATAT AAGATTTTAT   
  
  
+ TTAGAAATAA AAATTTAAAC ACTATGTGAT TTATAGTTAT TATAATAAAT AGTATTTTTT AAACTATTTC   
  
  
+ ATTGATGAGA ACAGGAAAGT AAATTGCCGA CATGTTAATG TTATTATATC TAAAAATTAA ATCATAAAAT   
  
  
+ TAATTATAAT ATTTTAAAAA TATTTCAACT AAAATTTTAG AACAATAAGT GCGTAATACG GACTTAAAGG   
  
  
+ CTAGTTTATC GTAAAAATGT AAGTTCCTTT TGGATCTTAT GGTTGAATAT CTTGGTTGAT AAACTGTGAT   
  
  
+ TGGTTTTACA CTTTTACCAC ATATTCATAG AAGAATTATG TGCACGTTGA TGGTTCAAGA TGGGACAAAA   
  
  
+ GGAAACATGT ATTCTTCCCC CTTTTGCCTT TTCCCCTAAG CATTTGGATT GAGGTGTTGT CATCTGAATA   
  
  
+ TCAAAAATTC TTTTTGAAAA TTCAGTCTCA ATCAAAATTC AAAATTAAAA CAAAGACTTA AGAGTTTTAT   
  
  
+ TTGTTCTTAA CCTTCTTAAC TTTCTACTTT TCTTTTTTTT CTCATTCCCC CTCTTCAAAT CTCATTTATT   
  
  
+ CTTAACCCCC TCTTCCGTTC ATGCTCTCTC TGTCTATCAA CTAATATTAA TCTACCCCGA CTTTCAATTT   
  
  
+ GATGAGGTCT AATCCTTATC ATCGTATTGT GGTTGGGTTA ATTATGCAAG AAGGCAATAA ACCCCAGTGA   
  
  
+ CAAAGTCCAC GTTAAGTAGG CACCTCACCA TTAAGACATG CTCAGAAAAC ACCAAAACCA TTGAACACAA   
  
  
+ GTCCCCCAAG TCCCTTCCCT AGCTAGCCCA TTCTCTCTCC TCCCCTGTCT ATATCTACCA ACTCTTGCTC   
  
  
+ TTTGCTGAGC CTGAGTTGAA AGCGATACAC CCATAGCCTG TTCAGTTTTC AGTTTTCACA TTTGTCTTCC   
  
  
+ TCTGTTCAGT TGAAAGCGAT ACACTCTCAT TAGCTTTCAT TTTAAATACG TTCAATTCAC ACATAAATGG   
  
  
+ CTTTCAAAGC ATTGCCATTT TCGTTTAAAT GATGACAAAA TATATAGTAT CTGGCTATCT GCCCATATCT   
  
  
+ TGACCTTATT TACAAAGGCT GAGCCATCTT TTAATTTTTT TAGCCTCTTT TTTTAATCAA AAATAGAAAT   
  
  
+ TTCAAGAAAG CAGAAATCGT CAGATGACAG TGCAGACAGC GTTTTCGTTT CATACAAATC TCAAAAGCTG   
  
  
+ AAGTGTCAAT TTCTTCGATT ATTTTTGTCA GAAAATATTT ACGTCTCACC TTCACGTTGT TATTATTCTT   
  
  
+ TCTTTTTTAT TATTCTCATA ACTAGTAATA AAGTTAGCAA TGATAAAAAA TTATTCGATC CGAATATTTT   
  
  
+ AATTTATCTG ACCTAAAAAC ATAAGTAAAG GCACAAATTT TTCATCCAAA TTTTAATTTT TGATGTAATA   
  
  
+ TTTTTTATAT TTTTATTGTT CAAATCTGAT TTTAATCTTA TTTAAATTAT CTGACCTAAA AAAATCAAAT   
  
  
+ AATAATAAAC GTTAATTTTT TATTTAAATT TTGACATTAG TCAATCTGAC TTAAATTCGA ACCGAGCTTG   
  
  
+ AATTTTTTTG CCAGTGCTGG TAGTACTCCT GCTTTTGTAG CTCCCAATGC CATCGTACCA CCCTCTTCTA   
  
  
+ GTCTCGTCTC TTTCTCTGGT CTCCAATTAA TCACACCATC ATATCATACC GTATGATGAT ACAGTCCACT   
  
  
+ GAAATCCCAT TCTCACCGCC AATCTCTCTC CGCCACCATC CAACACTCTT AACGATGAAC AGGGCCGCCG   
  
  
+ CCTCGTCCTC CACACTCAGG CCGTGGCCGG GCAGCTTTCC CACCCAATCA AAATCTCTCT CCTCCGCCAA   
  
  
+ CTTCGGTAAC GCCAATTGCA TGGAGCAGCT CTTAGTCCAC TGCGCCGAAG CCATCGACAA CAATGACGCC   
  
  
+ ACCCCGGCCC AGCAAATCTT ATGGGTCCTG AATAACATAG CCCGACCCGA CGGCGACTCC ACCCAACGCC   
  
  
+ TCACGTGCGC ATTCCTACGT GCCTTAATCT CACGCGCCGT CCTCACTAGC ACCTGCAAGA TGGTAATCCC   
  
  
+ TCATTTCAAC CCCATCAATT CACCCCACAA ATTCTCGCTC CTCGAACTTG CCCACTTCGT CGATTTAACC   
  
  
+ CCTTGGCATC GATTCGGATT CACCGCCGCC AATTCGATCA TTCTGGAAGC TATTTCCGAC CTACCCGTTG   
  
  
+ TACACATTGT CGACCTCAGC ATCTCCCACT GTATGCAGAT CCCCACGTTG ATCGACTCCA TTGCGACCCG   
  
  
+ GTTGGAAGCC CCGGGTCGAG TCCCTCCTAT TGTCAAGCTC ACCGTCGGGG CTATTTCCGA CGAAATCCCG   
  
  
+ CCGGTGTTCG ATCTTCTGTC GTACGATGAG CTCGGAATGA GACTAATCAA CTTCGCTCGT TTTAGAAACA   
  
  
+ TCGTCCTCGA ATTCCAAGCA ATACCCACCT CCCCTTCCGA CGGATTTGCT TCGCTGTTGG AGGAGATTCG   
  
  
+ ACAAAGCAAG CTCTACTCCA ACGATGCGGC GGCGGTTATT GTGAATTGTC AGATGAGTTT GCATTTATTG   
  
  
+ CAGGAAGAGG AGGTGTCTTC ATCGTCGCCG TCGTCGATGA GGGGGATGTT TTTGCAGGCG GTGAGGAGCT   
  
  
+ TGGAGCCGAG CATGGTGGTG GTGGTGGAGG AGGACGTGGA TTTCACGGCG AGGAGTCTGG TGGGGCGGCT   
  
  
+ GAGATCGGCG TTTAATCACA TGTGGATACC CTTCGACACG GTGGACACGT TCTTGCCACG TGGGAGCCAG   
  
  
+ CAGAGAGAGT GGTTCGAGGC CGAGGTGTGC TGGAAGATTG AGAATGTGAT CGCTCATGAG GGACCCGCGA   
  
  
+ GGGTCGAGAG GCAGGAGCCC AGGGCCAAGT GGGCCCTCCG AATGAGGGAG GCCGAGTTTC AAGGGATCGA   
  
  
+ GTTCGGTGAT GAAGGTACGA CCGAGGTCAA GGCCATGCTG GAGGAGCATG CCGCTGGGTG GGGGTCTAAG   
  
  
+ AAGGAAGAGG ATGATCTTGT GCTCACTTGG AAGGGACATA GTGTTGTCTT TGCTTCTGCT TGGGTACCCA   
  
  
+ CTTA  

- +Up\_Stream \_Len000TCTTCG ATTAATTTGG TTTGTACGTG CATACAGGAT TAATTGTACT GACAGGTAGT   
  
  
- AGAGGAGGGT ACAACAAGAC AACTGTTCGG ACGTGTGGGT ACGAGGAGAG AGTACAGATT GGAGCAGGGT   
  
  
- TGCTAGTTCT AGTGACAGAC ACTCCGGTCC CCTTCTGAAC GAAAATAAAG GAAAAGAAAA CAAATGAGAG   
  
  
- ACTAAGGTAA ATCGGTATAT ATGTTCCTCT TACAGTACAC ATAGAGTATA CATATATATA TTCTAAAATA   
  
  
- AATCTTTATT TTTAAATTTG TGATACACTA AATATCAATA ATATTATTTA TCATAAAAAA TTTGATAAAG   
  
  
- TAACTACTCT TGTCCTTTCA TTTAACGGCT GTACAATTAC AATAATATAG ATTTTTAATT TAGTATTTTA   
  
  
- ATTAATATTA TAAAATTTTT ATAAAGTTGA TTTTAAAATC TTGTTATTCA CGCATTATGC CTGAATTTCC   
  
  
- GATCAAATAG CATTTTTACA TTCAAGGAAA ACCTAGAATA CCAACTTATA GAACCAACTA TTTGACACTA   
  
  
- ACCAAAATGT GAAAATGGTG TATAAGTATC TTCTTAATAC ACGTGCAACT ACCAAGTTCT ACCCTGTTTT   
  
  
- CCTTTGTACA TAAGAAGGGG GAAAACGGAA AAGGGGATTC GTAAACCTAA CTCCACAACA GTAGACTTAT   
  
  
- AGTTTTTAAG AAAAACTTTT AAGTCAGAGT TAGTTTTAAG TTTTAATTTT GTTTCTGAAT TCTCAAAATA   
  
  
- AACAAGAATT GGAAGAATTG AAAGATGAAA AGAAAAAAAA GAGTAAGGGG GAGAAGTTTA GAGTAAATAA   
  
  
- GAATTGGGGG AGAAGGCAAG TACGAGAGAG ACAGATAGTT GATTATAATT AGATGGGGCT GAAAGTTAAA   
  
  
- CTACTCCAGA TTAGGAATAG TAGCATAACA CCAACCCAAT TAATACGTTC TTCCGTTATT TGGGGTCACT   
  
  
- GTTTCAGGTG CAATTCATCC GTGGAGTGGT AATTCTGTAC GAGTCTTTTG TGGTTTTGGT AACTTGTGTT   
  
  
- CAGGGGGTTC AGGGAAGGGA TCGATCGGGT AAGAGAGAGG AGGGGACAGA TATAGATGGT TGAGAACGAG   
  
  
- AAACGACTCG GACTCAACTT TCGCTATGTG GGTATCGGAC AAGTCAAAAG TCAAAAGTGT AAACAGAAGG   
  
  
- AGACAAGTCA ACTTTCGCTA TGTGAGAGTA ATCGAAAGTA AAATTTATGC AAGTTAAGTG TGTATTTACC   
  
  
- GAAAGTTTCG TAACGGTAAA AGCAAATTTA CTACTGTTTT ATATATCATA GACCGATAGA CGGGTATAGA   
  
  
- ACTGGAATAA ATGTTTCCGA CTCGGTAGAA AATTAAAAAA ATCGGAGAAA AAAATTAGTT TTTATCTTTA   
  
  
- AAGTTCTTTC GTCTTTAGCA GTCTACTGTC ACGTCTGTCG CAAAAGCAAA GTATGTTTAG AGTTTTCGAC   
  
  
- TTCACAGTTA AAGAAGCTAA TAAAAACAGT CTTTTATAAA TGCAGAGTGG AAGTGCAACA ATAATAAGAA   
  
  
- AGAAAAAATA ATAAGAGTAT TGATCATTAT TTCAATCGTT ACTATTTTTT AATAAGCTAG GCTTATAAAA   
  
  
- TTAAATAGAC TGGATTTTTG TATTCATTTC CGTGTTTAAA AAGTAGGTTT AAAATTAAAA ACTACATTAT   
  
  
- AAAAAATATA AAAATAACAA GTTTAGACTA AAATTAGAAT AAATTTAATA GACTGGATTT TTTTAGTTTA   
  
  
- TTATTATTTG CAATTAAAAA ATAAATTTAA AACTGTAATC AGTTAGACTG AATTTAAGCT TGGCTCGAAC   
  
  
- TTAAAAAAAC GGTCACGACC ATCATGAGGA CGAAAACATC GAGGGTTACG GTAGCATGGT GGGAGAAGAT   
  
  
- CAGAGCAGAG AAAGAGACCA GAGGTTAATT AGTGTGGTAG TATAGTATGG CATACTACTA TGTCAGGTGA   
  
  
- CTTTAGGGTA AGAGTGGCGG TTAGAGAGAG GCGGTGGTAG GTTGTGAGAA TTGCTACTTG TCCCGGCGGC   
  
  
- GGAGCAGGAG GTGTGAGTCC GGCACCGGCC CGTCGAAAGG GTGGGTTAGT TTTAGAGAGA GGAGGCGGTT   
  
  
- GAAGCCATTG CGGTTAACGT ACCTCGTCGA GAATCAGGTG ACGCGGCTTC GGTAGCTGTT GTTACTGCGG   
  
  
- TGGGGCCGGG TCGTTTAGAA TACCCAGGAC TTATTGTATC GGGCTGGGCT GCCGCTGAGG TGGGTTGCGG   
  
  
- AGTGCACGCG TAAGGATGCA CGGAATTAGA GTGCGCGGCA GGAGTGATCG TGGACGTTCT ACCATTAGGG   
  
  
- AGTAAAGTTG GGGTAGTTAA GTGGGGTGTT TAAGAGCGAG GAGCTTGAAC GGGTGAAGCA GCTAAATTGG   
  
  
- GGAACCGTAG CTAAGCCTAA GTGGCGGCGG TTAAGCTAGT AAGACCTTCG ATAAAGGCTG GATGGGCAAC   
  
  
- ATGTGTAACA GCTGGAGTCG TAGAGGGTGA CATACGTCTA GGGGTGCAAC TAGCTGAGGT AACGCTGGGC   
  
  
- CAACCTTCGG GGCCCAGCTC AGGGAGGATA ACAGTTCGAG TGGCAGCCCC GATAAAGGCT GCTTTAGGGC   
  
  
- GGCCACAAGC TAGAAGACAG CATGCTACTC GAGCCTTACT CTGATTAGTT GAAGCGAGCA AAATCTTTGT   
  
  
- AGCAGGAGCT TAAGGTTCGT TATGGGTGGA GGGGAAGGCT GCCTAAACGA AGCGACAACC TCCTCTAAGC   
  
  
- TGTTTCGTTC GAGATGAGGT TGCTACGCCG CCGCCAATAA CACTTAACAG TCTACTCAAA CGTAAATAAC   
  
  
- GTCCTTCTCC TCCACAGAAG TAGCAGCGGC AGCAGCTACT CCCCCTACAA AAACGTCCGC CACTCCTCGA   
  
  
- ACCTCGGCTC GTACCACCAC CACCACCTCC TCCTGCACCT AAAGTGCCGC TCCTCAGACC ACCCCGCCGA   
  
  
- CTCTAGCCGC AAATTAGTGT ACACCTATGG GAAGCTGTGC CACCTGTGCA AGAACGGTGC ACCCTCGGTC   
  
  
- GTCTCTCTCA CCAAGCTCCG GCTCCACACG ACCTTCTAAC TCTTACACTA GCGAGTACTC CCTGGGCGCT   
  
  
- CCCAGCTCTC CGTCCTCGGG TCCCGGTTCA CCCGGGAGGC TTACTCCCTC CGGCTCAAAG TTCCCTAGCT   
  
  
- CAAGCCACTA CTTCCATGCT GGCTCCAGTT CCGGTACGAC CTCCTCGTAC GGCGACCCAC CCCCAGATTC   
  
  
- TTCCTTCTCC TACTAGAACA CGAGTGAACC TTCCCTGTAT CACAACAGAA ACGAAGACGA ACCCATGGGT   
  
  
- GAAT

+     Box 4

| Site Name | Organism | Position | Strand | Matrix score. | sequence | function |
| --- | --- | --- | --- | --- | --- | --- |
| Box 4 | Petroselinum crispum | 1920 | - | 6 | ATTAAT | part of a conserved DNA module involved in light responsiveness |
| Box 4 | Petroselinum crispum | 890 | + | 6 | ATTAAT | part of a conserved DNA module involved in light responsiveness |
| Box 4 | Petroselinum crispum | 423 | + | 6 | ATTAAT | part of a conserved DNA module involved in light responsiveness |

>HU06G00376.1   
+ +Up\_Stream \_Len000AGAAGC TAATTAAACC AAACATGCAC GTATGTCCTA ATTAACATGA CTGTCCATCA   
  
  
+ TCTCCTCCCA TGTTGTTCTG TTGACAAGCC TGCACACCCA TGCTCCTCTC TCATGTCTAA CCTCGTCCCA   
  
  
+ ACGATCAAGA TCACTGTCTG TGAGGCCAGG GGAAGACTTG CTTTTATTTC CTTTTCTTTT GTTTACTCTC   
  
  
+ TGATTCCATT TAGCCATATA TACAAGGAGA ATGTCATGTG TATCTCATAT GTATATATAT AAGATTTTAT   
  
  
+ TTAGAAATAA AAATTTAAAC ACTATGTGAT TTATAGTTAT TATAATAAAT AGTATTTTTT AAACTATTTC   
  
  
+ ATTGATGAGA ACAGGAAAGT AAATTGCCGA CATGTTAATG TTATTATATC TAAAAATTAA ATCATAAAAT   
  
  
+ TAATTATAAT ATTTTAAAAA TATTTCAACT AAAATTTTAG AACAATAAGT GCGTAATACG GACTTAAAGG   
  
  
+ CTAGTTTATC GTAAAAATGT AAGTTCCTTT TGGATCTTAT GGTTGAATAT CTTGGTTGAT AAACTGTGAT   
  
  
+ TGGTTTTACA CTTTTACCAC ATATTCATAG AAGAATTATG TGCACGTTGA TGGTTCAAGA TGGGACAAAA   
  
  
+ GGAAACATGT ATTCTTCCCC CTTTTGCCTT TTCCCCTAAG CATTTGGATT GAGGTGTTGT CATCTGAATA   
  
  
+ TCAAAAATTC TTTTTGAAAA TTCAGTCTCA ATCAAAATTC AAAATTAAAA CAAAGACTTA AGAGTTTTAT   
  
  
+ TTGTTCTTAA CCTTCTTAAC TTTCTACTTT TCTTTTTTTT CTCATTCCCC CTCTTCAAAT CTCATTTATT   
  
  
+ CTTAACCCCC TCTTCCGTTC ATGCTCTCTC TGTCTATCAA CTAATATTAA TCTACCCCGA CTTTCAATTT   
  
  
+ GATGAGGTCT AATCCTTATC ATCGTATTGT GGTTGGGTTA ATTATGCAAG AAGGCAATAA ACCCCAGTGA   
  
  
+ CAAAGTCCAC GTTAAGTAGG CACCTCACCA TTAAGACATG CTCAGAAAAC ACCAAAACCA TTGAACACAA   
  
  
+ GTCCCCCAAG TCCCTTCCCT AGCTAGCCCA TTCTCTCTCC TCCCCTGTCT ATATCTACCA ACTCTTGCTC   
  
  
+ TTTGCTGAGC CTGAGTTGAA AGCGATACAC CCATAGCCTG TTCAGTTTTC AGTTTTCACA TTTGTCTTCC   
  
  
+ TCTGTTCAGT TGAAAGCGAT ACACTCTCAT TAGCTTTCAT TTTAAATACG TTCAATTCAC ACATAAATGG   
  
  
+ CTTTCAAAGC ATTGCCATTT TCGTTTAAAT GATGACAAAA TATATAGTAT CTGGCTATCT GCCCATATCT   
  
  
+ TGACCTTATT TACAAAGGCT GAGCCATCTT TTAATTTTTT TAGCCTCTTT TTTTAATCAA AAATAGAAAT   
  
  
+ TTCAAGAAAG CAGAAATCGT CAGATGACAG TGCAGACAGC GTTTTCGTTT CATACAAATC TCAAAAGCTG   
  
  
+ AAGTGTCAAT TTCTTCGATT ATTTTTGTCA GAAAATATTT ACGTCTCACC TTCACGTTGT TATTATTCTT   
  
  
+ TCTTTTTTAT TATTCTCATA ACTAGTAATA AAGTTAGCAA TGATAAAAAA TTATTCGATC CGAATATTTT   
  
  
+ AATTTATCTG ACCTAAAAAC ATAAGTAAAG GCACAAATTT TTCATCCAAA TTTTAATTTT TGATGTAATA   
  
  
+ TTTTTTATAT TTTTATTGTT CAAATCTGAT TTTAATCTTA TTTAAATTAT CTGACCTAAA AAAATCAAAT   
  
  
+ AATAATAAAC GTTAATTTTT TATTTAAATT TTGACATTAG TCAATCTGAC TTAAATTCGA ACCGAGCTTG   
  
  
+ AATTTTTTTG CCAGTGCTGG TAGTACTCCT GCTTTTGTAG CTCCCAATGC CATCGTACCA CCCTCTTCTA   
  
  
+ GTCTCGTCTC TTTCTCTGGT CTCCAATTAA TCACACCATC ATATCATACC GTATGATGAT ACAGTCCACT   
  
  
+ GAAATCCCAT TCTCACCGCC AATCTCTCTC CGCCACCATC CAACACTCTT AACGATGAAC AGGGCCGCCG   
  
  
+ CCTCGTCCTC CACACTCAGG CCGTGGCCGG GCAGCTTTCC CACCCAATCA AAATCTCTCT CCTCCGCCAA   
  
  
+ CTTCGGTAAC GCCAATTGCA TGGAGCAGCT CTTAGTCCAC TGCGCCGAAG CCATCGACAA CAATGACGCC   
  
  
+ ACCCCGGCCC AGCAAATCTT ATGGGTCCTG AATAACATAG CCCGACCCGA CGGCGACTCC ACCCAACGCC   
  
  
+ TCACGTGCGC ATTCCTACGT GCCTTAATCT CACGCGCCGT CCTCACTAGC ACCTGCAAGA TGGTAATCCC   
  
  
+ TCATTTCAAC CCCATCAATT CACCCCACAA ATTCTCGCTC CTCGAACTTG CCCACTTCGT CGATTTAACC   
  
  
+ CCTTGGCATC GATTCGGATT CACCGCCGCC AATTCGATCA TTCTGGAAGC TATTTCCGAC CTACCCGTTG   
  
  
+ TACACATTGT CGACCTCAGC ATCTCCCACT GTATGCAGAT CCCCACGTTG ATCGACTCCA TTGCGACCCG   
  
  
+ GTTGGAAGCC CCGGGTCGAG TCCCTCCTAT TGTCAAGCTC ACCGTCGGGG CTATTTCCGA CGAAATCCCG   
  
  
+ CCGGTGTTCG ATCTTCTGTC GTACGATGAG CTCGGAATGA GACTAATCAA CTTCGCTCGT TTTAGAAACA   
  
  
+ TCGTCCTCGA ATTCCAAGCA ATACCCACCT CCCCTTCCGA CGGATTTGCT TCGCTGTTGG AGGAGATTCG   
  
  
+ ACAAAGCAAG CTCTACTCCA ACGATGCGGC GGCGGTTATT GTGAATTGTC AGATGAGTTT GCATTTATTG   
  
  
+ CAGGAAGAGG AGGTGTCTTC ATCGTCGCCG TCGTCGATGA GGGGGATGTT TTTGCAGGCG GTGAGGAGCT   
  
  
+ TGGAGCCGAG CATGGTGGTG GTGGTGGAGG AGGACGTGGA TTTCACGGCG AGGAGTCTGG TGGGGCGGCT   
  
  
+ GAGATCGGCG TTTAATCACA TGTGGATACC CTTCGACACG GTGGACACGT TCTTGCCACG TGGGAGCCAG   
  
  
+ CAGAGAGAGT GGTTCGAGGC CGAGGTGTGC TGGAAGATTG AGAATGTGAT CGCTCATGAG GGACCCGCGA   
  
  
+ GGGTCGAGAG GCAGGAGCCC AGGGCCAAGT GGGCCCTCCG AATGAGGGAG GCCGAGTTTC AAGGGATCGA   
  
  
+ GTTCGGTGAT GAAGGTACGA CCGAGGTCAA GGCCATGCTG GAGGAGCATG CCGCTGGGTG GGGGTCTAAG   
  
  
+ AAGGAAGAGG ATGATCTTGT GCTCACTTGG AAGGGACATA GTGTTGTCTT TGCTTCTGCT TGGGTACCCA   
  
  
+ CTTA  

- +Up\_Stream \_Len000TCTTCG ATTAATTTGG TTTGTACGTG CATACAGGAT TAATTGTACT GACAGGTAGT   
  
  
- AGAGGAGGGT ACAACAAGAC AACTGTTCGG ACGTGTGGGT ACGAGGAGAG AGTACAGATT GGAGCAGGGT   
  
  
- TGCTAGTTCT AGTGACAGAC ACTCCGGTCC CCTTCTGAAC GAAAATAAAG GAAAAGAAAA CAAATGAGAG   
  
  
- ACTAAGGTAA ATCGGTATAT ATGTTCCTCT TACAGTACAC ATAGAGTATA CATATATATA TTCTAAAATA   
  
  
- AATCTTTATT TTTAAATTTG TGATACACTA AATATCAATA ATATTATTTA TCATAAAAAA TTTGATAAAG   
  
  
- TAACTACTCT TGTCCTTTCA TTTAACGGCT GTACAATTAC AATAATATAG ATTTTTAATT TAGTATTTTA   
  
  
- ATTAATATTA TAAAATTTTT ATAAAGTTGA TTTTAAAATC TTGTTATTCA CGCATTATGC CTGAATTTCC   
  
  
- GATCAAATAG CATTTTTACA TTCAAGGAAA ACCTAGAATA CCAACTTATA GAACCAACTA TTTGACACTA   
  
  
- ACCAAAATGT GAAAATGGTG TATAAGTATC TTCTTAATAC ACGTGCAACT ACCAAGTTCT ACCCTGTTTT   
  
  
- CCTTTGTACA TAAGAAGGGG GAAAACGGAA AAGGGGATTC GTAAACCTAA CTCCACAACA GTAGACTTAT   
  
  
- AGTTTTTAAG AAAAACTTTT AAGTCAGAGT TAGTTTTAAG TTTTAATTTT GTTTCTGAAT TCTCAAAATA   
  
  
- AACAAGAATT GGAAGAATTG AAAGATGAAA AGAAAAAAAA GAGTAAGGGG GAGAAGTTTA GAGTAAATAA   
  
  
- GAATTGGGGG AGAAGGCAAG TACGAGAGAG ACAGATAGTT GATTATAATT AGATGGGGCT GAAAGTTAAA   
  
  
- CTACTCCAGA TTAGGAATAG TAGCATAACA CCAACCCAAT TAATACGTTC TTCCGTTATT TGGGGTCACT   
  
  
- GTTTCAGGTG CAATTCATCC GTGGAGTGGT AATTCTGTAC GAGTCTTTTG TGGTTTTGGT AACTTGTGTT   
  
  
- CAGGGGGTTC AGGGAAGGGA TCGATCGGGT AAGAGAGAGG AGGGGACAGA TATAGATGGT TGAGAACGAG   
  
  
- AAACGACTCG GACTCAACTT TCGCTATGTG GGTATCGGAC AAGTCAAAAG TCAAAAGTGT AAACAGAAGG   
  
  
- AGACAAGTCA ACTTTCGCTA TGTGAGAGTA ATCGAAAGTA AAATTTATGC AAGTTAAGTG TGTATTTACC   
  
  
- GAAAGTTTCG TAACGGTAAA AGCAAATTTA CTACTGTTTT ATATATCATA GACCGATAGA CGGGTATAGA   
  
  
- ACTGGAATAA ATGTTTCCGA CTCGGTAGAA AATTAAAAAA ATCGGAGAAA AAAATTAGTT TTTATCTTTA   
  
  
- AAGTTCTTTC GTCTTTAGCA GTCTACTGTC ACGTCTGTCG CAAAAGCAAA GTATGTTTAG AGTTTTCGAC   
  
  
- TTCACAGTTA AAGAAGCTAA TAAAAACAGT CTTTTATAAA TGCAGAGTGG AAGTGCAACA ATAATAAGAA   
  
  
- AGAAAAAATA ATAAGAGTAT TGATCATTAT TTCAATCGTT ACTATTTTTT AATAAGCTAG GCTTATAAAA   
  
  
- TTAAATAGAC TGGATTTTTG TATTCATTTC CGTGTTTAAA AAGTAGGTTT AAAATTAAAA ACTACATTAT   
  
  
- AAAAAATATA AAAATAACAA GTTTAGACTA AAATTAGAAT AAATTTAATA GACTGGATTT TTTTAGTTTA   
  
  
- TTATTATTTG CAATTAAAAA ATAAATTTAA AACTGTAATC AGTTAGACTG AATTTAAGCT TGGCTCGAAC   
  
  
- TTAAAAAAAC GGTCACGACC ATCATGAGGA CGAAAACATC GAGGGTTACG GTAGCATGGT GGGAGAAGAT   
  
  
- CAGAGCAGAG AAAGAGACCA GAGGTTAATT AGTGTGGTAG TATAGTATGG CATACTACTA TGTCAGGTGA   
  
  
- CTTTAGGGTA AGAGTGGCGG TTAGAGAGAG GCGGTGGTAG GTTGTGAGAA TTGCTACTTG TCCCGGCGGC   
  
  
- GGAGCAGGAG GTGTGAGTCC GGCACCGGCC CGTCGAAAGG GTGGGTTAGT TTTAGAGAGA GGAGGCGGTT   
  
  
- GAAGCCATTG CGGTTAACGT ACCTCGTCGA GAATCAGGTG ACGCGGCTTC GGTAGCTGTT GTTACTGCGG   
  
  
- TGGGGCCGGG TCGTTTAGAA TACCCAGGAC TTATTGTATC GGGCTGGGCT GCCGCTGAGG TGGGTTGCGG   
  
  
- AGTGCACGCG TAAGGATGCA CGGAATTAGA GTGCGCGGCA GGAGTGATCG TGGACGTTCT ACCATTAGGG   
  
  
- AGTAAAGTTG GGGTAGTTAA GTGGGGTGTT TAAGAGCGAG GAGCTTGAAC GGGTGAAGCA GCTAAATTGG   
  
  
- GGAACCGTAG CTAAGCCTAA GTGGCGGCGG TTAAGCTAGT AAGACCTTCG ATAAAGGCTG GATGGGCAAC   
  
  
- ATGTGTAACA GCTGGAGTCG TAGAGGGTGA CATACGTCTA GGGGTGCAAC TAGCTGAGGT AACGCTGGGC   
  
  
- CAACCTTCGG GGCCCAGCTC AGGGAGGATA ACAGTTCGAG TGGCAGCCCC GATAAAGGCT GCTTTAGGGC   
  
  
- GGCCACAAGC TAGAAGACAG CATGCTACTC GAGCCTTACT CTGATTAGTT GAAGCGAGCA AAATCTTTGT   
  
  
- AGCAGGAGCT TAAGGTTCGT TATGGGTGGA GGGGAAGGCT GCCTAAACGA AGCGACAACC TCCTCTAAGC   
  
  
- TGTTTCGTTC GAGATGAGGT TGCTACGCCG CCGCCAATAA CACTTAACAG TCTACTCAAA CGTAAATAAC   
  
  
- GTCCTTCTCC TCCACAGAAG TAGCAGCGGC AGCAGCTACT CCCCCTACAA AAACGTCCGC CACTCCTCGA   
  
  
- ACCTCGGCTC GTACCACCAC CACCACCTCC TCCTGCACCT AAAGTGCCGC TCCTCAGACC ACCCCGCCGA   
  
  
- CTCTAGCCGC AAATTAGTGT ACACCTATGG GAAGCTGTGC CACCTGTGCA AGAACGGTGC ACCCTCGGTC   
  
  
- GTCTCTCTCA CCAAGCTCCG GCTCCACACG ACCTTCTAAC TCTTACACTA GCGAGTACTC CCTGGGCGCT   
  
  
- CCCAGCTCTC CGTCCTCGGG TCCCGGTTCA CCCGGGAGGC TTACTCCCTC CGGCTCAAAG TTCCCTAGCT   
  
  
- CAAGCCACTA CTTCCATGCT GGCTCCAGTT CCGGTACGAC CTCCTCGTAC GGCGACCCAC CCCCAGATTC   
  
  
- TTCCTTCTCC TACTAGAACA CGAGTGAACC TTCCCTGTAT CACAACAGAA ACGAAGACGA ACCCATGGGT   
  
  
- GAAT

+     Box II

| Site Name | Organism | Position | Strand | Matrix score. | sequence | function |
| --- | --- | --- | --- | --- | --- | --- |
| Box II | Petroselinum crispum | 2999 | - | 9 | CCACGTGGC | part of a light responsive element |

>HU06G00376.1   
+ +Up\_Stream \_Len000AGAAGC TAATTAAACC AAACATGCAC GTATGTCCTA ATTAACATGA CTGTCCATCA   
  
  
+ TCTCCTCCCA TGTTGTTCTG TTGACAAGCC TGCACACCCA TGCTCCTCTC TCATGTCTAA CCTCGTCCCA   
  
  
+ ACGATCAAGA TCACTGTCTG TGAGGCCAGG GGAAGACTTG CTTTTATTTC CTTTTCTTTT GTTTACTCTC   
  
  
+ TGATTCCATT TAGCCATATA TACAAGGAGA ATGTCATGTG TATCTCATAT GTATATATAT AAGATTTTAT   
  
  
+ TTAGAAATAA AAATTTAAAC ACTATGTGAT TTATAGTTAT TATAATAAAT AGTATTTTTT AAACTATTTC   
  
  
+ ATTGATGAGA ACAGGAAAGT AAATTGCCGA CATGTTAATG TTATTATATC TAAAAATTAA ATCATAAAAT   
  
  
+ TAATTATAAT ATTTTAAAAA TATTTCAACT AAAATTTTAG AACAATAAGT GCGTAATACG GACTTAAAGG   
  
  
+ CTAGTTTATC GTAAAAATGT AAGTTCCTTT TGGATCTTAT GGTTGAATAT CTTGGTTGAT AAACTGTGAT   
  
  
+ TGGTTTTACA CTTTTACCAC ATATTCATAG AAGAATTATG TGCACGTTGA TGGTTCAAGA TGGGACAAAA   
  
  
+ GGAAACATGT ATTCTTCCCC CTTTTGCCTT TTCCCCTAAG CATTTGGATT GAGGTGTTGT CATCTGAATA   
  
  
+ TCAAAAATTC TTTTTGAAAA TTCAGTCTCA ATCAAAATTC AAAATTAAAA CAAAGACTTA AGAGTTTTAT   
  
  
+ TTGTTCTTAA CCTTCTTAAC TTTCTACTTT TCTTTTTTTT CTCATTCCCC CTCTTCAAAT CTCATTTATT   
  
  
+ CTTAACCCCC TCTTCCGTTC ATGCTCTCTC TGTCTATCAA CTAATATTAA TCTACCCCGA CTTTCAATTT   
  
  
+ GATGAGGTCT AATCCTTATC ATCGTATTGT GGTTGGGTTA ATTATGCAAG AAGGCAATAA ACCCCAGTGA   
  
  
+ CAAAGTCCAC GTTAAGTAGG CACCTCACCA TTAAGACATG CTCAGAAAAC ACCAAAACCA TTGAACACAA   
  
  
+ GTCCCCCAAG TCCCTTCCCT AGCTAGCCCA TTCTCTCTCC TCCCCTGTCT ATATCTACCA ACTCTTGCTC   
  
  
+ TTTGCTGAGC CTGAGTTGAA AGCGATACAC CCATAGCCTG TTCAGTTTTC AGTTTTCACA TTTGTCTTCC   
  
  
+ TCTGTTCAGT TGAAAGCGAT ACACTCTCAT TAGCTTTCAT TTTAAATACG TTCAATTCAC ACATAAATGG   
  
  
+ CTTTCAAAGC ATTGCCATTT TCGTTTAAAT GATGACAAAA TATATAGTAT CTGGCTATCT GCCCATATCT   
  
  
+ TGACCTTATT TACAAAGGCT GAGCCATCTT TTAATTTTTT TAGCCTCTTT TTTTAATCAA AAATAGAAAT   
  
  
+ TTCAAGAAAG CAGAAATCGT CAGATGACAG TGCAGACAGC GTTTTCGTTT CATACAAATC TCAAAAGCTG   
  
  
+ AAGTGTCAAT TTCTTCGATT ATTTTTGTCA GAAAATATTT ACGTCTCACC TTCACGTTGT TATTATTCTT   
  
  
+ TCTTTTTTAT TATTCTCATA ACTAGTAATA AAGTTAGCAA TGATAAAAAA TTATTCGATC CGAATATTTT   
  
  
+ AATTTATCTG ACCTAAAAAC ATAAGTAAAG GCACAAATTT TTCATCCAAA TTTTAATTTT TGATGTAATA   
  
  
+ TTTTTTATAT TTTTATTGTT CAAATCTGAT TTTAATCTTA TTTAAATTAT CTGACCTAAA AAAATCAAAT   
  
  
+ AATAATAAAC GTTAATTTTT TATTTAAATT TTGACATTAG TCAATCTGAC TTAAATTCGA ACCGAGCTTG   
  
  
+ AATTTTTTTG CCAGTGCTGG TAGTACTCCT GCTTTTGTAG CTCCCAATGC CATCGTACCA CCCTCTTCTA   
  
  
+ GTCTCGTCTC TTTCTCTGGT CTCCAATTAA TCACACCATC ATATCATACC GTATGATGAT ACAGTCCACT   
  
  
+ GAAATCCCAT TCTCACCGCC AATCTCTCTC CGCCACCATC CAACACTCTT AACGATGAAC AGGGCCGCCG   
  
  
+ CCTCGTCCTC CACACTCAGG CCGTGGCCGG GCAGCTTTCC CACCCAATCA AAATCTCTCT CCTCCGCCAA   
  
  
+ CTTCGGTAAC GCCAATTGCA TGGAGCAGCT CTTAGTCCAC TGCGCCGAAG CCATCGACAA CAATGACGCC   
  
  
+ ACCCCGGCCC AGCAAATCTT ATGGGTCCTG AATAACATAG CCCGACCCGA CGGCGACTCC ACCCAACGCC   
  
  
+ TCACGTGCGC ATTCCTACGT GCCTTAATCT CACGCGCCGT CCTCACTAGC ACCTGCAAGA TGGTAATCCC   
  
  
+ TCATTTCAAC CCCATCAATT CACCCCACAA ATTCTCGCTC CTCGAACTTG CCCACTTCGT CGATTTAACC   
  
  
+ CCTTGGCATC GATTCGGATT CACCGCCGCC AATTCGATCA TTCTGGAAGC TATTTCCGAC CTACCCGTTG   
  
  
+ TACACATTGT CGACCTCAGC ATCTCCCACT GTATGCAGAT CCCCACGTTG ATCGACTCCA TTGCGACCCG   
  
  
+ GTTGGAAGCC CCGGGTCGAG TCCCTCCTAT TGTCAAGCTC ACCGTCGGGG CTATTTCCGA CGAAATCCCG   
  
  
+ CCGGTGTTCG ATCTTCTGTC GTACGATGAG CTCGGAATGA GACTAATCAA CTTCGCTCGT TTTAGAAACA   
  
  
+ TCGTCCTCGA ATTCCAAGCA ATACCCACCT CCCCTTCCGA CGGATTTGCT TCGCTGTTGG AGGAGATTCG   
  
  
+ ACAAAGCAAG CTCTACTCCA ACGATGCGGC GGCGGTTATT GTGAATTGTC AGATGAGTTT GCATTTATTG   
  
  
+ CAGGAAGAGG AGGTGTCTTC ATCGTCGCCG TCGTCGATGA GGGGGATGTT TTTGCAGGCG GTGAGGAGCT   
  
  
+ TGGAGCCGAG CATGGTGGTG GTGGTGGAGG AGGACGTGGA TTTCACGGCG AGGAGTCTGG TGGGGCGGCT   
  
  
+ GAGATCGGCG TTTAATCACA TGTGGATACC CTTCGACACG GTGGACACGT TCTTGCCACG TGGGAGCCAG   
  
  
+ CAGAGAGAGT GGTTCGAGGC CGAGGTGTGC TGGAAGATTG AGAATGTGAT CGCTCATGAG GGACCCGCGA   
  
  
+ GGGTCGAGAG GCAGGAGCCC AGGGCCAAGT GGGCCCTCCG AATGAGGGAG GCCGAGTTTC AAGGGATCGA   
  
  
+ GTTCGGTGAT GAAGGTACGA CCGAGGTCAA GGCCATGCTG GAGGAGCATG CCGCTGGGTG GGGGTCTAAG   
  
  
+ AAGGAAGAGG ATGATCTTGT GCTCACTTGG AAGGGACATA GTGTTGTCTT TGCTTCTGCT TGGGTACCCA   
  
  
+ CTTA  

- +Up\_Stream \_Len000TCTTCG ATTAATTTGG TTTGTACGTG CATACAGGAT TAATTGTACT GACAGGTAGT   
  
  
- AGAGGAGGGT ACAACAAGAC AACTGTTCGG ACGTGTGGGT ACGAGGAGAG AGTACAGATT GGAGCAGGGT   
  
  
- TGCTAGTTCT AGTGACAGAC ACTCCGGTCC CCTTCTGAAC GAAAATAAAG GAAAAGAAAA CAAATGAGAG   
  
  
- ACTAAGGTAA ATCGGTATAT ATGTTCCTCT TACAGTACAC ATAGAGTATA CATATATATA TTCTAAAATA   
  
  
- AATCTTTATT TTTAAATTTG TGATACACTA AATATCAATA ATATTATTTA TCATAAAAAA TTTGATAAAG   
  
  
- TAACTACTCT TGTCCTTTCA TTTAACGGCT GTACAATTAC AATAATATAG ATTTTTAATT TAGTATTTTA   
  
  
- ATTAATATTA TAAAATTTTT ATAAAGTTGA TTTTAAAATC TTGTTATTCA CGCATTATGC CTGAATTTCC   
  
  
- GATCAAATAG CATTTTTACA TTCAAGGAAA ACCTAGAATA CCAACTTATA GAACCAACTA TTTGACACTA   
  
  
- ACCAAAATGT GAAAATGGTG TATAAGTATC TTCTTAATAC ACGTGCAACT ACCAAGTTCT ACCCTGTTTT   
  
  
- CCTTTGTACA TAAGAAGGGG GAAAACGGAA AAGGGGATTC GTAAACCTAA CTCCACAACA GTAGACTTAT   
  
  
- AGTTTTTAAG AAAAACTTTT AAGTCAGAGT TAGTTTTAAG TTTTAATTTT GTTTCTGAAT TCTCAAAATA   
  
  
- AACAAGAATT GGAAGAATTG AAAGATGAAA AGAAAAAAAA GAGTAAGGGG GAGAAGTTTA GAGTAAATAA   
  
  
- GAATTGGGGG AGAAGGCAAG TACGAGAGAG ACAGATAGTT GATTATAATT AGATGGGGCT GAAAGTTAAA   
  
  
- CTACTCCAGA TTAGGAATAG TAGCATAACA CCAACCCAAT TAATACGTTC TTCCGTTATT TGGGGTCACT   
  
  
- GTTTCAGGTG CAATTCATCC GTGGAGTGGT AATTCTGTAC GAGTCTTTTG TGGTTTTGGT AACTTGTGTT   
  
  
- CAGGGGGTTC AGGGAAGGGA TCGATCGGGT AAGAGAGAGG AGGGGACAGA TATAGATGGT TGAGAACGAG   
  
  
- AAACGACTCG GACTCAACTT TCGCTATGTG GGTATCGGAC AAGTCAAAAG TCAAAAGTGT AAACAGAAGG   
  
  
- AGACAAGTCA ACTTTCGCTA TGTGAGAGTA ATCGAAAGTA AAATTTATGC AAGTTAAGTG TGTATTTACC   
  
  
- GAAAGTTTCG TAACGGTAAA AGCAAATTTA CTACTGTTTT ATATATCATA GACCGATAGA CGGGTATAGA   
  
  
- ACTGGAATAA ATGTTTCCGA CTCGGTAGAA AATTAAAAAA ATCGGAGAAA AAAATTAGTT TTTATCTTTA   
  
  
- AAGTTCTTTC GTCTTTAGCA GTCTACTGTC ACGTCTGTCG CAAAAGCAAA GTATGTTTAG AGTTTTCGAC   
  
  
- TTCACAGTTA AAGAAGCTAA TAAAAACAGT CTTTTATAAA TGCAGAGTGG AAGTGCAACA ATAATAAGAA   
  
  
- AGAAAAAATA ATAAGAGTAT TGATCATTAT TTCAATCGTT ACTATTTTTT AATAAGCTAG GCTTATAAAA   
  
  
- TTAAATAGAC TGGATTTTTG TATTCATTTC CGTGTTTAAA AAGTAGGTTT AAAATTAAAA ACTACATTAT   
  
  
- AAAAAATATA AAAATAACAA GTTTAGACTA AAATTAGAAT AAATTTAATA GACTGGATTT TTTTAGTTTA   
  
  
- TTATTATTTG CAATTAAAAA ATAAATTTAA AACTGTAATC AGTTAGACTG AATTTAAGCT TGGCTCGAAC   
  
  
- TTAAAAAAAC GGTCACGACC ATCATGAGGA CGAAAACATC GAGGGTTACG GTAGCATGGT GGGAGAAGAT   
  
  
- CAGAGCAGAG AAAGAGACCA GAGGTTAATT AGTGTGGTAG TATAGTATGG CATACTACTA TGTCAGGTGA   
  
  
- CTTTAGGGTA AGAGTGGCGG TTAGAGAGAG GCGGTGGTAG GTTGTGAGAA TTGCTACTTG TCCCGGCGGC   
  
  
- GGAGCAGGAG GTGTGAGTCC GGCACCGGCC CGTCGAAAGG GTGGGTTAGT TTTAGAGAGA GGAGGCGGTT   
  
  
- GAAGCCATTG CGGTTAACGT ACCTCGTCGA GAATCAGGTG ACGCGGCTTC GGTAGCTGTT GTTACTGCGG   
  
  
- TGGGGCCGGG TCGTTTAGAA TACCCAGGAC TTATTGTATC GGGCTGGGCT GCCGCTGAGG TGGGTTGCGG   
  
  
- AGTGCACGCG TAAGGATGCA CGGAATTAGA GTGCGCGGCA GGAGTGATCG TGGACGTTCT ACCATTAGGG   
  
  
- AGTAAAGTTG GGGTAGTTAA GTGGGGTGTT TAAGAGCGAG GAGCTTGAAC GGGTGAAGCA GCTAAATTGG   
  
  
- GGAACCGTAG CTAAGCCTAA GTGGCGGCGG TTAAGCTAGT AAGACCTTCG ATAAAGGCTG GATGGGCAAC   
  
  
- ATGTGTAACA GCTGGAGTCG TAGAGGGTGA CATACGTCTA GGGGTGCAAC TAGCTGAGGT AACGCTGGGC   
  
  
- CAACCTTCGG GGCCCAGCTC AGGGAGGATA ACAGTTCGAG TGGCAGCCCC GATAAAGGCT GCTTTAGGGC   
  
  
- GGCCACAAGC TAGAAGACAG CATGCTACTC GAGCCTTACT CTGATTAGTT GAAGCGAGCA AAATCTTTGT   
  
  
- AGCAGGAGCT TAAGGTTCGT TATGGGTGGA GGGGAAGGCT GCCTAAACGA AGCGACAACC TCCTCTAAGC   
  
  
- TGTTTCGTTC GAGATGAGGT TGCTACGCCG CCGCCAATAA CACTTAACAG TCTACTCAAA CGTAAATAAC   
  
  
- GTCCTTCTCC TCCACAGAAG TAGCAGCGGC AGCAGCTACT CCCCCTACAA AAACGTCCGC CACTCCTCGA   
  
  
- ACCTCGGCTC GTACCACCAC CACCACCTCC TCCTGCACCT AAAGTGCCGC TCCTCAGACC ACCCCGCCGA   
  
  
- CTCTAGCCGC AAATTAGTGT ACACCTATGG GAAGCTGTGC CACCTGTGCA AGAACGGTGC ACCCTCGGTC   
  
  
- GTCTCTCTCA CCAAGCTCCG GCTCCACACG ACCTTCTAAC TCTTACACTA GCGAGTACTC CCTGGGCGCT   
  
  
- CCCAGCTCTC CGTCCTCGGG TCCCGGTTCA CCCGGGAGGC TTACTCCCTC CGGCTCAAAG TTCCCTAGCT   
  
  
- CAAGCCACTA CTTCCATGCT GGCTCCAGTT CCGGTACGAC CTCCTCGTAC GGCGACCCAC CCCCAGATTC   
  
  
- TTCCTTCTCC TACTAGAACA CGAGTGAACC TTCCCTGTAT CACAACAGAA ACGAAGACGA ACCCATGGGT   
  
  
- GAAT

+     CAAT-box

| Site Name | Organism | Position | Strand | Matrix score. | sequence | function |
| --- | --- | --- | --- | --- | --- | --- |
| CAAT-box | Nicotiana glutinosa | 2514 | - | 4 | CAAT |  |
| CAAT-box | Arabidopsis thaliana | 1983 | + | 5 | CCAAT | common cis-acting element in promoter and enhancer regions |
| CAAT-box | Nicotiana glutinosa | 1582 | + | 4 | CAAT |  |
| CAAT-box | Pisum sativum | 1459 | + | 5 | CAAAT | common cis-acting element in promoter and enhancer regions |
| CAAT-box | Pisum sativum | 911 | - | 5 | CAAAT | common cis-acting element in promoter and enhancer regions |
| CAAT-box | Nicotiana glutinosa | 940 | - | 4 | CAAT |  |
| CAAT-box | Nicotiana glutinosa | 733 | + | 4 | CAAT |  |
| CAAT-box | Nicotiana glutinosa | 467 | + | 4 | CAAT |  |
| CAAT-box | Arabidopsis thaliana | 2078 | + | 5 | CCAAT | common cis-acting element in promoter and enhancer regions |
| CAAT-box | Nicotiana glutinosa | 909 | + | 4 | CAAT |  |
| CAAT-box | Pisum sativum | 773 | - | 5 | CAAAT | common cis-acting element in promoter and enhancer regions |
| CAAT-box | Pisum sativum | 676 | - | 5 | CAAAT | common cis-acting element in promoter and enhancer regions |
| CAAT-box | Nicotiana glutinosa | 377 | - | 4 | CAAT |  |
| CAAT-box | Pisum sativum | 830 | + | 5 | CAAAT | common cis-acting element in promoter and enhancer regions |
| CAAT-box | Nicotiana glutinosa | 2683 | + | 4 | CAAT |  |
| CAAT-box | Nicotiana glutinosa | 2553 | - | 4 | CAAT |  |
| CAAT-box | Nicotiana glutinosa | 355 | - | 4 | CAAT |  |
| CAAT-box | Nicotiana glutinosa | 1044 | - | 4 | CAAT |  |
| CAAT-box | Arabidopsis thaliana | 1868 | + | 5 | CCAAT | common cis-acting element in promoter and enhancer regions |
| CAAT-box | Nicotiana glutinosa | 2460 | - | 4 | CAAT |  |
| CAAT-box | Nicotiana glutinosa | 2079 | + | 4 | CAAT |  |
| CAAT-box | Nicotiana glutinosa | 682 | - | 4 | CAAT |  |
| CAAT-box | Arabidopsis thaliana | 563 | - | 5 | CCAAT | common cis-acting element in promoter and enhancer regions |
| CAAT-box | Nicotiana glutinosa | 969 | + | 4 | CAAT |  |
| CAAT-box | Nicotiana glutinosa | 2772 | - | 4 | CAAT |  |
| CAAT-box | Nicotiana glutinosa | 1481 | + | 4 | CAAT |  |
| CAAT-box | Nicotiana glutinosa | 1275 | - | 4 | CAAT |  |
| CAAT-box | Pisum sativum | 1184 | - | 5 | CAAAT | common cis-acting element in promoter and enhancer regions |
| CAAT-box | Nicotiana glutinosa | 2330 | + | 4 | CAAT |  |
| CAAT-box | Nicotiana glutinosa | 2414 | + | 4 | CAAT |  |
| CAAT-box | Pisum sativum | 1750 | + | 5 | CAAAT | common cis-acting element in promoter and enhancer regions |
| CAAT-box | Pisum sativum | 2342 | + | 5 | CAAAT | common cis-acting element in promoter and enhancer regions |
| CAAT-box | Nicotiana glutinosa | 1247 | + | 4 | CAAT |  |
| CAAT-box | Nicotiana glutinosa | 1796 | + | 4 | CAAT |  |
| CAAT-box | Arabidopsis thaliana | 1917 | + | 5 | CCAAT | common cis-acting element in promoter and enhancer regions |
| CAAT-box | Pisum sativum | 1648 | + | 5 | CAAAT | common cis-acting element in promoter and enhancer regions |
| CAAT-box | Nicotiana glutinosa | 1699 | - | 4 | CAAT |  |
| CAAT-box | Nicotiana glutinosa | 1918 | + | 4 | CAAT |  |
| CAAT-box | Pisum sativum | 1705 | + | 5 | CAAAT | common cis-acting element in promoter and enhancer regions |
| CAAT-box | Nicotiana glutinosa | 2801 | - | 4 | CAAT |  |
| CAAT-box | Pisum sativum | 1661 | + | 5 | CAAAT | common cis-acting element in promoter and enhancer regions |
| CAAT-box | Nicotiana glutinosa | 1984 | + | 4 | CAAT |  |
| CAAT-box | Arabidopsis thaliana | 2413 | + | 5 | CCAAT | common cis-acting element in promoter and enhancer regions |
| CAAT-box | Nicotiana glutinosa | 1869 | + | 4 | CAAT |  |
| CAAT-box | Pisum sativum | 2708 | - | 5 | CAAAT | common cis-acting element in promoter and enhancer regions |
| CAAT-box | Nicotiana glutinosa | 3051 | - | 4 | CAAT |  |
| CAAT-box | Pisum sativum | 2187 | + | 5 | CAAAT | common cis-acting element in promoter and enhancer regions |
| CAAT-box | Arabidopsis thaliana | 2116 | + | 5 | CCAAT | common cis-acting element in promoter and enhancer regions |
| CAAT-box | Nicotiana glutinosa | 2779 | - | 4 | CAAT |  |
| CAAT-box | Nicotiana glutinosa | 2117 | + | 4 | CAAT |  |
| CAAT-box | Nicotiana glutinosa | 2119 | - | 4 | CAAT |  |
| CAAT-box | Nicotiana glutinosa | 2165 | + | 4 | CAAT |  |

>HU06G00376.1   
+ +Up\_Stream \_Len000AGAAGC TAATTAAACC AAACATGCAC GTATGTCCTA ATTAACATGA CTGTCCATCA   
  
  
+ TCTCCTCCCA TGTTGTTCTG TTGACAAGCC TGCACACCCA TGCTCCTCTC TCATGTCTAA CCTCGTCCCA   
  
  
+ ACGATCAAGA TCACTGTCTG TGAGGCCAGG GGAAGACTTG CTTTTATTTC CTTTTCTTTT GTTTACTCTC   
  
  
+ TGATTCCATT TAGCCATATA TACAAGGAGA ATGTCATGTG TATCTCATAT GTATATATAT AAGATTTTAT   
  
  
+ TTAGAAATAA AAATTTAAAC ACTATGTGAT TTATAGTTAT TATAATAAAT AGTATTTTTT AAACTATTTC   
  
  
+ ATTGATGAGA ACAGGAAAGT AAATTGCCGA CATGTTAATG TTATTATATC TAAAAATTAA ATCATAAAAT   
  
  
+ TAATTATAAT ATTTTAAAAA TATTTCAACT AAAATTTTAG AACAATAAGT GCGTAATACG GACTTAAAGG   
  
  
+ CTAGTTTATC GTAAAAATGT AAGTTCCTTT TGGATCTTAT GGTTGAATAT CTTGGTTGAT AAACTGTGAT   
  
  
+ TGGTTTTACA CTTTTACCAC ATATTCATAG AAGAATTATG TGCACGTTGA TGGTTCAAGA TGGGACAAAA   
  
  
+ GGAAACATGT ATTCTTCCCC CTTTTGCCTT TTCCCCTAAG CATTTGGATT GAGGTGTTGT CATCTGAATA   
  
  
+ TCAAAAATTC TTTTTGAAAA TTCAGTCTCA ATCAAAATTC AAAATTAAAA CAAAGACTTA AGAGTTTTAT   
  
  
+ TTGTTCTTAA CCTTCTTAAC TTTCTACTTT TCTTTTTTTT CTCATTCCCC CTCTTCAAAT CTCATTTATT   
  
  
+ CTTAACCCCC TCTTCCGTTC ATGCTCTCTC TGTCTATCAA CTAATATTAA TCTACCCCGA CTTTCAATTT   
  
  
+ GATGAGGTCT AATCCTTATC ATCGTATTGT GGTTGGGTTA ATTATGCAAG AAGGCAATAA ACCCCAGTGA   
  
  
+ CAAAGTCCAC GTTAAGTAGG CACCTCACCA TTAAGACATG CTCAGAAAAC ACCAAAACCA TTGAACACAA   
  
  
+ GTCCCCCAAG TCCCTTCCCT AGCTAGCCCA TTCTCTCTCC TCCCCTGTCT ATATCTACCA ACTCTTGCTC   
  
  
+ TTTGCTGAGC CTGAGTTGAA AGCGATACAC CCATAGCCTG TTCAGTTTTC AGTTTTCACA TTTGTCTTCC   
  
  
+ TCTGTTCAGT TGAAAGCGAT ACACTCTCAT TAGCTTTCAT TTTAAATACG TTCAATTCAC ACATAAATGG   
  
  
+ CTTTCAAAGC ATTGCCATTT TCGTTTAAAT GATGACAAAA TATATAGTAT CTGGCTATCT GCCCATATCT   
  
  
+ TGACCTTATT TACAAAGGCT GAGCCATCTT TTAATTTTTT TAGCCTCTTT TTTTAATCAA AAATAGAAAT   
  
  
+ TTCAAGAAAG CAGAAATCGT CAGATGACAG TGCAGACAGC GTTTTCGTTT CATACAAATC TCAAAAGCTG   
  
  
+ AAGTGTCAAT TTCTTCGATT ATTTTTGTCA GAAAATATTT ACGTCTCACC TTCACGTTGT TATTATTCTT   
  
  
+ TCTTTTTTAT TATTCTCATA ACTAGTAATA AAGTTAGCAA TGATAAAAAA TTATTCGATC CGAATATTTT   
  
  
+ AATTTATCTG ACCTAAAAAC ATAAGTAAAG GCACAAATTT TTCATCCAAA TTTTAATTTT TGATGTAATA   
  
  
+ TTTTTTATAT TTTTATTGTT CAAATCTGAT TTTAATCTTA TTTAAATTAT CTGACCTAAA AAAATCAAAT   
  
  
+ AATAATAAAC GTTAATTTTT TATTTAAATT TTGACATTAG TCAATCTGAC TTAAATTCGA ACCGAGCTTG   
  
  
+ AATTTTTTTG CCAGTGCTGG TAGTACTCCT GCTTTTGTAG CTCCCAATGC CATCGTACCA CCCTCTTCTA   
  
  
+ GTCTCGTCTC TTTCTCTGGT CTCCAATTAA TCACACCATC ATATCATACC GTATGATGAT ACAGTCCACT   
  
  
+ GAAATCCCAT TCTCACCGCC AATCTCTCTC CGCCACCATC CAACACTCTT AACGATGAAC AGGGCCGCCG   
  
  
+ CCTCGTCCTC CACACTCAGG CCGTGGCCGG GCAGCTTTCC CACCCAATCA AAATCTCTCT CCTCCGCCAA   
  
  
+ CTTCGGTAAC GCCAATTGCA TGGAGCAGCT CTTAGTCCAC TGCGCCGAAG CCATCGACAA CAATGACGCC   
  
  
+ ACCCCGGCCC AGCAAATCTT ATGGGTCCTG AATAACATAG CCCGACCCGA CGGCGACTCC ACCCAACGCC   
  
  
+ TCACGTGCGC ATTCCTACGT GCCTTAATCT CACGCGCCGT CCTCACTAGC ACCTGCAAGA TGGTAATCCC   
  
  
+ TCATTTCAAC CCCATCAATT CACCCCACAA ATTCTCGCTC CTCGAACTTG CCCACTTCGT CGATTTAACC   
  
  
+ CCTTGGCATC GATTCGGATT CACCGCCGCC AATTCGATCA TTCTGGAAGC TATTTCCGAC CTACCCGTTG   
  
  
+ TACACATTGT CGACCTCAGC ATCTCCCACT GTATGCAGAT CCCCACGTTG ATCGACTCCA TTGCGACCCG   
  
  
+ GTTGGAAGCC CCGGGTCGAG TCCCTCCTAT TGTCAAGCTC ACCGTCGGGG CTATTTCCGA CGAAATCCCG   
  
  
+ CCGGTGTTCG ATCTTCTGTC GTACGATGAG CTCGGAATGA GACTAATCAA CTTCGCTCGT TTTAGAAACA   
  
  
+ TCGTCCTCGA ATTCCAAGCA ATACCCACCT CCCCTTCCGA CGGATTTGCT TCGCTGTTGG AGGAGATTCG   
  
  
+ ACAAAGCAAG CTCTACTCCA ACGATGCGGC GGCGGTTATT GTGAATTGTC AGATGAGTTT GCATTTATTG   
  
  
+ CAGGAAGAGG AGGTGTCTTC ATCGTCGCCG TCGTCGATGA GGGGGATGTT TTTGCAGGCG GTGAGGAGCT   
  
  
+ TGGAGCCGAG CATGGTGGTG GTGGTGGAGG AGGACGTGGA TTTCACGGCG AGGAGTCTGG TGGGGCGGCT   
  
  
+ GAGATCGGCG TTTAATCACA TGTGGATACC CTTCGACACG GTGGACACGT TCTTGCCACG TGGGAGCCAG   
  
  
+ CAGAGAGAGT GGTTCGAGGC CGAGGTGTGC TGGAAGATTG AGAATGTGAT CGCTCATGAG GGACCCGCGA   
  
  
+ GGGTCGAGAG GCAGGAGCCC AGGGCCAAGT GGGCCCTCCG AATGAGGGAG GCCGAGTTTC AAGGGATCGA   
  
  
+ GTTCGGTGAT GAAGGTACGA CCGAGGTCAA GGCCATGCTG GAGGAGCATG CCGCTGGGTG GGGGTCTAAG   
  
  
+ AAGGAAGAGG ATGATCTTGT GCTCACTTGG AAGGGACATA GTGTTGTCTT TGCTTCTGCT TGGGTACCCA   
  
  
+ CTTA  

- +Up\_Stream \_Len000TCTTCG ATTAATTTGG TTTGTACGTG CATACAGGAT TAATTGTACT GACAGGTAGT   
  
  
- AGAGGAGGGT ACAACAAGAC AACTGTTCGG ACGTGTGGGT ACGAGGAGAG AGTACAGATT GGAGCAGGGT   
  
  
- TGCTAGTTCT AGTGACAGAC ACTCCGGTCC CCTTCTGAAC GAAAATAAAG GAAAAGAAAA CAAATGAGAG   
  
  
- ACTAAGGTAA ATCGGTATAT ATGTTCCTCT TACAGTACAC ATAGAGTATA CATATATATA TTCTAAAATA   
  
  
- AATCTTTATT TTTAAATTTG TGATACACTA AATATCAATA ATATTATTTA TCATAAAAAA TTTGATAAAG   
  
  
- TAACTACTCT TGTCCTTTCA TTTAACGGCT GTACAATTAC AATAATATAG ATTTTTAATT TAGTATTTTA   
  
  
- ATTAATATTA TAAAATTTTT ATAAAGTTGA TTTTAAAATC TTGTTATTCA CGCATTATGC CTGAATTTCC   
  
  
- GATCAAATAG CATTTTTACA TTCAAGGAAA ACCTAGAATA CCAACTTATA GAACCAACTA TTTGACACTA   
  
  
- ACCAAAATGT GAAAATGGTG TATAAGTATC TTCTTAATAC ACGTGCAACT ACCAAGTTCT ACCCTGTTTT   
  
  
- CCTTTGTACA TAAGAAGGGG GAAAACGGAA AAGGGGATTC GTAAACCTAA CTCCACAACA GTAGACTTAT   
  
  
- AGTTTTTAAG AAAAACTTTT AAGTCAGAGT TAGTTTTAAG TTTTAATTTT GTTTCTGAAT TCTCAAAATA   
  
  
- AACAAGAATT GGAAGAATTG AAAGATGAAA AGAAAAAAAA GAGTAAGGGG GAGAAGTTTA GAGTAAATAA   
  
  
- GAATTGGGGG AGAAGGCAAG TACGAGAGAG ACAGATAGTT GATTATAATT AGATGGGGCT GAAAGTTAAA   
  
  
- CTACTCCAGA TTAGGAATAG TAGCATAACA CCAACCCAAT TAATACGTTC TTCCGTTATT TGGGGTCACT   
  
  
- GTTTCAGGTG CAATTCATCC GTGGAGTGGT AATTCTGTAC GAGTCTTTTG TGGTTTTGGT AACTTGTGTT   
  
  
- CAGGGGGTTC AGGGAAGGGA TCGATCGGGT AAGAGAGAGG AGGGGACAGA TATAGATGGT TGAGAACGAG   
  
  
- AAACGACTCG GACTCAACTT TCGCTATGTG GGTATCGGAC AAGTCAAAAG TCAAAAGTGT AAACAGAAGG   
  
  
- AGACAAGTCA ACTTTCGCTA TGTGAGAGTA ATCGAAAGTA AAATTTATGC AAGTTAAGTG TGTATTTACC   
  
  
- GAAAGTTTCG TAACGGTAAA AGCAAATTTA CTACTGTTTT ATATATCATA GACCGATAGA CGGGTATAGA   
  
  
- ACTGGAATAA ATGTTTCCGA CTCGGTAGAA AATTAAAAAA ATCGGAGAAA AAAATTAGTT TTTATCTTTA   
  
  
- AAGTTCTTTC GTCTTTAGCA GTCTACTGTC ACGTCTGTCG CAAAAGCAAA GTATGTTTAG AGTTTTCGAC   
  
  
- TTCACAGTTA AAGAAGCTAA TAAAAACAGT CTTTTATAAA TGCAGAGTGG AAGTGCAACA ATAATAAGAA   
  
  
- AGAAAAAATA ATAAGAGTAT TGATCATTAT TTCAATCGTT ACTATTTTTT AATAAGCTAG GCTTATAAAA   
  
  
- TTAAATAGAC TGGATTTTTG TATTCATTTC CGTGTTTAAA AAGTAGGTTT AAAATTAAAA ACTACATTAT   
  
  
- AAAAAATATA AAAATAACAA GTTTAGACTA AAATTAGAAT AAATTTAATA GACTGGATTT TTTTAGTTTA   
  
  
- TTATTATTTG CAATTAAAAA ATAAATTTAA AACTGTAATC AGTTAGACTG AATTTAAGCT TGGCTCGAAC   
  
  
- TTAAAAAAAC GGTCACGACC ATCATGAGGA CGAAAACATC GAGGGTTACG GTAGCATGGT GGGAGAAGAT   
  
  
- CAGAGCAGAG AAAGAGACCA GAGGTTAATT AGTGTGGTAG TATAGTATGG CATACTACTA TGTCAGGTGA   
  
  
- CTTTAGGGTA AGAGTGGCGG TTAGAGAGAG GCGGTGGTAG GTTGTGAGAA TTGCTACTTG TCCCGGCGGC   
  
  
- GGAGCAGGAG GTGTGAGTCC GGCACCGGCC CGTCGAAAGG GTGGGTTAGT TTTAGAGAGA GGAGGCGGTT   
  
  
- GAAGCCATTG CGGTTAACGT ACCTCGTCGA GAATCAGGTG ACGCGGCTTC GGTAGCTGTT GTTACTGCGG   
  
  
- TGGGGCCGGG TCGTTTAGAA TACCCAGGAC TTATTGTATC GGGCTGGGCT GCCGCTGAGG TGGGTTGCGG   
  
  
- AGTGCACGCG TAAGGATGCA CGGAATTAGA GTGCGCGGCA GGAGTGATCG TGGACGTTCT ACCATTAGGG   
  
  
- AGTAAAGTTG GGGTAGTTAA GTGGGGTGTT TAAGAGCGAG GAGCTTGAAC GGGTGAAGCA GCTAAATTGG   
  
  
- GGAACCGTAG CTAAGCCTAA GTGGCGGCGG TTAAGCTAGT AAGACCTTCG ATAAAGGCTG GATGGGCAAC   
  
  
- ATGTGTAACA GCTGGAGTCG TAGAGGGTGA CATACGTCTA GGGGTGCAAC TAGCTGAGGT AACGCTGGGC   
  
  
- CAACCTTCGG GGCCCAGCTC AGGGAGGATA ACAGTTCGAG TGGCAGCCCC GATAAAGGCT GCTTTAGGGC   
  
  
- GGCCACAAGC TAGAAGACAG CATGCTACTC GAGCCTTACT CTGATTAGTT GAAGCGAGCA AAATCTTTGT   
  
  
- AGCAGGAGCT TAAGGTTCGT TATGGGTGGA GGGGAAGGCT GCCTAAACGA AGCGACAACC TCCTCTAAGC   
  
  
- TGTTTCGTTC GAGATGAGGT TGCTACGCCG CCGCCAATAA CACTTAACAG TCTACTCAAA CGTAAATAAC   
  
  
- GTCCTTCTCC TCCACAGAAG TAGCAGCGGC AGCAGCTACT CCCCCTACAA AAACGTCCGC CACTCCTCGA   
  
  
- ACCTCGGCTC GTACCACCAC CACCACCTCC TCCTGCACCT AAAGTGCCGC TCCTCAGACC ACCCCGCCGA   
  
  
- CTCTAGCCGC AAATTAGTGT ACACCTATGG GAAGCTGTGC CACCTGTGCA AGAACGGTGC ACCCTCGGTC   
  
  
- GTCTCTCTCA CCAAGCTCCG GCTCCACACG ACCTTCTAAC TCTTACACTA GCGAGTACTC CCTGGGCGCT   
  
  
- CCCAGCTCTC CGTCCTCGGG TCCCGGTTCA CCCGGGAGGC TTACTCCCTC CGGCTCAAAG TTCCCTAGCT   
  
  
- CAAGCCACTA CTTCCATGCT GGCTCCAGTT CCGGTACGAC CTCCTCGTAC GGCGACCCAC CCCCAGATTC   
  
  
- TTCCTTCTCC TACTAGAACA CGAGTGAACC TTCCCTGTAT CACAACAGAA ACGAAGACGA ACCCATGGGT   
  
  
- GAAT

+     CCAAT-box

| Site Name | Organism | Position | Strand | Matrix score. | sequence | function |
| --- | --- | --- | --- | --- | --- | --- |
| CCAAT-box | Hordeum vulgare | 2449 | - | 6 | CAACGG | MYBHv1 binding site |

>HU06G00376.1   
+ +Up\_Stream \_Len000AGAAGC TAATTAAACC AAACATGCAC GTATGTCCTA ATTAACATGA CTGTCCATCA   
  
  
+ TCTCCTCCCA TGTTGTTCTG TTGACAAGCC TGCACACCCA TGCTCCTCTC TCATGTCTAA CCTCGTCCCA   
  
  
+ ACGATCAAGA TCACTGTCTG TGAGGCCAGG GGAAGACTTG CTTTTATTTC CTTTTCTTTT GTTTACTCTC   
  
  
+ TGATTCCATT TAGCCATATA TACAAGGAGA ATGTCATGTG TATCTCATAT GTATATATAT AAGATTTTAT   
  
  
+ TTAGAAATAA AAATTTAAAC ACTATGTGAT TTATAGTTAT TATAATAAAT AGTATTTTTT AAACTATTTC   
  
  
+ ATTGATGAGA ACAGGAAAGT AAATTGCCGA CATGTTAATG TTATTATATC TAAAAATTAA ATCATAAAAT   
  
  
+ TAATTATAAT ATTTTAAAAA TATTTCAACT AAAATTTTAG AACAATAAGT GCGTAATACG GACTTAAAGG   
  
  
+ CTAGTTTATC GTAAAAATGT AAGTTCCTTT TGGATCTTAT GGTTGAATAT CTTGGTTGAT AAACTGTGAT   
  
  
+ TGGTTTTACA CTTTTACCAC ATATTCATAG AAGAATTATG TGCACGTTGA TGGTTCAAGA TGGGACAAAA   
  
  
+ GGAAACATGT ATTCTTCCCC CTTTTGCCTT TTCCCCTAAG CATTTGGATT GAGGTGTTGT CATCTGAATA   
  
  
+ TCAAAAATTC TTTTTGAAAA TTCAGTCTCA ATCAAAATTC AAAATTAAAA CAAAGACTTA AGAGTTTTAT   
  
  
+ TTGTTCTTAA CCTTCTTAAC TTTCTACTTT TCTTTTTTTT CTCATTCCCC CTCTTCAAAT CTCATTTATT   
  
  
+ CTTAACCCCC TCTTCCGTTC ATGCTCTCTC TGTCTATCAA CTAATATTAA TCTACCCCGA CTTTCAATTT   
  
  
+ GATGAGGTCT AATCCTTATC ATCGTATTGT GGTTGGGTTA ATTATGCAAG AAGGCAATAA ACCCCAGTGA   
  
  
+ CAAAGTCCAC GTTAAGTAGG CACCTCACCA TTAAGACATG CTCAGAAAAC ACCAAAACCA TTGAACACAA   
  
  
+ GTCCCCCAAG TCCCTTCCCT AGCTAGCCCA TTCTCTCTCC TCCCCTGTCT ATATCTACCA ACTCTTGCTC   
  
  
+ TTTGCTGAGC CTGAGTTGAA AGCGATACAC CCATAGCCTG TTCAGTTTTC AGTTTTCACA TTTGTCTTCC   
  
  
+ TCTGTTCAGT TGAAAGCGAT ACACTCTCAT TAGCTTTCAT TTTAAATACG TTCAATTCAC ACATAAATGG   
  
  
+ CTTTCAAAGC ATTGCCATTT TCGTTTAAAT GATGACAAAA TATATAGTAT CTGGCTATCT GCCCATATCT   
  
  
+ TGACCTTATT TACAAAGGCT GAGCCATCTT TTAATTTTTT TAGCCTCTTT TTTTAATCAA AAATAGAAAT   
  
  
+ TTCAAGAAAG CAGAAATCGT CAGATGACAG TGCAGACAGC GTTTTCGTTT CATACAAATC TCAAAAGCTG   
  
  
+ AAGTGTCAAT TTCTTCGATT ATTTTTGTCA GAAAATATTT ACGTCTCACC TTCACGTTGT TATTATTCTT   
  
  
+ TCTTTTTTAT TATTCTCATA ACTAGTAATA AAGTTAGCAA TGATAAAAAA TTATTCGATC CGAATATTTT   
  
  
+ AATTTATCTG ACCTAAAAAC ATAAGTAAAG GCACAAATTT TTCATCCAAA TTTTAATTTT TGATGTAATA   
  
  
+ TTTTTTATAT TTTTATTGTT CAAATCTGAT TTTAATCTTA TTTAAATTAT CTGACCTAAA AAAATCAAAT   
  
  
+ AATAATAAAC GTTAATTTTT TATTTAAATT TTGACATTAG TCAATCTGAC TTAAATTCGA ACCGAGCTTG   
  
  
+ AATTTTTTTG CCAGTGCTGG TAGTACTCCT GCTTTTGTAG CTCCCAATGC CATCGTACCA CCCTCTTCTA   
  
  
+ GTCTCGTCTC TTTCTCTGGT CTCCAATTAA TCACACCATC ATATCATACC GTATGATGAT ACAGTCCACT   
  
  
+ GAAATCCCAT TCTCACCGCC AATCTCTCTC CGCCACCATC CAACACTCTT AACGATGAAC AGGGCCGCCG   
  
  
+ CCTCGTCCTC CACACTCAGG CCGTGGCCGG GCAGCTTTCC CACCCAATCA AAATCTCTCT CCTCCGCCAA   
  
  
+ CTTCGGTAAC GCCAATTGCA TGGAGCAGCT CTTAGTCCAC TGCGCCGAAG CCATCGACAA CAATGACGCC   
  
  
+ ACCCCGGCCC AGCAAATCTT ATGGGTCCTG AATAACATAG CCCGACCCGA CGGCGACTCC ACCCAACGCC   
  
  
+ TCACGTGCGC ATTCCTACGT GCCTTAATCT CACGCGCCGT CCTCACTAGC ACCTGCAAGA TGGTAATCCC   
  
  
+ TCATTTCAAC CCCATCAATT CACCCCACAA ATTCTCGCTC CTCGAACTTG CCCACTTCGT CGATTTAACC   
  
  
+ CCTTGGCATC GATTCGGATT CACCGCCGCC AATTCGATCA TTCTGGAAGC TATTTCCGAC CTACCCGTTG   
  
  
+ TACACATTGT CGACCTCAGC ATCTCCCACT GTATGCAGAT CCCCACGTTG ATCGACTCCA TTGCGACCCG   
  
  
+ GTTGGAAGCC CCGGGTCGAG TCCCTCCTAT TGTCAAGCTC ACCGTCGGGG CTATTTCCGA CGAAATCCCG   
  
  
+ CCGGTGTTCG ATCTTCTGTC GTACGATGAG CTCGGAATGA GACTAATCAA CTTCGCTCGT TTTAGAAACA   
  
  
+ TCGTCCTCGA ATTCCAAGCA ATACCCACCT CCCCTTCCGA CGGATTTGCT TCGCTGTTGG AGGAGATTCG   
  
  
+ ACAAAGCAAG CTCTACTCCA ACGATGCGGC GGCGGTTATT GTGAATTGTC AGATGAGTTT GCATTTATTG   
  
  
+ CAGGAAGAGG AGGTGTCTTC ATCGTCGCCG TCGTCGATGA GGGGGATGTT TTTGCAGGCG GTGAGGAGCT   
  
  
+ TGGAGCCGAG CATGGTGGTG GTGGTGGAGG AGGACGTGGA TTTCACGGCG AGGAGTCTGG TGGGGCGGCT   
  
  
+ GAGATCGGCG TTTAATCACA TGTGGATACC CTTCGACACG GTGGACACGT TCTTGCCACG TGGGAGCCAG   
  
  
+ CAGAGAGAGT GGTTCGAGGC CGAGGTGTGC TGGAAGATTG AGAATGTGAT CGCTCATGAG GGACCCGCGA   
  
  
+ GGGTCGAGAG GCAGGAGCCC AGGGCCAAGT GGGCCCTCCG AATGAGGGAG GCCGAGTTTC AAGGGATCGA   
  
  
+ GTTCGGTGAT GAAGGTACGA CCGAGGTCAA GGCCATGCTG GAGGAGCATG CCGCTGGGTG GGGGTCTAAG   
  
  
+ AAGGAAGAGG ATGATCTTGT GCTCACTTGG AAGGGACATA GTGTTGTCTT TGCTTCTGCT TGGGTACCCA   
  
  
+ CTTA  

- +Up\_Stream \_Len000TCTTCG ATTAATTTGG TTTGTACGTG CATACAGGAT TAATTGTACT GACAGGTAGT   
  
  
- AGAGGAGGGT ACAACAAGAC AACTGTTCGG ACGTGTGGGT ACGAGGAGAG AGTACAGATT GGAGCAGGGT   
  
  
- TGCTAGTTCT AGTGACAGAC ACTCCGGTCC CCTTCTGAAC GAAAATAAAG GAAAAGAAAA CAAATGAGAG   
  
  
- ACTAAGGTAA ATCGGTATAT ATGTTCCTCT TACAGTACAC ATAGAGTATA CATATATATA TTCTAAAATA   
  
  
- AATCTTTATT TTTAAATTTG TGATACACTA AATATCAATA ATATTATTTA TCATAAAAAA TTTGATAAAG   
  
  
- TAACTACTCT TGTCCTTTCA TTTAACGGCT GTACAATTAC AATAATATAG ATTTTTAATT TAGTATTTTA   
  
  
- ATTAATATTA TAAAATTTTT ATAAAGTTGA TTTTAAAATC TTGTTATTCA CGCATTATGC CTGAATTTCC   
  
  
- GATCAAATAG CATTTTTACA TTCAAGGAAA ACCTAGAATA CCAACTTATA GAACCAACTA TTTGACACTA   
  
  
- ACCAAAATGT GAAAATGGTG TATAAGTATC TTCTTAATAC ACGTGCAACT ACCAAGTTCT ACCCTGTTTT   
  
  
- CCTTTGTACA TAAGAAGGGG GAAAACGGAA AAGGGGATTC GTAAACCTAA CTCCACAACA GTAGACTTAT   
  
  
- AGTTTTTAAG AAAAACTTTT AAGTCAGAGT TAGTTTTAAG TTTTAATTTT GTTTCTGAAT TCTCAAAATA   
  
  
- AACAAGAATT GGAAGAATTG AAAGATGAAA AGAAAAAAAA GAGTAAGGGG GAGAAGTTTA GAGTAAATAA   
  
  
- GAATTGGGGG AGAAGGCAAG TACGAGAGAG ACAGATAGTT GATTATAATT AGATGGGGCT GAAAGTTAAA   
  
  
- CTACTCCAGA TTAGGAATAG TAGCATAACA CCAACCCAAT TAATACGTTC TTCCGTTATT TGGGGTCACT   
  
  
- GTTTCAGGTG CAATTCATCC GTGGAGTGGT AATTCTGTAC GAGTCTTTTG TGGTTTTGGT AACTTGTGTT   
  
  
- CAGGGGGTTC AGGGAAGGGA TCGATCGGGT AAGAGAGAGG AGGGGACAGA TATAGATGGT TGAGAACGAG   
  
  
- AAACGACTCG GACTCAACTT TCGCTATGTG GGTATCGGAC AAGTCAAAAG TCAAAAGTGT AAACAGAAGG   
  
  
- AGACAAGTCA ACTTTCGCTA TGTGAGAGTA ATCGAAAGTA AAATTTATGC AAGTTAAGTG TGTATTTACC   
  
  
- GAAAGTTTCG TAACGGTAAA AGCAAATTTA CTACTGTTTT ATATATCATA GACCGATAGA CGGGTATAGA   
  
  
- ACTGGAATAA ATGTTTCCGA CTCGGTAGAA AATTAAAAAA ATCGGAGAAA AAAATTAGTT TTTATCTTTA   
  
  
- AAGTTCTTTC GTCTTTAGCA GTCTACTGTC ACGTCTGTCG CAAAAGCAAA GTATGTTTAG AGTTTTCGAC   
  
  
- TTCACAGTTA AAGAAGCTAA TAAAAACAGT CTTTTATAAA TGCAGAGTGG AAGTGCAACA ATAATAAGAA   
  
  
- AGAAAAAATA ATAAGAGTAT TGATCATTAT TTCAATCGTT ACTATTTTTT AATAAGCTAG GCTTATAAAA   
  
  
- TTAAATAGAC TGGATTTTTG TATTCATTTC CGTGTTTAAA AAGTAGGTTT AAAATTAAAA ACTACATTAT   
  
  
- AAAAAATATA AAAATAACAA GTTTAGACTA AAATTAGAAT AAATTTAATA GACTGGATTT TTTTAGTTTA   
  
  
- TTATTATTTG CAATTAAAAA ATAAATTTAA AACTGTAATC AGTTAGACTG AATTTAAGCT TGGCTCGAAC   
  
  
- TTAAAAAAAC GGTCACGACC ATCATGAGGA CGAAAACATC GAGGGTTACG GTAGCATGGT GGGAGAAGAT   
  
  
- CAGAGCAGAG AAAGAGACCA GAGGTTAATT AGTGTGGTAG TATAGTATGG CATACTACTA TGTCAGGTGA   
  
  
- CTTTAGGGTA AGAGTGGCGG TTAGAGAGAG GCGGTGGTAG GTTGTGAGAA TTGCTACTTG TCCCGGCGGC   
  
  
- GGAGCAGGAG GTGTGAGTCC GGCACCGGCC CGTCGAAAGG GTGGGTTAGT TTTAGAGAGA GGAGGCGGTT   
  
  
- GAAGCCATTG CGGTTAACGT ACCTCGTCGA GAATCAGGTG ACGCGGCTTC GGTAGCTGTT GTTACTGCGG   
  
  
- TGGGGCCGGG TCGTTTAGAA TACCCAGGAC TTATTGTATC GGGCTGGGCT GCCGCTGAGG TGGGTTGCGG   
  
  
- AGTGCACGCG TAAGGATGCA CGGAATTAGA GTGCGCGGCA GGAGTGATCG TGGACGTTCT ACCATTAGGG   
  
  
- AGTAAAGTTG GGGTAGTTAA GTGGGGTGTT TAAGAGCGAG GAGCTTGAAC GGGTGAAGCA GCTAAATTGG   
  
  
- GGAACCGTAG CTAAGCCTAA GTGGCGGCGG TTAAGCTAGT AAGACCTTCG ATAAAGGCTG GATGGGCAAC   
  
  
- ATGTGTAACA GCTGGAGTCG TAGAGGGTGA CATACGTCTA GGGGTGCAAC TAGCTGAGGT AACGCTGGGC   
  
  
- CAACCTTCGG GGCCCAGCTC AGGGAGGATA ACAGTTCGAG TGGCAGCCCC GATAAAGGCT GCTTTAGGGC   
  
  
- GGCCACAAGC TAGAAGACAG CATGCTACTC GAGCCTTACT CTGATTAGTT GAAGCGAGCA AAATCTTTGT   
  
  
- AGCAGGAGCT TAAGGTTCGT TATGGGTGGA GGGGAAGGCT GCCTAAACGA AGCGACAACC TCCTCTAAGC   
  
  
- TGTTTCGTTC GAGATGAGGT TGCTACGCCG CCGCCAATAA CACTTAACAG TCTACTCAAA CGTAAATAAC   
  
  
- GTCCTTCTCC TCCACAGAAG TAGCAGCGGC AGCAGCTACT CCCCCTACAA AAACGTCCGC CACTCCTCGA   
  
  
- ACCTCGGCTC GTACCACCAC CACCACCTCC TCCTGCACCT AAAGTGCCGC TCCTCAGACC ACCCCGCCGA   
  
  
- CTCTAGCCGC AAATTAGTGT ACACCTATGG GAAGCTGTGC CACCTGTGCA AGAACGGTGC ACCCTCGGTC   
  
  
- GTCTCTCTCA CCAAGCTCCG GCTCCACACG ACCTTCTAAC TCTTACACTA GCGAGTACTC CCTGGGCGCT   
  
  
- CCCAGCTCTC CGTCCTCGGG TCCCGGTTCA CCCGGGAGGC TTACTCCCTC CGGCTCAAAG TTCCCTAGCT   
  
  
- CAAGCCACTA CTTCCATGCT GGCTCCAGTT CCGGTACGAC CTCCTCGTAC GGCGACCCAC CCCCAGATTC   
  
  
- TTCCTTCTCC TACTAGAACA CGAGTGAACC TTCCCTGTAT CACAACAGAA ACGAAGACGA ACCCATGGGT   
  
  
- GAAT

+     CCGTCC motif

| Site Name | Organism | Position | Strand | Matrix score. | sequence | function |
| --- | --- | --- | --- | --- | --- | --- |
| CCGTCC motif | Nicotiana tabacum | 2281 | + | 6 | CCGTCC |  |

>HU06G00376.1   
+ +Up\_Stream \_Len000AGAAGC TAATTAAACC AAACATGCAC GTATGTCCTA ATTAACATGA CTGTCCATCA   
  
  
+ TCTCCTCCCA TGTTGTTCTG TTGACAAGCC TGCACACCCA TGCTCCTCTC TCATGTCTAA CCTCGTCCCA   
  
  
+ ACGATCAAGA TCACTGTCTG TGAGGCCAGG GGAAGACTTG CTTTTATTTC CTTTTCTTTT GTTTACTCTC   
  
  
+ TGATTCCATT TAGCCATATA TACAAGGAGA ATGTCATGTG TATCTCATAT GTATATATAT AAGATTTTAT   
  
  
+ TTAGAAATAA AAATTTAAAC ACTATGTGAT TTATAGTTAT TATAATAAAT AGTATTTTTT AAACTATTTC   
  
  
+ ATTGATGAGA ACAGGAAAGT AAATTGCCGA CATGTTAATG TTATTATATC TAAAAATTAA ATCATAAAAT   
  
  
+ TAATTATAAT ATTTTAAAAA TATTTCAACT AAAATTTTAG AACAATAAGT GCGTAATACG GACTTAAAGG   
  
  
+ CTAGTTTATC GTAAAAATGT AAGTTCCTTT TGGATCTTAT GGTTGAATAT CTTGGTTGAT AAACTGTGAT   
  
  
+ TGGTTTTACA CTTTTACCAC ATATTCATAG AAGAATTATG TGCACGTTGA TGGTTCAAGA TGGGACAAAA   
  
  
+ GGAAACATGT ATTCTTCCCC CTTTTGCCTT TTCCCCTAAG CATTTGGATT GAGGTGTTGT CATCTGAATA   
  
  
+ TCAAAAATTC TTTTTGAAAA TTCAGTCTCA ATCAAAATTC AAAATTAAAA CAAAGACTTA AGAGTTTTAT   
  
  
+ TTGTTCTTAA CCTTCTTAAC TTTCTACTTT TCTTTTTTTT CTCATTCCCC CTCTTCAAAT CTCATTTATT   
  
  
+ CTTAACCCCC TCTTCCGTTC ATGCTCTCTC TGTCTATCAA CTAATATTAA TCTACCCCGA CTTTCAATTT   
  
  
+ GATGAGGTCT AATCCTTATC ATCGTATTGT GGTTGGGTTA ATTATGCAAG AAGGCAATAA ACCCCAGTGA   
  
  
+ CAAAGTCCAC GTTAAGTAGG CACCTCACCA TTAAGACATG CTCAGAAAAC ACCAAAACCA TTGAACACAA   
  
  
+ GTCCCCCAAG TCCCTTCCCT AGCTAGCCCA TTCTCTCTCC TCCCCTGTCT ATATCTACCA ACTCTTGCTC   
  
  
+ TTTGCTGAGC CTGAGTTGAA AGCGATACAC CCATAGCCTG TTCAGTTTTC AGTTTTCACA TTTGTCTTCC   
  
  
+ TCTGTTCAGT TGAAAGCGAT ACACTCTCAT TAGCTTTCAT TTTAAATACG TTCAATTCAC ACATAAATGG   
  
  
+ CTTTCAAAGC ATTGCCATTT TCGTTTAAAT GATGACAAAA TATATAGTAT CTGGCTATCT GCCCATATCT   
  
  
+ TGACCTTATT TACAAAGGCT GAGCCATCTT TTAATTTTTT TAGCCTCTTT TTTTAATCAA AAATAGAAAT   
  
  
+ TTCAAGAAAG CAGAAATCGT CAGATGACAG TGCAGACAGC GTTTTCGTTT CATACAAATC TCAAAAGCTG   
  
  
+ AAGTGTCAAT TTCTTCGATT ATTTTTGTCA GAAAATATTT ACGTCTCACC TTCACGTTGT TATTATTCTT   
  
  
+ TCTTTTTTAT TATTCTCATA ACTAGTAATA AAGTTAGCAA TGATAAAAAA TTATTCGATC CGAATATTTT   
  
  
+ AATTTATCTG ACCTAAAAAC ATAAGTAAAG GCACAAATTT TTCATCCAAA TTTTAATTTT TGATGTAATA   
  
  
+ TTTTTTATAT TTTTATTGTT CAAATCTGAT TTTAATCTTA TTTAAATTAT CTGACCTAAA AAAATCAAAT   
  
  
+ AATAATAAAC GTTAATTTTT TATTTAAATT TTGACATTAG TCAATCTGAC TTAAATTCGA ACCGAGCTTG   
  
  
+ AATTTTTTTG CCAGTGCTGG TAGTACTCCT GCTTTTGTAG CTCCCAATGC CATCGTACCA CCCTCTTCTA   
  
  
+ GTCTCGTCTC TTTCTCTGGT CTCCAATTAA TCACACCATC ATATCATACC GTATGATGAT ACAGTCCACT   
  
  
+ GAAATCCCAT TCTCACCGCC AATCTCTCTC CGCCACCATC CAACACTCTT AACGATGAAC AGGGCCGCCG   
  
  
+ CCTCGTCCTC CACACTCAGG CCGTGGCCGG GCAGCTTTCC CACCCAATCA AAATCTCTCT CCTCCGCCAA   
  
  
+ CTTCGGTAAC GCCAATTGCA TGGAGCAGCT CTTAGTCCAC TGCGCCGAAG CCATCGACAA CAATGACGCC   
  
  
+ ACCCCGGCCC AGCAAATCTT ATGGGTCCTG AATAACATAG CCCGACCCGA CGGCGACTCC ACCCAACGCC   
  
  
+ TCACGTGCGC ATTCCTACGT GCCTTAATCT CACGCGCCGT CCTCACTAGC ACCTGCAAGA TGGTAATCCC   
  
  
+ TCATTTCAAC CCCATCAATT CACCCCACAA ATTCTCGCTC CTCGAACTTG CCCACTTCGT CGATTTAACC   
  
  
+ CCTTGGCATC GATTCGGATT CACCGCCGCC AATTCGATCA TTCTGGAAGC TATTTCCGAC CTACCCGTTG   
  
  
+ TACACATTGT CGACCTCAGC ATCTCCCACT GTATGCAGAT CCCCACGTTG ATCGACTCCA TTGCGACCCG   
  
  
+ GTTGGAAGCC CCGGGTCGAG TCCCTCCTAT TGTCAAGCTC ACCGTCGGGG CTATTTCCGA CGAAATCCCG   
  
  
+ CCGGTGTTCG ATCTTCTGTC GTACGATGAG CTCGGAATGA GACTAATCAA CTTCGCTCGT TTTAGAAACA   
  
  
+ TCGTCCTCGA ATTCCAAGCA ATACCCACCT CCCCTTCCGA CGGATTTGCT TCGCTGTTGG AGGAGATTCG   
  
  
+ ACAAAGCAAG CTCTACTCCA ACGATGCGGC GGCGGTTATT GTGAATTGTC AGATGAGTTT GCATTTATTG   
  
  
+ CAGGAAGAGG AGGTGTCTTC ATCGTCGCCG TCGTCGATGA GGGGGATGTT TTTGCAGGCG GTGAGGAGCT   
  
  
+ TGGAGCCGAG CATGGTGGTG GTGGTGGAGG AGGACGTGGA TTTCACGGCG AGGAGTCTGG TGGGGCGGCT   
  
  
+ GAGATCGGCG TTTAATCACA TGTGGATACC CTTCGACACG GTGGACACGT TCTTGCCACG TGGGAGCCAG   
  
  
+ CAGAGAGAGT GGTTCGAGGC CGAGGTGTGC TGGAAGATTG AGAATGTGAT CGCTCATGAG GGACCCGCGA   
  
  
+ GGGTCGAGAG GCAGGAGCCC AGGGCCAAGT GGGCCCTCCG AATGAGGGAG GCCGAGTTTC AAGGGATCGA   
  
  
+ GTTCGGTGAT GAAGGTACGA CCGAGGTCAA GGCCATGCTG GAGGAGCATG CCGCTGGGTG GGGGTCTAAG   
  
  
+ AAGGAAGAGG ATGATCTTGT GCTCACTTGG AAGGGACATA GTGTTGTCTT TGCTTCTGCT TGGGTACCCA   
  
  
+ CTTA  

- +Up\_Stream \_Len000TCTTCG ATTAATTTGG TTTGTACGTG CATACAGGAT TAATTGTACT GACAGGTAGT   
  
  
- AGAGGAGGGT ACAACAAGAC AACTGTTCGG ACGTGTGGGT ACGAGGAGAG AGTACAGATT GGAGCAGGGT   
  
  
- TGCTAGTTCT AGTGACAGAC ACTCCGGTCC CCTTCTGAAC GAAAATAAAG GAAAAGAAAA CAAATGAGAG   
  
  
- ACTAAGGTAA ATCGGTATAT ATGTTCCTCT TACAGTACAC ATAGAGTATA CATATATATA TTCTAAAATA   
  
  
- AATCTTTATT TTTAAATTTG TGATACACTA AATATCAATA ATATTATTTA TCATAAAAAA TTTGATAAAG   
  
  
- TAACTACTCT TGTCCTTTCA TTTAACGGCT GTACAATTAC AATAATATAG ATTTTTAATT TAGTATTTTA   
  
  
- ATTAATATTA TAAAATTTTT ATAAAGTTGA TTTTAAAATC TTGTTATTCA CGCATTATGC CTGAATTTCC   
  
  
- GATCAAATAG CATTTTTACA TTCAAGGAAA ACCTAGAATA CCAACTTATA GAACCAACTA TTTGACACTA   
  
  
- ACCAAAATGT GAAAATGGTG TATAAGTATC TTCTTAATAC ACGTGCAACT ACCAAGTTCT ACCCTGTTTT   
  
  
- CCTTTGTACA TAAGAAGGGG GAAAACGGAA AAGGGGATTC GTAAACCTAA CTCCACAACA GTAGACTTAT   
  
  
- AGTTTTTAAG AAAAACTTTT AAGTCAGAGT TAGTTTTAAG TTTTAATTTT GTTTCTGAAT TCTCAAAATA   
  
  
- AACAAGAATT GGAAGAATTG AAAGATGAAA AGAAAAAAAA GAGTAAGGGG GAGAAGTTTA GAGTAAATAA   
  
  
- GAATTGGGGG AGAAGGCAAG TACGAGAGAG ACAGATAGTT GATTATAATT AGATGGGGCT GAAAGTTAAA   
  
  
- CTACTCCAGA TTAGGAATAG TAGCATAACA CCAACCCAAT TAATACGTTC TTCCGTTATT TGGGGTCACT   
  
  
- GTTTCAGGTG CAATTCATCC GTGGAGTGGT AATTCTGTAC GAGTCTTTTG TGGTTTTGGT AACTTGTGTT   
  
  
- CAGGGGGTTC AGGGAAGGGA TCGATCGGGT AAGAGAGAGG AGGGGACAGA TATAGATGGT TGAGAACGAG   
  
  
- AAACGACTCG GACTCAACTT TCGCTATGTG GGTATCGGAC AAGTCAAAAG TCAAAAGTGT AAACAGAAGG   
  
  
- AGACAAGTCA ACTTTCGCTA TGTGAGAGTA ATCGAAAGTA AAATTTATGC AAGTTAAGTG TGTATTTACC   
  
  
- GAAAGTTTCG TAACGGTAAA AGCAAATTTA CTACTGTTTT ATATATCATA GACCGATAGA CGGGTATAGA   
  
  
- ACTGGAATAA ATGTTTCCGA CTCGGTAGAA AATTAAAAAA ATCGGAGAAA AAAATTAGTT TTTATCTTTA   
  
  
- AAGTTCTTTC GTCTTTAGCA GTCTACTGTC ACGTCTGTCG CAAAAGCAAA GTATGTTTAG AGTTTTCGAC   
  
  
- TTCACAGTTA AAGAAGCTAA TAAAAACAGT CTTTTATAAA TGCAGAGTGG AAGTGCAACA ATAATAAGAA   
  
  
- AGAAAAAATA ATAAGAGTAT TGATCATTAT TTCAATCGTT ACTATTTTTT AATAAGCTAG GCTTATAAAA   
  
  
- TTAAATAGAC TGGATTTTTG TATTCATTTC CGTGTTTAAA AAGTAGGTTT AAAATTAAAA ACTACATTAT   
  
  
- AAAAAATATA AAAATAACAA GTTTAGACTA AAATTAGAAT AAATTTAATA GACTGGATTT TTTTAGTTTA   
  
  
- TTATTATTTG CAATTAAAAA ATAAATTTAA AACTGTAATC AGTTAGACTG AATTTAAGCT TGGCTCGAAC   
  
  
- TTAAAAAAAC GGTCACGACC ATCATGAGGA CGAAAACATC GAGGGTTACG GTAGCATGGT GGGAGAAGAT   
  
  
- CAGAGCAGAG AAAGAGACCA GAGGTTAATT AGTGTGGTAG TATAGTATGG CATACTACTA TGTCAGGTGA   
  
  
- CTTTAGGGTA AGAGTGGCGG TTAGAGAGAG GCGGTGGTAG GTTGTGAGAA TTGCTACTTG TCCCGGCGGC   
  
  
- GGAGCAGGAG GTGTGAGTCC GGCACCGGCC CGTCGAAAGG GTGGGTTAGT TTTAGAGAGA GGAGGCGGTT   
  
  
- GAAGCCATTG CGGTTAACGT ACCTCGTCGA GAATCAGGTG ACGCGGCTTC GGTAGCTGTT GTTACTGCGG   
  
  
- TGGGGCCGGG TCGTTTAGAA TACCCAGGAC TTATTGTATC GGGCTGGGCT GCCGCTGAGG TGGGTTGCGG   
  
  
- AGTGCACGCG TAAGGATGCA CGGAATTAGA GTGCGCGGCA GGAGTGATCG TGGACGTTCT ACCATTAGGG   
  
  
- AGTAAAGTTG GGGTAGTTAA GTGGGGTGTT TAAGAGCGAG GAGCTTGAAC GGGTGAAGCA GCTAAATTGG   
  
  
- GGAACCGTAG CTAAGCCTAA GTGGCGGCGG TTAAGCTAGT AAGACCTTCG ATAAAGGCTG GATGGGCAAC   
  
  
- ATGTGTAACA GCTGGAGTCG TAGAGGGTGA CATACGTCTA GGGGTGCAAC TAGCTGAGGT AACGCTGGGC   
  
  
- CAACCTTCGG GGCCCAGCTC AGGGAGGATA ACAGTTCGAG TGGCAGCCCC GATAAAGGCT GCTTTAGGGC   
  
  
- GGCCACAAGC TAGAAGACAG CATGCTACTC GAGCCTTACT CTGATTAGTT GAAGCGAGCA AAATCTTTGT   
  
  
- AGCAGGAGCT TAAGGTTCGT TATGGGTGGA GGGGAAGGCT GCCTAAACGA AGCGACAACC TCCTCTAAGC   
  
  
- TGTTTCGTTC GAGATGAGGT TGCTACGCCG CCGCCAATAA CACTTAACAG TCTACTCAAA CGTAAATAAC   
  
  
- GTCCTTCTCC TCCACAGAAG TAGCAGCGGC AGCAGCTACT CCCCCTACAA AAACGTCCGC CACTCCTCGA   
  
  
- ACCTCGGCTC GTACCACCAC CACCACCTCC TCCTGCACCT AAAGTGCCGC TCCTCAGACC ACCCCGCCGA   
  
  
- CTCTAGCCGC AAATTAGTGT ACACCTATGG GAAGCTGTGC CACCTGTGCA AGAACGGTGC ACCCTCGGTC   
  
  
- GTCTCTCTCA CCAAGCTCCG GCTCCACACG ACCTTCTAAC TCTTACACTA GCGAGTACTC CCTGGGCGCT   
  
  
- CCCAGCTCTC CGTCCTCGGG TCCCGGTTCA CCCGGGAGGC TTACTCCCTC CGGCTCAAAG TTCCCTAGCT   
  
  
- CAAGCCACTA CTTCCATGCT GGCTCCAGTT CCGGTACGAC CTCCTCGTAC GGCGACCCAC CCCCAGATTC   
  
  
- TTCCTTCTCC TACTAGAACA CGAGTGAACC TTCCCTGTAT CACAACAGAA ACGAAGACGA ACCCATGGGT   
  
  
- GAAT

+     CCGTCC-box

| Site Name | Organism | Position | Strand | Matrix score. | sequence | function |
| --- | --- | --- | --- | --- | --- | --- |
| CCGTCC-box | Petroselinum hortense | 2281 | + | 6 | CCGTCC |  |

>HU06G00376.1   
+ +Up\_Stream \_Len000AGAAGC TAATTAAACC AAACATGCAC GTATGTCCTA ATTAACATGA CTGTCCATCA   
  
  
+ TCTCCTCCCA TGTTGTTCTG TTGACAAGCC TGCACACCCA TGCTCCTCTC TCATGTCTAA CCTCGTCCCA   
  
  
+ ACGATCAAGA TCACTGTCTG TGAGGCCAGG GGAAGACTTG CTTTTATTTC CTTTTCTTTT GTTTACTCTC   
  
  
+ TGATTCCATT TAGCCATATA TACAAGGAGA ATGTCATGTG TATCTCATAT GTATATATAT AAGATTTTAT   
  
  
+ TTAGAAATAA AAATTTAAAC ACTATGTGAT TTATAGTTAT TATAATAAAT AGTATTTTTT AAACTATTTC   
  
  
+ ATTGATGAGA ACAGGAAAGT AAATTGCCGA CATGTTAATG TTATTATATC TAAAAATTAA ATCATAAAAT   
  
  
+ TAATTATAAT ATTTTAAAAA TATTTCAACT AAAATTTTAG AACAATAAGT GCGTAATACG GACTTAAAGG   
  
  
+ CTAGTTTATC GTAAAAATGT AAGTTCCTTT TGGATCTTAT GGTTGAATAT CTTGGTTGAT AAACTGTGAT   
  
  
+ TGGTTTTACA CTTTTACCAC ATATTCATAG AAGAATTATG TGCACGTTGA TGGTTCAAGA TGGGACAAAA   
  
  
+ GGAAACATGT ATTCTTCCCC CTTTTGCCTT TTCCCCTAAG CATTTGGATT GAGGTGTTGT CATCTGAATA   
  
  
+ TCAAAAATTC TTTTTGAAAA TTCAGTCTCA ATCAAAATTC AAAATTAAAA CAAAGACTTA AGAGTTTTAT   
  
  
+ TTGTTCTTAA CCTTCTTAAC TTTCTACTTT TCTTTTTTTT CTCATTCCCC CTCTTCAAAT CTCATTTATT   
  
  
+ CTTAACCCCC TCTTCCGTTC ATGCTCTCTC TGTCTATCAA CTAATATTAA TCTACCCCGA CTTTCAATTT   
  
  
+ GATGAGGTCT AATCCTTATC ATCGTATTGT GGTTGGGTTA ATTATGCAAG AAGGCAATAA ACCCCAGTGA   
  
  
+ CAAAGTCCAC GTTAAGTAGG CACCTCACCA TTAAGACATG CTCAGAAAAC ACCAAAACCA TTGAACACAA   
  
  
+ GTCCCCCAAG TCCCTTCCCT AGCTAGCCCA TTCTCTCTCC TCCCCTGTCT ATATCTACCA ACTCTTGCTC   
  
  
+ TTTGCTGAGC CTGAGTTGAA AGCGATACAC CCATAGCCTG TTCAGTTTTC AGTTTTCACA TTTGTCTTCC   
  
  
+ TCTGTTCAGT TGAAAGCGAT ACACTCTCAT TAGCTTTCAT TTTAAATACG TTCAATTCAC ACATAAATGG   
  
  
+ CTTTCAAAGC ATTGCCATTT TCGTTTAAAT GATGACAAAA TATATAGTAT CTGGCTATCT GCCCATATCT   
  
  
+ TGACCTTATT TACAAAGGCT GAGCCATCTT TTAATTTTTT TAGCCTCTTT TTTTAATCAA AAATAGAAAT   
  
  
+ TTCAAGAAAG CAGAAATCGT CAGATGACAG TGCAGACAGC GTTTTCGTTT CATACAAATC TCAAAAGCTG   
  
  
+ AAGTGTCAAT TTCTTCGATT ATTTTTGTCA GAAAATATTT ACGTCTCACC TTCACGTTGT TATTATTCTT   
  
  
+ TCTTTTTTAT TATTCTCATA ACTAGTAATA AAGTTAGCAA TGATAAAAAA TTATTCGATC CGAATATTTT   
  
  
+ AATTTATCTG ACCTAAAAAC ATAAGTAAAG GCACAAATTT TTCATCCAAA TTTTAATTTT TGATGTAATA   
  
  
+ TTTTTTATAT TTTTATTGTT CAAATCTGAT TTTAATCTTA TTTAAATTAT CTGACCTAAA AAAATCAAAT   
  
  
+ AATAATAAAC GTTAATTTTT TATTTAAATT TTGACATTAG TCAATCTGAC TTAAATTCGA ACCGAGCTTG   
  
  
+ AATTTTTTTG CCAGTGCTGG TAGTACTCCT GCTTTTGTAG CTCCCAATGC CATCGTACCA CCCTCTTCTA   
  
  
+ GTCTCGTCTC TTTCTCTGGT CTCCAATTAA TCACACCATC ATATCATACC GTATGATGAT ACAGTCCACT   
  
  
+ GAAATCCCAT TCTCACCGCC AATCTCTCTC CGCCACCATC CAACACTCTT AACGATGAAC AGGGCCGCCG   
  
  
+ CCTCGTCCTC CACACTCAGG CCGTGGCCGG GCAGCTTTCC CACCCAATCA AAATCTCTCT CCTCCGCCAA   
  
  
+ CTTCGGTAAC GCCAATTGCA TGGAGCAGCT CTTAGTCCAC TGCGCCGAAG CCATCGACAA CAATGACGCC   
  
  
+ ACCCCGGCCC AGCAAATCTT ATGGGTCCTG AATAACATAG CCCGACCCGA CGGCGACTCC ACCCAACGCC   
  
  
+ TCACGTGCGC ATTCCTACGT GCCTTAATCT CACGCGCCGT CCTCACTAGC ACCTGCAAGA TGGTAATCCC   
  
  
+ TCATTTCAAC CCCATCAATT CACCCCACAA ATTCTCGCTC CTCGAACTTG CCCACTTCGT CGATTTAACC   
  
  
+ CCTTGGCATC GATTCGGATT CACCGCCGCC AATTCGATCA TTCTGGAAGC TATTTCCGAC CTACCCGTTG   
  
  
+ TACACATTGT CGACCTCAGC ATCTCCCACT GTATGCAGAT CCCCACGTTG ATCGACTCCA TTGCGACCCG   
  
  
+ GTTGGAAGCC CCGGGTCGAG TCCCTCCTAT TGTCAAGCTC ACCGTCGGGG CTATTTCCGA CGAAATCCCG   
  
  
+ CCGGTGTTCG ATCTTCTGTC GTACGATGAG CTCGGAATGA GACTAATCAA CTTCGCTCGT TTTAGAAACA   
  
  
+ TCGTCCTCGA ATTCCAAGCA ATACCCACCT CCCCTTCCGA CGGATTTGCT TCGCTGTTGG AGGAGATTCG   
  
  
+ ACAAAGCAAG CTCTACTCCA ACGATGCGGC GGCGGTTATT GTGAATTGTC AGATGAGTTT GCATTTATTG   
  
  
+ CAGGAAGAGG AGGTGTCTTC ATCGTCGCCG TCGTCGATGA GGGGGATGTT TTTGCAGGCG GTGAGGAGCT   
  
  
+ TGGAGCCGAG CATGGTGGTG GTGGTGGAGG AGGACGTGGA TTTCACGGCG AGGAGTCTGG TGGGGCGGCT   
  
  
+ GAGATCGGCG TTTAATCACA TGTGGATACC CTTCGACACG GTGGACACGT TCTTGCCACG TGGGAGCCAG   
  
  
+ CAGAGAGAGT GGTTCGAGGC CGAGGTGTGC TGGAAGATTG AGAATGTGAT CGCTCATGAG GGACCCGCGA   
  
  
+ GGGTCGAGAG GCAGGAGCCC AGGGCCAAGT GGGCCCTCCG AATGAGGGAG GCCGAGTTTC AAGGGATCGA   
  
  
+ GTTCGGTGAT GAAGGTACGA CCGAGGTCAA GGCCATGCTG GAGGAGCATG CCGCTGGGTG GGGGTCTAAG   
  
  
+ AAGGAAGAGG ATGATCTTGT GCTCACTTGG AAGGGACATA GTGTTGTCTT TGCTTCTGCT TGGGTACCCA   
  
  
+ CTTA  

- +Up\_Stream \_Len000TCTTCG ATTAATTTGG TTTGTACGTG CATACAGGAT TAATTGTACT GACAGGTAGT   
  
  
- AGAGGAGGGT ACAACAAGAC AACTGTTCGG ACGTGTGGGT ACGAGGAGAG AGTACAGATT GGAGCAGGGT   
  
  
- TGCTAGTTCT AGTGACAGAC ACTCCGGTCC CCTTCTGAAC GAAAATAAAG GAAAAGAAAA CAAATGAGAG   
  
  
- ACTAAGGTAA ATCGGTATAT ATGTTCCTCT TACAGTACAC ATAGAGTATA CATATATATA TTCTAAAATA   
  
  
- AATCTTTATT TTTAAATTTG TGATACACTA AATATCAATA ATATTATTTA TCATAAAAAA TTTGATAAAG   
  
  
- TAACTACTCT TGTCCTTTCA TTTAACGGCT GTACAATTAC AATAATATAG ATTTTTAATT TAGTATTTTA   
  
  
- ATTAATATTA TAAAATTTTT ATAAAGTTGA TTTTAAAATC TTGTTATTCA CGCATTATGC CTGAATTTCC   
  
  
- GATCAAATAG CATTTTTACA TTCAAGGAAA ACCTAGAATA CCAACTTATA GAACCAACTA TTTGACACTA   
  
  
- ACCAAAATGT GAAAATGGTG TATAAGTATC TTCTTAATAC ACGTGCAACT ACCAAGTTCT ACCCTGTTTT   
  
  
- CCTTTGTACA TAAGAAGGGG GAAAACGGAA AAGGGGATTC GTAAACCTAA CTCCACAACA GTAGACTTAT   
  
  
- AGTTTTTAAG AAAAACTTTT AAGTCAGAGT TAGTTTTAAG TTTTAATTTT GTTTCTGAAT TCTCAAAATA   
  
  
- AACAAGAATT GGAAGAATTG AAAGATGAAA AGAAAAAAAA GAGTAAGGGG GAGAAGTTTA GAGTAAATAA   
  
  
- GAATTGGGGG AGAAGGCAAG TACGAGAGAG ACAGATAGTT GATTATAATT AGATGGGGCT GAAAGTTAAA   
  
  
- CTACTCCAGA TTAGGAATAG TAGCATAACA CCAACCCAAT TAATACGTTC TTCCGTTATT TGGGGTCACT   
  
  
- GTTTCAGGTG CAATTCATCC GTGGAGTGGT AATTCTGTAC GAGTCTTTTG TGGTTTTGGT AACTTGTGTT   
  
  
- CAGGGGGTTC AGGGAAGGGA TCGATCGGGT AAGAGAGAGG AGGGGACAGA TATAGATGGT TGAGAACGAG   
  
  
- AAACGACTCG GACTCAACTT TCGCTATGTG GGTATCGGAC AAGTCAAAAG TCAAAAGTGT AAACAGAAGG   
  
  
- AGACAAGTCA ACTTTCGCTA TGTGAGAGTA ATCGAAAGTA AAATTTATGC AAGTTAAGTG TGTATTTACC   
  
  
- GAAAGTTTCG TAACGGTAAA AGCAAATTTA CTACTGTTTT ATATATCATA GACCGATAGA CGGGTATAGA   
  
  
- ACTGGAATAA ATGTTTCCGA CTCGGTAGAA AATTAAAAAA ATCGGAGAAA AAAATTAGTT TTTATCTTTA   
  
  
- AAGTTCTTTC GTCTTTAGCA GTCTACTGTC ACGTCTGTCG CAAAAGCAAA GTATGTTTAG AGTTTTCGAC   
  
  
- TTCACAGTTA AAGAAGCTAA TAAAAACAGT CTTTTATAAA TGCAGAGTGG AAGTGCAACA ATAATAAGAA   
  
  
- AGAAAAAATA ATAAGAGTAT TGATCATTAT TTCAATCGTT ACTATTTTTT AATAAGCTAG GCTTATAAAA   
  
  
- TTAAATAGAC TGGATTTTTG TATTCATTTC CGTGTTTAAA AAGTAGGTTT AAAATTAAAA ACTACATTAT   
  
  
- AAAAAATATA AAAATAACAA GTTTAGACTA AAATTAGAAT AAATTTAATA GACTGGATTT TTTTAGTTTA   
  
  
- TTATTATTTG CAATTAAAAA ATAAATTTAA AACTGTAATC AGTTAGACTG AATTTAAGCT TGGCTCGAAC   
  
  
- TTAAAAAAAC GGTCACGACC ATCATGAGGA CGAAAACATC GAGGGTTACG GTAGCATGGT GGGAGAAGAT   
  
  
- CAGAGCAGAG AAAGAGACCA GAGGTTAATT AGTGTGGTAG TATAGTATGG CATACTACTA TGTCAGGTGA   
  
  
- CTTTAGGGTA AGAGTGGCGG TTAGAGAGAG GCGGTGGTAG GTTGTGAGAA TTGCTACTTG TCCCGGCGGC   
  
  
- GGAGCAGGAG GTGTGAGTCC GGCACCGGCC CGTCGAAAGG GTGGGTTAGT TTTAGAGAGA GGAGGCGGTT   
  
  
- GAAGCCATTG CGGTTAACGT ACCTCGTCGA GAATCAGGTG ACGCGGCTTC GGTAGCTGTT GTTACTGCGG   
  
  
- TGGGGCCGGG TCGTTTAGAA TACCCAGGAC TTATTGTATC GGGCTGGGCT GCCGCTGAGG TGGGTTGCGG   
  
  
- AGTGCACGCG TAAGGATGCA CGGAATTAGA GTGCGCGGCA GGAGTGATCG TGGACGTTCT ACCATTAGGG   
  
  
- AGTAAAGTTG GGGTAGTTAA GTGGGGTGTT TAAGAGCGAG GAGCTTGAAC GGGTGAAGCA GCTAAATTGG   
  
  
- GGAACCGTAG CTAAGCCTAA GTGGCGGCGG TTAAGCTAGT AAGACCTTCG ATAAAGGCTG GATGGGCAAC   
  
  
- ATGTGTAACA GCTGGAGTCG TAGAGGGTGA CATACGTCTA GGGGTGCAAC TAGCTGAGGT AACGCTGGGC   
  
  
- CAACCTTCGG GGCCCAGCTC AGGGAGGATA ACAGTTCGAG TGGCAGCCCC GATAAAGGCT GCTTTAGGGC   
  
  
- GGCCACAAGC TAGAAGACAG CATGCTACTC GAGCCTTACT CTGATTAGTT GAAGCGAGCA AAATCTTTGT   
  
  
- AGCAGGAGCT TAAGGTTCGT TATGGGTGGA GGGGAAGGCT GCCTAAACGA AGCGACAACC TCCTCTAAGC   
  
  
- TGTTTCGTTC GAGATGAGGT TGCTACGCCG CCGCCAATAA CACTTAACAG TCTACTCAAA CGTAAATAAC   
  
  
- GTCCTTCTCC TCCACAGAAG TAGCAGCGGC AGCAGCTACT CCCCCTACAA AAACGTCCGC CACTCCTCGA   
  
  
- ACCTCGGCTC GTACCACCAC CACCACCTCC TCCTGCACCT AAAGTGCCGC TCCTCAGACC ACCCCGCCGA   
  
  
- CTCTAGCCGC AAATTAGTGT ACACCTATGG GAAGCTGTGC CACCTGTGCA AGAACGGTGC ACCCTCGGTC   
  
  
- GTCTCTCTCA CCAAGCTCCG GCTCCACACG ACCTTCTAAC TCTTACACTA GCGAGTACTC CCTGGGCGCT   
  
  
- CCCAGCTCTC CGTCCTCGGG TCCCGGTTCA CCCGGGAGGC TTACTCCCTC CGGCTCAAAG TTCCCTAGCT   
  
  
- CAAGCCACTA CTTCCATGCT GGCTCCAGTT CCGGTACGAC CTCCTCGTAC GGCGACCCAC CCCCAGATTC   
  
  
- TTCCTTCTCC TACTAGAACA CGAGTGAACC TTCCCTGTAT CACAACAGAA ACGAAGACGA ACCCATGGGT   
  
  
- GAAT

+     CGTCA-motif

| Site Name | Organism | Position | Strand | Matrix score. | sequence | function |
| --- | --- | --- | --- | --- | --- | --- |
| CGTCA-motif | Hordeum vulgare | 2168 | - | 5 | CGTCA | cis-acting regulatory element involved in the MeJA-responsiveness |
| CGTCA-motif | Hordeum vulgare | 1422 | + | 5 | CGTCA | cis-acting regulatory element involved in the MeJA-responsiveness |

>HU06G00376.1   
+ +Up\_Stream \_Len000AGAAGC TAATTAAACC AAACATGCAC GTATGTCCTA ATTAACATGA CTGTCCATCA   
  
  
+ TCTCCTCCCA TGTTGTTCTG TTGACAAGCC TGCACACCCA TGCTCCTCTC TCATGTCTAA CCTCGTCCCA   
  
  
+ ACGATCAAGA TCACTGTCTG TGAGGCCAGG GGAAGACTTG CTTTTATTTC CTTTTCTTTT GTTTACTCTC   
  
  
+ TGATTCCATT TAGCCATATA TACAAGGAGA ATGTCATGTG TATCTCATAT GTATATATAT AAGATTTTAT   
  
  
+ TTAGAAATAA AAATTTAAAC ACTATGTGAT TTATAGTTAT TATAATAAAT AGTATTTTTT AAACTATTTC   
  
  
+ ATTGATGAGA ACAGGAAAGT AAATTGCCGA CATGTTAATG TTATTATATC TAAAAATTAA ATCATAAAAT   
  
  
+ TAATTATAAT ATTTTAAAAA TATTTCAACT AAAATTTTAG AACAATAAGT GCGTAATACG GACTTAAAGG   
  
  
+ CTAGTTTATC GTAAAAATGT AAGTTCCTTT TGGATCTTAT GGTTGAATAT CTTGGTTGAT AAACTGTGAT   
  
  
+ TGGTTTTACA CTTTTACCAC ATATTCATAG AAGAATTATG TGCACGTTGA TGGTTCAAGA TGGGACAAAA   
  
  
+ GGAAACATGT ATTCTTCCCC CTTTTGCCTT TTCCCCTAAG CATTTGGATT GAGGTGTTGT CATCTGAATA   
  
  
+ TCAAAAATTC TTTTTGAAAA TTCAGTCTCA ATCAAAATTC AAAATTAAAA CAAAGACTTA AGAGTTTTAT   
  
  
+ TTGTTCTTAA CCTTCTTAAC TTTCTACTTT TCTTTTTTTT CTCATTCCCC CTCTTCAAAT CTCATTTATT   
  
  
+ CTTAACCCCC TCTTCCGTTC ATGCTCTCTC TGTCTATCAA CTAATATTAA TCTACCCCGA CTTTCAATTT   
  
  
+ GATGAGGTCT AATCCTTATC ATCGTATTGT GGTTGGGTTA ATTATGCAAG AAGGCAATAA ACCCCAGTGA   
  
  
+ CAAAGTCCAC GTTAAGTAGG CACCTCACCA TTAAGACATG CTCAGAAAAC ACCAAAACCA TTGAACACAA   
  
  
+ GTCCCCCAAG TCCCTTCCCT AGCTAGCCCA TTCTCTCTCC TCCCCTGTCT ATATCTACCA ACTCTTGCTC   
  
  
+ TTTGCTGAGC CTGAGTTGAA AGCGATACAC CCATAGCCTG TTCAGTTTTC AGTTTTCACA TTTGTCTTCC   
  
  
+ TCTGTTCAGT TGAAAGCGAT ACACTCTCAT TAGCTTTCAT TTTAAATACG TTCAATTCAC ACATAAATGG   
  
  
+ CTTTCAAAGC ATTGCCATTT TCGTTTAAAT GATGACAAAA TATATAGTAT CTGGCTATCT GCCCATATCT   
  
  
+ TGACCTTATT TACAAAGGCT GAGCCATCTT TTAATTTTTT TAGCCTCTTT TTTTAATCAA AAATAGAAAT   
  
  
+ TTCAAGAAAG CAGAAATCGT CAGATGACAG TGCAGACAGC GTTTTCGTTT CATACAAATC TCAAAAGCTG   
  
  
+ AAGTGTCAAT TTCTTCGATT ATTTTTGTCA GAAAATATTT ACGTCTCACC TTCACGTTGT TATTATTCTT   
  
  
+ TCTTTTTTAT TATTCTCATA ACTAGTAATA AAGTTAGCAA TGATAAAAAA TTATTCGATC CGAATATTTT   
  
  
+ AATTTATCTG ACCTAAAAAC ATAAGTAAAG GCACAAATTT TTCATCCAAA TTTTAATTTT TGATGTAATA   
  
  
+ TTTTTTATAT TTTTATTGTT CAAATCTGAT TTTAATCTTA TTTAAATTAT CTGACCTAAA AAAATCAAAT   
  
  
+ AATAATAAAC GTTAATTTTT TATTTAAATT TTGACATTAG TCAATCTGAC TTAAATTCGA ACCGAGCTTG   
  
  
+ AATTTTTTTG CCAGTGCTGG TAGTACTCCT GCTTTTGTAG CTCCCAATGC CATCGTACCA CCCTCTTCTA   
  
  
+ GTCTCGTCTC TTTCTCTGGT CTCCAATTAA TCACACCATC ATATCATACC GTATGATGAT ACAGTCCACT   
  
  
+ GAAATCCCAT TCTCACCGCC AATCTCTCTC CGCCACCATC CAACACTCTT AACGATGAAC AGGGCCGCCG   
  
  
+ CCTCGTCCTC CACACTCAGG CCGTGGCCGG GCAGCTTTCC CACCCAATCA AAATCTCTCT CCTCCGCCAA   
  
  
+ CTTCGGTAAC GCCAATTGCA TGGAGCAGCT CTTAGTCCAC TGCGCCGAAG CCATCGACAA CAATGACGCC   
  
  
+ ACCCCGGCCC AGCAAATCTT ATGGGTCCTG AATAACATAG CCCGACCCGA CGGCGACTCC ACCCAACGCC   
  
  
+ TCACGTGCGC ATTCCTACGT GCCTTAATCT CACGCGCCGT CCTCACTAGC ACCTGCAAGA TGGTAATCCC   
  
  
+ TCATTTCAAC CCCATCAATT CACCCCACAA ATTCTCGCTC CTCGAACTTG CCCACTTCGT CGATTTAACC   
  
  
+ CCTTGGCATC GATTCGGATT CACCGCCGCC AATTCGATCA TTCTGGAAGC TATTTCCGAC CTACCCGTTG   
  
  
+ TACACATTGT CGACCTCAGC ATCTCCCACT GTATGCAGAT CCCCACGTTG ATCGACTCCA TTGCGACCCG   
  
  
+ GTTGGAAGCC CCGGGTCGAG TCCCTCCTAT TGTCAAGCTC ACCGTCGGGG CTATTTCCGA CGAAATCCCG   
  
  
+ CCGGTGTTCG ATCTTCTGTC GTACGATGAG CTCGGAATGA GACTAATCAA CTTCGCTCGT TTTAGAAACA   
  
  
+ TCGTCCTCGA ATTCCAAGCA ATACCCACCT CCCCTTCCGA CGGATTTGCT TCGCTGTTGG AGGAGATTCG   
  
  
+ ACAAAGCAAG CTCTACTCCA ACGATGCGGC GGCGGTTATT GTGAATTGTC AGATGAGTTT GCATTTATTG   
  
  
+ CAGGAAGAGG AGGTGTCTTC ATCGTCGCCG TCGTCGATGA GGGGGATGTT TTTGCAGGCG GTGAGGAGCT   
  
  
+ TGGAGCCGAG CATGGTGGTG GTGGTGGAGG AGGACGTGGA TTTCACGGCG AGGAGTCTGG TGGGGCGGCT   
  
  
+ GAGATCGGCG TTTAATCACA TGTGGATACC CTTCGACACG GTGGACACGT TCTTGCCACG TGGGAGCCAG   
  
  
+ CAGAGAGAGT GGTTCGAGGC CGAGGTGTGC TGGAAGATTG AGAATGTGAT CGCTCATGAG GGACCCGCGA   
  
  
+ GGGTCGAGAG GCAGGAGCCC AGGGCCAAGT GGGCCCTCCG AATGAGGGAG GCCGAGTTTC AAGGGATCGA   
  
  
+ GTTCGGTGAT GAAGGTACGA CCGAGGTCAA GGCCATGCTG GAGGAGCATG CCGCTGGGTG GGGGTCTAAG   
  
  
+ AAGGAAGAGG ATGATCTTGT GCTCACTTGG AAGGGACATA GTGTTGTCTT TGCTTCTGCT TGGGTACCCA   
  
  
+ CTTA  

- +Up\_Stream \_Len000TCTTCG ATTAATTTGG TTTGTACGTG CATACAGGAT TAATTGTACT GACAGGTAGT   
  
  
- AGAGGAGGGT ACAACAAGAC AACTGTTCGG ACGTGTGGGT ACGAGGAGAG AGTACAGATT GGAGCAGGGT   
  
  
- TGCTAGTTCT AGTGACAGAC ACTCCGGTCC CCTTCTGAAC GAAAATAAAG GAAAAGAAAA CAAATGAGAG   
  
  
- ACTAAGGTAA ATCGGTATAT ATGTTCCTCT TACAGTACAC ATAGAGTATA CATATATATA TTCTAAAATA   
  
  
- AATCTTTATT TTTAAATTTG TGATACACTA AATATCAATA ATATTATTTA TCATAAAAAA TTTGATAAAG   
  
  
- TAACTACTCT TGTCCTTTCA TTTAACGGCT GTACAATTAC AATAATATAG ATTTTTAATT TAGTATTTTA   
  
  
- ATTAATATTA TAAAATTTTT ATAAAGTTGA TTTTAAAATC TTGTTATTCA CGCATTATGC CTGAATTTCC   
  
  
- GATCAAATAG CATTTTTACA TTCAAGGAAA ACCTAGAATA CCAACTTATA GAACCAACTA TTTGACACTA   
  
  
- ACCAAAATGT GAAAATGGTG TATAAGTATC TTCTTAATAC ACGTGCAACT ACCAAGTTCT ACCCTGTTTT   
  
  
- CCTTTGTACA TAAGAAGGGG GAAAACGGAA AAGGGGATTC GTAAACCTAA CTCCACAACA GTAGACTTAT   
  
  
- AGTTTTTAAG AAAAACTTTT AAGTCAGAGT TAGTTTTAAG TTTTAATTTT GTTTCTGAAT TCTCAAAATA   
  
  
- AACAAGAATT GGAAGAATTG AAAGATGAAA AGAAAAAAAA GAGTAAGGGG GAGAAGTTTA GAGTAAATAA   
  
  
- GAATTGGGGG AGAAGGCAAG TACGAGAGAG ACAGATAGTT GATTATAATT AGATGGGGCT GAAAGTTAAA   
  
  
- CTACTCCAGA TTAGGAATAG TAGCATAACA CCAACCCAAT TAATACGTTC TTCCGTTATT TGGGGTCACT   
  
  
- GTTTCAGGTG CAATTCATCC GTGGAGTGGT AATTCTGTAC GAGTCTTTTG TGGTTTTGGT AACTTGTGTT   
  
  
- CAGGGGGTTC AGGGAAGGGA TCGATCGGGT AAGAGAGAGG AGGGGACAGA TATAGATGGT TGAGAACGAG   
  
  
- AAACGACTCG GACTCAACTT TCGCTATGTG GGTATCGGAC AAGTCAAAAG TCAAAAGTGT AAACAGAAGG   
  
  
- AGACAAGTCA ACTTTCGCTA TGTGAGAGTA ATCGAAAGTA AAATTTATGC AAGTTAAGTG TGTATTTACC   
  
  
- GAAAGTTTCG TAACGGTAAA AGCAAATTTA CTACTGTTTT ATATATCATA GACCGATAGA CGGGTATAGA   
  
  
- ACTGGAATAA ATGTTTCCGA CTCGGTAGAA AATTAAAAAA ATCGGAGAAA AAAATTAGTT TTTATCTTTA   
  
  
- AAGTTCTTTC GTCTTTAGCA GTCTACTGTC ACGTCTGTCG CAAAAGCAAA GTATGTTTAG AGTTTTCGAC   
  
  
- TTCACAGTTA AAGAAGCTAA TAAAAACAGT CTTTTATAAA TGCAGAGTGG AAGTGCAACA ATAATAAGAA   
  
  
- AGAAAAAATA ATAAGAGTAT TGATCATTAT TTCAATCGTT ACTATTTTTT AATAAGCTAG GCTTATAAAA   
  
  
- TTAAATAGAC TGGATTTTTG TATTCATTTC CGTGTTTAAA AAGTAGGTTT AAAATTAAAA ACTACATTAT   
  
  
- AAAAAATATA AAAATAACAA GTTTAGACTA AAATTAGAAT AAATTTAATA GACTGGATTT TTTTAGTTTA   
  
  
- TTATTATTTG CAATTAAAAA ATAAATTTAA AACTGTAATC AGTTAGACTG AATTTAAGCT TGGCTCGAAC   
  
  
- TTAAAAAAAC GGTCACGACC ATCATGAGGA CGAAAACATC GAGGGTTACG GTAGCATGGT GGGAGAAGAT   
  
  
- CAGAGCAGAG AAAGAGACCA GAGGTTAATT AGTGTGGTAG TATAGTATGG CATACTACTA TGTCAGGTGA   
  
  
- CTTTAGGGTA AGAGTGGCGG TTAGAGAGAG GCGGTGGTAG GTTGTGAGAA TTGCTACTTG TCCCGGCGGC   
  
  
- GGAGCAGGAG GTGTGAGTCC GGCACCGGCC CGTCGAAAGG GTGGGTTAGT TTTAGAGAGA GGAGGCGGTT   
  
  
- GAAGCCATTG CGGTTAACGT ACCTCGTCGA GAATCAGGTG ACGCGGCTTC GGTAGCTGTT GTTACTGCGG   
  
  
- TGGGGCCGGG TCGTTTAGAA TACCCAGGAC TTATTGTATC GGGCTGGGCT GCCGCTGAGG TGGGTTGCGG   
  
  
- AGTGCACGCG TAAGGATGCA CGGAATTAGA GTGCGCGGCA GGAGTGATCG TGGACGTTCT ACCATTAGGG   
  
  
- AGTAAAGTTG GGGTAGTTAA GTGGGGTGTT TAAGAGCGAG GAGCTTGAAC GGGTGAAGCA GCTAAATTGG   
  
  
- GGAACCGTAG CTAAGCCTAA GTGGCGGCGG TTAAGCTAGT AAGACCTTCG ATAAAGGCTG GATGGGCAAC   
  
  
- ATGTGTAACA GCTGGAGTCG TAGAGGGTGA CATACGTCTA GGGGTGCAAC TAGCTGAGGT AACGCTGGGC   
  
  
- CAACCTTCGG GGCCCAGCTC AGGGAGGATA ACAGTTCGAG TGGCAGCCCC GATAAAGGCT GCTTTAGGGC   
  
  
- GGCCACAAGC TAGAAGACAG CATGCTACTC GAGCCTTACT CTGATTAGTT GAAGCGAGCA AAATCTTTGT   
  
  
- AGCAGGAGCT TAAGGTTCGT TATGGGTGGA GGGGAAGGCT GCCTAAACGA AGCGACAACC TCCTCTAAGC   
  
  
- TGTTTCGTTC GAGATGAGGT TGCTACGCCG CCGCCAATAA CACTTAACAG TCTACTCAAA CGTAAATAAC   
  
  
- GTCCTTCTCC TCCACAGAAG TAGCAGCGGC AGCAGCTACT CCCCCTACAA AAACGTCCGC CACTCCTCGA   
  
  
- ACCTCGGCTC GTACCACCAC CACCACCTCC TCCTGCACCT AAAGTGCCGC TCCTCAGACC ACCCCGCCGA   
  
  
- CTCTAGCCGC AAATTAGTGT ACACCTATGG GAAGCTGTGC CACCTGTGCA AGAACGGTGC ACCCTCGGTC   
  
  
- GTCTCTCTCA CCAAGCTCCG GCTCCACACG ACCTTCTAAC TCTTACACTA GCGAGTACTC CCTGGGCGCT   
  
  
- CCCAGCTCTC CGTCCTCGGG TCCCGGTTCA CCCGGGAGGC TTACTCCCTC CGGCTCAAAG TTCCCTAGCT   
  
  
- CAAGCCACTA CTTCCATGCT GGCTCCAGTT CCGGTACGAC CTCCTCGTAC GGCGACCCAC CCCCAGATTC   
  
  
- TTCCTTCTCC TACTAGAACA CGAGTGAACC TTCCCTGTAT CACAACAGAA ACGAAGACGA ACCCATGGGT   
  
  
- GAAT

+     DRE core

| Site Name | Organism | Position | Strand | Matrix score. | sequence | function |
| --- | --- | --- | --- | --- | --- | --- |
| DRE core | Arabidopsis thaliana | 380 | + | 6 | GCCGAC |  |

>HU06G00376.1   
+ +Up\_Stream \_Len000AGAAGC TAATTAAACC AAACATGCAC GTATGTCCTA ATTAACATGA CTGTCCATCA   
  
  
+ TCTCCTCCCA TGTTGTTCTG TTGACAAGCC TGCACACCCA TGCTCCTCTC TCATGTCTAA CCTCGTCCCA   
  
  
+ ACGATCAAGA TCACTGTCTG TGAGGCCAGG GGAAGACTTG CTTTTATTTC CTTTTCTTTT GTTTACTCTC   
  
  
+ TGATTCCATT TAGCCATATA TACAAGGAGA ATGTCATGTG TATCTCATAT GTATATATAT AAGATTTTAT   
  
  
+ TTAGAAATAA AAATTTAAAC ACTATGTGAT TTATAGTTAT TATAATAAAT AGTATTTTTT AAACTATTTC   
  
  
+ ATTGATGAGA ACAGGAAAGT AAATTGCCGA CATGTTAATG TTATTATATC TAAAAATTAA ATCATAAAAT   
  
  
+ TAATTATAAT ATTTTAAAAA TATTTCAACT AAAATTTTAG AACAATAAGT GCGTAATACG GACTTAAAGG   
  
  
+ CTAGTTTATC GTAAAAATGT AAGTTCCTTT TGGATCTTAT GGTTGAATAT CTTGGTTGAT AAACTGTGAT   
  
  
+ TGGTTTTACA CTTTTACCAC ATATTCATAG AAGAATTATG TGCACGTTGA TGGTTCAAGA TGGGACAAAA   
  
  
+ GGAAACATGT ATTCTTCCCC CTTTTGCCTT TTCCCCTAAG CATTTGGATT GAGGTGTTGT CATCTGAATA   
  
  
+ TCAAAAATTC TTTTTGAAAA TTCAGTCTCA ATCAAAATTC AAAATTAAAA CAAAGACTTA AGAGTTTTAT   
  
  
+ TTGTTCTTAA CCTTCTTAAC TTTCTACTTT TCTTTTTTTT CTCATTCCCC CTCTTCAAAT CTCATTTATT   
  
  
+ CTTAACCCCC TCTTCCGTTC ATGCTCTCTC TGTCTATCAA CTAATATTAA TCTACCCCGA CTTTCAATTT   
  
  
+ GATGAGGTCT AATCCTTATC ATCGTATTGT GGTTGGGTTA ATTATGCAAG AAGGCAATAA ACCCCAGTGA   
  
  
+ CAAAGTCCAC GTTAAGTAGG CACCTCACCA TTAAGACATG CTCAGAAAAC ACCAAAACCA TTGAACACAA   
  
  
+ GTCCCCCAAG TCCCTTCCCT AGCTAGCCCA TTCTCTCTCC TCCCCTGTCT ATATCTACCA ACTCTTGCTC   
  
  
+ TTTGCTGAGC CTGAGTTGAA AGCGATACAC CCATAGCCTG TTCAGTTTTC AGTTTTCACA TTTGTCTTCC   
  
  
+ TCTGTTCAGT TGAAAGCGAT ACACTCTCAT TAGCTTTCAT TTTAAATACG TTCAATTCAC ACATAAATGG   
  
  
+ CTTTCAAAGC ATTGCCATTT TCGTTTAAAT GATGACAAAA TATATAGTAT CTGGCTATCT GCCCATATCT   
  
  
+ TGACCTTATT TACAAAGGCT GAGCCATCTT TTAATTTTTT TAGCCTCTTT TTTTAATCAA AAATAGAAAT   
  
  
+ TTCAAGAAAG CAGAAATCGT CAGATGACAG TGCAGACAGC GTTTTCGTTT CATACAAATC TCAAAAGCTG   
  
  
+ AAGTGTCAAT TTCTTCGATT ATTTTTGTCA GAAAATATTT ACGTCTCACC TTCACGTTGT TATTATTCTT   
  
  
+ TCTTTTTTAT TATTCTCATA ACTAGTAATA AAGTTAGCAA TGATAAAAAA TTATTCGATC CGAATATTTT   
  
  
+ AATTTATCTG ACCTAAAAAC ATAAGTAAAG GCACAAATTT TTCATCCAAA TTTTAATTTT TGATGTAATA   
  
  
+ TTTTTTATAT TTTTATTGTT CAAATCTGAT TTTAATCTTA TTTAAATTAT CTGACCTAAA AAAATCAAAT   
  
  
+ AATAATAAAC GTTAATTTTT TATTTAAATT TTGACATTAG TCAATCTGAC TTAAATTCGA ACCGAGCTTG   
  
  
+ AATTTTTTTG CCAGTGCTGG TAGTACTCCT GCTTTTGTAG CTCCCAATGC CATCGTACCA CCCTCTTCTA   
  
  
+ GTCTCGTCTC TTTCTCTGGT CTCCAATTAA TCACACCATC ATATCATACC GTATGATGAT ACAGTCCACT   
  
  
+ GAAATCCCAT TCTCACCGCC AATCTCTCTC CGCCACCATC CAACACTCTT AACGATGAAC AGGGCCGCCG   
  
  
+ CCTCGTCCTC CACACTCAGG CCGTGGCCGG GCAGCTTTCC CACCCAATCA AAATCTCTCT CCTCCGCCAA   
  
  
+ CTTCGGTAAC GCCAATTGCA TGGAGCAGCT CTTAGTCCAC TGCGCCGAAG CCATCGACAA CAATGACGCC   
  
  
+ ACCCCGGCCC AGCAAATCTT ATGGGTCCTG AATAACATAG CCCGACCCGA CGGCGACTCC ACCCAACGCC   
  
  
+ TCACGTGCGC ATTCCTACGT GCCTTAATCT CACGCGCCGT CCTCACTAGC ACCTGCAAGA TGGTAATCCC   
  
  
+ TCATTTCAAC CCCATCAATT CACCCCACAA ATTCTCGCTC CTCGAACTTG CCCACTTCGT CGATTTAACC   
  
  
+ CCTTGGCATC GATTCGGATT CACCGCCGCC AATTCGATCA TTCTGGAAGC TATTTCCGAC CTACCCGTTG   
  
  
+ TACACATTGT CGACCTCAGC ATCTCCCACT GTATGCAGAT CCCCACGTTG ATCGACTCCA TTGCGACCCG   
  
  
+ GTTGGAAGCC CCGGGTCGAG TCCCTCCTAT TGTCAAGCTC ACCGTCGGGG CTATTTCCGA CGAAATCCCG   
  
  
+ CCGGTGTTCG ATCTTCTGTC GTACGATGAG CTCGGAATGA GACTAATCAA CTTCGCTCGT TTTAGAAACA   
  
  
+ TCGTCCTCGA ATTCCAAGCA ATACCCACCT CCCCTTCCGA CGGATTTGCT TCGCTGTTGG AGGAGATTCG   
  
  
+ ACAAAGCAAG CTCTACTCCA ACGATGCGGC GGCGGTTATT GTGAATTGTC AGATGAGTTT GCATTTATTG   
  
  
+ CAGGAAGAGG AGGTGTCTTC ATCGTCGCCG TCGTCGATGA GGGGGATGTT TTTGCAGGCG GTGAGGAGCT   
  
  
+ TGGAGCCGAG CATGGTGGTG GTGGTGGAGG AGGACGTGGA TTTCACGGCG AGGAGTCTGG TGGGGCGGCT   
  
  
+ GAGATCGGCG TTTAATCACA TGTGGATACC CTTCGACACG GTGGACACGT TCTTGCCACG TGGGAGCCAG   
  
  
+ CAGAGAGAGT GGTTCGAGGC CGAGGTGTGC TGGAAGATTG AGAATGTGAT CGCTCATGAG GGACCCGCGA   
  
  
+ GGGTCGAGAG GCAGGAGCCC AGGGCCAAGT GGGCCCTCCG AATGAGGGAG GCCGAGTTTC AAGGGATCGA   
  
  
+ GTTCGGTGAT GAAGGTACGA CCGAGGTCAA GGCCATGCTG GAGGAGCATG CCGCTGGGTG GGGGTCTAAG   
  
  
+ AAGGAAGAGG ATGATCTTGT GCTCACTTGG AAGGGACATA GTGTTGTCTT TGCTTCTGCT TGGGTACCCA   
  
  
+ CTTA  

- +Up\_Stream \_Len000TCTTCG ATTAATTTGG TTTGTACGTG CATACAGGAT TAATTGTACT GACAGGTAGT   
  
  
- AGAGGAGGGT ACAACAAGAC AACTGTTCGG ACGTGTGGGT ACGAGGAGAG AGTACAGATT GGAGCAGGGT   
  
  
- TGCTAGTTCT AGTGACAGAC ACTCCGGTCC CCTTCTGAAC GAAAATAAAG GAAAAGAAAA CAAATGAGAG   
  
  
- ACTAAGGTAA ATCGGTATAT ATGTTCCTCT TACAGTACAC ATAGAGTATA CATATATATA TTCTAAAATA   
  
  
- AATCTTTATT TTTAAATTTG TGATACACTA AATATCAATA ATATTATTTA TCATAAAAAA TTTGATAAAG   
  
  
- TAACTACTCT TGTCCTTTCA TTTAACGGCT GTACAATTAC AATAATATAG ATTTTTAATT TAGTATTTTA   
  
  
- ATTAATATTA TAAAATTTTT ATAAAGTTGA TTTTAAAATC TTGTTATTCA CGCATTATGC CTGAATTTCC   
  
  
- GATCAAATAG CATTTTTACA TTCAAGGAAA ACCTAGAATA CCAACTTATA GAACCAACTA TTTGACACTA   
  
  
- ACCAAAATGT GAAAATGGTG TATAAGTATC TTCTTAATAC ACGTGCAACT ACCAAGTTCT ACCCTGTTTT   
  
  
- CCTTTGTACA TAAGAAGGGG GAAAACGGAA AAGGGGATTC GTAAACCTAA CTCCACAACA GTAGACTTAT   
  
  
- AGTTTTTAAG AAAAACTTTT AAGTCAGAGT TAGTTTTAAG TTTTAATTTT GTTTCTGAAT TCTCAAAATA   
  
  
- AACAAGAATT GGAAGAATTG AAAGATGAAA AGAAAAAAAA GAGTAAGGGG GAGAAGTTTA GAGTAAATAA   
  
  
- GAATTGGGGG AGAAGGCAAG TACGAGAGAG ACAGATAGTT GATTATAATT AGATGGGGCT GAAAGTTAAA   
  
  
- CTACTCCAGA TTAGGAATAG TAGCATAACA CCAACCCAAT TAATACGTTC TTCCGTTATT TGGGGTCACT   
  
  
- GTTTCAGGTG CAATTCATCC GTGGAGTGGT AATTCTGTAC GAGTCTTTTG TGGTTTTGGT AACTTGTGTT   
  
  
- CAGGGGGTTC AGGGAAGGGA TCGATCGGGT AAGAGAGAGG AGGGGACAGA TATAGATGGT TGAGAACGAG   
  
  
- AAACGACTCG GACTCAACTT TCGCTATGTG GGTATCGGAC AAGTCAAAAG TCAAAAGTGT AAACAGAAGG   
  
  
- AGACAAGTCA ACTTTCGCTA TGTGAGAGTA ATCGAAAGTA AAATTTATGC AAGTTAAGTG TGTATTTACC   
  
  
- GAAAGTTTCG TAACGGTAAA AGCAAATTTA CTACTGTTTT ATATATCATA GACCGATAGA CGGGTATAGA   
  
  
- ACTGGAATAA ATGTTTCCGA CTCGGTAGAA AATTAAAAAA ATCGGAGAAA AAAATTAGTT TTTATCTTTA   
  
  
- AAGTTCTTTC GTCTTTAGCA GTCTACTGTC ACGTCTGTCG CAAAAGCAAA GTATGTTTAG AGTTTTCGAC   
  
  
- TTCACAGTTA AAGAAGCTAA TAAAAACAGT CTTTTATAAA TGCAGAGTGG AAGTGCAACA ATAATAAGAA   
  
  
- AGAAAAAATA ATAAGAGTAT TGATCATTAT TTCAATCGTT ACTATTTTTT AATAAGCTAG GCTTATAAAA   
  
  
- TTAAATAGAC TGGATTTTTG TATTCATTTC CGTGTTTAAA AAGTAGGTTT AAAATTAAAA ACTACATTAT   
  
  
- AAAAAATATA AAAATAACAA GTTTAGACTA AAATTAGAAT AAATTTAATA GACTGGATTT TTTTAGTTTA   
  
  
- TTATTATTTG CAATTAAAAA ATAAATTTAA AACTGTAATC AGTTAGACTG AATTTAAGCT TGGCTCGAAC   
  
  
- TTAAAAAAAC GGTCACGACC ATCATGAGGA CGAAAACATC GAGGGTTACG GTAGCATGGT GGGAGAAGAT   
  
  
- CAGAGCAGAG AAAGAGACCA GAGGTTAATT AGTGTGGTAG TATAGTATGG CATACTACTA TGTCAGGTGA   
  
  
- CTTTAGGGTA AGAGTGGCGG TTAGAGAGAG GCGGTGGTAG GTTGTGAGAA TTGCTACTTG TCCCGGCGGC   
  
  
- GGAGCAGGAG GTGTGAGTCC GGCACCGGCC CGTCGAAAGG GTGGGTTAGT TTTAGAGAGA GGAGGCGGTT   
  
  
- GAAGCCATTG CGGTTAACGT ACCTCGTCGA GAATCAGGTG ACGCGGCTTC GGTAGCTGTT GTTACTGCGG   
  
  
- TGGGGCCGGG TCGTTTAGAA TACCCAGGAC TTATTGTATC GGGCTGGGCT GCCGCTGAGG TGGGTTGCGG   
  
  
- AGTGCACGCG TAAGGATGCA CGGAATTAGA GTGCGCGGCA GGAGTGATCG TGGACGTTCT ACCATTAGGG   
  
  
- AGTAAAGTTG GGGTAGTTAA GTGGGGTGTT TAAGAGCGAG GAGCTTGAAC GGGTGAAGCA GCTAAATTGG   
  
  
- GGAACCGTAG CTAAGCCTAA GTGGCGGCGG TTAAGCTAGT AAGACCTTCG ATAAAGGCTG GATGGGCAAC   
  
  
- ATGTGTAACA GCTGGAGTCG TAGAGGGTGA CATACGTCTA GGGGTGCAAC TAGCTGAGGT AACGCTGGGC   
  
  
- CAACCTTCGG GGCCCAGCTC AGGGAGGATA ACAGTTCGAG TGGCAGCCCC GATAAAGGCT GCTTTAGGGC   
  
  
- GGCCACAAGC TAGAAGACAG CATGCTACTC GAGCCTTACT CTGATTAGTT GAAGCGAGCA AAATCTTTGT   
  
  
- AGCAGGAGCT TAAGGTTCGT TATGGGTGGA GGGGAAGGCT GCCTAAACGA AGCGACAACC TCCTCTAAGC   
  
  
- TGTTTCGTTC GAGATGAGGT TGCTACGCCG CCGCCAATAA CACTTAACAG TCTACTCAAA CGTAAATAAC   
  
  
- GTCCTTCTCC TCCACAGAAG TAGCAGCGGC AGCAGCTACT CCCCCTACAA AAACGTCCGC CACTCCTCGA   
  
  
- ACCTCGGCTC GTACCACCAC CACCACCTCC TCCTGCACCT AAAGTGCCGC TCCTCAGACC ACCCCGCCGA   
  
  
- CTCTAGCCGC AAATTAGTGT ACACCTATGG GAAGCTGTGC CACCTGTGCA AGAACGGTGC ACCCTCGGTC   
  
  
- GTCTCTCTCA CCAAGCTCCG GCTCCACACG ACCTTCTAAC TCTTACACTA GCGAGTACTC CCTGGGCGCT   
  
  
- CCCAGCTCTC CGTCCTCGGG TCCCGGTTCA CCCGGGAGGC TTACTCCCTC CGGCTCAAAG TTCCCTAGCT   
  
  
- CAAGCCACTA CTTCCATGCT GGCTCCAGTT CCGGTACGAC CTCCTCGTAC GGCGACCCAC CCCCAGATTC   
  
  
- TTCCTTCTCC TACTAGAACA CGAGTGAACC TTCCCTGTAT CACAACAGAA ACGAAGACGA ACCCATGGGT   
  
  
- GAAT

+     ERE

| Site Name | Organism | Position | Strand | Matrix score. | sequence | function |
| --- | --- | --- | --- | --- | --- | --- |
| ERE | Nicotiana glutinos | 435 | + | 8 | ATTTTAAA |  |
| ERE | Nicotiana glutinos | 1233 | + | 8 | ATTTTAAA |  |

>HU06G00376.1   
+ +Up\_Stream \_Len000AGAAGC TAATTAAACC AAACATGCAC GTATGTCCTA ATTAACATGA CTGTCCATCA   
  
  
+ TCTCCTCCCA TGTTGTTCTG TTGACAAGCC TGCACACCCA TGCTCCTCTC TCATGTCTAA CCTCGTCCCA   
  
  
+ ACGATCAAGA TCACTGTCTG TGAGGCCAGG GGAAGACTTG CTTTTATTTC CTTTTCTTTT GTTTACTCTC   
  
  
+ TGATTCCATT TAGCCATATA TACAAGGAGA ATGTCATGTG TATCTCATAT GTATATATAT AAGATTTTAT   
  
  
+ TTAGAAATAA AAATTTAAAC ACTATGTGAT TTATAGTTAT TATAATAAAT AGTATTTTTT AAACTATTTC   
  
  
+ ATTGATGAGA ACAGGAAAGT AAATTGCCGA CATGTTAATG TTATTATATC TAAAAATTAA ATCATAAAAT   
  
  
+ TAATTATAAT ATTTTAAAAA TATTTCAACT AAAATTTTAG AACAATAAGT GCGTAATACG GACTTAAAGG   
  
  
+ CTAGTTTATC GTAAAAATGT AAGTTCCTTT TGGATCTTAT GGTTGAATAT CTTGGTTGAT AAACTGTGAT   
  
  
+ TGGTTTTACA CTTTTACCAC ATATTCATAG AAGAATTATG TGCACGTTGA TGGTTCAAGA TGGGACAAAA   
  
  
+ GGAAACATGT ATTCTTCCCC CTTTTGCCTT TTCCCCTAAG CATTTGGATT GAGGTGTTGT CATCTGAATA   
  
  
+ TCAAAAATTC TTTTTGAAAA TTCAGTCTCA ATCAAAATTC AAAATTAAAA CAAAGACTTA AGAGTTTTAT   
  
  
+ TTGTTCTTAA CCTTCTTAAC TTTCTACTTT TCTTTTTTTT CTCATTCCCC CTCTTCAAAT CTCATTTATT   
  
  
+ CTTAACCCCC TCTTCCGTTC ATGCTCTCTC TGTCTATCAA CTAATATTAA TCTACCCCGA CTTTCAATTT   
  
  
+ GATGAGGTCT AATCCTTATC ATCGTATTGT GGTTGGGTTA ATTATGCAAG AAGGCAATAA ACCCCAGTGA   
  
  
+ CAAAGTCCAC GTTAAGTAGG CACCTCACCA TTAAGACATG CTCAGAAAAC ACCAAAACCA TTGAACACAA   
  
  
+ GTCCCCCAAG TCCCTTCCCT AGCTAGCCCA TTCTCTCTCC TCCCCTGTCT ATATCTACCA ACTCTTGCTC   
  
  
+ TTTGCTGAGC CTGAGTTGAA AGCGATACAC CCATAGCCTG TTCAGTTTTC AGTTTTCACA TTTGTCTTCC   
  
  
+ TCTGTTCAGT TGAAAGCGAT ACACTCTCAT TAGCTTTCAT TTTAAATACG TTCAATTCAC ACATAAATGG   
  
  
+ CTTTCAAAGC ATTGCCATTT TCGTTTAAAT GATGACAAAA TATATAGTAT CTGGCTATCT GCCCATATCT   
  
  
+ TGACCTTATT TACAAAGGCT GAGCCATCTT TTAATTTTTT TAGCCTCTTT TTTTAATCAA AAATAGAAAT   
  
  
+ TTCAAGAAAG CAGAAATCGT CAGATGACAG TGCAGACAGC GTTTTCGTTT CATACAAATC TCAAAAGCTG   
  
  
+ AAGTGTCAAT TTCTTCGATT ATTTTTGTCA GAAAATATTT ACGTCTCACC TTCACGTTGT TATTATTCTT   
  
  
+ TCTTTTTTAT TATTCTCATA ACTAGTAATA AAGTTAGCAA TGATAAAAAA TTATTCGATC CGAATATTTT   
  
  
+ AATTTATCTG ACCTAAAAAC ATAAGTAAAG GCACAAATTT TTCATCCAAA TTTTAATTTT TGATGTAATA   
  
  
+ TTTTTTATAT TTTTATTGTT CAAATCTGAT TTTAATCTTA TTTAAATTAT CTGACCTAAA AAAATCAAAT   
  
  
+ AATAATAAAC GTTAATTTTT TATTTAAATT TTGACATTAG TCAATCTGAC TTAAATTCGA ACCGAGCTTG   
  
  
+ AATTTTTTTG CCAGTGCTGG TAGTACTCCT GCTTTTGTAG CTCCCAATGC CATCGTACCA CCCTCTTCTA   
  
  
+ GTCTCGTCTC TTTCTCTGGT CTCCAATTAA TCACACCATC ATATCATACC GTATGATGAT ACAGTCCACT   
  
  
+ GAAATCCCAT TCTCACCGCC AATCTCTCTC CGCCACCATC CAACACTCTT AACGATGAAC AGGGCCGCCG   
  
  
+ CCTCGTCCTC CACACTCAGG CCGTGGCCGG GCAGCTTTCC CACCCAATCA AAATCTCTCT CCTCCGCCAA   
  
  
+ CTTCGGTAAC GCCAATTGCA TGGAGCAGCT CTTAGTCCAC TGCGCCGAAG CCATCGACAA CAATGACGCC   
  
  
+ ACCCCGGCCC AGCAAATCTT ATGGGTCCTG AATAACATAG CCCGACCCGA CGGCGACTCC ACCCAACGCC   
  
  
+ TCACGTGCGC ATTCCTACGT GCCTTAATCT CACGCGCCGT CCTCACTAGC ACCTGCAAGA TGGTAATCCC   
  
  
+ TCATTTCAAC CCCATCAATT CACCCCACAA ATTCTCGCTC CTCGAACTTG CCCACTTCGT CGATTTAACC   
  
  
+ CCTTGGCATC GATTCGGATT CACCGCCGCC AATTCGATCA TTCTGGAAGC TATTTCCGAC CTACCCGTTG   
  
  
+ TACACATTGT CGACCTCAGC ATCTCCCACT GTATGCAGAT CCCCACGTTG ATCGACTCCA TTGCGACCCG   
  
  
+ GTTGGAAGCC CCGGGTCGAG TCCCTCCTAT TGTCAAGCTC ACCGTCGGGG CTATTTCCGA CGAAATCCCG   
  
  
+ CCGGTGTTCG ATCTTCTGTC GTACGATGAG CTCGGAATGA GACTAATCAA CTTCGCTCGT TTTAGAAACA   
  
  
+ TCGTCCTCGA ATTCCAAGCA ATACCCACCT CCCCTTCCGA CGGATTTGCT TCGCTGTTGG AGGAGATTCG   
  
  
+ ACAAAGCAAG CTCTACTCCA ACGATGCGGC GGCGGTTATT GTGAATTGTC AGATGAGTTT GCATTTATTG   
  
  
+ CAGGAAGAGG AGGTGTCTTC ATCGTCGCCG TCGTCGATGA GGGGGATGTT TTTGCAGGCG GTGAGGAGCT   
  
  
+ TGGAGCCGAG CATGGTGGTG GTGGTGGAGG AGGACGTGGA TTTCACGGCG AGGAGTCTGG TGGGGCGGCT   
  
  
+ GAGATCGGCG TTTAATCACA TGTGGATACC CTTCGACACG GTGGACACGT TCTTGCCACG TGGGAGCCAG   
  
  
+ CAGAGAGAGT GGTTCGAGGC CGAGGTGTGC TGGAAGATTG AGAATGTGAT CGCTCATGAG GGACCCGCGA   
  
  
+ GGGTCGAGAG GCAGGAGCCC AGGGCCAAGT GGGCCCTCCG AATGAGGGAG GCCGAGTTTC AAGGGATCGA   
  
  
+ GTTCGGTGAT GAAGGTACGA CCGAGGTCAA GGCCATGCTG GAGGAGCATG CCGCTGGGTG GGGGTCTAAG   
  
  
+ AAGGAAGAGG ATGATCTTGT GCTCACTTGG AAGGGACATA GTGTTGTCTT TGCTTCTGCT TGGGTACCCA   
  
  
+ CTTA  

- +Up\_Stream \_Len000TCTTCG ATTAATTTGG TTTGTACGTG CATACAGGAT TAATTGTACT GACAGGTAGT   
  
  
- AGAGGAGGGT ACAACAAGAC AACTGTTCGG ACGTGTGGGT ACGAGGAGAG AGTACAGATT GGAGCAGGGT   
  
  
- TGCTAGTTCT AGTGACAGAC ACTCCGGTCC CCTTCTGAAC GAAAATAAAG GAAAAGAAAA CAAATGAGAG   
  
  
- ACTAAGGTAA ATCGGTATAT ATGTTCCTCT TACAGTACAC ATAGAGTATA CATATATATA TTCTAAAATA   
  
  
- AATCTTTATT TTTAAATTTG TGATACACTA AATATCAATA ATATTATTTA TCATAAAAAA TTTGATAAAG   
  
  
- TAACTACTCT TGTCCTTTCA TTTAACGGCT GTACAATTAC AATAATATAG ATTTTTAATT TAGTATTTTA   
  
  
- ATTAATATTA TAAAATTTTT ATAAAGTTGA TTTTAAAATC TTGTTATTCA CGCATTATGC CTGAATTTCC   
  
  
- GATCAAATAG CATTTTTACA TTCAAGGAAA ACCTAGAATA CCAACTTATA GAACCAACTA TTTGACACTA   
  
  
- ACCAAAATGT GAAAATGGTG TATAAGTATC TTCTTAATAC ACGTGCAACT ACCAAGTTCT ACCCTGTTTT   
  
  
- CCTTTGTACA TAAGAAGGGG GAAAACGGAA AAGGGGATTC GTAAACCTAA CTCCACAACA GTAGACTTAT   
  
  
- AGTTTTTAAG AAAAACTTTT AAGTCAGAGT TAGTTTTAAG TTTTAATTTT GTTTCTGAAT TCTCAAAATA   
  
  
- AACAAGAATT GGAAGAATTG AAAGATGAAA AGAAAAAAAA GAGTAAGGGG GAGAAGTTTA GAGTAAATAA   
  
  
- GAATTGGGGG AGAAGGCAAG TACGAGAGAG ACAGATAGTT GATTATAATT AGATGGGGCT GAAAGTTAAA   
  
  
- CTACTCCAGA TTAGGAATAG TAGCATAACA CCAACCCAAT TAATACGTTC TTCCGTTATT TGGGGTCACT   
  
  
- GTTTCAGGTG CAATTCATCC GTGGAGTGGT AATTCTGTAC GAGTCTTTTG TGGTTTTGGT AACTTGTGTT   
  
  
- CAGGGGGTTC AGGGAAGGGA TCGATCGGGT AAGAGAGAGG AGGGGACAGA TATAGATGGT TGAGAACGAG   
  
  
- AAACGACTCG GACTCAACTT TCGCTATGTG GGTATCGGAC AAGTCAAAAG TCAAAAGTGT AAACAGAAGG   
  
  
- AGACAAGTCA ACTTTCGCTA TGTGAGAGTA ATCGAAAGTA AAATTTATGC AAGTTAAGTG TGTATTTACC   
  
  
- GAAAGTTTCG TAACGGTAAA AGCAAATTTA CTACTGTTTT ATATATCATA GACCGATAGA CGGGTATAGA   
  
  
- ACTGGAATAA ATGTTTCCGA CTCGGTAGAA AATTAAAAAA ATCGGAGAAA AAAATTAGTT TTTATCTTTA   
  
  
- AAGTTCTTTC GTCTTTAGCA GTCTACTGTC ACGTCTGTCG CAAAAGCAAA GTATGTTTAG AGTTTTCGAC   
  
  
- TTCACAGTTA AAGAAGCTAA TAAAAACAGT CTTTTATAAA TGCAGAGTGG AAGTGCAACA ATAATAAGAA   
  
  
- AGAAAAAATA ATAAGAGTAT TGATCATTAT TTCAATCGTT ACTATTTTTT AATAAGCTAG GCTTATAAAA   
  
  
- TTAAATAGAC TGGATTTTTG TATTCATTTC CGTGTTTAAA AAGTAGGTTT AAAATTAAAA ACTACATTAT   
  
  
- AAAAAATATA AAAATAACAA GTTTAGACTA AAATTAGAAT AAATTTAATA GACTGGATTT TTTTAGTTTA   
  
  
- TTATTATTTG CAATTAAAAA ATAAATTTAA AACTGTAATC AGTTAGACTG AATTTAAGCT TGGCTCGAAC   
  
  
- TTAAAAAAAC GGTCACGACC ATCATGAGGA CGAAAACATC GAGGGTTACG GTAGCATGGT GGGAGAAGAT   
  
  
- CAGAGCAGAG AAAGAGACCA GAGGTTAATT AGTGTGGTAG TATAGTATGG CATACTACTA TGTCAGGTGA   
  
  
- CTTTAGGGTA AGAGTGGCGG TTAGAGAGAG GCGGTGGTAG GTTGTGAGAA TTGCTACTTG TCCCGGCGGC   
  
  
- GGAGCAGGAG GTGTGAGTCC GGCACCGGCC CGTCGAAAGG GTGGGTTAGT TTTAGAGAGA GGAGGCGGTT   
  
  
- GAAGCCATTG CGGTTAACGT ACCTCGTCGA GAATCAGGTG ACGCGGCTTC GGTAGCTGTT GTTACTGCGG   
  
  
- TGGGGCCGGG TCGTTTAGAA TACCCAGGAC TTATTGTATC GGGCTGGGCT GCCGCTGAGG TGGGTTGCGG   
  
  
- AGTGCACGCG TAAGGATGCA CGGAATTAGA GTGCGCGGCA GGAGTGATCG TGGACGTTCT ACCATTAGGG   
  
  
- AGTAAAGTTG GGGTAGTTAA GTGGGGTGTT TAAGAGCGAG GAGCTTGAAC GGGTGAAGCA GCTAAATTGG   
  
  
- GGAACCGTAG CTAAGCCTAA GTGGCGGCGG TTAAGCTAGT AAGACCTTCG ATAAAGGCTG GATGGGCAAC   
  
  
- ATGTGTAACA GCTGGAGTCG TAGAGGGTGA CATACGTCTA GGGGTGCAAC TAGCTGAGGT AACGCTGGGC   
  
  
- CAACCTTCGG GGCCCAGCTC AGGGAGGATA ACAGTTCGAG TGGCAGCCCC GATAAAGGCT GCTTTAGGGC   
  
  
- GGCCACAAGC TAGAAGACAG CATGCTACTC GAGCCTTACT CTGATTAGTT GAAGCGAGCA AAATCTTTGT   
  
  
- AGCAGGAGCT TAAGGTTCGT TATGGGTGGA GGGGAAGGCT GCCTAAACGA AGCGACAACC TCCTCTAAGC   
  
  
- TGTTTCGTTC GAGATGAGGT TGCTACGCCG CCGCCAATAA CACTTAACAG TCTACTCAAA CGTAAATAAC   
  
  
- GTCCTTCTCC TCCACAGAAG TAGCAGCGGC AGCAGCTACT CCCCCTACAA AAACGTCCGC CACTCCTCGA   
  
  
- ACCTCGGCTC GTACCACCAC CACCACCTCC TCCTGCACCT AAAGTGCCGC TCCTCAGACC ACCCCGCCGA   
  
  
- CTCTAGCCGC AAATTAGTGT ACACCTATGG GAAGCTGTGC CACCTGTGCA AGAACGGTGC ACCCTCGGTC   
  
  
- GTCTCTCTCA CCAAGCTCCG GCTCCACACG ACCTTCTAAC TCTTACACTA GCGAGTACTC CCTGGGCGCT   
  
  
- CCCAGCTCTC CGTCCTCGGG TCCCGGTTCA CCCGGGAGGC TTACTCCCTC CGGCTCAAAG TTCCCTAGCT   
  
  
- CAAGCCACTA CTTCCATGCT GGCTCCAGTT CCGGTACGAC CTCCTCGTAC GGCGACCCAC CCCCAGATTC   
  
  
- TTCCTTCTCC TACTAGAACA CGAGTGAACC TTCCCTGTAT CACAACAGAA ACGAAGACGA ACCCATGGGT   
  
  
- GAAT

+     G-Box

| Site Name | Organism | Position | Strand | Matrix score. | sequence | function |
| --- | --- | --- | --- | --- | --- | --- |
| G-Box | Pisum sativum | 3001 | - | 6 | CACGTG | cis-acting regulatory element involved in light responsiveness |
| G-Box | Pisum sativum | 2246 | - | 6 | CACGTG | cis-acting regulatory element involved in light responsiveness |
| G-Box | Pisum sativum | 992 | + | 6 | CACGTT | cis-acting regulatory element involved in light responsiveness |
| G-Box | Pisum sativum | 2498 | + | 6 | CACGTT | cis-acting regulatory element involved in light responsiveness |
| G-Box | Pisum sativum | 607 | + | 6 | CACGTT | cis-acting regulatory element involved in light responsiveness |
| G-Box | Pisum sativum | 2990 | + | 6 | CACGTT | cis-acting regulatory element involved in light responsiveness |
| G-Box | Pisum sativum | 1527 | + | 6 | CACGTT | cis-acting regulatory element involved in light responsiveness |

>HU06G00376.1   
+ +Up\_Stream \_Len000AGAAGC TAATTAAACC AAACATGCAC GTATGTCCTA ATTAACATGA CTGTCCATCA   
  
  
+ TCTCCTCCCA TGTTGTTCTG TTGACAAGCC TGCACACCCA TGCTCCTCTC TCATGTCTAA CCTCGTCCCA   
  
  
+ ACGATCAAGA TCACTGTCTG TGAGGCCAGG GGAAGACTTG CTTTTATTTC CTTTTCTTTT GTTTACTCTC   
  
  
+ TGATTCCATT TAGCCATATA TACAAGGAGA ATGTCATGTG TATCTCATAT GTATATATAT AAGATTTTAT   
  
  
+ TTAGAAATAA AAATTTAAAC ACTATGTGAT TTATAGTTAT TATAATAAAT AGTATTTTTT AAACTATTTC   
  
  
+ ATTGATGAGA ACAGGAAAGT AAATTGCCGA CATGTTAATG TTATTATATC TAAAAATTAA ATCATAAAAT   
  
  
+ TAATTATAAT ATTTTAAAAA TATTTCAACT AAAATTTTAG AACAATAAGT GCGTAATACG GACTTAAAGG   
  
  
+ CTAGTTTATC GTAAAAATGT AAGTTCCTTT TGGATCTTAT GGTTGAATAT CTTGGTTGAT AAACTGTGAT   
  
  
+ TGGTTTTACA CTTTTACCAC ATATTCATAG AAGAATTATG TGCACGTTGA TGGTTCAAGA TGGGACAAAA   
  
  
+ GGAAACATGT ATTCTTCCCC CTTTTGCCTT TTCCCCTAAG CATTTGGATT GAGGTGTTGT CATCTGAATA   
  
  
+ TCAAAAATTC TTTTTGAAAA TTCAGTCTCA ATCAAAATTC AAAATTAAAA CAAAGACTTA AGAGTTTTAT   
  
  
+ TTGTTCTTAA CCTTCTTAAC TTTCTACTTT TCTTTTTTTT CTCATTCCCC CTCTTCAAAT CTCATTTATT   
  
  
+ CTTAACCCCC TCTTCCGTTC ATGCTCTCTC TGTCTATCAA CTAATATTAA TCTACCCCGA CTTTCAATTT   
  
  
+ GATGAGGTCT AATCCTTATC ATCGTATTGT GGTTGGGTTA ATTATGCAAG AAGGCAATAA ACCCCAGTGA   
  
  
+ CAAAGTCCAC GTTAAGTAGG CACCTCACCA TTAAGACATG CTCAGAAAAC ACCAAAACCA TTGAACACAA   
  
  
+ GTCCCCCAAG TCCCTTCCCT AGCTAGCCCA TTCTCTCTCC TCCCCTGTCT ATATCTACCA ACTCTTGCTC   
  
  
+ TTTGCTGAGC CTGAGTTGAA AGCGATACAC CCATAGCCTG TTCAGTTTTC AGTTTTCACA TTTGTCTTCC   
  
  
+ TCTGTTCAGT TGAAAGCGAT ACACTCTCAT TAGCTTTCAT TTTAAATACG TTCAATTCAC ACATAAATGG   
  
  
+ CTTTCAAAGC ATTGCCATTT TCGTTTAAAT GATGACAAAA TATATAGTAT CTGGCTATCT GCCCATATCT   
  
  
+ TGACCTTATT TACAAAGGCT GAGCCATCTT TTAATTTTTT TAGCCTCTTT TTTTAATCAA AAATAGAAAT   
  
  
+ TTCAAGAAAG CAGAAATCGT CAGATGACAG TGCAGACAGC GTTTTCGTTT CATACAAATC TCAAAAGCTG   
  
  
+ AAGTGTCAAT TTCTTCGATT ATTTTTGTCA GAAAATATTT ACGTCTCACC TTCACGTTGT TATTATTCTT   
  
  
+ TCTTTTTTAT TATTCTCATA ACTAGTAATA AAGTTAGCAA TGATAAAAAA TTATTCGATC CGAATATTTT   
  
  
+ AATTTATCTG ACCTAAAAAC ATAAGTAAAG GCACAAATTT TTCATCCAAA TTTTAATTTT TGATGTAATA   
  
  
+ TTTTTTATAT TTTTATTGTT CAAATCTGAT TTTAATCTTA TTTAAATTAT CTGACCTAAA AAAATCAAAT   
  
  
+ AATAATAAAC GTTAATTTTT TATTTAAATT TTGACATTAG TCAATCTGAC TTAAATTCGA ACCGAGCTTG   
  
  
+ AATTTTTTTG CCAGTGCTGG TAGTACTCCT GCTTTTGTAG CTCCCAATGC CATCGTACCA CCCTCTTCTA   
  
  
+ GTCTCGTCTC TTTCTCTGGT CTCCAATTAA TCACACCATC ATATCATACC GTATGATGAT ACAGTCCACT   
  
  
+ GAAATCCCAT TCTCACCGCC AATCTCTCTC CGCCACCATC CAACACTCTT AACGATGAAC AGGGCCGCCG   
  
  
+ CCTCGTCCTC CACACTCAGG CCGTGGCCGG GCAGCTTTCC CACCCAATCA AAATCTCTCT CCTCCGCCAA   
  
  
+ CTTCGGTAAC GCCAATTGCA TGGAGCAGCT CTTAGTCCAC TGCGCCGAAG CCATCGACAA CAATGACGCC   
  
  
+ ACCCCGGCCC AGCAAATCTT ATGGGTCCTG AATAACATAG CCCGACCCGA CGGCGACTCC ACCCAACGCC   
  
  
+ TCACGTGCGC ATTCCTACGT GCCTTAATCT CACGCGCCGT CCTCACTAGC ACCTGCAAGA TGGTAATCCC   
  
  
+ TCATTTCAAC CCCATCAATT CACCCCACAA ATTCTCGCTC CTCGAACTTG CCCACTTCGT CGATTTAACC   
  
  
+ CCTTGGCATC GATTCGGATT CACCGCCGCC AATTCGATCA TTCTGGAAGC TATTTCCGAC CTACCCGTTG   
  
  
+ TACACATTGT CGACCTCAGC ATCTCCCACT GTATGCAGAT CCCCACGTTG ATCGACTCCA TTGCGACCCG   
  
  
+ GTTGGAAGCC CCGGGTCGAG TCCCTCCTAT TGTCAAGCTC ACCGTCGGGG CTATTTCCGA CGAAATCCCG   
  
  
+ CCGGTGTTCG ATCTTCTGTC GTACGATGAG CTCGGAATGA GACTAATCAA CTTCGCTCGT TTTAGAAACA   
  
  
+ TCGTCCTCGA ATTCCAAGCA ATACCCACCT CCCCTTCCGA CGGATTTGCT TCGCTGTTGG AGGAGATTCG   
  
  
+ ACAAAGCAAG CTCTACTCCA ACGATGCGGC GGCGGTTATT GTGAATTGTC AGATGAGTTT GCATTTATTG   
  
  
+ CAGGAAGAGG AGGTGTCTTC ATCGTCGCCG TCGTCGATGA GGGGGATGTT TTTGCAGGCG GTGAGGAGCT   
  
  
+ TGGAGCCGAG CATGGTGGTG GTGGTGGAGG AGGACGTGGA TTTCACGGCG AGGAGTCTGG TGGGGCGGCT   
  
  
+ GAGATCGGCG TTTAATCACA TGTGGATACC CTTCGACACG GTGGACACGT TCTTGCCACG TGGGAGCCAG   
  
  
+ CAGAGAGAGT GGTTCGAGGC CGAGGTGTGC TGGAAGATTG AGAATGTGAT CGCTCATGAG GGACCCGCGA   
  
  
+ GGGTCGAGAG GCAGGAGCCC AGGGCCAAGT GGGCCCTCCG AATGAGGGAG GCCGAGTTTC AAGGGATCGA   
  
  
+ GTTCGGTGAT GAAGGTACGA CCGAGGTCAA GGCCATGCTG GAGGAGCATG CCGCTGGGTG GGGGTCTAAG   
  
  
+ AAGGAAGAGG ATGATCTTGT GCTCACTTGG AAGGGACATA GTGTTGTCTT TGCTTCTGCT TGGGTACCCA   
  
  
+ CTTA  

- +Up\_Stream \_Len000TCTTCG ATTAATTTGG TTTGTACGTG CATACAGGAT TAATTGTACT GACAGGTAGT   
  
  
- AGAGGAGGGT ACAACAAGAC AACTGTTCGG ACGTGTGGGT ACGAGGAGAG AGTACAGATT GGAGCAGGGT   
  
  
- TGCTAGTTCT AGTGACAGAC ACTCCGGTCC CCTTCTGAAC GAAAATAAAG GAAAAGAAAA CAAATGAGAG   
  
  
- ACTAAGGTAA ATCGGTATAT ATGTTCCTCT TACAGTACAC ATAGAGTATA CATATATATA TTCTAAAATA   
  
  
- AATCTTTATT TTTAAATTTG TGATACACTA AATATCAATA ATATTATTTA TCATAAAAAA TTTGATAAAG   
  
  
- TAACTACTCT TGTCCTTTCA TTTAACGGCT GTACAATTAC AATAATATAG ATTTTTAATT TAGTATTTTA   
  
  
- ATTAATATTA TAAAATTTTT ATAAAGTTGA TTTTAAAATC TTGTTATTCA CGCATTATGC CTGAATTTCC   
  
  
- GATCAAATAG CATTTTTACA TTCAAGGAAA ACCTAGAATA CCAACTTATA GAACCAACTA TTTGACACTA   
  
  
- ACCAAAATGT GAAAATGGTG TATAAGTATC TTCTTAATAC ACGTGCAACT ACCAAGTTCT ACCCTGTTTT   
  
  
- CCTTTGTACA TAAGAAGGGG GAAAACGGAA AAGGGGATTC GTAAACCTAA CTCCACAACA GTAGACTTAT   
  
  
- AGTTTTTAAG AAAAACTTTT AAGTCAGAGT TAGTTTTAAG TTTTAATTTT GTTTCTGAAT TCTCAAAATA   
  
  
- AACAAGAATT GGAAGAATTG AAAGATGAAA AGAAAAAAAA GAGTAAGGGG GAGAAGTTTA GAGTAAATAA   
  
  
- GAATTGGGGG AGAAGGCAAG TACGAGAGAG ACAGATAGTT GATTATAATT AGATGGGGCT GAAAGTTAAA   
  
  
- CTACTCCAGA TTAGGAATAG TAGCATAACA CCAACCCAAT TAATACGTTC TTCCGTTATT TGGGGTCACT   
  
  
- GTTTCAGGTG CAATTCATCC GTGGAGTGGT AATTCTGTAC GAGTCTTTTG TGGTTTTGGT AACTTGTGTT   
  
  
- CAGGGGGTTC AGGGAAGGGA TCGATCGGGT AAGAGAGAGG AGGGGACAGA TATAGATGGT TGAGAACGAG   
  
  
- AAACGACTCG GACTCAACTT TCGCTATGTG GGTATCGGAC AAGTCAAAAG TCAAAAGTGT AAACAGAAGG   
  
  
- AGACAAGTCA ACTTTCGCTA TGTGAGAGTA ATCGAAAGTA AAATTTATGC AAGTTAAGTG TGTATTTACC   
  
  
- GAAAGTTTCG TAACGGTAAA AGCAAATTTA CTACTGTTTT ATATATCATA GACCGATAGA CGGGTATAGA   
  
  
- ACTGGAATAA ATGTTTCCGA CTCGGTAGAA AATTAAAAAA ATCGGAGAAA AAAATTAGTT TTTATCTTTA   
  
  
- AAGTTCTTTC GTCTTTAGCA GTCTACTGTC ACGTCTGTCG CAAAAGCAAA GTATGTTTAG AGTTTTCGAC   
  
  
- TTCACAGTTA AAGAAGCTAA TAAAAACAGT CTTTTATAAA TGCAGAGTGG AAGTGCAACA ATAATAAGAA   
  
  
- AGAAAAAATA ATAAGAGTAT TGATCATTAT TTCAATCGTT ACTATTTTTT AATAAGCTAG GCTTATAAAA   
  
  
- TTAAATAGAC TGGATTTTTG TATTCATTTC CGTGTTTAAA AAGTAGGTTT AAAATTAAAA ACTACATTAT   
  
  
- AAAAAATATA AAAATAACAA GTTTAGACTA AAATTAGAAT AAATTTAATA GACTGGATTT TTTTAGTTTA   
  
  
- TTATTATTTG CAATTAAAAA ATAAATTTAA AACTGTAATC AGTTAGACTG AATTTAAGCT TGGCTCGAAC   
  
  
- TTAAAAAAAC GGTCACGACC ATCATGAGGA CGAAAACATC GAGGGTTACG GTAGCATGGT GGGAGAAGAT   
  
  
- CAGAGCAGAG AAAGAGACCA GAGGTTAATT AGTGTGGTAG TATAGTATGG CATACTACTA TGTCAGGTGA   
  
  
- CTTTAGGGTA AGAGTGGCGG TTAGAGAGAG GCGGTGGTAG GTTGTGAGAA TTGCTACTTG TCCCGGCGGC   
  
  
- GGAGCAGGAG GTGTGAGTCC GGCACCGGCC CGTCGAAAGG GTGGGTTAGT TTTAGAGAGA GGAGGCGGTT   
  
  
- GAAGCCATTG CGGTTAACGT ACCTCGTCGA GAATCAGGTG ACGCGGCTTC GGTAGCTGTT GTTACTGCGG   
  
  
- TGGGGCCGGG TCGTTTAGAA TACCCAGGAC TTATTGTATC GGGCTGGGCT GCCGCTGAGG TGGGTTGCGG   
  
  
- AGTGCACGCG TAAGGATGCA CGGAATTAGA GTGCGCGGCA GGAGTGATCG TGGACGTTCT ACCATTAGGG   
  
  
- AGTAAAGTTG GGGTAGTTAA GTGGGGTGTT TAAGAGCGAG GAGCTTGAAC GGGTGAAGCA GCTAAATTGG   
  
  
- GGAACCGTAG CTAAGCCTAA GTGGCGGCGG TTAAGCTAGT AAGACCTTCG ATAAAGGCTG GATGGGCAAC   
  
  
- ATGTGTAACA GCTGGAGTCG TAGAGGGTGA CATACGTCTA GGGGTGCAAC TAGCTGAGGT AACGCTGGGC   
  
  
- CAACCTTCGG GGCCCAGCTC AGGGAGGATA ACAGTTCGAG TGGCAGCCCC GATAAAGGCT GCTTTAGGGC   
  
  
- GGCCACAAGC TAGAAGACAG CATGCTACTC GAGCCTTACT CTGATTAGTT GAAGCGAGCA AAATCTTTGT   
  
  
- AGCAGGAGCT TAAGGTTCGT TATGGGTGGA GGGGAAGGCT GCCTAAACGA AGCGACAACC TCCTCTAAGC   
  
  
- TGTTTCGTTC GAGATGAGGT TGCTACGCCG CCGCCAATAA CACTTAACAG TCTACTCAAA CGTAAATAAC   
  
  
- GTCCTTCTCC TCCACAGAAG TAGCAGCGGC AGCAGCTACT CCCCCTACAA AAACGTCCGC CACTCCTCGA   
  
  
- ACCTCGGCTC GTACCACCAC CACCACCTCC TCCTGCACCT AAAGTGCCGC TCCTCAGACC ACCCCGCCGA   
  
  
- CTCTAGCCGC AAATTAGTGT ACACCTATGG GAAGCTGTGC CACCTGTGCA AGAACGGTGC ACCCTCGGTC   
  
  
- GTCTCTCTCA CCAAGCTCCG GCTCCACACG ACCTTCTAAC TCTTACACTA GCGAGTACTC CCTGGGCGCT   
  
  
- CCCAGCTCTC CGTCCTCGGG TCCCGGTTCA CCCGGGAGGC TTACTCCCTC CGGCTCAAAG TTCCCTAGCT   
  
  
- CAAGCCACTA CTTCCATGCT GGCTCCAGTT CCGGTACGAC CTCCTCGTAC GGCGACCCAC CCCCAGATTC   
  
  
- TTCCTTCTCC TACTAGAACA CGAGTGAACC TTCCCTGTAT CACAACAGAA ACGAAGACGA ACCCATGGGT   
  
  
- GAAT

+     G-box

| Site Name | Organism | Position | Strand | Matrix score. | sequence | function |
| --- | --- | --- | --- | --- | --- | --- |
| G-box | Arabidopsis thaliana | 3001 | - | 6 | CACGTG | cis-acting regulatory element involved in light responsiveness |
| G-box | Arabidopsis thaliana | 2246 | - | 6 | CACGTG | cis-acting regulatory element involved in light responsiveness |
| G-box | Arabidopsis thaliana | 42 | - | 6 | TACGTG | cis-acting regulatory element involved in light responsiveness |
| G-box | Arabidopsis thaliana | 2998 | - | 12 | CTTCCACGTGGCA | cis-acting regulatory element involved in light responsiveness |
| G-box | Zea mays | 2907 | - | 6 | CACGTC | cis-acting regulatory element involved in light responsiveness |
| G-box | Arabidopsis thaliana | 2999 | + | 9 | GCCACGTGGA | cis-acting regulatory element involved in light responsiveness |
| G-box | Arabidopsis thaliana | 2260 | + | 6 | TACGTG | cis-acting regulatory element involved in light responsiveness |

>HU06G00376.1   
+ +Up\_Stream \_Len000AGAAGC TAATTAAACC AAACATGCAC GTATGTCCTA ATTAACATGA CTGTCCATCA   
  
  
+ TCTCCTCCCA TGTTGTTCTG TTGACAAGCC TGCACACCCA TGCTCCTCTC TCATGTCTAA CCTCGTCCCA   
  
  
+ ACGATCAAGA TCACTGTCTG TGAGGCCAGG GGAAGACTTG CTTTTATTTC CTTTTCTTTT GTTTACTCTC   
  
  
+ TGATTCCATT TAGCCATATA TACAAGGAGA ATGTCATGTG TATCTCATAT GTATATATAT AAGATTTTAT   
  
  
+ TTAGAAATAA AAATTTAAAC ACTATGTGAT TTATAGTTAT TATAATAAAT AGTATTTTTT AAACTATTTC   
  
  
+ ATTGATGAGA ACAGGAAAGT AAATTGCCGA CATGTTAATG TTATTATATC TAAAAATTAA ATCATAAAAT   
  
  
+ TAATTATAAT ATTTTAAAAA TATTTCAACT AAAATTTTAG AACAATAAGT GCGTAATACG GACTTAAAGG   
  
  
+ CTAGTTTATC GTAAAAATGT AAGTTCCTTT TGGATCTTAT GGTTGAATAT CTTGGTTGAT AAACTGTGAT   
  
  
+ TGGTTTTACA CTTTTACCAC ATATTCATAG AAGAATTATG TGCACGTTGA TGGTTCAAGA TGGGACAAAA   
  
  
+ GGAAACATGT ATTCTTCCCC CTTTTGCCTT TTCCCCTAAG CATTTGGATT GAGGTGTTGT CATCTGAATA   
  
  
+ TCAAAAATTC TTTTTGAAAA TTCAGTCTCA ATCAAAATTC AAAATTAAAA CAAAGACTTA AGAGTTTTAT   
  
  
+ TTGTTCTTAA CCTTCTTAAC TTTCTACTTT TCTTTTTTTT CTCATTCCCC CTCTTCAAAT CTCATTTATT   
  
  
+ CTTAACCCCC TCTTCCGTTC ATGCTCTCTC TGTCTATCAA CTAATATTAA TCTACCCCGA CTTTCAATTT   
  
  
+ GATGAGGTCT AATCCTTATC ATCGTATTGT GGTTGGGTTA ATTATGCAAG AAGGCAATAA ACCCCAGTGA   
  
  
+ CAAAGTCCAC GTTAAGTAGG CACCTCACCA TTAAGACATG CTCAGAAAAC ACCAAAACCA TTGAACACAA   
  
  
+ GTCCCCCAAG TCCCTTCCCT AGCTAGCCCA TTCTCTCTCC TCCCCTGTCT ATATCTACCA ACTCTTGCTC   
  
  
+ TTTGCTGAGC CTGAGTTGAA AGCGATACAC CCATAGCCTG TTCAGTTTTC AGTTTTCACA TTTGTCTTCC   
  
  
+ TCTGTTCAGT TGAAAGCGAT ACACTCTCAT TAGCTTTCAT TTTAAATACG TTCAATTCAC ACATAAATGG   
  
  
+ CTTTCAAAGC ATTGCCATTT TCGTTTAAAT GATGACAAAA TATATAGTAT CTGGCTATCT GCCCATATCT   
  
  
+ TGACCTTATT TACAAAGGCT GAGCCATCTT TTAATTTTTT TAGCCTCTTT TTTTAATCAA AAATAGAAAT   
  
  
+ TTCAAGAAAG CAGAAATCGT CAGATGACAG TGCAGACAGC GTTTTCGTTT CATACAAATC TCAAAAGCTG   
  
  
+ AAGTGTCAAT TTCTTCGATT ATTTTTGTCA GAAAATATTT ACGTCTCACC TTCACGTTGT TATTATTCTT   
  
  
+ TCTTTTTTAT TATTCTCATA ACTAGTAATA AAGTTAGCAA TGATAAAAAA TTATTCGATC CGAATATTTT   
  
  
+ AATTTATCTG ACCTAAAAAC ATAAGTAAAG GCACAAATTT TTCATCCAAA TTTTAATTTT TGATGTAATA   
  
  
+ TTTTTTATAT TTTTATTGTT CAAATCTGAT TTTAATCTTA TTTAAATTAT CTGACCTAAA AAAATCAAAT   
  
  
+ AATAATAAAC GTTAATTTTT TATTTAAATT TTGACATTAG TCAATCTGAC TTAAATTCGA ACCGAGCTTG   
  
  
+ AATTTTTTTG CCAGTGCTGG TAGTACTCCT GCTTTTGTAG CTCCCAATGC CATCGTACCA CCCTCTTCTA   
  
  
+ GTCTCGTCTC TTTCTCTGGT CTCCAATTAA TCACACCATC ATATCATACC GTATGATGAT ACAGTCCACT   
  
  
+ GAAATCCCAT TCTCACCGCC AATCTCTCTC CGCCACCATC CAACACTCTT AACGATGAAC AGGGCCGCCG   
  
  
+ CCTCGTCCTC CACACTCAGG CCGTGGCCGG GCAGCTTTCC CACCCAATCA AAATCTCTCT CCTCCGCCAA   
  
  
+ CTTCGGTAAC GCCAATTGCA TGGAGCAGCT CTTAGTCCAC TGCGCCGAAG CCATCGACAA CAATGACGCC   
  
  
+ ACCCCGGCCC AGCAAATCTT ATGGGTCCTG AATAACATAG CCCGACCCGA CGGCGACTCC ACCCAACGCC   
  
  
+ TCACGTGCGC ATTCCTACGT GCCTTAATCT CACGCGCCGT CCTCACTAGC ACCTGCAAGA TGGTAATCCC   
  
  
+ TCATTTCAAC CCCATCAATT CACCCCACAA ATTCTCGCTC CTCGAACTTG CCCACTTCGT CGATTTAACC   
  
  
+ CCTTGGCATC GATTCGGATT CACCGCCGCC AATTCGATCA TTCTGGAAGC TATTTCCGAC CTACCCGTTG   
  
  
+ TACACATTGT CGACCTCAGC ATCTCCCACT GTATGCAGAT CCCCACGTTG ATCGACTCCA TTGCGACCCG   
  
  
+ GTTGGAAGCC CCGGGTCGAG TCCCTCCTAT TGTCAAGCTC ACCGTCGGGG CTATTTCCGA CGAAATCCCG   
  
  
+ CCGGTGTTCG ATCTTCTGTC GTACGATGAG CTCGGAATGA GACTAATCAA CTTCGCTCGT TTTAGAAACA   
  
  
+ TCGTCCTCGA ATTCCAAGCA ATACCCACCT CCCCTTCCGA CGGATTTGCT TCGCTGTTGG AGGAGATTCG   
  
  
+ ACAAAGCAAG CTCTACTCCA ACGATGCGGC GGCGGTTATT GTGAATTGTC AGATGAGTTT GCATTTATTG   
  
  
+ CAGGAAGAGG AGGTGTCTTC ATCGTCGCCG TCGTCGATGA GGGGGATGTT TTTGCAGGCG GTGAGGAGCT   
  
  
+ TGGAGCCGAG CATGGTGGTG GTGGTGGAGG AGGACGTGGA TTTCACGGCG AGGAGTCTGG TGGGGCGGCT   
  
  
+ GAGATCGGCG TTTAATCACA TGTGGATACC CTTCGACACG GTGGACACGT TCTTGCCACG TGGGAGCCAG   
  
  
+ CAGAGAGAGT GGTTCGAGGC CGAGGTGTGC TGGAAGATTG AGAATGTGAT CGCTCATGAG GGACCCGCGA   
  
  
+ GGGTCGAGAG GCAGGAGCCC AGGGCCAAGT GGGCCCTCCG AATGAGGGAG GCCGAGTTTC AAGGGATCGA   
  
  
+ GTTCGGTGAT GAAGGTACGA CCGAGGTCAA GGCCATGCTG GAGGAGCATG CCGCTGGGTG GGGGTCTAAG   
  
  
+ AAGGAAGAGG ATGATCTTGT GCTCACTTGG AAGGGACATA GTGTTGTCTT TGCTTCTGCT TGGGTACCCA   
  
  
+ CTTA  

- +Up\_Stream \_Len000TCTTCG ATTAATTTGG TTTGTACGTG CATACAGGAT TAATTGTACT GACAGGTAGT   
  
  
- AGAGGAGGGT ACAACAAGAC AACTGTTCGG ACGTGTGGGT ACGAGGAGAG AGTACAGATT GGAGCAGGGT   
  
  
- TGCTAGTTCT AGTGACAGAC ACTCCGGTCC CCTTCTGAAC GAAAATAAAG GAAAAGAAAA CAAATGAGAG   
  
  
- ACTAAGGTAA ATCGGTATAT ATGTTCCTCT TACAGTACAC ATAGAGTATA CATATATATA TTCTAAAATA   
  
  
- AATCTTTATT TTTAAATTTG TGATACACTA AATATCAATA ATATTATTTA TCATAAAAAA TTTGATAAAG   
  
  
- TAACTACTCT TGTCCTTTCA TTTAACGGCT GTACAATTAC AATAATATAG ATTTTTAATT TAGTATTTTA   
  
  
- ATTAATATTA TAAAATTTTT ATAAAGTTGA TTTTAAAATC TTGTTATTCA CGCATTATGC CTGAATTTCC   
  
  
- GATCAAATAG CATTTTTACA TTCAAGGAAA ACCTAGAATA CCAACTTATA GAACCAACTA TTTGACACTA   
  
  
- ACCAAAATGT GAAAATGGTG TATAAGTATC TTCTTAATAC ACGTGCAACT ACCAAGTTCT ACCCTGTTTT   
  
  
- CCTTTGTACA TAAGAAGGGG GAAAACGGAA AAGGGGATTC GTAAACCTAA CTCCACAACA GTAGACTTAT   
  
  
- AGTTTTTAAG AAAAACTTTT AAGTCAGAGT TAGTTTTAAG TTTTAATTTT GTTTCTGAAT TCTCAAAATA   
  
  
- AACAAGAATT GGAAGAATTG AAAGATGAAA AGAAAAAAAA GAGTAAGGGG GAGAAGTTTA GAGTAAATAA   
  
  
- GAATTGGGGG AGAAGGCAAG TACGAGAGAG ACAGATAGTT GATTATAATT AGATGGGGCT GAAAGTTAAA   
  
  
- CTACTCCAGA TTAGGAATAG TAGCATAACA CCAACCCAAT TAATACGTTC TTCCGTTATT TGGGGTCACT   
  
  
- GTTTCAGGTG CAATTCATCC GTGGAGTGGT AATTCTGTAC GAGTCTTTTG TGGTTTTGGT AACTTGTGTT   
  
  
- CAGGGGGTTC AGGGAAGGGA TCGATCGGGT AAGAGAGAGG AGGGGACAGA TATAGATGGT TGAGAACGAG   
  
  
- AAACGACTCG GACTCAACTT TCGCTATGTG GGTATCGGAC AAGTCAAAAG TCAAAAGTGT AAACAGAAGG   
  
  
- AGACAAGTCA ACTTTCGCTA TGTGAGAGTA ATCGAAAGTA AAATTTATGC AAGTTAAGTG TGTATTTACC   
  
  
- GAAAGTTTCG TAACGGTAAA AGCAAATTTA CTACTGTTTT ATATATCATA GACCGATAGA CGGGTATAGA   
  
  
- ACTGGAATAA ATGTTTCCGA CTCGGTAGAA AATTAAAAAA ATCGGAGAAA AAAATTAGTT TTTATCTTTA   
  
  
- AAGTTCTTTC GTCTTTAGCA GTCTACTGTC ACGTCTGTCG CAAAAGCAAA GTATGTTTAG AGTTTTCGAC   
  
  
- TTCACAGTTA AAGAAGCTAA TAAAAACAGT CTTTTATAAA TGCAGAGTGG AAGTGCAACA ATAATAAGAA   
  
  
- AGAAAAAATA ATAAGAGTAT TGATCATTAT TTCAATCGTT ACTATTTTTT AATAAGCTAG GCTTATAAAA   
  
  
- TTAAATAGAC TGGATTTTTG TATTCATTTC CGTGTTTAAA AAGTAGGTTT AAAATTAAAA ACTACATTAT   
  
  
- AAAAAATATA AAAATAACAA GTTTAGACTA AAATTAGAAT AAATTTAATA GACTGGATTT TTTTAGTTTA   
  
  
- TTATTATTTG CAATTAAAAA ATAAATTTAA AACTGTAATC AGTTAGACTG AATTTAAGCT TGGCTCGAAC   
  
  
- TTAAAAAAAC GGTCACGACC ATCATGAGGA CGAAAACATC GAGGGTTACG GTAGCATGGT GGGAGAAGAT   
  
  
- CAGAGCAGAG AAAGAGACCA GAGGTTAATT AGTGTGGTAG TATAGTATGG CATACTACTA TGTCAGGTGA   
  
  
- CTTTAGGGTA AGAGTGGCGG TTAGAGAGAG GCGGTGGTAG GTTGTGAGAA TTGCTACTTG TCCCGGCGGC   
  
  
- GGAGCAGGAG GTGTGAGTCC GGCACCGGCC CGTCGAAAGG GTGGGTTAGT TTTAGAGAGA GGAGGCGGTT   
  
  
- GAAGCCATTG CGGTTAACGT ACCTCGTCGA GAATCAGGTG ACGCGGCTTC GGTAGCTGTT GTTACTGCGG   
  
  
- TGGGGCCGGG TCGTTTAGAA TACCCAGGAC TTATTGTATC GGGCTGGGCT GCCGCTGAGG TGGGTTGCGG   
  
  
- AGTGCACGCG TAAGGATGCA CGGAATTAGA GTGCGCGGCA GGAGTGATCG TGGACGTTCT ACCATTAGGG   
  
  
- AGTAAAGTTG GGGTAGTTAA GTGGGGTGTT TAAGAGCGAG GAGCTTGAAC GGGTGAAGCA GCTAAATTGG   
  
  
- GGAACCGTAG CTAAGCCTAA GTGGCGGCGG TTAAGCTAGT AAGACCTTCG ATAAAGGCTG GATGGGCAAC   
  
  
- ATGTGTAACA GCTGGAGTCG TAGAGGGTGA CATACGTCTA GGGGTGCAAC TAGCTGAGGT AACGCTGGGC   
  
  
- CAACCTTCGG GGCCCAGCTC AGGGAGGATA ACAGTTCGAG TGGCAGCCCC GATAAAGGCT GCTTTAGGGC   
  
  
- GGCCACAAGC TAGAAGACAG CATGCTACTC GAGCCTTACT CTGATTAGTT GAAGCGAGCA AAATCTTTGT   
  
  
- AGCAGGAGCT TAAGGTTCGT TATGGGTGGA GGGGAAGGCT GCCTAAACGA AGCGACAACC TCCTCTAAGC   
  
  
- TGTTTCGTTC GAGATGAGGT TGCTACGCCG CCGCCAATAA CACTTAACAG TCTACTCAAA CGTAAATAAC   
  
  
- GTCCTTCTCC TCCACAGAAG TAGCAGCGGC AGCAGCTACT CCCCCTACAA AAACGTCCGC CACTCCTCGA   
  
  
- ACCTCGGCTC GTACCACCAC CACCACCTCC TCCTGCACCT AAAGTGCCGC TCCTCAGACC ACCCCGCCGA   
  
  
- CTCTAGCCGC AAATTAGTGT ACACCTATGG GAAGCTGTGC CACCTGTGCA AGAACGGTGC ACCCTCGGTC   
  
  
- GTCTCTCTCA CCAAGCTCCG GCTCCACACG ACCTTCTAAC TCTTACACTA GCGAGTACTC CCTGGGCGCT   
  
  
- CCCAGCTCTC CGTCCTCGGG TCCCGGTTCA CCCGGGAGGC TTACTCCCTC CGGCTCAAAG TTCCCTAGCT   
  
  
- CAAGCCACTA CTTCCATGCT GGCTCCAGTT CCGGTACGAC CTCCTCGTAC GGCGACCCAC CCCCAGATTC   
  
  
- TTCCTTCTCC TACTAGAACA CGAGTGAACC TTCCCTGTAT CACAACAGAA ACGAAGACGA ACCCATGGGT   
  
  
- GAAT

+     GARE-motif

| Site Name | Organism | Position | Strand | Matrix score. | sequence | function |
| --- | --- | --- | --- | --- | --- | --- |
| GARE-motif | Brassica oleracea | 91 | + | 7 | TCTGTTG | gibberellin-responsive element |

>HU06G00376.1   
+ +Up\_Stream \_Len000AGAAGC TAATTAAACC AAACATGCAC GTATGTCCTA ATTAACATGA CTGTCCATCA   
  
  
+ TCTCCTCCCA TGTTGTTCTG TTGACAAGCC TGCACACCCA TGCTCCTCTC TCATGTCTAA CCTCGTCCCA   
  
  
+ ACGATCAAGA TCACTGTCTG TGAGGCCAGG GGAAGACTTG CTTTTATTTC CTTTTCTTTT GTTTACTCTC   
  
  
+ TGATTCCATT TAGCCATATA TACAAGGAGA ATGTCATGTG TATCTCATAT GTATATATAT AAGATTTTAT   
  
  
+ TTAGAAATAA AAATTTAAAC ACTATGTGAT TTATAGTTAT TATAATAAAT AGTATTTTTT AAACTATTTC   
  
  
+ ATTGATGAGA ACAGGAAAGT AAATTGCCGA CATGTTAATG TTATTATATC TAAAAATTAA ATCATAAAAT   
  
  
+ TAATTATAAT ATTTTAAAAA TATTTCAACT AAAATTTTAG AACAATAAGT GCGTAATACG GACTTAAAGG   
  
  
+ CTAGTTTATC GTAAAAATGT AAGTTCCTTT TGGATCTTAT GGTTGAATAT CTTGGTTGAT AAACTGTGAT   
  
  
+ TGGTTTTACA CTTTTACCAC ATATTCATAG AAGAATTATG TGCACGTTGA TGGTTCAAGA TGGGACAAAA   
  
  
+ GGAAACATGT ATTCTTCCCC CTTTTGCCTT TTCCCCTAAG CATTTGGATT GAGGTGTTGT CATCTGAATA   
  
  
+ TCAAAAATTC TTTTTGAAAA TTCAGTCTCA ATCAAAATTC AAAATTAAAA CAAAGACTTA AGAGTTTTAT   
  
  
+ TTGTTCTTAA CCTTCTTAAC TTTCTACTTT TCTTTTTTTT CTCATTCCCC CTCTTCAAAT CTCATTTATT   
  
  
+ CTTAACCCCC TCTTCCGTTC ATGCTCTCTC TGTCTATCAA CTAATATTAA TCTACCCCGA CTTTCAATTT   
  
  
+ GATGAGGTCT AATCCTTATC ATCGTATTGT GGTTGGGTTA ATTATGCAAG AAGGCAATAA ACCCCAGTGA   
  
  
+ CAAAGTCCAC GTTAAGTAGG CACCTCACCA TTAAGACATG CTCAGAAAAC ACCAAAACCA TTGAACACAA   
  
  
+ GTCCCCCAAG TCCCTTCCCT AGCTAGCCCA TTCTCTCTCC TCCCCTGTCT ATATCTACCA ACTCTTGCTC   
  
  
+ TTTGCTGAGC CTGAGTTGAA AGCGATACAC CCATAGCCTG TTCAGTTTTC AGTTTTCACA TTTGTCTTCC   
  
  
+ TCTGTTCAGT TGAAAGCGAT ACACTCTCAT TAGCTTTCAT TTTAAATACG TTCAATTCAC ACATAAATGG   
  
  
+ CTTTCAAAGC ATTGCCATTT TCGTTTAAAT GATGACAAAA TATATAGTAT CTGGCTATCT GCCCATATCT   
  
  
+ TGACCTTATT TACAAAGGCT GAGCCATCTT TTAATTTTTT TAGCCTCTTT TTTTAATCAA AAATAGAAAT   
  
  
+ TTCAAGAAAG CAGAAATCGT CAGATGACAG TGCAGACAGC GTTTTCGTTT CATACAAATC TCAAAAGCTG   
  
  
+ AAGTGTCAAT TTCTTCGATT ATTTTTGTCA GAAAATATTT ACGTCTCACC TTCACGTTGT TATTATTCTT   
  
  
+ TCTTTTTTAT TATTCTCATA ACTAGTAATA AAGTTAGCAA TGATAAAAAA TTATTCGATC CGAATATTTT   
  
  
+ AATTTATCTG ACCTAAAAAC ATAAGTAAAG GCACAAATTT TTCATCCAAA TTTTAATTTT TGATGTAATA   
  
  
+ TTTTTTATAT TTTTATTGTT CAAATCTGAT TTTAATCTTA TTTAAATTAT CTGACCTAAA AAAATCAAAT   
  
  
+ AATAATAAAC GTTAATTTTT TATTTAAATT TTGACATTAG TCAATCTGAC TTAAATTCGA ACCGAGCTTG   
  
  
+ AATTTTTTTG CCAGTGCTGG TAGTACTCCT GCTTTTGTAG CTCCCAATGC CATCGTACCA CCCTCTTCTA   
  
  
+ GTCTCGTCTC TTTCTCTGGT CTCCAATTAA TCACACCATC ATATCATACC GTATGATGAT ACAGTCCACT   
  
  
+ GAAATCCCAT TCTCACCGCC AATCTCTCTC CGCCACCATC CAACACTCTT AACGATGAAC AGGGCCGCCG   
  
  
+ CCTCGTCCTC CACACTCAGG CCGTGGCCGG GCAGCTTTCC CACCCAATCA AAATCTCTCT CCTCCGCCAA   
  
  
+ CTTCGGTAAC GCCAATTGCA TGGAGCAGCT CTTAGTCCAC TGCGCCGAAG CCATCGACAA CAATGACGCC   
  
  
+ ACCCCGGCCC AGCAAATCTT ATGGGTCCTG AATAACATAG CCCGACCCGA CGGCGACTCC ACCCAACGCC   
  
  
+ TCACGTGCGC ATTCCTACGT GCCTTAATCT CACGCGCCGT CCTCACTAGC ACCTGCAAGA TGGTAATCCC   
  
  
+ TCATTTCAAC CCCATCAATT CACCCCACAA ATTCTCGCTC CTCGAACTTG CCCACTTCGT CGATTTAACC   
  
  
+ CCTTGGCATC GATTCGGATT CACCGCCGCC AATTCGATCA TTCTGGAAGC TATTTCCGAC CTACCCGTTG   
  
  
+ TACACATTGT CGACCTCAGC ATCTCCCACT GTATGCAGAT CCCCACGTTG ATCGACTCCA TTGCGACCCG   
  
  
+ GTTGGAAGCC CCGGGTCGAG TCCCTCCTAT TGTCAAGCTC ACCGTCGGGG CTATTTCCGA CGAAATCCCG   
  
  
+ CCGGTGTTCG ATCTTCTGTC GTACGATGAG CTCGGAATGA GACTAATCAA CTTCGCTCGT TTTAGAAACA   
  
  
+ TCGTCCTCGA ATTCCAAGCA ATACCCACCT CCCCTTCCGA CGGATTTGCT TCGCTGTTGG AGGAGATTCG   
  
  
+ ACAAAGCAAG CTCTACTCCA ACGATGCGGC GGCGGTTATT GTGAATTGTC AGATGAGTTT GCATTTATTG   
  
  
+ CAGGAAGAGG AGGTGTCTTC ATCGTCGCCG TCGTCGATGA GGGGGATGTT TTTGCAGGCG GTGAGGAGCT   
  
  
+ TGGAGCCGAG CATGGTGGTG GTGGTGGAGG AGGACGTGGA TTTCACGGCG AGGAGTCTGG TGGGGCGGCT   
  
  
+ GAGATCGGCG TTTAATCACA TGTGGATACC CTTCGACACG GTGGACACGT TCTTGCCACG TGGGAGCCAG   
  
  
+ CAGAGAGAGT GGTTCGAGGC CGAGGTGTGC TGGAAGATTG AGAATGTGAT CGCTCATGAG GGACCCGCGA   
  
  
+ GGGTCGAGAG GCAGGAGCCC AGGGCCAAGT GGGCCCTCCG AATGAGGGAG GCCGAGTTTC AAGGGATCGA   
  
  
+ GTTCGGTGAT GAAGGTACGA CCGAGGTCAA GGCCATGCTG GAGGAGCATG CCGCTGGGTG GGGGTCTAAG   
  
  
+ AAGGAAGAGG ATGATCTTGT GCTCACTTGG AAGGGACATA GTGTTGTCTT TGCTTCTGCT TGGGTACCCA   
  
  
+ CTTA  

- +Up\_Stream \_Len000TCTTCG ATTAATTTGG TTTGTACGTG CATACAGGAT TAATTGTACT GACAGGTAGT   
  
  
- AGAGGAGGGT ACAACAAGAC AACTGTTCGG ACGTGTGGGT ACGAGGAGAG AGTACAGATT GGAGCAGGGT   
  
  
- TGCTAGTTCT AGTGACAGAC ACTCCGGTCC CCTTCTGAAC GAAAATAAAG GAAAAGAAAA CAAATGAGAG   
  
  
- ACTAAGGTAA ATCGGTATAT ATGTTCCTCT TACAGTACAC ATAGAGTATA CATATATATA TTCTAAAATA   
  
  
- AATCTTTATT TTTAAATTTG TGATACACTA AATATCAATA ATATTATTTA TCATAAAAAA TTTGATAAAG   
  
  
- TAACTACTCT TGTCCTTTCA TTTAACGGCT GTACAATTAC AATAATATAG ATTTTTAATT TAGTATTTTA   
  
  
- ATTAATATTA TAAAATTTTT ATAAAGTTGA TTTTAAAATC TTGTTATTCA CGCATTATGC CTGAATTTCC   
  
  
- GATCAAATAG CATTTTTACA TTCAAGGAAA ACCTAGAATA CCAACTTATA GAACCAACTA TTTGACACTA   
  
  
- ACCAAAATGT GAAAATGGTG TATAAGTATC TTCTTAATAC ACGTGCAACT ACCAAGTTCT ACCCTGTTTT   
  
  
- CCTTTGTACA TAAGAAGGGG GAAAACGGAA AAGGGGATTC GTAAACCTAA CTCCACAACA GTAGACTTAT   
  
  
- AGTTTTTAAG AAAAACTTTT AAGTCAGAGT TAGTTTTAAG TTTTAATTTT GTTTCTGAAT TCTCAAAATA   
  
  
- AACAAGAATT GGAAGAATTG AAAGATGAAA AGAAAAAAAA GAGTAAGGGG GAGAAGTTTA GAGTAAATAA   
  
  
- GAATTGGGGG AGAAGGCAAG TACGAGAGAG ACAGATAGTT GATTATAATT AGATGGGGCT GAAAGTTAAA   
  
  
- CTACTCCAGA TTAGGAATAG TAGCATAACA CCAACCCAAT TAATACGTTC TTCCGTTATT TGGGGTCACT   
  
  
- GTTTCAGGTG CAATTCATCC GTGGAGTGGT AATTCTGTAC GAGTCTTTTG TGGTTTTGGT AACTTGTGTT   
  
  
- CAGGGGGTTC AGGGAAGGGA TCGATCGGGT AAGAGAGAGG AGGGGACAGA TATAGATGGT TGAGAACGAG   
  
  
- AAACGACTCG GACTCAACTT TCGCTATGTG GGTATCGGAC AAGTCAAAAG TCAAAAGTGT AAACAGAAGG   
  
  
- AGACAAGTCA ACTTTCGCTA TGTGAGAGTA ATCGAAAGTA AAATTTATGC AAGTTAAGTG TGTATTTACC   
  
  
- GAAAGTTTCG TAACGGTAAA AGCAAATTTA CTACTGTTTT ATATATCATA GACCGATAGA CGGGTATAGA   
  
  
- ACTGGAATAA ATGTTTCCGA CTCGGTAGAA AATTAAAAAA ATCGGAGAAA AAAATTAGTT TTTATCTTTA   
  
  
- AAGTTCTTTC GTCTTTAGCA GTCTACTGTC ACGTCTGTCG CAAAAGCAAA GTATGTTTAG AGTTTTCGAC   
  
  
- TTCACAGTTA AAGAAGCTAA TAAAAACAGT CTTTTATAAA TGCAGAGTGG AAGTGCAACA ATAATAAGAA   
  
  
- AGAAAAAATA ATAAGAGTAT TGATCATTAT TTCAATCGTT ACTATTTTTT AATAAGCTAG GCTTATAAAA   
  
  
- TTAAATAGAC TGGATTTTTG TATTCATTTC CGTGTTTAAA AAGTAGGTTT AAAATTAAAA ACTACATTAT   
  
  
- AAAAAATATA AAAATAACAA GTTTAGACTA AAATTAGAAT AAATTTAATA GACTGGATTT TTTTAGTTTA   
  
  
- TTATTATTTG CAATTAAAAA ATAAATTTAA AACTGTAATC AGTTAGACTG AATTTAAGCT TGGCTCGAAC   
  
  
- TTAAAAAAAC GGTCACGACC ATCATGAGGA CGAAAACATC GAGGGTTACG GTAGCATGGT GGGAGAAGAT   
  
  
- CAGAGCAGAG AAAGAGACCA GAGGTTAATT AGTGTGGTAG TATAGTATGG CATACTACTA TGTCAGGTGA   
  
  
- CTTTAGGGTA AGAGTGGCGG TTAGAGAGAG GCGGTGGTAG GTTGTGAGAA TTGCTACTTG TCCCGGCGGC   
  
  
- GGAGCAGGAG GTGTGAGTCC GGCACCGGCC CGTCGAAAGG GTGGGTTAGT TTTAGAGAGA GGAGGCGGTT   
  
  
- GAAGCCATTG CGGTTAACGT ACCTCGTCGA GAATCAGGTG ACGCGGCTTC GGTAGCTGTT GTTACTGCGG   
  
  
- TGGGGCCGGG TCGTTTAGAA TACCCAGGAC TTATTGTATC GGGCTGGGCT GCCGCTGAGG TGGGTTGCGG   
  
  
- AGTGCACGCG TAAGGATGCA CGGAATTAGA GTGCGCGGCA GGAGTGATCG TGGACGTTCT ACCATTAGGG   
  
  
- AGTAAAGTTG GGGTAGTTAA GTGGGGTGTT TAAGAGCGAG GAGCTTGAAC GGGTGAAGCA GCTAAATTGG   
  
  
- GGAACCGTAG CTAAGCCTAA GTGGCGGCGG TTAAGCTAGT AAGACCTTCG ATAAAGGCTG GATGGGCAAC   
  
  
- ATGTGTAACA GCTGGAGTCG TAGAGGGTGA CATACGTCTA GGGGTGCAAC TAGCTGAGGT AACGCTGGGC   
  
  
- CAACCTTCGG GGCCCAGCTC AGGGAGGATA ACAGTTCGAG TGGCAGCCCC GATAAAGGCT GCTTTAGGGC   
  
  
- GGCCACAAGC TAGAAGACAG CATGCTACTC GAGCCTTACT CTGATTAGTT GAAGCGAGCA AAATCTTTGT   
  
  
- AGCAGGAGCT TAAGGTTCGT TATGGGTGGA GGGGAAGGCT GCCTAAACGA AGCGACAACC TCCTCTAAGC   
  
  
- TGTTTCGTTC GAGATGAGGT TGCTACGCCG CCGCCAATAA CACTTAACAG TCTACTCAAA CGTAAATAAC   
  
  
- GTCCTTCTCC TCCACAGAAG TAGCAGCGGC AGCAGCTACT CCCCCTACAA AAACGTCCGC CACTCCTCGA   
  
  
- ACCTCGGCTC GTACCACCAC CACCACCTCC TCCTGCACCT AAAGTGCCGC TCCTCAGACC ACCCCGCCGA   
  
  
- CTCTAGCCGC AAATTAGTGT ACACCTATGG GAAGCTGTGC CACCTGTGCA AGAACGGTGC ACCCTCGGTC   
  
  
- GTCTCTCTCA CCAAGCTCCG GCTCCACACG ACCTTCTAAC TCTTACACTA GCGAGTACTC CCTGGGCGCT   
  
  
- CCCAGCTCTC CGTCCTCGGG TCCCGGTTCA CCCGGGAGGC TTACTCCCTC CGGCTCAAAG TTCCCTAGCT   
  
  
- CAAGCCACTA CTTCCATGCT GGCTCCAGTT CCGGTACGAC CTCCTCGTAC GGCGACCCAC CCCCAGATTC   
  
  
- TTCCTTCTCC TACTAGAACA CGAGTGAACC TTCCCTGTAT CACAACAGAA ACGAAGACGA ACCCATGGGT   
  
  
- GAAT

+     GT1-motif

| Site Name | Organism | Position | Strand | Matrix score. | sequence | function |
| --- | --- | --- | --- | --- | --- | --- |
| GT1-motif | Arabidopsis thaliana | 846 | - | 6 | GGTTAA | light responsive element |
| GT1-motif | Avena sativa | 950 | + | 7 | GGTTAAT | light responsive element |
| GT1-motif | Arabidopsis thaliana | 781 | - | 6 | GGTTAA | light responsive element |
| GT1-motif | Arabidopsis thaliana | 2379 | - | 6 | GGTTAA | light responsive element |

>HU06G00376.1   
+ +Up\_Stream \_Len000AGAAGC TAATTAAACC AAACATGCAC GTATGTCCTA ATTAACATGA CTGTCCATCA   
  
  
+ TCTCCTCCCA TGTTGTTCTG TTGACAAGCC TGCACACCCA TGCTCCTCTC TCATGTCTAA CCTCGTCCCA   
  
  
+ ACGATCAAGA TCACTGTCTG TGAGGCCAGG GGAAGACTTG CTTTTATTTC CTTTTCTTTT GTTTACTCTC   
  
  
+ TGATTCCATT TAGCCATATA TACAAGGAGA ATGTCATGTG TATCTCATAT GTATATATAT AAGATTTTAT   
  
  
+ TTAGAAATAA AAATTTAAAC ACTATGTGAT TTATAGTTAT TATAATAAAT AGTATTTTTT AAACTATTTC   
  
  
+ ATTGATGAGA ACAGGAAAGT AAATTGCCGA CATGTTAATG TTATTATATC TAAAAATTAA ATCATAAAAT   
  
  
+ TAATTATAAT ATTTTAAAAA TATTTCAACT AAAATTTTAG AACAATAAGT GCGTAATACG GACTTAAAGG   
  
  
+ CTAGTTTATC GTAAAAATGT AAGTTCCTTT TGGATCTTAT GGTTGAATAT CTTGGTTGAT AAACTGTGAT   
  
  
+ TGGTTTTACA CTTTTACCAC ATATTCATAG AAGAATTATG TGCACGTTGA TGGTTCAAGA TGGGACAAAA   
  
  
+ GGAAACATGT ATTCTTCCCC CTTTTGCCTT TTCCCCTAAG CATTTGGATT GAGGTGTTGT CATCTGAATA   
  
  
+ TCAAAAATTC TTTTTGAAAA TTCAGTCTCA ATCAAAATTC AAAATTAAAA CAAAGACTTA AGAGTTTTAT   
  
  
+ TTGTTCTTAA CCTTCTTAAC TTTCTACTTT TCTTTTTTTT CTCATTCCCC CTCTTCAAAT CTCATTTATT   
  
  
+ CTTAACCCCC TCTTCCGTTC ATGCTCTCTC TGTCTATCAA CTAATATTAA TCTACCCCGA CTTTCAATTT   
  
  
+ GATGAGGTCT AATCCTTATC ATCGTATTGT GGTTGGGTTA ATTATGCAAG AAGGCAATAA ACCCCAGTGA   
  
  
+ CAAAGTCCAC GTTAAGTAGG CACCTCACCA TTAAGACATG CTCAGAAAAC ACCAAAACCA TTGAACACAA   
  
  
+ GTCCCCCAAG TCCCTTCCCT AGCTAGCCCA TTCTCTCTCC TCCCCTGTCT ATATCTACCA ACTCTTGCTC   
  
  
+ TTTGCTGAGC CTGAGTTGAA AGCGATACAC CCATAGCCTG TTCAGTTTTC AGTTTTCACA TTTGTCTTCC   
  
  
+ TCTGTTCAGT TGAAAGCGAT ACACTCTCAT TAGCTTTCAT TTTAAATACG TTCAATTCAC ACATAAATGG   
  
  
+ CTTTCAAAGC ATTGCCATTT TCGTTTAAAT GATGACAAAA TATATAGTAT CTGGCTATCT GCCCATATCT   
  
  
+ TGACCTTATT TACAAAGGCT GAGCCATCTT TTAATTTTTT TAGCCTCTTT TTTTAATCAA AAATAGAAAT   
  
  
+ TTCAAGAAAG CAGAAATCGT CAGATGACAG TGCAGACAGC GTTTTCGTTT CATACAAATC TCAAAAGCTG   
  
  
+ AAGTGTCAAT TTCTTCGATT ATTTTTGTCA GAAAATATTT ACGTCTCACC TTCACGTTGT TATTATTCTT   
  
  
+ TCTTTTTTAT TATTCTCATA ACTAGTAATA AAGTTAGCAA TGATAAAAAA TTATTCGATC CGAATATTTT   
  
  
+ AATTTATCTG ACCTAAAAAC ATAAGTAAAG GCACAAATTT TTCATCCAAA TTTTAATTTT TGATGTAATA   
  
  
+ TTTTTTATAT TTTTATTGTT CAAATCTGAT TTTAATCTTA TTTAAATTAT CTGACCTAAA AAAATCAAAT   
  
  
+ AATAATAAAC GTTAATTTTT TATTTAAATT TTGACATTAG TCAATCTGAC TTAAATTCGA ACCGAGCTTG   
  
  
+ AATTTTTTTG CCAGTGCTGG TAGTACTCCT GCTTTTGTAG CTCCCAATGC CATCGTACCA CCCTCTTCTA   
  
  
+ GTCTCGTCTC TTTCTCTGGT CTCCAATTAA TCACACCATC ATATCATACC GTATGATGAT ACAGTCCACT   
  
  
+ GAAATCCCAT TCTCACCGCC AATCTCTCTC CGCCACCATC CAACACTCTT AACGATGAAC AGGGCCGCCG   
  
  
+ CCTCGTCCTC CACACTCAGG CCGTGGCCGG GCAGCTTTCC CACCCAATCA AAATCTCTCT CCTCCGCCAA   
  
  
+ CTTCGGTAAC GCCAATTGCA TGGAGCAGCT CTTAGTCCAC TGCGCCGAAG CCATCGACAA CAATGACGCC   
  
  
+ ACCCCGGCCC AGCAAATCTT ATGGGTCCTG AATAACATAG CCCGACCCGA CGGCGACTCC ACCCAACGCC   
  
  
+ TCACGTGCGC ATTCCTACGT GCCTTAATCT CACGCGCCGT CCTCACTAGC ACCTGCAAGA TGGTAATCCC   
  
  
+ TCATTTCAAC CCCATCAATT CACCCCACAA ATTCTCGCTC CTCGAACTTG CCCACTTCGT CGATTTAACC   
  
  
+ CCTTGGCATC GATTCGGATT CACCGCCGCC AATTCGATCA TTCTGGAAGC TATTTCCGAC CTACCCGTTG   
  
  
+ TACACATTGT CGACCTCAGC ATCTCCCACT GTATGCAGAT CCCCACGTTG ATCGACTCCA TTGCGACCCG   
  
  
+ GTTGGAAGCC CCGGGTCGAG TCCCTCCTAT TGTCAAGCTC ACCGTCGGGG CTATTTCCGA CGAAATCCCG   
  
  
+ CCGGTGTTCG ATCTTCTGTC GTACGATGAG CTCGGAATGA GACTAATCAA CTTCGCTCGT TTTAGAAACA   
  
  
+ TCGTCCTCGA ATTCCAAGCA ATACCCACCT CCCCTTCCGA CGGATTTGCT TCGCTGTTGG AGGAGATTCG   
  
  
+ ACAAAGCAAG CTCTACTCCA ACGATGCGGC GGCGGTTATT GTGAATTGTC AGATGAGTTT GCATTTATTG   
  
  
+ CAGGAAGAGG AGGTGTCTTC ATCGTCGCCG TCGTCGATGA GGGGGATGTT TTTGCAGGCG GTGAGGAGCT   
  
  
+ TGGAGCCGAG CATGGTGGTG GTGGTGGAGG AGGACGTGGA TTTCACGGCG AGGAGTCTGG TGGGGCGGCT   
  
  
+ GAGATCGGCG TTTAATCACA TGTGGATACC CTTCGACACG GTGGACACGT TCTTGCCACG TGGGAGCCAG   
  
  
+ CAGAGAGAGT GGTTCGAGGC CGAGGTGTGC TGGAAGATTG AGAATGTGAT CGCTCATGAG GGACCCGCGA   
  
  
+ GGGTCGAGAG GCAGGAGCCC AGGGCCAAGT GGGCCCTCCG AATGAGGGAG GCCGAGTTTC AAGGGATCGA   
  
  
+ GTTCGGTGAT GAAGGTACGA CCGAGGTCAA GGCCATGCTG GAGGAGCATG CCGCTGGGTG GGGGTCTAAG   
  
  
+ AAGGAAGAGG ATGATCTTGT GCTCACTTGG AAGGGACATA GTGTTGTCTT TGCTTCTGCT TGGGTACCCA   
  
  
+ CTTA  

- +Up\_Stream \_Len000TCTTCG ATTAATTTGG TTTGTACGTG CATACAGGAT TAATTGTACT GACAGGTAGT   
  
  
- AGAGGAGGGT ACAACAAGAC AACTGTTCGG ACGTGTGGGT ACGAGGAGAG AGTACAGATT GGAGCAGGGT   
  
  
- TGCTAGTTCT AGTGACAGAC ACTCCGGTCC CCTTCTGAAC GAAAATAAAG GAAAAGAAAA CAAATGAGAG   
  
  
- ACTAAGGTAA ATCGGTATAT ATGTTCCTCT TACAGTACAC ATAGAGTATA CATATATATA TTCTAAAATA   
  
  
- AATCTTTATT TTTAAATTTG TGATACACTA AATATCAATA ATATTATTTA TCATAAAAAA TTTGATAAAG   
  
  
- TAACTACTCT TGTCCTTTCA TTTAACGGCT GTACAATTAC AATAATATAG ATTTTTAATT TAGTATTTTA   
  
  
- ATTAATATTA TAAAATTTTT ATAAAGTTGA TTTTAAAATC TTGTTATTCA CGCATTATGC CTGAATTTCC   
  
  
- GATCAAATAG CATTTTTACA TTCAAGGAAA ACCTAGAATA CCAACTTATA GAACCAACTA TTTGACACTA   
  
  
- ACCAAAATGT GAAAATGGTG TATAAGTATC TTCTTAATAC ACGTGCAACT ACCAAGTTCT ACCCTGTTTT   
  
  
- CCTTTGTACA TAAGAAGGGG GAAAACGGAA AAGGGGATTC GTAAACCTAA CTCCACAACA GTAGACTTAT   
  
  
- AGTTTTTAAG AAAAACTTTT AAGTCAGAGT TAGTTTTAAG TTTTAATTTT GTTTCTGAAT TCTCAAAATA   
  
  
- AACAAGAATT GGAAGAATTG AAAGATGAAA AGAAAAAAAA GAGTAAGGGG GAGAAGTTTA GAGTAAATAA   
  
  
- GAATTGGGGG AGAAGGCAAG TACGAGAGAG ACAGATAGTT GATTATAATT AGATGGGGCT GAAAGTTAAA   
  
  
- CTACTCCAGA TTAGGAATAG TAGCATAACA CCAACCCAAT TAATACGTTC TTCCGTTATT TGGGGTCACT   
  
  
- GTTTCAGGTG CAATTCATCC GTGGAGTGGT AATTCTGTAC GAGTCTTTTG TGGTTTTGGT AACTTGTGTT   
  
  
- CAGGGGGTTC AGGGAAGGGA TCGATCGGGT AAGAGAGAGG AGGGGACAGA TATAGATGGT TGAGAACGAG   
  
  
- AAACGACTCG GACTCAACTT TCGCTATGTG GGTATCGGAC AAGTCAAAAG TCAAAAGTGT AAACAGAAGG   
  
  
- AGACAAGTCA ACTTTCGCTA TGTGAGAGTA ATCGAAAGTA AAATTTATGC AAGTTAAGTG TGTATTTACC   
  
  
- GAAAGTTTCG TAACGGTAAA AGCAAATTTA CTACTGTTTT ATATATCATA GACCGATAGA CGGGTATAGA   
  
  
- ACTGGAATAA ATGTTTCCGA CTCGGTAGAA AATTAAAAAA ATCGGAGAAA AAAATTAGTT TTTATCTTTA   
  
  
- AAGTTCTTTC GTCTTTAGCA GTCTACTGTC ACGTCTGTCG CAAAAGCAAA GTATGTTTAG AGTTTTCGAC   
  
  
- TTCACAGTTA AAGAAGCTAA TAAAAACAGT CTTTTATAAA TGCAGAGTGG AAGTGCAACA ATAATAAGAA   
  
  
- AGAAAAAATA ATAAGAGTAT TGATCATTAT TTCAATCGTT ACTATTTTTT AATAAGCTAG GCTTATAAAA   
  
  
- TTAAATAGAC TGGATTTTTG TATTCATTTC CGTGTTTAAA AAGTAGGTTT AAAATTAAAA ACTACATTAT   
  
  
- AAAAAATATA AAAATAACAA GTTTAGACTA AAATTAGAAT AAATTTAATA GACTGGATTT TTTTAGTTTA   
  
  
- TTATTATTTG CAATTAAAAA ATAAATTTAA AACTGTAATC AGTTAGACTG AATTTAAGCT TGGCTCGAAC   
  
  
- TTAAAAAAAC GGTCACGACC ATCATGAGGA CGAAAACATC GAGGGTTACG GTAGCATGGT GGGAGAAGAT   
  
  
- CAGAGCAGAG AAAGAGACCA GAGGTTAATT AGTGTGGTAG TATAGTATGG CATACTACTA TGTCAGGTGA   
  
  
- CTTTAGGGTA AGAGTGGCGG TTAGAGAGAG GCGGTGGTAG GTTGTGAGAA TTGCTACTTG TCCCGGCGGC   
  
  
- GGAGCAGGAG GTGTGAGTCC GGCACCGGCC CGTCGAAAGG GTGGGTTAGT TTTAGAGAGA GGAGGCGGTT   
  
  
- GAAGCCATTG CGGTTAACGT ACCTCGTCGA GAATCAGGTG ACGCGGCTTC GGTAGCTGTT GTTACTGCGG   
  
  
- TGGGGCCGGG TCGTTTAGAA TACCCAGGAC TTATTGTATC GGGCTGGGCT GCCGCTGAGG TGGGTTGCGG   
  
  
- AGTGCACGCG TAAGGATGCA CGGAATTAGA GTGCGCGGCA GGAGTGATCG TGGACGTTCT ACCATTAGGG   
  
  
- AGTAAAGTTG GGGTAGTTAA GTGGGGTGTT TAAGAGCGAG GAGCTTGAAC GGGTGAAGCA GCTAAATTGG   
  
  
- GGAACCGTAG CTAAGCCTAA GTGGCGGCGG TTAAGCTAGT AAGACCTTCG ATAAAGGCTG GATGGGCAAC   
  
  
- ATGTGTAACA GCTGGAGTCG TAGAGGGTGA CATACGTCTA GGGGTGCAAC TAGCTGAGGT AACGCTGGGC   
  
  
- CAACCTTCGG GGCCCAGCTC AGGGAGGATA ACAGTTCGAG TGGCAGCCCC GATAAAGGCT GCTTTAGGGC   
  
  
- GGCCACAAGC TAGAAGACAG CATGCTACTC GAGCCTTACT CTGATTAGTT GAAGCGAGCA AAATCTTTGT   
  
  
- AGCAGGAGCT TAAGGTTCGT TATGGGTGGA GGGGAAGGCT GCCTAAACGA AGCGACAACC TCCTCTAAGC   
  
  
- TGTTTCGTTC GAGATGAGGT TGCTACGCCG CCGCCAATAA CACTTAACAG TCTACTCAAA CGTAAATAAC   
  
  
- GTCCTTCTCC TCCACAGAAG TAGCAGCGGC AGCAGCTACT CCCCCTACAA AAACGTCCGC CACTCCTCGA   
  
  
- ACCTCGGCTC GTACCACCAC CACCACCTCC TCCTGCACCT AAAGTGCCGC TCCTCAGACC ACCCCGCCGA   
  
  
- CTCTAGCCGC AAATTAGTGT ACACCTATGG GAAGCTGTGC CACCTGTGCA AGAACGGTGC ACCCTCGGTC   
  
  
- GTCTCTCTCA CCAAGCTCCG GCTCCACACG ACCTTCTAAC TCTTACACTA GCGAGTACTC CCTGGGCGCT   
  
  
- CCCAGCTCTC CGTCCTCGGG TCCCGGTTCA CCCGGGAGGC TTACTCCCTC CGGCTCAAAG TTCCCTAGCT   
  
  
- CAAGCCACTA CTTCCATGCT GGCTCCAGTT CCGGTACGAC CTCCTCGTAC GGCGACCCAC CCCCAGATTC   
  
  
- TTCCTTCTCC TACTAGAACA CGAGTGAACC TTCCCTGTAT CACAACAGAA ACGAAGACGA ACCCATGGGT   
  
  
- GAAT

+     MBS

| Site Name | Organism | Position | Strand | Matrix score. | sequence | function |
| --- | --- | --- | --- | --- | --- | --- |
| MBS | Arabidopsis thaliana | 1201 | - | 6 | CAACTG | MYB binding site involved in drought-inducibility |

>HU06G00376.1   
+ +Up\_Stream \_Len000AGAAGC TAATTAAACC AAACATGCAC GTATGTCCTA ATTAACATGA CTGTCCATCA   
  
  
+ TCTCCTCCCA TGTTGTTCTG TTGACAAGCC TGCACACCCA TGCTCCTCTC TCATGTCTAA CCTCGTCCCA   
  
  
+ ACGATCAAGA TCACTGTCTG TGAGGCCAGG GGAAGACTTG CTTTTATTTC CTTTTCTTTT GTTTACTCTC   
  
  
+ TGATTCCATT TAGCCATATA TACAAGGAGA ATGTCATGTG TATCTCATAT GTATATATAT AAGATTTTAT   
  
  
+ TTAGAAATAA AAATTTAAAC ACTATGTGAT TTATAGTTAT TATAATAAAT AGTATTTTTT AAACTATTTC   
  
  
+ ATTGATGAGA ACAGGAAAGT AAATTGCCGA CATGTTAATG TTATTATATC TAAAAATTAA ATCATAAAAT   
  
  
+ TAATTATAAT ATTTTAAAAA TATTTCAACT AAAATTTTAG AACAATAAGT GCGTAATACG GACTTAAAGG   
  
  
+ CTAGTTTATC GTAAAAATGT AAGTTCCTTT TGGATCTTAT GGTTGAATAT CTTGGTTGAT AAACTGTGAT   
  
  
+ TGGTTTTACA CTTTTACCAC ATATTCATAG AAGAATTATG TGCACGTTGA TGGTTCAAGA TGGGACAAAA   
  
  
+ GGAAACATGT ATTCTTCCCC CTTTTGCCTT TTCCCCTAAG CATTTGGATT GAGGTGTTGT CATCTGAATA   
  
  
+ TCAAAAATTC TTTTTGAAAA TTCAGTCTCA ATCAAAATTC AAAATTAAAA CAAAGACTTA AGAGTTTTAT   
  
  
+ TTGTTCTTAA CCTTCTTAAC TTTCTACTTT TCTTTTTTTT CTCATTCCCC CTCTTCAAAT CTCATTTATT   
  
  
+ CTTAACCCCC TCTTCCGTTC ATGCTCTCTC TGTCTATCAA CTAATATTAA TCTACCCCGA CTTTCAATTT   
  
  
+ GATGAGGTCT AATCCTTATC ATCGTATTGT GGTTGGGTTA ATTATGCAAG AAGGCAATAA ACCCCAGTGA   
  
  
+ CAAAGTCCAC GTTAAGTAGG CACCTCACCA TTAAGACATG CTCAGAAAAC ACCAAAACCA TTGAACACAA   
  
  
+ GTCCCCCAAG TCCCTTCCCT AGCTAGCCCA TTCTCTCTCC TCCCCTGTCT ATATCTACCA ACTCTTGCTC   
  
  
+ TTTGCTGAGC CTGAGTTGAA AGCGATACAC CCATAGCCTG TTCAGTTTTC AGTTTTCACA TTTGTCTTCC   
  
  
+ TCTGTTCAGT TGAAAGCGAT ACACTCTCAT TAGCTTTCAT TTTAAATACG TTCAATTCAC ACATAAATGG   
  
  
+ CTTTCAAAGC ATTGCCATTT TCGTTTAAAT GATGACAAAA TATATAGTAT CTGGCTATCT GCCCATATCT   
  
  
+ TGACCTTATT TACAAAGGCT GAGCCATCTT TTAATTTTTT TAGCCTCTTT TTTTAATCAA AAATAGAAAT   
  
  
+ TTCAAGAAAG CAGAAATCGT CAGATGACAG TGCAGACAGC GTTTTCGTTT CATACAAATC TCAAAAGCTG   
  
  
+ AAGTGTCAAT TTCTTCGATT ATTTTTGTCA GAAAATATTT ACGTCTCACC TTCACGTTGT TATTATTCTT   
  
  
+ TCTTTTTTAT TATTCTCATA ACTAGTAATA AAGTTAGCAA TGATAAAAAA TTATTCGATC CGAATATTTT   
  
  
+ AATTTATCTG ACCTAAAAAC ATAAGTAAAG GCACAAATTT TTCATCCAAA TTTTAATTTT TGATGTAATA   
  
  
+ TTTTTTATAT TTTTATTGTT CAAATCTGAT TTTAATCTTA TTTAAATTAT CTGACCTAAA AAAATCAAAT   
  
  
+ AATAATAAAC GTTAATTTTT TATTTAAATT TTGACATTAG TCAATCTGAC TTAAATTCGA ACCGAGCTTG   
  
  
+ AATTTTTTTG CCAGTGCTGG TAGTACTCCT GCTTTTGTAG CTCCCAATGC CATCGTACCA CCCTCTTCTA   
  
  
+ GTCTCGTCTC TTTCTCTGGT CTCCAATTAA TCACACCATC ATATCATACC GTATGATGAT ACAGTCCACT   
  
  
+ GAAATCCCAT TCTCACCGCC AATCTCTCTC CGCCACCATC CAACACTCTT AACGATGAAC AGGGCCGCCG   
  
  
+ CCTCGTCCTC CACACTCAGG CCGTGGCCGG GCAGCTTTCC CACCCAATCA AAATCTCTCT CCTCCGCCAA   
  
  
+ CTTCGGTAAC GCCAATTGCA TGGAGCAGCT CTTAGTCCAC TGCGCCGAAG CCATCGACAA CAATGACGCC   
  
  
+ ACCCCGGCCC AGCAAATCTT ATGGGTCCTG AATAACATAG CCCGACCCGA CGGCGACTCC ACCCAACGCC   
  
  
+ TCACGTGCGC ATTCCTACGT GCCTTAATCT CACGCGCCGT CCTCACTAGC ACCTGCAAGA TGGTAATCCC   
  
  
+ TCATTTCAAC CCCATCAATT CACCCCACAA ATTCTCGCTC CTCGAACTTG CCCACTTCGT CGATTTAACC   
  
  
+ CCTTGGCATC GATTCGGATT CACCGCCGCC AATTCGATCA TTCTGGAAGC TATTTCCGAC CTACCCGTTG   
  
  
+ TACACATTGT CGACCTCAGC ATCTCCCACT GTATGCAGAT CCCCACGTTG ATCGACTCCA TTGCGACCCG   
  
  
+ GTTGGAAGCC CCGGGTCGAG TCCCTCCTAT TGTCAAGCTC ACCGTCGGGG CTATTTCCGA CGAAATCCCG   
  
  
+ CCGGTGTTCG ATCTTCTGTC GTACGATGAG CTCGGAATGA GACTAATCAA CTTCGCTCGT TTTAGAAACA   
  
  
+ TCGTCCTCGA ATTCCAAGCA ATACCCACCT CCCCTTCCGA CGGATTTGCT TCGCTGTTGG AGGAGATTCG   
  
  
+ ACAAAGCAAG CTCTACTCCA ACGATGCGGC GGCGGTTATT GTGAATTGTC AGATGAGTTT GCATTTATTG   
  
  
+ CAGGAAGAGG AGGTGTCTTC ATCGTCGCCG TCGTCGATGA GGGGGATGTT TTTGCAGGCG GTGAGGAGCT   
  
  
+ TGGAGCCGAG CATGGTGGTG GTGGTGGAGG AGGACGTGGA TTTCACGGCG AGGAGTCTGG TGGGGCGGCT   
  
  
+ GAGATCGGCG TTTAATCACA TGTGGATACC CTTCGACACG GTGGACACGT TCTTGCCACG TGGGAGCCAG   
  
  
+ CAGAGAGAGT GGTTCGAGGC CGAGGTGTGC TGGAAGATTG AGAATGTGAT CGCTCATGAG GGACCCGCGA   
  
  
+ GGGTCGAGAG GCAGGAGCCC AGGGCCAAGT GGGCCCTCCG AATGAGGGAG GCCGAGTTTC AAGGGATCGA   
  
  
+ GTTCGGTGAT GAAGGTACGA CCGAGGTCAA GGCCATGCTG GAGGAGCATG CCGCTGGGTG GGGGTCTAAG   
  
  
+ AAGGAAGAGG ATGATCTTGT GCTCACTTGG AAGGGACATA GTGTTGTCTT TGCTTCTGCT TGGGTACCCA   
  
  
+ CTTA  

- +Up\_Stream \_Len000TCTTCG ATTAATTTGG TTTGTACGTG CATACAGGAT TAATTGTACT GACAGGTAGT   
  
  
- AGAGGAGGGT ACAACAAGAC AACTGTTCGG ACGTGTGGGT ACGAGGAGAG AGTACAGATT GGAGCAGGGT   
  
  
- TGCTAGTTCT AGTGACAGAC ACTCCGGTCC CCTTCTGAAC GAAAATAAAG GAAAAGAAAA CAAATGAGAG   
  
  
- ACTAAGGTAA ATCGGTATAT ATGTTCCTCT TACAGTACAC ATAGAGTATA CATATATATA TTCTAAAATA   
  
  
- AATCTTTATT TTTAAATTTG TGATACACTA AATATCAATA ATATTATTTA TCATAAAAAA TTTGATAAAG   
  
  
- TAACTACTCT TGTCCTTTCA TTTAACGGCT GTACAATTAC AATAATATAG ATTTTTAATT TAGTATTTTA   
  
  
- ATTAATATTA TAAAATTTTT ATAAAGTTGA TTTTAAAATC TTGTTATTCA CGCATTATGC CTGAATTTCC   
  
  
- GATCAAATAG CATTTTTACA TTCAAGGAAA ACCTAGAATA CCAACTTATA GAACCAACTA TTTGACACTA   
  
  
- ACCAAAATGT GAAAATGGTG TATAAGTATC TTCTTAATAC ACGTGCAACT ACCAAGTTCT ACCCTGTTTT   
  
  
- CCTTTGTACA TAAGAAGGGG GAAAACGGAA AAGGGGATTC GTAAACCTAA CTCCACAACA GTAGACTTAT   
  
  
- AGTTTTTAAG AAAAACTTTT AAGTCAGAGT TAGTTTTAAG TTTTAATTTT GTTTCTGAAT TCTCAAAATA   
  
  
- AACAAGAATT GGAAGAATTG AAAGATGAAA AGAAAAAAAA GAGTAAGGGG GAGAAGTTTA GAGTAAATAA   
  
  
- GAATTGGGGG AGAAGGCAAG TACGAGAGAG ACAGATAGTT GATTATAATT AGATGGGGCT GAAAGTTAAA   
  
  
- CTACTCCAGA TTAGGAATAG TAGCATAACA CCAACCCAAT TAATACGTTC TTCCGTTATT TGGGGTCACT   
  
  
- GTTTCAGGTG CAATTCATCC GTGGAGTGGT AATTCTGTAC GAGTCTTTTG TGGTTTTGGT AACTTGTGTT   
  
  
- CAGGGGGTTC AGGGAAGGGA TCGATCGGGT AAGAGAGAGG AGGGGACAGA TATAGATGGT TGAGAACGAG   
  
  
- AAACGACTCG GACTCAACTT TCGCTATGTG GGTATCGGAC AAGTCAAAAG TCAAAAGTGT AAACAGAAGG   
  
  
- AGACAAGTCA ACTTTCGCTA TGTGAGAGTA ATCGAAAGTA AAATTTATGC AAGTTAAGTG TGTATTTACC   
  
  
- GAAAGTTTCG TAACGGTAAA AGCAAATTTA CTACTGTTTT ATATATCATA GACCGATAGA CGGGTATAGA   
  
  
- ACTGGAATAA ATGTTTCCGA CTCGGTAGAA AATTAAAAAA ATCGGAGAAA AAAATTAGTT TTTATCTTTA   
  
  
- AAGTTCTTTC GTCTTTAGCA GTCTACTGTC ACGTCTGTCG CAAAAGCAAA GTATGTTTAG AGTTTTCGAC   
  
  
- TTCACAGTTA AAGAAGCTAA TAAAAACAGT CTTTTATAAA TGCAGAGTGG AAGTGCAACA ATAATAAGAA   
  
  
- AGAAAAAATA ATAAGAGTAT TGATCATTAT TTCAATCGTT ACTATTTTTT AATAAGCTAG GCTTATAAAA   
  
  
- TTAAATAGAC TGGATTTTTG TATTCATTTC CGTGTTTAAA AAGTAGGTTT AAAATTAAAA ACTACATTAT   
  
  
- AAAAAATATA AAAATAACAA GTTTAGACTA AAATTAGAAT AAATTTAATA GACTGGATTT TTTTAGTTTA   
  
  
- TTATTATTTG CAATTAAAAA ATAAATTTAA AACTGTAATC AGTTAGACTG AATTTAAGCT TGGCTCGAAC   
  
  
- TTAAAAAAAC GGTCACGACC ATCATGAGGA CGAAAACATC GAGGGTTACG GTAGCATGGT GGGAGAAGAT   
  
  
- CAGAGCAGAG AAAGAGACCA GAGGTTAATT AGTGTGGTAG TATAGTATGG CATACTACTA TGTCAGGTGA   
  
  
- CTTTAGGGTA AGAGTGGCGG TTAGAGAGAG GCGGTGGTAG GTTGTGAGAA TTGCTACTTG TCCCGGCGGC   
  
  
- GGAGCAGGAG GTGTGAGTCC GGCACCGGCC CGTCGAAAGG GTGGGTTAGT TTTAGAGAGA GGAGGCGGTT   
  
  
- GAAGCCATTG CGGTTAACGT ACCTCGTCGA GAATCAGGTG ACGCGGCTTC GGTAGCTGTT GTTACTGCGG   
  
  
- TGGGGCCGGG TCGTTTAGAA TACCCAGGAC TTATTGTATC GGGCTGGGCT GCCGCTGAGG TGGGTTGCGG   
  
  
- AGTGCACGCG TAAGGATGCA CGGAATTAGA GTGCGCGGCA GGAGTGATCG TGGACGTTCT ACCATTAGGG   
  
  
- AGTAAAGTTG GGGTAGTTAA GTGGGGTGTT TAAGAGCGAG GAGCTTGAAC GGGTGAAGCA GCTAAATTGG   
  
  
- GGAACCGTAG CTAAGCCTAA GTGGCGGCGG TTAAGCTAGT AAGACCTTCG ATAAAGGCTG GATGGGCAAC   
  
  
- ATGTGTAACA GCTGGAGTCG TAGAGGGTGA CATACGTCTA GGGGTGCAAC TAGCTGAGGT AACGCTGGGC   
  
  
- CAACCTTCGG GGCCCAGCTC AGGGAGGATA ACAGTTCGAG TGGCAGCCCC GATAAAGGCT GCTTTAGGGC   
  
  
- GGCCACAAGC TAGAAGACAG CATGCTACTC GAGCCTTACT CTGATTAGTT GAAGCGAGCA AAATCTTTGT   
  
  
- AGCAGGAGCT TAAGGTTCGT TATGGGTGGA GGGGAAGGCT GCCTAAACGA AGCGACAACC TCCTCTAAGC   
  
  
- TGTTTCGTTC GAGATGAGGT TGCTACGCCG CCGCCAATAA CACTTAACAG TCTACTCAAA CGTAAATAAC   
  
  
- GTCCTTCTCC TCCACAGAAG TAGCAGCGGC AGCAGCTACT CCCCCTACAA AAACGTCCGC CACTCCTCGA   
  
  
- ACCTCGGCTC GTACCACCAC CACCACCTCC TCCTGCACCT AAAGTGCCGC TCCTCAGACC ACCCCGCCGA   
  
  
- CTCTAGCCGC AAATTAGTGT ACACCTATGG GAAGCTGTGC CACCTGTGCA AGAACGGTGC ACCCTCGGTC   
  
  
- GTCTCTCTCA CCAAGCTCCG GCTCCACACG ACCTTCTAAC TCTTACACTA GCGAGTACTC CCTGGGCGCT   
  
  
- CCCAGCTCTC CGTCCTCGGG TCCCGGTTCA CCCGGGAGGC TTACTCCCTC CGGCTCAAAG TTCCCTAGCT   
  
  
- CAAGCCACTA CTTCCATGCT GGCTCCAGTT CCGGTACGAC CTCCTCGTAC GGCGACCCAC CCCCAGATTC   
  
  
- TTCCTTCTCC TACTAGAACA CGAGTGAACC TTCCCTGTAT CACAACAGAA ACGAAGACGA ACCCATGGGT   
  
  
- GAAT

+     MYB

| Site Name | Organism | Position | Strand | Matrix score. | sequence | function |
| --- | --- | --- | --- | --- | --- | --- |
| MYB | Arabidopsis thaliana | 547 | - | 6 | CAACCA |  |
| MYB | Arabidopsis thaliana | 92 | - | 6 | CAACAG |  |
| MYB | Arabidopsis thaliana | 2718 | - | 6 | CAACAG |  |
| MYB | Arabidopsis thaliana | 534 | - | 6 | CAACCA |  |
| MYB | Arabidopsis thaliana | 944 | - | 6 | CAACCA |  |

>HU06G00376.1   
+ +Up\_Stream \_Len000AGAAGC TAATTAAACC AAACATGCAC GTATGTCCTA ATTAACATGA CTGTCCATCA   
  
  
+ TCTCCTCCCA TGTTGTTCTG TTGACAAGCC TGCACACCCA TGCTCCTCTC TCATGTCTAA CCTCGTCCCA   
  
  
+ ACGATCAAGA TCACTGTCTG TGAGGCCAGG GGAAGACTTG CTTTTATTTC CTTTTCTTTT GTTTACTCTC   
  
  
+ TGATTCCATT TAGCCATATA TACAAGGAGA ATGTCATGTG TATCTCATAT GTATATATAT AAGATTTTAT   
  
  
+ TTAGAAATAA AAATTTAAAC ACTATGTGAT TTATAGTTAT TATAATAAAT AGTATTTTTT AAACTATTTC   
  
  
+ ATTGATGAGA ACAGGAAAGT AAATTGCCGA CATGTTAATG TTATTATATC TAAAAATTAA ATCATAAAAT   
  
  
+ TAATTATAAT ATTTTAAAAA TATTTCAACT AAAATTTTAG AACAATAAGT GCGTAATACG GACTTAAAGG   
  
  
+ CTAGTTTATC GTAAAAATGT AAGTTCCTTT TGGATCTTAT GGTTGAATAT CTTGGTTGAT AAACTGTGAT   
  
  
+ TGGTTTTACA CTTTTACCAC ATATTCATAG AAGAATTATG TGCACGTTGA TGGTTCAAGA TGGGACAAAA   
  
  
+ GGAAACATGT ATTCTTCCCC CTTTTGCCTT TTCCCCTAAG CATTTGGATT GAGGTGTTGT CATCTGAATA   
  
  
+ TCAAAAATTC TTTTTGAAAA TTCAGTCTCA ATCAAAATTC AAAATTAAAA CAAAGACTTA AGAGTTTTAT   
  
  
+ TTGTTCTTAA CCTTCTTAAC TTTCTACTTT TCTTTTTTTT CTCATTCCCC CTCTTCAAAT CTCATTTATT   
  
  
+ CTTAACCCCC TCTTCCGTTC ATGCTCTCTC TGTCTATCAA CTAATATTAA TCTACCCCGA CTTTCAATTT   
  
  
+ GATGAGGTCT AATCCTTATC ATCGTATTGT GGTTGGGTTA ATTATGCAAG AAGGCAATAA ACCCCAGTGA   
  
  
+ CAAAGTCCAC GTTAAGTAGG CACCTCACCA TTAAGACATG CTCAGAAAAC ACCAAAACCA TTGAACACAA   
  
  
+ GTCCCCCAAG TCCCTTCCCT AGCTAGCCCA TTCTCTCTCC TCCCCTGTCT ATATCTACCA ACTCTTGCTC   
  
  
+ TTTGCTGAGC CTGAGTTGAA AGCGATACAC CCATAGCCTG TTCAGTTTTC AGTTTTCACA TTTGTCTTCC   
  
  
+ TCTGTTCAGT TGAAAGCGAT ACACTCTCAT TAGCTTTCAT TTTAAATACG TTCAATTCAC ACATAAATGG   
  
  
+ CTTTCAAAGC ATTGCCATTT TCGTTTAAAT GATGACAAAA TATATAGTAT CTGGCTATCT GCCCATATCT   
  
  
+ TGACCTTATT TACAAAGGCT GAGCCATCTT TTAATTTTTT TAGCCTCTTT TTTTAATCAA AAATAGAAAT   
  
  
+ TTCAAGAAAG CAGAAATCGT CAGATGACAG TGCAGACAGC GTTTTCGTTT CATACAAATC TCAAAAGCTG   
  
  
+ AAGTGTCAAT TTCTTCGATT ATTTTTGTCA GAAAATATTT ACGTCTCACC TTCACGTTGT TATTATTCTT   
  
  
+ TCTTTTTTAT TATTCTCATA ACTAGTAATA AAGTTAGCAA TGATAAAAAA TTATTCGATC CGAATATTTT   
  
  
+ AATTTATCTG ACCTAAAAAC ATAAGTAAAG GCACAAATTT TTCATCCAAA TTTTAATTTT TGATGTAATA   
  
  
+ TTTTTTATAT TTTTATTGTT CAAATCTGAT TTTAATCTTA TTTAAATTAT CTGACCTAAA AAAATCAAAT   
  
  
+ AATAATAAAC GTTAATTTTT TATTTAAATT TTGACATTAG TCAATCTGAC TTAAATTCGA ACCGAGCTTG   
  
  
+ AATTTTTTTG CCAGTGCTGG TAGTACTCCT GCTTTTGTAG CTCCCAATGC CATCGTACCA CCCTCTTCTA   
  
  
+ GTCTCGTCTC TTTCTCTGGT CTCCAATTAA TCACACCATC ATATCATACC GTATGATGAT ACAGTCCACT   
  
  
+ GAAATCCCAT TCTCACCGCC AATCTCTCTC CGCCACCATC CAACACTCTT AACGATGAAC AGGGCCGCCG   
  
  
+ CCTCGTCCTC CACACTCAGG CCGTGGCCGG GCAGCTTTCC CACCCAATCA AAATCTCTCT CCTCCGCCAA   
  
  
+ CTTCGGTAAC GCCAATTGCA TGGAGCAGCT CTTAGTCCAC TGCGCCGAAG CCATCGACAA CAATGACGCC   
  
  
+ ACCCCGGCCC AGCAAATCTT ATGGGTCCTG AATAACATAG CCCGACCCGA CGGCGACTCC ACCCAACGCC   
  
  
+ TCACGTGCGC ATTCCTACGT GCCTTAATCT CACGCGCCGT CCTCACTAGC ACCTGCAAGA TGGTAATCCC   
  
  
+ TCATTTCAAC CCCATCAATT CACCCCACAA ATTCTCGCTC CTCGAACTTG CCCACTTCGT CGATTTAACC   
  
  
+ CCTTGGCATC GATTCGGATT CACCGCCGCC AATTCGATCA TTCTGGAAGC TATTTCCGAC CTACCCGTTG   
  
  
+ TACACATTGT CGACCTCAGC ATCTCCCACT GTATGCAGAT CCCCACGTTG ATCGACTCCA TTGCGACCCG   
  
  
+ GTTGGAAGCC CCGGGTCGAG TCCCTCCTAT TGTCAAGCTC ACCGTCGGGG CTATTTCCGA CGAAATCCCG   
  
  
+ CCGGTGTTCG ATCTTCTGTC GTACGATGAG CTCGGAATGA GACTAATCAA CTTCGCTCGT TTTAGAAACA   
  
  
+ TCGTCCTCGA ATTCCAAGCA ATACCCACCT CCCCTTCCGA CGGATTTGCT TCGCTGTTGG AGGAGATTCG   
  
  
+ ACAAAGCAAG CTCTACTCCA ACGATGCGGC GGCGGTTATT GTGAATTGTC AGATGAGTTT GCATTTATTG   
  
  
+ CAGGAAGAGG AGGTGTCTTC ATCGTCGCCG TCGTCGATGA GGGGGATGTT TTTGCAGGCG GTGAGGAGCT   
  
  
+ TGGAGCCGAG CATGGTGGTG GTGGTGGAGG AGGACGTGGA TTTCACGGCG AGGAGTCTGG TGGGGCGGCT   
  
  
+ GAGATCGGCG TTTAATCACA TGTGGATACC CTTCGACACG GTGGACACGT TCTTGCCACG TGGGAGCCAG   
  
  
+ CAGAGAGAGT GGTTCGAGGC CGAGGTGTGC TGGAAGATTG AGAATGTGAT CGCTCATGAG GGACCCGCGA   
  
  
+ GGGTCGAGAG GCAGGAGCCC AGGGCCAAGT GGGCCCTCCG AATGAGGGAG GCCGAGTTTC AAGGGATCGA   
  
  
+ GTTCGGTGAT GAAGGTACGA CCGAGGTCAA GGCCATGCTG GAGGAGCATG CCGCTGGGTG GGGGTCTAAG   
  
  
+ AAGGAAGAGG ATGATCTTGT GCTCACTTGG AAGGGACATA GTGTTGTCTT TGCTTCTGCT TGGGTACCCA   
  
  
+ CTTA  

- +Up\_Stream \_Len000TCTTCG ATTAATTTGG TTTGTACGTG CATACAGGAT TAATTGTACT GACAGGTAGT   
  
  
- AGAGGAGGGT ACAACAAGAC AACTGTTCGG ACGTGTGGGT ACGAGGAGAG AGTACAGATT GGAGCAGGGT   
  
  
- TGCTAGTTCT AGTGACAGAC ACTCCGGTCC CCTTCTGAAC GAAAATAAAG GAAAAGAAAA CAAATGAGAG   
  
  
- ACTAAGGTAA ATCGGTATAT ATGTTCCTCT TACAGTACAC ATAGAGTATA CATATATATA TTCTAAAATA   
  
  
- AATCTTTATT TTTAAATTTG TGATACACTA AATATCAATA ATATTATTTA TCATAAAAAA TTTGATAAAG   
  
  
- TAACTACTCT TGTCCTTTCA TTTAACGGCT GTACAATTAC AATAATATAG ATTTTTAATT TAGTATTTTA   
  
  
- ATTAATATTA TAAAATTTTT ATAAAGTTGA TTTTAAAATC TTGTTATTCA CGCATTATGC CTGAATTTCC   
  
  
- GATCAAATAG CATTTTTACA TTCAAGGAAA ACCTAGAATA CCAACTTATA GAACCAACTA TTTGACACTA   
  
  
- ACCAAAATGT GAAAATGGTG TATAAGTATC TTCTTAATAC ACGTGCAACT ACCAAGTTCT ACCCTGTTTT   
  
  
- CCTTTGTACA TAAGAAGGGG GAAAACGGAA AAGGGGATTC GTAAACCTAA CTCCACAACA GTAGACTTAT   
  
  
- AGTTTTTAAG AAAAACTTTT AAGTCAGAGT TAGTTTTAAG TTTTAATTTT GTTTCTGAAT TCTCAAAATA   
  
  
- AACAAGAATT GGAAGAATTG AAAGATGAAA AGAAAAAAAA GAGTAAGGGG GAGAAGTTTA GAGTAAATAA   
  
  
- GAATTGGGGG AGAAGGCAAG TACGAGAGAG ACAGATAGTT GATTATAATT AGATGGGGCT GAAAGTTAAA   
  
  
- CTACTCCAGA TTAGGAATAG TAGCATAACA CCAACCCAAT TAATACGTTC TTCCGTTATT TGGGGTCACT   
  
  
- GTTTCAGGTG CAATTCATCC GTGGAGTGGT AATTCTGTAC GAGTCTTTTG TGGTTTTGGT AACTTGTGTT   
  
  
- CAGGGGGTTC AGGGAAGGGA TCGATCGGGT AAGAGAGAGG AGGGGACAGA TATAGATGGT TGAGAACGAG   
  
  
- AAACGACTCG GACTCAACTT TCGCTATGTG GGTATCGGAC AAGTCAAAAG TCAAAAGTGT AAACAGAAGG   
  
  
- AGACAAGTCA ACTTTCGCTA TGTGAGAGTA ATCGAAAGTA AAATTTATGC AAGTTAAGTG TGTATTTACC   
  
  
- GAAAGTTTCG TAACGGTAAA AGCAAATTTA CTACTGTTTT ATATATCATA GACCGATAGA CGGGTATAGA   
  
  
- ACTGGAATAA ATGTTTCCGA CTCGGTAGAA AATTAAAAAA ATCGGAGAAA AAAATTAGTT TTTATCTTTA   
  
  
- AAGTTCTTTC GTCTTTAGCA GTCTACTGTC ACGTCTGTCG CAAAAGCAAA GTATGTTTAG AGTTTTCGAC   
  
  
- TTCACAGTTA AAGAAGCTAA TAAAAACAGT CTTTTATAAA TGCAGAGTGG AAGTGCAACA ATAATAAGAA   
  
  
- AGAAAAAATA ATAAGAGTAT TGATCATTAT TTCAATCGTT ACTATTTTTT AATAAGCTAG GCTTATAAAA   
  
  
- TTAAATAGAC TGGATTTTTG TATTCATTTC CGTGTTTAAA AAGTAGGTTT AAAATTAAAA ACTACATTAT   
  
  
- AAAAAATATA AAAATAACAA GTTTAGACTA AAATTAGAAT AAATTTAATA GACTGGATTT TTTTAGTTTA   
  
  
- TTATTATTTG CAATTAAAAA ATAAATTTAA AACTGTAATC AGTTAGACTG AATTTAAGCT TGGCTCGAAC   
  
  
- TTAAAAAAAC GGTCACGACC ATCATGAGGA CGAAAACATC GAGGGTTACG GTAGCATGGT GGGAGAAGAT   
  
  
- CAGAGCAGAG AAAGAGACCA GAGGTTAATT AGTGTGGTAG TATAGTATGG CATACTACTA TGTCAGGTGA   
  
  
- CTTTAGGGTA AGAGTGGCGG TTAGAGAGAG GCGGTGGTAG GTTGTGAGAA TTGCTACTTG TCCCGGCGGC   
  
  
- GGAGCAGGAG GTGTGAGTCC GGCACCGGCC CGTCGAAAGG GTGGGTTAGT TTTAGAGAGA GGAGGCGGTT   
  
  
- GAAGCCATTG CGGTTAACGT ACCTCGTCGA GAATCAGGTG ACGCGGCTTC GGTAGCTGTT GTTACTGCGG   
  
  
- TGGGGCCGGG TCGTTTAGAA TACCCAGGAC TTATTGTATC GGGCTGGGCT GCCGCTGAGG TGGGTTGCGG   
  
  
- AGTGCACGCG TAAGGATGCA CGGAATTAGA GTGCGCGGCA GGAGTGATCG TGGACGTTCT ACCATTAGGG   
  
  
- AGTAAAGTTG GGGTAGTTAA GTGGGGTGTT TAAGAGCGAG GAGCTTGAAC GGGTGAAGCA GCTAAATTGG   
  
  
- GGAACCGTAG CTAAGCCTAA GTGGCGGCGG TTAAGCTAGT AAGACCTTCG ATAAAGGCTG GATGGGCAAC   
  
  
- ATGTGTAACA GCTGGAGTCG TAGAGGGTGA CATACGTCTA GGGGTGCAAC TAGCTGAGGT AACGCTGGGC   
  
  
- CAACCTTCGG GGCCCAGCTC AGGGAGGATA ACAGTTCGAG TGGCAGCCCC GATAAAGGCT GCTTTAGGGC   
  
  
- GGCCACAAGC TAGAAGACAG CATGCTACTC GAGCCTTACT CTGATTAGTT GAAGCGAGCA AAATCTTTGT   
  
  
- AGCAGGAGCT TAAGGTTCGT TATGGGTGGA GGGGAAGGCT GCCTAAACGA AGCGACAACC TCCTCTAAGC   
  
  
- TGTTTCGTTC GAGATGAGGT TGCTACGCCG CCGCCAATAA CACTTAACAG TCTACTCAAA CGTAAATAAC   
  
  
- GTCCTTCTCC TCCACAGAAG TAGCAGCGGC AGCAGCTACT CCCCCTACAA AAACGTCCGC CACTCCTCGA   
  
  
- ACCTCGGCTC GTACCACCAC CACCACCTCC TCCTGCACCT AAAGTGCCGC TCCTCAGACC ACCCCGCCGA   
  
  
- CTCTAGCCGC AAATTAGTGT ACACCTATGG GAAGCTGTGC CACCTGTGCA AGAACGGTGC ACCCTCGGTC   
  
  
- GTCTCTCTCA CCAAGCTCCG GCTCCACACG ACCTTCTAAC TCTTACACTA GCGAGTACTC CCTGGGCGCT   
  
  
- CCCAGCTCTC CGTCCTCGGG TCCCGGTTCA CCCGGGAGGC TTACTCCCTC CGGCTCAAAG TTCCCTAGCT   
  
  
- CAAGCCACTA CTTCCATGCT GGCTCCAGTT CCGGTACGAC CTCCTCGTAC GGCGACCCAC CCCCAGATTC   
  
  
- TTCCTTCTCC TACTAGAACA CGAGTGAACC TTCCCTGTAT CACAACAGAA ACGAAGACGA ACCCATGGGT   
  
  
- GAAT

+     MYB recognition site

| Site Name | Organism | Position | Strand | Matrix score. | sequence | function |
| --- | --- | --- | --- | --- | --- | --- |
| MYB recognition site | Arabidopsis thaliana | 2449 | + | 6 | CCGTTG |  |

>HU06G00376.1   
+ +Up\_Stream \_Len000AGAAGC TAATTAAACC AAACATGCAC GTATGTCCTA ATTAACATGA CTGTCCATCA   
  
  
+ TCTCCTCCCA TGTTGTTCTG TTGACAAGCC TGCACACCCA TGCTCCTCTC TCATGTCTAA CCTCGTCCCA   
  
  
+ ACGATCAAGA TCACTGTCTG TGAGGCCAGG GGAAGACTTG CTTTTATTTC CTTTTCTTTT GTTTACTCTC   
  
  
+ TGATTCCATT TAGCCATATA TACAAGGAGA ATGTCATGTG TATCTCATAT GTATATATAT AAGATTTTAT   
  
  
+ TTAGAAATAA AAATTTAAAC ACTATGTGAT TTATAGTTAT TATAATAAAT AGTATTTTTT AAACTATTTC   
  
  
+ ATTGATGAGA ACAGGAAAGT AAATTGCCGA CATGTTAATG TTATTATATC TAAAAATTAA ATCATAAAAT   
  
  
+ TAATTATAAT ATTTTAAAAA TATTTCAACT AAAATTTTAG AACAATAAGT GCGTAATACG GACTTAAAGG   
  
  
+ CTAGTTTATC GTAAAAATGT AAGTTCCTTT TGGATCTTAT GGTTGAATAT CTTGGTTGAT AAACTGTGAT   
  
  
+ TGGTTTTACA CTTTTACCAC ATATTCATAG AAGAATTATG TGCACGTTGA TGGTTCAAGA TGGGACAAAA   
  
  
+ GGAAACATGT ATTCTTCCCC CTTTTGCCTT TTCCCCTAAG CATTTGGATT GAGGTGTTGT CATCTGAATA   
  
  
+ TCAAAAATTC TTTTTGAAAA TTCAGTCTCA ATCAAAATTC AAAATTAAAA CAAAGACTTA AGAGTTTTAT   
  
  
+ TTGTTCTTAA CCTTCTTAAC TTTCTACTTT TCTTTTTTTT CTCATTCCCC CTCTTCAAAT CTCATTTATT   
  
  
+ CTTAACCCCC TCTTCCGTTC ATGCTCTCTC TGTCTATCAA CTAATATTAA TCTACCCCGA CTTTCAATTT   
  
  
+ GATGAGGTCT AATCCTTATC ATCGTATTGT GGTTGGGTTA ATTATGCAAG AAGGCAATAA ACCCCAGTGA   
  
  
+ CAAAGTCCAC GTTAAGTAGG CACCTCACCA TTAAGACATG CTCAGAAAAC ACCAAAACCA TTGAACACAA   
  
  
+ GTCCCCCAAG TCCCTTCCCT AGCTAGCCCA TTCTCTCTCC TCCCCTGTCT ATATCTACCA ACTCTTGCTC   
  
  
+ TTTGCTGAGC CTGAGTTGAA AGCGATACAC CCATAGCCTG TTCAGTTTTC AGTTTTCACA TTTGTCTTCC   
  
  
+ TCTGTTCAGT TGAAAGCGAT ACACTCTCAT TAGCTTTCAT TTTAAATACG TTCAATTCAC ACATAAATGG   
  
  
+ CTTTCAAAGC ATTGCCATTT TCGTTTAAAT GATGACAAAA TATATAGTAT CTGGCTATCT GCCCATATCT   
  
  
+ TGACCTTATT TACAAAGGCT GAGCCATCTT TTAATTTTTT TAGCCTCTTT TTTTAATCAA AAATAGAAAT   
  
  
+ TTCAAGAAAG CAGAAATCGT CAGATGACAG TGCAGACAGC GTTTTCGTTT CATACAAATC TCAAAAGCTG   
  
  
+ AAGTGTCAAT TTCTTCGATT ATTTTTGTCA GAAAATATTT ACGTCTCACC TTCACGTTGT TATTATTCTT   
  
  
+ TCTTTTTTAT TATTCTCATA ACTAGTAATA AAGTTAGCAA TGATAAAAAA TTATTCGATC CGAATATTTT   
  
  
+ AATTTATCTG ACCTAAAAAC ATAAGTAAAG GCACAAATTT TTCATCCAAA TTTTAATTTT TGATGTAATA   
  
  
+ TTTTTTATAT TTTTATTGTT CAAATCTGAT TTTAATCTTA TTTAAATTAT CTGACCTAAA AAAATCAAAT   
  
  
+ AATAATAAAC GTTAATTTTT TATTTAAATT TTGACATTAG TCAATCTGAC TTAAATTCGA ACCGAGCTTG   
  
  
+ AATTTTTTTG CCAGTGCTGG TAGTACTCCT GCTTTTGTAG CTCCCAATGC CATCGTACCA CCCTCTTCTA   
  
  
+ GTCTCGTCTC TTTCTCTGGT CTCCAATTAA TCACACCATC ATATCATACC GTATGATGAT ACAGTCCACT   
  
  
+ GAAATCCCAT TCTCACCGCC AATCTCTCTC CGCCACCATC CAACACTCTT AACGATGAAC AGGGCCGCCG   
  
  
+ CCTCGTCCTC CACACTCAGG CCGTGGCCGG GCAGCTTTCC CACCCAATCA AAATCTCTCT CCTCCGCCAA   
  
  
+ CTTCGGTAAC GCCAATTGCA TGGAGCAGCT CTTAGTCCAC TGCGCCGAAG CCATCGACAA CAATGACGCC   
  
  
+ ACCCCGGCCC AGCAAATCTT ATGGGTCCTG AATAACATAG CCCGACCCGA CGGCGACTCC ACCCAACGCC   
  
  
+ TCACGTGCGC ATTCCTACGT GCCTTAATCT CACGCGCCGT CCTCACTAGC ACCTGCAAGA TGGTAATCCC   
  
  
+ TCATTTCAAC CCCATCAATT CACCCCACAA ATTCTCGCTC CTCGAACTTG CCCACTTCGT CGATTTAACC   
  
  
+ CCTTGGCATC GATTCGGATT CACCGCCGCC AATTCGATCA TTCTGGAAGC TATTTCCGAC CTACCCGTTG   
  
  
+ TACACATTGT CGACCTCAGC ATCTCCCACT GTATGCAGAT CCCCACGTTG ATCGACTCCA TTGCGACCCG   
  
  
+ GTTGGAAGCC CCGGGTCGAG TCCCTCCTAT TGTCAAGCTC ACCGTCGGGG CTATTTCCGA CGAAATCCCG   
  
  
+ CCGGTGTTCG ATCTTCTGTC GTACGATGAG CTCGGAATGA GACTAATCAA CTTCGCTCGT TTTAGAAACA   
  
  
+ TCGTCCTCGA ATTCCAAGCA ATACCCACCT CCCCTTCCGA CGGATTTGCT TCGCTGTTGG AGGAGATTCG   
  
  
+ ACAAAGCAAG CTCTACTCCA ACGATGCGGC GGCGGTTATT GTGAATTGTC AGATGAGTTT GCATTTATTG   
  
  
+ CAGGAAGAGG AGGTGTCTTC ATCGTCGCCG TCGTCGATGA GGGGGATGTT TTTGCAGGCG GTGAGGAGCT   
  
  
+ TGGAGCCGAG CATGGTGGTG GTGGTGGAGG AGGACGTGGA TTTCACGGCG AGGAGTCTGG TGGGGCGGCT   
  
  
+ GAGATCGGCG TTTAATCACA TGTGGATACC CTTCGACACG GTGGACACGT TCTTGCCACG TGGGAGCCAG   
  
  
+ CAGAGAGAGT GGTTCGAGGC CGAGGTGTGC TGGAAGATTG AGAATGTGAT CGCTCATGAG GGACCCGCGA   
  
  
+ GGGTCGAGAG GCAGGAGCCC AGGGCCAAGT GGGCCCTCCG AATGAGGGAG GCCGAGTTTC AAGGGATCGA   
  
  
+ GTTCGGTGAT GAAGGTACGA CCGAGGTCAA GGCCATGCTG GAGGAGCATG CCGCTGGGTG GGGGTCTAAG   
  
  
+ AAGGAAGAGG ATGATCTTGT GCTCACTTGG AAGGGACATA GTGTTGTCTT TGCTTCTGCT TGGGTACCCA   
  
  
+ CTTA  

- +Up\_Stream \_Len000TCTTCG ATTAATTTGG TTTGTACGTG CATACAGGAT TAATTGTACT GACAGGTAGT   
  
  
- AGAGGAGGGT ACAACAAGAC AACTGTTCGG ACGTGTGGGT ACGAGGAGAG AGTACAGATT GGAGCAGGGT   
  
  
- TGCTAGTTCT AGTGACAGAC ACTCCGGTCC CCTTCTGAAC GAAAATAAAG GAAAAGAAAA CAAATGAGAG   
  
  
- ACTAAGGTAA ATCGGTATAT ATGTTCCTCT TACAGTACAC ATAGAGTATA CATATATATA TTCTAAAATA   
  
  
- AATCTTTATT TTTAAATTTG TGATACACTA AATATCAATA ATATTATTTA TCATAAAAAA TTTGATAAAG   
  
  
- TAACTACTCT TGTCCTTTCA TTTAACGGCT GTACAATTAC AATAATATAG ATTTTTAATT TAGTATTTTA   
  
  
- ATTAATATTA TAAAATTTTT ATAAAGTTGA TTTTAAAATC TTGTTATTCA CGCATTATGC CTGAATTTCC   
  
  
- GATCAAATAG CATTTTTACA TTCAAGGAAA ACCTAGAATA CCAACTTATA GAACCAACTA TTTGACACTA   
  
  
- ACCAAAATGT GAAAATGGTG TATAAGTATC TTCTTAATAC ACGTGCAACT ACCAAGTTCT ACCCTGTTTT   
  
  
- CCTTTGTACA TAAGAAGGGG GAAAACGGAA AAGGGGATTC GTAAACCTAA CTCCACAACA GTAGACTTAT   
  
  
- AGTTTTTAAG AAAAACTTTT AAGTCAGAGT TAGTTTTAAG TTTTAATTTT GTTTCTGAAT TCTCAAAATA   
  
  
- AACAAGAATT GGAAGAATTG AAAGATGAAA AGAAAAAAAA GAGTAAGGGG GAGAAGTTTA GAGTAAATAA   
  
  
- GAATTGGGGG AGAAGGCAAG TACGAGAGAG ACAGATAGTT GATTATAATT AGATGGGGCT GAAAGTTAAA   
  
  
- CTACTCCAGA TTAGGAATAG TAGCATAACA CCAACCCAAT TAATACGTTC TTCCGTTATT TGGGGTCACT   
  
  
- GTTTCAGGTG CAATTCATCC GTGGAGTGGT AATTCTGTAC GAGTCTTTTG TGGTTTTGGT AACTTGTGTT   
  
  
- CAGGGGGTTC AGGGAAGGGA TCGATCGGGT AAGAGAGAGG AGGGGACAGA TATAGATGGT TGAGAACGAG   
  
  
- AAACGACTCG GACTCAACTT TCGCTATGTG GGTATCGGAC AAGTCAAAAG TCAAAAGTGT AAACAGAAGG   
  
  
- AGACAAGTCA ACTTTCGCTA TGTGAGAGTA ATCGAAAGTA AAATTTATGC AAGTTAAGTG TGTATTTACC   
  
  
- GAAAGTTTCG TAACGGTAAA AGCAAATTTA CTACTGTTTT ATATATCATA GACCGATAGA CGGGTATAGA   
  
  
- ACTGGAATAA ATGTTTCCGA CTCGGTAGAA AATTAAAAAA ATCGGAGAAA AAAATTAGTT TTTATCTTTA   
  
  
- AAGTTCTTTC GTCTTTAGCA GTCTACTGTC ACGTCTGTCG CAAAAGCAAA GTATGTTTAG AGTTTTCGAC   
  
  
- TTCACAGTTA AAGAAGCTAA TAAAAACAGT CTTTTATAAA TGCAGAGTGG AAGTGCAACA ATAATAAGAA   
  
  
- AGAAAAAATA ATAAGAGTAT TGATCATTAT TTCAATCGTT ACTATTTTTT AATAAGCTAG GCTTATAAAA   
  
  
- TTAAATAGAC TGGATTTTTG TATTCATTTC CGTGTTTAAA AAGTAGGTTT AAAATTAAAA ACTACATTAT   
  
  
- AAAAAATATA AAAATAACAA GTTTAGACTA AAATTAGAAT AAATTTAATA GACTGGATTT TTTTAGTTTA   
  
  
- TTATTATTTG CAATTAAAAA ATAAATTTAA AACTGTAATC AGTTAGACTG AATTTAAGCT TGGCTCGAAC   
  
  
- TTAAAAAAAC GGTCACGACC ATCATGAGGA CGAAAACATC GAGGGTTACG GTAGCATGGT GGGAGAAGAT   
  
  
- CAGAGCAGAG AAAGAGACCA GAGGTTAATT AGTGTGGTAG TATAGTATGG CATACTACTA TGTCAGGTGA   
  
  
- CTTTAGGGTA AGAGTGGCGG TTAGAGAGAG GCGGTGGTAG GTTGTGAGAA TTGCTACTTG TCCCGGCGGC   
  
  
- GGAGCAGGAG GTGTGAGTCC GGCACCGGCC CGTCGAAAGG GTGGGTTAGT TTTAGAGAGA GGAGGCGGTT   
  
  
- GAAGCCATTG CGGTTAACGT ACCTCGTCGA GAATCAGGTG ACGCGGCTTC GGTAGCTGTT GTTACTGCGG   
  
  
- TGGGGCCGGG TCGTTTAGAA TACCCAGGAC TTATTGTATC GGGCTGGGCT GCCGCTGAGG TGGGTTGCGG   
  
  
- AGTGCACGCG TAAGGATGCA CGGAATTAGA GTGCGCGGCA GGAGTGATCG TGGACGTTCT ACCATTAGGG   
  
  
- AGTAAAGTTG GGGTAGTTAA GTGGGGTGTT TAAGAGCGAG GAGCTTGAAC GGGTGAAGCA GCTAAATTGG   
  
  
- GGAACCGTAG CTAAGCCTAA GTGGCGGCGG TTAAGCTAGT AAGACCTTCG ATAAAGGCTG GATGGGCAAC   
  
  
- ATGTGTAACA GCTGGAGTCG TAGAGGGTGA CATACGTCTA GGGGTGCAAC TAGCTGAGGT AACGCTGGGC   
  
  
- CAACCTTCGG GGCCCAGCTC AGGGAGGATA ACAGTTCGAG TGGCAGCCCC GATAAAGGCT GCTTTAGGGC   
  
  
- GGCCACAAGC TAGAAGACAG CATGCTACTC GAGCCTTACT CTGATTAGTT GAAGCGAGCA AAATCTTTGT   
  
  
- AGCAGGAGCT TAAGGTTCGT TATGGGTGGA GGGGAAGGCT GCCTAAACGA AGCGACAACC TCCTCTAAGC   
  
  
- TGTTTCGTTC GAGATGAGGT TGCTACGCCG CCGCCAATAA CACTTAACAG TCTACTCAAA CGTAAATAAC   
  
  
- GTCCTTCTCC TCCACAGAAG TAGCAGCGGC AGCAGCTACT CCCCCTACAA AAACGTCCGC CACTCCTCGA   
  
  
- ACCTCGGCTC GTACCACCAC CACCACCTCC TCCTGCACCT AAAGTGCCGC TCCTCAGACC ACCCCGCCGA   
  
  
- CTCTAGCCGC AAATTAGTGT ACACCTATGG GAAGCTGTGC CACCTGTGCA AGAACGGTGC ACCCTCGGTC   
  
  
- GTCTCTCTCA CCAAGCTCCG GCTCCACACG ACCTTCTAAC TCTTACACTA GCGAGTACTC CCTGGGCGCT   
  
  
- CCCAGCTCTC CGTCCTCGGG TCCCGGTTCA CCCGGGAGGC TTACTCCCTC CGGCTCAAAG TTCCCTAGCT   
  
  
- CAAGCCACTA CTTCCATGCT GGCTCCAGTT CCGGTACGAC CTCCTCGTAC GGCGACCCAC CCCCAGATTC   
  
  
- TTCCTTCTCC TACTAGAACA CGAGTGAACC TTCCCTGTAT CACAACAGAA ACGAAGACGA ACCCATGGGT   
  
  
- GAAT

+     MYC

| Site Name | Organism | Position | Strand | Matrix score. | sequence | function |
| --- | --- | --- | --- | --- | --- | --- |
| MYC | Arabidopsis thaliana | 675 | + | 6 | CATTTG |  |
| MYC | Arabidopsis thaliana | 2961 | - | 6 | CATGTG |  |
| MYC | Arabidopsis thaliana | 2963 | + | 6 | CATGTG |  |
| MYC | Arabidopsis thaliana | 249 | + | 6 | CATGTG |  |
| MYC | Arabidopsis thaliana | 1183 | + | 6 | CATTTG |  |
| MYC | Arabidopsis thaliana | 2117 | - | 6 | CAATTG |  |

>HU06G00376.1   
+ +Up\_Stream \_Len000AGAAGC TAATTAAACC AAACATGCAC GTATGTCCTA ATTAACATGA CTGTCCATCA   
  
  
+ TCTCCTCCCA TGTTGTTCTG TTGACAAGCC TGCACACCCA TGCTCCTCTC TCATGTCTAA CCTCGTCCCA   
  
  
+ ACGATCAAGA TCACTGTCTG TGAGGCCAGG GGAAGACTTG CTTTTATTTC CTTTTCTTTT GTTTACTCTC   
  
  
+ TGATTCCATT TAGCCATATA TACAAGGAGA ATGTCATGTG TATCTCATAT GTATATATAT AAGATTTTAT   
  
  
+ TTAGAAATAA AAATTTAAAC ACTATGTGAT TTATAGTTAT TATAATAAAT AGTATTTTTT AAACTATTTC   
  
  
+ ATTGATGAGA ACAGGAAAGT AAATTGCCGA CATGTTAATG TTATTATATC TAAAAATTAA ATCATAAAAT   
  
  
+ TAATTATAAT ATTTTAAAAA TATTTCAACT AAAATTTTAG AACAATAAGT GCGTAATACG GACTTAAAGG   
  
  
+ CTAGTTTATC GTAAAAATGT AAGTTCCTTT TGGATCTTAT GGTTGAATAT CTTGGTTGAT AAACTGTGAT   
  
  
+ TGGTTTTACA CTTTTACCAC ATATTCATAG AAGAATTATG TGCACGTTGA TGGTTCAAGA TGGGACAAAA   
  
  
+ GGAAACATGT ATTCTTCCCC CTTTTGCCTT TTCCCCTAAG CATTTGGATT GAGGTGTTGT CATCTGAATA   
  
  
+ TCAAAAATTC TTTTTGAAAA TTCAGTCTCA ATCAAAATTC AAAATTAAAA CAAAGACTTA AGAGTTTTAT   
  
  
+ TTGTTCTTAA CCTTCTTAAC TTTCTACTTT TCTTTTTTTT CTCATTCCCC CTCTTCAAAT CTCATTTATT   
  
  
+ CTTAACCCCC TCTTCCGTTC ATGCTCTCTC TGTCTATCAA CTAATATTAA TCTACCCCGA CTTTCAATTT   
  
  
+ GATGAGGTCT AATCCTTATC ATCGTATTGT GGTTGGGTTA ATTATGCAAG AAGGCAATAA ACCCCAGTGA   
  
  
+ CAAAGTCCAC GTTAAGTAGG CACCTCACCA TTAAGACATG CTCAGAAAAC ACCAAAACCA TTGAACACAA   
  
  
+ GTCCCCCAAG TCCCTTCCCT AGCTAGCCCA TTCTCTCTCC TCCCCTGTCT ATATCTACCA ACTCTTGCTC   
  
  
+ TTTGCTGAGC CTGAGTTGAA AGCGATACAC CCATAGCCTG TTCAGTTTTC AGTTTTCACA TTTGTCTTCC   
  
  
+ TCTGTTCAGT TGAAAGCGAT ACACTCTCAT TAGCTTTCAT TTTAAATACG TTCAATTCAC ACATAAATGG   
  
  
+ CTTTCAAAGC ATTGCCATTT TCGTTTAAAT GATGACAAAA TATATAGTAT CTGGCTATCT GCCCATATCT   
  
  
+ TGACCTTATT TACAAAGGCT GAGCCATCTT TTAATTTTTT TAGCCTCTTT TTTTAATCAA AAATAGAAAT   
  
  
+ TTCAAGAAAG CAGAAATCGT CAGATGACAG TGCAGACAGC GTTTTCGTTT CATACAAATC TCAAAAGCTG   
  
  
+ AAGTGTCAAT TTCTTCGATT ATTTTTGTCA GAAAATATTT ACGTCTCACC TTCACGTTGT TATTATTCTT   
  
  
+ TCTTTTTTAT TATTCTCATA ACTAGTAATA AAGTTAGCAA TGATAAAAAA TTATTCGATC CGAATATTTT   
  
  
+ AATTTATCTG ACCTAAAAAC ATAAGTAAAG GCACAAATTT TTCATCCAAA TTTTAATTTT TGATGTAATA   
  
  
+ TTTTTTATAT TTTTATTGTT CAAATCTGAT TTTAATCTTA TTTAAATTAT CTGACCTAAA AAAATCAAAT   
  
  
+ AATAATAAAC GTTAATTTTT TATTTAAATT TTGACATTAG TCAATCTGAC TTAAATTCGA ACCGAGCTTG   
  
  
+ AATTTTTTTG CCAGTGCTGG TAGTACTCCT GCTTTTGTAG CTCCCAATGC CATCGTACCA CCCTCTTCTA   
  
  
+ GTCTCGTCTC TTTCTCTGGT CTCCAATTAA TCACACCATC ATATCATACC GTATGATGAT ACAGTCCACT   
  
  
+ GAAATCCCAT TCTCACCGCC AATCTCTCTC CGCCACCATC CAACACTCTT AACGATGAAC AGGGCCGCCG   
  
  
+ CCTCGTCCTC CACACTCAGG CCGTGGCCGG GCAGCTTTCC CACCCAATCA AAATCTCTCT CCTCCGCCAA   
  
  
+ CTTCGGTAAC GCCAATTGCA TGGAGCAGCT CTTAGTCCAC TGCGCCGAAG CCATCGACAA CAATGACGCC   
  
  
+ ACCCCGGCCC AGCAAATCTT ATGGGTCCTG AATAACATAG CCCGACCCGA CGGCGACTCC ACCCAACGCC   
  
  
+ TCACGTGCGC ATTCCTACGT GCCTTAATCT CACGCGCCGT CCTCACTAGC ACCTGCAAGA TGGTAATCCC   
  
  
+ TCATTTCAAC CCCATCAATT CACCCCACAA ATTCTCGCTC CTCGAACTTG CCCACTTCGT CGATTTAACC   
  
  
+ CCTTGGCATC GATTCGGATT CACCGCCGCC AATTCGATCA TTCTGGAAGC TATTTCCGAC CTACCCGTTG   
  
  
+ TACACATTGT CGACCTCAGC ATCTCCCACT GTATGCAGAT CCCCACGTTG ATCGACTCCA TTGCGACCCG   
  
  
+ GTTGGAAGCC CCGGGTCGAG TCCCTCCTAT TGTCAAGCTC ACCGTCGGGG CTATTTCCGA CGAAATCCCG   
  
  
+ CCGGTGTTCG ATCTTCTGTC GTACGATGAG CTCGGAATGA GACTAATCAA CTTCGCTCGT TTTAGAAACA   
  
  
+ TCGTCCTCGA ATTCCAAGCA ATACCCACCT CCCCTTCCGA CGGATTTGCT TCGCTGTTGG AGGAGATTCG   
  
  
+ ACAAAGCAAG CTCTACTCCA ACGATGCGGC GGCGGTTATT GTGAATTGTC AGATGAGTTT GCATTTATTG   
  
  
+ CAGGAAGAGG AGGTGTCTTC ATCGTCGCCG TCGTCGATGA GGGGGATGTT TTTGCAGGCG GTGAGGAGCT   
  
  
+ TGGAGCCGAG CATGGTGGTG GTGGTGGAGG AGGACGTGGA TTTCACGGCG AGGAGTCTGG TGGGGCGGCT   
  
  
+ GAGATCGGCG TTTAATCACA TGTGGATACC CTTCGACACG GTGGACACGT TCTTGCCACG TGGGAGCCAG   
  
  
+ CAGAGAGAGT GGTTCGAGGC CGAGGTGTGC TGGAAGATTG AGAATGTGAT CGCTCATGAG GGACCCGCGA   
  
  
+ GGGTCGAGAG GCAGGAGCCC AGGGCCAAGT GGGCCCTCCG AATGAGGGAG GCCGAGTTTC AAGGGATCGA   
  
  
+ GTTCGGTGAT GAAGGTACGA CCGAGGTCAA GGCCATGCTG GAGGAGCATG CCGCTGGGTG GGGGTCTAAG   
  
  
+ AAGGAAGAGG ATGATCTTGT GCTCACTTGG AAGGGACATA GTGTTGTCTT TGCTTCTGCT TGGGTACCCA   
  
  
+ CTTA  

- +Up\_Stream \_Len000TCTTCG ATTAATTTGG TTTGTACGTG CATACAGGAT TAATTGTACT GACAGGTAGT   
  
  
- AGAGGAGGGT ACAACAAGAC AACTGTTCGG ACGTGTGGGT ACGAGGAGAG AGTACAGATT GGAGCAGGGT   
  
  
- TGCTAGTTCT AGTGACAGAC ACTCCGGTCC CCTTCTGAAC GAAAATAAAG GAAAAGAAAA CAAATGAGAG   
  
  
- ACTAAGGTAA ATCGGTATAT ATGTTCCTCT TACAGTACAC ATAGAGTATA CATATATATA TTCTAAAATA   
  
  
- AATCTTTATT TTTAAATTTG TGATACACTA AATATCAATA ATATTATTTA TCATAAAAAA TTTGATAAAG   
  
  
- TAACTACTCT TGTCCTTTCA TTTAACGGCT GTACAATTAC AATAATATAG ATTTTTAATT TAGTATTTTA   
  
  
- ATTAATATTA TAAAATTTTT ATAAAGTTGA TTTTAAAATC TTGTTATTCA CGCATTATGC CTGAATTTCC   
  
  
- GATCAAATAG CATTTTTACA TTCAAGGAAA ACCTAGAATA CCAACTTATA GAACCAACTA TTTGACACTA   
  
  
- ACCAAAATGT GAAAATGGTG TATAAGTATC TTCTTAATAC ACGTGCAACT ACCAAGTTCT ACCCTGTTTT   
  
  
- CCTTTGTACA TAAGAAGGGG GAAAACGGAA AAGGGGATTC GTAAACCTAA CTCCACAACA GTAGACTTAT   
  
  
- AGTTTTTAAG AAAAACTTTT AAGTCAGAGT TAGTTTTAAG TTTTAATTTT GTTTCTGAAT TCTCAAAATA   
  
  
- AACAAGAATT GGAAGAATTG AAAGATGAAA AGAAAAAAAA GAGTAAGGGG GAGAAGTTTA GAGTAAATAA   
  
  
- GAATTGGGGG AGAAGGCAAG TACGAGAGAG ACAGATAGTT GATTATAATT AGATGGGGCT GAAAGTTAAA   
  
  
- CTACTCCAGA TTAGGAATAG TAGCATAACA CCAACCCAAT TAATACGTTC TTCCGTTATT TGGGGTCACT   
  
  
- GTTTCAGGTG CAATTCATCC GTGGAGTGGT AATTCTGTAC GAGTCTTTTG TGGTTTTGGT AACTTGTGTT   
  
  
- CAGGGGGTTC AGGGAAGGGA TCGATCGGGT AAGAGAGAGG AGGGGACAGA TATAGATGGT TGAGAACGAG   
  
  
- AAACGACTCG GACTCAACTT TCGCTATGTG GGTATCGGAC AAGTCAAAAG TCAAAAGTGT AAACAGAAGG   
  
  
- AGACAAGTCA ACTTTCGCTA TGTGAGAGTA ATCGAAAGTA AAATTTATGC AAGTTAAGTG TGTATTTACC   
  
  
- GAAAGTTTCG TAACGGTAAA AGCAAATTTA CTACTGTTTT ATATATCATA GACCGATAGA CGGGTATAGA   
  
  
- ACTGGAATAA ATGTTTCCGA CTCGGTAGAA AATTAAAAAA ATCGGAGAAA AAAATTAGTT TTTATCTTTA   
  
  
- AAGTTCTTTC GTCTTTAGCA GTCTACTGTC ACGTCTGTCG CAAAAGCAAA GTATGTTTAG AGTTTTCGAC   
  
  
- TTCACAGTTA AAGAAGCTAA TAAAAACAGT CTTTTATAAA TGCAGAGTGG AAGTGCAACA ATAATAAGAA   
  
  
- AGAAAAAATA ATAAGAGTAT TGATCATTAT TTCAATCGTT ACTATTTTTT AATAAGCTAG GCTTATAAAA   
  
  
- TTAAATAGAC TGGATTTTTG TATTCATTTC CGTGTTTAAA AAGTAGGTTT AAAATTAAAA ACTACATTAT   
  
  
- AAAAAATATA AAAATAACAA GTTTAGACTA AAATTAGAAT AAATTTAATA GACTGGATTT TTTTAGTTTA   
  
  
- TTATTATTTG CAATTAAAAA ATAAATTTAA AACTGTAATC AGTTAGACTG AATTTAAGCT TGGCTCGAAC   
  
  
- TTAAAAAAAC GGTCACGACC ATCATGAGGA CGAAAACATC GAGGGTTACG GTAGCATGGT GGGAGAAGAT   
  
  
- CAGAGCAGAG AAAGAGACCA GAGGTTAATT AGTGTGGTAG TATAGTATGG CATACTACTA TGTCAGGTGA   
  
  
- CTTTAGGGTA AGAGTGGCGG TTAGAGAGAG GCGGTGGTAG GTTGTGAGAA TTGCTACTTG TCCCGGCGGC   
  
  
- GGAGCAGGAG GTGTGAGTCC GGCACCGGCC CGTCGAAAGG GTGGGTTAGT TTTAGAGAGA GGAGGCGGTT   
  
  
- GAAGCCATTG CGGTTAACGT ACCTCGTCGA GAATCAGGTG ACGCGGCTTC GGTAGCTGTT GTTACTGCGG   
  
  
- TGGGGCCGGG TCGTTTAGAA TACCCAGGAC TTATTGTATC GGGCTGGGCT GCCGCTGAGG TGGGTTGCGG   
  
  
- AGTGCACGCG TAAGGATGCA CGGAATTAGA GTGCGCGGCA GGAGTGATCG TGGACGTTCT ACCATTAGGG   
  
  
- AGTAAAGTTG GGGTAGTTAA GTGGGGTGTT TAAGAGCGAG GAGCTTGAAC GGGTGAAGCA GCTAAATTGG   
  
  
- GGAACCGTAG CTAAGCCTAA GTGGCGGCGG TTAAGCTAGT AAGACCTTCG ATAAAGGCTG GATGGGCAAC   
  
  
- ATGTGTAACA GCTGGAGTCG TAGAGGGTGA CATACGTCTA GGGGTGCAAC TAGCTGAGGT AACGCTGGGC   
  
  
- CAACCTTCGG GGCCCAGCTC AGGGAGGATA ACAGTTCGAG TGGCAGCCCC GATAAAGGCT GCTTTAGGGC   
  
  
- GGCCACAAGC TAGAAGACAG CATGCTACTC GAGCCTTACT CTGATTAGTT GAAGCGAGCA AAATCTTTGT   
  
  
- AGCAGGAGCT TAAGGTTCGT TATGGGTGGA GGGGAAGGCT GCCTAAACGA AGCGACAACC TCCTCTAAGC   
  
  
- TGTTTCGTTC GAGATGAGGT TGCTACGCCG CCGCCAATAA CACTTAACAG TCTACTCAAA CGTAAATAAC   
  
  
- GTCCTTCTCC TCCACAGAAG TAGCAGCGGC AGCAGCTACT CCCCCTACAA AAACGTCCGC CACTCCTCGA   
  
  
- ACCTCGGCTC GTACCACCAC CACCACCTCC TCCTGCACCT AAAGTGCCGC TCCTCAGACC ACCCCGCCGA   
  
  
- CTCTAGCCGC AAATTAGTGT ACACCTATGG GAAGCTGTGC CACCTGTGCA AGAACGGTGC ACCCTCGGTC   
  
  
- GTCTCTCTCA CCAAGCTCCG GCTCCACACG ACCTTCTAAC TCTTACACTA GCGAGTACTC CCTGGGCGCT   
  
  
- CCCAGCTCTC CGTCCTCGGG TCCCGGTTCA CCCGGGAGGC TTACTCCCTC CGGCTCAAAG TTCCCTAGCT   
  
  
- CAAGCCACTA CTTCCATGCT GGCTCCAGTT CCGGTACGAC CTCCTCGTAC GGCGACCCAC CCCCAGATTC   
  
  
- TTCCTTCTCC TACTAGAACA CGAGTGAACC TTCCCTGTAT CACAACAGAA ACGAAGACGA ACCCATGGGT   
  
  
- GAAT

+     Myb

| Site Name | Organism | Position | Strand | Matrix score. | sequence | function |
| --- | --- | --- | --- | --- | --- | --- |
| Myb | Arabidopsis thaliana | 1201 | - | 6 | CAACTG |  |

>HU06G00376.1   
+ +Up\_Stream \_Len000AGAAGC TAATTAAACC AAACATGCAC GTATGTCCTA ATTAACATGA CTGTCCATCA   
  
  
+ TCTCCTCCCA TGTTGTTCTG TTGACAAGCC TGCACACCCA TGCTCCTCTC TCATGTCTAA CCTCGTCCCA   
  
  
+ ACGATCAAGA TCACTGTCTG TGAGGCCAGG GGAAGACTTG CTTTTATTTC CTTTTCTTTT GTTTACTCTC   
  
  
+ TGATTCCATT TAGCCATATA TACAAGGAGA ATGTCATGTG TATCTCATAT GTATATATAT AAGATTTTAT   
  
  
+ TTAGAAATAA AAATTTAAAC ACTATGTGAT TTATAGTTAT TATAATAAAT AGTATTTTTT AAACTATTTC   
  
  
+ ATTGATGAGA ACAGGAAAGT AAATTGCCGA CATGTTAATG TTATTATATC TAAAAATTAA ATCATAAAAT   
  
  
+ TAATTATAAT ATTTTAAAAA TATTTCAACT AAAATTTTAG AACAATAAGT GCGTAATACG GACTTAAAGG   
  
  
+ CTAGTTTATC GTAAAAATGT AAGTTCCTTT TGGATCTTAT GGTTGAATAT CTTGGTTGAT AAACTGTGAT   
  
  
+ TGGTTTTACA CTTTTACCAC ATATTCATAG AAGAATTATG TGCACGTTGA TGGTTCAAGA TGGGACAAAA   
  
  
+ GGAAACATGT ATTCTTCCCC CTTTTGCCTT TTCCCCTAAG CATTTGGATT GAGGTGTTGT CATCTGAATA   
  
  
+ TCAAAAATTC TTTTTGAAAA TTCAGTCTCA ATCAAAATTC AAAATTAAAA CAAAGACTTA AGAGTTTTAT   
  
  
+ TTGTTCTTAA CCTTCTTAAC TTTCTACTTT TCTTTTTTTT CTCATTCCCC CTCTTCAAAT CTCATTTATT   
  
  
+ CTTAACCCCC TCTTCCGTTC ATGCTCTCTC TGTCTATCAA CTAATATTAA TCTACCCCGA CTTTCAATTT   
  
  
+ GATGAGGTCT AATCCTTATC ATCGTATTGT GGTTGGGTTA ATTATGCAAG AAGGCAATAA ACCCCAGTGA   
  
  
+ CAAAGTCCAC GTTAAGTAGG CACCTCACCA TTAAGACATG CTCAGAAAAC ACCAAAACCA TTGAACACAA   
  
  
+ GTCCCCCAAG TCCCTTCCCT AGCTAGCCCA TTCTCTCTCC TCCCCTGTCT ATATCTACCA ACTCTTGCTC   
  
  
+ TTTGCTGAGC CTGAGTTGAA AGCGATACAC CCATAGCCTG TTCAGTTTTC AGTTTTCACA TTTGTCTTCC   
  
  
+ TCTGTTCAGT TGAAAGCGAT ACACTCTCAT TAGCTTTCAT TTTAAATACG TTCAATTCAC ACATAAATGG   
  
  
+ CTTTCAAAGC ATTGCCATTT TCGTTTAAAT GATGACAAAA TATATAGTAT CTGGCTATCT GCCCATATCT   
  
  
+ TGACCTTATT TACAAAGGCT GAGCCATCTT TTAATTTTTT TAGCCTCTTT TTTTAATCAA AAATAGAAAT   
  
  
+ TTCAAGAAAG CAGAAATCGT CAGATGACAG TGCAGACAGC GTTTTCGTTT CATACAAATC TCAAAAGCTG   
  
  
+ AAGTGTCAAT TTCTTCGATT ATTTTTGTCA GAAAATATTT ACGTCTCACC TTCACGTTGT TATTATTCTT   
  
  
+ TCTTTTTTAT TATTCTCATA ACTAGTAATA AAGTTAGCAA TGATAAAAAA TTATTCGATC CGAATATTTT   
  
  
+ AATTTATCTG ACCTAAAAAC ATAAGTAAAG GCACAAATTT TTCATCCAAA TTTTAATTTT TGATGTAATA   
  
  
+ TTTTTTATAT TTTTATTGTT CAAATCTGAT TTTAATCTTA TTTAAATTAT CTGACCTAAA AAAATCAAAT   
  
  
+ AATAATAAAC GTTAATTTTT TATTTAAATT TTGACATTAG TCAATCTGAC TTAAATTCGA ACCGAGCTTG   
  
  
+ AATTTTTTTG CCAGTGCTGG TAGTACTCCT GCTTTTGTAG CTCCCAATGC CATCGTACCA CCCTCTTCTA   
  
  
+ GTCTCGTCTC TTTCTCTGGT CTCCAATTAA TCACACCATC ATATCATACC GTATGATGAT ACAGTCCACT   
  
  
+ GAAATCCCAT TCTCACCGCC AATCTCTCTC CGCCACCATC CAACACTCTT AACGATGAAC AGGGCCGCCG   
  
  
+ CCTCGTCCTC CACACTCAGG CCGTGGCCGG GCAGCTTTCC CACCCAATCA AAATCTCTCT CCTCCGCCAA   
  
  
+ CTTCGGTAAC GCCAATTGCA TGGAGCAGCT CTTAGTCCAC TGCGCCGAAG CCATCGACAA CAATGACGCC   
  
  
+ ACCCCGGCCC AGCAAATCTT ATGGGTCCTG AATAACATAG CCCGACCCGA CGGCGACTCC ACCCAACGCC   
  
  
+ TCACGTGCGC ATTCCTACGT GCCTTAATCT CACGCGCCGT CCTCACTAGC ACCTGCAAGA TGGTAATCCC   
  
  
+ TCATTTCAAC CCCATCAATT CACCCCACAA ATTCTCGCTC CTCGAACTTG CCCACTTCGT CGATTTAACC   
  
  
+ CCTTGGCATC GATTCGGATT CACCGCCGCC AATTCGATCA TTCTGGAAGC TATTTCCGAC CTACCCGTTG   
  
  
+ TACACATTGT CGACCTCAGC ATCTCCCACT GTATGCAGAT CCCCACGTTG ATCGACTCCA TTGCGACCCG   
  
  
+ GTTGGAAGCC CCGGGTCGAG TCCCTCCTAT TGTCAAGCTC ACCGTCGGGG CTATTTCCGA CGAAATCCCG   
  
  
+ CCGGTGTTCG ATCTTCTGTC GTACGATGAG CTCGGAATGA GACTAATCAA CTTCGCTCGT TTTAGAAACA   
  
  
+ TCGTCCTCGA ATTCCAAGCA ATACCCACCT CCCCTTCCGA CGGATTTGCT TCGCTGTTGG AGGAGATTCG   
  
  
+ ACAAAGCAAG CTCTACTCCA ACGATGCGGC GGCGGTTATT GTGAATTGTC AGATGAGTTT GCATTTATTG   
  
  
+ CAGGAAGAGG AGGTGTCTTC ATCGTCGCCG TCGTCGATGA GGGGGATGTT TTTGCAGGCG GTGAGGAGCT   
  
  
+ TGGAGCCGAG CATGGTGGTG GTGGTGGAGG AGGACGTGGA TTTCACGGCG AGGAGTCTGG TGGGGCGGCT   
  
  
+ GAGATCGGCG TTTAATCACA TGTGGATACC CTTCGACACG GTGGACACGT TCTTGCCACG TGGGAGCCAG   
  
  
+ CAGAGAGAGT GGTTCGAGGC CGAGGTGTGC TGGAAGATTG AGAATGTGAT CGCTCATGAG GGACCCGCGA   
  
  
+ GGGTCGAGAG GCAGGAGCCC AGGGCCAAGT GGGCCCTCCG AATGAGGGAG GCCGAGTTTC AAGGGATCGA   
  
  
+ GTTCGGTGAT GAAGGTACGA CCGAGGTCAA GGCCATGCTG GAGGAGCATG CCGCTGGGTG GGGGTCTAAG   
  
  
+ AAGGAAGAGG ATGATCTTGT GCTCACTTGG AAGGGACATA GTGTTGTCTT TGCTTCTGCT TGGGTACCCA   
  
  
+ CTTA  

- +Up\_Stream \_Len000TCTTCG ATTAATTTGG TTTGTACGTG CATACAGGAT TAATTGTACT GACAGGTAGT   
  
  
- AGAGGAGGGT ACAACAAGAC AACTGTTCGG ACGTGTGGGT ACGAGGAGAG AGTACAGATT GGAGCAGGGT   
  
  
- TGCTAGTTCT AGTGACAGAC ACTCCGGTCC CCTTCTGAAC GAAAATAAAG GAAAAGAAAA CAAATGAGAG   
  
  
- ACTAAGGTAA ATCGGTATAT ATGTTCCTCT TACAGTACAC ATAGAGTATA CATATATATA TTCTAAAATA   
  
  
- AATCTTTATT TTTAAATTTG TGATACACTA AATATCAATA ATATTATTTA TCATAAAAAA TTTGATAAAG   
  
  
- TAACTACTCT TGTCCTTTCA TTTAACGGCT GTACAATTAC AATAATATAG ATTTTTAATT TAGTATTTTA   
  
  
- ATTAATATTA TAAAATTTTT ATAAAGTTGA TTTTAAAATC TTGTTATTCA CGCATTATGC CTGAATTTCC   
  
  
- GATCAAATAG CATTTTTACA TTCAAGGAAA ACCTAGAATA CCAACTTATA GAACCAACTA TTTGACACTA   
  
  
- ACCAAAATGT GAAAATGGTG TATAAGTATC TTCTTAATAC ACGTGCAACT ACCAAGTTCT ACCCTGTTTT   
  
  
- CCTTTGTACA TAAGAAGGGG GAAAACGGAA AAGGGGATTC GTAAACCTAA CTCCACAACA GTAGACTTAT   
  
  
- AGTTTTTAAG AAAAACTTTT AAGTCAGAGT TAGTTTTAAG TTTTAATTTT GTTTCTGAAT TCTCAAAATA   
  
  
- AACAAGAATT GGAAGAATTG AAAGATGAAA AGAAAAAAAA GAGTAAGGGG GAGAAGTTTA GAGTAAATAA   
  
  
- GAATTGGGGG AGAAGGCAAG TACGAGAGAG ACAGATAGTT GATTATAATT AGATGGGGCT GAAAGTTAAA   
  
  
- CTACTCCAGA TTAGGAATAG TAGCATAACA CCAACCCAAT TAATACGTTC TTCCGTTATT TGGGGTCACT   
  
  
- GTTTCAGGTG CAATTCATCC GTGGAGTGGT AATTCTGTAC GAGTCTTTTG TGGTTTTGGT AACTTGTGTT   
  
  
- CAGGGGGTTC AGGGAAGGGA TCGATCGGGT AAGAGAGAGG AGGGGACAGA TATAGATGGT TGAGAACGAG   
  
  
- AAACGACTCG GACTCAACTT TCGCTATGTG GGTATCGGAC AAGTCAAAAG TCAAAAGTGT AAACAGAAGG   
  
  
- AGACAAGTCA ACTTTCGCTA TGTGAGAGTA ATCGAAAGTA AAATTTATGC AAGTTAAGTG TGTATTTACC   
  
  
- GAAAGTTTCG TAACGGTAAA AGCAAATTTA CTACTGTTTT ATATATCATA GACCGATAGA CGGGTATAGA   
  
  
- ACTGGAATAA ATGTTTCCGA CTCGGTAGAA AATTAAAAAA ATCGGAGAAA AAAATTAGTT TTTATCTTTA   
  
  
- AAGTTCTTTC GTCTTTAGCA GTCTACTGTC ACGTCTGTCG CAAAAGCAAA GTATGTTTAG AGTTTTCGAC   
  
  
- TTCACAGTTA AAGAAGCTAA TAAAAACAGT CTTTTATAAA TGCAGAGTGG AAGTGCAACA ATAATAAGAA   
  
  
- AGAAAAAATA ATAAGAGTAT TGATCATTAT TTCAATCGTT ACTATTTTTT AATAAGCTAG GCTTATAAAA   
  
  
- TTAAATAGAC TGGATTTTTG TATTCATTTC CGTGTTTAAA AAGTAGGTTT AAAATTAAAA ACTACATTAT   
  
  
- AAAAAATATA AAAATAACAA GTTTAGACTA AAATTAGAAT AAATTTAATA GACTGGATTT TTTTAGTTTA   
  
  
- TTATTATTTG CAATTAAAAA ATAAATTTAA AACTGTAATC AGTTAGACTG AATTTAAGCT TGGCTCGAAC   
  
  
- TTAAAAAAAC GGTCACGACC ATCATGAGGA CGAAAACATC GAGGGTTACG GTAGCATGGT GGGAGAAGAT   
  
  
- CAGAGCAGAG AAAGAGACCA GAGGTTAATT AGTGTGGTAG TATAGTATGG CATACTACTA TGTCAGGTGA   
  
  
- CTTTAGGGTA AGAGTGGCGG TTAGAGAGAG GCGGTGGTAG GTTGTGAGAA TTGCTACTTG TCCCGGCGGC   
  
  
- GGAGCAGGAG GTGTGAGTCC GGCACCGGCC CGTCGAAAGG GTGGGTTAGT TTTAGAGAGA GGAGGCGGTT   
  
  
- GAAGCCATTG CGGTTAACGT ACCTCGTCGA GAATCAGGTG ACGCGGCTTC GGTAGCTGTT GTTACTGCGG   
  
  
- TGGGGCCGGG TCGTTTAGAA TACCCAGGAC TTATTGTATC GGGCTGGGCT GCCGCTGAGG TGGGTTGCGG   
  
  
- AGTGCACGCG TAAGGATGCA CGGAATTAGA GTGCGCGGCA GGAGTGATCG TGGACGTTCT ACCATTAGGG   
  
  
- AGTAAAGTTG GGGTAGTTAA GTGGGGTGTT TAAGAGCGAG GAGCTTGAAC GGGTGAAGCA GCTAAATTGG   
  
  
- GGAACCGTAG CTAAGCCTAA GTGGCGGCGG TTAAGCTAGT AAGACCTTCG ATAAAGGCTG GATGGGCAAC   
  
  
- ATGTGTAACA GCTGGAGTCG TAGAGGGTGA CATACGTCTA GGGGTGCAAC TAGCTGAGGT AACGCTGGGC   
  
  
- CAACCTTCGG GGCCCAGCTC AGGGAGGATA ACAGTTCGAG TGGCAGCCCC GATAAAGGCT GCTTTAGGGC   
  
  
- GGCCACAAGC TAGAAGACAG CATGCTACTC GAGCCTTACT CTGATTAGTT GAAGCGAGCA AAATCTTTGT   
  
  
- AGCAGGAGCT TAAGGTTCGT TATGGGTGGA GGGGAAGGCT GCCTAAACGA AGCGACAACC TCCTCTAAGC   
  
  
- TGTTTCGTTC GAGATGAGGT TGCTACGCCG CCGCCAATAA CACTTAACAG TCTACTCAAA CGTAAATAAC   
  
  
- GTCCTTCTCC TCCACAGAAG TAGCAGCGGC AGCAGCTACT CCCCCTACAA AAACGTCCGC CACTCCTCGA   
  
  
- ACCTCGGCTC GTACCACCAC CACCACCTCC TCCTGCACCT AAAGTGCCGC TCCTCAGACC ACCCCGCCGA   
  
  
- CTCTAGCCGC AAATTAGTGT ACACCTATGG GAAGCTGTGC CACCTGTGCA AGAACGGTGC ACCCTCGGTC   
  
  
- GTCTCTCTCA CCAAGCTCCG GCTCCACACG ACCTTCTAAC TCTTACACTA GCGAGTACTC CCTGGGCGCT   
  
  
- CCCAGCTCTC CGTCCTCGGG TCCCGGTTCA CCCGGGAGGC TTACTCCCTC CGGCTCAAAG TTCCCTAGCT   
  
  
- CAAGCCACTA CTTCCATGCT GGCTCCAGTT CCGGTACGAC CTCCTCGTAC GGCGACCCAC CCCCAGATTC   
  
  
- TTCCTTCTCC TACTAGAACA CGAGTGAACC TTCCCTGTAT CACAACAGAA ACGAAGACGA ACCCATGGGT   
  
  
- GAAT

+     Myb-binding site

| Site Name | Organism | Position | Strand | Matrix score. | sequence | function |
| --- | --- | --- | --- | --- | --- | --- |
| Myb-binding site | Nicotiana tabacum | 2718 | - | 6 | CAACAG |  |
| Myb-binding site | Nicotiana tabacum | 92 | - | 6 | CAACAG |  |

>HU06G00376.1   
+ +Up\_Stream \_Len000AGAAGC TAATTAAACC AAACATGCAC GTATGTCCTA ATTAACATGA CTGTCCATCA   
  
  
+ TCTCCTCCCA TGTTGTTCTG TTGACAAGCC TGCACACCCA TGCTCCTCTC TCATGTCTAA CCTCGTCCCA   
  
  
+ ACGATCAAGA TCACTGTCTG TGAGGCCAGG GGAAGACTTG CTTTTATTTC CTTTTCTTTT GTTTACTCTC   
  
  
+ TGATTCCATT TAGCCATATA TACAAGGAGA ATGTCATGTG TATCTCATAT GTATATATAT AAGATTTTAT   
  
  
+ TTAGAAATAA AAATTTAAAC ACTATGTGAT TTATAGTTAT TATAATAAAT AGTATTTTTT AAACTATTTC   
  
  
+ ATTGATGAGA ACAGGAAAGT AAATTGCCGA CATGTTAATG TTATTATATC TAAAAATTAA ATCATAAAAT   
  
  
+ TAATTATAAT ATTTTAAAAA TATTTCAACT AAAATTTTAG AACAATAAGT GCGTAATACG GACTTAAAGG   
  
  
+ CTAGTTTATC GTAAAAATGT AAGTTCCTTT TGGATCTTAT GGTTGAATAT CTTGGTTGAT AAACTGTGAT   
  
  
+ TGGTTTTACA CTTTTACCAC ATATTCATAG AAGAATTATG TGCACGTTGA TGGTTCAAGA TGGGACAAAA   
  
  
+ GGAAACATGT ATTCTTCCCC CTTTTGCCTT TTCCCCTAAG CATTTGGATT GAGGTGTTGT CATCTGAATA   
  
  
+ TCAAAAATTC TTTTTGAAAA TTCAGTCTCA ATCAAAATTC AAAATTAAAA CAAAGACTTA AGAGTTTTAT   
  
  
+ TTGTTCTTAA CCTTCTTAAC TTTCTACTTT TCTTTTTTTT CTCATTCCCC CTCTTCAAAT CTCATTTATT   
  
  
+ CTTAACCCCC TCTTCCGTTC ATGCTCTCTC TGTCTATCAA CTAATATTAA TCTACCCCGA CTTTCAATTT   
  
  
+ GATGAGGTCT AATCCTTATC ATCGTATTGT GGTTGGGTTA ATTATGCAAG AAGGCAATAA ACCCCAGTGA   
  
  
+ CAAAGTCCAC GTTAAGTAGG CACCTCACCA TTAAGACATG CTCAGAAAAC ACCAAAACCA TTGAACACAA   
  
  
+ GTCCCCCAAG TCCCTTCCCT AGCTAGCCCA TTCTCTCTCC TCCCCTGTCT ATATCTACCA ACTCTTGCTC   
  
  
+ TTTGCTGAGC CTGAGTTGAA AGCGATACAC CCATAGCCTG TTCAGTTTTC AGTTTTCACA TTTGTCTTCC   
  
  
+ TCTGTTCAGT TGAAAGCGAT ACACTCTCAT TAGCTTTCAT TTTAAATACG TTCAATTCAC ACATAAATGG   
  
  
+ CTTTCAAAGC ATTGCCATTT TCGTTTAAAT GATGACAAAA TATATAGTAT CTGGCTATCT GCCCATATCT   
  
  
+ TGACCTTATT TACAAAGGCT GAGCCATCTT TTAATTTTTT TAGCCTCTTT TTTTAATCAA AAATAGAAAT   
  
  
+ TTCAAGAAAG CAGAAATCGT CAGATGACAG TGCAGACAGC GTTTTCGTTT CATACAAATC TCAAAAGCTG   
  
  
+ AAGTGTCAAT TTCTTCGATT ATTTTTGTCA GAAAATATTT ACGTCTCACC TTCACGTTGT TATTATTCTT   
  
  
+ TCTTTTTTAT TATTCTCATA ACTAGTAATA AAGTTAGCAA TGATAAAAAA TTATTCGATC CGAATATTTT   
  
  
+ AATTTATCTG ACCTAAAAAC ATAAGTAAAG GCACAAATTT TTCATCCAAA TTTTAATTTT TGATGTAATA   
  
  
+ TTTTTTATAT TTTTATTGTT CAAATCTGAT TTTAATCTTA TTTAAATTAT CTGACCTAAA AAAATCAAAT   
  
  
+ AATAATAAAC GTTAATTTTT TATTTAAATT TTGACATTAG TCAATCTGAC TTAAATTCGA ACCGAGCTTG   
  
  
+ AATTTTTTTG CCAGTGCTGG TAGTACTCCT GCTTTTGTAG CTCCCAATGC CATCGTACCA CCCTCTTCTA   
  
  
+ GTCTCGTCTC TTTCTCTGGT CTCCAATTAA TCACACCATC ATATCATACC GTATGATGAT ACAGTCCACT   
  
  
+ GAAATCCCAT TCTCACCGCC AATCTCTCTC CGCCACCATC CAACACTCTT AACGATGAAC AGGGCCGCCG   
  
  
+ CCTCGTCCTC CACACTCAGG CCGTGGCCGG GCAGCTTTCC CACCCAATCA AAATCTCTCT CCTCCGCCAA   
  
  
+ CTTCGGTAAC GCCAATTGCA TGGAGCAGCT CTTAGTCCAC TGCGCCGAAG CCATCGACAA CAATGACGCC   
  
  
+ ACCCCGGCCC AGCAAATCTT ATGGGTCCTG AATAACATAG CCCGACCCGA CGGCGACTCC ACCCAACGCC   
  
  
+ TCACGTGCGC ATTCCTACGT GCCTTAATCT CACGCGCCGT CCTCACTAGC ACCTGCAAGA TGGTAATCCC   
  
  
+ TCATTTCAAC CCCATCAATT CACCCCACAA ATTCTCGCTC CTCGAACTTG CCCACTTCGT CGATTTAACC   
  
  
+ CCTTGGCATC GATTCGGATT CACCGCCGCC AATTCGATCA TTCTGGAAGC TATTTCCGAC CTACCCGTTG   
  
  
+ TACACATTGT CGACCTCAGC ATCTCCCACT GTATGCAGAT CCCCACGTTG ATCGACTCCA TTGCGACCCG   
  
  
+ GTTGGAAGCC CCGGGTCGAG TCCCTCCTAT TGTCAAGCTC ACCGTCGGGG CTATTTCCGA CGAAATCCCG   
  
  
+ CCGGTGTTCG ATCTTCTGTC GTACGATGAG CTCGGAATGA GACTAATCAA CTTCGCTCGT TTTAGAAACA   
  
  
+ TCGTCCTCGA ATTCCAAGCA ATACCCACCT CCCCTTCCGA CGGATTTGCT TCGCTGTTGG AGGAGATTCG   
  
  
+ ACAAAGCAAG CTCTACTCCA ACGATGCGGC GGCGGTTATT GTGAATTGTC AGATGAGTTT GCATTTATTG   
  
  
+ CAGGAAGAGG AGGTGTCTTC ATCGTCGCCG TCGTCGATGA GGGGGATGTT TTTGCAGGCG GTGAGGAGCT   
  
  
+ TGGAGCCGAG CATGGTGGTG GTGGTGGAGG AGGACGTGGA TTTCACGGCG AGGAGTCTGG TGGGGCGGCT   
  
  
+ GAGATCGGCG TTTAATCACA TGTGGATACC CTTCGACACG GTGGACACGT TCTTGCCACG TGGGAGCCAG   
  
  
+ CAGAGAGAGT GGTTCGAGGC CGAGGTGTGC TGGAAGATTG AGAATGTGAT CGCTCATGAG GGACCCGCGA   
  
  
+ GGGTCGAGAG GCAGGAGCCC AGGGCCAAGT GGGCCCTCCG AATGAGGGAG GCCGAGTTTC AAGGGATCGA   
  
  
+ GTTCGGTGAT GAAGGTACGA CCGAGGTCAA GGCCATGCTG GAGGAGCATG CCGCTGGGTG GGGGTCTAAG   
  
  
+ AAGGAAGAGG ATGATCTTGT GCTCACTTGG AAGGGACATA GTGTTGTCTT TGCTTCTGCT TGGGTACCCA   
  
  
+ CTTA  

- +Up\_Stream \_Len000TCTTCG ATTAATTTGG TTTGTACGTG CATACAGGAT TAATTGTACT GACAGGTAGT   
  
  
- AGAGGAGGGT ACAACAAGAC AACTGTTCGG ACGTGTGGGT ACGAGGAGAG AGTACAGATT GGAGCAGGGT   
  
  
- TGCTAGTTCT AGTGACAGAC ACTCCGGTCC CCTTCTGAAC GAAAATAAAG GAAAAGAAAA CAAATGAGAG   
  
  
- ACTAAGGTAA ATCGGTATAT ATGTTCCTCT TACAGTACAC ATAGAGTATA CATATATATA TTCTAAAATA   
  
  
- AATCTTTATT TTTAAATTTG TGATACACTA AATATCAATA ATATTATTTA TCATAAAAAA TTTGATAAAG   
  
  
- TAACTACTCT TGTCCTTTCA TTTAACGGCT GTACAATTAC AATAATATAG ATTTTTAATT TAGTATTTTA   
  
  
- ATTAATATTA TAAAATTTTT ATAAAGTTGA TTTTAAAATC TTGTTATTCA CGCATTATGC CTGAATTTCC   
  
  
- GATCAAATAG CATTTTTACA TTCAAGGAAA ACCTAGAATA CCAACTTATA GAACCAACTA TTTGACACTA   
  
  
- ACCAAAATGT GAAAATGGTG TATAAGTATC TTCTTAATAC ACGTGCAACT ACCAAGTTCT ACCCTGTTTT   
  
  
- CCTTTGTACA TAAGAAGGGG GAAAACGGAA AAGGGGATTC GTAAACCTAA CTCCACAACA GTAGACTTAT   
  
  
- AGTTTTTAAG AAAAACTTTT AAGTCAGAGT TAGTTTTAAG TTTTAATTTT GTTTCTGAAT TCTCAAAATA   
  
  
- AACAAGAATT GGAAGAATTG AAAGATGAAA AGAAAAAAAA GAGTAAGGGG GAGAAGTTTA GAGTAAATAA   
  
  
- GAATTGGGGG AGAAGGCAAG TACGAGAGAG ACAGATAGTT GATTATAATT AGATGGGGCT GAAAGTTAAA   
  
  
- CTACTCCAGA TTAGGAATAG TAGCATAACA CCAACCCAAT TAATACGTTC TTCCGTTATT TGGGGTCACT   
  
  
- GTTTCAGGTG CAATTCATCC GTGGAGTGGT AATTCTGTAC GAGTCTTTTG TGGTTTTGGT AACTTGTGTT   
  
  
- CAGGGGGTTC AGGGAAGGGA TCGATCGGGT AAGAGAGAGG AGGGGACAGA TATAGATGGT TGAGAACGAG   
  
  
- AAACGACTCG GACTCAACTT TCGCTATGTG GGTATCGGAC AAGTCAAAAG TCAAAAGTGT AAACAGAAGG   
  
  
- AGACAAGTCA ACTTTCGCTA TGTGAGAGTA ATCGAAAGTA AAATTTATGC AAGTTAAGTG TGTATTTACC   
  
  
- GAAAGTTTCG TAACGGTAAA AGCAAATTTA CTACTGTTTT ATATATCATA GACCGATAGA CGGGTATAGA   
  
  
- ACTGGAATAA ATGTTTCCGA CTCGGTAGAA AATTAAAAAA ATCGGAGAAA AAAATTAGTT TTTATCTTTA   
  
  
- AAGTTCTTTC GTCTTTAGCA GTCTACTGTC ACGTCTGTCG CAAAAGCAAA GTATGTTTAG AGTTTTCGAC   
  
  
- TTCACAGTTA AAGAAGCTAA TAAAAACAGT CTTTTATAAA TGCAGAGTGG AAGTGCAACA ATAATAAGAA   
  
  
- AGAAAAAATA ATAAGAGTAT TGATCATTAT TTCAATCGTT ACTATTTTTT AATAAGCTAG GCTTATAAAA   
  
  
- TTAAATAGAC TGGATTTTTG TATTCATTTC CGTGTTTAAA AAGTAGGTTT AAAATTAAAA ACTACATTAT   
  
  
- AAAAAATATA AAAATAACAA GTTTAGACTA AAATTAGAAT AAATTTAATA GACTGGATTT TTTTAGTTTA   
  
  
- TTATTATTTG CAATTAAAAA ATAAATTTAA AACTGTAATC AGTTAGACTG AATTTAAGCT TGGCTCGAAC   
  
  
- TTAAAAAAAC GGTCACGACC ATCATGAGGA CGAAAACATC GAGGGTTACG GTAGCATGGT GGGAGAAGAT   
  
  
- CAGAGCAGAG AAAGAGACCA GAGGTTAATT AGTGTGGTAG TATAGTATGG CATACTACTA TGTCAGGTGA   
  
  
- CTTTAGGGTA AGAGTGGCGG TTAGAGAGAG GCGGTGGTAG GTTGTGAGAA TTGCTACTTG TCCCGGCGGC   
  
  
- GGAGCAGGAG GTGTGAGTCC GGCACCGGCC CGTCGAAAGG GTGGGTTAGT TTTAGAGAGA GGAGGCGGTT   
  
  
- GAAGCCATTG CGGTTAACGT ACCTCGTCGA GAATCAGGTG ACGCGGCTTC GGTAGCTGTT GTTACTGCGG   
  
  
- TGGGGCCGGG TCGTTTAGAA TACCCAGGAC TTATTGTATC GGGCTGGGCT GCCGCTGAGG TGGGTTGCGG   
  
  
- AGTGCACGCG TAAGGATGCA CGGAATTAGA GTGCGCGGCA GGAGTGATCG TGGACGTTCT ACCATTAGGG   
  
  
- AGTAAAGTTG GGGTAGTTAA GTGGGGTGTT TAAGAGCGAG GAGCTTGAAC GGGTGAAGCA GCTAAATTGG   
  
  
- GGAACCGTAG CTAAGCCTAA GTGGCGGCGG TTAAGCTAGT AAGACCTTCG ATAAAGGCTG GATGGGCAAC   
  
  
- ATGTGTAACA GCTGGAGTCG TAGAGGGTGA CATACGTCTA GGGGTGCAAC TAGCTGAGGT AACGCTGGGC   
  
  
- CAACCTTCGG GGCCCAGCTC AGGGAGGATA ACAGTTCGAG TGGCAGCCCC GATAAAGGCT GCTTTAGGGC   
  
  
- GGCCACAAGC TAGAAGACAG CATGCTACTC GAGCCTTACT CTGATTAGTT GAAGCGAGCA AAATCTTTGT   
  
  
- AGCAGGAGCT TAAGGTTCGT TATGGGTGGA GGGGAAGGCT GCCTAAACGA AGCGACAACC TCCTCTAAGC   
  
  
- TGTTTCGTTC GAGATGAGGT TGCTACGCCG CCGCCAATAA CACTTAACAG TCTACTCAAA CGTAAATAAC   
  
  
- GTCCTTCTCC TCCACAGAAG TAGCAGCGGC AGCAGCTACT CCCCCTACAA AAACGTCCGC CACTCCTCGA   
  
  
- ACCTCGGCTC GTACCACCAC CACCACCTCC TCCTGCACCT AAAGTGCCGC TCCTCAGACC ACCCCGCCGA   
  
  
- CTCTAGCCGC AAATTAGTGT ACACCTATGG GAAGCTGTGC CACCTGTGCA AGAACGGTGC ACCCTCGGTC   
  
  
- GTCTCTCTCA CCAAGCTCCG GCTCCACACG ACCTTCTAAC TCTTACACTA GCGAGTACTC CCTGGGCGCT   
  
  
- CCCAGCTCTC CGTCCTCGGG TCCCGGTTCA CCCGGGAGGC TTACTCCCTC CGGCTCAAAG TTCCCTAGCT   
  
  
- CAAGCCACTA CTTCCATGCT GGCTCCAGTT CCGGTACGAC CTCCTCGTAC GGCGACCCAC CCCCAGATTC   
  
  
- TTCCTTCTCC TACTAGAACA CGAGTGAACC TTCCCTGTAT CACAACAGAA ACGAAGACGA ACCCATGGGT   
  
  
- GAAT

+     P-box

| Site Name | Organism | Position | Strand | Matrix score. | sequence | function |
| --- | --- | --- | --- | --- | --- | --- |
| P-box | Oryza sativa | 630 | - | 7 | CCTTTTG | gibberellin-responsive element |
| P-box | Oryza sativa | 654 | + | 7 | CCTTTTG | gibberellin-responsive element |
| P-box | Oryza sativa | 520 | + | 7 | CCTTTTG | gibberellin-responsive element |

>HU06G00376.1   
+ +Up\_Stream \_Len000AGAAGC TAATTAAACC AAACATGCAC GTATGTCCTA ATTAACATGA CTGTCCATCA   
  
  
+ TCTCCTCCCA TGTTGTTCTG TTGACAAGCC TGCACACCCA TGCTCCTCTC TCATGTCTAA CCTCGTCCCA   
  
  
+ ACGATCAAGA TCACTGTCTG TGAGGCCAGG GGAAGACTTG CTTTTATTTC CTTTTCTTTT GTTTACTCTC   
  
  
+ TGATTCCATT TAGCCATATA TACAAGGAGA ATGTCATGTG TATCTCATAT GTATATATAT AAGATTTTAT   
  
  
+ TTAGAAATAA AAATTTAAAC ACTATGTGAT TTATAGTTAT TATAATAAAT AGTATTTTTT AAACTATTTC   
  
  
+ ATTGATGAGA ACAGGAAAGT AAATTGCCGA CATGTTAATG TTATTATATC TAAAAATTAA ATCATAAAAT   
  
  
+ TAATTATAAT ATTTTAAAAA TATTTCAACT AAAATTTTAG AACAATAAGT GCGTAATACG GACTTAAAGG   
  
  
+ CTAGTTTATC GTAAAAATGT AAGTTCCTTT TGGATCTTAT GGTTGAATAT CTTGGTTGAT AAACTGTGAT   
  
  
+ TGGTTTTACA CTTTTACCAC ATATTCATAG AAGAATTATG TGCACGTTGA TGGTTCAAGA TGGGACAAAA   
  
  
+ GGAAACATGT ATTCTTCCCC CTTTTGCCTT TTCCCCTAAG CATTTGGATT GAGGTGTTGT CATCTGAATA   
  
  
+ TCAAAAATTC TTTTTGAAAA TTCAGTCTCA ATCAAAATTC AAAATTAAAA CAAAGACTTA AGAGTTTTAT   
  
  
+ TTGTTCTTAA CCTTCTTAAC TTTCTACTTT TCTTTTTTTT CTCATTCCCC CTCTTCAAAT CTCATTTATT   
  
  
+ CTTAACCCCC TCTTCCGTTC ATGCTCTCTC TGTCTATCAA CTAATATTAA TCTACCCCGA CTTTCAATTT   
  
  
+ GATGAGGTCT AATCCTTATC ATCGTATTGT GGTTGGGTTA ATTATGCAAG AAGGCAATAA ACCCCAGTGA   
  
  
+ CAAAGTCCAC GTTAAGTAGG CACCTCACCA TTAAGACATG CTCAGAAAAC ACCAAAACCA TTGAACACAA   
  
  
+ GTCCCCCAAG TCCCTTCCCT AGCTAGCCCA TTCTCTCTCC TCCCCTGTCT ATATCTACCA ACTCTTGCTC   
  
  
+ TTTGCTGAGC CTGAGTTGAA AGCGATACAC CCATAGCCTG TTCAGTTTTC AGTTTTCACA TTTGTCTTCC   
  
  
+ TCTGTTCAGT TGAAAGCGAT ACACTCTCAT TAGCTTTCAT TTTAAATACG TTCAATTCAC ACATAAATGG   
  
  
+ CTTTCAAAGC ATTGCCATTT TCGTTTAAAT GATGACAAAA TATATAGTAT CTGGCTATCT GCCCATATCT   
  
  
+ TGACCTTATT TACAAAGGCT GAGCCATCTT TTAATTTTTT TAGCCTCTTT TTTTAATCAA AAATAGAAAT   
  
  
+ TTCAAGAAAG CAGAAATCGT CAGATGACAG TGCAGACAGC GTTTTCGTTT CATACAAATC TCAAAAGCTG   
  
  
+ AAGTGTCAAT TTCTTCGATT ATTTTTGTCA GAAAATATTT ACGTCTCACC TTCACGTTGT TATTATTCTT   
  
  
+ TCTTTTTTAT TATTCTCATA ACTAGTAATA AAGTTAGCAA TGATAAAAAA TTATTCGATC CGAATATTTT   
  
  
+ AATTTATCTG ACCTAAAAAC ATAAGTAAAG GCACAAATTT TTCATCCAAA TTTTAATTTT TGATGTAATA   
  
  
+ TTTTTTATAT TTTTATTGTT CAAATCTGAT TTTAATCTTA TTTAAATTAT CTGACCTAAA AAAATCAAAT   
  
  
+ AATAATAAAC GTTAATTTTT TATTTAAATT TTGACATTAG TCAATCTGAC TTAAATTCGA ACCGAGCTTG   
  
  
+ AATTTTTTTG CCAGTGCTGG TAGTACTCCT GCTTTTGTAG CTCCCAATGC CATCGTACCA CCCTCTTCTA   
  
  
+ GTCTCGTCTC TTTCTCTGGT CTCCAATTAA TCACACCATC ATATCATACC GTATGATGAT ACAGTCCACT   
  
  
+ GAAATCCCAT TCTCACCGCC AATCTCTCTC CGCCACCATC CAACACTCTT AACGATGAAC AGGGCCGCCG   
  
  
+ CCTCGTCCTC CACACTCAGG CCGTGGCCGG GCAGCTTTCC CACCCAATCA AAATCTCTCT CCTCCGCCAA   
  
  
+ CTTCGGTAAC GCCAATTGCA TGGAGCAGCT CTTAGTCCAC TGCGCCGAAG CCATCGACAA CAATGACGCC   
  
  
+ ACCCCGGCCC AGCAAATCTT ATGGGTCCTG AATAACATAG CCCGACCCGA CGGCGACTCC ACCCAACGCC   
  
  
+ TCACGTGCGC ATTCCTACGT GCCTTAATCT CACGCGCCGT CCTCACTAGC ACCTGCAAGA TGGTAATCCC   
  
  
+ TCATTTCAAC CCCATCAATT CACCCCACAA ATTCTCGCTC CTCGAACTTG CCCACTTCGT CGATTTAACC   
  
  
+ CCTTGGCATC GATTCGGATT CACCGCCGCC AATTCGATCA TTCTGGAAGC TATTTCCGAC CTACCCGTTG   
  
  
+ TACACATTGT CGACCTCAGC ATCTCCCACT GTATGCAGAT CCCCACGTTG ATCGACTCCA TTGCGACCCG   
  
  
+ GTTGGAAGCC CCGGGTCGAG TCCCTCCTAT TGTCAAGCTC ACCGTCGGGG CTATTTCCGA CGAAATCCCG   
  
  
+ CCGGTGTTCG ATCTTCTGTC GTACGATGAG CTCGGAATGA GACTAATCAA CTTCGCTCGT TTTAGAAACA   
  
  
+ TCGTCCTCGA ATTCCAAGCA ATACCCACCT CCCCTTCCGA CGGATTTGCT TCGCTGTTGG AGGAGATTCG   
  
  
+ ACAAAGCAAG CTCTACTCCA ACGATGCGGC GGCGGTTATT GTGAATTGTC AGATGAGTTT GCATTTATTG   
  
  
+ CAGGAAGAGG AGGTGTCTTC ATCGTCGCCG TCGTCGATGA GGGGGATGTT TTTGCAGGCG GTGAGGAGCT   
  
  
+ TGGAGCCGAG CATGGTGGTG GTGGTGGAGG AGGACGTGGA TTTCACGGCG AGGAGTCTGG TGGGGCGGCT   
  
  
+ GAGATCGGCG TTTAATCACA TGTGGATACC CTTCGACACG GTGGACACGT TCTTGCCACG TGGGAGCCAG   
  
  
+ CAGAGAGAGT GGTTCGAGGC CGAGGTGTGC TGGAAGATTG AGAATGTGAT CGCTCATGAG GGACCCGCGA   
  
  
+ GGGTCGAGAG GCAGGAGCCC AGGGCCAAGT GGGCCCTCCG AATGAGGGAG GCCGAGTTTC AAGGGATCGA   
  
  
+ GTTCGGTGAT GAAGGTACGA CCGAGGTCAA GGCCATGCTG GAGGAGCATG CCGCTGGGTG GGGGTCTAAG   
  
  
+ AAGGAAGAGG ATGATCTTGT GCTCACTTGG AAGGGACATA GTGTTGTCTT TGCTTCTGCT TGGGTACCCA   
  
  
+ CTTA  

- +Up\_Stream \_Len000TCTTCG ATTAATTTGG TTTGTACGTG CATACAGGAT TAATTGTACT GACAGGTAGT   
  
  
- AGAGGAGGGT ACAACAAGAC AACTGTTCGG ACGTGTGGGT ACGAGGAGAG AGTACAGATT GGAGCAGGGT   
  
  
- TGCTAGTTCT AGTGACAGAC ACTCCGGTCC CCTTCTGAAC GAAAATAAAG GAAAAGAAAA CAAATGAGAG   
  
  
- ACTAAGGTAA ATCGGTATAT ATGTTCCTCT TACAGTACAC ATAGAGTATA CATATATATA TTCTAAAATA   
  
  
- AATCTTTATT TTTAAATTTG TGATACACTA AATATCAATA ATATTATTTA TCATAAAAAA TTTGATAAAG   
  
  
- TAACTACTCT TGTCCTTTCA TTTAACGGCT GTACAATTAC AATAATATAG ATTTTTAATT TAGTATTTTA   
  
  
- ATTAATATTA TAAAATTTTT ATAAAGTTGA TTTTAAAATC TTGTTATTCA CGCATTATGC CTGAATTTCC   
  
  
- GATCAAATAG CATTTTTACA TTCAAGGAAA ACCTAGAATA CCAACTTATA GAACCAACTA TTTGACACTA   
  
  
- ACCAAAATGT GAAAATGGTG TATAAGTATC TTCTTAATAC ACGTGCAACT ACCAAGTTCT ACCCTGTTTT   
  
  
- CCTTTGTACA TAAGAAGGGG GAAAACGGAA AAGGGGATTC GTAAACCTAA CTCCACAACA GTAGACTTAT   
  
  
- AGTTTTTAAG AAAAACTTTT AAGTCAGAGT TAGTTTTAAG TTTTAATTTT GTTTCTGAAT TCTCAAAATA   
  
  
- AACAAGAATT GGAAGAATTG AAAGATGAAA AGAAAAAAAA GAGTAAGGGG GAGAAGTTTA GAGTAAATAA   
  
  
- GAATTGGGGG AGAAGGCAAG TACGAGAGAG ACAGATAGTT GATTATAATT AGATGGGGCT GAAAGTTAAA   
  
  
- CTACTCCAGA TTAGGAATAG TAGCATAACA CCAACCCAAT TAATACGTTC TTCCGTTATT TGGGGTCACT   
  
  
- GTTTCAGGTG CAATTCATCC GTGGAGTGGT AATTCTGTAC GAGTCTTTTG TGGTTTTGGT AACTTGTGTT   
  
  
- CAGGGGGTTC AGGGAAGGGA TCGATCGGGT AAGAGAGAGG AGGGGACAGA TATAGATGGT TGAGAACGAG   
  
  
- AAACGACTCG GACTCAACTT TCGCTATGTG GGTATCGGAC AAGTCAAAAG TCAAAAGTGT AAACAGAAGG   
  
  
- AGACAAGTCA ACTTTCGCTA TGTGAGAGTA ATCGAAAGTA AAATTTATGC AAGTTAAGTG TGTATTTACC   
  
  
- GAAAGTTTCG TAACGGTAAA AGCAAATTTA CTACTGTTTT ATATATCATA GACCGATAGA CGGGTATAGA   
  
  
- ACTGGAATAA ATGTTTCCGA CTCGGTAGAA AATTAAAAAA ATCGGAGAAA AAAATTAGTT TTTATCTTTA   
  
  
- AAGTTCTTTC GTCTTTAGCA GTCTACTGTC ACGTCTGTCG CAAAAGCAAA GTATGTTTAG AGTTTTCGAC   
  
  
- TTCACAGTTA AAGAAGCTAA TAAAAACAGT CTTTTATAAA TGCAGAGTGG AAGTGCAACA ATAATAAGAA   
  
  
- AGAAAAAATA ATAAGAGTAT TGATCATTAT TTCAATCGTT ACTATTTTTT AATAAGCTAG GCTTATAAAA   
  
  
- TTAAATAGAC TGGATTTTTG TATTCATTTC CGTGTTTAAA AAGTAGGTTT AAAATTAAAA ACTACATTAT   
  
  
- AAAAAATATA AAAATAACAA GTTTAGACTA AAATTAGAAT AAATTTAATA GACTGGATTT TTTTAGTTTA   
  
  
- TTATTATTTG CAATTAAAAA ATAAATTTAA AACTGTAATC AGTTAGACTG AATTTAAGCT TGGCTCGAAC   
  
  
- TTAAAAAAAC GGTCACGACC ATCATGAGGA CGAAAACATC GAGGGTTACG GTAGCATGGT GGGAGAAGAT   
  
  
- CAGAGCAGAG AAAGAGACCA GAGGTTAATT AGTGTGGTAG TATAGTATGG CATACTACTA TGTCAGGTGA   
  
  
- CTTTAGGGTA AGAGTGGCGG TTAGAGAGAG GCGGTGGTAG GTTGTGAGAA TTGCTACTTG TCCCGGCGGC   
  
  
- GGAGCAGGAG GTGTGAGTCC GGCACCGGCC CGTCGAAAGG GTGGGTTAGT TTTAGAGAGA GGAGGCGGTT   
  
  
- GAAGCCATTG CGGTTAACGT ACCTCGTCGA GAATCAGGTG ACGCGGCTTC GGTAGCTGTT GTTACTGCGG   
  
  
- TGGGGCCGGG TCGTTTAGAA TACCCAGGAC TTATTGTATC GGGCTGGGCT GCCGCTGAGG TGGGTTGCGG   
  
  
- AGTGCACGCG TAAGGATGCA CGGAATTAGA GTGCGCGGCA GGAGTGATCG TGGACGTTCT ACCATTAGGG   
  
  
- AGTAAAGTTG GGGTAGTTAA GTGGGGTGTT TAAGAGCGAG GAGCTTGAAC GGGTGAAGCA GCTAAATTGG   
  
  
- GGAACCGTAG CTAAGCCTAA GTGGCGGCGG TTAAGCTAGT AAGACCTTCG ATAAAGGCTG GATGGGCAAC   
  
  
- ATGTGTAACA GCTGGAGTCG TAGAGGGTGA CATACGTCTA GGGGTGCAAC TAGCTGAGGT AACGCTGGGC   
  
  
- CAACCTTCGG GGCCCAGCTC AGGGAGGATA ACAGTTCGAG TGGCAGCCCC GATAAAGGCT GCTTTAGGGC   
  
  
- GGCCACAAGC TAGAAGACAG CATGCTACTC GAGCCTTACT CTGATTAGTT GAAGCGAGCA AAATCTTTGT   
  
  
- AGCAGGAGCT TAAGGTTCGT TATGGGTGGA GGGGAAGGCT GCCTAAACGA AGCGACAACC TCCTCTAAGC   
  
  
- TGTTTCGTTC GAGATGAGGT TGCTACGCCG CCGCCAATAA CACTTAACAG TCTACTCAAA CGTAAATAAC   
  
  
- GTCCTTCTCC TCCACAGAAG TAGCAGCGGC AGCAGCTACT CCCCCTACAA AAACGTCCGC CACTCCTCGA   
  
  
- ACCTCGGCTC GTACCACCAC CACCACCTCC TCCTGCACCT AAAGTGCCGC TCCTCAGACC ACCCCGCCGA   
  
  
- CTCTAGCCGC AAATTAGTGT ACACCTATGG GAAGCTGTGC CACCTGTGCA AGAACGGTGC ACCCTCGGTC   
  
  
- GTCTCTCTCA CCAAGCTCCG GCTCCACACG ACCTTCTAAC TCTTACACTA GCGAGTACTC CCTGGGCGCT   
  
  
- CCCAGCTCTC CGTCCTCGGG TCCCGGTTCA CCCGGGAGGC TTACTCCCTC CGGCTCAAAG TTCCCTAGCT   
  
  
- CAAGCCACTA CTTCCATGCT GGCTCCAGTT CCGGTACGAC CTCCTCGTAC GGCGACCCAC CCCCAGATTC   
  
  
- TTCCTTCTCC TACTAGAACA CGAGTGAACC TTCCCTGTAT CACAACAGAA ACGAAGACGA ACCCATGGGT   
  
  
- GAAT

+     STRE

| Site Name | Organism | Position | Strand | Matrix score. | sequence | function |
| --- | --- | --- | --- | --- | --- | --- |
| STRE | Arabidopsis thaliana | 2383 | - | 5 | AGGGG |  |
| STRE | Arabidopsis thaliana | 652 | - | 5 | AGGGG |  |
| STRE | Arabidopsis thaliana | 851 | - | 5 | AGGGG |  |
| STRE | Arabidopsis thaliana | 2844 | + | 5 | AGGGG |  |
| STRE | Arabidopsis thaliana | 667 | - | 5 | AGGGG |  |
| STRE | Arabidopsis thaliana | 2695 | - | 5 | AGGGG |  |
| STRE | Arabidopsis thaliana | 172 | + | 5 | AGGGG |  |
| STRE | Arabidopsis thaliana | 1096 | - | 5 | AGGGG |  |
| STRE | Arabidopsis thaliana | 822 | - | 5 | AGGGG |  |

>HU06G00376.1   
+ +Up\_Stream \_Len000AGAAGC TAATTAAACC AAACATGCAC GTATGTCCTA ATTAACATGA CTGTCCATCA   
  
  
+ TCTCCTCCCA TGTTGTTCTG TTGACAAGCC TGCACACCCA TGCTCCTCTC TCATGTCTAA CCTCGTCCCA   
  
  
+ ACGATCAAGA TCACTGTCTG TGAGGCCAGG GGAAGACTTG CTTTTATTTC CTTTTCTTTT GTTTACTCTC   
  
  
+ TGATTCCATT TAGCCATATA TACAAGGAGA ATGTCATGTG TATCTCATAT GTATATATAT AAGATTTTAT   
  
  
+ TTAGAAATAA AAATTTAAAC ACTATGTGAT TTATAGTTAT TATAATAAAT AGTATTTTTT AAACTATTTC   
  
  
+ ATTGATGAGA ACAGGAAAGT AAATTGCCGA CATGTTAATG TTATTATATC TAAAAATTAA ATCATAAAAT   
  
  
+ TAATTATAAT ATTTTAAAAA TATTTCAACT AAAATTTTAG AACAATAAGT GCGTAATACG GACTTAAAGG   
  
  
+ CTAGTTTATC GTAAAAATGT AAGTTCCTTT TGGATCTTAT GGTTGAATAT CTTGGTTGAT AAACTGTGAT   
  
  
+ TGGTTTTACA CTTTTACCAC ATATTCATAG AAGAATTATG TGCACGTTGA TGGTTCAAGA TGGGACAAAA   
  
  
+ GGAAACATGT ATTCTTCCCC CTTTTGCCTT TTCCCCTAAG CATTTGGATT GAGGTGTTGT CATCTGAATA   
  
  
+ TCAAAAATTC TTTTTGAAAA TTCAGTCTCA ATCAAAATTC AAAATTAAAA CAAAGACTTA AGAGTTTTAT   
  
  
+ TTGTTCTTAA CCTTCTTAAC TTTCTACTTT TCTTTTTTTT CTCATTCCCC CTCTTCAAAT CTCATTTATT   
  
  
+ CTTAACCCCC TCTTCCGTTC ATGCTCTCTC TGTCTATCAA CTAATATTAA TCTACCCCGA CTTTCAATTT   
  
  
+ GATGAGGTCT AATCCTTATC ATCGTATTGT GGTTGGGTTA ATTATGCAAG AAGGCAATAA ACCCCAGTGA   
  
  
+ CAAAGTCCAC GTTAAGTAGG CACCTCACCA TTAAGACATG CTCAGAAAAC ACCAAAACCA TTGAACACAA   
  
  
+ GTCCCCCAAG TCCCTTCCCT AGCTAGCCCA TTCTCTCTCC TCCCCTGTCT ATATCTACCA ACTCTTGCTC   
  
  
+ TTTGCTGAGC CTGAGTTGAA AGCGATACAC CCATAGCCTG TTCAGTTTTC AGTTTTCACA TTTGTCTTCC   
  
  
+ TCTGTTCAGT TGAAAGCGAT ACACTCTCAT TAGCTTTCAT TTTAAATACG TTCAATTCAC ACATAAATGG   
  
  
+ CTTTCAAAGC ATTGCCATTT TCGTTTAAAT GATGACAAAA TATATAGTAT CTGGCTATCT GCCCATATCT   
  
  
+ TGACCTTATT TACAAAGGCT GAGCCATCTT TTAATTTTTT TAGCCTCTTT TTTTAATCAA AAATAGAAAT   
  
  
+ TTCAAGAAAG CAGAAATCGT CAGATGACAG TGCAGACAGC GTTTTCGTTT CATACAAATC TCAAAAGCTG   
  
  
+ AAGTGTCAAT TTCTTCGATT ATTTTTGTCA GAAAATATTT ACGTCTCACC TTCACGTTGT TATTATTCTT   
  
  
+ TCTTTTTTAT TATTCTCATA ACTAGTAATA AAGTTAGCAA TGATAAAAAA TTATTCGATC CGAATATTTT   
  
  
+ AATTTATCTG ACCTAAAAAC ATAAGTAAAG GCACAAATTT TTCATCCAAA TTTTAATTTT TGATGTAATA   
  
  
+ TTTTTTATAT TTTTATTGTT CAAATCTGAT TTTAATCTTA TTTAAATTAT CTGACCTAAA AAAATCAAAT   
  
  
+ AATAATAAAC GTTAATTTTT TATTTAAATT TTGACATTAG TCAATCTGAC TTAAATTCGA ACCGAGCTTG   
  
  
+ AATTTTTTTG CCAGTGCTGG TAGTACTCCT GCTTTTGTAG CTCCCAATGC CATCGTACCA CCCTCTTCTA   
  
  
+ GTCTCGTCTC TTTCTCTGGT CTCCAATTAA TCACACCATC ATATCATACC GTATGATGAT ACAGTCCACT   
  
  
+ GAAATCCCAT TCTCACCGCC AATCTCTCTC CGCCACCATC CAACACTCTT AACGATGAAC AGGGCCGCCG   
  
  
+ CCTCGTCCTC CACACTCAGG CCGTGGCCGG GCAGCTTTCC CACCCAATCA AAATCTCTCT CCTCCGCCAA   
  
  
+ CTTCGGTAAC GCCAATTGCA TGGAGCAGCT CTTAGTCCAC TGCGCCGAAG CCATCGACAA CAATGACGCC   
  
  
+ ACCCCGGCCC AGCAAATCTT ATGGGTCCTG AATAACATAG CCCGACCCGA CGGCGACTCC ACCCAACGCC   
  
  
+ TCACGTGCGC ATTCCTACGT GCCTTAATCT CACGCGCCGT CCTCACTAGC ACCTGCAAGA TGGTAATCCC   
  
  
+ TCATTTCAAC CCCATCAATT CACCCCACAA ATTCTCGCTC CTCGAACTTG CCCACTTCGT CGATTTAACC   
  
  
+ CCTTGGCATC GATTCGGATT CACCGCCGCC AATTCGATCA TTCTGGAAGC TATTTCCGAC CTACCCGTTG   
  
  
+ TACACATTGT CGACCTCAGC ATCTCCCACT GTATGCAGAT CCCCACGTTG ATCGACTCCA TTGCGACCCG   
  
  
+ GTTGGAAGCC CCGGGTCGAG TCCCTCCTAT TGTCAAGCTC ACCGTCGGGG CTATTTCCGA CGAAATCCCG   
  
  
+ CCGGTGTTCG ATCTTCTGTC GTACGATGAG CTCGGAATGA GACTAATCAA CTTCGCTCGT TTTAGAAACA   
  
  
+ TCGTCCTCGA ATTCCAAGCA ATACCCACCT CCCCTTCCGA CGGATTTGCT TCGCTGTTGG AGGAGATTCG   
  
  
+ ACAAAGCAAG CTCTACTCCA ACGATGCGGC GGCGGTTATT GTGAATTGTC AGATGAGTTT GCATTTATTG   
  
  
+ CAGGAAGAGG AGGTGTCTTC ATCGTCGCCG TCGTCGATGA GGGGGATGTT TTTGCAGGCG GTGAGGAGCT   
  
  
+ TGGAGCCGAG CATGGTGGTG GTGGTGGAGG AGGACGTGGA TTTCACGGCG AGGAGTCTGG TGGGGCGGCT   
  
  
+ GAGATCGGCG TTTAATCACA TGTGGATACC CTTCGACACG GTGGACACGT TCTTGCCACG TGGGAGCCAG   
  
  
+ CAGAGAGAGT GGTTCGAGGC CGAGGTGTGC TGGAAGATTG AGAATGTGAT CGCTCATGAG GGACCCGCGA   
  
  
+ GGGTCGAGAG GCAGGAGCCC AGGGCCAAGT GGGCCCTCCG AATGAGGGAG GCCGAGTTTC AAGGGATCGA   
  
  
+ GTTCGGTGAT GAAGGTACGA CCGAGGTCAA GGCCATGCTG GAGGAGCATG CCGCTGGGTG GGGGTCTAAG   
  
  
+ AAGGAAGAGG ATGATCTTGT GCTCACTTGG AAGGGACATA GTGTTGTCTT TGCTTCTGCT TGGGTACCCA   
  
  
+ CTTA  

- +Up\_Stream \_Len000TCTTCG ATTAATTTGG TTTGTACGTG CATACAGGAT TAATTGTACT GACAGGTAGT   
  
  
- AGAGGAGGGT ACAACAAGAC AACTGTTCGG ACGTGTGGGT ACGAGGAGAG AGTACAGATT GGAGCAGGGT   
  
  
- TGCTAGTTCT AGTGACAGAC ACTCCGGTCC CCTTCTGAAC GAAAATAAAG GAAAAGAAAA CAAATGAGAG   
  
  
- ACTAAGGTAA ATCGGTATAT ATGTTCCTCT TACAGTACAC ATAGAGTATA CATATATATA TTCTAAAATA   
  
  
- AATCTTTATT TTTAAATTTG TGATACACTA AATATCAATA ATATTATTTA TCATAAAAAA TTTGATAAAG   
  
  
- TAACTACTCT TGTCCTTTCA TTTAACGGCT GTACAATTAC AATAATATAG ATTTTTAATT TAGTATTTTA   
  
  
- ATTAATATTA TAAAATTTTT ATAAAGTTGA TTTTAAAATC TTGTTATTCA CGCATTATGC CTGAATTTCC   
  
  
- GATCAAATAG CATTTTTACA TTCAAGGAAA ACCTAGAATA CCAACTTATA GAACCAACTA TTTGACACTA   
  
  
- ACCAAAATGT GAAAATGGTG TATAAGTATC TTCTTAATAC ACGTGCAACT ACCAAGTTCT ACCCTGTTTT   
  
  
- CCTTTGTACA TAAGAAGGGG GAAAACGGAA AAGGGGATTC GTAAACCTAA CTCCACAACA GTAGACTTAT   
  
  
- AGTTTTTAAG AAAAACTTTT AAGTCAGAGT TAGTTTTAAG TTTTAATTTT GTTTCTGAAT TCTCAAAATA   
  
  
- AACAAGAATT GGAAGAATTG AAAGATGAAA AGAAAAAAAA GAGTAAGGGG GAGAAGTTTA GAGTAAATAA   
  
  
- GAATTGGGGG AGAAGGCAAG TACGAGAGAG ACAGATAGTT GATTATAATT AGATGGGGCT GAAAGTTAAA   
  
  
- CTACTCCAGA TTAGGAATAG TAGCATAACA CCAACCCAAT TAATACGTTC TTCCGTTATT TGGGGTCACT   
  
  
- GTTTCAGGTG CAATTCATCC GTGGAGTGGT AATTCTGTAC GAGTCTTTTG TGGTTTTGGT AACTTGTGTT   
  
  
- CAGGGGGTTC AGGGAAGGGA TCGATCGGGT AAGAGAGAGG AGGGGACAGA TATAGATGGT TGAGAACGAG   
  
  
- AAACGACTCG GACTCAACTT TCGCTATGTG GGTATCGGAC AAGTCAAAAG TCAAAAGTGT AAACAGAAGG   
  
  
- AGACAAGTCA ACTTTCGCTA TGTGAGAGTA ATCGAAAGTA AAATTTATGC AAGTTAAGTG TGTATTTACC   
  
  
- GAAAGTTTCG TAACGGTAAA AGCAAATTTA CTACTGTTTT ATATATCATA GACCGATAGA CGGGTATAGA   
  
  
- ACTGGAATAA ATGTTTCCGA CTCGGTAGAA AATTAAAAAA ATCGGAGAAA AAAATTAGTT TTTATCTTTA   
  
  
- AAGTTCTTTC GTCTTTAGCA GTCTACTGTC ACGTCTGTCG CAAAAGCAAA GTATGTTTAG AGTTTTCGAC   
  
  
- TTCACAGTTA AAGAAGCTAA TAAAAACAGT CTTTTATAAA TGCAGAGTGG AAGTGCAACA ATAATAAGAA   
  
  
- AGAAAAAATA ATAAGAGTAT TGATCATTAT TTCAATCGTT ACTATTTTTT AATAAGCTAG GCTTATAAAA   
  
  
- TTAAATAGAC TGGATTTTTG TATTCATTTC CGTGTTTAAA AAGTAGGTTT AAAATTAAAA ACTACATTAT   
  
  
- AAAAAATATA AAAATAACAA GTTTAGACTA AAATTAGAAT AAATTTAATA GACTGGATTT TTTTAGTTTA   
  
  
- TTATTATTTG CAATTAAAAA ATAAATTTAA AACTGTAATC AGTTAGACTG AATTTAAGCT TGGCTCGAAC   
  
  
- TTAAAAAAAC GGTCACGACC ATCATGAGGA CGAAAACATC GAGGGTTACG GTAGCATGGT GGGAGAAGAT   
  
  
- CAGAGCAGAG AAAGAGACCA GAGGTTAATT AGTGTGGTAG TATAGTATGG CATACTACTA TGTCAGGTGA   
  
  
- CTTTAGGGTA AGAGTGGCGG TTAGAGAGAG GCGGTGGTAG GTTGTGAGAA TTGCTACTTG TCCCGGCGGC   
  
  
- GGAGCAGGAG GTGTGAGTCC GGCACCGGCC CGTCGAAAGG GTGGGTTAGT TTTAGAGAGA GGAGGCGGTT   
  
  
- GAAGCCATTG CGGTTAACGT ACCTCGTCGA GAATCAGGTG ACGCGGCTTC GGTAGCTGTT GTTACTGCGG   
  
  
- TGGGGCCGGG TCGTTTAGAA TACCCAGGAC TTATTGTATC GGGCTGGGCT GCCGCTGAGG TGGGTTGCGG   
  
  
- AGTGCACGCG TAAGGATGCA CGGAATTAGA GTGCGCGGCA GGAGTGATCG TGGACGTTCT ACCATTAGGG   
  
  
- AGTAAAGTTG GGGTAGTTAA GTGGGGTGTT TAAGAGCGAG GAGCTTGAAC GGGTGAAGCA GCTAAATTGG   
  
  
- GGAACCGTAG CTAAGCCTAA GTGGCGGCGG TTAAGCTAGT AAGACCTTCG ATAAAGGCTG GATGGGCAAC   
  
  
- ATGTGTAACA GCTGGAGTCG TAGAGGGTGA CATACGTCTA GGGGTGCAAC TAGCTGAGGT AACGCTGGGC   
  
  
- CAACCTTCGG GGCCCAGCTC AGGGAGGATA ACAGTTCGAG TGGCAGCCCC GATAAAGGCT GCTTTAGGGC   
  
  
- GGCCACAAGC TAGAAGACAG CATGCTACTC GAGCCTTACT CTGATTAGTT GAAGCGAGCA AAATCTTTGT   
  
  
- AGCAGGAGCT TAAGGTTCGT TATGGGTGGA GGGGAAGGCT GCCTAAACGA AGCGACAACC TCCTCTAAGC   
  
  
- TGTTTCGTTC GAGATGAGGT TGCTACGCCG CCGCCAATAA CACTTAACAG TCTACTCAAA CGTAAATAAC   
  
  
- GTCCTTCTCC TCCACAGAAG TAGCAGCGGC AGCAGCTACT CCCCCTACAA AAACGTCCGC CACTCCTCGA   
  
  
- ACCTCGGCTC GTACCACCAC CACCACCTCC TCCTGCACCT AAAGTGCCGC TCCTCAGACC ACCCCGCCGA   
  
  
- CTCTAGCCGC AAATTAGTGT ACACCTATGG GAAGCTGTGC CACCTGTGCA AGAACGGTGC ACCCTCGGTC   
  
  
- GTCTCTCTCA CCAAGCTCCG GCTCCACACG ACCTTCTAAC TCTTACACTA GCGAGTACTC CCTGGGCGCT   
  
  
- CCCAGCTCTC CGTCCTCGGG TCCCGGTTCA CCCGGGAGGC TTACTCCCTC CGGCTCAAAG TTCCCTAGCT   
  
  
- CAAGCCACTA CTTCCATGCT GGCTCCAGTT CCGGTACGAC CTCCTCGTAC GGCGACCCAC CCCCAGATTC   
  
  
- TTCCTTCTCC TACTAGAACA CGAGTGAACC TTCCCTGTAT CACAACAGAA ACGAAGACGA ACCCATGGGT   
  
  
- GAAT

+     Sp1

| Site Name | Organism | Position | Strand | Matrix score. | sequence | function |
| --- | --- | --- | --- | --- | --- | --- |
| Sp1 | Oryza sativa | 2937 | + | 6 | GGGCGG | light responsive element |

>HU06G00376.1   
+ +Up\_Stream \_Len000AGAAGC TAATTAAACC AAACATGCAC GTATGTCCTA ATTAACATGA CTGTCCATCA   
  
  
+ TCTCCTCCCA TGTTGTTCTG TTGACAAGCC TGCACACCCA TGCTCCTCTC TCATGTCTAA CCTCGTCCCA   
  
  
+ ACGATCAAGA TCACTGTCTG TGAGGCCAGG GGAAGACTTG CTTTTATTTC CTTTTCTTTT GTTTACTCTC   
  
  
+ TGATTCCATT TAGCCATATA TACAAGGAGA ATGTCATGTG TATCTCATAT GTATATATAT AAGATTTTAT   
  
  
+ TTAGAAATAA AAATTTAAAC ACTATGTGAT TTATAGTTAT TATAATAAAT AGTATTTTTT AAACTATTTC   
  
  
+ ATTGATGAGA ACAGGAAAGT AAATTGCCGA CATGTTAATG TTATTATATC TAAAAATTAA ATCATAAAAT   
  
  
+ TAATTATAAT ATTTTAAAAA TATTTCAACT AAAATTTTAG AACAATAAGT GCGTAATACG GACTTAAAGG   
  
  
+ CTAGTTTATC GTAAAAATGT AAGTTCCTTT TGGATCTTAT GGTTGAATAT CTTGGTTGAT AAACTGTGAT   
  
  
+ TGGTTTTACA CTTTTACCAC ATATTCATAG AAGAATTATG TGCACGTTGA TGGTTCAAGA TGGGACAAAA   
  
  
+ GGAAACATGT ATTCTTCCCC CTTTTGCCTT TTCCCCTAAG CATTTGGATT GAGGTGTTGT CATCTGAATA   
  
  
+ TCAAAAATTC TTTTTGAAAA TTCAGTCTCA ATCAAAATTC AAAATTAAAA CAAAGACTTA AGAGTTTTAT   
  
  
+ TTGTTCTTAA CCTTCTTAAC TTTCTACTTT TCTTTTTTTT CTCATTCCCC CTCTTCAAAT CTCATTTATT   
  
  
+ CTTAACCCCC TCTTCCGTTC ATGCTCTCTC TGTCTATCAA CTAATATTAA TCTACCCCGA CTTTCAATTT   
  
  
+ GATGAGGTCT AATCCTTATC ATCGTATTGT GGTTGGGTTA ATTATGCAAG AAGGCAATAA ACCCCAGTGA   
  
  
+ CAAAGTCCAC GTTAAGTAGG CACCTCACCA TTAAGACATG CTCAGAAAAC ACCAAAACCA TTGAACACAA   
  
  
+ GTCCCCCAAG TCCCTTCCCT AGCTAGCCCA TTCTCTCTCC TCCCCTGTCT ATATCTACCA ACTCTTGCTC   
  
  
+ TTTGCTGAGC CTGAGTTGAA AGCGATACAC CCATAGCCTG TTCAGTTTTC AGTTTTCACA TTTGTCTTCC   
  
  
+ TCTGTTCAGT TGAAAGCGAT ACACTCTCAT TAGCTTTCAT TTTAAATACG TTCAATTCAC ACATAAATGG   
  
  
+ CTTTCAAAGC ATTGCCATTT TCGTTTAAAT GATGACAAAA TATATAGTAT CTGGCTATCT GCCCATATCT   
  
  
+ TGACCTTATT TACAAAGGCT GAGCCATCTT TTAATTTTTT TAGCCTCTTT TTTTAATCAA AAATAGAAAT   
  
  
+ TTCAAGAAAG CAGAAATCGT CAGATGACAG TGCAGACAGC GTTTTCGTTT CATACAAATC TCAAAAGCTG   
  
  
+ AAGTGTCAAT TTCTTCGATT ATTTTTGTCA GAAAATATTT ACGTCTCACC TTCACGTTGT TATTATTCTT   
  
  
+ TCTTTTTTAT TATTCTCATA ACTAGTAATA AAGTTAGCAA TGATAAAAAA TTATTCGATC CGAATATTTT   
  
  
+ AATTTATCTG ACCTAAAAAC ATAAGTAAAG GCACAAATTT TTCATCCAAA TTTTAATTTT TGATGTAATA   
  
  
+ TTTTTTATAT TTTTATTGTT CAAATCTGAT TTTAATCTTA TTTAAATTAT CTGACCTAAA AAAATCAAAT   
  
  
+ AATAATAAAC GTTAATTTTT TATTTAAATT TTGACATTAG TCAATCTGAC TTAAATTCGA ACCGAGCTTG   
  
  
+ AATTTTTTTG CCAGTGCTGG TAGTACTCCT GCTTTTGTAG CTCCCAATGC CATCGTACCA CCCTCTTCTA   
  
  
+ GTCTCGTCTC TTTCTCTGGT CTCCAATTAA TCACACCATC ATATCATACC GTATGATGAT ACAGTCCACT   
  
  
+ GAAATCCCAT TCTCACCGCC AATCTCTCTC CGCCACCATC CAACACTCTT AACGATGAAC AGGGCCGCCG   
  
  
+ CCTCGTCCTC CACACTCAGG CCGTGGCCGG GCAGCTTTCC CACCCAATCA AAATCTCTCT CCTCCGCCAA   
  
  
+ CTTCGGTAAC GCCAATTGCA TGGAGCAGCT CTTAGTCCAC TGCGCCGAAG CCATCGACAA CAATGACGCC   
  
  
+ ACCCCGGCCC AGCAAATCTT ATGGGTCCTG AATAACATAG CCCGACCCGA CGGCGACTCC ACCCAACGCC   
  
  
+ TCACGTGCGC ATTCCTACGT GCCTTAATCT CACGCGCCGT CCTCACTAGC ACCTGCAAGA TGGTAATCCC   
  
  
+ TCATTTCAAC CCCATCAATT CACCCCACAA ATTCTCGCTC CTCGAACTTG CCCACTTCGT CGATTTAACC   
  
  
+ CCTTGGCATC GATTCGGATT CACCGCCGCC AATTCGATCA TTCTGGAAGC TATTTCCGAC CTACCCGTTG   
  
  
+ TACACATTGT CGACCTCAGC ATCTCCCACT GTATGCAGAT CCCCACGTTG ATCGACTCCA TTGCGACCCG   
  
  
+ GTTGGAAGCC CCGGGTCGAG TCCCTCCTAT TGTCAAGCTC ACCGTCGGGG CTATTTCCGA CGAAATCCCG   
  
  
+ CCGGTGTTCG ATCTTCTGTC GTACGATGAG CTCGGAATGA GACTAATCAA CTTCGCTCGT TTTAGAAACA   
  
  
+ TCGTCCTCGA ATTCCAAGCA ATACCCACCT CCCCTTCCGA CGGATTTGCT TCGCTGTTGG AGGAGATTCG   
  
  
+ ACAAAGCAAG CTCTACTCCA ACGATGCGGC GGCGGTTATT GTGAATTGTC AGATGAGTTT GCATTTATTG   
  
  
+ CAGGAAGAGG AGGTGTCTTC ATCGTCGCCG TCGTCGATGA GGGGGATGTT TTTGCAGGCG GTGAGGAGCT   
  
  
+ TGGAGCCGAG CATGGTGGTG GTGGTGGAGG AGGACGTGGA TTTCACGGCG AGGAGTCTGG TGGGGCGGCT   
  
  
+ GAGATCGGCG TTTAATCACA TGTGGATACC CTTCGACACG GTGGACACGT TCTTGCCACG TGGGAGCCAG   
  
  
+ CAGAGAGAGT GGTTCGAGGC CGAGGTGTGC TGGAAGATTG AGAATGTGAT CGCTCATGAG GGACCCGCGA   
  
  
+ GGGTCGAGAG GCAGGAGCCC AGGGCCAAGT GGGCCCTCCG AATGAGGGAG GCCGAGTTTC AAGGGATCGA   
  
  
+ GTTCGGTGAT GAAGGTACGA CCGAGGTCAA GGCCATGCTG GAGGAGCATG CCGCTGGGTG GGGGTCTAAG   
  
  
+ AAGGAAGAGG ATGATCTTGT GCTCACTTGG AAGGGACATA GTGTTGTCTT TGCTTCTGCT TGGGTACCCA   
  
  
+ CTTA  

- +Up\_Stream \_Len000TCTTCG ATTAATTTGG TTTGTACGTG CATACAGGAT TAATTGTACT GACAGGTAGT   
  
  
- AGAGGAGGGT ACAACAAGAC AACTGTTCGG ACGTGTGGGT ACGAGGAGAG AGTACAGATT GGAGCAGGGT   
  
  
- TGCTAGTTCT AGTGACAGAC ACTCCGGTCC CCTTCTGAAC GAAAATAAAG GAAAAGAAAA CAAATGAGAG   
  
  
- ACTAAGGTAA ATCGGTATAT ATGTTCCTCT TACAGTACAC ATAGAGTATA CATATATATA TTCTAAAATA   
  
  
- AATCTTTATT TTTAAATTTG TGATACACTA AATATCAATA ATATTATTTA TCATAAAAAA TTTGATAAAG   
  
  
- TAACTACTCT TGTCCTTTCA TTTAACGGCT GTACAATTAC AATAATATAG ATTTTTAATT TAGTATTTTA   
  
  
- ATTAATATTA TAAAATTTTT ATAAAGTTGA TTTTAAAATC TTGTTATTCA CGCATTATGC CTGAATTTCC   
  
  
- GATCAAATAG CATTTTTACA TTCAAGGAAA ACCTAGAATA CCAACTTATA GAACCAACTA TTTGACACTA   
  
  
- ACCAAAATGT GAAAATGGTG TATAAGTATC TTCTTAATAC ACGTGCAACT ACCAAGTTCT ACCCTGTTTT   
  
  
- CCTTTGTACA TAAGAAGGGG GAAAACGGAA AAGGGGATTC GTAAACCTAA CTCCACAACA GTAGACTTAT   
  
  
- AGTTTTTAAG AAAAACTTTT AAGTCAGAGT TAGTTTTAAG TTTTAATTTT GTTTCTGAAT TCTCAAAATA   
  
  
- AACAAGAATT GGAAGAATTG AAAGATGAAA AGAAAAAAAA GAGTAAGGGG GAGAAGTTTA GAGTAAATAA   
  
  
- GAATTGGGGG AGAAGGCAAG TACGAGAGAG ACAGATAGTT GATTATAATT AGATGGGGCT GAAAGTTAAA   
  
  
- CTACTCCAGA TTAGGAATAG TAGCATAACA CCAACCCAAT TAATACGTTC TTCCGTTATT TGGGGTCACT   
  
  
- GTTTCAGGTG CAATTCATCC GTGGAGTGGT AATTCTGTAC GAGTCTTTTG TGGTTTTGGT AACTTGTGTT   
  
  
- CAGGGGGTTC AGGGAAGGGA TCGATCGGGT AAGAGAGAGG AGGGGACAGA TATAGATGGT TGAGAACGAG   
  
  
- AAACGACTCG GACTCAACTT TCGCTATGTG GGTATCGGAC AAGTCAAAAG TCAAAAGTGT AAACAGAAGG   
  
  
- AGACAAGTCA ACTTTCGCTA TGTGAGAGTA ATCGAAAGTA AAATTTATGC AAGTTAAGTG TGTATTTACC   
  
  
- GAAAGTTTCG TAACGGTAAA AGCAAATTTA CTACTGTTTT ATATATCATA GACCGATAGA CGGGTATAGA   
  
  
- ACTGGAATAA ATGTTTCCGA CTCGGTAGAA AATTAAAAAA ATCGGAGAAA AAAATTAGTT TTTATCTTTA   
  
  
- AAGTTCTTTC GTCTTTAGCA GTCTACTGTC ACGTCTGTCG CAAAAGCAAA GTATGTTTAG AGTTTTCGAC   
  
  
- TTCACAGTTA AAGAAGCTAA TAAAAACAGT CTTTTATAAA TGCAGAGTGG AAGTGCAACA ATAATAAGAA   
  
  
- AGAAAAAATA ATAAGAGTAT TGATCATTAT TTCAATCGTT ACTATTTTTT AATAAGCTAG GCTTATAAAA   
  
  
- TTAAATAGAC TGGATTTTTG TATTCATTTC CGTGTTTAAA AAGTAGGTTT AAAATTAAAA ACTACATTAT   
  
  
- AAAAAATATA AAAATAACAA GTTTAGACTA AAATTAGAAT AAATTTAATA GACTGGATTT TTTTAGTTTA   
  
  
- TTATTATTTG CAATTAAAAA ATAAATTTAA AACTGTAATC AGTTAGACTG AATTTAAGCT TGGCTCGAAC   
  
  
- TTAAAAAAAC GGTCACGACC ATCATGAGGA CGAAAACATC GAGGGTTACG GTAGCATGGT GGGAGAAGAT   
  
  
- CAGAGCAGAG AAAGAGACCA GAGGTTAATT AGTGTGGTAG TATAGTATGG CATACTACTA TGTCAGGTGA   
  
  
- CTTTAGGGTA AGAGTGGCGG TTAGAGAGAG GCGGTGGTAG GTTGTGAGAA TTGCTACTTG TCCCGGCGGC   
  
  
- GGAGCAGGAG GTGTGAGTCC GGCACCGGCC CGTCGAAAGG GTGGGTTAGT TTTAGAGAGA GGAGGCGGTT   
  
  
- GAAGCCATTG CGGTTAACGT ACCTCGTCGA GAATCAGGTG ACGCGGCTTC GGTAGCTGTT GTTACTGCGG   
  
  
- TGGGGCCGGG TCGTTTAGAA TACCCAGGAC TTATTGTATC GGGCTGGGCT GCCGCTGAGG TGGGTTGCGG   
  
  
- AGTGCACGCG TAAGGATGCA CGGAATTAGA GTGCGCGGCA GGAGTGATCG TGGACGTTCT ACCATTAGGG   
  
  
- AGTAAAGTTG GGGTAGTTAA GTGGGGTGTT TAAGAGCGAG GAGCTTGAAC GGGTGAAGCA GCTAAATTGG   
  
  
- GGAACCGTAG CTAAGCCTAA GTGGCGGCGG TTAAGCTAGT AAGACCTTCG ATAAAGGCTG GATGGGCAAC   
  
  
- ATGTGTAACA GCTGGAGTCG TAGAGGGTGA CATACGTCTA GGGGTGCAAC TAGCTGAGGT AACGCTGGGC   
  
  
- CAACCTTCGG GGCCCAGCTC AGGGAGGATA ACAGTTCGAG TGGCAGCCCC GATAAAGGCT GCTTTAGGGC   
  
  
- GGCCACAAGC TAGAAGACAG CATGCTACTC GAGCCTTACT CTGATTAGTT GAAGCGAGCA AAATCTTTGT   
  
  
- AGCAGGAGCT TAAGGTTCGT TATGGGTGGA GGGGAAGGCT GCCTAAACGA AGCGACAACC TCCTCTAAGC   
  
  
- TGTTTCGTTC GAGATGAGGT TGCTACGCCG CCGCCAATAA CACTTAACAG TCTACTCAAA CGTAAATAAC   
  
  
- GTCCTTCTCC TCCACAGAAG TAGCAGCGGC AGCAGCTACT CCCCCTACAA AAACGTCCGC CACTCCTCGA   
  
  
- ACCTCGGCTC GTACCACCAC CACCACCTCC TCCTGCACCT AAAGTGCCGC TCCTCAGACC ACCCCGCCGA   
  
  
- CTCTAGCCGC AAATTAGTGT ACACCTATGG GAAGCTGTGC CACCTGTGCA AGAACGGTGC ACCCTCGGTC   
  
  
- GTCTCTCTCA CCAAGCTCCG GCTCCACACG ACCTTCTAAC TCTTACACTA GCGAGTACTC CCTGGGCGCT   
  
  
- CCCAGCTCTC CGTCCTCGGG TCCCGGTTCA CCCGGGAGGC TTACTCCCTC CGGCTCAAAG TTCCCTAGCT   
  
  
- CAAGCCACTA CTTCCATGCT GGCTCCAGTT CCGGTACGAC CTCCTCGTAC GGCGACCCAC CCCCAGATTC   
  
  
- TTCCTTCTCC TACTAGAACA CGAGTGAACC TTCCCTGTAT CACAACAGAA ACGAAGACGA ACCCATGGGT   
  
  
- GAAT

+     TATA-box

| Site Name | Organism | Position | Strand | Matrix score. | sequence | function |
| --- | --- | --- | --- | --- | --- | --- |
| TATA-box | Oryza sativa | 1857 | - | 7 | TACAAAA | core promoter element around -30 of transcription start |
| TATA-box | Arabidopsis thaliana | 1690 | - | 4 | TATA | core promoter element around -30 of transcription start |
| TATA-box | Arabidopsis thaliana | 1689 | - | 5 | TATAA | core promoter element around -30 of transcription start |
| TATA-box | Arabidopsis thaliana | 1307 | + | 4 | TATA | core promoter element around -30 of transcription start |
| TATA-box | Helianthus annuus | 264 | - | 6 | TATACA | core promoter element around -30 of transcription start |
| TATA-box | Helianthus annuus | 1688 | - | 6 | TATAAA | core promoter element around -30 of transcription start |
| TATA-box | Arabidopsis thaliana | 1775 | + | 8 | TATTTAAA | core promoter element around -30 of transcription start |
| TATA-box | Brassica napus | 1304 | + | 6 | ATATAT | core promoter element around -30 of transcription start |
| TATA-box | Arabidopsis thaliana | 1235 | - | 8 | TATTTAAA | core promoter element around -30 of transcription start |
| TATA-box | Arabidopsis thaliana | 231 | + | 6 | TATATA | core promoter element around -30 of transcription start |
| TATA-box | Arabidopsis thaliana | 268 | + | 6 | TATATA | core promoter element around -30 of transcription start |
| TATA-box | Arabidopsis thaliana | 1104 | + | 4 | TATA | core promoter element around -30 of transcription start |
| TATA-box | Arabidopsis thaliana | 429 | + | 4 | TATA | core promoter element around -30 of transcription start |
| TATA-box | Arabidopsis thaliana | 428 | - | 5 | TATAA | core promoter element around -30 of transcription start |
| TATA-box | Brassica napus | 230 | + | 6 | ATATAT | core promoter element around -30 of transcription start |
| TATA-box | Brassica juncea | 313 | - | 7 | TATAAAT | core promoter element around -30 of transcription start |
| TATA-box | Arabidopsis thaliana | 1723 | + | 8 | TATTTAAA | core promoter element around -30 of transcription start |
| TATA-box | Brassica napus | 427 | + | 6 | ATTATA | core promoter element around -30 of transcription start |
| TATA-box | Arabidopsis thaliana | 272 | + | 4 | TATA | core promoter element around -30 of transcription start |
| TATA-box | Helianthus annuus | 314 | - | 6 | TATAAA | core promoter element around -30 of transcription start |
| TATA-box | Arabidopsis thaliana | 1739 | + | 9 | ccTATAAAaa | core promoter element around -30 of transcription start |
| TATA-box | Arabidopsis thaliana | 398 | - | 5 | TATAA | core promoter element around -30 of transcription start |
| TATA-box | Arabidopsis thaliana | 1305 | + | 6 | TATATA | core promoter element around -30 of transcription start |
| TATA-box | Arabidopsis thaliana | 270 | + | 6 | TATATA | core promoter element around -30 of transcription start |
| TATA-box | Arabidopsis thaliana | 399 | + | 4 | TATA | core promoter element around -30 of transcription start |
| TATA-box | Brassica napus | 269 | + | 6 | ATATAT | core promoter element around -30 of transcription start |
| TATA-box | Pisum sativum | 1687 | - | 7 | TATAAAA | core promoter element around -30 of transcription start |
| TATA-box | Arabidopsis thaliana | 315 | - | 5 | TATAA | core promoter element around -30 of transcription start |
| TATA-box | Arabidopsis thaliana | 324 | - | 5 | TATAA | core promoter element around -30 of transcription start |
| TATA-box | Arabidopsis thaliana | 325 | + | 4 | TATA | core promoter element around -30 of transcription start |
| TATA-box | Brassica oleracea | 271 | + | 6 | ATATAA | core promoter element around -30 of transcription start |
| TATA-box | Arabidopsis thaliana | 266 | + | 6 | TATATA | core promoter element around -30 of transcription start |
| TATA-box | Arabidopsis thaliana | 316 | + | 4 | TATA | core promoter element around -30 of transcription start |
| TATA-box | Brassica napus | 323 | + | 6 | ATTATA | core promoter element around -30 of transcription start |
| TATA-box | Arabidopsis thaliana | 233 | + | 4 | TATA | core promoter element around -30 of transcription start |
| TATA-box | Brassica napus | 397 | + | 6 | ATTATA | core promoter element around -30 of transcription start |
| TATA-box | Brassica napus | 267 | + | 6 | ATATAT | core promoter element around -30 of transcription start |

>HU06G00376.1   
+ +Up\_Stream \_Len000AGAAGC TAATTAAACC AAACATGCAC GTATGTCCTA ATTAACATGA CTGTCCATCA   
  
  
+ TCTCCTCCCA TGTTGTTCTG TTGACAAGCC TGCACACCCA TGCTCCTCTC TCATGTCTAA CCTCGTCCCA   
  
  
+ ACGATCAAGA TCACTGTCTG TGAGGCCAGG GGAAGACTTG CTTTTATTTC CTTTTCTTTT GTTTACTCTC   
  
  
+ TGATTCCATT TAGCCATATA TACAAGGAGA ATGTCATGTG TATCTCATAT GTATATATAT AAGATTTTAT   
  
  
+ TTAGAAATAA AAATTTAAAC ACTATGTGAT TTATAGTTAT TATAATAAAT AGTATTTTTT AAACTATTTC   
  
  
+ ATTGATGAGA ACAGGAAAGT AAATTGCCGA CATGTTAATG TTATTATATC TAAAAATTAA ATCATAAAAT   
  
  
+ TAATTATAAT ATTTTAAAAA TATTTCAACT AAAATTTTAG AACAATAAGT GCGTAATACG GACTTAAAGG   
  
  
+ CTAGTTTATC GTAAAAATGT AAGTTCCTTT TGGATCTTAT GGTTGAATAT CTTGGTTGAT AAACTGTGAT   
  
  
+ TGGTTTTACA CTTTTACCAC ATATTCATAG AAGAATTATG TGCACGTTGA TGGTTCAAGA TGGGACAAAA   
  
  
+ GGAAACATGT ATTCTTCCCC CTTTTGCCTT TTCCCCTAAG CATTTGGATT GAGGTGTTGT CATCTGAATA   
  
  
+ TCAAAAATTC TTTTTGAAAA TTCAGTCTCA ATCAAAATTC AAAATTAAAA CAAAGACTTA AGAGTTTTAT   
  
  
+ TTGTTCTTAA CCTTCTTAAC TTTCTACTTT TCTTTTTTTT CTCATTCCCC CTCTTCAAAT CTCATTTATT   
  
  
+ CTTAACCCCC TCTTCCGTTC ATGCTCTCTC TGTCTATCAA CTAATATTAA TCTACCCCGA CTTTCAATTT   
  
  
+ GATGAGGTCT AATCCTTATC ATCGTATTGT GGTTGGGTTA ATTATGCAAG AAGGCAATAA ACCCCAGTGA   
  
  
+ CAAAGTCCAC GTTAAGTAGG CACCTCACCA TTAAGACATG CTCAGAAAAC ACCAAAACCA TTGAACACAA   
  
  
+ GTCCCCCAAG TCCCTTCCCT AGCTAGCCCA TTCTCTCTCC TCCCCTGTCT ATATCTACCA ACTCTTGCTC   
  
  
+ TTTGCTGAGC CTGAGTTGAA AGCGATACAC CCATAGCCTG TTCAGTTTTC AGTTTTCACA TTTGTCTTCC   
  
  
+ TCTGTTCAGT TGAAAGCGAT ACACTCTCAT TAGCTTTCAT TTTAAATACG TTCAATTCAC ACATAAATGG   
  
  
+ CTTTCAAAGC ATTGCCATTT TCGTTTAAAT GATGACAAAA TATATAGTAT CTGGCTATCT GCCCATATCT   
  
  
+ TGACCTTATT TACAAAGGCT GAGCCATCTT TTAATTTTTT TAGCCTCTTT TTTTAATCAA AAATAGAAAT   
  
  
+ TTCAAGAAAG CAGAAATCGT CAGATGACAG TGCAGACAGC GTTTTCGTTT CATACAAATC TCAAAAGCTG   
  
  
+ AAGTGTCAAT TTCTTCGATT ATTTTTGTCA GAAAATATTT ACGTCTCACC TTCACGTTGT TATTATTCTT   
  
  
+ TCTTTTTTAT TATTCTCATA ACTAGTAATA AAGTTAGCAA TGATAAAAAA TTATTCGATC CGAATATTTT   
  
  
+ AATTTATCTG ACCTAAAAAC ATAAGTAAAG GCACAAATTT TTCATCCAAA TTTTAATTTT TGATGTAATA   
  
  
+ TTTTTTATAT TTTTATTGTT CAAATCTGAT TTTAATCTTA TTTAAATTAT CTGACCTAAA AAAATCAAAT   
  
  
+ AATAATAAAC GTTAATTTTT TATTTAAATT TTGACATTAG TCAATCTGAC TTAAATTCGA ACCGAGCTTG   
  
  
+ AATTTTTTTG CCAGTGCTGG TAGTACTCCT GCTTTTGTAG CTCCCAATGC CATCGTACCA CCCTCTTCTA   
  
  
+ GTCTCGTCTC TTTCTCTGGT CTCCAATTAA TCACACCATC ATATCATACC GTATGATGAT ACAGTCCACT   
  
  
+ GAAATCCCAT TCTCACCGCC AATCTCTCTC CGCCACCATC CAACACTCTT AACGATGAAC AGGGCCGCCG   
  
  
+ CCTCGTCCTC CACACTCAGG CCGTGGCCGG GCAGCTTTCC CACCCAATCA AAATCTCTCT CCTCCGCCAA   
  
  
+ CTTCGGTAAC GCCAATTGCA TGGAGCAGCT CTTAGTCCAC TGCGCCGAAG CCATCGACAA CAATGACGCC   
  
  
+ ACCCCGGCCC AGCAAATCTT ATGGGTCCTG AATAACATAG CCCGACCCGA CGGCGACTCC ACCCAACGCC   
  
  
+ TCACGTGCGC ATTCCTACGT GCCTTAATCT CACGCGCCGT CCTCACTAGC ACCTGCAAGA TGGTAATCCC   
  
  
+ TCATTTCAAC CCCATCAATT CACCCCACAA ATTCTCGCTC CTCGAACTTG CCCACTTCGT CGATTTAACC   
  
  
+ CCTTGGCATC GATTCGGATT CACCGCCGCC AATTCGATCA TTCTGGAAGC TATTTCCGAC CTACCCGTTG   
  
  
+ TACACATTGT CGACCTCAGC ATCTCCCACT GTATGCAGAT CCCCACGTTG ATCGACTCCA TTGCGACCCG   
  
  
+ GTTGGAAGCC CCGGGTCGAG TCCCTCCTAT TGTCAAGCTC ACCGTCGGGG CTATTTCCGA CGAAATCCCG   
  
  
+ CCGGTGTTCG ATCTTCTGTC GTACGATGAG CTCGGAATGA GACTAATCAA CTTCGCTCGT TTTAGAAACA   
  
  
+ TCGTCCTCGA ATTCCAAGCA ATACCCACCT CCCCTTCCGA CGGATTTGCT TCGCTGTTGG AGGAGATTCG   
  
  
+ ACAAAGCAAG CTCTACTCCA ACGATGCGGC GGCGGTTATT GTGAATTGTC AGATGAGTTT GCATTTATTG   
  
  
+ CAGGAAGAGG AGGTGTCTTC ATCGTCGCCG TCGTCGATGA GGGGGATGTT TTTGCAGGCG GTGAGGAGCT   
  
  
+ TGGAGCCGAG CATGGTGGTG GTGGTGGAGG AGGACGTGGA TTTCACGGCG AGGAGTCTGG TGGGGCGGCT   
  
  
+ GAGATCGGCG TTTAATCACA TGTGGATACC CTTCGACACG GTGGACACGT TCTTGCCACG TGGGAGCCAG   
  
  
+ CAGAGAGAGT GGTTCGAGGC CGAGGTGTGC TGGAAGATTG AGAATGTGAT CGCTCATGAG GGACCCGCGA   
  
  
+ GGGTCGAGAG GCAGGAGCCC AGGGCCAAGT GGGCCCTCCG AATGAGGGAG GCCGAGTTTC AAGGGATCGA   
  
  
+ GTTCGGTGAT GAAGGTACGA CCGAGGTCAA GGCCATGCTG GAGGAGCATG CCGCTGGGTG GGGGTCTAAG   
  
  
+ AAGGAAGAGG ATGATCTTGT GCTCACTTGG AAGGGACATA GTGTTGTCTT TGCTTCTGCT TGGGTACCCA   
  
  
+ CTTA  

- +Up\_Stream \_Len000TCTTCG ATTAATTTGG TTTGTACGTG CATACAGGAT TAATTGTACT GACAGGTAGT   
  
  
- AGAGGAGGGT ACAACAAGAC AACTGTTCGG ACGTGTGGGT ACGAGGAGAG AGTACAGATT GGAGCAGGGT   
  
  
- TGCTAGTTCT AGTGACAGAC ACTCCGGTCC CCTTCTGAAC GAAAATAAAG GAAAAGAAAA CAAATGAGAG   
  
  
- ACTAAGGTAA ATCGGTATAT ATGTTCCTCT TACAGTACAC ATAGAGTATA CATATATATA TTCTAAAATA   
  
  
- AATCTTTATT TTTAAATTTG TGATACACTA AATATCAATA ATATTATTTA TCATAAAAAA TTTGATAAAG   
  
  
- TAACTACTCT TGTCCTTTCA TTTAACGGCT GTACAATTAC AATAATATAG ATTTTTAATT TAGTATTTTA   
  
  
- ATTAATATTA TAAAATTTTT ATAAAGTTGA TTTTAAAATC TTGTTATTCA CGCATTATGC CTGAATTTCC   
  
  
- GATCAAATAG CATTTTTACA TTCAAGGAAA ACCTAGAATA CCAACTTATA GAACCAACTA TTTGACACTA   
  
  
- ACCAAAATGT GAAAATGGTG TATAAGTATC TTCTTAATAC ACGTGCAACT ACCAAGTTCT ACCCTGTTTT   
  
  
- CCTTTGTACA TAAGAAGGGG GAAAACGGAA AAGGGGATTC GTAAACCTAA CTCCACAACA GTAGACTTAT   
  
  
- AGTTTTTAAG AAAAACTTTT AAGTCAGAGT TAGTTTTAAG TTTTAATTTT GTTTCTGAAT TCTCAAAATA   
  
  
- AACAAGAATT GGAAGAATTG AAAGATGAAA AGAAAAAAAA GAGTAAGGGG GAGAAGTTTA GAGTAAATAA   
  
  
- GAATTGGGGG AGAAGGCAAG TACGAGAGAG ACAGATAGTT GATTATAATT AGATGGGGCT GAAAGTTAAA   
  
  
- CTACTCCAGA TTAGGAATAG TAGCATAACA CCAACCCAAT TAATACGTTC TTCCGTTATT TGGGGTCACT   
  
  
- GTTTCAGGTG CAATTCATCC GTGGAGTGGT AATTCTGTAC GAGTCTTTTG TGGTTTTGGT AACTTGTGTT   
  
  
- CAGGGGGTTC AGGGAAGGGA TCGATCGGGT AAGAGAGAGG AGGGGACAGA TATAGATGGT TGAGAACGAG   
  
  
- AAACGACTCG GACTCAACTT TCGCTATGTG GGTATCGGAC AAGTCAAAAG TCAAAAGTGT AAACAGAAGG   
  
  
- AGACAAGTCA ACTTTCGCTA TGTGAGAGTA ATCGAAAGTA AAATTTATGC AAGTTAAGTG TGTATTTACC   
  
  
- GAAAGTTTCG TAACGGTAAA AGCAAATTTA CTACTGTTTT ATATATCATA GACCGATAGA CGGGTATAGA   
  
  
- ACTGGAATAA ATGTTTCCGA CTCGGTAGAA AATTAAAAAA ATCGGAGAAA AAAATTAGTT TTTATCTTTA   
  
  
- AAGTTCTTTC GTCTTTAGCA GTCTACTGTC ACGTCTGTCG CAAAAGCAAA GTATGTTTAG AGTTTTCGAC   
  
  
- TTCACAGTTA AAGAAGCTAA TAAAAACAGT CTTTTATAAA TGCAGAGTGG AAGTGCAACA ATAATAAGAA   
  
  
- AGAAAAAATA ATAAGAGTAT TGATCATTAT TTCAATCGTT ACTATTTTTT AATAAGCTAG GCTTATAAAA   
  
  
- TTAAATAGAC TGGATTTTTG TATTCATTTC CGTGTTTAAA AAGTAGGTTT AAAATTAAAA ACTACATTAT   
  
  
- AAAAAATATA AAAATAACAA GTTTAGACTA AAATTAGAAT AAATTTAATA GACTGGATTT TTTTAGTTTA   
  
  
- TTATTATTTG CAATTAAAAA ATAAATTTAA AACTGTAATC AGTTAGACTG AATTTAAGCT TGGCTCGAAC   
  
  
- TTAAAAAAAC GGTCACGACC ATCATGAGGA CGAAAACATC GAGGGTTACG GTAGCATGGT GGGAGAAGAT   
  
  
- CAGAGCAGAG AAAGAGACCA GAGGTTAATT AGTGTGGTAG TATAGTATGG CATACTACTA TGTCAGGTGA   
  
  
- CTTTAGGGTA AGAGTGGCGG TTAGAGAGAG GCGGTGGTAG GTTGTGAGAA TTGCTACTTG TCCCGGCGGC   
  
  
- GGAGCAGGAG GTGTGAGTCC GGCACCGGCC CGTCGAAAGG GTGGGTTAGT TTTAGAGAGA GGAGGCGGTT   
  
  
- GAAGCCATTG CGGTTAACGT ACCTCGTCGA GAATCAGGTG ACGCGGCTTC GGTAGCTGTT GTTACTGCGG   
  
  
- TGGGGCCGGG TCGTTTAGAA TACCCAGGAC TTATTGTATC GGGCTGGGCT GCCGCTGAGG TGGGTTGCGG   
  
  
- AGTGCACGCG TAAGGATGCA CGGAATTAGA GTGCGCGGCA GGAGTGATCG TGGACGTTCT ACCATTAGGG   
  
  
- AGTAAAGTTG GGGTAGTTAA GTGGGGTGTT TAAGAGCGAG GAGCTTGAAC GGGTGAAGCA GCTAAATTGG   
  
  
- GGAACCGTAG CTAAGCCTAA GTGGCGGCGG TTAAGCTAGT AAGACCTTCG ATAAAGGCTG GATGGGCAAC   
  
  
- ATGTGTAACA GCTGGAGTCG TAGAGGGTGA CATACGTCTA GGGGTGCAAC TAGCTGAGGT AACGCTGGGC   
  
  
- CAACCTTCGG GGCCCAGCTC AGGGAGGATA ACAGTTCGAG TGGCAGCCCC GATAAAGGCT GCTTTAGGGC   
  
  
- GGCCACAAGC TAGAAGACAG CATGCTACTC GAGCCTTACT CTGATTAGTT GAAGCGAGCA AAATCTTTGT   
  
  
- AGCAGGAGCT TAAGGTTCGT TATGGGTGGA GGGGAAGGCT GCCTAAACGA AGCGACAACC TCCTCTAAGC   
  
  
- TGTTTCGTTC GAGATGAGGT TGCTACGCCG CCGCCAATAA CACTTAACAG TCTACTCAAA CGTAAATAAC   
  
  
- GTCCTTCTCC TCCACAGAAG TAGCAGCGGC AGCAGCTACT CCCCCTACAA AAACGTCCGC CACTCCTCGA   
  
  
- ACCTCGGCTC GTACCACCAC CACCACCTCC TCCTGCACCT AAAGTGCCGC TCCTCAGACC ACCCCGCCGA   
  
  
- CTCTAGCCGC AAATTAGTGT ACACCTATGG GAAGCTGTGC CACCTGTGCA AGAACGGTGC ACCCTCGGTC   
  
  
- GTCTCTCTCA CCAAGCTCCG GCTCCACACG ACCTTCTAAC TCTTACACTA GCGAGTACTC CCTGGGCGCT   
  
  
- CCCAGCTCTC CGTCCTCGGG TCCCGGTTCA CCCGGGAGGC TTACTCCCTC CGGCTCAAAG TTCCCTAGCT   
  
  
- CAAGCCACTA CTTCCATGCT GGCTCCAGTT CCGGTACGAC CTCCTCGTAC GGCGACCCAC CCCCAGATTC   
  
  
- TTCCTTCTCC TACTAGAACA CGAGTGAACC TTCCCTGTAT CACAACAGAA ACGAAGACGA ACCCATGGGT   
  
  
- GAAT

+     TCA-element

| Site Name | Organism | Position | Strand | Matrix score. | sequence | function |
| --- | --- | --- | --- | --- | --- | --- |
| TCA-element | Nicotiana tabacum | 1358 | + | 9 | CCATCTTTTT | cis-acting element involved in salicylic acid responsiveness |

>HU06G00376.1   
+ +Up\_Stream \_Len000AGAAGC TAATTAAACC AAACATGCAC GTATGTCCTA ATTAACATGA CTGTCCATCA   
  
  
+ TCTCCTCCCA TGTTGTTCTG TTGACAAGCC TGCACACCCA TGCTCCTCTC TCATGTCTAA CCTCGTCCCA   
  
  
+ ACGATCAAGA TCACTGTCTG TGAGGCCAGG GGAAGACTTG CTTTTATTTC CTTTTCTTTT GTTTACTCTC   
  
  
+ TGATTCCATT TAGCCATATA TACAAGGAGA ATGTCATGTG TATCTCATAT GTATATATAT AAGATTTTAT   
  
  
+ TTAGAAATAA AAATTTAAAC ACTATGTGAT TTATAGTTAT TATAATAAAT AGTATTTTTT AAACTATTTC   
  
  
+ ATTGATGAGA ACAGGAAAGT AAATTGCCGA CATGTTAATG TTATTATATC TAAAAATTAA ATCATAAAAT   
  
  
+ TAATTATAAT ATTTTAAAAA TATTTCAACT AAAATTTTAG AACAATAAGT GCGTAATACG GACTTAAAGG   
  
  
+ CTAGTTTATC GTAAAAATGT AAGTTCCTTT TGGATCTTAT GGTTGAATAT CTTGGTTGAT AAACTGTGAT   
  
  
+ TGGTTTTACA CTTTTACCAC ATATTCATAG AAGAATTATG TGCACGTTGA TGGTTCAAGA TGGGACAAAA   
  
  
+ GGAAACATGT ATTCTTCCCC CTTTTGCCTT TTCCCCTAAG CATTTGGATT GAGGTGTTGT CATCTGAATA   
  
  
+ TCAAAAATTC TTTTTGAAAA TTCAGTCTCA ATCAAAATTC AAAATTAAAA CAAAGACTTA AGAGTTTTAT   
  
  
+ TTGTTCTTAA CCTTCTTAAC TTTCTACTTT TCTTTTTTTT CTCATTCCCC CTCTTCAAAT CTCATTTATT   
  
  
+ CTTAACCCCC TCTTCCGTTC ATGCTCTCTC TGTCTATCAA CTAATATTAA TCTACCCCGA CTTTCAATTT   
  
  
+ GATGAGGTCT AATCCTTATC ATCGTATTGT GGTTGGGTTA ATTATGCAAG AAGGCAATAA ACCCCAGTGA   
  
  
+ CAAAGTCCAC GTTAAGTAGG CACCTCACCA TTAAGACATG CTCAGAAAAC ACCAAAACCA TTGAACACAA   
  
  
+ GTCCCCCAAG TCCCTTCCCT AGCTAGCCCA TTCTCTCTCC TCCCCTGTCT ATATCTACCA ACTCTTGCTC   
  
  
+ TTTGCTGAGC CTGAGTTGAA AGCGATACAC CCATAGCCTG TTCAGTTTTC AGTTTTCACA TTTGTCTTCC   
  
  
+ TCTGTTCAGT TGAAAGCGAT ACACTCTCAT TAGCTTTCAT TTTAAATACG TTCAATTCAC ACATAAATGG   
  
  
+ CTTTCAAAGC ATTGCCATTT TCGTTTAAAT GATGACAAAA TATATAGTAT CTGGCTATCT GCCCATATCT   
  
  
+ TGACCTTATT TACAAAGGCT GAGCCATCTT TTAATTTTTT TAGCCTCTTT TTTTAATCAA AAATAGAAAT   
  
  
+ TTCAAGAAAG CAGAAATCGT CAGATGACAG TGCAGACAGC GTTTTCGTTT CATACAAATC TCAAAAGCTG   
  
  
+ AAGTGTCAAT TTCTTCGATT ATTTTTGTCA GAAAATATTT ACGTCTCACC TTCACGTTGT TATTATTCTT   
  
  
+ TCTTTTTTAT TATTCTCATA ACTAGTAATA AAGTTAGCAA TGATAAAAAA TTATTCGATC CGAATATTTT   
  
  
+ AATTTATCTG ACCTAAAAAC ATAAGTAAAG GCACAAATTT TTCATCCAAA TTTTAATTTT TGATGTAATA   
  
  
+ TTTTTTATAT TTTTATTGTT CAAATCTGAT TTTAATCTTA TTTAAATTAT CTGACCTAAA AAAATCAAAT   
  
  
+ AATAATAAAC GTTAATTTTT TATTTAAATT TTGACATTAG TCAATCTGAC TTAAATTCGA ACCGAGCTTG   
  
  
+ AATTTTTTTG CCAGTGCTGG TAGTACTCCT GCTTTTGTAG CTCCCAATGC CATCGTACCA CCCTCTTCTA   
  
  
+ GTCTCGTCTC TTTCTCTGGT CTCCAATTAA TCACACCATC ATATCATACC GTATGATGAT ACAGTCCACT   
  
  
+ GAAATCCCAT TCTCACCGCC AATCTCTCTC CGCCACCATC CAACACTCTT AACGATGAAC AGGGCCGCCG   
  
  
+ CCTCGTCCTC CACACTCAGG CCGTGGCCGG GCAGCTTTCC CACCCAATCA AAATCTCTCT CCTCCGCCAA   
  
  
+ CTTCGGTAAC GCCAATTGCA TGGAGCAGCT CTTAGTCCAC TGCGCCGAAG CCATCGACAA CAATGACGCC   
  
  
+ ACCCCGGCCC AGCAAATCTT ATGGGTCCTG AATAACATAG CCCGACCCGA CGGCGACTCC ACCCAACGCC   
  
  
+ TCACGTGCGC ATTCCTACGT GCCTTAATCT CACGCGCCGT CCTCACTAGC ACCTGCAAGA TGGTAATCCC   
  
  
+ TCATTTCAAC CCCATCAATT CACCCCACAA ATTCTCGCTC CTCGAACTTG CCCACTTCGT CGATTTAACC   
  
  
+ CCTTGGCATC GATTCGGATT CACCGCCGCC AATTCGATCA TTCTGGAAGC TATTTCCGAC CTACCCGTTG   
  
  
+ TACACATTGT CGACCTCAGC ATCTCCCACT GTATGCAGAT CCCCACGTTG ATCGACTCCA TTGCGACCCG   
  
  
+ GTTGGAAGCC CCGGGTCGAG TCCCTCCTAT TGTCAAGCTC ACCGTCGGGG CTATTTCCGA CGAAATCCCG   
  
  
+ CCGGTGTTCG ATCTTCTGTC GTACGATGAG CTCGGAATGA GACTAATCAA CTTCGCTCGT TTTAGAAACA   
  
  
+ TCGTCCTCGA ATTCCAAGCA ATACCCACCT CCCCTTCCGA CGGATTTGCT TCGCTGTTGG AGGAGATTCG   
  
  
+ ACAAAGCAAG CTCTACTCCA ACGATGCGGC GGCGGTTATT GTGAATTGTC AGATGAGTTT GCATTTATTG   
  
  
+ CAGGAAGAGG AGGTGTCTTC ATCGTCGCCG TCGTCGATGA GGGGGATGTT TTTGCAGGCG GTGAGGAGCT   
  
  
+ TGGAGCCGAG CATGGTGGTG GTGGTGGAGG AGGACGTGGA TTTCACGGCG AGGAGTCTGG TGGGGCGGCT   
  
  
+ GAGATCGGCG TTTAATCACA TGTGGATACC CTTCGACACG GTGGACACGT TCTTGCCACG TGGGAGCCAG   
  
  
+ CAGAGAGAGT GGTTCGAGGC CGAGGTGTGC TGGAAGATTG AGAATGTGAT CGCTCATGAG GGACCCGCGA   
  
  
+ GGGTCGAGAG GCAGGAGCCC AGGGCCAAGT GGGCCCTCCG AATGAGGGAG GCCGAGTTTC AAGGGATCGA   
  
  
+ GTTCGGTGAT GAAGGTACGA CCGAGGTCAA GGCCATGCTG GAGGAGCATG CCGCTGGGTG GGGGTCTAAG   
  
  
+ AAGGAAGAGG ATGATCTTGT GCTCACTTGG AAGGGACATA GTGTTGTCTT TGCTTCTGCT TGGGTACCCA   
  
  
+ CTTA  

- +Up\_Stream \_Len000TCTTCG ATTAATTTGG TTTGTACGTG CATACAGGAT TAATTGTACT GACAGGTAGT   
  
  
- AGAGGAGGGT ACAACAAGAC AACTGTTCGG ACGTGTGGGT ACGAGGAGAG AGTACAGATT GGAGCAGGGT   
  
  
- TGCTAGTTCT AGTGACAGAC ACTCCGGTCC CCTTCTGAAC GAAAATAAAG GAAAAGAAAA CAAATGAGAG   
  
  
- ACTAAGGTAA ATCGGTATAT ATGTTCCTCT TACAGTACAC ATAGAGTATA CATATATATA TTCTAAAATA   
  
  
- AATCTTTATT TTTAAATTTG TGATACACTA AATATCAATA ATATTATTTA TCATAAAAAA TTTGATAAAG   
  
  
- TAACTACTCT TGTCCTTTCA TTTAACGGCT GTACAATTAC AATAATATAG ATTTTTAATT TAGTATTTTA   
  
  
- ATTAATATTA TAAAATTTTT ATAAAGTTGA TTTTAAAATC TTGTTATTCA CGCATTATGC CTGAATTTCC   
  
  
- GATCAAATAG CATTTTTACA TTCAAGGAAA ACCTAGAATA CCAACTTATA GAACCAACTA TTTGACACTA   
  
  
- ACCAAAATGT GAAAATGGTG TATAAGTATC TTCTTAATAC ACGTGCAACT ACCAAGTTCT ACCCTGTTTT   
  
  
- CCTTTGTACA TAAGAAGGGG GAAAACGGAA AAGGGGATTC GTAAACCTAA CTCCACAACA GTAGACTTAT   
  
  
- AGTTTTTAAG AAAAACTTTT AAGTCAGAGT TAGTTTTAAG TTTTAATTTT GTTTCTGAAT TCTCAAAATA   
  
  
- AACAAGAATT GGAAGAATTG AAAGATGAAA AGAAAAAAAA GAGTAAGGGG GAGAAGTTTA GAGTAAATAA   
  
  
- GAATTGGGGG AGAAGGCAAG TACGAGAGAG ACAGATAGTT GATTATAATT AGATGGGGCT GAAAGTTAAA   
  
  
- CTACTCCAGA TTAGGAATAG TAGCATAACA CCAACCCAAT TAATACGTTC TTCCGTTATT TGGGGTCACT   
  
  
- GTTTCAGGTG CAATTCATCC GTGGAGTGGT AATTCTGTAC GAGTCTTTTG TGGTTTTGGT AACTTGTGTT   
  
  
- CAGGGGGTTC AGGGAAGGGA TCGATCGGGT AAGAGAGAGG AGGGGACAGA TATAGATGGT TGAGAACGAG   
  
  
- AAACGACTCG GACTCAACTT TCGCTATGTG GGTATCGGAC AAGTCAAAAG TCAAAAGTGT AAACAGAAGG   
  
  
- AGACAAGTCA ACTTTCGCTA TGTGAGAGTA ATCGAAAGTA AAATTTATGC AAGTTAAGTG TGTATTTACC   
  
  
- GAAAGTTTCG TAACGGTAAA AGCAAATTTA CTACTGTTTT ATATATCATA GACCGATAGA CGGGTATAGA   
  
  
- ACTGGAATAA ATGTTTCCGA CTCGGTAGAA AATTAAAAAA ATCGGAGAAA AAAATTAGTT TTTATCTTTA   
  
  
- AAGTTCTTTC GTCTTTAGCA GTCTACTGTC ACGTCTGTCG CAAAAGCAAA GTATGTTTAG AGTTTTCGAC   
  
  
- TTCACAGTTA AAGAAGCTAA TAAAAACAGT CTTTTATAAA TGCAGAGTGG AAGTGCAACA ATAATAAGAA   
  
  
- AGAAAAAATA ATAAGAGTAT TGATCATTAT TTCAATCGTT ACTATTTTTT AATAAGCTAG GCTTATAAAA   
  
  
- TTAAATAGAC TGGATTTTTG TATTCATTTC CGTGTTTAAA AAGTAGGTTT AAAATTAAAA ACTACATTAT   
  
  
- AAAAAATATA AAAATAACAA GTTTAGACTA AAATTAGAAT AAATTTAATA GACTGGATTT TTTTAGTTTA   
  
  
- TTATTATTTG CAATTAAAAA ATAAATTTAA AACTGTAATC AGTTAGACTG AATTTAAGCT TGGCTCGAAC   
  
  
- TTAAAAAAAC GGTCACGACC ATCATGAGGA CGAAAACATC GAGGGTTACG GTAGCATGGT GGGAGAAGAT   
  
  
- CAGAGCAGAG AAAGAGACCA GAGGTTAATT AGTGTGGTAG TATAGTATGG CATACTACTA TGTCAGGTGA   
  
  
- CTTTAGGGTA AGAGTGGCGG TTAGAGAGAG GCGGTGGTAG GTTGTGAGAA TTGCTACTTG TCCCGGCGGC   
  
  
- GGAGCAGGAG GTGTGAGTCC GGCACCGGCC CGTCGAAAGG GTGGGTTAGT TTTAGAGAGA GGAGGCGGTT   
  
  
- GAAGCCATTG CGGTTAACGT ACCTCGTCGA GAATCAGGTG ACGCGGCTTC GGTAGCTGTT GTTACTGCGG   
  
  
- TGGGGCCGGG TCGTTTAGAA TACCCAGGAC TTATTGTATC GGGCTGGGCT GCCGCTGAGG TGGGTTGCGG   
  
  
- AGTGCACGCG TAAGGATGCA CGGAATTAGA GTGCGCGGCA GGAGTGATCG TGGACGTTCT ACCATTAGGG   
  
  
- AGTAAAGTTG GGGTAGTTAA GTGGGGTGTT TAAGAGCGAG GAGCTTGAAC GGGTGAAGCA GCTAAATTGG   
  
  
- GGAACCGTAG CTAAGCCTAA GTGGCGGCGG TTAAGCTAGT AAGACCTTCG ATAAAGGCTG GATGGGCAAC   
  
  
- ATGTGTAACA GCTGGAGTCG TAGAGGGTGA CATACGTCTA GGGGTGCAAC TAGCTGAGGT AACGCTGGGC   
  
  
- CAACCTTCGG GGCCCAGCTC AGGGAGGATA ACAGTTCGAG TGGCAGCCCC GATAAAGGCT GCTTTAGGGC   
  
  
- GGCCACAAGC TAGAAGACAG CATGCTACTC GAGCCTTACT CTGATTAGTT GAAGCGAGCA AAATCTTTGT   
  
  
- AGCAGGAGCT TAAGGTTCGT TATGGGTGGA GGGGAAGGCT GCCTAAACGA AGCGACAACC TCCTCTAAGC   
  
  
- TGTTTCGTTC GAGATGAGGT TGCTACGCCG CCGCCAATAA CACTTAACAG TCTACTCAAA CGTAAATAAC   
  
  
- GTCCTTCTCC TCCACAGAAG TAGCAGCGGC AGCAGCTACT CCCCCTACAA AAACGTCCGC CACTCCTCGA   
  
  
- ACCTCGGCTC GTACCACCAC CACCACCTCC TCCTGCACCT AAAGTGCCGC TCCTCAGACC ACCCCGCCGA   
  
  
- CTCTAGCCGC AAATTAGTGT ACACCTATGG GAAGCTGTGC CACCTGTGCA AGAACGGTGC ACCCTCGGTC   
  
  
- GTCTCTCTCA CCAAGCTCCG GCTCCACACG ACCTTCTAAC TCTTACACTA GCGAGTACTC CCTGGGCGCT   
  
  
- CCCAGCTCTC CGTCCTCGGG TCCCGGTTCA CCCGGGAGGC TTACTCCCTC CGGCTCAAAG TTCCCTAGCT   
  
  
- CAAGCCACTA CTTCCATGCT GGCTCCAGTT CCGGTACGAC CTCCTCGTAC GGCGACCCAC CCCCAGATTC   
  
  
- TTCCTTCTCC TACTAGAACA CGAGTGAACC TTCCCTGTAT CACAACAGAA ACGAAGACGA ACCCATGGGT   
  
  
- GAAT

+     TGACG-motif

| Site Name | Organism | Position | Strand | Matrix score. | sequence | function |
| --- | --- | --- | --- | --- | --- | --- |
| TGACG-motif | Hordeum vulgare | 1422 | - | 5 | TGACG | cis-acting regulatory element involved in the MeJA-responsiveness |
| TGACG-motif | Hordeum vulgare | 2168 | + | 5 | TGACG | cis-acting regulatory element involved in the MeJA-responsiveness |

>HU06G00376.1   
+ +Up\_Stream \_Len000AGAAGC TAATTAAACC AAACATGCAC GTATGTCCTA ATTAACATGA CTGTCCATCA   
  
  
+ TCTCCTCCCA TGTTGTTCTG TTGACAAGCC TGCACACCCA TGCTCCTCTC TCATGTCTAA CCTCGTCCCA   
  
  
+ ACGATCAAGA TCACTGTCTG TGAGGCCAGG GGAAGACTTG CTTTTATTTC CTTTTCTTTT GTTTACTCTC   
  
  
+ TGATTCCATT TAGCCATATA TACAAGGAGA ATGTCATGTG TATCTCATAT GTATATATAT AAGATTTTAT   
  
  
+ TTAGAAATAA AAATTTAAAC ACTATGTGAT TTATAGTTAT TATAATAAAT AGTATTTTTT AAACTATTTC   
  
  
+ ATTGATGAGA ACAGGAAAGT AAATTGCCGA CATGTTAATG TTATTATATC TAAAAATTAA ATCATAAAAT   
  
  
+ TAATTATAAT ATTTTAAAAA TATTTCAACT AAAATTTTAG AACAATAAGT GCGTAATACG GACTTAAAGG   
  
  
+ CTAGTTTATC GTAAAAATGT AAGTTCCTTT TGGATCTTAT GGTTGAATAT CTTGGTTGAT AAACTGTGAT   
  
  
+ TGGTTTTACA CTTTTACCAC ATATTCATAG AAGAATTATG TGCACGTTGA TGGTTCAAGA TGGGACAAAA   
  
  
+ GGAAACATGT ATTCTTCCCC CTTTTGCCTT TTCCCCTAAG CATTTGGATT GAGGTGTTGT CATCTGAATA   
  
  
+ TCAAAAATTC TTTTTGAAAA TTCAGTCTCA ATCAAAATTC AAAATTAAAA CAAAGACTTA AGAGTTTTAT   
  
  
+ TTGTTCTTAA CCTTCTTAAC TTTCTACTTT TCTTTTTTTT CTCATTCCCC CTCTTCAAAT CTCATTTATT   
  
  
+ CTTAACCCCC TCTTCCGTTC ATGCTCTCTC TGTCTATCAA CTAATATTAA TCTACCCCGA CTTTCAATTT   
  
  
+ GATGAGGTCT AATCCTTATC ATCGTATTGT GGTTGGGTTA ATTATGCAAG AAGGCAATAA ACCCCAGTGA   
  
  
+ CAAAGTCCAC GTTAAGTAGG CACCTCACCA TTAAGACATG CTCAGAAAAC ACCAAAACCA TTGAACACAA   
  
  
+ GTCCCCCAAG TCCCTTCCCT AGCTAGCCCA TTCTCTCTCC TCCCCTGTCT ATATCTACCA ACTCTTGCTC   
  
  
+ TTTGCTGAGC CTGAGTTGAA AGCGATACAC CCATAGCCTG TTCAGTTTTC AGTTTTCACA TTTGTCTTCC   
  
  
+ TCTGTTCAGT TGAAAGCGAT ACACTCTCAT TAGCTTTCAT TTTAAATACG TTCAATTCAC ACATAAATGG   
  
  
+ CTTTCAAAGC ATTGCCATTT TCGTTTAAAT GATGACAAAA TATATAGTAT CTGGCTATCT GCCCATATCT   
  
  
+ TGACCTTATT TACAAAGGCT GAGCCATCTT TTAATTTTTT TAGCCTCTTT TTTTAATCAA AAATAGAAAT   
  
  
+ TTCAAGAAAG CAGAAATCGT CAGATGACAG TGCAGACAGC GTTTTCGTTT CATACAAATC TCAAAAGCTG   
  
  
+ AAGTGTCAAT TTCTTCGATT ATTTTTGTCA GAAAATATTT ACGTCTCACC TTCACGTTGT TATTATTCTT   
  
  
+ TCTTTTTTAT TATTCTCATA ACTAGTAATA AAGTTAGCAA TGATAAAAAA TTATTCGATC CGAATATTTT   
  
  
+ AATTTATCTG ACCTAAAAAC ATAAGTAAAG GCACAAATTT TTCATCCAAA TTTTAATTTT TGATGTAATA   
  
  
+ TTTTTTATAT TTTTATTGTT CAAATCTGAT TTTAATCTTA TTTAAATTAT CTGACCTAAA AAAATCAAAT   
  
  
+ AATAATAAAC GTTAATTTTT TATTTAAATT TTGACATTAG TCAATCTGAC TTAAATTCGA ACCGAGCTTG   
  
  
+ AATTTTTTTG CCAGTGCTGG TAGTACTCCT GCTTTTGTAG CTCCCAATGC CATCGTACCA CCCTCTTCTA   
  
  
+ GTCTCGTCTC TTTCTCTGGT CTCCAATTAA TCACACCATC ATATCATACC GTATGATGAT ACAGTCCACT   
  
  
+ GAAATCCCAT TCTCACCGCC AATCTCTCTC CGCCACCATC CAACACTCTT AACGATGAAC AGGGCCGCCG   
  
  
+ CCTCGTCCTC CACACTCAGG CCGTGGCCGG GCAGCTTTCC CACCCAATCA AAATCTCTCT CCTCCGCCAA   
  
  
+ CTTCGGTAAC GCCAATTGCA TGGAGCAGCT CTTAGTCCAC TGCGCCGAAG CCATCGACAA CAATGACGCC   
  
  
+ ACCCCGGCCC AGCAAATCTT ATGGGTCCTG AATAACATAG CCCGACCCGA CGGCGACTCC ACCCAACGCC   
  
  
+ TCACGTGCGC ATTCCTACGT GCCTTAATCT CACGCGCCGT CCTCACTAGC ACCTGCAAGA TGGTAATCCC   
  
  
+ TCATTTCAAC CCCATCAATT CACCCCACAA ATTCTCGCTC CTCGAACTTG CCCACTTCGT CGATTTAACC   
  
  
+ CCTTGGCATC GATTCGGATT CACCGCCGCC AATTCGATCA TTCTGGAAGC TATTTCCGAC CTACCCGTTG   
  
  
+ TACACATTGT CGACCTCAGC ATCTCCCACT GTATGCAGAT CCCCACGTTG ATCGACTCCA TTGCGACCCG   
  
  
+ GTTGGAAGCC CCGGGTCGAG TCCCTCCTAT TGTCAAGCTC ACCGTCGGGG CTATTTCCGA CGAAATCCCG   
  
  
+ CCGGTGTTCG ATCTTCTGTC GTACGATGAG CTCGGAATGA GACTAATCAA CTTCGCTCGT TTTAGAAACA   
  
  
+ TCGTCCTCGA ATTCCAAGCA ATACCCACCT CCCCTTCCGA CGGATTTGCT TCGCTGTTGG AGGAGATTCG   
  
  
+ ACAAAGCAAG CTCTACTCCA ACGATGCGGC GGCGGTTATT GTGAATTGTC AGATGAGTTT GCATTTATTG   
  
  
+ CAGGAAGAGG AGGTGTCTTC ATCGTCGCCG TCGTCGATGA GGGGGATGTT TTTGCAGGCG GTGAGGAGCT   
  
  
+ TGGAGCCGAG CATGGTGGTG GTGGTGGAGG AGGACGTGGA TTTCACGGCG AGGAGTCTGG TGGGGCGGCT   
  
  
+ GAGATCGGCG TTTAATCACA TGTGGATACC CTTCGACACG GTGGACACGT TCTTGCCACG TGGGAGCCAG   
  
  
+ CAGAGAGAGT GGTTCGAGGC CGAGGTGTGC TGGAAGATTG AGAATGTGAT CGCTCATGAG GGACCCGCGA   
  
  
+ GGGTCGAGAG GCAGGAGCCC AGGGCCAAGT GGGCCCTCCG AATGAGGGAG GCCGAGTTTC AAGGGATCGA   
  
  
+ GTTCGGTGAT GAAGGTACGA CCGAGGTCAA GGCCATGCTG GAGGAGCATG CCGCTGGGTG GGGGTCTAAG   
  
  
+ AAGGAAGAGG ATGATCTTGT GCTCACTTGG AAGGGACATA GTGTTGTCTT TGCTTCTGCT TGGGTACCCA   
  
  
+ CTTA  

- +Up\_Stream \_Len000TCTTCG ATTAATTTGG TTTGTACGTG CATACAGGAT TAATTGTACT GACAGGTAGT   
  
  
- AGAGGAGGGT ACAACAAGAC AACTGTTCGG ACGTGTGGGT ACGAGGAGAG AGTACAGATT GGAGCAGGGT   
  
  
- TGCTAGTTCT AGTGACAGAC ACTCCGGTCC CCTTCTGAAC GAAAATAAAG GAAAAGAAAA CAAATGAGAG   
  
  
- ACTAAGGTAA ATCGGTATAT ATGTTCCTCT TACAGTACAC ATAGAGTATA CATATATATA TTCTAAAATA   
  
  
- AATCTTTATT TTTAAATTTG TGATACACTA AATATCAATA ATATTATTTA TCATAAAAAA TTTGATAAAG   
  
  
- TAACTACTCT TGTCCTTTCA TTTAACGGCT GTACAATTAC AATAATATAG ATTTTTAATT TAGTATTTTA   
  
  
- ATTAATATTA TAAAATTTTT ATAAAGTTGA TTTTAAAATC TTGTTATTCA CGCATTATGC CTGAATTTCC   
  
  
- GATCAAATAG CATTTTTACA TTCAAGGAAA ACCTAGAATA CCAACTTATA GAACCAACTA TTTGACACTA   
  
  
- ACCAAAATGT GAAAATGGTG TATAAGTATC TTCTTAATAC ACGTGCAACT ACCAAGTTCT ACCCTGTTTT   
  
  
- CCTTTGTACA TAAGAAGGGG GAAAACGGAA AAGGGGATTC GTAAACCTAA CTCCACAACA GTAGACTTAT   
  
  
- AGTTTTTAAG AAAAACTTTT AAGTCAGAGT TAGTTTTAAG TTTTAATTTT GTTTCTGAAT TCTCAAAATA   
  
  
- AACAAGAATT GGAAGAATTG AAAGATGAAA AGAAAAAAAA GAGTAAGGGG GAGAAGTTTA GAGTAAATAA   
  
  
- GAATTGGGGG AGAAGGCAAG TACGAGAGAG ACAGATAGTT GATTATAATT AGATGGGGCT GAAAGTTAAA   
  
  
- CTACTCCAGA TTAGGAATAG TAGCATAACA CCAACCCAAT TAATACGTTC TTCCGTTATT TGGGGTCACT   
  
  
- GTTTCAGGTG CAATTCATCC GTGGAGTGGT AATTCTGTAC GAGTCTTTTG TGGTTTTGGT AACTTGTGTT   
  
  
- CAGGGGGTTC AGGGAAGGGA TCGATCGGGT AAGAGAGAGG AGGGGACAGA TATAGATGGT TGAGAACGAG   
  
  
- AAACGACTCG GACTCAACTT TCGCTATGTG GGTATCGGAC AAGTCAAAAG TCAAAAGTGT AAACAGAAGG   
  
  
- AGACAAGTCA ACTTTCGCTA TGTGAGAGTA ATCGAAAGTA AAATTTATGC AAGTTAAGTG TGTATTTACC   
  
  
- GAAAGTTTCG TAACGGTAAA AGCAAATTTA CTACTGTTTT ATATATCATA GACCGATAGA CGGGTATAGA   
  
  
- ACTGGAATAA ATGTTTCCGA CTCGGTAGAA AATTAAAAAA ATCGGAGAAA AAAATTAGTT TTTATCTTTA   
  
  
- AAGTTCTTTC GTCTTTAGCA GTCTACTGTC ACGTCTGTCG CAAAAGCAAA GTATGTTTAG AGTTTTCGAC   
  
  
- TTCACAGTTA AAGAAGCTAA TAAAAACAGT CTTTTATAAA TGCAGAGTGG AAGTGCAACA ATAATAAGAA   
  
  
- AGAAAAAATA ATAAGAGTAT TGATCATTAT TTCAATCGTT ACTATTTTTT AATAAGCTAG GCTTATAAAA   
  
  
- TTAAATAGAC TGGATTTTTG TATTCATTTC CGTGTTTAAA AAGTAGGTTT AAAATTAAAA ACTACATTAT   
  
  
- AAAAAATATA AAAATAACAA GTTTAGACTA AAATTAGAAT AAATTTAATA GACTGGATTT TTTTAGTTTA   
  
  
- TTATTATTTG CAATTAAAAA ATAAATTTAA AACTGTAATC AGTTAGACTG AATTTAAGCT TGGCTCGAAC   
  
  
- TTAAAAAAAC GGTCACGACC ATCATGAGGA CGAAAACATC GAGGGTTACG GTAGCATGGT GGGAGAAGAT   
  
  
- CAGAGCAGAG AAAGAGACCA GAGGTTAATT AGTGTGGTAG TATAGTATGG CATACTACTA TGTCAGGTGA   
  
  
- CTTTAGGGTA AGAGTGGCGG TTAGAGAGAG GCGGTGGTAG GTTGTGAGAA TTGCTACTTG TCCCGGCGGC   
  
  
- GGAGCAGGAG GTGTGAGTCC GGCACCGGCC CGTCGAAAGG GTGGGTTAGT TTTAGAGAGA GGAGGCGGTT   
  
  
- GAAGCCATTG CGGTTAACGT ACCTCGTCGA GAATCAGGTG ACGCGGCTTC GGTAGCTGTT GTTACTGCGG   
  
  
- TGGGGCCGGG TCGTTTAGAA TACCCAGGAC TTATTGTATC GGGCTGGGCT GCCGCTGAGG TGGGTTGCGG   
  
  
- AGTGCACGCG TAAGGATGCA CGGAATTAGA GTGCGCGGCA GGAGTGATCG TGGACGTTCT ACCATTAGGG   
  
  
- AGTAAAGTTG GGGTAGTTAA GTGGGGTGTT TAAGAGCGAG GAGCTTGAAC GGGTGAAGCA GCTAAATTGG   
  
  
- GGAACCGTAG CTAAGCCTAA GTGGCGGCGG TTAAGCTAGT AAGACCTTCG ATAAAGGCTG GATGGGCAAC   
  
  
- ATGTGTAACA GCTGGAGTCG TAGAGGGTGA CATACGTCTA GGGGTGCAAC TAGCTGAGGT AACGCTGGGC   
  
  
- CAACCTTCGG GGCCCAGCTC AGGGAGGATA ACAGTTCGAG TGGCAGCCCC GATAAAGGCT GCTTTAGGGC   
  
  
- GGCCACAAGC TAGAAGACAG CATGCTACTC GAGCCTTACT CTGATTAGTT GAAGCGAGCA AAATCTTTGT   
  
  
- AGCAGGAGCT TAAGGTTCGT TATGGGTGGA GGGGAAGGCT GCCTAAACGA AGCGACAACC TCCTCTAAGC   
  
  
- TGTTTCGTTC GAGATGAGGT TGCTACGCCG CCGCCAATAA CACTTAACAG TCTACTCAAA CGTAAATAAC   
  
  
- GTCCTTCTCC TCCACAGAAG TAGCAGCGGC AGCAGCTACT CCCCCTACAA AAACGTCCGC CACTCCTCGA   
  
  
- ACCTCGGCTC GTACCACCAC CACCACCTCC TCCTGCACCT AAAGTGCCGC TCCTCAGACC ACCCCGCCGA   
  
  
- CTCTAGCCGC AAATTAGTGT ACACCTATGG GAAGCTGTGC CACCTGTGCA AGAACGGTGC ACCCTCGGTC   
  
  
- GTCTCTCTCA CCAAGCTCCG GCTCCACACG ACCTTCTAAC TCTTACACTA GCGAGTACTC CCTGGGCGCT   
  
  
- CCCAGCTCTC CGTCCTCGGG TCCCGGTTCA CCCGGGAGGC TTACTCCCTC CGGCTCAAAG TTCCCTAGCT   
  
  
- CAAGCCACTA CTTCCATGCT GGCTCCAGTT CCGGTACGAC CTCCTCGTAC GGCGACCCAC CCCCAGATTC   
  
  
- TTCCTTCTCC TACTAGAACA CGAGTGAACC TTCCCTGTAT CACAACAGAA ACGAAGACGA ACCCATGGGT   
  
  
- GAAT

+     Unnamed\_\_1

| Site Name | Organism | Position | Strand | Matrix score. | sequence | function |
| --- | --- | --- | --- | --- | --- | --- |
| Unnamed\_\_1 | Petunia sp. | 2999 | - | 9 | GCCACGTGGC |  |
| Unnamed\_\_1 | Zea mays | 2056 | + | 5 | CGTGG |  |
| Unnamed\_\_1 | Zea mays | 2909 | + | 5 | CGTGG |  |
| Unnamed\_\_1 | Zea mays | 3003 | + | 5 | CGTGG |  |
| Unnamed\_\_1 | Zea mays | 3000 | - | 5 | CGTGG |  |
| Unnamed\_\_1 | Zea mays | 991 | - | 5 | CGTGG |  |
| Unnamed\_\_1 | Zea mays | 2497 | - | 5 | CGTGG |  |

>HU06G00376.1   
+ +Up\_Stream \_Len000AGAAGC TAATTAAACC AAACATGCAC GTATGTCCTA ATTAACATGA CTGTCCATCA   
  
  
+ TCTCCTCCCA TGTTGTTCTG TTGACAAGCC TGCACACCCA TGCTCCTCTC TCATGTCTAA CCTCGTCCCA   
  
  
+ ACGATCAAGA TCACTGTCTG TGAGGCCAGG GGAAGACTTG CTTTTATTTC CTTTTCTTTT GTTTACTCTC   
  
  
+ TGATTCCATT TAGCCATATA TACAAGGAGA ATGTCATGTG TATCTCATAT GTATATATAT AAGATTTTAT   
  
  
+ TTAGAAATAA AAATTTAAAC ACTATGTGAT TTATAGTTAT TATAATAAAT AGTATTTTTT AAACTATTTC   
  
  
+ ATTGATGAGA ACAGGAAAGT AAATTGCCGA CATGTTAATG TTATTATATC TAAAAATTAA ATCATAAAAT   
  
  
+ TAATTATAAT ATTTTAAAAA TATTTCAACT AAAATTTTAG AACAATAAGT GCGTAATACG GACTTAAAGG   
  
  
+ CTAGTTTATC GTAAAAATGT AAGTTCCTTT TGGATCTTAT GGTTGAATAT CTTGGTTGAT AAACTGTGAT   
  
  
+ TGGTTTTACA CTTTTACCAC ATATTCATAG AAGAATTATG TGCACGTTGA TGGTTCAAGA TGGGACAAAA   
  
  
+ GGAAACATGT ATTCTTCCCC CTTTTGCCTT TTCCCCTAAG CATTTGGATT GAGGTGTTGT CATCTGAATA   
  
  
+ TCAAAAATTC TTTTTGAAAA TTCAGTCTCA ATCAAAATTC AAAATTAAAA CAAAGACTTA AGAGTTTTAT   
  
  
+ TTGTTCTTAA CCTTCTTAAC TTTCTACTTT TCTTTTTTTT CTCATTCCCC CTCTTCAAAT CTCATTTATT   
  
  
+ CTTAACCCCC TCTTCCGTTC ATGCTCTCTC TGTCTATCAA CTAATATTAA TCTACCCCGA CTTTCAATTT   
  
  
+ GATGAGGTCT AATCCTTATC ATCGTATTGT GGTTGGGTTA ATTATGCAAG AAGGCAATAA ACCCCAGTGA   
  
  
+ CAAAGTCCAC GTTAAGTAGG CACCTCACCA TTAAGACATG CTCAGAAAAC ACCAAAACCA TTGAACACAA   
  
  
+ GTCCCCCAAG TCCCTTCCCT AGCTAGCCCA TTCTCTCTCC TCCCCTGTCT ATATCTACCA ACTCTTGCTC   
  
  
+ TTTGCTGAGC CTGAGTTGAA AGCGATACAC CCATAGCCTG TTCAGTTTTC AGTTTTCACA TTTGTCTTCC   
  
  
+ TCTGTTCAGT TGAAAGCGAT ACACTCTCAT TAGCTTTCAT TTTAAATACG TTCAATTCAC ACATAAATGG   
  
  
+ CTTTCAAAGC ATTGCCATTT TCGTTTAAAT GATGACAAAA TATATAGTAT CTGGCTATCT GCCCATATCT   
  
  
+ TGACCTTATT TACAAAGGCT GAGCCATCTT TTAATTTTTT TAGCCTCTTT TTTTAATCAA AAATAGAAAT   
  
  
+ TTCAAGAAAG CAGAAATCGT CAGATGACAG TGCAGACAGC GTTTTCGTTT CATACAAATC TCAAAAGCTG   
  
  
+ AAGTGTCAAT TTCTTCGATT ATTTTTGTCA GAAAATATTT ACGTCTCACC TTCACGTTGT TATTATTCTT   
  
  
+ TCTTTTTTAT TATTCTCATA ACTAGTAATA AAGTTAGCAA TGATAAAAAA TTATTCGATC CGAATATTTT   
  
  
+ AATTTATCTG ACCTAAAAAC ATAAGTAAAG GCACAAATTT TTCATCCAAA TTTTAATTTT TGATGTAATA   
  
  
+ TTTTTTATAT TTTTATTGTT CAAATCTGAT TTTAATCTTA TTTAAATTAT CTGACCTAAA AAAATCAAAT   
  
  
+ AATAATAAAC GTTAATTTTT TATTTAAATT TTGACATTAG TCAATCTGAC TTAAATTCGA ACCGAGCTTG   
  
  
+ AATTTTTTTG CCAGTGCTGG TAGTACTCCT GCTTTTGTAG CTCCCAATGC CATCGTACCA CCCTCTTCTA   
  
  
+ GTCTCGTCTC TTTCTCTGGT CTCCAATTAA TCACACCATC ATATCATACC GTATGATGAT ACAGTCCACT   
  
  
+ GAAATCCCAT TCTCACCGCC AATCTCTCTC CGCCACCATC CAACACTCTT AACGATGAAC AGGGCCGCCG   
  
  
+ CCTCGTCCTC CACACTCAGG CCGTGGCCGG GCAGCTTTCC CACCCAATCA AAATCTCTCT CCTCCGCCAA   
  
  
+ CTTCGGTAAC GCCAATTGCA TGGAGCAGCT CTTAGTCCAC TGCGCCGAAG CCATCGACAA CAATGACGCC   
  
  
+ ACCCCGGCCC AGCAAATCTT ATGGGTCCTG AATAACATAG CCCGACCCGA CGGCGACTCC ACCCAACGCC   
  
  
+ TCACGTGCGC ATTCCTACGT GCCTTAATCT CACGCGCCGT CCTCACTAGC ACCTGCAAGA TGGTAATCCC   
  
  
+ TCATTTCAAC CCCATCAATT CACCCCACAA ATTCTCGCTC CTCGAACTTG CCCACTTCGT CGATTTAACC   
  
  
+ CCTTGGCATC GATTCGGATT CACCGCCGCC AATTCGATCA TTCTGGAAGC TATTTCCGAC CTACCCGTTG   
  
  
+ TACACATTGT CGACCTCAGC ATCTCCCACT GTATGCAGAT CCCCACGTTG ATCGACTCCA TTGCGACCCG   
  
  
+ GTTGGAAGCC CCGGGTCGAG TCCCTCCTAT TGTCAAGCTC ACCGTCGGGG CTATTTCCGA CGAAATCCCG   
  
  
+ CCGGTGTTCG ATCTTCTGTC GTACGATGAG CTCGGAATGA GACTAATCAA CTTCGCTCGT TTTAGAAACA   
  
  
+ TCGTCCTCGA ATTCCAAGCA ATACCCACCT CCCCTTCCGA CGGATTTGCT TCGCTGTTGG AGGAGATTCG   
  
  
+ ACAAAGCAAG CTCTACTCCA ACGATGCGGC GGCGGTTATT GTGAATTGTC AGATGAGTTT GCATTTATTG   
  
  
+ CAGGAAGAGG AGGTGTCTTC ATCGTCGCCG TCGTCGATGA GGGGGATGTT TTTGCAGGCG GTGAGGAGCT   
  
  
+ TGGAGCCGAG CATGGTGGTG GTGGTGGAGG AGGACGTGGA TTTCACGGCG AGGAGTCTGG TGGGGCGGCT   
  
  
+ GAGATCGGCG TTTAATCACA TGTGGATACC CTTCGACACG GTGGACACGT TCTTGCCACG TGGGAGCCAG   
  
  
+ CAGAGAGAGT GGTTCGAGGC CGAGGTGTGC TGGAAGATTG AGAATGTGAT CGCTCATGAG GGACCCGCGA   
  
  
+ GGGTCGAGAG GCAGGAGCCC AGGGCCAAGT GGGCCCTCCG AATGAGGGAG GCCGAGTTTC AAGGGATCGA   
  
  
+ GTTCGGTGAT GAAGGTACGA CCGAGGTCAA GGCCATGCTG GAGGAGCATG CCGCTGGGTG GGGGTCTAAG   
  
  
+ AAGGAAGAGG ATGATCTTGT GCTCACTTGG AAGGGACATA GTGTTGTCTT TGCTTCTGCT TGGGTACCCA   
  
  
+ CTTA  

- +Up\_Stream \_Len000TCTTCG ATTAATTTGG TTTGTACGTG CATACAGGAT TAATTGTACT GACAGGTAGT   
  
  
- AGAGGAGGGT ACAACAAGAC AACTGTTCGG ACGTGTGGGT ACGAGGAGAG AGTACAGATT GGAGCAGGGT   
  
  
- TGCTAGTTCT AGTGACAGAC ACTCCGGTCC CCTTCTGAAC GAAAATAAAG GAAAAGAAAA CAAATGAGAG   
  
  
- ACTAAGGTAA ATCGGTATAT ATGTTCCTCT TACAGTACAC ATAGAGTATA CATATATATA TTCTAAAATA   
  
  
- AATCTTTATT TTTAAATTTG TGATACACTA AATATCAATA ATATTATTTA TCATAAAAAA TTTGATAAAG   
  
  
- TAACTACTCT TGTCCTTTCA TTTAACGGCT GTACAATTAC AATAATATAG ATTTTTAATT TAGTATTTTA   
  
  
- ATTAATATTA TAAAATTTTT ATAAAGTTGA TTTTAAAATC TTGTTATTCA CGCATTATGC CTGAATTTCC   
  
  
- GATCAAATAG CATTTTTACA TTCAAGGAAA ACCTAGAATA CCAACTTATA GAACCAACTA TTTGACACTA   
  
  
- ACCAAAATGT GAAAATGGTG TATAAGTATC TTCTTAATAC ACGTGCAACT ACCAAGTTCT ACCCTGTTTT   
  
  
- CCTTTGTACA TAAGAAGGGG GAAAACGGAA AAGGGGATTC GTAAACCTAA CTCCACAACA GTAGACTTAT   
  
  
- AGTTTTTAAG AAAAACTTTT AAGTCAGAGT TAGTTTTAAG TTTTAATTTT GTTTCTGAAT TCTCAAAATA   
  
  
- AACAAGAATT GGAAGAATTG AAAGATGAAA AGAAAAAAAA GAGTAAGGGG GAGAAGTTTA GAGTAAATAA   
  
  
- GAATTGGGGG AGAAGGCAAG TACGAGAGAG ACAGATAGTT GATTATAATT AGATGGGGCT GAAAGTTAAA   
  
  
- CTACTCCAGA TTAGGAATAG TAGCATAACA CCAACCCAAT TAATACGTTC TTCCGTTATT TGGGGTCACT   
  
  
- GTTTCAGGTG CAATTCATCC GTGGAGTGGT AATTCTGTAC GAGTCTTTTG TGGTTTTGGT AACTTGTGTT   
  
  
- CAGGGGGTTC AGGGAAGGGA TCGATCGGGT AAGAGAGAGG AGGGGACAGA TATAGATGGT TGAGAACGAG   
  
  
- AAACGACTCG GACTCAACTT TCGCTATGTG GGTATCGGAC AAGTCAAAAG TCAAAAGTGT AAACAGAAGG   
  
  
- AGACAAGTCA ACTTTCGCTA TGTGAGAGTA ATCGAAAGTA AAATTTATGC AAGTTAAGTG TGTATTTACC   
  
  
- GAAAGTTTCG TAACGGTAAA AGCAAATTTA CTACTGTTTT ATATATCATA GACCGATAGA CGGGTATAGA   
  
  
- ACTGGAATAA ATGTTTCCGA CTCGGTAGAA AATTAAAAAA ATCGGAGAAA AAAATTAGTT TTTATCTTTA   
  
  
- AAGTTCTTTC GTCTTTAGCA GTCTACTGTC ACGTCTGTCG CAAAAGCAAA GTATGTTTAG AGTTTTCGAC   
  
  
- TTCACAGTTA AAGAAGCTAA TAAAAACAGT CTTTTATAAA TGCAGAGTGG AAGTGCAACA ATAATAAGAA   
  
  
- AGAAAAAATA ATAAGAGTAT TGATCATTAT TTCAATCGTT ACTATTTTTT AATAAGCTAG GCTTATAAAA   
  
  
- TTAAATAGAC TGGATTTTTG TATTCATTTC CGTGTTTAAA AAGTAGGTTT AAAATTAAAA ACTACATTAT   
  
  
- AAAAAATATA AAAATAACAA GTTTAGACTA AAATTAGAAT AAATTTAATA GACTGGATTT TTTTAGTTTA   
  
  
- TTATTATTTG CAATTAAAAA ATAAATTTAA AACTGTAATC AGTTAGACTG AATTTAAGCT TGGCTCGAAC   
  
  
- TTAAAAAAAC GGTCACGACC ATCATGAGGA CGAAAACATC GAGGGTTACG GTAGCATGGT GGGAGAAGAT   
  
  
- CAGAGCAGAG AAAGAGACCA GAGGTTAATT AGTGTGGTAG TATAGTATGG CATACTACTA TGTCAGGTGA   
  
  
- CTTTAGGGTA AGAGTGGCGG TTAGAGAGAG GCGGTGGTAG GTTGTGAGAA TTGCTACTTG TCCCGGCGGC   
  
  
- GGAGCAGGAG GTGTGAGTCC GGCACCGGCC CGTCGAAAGG GTGGGTTAGT TTTAGAGAGA GGAGGCGGTT   
  
  
- GAAGCCATTG CGGTTAACGT ACCTCGTCGA GAATCAGGTG ACGCGGCTTC GGTAGCTGTT GTTACTGCGG   
  
  
- TGGGGCCGGG TCGTTTAGAA TACCCAGGAC TTATTGTATC GGGCTGGGCT GCCGCTGAGG TGGGTTGCGG   
  
  
- AGTGCACGCG TAAGGATGCA CGGAATTAGA GTGCGCGGCA GGAGTGATCG TGGACGTTCT ACCATTAGGG   
  
  
- AGTAAAGTTG GGGTAGTTAA GTGGGGTGTT TAAGAGCGAG GAGCTTGAAC GGGTGAAGCA GCTAAATTGG   
  
  
- GGAACCGTAG CTAAGCCTAA GTGGCGGCGG TTAAGCTAGT AAGACCTTCG ATAAAGGCTG GATGGGCAAC   
  
  
- ATGTGTAACA GCTGGAGTCG TAGAGGGTGA CATACGTCTA GGGGTGCAAC TAGCTGAGGT AACGCTGGGC   
  
  
- CAACCTTCGG GGCCCAGCTC AGGGAGGATA ACAGTTCGAG TGGCAGCCCC GATAAAGGCT GCTTTAGGGC   
  
  
- GGCCACAAGC TAGAAGACAG CATGCTACTC GAGCCTTACT CTGATTAGTT GAAGCGAGCA AAATCTTTGT   
  
  
- AGCAGGAGCT TAAGGTTCGT TATGGGTGGA GGGGAAGGCT GCCTAAACGA AGCGACAACC TCCTCTAAGC   
  
  
- TGTTTCGTTC GAGATGAGGT TGCTACGCCG CCGCCAATAA CACTTAACAG TCTACTCAAA CGTAAATAAC   
  
  
- GTCCTTCTCC TCCACAGAAG TAGCAGCGGC AGCAGCTACT CCCCCTACAA AAACGTCCGC CACTCCTCGA   
  
  
- ACCTCGGCTC GTACCACCAC CACCACCTCC TCCTGCACCT AAAGTGCCGC TCCTCAGACC ACCCCGCCGA   
  
  
- CTCTAGCCGC AAATTAGTGT ACACCTATGG GAAGCTGTGC CACCTGTGCA AGAACGGTGC ACCCTCGGTC   
  
  
- GTCTCTCTCA CCAAGCTCCG GCTCCACACG ACCTTCTAAC TCTTACACTA GCGAGTACTC CCTGGGCGCT   
  
  
- CCCAGCTCTC CGTCCTCGGG TCCCGGTTCA CCCGGGAGGC TTACTCCCTC CGGCTCAAAG TTCCCTAGCT   
  
  
- CAAGCCACTA CTTCCATGCT GGCTCCAGTT CCGGTACGAC CTCCTCGTAC GGCGACCCAC CCCCAGATTC   
  
  
- TTCCTTCTCC TACTAGAACA CGAGTGAACC TTCCCTGTAT CACAACAGAA ACGAAGACGA ACCCATGGGT   
  
  
- GAAT

+     Unnamed\_\_16

| Site Name | Organism | Position | Strand | Matrix score. | sequence | function |
| --- | --- | --- | --- | --- | --- | --- |
| Unnamed\_\_16 | Zea mays | 2060 | - | 9 | GCTGCCCGTC |  |

>HU06G00376.1   
+ +Up\_Stream \_Len000AGAAGC TAATTAAACC AAACATGCAC GTATGTCCTA ATTAACATGA CTGTCCATCA   
  
  
+ TCTCCTCCCA TGTTGTTCTG TTGACAAGCC TGCACACCCA TGCTCCTCTC TCATGTCTAA CCTCGTCCCA   
  
  
+ ACGATCAAGA TCACTGTCTG TGAGGCCAGG GGAAGACTTG CTTTTATTTC CTTTTCTTTT GTTTACTCTC   
  
  
+ TGATTCCATT TAGCCATATA TACAAGGAGA ATGTCATGTG TATCTCATAT GTATATATAT AAGATTTTAT   
  
  
+ TTAGAAATAA AAATTTAAAC ACTATGTGAT TTATAGTTAT TATAATAAAT AGTATTTTTT AAACTATTTC   
  
  
+ ATTGATGAGA ACAGGAAAGT AAATTGCCGA CATGTTAATG TTATTATATC TAAAAATTAA ATCATAAAAT   
  
  
+ TAATTATAAT ATTTTAAAAA TATTTCAACT AAAATTTTAG AACAATAAGT GCGTAATACG GACTTAAAGG   
  
  
+ CTAGTTTATC GTAAAAATGT AAGTTCCTTT TGGATCTTAT GGTTGAATAT CTTGGTTGAT AAACTGTGAT   
  
  
+ TGGTTTTACA CTTTTACCAC ATATTCATAG AAGAATTATG TGCACGTTGA TGGTTCAAGA TGGGACAAAA   
  
  
+ GGAAACATGT ATTCTTCCCC CTTTTGCCTT TTCCCCTAAG CATTTGGATT GAGGTGTTGT CATCTGAATA   
  
  
+ TCAAAAATTC TTTTTGAAAA TTCAGTCTCA ATCAAAATTC AAAATTAAAA CAAAGACTTA AGAGTTTTAT   
  
  
+ TTGTTCTTAA CCTTCTTAAC TTTCTACTTT TCTTTTTTTT CTCATTCCCC CTCTTCAAAT CTCATTTATT   
  
  
+ CTTAACCCCC TCTTCCGTTC ATGCTCTCTC TGTCTATCAA CTAATATTAA TCTACCCCGA CTTTCAATTT   
  
  
+ GATGAGGTCT AATCCTTATC ATCGTATTGT GGTTGGGTTA ATTATGCAAG AAGGCAATAA ACCCCAGTGA   
  
  
+ CAAAGTCCAC GTTAAGTAGG CACCTCACCA TTAAGACATG CTCAGAAAAC ACCAAAACCA TTGAACACAA   
  
  
+ GTCCCCCAAG TCCCTTCCCT AGCTAGCCCA TTCTCTCTCC TCCCCTGTCT ATATCTACCA ACTCTTGCTC   
  
  
+ TTTGCTGAGC CTGAGTTGAA AGCGATACAC CCATAGCCTG TTCAGTTTTC AGTTTTCACA TTTGTCTTCC   
  
  
+ TCTGTTCAGT TGAAAGCGAT ACACTCTCAT TAGCTTTCAT TTTAAATACG TTCAATTCAC ACATAAATGG   
  
  
+ CTTTCAAAGC ATTGCCATTT TCGTTTAAAT GATGACAAAA TATATAGTAT CTGGCTATCT GCCCATATCT   
  
  
+ TGACCTTATT TACAAAGGCT GAGCCATCTT TTAATTTTTT TAGCCTCTTT TTTTAATCAA AAATAGAAAT   
  
  
+ TTCAAGAAAG CAGAAATCGT CAGATGACAG TGCAGACAGC GTTTTCGTTT CATACAAATC TCAAAAGCTG   
  
  
+ AAGTGTCAAT TTCTTCGATT ATTTTTGTCA GAAAATATTT ACGTCTCACC TTCACGTTGT TATTATTCTT   
  
  
+ TCTTTTTTAT TATTCTCATA ACTAGTAATA AAGTTAGCAA TGATAAAAAA TTATTCGATC CGAATATTTT   
  
  
+ AATTTATCTG ACCTAAAAAC ATAAGTAAAG GCACAAATTT TTCATCCAAA TTTTAATTTT TGATGTAATA   
  
  
+ TTTTTTATAT TTTTATTGTT CAAATCTGAT TTTAATCTTA TTTAAATTAT CTGACCTAAA AAAATCAAAT   
  
  
+ AATAATAAAC GTTAATTTTT TATTTAAATT TTGACATTAG TCAATCTGAC TTAAATTCGA ACCGAGCTTG   
  
  
+ AATTTTTTTG CCAGTGCTGG TAGTACTCCT GCTTTTGTAG CTCCCAATGC CATCGTACCA CCCTCTTCTA   
  
  
+ GTCTCGTCTC TTTCTCTGGT CTCCAATTAA TCACACCATC ATATCATACC GTATGATGAT ACAGTCCACT   
  
  
+ GAAATCCCAT TCTCACCGCC AATCTCTCTC CGCCACCATC CAACACTCTT AACGATGAAC AGGGCCGCCG   
  
  
+ CCTCGTCCTC CACACTCAGG CCGTGGCCGG GCAGCTTTCC CACCCAATCA AAATCTCTCT CCTCCGCCAA   
  
  
+ CTTCGGTAAC GCCAATTGCA TGGAGCAGCT CTTAGTCCAC TGCGCCGAAG CCATCGACAA CAATGACGCC   
  
  
+ ACCCCGGCCC AGCAAATCTT ATGGGTCCTG AATAACATAG CCCGACCCGA CGGCGACTCC ACCCAACGCC   
  
  
+ TCACGTGCGC ATTCCTACGT GCCTTAATCT CACGCGCCGT CCTCACTAGC ACCTGCAAGA TGGTAATCCC   
  
  
+ TCATTTCAAC CCCATCAATT CACCCCACAA ATTCTCGCTC CTCGAACTTG CCCACTTCGT CGATTTAACC   
  
  
+ CCTTGGCATC GATTCGGATT CACCGCCGCC AATTCGATCA TTCTGGAAGC TATTTCCGAC CTACCCGTTG   
  
  
+ TACACATTGT CGACCTCAGC ATCTCCCACT GTATGCAGAT CCCCACGTTG ATCGACTCCA TTGCGACCCG   
  
  
+ GTTGGAAGCC CCGGGTCGAG TCCCTCCTAT TGTCAAGCTC ACCGTCGGGG CTATTTCCGA CGAAATCCCG   
  
  
+ CCGGTGTTCG ATCTTCTGTC GTACGATGAG CTCGGAATGA GACTAATCAA CTTCGCTCGT TTTAGAAACA   
  
  
+ TCGTCCTCGA ATTCCAAGCA ATACCCACCT CCCCTTCCGA CGGATTTGCT TCGCTGTTGG AGGAGATTCG   
  
  
+ ACAAAGCAAG CTCTACTCCA ACGATGCGGC GGCGGTTATT GTGAATTGTC AGATGAGTTT GCATTTATTG   
  
  
+ CAGGAAGAGG AGGTGTCTTC ATCGTCGCCG TCGTCGATGA GGGGGATGTT TTTGCAGGCG GTGAGGAGCT   
  
  
+ TGGAGCCGAG CATGGTGGTG GTGGTGGAGG AGGACGTGGA TTTCACGGCG AGGAGTCTGG TGGGGCGGCT   
  
  
+ GAGATCGGCG TTTAATCACA TGTGGATACC CTTCGACACG GTGGACACGT TCTTGCCACG TGGGAGCCAG   
  
  
+ CAGAGAGAGT GGTTCGAGGC CGAGGTGTGC TGGAAGATTG AGAATGTGAT CGCTCATGAG GGACCCGCGA   
  
  
+ GGGTCGAGAG GCAGGAGCCC AGGGCCAAGT GGGCCCTCCG AATGAGGGAG GCCGAGTTTC AAGGGATCGA   
  
  
+ GTTCGGTGAT GAAGGTACGA CCGAGGTCAA GGCCATGCTG GAGGAGCATG CCGCTGGGTG GGGGTCTAAG   
  
  
+ AAGGAAGAGG ATGATCTTGT GCTCACTTGG AAGGGACATA GTGTTGTCTT TGCTTCTGCT TGGGTACCCA   
  
  
+ CTTA  

- +Up\_Stream \_Len000TCTTCG ATTAATTTGG TTTGTACGTG CATACAGGAT TAATTGTACT GACAGGTAGT   
  
  
- AGAGGAGGGT ACAACAAGAC AACTGTTCGG ACGTGTGGGT ACGAGGAGAG AGTACAGATT GGAGCAGGGT   
  
  
- TGCTAGTTCT AGTGACAGAC ACTCCGGTCC CCTTCTGAAC GAAAATAAAG GAAAAGAAAA CAAATGAGAG   
  
  
- ACTAAGGTAA ATCGGTATAT ATGTTCCTCT TACAGTACAC ATAGAGTATA CATATATATA TTCTAAAATA   
  
  
- AATCTTTATT TTTAAATTTG TGATACACTA AATATCAATA ATATTATTTA TCATAAAAAA TTTGATAAAG   
  
  
- TAACTACTCT TGTCCTTTCA TTTAACGGCT GTACAATTAC AATAATATAG ATTTTTAATT TAGTATTTTA   
  
  
- ATTAATATTA TAAAATTTTT ATAAAGTTGA TTTTAAAATC TTGTTATTCA CGCATTATGC CTGAATTTCC   
  
  
- GATCAAATAG CATTTTTACA TTCAAGGAAA ACCTAGAATA CCAACTTATA GAACCAACTA TTTGACACTA   
  
  
- ACCAAAATGT GAAAATGGTG TATAAGTATC TTCTTAATAC ACGTGCAACT ACCAAGTTCT ACCCTGTTTT   
  
  
- CCTTTGTACA TAAGAAGGGG GAAAACGGAA AAGGGGATTC GTAAACCTAA CTCCACAACA GTAGACTTAT   
  
  
- AGTTTTTAAG AAAAACTTTT AAGTCAGAGT TAGTTTTAAG TTTTAATTTT GTTTCTGAAT TCTCAAAATA   
  
  
- AACAAGAATT GGAAGAATTG AAAGATGAAA AGAAAAAAAA GAGTAAGGGG GAGAAGTTTA GAGTAAATAA   
  
  
- GAATTGGGGG AGAAGGCAAG TACGAGAGAG ACAGATAGTT GATTATAATT AGATGGGGCT GAAAGTTAAA   
  
  
- CTACTCCAGA TTAGGAATAG TAGCATAACA CCAACCCAAT TAATACGTTC TTCCGTTATT TGGGGTCACT   
  
  
- GTTTCAGGTG CAATTCATCC GTGGAGTGGT AATTCTGTAC GAGTCTTTTG TGGTTTTGGT AACTTGTGTT   
  
  
- CAGGGGGTTC AGGGAAGGGA TCGATCGGGT AAGAGAGAGG AGGGGACAGA TATAGATGGT TGAGAACGAG   
  
  
- AAACGACTCG GACTCAACTT TCGCTATGTG GGTATCGGAC AAGTCAAAAG TCAAAAGTGT AAACAGAAGG   
  
  
- AGACAAGTCA ACTTTCGCTA TGTGAGAGTA ATCGAAAGTA AAATTTATGC AAGTTAAGTG TGTATTTACC   
  
  
- GAAAGTTTCG TAACGGTAAA AGCAAATTTA CTACTGTTTT ATATATCATA GACCGATAGA CGGGTATAGA   
  
  
- ACTGGAATAA ATGTTTCCGA CTCGGTAGAA AATTAAAAAA ATCGGAGAAA AAAATTAGTT TTTATCTTTA   
  
  
- AAGTTCTTTC GTCTTTAGCA GTCTACTGTC ACGTCTGTCG CAAAAGCAAA GTATGTTTAG AGTTTTCGAC   
  
  
- TTCACAGTTA AAGAAGCTAA TAAAAACAGT CTTTTATAAA TGCAGAGTGG AAGTGCAACA ATAATAAGAA   
  
  
- AGAAAAAATA ATAAGAGTAT TGATCATTAT TTCAATCGTT ACTATTTTTT AATAAGCTAG GCTTATAAAA   
  
  
- TTAAATAGAC TGGATTTTTG TATTCATTTC CGTGTTTAAA AAGTAGGTTT AAAATTAAAA ACTACATTAT   
  
  
- AAAAAATATA AAAATAACAA GTTTAGACTA AAATTAGAAT AAATTTAATA GACTGGATTT TTTTAGTTTA   
  
  
- TTATTATTTG CAATTAAAAA ATAAATTTAA AACTGTAATC AGTTAGACTG AATTTAAGCT TGGCTCGAAC   
  
  
- TTAAAAAAAC GGTCACGACC ATCATGAGGA CGAAAACATC GAGGGTTACG GTAGCATGGT GGGAGAAGAT   
  
  
- CAGAGCAGAG AAAGAGACCA GAGGTTAATT AGTGTGGTAG TATAGTATGG CATACTACTA TGTCAGGTGA   
  
  
- CTTTAGGGTA AGAGTGGCGG TTAGAGAGAG GCGGTGGTAG GTTGTGAGAA TTGCTACTTG TCCCGGCGGC   
  
  
- GGAGCAGGAG GTGTGAGTCC GGCACCGGCC CGTCGAAAGG GTGGGTTAGT TTTAGAGAGA GGAGGCGGTT   
  
  
- GAAGCCATTG CGGTTAACGT ACCTCGTCGA GAATCAGGTG ACGCGGCTTC GGTAGCTGTT GTTACTGCGG   
  
  
- TGGGGCCGGG TCGTTTAGAA TACCCAGGAC TTATTGTATC GGGCTGGGCT GCCGCTGAGG TGGGTTGCGG   
  
  
- AGTGCACGCG TAAGGATGCA CGGAATTAGA GTGCGCGGCA GGAGTGATCG TGGACGTTCT ACCATTAGGG   
  
  
- AGTAAAGTTG GGGTAGTTAA GTGGGGTGTT TAAGAGCGAG GAGCTTGAAC GGGTGAAGCA GCTAAATTGG   
  
  
- GGAACCGTAG CTAAGCCTAA GTGGCGGCGG TTAAGCTAGT AAGACCTTCG ATAAAGGCTG GATGGGCAAC   
  
  
- ATGTGTAACA GCTGGAGTCG TAGAGGGTGA CATACGTCTA GGGGTGCAAC TAGCTGAGGT AACGCTGGGC   
  
  
- CAACCTTCGG GGCCCAGCTC AGGGAGGATA ACAGTTCGAG TGGCAGCCCC GATAAAGGCT GCTTTAGGGC   
  
  
- GGCCACAAGC TAGAAGACAG CATGCTACTC GAGCCTTACT CTGATTAGTT GAAGCGAGCA AAATCTTTGT   
  
  
- AGCAGGAGCT TAAGGTTCGT TATGGGTGGA GGGGAAGGCT GCCTAAACGA AGCGACAACC TCCTCTAAGC   
  
  
- TGTTTCGTTC GAGATGAGGT TGCTACGCCG CCGCCAATAA CACTTAACAG TCTACTCAAA CGTAAATAAC   
  
  
- GTCCTTCTCC TCCACAGAAG TAGCAGCGGC AGCAGCTACT CCCCCTACAA AAACGTCCGC CACTCCTCGA   
  
  
- ACCTCGGCTC GTACCACCAC CACCACCTCC TCCTGCACCT AAAGTGCCGC TCCTCAGACC ACCCCGCCGA   
  
  
- CTCTAGCCGC AAATTAGTGT ACACCTATGG GAAGCTGTGC CACCTGTGCA AGAACGGTGC ACCCTCGGTC   
  
  
- GTCTCTCTCA CCAAGCTCCG GCTCCACACG ACCTTCTAAC TCTTACACTA GCGAGTACTC CCTGGGCGCT   
  
  
- CCCAGCTCTC CGTCCTCGGG TCCCGGTTCA CCCGGGAGGC TTACTCCCTC CGGCTCAAAG TTCCCTAGCT   
  
  
- CAAGCCACTA CTTCCATGCT GGCTCCAGTT CCGGTACGAC CTCCTCGTAC GGCGACCCAC CCCCAGATTC   
  
  
- TTCCTTCTCC TACTAGAACA CGAGTGAACC TTCCCTGTAT CACAACAGAA ACGAAGACGA ACCCATGGGT   
  
  
- GAAT

+     Unnamed\_\_2

| Site Name | Organism | Position | Strand | Matrix score. | sequence | function |
| --- | --- | --- | --- | --- | --- | --- |
| Unnamed\_\_2 | Zea mays | 2533 | + | 6 | CCCCGG |  |
| Unnamed\_\_2 | Zea mays | 2176 | + | 6 | CCCCGG |  |

>HU06G00376.1   
+ +Up\_Stream \_Len000AGAAGC TAATTAAACC AAACATGCAC GTATGTCCTA ATTAACATGA CTGTCCATCA   
  
  
+ TCTCCTCCCA TGTTGTTCTG TTGACAAGCC TGCACACCCA TGCTCCTCTC TCATGTCTAA CCTCGTCCCA   
  
  
+ ACGATCAAGA TCACTGTCTG TGAGGCCAGG GGAAGACTTG CTTTTATTTC CTTTTCTTTT GTTTACTCTC   
  
  
+ TGATTCCATT TAGCCATATA TACAAGGAGA ATGTCATGTG TATCTCATAT GTATATATAT AAGATTTTAT   
  
  
+ TTAGAAATAA AAATTTAAAC ACTATGTGAT TTATAGTTAT TATAATAAAT AGTATTTTTT AAACTATTTC   
  
  
+ ATTGATGAGA ACAGGAAAGT AAATTGCCGA CATGTTAATG TTATTATATC TAAAAATTAA ATCATAAAAT   
  
  
+ TAATTATAAT ATTTTAAAAA TATTTCAACT AAAATTTTAG AACAATAAGT GCGTAATACG GACTTAAAGG   
  
  
+ CTAGTTTATC GTAAAAATGT AAGTTCCTTT TGGATCTTAT GGTTGAATAT CTTGGTTGAT AAACTGTGAT   
  
  
+ TGGTTTTACA CTTTTACCAC ATATTCATAG AAGAATTATG TGCACGTTGA TGGTTCAAGA TGGGACAAAA   
  
  
+ GGAAACATGT ATTCTTCCCC CTTTTGCCTT TTCCCCTAAG CATTTGGATT GAGGTGTTGT CATCTGAATA   
  
  
+ TCAAAAATTC TTTTTGAAAA TTCAGTCTCA ATCAAAATTC AAAATTAAAA CAAAGACTTA AGAGTTTTAT   
  
  
+ TTGTTCTTAA CCTTCTTAAC TTTCTACTTT TCTTTTTTTT CTCATTCCCC CTCTTCAAAT CTCATTTATT   
  
  
+ CTTAACCCCC TCTTCCGTTC ATGCTCTCTC TGTCTATCAA CTAATATTAA TCTACCCCGA CTTTCAATTT   
  
  
+ GATGAGGTCT AATCCTTATC ATCGTATTGT GGTTGGGTTA ATTATGCAAG AAGGCAATAA ACCCCAGTGA   
  
  
+ CAAAGTCCAC GTTAAGTAGG CACCTCACCA TTAAGACATG CTCAGAAAAC ACCAAAACCA TTGAACACAA   
  
  
+ GTCCCCCAAG TCCCTTCCCT AGCTAGCCCA TTCTCTCTCC TCCCCTGTCT ATATCTACCA ACTCTTGCTC   
  
  
+ TTTGCTGAGC CTGAGTTGAA AGCGATACAC CCATAGCCTG TTCAGTTTTC AGTTTTCACA TTTGTCTTCC   
  
  
+ TCTGTTCAGT TGAAAGCGAT ACACTCTCAT TAGCTTTCAT TTTAAATACG TTCAATTCAC ACATAAATGG   
  
  
+ CTTTCAAAGC ATTGCCATTT TCGTTTAAAT GATGACAAAA TATATAGTAT CTGGCTATCT GCCCATATCT   
  
  
+ TGACCTTATT TACAAAGGCT GAGCCATCTT TTAATTTTTT TAGCCTCTTT TTTTAATCAA AAATAGAAAT   
  
  
+ TTCAAGAAAG CAGAAATCGT CAGATGACAG TGCAGACAGC GTTTTCGTTT CATACAAATC TCAAAAGCTG   
  
  
+ AAGTGTCAAT TTCTTCGATT ATTTTTGTCA GAAAATATTT ACGTCTCACC TTCACGTTGT TATTATTCTT   
  
  
+ TCTTTTTTAT TATTCTCATA ACTAGTAATA AAGTTAGCAA TGATAAAAAA TTATTCGATC CGAATATTTT   
  
  
+ AATTTATCTG ACCTAAAAAC ATAAGTAAAG GCACAAATTT TTCATCCAAA TTTTAATTTT TGATGTAATA   
  
  
+ TTTTTTATAT TTTTATTGTT CAAATCTGAT TTTAATCTTA TTTAAATTAT CTGACCTAAA AAAATCAAAT   
  
  
+ AATAATAAAC GTTAATTTTT TATTTAAATT TTGACATTAG TCAATCTGAC TTAAATTCGA ACCGAGCTTG   
  
  
+ AATTTTTTTG CCAGTGCTGG TAGTACTCCT GCTTTTGTAG CTCCCAATGC CATCGTACCA CCCTCTTCTA   
  
  
+ GTCTCGTCTC TTTCTCTGGT CTCCAATTAA TCACACCATC ATATCATACC GTATGATGAT ACAGTCCACT   
  
  
+ GAAATCCCAT TCTCACCGCC AATCTCTCTC CGCCACCATC CAACACTCTT AACGATGAAC AGGGCCGCCG   
  
  
+ CCTCGTCCTC CACACTCAGG CCGTGGCCGG GCAGCTTTCC CACCCAATCA AAATCTCTCT CCTCCGCCAA   
  
  
+ CTTCGGTAAC GCCAATTGCA TGGAGCAGCT CTTAGTCCAC TGCGCCGAAG CCATCGACAA CAATGACGCC   
  
  
+ ACCCCGGCCC AGCAAATCTT ATGGGTCCTG AATAACATAG CCCGACCCGA CGGCGACTCC ACCCAACGCC   
  
  
+ TCACGTGCGC ATTCCTACGT GCCTTAATCT CACGCGCCGT CCTCACTAGC ACCTGCAAGA TGGTAATCCC   
  
  
+ TCATTTCAAC CCCATCAATT CACCCCACAA ATTCTCGCTC CTCGAACTTG CCCACTTCGT CGATTTAACC   
  
  
+ CCTTGGCATC GATTCGGATT CACCGCCGCC AATTCGATCA TTCTGGAAGC TATTTCCGAC CTACCCGTTG   
  
  
+ TACACATTGT CGACCTCAGC ATCTCCCACT GTATGCAGAT CCCCACGTTG ATCGACTCCA TTGCGACCCG   
  
  
+ GTTGGAAGCC CCGGGTCGAG TCCCTCCTAT TGTCAAGCTC ACCGTCGGGG CTATTTCCGA CGAAATCCCG   
  
  
+ CCGGTGTTCG ATCTTCTGTC GTACGATGAG CTCGGAATGA GACTAATCAA CTTCGCTCGT TTTAGAAACA   
  
  
+ TCGTCCTCGA ATTCCAAGCA ATACCCACCT CCCCTTCCGA CGGATTTGCT TCGCTGTTGG AGGAGATTCG   
  
  
+ ACAAAGCAAG CTCTACTCCA ACGATGCGGC GGCGGTTATT GTGAATTGTC AGATGAGTTT GCATTTATTG   
  
  
+ CAGGAAGAGG AGGTGTCTTC ATCGTCGCCG TCGTCGATGA GGGGGATGTT TTTGCAGGCG GTGAGGAGCT   
  
  
+ TGGAGCCGAG CATGGTGGTG GTGGTGGAGG AGGACGTGGA TTTCACGGCG AGGAGTCTGG TGGGGCGGCT   
  
  
+ GAGATCGGCG TTTAATCACA TGTGGATACC CTTCGACACG GTGGACACGT TCTTGCCACG TGGGAGCCAG   
  
  
+ CAGAGAGAGT GGTTCGAGGC CGAGGTGTGC TGGAAGATTG AGAATGTGAT CGCTCATGAG GGACCCGCGA   
  
  
+ GGGTCGAGAG GCAGGAGCCC AGGGCCAAGT GGGCCCTCCG AATGAGGGAG GCCGAGTTTC AAGGGATCGA   
  
  
+ GTTCGGTGAT GAAGGTACGA CCGAGGTCAA GGCCATGCTG GAGGAGCATG CCGCTGGGTG GGGGTCTAAG   
  
  
+ AAGGAAGAGG ATGATCTTGT GCTCACTTGG AAGGGACATA GTGTTGTCTT TGCTTCTGCT TGGGTACCCA   
  
  
+ CTTA  

- +Up\_Stream \_Len000TCTTCG ATTAATTTGG TTTGTACGTG CATACAGGAT TAATTGTACT GACAGGTAGT   
  
  
- AGAGGAGGGT ACAACAAGAC AACTGTTCGG ACGTGTGGGT ACGAGGAGAG AGTACAGATT GGAGCAGGGT   
  
  
- TGCTAGTTCT AGTGACAGAC ACTCCGGTCC CCTTCTGAAC GAAAATAAAG GAAAAGAAAA CAAATGAGAG   
  
  
- ACTAAGGTAA ATCGGTATAT ATGTTCCTCT TACAGTACAC ATAGAGTATA CATATATATA TTCTAAAATA   
  
  
- AATCTTTATT TTTAAATTTG TGATACACTA AATATCAATA ATATTATTTA TCATAAAAAA TTTGATAAAG   
  
  
- TAACTACTCT TGTCCTTTCA TTTAACGGCT GTACAATTAC AATAATATAG ATTTTTAATT TAGTATTTTA   
  
  
- ATTAATATTA TAAAATTTTT ATAAAGTTGA TTTTAAAATC TTGTTATTCA CGCATTATGC CTGAATTTCC   
  
  
- GATCAAATAG CATTTTTACA TTCAAGGAAA ACCTAGAATA CCAACTTATA GAACCAACTA TTTGACACTA   
  
  
- ACCAAAATGT GAAAATGGTG TATAAGTATC TTCTTAATAC ACGTGCAACT ACCAAGTTCT ACCCTGTTTT   
  
  
- CCTTTGTACA TAAGAAGGGG GAAAACGGAA AAGGGGATTC GTAAACCTAA CTCCACAACA GTAGACTTAT   
  
  
- AGTTTTTAAG AAAAACTTTT AAGTCAGAGT TAGTTTTAAG TTTTAATTTT GTTTCTGAAT TCTCAAAATA   
  
  
- AACAAGAATT GGAAGAATTG AAAGATGAAA AGAAAAAAAA GAGTAAGGGG GAGAAGTTTA GAGTAAATAA   
  
  
- GAATTGGGGG AGAAGGCAAG TACGAGAGAG ACAGATAGTT GATTATAATT AGATGGGGCT GAAAGTTAAA   
  
  
- CTACTCCAGA TTAGGAATAG TAGCATAACA CCAACCCAAT TAATACGTTC TTCCGTTATT TGGGGTCACT   
  
  
- GTTTCAGGTG CAATTCATCC GTGGAGTGGT AATTCTGTAC GAGTCTTTTG TGGTTTTGGT AACTTGTGTT   
  
  
- CAGGGGGTTC AGGGAAGGGA TCGATCGGGT AAGAGAGAGG AGGGGACAGA TATAGATGGT TGAGAACGAG   
  
  
- AAACGACTCG GACTCAACTT TCGCTATGTG GGTATCGGAC AAGTCAAAAG TCAAAAGTGT AAACAGAAGG   
  
  
- AGACAAGTCA ACTTTCGCTA TGTGAGAGTA ATCGAAAGTA AAATTTATGC AAGTTAAGTG TGTATTTACC   
  
  
- GAAAGTTTCG TAACGGTAAA AGCAAATTTA CTACTGTTTT ATATATCATA GACCGATAGA CGGGTATAGA   
  
  
- ACTGGAATAA ATGTTTCCGA CTCGGTAGAA AATTAAAAAA ATCGGAGAAA AAAATTAGTT TTTATCTTTA   
  
  
- AAGTTCTTTC GTCTTTAGCA GTCTACTGTC ACGTCTGTCG CAAAAGCAAA GTATGTTTAG AGTTTTCGAC   
  
  
- TTCACAGTTA AAGAAGCTAA TAAAAACAGT CTTTTATAAA TGCAGAGTGG AAGTGCAACA ATAATAAGAA   
  
  
- AGAAAAAATA ATAAGAGTAT TGATCATTAT TTCAATCGTT ACTATTTTTT AATAAGCTAG GCTTATAAAA   
  
  
- TTAAATAGAC TGGATTTTTG TATTCATTTC CGTGTTTAAA AAGTAGGTTT AAAATTAAAA ACTACATTAT   
  
  
- AAAAAATATA AAAATAACAA GTTTAGACTA AAATTAGAAT AAATTTAATA GACTGGATTT TTTTAGTTTA   
  
  
- TTATTATTTG CAATTAAAAA ATAAATTTAA AACTGTAATC AGTTAGACTG AATTTAAGCT TGGCTCGAAC   
  
  
- TTAAAAAAAC GGTCACGACC ATCATGAGGA CGAAAACATC GAGGGTTACG GTAGCATGGT GGGAGAAGAT   
  
  
- CAGAGCAGAG AAAGAGACCA GAGGTTAATT AGTGTGGTAG TATAGTATGG CATACTACTA TGTCAGGTGA   
  
  
- CTTTAGGGTA AGAGTGGCGG TTAGAGAGAG GCGGTGGTAG GTTGTGAGAA TTGCTACTTG TCCCGGCGGC   
  
  
- GGAGCAGGAG GTGTGAGTCC GGCACCGGCC CGTCGAAAGG GTGGGTTAGT TTTAGAGAGA GGAGGCGGTT   
  
  
- GAAGCCATTG CGGTTAACGT ACCTCGTCGA GAATCAGGTG ACGCGGCTTC GGTAGCTGTT GTTACTGCGG   
  
  
- TGGGGCCGGG TCGTTTAGAA TACCCAGGAC TTATTGTATC GGGCTGGGCT GCCGCTGAGG TGGGTTGCGG   
  
  
- AGTGCACGCG TAAGGATGCA CGGAATTAGA GTGCGCGGCA GGAGTGATCG TGGACGTTCT ACCATTAGGG   
  
  
- AGTAAAGTTG GGGTAGTTAA GTGGGGTGTT TAAGAGCGAG GAGCTTGAAC GGGTGAAGCA GCTAAATTGG   
  
  
- GGAACCGTAG CTAAGCCTAA GTGGCGGCGG TTAAGCTAGT AAGACCTTCG ATAAAGGCTG GATGGGCAAC   
  
  
- ATGTGTAACA GCTGGAGTCG TAGAGGGTGA CATACGTCTA GGGGTGCAAC TAGCTGAGGT AACGCTGGGC   
  
  
- CAACCTTCGG GGCCCAGCTC AGGGAGGATA ACAGTTCGAG TGGCAGCCCC GATAAAGGCT GCTTTAGGGC   
  
  
- GGCCACAAGC TAGAAGACAG CATGCTACTC GAGCCTTACT CTGATTAGTT GAAGCGAGCA AAATCTTTGT   
  
  
- AGCAGGAGCT TAAGGTTCGT TATGGGTGGA GGGGAAGGCT GCCTAAACGA AGCGACAACC TCCTCTAAGC   
  
  
- TGTTTCGTTC GAGATGAGGT TGCTACGCCG CCGCCAATAA CACTTAACAG TCTACTCAAA CGTAAATAAC   
  
  
- GTCCTTCTCC TCCACAGAAG TAGCAGCGGC AGCAGCTACT CCCCCTACAA AAACGTCCGC CACTCCTCGA   
  
  
- ACCTCGGCTC GTACCACCAC CACCACCTCC TCCTGCACCT AAAGTGCCGC TCCTCAGACC ACCCCGCCGA   
  
  
- CTCTAGCCGC AAATTAGTGT ACACCTATGG GAAGCTGTGC CACCTGTGCA AGAACGGTGC ACCCTCGGTC   
  
  
- GTCTCTCTCA CCAAGCTCCG GCTCCACACG ACCTTCTAAC TCTTACACTA GCGAGTACTC CCTGGGCGCT   
  
  
- CCCAGCTCTC CGTCCTCGGG TCCCGGTTCA CCCGGGAGGC TTACTCCCTC CGGCTCAAAG TTCCCTAGCT   
  
  
- CAAGCCACTA CTTCCATGCT GGCTCCAGTT CCGGTACGAC CTCCTCGTAC GGCGACCCAC CCCCAGATTC   
  
  
- TTCCTTCTCC TACTAGAACA CGAGTGAACC TTCCCTGTAT CACAACAGAA ACGAAGACGA ACCCATGGGT   
  
  
- GAAT

+     Unnamed\_\_4

| Site Name | Organism | Position | Strand | Matrix score. | sequence | function |
| --- | --- | --- | --- | --- | --- | --- |
| Unnamed\_\_4 | Petroselinum hortense | 1850 | + | 4 | CTCC |  |
| Unnamed\_\_4 | Petroselinum hortense | 3197 | - | 4 | CTCC |  |
| Unnamed\_\_4 | Petroselinum hortense | 3007 | - | 4 | CTCC |  |
| Unnamed\_\_4 | Petroselinum hortense | 2926 | - | 4 | CTCC |  |
| Unnamed\_\_4 | Petroselinum hortense | 2352 | + | 4 | CTCC |  |
| Unnamed\_\_4 | Petroselinum hortense | 1865 | + | 4 | CTCC |  |
| Unnamed\_\_4 | Petroselinum hortense | 79 | + | 4 | CTCC |  |
| Unnamed\_\_4 | Petroselinum hortense | 3131 | - | 4 | CTCC |  |
| Unnamed\_\_4 | Petroselinum hortense | 2477 | + | 4 | CTCC |  |
| Unnamed\_\_4 | Petroselinum hortense | 2093 | + | 4 | CTCC |  |
| Unnamed\_\_4 | Petroselinum hortense | 117 | + | 4 | CTCC |  |
| Unnamed\_\_4 | Petroselinum hortense | 2869 | - | 4 | CTCC |  |
| Unnamed\_\_4 | Petroselinum hortense | 1915 | + | 4 | CTCC |  |
| Unnamed\_\_4 | Petroselinum hortense | 2876 | - | 4 | CTCC |  |
| Unnamed\_\_4 | Petroselinum hortense | 2096 | + | 4 | CTCC |  |
| Unnamed\_\_4 | Petroselinum hortense | 1992 | + | 4 | CTCC |  |
| Unnamed\_\_4 | Petroselinum hortense | 3098 | - | 4 | CTCC |  |
| Unnamed\_\_4 | Petroselinum hortense | 2693 | + | 4 | CTCC |  |
| Unnamed\_\_4 | Petroselinum hortense | 2900 | - | 4 | CTCC |  |
| Unnamed\_\_4 | Petroselinum hortense | 2726 | - | 4 | CTCC |  |
| Unnamed\_\_4 | Petroselinum hortense | 2231 | + | 4 | CTCC |  |
| Unnamed\_\_4 | Petroselinum hortense | 2903 | - | 4 | CTCC |  |
| Unnamed\_\_4 | Petroselinum hortense | 3194 | - | 4 | CTCC |  |
| Unnamed\_\_4 | Petroselinum hortense | 240 | - | 4 | CTCC |  |
| Unnamed\_\_4 | Petroselinum hortense | 76 | + | 4 | CTCC |  |
| Unnamed\_\_4 | Petroselinum hortense | 1091 | + | 4 | CTCC |  |
| Unnamed\_\_4 | Petroselinum hortense | 2042 | + | 4 | CTCC |  |
| Unnamed\_\_4 | Petroselinum hortense | 1094 | + | 4 | CTCC |  |
| Unnamed\_\_4 | Petroselinum hortense | 2750 | + | 4 | CTCC |  |
| Unnamed\_\_4 | Petroselinum hortense | 2723 | - | 4 | CTCC |  |
| Unnamed\_\_4 | Petroselinum hortense | 2813 | - | 4 | CTCC |  |
| Unnamed\_\_4 | Petroselinum hortense | 2510 | + | 4 | CTCC |  |
| Unnamed\_\_4 | Petroselinum hortense | 2126 | - | 4 | CTCC |  |
| Unnamed\_\_4 | Petroselinum hortense | 2548 | + | 4 | CTCC |  |
| Unnamed\_\_4 | Petroselinum hortense | 3120 | + | 4 | CTCC |  |

>HU06G00376.1   
+ +Up\_Stream \_Len000AGAAGC TAATTAAACC AAACATGCAC GTATGTCCTA ATTAACATGA CTGTCCATCA   
  
  
+ TCTCCTCCCA TGTTGTTCTG TTGACAAGCC TGCACACCCA TGCTCCTCTC TCATGTCTAA CCTCGTCCCA   
  
  
+ ACGATCAAGA TCACTGTCTG TGAGGCCAGG GGAAGACTTG CTTTTATTTC CTTTTCTTTT GTTTACTCTC   
  
  
+ TGATTCCATT TAGCCATATA TACAAGGAGA ATGTCATGTG TATCTCATAT GTATATATAT AAGATTTTAT   
  
  
+ TTAGAAATAA AAATTTAAAC ACTATGTGAT TTATAGTTAT TATAATAAAT AGTATTTTTT AAACTATTTC   
  
  
+ ATTGATGAGA ACAGGAAAGT AAATTGCCGA CATGTTAATG TTATTATATC TAAAAATTAA ATCATAAAAT   
  
  
+ TAATTATAAT ATTTTAAAAA TATTTCAACT AAAATTTTAG AACAATAAGT GCGTAATACG GACTTAAAGG   
  
  
+ CTAGTTTATC GTAAAAATGT AAGTTCCTTT TGGATCTTAT GGTTGAATAT CTTGGTTGAT AAACTGTGAT   
  
  
+ TGGTTTTACA CTTTTACCAC ATATTCATAG AAGAATTATG TGCACGTTGA TGGTTCAAGA TGGGACAAAA   
  
  
+ GGAAACATGT ATTCTTCCCC CTTTTGCCTT TTCCCCTAAG CATTTGGATT GAGGTGTTGT CATCTGAATA   
  
  
+ TCAAAAATTC TTTTTGAAAA TTCAGTCTCA ATCAAAATTC AAAATTAAAA CAAAGACTTA AGAGTTTTAT   
  
  
+ TTGTTCTTAA CCTTCTTAAC TTTCTACTTT TCTTTTTTTT CTCATTCCCC CTCTTCAAAT CTCATTTATT   
  
  
+ CTTAACCCCC TCTTCCGTTC ATGCTCTCTC TGTCTATCAA CTAATATTAA TCTACCCCGA CTTTCAATTT   
  
  
+ GATGAGGTCT AATCCTTATC ATCGTATTGT GGTTGGGTTA ATTATGCAAG AAGGCAATAA ACCCCAGTGA   
  
  
+ CAAAGTCCAC GTTAAGTAGG CACCTCACCA TTAAGACATG CTCAGAAAAC ACCAAAACCA TTGAACACAA   
  
  
+ GTCCCCCAAG TCCCTTCCCT AGCTAGCCCA TTCTCTCTCC TCCCCTGTCT ATATCTACCA ACTCTTGCTC   
  
  
+ TTTGCTGAGC CTGAGTTGAA AGCGATACAC CCATAGCCTG TTCAGTTTTC AGTTTTCACA TTTGTCTTCC   
  
  
+ TCTGTTCAGT TGAAAGCGAT ACACTCTCAT TAGCTTTCAT TTTAAATACG TTCAATTCAC ACATAAATGG   
  
  
+ CTTTCAAAGC ATTGCCATTT TCGTTTAAAT GATGACAAAA TATATAGTAT CTGGCTATCT GCCCATATCT   
  
  
+ TGACCTTATT TACAAAGGCT GAGCCATCTT TTAATTTTTT TAGCCTCTTT TTTTAATCAA AAATAGAAAT   
  
  
+ TTCAAGAAAG CAGAAATCGT CAGATGACAG TGCAGACAGC GTTTTCGTTT CATACAAATC TCAAAAGCTG   
  
  
+ AAGTGTCAAT TTCTTCGATT ATTTTTGTCA GAAAATATTT ACGTCTCACC TTCACGTTGT TATTATTCTT   
  
  
+ TCTTTTTTAT TATTCTCATA ACTAGTAATA AAGTTAGCAA TGATAAAAAA TTATTCGATC CGAATATTTT   
  
  
+ AATTTATCTG ACCTAAAAAC ATAAGTAAAG GCACAAATTT TTCATCCAAA TTTTAATTTT TGATGTAATA   
  
  
+ TTTTTTATAT TTTTATTGTT CAAATCTGAT TTTAATCTTA TTTAAATTAT CTGACCTAAA AAAATCAAAT   
  
  
+ AATAATAAAC GTTAATTTTT TATTTAAATT TTGACATTAG TCAATCTGAC TTAAATTCGA ACCGAGCTTG   
  
  
+ AATTTTTTTG CCAGTGCTGG TAGTACTCCT GCTTTTGTAG CTCCCAATGC CATCGTACCA CCCTCTTCTA   
  
  
+ GTCTCGTCTC TTTCTCTGGT CTCCAATTAA TCACACCATC ATATCATACC GTATGATGAT ACAGTCCACT   
  
  
+ GAAATCCCAT TCTCACCGCC AATCTCTCTC CGCCACCATC CAACACTCTT AACGATGAAC AGGGCCGCCG   
  
  
+ CCTCGTCCTC CACACTCAGG CCGTGGCCGG GCAGCTTTCC CACCCAATCA AAATCTCTCT CCTCCGCCAA   
  
  
+ CTTCGGTAAC GCCAATTGCA TGGAGCAGCT CTTAGTCCAC TGCGCCGAAG CCATCGACAA CAATGACGCC   
  
  
+ ACCCCGGCCC AGCAAATCTT ATGGGTCCTG AATAACATAG CCCGACCCGA CGGCGACTCC ACCCAACGCC   
  
  
+ TCACGTGCGC ATTCCTACGT GCCTTAATCT CACGCGCCGT CCTCACTAGC ACCTGCAAGA TGGTAATCCC   
  
  
+ TCATTTCAAC CCCATCAATT CACCCCACAA ATTCTCGCTC CTCGAACTTG CCCACTTCGT CGATTTAACC   
  
  
+ CCTTGGCATC GATTCGGATT CACCGCCGCC AATTCGATCA TTCTGGAAGC TATTTCCGAC CTACCCGTTG   
  
  
+ TACACATTGT CGACCTCAGC ATCTCCCACT GTATGCAGAT CCCCACGTTG ATCGACTCCA TTGCGACCCG   
  
  
+ GTTGGAAGCC CCGGGTCGAG TCCCTCCTAT TGTCAAGCTC ACCGTCGGGG CTATTTCCGA CGAAATCCCG   
  
  
+ CCGGTGTTCG ATCTTCTGTC GTACGATGAG CTCGGAATGA GACTAATCAA CTTCGCTCGT TTTAGAAACA   
  
  
+ TCGTCCTCGA ATTCCAAGCA ATACCCACCT CCCCTTCCGA CGGATTTGCT TCGCTGTTGG AGGAGATTCG   
  
  
+ ACAAAGCAAG CTCTACTCCA ACGATGCGGC GGCGGTTATT GTGAATTGTC AGATGAGTTT GCATTTATTG   
  
  
+ CAGGAAGAGG AGGTGTCTTC ATCGTCGCCG TCGTCGATGA GGGGGATGTT TTTGCAGGCG GTGAGGAGCT   
  
  
+ TGGAGCCGAG CATGGTGGTG GTGGTGGAGG AGGACGTGGA TTTCACGGCG AGGAGTCTGG TGGGGCGGCT   
  
  
+ GAGATCGGCG TTTAATCACA TGTGGATACC CTTCGACACG GTGGACACGT TCTTGCCACG TGGGAGCCAG   
  
  
+ CAGAGAGAGT GGTTCGAGGC CGAGGTGTGC TGGAAGATTG AGAATGTGAT CGCTCATGAG GGACCCGCGA   
  
  
+ GGGTCGAGAG GCAGGAGCCC AGGGCCAAGT GGGCCCTCCG AATGAGGGAG GCCGAGTTTC AAGGGATCGA   
  
  
+ GTTCGGTGAT GAAGGTACGA CCGAGGTCAA GGCCATGCTG GAGGAGCATG CCGCTGGGTG GGGGTCTAAG   
  
  
+ AAGGAAGAGG ATGATCTTGT GCTCACTTGG AAGGGACATA GTGTTGTCTT TGCTTCTGCT TGGGTACCCA   
  
  
+ CTTA  

- +Up\_Stream \_Len000TCTTCG ATTAATTTGG TTTGTACGTG CATACAGGAT TAATTGTACT GACAGGTAGT   
  
  
- AGAGGAGGGT ACAACAAGAC AACTGTTCGG ACGTGTGGGT ACGAGGAGAG AGTACAGATT GGAGCAGGGT   
  
  
- TGCTAGTTCT AGTGACAGAC ACTCCGGTCC CCTTCTGAAC GAAAATAAAG GAAAAGAAAA CAAATGAGAG   
  
  
- ACTAAGGTAA ATCGGTATAT ATGTTCCTCT TACAGTACAC ATAGAGTATA CATATATATA TTCTAAAATA   
  
  
- AATCTTTATT TTTAAATTTG TGATACACTA AATATCAATA ATATTATTTA TCATAAAAAA TTTGATAAAG   
  
  
- TAACTACTCT TGTCCTTTCA TTTAACGGCT GTACAATTAC AATAATATAG ATTTTTAATT TAGTATTTTA   
  
  
- ATTAATATTA TAAAATTTTT ATAAAGTTGA TTTTAAAATC TTGTTATTCA CGCATTATGC CTGAATTTCC   
  
  
- GATCAAATAG CATTTTTACA TTCAAGGAAA ACCTAGAATA CCAACTTATA GAACCAACTA TTTGACACTA   
  
  
- ACCAAAATGT GAAAATGGTG TATAAGTATC TTCTTAATAC ACGTGCAACT ACCAAGTTCT ACCCTGTTTT   
  
  
- CCTTTGTACA TAAGAAGGGG GAAAACGGAA AAGGGGATTC GTAAACCTAA CTCCACAACA GTAGACTTAT   
  
  
- AGTTTTTAAG AAAAACTTTT AAGTCAGAGT TAGTTTTAAG TTTTAATTTT GTTTCTGAAT TCTCAAAATA   
  
  
- AACAAGAATT GGAAGAATTG AAAGATGAAA AGAAAAAAAA GAGTAAGGGG GAGAAGTTTA GAGTAAATAA   
  
  
- GAATTGGGGG AGAAGGCAAG TACGAGAGAG ACAGATAGTT GATTATAATT AGATGGGGCT GAAAGTTAAA   
  
  
- CTACTCCAGA TTAGGAATAG TAGCATAACA CCAACCCAAT TAATACGTTC TTCCGTTATT TGGGGTCACT   
  
  
- GTTTCAGGTG CAATTCATCC GTGGAGTGGT AATTCTGTAC GAGTCTTTTG TGGTTTTGGT AACTTGTGTT   
  
  
- CAGGGGGTTC AGGGAAGGGA TCGATCGGGT AAGAGAGAGG AGGGGACAGA TATAGATGGT TGAGAACGAG   
  
  
- AAACGACTCG GACTCAACTT TCGCTATGTG GGTATCGGAC AAGTCAAAAG TCAAAAGTGT AAACAGAAGG   
  
  
- AGACAAGTCA ACTTTCGCTA TGTGAGAGTA ATCGAAAGTA AAATTTATGC AAGTTAAGTG TGTATTTACC   
  
  
- GAAAGTTTCG TAACGGTAAA AGCAAATTTA CTACTGTTTT ATATATCATA GACCGATAGA CGGGTATAGA   
  
  
- ACTGGAATAA ATGTTTCCGA CTCGGTAGAA AATTAAAAAA ATCGGAGAAA AAAATTAGTT TTTATCTTTA   
  
  
- AAGTTCTTTC GTCTTTAGCA GTCTACTGTC ACGTCTGTCG CAAAAGCAAA GTATGTTTAG AGTTTTCGAC   
  
  
- TTCACAGTTA AAGAAGCTAA TAAAAACAGT CTTTTATAAA TGCAGAGTGG AAGTGCAACA ATAATAAGAA   
  
  
- AGAAAAAATA ATAAGAGTAT TGATCATTAT TTCAATCGTT ACTATTTTTT AATAAGCTAG GCTTATAAAA   
  
  
- TTAAATAGAC TGGATTTTTG TATTCATTTC CGTGTTTAAA AAGTAGGTTT AAAATTAAAA ACTACATTAT   
  
  
- AAAAAATATA AAAATAACAA GTTTAGACTA AAATTAGAAT AAATTTAATA GACTGGATTT TTTTAGTTTA   
  
  
- TTATTATTTG CAATTAAAAA ATAAATTTAA AACTGTAATC AGTTAGACTG AATTTAAGCT TGGCTCGAAC   
  
  
- TTAAAAAAAC GGTCACGACC ATCATGAGGA CGAAAACATC GAGGGTTACG GTAGCATGGT GGGAGAAGAT   
  
  
- CAGAGCAGAG AAAGAGACCA GAGGTTAATT AGTGTGGTAG TATAGTATGG CATACTACTA TGTCAGGTGA   
  
  
- CTTTAGGGTA AGAGTGGCGG TTAGAGAGAG GCGGTGGTAG GTTGTGAGAA TTGCTACTTG TCCCGGCGGC   
  
  
- GGAGCAGGAG GTGTGAGTCC GGCACCGGCC CGTCGAAAGG GTGGGTTAGT TTTAGAGAGA GGAGGCGGTT   
  
  
- GAAGCCATTG CGGTTAACGT ACCTCGTCGA GAATCAGGTG ACGCGGCTTC GGTAGCTGTT GTTACTGCGG   
  
  
- TGGGGCCGGG TCGTTTAGAA TACCCAGGAC TTATTGTATC GGGCTGGGCT GCCGCTGAGG TGGGTTGCGG   
  
  
- AGTGCACGCG TAAGGATGCA CGGAATTAGA GTGCGCGGCA GGAGTGATCG TGGACGTTCT ACCATTAGGG   
  
  
- AGTAAAGTTG GGGTAGTTAA GTGGGGTGTT TAAGAGCGAG GAGCTTGAAC GGGTGAAGCA GCTAAATTGG   
  
  
- GGAACCGTAG CTAAGCCTAA GTGGCGGCGG TTAAGCTAGT AAGACCTTCG ATAAAGGCTG GATGGGCAAC   
  
  
- ATGTGTAACA GCTGGAGTCG TAGAGGGTGA CATACGTCTA GGGGTGCAAC TAGCTGAGGT AACGCTGGGC   
  
  
- CAACCTTCGG GGCCCAGCTC AGGGAGGATA ACAGTTCGAG TGGCAGCCCC GATAAAGGCT GCTTTAGGGC   
  
  
- GGCCACAAGC TAGAAGACAG CATGCTACTC GAGCCTTACT CTGATTAGTT GAAGCGAGCA AAATCTTTGT   
  
  
- AGCAGGAGCT TAAGGTTCGT TATGGGTGGA GGGGAAGGCT GCCTAAACGA AGCGACAACC TCCTCTAAGC   
  
  
- TGTTTCGTTC GAGATGAGGT TGCTACGCCG CCGCCAATAA CACTTAACAG TCTACTCAAA CGTAAATAAC   
  
  
- GTCCTTCTCC TCCACAGAAG TAGCAGCGGC AGCAGCTACT CCCCCTACAA AAACGTCCGC CACTCCTCGA   
  
  
- ACCTCGGCTC GTACCACCAC CACCACCTCC TCCTGCACCT AAAGTGCCGC TCCTCAGACC ACCCCGCCGA   
  
  
- CTCTAGCCGC AAATTAGTGT ACACCTATGG GAAGCTGTGC CACCTGTGCA AGAACGGTGC ACCCTCGGTC   
  
  
- GTCTCTCTCA CCAAGCTCCG GCTCCACACG ACCTTCTAAC TCTTACACTA GCGAGTACTC CCTGGGCGCT   
  
  
- CCCAGCTCTC CGTCCTCGGG TCCCGGTTCA CCCGGGAGGC TTACTCCCTC CGGCTCAAAG TTCCCTAGCT   
  
  
- CAAGCCACTA CTTCCATGCT GGCTCCAGTT CCGGTACGAC CTCCTCGTAC GGCGACCCAC CCCCAGATTC   
  
  
- TTCCTTCTCC TACTAGAACA CGAGTGAACC TTCCCTGTAT CACAACAGAA ACGAAGACGA ACCCATGGGT   
  
  
- GAAT

+     W box

| Site Name | Organism | Position | Strand | Matrix score. | sequence | function |
| --- | --- | --- | --- | --- | --- | --- |
| W box | Arabidopsis thaliana | 3179 | - | 6 | TTGACC |  |
| W box | Arabidopsis thaliana | 1334 | + | 6 | TTGACC |  |

>HU06G00376.1   
+ +Up\_Stream \_Len000AGAAGC TAATTAAACC AAACATGCAC GTATGTCCTA ATTAACATGA CTGTCCATCA   
  
  
+ TCTCCTCCCA TGTTGTTCTG TTGACAAGCC TGCACACCCA TGCTCCTCTC TCATGTCTAA CCTCGTCCCA   
  
  
+ ACGATCAAGA TCACTGTCTG TGAGGCCAGG GGAAGACTTG CTTTTATTTC CTTTTCTTTT GTTTACTCTC   
  
  
+ TGATTCCATT TAGCCATATA TACAAGGAGA ATGTCATGTG TATCTCATAT GTATATATAT AAGATTTTAT   
  
  
+ TTAGAAATAA AAATTTAAAC ACTATGTGAT TTATAGTTAT TATAATAAAT AGTATTTTTT AAACTATTTC   
  
  
+ ATTGATGAGA ACAGGAAAGT AAATTGCCGA CATGTTAATG TTATTATATC TAAAAATTAA ATCATAAAAT   
  
  
+ TAATTATAAT ATTTTAAAAA TATTTCAACT AAAATTTTAG AACAATAAGT GCGTAATACG GACTTAAAGG   
  
  
+ CTAGTTTATC GTAAAAATGT AAGTTCCTTT TGGATCTTAT GGTTGAATAT CTTGGTTGAT AAACTGTGAT   
  
  
+ TGGTTTTACA CTTTTACCAC ATATTCATAG AAGAATTATG TGCACGTTGA TGGTTCAAGA TGGGACAAAA   
  
  
+ GGAAACATGT ATTCTTCCCC CTTTTGCCTT TTCCCCTAAG CATTTGGATT GAGGTGTTGT CATCTGAATA   
  
  
+ TCAAAAATTC TTTTTGAAAA TTCAGTCTCA ATCAAAATTC AAAATTAAAA CAAAGACTTA AGAGTTTTAT   
  
  
+ TTGTTCTTAA CCTTCTTAAC TTTCTACTTT TCTTTTTTTT CTCATTCCCC CTCTTCAAAT CTCATTTATT   
  
  
+ CTTAACCCCC TCTTCCGTTC ATGCTCTCTC TGTCTATCAA CTAATATTAA TCTACCCCGA CTTTCAATTT   
  
  
+ GATGAGGTCT AATCCTTATC ATCGTATTGT GGTTGGGTTA ATTATGCAAG AAGGCAATAA ACCCCAGTGA   
  
  
+ CAAAGTCCAC GTTAAGTAGG CACCTCACCA TTAAGACATG CTCAGAAAAC ACCAAAACCA TTGAACACAA   
  
  
+ GTCCCCCAAG TCCCTTCCCT AGCTAGCCCA TTCTCTCTCC TCCCCTGTCT ATATCTACCA ACTCTTGCTC   
  
  
+ TTTGCTGAGC CTGAGTTGAA AGCGATACAC CCATAGCCTG TTCAGTTTTC AGTTTTCACA TTTGTCTTCC   
  
  
+ TCTGTTCAGT TGAAAGCGAT ACACTCTCAT TAGCTTTCAT TTTAAATACG TTCAATTCAC ACATAAATGG   
  
  
+ CTTTCAAAGC ATTGCCATTT TCGTTTAAAT GATGACAAAA TATATAGTAT CTGGCTATCT GCCCATATCT   
  
  
+ TGACCTTATT TACAAAGGCT GAGCCATCTT TTAATTTTTT TAGCCTCTTT TTTTAATCAA AAATAGAAAT   
  
  
+ TTCAAGAAAG CAGAAATCGT CAGATGACAG TGCAGACAGC GTTTTCGTTT CATACAAATC TCAAAAGCTG   
  
  
+ AAGTGTCAAT TTCTTCGATT ATTTTTGTCA GAAAATATTT ACGTCTCACC TTCACGTTGT TATTATTCTT   
  
  
+ TCTTTTTTAT TATTCTCATA ACTAGTAATA AAGTTAGCAA TGATAAAAAA TTATTCGATC CGAATATTTT   
  
  
+ AATTTATCTG ACCTAAAAAC ATAAGTAAAG GCACAAATTT TTCATCCAAA TTTTAATTTT TGATGTAATA   
  
  
+ TTTTTTATAT TTTTATTGTT CAAATCTGAT TTTAATCTTA TTTAAATTAT CTGACCTAAA AAAATCAAAT   
  
  
+ AATAATAAAC GTTAATTTTT TATTTAAATT TTGACATTAG TCAATCTGAC TTAAATTCGA ACCGAGCTTG   
  
  
+ AATTTTTTTG CCAGTGCTGG TAGTACTCCT GCTTTTGTAG CTCCCAATGC CATCGTACCA CCCTCTTCTA   
  
  
+ GTCTCGTCTC TTTCTCTGGT CTCCAATTAA TCACACCATC ATATCATACC GTATGATGAT ACAGTCCACT   
  
  
+ GAAATCCCAT TCTCACCGCC AATCTCTCTC CGCCACCATC CAACACTCTT AACGATGAAC AGGGCCGCCG   
  
  
+ CCTCGTCCTC CACACTCAGG CCGTGGCCGG GCAGCTTTCC CACCCAATCA AAATCTCTCT CCTCCGCCAA   
  
  
+ CTTCGGTAAC GCCAATTGCA TGGAGCAGCT CTTAGTCCAC TGCGCCGAAG CCATCGACAA CAATGACGCC   
  
  
+ ACCCCGGCCC AGCAAATCTT ATGGGTCCTG AATAACATAG CCCGACCCGA CGGCGACTCC ACCCAACGCC   
  
  
+ TCACGTGCGC ATTCCTACGT GCCTTAATCT CACGCGCCGT CCTCACTAGC ACCTGCAAGA TGGTAATCCC   
  
  
+ TCATTTCAAC CCCATCAATT CACCCCACAA ATTCTCGCTC CTCGAACTTG CCCACTTCGT CGATTTAACC   
  
  
+ CCTTGGCATC GATTCGGATT CACCGCCGCC AATTCGATCA TTCTGGAAGC TATTTCCGAC CTACCCGTTG   
  
  
+ TACACATTGT CGACCTCAGC ATCTCCCACT GTATGCAGAT CCCCACGTTG ATCGACTCCA TTGCGACCCG   
  
  
+ GTTGGAAGCC CCGGGTCGAG TCCCTCCTAT TGTCAAGCTC ACCGTCGGGG CTATTTCCGA CGAAATCCCG   
  
  
+ CCGGTGTTCG ATCTTCTGTC GTACGATGAG CTCGGAATGA GACTAATCAA CTTCGCTCGT TTTAGAAACA   
  
  
+ TCGTCCTCGA ATTCCAAGCA ATACCCACCT CCCCTTCCGA CGGATTTGCT TCGCTGTTGG AGGAGATTCG   
  
  
+ ACAAAGCAAG CTCTACTCCA ACGATGCGGC GGCGGTTATT GTGAATTGTC AGATGAGTTT GCATTTATTG   
  
  
+ CAGGAAGAGG AGGTGTCTTC ATCGTCGCCG TCGTCGATGA GGGGGATGTT TTTGCAGGCG GTGAGGAGCT   
  
  
+ TGGAGCCGAG CATGGTGGTG GTGGTGGAGG AGGACGTGGA TTTCACGGCG AGGAGTCTGG TGGGGCGGCT   
  
  
+ GAGATCGGCG TTTAATCACA TGTGGATACC CTTCGACACG GTGGACACGT TCTTGCCACG TGGGAGCCAG   
  
  
+ CAGAGAGAGT GGTTCGAGGC CGAGGTGTGC TGGAAGATTG AGAATGTGAT CGCTCATGAG GGACCCGCGA   
  
  
+ GGGTCGAGAG GCAGGAGCCC AGGGCCAAGT GGGCCCTCCG AATGAGGGAG GCCGAGTTTC AAGGGATCGA   
  
  
+ GTTCGGTGAT GAAGGTACGA CCGAGGTCAA GGCCATGCTG GAGGAGCATG CCGCTGGGTG GGGGTCTAAG   
  
  
+ AAGGAAGAGG ATGATCTTGT GCTCACTTGG AAGGGACATA GTGTTGTCTT TGCTTCTGCT TGGGTACCCA   
  
  
+ CTTA  

- +Up\_Stream \_Len000TCTTCG ATTAATTTGG TTTGTACGTG CATACAGGAT TAATTGTACT GACAGGTAGT   
  
  
- AGAGGAGGGT ACAACAAGAC AACTGTTCGG ACGTGTGGGT ACGAGGAGAG AGTACAGATT GGAGCAGGGT   
  
  
- TGCTAGTTCT AGTGACAGAC ACTCCGGTCC CCTTCTGAAC GAAAATAAAG GAAAAGAAAA CAAATGAGAG   
  
  
- ACTAAGGTAA ATCGGTATAT ATGTTCCTCT TACAGTACAC ATAGAGTATA CATATATATA TTCTAAAATA   
  
  
- AATCTTTATT TTTAAATTTG TGATACACTA AATATCAATA ATATTATTTA TCATAAAAAA TTTGATAAAG   
  
  
- TAACTACTCT TGTCCTTTCA TTTAACGGCT GTACAATTAC AATAATATAG ATTTTTAATT TAGTATTTTA   
  
  
- ATTAATATTA TAAAATTTTT ATAAAGTTGA TTTTAAAATC TTGTTATTCA CGCATTATGC CTGAATTTCC   
  
  
- GATCAAATAG CATTTTTACA TTCAAGGAAA ACCTAGAATA CCAACTTATA GAACCAACTA TTTGACACTA   
  
  
- ACCAAAATGT GAAAATGGTG TATAAGTATC TTCTTAATAC ACGTGCAACT ACCAAGTTCT ACCCTGTTTT   
  
  
- CCTTTGTACA TAAGAAGGGG GAAAACGGAA AAGGGGATTC GTAAACCTAA CTCCACAACA GTAGACTTAT   
  
  
- AGTTTTTAAG AAAAACTTTT AAGTCAGAGT TAGTTTTAAG TTTTAATTTT GTTTCTGAAT TCTCAAAATA   
  
  
- AACAAGAATT GGAAGAATTG AAAGATGAAA AGAAAAAAAA GAGTAAGGGG GAGAAGTTTA GAGTAAATAA   
  
  
- GAATTGGGGG AGAAGGCAAG TACGAGAGAG ACAGATAGTT GATTATAATT AGATGGGGCT GAAAGTTAAA   
  
  
- CTACTCCAGA TTAGGAATAG TAGCATAACA CCAACCCAAT TAATACGTTC TTCCGTTATT TGGGGTCACT   
  
  
- GTTTCAGGTG CAATTCATCC GTGGAGTGGT AATTCTGTAC GAGTCTTTTG TGGTTTTGGT AACTTGTGTT   
  
  
- CAGGGGGTTC AGGGAAGGGA TCGATCGGGT AAGAGAGAGG AGGGGACAGA TATAGATGGT TGAGAACGAG   
  
  
- AAACGACTCG GACTCAACTT TCGCTATGTG GGTATCGGAC AAGTCAAAAG TCAAAAGTGT AAACAGAAGG   
  
  
- AGACAAGTCA ACTTTCGCTA TGTGAGAGTA ATCGAAAGTA AAATTTATGC AAGTTAAGTG TGTATTTACC   
  
  
- GAAAGTTTCG TAACGGTAAA AGCAAATTTA CTACTGTTTT ATATATCATA GACCGATAGA CGGGTATAGA   
  
  
- ACTGGAATAA ATGTTTCCGA CTCGGTAGAA AATTAAAAAA ATCGGAGAAA AAAATTAGTT TTTATCTTTA   
  
  
- AAGTTCTTTC GTCTTTAGCA GTCTACTGTC ACGTCTGTCG CAAAAGCAAA GTATGTTTAG AGTTTTCGAC   
  
  
- TTCACAGTTA AAGAAGCTAA TAAAAACAGT CTTTTATAAA TGCAGAGTGG AAGTGCAACA ATAATAAGAA   
  
  
- AGAAAAAATA ATAAGAGTAT TGATCATTAT TTCAATCGTT ACTATTTTTT AATAAGCTAG GCTTATAAAA   
  
  
- TTAAATAGAC TGGATTTTTG TATTCATTTC CGTGTTTAAA AAGTAGGTTT AAAATTAAAA ACTACATTAT   
  
  
- AAAAAATATA AAAATAACAA GTTTAGACTA AAATTAGAAT AAATTTAATA GACTGGATTT TTTTAGTTTA   
  
  
- TTATTATTTG CAATTAAAAA ATAAATTTAA AACTGTAATC AGTTAGACTG AATTTAAGCT TGGCTCGAAC   
  
  
- TTAAAAAAAC GGTCACGACC ATCATGAGGA CGAAAACATC GAGGGTTACG GTAGCATGGT GGGAGAAGAT   
  
  
- CAGAGCAGAG AAAGAGACCA GAGGTTAATT AGTGTGGTAG TATAGTATGG CATACTACTA TGTCAGGTGA   
  
  
- CTTTAGGGTA AGAGTGGCGG TTAGAGAGAG GCGGTGGTAG GTTGTGAGAA TTGCTACTTG TCCCGGCGGC   
  
  
- GGAGCAGGAG GTGTGAGTCC GGCACCGGCC CGTCGAAAGG GTGGGTTAGT TTTAGAGAGA GGAGGCGGTT   
  
  
- GAAGCCATTG CGGTTAACGT ACCTCGTCGA GAATCAGGTG ACGCGGCTTC GGTAGCTGTT GTTACTGCGG   
  
  
- TGGGGCCGGG TCGTTTAGAA TACCCAGGAC TTATTGTATC GGGCTGGGCT GCCGCTGAGG TGGGTTGCGG   
  
  
- AGTGCACGCG TAAGGATGCA CGGAATTAGA GTGCGCGGCA GGAGTGATCG TGGACGTTCT ACCATTAGGG   
  
  
- AGTAAAGTTG GGGTAGTTAA GTGGGGTGTT TAAGAGCGAG GAGCTTGAAC GGGTGAAGCA GCTAAATTGG   
  
  
- GGAACCGTAG CTAAGCCTAA GTGGCGGCGG TTAAGCTAGT AAGACCTTCG ATAAAGGCTG GATGGGCAAC   
  
  
- ATGTGTAACA GCTGGAGTCG TAGAGGGTGA CATACGTCTA GGGGTGCAAC TAGCTGAGGT AACGCTGGGC   
  
  
- CAACCTTCGG GGCCCAGCTC AGGGAGGATA ACAGTTCGAG TGGCAGCCCC GATAAAGGCT GCTTTAGGGC   
  
  
- GGCCACAAGC TAGAAGACAG CATGCTACTC GAGCCTTACT CTGATTAGTT GAAGCGAGCA AAATCTTTGT   
  
  
- AGCAGGAGCT TAAGGTTCGT TATGGGTGGA GGGGAAGGCT GCCTAAACGA AGCGACAACC TCCTCTAAGC   
  
  
- TGTTTCGTTC GAGATGAGGT TGCTACGCCG CCGCCAATAA CACTTAACAG TCTACTCAAA CGTAAATAAC   
  
  
- GTCCTTCTCC TCCACAGAAG TAGCAGCGGC AGCAGCTACT CCCCCTACAA AAACGTCCGC CACTCCTCGA   
  
  
- ACCTCGGCTC GTACCACCAC CACCACCTCC TCCTGCACCT AAAGTGCCGC TCCTCAGACC ACCCCGCCGA   
  
  
- CTCTAGCCGC AAATTAGTGT ACACCTATGG GAAGCTGTGC CACCTGTGCA AGAACGGTGC ACCCTCGGTC   
  
  
- GTCTCTCTCA CCAAGCTCCG GCTCCACACG ACCTTCTAAC TCTTACACTA GCGAGTACTC CCTGGGCGCT   
  
  
- CCCAGCTCTC CGTCCTCGGG TCCCGGTTCA CCCGGGAGGC TTACTCCCTC CGGCTCAAAG TTCCCTAGCT   
  
  
- CAAGCCACTA CTTCCATGCT GGCTCCAGTT CCGGTACGAC CTCCTCGTAC GGCGACCCAC CCCCAGATTC   
  
  
- TTCCTTCTCC TACTAGAACA CGAGTGAACC TTCCCTGTAT CACAACAGAA ACGAAGACGA ACCCATGGGT   
  
  
- GAAT

+     WRE3

| Site Name | Organism | Position | Strand | Matrix score. | sequence | function |
| --- | --- | --- | --- | --- | --- | --- |
| WRE3 | Pisum sativum | 2689 | + | 6 | CCACCT |  |

>HU06G00376.1   
+ +Up\_Stream \_Len000AGAAGC TAATTAAACC AAACATGCAC GTATGTCCTA ATTAACATGA CTGTCCATCA   
  
  
+ TCTCCTCCCA TGTTGTTCTG TTGACAAGCC TGCACACCCA TGCTCCTCTC TCATGTCTAA CCTCGTCCCA   
  
  
+ ACGATCAAGA TCACTGTCTG TGAGGCCAGG GGAAGACTTG CTTTTATTTC CTTTTCTTTT GTTTACTCTC   
  
  
+ TGATTCCATT TAGCCATATA TACAAGGAGA ATGTCATGTG TATCTCATAT GTATATATAT AAGATTTTAT   
  
  
+ TTAGAAATAA AAATTTAAAC ACTATGTGAT TTATAGTTAT TATAATAAAT AGTATTTTTT AAACTATTTC   
  
  
+ ATTGATGAGA ACAGGAAAGT AAATTGCCGA CATGTTAATG TTATTATATC TAAAAATTAA ATCATAAAAT   
  
  
+ TAATTATAAT ATTTTAAAAA TATTTCAACT AAAATTTTAG AACAATAAGT GCGTAATACG GACTTAAAGG   
  
  
+ CTAGTTTATC GTAAAAATGT AAGTTCCTTT TGGATCTTAT GGTTGAATAT CTTGGTTGAT AAACTGTGAT   
  
  
+ TGGTTTTACA CTTTTACCAC ATATTCATAG AAGAATTATG TGCACGTTGA TGGTTCAAGA TGGGACAAAA   
  
  
+ GGAAACATGT ATTCTTCCCC CTTTTGCCTT TTCCCCTAAG CATTTGGATT GAGGTGTTGT CATCTGAATA   
  
  
+ TCAAAAATTC TTTTTGAAAA TTCAGTCTCA ATCAAAATTC AAAATTAAAA CAAAGACTTA AGAGTTTTAT   
  
  
+ TTGTTCTTAA CCTTCTTAAC TTTCTACTTT TCTTTTTTTT CTCATTCCCC CTCTTCAAAT CTCATTTATT   
  
  
+ CTTAACCCCC TCTTCCGTTC ATGCTCTCTC TGTCTATCAA CTAATATTAA TCTACCCCGA CTTTCAATTT   
  
  
+ GATGAGGTCT AATCCTTATC ATCGTATTGT GGTTGGGTTA ATTATGCAAG AAGGCAATAA ACCCCAGTGA   
  
  
+ CAAAGTCCAC GTTAAGTAGG CACCTCACCA TTAAGACATG CTCAGAAAAC ACCAAAACCA TTGAACACAA   
  
  
+ GTCCCCCAAG TCCCTTCCCT AGCTAGCCCA TTCTCTCTCC TCCCCTGTCT ATATCTACCA ACTCTTGCTC   
  
  
+ TTTGCTGAGC CTGAGTTGAA AGCGATACAC CCATAGCCTG TTCAGTTTTC AGTTTTCACA TTTGTCTTCC   
  
  
+ TCTGTTCAGT TGAAAGCGAT ACACTCTCAT TAGCTTTCAT TTTAAATACG TTCAATTCAC ACATAAATGG   
  
  
+ CTTTCAAAGC ATTGCCATTT TCGTTTAAAT GATGACAAAA TATATAGTAT CTGGCTATCT GCCCATATCT   
  
  
+ TGACCTTATT TACAAAGGCT GAGCCATCTT TTAATTTTTT TAGCCTCTTT TTTTAATCAA AAATAGAAAT   
  
  
+ TTCAAGAAAG CAGAAATCGT CAGATGACAG TGCAGACAGC GTTTTCGTTT CATACAAATC TCAAAAGCTG   
  
  
+ AAGTGTCAAT TTCTTCGATT ATTTTTGTCA GAAAATATTT ACGTCTCACC TTCACGTTGT TATTATTCTT   
  
  
+ TCTTTTTTAT TATTCTCATA ACTAGTAATA AAGTTAGCAA TGATAAAAAA TTATTCGATC CGAATATTTT   
  
  
+ AATTTATCTG ACCTAAAAAC ATAAGTAAAG GCACAAATTT TTCATCCAAA TTTTAATTTT TGATGTAATA   
  
  
+ TTTTTTATAT TTTTATTGTT CAAATCTGAT TTTAATCTTA TTTAAATTAT CTGACCTAAA AAAATCAAAT   
  
  
+ AATAATAAAC GTTAATTTTT TATTTAAATT TTGACATTAG TCAATCTGAC TTAAATTCGA ACCGAGCTTG   
  
  
+ AATTTTTTTG CCAGTGCTGG TAGTACTCCT GCTTTTGTAG CTCCCAATGC CATCGTACCA CCCTCTTCTA   
  
  
+ GTCTCGTCTC TTTCTCTGGT CTCCAATTAA TCACACCATC ATATCATACC GTATGATGAT ACAGTCCACT   
  
  
+ GAAATCCCAT TCTCACCGCC AATCTCTCTC CGCCACCATC CAACACTCTT AACGATGAAC AGGGCCGCCG   
  
  
+ CCTCGTCCTC CACACTCAGG CCGTGGCCGG GCAGCTTTCC CACCCAATCA AAATCTCTCT CCTCCGCCAA   
  
  
+ CTTCGGTAAC GCCAATTGCA TGGAGCAGCT CTTAGTCCAC TGCGCCGAAG CCATCGACAA CAATGACGCC   
  
  
+ ACCCCGGCCC AGCAAATCTT ATGGGTCCTG AATAACATAG CCCGACCCGA CGGCGACTCC ACCCAACGCC   
  
  
+ TCACGTGCGC ATTCCTACGT GCCTTAATCT CACGCGCCGT CCTCACTAGC ACCTGCAAGA TGGTAATCCC   
  
  
+ TCATTTCAAC CCCATCAATT CACCCCACAA ATTCTCGCTC CTCGAACTTG CCCACTTCGT CGATTTAACC   
  
  
+ CCTTGGCATC GATTCGGATT CACCGCCGCC AATTCGATCA TTCTGGAAGC TATTTCCGAC CTACCCGTTG   
  
  
+ TACACATTGT CGACCTCAGC ATCTCCCACT GTATGCAGAT CCCCACGTTG ATCGACTCCA TTGCGACCCG   
  
  
+ GTTGGAAGCC CCGGGTCGAG TCCCTCCTAT TGTCAAGCTC ACCGTCGGGG CTATTTCCGA CGAAATCCCG   
  
  
+ CCGGTGTTCG ATCTTCTGTC GTACGATGAG CTCGGAATGA GACTAATCAA CTTCGCTCGT TTTAGAAACA   
  
  
+ TCGTCCTCGA ATTCCAAGCA ATACCCACCT CCCCTTCCGA CGGATTTGCT TCGCTGTTGG AGGAGATTCG   
  
  
+ ACAAAGCAAG CTCTACTCCA ACGATGCGGC GGCGGTTATT GTGAATTGTC AGATGAGTTT GCATTTATTG   
  
  
+ CAGGAAGAGG AGGTGTCTTC ATCGTCGCCG TCGTCGATGA GGGGGATGTT TTTGCAGGCG GTGAGGAGCT   
  
  
+ TGGAGCCGAG CATGGTGGTG GTGGTGGAGG AGGACGTGGA TTTCACGGCG AGGAGTCTGG TGGGGCGGCT   
  
  
+ GAGATCGGCG TTTAATCACA TGTGGATACC CTTCGACACG GTGGACACGT TCTTGCCACG TGGGAGCCAG   
  
  
+ CAGAGAGAGT GGTTCGAGGC CGAGGTGTGC TGGAAGATTG AGAATGTGAT CGCTCATGAG GGACCCGCGA   
  
  
+ GGGTCGAGAG GCAGGAGCCC AGGGCCAAGT GGGCCCTCCG AATGAGGGAG GCCGAGTTTC AAGGGATCGA   
  
  
+ GTTCGGTGAT GAAGGTACGA CCGAGGTCAA GGCCATGCTG GAGGAGCATG CCGCTGGGTG GGGGTCTAAG   
  
  
+ AAGGAAGAGG ATGATCTTGT GCTCACTTGG AAGGGACATA GTGTTGTCTT TGCTTCTGCT TGGGTACCCA   
  
  
+ CTTA  

- +Up\_Stream \_Len000TCTTCG ATTAATTTGG TTTGTACGTG CATACAGGAT TAATTGTACT GACAGGTAGT   
  
  
- AGAGGAGGGT ACAACAAGAC AACTGTTCGG ACGTGTGGGT ACGAGGAGAG AGTACAGATT GGAGCAGGGT   
  
  
- TGCTAGTTCT AGTGACAGAC ACTCCGGTCC CCTTCTGAAC GAAAATAAAG GAAAAGAAAA CAAATGAGAG   
  
  
- ACTAAGGTAA ATCGGTATAT ATGTTCCTCT TACAGTACAC ATAGAGTATA CATATATATA TTCTAAAATA   
  
  
- AATCTTTATT TTTAAATTTG TGATACACTA AATATCAATA ATATTATTTA TCATAAAAAA TTTGATAAAG   
  
  
- TAACTACTCT TGTCCTTTCA TTTAACGGCT GTACAATTAC AATAATATAG ATTTTTAATT TAGTATTTTA   
  
  
- ATTAATATTA TAAAATTTTT ATAAAGTTGA TTTTAAAATC TTGTTATTCA CGCATTATGC CTGAATTTCC   
  
  
- GATCAAATAG CATTTTTACA TTCAAGGAAA ACCTAGAATA CCAACTTATA GAACCAACTA TTTGACACTA   
  
  
- ACCAAAATGT GAAAATGGTG TATAAGTATC TTCTTAATAC ACGTGCAACT ACCAAGTTCT ACCCTGTTTT   
  
  
- CCTTTGTACA TAAGAAGGGG GAAAACGGAA AAGGGGATTC GTAAACCTAA CTCCACAACA GTAGACTTAT   
  
  
- AGTTTTTAAG AAAAACTTTT AAGTCAGAGT TAGTTTTAAG TTTTAATTTT GTTTCTGAAT TCTCAAAATA   
  
  
- AACAAGAATT GGAAGAATTG AAAGATGAAA AGAAAAAAAA GAGTAAGGGG GAGAAGTTTA GAGTAAATAA   
  
  
- GAATTGGGGG AGAAGGCAAG TACGAGAGAG ACAGATAGTT GATTATAATT AGATGGGGCT GAAAGTTAAA   
  
  
- CTACTCCAGA TTAGGAATAG TAGCATAACA CCAACCCAAT TAATACGTTC TTCCGTTATT TGGGGTCACT   
  
  
- GTTTCAGGTG CAATTCATCC GTGGAGTGGT AATTCTGTAC GAGTCTTTTG TGGTTTTGGT AACTTGTGTT   
  
  
- CAGGGGGTTC AGGGAAGGGA TCGATCGGGT AAGAGAGAGG AGGGGACAGA TATAGATGGT TGAGAACGAG   
  
  
- AAACGACTCG GACTCAACTT TCGCTATGTG GGTATCGGAC AAGTCAAAAG TCAAAAGTGT AAACAGAAGG   
  
  
- AGACAAGTCA ACTTTCGCTA TGTGAGAGTA ATCGAAAGTA AAATTTATGC AAGTTAAGTG TGTATTTACC   
  
  
- GAAAGTTTCG TAACGGTAAA AGCAAATTTA CTACTGTTTT ATATATCATA GACCGATAGA CGGGTATAGA   
  
  
- ACTGGAATAA ATGTTTCCGA CTCGGTAGAA AATTAAAAAA ATCGGAGAAA AAAATTAGTT TTTATCTTTA   
  
  
- AAGTTCTTTC GTCTTTAGCA GTCTACTGTC ACGTCTGTCG CAAAAGCAAA GTATGTTTAG AGTTTTCGAC   
  
  
- TTCACAGTTA AAGAAGCTAA TAAAAACAGT CTTTTATAAA TGCAGAGTGG AAGTGCAACA ATAATAAGAA   
  
  
- AGAAAAAATA ATAAGAGTAT TGATCATTAT TTCAATCGTT ACTATTTTTT AATAAGCTAG GCTTATAAAA   
  
  
- TTAAATAGAC TGGATTTTTG TATTCATTTC CGTGTTTAAA AAGTAGGTTT AAAATTAAAA ACTACATTAT   
  
  
- AAAAAATATA AAAATAACAA GTTTAGACTA AAATTAGAAT AAATTTAATA GACTGGATTT TTTTAGTTTA   
  
  
- TTATTATTTG CAATTAAAAA ATAAATTTAA AACTGTAATC AGTTAGACTG AATTTAAGCT TGGCTCGAAC   
  
  
- TTAAAAAAAC GGTCACGACC ATCATGAGGA CGAAAACATC GAGGGTTACG GTAGCATGGT GGGAGAAGAT   
  
  
- CAGAGCAGAG AAAGAGACCA GAGGTTAATT AGTGTGGTAG TATAGTATGG CATACTACTA TGTCAGGTGA   
  
  
- CTTTAGGGTA AGAGTGGCGG TTAGAGAGAG GCGGTGGTAG GTTGTGAGAA TTGCTACTTG TCCCGGCGGC   
  
  
- GGAGCAGGAG GTGTGAGTCC GGCACCGGCC CGTCGAAAGG GTGGGTTAGT TTTAGAGAGA GGAGGCGGTT   
  
  
- GAAGCCATTG CGGTTAACGT ACCTCGTCGA GAATCAGGTG ACGCGGCTTC GGTAGCTGTT GTTACTGCGG   
  
  
- TGGGGCCGGG TCGTTTAGAA TACCCAGGAC TTATTGTATC GGGCTGGGCT GCCGCTGAGG TGGGTTGCGG   
  
  
- AGTGCACGCG TAAGGATGCA CGGAATTAGA GTGCGCGGCA GGAGTGATCG TGGACGTTCT ACCATTAGGG   
  
  
- AGTAAAGTTG GGGTAGTTAA GTGGGGTGTT TAAGAGCGAG GAGCTTGAAC GGGTGAAGCA GCTAAATTGG   
  
  
- GGAACCGTAG CTAAGCCTAA GTGGCGGCGG TTAAGCTAGT AAGACCTTCG ATAAAGGCTG GATGGGCAAC   
  
  
- ATGTGTAACA GCTGGAGTCG TAGAGGGTGA CATACGTCTA GGGGTGCAAC TAGCTGAGGT AACGCTGGGC   
  
  
- CAACCTTCGG GGCCCAGCTC AGGGAGGATA ACAGTTCGAG TGGCAGCCCC GATAAAGGCT GCTTTAGGGC   
  
  
- GGCCACAAGC TAGAAGACAG CATGCTACTC GAGCCTTACT CTGATTAGTT GAAGCGAGCA AAATCTTTGT   
  
  
- AGCAGGAGCT TAAGGTTCGT TATGGGTGGA GGGGAAGGCT GCCTAAACGA AGCGACAACC TCCTCTAAGC   
  
  
- TGTTTCGTTC GAGATGAGGT TGCTACGCCG CCGCCAATAA CACTTAACAG TCTACTCAAA CGTAAATAAC   
  
  
- GTCCTTCTCC TCCACAGAAG TAGCAGCGGC AGCAGCTACT CCCCCTACAA AAACGTCCGC CACTCCTCGA   
  
  
- ACCTCGGCTC GTACCACCAC CACCACCTCC TCCTGCACCT AAAGTGCCGC TCCTCAGACC ACCCCGCCGA   
  
  
- CTCTAGCCGC AAATTAGTGT ACACCTATGG GAAGCTGTGC CACCTGTGCA AGAACGGTGC ACCCTCGGTC   
  
  
- GTCTCTCTCA CCAAGCTCCG GCTCCACACG ACCTTCTAAC TCTTACACTA GCGAGTACTC CCTGGGCGCT   
  
  
- CCCAGCTCTC CGTCCTCGGG TCCCGGTTCA CCCGGGAGGC TTACTCCCTC CGGCTCAAAG TTCCCTAGCT   
  
  
- CAAGCCACTA CTTCCATGCT GGCTCCAGTT CCGGTACGAC CTCCTCGTAC GGCGACCCAC CCCCAGATTC   
  
  
- TTCCTTCTCC TACTAGAACA CGAGTGAACC TTCCCTGTAT CACAACAGAA ACGAAGACGA ACCCATGGGT   
  
  
- GAAT

+     as-1

| Site Name | Organism | Position | Strand | Matrix score. | sequence | function |
| --- | --- | --- | --- | --- | --- | --- |
| as-1 | Arabidopsis thaliana | 2168 | + | 5 | TGACG |  |
| as-1 | Arabidopsis thaliana | 1422 | - | 5 | TGACG |  |

>HU06G00376.1   
+ +Up\_Stream \_Len000AGAAGC TAATTAAACC AAACATGCAC GTATGTCCTA ATTAACATGA CTGTCCATCA   
  
  
+ TCTCCTCCCA TGTTGTTCTG TTGACAAGCC TGCACACCCA TGCTCCTCTC TCATGTCTAA CCTCGTCCCA   
  
  
+ ACGATCAAGA TCACTGTCTG TGAGGCCAGG GGAAGACTTG CTTTTATTTC CTTTTCTTTT GTTTACTCTC   
  
  
+ TGATTCCATT TAGCCATATA TACAAGGAGA ATGTCATGTG TATCTCATAT GTATATATAT AAGATTTTAT   
  
  
+ TTAGAAATAA AAATTTAAAC ACTATGTGAT TTATAGTTAT TATAATAAAT AGTATTTTTT AAACTATTTC   
  
  
+ ATTGATGAGA ACAGGAAAGT AAATTGCCGA CATGTTAATG TTATTATATC TAAAAATTAA ATCATAAAAT   
  
  
+ TAATTATAAT ATTTTAAAAA TATTTCAACT AAAATTTTAG AACAATAAGT GCGTAATACG GACTTAAAGG   
  
  
+ CTAGTTTATC GTAAAAATGT AAGTTCCTTT TGGATCTTAT GGTTGAATAT CTTGGTTGAT AAACTGTGAT   
  
  
+ TGGTTTTACA CTTTTACCAC ATATTCATAG AAGAATTATG TGCACGTTGA TGGTTCAAGA TGGGACAAAA   
  
  
+ GGAAACATGT ATTCTTCCCC CTTTTGCCTT TTCCCCTAAG CATTTGGATT GAGGTGTTGT CATCTGAATA   
  
  
+ TCAAAAATTC TTTTTGAAAA TTCAGTCTCA ATCAAAATTC AAAATTAAAA CAAAGACTTA AGAGTTTTAT   
  
  
+ TTGTTCTTAA CCTTCTTAAC TTTCTACTTT TCTTTTTTTT CTCATTCCCC CTCTTCAAAT CTCATTTATT   
  
  
+ CTTAACCCCC TCTTCCGTTC ATGCTCTCTC TGTCTATCAA CTAATATTAA TCTACCCCGA CTTTCAATTT   
  
  
+ GATGAGGTCT AATCCTTATC ATCGTATTGT GGTTGGGTTA ATTATGCAAG AAGGCAATAA ACCCCAGTGA   
  
  
+ CAAAGTCCAC GTTAAGTAGG CACCTCACCA TTAAGACATG CTCAGAAAAC ACCAAAACCA TTGAACACAA   
  
  
+ GTCCCCCAAG TCCCTTCCCT AGCTAGCCCA TTCTCTCTCC TCCCCTGTCT ATATCTACCA ACTCTTGCTC   
  
  
+ TTTGCTGAGC CTGAGTTGAA AGCGATACAC CCATAGCCTG TTCAGTTTTC AGTTTTCACA TTTGTCTTCC   
  
  
+ TCTGTTCAGT TGAAAGCGAT ACACTCTCAT TAGCTTTCAT TTTAAATACG TTCAATTCAC ACATAAATGG   
  
  
+ CTTTCAAAGC ATTGCCATTT TCGTTTAAAT GATGACAAAA TATATAGTAT CTGGCTATCT GCCCATATCT   
  
  
+ TGACCTTATT TACAAAGGCT GAGCCATCTT TTAATTTTTT TAGCCTCTTT TTTTAATCAA AAATAGAAAT   
  
  
+ TTCAAGAAAG CAGAAATCGT CAGATGACAG TGCAGACAGC GTTTTCGTTT CATACAAATC TCAAAAGCTG   
  
  
+ AAGTGTCAAT TTCTTCGATT ATTTTTGTCA GAAAATATTT ACGTCTCACC TTCACGTTGT TATTATTCTT   
  
  
+ TCTTTTTTAT TATTCTCATA ACTAGTAATA AAGTTAGCAA TGATAAAAAA TTATTCGATC CGAATATTTT   
  
  
+ AATTTATCTG ACCTAAAAAC ATAAGTAAAG GCACAAATTT TTCATCCAAA TTTTAATTTT TGATGTAATA   
  
  
+ TTTTTTATAT TTTTATTGTT CAAATCTGAT TTTAATCTTA TTTAAATTAT CTGACCTAAA AAAATCAAAT   
  
  
+ AATAATAAAC GTTAATTTTT TATTTAAATT TTGACATTAG TCAATCTGAC TTAAATTCGA ACCGAGCTTG   
  
  
+ AATTTTTTTG CCAGTGCTGG TAGTACTCCT GCTTTTGTAG CTCCCAATGC CATCGTACCA CCCTCTTCTA   
  
  
+ GTCTCGTCTC TTTCTCTGGT CTCCAATTAA TCACACCATC ATATCATACC GTATGATGAT ACAGTCCACT   
  
  
+ GAAATCCCAT TCTCACCGCC AATCTCTCTC CGCCACCATC CAACACTCTT AACGATGAAC AGGGCCGCCG   
  
  
+ CCTCGTCCTC CACACTCAGG CCGTGGCCGG GCAGCTTTCC CACCCAATCA AAATCTCTCT CCTCCGCCAA   
  
  
+ CTTCGGTAAC GCCAATTGCA TGGAGCAGCT CTTAGTCCAC TGCGCCGAAG CCATCGACAA CAATGACGCC   
  
  
+ ACCCCGGCCC AGCAAATCTT ATGGGTCCTG AATAACATAG CCCGACCCGA CGGCGACTCC ACCCAACGCC   
  
  
+ TCACGTGCGC ATTCCTACGT GCCTTAATCT CACGCGCCGT CCTCACTAGC ACCTGCAAGA TGGTAATCCC   
  
  
+ TCATTTCAAC CCCATCAATT CACCCCACAA ATTCTCGCTC CTCGAACTTG CCCACTTCGT CGATTTAACC   
  
  
+ CCTTGGCATC GATTCGGATT CACCGCCGCC AATTCGATCA TTCTGGAAGC TATTTCCGAC CTACCCGTTG   
  
  
+ TACACATTGT CGACCTCAGC ATCTCCCACT GTATGCAGAT CCCCACGTTG ATCGACTCCA TTGCGACCCG   
  
  
+ GTTGGAAGCC CCGGGTCGAG TCCCTCCTAT TGTCAAGCTC ACCGTCGGGG CTATTTCCGA CGAAATCCCG   
  
  
+ CCGGTGTTCG ATCTTCTGTC GTACGATGAG CTCGGAATGA GACTAATCAA CTTCGCTCGT TTTAGAAACA   
  
  
+ TCGTCCTCGA ATTCCAAGCA ATACCCACCT CCCCTTCCGA CGGATTTGCT TCGCTGTTGG AGGAGATTCG   
  
  
+ ACAAAGCAAG CTCTACTCCA ACGATGCGGC GGCGGTTATT GTGAATTGTC AGATGAGTTT GCATTTATTG   
  
  
+ CAGGAAGAGG AGGTGTCTTC ATCGTCGCCG TCGTCGATGA GGGGGATGTT TTTGCAGGCG GTGAGGAGCT   
  
  
+ TGGAGCCGAG CATGGTGGTG GTGGTGGAGG AGGACGTGGA TTTCACGGCG AGGAGTCTGG TGGGGCGGCT   
  
  
+ GAGATCGGCG TTTAATCACA TGTGGATACC CTTCGACACG GTGGACACGT TCTTGCCACG TGGGAGCCAG   
  
  
+ CAGAGAGAGT GGTTCGAGGC CGAGGTGTGC TGGAAGATTG AGAATGTGAT CGCTCATGAG GGACCCGCGA   
  
  
+ GGGTCGAGAG GCAGGAGCCC AGGGCCAAGT GGGCCCTCCG AATGAGGGAG GCCGAGTTTC AAGGGATCGA   
  
  
+ GTTCGGTGAT GAAGGTACGA CCGAGGTCAA GGCCATGCTG GAGGAGCATG CCGCTGGGTG GGGGTCTAAG   
  
  
+ AAGGAAGAGG ATGATCTTGT GCTCACTTGG AAGGGACATA GTGTTGTCTT TGCTTCTGCT TGGGTACCCA   
  
  
+ CTTA  

- +Up\_Stream \_Len000TCTTCG ATTAATTTGG TTTGTACGTG CATACAGGAT TAATTGTACT GACAGGTAGT   
  
  
- AGAGGAGGGT ACAACAAGAC AACTGTTCGG ACGTGTGGGT ACGAGGAGAG AGTACAGATT GGAGCAGGGT   
  
  
- TGCTAGTTCT AGTGACAGAC ACTCCGGTCC CCTTCTGAAC GAAAATAAAG GAAAAGAAAA CAAATGAGAG   
  
  
- ACTAAGGTAA ATCGGTATAT ATGTTCCTCT TACAGTACAC ATAGAGTATA CATATATATA TTCTAAAATA   
  
  
- AATCTTTATT TTTAAATTTG TGATACACTA AATATCAATA ATATTATTTA TCATAAAAAA TTTGATAAAG   
  
  
- TAACTACTCT TGTCCTTTCA TTTAACGGCT GTACAATTAC AATAATATAG ATTTTTAATT TAGTATTTTA   
  
  
- ATTAATATTA TAAAATTTTT ATAAAGTTGA TTTTAAAATC TTGTTATTCA CGCATTATGC CTGAATTTCC   
  
  
- GATCAAATAG CATTTTTACA TTCAAGGAAA ACCTAGAATA CCAACTTATA GAACCAACTA TTTGACACTA   
  
  
- ACCAAAATGT GAAAATGGTG TATAAGTATC TTCTTAATAC ACGTGCAACT ACCAAGTTCT ACCCTGTTTT   
  
  
- CCTTTGTACA TAAGAAGGGG GAAAACGGAA AAGGGGATTC GTAAACCTAA CTCCACAACA GTAGACTTAT   
  
  
- AGTTTTTAAG AAAAACTTTT AAGTCAGAGT TAGTTTTAAG TTTTAATTTT GTTTCTGAAT TCTCAAAATA   
  
  
- AACAAGAATT GGAAGAATTG AAAGATGAAA AGAAAAAAAA GAGTAAGGGG GAGAAGTTTA GAGTAAATAA   
  
  
- GAATTGGGGG AGAAGGCAAG TACGAGAGAG ACAGATAGTT GATTATAATT AGATGGGGCT GAAAGTTAAA   
  
  
- CTACTCCAGA TTAGGAATAG TAGCATAACA CCAACCCAAT TAATACGTTC TTCCGTTATT TGGGGTCACT   
  
  
- GTTTCAGGTG CAATTCATCC GTGGAGTGGT AATTCTGTAC GAGTCTTTTG TGGTTTTGGT AACTTGTGTT   
  
  
- CAGGGGGTTC AGGGAAGGGA TCGATCGGGT AAGAGAGAGG AGGGGACAGA TATAGATGGT TGAGAACGAG   
  
  
- AAACGACTCG GACTCAACTT TCGCTATGTG GGTATCGGAC AAGTCAAAAG TCAAAAGTGT AAACAGAAGG   
  
  
- AGACAAGTCA ACTTTCGCTA TGTGAGAGTA ATCGAAAGTA AAATTTATGC AAGTTAAGTG TGTATTTACC   
  
  
- GAAAGTTTCG TAACGGTAAA AGCAAATTTA CTACTGTTTT ATATATCATA GACCGATAGA CGGGTATAGA   
  
  
- ACTGGAATAA ATGTTTCCGA CTCGGTAGAA AATTAAAAAA ATCGGAGAAA AAAATTAGTT TTTATCTTTA   
  
  
- AAGTTCTTTC GTCTTTAGCA GTCTACTGTC ACGTCTGTCG CAAAAGCAAA GTATGTTTAG AGTTTTCGAC   
  
  
- TTCACAGTTA AAGAAGCTAA TAAAAACAGT CTTTTATAAA TGCAGAGTGG AAGTGCAACA ATAATAAGAA   
  
  
- AGAAAAAATA ATAAGAGTAT TGATCATTAT TTCAATCGTT ACTATTTTTT AATAAGCTAG GCTTATAAAA   
  
  
- TTAAATAGAC TGGATTTTTG TATTCATTTC CGTGTTTAAA AAGTAGGTTT AAAATTAAAA ACTACATTAT   
  
  
- AAAAAATATA AAAATAACAA GTTTAGACTA AAATTAGAAT AAATTTAATA GACTGGATTT TTTTAGTTTA   
  
  
- TTATTATTTG CAATTAAAAA ATAAATTTAA AACTGTAATC AGTTAGACTG AATTTAAGCT TGGCTCGAAC   
  
  
- TTAAAAAAAC GGTCACGACC ATCATGAGGA CGAAAACATC GAGGGTTACG GTAGCATGGT GGGAGAAGAT   
  
  
- CAGAGCAGAG AAAGAGACCA GAGGTTAATT AGTGTGGTAG TATAGTATGG CATACTACTA TGTCAGGTGA   
  
  
- CTTTAGGGTA AGAGTGGCGG TTAGAGAGAG GCGGTGGTAG GTTGTGAGAA TTGCTACTTG TCCCGGCGGC   
  
  
- GGAGCAGGAG GTGTGAGTCC GGCACCGGCC CGTCGAAAGG GTGGGTTAGT TTTAGAGAGA GGAGGCGGTT   
  
  
- GAAGCCATTG CGGTTAACGT ACCTCGTCGA GAATCAGGTG ACGCGGCTTC GGTAGCTGTT GTTACTGCGG   
  
  
- TGGGGCCGGG TCGTTTAGAA TACCCAGGAC TTATTGTATC GGGCTGGGCT GCCGCTGAGG TGGGTTGCGG   
  
  
- AGTGCACGCG TAAGGATGCA CGGAATTAGA GTGCGCGGCA GGAGTGATCG TGGACGTTCT ACCATTAGGG   
  
  
- AGTAAAGTTG GGGTAGTTAA GTGGGGTGTT TAAGAGCGAG GAGCTTGAAC GGGTGAAGCA GCTAAATTGG   
  
  
- GGAACCGTAG CTAAGCCTAA GTGGCGGCGG TTAAGCTAGT AAGACCTTCG ATAAAGGCTG GATGGGCAAC   
  
  
- ATGTGTAACA GCTGGAGTCG TAGAGGGTGA CATACGTCTA GGGGTGCAAC TAGCTGAGGT AACGCTGGGC   
  
  
- CAACCTTCGG GGCCCAGCTC AGGGAGGATA ACAGTTCGAG TGGCAGCCCC GATAAAGGCT GCTTTAGGGC   
  
  
- GGCCACAAGC TAGAAGACAG CATGCTACTC GAGCCTTACT CTGATTAGTT GAAGCGAGCA AAATCTTTGT   
  
  
- AGCAGGAGCT TAAGGTTCGT TATGGGTGGA GGGGAAGGCT GCCTAAACGA AGCGACAACC TCCTCTAAGC   
  
  
- TGTTTCGTTC GAGATGAGGT TGCTACGCCG CCGCCAATAA CACTTAACAG TCTACTCAAA CGTAAATAAC   
  
  
- GTCCTTCTCC TCCACAGAAG TAGCAGCGGC AGCAGCTACT CCCCCTACAA AAACGTCCGC CACTCCTCGA   
  
  
- ACCTCGGCTC GTACCACCAC CACCACCTCC TCCTGCACCT AAAGTGCCGC TCCTCAGACC ACCCCGCCGA   
  
  
- CTCTAGCCGC AAATTAGTGT ACACCTATGG GAAGCTGTGC CACCTGTGCA AGAACGGTGC ACCCTCGGTC   
  
  
- GTCTCTCTCA CCAAGCTCCG GCTCCACACG ACCTTCTAAC TCTTACACTA GCGAGTACTC CCTGGGCGCT   
  
  
- CCCAGCTCTC CGTCCTCGGG TCCCGGTTCA CCCGGGAGGC TTACTCCCTC CGGCTCAAAG TTCCCTAGCT   
  
  
- CAAGCCACTA CTTCCATGCT GGCTCCAGTT CCGGTACGAC CTCCTCGTAC GGCGACCCAC CCCCAGATTC   
  
  
- TTCCTTCTCC TACTAGAACA CGAGTGAACC TTCCCTGTAT CACAACAGAA ACGAAGACGA ACCCATGGGT   
  
  
- GAAT
